# Supplementary material for: Nanoparticle enrichment mass-spectrometry proteomics identifies protein-altering variants for precise pQTL mapping
Source: Nat Commun. 2024 Feb 2;15:989. doi: 10.1038/s41467-024-45233-y (PMC10837160; doi:10.1038/s41467-024-45233-y)
Supplement: Supplementary file 1 — Supplementary Information [file 41467_2024_45233_MOESM1_ESM.pdf]

SUPPLEMENTARY DATA

Nanoparticle Enrichment Mass-Spectrometry Proteomics Identifies Protein-Altering Variants for Precise pQTL Mapping

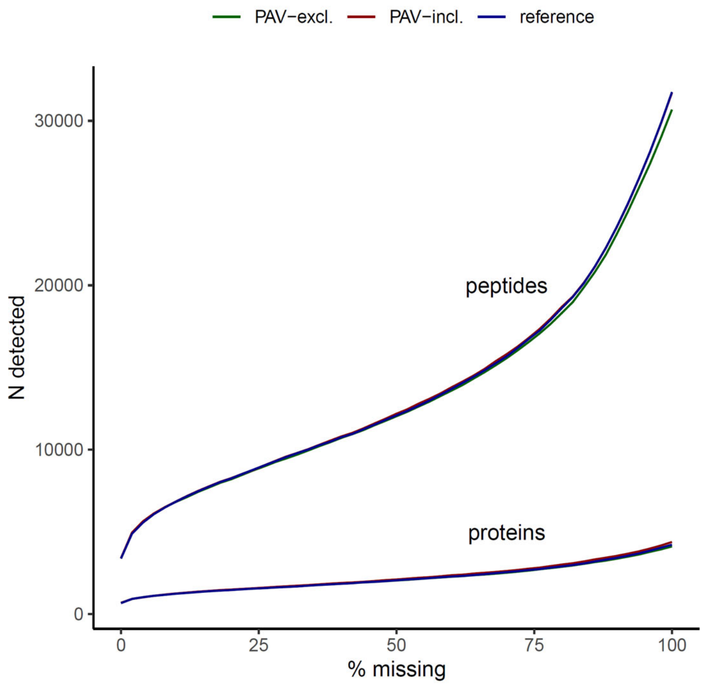

Supplementary Figure 1: Number of detected proteins and peptides as a function of % missingness.

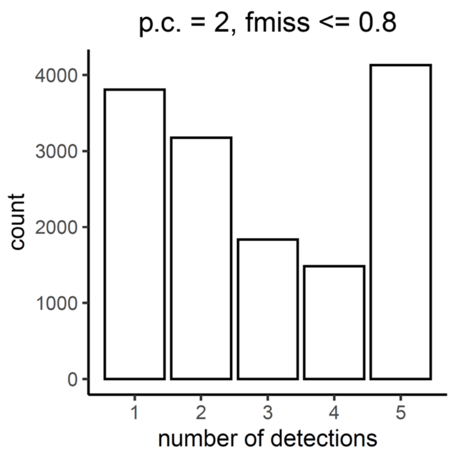

(a)

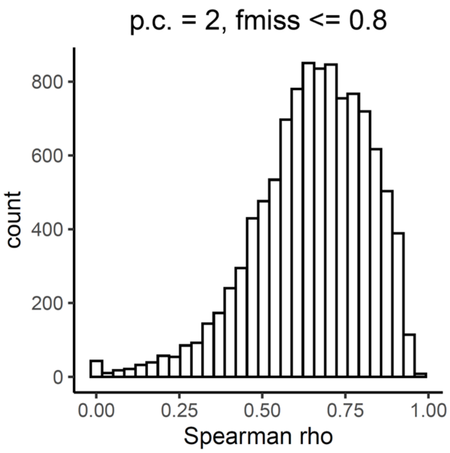

(b)

Supplementary Figure 2: Detection of the same peptide by multiple nanoparticles. (a) Histogram of the number of detections, (b) Histogram of the largest Spearman correlation observed between two peptides when they were detected by more than one nanoparticle. Analysis was limited to 14,430 unique peptides (42,238 detections) that had doubly-charged precursor ions and that were detected in > 20% of the samples. 3,808 (26.4%) of these peptides were detected in a single nano particle fraction while 4,129 (28.6%) were detected in all five. 73.6% were detected more than once. The median Spearman correlation between a peptide measured in two or more nano particle fractions was rho = 0.67, and the Spearman correlation of peptides measured in exactly two fractions was rho = 0.56 (Supplementary Data 1).

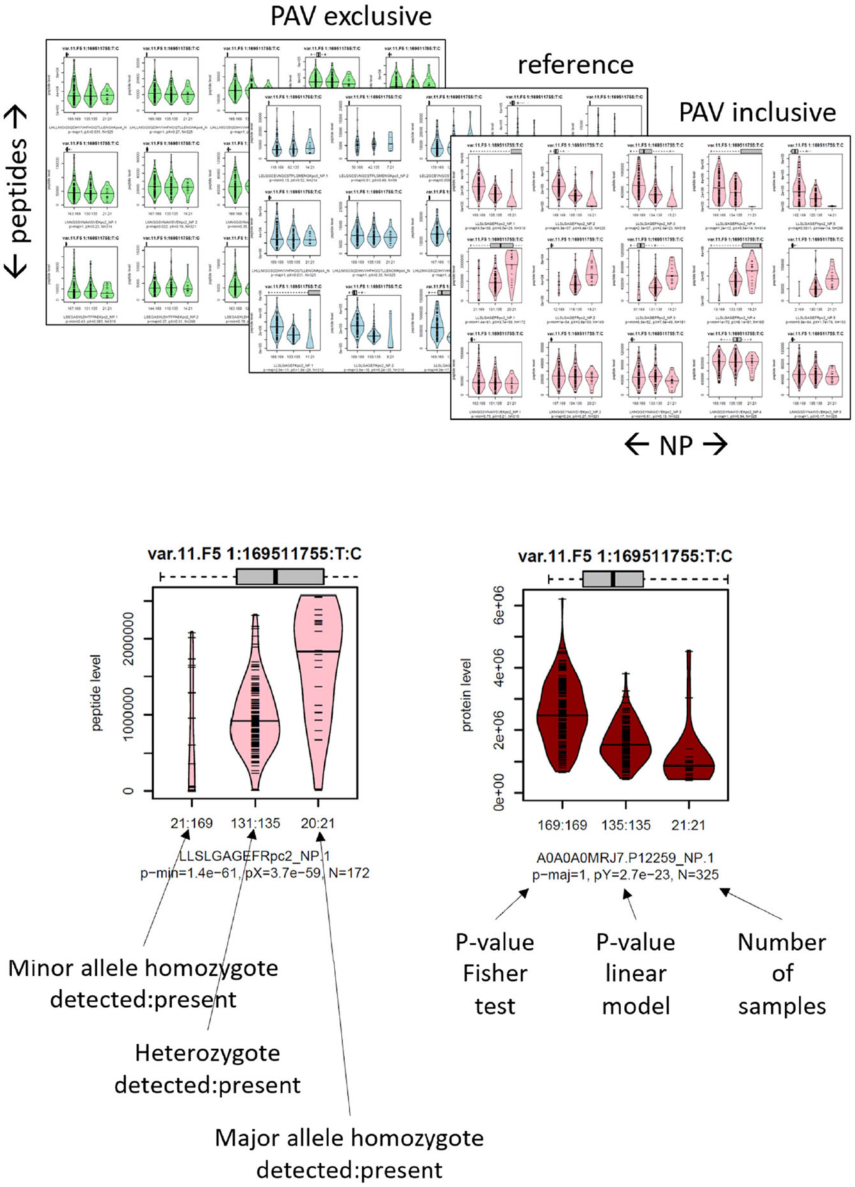

Supplementary Figure 3: Summary violin plots for the MS-PAVs. These plots are provided for all 184 MS-PAVs reported in Supplementary Data 3 and are available with this paper as Source Data. The plots are labeled above the plots, indicating the index of the association (var.N, with N given in column A in Supplementary Data 3), the protein symbol, and the SNP (chr, position, allele 1, allele 2). The libraries that have been used are color-coded as follows: the *PAV-exclusive* library (green), the *reference* library (blue), and the *PAV-inclusive* library (red). Protein intensities are in dark colors, and peptide intensities are in light colors.

The violin plots are ordered alphabetically in rows by peptide sequence, followed by the proteins, and in column by nanoparticle number. The PDF file is searchable; i.e. the sequence of the variant peptide provided in Supplementary Data 3 can be used to locate it. Missing plots indicate that no or insufficient data were available to create that individual plot.

The grey horizontal boxplots on top of the violin plots represent the range of the data shown in that plot compared to the 5%-95% quantile range of the entire data for that protein. Units on the *y*-axis are engine-normalized intensities as provided by DIA-NN. The *x*-axis labels indicate the number of detected peptides or proteins, followed by a colon and the number of samples with the given genotype, in the order: reference/major allele homozygote, heterozygote, alternate/minor allele homozygote. The width of the violins is proportional to the fraction of peptides or proteins detected for a given genotype.

The first line of the subtitle identifies the protein (Uniprot ID and rsID, where applicable) or the peptide sequence followed by the nanoparticle number. The second line shows the number of data points included in generating the plot (N). Significance levels (p-values) for the following hypothesis tests are provided: (1) Fisher's Exact test on detected/non-detected versus presence/absence of the major (*p-maj*) or minor (*p-min*) allele, where the stronger of the two associations is shown (indicating MS-PAV detection significance), and (2) a linear regression of peptide intensity versus genotype (coded 0-1-2) with missing values set to zero (*pX*), and for proteins, a linear model including relevant covariates using inverse-normalized protein intensities (excluding missing values) against genotype (*pY*; indicating pQTL significance). Protein name, chromosome, chromosome position (GRCh37), and major and minor alleles are indicated in boldface on top of the boxplots, matching information given in the PDF file name.

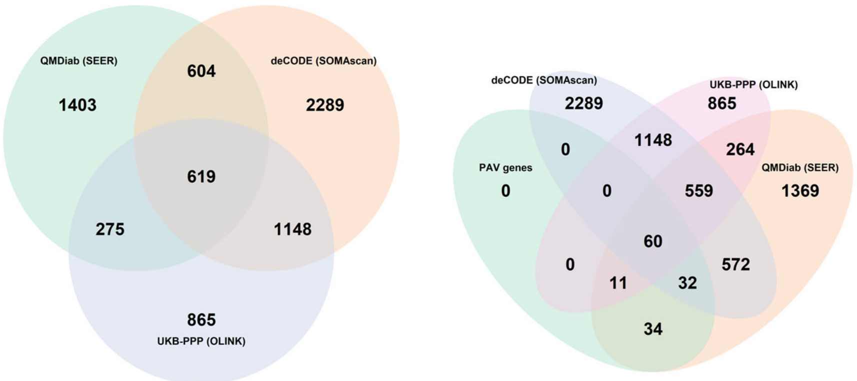

(a) (b)

Group	Detected
QMDiab	2,901
deCODE	4,660
UKB-PPP	2,907
PAV genes	137
Joint	7,203

(c)

Supplementary Figure 4: Protein-coding genes reported by the QMdiab, deCODE, and UKB-PPP studies. (a) Venn diagram representing the overlap of the three studies, (b) the three studies plus the 137 genes that correspond to the 184 PAVs identified here, and (c) gene count by study. Proteins reported by the Seer Proteograph workflow are limited to proteins detected in >20% of the samples. Matching of equivalent proteins was made on the basis of gene annotations rather than UniProt IDs to avoid issues when multiple or different protein isoforms are involved.

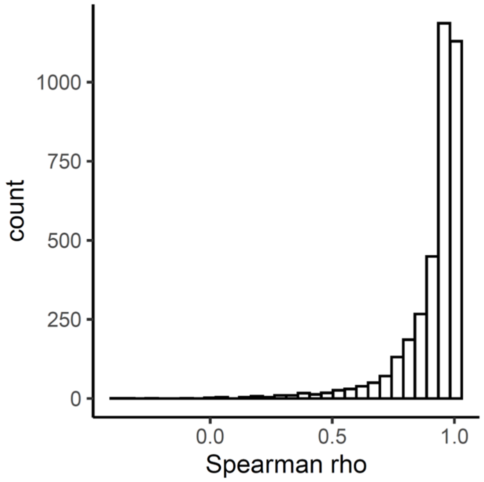

Supplementary Figure 5: Histogram of Spearman correlation between protein levels derived using the reference and the *PAV-exclusive* library, respectively. The figure is limited to 3,657 protein group / nanoparticle combinations that were detected in >80% of the samples. Most proteins (3,183, 87.0%) correlated strongly (Spearman rho > 0.8) and only few (91, 2.5%) showed weak correlation (Spearman rho < 0.5). Full association data is in Supplementary Data 4.

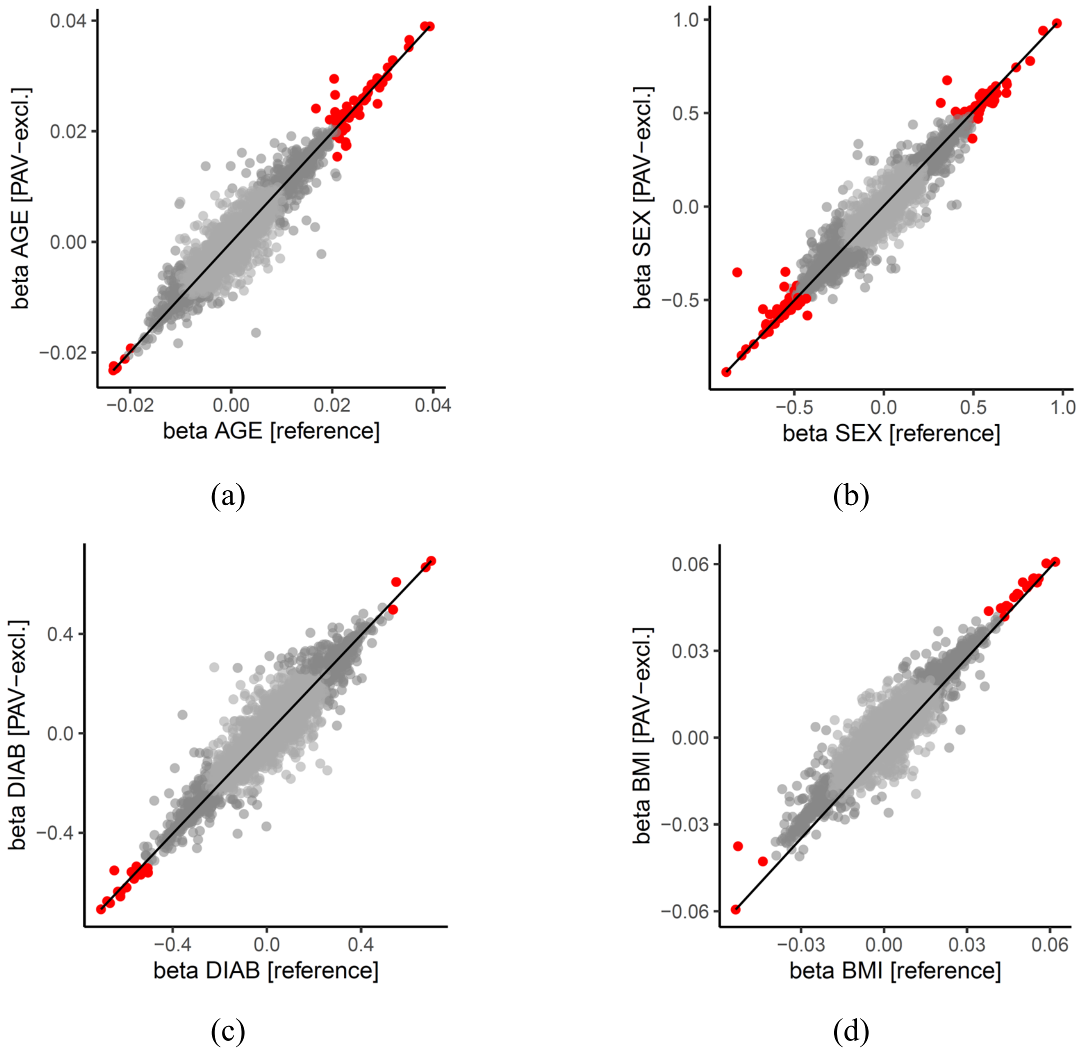

Supplementary Figure 6: Scatterplot of the effect size (beta) for the associations of (a) age, (b) sex, (c) diabetes state, and (d) BMI using the reference and the *PAV-exclusive* library. Statistics were computed using a linear model including age, sex, diabetes status, BMI, and the first three genotype principal components. Analysis was limited to 3,657 protein group / nanoparticle combinations detected in > 20% of the samples. Bonferroni significant associations with the non-genetic determinants are in red ($p < 1.4 \times 10^{-5} = 0.05 / 3657$), nominal associations ($p < 0.05$) are in dark grey, and non-significant associations ($p > 0.05$) are in light grey. Full association data is in Supplementary Data 4.

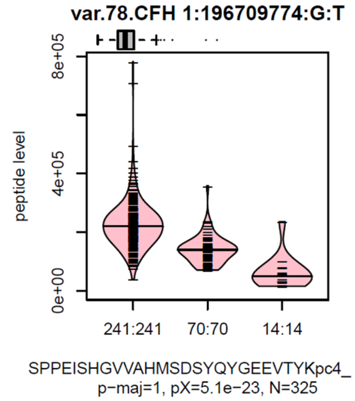

(a)

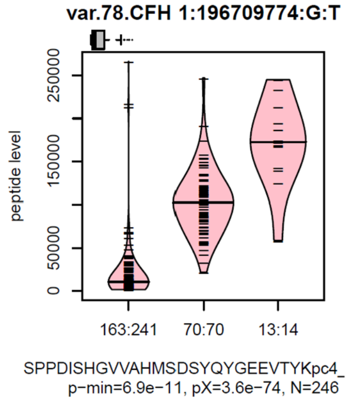

(b)

Supplementary Figure 7: Example of E>D substitution in Complement Factor H (CFH). Shown is the data for the peptides SPP[E/D]ISHGVVAHMSDSYQYGEEVTYK with a precursor charge of 4. Refer to the legend of Supplementary Figure 3 for details. Panel (a) is for the wild type (E variant) and panel (b) shows the D variant.
