# Supplementary material for: Nanoparticle enrichment mass-spectrometry proteomics identifies protein-altering variants for precise pQTL mapping
Source: Nat Commun. 2024 Feb 2;15:989. doi: 10.1038/s41467-024-45233-y (PMC10837160; doi:10.1038/s41467-024-45233-y)
Supplement: Supplementary file 3 — Description of Additional Supplementary Files [file 41467_2024_45233_MOESM3_ESM.pdf]

Description of Additional Supplementary Files:

Supplementary Data 1: Spearman correlations between peptides measured in more than one nanoparticle fraction; limited to doubly-charged precursor ions and peptides detected in > 20% of the samples

Supplementary Data 2: List of all detected variant-peptides (2,341 detections, often on multiple nano particles and with varying precursor charges, for a total of 492 unique peptides)

Supplementary Data 3: List of significant lead variant to variant-peptide associations (N = 184)

Supplementary Data 4: Spearman correlations between protein levels derived using the reference and the PAV-exclusive. libraries; associations with non-genetic determinants (age, sex, diabetes state, BMI, genotype principal components 1-3)
