# Supplementary material for: Nanoparticle enrichment mass-spectrometry proteomics identifies protein-altering variants for precise pQTL mapping
Source: Nat Commun. 2024 Feb 2;15:989. doi: 10.1038/s41467-024-45233-y (PMC10837160; doi:10.1038/s41467-024-45233-y)
Supplement: Supplementary file 9 — Source Data [file 41467_2024_45233_MOESM9_ESM.zip › Source_Data.pdf]

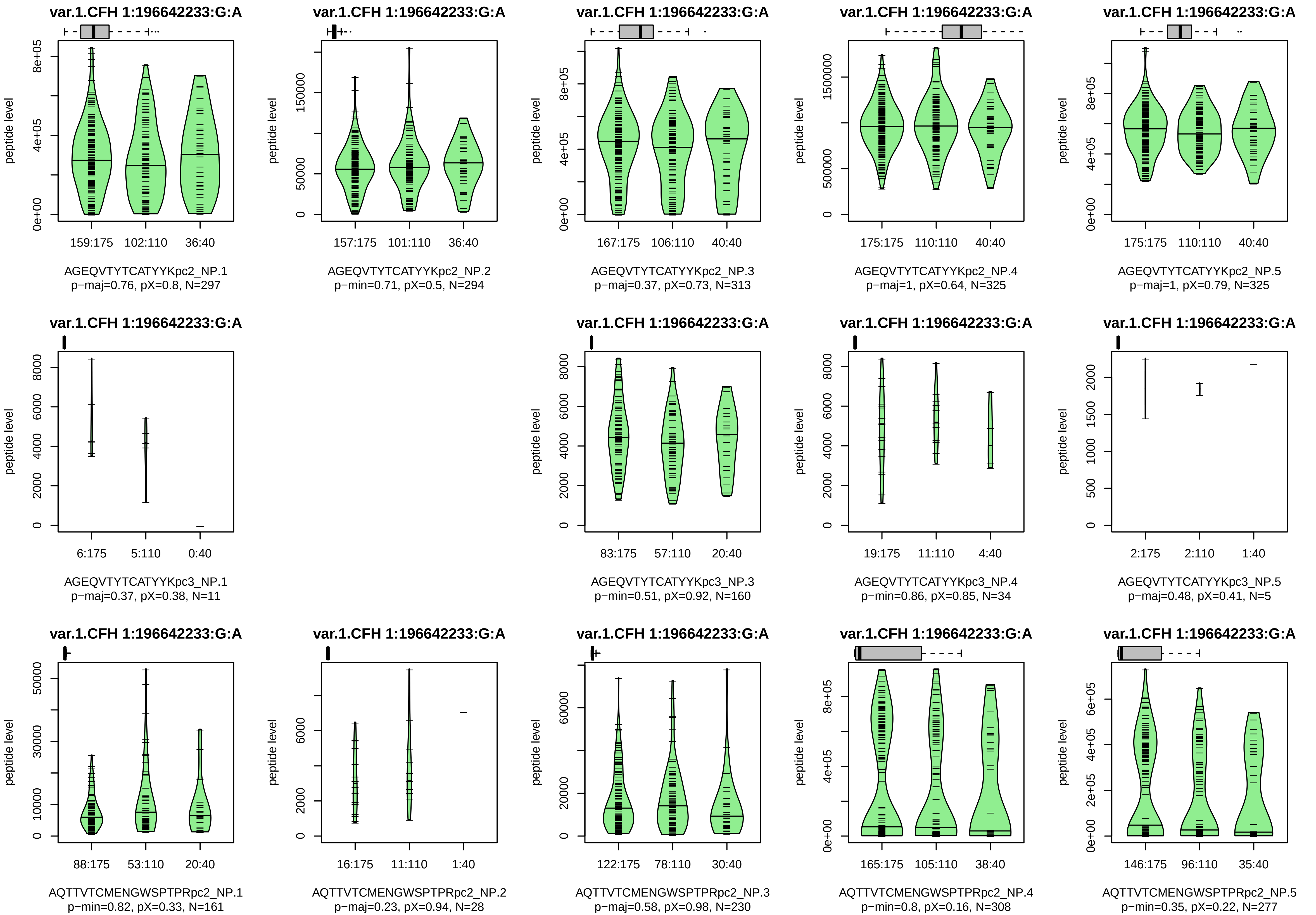
var.1.CFH 1:196642233:G:A
peptide level
0e+00
4e+05
8e+05
159:175
102:110
36:40
AGEQVTYTCATYYKpc2_NP.1
p−maj=0.76, pX=0.8, N=297
var.1.CFH 1:196642233:G:A
peptide level
0
50000
150000
157:175
101:110
36:40
AGEQVTYTCATYYKpc2_NP.2
p−min=0.71, pX=0.5, N=294
var.1.CFH 1:196642233:G:A
peptide level
0e+00
4e+05
8e+05
167:175
106:110
40:40
AGEQVTYTCATYYKpc2_NP.3
p−maj=0.37, pX=0.73, N=313
var.1.CFH 1:196642233:G:A
peptide level
0
500000
1500000
175:175
110:110
40:40
AGEQVTYTCATYYKpc2_NP.4
p−maj=1, pX=0.64, N=325
var.1.CFH 1:196642233:G:A
peptide level
0e+00
4e+05
8e+05
175:175
110:110
40:40
AGEQVTYTCATYYKpc2_NP.5
p−maj=1, pX=0.79, N=325
var.1.CFH 1:196642233:G:A
peptide level
0
2000
4000
6000
8000
6:175
5:110
0:40
AGEQVTYTCATYYKpc3_NP.1
p−maj=0.37, pX=0.38, N=11
var.1.CFH 1:196642233:G:A
peptide level
0
2000
4000
6000
8000
83:175
57:110
20:40
AGEQVTYTCATYYKpc3_NP.3
p−min=0.51, pX=0.92, N=160
var.1.CFH 1:196642233:G:A
peptide level
0
2000
4000
6000
8000
19:175
11:110
4:40
AGEQVTYTCATYYKpc3_NP.4
p−min=0.86, pX=0.85, N=34
var.1.CFH 1:196642233:G:A
peptide level
0
500
1000
1500
2000
2:175
2:110
1:40
AGEQVTYTCATYYKpc3_NP.5
p−maj=0.48, pX=0.41, N=5
var.1.CFH 1:196642233:G:A
peptide level
0
10000
30000
50000
88:175
53:110
20:40
AQTTVTCMENGWSPTPRpc2_NP.1
p−min=0.82, pX=0.33, N=161
var.1.CFH 1:196642233:G:A
peptide level
0
2000
6000
16:175
11:110
1:40
AQTTVTCMENGWSPTPRpc2_NP.2
p−maj=0.23, pX=0.94, N=28
var.1.CFH 1:196642233:G:A
peptide level
0
20000
60000
122:175
78:110
30:40
AQTTVTCMENGWSPTPRpc2_NP.3
p−maj=0.58, pX=0.98, N=230
var.1.CFH 1:196642233:G:A
peptide level
0e+00
4e+05
8e+05
165:175
105:110
38:40
AQTTVTCMENGWSPTPRpc2_NP.4
p−min=0.8, pX=0.16, N=308
var.1.CFH 1:196642233:G:A
peptide level
0e+00
2e+05
4e+05
6e+05
146:175
96:110
35:40
AQTTVTCMENGWSPTPRpc2_NP.5
p−min=0.35, pX=0.22, N=277

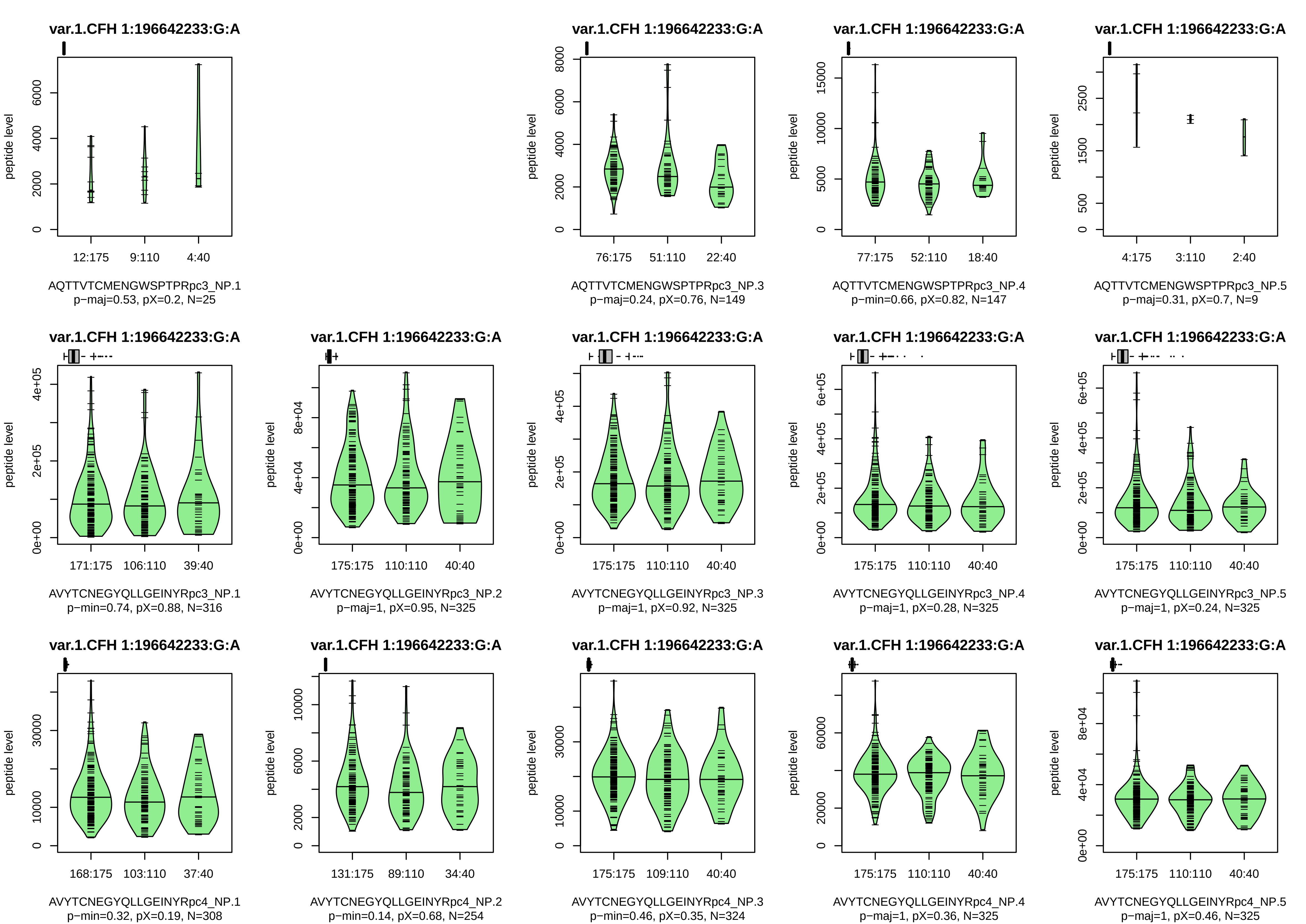
var.1.CFH 1:196642233:G:A
peptide level
12:175
9:110
4:40
AQTTVTCMENGWSPTPRpc3_NP.1
p−maj=0.53, pX=0.2, N=25
var.1.CFH 1:196642233:G:A
peptide level
76:175
51:110
22:40
AQTTVTCMENGWSPTPRpc3_NP.3
p−maj=0.24, pX=0.76, N=149
var.1.CFH 1:196642233:G:A
peptide level
77:175
52:110
18:40
AQTTVTCMENGWSPTPRpc3_NP.4
p−min=0.66, pX=0.82, N=147
var.1.CFH 1:196642233:G:A
peptide level
4:175
3:110
2:40
AQTTVTCMENGWSPTPRpc3_NP.5
p−maj=0.31, pX=0.7, N=9
var.1.CFH 1:196642233:G:A
peptide level
171:175
106:110
39:40
AVYTCNEGYQLLGEINYRpc3_NP.1
p−min=0.74, pX=0.88, N=316
var.1.CFH 1:196642233:G:A
peptide level
175:175
110:110
40:40
AVYTCNEGYQLLGEINYRpc3_NP.2
p−maj=1, pX=0.95, N=325
var.1.CFH 1:196642233:G:A
peptide level
175:175
110:110
40:40
AVYTCNEGYQLLGEINYRpc3_NP.3
p−maj=1, pX=0.92, N=325
var.1.CFH 1:196642233:G:A
peptide level
175:175
110:110
40:40
AVYTCNEGYQLLGEINYRpc3_NP.4
p−maj=1, pX=0.28, N=325
var.1.CFH 1:196642233:G:A
peptide level
175:175
110:110
40:40
AVYTCNEGYQLLGEINYRpc3_NP.5
p−maj=1, pX=0.24, N=325
var.1.CFH 1:196642233:G:A
peptide level
168:175
103:110
37:40
AVYTCNEGYQLLGEINYRpc4_NP.1
p−min=0.32, pX=0.19, N=308
var.1.CFH 1:196642233:G:A
peptide level
131:175
89:110
34:40
AVYTCNEGYQLLGEINYRpc4_NP.2
p−min=0.14, pX=0.68, N=254
var.1.CFH 1:196642233:G:A
peptide level
175:175
109:110
40:40
AVYTCNEGYQLLGEINYRpc4_NP.3
p−min=0.46, pX=0.35, N=324
var.1.CFH 1:196642233:G:A
peptide level
175:175
110:110
40:40
AVYTCNEGYQLLGEINYRpc4_NP.4
p−maj=1, pX=0.36, N=325
var.1.CFH 1:196642233:G:A
peptide level
175:175
110:110
40:40
AVYTCNEGYQLLGEINYRpc4_NP.5
p−maj=1, pX=0.46, N=325

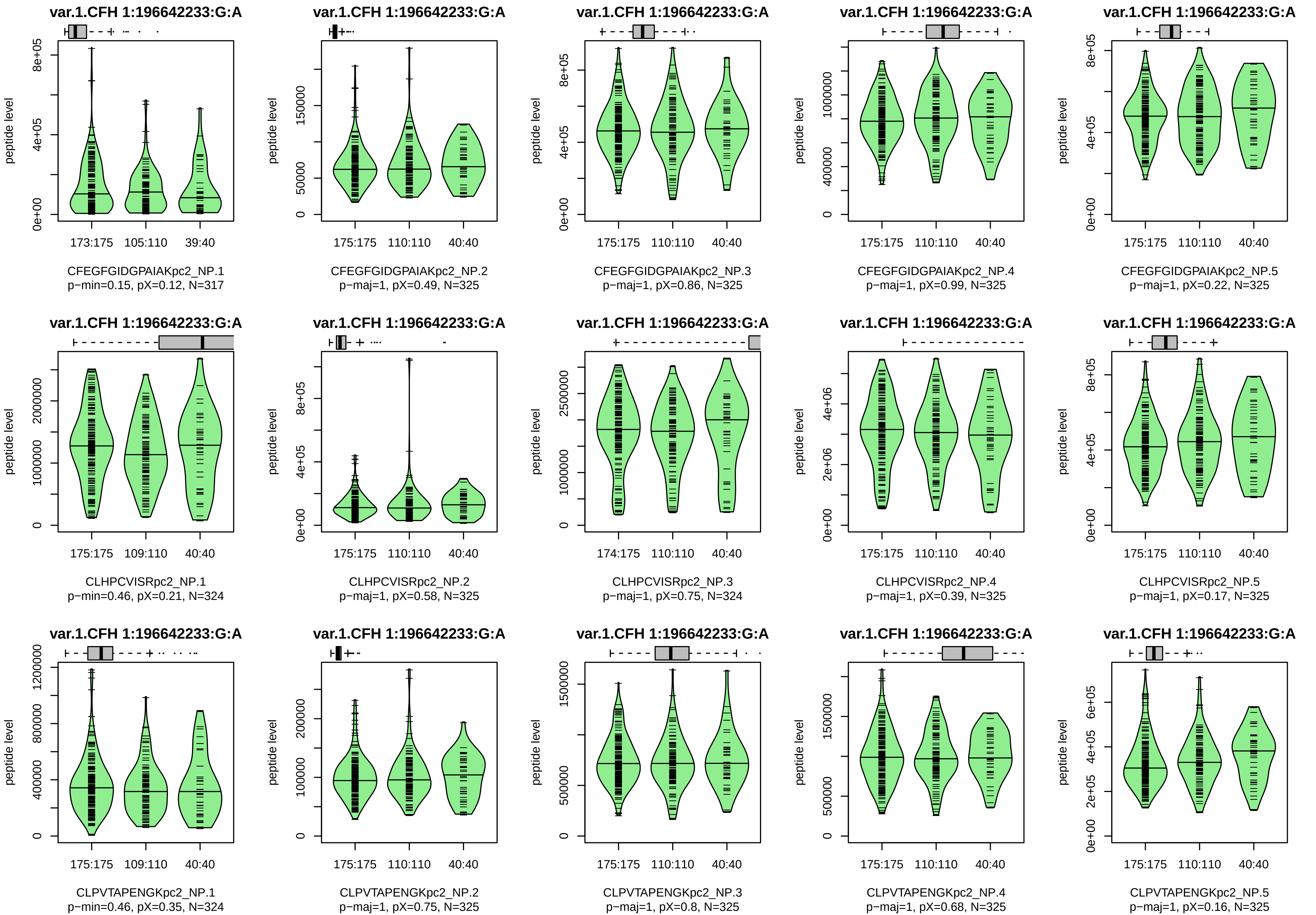
var.1.CFH 1:196642233:G:A
peptide level
173:175
105:110
39:40
CFEGFGIDGPAIAKpc2_NP.1
p−min=0.15, pX=0.12, N=317
var.1.CFH 1:196642233:G:A
peptide level
175:175
110:110
40:40
CFEGFGIDGPAIAKpc2_NP.2
p−maj=1, pX=0.49, N=325
var.1.CFH 1:196642233:G:A
peptide level
175:175
110:110
40:40
CFEGFGIDGPAIAKpc2_NP.3
p−maj=1, pX=0.86, N=325
var.1.CFH 1:196642233:G:A
peptide level
175:175
110:110
40:40
CFEGFGIDGPAIAKpc2_NP.4
p−maj=1, pX=0.99, N=325
var.1.CFH 1:196642233:G:A
peptide level
175:175
110:110
40:40
CFEGFGIDGPAIAKpc2_NP.5
p−maj=1, pX=0.22, N=325
var.1.CFH 1:196642233:G:A
peptide level
175:175
109:110
40:40
CLHPCVISRpc2_NP.1
p−min=0.46, pX=0.21, N=324
var.1.CFH 1:196642233:G:A
peptide level
175:175
110:110
40:40
CLHPCVISRpc2_NP.2
p−maj=1, pX=0.58, N=325
var.1.CFH 1:196642233:G:A
peptide level
174:175
110:110
40:40
CLHPCVISRpc2_NP.3
p−maj=1, pX=0.75, N=324
var.1.CFH 1:196642233:G:A
peptide level
175:175
110:110
40:40
CLHPCVISRpc2_NP.4
p−maj=1, pX=0.39, N=325
var.1.CFH 1:196642233:G:A
peptide level
175:175
110:110
40:40
CLHPCVISRpc2_NP.5
p−maj=1, pX=0.17, N=325
var.1.CFH 1:196642233:G:A
peptide level
175:175
109:110
40:40
CLPVTAPENGKpc2_NP.1
p−min=0.46, pX=0.35, N=324
var.1.CFH 1:196642233:G:A
peptide level
175:175
110:110
40:40
CLPVTAPENGKpc2_NP.2
p−maj=1, pX=0.75, N=325
var.1.CFH 1:196642233:G:A
peptide level
175:175
110:110
40:40
CLPVTAPENGKpc2_NP.3
p−maj=1, pX=0.8, N=325
var.1.CFH 1:196642233:G:A
peptide level
175:175
110:110
40:40
CLPVTAPENGKpc2_NP.4
p−maj=1, pX=0.68, N=325
var.1.CFH 1:196642233:G:A
peptide level
175:175
110:110
40:40
CLPVTAPENGKpc2_NP.5
p−maj=1, pX=0.16, N=325

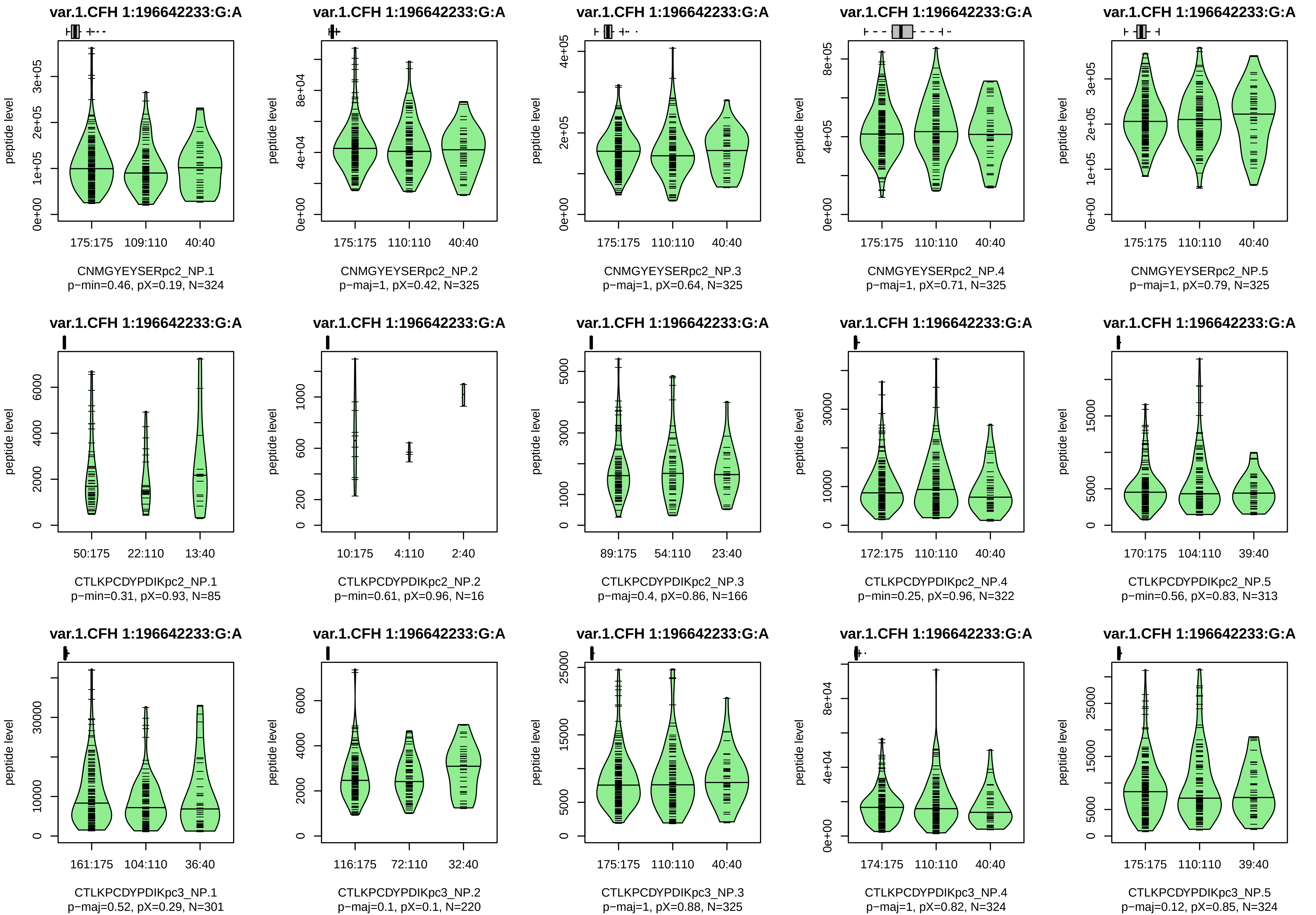
var.1.CFH 1:196642233:G:A
peptide level
175:175
109:110
40:40
CNMGYEYSERpc2_NP.1
p−min=0.46, pX=0.19, N=324
var.1.CFH 1:196642233:G:A
peptide level
175:175
110:110
40:40
CNMGYEYSERpc2_NP.2
p−maj=1, pX=0.42, N=325
var.1.CFH 1:196642233:G:A
peptide level
175:175
110:110
40:40
CNMGYEYSERpc2_NP.3
p−maj=1, pX=0.64, N=325
var.1.CFH 1:196642233:G:A
peptide level
175:175
110:110
40:40
CNMGYEYSERpc2_NP.4
p−maj=1, pX=0.71, N=325
var.1.CFH 1:196642233:G:A
peptide level
175:175
110:110
40:40
CNMGYEYSERpc2_NP.5
p−maj=1, pX=0.79, N=325
var.1.CFH 1:196642233:G:A
peptide level
50:175
22:110
13:40
CTLKPCDYPDIKpc2_NP.1
p−min=0.31, pX=0.93, N=85
var.1.CFH 1:196642233:G:A
peptide level
10:175
4:110
2:40
CTLKPCDYPDIKpc2_NP.2
p−min=0.61, pX=0.96, N=16
var.1.CFH 1:196642233:G:A
peptide level
89:175
54:110
23:40
CTLKPCDYPDIKpc2_NP.3
p−maj=0.4, pX=0.86, N=166
var.1.CFH 1:196642233:G:A
peptide level
172:175
110:110
40:40
CTLKPCDYPDIKpc2_NP.4
p−min=0.25, pX=0.96, N=322
var.1.CFH 1:196642233:G:A
peptide level
170:175
104:110
39:40
CTLKPCDYPDIKpc2_NP.5
p−min=0.56, pX=0.83, N=313
var.1.CFH 1:196642233:G:A
peptide level
161:175
104:110
36:40
CTLKPCDYPDIKpc3_NP.1
p−maj=0.52, pX=0.29, N=301
var.1.CFH 1:196642233:G:A
peptide level
116:175
72:110
32:40
CTLKPCDYPDIKpc3_NP.2
p−maj=0.1, pX=0.1, N=220
var.1.CFH 1:196642233:G:A
peptide level
175:175
110:110
40:40
CTLKPCDYPDIKpc3_NP.3
p−maj=1, pX=0.88, N=325
var.1.CFH 1:196642233:G:A
peptide level
174:175
110:110
40:40
CTLKPCDYPDIKpc3_NP.4
p−maj=1, pX=0.82, N=324
var.1.CFH 1:196642233:G:A
peptide level
175:175
110:110
39:40
CTLKPCDYPDIKpc3_NP.5
p−maj=0.12, pX=0.85, N=324

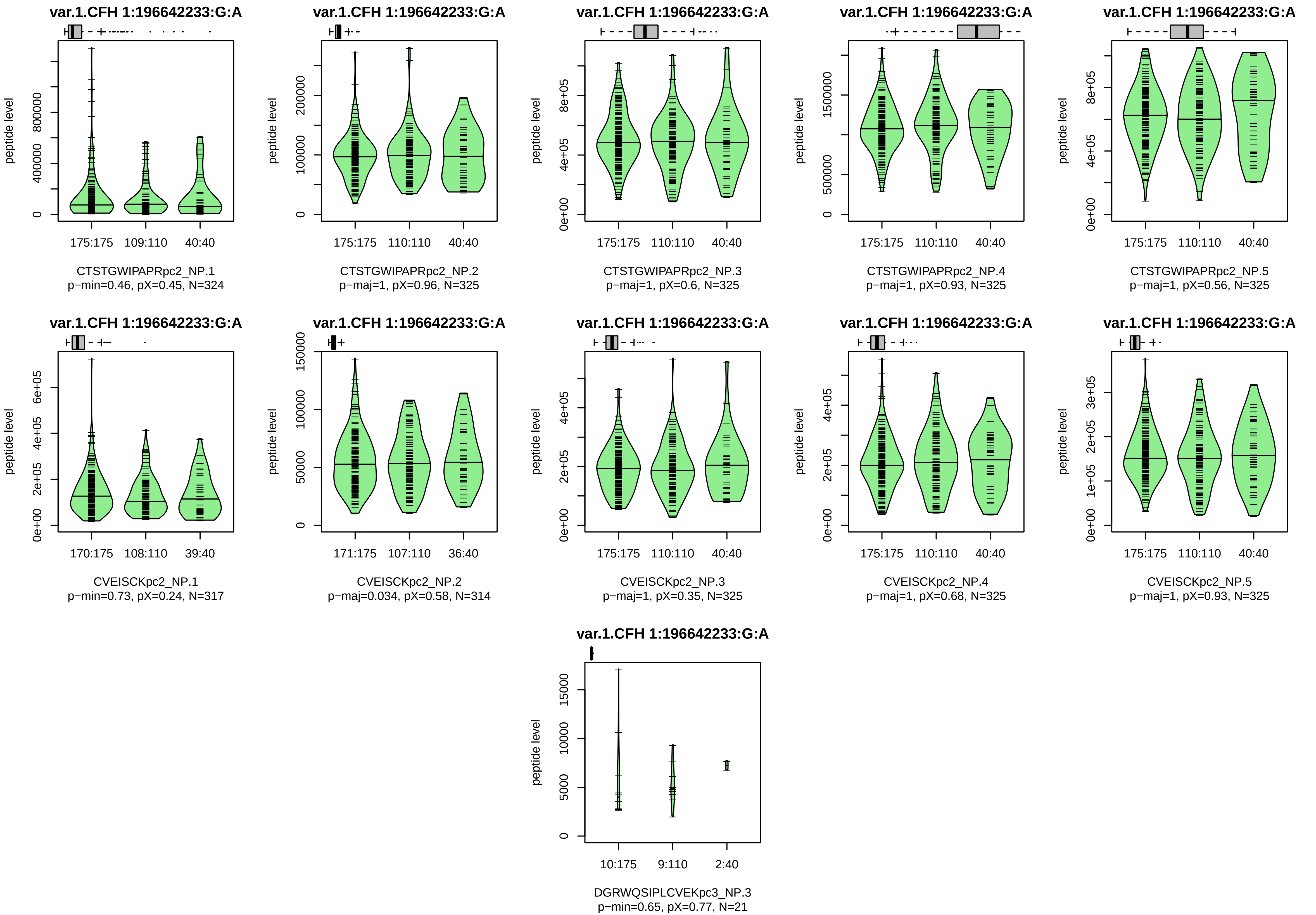
var.1.CFH 1:196642233:G:A
peptide level
175:175 109:110 40:40
CTSTGWIPAPRpc2_NP.1
p−min=0.46, pX=0.45, N=324
var.1.CFH 1:196642233:G:A
peptide level
175:175 110:110 40:40
CTSTGWIPAPRpc2_NP.2
p−maj=1, pX=0.96, N=325
var.1.CFH 1:196642233:G:A
peptide level
175:175 110:110 40:40
CTSTGWIPAPRpc2_NP.3
p−maj=1, pX=0.6, N=325
var.1.CFH 1:196642233:G:A
peptide level
175:175 110:110 40:40
CTSTGWIPAPRpc2_NP.4
p−maj=1, pX=0.93, N=325
var.1.CFH 1:196642233:G:A
peptide level
175:175 110:110 40:40
CTSTGWIPAPRpc2_NP.5
p−maj=1, pX=0.56, N=325
var.1.CFH 1:196642233:G:A
peptide level
170:175 108:110 39:40
CVEISCKpc2_NP.1
p−min=0.73, pX=0.24, N=317
var.1.CFH 1:196642233:G:A
peptide level
171:175 107:110 36:40
CVEISCKpc2_NP.2
p−maj=0.034, pX=0.58, N=314
var.1.CFH 1:196642233:G:A
peptide level
175:175 110:110 40:40
CVEISCKpc2_NP.3
p−maj=1, pX=0.35, N=325
var.1.CFH 1:196642233:G:A
peptide level
175:175 110:110 40:40
CVEISCKpc2_NP.4
p−maj=1, pX=0.68, N=325
var.1.CFH 1:196642233:G:A
peptide level
175:175 110:110 40:40
CVEISCKpc2_NP.5
p−maj=1, pX=0.93, N=325
var.1.CFH 1:196642233:G:A
peptide level
10:175 9:110 2:40
DGRWQSIPLCVEKpc3_NP.3
p−min=0.65, pX=0.77, N=21

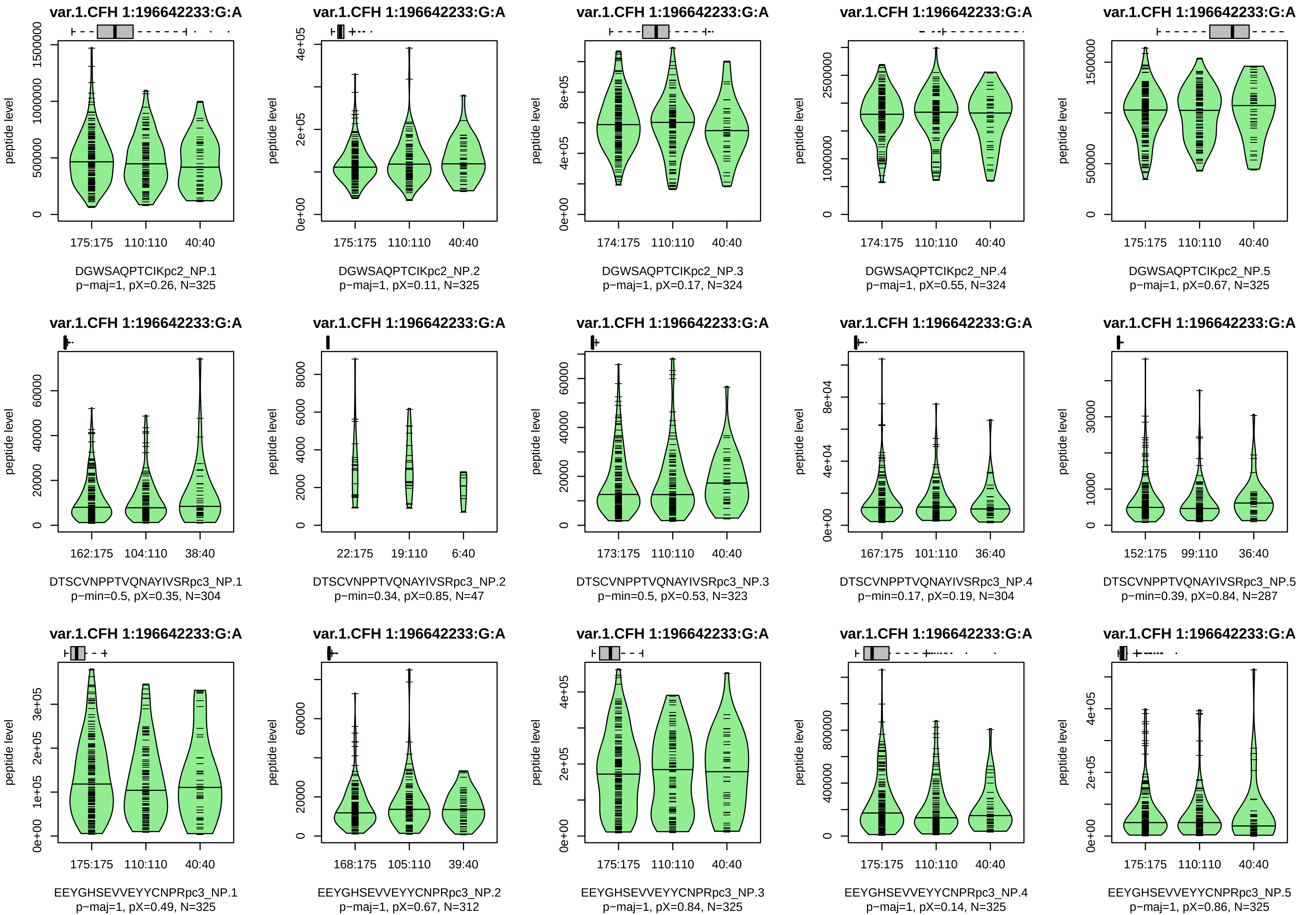

var.1.CFH 1:196642233:G:A
peptide level
175:175 110:110 40:40
DGWSAQPTCIKpc2_NP.1
p−maj=1, pX=0.26, N=325
var.1.CFH 1:196642233:G:A
peptide level
175:175 110:110 40:40
DGWSAQPTCIKpc2_NP.2
p−maj=1, pX=0.11, N=325
var.1.CFH 1:196642233:G:A
peptide level
174:175 110:110 40:40
DGWSAQPTCIKpc2_NP.3
p−maj=1, pX=0.17, N=324
var.1.CFH 1:196642233:G:A
peptide level
174:175 110:110 40:40
DGWSAQPTCIKpc2_NP.4
p−maj=1, pX=0.55, N=324
var.1.CFH 1:196642233:G:A
peptide level
175:175 110:110 40:40
DGWSAQPTCIKpc2_NP.5
p−maj=1, pX=0.67, N=325
var.1.CFH 1:196642233:G:A
peptide level
162:175 104:110 38:40
DTSCVNPPTVQNAYIVSRpc3_NP.1
p−min=0.5, pX=0.35, N=304
var.1.CFH 1:196642233:G:A
peptide level
22:175 19:110 6:40
DTSCVNPPTVQNAYIVSRpc3_NP.2
p−min=0.34, pX=0.85, N=47
var.1.CFH 1:196642233:G:A
peptide level
173:175 110:110 40:40
DTSCVNPPTVQNAYIVSRpc3_NP.3
p−min=0.5, pX=0.53, N=323
var.1.CFH 1:196642233:G:A
peptide level
167:175 101:110 36:40
DTSCVNPPTVQNAYIVSRpc3_NP.4
p−min=0.17, pX=0.19, N=304
var.1.CFH 1:196642233:G:A
peptide level
152:175 99:110 36:40
DTSCVNPPTVQNAYIVSRpc3_NP.5
p−min=0.39, pX=0.84, N=287
var.1.CFH 1:196642233:G:A
peptide level
175:175 110:110 40:40
EEYGHSEVVEYYCNPRpc3_NP.1
p−maj=1, pX=0.49, N=325
var.1.CFH 1:196642233:G:A
peptide level
168:175 105:110 39:40
EEYGHSEVVEYYCNPRpc3_NP.2
p−maj=1, pX=0.67, N=312
var.1.CFH 1:196642233:G:A
peptide level
175:175 110:110 40:40
EEYGHSEVVEYYCNPRpc3_NP.3
p−maj=1, pX=0.84, N=325
var.1.CFH 1:196642233:G:A
peptide level
175:175 110:110 40:40
EEYGHSEVVEYYCNPRpc3_NP.4
p−maj=1, pX=0.14, N=325
var.1.CFH 1:196642233:G:A
peptide level
175:175 110:110 40:40
EEYGHSEVVEYYCNPRpc3_NP.5
p−maj=1, pX=0.86, N=325

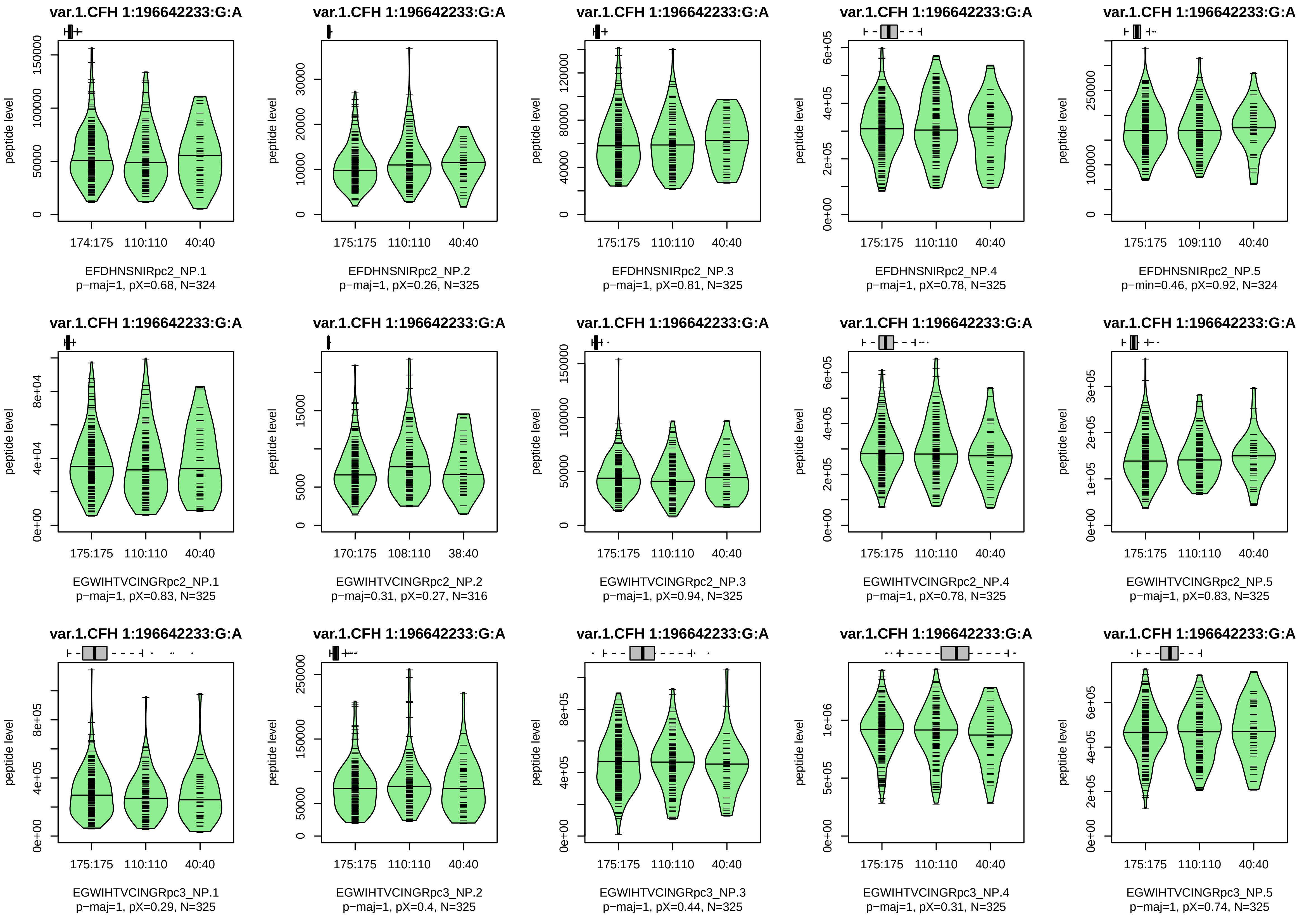
var.1.CFH 1:196642233:G:A
peptide level
174:175 110:110 40:40
EFDHNSNIRpc2_NP.1
p−maj=1, pX=0.68, N=324
var.1.CFH 1:196642233:G:A
175:175 110:110 40:40
EFDHNSNIRpc2_NP.2
p−maj=1, pX=0.26, N=325
var.1.CFH 1:196642233:G:A
175:175 110:110 40:40
EFDHNSNIRpc2_NP.3
p−maj=1, pX=0.81, N=325
var.1.CFH 1:196642233:G:A
175:175 110:110 40:40
EFDHNSNIRpc2_NP.4
p−maj=1, pX=0.78, N=325
var.1.CFH 1:196642233:G:A
175:175 109:110 40:40
EFDHNSNIRpc2_NP.5
p−min=0.46, pX=0.92, N=324
var.1.CFH 1:196642233:G:A
175:175 110:110 40:40
EGWIHTVCINGRpc2_NP.1
p−maj=1, pX=0.83, N=325
var.1.CFH 1:196642233:G:A
170:175 108:110 38:40
EGWIHTVCINGRpc2_NP.2
p−maj=0.31, pX=0.27, N=316
var.1.CFH 1:196642233:G:A
175:175 110:110 40:40
EGWIHTVCINGRpc2_NP.3
p−maj=1, pX=0.94, N=325
var.1.CFH 1:196642233:G:A
175:175 110:110 40:40
EGWIHTVCINGRpc2_NP.4
p−maj=1, pX=0.78, N=325
var.1.CFH 1:196642233:G:A
175:175 110:110 40:40
EGWIHTVCINGRpc2_NP.5
p−maj=1, pX=0.83, N=325
var.1.CFH 1:196642233:G:A
175:175 110:110 40:40
EGWIHTVCINGRpc3_NP.1
p−maj=1, pX=0.29, N=325
var.1.CFH 1:196642233:G:A
175:175 110:110 40:40
EGWIHTVCINGRpc3_NP.2
p−maj=1, pX=0.4, N=325
var.1.CFH 1:196642233:G:A
175:175 110:110 40:40
EGWIHTVCINGRpc3_NP.3
p−maj=1, pX=0.44, N=325
var.1.CFH 1:196642233:G:A
175:175 110:110 40:40
EGWIHTVCINGRpc3_NP.4
p−maj=1, pX=0.31, N=325
var.1.CFH 1:196642233:G:A
175:175 110:110 40:40
EGWIHTVCINGRpc3_NP.5
p−maj=1, pX=0.74, N=325

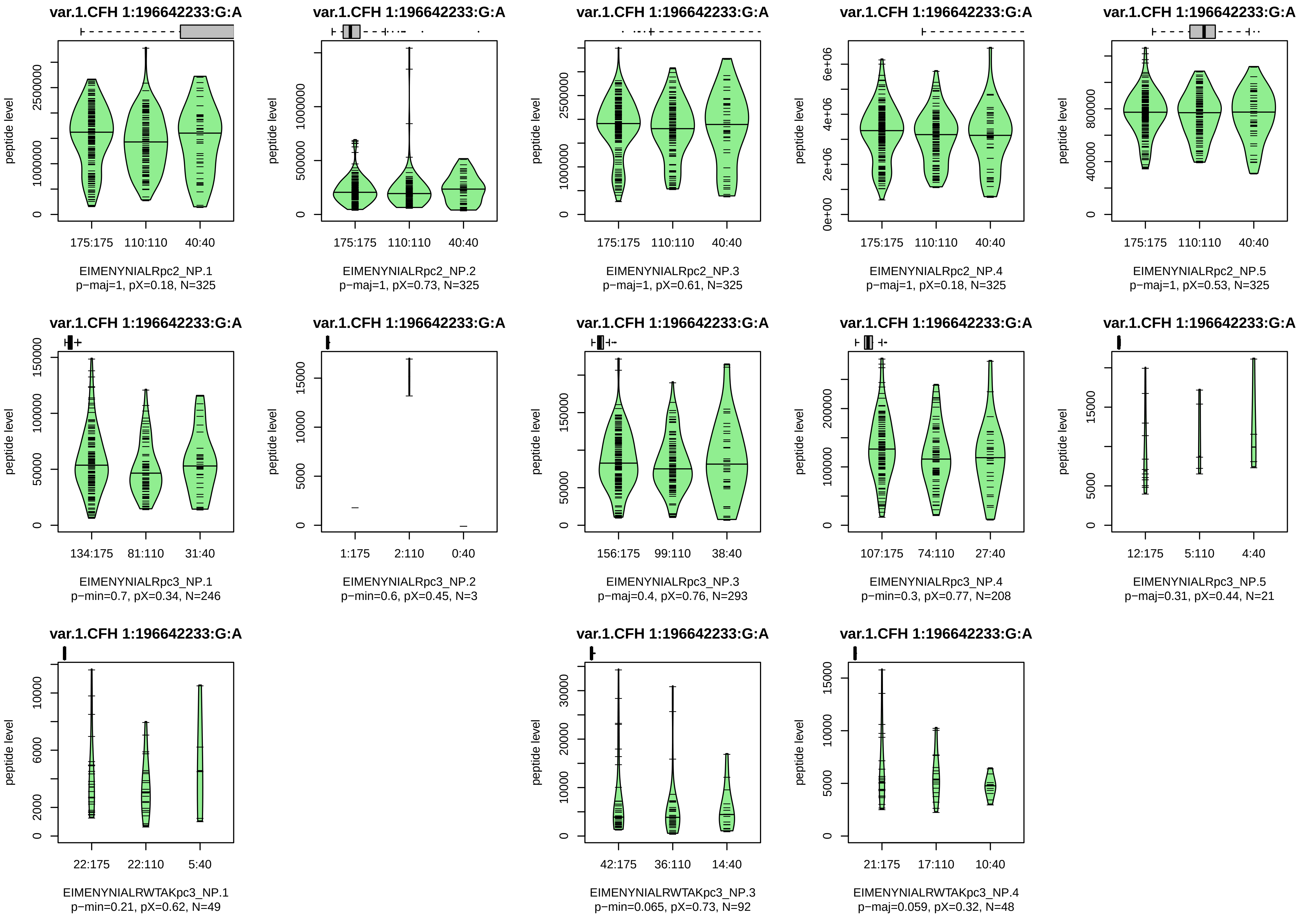
var.1.CFH 1:196642233:G:A
peptide level
175:175
110:110
40:40
EIMENYNIALRpc2_NP.1
p−maj=1, pX=0.18, N=325
EIMENYNIALRpc2_NP.2
p−maj=1, pX=0.73, N=325
EIMENYNIALRpc2_NP.3
p−maj=1, pX=0.61, N=325
EIMENYNIALRpc2_NP.4
p−maj=1, pX=0.18, N=325
EIMENYNIALRpc2_NP.5
p−maj=1, pX=0.53, N=325
134:175
81:110
31:40
EIMENYNIALRpc3_NP.1
p−min=0.7, pX=0.34, N=246
1:175
2:110
0:40
EIMENYNIALRpc3_NP.2
p−min=0.6, pX=0.45, N=3
156:175
99:110
38:40
EIMENYNIALRpc3_NP.3
p−maj=0.4, pX=0.76, N=293
107:175
74:110
27:40
EIMENYNIALRpc3_NP.4
p−min=0.3, pX=0.77, N=208
12:175
5:110
4:40
EIMENYNIALRpc3_NP.5
p−maj=0.31, pX=0.44, N=21
22:175
22:110
5:40
EIMENYNIALRWTAKpc3_NP.1
p−min=0.21, pX=0.62, N=49
42:175
36:110
14:40
EIMENYNIALRWTAKpc3_NP.3
p−min=0.065, pX=0.73, N=92
21:175
17:110
10:40
EIMENYNIALRWTAKpc3_NP.4
p−maj=0.059, pX=0.32, N=48

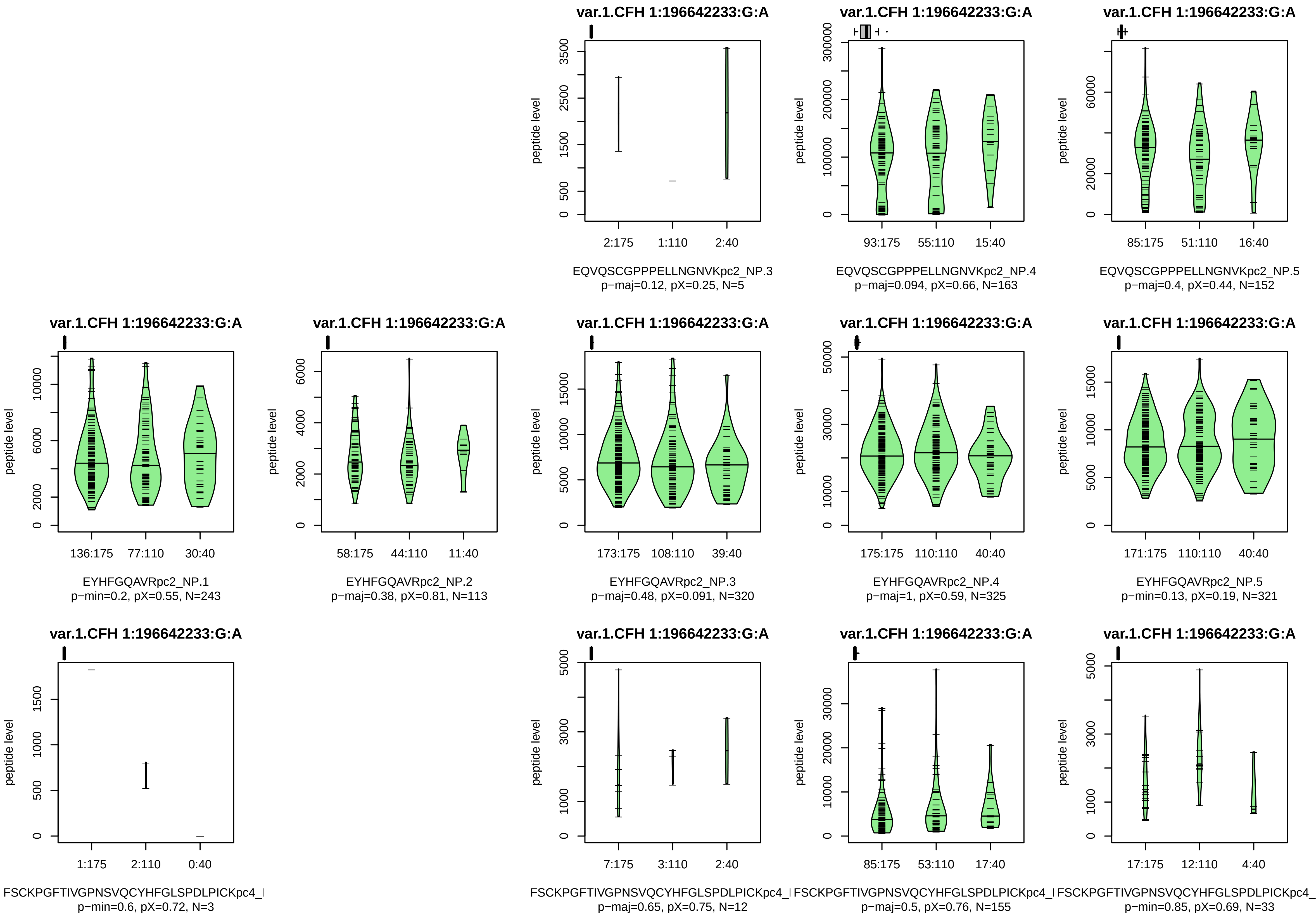
var.1.CFH 1:196642233:G:A
peptide level
2:175
1:110
2:40
EQVQSCGPPPELLNGNVKpc2_NP.3
p−maj=0.12, pX=0.25, N=5
var.1.CFH 1:196642233:G:A
peptide level
93:175
55:110
15:40
EQVQSCGPPPELLNGNVKpc2_NP.4
p−maj=0.094, pX=0.66, N=163
var.1.CFH 1:196642233:G:A
peptide level
85:175
51:110
16:40
EQVQSCGPPPELLNGNVKpc2_NP.5
p−maj=0.4, pX=0.44, N=152
var.1.CFH 1:196642233:G:A
peptide level
136:175
77:110
30:40
EYHFGQAVRpc2_NP.1
p−min=0.2, pX=0.55, N=243
var.1.CFH 1:196642233:G:A
peptide level
58:175
44:110
11:40
EYHFGQAVRpc2_NP.2
p−maj=0.38, pX=0.81, N=113
var.1.CFH 1:196642233:G:A
peptide level
173:175
108:110
39:40
EYHFGQAVRpc2_NP.3
p−maj=0.48, pX=0.091, N=320
var.1.CFH 1:196642233:G:A
peptide level
175:175
110:110
40:40
EYHFGQAVRpc2_NP.4
p−maj=1, pX=0.59, N=325
var.1.CFH 1:196642233:G:A
peptide level
171:175
110:110
40:40
EYHFGQAVRpc2_NP.5
p−min=0.13, pX=0.19, N=321
var.1.CFH 1:196642233:G:A
peptide level
1:175
2:110
0:40
FSCKPGFTIVGPNSVQCYHFGLSPDLPICKpc4_l
p−min=0.6, pX=0.72, N=3
var.1.CFH 1:196642233:G:A
peptide level
7:175
3:110
2:40
FSCKPGFTIVGPNSVQCYHFGLSPDLPICKpc4_l
p−maj=0.65, pX=0.75, N=12
var.1.CFH 1:196642233:G:A
peptide level
85:175
53:110
17:40
FSCKPGFTIVGPNSVQCYHFGLSPDLPICKpc4_l
p−maj=0.5, pX=0.76, N=155
var.1.CFH 1:196642233:G:A
peptide level
17:175
12:110
4:40
FSCKPGFTIVGPNSVQCYHFGLSPDLPICKpc4_
p−min=0.85, pX=0.69, N=33

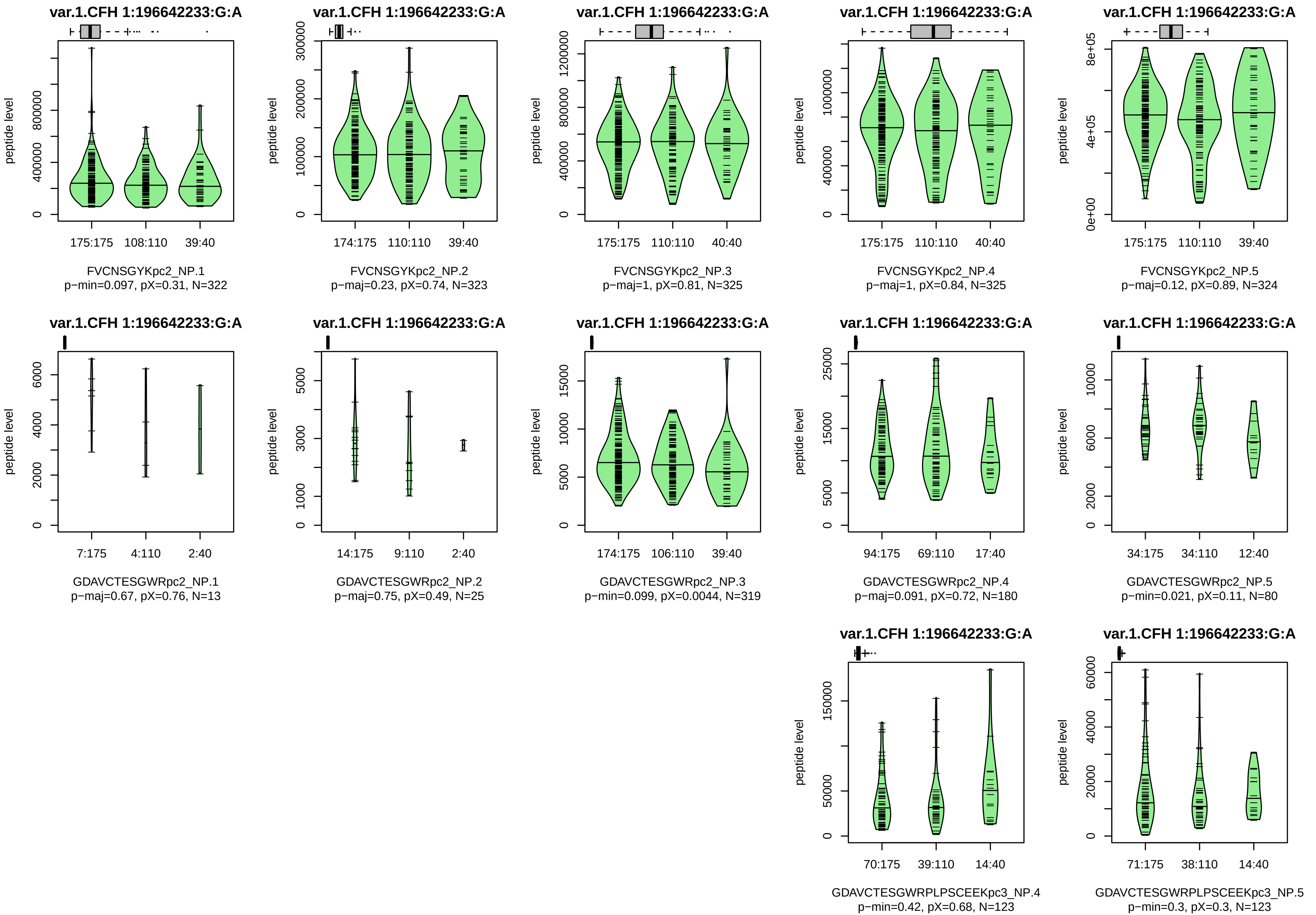
var.1.CFH 1:196642233:G:A
peptide level
0
400000
800000
175:175
108:110
39:40
FVCNSGYKpc2_NP.1
p−min=0.097, pX=0.31, N=322
var.1.CFH 1:196642233:G:A
peptide level
0
100000
200000
300000
174:175
110:110
39:40
FVCNSGYKpc2_NP.2
p−maj=0.23, pX=0.74, N=323
var.1.CFH 1:196642233:G:A
peptide level
0
400000
800000
1200000
175:175
110:110
40:40
FVCNSGYKpc2_NP.3
p−maj=1, pX=0.81, N=325
var.1.CFH 1:196642233:G:A
peptide level
0
400000
1000000
175:175
110:110
40:40
FVCNSGYKpc2_NP.4
p−maj=1, pX=0.84, N=325
var.1.CFH 1:196642233:G:A
peptide level
0e+00
4e+05
8e+05
175:175
110:110
39:40
FVCNSGYKpc2_NP.5
p−maj=0.12, pX=0.89, N=324
var.1.CFH 1:196642233:G:A
peptide level
0
2000
4000
6000
7:175
4:110
2:40
GDAVCTESGWRpc2_NP.1
p−maj=0.67, pX=0.76, N=13
var.1.CFH 1:196642233:G:A
peptide level
0
1000
3000
5000
14:175
9:110
2:40
GDAVCTESGWRpc2_NP.2
p−maj=0.75, pX=0.49, N=25
var.1.CFH 1:196642233:G:A
peptide level
0
5000
10000
15000
174:175
106:110
39:40
GDAVCTESGWRpc2_NP.3
p−min=0.099, pX=0.0044, N=319
var.1.CFH 1:196642233:G:A
peptide level
0
5000
15000
25000
94:175
69:110
17:40
GDAVCTESGWRpc2_NP.4
p−maj=0.091, pX=0.72, N=180
var.1.CFH 1:196642233:G:A
peptide level
0
2000
6000
10000
34:175
34:110
12:40
GDAVCTESGWRpc2_NP.5
p−min=0.021, pX=0.11, N=80
var.1.CFH 1:196642233:G:A
peptide level
0
50000
150000
70:175
39:110
14:40
GDAVCTESGWRPLPSCEEKpc3_NP.4
p−min=0.42, pX=0.68, N=123
var.1.CFH 1:196642233:G:A
peptide level
0
20000
40000
60000
71:175
38:110
14:40
GDAVCTESGWRPLPSCEEKpc3_NP.5
p−min=0.3, pX=0.3, N=123

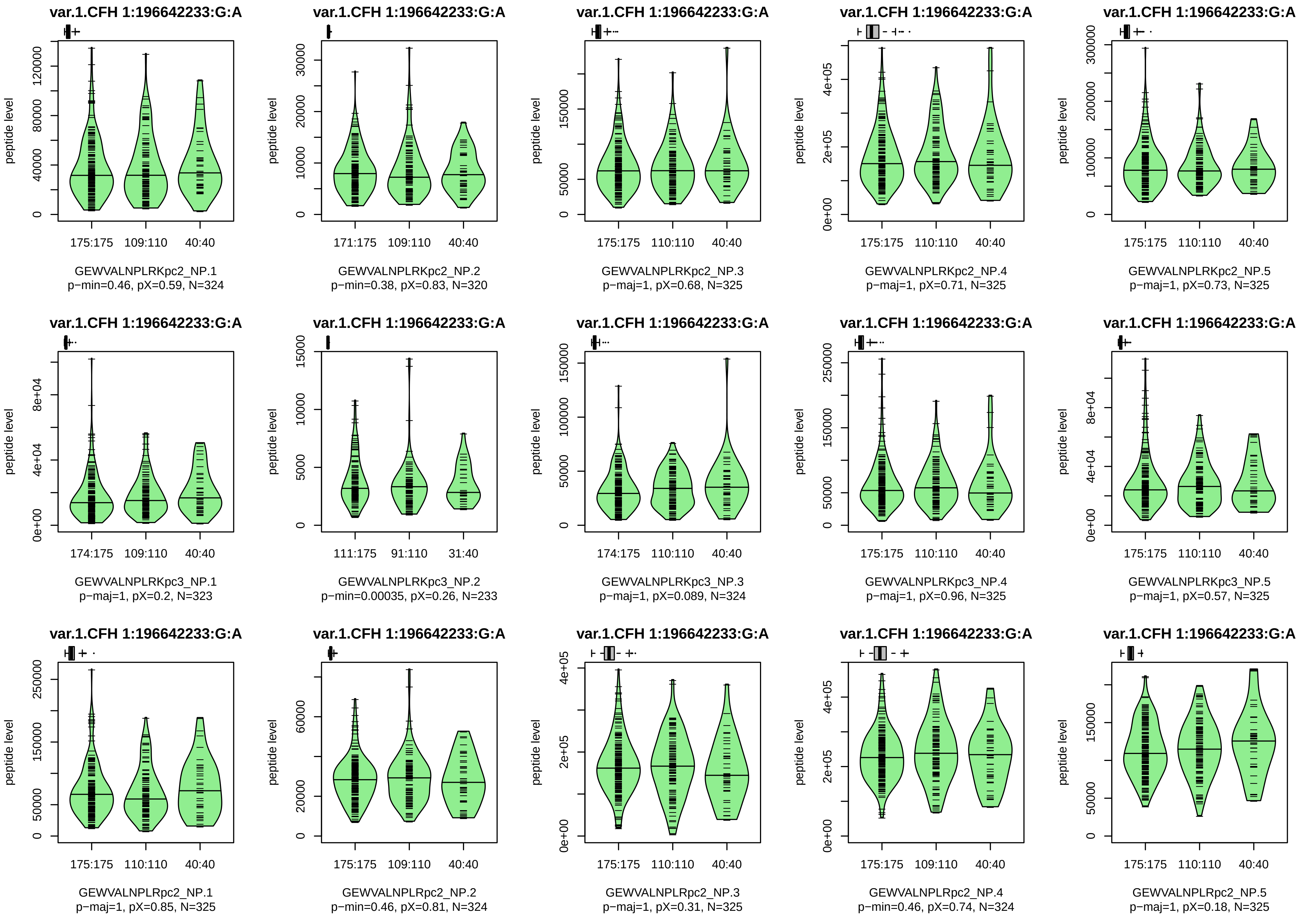
var.1.CFH 1:196642233:G:A
peptide level
175:175 109:110 40:40
GEWVALNPLRKpc2_NP.1
p−min=0.46, pX=0.59, N=324
var.1.CFH 1:196642233:G:A
peptide level
171:175 109:110 40:40
GEWVALNPLRKpc2_NP.2
p−min=0.38, pX=0.83, N=320
var.1.CFH 1:196642233:G:A
peptide level
175:175 110:110 40:40
GEWVALNPLRKpc2_NP.3
p−maj=1, pX=0.68, N=325
var.1.CFH 1:196642233:G:A
peptide level
175:175 110:110 40:40
GEWVALNPLRKpc2_NP.4
p−maj=1, pX=0.71, N=325
var.1.CFH 1:196642233:G:A
peptide level
175:175 110:110 40:40
GEWVALNPLRKpc2_NP.5
p−maj=1, pX=0.73, N=325
var.1.CFH 1:196642233:G:A
peptide level
174:175 109:110 40:40
GEWVALNPLRKpc3_NP.1
p−maj=1, pX=0.2, N=323
var.1.CFH 1:196642233:G:A
peptide level
111:175 91:110 31:40
GEWVALNPLRKpc3_NP.2
p−min=0.00035, pX=0.26, N=233
var.1.CFH 1:196642233:G:A
peptide level
174:175 110:110 40:40
GEWVALNPLRKpc3_NP.3
p−maj=1, pX=0.089, N=324
var.1.CFH 1:196642233:G:A
peptide level
175:175 110:110 40:40
GEWVALNPLRKpc3_NP.4
p−maj=1, pX=0.96, N=325
var.1.CFH 1:196642233:G:A
peptide level
175:175 110:110 40:40
GEWVALNPLRKpc3_NP.5
p−maj=1, pX=0.57, N=325
var.1.CFH 1:196642233:G:A
peptide level
175:175 110:110 40:40
GEWVALNPLRpc2_NP.1
p−maj=1, pX=0.85, N=325
var.1.CFH 1:196642233:G:A
peptide level
175:175 109:110 40:40
GEWVALNPLRpc2_NP.2
p−min=0.46, pX=0.81, N=324
var.1.CFH 1:196642233:G:A
peptide level
175:175 110:110 40:40
GEWVALNPLRpc2_NP.3
p−maj=1, pX=0.31, N=325
var.1.CFH 1:196642233:G:A
peptide level
175:175 109:110 40:40
GEWVALNPLRpc2_NP.4
p−min=0.46, pX=0.74, N=324
var.1.CFH 1:196642233:G:A
peptide level
175:175 110:110 40:40
GEWVALNPLRpc2_NP.5
p−maj=1, pX=0.18, N=325

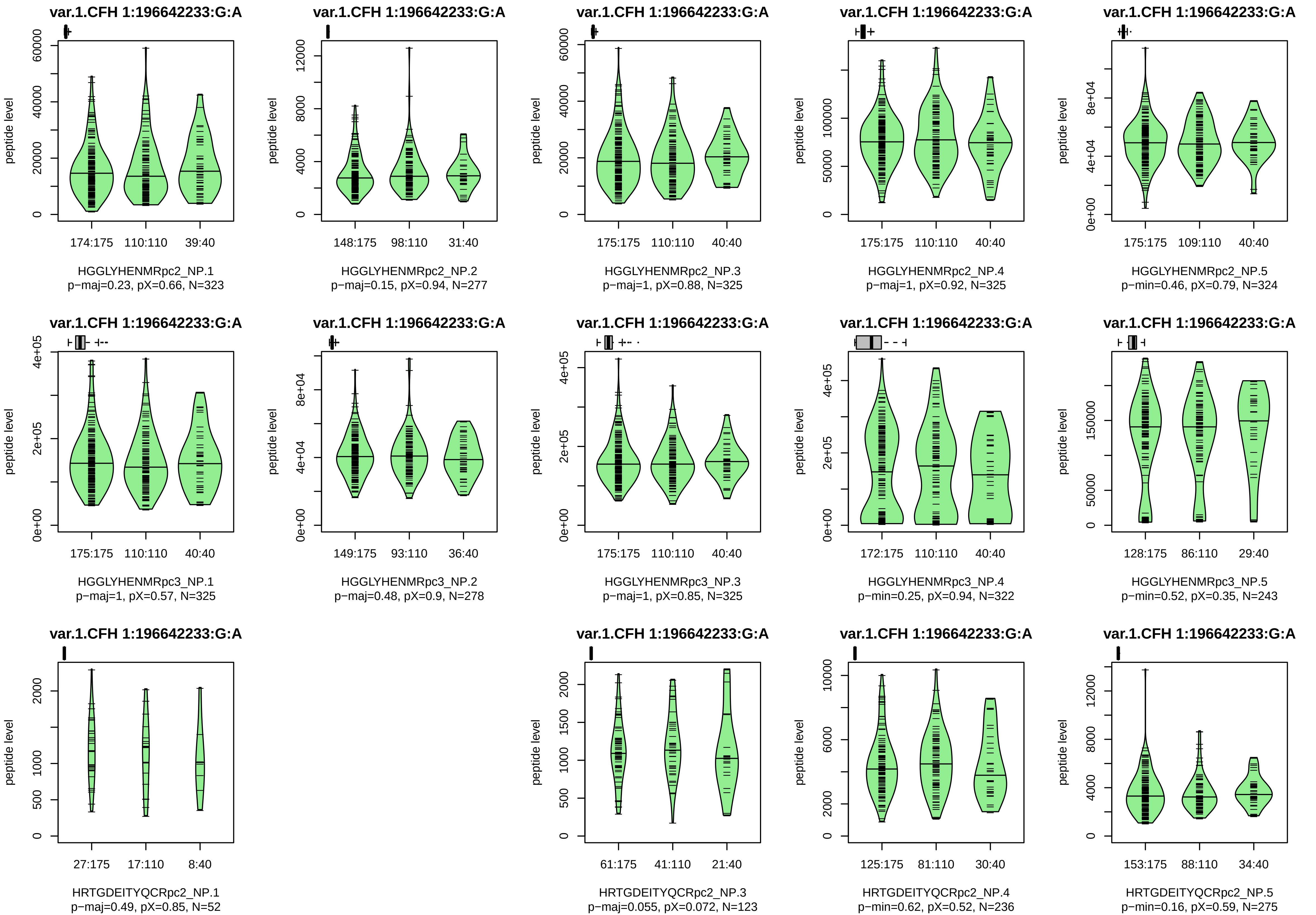
var.1.CFH 1:196642233:G:A
peptide level
174:175
110:110
39:40
HGGLYHENMRpc2_NP.1
p−maj=0.23, pX=0.66, N=323
var.1.CFH 1:196642233:G:A
peptide level
148:175
98:110
31:40
HGGLYHENMRpc2_NP.2
p−maj=0.15, pX=0.94, N=277
var.1.CFH 1:196642233:G:A
peptide level
175:175
110:110
40:40
HGGLYHENMRpc2_NP.3
p−maj=1, pX=0.88, N=325
var.1.CFH 1:196642233:G:A
peptide level
175:175
110:110
40:40
HGGLYHENMRpc2_NP.4
p−maj=1, pX=0.92, N=325
var.1.CFH 1:196642233:G:A
peptide level
175:175
109:110
40:40
HGGLYHENMRpc2_NP.5
p−min=0.46, pX=0.79, N=324
var.1.CFH 1:196642233:G:A
peptide level
175:175
110:110
40:40
HGGLYHENMRpc3_NP.1
p−maj=1, pX=0.57, N=325
var.1.CFH 1:196642233:G:A
peptide level
149:175
93:110
36:40
HGGLYHENMRpc3_NP.2
p−maj=0.48, pX=0.9, N=278
var.1.CFH 1:196642233:G:A
peptide level
175:175
110:110
40:40
HGGLYHENMRpc3_NP.3
p−maj=1, pX=0.85, N=325
var.1.CFH 1:196642233:G:A
peptide level
172:175
110:110
40:40
HGGLYHENMRpc3_NP.4
p−min=0.25, pX=0.94, N=322
var.1.CFH 1:196642233:G:A
peptide level
128:175
86:110
29:40
HGGLYHENMRpc3_NP.5
p−min=0.52, pX=0.35, N=243
var.1.CFH 1:196642233:G:A
peptide level
27:175
17:110
8:40
HRTGDEITYQCRpc2_NP.1
p−maj=0.49, pX=0.85, N=52
var.1.CFH 1:196642233:G:A
peptide level
61:175
41:110
21:40
HRTGDEITYQCRpc2_NP.3
p−maj=0.055, pX=0.072, N=123
var.1.CFH 1:196642233:G:A
peptide level
125:175
81:110
30:40
HRTGDEITYQCRpc2_NP.4
p−min=0.62, pX=0.52, N=236
var.1.CFH 1:196642233:G:A
peptide level
153:175
88:110
34:40
HRTGDEITYQCRpc2_NP.5
p−min=0.16, pX=0.59, N=275

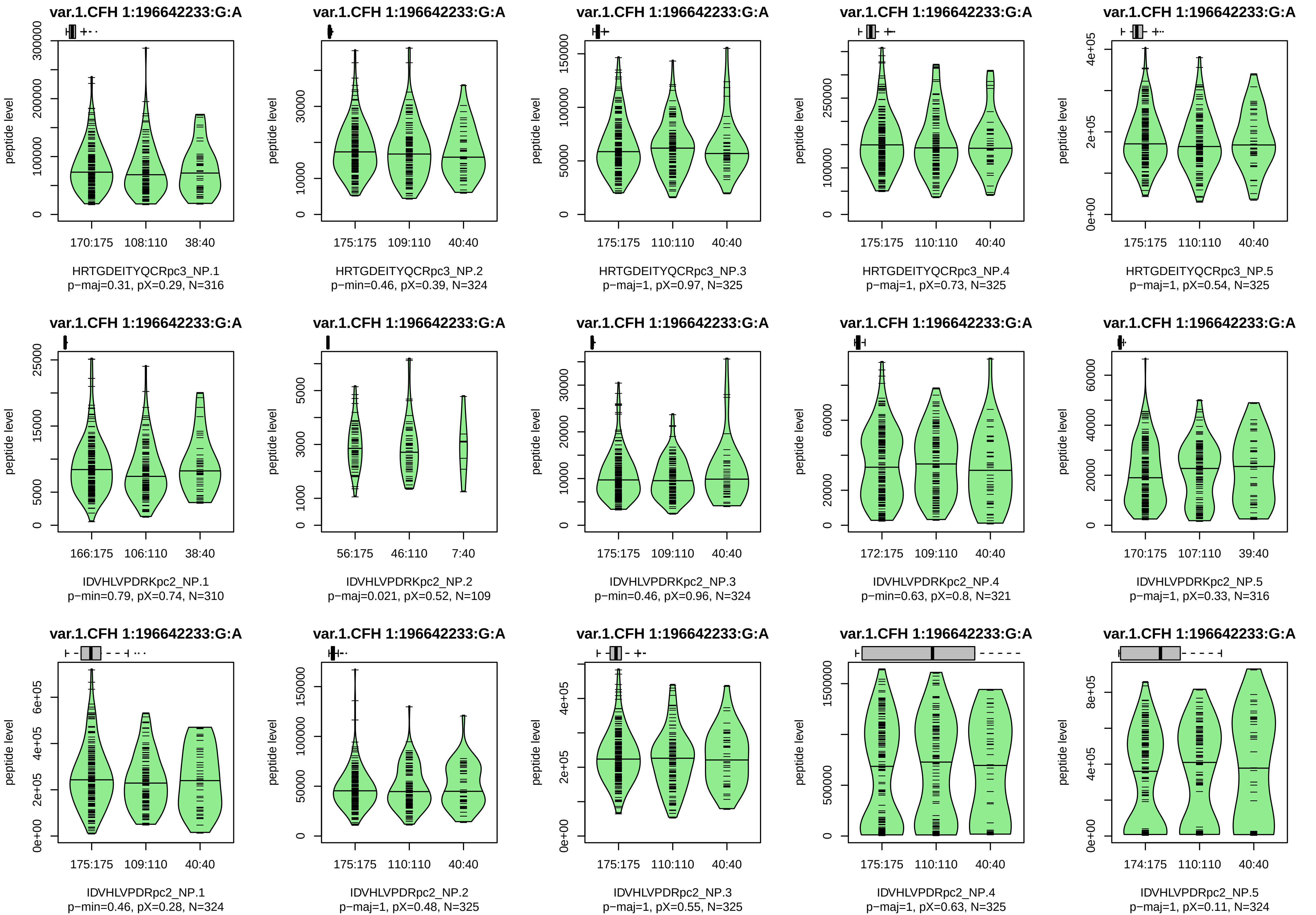
var.1.CFH 1:196642233:G:A
peptide level
170:175 108:110 38:40
HRTGDEITYQCRpc3_NP.1
p−maj=0.31, pX=0.29, N=316
var.1.CFH 1:196642233:G:A
peptide level
175:175 109:110 40:40
HRTGDEITYQCRpc3_NP.2
p−min=0.46, pX=0.39, N=324
var.1.CFH 1:196642233:G:A
peptide level
175:175 110:110 40:40
HRTGDEITYQCRpc3_NP.3
p−maj=1, pX=0.97, N=325
var.1.CFH 1:196642233:G:A
peptide level
175:175 110:110 40:40
HRTGDEITYQCRpc3_NP.4
p−maj=1, pX=0.73, N=325
var.1.CFH 1:196642233:G:A
peptide level
175:175 110:110 40:40
HRTGDEITYQCRpc3_NP.5
p−maj=1, pX=0.54, N=325
var.1.CFH 1:196642233:G:A
peptide level
166:175 106:110 38:40
IDVHLVPDRKpc2_NP.1
p−min=0.79, pX=0.74, N=310
var.1.CFH 1:196642233:G:A
peptide level
56:175 46:110 7:40
IDVHLVPDRKpc2_NP.2
p−maj=0.021, pX=0.52, N=109
var.1.CFH 1:196642233:G:A
peptide level
175:175 109:110 40:40
IDVHLVPDRKpc2_NP.3
p−min=0.46, pX=0.96, N=324
var.1.CFH 1:196642233:G:A
peptide level
172:175 109:110 40:40
IDVHLVPDRKpc2_NP.4
p−min=0.63, pX=0.8, N=321
var.1.CFH 1:196642233:G:A
peptide level
170:175 107:110 39:40
IDVHLVPDRKpc2_NP.5
p−maj=1, pX=0.33, N=316
var.1.CFH 1:196642233:G:A
peptide level
175:175 109:110 40:40
IDVHLVPDRpc2_NP.1
p−min=0.46, pX=0.28, N=324
var.1.CFH 1:196642233:G:A
peptide level
175:175 110:110 40:40
IDVHLVPDRpc2_NP.2
p−maj=1, pX=0.48, N=325
var.1.CFH 1:196642233:G:A
peptide level
175:175 110:110 40:40
IDVHLVPDRpc2_NP.3
p−maj=1, pX=0.55, N=325
var.1.CFH 1:196642233:G:A
peptide level
175:175 110:110 40:40
IDVHLVPDRpc2_NP.4
p−maj=1, pX=0.63, N=325
var.1.CFH 1:196642233:G:A
peptide level
174:175 110:110 40:40
IDVHLVPDRpc2_NP.5
p−maj=1, pX=0.11, N=324

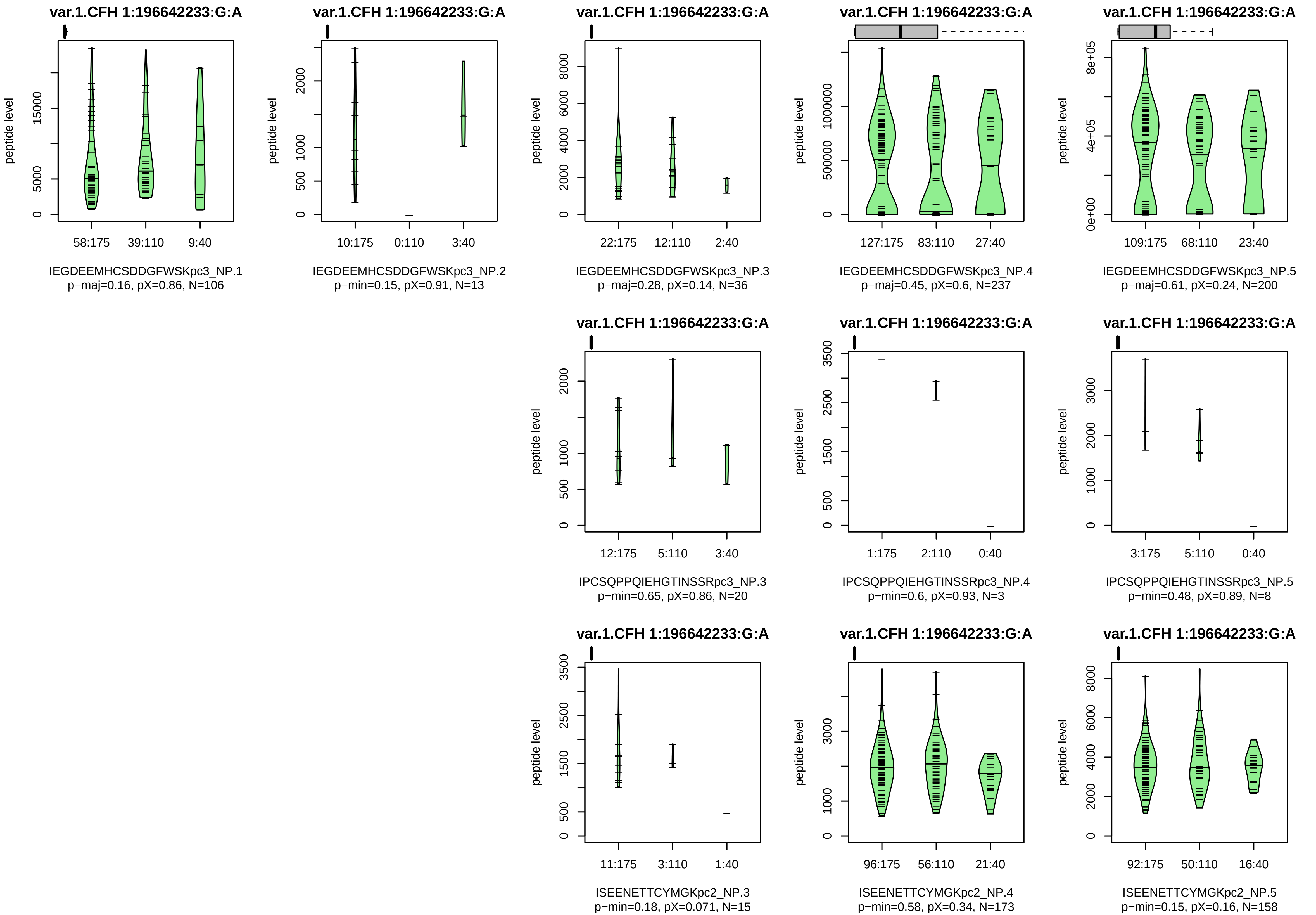
var.1.CFH 1:196642233:G:A
peptide level
0
5000
15000
58:175
39:110
9:40
IEGDEEMHCSDDGFWSKpc3_NP.1
p−maj=0.16, pX=0.86, N=106
var.1.CFH 1:196642233:G:A
peptide level
0
500
1000
2000
10:175
0:110
3:40
IEGDEEMHCSDDGFWSKpc3_NP.2
p−min=0.15, pX=0.91, N=13
var.1.CFH 1:196642233:G:A
peptide level
0
2000
4000
6000
8000
22:175
12:110
2:40
IEGDEEMHCSDDGFWSKpc3_NP.3
p−maj=0.28, pX=0.14, N=36
var.1.CFH 1:196642233:G:A
peptide level
0
500000
1000000
127:175
83:110
27:40
IEGDEEMHCSDDGFWSKpc3_NP.4
p−maj=0.45, pX=0.6, N=237
var.1.CFH 1:196642233:G:A
peptide level
0e+00
4e+05
8e+05
109:175
68:110
23:40
IEGDEEMHCSDDGFWSKpc3_NP.5
p−maj=0.61, pX=0.24, N=200
var.1.CFH 1:196642233:G:A
peptide level
0
500
1000
2000
12:175
5:110
3:40
IPCSQPPQIEHGTINSSRpc3_NP.3
p−min=0.65, pX=0.86, N=20
var.1.CFH 1:196642233:G:A
peptide level
0
500
1500
2500
3500
1:175
2:110
0:40
IPCSQPPQIEHGTINSSRpc3_NP.4
p−min=0.6, pX=0.93, N=3
var.1.CFH 1:196642233:G:A
peptide level
0
1000
2000
3000
3:175
5:110
0:40
IPCSQPPQIEHGTINSSRpc3_NP.5
p−min=0.48, pX=0.89, N=8
var.1.CFH 1:196642233:G:A
peptide level
0
500
1500
2500
3500
11:175
3:110
1:40
ISEENETTCYMGKpc2_NP.3
p−min=0.18, pX=0.071, N=15
var.1.CFH 1:196642233:G:A
peptide level
0
1000
2000
3000
96:175
56:110
21:40
ISEENETTCYMGKpc2_NP.4
p−min=0.58, pX=0.34, N=173
var.1.CFH 1:196642233:G:A
peptide level
0
2000
4000
6000
8000
92:175
50:110
16:40
ISEENETTCYMGKpc2_NP.5
p−min=0.15, pX=0.16, N=158

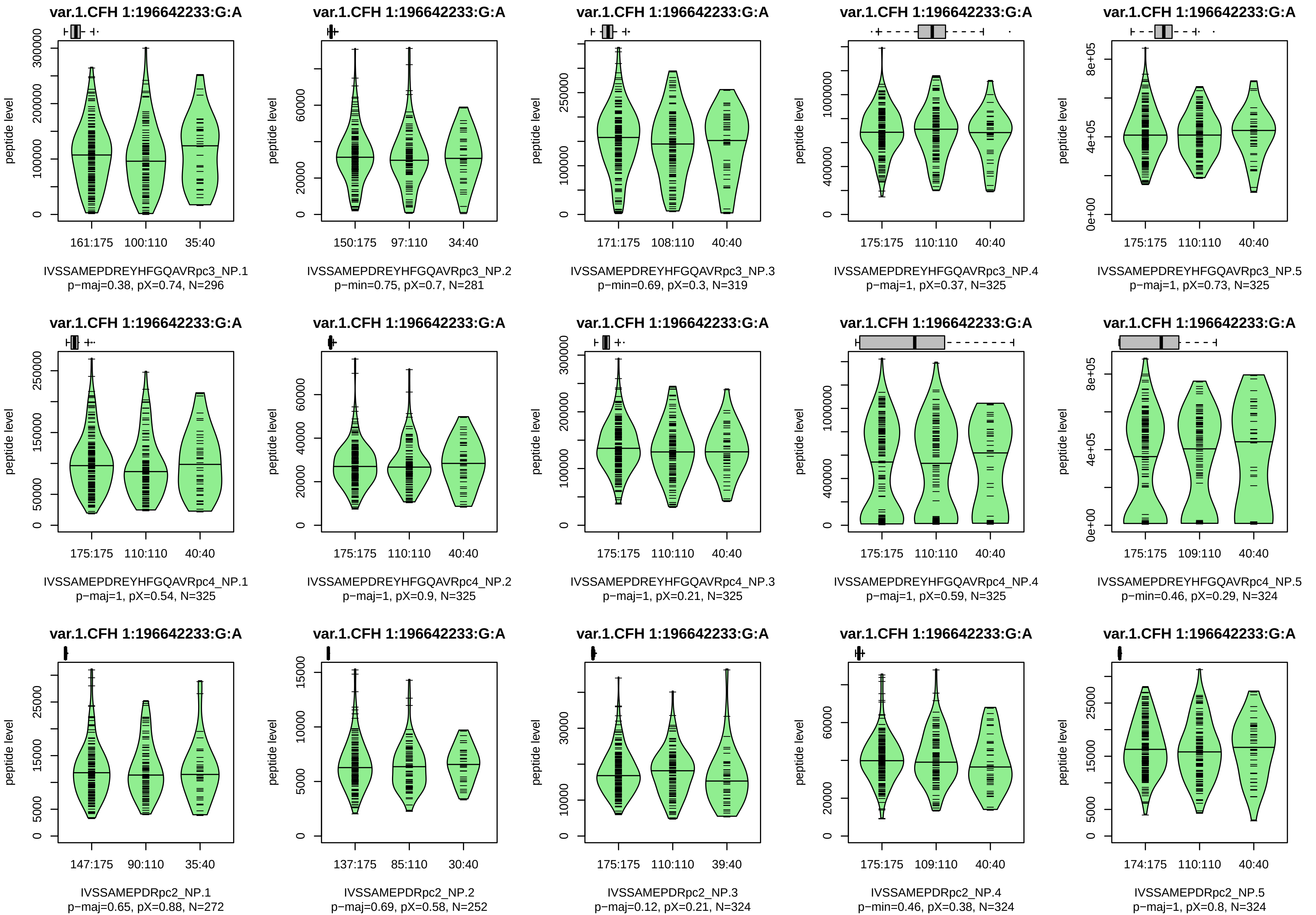
var.1.CFH 1:196642233:G:A
peptide level
161:175
100:110
35:40
IVSSAMEPDREYHFGQAVRpc3_NP.1
p−maj=0.38, pX=0.74, N=296
var.1.CFH 1:196642233:G:A
150:175
97:110
34:40
IVSSAMEPDREYHFGQAVRpc3_NP.2
p−min=0.75, pX=0.7, N=281
var.1.CFH 1:196642233:G:A
171:175
108:110
40:40
IVSSAMEPDREYHFGQAVRpc3_NP.3
p−min=0.69, pX=0.3, N=319
var.1.CFH 1:196642233:G:A
175:175
110:110
40:40
IVSSAMEPDREYHFGQAVRpc3_NP.4
p−maj=1, pX=0.37, N=325
var.1.CFH 1:196642233:G:A
175:175
110:110
40:40
IVSSAMEPDREYHFGQAVRpc3_NP.5
p−maj=1, pX=0.73, N=325
var.1.CFH 1:196642233:G:A
175:175
110:110
40:40
IVSSAMEPDREYHFGQAVRpc4_NP.1
p−maj=1, pX=0.54, N=325
var.1.CFH 1:196642233:G:A
175:175
110:110
40:40
IVSSAMEPDREYHFGQAVRpc4_NP.2
p−maj=1, pX=0.9, N=325
var.1.CFH 1:196642233:G:A
175:175
110:110
40:40
IVSSAMEPDREYHFGQAVRpc4_NP.3
p−maj=1, pX=0.21, N=325
var.1.CFH 1:196642233:G:A
175:175
110:110
40:40
IVSSAMEPDREYHFGQAVRpc4_NP.4
p−maj=1, pX=0.59, N=325
var.1.CFH 1:196642233:G:A
175:175
109:110
40:40
IVSSAMEPDREYHFGQAVRpc4_NP.5
p−min=0.46, pX=0.29, N=324
var.1.CFH 1:196642233:G:A
147:175
90:110
35:40
IVSSAMEPDRpc2_NP.1
p−maj=0.65, pX=0.88, N=272
var.1.CFH 1:196642233:G:A
137:175
85:110
30:40
IVSSAMEPDRpc2_NP.2
p−maj=0.69, pX=0.58, N=252
var.1.CFH 1:196642233:G:A
175:175
110:110
39:40
IVSSAMEPDRpc2_NP.3
p−maj=0.12, pX=0.21, N=324
var.1.CFH 1:196642233:G:A
175:175
109:110
40:40
IVSSAMEPDRpc2_NP.4
p−min=0.46, pX=0.38, N=324
var.1.CFH 1:196642233:G:A
174:175
110:110
40:40
IVSSAMEPDRpc2_NP.5
p−maj=1, pX=0.8, N=324

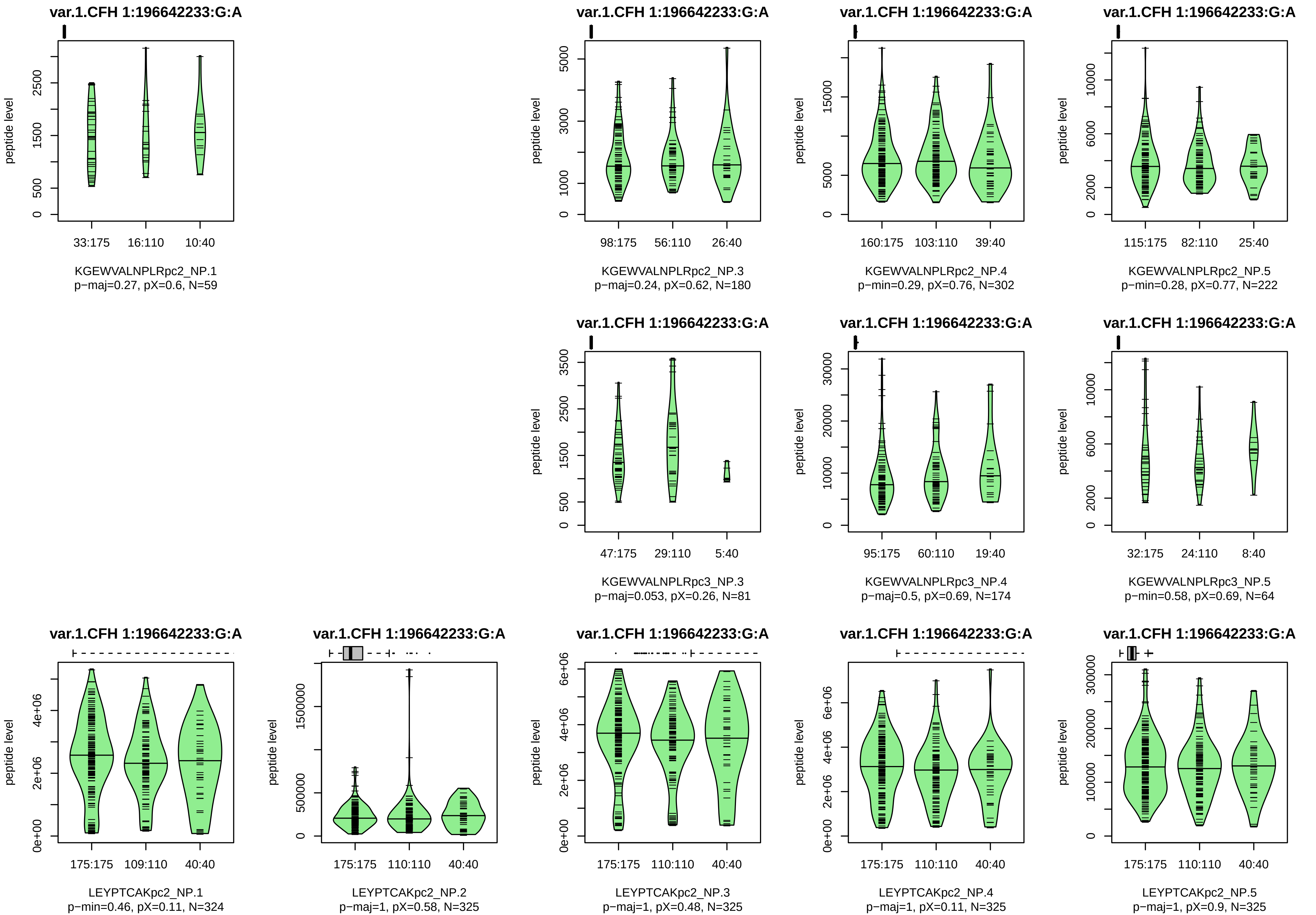
var.1.CFH 1:196642233:G:A
peptide level
33:175
16:110
10:40
KGEWVALNPLRpc2_NP.1
p−maj=0.27, pX=0.6, N=59
var.1.CFH 1:196642233:G:A
peptide level
98:175
56:110
26:40
KGEWVALNPLRpc2_NP.3
p−maj=0.24, pX=0.62, N=180
var.1.CFH 1:196642233:G:A
peptide level
160:175
103:110
39:40
KGEWVALNPLRpc2_NP.4
p−min=0.29, pX=0.76, N=302
var.1.CFH 1:196642233:G:A
peptide level
115:175
82:110
25:40
KGEWVALNPLRpc2_NP.5
p−min=0.28, pX=0.77, N=222
var.1.CFH 1:196642233:G:A
peptide level
47:175
29:110
5:40
KGEWVALNPLRpc3_NP.3
p−maj=0.053, pX=0.26, N=81
var.1.CFH 1:196642233:G:A
peptide level
95:175
60:110
19:40
KGEWVALNPLRpc3_NP.4
p−maj=0.5, pX=0.69, N=174
var.1.CFH 1:196642233:G:A
peptide level
32:175
24:110
8:40
KGEWVALNPLRpc3_NP.5
p−min=0.58, pX=0.69, N=64
var.1.CFH 1:196642233:G:A
peptide level
175:175
109:110
40:40
LEYPTCAKpc2_NP.1
p−min=0.46, pX=0.11, N=324
var.1.CFH 1:196642233:G:A
peptide level
175:175
110:110
40:40
LEYPTCAKpc2_NP.2
p−maj=1, pX=0.58, N=325
var.1.CFH 1:196642233:G:A
peptide level
175:175
110:110
40:40
LEYPTCAKpc2_NP.3
p−maj=1, pX=0.48, N=325
var.1.CFH 1:196642233:G:A
peptide level
175:175
110:110
40:40
LEYPTCAKpc2_NP.4
p−maj=1, pX=0.11, N=325
var.1.CFH 1:196642233:G:A
peptide level
175:175
110:110
40:40
LEYPTCAKpc2_NP.5
p−maj=1, pX=0.9, N=325

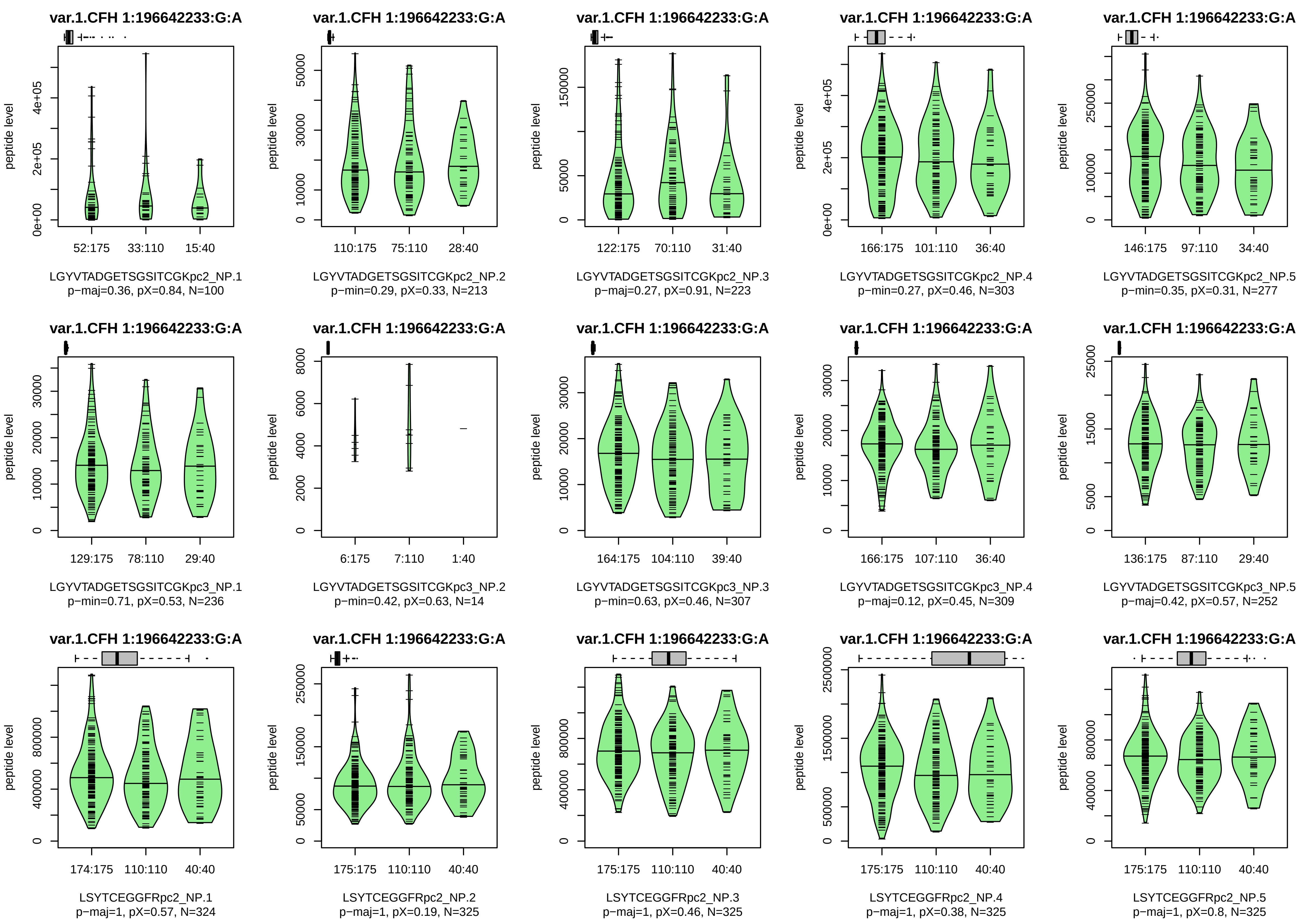
var.1.CFH 1:196642233:G:A
peptide level
52:175 33:110 15:40
LGYVTADGETSGSITCGKpc2_NP.1
p−maj=0.36, pX=0.84, N=100
var.1.CFH 1:196642233:G:A
peptide level
110:175 75:110 28:40
LGYVTADGETSGSITCGKpc2_NP.2
p−min=0.29, pX=0.33, N=213
var.1.CFH 1:196642233:G:A
peptide level
122:175 70:110 31:40
LGYVTADGETSGSITCGKpc2_NP.3
p−maj=0.27, pX=0.91, N=223
var.1.CFH 1:196642233:G:A
peptide level
166:175 101:110 36:40
LGYVTADGETSGSITCGKpc2_NP.4
p−min=0.27, pX=0.46, N=303
var.1.CFH 1:196642233:G:A
peptide level
146:175 97:110 34:40
LGYVTADGETSGSITCGKpc2_NP.5
p−min=0.35, pX=0.31, N=277
var.1.CFH 1:196642233:G:A
peptide level
129:175 78:110 29:40
LGYVTADGETSGSITCGKpc3_NP.1
p−min=0.71, pX=0.53, N=236
var.1.CFH 1:196642233:G:A
peptide level
6:175 7:110 1:40
LGYVTADGETSGSITCGKpc3_NP.2
p−min=0.42, pX=0.63, N=14
var.1.CFH 1:196642233:G:A
peptide level
164:175 104:110 39:40
LGYVTADGETSGSITCGKpc3_NP.3
p−min=0.63, pX=0.46, N=307
var.1.CFH 1:196642233:G:A
peptide level
166:175 107:110 36:40
LGYVTADGETSGSITCGKpc3_NP.4
p−maj=0.12, pX=0.45, N=309
var.1.CFH 1:196642233:G:A
peptide level
136:175 87:110 29:40
LGYVTADGETSGSITCGKpc3_NP.5
p−maj=0.42, pX=0.57, N=252
var.1.CFH 1:196642233:G:A
peptide level
174:175 110:110 40:40
LSYTCEGGFRpc2_NP.1
p−maj=1, pX=0.57, N=324
var.1.CFH 1:196642233:G:A
peptide level
175:175 110:110 40:40
LSYTCEGGFRpc2_NP.2
p−maj=1, pX=0.19, N=325
var.1.CFH 1:196642233:G:A
peptide level
175:175 110:110 40:40
LSYTCEGGFRpc2_NP.3
p−maj=1, pX=0.46, N=325
var.1.CFH 1:196642233:G:A
peptide level
175:175 110:110 40:40
LSYTCEGGFRpc2_NP.4
p−maj=1, pX=0.38, N=325
var.1.CFH 1:196642233:G:A
peptide level
175:175 110:110 40:40
LSYTCEGGFRpc2_NP.5
p−maj=1, pX=0.8, N=325

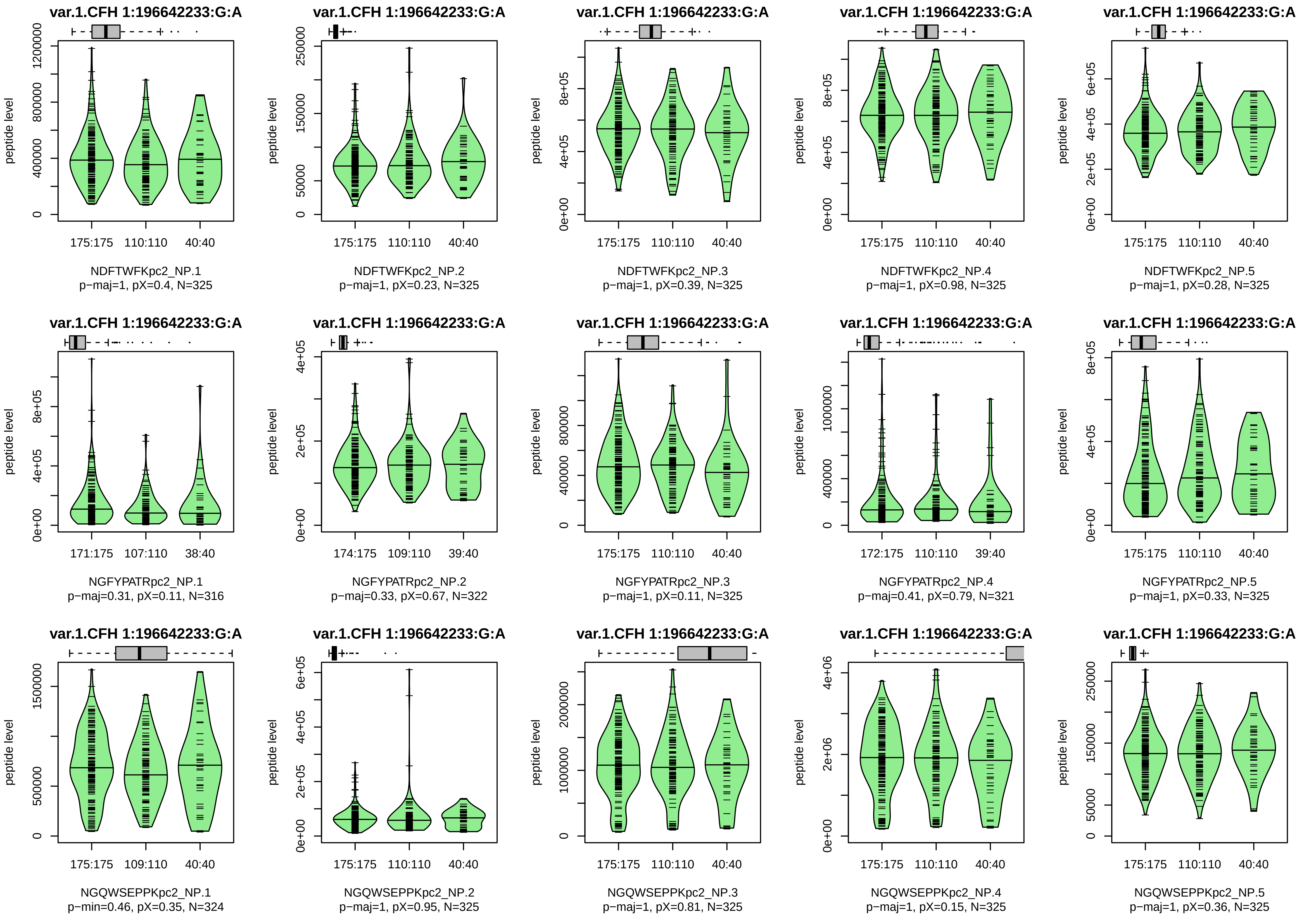
var.1.CFH 1:196642233:G:A
peptide level
175:175 110:110 40:40
NDFTWFKpc2_NP.1
p−maj=1, pX=0.4, N=325
var.1.CFH 1:196642233:G:A
peptide level
175:175 110:110 40:40
NDFTWFKpc2_NP.2
p−maj=1, pX=0.23, N=325
var.1.CFH 1:196642233:G:A
peptide level
175:175 110:110 40:40
NDFTWFKpc2_NP.3
p−maj=1, pX=0.39, N=325
var.1.CFH 1:196642233:G:A
peptide level
175:175 110:110 40:40
NDFTWFKpc2_NP.4
p−maj=1, pX=0.98, N=325
var.1.CFH 1:196642233:G:A
peptide level
175:175 110:110 40:40
NDFTWFKpc2_NP.5
p−maj=1, pX=0.28, N=325
var.1.CFH 1:196642233:G:A
peptide level
171:175 107:110 38:40
NGFYPATRpc2_NP.1
p−maj=0.31, pX=0.11, N=316
var.1.CFH 1:196642233:G:A
peptide level
174:175 109:110 39:40
NGFYPATRpc2_NP.2
p−maj=0.33, pX=0.67, N=322
var.1.CFH 1:196642233:G:A
peptide level
175:175 110:110 40:40
NGFYPATRpc2_NP.3
p−maj=1, pX=0.11, N=325
var.1.CFH 1:196642233:G:A
peptide level
172:175 110:110 39:40
NGFYPATRpc2_NP.4
p−maj=0.41, pX=0.79, N=321
var.1.CFH 1:196642233:G:A
peptide level
175:175 110:110 40:40
NGFYPATRpc2_NP.5
p−maj=1, pX=0.33, N=325
var.1.CFH 1:196642233:G:A
peptide level
175:175 109:110 40:40
NGQWSEPPKpc2_NP.1
p−min=0.46, pX=0.35, N=324
var.1.CFH 1:196642233:G:A
peptide level
175:175 110:110 40:40
NGQWSEPPKpc2_NP.2
p−maj=1, pX=0.95, N=325
var.1.CFH 1:196642233:G:A
peptide level
175:175 110:110 40:40
NGQWSEPPKpc2_NP.3
p−maj=1, pX=0.81, N=325
var.1.CFH 1:196642233:G:A
peptide level
175:175 110:110 40:40
NGQWSEPPKpc2_NP.4
p−maj=1, pX=0.15, N=325
var.1.CFH 1:196642233:G:A
peptide level
175:175 110:110 40:40
NGQWSEPPKpc2_NP.5
p−maj=1, pX=0.36, N=325

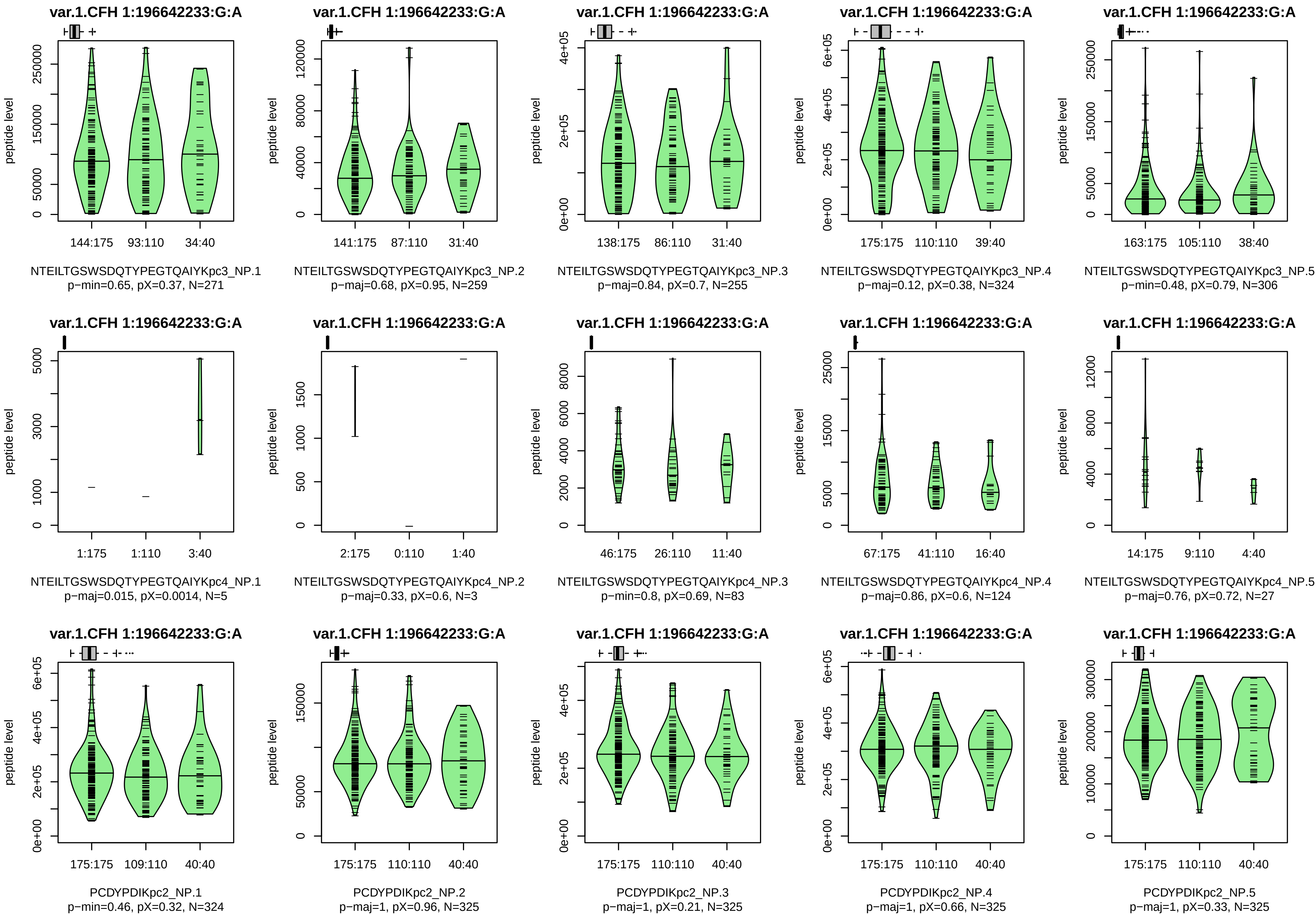
var.1.CFH 1:196642233:G:A
peptide level
144:175
93:110
34:40
NTEILTGSWSDQTYPEGTQAIYKpc3_NP.1
p−min=0.65, pX=0.37, N=271
var.1.CFH 1:196642233:G:A
peptide level
141:175
87:110
31:40
NTEILTGSWSDQTYPEGTQAIYKpc3_NP.2
p−maj=0.68, pX=0.95, N=259
var.1.CFH 1:196642233:G:A
peptide level
138:175
86:110
31:40
NTEILTGSWSDQTYPEGTQAIYKpc3_NP.3
p−maj=0.84, pX=0.7, N=255
var.1.CFH 1:196642233:G:A
peptide level
175:175
110:110
39:40
NTEILTGSWSDQTYPEGTQAIYKpc3_NP.4
p−maj=0.12, pX=0.38, N=324
var.1.CFH 1:196642233:G:A
peptide level
163:175
105:110
38:40
NTEILTGSWSDQTYPEGTQAIYKpc3_NP.5
p−min=0.48, pX=0.79, N=306
var.1.CFH 1:196642233:G:A
peptide level
1:175
1:110
3:40
NTEILTGSWSDQTYPEGTQAIYKpc4_NP.1
p−maj=0.015, pX=0.0014, N=5
var.1.CFH 1:196642233:G:A
peptide level
2:175
0:110
1:40
NTEILTGSWSDQTYPEGTQAIYKpc4_NP.2
p−maj=0.33, pX=0.6, N=3
var.1.CFH 1:196642233:G:A
peptide level
46:175
26:110
11:40
NTEILTGSWSDQTYPEGTQAIYKpc4_NP.3
p−min=0.8, pX=0.69, N=83
var.1.CFH 1:196642233:G:A
peptide level
67:175
41:110
16:40
NTEILTGSWSDQTYPEGTQAIYKpc4_NP.4
p−maj=0.86, pX=0.6, N=124
var.1.CFH 1:196642233:G:A
peptide level
14:175
9:110
4:40
NTEILTGSWSDQTYPEGTQAIYKpc4_NP.5
p−maj=0.76, pX=0.72, N=27
var.1.CFH 1:196642233:G:A
peptide level
175:175
109:110
40:40
PCDYPDIKpc2_NP.1
p−min=0.46, pX=0.32, N=324
var.1.CFH 1:196642233:G:A
peptide level
175:175
110:110
40:40
PCDYPDIKpc2_NP.2
p−maj=1, pX=0.96, N=325
var.1.CFH 1:196642233:G:A
peptide level
175:175
110:110
40:40
PCDYPDIKpc2_NP.3
p−maj=1, pX=0.21, N=325
var.1.CFH 1:196642233:G:A
peptide level
175:175
110:110
40:40
PCDYPDIKpc2_NP.4
p−maj=1, pX=0.66, N=325
var.1.CFH 1:196642233:G:A
peptide level
175:175
110:110
40:40
PCDYPDIKpc2_NP.5
p−maj=1, pX=0.33, N=325

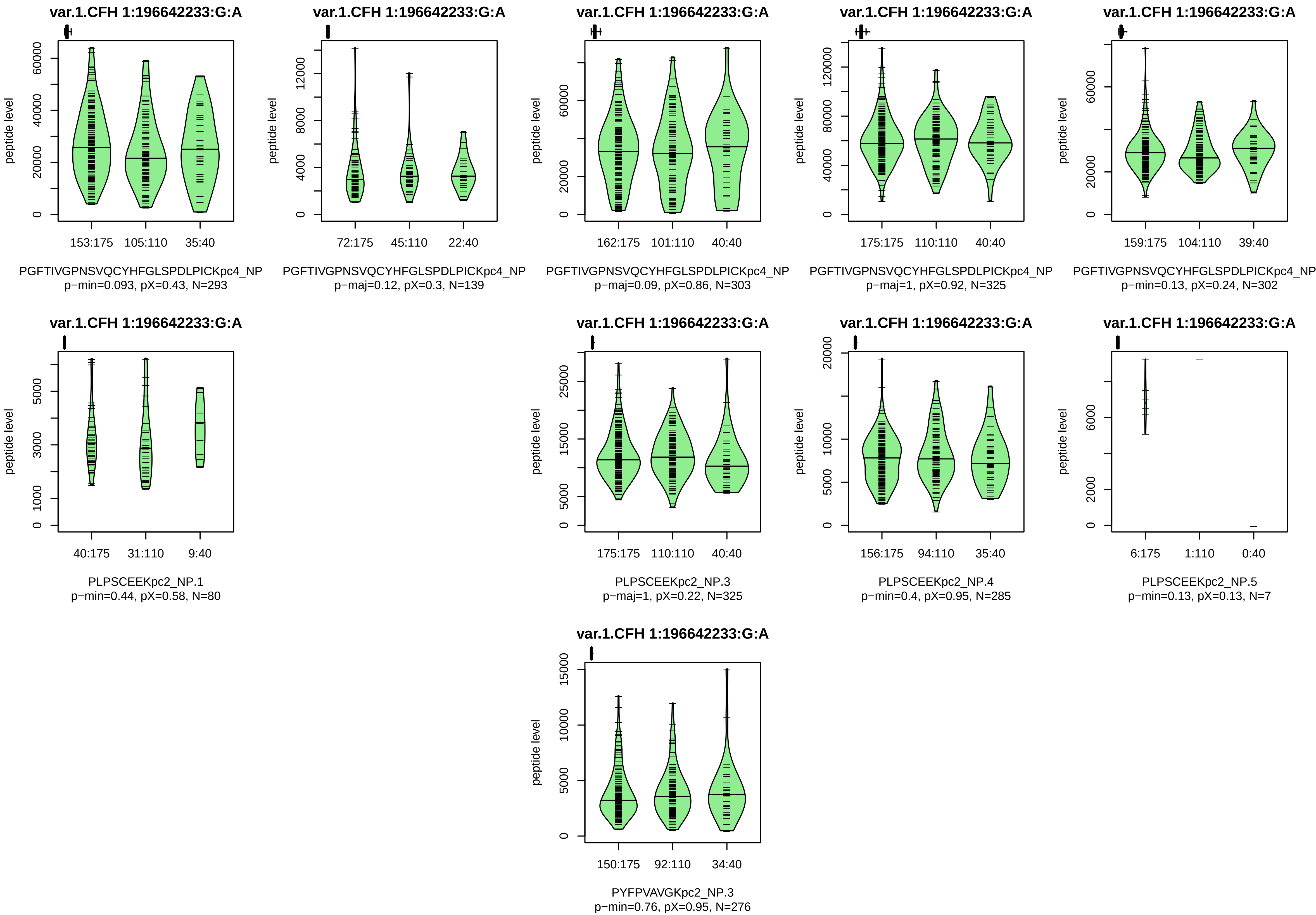

var.1.CFH 1:196642233:G:A
peptide level
153:175
105:110
35:40
PGFTIVGPNSVQCYHFGLSPDLPICKpc4_NP
p−min=0.093, pX=0.43, N=293
var.1.CFH 1:196642233:G:A
peptide level
72:175
45:110
22:40
PGFTIVGPNSVQCYHFGLSPDLPICKpc4_NP
p−maj=0.12, pX=0.3, N=139
var.1.CFH 1:196642233:G:A
peptide level
162:175
101:110
40:40
PGFTIVGPNSVQCYHFGLSPDLPICKpc4_NP
p−maj=0.09, pX=0.86, N=303
var.1.CFH 1:196642233:G:A
peptide level
175:175
110:110
40:40
PGFTIVGPNSVQCYHFGLSPDLPICKpc4_NP
p−maj=1, pX=0.92, N=325
var.1.CFH 1:196642233:G:A
peptide level
159:175
104:110
39:40
PGFTIVGPNSVQCYHFGLSPDLPICKpc4_NP
p−min=0.13, pX=0.24, N=302
var.1.CFH 1:196642233:G:A
peptide level
40:175
31:110
9:40
PLPSCEEKpc2_NP.1
p−min=0.44, pX=0.58, N=80
var.1.CFH 1:196642233:G:A
peptide level
175:175
110:110
40:40
PLPSCEEKpc2_NP.3
p−maj=1, pX=0.22, N=325
var.1.CFH 1:196642233:G:A
peptide level
156:175
94:110
35:40
PLPSCEEKpc2_NP.4
p−min=0.4, pX=0.95, N=285
var.1.CFH 1:196642233:G:A
peptide level
6:175
1:110
0:40
PLPSCEEKpc2_NP.5
p−min=0.13, pX=0.13, N=7
var.1.CFH 1:196642233:G:A
peptide level
150:175
92:110
34:40
PYFPVAVGKpc2_NP.3
p−min=0.76, pX=0.95, N=276

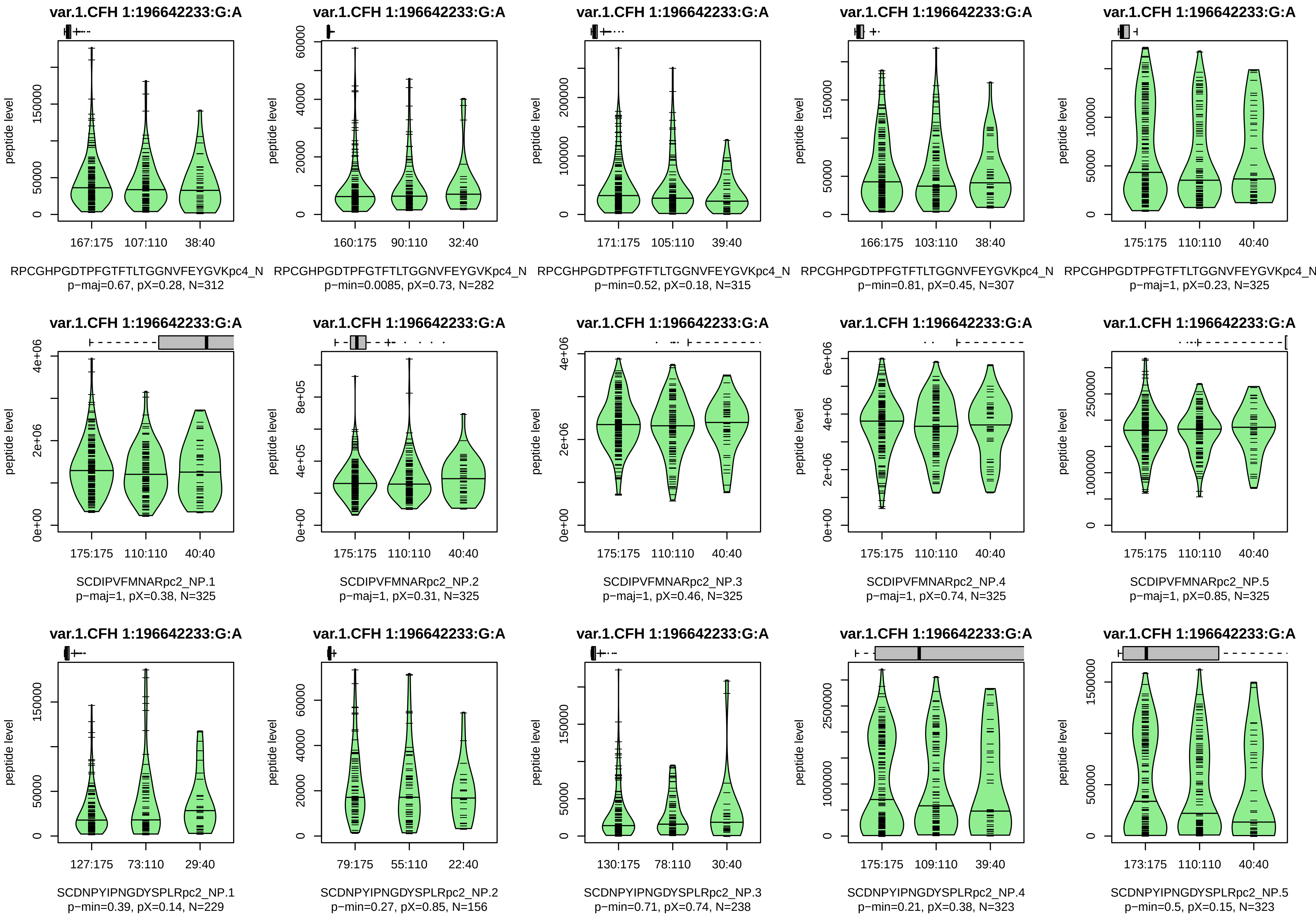
var.1.CFH 1:196642233:G:A
peptide level
167:175
107:110
38:40
RPCGHPGDTPFGTFTLTGGNVFEYGVKpc4_N
p−maj=0.67, pX=0.28, N=312
var.1.CFH 1:196642233:G:A
peptide level
160:175
90:110
32:40
RPCGHPGDTPFGTFTLTGGNVFEYGVKpc4_N
p−min=0.0085, pX=0.73, N=282
var.1.CFH 1:196642233:G:A
peptide level
171:175
105:110
39:40
RPCGHPGDTPFGTFTLTGGNVFEYGVKpc4_N
p−min=0.52, pX=0.18, N=315
var.1.CFH 1:196642233:G:A
peptide level
166:175
103:110
38:40
RPCGHPGDTPFGTFTLTGGNVFEYGVKpc4_N
p−min=0.81, pX=0.45, N=307
var.1.CFH 1:196642233:G:A
peptide level
175:175
110:110
40:40
RPCGHPGDTPFGTFTLTGGNVFEYGVKpc4_N
p−maj=1, pX=0.23, N=325
var.1.CFH 1:196642233:G:A
peptide level
175:175
110:110
40:40
SCDIPVFMNARpc2_NP.1
p−maj=1, pX=0.38, N=325
var.1.CFH 1:196642233:G:A
peptide level
175:175
110:110
40:40
SCDIPVFMNARpc2_NP.2
p−maj=1, pX=0.31, N=325
var.1.CFH 1:196642233:G:A
peptide level
175:175
110:110
40:40
SCDIPVFMNARpc2_NP.3
p−maj=1, pX=0.46, N=325
var.1.CFH 1:196642233:G:A
peptide level
175:175
110:110
40:40
SCDIPVFMNARpc2_NP.4
p−maj=1, pX=0.74, N=325
var.1.CFH 1:196642233:G:A
peptide level
175:175
110:110
40:40
SCDIPVFMNARpc2_NP.5
p−maj=1, pX=0.85, N=325
var.1.CFH 1:196642233:G:A
peptide level
127:175
73:110
29:40
SCDNPYIPNGDYSPLRpc2_NP.1
p−min=0.39, pX=0.14, N=229
var.1.CFH 1:196642233:G:A
peptide level
79:175
55:110
22:40
SCDNPYIPNGDYSPLRpc2_NP.2
p−min=0.27, pX=0.85, N=156
var.1.CFH 1:196642233:G:A
peptide level
130:175
78:110
30:40
SCDNPYIPNGDYSPLRpc2_NP.3
p−min=0.71, pX=0.74, N=238
var.1.CFH 1:196642233:G:A
peptide level
175:175
109:110
39:40
SCDNPYIPNGDYSPLRpc2_NP.4
p−min=0.21, pX=0.38, N=323
var.1.CFH 1:196642233:G:A
peptide level
173:175
110:110
40:40
SCDNPYIPNGDYSPLRpc2_NP.5
p−min=0.5, pX=0.15, N=323

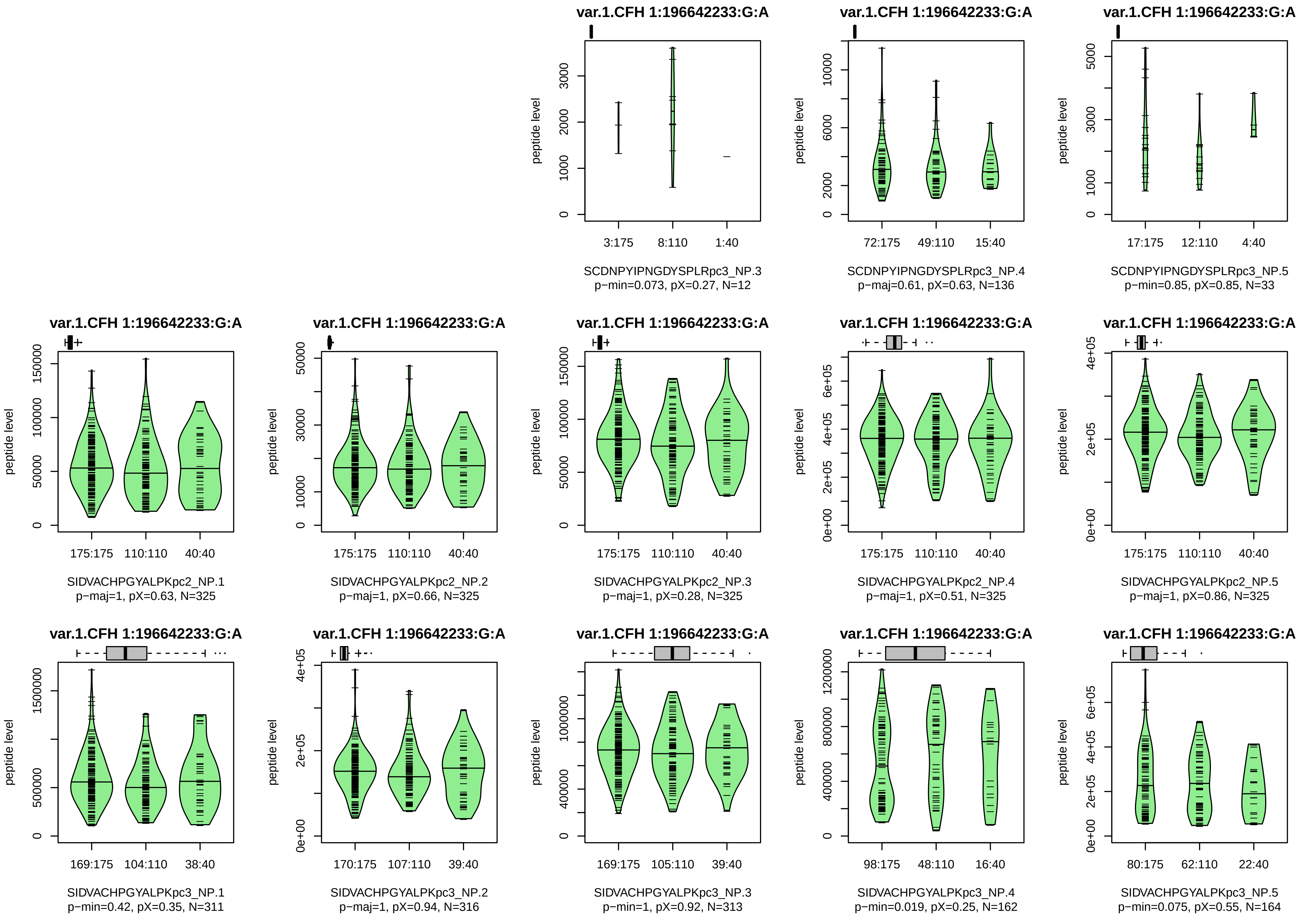
var.1.CFH 1:196642233:G:A
peptide level
3:175
8:110
1:40
SCDNPYIPNGDYSPLRpc3_NP.3
p−min=0.073, pX=0.27, N=12
var.1.CFH 1:196642233:G:A
peptide level
72:175
49:110
15:40
SCDNPYIPNGDYSPLRpc3_NP.4
p−maj=0.61, pX=0.63, N=136
var.1.CFH 1:196642233:G:A
peptide level
17:175
12:110
4:40
SCDNPYIPNGDYSPLRpc3_NP.5
p−min=0.85, pX=0.85, N=33
var.1.CFH 1:196642233:G:A
peptide level
175:175
110:110
40:40
SIDVACHPGYALPKpc2_NP.1
p−maj=1, pX=0.63, N=325
var.1.CFH 1:196642233:G:A
peptide level
175:175
110:110
40:40
SIDVACHPGYALPKpc2_NP.2
p−maj=1, pX=0.66, N=325
var.1.CFH 1:196642233:G:A
peptide level
175:175
110:110
40:40
SIDVACHPGYALPKpc2_NP.3
p−maj=1, pX=0.28, N=325
var.1.CFH 1:196642233:G:A
peptide level
175:175
110:110
40:40
SIDVACHPGYALPKpc2_NP.4
p−maj=1, pX=0.51, N=325
var.1.CFH 1:196642233:G:A
peptide level
175:175
110:110
40:40
SIDVACHPGYALPKpc2_NP.5
p−maj=1, pX=0.86, N=325
var.1.CFH 1:196642233:G:A
peptide level
169:175
104:110
38:40
SIDVACHPGYALPKpc3_NP.1
p−min=0.42, pX=0.35, N=311
var.1.CFH 1:196642233:G:A
peptide level
170:175
107:110
39:40
SIDVACHPGYALPKpc3_NP.2
p−maj=1, pX=0.94, N=316
var.1.CFH 1:196642233:G:A
peptide level
169:175
105:110
39:40
SIDVACHPGYALPKpc3_NP.3
p−min=1, pX=0.92, N=313
var.1.CFH 1:196642233:G:A
peptide level
98:175
48:110
16:40
SIDVACHPGYALPKpc3_NP.4
p−min=0.019, pX=0.25, N=162
var.1.CFH 1:196642233:G:A
peptide level
80:175
62:110
22:40
SIDVACHPGYALPKpc3_NP.5
p−min=0.075, pX=0.55, N=164

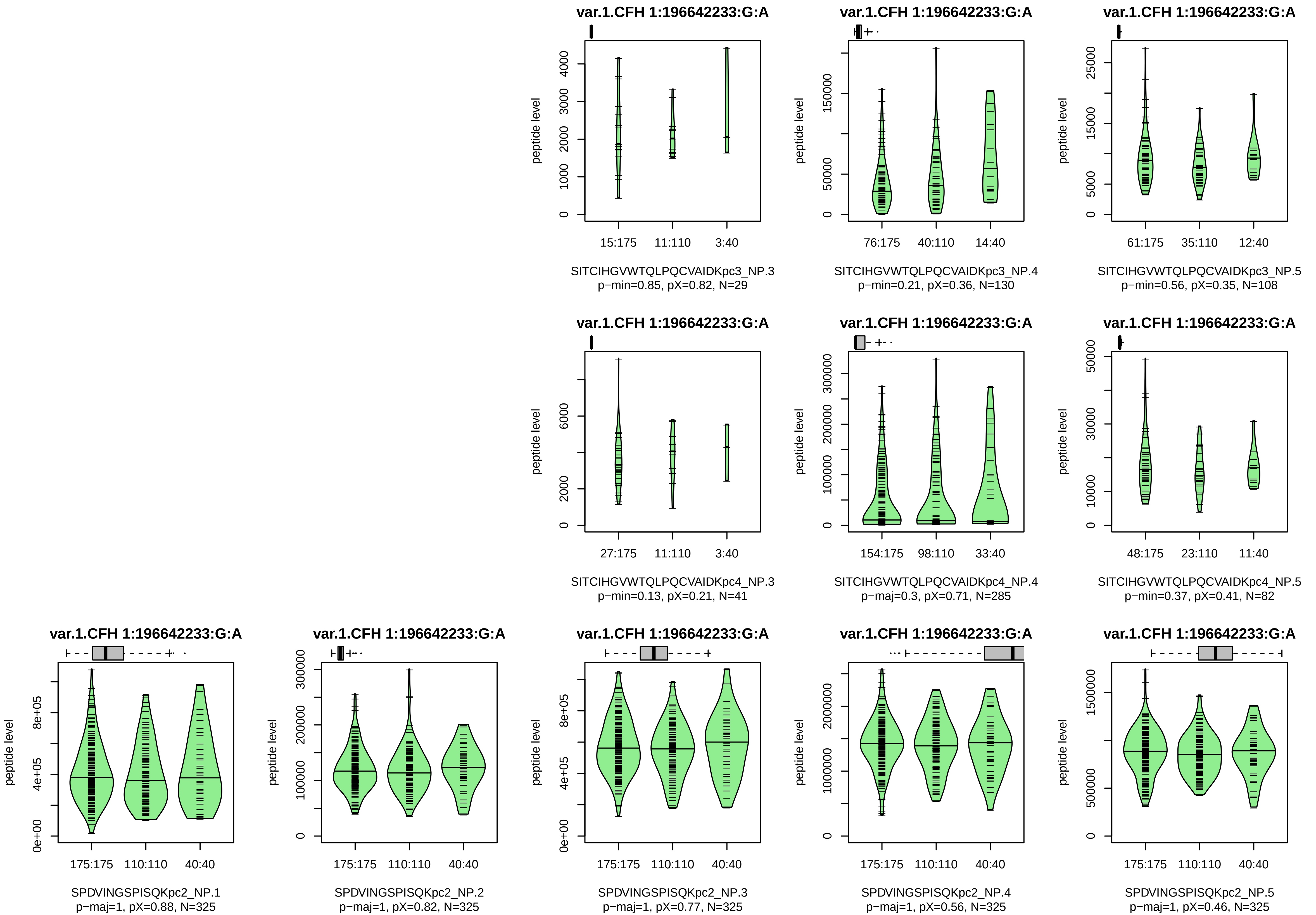
var.1.CFH 1:196642233:G:A
peptide level
15:175
11:110
3:40
SITCIHGVWTQLPQCVAIDKpc3_NP.3
p−min=0.85, pX=0.82, N=29
var.1.CFH 1:196642233:G:A
peptide level
76:175
40:110
14:40
SITCIHGVWTQLPQCVAIDKpc3_NP.4
p−min=0.21, pX=0.36, N=130
var.1.CFH 1:196642233:G:A
peptide level
61:175
35:110
12:40
SITCIHGVWTQLPQCVAIDKpc3_NP.5
p−min=0.56, pX=0.35, N=108
var.1.CFH 1:196642233:G:A
peptide level
27:175
11:110
3:40
SITCIHGVWTQLPQCVAIDKpc4_NP.3
p−min=0.13, pX=0.21, N=41
var.1.CFH 1:196642233:G:A
peptide level
154:175
98:110
33:40
SITCIHGVWTQLPQCVAIDKpc4_NP.4
p−maj=0.3, pX=0.71, N=285
var.1.CFH 1:196642233:G:A
peptide level
48:175
23:110
11:40
SITCIHGVWTQLPQCVAIDKpc4_NP.5
p−min=0.37, pX=0.41, N=82
var.1.CFH 1:196642233:G:A
peptide level
175:175
110:110
40:40
SPDVINGSPISQKpc2_NP.1
p−maj=1, pX=0.88, N=325
var.1.CFH 1:196642233:G:A
peptide level
175:175
110:110
40:40
SPDVINGSPISQKpc2_NP.2
p−maj=1, pX=0.82, N=325
var.1.CFH 1:196642233:G:A
peptide level
175:175
110:110
40:40
SPDVINGSPISQKpc2_NP.3
p−maj=1, pX=0.77, N=325
var.1.CFH 1:196642233:G:A
peptide level
175:175
110:110
40:40
SPDVINGSPISQKpc2_NP.4
p−maj=1, pX=0.56, N=325
var.1.CFH 1:196642233:G:A
peptide level
175:175
110:110
40:40
SPDVINGSPISQKpc2_NP.5
p−maj=1, pX=0.46, N=325

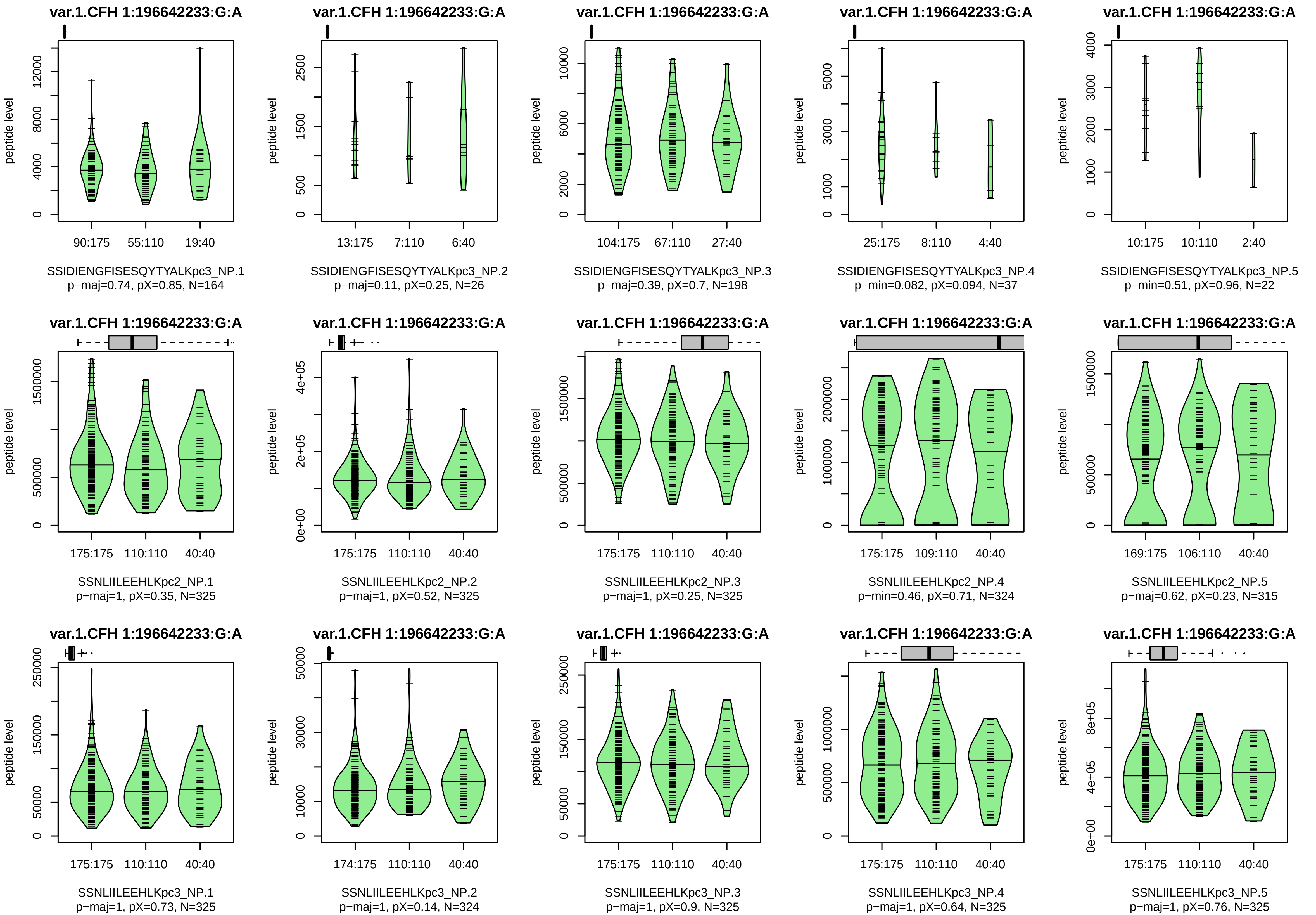
var.1.CFH 1:196642233:G:A
peptide level
90:175
55:110
19:40
SSIDIENGFISESQYTYALKpc3_NP.1
p−maj=0.74, pX=0.85, N=164
var.1.CFH 1:196642233:G:A
peptide level
13:175
7:110
6:40
SSIDIENGFISESQYTYALKpc3_NP.2
p−maj=0.11, pX=0.25, N=26
var.1.CFH 1:196642233:G:A
peptide level
104:175
67:110
27:40
SSIDIENGFISESQYTYALKpc3_NP.3
p−maj=0.39, pX=0.7, N=198
var.1.CFH 1:196642233:G:A
peptide level
25:175
8:110
4:40
SSIDIENGFISESQYTYALKpc3_NP.4
p−min=0.082, pX=0.094, N=37
var.1.CFH 1:196642233:G:A
peptide level
10:175
10:110
2:40
SSIDIENGFISESQYTYALKpc3_NP.5
p−min=0.51, pX=0.96, N=22
var.1.CFH 1:196642233:G:A
peptide level
175:175
110:110
40:40
SSNLIILEEHLKpc2_NP.1
p−maj=1, pX=0.35, N=325
var.1.CFH 1:196642233:G:A
peptide level
175:175
110:110
40:40
SSNLIILEEHLKpc2_NP.2
p−maj=1, pX=0.52, N=325
var.1.CFH 1:196642233:G:A
peptide level
175:175
110:110
40:40
SSNLIILEEHLKpc2_NP.3
p−maj=1, pX=0.25, N=325
var.1.CFH 1:196642233:G:A
peptide level
175:175
109:110
40:40
SSNLIILEEHLKpc2_NP.4
p−min=0.46, pX=0.71, N=324
var.1.CFH 1:196642233:G:A
peptide level
169:175
106:110
40:40
SSNLIILEEHLKpc2_NP.5
p−maj=0.62, pX=0.23, N=315
var.1.CFH 1:196642233:G:A
peptide level
175:175
110:110
40:40
SSNLIILEEHLKpc3_NP.1
p−maj=1, pX=0.73, N=325
var.1.CFH 1:196642233:G:A
peptide level
174:175
110:110
40:40
SSNLIILEEHLKpc3_NP.2
p−maj=1, pX=0.14, N=324
var.1.CFH 1:196642233:G:A
peptide level
175:175
110:110
40:40
SSNLIILEEHLKpc3_NP.3
p−maj=1, pX=0.9, N=325
var.1.CFH 1:196642233:G:A
peptide level
175:175
110:110
40:40
SSNLIILEEHLKpc3_NP.4
p−maj=1, pX=0.64, N=325
var.1.CFH 1:196642233:G:A
peptide level
175:175
110:110
40:40
SSNLIILEEHLKpc3_NP.5
p−maj=1, pX=0.76, N=325

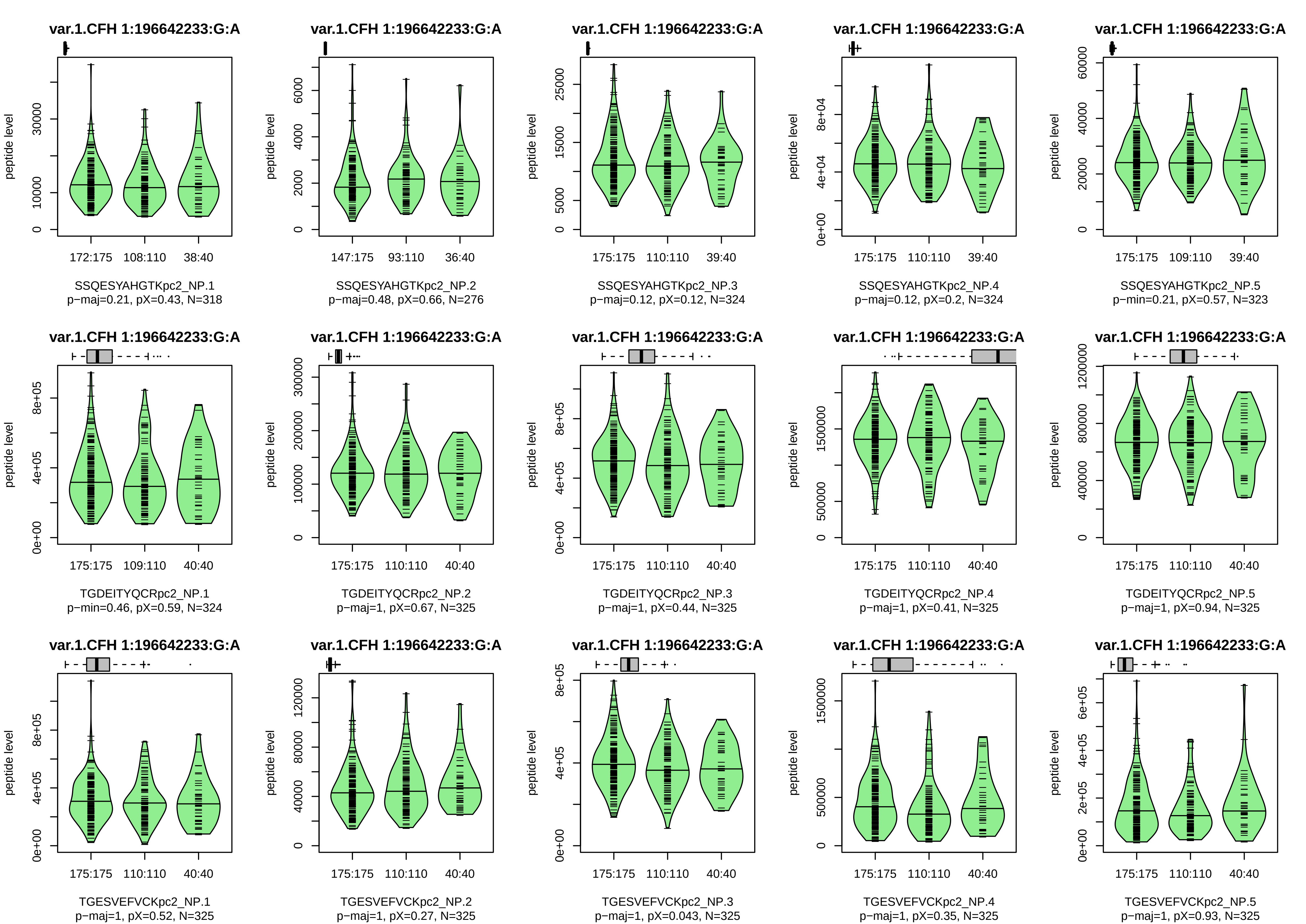
var.1.CFH 1:196642233:G:A
peptide level
172:175 108:110 38:40
SSQESYAHGTKpc2_NP.1
p−maj=0.21, pX=0.43, N=318
var.1.CFH 1:196642233:G:A
peptide level
147:175 93:110 36:40
SSQESYAHGTKpc2_NP.2
p−maj=0.48, pX=0.66, N=276
var.1.CFH 1:196642233:G:A
peptide level
175:175 110:110 39:40
SSQESYAHGTKpc2_NP.3
p−maj=0.12, pX=0.12, N=324
var.1.CFH 1:196642233:G:A
peptide level
175:175 110:110 39:40
SSQESYAHGTKpc2_NP.4
p−maj=0.12, pX=0.2, N=324
var.1.CFH 1:196642233:G:A
peptide level
175:175 109:110 39:40
SSQESYAHGTKpc2_NP.5
p−min=0.21, pX=0.57, N=323
var.1.CFH 1:196642233:G:A
peptide level
175:175 109:110 40:40
TGDEITYQCRpc2_NP.1
p−min=0.46, pX=0.59, N=324
var.1.CFH 1:196642233:G:A
peptide level
175:175 110:110 40:40
TGDEITYQCRpc2_NP.2
p−maj=1, pX=0.67, N=325
var.1.CFH 1:196642233:G:A
peptide level
175:175 110:110 40:40
TGDEITYQCRpc2_NP.3
p−maj=1, pX=0.44, N=325
var.1.CFH 1:196642233:G:A
peptide level
175:175 110:110 40:40
TGDEITYQCRpc2_NP.4
p−maj=1, pX=0.41, N=325
var.1.CFH 1:196642233:G:A
peptide level
175:175 110:110 40:40
TGDEITYQCRpc2_NP.5
p−maj=1, pX=0.94, N=325
var.1.CFH 1:196642233:G:A
peptide level
175:175 110:110 40:40
TGESVEFVCKpc2_NP.1
p−maj=1, pX=0.52, N=325
var.1.CFH 1:196642233:G:A
peptide level
175:175 110:110 40:40
TGESVEFVCKpc2_NP.2
p−maj=1, pX=0.27, N=325
var.1.CFH 1:196642233:G:A
peptide level
175:175 110:110 40:40
TGESVEFVCKpc2_NP.3
p−maj=1, pX=0.043, N=325
var.1.CFH 1:196642233:G:A
peptide level
175:175 110:110 40:40
TGESVEFVCKpc2_NP.4
p−maj=1, pX=0.35, N=325
var.1.CFH 1:196642233:G:A
peptide level
175:175 110:110 40:40
TGESVEFVCKpc2_NP.5
p−maj=1, pX=0.93, N=325

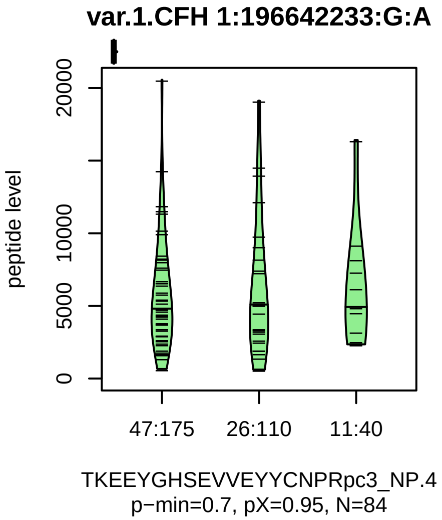
var.1.CFH 1:196642233:G:A
peptide level
0
5000
10000
20000
47:175
26:110
11:40
TKEEYGHSEVVEYYCNPRpc3_NP.4
p−min=0.7, pX=0.95, N=84

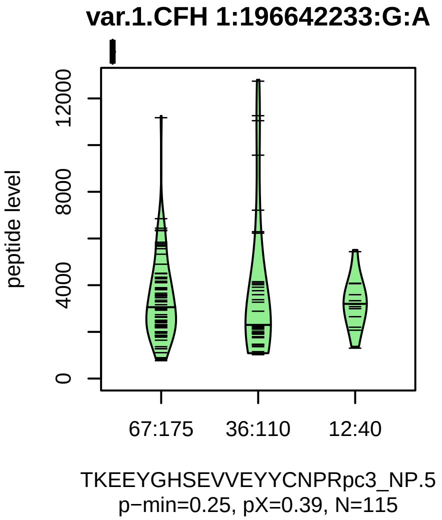
var.1.CFH 1:196642233:G:A
peptide level
0
4000
8000
12000
67:175
36:110
12:40
TKEEYGHSEVVEYYCNPRpc3_NP.5
p−min=0.25, pX=0.39, N=115

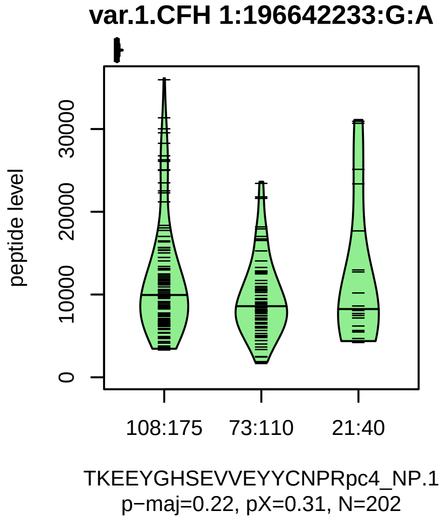
var.1.CFH 1:196642233:G:A
peptide level
0
10000
20000
30000
108:175
73:110
21:40
TKEEYGHSEVVEYYCNPRpc4_NP.1
p−maj=0.22, pX=0.31, N=202

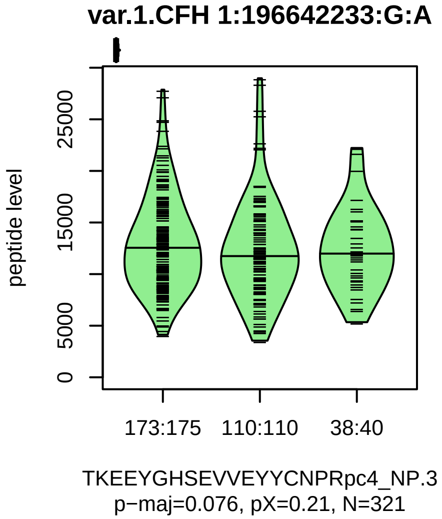
var.1.CFH 1:196642233:G:A
peptide level
0
5000
15000
25000
173:175
110:110
38:40
TKEEYGHSEVVEYYCNPRpc4_NP.3
p−maj=0.076, pX=0.21, N=321

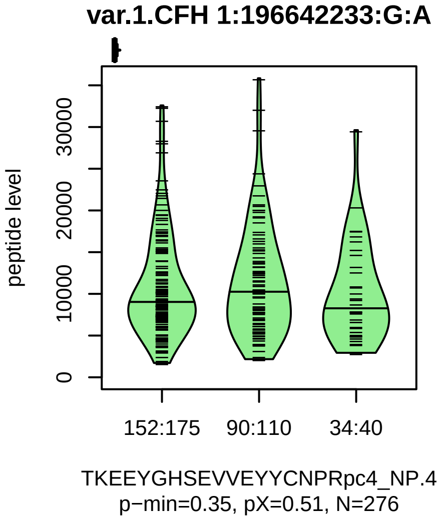
var.1.CFH 1:196642233:G:A
peptide level
0
10000
20000
30000
152:175
90:110
34:40
TKEEYGHSEVVEYYCNPRpc4_NP.4
p−min=0.35, pX=0.51, N=276

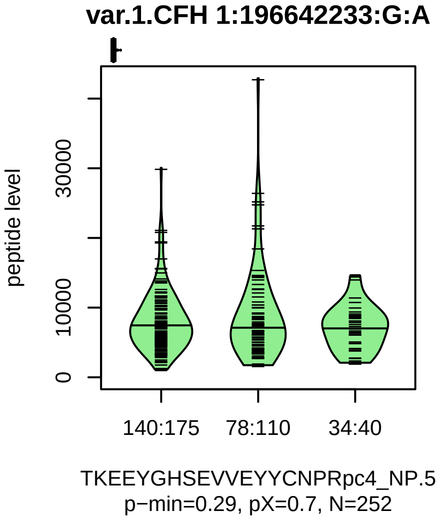
var.1.CFH 1:196642233:G:A
peptide level
0
10000
30000
140:175
78:110
34:40
TKEEYGHSEVVEYYCNPRpc4_NP.5
p−min=0.29, pX=0.7, N=252

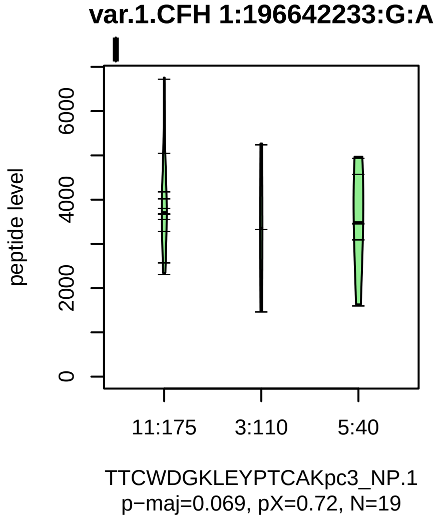
var.1.CFH 1:196642233:G:A
peptide level
0
2000
4000
6000
11:175
3:110
5:40
TTCWDGKLEYPTCAKpc3_NP.1
p−maj=0.069, pX=0.72, N=19

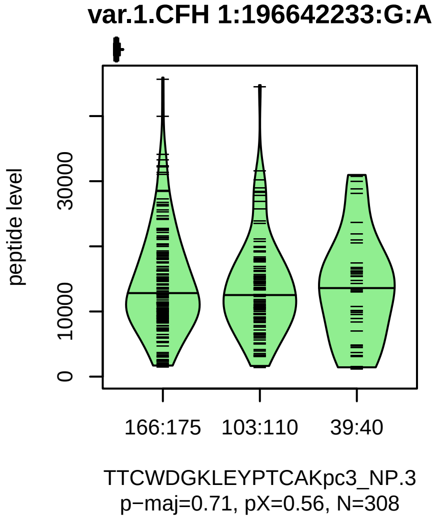
var.1.CFH 1:196642233:G:A
peptide level
0
10000
30000
166:175
103:110
39:40
TTCWDGKLEYPTCAKpc3_NP.3
p−maj=0.71, pX=0.56, N=308

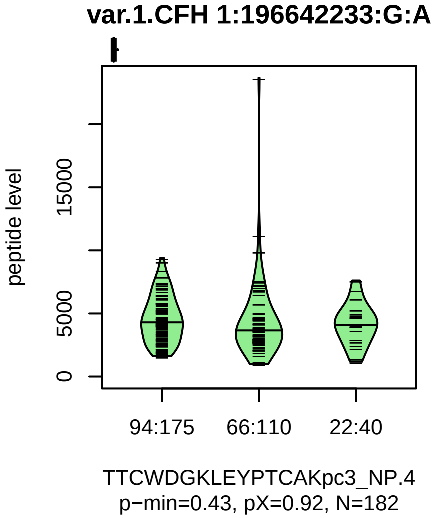
var.1.CFH 1:196642233:G:A
peptide level
0
5000
15000
94:175
66:110
22:40
TTCWDGKLEYPTCAKpc3_NP.4
p−min=0.43, pX=0.92, N=182

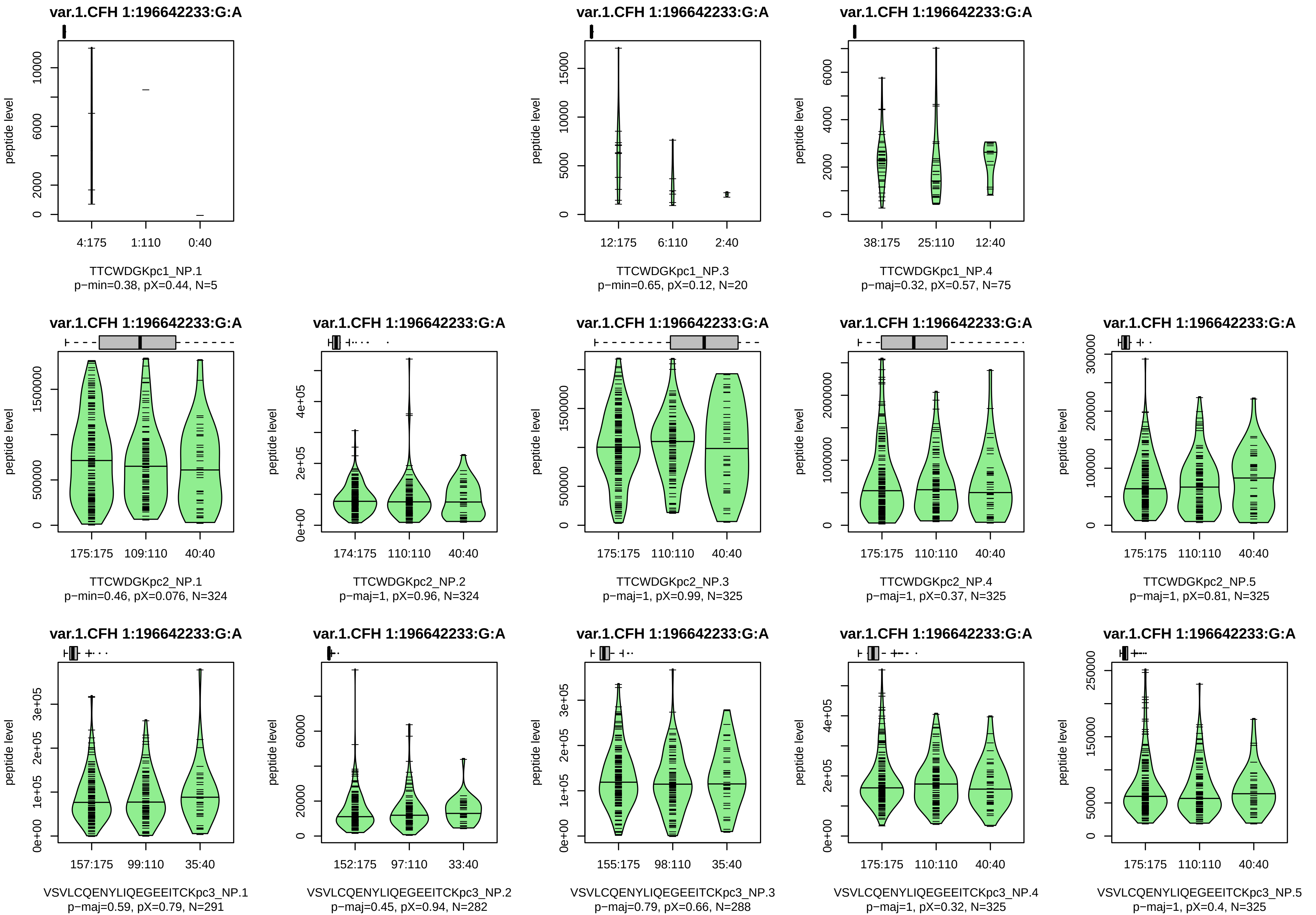

var.1.CFH 1:196642233:G:A
peptide level
4:175
1:110
0:40
TTCWDGKpc1_NP.1
p−min=0.38, pX=0.44, N=5
var.1.CFH 1:196642233:G:A
12:175
6:110
2:40
TTCWDGKpc1_NP.3
p−min=0.65, pX=0.12, N=20
var.1.CFH 1:196642233:G:A
38:175
25:110
12:40
TTCWDGKpc1_NP.4
p−maj=0.32, pX=0.57, N=75
var.1.CFH 1:196642233:G:A
175:175
109:110
40:40
TTCWDGKpc2_NP.1
p−min=0.46, pX=0.076, N=324
var.1.CFH 1:196642233:G:A
174:175
110:110
40:40
TTCWDGKpc2_NP.2
p−maj=1, pX=0.96, N=324
var.1.CFH 1:196642233:G:A
175:175
110:110
40:40
TTCWDGKpc2_NP.3
p−maj=1, pX=0.99, N=325
var.1.CFH 1:196642233:G:A
175:175
110:110
40:40
TTCWDGKpc2_NP.4
p−maj=1, pX=0.37, N=325
var.1.CFH 1:196642233:G:A
175:175
110:110
40:40
TTCWDGKpc2_NP.5
p−maj=1, pX=0.81, N=325
var.1.CFH 1:196642233:G:A
157:175
99:110
35:40
VSVLCQENYLIQEGEEITCKpc3_NP.1
p−maj=0.59, pX=0.79, N=291
var.1.CFH 1:196642233:G:A
152:175
97:110
33:40
VSVLCQENYLIQEGEEITCKpc3_NP.2
p−maj=0.45, pX=0.94, N=282
var.1.CFH 1:196642233:G:A
155:175
98:110
35:40
VSVLCQENYLIQEGEEITCKpc3_NP.3
p−maj=0.79, pX=0.66, N=288
var.1.CFH 1:196642233:G:A
175:175
110:110
40:40
VSVLCQENYLIQEGEEITCKpc3_NP.4
p−maj=1, pX=0.32, N=325
var.1.CFH 1:196642233:G:A
175:175
110:110
40:40
VSVLCQENYLIQEGEEITCKpc3_NP.5
p−maj=1, pX=0.4, N=325

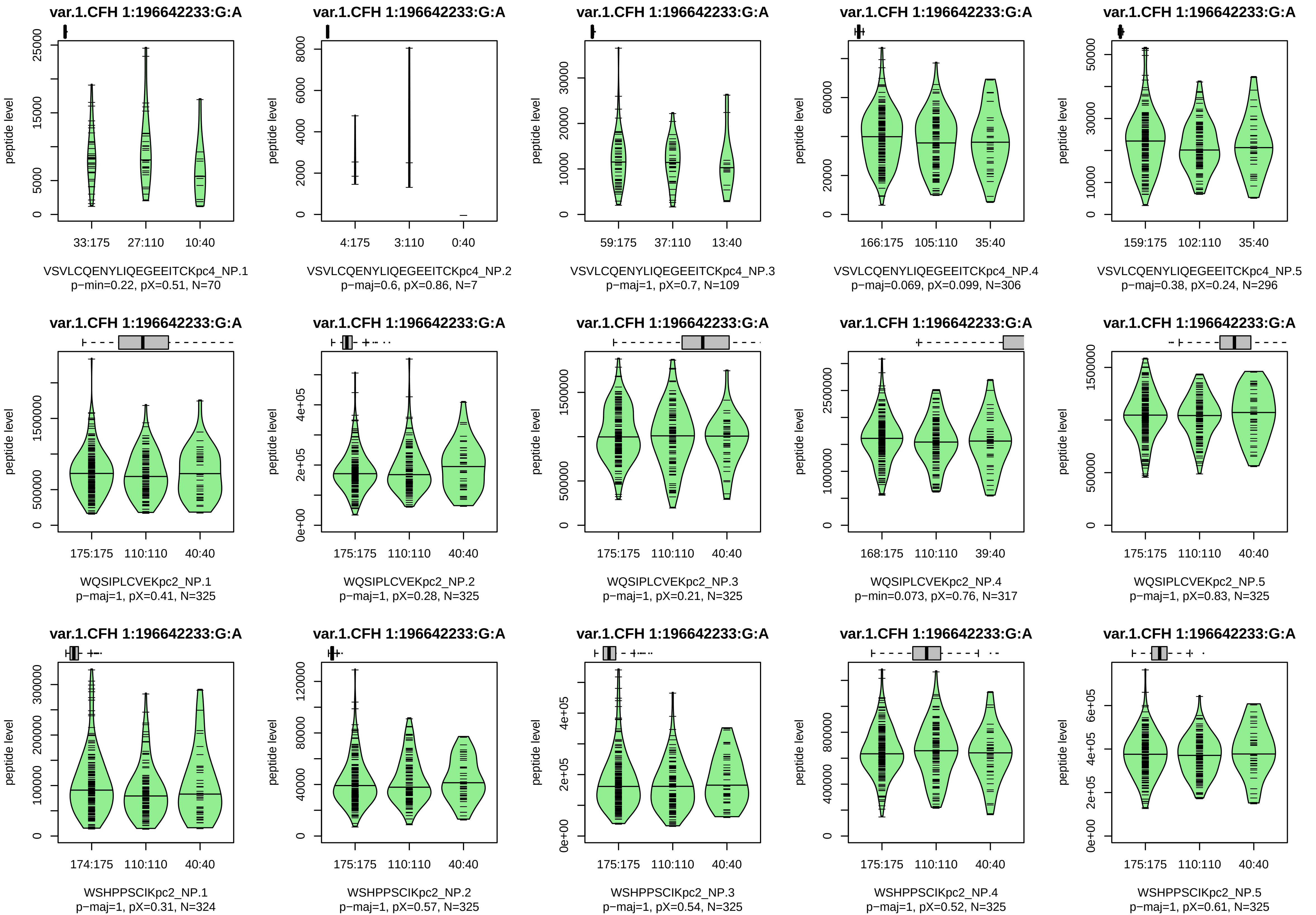
var.1.CFH 1:196642233:G:A
peptide level
33:175
27:110
10:40
VSVLCQENYLIQEGEEITCKpc4_NP.1
p−min=0.22, pX=0.51, N=70
var.1.CFH 1:196642233:G:A
4:175
3:110
0:40
VSVLCQENYLIQEGEEITCKpc4_NP.2
p−maj=0.6, pX=0.86, N=7
var.1.CFH 1:196642233:G:A
59:175
37:110
13:40
VSVLCQENYLIQEGEEITCKpc4_NP.3
p−maj=1, pX=0.7, N=109
var.1.CFH 1:196642233:G:A
166:175
105:110
35:40
VSVLCQENYLIQEGEEITCKpc4_NP.4
p−maj=0.069, pX=0.099, N=306
var.1.CFH 1:196642233:G:A
159:175
102:110
35:40
VSVLCQENYLIQEGEEITCKpc4_NP.5
p−maj=0.38, pX=0.24, N=296
var.1.CFH 1:196642233:G:A
175:175
110:110
40:40
WQSIPLCVEKpc2_NP.1
p−maj=1, pX=0.41, N=325
var.1.CFH 1:196642233:G:A
175:175
110:110
40:40
WQSIPLCVEKpc2_NP.2
p−maj=1, pX=0.28, N=325
var.1.CFH 1:196642233:G:A
175:175
110:110
40:40
WQSIPLCVEKpc2_NP.3
p−maj=1, pX=0.21, N=325
var.1.CFH 1:196642233:G:A
168:175
110:110
39:40
WQSIPLCVEKpc2_NP.4
p−min=0.073, pX=0.76, N=317
var.1.CFH 1:196642233:G:A
175:175
110:110
40:40
WQSIPLCVEKpc2_NP.5
p−maj=1, pX=0.83, N=325
var.1.CFH 1:196642233:G:A
174:175
110:110
40:40
WSHPPSCIKpc2_NP.1
p−maj=1, pX=0.31, N=324
var.1.CFH 1:196642233:G:A
175:175
110:110
40:40
WSHPPSCIKpc2_NP.2
p−maj=1, pX=0.57, N=325
var.1.CFH 1:196642233:G:A
175:175
110:110
40:40
WSHPPSCIKpc2_NP.3
p−maj=1, pX=0.54, N=325
var.1.CFH 1:196642233:G:A
175:175
110:110
40:40
WSHPPSCIKpc2_NP.4
p−maj=1, pX=0.52, N=325
var.1.CFH 1:196642233:G:A
175:175
110:110
40:40
WSHPPSCIKpc2_NP.5
p−maj=1, pX=0.61, N=325

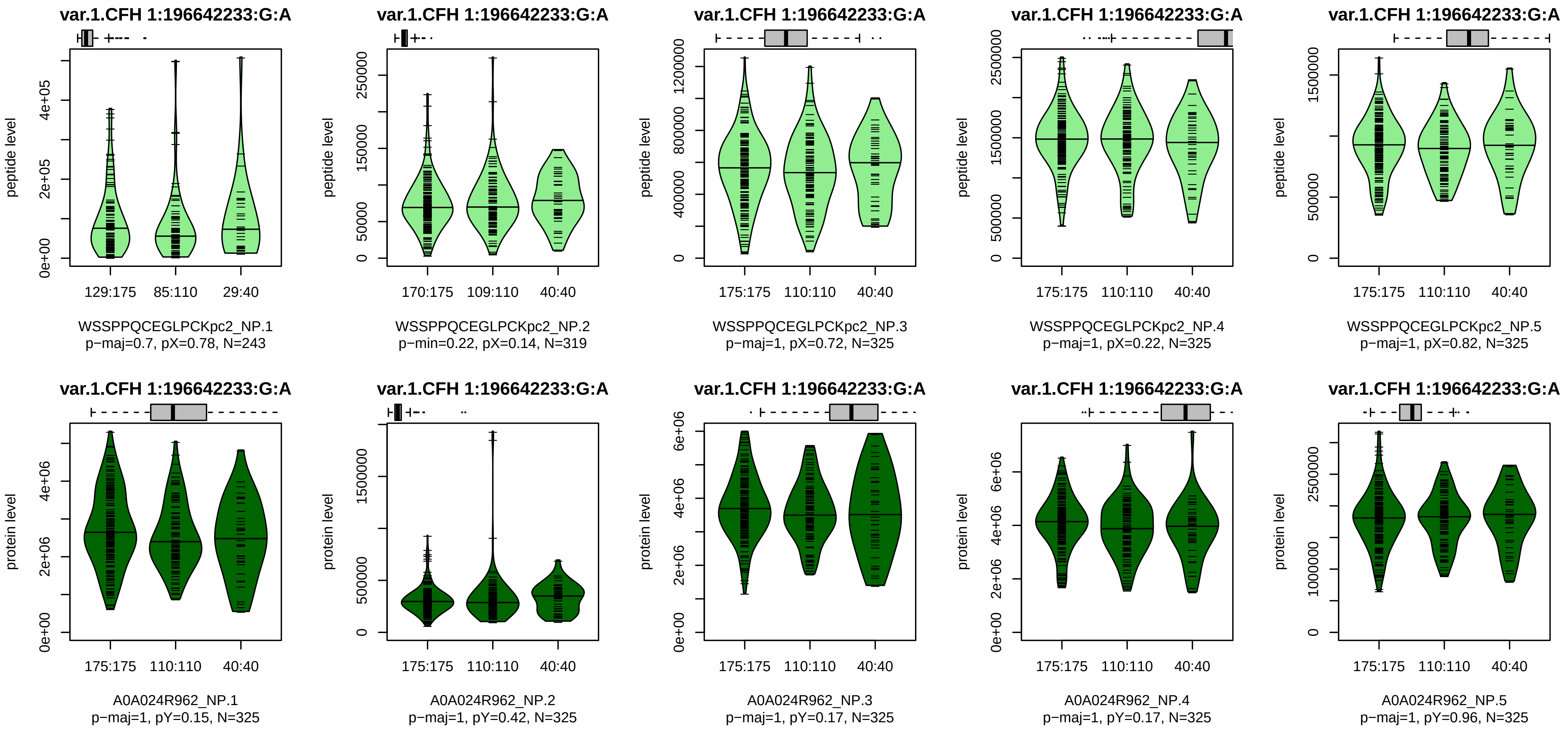

var.1.CFH 1:196642233:G:A
peptide level
0e+00
2e+05
4e+05
129:175
85:110
29:40
WSSPPQCEGLPCKpc2_NP.1
p−maj=0.7, pX=0.78, N=243
var.1.CFH 1:196642233:G:A
peptide level
0
50000
150000
250000
170:175
109:110
40:40
WSSPPQCEGLPCKpc2_NP.2
p−min=0.22, pX=0.14, N=319
var.1.CFH 1:196642233:G:A
peptide level
0
400000
800000
1200000
175:175
110:110
40:40
WSSPPQCEGLPCKpc2_NP.3
p−maj=1, pX=0.72, N=325
var.1.CFH 1:196642233:G:A
peptide level
0
500000
1500000
2500000
175:175
110:110
40:40
WSSPPQCEGLPCKpc2_NP.4
p−maj=1, pX=0.22, N=325
var.1.CFH 1:196642233:G:A
peptide level
0
500000
1500000
175:175
110:110
40:40
WSSPPQCEGLPCKpc2_NP.5
p−maj=1, pX=0.82, N=325
var.1.CFH 1:196642233:G:A
protein level
0e+00
2e+06
4e+06
175:175
110:110
40:40
A0A024R962_NP.1
p−maj=1, pY=0.15, N=325
var.1.CFH 1:196642233:G:A
protein level
0
500000
1500000
175:175
110:110
40:40
A0A024R962_NP.2
p−maj=1, pY=0.42, N=325
var.1.CFH 1:196642233:G:A
protein level
0e+00
2e+06
4e+06
6e+06
175:175
110:110
40:40
A0A024R962_NP.3
p−maj=1, pY=0.17, N=325
var.1.CFH 1:196642233:G:A
protein level
0e+00
2e+06
4e+06
6e+06
175:175
110:110
40:40
A0A024R962_NP.4
p−maj=1, pY=0.17, N=325
var.1.CFH 1:196642233:G:A
protein level
0
1000000
2500000
175:175
110:110
40:40
A0A024R962_NP.5
p−maj=1, pY=0.96, N=325

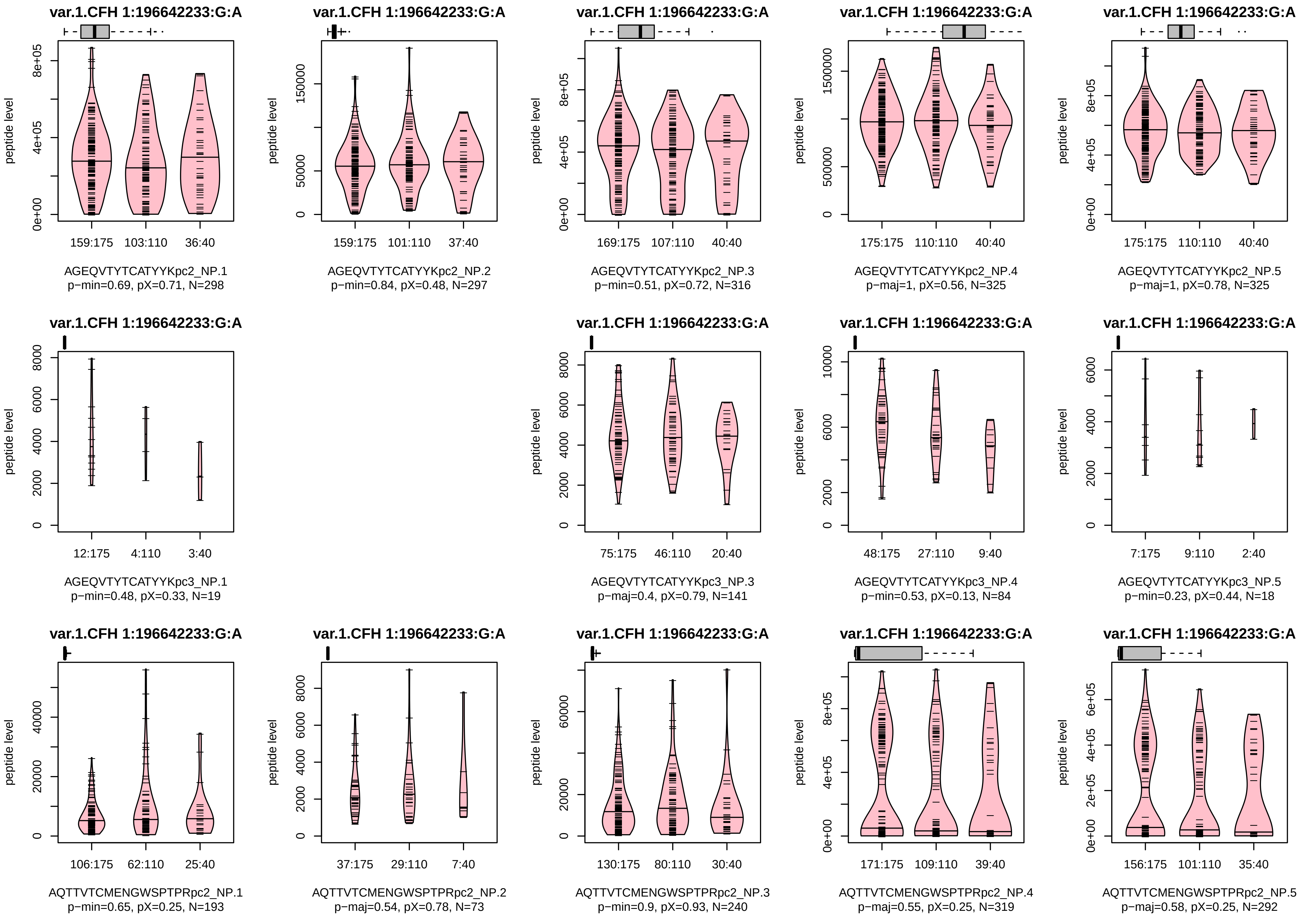
var.1.CFH 1:196642233:G:A
peptide level
159:175
103:110
36:40
AGEQVTYTCATYYKpc2_NP.1
p−min=0.69, pX=0.71, N=298
var.1.CFH 1:196642233:G:A
peptide level
159:175
101:110
37:40
AGEQVTYTCATYYKpc2_NP.2
p−min=0.84, pX=0.48, N=297
var.1.CFH 1:196642233:G:A
peptide level
169:175
107:110
40:40
AGEQVTYTCATYYKpc2_NP.3
p−min=0.51, pX=0.72, N=316
var.1.CFH 1:196642233:G:A
peptide level
175:175
110:110
40:40
AGEQVTYTCATYYKpc2_NP.4
p−maj=1, pX=0.56, N=325
var.1.CFH 1:196642233:G:A
peptide level
175:175
110:110
40:40
AGEQVTYTCATYYKpc2_NP.5
p−maj=1, pX=0.78, N=325
var.1.CFH 1:196642233:G:A
peptide level
12:175
4:110
3:40
AGEQVTYTCATYYKpc3_NP.1
p−min=0.48, pX=0.33, N=19
var.1.CFH 1:196642233:G:A
peptide level
75:175
46:110
20:40
AGEQVTYTCATYYKpc3_NP.3
p−maj=0.4, pX=0.79, N=141
var.1.CFH 1:196642233:G:A
peptide level
48:175
27:110
9:40
AGEQVTYTCATYYKpc3_NP.4
p−min=0.53, pX=0.13, N=84
var.1.CFH 1:196642233:G:A
peptide level
7:175
9:110
2:40
AGEQVTYTCATYYKpc3_NP.5
p−min=0.23, pX=0.44, N=18
var.1.CFH 1:196642233:G:A
peptide level
106:175
62:110
25:40
AQTTVTCMENGWSPTPRpc2_NP.1
p−min=0.65, pX=0.25, N=193
var.1.CFH 1:196642233:G:A
peptide level
37:175
29:110
7:40
AQTTVTCMENGWSPTPRpc2_NP.2
p−maj=0.54, pX=0.78, N=73
var.1.CFH 1:196642233:G:A
peptide level
130:175
80:110
30:40
AQTTVTCMENGWSPTPRpc2_NP.3
p−min=0.9, pX=0.93, N=240
var.1.CFH 1:196642233:G:A
peptide level
171:175
109:110
39:40
AQTTVTCMENGWSPTPRpc2_NP.4
p−maj=0.55, pX=0.25, N=319
var.1.CFH 1:196642233:G:A
peptide level
156:175
101:110
35:40
AQTTVTCMENGWSPTPRpc2_NP.5
p−maj=0.58, pX=0.25, N=292

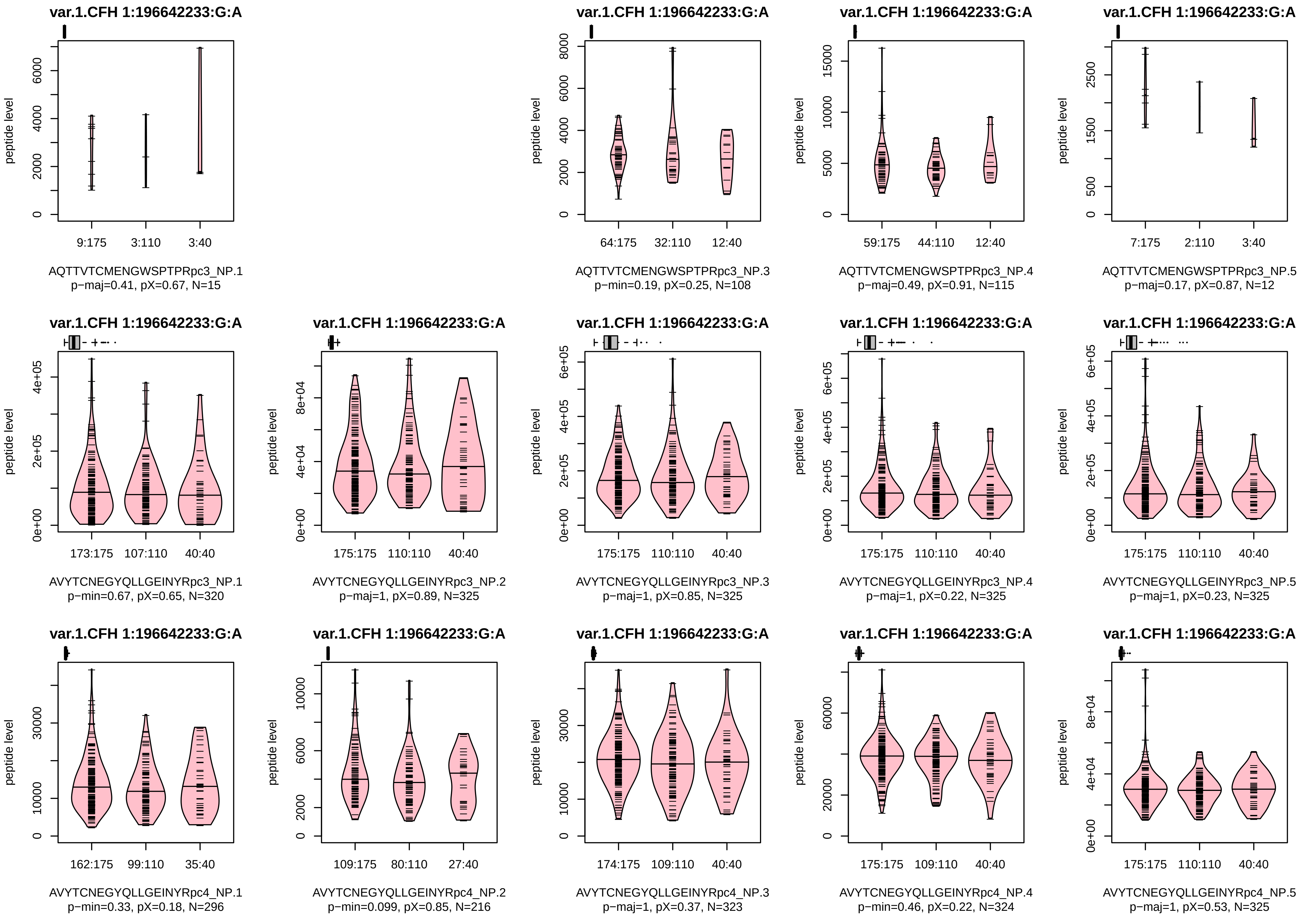
var.1.CFH 1:196642233:G:A
peptide level
9:175
3:110
3:40
AQTTVTCMENGWSPTPRpc3_NP.1
p−maj=0.41, pX=0.67, N=15
var.1.CFH 1:196642233:G:A
peptide level
64:175
32:110
12:40
AQTTVTCMENGWSPTPRpc3_NP.3
p−min=0.19, pX=0.25, N=108
var.1.CFH 1:196642233:G:A
peptide level
59:175
44:110
12:40
AQTTVTCMENGWSPTPRpc3_NP.4
p−maj=0.49, pX=0.91, N=115
var.1.CFH 1:196642233:G:A
peptide level
7:175
2:110
3:40
AQTTVTCMENGWSPTPRpc3_NP.5
p−maj=0.17, pX=0.87, N=12
var.1.CFH 1:196642233:G:A
peptide level
173:175
107:110
40:40
AVYTCNEGYQLLGEINYRpc3_NP.1
p−min=0.67, pX=0.65, N=320
var.1.CFH 1:196642233:G:A
peptide level
175:175
110:110
40:40
AVYTCNEGYQLLGEINYRpc3_NP.2
p−maj=1, pX=0.89, N=325
var.1.CFH 1:196642233:G:A
peptide level
175:175
110:110
40:40
AVYTCNEGYQLLGEINYRpc3_NP.3
p−maj=1, pX=0.85, N=325
var.1.CFH 1:196642233:G:A
peptide level
175:175
110:110
40:40
AVYTCNEGYQLLGEINYRpc3_NP.4
p−maj=1, pX=0.22, N=325
var.1.CFH 1:196642233:G:A
peptide level
175:175
110:110
40:40
AVYTCNEGYQLLGEINYRpc3_NP.5
p−maj=1, pX=0.23, N=325
var.1.CFH 1:196642233:G:A
peptide level
162:175
99:110
35:40
AVYTCNEGYQLLGEINYRpc4_NP.1
p−min=0.33, pX=0.18, N=296
var.1.CFH 1:196642233:G:A
peptide level
109:175
80:110
27:40
AVYTCNEGYQLLGEINYRpc4_NP.2
p−min=0.099, pX=0.85, N=216
var.1.CFH 1:196642233:G:A
peptide level
174:175
109:110
40:40
AVYTCNEGYQLLGEINYRpc4_NP.3
p−maj=1, pX=0.37, N=323
var.1.CFH 1:196642233:G:A
peptide level
175:175
109:110
40:40
AVYTCNEGYQLLGEINYRpc4_NP.4
p−min=0.46, pX=0.22, N=324
var.1.CFH 1:196642233:G:A
peptide level
175:175
110:110
40:40
AVYTCNEGYQLLGEINYRpc4_NP.5
p−maj=1, pX=0.53, N=325

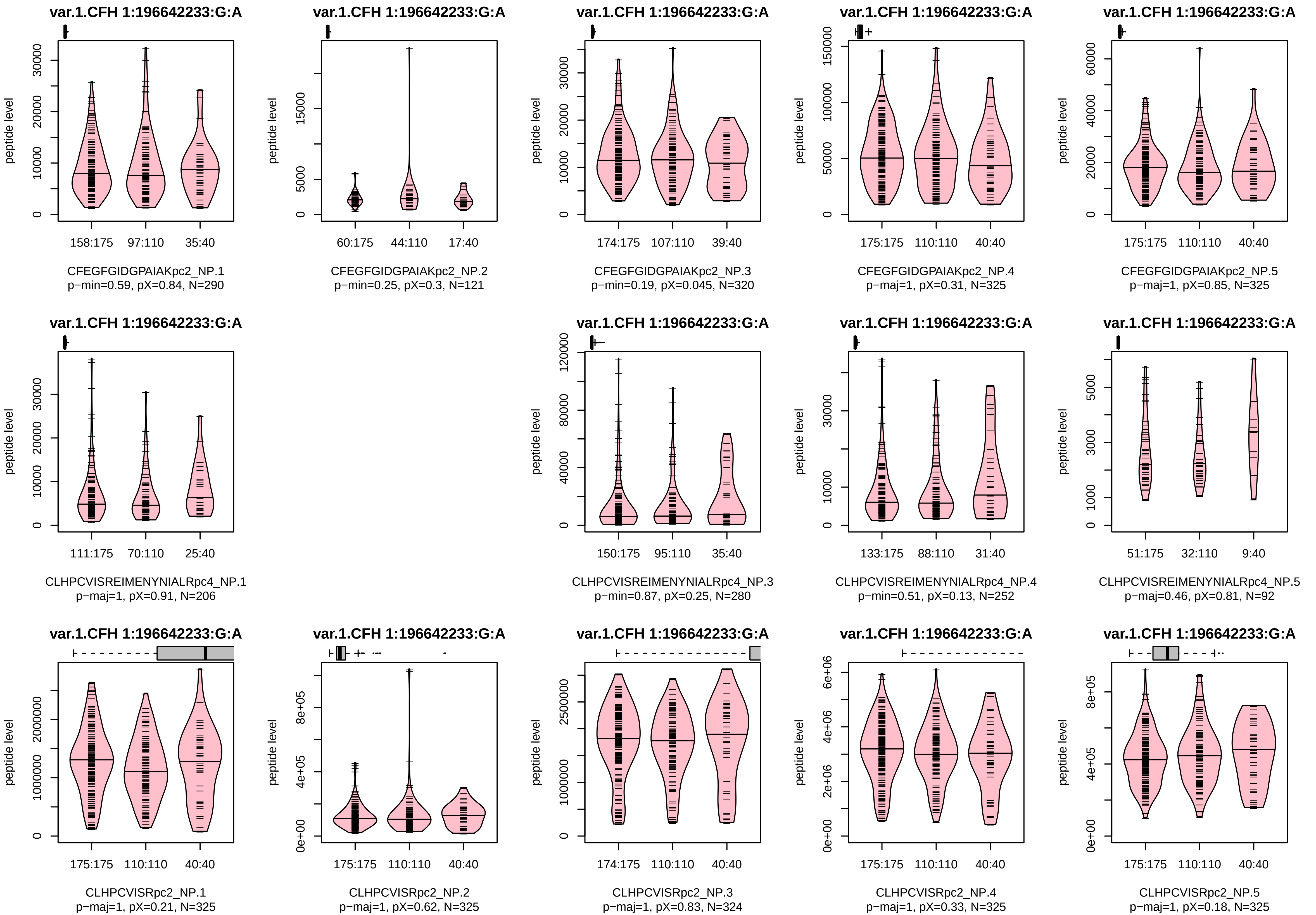
var.1.CFH 1:196642233:G:A
peptide level
158:175
97:110
35:40
CFEGFGIDGPAIAKpc2_NP.1
p−min=0.59, pX=0.84, N=290
var.1.CFH 1:196642233:G:A
peptide level
60:175
44:110
17:40
CFEGFGIDGPAIAKpc2_NP.2
p−min=0.25, pX=0.3, N=121
var.1.CFH 1:196642233:G:A
peptide level
174:175
107:110
39:40
CFEGFGIDGPAIAKpc2_NP.3
p−min=0.19, pX=0.045, N=320
var.1.CFH 1:196642233:G:A
peptide level
175:175
110:110
40:40
CFEGFGIDGPAIAKpc2_NP.4
p−maj=1, pX=0.31, N=325
var.1.CFH 1:196642233:G:A
peptide level
175:175
110:110
40:40
CFEGFGIDGPAIAKpc2_NP.5
p−maj=1, pX=0.85, N=325
var.1.CFH 1:196642233:G:A
peptide level
111:175
70:110
25:40
CLHPCVISREIMENYNIALRpc4_NP.1
p−maj=1, pX=0.91, N=206
var.1.CFH 1:196642233:G:A
peptide level
150:175
95:110
35:40
CLHPCVISREIMENYNIALRpc4_NP.3
p−min=0.87, pX=0.25, N=280
var.1.CFH 1:196642233:G:A
peptide level
133:175
88:110
31:40
CLHPCVISREIMENYNIALRpc4_NP.4
p−min=0.51, pX=0.13, N=252
var.1.CFH 1:196642233:G:A
peptide level
51:175
32:110
9:40
CLHPCVISREIMENYNIALRpc4_NP.5
p−maj=0.46, pX=0.81, N=92
var.1.CFH 1:196642233:G:A
peptide level
175:175
110:110
40:40
CLHPCVISRpc2_NP.1
p−maj=1, pX=0.21, N=325
var.1.CFH 1:196642233:G:A
peptide level
175:175
110:110
40:40
CLHPCVISRpc2_NP.2
p−maj=1, pX=0.62, N=325
var.1.CFH 1:196642233:G:A
peptide level
174:175
110:110
40:40
CLHPCVISRpc2_NP.3
p−maj=1, pX=0.83, N=324
var.1.CFH 1:196642233:G:A
peptide level
175:175
110:110
40:40
CLHPCVISRpc2_NP.4
p−maj=1, pX=0.33, N=325
var.1.CFH 1:196642233:G:A
peptide level
175:175
110:110
40:40
CLHPCVISRpc2_NP.5
p−maj=1, pX=0.18, N=325

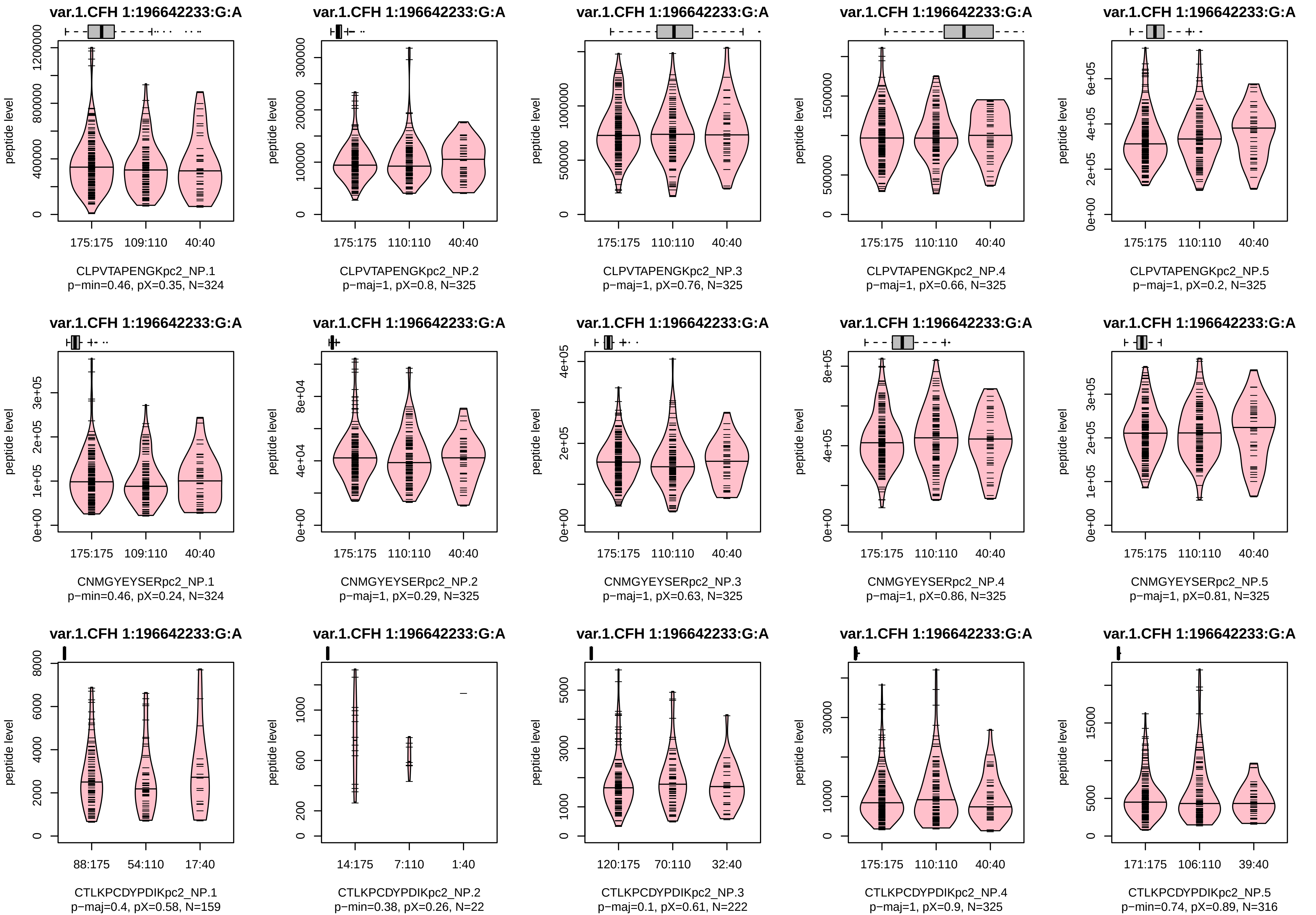
var.1.CFH 1:196642233:G:A
peptide level
0
400000
800000
1200000
175:175
109:110
40:40
CLPVTAPENGKpc2_NP.1
p−min=0.46, pX=0.35, N=324
var.1.CFH 1:196642233:G:A
peptide level
0
100000
200000
300000
175:175
110:110
40:40
CLPVTAPENGKpc2_NP.2
p−maj=1, pX=0.8, N=325
var.1.CFH 1:196642233:G:A
peptide level
0
500000
1000000
175:175
110:110
40:40
CLPVTAPENGKpc2_NP.3
p−maj=1, pX=0.76, N=325
var.1.CFH 1:196642233:G:A
peptide level
0
500000
1500000
175:175
110:110
40:40
CLPVTAPENGKpc2_NP.4
p−maj=1, pX=0.66, N=325
var.1.CFH 1:196642233:G:A
peptide level
0e+00
2e+05
4e+05
6e+05
175:175
110:110
40:40
CLPVTAPENGKpc2_NP.5
p−maj=1, pX=0.2, N=325
var.1.CFH 1:196642233:G:A
peptide level
0e+00
1e+05
2e+05
3e+05
175:175
109:110
40:40
CNMGYEYSERpc2_NP.1
p−min=0.46, pX=0.24, N=324
var.1.CFH 1:196642233:G:A
peptide level
0e+00
4e+04
8e+04
175:175
110:110
40:40
CNMGYEYSERpc2_NP.2
p−maj=1, pX=0.29, N=325
var.1.CFH 1:196642233:G:A
peptide level
0e+00
2e+05
4e+05
175:175
110:110
40:40
CNMGYEYSERpc2_NP.3
p−maj=1, pX=0.63, N=325
var.1.CFH 1:196642233:G:A
peptide level
0e+00
4e+05
8e+05
175:175
110:110
40:40
CNMGYEYSERpc2_NP.4
p−maj=1, pX=0.86, N=325
var.1.CFH 1:196642233:G:A
peptide level
0e+00
1e+05
2e+05
3e+05
175:175
110:110
40:40
CNMGYEYSERpc2_NP.5
p−maj=1, pX=0.81, N=325
var.1.CFH 1:196642233:G:A
peptide level
0
2000
4000
6000
8000
88:175
54:110
17:40
CTLKPCDYPDIKpc2_NP.1
p−maj=0.4, pX=0.58, N=159
var.1.CFH 1:196642233:G:A
peptide level
0
200
600
1000
14:175
7:110
1:40
CTLKPCDYPDIKpc2_NP.2
p−min=0.38, pX=0.26, N=22
var.1.CFH 1:196642233:G:A
peptide level
0
1000
3000
5000
120:175
70:110
32:40
CTLKPCDYPDIKpc2_NP.3
p−maj=0.1, pX=0.61, N=222
var.1.CFH 1:196642233:G:A
peptide level
0
10000
20000
30000
175:175
110:110
40:40
CTLKPCDYPDIKpc2_NP.4
p−maj=1, pX=0.9, N=325
var.1.CFH 1:196642233:G:A
peptide level
0
5000
15000
171:175
106:110
39:40
CTLKPCDYPDIKpc2_NP.5
p−min=0.74, pX=0.89, N=316

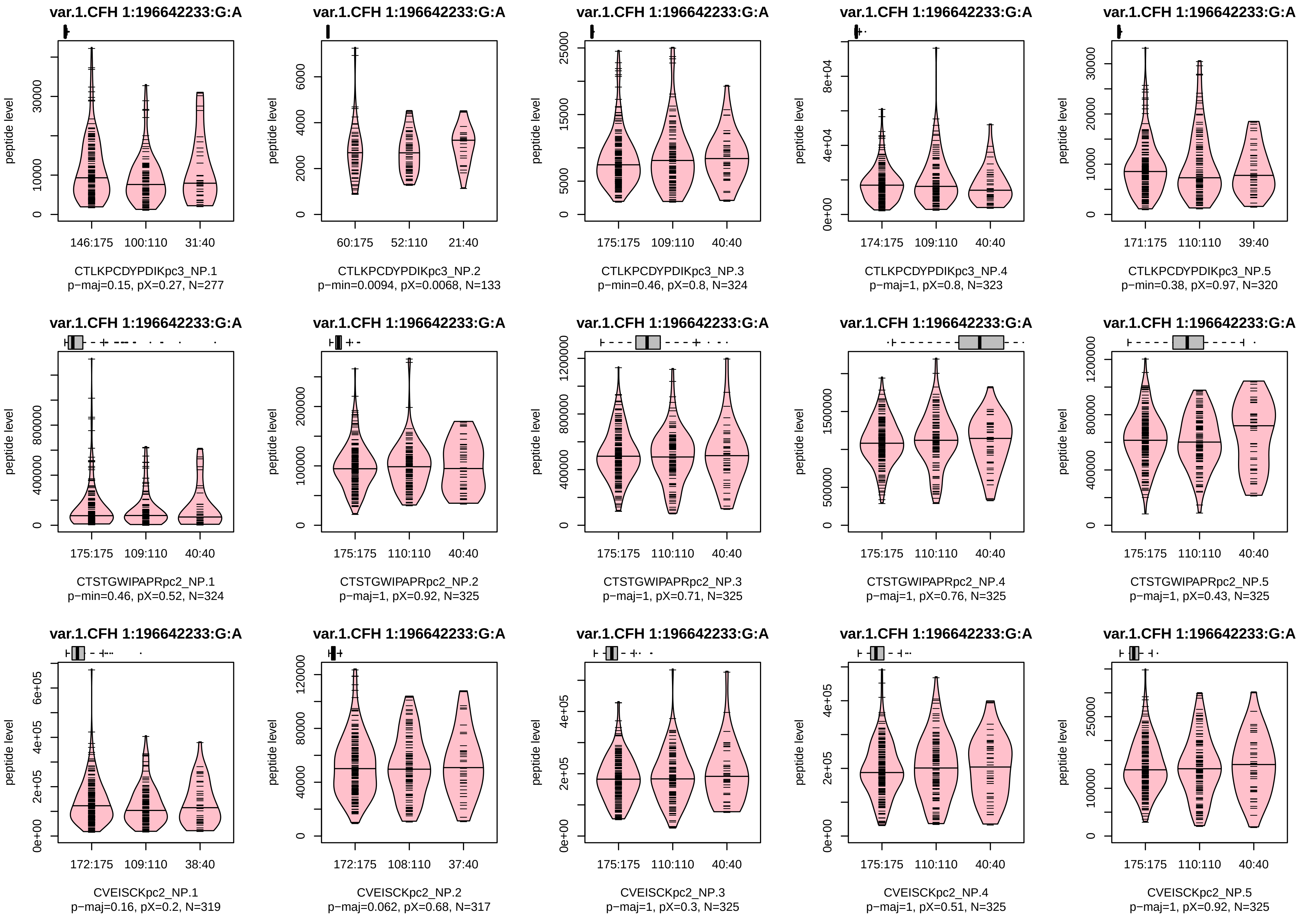
var.1.CFH 1:196642233:G:A
peptide level
146:175
100:110
31:40
CTLKPCDYPDIKpc3_NP.1
p−maj=0.15, pX=0.27, N=277
var.1.CFH 1:196642233:G:A
peptide level
60:175
52:110
21:40
CTLKPCDYPDIKpc3_NP.2
p−min=0.0094, pX=0.0068, N=133
var.1.CFH 1:196642233:G:A
peptide level
175:175
109:110
40:40
CTLKPCDYPDIKpc3_NP.3
p−min=0.46, pX=0.8, N=324
var.1.CFH 1:196642233:G:A
peptide level
174:175
109:110
40:40
CTLKPCDYPDIKpc3_NP.4
p−maj=1, pX=0.8, N=323
var.1.CFH 1:196642233:G:A
peptide level
171:175
110:110
39:40
CTLKPCDYPDIKpc3_NP.5
p−min=0.38, pX=0.97, N=320
var.1.CFH 1:196642233:G:A
peptide level
175:175
109:110
40:40
CTSTGWIPAPRpc2_NP.1
p−min=0.46, pX=0.52, N=324
var.1.CFH 1:196642233:G:A
peptide level
175:175
110:110
40:40
CTSTGWIPAPRpc2_NP.2
p−maj=1, pX=0.92, N=325
var.1.CFH 1:196642233:G:A
peptide level
175:175
110:110
40:40
CTSTGWIPAPRpc2_NP.3
p−maj=1, pX=0.71, N=325
var.1.CFH 1:196642233:G:A
peptide level
175:175
110:110
40:40
CTSTGWIPAPRpc2_NP.4
p−maj=1, pX=0.76, N=325
var.1.CFH 1:196642233:G:A
peptide level
175:175
110:110
40:40
CTSTGWIPAPRpc2_NP.5
p−maj=1, pX=0.43, N=325
var.1.CFH 1:196642233:G:A
peptide level
172:175
109:110
38:40
CVEISCKpc2_NP.1
p−maj=0.16, pX=0.2, N=319
var.1.CFH 1:196642233:G:A
peptide level
172:175
108:110
37:40
CVEISCKpc2_NP.2
p−maj=0.062, pX=0.68, N=317
var.1.CFH 1:196642233:G:A
peptide level
175:175
110:110
40:40
CVEISCKpc2_NP.3
p−maj=1, pX=0.3, N=325
var.1.CFH 1:196642233:G:A
peptide level
175:175
110:110
40:40
CVEISCKpc2_NP.4
p−maj=1, pX=0.51, N=325
var.1.CFH 1:196642233:G:A
peptide level
175:175
110:110
40:40
CVEISCKpc2_NP.5
p−maj=1, pX=0.92, N=325

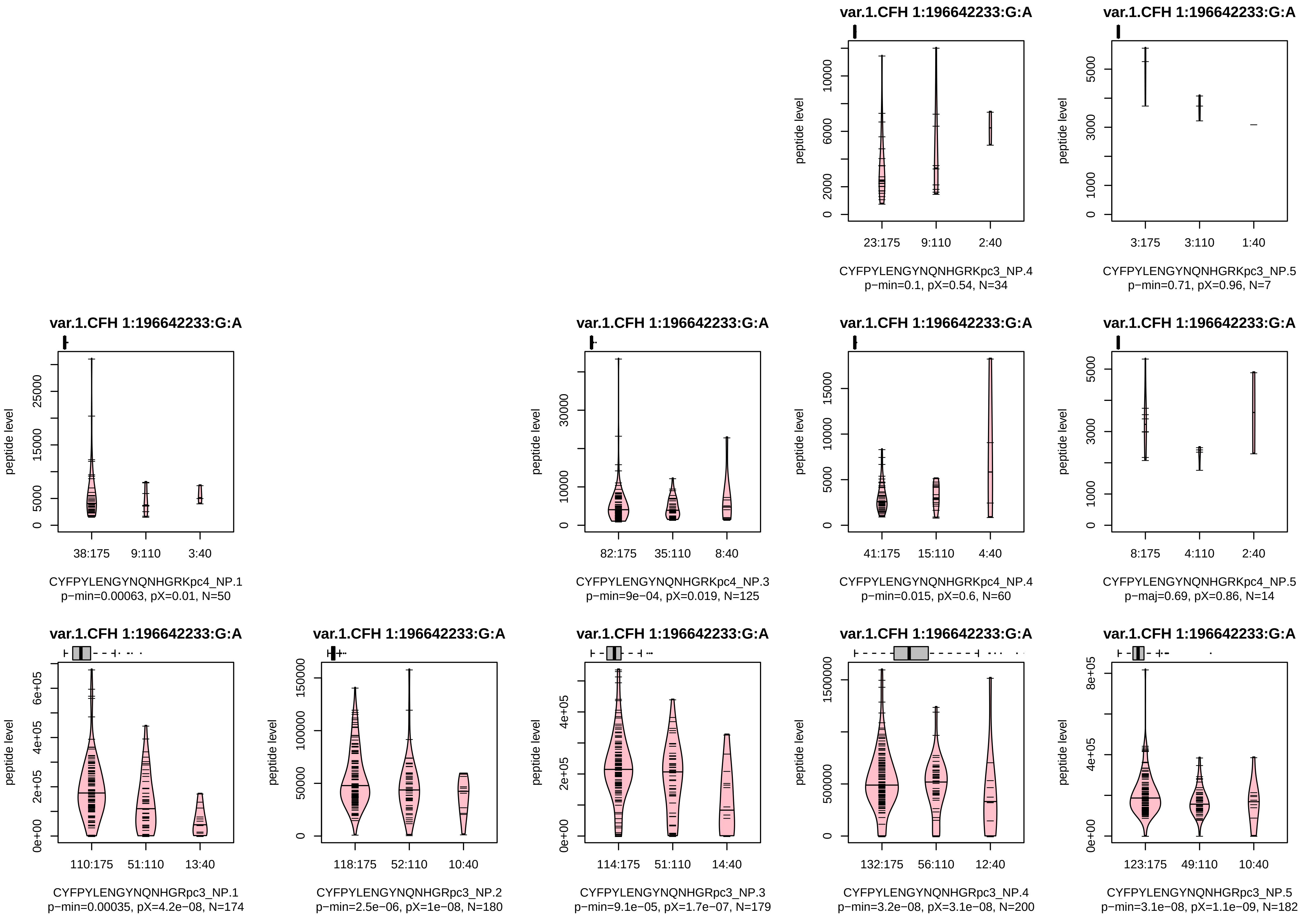
var.1.CFH 1:196642233:G:A
peptide level
23:175
9:110
2:40
CYFPYLENGYNQNHGRKpc3_NP.4
p−min=0.1, pX=0.54, N=34
var.1.CFH 1:196642233:G:A
peptide level
3:175
3:110
1:40
CYFPYLENGYNQNHGRKpc3_NP.5
p−min=0.71, pX=0.96, N=7
var.1.CFH 1:196642233:G:A
peptide level
38:175
9:110
3:40
CYFPYLENGYNQNHGRKpc4_NP.1
p−min=0.00063, pX=0.01, N=50
var.1.CFH 1:196642233:G:A
peptide level
82:175
35:110
8:40
CYFPYLENGYNQNHGRKpc4_NP.3
p−min=9e−04, pX=0.019, N=125
var.1.CFH 1:196642233:G:A
peptide level
41:175
15:110
4:40
CYFPYLENGYNQNHGRKpc4_NP.4
p−min=0.015, pX=0.6, N=60
var.1.CFH 1:196642233:G:A
peptide level
8:175
4:110
2:40
CYFPYLENGYNQNHGRKpc4_NP.5
p−maj=0.69, pX=0.86, N=14
var.1.CFH 1:196642233:G:A
peptide level
110:175
51:110
13:40
CYFPYLENGYNQNHGRpc3_NP.1
p−min=0.00035, pX=4.2e−08, N=174
var.1.CFH 1:196642233:G:A
peptide level
118:175
52:110
10:40
CYFPYLENGYNQNHGRpc3_NP.2
p−min=2.5e−06, pX=1e−08, N=180
var.1.CFH 1:196642233:G:A
peptide level
114:175
51:110
14:40
CYFPYLENGYNQNHGRpc3_NP.3
p−min=9.1e−05, pX=1.7e−07, N=179
var.1.CFH 1:196642233:G:A
peptide level
132:175
56:110
12:40
CYFPYLENGYNQNHGRpc3_NP.4
p−min=3.2e−08, pX=3.1e−08, N=200
var.1.CFH 1:196642233:G:A
peptide level
123:175
49:110
10:40
CYFPYLENGYNQNHGRpc3_NP.5
p−min=3.1e−08, pX=1.1e−09, N=182

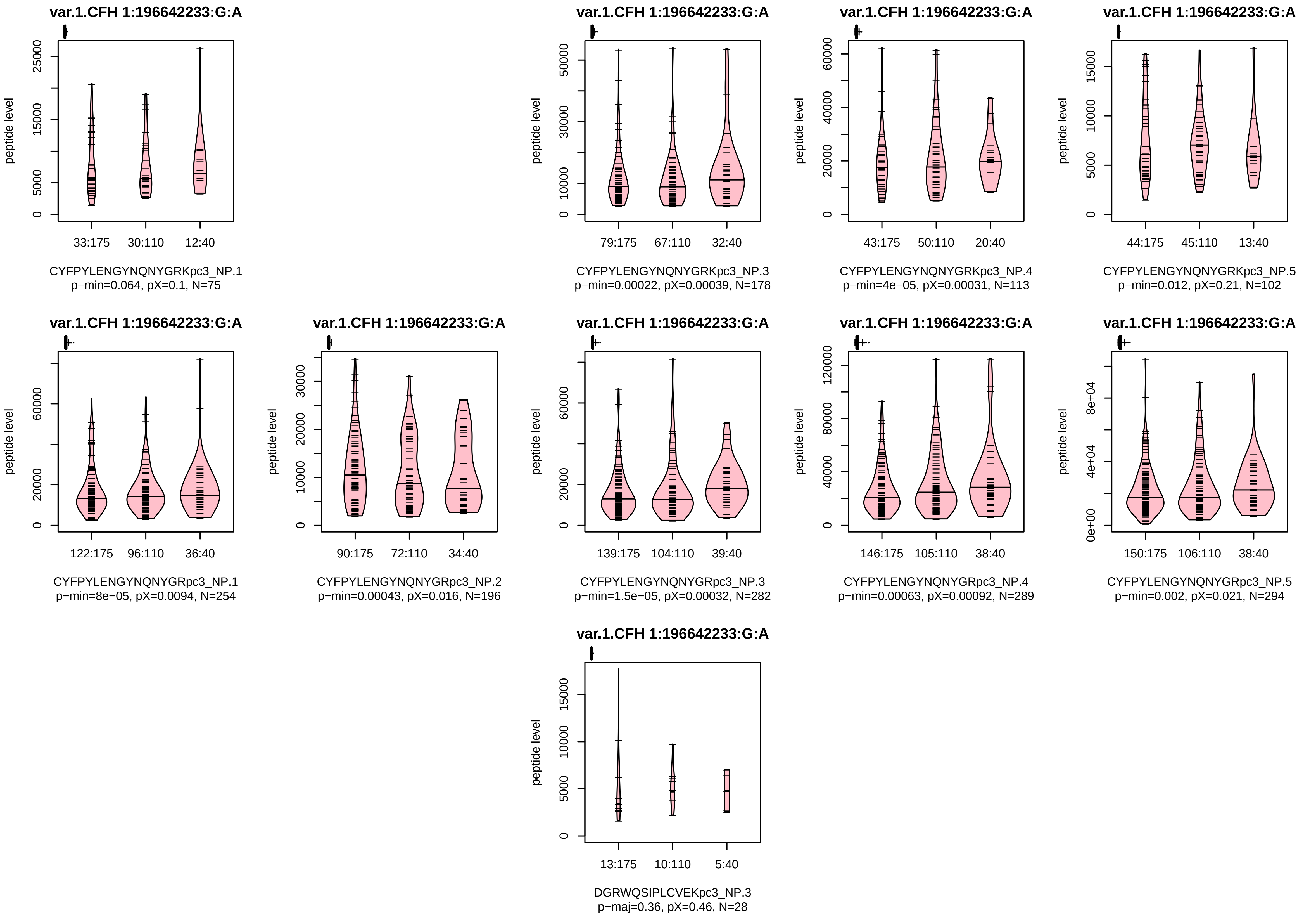
var.1.CFH 1:196642233:G:A
peptide level
33:175
30:110
12:40
CYFPYLENGYNQNYGRKpc3_NP.1
p−min=0.064, pX=0.1, N=75
var.1.CFH 1:196642233:G:A
peptide level
79:175
67:110
32:40
CYFPYLENGYNQNYGRKpc3_NP.3
p−min=0.00022, pX=0.00039, N=178
var.1.CFH 1:196642233:G:A
peptide level
43:175
50:110
20:40
CYFPYLENGYNQNYGRKpc3_NP.4
p−min=4e−05, pX=0.00031, N=113
var.1.CFH 1:196642233:G:A
peptide level
44:175
45:110
13:40
CYFPYLENGYNQNYGRKpc3_NP.5
p−min=0.012, pX=0.21, N=102
var.1.CFH 1:196642233:G:A
peptide level
122:175
96:110
36:40
CYFPYLENGYNQNYGRpc3_NP.1
p−min=8e−05, pX=0.0094, N=254
var.1.CFH 1:196642233:G:A
peptide level
90:175
72:110
34:40
CYFPYLENGYNQNYGRpc3_NP.2
p−min=0.00043, pX=0.016, N=196
var.1.CFH 1:196642233:G:A
peptide level
139:175
104:110
39:40
CYFPYLENGYNQNYGRpc3_NP.3
p−min=1.5e−05, pX=0.00032, N=282
var.1.CFH 1:196642233:G:A
peptide level
146:175
105:110
38:40
CYFPYLENGYNQNYGRpc3_NP.4
p−min=0.00063, pX=0.00092, N=289
var.1.CFH 1:196642233:G:A
peptide level
150:175
106:110
38:40
CYFPYLENGYNQNYGRpc3_NP.5
p−min=0.002, pX=0.021, N=294
var.1.CFH 1:196642233:G:A
peptide level
13:175
10:110
5:40
DGRWQSIPLCVEKpc3_NP.3
p−maj=0.36, pX=0.46, N=28

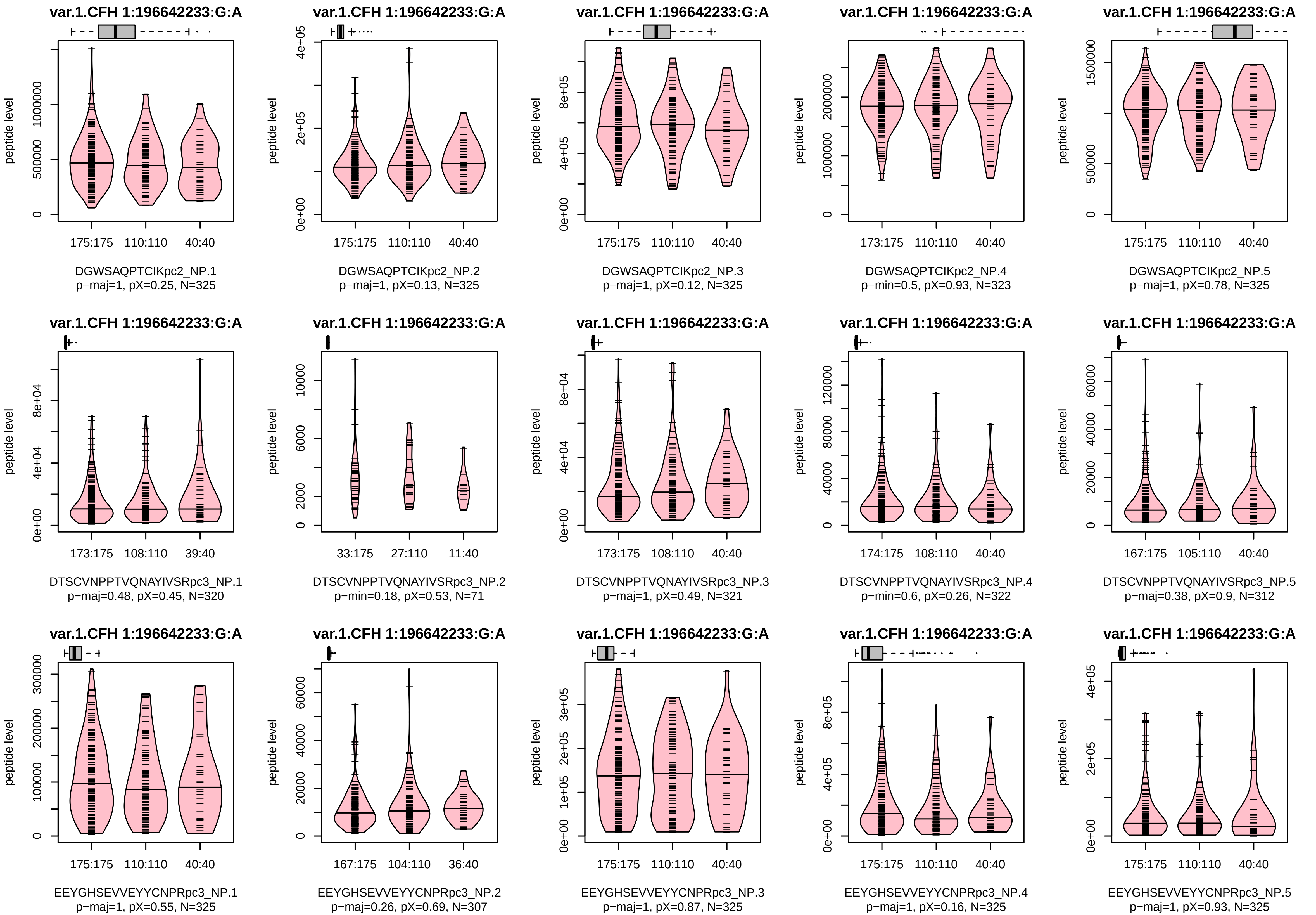
var.1.CFH 1:196642233:G:A
peptide level
175:175 110:110 40:40
DGWSAQPTCIKpc2_NP.1
p−maj=1, pX=0.25, N=325
var.1.CFH 1:196642233:G:A
175:175 110:110 40:40
DGWSAQPTCIKpc2_NP.2
p−maj=1, pX=0.13, N=325
var.1.CFH 1:196642233:G:A
175:175 110:110 40:40
DGWSAQPTCIKpc2_NP.3
p−maj=1, pX=0.12, N=325
var.1.CFH 1:196642233:G:A
173:175 110:110 40:40
DGWSAQPTCIKpc2_NP.4
p−min=0.5, pX=0.93, N=323
var.1.CFH 1:196642233:G:A
175:175 110:110 40:40
DGWSAQPTCIKpc2_NP.5
p−maj=1, pX=0.78, N=325
var.1.CFH 1:196642233:G:A
173:175 108:110 39:40
DTSCVNPPTVQNAYIVSRpc3_NP.1
p−maj=0.48, pX=0.45, N=320
var.1.CFH 1:196642233:G:A
33:175 27:110 11:40
DTSCVNPPTVQNAYIVSRpc3_NP.2
p−min=0.18, pX=0.53, N=71
var.1.CFH 1:196642233:G:A
173:175 108:110 40:40
DTSCVNPPTVQNAYIVSRpc3_NP.3
p−maj=1, pX=0.49, N=321
var.1.CFH 1:196642233:G:A
174:175 108:110 40:40
DTSCVNPPTVQNAYIVSRpc3_NP.4
p−min=0.6, pX=0.26, N=322
var.1.CFH 1:196642233:G:A
167:175 105:110 40:40
DTSCVNPPTVQNAYIVSRpc3_NP.5
p−maj=0.38, pX=0.9, N=312
var.1.CFH 1:196642233:G:A
175:175 110:110 40:40
EEYGHSEVVEYYCNPRpc3_NP.1
p−maj=1, pX=0.55, N=325
var.1.CFH 1:196642233:G:A
167:175 104:110 36:40
EEYGHSEVVEYYCNPRpc3_NP.2
p−maj=0.26, pX=0.69, N=307
var.1.CFH 1:196642233:G:A
175:175 110:110 40:40
EEYGHSEVVEYYCNPRpc3_NP.3
p−maj=1, pX=0.87, N=325
var.1.CFH 1:196642233:G:A
175:175 110:110 40:40
EEYGHSEVVEYYCNPRpc3_NP.4
p−maj=1, pX=0.16, N=325
var.1.CFH 1:196642233:G:A
175:175 110:110 40:40
EEYGHSEVVEYYCNPRpc3_NP.5
p−maj=1, pX=0.93, N=325

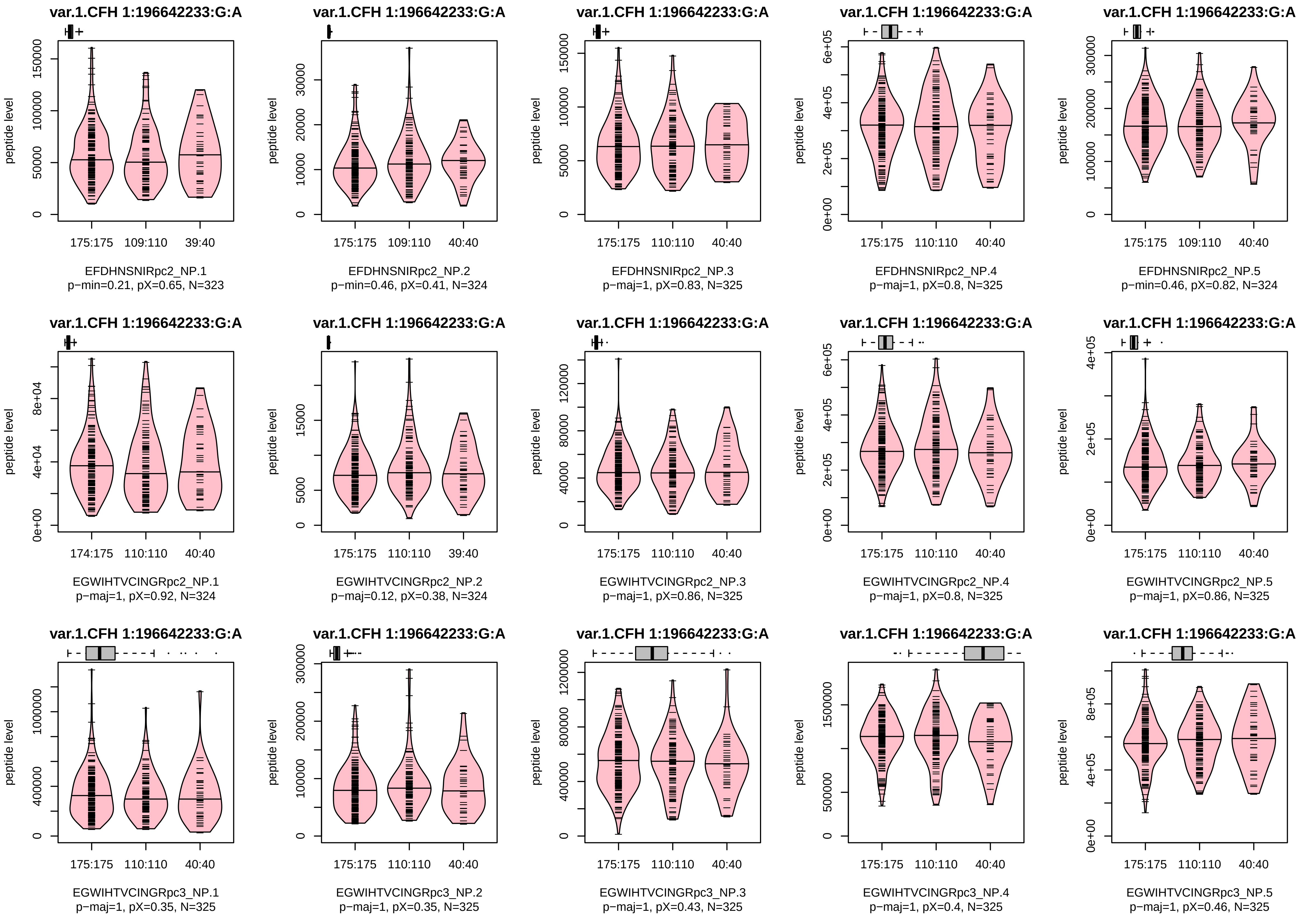

var.1.CFH 1:196642233:G:A
peptide level
175:175
109:110
39:40
EFDHNSNIRpc2_NP.1
p−min=0.21, pX=0.65, N=323
var.1.CFH 1:196642233:G:A
peptide level
175:175
109:110
40:40
EFDHNSNIRpc2_NP.2
p−min=0.46, pX=0.41, N=324
var.1.CFH 1:196642233:G:A
peptide level
175:175
110:110
40:40
EFDHNSNIRpc2_NP.3
p−maj=1, pX=0.83, N=325
var.1.CFH 1:196642233:G:A
peptide level
175:175
110:110
40:40
EFDHNSNIRpc2_NP.4
p−maj=1, pX=0.8, N=325
var.1.CFH 1:196642233:G:A
peptide level
175:175
109:110
40:40
EFDHNSNIRpc2_NP.5
p−min=0.46, pX=0.82, N=324
var.1.CFH 1:196642233:G:A
peptide level
174:175
110:110
40:40
EGWIHTVCINGRpc2_NP.1
p−maj=1, pX=0.92, N=324
var.1.CFH 1:196642233:G:A
peptide level
175:175
110:110
39:40
EGWIHTVCINGRpc2_NP.2
p−maj=0.12, pX=0.38, N=324
var.1.CFH 1:196642233:G:A
peptide level
175:175
110:110
40:40
EGWIHTVCINGRpc2_NP.3
p−maj=1, pX=0.86, N=325
var.1.CFH 1:196642233:G:A
peptide level
175:175
110:110
40:40
EGWIHTVCINGRpc2_NP.4
p−maj=1, pX=0.8, N=325
var.1.CFH 1:196642233:G:A
peptide level
175:175
110:110
40:40
EGWIHTVCINGRpc2_NP.5
p−maj=1, pX=0.86, N=325
var.1.CFH 1:196642233:G:A
peptide level
175:175
110:110
40:40
EGWIHTVCINGRpc3_NP.1
p−maj=1, pX=0.35, N=325
var.1.CFH 1:196642233:G:A
peptide level
175:175
110:110
40:40
EGWIHTVCINGRpc3_NP.2
p−maj=1, pX=0.35, N=325
var.1.CFH 1:196642233:G:A
peptide level
175:175
110:110
40:40
EGWIHTVCINGRpc3_NP.3
p−maj=1, pX=0.43, N=325
var.1.CFH 1:196642233:G:A
peptide level
175:175
110:110
40:40
EGWIHTVCINGRpc3_NP.4
p−maj=1, pX=0.4, N=325
var.1.CFH 1:196642233:G:A
peptide level
175:175
110:110
40:40
EGWIHTVCINGRpc3_NP.5
p−maj=1, pX=0.46, N=325

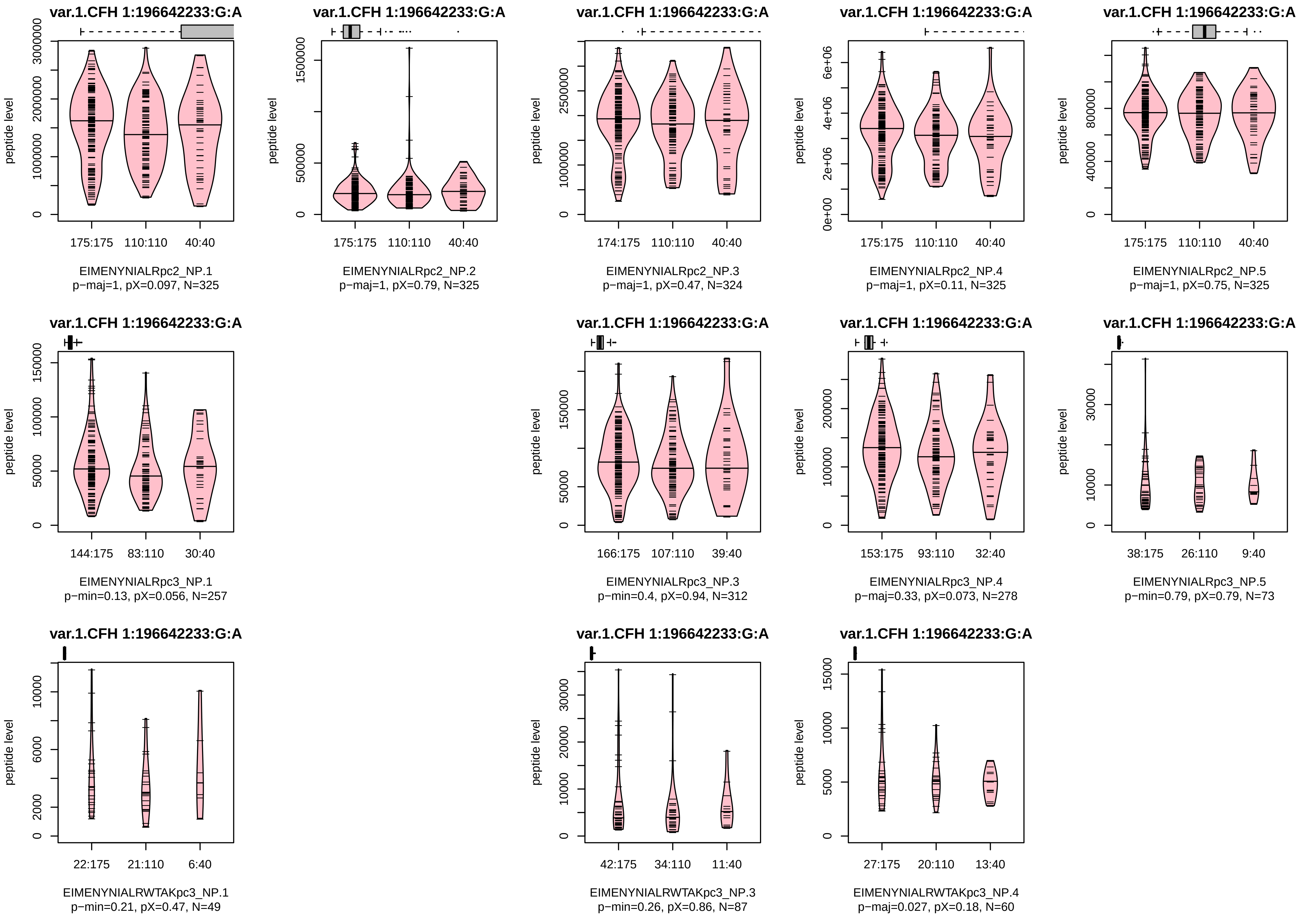
var.1.CFH 1:196642233:G:A
peptide level
175:175
110:110
40:40
EIMENYNIALRpc2_NP.1
p−maj=1, pX=0.097, N=325
var.1.CFH 1:196642233:G:A
175:175
110:110
40:40
EIMENYNIALRpc2_NP.2
p−maj=1, pX=0.79, N=325
var.1.CFH 1:196642233:G:A
174:175
110:110
40:40
EIMENYNIALRpc2_NP.3
p−maj=1, pX=0.47, N=324
var.1.CFH 1:196642233:G:A
175:175
110:110
40:40
EIMENYNIALRpc2_NP.4
p−maj=1, pX=0.11, N=325
var.1.CFH 1:196642233:G:A
175:175
110:110
40:40
EIMENYNIALRpc2_NP.5
p−maj=1, pX=0.75, N=325
var.1.CFH 1:196642233:G:A
144:175
83:110
30:40
EIMENYNIALRpc3_NP.1
p−min=0.13, pX=0.056, N=257
var.1.CFH 1:196642233:G:A
166:175
107:110
39:40
EIMENYNIALRpc3_NP.3
p−min=0.4, pX=0.94, N=312
var.1.CFH 1:196642233:G:A
153:175
93:110
32:40
EIMENYNIALRpc3_NP.4
p−maj=0.33, pX=0.073, N=278
var.1.CFH 1:196642233:G:A
38:175
26:110
9:40
EIMENYNIALRpc3_NP.5
p−min=0.79, pX=0.79, N=73
var.1.CFH 1:196642233:G:A
22:175
21:110
6:40
EIMENYNIALRWTAKpc3_NP.1
p−min=0.21, pX=0.47, N=49
var.1.CFH 1:196642233:G:A
42:175
34:110
11:40
EIMENYNIALRWTAKpc3_NP.3
p−min=0.26, pX=0.86, N=87
var.1.CFH 1:196642233:G:A
27:175
20:110
13:40
EIMENYNIALRWTAKpc3_NP.4
p−maj=0.027, pX=0.18, N=60

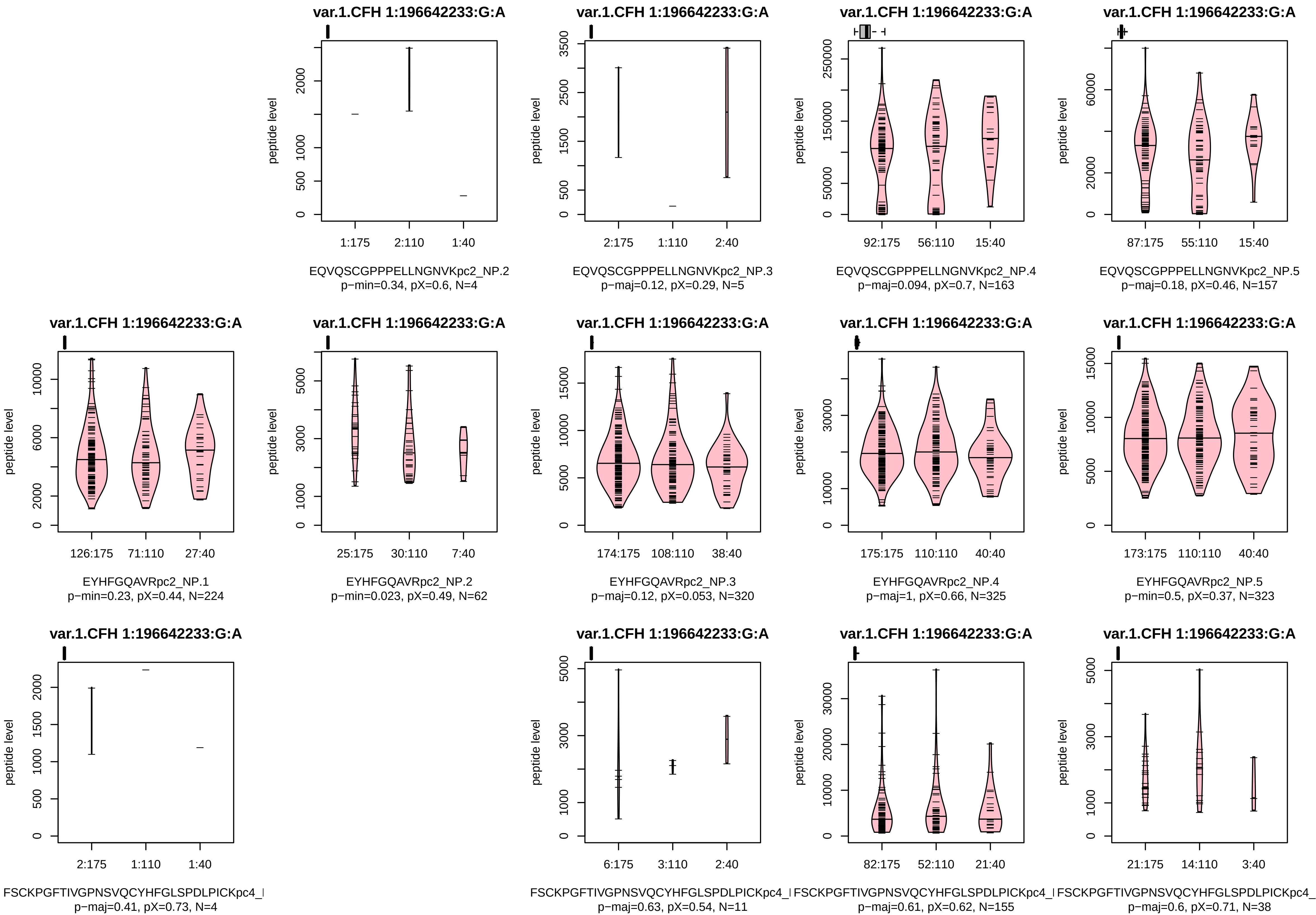
var.1.CFH 1:196642233:G:A
peptide level
1:175
2:110
1:40
EQVQSCGPPPELLNGNVKpc2_NP.2
p−min=0.34, pX=0.6, N=4
var.1.CFH 1:196642233:G:A
peptide level
2:175
1:110
2:40
EQVQSCGPPPELLNGNVKpc2_NP.3
p−maj=0.12, pX=0.29, N=5
var.1.CFH 1:196642233:G:A
peptide level
92:175
56:110
15:40
EQVQSCGPPPELLNGNVKpc2_NP.4
p−maj=0.094, pX=0.7, N=163
var.1.CFH 1:196642233:G:A
peptide level
87:175
55:110
15:40
EQVQSCGPPPELLNGNVKpc2_NP.5
p−maj=0.18, pX=0.46, N=157
var.1.CFH 1:196642233:G:A
peptide level
126:175
71:110
27:40
EYHFGQAVRpc2_NP.1
p−min=0.23, pX=0.44, N=224
var.1.CFH 1:196642233:G:A
peptide level
25:175
30:110
7:40
EYHFGQAVRpc2_NP.2
p−min=0.023, pX=0.49, N=62
var.1.CFH 1:196642233:G:A
peptide level
174:175
108:110
38:40
EYHFGQAVRpc2_NP.3
p−maj=0.12, pX=0.053, N=320
var.1.CFH 1:196642233:G:A
peptide level
175:175
110:110
40:40
EYHFGQAVRpc2_NP.4
p−maj=1, pX=0.66, N=325
var.1.CFH 1:196642233:G:A
peptide level
173:175
110:110
40:40
EYHFGQAVRpc2_NP.5
p−min=0.5, pX=0.37, N=323
var.1.CFH 1:196642233:G:A
peptide level
2:175
1:110
1:40
FSCKPGFTIVGPNSVQCYHFGLSPDLPICKpc4_I
p−maj=0.41, pX=0.73, N=4
var.1.CFH 1:196642233:G:A
peptide level
6:175
3:110
2:40
FSCKPGFTIVGPNSVQCYHFGLSPDLPICKpc4_I
p−maj=0.63, pX=0.54, N=11
var.1.CFH 1:196642233:G:A
peptide level
82:175
52:110
21:40
FSCKPGFTIVGPNSVQCYHFGLSPDLPICKpc4_I
p−maj=0.61, pX=0.62, N=155
var.1.CFH 1:196642233:G:A
peptide level
21:175
14:110
3:40
FSCKPGFTIVGPNSVQCYHFGLSPDLPICKpc4_
p−maj=0.6, pX=0.71, N=38

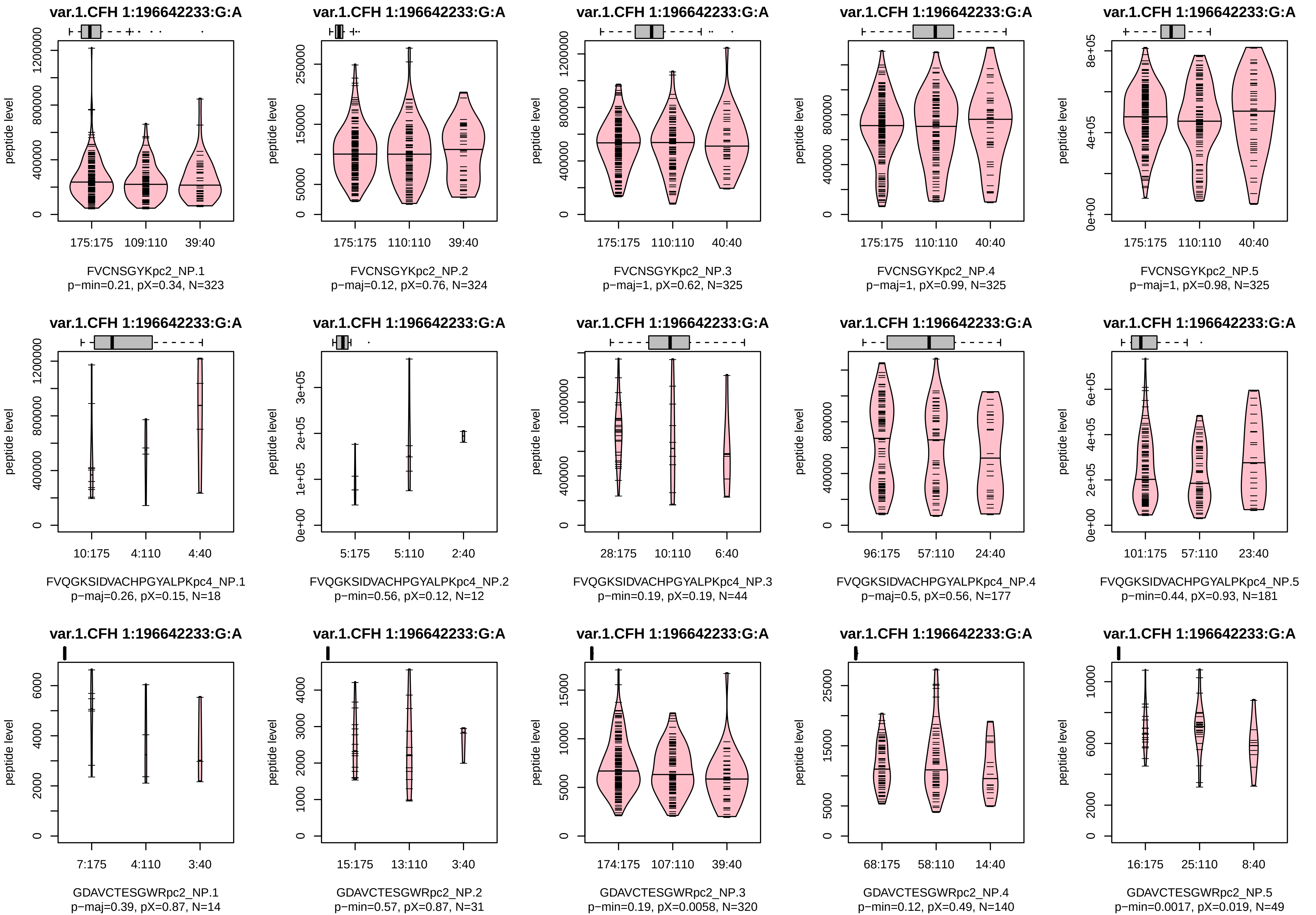
var.1.CFH 1:196642233:G:A
peptide level
175:175
109:110
39:40
FVCNSGYKpc2_NP.1
p−min=0.21, pX=0.34, N=323
var.1.CFH 1:196642233:G:A
peptide level
175:175
110:110
39:40
FVCNSGYKpc2_NP.2
p−maj=0.12, pX=0.76, N=324
var.1.CFH 1:196642233:G:A
peptide level
175:175
110:110
40:40
FVCNSGYKpc2_NP.3
p−maj=1, pX=0.62, N=325
var.1.CFH 1:196642233:G:A
peptide level
175:175
110:110
40:40
FVCNSGYKpc2_NP.4
p−maj=1, pX=0.99, N=325
var.1.CFH 1:196642233:G:A
peptide level
175:175
110:110
40:40
FVCNSGYKpc2_NP.5
p−maj=1, pX=0.98, N=325
var.1.CFH 1:196642233:G:A
peptide level
10:175
4:110
4:40
FVQGKSIDVACHPGYALPKpc4_NP.1
p−maj=0.26, pX=0.15, N=18
var.1.CFH 1:196642233:G:A
peptide level
5:175
5:110
2:40
FVQGKSIDVACHPGYALPKpc4_NP.2
p−min=0.56, pX=0.12, N=12
var.1.CFH 1:196642233:G:A
peptide level
28:175
10:110
6:40
FVQGKSIDVACHPGYALPKpc4_NP.3
p−min=0.19, pX=0.19, N=44
var.1.CFH 1:196642233:G:A
peptide level
96:175
57:110
24:40
FVQGKSIDVACHPGYALPKpc4_NP.4
p−maj=0.5, pX=0.56, N=177
var.1.CFH 1:196642233:G:A
peptide level
101:175
57:110
23:40
FVQGKSIDVACHPGYALPKpc4_NP.5
p−min=0.44, pX=0.93, N=181
var.1.CFH 1:196642233:G:A
peptide level
7:175
4:110
3:40
GDAVCTESGWRpc2_NP.1
p−maj=0.39, pX=0.87, N=14
var.1.CFH 1:196642233:G:A
peptide level
15:175
13:110
3:40
GDAVCTESGWRpc2_NP.2
p−min=0.57, pX=0.87, N=31
var.1.CFH 1:196642233:G:A
peptide level
174:175
107:110
39:40
GDAVCTESGWRpc2_NP.3
p−min=0.19, pX=0.0058, N=320
var.1.CFH 1:196642233:G:A
peptide level
68:175
58:110
14:40
GDAVCTESGWRpc2_NP.4
p−min=0.12, pX=0.49, N=140
var.1.CFH 1:196642233:G:A
peptide level
16:175
25:110
8:40
GDAVCTESGWRpc2_NP.5
p−min=0.0017, pX=0.019, N=49

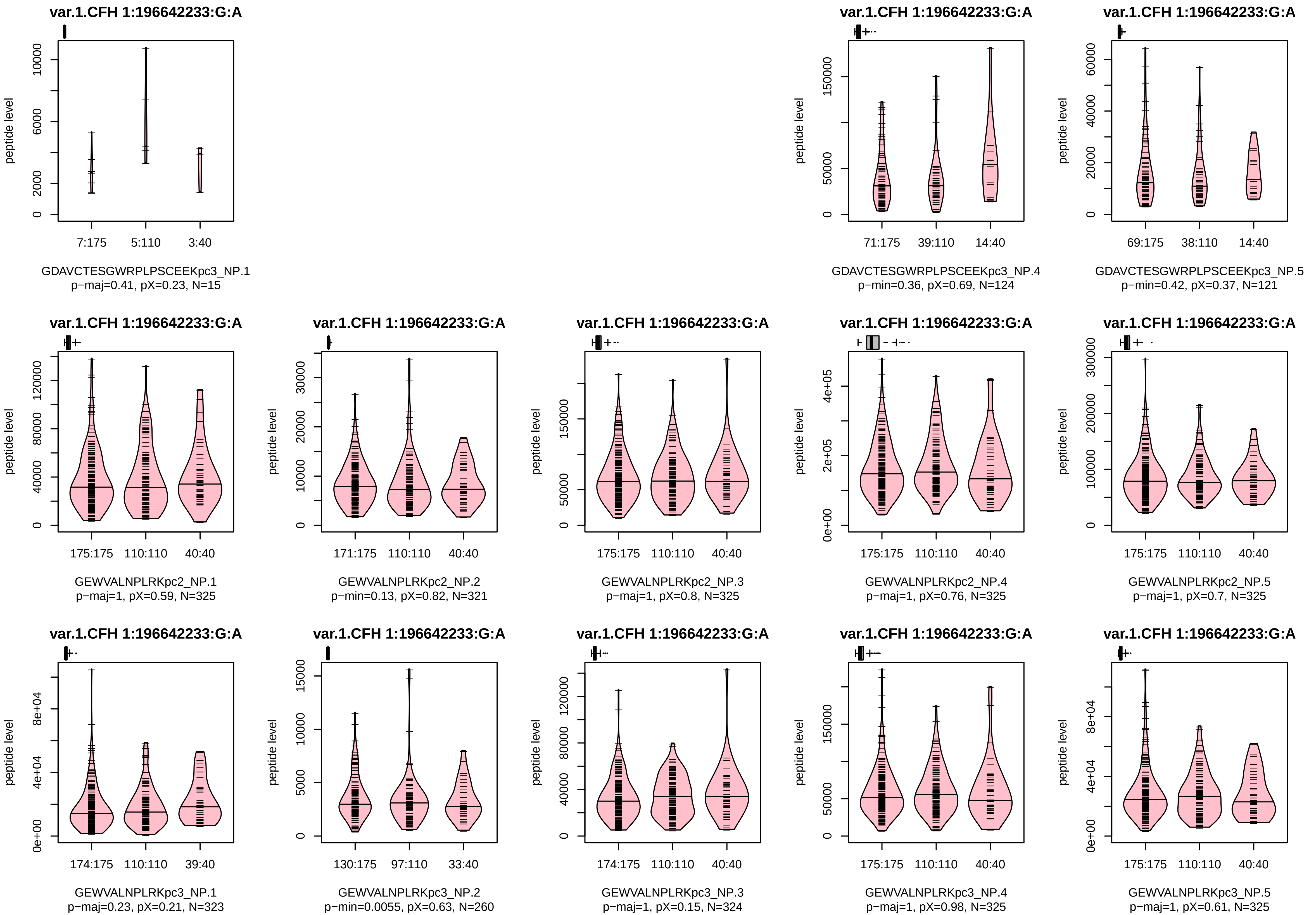
var.1.CFH 1:196642233:G:A
peptide level
7:175
5:110
3:40
GDAVCTESGWRPLPSCEEKpc3_NP.1
p−maj=0.41, pX=0.23, N=15
var.1.CFH 1:196642233:G:A
peptide level
71:175
39:110
14:40
GDAVCTESGWRPLPSCEEKpc3_NP.4
p−min=0.36, pX=0.69, N=124
var.1.CFH 1:196642233:G:A
peptide level
69:175
38:110
14:40
GDAVCTESGWRPLPSCEEKpc3_NP.5
p−min=0.42, pX=0.37, N=121
var.1.CFH 1:196642233:G:A
peptide level
175:175
110:110
40:40
GEWVALNPLRKpc2_NP.1
p−maj=1, pX=0.59, N=325
var.1.CFH 1:196642233:G:A
peptide level
171:175
110:110
40:40
GEWVALNPLRKpc2_NP.2
p−min=0.13, pX=0.82, N=321
var.1.CFH 1:196642233:G:A
peptide level
175:175
110:110
40:40
GEWVALNPLRKpc2_NP.3
p−maj=1, pX=0.8, N=325
var.1.CFH 1:196642233:G:A
peptide level
175:175
110:110
40:40
GEWVALNPLRKpc2_NP.4
p−maj=1, pX=0.76, N=325
var.1.CFH 1:196642233:G:A
peptide level
175:175
110:110
40:40
GEWVALNPLRKpc2_NP.5
p−maj=1, pX=0.7, N=325
var.1.CFH 1:196642233:G:A
peptide level
174:175
110:110
39:40
GEWVALNPLRKpc3_NP.1
p−maj=0.23, pX=0.21, N=323
var.1.CFH 1:196642233:G:A
peptide level
130:175
97:110
33:40
GEWVALNPLRKpc3_NP.2
p−min=0.0055, pX=0.63, N=260
var.1.CFH 1:196642233:G:A
peptide level
174:175
110:110
40:40
GEWVALNPLRKpc3_NP.3
p−maj=1, pX=0.15, N=324
var.1.CFH 1:196642233:G:A
peptide level
175:175
110:110
40:40
GEWVALNPLRKpc3_NP.4
p−maj=1, pX=0.98, N=325
var.1.CFH 1:196642233:G:A
peptide level
175:175
110:110
40:40
GEWVALNPLRKpc3_NP.5
p−maj=1, pX=0.61, N=325

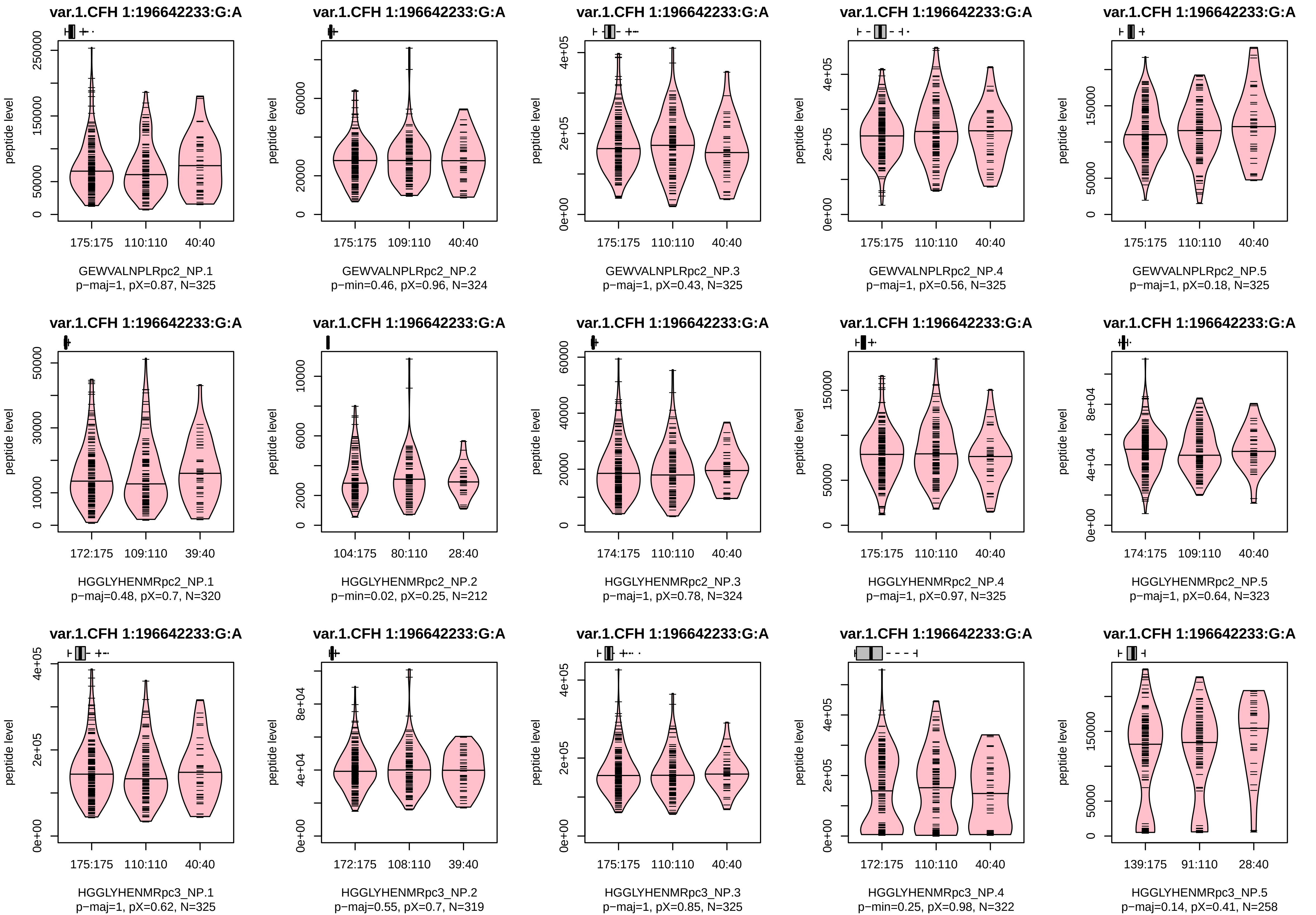
var.1.CFH 1:196642233:G:A
peptide level
175:175 110:110 40:40
GEWVALNPLRpc2_NP.1
p−maj=1, pX=0.87, N=325
var.1.CFH 1:196642233:G:A
peptide level
175:175 109:110 40:40
GEWVALNPLRpc2_NP.2
p−min=0.46, pX=0.96, N=324
var.1.CFH 1:196642233:G:A
peptide level
175:175 110:110 40:40
GEWVALNPLRpc2_NP.3
p−maj=1, pX=0.43, N=325
var.1.CFH 1:196642233:G:A
peptide level
175:175 110:110 40:40
GEWVALNPLRpc2_NP.4
p−maj=1, pX=0.56, N=325
var.1.CFH 1:196642233:G:A
peptide level
175:175 110:110 40:40
GEWVALNPLRpc2_NP.5
p−maj=1, pX=0.18, N=325
var.1.CFH 1:196642233:G:A
peptide level
172:175 109:110 39:40
HGGLYHENMRpc2_NP.1
p−maj=0.48, pX=0.7, N=320
var.1.CFH 1:196642233:G:A
peptide level
104:175 80:110 28:40
HGGLYHENMRpc2_NP.2
p−min=0.02, pX=0.25, N=212
var.1.CFH 1:196642233:G:A
peptide level
174:175 110:110 40:40
HGGLYHENMRpc2_NP.3
p−maj=1, pX=0.78, N=324
var.1.CFH 1:196642233:G:A
peptide level
175:175 110:110 40:40
HGGLYHENMRpc2_NP.4
p−maj=1, pX=0.97, N=325
var.1.CFH 1:196642233:G:A
peptide level
174:175 109:110 40:40
HGGLYHENMRpc2_NP.5
p−maj=1, pX=0.64, N=323
var.1.CFH 1:196642233:G:A
peptide level
175:175 110:110 40:40
HGGLYHENMRpc3_NP.1
p−maj=1, pX=0.62, N=325
var.1.CFH 1:196642233:G:A
peptide level
172:175 108:110 39:40
HGGLYHENMRpc3_NP.2
p−maj=0.55, pX=0.7, N=319
var.1.CFH 1:196642233:G:A
peptide level
175:175 110:110 40:40
HGGLYHENMRpc3_NP.3
p−maj=1, pX=0.85, N=325
var.1.CFH 1:196642233:G:A
peptide level
172:175 110:110 40:40
HGGLYHENMRpc3_NP.4
p−min=0.25, pX=0.98, N=322
var.1.CFH 1:196642233:G:A
peptide level
139:175 91:110 28:40
HGGLYHENMRpc3_NP.5
p−maj=0.14, pX=0.41, N=258

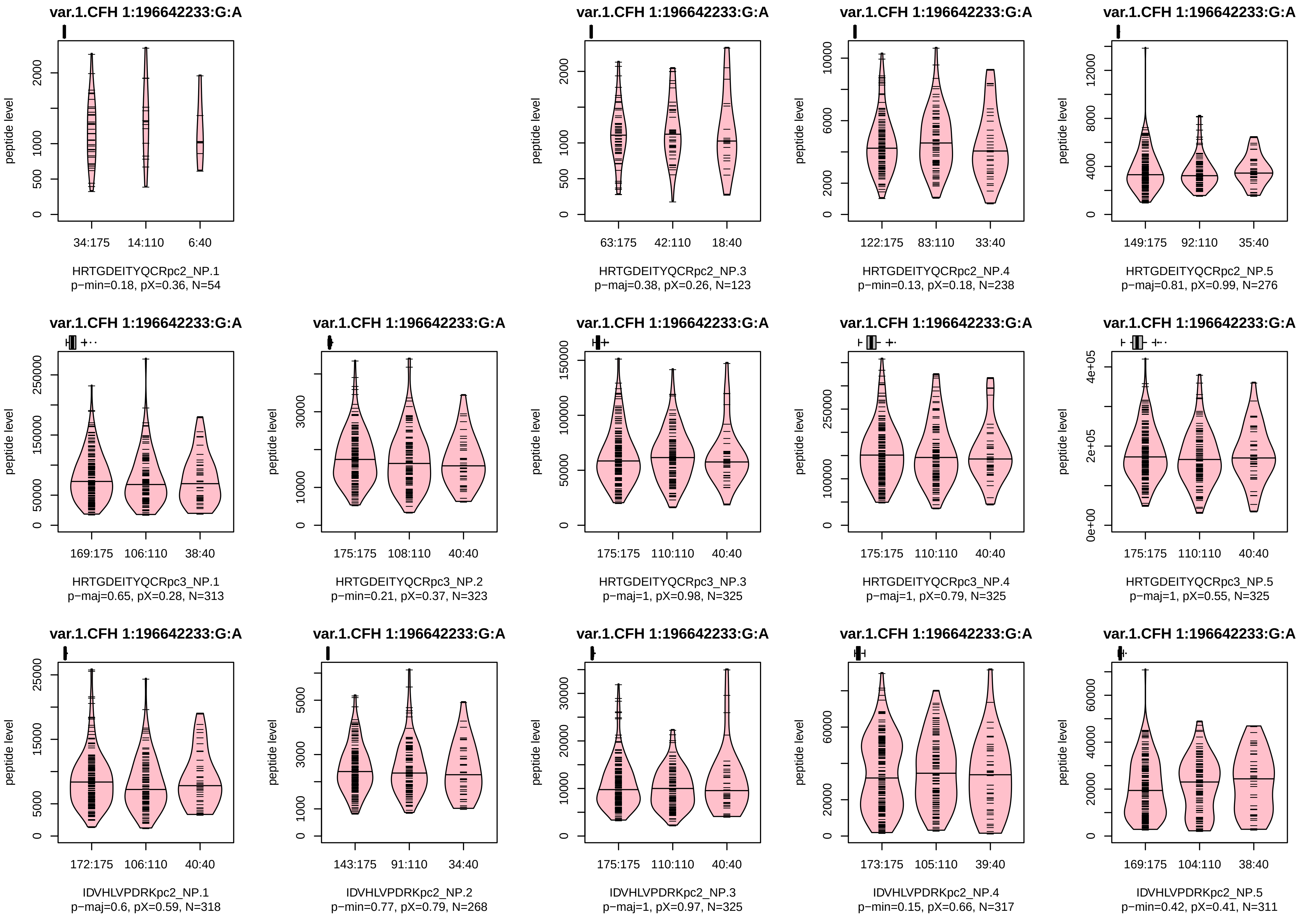
var.1.CFH 1:196642233:G:A
peptide level
34:175 14:110 6:40
HRTGDEITYQCRpc2_NP.1
p−min=0.18, pX=0.36, N=54
var.1.CFH 1:196642233:G:A
peptide level
63:175 42:110 18:40
HRTGDEITYQCRpc2_NP.3
p−maj=0.38, pX=0.26, N=123
var.1.CFH 1:196642233:G:A
peptide level
122:175 83:110 33:40
HRTGDEITYQCRpc2_NP.4
p−min=0.13, pX=0.18, N=238
var.1.CFH 1:196642233:G:A
peptide level
149:175 92:110 35:40
HRTGDEITYQCRpc2_NP.5
p−maj=0.81, pX=0.99, N=276
var.1.CFH 1:196642233:G:A
peptide level
169:175 106:110 38:40
HRTGDEITYQCRpc3_NP.1
p−maj=0.65, pX=0.28, N=313
var.1.CFH 1:196642233:G:A
peptide level
175:175 108:110 40:40
HRTGDEITYQCRpc3_NP.2
p−min=0.21, pX=0.37, N=323
var.1.CFH 1:196642233:G:A
peptide level
175:175 110:110 40:40
HRTGDEITYQCRpc3_NP.3
p−maj=1, pX=0.98, N=325
var.1.CFH 1:196642233:G:A
peptide level
175:175 110:110 40:40
HRTGDEITYQCRpc3_NP.4
p−maj=1, pX=0.79, N=325
var.1.CFH 1:196642233:G:A
peptide level
175:175 110:110 40:40
HRTGDEITYQCRpc3_NP.5
p−maj=1, pX=0.55, N=325
var.1.CFH 1:196642233:G:A
peptide level
172:175 106:110 40:40
IDVHLVPDRKpc2_NP.1
p−maj=0.6, pX=0.59, N=318
var.1.CFH 1:196642233:G:A
peptide level
143:175 91:110 34:40
IDVHLVPDRKpc2_NP.2
p−min=0.77, pX=0.79, N=268
var.1.CFH 1:196642233:G:A
peptide level
175:175 110:110 40:40
IDVHLVPDRKpc2_NP.3
p−maj=1, pX=0.97, N=325
var.1.CFH 1:196642233:G:A
peptide level
173:175 105:110 39:40
IDVHLVPDRKpc2_NP.4
p−min=0.15, pX=0.66, N=317
var.1.CFH 1:196642233:G:A
peptide level
169:175 104:110 38:40
IDVHLVPDRKpc2_NP.5
p−min=0.42, pX=0.41, N=311

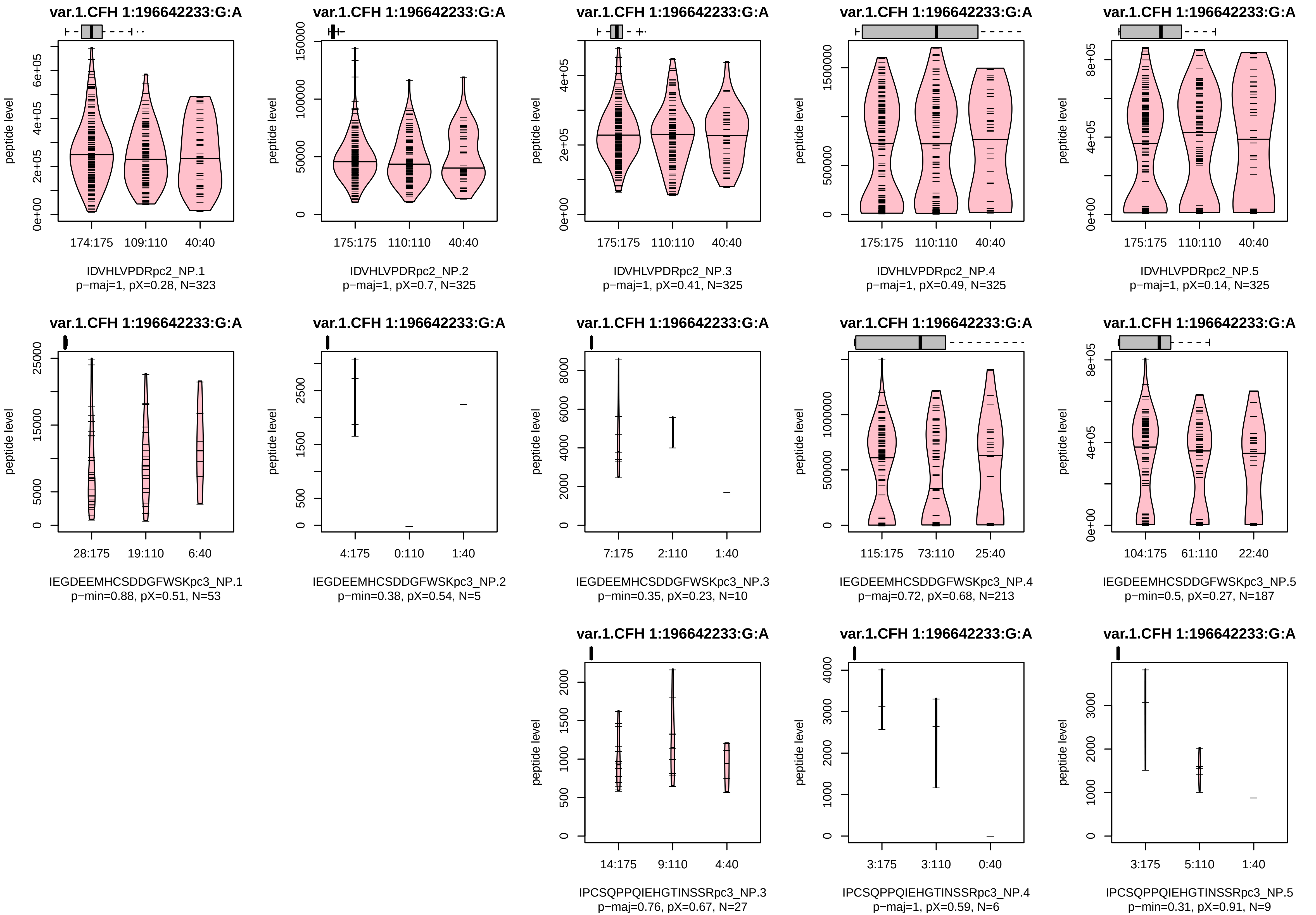
var.1.CFH 1:196642233:G:A
peptide level
174:175
109:110
40:40
IDVHLVPDRpc2_NP.1
p−maj=1, pX=0.28, N=323
var.1.CFH 1:196642233:G:A
175:175
110:110
40:40
IDVHLVPDRpc2_NP.2
p−maj=1, pX=0.7, N=325
var.1.CFH 1:196642233:G:A
175:175
110:110
40:40
IDVHLVPDRpc2_NP.3
p−maj=1, pX=0.41, N=325
var.1.CFH 1:196642233:G:A
175:175
110:110
40:40
IDVHLVPDRpc2_NP.4
p−maj=1, pX=0.49, N=325
var.1.CFH 1:196642233:G:A
175:175
110:110
40:40
IDVHLVPDRpc2_NP.5
p−maj=1, pX=0.14, N=325
var.1.CFH 1:196642233:G:A
28:175
19:110
6:40
IEGDEEMHCSDDGFWSKpc3_NP.1
p−min=0.88, pX=0.51, N=53
var.1.CFH 1:196642233:G:A
4:175
0:110
1:40
IEGDEEMHCSDDGFWSKpc3_NP.2
p−min=0.38, pX=0.54, N=5
var.1.CFH 1:196642233:G:A
7:175
2:110
1:40
IEGDEEMHCSDDGFWSKpc3_NP.3
p−min=0.35, pX=0.23, N=10
var.1.CFH 1:196642233:G:A
115:175
73:110
25:40
IEGDEEMHCSDDGFWSKpc3_NP.4
p−maj=0.72, pX=0.68, N=213
var.1.CFH 1:196642233:G:A
104:175
61:110
22:40
IEGDEEMHCSDDGFWSKpc3_NP.5
p−min=0.5, pX=0.27, N=187
var.1.CFH 1:196642233:G:A
14:175
9:110
4:40
IPCSQPPQIEHGTINSSRpc3_NP.3
p−maj=0.76, pX=0.67, N=27
var.1.CFH 1:196642233:G:A
3:175
3:110
0:40
IPCSQPPQIEHGTINSSRpc3_NP.4
p−maj=1, pX=0.59, N=6
var.1.CFH 1:196642233:G:A
3:175
5:110
1:40
IPCSQPPQIEHGTINSSRpc3_NP.5
p−min=0.31, pX=0.91, N=9

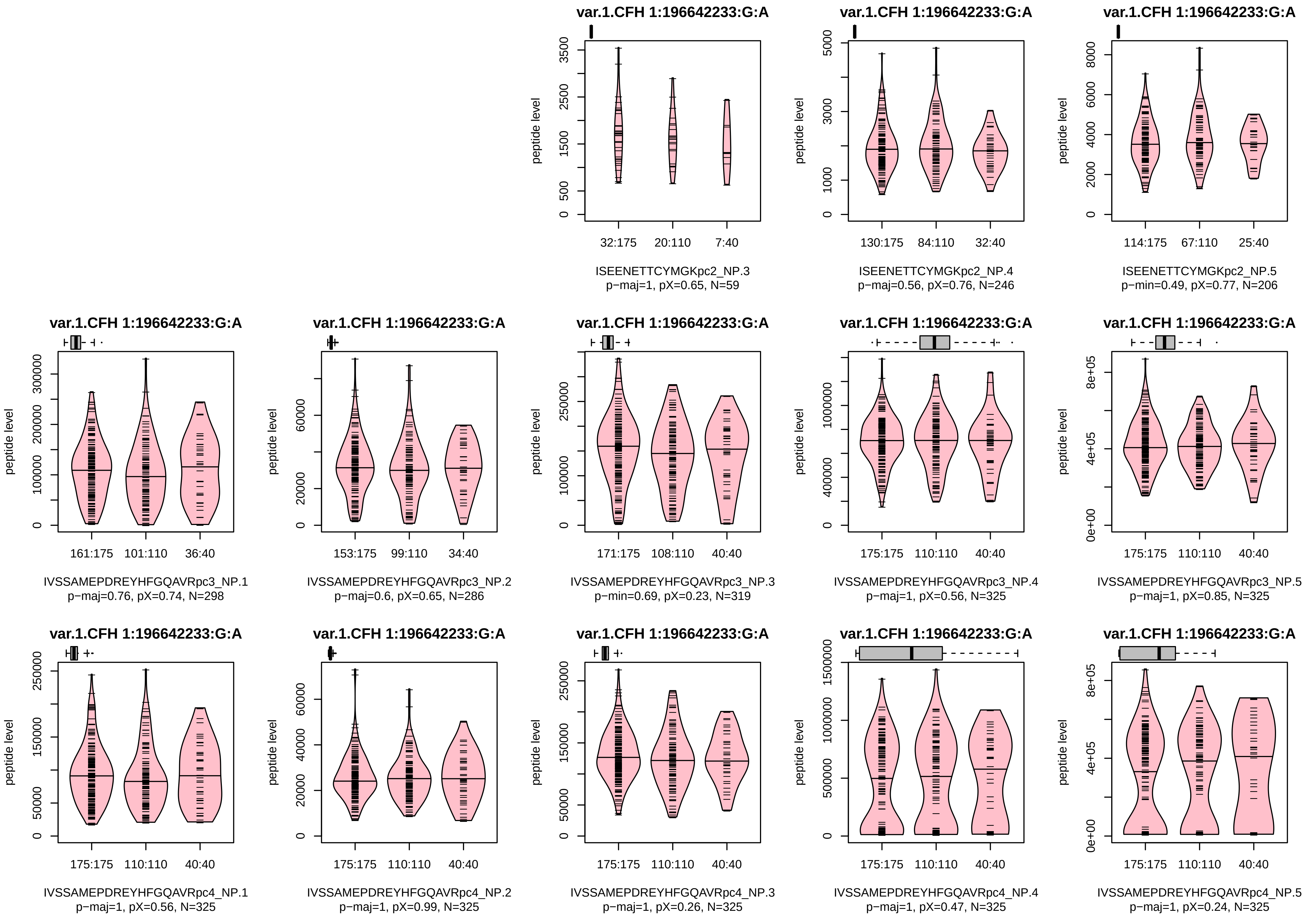

var.1.CFH 1:196642233:G:A
peptide level
32:175
20:110
7:40
ISEENETTCYMGKpc2_NP.3
p−maj=1, pX=0.65, N=59
var.1.CFH 1:196642233:G:A
peptide level
130:175
84:110
32:40
ISEENETTCYMGKpc2_NP.4
p−maj=0.56, pX=0.76, N=246
var.1.CFH 1:196642233:G:A
peptide level
114:175
67:110
25:40
ISEENETTCYMGKpc2_NP.5
p−min=0.49, pX=0.77, N=206
var.1.CFH 1:196642233:G:A
peptide level
161:175
101:110
36:40
IVSSAMEPDREYHFGQAVRpc3_NP.1
p−maj=0.76, pX=0.74, N=298
var.1.CFH 1:196642233:G:A
peptide level
153:175
99:110
34:40
IVSSAMEPDREYHFGQAVRpc3_NP.2
p−maj=0.6, pX=0.65, N=286
var.1.CFH 1:196642233:G:A
peptide level
171:175
108:110
40:40
IVSSAMEPDREYHFGQAVRpc3_NP.3
p−min=0.69, pX=0.23, N=319
var.1.CFH 1:196642233:G:A
peptide level
175:175
110:110
40:40
IVSSAMEPDREYHFGQAVRpc3_NP.4
p−maj=1, pX=0.56, N=325
var.1.CFH 1:196642233:G:A
peptide level
175:175
110:110
40:40
IVSSAMEPDREYHFGQAVRpc3_NP.5
p−maj=1, pX=0.85, N=325
var.1.CFH 1:196642233:G:A
peptide level
175:175
110:110
40:40
IVSSAMEPDREYHFGQAVRpc4_NP.1
p−maj=1, pX=0.56, N=325
var.1.CFH 1:196642233:G:A
peptide level
175:175
110:110
40:40
IVSSAMEPDREYHFGQAVRpc4_NP.2
p−maj=1, pX=0.99, N=325
var.1.CFH 1:196642233:G:A
peptide level
175:175
110:110
40:40
IVSSAMEPDREYHFGQAVRpc4_NP.3
p−maj=1, pX=0.26, N=325
var.1.CFH 1:196642233:G:A
peptide level
175:175
110:110
40:40
IVSSAMEPDREYHFGQAVRpc4_NP.4
p−maj=1, pX=0.47, N=325
var.1.CFH 1:196642233:G:A
peptide level
175:175
110:110
40:40
IVSSAMEPDREYHFGQAVRpc4_NP.5
p−maj=1, pX=0.24, N=325

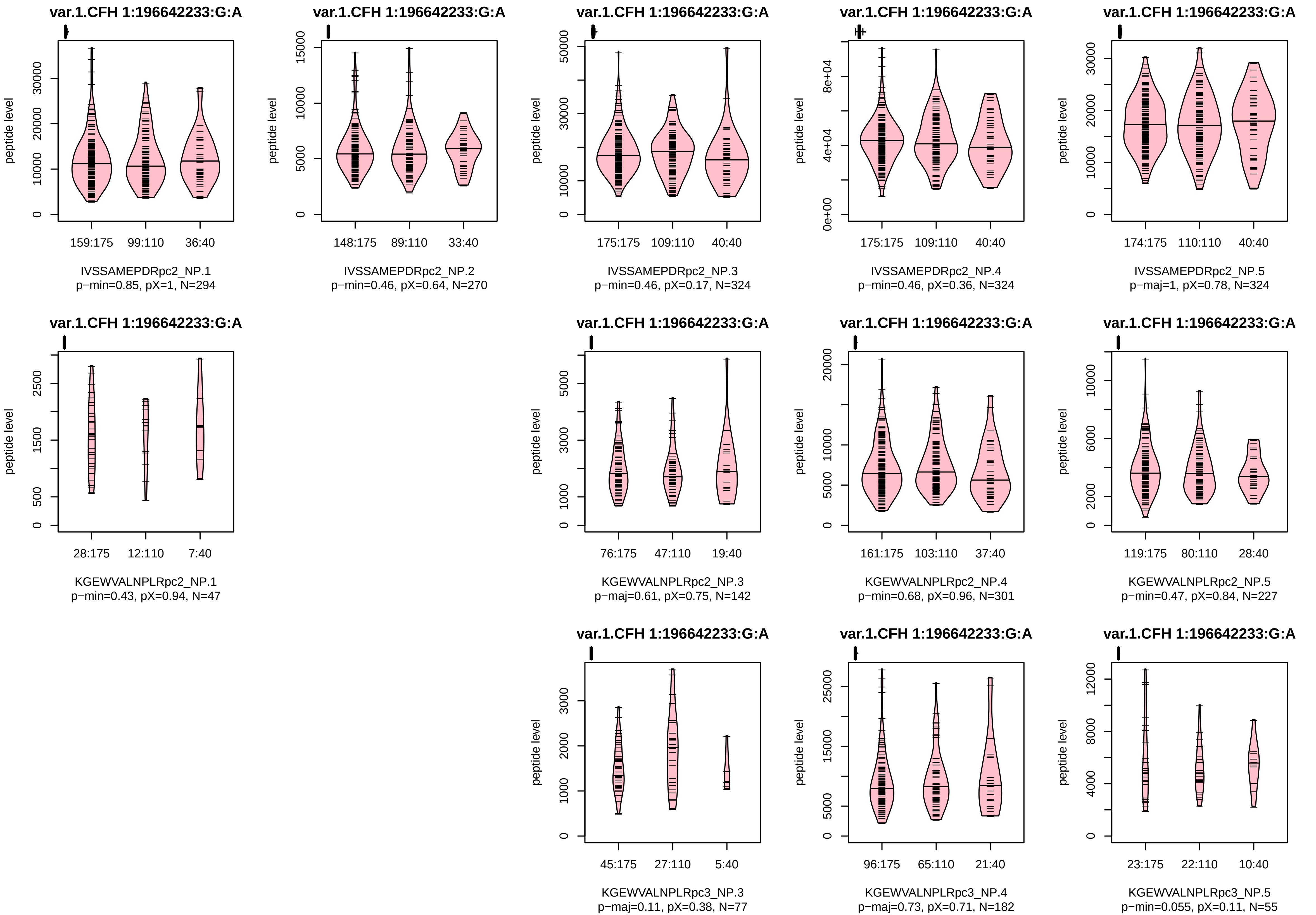

var.1.CFH 1:196642233:G:A
peptide level
159:175
99:110
36:40
IVSSAMEPDRpc2_NP.1
p−min=0.85, pX=1, N=294
var.1.CFH 1:196642233:G:A
peptide level
148:175
89:110
33:40
IVSSAMEPDRpc2_NP.2
p−min=0.46, pX=0.64, N=270
var.1.CFH 1:196642233:G:A
peptide level
175:175
109:110
40:40
IVSSAMEPDRpc2_NP.3
p−min=0.46, pX=0.17, N=324
var.1.CFH 1:196642233:G:A
peptide level
175:175
109:110
40:40
IVSSAMEPDRpc2_NP.4
p−min=0.46, pX=0.36, N=324
var.1.CFH 1:196642233:G:A
peptide level
174:175
110:110
40:40
IVSSAMEPDRpc2_NP.5
p−maj=1, pX=0.78, N=324
var.1.CFH 1:196642233:G:A
peptide level
28:175
12:110
7:40
KGEWVALNPLRpc2_NP.1
p−min=0.43, pX=0.94, N=47
var.1.CFH 1:196642233:G:A
peptide level
76:175
47:110
19:40
KGEWVALNPLRpc2_NP.3
p−maj=0.61, pX=0.75, N=142
var.1.CFH 1:196642233:G:A
peptide level
161:175
103:110
37:40
KGEWVALNPLRpc2_NP.4
p−min=0.68, pX=0.96, N=301
var.1.CFH 1:196642233:G:A
peptide level
119:175
80:110
28:40
KGEWVALNPLRpc2_NP.5
p−min=0.47, pX=0.84, N=227
var.1.CFH 1:196642233:G:A
peptide level
45:175
27:110
5:40
KGEWVALNPLRpc3_NP.3
p−maj=0.11, pX=0.38, N=77
var.1.CFH 1:196642233:G:A
peptide level
96:175
65:110
21:40
KGEWVALNPLRpc3_NP.4
p−maj=0.73, pX=0.71, N=182
var.1.CFH 1:196642233:G:A
peptide level
23:175
22:110
10:40
KGEWVALNPLRpc3_NP.5
p−min=0.055, pX=0.11, N=55

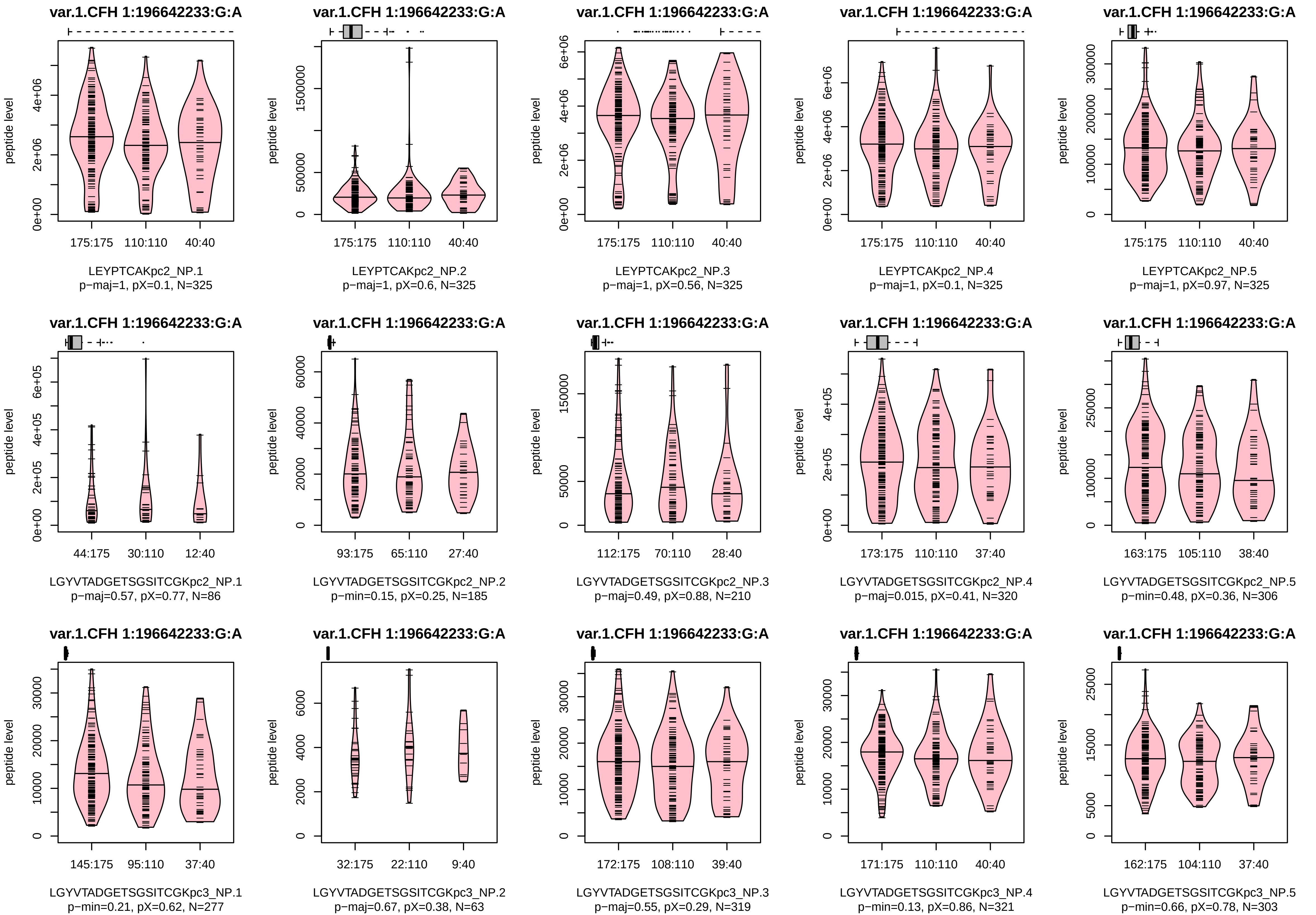
var.1.CFH 1:196642233:G:A
peptide level
175:175
110:110
40:40
LEYPTCAKpc2_NP.1
p−maj=1, pX=0.1, N=325
var.1.CFH 1:196642233:G:A
peptide level
175:175
110:110
40:40
LEYPTCAKpc2_NP.2
p−maj=1, pX=0.6, N=325
var.1.CFH 1:196642233:G:A
peptide level
175:175
110:110
40:40
LEYPTCAKpc2_NP.3
p−maj=1, pX=0.56, N=325
var.1.CFH 1:196642233:G:A
peptide level
175:175
110:110
40:40
LEYPTCAKpc2_NP.4
p−maj=1, pX=0.1, N=325
var.1.CFH 1:196642233:G:A
peptide level
175:175
110:110
40:40
LEYPTCAKpc2_NP.5
p−maj=1, pX=0.97, N=325
var.1.CFH 1:196642233:G:A
peptide level
44:175
30:110
12:40
LGYVTADGETSGSITCGKpc2_NP.1
p−maj=0.57, pX=0.77, N=86
var.1.CFH 1:196642233:G:A
peptide level
93:175
65:110
27:40
LGYVTADGETSGSITCGKpc2_NP.2
p−min=0.15, pX=0.25, N=185
var.1.CFH 1:196642233:G:A
peptide level
112:175
70:110
28:40
LGYVTADGETSGSITCGKpc2_NP.3
p−maj=0.49, pX=0.88, N=210
var.1.CFH 1:196642233:G:A
peptide level
173:175
110:110
37:40
LGYVTADGETSGSITCGKpc2_NP.4
p−maj=0.015, pX=0.41, N=320
var.1.CFH 1:196642233:G:A
peptide level
163:175
105:110
38:40
LGYVTADGETSGSITCGKpc2_NP.5
p−min=0.48, pX=0.36, N=306
var.1.CFH 1:196642233:G:A
peptide level
145:175
95:110
37:40
LGYVTADGETSGSITCGKpc3_NP.1
p−min=0.21, pX=0.62, N=277
var.1.CFH 1:196642233:G:A
peptide level
32:175
22:110
9:40
LGYVTADGETSGSITCGKpc3_NP.2
p−maj=0.67, pX=0.38, N=63
var.1.CFH 1:196642233:G:A
peptide level
172:175
108:110
39:40
LGYVTADGETSGSITCGKpc3_NP.3
p−maj=0.55, pX=0.29, N=319
var.1.CFH 1:196642233:G:A
peptide level
171:175
110:110
40:40
LGYVTADGETSGSITCGKpc3_NP.4
p−min=0.13, pX=0.86, N=321
var.1.CFH 1:196642233:G:A
peptide level
162:175
104:110
37:40
LGYVTADGETSGSITCGKpc3_NP.5
p−min=0.66, pX=0.78, N=303

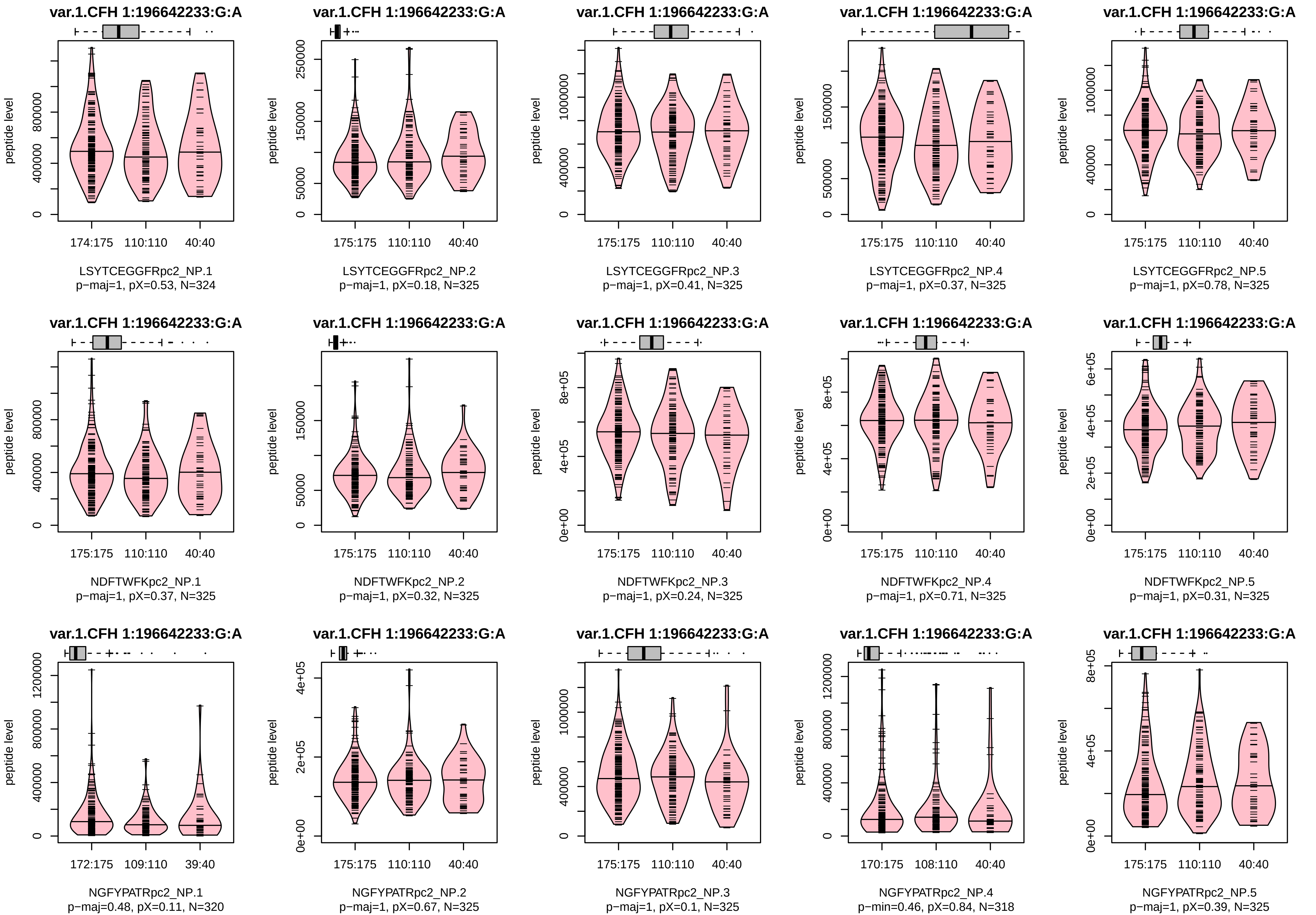
var.1.CFH 1:196642233:G:A
peptide level
174:175
110:110
40:40
LSYTCEGGFRpc2_NP.1
p−maj=1, pX=0.53, N=324
var.1.CFH 1:196642233:G:A
peptide level
175:175
110:110
40:40
LSYTCEGGFRpc2_NP.2
p−maj=1, pX=0.18, N=325
var.1.CFH 1:196642233:G:A
peptide level
175:175
110:110
40:40
LSYTCEGGFRpc2_NP.3
p−maj=1, pX=0.41, N=325
var.1.CFH 1:196642233:G:A
peptide level
175:175
110:110
40:40
LSYTCEGGFRpc2_NP.4
p−maj=1, pX=0.37, N=325
var.1.CFH 1:196642233:G:A
peptide level
175:175
110:110
40:40
LSYTCEGGFRpc2_NP.5
p−maj=1, pX=0.78, N=325
var.1.CFH 1:196642233:G:A
peptide level
175:175
110:110
40:40
NDFTWFKpc2_NP.1
p−maj=1, pX=0.37, N=325
var.1.CFH 1:196642233:G:A
peptide level
175:175
110:110
40:40
NDFTWFKpc2_NP.2
p−maj=1, pX=0.32, N=325
var.1.CFH 1:196642233:G:A
peptide level
175:175
110:110
40:40
NDFTWFKpc2_NP.3
p−maj=1, pX=0.24, N=325
var.1.CFH 1:196642233:G:A
peptide level
175:175
110:110
40:40
NDFTWFKpc2_NP.4
p−maj=1, pX=0.71, N=325
var.1.CFH 1:196642233:G:A
peptide level
175:175
110:110
40:40
NDFTWFKpc2_NP.5
p−maj=1, pX=0.31, N=325
var.1.CFH 1:196642233:G:A
peptide level
172:175
109:110
39:40
NGFYPATRpc2_NP.1
p−maj=0.48, pX=0.11, N=320
var.1.CFH 1:196642233:G:A
peptide level
175:175
110:110
40:40
NGFYPATRpc2_NP.2
p−maj=1, pX=0.67, N=325
var.1.CFH 1:196642233:G:A
peptide level
175:175
110:110
40:40
NGFYPATRpc2_NP.3
p−maj=1, pX=0.1, N=325
var.1.CFH 1:196642233:G:A
peptide level
170:175
108:110
40:40
NGFYPATRpc2_NP.4
p−min=0.46, pX=0.84, N=318
var.1.CFH 1:196642233:G:A
peptide level
175:175
110:110
40:40
NGFYPATRpc2_NP.5
p−maj=1, pX=0.39, N=325

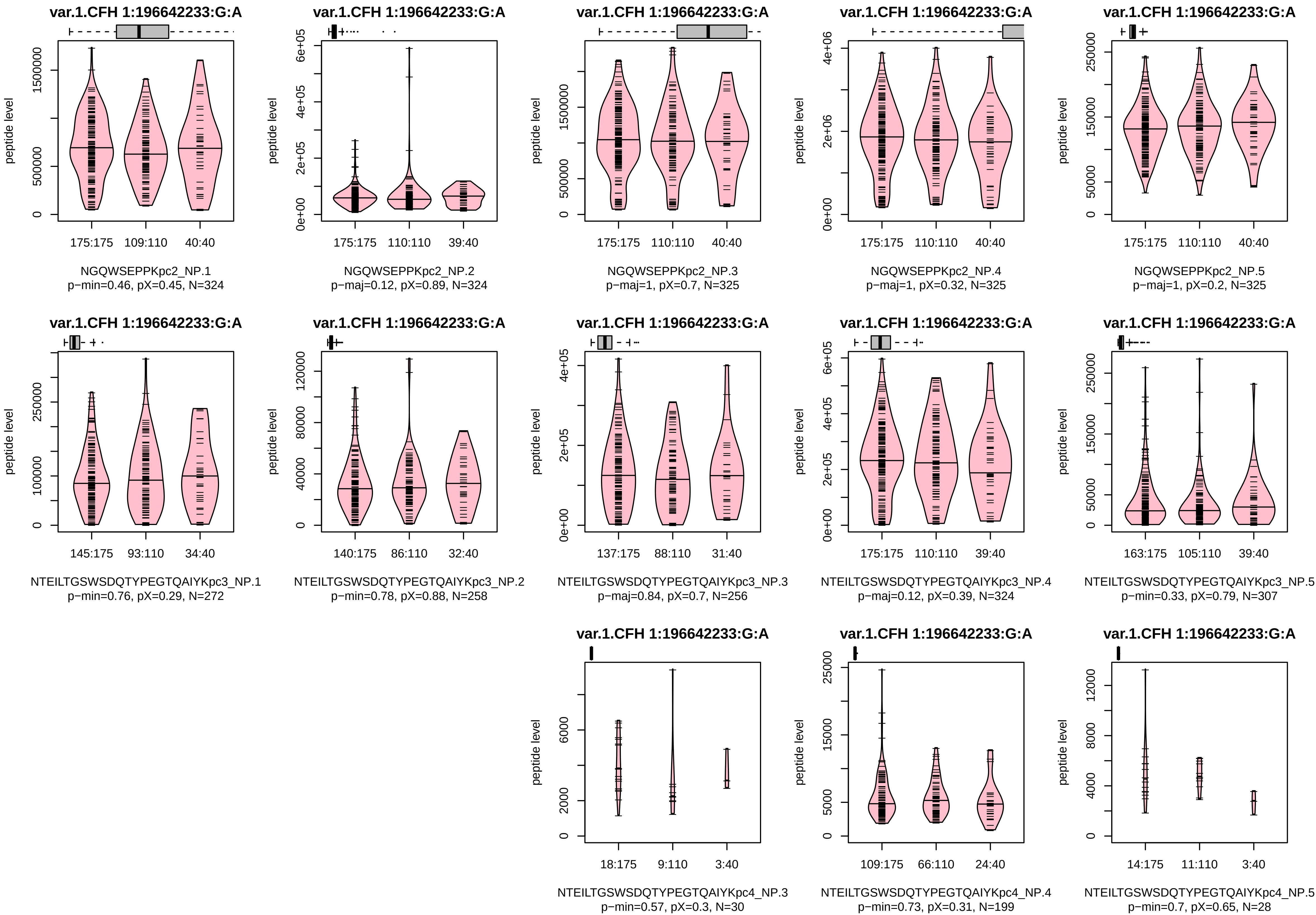
var.1.CFH 1:196642233:G:A
peptide level
175:175
109:110
40:40
NGQWSEPPKpc2_NP.1
p−min=0.46, pX=0.45, N=324
var.1.CFH 1:196642233:G:A
peptide level
175:175
110:110
39:40
NGQWSEPPKpc2_NP.2
p−maj=0.12, pX=0.89, N=324
var.1.CFH 1:196642233:G:A
peptide level
175:175
110:110
40:40
NGQWSEPPKpc2_NP.3
p−maj=1, pX=0.7, N=325
var.1.CFH 1:196642233:G:A
peptide level
175:175
110:110
40:40
NGQWSEPPKpc2_NP.4
p−maj=1, pX=0.32, N=325
var.1.CFH 1:196642233:G:A
peptide level
175:175
110:110
40:40
NGQWSEPPKpc2_NP.5
p−maj=1, pX=0.2, N=325
var.1.CFH 1:196642233:G:A
peptide level
145:175
93:110
34:40
NTEILTGSWSDQTYPEGTQAIYKpc3_NP.1
p−min=0.76, pX=0.29, N=272
var.1.CFH 1:196642233:G:A
peptide level
140:175
86:110
32:40
NTEILTGSWSDQTYPEGTQAIYKpc3_NP.2
p−min=0.78, pX=0.88, N=258
var.1.CFH 1:196642233:G:A
peptide level
137:175
88:110
31:40
NTEILTGSWSDQTYPEGTQAIYKpc3_NP.3
p−maj=0.84, pX=0.7, N=256
var.1.CFH 1:196642233:G:A
peptide level
175:175
110:110
39:40
NTEILTGSWSDQTYPEGTQAIYKpc3_NP.4
p−maj=0.12, pX=0.39, N=324
var.1.CFH 1:196642233:G:A
peptide level
163:175
105:110
39:40
NTEILTGSWSDQTYPEGTQAIYKpc3_NP.5
p−min=0.33, pX=0.79, N=307
var.1.CFH 1:196642233:G:A
peptide level
18:175
9:110
3:40
NTEILTGSWSDQTYPEGTQAIYKpc4_NP.3
p−min=0.57, pX=0.3, N=30
var.1.CFH 1:196642233:G:A
peptide level
109:175
66:110
24:40
NTEILTGSWSDQTYPEGTQAIYKpc4_NP.4
p−min=0.73, pX=0.31, N=199
var.1.CFH 1:196642233:G:A
peptide level
14:175
11:110
3:40
NTEILTGSWSDQTYPEGTQAIYKpc4_NP.5
p−min=0.7, pX=0.65, N=28

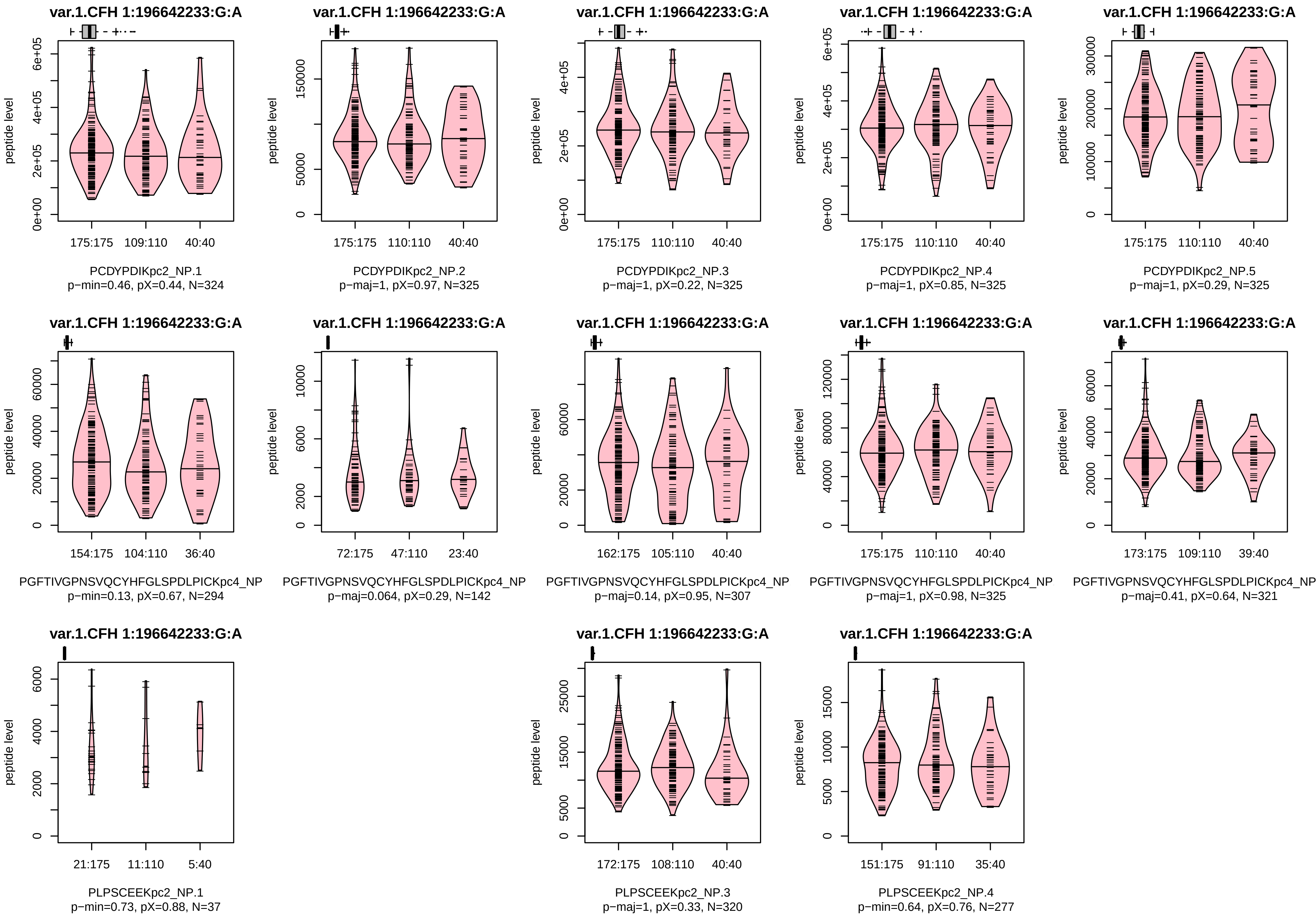
var.1.CFH 1:196642233:G:A
peptide level
175:175
109:110
40:40
PCDYPDIKpc2_NP.1
p−min=0.46, pX=0.44, N=324
var.1.CFH 1:196642233:G:A
peptide level
175:175
110:110
40:40
PCDYPDIKpc2_NP.2
p−maj=1, pX=0.97, N=325
var.1.CFH 1:196642233:G:A
peptide level
175:175
110:110
40:40
PCDYPDIKpc2_NP.3
p−maj=1, pX=0.22, N=325
var.1.CFH 1:196642233:G:A
peptide level
175:175
110:110
40:40
PCDYPDIKpc2_NP.4
p−maj=1, pX=0.85, N=325
var.1.CFH 1:196642233:G:A
peptide level
175:175
110:110
40:40
PCDYPDIKpc2_NP.5
p−maj=1, pX=0.29, N=325
var.1.CFH 1:196642233:G:A
peptide level
154:175
104:110
36:40
PGFTIVGPNSVQCYHFGLSPDLPICKpc4_NP
p−min=0.13, pX=0.67, N=294
var.1.CFH 1:196642233:G:A
peptide level
72:175
47:110
23:40
PGFTIVGPNSVQCYHFGLSPDLPICKpc4_NP
p−maj=0.064, pX=0.29, N=142
var.1.CFH 1:196642233:G:A
peptide level
162:175
105:110
40:40
PGFTIVGPNSVQCYHFGLSPDLPICKpc4_NP
p−maj=0.14, pX=0.95, N=307
var.1.CFH 1:196642233:G:A
peptide level
175:175
110:110
40:40
PGFTIVGPNSVQCYHFGLSPDLPICKpc4_NP
p−maj=1, pX=0.98, N=325
var.1.CFH 1:196642233:G:A
peptide level
173:175
109:110
39:40
PGFTIVGPNSVQCYHFGLSPDLPICKpc4_NP
p−maj=0.41, pX=0.64, N=321
var.1.CFH 1:196642233:G:A
peptide level
21:175
11:110
5:40
PLPSCEEKpc2_NP.1
p−min=0.73, pX=0.88, N=37
var.1.CFH 1:196642233:G:A
peptide level
172:175
108:110
40:40
PLPSCEEKpc2_NP.3
p−maj=1, pX=0.33, N=320
var.1.CFH 1:196642233:G:A
peptide level
151:175
91:110
35:40
PLPSCEEKpc2_NP.4
p−min=0.64, pX=0.76, N=277

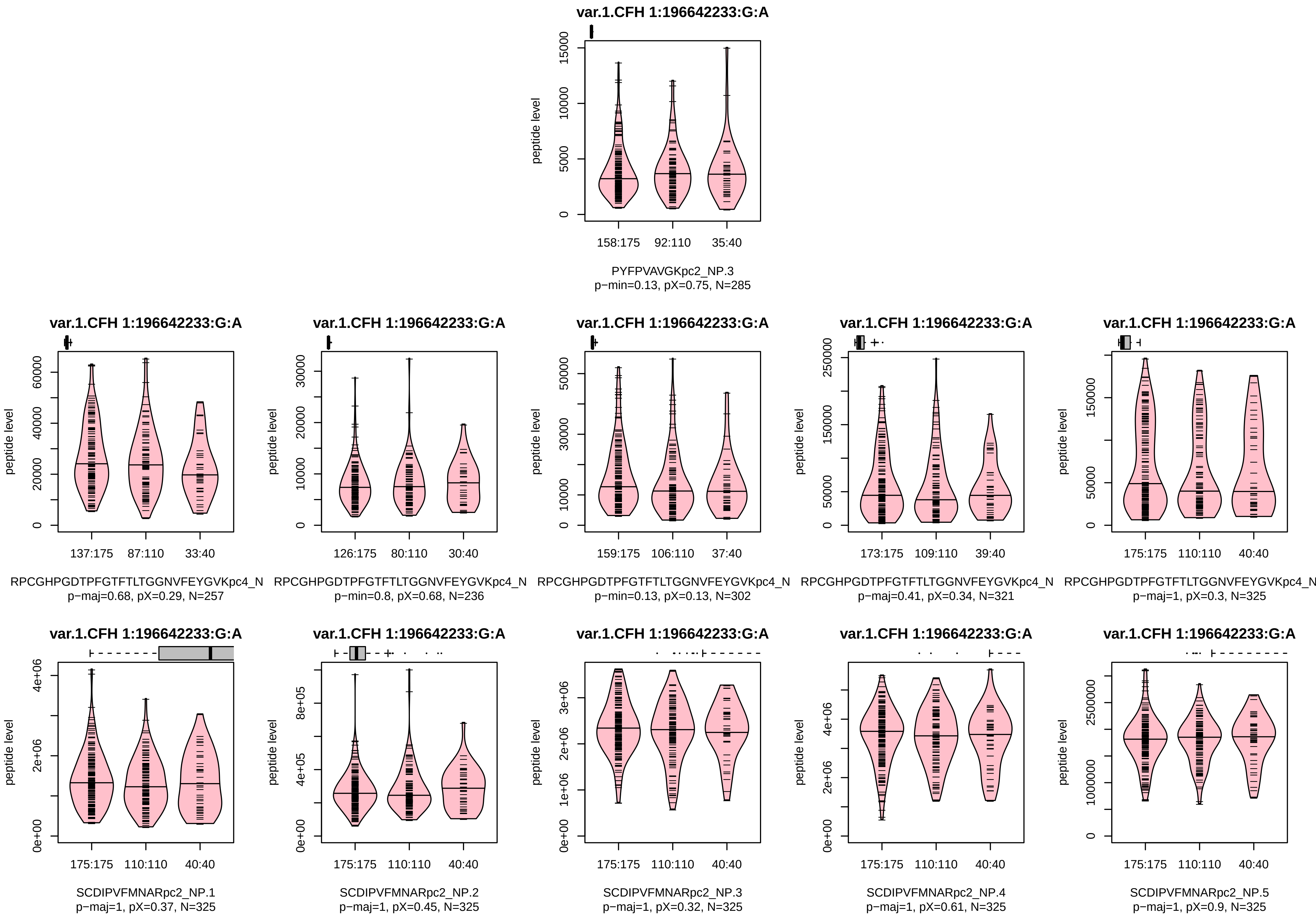

var.1.CFH 1:196642233:G:A
peptide level
158:175
92:110
35:40
PYFPVAVGKpc2_NP.3
p−min=0.13, pX=0.75, N=285
var.1.CFH 1:196642233:G:A
137:175
87:110
33:40
RPCGHPGDTPFGTFTLTGGNVFEYGVKpc4_N
p−maj=0.68, pX=0.29, N=257
var.1.CFH 1:196642233:G:A
126:175
80:110
30:40
RPCGHPGDTPFGTFTLTGGNVFEYGVKpc4_N
p−min=0.8, pX=0.68, N=236
var.1.CFH 1:196642233:G:A
159:175
106:110
37:40
RPCGHPGDTPFGTFTLTGGNVFEYGVKpc4_N
p−min=0.13, pX=0.13, N=302
var.1.CFH 1:196642233:G:A
173:175
109:110
39:40
RPCGHPGDTPFGTFTLTGGNVFEYGVKpc4_N
p−maj=0.41, pX=0.34, N=321
var.1.CFH 1:196642233:G:A
175:175
110:110
40:40
RPCGHPGDTPFGTFTLTGGNVFEYGVKpc4_N
p−maj=1, pX=0.3, N=325
var.1.CFH 1:196642233:G:A
175:175
110:110
40:40
SCDIPVFMNARpc2_NP.1
p−maj=1, pX=0.37, N=325
var.1.CFH 1:196642233:G:A
175:175
110:110
40:40
SCDIPVFMNARpc2_NP.2
p−maj=1, pX=0.45, N=325
var.1.CFH 1:196642233:G:A
175:175
110:110
40:40
SCDIPVFMNARpc2_NP.3
p−maj=1, pX=0.32, N=325
var.1.CFH 1:196642233:G:A
175:175
110:110
40:40
SCDIPVFMNARpc2_NP.4
p−maj=1, pX=0.61, N=325
var.1.CFH 1:196642233:G:A
175:175
110:110
40:40
SCDIPVFMNARpc2_NP.5
p−maj=1, pX=0.9, N=325

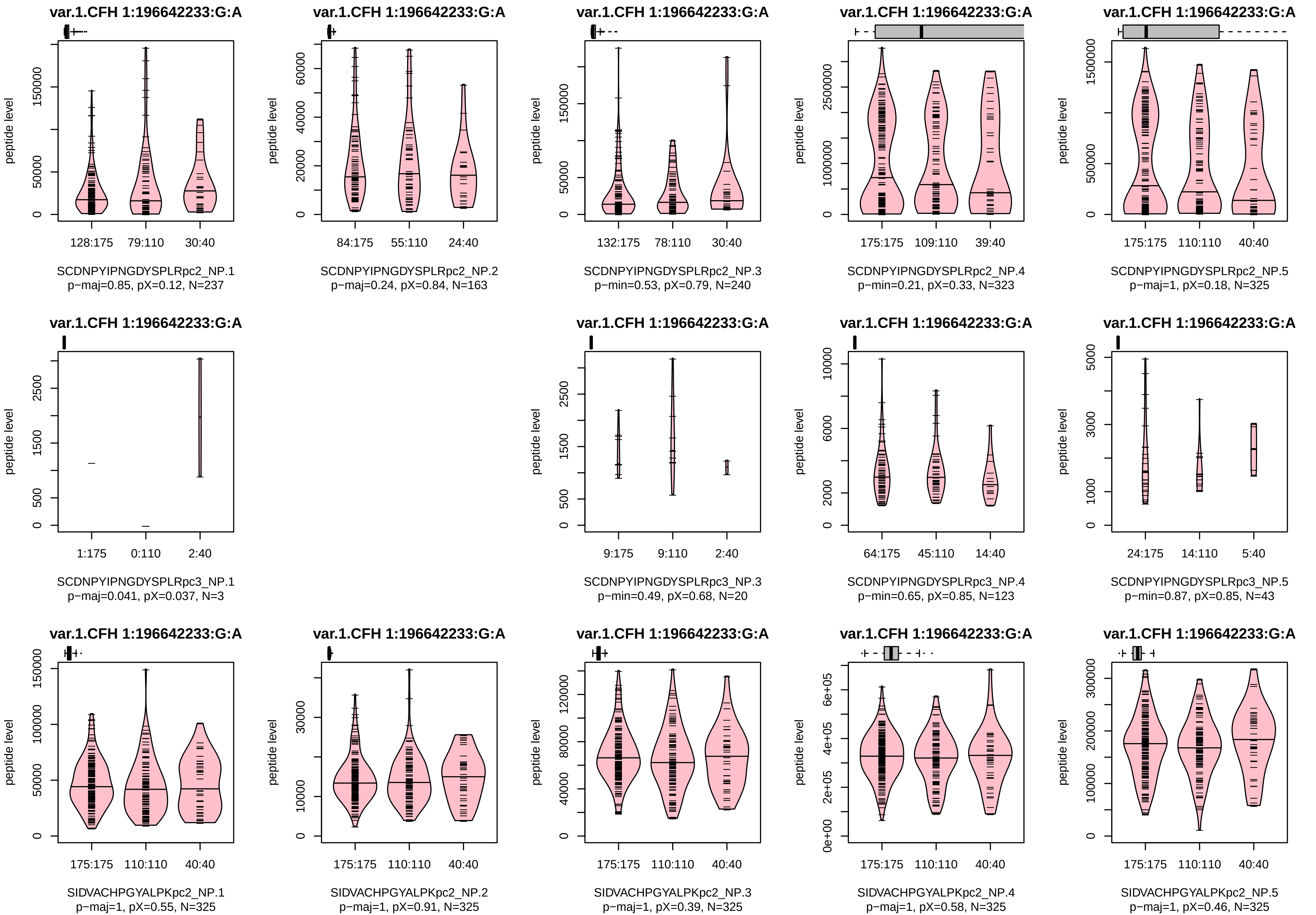

var.1.CFH 1:196642233:G:A
peptide level
128:175
79:110
30:40
SCDNPYIPNGDYSPLRpc2_NP.1
p−maj=0.85, pX=0.12, N=237
var.1.CFH 1:196642233:G:A
peptide level
84:175
55:110
24:40
SCDNPYIPNGDYSPLRpc2_NP.2
p−maj=0.24, pX=0.84, N=163
var.1.CFH 1:196642233:G:A
peptide level
132:175
78:110
30:40
SCDNPYIPNGDYSPLRpc2_NP.3
p−min=0.53, pX=0.79, N=240
var.1.CFH 1:196642233:G:A
peptide level
175:175
109:110
39:40
SCDNPYIPNGDYSPLRpc2_NP.4
p−min=0.21, pX=0.33, N=323
var.1.CFH 1:196642233:G:A
peptide level
175:175
110:110
40:40
SCDNPYIPNGDYSPLRpc2_NP.5
p−maj=1, pX=0.18, N=325
var.1.CFH 1:196642233:G:A
peptide level
1:175
0:110
2:40
SCDNPYIPNGDYSPLRpc3_NP.1
p−maj=0.041, pX=0.037, N=3
var.1.CFH 1:196642233:G:A
peptide level
9:175
9:110
2:40
SCDNPYIPNGDYSPLRpc3_NP.3
p−min=0.49, pX=0.68, N=20
var.1.CFH 1:196642233:G:A
peptide level
64:175
45:110
14:40
SCDNPYIPNGDYSPLRpc3_NP.4
p−min=0.65, pX=0.85, N=123
var.1.CFH 1:196642233:G:A
peptide level
24:175
14:110
5:40
SCDNPYIPNGDYSPLRpc3_NP.5
p−min=0.87, pX=0.85, N=43
var.1.CFH 1:196642233:G:A
peptide level
175:175
110:110
40:40
SIDVACHPGYALPKpc2_NP.1
p−maj=1, pX=0.55, N=325
var.1.CFH 1:196642233:G:A
peptide level
175:175
110:110
40:40
SIDVACHPGYALPKpc2_NP.2
p−maj=1, pX=0.91, N=325
var.1.CFH 1:196642233:G:A
peptide level
175:175
110:110
40:40
SIDVACHPGYALPKpc2_NP.3
p−maj=1, pX=0.39, N=325
var.1.CFH 1:196642233:G:A
peptide level
175:175
110:110
40:40
SIDVACHPGYALPKpc2_NP.4
p−maj=1, pX=0.58, N=325
var.1.CFH 1:196642233:G:A
peptide level
175:175
110:110
40:40
SIDVACHPGYALPKpc2_NP.5
p−maj=1, pX=0.46, N=325

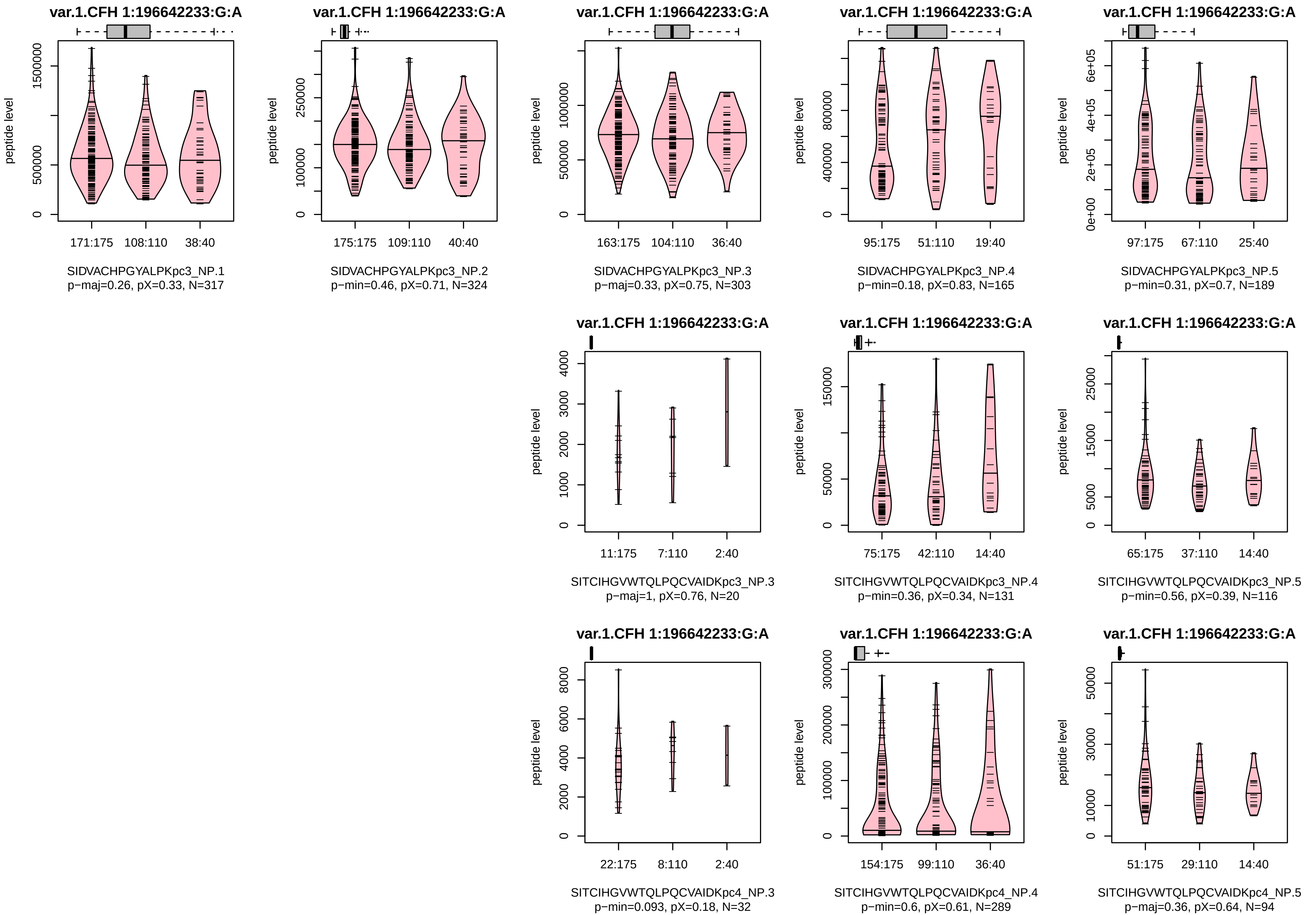
var.1.CFH 1:196642233:G:A
peptide level
171:175
108:110
38:40
SIDVACHPGYALPKpc3_NP.1
p−maj=0.26, pX=0.33, N=317
var.1.CFH 1:196642233:G:A
peptide level
175:175
109:110
40:40
SIDVACHPGYALPKpc3_NP.2
p−min=0.46, pX=0.71, N=324
var.1.CFH 1:196642233:G:A
peptide level
163:175
104:110
36:40
SIDVACHPGYALPKpc3_NP.3
p−maj=0.33, pX=0.75, N=303
var.1.CFH 1:196642233:G:A
peptide level
95:175
51:110
19:40
SIDVACHPGYALPKpc3_NP.4
p−min=0.18, pX=0.83, N=165
var.1.CFH 1:196642233:G:A
peptide level
97:175
67:110
25:40
SIDVACHPGYALPKpc3_NP.5
p−min=0.31, pX=0.7, N=189
var.1.CFH 1:196642233:G:A
peptide level
11:175
7:110
2:40
SITCIHGVWTQLPQCVAIDKpc3_NP.3
p−maj=1, pX=0.76, N=20
var.1.CFH 1:196642233:G:A
peptide level
75:175
42:110
14:40
SITCIHGVWTQLPQCVAIDKpc3_NP.4
p−min=0.36, pX=0.34, N=131
var.1.CFH 1:196642233:G:A
peptide level
65:175
37:110
14:40
SITCIHGVWTQLPQCVAIDKpc3_NP.5
p−min=0.56, pX=0.39, N=116
var.1.CFH 1:196642233:G:A
peptide level
22:175
8:110
2:40
SITCIHGVWTQLPQCVAIDKpc4_NP.3
p−min=0.093, pX=0.18, N=32
var.1.CFH 1:196642233:G:A
peptide level
154:175
99:110
36:40
SITCIHGVWTQLPQCVAIDKpc4_NP.4
p−min=0.6, pX=0.61, N=289
var.1.CFH 1:196642233:G:A
peptide level
51:175
29:110
14:40
SITCIHGVWTQLPQCVAIDKpc4_NP.5
p−maj=0.36, pX=0.64, N=94

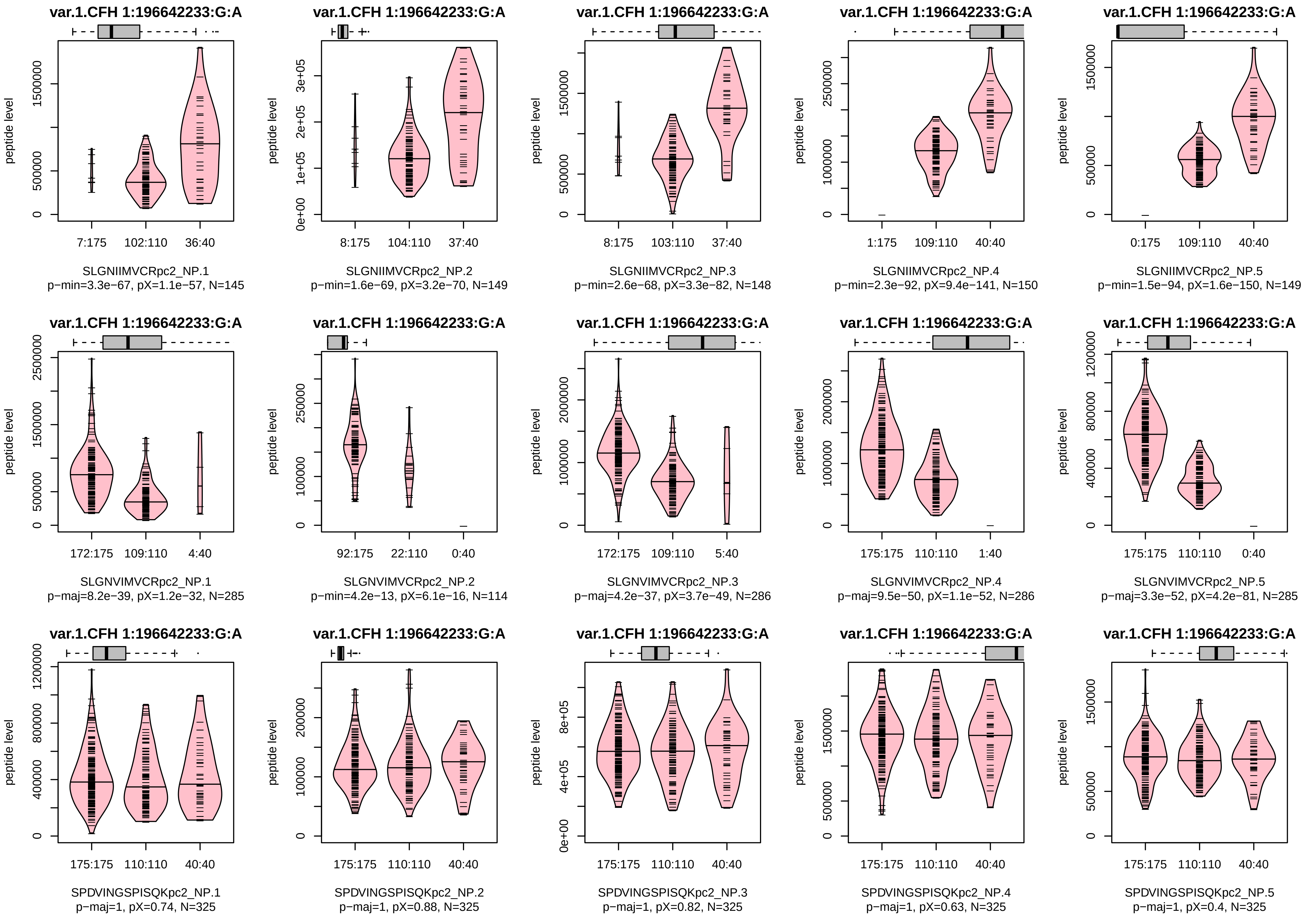
var.1.CFH 1:196642233:G:A
peptide level
7:175
102:110
36:40
SLGNIIMVCRpc2_NP.1
p−min=3.3e−67, pX=1.1e−57, N=145
var.1.CFH 1:196642233:G:A
peptide level
8:175
104:110
37:40
SLGNIIMVCRpc2_NP.2
p−min=1.6e−69, pX=3.2e−70, N=149
var.1.CFH 1:196642233:G:A
peptide level
8:175
103:110
37:40
SLGNIIMVCRpc2_NP.3
p−min=2.6e−68, pX=3.3e−82, N=148
var.1.CFH 1:196642233:G:A
peptide level
1:175
109:110
40:40
SLGNIIMVCRpc2_NP.4
p−min=2.3e−92, pX=9.4e−141, N=150
var.1.CFH 1:196642233:G:A
peptide level
0:175
109:110
40:40
SLGNIIMVCRpc2_NP.5
p−min=1.5e−94, pX=1.6e−150, N=149
var.1.CFH 1:196642233:G:A
peptide level
172:175
109:110
4:40
SLGNVIMVCRpc2_NP.1
p−maj=8.2e−39, pX=1.2e−32, N=285
var.1.CFH 1:196642233:G:A
peptide level
92:175
22:110
0:40
SLGNVIMVCRpc2_NP.2
p−min=4.2e−13, pX=6.1e−16, N=114
var.1.CFH 1:196642233:G:A
peptide level
172:175
109:110
5:40
SLGNVIMVCRpc2_NP.3
p−maj=4.2e−37, pX=3.7e−49, N=286
var.1.CFH 1:196642233:G:A
peptide level
175:175
110:110
1:40
SLGNVIMVCRpc2_NP.4
p−maj=9.5e−50, pX=1.1e−52, N=286
var.1.CFH 1:196642233:G:A
peptide level
175:175
110:110
0:40
SLGNVIMVCRpc2_NP.5
p−maj=3.3e−52, pX=4.2e−81, N=285
var.1.CFH 1:196642233:G:A
peptide level
175:175
110:110
40:40
SPDVINGSPISQKpc2_NP.1
p−maj=1, pX=0.74, N=325
var.1.CFH 1:196642233:G:A
peptide level
175:175
110:110
40:40
SPDVINGSPISQKpc2_NP.2
p−maj=1, pX=0.88, N=325
var.1.CFH 1:196642233:G:A
peptide level
175:175
110:110
40:40
SPDVINGSPISQKpc2_NP.3
p−maj=1, pX=0.82, N=325
var.1.CFH 1:196642233:G:A
peptide level
175:175
110:110
40:40
SPDVINGSPISQKpc2_NP.4
p−maj=1, pX=0.63, N=325
var.1.CFH 1:196642233:G:A
peptide level
175:175
110:110
40:40
SPDVINGSPISQKpc2_NP.5
p−maj=1, pX=0.4, N=325

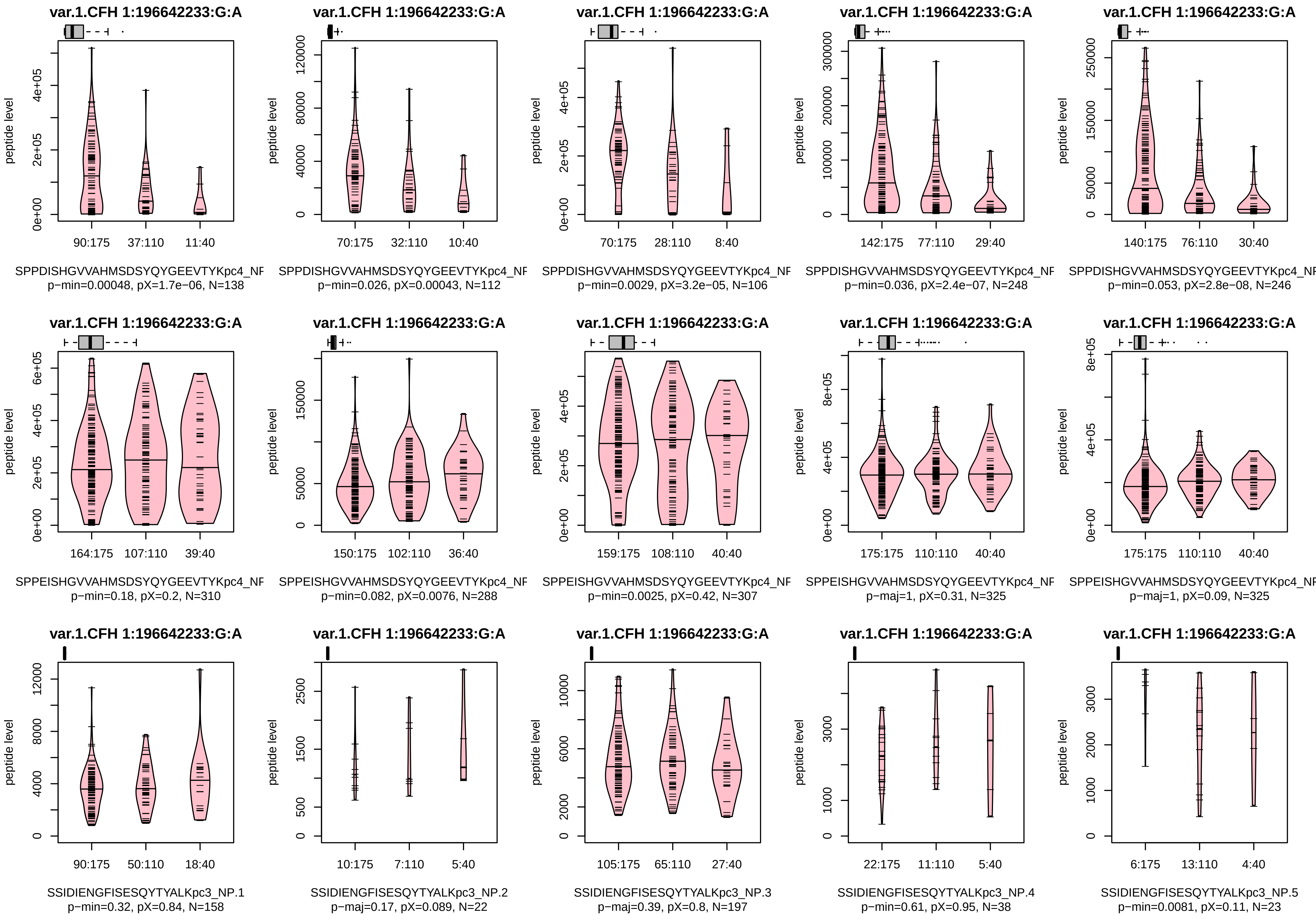
var.1.CFH 1:196642233:G:A
peptide level
90:175
37:110
11:40
SPPDISHGVVAHMSDSYQYGEEVTYKpc4_NF
p−min=0.00048, pX=1.7e−06, N=138
var.1.CFH 1:196642233:G:A
peptide level
70:175
32:110
10:40
SPPDISHGVVAHMSDSYQYGEEVTYKpc4_NF
p−min=0.026, pX=0.00043, N=112
var.1.CFH 1:196642233:G:A
peptide level
70:175
28:110
8:40
SPPDISHGVVAHMSDSYQYGEEVTYKpc4_NF
p−min=0.0029, pX=3.2e−05, N=106
var.1.CFH 1:196642233:G:A
peptide level
142:175
77:110
29:40
SPPDISHGVVAHMSDSYQYGEEVTYKpc4_NF
p−min=0.036, pX=2.4e−07, N=248
var.1.CFH 1:196642233:G:A
peptide level
140:175
76:110
30:40
SPPDISHGVVAHMSDSYQYGEEVTYKpc4_NF
p−min=0.053, pX=2.8e−08, N=246
var.1.CFH 1:196642233:G:A
peptide level
164:175
107:110
39:40
SPPEISHGVVAHMSDSYQYGEEVTYKpc4_NF
p−min=0.18, pX=0.2, N=310
var.1.CFH 1:196642233:G:A
peptide level
150:175
102:110
36:40
SPPEISHGVVAHMSDSYQYGEEVTYKpc4_NF
p−min=0.082, pX=0.0076, N=288
var.1.CFH 1:196642233:G:A
peptide level
159:175
108:110
40:40
SPPEISHGVVAHMSDSYQYGEEVTYKpc4_NF
p−min=0.0025, pX=0.42, N=307
var.1.CFH 1:196642233:G:A
peptide level
175:175
110:110
40:40
SPPEISHGVVAHMSDSYQYGEEVTYKpc4_NF
p−maj=1, pX=0.31, N=325
var.1.CFH 1:196642233:G:A
peptide level
175:175
110:110
40:40
SPPEISHGVVAHMSDSYQYGEEVTYKpc4_NF
p−maj=1, pX=0.09, N=325
var.1.CFH 1:196642233:G:A
peptide level
90:175
50:110
18:40
SSIDIENGFISESQYTYALKpc3_NP.1
p−min=0.32, pX=0.84, N=158
var.1.CFH 1:196642233:G:A
peptide level
10:175
7:110
5:40
SSIDIENGFISESQYTYALKpc3_NP.2
p−maj=0.17, pX=0.089, N=22
var.1.CFH 1:196642233:G:A
peptide level
105:175
65:110
27:40
SSIDIENGFISESQYTYALKpc3_NP.3
p−maj=0.39, pX=0.8, N=197
var.1.CFH 1:196642233:G:A
peptide level
22:175
11:110
5:40
SSIDIENGFISESQYTYALKpc3_NP.4
p−min=0.61, pX=0.95, N=38
var.1.CFH 1:196642233:G:A
peptide level
6:175
13:110
4:40
SSIDIENGFISESQYTYALKpc3_NP.5
p−min=0.0081, pX=0.11, N=23

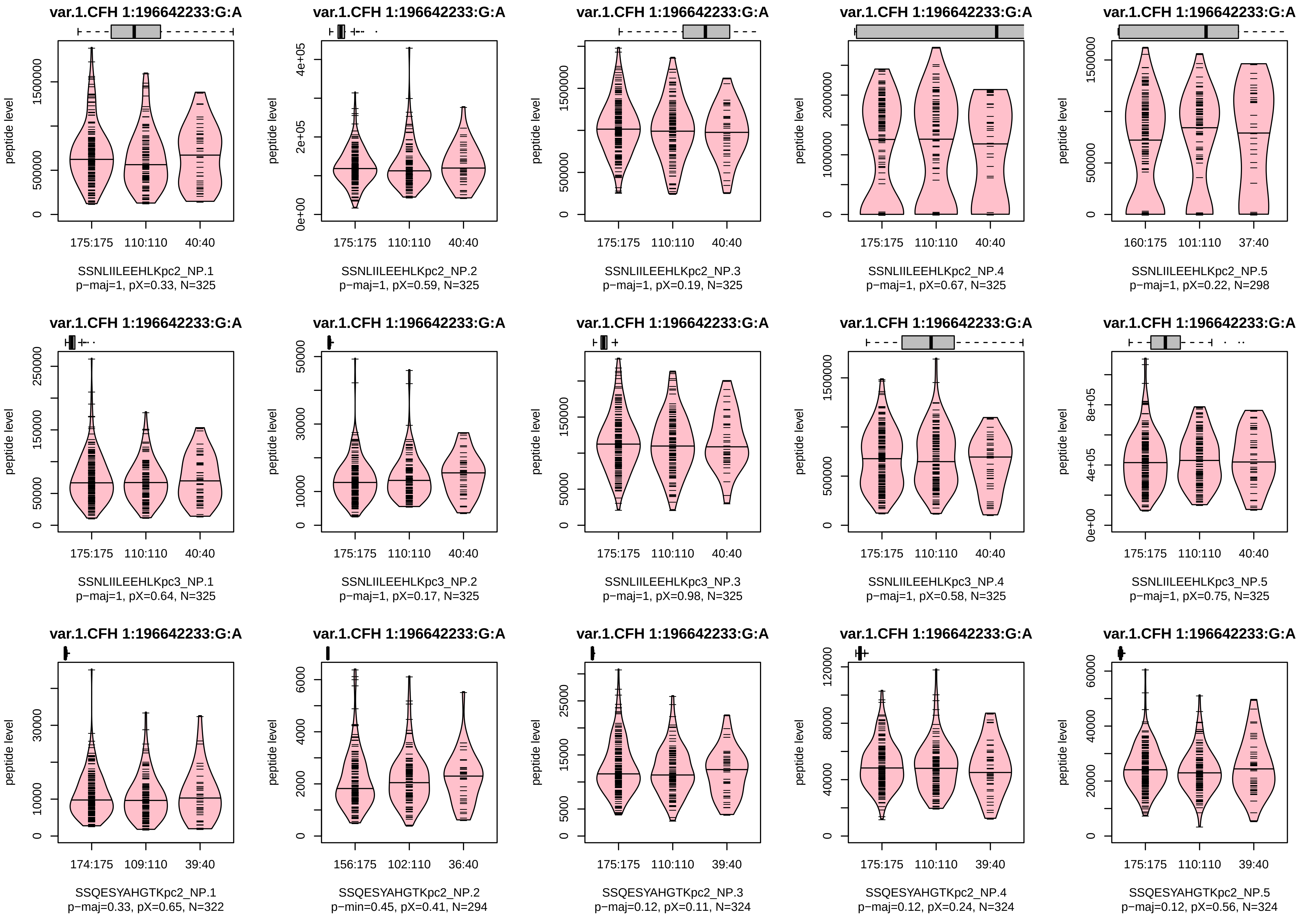
var.1.CFH 1:196642233:G:A
peptide level
175:175 110:110 40:40
SSNLIILEEHLKpc2_NP.1
p−maj=1, pX=0.33, N=325
var.1.CFH 1:196642233:G:A
peptide level
175:175 110:110 40:40
SSNLIILEEHLKpc2_NP.2
p−maj=1, pX=0.59, N=325
var.1.CFH 1:196642233:G:A
peptide level
175:175 110:110 40:40
SSNLIILEEHLKpc2_NP.3
p−maj=1, pX=0.19, N=325
var.1.CFH 1:196642233:G:A
peptide level
175:175 110:110 40:40
SSNLIILEEHLKpc2_NP.4
p−maj=1, pX=0.67, N=325
var.1.CFH 1:196642233:G:A
peptide level
160:175 101:110 37:40
SSNLIILEEHLKpc2_NP.5
p−maj=1, pX=0.22, N=298
var.1.CFH 1:196642233:G:A
peptide level
175:175 110:110 40:40
SSNLIILEEHLKpc3_NP.1
p−maj=1, pX=0.64, N=325
var.1.CFH 1:196642233:G:A
peptide level
175:175 110:110 40:40
SSNLIILEEHLKpc3_NP.2
p−maj=1, pX=0.17, N=325
var.1.CFH 1:196642233:G:A
peptide level
175:175 110:110 40:40
SSNLIILEEHLKpc3_NP.3
p−maj=1, pX=0.98, N=325
var.1.CFH 1:196642233:G:A
peptide level
175:175 110:110 40:40
SSNLIILEEHLKpc3_NP.4
p−maj=1, pX=0.58, N=325
var.1.CFH 1:196642233:G:A
peptide level
175:175 110:110 40:40
SSNLIILEEHLKpc3_NP.5
p−maj=1, pX=0.75, N=325
var.1.CFH 1:196642233:G:A
peptide level
174:175 109:110 39:40
SSQESYAHGTKpc2_NP.1
p−maj=0.33, pX=0.65, N=322
var.1.CFH 1:196642233:G:A
peptide level
156:175 102:110 36:40
SSQESYAHGTKpc2_NP.2
p−min=0.45, pX=0.41, N=294
var.1.CFH 1:196642233:G:A
peptide level
175:175 110:110 39:40
SSQESYAHGTKpc2_NP.3
p−maj=0.12, pX=0.11, N=324
var.1.CFH 1:196642233:G:A
peptide level
175:175 110:110 39:40
SSQESYAHGTKpc2_NP.4
p−maj=0.12, pX=0.24, N=324
var.1.CFH 1:196642233:G:A
peptide level
175:175 110:110 39:40
SSQESYAHGTKpc2_NP.5
p−maj=0.12, pX=0.56, N=324

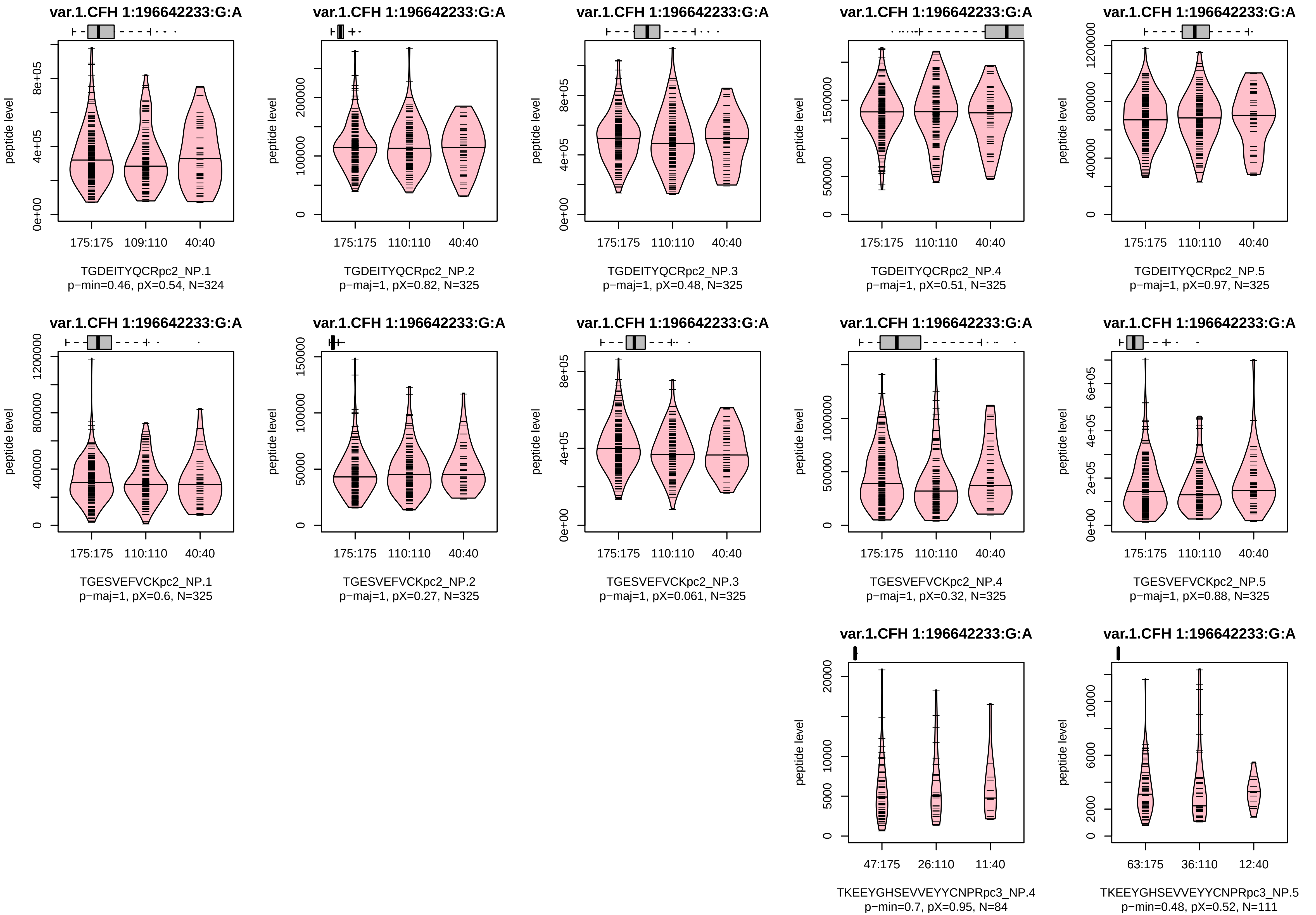
var.1.CFH 1:196642233:G:A
peptide level
175:175
109:110
40:40
TGDEITYQCRpc2_NP.1
p−min=0.46, pX=0.54, N=324
var.1.CFH 1:196642233:G:A
peptide level
175:175
110:110
40:40
TGDEITYQCRpc2_NP.2
p−maj=1, pX=0.82, N=325
var.1.CFH 1:196642233:G:A
peptide level
175:175
110:110
40:40
TGDEITYQCRpc2_NP.3
p−maj=1, pX=0.48, N=325
var.1.CFH 1:196642233:G:A
peptide level
175:175
110:110
40:40
TGDEITYQCRpc2_NP.4
p−maj=1, pX=0.51, N=325
var.1.CFH 1:196642233:G:A
peptide level
175:175
110:110
40:40
TGDEITYQCRpc2_NP.5
p−maj=1, pX=0.97, N=325
var.1.CFH 1:196642233:G:A
peptide level
175:175
110:110
40:40
TGESVEFVCKpc2_NP.1
p−maj=1, pX=0.6, N=325
var.1.CFH 1:196642233:G:A
peptide level
175:175
110:110
40:40
TGESVEFVCKpc2_NP.2
p−maj=1, pX=0.27, N=325
var.1.CFH 1:196642233:G:A
peptide level
175:175
110:110
40:40
TGESVEFVCKpc2_NP.3
p−maj=1, pX=0.061, N=325
var.1.CFH 1:196642233:G:A
peptide level
175:175
110:110
40:40
TGESVEFVCKpc2_NP.4
p−maj=1, pX=0.32, N=325
var.1.CFH 1:196642233:G:A
peptide level
175:175
110:110
40:40
TGESVEFVCKpc2_NP.5
p−maj=1, pX=0.88, N=325
var.1.CFH 1:196642233:G:A
peptide level
47:175
26:110
11:40
TKEEYGHSEVVEYYCNPRpc3_NP.4
p−min=0.7, pX=0.95, N=84
var.1.CFH 1:196642233:G:A
peptide level
63:175
36:110
12:40
TKEEYGHSEVVEYYCNPRpc3_NP.5
p−min=0.48, pX=0.52, N=111

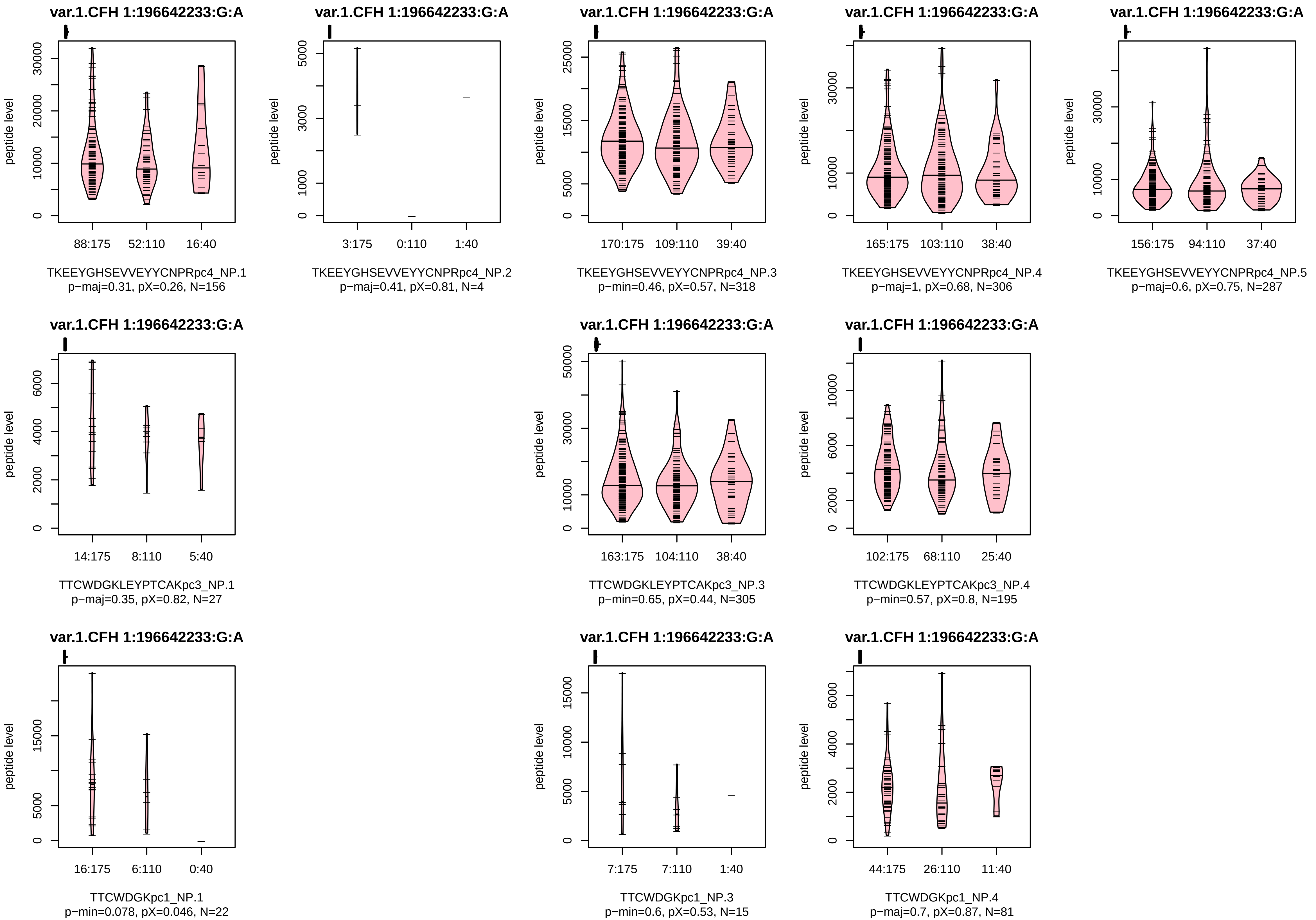
var.1.CFH 1:196642233:G:A
peptide level
88:175
52:110
16:40
TKEEYGHSEVVEYYCNPRpc4_NP.1
p−maj=0.31, pX=0.26, N=156
var.1.CFH 1:196642233:G:A
peptide level
3:175
0:110
1:40
TKEEYGHSEVVEYYCNPRpc4_NP.2
p−maj=0.41, pX=0.81, N=4
var.1.CFH 1:196642233:G:A
peptide level
170:175
109:110
39:40
TKEEYGHSEVVEYYCNPRpc4_NP.3
p−min=0.46, pX=0.57, N=318
var.1.CFH 1:196642233:G:A
peptide level
165:175
103:110
38:40
TKEEYGHSEVVEYYCNPRpc4_NP.4
p−maj=1, pX=0.68, N=306
var.1.CFH 1:196642233:G:A
peptide level
156:175
94:110
37:40
TKEEYGHSEVVEYYCNPRpc4_NP.5
p−maj=0.6, pX=0.75, N=287
var.1.CFH 1:196642233:G:A
peptide level
14:175
8:110
5:40
TTCWDGKLEYPTCAKpc3_NP.1
p−maj=0.35, pX=0.82, N=27
var.1.CFH 1:196642233:G:A
peptide level
163:175
104:110
38:40
TTCWDGKLEYPTCAKpc3_NP.3
p−min=0.65, pX=0.44, N=305
var.1.CFH 1:196642233:G:A
peptide level
102:175
68:110
25:40
TTCWDGKLEYPTCAKpc3_NP.4
p−min=0.57, pX=0.8, N=195
var.1.CFH 1:196642233:G:A
peptide level
16:175
6:110
0:40
TTCWDGKpc1_NP.1
p−min=0.078, pX=0.046, N=22
var.1.CFH 1:196642233:G:A
peptide level
7:175
7:110
1:40
TTCWDGKpc1_NP.3
p−min=0.6, pX=0.53, N=15
var.1.CFH 1:196642233:G:A
peptide level
44:175
26:110
11:40
TTCWDGKpc1_NP.4
p−maj=0.7, pX=0.87, N=81

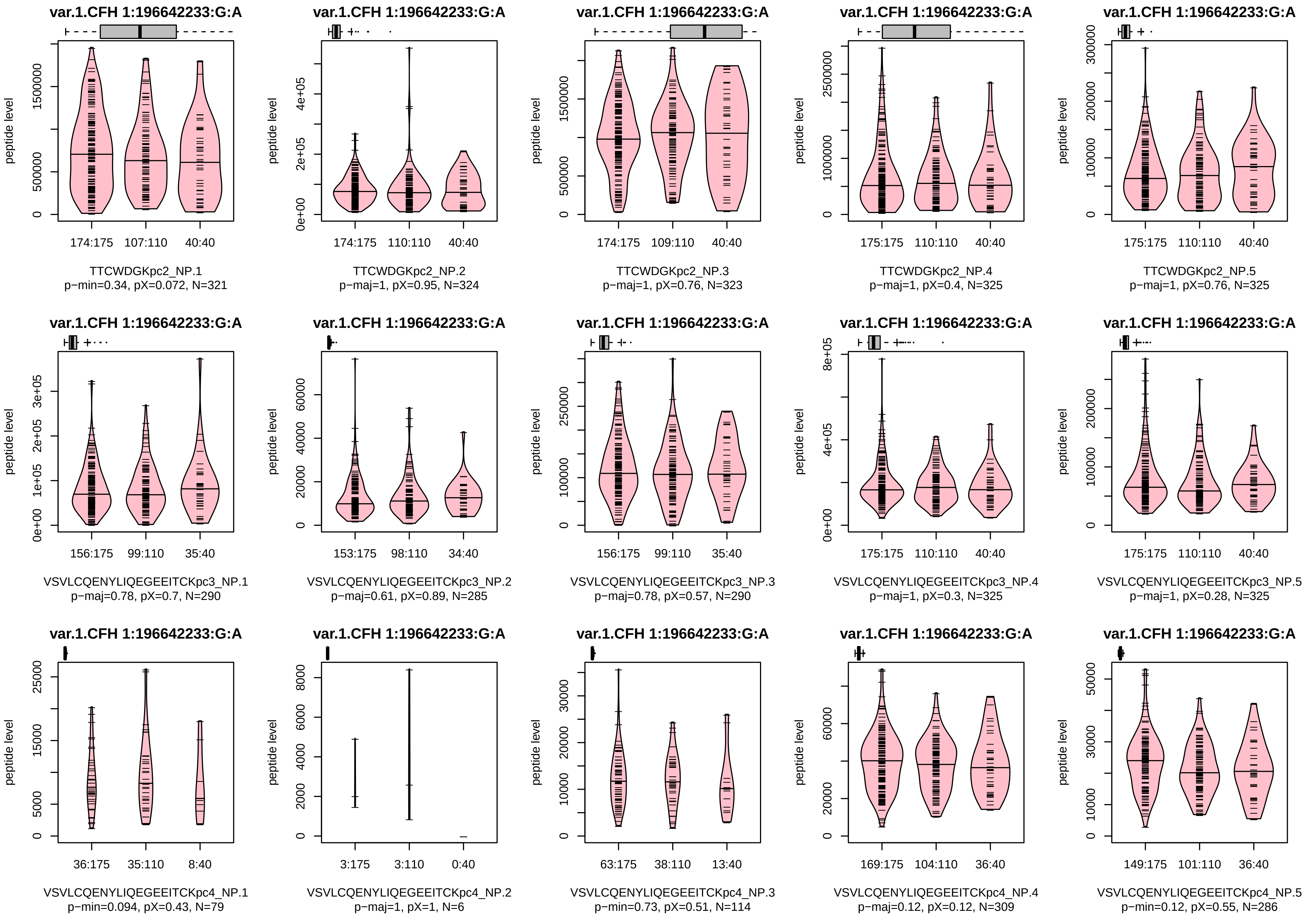
var.1.CFH 1:196642233:G:A
peptide level
174:175
107:110
40:40
TTCWDGKpc2_NP.1
p−min=0.34, pX=0.072, N=321
var.1.CFH 1:196642233:G:A
peptide level
174:175
110:110
40:40
TTCWDGKpc2_NP.2
p−maj=1, pX=0.95, N=324
var.1.CFH 1:196642233:G:A
peptide level
174:175
109:110
40:40
TTCWDGKpc2_NP.3
p−maj=1, pX=0.76, N=323
var.1.CFH 1:196642233:G:A
peptide level
175:175
110:110
40:40
TTCWDGKpc2_NP.4
p−maj=1, pX=0.4, N=325
var.1.CFH 1:196642233:G:A
peptide level
175:175
110:110
40:40
TTCWDGKpc2_NP.5
p−maj=1, pX=0.76, N=325
var.1.CFH 1:196642233:G:A
peptide level
156:175
99:110
35:40
VSVLCQENYLIQEGEEITCKpc3_NP.1
p−maj=0.78, pX=0.7, N=290
var.1.CFH 1:196642233:G:A
peptide level
153:175
98:110
34:40
VSVLCQENYLIQEGEEITCKpc3_NP.2
p−maj=0.61, pX=0.89, N=285
var.1.CFH 1:196642233:G:A
peptide level
156:175
99:110
35:40
VSVLCQENYLIQEGEEITCKpc3_NP.3
p−maj=0.78, pX=0.57, N=290
var.1.CFH 1:196642233:G:A
peptide level
175:175
110:110
40:40
VSVLCQENYLIQEGEEITCKpc3_NP.4
p−maj=1, pX=0.3, N=325
var.1.CFH 1:196642233:G:A
peptide level
175:175
110:110
40:40
VSVLCQENYLIQEGEEITCKpc3_NP.5
p−maj=1, pX=0.28, N=325
var.1.CFH 1:196642233:G:A
peptide level
36:175
35:110
8:40
VSVLCQENYLIQEGEEITCKpc4_NP.1
p−min=0.094, pX=0.43, N=79
var.1.CFH 1:196642233:G:A
peptide level
3:175
3:110
0:40
VSVLCQENYLIQEGEEITCKpc4_NP.2
p−maj=1, pX=1, N=6
var.1.CFH 1:196642233:G:A
peptide level
63:175
38:110
13:40
VSVLCQENYLIQEGEEITCKpc4_NP.3
p−min=0.73, pX=0.51, N=114
var.1.CFH 1:196642233:G:A
peptide level
169:175
104:110
36:40
VSVLCQENYLIQEGEEITCKpc4_NP.4
p−maj=0.12, pX=0.12, N=309
var.1.CFH 1:196642233:G:A
peptide level
149:175
101:110
36:40
VSVLCQENYLIQEGEEITCKpc4_NP.5
p−min=0.12, pX=0.55, N=286

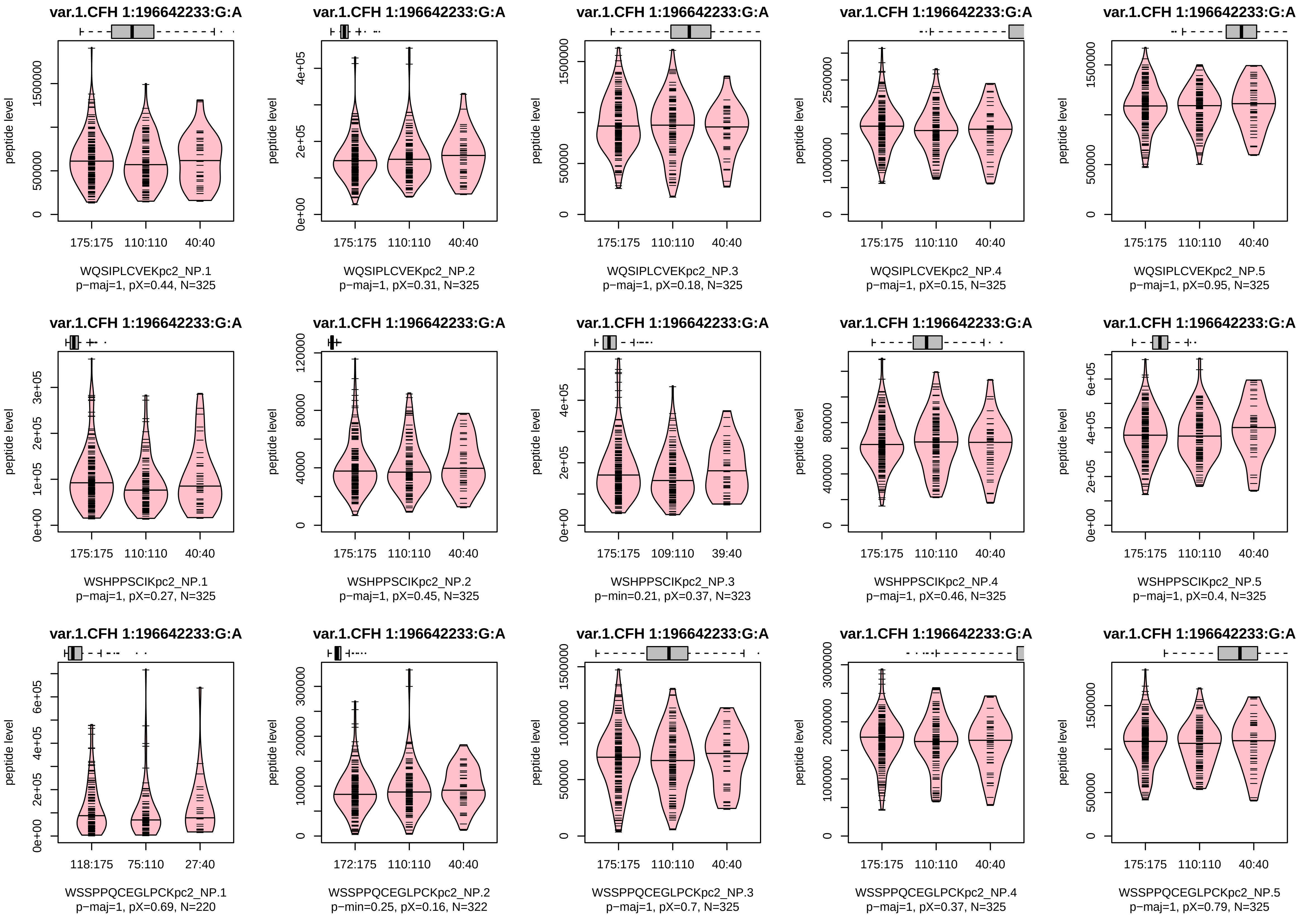
var.1.CFH 1:196642233:G:A
peptide level
175:175 110:110 40:40
WQSIPLCVEKpc2_NP.1
p−maj=1, pX=0.44, N=325
var.1.CFH 1:196642233:G:A
175:175 110:110 40:40
WQSIPLCVEKpc2_NP.2
p−maj=1, pX=0.31, N=325
var.1.CFH 1:196642233:G:A
175:175 110:110 40:40
WQSIPLCVEKpc2_NP.3
p−maj=1, pX=0.18, N=325
var.1.CFH 1:196642233:G:A
175:175 110:110 40:40
WQSIPLCVEKpc2_NP.4
p−maj=1, pX=0.15, N=325
var.1.CFH 1:196642233:G:A
175:175 110:110 40:40
WQSIPLCVEKpc2_NP.5
p−maj=1, pX=0.95, N=325
var.1.CFH 1:196642233:G:A
175:175 110:110 40:40
WSHPPSCIKpc2_NP.1
p−maj=1, pX=0.27, N=325
var.1.CFH 1:196642233:G:A
175:175 110:110 40:40
WSHPPSCIKpc2_NP.2
p−maj=1, pX=0.45, N=325
var.1.CFH 1:196642233:G:A
175:175 109:110 39:40
WSHPPSCIKpc2_NP.3
p−min=0.21, pX=0.37, N=323
var.1.CFH 1:196642233:G:A
175:175 110:110 40:40
WSHPPSCIKpc2_NP.4
p−maj=1, pX=0.46, N=325
var.1.CFH 1:196642233:G:A
175:175 110:110 40:40
WSHPPSCIKpc2_NP.5
p−maj=1, pX=0.4, N=325
var.1.CFH 1:196642233:G:A
118:175 75:110 27:40
WSSPPQCEGLPCKpc2_NP.1
p−maj=1, pX=0.69, N=220
var.1.CFH 1:196642233:G:A
172:175 110:110 40:40
WSSPPQCEGLPCKpc2_NP.2
p−min=0.25, pX=0.16, N=322
var.1.CFH 1:196642233:G:A
175:175 110:110 40:40
WSSPPQCEGLPCKpc2_NP.3
p−maj=1, pX=0.7, N=325
var.1.CFH 1:196642233:G:A
175:175 110:110 40:40
WSSPPQCEGLPCKpc2_NP.4
p−maj=1, pX=0.37, N=325
var.1.CFH 1:196642233:G:A
175:175 110:110 40:40
WSSPPQCEGLPCKpc2_NP.5
p−maj=1, pX=0.79, N=325

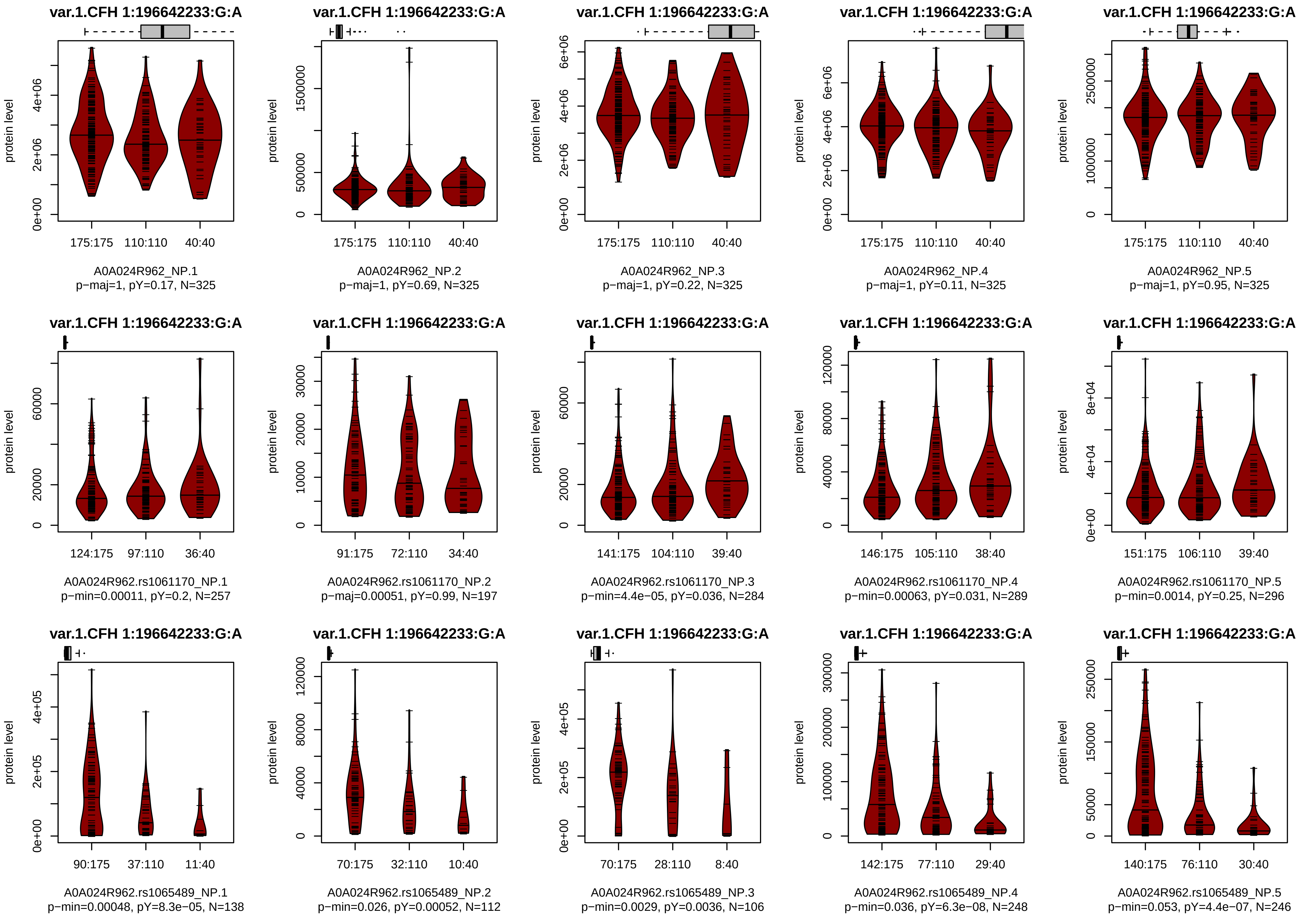
var.1.CFH 1:196642233:G:A
protein level
175:175
110:110
40:40
A0A024R962_NP.1
p−maj=1, pY=0.17, N=325
A0A024R962_NP.2
p−maj=1, pY=0.69, N=325
A0A024R962_NP.3
p−maj=1, pY=0.22, N=325
A0A024R962_NP.4
p−maj=1, pY=0.11, N=325
A0A024R962_NP.5
p−maj=1, pY=0.95, N=325
124:175
97:110
36:40
A0A024R962.rs1061170_NP.1
p−min=0.00011, pY=0.2, N=257
91:175
72:110
34:40
A0A024R962.rs1061170_NP.2
p−maj=0.00051, pY=0.99, N=197
141:175
104:110
39:40
A0A024R962.rs1061170_NP.3
p−min=4.4e−05, pY=0.036, N=284
146:175
105:110
38:40
A0A024R962.rs1061170_NP.4
p−min=0.00063, pY=0.031, N=289
151:175
106:110
39:40
A0A024R962.rs1061170_NP.5
p−min=0.0014, pY=0.25, N=296
90:175
37:110
11:40
A0A024R962.rs1065489_NP.1
p−min=0.00048, pY=8.3e−05, N=138
70:175
32:110
10:40
A0A024R962.rs1065489_NP.2
p−min=0.026, pY=0.00052, N=112
70:175
28:110
8:40
A0A024R962.rs1065489_NP.3
p−min=0.0029, pY=0.0036, N=106
142:175
77:110
29:40
A0A024R962.rs1065489_NP.4
p−min=0.036, pY=6.3e−08, N=248
140:175
76:110
30:40
A0A024R962.rs1065489_NP.5
p−min=0.053, pY=4.4e−07, N=246

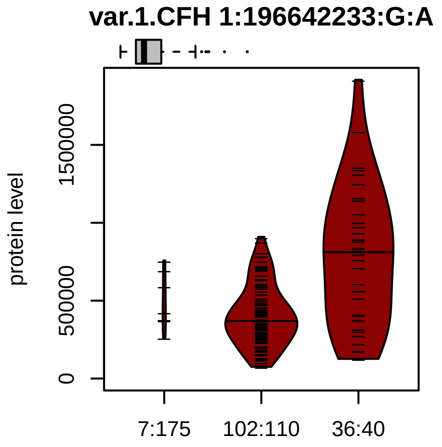

A0A024R962.rs800292_NP.1
p−min=3.3e−67, pY=3e−06, N=145

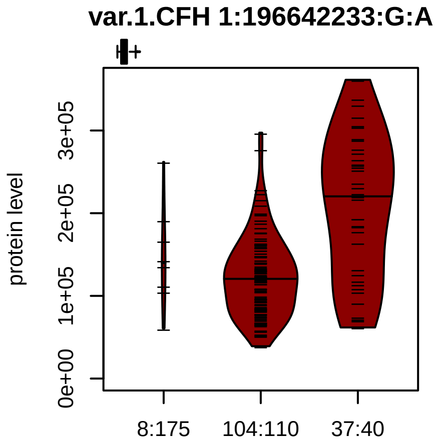

A0A024R962.rs800292_NP.2
p−min=1.6e−69, pY=3.3e−06, N=149

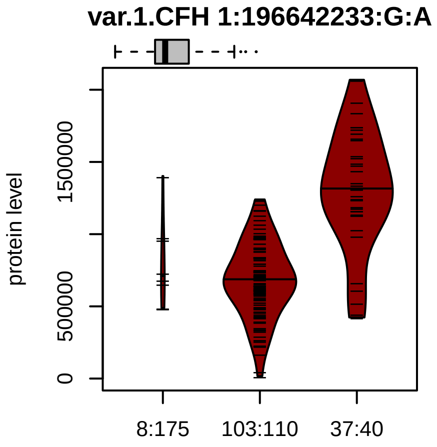

A0A024R962.rs800292_NP.3
p−min=2.6e−68, pY=3.9e−12, N=148

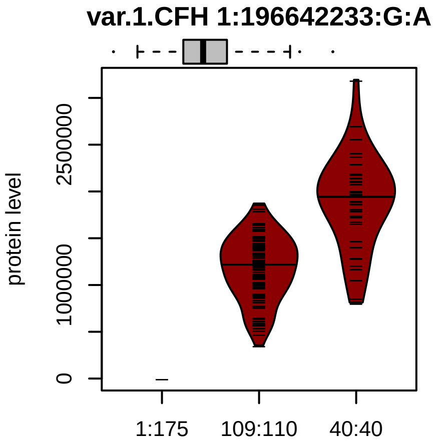

A0A024R962.rs800292_NP.4
p−min=2.3e−92, pY=1.5e−17, N=150

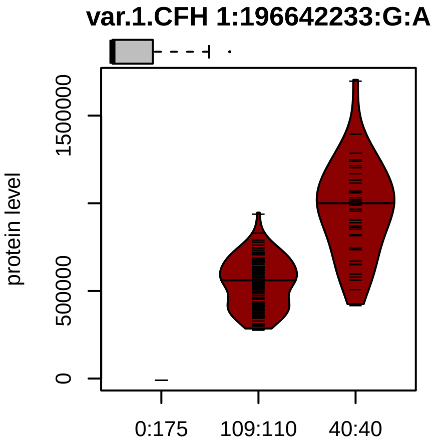

A0A024R962.rs800292_NP.5
p−min=1.5e−94, pY=5.5e−17, N=149

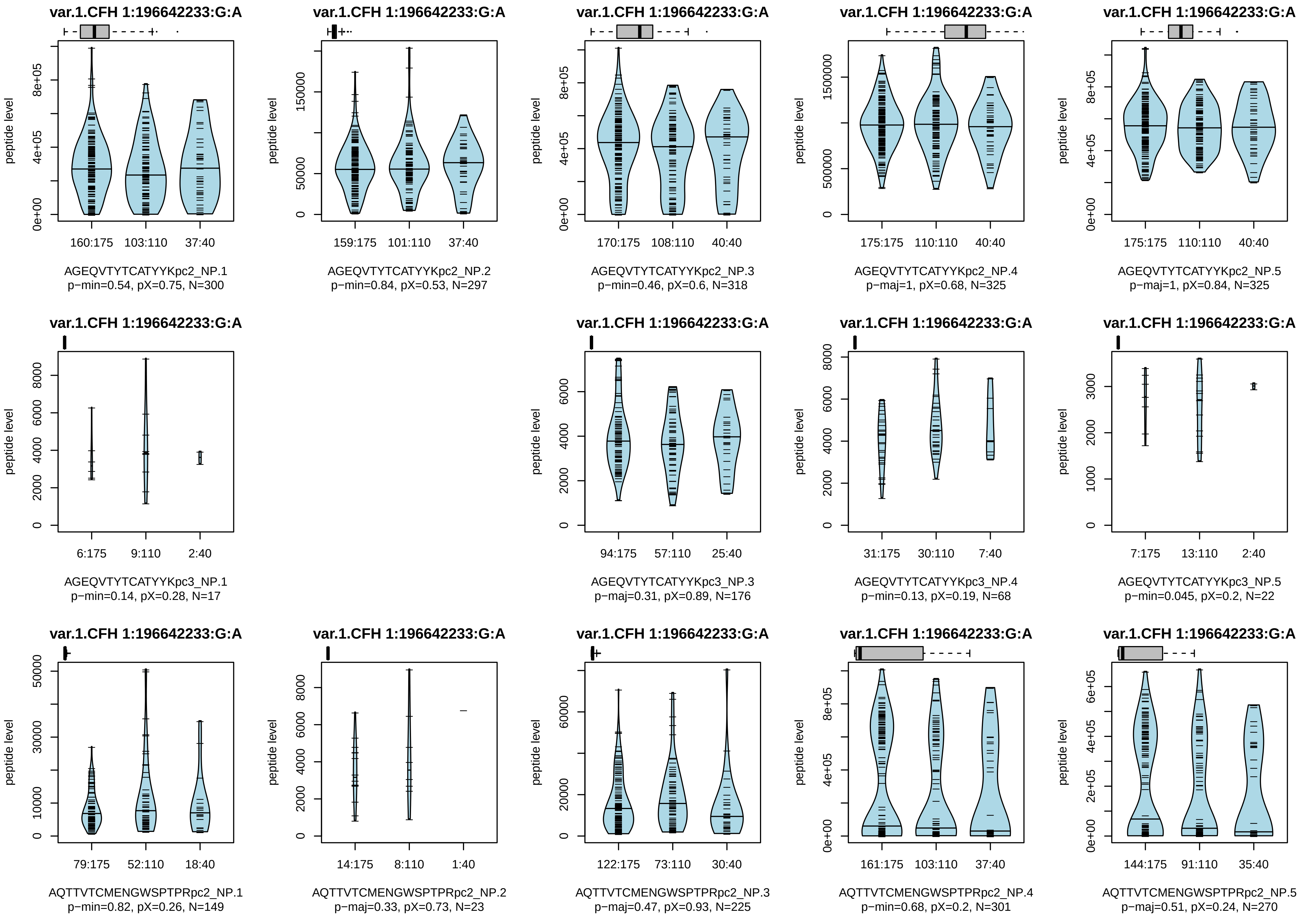
var.1.CFH 1:196642233:G:A
160:175
103:110
37:40
AGEQVTYTCATYYKpc2_NP.1
p−min=0.54, pX=0.75, N=300
var.1.CFH 1:196642233:G:A
159:175
101:110
37:40
AGEQVTYTCATYYKpc2_NP.2
p−min=0.84, pX=0.53, N=297
var.1.CFH 1:196642233:G:A
170:175
108:110
40:40
AGEQVTYTCATYYKpc2_NP.3
p−min=0.46, pX=0.6, N=318
var.1.CFH 1:196642233:G:A
175:175
110:110
40:40
AGEQVTYTCATYYKpc2_NP.4
p−maj=1, pX=0.68, N=325
var.1.CFH 1:196642233:G:A
175:175
110:110
40:40
AGEQVTYTCATYYKpc2_NP.5
p−maj=1, pX=0.84, N=325
var.1.CFH 1:196642233:G:A
6:175
9:110
2:40
AGEQVTYTCATYYKpc3_NP.1
p−min=0.14, pX=0.28, N=17
var.1.CFH 1:196642233:G:A
94:175
57:110
25:40
AGEQVTYTCATYYKpc3_NP.3
p−maj=0.31, pX=0.89, N=176
var.1.CFH 1:196642233:G:A
31:175
30:110
7:40
AGEQVTYTCATYYKpc3_NP.4
p−min=0.13, pX=0.19, N=68
var.1.CFH 1:196642233:G:A
7:175
13:110
2:40
AGEQVTYTCATYYKpc3_NP.5
p−min=0.045, pX=0.2, N=22
var.1.CFH 1:196642233:G:A
79:175
52:110
18:40
AQTTVTCMENGWSPTPRpc2_NP.1
p−min=0.82, pX=0.26, N=149
var.1.CFH 1:196642233:G:A
14:175
8:110
1:40
AQTTVTCMENGWSPTPRpc2_NP.2
p−maj=0.33, pX=0.73, N=23
var.1.CFH 1:196642233:G:A
122:175
73:110
30:40
AQTTVTCMENGWSPTPRpc2_NP.3
p−maj=0.47, pX=0.93, N=225
var.1.CFH 1:196642233:G:A
161:175
103:110
37:40
AQTTVTCMENGWSPTPRpc2_NP.4
p−min=0.68, pX=0.2, N=301
var.1.CFH 1:196642233:G:A
144:175
91:110
35:40
AQTTVTCMENGWSPTPRpc2_NP.5
p−maj=0.51, pX=0.24, N=270
peptide level

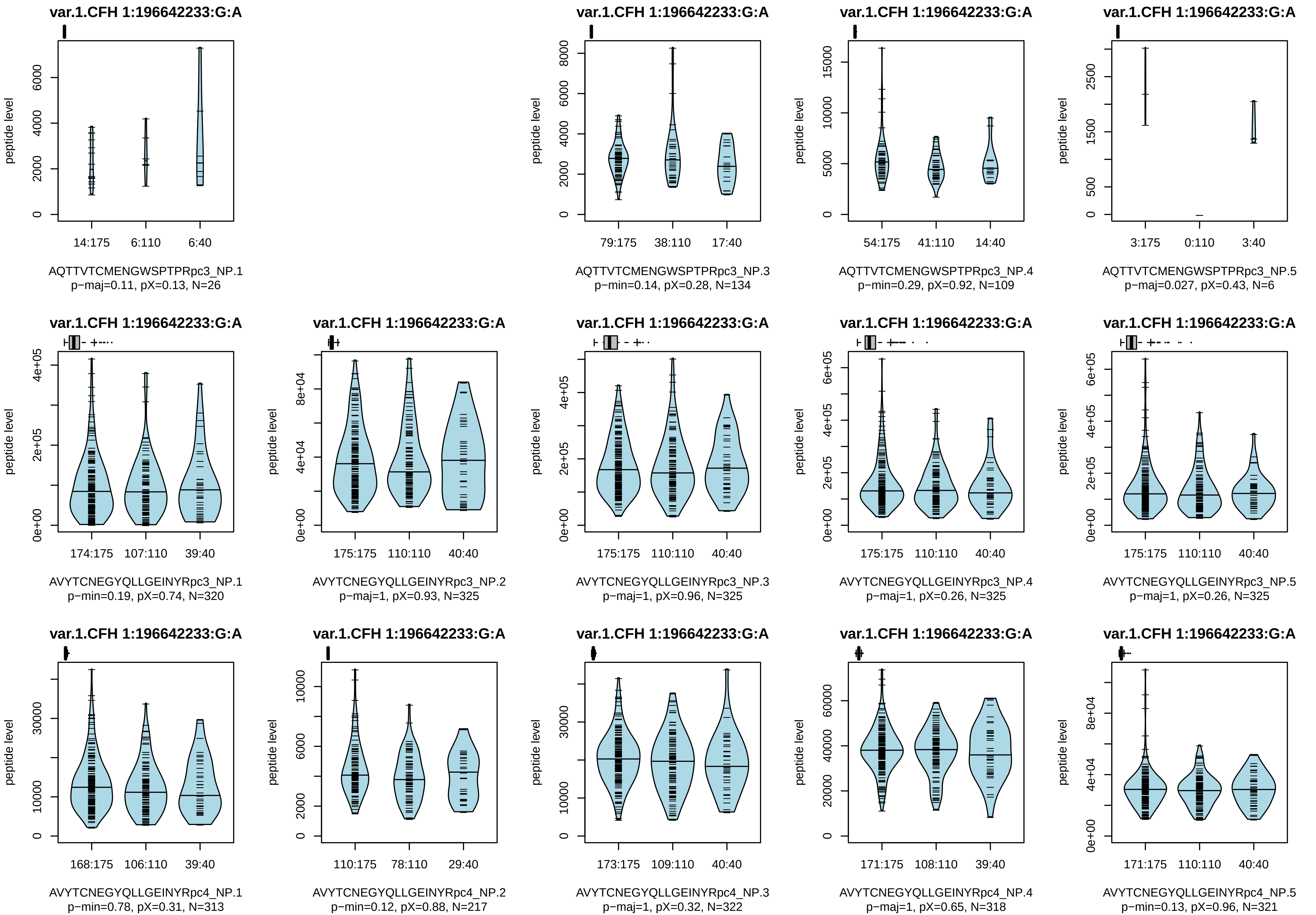

var.1.CFH 1:196642233:G:A
peptide level
14:175
6:110
6:40
AQTTVTCMENGWSPTPRpc3_NP.1
p−maj=0.11, pX=0.13, N=26
var.1.CFH 1:196642233:G:A
peptide level
79:175
38:110
17:40
AQTTVTCMENGWSPTPRpc3_NP.3
p−min=0.14, pX=0.28, N=134
var.1.CFH 1:196642233:G:A
peptide level
54:175
41:110
14:40
AQTTVTCMENGWSPTPRpc3_NP.4
p−min=0.29, pX=0.92, N=109
var.1.CFH 1:196642233:G:A
peptide level
3:175
0:110
3:40
AQTTVTCMENGWSPTPRpc3_NP.5
p−maj=0.027, pX=0.43, N=6
var.1.CFH 1:196642233:G:A
peptide level
174:175
107:110
39:40
AVYTCNEGYQLLGEINYRpc3_NP.1
p−min=0.19, pX=0.74, N=320
var.1.CFH 1:196642233:G:A
peptide level
175:175
110:110
40:40
AVYTCNEGYQLLGEINYRpc3_NP.2
p−maj=1, pX=0.93, N=325
var.1.CFH 1:196642233:G:A
peptide level
175:175
110:110
40:40
AVYTCNEGYQLLGEINYRpc3_NP.3
p−maj=1, pX=0.96, N=325
var.1.CFH 1:196642233:G:A
peptide level
175:175
110:110
40:40
AVYTCNEGYQLLGEINYRpc3_NP.4
p−maj=1, pX=0.26, N=325
var.1.CFH 1:196642233:G:A
peptide level
175:175
110:110
40:40
AVYTCNEGYQLLGEINYRpc3_NP.5
p−maj=1, pX=0.26, N=325
var.1.CFH 1:196642233:G:A
peptide level
168:175
106:110
39:40
AVYTCNEGYQLLGEINYRpc4_NP.1
p−min=0.78, pX=0.31, N=313
var.1.CFH 1:196642233:G:A
peptide level
110:175
78:110
29:40
AVYTCNEGYQLLGEINYRpc4_NP.2
p−min=0.12, pX=0.88, N=217
var.1.CFH 1:196642233:G:A
peptide level
173:175
109:110
40:40
AVYTCNEGYQLLGEINYRpc4_NP.3
p−maj=1, pX=0.32, N=322
var.1.CFH 1:196642233:G:A
peptide level
171:175
108:110
39:40
AVYTCNEGYQLLGEINYRpc4_NP.4
p−maj=1, pX=0.65, N=318
var.1.CFH 1:196642233:G:A
peptide level
171:175
110:110
40:40
AVYTCNEGYQLLGEINYRpc4_NP.5
p−min=0.13, pX=0.96, N=321

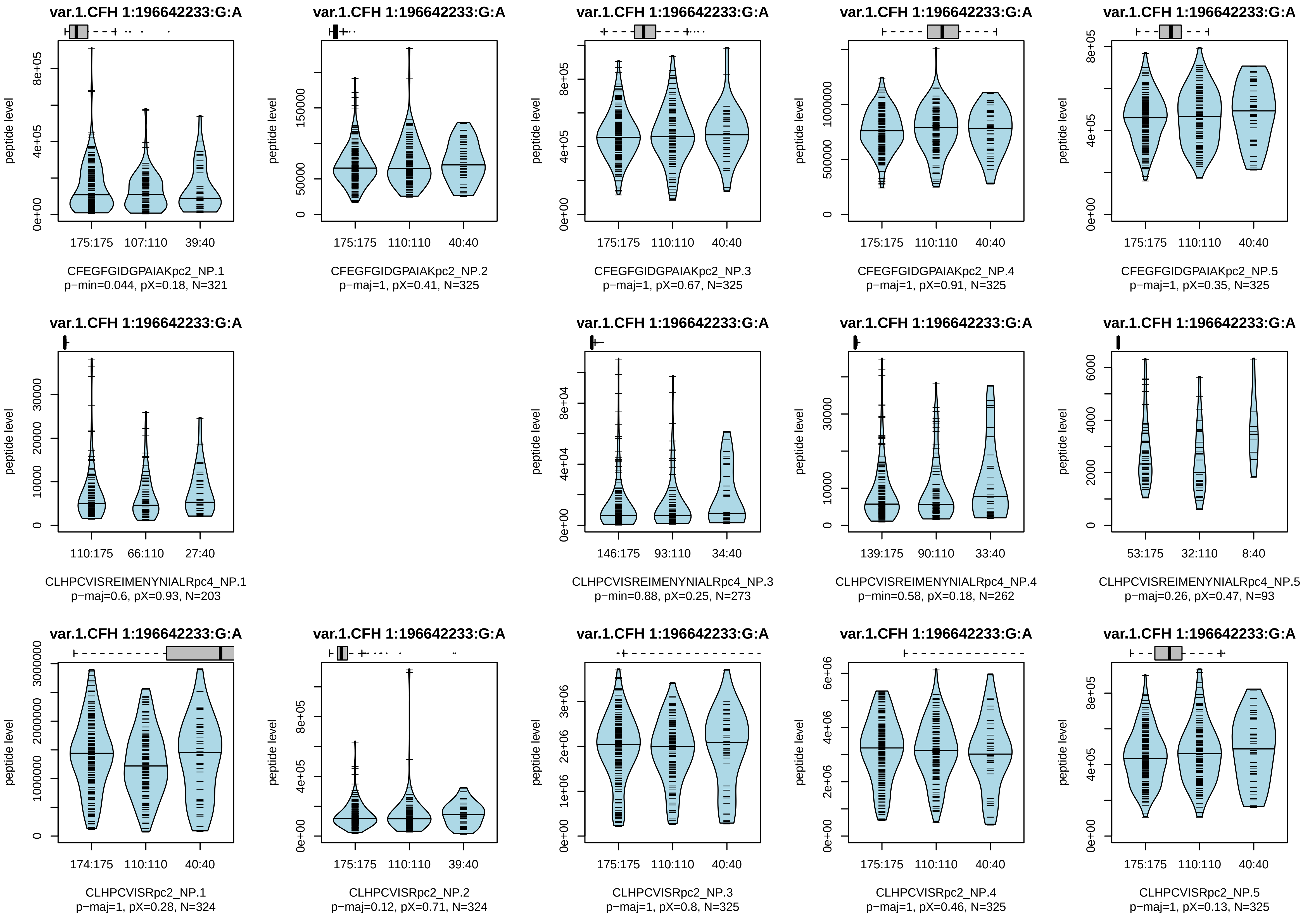
var.1.CFH 1:196642233:G:A
peptide level
175:175
107:110
39:40
CFEGFGIDGPAIAKpc2_NP.1
p−min=0.044, pX=0.18, N=321
110:110
40:40
CFEGFGIDGPAIAKpc2_NP.2
p−maj=1, pX=0.41, N=325
CFEGFGIDGPAIAKpc2_NP.3
p−maj=1, pX=0.67, N=325
CFEGFGIDGPAIAKpc2_NP.4
p−maj=1, pX=0.91, N=325
CFEGFGIDGPAIAKpc2_NP.5
p−maj=1, pX=0.35, N=325
110:175
66:110
27:40
CLHPCVISREIMENYNIALRpc4_NP.1
p−maj=0.6, pX=0.93, N=203
146:175
93:110
34:40
CLHPCVISREIMENYNIALRpc4_NP.3
p−min=0.88, pX=0.25, N=273
139:175
90:110
33:40
CLHPCVISREIMENYNIALRpc4_NP.4
p−min=0.58, pX=0.18, N=262
53:175
32:110
8:40
CLHPCVISREIMENYNIALRpc4_NP.5
p−maj=0.26, pX=0.47, N=93
174:175
CLHPCVISRpc2_NP.1
p−maj=1, pX=0.28, N=324
39:40
CLHPCVISRpc2_NP.2
p−maj=0.12, pX=0.71, N=324
CLHPCVISRpc2_NP.3
p−maj=1, pX=0.8, N=325
CLHPCVISRpc2_NP.4
p−maj=1, pX=0.46, N=325
CLHPCVISRpc2_NP.5
p−maj=1, pX=0.13, N=325

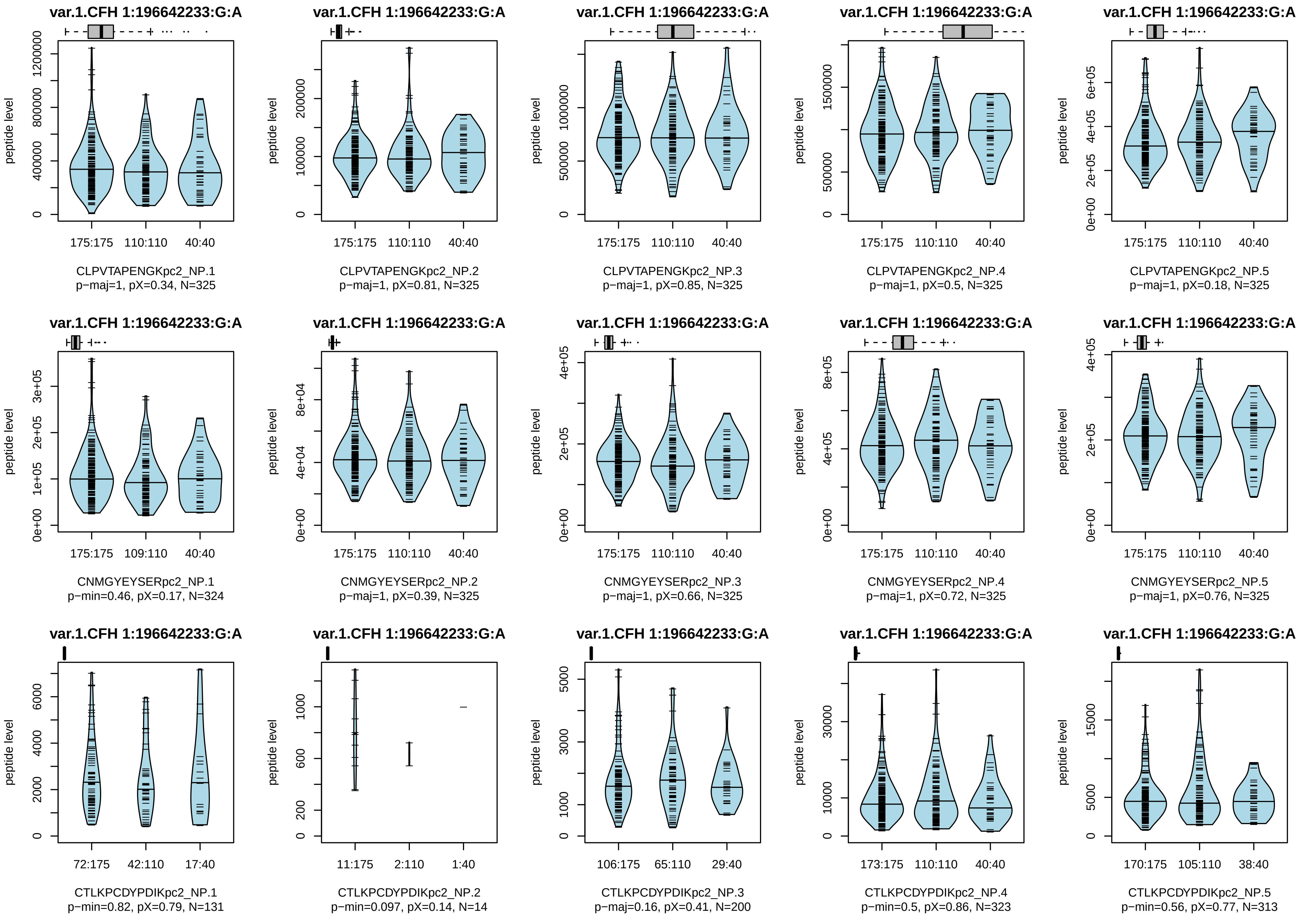

var.1.CFH 1:196642233:G:A
peptide level
175:175 110:110 40:40
CLPVTAPENGKpc2_NP.1
p−maj=1, pX=0.34, N=325
var.1.CFH 1:196642233:G:A
175:175 110:110 40:40
CLPVTAPENGKpc2_NP.2
p−maj=1, pX=0.81, N=325
var.1.CFH 1:196642233:G:A
175:175 110:110 40:40
CLPVTAPENGKpc2_NP.3
p−maj=1, pX=0.85, N=325
var.1.CFH 1:196642233:G:A
175:175 110:110 40:40
CLPVTAPENGKpc2_NP.4
p−maj=1, pX=0.5, N=325
var.1.CFH 1:196642233:G:A
175:175 110:110 40:40
CLPVTAPENGKpc2_NP.5
p−maj=1, pX=0.18, N=325
var.1.CFH 1:196642233:G:A
175:175 109:110 40:40
CNMGYEYSERpc2_NP.1
p−min=0.46, pX=0.17, N=324
var.1.CFH 1:196642233:G:A
175:175 110:110 40:40
CNMGYEYSERpc2_NP.2
p−maj=1, pX=0.39, N=325
var.1.CFH 1:196642233:G:A
175:175 110:110 40:40
CNMGYEYSERpc2_NP.3
p−maj=1, pX=0.66, N=325
var.1.CFH 1:196642233:G:A
175:175 110:110 40:40
CNMGYEYSERpc2_NP.4
p−maj=1, pX=0.72, N=325
var.1.CFH 1:196642233:G:A
175:175 110:110 40:40
CNMGYEYSERpc2_NP.5
p−maj=1, pX=0.76, N=325
var.1.CFH 1:196642233:G:A
72:175 42:110 17:40
CTLKPCDYPDIKpc2_NP.1
p−min=0.82, pX=0.79, N=131
var.1.CFH 1:196642233:G:A
11:175 2:110 1:40
CTLKPCDYPDIKpc2_NP.2
p−min=0.097, pX=0.14, N=14
var.1.CFH 1:196642233:G:A
106:175 65:110 29:40
CTLKPCDYPDIKpc2_NP.3
p−maj=0.16, pX=0.41, N=200
var.1.CFH 1:196642233:G:A
173:175 110:110 40:40
CTLKPCDYPDIKpc2_NP.4
p−min=0.5, pX=0.86, N=323
var.1.CFH 1:196642233:G:A
170:175 105:110 38:40
CTLKPCDYPDIKpc2_NP.5
p−min=0.56, pX=0.77, N=313

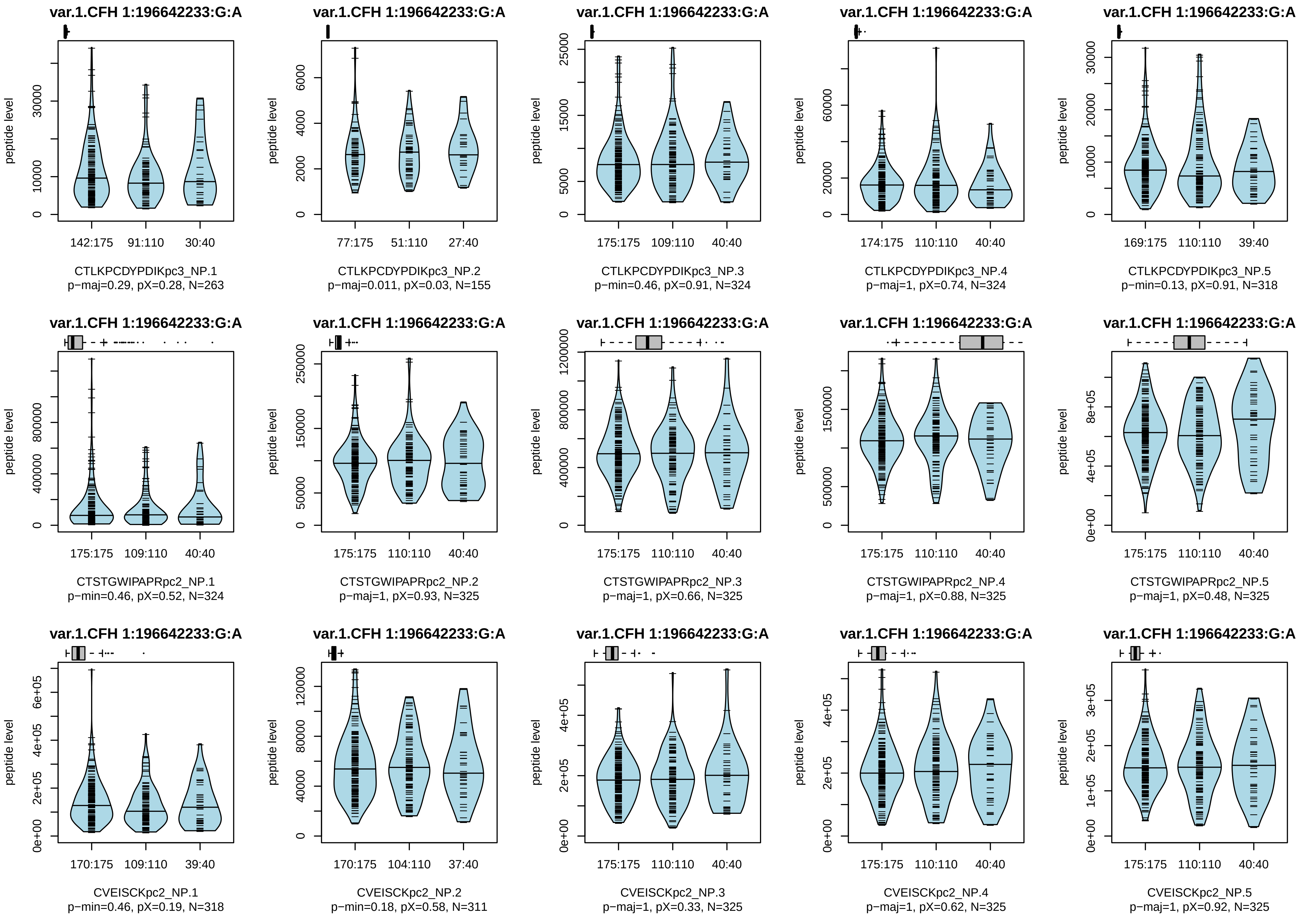
var.1.CFH 1:196642233:G:A
peptide level
142:175
91:110
30:40
CTLKPCDYPDIKpc3_NP.1
p−maj=0.29, pX=0.28, N=263
77:175
51:110
27:40
CTLKPCDYPDIKpc3_NP.2
p−maj=0.011, pX=0.03, N=155
175:175
109:110
40:40
CTLKPCDYPDIKpc3_NP.3
p−min=0.46, pX=0.91, N=324
174:175
110:110
40:40
CTLKPCDYPDIKpc3_NP.4
p−maj=1, pX=0.74, N=324
169:175
110:110
39:40
CTLKPCDYPDIKpc3_NP.5
p−min=0.13, pX=0.91, N=318
175:175
109:110
40:40
CTSTGWIPAPRpc2_NP.1
p−min=0.46, pX=0.52, N=324
175:175
110:110
40:40
CTSTGWIPAPRpc2_NP.2
p−maj=1, pX=0.93, N=325
CTSTGWIPAPRpc2_NP.3
p−maj=1, pX=0.66, N=325
CTSTGWIPAPRpc2_NP.4
p−maj=1, pX=0.88, N=325
CTSTGWIPAPRpc2_NP.5
p−maj=1, pX=0.48, N=325
170:175
109:110
39:40
CVEISCKpc2_NP.1
p−min=0.46, pX=0.19, N=318
170:175
104:110
37:40
CVEISCKpc2_NP.2
p−min=0.18, pX=0.58, N=311
CVEISCKpc2_NP.3
p−maj=1, pX=0.33, N=325
CVEISCKpc2_NP.4
p−maj=1, pX=0.62, N=325
CVEISCKpc2_NP.5
p−maj=1, pX=0.92, N=325

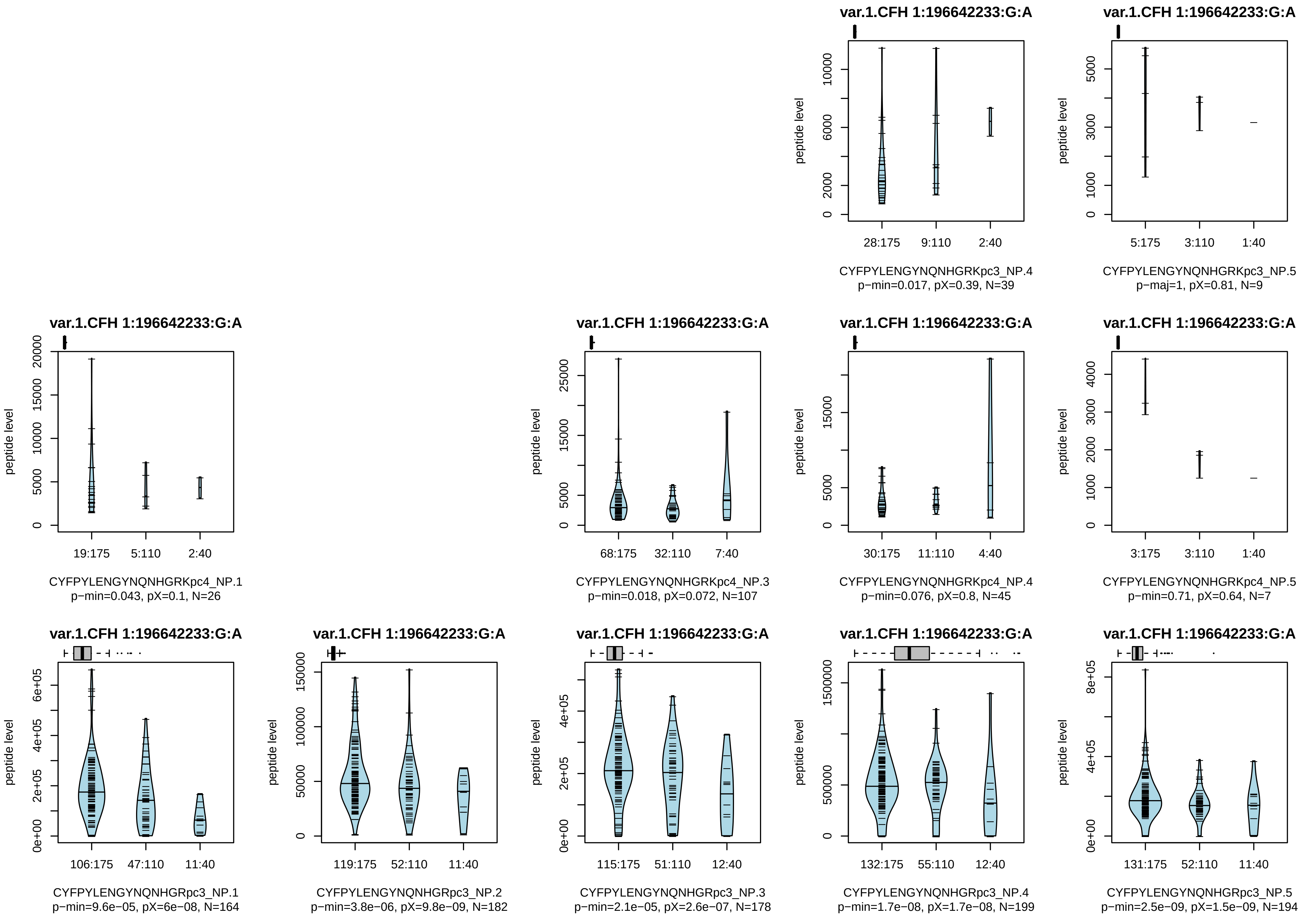
var.1.CFH 1:196642233:G:A
peptide level
28:175
9:110
2:40
CYFPYLENGYNQNHGRKpc3_NP.4
p−min=0.017, pX=0.39, N=39
var.1.CFH 1:196642233:G:A
peptide level
5:175
3:110
1:40
CYFPYLENGYNQNHGRKpc3_NP.5
p−maj=1, pX=0.81, N=9
var.1.CFH 1:196642233:G:A
peptide level
19:175
5:110
2:40
CYFPYLENGYNQNHGRKpc4_NP.1
p−min=0.043, pX=0.1, N=26
var.1.CFH 1:196642233:G:A
peptide level
68:175
32:110
7:40
CYFPYLENGYNQNHGRKpc4_NP.3
p−min=0.018, pX=0.072, N=107
var.1.CFH 1:196642233:G:A
peptide level
30:175
11:110
4:40
CYFPYLENGYNQNHGRKpc4_NP.4
p−min=0.076, pX=0.8, N=45
var.1.CFH 1:196642233:G:A
peptide level
3:175
3:110
1:40
CYFPYLENGYNQNHGRKpc4_NP.5
p−min=0.71, pX=0.64, N=7
var.1.CFH 1:196642233:G:A
peptide level
106:175
47:110
11:40
CYFPYLENGYNQNHGRpc3_NP.1
p−min=9.6e−05, pX=6e−08, N=164
var.1.CFH 1:196642233:G:A
peptide level
119:175
52:110
11:40
CYFPYLENGYNQNHGRpc3_NP.2
p−min=3.8e−06, pX=9.8e−09, N=182
var.1.CFH 1:196642233:G:A
peptide level
115:175
51:110
12:40
CYFPYLENGYNQNHGRpc3_NP.3
p−min=2.1e−05, pX=2.6e−07, N=178
var.1.CFH 1:196642233:G:A
peptide level
132:175
55:110
12:40
CYFPYLENGYNQNHGRpc3_NP.4
p−min=1.7e−08, pX=1.7e−08, N=199
var.1.CFH 1:196642233:G:A
peptide level
131:175
52:110
11:40
CYFPYLENGYNQNHGRpc3_NP.5
p−min=2.5e−09, pX=1.5e−09, N=194

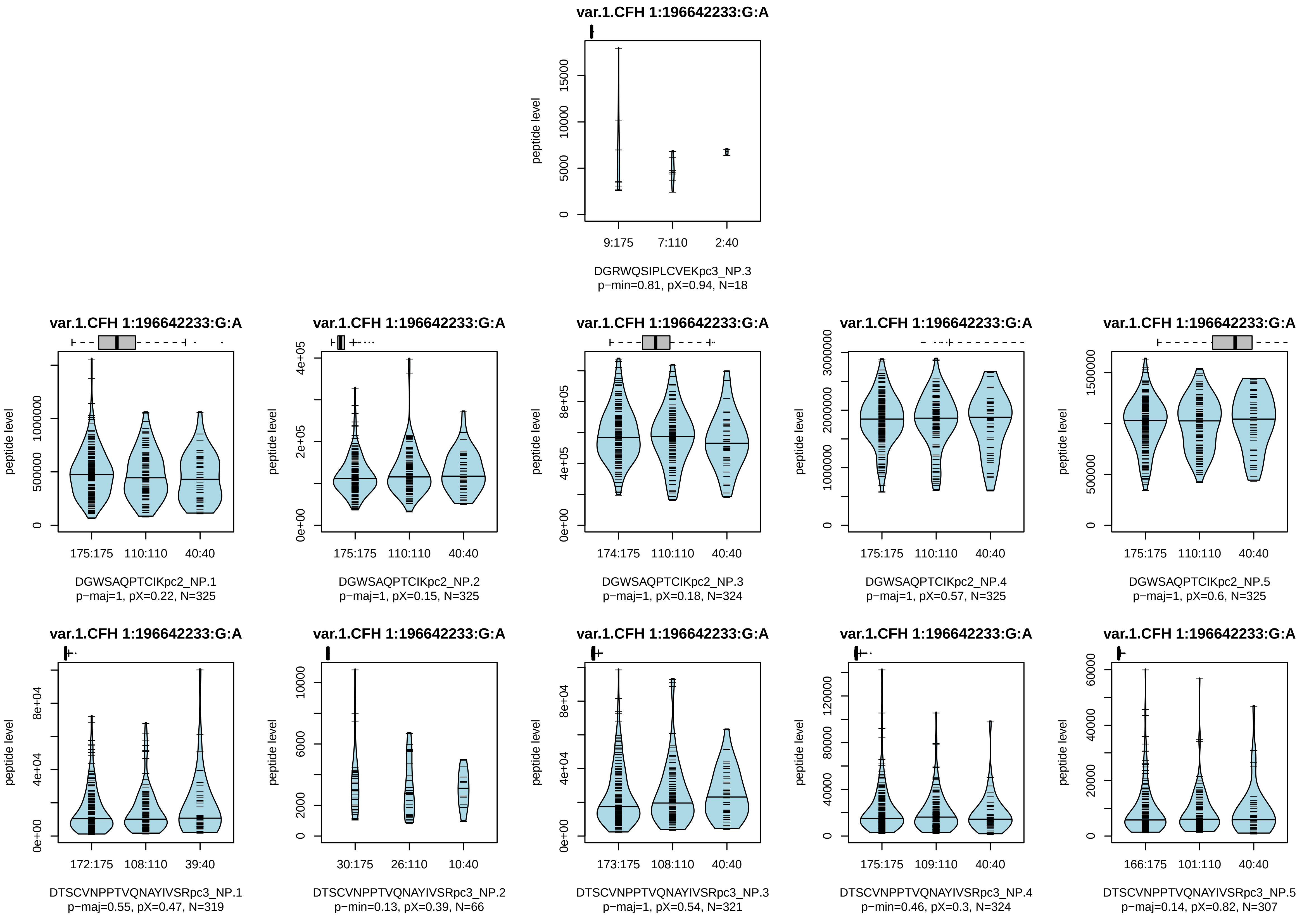
var.1.CFH 1:196642233:G:A
peptide level
0
5000
10000
15000
9:175
7:110
2:40
DGRWQSIPLCVEKpc3_NP.3
p−min=0.81, pX=0.94, N=18
var.1.CFH 1:196642233:G:A
peptide level
0
500000
1000000
175:175
110:110
40:40
DGWSAQPTCIKpc2_NP.1
p−maj=1, pX=0.22, N=325
var.1.CFH 1:196642233:G:A
peptide level
0e+00
2e+05
4e+05
175:175
110:110
40:40
DGWSAQPTCIKpc2_NP.2
p−maj=1, pX=0.15, N=325
var.1.CFH 1:196642233:G:A
peptide level
0e+00
4e+05
8e+05
174:175
110:110
40:40
DGWSAQPTCIKpc2_NP.3
p−maj=1, pX=0.18, N=324
var.1.CFH 1:196642233:G:A
peptide level
0
1000000
2000000
3000000
175:175
110:110
40:40
DGWSAQPTCIKpc2_NP.4
p−maj=1, pX=0.57, N=325
var.1.CFH 1:196642233:G:A
peptide level
0
500000
1500000
175:175
110:110
40:40
DGWSAQPTCIKpc2_NP.5
p−maj=1, pX=0.6, N=325
var.1.CFH 1:196642233:G:A
peptide level
0e+00
4e+04
8e+04
172:175
108:110
39:40
DTSCVNPPTVQNAYIVSRpc3_NP.1
p−maj=0.55, pX=0.47, N=319
var.1.CFH 1:196642233:G:A
peptide level
0
2000
6000
10000
30:175
26:110
10:40
DTSCVNPPTVQNAYIVSRpc3_NP.2
p−min=0.13, pX=0.39, N=66
var.1.CFH 1:196642233:G:A
peptide level
0e+00
4e+04
8e+04
173:175
108:110
40:40
DTSCVNPPTVQNAYIVSRpc3_NP.3
p−maj=1, pX=0.54, N=321
var.1.CFH 1:196642233:G:A
peptide level
0
40000
80000
120000
175:175
109:110
40:40
DTSCVNPPTVQNAYIVSRpc3_NP.4
p−min=0.46, pX=0.3, N=324
var.1.CFH 1:196642233:G:A
peptide level
0
20000
40000
60000
166:175
101:110
40:40
DTSCVNPPTVQNAYIVSRpc3_NP.5
p−maj=0.14, pX=0.82, N=307

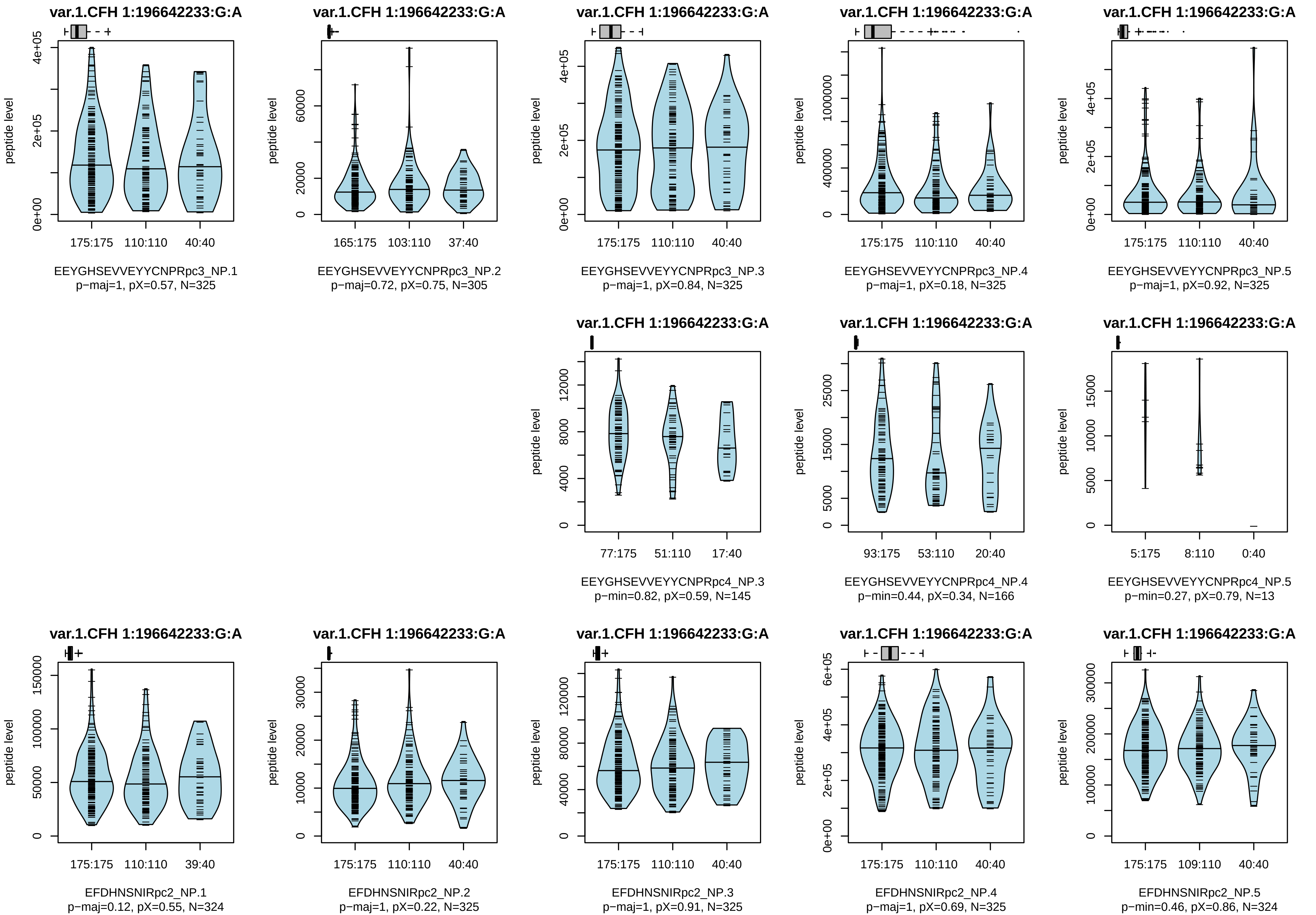
var.1.CFH 1:196642233:G:A
peptide level
0e+00
2e+05
4e+05
175:175
110:110
40:40
EEYGHSEVVEYYCNPRpc3_NP.1
p−maj=1, pX=0.57, N=325
var.1.CFH 1:196642233:G:A
peptide level
0
20000
60000
165:175
103:110
37:40
EEYGHSEVVEYYCNPRpc3_NP.2
p−maj=0.72, pX=0.75, N=305
var.1.CFH 1:196642233:G:A
peptide level
0e+00
2e+05
4e+05
175:175
110:110
40:40
EEYGHSEVVEYYCNPRpc3_NP.3
p−maj=1, pX=0.84, N=325
var.1.CFH 1:196642233:G:A
peptide level
0
400000
1000000
175:175
110:110
40:40
EEYGHSEVVEYYCNPRpc3_NP.4
p−maj=1, pX=0.18, N=325
var.1.CFH 1:196642233:G:A
peptide level
0e+00
2e+05
4e+05
175:175
110:110
40:40
EEYGHSEVVEYYCNPRpc3_NP.5
p−maj=1, pX=0.92, N=325
var.1.CFH 1:196642233:G:A
peptide level
0
4000
8000
12000
77:175
51:110
17:40
EEYGHSEVVEYYCNPRpc4_NP.3
p−min=0.82, pX=0.59, N=145
var.1.CFH 1:196642233:G:A
peptide level
0
5000
15000
25000
93:175
53:110
20:40
EEYGHSEVVEYYCNPRpc4_NP.4
p−min=0.44, pX=0.34, N=166
var.1.CFH 1:196642233:G:A
peptide level
0
5000
10000
15000
5:175
8:110
0:40
EEYGHSEVVEYYCNPRpc4_NP.5
p−min=0.27, pX=0.79, N=13
var.1.CFH 1:196642233:G:A
peptide level
0
50000
100000
150000
175:175
110:110
39:40
EFDHNSNIRpc2_NP.1
p−maj=0.12, pX=0.55, N=324
var.1.CFH 1:196642233:G:A
peptide level
0
10000
20000
30000
175:175
110:110
40:40
EFDHNSNIRpc2_NP.2
p−maj=1, pX=0.22, N=325
var.1.CFH 1:196642233:G:A
peptide level
0
40000
80000
120000
175:175
110:110
40:40
EFDHNSNIRpc2_NP.3
p−maj=1, pX=0.91, N=325
var.1.CFH 1:196642233:G:A
peptide level
0e+00
2e+05
4e+05
6e+05
175:175
110:110
40:40
EFDHNSNIRpc2_NP.4
p−maj=1, pX=0.69, N=325
var.1.CFH 1:196642233:G:A
peptide level
0
100000
200000
300000
175:175
109:110
40:40
EFDHNSNIRpc2_NP.5
p−min=0.46, pX=0.86, N=324

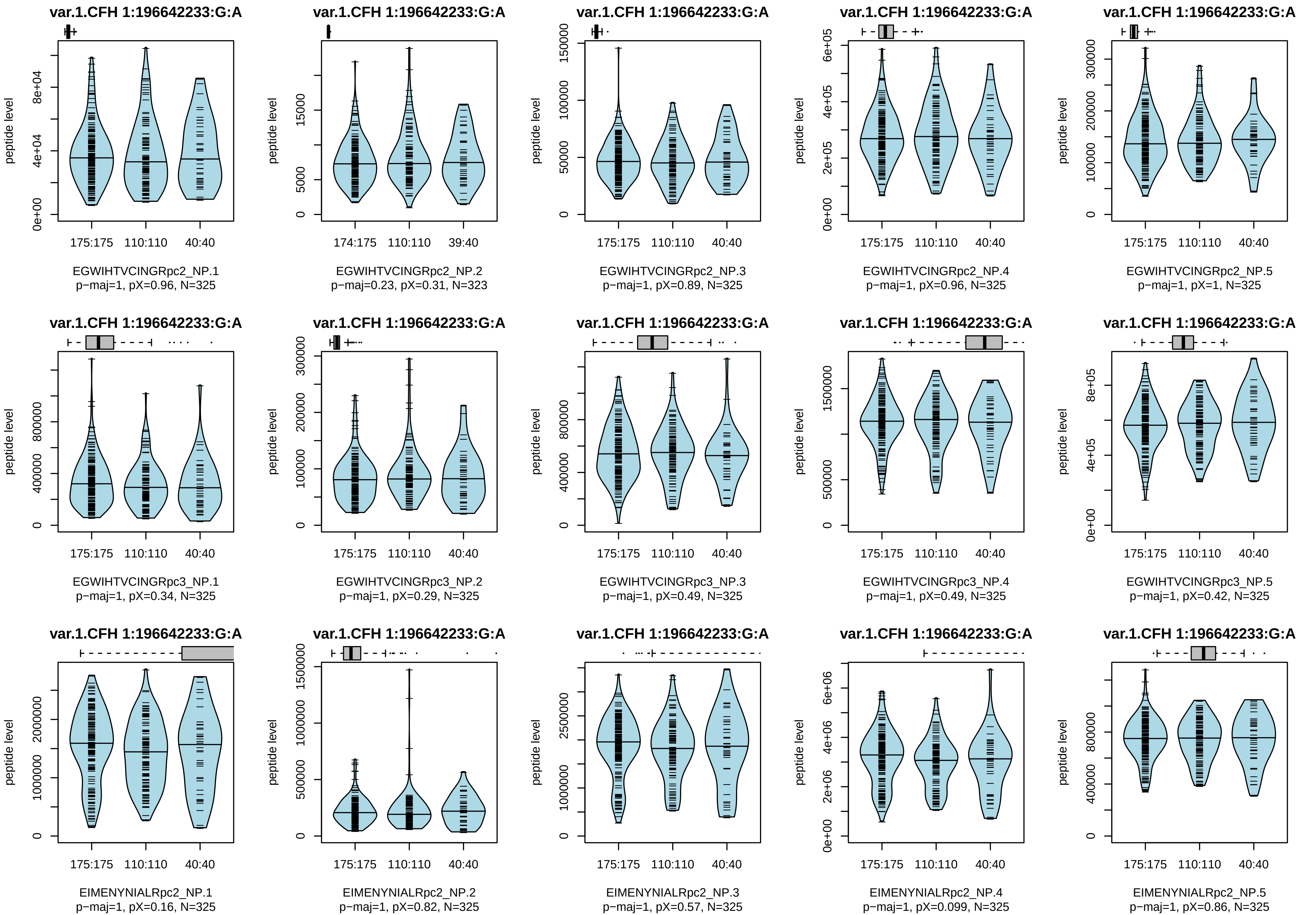

var.1.CFH 1:196642233:G:A
peptide level
0e+00
4e+04
8e+04
175:175
110:110
40:40
EGWIHTVCINGRpc2_NP.1
p−maj=1, pX=0.96, N=325
var.1.CFH 1:196642233:G:A
peptide level
0
5000
15000
174:175
110:110
39:40
EGWIHTVCINGRpc2_NP.2
p−maj=0.23, pX=0.31, N=323
var.1.CFH 1:196642233:G:A
peptide level
0
50000
100000
150000
175:175
110:110
40:40
EGWIHTVCINGRpc2_NP.3
p−maj=1, pX=0.89, N=325
var.1.CFH 1:196642233:G:A
peptide level
0e+00
2e+05
4e+05
6e+05
175:175
110:110
40:40
EGWIHTVCINGRpc2_NP.4
p−maj=1, pX=0.96, N=325
var.1.CFH 1:196642233:G:A
peptide level
0
100000
200000
300000
175:175
110:110
40:40
EGWIHTVCINGRpc2_NP.5
p−maj=1, pX=1, N=325
var.1.CFH 1:196642233:G:A
peptide level
0
400000
800000
175:175
110:110
40:40
EGWIHTVCINGRpc3_NP.1
p−maj=1, pX=0.34, N=325
var.1.CFH 1:196642233:G:A
peptide level
0
100000
200000
300000
175:175
110:110
40:40
EGWIHTVCINGRpc3_NP.2
p−maj=1, pX=0.29, N=325
var.1.CFH 1:196642233:G:A
peptide level
0
400000
800000
175:175
110:110
40:40
EGWIHTVCINGRpc3_NP.3
p−maj=1, pX=0.49, N=325
var.1.CFH 1:196642233:G:A
peptide level
0
500000
1500000
175:175
110:110
40:40
EGWIHTVCINGRpc3_NP.4
p−maj=1, pX=0.49, N=325
var.1.CFH 1:196642233:G:A
peptide level
0e+00
4e+05
8e+05
175:175
110:110
40:40
EGWIHTVCINGRpc3_NP.5
p−maj=1, pX=0.42, N=325
var.1.CFH 1:196642233:G:A
peptide level
0
1000000
2000000
175:175
110:110
40:40
EIMENYNIALRpc2_NP.1
p−maj=1, pX=0.16, N=325
var.1.CFH 1:196642233:G:A
peptide level
0
500000
1000000
1500000
175:175
110:110
40:40
EIMENYNIALRpc2_NP.2
p−maj=1, pX=0.82, N=325
var.1.CFH 1:196642233:G:A
peptide level
0
1000000
2500000
175:175
110:110
40:40
EIMENYNIALRpc2_NP.3
p−maj=1, pX=0.57, N=325
var.1.CFH 1:196642233:G:A
peptide level
0e+00
2e+06
4e+06
6e+06
175:175
110:110
40:40
EIMENYNIALRpc2_NP.4
p−maj=1, pX=0.099, N=325
var.1.CFH 1:196642233:G:A
peptide level
0
400000
800000
175:175
110:110
40:40
EIMENYNIALRpc2_NP.5
p−maj=1, pX=0.86, N=325

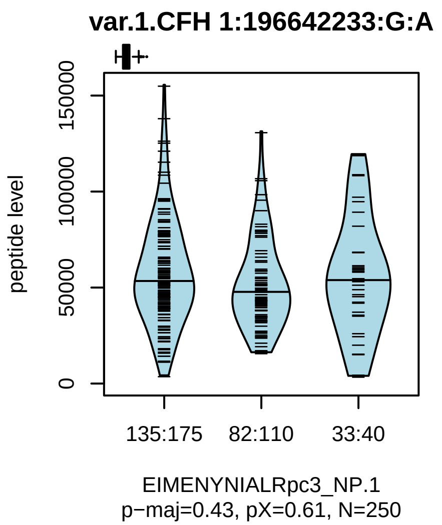
var.1.CFH 1:196642233:G:A
peptide level
0
50000
100000
150000
135:175
82:110
33:40
EIMENYNIALRpc3_NP.1
p−maj=0.43, pX=0.61, N=250

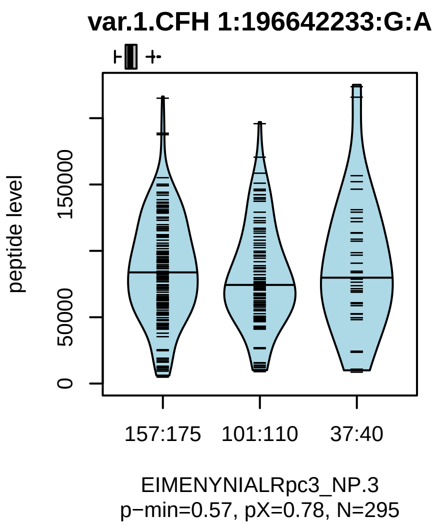
var.1.CFH 1:196642233:G:A
peptide level
0
50000
150000
157:175
101:110
37:40
EIMENYNIALRpc3_NP.3
p−min=0.57, pX=0.78, N=295

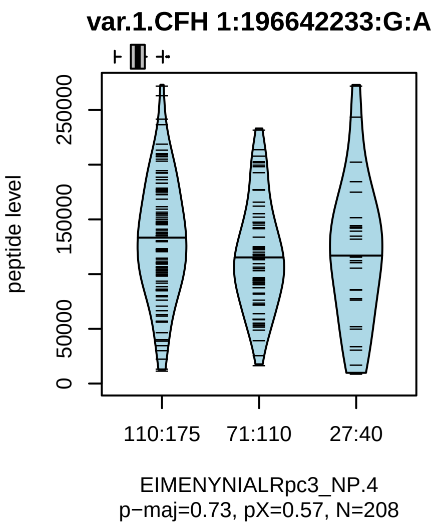
var.1.CFH 1:196642233:G:A
peptide level
0
50000
150000
250000
110:175
71:110
27:40
EIMENYNIALRpc3_NP.4
p−maj=0.73, pX=0.57, N=208

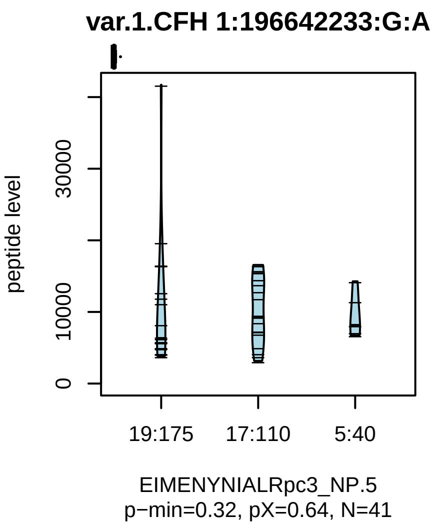
var.1.CFH 1:196642233:G:A
peptide level
0
10000
30000
19:175
17:110
5:40
EIMENYNIALRpc3_NP.5
p−min=0.32, pX=0.64, N=41

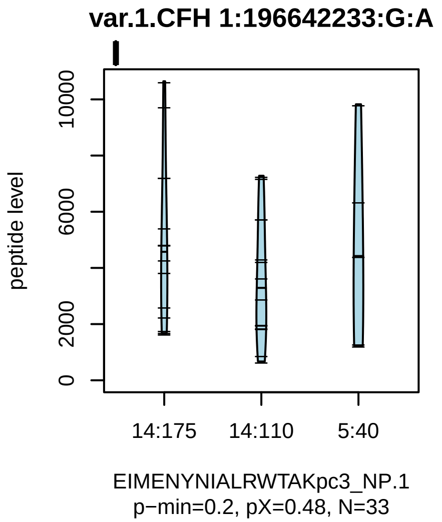
var.1.CFH 1:196642233:G:A
peptide level
0
2000
6000
10000
14:175
14:110
5:40
EIMENYNIALRWTAKpc3_NP.1
p−min=0.2, pX=0.48, N=33

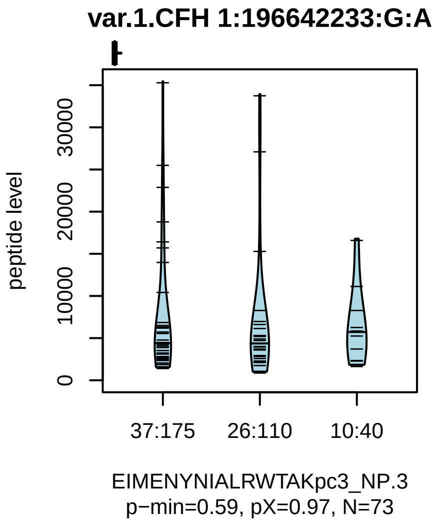
var.1.CFH 1:196642233:G:A
peptide level
0
10000
20000
30000
37:175
26:110
10:40
EIMENYNIALRWTAKpc3_NP.3
p−min=0.59, pX=0.97, N=73

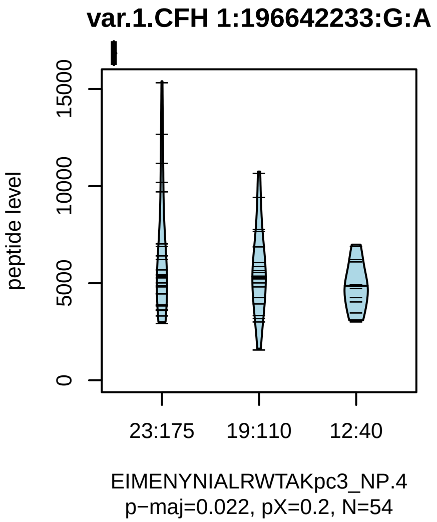
var.1.CFH 1:196642233:G:A
peptide level
0
5000
10000
15000
23:175
19:110
12:40
EIMENYNIALRWTAKpc3_NP.4
p−maj=0.022, pX=0.2, N=54

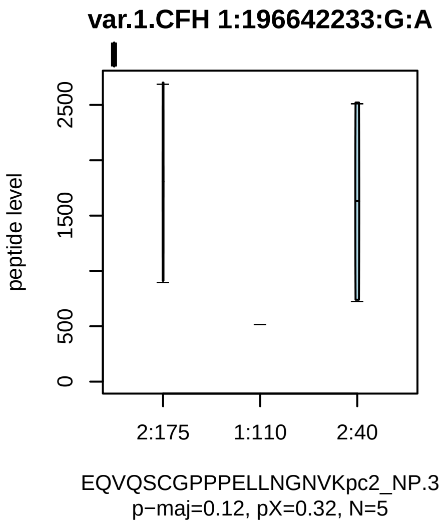
var.1.CFH 1:196642233:G:A
peptide level
0
500
1500
2500
2:175
1:110
2:40
EQVQSCGPPPELLNGNVKpc2_NP.3
p−maj=0.12, pX=0.32, N=5

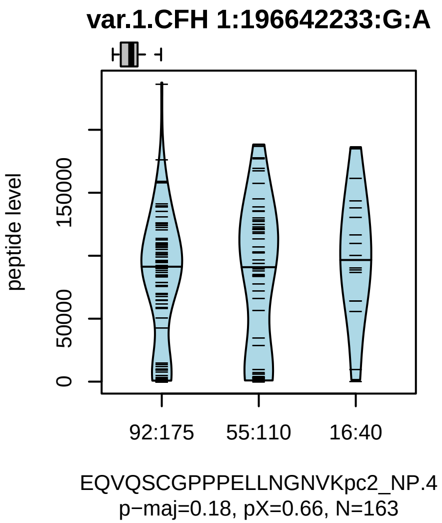
var.1.CFH 1:196642233:G:A
peptide level
0
50000
150000
92:175
55:110
16:40
EQVQSCGPPPELLNGNVKpc2_NP.4
p−maj=0.18, pX=0.66, N=163

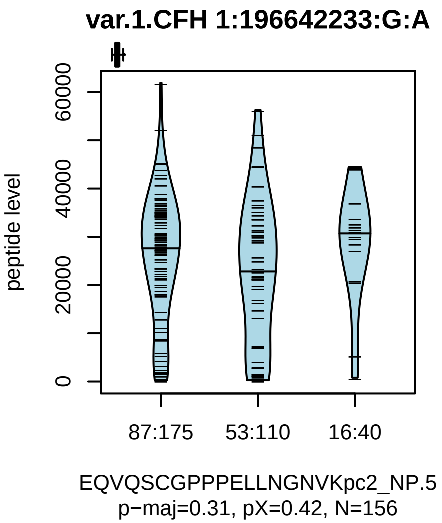
var.1.CFH 1:196642233:G:A
peptide level
0
20000
40000
60000
87:175
53:110
16:40
EQVQSCGPPPELLNGNVKpc2_NP.5
p−maj=0.31, pX=0.42, N=156

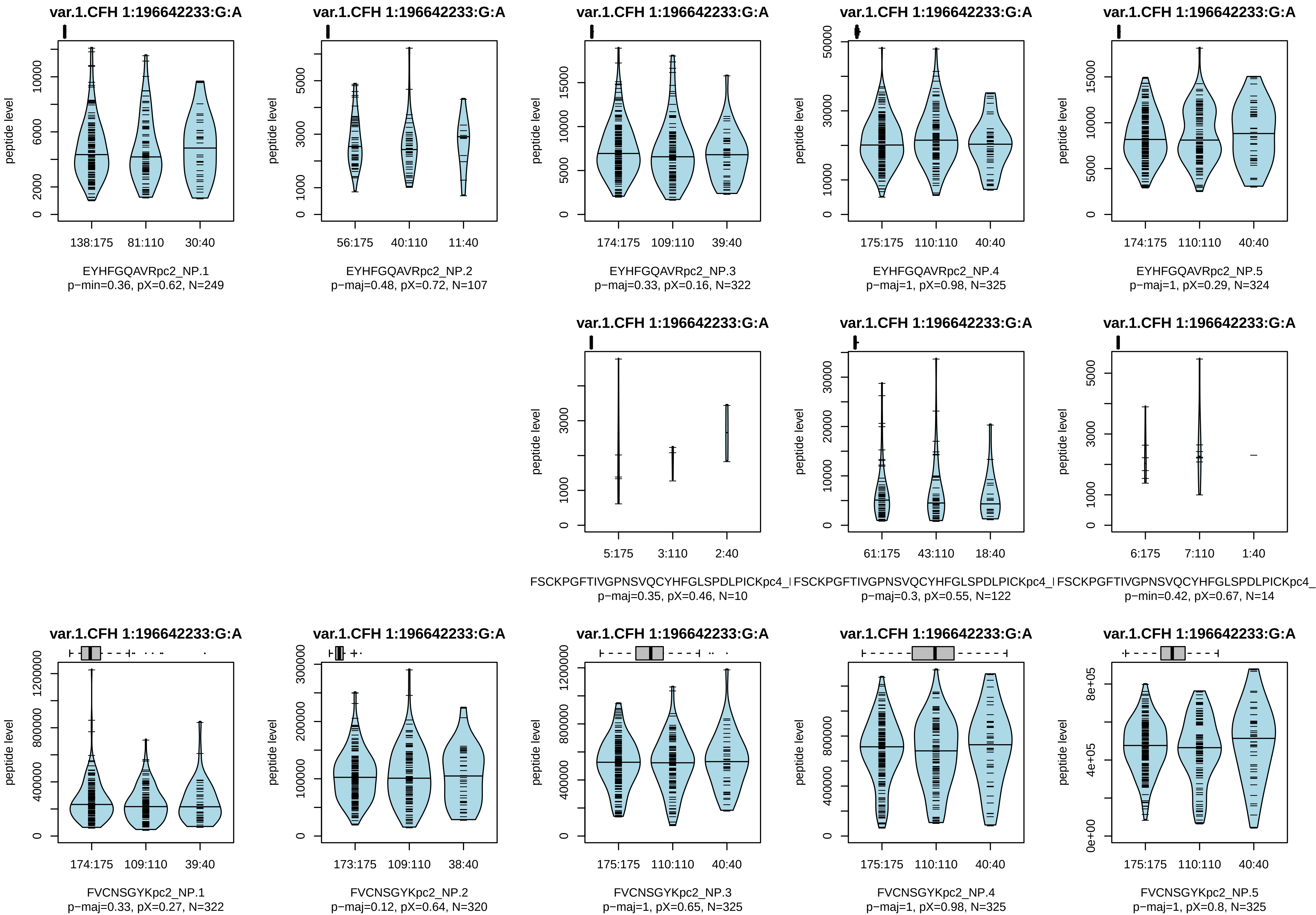
var.1.CFH 1:196642233:G:A
peptide level
138:175
81:110
30:40
EYHFGQAVRpc2_NP.1
p−min=0.36, pX=0.62, N=249
var.1.CFH 1:196642233:G:A
56:175
40:110
11:40
EYHFGQAVRpc2_NP.2
p−maj=0.48, pX=0.72, N=107
var.1.CFH 1:196642233:G:A
174:175
109:110
39:40
EYHFGQAVRpc2_NP.3
p−maj=0.33, pX=0.16, N=322
var.1.CFH 1:196642233:G:A
175:175
110:110
40:40
EYHFGQAVRpc2_NP.4
p−maj=1, pX=0.98, N=325
var.1.CFH 1:196642233:G:A
174:175
110:110
40:40
EYHFGQAVRpc2_NP.5
p−maj=1, pX=0.29, N=324
var.1.CFH 1:196642233:G:A
5:175
3:110
2:40
FSCKPGFTIVGPNSVQCYHFGLSPDLPICKpc4_I
p−maj=0.35, pX=0.46, N=10
var.1.CFH 1:196642233:G:A
61:175
43:110
18:40
FSCKPGFTIVGPNSVQCYHFGLSPDLPICKpc4_I
p−maj=0.3, pX=0.55, N=122
var.1.CFH 1:196642233:G:A
6:175
7:110
1:40
FSCKPGFTIVGPNSVQCYHFGLSPDLPICKpc4_
p−min=0.42, pX=0.67, N=14
var.1.CFH 1:196642233:G:A
174:175
109:110
39:40
FVCNSGYKpc2_NP.1
p−maj=0.33, pX=0.27, N=322
var.1.CFH 1:196642233:G:A
173:175
109:110
38:40
FVCNSGYKpc2_NP.2
p−maj=0.12, pX=0.64, N=320
var.1.CFH 1:196642233:G:A
175:175
110:110
40:40
FVCNSGYKpc2_NP.3
p−maj=1, pX=0.65, N=325
var.1.CFH 1:196642233:G:A
175:175
110:110
40:40
FVCNSGYKpc2_NP.4
p−maj=1, pX=0.98, N=325
var.1.CFH 1:196642233:G:A
175:175
110:110
40:40
FVCNSGYKpc2_NP.5
p−maj=1, pX=0.8, N=325

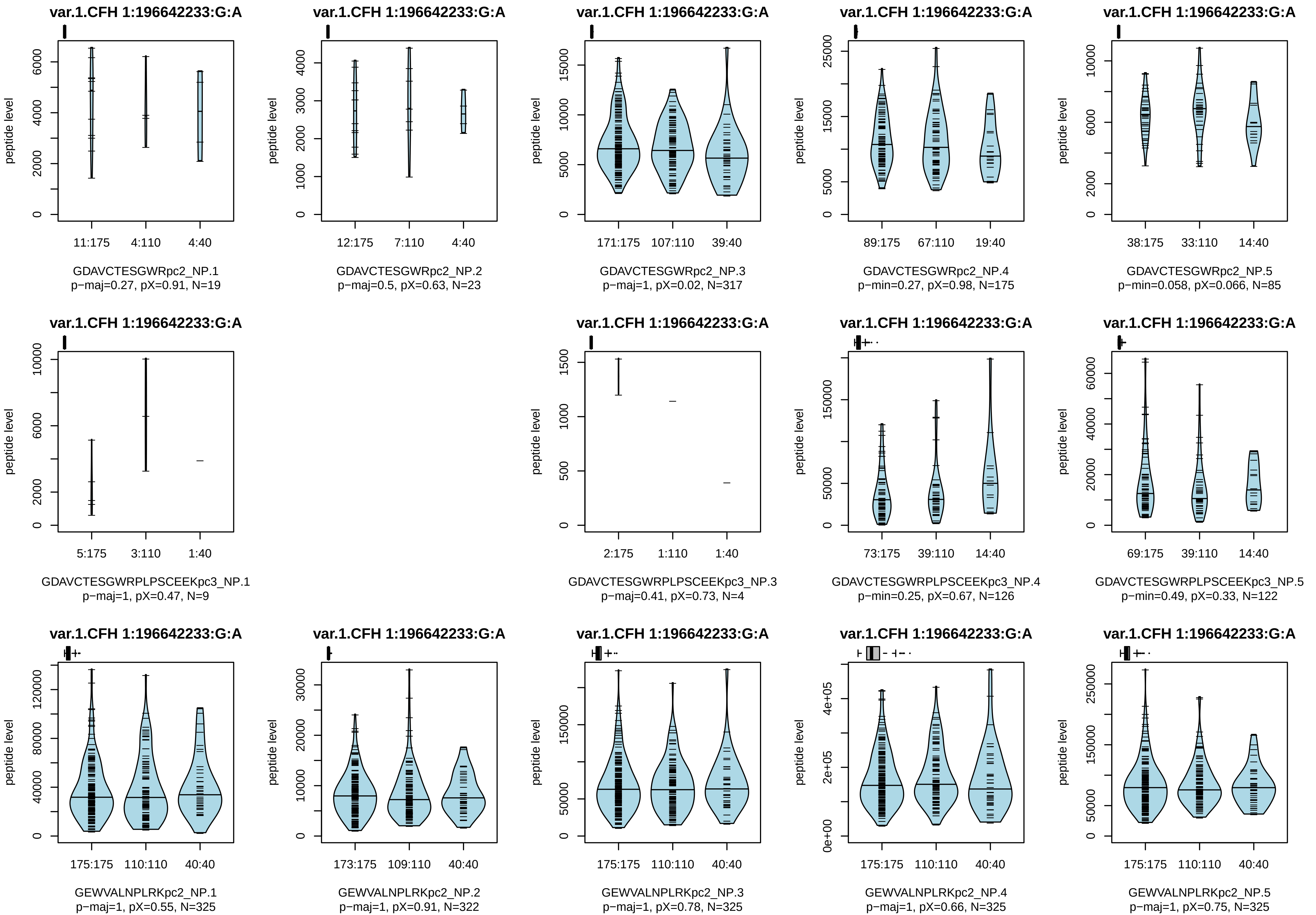

var.1.CFH 1:196642233:G:A
peptide level
11:175
4:110
4:40
GDAVCTESGWRpc2_NP.1
p−maj=0.27, pX=0.91, N=19
var.1.CFH 1:196642233:G:A
peptide level
12:175
7:110
4:40
GDAVCTESGWRpc2_NP.2
p−maj=0.5, pX=0.63, N=23
var.1.CFH 1:196642233:G:A
peptide level
171:175
107:110
39:40
GDAVCTESGWRpc2_NP.3
p−maj=1, pX=0.02, N=317
var.1.CFH 1:196642233:G:A
peptide level
89:175
67:110
19:40
GDAVCTESGWRpc2_NP.4
p−min=0.27, pX=0.98, N=175
var.1.CFH 1:196642233:G:A
peptide level
38:175
33:110
14:40
GDAVCTESGWRpc2_NP.5
p−min=0.058, pX=0.066, N=85
var.1.CFH 1:196642233:G:A
peptide level
5:175
3:110
1:40
GDAVCTESGWRPLPSCEEKpc3_NP.1
p−maj=1, pX=0.47, N=9
var.1.CFH 1:196642233:G:A
peptide level
2:175
1:110
1:40
GDAVCTESGWRPLPSCEEKpc3_NP.3
p−maj=0.41, pX=0.73, N=4
var.1.CFH 1:196642233:G:A
peptide level
73:175
39:110
14:40
GDAVCTESGWRPLPSCEEKpc3_NP.4
p−min=0.25, pX=0.67, N=126
var.1.CFH 1:196642233:G:A
peptide level
69:175
39:110
14:40
GDAVCTESGWRPLPSCEEKpc3_NP.5
p−min=0.49, pX=0.33, N=122
var.1.CFH 1:196642233:G:A
peptide level
175:175
110:110
40:40
GEWVALNPLRKpc2_NP.1
p−maj=1, pX=0.55, N=325
var.1.CFH 1:196642233:G:A
peptide level
173:175
109:110
40:40
GEWVALNPLRKpc2_NP.2
p−maj=1, pX=0.91, N=322
var.1.CFH 1:196642233:G:A
peptide level
175:175
110:110
40:40
GEWVALNPLRKpc2_NP.3
p−maj=1, pX=0.78, N=325
var.1.CFH 1:196642233:G:A
peptide level
175:175
110:110
40:40
GEWVALNPLRKpc2_NP.4
p−maj=1, pX=0.66, N=325
var.1.CFH 1:196642233:G:A
peptide level
175:175
110:110
40:40
GEWVALNPLRKpc2_NP.5
p−maj=1, pX=0.75, N=325

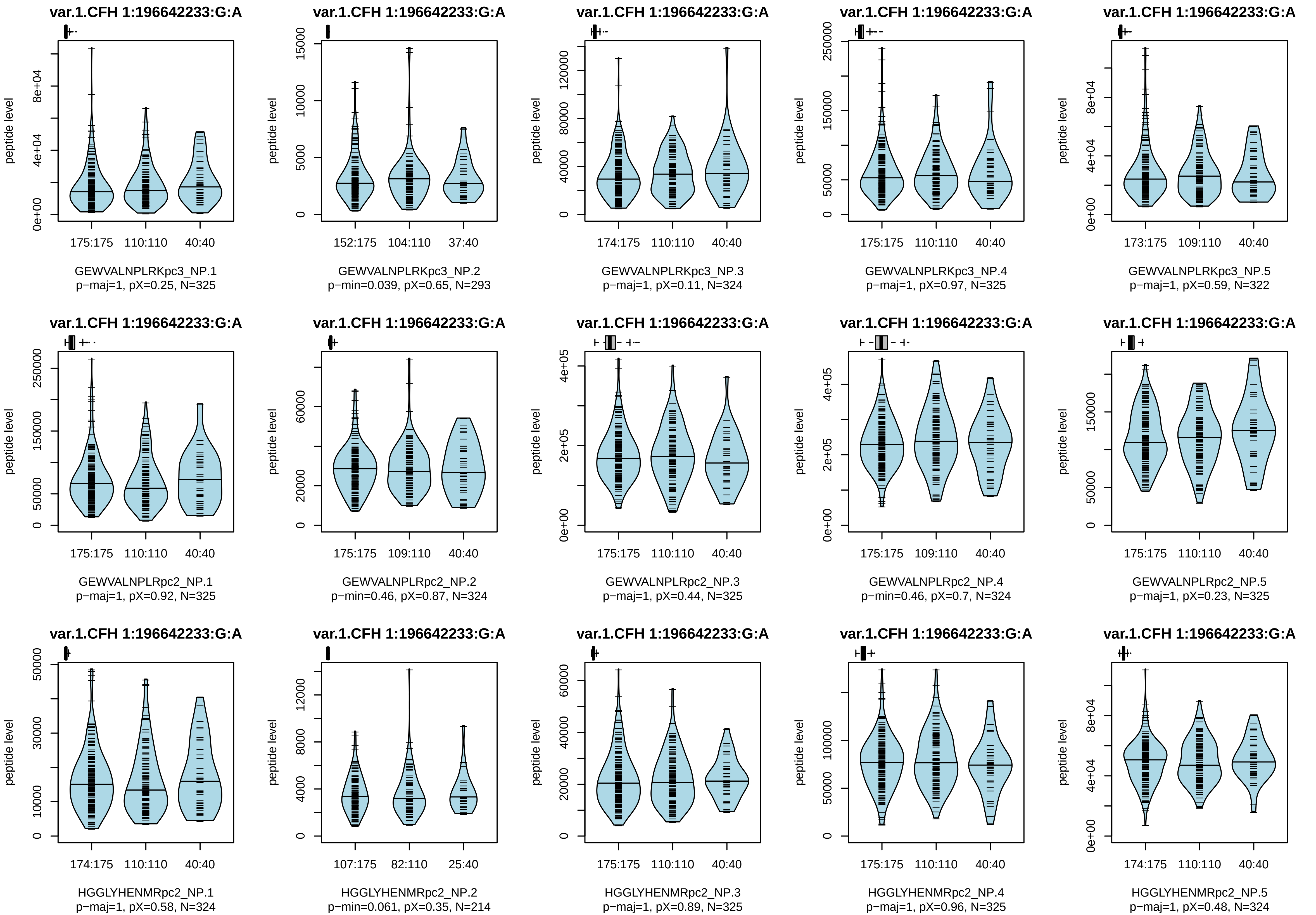
var.1.CFH 1:196642233:G:A
peptide level
175:175 110:110 40:40
GEWVALNPLRKpc3_NP.1
p−maj=1, pX=0.25, N=325
var.1.CFH 1:196642233:G:A
peptide level
152:175 104:110 37:40
GEWVALNPLRKpc3_NP.2
p−min=0.039, pX=0.65, N=293
var.1.CFH 1:196642233:G:A
peptide level
174:175 110:110 40:40
GEWVALNPLRKpc3_NP.3
p−maj=1, pX=0.11, N=324
var.1.CFH 1:196642233:G:A
peptide level
175:175 110:110 40:40
GEWVALNPLRKpc3_NP.4
p−maj=1, pX=0.97, N=325
var.1.CFH 1:196642233:G:A
peptide level
173:175 109:110 40:40
GEWVALNPLRKpc3_NP.5
p−maj=1, pX=0.59, N=322
var.1.CFH 1:196642233:G:A
peptide level
175:175 110:110 40:40
GEWVALNPLRpc2_NP.1
p−maj=1, pX=0.92, N=325
var.1.CFH 1:196642233:G:A
peptide level
175:175 109:110 40:40
GEWVALNPLRpc2_NP.2
p−min=0.46, pX=0.87, N=324
var.1.CFH 1:196642233:G:A
peptide level
175:175 110:110 40:40
GEWVALNPLRpc2_NP.3
p−maj=1, pX=0.44, N=325
var.1.CFH 1:196642233:G:A
peptide level
175:175 109:110 40:40
GEWVALNPLRpc2_NP.4
p−min=0.46, pX=0.7, N=324
var.1.CFH 1:196642233:G:A
peptide level
175:175 110:110 40:40
GEWVALNPLRpc2_NP.5
p−maj=1, pX=0.23, N=325
var.1.CFH 1:196642233:G:A
peptide level
174:175 110:110 40:40
HGGLYHENMRpc2_NP.1
p−maj=1, pX=0.58, N=324
var.1.CFH 1:196642233:G:A
peptide level
107:175 82:110 25:40
HGGLYHENMRpc2_NP.2
p−min=0.061, pX=0.35, N=214
var.1.CFH 1:196642233:G:A
peptide level
175:175 110:110 40:40
HGGLYHENMRpc2_NP.3
p−maj=1, pX=0.89, N=325
var.1.CFH 1:196642233:G:A
peptide level
175:175 110:110 40:40
HGGLYHENMRpc2_NP.4
p−maj=1, pX=0.96, N=325
var.1.CFH 1:196642233:G:A
peptide level
174:175 110:110 40:40
HGGLYHENMRpc2_NP.5
p−maj=1, pX=0.48, N=324

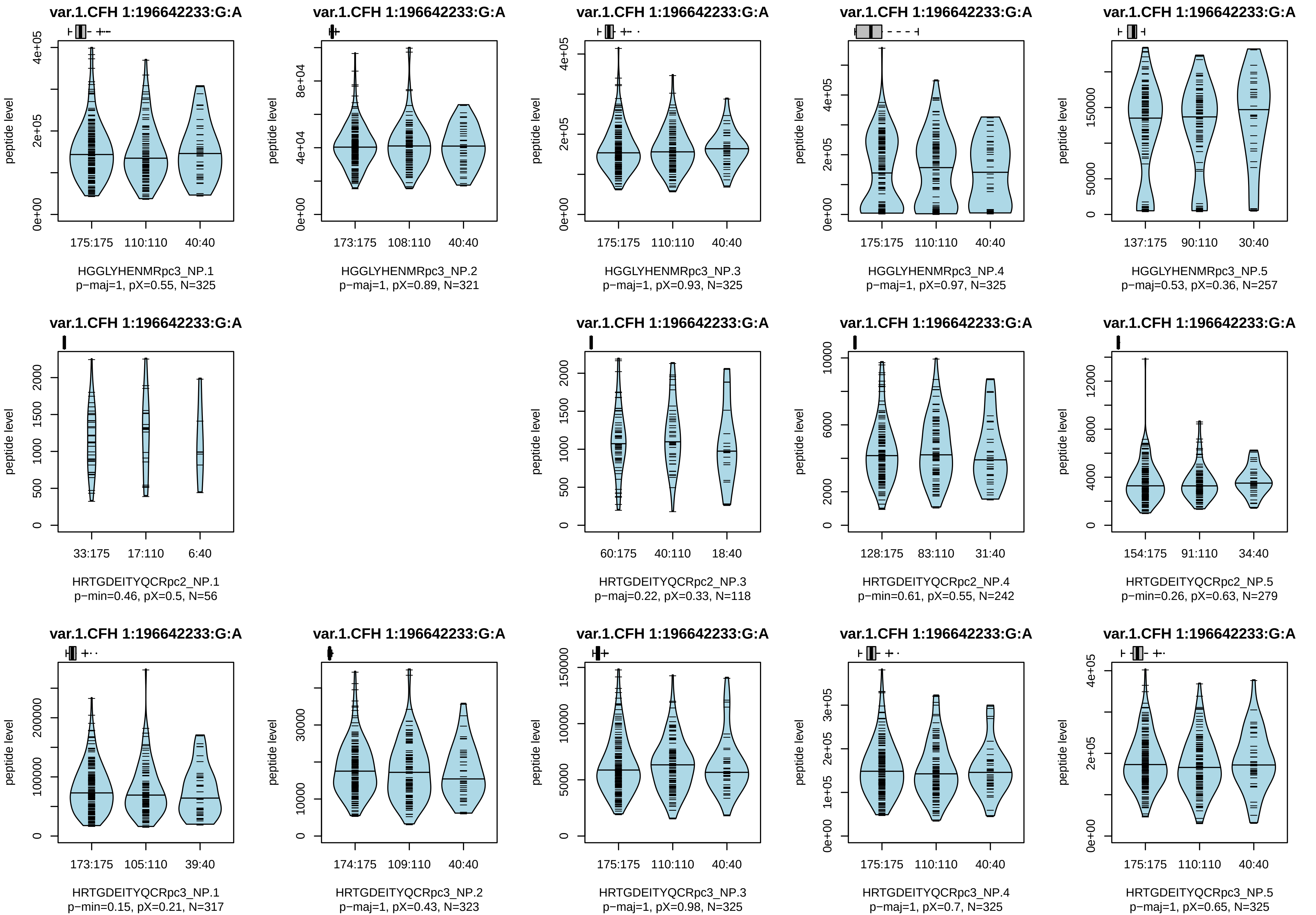
var.1.CFH 1:196642233:G:A
peptide level
175:175
110:110
40:40
HGGLYHENMRpc3_NP.1
p−maj=1, pX=0.55, N=325
var.1.CFH 1:196642233:G:A
peptide level
173:175
108:110
40:40
HGGLYHENMRpc3_NP.2
p−maj=1, pX=0.89, N=321
var.1.CFH 1:196642233:G:A
peptide level
175:175
110:110
40:40
HGGLYHENMRpc3_NP.3
p−maj=1, pX=0.93, N=325
var.1.CFH 1:196642233:G:A
peptide level
175:175
110:110
40:40
HGGLYHENMRpc3_NP.4
p−maj=1, pX=0.97, N=325
var.1.CFH 1:196642233:G:A
peptide level
137:175
90:110
30:40
HGGLYHENMRpc3_NP.5
p−maj=0.53, pX=0.36, N=257
var.1.CFH 1:196642233:G:A
peptide level
33:175
17:110
6:40
HRTGDEITYQCRpc2_NP.1
p−min=0.46, pX=0.5, N=56
var.1.CFH 1:196642233:G:A
peptide level
60:175
40:110
18:40
HRTGDEITYQCRpc2_NP.3
p−maj=0.22, pX=0.33, N=118
var.1.CFH 1:196642233:G:A
peptide level
128:175
83:110
31:40
HRTGDEITYQCRpc2_NP.4
p−min=0.61, pX=0.55, N=242
var.1.CFH 1:196642233:G:A
peptide level
154:175
91:110
34:40
HRTGDEITYQCRpc2_NP.5
p−min=0.26, pX=0.63, N=279
var.1.CFH 1:196642233:G:A
peptide level
173:175
105:110
39:40
HRTGDEITYQCRpc3_NP.1
p−min=0.15, pX=0.21, N=317
var.1.CFH 1:196642233:G:A
peptide level
174:175
109:110
40:40
HRTGDEITYQCRpc3_NP.2
p−maj=1, pX=0.43, N=323
var.1.CFH 1:196642233:G:A
peptide level
175:175
110:110
40:40
HRTGDEITYQCRpc3_NP.3
p−maj=1, pX=0.98, N=325
var.1.CFH 1:196642233:G:A
peptide level
175:175
110:110
40:40
HRTGDEITYQCRpc3_NP.4
p−maj=1, pX=0.7, N=325
var.1.CFH 1:196642233:G:A
peptide level
175:175
110:110
40:40
HRTGDEITYQCRpc3_NP.5
p−maj=1, pX=0.65, N=325

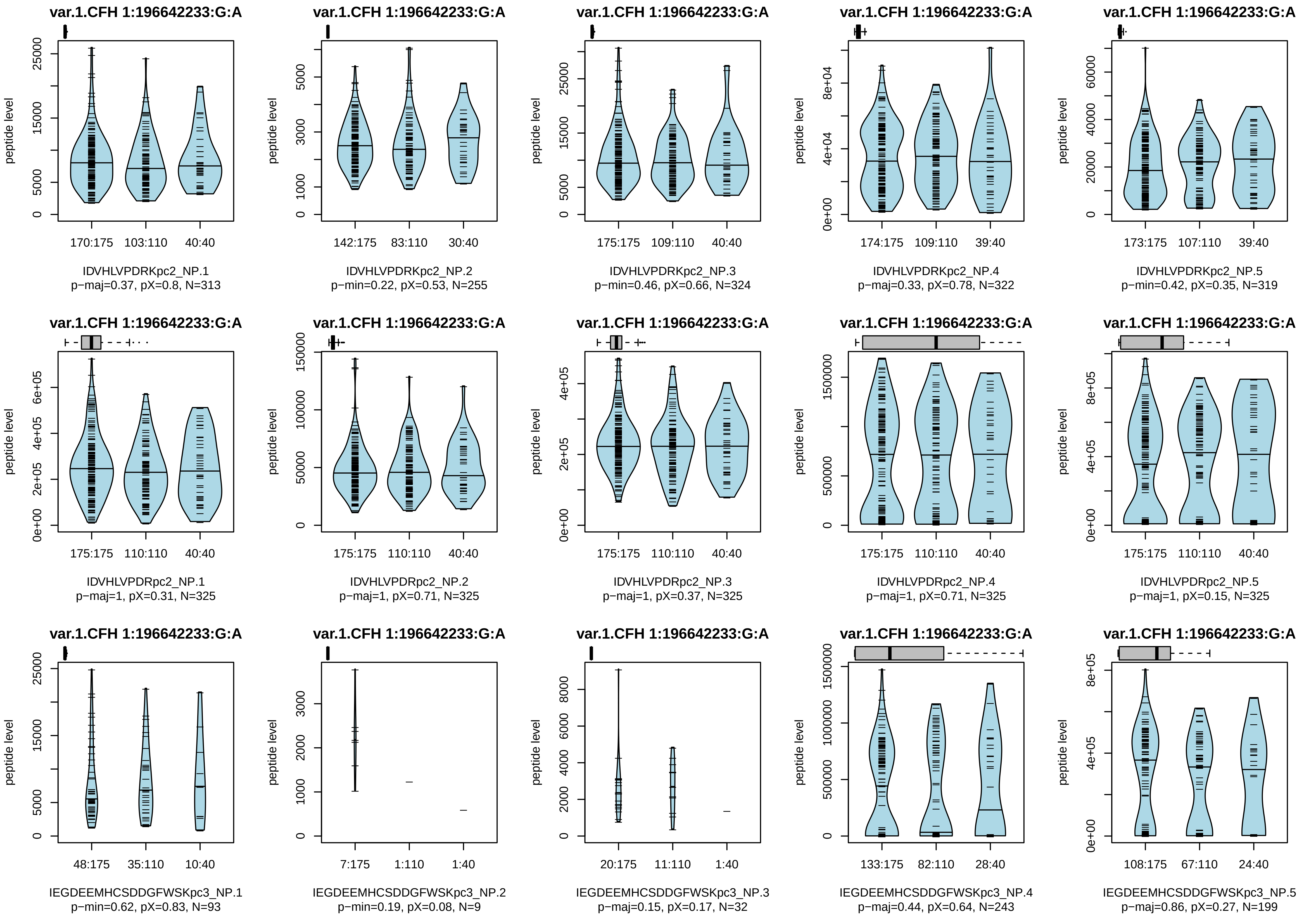
var.1.CFH 1:196642233:G:A
peptide level
170:175
103:110
40:40
IDVHLVPDRKpc2_NP.1
p−maj=0.37, pX=0.8, N=313
var.1.CFH 1:196642233:G:A
peptide level
142:175
83:110
30:40
IDVHLVPDRKpc2_NP.2
p−min=0.22, pX=0.53, N=255
var.1.CFH 1:196642233:G:A
peptide level
175:175
109:110
40:40
IDVHLVPDRKpc2_NP.3
p−min=0.46, pX=0.66, N=324
var.1.CFH 1:196642233:G:A
peptide level
174:175
109:110
39:40
IDVHLVPDRKpc2_NP.4
p−maj=0.33, pX=0.78, N=322
var.1.CFH 1:196642233:G:A
peptide level
173:175
107:110
39:40
IDVHLVPDRKpc2_NP.5
p−min=0.42, pX=0.35, N=319
var.1.CFH 1:196642233:G:A
peptide level
175:175
110:110
40:40
IDVHLVPDRpc2_NP.1
p−maj=1, pX=0.31, N=325
var.1.CFH 1:196642233:G:A
peptide level
175:175
110:110
40:40
IDVHLVPDRpc2_NP.2
p−maj=1, pX=0.71, N=325
var.1.CFH 1:196642233:G:A
peptide level
175:175
110:110
40:40
IDVHLVPDRpc2_NP.3
p−maj=1, pX=0.37, N=325
var.1.CFH 1:196642233:G:A
peptide level
175:175
110:110
40:40
IDVHLVPDRpc2_NP.4
p−maj=1, pX=0.71, N=325
var.1.CFH 1:196642233:G:A
peptide level
175:175
110:110
40:40
IDVHLVPDRpc2_NP.5
p−maj=1, pX=0.15, N=325
var.1.CFH 1:196642233:G:A
peptide level
48:175
35:110
10:40
IEGDEEMHCSDDGFWSKpc3_NP.1
p−min=0.62, pX=0.83, N=93
var.1.CFH 1:196642233:G:A
peptide level
7:175
1:110
1:40
IEGDEEMHCSDDGFWSKpc3_NP.2
p−min=0.19, pX=0.08, N=9
var.1.CFH 1:196642233:G:A
peptide level
20:175
11:110
1:40
IEGDEEMHCSDDGFWSKpc3_NP.3
p−maj=0.15, pX=0.17, N=32
var.1.CFH 1:196642233:G:A
peptide level
133:175
82:110
28:40
IEGDEEMHCSDDGFWSKpc3_NP.4
p−maj=0.44, pX=0.64, N=243
var.1.CFH 1:196642233:G:A
peptide level
108:175
67:110
24:40
IEGDEEMHCSDDGFWSKpc3_NP.5
p−maj=0.86, pX=0.27, N=199

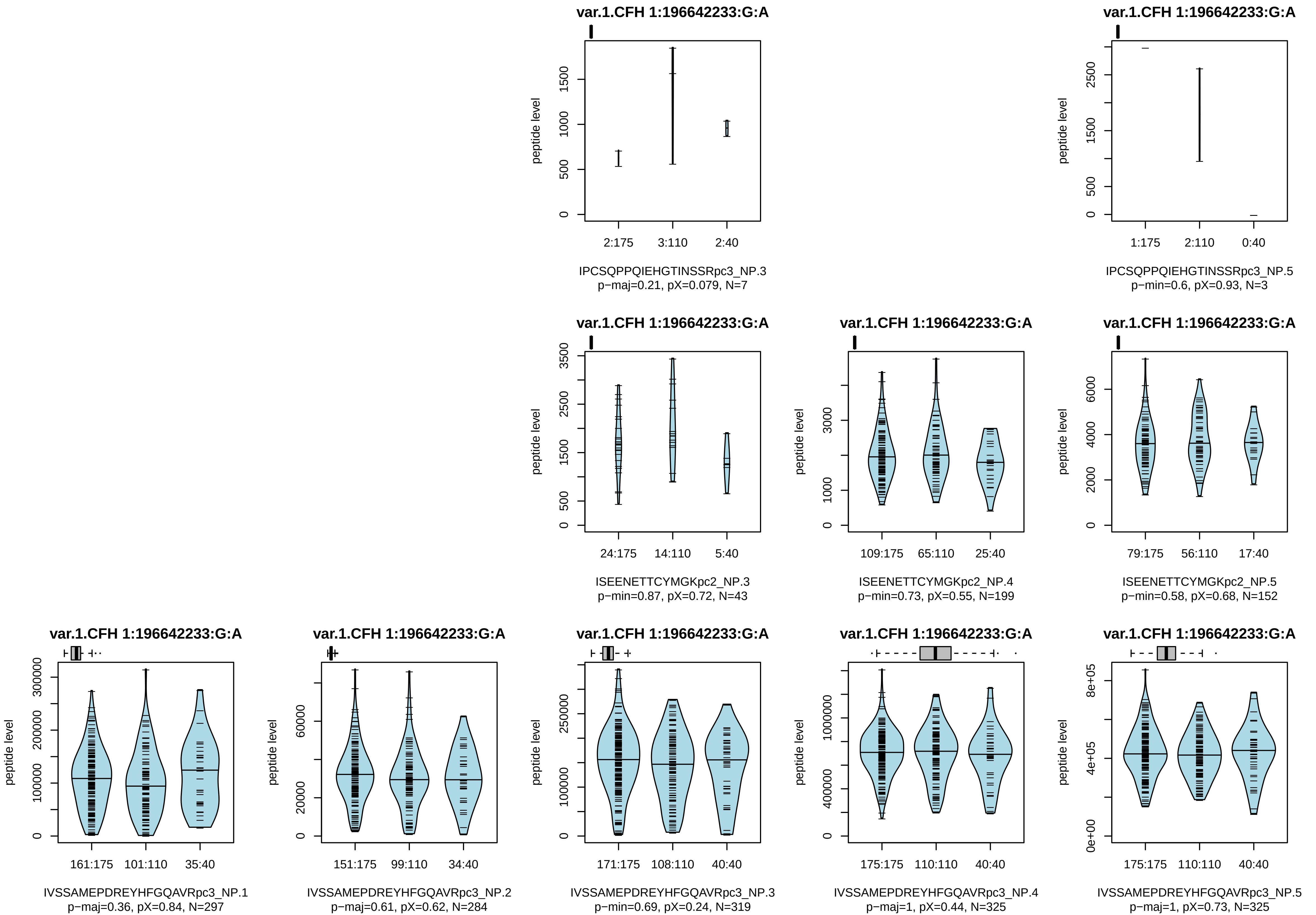

var.1.CFH 1:196642233:G:A
peptide level
0
500
1000
1500
2:175
3:110
2:40
IPCSQPPQIEHGTINSSRpc3_NP.3
p−maj=0.21, pX=0.079, N=7
var.1.CFH 1:196642233:G:A
peptide level
0
500
1500
2500
1:175
2:110
0:40
IPCSQPPQIEHGTINSSRpc3_NP.5
p−min=0.6, pX=0.93, N=3
var.1.CFH 1:196642233:G:A
peptide level
0
500
1500
2500
3500
24:175
14:110
5:40
ISEENETTCYMGKpc2_NP.3
p−min=0.87, pX=0.72, N=43
var.1.CFH 1:196642233:G:A
peptide level
0
1000
3000
109:175
65:110
25:40
ISEENETTCYMGKpc2_NP.4
p−min=0.73, pX=0.55, N=199
var.1.CFH 1:196642233:G:A
peptide level
0
2000
4000
6000
79:175
56:110
17:40
ISEENETTCYMGKpc2_NP.5
p−min=0.58, pX=0.68, N=152
var.1.CFH 1:196642233:G:A
peptide level
0
100000
200000
300000
161:175
101:110
35:40
IVSSAMEPDREYHFGQAVRpc3_NP.1
p−maj=0.36, pX=0.84, N=297
var.1.CFH 1:196642233:G:A
peptide level
0
20000
60000
151:175
99:110
34:40
IVSSAMEPDREYHFGQAVRpc3_NP.2
p−maj=0.61, pX=0.62, N=284
var.1.CFH 1:196642233:G:A
peptide level
0
100000
250000
171:175
108:110
40:40
IVSSAMEPDREYHFGQAVRpc3_NP.3
p−min=0.69, pX=0.24, N=319
var.1.CFH 1:196642233:G:A
peptide level
0
400000
1000000
175:175
110:110
40:40
IVSSAMEPDREYHFGQAVRpc3_NP.4
p−maj=1, pX=0.44, N=325
var.1.CFH 1:196642233:G:A
peptide level
0e+00
4e+05
8e+05
175:175
110:110
40:40
IVSSAMEPDREYHFGQAVRpc3_NP.5
p−maj=1, pX=0.73, N=325

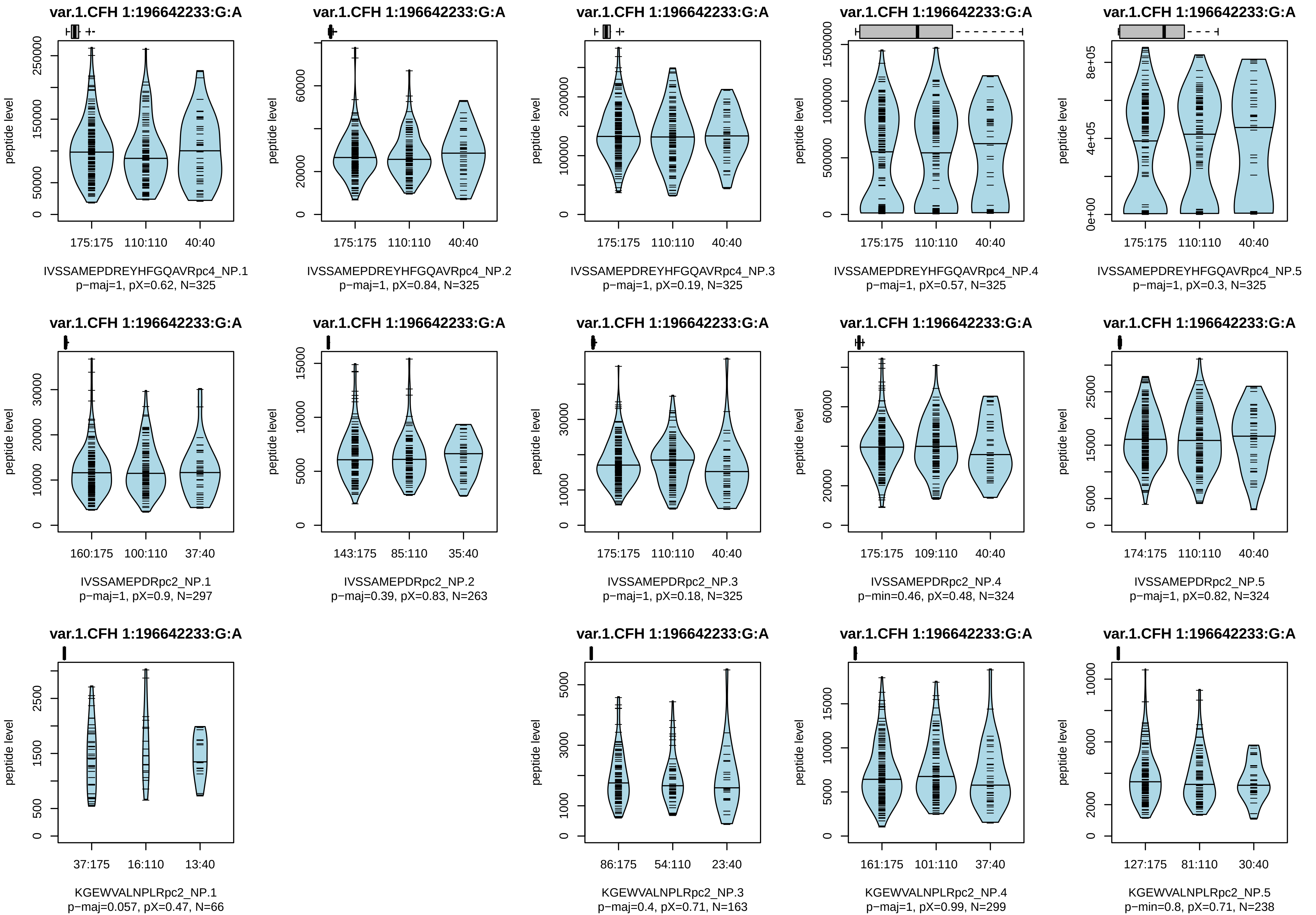
var.1.CFH 1:196642233:G:A
peptide level
175:175
110:110
40:40
IVSSAMEPDREYHFGQAVRpc4_NP.1
p−maj=1, pX=0.62, N=325
IVSSAMEPDREYHFGQAVRpc4_NP.2
p−maj=1, pX=0.84, N=325
IVSSAMEPDREYHFGQAVRpc4_NP.3
p−maj=1, pX=0.19, N=325
IVSSAMEPDREYHFGQAVRpc4_NP.4
p−maj=1, pX=0.57, N=325
IVSSAMEPDREYHFGQAVRpc4_NP.5
p−maj=1, pX=0.3, N=325
160:175
100:110
37:40
IVSSAMEPDRpc2_NP.1
p−maj=1, pX=0.9, N=297
143:175
85:110
35:40
IVSSAMEPDRpc2_NP.2
p−maj=0.39, pX=0.83, N=263
IVSSAMEPDRpc2_NP.3
p−maj=1, pX=0.18, N=325
109:110
IVSSAMEPDRpc2_NP.4
p−min=0.46, pX=0.48, N=324
174:175
IVSSAMEPDRpc2_NP.5
p−maj=1, pX=0.82, N=324
37:175
16:110
13:40
KGEWVALNPLRpc2_NP.1
p−maj=0.057, pX=0.47, N=66
86:175
54:110
23:40
KGEWVALNPLRpc2_NP.3
p−maj=0.4, pX=0.71, N=163
161:175
101:110
KGEWVALNPLRpc2_NP.4
p−maj=1, pX=0.99, N=299
127:175
81:110
30:40
KGEWVALNPLRpc2_NP.5
p−min=0.8, pX=0.71, N=238

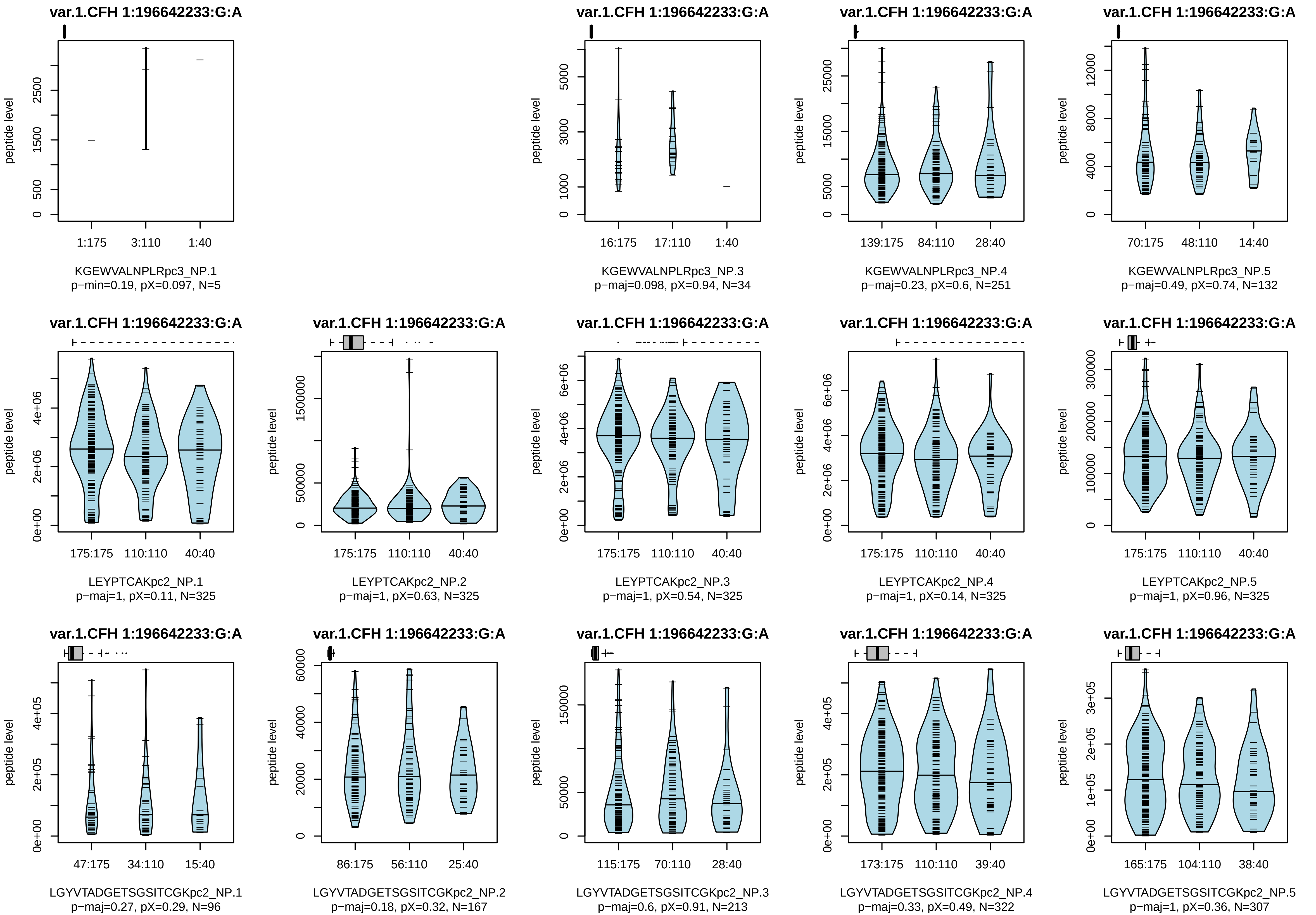

var.1.CFH 1:196642233:G:A
peptide level
1:175
3:110
1:40
KGEWVALNPLRpc3_NP.1
p−min=0.19, pX=0.097, N=5
var.1.CFH 1:196642233:G:A
peptide level
16:175
17:110
1:40
KGEWVALNPLRpc3_NP.3
p−maj=0.098, pX=0.94, N=34
var.1.CFH 1:196642233:G:A
peptide level
139:175
84:110
28:40
KGEWVALNPLRpc3_NP.4
p−maj=0.23, pX=0.6, N=251
var.1.CFH 1:196642233:G:A
peptide level
70:175
48:110
14:40
KGEWVALNPLRpc3_NP.5
p−maj=0.49, pX=0.74, N=132
var.1.CFH 1:196642233:G:A
peptide level
175:175
110:110
40:40
LEYPTCAKpc2_NP.1
p−maj=1, pX=0.11, N=325
var.1.CFH 1:196642233:G:A
peptide level
175:175
110:110
40:40
LEYPTCAKpc2_NP.2
p−maj=1, pX=0.63, N=325
var.1.CFH 1:196642233:G:A
peptide level
175:175
110:110
40:40
LEYPTCAKpc2_NP.3
p−maj=1, pX=0.54, N=325
var.1.CFH 1:196642233:G:A
peptide level
175:175
110:110
40:40
LEYPTCAKpc2_NP.4
p−maj=1, pX=0.14, N=325
var.1.CFH 1:196642233:G:A
peptide level
175:175
110:110
40:40
LEYPTCAKpc2_NP.5
p−maj=1, pX=0.96, N=325
var.1.CFH 1:196642233:G:A
peptide level
47:175
34:110
15:40
LGYVTADGETSGSITCGKpc2_NP.1
p−maj=0.27, pX=0.29, N=96
var.1.CFH 1:196642233:G:A
peptide level
86:175
56:110
25:40
LGYVTADGETSGSITCGKpc2_NP.2
p−maj=0.18, pX=0.32, N=167
var.1.CFH 1:196642233:G:A
peptide level
115:175
70:110
28:40
LGYVTADGETSGSITCGKpc2_NP.3
p−maj=0.6, pX=0.91, N=213
var.1.CFH 1:196642233:G:A
peptide level
173:175
110:110
39:40
LGYVTADGETSGSITCGKpc2_NP.4
p−maj=0.33, pX=0.49, N=322
var.1.CFH 1:196642233:G:A
peptide level
165:175
104:110
38:40
LGYVTADGETSGSITCGKpc2_NP.5
p−maj=1, pX=0.36, N=307

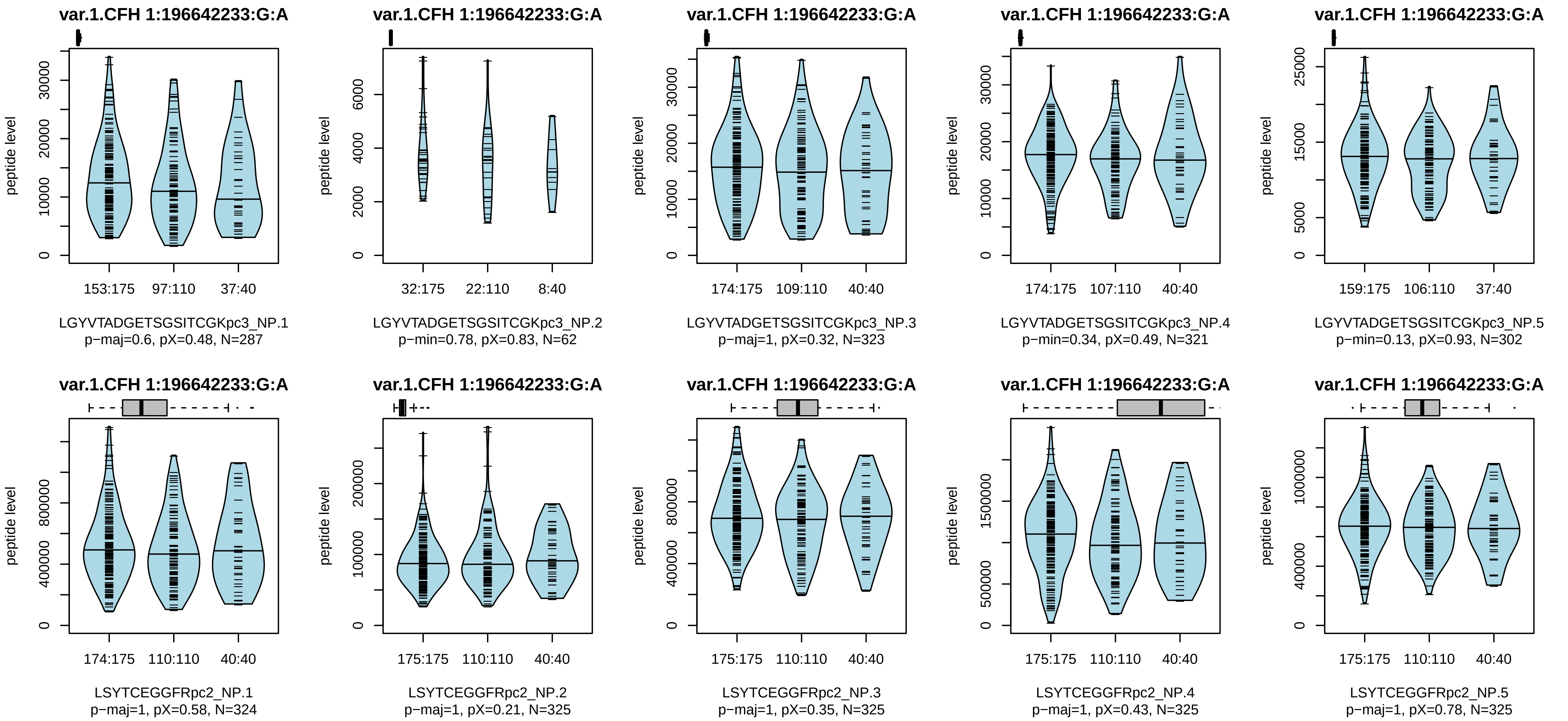
var.1.CFH 1:196642233:G:A
peptide level
153:175
97:110
37:40
LGYVTADGETSGSITCGKpc3_NP.1
p−maj=0.6, pX=0.48, N=287
var.1.CFH 1:196642233:G:A
peptide level
32:175
22:110
8:40
LGYVTADGETSGSITCGKpc3_NP.2
p−min=0.78, pX=0.83, N=62
var.1.CFH 1:196642233:G:A
peptide level
174:175
109:110
40:40
LGYVTADGETSGSITCGKpc3_NP.3
p−maj=1, pX=0.32, N=323
var.1.CFH 1:196642233:G:A
peptide level
174:175
107:110
40:40
LGYVTADGETSGSITCGKpc3_NP.4
p−min=0.34, pX=0.49, N=321
var.1.CFH 1:196642233:G:A
peptide level
159:175
106:110
37:40
LGYVTADGETSGSITCGKpc3_NP.5
p−min=0.13, pX=0.93, N=302
var.1.CFH 1:196642233:G:A
peptide level
174:175
110:110
40:40
LSYTCEGGFRpc2_NP.1
p−maj=1, pX=0.58, N=324
var.1.CFH 1:196642233:G:A
peptide level
175:175
110:110
40:40
LSYTCEGGFRpc2_NP.2
p−maj=1, pX=0.21, N=325
var.1.CFH 1:196642233:G:A
peptide level
175:175
110:110
40:40
LSYTCEGGFRpc2_NP.3
p−maj=1, pX=0.35, N=325
var.1.CFH 1:196642233:G:A
peptide level
175:175
110:110
40:40
LSYTCEGGFRpc2_NP.4
p−maj=1, pX=0.43, N=325
var.1.CFH 1:196642233:G:A
peptide level
175:175
110:110
40:40
LSYTCEGGFRpc2_NP.5
p−maj=1, pX=0.78, N=325

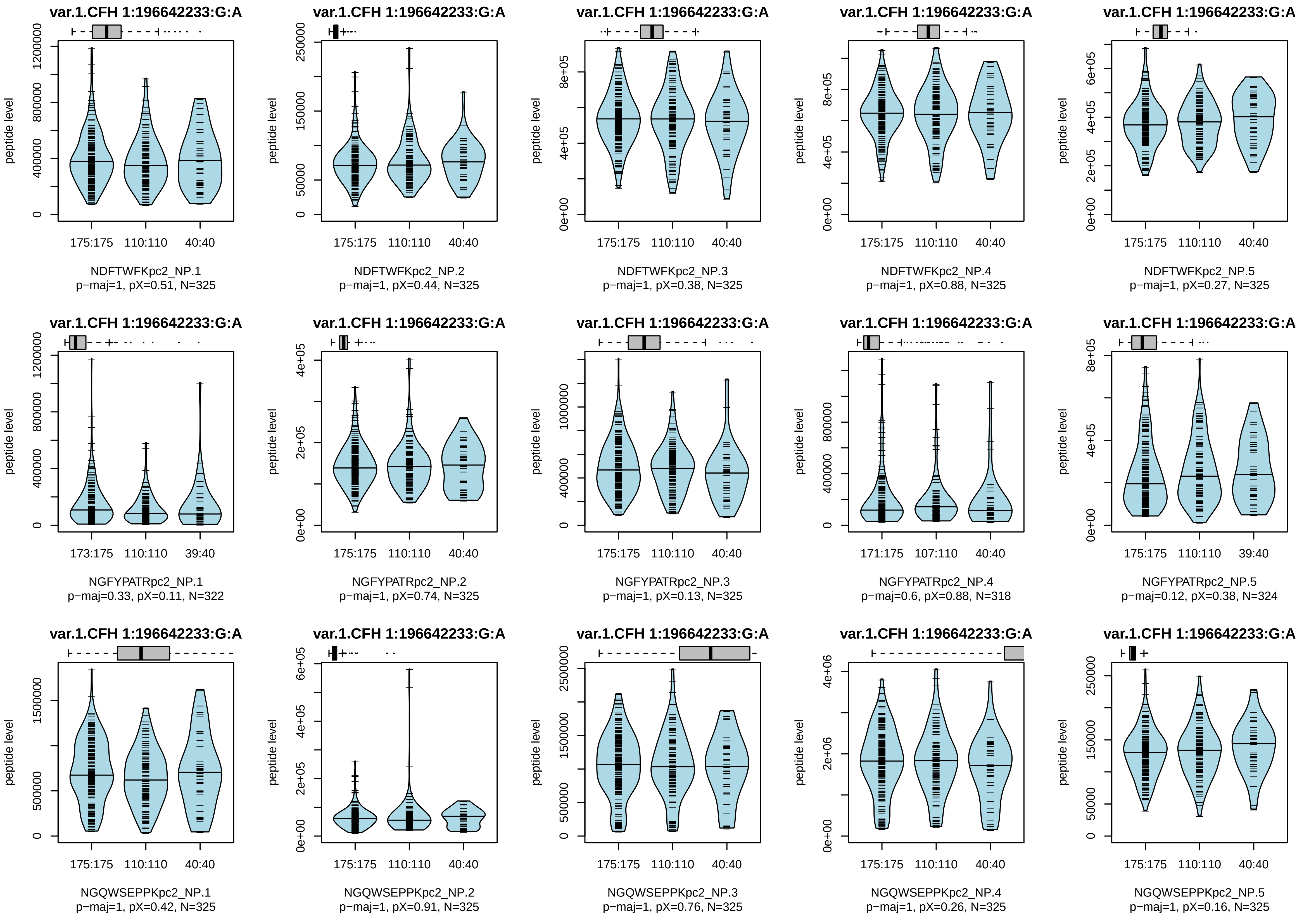
var.1.CFH 1:196642233:G:A
peptide level
175:175 110:110 40:40
NDFTWFKpc2_NP.1
p−maj=1, pX=0.51, N=325
var.1.CFH 1:196642233:G:A
peptide level
175:175 110:110 40:40
NDFTWFKpc2_NP.2
p−maj=1, pX=0.44, N=325
var.1.CFH 1:196642233:G:A
peptide level
175:175 110:110 40:40
NDFTWFKpc2_NP.3
p−maj=1, pX=0.38, N=325
var.1.CFH 1:196642233:G:A
peptide level
175:175 110:110 40:40
NDFTWFKpc2_NP.4
p−maj=1, pX=0.88, N=325
var.1.CFH 1:196642233:G:A
peptide level
175:175 110:110 40:40
NDFTWFKpc2_NP.5
p−maj=1, pX=0.27, N=325
var.1.CFH 1:196642233:G:A
peptide level
173:175 110:110 39:40
NGFYPATRpc2_NP.1
p−maj=0.33, pX=0.11, N=322
var.1.CFH 1:196642233:G:A
peptide level
175:175 110:110 40:40
NGFYPATRpc2_NP.2
p−maj=1, pX=0.74, N=325
var.1.CFH 1:196642233:G:A
peptide level
175:175 110:110 40:40
NGFYPATRpc2_NP.3
p−maj=1, pX=0.13, N=325
var.1.CFH 1:196642233:G:A
peptide level
171:175 107:110 40:40
NGFYPATRpc2_NP.4
p−maj=0.6, pX=0.88, N=318
var.1.CFH 1:196642233:G:A
peptide level
175:175 110:110 39:40
NGFYPATRpc2_NP.5
p−maj=0.12, pX=0.38, N=324
var.1.CFH 1:196642233:G:A
peptide level
175:175 110:110 40:40
NGQWSEPPKpc2_NP.1
p−maj=1, pX=0.42, N=325
var.1.CFH 1:196642233:G:A
peptide level
175:175 110:110 40:40
NGQWSEPPKpc2_NP.2
p−maj=1, pX=0.91, N=325
var.1.CFH 1:196642233:G:A
peptide level
175:175 110:110 40:40
NGQWSEPPKpc2_NP.3
p−maj=1, pX=0.76, N=325
var.1.CFH 1:196642233:G:A
peptide level
175:175 110:110 40:40
NGQWSEPPKpc2_NP.4
p−maj=1, pX=0.26, N=325
var.1.CFH 1:196642233:G:A
peptide level
175:175 110:110 40:40
NGQWSEPPKpc2_NP.5
p−maj=1, pX=0.16, N=325

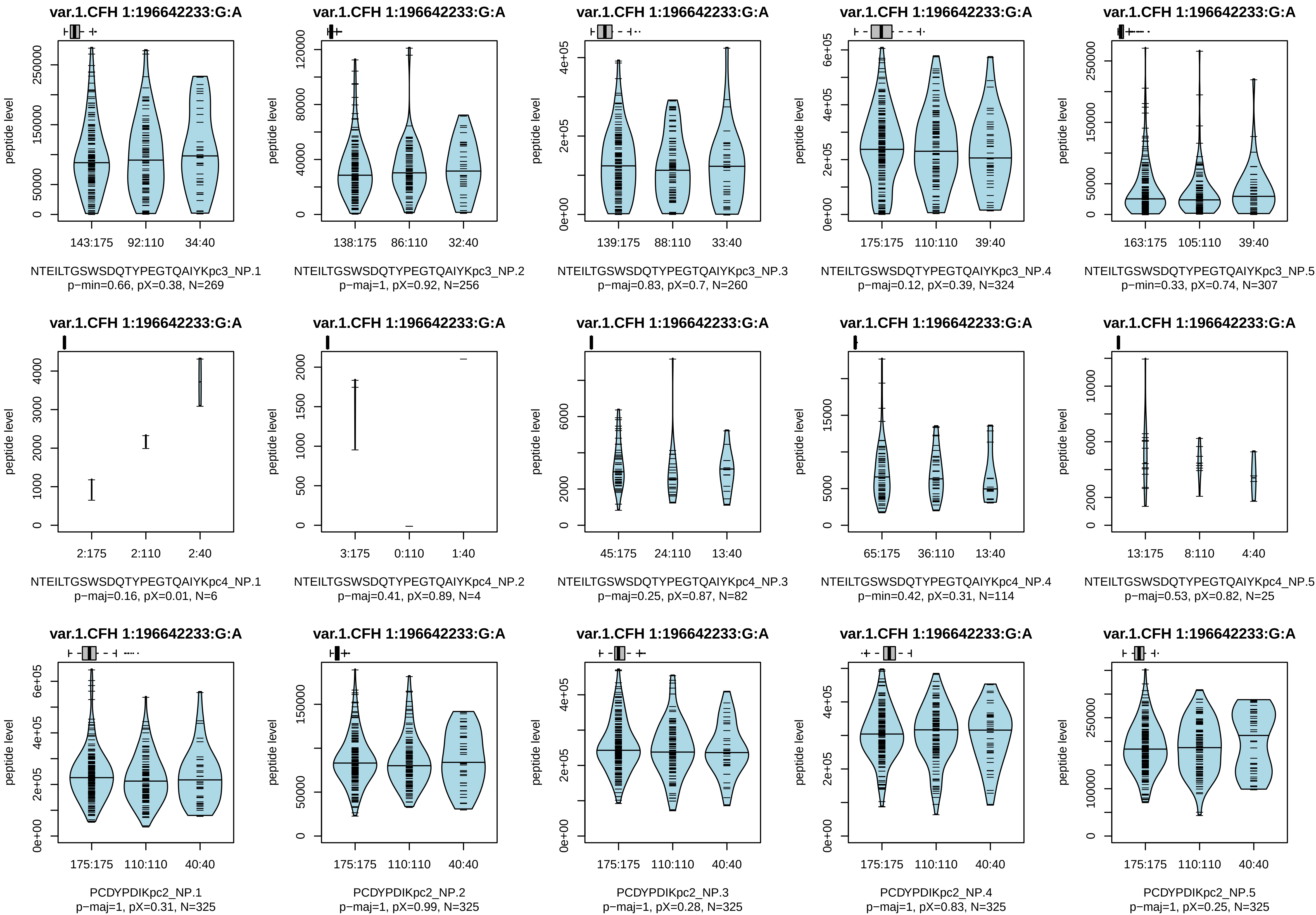
var.1.CFH 1:196642233:G:A
peptide level
143:175
92:110
34:40
NTEILTGSWSDQTYPEGTQAIYKpc3_NP.1
p−min=0.66, pX=0.38, N=269
138:175
86:110
32:40
NTEILTGSWSDQTYPEGTQAIYKpc3_NP.2
p−maj=1, pX=0.92, N=256
139:175
88:110
33:40
NTEILTGSWSDQTYPEGTQAIYKpc3_NP.3
p−maj=0.83, pX=0.7, N=260
175:175
110:110
39:40
NTEILTGSWSDQTYPEGTQAIYKpc3_NP.4
p−maj=0.12, pX=0.39, N=324
163:175
105:110
39:40
NTEILTGSWSDQTYPEGTQAIYKpc3_NP.5
p−min=0.33, pX=0.74, N=307
2:175
2:110
2:40
NTEILTGSWSDQTYPEGTQAIYKpc4_NP.1
p−maj=0.16, pX=0.01, N=6
3:175
0:110
1:40
NTEILTGSWSDQTYPEGTQAIYKpc4_NP.2
p−maj=0.41, pX=0.89, N=4
45:175
24:110
13:40
NTEILTGSWSDQTYPEGTQAIYKpc4_NP.3
p−maj=0.25, pX=0.87, N=82
65:175
36:110
13:40
NTEILTGSWSDQTYPEGTQAIYKpc4_NP.4
p−min=0.42, pX=0.31, N=114
13:175
8:110
4:40
NTEILTGSWSDQTYPEGTQAIYKpc4_NP.5
p−maj=0.53, pX=0.82, N=25
175:175
110:110
40:40
PCDYPDIKpc2_NP.1
p−maj=1, pX=0.31, N=325
PCDYPDIKpc2_NP.2
p−maj=1, pX=0.99, N=325
PCDYPDIKpc2_NP.3
p−maj=1, pX=0.28, N=325
PCDYPDIKpc2_NP.4
p−maj=1, pX=0.83, N=325
PCDYPDIKpc2_NP.5
p−maj=1, pX=0.25, N=325

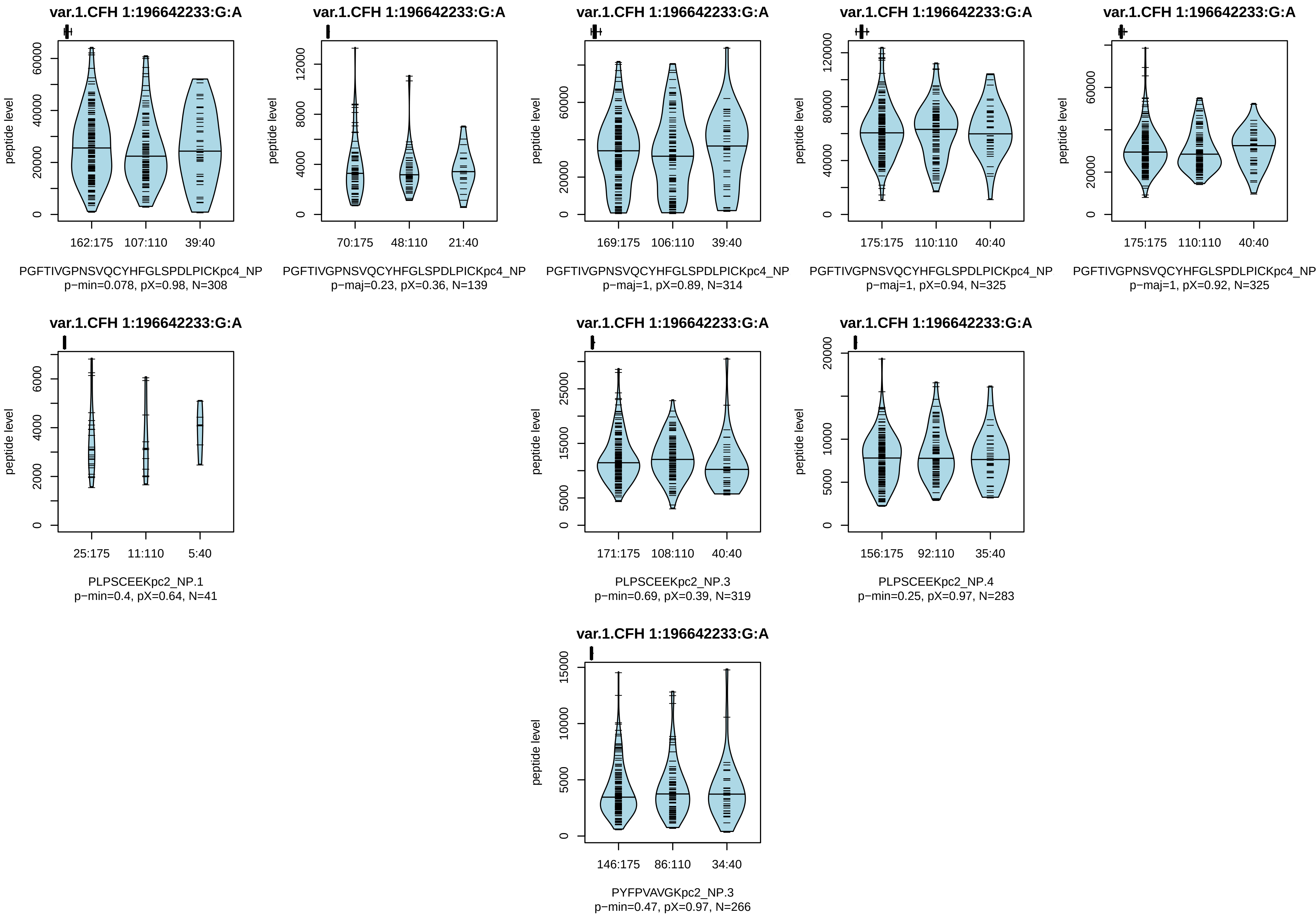
var.1.CFH 1:196642233:G:A
peptide level
162:175
107:110
39:40
PGFTIVGPNSVQCYHFGLSPDLPICKpc4_NP
p−min=0.078, pX=0.98, N=308
var.1.CFH 1:196642233:G:A
peptide level
70:175
48:110
21:40
PGFTIVGPNSVQCYHFGLSPDLPICKpc4_NP
p−maj=0.23, pX=0.36, N=139
var.1.CFH 1:196642233:G:A
peptide level
169:175
106:110
39:40
PGFTIVGPNSVQCYHFGLSPDLPICKpc4_NP
p−maj=1, pX=0.89, N=314
var.1.CFH 1:196642233:G:A
peptide level
175:175
110:110
40:40
PGFTIVGPNSVQCYHFGLSPDLPICKpc4_NP
p−maj=1, pX=0.94, N=325
var.1.CFH 1:196642233:G:A
peptide level
175:175
110:110
40:40
PGFTIVGPNSVQCYHFGLSPDLPICKpc4_NP
p−maj=1, pX=0.92, N=325
var.1.CFH 1:196642233:G:A
peptide level
25:175
11:110
5:40
PLPSCEEKpc2_NP.1
p−min=0.4, pX=0.64, N=41
var.1.CFH 1:196642233:G:A
peptide level
171:175
108:110
40:40
PLPSCEEKpc2_NP.3
p−min=0.69, pX=0.39, N=319
var.1.CFH 1:196642233:G:A
peptide level
156:175
92:110
35:40
PLPSCEEKpc2_NP.4
p−min=0.25, pX=0.97, N=283
var.1.CFH 1:196642233:G:A
peptide level
146:175
86:110
34:40
PYFPVAVGKpc2_NP.3
p−min=0.47, pX=0.97, N=266

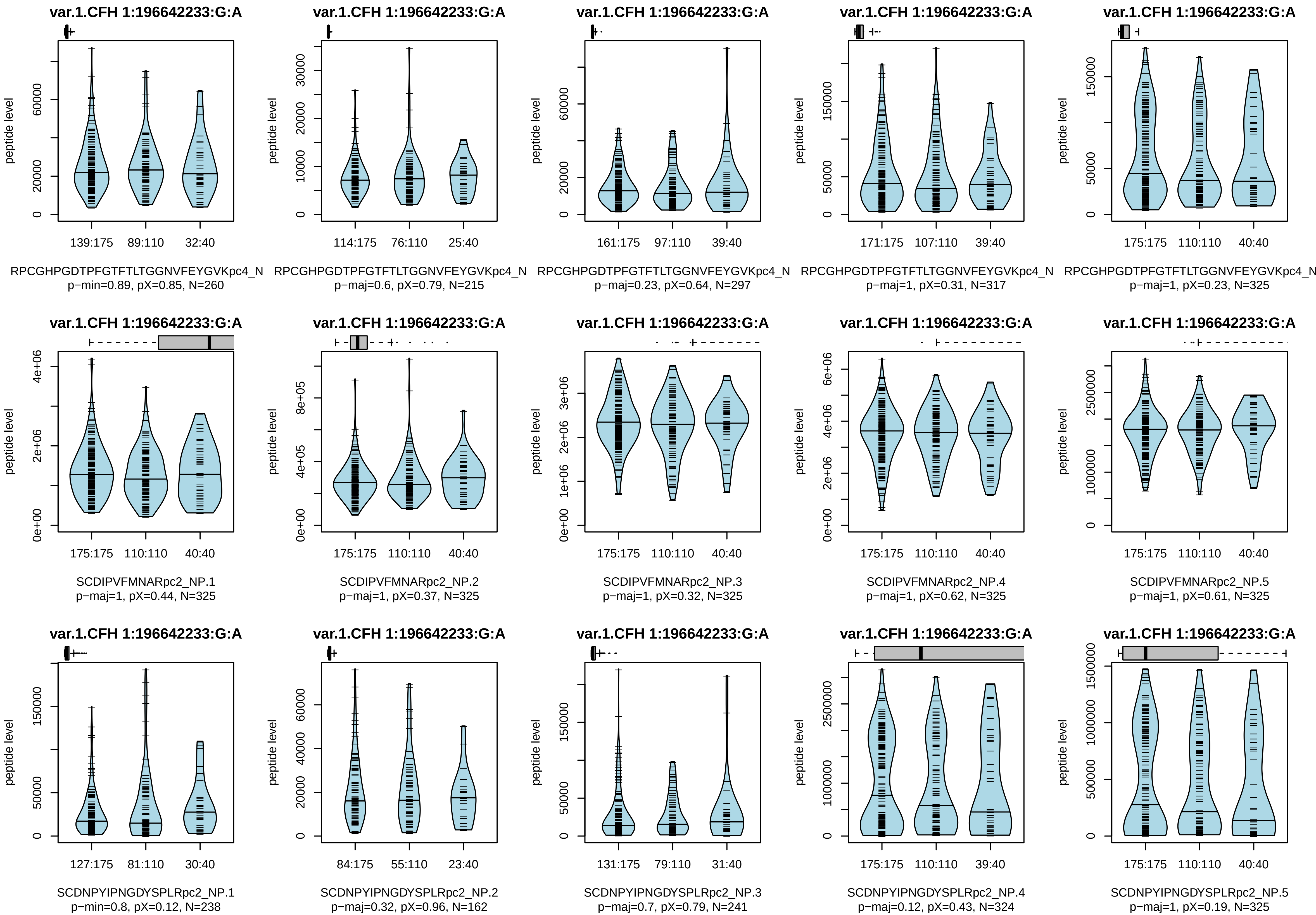
var.1.CFH 1:196642233:G:A
peptide level
139:175
89:110
32:40
RPCGHPGDTPFGTFTLTGGNVFEYGVKpc4_N
p−min=0.89, pX=0.85, N=260
var.1.CFH 1:196642233:G:A
peptide level
114:175
76:110
25:40
RPCGHPGDTPFGTFTLTGGNVFEYGVKpc4_N
p−maj=0.6, pX=0.79, N=215
var.1.CFH 1:196642233:G:A
peptide level
161:175
97:110
39:40
RPCGHPGDTPFGTFTLTGGNVFEYGVKpc4_N
p−maj=0.23, pX=0.64, N=297
var.1.CFH 1:196642233:G:A
peptide level
171:175
107:110
39:40
RPCGHPGDTPFGTFTLTGGNVFEYGVKpc4_N
p−maj=1, pX=0.31, N=317
var.1.CFH 1:196642233:G:A
peptide level
175:175
110:110
40:40
RPCGHPGDTPFGTFTLTGGNVFEYGVKpc4_N
p−maj=1, pX=0.23, N=325
var.1.CFH 1:196642233:G:A
peptide level
175:175
110:110
40:40
SCDIPVFMNARpc2_NP.1
p−maj=1, pX=0.44, N=325
var.1.CFH 1:196642233:G:A
peptide level
175:175
110:110
40:40
SCDIPVFMNARpc2_NP.2
p−maj=1, pX=0.37, N=325
var.1.CFH 1:196642233:G:A
peptide level
175:175
110:110
40:40
SCDIPVFMNARpc2_NP.3
p−maj=1, pX=0.32, N=325
var.1.CFH 1:196642233:G:A
peptide level
175:175
110:110
40:40
SCDIPVFMNARpc2_NP.4
p−maj=1, pX=0.62, N=325
var.1.CFH 1:196642233:G:A
peptide level
175:175
110:110
40:40
SCDIPVFMNARpc2_NP.5
p−maj=1, pX=0.61, N=325
var.1.CFH 1:196642233:G:A
peptide level
127:175
81:110
30:40
SCDNPYIPNGDYSPLRpc2_NP.1
p−min=0.8, pX=0.12, N=238
var.1.CFH 1:196642233:G:A
peptide level
84:175
55:110
23:40
SCDNPYIPNGDYSPLRpc2_NP.2
p−maj=0.32, pX=0.96, N=162
var.1.CFH 1:196642233:G:A
peptide level
131:175
79:110
31:40
SCDNPYIPNGDYSPLRpc2_NP.3
p−maj=0.7, pX=0.79, N=241
var.1.CFH 1:196642233:G:A
peptide level
175:175
110:110
39:40
SCDNPYIPNGDYSPLRpc2_NP.4
p−maj=0.12, pX=0.43, N=324
var.1.CFH 1:196642233:G:A
peptide level
175:175
110:110
40:40
SCDNPYIPNGDYSPLRpc2_NP.5
p−maj=1, pX=0.19, N=325

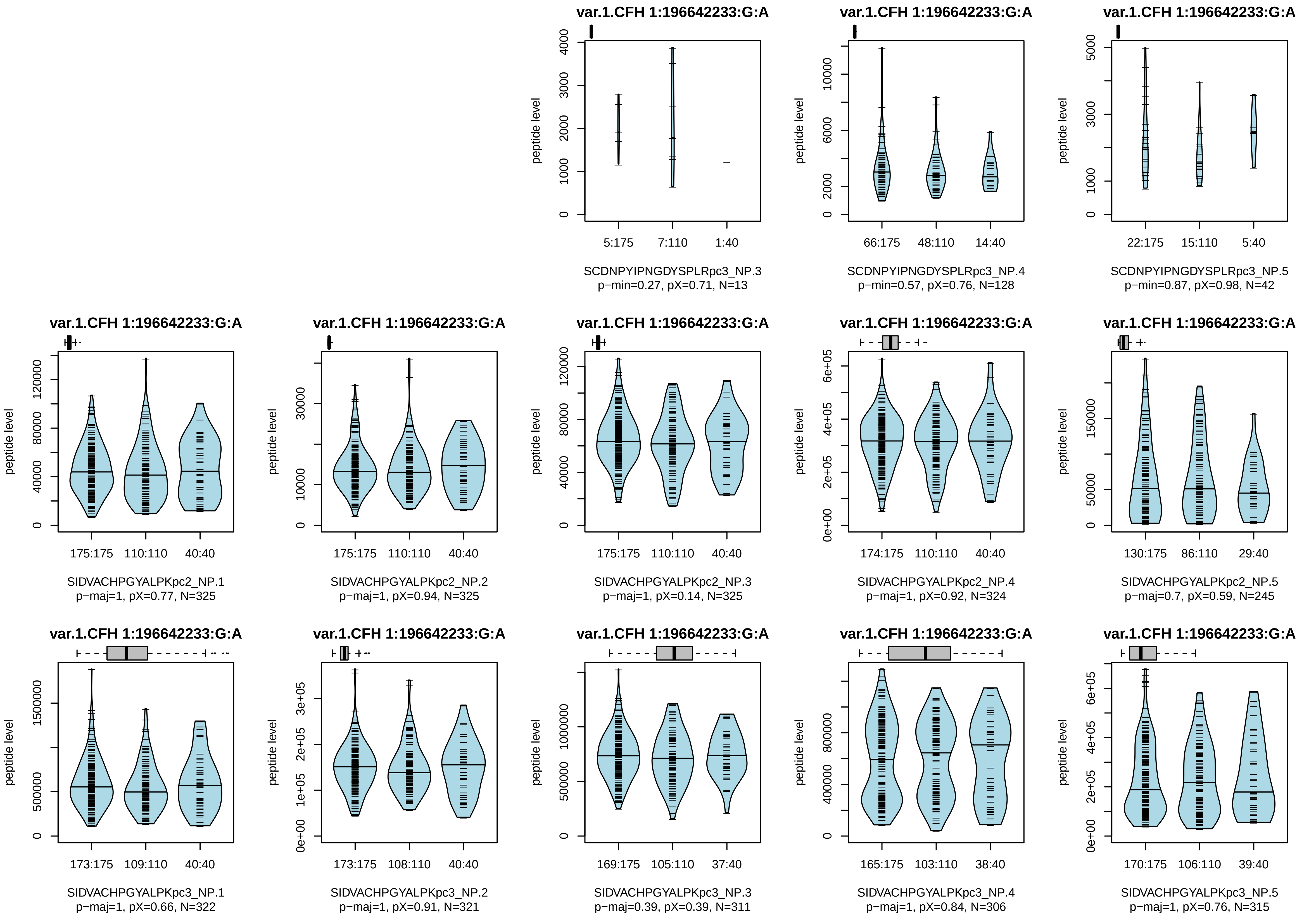
var.1.CFH 1:196642233:G:A
peptide level
5:175
7:110
1:40
SCDNPYIPNGDYSPLRpc3_NP.3
p−min=0.27, pX=0.71, N=13
var.1.CFH 1:196642233:G:A
peptide level
66:175
48:110
14:40
SCDNPYIPNGDYSPLRpc3_NP.4
p−min=0.57, pX=0.76, N=128
var.1.CFH 1:196642233:G:A
peptide level
22:175
15:110
5:40
SCDNPYIPNGDYSPLRpc3_NP.5
p−min=0.87, pX=0.98, N=42
var.1.CFH 1:196642233:G:A
peptide level
175:175
110:110
40:40
SIDVACHPGYALPKpc2_NP.1
p−maj=1, pX=0.77, N=325
var.1.CFH 1:196642233:G:A
peptide level
175:175
110:110
40:40
SIDVACHPGYALPKpc2_NP.2
p−maj=1, pX=0.94, N=325
var.1.CFH 1:196642233:G:A
peptide level
175:175
110:110
40:40
SIDVACHPGYALPKpc2_NP.3
p−maj=1, pX=0.14, N=325
var.1.CFH 1:196642233:G:A
peptide level
174:175
110:110
40:40
SIDVACHPGYALPKpc2_NP.4
p−maj=1, pX=0.92, N=324
var.1.CFH 1:196642233:G:A
peptide level
130:175
86:110
29:40
SIDVACHPGYALPKpc2_NP.5
p−maj=0.7, pX=0.59, N=245
var.1.CFH 1:196642233:G:A
peptide level
173:175
109:110
40:40
SIDVACHPGYALPKpc3_NP.1
p−maj=1, pX=0.66, N=322
var.1.CFH 1:196642233:G:A
peptide level
173:175
108:110
40:40
SIDVACHPGYALPKpc3_NP.2
p−maj=1, pX=0.91, N=321
var.1.CFH 1:196642233:G:A
peptide level
169:175
105:110
37:40
SIDVACHPGYALPKpc3_NP.3
p−maj=0.39, pX=0.39, N=311
var.1.CFH 1:196642233:G:A
peptide level
165:175
103:110
38:40
SIDVACHPGYALPKpc3_NP.4
p−maj=1, pX=0.84, N=306
var.1.CFH 1:196642233:G:A
peptide level
170:175
106:110
39:40
SIDVACHPGYALPKpc3_NP.5
p−maj=1, pX=0.76, N=315

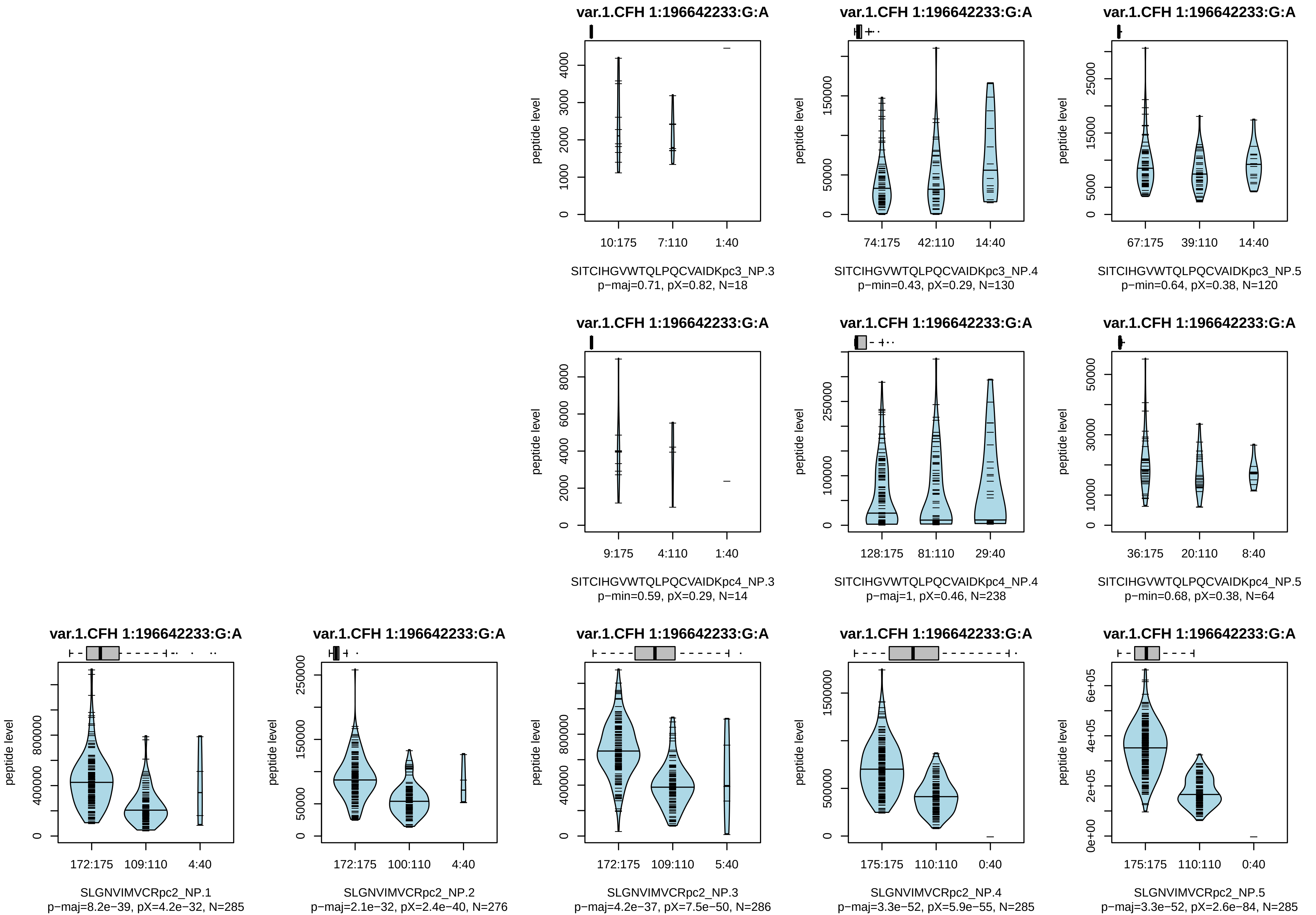
var.1.CFH 1:196642233:G:A
peptide level
0
1000
2000
3000
4000
10:175
7:110
1:40
SITCIHGVWTQLPQCVAIDKpc3_NP.3
p−maj=0.71, pX=0.82, N=18
var.1.CFH 1:196642233:G:A
peptide level
0
50000
150000
74:175
42:110
14:40
SITCIHGVWTQLPQCVAIDKpc3_NP.4
p−min=0.43, pX=0.29, N=130
var.1.CFH 1:196642233:G:A
peptide level
0
5000
15000
25000
67:175
39:110
14:40
SITCIHGVWTQLPQCVAIDKpc3_NP.5
p−min=0.64, pX=0.38, N=120
var.1.CFH 1:196642233:G:A
peptide level
0
2000
4000
6000
8000
9:175
4:110
1:40
SITCIHGVWTQLPQCVAIDKpc4_NP.3
p−min=0.59, pX=0.29, N=14
var.1.CFH 1:196642233:G:A
peptide level
0
100000
250000
128:175
81:110
29:40
SITCIHGVWTQLPQCVAIDKpc4_NP.4
p−maj=1, pX=0.46, N=238
var.1.CFH 1:196642233:G:A
peptide level
10000
30000
50000
36:175
20:110
8:40
SITCIHGVWTQLPQCVAIDKpc4_NP.5
p−min=0.68, pX=0.38, N=64
var.1.CFH 1:196642233:G:A
peptide level
0
400000
800000
172:175
109:110
4:40
SLGNVIMVCRpc2_NP.1
p−maj=8.2e−39, pX=4.2e−32, N=285
var.1.CFH 1:196642233:G:A
peptide level
0
50000
150000
250000
172:175
100:110
4:40
SLGNVIMVCRpc2_NP.2
p−maj=2.1e−32, pX=2.4e−40, N=276
var.1.CFH 1:196642233:G:A
peptide level
0
400000
800000
172:175
109:110
5:40
SLGNVIMVCRpc2_NP.3
p−maj=4.2e−37, pX=7.5e−50, N=286
var.1.CFH 1:196642233:G:A
peptide level
0
500000
1500000
175:175
110:110
0:40
SLGNVIMVCRpc2_NP.4
p−maj=3.3e−52, pX=5.9e−55, N=285
var.1.CFH 1:196642233:G:A
peptide level
0e+00
2e+05
4e+05
6e+05
175:175
110:110
0:40
SLGNVIMVCRpc2_NP.5
p−maj=3.3e−52, pX=2.6e−84, N=285

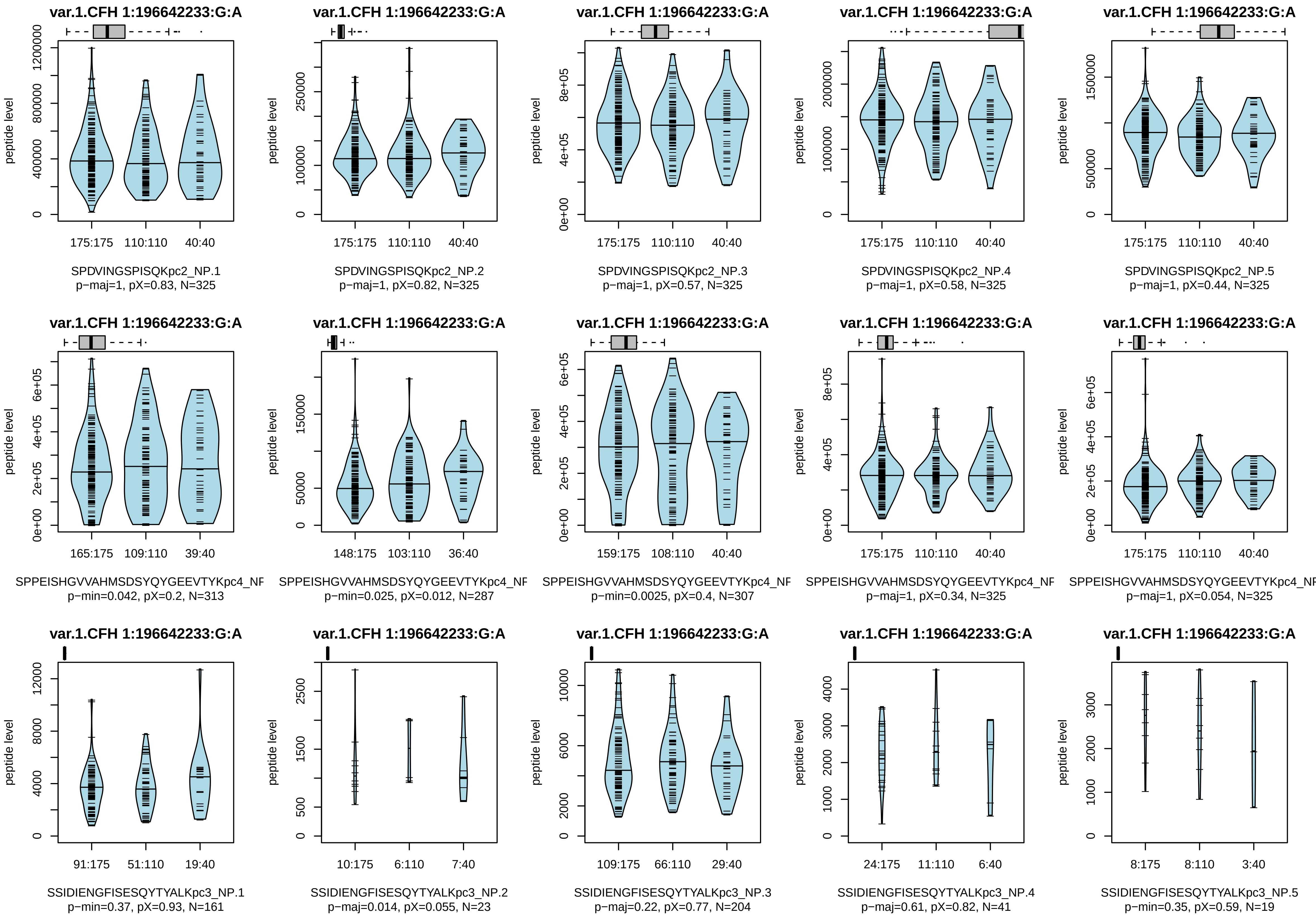
var.1.CFH 1:196642233:G:A
peptide level
175:175
110:110
40:40
SPDVINGSPISQKpc2_NP.1
p−maj=1, pX=0.83, N=325
var.1.CFH 1:196642233:G:A
peptide level
175:175
110:110
40:40
SPDVINGSPISQKpc2_NP.2
p−maj=1, pX=0.82, N=325
var.1.CFH 1:196642233:G:A
peptide level
175:175
110:110
40:40
SPDVINGSPISQKpc2_NP.3
p−maj=1, pX=0.57, N=325
var.1.CFH 1:196642233:G:A
peptide level
175:175
110:110
40:40
SPDVINGSPISQKpc2_NP.4
p−maj=1, pX=0.58, N=325
var.1.CFH 1:196642233:G:A
peptide level
175:175
110:110
40:40
SPDVINGSPISQKpc2_NP.5
p−maj=1, pX=0.44, N=325
var.1.CFH 1:196642233:G:A
peptide level
165:175
109:110
39:40
SPPEISHGVVAHMSDSYQYGEEVTYKpc4_NF
p−min=0.042, pX=0.2, N=313
var.1.CFH 1:196642233:G:A
peptide level
148:175
103:110
36:40
SPPEISHGVVAHMSDSYQYGEEVTYKpc4_NF
p−min=0.025, pX=0.012, N=287
var.1.CFH 1:196642233:G:A
peptide level
159:175
108:110
40:40
SPPEISHGVVAHMSDSYQYGEEVTYKpc4_NF
p−min=0.0025, pX=0.4, N=307
var.1.CFH 1:196642233:G:A
peptide level
175:175
110:110
40:40
SPPEISHGVVAHMSDSYQYGEEVTYKpc4_NF
p−maj=1, pX=0.34, N=325
var.1.CFH 1:196642233:G:A
peptide level
175:175
110:110
40:40
SPPEISHGVVAHMSDSYQYGEEVTYKpc4_NF
p−maj=1, pX=0.054, N=325
var.1.CFH 1:196642233:G:A
peptide level
91:175
51:110
19:40
SSIDIENGFISESQYTYALKpc3_NP.1
p−min=0.37, pX=0.93, N=161
var.1.CFH 1:196642233:G:A
peptide level
10:175
6:110
7:40
SSIDIENGFISESQYTYALKpc3_NP.2
p−maj=0.014, pX=0.055, N=23
var.1.CFH 1:196642233:G:A
peptide level
109:175
66:110
29:40
SSIDIENGFISESQYTYALKpc3_NP.3
p−maj=0.22, pX=0.77, N=204
var.1.CFH 1:196642233:G:A
peptide level
24:175
11:110
6:40
SSIDIENGFISESQYTYALKpc3_NP.4
p−maj=0.61, pX=0.82, N=41
var.1.CFH 1:196642233:G:A
peptide level
8:175
8:110
3:40
SSIDIENGFISESQYTYALKpc3_NP.5
p−min=0.35, pX=0.59, N=19

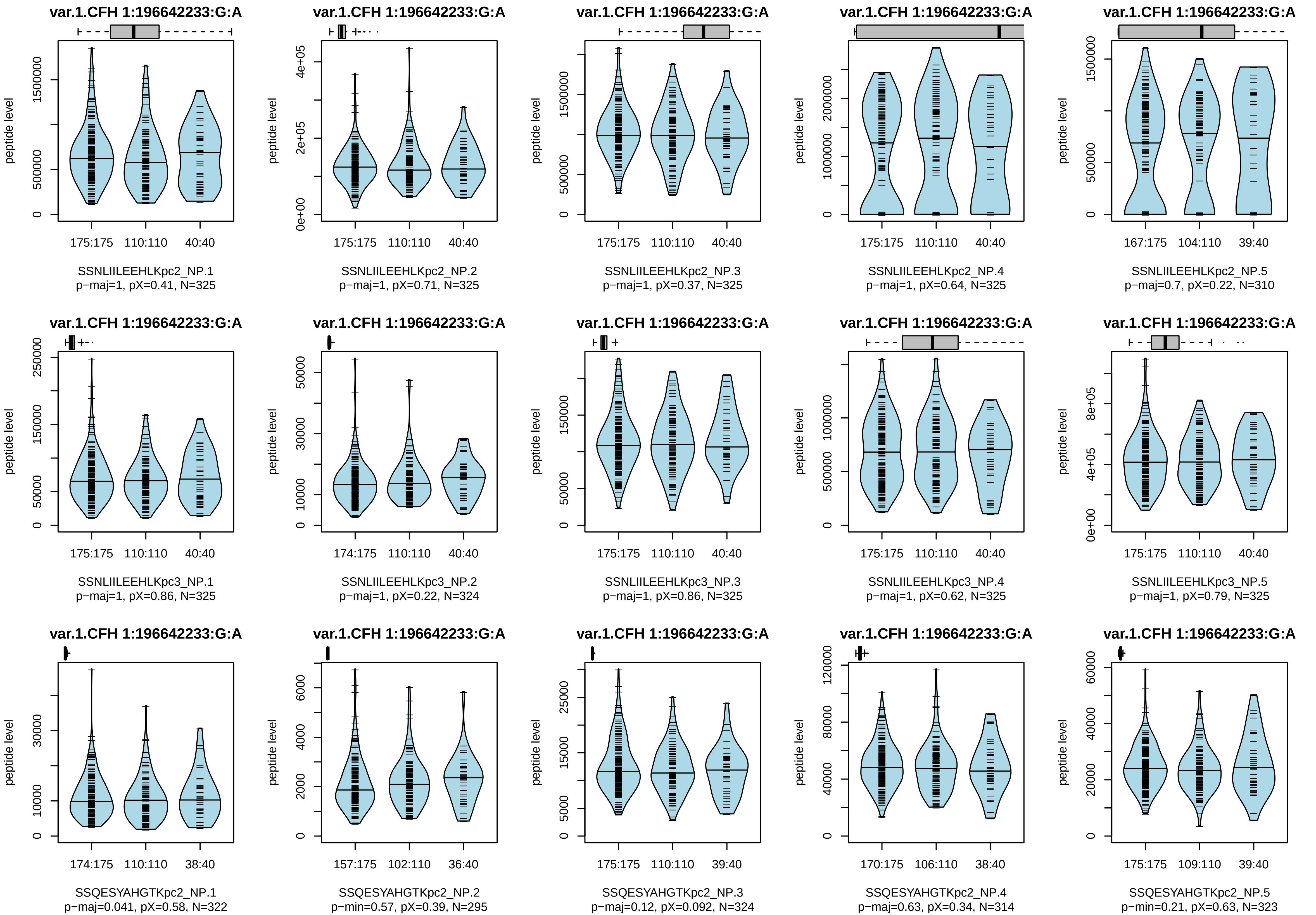
var.1.CFH 1:196642233:G:A
peptide level
175:175
110:110
40:40
SSNLIILEEHLKpc2_NP.1
p−maj=1, pX=0.41, N=325
var.1.CFH 1:196642233:G:A
175:175
110:110
40:40
SSNLIILEEHLKpc2_NP.2
p−maj=1, pX=0.71, N=325
var.1.CFH 1:196642233:G:A
175:175
110:110
40:40
SSNLIILEEHLKpc2_NP.3
p−maj=1, pX=0.37, N=325
var.1.CFH 1:196642233:G:A
175:175
110:110
40:40
SSNLIILEEHLKpc2_NP.4
p−maj=1, pX=0.64, N=325
var.1.CFH 1:196642233:G:A
167:175
104:110
39:40
SSNLIILEEHLKpc2_NP.5
p−maj=0.7, pX=0.22, N=310
var.1.CFH 1:196642233:G:A
175:175
110:110
40:40
SSNLIILEEHLKpc3_NP.1
p−maj=1, pX=0.86, N=325
var.1.CFH 1:196642233:G:A
174:175
110:110
40:40
SSNLIILEEHLKpc3_NP.2
p−maj=1, pX=0.22, N=324
var.1.CFH 1:196642233:G:A
175:175
110:110
40:40
SSNLIILEEHLKpc3_NP.3
p−maj=1, pX=0.86, N=325
var.1.CFH 1:196642233:G:A
175:175
110:110
40:40
SSNLIILEEHLKpc3_NP.4
p−maj=1, pX=0.62, N=325
var.1.CFH 1:196642233:G:A
175:175
110:110
40:40
SSNLIILEEHLKpc3_NP.5
p−maj=1, pX=0.79, N=325
var.1.CFH 1:196642233:G:A
174:175
110:110
38:40
SSQESYAHGTKpc2_NP.1
p−maj=0.041, pX=0.58, N=322
var.1.CFH 1:196642233:G:A
157:175
102:110
36:40
SSQESYAHGTKpc2_NP.2
p−min=0.57, pX=0.39, N=295
var.1.CFH 1:196642233:G:A
175:175
110:110
39:40
SSQESYAHGTKpc2_NP.3
p−maj=0.12, pX=0.092, N=324
var.1.CFH 1:196642233:G:A
170:175
106:110
38:40
SSQESYAHGTKpc2_NP.4
p−maj=0.63, pX=0.34, N=314
var.1.CFH 1:196642233:G:A
175:175
109:110
39:40
SSQESYAHGTKpc2_NP.5
p−min=0.21, pX=0.63, N=323

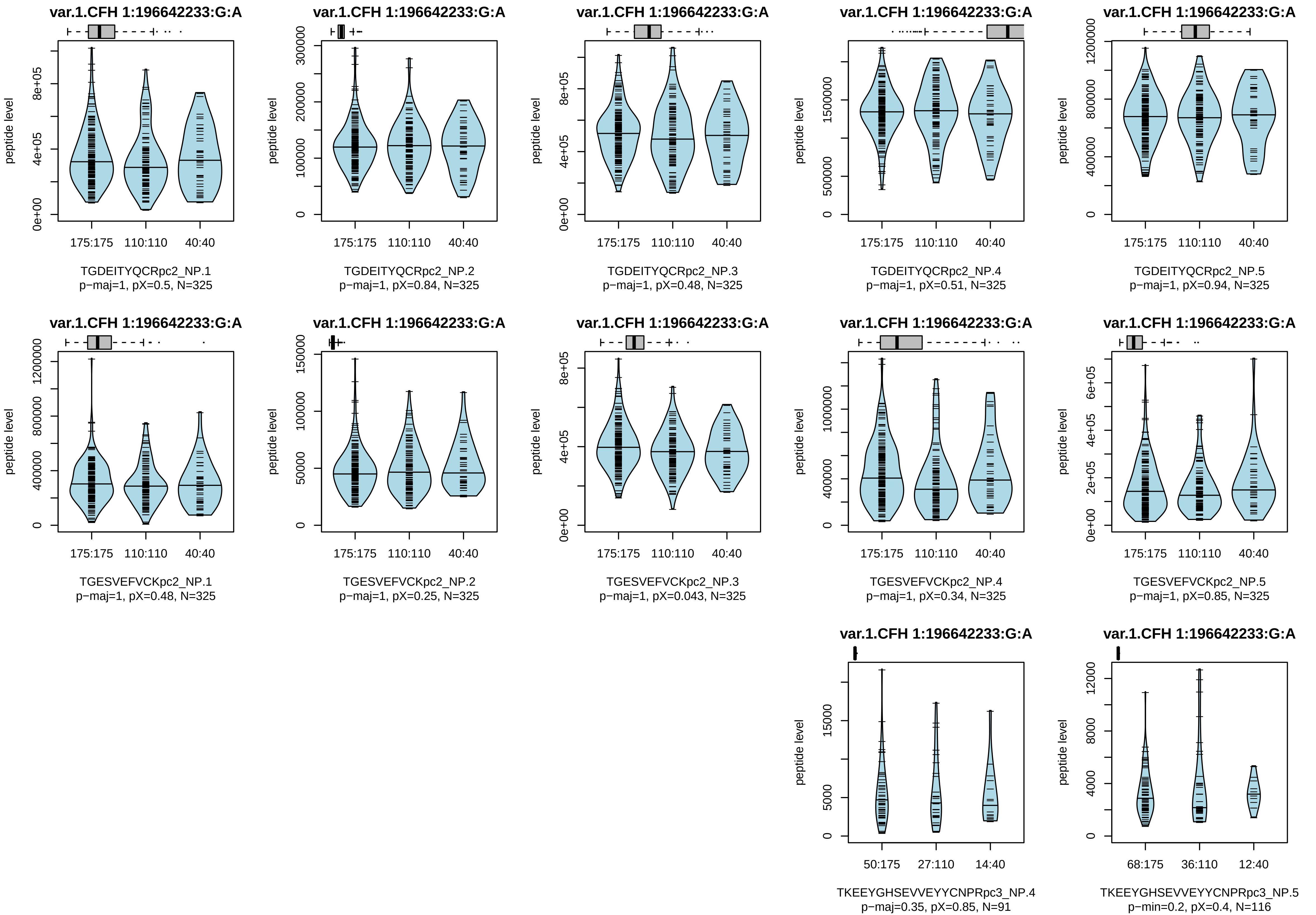
var.1.CFH 1:196642233:G:A
peptide level
175:175
110:110
40:40
TGDEITYQCRpc2_NP.1
p−maj=1, pX=0.5, N=325
var.1.CFH 1:196642233:G:A
TGDEITYQCRpc2_NP.2
p−maj=1, pX=0.84, N=325
var.1.CFH 1:196642233:G:A
TGDEITYQCRpc2_NP.3
p−maj=1, pX=0.48, N=325
var.1.CFH 1:196642233:G:A
TGDEITYQCRpc2_NP.4
p−maj=1, pX=0.51, N=325
var.1.CFH 1:196642233:G:A
TGDEITYQCRpc2_NP.5
p−maj=1, pX=0.94, N=325
var.1.CFH 1:196642233:G:A
TGESVEFVCKpc2_NP.1
p−maj=1, pX=0.48, N=325
var.1.CFH 1:196642233:G:A
TGESVEFVCKpc2_NP.2
p−maj=1, pX=0.25, N=325
var.1.CFH 1:196642233:G:A
TGESVEFVCKpc2_NP.3
p−maj=1, pX=0.043, N=325
var.1.CFH 1:196642233:G:A
TGESVEFVCKpc2_NP.4
p−maj=1, pX=0.34, N=325
var.1.CFH 1:196642233:G:A
TGESVEFVCKpc2_NP.5
p−maj=1, pX=0.85, N=325
var.1.CFH 1:196642233:G:A
50:175
27:110
14:40
TKEEYGHSEVVEYYCNPRpc3_NP.4
p−maj=0.35, pX=0.85, N=91
var.1.CFH 1:196642233:G:A
68:175
36:110
12:40
TKEEYGHSEVVEYYCNPRpc3_NP.5
p−min=0.2, pX=0.4, N=116

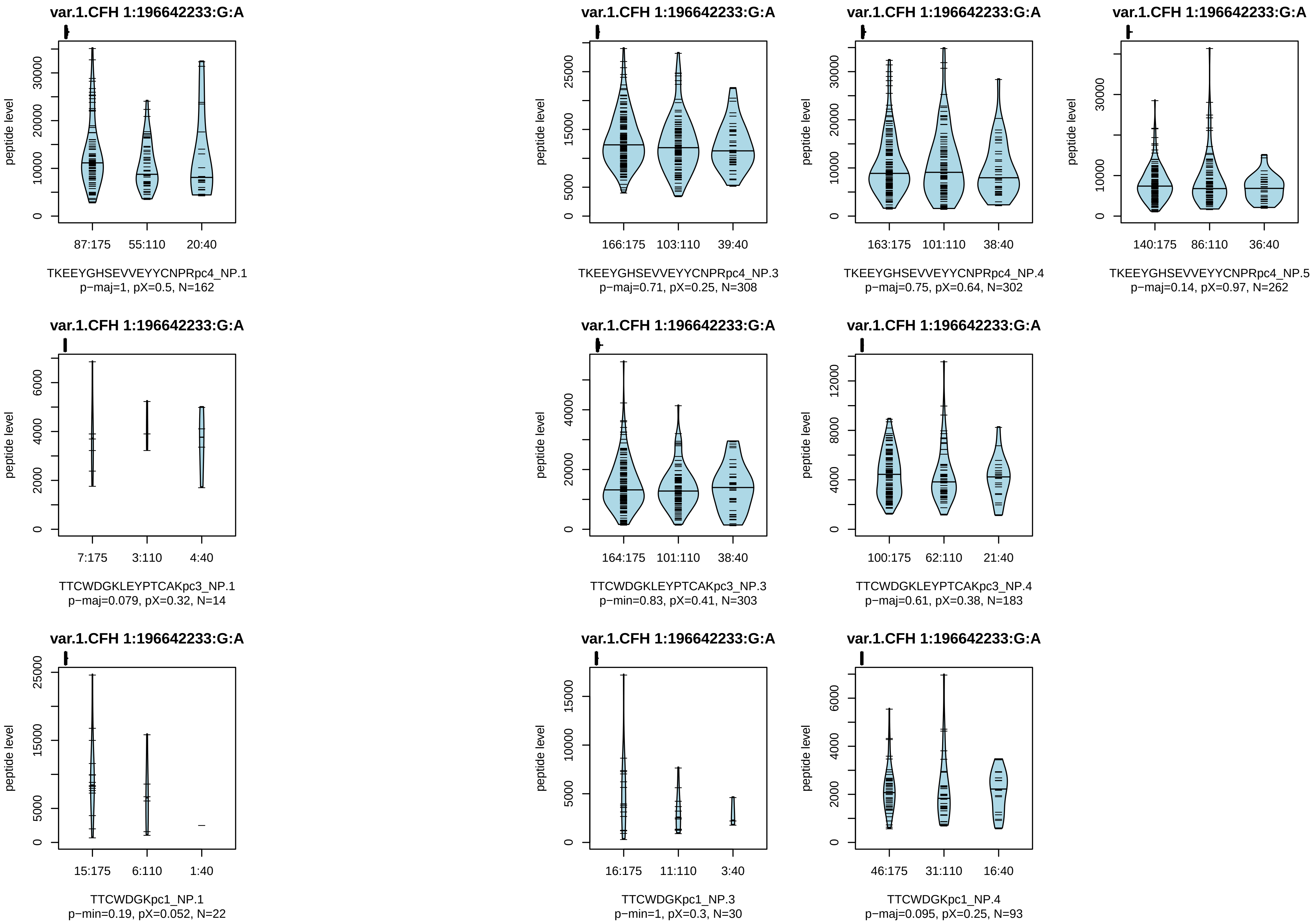
var.1.CFH 1:196642233:G:A
peptide level
87:175
55:110
20:40
TKEEYGHSEVVEYYCNPRpc4_NP.1
p−maj=1, pX=0.5, N=162
var.1.CFH 1:196642233:G:A
peptide level
166:175
103:110
39:40
TKEEYGHSEVVEYYCNPRpc4_NP.3
p−maj=0.71, pX=0.25, N=308
var.1.CFH 1:196642233:G:A
peptide level
163:175
101:110
38:40
TKEEYGHSEVVEYYCNPRpc4_NP.4
p−maj=0.75, pX=0.64, N=302
var.1.CFH 1:196642233:G:A
peptide level
140:175
86:110
36:40
TKEEYGHSEVVEYYCNPRpc4_NP.5
p−maj=0.14, pX=0.97, N=262
var.1.CFH 1:196642233:G:A
peptide level
7:175
3:110
4:40
TTCWDGKLEYPTCAKpc3_NP.1
p−maj=0.079, pX=0.32, N=14
var.1.CFH 1:196642233:G:A
peptide level
164:175
101:110
38:40
TTCWDGKLEYPTCAKpc3_NP.3
p−min=0.83, pX=0.41, N=303
var.1.CFH 1:196642233:G:A
peptide level
100:175
62:110
21:40
TTCWDGKLEYPTCAKpc3_NP.4
p−maj=0.61, pX=0.38, N=183
var.1.CFH 1:196642233:G:A
peptide level
15:175
6:110
1:40
TTCWDGKpc1_NP.1
p−min=0.19, pX=0.052, N=22
var.1.CFH 1:196642233:G:A
peptide level
16:175
11:110
3:40
TTCWDGKpc1_NP.3
p−min=1, pX=0.3, N=30
var.1.CFH 1:196642233:G:A
peptide level
46:175
31:110
16:40
TTCWDGKpc1_NP.4
p−maj=0.095, pX=0.25, N=93

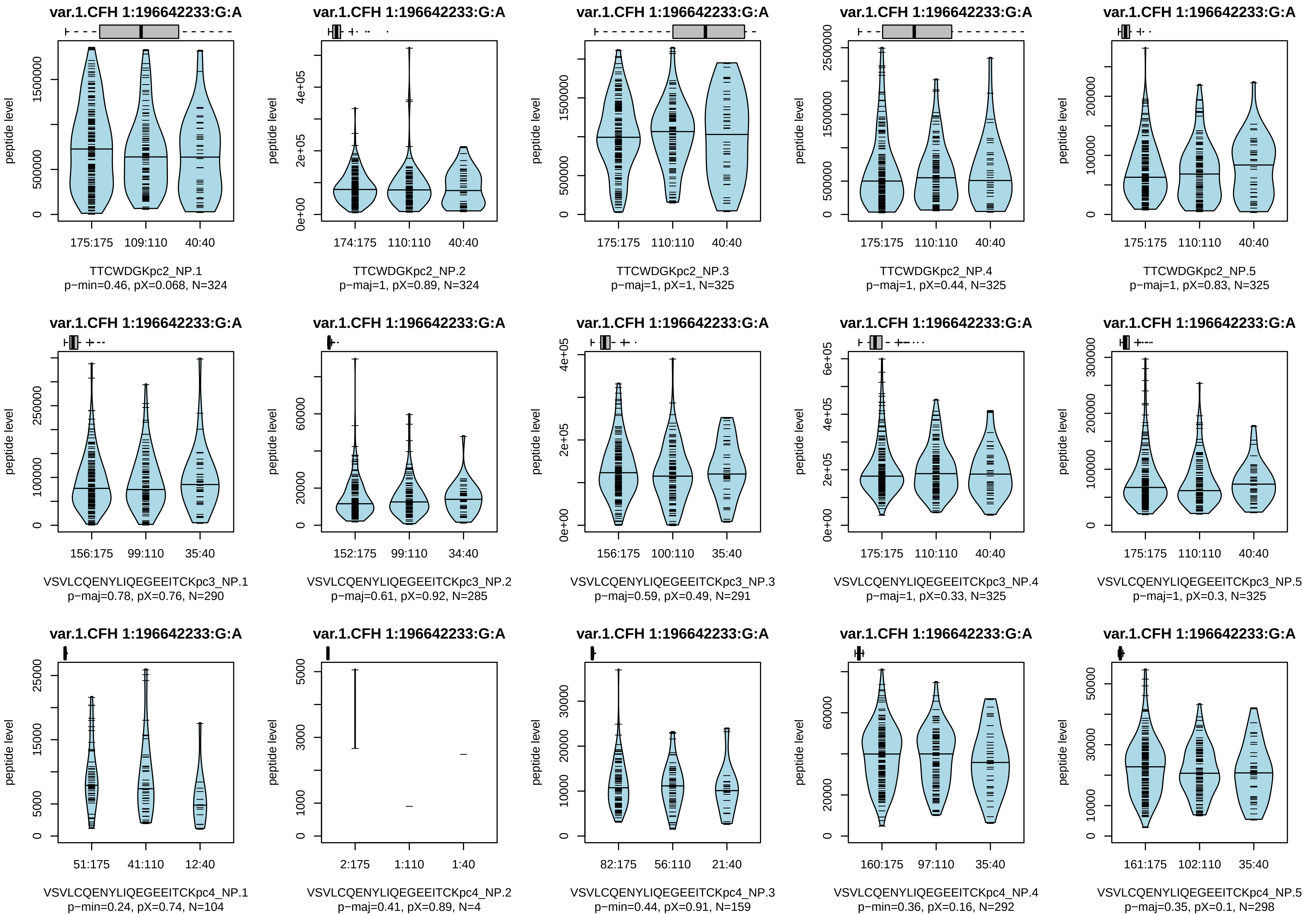

var.1.CFH 1:196642233:G:A
peptide level
0
500000
1500000
175:175
109:110
40:40
TTCWDGKpc2_NP.1
p−min=0.46, pX=0.068, N=324
var.1.CFH 1:196642233:G:A
peptide level
0e+00
2e+05
4e+05
174:175
110:110
40:40
TTCWDGKpc2_NP.2
p−maj=1, pX=0.89, N=324
var.1.CFH 1:196642233:G:A
peptide level
0
500000
1500000
175:175
110:110
40:40
TTCWDGKpc2_NP.3
p−maj=1, pX=1, N=325
var.1.CFH 1:196642233:G:A
peptide level
0
500000
1500000
2500000
175:175
110:110
40:40
TTCWDGKpc2_NP.4
p−maj=1, pX=0.44, N=325
var.1.CFH 1:196642233:G:A
peptide level
0
100000
200000
175:175
110:110
40:40
TTCWDGKpc2_NP.5
p−maj=1, pX=0.83, N=325
var.1.CFH 1:196642233:G:A
peptide level
0
100000
250000
156:175
99:110
35:40
VSVLCQENYLIQEGEEITCKpc3_NP.1
p−maj=0.78, pX=0.76, N=290
var.1.CFH 1:196642233:G:A
peptide level
0
20000
60000
152:175
99:110
34:40
VSVLCQENYLIQEGEEITCKpc3_NP.2
p−maj=0.61, pX=0.92, N=285
var.1.CFH 1:196642233:G:A
peptide level
0e+00
2e+05
4e+05
156:175
100:110
35:40
VSVLCQENYLIQEGEEITCKpc3_NP.3
p−maj=0.59, pX=0.49, N=291
var.1.CFH 1:196642233:G:A
peptide level
0e+00
2e+05
4e+05
6e+05
175:175
110:110
40:40
VSVLCQENYLIQEGEEITCKpc3_NP.4
p−maj=1, pX=0.33, N=325
var.1.CFH 1:196642233:G:A
peptide level
0
100000
200000
300000
175:175
110:110
40:40
VSVLCQENYLIQEGEEITCKpc3_NP.5
p−maj=1, pX=0.3, N=325
var.1.CFH 1:196642233:G:A
peptide level
0
5000
15000
25000
51:175
41:110
12:40
VSVLCQENYLIQEGEEITCKpc4_NP.1
p−min=0.24, pX=0.74, N=104
var.1.CFH 1:196642233:G:A
peptide level
0
1000
3000
5000
2:175
1:110
1:40
VSVLCQENYLIQEGEEITCKpc4_NP.2
p−maj=0.41, pX=0.89, N=4
var.1.CFH 1:196642233:G:A
peptide level
0
10000
20000
30000
82:175
56:110
21:40
VSVLCQENYLIQEGEEITCKpc4_NP.3
p−min=0.44, pX=0.91, N=159
var.1.CFH 1:196642233:G:A
peptide level
0
20000
60000
160:175
97:110
35:40
VSVLCQENYLIQEGEEITCKpc4_NP.4
p−min=0.36, pX=0.16, N=292
var.1.CFH 1:196642233:G:A
peptide level
0
10000
30000
50000
161:175
102:110
35:40
VSVLCQENYLIQEGEEITCKpc4_NP.5
p−maj=0.35, pX=0.1, N=298

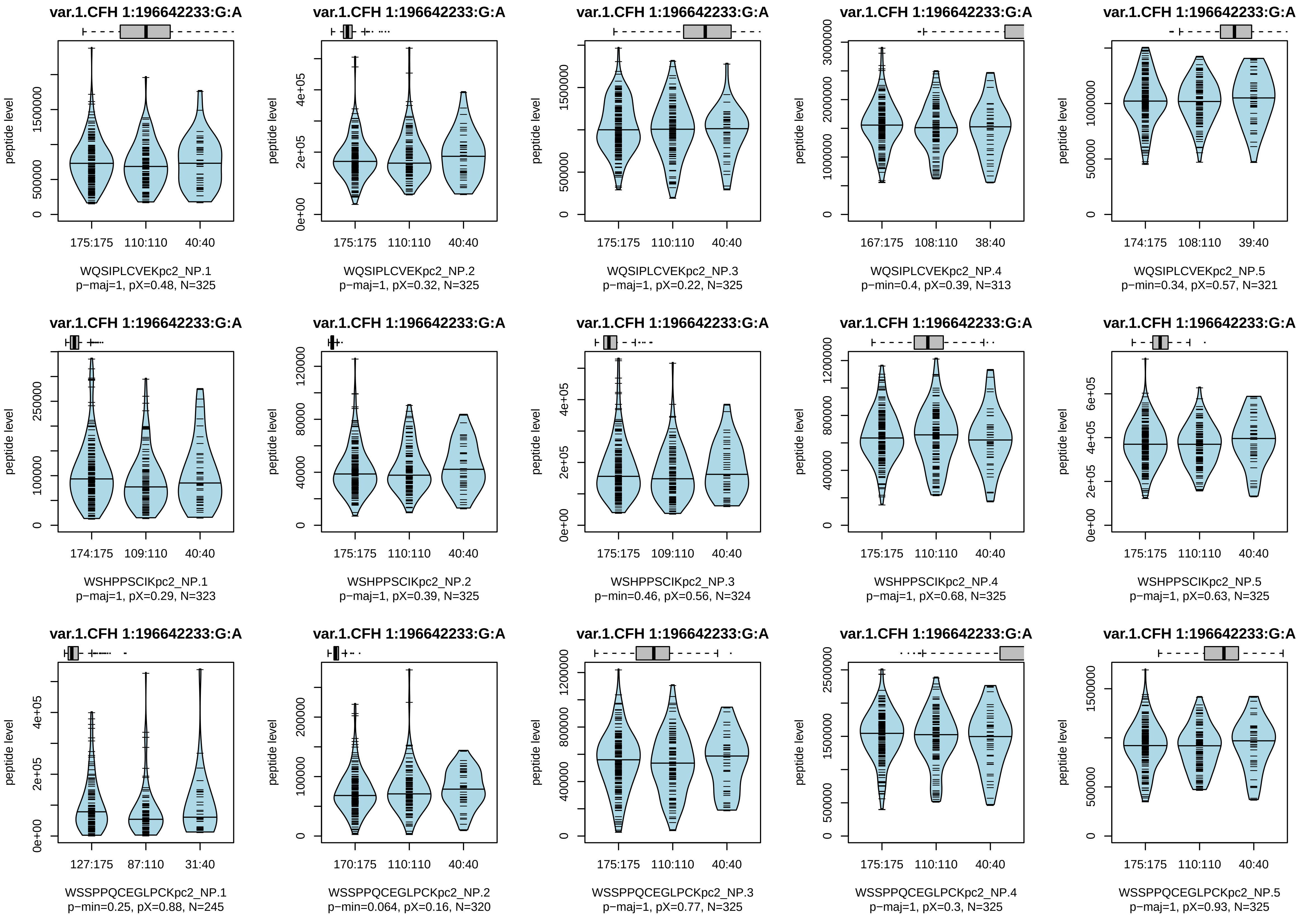
var.1.CFH 1:196642233:G:A
peptide level
175:175 110:110 40:40
WQSIPLCVEKpc2_NP.1
p−maj=1, pX=0.48, N=325
var.1.CFH 1:196642233:G:A
peptide level
175:175 110:110 40:40
WQSIPLCVEKpc2_NP.2
p−maj=1, pX=0.32, N=325
var.1.CFH 1:196642233:G:A
peptide level
175:175 110:110 40:40
WQSIPLCVEKpc2_NP.3
p−maj=1, pX=0.22, N=325
var.1.CFH 1:196642233:G:A
peptide level
167:175 108:110 38:40
WQSIPLCVEKpc2_NP.4
p−min=0.4, pX=0.39, N=313
var.1.CFH 1:196642233:G:A
peptide level
174:175 108:110 39:40
WQSIPLCVEKpc2_NP.5
p−min=0.34, pX=0.57, N=321
var.1.CFH 1:196642233:G:A
peptide level
174:175 109:110 40:40
WSHPPSCIKpc2_NP.1
p−maj=1, pX=0.29, N=323
var.1.CFH 1:196642233:G:A
peptide level
175:175 110:110 40:40
WSHPPSCIKpc2_NP.2
p−maj=1, pX=0.39, N=325
var.1.CFH 1:196642233:G:A
peptide level
175:175 109:110 40:40
WSHPPSCIKpc2_NP.3
p−min=0.46, pX=0.56, N=324
var.1.CFH 1:196642233:G:A
peptide level
175:175 110:110 40:40
WSHPPSCIKpc2_NP.4
p−maj=1, pX=0.68, N=325
var.1.CFH 1:196642233:G:A
peptide level
175:175 110:110 40:40
WSHPPSCIKpc2_NP.5
p−maj=1, pX=0.63, N=325
var.1.CFH 1:196642233:G:A
peptide level
127:175 87:110 31:40
WSSPPQCEGLPCKpc2_NP.1
p−min=0.25, pX=0.88, N=245
var.1.CFH 1:196642233:G:A
peptide level
170:175 110:110 40:40
WSSPPQCEGLPCKpc2_NP.2
p−min=0.064, pX=0.16, N=320
var.1.CFH 1:196642233:G:A
peptide level
175:175 110:110 40:40
WSSPPQCEGLPCKpc2_NP.3
p−maj=1, pX=0.77, N=325
var.1.CFH 1:196642233:G:A
peptide level
175:175 110:110 40:40
WSSPPQCEGLPCKpc2_NP.4
p−maj=1, pX=0.3, N=325
var.1.CFH 1:196642233:G:A
peptide level
175:175 110:110 40:40
WSSPPQCEGLPCKpc2_NP.5
p−maj=1, pX=0.93, N=325

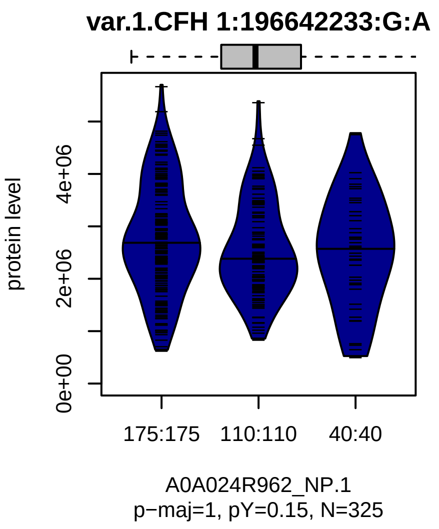
var.1.CFH 1:196642233:G:A
protein level
0e+00
2e+06
4e+06
175:175
110:110
40:40
A0A024R962_NP.1
p−maj=1, pY=0.15, N=325

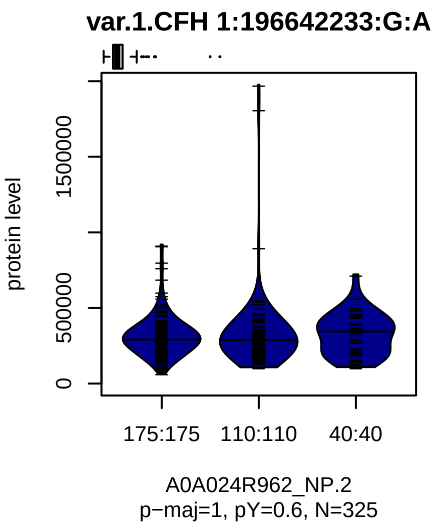
var.1.CFH 1:196642233:G:A
protein level
0
500000
1500000
175:175
110:110
40:40
A0A024R962_NP.2
p−maj=1, pY=0.6, N=325

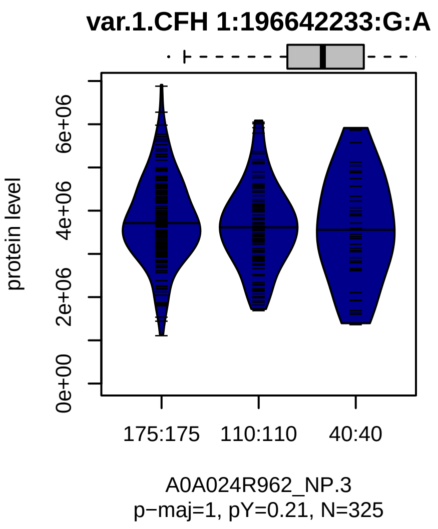
var.1.CFH 1:196642233:G:A
protein level
0e+00
2e+06
4e+06
6e+06
175:175
110:110
40:40
A0A024R962_NP.3
p−maj=1, pY=0.21, N=325

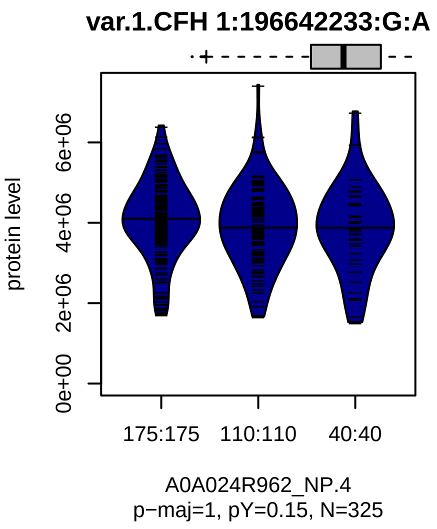
var.1.CFH 1:196642233:G:A
protein level
0e+00
2e+06
4e+06
6e+06
175:175
110:110
40:40
A0A024R962_NP.4
p−maj=1, pY=0.15, N=325

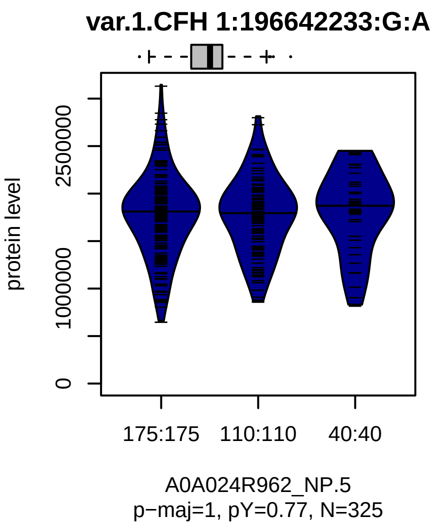
var.1.CFH 1:196642233:G:A
protein level
0
1000000
2500000
175:175
110:110
40:40
A0A024R962_NP.5
p−maj=1, pY=0.77, N=325

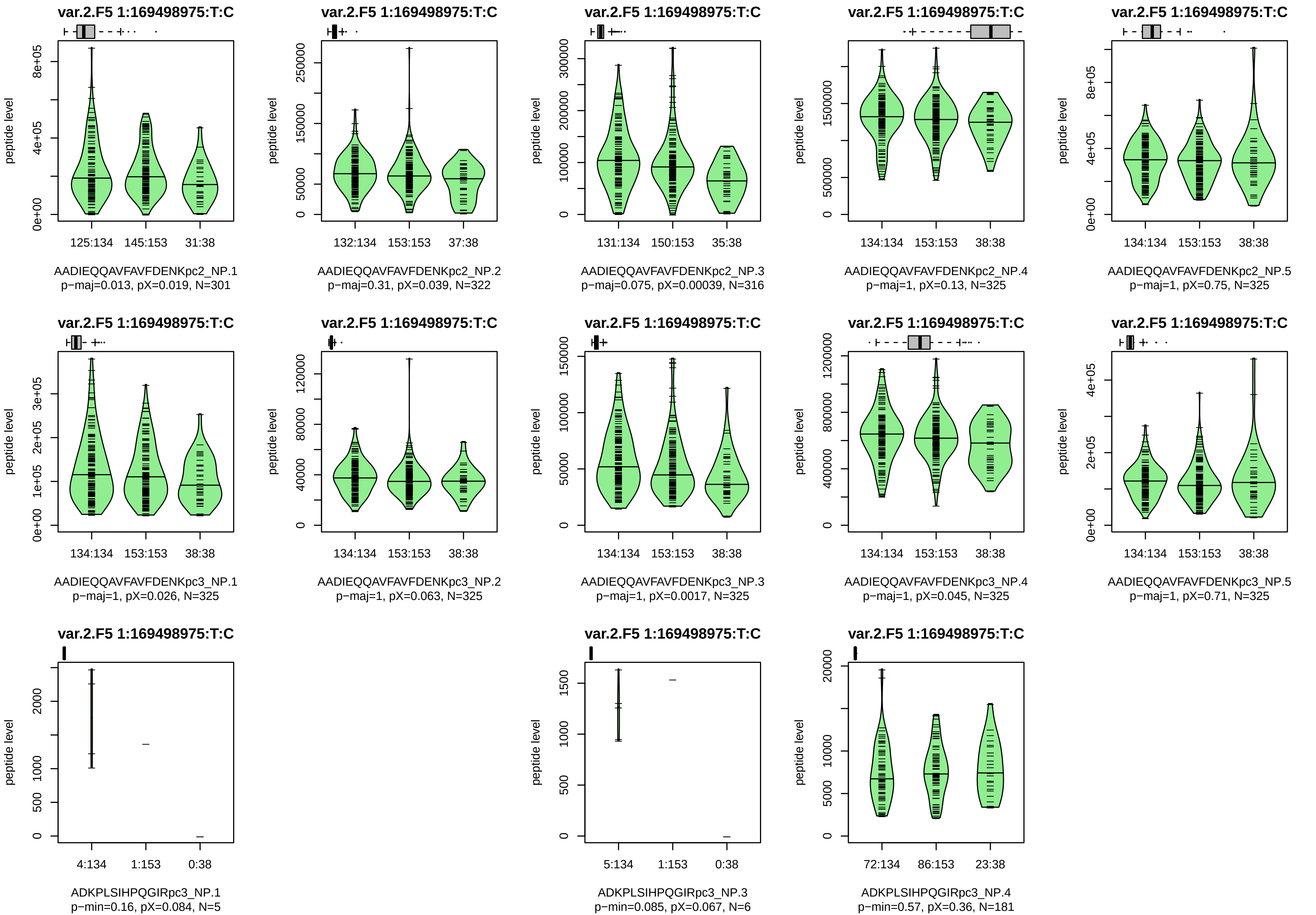
var.2.F5 1:169498975:T:C
peptide level
125:134
145:153
31:38
AADIEQQAVFAVFDENKpc2_NP.1
p−maj=0.013, pX=0.019, N=301
132:134
153:153
37:38
AADIEQQAVFAVFDENKpc2_NP.2
p−maj=0.31, pX=0.039, N=322
131:134
150:153
35:38
AADIEQQAVFAVFDENKpc2_NP.3
p−maj=0.075, pX=0.00039, N=316
134:134
153:153
38:38
AADIEQQAVFAVFDENKpc2_NP.4
p−maj=1, pX=0.13, N=325
AADIEQQAVFAVFDENKpc2_NP.5
p−maj=1, pX=0.75, N=325
AADIEQQAVFAVFDENKpc3_NP.1
p−maj=1, pX=0.026, N=325
AADIEQQAVFAVFDENKpc3_NP.2
p−maj=1, pX=0.063, N=325
AADIEQQAVFAVFDENKpc3_NP.3
p−maj=1, pX=0.0017, N=325
AADIEQQAVFAVFDENKpc3_NP.4
p−maj=1, pX=0.045, N=325
AADIEQQAVFAVFDENKpc3_NP.5
p−maj=1, pX=0.71, N=325
4:134
1:153
0:38
ADKPLSIHPQGIRpc3_NP.1
p−min=0.16, pX=0.084, N=5
5:134
1:153
0:38
ADKPLSIHPQGIRpc3_NP.3
p−min=0.085, pX=0.067, N=6
72:134
86:153
23:38
ADKPLSIHPQGIRpc3_NP.4
p−min=0.57, pX=0.36, N=181

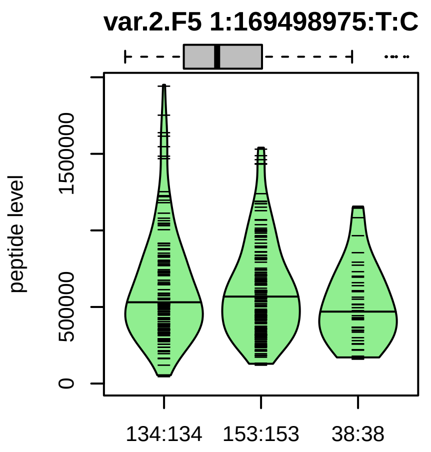

AEVDDVIQVRpc2_NP.1
p−maj=1, pX=0.091, N=325

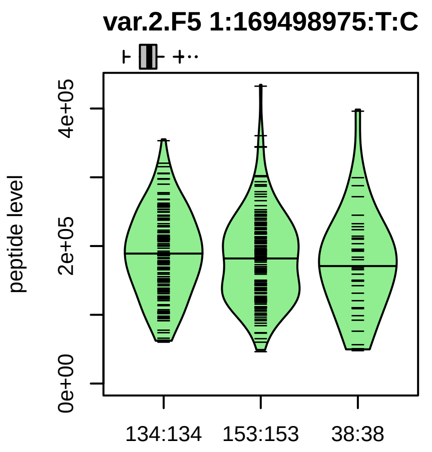

AEVDDVIQVRpc2_NP.2
p−maj=1, pX=0.2, N=325

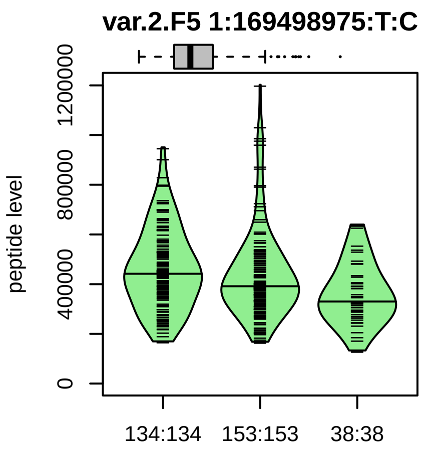

AEVDDVIQVRpc2_NP.3
p−maj=1, pX=0.0056, N=325

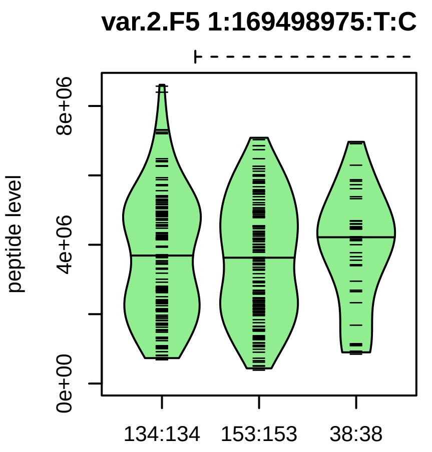

AEVDDVIQVRpc2_NP.4
p−maj=1, pX=0.85, N=325

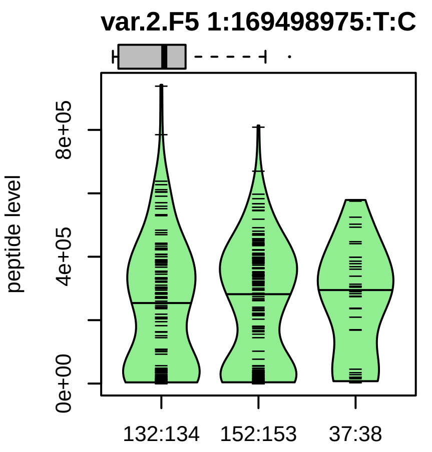

AEVDDVIQVRpc2_NP.5
p−maj=0.39, pX=0.69, N=321

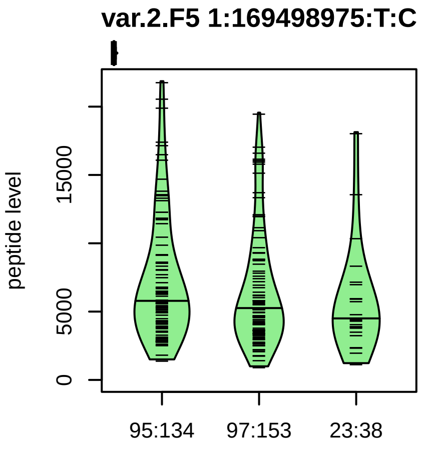

AGMQTPFLIMDRDCRpc2_NP.4
p−min=0.15, pX=0.021, N=215

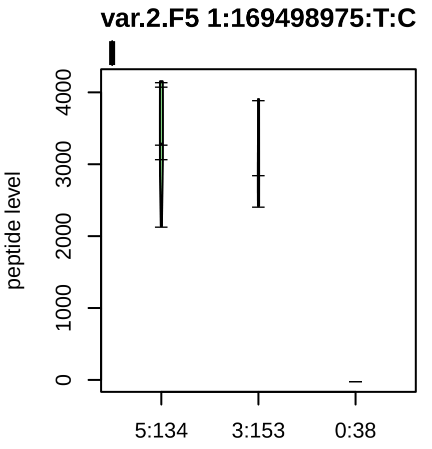

AGMQTPFLIMDRDCRpc2_NP.5
p−min=0.28, pX=0.14, N=8

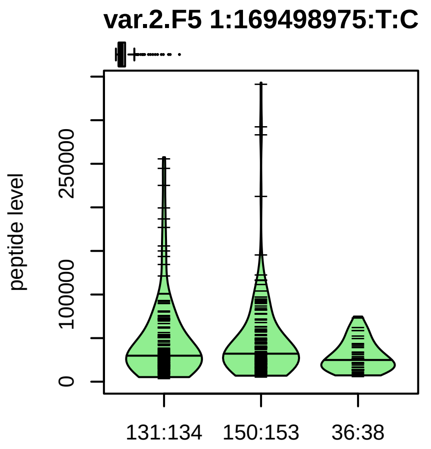

AGMQTPFLIMDRDCRpc3_NP.1
p−maj=0.24, pX=0.058, N=317

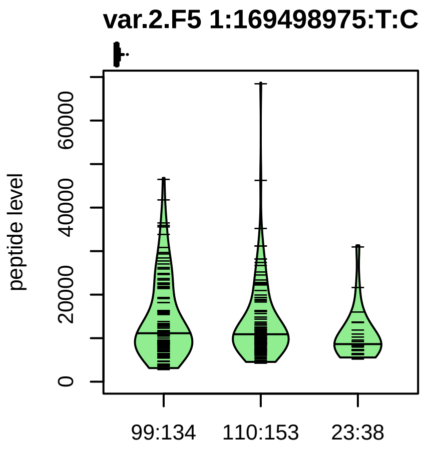

AGMQTPFLIMDRDCRpc3_NP.2
p−maj=0.13, pX=0.038, N=232

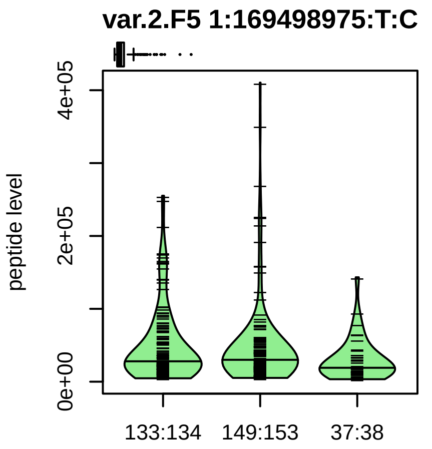

AGMQTPFLIMDRDCRpc3_NP.3
p−min=0.41, pX=0.082, N=319

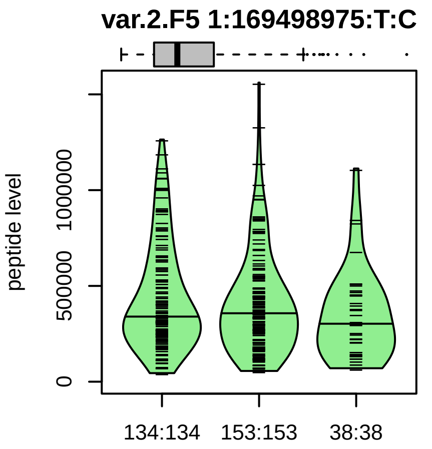

AGMQTPFLIMDRDCRpc3_NP.4
p−maj=1, pX=0.063, N=325

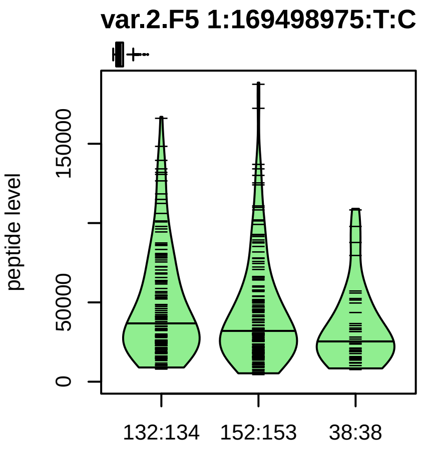

AGMQTPFLIMDRDCRpc3_NP.5
p−min=0.57, pX=0.03, N=322

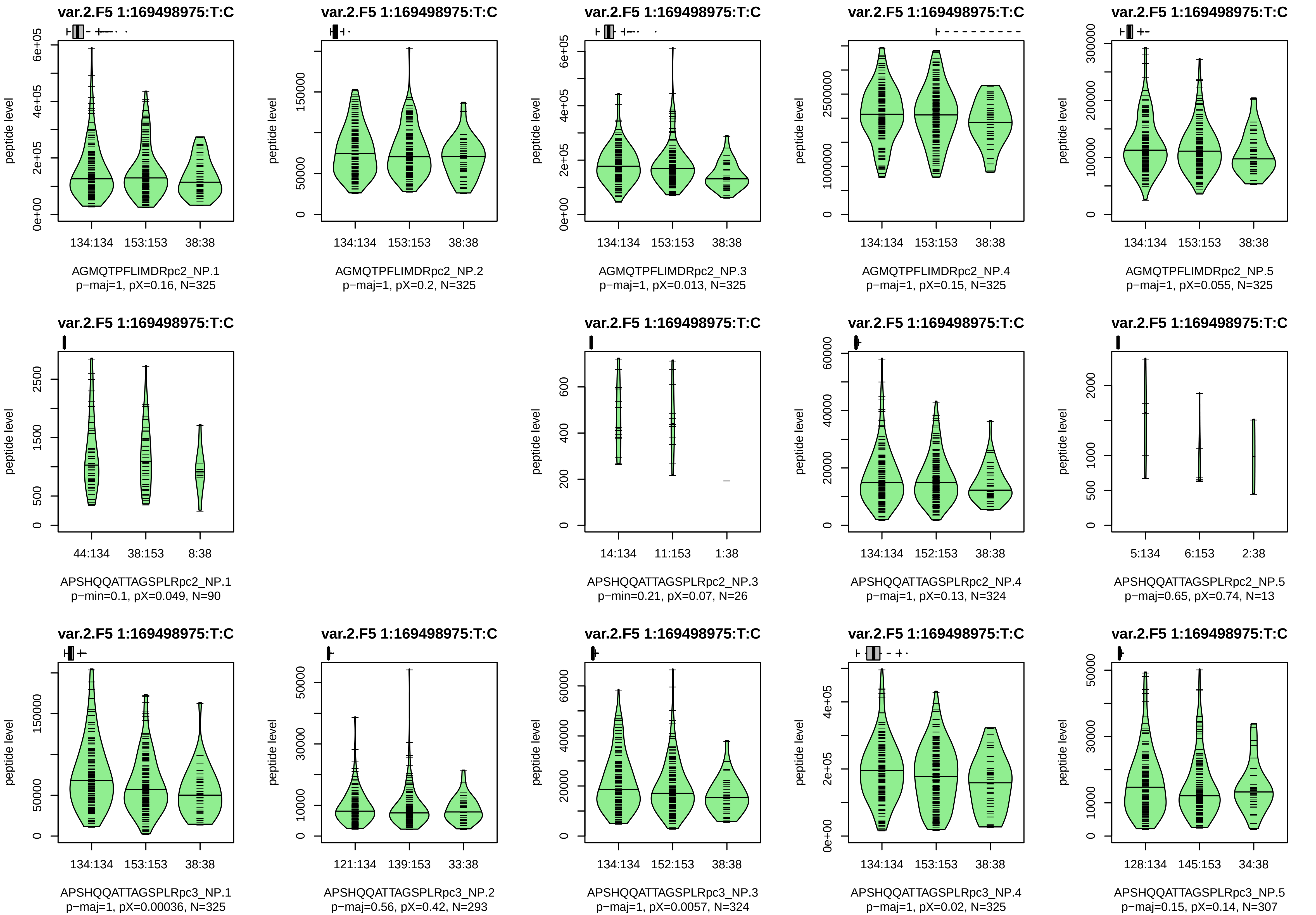

var.2.F5 1:169498975:T:C
peptide level
0e+00
2e+05
4e+05
6e+05
134:134
153:153
38:38
AGMQTPFLIMDRpc2_NP.1
p−maj=1, pX=0.16, N=325
var.2.F5 1:169498975:T:C
peptide level
0
50000
150000
134:134
153:153
38:38
AGMQTPFLIMDRpc2_NP.2
p−maj=1, pX=0.2, N=325
var.2.F5 1:169498975:T:C
peptide level
0e+00
2e+05
4e+05
6e+05
134:134
153:153
38:38
AGMQTPFLIMDRpc2_NP.3
p−maj=1, pX=0.013, N=325
var.2.F5 1:169498975:T:C
peptide level
0
1000000
2500000
134:134
153:153
38:38
AGMQTPFLIMDRpc2_NP.4
p−maj=1, pX=0.15, N=325
var.2.F5 1:169498975:T:C
peptide level
0
100000
200000
300000
134:134
153:153
38:38
AGMQTPFLIMDRpc2_NP.5
p−maj=1, pX=0.055, N=325
var.2.F5 1:169498975:T:C
peptide level
0
500
1500
2500
44:134
38:153
8:38
APSHQQATTAGSPLRpc2_NP.1
p−min=0.1, pX=0.049, N=90
var.2.F5 1:169498975:T:C
peptide level
0
200
400
600
14:134
11:153
1:38
APSHQQATTAGSPLRpc2_NP.3
p−min=0.21, pX=0.07, N=26
var.2.F5 1:169498975:T:C
peptide level
0
20000
40000
60000
134:134
152:153
38:38
APSHQQATTAGSPLRpc2_NP.4
p−maj=1, pX=0.13, N=324
var.2.F5 1:169498975:T:C
peptide level
0
500
1000
2000
5:134
6:153
2:38
APSHQQATTAGSPLRpc2_NP.5
p−maj=0.65, pX=0.74, N=13
var.2.F5 1:169498975:T:C
peptide level
0
50000
150000
134:134
153:153
38:38
APSHQQATTAGSPLRpc3_NP.1
p−maj=1, pX=0.00036, N=325
var.2.F5 1:169498975:T:C
peptide level
0
10000
30000
50000
121:134
139:153
33:38
APSHQQATTAGSPLRpc3_NP.2
p−maj=0.56, pX=0.42, N=293
var.2.F5 1:169498975:T:C
peptide level
0
20000
40000
60000
134:134
152:153
38:38
APSHQQATTAGSPLRpc3_NP.3
p−maj=1, pX=0.0057, N=324
var.2.F5 1:169498975:T:C
peptide level
0e+00
2e+05
4e+05
134:134
153:153
38:38
APSHQQATTAGSPLRpc3_NP.4
p−maj=1, pX=0.02, N=325
var.2.F5 1:169498975:T:C
peptide level
0
10000
30000
50000
128:134
145:153
34:38
APSHQQATTAGSPLRpc3_NP.5
p−maj=0.15, pX=0.14, N=307

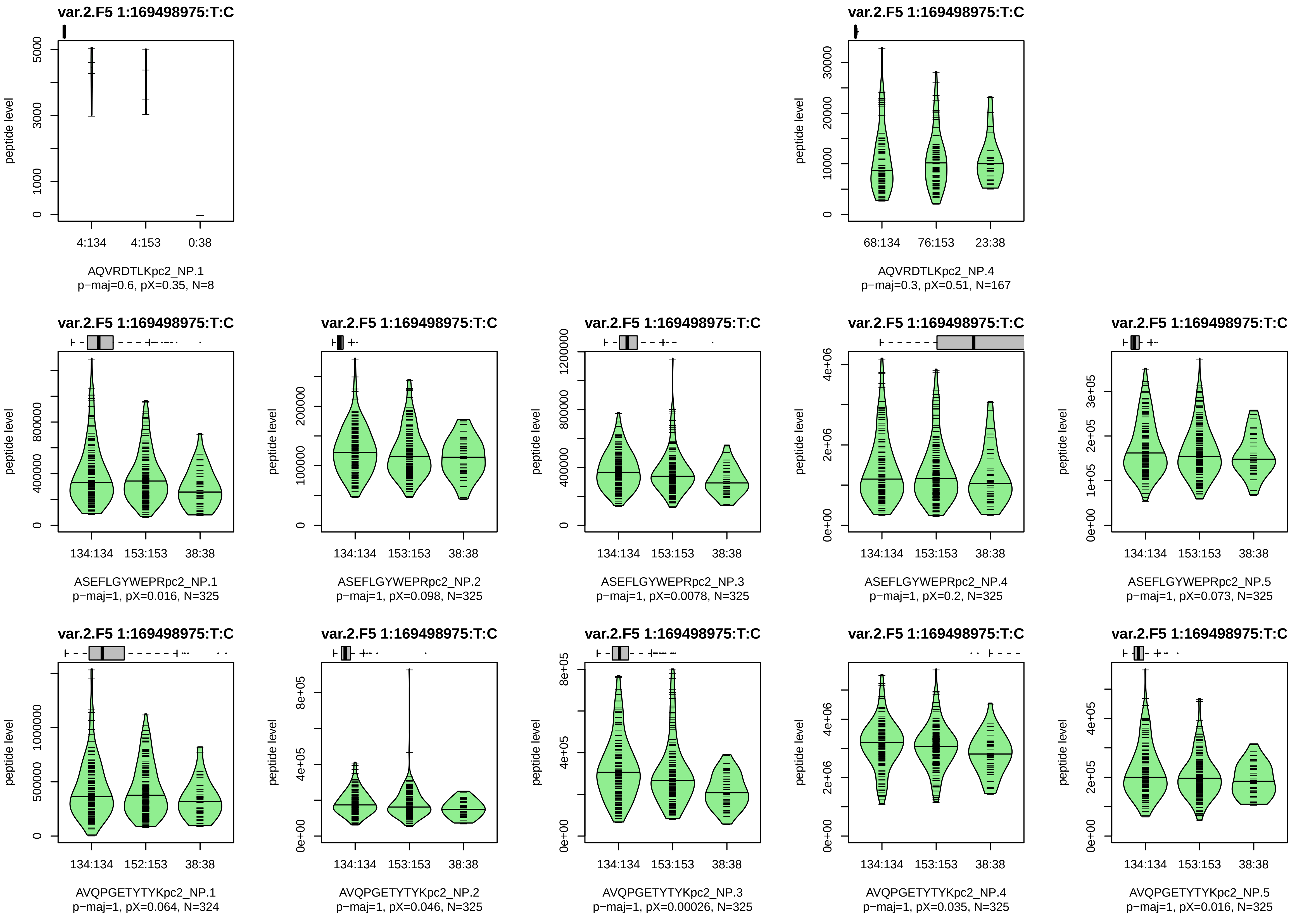

var.2.F5 1:169498975:T:C
peptide level
0 1000 3000 5000
4:134 4:153 0:38
AQVRDTLKpc2_NP.1
p−maj=0.6, pX=0.35, N=8
var.2.F5 1:169498975:T:C
peptide level
0 10000 20000 30000
68:134 76:153 23:38
AQVRDTLKpc2_NP.4
p−maj=0.3, pX=0.51, N=167
var.2.F5 1:169498975:T:C
peptide level
0 400000 800000
134:134 153:153 38:38
ASEFLGYWEPRpc2_NP.1
p−maj=1, pX=0.016, N=325
var.2.F5 1:169498975:T:C
peptide level
0 100000 200000
134:134 153:153 38:38
ASEFLGYWEPRpc2_NP.2
p−maj=1, pX=0.098, N=325
var.2.F5 1:169498975:T:C
peptide level
0 400000 800000 1200000
134:134 153:153 38:38
ASEFLGYWEPRpc2_NP.3
p−maj=1, pX=0.0078, N=325
var.2.F5 1:169498975:T:C
peptide level
0e+00 2e+06 4e+06
134:134 153:153 38:38
ASEFLGYWEPRpc2_NP.4
p−maj=1, pX=0.2, N=325
var.2.F5 1:169498975:T:C
peptide level
0e+00 1e+05 2e+05 3e+05
134:134 153:153 38:38
ASEFLGYWEPRpc2_NP.5
p−maj=1, pX=0.073, N=325
var.2.F5 1:169498975:T:C
peptide level
0 500000 1000000
134:134 152:153 38:38
AVQPGETYTYKpc2_NP.1
p−maj=1, pX=0.064, N=324
var.2.F5 1:169498975:T:C
peptide level
0e+00 4e+05 8e+05
134:134 153:153 38:38
AVQPGETYTYKpc2_NP.2
p−maj=1, pX=0.046, N=325
var.2.F5 1:169498975:T:C
peptide level
0e+00 4e+05 8e+05
134:134 153:153 38:38
AVQPGETYTYKpc2_NP.3
p−maj=1, pX=0.00026, N=325
var.2.F5 1:169498975:T:C
peptide level
0e+00 2e+06 4e+06
134:134 153:153 38:38
AVQPGETYTYKpc2_NP.4
p−maj=1, pX=0.035, N=325
var.2.F5 1:169498975:T:C
peptide level
0e+00 2e+05 4e+05
134:134 153:153 38:38
AVQPGETYTYKpc2_NP.5
p−maj=1, pX=0.016, N=325

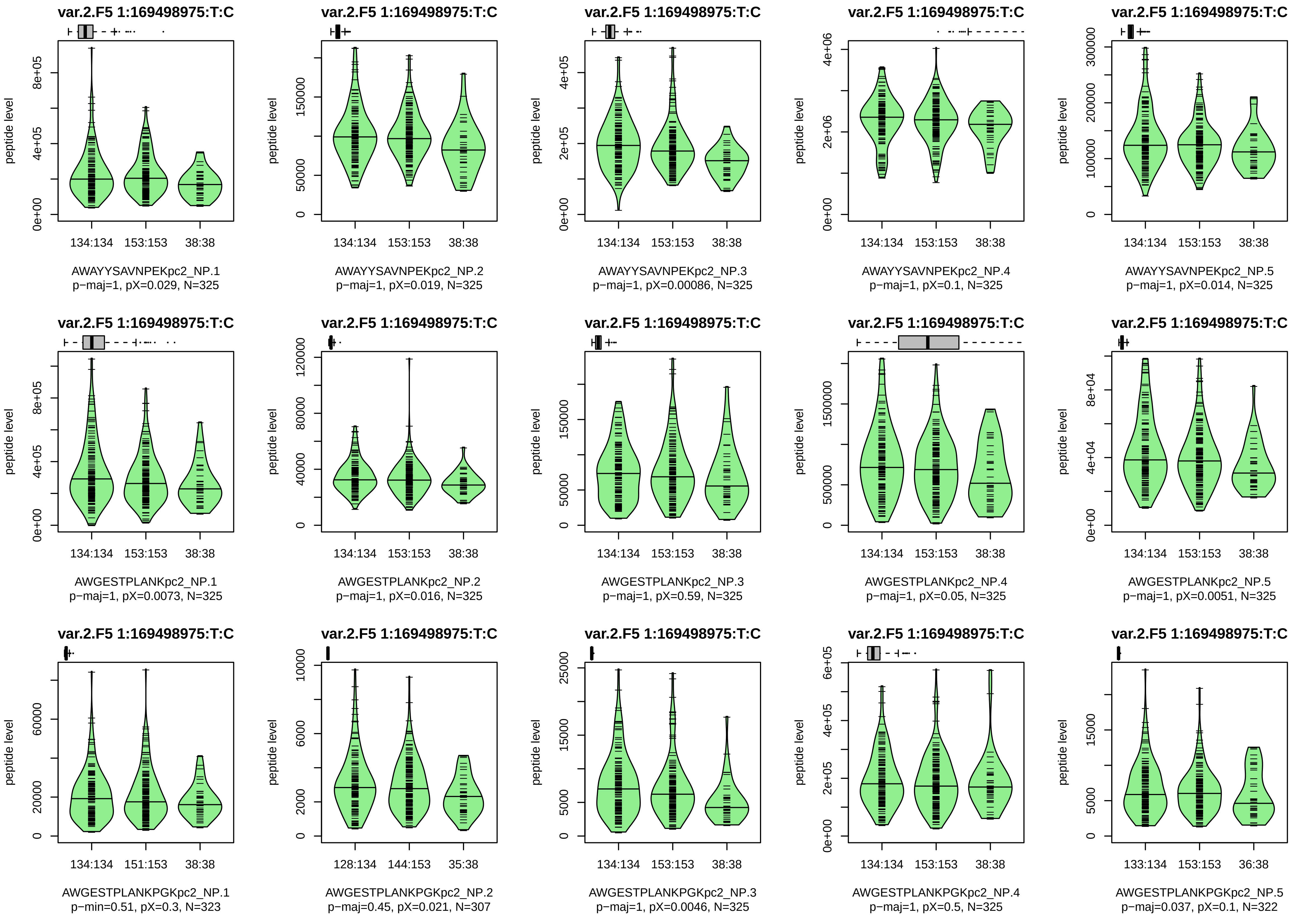

var.2.F5 1:169498975:T:C
peptide level
134:134
153:153
38:38
AWAYYSAVNPEKpc2_NP.1
p−maj=1, pX=0.029, N=325
var.2.F5 1:169498975:T:C
peptide level
134:134
153:153
38:38
AWAYYSAVNPEKpc2_NP.2
p−maj=1, pX=0.019, N=325
var.2.F5 1:169498975:T:C
peptide level
134:134
153:153
38:38
AWAYYSAVNPEKpc2_NP.3
p−maj=1, pX=0.00086, N=325
var.2.F5 1:169498975:T:C
peptide level
134:134
153:153
38:38
AWAYYSAVNPEKpc2_NP.4
p−maj=1, pX=0.1, N=325
var.2.F5 1:169498975:T:C
peptide level
134:134
153:153
38:38
AWAYYSAVNPEKpc2_NP.5
p−maj=1, pX=0.014, N=325
var.2.F5 1:169498975:T:C
peptide level
134:134
153:153
38:38
AWGESTPLANKpc2_NP.1
p−maj=1, pX=0.0073, N=325
var.2.F5 1:169498975:T:C
peptide level
134:134
153:153
38:38
AWGESTPLANKpc2_NP.2
p−maj=1, pX=0.016, N=325
var.2.F5 1:169498975:T:C
peptide level
134:134
153:153
38:38
AWGESTPLANKpc2_NP.3
p−maj=1, pX=0.59, N=325
var.2.F5 1:169498975:T:C
peptide level
134:134
153:153
38:38
AWGESTPLANKpc2_NP.4
p−maj=1, pX=0.05, N=325
var.2.F5 1:169498975:T:C
peptide level
134:134
153:153
38:38
AWGESTPLANKpc2_NP.5
p−maj=1, pX=0.0051, N=325
var.2.F5 1:169498975:T:C
peptide level
134:134
151:153
38:38
AWGESTPLANKPGKpc2_NP.1
p−min=0.51, pX=0.3, N=323
var.2.F5 1:169498975:T:C
peptide level
128:134
144:153
35:38
AWGESTPLANKPGKpc2_NP.2
p−maj=0.45, pX=0.021, N=307
var.2.F5 1:169498975:T:C
peptide level
134:134
153:153
38:38
AWGESTPLANKPGKpc2_NP.3
p−maj=1, pX=0.0046, N=325
var.2.F5 1:169498975:T:C
peptide level
134:134
153:153
38:38
AWGESTPLANKPGKpc2_NP.4
p−maj=1, pX=0.5, N=325
var.2.F5 1:169498975:T:C
peptide level
133:134
153:153
36:38
AWGESTPLANKPGKpc2_NP.5
p−maj=0.037, pX=0.1, N=322

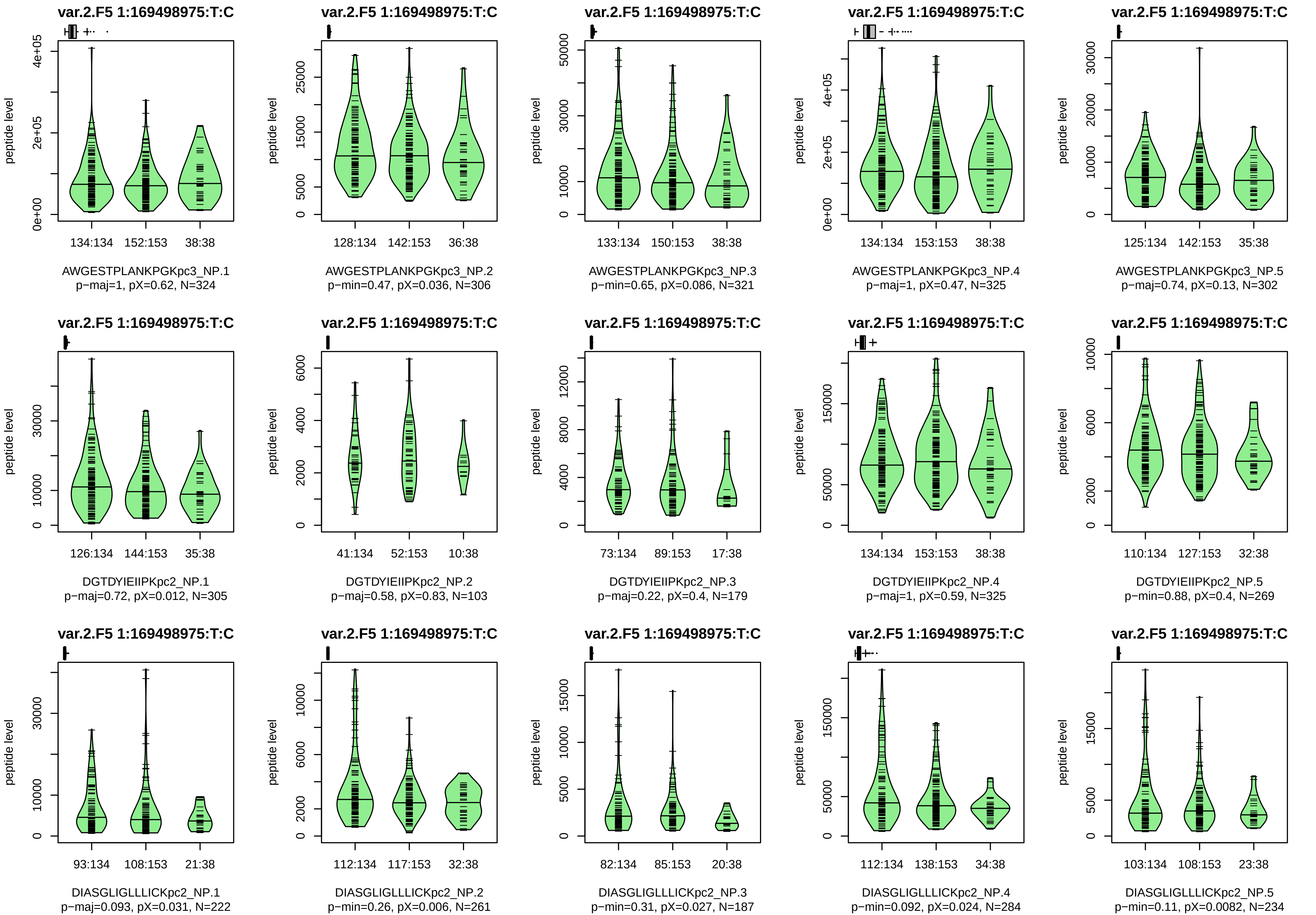
var.2.F5 1:169498975:T:C
134:134
152:153
38:38
AWGESTPLANKPGKpc3_NP.1
p−maj=1, pX=0.62, N=324
var.2.F5 1:169498975:T:C
128:134
142:153
36:38
AWGESTPLANKPGKpc3_NP.2
p−min=0.47, pX=0.036, N=306
var.2.F5 1:169498975:T:C
133:134
150:153
38:38
AWGESTPLANKPGKpc3_NP.3
p−min=0.65, pX=0.086, N=321
var.2.F5 1:169498975:T:C
134:134
153:153
38:38
AWGESTPLANKPGKpc3_NP.4
p−maj=1, pX=0.47, N=325
var.2.F5 1:169498975:T:C
125:134
142:153
35:38
AWGESTPLANKPGKpc3_NP.5
p−maj=0.74, pX=0.13, N=302
var.2.F5 1:169498975:T:C
126:134
144:153
35:38
DGTDYIEIIPKpc2_NP.1
p−maj=0.72, pX=0.012, N=305
var.2.F5 1:169498975:T:C
41:134
52:153
10:38
DGTDYIEIIPKpc2_NP.2
p−maj=0.58, pX=0.83, N=103
var.2.F5 1:169498975:T:C
73:134
89:153
17:38
DGTDYIEIIPKpc2_NP.3
p−maj=0.22, pX=0.4, N=179
var.2.F5 1:169498975:T:C
134:134
153:153
38:38
DGTDYIEIIPKpc2_NP.4
p−maj=1, pX=0.59, N=325
var.2.F5 1:169498975:T:C
110:134
127:153
32:38
DGTDYIEIIPKpc2_NP.5
p−min=0.88, pX=0.4, N=269
var.2.F5 1:169498975:T:C
93:134
108:153
21:38
DIASGLIGLLLICKpc2_NP.1
p−maj=0.093, pX=0.031, N=222
var.2.F5 1:169498975:T:C
112:134
117:153
32:38
DIASGLIGLLLICKpc2_NP.2
p−min=0.26, pX=0.006, N=261
var.2.F5 1:169498975:T:C
82:134
85:153
20:38
DIASGLIGLLLICKpc2_NP.3
p−min=0.31, pX=0.027, N=187
var.2.F5 1:169498975:T:C
112:134
138:153
34:38
DIASGLIGLLLICKpc2_NP.4
p−min=0.092, pX=0.024, N=284
var.2.F5 1:169498975:T:C
103:134
108:153
23:38
DIASGLIGLLLICKpc2_NP.5
p−min=0.11, pX=0.0082, N=234

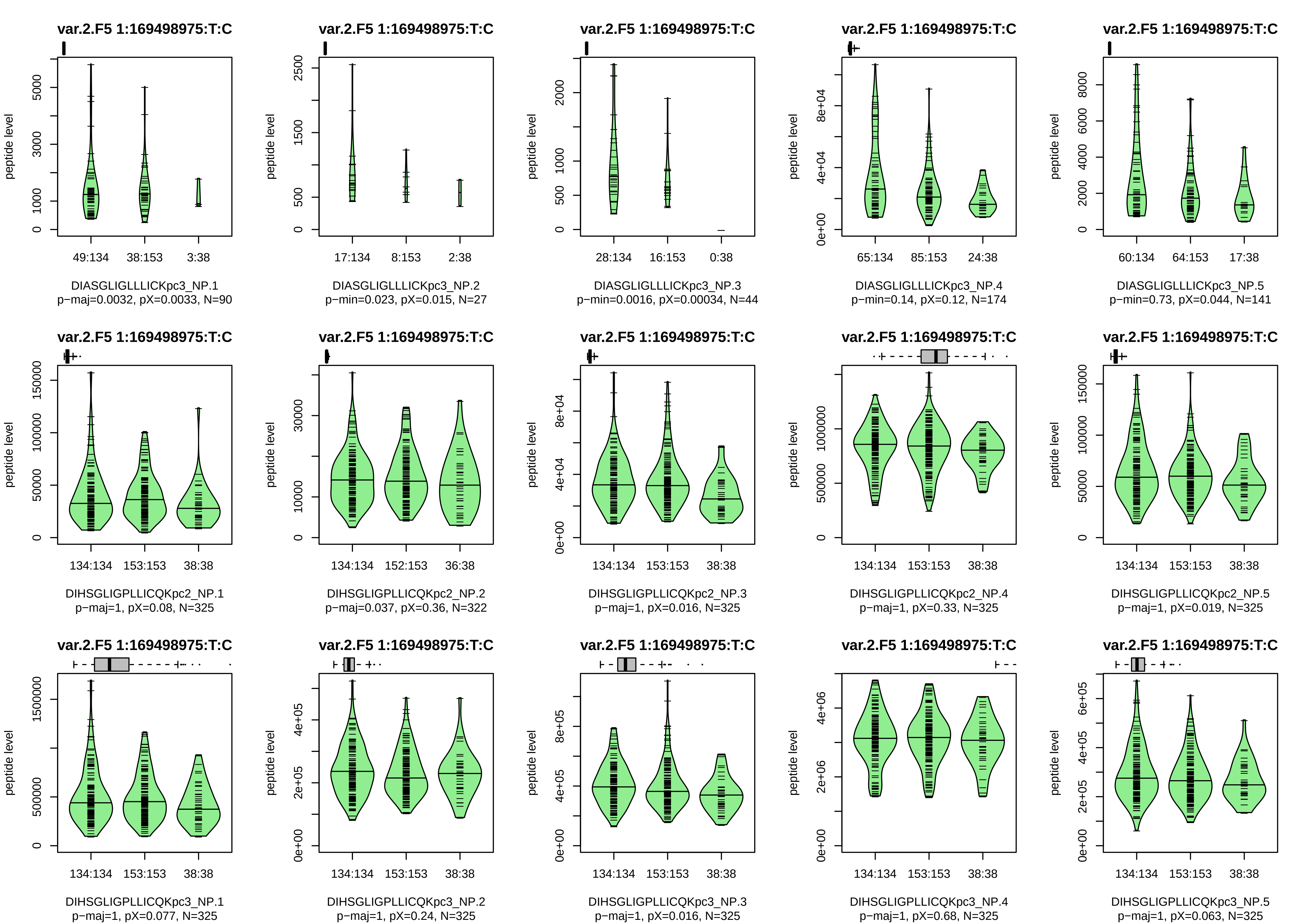
var.2.F5 1:169498975:T:C
49:134 38:153 3:38
DIASGLIGLLLICKpc3_NP.1
p−maj=0.0032, pX=0.0033, N=90
var.2.F5 1:169498975:T:C
17:134 8:153 2:38
DIASGLIGLLLICKpc3_NP.2
p−min=0.023, pX=0.015, N=27
var.2.F5 1:169498975:T:C
28:134 16:153 0:38
DIASGLIGLLLICKpc3_NP.3
p−min=0.0016, pX=0.00034, N=44
var.2.F5 1:169498975:T:C
65:134 85:153 24:38
DIASGLIGLLLICKpc3_NP.4
p−min=0.14, pX=0.12, N=174
var.2.F5 1:169498975:T:C
60:134 64:153 17:38
DIASGLIGLLLICKpc3_NP.5
p−min=0.73, pX=0.044, N=141
var.2.F5 1:169498975:T:C
134:134 153:153 38:38
DIHSGLIGPLLICQKpc2_NP.1
p−maj=1, pX=0.08, N=325
var.2.F5 1:169498975:T:C
134:134 152:153 36:38
DIHSGLIGPLLICQKpc2_NP.2
p−maj=0.037, pX=0.36, N=322
var.2.F5 1:169498975:T:C
134:134 153:153 38:38
DIHSGLIGPLLICQKpc2_NP.3
p−maj=1, pX=0.016, N=325
var.2.F5 1:169498975:T:C
134:134 153:153 38:38
DIHSGLIGPLLICQKpc2_NP.4
p−maj=1, pX=0.33, N=325
var.2.F5 1:169498975:T:C
134:134 153:153 38:38
DIHSGLIGPLLICQKpc2_NP.5
p−maj=1, pX=0.019, N=325
var.2.F5 1:169498975:T:C
134:134 153:153 38:38
DIHSGLIGPLLICQKpc3_NP.1
p−maj=1, pX=0.077, N=325
var.2.F5 1:169498975:T:C
134:134 153:153 38:38
DIHSGLIGPLLICQKpc3_NP.2
p−maj=1, pX=0.24, N=325
var.2.F5 1:169498975:T:C
134:134 153:153 38:38
DIHSGLIGPLLICQKpc3_NP.3
p−maj=1, pX=0.016, N=325
var.2.F5 1:169498975:T:C
134:134 153:153 38:38
DIHSGLIGPLLICQKpc3_NP.4
p−maj=1, pX=0.68, N=325
var.2.F5 1:169498975:T:C
134:134 153:153 38:38
DIHSGLIGPLLICQKpc3_NP.5
p−maj=1, pX=0.063, N=325

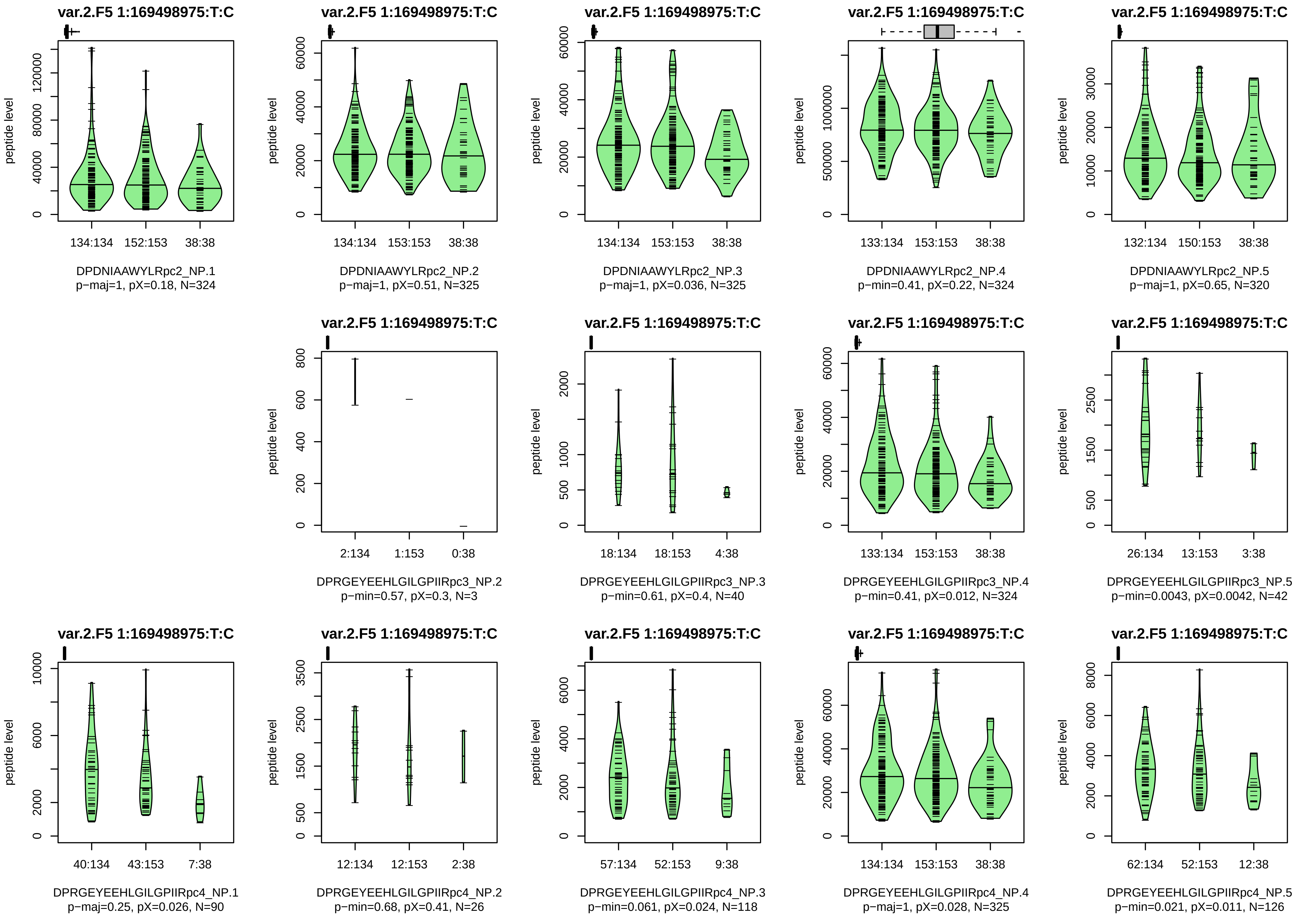
var.2.F5 1:169498975:T:C
peptide level
0
40000
80000
120000
134:134
152:153
38:38
DPDNIAAWYLRpc2_NP.1
p−maj=1, pX=0.18, N=324
var.2.F5 1:169498975:T:C
peptide level
0
20000
40000
60000
134:134
153:153
38:38
DPDNIAAWYLRpc2_NP.2
p−maj=1, pX=0.51, N=325
var.2.F5 1:169498975:T:C
peptide level
0
20000
40000
60000
134:134
153:153
38:38
DPDNIAAWYLRpc2_NP.3
p−maj=1, pX=0.036, N=325
var.2.F5 1:169498975:T:C
peptide level
0
500000
1000000
133:134
153:153
38:38
DPDNIAAWYLRpc2_NP.4
p−min=0.41, pX=0.22, N=324
var.2.F5 1:169498975:T:C
peptide level
0
10000
20000
30000
132:134
150:153
38:38
DPDNIAAWYLRpc2_NP.5
p−maj=1, pX=0.65, N=320
var.2.F5 1:169498975:T:C
peptide level
0
200
400
600
800
2:134
1:153
0:38
DPRGEYEEHLGILGPIIRpc3_NP.2
p−min=0.57, pX=0.3, N=3
var.2.F5 1:169498975:T:C
peptide level
0
500
1000
2000
18:134
18:153
4:38
DPRGEYEEHLGILGPIIRpc3_NP.3
p−min=0.61, pX=0.4, N=40
var.2.F5 1:169498975:T:C
peptide level
0
20000
40000
60000
133:134
153:153
38:38
DPRGEYEEHLGILGPIIRpc3_NP.4
p−min=0.41, pX=0.012, N=324
var.2.F5 1:169498975:T:C
peptide level
0
500
1500
2500
26:134
13:153
3:38
DPRGEYEEHLGILGPIIRpc3_NP.5
p−min=0.0043, pX=0.0042, N=42
var.2.F5 1:169498975:T:C
peptide level
0
2000
6000
10000
40:134
43:153
7:38
DPRGEYEEHLGILGPIIRpc4_NP.1
p−maj=0.25, pX=0.026, N=90
var.2.F5 1:169498975:T:C
peptide level
0
500
1500
2500
3500
12:134
12:153
2:38
DPRGEYEEHLGILGPIIRpc4_NP.2
p−min=0.68, pX=0.41, N=26
var.2.F5 1:169498975:T:C
peptide level
0
2000
4000
6000
57:134
52:153
9:38
DPRGEYEEHLGILGPIIRpc4_NP.3
p−min=0.061, pX=0.024, N=118
var.2.F5 1:169498975:T:C
peptide level
0
20000
40000
60000
134:134
153:153
38:38
DPRGEYEEHLGILGPIIRpc4_NP.4
p−maj=1, pX=0.028, N=325
var.2.F5 1:169498975:T:C
peptide level
0
2000
4000
6000
8000
62:134
52:153
12:38
DPRGEYEEHLGILGPIIRpc4_NP.5
p−min=0.021, pX=0.011, N=126

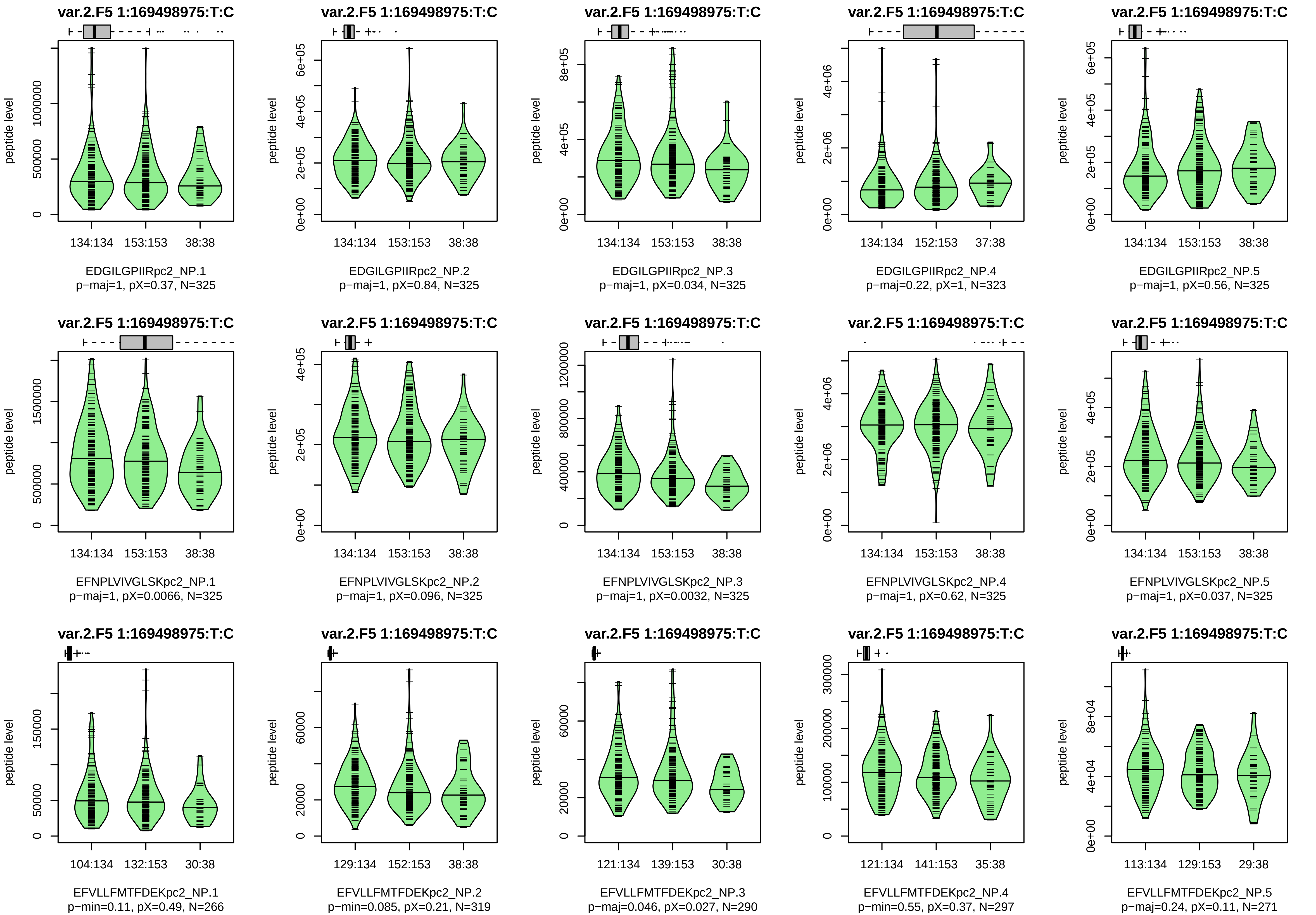

var.2.F5 1:169498975:T:C
peptide level
134:134
153:153
38:38
EDGILGPIIRpc2_NP.1
p−maj=1, pX=0.37, N=325
var.2.F5 1:169498975:T:C
134:134
153:153
38:38
EDGILGPIIRpc2_NP.2
p−maj=1, pX=0.84, N=325
var.2.F5 1:169498975:T:C
134:134
153:153
38:38
EDGILGPIIRpc2_NP.3
p−maj=1, pX=0.034, N=325
var.2.F5 1:169498975:T:C
134:134
152:153
37:38
EDGILGPIIRpc2_NP.4
p−maj=0.22, pX=1, N=323
var.2.F5 1:169498975:T:C
134:134
153:153
38:38
EDGILGPIIRpc2_NP.5
p−maj=1, pX=0.56, N=325
var.2.F5 1:169498975:T:C
134:134
153:153
38:38
EFNPLVIVGLSKpc2_NP.1
p−maj=1, pX=0.0066, N=325
var.2.F5 1:169498975:T:C
134:134
153:153
38:38
EFNPLVIVGLSKpc2_NP.2
p−maj=1, pX=0.096, N=325
var.2.F5 1:169498975:T:C
134:134
153:153
38:38
EFNPLVIVGLSKpc2_NP.3
p−maj=1, pX=0.0032, N=325
var.2.F5 1:169498975:T:C
134:134
153:153
38:38
EFNPLVIVGLSKpc2_NP.4
p−maj=1, pX=0.62, N=325
var.2.F5 1:169498975:T:C
134:134
153:153
38:38
EFNPLVIVGLSKpc2_NP.5
p−maj=1, pX=0.037, N=325
var.2.F5 1:169498975:T:C
104:134
132:153
30:38
EFVLLFMTFDEKpc2_NP.1
p−min=0.11, pX=0.49, N=266
var.2.F5 1:169498975:T:C
129:134
152:153
38:38
EFVLLFMTFDEKpc2_NP.2
p−min=0.085, pX=0.21, N=319
var.2.F5 1:169498975:T:C
121:134
139:153
30:38
EFVLLFMTFDEKpc2_NP.3
p−maj=0.046, pX=0.027, N=290
var.2.F5 1:169498975:T:C
121:134
141:153
35:38
EFVLLFMTFDEKpc2_NP.4
p−min=0.55, pX=0.37, N=297
var.2.F5 1:169498975:T:C
113:134
129:153
29:38
EFVLLFMTFDEKpc2_NP.5
p−maj=0.24, pX=0.11, N=271

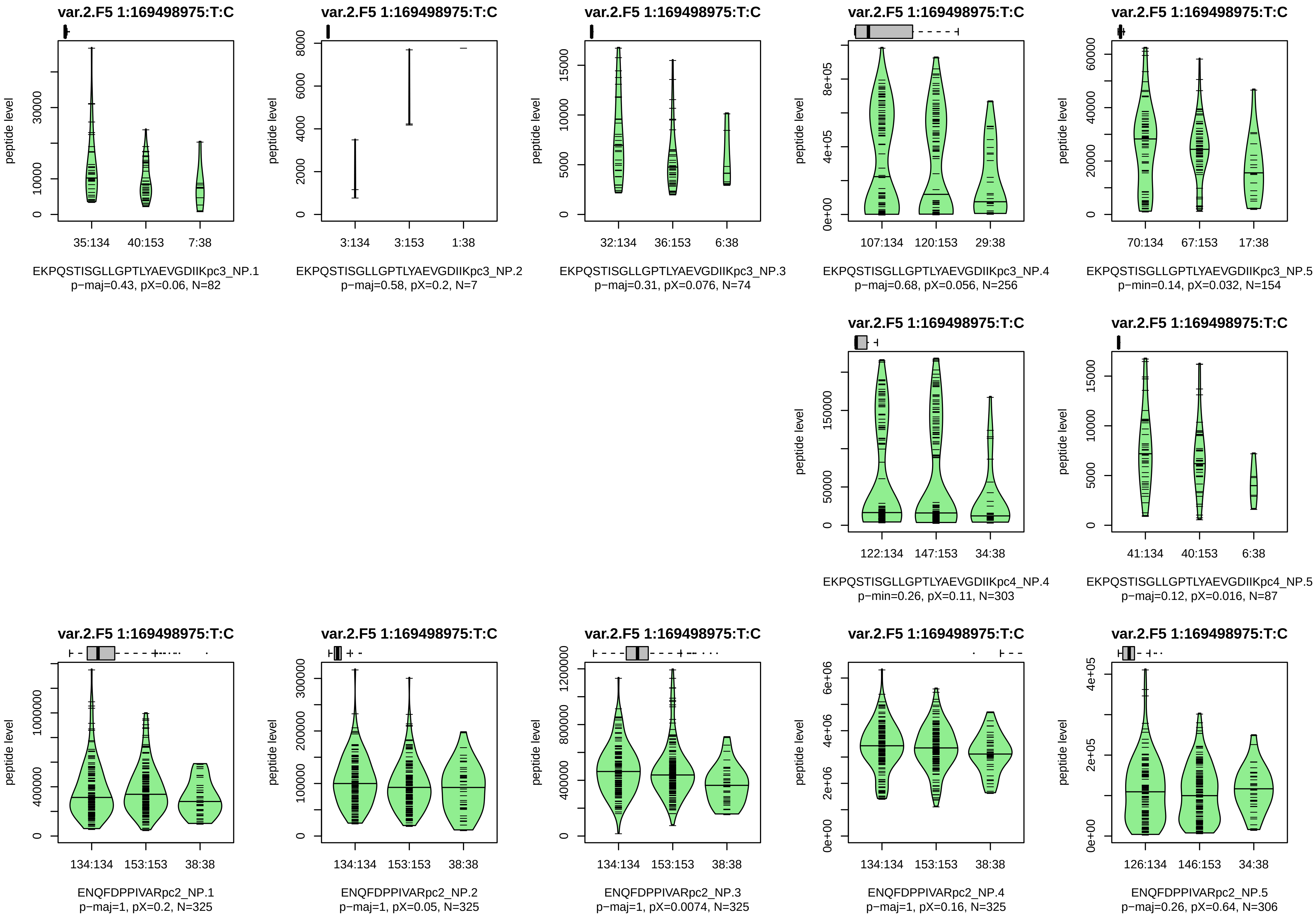

var.2.F5 1:169498975:T:C
peptide level
35:134
40:153
7:38
EKPQSTISGLLGPTLYAEVGDIIKpc3_NP.1
p−maj=0.43, pX=0.06, N=82
var.2.F5 1:169498975:T:C
peptide level
3:134
3:153
1:38
EKPQSTISGLLGPTLYAEVGDIIKpc3_NP.2
p−maj=0.58, pX=0.2, N=7
var.2.F5 1:169498975:T:C
peptide level
32:134
36:153
6:38
EKPQSTISGLLGPTLYAEVGDIIKpc3_NP.3
p−maj=0.31, pX=0.076, N=74
var.2.F5 1:169498975:T:C
peptide level
107:134
120:153
29:38
EKPQSTISGLLGPTLYAEVGDIIKpc3_NP.4
p−maj=0.68, pX=0.056, N=256
var.2.F5 1:169498975:T:C
peptide level
70:134
67:153
17:38
EKPQSTISGLLGPTLYAEVGDIIKpc3_NP.5
p−min=0.14, pX=0.032, N=154
var.2.F5 1:169498975:T:C
peptide level
122:134
147:153
34:38
EKPQSTISGLLGPTLYAEVGDIIKpc4_NP.4
p−min=0.26, pX=0.11, N=303
var.2.F5 1:169498975:T:C
peptide level
41:134
40:153
6:38
EKPQSTISGLLGPTLYAEVGDIIKpc4_NP.5
p−maj=0.12, pX=0.016, N=87
var.2.F5 1:169498975:T:C
peptide level
134:134
153:153
38:38
ENQFDPPIVARpc2_NP.1
p−maj=1, pX=0.2, N=325
var.2.F5 1:169498975:T:C
peptide level
134:134
153:153
38:38
ENQFDPPIVARpc2_NP.2
p−maj=1, pX=0.05, N=325
var.2.F5 1:169498975:T:C
peptide level
134:134
153:153
38:38
ENQFDPPIVARpc2_NP.3
p−maj=1, pX=0.0074, N=325
var.2.F5 1:169498975:T:C
peptide level
134:134
153:153
38:38
ENQFDPPIVARpc2_NP.4
p−maj=1, pX=0.16, N=325
var.2.F5 1:169498975:T:C
peptide level
126:134
146:153
34:38
ENQFDPPIVARpc2_NP.5
p−maj=0.26, pX=0.64, N=306

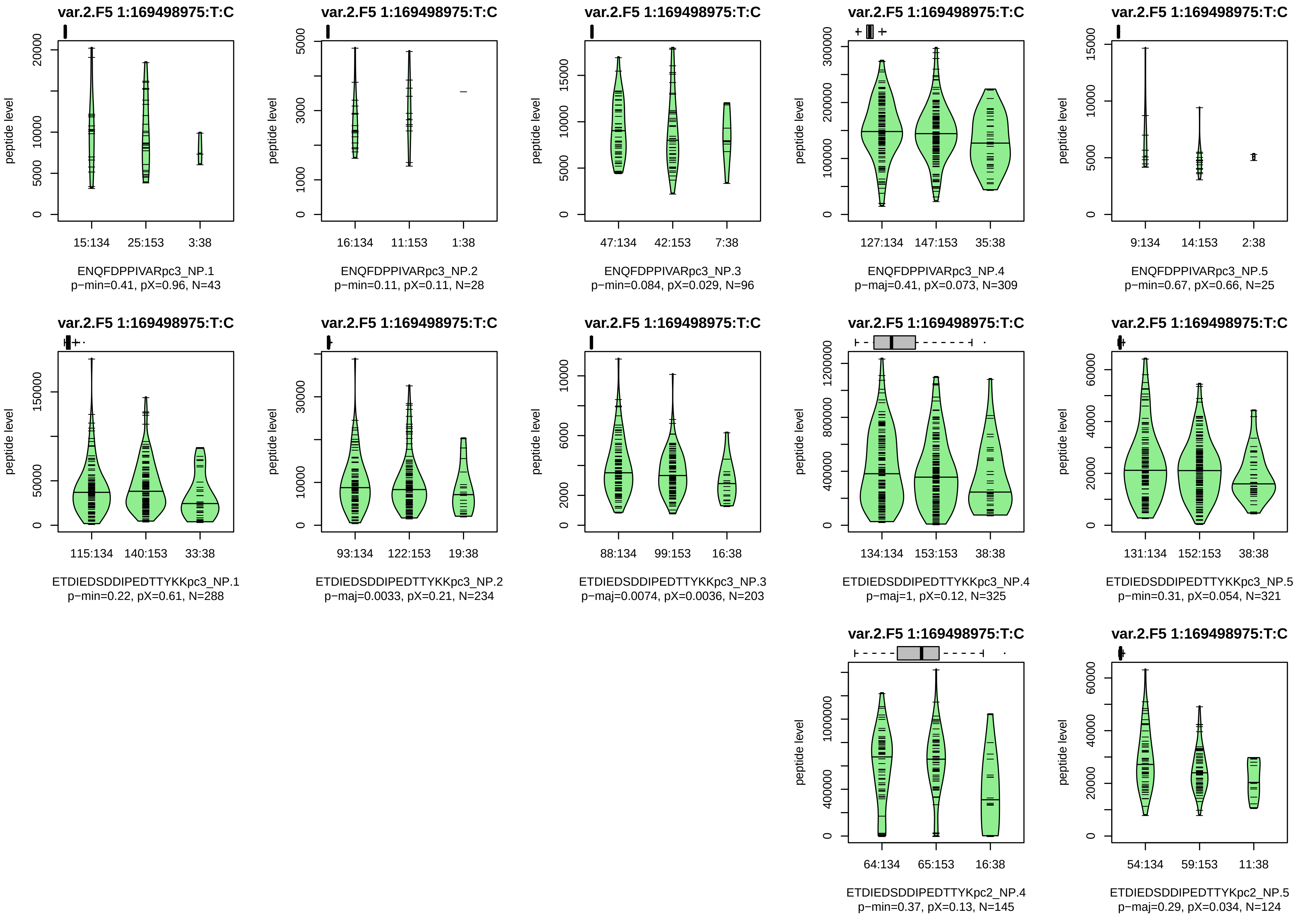
var.2.F5 1:169498975:T:C
peptide level
15:134
25:153
3:38
ENQFDPPIVARpc3_NP.1
p−min=0.41, pX=0.96, N=43
var.2.F5 1:169498975:T:C
peptide level
16:134
11:153
1:38
ENQFDPPIVARpc3_NP.2
p−min=0.11, pX=0.11, N=28
var.2.F5 1:169498975:T:C
peptide level
47:134
42:153
7:38
ENQFDPPIVARpc3_NP.3
p−min=0.084, pX=0.029, N=96
var.2.F5 1:169498975:T:C
peptide level
127:134
147:153
35:38
ENQFDPPIVARpc3_NP.4
p−maj=0.41, pX=0.073, N=309
var.2.F5 1:169498975:T:C
peptide level
9:134
14:153
2:38
ENQFDPPIVARpc3_NP.5
p−min=0.67, pX=0.66, N=25
var.2.F5 1:169498975:T:C
peptide level
115:134
140:153
33:38
ETDIEDSDDIPEDTTYKKpc3_NP.1
p−min=0.22, pX=0.61, N=288
var.2.F5 1:169498975:T:C
peptide level
93:134
122:153
19:38
ETDIEDSDDIPEDTTYKKpc3_NP.2
p−maj=0.0033, pX=0.21, N=234
var.2.F5 1:169498975:T:C
peptide level
88:134
99:153
16:38
ETDIEDSDDIPEDTTYKKpc3_NP.3
p−maj=0.0074, pX=0.0036, N=203
var.2.F5 1:169498975:T:C
peptide level
134:134
153:153
38:38
ETDIEDSDDIPEDTTYKKpc3_NP.4
p−maj=1, pX=0.12, N=325
var.2.F5 1:169498975:T:C
peptide level
131:134
152:153
38:38
ETDIEDSDDIPEDTTYKKpc3_NP.5
p−min=0.31, pX=0.054, N=321
var.2.F5 1:169498975:T:C
peptide level
64:134
65:153
16:38
ETDIEDSDDIPEDTTYKpc2_NP.4
p−min=0.37, pX=0.13, N=145
var.2.F5 1:169498975:T:C
peptide level
54:134
59:153
11:38
ETDIEDSDDIPEDTTYKpc2_NP.5
p−maj=0.29, pX=0.034, N=124

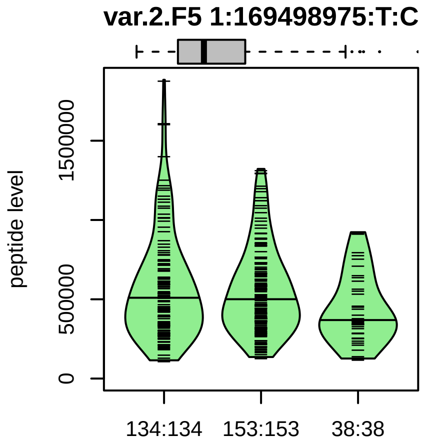

EVIITGIQTQGAKpc2_NP.1
p−maj=1, pX=0.038, N=325

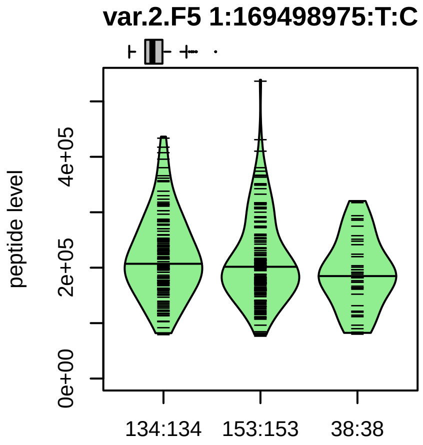

EVIITGIQTQGAKpc2_NP.2
p−maj=1, pX=0.052, N=325

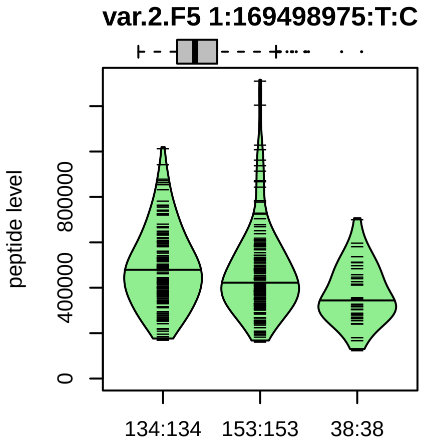

EVIITGIQTQGAKpc2_NP.3
p−maj=1, pX=0.0032, N=325

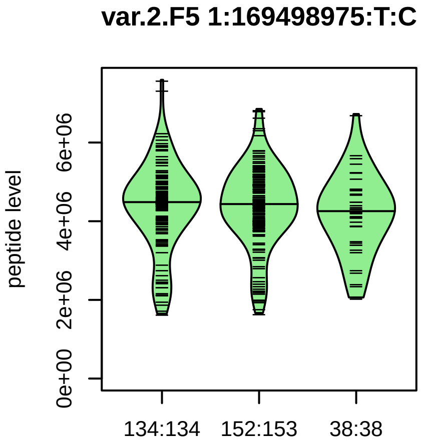

EVIITGIQTQGAKpc2_NP.4
p−maj=1, pX=0.49, N=324

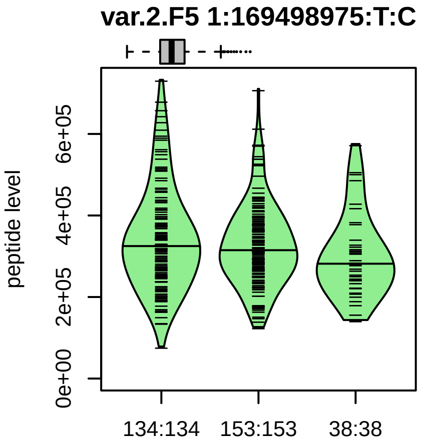

EVIITGIQTQGAKpc2_NP.5
p−maj=1, pX=0.031, N=325

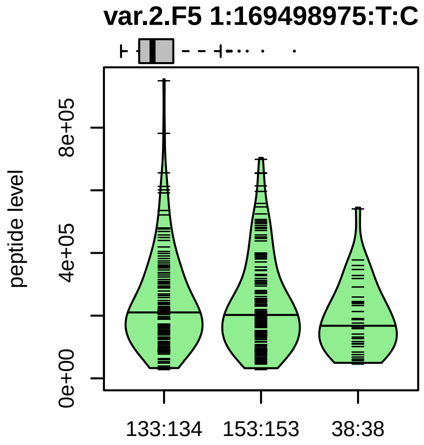

EYEPYFKpc2_NP.1
p−min=0.41, pX=0.13, N=324

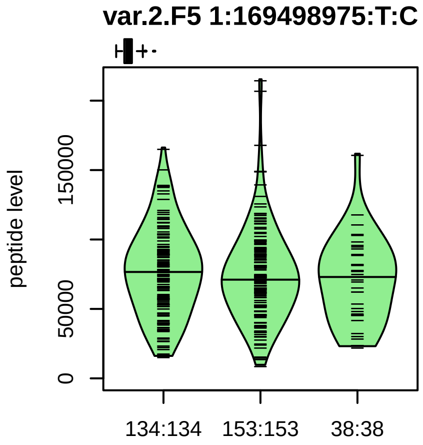

EYEPYFKpc2_NP.2
p−maj=1, pX=0.24, N=325

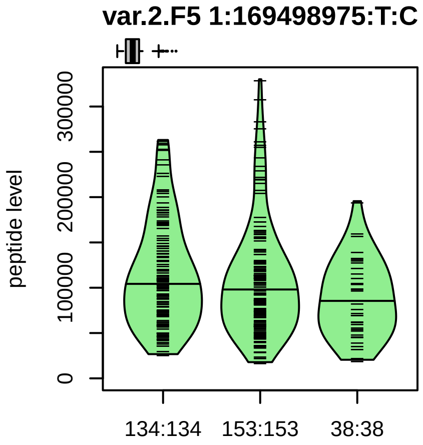

EYEPYFKpc2_NP.3
p−maj=1, pX=0.026, N=325

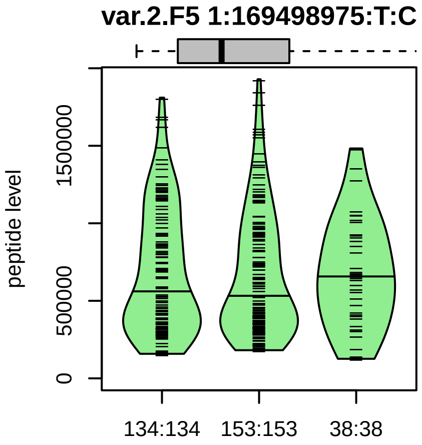

EYEPYFKpc2_NP.4
p−maj=1, pX=0.84, N=325

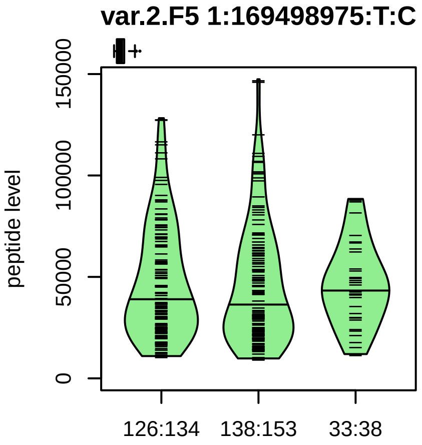

EYEPYFKpc2_NP.5
p−min=0.17, pX=0.28, N=297

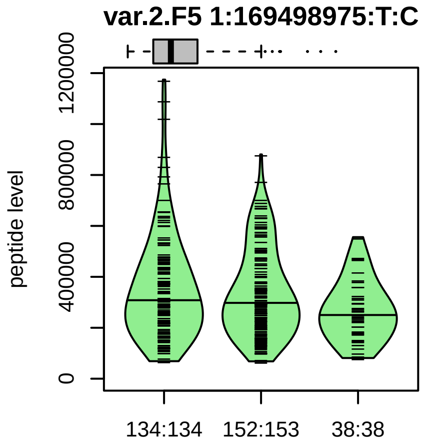

FCENPDEVKpc2_NP.1
p−maj=1, pX=0.015, N=324

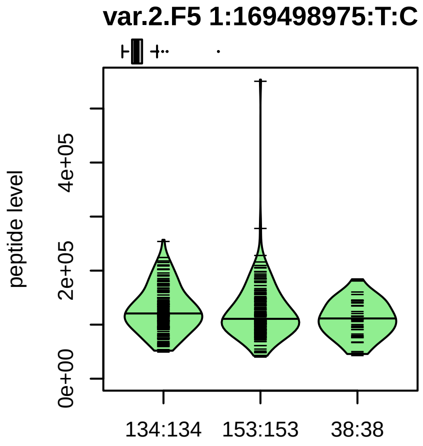

FCENPDEVKpc2_NP.2
p−maj=1, pX=0.089, N=325

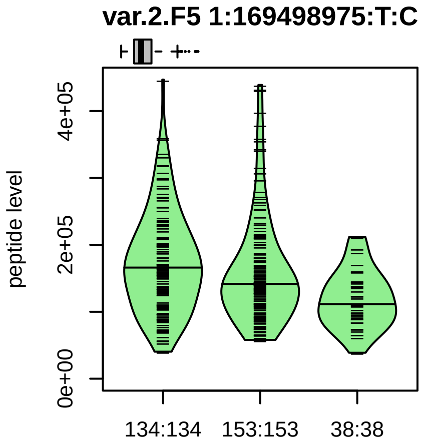

FCENPDEVKpc2_NP.3
p−maj=1, pX=0.00018, N=325

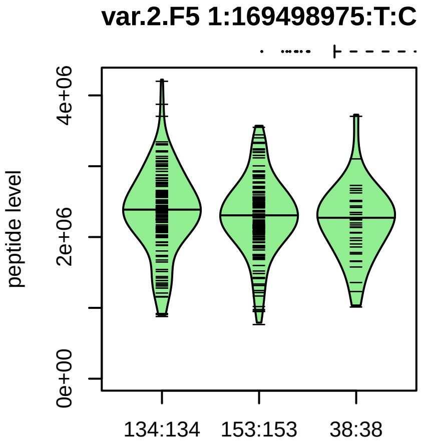

FCENPDEVKpc2_NP.4
p−maj=1, pX=0.14, N=325

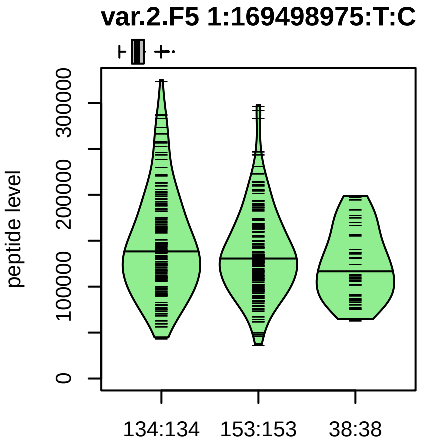

FCENPDEVKpc2_NP.5
p−maj=1, pX=0.015, N=325

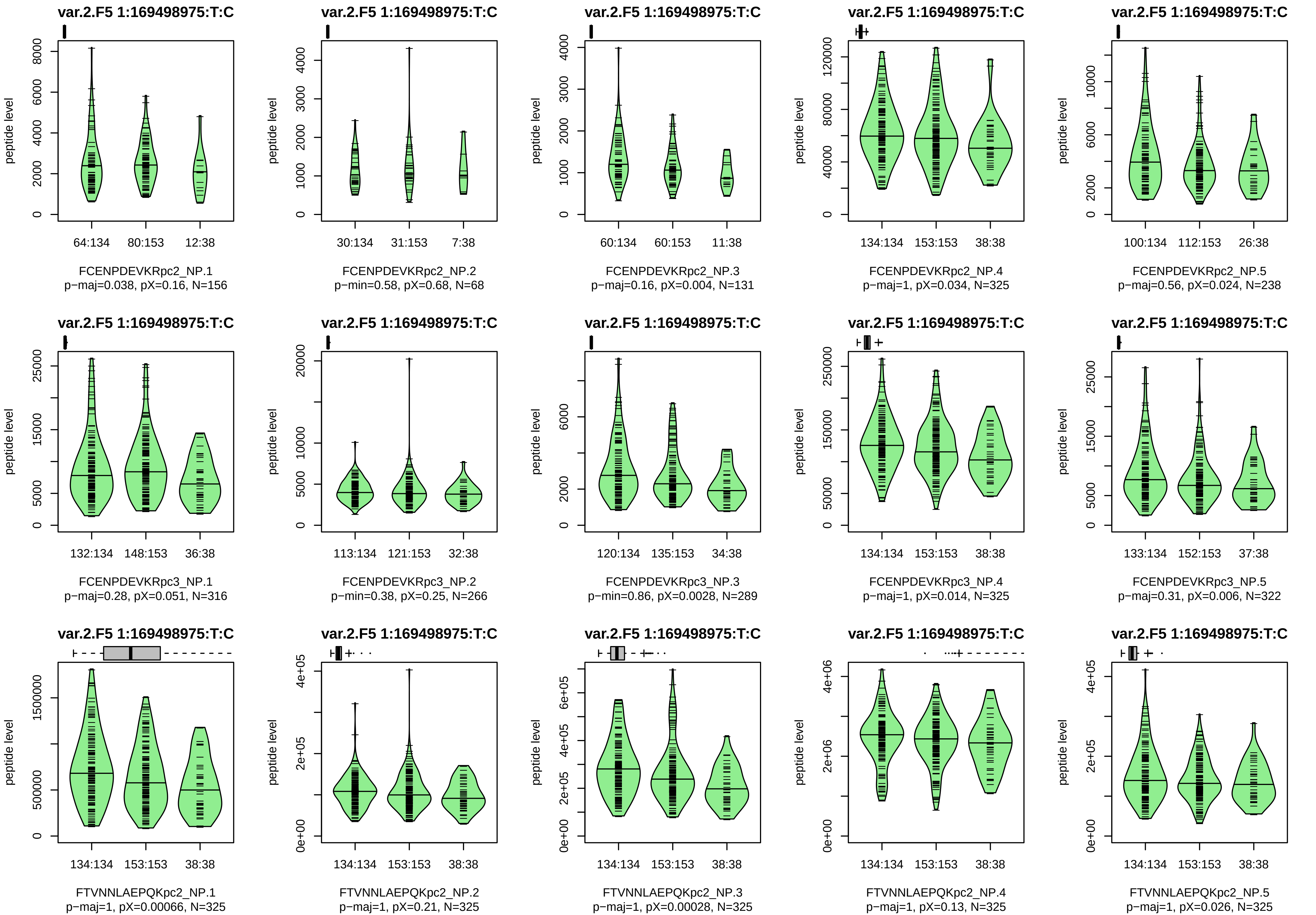

var.2.F5 1:169498975:T:C
peptide level
64:134
80:153
12:38
FCENPDEVKRpc2_NP.1
p−maj=0.038, pX=0.16, N=156
var.2.F5 1:169498975:T:C
peptide level
30:134
31:153
7:38
FCENPDEVKRpc2_NP.2
p−min=0.58, pX=0.68, N=68
var.2.F5 1:169498975:T:C
peptide level
60:134
60:153
11:38
FCENPDEVKRpc2_NP.3
p−maj=0.16, pX=0.004, N=131
var.2.F5 1:169498975:T:C
peptide level
134:134
153:153
38:38
FCENPDEVKRpc2_NP.4
p−maj=1, pX=0.034, N=325
var.2.F5 1:169498975:T:C
peptide level
100:134
112:153
26:38
FCENPDEVKRpc2_NP.5
p−maj=0.56, pX=0.024, N=238
var.2.F5 1:169498975:T:C
peptide level
132:134
148:153
36:38
FCENPDEVKRpc3_NP.1
p−maj=0.28, pX=0.051, N=316
var.2.F5 1:169498975:T:C
peptide level
113:134
121:153
32:38
FCENPDEVKRpc3_NP.2
p−min=0.38, pX=0.25, N=266
var.2.F5 1:169498975:T:C
peptide level
120:134
135:153
34:38
FCENPDEVKRpc3_NP.3
p−min=0.86, pX=0.0028, N=289
var.2.F5 1:169498975:T:C
peptide level
134:134
153:153
38:38
FCENPDEVKRpc3_NP.4
p−maj=1, pX=0.014, N=325
var.2.F5 1:169498975:T:C
peptide level
133:134
152:153
37:38
FCENPDEVKRpc3_NP.5
p−maj=0.31, pX=0.006, N=322
var.2.F5 1:169498975:T:C
peptide level
134:134
153:153
38:38
FTVNNLAEPQKpc2_NP.1
p−maj=1, pX=0.00066, N=325
var.2.F5 1:169498975:T:C
peptide level
134:134
153:153
38:38
FTVNNLAEPQKpc2_NP.2
p−maj=1, pX=0.21, N=325
var.2.F5 1:169498975:T:C
peptide level
134:134
153:153
38:38
FTVNNLAEPQKpc2_NP.3
p−maj=1, pX=0.00028, N=325
var.2.F5 1:169498975:T:C
peptide level
134:134
153:153
38:38
FTVNNLAEPQKpc2_NP.4
p−maj=1, pX=0.13, N=325
var.2.F5 1:169498975:T:C
peptide level
134:134
153:153
38:38
FTVNNLAEPQKpc2_NP.5
p−maj=1, pX=0.026, N=325

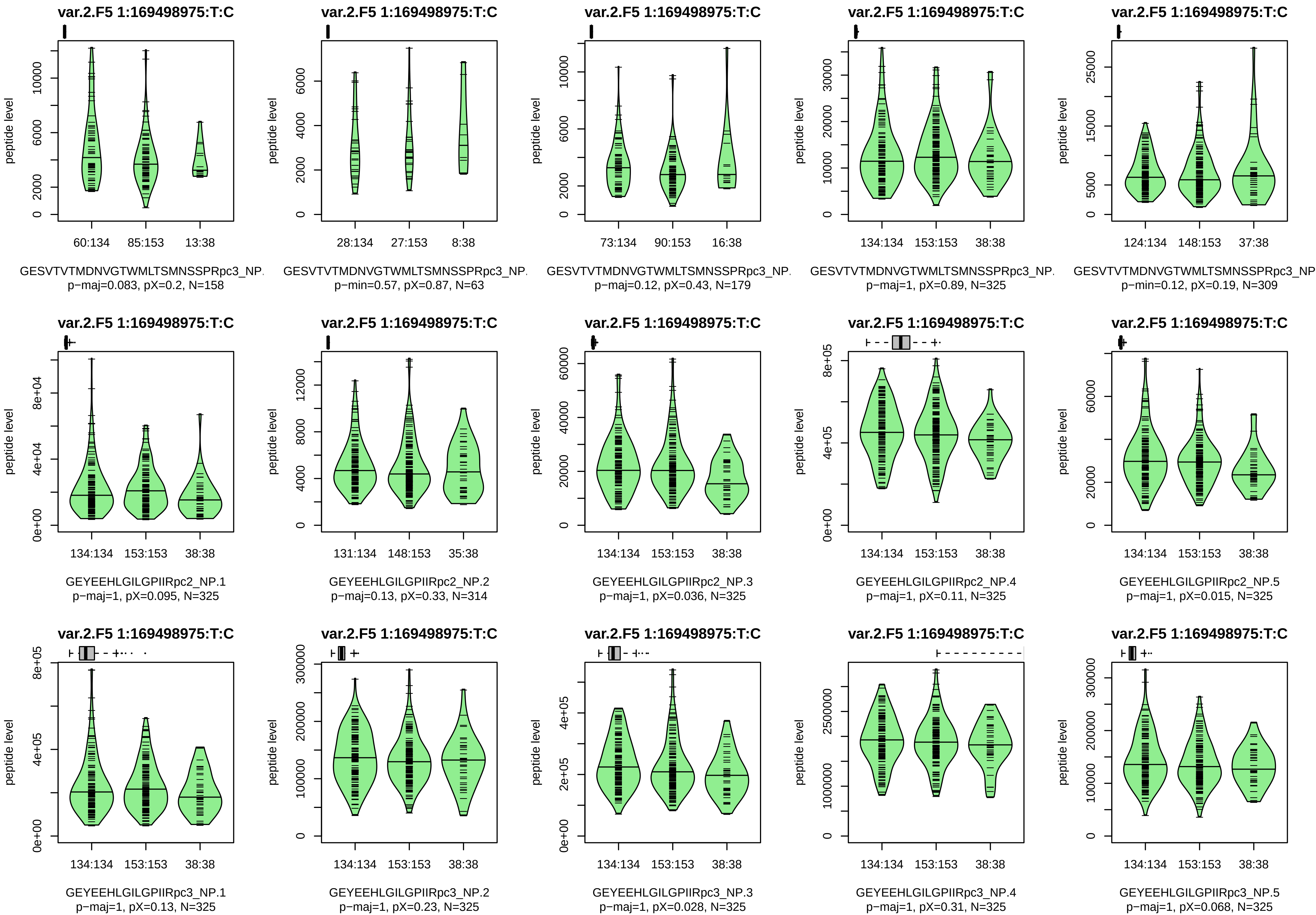

var.2.F5 1:169498975:T:C
peptide level
60:134
85:153
13:38
GESVTVTMDNVGTWMLTSMNSSPRpc3_NP.
p−maj=0.083, pX=0.2, N=158
var.2.F5 1:169498975:T:C
peptide level
28:134
27:153
8:38
GESVTVTMDNVGTWMLTSMNSSPRpc3_NP.
p−min=0.57, pX=0.87, N=63
var.2.F5 1:169498975:T:C
peptide level
73:134
90:153
16:38
GESVTVTMDNVGTWMLTSMNSSPRpc3_NP.
p−maj=0.12, pX=0.43, N=179
var.2.F5 1:169498975:T:C
peptide level
134:134
153:153
38:38
GESVTVTMDNVGTWMLTSMNSSPRpc3_NP.
p−maj=1, pX=0.89, N=325
var.2.F5 1:169498975:T:C
peptide level
124:134
148:153
37:38
GESVTVTMDNVGTWMLTSMNSSPRpc3_NP
p−min=0.12, pX=0.19, N=309
var.2.F5 1:169498975:T:C
peptide level
134:134
153:153
38:38
GEYEEHLGILGPIIRpc2_NP.1
p−maj=1, pX=0.095, N=325
var.2.F5 1:169498975:T:C
peptide level
131:134
148:153
35:38
GEYEEHLGILGPIIRpc2_NP.2
p−maj=0.13, pX=0.33, N=314
var.2.F5 1:169498975:T:C
peptide level
134:134
153:153
38:38
GEYEEHLGILGPIIRpc2_NP.3
p−maj=1, pX=0.036, N=325
var.2.F5 1:169498975:T:C
peptide level
134:134
153:153
38:38
GEYEEHLGILGPIIRpc2_NP.4
p−maj=1, pX=0.11, N=325
var.2.F5 1:169498975:T:C
peptide level
134:134
153:153
38:38
GEYEEHLGILGPIIRpc2_NP.5
p−maj=1, pX=0.015, N=325
var.2.F5 1:169498975:T:C
peptide level
134:134
153:153
38:38
GEYEEHLGILGPIIRpc3_NP.1
p−maj=1, pX=0.13, N=325
var.2.F5 1:169498975:T:C
peptide level
134:134
153:153
38:38
GEYEEHLGILGPIIRpc3_NP.2
p−maj=1, pX=0.23, N=325
var.2.F5 1:169498975:T:C
peptide level
134:134
153:153
38:38
GEYEEHLGILGPIIRpc3_NP.3
p−maj=1, pX=0.028, N=325
var.2.F5 1:169498975:T:C
peptide level
134:134
153:153
38:38
GEYEEHLGILGPIIRpc3_NP.4
p−maj=1, pX=0.31, N=325
var.2.F5 1:169498975:T:C
peptide level
134:134
153:153
38:38
GEYEEHLGILGPIIRpc3_NP.5
p−maj=1, pX=0.068, N=325

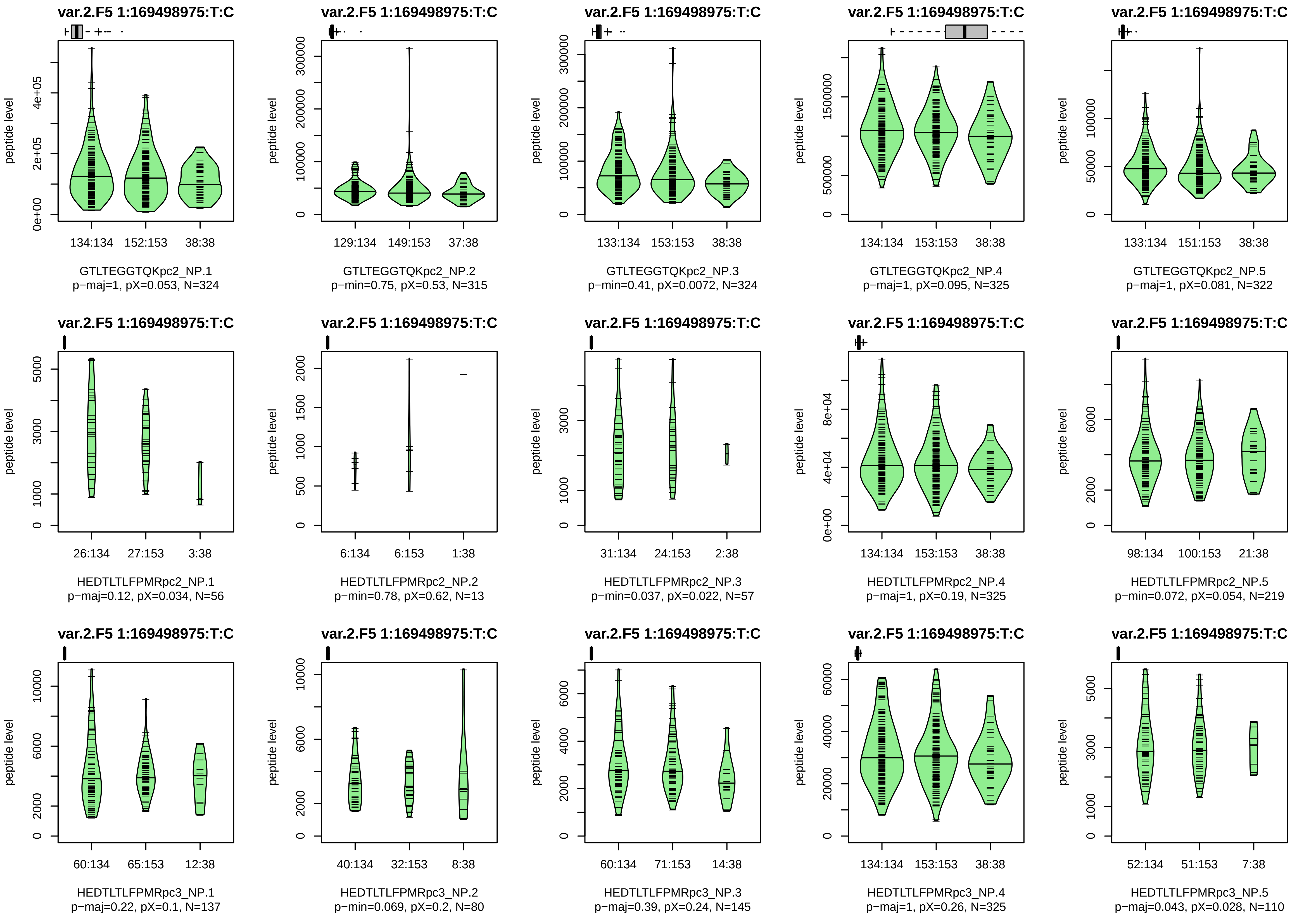
var.2.F5 1:169498975:T:C
peptide level
134:134
152:153
38:38
GTLTEGGTQKpc2_NP.1
p−maj=1, pX=0.053, N=324
129:134
149:153
37:38
GTLTEGGTQKpc2_NP.2
p−min=0.75, pX=0.53, N=315
133:134
153:153
38:38
GTLTEGGTQKpc2_NP.3
p−min=0.41, pX=0.0072, N=324
134:134
153:153
38:38
GTLTEGGTQKpc2_NP.4
p−maj=1, pX=0.095, N=325
133:134
151:153
38:38
GTLTEGGTQKpc2_NP.5
p−maj=1, pX=0.081, N=322
26:134
27:153
3:38
HEDTLTLFPMRpc2_NP.1
p−maj=0.12, pX=0.034, N=56
6:134
6:153
1:38
HEDTLTLFPMRpc2_NP.2
p−min=0.78, pX=0.62, N=13
31:134
24:153
2:38
HEDTLTLFPMRpc2_NP.3
p−min=0.037, pX=0.022, N=57
134:134
153:153
38:38
HEDTLTLFPMRpc2_NP.4
p−maj=1, pX=0.19, N=325
98:134
100:153
21:38
HEDTLTLFPMRpc2_NP.5
p−min=0.072, pX=0.054, N=219
60:134
65:153
12:38
HEDTLTLFPMRpc3_NP.1
p−maj=0.22, pX=0.1, N=137
40:134
32:153
8:38
HEDTLTLFPMRpc3_NP.2
p−min=0.069, pX=0.2, N=80
60:134
71:153
14:38
HEDTLTLFPMRpc3_NP.3
p−maj=0.39, pX=0.24, N=145
134:134
153:153
38:38
HEDTLTLFPMRpc3_NP.4
p−maj=1, pX=0.26, N=325
52:134
51:153
7:38
HEDTLTLFPMRpc3_NP.5
p−maj=0.043, pX=0.028, N=110

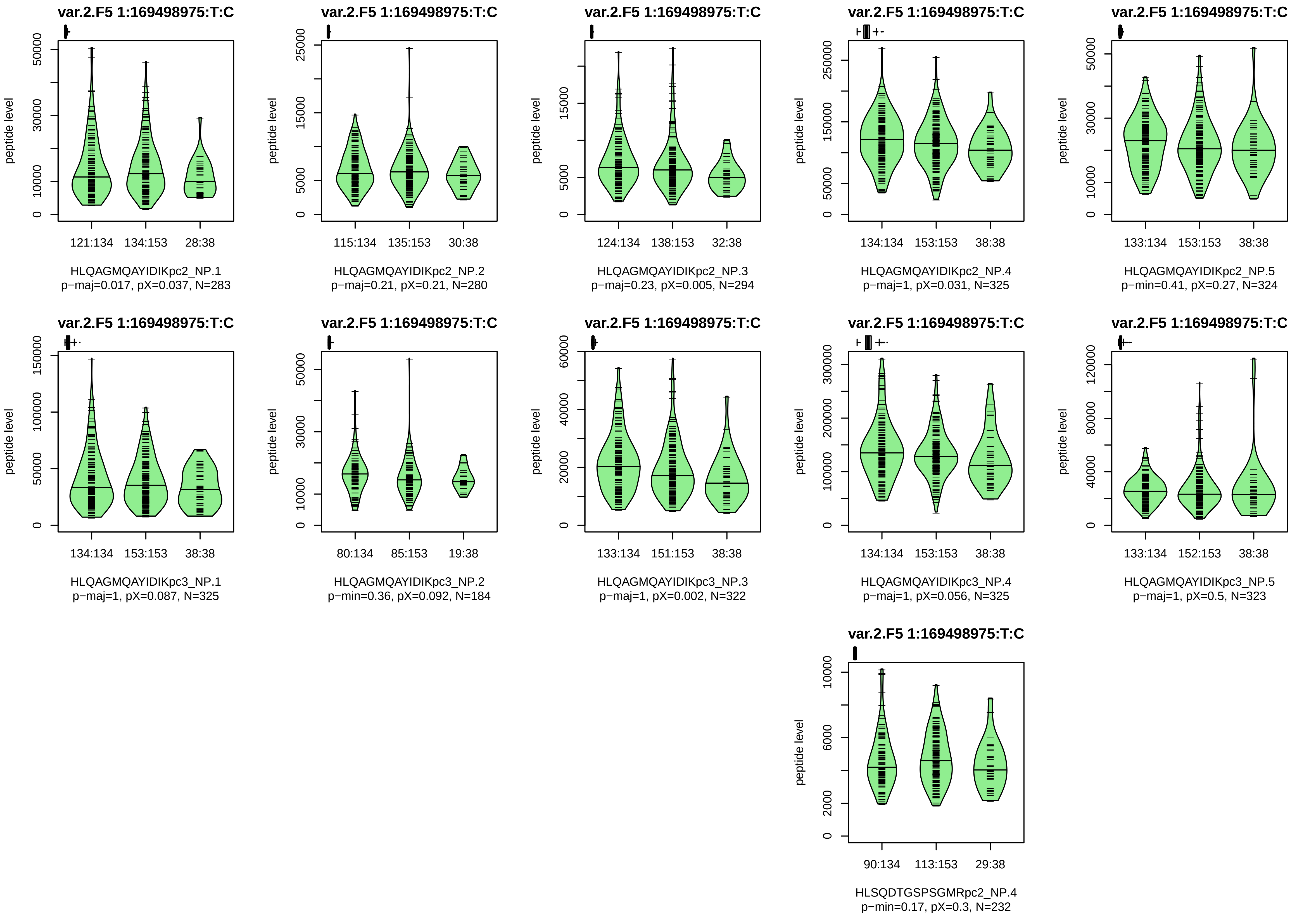

var.2.F5 1:169498975:T:C
peptide level
121:134
134:153
28:38
HLQAGMQAYIDIKpc2_NP.1
p−maj=0.017, pX=0.037, N=283
var.2.F5 1:169498975:T:C
peptide level
115:134
135:153
30:38
HLQAGMQAYIDIKpc2_NP.2
p−maj=0.21, pX=0.21, N=280
var.2.F5 1:169498975:T:C
peptide level
124:134
138:153
32:38
HLQAGMQAYIDIKpc2_NP.3
p−maj=0.23, pX=0.005, N=294
var.2.F5 1:169498975:T:C
peptide level
134:134
153:153
38:38
HLQAGMQAYIDIKpc2_NP.4
p−maj=1, pX=0.031, N=325
var.2.F5 1:169498975:T:C
peptide level
133:134
153:153
38:38
HLQAGMQAYIDIKpc2_NP.5
p−min=0.41, pX=0.27, N=324
var.2.F5 1:169498975:T:C
peptide level
134:134
153:153
38:38
HLQAGMQAYIDIKpc3_NP.1
p−maj=1, pX=0.087, N=325
var.2.F5 1:169498975:T:C
peptide level
80:134
85:153
19:38
HLQAGMQAYIDIKpc3_NP.2
p−min=0.36, pX=0.092, N=184
var.2.F5 1:169498975:T:C
peptide level
133:134
151:153
38:38
HLQAGMQAYIDIKpc3_NP.3
p−maj=1, pX=0.002, N=322
var.2.F5 1:169498975:T:C
peptide level
134:134
153:153
38:38
HLQAGMQAYIDIKpc3_NP.4
p−maj=1, pX=0.056, N=325
var.2.F5 1:169498975:T:C
peptide level
133:134
152:153
38:38
HLQAGMQAYIDIKpc3_NP.5
p−maj=1, pX=0.5, N=323
var.2.F5 1:169498975:T:C
peptide level
90:134
113:153
29:38
HLSQDTGSPSGMRpc2_NP.4
p−min=0.17, pX=0.3, N=232

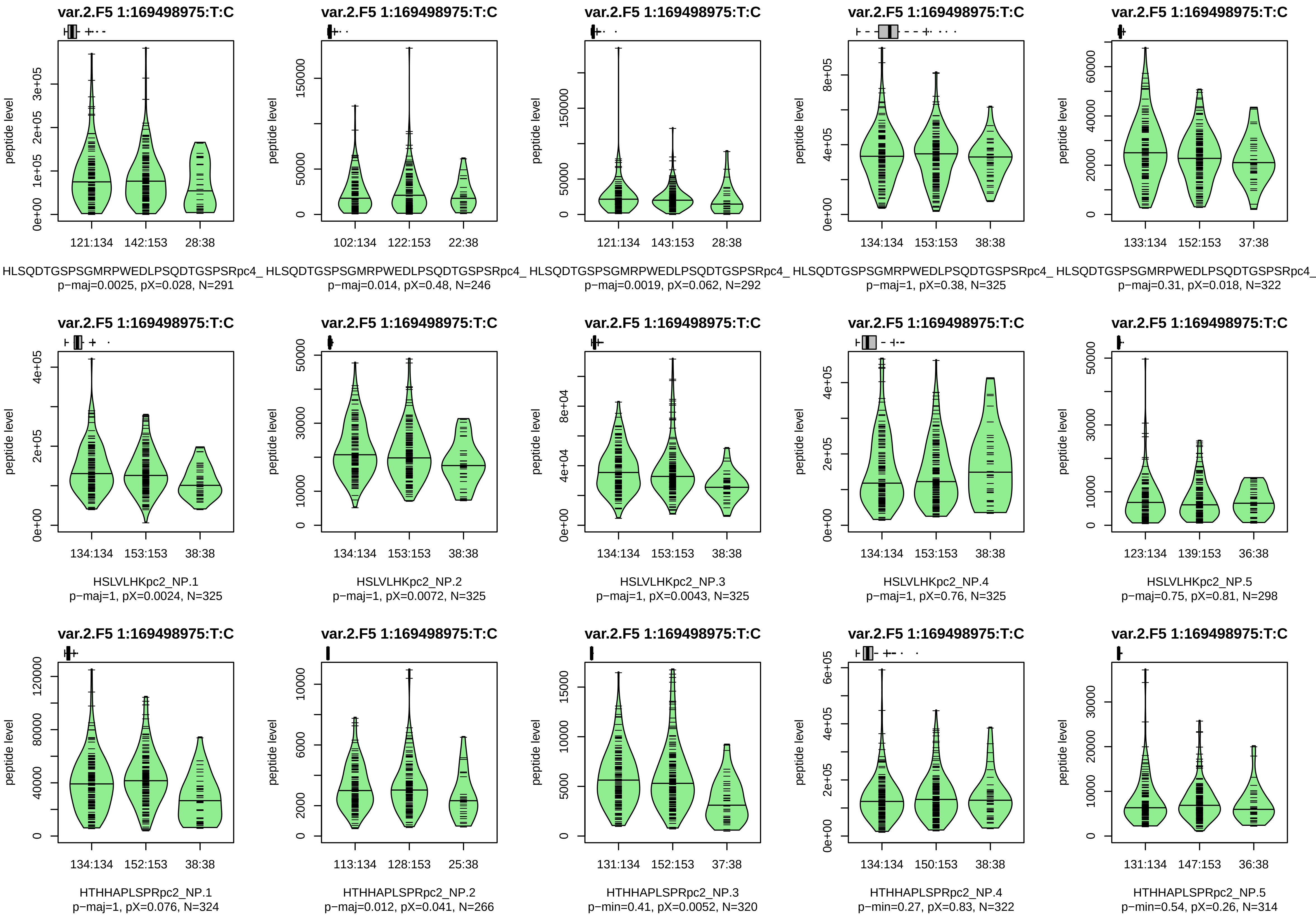
var.2.F5 1:169498975:T:C
peptide level
121:134
142:153
28:38
HLSQDTGSPSGMRPWEDLPSQDTGSPSRpc4_
p−maj=0.0025, pX=0.028, N=291
var.2.F5 1:169498975:T:C
102:134
122:153
22:38
HLSQDTGSPSGMRPWEDLPSQDTGSPSRpc4_
p−maj=0.014, pX=0.48, N=246
var.2.F5 1:169498975:T:C
121:134
143:153
28:38
HLSQDTGSPSGMRPWEDLPSQDTGSPSRpc4_
p−maj=0.0019, pX=0.062, N=292
var.2.F5 1:169498975:T:C
134:134
153:153
38:38
HLSQDTGSPSGMRPWEDLPSQDTGSPSRpc4_
p−maj=1, pX=0.38, N=325
var.2.F5 1:169498975:T:C
133:134
152:153
37:38
HLSQDTGSPSGMRPWEDLPSQDTGSPSRpc4_
p−maj=0.31, pX=0.018, N=322
var.2.F5 1:169498975:T:C
134:134
153:153
38:38
HSLVLHKpc2_NP.1
p−maj=1, pX=0.0024, N=325
var.2.F5 1:169498975:T:C
134:134
153:153
38:38
HSLVLHKpc2_NP.2
p−maj=1, pX=0.0072, N=325
var.2.F5 1:169498975:T:C
134:134
153:153
38:38
HSLVLHKpc2_NP.3
p−maj=1, pX=0.0043, N=325
var.2.F5 1:169498975:T:C
134:134
153:153
38:38
HSLVLHKpc2_NP.4
p−maj=1, pX=0.76, N=325
var.2.F5 1:169498975:T:C
123:134
139:153
36:38
HSLVLHKpc2_NP.5
p−maj=0.75, pX=0.81, N=298
var.2.F5 1:169498975:T:C
134:134
152:153
38:38
HTHHAPLSPRpc2_NP.1
p−maj=1, pX=0.076, N=324
var.2.F5 1:169498975:T:C
113:134
128:153
25:38
HTHHAPLSPRpc2_NP.2
p−maj=0.012, pX=0.041, N=266
var.2.F5 1:169498975:T:C
131:134
152:153
37:38
HTHHAPLSPRpc2_NP.3
p−min=0.41, pX=0.0052, N=320
var.2.F5 1:169498975:T:C
134:134
150:153
38:38
HTHHAPLSPRpc2_NP.4
p−min=0.27, pX=0.83, N=322
var.2.F5 1:169498975:T:C
131:134
147:153
36:38
HTHHAPLSPRpc2_NP.5
p−min=0.54, pX=0.26, N=314

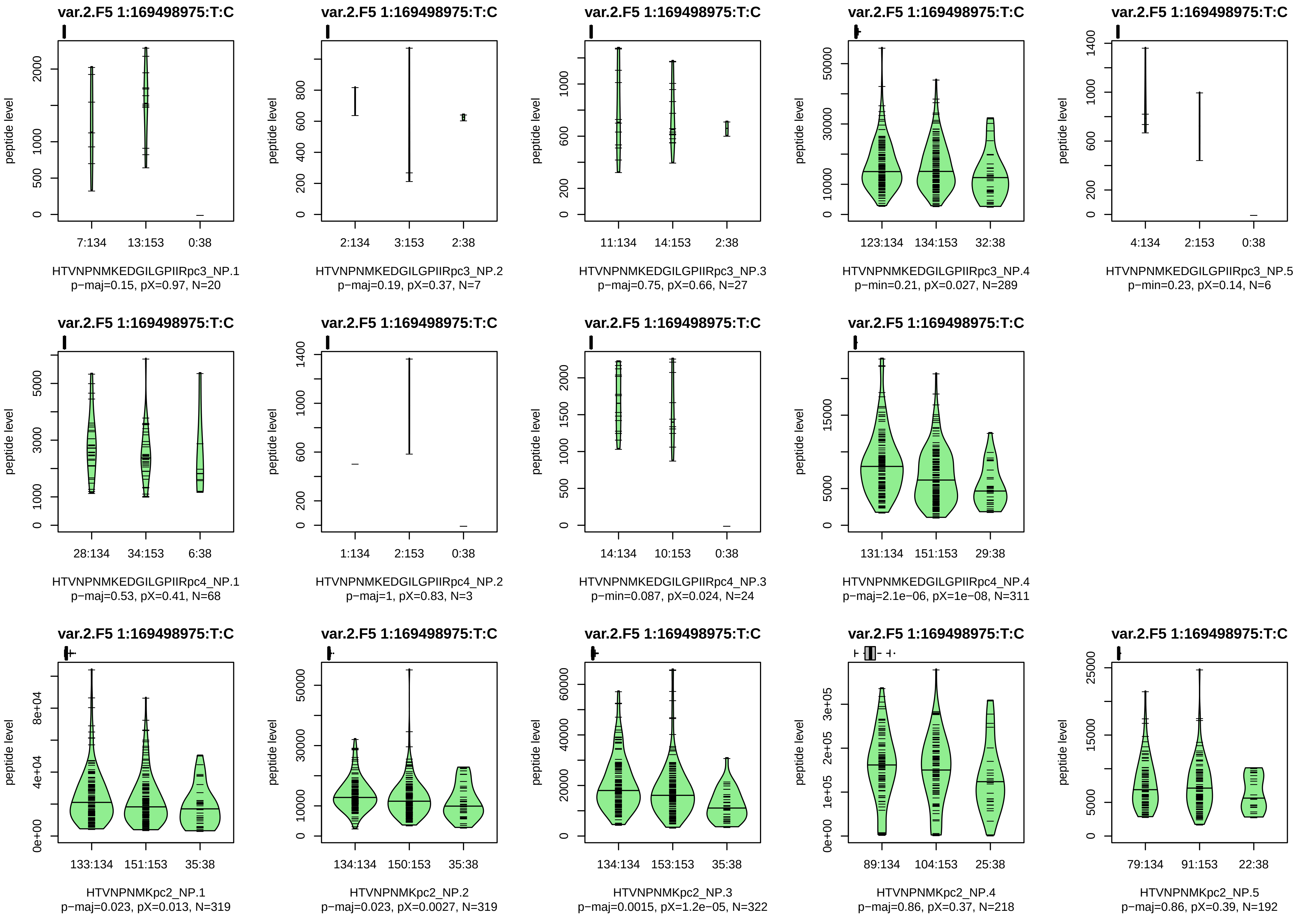

var.2.F5 1:169498975:T:C
peptide level
7:134
13:153
0:38
HTVNPNMKEDGILGPIIRpc3_NP.1
p−maj=0.15, pX=0.97, N=20
var.2.F5 1:169498975:T:C
peptide level
2:134
3:153
2:38
HTVNPNMKEDGILGPIIRpc3_NP.2
p−maj=0.19, pX=0.37, N=7
var.2.F5 1:169498975:T:C
peptide level
11:134
14:153
2:38
HTVNPNMKEDGILGPIIRpc3_NP.3
p−maj=0.75, pX=0.66, N=27
var.2.F5 1:169498975:T:C
peptide level
123:134
134:153
32:38
HTVNPNMKEDGILGPIIRpc3_NP.4
p−min=0.21, pX=0.027, N=289
var.2.F5 1:169498975:T:C
peptide level
4:134
2:153
0:38
HTVNPNMKEDGILGPIIRpc3_NP.5
p−min=0.23, pX=0.14, N=6
var.2.F5 1:169498975:T:C
peptide level
28:134
34:153
6:38
HTVNPNMKEDGILGPIIRpc4_NP.1
p−maj=0.53, pX=0.41, N=68
var.2.F5 1:169498975:T:C
peptide level
1:134
2:153
0:38
HTVNPNMKEDGILGPIIRpc4_NP.2
p−maj=1, pX=0.83, N=3
var.2.F5 1:169498975:T:C
peptide level
14:134
10:153
0:38
HTVNPNMKEDGILGPIIRpc4_NP.3
p−min=0.087, pX=0.024, N=24
var.2.F5 1:169498975:T:C
peptide level
131:134
151:153
29:38
HTVNPNMKEDGILGPIIRpc4_NP.4
p−maj=2.1e−06, pX=1e−08, N=311
var.2.F5 1:169498975:T:C
peptide level
133:134
151:153
35:38
HTVNPNMKpc2_NP.1
p−maj=0.023, pX=0.013, N=319
var.2.F5 1:169498975:T:C
peptide level
134:134
150:153
35:38
HTVNPNMKpc2_NP.2
p−maj=0.023, pX=0.0027, N=319
var.2.F5 1:169498975:T:C
peptide level
134:134
153:153
35:38
HTVNPNMKpc2_NP.3
p−maj=0.0015, pX=1.2e−05, N=322
var.2.F5 1:169498975:T:C
peptide level
89:134
104:153
25:38
HTVNPNMKpc2_NP.4
p−maj=0.86, pX=0.37, N=218
var.2.F5 1:169498975:T:C
peptide level
79:134
91:153
22:38
HTVNPNMKpc2_NP.5
p−maj=0.86, pX=0.39, N=192

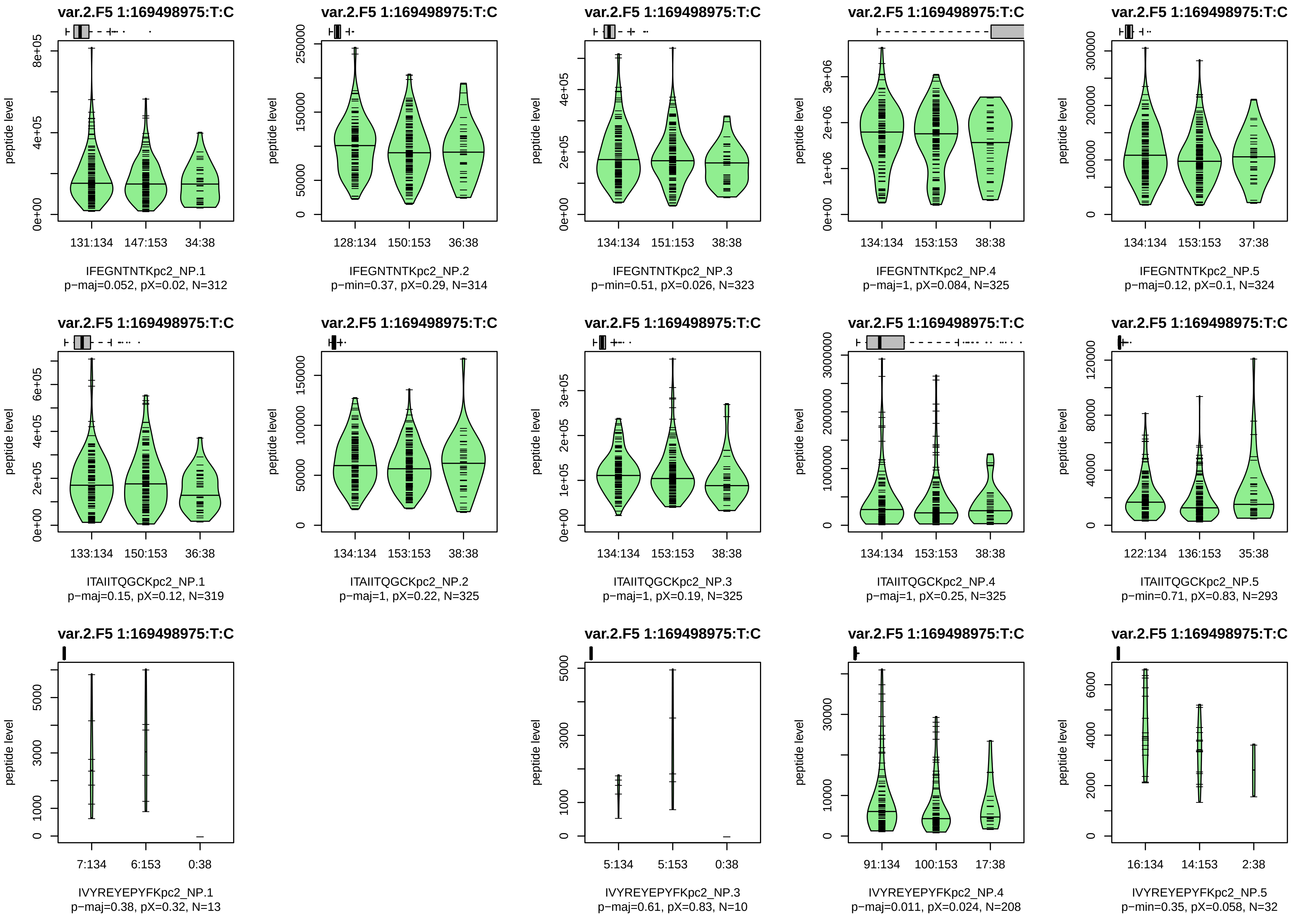
var.2.F5 1:169498975:T:C
peptide level
131:134 147:153 34:38
IFEGNTNTKpc2_NP.1
p−maj=0.052, pX=0.02, N=312
var.2.F5 1:169498975:T:C
peptide level
128:134 150:153 36:38
IFEGNTNTKpc2_NP.2
p−min=0.37, pX=0.29, N=314
var.2.F5 1:169498975:T:C
peptide level
134:134 151:153 38:38
IFEGNTNTKpc2_NP.3
p−min=0.51, pX=0.026, N=323
var.2.F5 1:169498975:T:C
peptide level
134:134 153:153 38:38
IFEGNTNTKpc2_NP.4
p−maj=1, pX=0.084, N=325
var.2.F5 1:169498975:T:C
peptide level
134:134 153:153 37:38
IFEGNTNTKpc2_NP.5
p−maj=0.12, pX=0.1, N=324
var.2.F5 1:169498975:T:C
peptide level
133:134 150:153 36:38
ITAIITQGCKpc2_NP.1
p−maj=0.15, pX=0.12, N=319
var.2.F5 1:169498975:T:C
peptide level
134:134 153:153 38:38
ITAIITQGCKpc2_NP.2
p−maj=1, pX=0.22, N=325
var.2.F5 1:169498975:T:C
peptide level
134:134 153:153 38:38
ITAIITQGCKpc2_NP.3
p−maj=1, pX=0.19, N=325
var.2.F5 1:169498975:T:C
peptide level
134:134 153:153 38:38
ITAIITQGCKpc2_NP.4
p−maj=1, pX=0.25, N=325
var.2.F5 1:169498975:T:C
peptide level
122:134 136:153 35:38
ITAIITQGCKpc2_NP.5
p−min=0.71, pX=0.83, N=293
var.2.F5 1:169498975:T:C
peptide level
7:134 6:153 0:38
IVYREYEPYFKpc2_NP.1
p−maj=0.38, pX=0.32, N=13
var.2.F5 1:169498975:T:C
peptide level
5:134 5:153 0:38
IVYREYEPYFKpc2_NP.3
p−maj=0.61, pX=0.83, N=10
var.2.F5 1:169498975:T:C
peptide level
91:134 100:153 17:38
IVYREYEPYFKpc2_NP.4
p−maj=0.011, pX=0.024, N=208
var.2.F5 1:169498975:T:C
peptide level
16:134 14:153 2:38
IVYREYEPYFKpc2_NP.5
p−min=0.35, pX=0.058, N=32

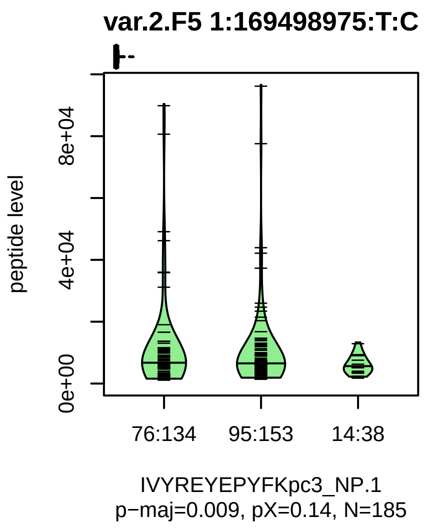
var.2.F5 1:169498975:T:C
peptide level
0e+00
4e+04
8e+04
76:134
95:153
14:38
IVYREYEPYFKpc3_NP.1
p−maj=0.009, pX=0.14, N=185

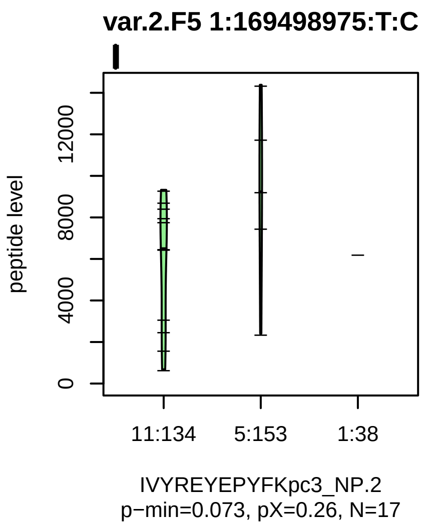
var.2.F5 1:169498975:T:C
peptide level
0
4000
8000
12000
11:134
5:153
1:38
IVYREYEPYFKpc3_NP.2
p−min=0.073, pX=0.26, N=17

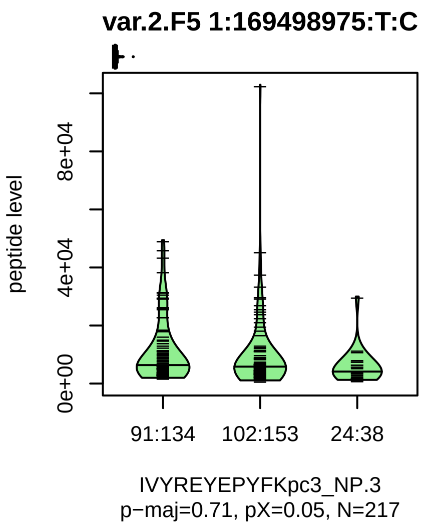
var.2.F5 1:169498975:T:C
peptide level
0e+00
4e+04
8e+04
91:134
102:153
24:38
IVYREYEPYFKpc3_NP.3
p−maj=0.71, pX=0.05, N=217

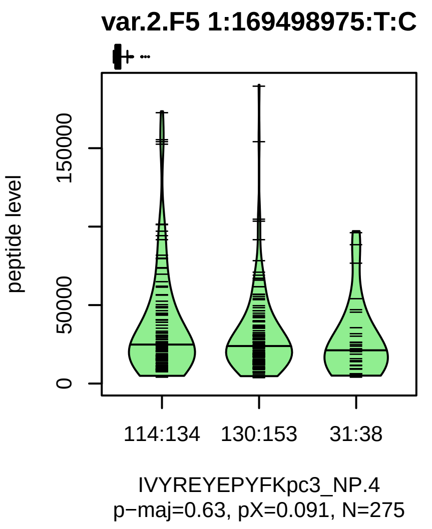
var.2.F5 1:169498975:T:C
peptide level
0
50000
150000
114:134
130:153
31:38
IVYREYEPYFKpc3_NP.4
p−maj=0.63, pX=0.091, N=275

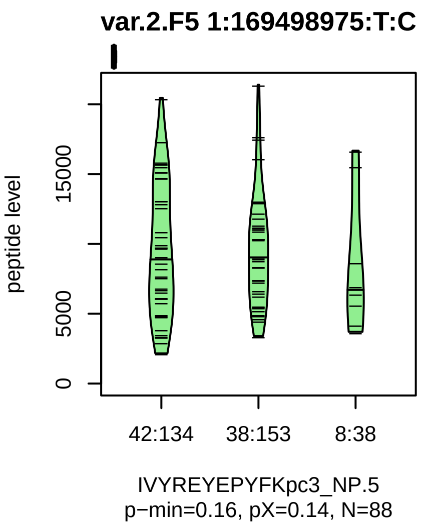
var.2.F5 1:169498975:T:C
peptide level
0
5000
15000
42:134
38:153
8:38
IVYREYEPYFKpc3_NP.5
p−min=0.16, pX=0.14, N=88

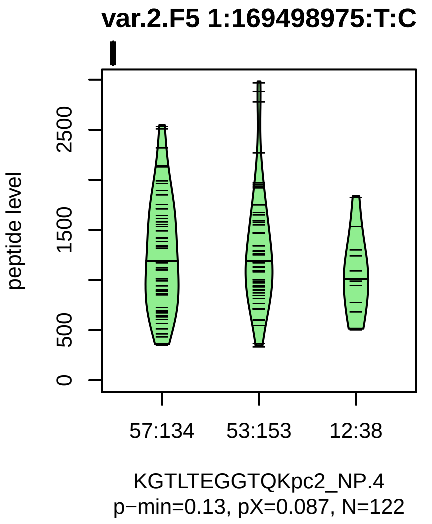
var.2.F5 1:169498975:T:C
peptide level
0
500
1500
2500
57:134
53:153
12:38
KGTLTEGGTQKpc2_NP.4
p−min=0.13, pX=0.087, N=122

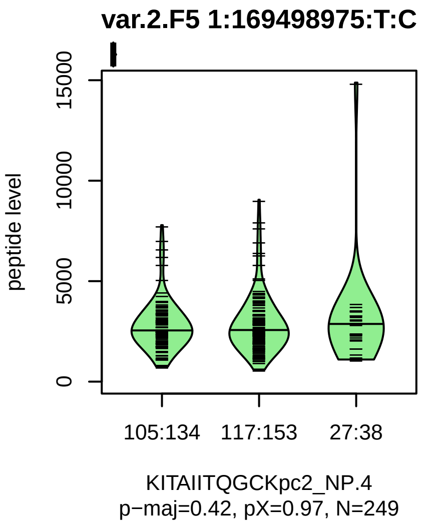
var.2.F5 1:169498975:T:C
peptide level
0
5000
10000
15000
105:134
117:153
27:38
KITAIITQGCKpc2_NP.4
p−maj=0.42, pX=0.97, N=249

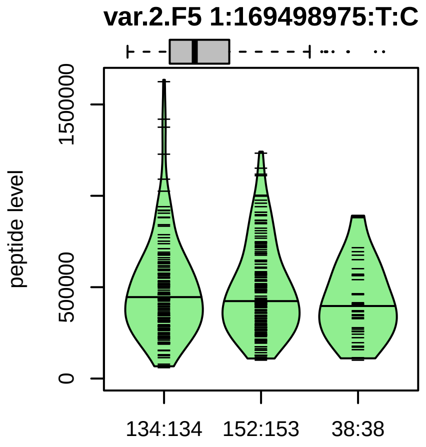

LAAEFASKpc2_NP.1
p−maj=1, pX=0.23, N=324

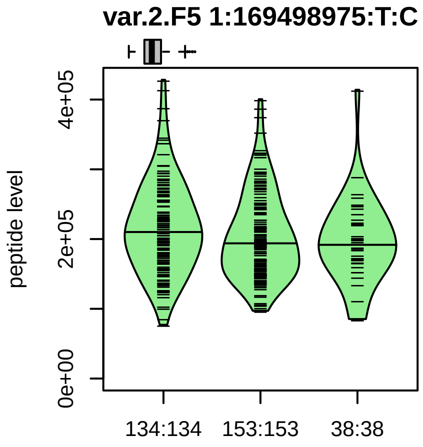

LAAEFASKpc2_NP.2
p−maj=1, pX=0.033, N=325

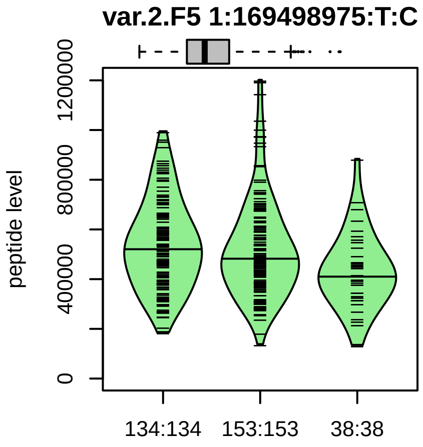

LAAEFASKpc2_NP.3
p−maj=1, pX=0.007, N=325

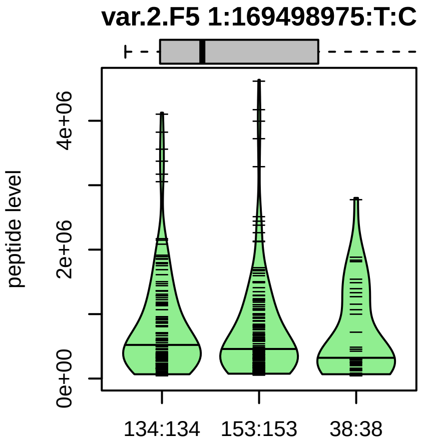

LAAEFASKpc2_NP.4
p−maj=1, pX=0.32, N=325

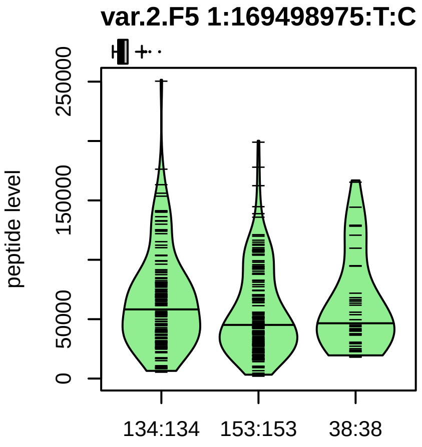

LAAEFASKpc2_NP.5
p−maj=1, pX=0.23, N=325

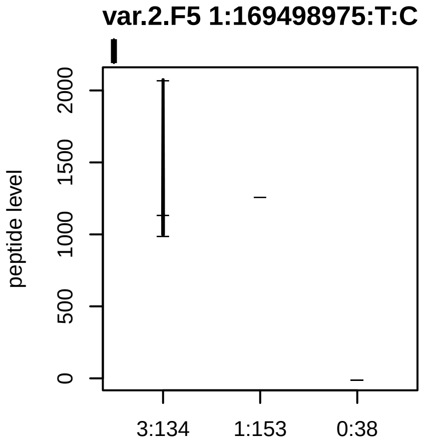

LAAEFASKPWIQVDMQKpc3_NP.3
p−min=0.31, pX=0.17, N=4

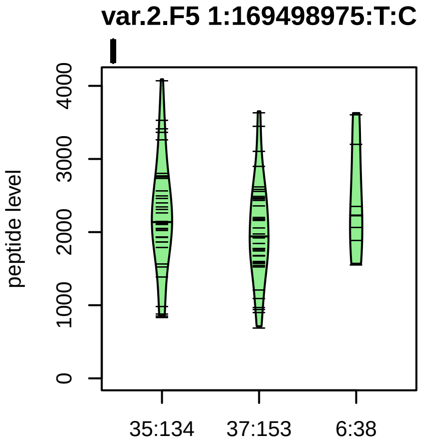

LAAEFASKPWIQVDMQKpc3_NP.4
p−maj=0.23, pX=0.24, N=78

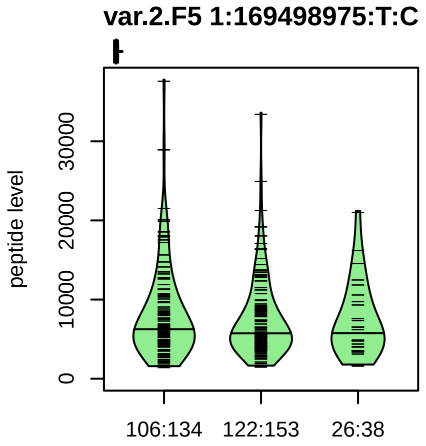

LELQGCEVNGCSTPLGMENGKpc3_NP.1
p−maj=0.14, pX=0.25, N=254

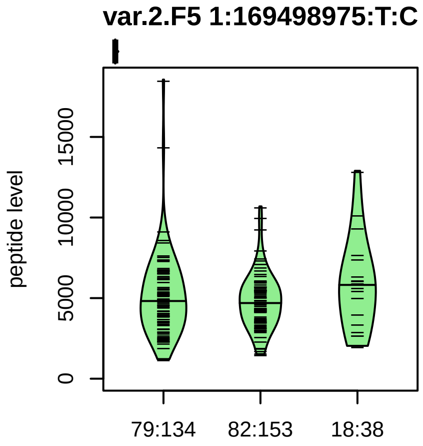

LELQGCEVNGCSTPLGMENGKpc3_NP.2
p−min=0.26, pX=0.35, N=179

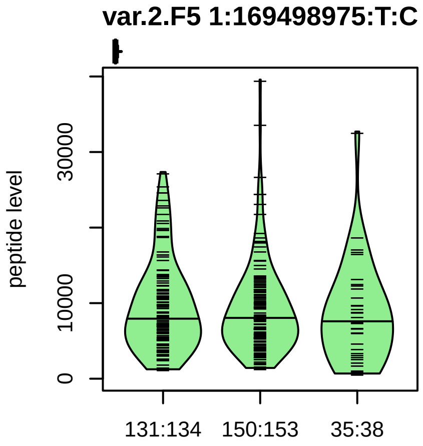

LELQGCEVNGCSTPLGMENGKpc3_NP.3
p−maj=0.075, pX=0.2, N=316

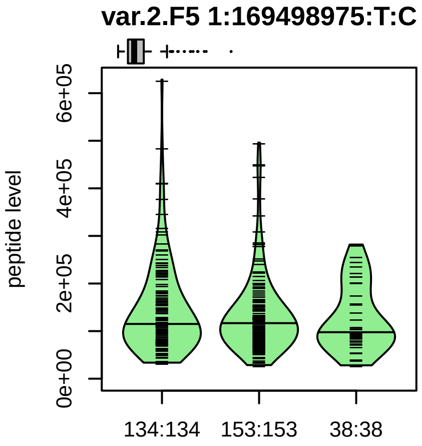

LELQGCEVNGCSTPLGMENGKpc3_NP.4
p−maj=1, pX=0.2, N=325

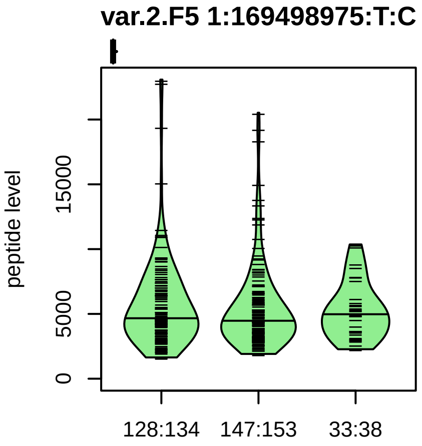

LELQGCEVNGCSTPLGMENGKpc3_NP.5
p−maj=0.036, pX=0.18, N=308

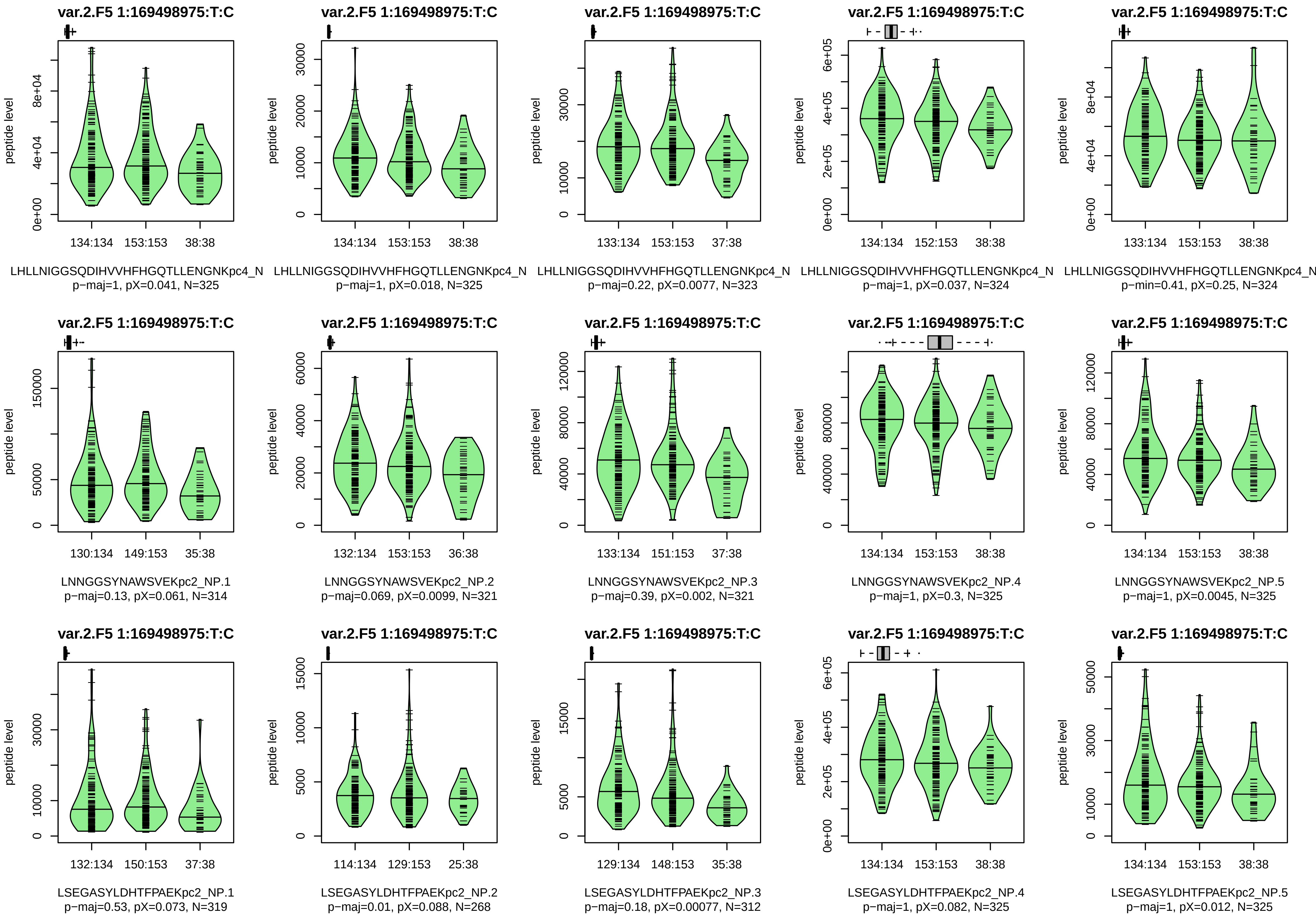

var.2.F5 1:169498975:T:C
peptide level
0e+00
4e+04
8e+04
134:134
153:153
38:38
LHLLNIGGSQDIHVVHFHGQTLLENGNKpc4_N
p−maj=1, pX=0.041, N=325
var.2.F5 1:169498975:T:C
peptide level
0
10000
20000
30000
134:134
153:153
38:38
LHLLNIGGSQDIHVVHFHGQTLLENGNKpc4_N
p−maj=1, pX=0.018, N=325
var.2.F5 1:169498975:T:C
peptide level
0
10000
30000
133:134
153:153
37:38
LHLLNIGGSQDIHVVHFHGQTLLENGNKpc4_N
p−maj=0.22, pX=0.0077, N=323
var.2.F5 1:169498975:T:C
peptide level
0e+00
2e+05
4e+05
6e+05
134:134
152:153
38:38
LHLLNIGGSQDIHVVHFHGQTLLENGNKpc4_N
p−maj=1, pX=0.037, N=324
var.2.F5 1:169498975:T:C
peptide level
0e+00
4e+04
8e+04
133:134
153:153
38:38
LHLLNIGGSQDIHVVHFHGQTLLENGNKpc4_N
p−min=0.41, pX=0.25, N=324
var.2.F5 1:169498975:T:C
peptide level
0
50000
150000
130:134
149:153
35:38
LNNGGSYNAWSVEKpc2_NP.1
p−maj=0.13, pX=0.061, N=314
var.2.F5 1:169498975:T:C
peptide level
0
20000
40000
60000
132:134
153:153
36:38
LNNGGSYNAWSVEKpc2_NP.2
p−maj=0.069, pX=0.0099, N=321
var.2.F5 1:169498975:T:C
peptide level
0
40000
80000
120000
133:134
151:153
37:38
LNNGGSYNAWSVEKpc2_NP.3
p−maj=0.39, pX=0.002, N=321
var.2.F5 1:169498975:T:C
peptide level
0
400000
800000
134:134
153:153
38:38
LNNGGSYNAWSVEKpc2_NP.4
p−maj=1, pX=0.3, N=325
var.2.F5 1:169498975:T:C
peptide level
0
40000
80000
120000
134:134
153:153
38:38
LNNGGSYNAWSVEKpc2_NP.5
p−maj=1, pX=0.0045, N=325
var.2.F5 1:169498975:T:C
peptide level
0
10000
30000
132:134
150:153
37:38
LSEGASYLDHTFPAEKpc2_NP.1
p−maj=0.53, pX=0.073, N=319
var.2.F5 1:169498975:T:C
peptide level
0
5000
10000
15000
114:134
129:153
25:38
LSEGASYLDHTFPAEKpc2_NP.2
p−maj=0.01, pX=0.088, N=268
var.2.F5 1:169498975:T:C
peptide level
0
5000
15000
129:134
148:153
35:38
LSEGASYLDHTFPAEKpc2_NP.3
p−maj=0.18, pX=0.00077, N=312
var.2.F5 1:169498975:T:C
peptide level
0e+00
2e+05
4e+05
6e+05
134:134
153:153
38:38
LSEGASYLDHTFPAEKpc2_NP.4
p−maj=1, pX=0.082, N=325
var.2.F5 1:169498975:T:C
peptide level
0
10000
30000
50000
134:134
153:153
38:38
LSEGASYLDHTFPAEKpc2_NP.5
p−maj=1, pX=0.012, N=325

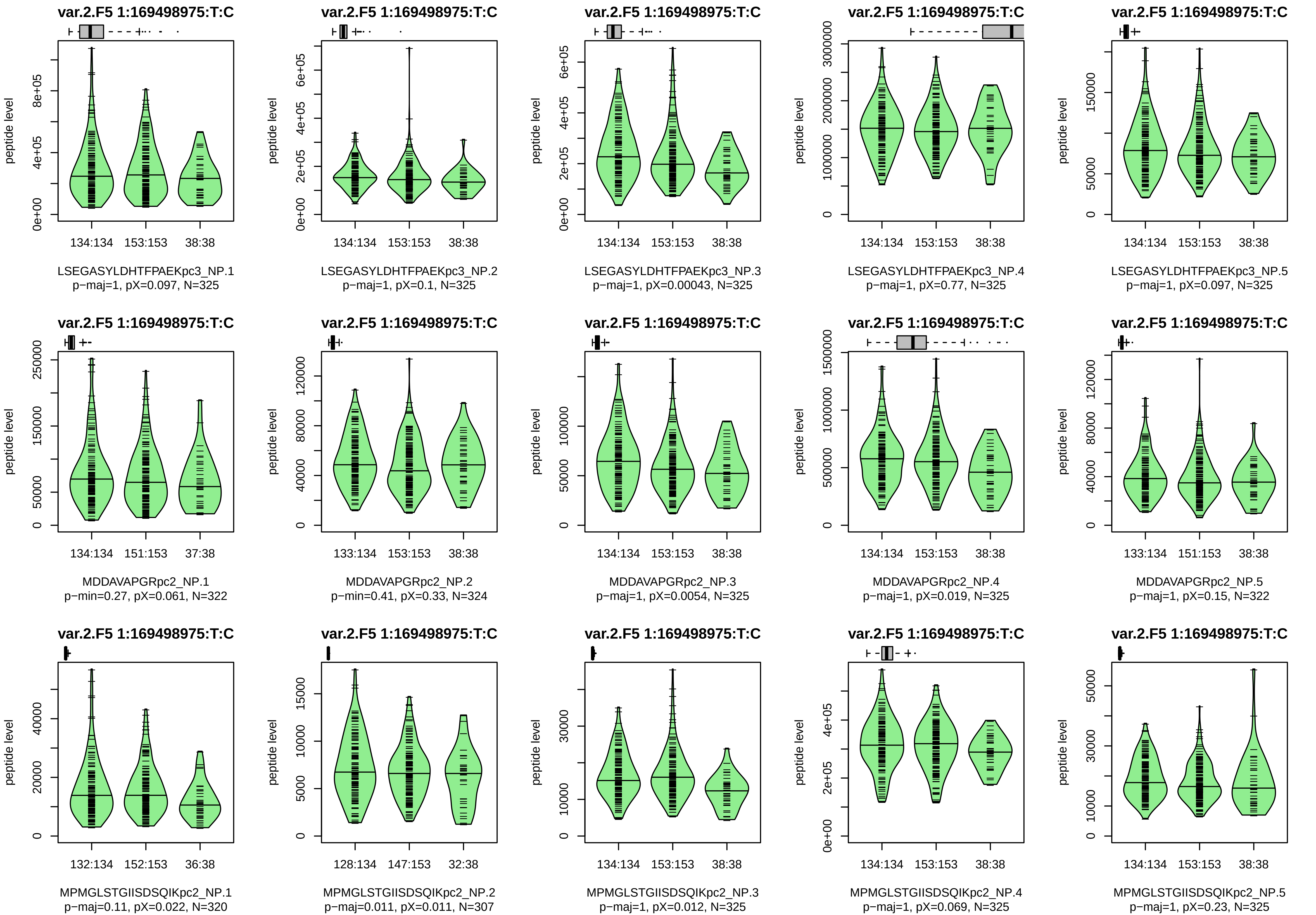

var.2.F5 1:169498975:T:C
peptide level
134:134
153:153
38:38
LSEGASYLDHTFPAEKpc3_NP.1
p−maj=1, pX=0.097, N=325
LSEGASYLDHTFPAEKpc3_NP.2
p−maj=1, pX=0.1, N=325
LSEGASYLDHTFPAEKpc3_NP.3
p−maj=1, pX=0.00043, N=325
LSEGASYLDHTFPAEKpc3_NP.4
p−maj=1, pX=0.77, N=325
LSEGASYLDHTFPAEKpc3_NP.5
p−maj=1, pX=0.097, N=325
134:134
151:153
37:38
MDDAVAPGRpc2_NP.1
p−min=0.27, pX=0.061, N=322
133:134
153:153
38:38
MDDAVAPGRpc2_NP.2
p−min=0.41, pX=0.33, N=324
MDDAVAPGRpc2_NP.3
p−maj=1, pX=0.0054, N=325
MDDAVAPGRpc2_NP.4
p−maj=1, pX=0.019, N=325
133:134
151:153
38:38
MDDAVAPGRpc2_NP.5
p−maj=1, pX=0.15, N=322
132:134
152:153
36:38
MPMGLSTGIISDSQIKpc2_NP.1
p−maj=0.11, pX=0.022, N=320
128:134
147:153
32:38
MPMGLSTGIISDSQIKpc2_NP.2
p−maj=0.011, pX=0.011, N=307
MPMGLSTGIISDSQIKpc2_NP.3
p−maj=1, pX=0.012, N=325
MPMGLSTGIISDSQIKpc2_NP.4
p−maj=1, pX=0.069, N=325
MPMGLSTGIISDSQIKpc2_NP.5
p−maj=1, pX=0.23, N=325

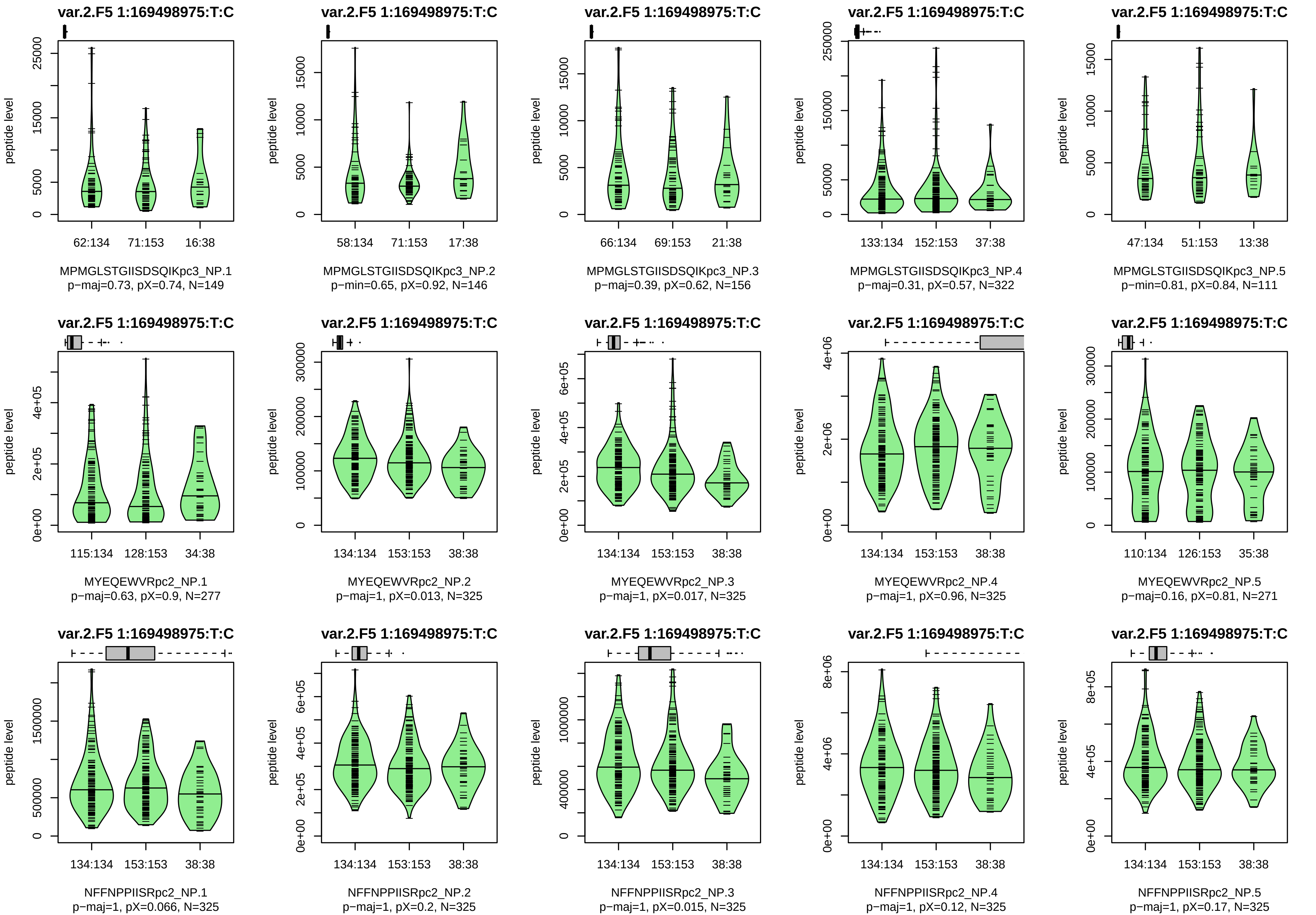
var.2.F5 1:169498975:T:C
peptide level
62:134
71:153
16:38
MPMGLSTGIISDSQIKpc3_NP.1
p−maj=0.73, pX=0.74, N=149
var.2.F5 1:169498975:T:C
peptide level
58:134
71:153
17:38
MPMGLSTGIISDSQIKpc3_NP.2
p−min=0.65, pX=0.92, N=146
var.2.F5 1:169498975:T:C
peptide level
66:134
69:153
21:38
MPMGLSTGIISDSQIKpc3_NP.3
p−maj=0.39, pX=0.62, N=156
var.2.F5 1:169498975:T:C
peptide level
133:134
152:153
37:38
MPMGLSTGIISDSQIKpc3_NP.4
p−maj=0.31, pX=0.57, N=322
var.2.F5 1:169498975:T:C
peptide level
47:134
51:153
13:38
MPMGLSTGIISDSQIKpc3_NP.5
p−min=0.81, pX=0.84, N=111
var.2.F5 1:169498975:T:C
peptide level
115:134
128:153
34:38
MYEQEWVRpc2_NP.1
p−maj=0.63, pX=0.9, N=277
var.2.F5 1:169498975:T:C
peptide level
134:134
153:153
38:38
MYEQEWVRpc2_NP.2
p−maj=1, pX=0.013, N=325
var.2.F5 1:169498975:T:C
peptide level
134:134
153:153
38:38
MYEQEWVRpc2_NP.3
p−maj=1, pX=0.017, N=325
var.2.F5 1:169498975:T:C
peptide level
134:134
153:153
38:38
MYEQEWVRpc2_NP.4
p−maj=1, pX=0.96, N=325
var.2.F5 1:169498975:T:C
peptide level
110:134
126:153
35:38
MYEQEWVRpc2_NP.5
p−maj=0.16, pX=0.81, N=271
var.2.F5 1:169498975:T:C
peptide level
134:134
153:153
38:38
NFFNPPIISRpc2_NP.1
p−maj=1, pX=0.066, N=325
var.2.F5 1:169498975:T:C
peptide level
134:134
153:153
38:38
NFFNPPIISRpc2_NP.2
p−maj=1, pX=0.2, N=325
var.2.F5 1:169498975:T:C
peptide level
134:134
153:153
38:38
NFFNPPIISRpc2_NP.3
p−maj=1, pX=0.015, N=325
var.2.F5 1:169498975:T:C
peptide level
134:134
153:153
38:38
NFFNPPIISRpc2_NP.4
p−maj=1, pX=0.12, N=325
var.2.F5 1:169498975:T:C
peptide level
134:134
153:153
38:38
NFFNPPIISRpc2_NP.5
p−maj=1, pX=0.17, N=325

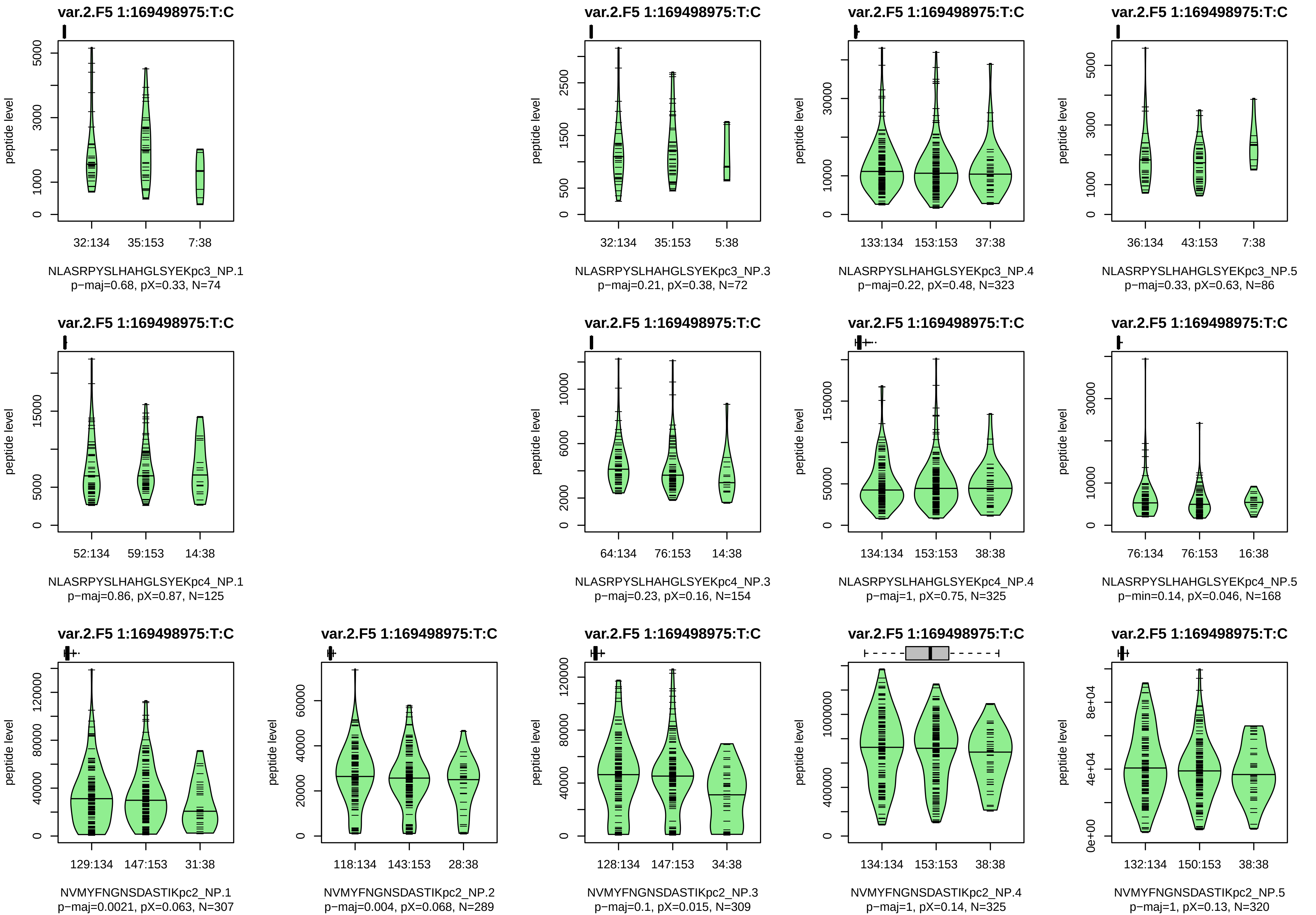
var.2.F5 1:169498975:T:C
peptide level
32:134
35:153
7:38
NLASRPYSLHAHGLSYEKpc3_NP.1
p−maj=0.68, pX=0.33, N=74
var.2.F5 1:169498975:T:C
peptide level
32:134
35:153
5:38
NLASRPYSLHAHGLSYEKpc3_NP.3
p−maj=0.21, pX=0.38, N=72
var.2.F5 1:169498975:T:C
peptide level
133:134
153:153
37:38
NLASRPYSLHAHGLSYEKpc3_NP.4
p−maj=0.22, pX=0.48, N=323
var.2.F5 1:169498975:T:C
peptide level
36:134
43:153
7:38
NLASRPYSLHAHGLSYEKpc3_NP.5
p−maj=0.33, pX=0.63, N=86
var.2.F5 1:169498975:T:C
peptide level
52:134
59:153
14:38
NLASRPYSLHAHGLSYEKpc4_NP.1
p−maj=0.86, pX=0.87, N=125
var.2.F5 1:169498975:T:C
peptide level
64:134
76:153
14:38
NLASRPYSLHAHGLSYEKpc4_NP.3
p−maj=0.23, pX=0.16, N=154
var.2.F5 1:169498975:T:C
peptide level
134:134
153:153
38:38
NLASRPYSLHAHGLSYEKpc4_NP.4
p−maj=1, pX=0.75, N=325
var.2.F5 1:169498975:T:C
peptide level
76:134
76:153
16:38
NLASRPYSLHAHGLSYEKpc4_NP.5
p−min=0.14, pX=0.046, N=168
var.2.F5 1:169498975:T:C
peptide level
129:134
147:153
31:38
NVMYFNGNSDASTIKpc2_NP.1
p−maj=0.0021, pX=0.063, N=307
var.2.F5 1:169498975:T:C
peptide level
118:134
143:153
28:38
NVMYFNGNSDASTIKpc2_NP.2
p−maj=0.004, pX=0.068, N=289
var.2.F5 1:169498975:T:C
peptide level
128:134
147:153
34:38
NVMYFNGNSDASTIKpc2_NP.3
p−maj=0.1, pX=0.015, N=309
var.2.F5 1:169498975:T:C
peptide level
134:134
153:153
38:38
NVMYFNGNSDASTIKpc2_NP.4
p−maj=1, pX=0.14, N=325
var.2.F5 1:169498975:T:C
peptide level
132:134
150:153
38:38
NVMYFNGNSDASTIKpc2_NP.5
p−maj=1, pX=0.13, N=320

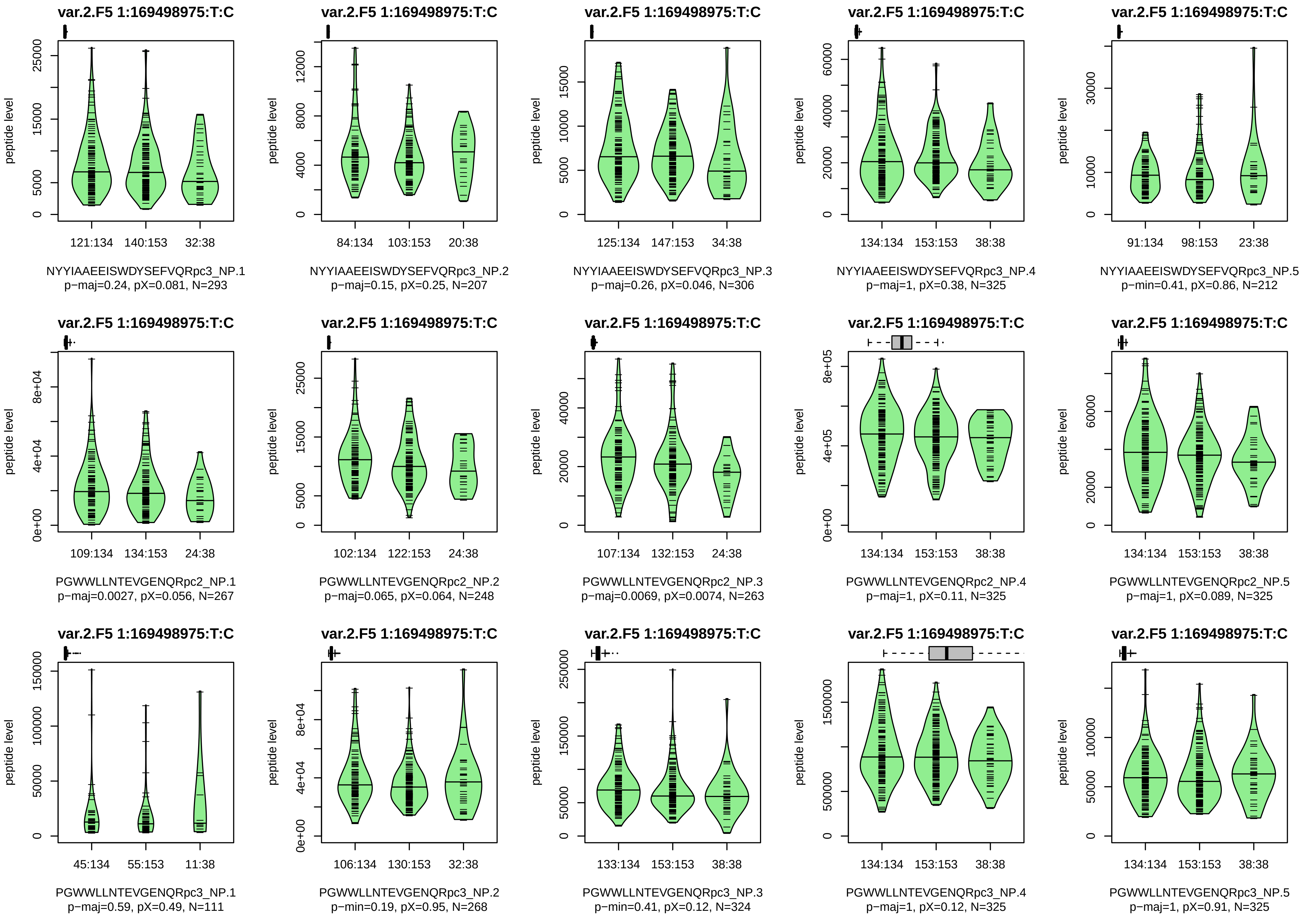
var.2.F5 1:169498975:T:C
peptide level
121:134
140:153
32:38
NYYIAAEEISWDYSEFVQRpc3_NP.1
p−maj=0.24, pX=0.081, N=293
var.2.F5 1:169498975:T:C
peptide level
84:134
103:153
20:38
NYYIAAEEISWDYSEFVQRpc3_NP.2
p−maj=0.15, pX=0.25, N=207
var.2.F5 1:169498975:T:C
peptide level
125:134
147:153
34:38
NYYIAAEEISWDYSEFVQRpc3_NP.3
p−maj=0.26, pX=0.046, N=306
var.2.F5 1:169498975:T:C
peptide level
134:134
153:153
38:38
NYYIAAEEISWDYSEFVQRpc3_NP.4
p−maj=1, pX=0.38, N=325
var.2.F5 1:169498975:T:C
peptide level
91:134
98:153
23:38
NYYIAAEEISWDYSEFVQRpc3_NP.5
p−min=0.41, pX=0.86, N=212
var.2.F5 1:169498975:T:C
peptide level
109:134
134:153
24:38
PGWWLLNTEVGENQRpc2_NP.1
p−maj=0.0027, pX=0.056, N=267
var.2.F5 1:169498975:T:C
peptide level
102:134
122:153
24:38
PGWWLLNTEVGENQRpc2_NP.2
p−maj=0.065, pX=0.064, N=248
var.2.F5 1:169498975:T:C
peptide level
107:134
132:153
24:38
PGWWLLNTEVGENQRpc2_NP.3
p−maj=0.0069, pX=0.0074, N=263
var.2.F5 1:169498975:T:C
peptide level
134:134
153:153
38:38
PGWWLLNTEVGENQRpc2_NP.4
p−maj=1, pX=0.11, N=325
var.2.F5 1:169498975:T:C
peptide level
134:134
153:153
38:38
PGWWLLNTEVGENQRpc2_NP.5
p−maj=1, pX=0.089, N=325
var.2.F5 1:169498975:T:C
peptide level
45:134
55:153
11:38
PGWWLLNTEVGENQRpc3_NP.1
p−maj=0.59, pX=0.49, N=111
var.2.F5 1:169498975:T:C
peptide level
106:134
130:153
32:38
PGWWLLNTEVGENQRpc3_NP.2
p−min=0.19, pX=0.95, N=268
var.2.F5 1:169498975:T:C
peptide level
133:134
153:153
38:38
PGWWLLNTEVGENQRpc3_NP.3
p−min=0.41, pX=0.12, N=324
var.2.F5 1:169498975:T:C
peptide level
134:134
153:153
38:38
PGWWLLNTEVGENQRpc3_NP.4
p−maj=1, pX=0.12, N=325
var.2.F5 1:169498975:T:C
peptide level
134:134
153:153
38:38
PGWWLLNTEVGENQRpc3_NP.5
p−maj=1, pX=0.91, N=325

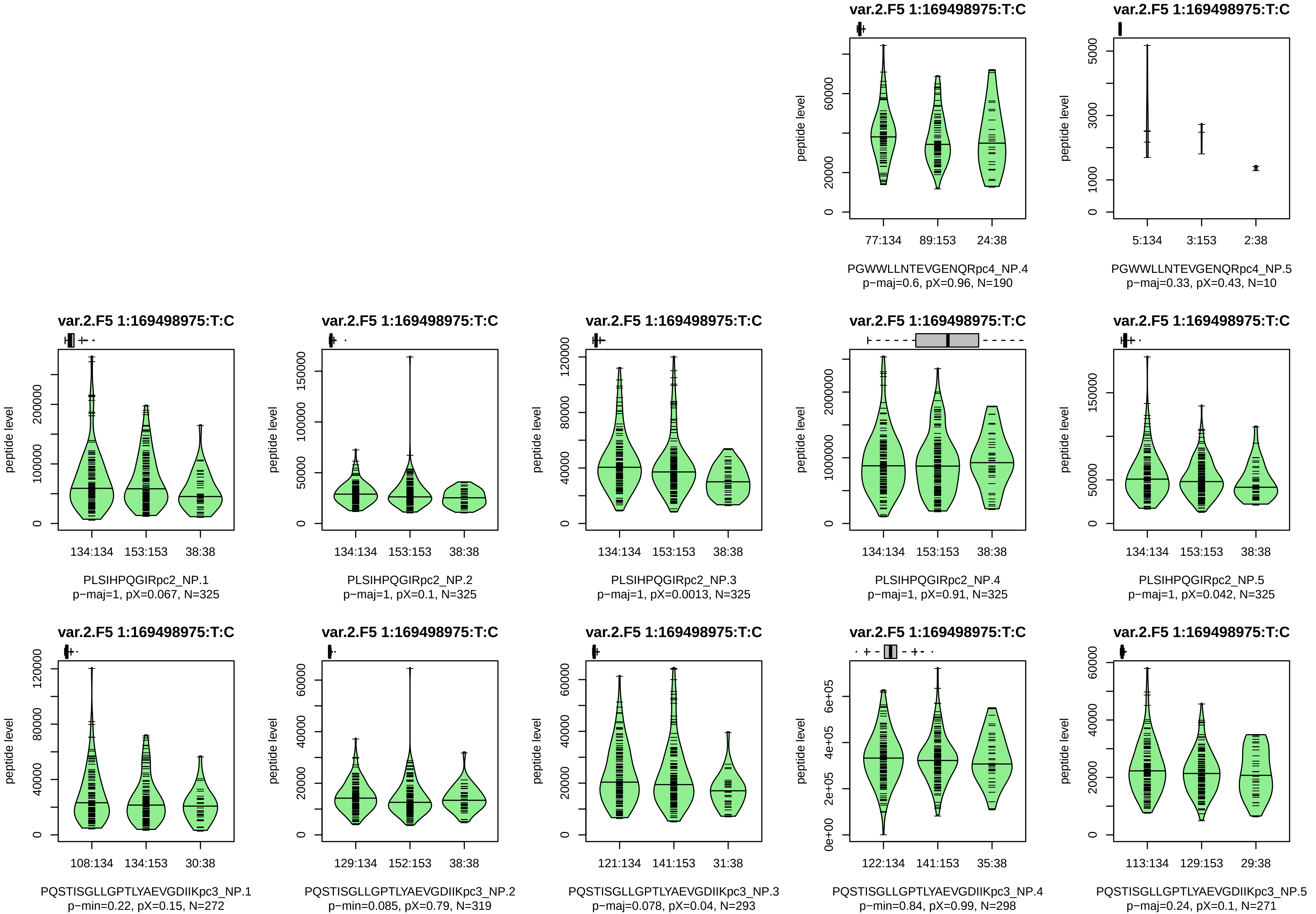
var.2.F5 1:169498975:T:C
peptide level
77:134
89:153
24:38
PGWWLLNTEVGENQRpc4_NP.4
p−maj=0.6, pX=0.96, N=190
var.2.F5 1:169498975:T:C
peptide level
5:134
3:153
2:38
PGWWLLNTEVGENQRpc4_NP.5
p−maj=0.33, pX=0.43, N=10
var.2.F5 1:169498975:T:C
peptide level
134:134
153:153
38:38
PLSIHPQGIRpc2_NP.1
p−maj=1, pX=0.067, N=325
var.2.F5 1:169498975:T:C
peptide level
134:134
153:153
38:38
PLSIHPQGIRpc2_NP.2
p−maj=1, pX=0.1, N=325
var.2.F5 1:169498975:T:C
peptide level
134:134
153:153
38:38
PLSIHPQGIRpc2_NP.3
p−maj=1, pX=0.0013, N=325
var.2.F5 1:169498975:T:C
peptide level
134:134
153:153
38:38
PLSIHPQGIRpc2_NP.4
p−maj=1, pX=0.91, N=325
var.2.F5 1:169498975:T:C
peptide level
134:134
153:153
38:38
PLSIHPQGIRpc2_NP.5
p−maj=1, pX=0.042, N=325
var.2.F5 1:169498975:T:C
peptide level
108:134
134:153
30:38
PQSTISGLLGPTLYAEVGDIIKpc3_NP.1
p−min=0.22, pX=0.15, N=272
var.2.F5 1:169498975:T:C
peptide level
129:134
152:153
38:38
PQSTISGLLGPTLYAEVGDIIKpc3_NP.2
p−min=0.085, pX=0.79, N=319
var.2.F5 1:169498975:T:C
peptide level
121:134
141:153
31:38
PQSTISGLLGPTLYAEVGDIIKpc3_NP.3
p−maj=0.078, pX=0.04, N=293
var.2.F5 1:169498975:T:C
peptide level
122:134
141:153
35:38
PQSTISGLLGPTLYAEVGDIIKpc3_NP.4
p−min=0.84, pX=0.99, N=298
var.2.F5 1:169498975:T:C
peptide level
113:134
129:153
29:38
PQSTISGLLGPTLYAEVGDIIKpc3_NP.5
p−maj=0.24, pX=0.1, N=271

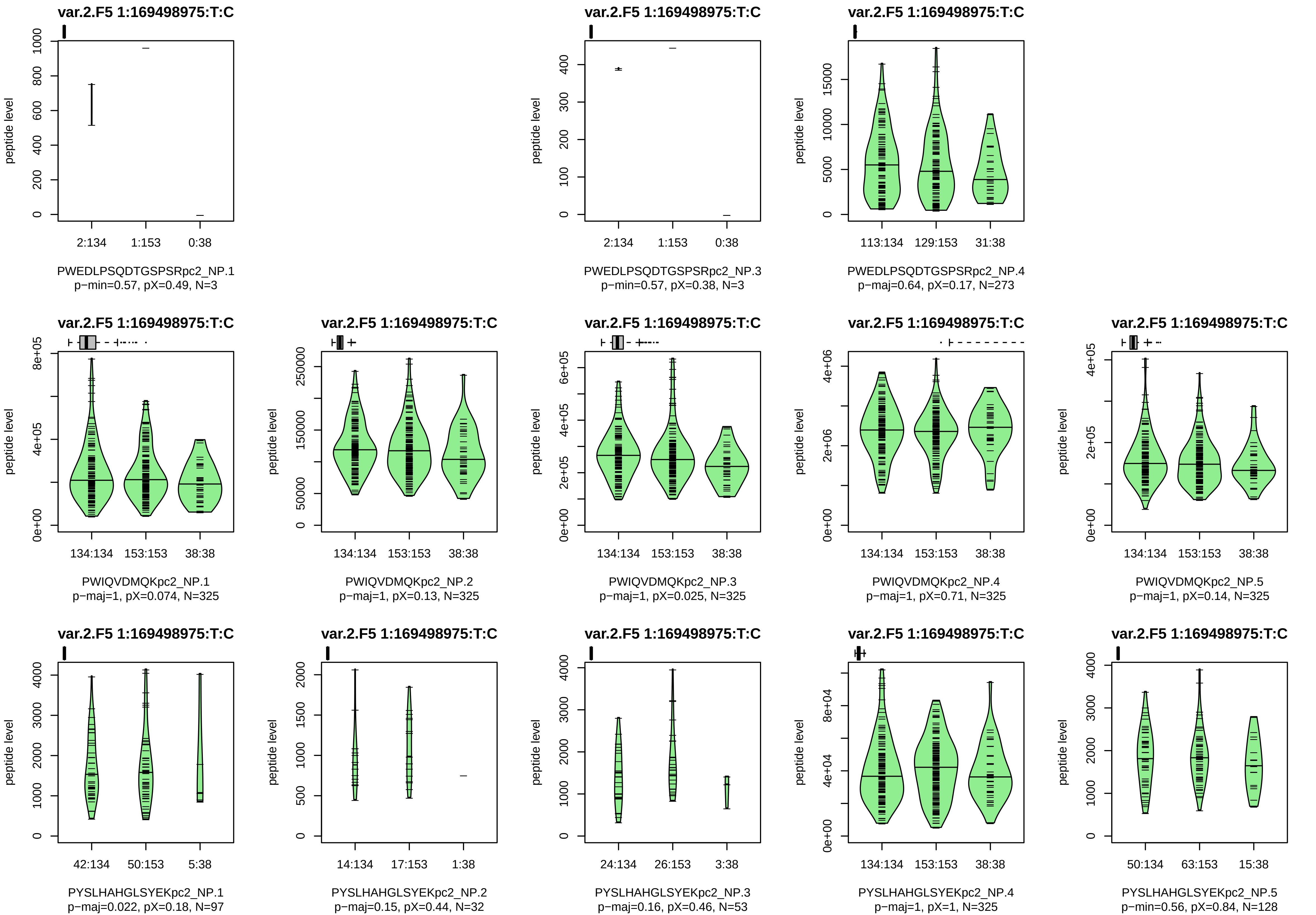
var.2.F5 1:169498975:T:C
peptide level
2:134
1:153
0:38
PWEDLPSQDTGSPSRpc2_NP.1
p−min=0.57, pX=0.49, N=3
var.2.F5 1:169498975:T:C
peptide level
2:134
1:153
0:38
PWEDLPSQDTGSPSRpc2_NP.3
p−min=0.57, pX=0.38, N=3
var.2.F5 1:169498975:T:C
peptide level
113:134
129:153
31:38
PWEDLPSQDTGSPSRpc2_NP.4
p−maj=0.64, pX=0.17, N=273
var.2.F5 1:169498975:T:C
peptide level
134:134
153:153
38:38
PWIQVDMQKpc2_NP.1
p−maj=1, pX=0.074, N=325
var.2.F5 1:169498975:T:C
peptide level
134:134
153:153
38:38
PWIQVDMQKpc2_NP.2
p−maj=1, pX=0.13, N=325
var.2.F5 1:169498975:T:C
peptide level
134:134
153:153
38:38
PWIQVDMQKpc2_NP.3
p−maj=1, pX=0.025, N=325
var.2.F5 1:169498975:T:C
peptide level
134:134
153:153
38:38
PWIQVDMQKpc2_NP.4
p−maj=1, pX=0.71, N=325
var.2.F5 1:169498975:T:C
peptide level
134:134
153:153
38:38
PWIQVDMQKpc2_NP.5
p−maj=1, pX=0.14, N=325
var.2.F5 1:169498975:T:C
peptide level
42:134
50:153
5:38
PYSLHAHGLSYEKpc2_NP.1
p−maj=0.022, pX=0.18, N=97
var.2.F5 1:169498975:T:C
peptide level
14:134
17:153
1:38
PYSLHAHGLSYEKpc2_NP.2
p−maj=0.15, pX=0.44, N=32
var.2.F5 1:169498975:T:C
peptide level
24:134
26:153
3:38
PYSLHAHGLSYEKpc2_NP.3
p−maj=0.16, pX=0.46, N=53
var.2.F5 1:169498975:T:C
peptide level
134:134
153:153
38:38
PYSLHAHGLSYEKpc2_NP.4
p−maj=1, pX=1, N=325
var.2.F5 1:169498975:T:C
peptide level
50:134
63:153
15:38
PYSLHAHGLSYEKpc2_NP.5
p−min=0.56, pX=0.84, N=128

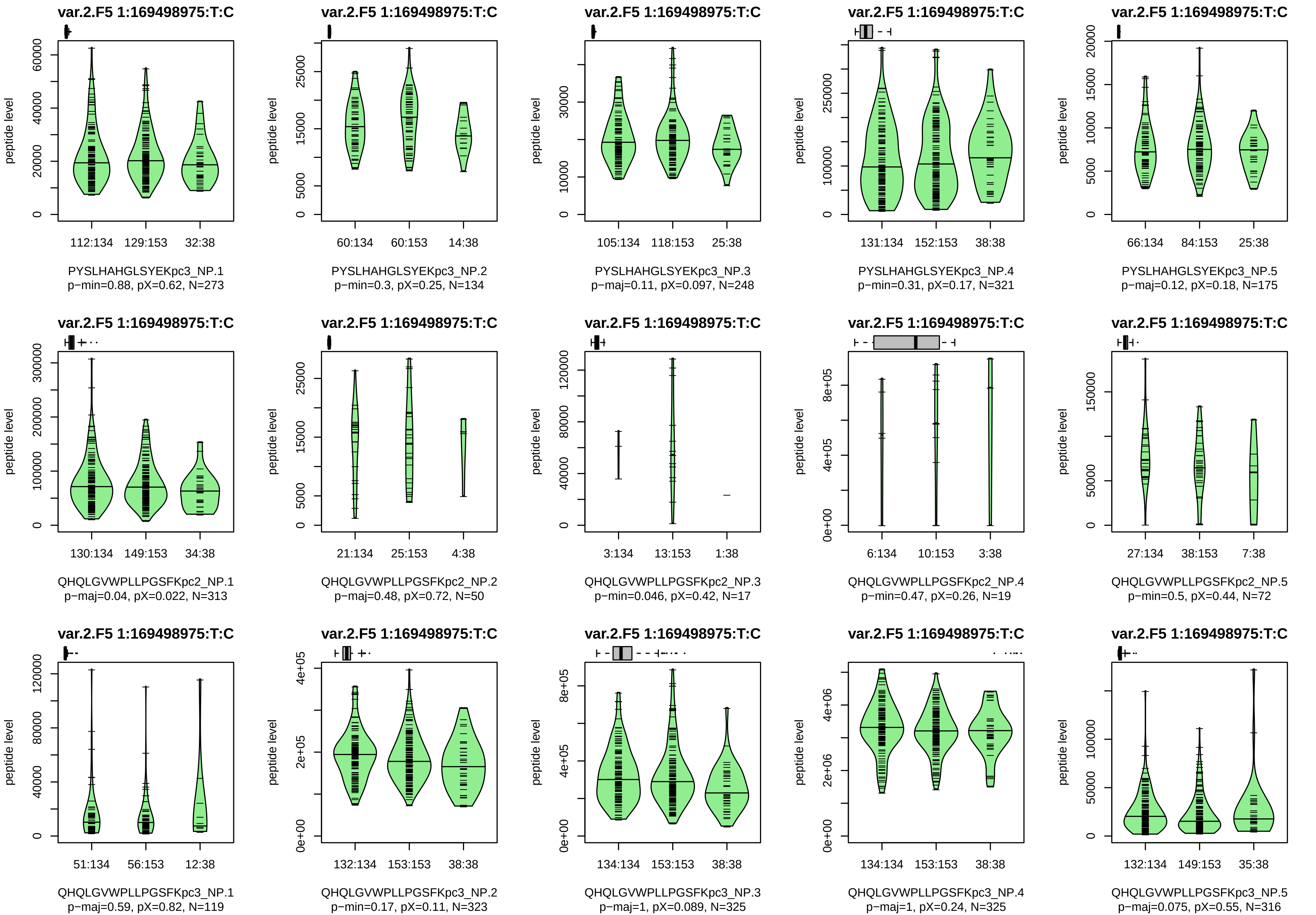
var.2.F5 1:169498975:T:C
peptide level
112:134
129:153
32:38
PYSLHAHGLSYEKpc3_NP.1
p−min=0.88, pX=0.62, N=273
var.2.F5 1:169498975:T:C
peptide level
60:134
60:153
14:38
PYSLHAHGLSYEKpc3_NP.2
p−min=0.3, pX=0.25, N=134
var.2.F5 1:169498975:T:C
peptide level
105:134
118:153
25:38
PYSLHAHGLSYEKpc3_NP.3
p−maj=0.11, pX=0.097, N=248
var.2.F5 1:169498975:T:C
peptide level
131:134
152:153
38:38
PYSLHAHGLSYEKpc3_NP.4
p−min=0.31, pX=0.17, N=321
var.2.F5 1:169498975:T:C
peptide level
66:134
84:153
25:38
PYSLHAHGLSYEKpc3_NP.5
p−maj=0.12, pX=0.18, N=175
var.2.F5 1:169498975:T:C
peptide level
130:134
149:153
34:38
QHQLGVWPLLPGSFKpc2_NP.1
p−maj=0.04, pX=0.022, N=313
var.2.F5 1:169498975:T:C
peptide level
21:134
25:153
4:38
QHQLGVWPLLPGSFKpc2_NP.2
p−maj=0.48, pX=0.72, N=50
var.2.F5 1:169498975:T:C
peptide level
3:134
13:153
1:38
QHQLGVWPLLPGSFKpc2_NP.3
p−min=0.046, pX=0.42, N=17
var.2.F5 1:169498975:T:C
peptide level
6:134
10:153
3:38
QHQLGVWPLLPGSFKpc2_NP.4
p−min=0.47, pX=0.26, N=19
var.2.F5 1:169498975:T:C
peptide level
27:134
38:153
7:38
QHQLGVWPLLPGSFKpc2_NP.5
p−min=0.5, pX=0.44, N=72
var.2.F5 1:169498975:T:C
peptide level
51:134
56:153
12:38
QHQLGVWPLLPGSFKpc3_NP.1
p−maj=0.59, pX=0.82, N=119
var.2.F5 1:169498975:T:C
peptide level
132:134
153:153
38:38
QHQLGVWPLLPGSFKpc3_NP.2
p−min=0.17, pX=0.11, N=323
var.2.F5 1:169498975:T:C
peptide level
134:134
153:153
38:38
QHQLGVWPLLPGSFKpc3_NP.3
p−maj=1, pX=0.089, N=325
var.2.F5 1:169498975:T:C
peptide level
134:134
153:153
38:38
QHQLGVWPLLPGSFKpc3_NP.4
p−maj=1, pX=0.24, N=325
var.2.F5 1:169498975:T:C
peptide level
132:134
149:153
35:38
QHQLGVWPLLPGSFKpc3_NP.5
p−maj=0.075, pX=0.55, N=316

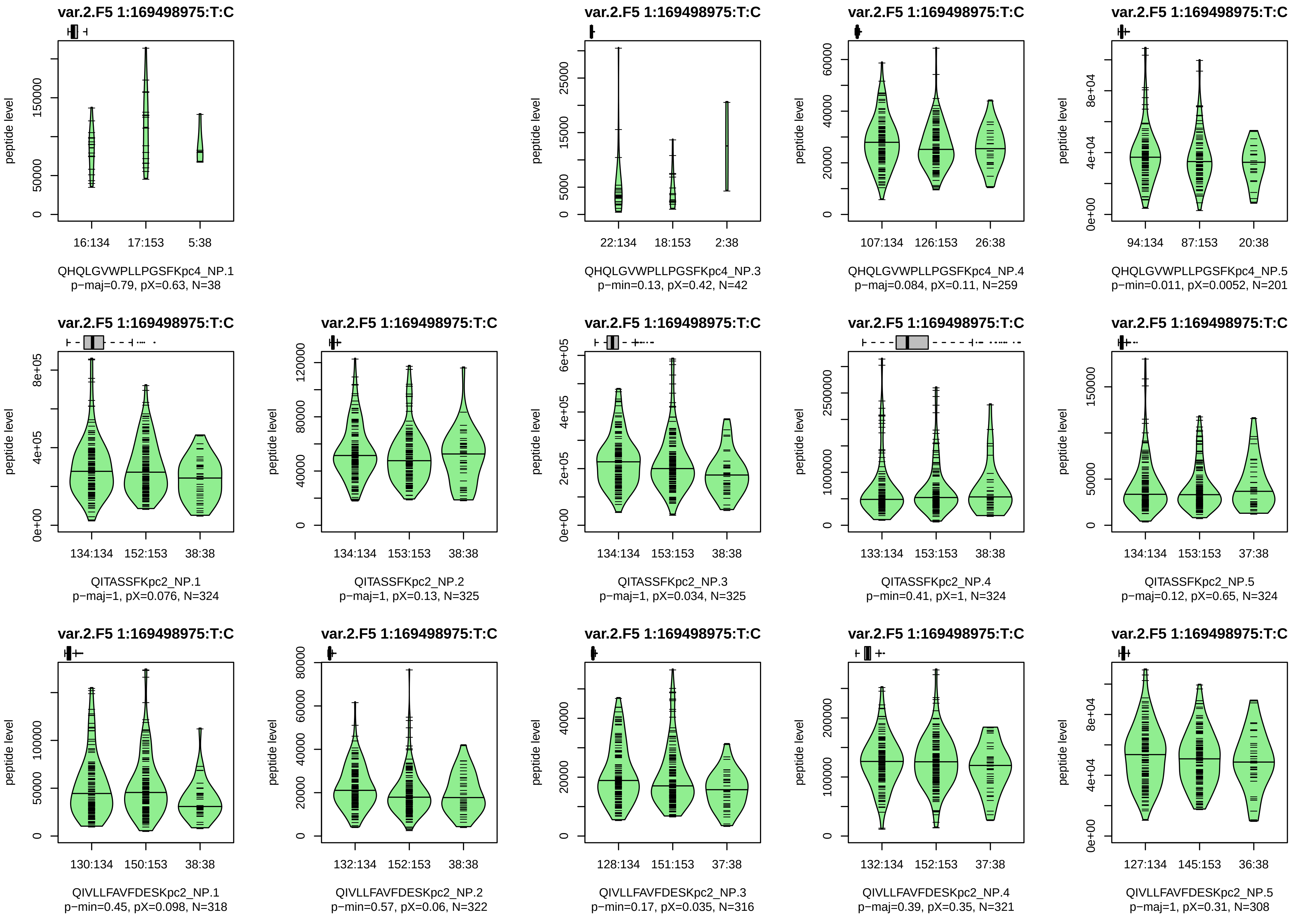
var.2.F5 1:169498975:T:C
peptide level
0
50000
150000
16:134
17:153
5:38
QHQLGVWPLLPGSFKpc4_NP.1
p−maj=0.79, pX=0.63, N=38
var.2.F5 1:169498975:T:C
peptide level
0
5000
15000
25000
22:134
18:153
2:38
QHQLGVWPLLPGSFKpc4_NP.3
p−min=0.13, pX=0.42, N=42
var.2.F5 1:169498975:T:C
peptide level
0
20000
40000
60000
107:134
126:153
26:38
QHQLGVWPLLPGSFKpc4_NP.4
p−maj=0.084, pX=0.11, N=259
var.2.F5 1:169498975:T:C
peptide level
0e+00
4e+04
8e+04
94:134
87:153
20:38
QHQLGVWPLLPGSFKpc4_NP.5
p−min=0.011, pX=0.0052, N=201
var.2.F5 1:169498975:T:C
peptide level
0e+00
4e+05
8e+05
134:134
152:153
38:38
QITASSFKpc2_NP.1
p−maj=1, pX=0.076, N=324
var.2.F5 1:169498975:T:C
peptide level
0
40000
80000
120000
134:134
153:153
38:38
QITASSFKpc2_NP.2
p−maj=1, pX=0.13, N=325
var.2.F5 1:169498975:T:C
peptide level
0e+00
2e+05
4e+05
6e+05
134:134
153:153
38:38
QITASSFKpc2_NP.3
p−maj=1, pX=0.034, N=325
var.2.F5 1:169498975:T:C
peptide level
0
1000000
2500000
133:134
153:153
38:38
QITASSFKpc2_NP.4
p−min=0.41, pX=1, N=324
var.2.F5 1:169498975:T:C
peptide level
50000
150000
134:134
153:153
37:38
QITASSFKpc2_NP.5
p−maj=0.12, pX=0.65, N=324
var.2.F5 1:169498975:T:C
peptide level
0
50000
100000
130:134
150:153
38:38
QIVLLFAVFDESKpc2_NP.1
p−min=0.45, pX=0.098, N=318
var.2.F5 1:169498975:T:C
peptide level
0
20000
40000
60000
80000
132:134
152:153
38:38
QIVLLFAVFDESKpc2_NP.2
p−min=0.57, pX=0.06, N=322
var.2.F5 1:169498975:T:C
peptide level
0
20000
40000
128:134
151:153
37:38
QIVLLFAVFDESKpc2_NP.3
p−min=0.17, pX=0.035, N=316
var.2.F5 1:169498975:T:C
peptide level
0
100000
200000
132:134
152:153
37:38
QIVLLFAVFDESKpc2_NP.4
p−maj=0.39, pX=0.35, N=321
var.2.F5 1:169498975:T:C
peptide level
0e+00
4e+04
8e+04
127:134
145:153
36:38
QIVLLFAVFDESKpc2_NP.5
p−maj=1, pX=0.31, N=308

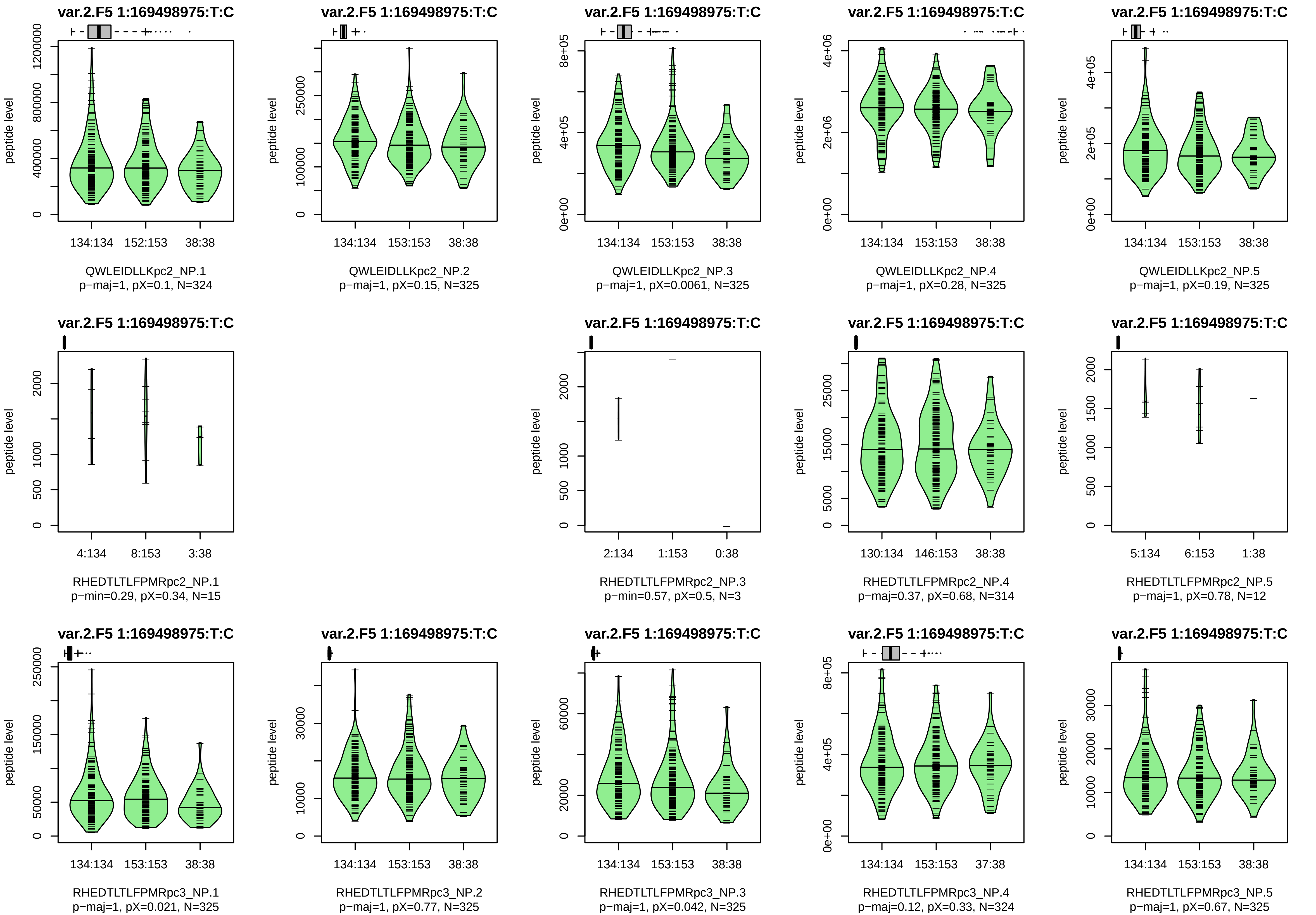

var.2.F5 1:169498975:T:C
peptide level
134:134
152:153
38:38
QWLEIDLLKpc2_NP.1
p−maj=1, pX=0.1, N=324
var.2.F5 1:169498975:T:C
134:134
153:153
38:38
QWLEIDLLKpc2_NP.2
p−maj=1, pX=0.15, N=325
var.2.F5 1:169498975:T:C
134:134
153:153
38:38
QWLEIDLLKpc2_NP.3
p−maj=1, pX=0.0061, N=325
var.2.F5 1:169498975:T:C
134:134
153:153
38:38
QWLEIDLLKpc2_NP.4
p−maj=1, pX=0.28, N=325
var.2.F5 1:169498975:T:C
134:134
153:153
38:38
QWLEIDLLKpc2_NP.5
p−maj=1, pX=0.19, N=325
var.2.F5 1:169498975:T:C
4:134
8:153
3:38
RHEDTLTLFPMRpc2_NP.1
p−min=0.29, pX=0.34, N=15
var.2.F5 1:169498975:T:C
2:134
1:153
0:38
RHEDTLTLFPMRpc2_NP.3
p−min=0.57, pX=0.5, N=3
var.2.F5 1:169498975:T:C
130:134
146:153
38:38
RHEDTLTLFPMRpc2_NP.4
p−maj=0.37, pX=0.68, N=314
var.2.F5 1:169498975:T:C
5:134
6:153
1:38
RHEDTLTLFPMRpc2_NP.5
p−maj=1, pX=0.78, N=12
var.2.F5 1:169498975:T:C
134:134
153:153
38:38
RHEDTLTLFPMRpc3_NP.1
p−maj=1, pX=0.021, N=325
var.2.F5 1:169498975:T:C
134:134
153:153
38:38
RHEDTLTLFPMRpc3_NP.2
p−maj=1, pX=0.77, N=325
var.2.F5 1:169498975:T:C
134:134
153:153
38:38
RHEDTLTLFPMRpc3_NP.3
p−maj=1, pX=0.042, N=325
var.2.F5 1:169498975:T:C
134:134
153:153
37:38
RHEDTLTLFPMRpc3_NP.4
p−maj=0.12, pX=0.33, N=324
var.2.F5 1:169498975:T:C
134:134
153:153
38:38
RHEDTLTLFPMRpc3_NP.5
p−maj=1, pX=0.67, N=325

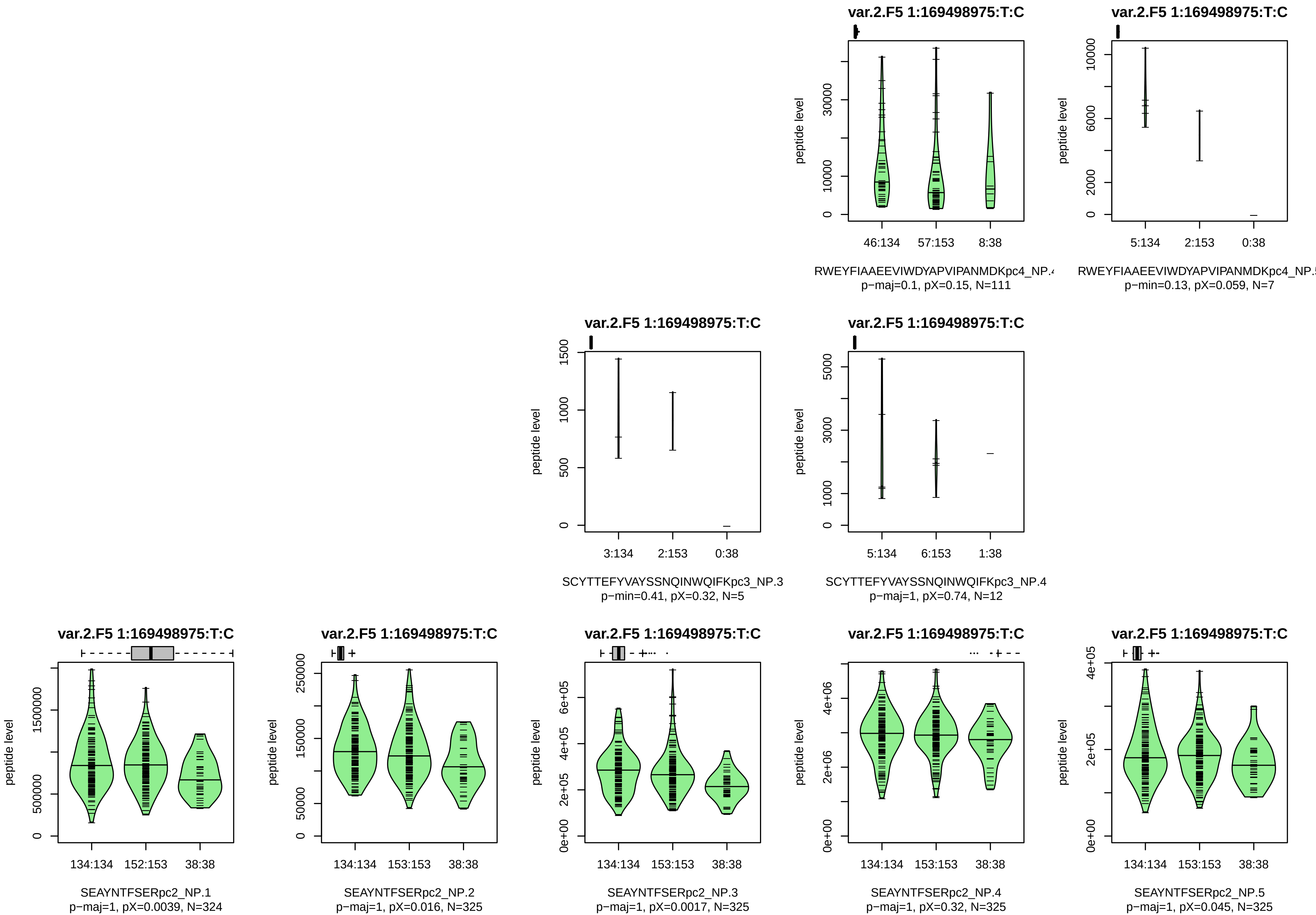
var.2.F5 1:169498975:T:C
peptide level
46:134
57:153
8:38
RWEYFIAAEEVIWDYAPVIPANMDKpc4_NP.4
p−maj=0.1, pX=0.15, N=111
var.2.F5 1:169498975:T:C
peptide level
5:134
2:153
0:38
RWEYFIAAEEVIWDYAPVIPANMDKpc4_NP.5
p−min=0.13, pX=0.059, N=7
var.2.F5 1:169498975:T:C
peptide level
3:134
2:153
0:38
SCYTTEFYVAYSSNQINWQIFKpc3_NP.3
p−min=0.41, pX=0.32, N=5
var.2.F5 1:169498975:T:C
peptide level
5:134
6:153
1:38
SCYTTEFYVAYSSNQINWQIFKpc3_NP.4
p−maj=1, pX=0.74, N=12
var.2.F5 1:169498975:T:C
peptide level
134:134
152:153
38:38
SEAYNTFSERpc2_NP.1
p−maj=1, pX=0.0039, N=324
var.2.F5 1:169498975:T:C
peptide level
134:134
153:153
38:38
SEAYNTFSERpc2_NP.2
p−maj=1, pX=0.016, N=325
var.2.F5 1:169498975:T:C
peptide level
134:134
153:153
38:38
SEAYNTFSERpc2_NP.3
p−maj=1, pX=0.0017, N=325
var.2.F5 1:169498975:T:C
peptide level
134:134
153:153
38:38
SEAYNTFSERpc2_NP.4
p−maj=1, pX=0.32, N=325
var.2.F5 1:169498975:T:C
peptide level
134:134
153:153
38:38
SEAYNTFSERpc2_NP.5
p−maj=1, pX=0.045, N=325

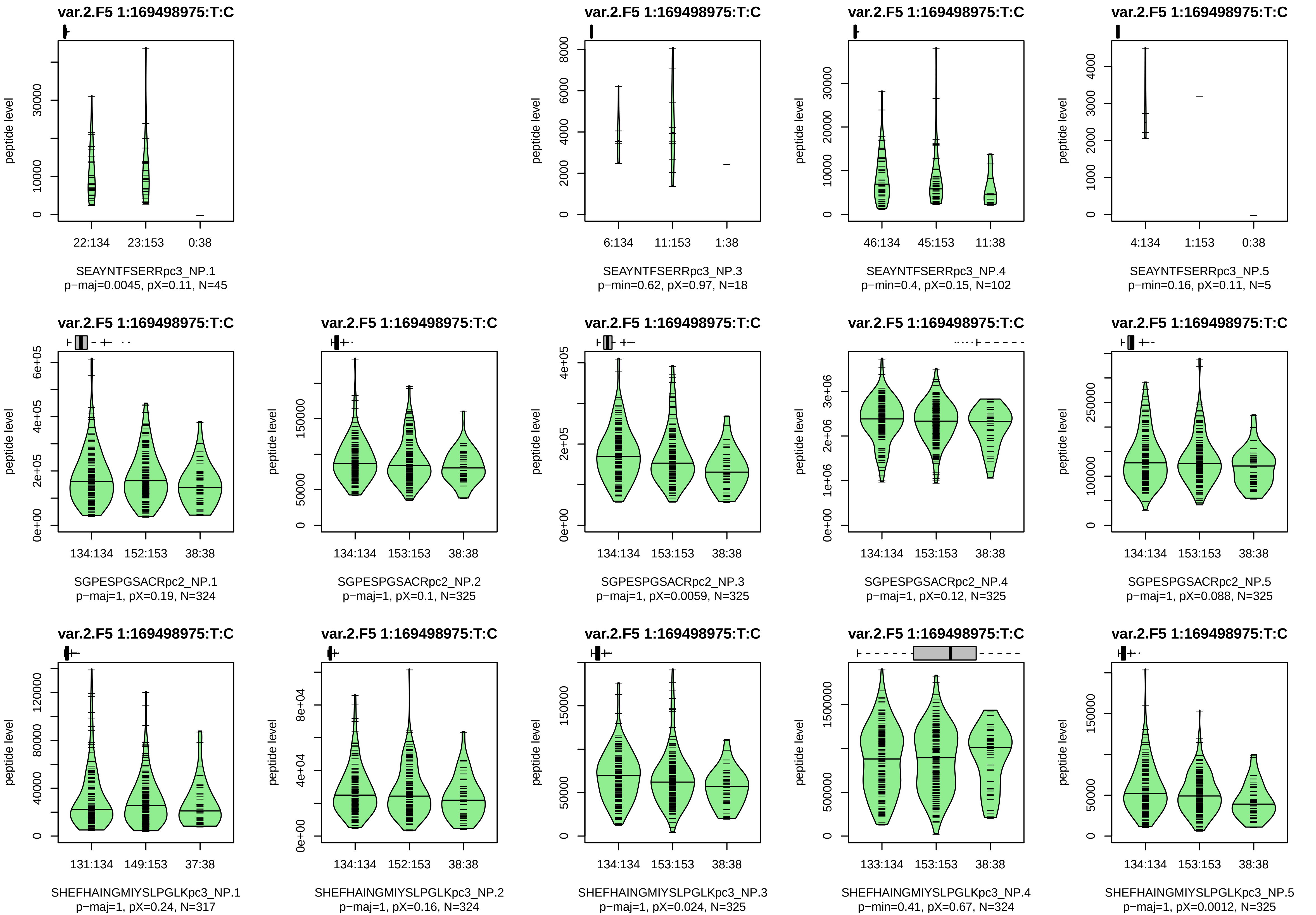
var.2.F5 1:169498975:T:C
peptide level
22:134
23:153
0:38
SEAYNTFSERRpc3_NP.1
p−maj=0.0045, pX=0.11, N=45
var.2.F5 1:169498975:T:C
peptide level
6:134
11:153
1:38
SEAYNTFSERRpc3_NP.3
p−min=0.62, pX=0.97, N=18
var.2.F5 1:169498975:T:C
peptide level
46:134
45:153
11:38
SEAYNTFSERRpc3_NP.4
p−min=0.4, pX=0.15, N=102
var.2.F5 1:169498975:T:C
peptide level
4:134
1:153
0:38
SEAYNTFSERRpc3_NP.5
p−min=0.16, pX=0.11, N=5
var.2.F5 1:169498975:T:C
peptide level
134:134
152:153
38:38
SGPESPGSACRpc2_NP.1
p−maj=1, pX=0.19, N=324
var.2.F5 1:169498975:T:C
peptide level
134:134
153:153
38:38
SGPESPGSACRpc2_NP.2
p−maj=1, pX=0.1, N=325
var.2.F5 1:169498975:T:C
peptide level
134:134
153:153
38:38
SGPESPGSACRpc2_NP.3
p−maj=1, pX=0.0059, N=325
var.2.F5 1:169498975:T:C
peptide level
134:134
153:153
38:38
SGPESPGSACRpc2_NP.4
p−maj=1, pX=0.12, N=325
var.2.F5 1:169498975:T:C
peptide level
134:134
153:153
38:38
SGPESPGSACRpc2_NP.5
p−maj=1, pX=0.088, N=325
var.2.F5 1:169498975:T:C
peptide level
131:134
149:153
37:38
SHEFHAINGMIYSLPGLKpc3_NP.1
p−maj=1, pX=0.24, N=317
var.2.F5 1:169498975:T:C
peptide level
134:134
152:153
38:38
SHEFHAINGMIYSLPGLKpc3_NP.2
p−maj=1, pX=0.16, N=324
var.2.F5 1:169498975:T:C
peptide level
134:134
153:153
38:38
SHEFHAINGMIYSLPGLKpc3_NP.3
p−maj=1, pX=0.024, N=325
var.2.F5 1:169498975:T:C
peptide level
133:134
153:153
38:38
SHEFHAINGMIYSLPGLKpc3_NP.4
p−min=0.41, pX=0.67, N=324
var.2.F5 1:169498975:T:C
peptide level
134:134
153:153
38:38
SHEFHAINGMIYSLPGLKpc3_NP.5
p−maj=1, pX=0.0012, N=325

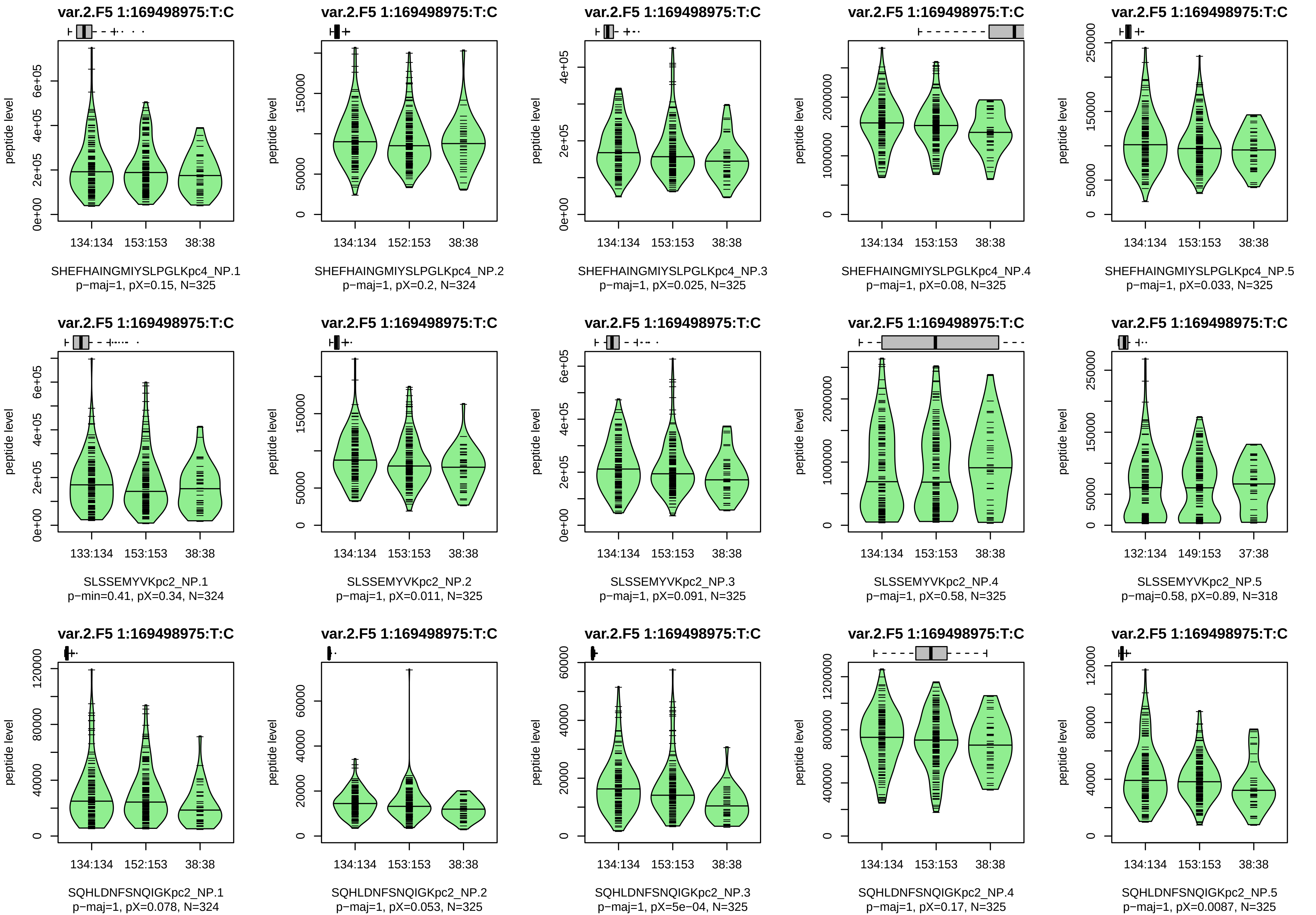
var.2.F5 1:169498975:T:C
peptide level
0e+00 2e+05 4e+05 6e+05
134:134 153:153 38:38
SHEFHAINGMIYSLPGLKpc4_NP.1
p−maj=1, pX=0.15, N=325
var.2.F5 1:169498975:T:C
peptide level
0 50000 150000
134:134 152:153 38:38
SHEFHAINGMIYSLPGLKpc4_NP.2
p−maj=1, pX=0.2, N=324
var.2.F5 1:169498975:T:C
peptide level
0e+00 2e+05 4e+05
134:134 153:153 38:38
SHEFHAINGMIYSLPGLKpc4_NP.3
p−maj=1, pX=0.025, N=325
var.2.F5 1:169498975:T:C
peptide level
0 1000000 2000000
134:134 153:153 38:38
SHEFHAINGMIYSLPGLKpc4_NP.4
p−maj=1, pX=0.08, N=325
var.2.F5 1:169498975:T:C
peptide level
0 50000 150000 250000
134:134 153:153 38:38
SHEFHAINGMIYSLPGLKpc4_NP.5
p−maj=1, pX=0.033, N=325
var.2.F5 1:169498975:T:C
peptide level
0e+00 2e+05 4e+05 6e+05
133:134 153:153 38:38
SLSSEMYVKpc2_NP.1
p−min=0.41, pX=0.34, N=324
var.2.F5 1:169498975:T:C
peptide level
0 50000 150000
134:134 153:153 38:38
SLSSEMYVKpc2_NP.2
p−maj=1, pX=0.011, N=325
var.2.F5 1:169498975:T:C
peptide level
0e+00 2e+05 4e+05 6e+05
134:134 153:153 38:38
SLSSEMYVKpc2_NP.3
p−maj=1, pX=0.091, N=325
var.2.F5 1:169498975:T:C
peptide level
0 1000000 2000000
134:134 153:153 38:38
SLSSEMYVKpc2_NP.4
p−maj=1, pX=0.58, N=325
var.2.F5 1:169498975:T:C
peptide level
0 50000 150000 250000
132:134 149:153 37:38
SLSSEMYVKpc2_NP.5
p−maj=0.58, pX=0.89, N=318
var.2.F5 1:169498975:T:C
peptide level
0 40000 80000 120000
134:134 152:153 38:38
SQHLDNFSNQIGKpc2_NP.1
p−maj=1, pX=0.078, N=324
var.2.F5 1:169498975:T:C
peptide level
0 20000 40000 60000
134:134 153:153 38:38
SQHLDNFSNQIGKpc2_NP.2
p−maj=1, pX=0.053, N=325
var.2.F5 1:169498975:T:C
peptide level
0 20000 40000 60000
134:134 153:153 38:38
SQHLDNFSNQIGKpc2_NP.3
p−maj=1, pX=5e−04, N=325
var.2.F5 1:169498975:T:C
peptide level
0 400000 800000 1200000
134:134 153:153 38:38
SQHLDNFSNQIGKpc2_NP.4
p−maj=1, pX=0.17, N=325
var.2.F5 1:169498975:T:C
peptide level
0 40000 80000 120000
134:134 153:153 38:38
SQHLDNFSNQIGKpc2_NP.5
p−maj=1, pX=0.0087, N=325

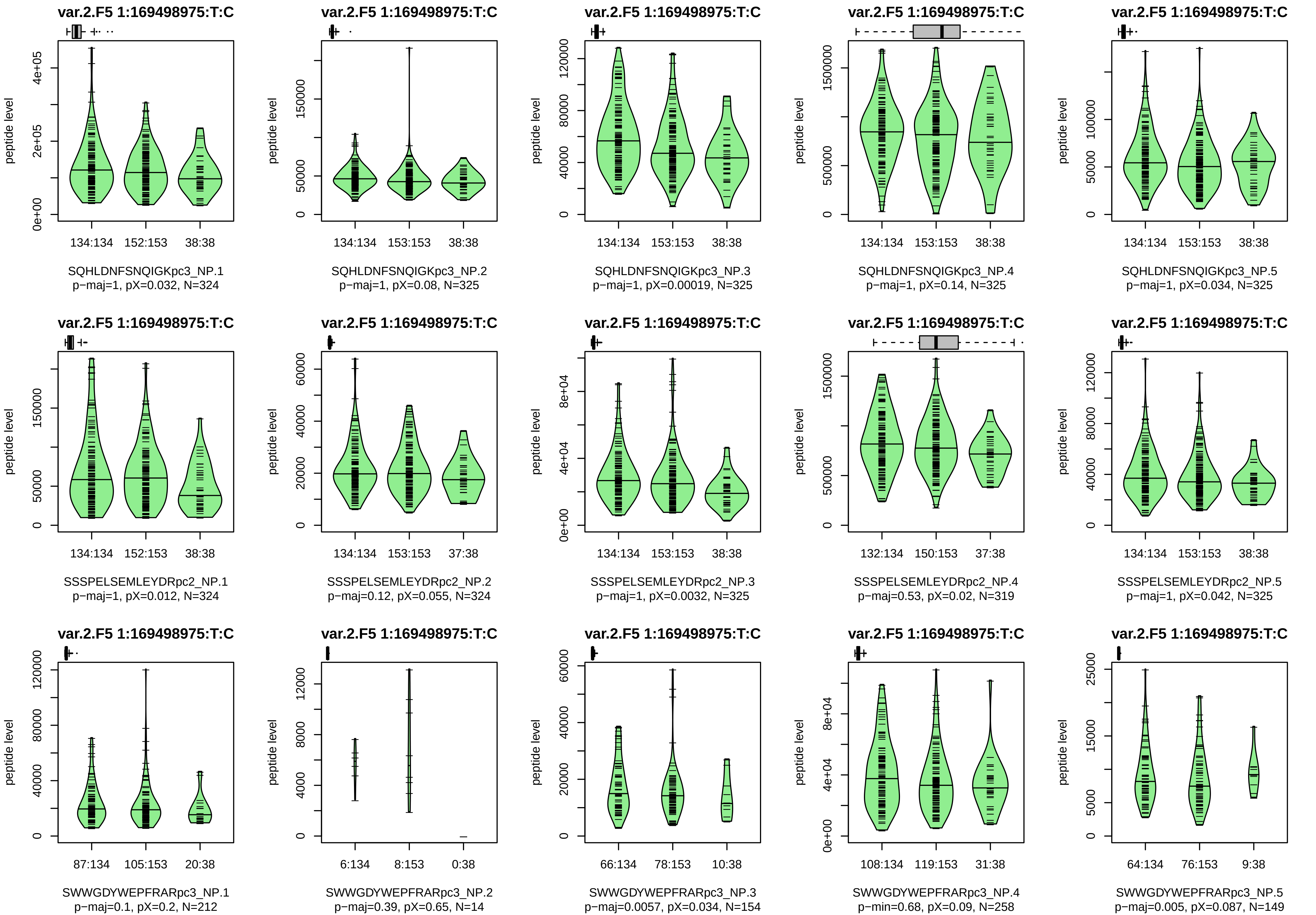
var.2.F5 1:169498975:T:C
peptide level
134:134 152:153 38:38
SQHLDNFSNQIGKpc3_NP.1
p−maj=1, pX=0.032, N=324
var.2.F5 1:169498975:T:C
peptide level
134:134 153:153 38:38
SQHLDNFSNQIGKpc3_NP.2
p−maj=1, pX=0.08, N=325
var.2.F5 1:169498975:T:C
peptide level
134:134 153:153 38:38
SQHLDNFSNQIGKpc3_NP.3
p−maj=1, pX=0.00019, N=325
var.2.F5 1:169498975:T:C
peptide level
134:134 153:153 38:38
SQHLDNFSNQIGKpc3_NP.4
p−maj=1, pX=0.14, N=325
var.2.F5 1:169498975:T:C
peptide level
134:134 153:153 38:38
SQHLDNFSNQIGKpc3_NP.5
p−maj=1, pX=0.034, N=325
var.2.F5 1:169498975:T:C
peptide level
134:134 152:153 38:38
SSSPELSEMLEYDRpc2_NP.1
p−maj=1, pX=0.012, N=324
var.2.F5 1:169498975:T:C
peptide level
134:134 153:153 37:38
SSSPELSEMLEYDRpc2_NP.2
p−maj=0.12, pX=0.055, N=324
var.2.F5 1:169498975:T:C
peptide level
134:134 153:153 38:38
SSSPELSEMLEYDRpc2_NP.3
p−maj=1, pX=0.0032, N=325
var.2.F5 1:169498975:T:C
peptide level
132:134 150:153 37:38
SSSPELSEMLEYDRpc2_NP.4
p−maj=0.53, pX=0.02, N=319
var.2.F5 1:169498975:T:C
peptide level
134:134 153:153 38:38
SSSPELSEMLEYDRpc2_NP.5
p−maj=1, pX=0.042, N=325
var.2.F5 1:169498975:T:C
peptide level
87:134 105:153 20:38
SWWGDYWEPFRARpc3_NP.1
p−maj=0.1, pX=0.2, N=212
var.2.F5 1:169498975:T:C
peptide level
6:134 8:153 0:38
SWWGDYWEPFRARpc3_NP.2
p−maj=0.39, pX=0.65, N=14
var.2.F5 1:169498975:T:C
peptide level
66:134 78:153 10:38
SWWGDYWEPFRARpc3_NP.3
p−maj=0.0057, pX=0.034, N=154
var.2.F5 1:169498975:T:C
peptide level
108:134 119:153 31:38
SWWGDYWEPFRARpc3_NP.4
p−min=0.68, pX=0.09, N=258
var.2.F5 1:169498975:T:C
peptide level
64:134 76:153 9:38
SWWGDYWEPFRARpc3_NP.5
p−maj=0.005, pX=0.087, N=149

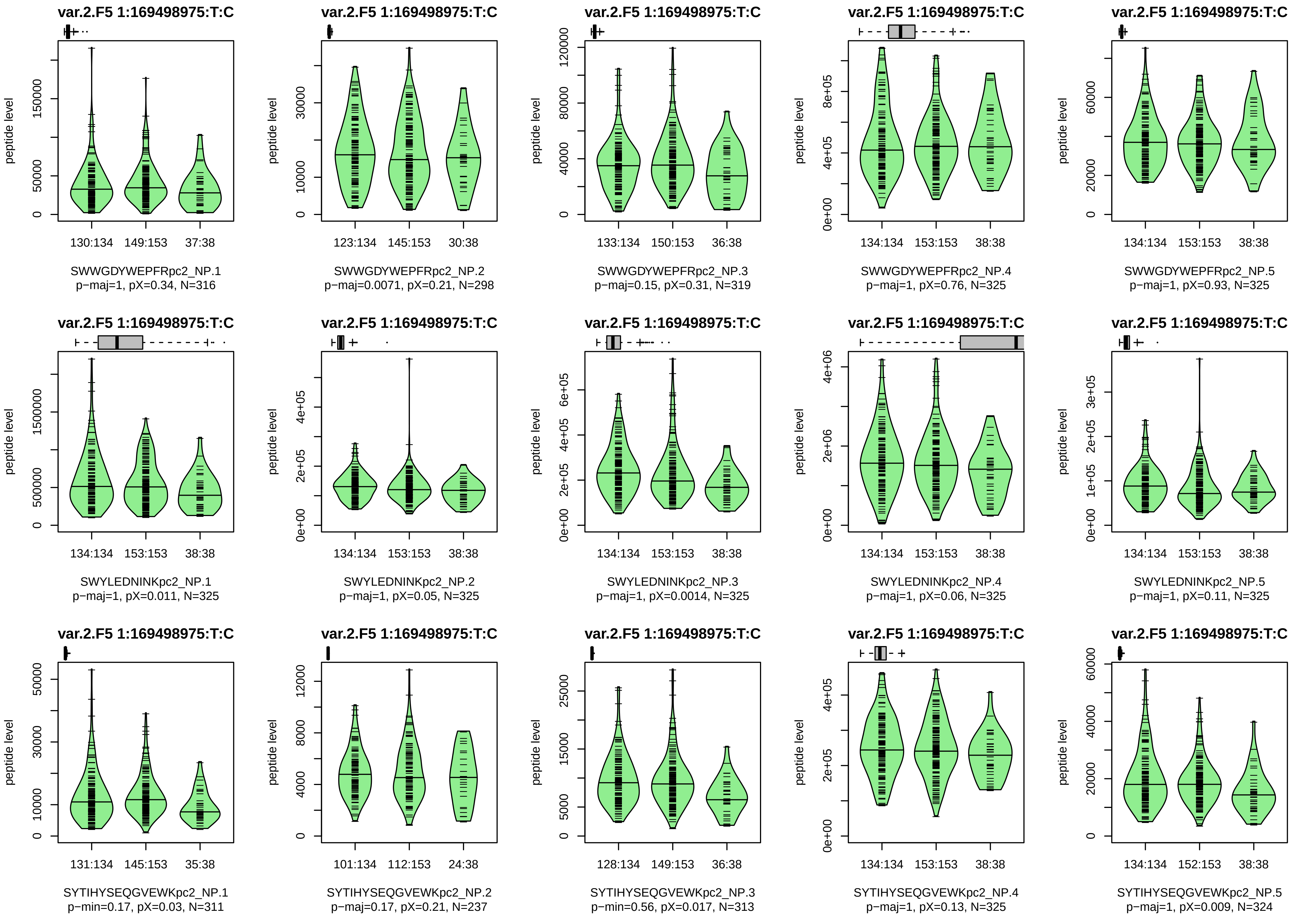
var.2.F5 1:169498975:T:C
peptide level
130:134 149:153 37:38
SWWGDYWEPFRpc2_NP.1
p−maj=1, pX=0.34, N=316
var.2.F5 1:169498975:T:C
123:134 145:153 30:38
SWWGDYWEPFRpc2_NP.2
p−maj=0.0071, pX=0.21, N=298
var.2.F5 1:169498975:T:C
133:134 150:153 36:38
SWWGDYWEPFRpc2_NP.3
p−maj=0.15, pX=0.31, N=319
var.2.F5 1:169498975:T:C
134:134 153:153 38:38
SWWGDYWEPFRpc2_NP.4
p−maj=1, pX=0.76, N=325
var.2.F5 1:169498975:T:C
134:134 153:153 38:38
SWWGDYWEPFRpc2_NP.5
p−maj=1, pX=0.93, N=325
var.2.F5 1:169498975:T:C
134:134 153:153 38:38
SWYLEDNINKpc2_NP.1
p−maj=1, pX=0.011, N=325
var.2.F5 1:169498975:T:C
134:134 153:153 38:38
SWYLEDNINKpc2_NP.2
p−maj=1, pX=0.05, N=325
var.2.F5 1:169498975:T:C
134:134 153:153 38:38
SWYLEDNINKpc2_NP.3
p−maj=1, pX=0.0014, N=325
var.2.F5 1:169498975:T:C
134:134 153:153 38:38
SWYLEDNINKpc2_NP.4
p−maj=1, pX=0.06, N=325
var.2.F5 1:169498975:T:C
134:134 153:153 38:38
SWYLEDNINKpc2_NP.5
p−maj=1, pX=0.11, N=325
var.2.F5 1:169498975:T:C
131:134 145:153 35:38
SYTIHYSEQGVEWKpc2_NP.1
p−min=0.17, pX=0.03, N=311
var.2.F5 1:169498975:T:C
101:134 112:153 24:38
SYTIHYSEQGVEWKpc2_NP.2
p−maj=0.17, pX=0.21, N=237
var.2.F5 1:169498975:T:C
128:134 149:153 36:38
SYTIHYSEQGVEWKpc2_NP.3
p−min=0.56, pX=0.017, N=313
var.2.F5 1:169498975:T:C
134:134 153:153 38:38
SYTIHYSEQGVEWKpc2_NP.4
p−maj=1, pX=0.13, N=325
var.2.F5 1:169498975:T:C
134:134 152:153 38:38
SYTIHYSEQGVEWKpc2_NP.5
p−maj=1, pX=0.009, N=324

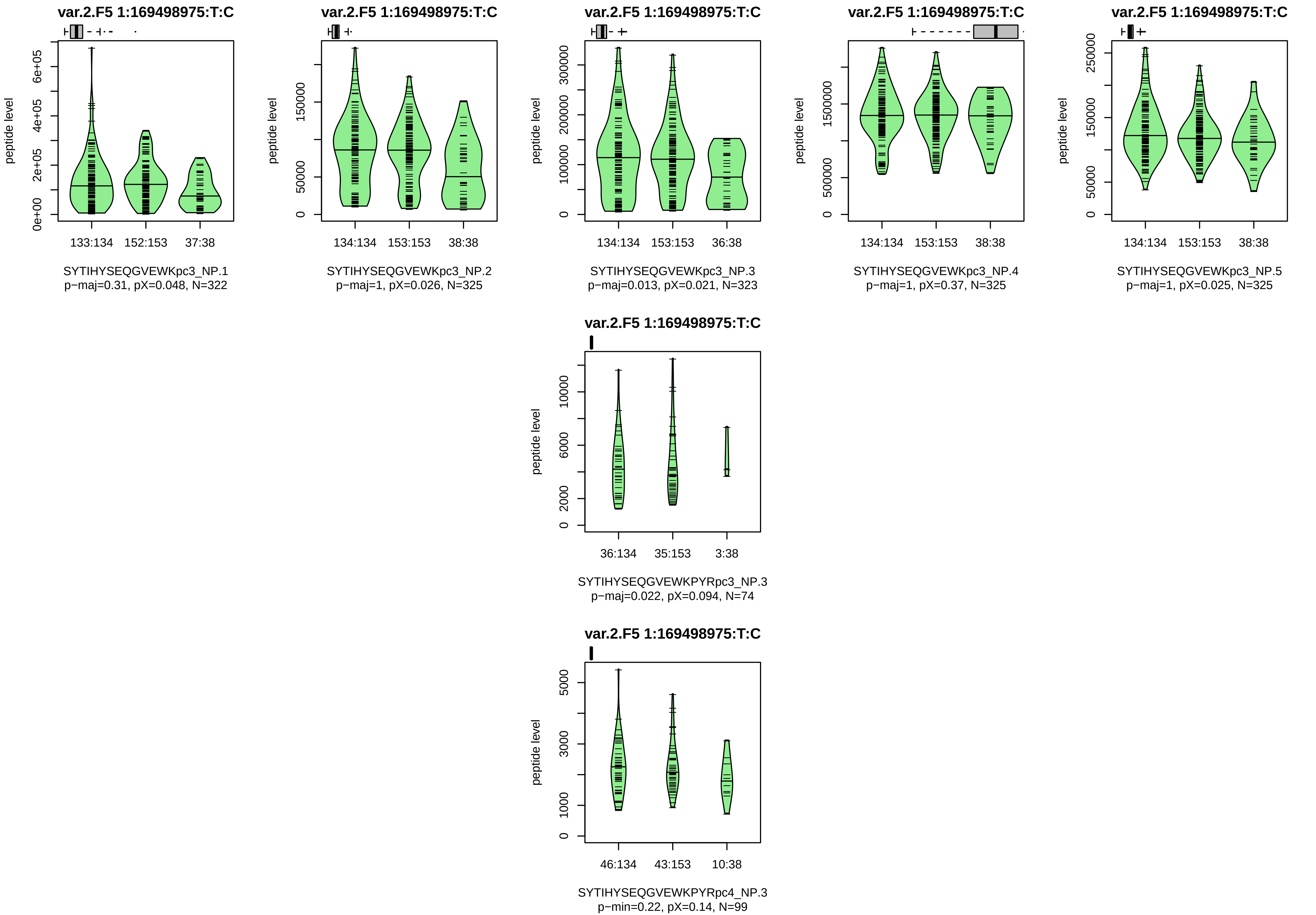

var.2.F5 1:169498975:T:C
peptide level
0e+00
2e+05
4e+05
6e+05
133:134
152:153
37:38
SYTIHYSEQGVEWKpc3_NP.1
p−maj=0.31, pX=0.048, N=322
var.2.F5 1:169498975:T:C
peptide level
0
50000
150000
134:134
153:153
38:38
SYTIHYSEQGVEWKpc3_NP.2
p−maj=1, pX=0.026, N=325
var.2.F5 1:169498975:T:C
peptide level
0
100000
200000
300000
134:134
153:153
36:38
SYTIHYSEQGVEWKpc3_NP.3
p−maj=0.013, pX=0.021, N=323
var.2.F5 1:169498975:T:C
peptide level
0
500000
1500000
134:134
153:153
38:38
SYTIHYSEQGVEWKpc3_NP.4
p−maj=1, pX=0.37, N=325
var.2.F5 1:169498975:T:C
peptide level
0
50000
150000
250000
134:134
153:153
38:38
SYTIHYSEQGVEWKpc3_NP.5
p−maj=1, pX=0.025, N=325
var.2.F5 1:169498975:T:C
peptide level
0
2000
6000
10000
36:134
35:153
3:38
SYTIHYSEQGVEWKPYRpc3_NP.3
p−maj=0.022, pX=0.094, N=74
var.2.F5 1:169498975:T:C
peptide level
0
1000
3000
5000
46:134
43:153
10:38
SYTIHYSEQGVEWKPYRpc4_NP.3
p−min=0.22, pX=0.14, N=99

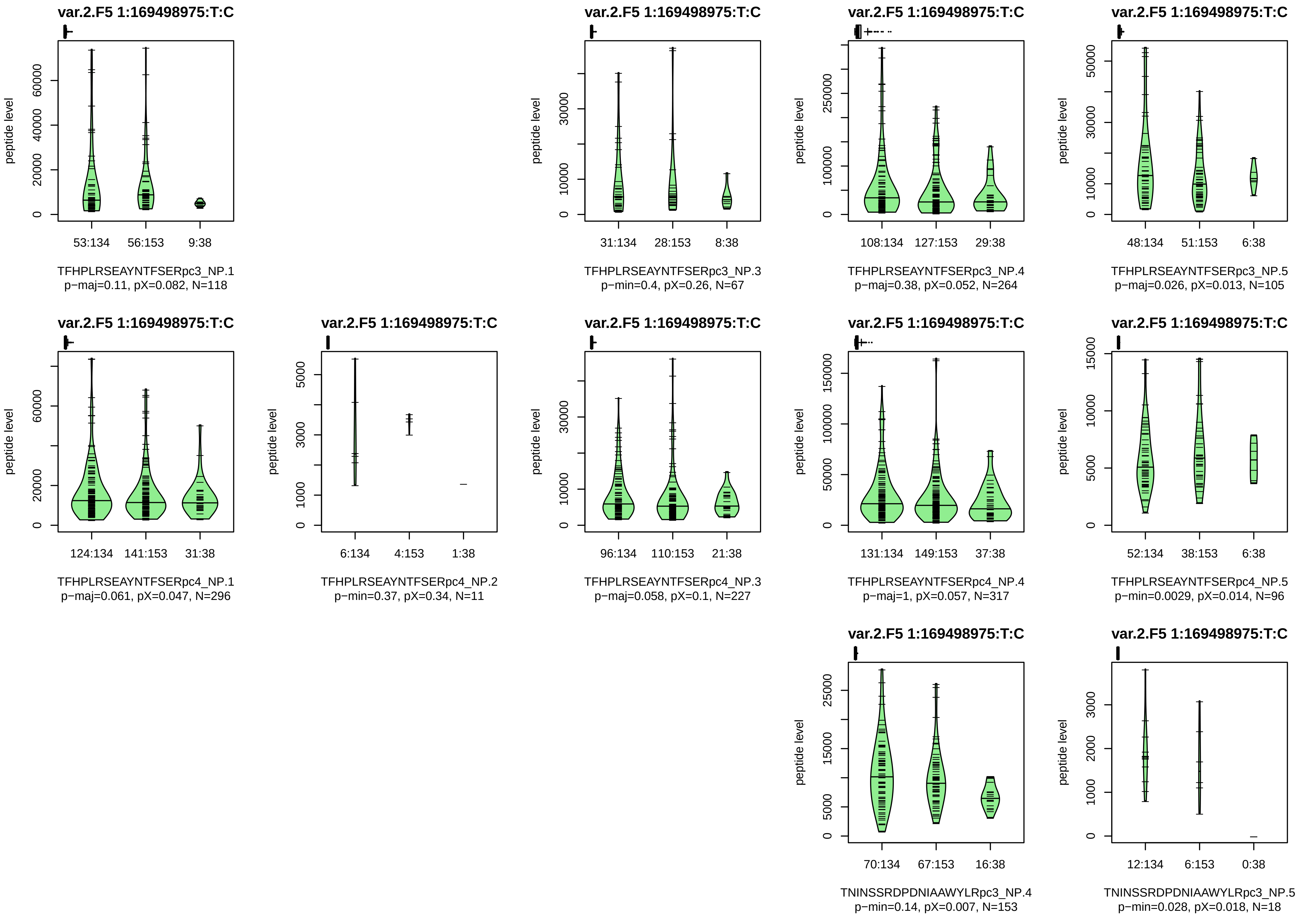
var.2.F5 1:169498975:T:C
peptide level
0
20000
40000
60000
53:134
56:153
9:38
TFHPLRSEAYNTFSERpc3_NP.1
p−maj=0.11, pX=0.082, N=118
var.2.F5 1:169498975:T:C
peptide level
0
10000
30000
31:134
28:153
8:38
TFHPLRSEAYNTFSERpc3_NP.3
p−min=0.4, pX=0.26, N=67
var.2.F5 1:169498975:T:C
peptide level
0
100000
250000
108:134
127:153
29:38
TFHPLRSEAYNTFSERpc3_NP.4
p−maj=0.38, pX=0.052, N=264
var.2.F5 1:169498975:T:C
peptide level
0
10000
30000
50000
48:134
51:153
6:38
TFHPLRSEAYNTFSERpc3_NP.5
p−maj=0.026, pX=0.013, N=105
var.2.F5 1:169498975:T:C
peptide level
0
20000
60000
124:134
141:153
31:38
TFHPLRSEAYNTFSERpc4_NP.1
p−maj=0.061, pX=0.047, N=296
var.2.F5 1:169498975:T:C
peptide level
0
1000
3000
5000
6:134
4:153
1:38
TFHPLRSEAYNTFSERpc4_NP.2
p−min=0.37, pX=0.34, N=11
var.2.F5 1:169498975:T:C
peptide level
0
10000
30000
96:134
110:153
21:38
TFHPLRSEAYNTFSERpc4_NP.3
p−maj=0.058, pX=0.1, N=227
var.2.F5 1:169498975:T:C
peptide level
0
50000
100000
150000
131:134
149:153
37:38
TFHPLRSEAYNTFSERpc4_NP.4
p−maj=1, pX=0.057, N=317
var.2.F5 1:169498975:T:C
peptide level
0
5000
10000
15000
52:134
38:153
6:38
TFHPLRSEAYNTFSERpc4_NP.5
p−min=0.0029, pX=0.014, N=96
var.2.F5 1:169498975:T:C
peptide level
0
5000
15000
25000
70:134
67:153
16:38
TNINSSRDPDNIAAWYLRpc3_NP.4
p−min=0.14, pX=0.007, N=153
var.2.F5 1:169498975:T:C
peptide level
0
1000
2000
3000
12:134
6:153
0:38
TNINSSRDPDNIAAWYLRpc3_NP.5
p−min=0.028, pX=0.018, N=18

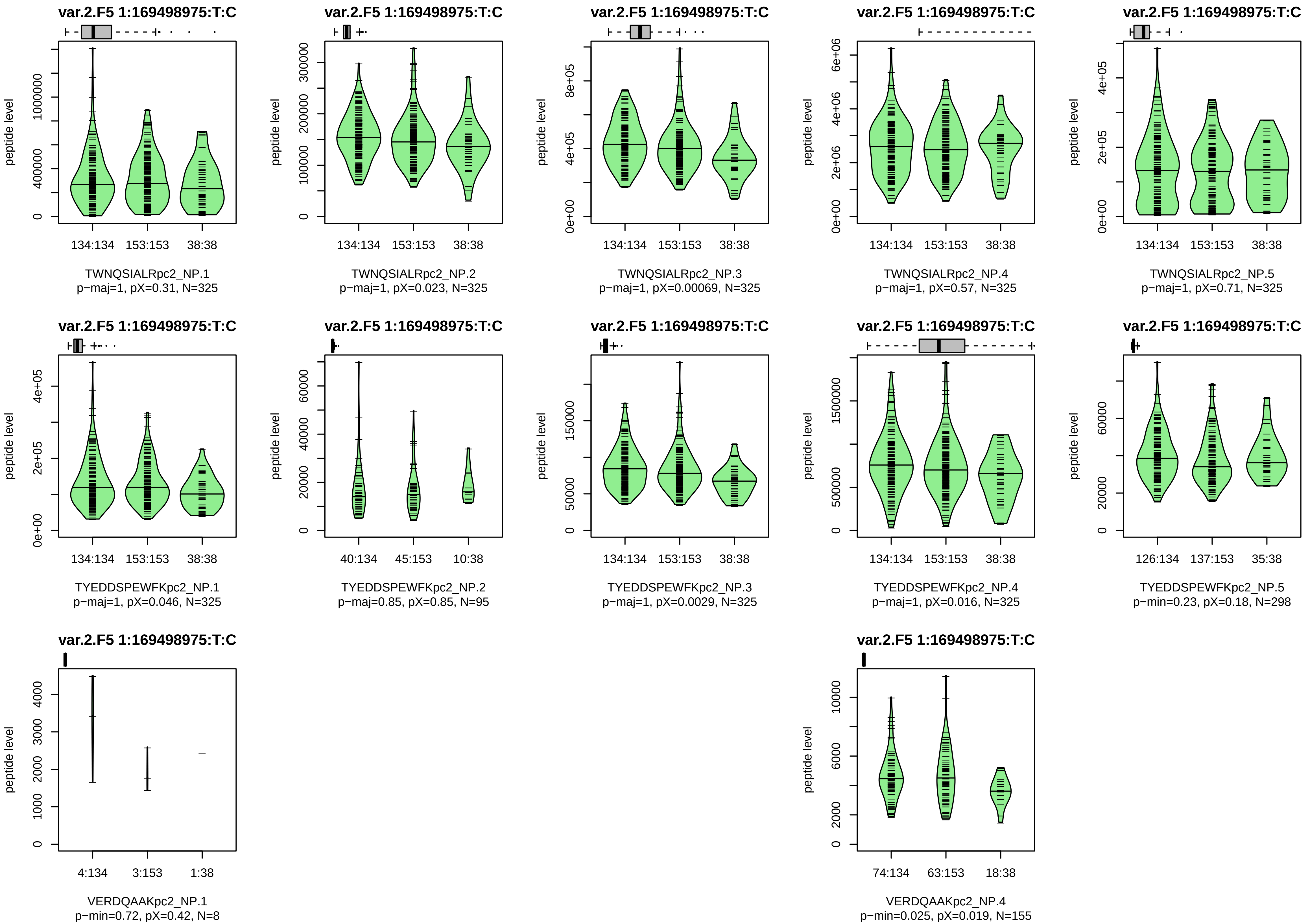
var.2.F5 1:169498975:T:C
peptide level
134:134
153:153
38:38
TWNQSIALRpc2_NP.1
p−maj=1, pX=0.31, N=325
var.2.F5 1:169498975:T:C
peptide level
134:134
153:153
38:38
TWNQSIALRpc2_NP.2
p−maj=1, pX=0.023, N=325
var.2.F5 1:169498975:T:C
peptide level
134:134
153:153
38:38
TWNQSIALRpc2_NP.3
p−maj=1, pX=0.00069, N=325
var.2.F5 1:169498975:T:C
peptide level
134:134
153:153
38:38
TWNQSIALRpc2_NP.4
p−maj=1, pX=0.57, N=325
var.2.F5 1:169498975:T:C
peptide level
134:134
153:153
38:38
TWNQSIALRpc2_NP.5
p−maj=1, pX=0.71, N=325
var.2.F5 1:169498975:T:C
peptide level
134:134
153:153
38:38
TYEDDSPEWFKpc2_NP.1
p−maj=1, pX=0.046, N=325
var.2.F5 1:169498975:T:C
peptide level
40:134
45:153
10:38
TYEDDSPEWFKpc2_NP.2
p−maj=0.85, pX=0.85, N=95
var.2.F5 1:169498975:T:C
peptide level
134:134
153:153
38:38
TYEDDSPEWFKpc2_NP.3
p−maj=1, pX=0.0029, N=325
var.2.F5 1:169498975:T:C
peptide level
134:134
153:153
38:38
TYEDDSPEWFKpc2_NP.4
p−maj=1, pX=0.016, N=325
var.2.F5 1:169498975:T:C
peptide level
126:134
137:153
35:38
TYEDDSPEWFKpc2_NP.5
p−min=0.23, pX=0.18, N=298
var.2.F5 1:169498975:T:C
peptide level
4:134
3:153
1:38
VERDQAAKpc2_NP.1
p−min=0.72, pX=0.42, N=8
var.2.F5 1:169498975:T:C
peptide level
74:134
63:153
18:38
VERDQAAKpc2_NP.4
p−min=0.025, pX=0.019, N=155

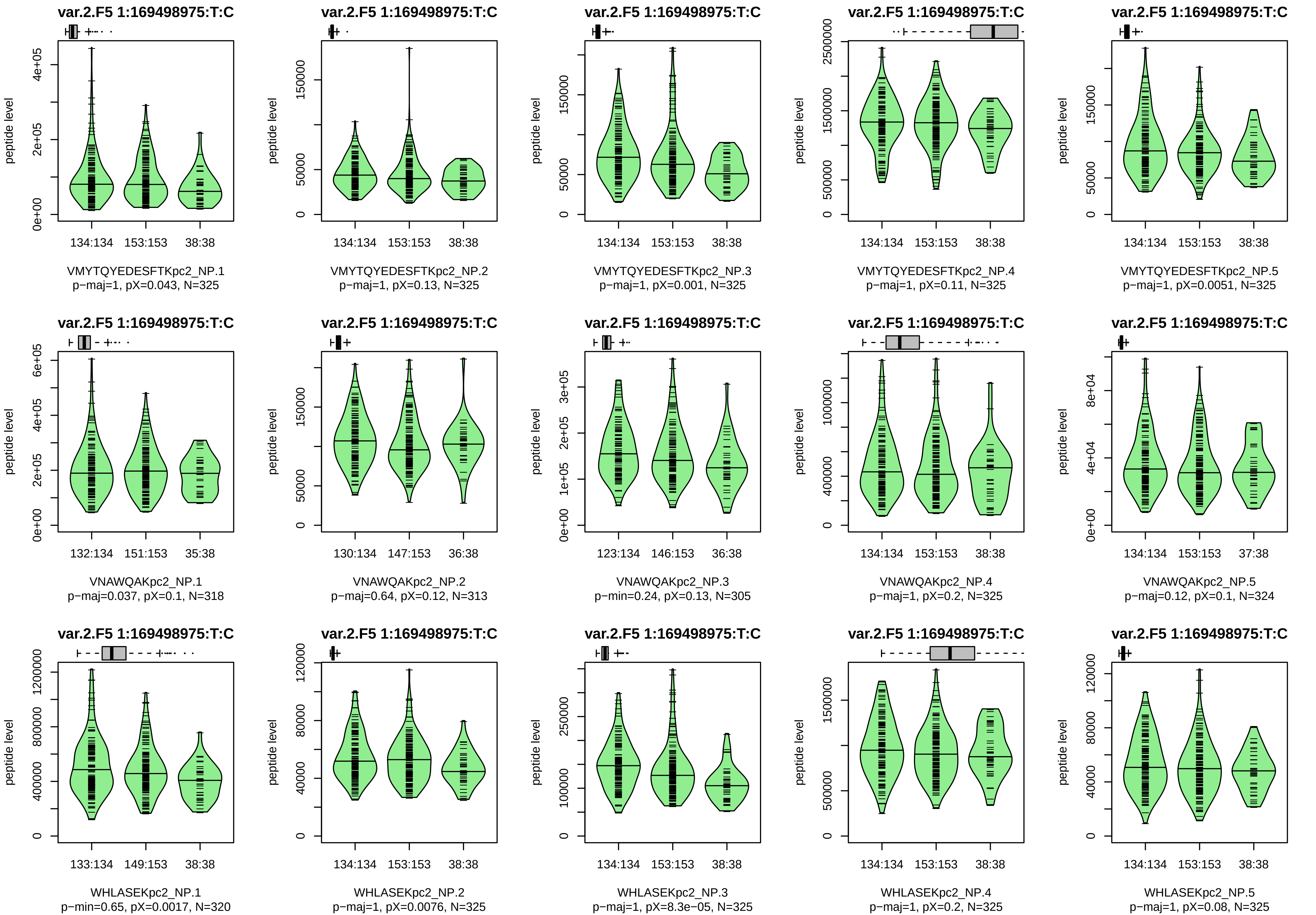
var.2.F5 1:169498975:T:C
peptide level
134:134
153:153
38:38
VMYTQYEDESFTKpc2_NP.1
p−maj=1, pX=0.043, N=325
var.2.F5 1:169498975:T:C
134:134
153:153
38:38
VMYTQYEDESFTKpc2_NP.2
p−maj=1, pX=0.13, N=325
var.2.F5 1:169498975:T:C
134:134
153:153
38:38
VMYTQYEDESFTKpc2_NP.3
p−maj=1, pX=0.001, N=325
var.2.F5 1:169498975:T:C
134:134
153:153
38:38
VMYTQYEDESFTKpc2_NP.4
p−maj=1, pX=0.11, N=325
var.2.F5 1:169498975:T:C
134:134
153:153
38:38
VMYTQYEDESFTKpc2_NP.5
p−maj=1, pX=0.0051, N=325
var.2.F5 1:169498975:T:C
132:134
151:153
35:38
VNAWQAKpc2_NP.1
p−maj=0.037, pX=0.1, N=318
var.2.F5 1:169498975:T:C
130:134
147:153
36:38
VNAWQAKpc2_NP.2
p−maj=0.64, pX=0.12, N=313
var.2.F5 1:169498975:T:C
123:134
146:153
36:38
VNAWQAKpc2_NP.3
p−min=0.24, pX=0.13, N=305
var.2.F5 1:169498975:T:C
134:134
153:153
38:38
VNAWQAKpc2_NP.4
p−maj=1, pX=0.2, N=325
var.2.F5 1:169498975:T:C
134:134
153:153
37:38
VNAWQAKpc2_NP.5
p−maj=0.12, pX=0.1, N=324
var.2.F5 1:169498975:T:C
133:134
149:153
38:38
WHLASEKpc2_NP.1
p−min=0.65, pX=0.0017, N=320
var.2.F5 1:169498975:T:C
134:134
153:153
38:38
WHLASEKpc2_NP.2
p−maj=1, pX=0.0076, N=325
var.2.F5 1:169498975:T:C
134:134
153:153
38:38
WHLASEKpc2_NP.3
p−maj=1, pX=8.3e−05, N=325
var.2.F5 1:169498975:T:C
134:134
153:153
38:38
WHLASEKpc2_NP.4
p−maj=1, pX=0.2, N=325
var.2.F5 1:169498975:T:C
134:134
153:153
38:38
WHLASEKpc2_NP.5
p−maj=1, pX=0.08, N=325

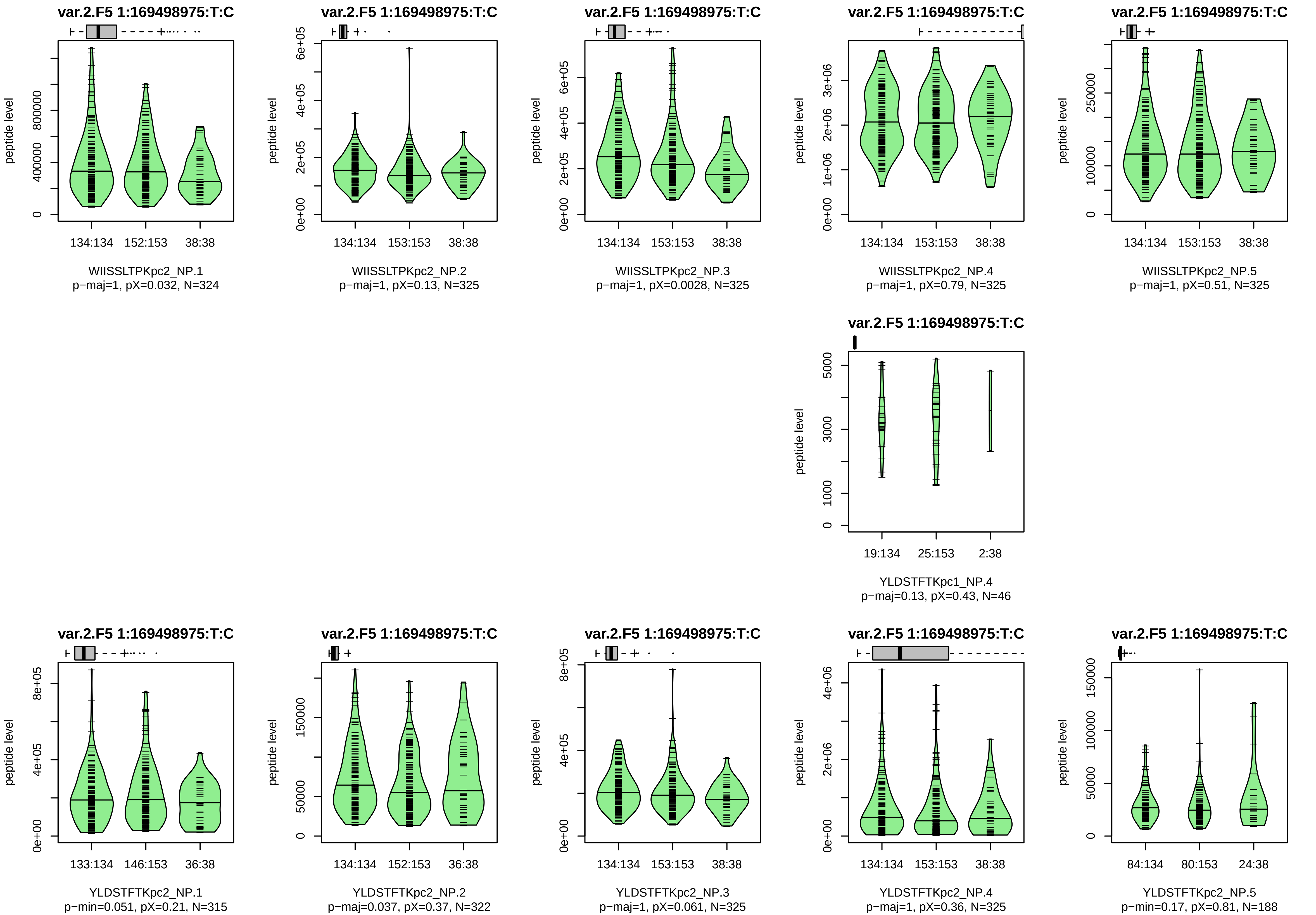

var.2.F5 1:169498975:T:C
peptide level
0
400000
800000
134:134
152:153
38:38
WIISSLTPKpc2_NP.1
p−maj=1, pX=0.032, N=324
var.2.F5 1:169498975:T:C
peptide level
0e+00
2e+05
4e+05
6e+05
134:134
153:153
38:38
WIISSLTPKpc2_NP.2
p−maj=1, pX=0.13, N=325
var.2.F5 1:169498975:T:C
peptide level
0e+00
2e+05
4e+05
6e+05
134:134
153:153
38:38
WIISSLTPKpc2_NP.3
p−maj=1, pX=0.0028, N=325
var.2.F5 1:169498975:T:C
peptide level
0e+00
1e+06
2e+06
3e+06
134:134
153:153
38:38
WIISSLTPKpc2_NP.4
p−maj=1, pX=0.79, N=325
var.2.F5 1:169498975:T:C
peptide level
0
100000
250000
134:134
153:153
38:38
WIISSLTPKpc2_NP.5
p−maj=1, pX=0.51, N=325
var.2.F5 1:169498975:T:C
peptide level
0
1000
3000
5000
19:134
25:153
2:38
YLDSTFTKpc1_NP.4
p−maj=0.13, pX=0.43, N=46
var.2.F5 1:169498975:T:C
peptide level
0e+00
4e+05
8e+05
133:134
146:153
36:38
YLDSTFTKpc2_NP.1
p−min=0.051, pX=0.21, N=315
var.2.F5 1:169498975:T:C
peptide level
0
50000
150000
134:134
152:153
36:38
YLDSTFTKpc2_NP.2
p−maj=0.037, pX=0.37, N=322
var.2.F5 1:169498975:T:C
peptide level
0e+00
4e+05
8e+05
134:134
153:153
38:38
YLDSTFTKpc2_NP.3
p−maj=1, pX=0.061, N=325
var.2.F5 1:169498975:T:C
peptide level
0e+00
2e+06
4e+06
134:134
153:153
38:38
YLDSTFTKpc2_NP.4
p−maj=1, pX=0.36, N=325
var.2.F5 1:169498975:T:C
peptide level
0
50000
100000
150000
84:134
80:153
24:38
YLDSTFTKpc2_NP.5
p−min=0.17, pX=0.81, N=188

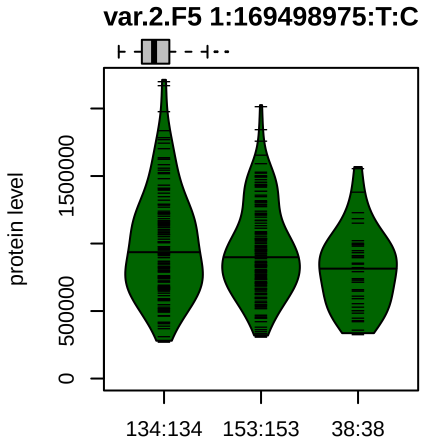

A0A0A0MRJ7.P12259_NP.1
p−maj=1, pY=0.015, N=325

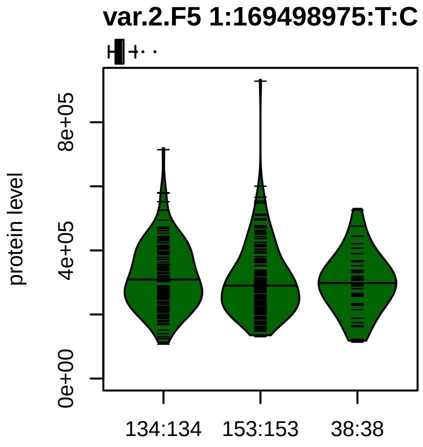

A0A0A0MRJ7.P12259_NP.2
p−maj=1, pY=0.38, N=325

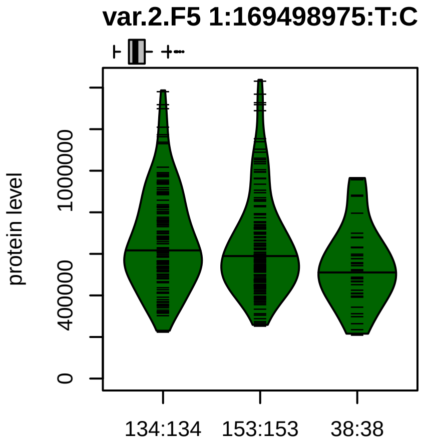

A0A0A0MRJ7.P12259_NP.3
p−maj=1, pY=0.002, N=325

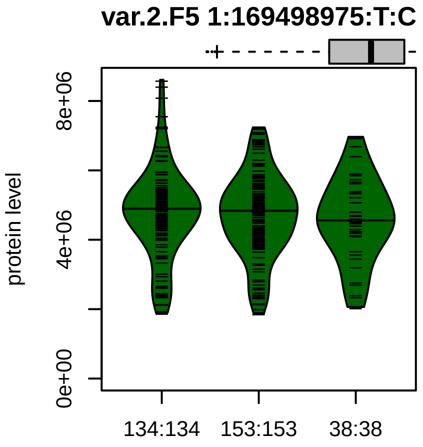

A0A0A0MRJ7.P12259_NP.4
p−maj=1, pY=0.52, N=325

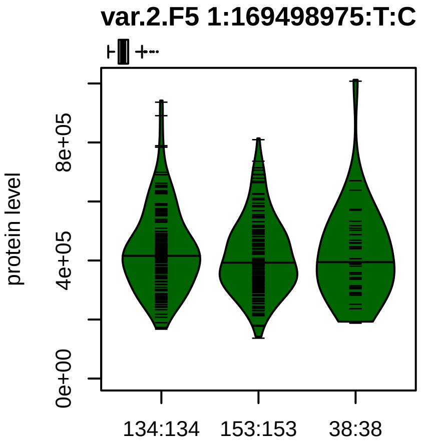

A0A0A0MRJ7.P12259_NP.5
p−maj=1, pY=0.47, N=325

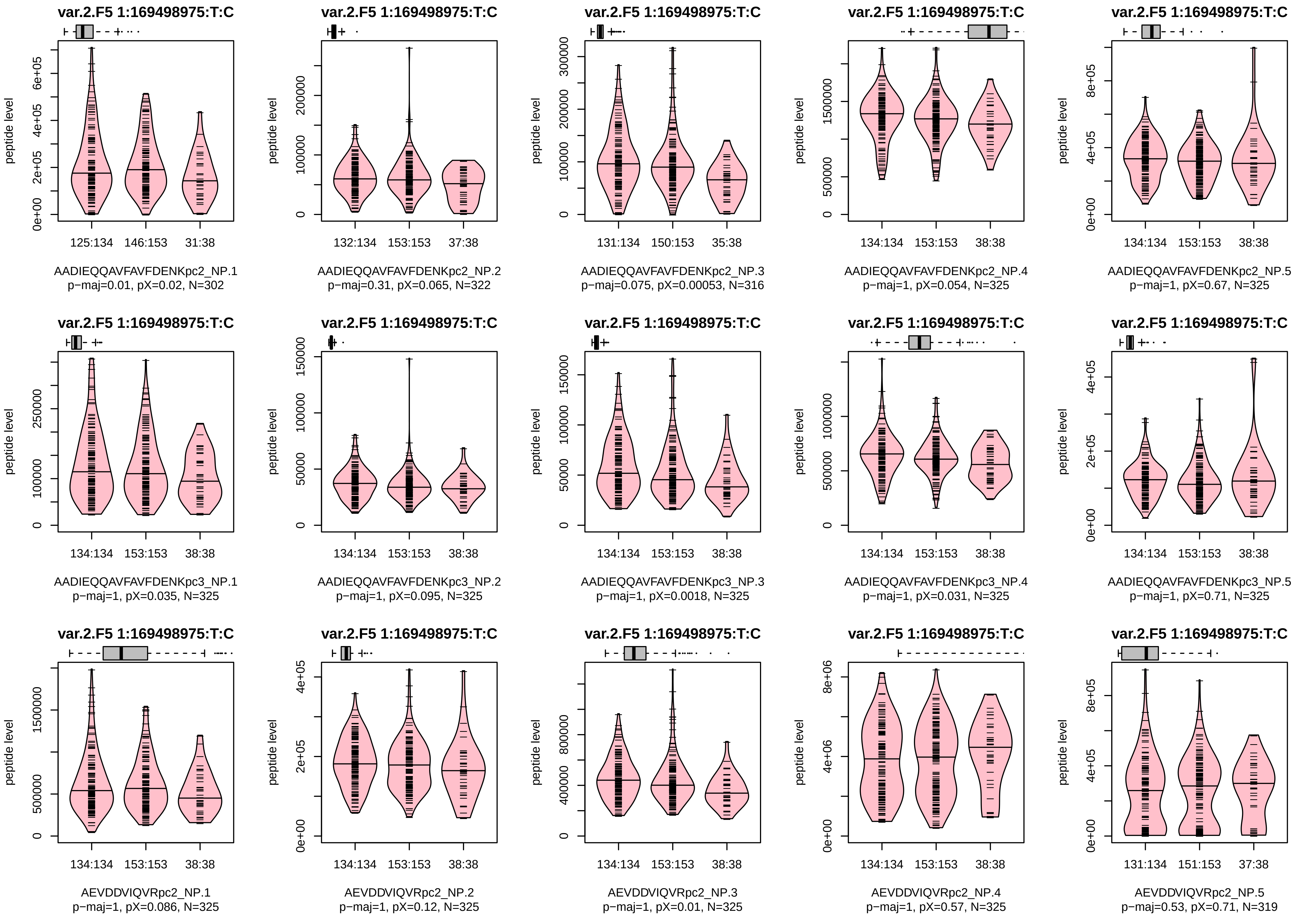
var.2.F5 1:169498975:T:C
peptide level
0e+00 2e+05 4e+05 6e+05
125:134 146:153 31:38
AADIEQQAVFAVFDENKpc2_NP.1
p−maj=0.01, pX=0.02, N=302
var.2.F5 1:169498975:T:C
peptide level
0 100000 200000
132:134 153:153 37:38
AADIEQQAVFAVFDENKpc2_NP.2
p−maj=0.31, pX=0.065, N=322
var.2.F5 1:169498975:T:C
peptide level
0 100000 200000 300000
131:134 150:153 35:38
AADIEQQAVFAVFDENKpc2_NP.3
p−maj=0.075, pX=0.00053, N=316
var.2.F5 1:169498975:T:C
peptide level
0 500000 1500000
134:134 153:153 38:38
AADIEQQAVFAVFDENKpc2_NP.4
p−maj=1, pX=0.054, N=325
var.2.F5 1:169498975:T:C
peptide level
0e+00 4e+05 8e+05
134:134 153:153 38:38
AADIEQQAVFAVFDENKpc2_NP.5
p−maj=1, pX=0.67, N=325
var.2.F5 1:169498975:T:C
peptide level
0 100000 250000
134:134 153:153 38:38
AADIEQQAVFAVFDENKpc3_NP.1
p−maj=1, pX=0.035, N=325
var.2.F5 1:169498975:T:C
peptide level
0 50000 100000 150000
134:134 153:153 38:38
AADIEQQAVFAVFDENKpc3_NP.2
p−maj=1, pX=0.095, N=325
var.2.F5 1:169498975:T:C
peptide level
0 50000 100000 150000
134:134 153:153 38:38
AADIEQQAVFAVFDENKpc3_NP.3
p−maj=1, pX=0.0018, N=325
var.2.F5 1:169498975:T:C
peptide level
0 500000 1000000
134:134 153:153 38:38
AADIEQQAVFAVFDENKpc3_NP.4
p−maj=1, pX=0.031, N=325
var.2.F5 1:169498975:T:C
peptide level
0e+00 2e+05 4e+05
134:134 153:153 38:38
AADIEQQAVFAVFDENKpc3_NP.5
p−maj=1, pX=0.71, N=325
var.2.F5 1:169498975:T:C
peptide level
0 500000 1500000
134:134 153:153 38:38
AEVDDVIQVRpc2_NP.1
p−maj=1, pX=0.086, N=325
var.2.F5 1:169498975:T:C
peptide level
0e+00 2e+05 4e+05
134:134 153:153 38:38
AEVDDVIQVRpc2_NP.2
p−maj=1, pX=0.12, N=325
var.2.F5 1:169498975:T:C
peptide level
0 400000 800000
134:134 153:153 38:38
AEVDDVIQVRpc2_NP.3
p−maj=1, pX=0.01, N=325
var.2.F5 1:169498975:T:C
peptide level
0e+00 4e+06 8e+06
134:134 153:153 38:38
AEVDDVIQVRpc2_NP.4
p−maj=1, pX=0.57, N=325
var.2.F5 1:169498975:T:C
peptide level
0e+00 4e+05 8e+05
131:134 151:153 37:38
AEVDDVIQVRpc2_NP.5
p−maj=0.53, pX=0.71, N=319

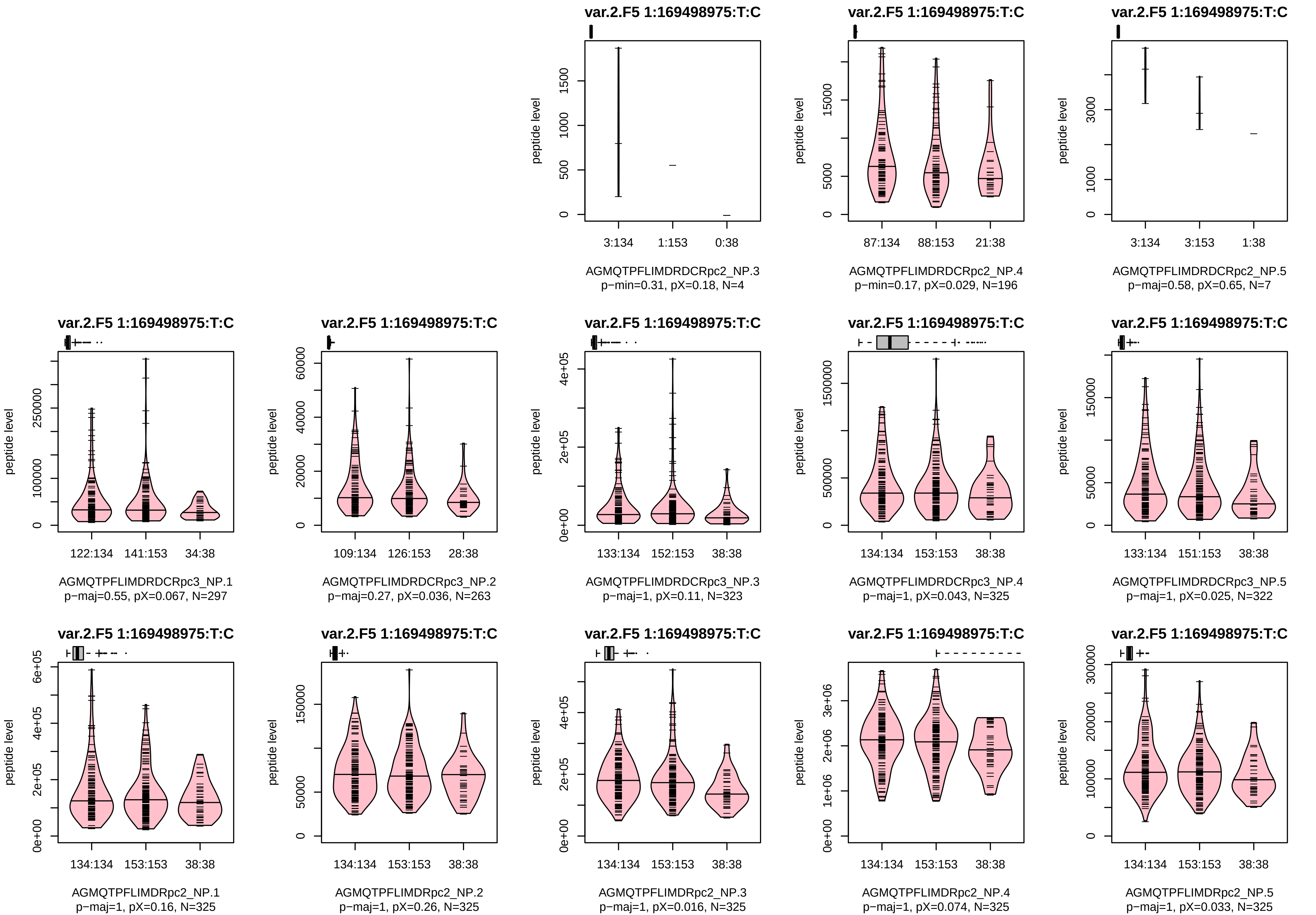
var.2.F5 1:169498975:T:C
peptide level
3:134 1:153 0:38
AGMQTPFLIMDRDCRpc2_NP.3
p−min=0.31, pX=0.18, N=4
var.2.F5 1:169498975:T:C
peptide level
87:134 88:153 21:38
AGMQTPFLIMDRDCRpc2_NP.4
p−min=0.17, pX=0.029, N=196
var.2.F5 1:169498975:T:C
peptide level
3:134 3:153 1:38
AGMQTPFLIMDRDCRpc2_NP.5
p−maj=0.58, pX=0.65, N=7
var.2.F5 1:169498975:T:C
peptide level
122:134 141:153 34:38
AGMQTPFLIMDRDCRpc3_NP.1
p−maj=0.55, pX=0.067, N=297
var.2.F5 1:169498975:T:C
peptide level
109:134 126:153 28:38
AGMQTPFLIMDRDCRpc3_NP.2
p−maj=0.27, pX=0.036, N=263
var.2.F5 1:169498975:T:C
peptide level
133:134 152:153 38:38
AGMQTPFLIMDRDCRpc3_NP.3
p−maj=1, pX=0.11, N=323
var.2.F5 1:169498975:T:C
peptide level
134:134 153:153 38:38
AGMQTPFLIMDRDCRpc3_NP.4
p−maj=1, pX=0.043, N=325
var.2.F5 1:169498975:T:C
peptide level
133:134 151:153 38:38
AGMQTPFLIMDRDCRpc3_NP.5
p−maj=1, pX=0.025, N=322
var.2.F5 1:169498975:T:C
peptide level
134:134 153:153 38:38
AGMQTPFLIMDRpc2_NP.1
p−maj=1, pX=0.16, N=325
var.2.F5 1:169498975:T:C
peptide level
134:134 153:153 38:38
AGMQTPFLIMDRpc2_NP.2
p−maj=1, pX=0.26, N=325
var.2.F5 1:169498975:T:C
peptide level
134:134 153:153 38:38
AGMQTPFLIMDRpc2_NP.3
p−maj=1, pX=0.016, N=325
var.2.F5 1:169498975:T:C
peptide level
134:134 153:153 38:38
AGMQTPFLIMDRpc2_NP.4
p−maj=1, pX=0.074, N=325
var.2.F5 1:169498975:T:C
peptide level
134:134 153:153 38:38
AGMQTPFLIMDRpc2_NP.5
p−maj=1, pX=0.033, N=325

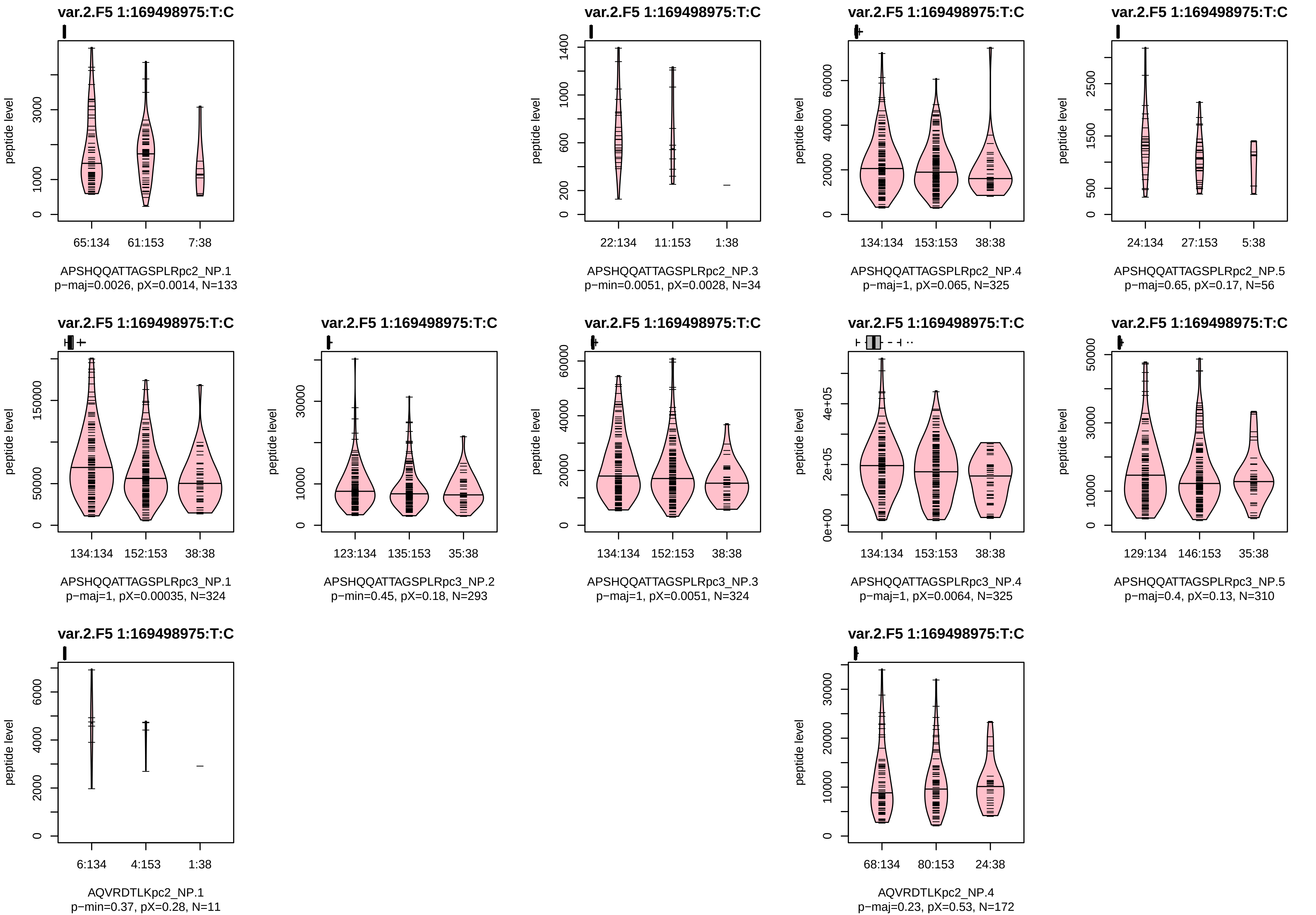

var.2.F5 1:169498975:T:C
peptide level
65:134
61:153
7:38
APSHQQATTAGSPLRpc2_NP.1
p−maj=0.0026, pX=0.0014, N=133
var.2.F5 1:169498975:T:C
peptide level
22:134
11:153
1:38
APSHQQATTAGSPLRpc2_NP.3
p−min=0.0051, pX=0.0028, N=34
var.2.F5 1:169498975:T:C
peptide level
134:134
153:153
38:38
APSHQQATTAGSPLRpc2_NP.4
p−maj=1, pX=0.065, N=325
var.2.F5 1:169498975:T:C
peptide level
24:134
27:153
5:38
APSHQQATTAGSPLRpc2_NP.5
p−maj=0.65, pX=0.17, N=56
var.2.F5 1:169498975:T:C
peptide level
134:134
152:153
38:38
APSHQQATTAGSPLRpc3_NP.1
p−maj=1, pX=0.00035, N=324
var.2.F5 1:169498975:T:C
peptide level
123:134
135:153
35:38
APSHQQATTAGSPLRpc3_NP.2
p−min=0.45, pX=0.18, N=293
var.2.F5 1:169498975:T:C
peptide level
134:134
152:153
38:38
APSHQQATTAGSPLRpc3_NP.3
p−maj=1, pX=0.0051, N=324
var.2.F5 1:169498975:T:C
peptide level
134:134
153:153
38:38
APSHQQATTAGSPLRpc3_NP.4
p−maj=1, pX=0.0064, N=325
var.2.F5 1:169498975:T:C
peptide level
129:134
146:153
35:38
APSHQQATTAGSPLRpc3_NP.5
p−maj=0.4, pX=0.13, N=310
var.2.F5 1:169498975:T:C
peptide level
6:134
4:153
1:38
AQVRDTLKpc2_NP.1
p−min=0.37, pX=0.28, N=11
var.2.F5 1:169498975:T:C
peptide level
68:134
80:153
24:38
AQVRDTLKpc2_NP.4
p−maj=0.23, pX=0.53, N=172

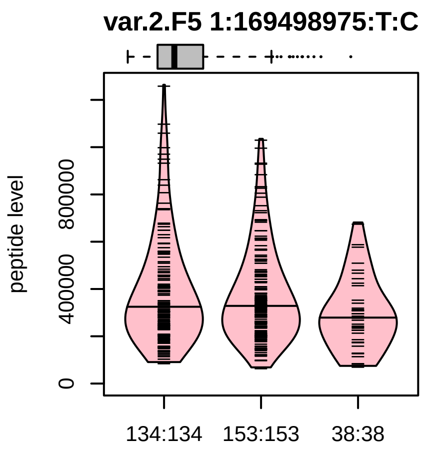

ASEFLGYWEPRpc2_NP.1
p−maj=1, pX=0.026, N=325

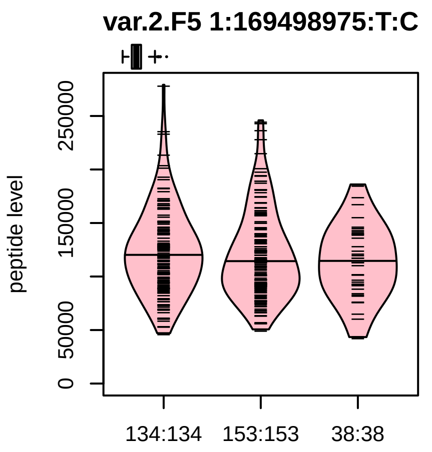

ASEFLGYWEPRpc2_NP.2
p−maj=1, pX=0.16, N=325

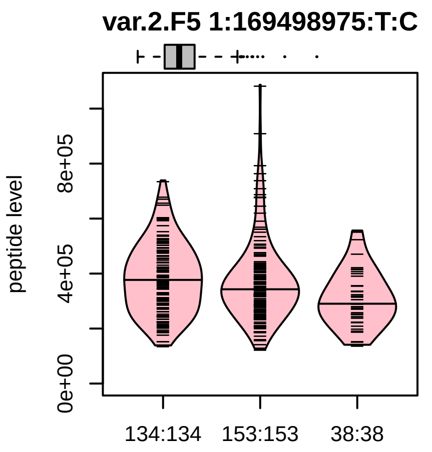

ASEFLGYWEPRpc2_NP.3
p−maj=1, pX=0.012, N=325

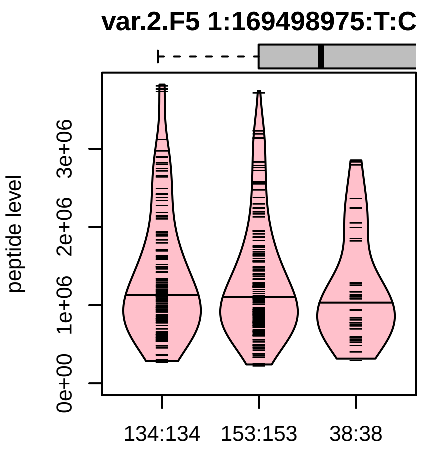

ASEFLGYWEPRpc2_NP.4
p−maj=1, pX=0.2, N=325

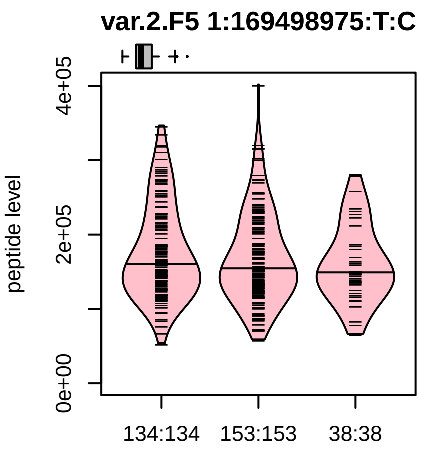

ASEFLGYWEPRpc2_NP.5
p−maj=1, pX=0.12, N=325

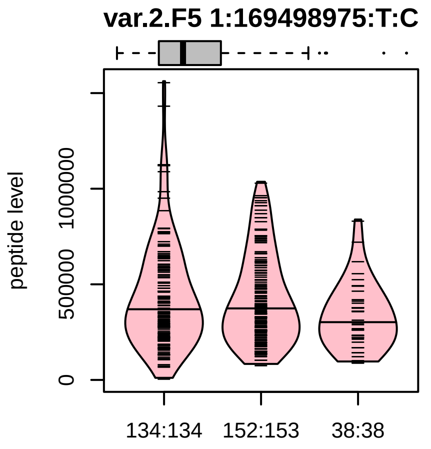

AVQPGETYTYKpc2_NP.1
p−maj=1, pX=0.058, N=324

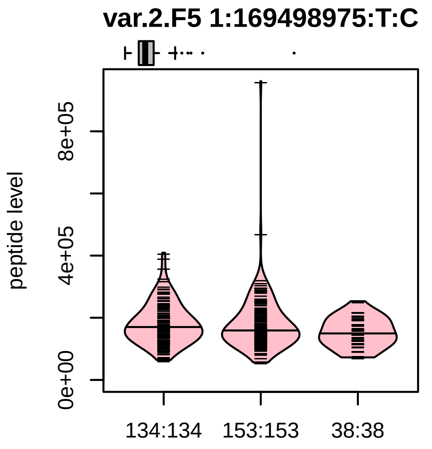

AVQPGETYTYKpc2_NP.2
p−maj=1, pX=0.099, N=325

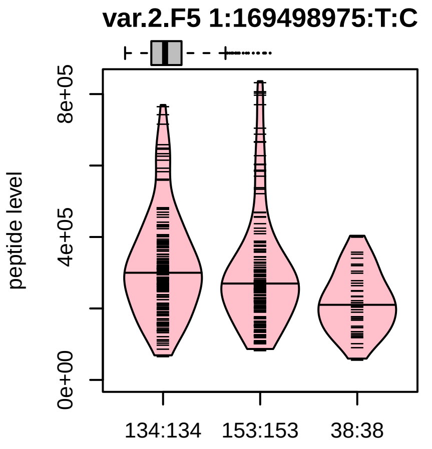

AVQPGETYTYKpc2_NP.3
p−maj=1, pX=0.00071, N=325

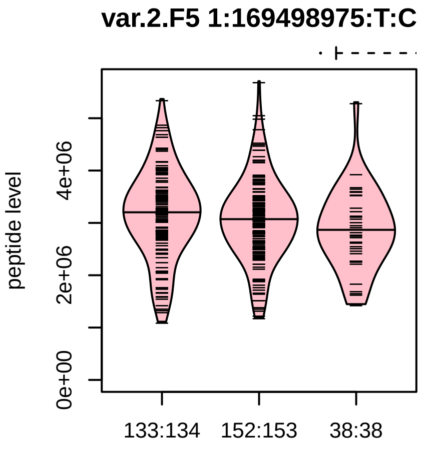

AVQPGETYTYKpc2_NP.4
p−maj=1, pX=0.097, N=323

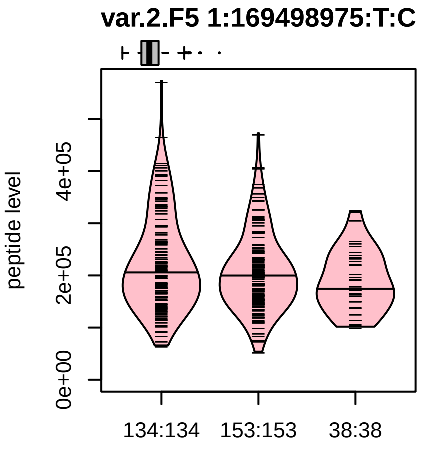

AVQPGETYTYKpc2_NP.5
p−maj=1, pX=0.011, N=325

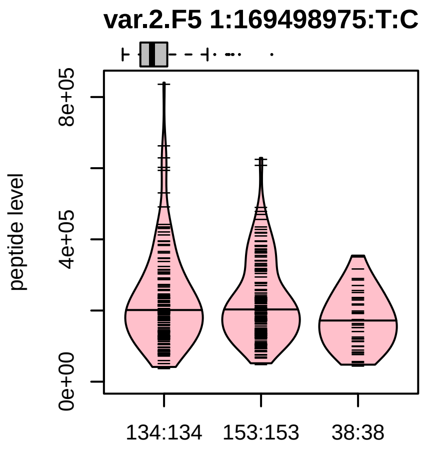

AWAYYSAVNPEKpc2_NP.1
p−maj=1, pX=0.031, N=325

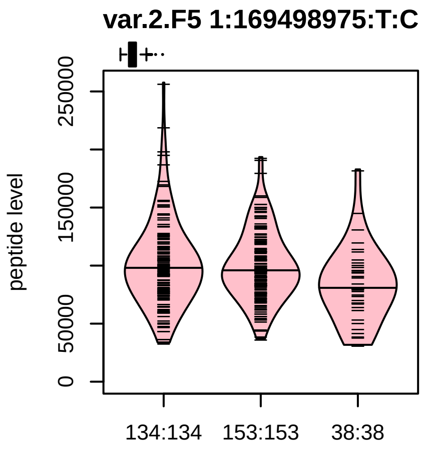

AWAYYSAVNPEKpc2_NP.2
p−maj=1, pX=0.013, N=325

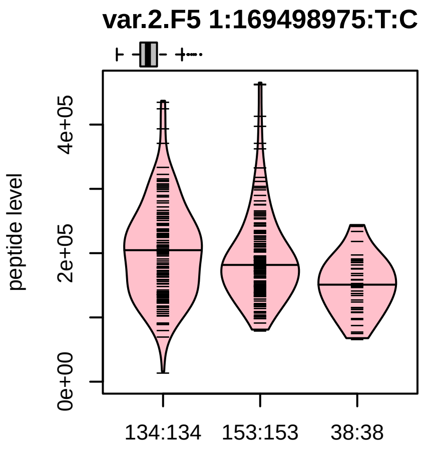

AWAYYSAVNPEKpc2_NP.3
p−maj=1, pX=0.00042, N=325

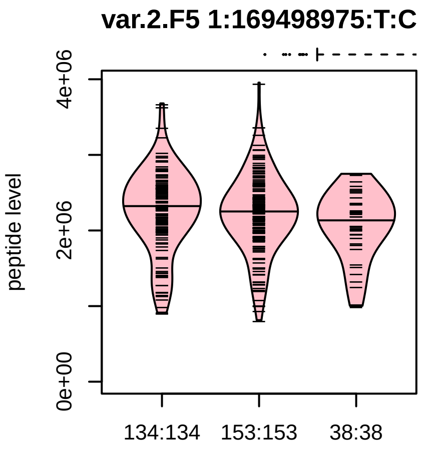

AWAYYSAVNPEKpc2_NP.4
p−maj=1, pX=0.075, N=325

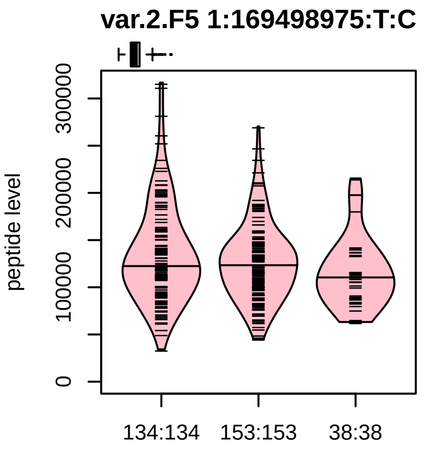

AWAYYSAVNPEKpc2_NP.5
p−maj=1, pX=0.014, N=325

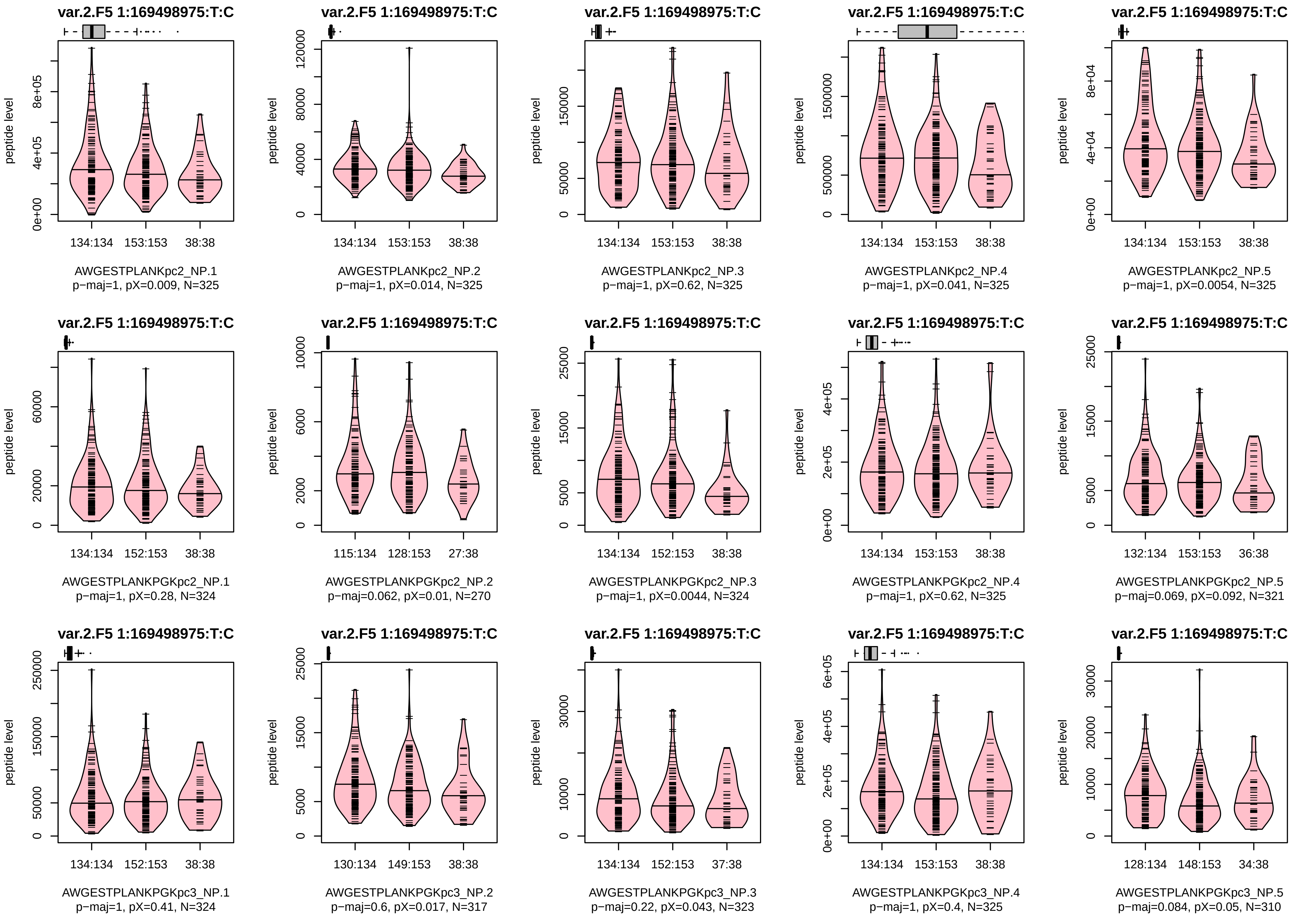
var.2.F5 1:169498975:T:C
peptide level
134:134
153:153
38:38
AWGESTPLANKpc2_NP.1
p−maj=1, pX=0.009, N=325
var.2.F5 1:169498975:T:C
peptide level
134:134
153:153
38:38
AWGESTPLANKpc2_NP.2
p−maj=1, pX=0.014, N=325
var.2.F5 1:169498975:T:C
peptide level
134:134
153:153
38:38
AWGESTPLANKpc2_NP.3
p−maj=1, pX=0.62, N=325
var.2.F5 1:169498975:T:C
peptide level
134:134
153:153
38:38
AWGESTPLANKpc2_NP.4
p−maj=1, pX=0.041, N=325
var.2.F5 1:169498975:T:C
peptide level
134:134
153:153
38:38
AWGESTPLANKpc2_NP.5
p−maj=1, pX=0.0054, N=325
var.2.F5 1:169498975:T:C
peptide level
134:134
152:153
38:38
AWGESTPLANKPGKpc2_NP.1
p−maj=1, pX=0.28, N=324
var.2.F5 1:169498975:T:C
peptide level
115:134
128:153
27:38
AWGESTPLANKPGKpc2_NP.2
p−maj=0.062, pX=0.01, N=270
var.2.F5 1:169498975:T:C
peptide level
134:134
152:153
38:38
AWGESTPLANKPGKpc2_NP.3
p−maj=1, pX=0.0044, N=324
var.2.F5 1:169498975:T:C
peptide level
134:134
153:153
38:38
AWGESTPLANKPGKpc2_NP.4
p−maj=1, pX=0.62, N=325
var.2.F5 1:169498975:T:C
peptide level
132:134
153:153
36:38
AWGESTPLANKPGKpc2_NP.5
p−maj=0.069, pX=0.092, N=321
var.2.F5 1:169498975:T:C
peptide level
134:134
152:153
38:38
AWGESTPLANKPGKpc3_NP.1
p−maj=1, pX=0.41, N=324
var.2.F5 1:169498975:T:C
peptide level
130:134
149:153
38:38
AWGESTPLANKPGKpc3_NP.2
p−maj=0.6, pX=0.017, N=317
var.2.F5 1:169498975:T:C
peptide level
134:134
152:153
37:38
AWGESTPLANKPGKpc3_NP.3
p−maj=0.22, pX=0.043, N=323
var.2.F5 1:169498975:T:C
peptide level
134:134
153:153
38:38
AWGESTPLANKPGKpc3_NP.4
p−maj=1, pX=0.4, N=325
var.2.F5 1:169498975:T:C
peptide level
128:134
148:153
34:38
AWGESTPLANKPGKpc3_NP.5
p−maj=0.084, pX=0.05, N=310

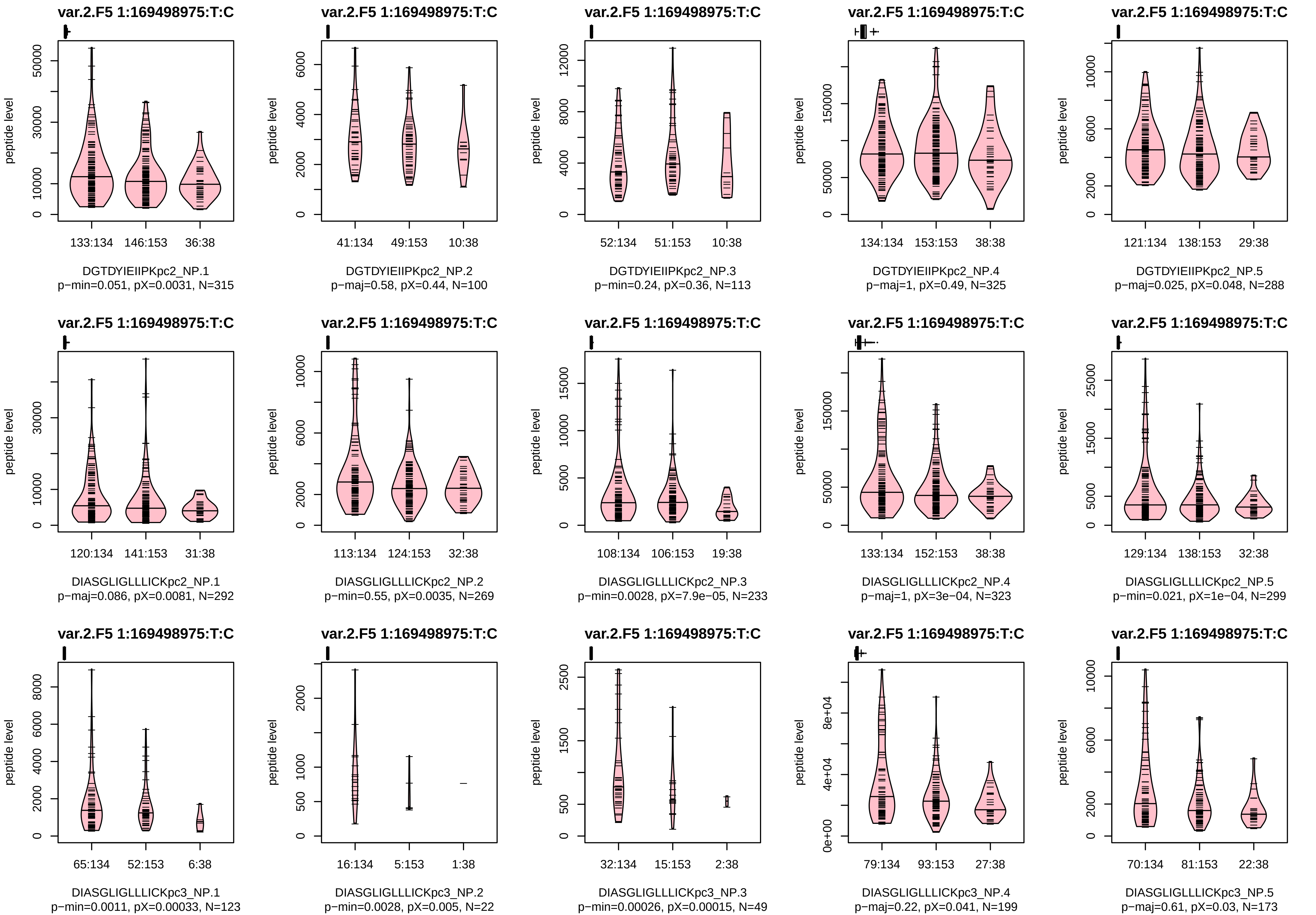

var.2.F5 1:169498975:T:C
peptide level
0
10000
30000
50000
133:134
146:153
36:38
DGTDYIEIIPKpc2_NP.1
p−min=0.051, pX=0.0031, N=315
var.2.F5 1:169498975:T:C
peptide level
0
2000
4000
6000
41:134
49:153
10:38
DGTDYIEIIPKpc2_NP.2
p−maj=0.58, pX=0.44, N=100
var.2.F5 1:169498975:T:C
peptide level
0
4000
8000
12000
52:134
51:153
10:38
DGTDYIEIIPKpc2_NP.3
p−min=0.24, pX=0.36, N=113
var.2.F5 1:169498975:T:C
peptide level
0
50000
150000
134:134
153:153
38:38
DGTDYIEIIPKpc2_NP.4
p−maj=1, pX=0.49, N=325
var.2.F5 1:169498975:T:C
peptide level
0
2000
6000
10000
121:134
138:153
29:38
DGTDYIEIIPKpc2_NP.5
p−maj=0.025, pX=0.048, N=288
var.2.F5 1:169498975:T:C
peptide level
0
10000
30000
120:134
141:153
31:38
DIASGLIGLLLICKpc2_NP.1
p−maj=0.086, pX=0.0081, N=292
var.2.F5 1:169498975:T:C
peptide level
0
2000
6000
10000
113:134
124:153
32:38
DIASGLIGLLLICKpc2_NP.2
p−min=0.55, pX=0.0035, N=269
var.2.F5 1:169498975:T:C
peptide level
0
5000
10000
15000
108:134
106:153
19:38
DIASGLIGLLLICKpc2_NP.3
p−min=0.0028, pX=7.9e−05, N=233
var.2.F5 1:169498975:T:C
peptide level
0
50000
150000
133:134
152:153
38:38
DIASGLIGLLLICKpc2_NP.4
p−maj=1, pX=3e−04, N=323
var.2.F5 1:169498975:T:C
peptide level
0
5000
15000
25000
129:134
138:153
32:38
DIASGLIGLLLICKpc2_NP.5
p−min=0.021, pX=1e−04, N=299
var.2.F5 1:169498975:T:C
peptide level
0
2000
4000
6000
8000
65:134
52:153
6:38
DIASGLIGLLLICKpc3_NP.1
p−min=0.0011, pX=0.00033, N=123
var.2.F5 1:169498975:T:C
peptide level
0
500
1000
2000
16:134
5:153
1:38
DIASGLIGLLLICKpc3_NP.2
p−min=0.0028, pX=0.005, N=22
var.2.F5 1:169498975:T:C
peptide level
0
500
1500
2500
32:134
15:153
2:38
DIASGLIGLLLICKpc3_NP.3
p−min=0.00026, pX=0.00015, N=49
var.2.F5 1:169498975:T:C
peptide level
0e+00
4e+04
8e+04
79:134
93:153
27:38
DIASGLIGLLLICKpc3_NP.4
p−maj=0.22, pX=0.041, N=199
var.2.F5 1:169498975:T:C
peptide level
0
2000
6000
10000
70:134
81:153
22:38
DIASGLIGLLLICKpc3_NP.5
p−maj=0.61, pX=0.03, N=173

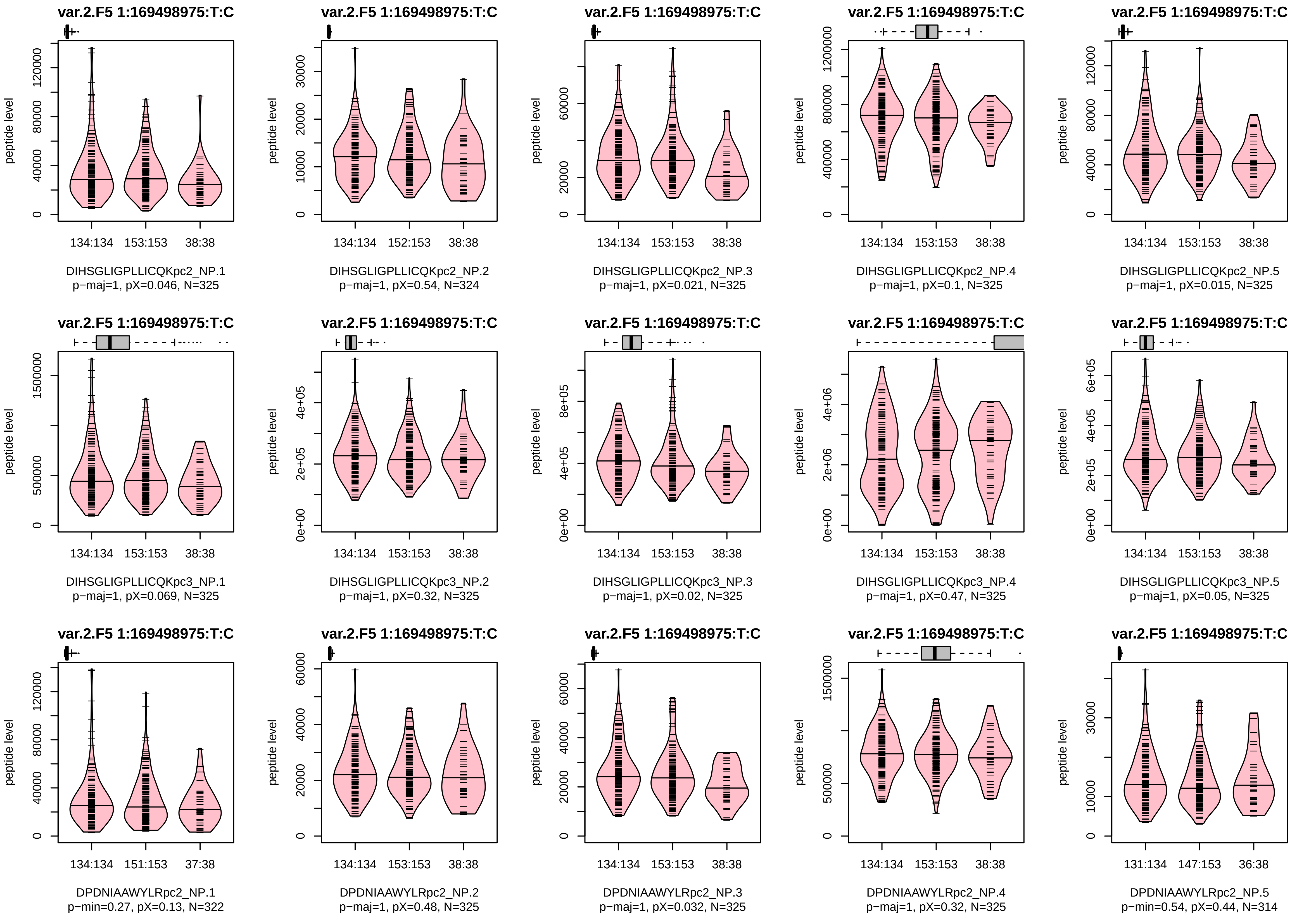
var.2.F5 1:169498975:T:C
peptide level
134:134
153:153
38:38
DIHSGLIGPLLICQKpc2_NP.1
p−maj=1, pX=0.046, N=325
var.2.F5 1:169498975:T:C
peptide level
134:134
152:153
38:38
DIHSGLIGPLLICQKpc2_NP.2
p−maj=1, pX=0.54, N=324
var.2.F5 1:169498975:T:C
peptide level
134:134
153:153
38:38
DIHSGLIGPLLICQKpc2_NP.3
p−maj=1, pX=0.021, N=325
var.2.F5 1:169498975:T:C
peptide level
134:134
153:153
38:38
DIHSGLIGPLLICQKpc2_NP.4
p−maj=1, pX=0.1, N=325
var.2.F5 1:169498975:T:C
peptide level
134:134
153:153
38:38
DIHSGLIGPLLICQKpc2_NP.5
p−maj=1, pX=0.015, N=325
var.2.F5 1:169498975:T:C
peptide level
134:134
153:153
38:38
DIHSGLIGPLLICQKpc3_NP.1
p−maj=1, pX=0.069, N=325
var.2.F5 1:169498975:T:C
peptide level
134:134
153:153
38:38
DIHSGLIGPLLICQKpc3_NP.2
p−maj=1, pX=0.32, N=325
var.2.F5 1:169498975:T:C
peptide level
134:134
153:153
38:38
DIHSGLIGPLLICQKpc3_NP.3
p−maj=1, pX=0.02, N=325
var.2.F5 1:169498975:T:C
peptide level
134:134
153:153
38:38
DIHSGLIGPLLICQKpc3_NP.4
p−maj=1, pX=0.47, N=325
var.2.F5 1:169498975:T:C
peptide level
134:134
153:153
38:38
DIHSGLIGPLLICQKpc3_NP.5
p−maj=1, pX=0.05, N=325
var.2.F5 1:169498975:T:C
peptide level
134:134
151:153
37:38
DPDNIAAWYLRpc2_NP.1
p−min=0.27, pX=0.13, N=322
var.2.F5 1:169498975:T:C
peptide level
134:134
153:153
38:38
DPDNIAAWYLRpc2_NP.2
p−maj=1, pX=0.48, N=325
var.2.F5 1:169498975:T:C
peptide level
134:134
153:153
38:38
DPDNIAAWYLRpc2_NP.3
p−maj=1, pX=0.032, N=325
var.2.F5 1:169498975:T:C
peptide level
134:134
153:153
38:38
DPDNIAAWYLRpc2_NP.4
p−maj=1, pX=0.32, N=325
var.2.F5 1:169498975:T:C
peptide level
131:134
147:153
36:38
DPDNIAAWYLRpc2_NP.5
p−min=0.54, pX=0.44, N=314

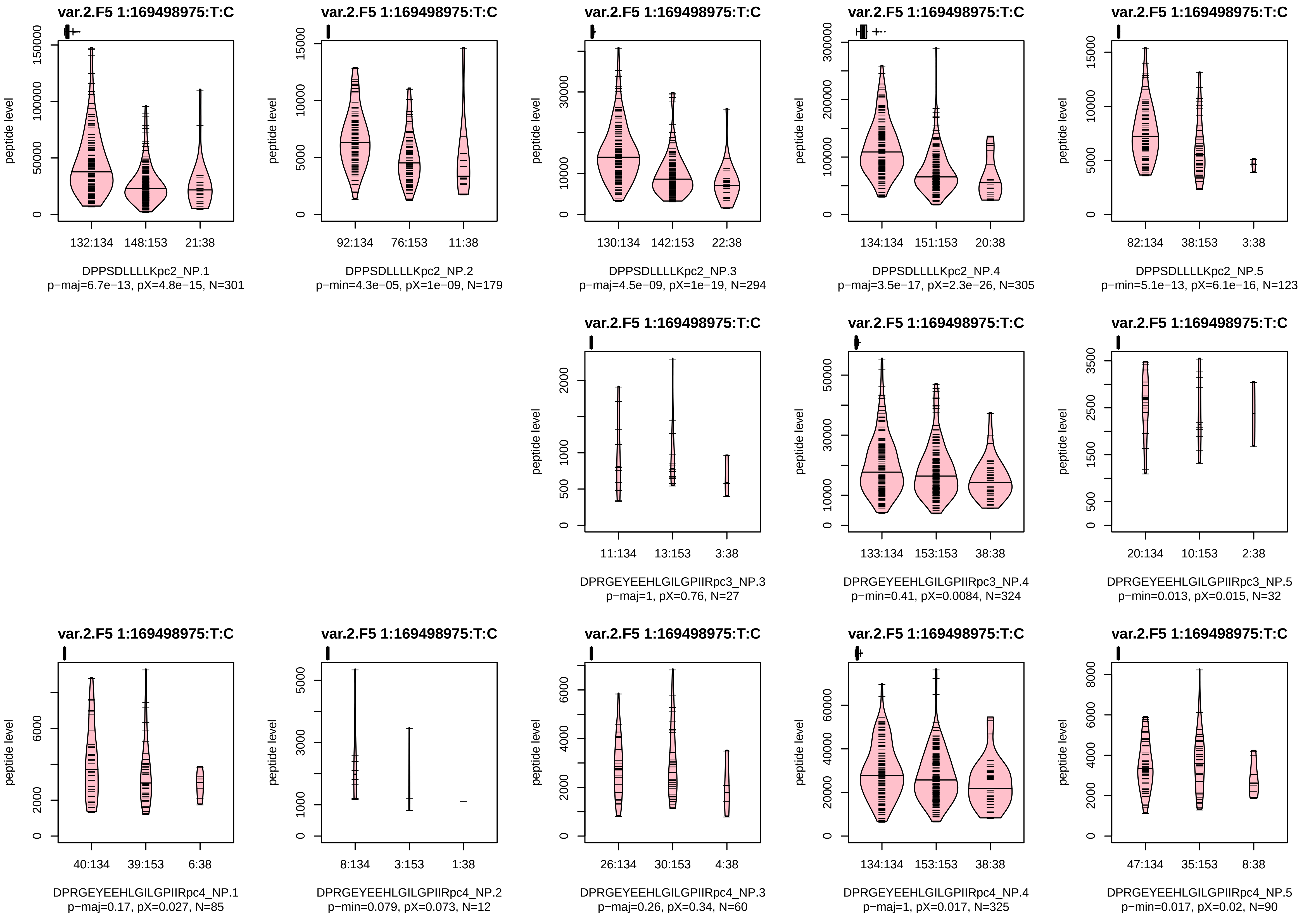
var.2.F5 1:169498975:T:C
peptide level
132:134
148:153
21:38
DPPSDLLLLKpc2_NP.1
p−maj=6.7e−13, pX=4.8e−15, N=301
var.2.F5 1:169498975:T:C
peptide level
92:134
76:153
11:38
DPPSDLLLLKpc2_NP.2
p−min=4.3e−05, pX=1e−09, N=179
var.2.F5 1:169498975:T:C
peptide level
130:134
142:153
22:38
DPPSDLLLLKpc2_NP.3
p−maj=4.5e−09, pX=1e−19, N=294
var.2.F5 1:169498975:T:C
peptide level
134:134
151:153
20:38
DPPSDLLLLKpc2_NP.4
p−maj=3.5e−17, pX=2.3e−26, N=305
var.2.F5 1:169498975:T:C
peptide level
82:134
38:153
3:38
DPPSDLLLLKpc2_NP.5
p−min=5.1e−13, pX=6.1e−16, N=123
var.2.F5 1:169498975:T:C
peptide level
11:134
13:153
3:38
DPRGEYEEHLGILGPIIRpc3_NP.3
p−maj=1, pX=0.76, N=27
var.2.F5 1:169498975:T:C
peptide level
133:134
153:153
38:38
DPRGEYEEHLGILGPIIRpc3_NP.4
p−min=0.41, pX=0.0084, N=324
var.2.F5 1:169498975:T:C
peptide level
20:134
10:153
2:38
DPRGEYEEHLGILGPIIRpc3_NP.5
p−min=0.013, pX=0.015, N=32
var.2.F5 1:169498975:T:C
peptide level
40:134
39:153
6:38
DPRGEYEEHLGILGPIIRpc4_NP.1
p−maj=0.17, pX=0.027, N=85
var.2.F5 1:169498975:T:C
peptide level
8:134
3:153
1:38
DPRGEYEEHLGILGPIIRpc4_NP.2
p−min=0.079, pX=0.073, N=12
var.2.F5 1:169498975:T:C
peptide level
26:134
30:153
4:38
DPRGEYEEHLGILGPIIRpc4_NP.3
p−maj=0.26, pX=0.34, N=60
var.2.F5 1:169498975:T:C
peptide level
134:134
153:153
38:38
DPRGEYEEHLGILGPIIRpc4_NP.4
p−maj=1, pX=0.017, N=325
var.2.F5 1:169498975:T:C
peptide level
47:134
35:153
8:38
DPRGEYEEHLGILGPIIRpc4_NP.5
p−min=0.017, pX=0.02, N=90

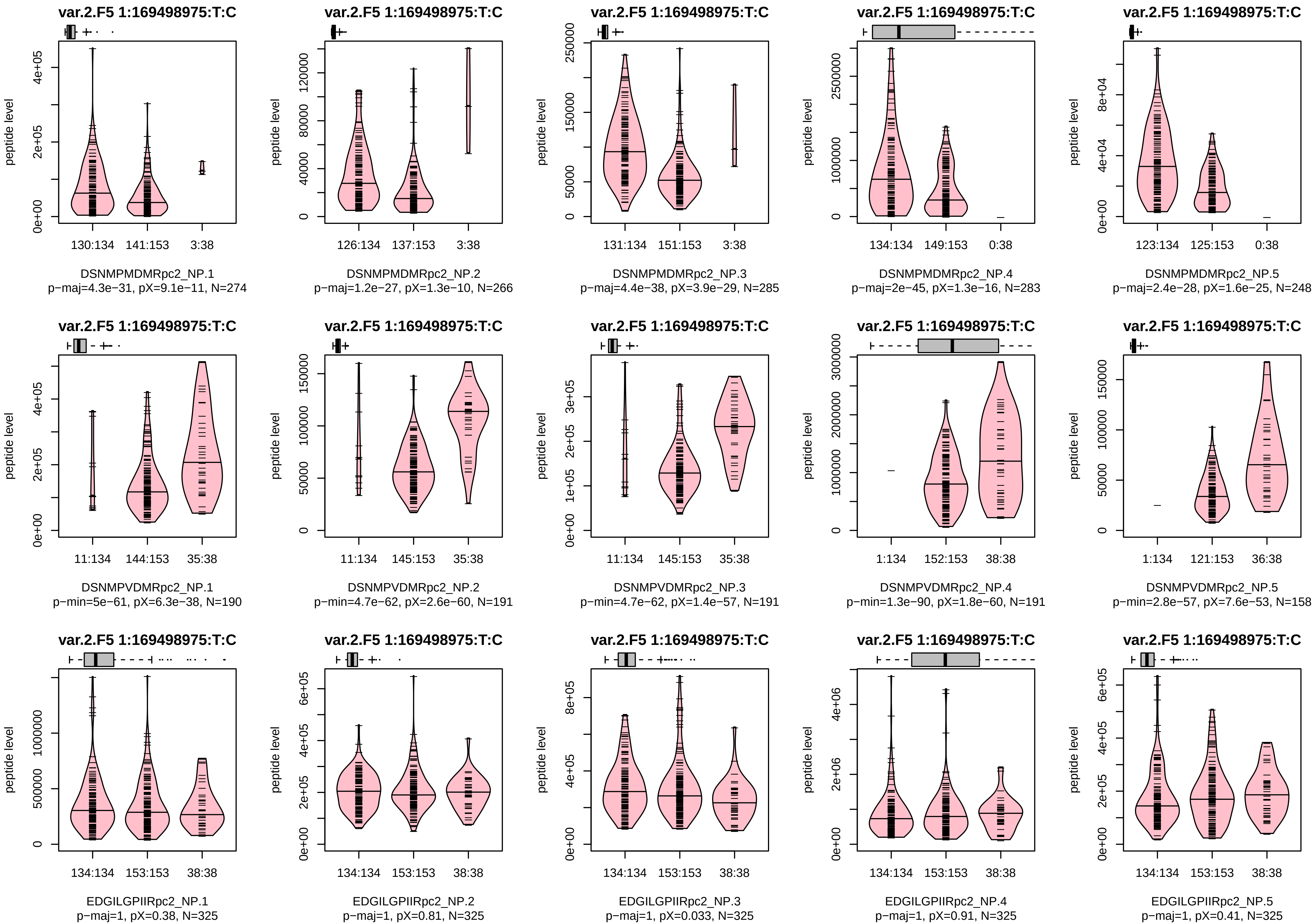

var.2.F5 1:169498975:T:C
peptide level
130:134
141:153
3:38
DSNMPMDMRpc2_NP.1
p−maj=4.3e−31, pX=9.1e−11, N=274
var.2.F5 1:169498975:T:C
peptide level
126:134
137:153
3:38
DSNMPMDMRpc2_NP.2
p−maj=1.2e−27, pX=1.3e−10, N=266
var.2.F5 1:169498975:T:C
peptide level
131:134
151:153
3:38
DSNMPMDMRpc2_NP.3
p−maj=4.4e−38, pX=3.9e−29, N=285
var.2.F5 1:169498975:T:C
peptide level
134:134
149:153
0:38
DSNMPMDMRpc2_NP.4
p−maj=2e−45, pX=1.3e−16, N=283
var.2.F5 1:169498975:T:C
peptide level
123:134
125:153
0:38
DSNMPMDMRpc2_NP.5
p−maj=2.4e−28, pX=1.6e−25, N=248
var.2.F5 1:169498975:T:C
peptide level
11:134
144:153
35:38
DSNMPVDMRpc2_NP.1
p−min=5e−61, pX=6.3e−38, N=190
var.2.F5 1:169498975:T:C
peptide level
11:134
145:153
35:38
DSNMPVDMRpc2_NP.2
p−min=4.7e−62, pX=2.6e−60, N=191
var.2.F5 1:169498975:T:C
peptide level
11:134
145:153
35:38
DSNMPVDMRpc2_NP.3
p−min=4.7e−62, pX=1.4e−57, N=191
var.2.F5 1:169498975:T:C
peptide level
1:134
152:153
38:38
DSNMPVDMRpc2_NP.4
p−min=1.3e−90, pX=1.8e−60, N=191
var.2.F5 1:169498975:T:C
peptide level
1:134
121:153
36:38
DSNMPVDMRpc2_NP.5
p−min=2.8e−57, pX=7.6e−53, N=158
var.2.F5 1:169498975:T:C
peptide level
134:134
153:153
38:38
EDGILGPIIRpc2_NP.1
p−maj=1, pX=0.38, N=325
var.2.F5 1:169498975:T:C
peptide level
134:134
153:153
38:38
EDGILGPIIRpc2_NP.2
p−maj=1, pX=0.81, N=325
var.2.F5 1:169498975:T:C
peptide level
134:134
153:153
38:38
EDGILGPIIRpc2_NP.3
p−maj=1, pX=0.033, N=325
var.2.F5 1:169498975:T:C
peptide level
134:134
153:153
38:38
EDGILGPIIRpc2_NP.4
p−maj=1, pX=0.91, N=325
var.2.F5 1:169498975:T:C
peptide level
134:134
153:153
38:38
EDGILGPIIRpc2_NP.5
p−maj=1, pX=0.41, N=325

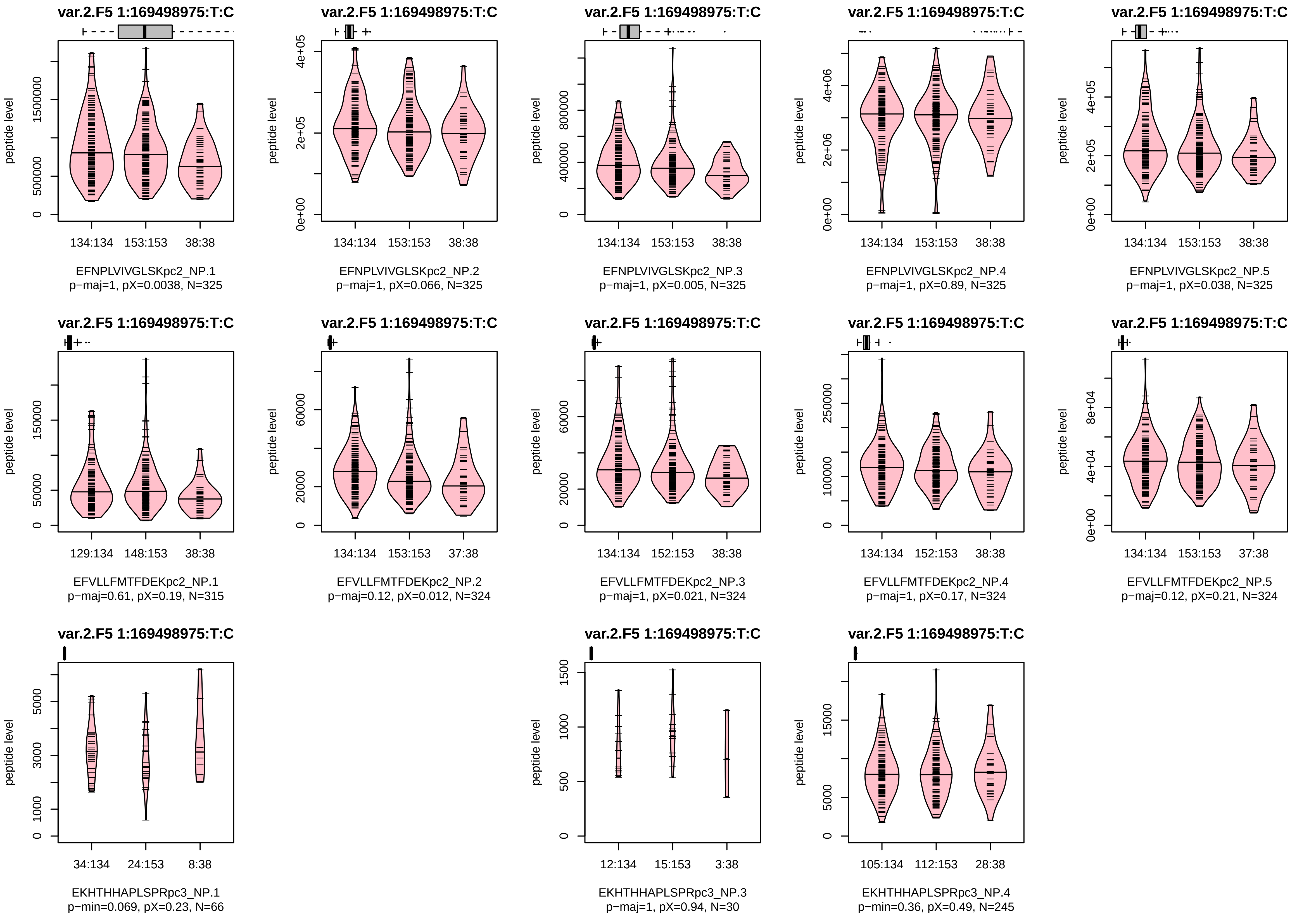
var.2.F5 1:169498975:T:C
peptide level
0
500000
1500000
134:134
153:153
38:38
EFNPLVIVGLSKpc2_NP.1
p−maj=1, pX=0.0038, N=325
var.2.F5 1:169498975:T:C
peptide level
0e+00
2e+05
4e+05
134:134
153:153
38:38
EFNPLVIVGLSKpc2_NP.2
p−maj=1, pX=0.066, N=325
var.2.F5 1:169498975:T:C
peptide level
0
400000
800000
134:134
153:153
38:38
EFNPLVIVGLSKpc2_NP.3
p−maj=1, pX=0.005, N=325
var.2.F5 1:169498975:T:C
peptide level
0e+00
2e+06
4e+06
134:134
153:153
38:38
EFNPLVIVGLSKpc2_NP.4
p−maj=1, pX=0.89, N=325
var.2.F5 1:169498975:T:C
peptide level
0e+00
2e+05
4e+05
134:134
153:153
38:38
EFNPLVIVGLSKpc2_NP.5
p−maj=1, pX=0.038, N=325
var.2.F5 1:169498975:T:C
peptide level
0
50000
150000
129:134
148:153
38:38
EFVLLFMTFDEKpc2_NP.1
p−maj=0.61, pX=0.19, N=315
var.2.F5 1:169498975:T:C
peptide level
0
20000
60000
134:134
153:153
37:38
EFVLLFMTFDEKpc2_NP.2
p−maj=0.12, pX=0.012, N=324
var.2.F5 1:169498975:T:C
peptide level
0
20000
60000
134:134
152:153
38:38
EFVLLFMTFDEKpc2_NP.3
p−maj=1, pX=0.021, N=324
var.2.F5 1:169498975:T:C
peptide level
0
100000
250000
134:134
152:153
38:38
EFVLLFMTFDEKpc2_NP.4
p−maj=1, pX=0.17, N=324
var.2.F5 1:169498975:T:C
peptide level
0e+00
4e+04
8e+04
134:134
153:153
37:38
EFVLLFMTFDEKpc2_NP.5
p−maj=0.12, pX=0.21, N=324
var.2.F5 1:169498975:T:C
peptide level
0
1000
3000
5000
34:134
24:153
8:38
EKHTHHAPLSPRpc3_NP.1
p−min=0.069, pX=0.23, N=66
var.2.F5 1:169498975:T:C
peptide level
0
500
1000
1500
12:134
15:153
3:38
EKHTHHAPLSPRpc3_NP.3
p−maj=1, pX=0.94, N=30
var.2.F5 1:169498975:T:C
peptide level
0
5000
15000
105:134
112:153
28:38
EKHTHHAPLSPRpc3_NP.4
p−min=0.36, pX=0.49, N=245

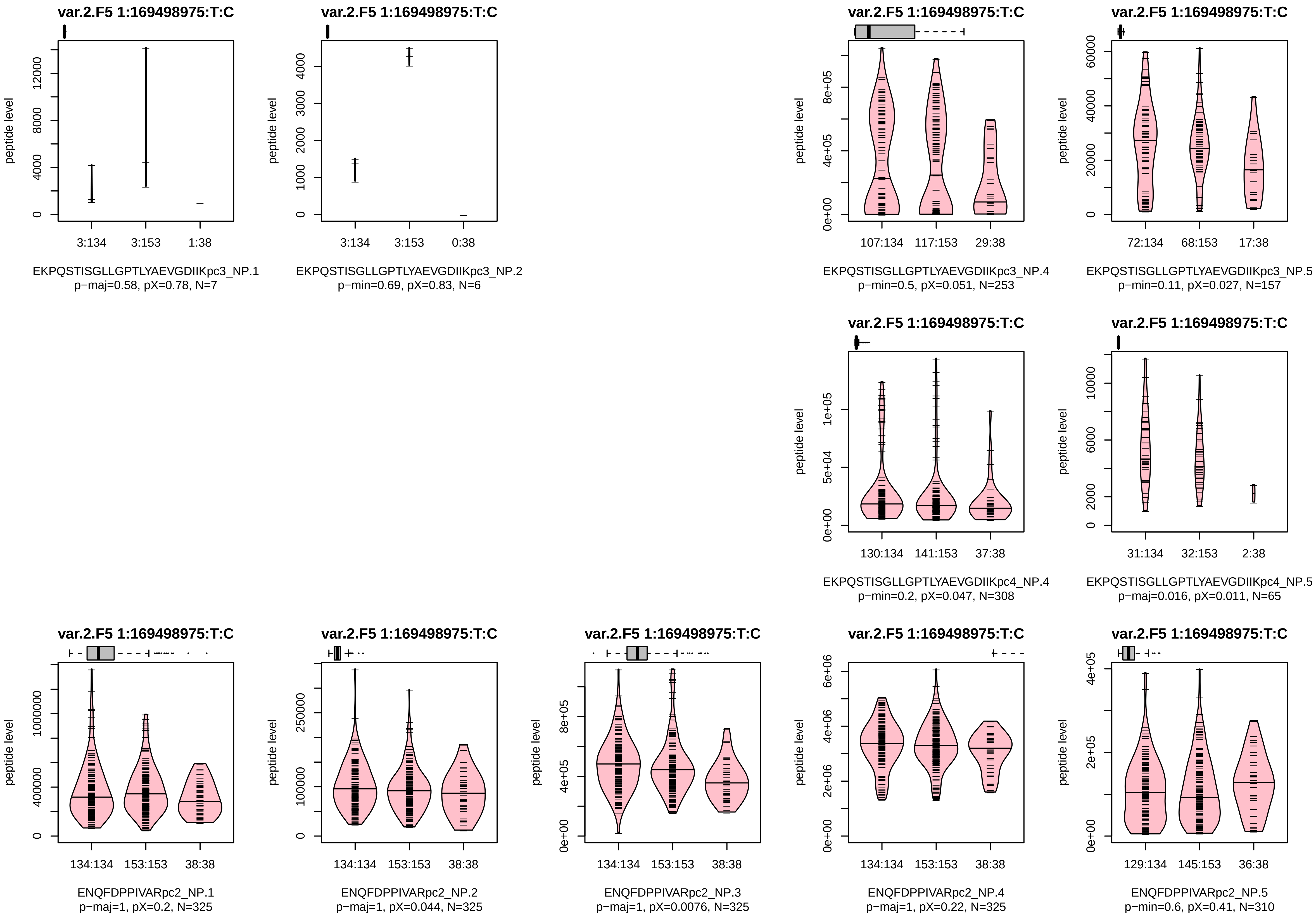
var.2.F5 1:169498975:T:C
peptide level
3:134
3:153
1:38
EKPQSTISGLLGPTLYAEVGDIIKpc3_NP.1
p−maj=0.58, pX=0.78, N=7
var.2.F5 1:169498975:T:C
peptide level
3:134
3:153
0:38
EKPQSTISGLLGPTLYAEVGDIIKpc3_NP.2
p−min=0.69, pX=0.83, N=6
var.2.F5 1:169498975:T:C
peptide level
107:134
117:153
29:38
EKPQSTISGLLGPTLYAEVGDIIKpc3_NP.4
p−min=0.5, pX=0.051, N=253
var.2.F5 1:169498975:T:C
peptide level
72:134
68:153
17:38
EKPQSTISGLLGPTLYAEVGDIIKpc3_NP.5
p−min=0.11, pX=0.027, N=157
var.2.F5 1:169498975:T:C
peptide level
130:134
141:153
37:38
EKPQSTISGLLGPTLYAEVGDIIKpc4_NP.4
p−min=0.2, pX=0.047, N=308
var.2.F5 1:169498975:T:C
peptide level
31:134
32:153
2:38
EKPQSTISGLLGPTLYAEVGDIIKpc4_NP.5
p−maj=0.016, pX=0.011, N=65
var.2.F5 1:169498975:T:C
peptide level
134:134
153:153
38:38
ENQFDPPIVARpc2_NP.1
p−maj=1, pX=0.2, N=325
var.2.F5 1:169498975:T:C
peptide level
134:134
153:153
38:38
ENQFDPPIVARpc2_NP.2
p−maj=1, pX=0.044, N=325
var.2.F5 1:169498975:T:C
peptide level
134:134
153:153
38:38
ENQFDPPIVARpc2_NP.3
p−maj=1, pX=0.0076, N=325
var.2.F5 1:169498975:T:C
peptide level
134:134
153:153
38:38
ENQFDPPIVARpc2_NP.4
p−maj=1, pX=0.22, N=325
var.2.F5 1:169498975:T:C
peptide level
129:134
145:153
36:38
ENQFDPPIVARpc2_NP.5
p−min=0.6, pX=0.41, N=310

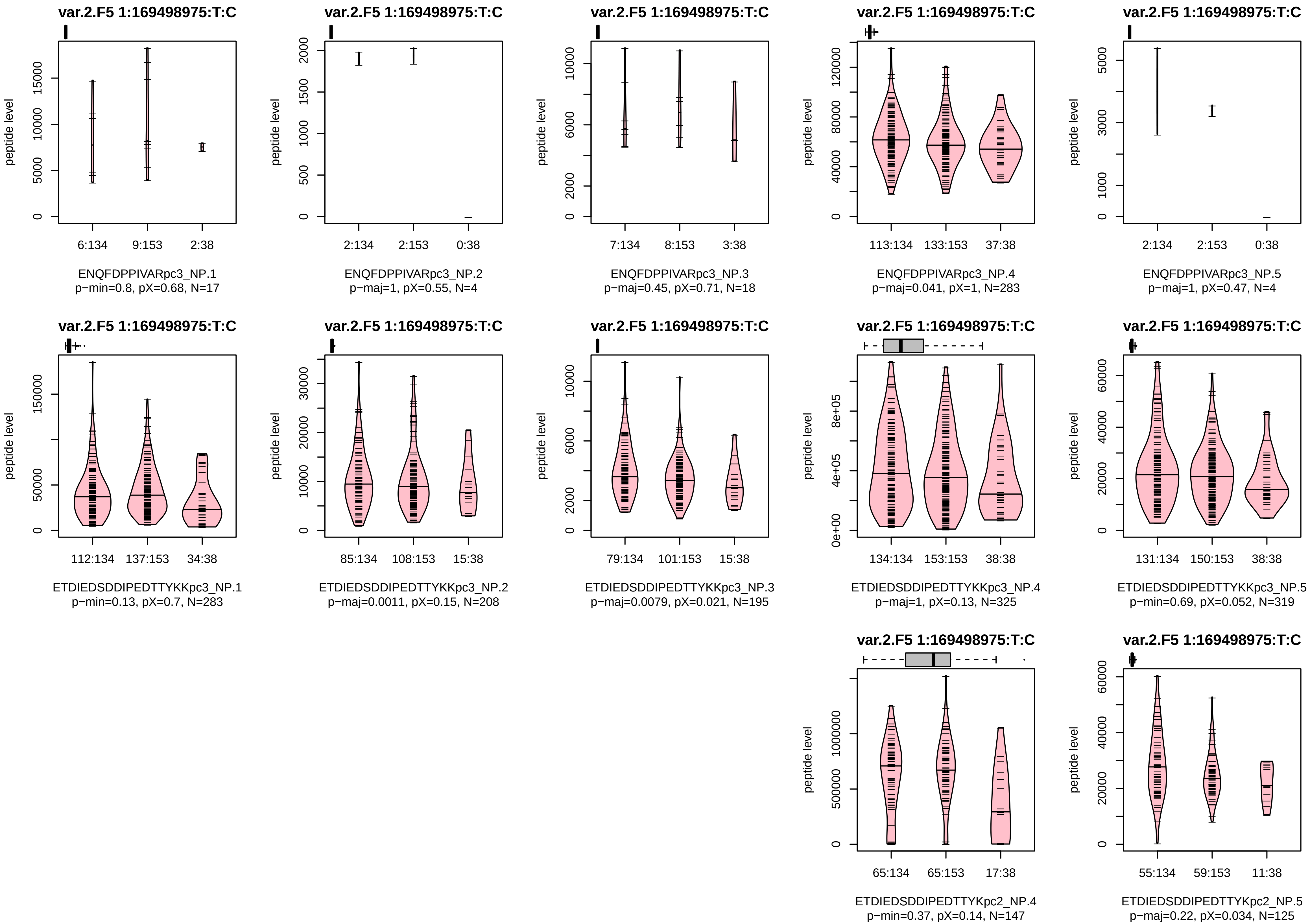

var.2.F5 1:169498975:T:C
peptide level
6:134
9:153
2:38
ENQFDPPIVARpc3_NP.1
p−min=0.8, pX=0.68, N=17
var.2.F5 1:169498975:T:C
peptide level
2:134
2:153
0:38
ENQFDPPIVARpc3_NP.2
p−maj=1, pX=0.55, N=4
var.2.F5 1:169498975:T:C
peptide level
7:134
8:153
3:38
ENQFDPPIVARpc3_NP.3
p−maj=0.45, pX=0.71, N=18
var.2.F5 1:169498975:T:C
peptide level
113:134
133:153
37:38
ENQFDPPIVARpc3_NP.4
p−maj=0.041, pX=1, N=283
var.2.F5 1:169498975:T:C
peptide level
2:134
2:153
0:38
ENQFDPPIVARpc3_NP.5
p−maj=1, pX=0.47, N=4
var.2.F5 1:169498975:T:C
peptide level
112:134
137:153
34:38
ETDIEDSDDIPEDTTYKKpc3_NP.1
p−min=0.13, pX=0.7, N=283
var.2.F5 1:169498975:T:C
peptide level
85:134
108:153
15:38
ETDIEDSDDIPEDTTYKKpc3_NP.2
p−maj=0.0011, pX=0.15, N=208
var.2.F5 1:169498975:T:C
peptide level
79:134
101:153
15:38
ETDIEDSDDIPEDTTYKKpc3_NP.3
p−maj=0.0079, pX=0.021, N=195
var.2.F5 1:169498975:T:C
peptide level
134:134
153:153
38:38
ETDIEDSDDIPEDTTYKKpc3_NP.4
p−maj=1, pX=0.13, N=325
var.2.F5 1:169498975:T:C
peptide level
131:134
150:153
38:38
ETDIEDSDDIPEDTTYKKpc3_NP.5
p−min=0.69, pX=0.052, N=319
var.2.F5 1:169498975:T:C
peptide level
65:134
65:153
17:38
ETDIEDSDDIPEDTTYKpc2_NP.4
p−min=0.37, pX=0.14, N=147
var.2.F5 1:169498975:T:C
peptide level
55:134
59:153
11:38
ETDIEDSDDIPEDTTYKpc2_NP.5
p−maj=0.22, pX=0.034, N=125

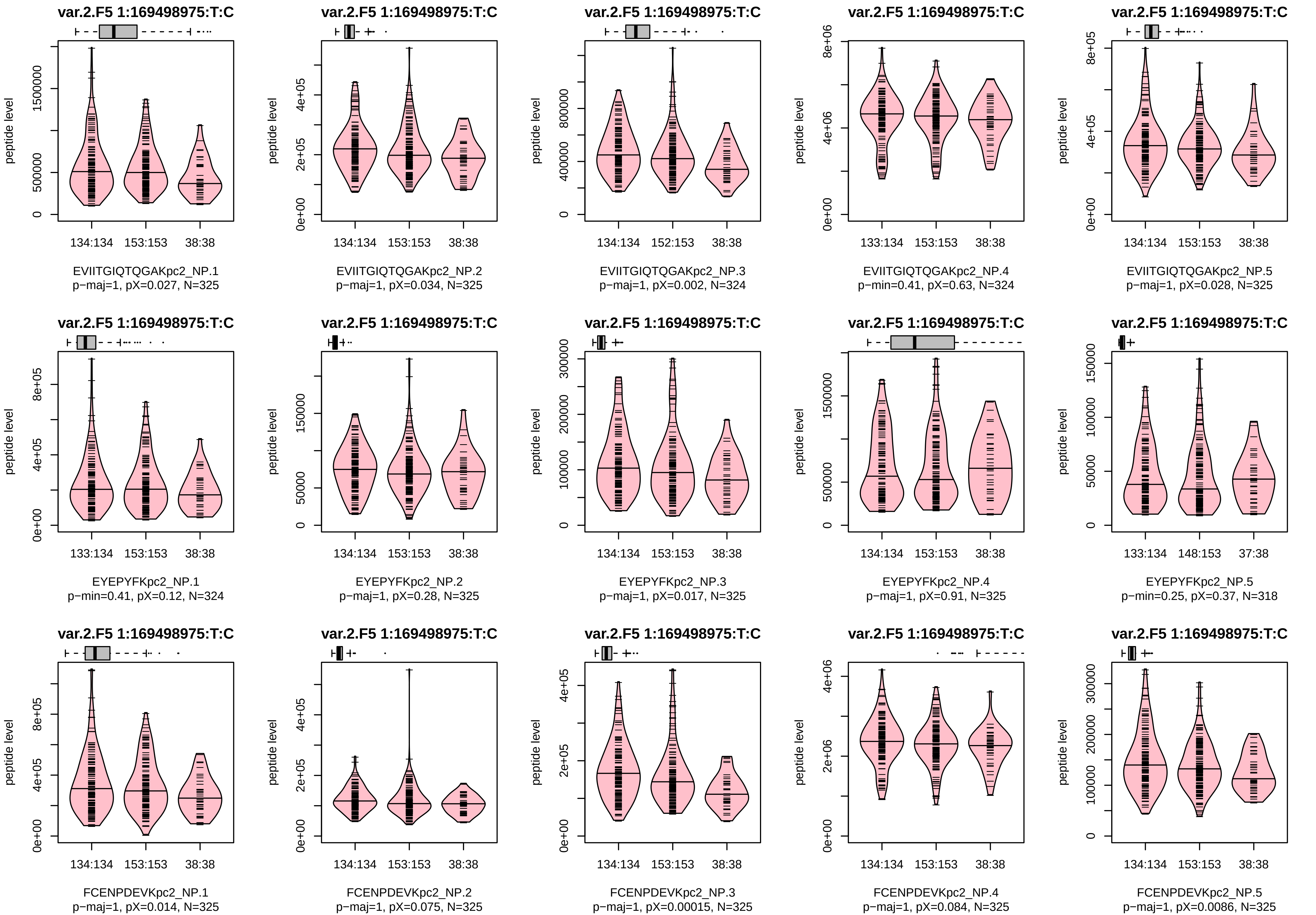
var.2.F5 1:169498975:T:C
peptide level
134:134 153:153 38:38
EVIITGIQTQGAKpc2_NP.1
p−maj=1, pX=0.027, N=325
var.2.F5 1:169498975:T:C
peptide level
134:134 153:153 38:38
EVIITGIQTQGAKpc2_NP.2
p−maj=1, pX=0.034, N=325
var.2.F5 1:169498975:T:C
peptide level
134:134 152:153 38:38
EVIITGIQTQGAKpc2_NP.3
p−maj=1, pX=0.002, N=324
var.2.F5 1:169498975:T:C
peptide level
133:134 153:153 38:38
EVIITGIQTQGAKpc2_NP.4
p−min=0.41, pX=0.63, N=324
var.2.F5 1:169498975:T:C
peptide level
134:134 153:153 38:38
EVIITGIQTQGAKpc2_NP.5
p−maj=1, pX=0.028, N=325
var.2.F5 1:169498975:T:C
peptide level
133:134 153:153 38:38
EYEPYFKpc2_NP.1
p−min=0.41, pX=0.12, N=324
var.2.F5 1:169498975:T:C
peptide level
134:134 153:153 38:38
EYEPYFKpc2_NP.2
p−maj=1, pX=0.28, N=325
var.2.F5 1:169498975:T:C
peptide level
134:134 153:153 38:38
EYEPYFKpc2_NP.3
p−maj=1, pX=0.017, N=325
var.2.F5 1:169498975:T:C
peptide level
134:134 153:153 38:38
EYEPYFKpc2_NP.4
p−maj=1, pX=0.91, N=325
var.2.F5 1:169498975:T:C
peptide level
133:134 148:153 37:38
EYEPYFKpc2_NP.5
p−min=0.25, pX=0.37, N=318
var.2.F5 1:169498975:T:C
peptide level
134:134 153:153 38:38
FCENPDEVKpc2_NP.1
p−maj=1, pX=0.014, N=325
var.2.F5 1:169498975:T:C
peptide level
134:134 153:153 38:38
FCENPDEVKpc2_NP.2
p−maj=1, pX=0.075, N=325
var.2.F5 1:169498975:T:C
peptide level
134:134 153:153 38:38
FCENPDEVKpc2_NP.3
p−maj=1, pX=0.00015, N=325
var.2.F5 1:169498975:T:C
peptide level
134:134 153:153 38:38
FCENPDEVKpc2_NP.4
p−maj=1, pX=0.084, N=325
var.2.F5 1:169498975:T:C
peptide level
134:134 153:153 38:38
FCENPDEVKpc2_NP.5
p−maj=1, pX=0.0086, N=325

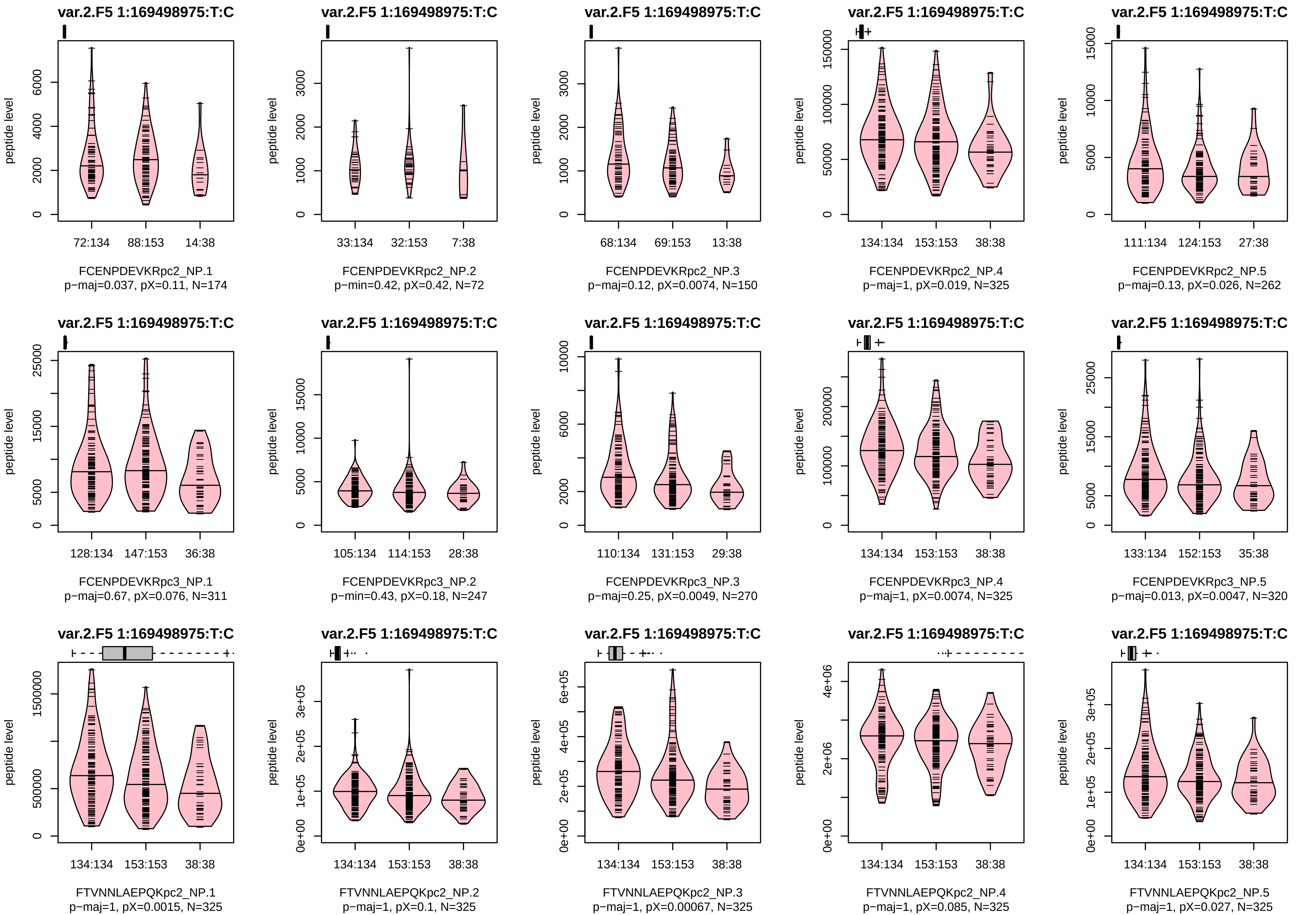
var.2.F5 1:169498975:T:C
peptide level
72:134
88:153
14:38
FCENPDEVKRpc2_NP.1
p−maj=0.037, pX=0.11, N=174
var.2.F5 1:169498975:T:C
peptide level
33:134
32:153
7:38
FCENPDEVKRpc2_NP.2
p−min=0.42, pX=0.42, N=72
var.2.F5 1:169498975:T:C
peptide level
68:134
69:153
13:38
FCENPDEVKRpc2_NP.3
p−maj=0.12, pX=0.0074, N=150
var.2.F5 1:169498975:T:C
peptide level
134:134
153:153
38:38
FCENPDEVKRpc2_NP.4
p−maj=1, pX=0.019, N=325
var.2.F5 1:169498975:T:C
peptide level
111:134
124:153
27:38
FCENPDEVKRpc2_NP.5
p−maj=0.13, pX=0.026, N=262
var.2.F5 1:169498975:T:C
peptide level
128:134
147:153
36:38
FCENPDEVKRpc3_NP.1
p−maj=0.67, pX=0.076, N=311
var.2.F5 1:169498975:T:C
peptide level
105:134
114:153
28:38
FCENPDEVKRpc3_NP.2
p−min=0.43, pX=0.18, N=247
var.2.F5 1:169498975:T:C
peptide level
110:134
131:153
29:38
FCENPDEVKRpc3_NP.3
p−maj=0.25, pX=0.0049, N=270
var.2.F5 1:169498975:T:C
peptide level
134:134
153:153
38:38
FCENPDEVKRpc3_NP.4
p−maj=1, pX=0.0074, N=325
var.2.F5 1:169498975:T:C
peptide level
133:134
152:153
35:38
FCENPDEVKRpc3_NP.5
p−maj=0.013, pX=0.0047, N=320
var.2.F5 1:169498975:T:C
peptide level
134:134
153:153
38:38
FTVNNLAEPQKpc2_NP.1
p−maj=1, pX=0.0015, N=325
var.2.F5 1:169498975:T:C
peptide level
134:134
153:153
38:38
FTVNNLAEPQKpc2_NP.2
p−maj=1, pX=0.1, N=325
var.2.F5 1:169498975:T:C
peptide level
134:134
153:153
38:38
FTVNNLAEPQKpc2_NP.3
p−maj=1, pX=0.00067, N=325
var.2.F5 1:169498975:T:C
peptide level
134:134
153:153
38:38
FTVNNLAEPQKpc2_NP.4
p−maj=1, pX=0.085, N=325
var.2.F5 1:169498975:T:C
peptide level
134:134
153:153
38:38
FTVNNLAEPQKpc2_NP.5
p−maj=1, pX=0.027, N=325

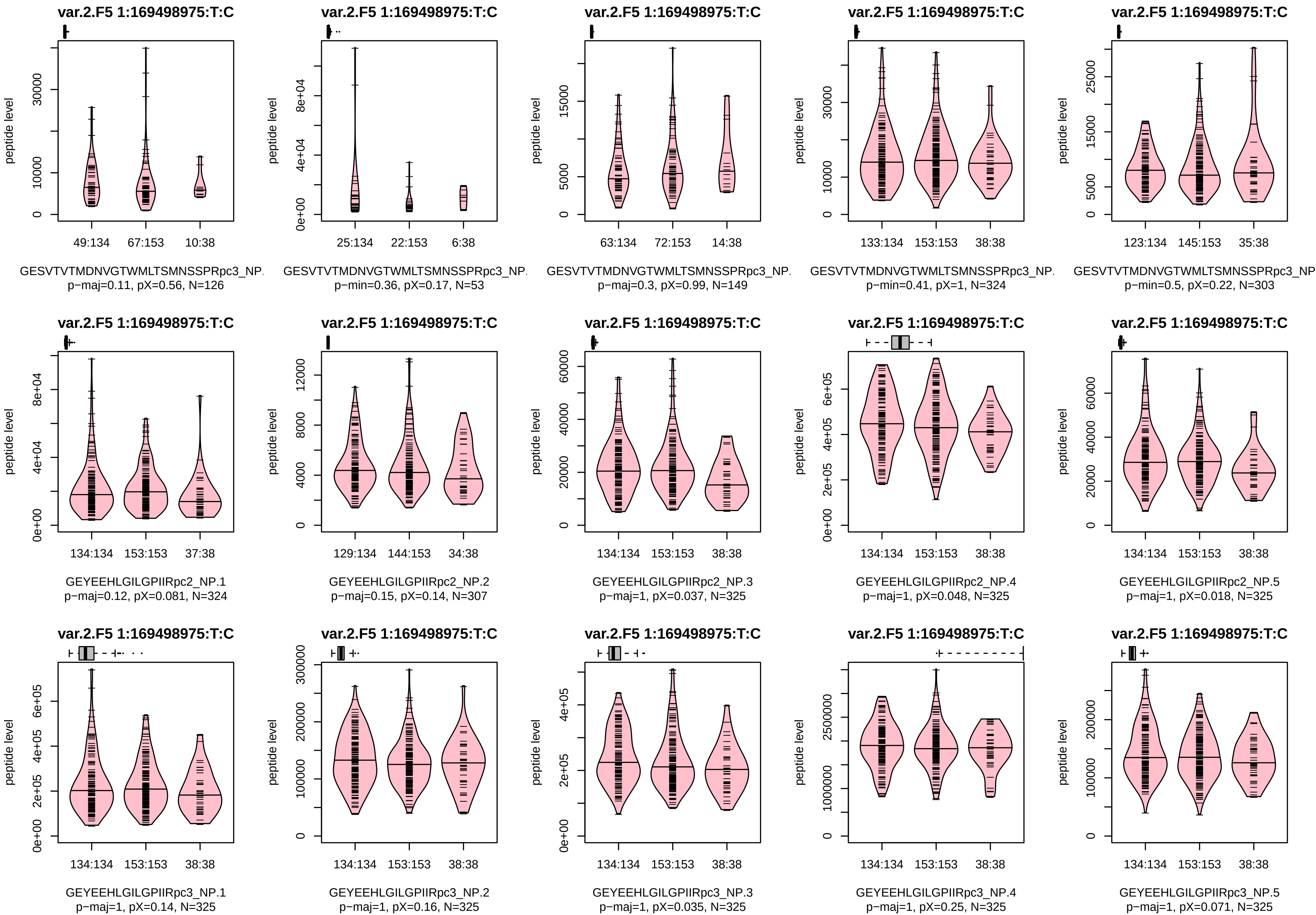
var.2.F5 1:169498975:T:C
peptide level
0
10000
20000
30000
49:134
67:153
10:38
GESVTVTMDNVGTWMLTSMNSSPRpc3_NP.
p−maj=0.11, pX=0.56, N=126
var.2.F5 1:169498975:T:C
peptide level
0e+00
4e+04
8e+04
25:134
22:153
6:38
GESVTVTMDNVGTWMLTSMNSSPRpc3_NP.
p−min=0.36, pX=0.17, N=53
var.2.F5 1:169498975:T:C
peptide level
0
5000
15000
63:134
72:153
14:38
GESVTVTMDNVGTWMLTSMNSSPRpc3_NP.
p−maj=0.3, pX=0.99, N=149
var.2.F5 1:169498975:T:C
peptide level
0
10000
20000
30000
133:134
153:153
38:38
GESVTVTMDNVGTWMLTSMNSSPRpc3_NP.
p−min=0.41, pX=1, N=324
var.2.F5 1:169498975:T:C
peptide level
0
5000
15000
25000
123:134
145:153
35:38
GESVTVTMDNVGTWMLTSMNSSPRpc3_NP
p−min=0.5, pX=0.22, N=303
var.2.F5 1:169498975:T:C
peptide level
0e+00
4e+04
8e+04
134:134
153:153
37:38
GEYEEHLGILGPIIRpc2_NP.1
p−maj=0.12, pX=0.081, N=324
var.2.F5 1:169498975:T:C
peptide level
0
4000
8000
12000
129:134
144:153
34:38
GEYEEHLGILGPIIRpc2_NP.2
p−maj=0.15, pX=0.14, N=307
var.2.F5 1:169498975:T:C
peptide level
0
20000
40000
60000
134:134
153:153
38:38
GEYEEHLGILGPIIRpc2_NP.3
p−maj=1, pX=0.037, N=325
var.2.F5 1:169498975:T:C
peptide level
0e+00
2e+05
4e+05
6e+05
134:134
153:153
38:38
GEYEEHLGILGPIIRpc2_NP.4
p−maj=1, pX=0.048, N=325
var.2.F5 1:169498975:T:C
peptide level
0
20000
40000
60000
134:134
153:153
38:38
GEYEEHLGILGPIIRpc2_NP.5
p−maj=1, pX=0.018, N=325
var.2.F5 1:169498975:T:C
peptide level
0e+00
2e+05
4e+05
6e+05
134:134
153:153
38:38
GEYEEHLGILGPIIRpc3_NP.1
p−maj=1, pX=0.14, N=325
var.2.F5 1:169498975:T:C
peptide level
0
100000
200000
300000
134:134
153:153
38:38
GEYEEHLGILGPIIRpc3_NP.2
p−maj=1, pX=0.16, N=325
var.2.F5 1:169498975:T:C
peptide level
0e+00
2e+05
4e+05
134:134
153:153
38:38
GEYEEHLGILGPIIRpc3_NP.3
p−maj=1, pX=0.035, N=325
var.2.F5 1:169498975:T:C
peptide level
0
1000000
2500000
134:134
153:153
38:38
GEYEEHLGILGPIIRpc3_NP.4
p−maj=1, pX=0.25, N=325
var.2.F5 1:169498975:T:C
peptide level
0
100000
200000
134:134
153:153
38:38
GEYEEHLGILGPIIRpc3_NP.5
p−maj=1, pX=0.071, N=325

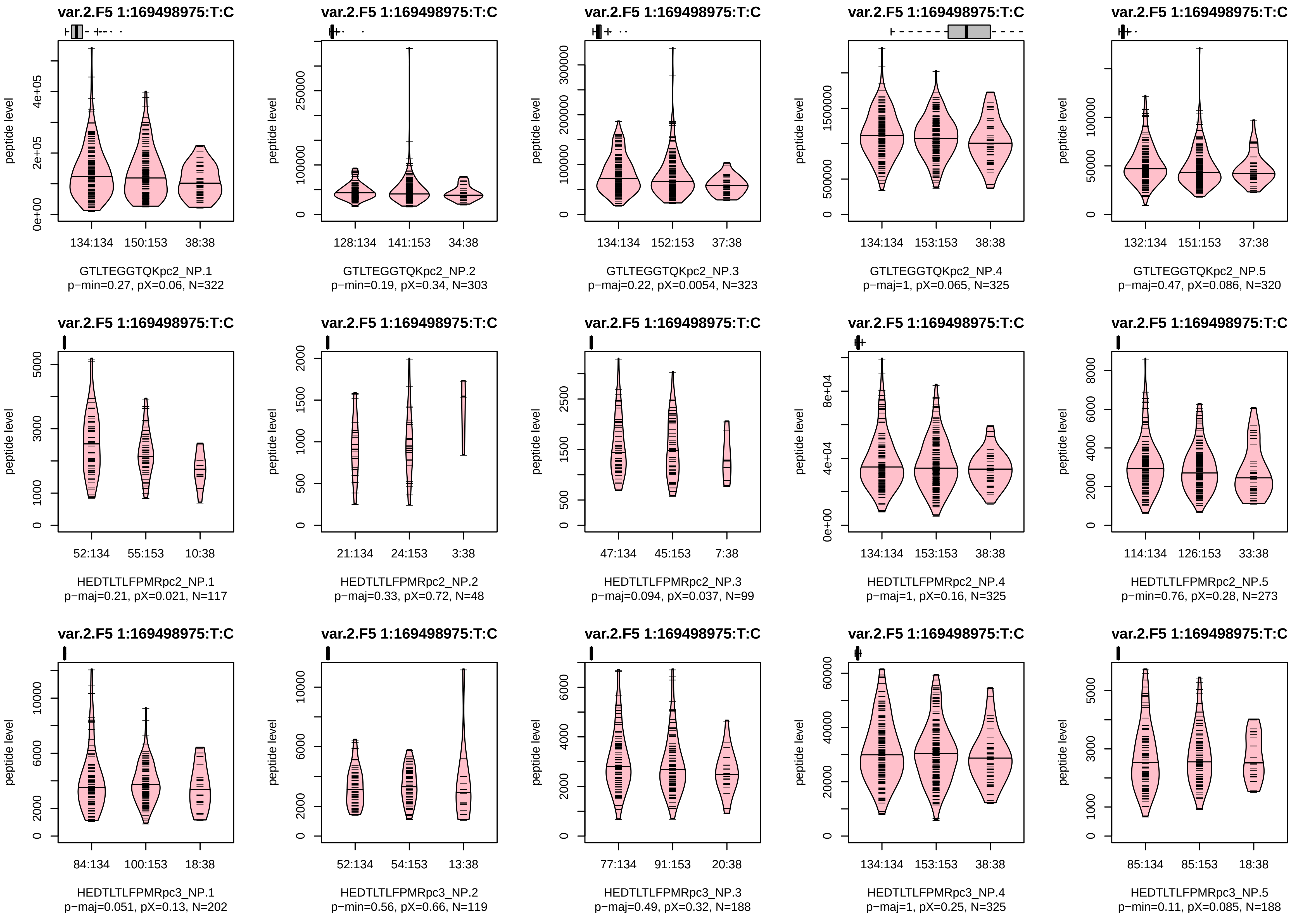
var.2.F5 1:169498975:T:C
peptide level
0e+00 2e+05 4e+05
134:134 150:153 38:38
GTLTEGGTQKpc2_NP.1
p−min=0.27, pX=0.06, N=322
var.2.F5 1:169498975:T:C
peptide level
0 100000 250000
128:134 141:153 34:38
GTLTEGGTQKpc2_NP.2
p−min=0.19, pX=0.34, N=303
var.2.F5 1:169498975:T:C
peptide level
0 100000 200000 300000
134:134 152:153 37:38
GTLTEGGTQKpc2_NP.3
p−maj=0.22, pX=0.0054, N=323
var.2.F5 1:169498975:T:C
peptide level
0 500000 1500000
134:134 153:153 38:38
GTLTEGGTQKpc2_NP.4
p−maj=1, pX=0.065, N=325
var.2.F5 1:169498975:T:C
peptide level
0 50000 100000
132:134 151:153 37:38
GTLTEGGTQKpc2_NP.5
p−maj=0.47, pX=0.086, N=320
var.2.F5 1:169498975:T:C
peptide level
0 1000 3000 5000
52:134 55:153 10:38
HEDTLTLFPMRpc2_NP.1
p−maj=0.21, pX=0.021, N=117
var.2.F5 1:169498975:T:C
peptide level
0 500 1000 1500 2000
21:134 24:153 3:38
HEDTLTLFPMRpc2_NP.2
p−maj=0.33, pX=0.72, N=48
var.2.F5 1:169498975:T:C
peptide level
0 500 1500 2500
47:134 45:153 7:38
HEDTLTLFPMRpc2_NP.3
p−maj=0.094, pX=0.037, N=99
var.2.F5 1:169498975:T:C
peptide level
0e+00 4e+04 8e+04
134:134 153:153 38:38
HEDTLTLFPMRpc2_NP.4
p−maj=1, pX=0.16, N=325
var.2.F5 1:169498975:T:C
peptide level
0 2000 4000 6000 8000
114:134 126:153 33:38
HEDTLTLFPMRpc2_NP.5
p−min=0.76, pX=0.28, N=273
var.2.F5 1:169498975:T:C
peptide level
0 2000 6000 10000
84:134 100:153 18:38
HEDTLTLFPMRpc3_NP.1
p−maj=0.051, pX=0.13, N=202
var.2.F5 1:169498975:T:C
peptide level
0 2000 6000 10000
52:134 54:153 13:38
HEDTLTLFPMRpc3_NP.2
p−min=0.56, pX=0.66, N=119
var.2.F5 1:169498975:T:C
peptide level
0 2000 4000 6000
77:134 91:153 20:38
HEDTLTLFPMRpc3_NP.3
p−maj=0.49, pX=0.32, N=188
var.2.F5 1:169498975:T:C
peptide level
0 20000 40000 60000
134:134 153:153 38:38
HEDTLTLFPMRpc3_NP.4
p−maj=1, pX=0.25, N=325
var.2.F5 1:169498975:T:C
peptide level
0 1000 3000 5000
85:134 85:153 18:38
HEDTLTLFPMRpc3_NP.5
p−min=0.11, pX=0.085, N=188

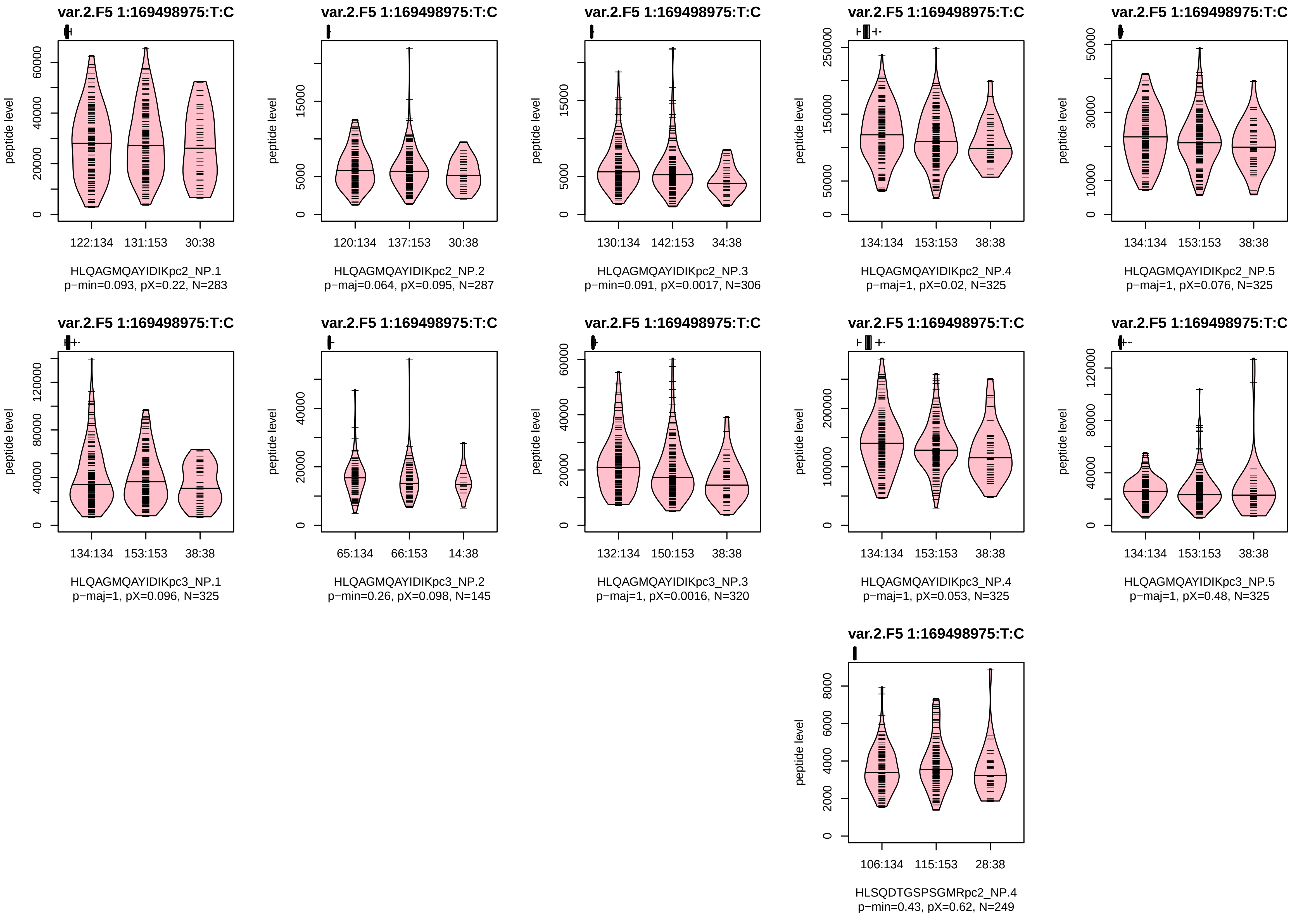
var.2.F5 1:169498975:T:C
peptide level
122:134
131:153
30:38
HLQAGMQAYIDIKpc2_NP.1
p−min=0.093, pX=0.22, N=283
var.2.F5 1:169498975:T:C
peptide level
120:134
137:153
30:38
HLQAGMQAYIDIKpc2_NP.2
p−maj=0.064, pX=0.095, N=287
var.2.F5 1:169498975:T:C
peptide level
130:134
142:153
34:38
HLQAGMQAYIDIKpc2_NP.3
p−min=0.091, pX=0.0017, N=306
var.2.F5 1:169498975:T:C
peptide level
134:134
153:153
38:38
HLQAGMQAYIDIKpc2_NP.4
p−maj=1, pX=0.02, N=325
var.2.F5 1:169498975:T:C
peptide level
134:134
153:153
38:38
HLQAGMQAYIDIKpc2_NP.5
p−maj=1, pX=0.076, N=325
var.2.F5 1:169498975:T:C
peptide level
134:134
153:153
38:38
HLQAGMQAYIDIKpc3_NP.1
p−maj=1, pX=0.096, N=325
var.2.F5 1:169498975:T:C
peptide level
65:134
66:153
14:38
HLQAGMQAYIDIKpc3_NP.2
p−min=0.26, pX=0.098, N=145
var.2.F5 1:169498975:T:C
peptide level
132:134
150:153
38:38
HLQAGMQAYIDIKpc3_NP.3
p−maj=1, pX=0.0016, N=320
var.2.F5 1:169498975:T:C
peptide level
134:134
153:153
38:38
HLQAGMQAYIDIKpc3_NP.4
p−maj=1, pX=0.053, N=325
var.2.F5 1:169498975:T:C
peptide level
134:134
153:153
38:38
HLQAGMQAYIDIKpc3_NP.5
p−maj=1, pX=0.48, N=325
var.2.F5 1:169498975:T:C
peptide level
106:134
115:153
28:38
HLSQDTGSPSGMRpc2_NP.4
p−min=0.43, pX=0.62, N=249

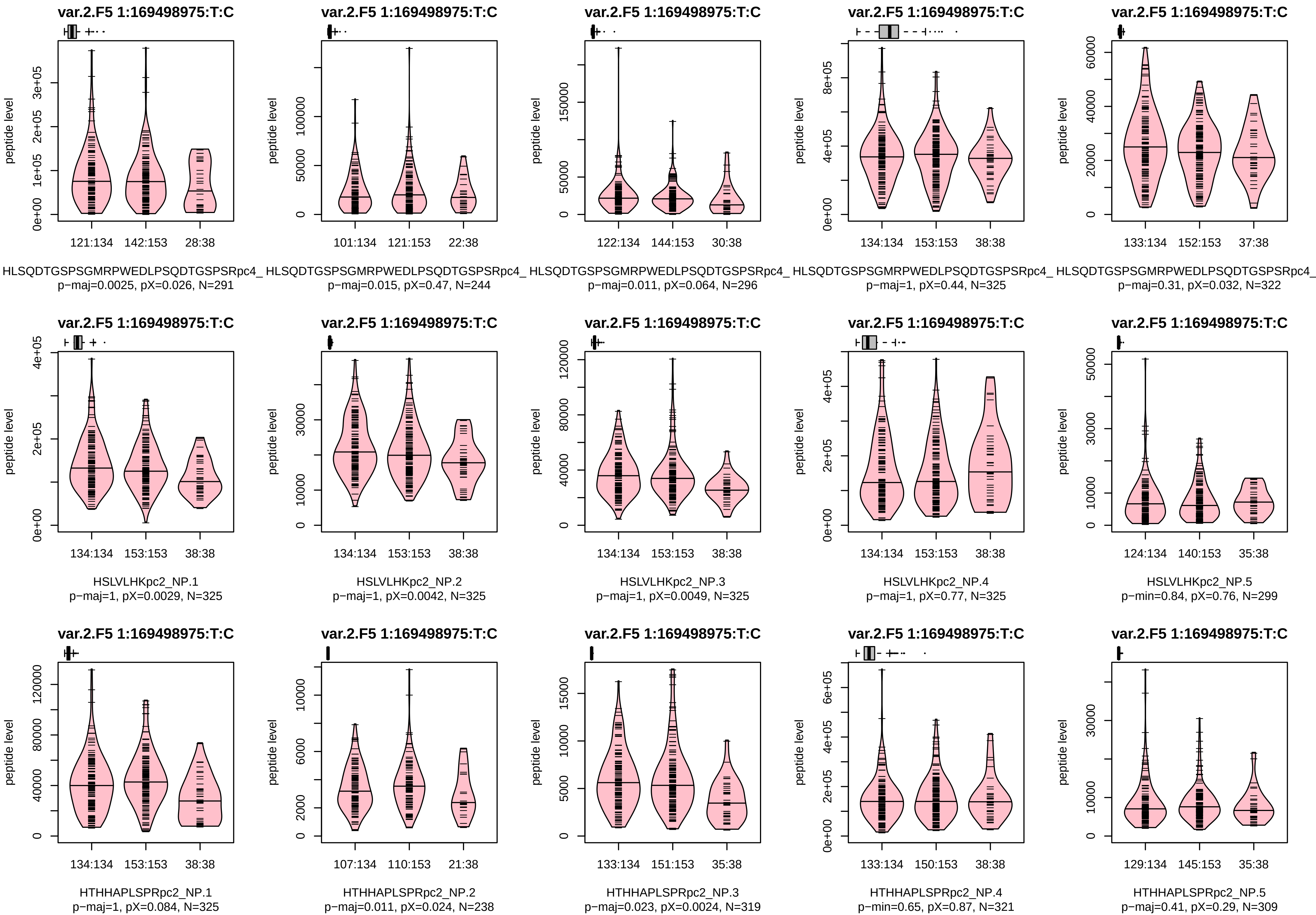

var.2.F5 1:169498975:T:C
peptide level
121:134
142:153
28:38
HLSQDTGSPSGMRPWEDLPSQDTGSPSRpc4_
p−maj=0.0025, pX=0.026, N=291
var.2.F5 1:169498975:T:C
peptide level
101:134
121:153
22:38
HLSQDTGSPSGMRPWEDLPSQDTGSPSRpc4_
p−maj=0.015, pX=0.47, N=244
var.2.F5 1:169498975:T:C
peptide level
122:134
144:153
30:38
HLSQDTGSPSGMRPWEDLPSQDTGSPSRpc4_
p−maj=0.011, pX=0.064, N=296
var.2.F5 1:169498975:T:C
peptide level
134:134
153:153
38:38
HLSQDTGSPSGMRPWEDLPSQDTGSPSRpc4_
p−maj=1, pX=0.44, N=325
var.2.F5 1:169498975:T:C
peptide level
133:134
152:153
37:38
HLSQDTGSPSGMRPWEDLPSQDTGSPSRpc4_
p−maj=0.31, pX=0.032, N=322
var.2.F5 1:169498975:T:C
peptide level
134:134
153:153
38:38
HSLVLHKpc2_NP.1
p−maj=1, pX=0.0029, N=325
var.2.F5 1:169498975:T:C
peptide level
134:134
153:153
38:38
HSLVLHKpc2_NP.2
p−maj=1, pX=0.0042, N=325
var.2.F5 1:169498975:T:C
peptide level
134:134
153:153
38:38
HSLVLHKpc2_NP.3
p−maj=1, pX=0.0049, N=325
var.2.F5 1:169498975:T:C
peptide level
134:134
153:153
38:38
HSLVLHKpc2_NP.4
p−maj=1, pX=0.77, N=325
var.2.F5 1:169498975:T:C
peptide level
124:134
140:153
35:38
HSLVLHKpc2_NP.5
p−min=0.84, pX=0.76, N=299
var.2.F5 1:169498975:T:C
peptide level
134:134
153:153
38:38
HTHHAPLSPRpc2_NP.1
p−maj=1, pX=0.084, N=325
var.2.F5 1:169498975:T:C
peptide level
107:134
110:153
21:38
HTHHAPLSPRpc2_NP.2
p−maj=0.011, pX=0.024, N=238
var.2.F5 1:169498975:T:C
peptide level
133:134
151:153
35:38
HTHHAPLSPRpc2_NP.3
p−maj=0.023, pX=0.0024, N=319
var.2.F5 1:169498975:T:C
peptide level
133:134
150:153
38:38
HTHHAPLSPRpc2_NP.4
p−min=0.65, pX=0.87, N=321
var.2.F5 1:169498975:T:C
peptide level
129:134
145:153
35:38
HTHHAPLSPRpc2_NP.5
p−maj=0.41, pX=0.29, N=309

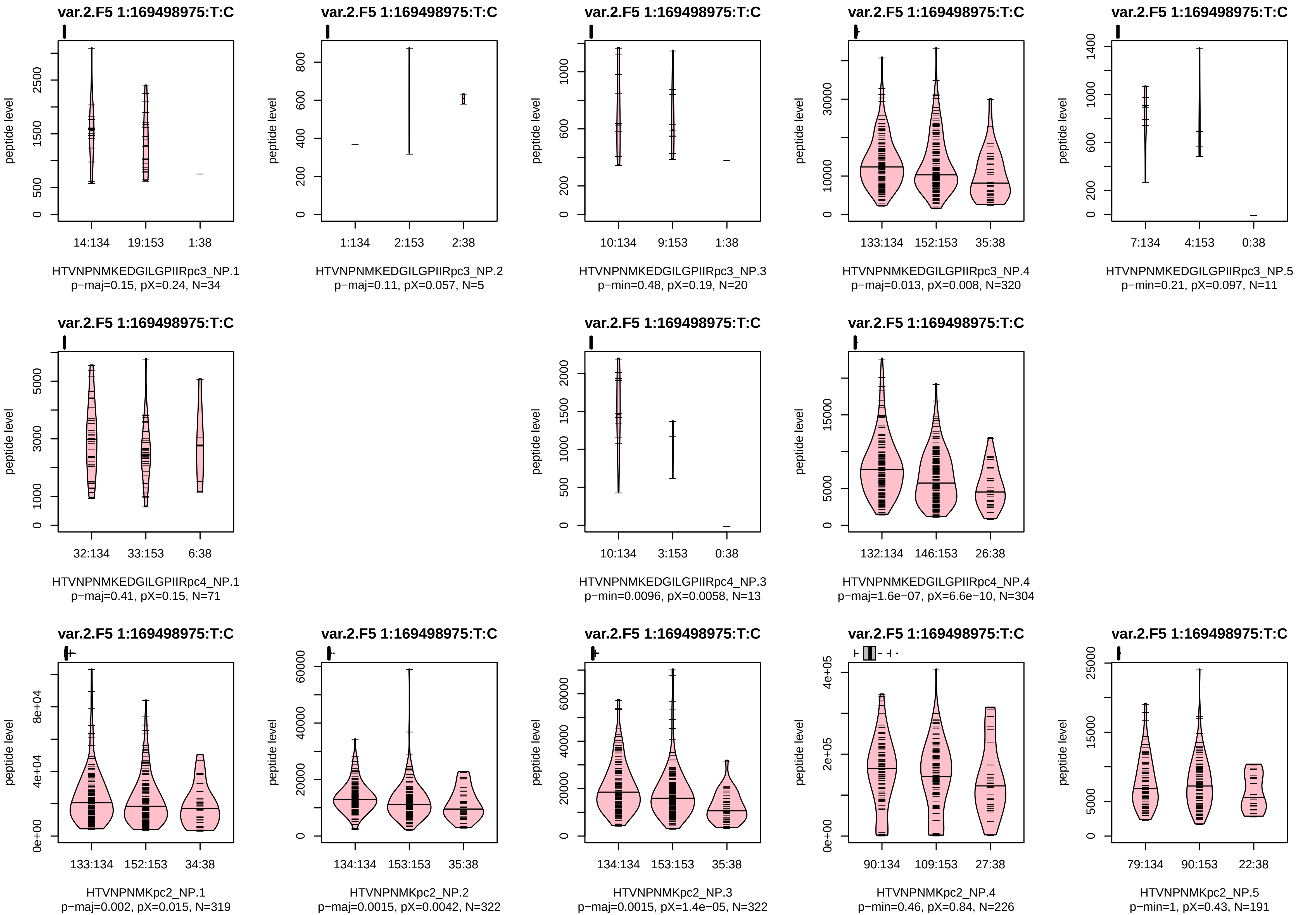

var.2.F5 1:169498975:T:C
peptide level
14:134
19:153
1:38
HTVNPNMKEDGILGPIIRpc3_NP.1
p−maj=0.15, pX=0.24, N=34
var.2.F5 1:169498975:T:C
peptide level
1:134
2:153
2:38
HTVNPNMKEDGILGPIIRpc3_NP.2
p−maj=0.11, pX=0.057, N=5
var.2.F5 1:169498975:T:C
peptide level
10:134
9:153
1:38
HTVNPNMKEDGILGPIIRpc3_NP.3
p−min=0.48, pX=0.19, N=20
var.2.F5 1:169498975:T:C
peptide level
133:134
152:153
35:38
HTVNPNMKEDGILGPIIRpc3_NP.4
p−maj=0.013, pX=0.008, N=320
var.2.F5 1:169498975:T:C
peptide level
7:134
4:153
0:38
HTVNPNMKEDGILGPIIRpc3_NP.5
p−min=0.21, pX=0.097, N=11
var.2.F5 1:169498975:T:C
peptide level
32:134
33:153
6:38
HTVNPNMKEDGILGPIIRpc4_NP.1
p−maj=0.41, pX=0.15, N=71
var.2.F5 1:169498975:T:C
peptide level
10:134
3:153
0:38
HTVNPNMKEDGILGPIIRpc4_NP.3
p−min=0.0096, pX=0.0058, N=13
var.2.F5 1:169498975:T:C
peptide level
132:134
146:153
26:38
HTVNPNMKEDGILGPIIRpc4_NP.4
p−maj=1.6e−07, pX=6.6e−10, N=304
var.2.F5 1:169498975:T:C
peptide level
133:134
152:153
34:38
HTVNPNMKpc2_NP.1
p−maj=0.002, pX=0.015, N=319
var.2.F5 1:169498975:T:C
peptide level
134:134
153:153
35:38
HTVNPNMKpc2_NP.2
p−maj=0.0015, pX=0.0042, N=322
var.2.F5 1:169498975:T:C
peptide level
134:134
153:153
35:38
HTVNPNMKpc2_NP.3
p−maj=0.0015, pX=1.4e−05, N=322
var.2.F5 1:169498975:T:C
peptide level
90:134
109:153
27:38
HTVNPNMKpc2_NP.4
p−min=0.46, pX=0.84, N=226
var.2.F5 1:169498975:T:C
peptide level
79:134
90:153
22:38
HTVNPNMKpc2_NP.5
p−min=1, pX=0.43, N=191

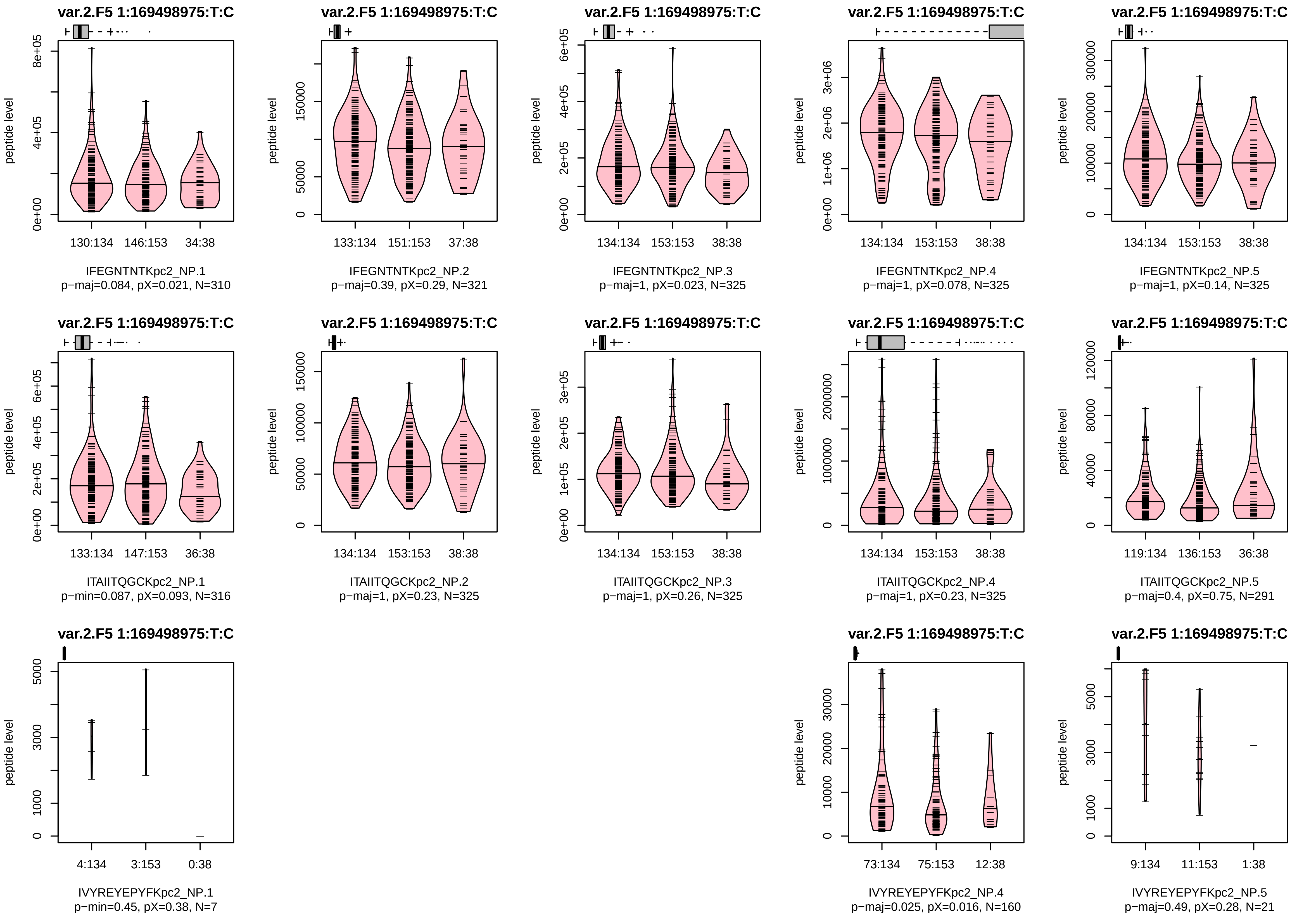

var.2.F5 1:169498975:T:C
peptide level
130:134
146:153
34:38
IFEGNTNTKpc2_NP.1
p−maj=0.084, pX=0.021, N=310
var.2.F5 1:169498975:T:C
peptide level
133:134
151:153
37:38
IFEGNTNTKpc2_NP.2
p−maj=0.39, pX=0.29, N=321
var.2.F5 1:169498975:T:C
peptide level
134:134
153:153
38:38
IFEGNTNTKpc2_NP.3
p−maj=1, pX=0.023, N=325
var.2.F5 1:169498975:T:C
peptide level
134:134
153:153
38:38
IFEGNTNTKpc2_NP.4
p−maj=1, pX=0.078, N=325
var.2.F5 1:169498975:T:C
peptide level
134:134
153:153
38:38
IFEGNTNTKpc2_NP.5
p−maj=1, pX=0.14, N=325
var.2.F5 1:169498975:T:C
peptide level
133:134
147:153
36:38
ITAIITQGCKpc2_NP.1
p−min=0.087, pX=0.093, N=316
var.2.F5 1:169498975:T:C
peptide level
134:134
153:153
38:38
ITAIITQGCKpc2_NP.2
p−maj=1, pX=0.23, N=325
var.2.F5 1:169498975:T:C
peptide level
134:134
153:153
38:38
ITAIITQGCKpc2_NP.3
p−maj=1, pX=0.26, N=325
var.2.F5 1:169498975:T:C
peptide level
134:134
153:153
38:38
ITAIITQGCKpc2_NP.4
p−maj=1, pX=0.23, N=325
var.2.F5 1:169498975:T:C
peptide level
119:134
136:153
36:38
ITAIITQGCKpc2_NP.5
p−maj=0.4, pX=0.75, N=291
var.2.F5 1:169498975:T:C
peptide level
4:134
3:153
0:38
IVYREYEPYFKpc2_NP.1
p−min=0.45, pX=0.38, N=7
var.2.F5 1:169498975:T:C
peptide level
73:134
75:153
12:38
IVYREYEPYFKpc2_NP.4
p−maj=0.025, pX=0.016, N=160
var.2.F5 1:169498975:T:C
peptide level
9:134
11:153
1:38
IVYREYEPYFKpc2_NP.5
p−maj=0.49, pX=0.28, N=21

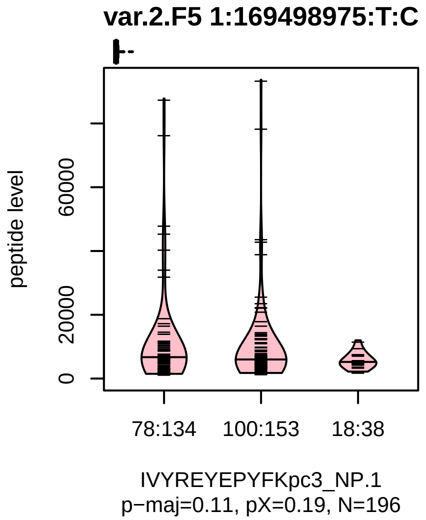
var.2.F5 1:169498975:T:C
peptide level
0
20000
60000
78:134
100:153
18:38
IVYREYEPYFKpc3_NP.1
p−maj=0.11, pX=0.19, N=196

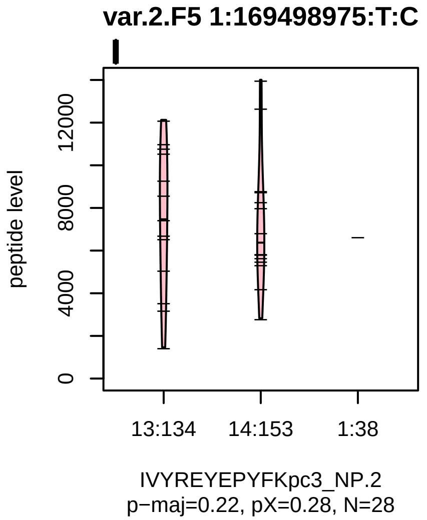
var.2.F5 1:169498975:T:C
peptide level
0
4000
8000
12000
13:134
14:153
1:38
IVYREYEPYFKpc3_NP.2
p−maj=0.22, pX=0.28, N=28

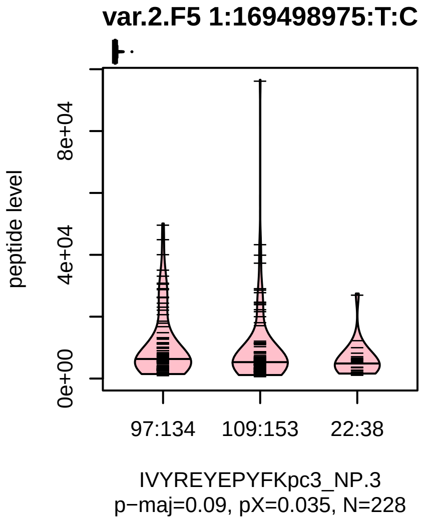
var.2.F5 1:169498975:T:C
peptide level
0e+00
4e+04
8e+04
97:134
109:153
22:38
IVYREYEPYFKpc3_NP.3
p−maj=0.09, pX=0.035, N=228

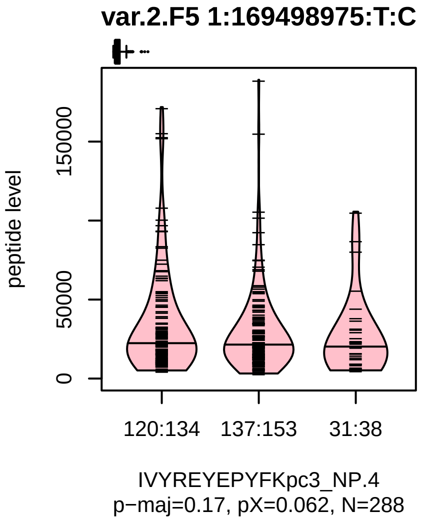
var.2.F5 1:169498975:T:C
peptide level
0
50000
150000
120:134
137:153
31:38
IVYREYEPYFKpc3_NP.4
p−maj=0.17, pX=0.062, N=288

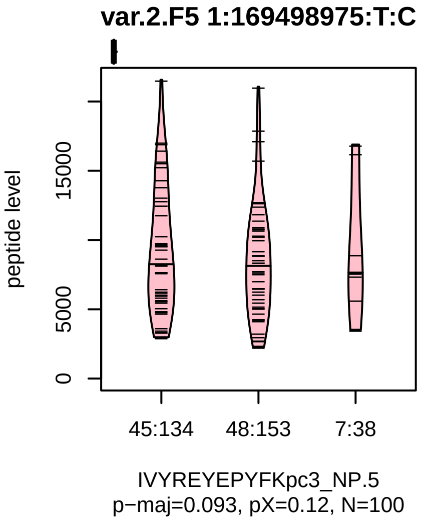
var.2.F5 1:169498975:T:C
peptide level
0
5000
15000
45:134
48:153
7:38
IVYREYEPYFKpc3_NP.5
p−maj=0.093, pX=0.12, N=100

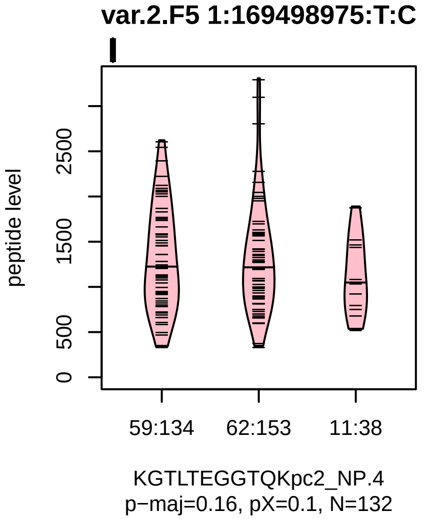
var.2.F5 1:169498975:T:C
peptide level
0
500
1500
2500
59:134
62:153
11:38
KGTLTEGGTQKpc2_NP.4
p−maj=0.16, pX=0.1, N=132

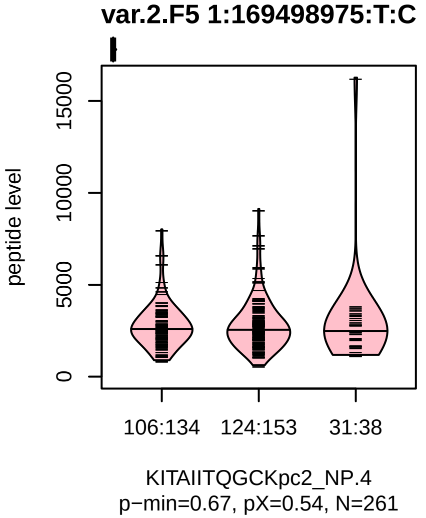
var.2.F5 1:169498975:T:C
peptide level
0
5000
10000
15000
106:134
124:153
31:38
KITAIITQGCKpc2_NP.4
p−min=0.67, pX=0.54, N=261

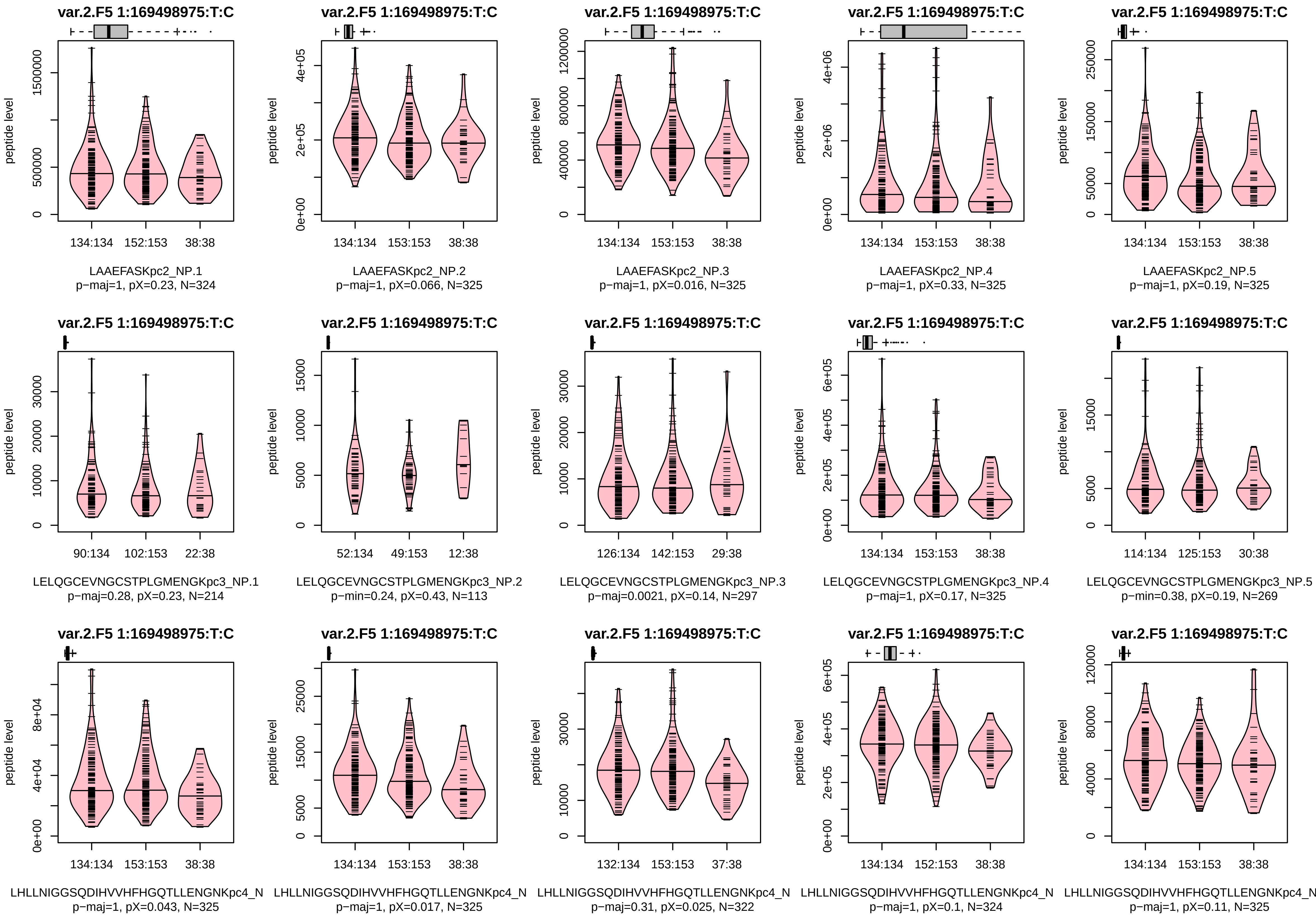

var.2.F5 1:169498975:T:C
peptide level
134:134 152:153 38:38
LAAEFASKpc2_NP.1
p−maj=1, pX=0.23, N=324
var.2.F5 1:169498975:T:C
peptide level
134:134 153:153 38:38
LAAEFASKpc2_NP.2
p−maj=1, pX=0.066, N=325
var.2.F5 1:169498975:T:C
peptide level
134:134 153:153 38:38
LAAEFASKpc2_NP.3
p−maj=1, pX=0.016, N=325
var.2.F5 1:169498975:T:C
peptide level
134:134 153:153 38:38
LAAEFASKpc2_NP.4
p−maj=1, pX=0.33, N=325
var.2.F5 1:169498975:T:C
peptide level
134:134 153:153 38:38
LAAEFASKpc2_NP.5
p−maj=1, pX=0.19, N=325
var.2.F5 1:169498975:T:C
peptide level
90:134 102:153 22:38
LELQGCEVNGCSTPLGMENGKpc3_NP.1
p−maj=0.28, pX=0.23, N=214
var.2.F5 1:169498975:T:C
peptide level
52:134 49:153 12:38
LELQGCEVNGCSTPLGMENGKpc3_NP.2
p−min=0.24, pX=0.43, N=113
var.2.F5 1:169498975:T:C
peptide level
126:134 142:153 29:38
LELQGCEVNGCSTPLGMENGKpc3_NP.3
p−maj=0.0021, pX=0.14, N=297
var.2.F5 1:169498975:T:C
peptide level
134:134 153:153 38:38
LELQGCEVNGCSTPLGMENGKpc3_NP.4
p−maj=1, pX=0.17, N=325
var.2.F5 1:169498975:T:C
peptide level
114:134 125:153 30:38
LELQGCEVNGCSTPLGMENGKpc3_NP.5
p−min=0.38, pX=0.19, N=269
var.2.F5 1:169498975:T:C
peptide level
134:134 153:153 38:38
LHLLNIGGSQDIHVVHFHGQTLLENGNKpc4_N
p−maj=1, pX=0.043, N=325
var.2.F5 1:169498975:T:C
peptide level
134:134 153:153 38:38
LHLLNIGGSQDIHVVHFHGQTLLENGNKpc4_N
p−maj=1, pX=0.017, N=325
var.2.F5 1:169498975:T:C
peptide level
132:134 153:153 37:38
LHLLNIGGSQDIHVVHFHGQTLLENGNKpc4_N
p−maj=0.31, pX=0.025, N=322
var.2.F5 1:169498975:T:C
peptide level
134:134 152:153 38:38
LHLLNIGGSQDIHVVHFHGQTLLENGNKpc4_N
p−maj=1, pX=0.1, N=324
var.2.F5 1:169498975:T:C
peptide level
134:134 153:153 38:38
LHLLNIGGSQDIHVVHFHGQTLLENGNKpc4_N
p−maj=1, pX=0.11, N=325

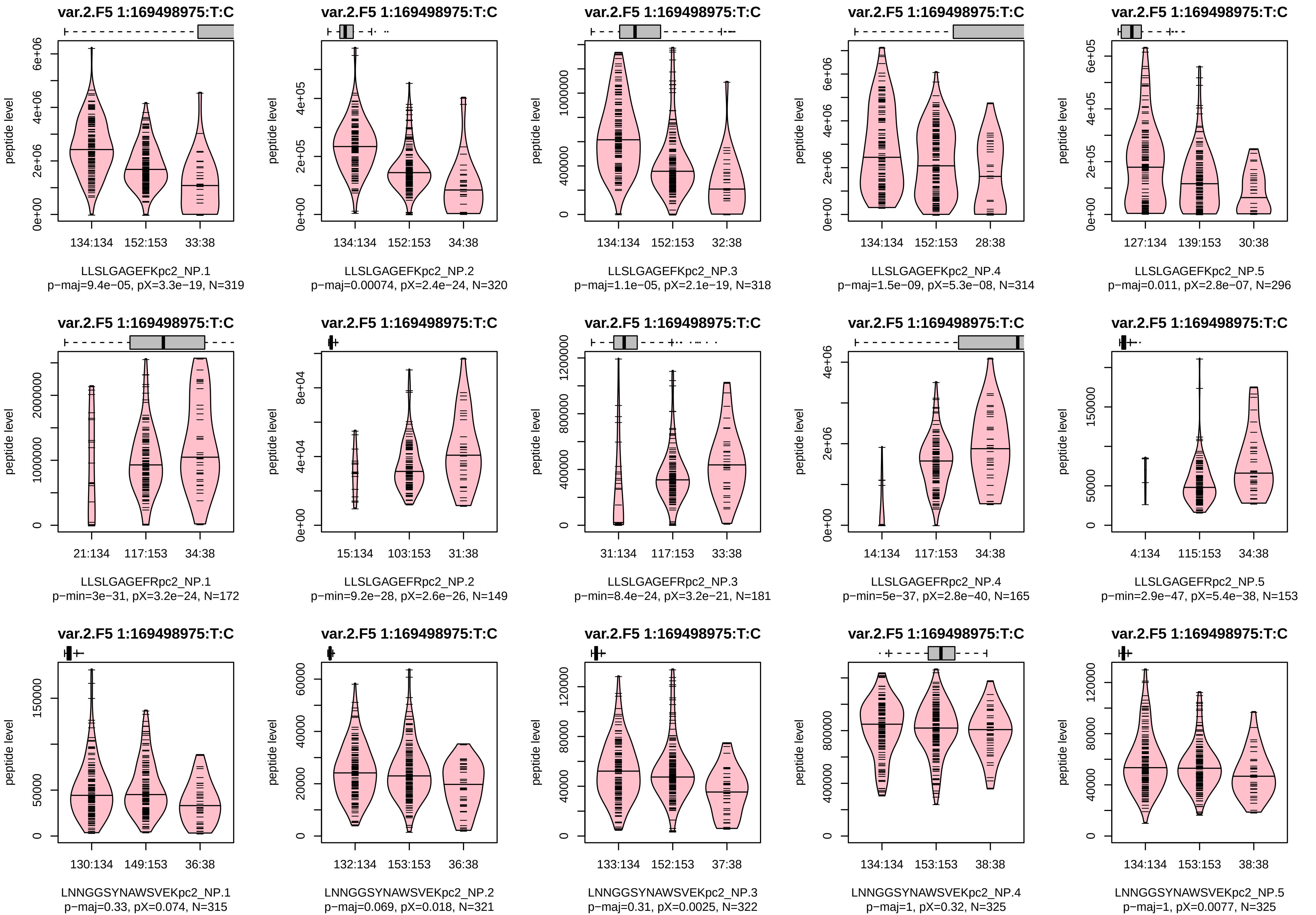
var.2.F5 1:169498975:T:C
peptide level
134:134
152:153
33:38
LLSLGAGEFKpc2_NP.1
p−maj=9.4e−05, pX=3.3e−19, N=319
var.2.F5 1:169498975:T:C
134:134
152:153
34:38
LLSLGAGEFKpc2_NP.2
p−maj=0.00074, pX=2.4e−24, N=320
var.2.F5 1:169498975:T:C
134:134
152:153
32:38
LLSLGAGEFKpc2_NP.3
p−maj=1.1e−05, pX=2.1e−19, N=318
var.2.F5 1:169498975:T:C
134:134
152:153
28:38
LLSLGAGEFKpc2_NP.4
p−maj=1.5e−09, pX=5.3e−08, N=314
var.2.F5 1:169498975:T:C
127:134
139:153
30:38
LLSLGAGEFKpc2_NP.5
p−maj=0.011, pX=2.8e−07, N=296
var.2.F5 1:169498975:T:C
21:134
117:153
34:38
LLSLGAGEFRpc2_NP.1
p−min=3e−31, pX=3.2e−24, N=172
var.2.F5 1:169498975:T:C
15:134
103:153
31:38
LLSLGAGEFRpc2_NP.2
p−min=9.2e−28, pX=2.6e−26, N=149
var.2.F5 1:169498975:T:C
31:134
117:153
33:38
LLSLGAGEFRpc2_NP.3
p−min=8.4e−24, pX=3.2e−21, N=181
var.2.F5 1:169498975:T:C
14:134
117:153
34:38
LLSLGAGEFRpc2_NP.4
p−min=5e−37, pX=2.8e−40, N=165
var.2.F5 1:169498975:T:C
4:134
115:153
34:38
LLSLGAGEFRpc2_NP.5
p−min=2.9e−47, pX=5.4e−38, N=153
var.2.F5 1:169498975:T:C
130:134
149:153
36:38
LNNGGSYNAWSVEKpc2_NP.1
p−maj=0.33, pX=0.074, N=315
var.2.F5 1:169498975:T:C
132:134
153:153
36:38
LNNGGSYNAWSVEKpc2_NP.2
p−maj=0.069, pX=0.018, N=321
var.2.F5 1:169498975:T:C
133:134
152:153
37:38
LNNGGSYNAWSVEKpc2_NP.3
p−maj=0.31, pX=0.0025, N=322
var.2.F5 1:169498975:T:C
134:134
153:153
38:38
LNNGGSYNAWSVEKpc2_NP.4
p−maj=1, pX=0.32, N=325
var.2.F5 1:169498975:T:C
134:134
153:153
38:38
LNNGGSYNAWSVEKpc2_NP.5
p−maj=1, pX=0.0077, N=325

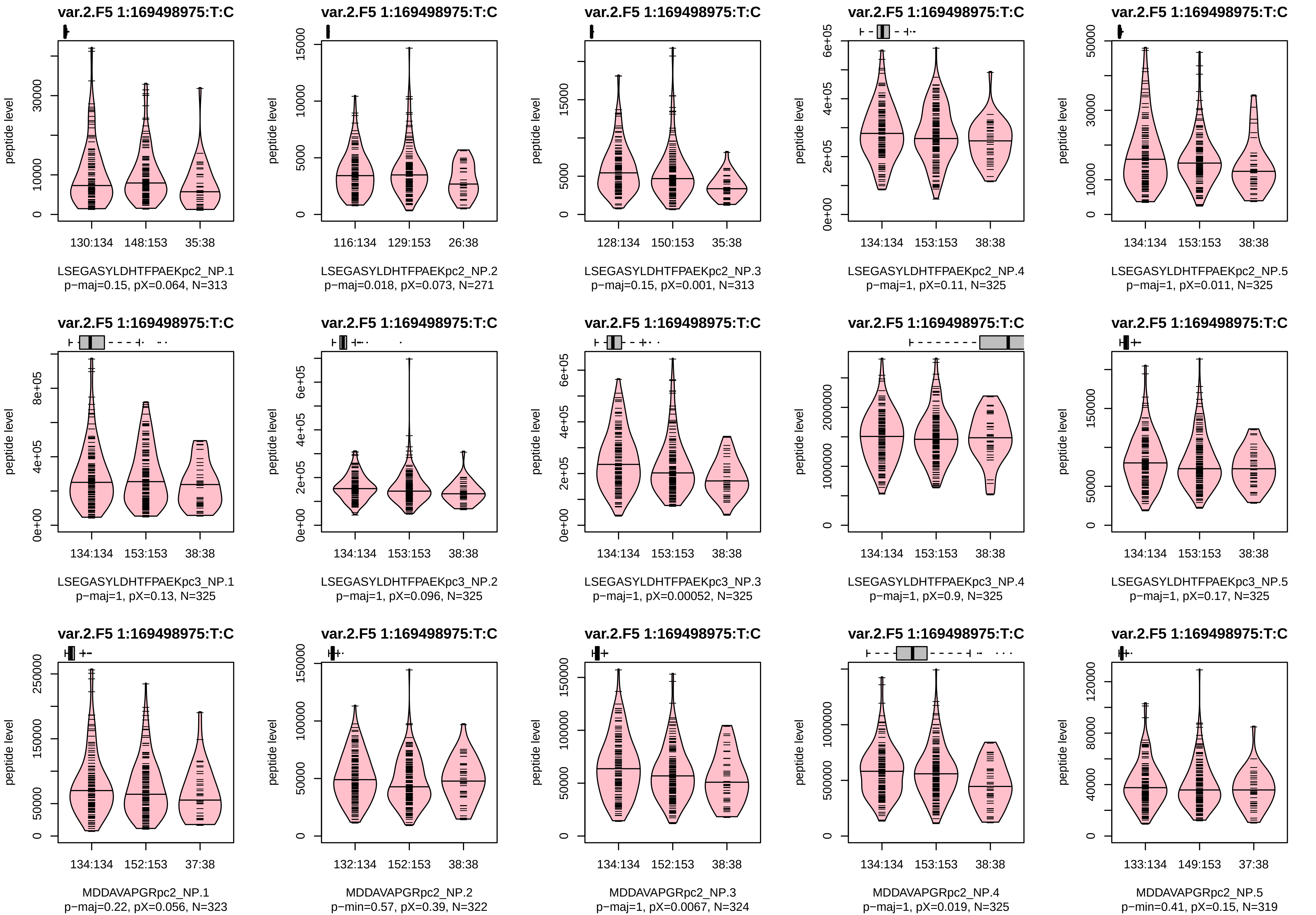
var.2.F5 1:169498975:T:C
peptide level
130:134 148:153 35:38
LSEGASYLDHTFPAEKpc2_NP.1
p−maj=0.15, pX=0.064, N=313
var.2.F5 1:169498975:T:C
peptide level
116:134 129:153 26:38
LSEGASYLDHTFPAEKpc2_NP.2
p−maj=0.018, pX=0.073, N=271
var.2.F5 1:169498975:T:C
peptide level
128:134 150:153 35:38
LSEGASYLDHTFPAEKpc2_NP.3
p−maj=0.15, pX=0.001, N=313
var.2.F5 1:169498975:T:C
peptide level
134:134 153:153 38:38
LSEGASYLDHTFPAEKpc2_NP.4
p−maj=1, pX=0.11, N=325
var.2.F5 1:169498975:T:C
peptide level
134:134 153:153 38:38
LSEGASYLDHTFPAEKpc2_NP.5
p−maj=1, pX=0.011, N=325
var.2.F5 1:169498975:T:C
peptide level
134:134 153:153 38:38
LSEGASYLDHTFPAEKpc3_NP.1
p−maj=1, pX=0.13, N=325
var.2.F5 1:169498975:T:C
peptide level
134:134 153:153 38:38
LSEGASYLDHTFPAEKpc3_NP.2
p−maj=1, pX=0.096, N=325
var.2.F5 1:169498975:T:C
peptide level
134:134 153:153 38:38
LSEGASYLDHTFPAEKpc3_NP.3
p−maj=1, pX=0.00052, N=325
var.2.F5 1:169498975:T:C
peptide level
134:134 153:153 38:38
LSEGASYLDHTFPAEKpc3_NP.4
p−maj=1, pX=0.9, N=325
var.2.F5 1:169498975:T:C
peptide level
134:134 153:153 38:38
LSEGASYLDHTFPAEKpc3_NP.5
p−maj=1, pX=0.17, N=325
var.2.F5 1:169498975:T:C
peptide level
134:134 152:153 37:38
MDDAVAPGRpc2_NP.1
p−maj=0.22, pX=0.056, N=323
var.2.F5 1:169498975:T:C
peptide level
132:134 152:153 38:38
MDDAVAPGRpc2_NP.2
p−min=0.57, pX=0.39, N=322
var.2.F5 1:169498975:T:C
peptide level
134:134 152:153 38:38
MDDAVAPGRpc2_NP.3
p−maj=1, pX=0.0067, N=324
var.2.F5 1:169498975:T:C
peptide level
134:134 153:153 38:38
MDDAVAPGRpc2_NP.4
p−maj=1, pX=0.019, N=325
var.2.F5 1:169498975:T:C
peptide level
133:134 149:153 37:38
MDDAVAPGRpc2_NP.5
p−min=0.41, pX=0.15, N=319

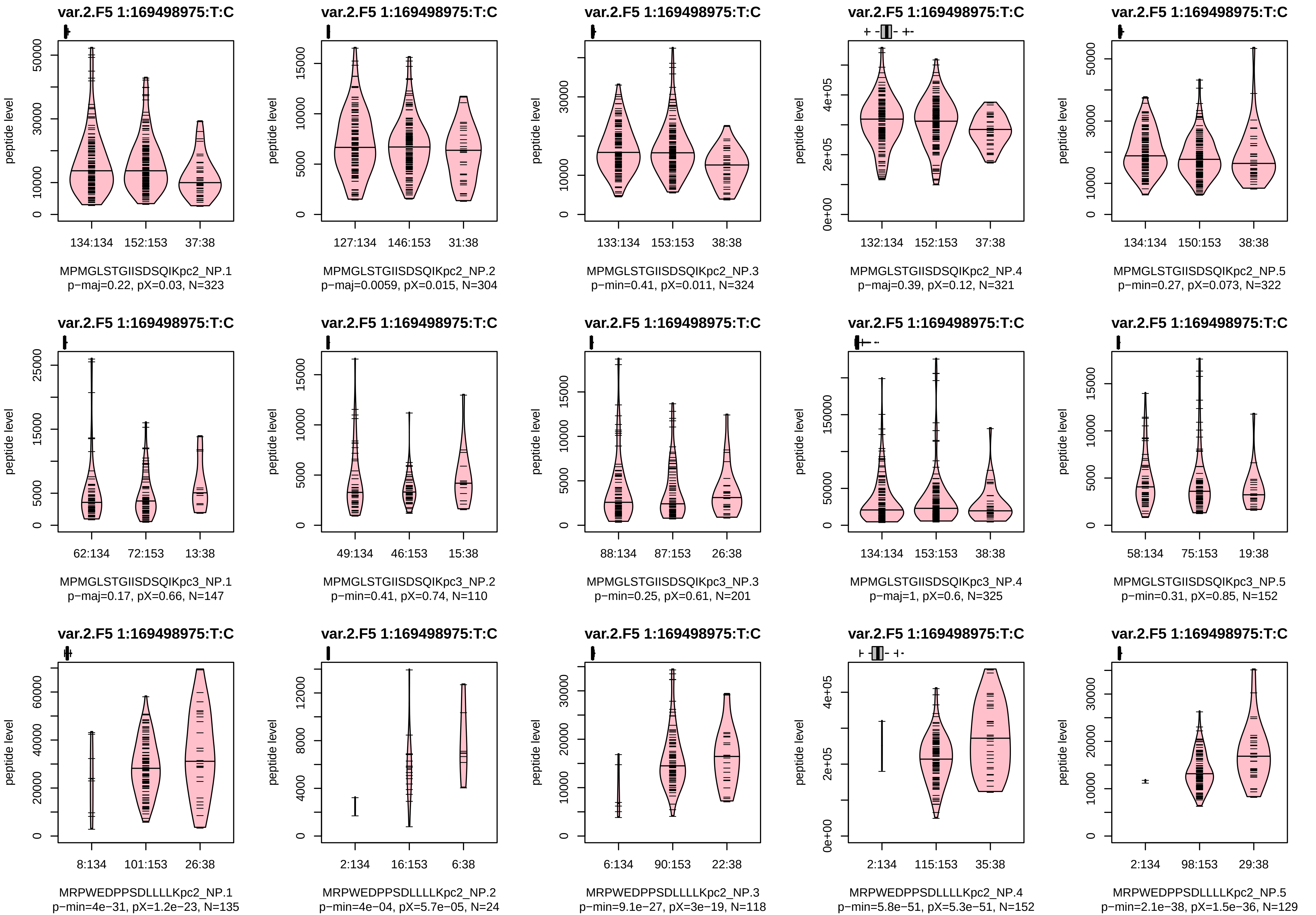
var.2.F5 1:169498975:T:C
peptide level
134:134
152:153
37:38
MPMGLSTGIISDSQIKpc2_NP.1
p−maj=0.22, pX=0.03, N=323
var.2.F5 1:169498975:T:C
peptide level
127:134
146:153
31:38
MPMGLSTGIISDSQIKpc2_NP.2
p−maj=0.0059, pX=0.015, N=304
var.2.F5 1:169498975:T:C
peptide level
133:134
153:153
38:38
MPMGLSTGIISDSQIKpc2_NP.3
p−min=0.41, pX=0.011, N=324
var.2.F5 1:169498975:T:C
peptide level
132:134
152:153
37:38
MPMGLSTGIISDSQIKpc2_NP.4
p−maj=0.39, pX=0.12, N=321
var.2.F5 1:169498975:T:C
peptide level
134:134
150:153
38:38
MPMGLSTGIISDSQIKpc2_NP.5
p−min=0.27, pX=0.073, N=322
var.2.F5 1:169498975:T:C
peptide level
62:134
72:153
13:38
MPMGLSTGIISDSQIKpc3_NP.1
p−maj=0.17, pX=0.66, N=147
var.2.F5 1:169498975:T:C
peptide level
49:134
46:153
15:38
MPMGLSTGIISDSQIKpc3_NP.2
p−min=0.41, pX=0.74, N=110
var.2.F5 1:169498975:T:C
peptide level
88:134
87:153
26:38
MPMGLSTGIISDSQIKpc3_NP.3
p−min=0.25, pX=0.61, N=201
var.2.F5 1:169498975:T:C
peptide level
134:134
153:153
38:38
MPMGLSTGIISDSQIKpc3_NP.4
p−maj=1, pX=0.6, N=325
var.2.F5 1:169498975:T:C
peptide level
58:134
75:153
19:38
MPMGLSTGIISDSQIKpc3_NP.5
p−min=0.31, pX=0.85, N=152
var.2.F5 1:169498975:T:C
peptide level
8:134
101:153
26:38
MRPWEDPPSDLLLLKpc2_NP.1
p−min=4e−31, pX=1.2e−23, N=135
var.2.F5 1:169498975:T:C
peptide level
2:134
16:153
6:38
MRPWEDPPSDLLLLKpc2_NP.2
p−min=4e−04, pX=5.7e−05, N=24
var.2.F5 1:169498975:T:C
peptide level
6:134
90:153
22:38
MRPWEDPPSDLLLLKpc2_NP.3
p−min=9.1e−27, pX=3e−19, N=118
var.2.F5 1:169498975:T:C
peptide level
2:134
115:153
35:38
MRPWEDPPSDLLLLKpc2_NP.4
p−min=5.8e−51, pX=5.3e−51, N=152
var.2.F5 1:169498975:T:C
peptide level
2:134
98:153
29:38
MRPWEDPPSDLLLLKpc2_NP.5
p−min=2.1e−38, pX=1.5e−36, N=129

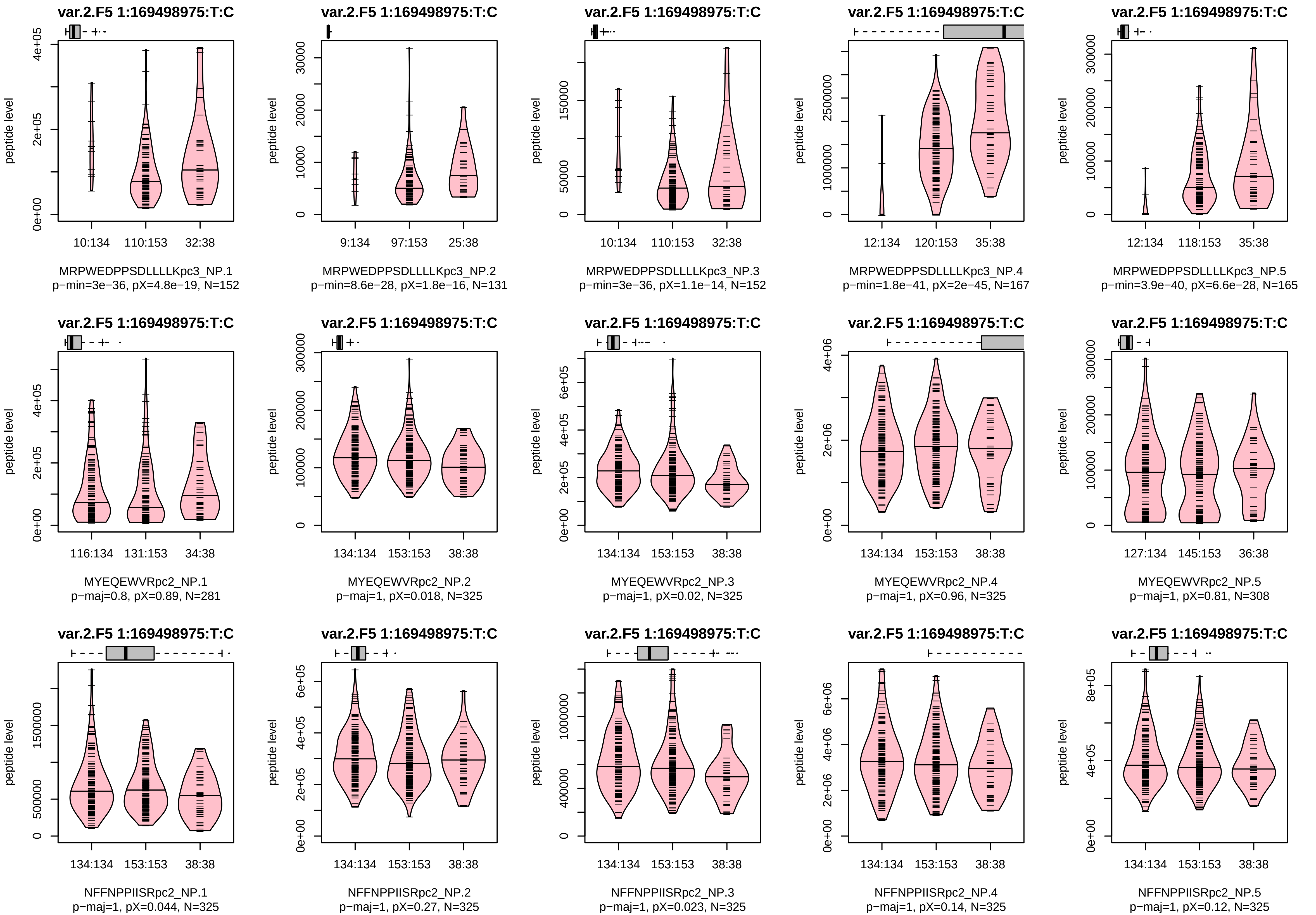
var.2.F5 1:169498975:T:C
peptide level
10:134
110:153
32:38
MRPWEDPPSDLLLLKpc3_NP.1
p−min=3e−36, pX=4.8e−19, N=152
9:134
97:153
25:38
MRPWEDPPSDLLLLKpc3_NP.2
p−min=8.6e−28, pX=1.8e−16, N=131
10:134
110:153
32:38
MRPWEDPPSDLLLLKpc3_NP.3
p−min=3e−36, pX=1.1e−14, N=152
12:134
120:153
35:38
MRPWEDPPSDLLLLKpc3_NP.4
p−min=1.8e−41, pX=2e−45, N=167
12:134
118:153
35:38
MRPWEDPPSDLLLLKpc3_NP.5
p−min=3.9e−40, pX=6.6e−28, N=165
116:134
131:153
34:38
MYEQEWVRpc2_NP.1
p−maj=0.8, pX=0.89, N=281
134:134
153:153
38:38
MYEQEWVRpc2_NP.2
p−maj=1, pX=0.018, N=325
MYEQEWVRpc2_NP.3
p−maj=1, pX=0.02, N=325
MYEQEWVRpc2_NP.4
p−maj=1, pX=0.96, N=325
127:134
145:153
36:38
MYEQEWVRpc2_NP.5
p−maj=1, pX=0.81, N=308
NFFNPPIISRpc2_NP.1
p−maj=1, pX=0.044, N=325
NFFNPPIISRpc2_NP.2
p−maj=1, pX=0.27, N=325
NFFNPPIISRpc2_NP.3
p−maj=1, pX=0.023, N=325
NFFNPPIISRpc2_NP.4
p−maj=1, pX=0.14, N=325
NFFNPPIISRpc2_NP.5
p−maj=1, pX=0.12, N=325

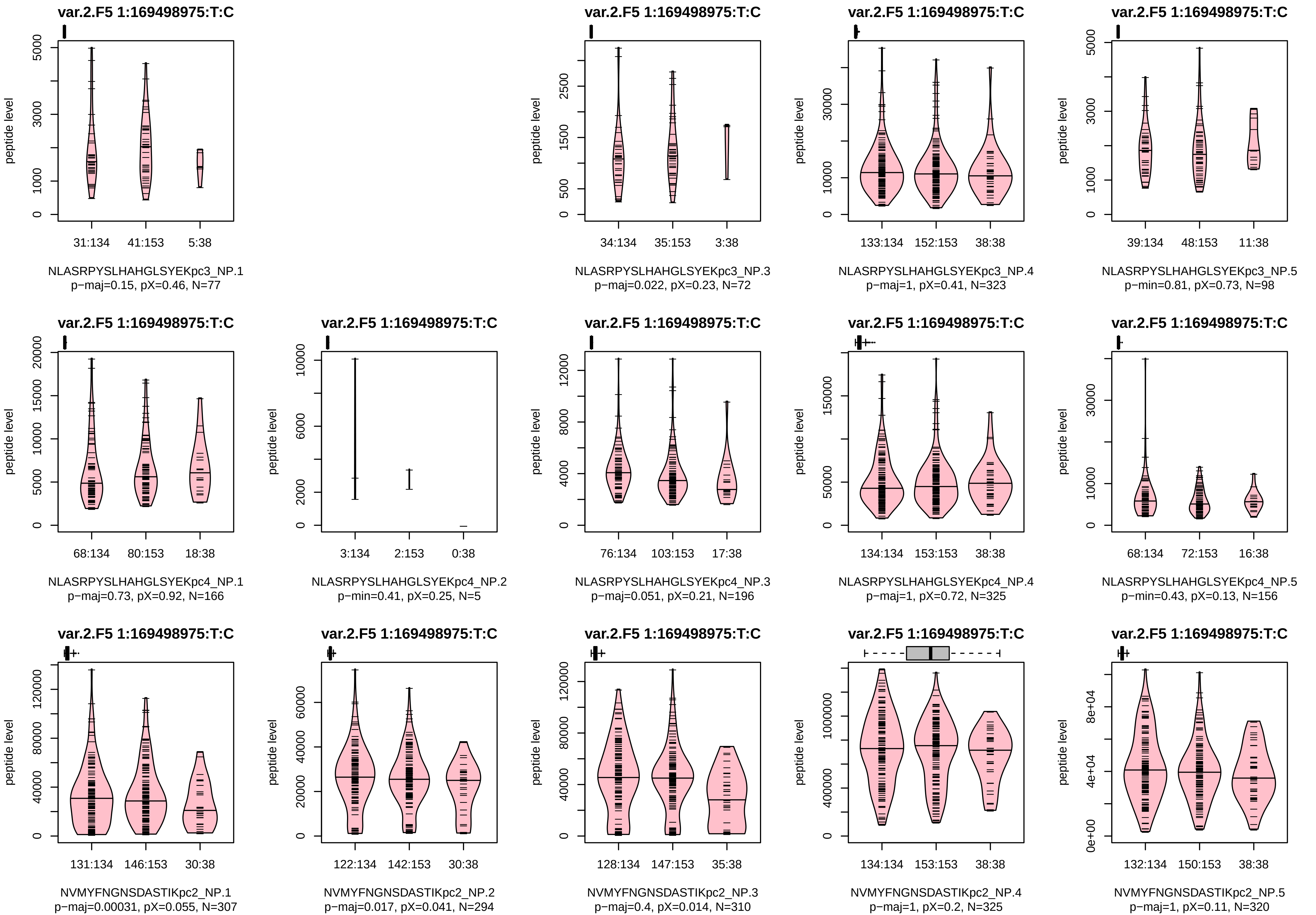
var.2.F5 1:169498975:T:C
peptide level
31:134
41:153
5:38
NLASRPYSLHAHGLSYEKpc3_NP.1
p−maj=0.15, pX=0.46, N=77
var.2.F5 1:169498975:T:C
peptide level
34:134
35:153
3:38
NLASRPYSLHAHGLSYEKpc3_NP.3
p−maj=0.022, pX=0.23, N=72
var.2.F5 1:169498975:T:C
peptide level
133:134
152:153
38:38
NLASRPYSLHAHGLSYEKpc3_NP.4
p−maj=1, pX=0.41, N=323
var.2.F5 1:169498975:T:C
peptide level
39:134
48:153
11:38
NLASRPYSLHAHGLSYEKpc3_NP.5
p−min=0.81, pX=0.73, N=98
var.2.F5 1:169498975:T:C
peptide level
68:134
80:153
18:38
NLASRPYSLHAHGLSYEKpc4_NP.1
p−maj=0.73, pX=0.92, N=166
var.2.F5 1:169498975:T:C
peptide level
3:134
2:153
0:38
NLASRPYSLHAHGLSYEKpc4_NP.2
p−min=0.41, pX=0.25, N=5
var.2.F5 1:169498975:T:C
peptide level
76:134
103:153
17:38
NLASRPYSLHAHGLSYEKpc4_NP.3
p−maj=0.051, pX=0.21, N=196
var.2.F5 1:169498975:T:C
peptide level
134:134
153:153
38:38
NLASRPYSLHAHGLSYEKpc4_NP.4
p−maj=1, pX=0.72, N=325
var.2.F5 1:169498975:T:C
peptide level
68:134
72:153
16:38
NLASRPYSLHAHGLSYEKpc4_NP.5
p−min=0.43, pX=0.13, N=156
var.2.F5 1:169498975:T:C
peptide level
131:134
146:153
30:38
NVMYFNGNSDASTIKpc2_NP.1
p−maj=0.00031, pX=0.055, N=307
var.2.F5 1:169498975:T:C
peptide level
122:134
142:153
30:38
NVMYFNGNSDASTIKpc2_NP.2
p−maj=0.017, pX=0.041, N=294
var.2.F5 1:169498975:T:C
peptide level
128:134
147:153
35:38
NVMYFNGNSDASTIKpc2_NP.3
p−maj=0.4, pX=0.014, N=310
var.2.F5 1:169498975:T:C
peptide level
134:134
153:153
38:38
NVMYFNGNSDASTIKpc2_NP.4
p−maj=1, pX=0.2, N=325
var.2.F5 1:169498975:T:C
peptide level
132:134
150:153
38:38
NVMYFNGNSDASTIKpc2_NP.5
p−maj=1, pX=0.11, N=320

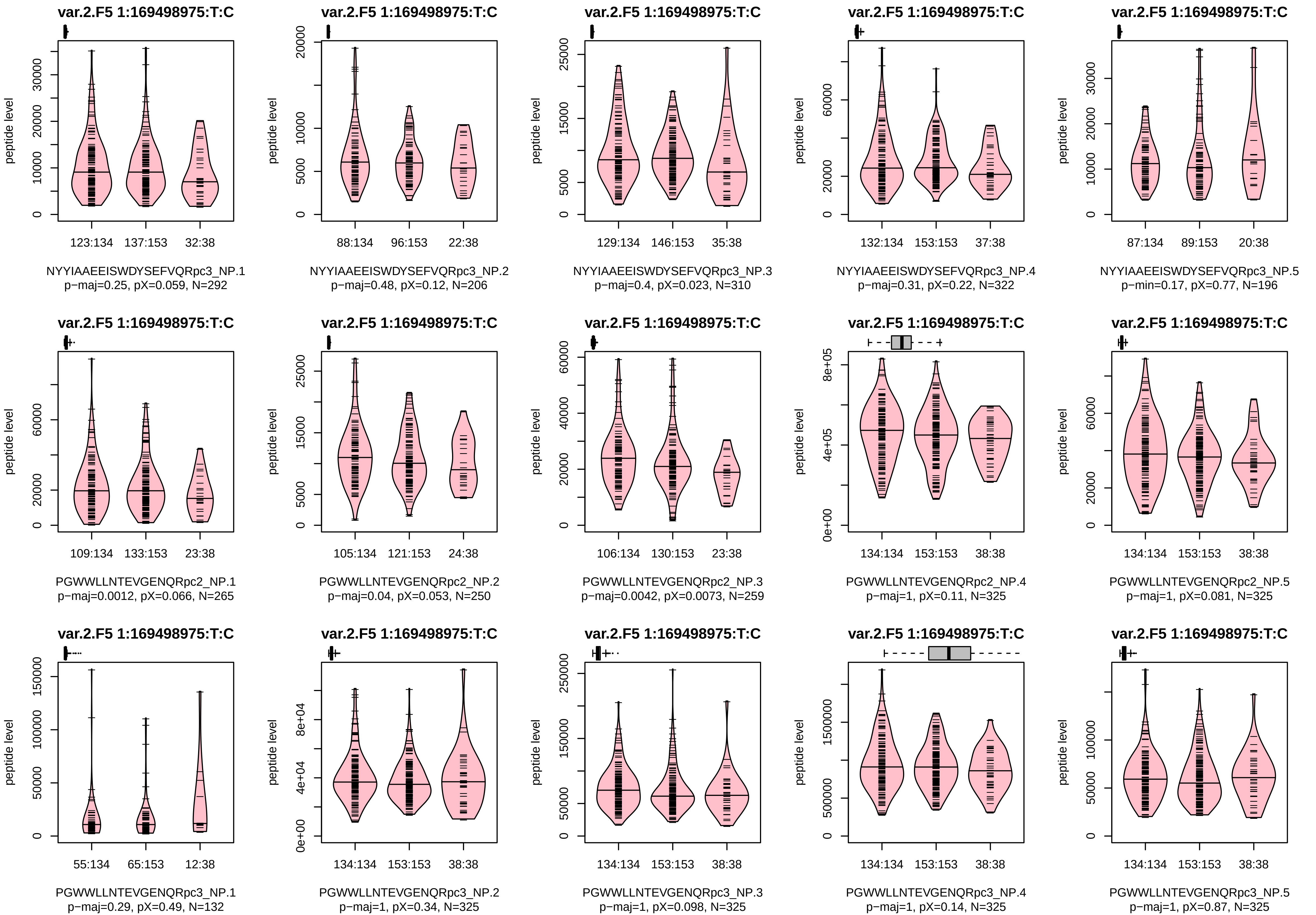
var.2.F5 1:169498975:T:C
peptide level
123:134
137:153
32:38
NYYIAAEEISWDYSEFVQRpc3_NP.1
p−maj=0.25, pX=0.059, N=292
var.2.F5 1:169498975:T:C
88:134
96:153
22:38
NYYIAAEEISWDYSEFVQRpc3_NP.2
p−maj=0.48, pX=0.12, N=206
var.2.F5 1:169498975:T:C
129:134
146:153
35:38
NYYIAAEEISWDYSEFVQRpc3_NP.3
p−maj=0.4, pX=0.023, N=310
var.2.F5 1:169498975:T:C
132:134
153:153
37:38
NYYIAAEEISWDYSEFVQRpc3_NP.4
p−maj=0.31, pX=0.22, N=322
var.2.F5 1:169498975:T:C
87:134
89:153
20:38
NYYIAAEEISWDYSEFVQRpc3_NP.5
p−min=0.17, pX=0.77, N=196
var.2.F5 1:169498975:T:C
109:134
133:153
23:38
PGWWLLNTEVGENQRpc2_NP.1
p−maj=0.0012, pX=0.066, N=265
var.2.F5 1:169498975:T:C
105:134
121:153
24:38
PGWWLLNTEVGENQRpc2_NP.2
p−maj=0.04, pX=0.053, N=250
var.2.F5 1:169498975:T:C
106:134
130:153
23:38
PGWWLLNTEVGENQRpc2_NP.3
p−maj=0.0042, pX=0.0073, N=259
var.2.F5 1:169498975:T:C
134:134
153:153
38:38
PGWWLLNTEVGENQRpc2_NP.4
p−maj=1, pX=0.11, N=325
var.2.F5 1:169498975:T:C
134:134
153:153
38:38
PGWWLLNTEVGENQRpc2_NP.5
p−maj=1, pX=0.081, N=325
var.2.F5 1:169498975:T:C
55:134
65:153
12:38
PGWWLLNTEVGENQRpc3_NP.1
p−maj=0.29, pX=0.49, N=132
var.2.F5 1:169498975:T:C
134:134
153:153
38:38
PGWWLLNTEVGENQRpc3_NP.2
p−maj=1, pX=0.34, N=325
var.2.F5 1:169498975:T:C
134:134
153:153
38:38
PGWWLLNTEVGENQRpc3_NP.3
p−maj=1, pX=0.098, N=325
var.2.F5 1:169498975:T:C
134:134
153:153
38:38
PGWWLLNTEVGENQRpc3_NP.4
p−maj=1, pX=0.14, N=325
var.2.F5 1:169498975:T:C
134:134
153:153
38:38
PGWWLLNTEVGENQRpc3_NP.5
p−maj=1, pX=0.87, N=325

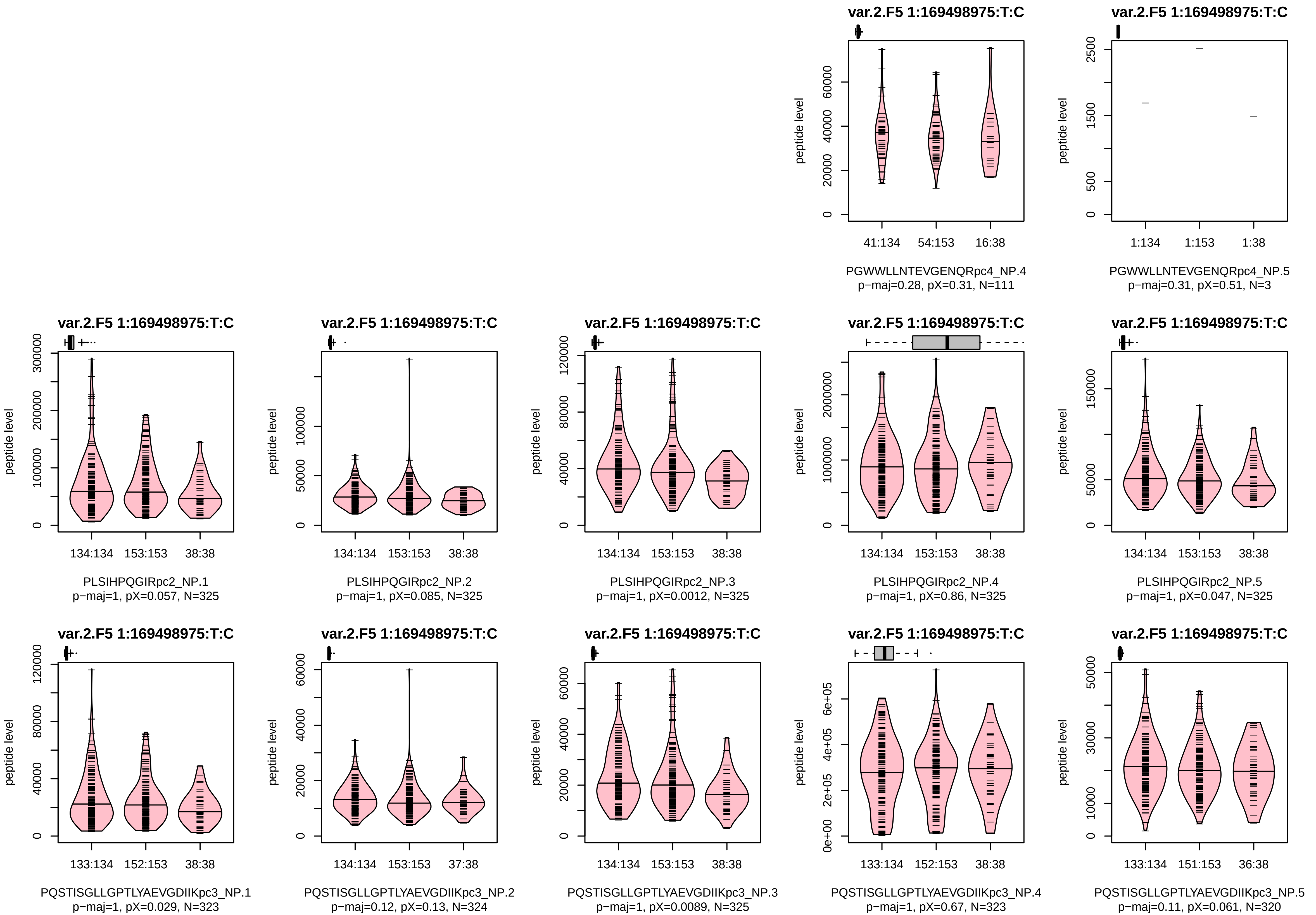
var.2.F5 1:169498975:T:C
peptide level
41:134
54:153
16:38
PGWWLLNTEVGENQRpc4_NP.4
p−maj=0.28, pX=0.31, N=111
var.2.F5 1:169498975:T:C
peptide level
1:134
1:153
1:38
PGWWLLNTEVGENQRpc4_NP.5
p−maj=0.31, pX=0.51, N=3
var.2.F5 1:169498975:T:C
peptide level
134:134
153:153
38:38
PLSIHPQGIRpc2_NP.1
p−maj=1, pX=0.057, N=325
var.2.F5 1:169498975:T:C
peptide level
134:134
153:153
38:38
PLSIHPQGIRpc2_NP.2
p−maj=1, pX=0.085, N=325
var.2.F5 1:169498975:T:C
peptide level
134:134
153:153
38:38
PLSIHPQGIRpc2_NP.3
p−maj=1, pX=0.0012, N=325
var.2.F5 1:169498975:T:C
peptide level
134:134
153:153
38:38
PLSIHPQGIRpc2_NP.4
p−maj=1, pX=0.86, N=325
var.2.F5 1:169498975:T:C
peptide level
134:134
153:153
38:38
PLSIHPQGIRpc2_NP.5
p−maj=1, pX=0.047, N=325
var.2.F5 1:169498975:T:C
peptide level
133:134
152:153
38:38
PQSTISGLLGPTLYAEVGDIIKpc3_NP.1
p−maj=1, pX=0.029, N=323
var.2.F5 1:169498975:T:C
peptide level
134:134
153:153
37:38
PQSTISGLLGPTLYAEVGDIIKpc3_NP.2
p−maj=0.12, pX=0.13, N=324
var.2.F5 1:169498975:T:C
peptide level
134:134
153:153
38:38
PQSTISGLLGPTLYAEVGDIIKpc3_NP.3
p−maj=1, pX=0.0089, N=325
var.2.F5 1:169498975:T:C
peptide level
133:134
152:153
38:38
PQSTISGLLGPTLYAEVGDIIKpc3_NP.4
p−maj=1, pX=0.67, N=323
var.2.F5 1:169498975:T:C
peptide level
133:134
151:153
36:38
PQSTISGLLGPTLYAEVGDIIKpc3_NP.5
p−maj=0.11, pX=0.061, N=320

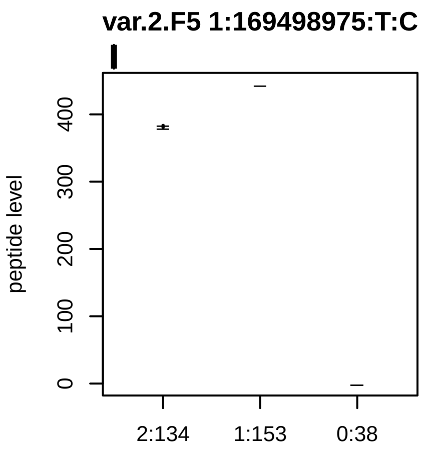

PWEDLPSQDTGSPSRpc2_NP.3
p−min=0.57, pX=0.38, N=3

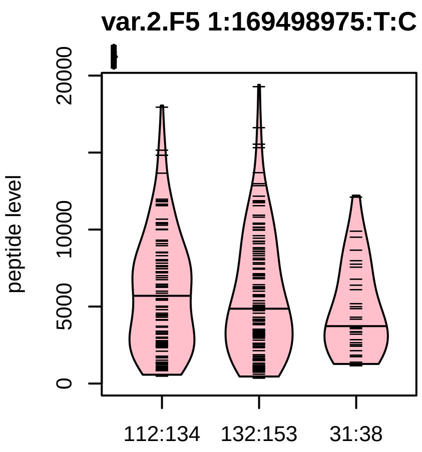

PWEDLPSQDTGSPSRpc2_NP.4
p−maj=0.63, pX=0.25, N=275

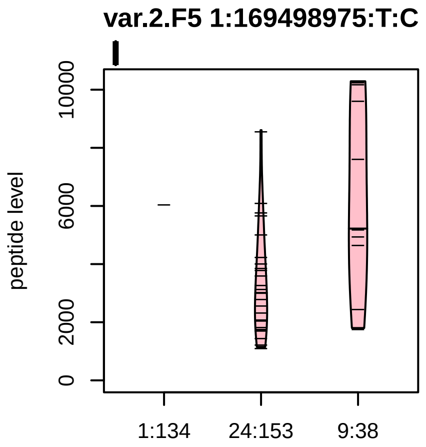

PWEDPPSDLLLLKpc2_NP.1
p−min=1.4e−07, pX=2.6e−07, N=34

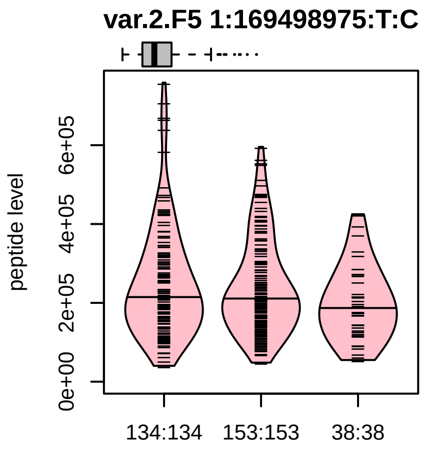

PWIQVDMQKpc2_NP.1
p−maj=1, pX=0.068, N=325

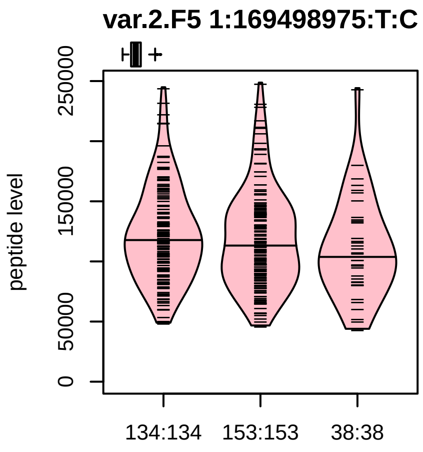

PWIQVDMQKpc2_NP.2
p−maj=1, pX=0.14, N=325

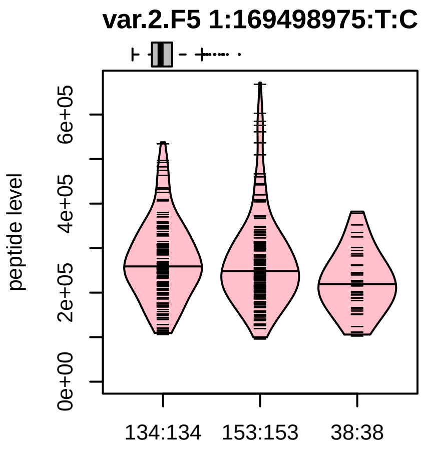

PWIQVDMQKpc2_NP.3
p−maj=1, pX=0.029, N=325

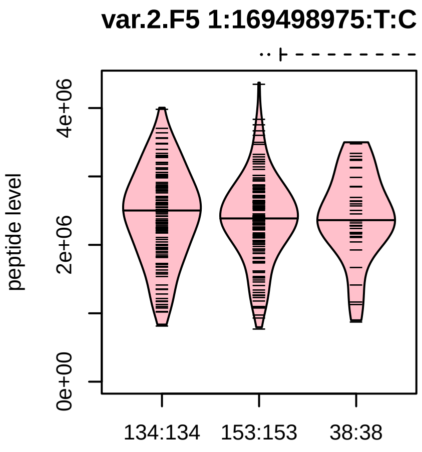

PWIQVDMQKpc2_NP.4
p−maj=1, pX=0.67, N=325

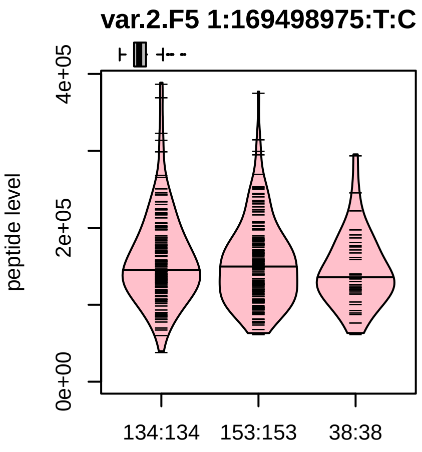

PWIQVDMQKpc2_NP.5
p−maj=1, pX=0.14, N=325

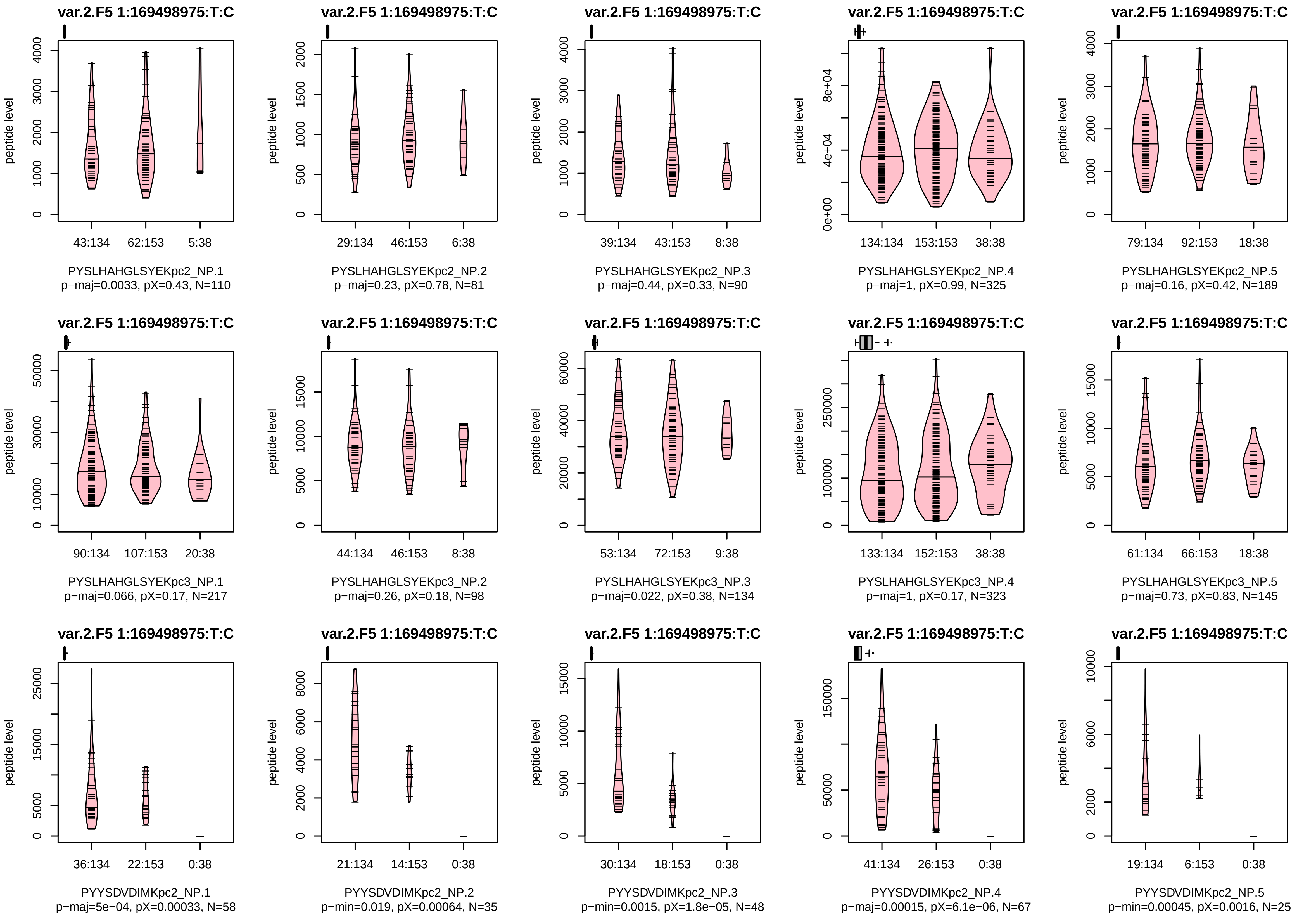

var.2.F5 1:169498975:T:C
peptide level
43:134
62:153
5:38
PYSLHAHGLSYEKpc2_NP.1
p−maj=0.0033, pX=0.43, N=110
29:134
46:153
6:38
PYSLHAHGLSYEKpc2_NP.2
p−maj=0.23, pX=0.78, N=81
39:134
43:153
8:38
PYSLHAHGLSYEKpc2_NP.3
p−maj=0.44, pX=0.33, N=90
134:134
153:153
38:38
PYSLHAHGLSYEKpc2_NP.4
p−maj=1, pX=0.99, N=325
79:134
92:153
18:38
PYSLHAHGLSYEKpc2_NP.5
p−maj=0.16, pX=0.42, N=189
90:134
107:153
20:38
PYSLHAHGLSYEKpc3_NP.1
p−maj=0.066, pX=0.17, N=217
44:134
46:153
8:38
PYSLHAHGLSYEKpc3_NP.2
p−maj=0.26, pX=0.18, N=98
53:134
72:153
9:38
PYSLHAHGLSYEKpc3_NP.3
p−maj=0.022, pX=0.38, N=134
133:134
152:153
38:38
PYSLHAHGLSYEKpc3_NP.4
p−maj=1, pX=0.17, N=323
61:134
66:153
18:38
PYSLHAHGLSYEKpc3_NP.5
p−maj=0.73, pX=0.83, N=145
36:134
22:153
0:38
PYYSDVDIMKpc2_NP.1
p−maj=5e−04, pX=0.00033, N=58
21:134
14:153
0:38
PYYSDVDIMKpc2_NP.2
p−min=0.019, pX=0.00064, N=35
30:134
18:153
0:38
PYYSDVDIMKpc2_NP.3
p−min=0.0015, pX=1.8e−05, N=48
41:134
26:153
0:38
PYYSDVDIMKpc2_NP.4
p−maj=0.00015, pX=6.1e−06, N=67
19:134
6:153
0:38
PYYSDVDIMKpc2_NP.5
p−min=0.00045, pX=0.0016, N=25

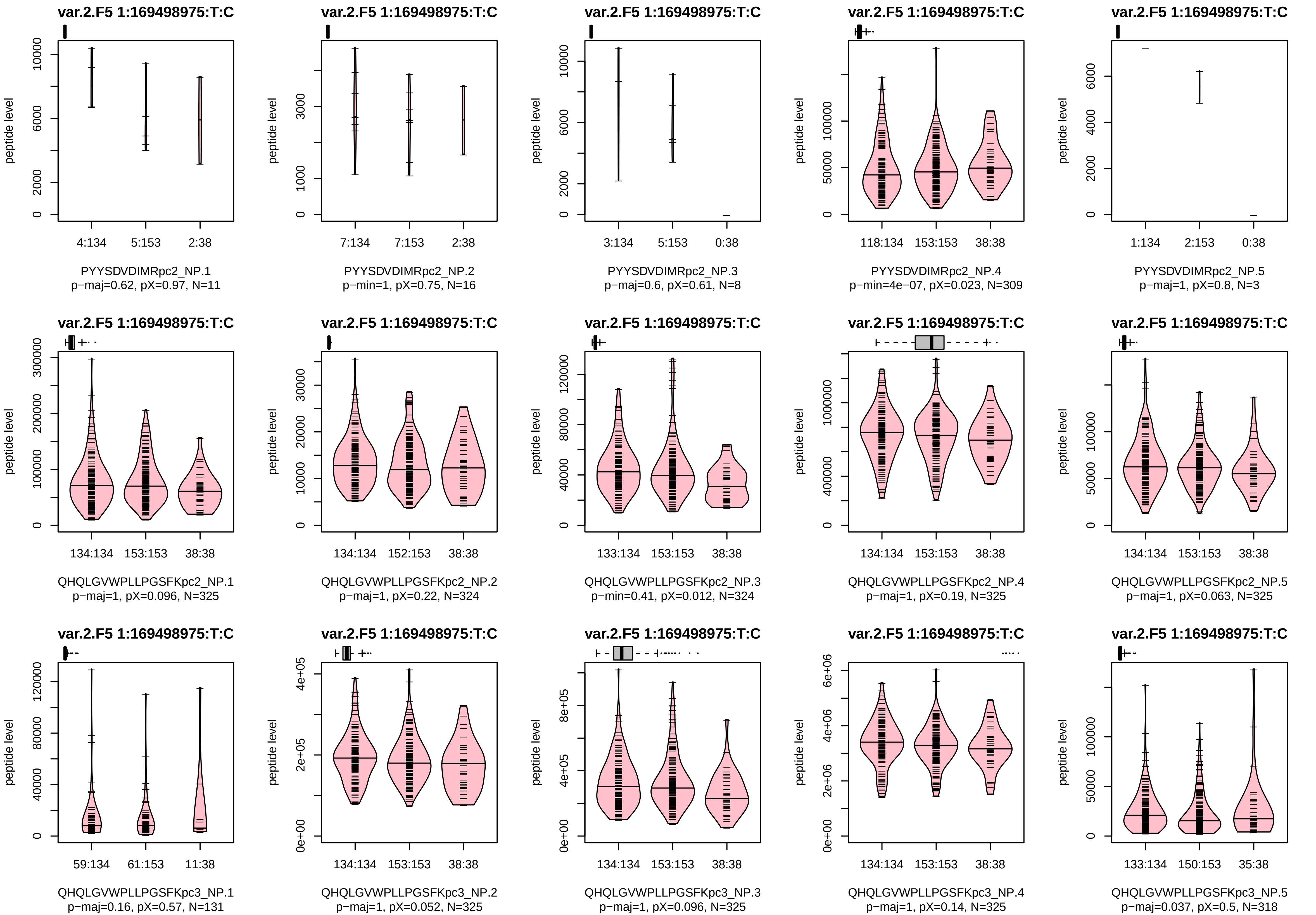
var.2.F5 1:169498975:T:C
peptide level
4:134
5:153
2:38
PYYSDVDIMRpc2_NP.1
p−maj=0.62, pX=0.97, N=11
var.2.F5 1:169498975:T:C
peptide level
7:134
7:153
2:38
PYYSDVDIMRpc2_NP.2
p−min=1, pX=0.75, N=16
var.2.F5 1:169498975:T:C
peptide level
3:134
5:153
0:38
PYYSDVDIMRpc2_NP.3
p−maj=0.6, pX=0.61, N=8
var.2.F5 1:169498975:T:C
peptide level
118:134
153:153
38:38
PYYSDVDIMRpc2_NP.4
p−min=4e−07, pX=0.023, N=309
var.2.F5 1:169498975:T:C
peptide level
1:134
2:153
0:38
PYYSDVDIMRpc2_NP.5
p−maj=1, pX=0.8, N=3
var.2.F5 1:169498975:T:C
peptide level
134:134
153:153
38:38
QHQLGVWPLLPGSFKpc2_NP.1
p−maj=1, pX=0.096, N=325
var.2.F5 1:169498975:T:C
peptide level
134:134
152:153
38:38
QHQLGVWPLLPGSFKpc2_NP.2
p−maj=1, pX=0.22, N=324
var.2.F5 1:169498975:T:C
peptide level
133:134
153:153
38:38
QHQLGVWPLLPGSFKpc2_NP.3
p−min=0.41, pX=0.012, N=324
var.2.F5 1:169498975:T:C
peptide level
134:134
153:153
38:38
QHQLGVWPLLPGSFKpc2_NP.4
p−maj=1, pX=0.19, N=325
var.2.F5 1:169498975:T:C
peptide level
134:134
153:153
38:38
QHQLGVWPLLPGSFKpc2_NP.5
p−maj=1, pX=0.063, N=325
var.2.F5 1:169498975:T:C
peptide level
59:134
61:153
11:38
QHQLGVWPLLPGSFKpc3_NP.1
p−maj=0.16, pX=0.57, N=131
var.2.F5 1:169498975:T:C
peptide level
134:134
153:153
38:38
QHQLGVWPLLPGSFKpc3_NP.2
p−maj=1, pX=0.052, N=325
var.2.F5 1:169498975:T:C
peptide level
134:134
153:153
38:38
QHQLGVWPLLPGSFKpc3_NP.3
p−maj=1, pX=0.096, N=325
var.2.F5 1:169498975:T:C
peptide level
134:134
153:153
38:38
QHQLGVWPLLPGSFKpc3_NP.4
p−maj=1, pX=0.14, N=325
var.2.F5 1:169498975:T:C
peptide level
133:134
150:153
35:38
QHQLGVWPLLPGSFKpc3_NP.5
p−maj=0.037, pX=0.5, N=318

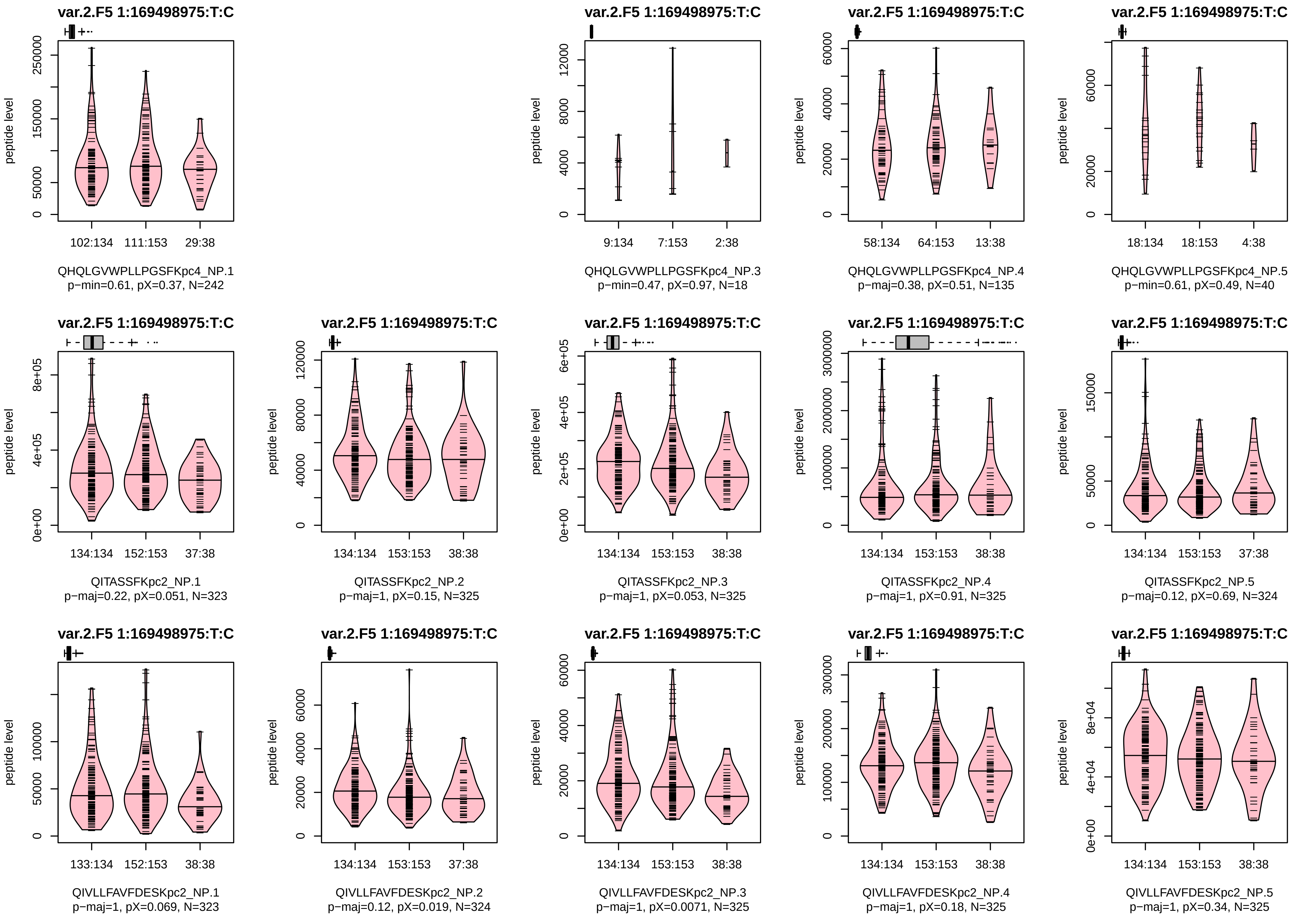
var.2.F5 1:169498975:T:C
peptide level
102:134
111:153
29:38
QHQLGVWPLLPGSFKpc4_NP.1
p−min=0.61, pX=0.37, N=242
var.2.F5 1:169498975:T:C
9:134
7:153
2:38
QHQLGVWPLLPGSFKpc4_NP.3
p−min=0.47, pX=0.97, N=18
var.2.F5 1:169498975:T:C
58:134
64:153
13:38
QHQLGVWPLLPGSFKpc4_NP.4
p−maj=0.38, pX=0.51, N=135
var.2.F5 1:169498975:T:C
18:134
18:153
4:38
QHQLGVWPLLPGSFKpc4_NP.5
p−min=0.61, pX=0.49, N=40
var.2.F5 1:169498975:T:C
134:134
152:153
37:38
QITASSFKpc2_NP.1
p−maj=0.22, pX=0.051, N=323
var.2.F5 1:169498975:T:C
134:134
153:153
38:38
QITASSFKpc2_NP.2
p−maj=1, pX=0.15, N=325
var.2.F5 1:169498975:T:C
134:134
153:153
38:38
QITASSFKpc2_NP.3
p−maj=1, pX=0.053, N=325
var.2.F5 1:169498975:T:C
134:134
153:153
38:38
QITASSFKpc2_NP.4
p−maj=1, pX=0.91, N=325
var.2.F5 1:169498975:T:C
134:134
153:153
37:38
QITASSFKpc2_NP.5
p−maj=0.12, pX=0.69, N=324
var.2.F5 1:169498975:T:C
133:134
152:153
38:38
QIVLLFAVFDESKpc2_NP.1
p−maj=1, pX=0.069, N=323
var.2.F5 1:169498975:T:C
134:134
153:153
37:38
QIVLLFAVFDESKpc2_NP.2
p−maj=0.12, pX=0.019, N=324
var.2.F5 1:169498975:T:C
134:134
153:153
38:38
QIVLLFAVFDESKpc2_NP.3
p−maj=1, pX=0.0071, N=325
var.2.F5 1:169498975:T:C
134:134
153:153
38:38
QIVLLFAVFDESKpc2_NP.4
p−maj=1, pX=0.18, N=325
var.2.F5 1:169498975:T:C
134:134
153:153
38:38
QIVLLFAVFDESKpc2_NP.5
p−maj=1, pX=0.34, N=325

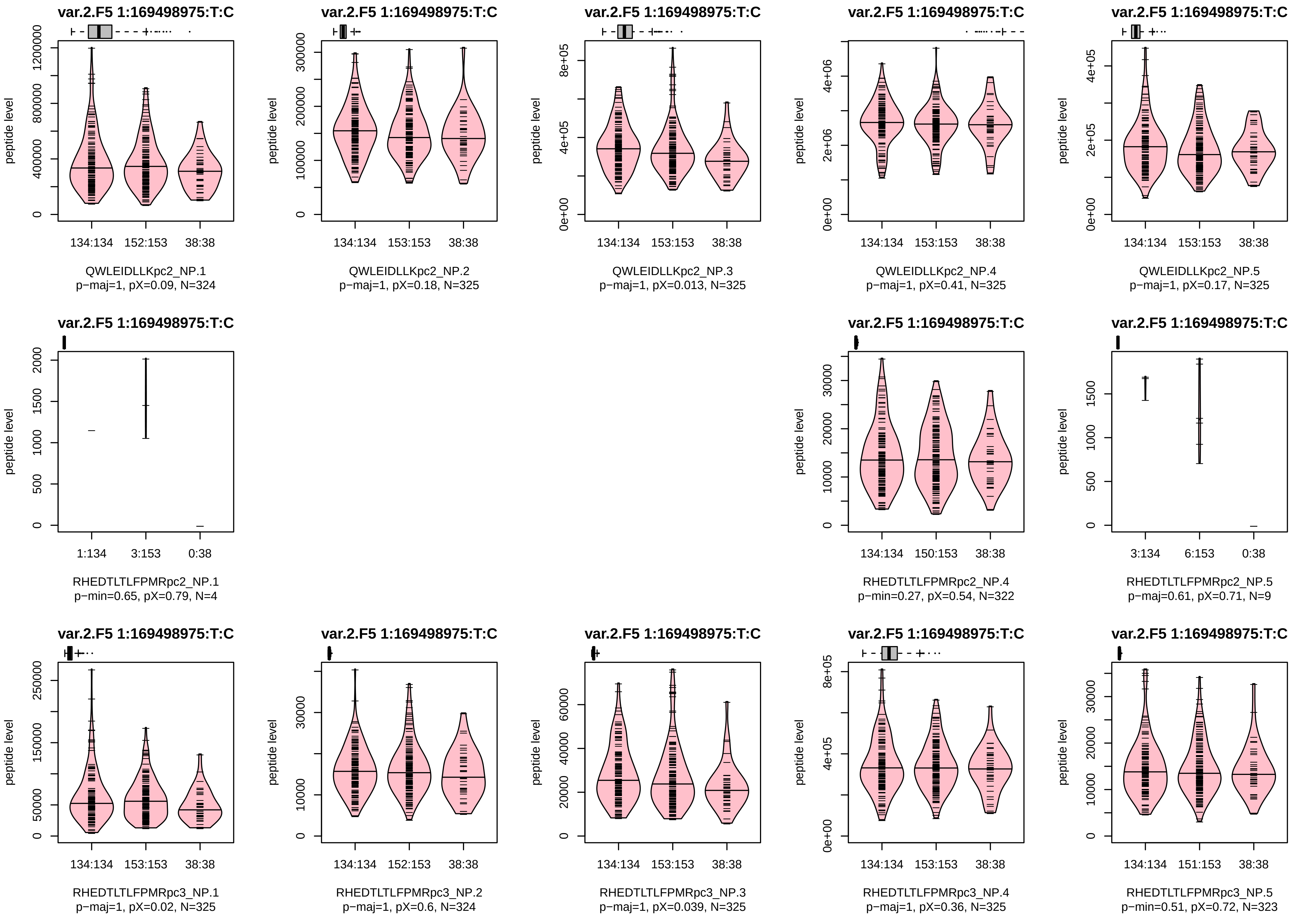

var.2.F5 1:169498975:T:C
peptide level
0
400000
800000
1200000
134:134
152:154
38:38
QWLEIDLLKpc2_NP.1
p−maj=1, pX=0.09, N=324
var.2.F5 1:169498975:T:C
peptide level
0
100000
200000
300000
134:134
153:153
38:38
QWLEIDLLKpc2_NP.2
p−maj=1, pX=0.18, N=325
var.2.F5 1:169498975:T:C
peptide level
0e+00
4e+05
8e+05
134:134
153:153
38:38
QWLEIDLLKpc2_NP.3
p−maj=1, pX=0.013, N=325
var.2.F5 1:169498975:T:C
peptide level
0e+00
2e+06
4e+06
134:134
153:153
38:38
QWLEIDLLKpc2_NP.4
p−maj=1, pX=0.41, N=325
var.2.F5 1:169498975:T:C
peptide level
0e+00
2e+05
4e+05
134:134
153:153
38:38
QWLEIDLLKpc2_NP.5
p−maj=1, pX=0.17, N=325
var.2.F5 1:169498975:T:C
peptide level
0
500
1000
1500
2000
1:134
3:153
0:38
RHEDTLTLFPMRpc2_NP.1
p−min=0.65, pX=0.79, N=4
var.2.F5 1:169498975:T:C
peptide level
0
10000
20000
30000
134:134
150:153
38:38
RHEDTLTLFPMRpc2_NP.4
p−min=0.27, pX=0.54, N=322
var.2.F5 1:169498975:T:C
peptide level
0
500
1000
1500
3:134
6:153
0:38
RHEDTLTLFPMRpc2_NP.5
p−maj=0.61, pX=0.71, N=9
var.2.F5 1:169498975:T:C
peptide level
0
50000
150000
250000
134:134
153:153
38:38
RHEDTLTLFPMRpc3_NP.1
p−maj=1, pX=0.02, N=325
var.2.F5 1:169498975:T:C
peptide level
0
10000
30000
134:134
152:153
38:38
RHEDTLTLFPMRpc3_NP.2
p−maj=1, pX=0.6, N=324
var.2.F5 1:169498975:T:C
peptide level
0
20000
40000
60000
134:134
153:153
38:38
RHEDTLTLFPMRpc3_NP.3
p−maj=1, pX=0.039, N=325
var.2.F5 1:169498975:T:C
peptide level
0e+00
4e+05
8e+05
134:134
153:153
38:38
RHEDTLTLFPMRpc3_NP.4
p−maj=1, pX=0.36, N=325
var.2.F5 1:169498975:T:C
peptide level
0
10000
20000
30000
134:134
151:153
38:38
RHEDTLTLFPMRpc3_NP.5
p−min=0.51, pX=0.72, N=323

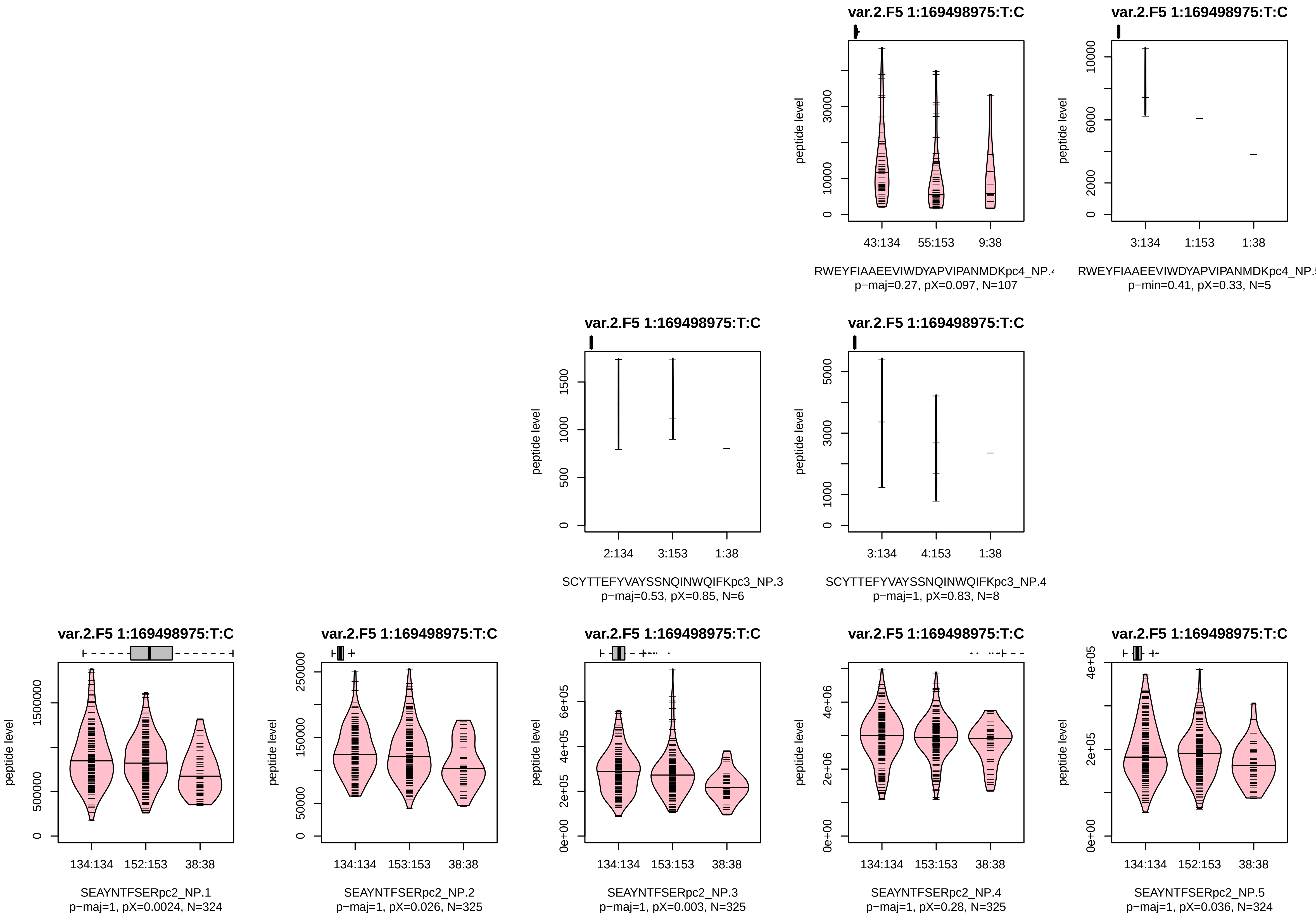

var.2.F5 1:169498975:T:C
peptide level
43:134
55:153
9:38
RWEYFIAAEEVIWDYAPVIPANMDKpc4_NP.4
p−maj=0.27, pX=0.097, N=107
var.2.F5 1:169498975:T:C
peptide level
3:134
1:153
1:38
RWEYFIAAEEVIWDYAPVIPANMDKpc4_NP.5
p−min=0.41, pX=0.33, N=5
var.2.F5 1:169498975:T:C
peptide level
2:134
3:153
1:38
SCYTTEFYVAYSSNQINWQIFKpc3_NP.3
p−maj=0.53, pX=0.85, N=6
var.2.F5 1:169498975:T:C
peptide level
3:134
4:153
1:38
SCYTTEFYVAYSSNQINWQIFKpc3_NP.4
p−maj=1, pX=0.83, N=8
var.2.F5 1:169498975:T:C
peptide level
134:134
152:153
38:38
SEAYNTFSERpc2_NP.1
p−maj=1, pX=0.0024, N=324
var.2.F5 1:169498975:T:C
peptide level
134:134
153:153
38:38
SEAYNTFSERpc2_NP.2
p−maj=1, pX=0.026, N=325
var.2.F5 1:169498975:T:C
peptide level
134:134
153:153
38:38
SEAYNTFSERpc2_NP.3
p−maj=1, pX=0.003, N=325
var.2.F5 1:169498975:T:C
peptide level
134:134
153:153
38:38
SEAYNTFSERpc2_NP.4
p−maj=1, pX=0.28, N=325
var.2.F5 1:169498975:T:C
peptide level
134:134
152:153
38:38
SEAYNTFSERpc2_NP.5
p−maj=1, pX=0.036, N=324

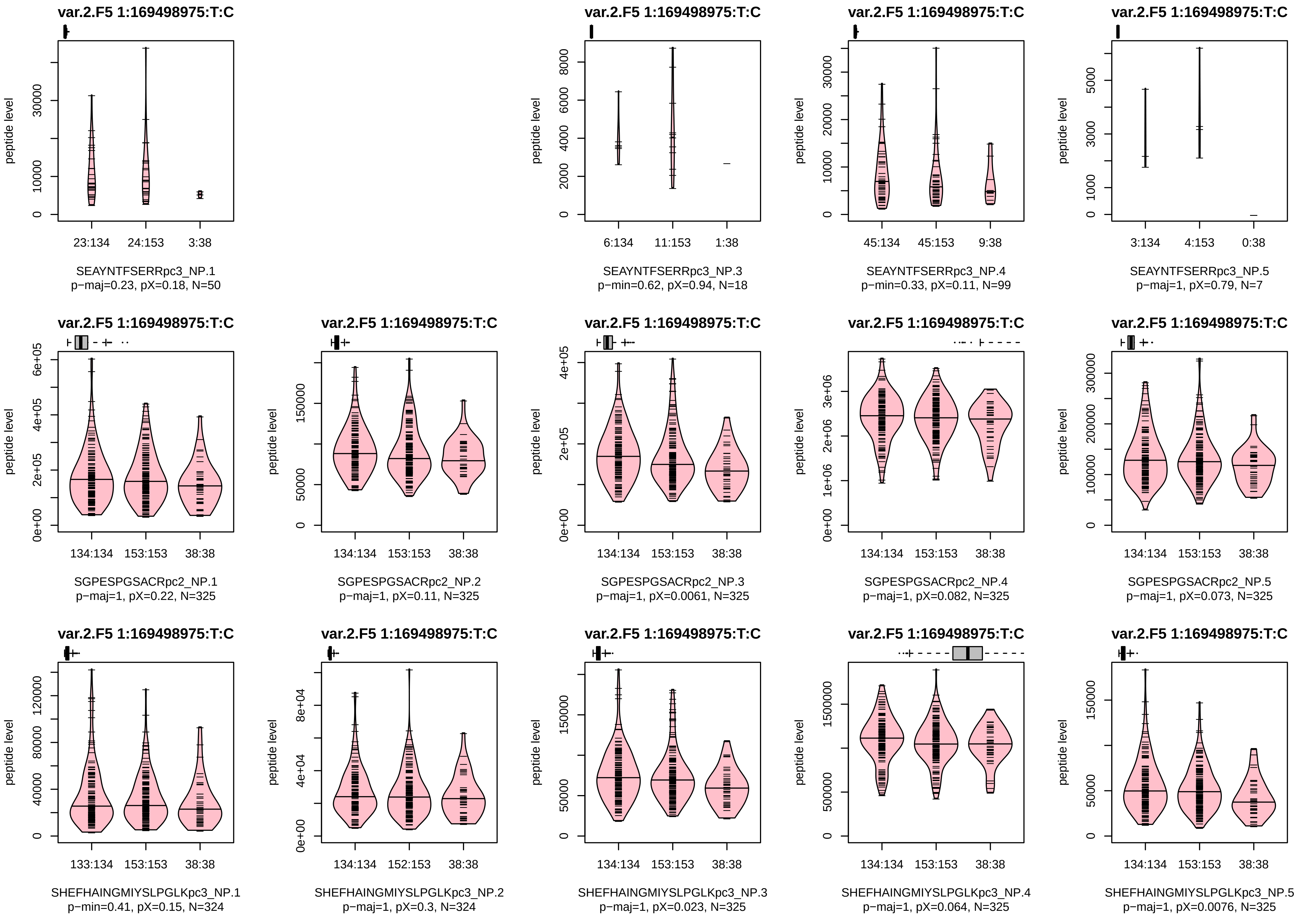

var.2.F5 1:169498975:T:C
peptide level
23:134
24:153
3:38
SEAYNTFSERRpc3_NP.1
p−maj=0.23, pX=0.18, N=50
var.2.F5 1:169498975:T:C
peptide level
6:134
11:153
1:38
SEAYNTFSERRpc3_NP.3
p−min=0.62, pX=0.94, N=18
var.2.F5 1:169498975:T:C
peptide level
45:134
45:153
9:38
SEAYNTFSERRpc3_NP.4
p−min=0.33, pX=0.11, N=99
var.2.F5 1:169498975:T:C
peptide level
3:134
4:153
0:38
SEAYNTFSERRpc3_NP.5
p−maj=1, pX=0.79, N=7
var.2.F5 1:169498975:T:C
peptide level
134:134
153:153
38:38
SGPESPGSACRpc2_NP.1
p−maj=1, pX=0.22, N=325
var.2.F5 1:169498975:T:C
peptide level
134:134
153:153
38:38
SGPESPGSACRpc2_NP.2
p−maj=1, pX=0.11, N=325
var.2.F5 1:169498975:T:C
peptide level
134:134
153:153
38:38
SGPESPGSACRpc2_NP.3
p−maj=1, pX=0.0061, N=325
var.2.F5 1:169498975:T:C
peptide level
134:134
153:153
38:38
SGPESPGSACRpc2_NP.4
p−maj=1, pX=0.082, N=325
var.2.F5 1:169498975:T:C
peptide level
134:134
153:153
38:38
SGPESPGSACRpc2_NP.5
p−maj=1, pX=0.073, N=325
var.2.F5 1:169498975:T:C
peptide level
133:134
153:153
38:38
SHEFHAINGMIYSLPGLKpc3_NP.1
p−min=0.41, pX=0.15, N=324
var.2.F5 1:169498975:T:C
peptide level
134:134
152:153
38:38
SHEFHAINGMIYSLPGLKpc3_NP.2
p−maj=1, pX=0.3, N=324
var.2.F5 1:169498975:T:C
peptide level
134:134
153:153
38:38
SHEFHAINGMIYSLPGLKpc3_NP.3
p−maj=1, pX=0.023, N=325
var.2.F5 1:169498975:T:C
peptide level
134:134
153:153
38:38
SHEFHAINGMIYSLPGLKpc3_NP.4
p−maj=1, pX=0.064, N=325
var.2.F5 1:169498975:T:C
peptide level
134:134
153:153
38:38
SHEFHAINGMIYSLPGLKpc3_NP.5
p−maj=1, pX=0.0076, N=325

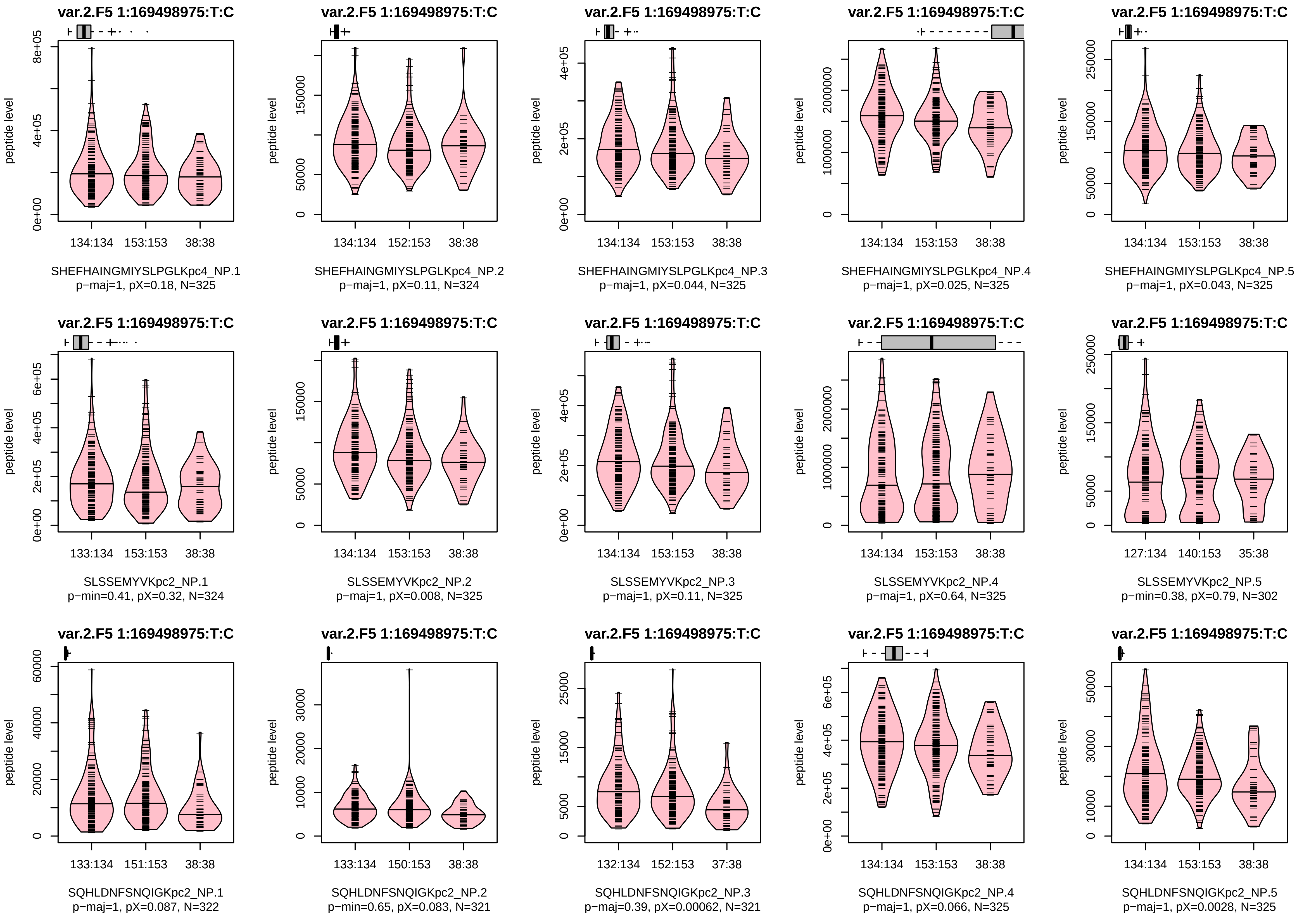
var.2.F5 1:169498975:T:C
peptide level
0e+00 4e+05 8e+05
134:134 153:153 38:38
SHEFHAINGMIYSLPGLKpc4_NP.1
p−maj=1, pX=0.18, N=325
var.2.F5 1:169498975:T:C
peptide level
0 50000 150000
134:134 152:153 38:38
SHEFHAINGMIYSLPGLKpc4_NP.2
p−maj=1, pX=0.11, N=324
var.2.F5 1:169498975:T:C
peptide level
0e+00 2e+05 4e+05
134:134 153:153 38:38
SHEFHAINGMIYSLPGLKpc4_NP.3
p−maj=1, pX=0.044, N=325
var.2.F5 1:169498975:T:C
peptide level
0 1000000 2000000
134:134 153:153 38:38
SHEFHAINGMIYSLPGLKpc4_NP.4
p−maj=1, pX=0.025, N=325
var.2.F5 1:169498975:T:C
peptide level
0 50000 150000 250000
134:134 153:153 38:38
SHEFHAINGMIYSLPGLKpc4_NP.5
p−maj=1, pX=0.043, N=325
var.2.F5 1:169498975:T:C
peptide level
0e+00 2e+05 4e+05 6e+05
133:134 153:153 38:38
SLSSEMYVKpc2_NP.1
p−min=0.41, pX=0.32, N=324
var.2.F5 1:169498975:T:C
peptide level
0 50000 150000
134:134 153:153 38:38
SLSSEMYVKpc2_NP.2
p−maj=1, pX=0.008, N=325
var.2.F5 1:169498975:T:C
peptide level
0e+00 2e+05 4e+05
134:134 153:153 38:38
SLSSEMYVKpc2_NP.3
p−maj=1, pX=0.11, N=325
var.2.F5 1:169498975:T:C
peptide level
0 1000000 2000000
134:134 153:153 38:38
SLSSEMYVKpc2_NP.4
p−maj=1, pX=0.64, N=325
var.2.F5 1:169498975:T:C
peptide level
0 50000 150000 250000
127:134 140:153 35:38
SLSSEMYVKpc2_NP.5
p−min=0.38, pX=0.79, N=302
var.2.F5 1:169498975:T:C
peptide level
0 20000 40000 60000
133:134 151:153 38:38
SQHLDNFSNQIGKpc2_NP.1
p−maj=1, pX=0.087, N=322
var.2.F5 1:169498975:T:C
peptide level
0 10000 20000 30000
133:134 150:153 38:38
SQHLDNFSNQIGKpc2_NP.2
p−min=0.65, pX=0.083, N=321
var.2.F5 1:169498975:T:C
peptide level
0 5000 15000 25000
132:134 152:153 37:38
SQHLDNFSNQIGKpc2_NP.3
p−maj=0.39, pX=0.00062, N=321
var.2.F5 1:169498975:T:C
peptide level
0e+00 2e+05 4e+05 6e+05
134:134 153:153 38:38
SQHLDNFSNQIGKpc2_NP.4
p−maj=1, pX=0.066, N=325
var.2.F5 1:169498975:T:C
peptide level
0 10000 30000 50000
134:134 153:153 38:38
SQHLDNFSNQIGKpc2_NP.5
p−maj=1, pX=0.0028, N=325

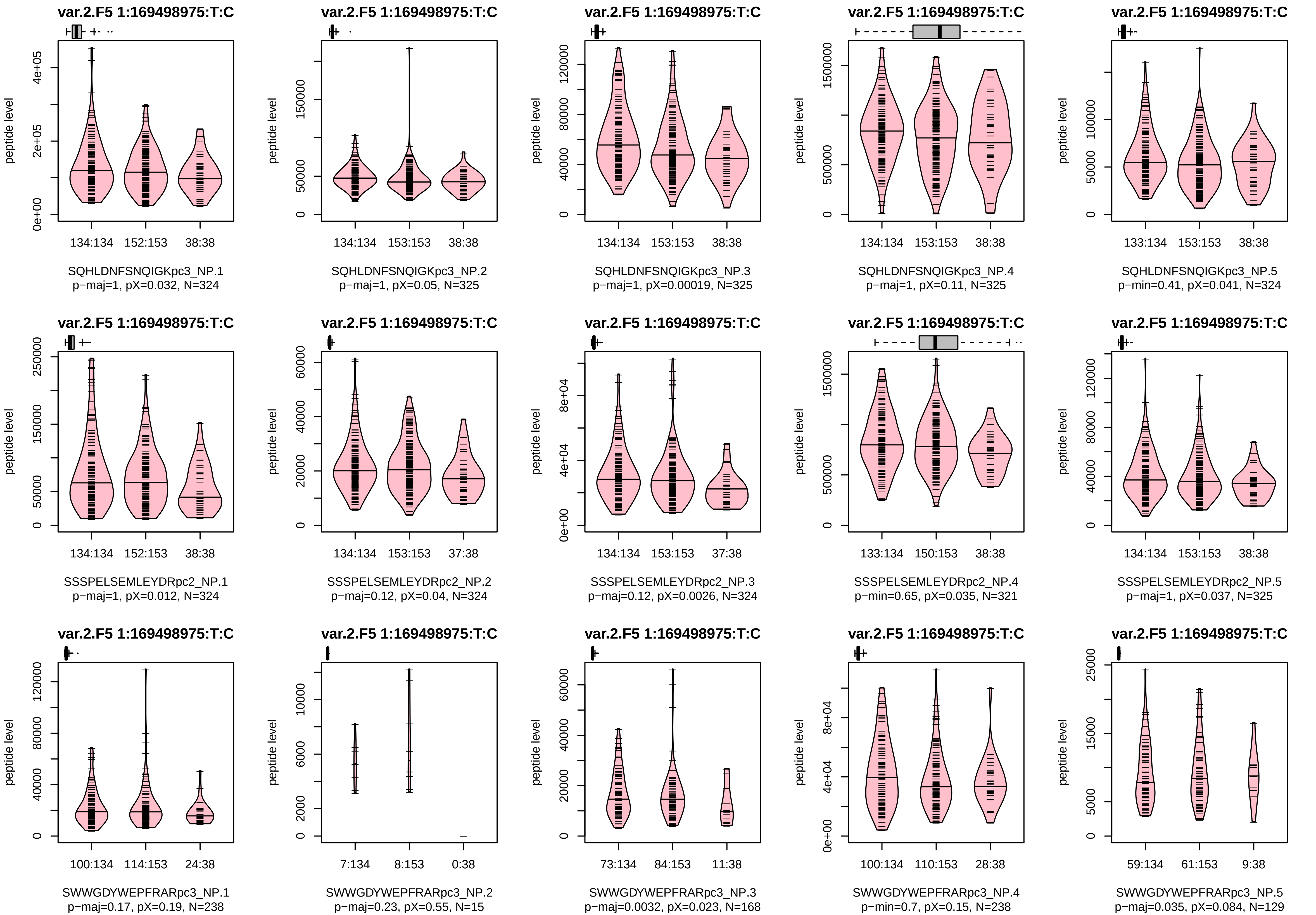
var.2.F5 1:169498975:T:C
peptide level
0e+00
2e+05
4e+05
134:134
152:153
38:38
SQHLDNFSNQIGKpc3_NP.1
p−maj=1, pX=0.032, N=324
var.2.F5 1:169498975:T:C
peptide level
0
50000
150000
134:134
153:153
38:38
SQHLDNFSNQIGKpc3_NP.2
p−maj=1, pX=0.05, N=325
var.2.F5 1:169498975:T:C
peptide level
0
40000
80000
120000
134:134
153:153
38:38
SQHLDNFSNQIGKpc3_NP.3
p−maj=1, pX=0.00019, N=325
var.2.F5 1:169498975:T:C
peptide level
0
500000
1500000
134:134
153:153
38:38
SQHLDNFSNQIGKpc3_NP.4
p−maj=1, pX=0.11, N=325
var.2.F5 1:169498975:T:C
peptide level
0
50000
100000
133:134
153:153
38:38
SQHLDNFSNQIGKpc3_NP.5
p−min=0.41, pX=0.041, N=324
var.2.F5 1:169498975:T:C
peptide level
0
50000
150000
250000
134:134
152:153
38:38
SSSPELSEMLEYDRpc2_NP.1
p−maj=1, pX=0.012, N=324
var.2.F5 1:169498975:T:C
peptide level
0
20000
40000
60000
134:134
153:153
37:38
SSSPELSEMLEYDRpc2_NP.2
p−maj=0.12, pX=0.04, N=324
var.2.F5 1:169498975:T:C
peptide level
0e+00
4e+04
8e+04
134:134
153:153
37:38
SSSPELSEMLEYDRpc2_NP.3
p−maj=0.12, pX=0.0026, N=324
var.2.F5 1:169498975:T:C
peptide level
0
500000
1500000
133:134
150:153
38:38
SSSPELSEMLEYDRpc2_NP.4
p−min=0.65, pX=0.035, N=321
var.2.F5 1:169498975:T:C
peptide level
0
40000
80000
120000
134:134
153:153
38:38
SSSPELSEMLEYDRpc2_NP.5
p−maj=1, pX=0.037, N=325
var.2.F5 1:169498975:T:C
peptide level
0
40000
80000
120000
100:134
114:153
24:38
SWWGDYWEPFRARpc3_NP.1
p−maj=0.17, pX=0.19, N=238
var.2.F5 1:169498975:T:C
peptide level
0
2000
6000
10000
7:134
8:153
0:38
SWWGDYWEPFRARpc3_NP.2
p−maj=0.23, pX=0.55, N=15
var.2.F5 1:169498975:T:C
peptide level
0
20000
40000
60000
73:134
84:153
11:38
SWWGDYWEPFRARpc3_NP.3
p−maj=0.0032, pX=0.023, N=168
var.2.F5 1:169498975:T:C
peptide level
0e+00
4e+04
8e+04
100:134
110:153
28:38
SWWGDYWEPFRARpc3_NP.4
p−min=0.7, pX=0.15, N=238
var.2.F5 1:169498975:T:C
peptide level
0
5000
15000
25000
59:134
61:153
9:38
SWWGDYWEPFRARpc3_NP.5
p−maj=0.035, pX=0.084, N=129

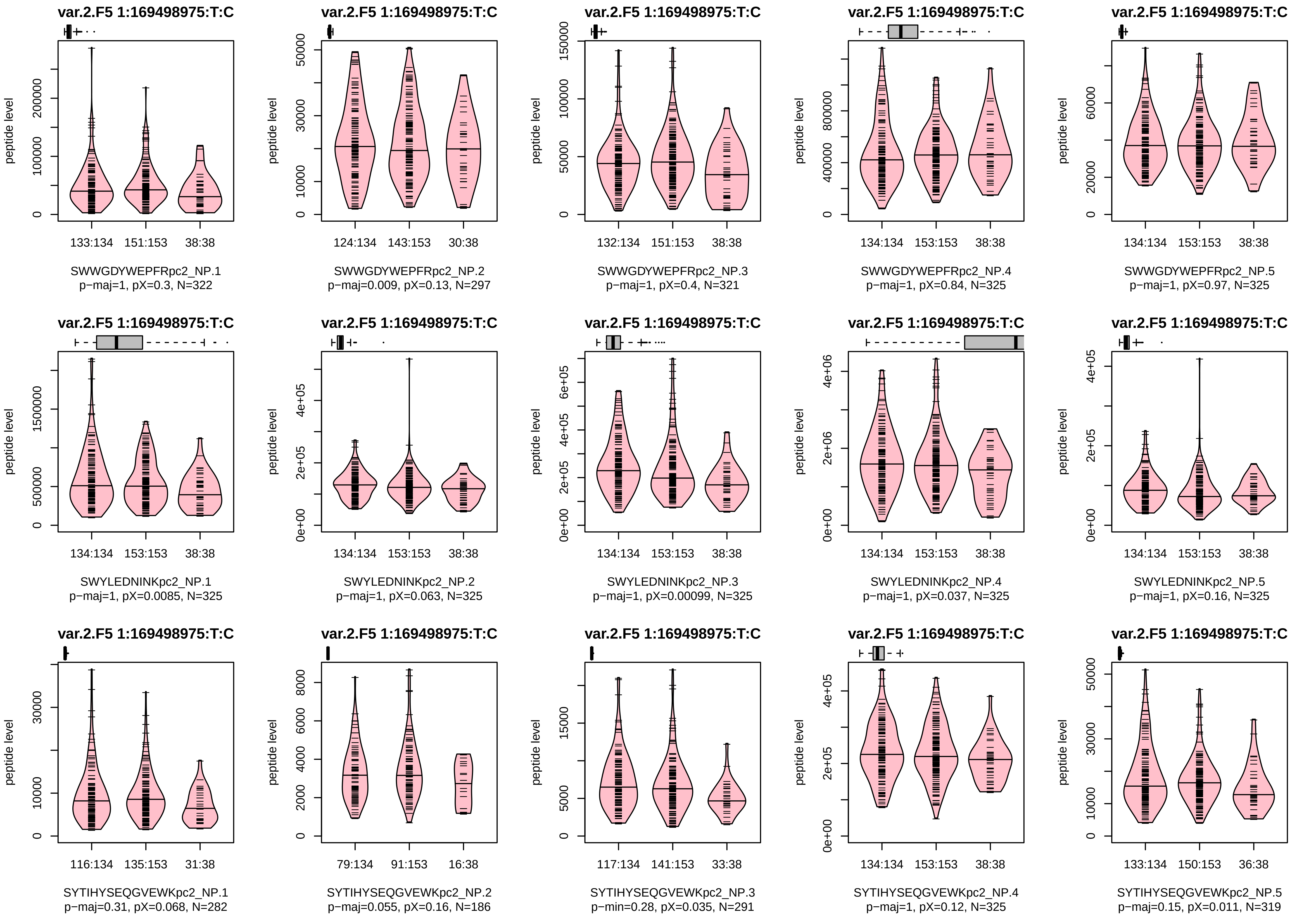
var.2.F5 1:169498975:T:C
peptide level
133:134
151:153
38:38
SWWGDYWEPFRpc2_NP.1
p−maj=1, pX=0.3, N=322
var.2.F5 1:169498975:T:C
peptide level
124:134
143:153
30:38
SWWGDYWEPFRpc2_NP.2
p−maj=0.009, pX=0.13, N=297
var.2.F5 1:169498975:T:C
peptide level
132:134
151:153
38:38
SWWGDYWEPFRpc2_NP.3
p−maj=1, pX=0.4, N=321
var.2.F5 1:169498975:T:C
peptide level
134:134
153:153
38:38
SWWGDYWEPFRpc2_NP.4
p−maj=1, pX=0.84, N=325
var.2.F5 1:169498975:T:C
peptide level
134:134
153:153
38:38
SWWGDYWEPFRpc2_NP.5
p−maj=1, pX=0.97, N=325
var.2.F5 1:169498975:T:C
peptide level
134:134
153:153
38:38
SWYLEDNINKpc2_NP.1
p−maj=1, pX=0.0085, N=325
var.2.F5 1:169498975:T:C
peptide level
134:134
153:153
38:38
SWYLEDNINKpc2_NP.2
p−maj=1, pX=0.063, N=325
var.2.F5 1:169498975:T:C
peptide level
134:134
153:153
38:38
SWYLEDNINKpc2_NP.3
p−maj=1, pX=0.00099, N=325
var.2.F5 1:169498975:T:C
peptide level
134:134
153:153
38:38
SWYLEDNINKpc2_NP.4
p−maj=1, pX=0.037, N=325
var.2.F5 1:169498975:T:C
peptide level
134:134
153:153
38:38
SWYLEDNINKpc2_NP.5
p−maj=1, pX=0.16, N=325
var.2.F5 1:169498975:T:C
peptide level
116:134
135:153
31:38
SYTIHYSEQGVEWKpc2_NP.1
p−maj=0.31, pX=0.068, N=282
var.2.F5 1:169498975:T:C
peptide level
79:134
91:153
16:38
SYTIHYSEQGVEWKpc2_NP.2
p−maj=0.055, pX=0.16, N=186
var.2.F5 1:169498975:T:C
peptide level
117:134
141:153
33:38
SYTIHYSEQGVEWKpc2_NP.3
p−min=0.28, pX=0.035, N=291
var.2.F5 1:169498975:T:C
peptide level
134:134
153:153
38:38
SYTIHYSEQGVEWKpc2_NP.4
p−maj=1, pX=0.12, N=325
var.2.F5 1:169498975:T:C
peptide level
133:134
150:153
36:38
SYTIHYSEQGVEWKpc2_NP.5
p−maj=0.15, pX=0.011, N=319

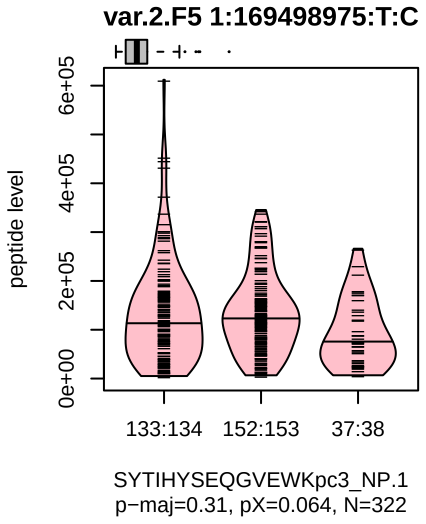
var.2.F5 1:169498975:T:C
peptide level
0e+00
2e+05
4e+05
6e+05
133:134
152:153
37:38
SYTIHYSEQGVEWKpc3_NP.1
p−maj=0.31, pX=0.064, N=322

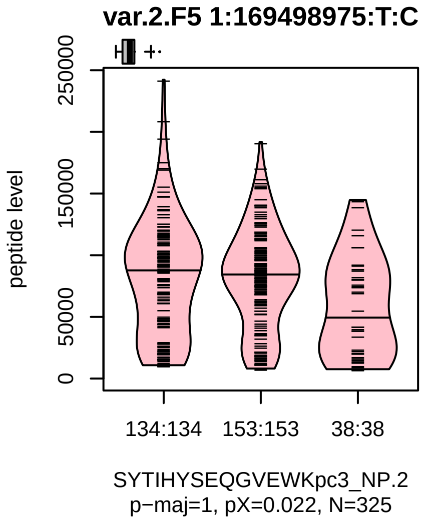
var.2.F5 1:169498975:T:C
peptide level
0
50000
150000
250000
134:134
153:153
38:38
SYTIHYSEQGVEWKpc3_NP.2
p−maj=1, pX=0.022, N=325

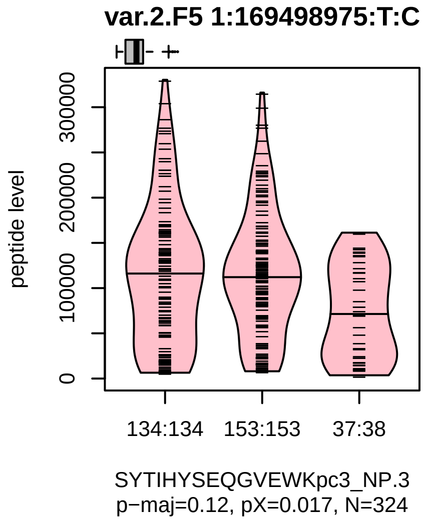
var.2.F5 1:169498975:T:C
peptide level
0
100000
200000
300000
134:134
153:153
37:38
SYTIHYSEQGVEWKpc3_NP.3
p−maj=0.12, pX=0.017, N=324

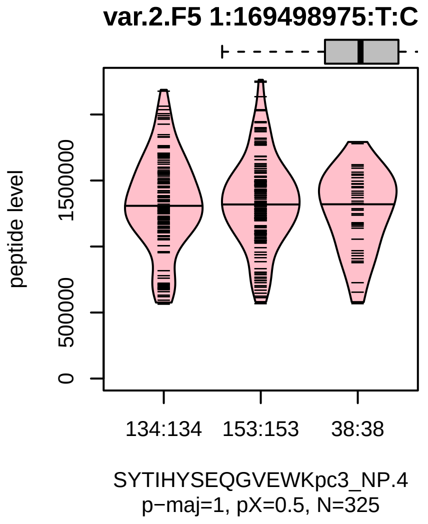
var.2.F5 1:169498975:T:C
peptide level
0
500000
1500000
134:134
153:153
38:38
SYTIHYSEQGVEWKpc3_NP.4
p−maj=1, pX=0.5, N=325

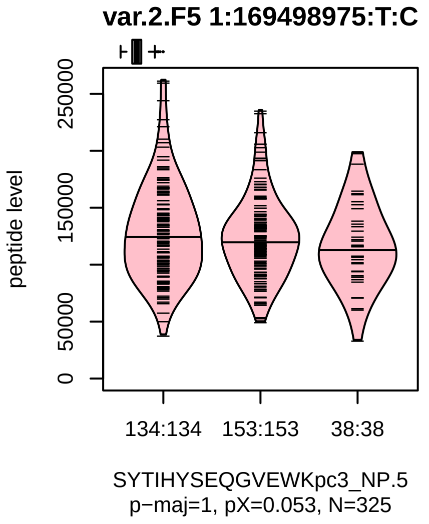
var.2.F5 1:169498975:T:C
peptide level
0
50000
150000
250000
134:134
153:153
38:38
SYTIHYSEQGVEWKpc3_NP.5
p−maj=1, pX=0.053, N=325

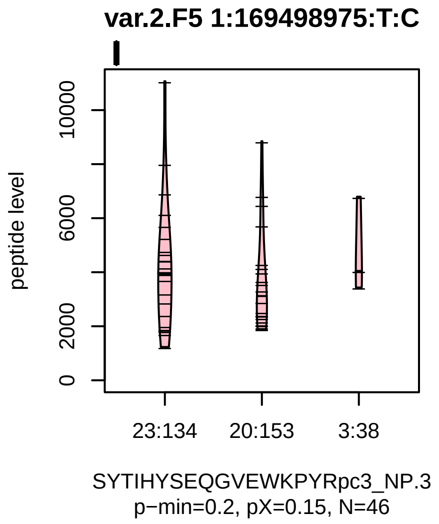
var.2.F5 1:169498975:T:C
peptide level
0
2000
6000
10000
23:134
20:153
3:38
SYTIHYSEQGVEWKPYRpc3_NP.3
p−min=0.2, pX=0.15, N=46

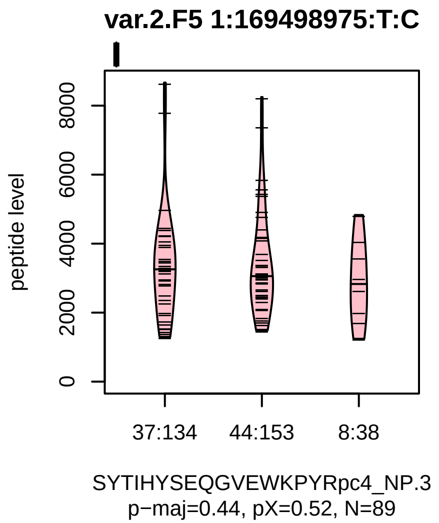
var.2.F5 1:169498975:T:C
peptide level
0
2000
4000
6000
8000
37:134
44:153
8:38
SYTIHYSEQGVEWKPYRpc4_NP.3
p−maj=0.44, pX=0.52, N=89

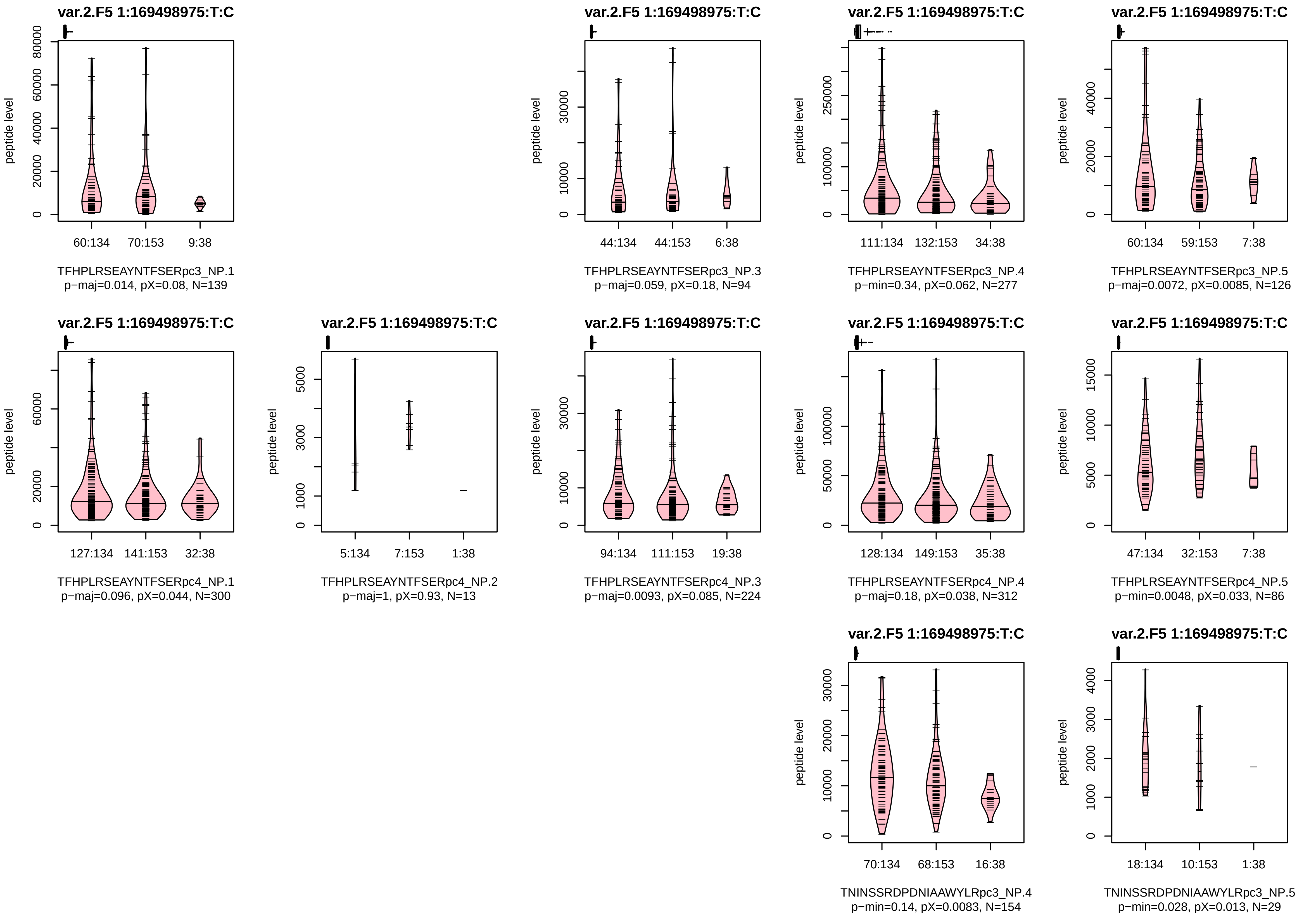

var.2.F5 1:169498975:T:C
peptide level
60:134
70:153
9:38
TFHPLRSEAYNTFSERpc3_NP.1
p−maj=0.014, pX=0.08, N=139
var.2.F5 1:169498975:T:C
peptide level
44:134
44:153
6:38
TFHPLRSEAYNTFSERpc3_NP.3
p−maj=0.059, pX=0.18, N=94
var.2.F5 1:169498975:T:C
peptide level
111:134
132:153
34:38
TFHPLRSEAYNTFSERpc3_NP.4
p−min=0.34, pX=0.062, N=277
var.2.F5 1:169498975:T:C
peptide level
60:134
59:153
7:38
TFHPLRSEAYNTFSERpc3_NP.5
p−maj=0.0072, pX=0.0085, N=126
var.2.F5 1:169498975:T:C
peptide level
127:134
141:153
32:38
TFHPLRSEAYNTFSERpc4_NP.1
p−maj=0.096, pX=0.044, N=300
var.2.F5 1:169498975:T:C
peptide level
5:134
7:153
1:38
TFHPLRSEAYNTFSERpc4_NP.2
p−maj=1, pX=0.93, N=13
var.2.F5 1:169498975:T:C
peptide level
94:134
111:153
19:38
TFHPLRSEAYNTFSERpc4_NP.3
p−maj=0.0093, pX=0.085, N=224
var.2.F5 1:169498975:T:C
peptide level
128:134
149:153
35:38
TFHPLRSEAYNTFSERpc4_NP.4
p−maj=0.18, pX=0.038, N=312
var.2.F5 1:169498975:T:C
peptide level
47:134
32:153
7:38
TFHPLRSEAYNTFSERpc4_NP.5
p−min=0.0048, pX=0.033, N=86
var.2.F5 1:169498975:T:C
peptide level
70:134
68:153
16:38
TNINSSRDPDNIAAWYLRpc3_NP.4
p−min=0.14, pX=0.0083, N=154
var.2.F5 1:169498975:T:C
peptide level
18:134
10:153
1:38
TNINSSRDPDNIAAWYLRpc3_NP.5
p−min=0.028, pX=0.013, N=29

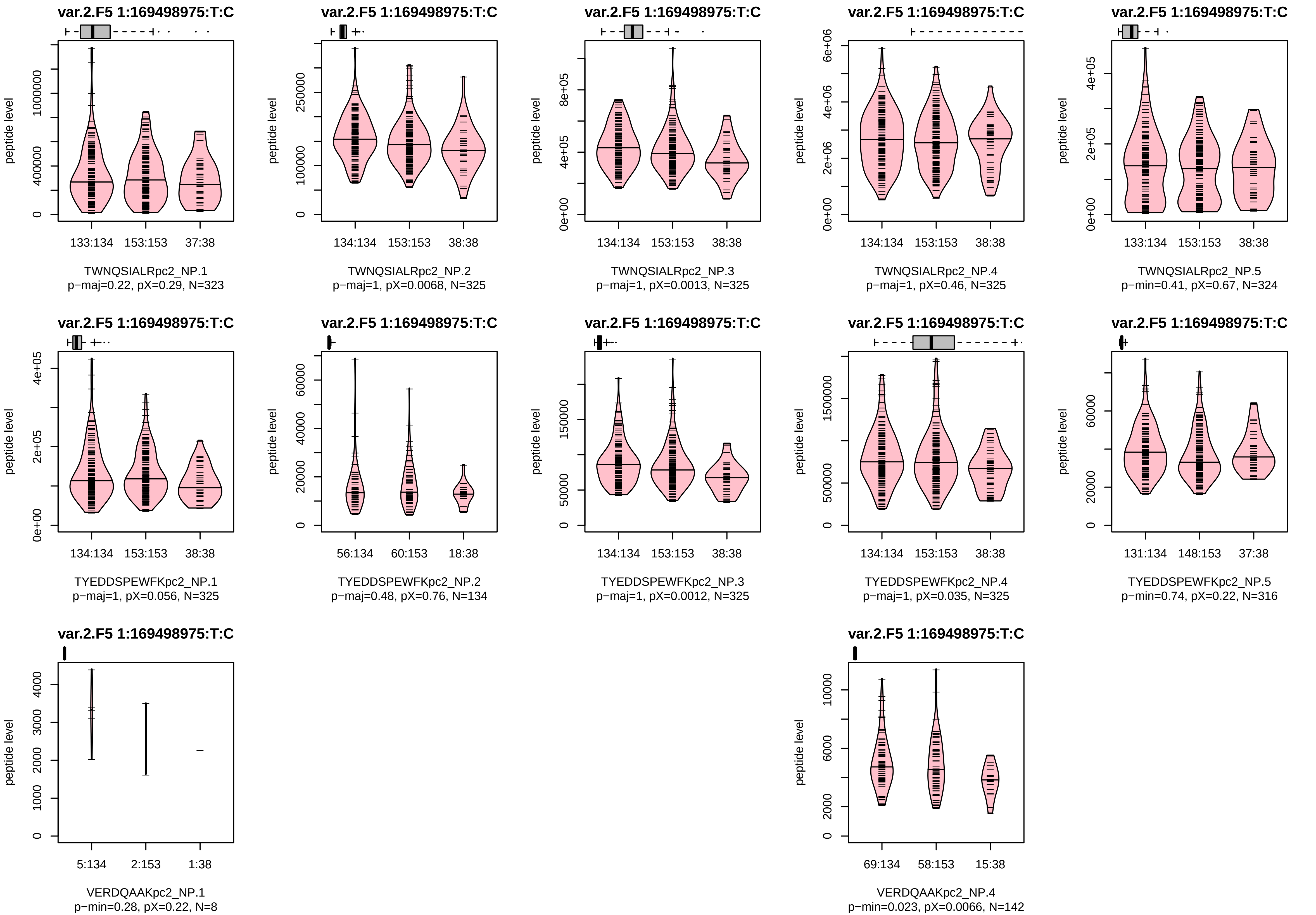
var.2.F5 1:169498975:T:C
peptide level
133:134
153:153
37:38
TWNQSIALRpc2_NP.1
p−maj=0.22, pX=0.29, N=323
var.2.F5 1:169498975:T:C
134:134
153:153
38:38
TWNQSIALRpc2_NP.2
p−maj=1, pX=0.0068, N=325
var.2.F5 1:169498975:T:C
134:134
153:153
38:38
TWNQSIALRpc2_NP.3
p−maj=1, pX=0.0013, N=325
var.2.F5 1:169498975:T:C
134:134
153:153
38:38
TWNQSIALRpc2_NP.4
p−maj=1, pX=0.46, N=325
var.2.F5 1:169498975:T:C
133:134
153:153
38:38
TWNQSIALRpc2_NP.5
p−min=0.41, pX=0.67, N=324
var.2.F5 1:169498975:T:C
134:134
153:153
38:38
TYEDDSPEWFKpc2_NP.1
p−maj=1, pX=0.056, N=325
var.2.F5 1:169498975:T:C
56:134
60:153
18:38
TYEDDSPEWFKpc2_NP.2
p−maj=0.48, pX=0.76, N=134
var.2.F5 1:169498975:T:C
134:134
153:153
38:38
TYEDDSPEWFKpc2_NP.3
p−maj=1, pX=0.0012, N=325
var.2.F5 1:169498975:T:C
134:134
153:153
38:38
TYEDDSPEWFKpc2_NP.4
p−maj=1, pX=0.035, N=325
var.2.F5 1:169498975:T:C
131:134
148:153
37:38
TYEDDSPEWFKpc2_NP.5
p−min=0.74, pX=0.22, N=316
var.2.F5 1:169498975:T:C
5:134
2:153
1:38
VERDQAAKpc2_NP.1
p−min=0.28, pX=0.22, N=8
var.2.F5 1:169498975:T:C
69:134
58:153
15:38
VERDQAAKpc2_NP.4
p−min=0.023, pX=0.0066, N=142

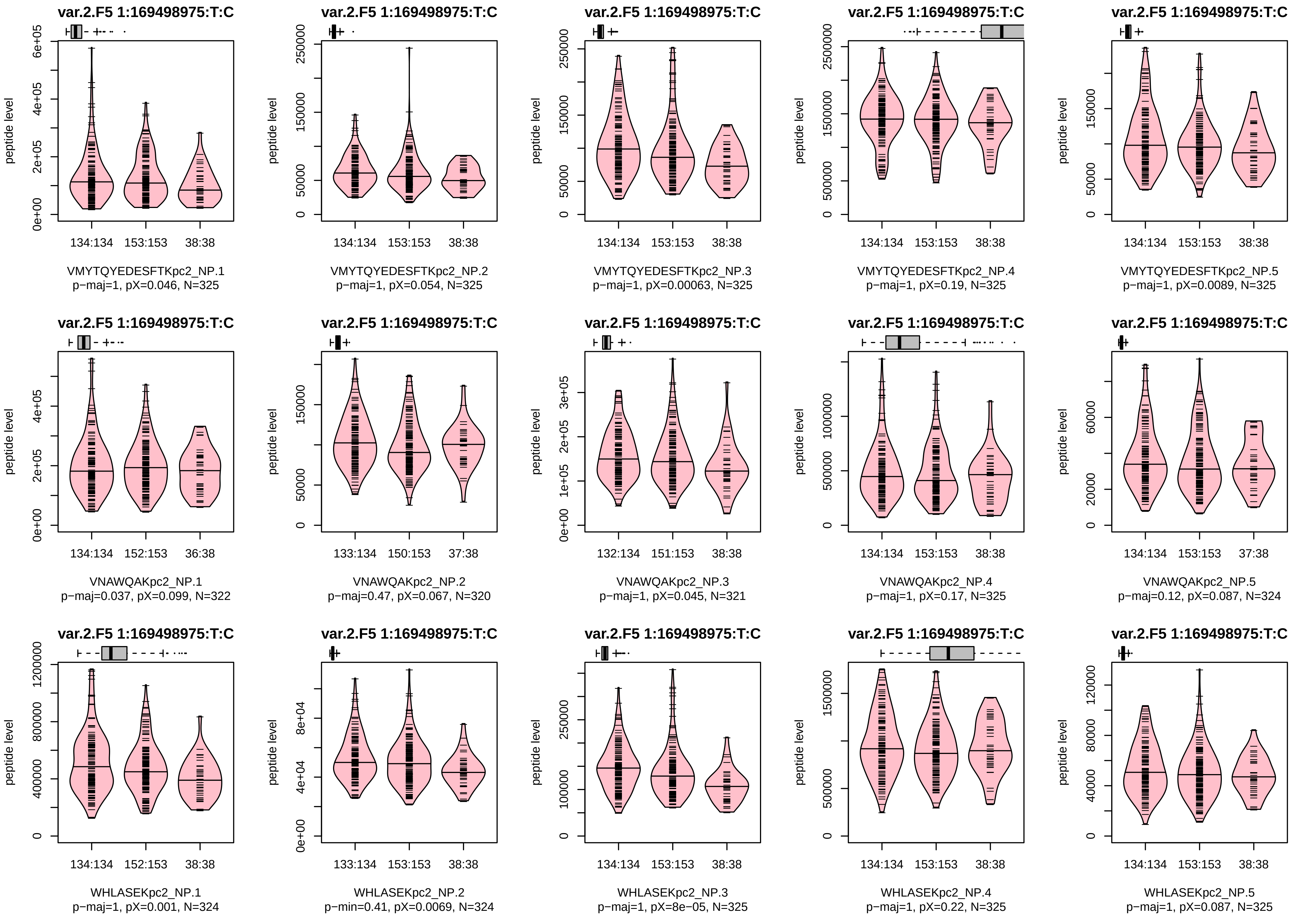
var.2.F5 1:169498975:T:C
peptide level
134:134
153:153
38:38
VMYTQYEDESFTKpc2_NP.1
p−maj=1, pX=0.046, N=325
var.2.F5 1:169498975:T:C
peptide level
134:134
153:153
38:38
VMYTQYEDESFTKpc2_NP.2
p−maj=1, pX=0.054, N=325
var.2.F5 1:169498975:T:C
peptide level
134:134
153:153
38:38
VMYTQYEDESFTKpc2_NP.3
p−maj=1, pX=0.00063, N=325
var.2.F5 1:169498975:T:C
peptide level
134:134
153:153
38:38
VMYTQYEDESFTKpc2_NP.4
p−maj=1, pX=0.19, N=325
var.2.F5 1:169498975:T:C
peptide level
134:134
153:153
38:38
VMYTQYEDESFTKpc2_NP.5
p−maj=1, pX=0.0089, N=325
var.2.F5 1:169498975:T:C
peptide level
134:134
152:153
36:38
VNAWQAKpc2_NP.1
p−maj=0.037, pX=0.099, N=322
var.2.F5 1:169498975:T:C
peptide level
133:134
150:153
37:38
VNAWQAKpc2_NP.2
p−maj=0.47, pX=0.067, N=320
var.2.F5 1:169498975:T:C
peptide level
132:134
151:153
38:38
VNAWQAKpc2_NP.3
p−maj=1, pX=0.045, N=321
var.2.F5 1:169498975:T:C
peptide level
134:134
153:153
38:38
VNAWQAKpc2_NP.4
p−maj=1, pX=0.17, N=325
var.2.F5 1:169498975:T:C
peptide level
134:134
153:153
37:38
VNAWQAKpc2_NP.5
p−maj=0.12, pX=0.087, N=324
var.2.F5 1:169498975:T:C
peptide level
134:134
152:153
38:38
WHLASEKpc2_NP.1
p−maj=1, pX=0.001, N=324
var.2.F5 1:169498975:T:C
peptide level
133:134
153:153
38:38
WHLASEKpc2_NP.2
p−min=0.41, pX=0.0069, N=324
var.2.F5 1:169498975:T:C
peptide level
134:134
153:153
38:38
WHLASEKpc2_NP.3
p−maj=1, pX=8e−05, N=325
var.2.F5 1:169498975:T:C
peptide level
134:134
153:153
38:38
WHLASEKpc2_NP.4
p−maj=1, pX=0.22, N=325
var.2.F5 1:169498975:T:C
peptide level
134:134
153:153
38:38
WHLASEKpc2_NP.5
p−maj=1, pX=0.087, N=325

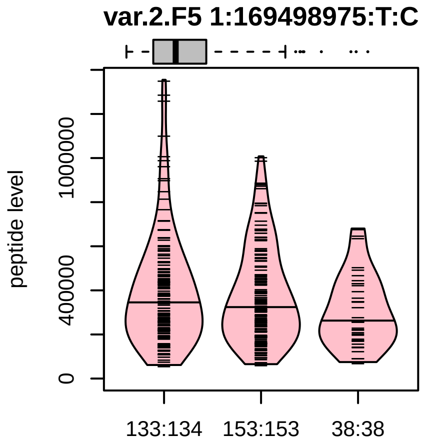

WIISSLTPKpc2_NP.1
p−min=0.41, pX=0.057, N=324

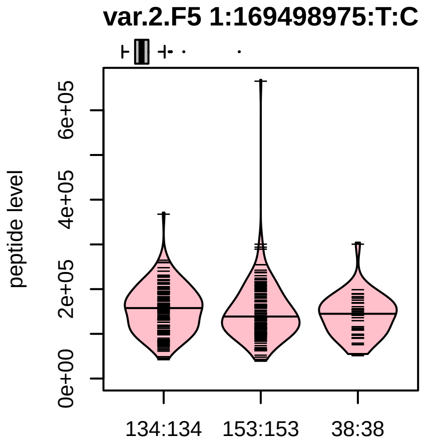

WIISSLTPKpc2_NP.2
p−maj=1, pX=0.17, N=325

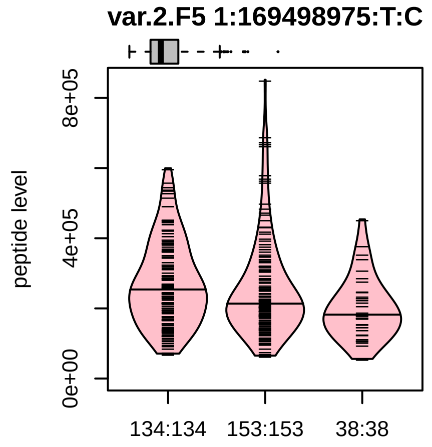

WIISSLTPKpc2_NP.3
p−maj=1, pX=0.0049, N=325

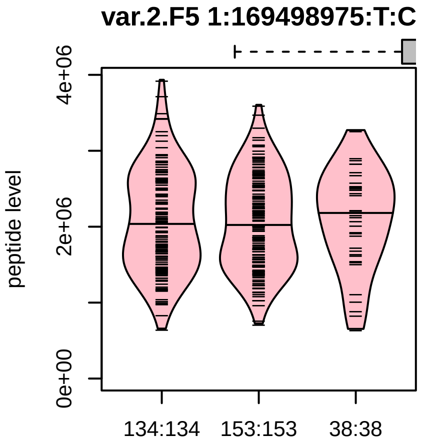

WIISSLTPKpc2_NP.4
p−maj=1, pX=0.91, N=325

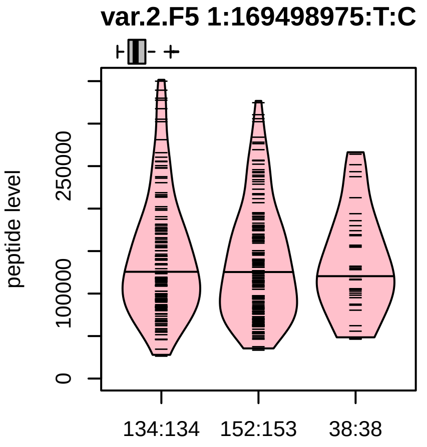

WIISSLTPKpc2_NP.5
p−maj=1, pX=0.37, N=324

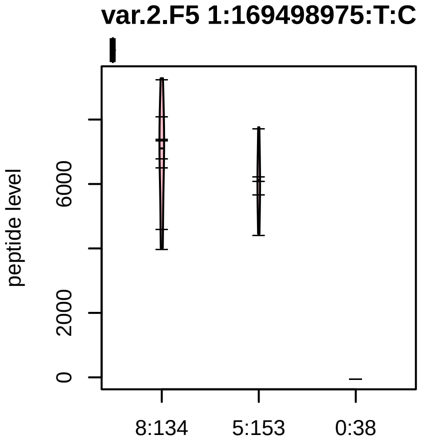

WNILEFDEPTENDAQCLTRPYYSDVDIMKpc4_N
p−min=0.16, pX=0.062, N=13

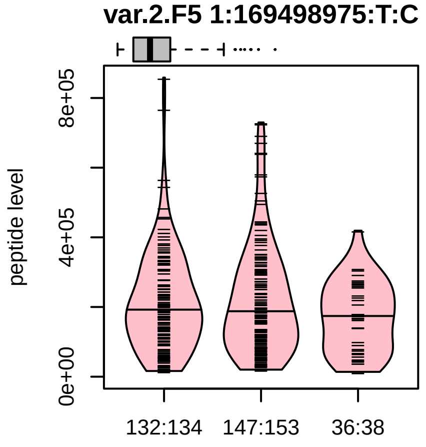

YLDSTFTKpc2_NP.1
p−min=0.21, pX=0.21, N=315

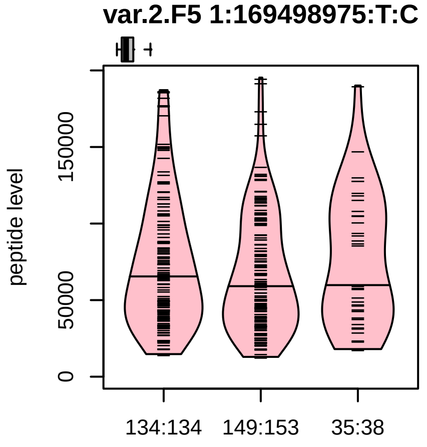

YLDSTFTKpc2_NP.2
p−maj=0.037, pX=0.31, N=318

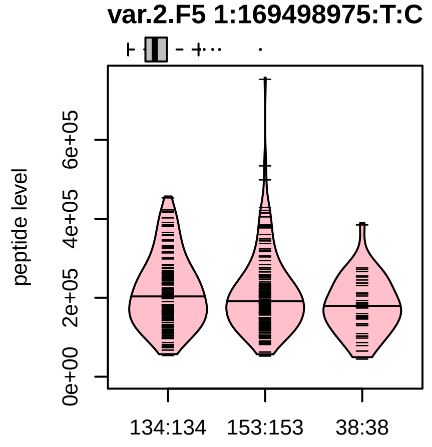

YLDSTFTKpc2_NP.3
p−maj=1, pX=0.073, N=325

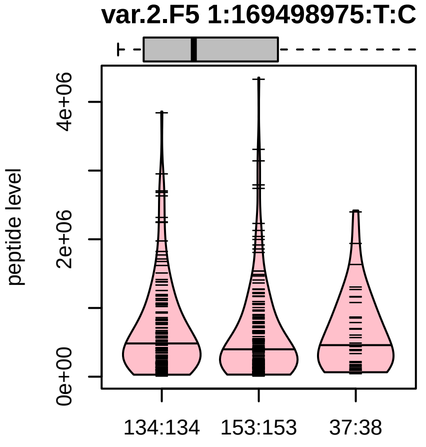

YLDSTFTKpc2_NP.4
p−maj=0.12, pX=0.38, N=324

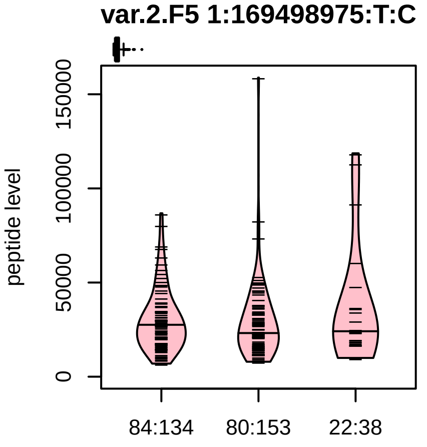

YLDSTFTKpc2_NP.5
p−min=0.11, pX=0.9, N=186

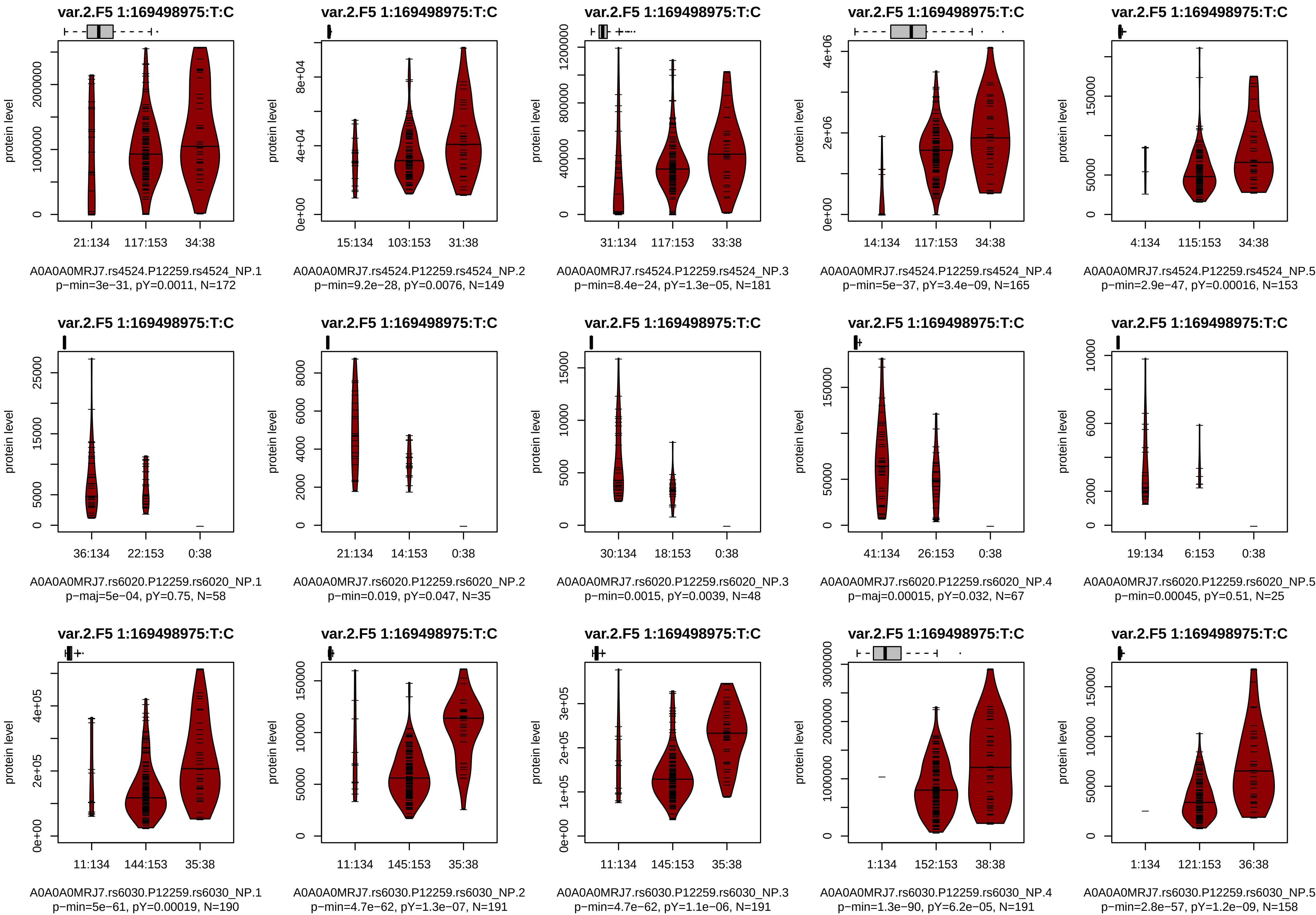
var.2.F5 1:169498975:T:C
protein level
21:134
117:153
34:38
A0A0A0MRJ7.rs4524.P12259.rs4524_NP.1
p−min=3e−31, pY=0.0011, N=172
var.2.F5 1:169498975:T:C
protein level
15:134
103:153
31:38
A0A0A0MRJ7.rs4524.P12259.rs4524_NP.2
p−min=9.2e−28, pY=0.0076, N=149
var.2.F5 1:169498975:T:C
protein level
31:134
117:153
33:38
A0A0A0MRJ7.rs4524.P12259.rs4524_NP.3
p−min=8.4e−24, pY=1.3e−05, N=181
var.2.F5 1:169498975:T:C
protein level
14:134
117:153
34:38
A0A0A0MRJ7.rs4524.P12259.rs4524_NP.4
p−min=5e−37, pY=3.4e−09, N=165
var.2.F5 1:169498975:T:C
protein level
4:134
115:153
34:38
A0A0A0MRJ7.rs4524.P12259.rs4524_NP.5
p−min=2.9e−47, pY=0.00016, N=153
var.2.F5 1:169498975:T:C
protein level
36:134
22:153
0:38
A0A0A0MRJ7.rs6020.P12259.rs6020_NP.1
p−maj=5e−04, pY=0.75, N=58
var.2.F5 1:169498975:T:C
protein level
21:134
14:153
0:38
A0A0A0MRJ7.rs6020.P12259.rs6020_NP.2
p−min=0.019, pY=0.047, N=35
var.2.F5 1:169498975:T:C
protein level
30:134
18:153
0:38
A0A0A0MRJ7.rs6020.P12259.rs6020_NP.3
p−min=0.0015, pY=0.0039, N=48
var.2.F5 1:169498975:T:C
protein level
41:134
26:153
0:38
A0A0A0MRJ7.rs6020.P12259.rs6020_NP.4
p−maj=0.00015, pY=0.032, N=67
var.2.F5 1:169498975:T:C
protein level
19:134
6:153
0:38
A0A0A0MRJ7.rs6020.P12259.rs6020_NP.5
p−min=0.00045, pY=0.51, N=25
var.2.F5 1:169498975:T:C
protein level
11:134
144:153
35:38
A0A0A0MRJ7.rs6030.P12259.rs6030_NP.1
p−min=5e−61, pY=0.00019, N=190
var.2.F5 1:169498975:T:C
protein level
11:134
145:153
35:38
A0A0A0MRJ7.rs6030.P12259.rs6030_NP.2
p−min=4.7e−62, pY=1.3e−07, N=191
var.2.F5 1:169498975:T:C
protein level
11:134
145:153
35:38
A0A0A0MRJ7.rs6030.P12259.rs6030_NP.3
p−min=4.7e−62, pY=1.1e−06, N=191
var.2.F5 1:169498975:T:C
protein level
1:134
152:153
38:38
A0A0A0MRJ7.rs6030.P12259.rs6030_NP.4
p−min=1.3e−90, pY=6.2e−05, N=191
var.2.F5 1:169498975:T:C
protein level
1:134
121:153
36:38
A0A0A0MRJ7.rs6030.P12259.rs6030_NP.5
p−min=2.8e−57, pY=1.2e−09, N=158

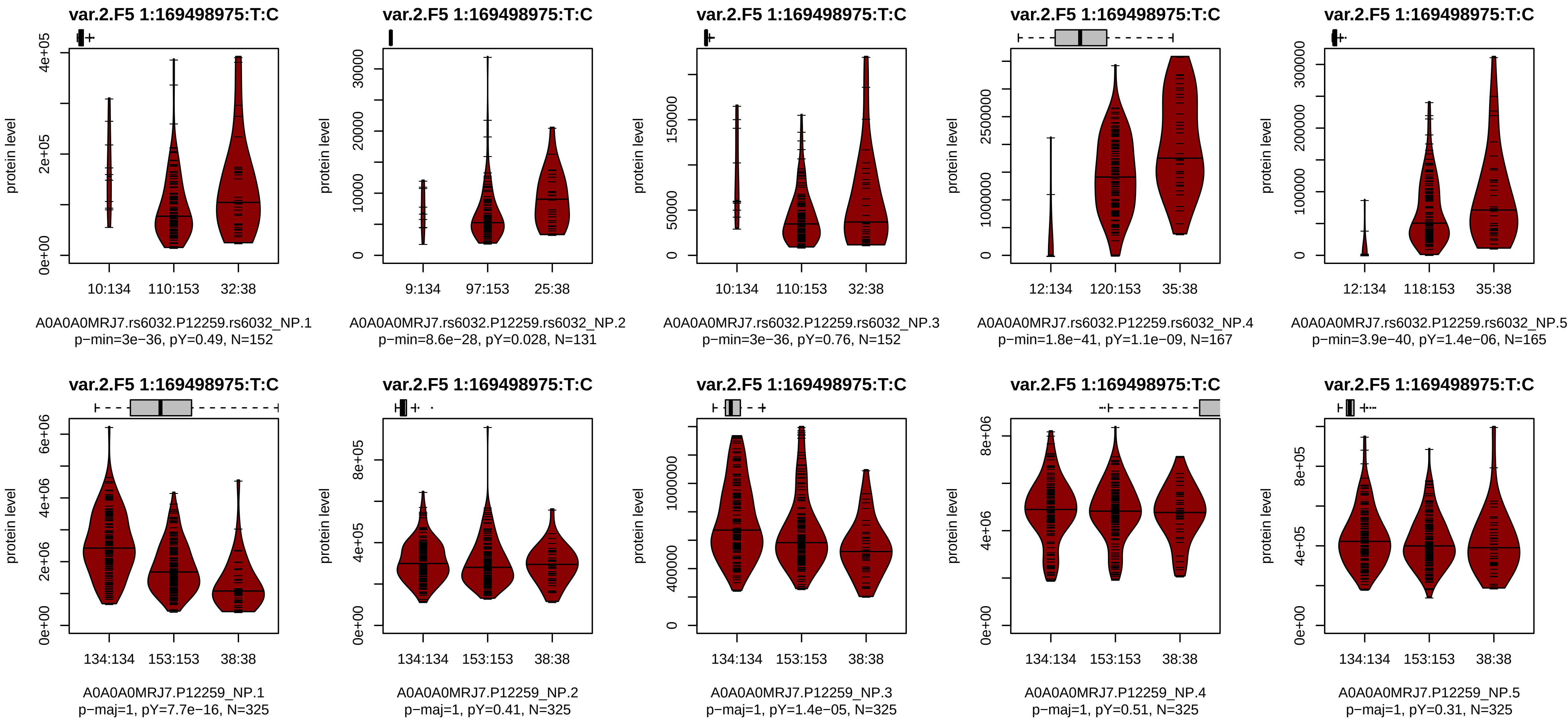
var.2.F5 1:169498975:T:C
protein level
10:134
110:153
32:38
A0A0A0MRJ7.rs6032.P12259.rs6032_NP.1
p−min=3e−36, pY=0.49, N=152
9:134
97:153
25:38
A0A0A0MRJ7.rs6032.P12259.rs6032_NP.2
p−min=8.6e−28, pY=0.028, N=131
A0A0A0MRJ7.rs6032.P12259.rs6032_NP.3
p−min=3e−36, pY=0.76, N=152
12:134
120:153
35:38
A0A0A0MRJ7.rs6032.P12259.rs6032_NP.4
p−min=1.8e−41, pY=1.1e−09, N=167
118:153
A0A0A0MRJ7.rs6032.P12259.rs6032_NP.5
p−min=3.9e−40, pY=1.4e−06, N=165
134:134
153:153
38:38
A0A0A0MRJ7.P12259_NP.1
p−maj=1, pY=7.7e−16, N=325
A0A0A0MRJ7.P12259_NP.2
p−maj=1, pY=0.41, N=325
A0A0A0MRJ7.P12259_NP.3
p−maj=1, pY=1.4e−05, N=325
A0A0A0MRJ7.P12259_NP.4
p−maj=1, pY=0.51, N=325
A0A0A0MRJ7.P12259_NP.5
p−maj=1, pY=0.31, N=325

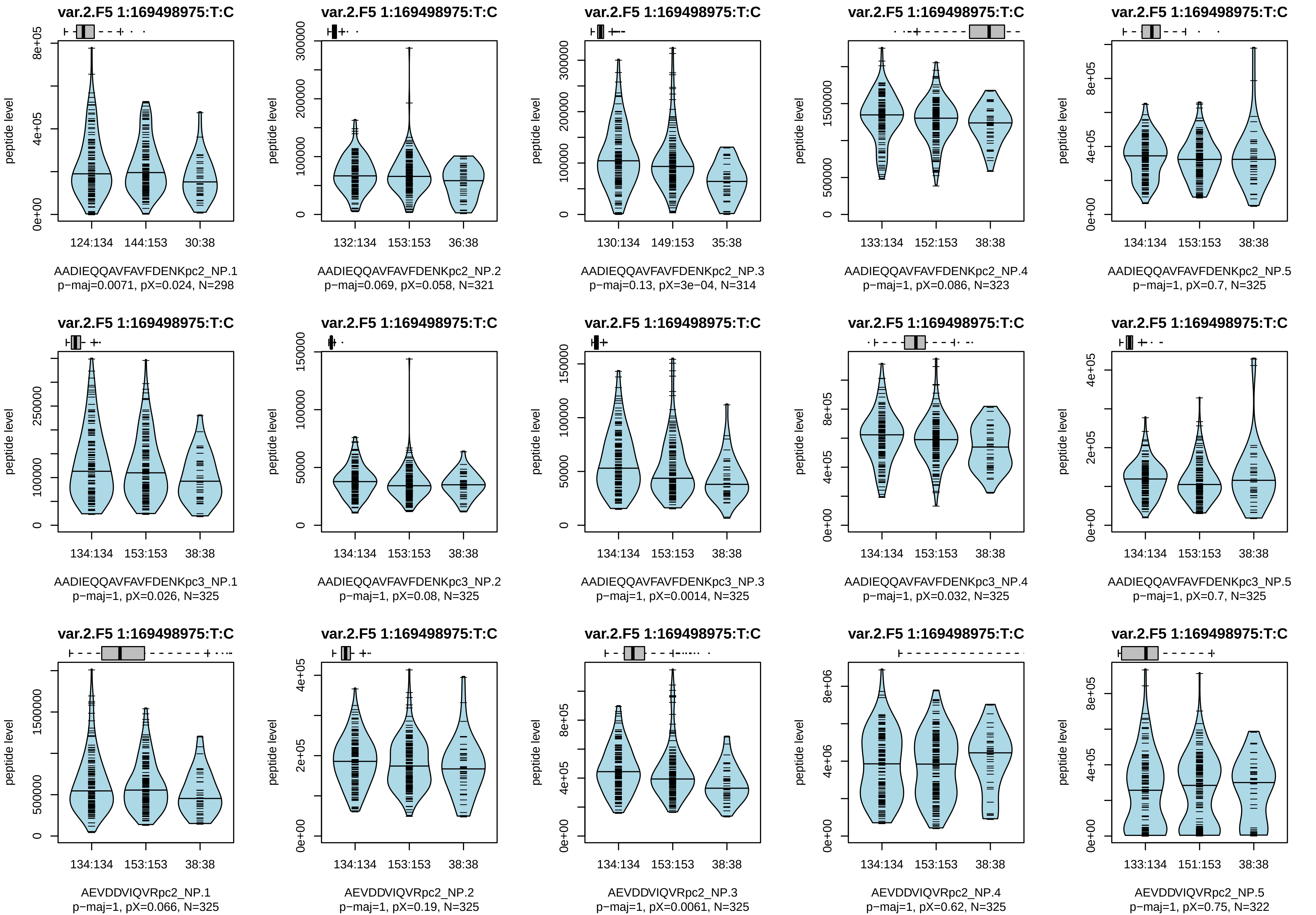
var.2.F5 1:169498975:T:C
peptide level
124:134
144:153
30:38
AADIEQQAVFAVFDENKpc2_NP.1
p−maj=0.0071, pX=0.024, N=298
var.2.F5 1:169498975:T:C
peptide level
132:134
153:153
36:38
AADIEQQAVFAVFDENKpc2_NP.2
p−maj=0.069, pX=0.058, N=321
var.2.F5 1:169498975:T:C
peptide level
130:134
149:153
35:38
AADIEQQAVFAVFDENKpc2_NP.3
p−maj=0.13, pX=3e−04, N=314
var.2.F5 1:169498975:T:C
peptide level
133:134
152:153
38:38
AADIEQQAVFAVFDENKpc2_NP.4
p−maj=1, pX=0.086, N=323
var.2.F5 1:169498975:T:C
peptide level
134:134
153:153
38:38
AADIEQQAVFAVFDENKpc2_NP.5
p−maj=1, pX=0.7, N=325
var.2.F5 1:169498975:T:C
peptide level
134:134
153:153
38:38
AADIEQQAVFAVFDENKpc3_NP.1
p−maj=1, pX=0.026, N=325
var.2.F5 1:169498975:T:C
peptide level
134:134
153:153
38:38
AADIEQQAVFAVFDENKpc3_NP.2
p−maj=1, pX=0.08, N=325
var.2.F5 1:169498975:T:C
peptide level
134:134
153:153
38:38
AADIEQQAVFAVFDENKpc3_NP.3
p−maj=1, pX=0.0014, N=325
var.2.F5 1:169498975:T:C
peptide level
134:134
153:153
38:38
AADIEQQAVFAVFDENKpc3_NP.4
p−maj=1, pX=0.032, N=325
var.2.F5 1:169498975:T:C
peptide level
134:134
153:153
38:38
AADIEQQAVFAVFDENKpc3_NP.5
p−maj=1, pX=0.7, N=325
var.2.F5 1:169498975:T:C
peptide level
134:134
153:153
38:38
AEVDDVIQVRpc2_NP.1
p−maj=1, pX=0.066, N=325
var.2.F5 1:169498975:T:C
peptide level
134:134
153:153
38:38
AEVDDVIQVRpc2_NP.2
p−maj=1, pX=0.19, N=325
var.2.F5 1:169498975:T:C
peptide level
134:134
153:153
38:38
AEVDDVIQVRpc2_NP.3
p−maj=1, pX=0.0061, N=325
var.2.F5 1:169498975:T:C
peptide level
134:134
153:153
38:38
AEVDDVIQVRpc2_NP.4
p−maj=1, pX=0.62, N=325
var.2.F5 1:169498975:T:C
peptide level
133:134
151:153
38:38
AEVDDVIQVRpc2_NP.5
p−maj=1, pX=0.75, N=322

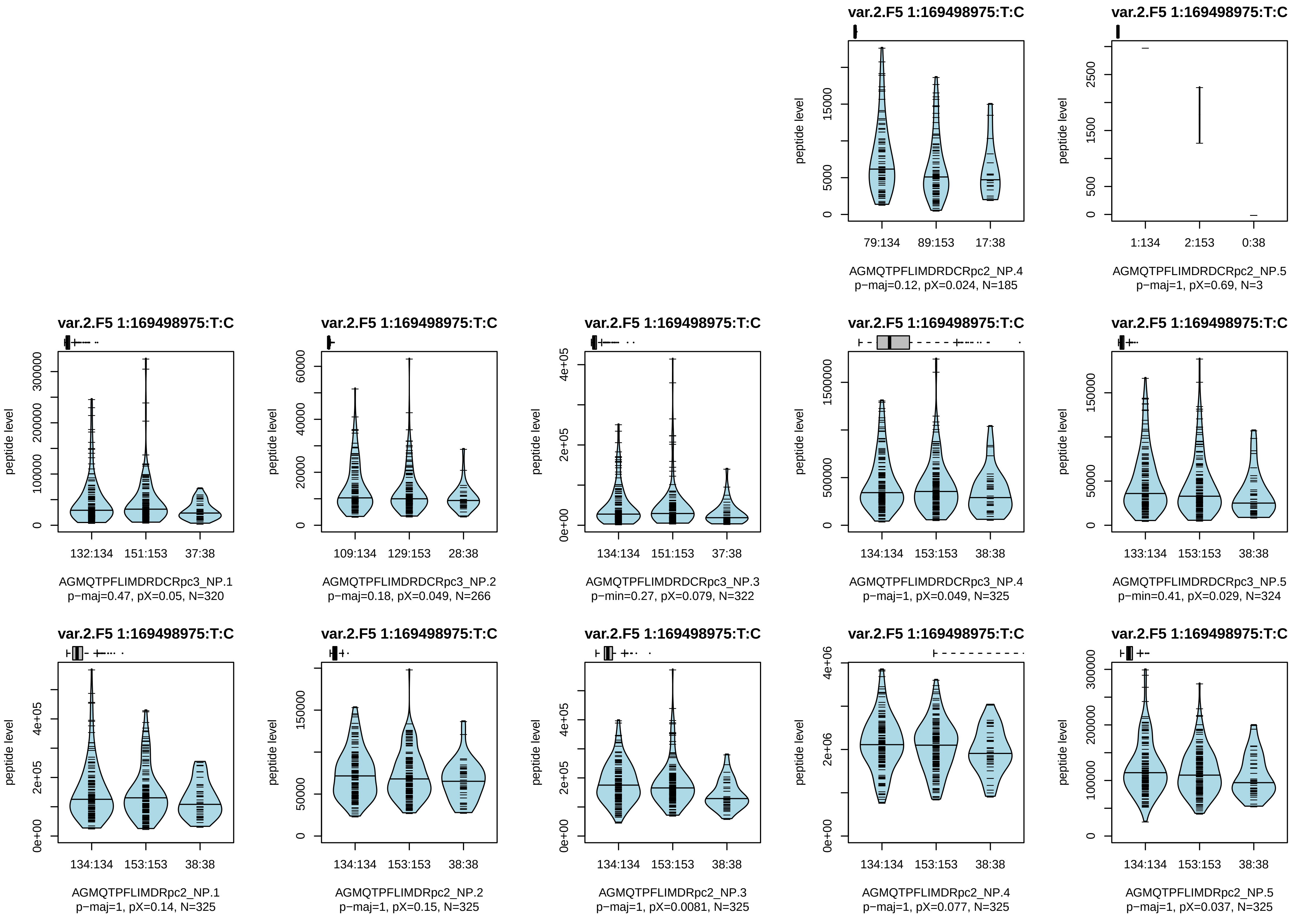
var.2.F5 1:169498975:T:C
peptide level
79:134
89:153
17:38
AGMQTPFLIMDRDCRpc2_NP.4
p−maj=0.12, pX=0.024, N=185
var.2.F5 1:169498975:T:C
peptide level
1:134
2:153
0:38
AGMQTPFLIMDRDCRpc2_NP.5
p−maj=1, pX=0.69, N=3
var.2.F5 1:169498975:T:C
peptide level
132:134
151:153
37:38
AGMQTPFLIMDRDCRpc3_NP.1
p−maj=0.47, pX=0.05, N=320
var.2.F5 1:169498975:T:C
peptide level
109:134
129:153
28:38
AGMQTPFLIMDRDCRpc3_NP.2
p−maj=0.18, pX=0.049, N=266
var.2.F5 1:169498975:T:C
peptide level
134:134
151:153
37:38
AGMQTPFLIMDRDCRpc3_NP.3
p−min=0.27, pX=0.079, N=322
var.2.F5 1:169498975:T:C
peptide level
134:134
153:153
38:38
AGMQTPFLIMDRDCRpc3_NP.4
p−maj=1, pX=0.049, N=325
var.2.F5 1:169498975:T:C
peptide level
133:134
153:153
38:38
AGMQTPFLIMDRDCRpc3_NP.5
p−min=0.41, pX=0.029, N=324
var.2.F5 1:169498975:T:C
peptide level
134:134
153:153
38:38
AGMQTPFLIMDRpc2_NP.1
p−maj=1, pX=0.14, N=325
var.2.F5 1:169498975:T:C
peptide level
134:134
153:153
38:38
AGMQTPFLIMDRpc2_NP.2
p−maj=1, pX=0.15, N=325
var.2.F5 1:169498975:T:C
peptide level
134:134
153:153
38:38
AGMQTPFLIMDRpc2_NP.3
p−maj=1, pX=0.0081, N=325
var.2.F5 1:169498975:T:C
peptide level
134:134
153:153
38:38
AGMQTPFLIMDRpc2_NP.4
p−maj=1, pX=0.077, N=325
var.2.F5 1:169498975:T:C
peptide level
134:134
153:153
38:38
AGMQTPFLIMDRpc2_NP.5
p−maj=1, pX=0.037, N=325

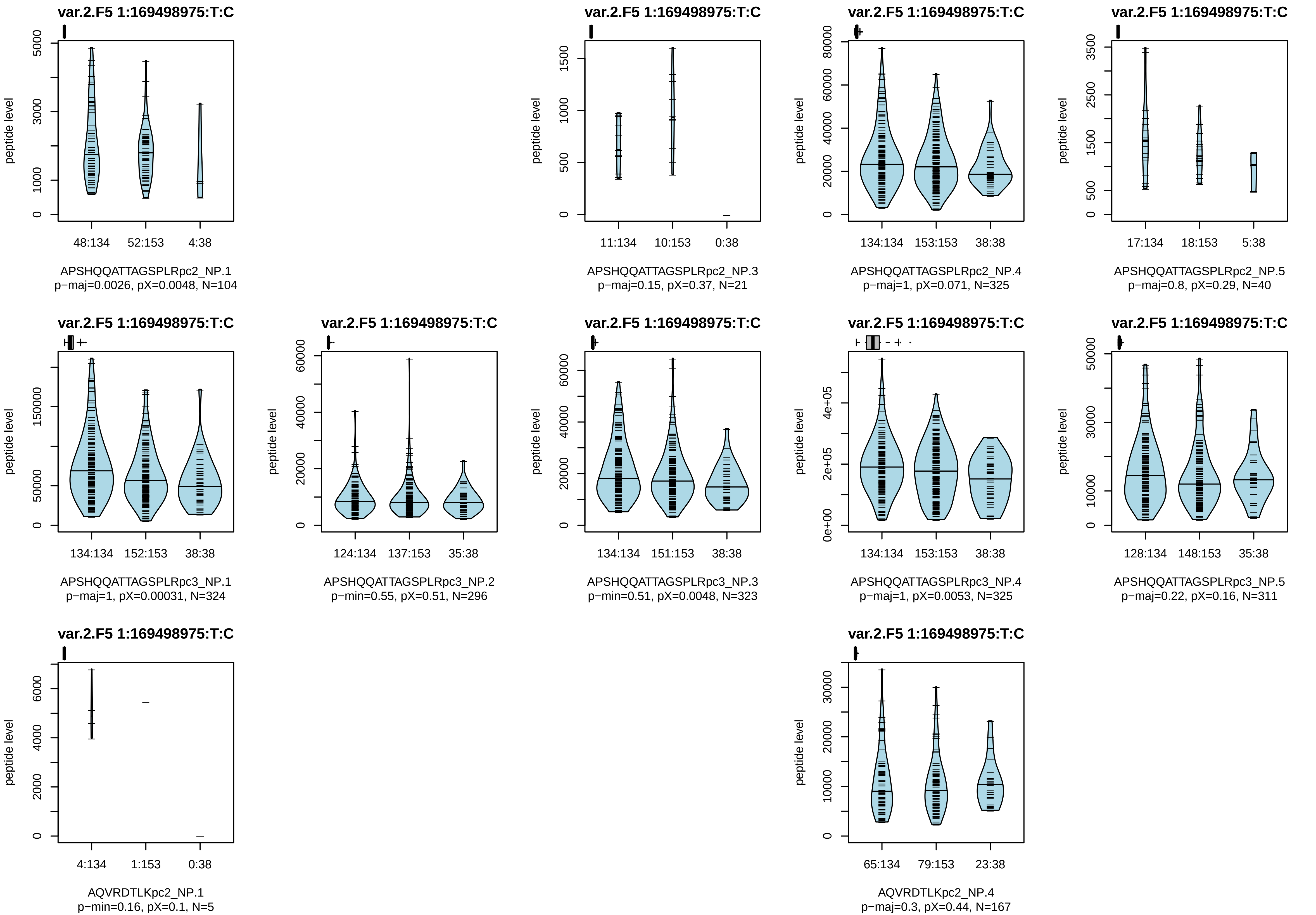
var.2.F5 1:169498975:T:C
peptide level
48:134
52:153
4:38
APSHQQATTAGSPLRpc2_NP.1
p−maj=0.0026, pX=0.0048, N=104
var.2.F5 1:169498975:T:C
peptide level
11:134
10:153
0:38
APSHQQATTAGSPLRpc2_NP.3
p−maj=0.15, pX=0.37, N=21
var.2.F5 1:169498975:T:C
peptide level
134:134
153:153
38:38
APSHQQATTAGSPLRpc2_NP.4
p−maj=1, pX=0.071, N=325
var.2.F5 1:169498975:T:C
peptide level
17:134
18:153
5:38
APSHQQATTAGSPLRpc2_NP.5
p−maj=0.8, pX=0.29, N=40
var.2.F5 1:169498975:T:C
peptide level
134:134
152:153
38:38
APSHQQATTAGSPLRpc3_NP.1
p−maj=1, pX=0.00031, N=324
var.2.F5 1:169498975:T:C
peptide level
124:134
137:153
35:38
APSHQQATTAGSPLRpc3_NP.2
p−min=0.55, pX=0.51, N=296
var.2.F5 1:169498975:T:C
peptide level
134:134
151:153
38:38
APSHQQATTAGSPLRpc3_NP.3
p−min=0.51, pX=0.0048, N=323
var.2.F5 1:169498975:T:C
peptide level
134:134
153:153
38:38
APSHQQATTAGSPLRpc3_NP.4
p−maj=1, pX=0.0053, N=325
var.2.F5 1:169498975:T:C
peptide level
128:134
148:153
35:38
APSHQQATTAGSPLRpc3_NP.5
p−maj=0.22, pX=0.16, N=311
var.2.F5 1:169498975:T:C
peptide level
4:134
1:153
0:38
AQVRDTLKpc2_NP.1
p−min=0.16, pX=0.1, N=5
var.2.F5 1:169498975:T:C
peptide level
65:134
79:153
23:38
AQVRDTLKpc2_NP.4
p−maj=0.3, pX=0.44, N=167

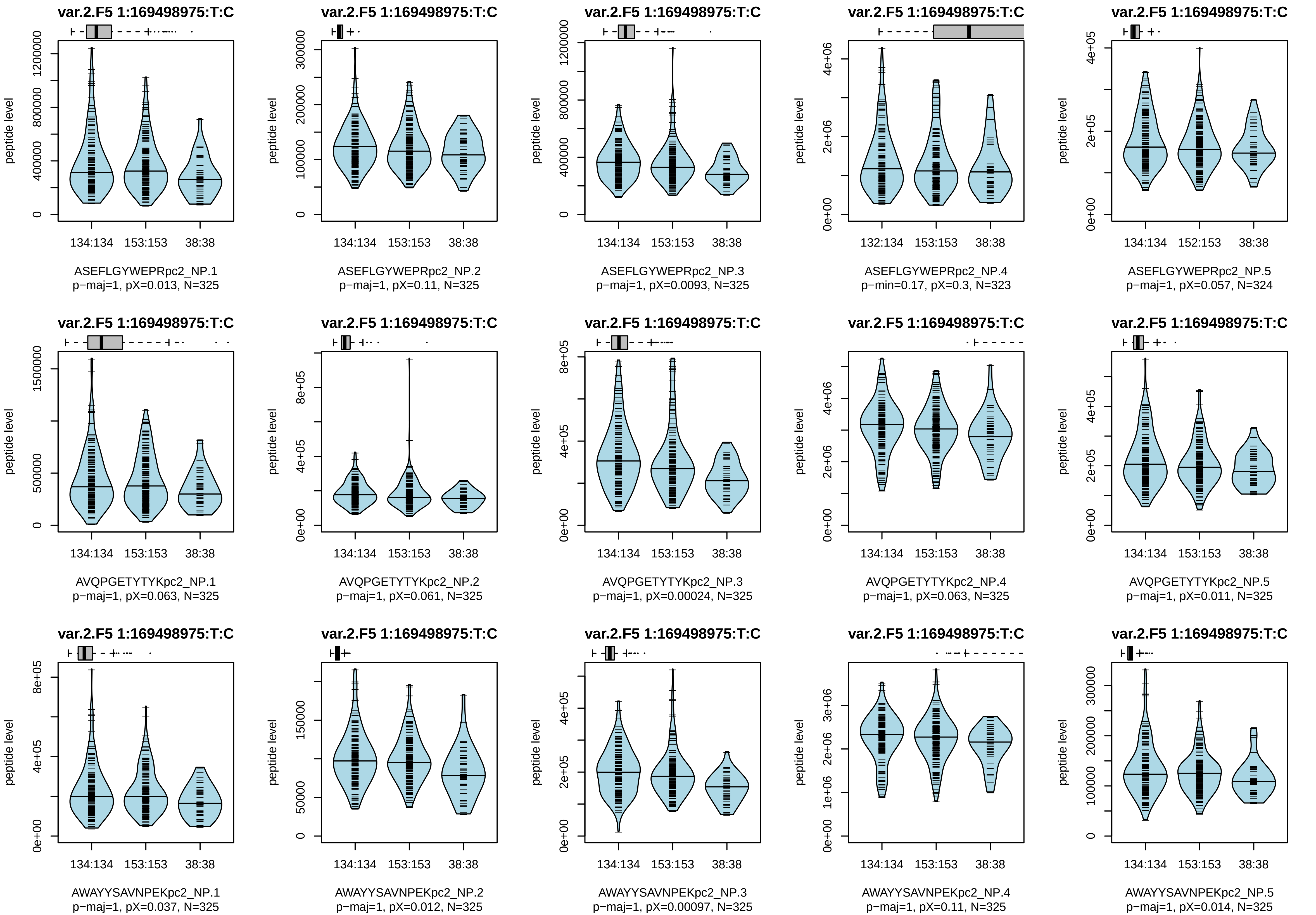

var.2.F5 1:169498975:T:C
peptide level
134:134
153:153
38:38
ASEFLGYWEPRpc2_NP.1
p−maj=1, pX=0.013, N=325
var.2.F5 1:169498975:T:C
peptide level
134:134
153:153
38:38
ASEFLGYWEPRpc2_NP.2
p−maj=1, pX=0.11, N=325
var.2.F5 1:169498975:T:C
peptide level
134:134
153:153
38:38
ASEFLGYWEPRpc2_NP.3
p−maj=1, pX=0.0093, N=325
var.2.F5 1:169498975:T:C
peptide level
132:134
153:153
38:38
ASEFLGYWEPRpc2_NP.4
p−min=0.17, pX=0.3, N=323
var.2.F5 1:169498975:T:C
peptide level
134:134
152:153
38:38
ASEFLGYWEPRpc2_NP.5
p−maj=1, pX=0.057, N=324
var.2.F5 1:169498975:T:C
peptide level
134:134
153:153
38:38
AVQPGETYTYKpc2_NP.1
p−maj=1, pX=0.063, N=325
var.2.F5 1:169498975:T:C
peptide level
134:134
153:153
38:38
AVQPGETYTYKpc2_NP.2
p−maj=1, pX=0.061, N=325
var.2.F5 1:169498975:T:C
peptide level
134:134
153:153
38:38
AVQPGETYTYKpc2_NP.3
p−maj=1, pX=0.00024, N=325
var.2.F5 1:169498975:T:C
peptide level
134:134
153:153
38:38
AVQPGETYTYKpc2_NP.4
p−maj=1, pX=0.063, N=325
var.2.F5 1:169498975:T:C
peptide level
134:134
153:153
38:38
AVQPGETYTYKpc2_NP.5
p−maj=1, pX=0.011, N=325
var.2.F5 1:169498975:T:C
peptide level
134:134
153:153
38:38
AWAYYSAVNPEKpc2_NP.1
p−maj=1, pX=0.037, N=325
var.2.F5 1:169498975:T:C
peptide level
134:134
153:153
38:38
AWAYYSAVNPEKpc2_NP.2
p−maj=1, pX=0.012, N=325
var.2.F5 1:169498975:T:C
peptide level
134:134
153:153
38:38
AWAYYSAVNPEKpc2_NP.3
p−maj=1, pX=0.00097, N=325
var.2.F5 1:169498975:T:C
peptide level
134:134
153:153
38:38
AWAYYSAVNPEKpc2_NP.4
p−maj=1, pX=0.11, N=325
var.2.F5 1:169498975:T:C
peptide level
134:134
153:153
38:38
AWAYYSAVNPEKpc2_NP.5
p−maj=1, pX=0.014, N=325

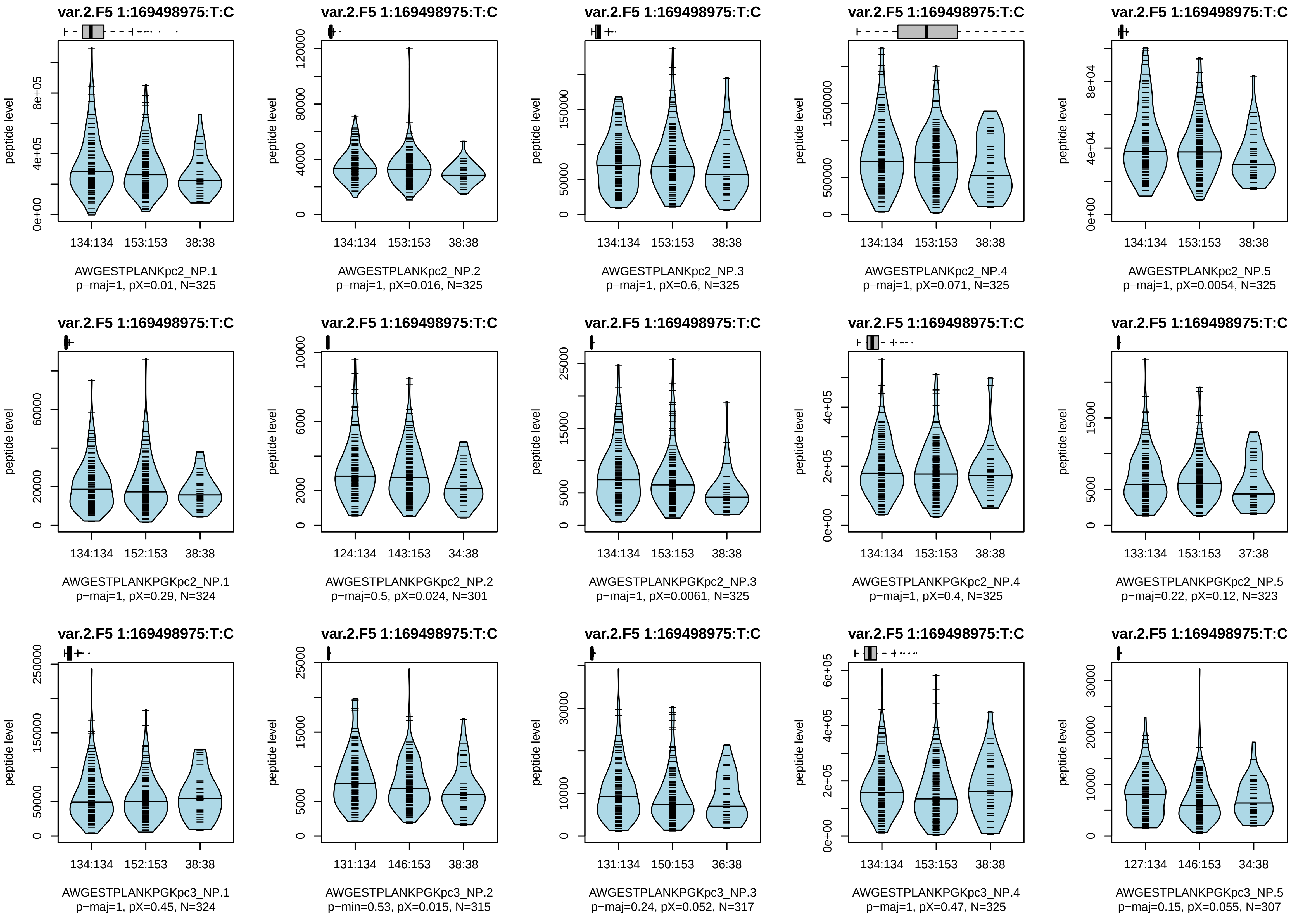

var.2.F5 1:169498975:T:C
peptide level
0e+00 4e+05 8e+05
134:134 153:153 38:38
AWGESTPLANKpc2_NP.1
p−maj=1, pX=0.01, N=325
var.2.F5 1:169498975:T:C
peptide level
0 40000 80000 120000
134:134 153:153 38:38
AWGESTPLANKpc2_NP.2
p−maj=1, pX=0.016, N=325
var.2.F5 1:169498975:T:C
peptide level
0 50000 150000
134:134 153:153 38:38
AWGESTPLANKpc2_NP.3
p−maj=1, pX=0.6, N=325
var.2.F5 1:169498975:T:C
peptide level
0 500000 1500000
134:134 153:153 38:38
AWGESTPLANKpc2_NP.4
p−maj=1, pX=0.071, N=325
var.2.F5 1:169498975:T:C
peptide level
0e+00 4e+04 8e+04
134:134 153:153 38:38
AWGESTPLANKpc2_NP.5
p−maj=1, pX=0.0054, N=325
var.2.F5 1:169498975:T:C
peptide level
0 20000 60000
134:134 152:153 38:38
AWGESTPLANKPGKpc2_NP.1
p−maj=1, pX=0.29, N=324
var.2.F5 1:169498975:T:C
peptide level
0 2000 6000 10000
124:134 143:153 34:38
AWGESTPLANKPGKpc2_NP.2
p−maj=0.5, pX=0.024, N=301
var.2.F5 1:169498975:T:C
peptide level
0 5000 15000 25000
134:134 153:153 38:38
AWGESTPLANKPGKpc2_NP.3
p−maj=1, pX=0.0061, N=325
var.2.F5 1:169498975:T:C
peptide level
0e+00 2e+05 4e+05
134:134 153:153 38:38
AWGESTPLANKPGKpc2_NP.4
p−maj=1, pX=0.4, N=325
var.2.F5 1:169498975:T:C
peptide level
0 5000 15000
133:134 153:153 37:38
AWGESTPLANKPGKpc2_NP.5
p−maj=0.22, pX=0.12, N=323
var.2.F5 1:169498975:T:C
peptide level
0 50000 150000 250000
134:134 152:153 38:38
AWGESTPLANKPGKpc3_NP.1
p−maj=1, pX=0.45, N=324
var.2.F5 1:169498975:T:C
peptide level
0 5000 15000 25000
131:134 146:153 38:38
AWGESTPLANKPGKpc3_NP.2
p−min=0.53, pX=0.015, N=315
var.2.F5 1:169498975:T:C
peptide level
0 10000 30000
131:134 150:153 36:38
AWGESTPLANKPGKpc3_NP.3
p−maj=0.24, pX=0.052, N=317
var.2.F5 1:169498975:T:C
peptide level
0e+00 2e+05 4e+05 6e+05
134:134 153:153 38:38
AWGESTPLANKPGKpc3_NP.4
p−maj=1, pX=0.47, N=325
var.2.F5 1:169498975:T:C
peptide level
0 10000 20000 30000
127:134 146:153 34:38
AWGESTPLANKPGKpc3_NP.5
p−maj=0.15, pX=0.055, N=307

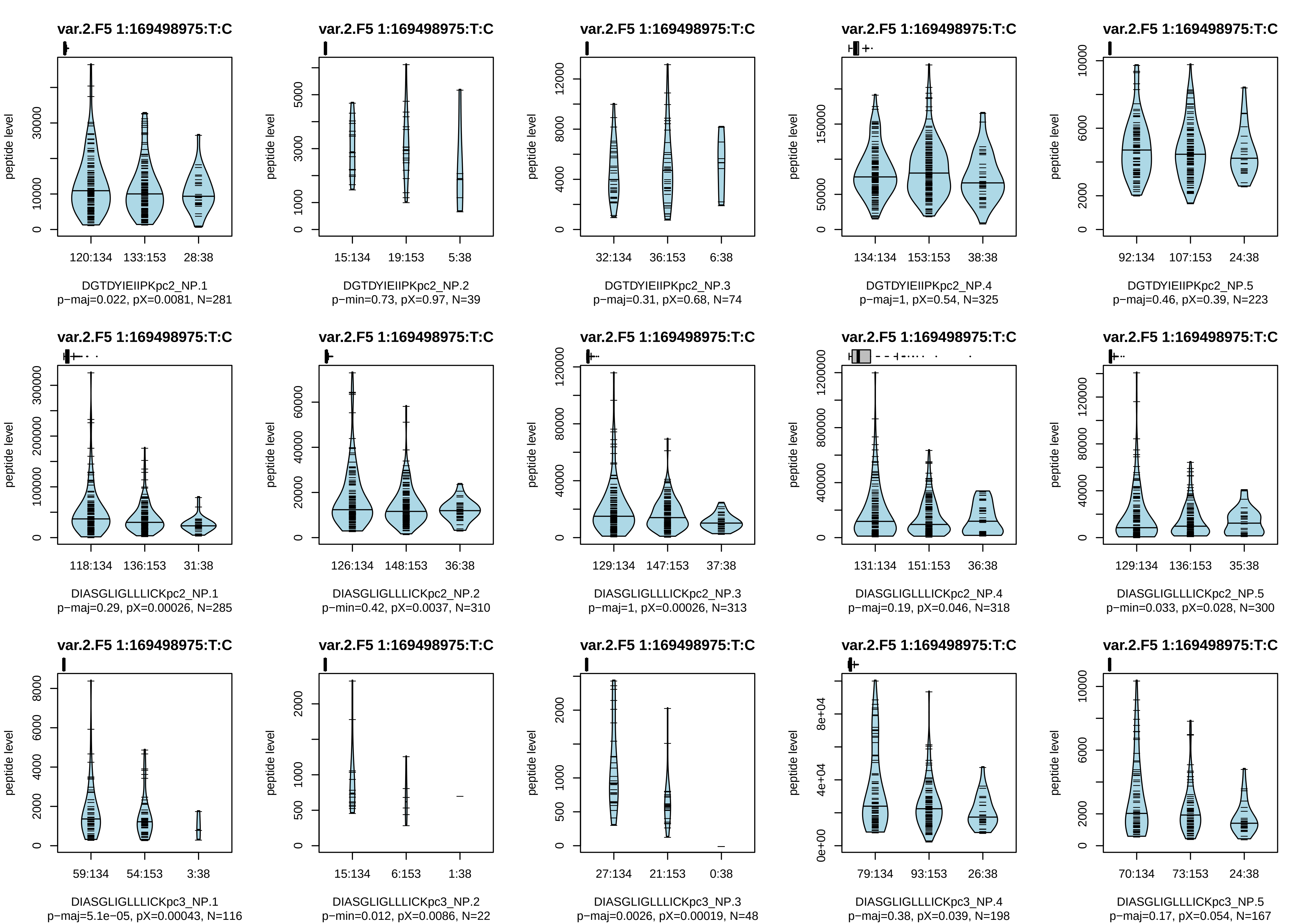
var.2.F5 1:169498975:T:C
120:134 133:153 28:38
DGTDYIEIIPKpc2_NP.1
p−maj=0.022, pX=0.0081, N=281
var.2.F5 1:169498975:T:C
15:134 19:153 5:38
DGTDYIEIIPKpc2_NP.2
p−min=0.73, pX=0.97, N=39
var.2.F5 1:169498975:T:C
32:134 36:153 6:38
DGTDYIEIIPKpc2_NP.3
p−maj=0.31, pX=0.68, N=74
var.2.F5 1:169498975:T:C
134:134 153:153 38:38
DGTDYIEIIPKpc2_NP.4
p−maj=1, pX=0.54, N=325
var.2.F5 1:169498975:T:C
92:134 107:153 24:38
DGTDYIEIIPKpc2_NP.5
p−maj=0.46, pX=0.39, N=223
var.2.F5 1:169498975:T:C
118:134 136:153 31:38
DIASGLIGLLLICKpc2_NP.1
p−maj=0.29, pX=0.00026, N=285
var.2.F5 1:169498975:T:C
126:134 148:153 36:38
DIASGLIGLLLICKpc2_NP.2
p−min=0.42, pX=0.0037, N=310
var.2.F5 1:169498975:T:C
129:134 147:153 37:38
DIASGLIGLLLICKpc2_NP.3
p−maj=1, pX=0.00026, N=313
var.2.F5 1:169498975:T:C
131:134 151:153 36:38
DIASGLIGLLLICKpc2_NP.4
p−maj=0.19, pX=0.046, N=318
var.2.F5 1:169498975:T:C
129:134 136:153 35:38
DIASGLIGLLLICKpc2_NP.5
p−min=0.033, pX=0.028, N=300
var.2.F5 1:169498975:T:C
59:134 54:153 3:38
DIASGLIGLLLICKpc3_NP.1
p−maj=5.1e−05, pX=0.00043, N=116
var.2.F5 1:169498975:T:C
15:134 6:153 1:38
DIASGLIGLLLICKpc3_NP.2
p−min=0.012, pX=0.0086, N=22
var.2.F5 1:169498975:T:C
27:134 21:153 0:38
DIASGLIGLLLICKpc3_NP.3
p−maj=0.0026, pX=0.00019, N=48
var.2.F5 1:169498975:T:C
79:134 93:153 26:38
DIASGLIGLLLICKpc3_NP.4
p−maj=0.38, pX=0.039, N=198
var.2.F5 1:169498975:T:C
70:134 73:153 24:38
DIASGLIGLLLICKpc3_NP.5
p−maj=0.17, pX=0.054, N=167

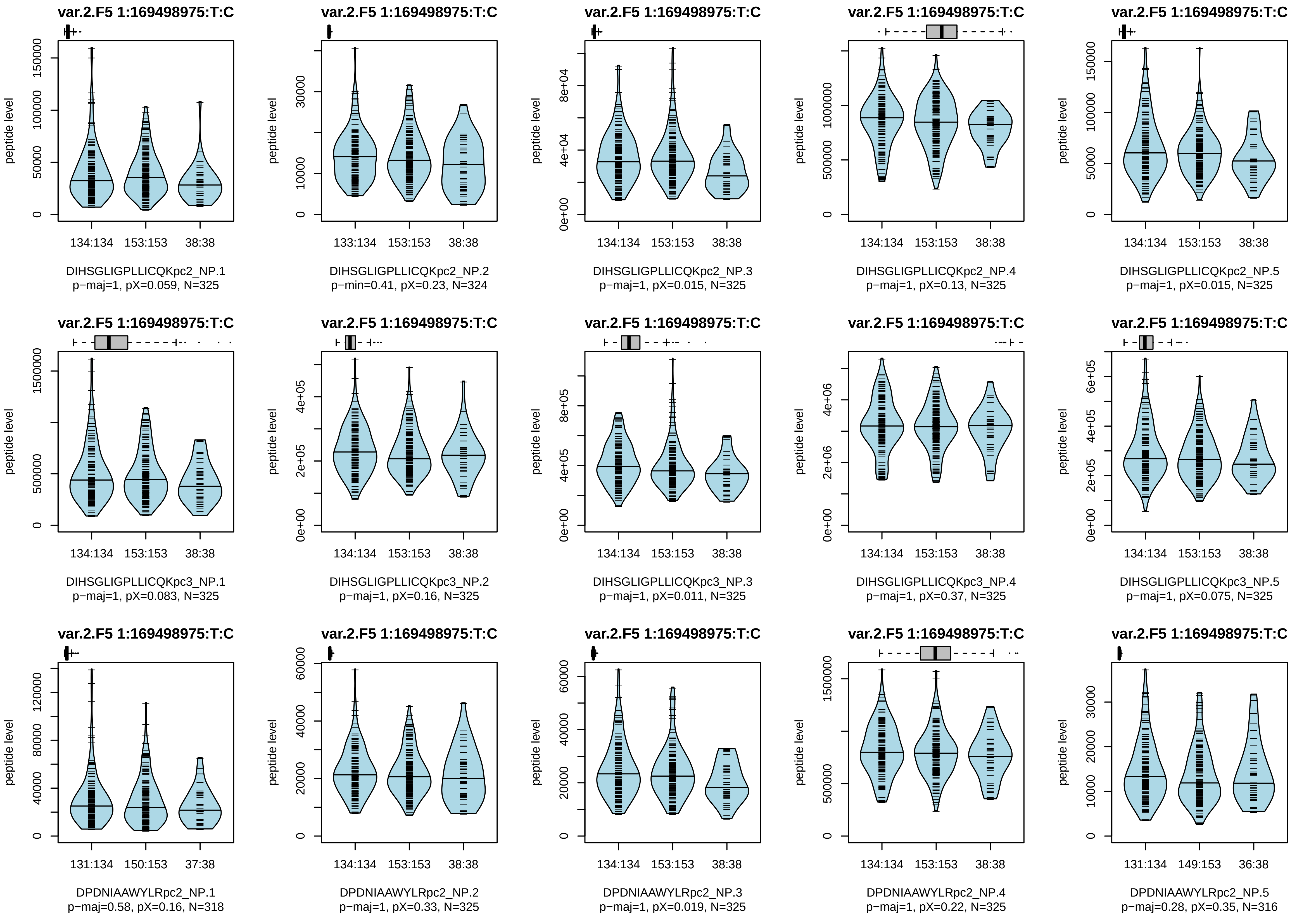
var.2.F5 1:169498975:T:C
peptide level
134:134 153:153 38:38
DIHSGLIGPLLICQKpc2_NP.1
p−maj=1, pX=0.059, N=325
var.2.F5 1:169498975:T:C
peptide level
133:134 153:153 38:38
DIHSGLIGPLLICQKpc2_NP.2
p−min=0.41, pX=0.23, N=324
var.2.F5 1:169498975:T:C
peptide level
134:134 153:153 38:38
DIHSGLIGPLLICQKpc2_NP.3
p−maj=1, pX=0.015, N=325
var.2.F5 1:169498975:T:C
peptide level
134:134 153:153 38:38
DIHSGLIGPLLICQKpc2_NP.4
p−maj=1, pX=0.13, N=325
var.2.F5 1:169498975:T:C
peptide level
134:134 153:153 38:38
DIHSGLIGPLLICQKpc2_NP.5
p−maj=1, pX=0.015, N=325
var.2.F5 1:169498975:T:C
peptide level
134:134 153:153 38:38
DIHSGLIGPLLICQKpc3_NP.1
p−maj=1, pX=0.083, N=325
var.2.F5 1:169498975:T:C
peptide level
134:134 153:153 38:38
DIHSGLIGPLLICQKpc3_NP.2
p−maj=1, pX=0.16, N=325
var.2.F5 1:169498975:T:C
peptide level
134:134 153:153 38:38
DIHSGLIGPLLICQKpc3_NP.3
p−maj=1, pX=0.011, N=325
var.2.F5 1:169498975:T:C
peptide level
134:134 153:153 38:38
DIHSGLIGPLLICQKpc3_NP.4
p−maj=1, pX=0.37, N=325
var.2.F5 1:169498975:T:C
peptide level
134:134 153:153 38:38
DIHSGLIGPLLICQKpc3_NP.5
p−maj=1, pX=0.075, N=325
var.2.F5 1:169498975:T:C
peptide level
131:134 150:153 37:38
DPDNIAAWYLRpc2_NP.1
p−maj=0.58, pX=0.16, N=318
var.2.F5 1:169498975:T:C
peptide level
134:134 153:153 38:38
DPDNIAAWYLRpc2_NP.2
p−maj=1, pX=0.33, N=325
var.2.F5 1:169498975:T:C
peptide level
134:134 153:153 38:38
DPDNIAAWYLRpc2_NP.3
p−maj=1, pX=0.019, N=325
var.2.F5 1:169498975:T:C
peptide level
134:134 153:153 38:38
DPDNIAAWYLRpc2_NP.4
p−maj=1, pX=0.22, N=325
var.2.F5 1:169498975:T:C
peptide level
131:134 149:153 36:38
DPDNIAAWYLRpc2_NP.5
p−maj=0.28, pX=0.35, N=316

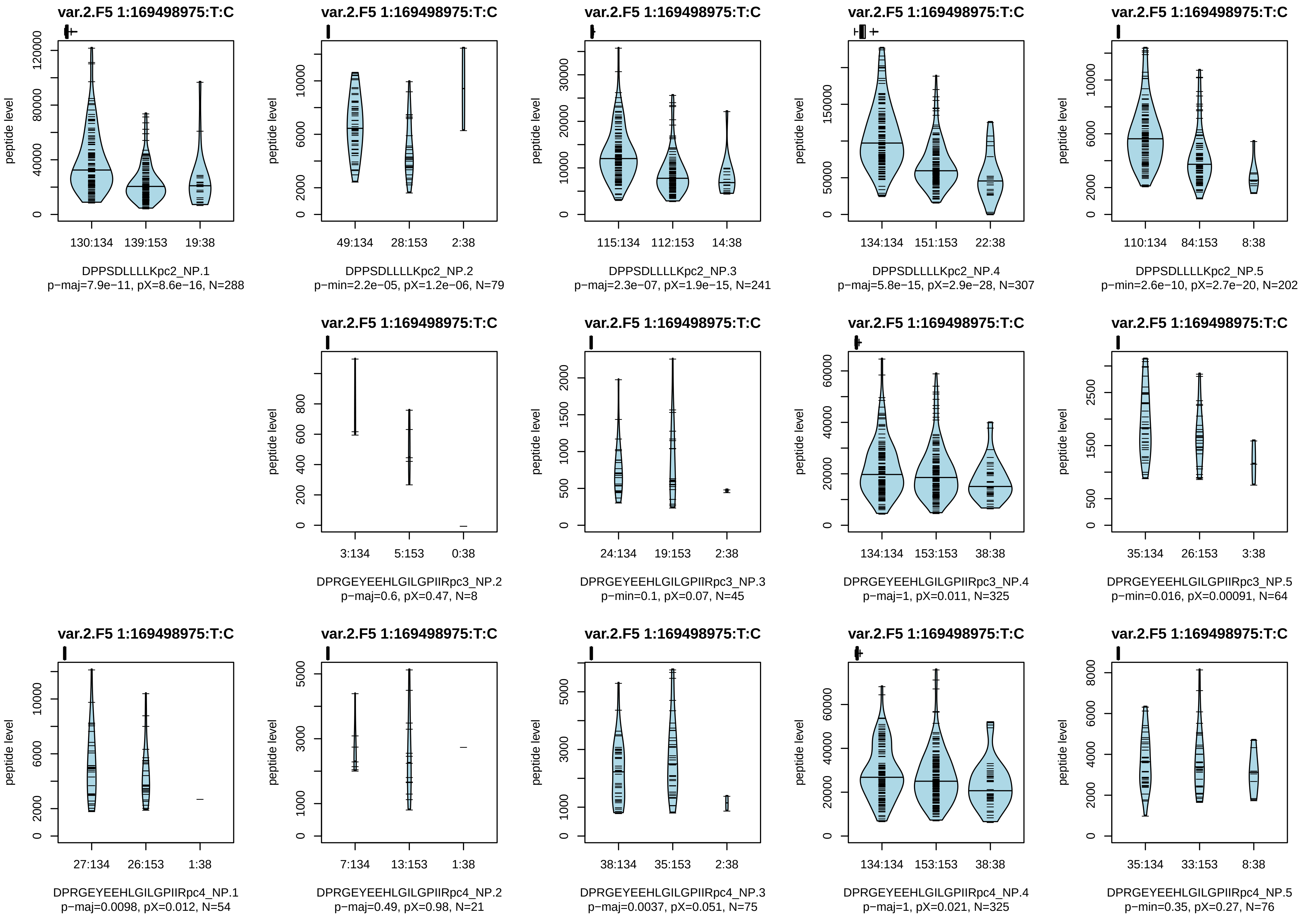
var.2.F5 1:169498975:T:C
peptide level
130:134
139:153
19:38
DPPSDLLLLKpc2_NP.1
p−maj=7.9e−11, pX=8.6e−16, N=288
var.2.F5 1:169498975:T:C
peptide level
49:134
28:153
2:38
DPPSDLLLLKpc2_NP.2
p−min=2.2e−05, pX=1.2e−06, N=79
var.2.F5 1:169498975:T:C
peptide level
115:134
112:153
14:38
DPPSDLLLLKpc2_NP.3
p−maj=2.3e−07, pX=1.9e−15, N=241
var.2.F5 1:169498975:T:C
peptide level
134:134
151:153
22:38
DPPSDLLLLKpc2_NP.4
p−maj=5.8e−15, pX=2.9e−28, N=307
var.2.F5 1:169498975:T:C
peptide level
110:134
84:153
8:38
DPPSDLLLLKpc2_NP.5
p−min=2.6e−10, pX=2.7e−20, N=202
var.2.F5 1:169498975:T:C
peptide level
3:134
5:153
0:38
DPRGEYEEHLGILGPIIRpc3_NP.2
p−maj=0.6, pX=0.47, N=8
var.2.F5 1:169498975:T:C
peptide level
24:134
19:153
2:38
DPRGEYEEHLGILGPIIRpc3_NP.3
p−min=0.1, pX=0.07, N=45
var.2.F5 1:169498975:T:C
peptide level
134:134
153:153
38:38
DPRGEYEEHLGILGPIIRpc3_NP.4
p−maj=1, pX=0.011, N=325
var.2.F5 1:169498975:T:C
peptide level
35:134
26:153
3:38
DPRGEYEEHLGILGPIIRpc3_NP.5
p−min=0.016, pX=0.00091, N=64
var.2.F5 1:169498975:T:C
peptide level
27:134
26:153
1:38
DPRGEYEEHLGILGPIIRpc4_NP.1
p−maj=0.0098, pX=0.012, N=54
var.2.F5 1:169498975:T:C
peptide level
7:134
13:153
1:38
DPRGEYEEHLGILGPIIRpc4_NP.2
p−maj=0.49, pX=0.98, N=21
var.2.F5 1:169498975:T:C
peptide level
38:134
35:153
2:38
DPRGEYEEHLGILGPIIRpc4_NP.3
p−maj=0.0037, pX=0.051, N=75
var.2.F5 1:169498975:T:C
peptide level
134:134
153:153
38:38
DPRGEYEEHLGILGPIIRpc4_NP.4
p−maj=1, pX=0.021, N=325
var.2.F5 1:169498975:T:C
peptide level
35:134
33:153
8:38
DPRGEYEEHLGILGPIIRpc4_NP.5
p−min=0.35, pX=0.27, N=76

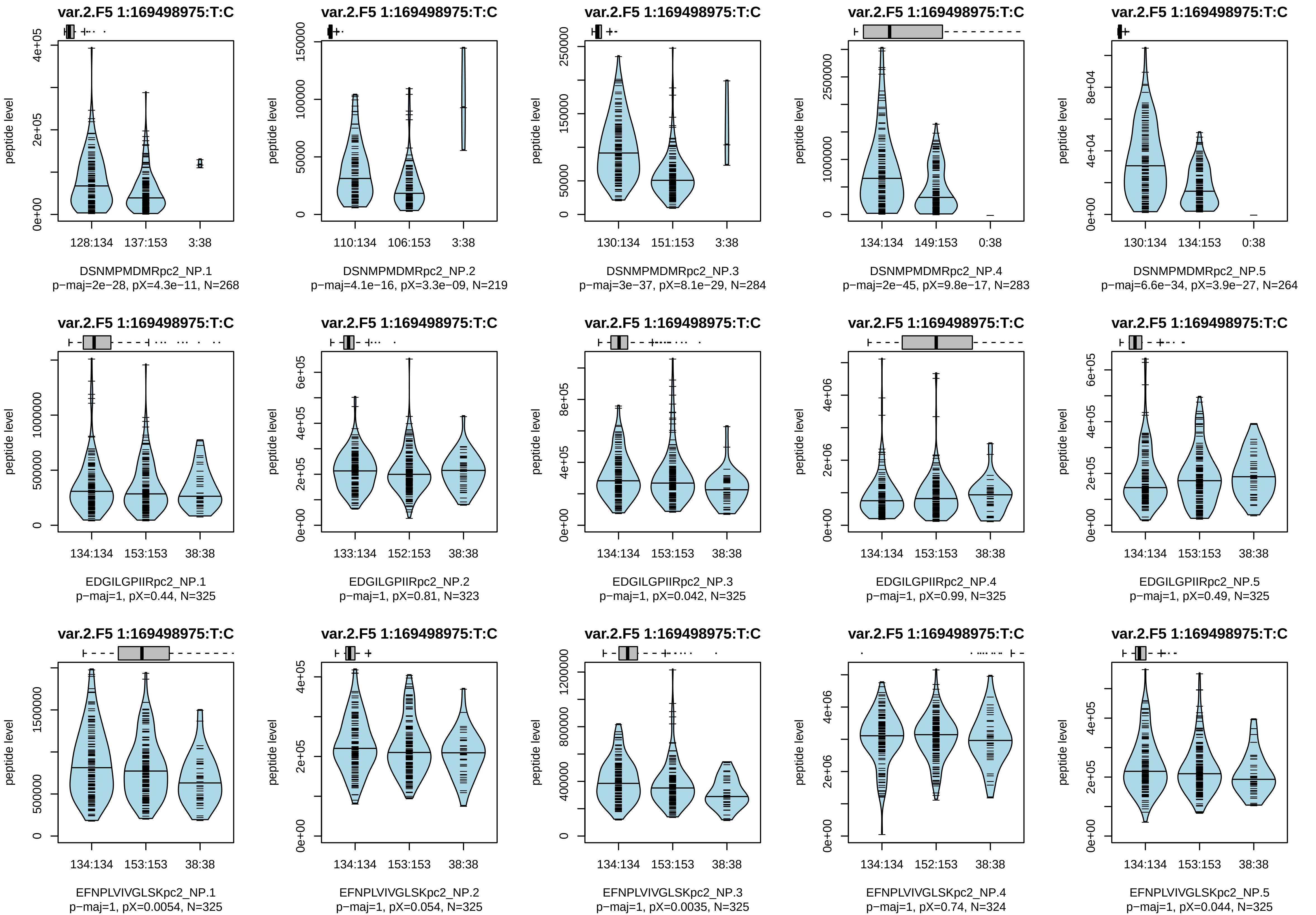
var.2.F5 1:169498975:T:C
peptide level
128:134
137:153
3:38
DSNMPMDMRpc2_NP.1
p−maj=2e−28, pX=4.3e−11, N=268
110:134
106:153
3:38
DSNMPMDMRpc2_NP.2
p−maj=4.1e−16, pX=3.3e−09, N=219
130:134
151:153
3:38
DSNMPMDMRpc2_NP.3
p−maj=3e−37, pX=8.1e−29, N=284
134:134
149:153
0:38
DSNMPMDMRpc2_NP.4
p−maj=2e−45, pX=9.8e−17, N=283
130:134
134:153
0:38
DSNMPMDMRpc2_NP.5
p−maj=6.6e−34, pX=3.9e−27, N=264
134:134
153:153
38:38
EDGILGPIIRpc2_NP.1
p−maj=1, pX=0.44, N=325
133:134
152:153
38:38
EDGILGPIIRpc2_NP.2
p−maj=1, pX=0.81, N=323
134:134
153:153
38:38
EDGILGPIIRpc2_NP.3
p−maj=1, pX=0.042, N=325
134:134
153:153
38:38
EDGILGPIIRpc2_NP.4
p−maj=1, pX=0.99, N=325
134:134
153:153
38:38
EDGILGPIIRpc2_NP.5
p−maj=1, pX=0.49, N=325
134:134
153:153
38:38
EFNPLVIVGLSKpc2_NP.1
p−maj=1, pX=0.0054, N=325
134:134
153:153
38:38
EFNPLVIVGLSKpc2_NP.2
p−maj=1, pX=0.054, N=325
134:134
153:153
38:38
EFNPLVIVGLSKpc2_NP.3
p−maj=1, pX=0.0035, N=325
134:134
152:153
38:38
EFNPLVIVGLSKpc2_NP.4
p−maj=1, pX=0.74, N=324
134:134
153:153
38:38
EFNPLVIVGLSKpc2_NP.5
p−maj=1, pX=0.044, N=325

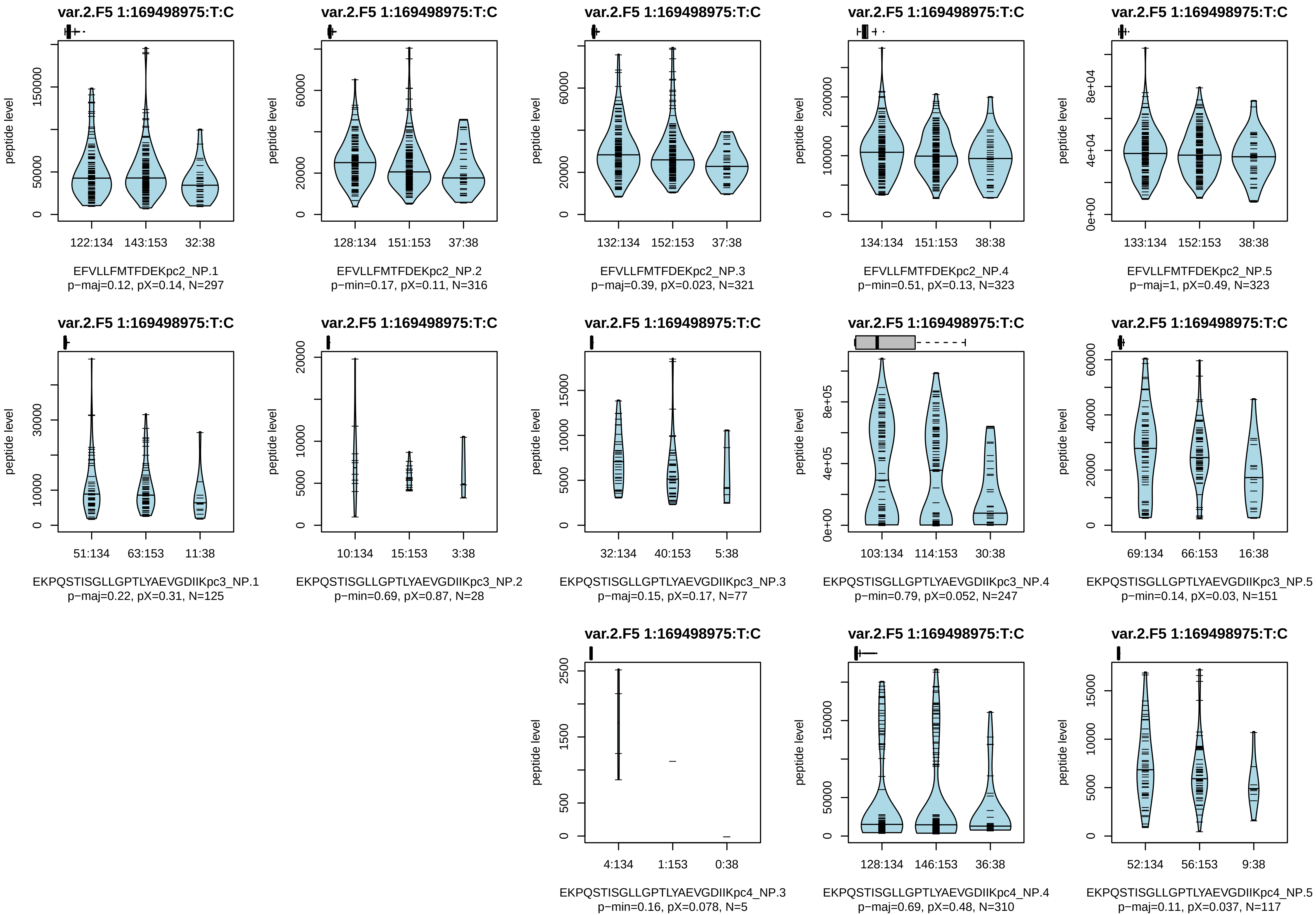
var.2.F5 1:169498975:T:C
peptide level
122:134 143:153 32:38
EFVLLFMTFDEKpc2_NP.1
p−maj=0.12, pX=0.14, N=297
128:134 151:153 37:38
EFVLLFMTFDEKpc2_NP.2
p−min=0.17, pX=0.11, N=316
132:134 152:153 37:38
EFVLLFMTFDEKpc2_NP.3
p−maj=0.39, pX=0.023, N=321
134:134 151:153 38:38
EFVLLFMTFDEKpc2_NP.4
p−min=0.51, pX=0.13, N=323
133:134 152:153 38:38
EFVLLFMTFDEKpc2_NP.5
p−maj=1, pX=0.49, N=323
51:134 63:153 11:38
EKPQSTISGLLGPTLYAEVGDIIKpc3_NP.1
p−maj=0.22, pX=0.31, N=125
10:134 15:153 3:38
EKPQSTISGLLGPTLYAEVGDIIKpc3_NP.2
p−min=0.69, pX=0.87, N=28
32:134 40:153 5:38
EKPQSTISGLLGPTLYAEVGDIIKpc3_NP.3
p−maj=0.15, pX=0.17, N=77
103:134 114:153 30:38
EKPQSTISGLLGPTLYAEVGDIIKpc3_NP.4
p−min=0.79, pX=0.052, N=247
69:134 66:153 16:38
EKPQSTISGLLGPTLYAEVGDIIKpc3_NP.5
p−min=0.14, pX=0.03, N=151
4:134 1:153 0:38
EKPQSTISGLLGPTLYAEVGDIIKpc4_NP.3
p−min=0.16, pX=0.078, N=5
128:134 146:153 36:38
EKPQSTISGLLGPTLYAEVGDIIKpc4_NP.4
p−maj=0.69, pX=0.48, N=310
52:134 56:153 9:38
EKPQSTISGLLGPTLYAEVGDIIKpc4_NP.5
p−maj=0.11, pX=0.037, N=117

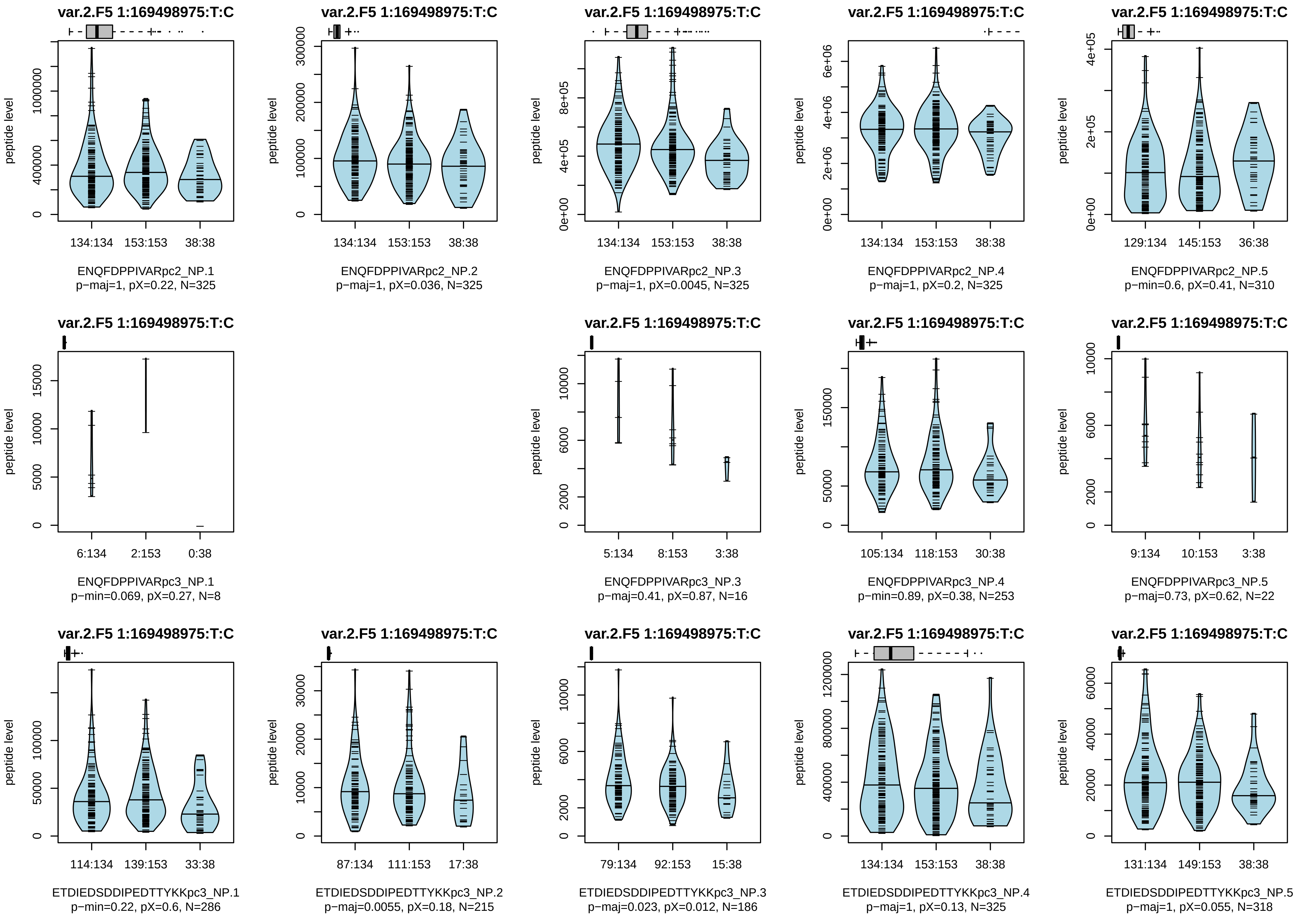
var.2.F5 1:169498975:T:C
peptide level
134:134
153:153
38:38
ENQFDPPIVARpc2_NP.1
p−maj=1, pX=0.22, N=325
var.2.F5 1:169498975:T:C
peptide level
134:134
153:153
38:38
ENQFDPPIVARpc2_NP.2
p−maj=1, pX=0.036, N=325
var.2.F5 1:169498975:T:C
peptide level
134:134
153:153
38:38
ENQFDPPIVARpc2_NP.3
p−maj=1, pX=0.0045, N=325
var.2.F5 1:169498975:T:C
peptide level
134:134
153:153
38:38
ENQFDPPIVARpc2_NP.4
p−maj=1, pX=0.2, N=325
var.2.F5 1:169498975:T:C
peptide level
129:134
145:153
36:38
ENQFDPPIVARpc2_NP.5
p−min=0.6, pX=0.41, N=310
var.2.F5 1:169498975:T:C
peptide level
6:134
2:153
0:38
ENQFDPPIVARpc3_NP.1
p−min=0.069, pX=0.27, N=8
var.2.F5 1:169498975:T:C
peptide level
5:134
8:153
3:38
ENQFDPPIVARpc3_NP.3
p−maj=0.41, pX=0.87, N=16
var.2.F5 1:169498975:T:C
peptide level
105:134
118:153
30:38
ENQFDPPIVARpc3_NP.4
p−min=0.89, pX=0.38, N=253
var.2.F5 1:169498975:T:C
peptide level
9:134
10:153
3:38
ENQFDPPIVARpc3_NP.5
p−maj=0.73, pX=0.62, N=22
var.2.F5 1:169498975:T:C
peptide level
114:134
139:153
33:38
ETDIEDSDDIPEDTTYKKpc3_NP.1
p−min=0.22, pX=0.6, N=286
var.2.F5 1:169498975:T:C
peptide level
87:134
111:153
17:38
ETDIEDSDDIPEDTTYKKpc3_NP.2
p−maj=0.0055, pX=0.18, N=215
var.2.F5 1:169498975:T:C
peptide level
79:134
92:153
15:38
ETDIEDSDDIPEDTTYKKpc3_NP.3
p−maj=0.023, pX=0.012, N=186
var.2.F5 1:169498975:T:C
peptide level
134:134
153:153
38:38
ETDIEDSDDIPEDTTYKKpc3_NP.4
p−maj=1, pX=0.13, N=325
var.2.F5 1:169498975:T:C
peptide level
131:134
149:153
38:38
ETDIEDSDDIPEDTTYKKpc3_NP.5
p−maj=1, pX=0.055, N=318

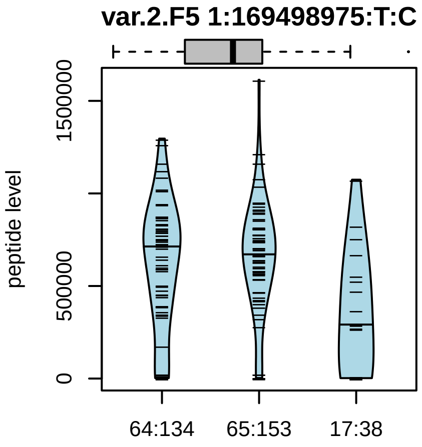

ETDIEDSDDIPEDTTYKpc2_NP.4
p−min=0.43, pX=0.12, N=146

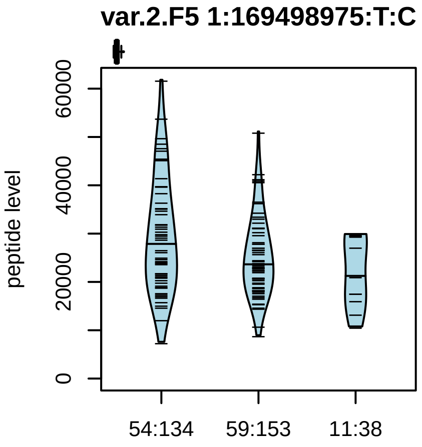

ETDIEDSDDIPEDTTYKpc2_NP.5
p−maj=0.29, pX=0.033, N=124

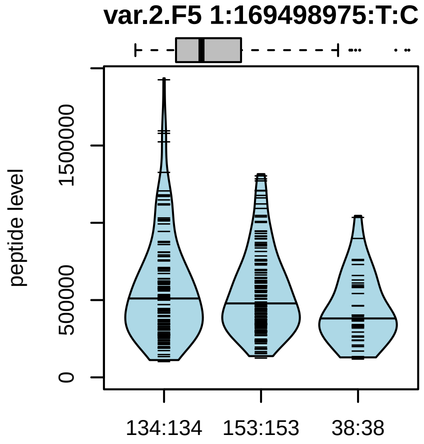

EVIITGIQTQGAKpc2_NP.1
p−maj=1, pX=0.032, N=325

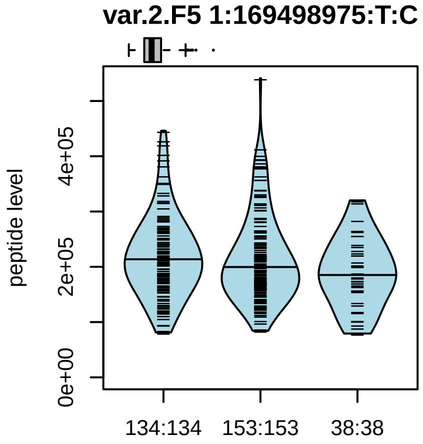

EVIITGIQTQGAKpc2_NP.2
p−maj=1, pX=0.067, N=325

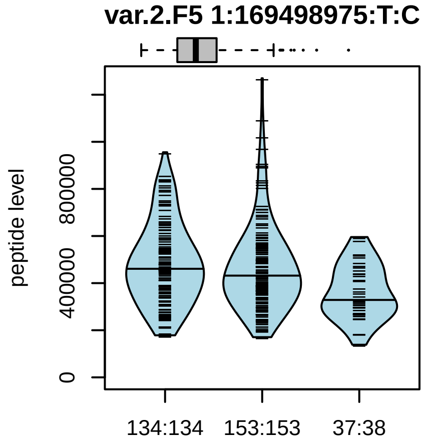

EVIITGIQTQGAKpc2_NP.3
p−maj=0.12, pX=0.00075, N=324

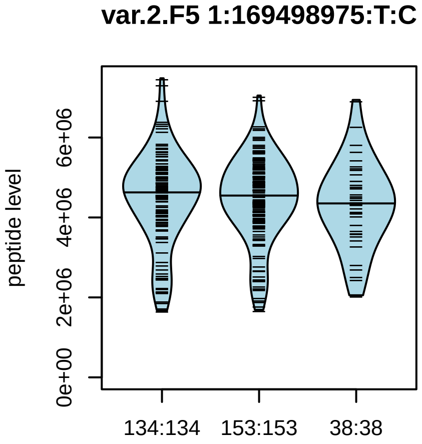

EVIITGIQTQGAKpc2_NP.4
p−maj=1, pX=0.56, N=325

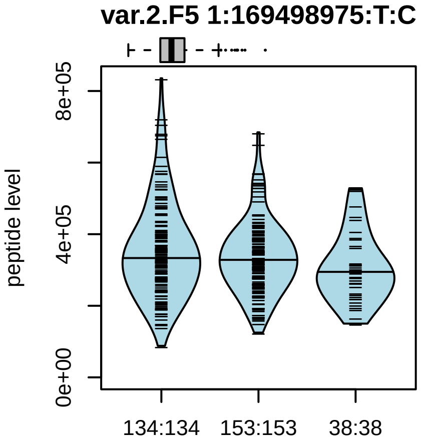

EVIITGIQTQGAKpc2_NP.5
p−maj=1, pX=0.015, N=325

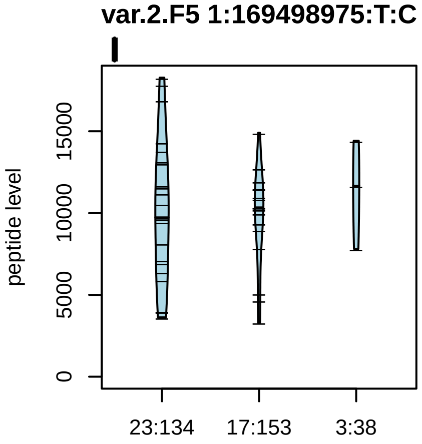

EVIITGIQTQGAKpc3_NP.4
p−min=0.096, pX=0.11, N=43

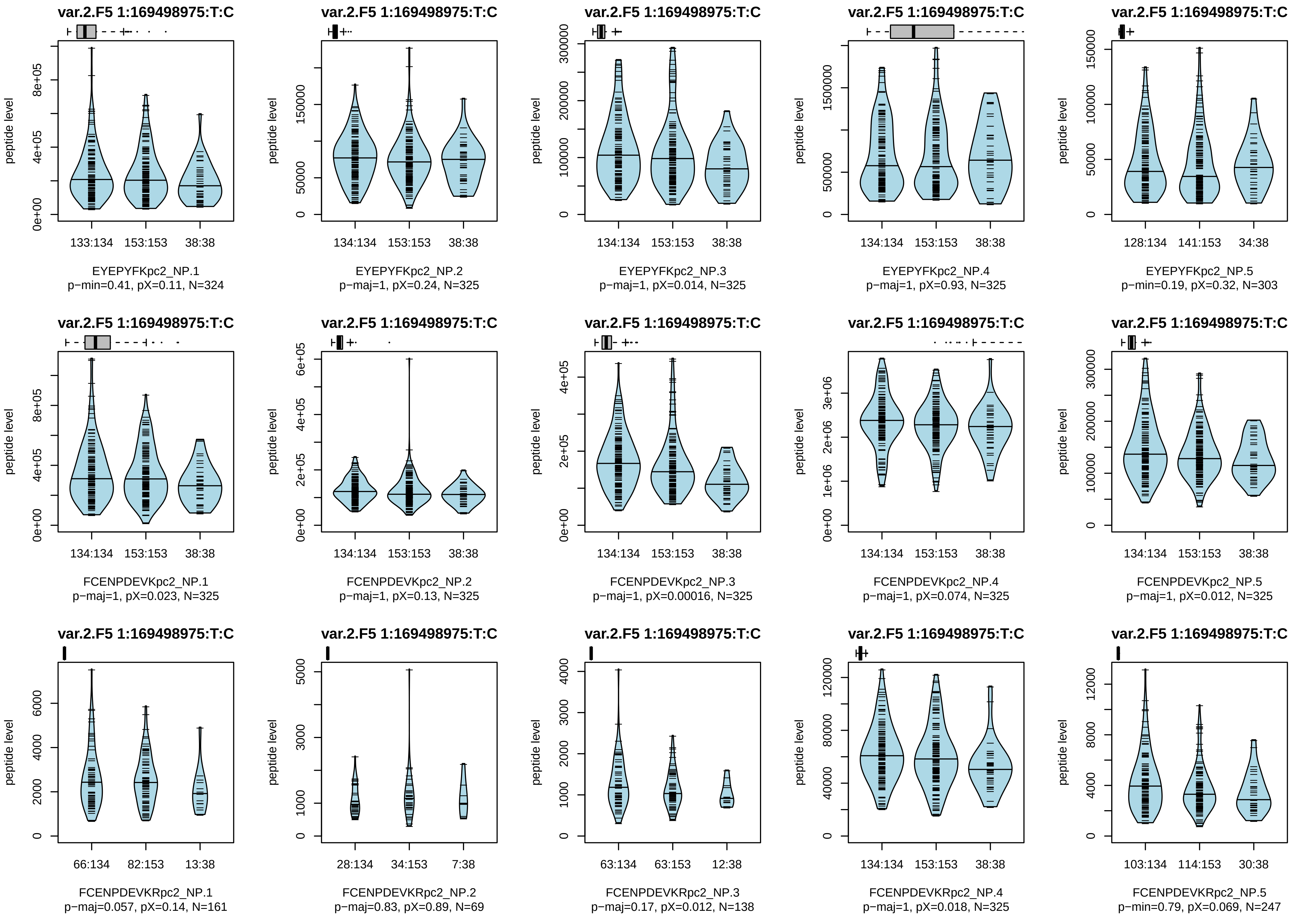

var.2.F5 1:169498975:T:C
peptide level
133:134
153:153
38:38
EYEPYFKpc2_NP.1
p−min=0.41, pX=0.11, N=324
var.2.F5 1:169498975:T:C
peptide level
134:134
153:153
38:38
EYEPYFKpc2_NP.2
p−maj=1, pX=0.24, N=325
var.2.F5 1:169498975:T:C
peptide level
134:134
153:153
38:38
EYEPYFKpc2_NP.3
p−maj=1, pX=0.014, N=325
var.2.F5 1:169498975:T:C
peptide level
134:134
153:153
38:38
EYEPYFKpc2_NP.4
p−maj=1, pX=0.93, N=325
var.2.F5 1:169498975:T:C
peptide level
128:134
141:153
34:38
EYEPYFKpc2_NP.5
p−min=0.19, pX=0.32, N=303
var.2.F5 1:169498975:T:C
peptide level
134:134
153:153
38:38
FCENPDEVKpc2_NP.1
p−maj=1, pX=0.023, N=325
var.2.F5 1:169498975:T:C
peptide level
134:134
153:153
38:38
FCENPDEVKpc2_NP.2
p−maj=1, pX=0.13, N=325
var.2.F5 1:169498975:T:C
peptide level
134:134
153:153
38:38
FCENPDEVKpc2_NP.3
p−maj=1, pX=0.00016, N=325
var.2.F5 1:169498975:T:C
peptide level
134:134
153:153
38:38
FCENPDEVKpc2_NP.4
p−maj=1, pX=0.074, N=325
var.2.F5 1:169498975:T:C
peptide level
134:134
153:153
38:38
FCENPDEVKpc2_NP.5
p−maj=1, pX=0.012, N=325
var.2.F5 1:169498975:T:C
peptide level
66:134
82:153
13:38
FCENPDEVKRpc2_NP.1
p−maj=0.057, pX=0.14, N=161
var.2.F5 1:169498975:T:C
peptide level
28:134
34:153
7:38
FCENPDEVKRpc2_NP.2
p−maj=0.83, pX=0.89, N=69
var.2.F5 1:169498975:T:C
peptide level
63:134
63:153
12:38
FCENPDEVKRpc2_NP.3
p−maj=0.17, pX=0.012, N=138
var.2.F5 1:169498975:T:C
peptide level
134:134
153:153
38:38
FCENPDEVKRpc2_NP.4
p−maj=1, pX=0.018, N=325
var.2.F5 1:169498975:T:C
peptide level
103:134
114:153
30:38
FCENPDEVKRpc2_NP.5
p−min=0.79, pX=0.069, N=247

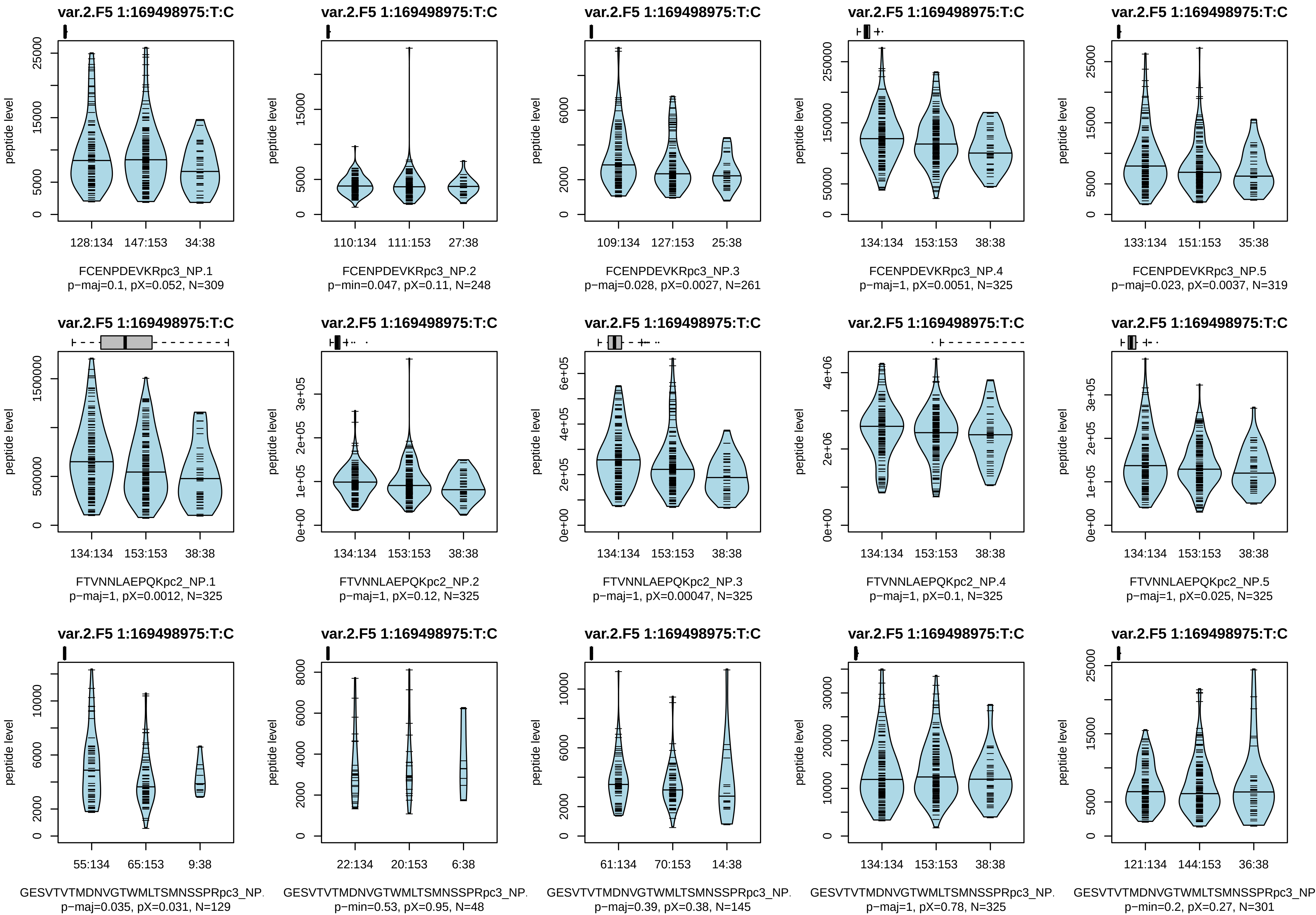
var.2.F5 1:169498975:T:C
peptide level
128:134
147:153
34:38
FCENPDEVKRpc3_NP.1
p−maj=0.1, pX=0.052, N=309
110:134
111:153
27:38
FCENPDEVKRpc3_NP.2
p−min=0.047, pX=0.11, N=248
109:134
127:153
25:38
FCENPDEVKRpc3_NP.3
p−maj=0.028, pX=0.0027, N=261
134:134
153:153
38:38
FCENPDEVKRpc3_NP.4
p−maj=1, pX=0.0051, N=325
133:134
151:153
35:38
FCENPDEVKRpc3_NP.5
p−maj=0.023, pX=0.0037, N=319
FTVNNLAEPQKpc2_NP.1
p−maj=1, pX=0.0012, N=325
FTVNNLAEPQKpc2_NP.2
p−maj=1, pX=0.12, N=325
FTVNNLAEPQKpc2_NP.3
p−maj=1, pX=0.00047, N=325
FTVNNLAEPQKpc2_NP.4
p−maj=1, pX=0.1, N=325
FTVNNLAEPQKpc2_NP.5
p−maj=1, pX=0.025, N=325
55:134
65:153
9:38
GESVTVTMDNVGTWMLTSMNSSPRpc3_NP.
p−maj=0.035, pX=0.031, N=129
22:134
20:153
6:38
GESVTVTMDNVGTWMLTSMNSSPRpc3_NP.
p−min=0.53, pX=0.95, N=48
61:134
70:153
14:38
GESVTVTMDNVGTWMLTSMNSSPRpc3_NP.
p−maj=0.39, pX=0.38, N=145
GESVTVTMDNVGTWMLTSMNSSPRpc3_NP.
p−maj=1, pX=0.78, N=325
121:134
144:153
36:38
GESVTVTMDNVGTWMLTSMNSSPRpc3_NP
p−min=0.2, pX=0.27, N=301

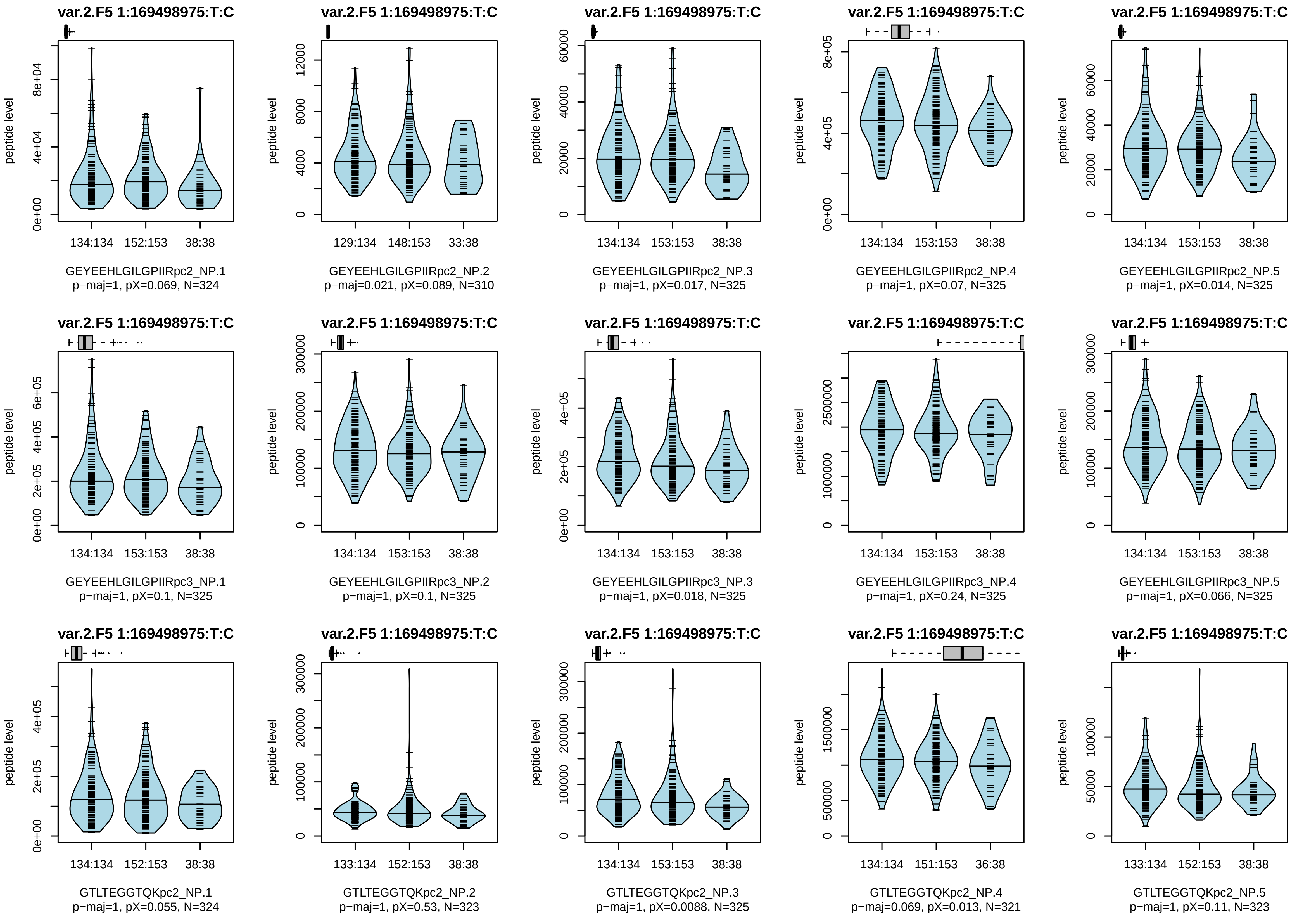

var.2.F5 1:169498975:T:C
peptide level
134:134
152:153
38:38
GEYEEHLGILGPIIRpc2_NP.1
p−maj=1, pX=0.069, N=324
var.2.F5 1:169498975:T:C
peptide level
129:134
148:153
33:38
GEYEEHLGILGPIIRpc2_NP.2
p−maj=0.021, pX=0.089, N=310
var.2.F5 1:169498975:T:C
peptide level
134:134
153:153
38:38
GEYEEHLGILGPIIRpc2_NP.3
p−maj=1, pX=0.017, N=325
var.2.F5 1:169498975:T:C
peptide level
134:134
153:153
38:38
GEYEEHLGILGPIIRpc2_NP.4
p−maj=1, pX=0.07, N=325
var.2.F5 1:169498975:T:C
peptide level
134:134
153:153
38:38
GEYEEHLGILGPIIRpc2_NP.5
p−maj=1, pX=0.014, N=325
var.2.F5 1:169498975:T:C
peptide level
134:134
153:153
38:38
GEYEEHLGILGPIIRpc3_NP.1
p−maj=1, pX=0.1, N=325
var.2.F5 1:169498975:T:C
peptide level
134:134
153:153
38:38
GEYEEHLGILGPIIRpc3_NP.2
p−maj=1, pX=0.1, N=325
var.2.F5 1:169498975:T:C
peptide level
134:134
153:153
38:38
GEYEEHLGILGPIIRpc3_NP.3
p−maj=1, pX=0.018, N=325
var.2.F5 1:169498975:T:C
peptide level
134:134
153:153
38:38
GEYEEHLGILGPIIRpc3_NP.4
p−maj=1, pX=0.24, N=325
var.2.F5 1:169498975:T:C
peptide level
134:134
153:153
38:38
GEYEEHLGILGPIIRpc3_NP.5
p−maj=1, pX=0.066, N=325
var.2.F5 1:169498975:T:C
peptide level
134:134
152:153
38:38
GTLTEGGTQKpc2_NP.1
p−maj=1, pX=0.055, N=324
var.2.F5 1:169498975:T:C
peptide level
133:134
152:153
38:38
GTLTEGGTQKpc2_NP.2
p−maj=1, pX=0.53, N=323
var.2.F5 1:169498975:T:C
peptide level
134:134
153:153
38:38
GTLTEGGTQKpc2_NP.3
p−maj=1, pX=0.0088, N=325
var.2.F5 1:169498975:T:C
peptide level
134:134
151:153
36:38
GTLTEGGTQKpc2_NP.4
p−maj=0.069, pX=0.013, N=321
var.2.F5 1:169498975:T:C
peptide level
133:134
152:153
38:38
GTLTEGGTQKpc2_NP.5
p−maj=1, pX=0.11, N=323

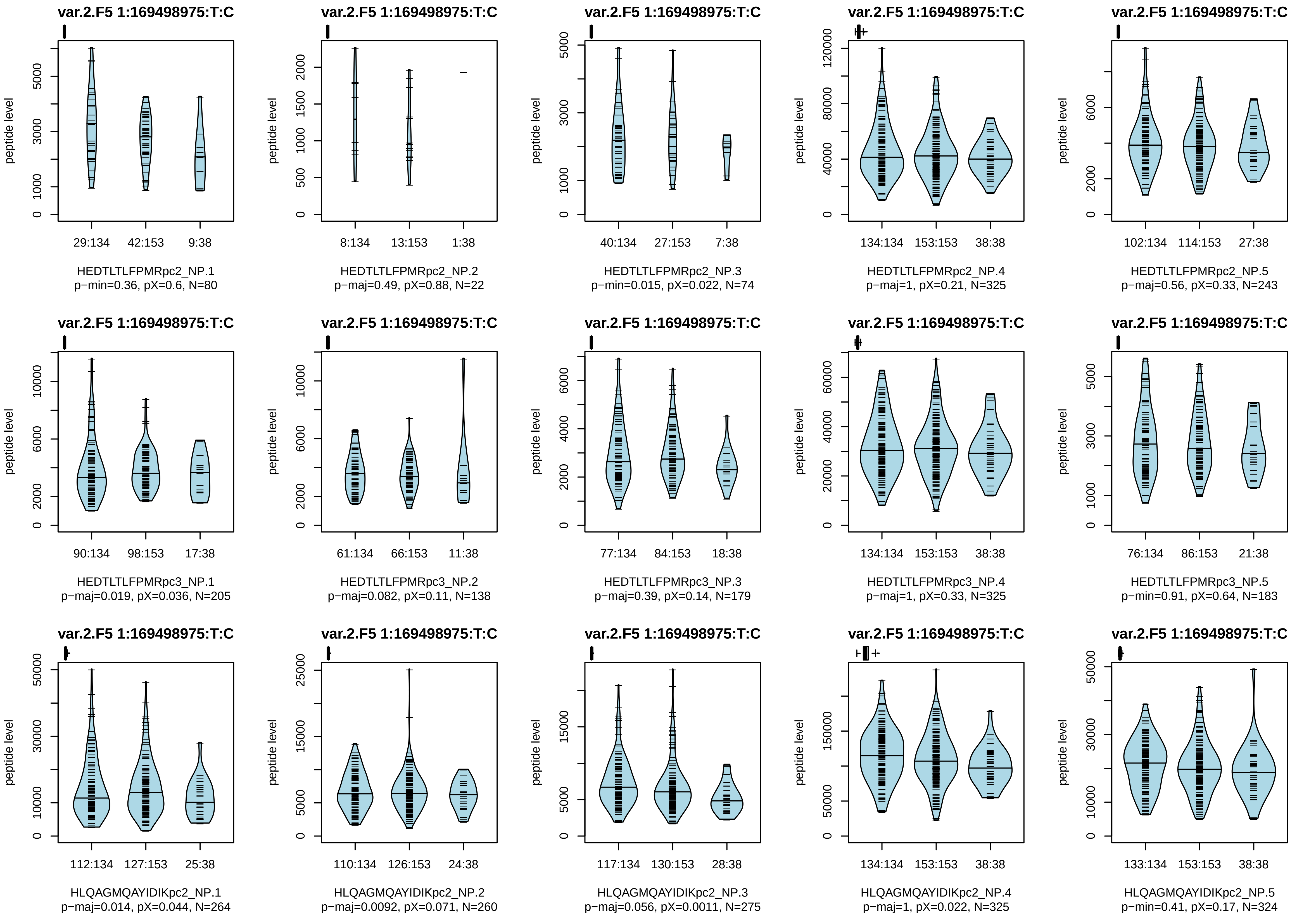
var.2.F5 1:169498975:T:C
peptide level
29:134
42:153
9:38
HEDTLTLFPMRpc2_NP.1
p−min=0.36, pX=0.6, N=80
var.2.F5 1:169498975:T:C
8:134
13:153
1:38
HEDTLTLFPMRpc2_NP.2
p−maj=0.49, pX=0.88, N=22
var.2.F5 1:169498975:T:C
40:134
27:153
7:38
HEDTLTLFPMRpc2_NP.3
p−min=0.015, pX=0.022, N=74
var.2.F5 1:169498975:T:C
134:134
153:153
38:38
HEDTLTLFPMRpc2_NP.4
p−maj=1, pX=0.21, N=325
var.2.F5 1:169498975:T:C
102:134
114:153
27:38
HEDTLTLFPMRpc2_NP.5
p−maj=0.56, pX=0.33, N=243
var.2.F5 1:169498975:T:C
90:134
98:153
17:38
HEDTLTLFPMRpc3_NP.1
p−maj=0.019, pX=0.036, N=205
var.2.F5 1:169498975:T:C
61:134
66:153
11:38
HEDTLTLFPMRpc3_NP.2
p−maj=0.082, pX=0.11, N=138
var.2.F5 1:169498975:T:C
77:134
84:153
18:38
HEDTLTLFPMRpc3_NP.3
p−maj=0.39, pX=0.14, N=179
var.2.F5 1:169498975:T:C
134:134
153:153
38:38
HEDTLTLFPMRpc3_NP.4
p−maj=1, pX=0.33, N=325
var.2.F5 1:169498975:T:C
76:134
86:153
21:38
HEDTLTLFPMRpc3_NP.5
p−min=0.91, pX=0.64, N=183
var.2.F5 1:169498975:T:C
112:134
127:153
25:38
HLQAGMQAYIDIKpc2_NP.1
p−maj=0.014, pX=0.044, N=264
var.2.F5 1:169498975:T:C
110:134
126:153
24:38
HLQAGMQAYIDIKpc2_NP.2
p−maj=0.0092, pX=0.071, N=260
var.2.F5 1:169498975:T:C
117:134
130:153
28:38
HLQAGMQAYIDIKpc2_NP.3
p−maj=0.056, pX=0.0011, N=275
var.2.F5 1:169498975:T:C
134:134
153:153
38:38
HLQAGMQAYIDIKpc2_NP.4
p−maj=1, pX=0.022, N=325
var.2.F5 1:169498975:T:C
133:134
153:153
38:38
HLQAGMQAYIDIKpc2_NP.5
p−min=0.41, pX=0.17, N=324

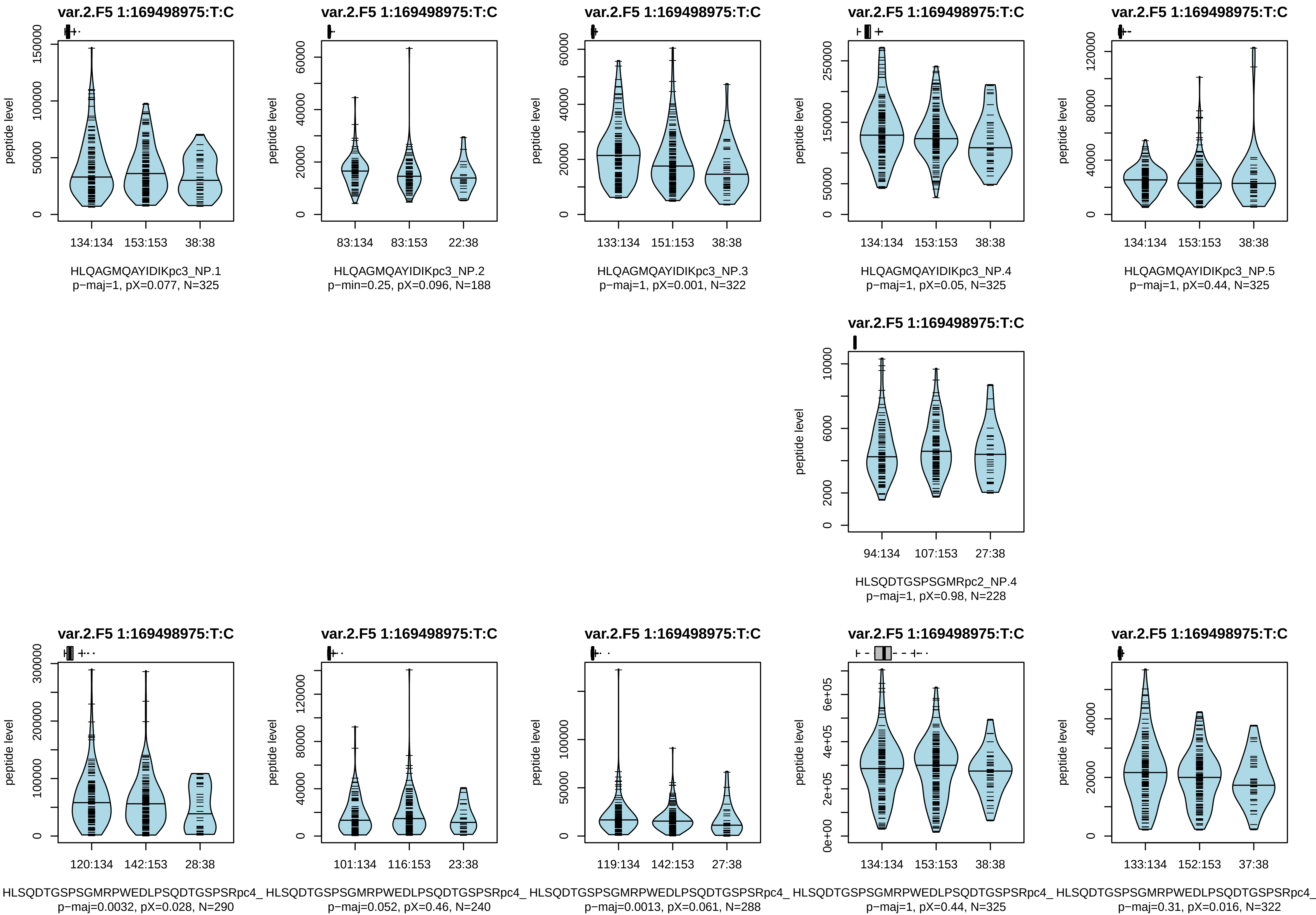
var.2.F5 1:169498975:T:C
peptide level
134:134
153:153
38:38
HLQAGMQAYIDIKpc3_NP.1
p−maj=1, pX=0.077, N=325
var.2.F5 1:169498975:T:C
83:134
83:153
22:38
HLQAGMQAYIDIKpc3_NP.2
p−min=0.25, pX=0.096, N=188
var.2.F5 1:169498975:T:C
133:134
151:153
38:38
HLQAGMQAYIDIKpc3_NP.3
p−maj=1, pX=0.001, N=322
var.2.F5 1:169498975:T:C
134:134
153:153
38:38
HLQAGMQAYIDIKpc3_NP.4
p−maj=1, pX=0.05, N=325
var.2.F5 1:169498975:T:C
134:134
153:153
38:38
HLQAGMQAYIDIKpc3_NP.5
p−maj=1, pX=0.44, N=325
var.2.F5 1:169498975:T:C
94:134
107:153
27:38
HLSQDTGSPSGMRpc2_NP.4
p−maj=1, pX=0.98, N=228
var.2.F5 1:169498975:T:C
120:134
142:153
28:38
HLSQDTGSPSGMRPWEDLPSQDTGSPSRpc4_
p−maj=0.0032, pX=0.028, N=290
var.2.F5 1:169498975:T:C
101:134
116:153
23:38
HLSQDTGSPSGMRPWEDLPSQDTGSPSRpc4_
p−maj=0.052, pX=0.46, N=240
var.2.F5 1:169498975:T:C
119:134
142:153
27:38
HLSQDTGSPSGMRPWEDLPSQDTGSPSRpc4_
p−maj=0.0013, pX=0.061, N=288
var.2.F5 1:169498975:T:C
134:134
153:153
38:38
HLSQDTGSPSGMRPWEDLPSQDTGSPSRpc4_
p−maj=1, pX=0.44, N=325
var.2.F5 1:169498975:T:C
133:134
152:153
37:38
HLSQDTGSPSGMRPWEDLPSQDTGSPSRpc4_
p−maj=0.31, pX=0.016, N=322

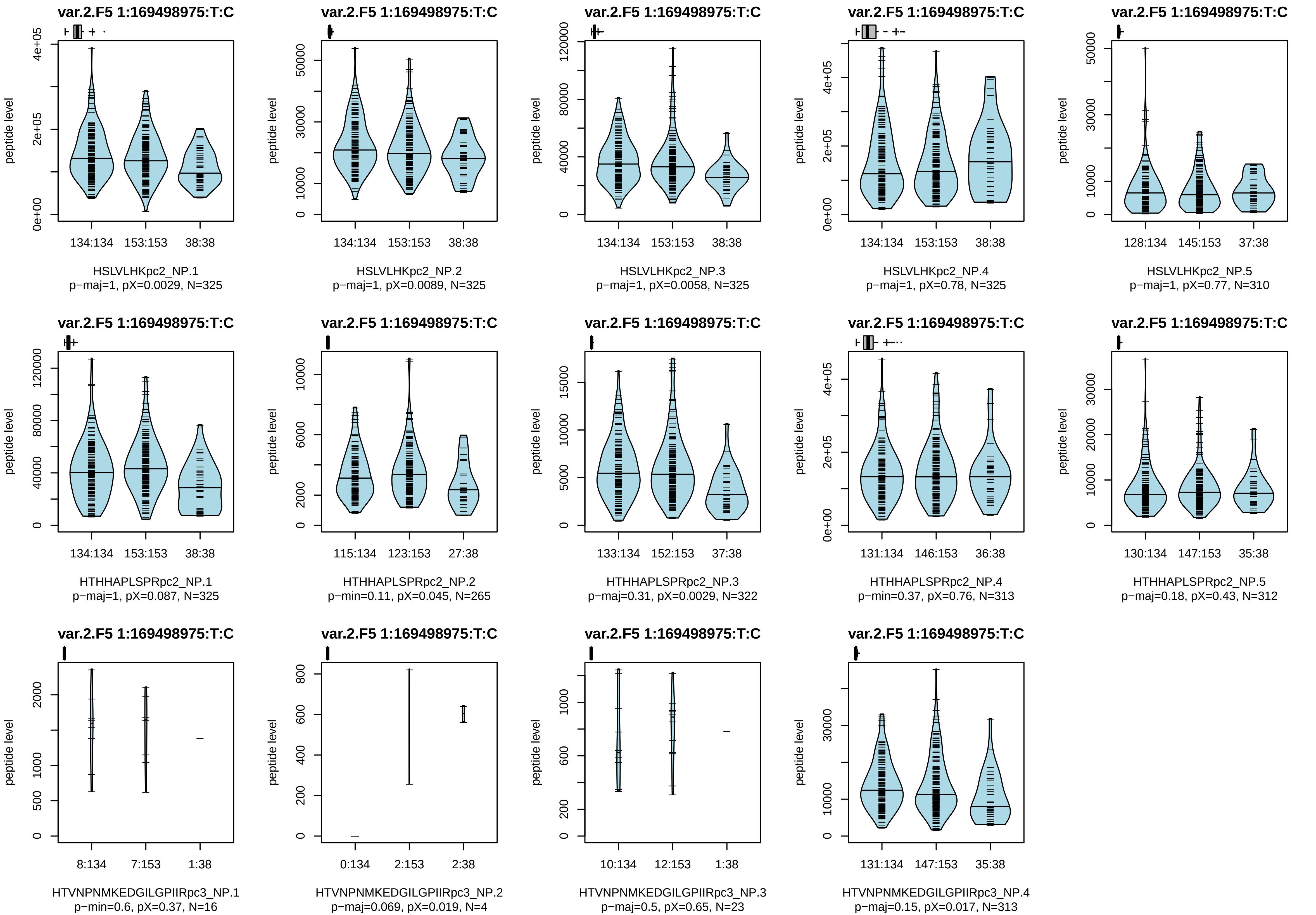
var.2.F5 1:169498975:T:C
peptide level
134:134
153:153
38:38
HSLVLHKpc2_NP.1
p−maj=1, pX=0.0029, N=325
var.2.F5 1:169498975:T:C
peptide level
134:134
153:153
38:38
HSLVLHKpc2_NP.2
p−maj=1, pX=0.0089, N=325
var.2.F5 1:169498975:T:C
peptide level
134:134
153:153
38:38
HSLVLHKpc2_NP.3
p−maj=1, pX=0.0058, N=325
var.2.F5 1:169498975:T:C
peptide level
134:134
153:153
38:38
HSLVLHKpc2_NP.4
p−maj=1, pX=0.78, N=325
var.2.F5 1:169498975:T:C
peptide level
128:134
145:153
37:38
HSLVLHKpc2_NP.5
p−maj=1, pX=0.77, N=310
var.2.F5 1:169498975:T:C
peptide level
134:134
153:153
38:38
HTHHAPLSPRpc2_NP.1
p−maj=1, pX=0.087, N=325
var.2.F5 1:169498975:T:C
peptide level
115:134
123:153
27:38
HTHHAPLSPRpc2_NP.2
p−min=0.11, pX=0.045, N=265
var.2.F5 1:169498975:T:C
peptide level
133:134
152:153
37:38
HTHHAPLSPRpc2_NP.3
p−maj=0.31, pX=0.0029, N=322
var.2.F5 1:169498975:T:C
peptide level
131:134
146:153
36:38
HTHHAPLSPRpc2_NP.4
p−min=0.37, pX=0.76, N=313
var.2.F5 1:169498975:T:C
peptide level
130:134
147:153
35:38
HTHHAPLSPRpc2_NP.5
p−maj=0.18, pX=0.43, N=312
var.2.F5 1:169498975:T:C
peptide level
8:134
7:153
1:38
HTVNPNMKEDGILGPIIRpc3_NP.1
p−min=0.6, pX=0.37, N=16
var.2.F5 1:169498975:T:C
peptide level
0:134
2:153
2:38
HTVNPNMKEDGILGPIIRpc3_NP.2
p−maj=0.069, pX=0.019, N=4
var.2.F5 1:169498975:T:C
peptide level
10:134
12:153
1:38
HTVNPNMKEDGILGPIIRpc3_NP.3
p−maj=0.5, pX=0.65, N=23
var.2.F5 1:169498975:T:C
peptide level
131:134
147:153
35:38
HTVNPNMKEDGILGPIIRpc3_NP.4
p−maj=0.15, pX=0.017, N=313

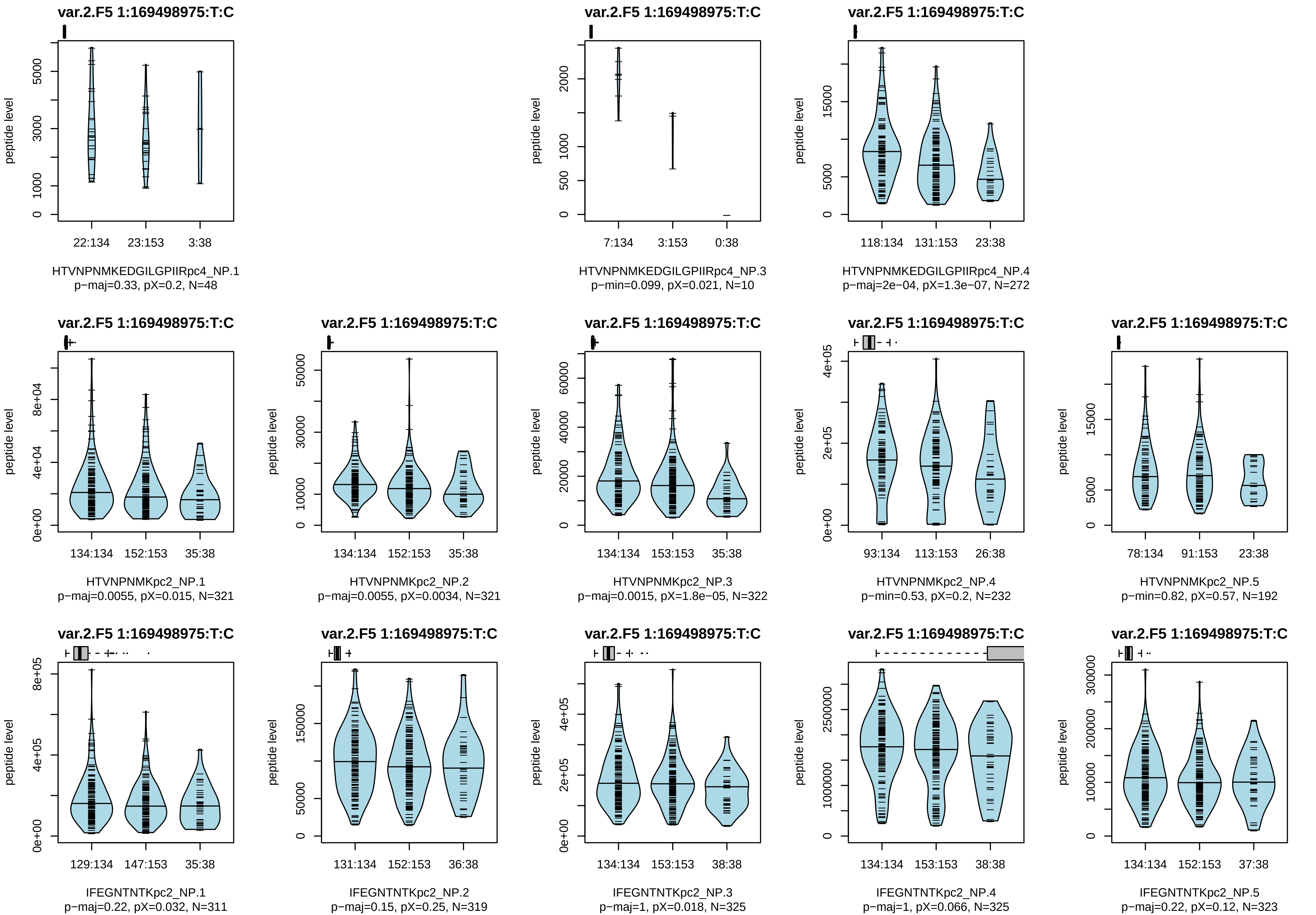
var.2.F5 1:169498975:T:C
peptide level
22:134
23:153
3:38
HTVNPNMKEDGILGPIIRpc4_NP.1
p−maj=0.33, pX=0.2, N=48
var.2.F5 1:169498975:T:C
peptide level
7:134
3:153
0:38
HTVNPNMKEDGILGPIIRpc4_NP.3
p−min=0.099, pX=0.021, N=10
var.2.F5 1:169498975:T:C
peptide level
118:134
131:153
23:38
HTVNPNMKEDGILGPIIRpc4_NP.4
p−maj=2e−04, pX=1.3e−07, N=272
var.2.F5 1:169498975:T:C
peptide level
134:134
152:153
35:38
HTVNPNMKpc2_NP.1
p−maj=0.0055, pX=0.015, N=321
var.2.F5 1:169498975:T:C
peptide level
134:134
152:153
35:38
HTVNPNMKpc2_NP.2
p−maj=0.0055, pX=0.0034, N=321
var.2.F5 1:169498975:T:C
peptide level
134:134
153:153
35:38
HTVNPNMKpc2_NP.3
p−maj=0.0015, pX=1.8e−05, N=322
var.2.F5 1:169498975:T:C
peptide level
93:134
113:153
26:38
HTVNPNMKpc2_NP.4
p−min=0.53, pX=0.2, N=232
var.2.F5 1:169498975:T:C
peptide level
78:134
91:153
23:38
HTVNPNMKpc2_NP.5
p−min=0.82, pX=0.57, N=192
var.2.F5 1:169498975:T:C
peptide level
129:134
147:153
35:38
IFEGNTNTKpc2_NP.1
p−maj=0.22, pX=0.032, N=311
var.2.F5 1:169498975:T:C
peptide level
131:134
152:153
36:38
IFEGNTNTKpc2_NP.2
p−maj=0.15, pX=0.25, N=319
var.2.F5 1:169498975:T:C
peptide level
134:134
153:153
38:38
IFEGNTNTKpc2_NP.3
p−maj=1, pX=0.018, N=325
var.2.F5 1:169498975:T:C
peptide level
134:134
153:153
38:38
IFEGNTNTKpc2_NP.4
p−maj=1, pX=0.066, N=325
var.2.F5 1:169498975:T:C
peptide level
134:134
152:153
37:38
IFEGNTNTKpc2_NP.5
p−maj=0.22, pX=0.12, N=323

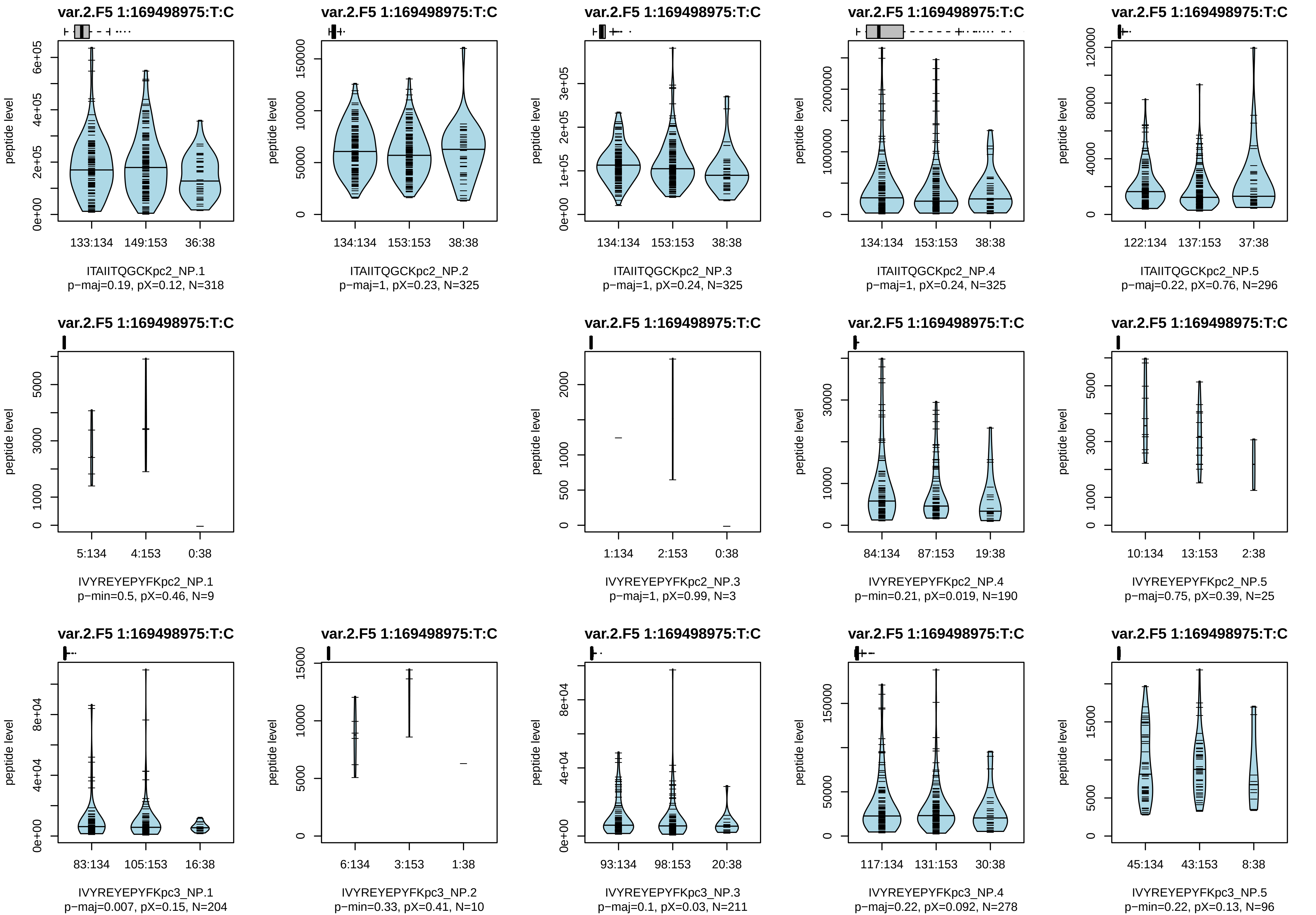

var.2.F5 1:169498975:T:C
peptide level
133:134
149:153
36:38
ITAIITQGCKpc2_NP.1
p−maj=0.19, pX=0.12, N=318
var.2.F5 1:169498975:T:C
peptide level
134:134
153:153
38:38
ITAIITQGCKpc2_NP.2
p−maj=1, pX=0.23, N=325
var.2.F5 1:169498975:T:C
peptide level
134:134
153:153
38:38
ITAIITQGCKpc2_NP.3
p−maj=1, pX=0.24, N=325
var.2.F5 1:169498975:T:C
peptide level
134:134
153:153
38:38
ITAIITQGCKpc2_NP.4
p−maj=1, pX=0.24, N=325
var.2.F5 1:169498975:T:C
peptide level
122:134
137:153
37:38
ITAIITQGCKpc2_NP.5
p−maj=0.22, pX=0.76, N=296
var.2.F5 1:169498975:T:C
peptide level
5:134
4:153
0:38
IVYREYEPYFKpc2_NP.1
p−min=0.5, pX=0.46, N=9
var.2.F5 1:169498975:T:C
peptide level
1:134
2:153
0:38
IVYREYEPYFKpc2_NP.3
p−maj=1, pX=0.99, N=3
var.2.F5 1:169498975:T:C
peptide level
84:134
87:153
19:38
IVYREYEPYFKpc2_NP.4
p−min=0.21, pX=0.019, N=190
var.2.F5 1:169498975:T:C
peptide level
10:134
13:153
2:38
IVYREYEPYFKpc2_NP.5
p−maj=0.75, pX=0.39, N=25
var.2.F5 1:169498975:T:C
peptide level
83:134
105:153
16:38
IVYREYEPYFKpc3_NP.1
p−maj=0.007, pX=0.15, N=204
var.2.F5 1:169498975:T:C
peptide level
6:134
3:153
1:38
IVYREYEPYFKpc3_NP.2
p−min=0.33, pX=0.41, N=10
var.2.F5 1:169498975:T:C
peptide level
93:134
98:153
20:38
IVYREYEPYFKpc3_NP.3
p−maj=0.1, pX=0.03, N=211
var.2.F5 1:169498975:T:C
peptide level
117:134
131:153
30:38
IVYREYEPYFKpc3_NP.4
p−maj=0.22, pX=0.092, N=278
var.2.F5 1:169498975:T:C
peptide level
45:134
43:153
8:38
IVYREYEPYFKpc3_NP.5
p−min=0.22, pX=0.13, N=96

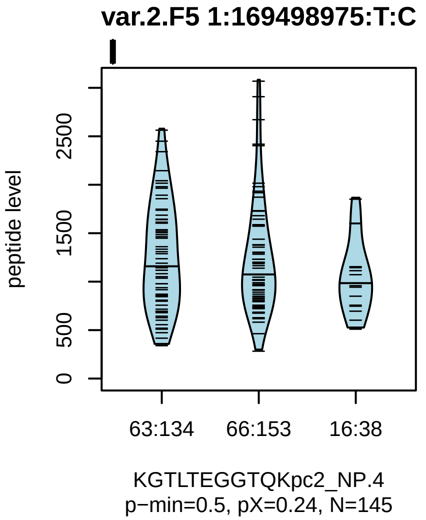
var.2.F5 1:169498975:T:C
peptide level
0
500
1500
2500
63:134
66:153
16:38
KGTLTEGGTQKpc2_NP.4
p−min=0.5, pX=0.24, N=145

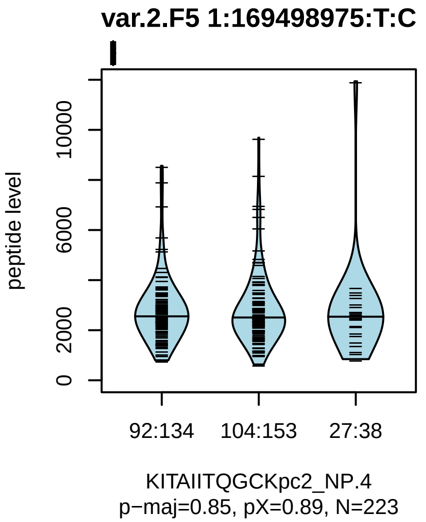
var.2.F5 1:169498975:T:C
peptide level
0
2000
6000
10000
92:134
104:153
27:38
KITAIITQGCKpc2_NP.4
p−maj=0.85, pX=0.89, N=223

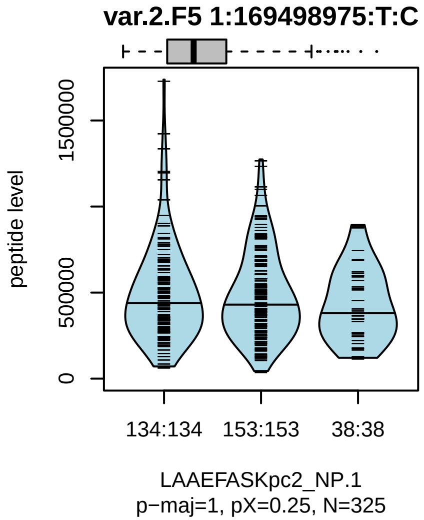
var.2.F5 1:169498975:T:C
peptide level
0
500000
1500000
134:134
153:153
38:38
LAAEFASKpc2_NP.1
p−maj=1, pX=0.25, N=325

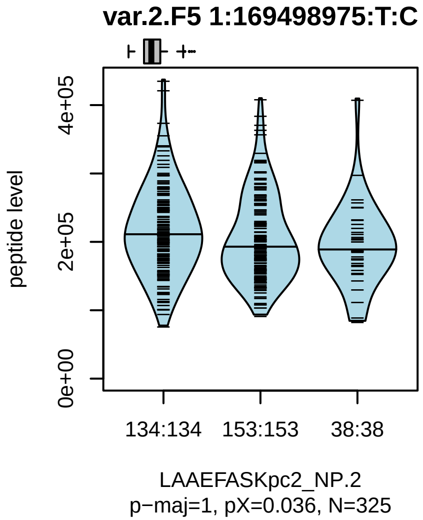
var.2.F5 1:169498975:T:C
peptide level
0e+00
2e+05
4e+05
134:134
153:153
38:38
LAAEFASKpc2_NP.2
p−maj=1, pX=0.036, N=325

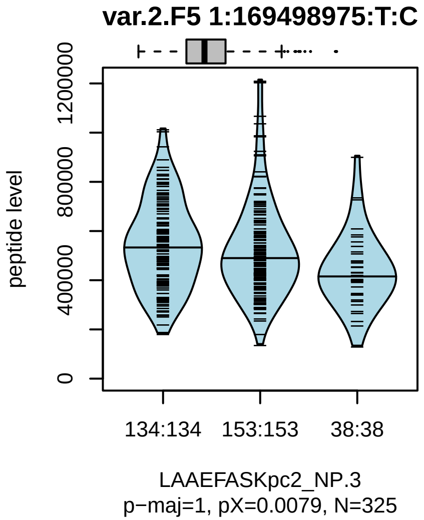
var.2.F5 1:169498975:T:C
peptide level
0
400000
800000
1200000
134:134
153:153
38:38
LAAEFASKpc2_NP.3
p−maj=1, pX=0.0079, N=325

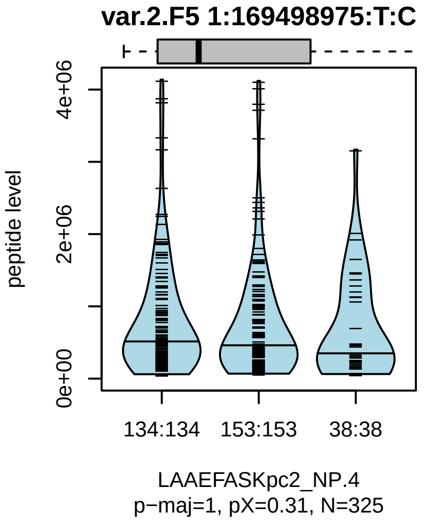
var.2.F5 1:169498975:T:C
peptide level
0e+00
2e+06
4e+06
134:134
153:153
38:38
LAAEFASKpc2_NP.4
p−maj=1, pX=0.31, N=325

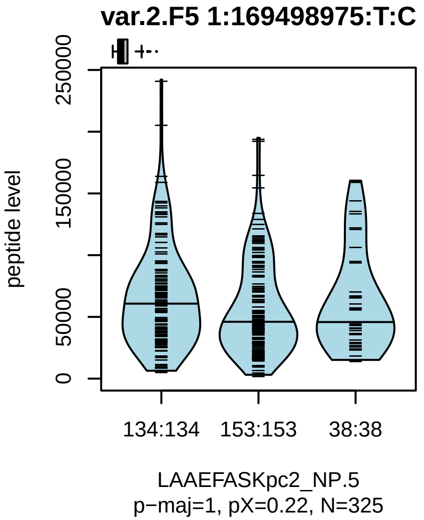
var.2.F5 1:169498975:T:C
peptide level
0
50000
150000
250000
134:134
153:153
38:38
LAAEFASKpc2_NP.5
p−maj=1, pX=0.22, N=325

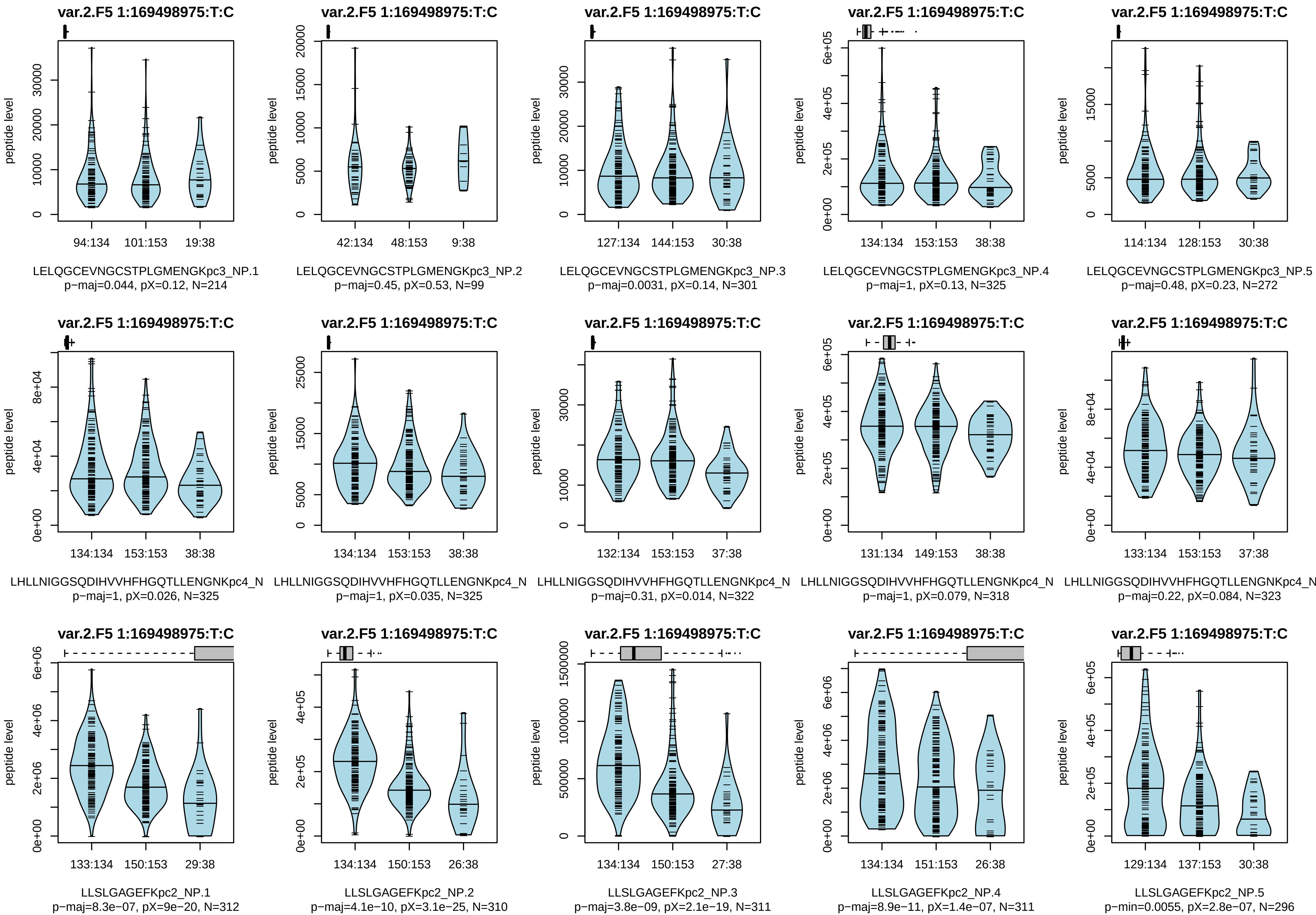

var.2.F5 1:169498975:T:C
peptide level
94:134
101:153
19:38
LELQGCEVNGCSTPLGMENGKpc3_NP.1
p−maj=0.044, pX=0.12, N=214
var.2.F5 1:169498975:T:C
peptide level
42:134
48:153
9:38
LELQGCEVNGCSTPLGMENGKpc3_NP.2
p−maj=0.45, pX=0.53, N=99
var.2.F5 1:169498975:T:C
peptide level
127:134
144:153
30:38
LELQGCEVNGCSTPLGMENGKpc3_NP.3
p−maj=0.0031, pX=0.14, N=301
var.2.F5 1:169498975:T:C
peptide level
134:134
153:153
38:38
LELQGCEVNGCSTPLGMENGKpc3_NP.4
p−maj=1, pX=0.13, N=325
var.2.F5 1:169498975:T:C
peptide level
114:134
128:153
30:38
LELQGCEVNGCSTPLGMENGKpc3_NP.5
p−maj=0.48, pX=0.23, N=272
var.2.F5 1:169498975:T:C
peptide level
134:134
153:153
38:38
LHLLNIGGSQDIHVVHFHGQTLLENGNKpc4_N
p−maj=1, pX=0.026, N=325
var.2.F5 1:169498975:T:C
peptide level
134:134
153:153
38:38
LHLLNIGGSQDIHVVHFHGQTLLENGNKpc4_N
p−maj=1, pX=0.035, N=325
var.2.F5 1:169498975:T:C
peptide level
132:134
153:153
37:38
LHLLNIGGSQDIHVVHFHGQTLLENGNKpc4_N
p−maj=0.31, pX=0.014, N=322
var.2.F5 1:169498975:T:C
peptide level
131:134
149:153
38:38
LHLLNIGGSQDIHVVHFHGQTLLENGNKpc4_N
p−maj=1, pX=0.079, N=318
var.2.F5 1:169498975:T:C
peptide level
133:134
153:153
37:38
LHLLNIGGSQDIHVVHFHGQTLLENGNKpc4_N
p−maj=0.22, pX=0.084, N=323
var.2.F5 1:169498975:T:C
peptide level
133:134
150:153
29:38
LLSLGAGEFKpc2_NP.1
p−maj=8.3e−07, pX=9e−20, N=312
var.2.F5 1:169498975:T:C
peptide level
134:134
150:153
26:38
LLSLGAGEFKpc2_NP.2
p−maj=4.1e−10, pX=3.1e−25, N=310
var.2.F5 1:169498975:T:C
peptide level
134:134
150:153
27:38
LLSLGAGEFKpc2_NP.3
p−maj=3.8e−09, pX=2.1e−19, N=311
var.2.F5 1:169498975:T:C
peptide level
134:134
151:153
26:38
LLSLGAGEFKpc2_NP.4
p−maj=8.9e−11, pX=1.4e−07, N=311
var.2.F5 1:169498975:T:C
peptide level
129:134
137:153
30:38
LLSLGAGEFKpc2_NP.5
p−min=0.0055, pX=2.8e−07, N=296

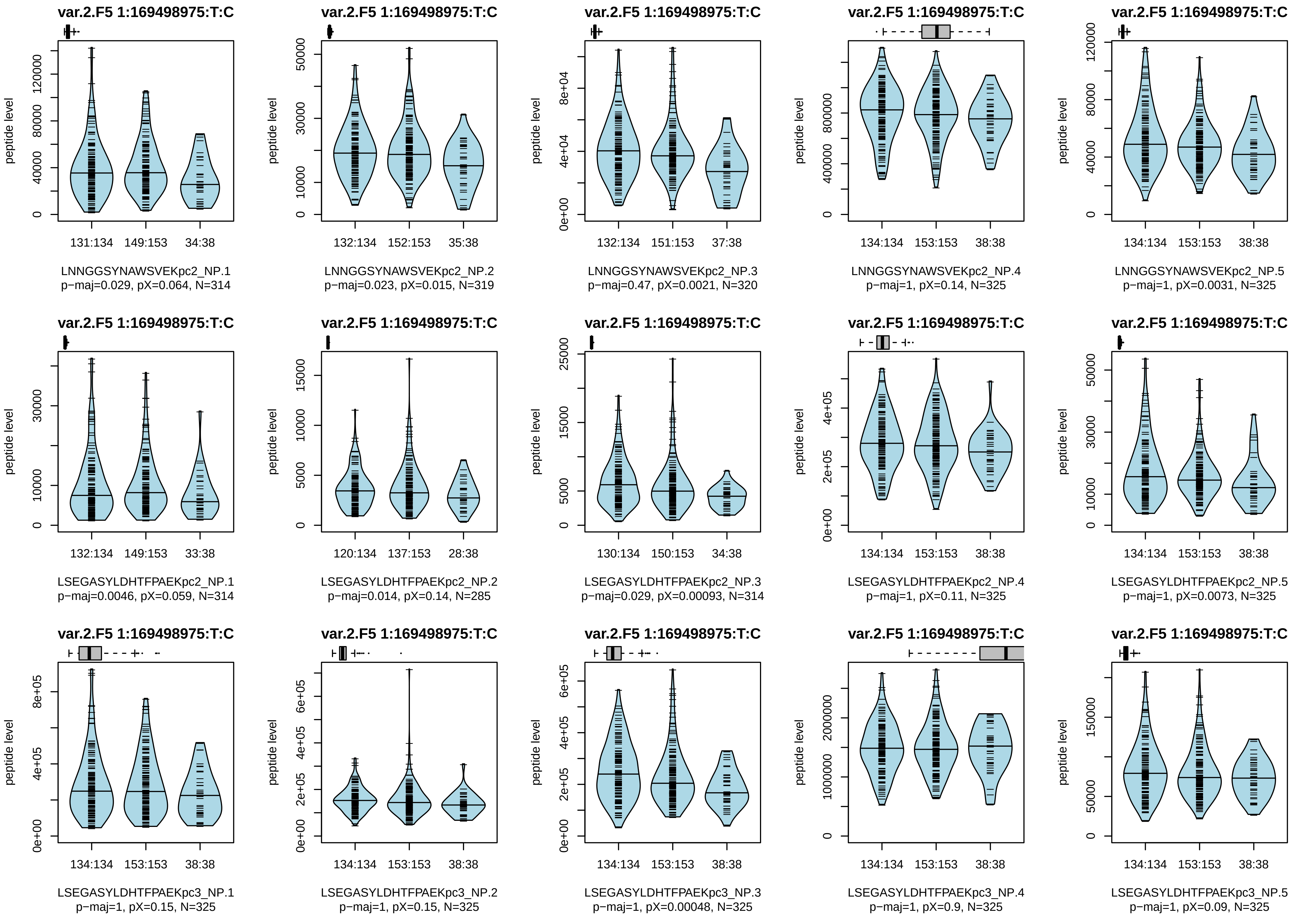
var.2.F5 1:169498975:T:C
peptide level
131:134 149:153 34:38
LNNGGSYNAWSVEKpc2_NP.1
p−maj=0.029, pX=0.064, N=314
var.2.F5 1:169498975:T:C
peptide level
132:134 152:153 35:38
LNNGGSYNAWSVEKpc2_NP.2
p−maj=0.023, pX=0.015, N=319
var.2.F5 1:169498975:T:C
peptide level
132:134 151:153 37:38
LNNGGSYNAWSVEKpc2_NP.3
p−maj=0.47, pX=0.0021, N=320
var.2.F5 1:169498975:T:C
peptide level
134:134 153:153 38:38
LNNGGSYNAWSVEKpc2_NP.4
p−maj=1, pX=0.14, N=325
var.2.F5 1:169498975:T:C
peptide level
134:134 153:153 38:38
LNNGGSYNAWSVEKpc2_NP.5
p−maj=1, pX=0.0031, N=325
var.2.F5 1:169498975:T:C
peptide level
132:134 149:153 33:38
LSEGASYLDHTFPAEKpc2_NP.1
p−maj=0.0046, pX=0.059, N=314
var.2.F5 1:169498975:T:C
peptide level
120:134 137:153 28:38
LSEGASYLDHTFPAEKpc2_NP.2
p−maj=0.014, pX=0.14, N=285
var.2.F5 1:169498975:T:C
peptide level
130:134 150:153 34:38
LSEGASYLDHTFPAEKpc2_NP.3
p−maj=0.029, pX=0.00093, N=314
var.2.F5 1:169498975:T:C
peptide level
134:134 153:153 38:38
LSEGASYLDHTFPAEKpc2_NP.4
p−maj=1, pX=0.11, N=325
var.2.F5 1:169498975:T:C
peptide level
134:134 153:153 38:38
LSEGASYLDHTFPAEKpc2_NP.5
p−maj=1, pX=0.0073, N=325
var.2.F5 1:169498975:T:C
peptide level
134:134 153:153 38:38
LSEGASYLDHTFPAEKpc3_NP.1
p−maj=1, pX=0.15, N=325
var.2.F5 1:169498975:T:C
peptide level
134:134 153:153 38:38
LSEGASYLDHTFPAEKpc3_NP.2
p−maj=1, pX=0.15, N=325
var.2.F5 1:169498975:T:C
peptide level
134:134 153:153 38:38
LSEGASYLDHTFPAEKpc3_NP.3
p−maj=1, pX=0.00048, N=325
var.2.F5 1:169498975:T:C
peptide level
134:134 153:153 38:38
LSEGASYLDHTFPAEKpc3_NP.4
p−maj=1, pX=0.9, N=325
var.2.F5 1:169498975:T:C
peptide level
134:134 153:153 38:38
LSEGASYLDHTFPAEKpc3_NP.5
p−maj=1, pX=0.09, N=325

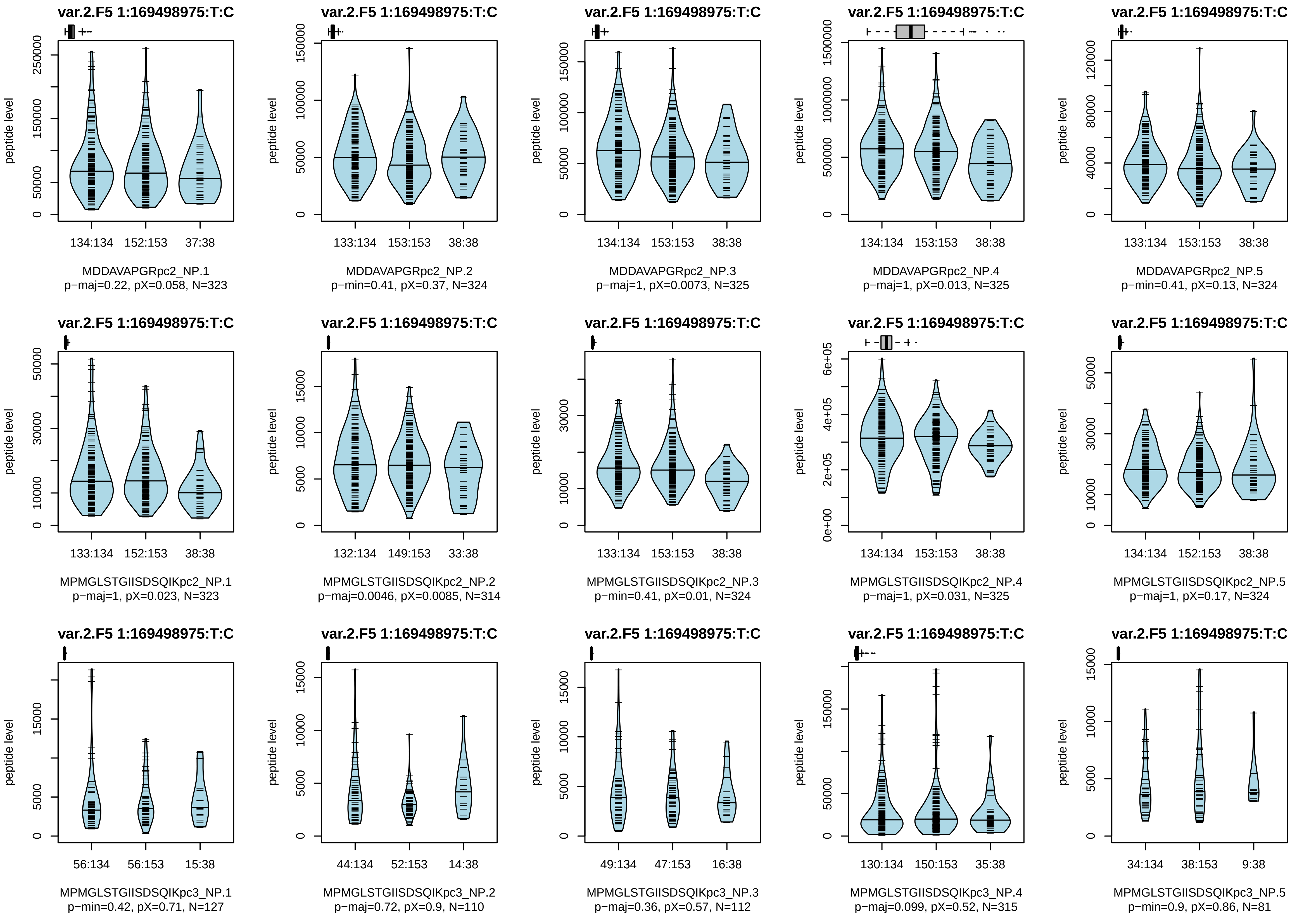
var.2.F5 1:169498975:T:C
peptide level
134:134 152:153 37:38
MDDAVAPGRpc2_NP.1
p−maj=0.22, pX=0.058, N=323
var.2.F5 1:169498975:T:C
peptide level
133:134 153:153 38:38
MDDAVAPGRpc2_NP.2
p−min=0.41, pX=0.37, N=324
var.2.F5 1:169498975:T:C
peptide level
134:134 153:153 38:38
MDDAVAPGRpc2_NP.3
p−maj=1, pX=0.0073, N=325
var.2.F5 1:169498975:T:C
peptide level
134:134 153:153 38:38
MDDAVAPGRpc2_NP.4
p−maj=1, pX=0.013, N=325
var.2.F5 1:169498975:T:C
peptide level
133:134 153:153 38:38
MDDAVAPGRpc2_NP.5
p−min=0.41, pX=0.13, N=324
var.2.F5 1:169498975:T:C
peptide level
133:134 152:153 38:38
MPMGLSTGIISDSQIKpc2_NP.1
p−maj=1, pX=0.023, N=323
var.2.F5 1:169498975:T:C
peptide level
132:134 149:153 33:38
MPMGLSTGIISDSQIKpc2_NP.2
p−maj=0.0046, pX=0.0085, N=314
var.2.F5 1:169498975:T:C
peptide level
133:134 153:153 38:38
MPMGLSTGIISDSQIKpc2_NP.3
p−min=0.41, pX=0.01, N=324
var.2.F5 1:169498975:T:C
peptide level
134:134 153:153 38:38
MPMGLSTGIISDSQIKpc2_NP.4
p−maj=1, pX=0.031, N=325
var.2.F5 1:169498975:T:C
peptide level
134:134 152:153 38:38
MPMGLSTGIISDSQIKpc2_NP.5
p−maj=1, pX=0.17, N=324
var.2.F5 1:169498975:T:C
peptide level
56:134 56:153 15:38
MPMGLSTGIISDSQIKpc3_NP.1
p−min=0.42, pX=0.71, N=127
var.2.F5 1:169498975:T:C
peptide level
44:134 52:153 14:38
MPMGLSTGIISDSQIKpc3_NP.2
p−maj=0.72, pX=0.9, N=110
var.2.F5 1:169498975:T:C
peptide level
49:134 47:153 16:38
MPMGLSTGIISDSQIKpc3_NP.3
p−maj=0.36, pX=0.57, N=112
var.2.F5 1:169498975:T:C
peptide level
130:134 150:153 35:38
MPMGLSTGIISDSQIKpc3_NP.4
p−maj=0.099, pX=0.52, N=315
var.2.F5 1:169498975:T:C
peptide level
34:134 38:153 9:38
MPMGLSTGIISDSQIKpc3_NP.5
p−min=0.9, pX=0.86, N=81

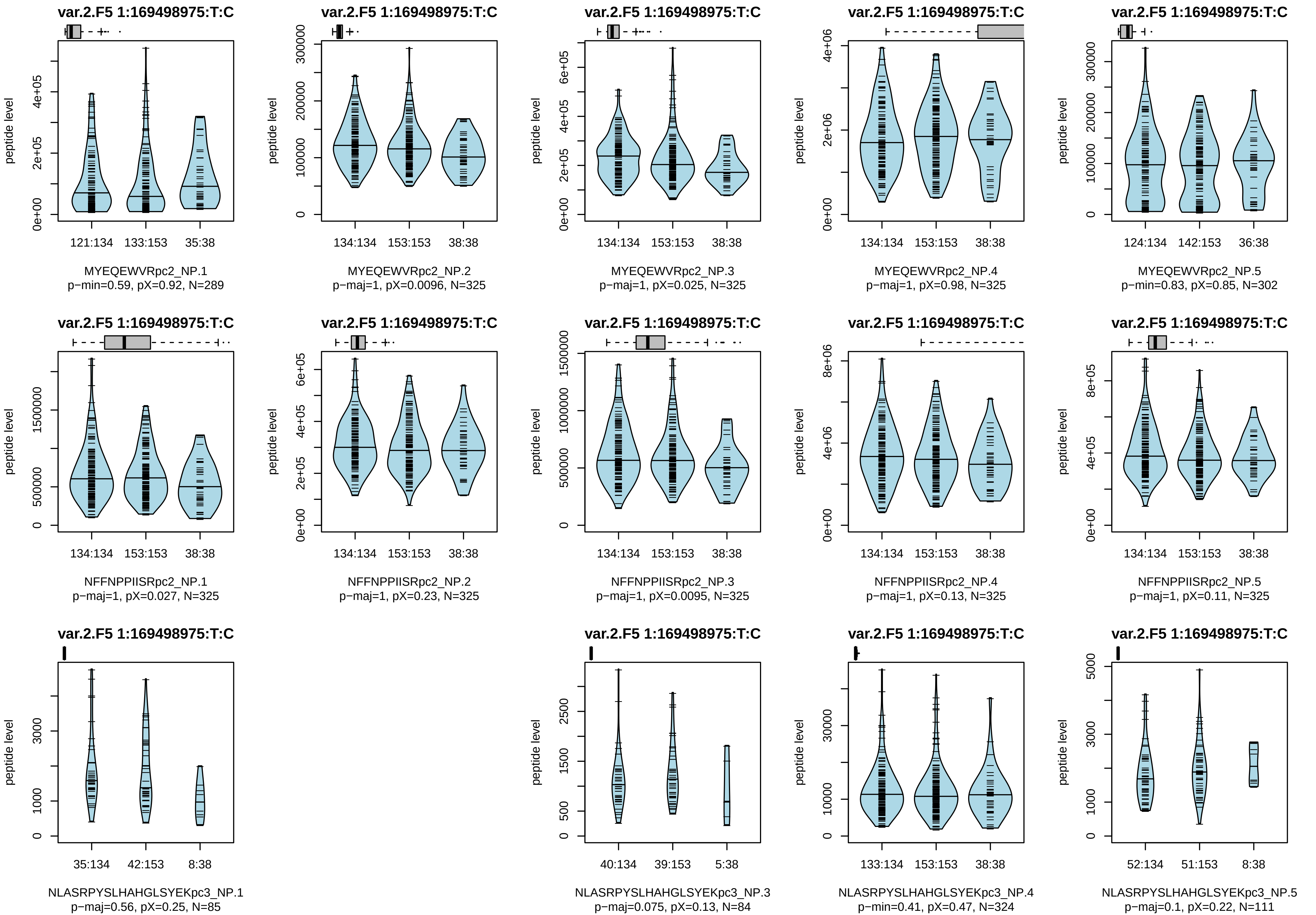

var.2.F5 1:169498975:T:C
peptide level
121:134
133:153
35:38
MYEQEWVRpc2_NP.1
p−min=0.59, pX=0.92, N=289
var.2.F5 1:169498975:T:C
peptide level
134:134
153:153
38:38
MYEQEWVRpc2_NP.2
p−maj=1, pX=0.0096, N=325
var.2.F5 1:169498975:T:C
peptide level
134:134
153:153
38:38
MYEQEWVRpc2_NP.3
p−maj=1, pX=0.025, N=325
var.2.F5 1:169498975:T:C
peptide level
134:134
153:153
38:38
MYEQEWVRpc2_NP.4
p−maj=1, pX=0.98, N=325
var.2.F5 1:169498975:T:C
peptide level
124:134
142:153
36:38
MYEQEWVRpc2_NP.5
p−min=0.83, pX=0.85, N=302
var.2.F5 1:169498975:T:C
peptide level
134:134
153:153
38:38
NFFNPPIISRpc2_NP.1
p−maj=1, pX=0.027, N=325
var.2.F5 1:169498975:T:C
peptide level
134:134
153:153
38:38
NFFNPPIISRpc2_NP.2
p−maj=1, pX=0.23, N=325
var.2.F5 1:169498975:T:C
peptide level
134:134
153:153
38:38
NFFNPPIISRpc2_NP.3
p−maj=1, pX=0.0095, N=325
var.2.F5 1:169498975:T:C
peptide level
134:134
153:153
38:38
NFFNPPIISRpc2_NP.4
p−maj=1, pX=0.13, N=325
var.2.F5 1:169498975:T:C
peptide level
134:134
153:153
38:38
NFFNPPIISRpc2_NP.5
p−maj=1, pX=0.11, N=325
var.2.F5 1:169498975:T:C
peptide level
35:134
42:153
8:38
NLASRPYSLHAHGLSYEKpc3_NP.1
p−maj=0.56, pX=0.25, N=85
var.2.F5 1:169498975:T:C
peptide level
40:134
39:153
5:38
NLASRPYSLHAHGLSYEKpc3_NP.3
p−maj=0.075, pX=0.13, N=84
var.2.F5 1:169498975:T:C
peptide level
133:134
153:153
38:38
NLASRPYSLHAHGLSYEKpc3_NP.4
p−min=0.41, pX=0.47, N=324
var.2.F5 1:169498975:T:C
peptide level
52:134
51:153
8:38
NLASRPYSLHAHGLSYEKpc3_NP.5
p−maj=0.1, pX=0.22, N=111

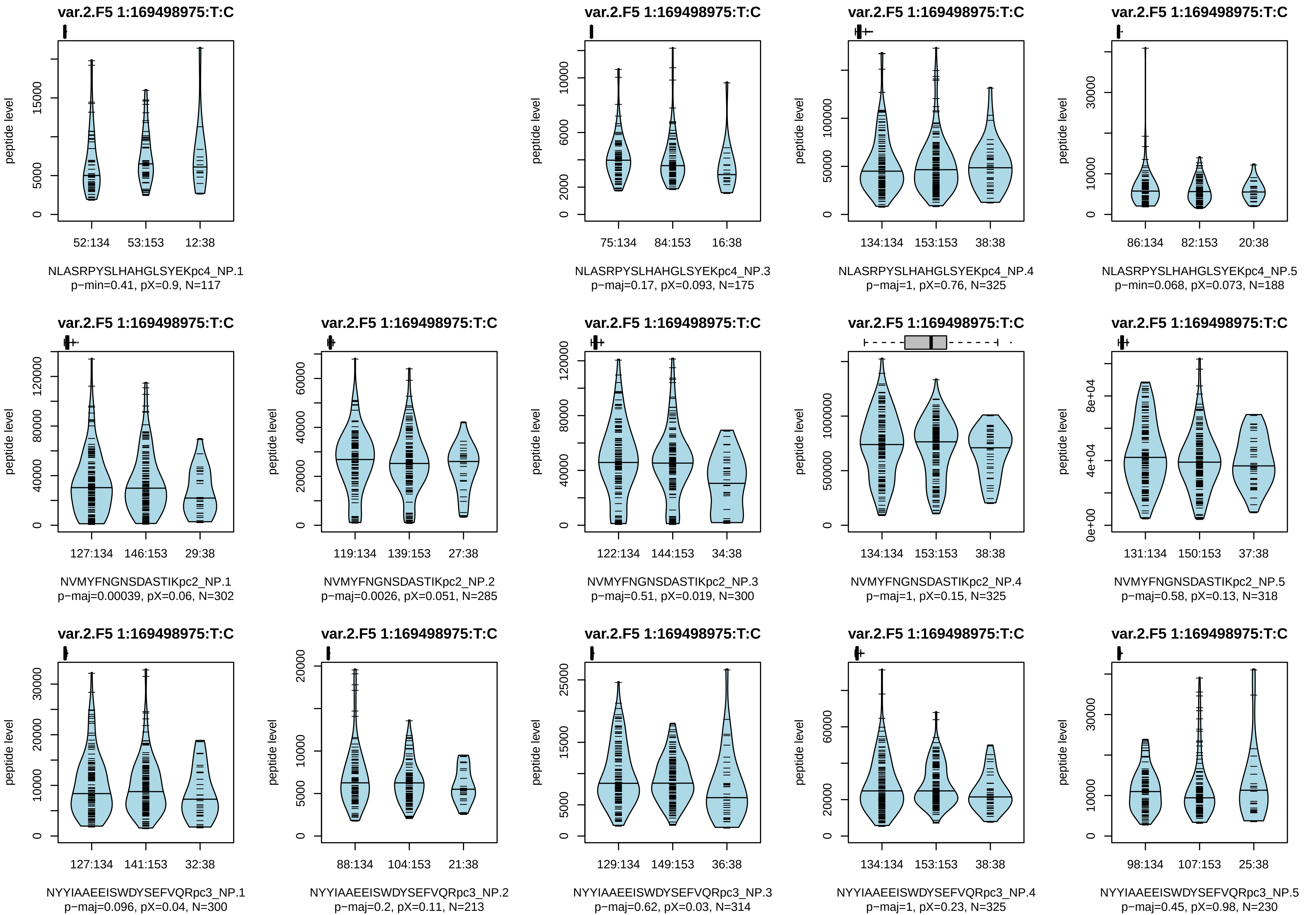
var.2.F5 1:169498975:T:C
peptide level
52:134
53:153
12:38
NLASRPYSLHAHGLSYEKpc4_NP.1
p−min=0.41, pX=0.9, N=117
var.2.F5 1:169498975:T:C
peptide level
75:134
84:153
16:38
NLASRPYSLHAHGLSYEKpc4_NP.3
p−maj=0.17, pX=0.093, N=175
var.2.F5 1:169498975:T:C
peptide level
134:134
153:153
38:38
NLASRPYSLHAHGLSYEKpc4_NP.4
p−maj=1, pX=0.76, N=325
var.2.F5 1:169498975:T:C
peptide level
86:134
82:153
20:38
NLASRPYSLHAHGLSYEKpc4_NP.5
p−min=0.068, pX=0.073, N=188
var.2.F5 1:169498975:T:C
peptide level
127:134
146:153
29:38
NVMYFNGNSDASTIKpc2_NP.1
p−maj=0.00039, pX=0.06, N=302
var.2.F5 1:169498975:T:C
peptide level
119:134
139:153
27:38
NVMYFNGNSDASTIKpc2_NP.2
p−maj=0.0026, pX=0.051, N=285
var.2.F5 1:169498975:T:C
peptide level
122:134
144:153
34:38
NVMYFNGNSDASTIKpc2_NP.3
p−maj=0.51, pX=0.019, N=300
var.2.F5 1:169498975:T:C
peptide level
134:134
153:153
38:38
NVMYFNGNSDASTIKpc2_NP.4
p−maj=1, pX=0.15, N=325
var.2.F5 1:169498975:T:C
peptide level
131:134
150:153
37:38
NVMYFNGNSDASTIKpc2_NP.5
p−maj=0.58, pX=0.13, N=318
var.2.F5 1:169498975:T:C
peptide level
127:134
141:153
32:38
NYYIAAEEISWDYSEFVQRpc3_NP.1
p−maj=0.096, pX=0.04, N=300
var.2.F5 1:169498975:T:C
peptide level
88:134
104:153
21:38
NYYIAAEEISWDYSEFVQRpc3_NP.2
p−maj=0.2, pX=0.11, N=213
var.2.F5 1:169498975:T:C
peptide level
129:134
149:153
36:38
NYYIAAEEISWDYSEFVQRpc3_NP.3
p−maj=0.62, pX=0.03, N=314
var.2.F5 1:169498975:T:C
peptide level
134:134
153:153
38:38
NYYIAAEEISWDYSEFVQRpc3_NP.4
p−maj=1, pX=0.23, N=325
var.2.F5 1:169498975:T:C
peptide level
98:134
107:153
25:38
NYYIAAEEISWDYSEFVQRpc3_NP.5
p−maj=0.45, pX=0.98, N=230

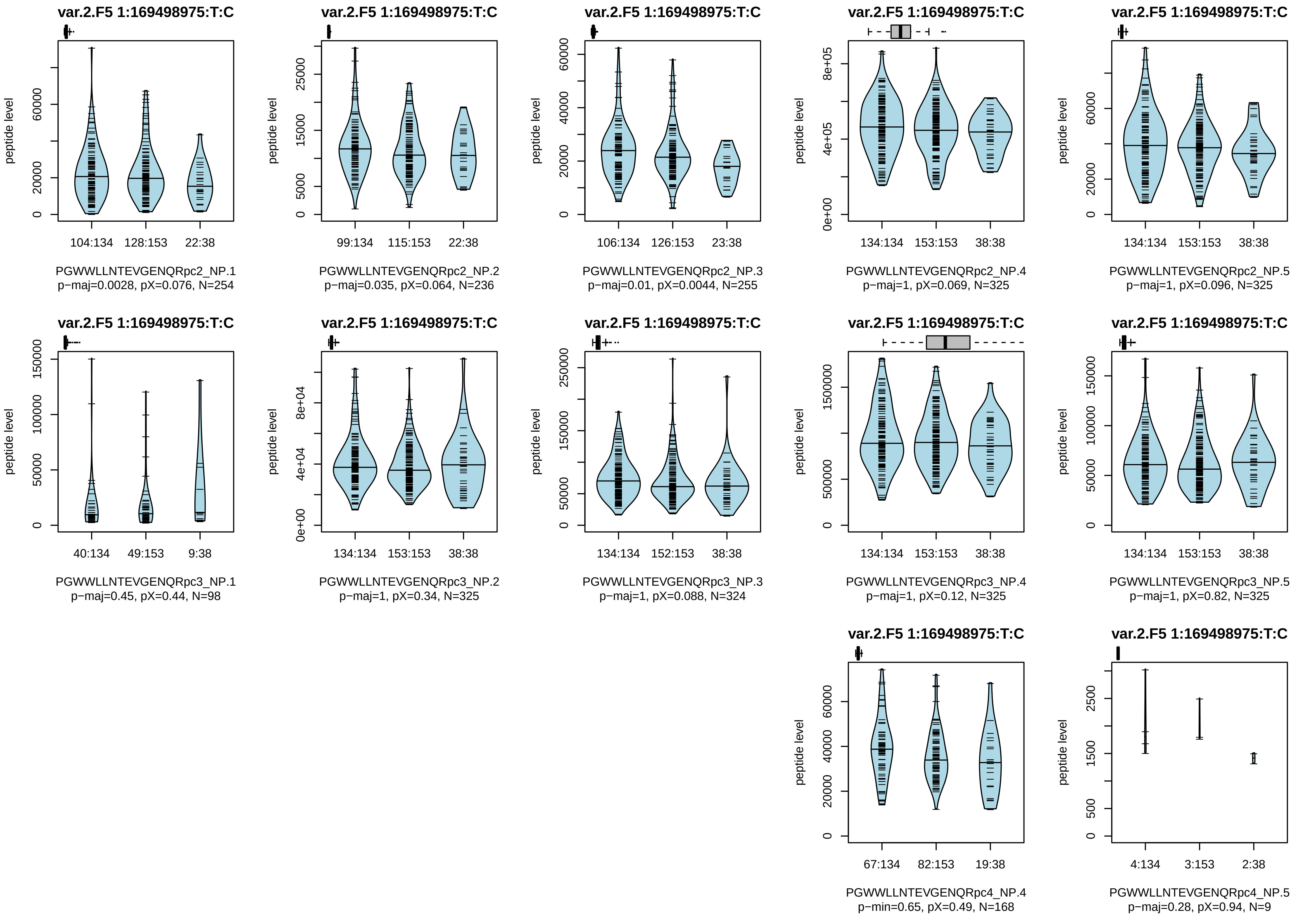
var.2.F5 1:169498975:T:C
peptide level
104:134
128:153
22:38
PGWWLLNTEVGENQRpc2_NP.1
p−maj=0.0028, pX=0.076, N=254
var.2.F5 1:169498975:T:C
peptide level
99:134
115:153
22:38
PGWWLLNTEVGENQRpc2_NP.2
p−maj=0.035, pX=0.064, N=236
var.2.F5 1:169498975:T:C
peptide level
106:134
126:153
23:38
PGWWLLNTEVGENQRpc2_NP.3
p−maj=0.01, pX=0.0044, N=255
var.2.F5 1:169498975:T:C
peptide level
134:134
153:153
38:38
PGWWLLNTEVGENQRpc2_NP.4
p−maj=1, pX=0.069, N=325
var.2.F5 1:169498975:T:C
peptide level
134:134
153:153
38:38
PGWWLLNTEVGENQRpc2_NP.5
p−maj=1, pX=0.096, N=325
var.2.F5 1:169498975:T:C
peptide level
40:134
49:153
9:38
PGWWLLNTEVGENQRpc3_NP.1
p−maj=0.45, pX=0.44, N=98
var.2.F5 1:169498975:T:C
peptide level
134:134
153:153
38:38
PGWWLLNTEVGENQRpc3_NP.2
p−maj=1, pX=0.34, N=325
var.2.F5 1:169498975:T:C
peptide level
134:134
152:153
38:38
PGWWLLNTEVGENQRpc3_NP.3
p−maj=1, pX=0.088, N=324
var.2.F5 1:169498975:T:C
peptide level
134:134
153:153
38:38
PGWWLLNTEVGENQRpc3_NP.4
p−maj=1, pX=0.12, N=325
var.2.F5 1:169498975:T:C
peptide level
134:134
153:153
38:38
PGWWLLNTEVGENQRpc3_NP.5
p−maj=1, pX=0.82, N=325
var.2.F5 1:169498975:T:C
peptide level
67:134
82:153
19:38
PGWWLLNTEVGENQRpc4_NP.4
p−min=0.65, pX=0.49, N=168
var.2.F5 1:169498975:T:C
peptide level
4:134
3:153
2:38
PGWWLLNTEVGENQRpc4_NP.5
p−maj=0.28, pX=0.94, N=9

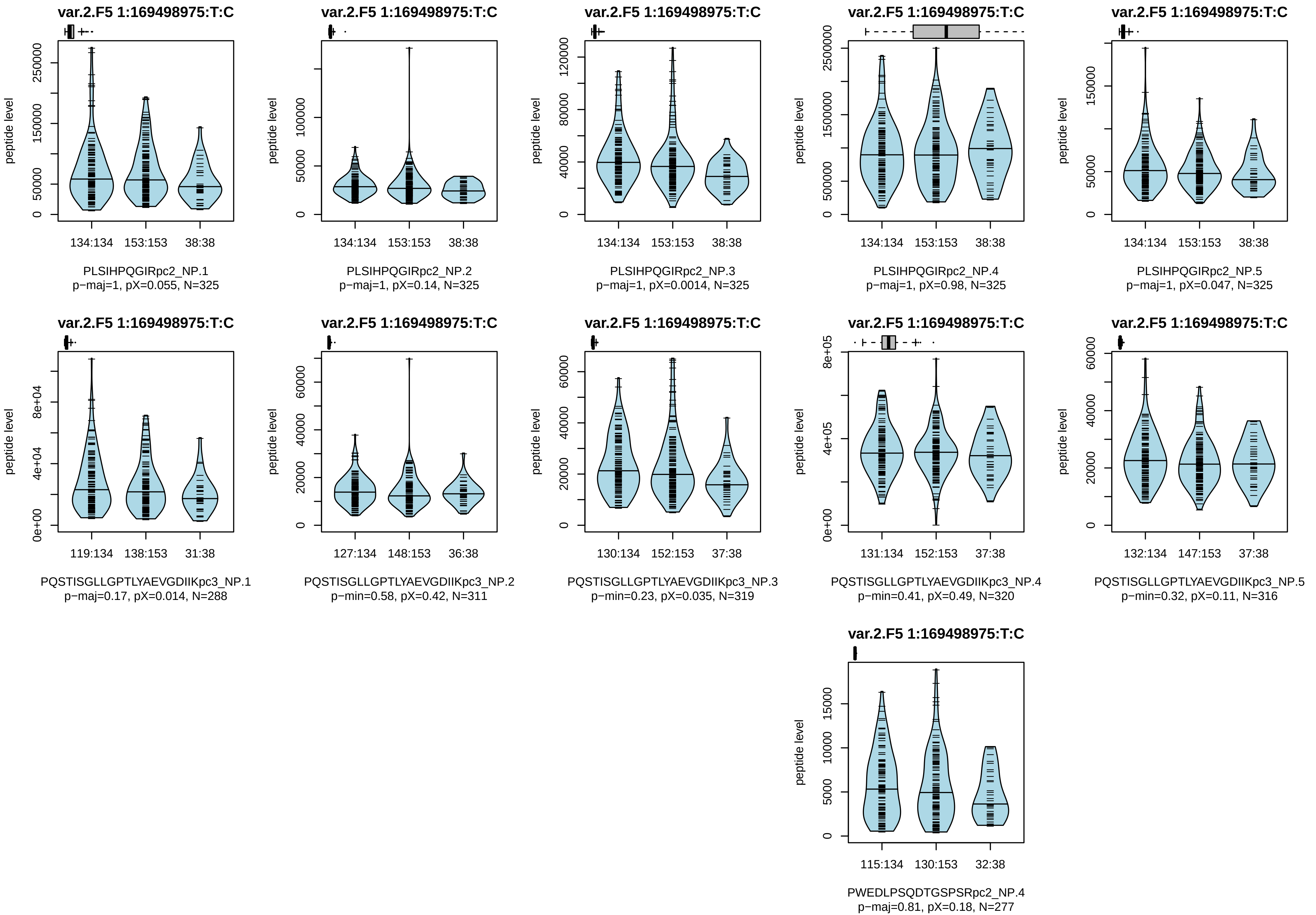

var.2.F5 1:169498975:T:C
peptide level
134:134
153:153
38:38
PLSIHPQGIRpc2_NP.1
p−maj=1, pX=0.055, N=325
var.2.F5 1:169498975:T:C
PLSIHPQGIRpc2_NP.2
p−maj=1, pX=0.14, N=325
var.2.F5 1:169498975:T:C
PLSIHPQGIRpc2_NP.3
p−maj=1, pX=0.0014, N=325
var.2.F5 1:169498975:T:C
PLSIHPQGIRpc2_NP.4
p−maj=1, pX=0.98, N=325
var.2.F5 1:169498975:T:C
PLSIHPQGIRpc2_NP.5
p−maj=1, pX=0.047, N=325
var.2.F5 1:169498975:T:C
119:134
138:153
31:38
PQSTISGLLGPTLYAEVGDIIKpc3_NP.1
p−maj=0.17, pX=0.014, N=288
var.2.F5 1:169498975:T:C
127:134
148:153
36:38
PQSTISGLLGPTLYAEVGDIIKpc3_NP.2
p−min=0.58, pX=0.42, N=311
var.2.F5 1:169498975:T:C
130:134
152:153
37:38
PQSTISGLLGPTLYAEVGDIIKpc3_NP.3
p−min=0.23, pX=0.035, N=319
var.2.F5 1:169498975:T:C
131:134
152:153
37:38
PQSTISGLLGPTLYAEVGDIIKpc3_NP.4
p−min=0.41, pX=0.49, N=320
var.2.F5 1:169498975:T:C
132:134
147:153
37:38
PQSTISGLLGPTLYAEVGDIIKpc3_NP.5
p−min=0.32, pX=0.11, N=316
var.2.F5 1:169498975:T:C
115:134
130:153
32:38
PWEDLPSQDTGSPSRpc2_NP.4
p−maj=0.81, pX=0.18, N=277

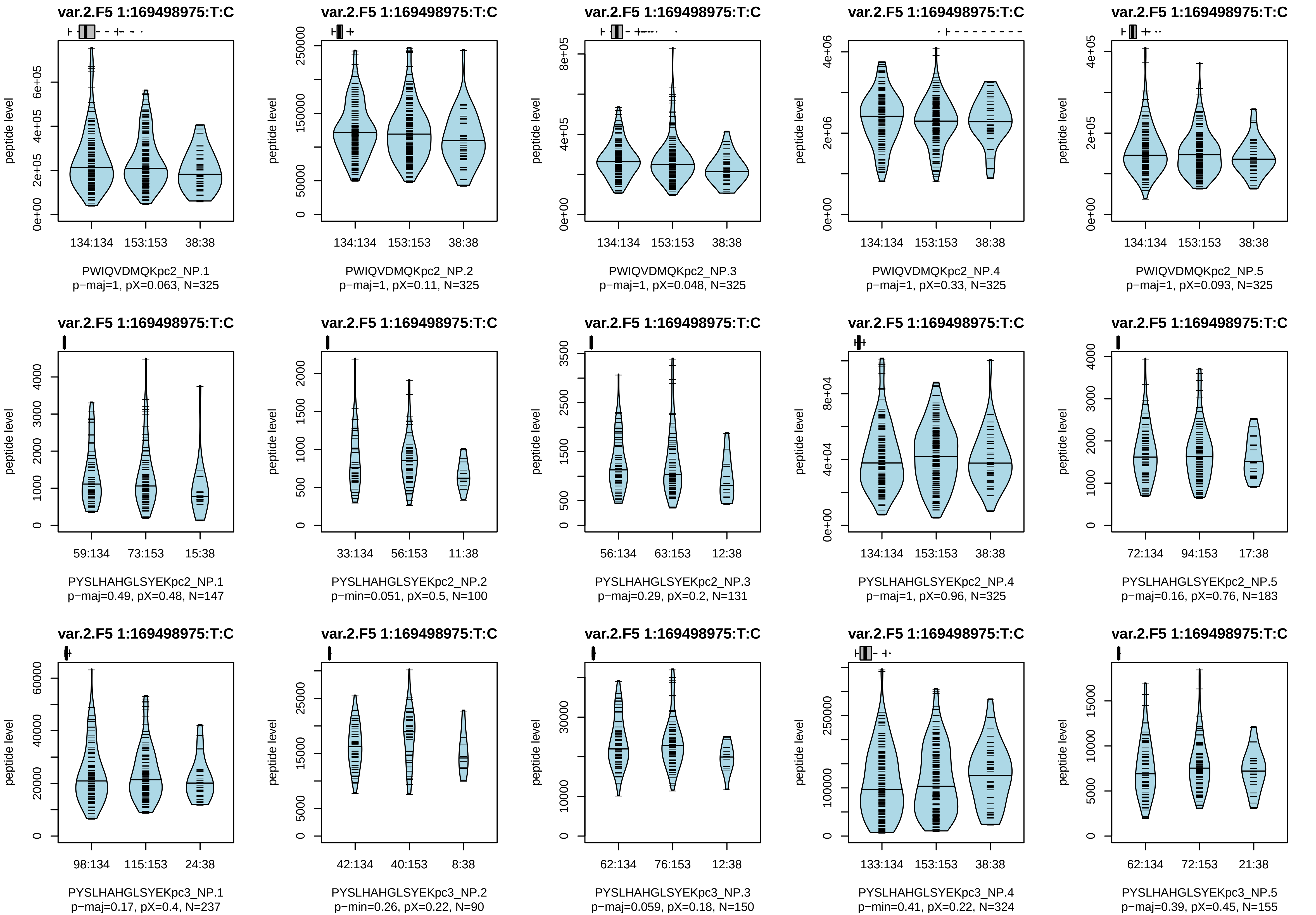
var.2.F5 1:169498975:T:C
peptide level
134:134
153:153
38:38
PWIQVDMQKpc2_NP.1
p−maj=1, pX=0.063, N=325
PWIQVDMQKpc2_NP.2
p−maj=1, pX=0.11, N=325
PWIQVDMQKpc2_NP.3
p−maj=1, pX=0.048, N=325
PWIQVDMQKpc2_NP.4
p−maj=1, pX=0.33, N=325
PWIQVDMQKpc2_NP.5
p−maj=1, pX=0.093, N=325
59:134
73:153
15:38
PYSLHAHGLSYEKpc2_NP.1
p−maj=0.49, pX=0.48, N=147
33:134
56:153
11:38
PYSLHAHGLSYEKpc2_NP.2
p−min=0.051, pX=0.5, N=100
56:134
63:153
12:38
PYSLHAHGLSYEKpc2_NP.3
p−maj=0.29, pX=0.2, N=131
PYSLHAHGLSYEKpc2_NP.4
p−maj=1, pX=0.96, N=325
72:134
94:153
17:38
PYSLHAHGLSYEKpc2_NP.5
p−maj=0.16, pX=0.76, N=183
98:134
115:153
24:38
PYSLHAHGLSYEKpc3_NP.1
p−maj=0.17, pX=0.4, N=237
42:134
40:153
8:38
PYSLHAHGLSYEKpc3_NP.2
p−min=0.26, pX=0.22, N=90
62:134
76:153
12:38
PYSLHAHGLSYEKpc3_NP.3
p−maj=0.059, pX=0.18, N=150
133:134
153:153
38:38
PYSLHAHGLSYEKpc3_NP.4
p−min=0.41, pX=0.22, N=324
62:134
72:153
21:38
PYSLHAHGLSYEKpc3_NP.5
p−maj=0.39, pX=0.45, N=155

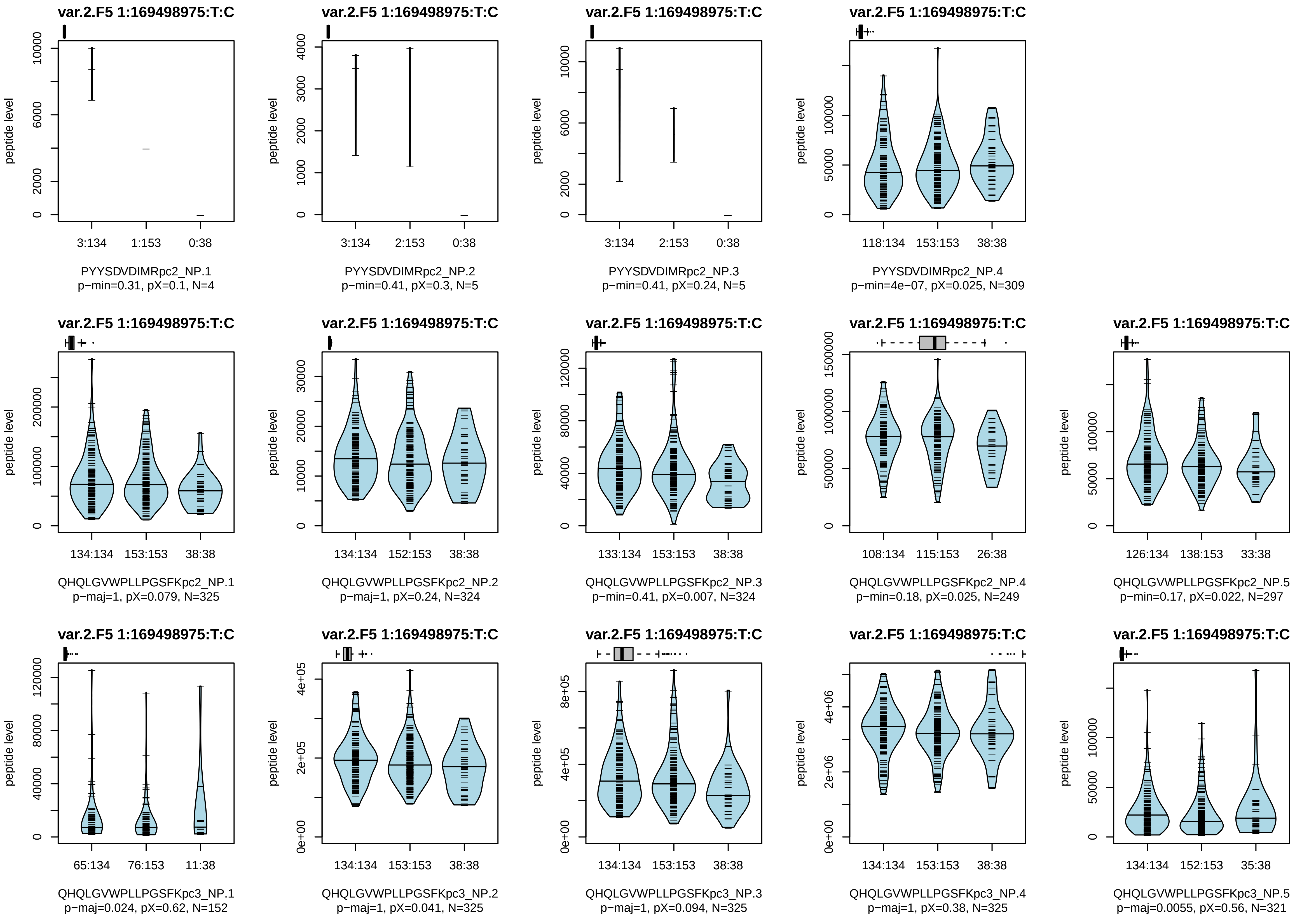
var.2.F5 1:169498975:T:C
peptide level
3:134 1:153 0:38
PYYSDVDIMRpc2_NP.1
p−min=0.31, pX=0.1, N=4
var.2.F5 1:169498975:T:C
peptide level
3:134 2:153 0:38
PYYSDVDIMRpc2_NP.2
p−min=0.41, pX=0.3, N=5
var.2.F5 1:169498975:T:C
peptide level
3:134 2:153 0:38
PYYSDVDIMRpc2_NP.3
p−min=0.41, pX=0.24, N=5
var.2.F5 1:169498975:T:C
peptide level
118:134 153:153 38:38
PYYSDVDIMRpc2_NP.4
p−min=4e−07, pX=0.025, N=309
var.2.F5 1:169498975:T:C
peptide level
134:134 153:153 38:38
QHQLGVWPLLPGSFKpc2_NP.1
p−maj=1, pX=0.079, N=325
var.2.F5 1:169498975:T:C
peptide level
134:134 152:153 38:38
QHQLGVWPLLPGSFKpc2_NP.2
p−maj=1, pX=0.24, N=324
var.2.F5 1:169498975:T:C
peptide level
133:134 153:153 38:38
QHQLGVWPLLPGSFKpc2_NP.3
p−min=0.41, pX=0.007, N=324
var.2.F5 1:169498975:T:C
peptide level
108:134 115:153 26:38
QHQLGVWPLLPGSFKpc2_NP.4
p−min=0.18, pX=0.025, N=249
var.2.F5 1:169498975:T:C
peptide level
126:134 138:153 33:38
QHQLGVWPLLPGSFKpc2_NP.5
p−min=0.17, pX=0.022, N=297
var.2.F5 1:169498975:T:C
peptide level
65:134 76:153 11:38
QHQLGVWPLLPGSFKpc3_NP.1
p−maj=0.024, pX=0.62, N=152
var.2.F5 1:169498975:T:C
peptide level
134:134 153:153 38:38
QHQLGVWPLLPGSFKpc3_NP.2
p−maj=1, pX=0.041, N=325
var.2.F5 1:169498975:T:C
peptide level
134:134 153:153 38:38
QHQLGVWPLLPGSFKpc3_NP.3
p−maj=1, pX=0.094, N=325
var.2.F5 1:169498975:T:C
peptide level
134:134 153:153 38:38
QHQLGVWPLLPGSFKpc3_NP.4
p−maj=1, pX=0.38, N=325
var.2.F5 1:169498975:T:C
peptide level
134:134 152:153 35:38
QHQLGVWPLLPGSFKpc3_NP.5
p−maj=0.0055, pX=0.56, N=321

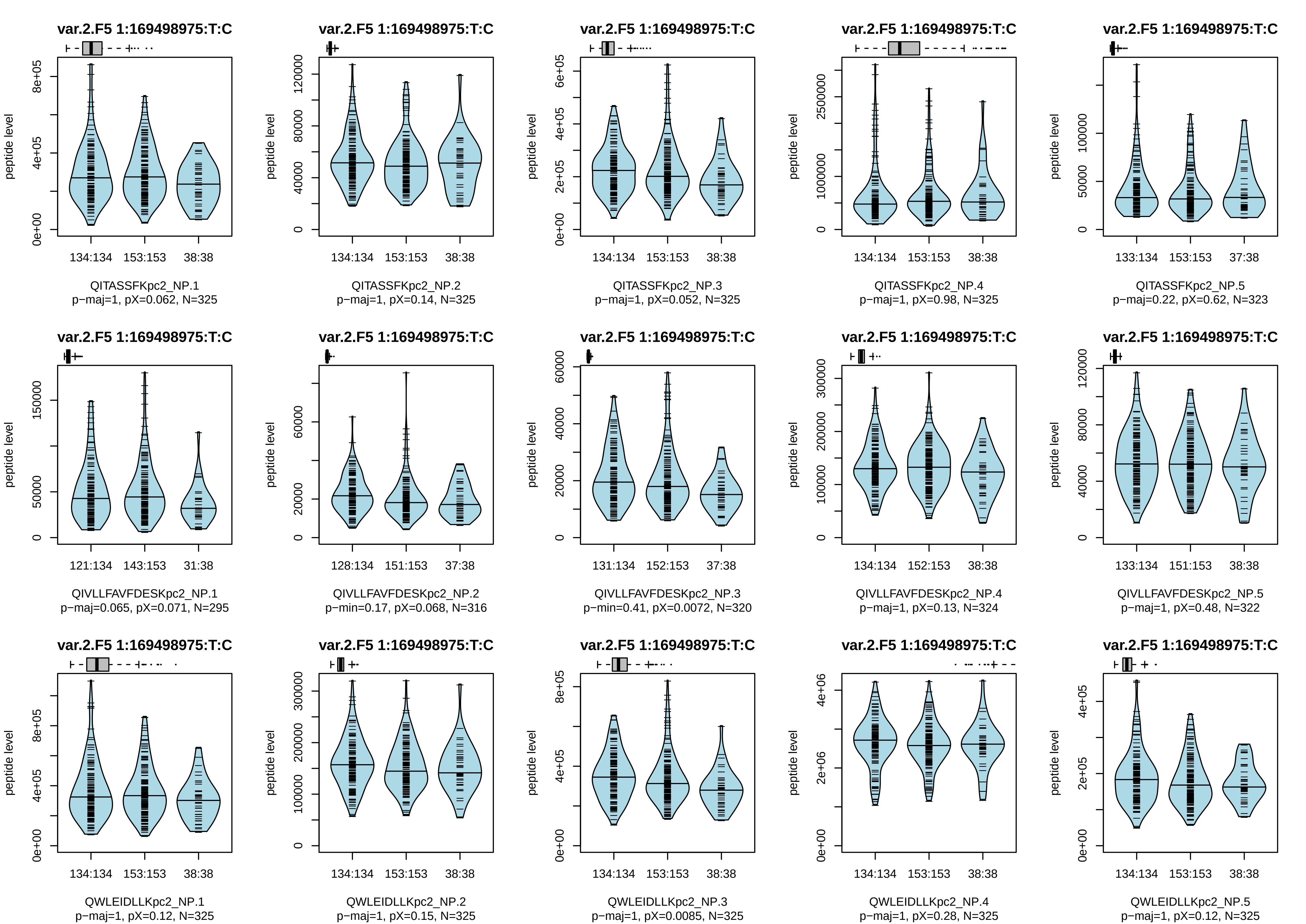

var.2.F5 1:169498975:T:C
peptide level
134:134
153:153
38:38
QITASSFKpc2_NP.1
p−maj=1, pX=0.062, N=325
var.2.F5 1:169498975:T:C
peptide level
134:134
153:153
38:38
QITASSFKpc2_NP.2
p−maj=1, pX=0.14, N=325
var.2.F5 1:169498975:T:C
peptide level
134:134
153:153
38:38
QITASSFKpc2_NP.3
p−maj=1, pX=0.052, N=325
var.2.F5 1:169498975:T:C
peptide level
134:134
153:153
38:38
QITASSFKpc2_NP.4
p−maj=1, pX=0.98, N=325
var.2.F5 1:169498975:T:C
peptide level
133:134
153:153
37:38
QITASSFKpc2_NP.5
p−maj=0.22, pX=0.62, N=323
var.2.F5 1:169498975:T:C
peptide level
121:134
143:153
31:38
QIVLLFAVFDESKpc2_NP.1
p−maj=0.065, pX=0.071, N=295
var.2.F5 1:169498975:T:C
peptide level
128:134
151:153
37:38
QIVLLFAVFDESKpc2_NP.2
p−min=0.17, pX=0.068, N=316
var.2.F5 1:169498975:T:C
peptide level
131:134
152:153
37:38
QIVLLFAVFDESKpc2_NP.3
p−min=0.41, pX=0.0072, N=320
var.2.F5 1:169498975:T:C
peptide level
134:134
152:153
38:38
QIVLLFAVFDESKpc2_NP.4
p−maj=1, pX=0.13, N=324
var.2.F5 1:169498975:T:C
peptide level
133:134
151:153
38:38
QIVLLFAVFDESKpc2_NP.5
p−maj=1, pX=0.48, N=322
var.2.F5 1:169498975:T:C
peptide level
134:134
153:153
38:38
QWLEIDLLKpc2_NP.1
p−maj=1, pX=0.12, N=325
var.2.F5 1:169498975:T:C
peptide level
134:134
153:153
38:38
QWLEIDLLKpc2_NP.2
p−maj=1, pX=0.15, N=325
var.2.F5 1:169498975:T:C
peptide level
134:134
153:153
38:38
QWLEIDLLKpc2_NP.3
p−maj=1, pX=0.0085, N=325
var.2.F5 1:169498975:T:C
peptide level
134:134
153:153
38:38
QWLEIDLLKpc2_NP.4
p−maj=1, pX=0.28, N=325
var.2.F5 1:169498975:T:C
peptide level
134:134
153:153
38:38
QWLEIDLLKpc2_NP.5
p−maj=1, pX=0.12, N=325

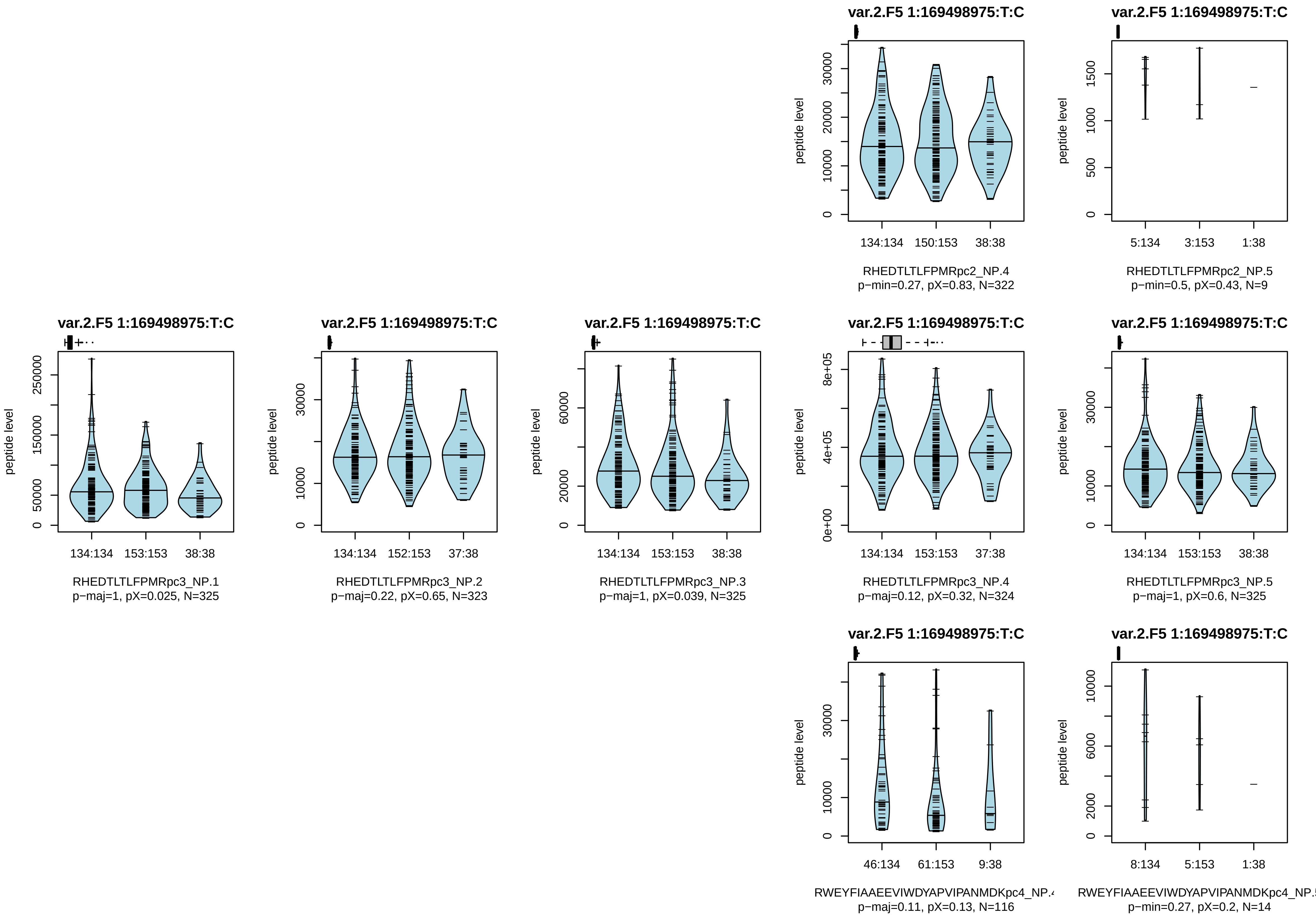
var.2.F5 1:169498975:T:C
peptide level
134:134
150:153
38:38
RHEDTLTLFPMRpc2_NP.4
p−min=0.27, pX=0.83, N=322
var.2.F5 1:169498975:T:C
peptide level
5:134
3:153
1:38
RHEDTLTLFPMRpc2_NP.5
p−min=0.5, pX=0.43, N=9
var.2.F5 1:169498975:T:C
peptide level
134:134
153:153
38:38
RHEDTLTLFPMRpc3_NP.1
p−maj=1, pX=0.025, N=325
var.2.F5 1:169498975:T:C
peptide level
134:134
152:153
37:38
RHEDTLTLFPMRpc3_NP.2
p−maj=0.22, pX=0.65, N=323
var.2.F5 1:169498975:T:C
peptide level
134:134
153:153
38:38
RHEDTLTLFPMRpc3_NP.3
p−maj=1, pX=0.039, N=325
var.2.F5 1:169498975:T:C
peptide level
134:134
153:153
37:38
RHEDTLTLFPMRpc3_NP.4
p−maj=0.12, pX=0.32, N=324
var.2.F5 1:169498975:T:C
peptide level
134:134
153:153
38:38
RHEDTLTLFPMRpc3_NP.5
p−maj=1, pX=0.6, N=325
var.2.F5 1:169498975:T:C
peptide level
46:134
61:153
9:38
RWEYFIAAEEVIWDYAPVIPANMDKpc4_NP.4
p−maj=0.11, pX=0.13, N=116
var.2.F5 1:169498975:T:C
peptide level
8:134
5:153
1:38
RWEYFIAAEEVIWDYAPVIPANMDKpc4_NP.5
p−min=0.27, pX=0.2, N=14

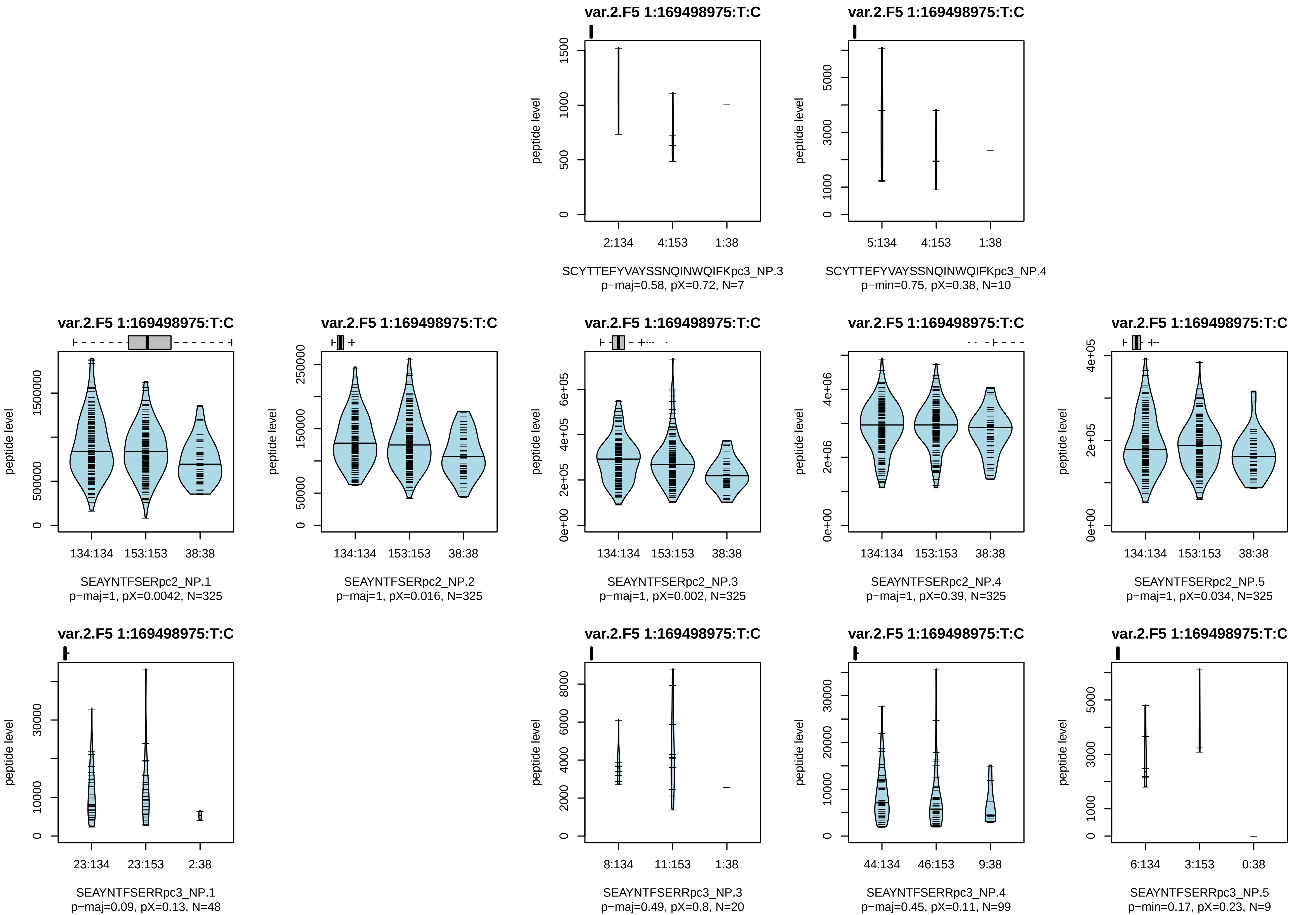
var.2.F5 1:169498975:T:C
peptide level
2:134
4:153
1:38
SCYTTEFYVAYSSNQINWQIFKpc3_NP.3
p−maj=0.58, pX=0.72, N=7
var.2.F5 1:169498975:T:C
peptide level
5:134
4:153
1:38
SCYTTEFYVAYSSNQINWQIFKpc3_NP.4
p−min=0.75, pX=0.38, N=10
var.2.F5 1:169498975:T:C
peptide level
134:134
153:153
38:38
SEAYNTFSERpc2_NP.1
p−maj=1, pX=0.0042, N=325
var.2.F5 1:169498975:T:C
peptide level
134:134
153:153
38:38
SEAYNTFSERpc2_NP.2
p−maj=1, pX=0.016, N=325
var.2.F5 1:169498975:T:C
peptide level
134:134
153:153
38:38
SEAYNTFSERpc2_NP.3
p−maj=1, pX=0.002, N=325
var.2.F5 1:169498975:T:C
peptide level
134:134
153:153
38:38
SEAYNTFSERpc2_NP.4
p−maj=1, pX=0.39, N=325
var.2.F5 1:169498975:T:C
peptide level
134:134
153:153
38:38
SEAYNTFSERpc2_NP.5
p−maj=1, pX=0.034, N=325
var.2.F5 1:169498975:T:C
peptide level
23:134
23:153
2:38
SEAYNTFSERRpc3_NP.1
p−maj=0.09, pX=0.13, N=48
var.2.F5 1:169498975:T:C
peptide level
8:134
11:153
1:38
SEAYNTFSERRpc3_NP.3
p−maj=0.49, pX=0.8, N=20
var.2.F5 1:169498975:T:C
peptide level
44:134
46:153
9:38
SEAYNTFSERRpc3_NP.4
p−maj=0.45, pX=0.11, N=99
var.2.F5 1:169498975:T:C
peptide level
6:134
3:153
0:38
SEAYNTFSERRpc3_NP.5
p−min=0.17, pX=0.23, N=9

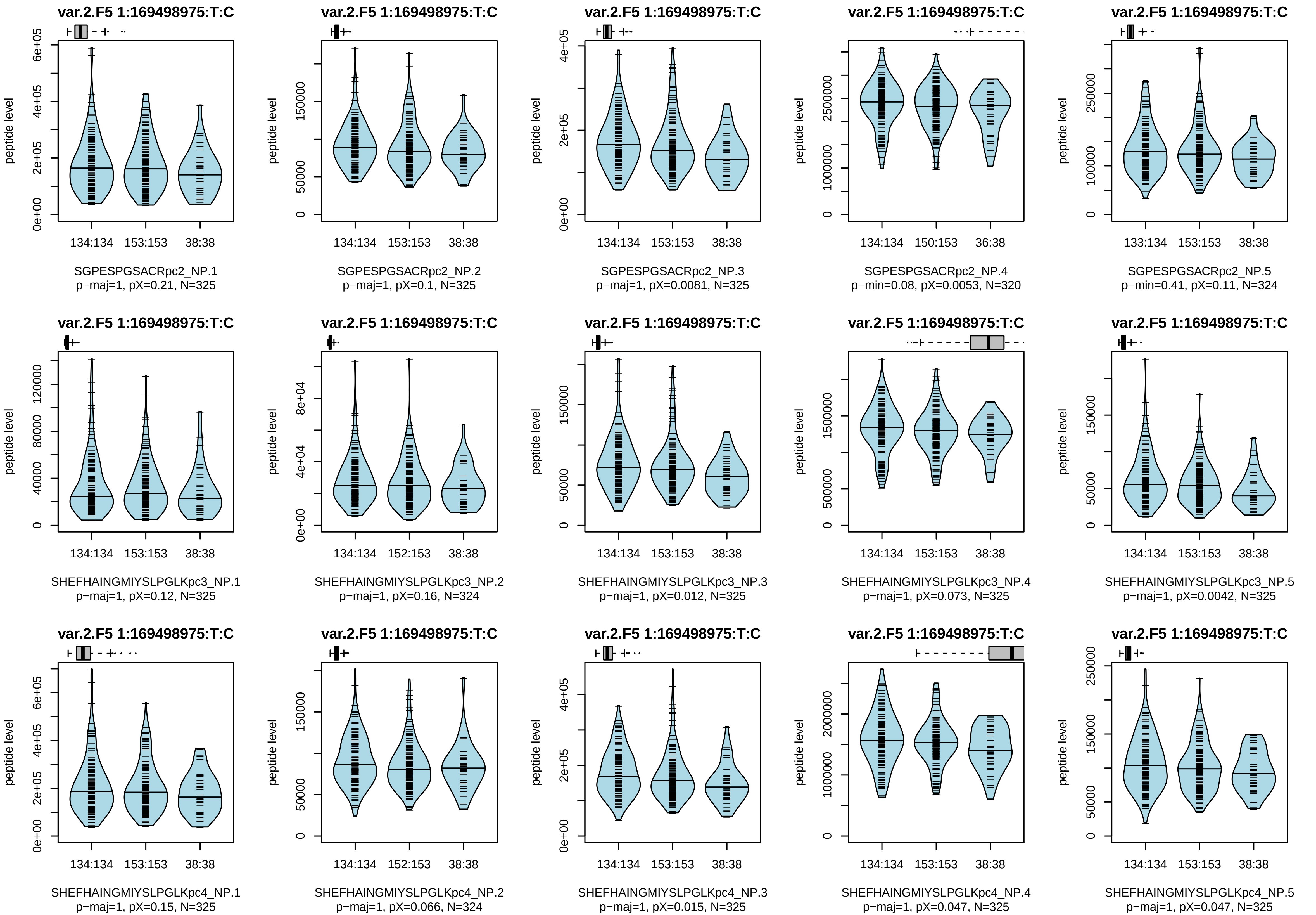
var.2.F5 1:169498975:T:C
peptide level
134:134 153:153 38:38
SGPESPGSACRpc2_NP.1
p−maj=1, pX=0.21, N=325
134:134 153:153 38:38
SGPESPGSACRpc2_NP.2
p−maj=1, pX=0.1, N=325
134:134 153:153 38:38
SGPESPGSACRpc2_NP.3
p−maj=1, pX=0.0081, N=325
134:134 150:153 36:38
SGPESPGSACRpc2_NP.4
p−min=0.08, pX=0.0053, N=320
133:134 153:153 38:38
SGPESPGSACRpc2_NP.5
p−min=0.41, pX=0.11, N=324
134:134 153:153 38:38
SHEFHAINGMIYSLPGLKpc3_NP.1
p−maj=1, pX=0.12, N=325
134:134 152:153 38:38
SHEFHAINGMIYSLPGLKpc3_NP.2
p−maj=1, pX=0.16, N=324
134:134 153:153 38:38
SHEFHAINGMIYSLPGLKpc3_NP.3
p−maj=1, pX=0.012, N=325
134:134 153:153 38:38
SHEFHAINGMIYSLPGLKpc3_NP.4
p−maj=1, pX=0.073, N=325
134:134 153:153 38:38
SHEFHAINGMIYSLPGLKpc3_NP.5
p−maj=1, pX=0.0042, N=325
134:134 153:153 38:38
SHEFHAINGMIYSLPGLKpc4_NP.1
p−maj=1, pX=0.15, N=325
134:134 152:153 38:38
SHEFHAINGMIYSLPGLKpc4_NP.2
p−maj=1, pX=0.066, N=324
134:134 153:153 38:38
SHEFHAINGMIYSLPGLKpc4_NP.3
p−maj=1, pX=0.015, N=325
134:134 153:153 38:38
SHEFHAINGMIYSLPGLKpc4_NP.4
p−maj=1, pX=0.047, N=325
134:134 153:153 38:38
SHEFHAINGMIYSLPGLKpc4_NP.5
p−maj=1, pX=0.047, N=325

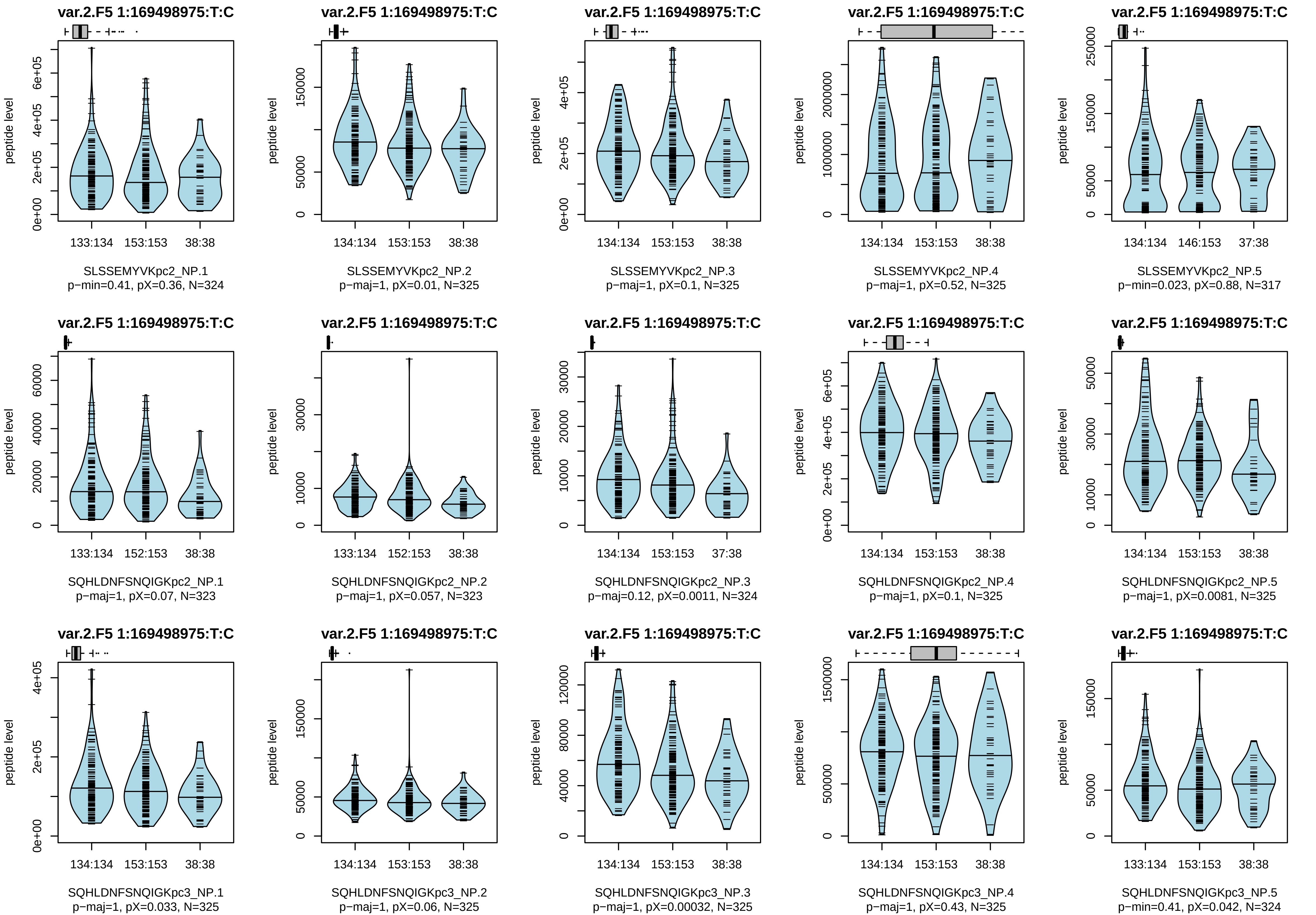
var.2.F5 1:169498975:T:C
peptide level
133:134
153:153
38:38
SLSSEMYVKpc2_NP.1
p−min=0.41, pX=0.36, N=324
var.2.F5 1:169498975:T:C
peptide level
134:134
153:153
38:38
SLSSEMYVKpc2_NP.2
p−maj=1, pX=0.01, N=325
var.2.F5 1:169498975:T:C
peptide level
134:134
153:153
38:38
SLSSEMYVKpc2_NP.3
p−maj=1, pX=0.1, N=325
var.2.F5 1:169498975:T:C
peptide level
134:134
153:153
38:38
SLSSEMYVKpc2_NP.4
p−maj=1, pX=0.52, N=325
var.2.F5 1:169498975:T:C
peptide level
134:134
146:153
37:38
SLSSEMYVKpc2_NP.5
p−min=0.023, pX=0.88, N=317
var.2.F5 1:169498975:T:C
peptide level
133:134
152:153
38:38
SQHLDNFSNQIGKpc2_NP.1
p−maj=1, pX=0.07, N=323
var.2.F5 1:169498975:T:C
peptide level
133:134
152:153
38:38
SQHLDNFSNQIGKpc2_NP.2
p−maj=1, pX=0.057, N=323
var.2.F5 1:169498975:T:C
peptide level
134:134
153:153
37:38
SQHLDNFSNQIGKpc2_NP.3
p−maj=0.12, pX=0.0011, N=324
var.2.F5 1:169498975:T:C
peptide level
134:134
153:153
38:38
SQHLDNFSNQIGKpc2_NP.4
p−maj=1, pX=0.1, N=325
var.2.F5 1:169498975:T:C
peptide level
134:134
153:153
38:38
SQHLDNFSNQIGKpc2_NP.5
p−maj=1, pX=0.0081, N=325
var.2.F5 1:169498975:T:C
peptide level
134:134
153:153
38:38
SQHLDNFSNQIGKpc3_NP.1
p−maj=1, pX=0.033, N=325
var.2.F5 1:169498975:T:C
peptide level
134:134
153:153
38:38
SQHLDNFSNQIGKpc3_NP.2
p−maj=1, pX=0.06, N=325
var.2.F5 1:169498975:T:C
peptide level
134:134
153:153
38:38
SQHLDNFSNQIGKpc3_NP.3
p−maj=1, pX=0.00032, N=325
var.2.F5 1:169498975:T:C
peptide level
134:134
153:153
38:38
SQHLDNFSNQIGKpc3_NP.4
p−maj=1, pX=0.43, N=325
var.2.F5 1:169498975:T:C
peptide level
133:134
153:153
38:38
SQHLDNFSNQIGKpc3_NP.5
p−min=0.41, pX=0.042, N=324

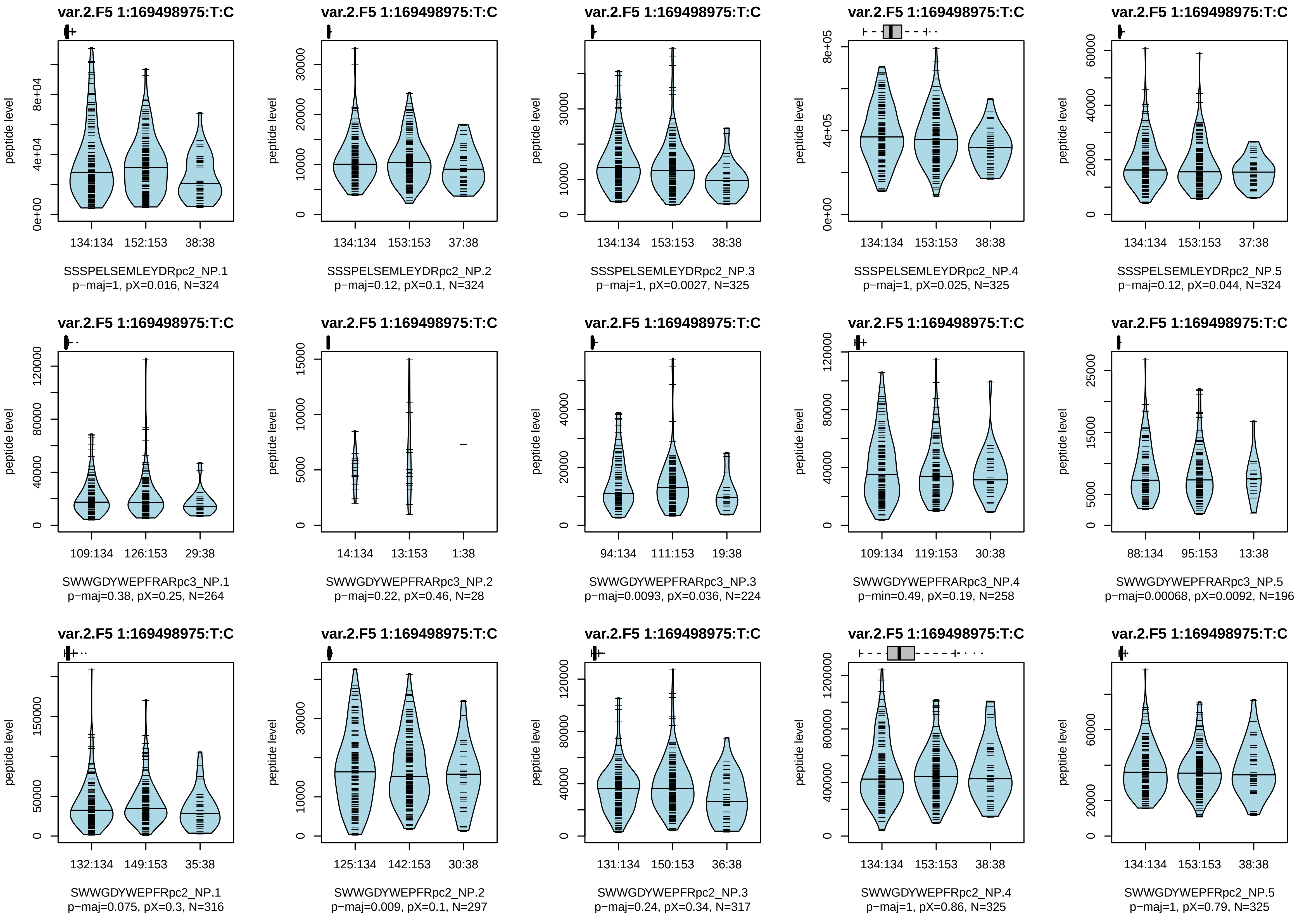
var.2.F5 1:169498975:T:C
peptide level
134:134
152:153
38:38
SSSPELSEMLEYDRpc2_NP.1
p−maj=1, pX=0.016, N=324
var.2.F5 1:169498975:T:C
peptide level
134:134
153:153
37:38
SSSPELSEMLEYDRpc2_NP.2
p−maj=0.12, pX=0.1, N=324
var.2.F5 1:169498975:T:C
peptide level
134:134
153:153
38:38
SSSPELSEMLEYDRpc2_NP.3
p−maj=1, pX=0.0027, N=325
var.2.F5 1:169498975:T:C
peptide level
134:134
153:153
38:38
SSSPELSEMLEYDRpc2_NP.4
p−maj=1, pX=0.025, N=325
var.2.F5 1:169498975:T:C
peptide level
134:134
153:153
37:38
SSSPELSEMLEYDRpc2_NP.5
p−maj=0.12, pX=0.044, N=324
var.2.F5 1:169498975:T:C
peptide level
109:134
126:153
29:38
SWWGDYWEPFRARpc3_NP.1
p−maj=0.38, pX=0.25, N=264
var.2.F5 1:169498975:T:C
peptide level
14:134
13:153
1:38
SWWGDYWEPFRARpc3_NP.2
p−maj=0.22, pX=0.46, N=28
var.2.F5 1:169498975:T:C
peptide level
94:134
111:153
19:38
SWWGDYWEPFRARpc3_NP.3
p−maj=0.0093, pX=0.036, N=224
var.2.F5 1:169498975:T:C
peptide level
109:134
119:153
30:38
SWWGDYWEPFRARpc3_NP.4
p−min=0.49, pX=0.19, N=258
var.2.F5 1:169498975:T:C
peptide level
88:134
95:153
13:38
SWWGDYWEPFRARpc3_NP.5
p−maj=0.00068, pX=0.0092, N=196
var.2.F5 1:169498975:T:C
peptide level
132:134
149:153
35:38
SWWGDYWEPFRpc2_NP.1
p−maj=0.075, pX=0.3, N=316
var.2.F5 1:169498975:T:C
peptide level
125:134
142:153
30:38
SWWGDYWEPFRpc2_NP.2
p−maj=0.009, pX=0.1, N=297
var.2.F5 1:169498975:T:C
peptide level
131:134
150:153
36:38
SWWGDYWEPFRpc2_NP.3
p−maj=0.24, pX=0.34, N=317
var.2.F5 1:169498975:T:C
peptide level
134:134
153:153
38:38
SWWGDYWEPFRpc2_NP.4
p−maj=1, pX=0.86, N=325
var.2.F5 1:169498975:T:C
peptide level
134:134
153:153
38:38
SWWGDYWEPFRpc2_NP.5
p−maj=1, pX=0.79, N=325

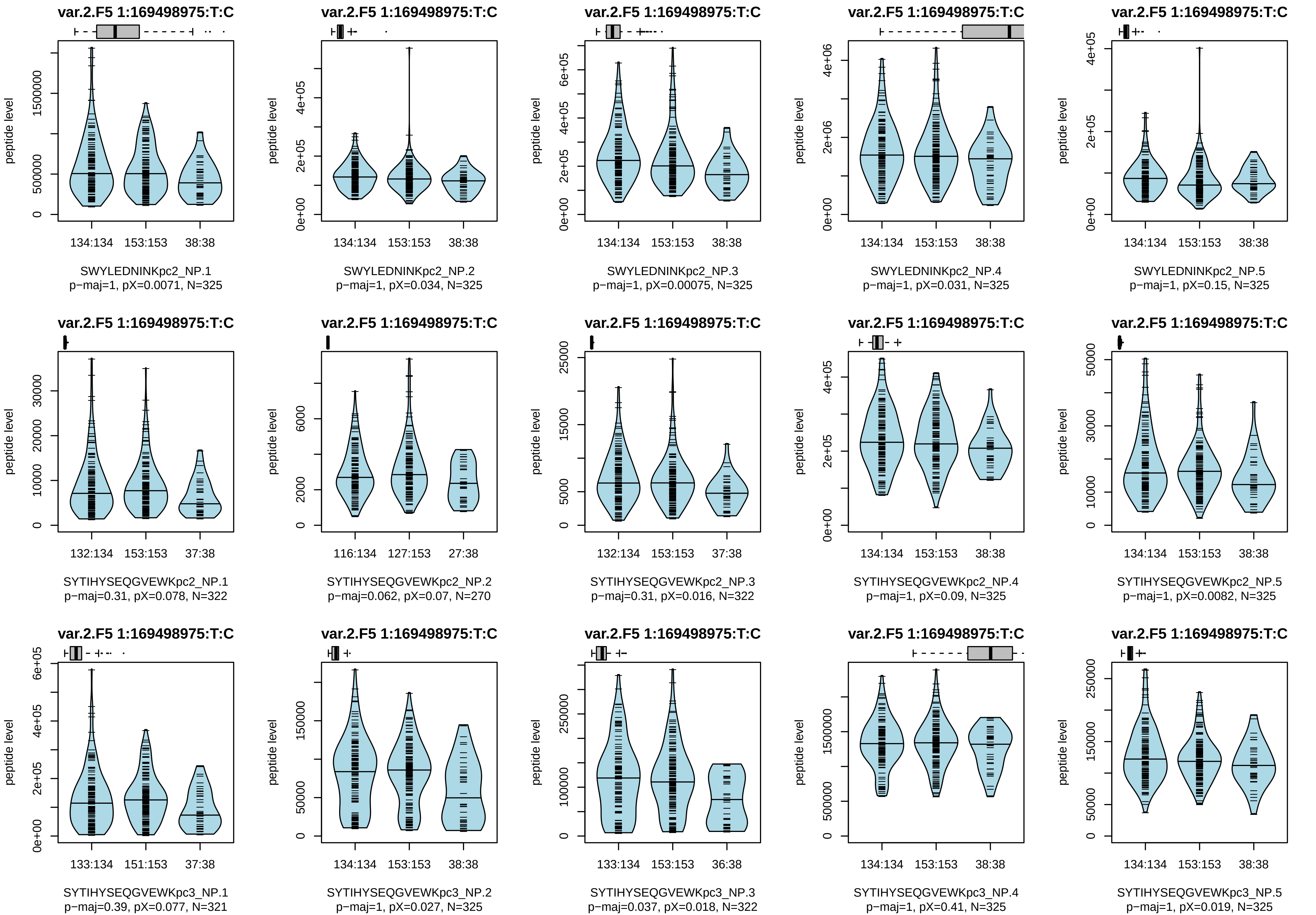
var.2.F5 1:169498975:T:C
peptide level
134:134
153:153
38:38
SWYLEDNINKpc2_NP.1
p−maj=1, pX=0.0071, N=325
var.2.F5 1:169498975:T:C
peptide level
134:134
153:153
38:38
SWYLEDNINKpc2_NP.2
p−maj=1, pX=0.034, N=325
var.2.F5 1:169498975:T:C
peptide level
134:134
153:153
38:38
SWYLEDNINKpc2_NP.3
p−maj=1, pX=0.00075, N=325
var.2.F5 1:169498975:T:C
peptide level
134:134
153:153
38:38
SWYLEDNINKpc2_NP.4
p−maj=1, pX=0.031, N=325
var.2.F5 1:169498975:T:C
peptide level
134:134
153:153
38:38
SWYLEDNINKpc2_NP.5
p−maj=1, pX=0.15, N=325
var.2.F5 1:169498975:T:C
peptide level
132:134
153:153
37:38
SYTIHYSEQGVEWKpc2_NP.1
p−maj=0.31, pX=0.078, N=322
var.2.F5 1:169498975:T:C
peptide level
116:134
127:153
27:38
SYTIHYSEQGVEWKpc2_NP.2
p−maj=0.062, pX=0.07, N=270
var.2.F5 1:169498975:T:C
peptide level
132:134
153:153
37:38
SYTIHYSEQGVEWKpc2_NP.3
p−maj=0.31, pX=0.016, N=322
var.2.F5 1:169498975:T:C
peptide level
134:134
153:153
38:38
SYTIHYSEQGVEWKpc2_NP.4
p−maj=1, pX=0.09, N=325
var.2.F5 1:169498975:T:C
peptide level
134:134
153:153
38:38
SYTIHYSEQGVEWKpc2_NP.5
p−maj=1, pX=0.0082, N=325
var.2.F5 1:169498975:T:C
peptide level
133:134
151:153
37:38
SYTIHYSEQGVEWKpc3_NP.1
p−maj=0.39, pX=0.077, N=321
var.2.F5 1:169498975:T:C
peptide level
134:134
153:153
38:38
SYTIHYSEQGVEWKpc3_NP.2
p−maj=1, pX=0.027, N=325
var.2.F5 1:169498975:T:C
peptide level
133:134
153:153
36:38
SYTIHYSEQGVEWKpc3_NP.3
p−maj=0.037, pX=0.018, N=322
var.2.F5 1:169498975:T:C
peptide level
134:134
153:153
38:38
SYTIHYSEQGVEWKpc3_NP.4
p−maj=1, pX=0.41, N=325
var.2.F5 1:169498975:T:C
peptide level
134:134
153:153
38:38
SYTIHYSEQGVEWKpc3_NP.5
p−maj=1, pX=0.019, N=325

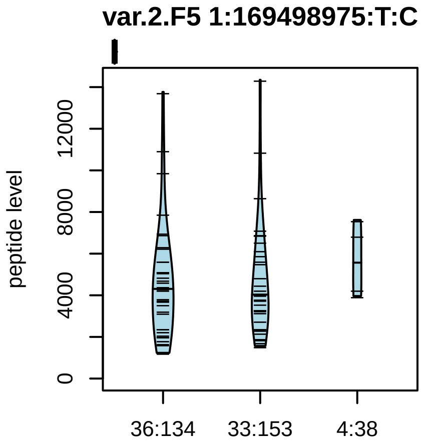

SYTIHYSEQGVEWKPYRpc3_NP.3
p−maj=0.065, pX=0.14, N=73

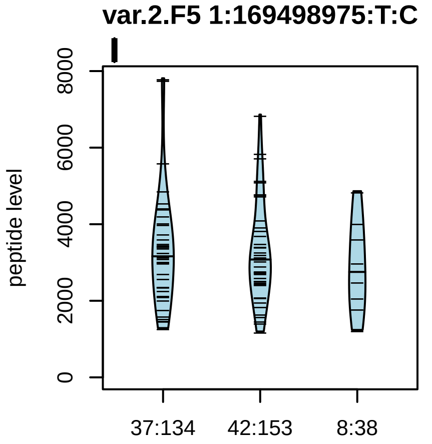

SYTIHYSEQGVEWKPYRpc4_NP.3
p−maj=0.44, pX=0.39, N=87

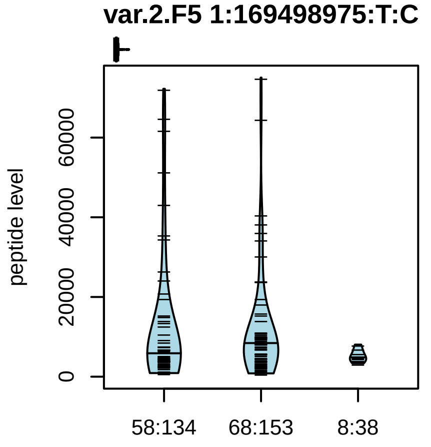

TFHPLRSEAYNTFSERpc3_NP.1
p−maj=0.008, pX=0.078, N=134

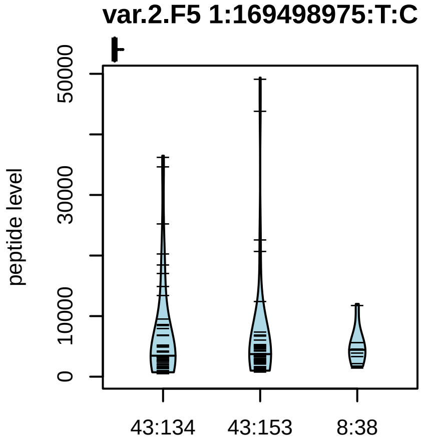

TFHPLRSEAYNTFSERpc3_NP.3
p−min=0.32, pX=0.24, N=94

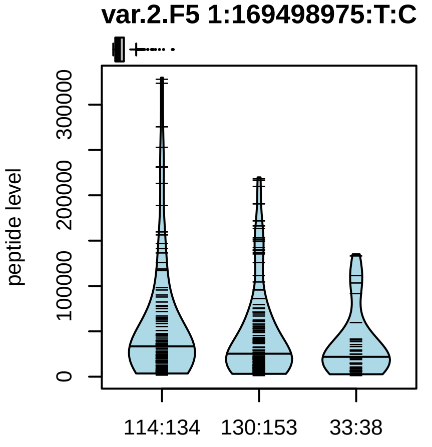

TFHPLRSEAYNTFSERpc3_NP.4
p−maj=1, pX=0.061, N=277

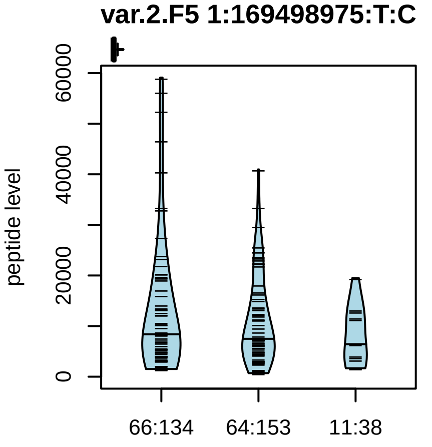

TFHPLRSEAYNTFSERpc3_NP.5
p−maj=0.08, pX=0.0095, N=141

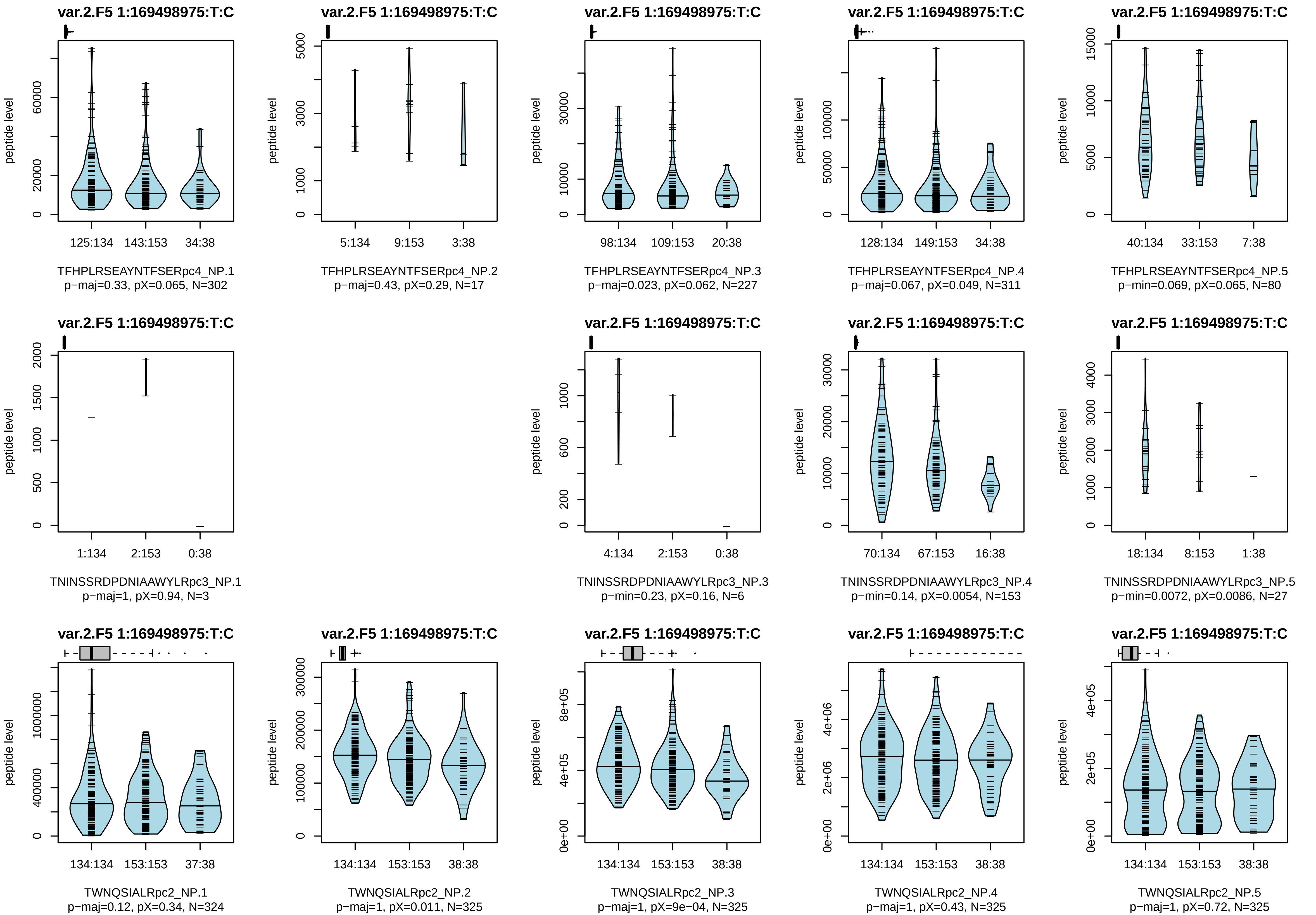
var.2.F5 1:169498975:T:C
peptide level
125:134
143:153
34:38
TFHPLRSEAYNTFSERpc4_NP.1
p−maj=0.33, pX=0.065, N=302
var.2.F5 1:169498975:T:C
peptide level
5:134
9:153
3:38
TFHPLRSEAYNTFSERpc4_NP.2
p−maj=0.43, pX=0.29, N=17
var.2.F5 1:169498975:T:C
peptide level
98:134
109:153
20:38
TFHPLRSEAYNTFSERpc4_NP.3
p−maj=0.023, pX=0.062, N=227
var.2.F5 1:169498975:T:C
peptide level
128:134
149:153
34:38
TFHPLRSEAYNTFSERpc4_NP.4
p−maj=0.067, pX=0.049, N=311
var.2.F5 1:169498975:T:C
peptide level
40:134
33:153
7:38
TFHPLRSEAYNTFSERpc4_NP.5
p−min=0.069, pX=0.065, N=80
var.2.F5 1:169498975:T:C
peptide level
1:134
2:153
0:38
TNINSSRDPDNIAAWYLRpc3_NP.1
p−maj=1, pX=0.94, N=3
var.2.F5 1:169498975:T:C
peptide level
4:134
2:153
0:38
TNINSSRDPDNIAAWYLRpc3_NP.3
p−min=0.23, pX=0.16, N=6
var.2.F5 1:169498975:T:C
peptide level
70:134
67:153
16:38
TNINSSRDPDNIAAWYLRpc3_NP.4
p−min=0.14, pX=0.0054, N=153
var.2.F5 1:169498975:T:C
peptide level
18:134
8:153
1:38
TNINSSRDPDNIAAWYLRpc3_NP.5
p−min=0.0072, pX=0.0086, N=27
var.2.F5 1:169498975:T:C
peptide level
134:134
153:153
37:38
TWNQSIALRpc2_NP.1
p−maj=0.12, pX=0.34, N=324
var.2.F5 1:169498975:T:C
peptide level
134:134
153:153
38:38
TWNQSIALRpc2_NP.2
p−maj=1, pX=0.011, N=325
var.2.F5 1:169498975:T:C
peptide level
134:134
153:153
38:38
TWNQSIALRpc2_NP.3
p−maj=1, pX=9e−04, N=325
var.2.F5 1:169498975:T:C
peptide level
134:134
153:153
38:38
TWNQSIALRpc2_NP.4
p−maj=1, pX=0.43, N=325
var.2.F5 1:169498975:T:C
peptide level
134:134
153:153
38:38
TWNQSIALRpc2_NP.5
p−maj=1, pX=0.72, N=325

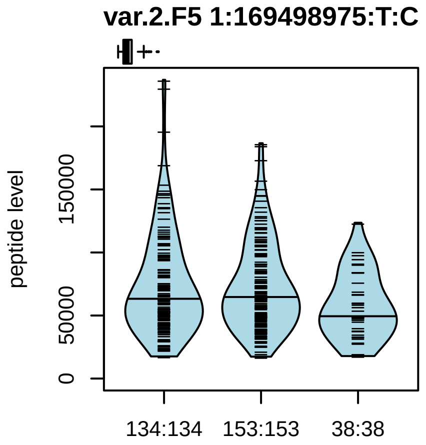

TYEDDSPEWFKpc2_NP.1
p−maj=1, pX=0.046, N=325

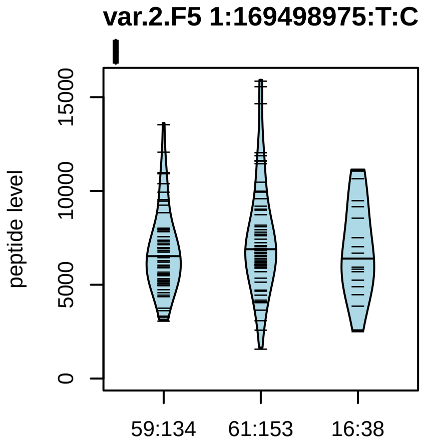

TYEDDSPEWFKpc2_NP.2
p−min=0.57, pX=0.93, N=136

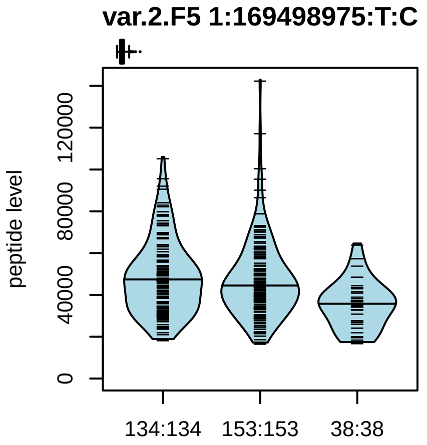

TYEDDSPEWFKpc2_NP.3
p−maj=1, pX=0.0012, N=325

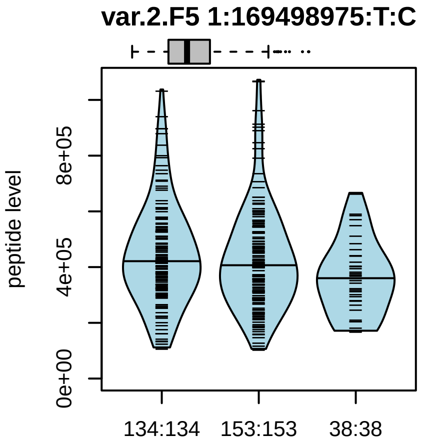

TYEDDSPEWFKpc2_NP.4
p−maj=1, pX=0.024, N=325

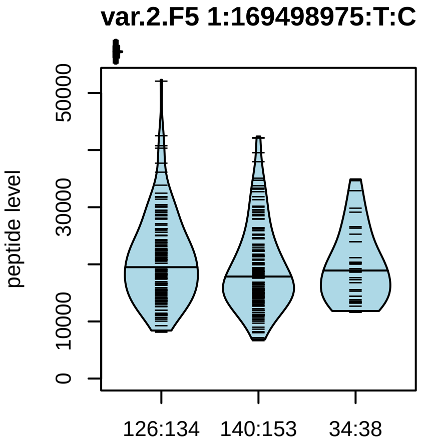

TYEDDSPEWFKpc2_NP.5
p−min=0.4, pX=0.072, N=300

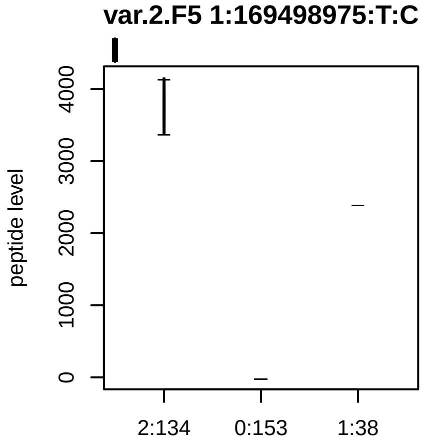

VERDQAAKpc2_NP.1
p−maj=0.31, pX=0.57, N=3

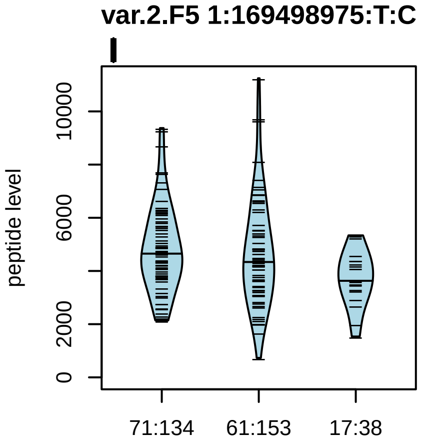

VERDQAAKpc2_NP.4
p−min=0.032, pX=0.016, N=149

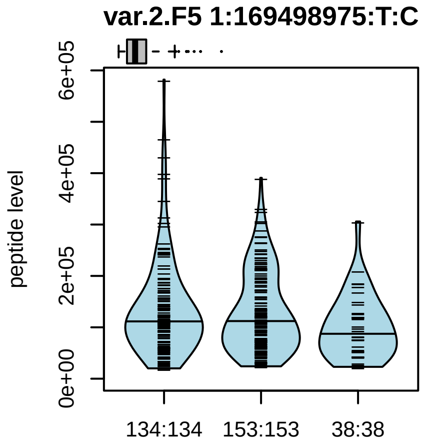

VMYTQYEDESFTKpc2_NP.1
p−maj=1, pX=0.046, N=325

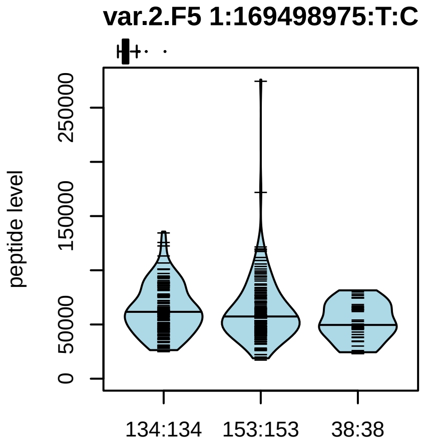

VMYTQYEDESFTKpc2_NP.2
p−maj=1, pX=0.07, N=325

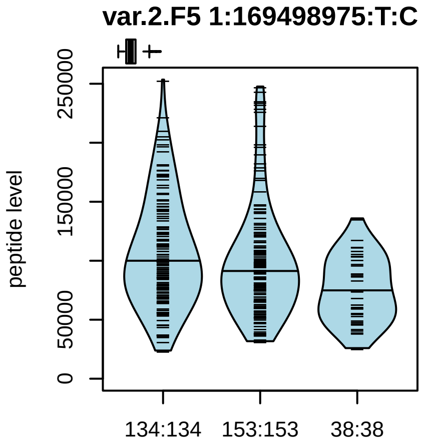

VMYTQYEDESFTKpc2_NP.3
p−maj=1, pX=0.00047, N=325

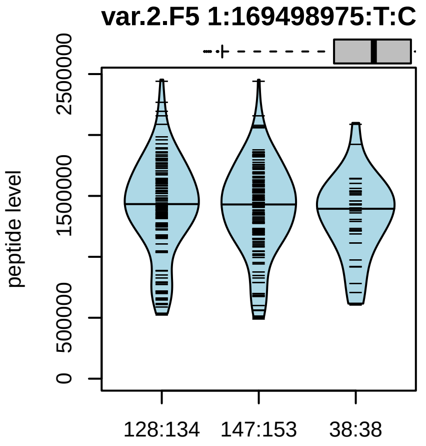

VMYTQYEDESFTKpc2_NP.4
p−maj=0.37, pX=0.73, N=313

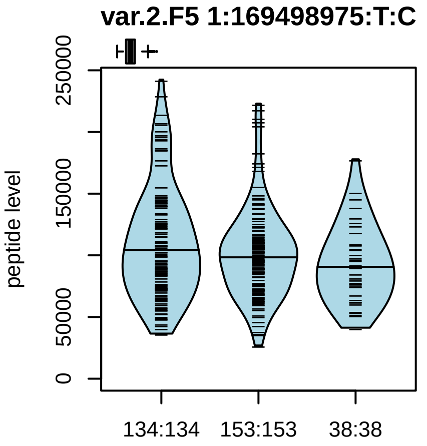

VMYTQYEDESFTKpc2_NP.5
p−maj=1, pX=0.0084, N=325

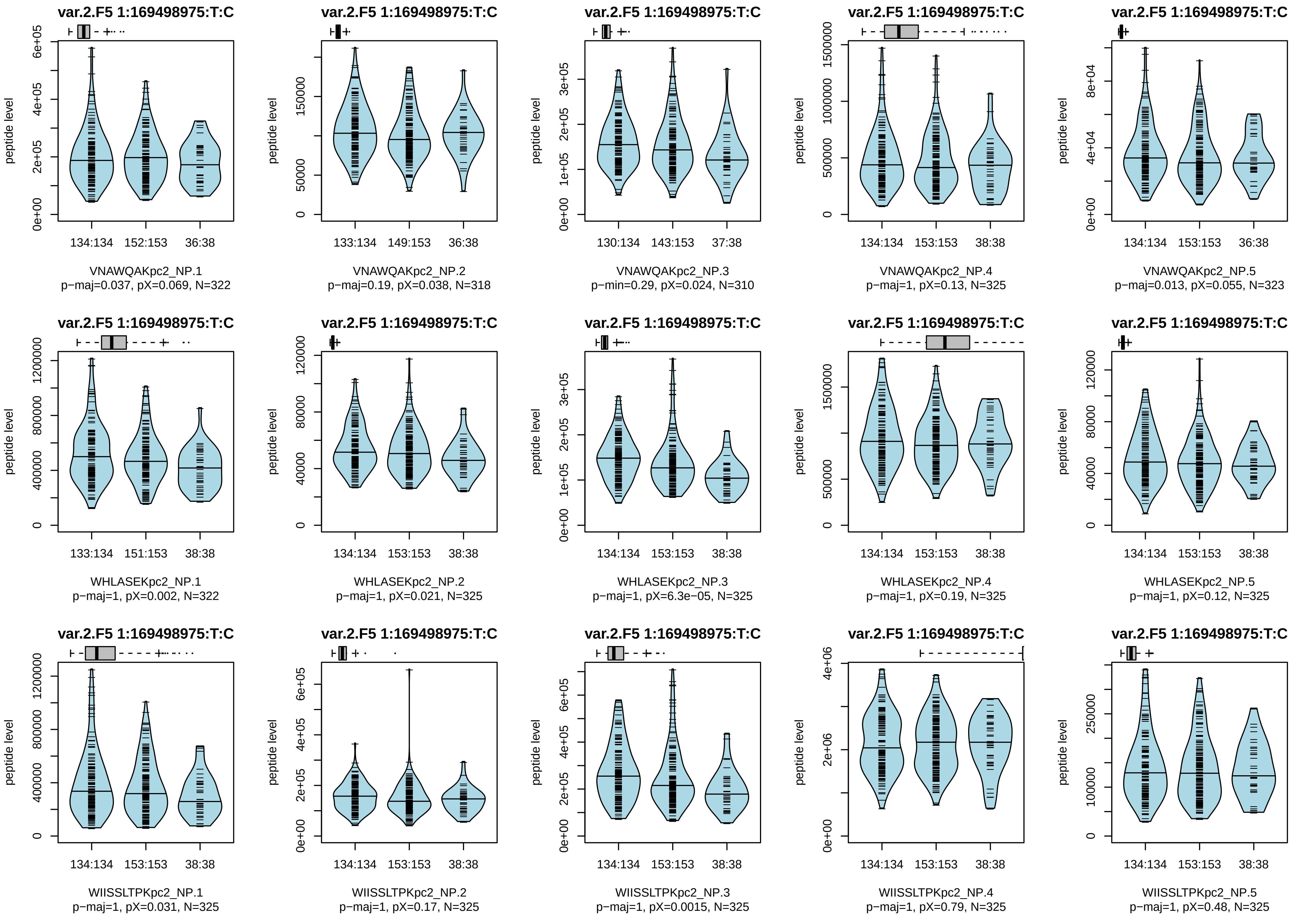
var.2.F5 1:169498975:T:C
peptide level
134:134
152:153
36:38
VNAWQAKpc2_NP.1
p−maj=0.037, pX=0.069, N=322
var.2.F5 1:169498975:T:C
133:134
149:153
36:38
VNAWQAKpc2_NP.2
p−maj=0.19, pX=0.038, N=318
var.2.F5 1:169498975:T:C
130:134
143:153
37:38
VNAWQAKpc2_NP.3
p−min=0.29, pX=0.024, N=310
var.2.F5 1:169498975:T:C
134:134
153:153
38:38
VNAWQAKpc2_NP.4
p−maj=1, pX=0.13, N=325
var.2.F5 1:169498975:T:C
134:134
153:153
36:38
VNAWQAKpc2_NP.5
p−maj=0.013, pX=0.055, N=323
var.2.F5 1:169498975:T:C
133:134
151:153
38:38
WHLASEKpc2_NP.1
p−maj=1, pX=0.002, N=322
var.2.F5 1:169498975:T:C
134:134
153:153
38:38
WHLASEKpc2_NP.2
p−maj=1, pX=0.021, N=325
var.2.F5 1:169498975:T:C
134:134
153:153
38:38
WHLASEKpc2_NP.3
p−maj=1, pX=6.3e−05, N=325
var.2.F5 1:169498975:T:C
134:134
153:153
38:38
WHLASEKpc2_NP.4
p−maj=1, pX=0.19, N=325
var.2.F5 1:169498975:T:C
134:134
153:153
38:38
WHLASEKpc2_NP.5
p−maj=1, pX=0.12, N=325
var.2.F5 1:169498975:T:C
134:134
153:153
38:38
WIISSLTPKpc2_NP.1
p−maj=1, pX=0.031, N=325
var.2.F5 1:169498975:T:C
134:134
153:153
38:38
WIISSLTPKpc2_NP.2
p−maj=1, pX=0.17, N=325
var.2.F5 1:169498975:T:C
134:134
153:153
38:38
WIISSLTPKpc2_NP.3
p−maj=1, pX=0.0015, N=325
var.2.F5 1:169498975:T:C
134:134
153:153
38:38
WIISSLTPKpc2_NP.4
p−maj=1, pX=0.79, N=325
var.2.F5 1:169498975:T:C
134:134
153:153
38:38
WIISSLTPKpc2_NP.5
p−maj=1, pX=0.48, N=325

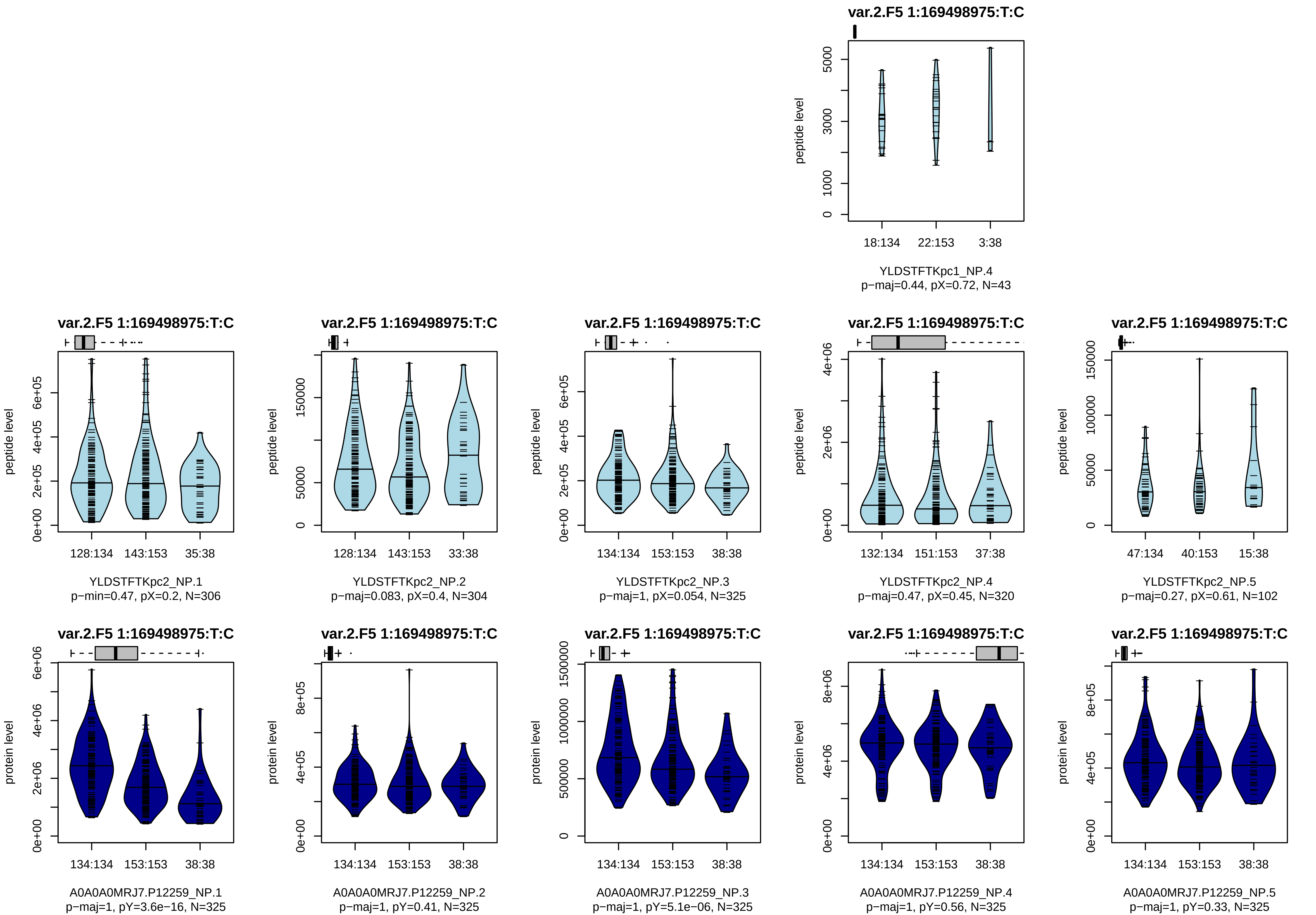

var.2.F5 1:169498975:T:C
peptide level
0
1000
3000
5000
18:134
22:153
3:38
YLDSTFTKpc1_NP.4
p−maj=0.44, pX=0.72, N=43
var.2.F5 1:169498975:T:C
peptide level
0e+00
2e+05
4e+05
6e+05
128:134
143:153
35:38
YLDSTFTKpc2_NP.1
p−min=0.47, pX=0.2, N=306
var.2.F5 1:169498975:T:C
peptide level
0
50000
150000
128:134
143:153
33:38
YLDSTFTKpc2_NP.2
p−maj=0.083, pX=0.4, N=304
var.2.F5 1:169498975:T:C
peptide level
0e+00
2e+05
4e+05
6e+05
134:134
153:153
38:38
YLDSTFTKpc2_NP.3
p−maj=1, pX=0.054, N=325
var.2.F5 1:169498975:T:C
peptide level
0e+00
2e+06
4e+06
132:134
151:153
37:38
YLDSTFTKpc2_NP.4
p−maj=0.47, pX=0.45, N=320
var.2.F5 1:169498975:T:C
peptide level
0
50000
100000
150000
47:134
40:153
15:38
YLDSTFTKpc2_NP.5
p−maj=0.27, pX=0.61, N=102
var.2.F5 1:169498975:T:C
protein level
0e+00
2e+06
4e+06
6e+06
134:134
153:153
38:38
A0A0A0MRJ7.P12259_NP.1
p−maj=1, pY=3.6e−16, N=325
var.2.F5 1:169498975:T:C
protein level
0e+00
4e+05
8e+05
134:134
153:153
38:38
A0A0A0MRJ7.P12259_NP.2
p−maj=1, pY=0.41, N=325
var.2.F5 1:169498975:T:C
protein level
0
500000
1000000
1500000
134:134
153:153
38:38
A0A0A0MRJ7.P12259_NP.3
p−maj=1, pY=5.1e−06, N=325
var.2.F5 1:169498975:T:C
protein level
0e+00
4e+06
8e+06
134:134
153:153
38:38
A0A0A0MRJ7.P12259_NP.4
p−maj=1, pY=0.56, N=325
var.2.F5 1:169498975:T:C
protein level
0e+00
4e+05
8e+05
134:134
153:153
38:38
A0A0A0MRJ7.P12259_NP.5
p−maj=1, pY=0.33, N=325

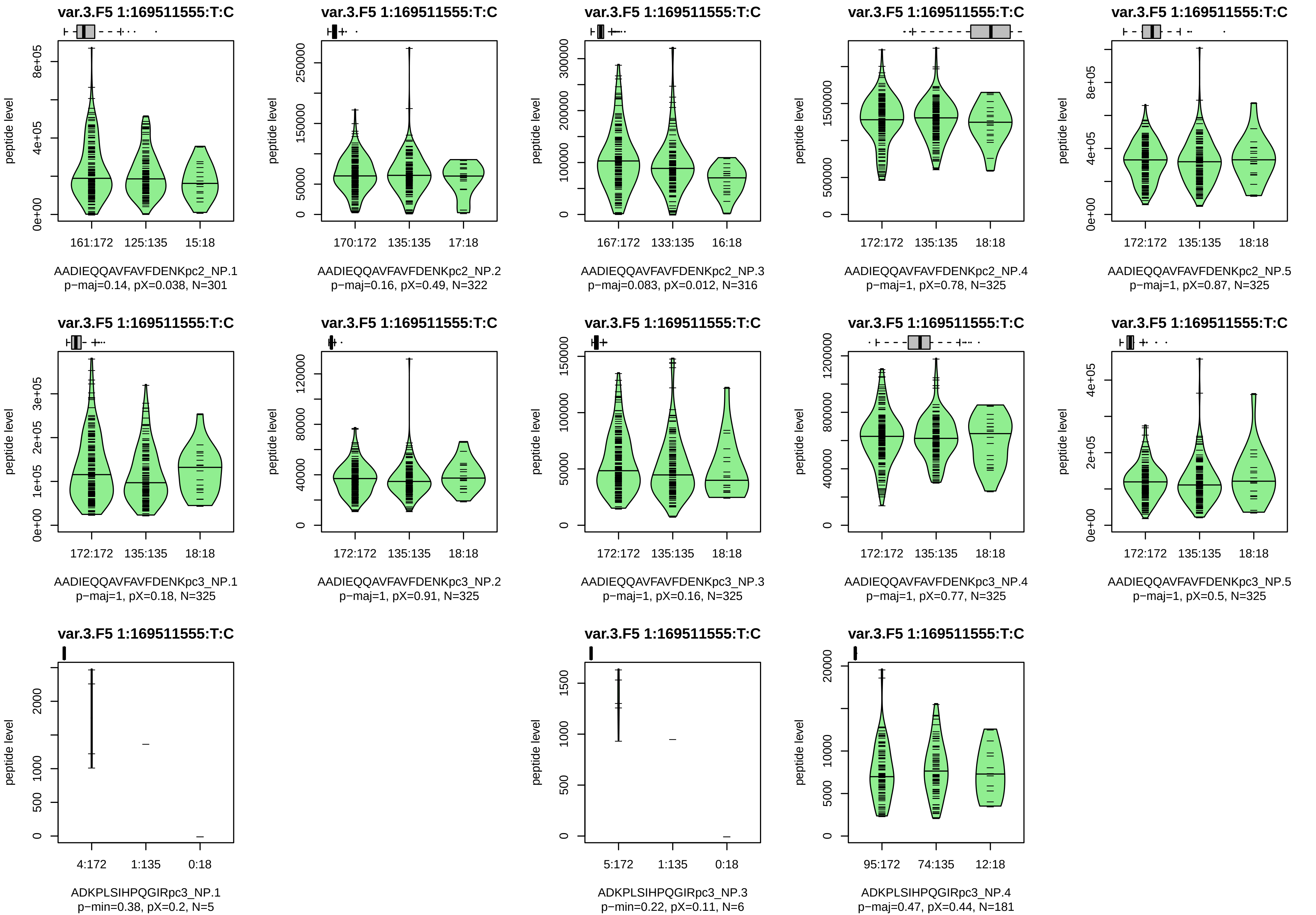

var.3.F5 1:169511555:T:C
peptide level
161:172
125:135
15:18
AADIEQQAVFAVFDENKpc2_NP.1
p−maj=0.14, pX=0.038, N=301
var.3.F5 1:169511555:T:C
peptide level
170:172
135:135
17:18
AADIEQQAVFAVFDENKpc2_NP.2
p−maj=0.16, pX=0.49, N=322
var.3.F5 1:169511555:T:C
peptide level
167:172
133:135
16:18
AADIEQQAVFAVFDENKpc2_NP.3
p−maj=0.083, pX=0.012, N=316
var.3.F5 1:169511555:T:C
peptide level
172:172
135:135
18:18
AADIEQQAVFAVFDENKpc2_NP.4
p−maj=1, pX=0.78, N=325
var.3.F5 1:169511555:T:C
peptide level
172:172
135:135
18:18
AADIEQQAVFAVFDENKpc2_NP.5
p−maj=1, pX=0.87, N=325
var.3.F5 1:169511555:T:C
peptide level
172:172
135:135
18:18
AADIEQQAVFAVFDENKpc3_NP.1
p−maj=1, pX=0.18, N=325
var.3.F5 1:169511555:T:C
peptide level
172:172
135:135
18:18
AADIEQQAVFAVFDENKpc3_NP.2
p−maj=1, pX=0.91, N=325
var.3.F5 1:169511555:T:C
peptide level
172:172
135:135
18:18
AADIEQQAVFAVFDENKpc3_NP.3
p−maj=1, pX=0.16, N=325
var.3.F5 1:169511555:T:C
peptide level
172:172
135:135
18:18
AADIEQQAVFAVFDENKpc3_NP.4
p−maj=1, pX=0.77, N=325
var.3.F5 1:169511555:T:C
peptide level
172:172
135:135
18:18
AADIEQQAVFAVFDENKpc3_NP.5
p−maj=1, pX=0.5, N=325
var.3.F5 1:169511555:T:C
peptide level
4:172
1:135
0:18
ADKPLSIHPQGIRpc3_NP.1
p−min=0.38, pX=0.2, N=5
var.3.F5 1:169511555:T:C
peptide level
5:172
1:135
0:18
ADKPLSIHPQGIRpc3_NP.3
p−min=0.22, pX=0.11, N=6
var.3.F5 1:169511555:T:C
peptide level
95:172
74:135
12:18
ADKPLSIHPQGIRpc3_NP.4
p−maj=0.47, pX=0.44, N=181

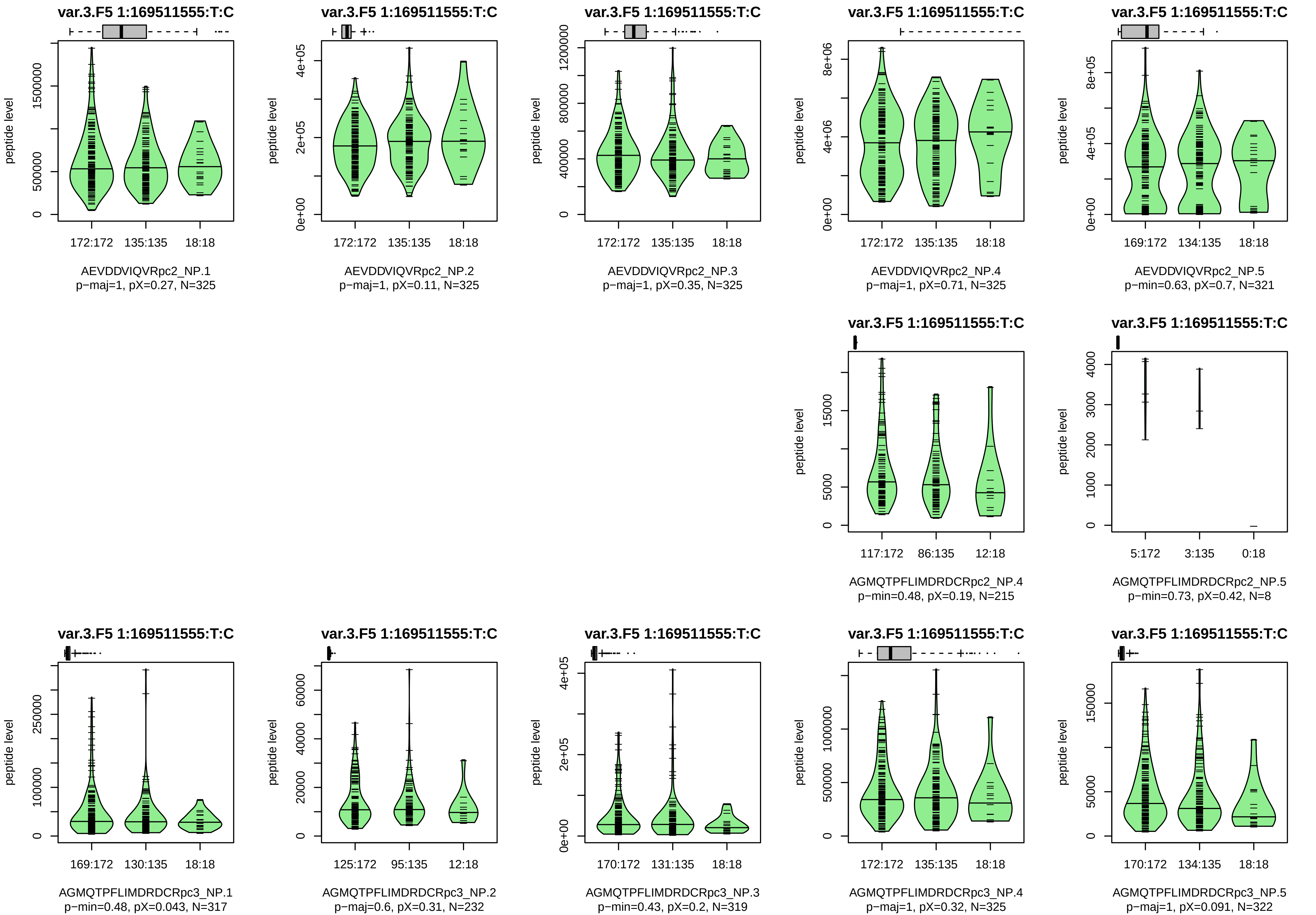

var.3.F5 1:169511555:T:C
peptide level
172:172
135:135
18:18
AEVDDVIQVRpc2_NP.1
p−maj=1, pX=0.27, N=325
var.3.F5 1:169511555:T:C
peptide level
172:172
135:135
18:18
AEVDDVIQVRpc2_NP.2
p−maj=1, pX=0.11, N=325
var.3.F5 1:169511555:T:C
peptide level
172:172
135:135
18:18
AEVDDVIQVRpc2_NP.3
p−maj=1, pX=0.35, N=325
var.3.F5 1:169511555:T:C
peptide level
172:172
135:135
18:18
AEVDDVIQVRpc2_NP.4
p−maj=1, pX=0.71, N=325
var.3.F5 1:169511555:T:C
peptide level
169:172
134:135
18:18
AEVDDVIQVRpc2_NP.5
p−min=0.63, pX=0.7, N=321
var.3.F5 1:169511555:T:C
peptide level
117:172
86:135
12:18
AGMQTPFLIMDRDCRpc2_NP.4
p−min=0.48, pX=0.19, N=215
var.3.F5 1:169511555:T:C
peptide level
5:172
3:135
0:18
AGMQTPFLIMDRDCRpc2_NP.5
p−min=0.73, pX=0.42, N=8
var.3.F5 1:169511555:T:C
peptide level
169:172
130:135
18:18
AGMQTPFLIMDRDCRpc3_NP.1
p−min=0.48, pX=0.043, N=317
var.3.F5 1:169511555:T:C
peptide level
125:172
95:135
12:18
AGMQTPFLIMDRDCRpc3_NP.2
p−maj=0.6, pX=0.31, N=232
var.3.F5 1:169511555:T:C
peptide level
170:172
131:135
18:18
AGMQTPFLIMDRDCRpc3_NP.3
p−min=0.43, pX=0.2, N=319
var.3.F5 1:169511555:T:C
peptide level
172:172
135:135
18:18
AGMQTPFLIMDRDCRpc3_NP.4
p−maj=1, pX=0.32, N=325
var.3.F5 1:169511555:T:C
peptide level
170:172
134:135
18:18
AGMQTPFLIMDRDCRpc3_NP.5
p−maj=1, pX=0.091, N=322

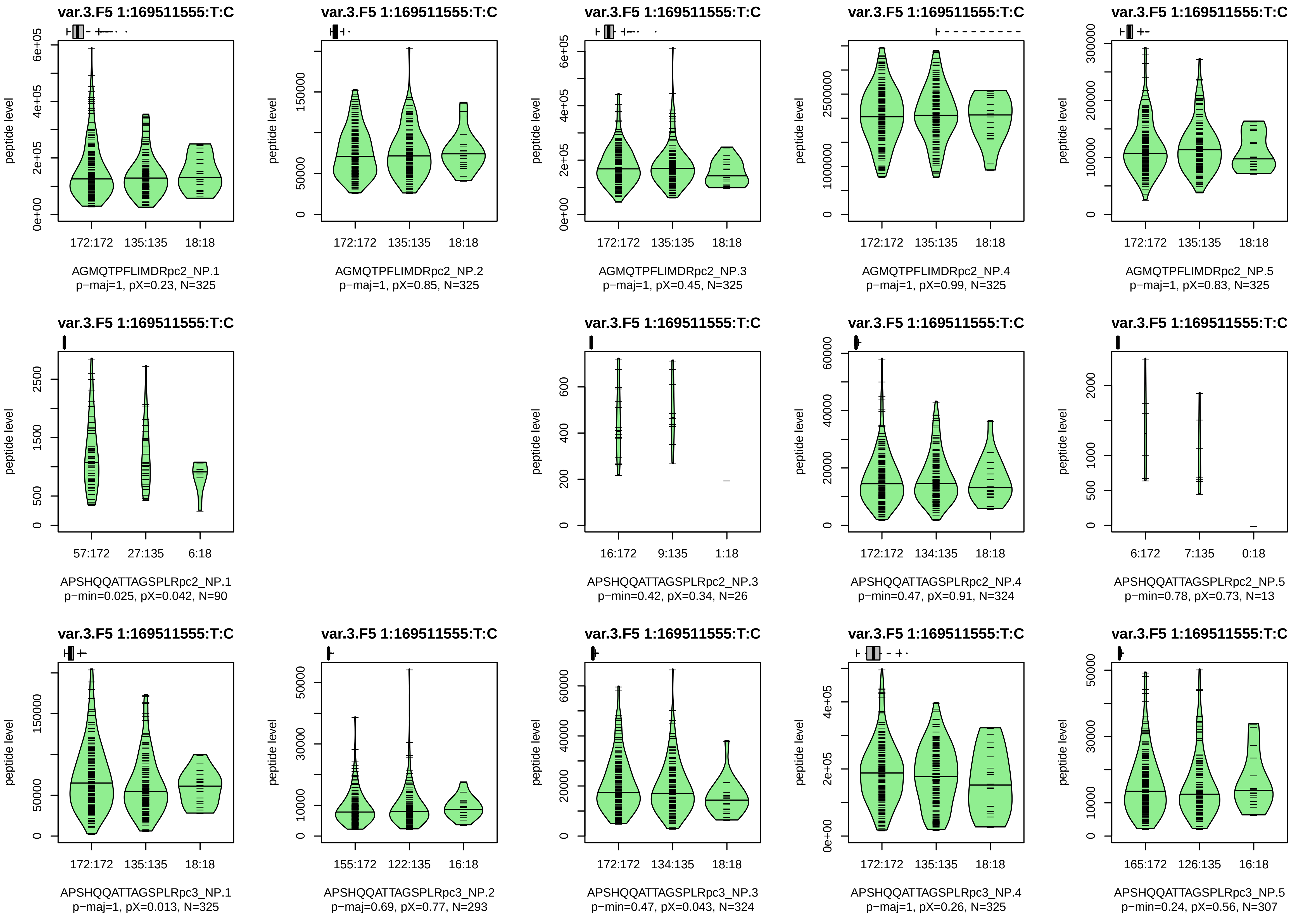

var.3.F5 1:169511555:T:C
peptide level
172:172 135:135 18:18
AGMQTPFLIMDRpc2_NP.1
p−maj=1, pX=0.23, N=325
var.3.F5 1:169511555:T:C
peptide level
172:172 135:135 18:18
AGMQTPFLIMDRpc2_NP.2
p−maj=1, pX=0.85, N=325
var.3.F5 1:169511555:T:C
peptide level
172:172 135:135 18:18
AGMQTPFLIMDRpc2_NP.3
p−maj=1, pX=0.45, N=325
var.3.F5 1:169511555:T:C
peptide level
172:172 135:135 18:18
AGMQTPFLIMDRpc2_NP.4
p−maj=1, pX=0.99, N=325
var.3.F5 1:169511555:T:C
peptide level
172:172 135:135 18:18
AGMQTPFLIMDRpc2_NP.5
p−maj=1, pX=0.83, N=325
var.3.F5 1:169511555:T:C
peptide level
57:172 27:135 6:18
APSHQQATTAGSPLRpc2_NP.1
p−min=0.025, pX=0.042, N=90
var.3.F5 1:169511555:T:C
peptide level
16:172 9:135 1:18
APSHQQATTAGSPLRpc2_NP.3
p−min=0.42, pX=0.34, N=26
var.3.F5 1:169511555:T:C
peptide level
172:172 134:135 18:18
APSHQQATTAGSPLRpc2_NP.4
p−min=0.47, pX=0.91, N=324
var.3.F5 1:169511555:T:C
peptide level
6:172 7:135 0:18
APSHQQATTAGSPLRpc2_NP.5
p−min=0.78, pX=0.73, N=13
var.3.F5 1:169511555:T:C
peptide level
172:172 135:135 18:18
APSHQQATTAGSPLRpc3_NP.1
p−maj=1, pX=0.013, N=325
var.3.F5 1:169511555:T:C
peptide level
155:172 122:135 16:18
APSHQQATTAGSPLRpc3_NP.2
p−maj=0.69, pX=0.77, N=293
var.3.F5 1:169511555:T:C
peptide level
172:172 134:135 18:18
APSHQQATTAGSPLRpc3_NP.3
p−min=0.47, pX=0.043, N=324
var.3.F5 1:169511555:T:C
peptide level
172:172 135:135 18:18
APSHQQATTAGSPLRpc3_NP.4
p−maj=1, pX=0.26, N=325
var.3.F5 1:169511555:T:C
peptide level
165:172 126:135 16:18
APSHQQATTAGSPLRpc3_NP.5
p−min=0.24, pX=0.56, N=307

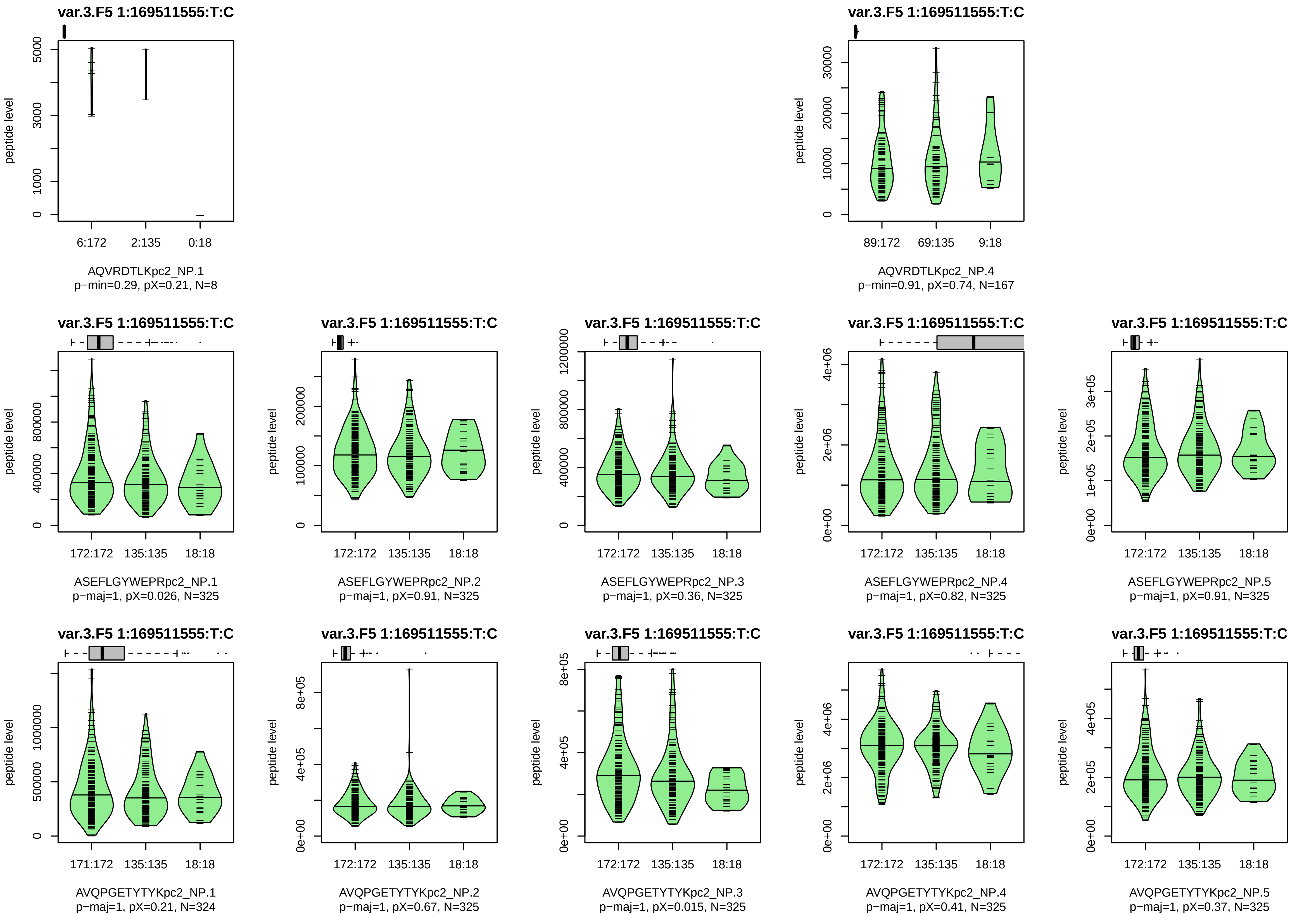
var.3.F5 1:169511555:T:C
peptide level
6:172
2:135
0:18
AQVRDTLKpc2_NP.1
p−min=0.29, pX=0.21, N=8
var.3.F5 1:169511555:T:C
peptide level
89:172
69:135
9:18
AQVRDTLKpc2_NP.4
p−min=0.91, pX=0.74, N=167
var.3.F5 1:169511555:T:C
172:172
135:135
18:18
ASEFLGYWEPRpc2_NP.1
p−maj=1, pX=0.026, N=325
var.3.F5 1:169511555:T:C
ASEFLGYWEPRpc2_NP.2
p−maj=1, pX=0.91, N=325
var.3.F5 1:169511555:T:C
ASEFLGYWEPRpc2_NP.3
p−maj=1, pX=0.36, N=325
var.3.F5 1:169511555:T:C
ASEFLGYWEPRpc2_NP.4
p−maj=1, pX=0.82, N=325
var.3.F5 1:169511555:T:C
ASEFLGYWEPRpc2_NP.5
p−maj=1, pX=0.91, N=325
var.3.F5 1:169511555:T:C
171:172
135:135
18:18
AVQPGETYTYKpc2_NP.1
p−maj=1, pX=0.21, N=324
var.3.F5 1:169511555:T:C
AVQPGETYTYKpc2_NP.2
p−maj=1, pX=0.67, N=325
var.3.F5 1:169511555:T:C
AVQPGETYTYKpc2_NP.3
p−maj=1, pX=0.015, N=325
var.3.F5 1:169511555:T:C
AVQPGETYTYKpc2_NP.4
p−maj=1, pX=0.41, N=325
var.3.F5 1:169511555:T:C
AVQPGETYTYKpc2_NP.5
p−maj=1, pX=0.37, N=325

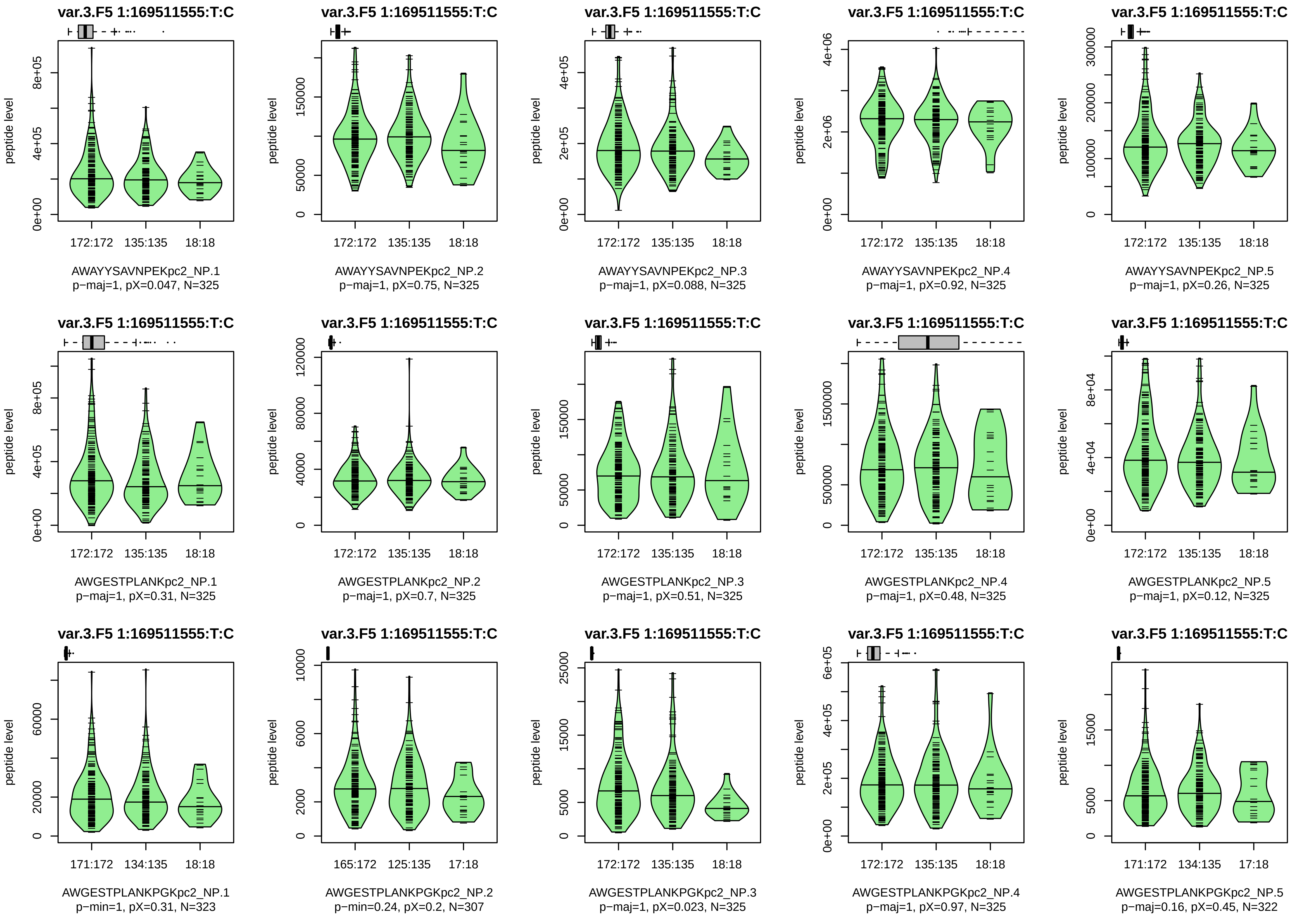
var.3.F5 1:169511555:T:C
peptide level
172:172
135:135
18:18
AWAYYSAVNPEKpc2_NP.1
p−maj=1, pX=0.047, N=325
AWAYYSAVNPEKpc2_NP.2
p−maj=1, pX=0.75, N=325
AWAYYSAVNPEKpc2_NP.3
p−maj=1, pX=0.088, N=325
AWAYYSAVNPEKpc2_NP.4
p−maj=1, pX=0.92, N=325
AWAYYSAVNPEKpc2_NP.5
p−maj=1, pX=0.26, N=325
AWGESTPLANKpc2_NP.1
p−maj=1, pX=0.31, N=325
AWGESTPLANKpc2_NP.2
p−maj=1, pX=0.7, N=325
AWGESTPLANKpc2_NP.3
p−maj=1, pX=0.51, N=325
AWGESTPLANKpc2_NP.4
p−maj=1, pX=0.48, N=325
AWGESTPLANKpc2_NP.5
p−maj=1, pX=0.12, N=325
171:172
134:135
165:172
125:135
17:18
AWGESTPLANKPGKpc2_NP.1
p−min=1, pX=0.31, N=323
AWGESTPLANKPGKpc2_NP.2
p−min=0.24, pX=0.2, N=307
AWGESTPLANKPGKpc2_NP.3
p−maj=1, pX=0.023, N=325
AWGESTPLANKPGKpc2_NP.4
p−maj=1, pX=0.97, N=325
AWGESTPLANKPGKpc2_NP.5
p−maj=0.16, pX=0.45, N=322

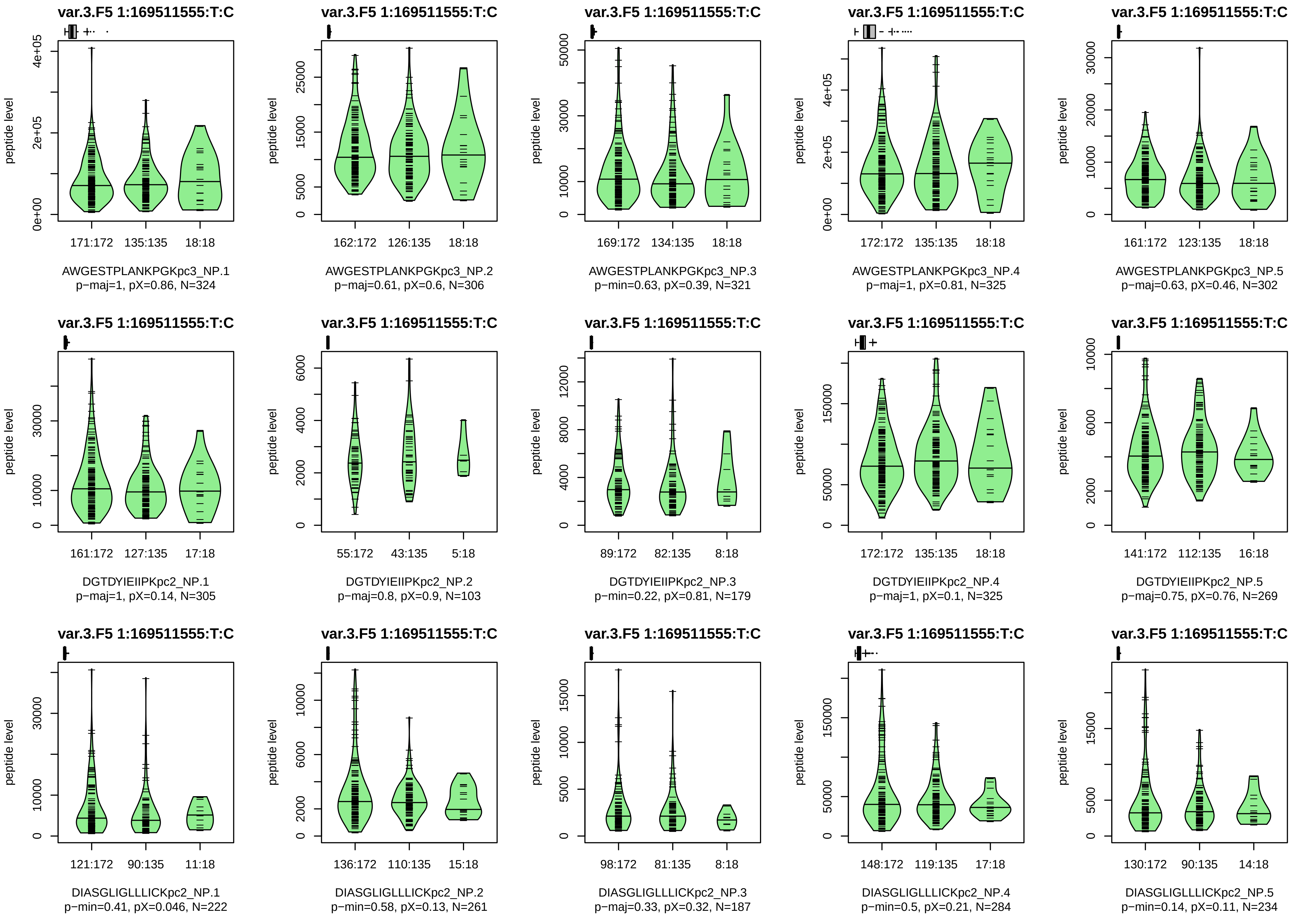
var.3.F5 1:169511555:T:C
peptide level
171:172 135:135 18:18
AWGESTPLANKPGKpc3_NP.1
p−maj=1, pX=0.86, N=324
var.3.F5 1:169511555:T:C
peptide level
162:172 126:135 18:18
AWGESTPLANKPGKpc3_NP.2
p−maj=0.61, pX=0.6, N=306
var.3.F5 1:169511555:T:C
peptide level
169:172 134:135 18:18
AWGESTPLANKPGKpc3_NP.3
p−min=0.63, pX=0.39, N=321
var.3.F5 1:169511555:T:C
peptide level
172:172 135:135 18:18
AWGESTPLANKPGKpc3_NP.4
p−maj=1, pX=0.81, N=325
var.3.F5 1:169511555:T:C
peptide level
161:172 123:135 18:18
AWGESTPLANKPGKpc3_NP.5
p−maj=0.63, pX=0.46, N=302
var.3.F5 1:169511555:T:C
peptide level
161:172 127:135 17:18
DGTDYIEIIPKpc2_NP.1
p−maj=1, pX=0.14, N=305
var.3.F5 1:169511555:T:C
peptide level
55:172 43:135 5:18
DGTDYIEIIPKpc2_NP.2
p−maj=0.8, pX=0.9, N=103
var.3.F5 1:169511555:T:C
peptide level
89:172 82:135 8:18
DGTDYIEIIPKpc2_NP.3
p−min=0.22, pX=0.81, N=179
var.3.F5 1:169511555:T:C
peptide level
172:172 135:135 18:18
DGTDYIEIIPKpc2_NP.4
p−maj=1, pX=0.1, N=325
var.3.F5 1:169511555:T:C
peptide level
141:172 112:135 16:18
DGTDYIEIIPKpc2_NP.5
p−maj=0.75, pX=0.76, N=269
var.3.F5 1:169511555:T:C
peptide level
121:172 90:135 11:18
DIASGLIGLLLICKpc2_NP.1
p−min=0.41, pX=0.046, N=222
var.3.F5 1:169511555:T:C
peptide level
136:172 110:135 15:18
DIASGLIGLLLICKpc2_NP.2
p−min=0.58, pX=0.13, N=261
var.3.F5 1:169511555:T:C
peptide level
98:172 81:135 8:18
DIASGLIGLLLICKpc2_NP.3
p−maj=0.33, pX=0.32, N=187
var.3.F5 1:169511555:T:C
peptide level
148:172 119:135 17:18
DIASGLIGLLLICKpc2_NP.4
p−min=0.5, pX=0.21, N=284
var.3.F5 1:169511555:T:C
peptide level
130:172 90:135 14:18
DIASGLIGLLLICKpc2_NP.5
p−min=0.14, pX=0.11, N=234

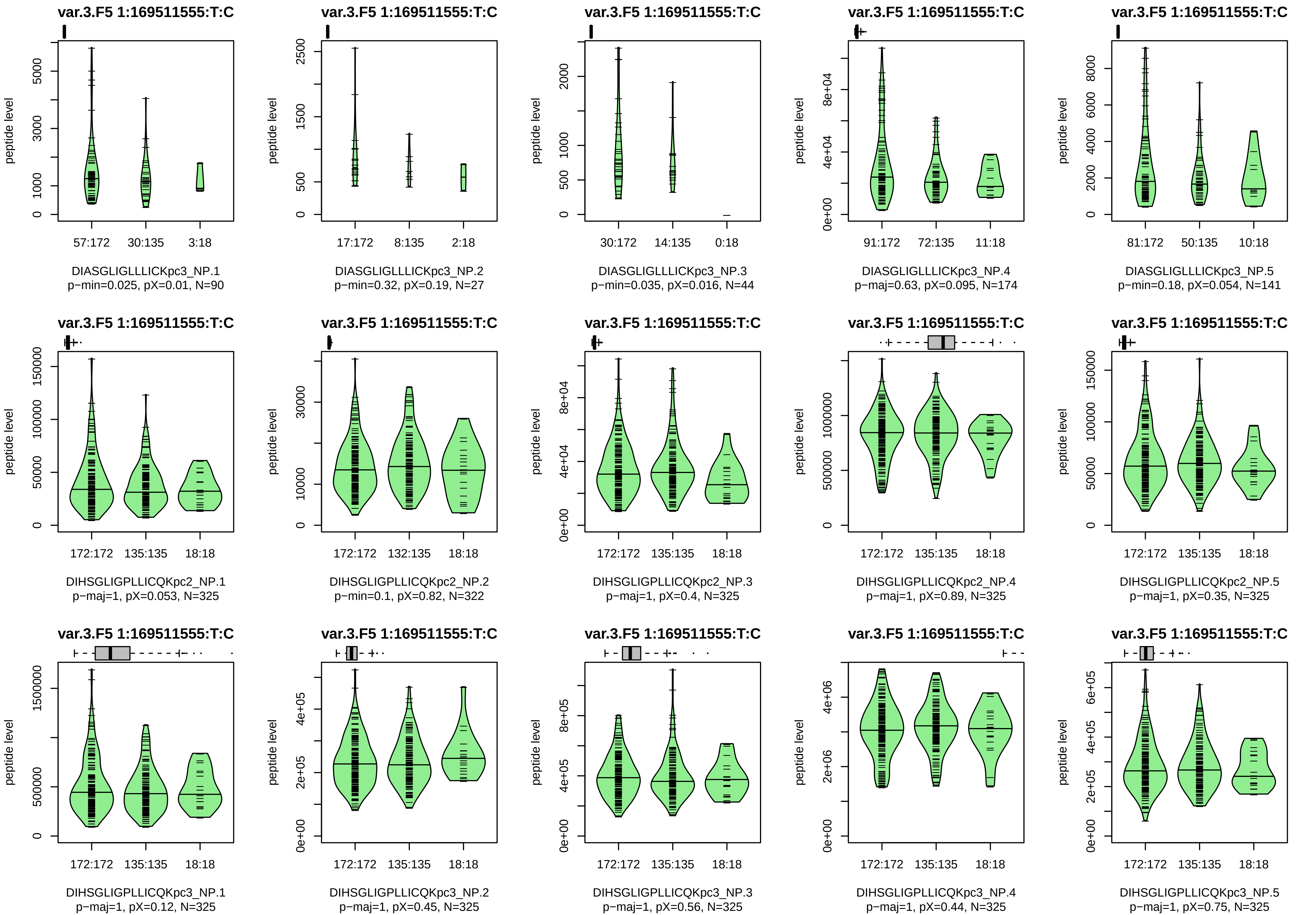

var.3.F5 1:169511555:T:C
peptide level
57:172
30:135
3:18
DIASGLIGLLLICKpc3_NP.1
p−min=0.025, pX=0.01, N=90
var.3.F5 1:169511555:T:C
peptide level
17:172
8:135
2:18
DIASGLIGLLLICKpc3_NP.2
p−min=0.32, pX=0.19, N=27
var.3.F5 1:169511555:T:C
peptide level
30:172
14:135
0:18
DIASGLIGLLLICKpc3_NP.3
p−min=0.035, pX=0.016, N=44
var.3.F5 1:169511555:T:C
peptide level
91:172
72:135
11:18
DIASGLIGLLLICKpc3_NP.4
p−maj=0.63, pX=0.095, N=174
var.3.F5 1:169511555:T:C
peptide level
81:172
50:135
10:18
DIASGLIGLLLICKpc3_NP.5
p−min=0.18, pX=0.054, N=141
var.3.F5 1:169511555:T:C
peptide level
172:172
135:135
18:18
DIHSGLIGPLLICQKpc2_NP.1
p−maj=1, pX=0.053, N=325
var.3.F5 1:169511555:T:C
peptide level
172:172
132:135
18:18
DIHSGLIGPLLICQKpc2_NP.2
p−min=0.1, pX=0.82, N=322
var.3.F5 1:169511555:T:C
peptide level
172:172
135:135
18:18
DIHSGLIGPLLICQKpc2_NP.3
p−maj=1, pX=0.4, N=325
var.3.F5 1:169511555:T:C
peptide level
172:172
135:135
18:18
DIHSGLIGPLLICQKpc2_NP.4
p−maj=1, pX=0.89, N=325
var.3.F5 1:169511555:T:C
peptide level
172:172
135:135
18:18
DIHSGLIGPLLICQKpc2_NP.5
p−maj=1, pX=0.35, N=325
var.3.F5 1:169511555:T:C
peptide level
172:172
135:135
18:18
DIHSGLIGPLLICQKpc3_NP.1
p−maj=1, pX=0.12, N=325
var.3.F5 1:169511555:T:C
peptide level
172:172
135:135
18:18
DIHSGLIGPLLICQKpc3_NP.2
p−maj=1, pX=0.45, N=325
var.3.F5 1:169511555:T:C
peptide level
172:172
135:135
18:18
DIHSGLIGPLLICQKpc3_NP.3
p−maj=1, pX=0.56, N=325
var.3.F5 1:169511555:T:C
peptide level
172:172
135:135
18:18
DIHSGLIGPLLICQKpc3_NP.4
p−maj=1, pX=0.44, N=325
var.3.F5 1:169511555:T:C
peptide level
172:172
135:135
18:18
DIHSGLIGPLLICQKpc3_NP.5
p−maj=1, pX=0.75, N=325

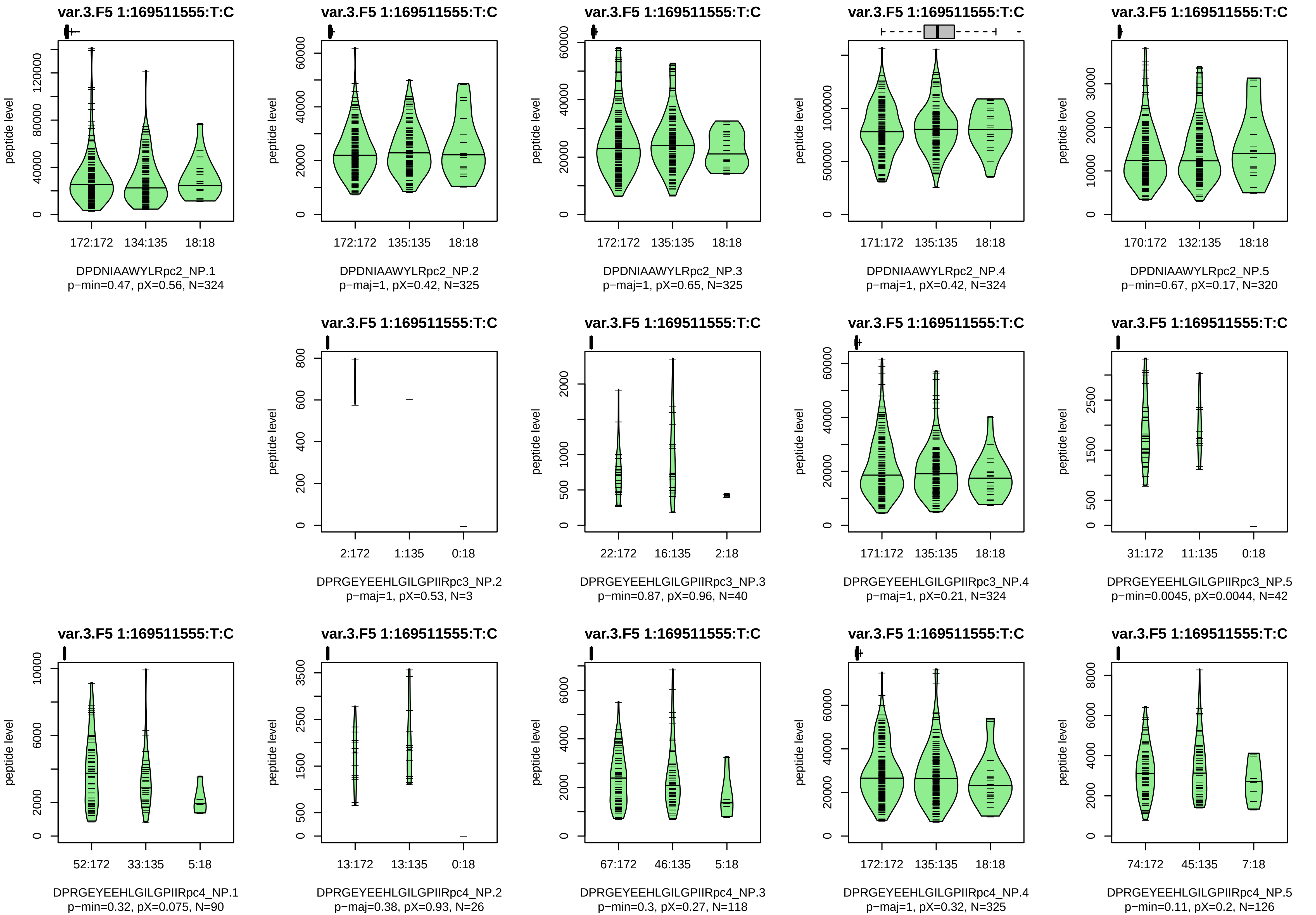
var.3.F5 1:169511555:T:C
peptide level
172:172
134:135
18:18
DPDNIAAWYLRpc2_NP.1
p−min=0.47, pX=0.56, N=324
var.3.F5 1:169511555:T:C
peptide level
172:172
135:135
18:18
DPDNIAAWYLRpc2_NP.2
p−maj=1, pX=0.42, N=325
var.3.F5 1:169511555:T:C
peptide level
172:172
135:135
18:18
DPDNIAAWYLRpc2_NP.3
p−maj=1, pX=0.65, N=325
var.3.F5 1:169511555:T:C
peptide level
171:172
135:135
18:18
DPDNIAAWYLRpc2_NP.4
p−maj=1, pX=0.42, N=324
var.3.F5 1:169511555:T:C
peptide level
170:172
132:135
18:18
DPDNIAAWYLRpc2_NP.5
p−min=0.67, pX=0.17, N=320
var.3.F5 1:169511555:T:C
peptide level
2:172
1:135
0:18
DPRGEYEEHLGILGPIIRpc3_NP.2
p−maj=1, pX=0.53, N=3
var.3.F5 1:169511555:T:C
peptide level
22:172
16:135
2:18
DPRGEYEEHLGILGPIIRpc3_NP.3
p−min=0.87, pX=0.96, N=40
var.3.F5 1:169511555:T:C
peptide level
171:172
135:135
18:18
DPRGEYEEHLGILGPIIRpc3_NP.4
p−maj=1, pX=0.21, N=324
var.3.F5 1:169511555:T:C
peptide level
31:172
11:135
0:18
DPRGEYEEHLGILGPIIRpc3_NP.5
p−min=0.0045, pX=0.0044, N=42
var.3.F5 1:169511555:T:C
peptide level
52:172
33:135
5:18
DPRGEYEEHLGILGPIIRpc4_NP.1
p−min=0.32, pX=0.075, N=90
var.3.F5 1:169511555:T:C
peptide level
13:172
13:135
0:18
DPRGEYEEHLGILGPIIRpc4_NP.2
p−maj=0.38, pX=0.93, N=26
var.3.F5 1:169511555:T:C
peptide level
67:172
46:135
5:18
DPRGEYEEHLGILGPIIRpc4_NP.3
p−min=0.3, pX=0.27, N=118
var.3.F5 1:169511555:T:C
peptide level
172:172
135:135
18:18
DPRGEYEEHLGILGPIIRpc4_NP.4
p−maj=1, pX=0.32, N=325
var.3.F5 1:169511555:T:C
peptide level
74:172
45:135
7:18
DPRGEYEEHLGILGPIIRpc4_NP.5
p−min=0.11, pX=0.2, N=126

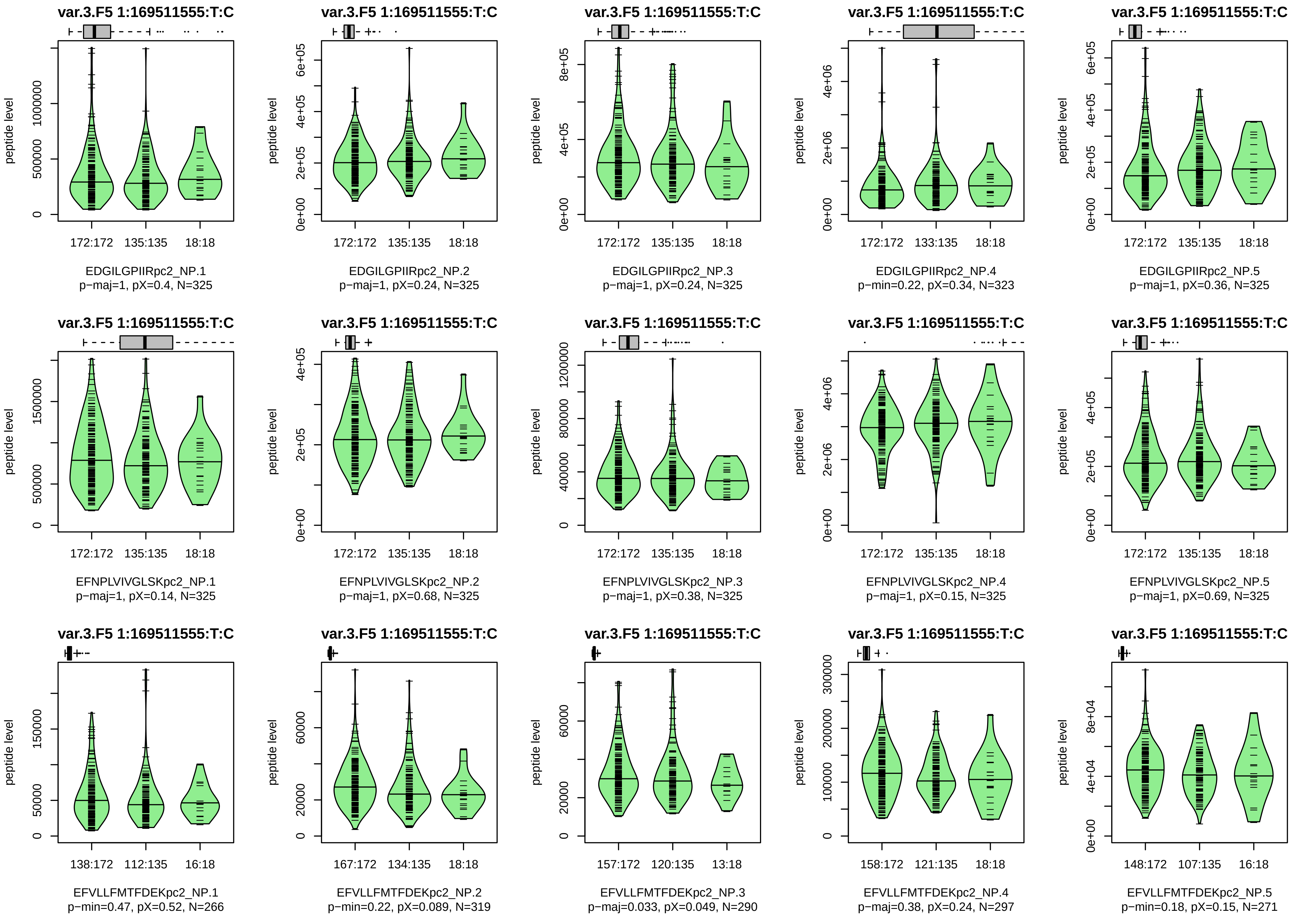
var.3.F5 1:169511555:T:C
peptide level
172:172
135:135
18:18
EDGILGPIIRpc2_NP.1
p−maj=1, pX=0.4, N=325
var.3.F5 1:169511555:T:C
172:172
135:135
18:18
EDGILGPIIRpc2_NP.2
p−maj=1, pX=0.24, N=325
var.3.F5 1:169511555:T:C
172:172
135:135
18:18
EDGILGPIIRpc2_NP.3
p−maj=1, pX=0.24, N=325
var.3.F5 1:169511555:T:C
172:172
133:135
18:18
EDGILGPIIRpc2_NP.4
p−min=0.22, pX=0.34, N=323
var.3.F5 1:169511555:T:C
172:172
135:135
18:18
EDGILGPIIRpc2_NP.5
p−maj=1, pX=0.36, N=325
var.3.F5 1:169511555:T:C
172:172
135:135
18:18
EFNPLVIVGLSKpc2_NP.1
p−maj=1, pX=0.14, N=325
var.3.F5 1:169511555:T:C
172:172
135:135
18:18
EFNPLVIVGLSKpc2_NP.2
p−maj=1, pX=0.68, N=325
var.3.F5 1:169511555:T:C
172:172
135:135
18:18
EFNPLVIVGLSKpc2_NP.3
p−maj=1, pX=0.38, N=325
var.3.F5 1:169511555:T:C
172:172
135:135
18:18
EFNPLVIVGLSKpc2_NP.4
p−maj=1, pX=0.15, N=325
var.3.F5 1:169511555:T:C
172:172
135:135
18:18
EFNPLVIVGLSKpc2_NP.5
p−maj=1, pX=0.69, N=325
var.3.F5 1:169511555:T:C
138:172
112:135
16:18
EFVLLFMTFDEKpc2_NP.1
p−min=0.47, pX=0.52, N=266
var.3.F5 1:169511555:T:C
167:172
134:135
18:18
EFVLLFMTFDEKpc2_NP.2
p−min=0.22, pX=0.089, N=319
var.3.F5 1:169511555:T:C
157:172
120:135
13:18
EFVLLFMTFDEKpc2_NP.3
p−maj=0.033, pX=0.049, N=290
var.3.F5 1:169511555:T:C
158:172
121:135
18:18
EFVLLFMTFDEKpc2_NP.4
p−maj=0.38, pX=0.24, N=297
var.3.F5 1:169511555:T:C
148:172
107:135
16:18
EFVLLFMTFDEKpc2_NP.5
p−min=0.18, pX=0.15, N=271

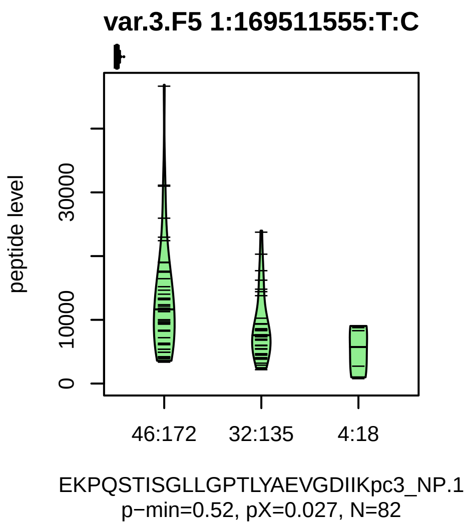
var.3.F5 1:169511555:T:C
peptide level
0
10000
30000
46:172
32:135
4:18
EKPQSTISGLLGPTLYAEVGDIIKpc3_NP.1
p−min=0.52, pX=0.027, N=82

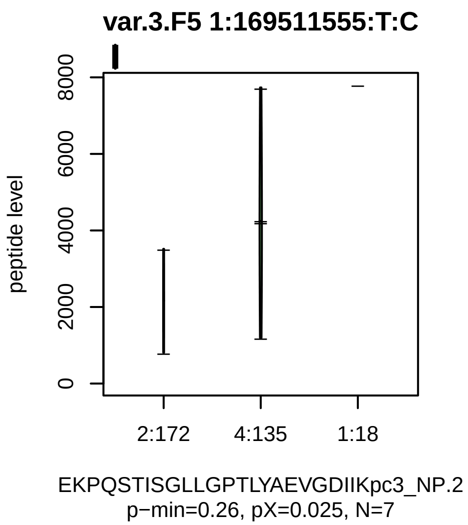
var.3.F5 1:169511555:T:C
peptide level
0
2000
4000
6000
8000
2:172
4:135
1:18
EKPQSTISGLLGPTLYAEVGDIIKpc3_NP.2
p−min=0.26, pX=0.025, N=7

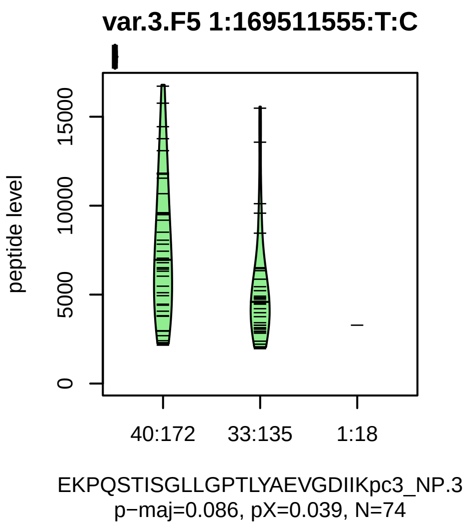
var.3.F5 1:169511555:T:C
peptide level
0
5000
10000
15000
40:172
33:135
1:18
EKPQSTISGLLGPTLYAEVGDIIKpc3_NP.3
p−maj=0.086, pX=0.039, N=74

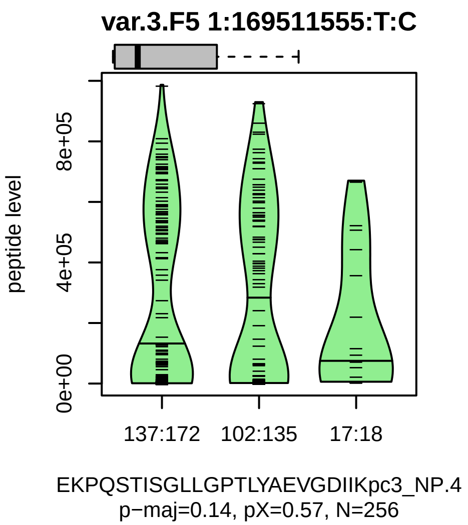
var.3.F5 1:169511555:T:C
peptide level
0e+00
4e+05
8e+05
137:172
102:135
17:18
EKPQSTISGLLGPTLYAEVGDIIKpc3_NP.4
p−maj=0.14, pX=0.57, N=256

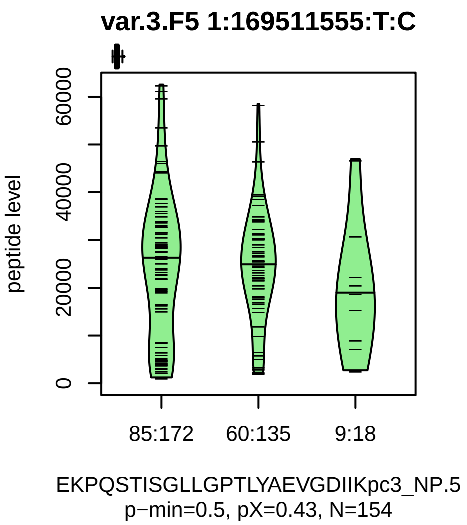
var.3.F5 1:169511555:T:C
peptide level
0
20000
40000
60000
85:172
60:135
9:18
EKPQSTISGLLGPTLYAEVGDIIKpc3_NP.5
p−min=0.5, pX=0.43, N=154

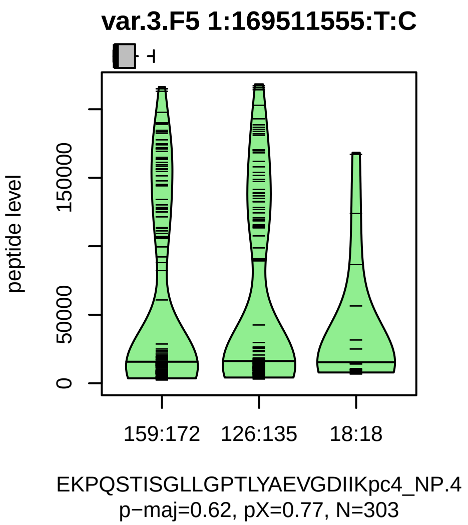
var.3.F5 1:169511555:T:C
peptide level
0
50000
150000
159:172
126:135
18:18
EKPQSTISGLLGPTLYAEVGDIIKpc4_NP.4
p−maj=0.62, pX=0.77, N=303

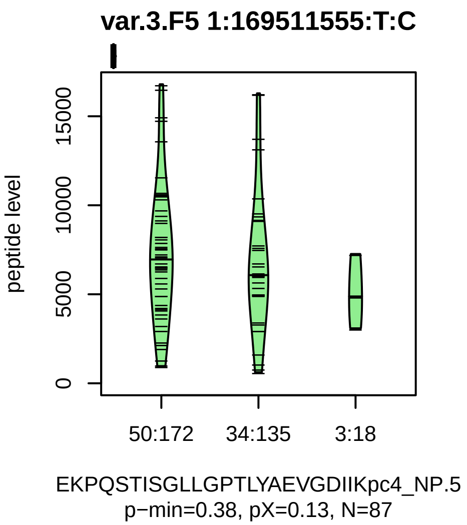
var.3.F5 1:169511555:T:C
peptide level
0
5000
10000
15000
50:172
34:135
3:18
EKPQSTISGLLGPTLYAEVGDIIKpc4_NP.5
p−min=0.38, pX=0.13, N=87

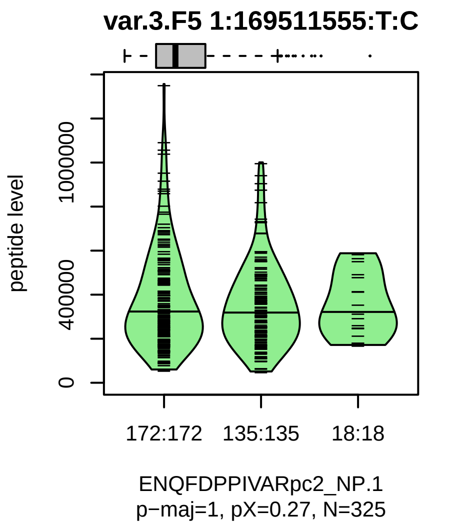
var.3.F5 1:169511555:T:C
peptide level
0
400000
1000000
172:172
135:135
18:18
ENQFDPPIVARpc2_NP.1
p−maj=1, pX=0.27, N=325

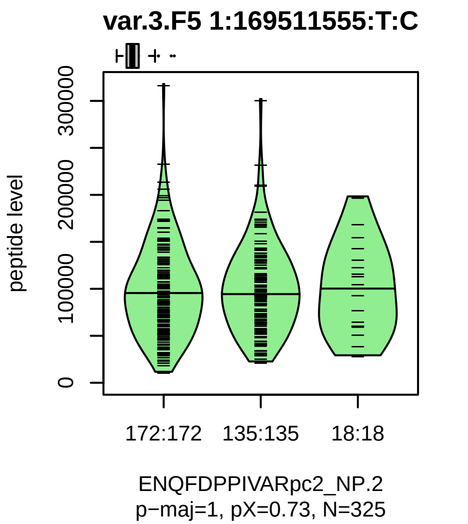
var.3.F5 1:169511555:T:C
peptide level
0
100000
200000
300000
172:172
135:135
18:18
ENQFDPPIVARpc2_NP.2
p−maj=1, pX=0.73, N=325

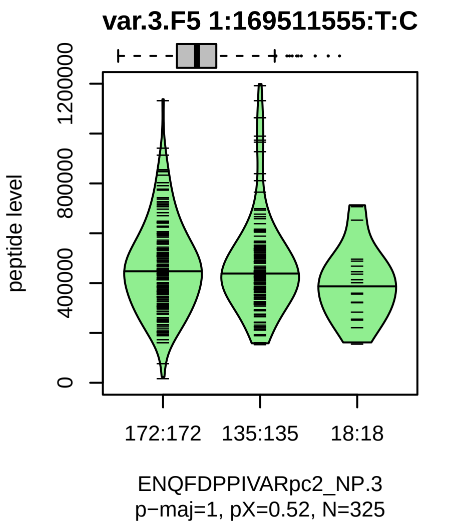
var.3.F5 1:169511555:T:C
peptide level
0
400000
800000
1200000
172:172
135:135
18:18
ENQFDPPIVARpc2_NP.3
p−maj=1, pX=0.52, N=325

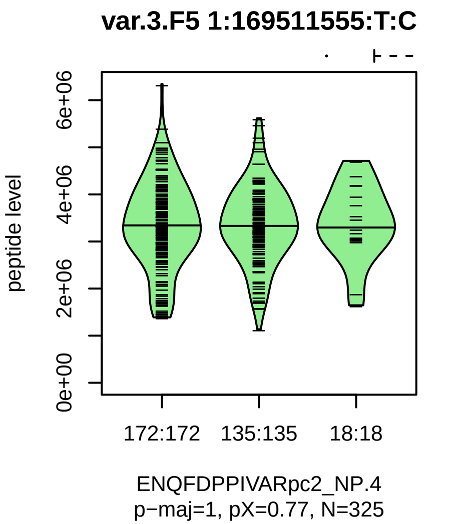
var.3.F5 1:169511555:T:C
peptide level
0e+00
2e+06
4e+06
6e+06
172:172
135:135
18:18
ENQFDPPIVARpc2_NP.4
p−maj=1, pX=0.77, N=325

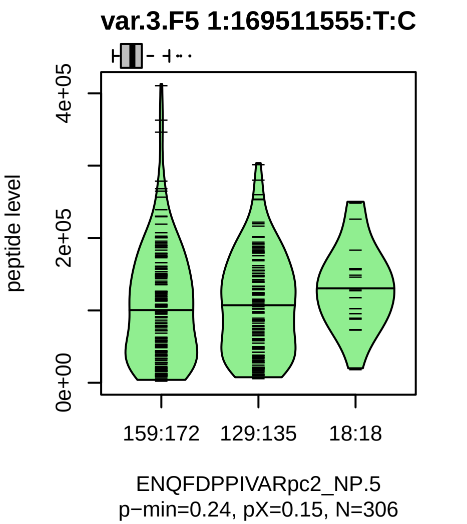
var.3.F5 1:169511555:T:C
peptide level
0e+00
2e+05
4e+05
159:172
129:135
18:18
ENQFDPPIVARpc2_NP.5
p−min=0.24, pX=0.15, N=306

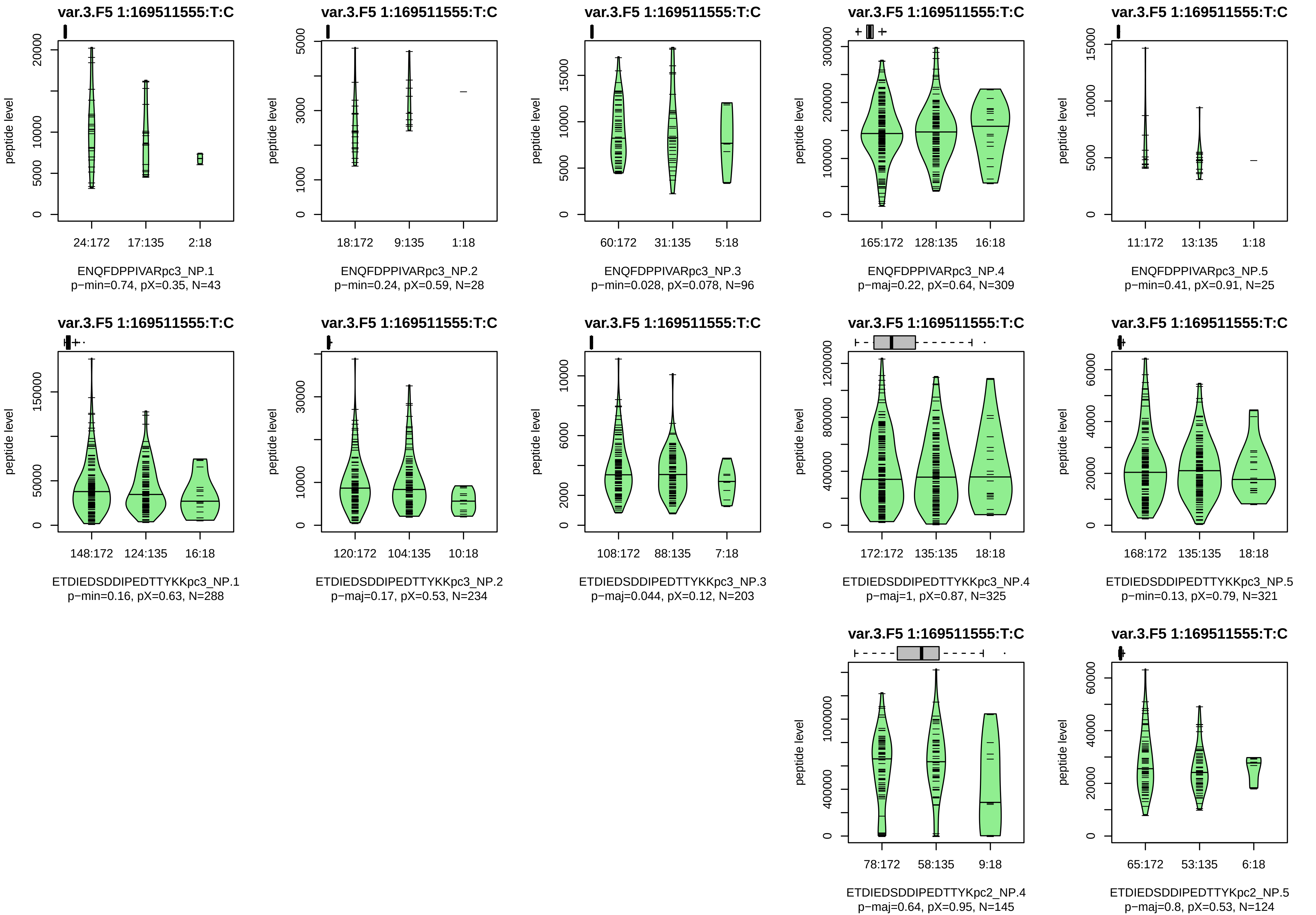
var.3.F5 1:169511555:T:C
peptide level
24:172
17:135
2:18
ENQFDPPIVARpc3_NP.1
p−min=0.74, pX=0.35, N=43
var.3.F5 1:169511555:T:C
peptide level
18:172
9:135
1:18
ENQFDPPIVARpc3_NP.2
p−min=0.24, pX=0.59, N=28
var.3.F5 1:169511555:T:C
peptide level
60:172
31:135
5:18
ENQFDPPIVARpc3_NP.3
p−min=0.028, pX=0.078, N=96
var.3.F5 1:169511555:T:C
peptide level
165:172
128:135
16:18
ENQFDPPIVARpc3_NP.4
p−maj=0.22, pX=0.64, N=309
var.3.F5 1:169511555:T:C
peptide level
11:172
13:135
1:18
ENQFDPPIVARpc3_NP.5
p−min=0.41, pX=0.91, N=25
var.3.F5 1:169511555:T:C
peptide level
148:172
124:135
16:18
ETDIEDSDDIPEDTTYKKpc3_NP.1
p−min=0.16, pX=0.63, N=288
var.3.F5 1:169511555:T:C
peptide level
120:172
104:135
10:18
ETDIEDSDDIPEDTTYKKpc3_NP.2
p−maj=0.17, pX=0.53, N=234
var.3.F5 1:169511555:T:C
peptide level
108:172
88:135
7:18
ETDIEDSDDIPEDTTYKKpc3_NP.3
p−maj=0.044, pX=0.12, N=203
var.3.F5 1:169511555:T:C
peptide level
172:172
135:135
18:18
ETDIEDSDDIPEDTTYKKpc3_NP.4
p−maj=1, pX=0.87, N=325
var.3.F5 1:169511555:T:C
peptide level
168:172
135:135
18:18
ETDIEDSDDIPEDTTYKKpc3_NP.5
p−min=0.13, pX=0.79, N=321
var.3.F5 1:169511555:T:C
peptide level
78:172
58:135
9:18
ETDIEDSDDIPEDTTYKpc2_NP.4
p−maj=0.64, pX=0.95, N=145
var.3.F5 1:169511555:T:C
peptide level
65:172
53:135
6:18
ETDIEDSDDIPEDTTYKpc2_NP.5
p−maj=0.8, pX=0.53, N=124

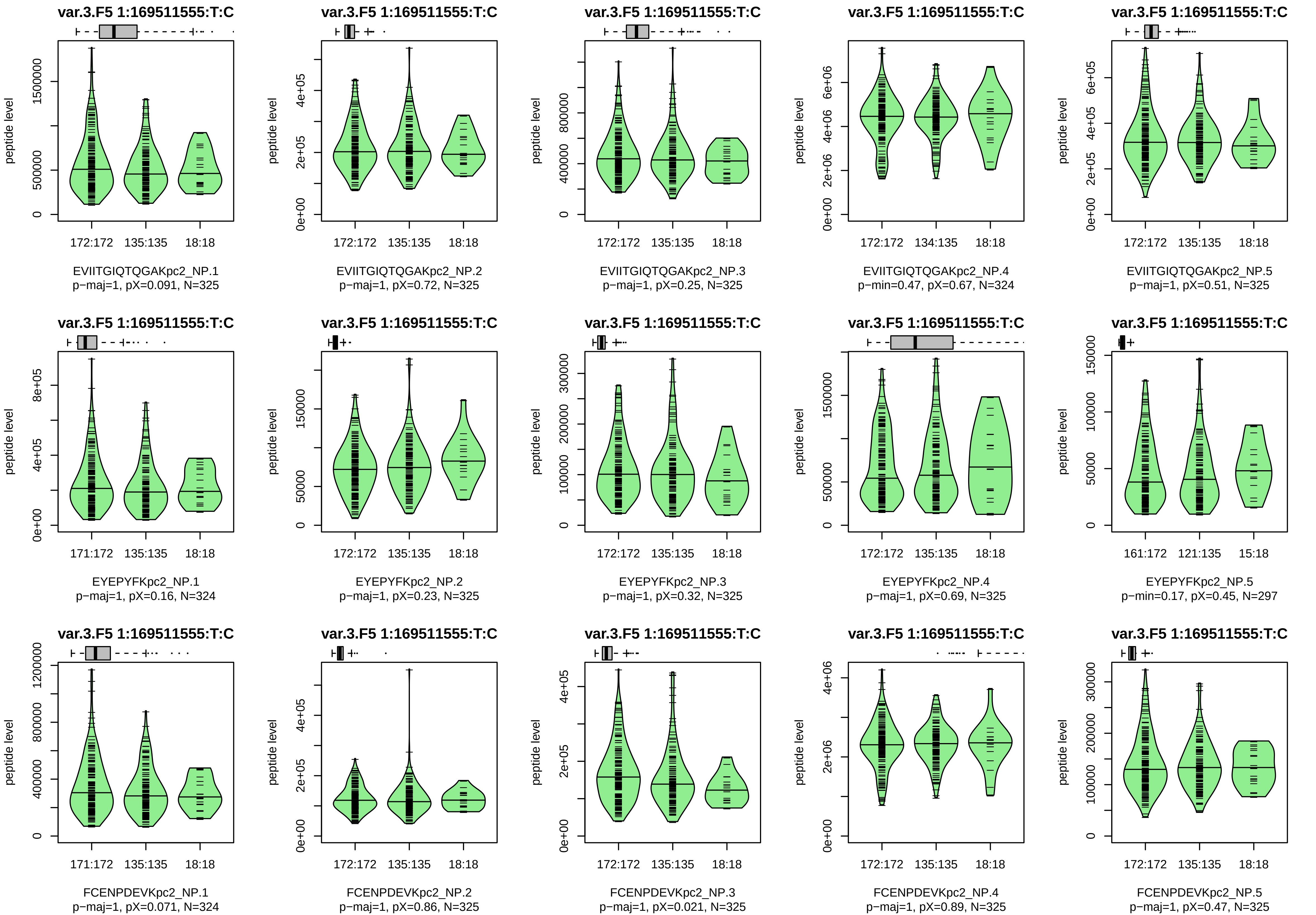

var.3.F5 1:169511555:T:C
peptide level
0
500000
1500000
172:172
135:135
18:18
EVIITGIQTQGAKpc2_NP.1
p−maj=1, pX=0.091, N=325
var.3.F5 1:169511555:T:C
peptide level
0e+00
2e+05
4e+05
172:172
135:135
18:18
EVIITGIQTQGAKpc2_NP.2
p−maj=1, pX=0.72, N=325
var.3.F5 1:169511555:T:C
peptide level
0
400000
800000
172:172
135:135
18:18
EVIITGIQTQGAKpc2_NP.3
p−maj=1, pX=0.25, N=325
var.3.F5 1:169511555:T:C
peptide level
0e+00
2e+06
4e+06
6e+06
172:172
134:135
18:18
EVIITGIQTQGAKpc2_NP.4
p−min=0.47, pX=0.67, N=324
var.3.F5 1:169511555:T:C
peptide level
0e+00
2e+05
4e+05
6e+05
172:172
135:135
18:18
EVIITGIQTQGAKpc2_NP.5
p−maj=1, pX=0.51, N=325
var.3.F5 1:169511555:T:C
peptide level
0e+00
4e+05
8e+05
171:172
135:135
18:18
EYEPYFKpc2_NP.1
p−maj=1, pX=0.16, N=324
var.3.F5 1:169511555:T:C
peptide level
0
50000
150000
172:172
135:135
18:18
EYEPYFKpc2_NP.2
p−maj=1, pX=0.23, N=325
var.3.F5 1:169511555:T:C
peptide level
0
100000
200000
300000
172:172
135:135
18:18
EYEPYFKpc2_NP.3
p−maj=1, pX=0.32, N=325
var.3.F5 1:169511555:T:C
peptide level
0
500000
1500000
172:172
135:135
18:18
EYEPYFKpc2_NP.4
p−maj=1, pX=0.69, N=325
var.3.F5 1:169511555:T:C
peptide level
0
50000
100000
150000
161:172
121:135
15:18
EYEPYFKpc2_NP.5
p−min=0.17, pX=0.45, N=297
var.3.F5 1:169511555:T:C
peptide level
0
400000
800000
1200000
171:172
135:135
18:18
FCENPDEVKpc2_NP.1
p−maj=1, pX=0.071, N=324
var.3.F5 1:169511555:T:C
peptide level
0e+00
2e+05
4e+05
172:172
135:135
18:18
FCENPDEVKpc2_NP.2
p−maj=1, pX=0.86, N=325
var.3.F5 1:169511555:T:C
peptide level
0e+00
2e+05
4e+05
172:172
135:135
18:18
FCENPDEVKpc2_NP.3
p−maj=1, pX=0.021, N=325
var.3.F5 1:169511555:T:C
peptide level
0e+00
2e+06
4e+06
172:172
135:135
18:18
FCENPDEVKpc2_NP.4
p−maj=1, pX=0.89, N=325
var.3.F5 1:169511555:T:C
peptide level
0
100000
200000
300000
172:172
135:135
18:18
FCENPDEVKpc2_NP.5
p−maj=1, pX=0.47, N=325

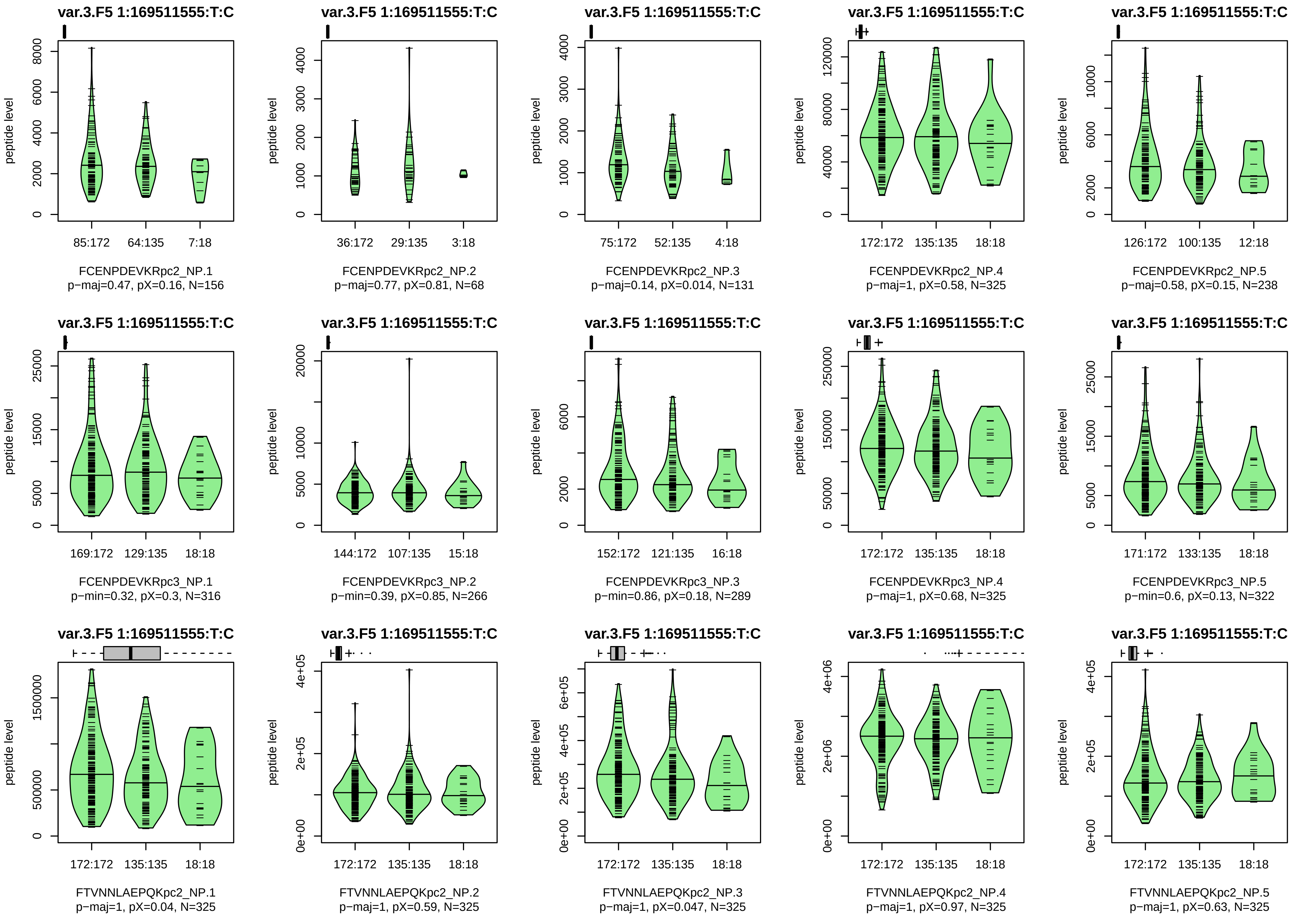

var.3.F5 1:169511555:T:C
peptide level
85:172
64:135
7:18
FCENPDEVKRpc2_NP.1
p−maj=0.47, pX=0.16, N=156
var.3.F5 1:169511555:T:C
peptide level
36:172
29:135
3:18
FCENPDEVKRpc2_NP.2
p−maj=0.77, pX=0.81, N=68
var.3.F5 1:169511555:T:C
peptide level
75:172
52:135
4:18
FCENPDEVKRpc2_NP.3
p−maj=0.14, pX=0.014, N=131
var.3.F5 1:169511555:T:C
peptide level
172:172
135:135
18:18
FCENPDEVKRpc2_NP.4
p−maj=1, pX=0.58, N=325
var.3.F5 1:169511555:T:C
peptide level
126:172
100:135
12:18
FCENPDEVKRpc2_NP.5
p−maj=0.58, pX=0.15, N=238
var.3.F5 1:169511555:T:C
peptide level
169:172
129:135
18:18
FCENPDEVKRpc3_NP.1
p−min=0.32, pX=0.3, N=316
var.3.F5 1:169511555:T:C
peptide level
144:172
107:135
15:18
FCENPDEVKRpc3_NP.2
p−min=0.39, pX=0.85, N=266
var.3.F5 1:169511555:T:C
peptide level
152:172
121:135
16:18
FCENPDEVKRpc3_NP.3
p−min=0.86, pX=0.18, N=289
var.3.F5 1:169511555:T:C
peptide level
172:172
135:135
18:18
FCENPDEVKRpc3_NP.4
p−maj=1, pX=0.68, N=325
var.3.F5 1:169511555:T:C
peptide level
171:172
133:135
18:18
FCENPDEVKRpc3_NP.5
p−min=0.6, pX=0.13, N=322
var.3.F5 1:169511555:T:C
peptide level
172:172
135:135
18:18
FTVNNLAEPQKpc2_NP.1
p−maj=1, pX=0.04, N=325
var.3.F5 1:169511555:T:C
peptide level
172:172
135:135
18:18
FTVNNLAEPQKpc2_NP.2
p−maj=1, pX=0.59, N=325
var.3.F5 1:169511555:T:C
peptide level
172:172
135:135
18:18
FTVNNLAEPQKpc2_NP.3
p−maj=1, pX=0.047, N=325
var.3.F5 1:169511555:T:C
peptide level
172:172
135:135
18:18
FTVNNLAEPQKpc2_NP.4
p−maj=1, pX=0.97, N=325
var.3.F5 1:169511555:T:C
peptide level
172:172
135:135
18:18
FTVNNLAEPQKpc2_NP.5
p−maj=1, pX=0.63, N=325

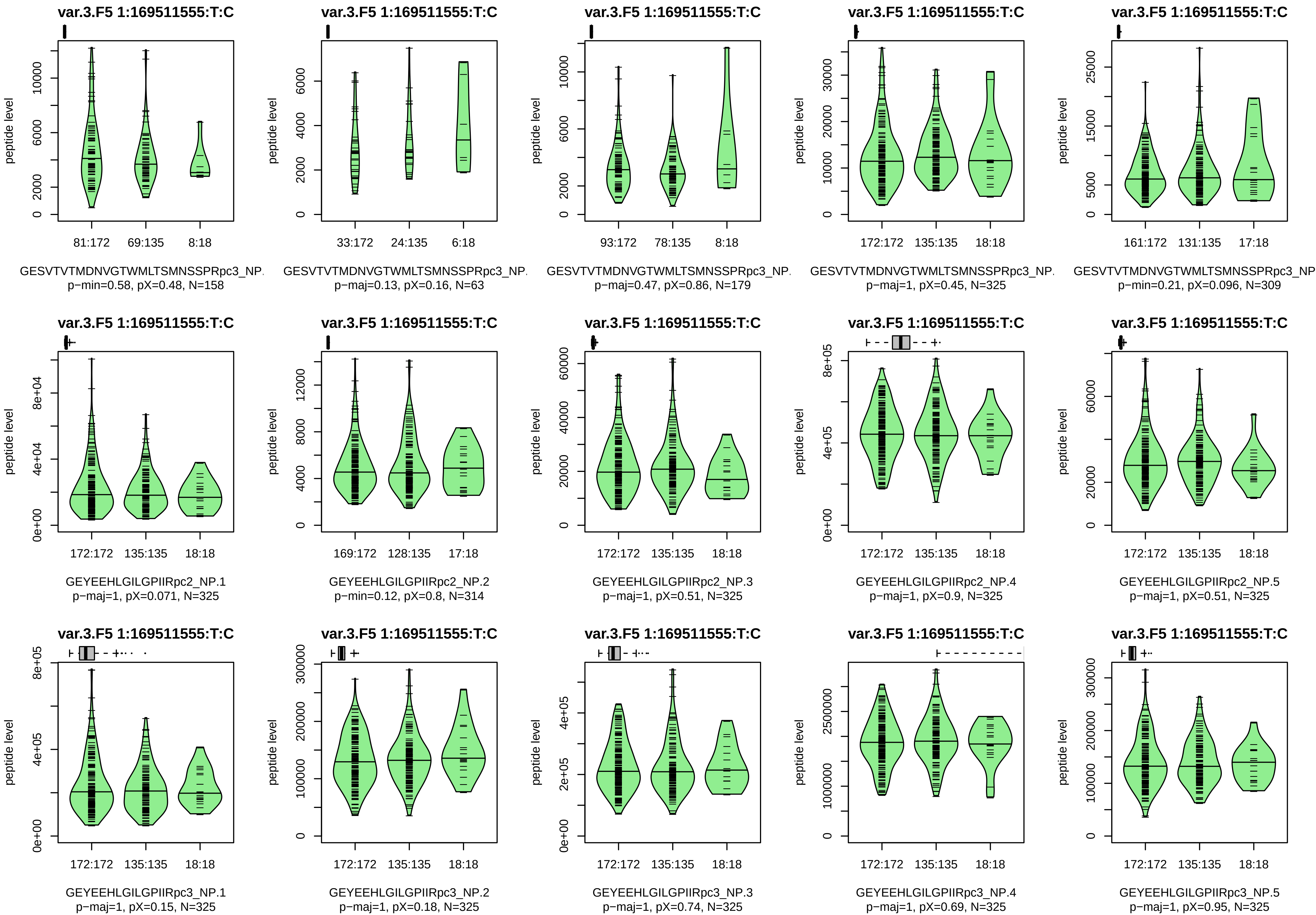
var.3.F5 1:169511555:T:C
peptide level
81:172
69:135
8:18
GESVTVTMDNVGTWMLTSMNSSPRpc3_NP.
p−min=0.58, pX=0.48, N=158
var.3.F5 1:169511555:T:C
peptide level
33:172
24:135
6:18
GESVTVTMDNVGTWMLTSMNSSPRpc3_NP.
p−maj=0.13, pX=0.16, N=63
var.3.F5 1:169511555:T:C
peptide level
93:172
78:135
8:18
GESVTVTMDNVGTWMLTSMNSSPRpc3_NP.
p−maj=0.47, pX=0.86, N=179
var.3.F5 1:169511555:T:C
peptide level
172:172
135:135
18:18
GESVTVTMDNVGTWMLTSMNSSPRpc3_NP.
p−maj=1, pX=0.45, N=325
var.3.F5 1:169511555:T:C
peptide level
161:172
131:135
17:18
GESVTVTMDNVGTWMLTSMNSSPRpc3_NP
p−min=0.21, pX=0.096, N=309
var.3.F5 1:169511555:T:C
peptide level
172:172
135:135
18:18
GEYEEHLGILGPIIRpc2_NP.1
p−maj=1, pX=0.071, N=325
var.3.F5 1:169511555:T:C
peptide level
169:172
128:135
17:18
GEYEEHLGILGPIIRpc2_NP.2
p−min=0.12, pX=0.8, N=314
var.3.F5 1:169511555:T:C
peptide level
172:172
135:135
18:18
GEYEEHLGILGPIIRpc2_NP.3
p−maj=1, pX=0.51, N=325
var.3.F5 1:169511555:T:C
peptide level
172:172
135:135
18:18
GEYEEHLGILGPIIRpc2_NP.4
p−maj=1, pX=0.9, N=325
var.3.F5 1:169511555:T:C
peptide level
172:172
135:135
18:18
GEYEEHLGILGPIIRpc2_NP.5
p−maj=1, pX=0.51, N=325
var.3.F5 1:169511555:T:C
peptide level
172:172
135:135
18:18
GEYEEHLGILGPIIRpc3_NP.1
p−maj=1, pX=0.15, N=325
var.3.F5 1:169511555:T:C
peptide level
172:172
135:135
18:18
GEYEEHLGILGPIIRpc3_NP.2
p−maj=1, pX=0.18, N=325
var.3.F5 1:169511555:T:C
peptide level
172:172
135:135
18:18
GEYEEHLGILGPIIRpc3_NP.3
p−maj=1, pX=0.74, N=325
var.3.F5 1:169511555:T:C
peptide level
172:172
135:135
18:18
GEYEEHLGILGPIIRpc3_NP.4
p−maj=1, pX=0.69, N=325
var.3.F5 1:169511555:T:C
peptide level
172:172
135:135
18:18
GEYEEHLGILGPIIRpc3_NP.5
p−maj=1, pX=0.95, N=325

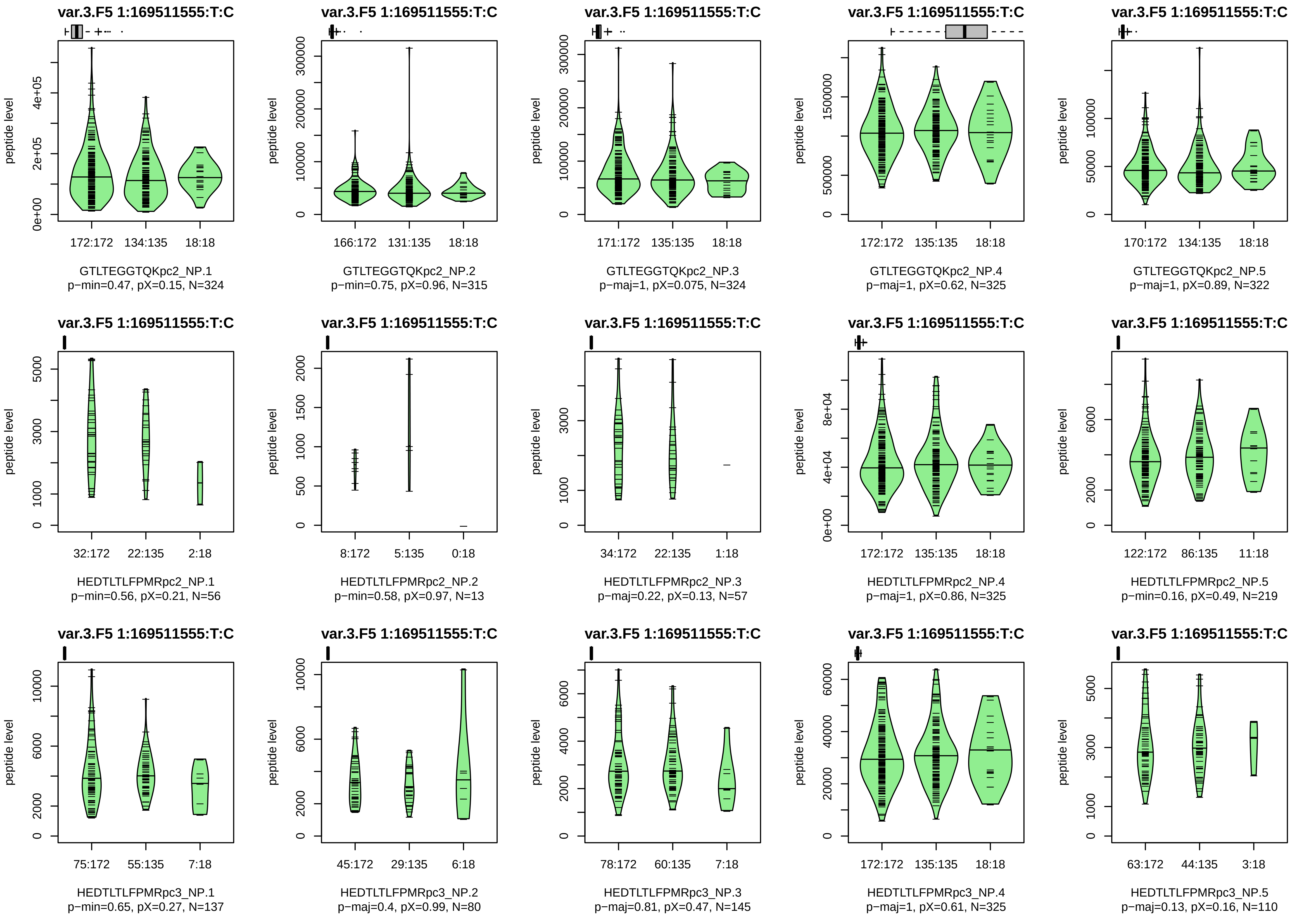

var.3.F5 1:169511555:T:C
peptide level
0e+00
2e+05
4e+05
172:172
134:135
18:18
GTLTEGGTQKpc2_NP.1
p−min=0.47, pX=0.15, N=324
var.3.F5 1:169511555:T:C
peptide level
0
100000
200000
300000
166:172
131:135
18:18
GTLTEGGTQKpc2_NP.2
p−min=0.75, pX=0.96, N=315
var.3.F5 1:169511555:T:C
peptide level
0
100000
200000
300000
171:172
135:135
18:18
GTLTEGGTQKpc2_NP.3
p−maj=1, pX=0.075, N=324
var.3.F5 1:169511555:T:C
peptide level
0
500000
1500000
172:172
135:135
18:18
GTLTEGGTQKpc2_NP.4
p−maj=1, pX=0.62, N=325
var.3.F5 1:169511555:T:C
peptide level
0
50000
100000
170:172
134:135
18:18
GTLTEGGTQKpc2_NP.5
p−maj=1, pX=0.89, N=322
var.3.F5 1:169511555:T:C
peptide level
0
1000
3000
5000
32:172
22:135
2:18
HEDTLTLFPMRpc2_NP.1
p−min=0.56, pX=0.21, N=56
var.3.F5 1:169511555:T:C
peptide level
0
500
1000
1500
2000
8:172
5:135
0:18
HEDTLTLFPMRpc2_NP.2
p−min=0.58, pX=0.97, N=13
var.3.F5 1:169511555:T:C
peptide level
0
1000
3000
34:172
22:135
1:18
HEDTLTLFPMRpc2_NP.3
p−maj=0.22, pX=0.13, N=57
var.3.F5 1:169511555:T:C
peptide level
0e+00
4e+04
8e+04
172:172
135:135
18:18
HEDTLTLFPMRpc2_NP.4
p−maj=1, pX=0.86, N=325
var.3.F5 1:169511555:T:C
peptide level
0
2000
6000
122:172
86:135
11:18
HEDTLTLFPMRpc2_NP.5
p−min=0.16, pX=0.49, N=219
var.3.F5 1:169511555:T:C
peptide level
0
2000
6000
10000
75:172
55:135
7:18
HEDTLTLFPMRpc3_NP.1
p−min=0.65, pX=0.27, N=137
var.3.F5 1:169511555:T:C
peptide level
0
2000
6000
10000
45:172
29:135
6:18
HEDTLTLFPMRpc3_NP.2
p−maj=0.4, pX=0.99, N=80
var.3.F5 1:169511555:T:C
peptide level
0
2000
4000
6000
78:172
60:135
7:18
HEDTLTLFPMRpc3_NP.3
p−maj=0.81, pX=0.47, N=145
var.3.F5 1:169511555:T:C
peptide level
0
20000
40000
60000
172:172
135:135
18:18
HEDTLTLFPMRpc3_NP.4
p−maj=1, pX=0.61, N=325
var.3.F5 1:169511555:T:C
peptide level
0
1000
3000
5000
63:172
44:135
3:18
HEDTLTLFPMRpc3_NP.5
p−maj=0.13, pX=0.16, N=110

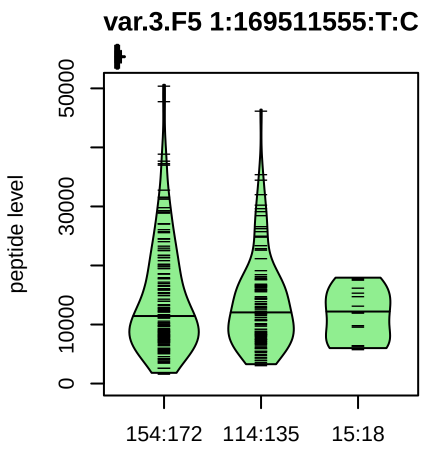

HLQAGMQAYIDIKpc2_NP.1
p−min=0.19, pX=0.085, N=283

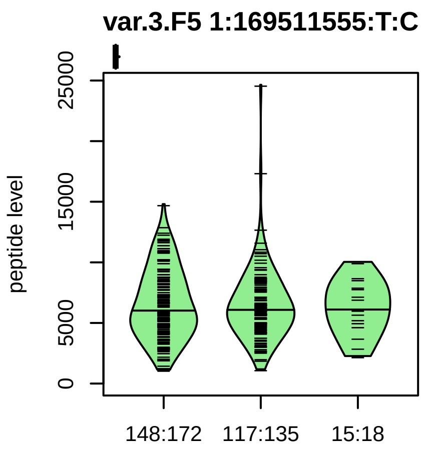

HLQAGMQAYIDIKpc2_NP.2
p−maj=0.72, pX=0.75, N=280

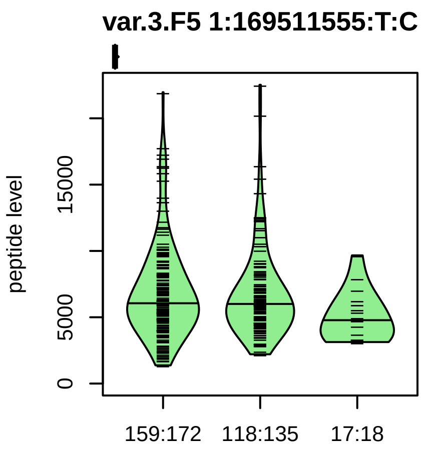

HLQAGMQAYIDIKpc2_NP.3
p−min=0.26, pX=0.073, N=294

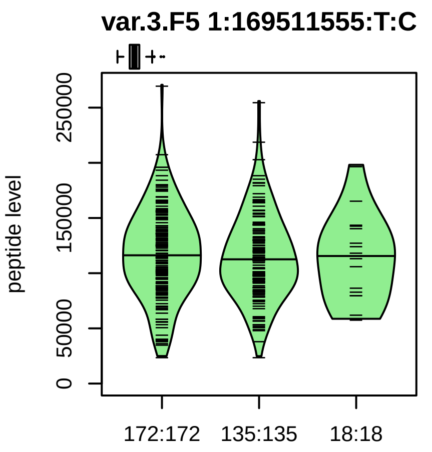

HLQAGMQAYIDIKpc2_NP.4
p−maj=1, pX=0.45, N=325

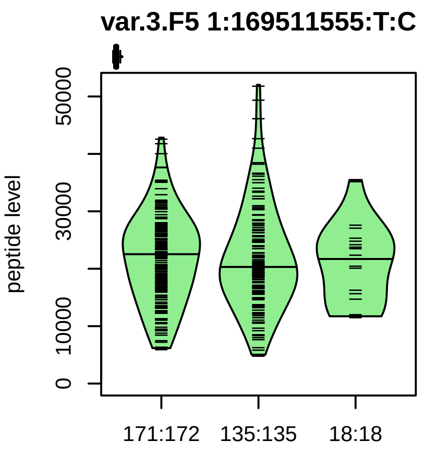

HLQAGMQAYIDIKpc2_NP.5
p−maj=1, pX=0.82, N=324

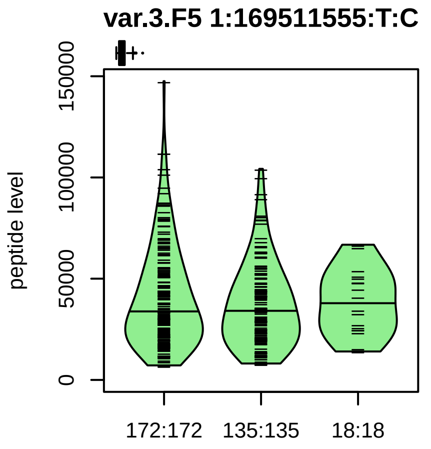

HLQAGMQAYIDIKpc3_NP.1
p−maj=1, pX=0.24, N=325

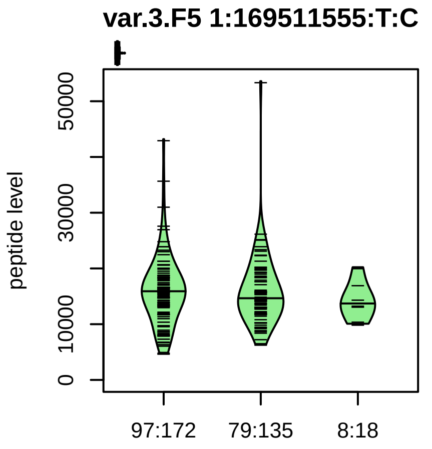

HLQAGMQAYIDIKpc3_NP.2
p−maj=0.33, pX=0.58, N=184

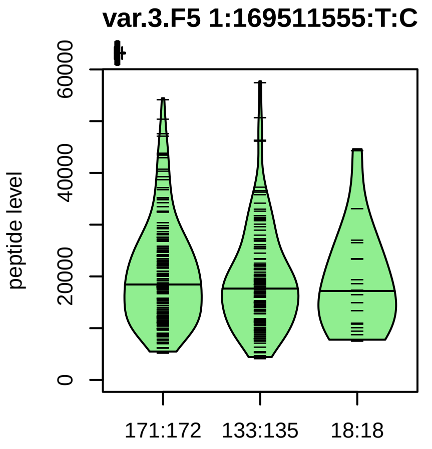

HLQAGMQAYIDIKpc3_NP.3
p−min=0.6, pX=0.19, N=322

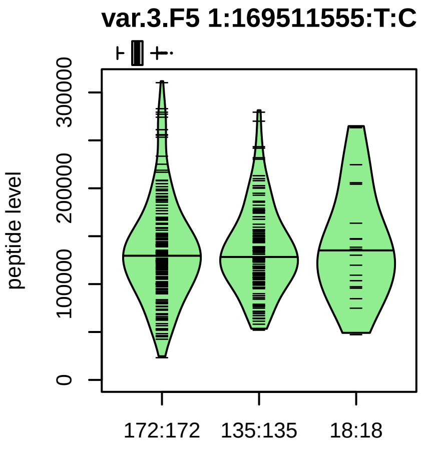

HLQAGMQAYIDIKpc3_NP.4
p−maj=1, pX=0.75, N=325

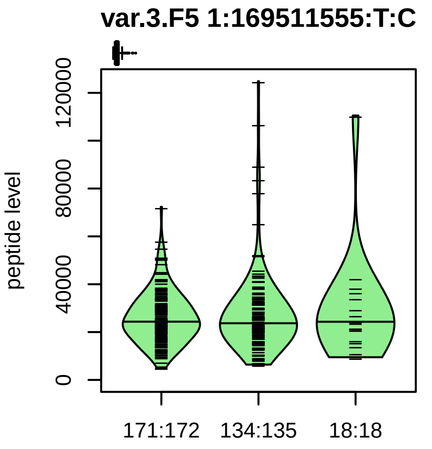

HLQAGMQAYIDIKpc3_NP.5
p−min=1, pX=0.23, N=323

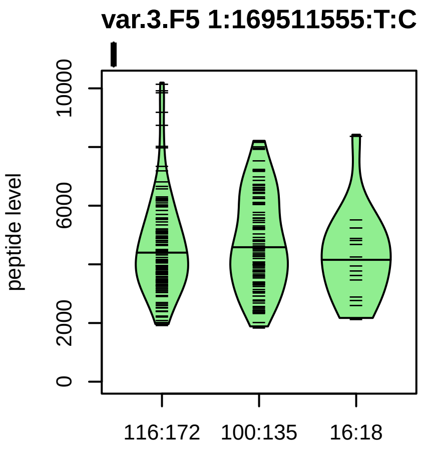

HLSQDTGSPSGMRpc2_NP.4
p−min=0.11, pX=0.11, N=232

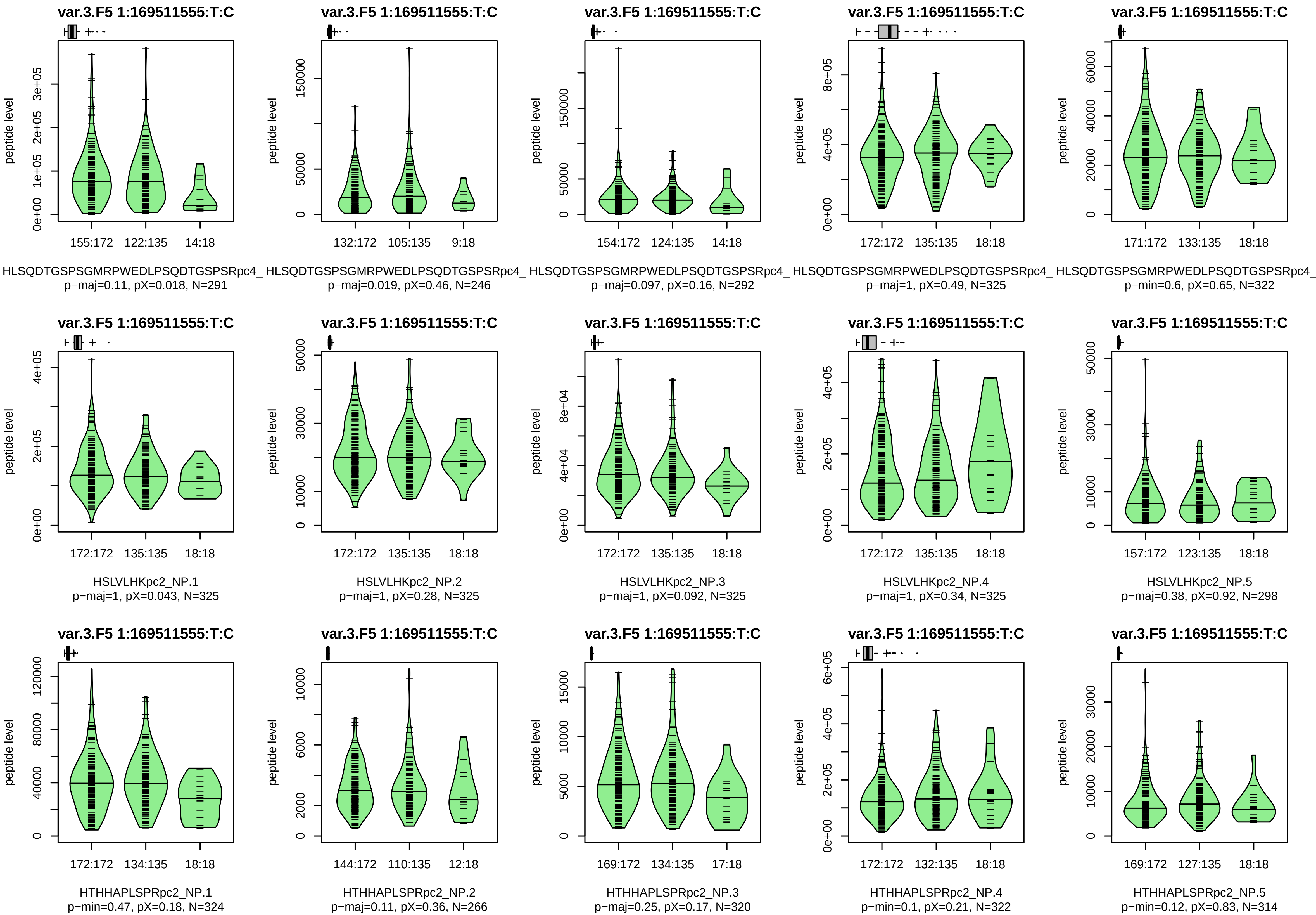

var.3.F5 1:169511555:T:C
peptide level
155:172
122:135
14:18
HLSQDTGSPSGMRPWEDLPSQDTGSPSRpc4_
p−maj=0.11, pX=0.018, N=291
var.3.F5 1:169511555:T:C
peptide level
132:172
105:135
9:18
HLSQDTGSPSGMRPWEDLPSQDTGSPSRpc4_
p−maj=0.019, pX=0.46, N=246
var.3.F5 1:169511555:T:C
peptide level
154:172
124:135
14:18
HLSQDTGSPSGMRPWEDLPSQDTGSPSRpc4_
p−maj=0.097, pX=0.16, N=292
var.3.F5 1:169511555:T:C
peptide level
172:172
135:135
18:18
HLSQDTGSPSGMRPWEDLPSQDTGSPSRpc4_
p−maj=1, pX=0.49, N=325
var.3.F5 1:169511555:T:C
peptide level
171:172
133:135
18:18
HLSQDTGSPSGMRPWEDLPSQDTGSPSRpc4_
p−min=0.6, pX=0.65, N=322
var.3.F5 1:169511555:T:C
peptide level
172:172
135:135
18:18
HSLVLHKpc2_NP.1
p−maj=1, pX=0.043, N=325
var.3.F5 1:169511555:T:C
peptide level
172:172
135:135
18:18
HSLVLHKpc2_NP.2
p−maj=1, pX=0.28, N=325
var.3.F5 1:169511555:T:C
peptide level
172:172
135:135
18:18
HSLVLHKpc2_NP.3
p−maj=1, pX=0.092, N=325
var.3.F5 1:169511555:T:C
peptide level
172:172
135:135
18:18
HSLVLHKpc2_NP.4
p−maj=1, pX=0.34, N=325
var.3.F5 1:169511555:T:C
peptide level
157:172
123:135
18:18
HSLVLHKpc2_NP.5
p−maj=0.38, pX=0.92, N=298
var.3.F5 1:169511555:T:C
peptide level
172:172
134:135
18:18
HTHHAPLSPRpc2_NP.1
p−min=0.47, pX=0.18, N=324
var.3.F5 1:169511555:T:C
peptide level
144:172
110:135
12:18
HTHHAPLSPRpc2_NP.2
p−maj=0.11, pX=0.36, N=266
var.3.F5 1:169511555:T:C
peptide level
169:172
134:135
17:18
HTHHAPLSPRpc2_NP.3
p−maj=0.25, pX=0.17, N=320
var.3.F5 1:169511555:T:C
peptide level
172:172
132:135
18:18
HTHHAPLSPRpc2_NP.4
p−min=0.1, pX=0.21, N=322
var.3.F5 1:169511555:T:C
peptide level
169:172
127:135
18:18
HTHHAPLSPRpc2_NP.5
p−min=0.12, pX=0.83, N=314

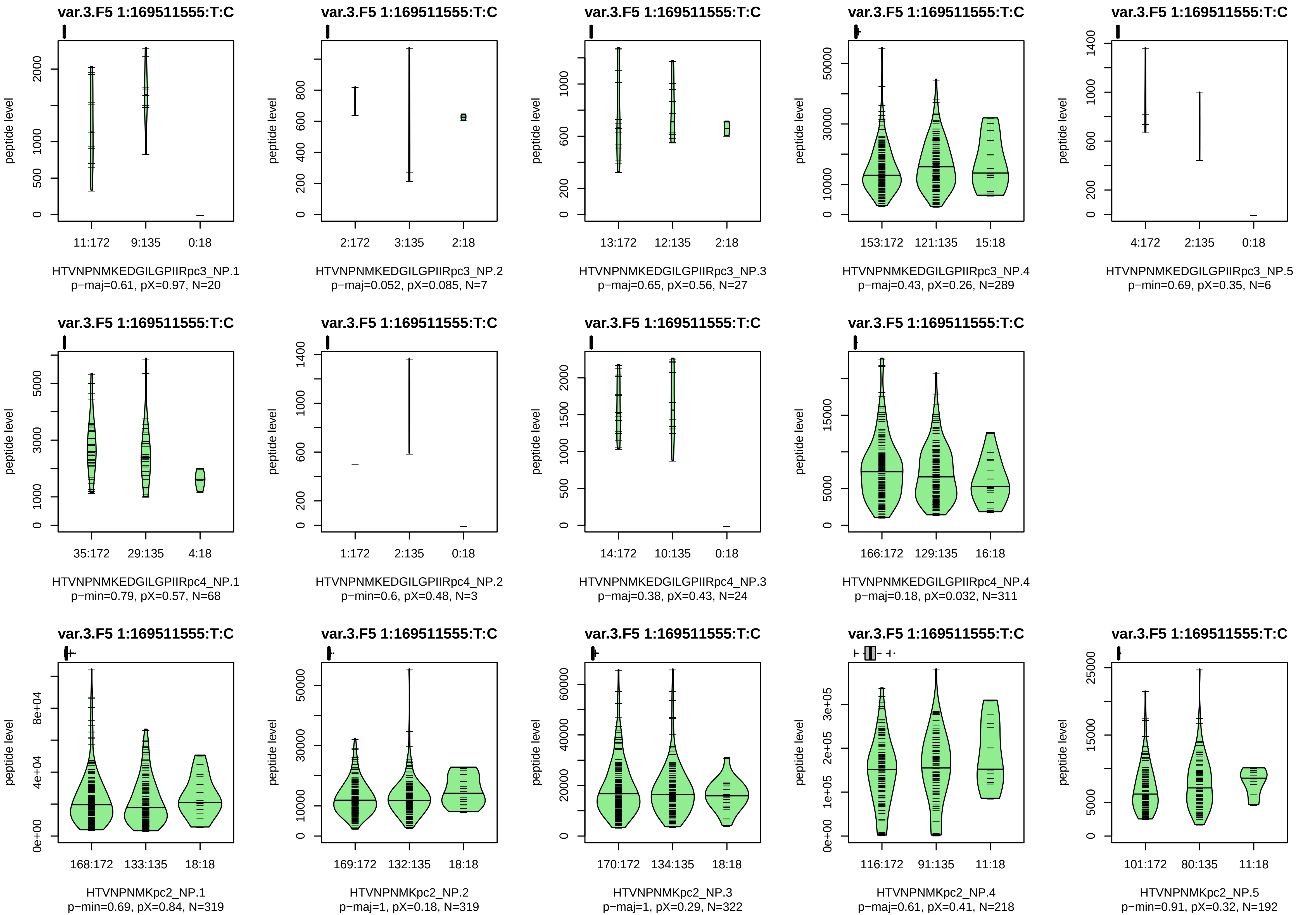
var.3.F5 1:169511555:T:C
peptide level
11:172
9:135
0:18
HTVNPNMKEDGILGPIIRpc3_NP.1
p−maj=0.61, pX=0.97, N=20
var.3.F5 1:169511555:T:C
peptide level
2:172
3:135
2:18
HTVNPNMKEDGILGPIIRpc3_NP.2
p−maj=0.052, pX=0.085, N=7
var.3.F5 1:169511555:T:C
peptide level
13:172
12:135
2:18
HTVNPNMKEDGILGPIIRpc3_NP.3
p−maj=0.65, pX=0.56, N=27
var.3.F5 1:169511555:T:C
peptide level
153:172
121:135
15:18
HTVNPNMKEDGILGPIIRpc3_NP.4
p−maj=0.43, pX=0.26, N=289
var.3.F5 1:169511555:T:C
peptide level
4:172
2:135
0:18
HTVNPNMKEDGILGPIIRpc3_NP.5
p−min=0.69, pX=0.35, N=6
var.3.F5 1:169511555:T:C
peptide level
35:172
29:135
4:18
HTVNPNMKEDGILGPIIRpc4_NP.1
p−min=0.79, pX=0.57, N=68
var.3.F5 1:169511555:T:C
peptide level
1:172
2:135
0:18
HTVNPNMKEDGILGPIIRpc4_NP.2
p−min=0.6, pX=0.48, N=3
var.3.F5 1:169511555:T:C
peptide level
14:172
10:135
0:18
HTVNPNMKEDGILGPIIRpc4_NP.3
p−maj=0.38, pX=0.43, N=24
var.3.F5 1:169511555:T:C
peptide level
166:172
129:135
16:18
HTVNPNMKEDGILGPIIRpc4_NP.4
p−maj=0.18, pX=0.032, N=311
var.3.F5 1:169511555:T:C
peptide level
168:172
133:135
18:18
HTVNPNMKpc2_NP.1
p−min=0.69, pX=0.84, N=319
var.3.F5 1:169511555:T:C
peptide level
169:172
132:135
18:18
HTVNPNMKpc2_NP.2
p−maj=1, pX=0.18, N=319
var.3.F5 1:169511555:T:C
peptide level
170:172
134:135
18:18
HTVNPNMKpc2_NP.3
p−maj=1, pX=0.29, N=322
var.3.F5 1:169511555:T:C
peptide level
116:172
91:135
11:18
HTVNPNMKpc2_NP.4
p−maj=0.61, pX=0.41, N=218
var.3.F5 1:169511555:T:C
peptide level
101:172
80:135
11:18
HTVNPNMKpc2_NP.5
p−min=0.91, pX=0.32, N=192

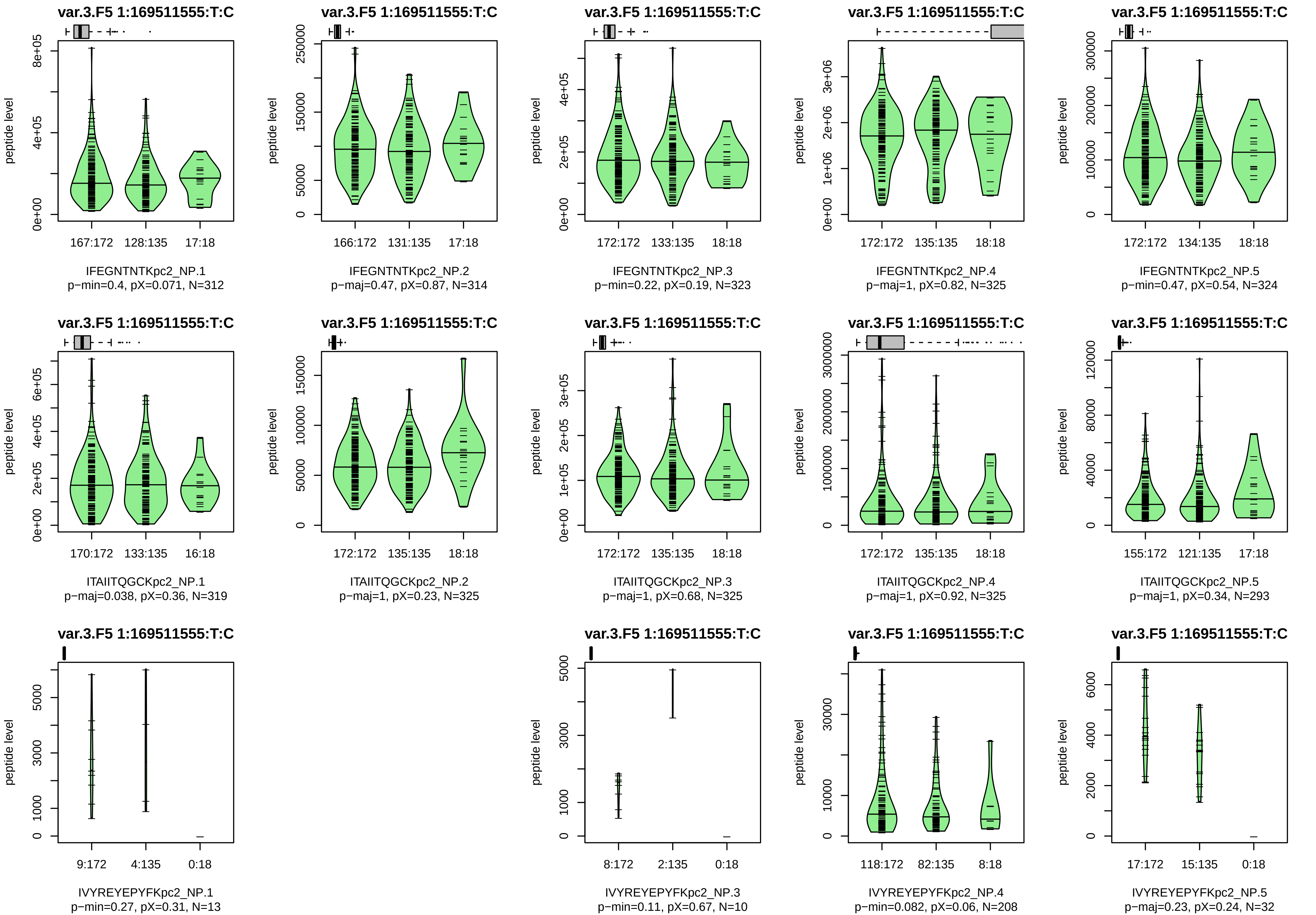

var.3.F5 1:169511555:T:C
peptide level
167:172
128:135
17:18
IFEGNTNTKpc2_NP.1
p−min=0.4, pX=0.071, N=312
var.3.F5 1:169511555:T:C
peptide level
166:172
131:135
17:18
IFEGNTNTKpc2_NP.2
p−maj=0.47, pX=0.87, N=314
var.3.F5 1:169511555:T:C
peptide level
172:172
133:135
18:18
IFEGNTNTKpc2_NP.3
p−min=0.22, pX=0.19, N=323
var.3.F5 1:169511555:T:C
peptide level
172:172
135:135
18:18
IFEGNTNTKpc2_NP.4
p−maj=1, pX=0.82, N=325
var.3.F5 1:169511555:T:C
peptide level
172:172
134:135
18:18
IFEGNTNTKpc2_NP.5
p−min=0.47, pX=0.54, N=324
var.3.F5 1:169511555:T:C
peptide level
170:172
133:135
16:18
ITAIITQGCKpc2_NP.1
p−maj=0.038, pX=0.36, N=319
var.3.F5 1:169511555:T:C
peptide level
172:172
135:135
18:18
ITAIITQGCKpc2_NP.2
p−maj=1, pX=0.23, N=325
var.3.F5 1:169511555:T:C
peptide level
172:172
135:135
18:18
ITAIITQGCKpc2_NP.3
p−maj=1, pX=0.68, N=325
var.3.F5 1:169511555:T:C
peptide level
172:172
135:135
18:18
ITAIITQGCKpc2_NP.4
p−maj=1, pX=0.92, N=325
var.3.F5 1:169511555:T:C
peptide level
155:172
121:135
17:18
ITAIITQGCKpc2_NP.5
p−maj=1, pX=0.34, N=293
var.3.F5 1:169511555:T:C
peptide level
9:172
4:135
0:18
IVYREYEPYFKpc2_NP.1
p−min=0.27, pX=0.31, N=13
var.3.F5 1:169511555:T:C
peptide level
8:172
2:135
0:18
IVYREYEPYFKpc2_NP.3
p−min=0.11, pX=0.67, N=10
var.3.F5 1:169511555:T:C
peptide level
118:172
82:135
8:18
IVYREYEPYFKpc2_NP.4
p−min=0.082, pX=0.06, N=208
var.3.F5 1:169511555:T:C
peptide level
17:172
15:135
0:18
IVYREYEPYFKpc2_NP.5
p−maj=0.23, pX=0.24, N=32

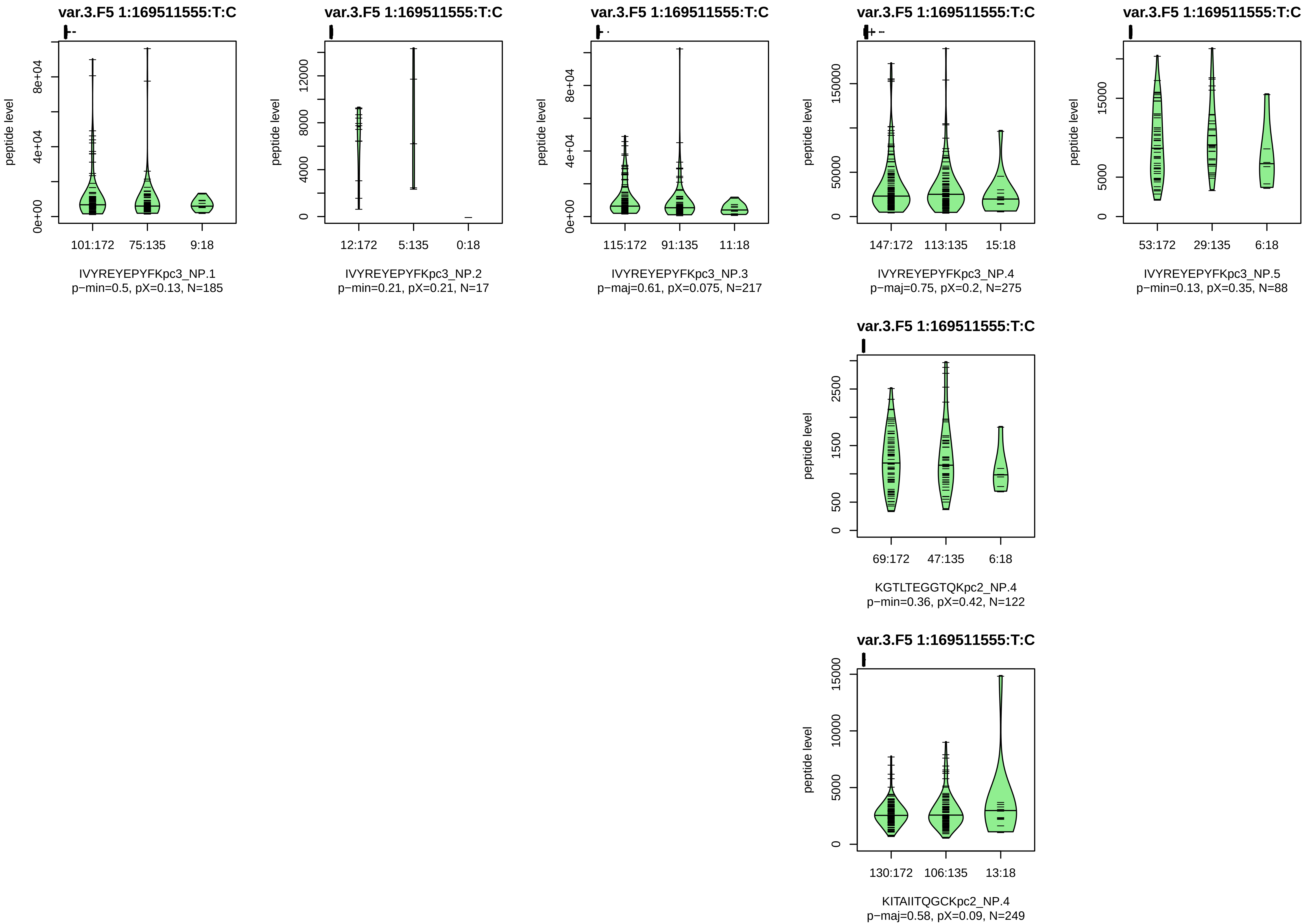
var.3.F5 1:169511555:T:C
peptide level
0e+00
4e+04
8e+04
101:172
75:135
9:18
IVYREYEPYFKpc3_NP.1
p−min=0.5, pX=0.13, N=185
var.3.F5 1:169511555:T:C
peptide level
0
4000
8000
12000
12:172
5:135
0:18
IVYREYEPYFKpc3_NP.2
p−min=0.21, pX=0.21, N=17
var.3.F5 1:169511555:T:C
peptide level
0e+00
4e+04
8e+04
115:172
91:135
11:18
IVYREYEPYFKpc3_NP.3
p−maj=0.61, pX=0.075, N=217
var.3.F5 1:169511555:T:C
peptide level
0
50000
150000
147:172
113:135
15:18
IVYREYEPYFKpc3_NP.4
p−maj=0.75, pX=0.2, N=275
var.3.F5 1:169511555:T:C
peptide level
0
5000
15000
53:172
29:135
6:18
IVYREYEPYFKpc3_NP.5
p−min=0.13, pX=0.35, N=88
var.3.F5 1:169511555:T:C
peptide level
0
500
1500
2500
69:172
47:135
6:18
KGTLTEGGTQKpc2_NP.4
p−min=0.36, pX=0.42, N=122
var.3.F5 1:169511555:T:C
peptide level
0
5000
10000
15000
130:172
106:135
13:18
KITAIITQGCKpc2_NP.4
p−maj=0.58, pX=0.09, N=249

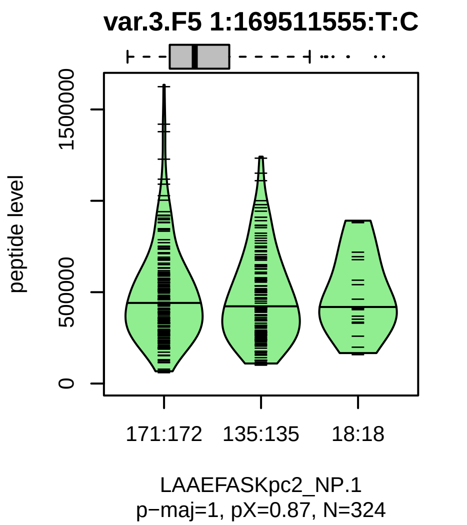
var.3.F5 1:169511555:T:C
peptide level
171:172
135:135
18:18
LAAEFASKpc2_NP.1
p−maj=1, pX=0.87, N=324

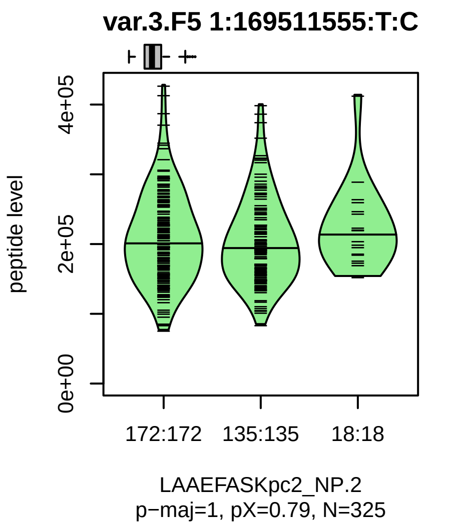
var.3.F5 1:169511555:T:C
peptide level
172:172
135:135
18:18
LAAEFASKpc2_NP.2
p−maj=1, pX=0.79, N=325

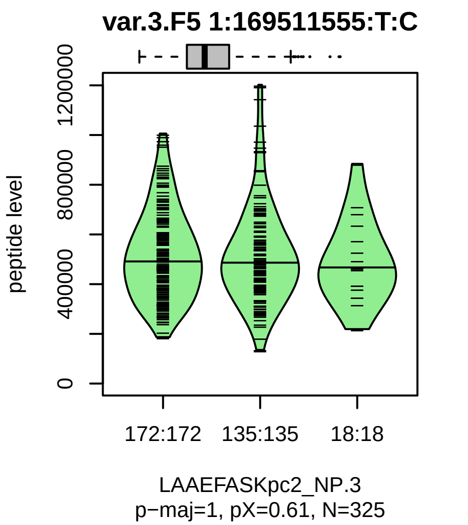
var.3.F5 1:169511555:T:C
peptide level
172:172
135:135
18:18
LAAEFASKpc2_NP.3
p−maj=1, pX=0.61, N=325

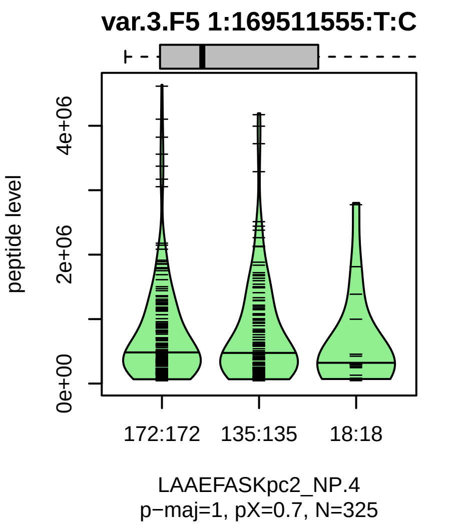
var.3.F5 1:169511555:T:C
peptide level
172:172
135:135
18:18
LAAEFASKpc2_NP.4
p−maj=1, pX=0.7, N=325

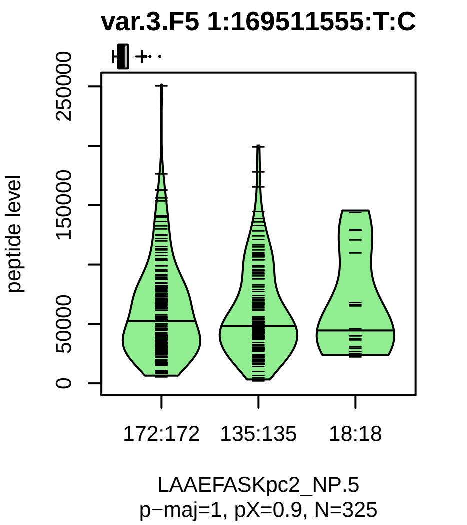
var.3.F5 1:169511555:T:C
peptide level
172:172
135:135
18:18
LAAEFASKpc2_NP.5
p−maj=1, pX=0.9, N=325

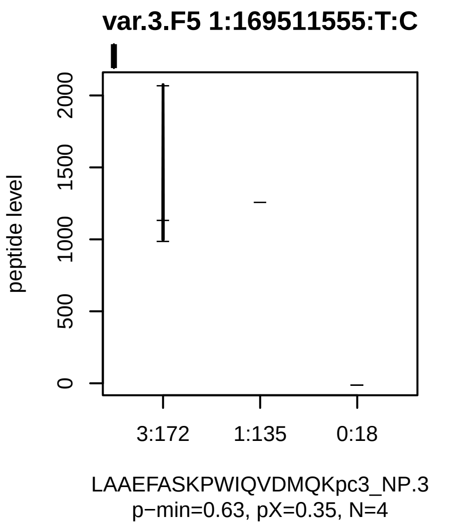
var.3.F5 1:169511555:T:C
peptide level
3:172
1:135
0:18
LAAEFASKPWIQVDMQKpc3_NP.3
p−min=0.63, pX=0.35, N=4

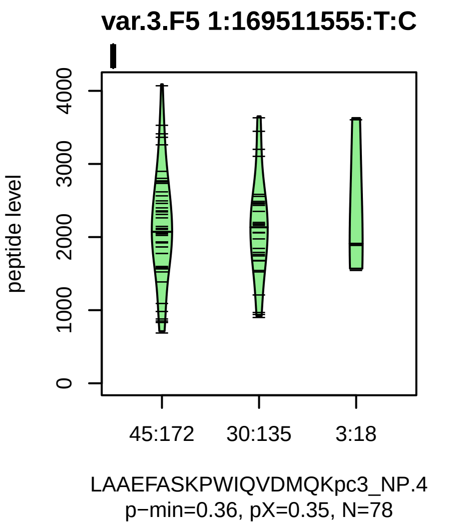
var.3.F5 1:169511555:T:C
peptide level
45:172
30:135
3:18
LAAEFASKPWIQVDMQKpc3_NP.4
p−min=0.36, pX=0.35, N=78

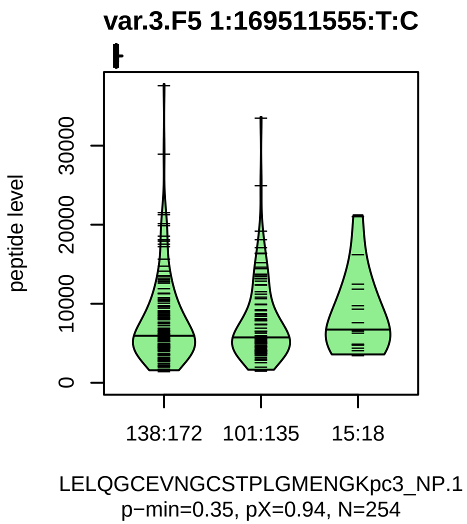
var.3.F5 1:169511555:T:C
peptide level
138:172
101:135
15:18
LELQGCEVNGCSTPLGMENGKpc3_NP.1
p−min=0.35, pX=0.94, N=254

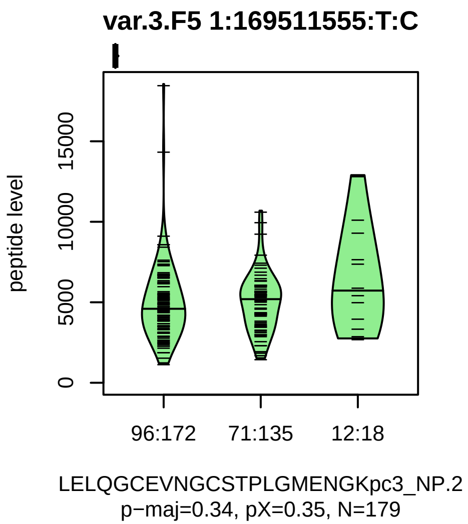
var.3.F5 1:169511555:T:C
peptide level
96:172
71:135
12:18
LELQGCEVNGCSTPLGMENGKpc3_NP.2
p−maj=0.34, pX=0.35, N=179

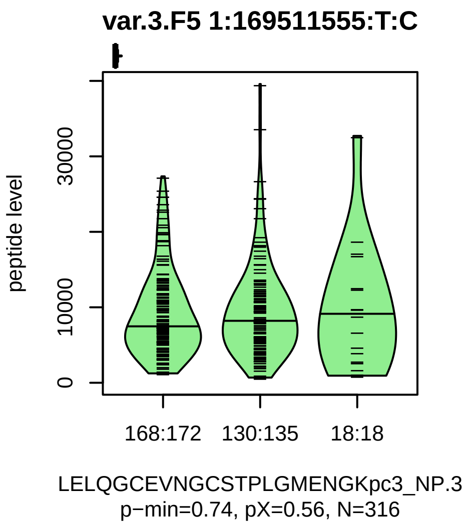
var.3.F5 1:169511555:T:C
peptide level
168:172
130:135
18:18
LELQGCEVNGCSTPLGMENGKpc3_NP.3
p−min=0.74, pX=0.56, N=316

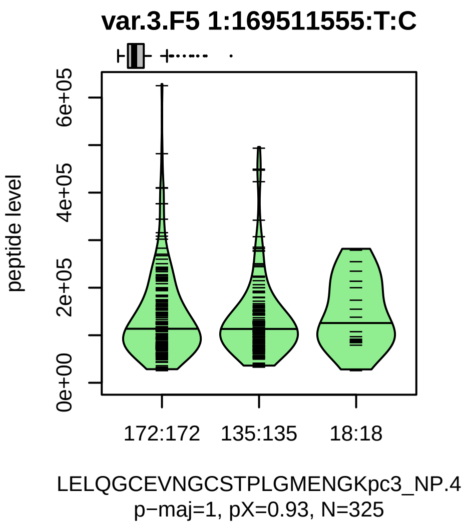
var.3.F5 1:169511555:T:C
peptide level
172:172
135:135
18:18
LELQGCEVNGCSTPLGMENGKpc3_NP.4
p−maj=1, pX=0.93, N=325

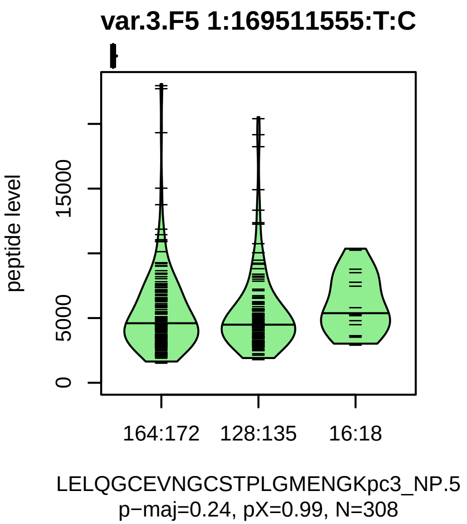
var.3.F5 1:169511555:T:C
peptide level
164:172
128:135
16:18
LELQGCEVNGCSTPLGMENGKpc3_NP.5
p−maj=0.24, pX=0.99, N=308

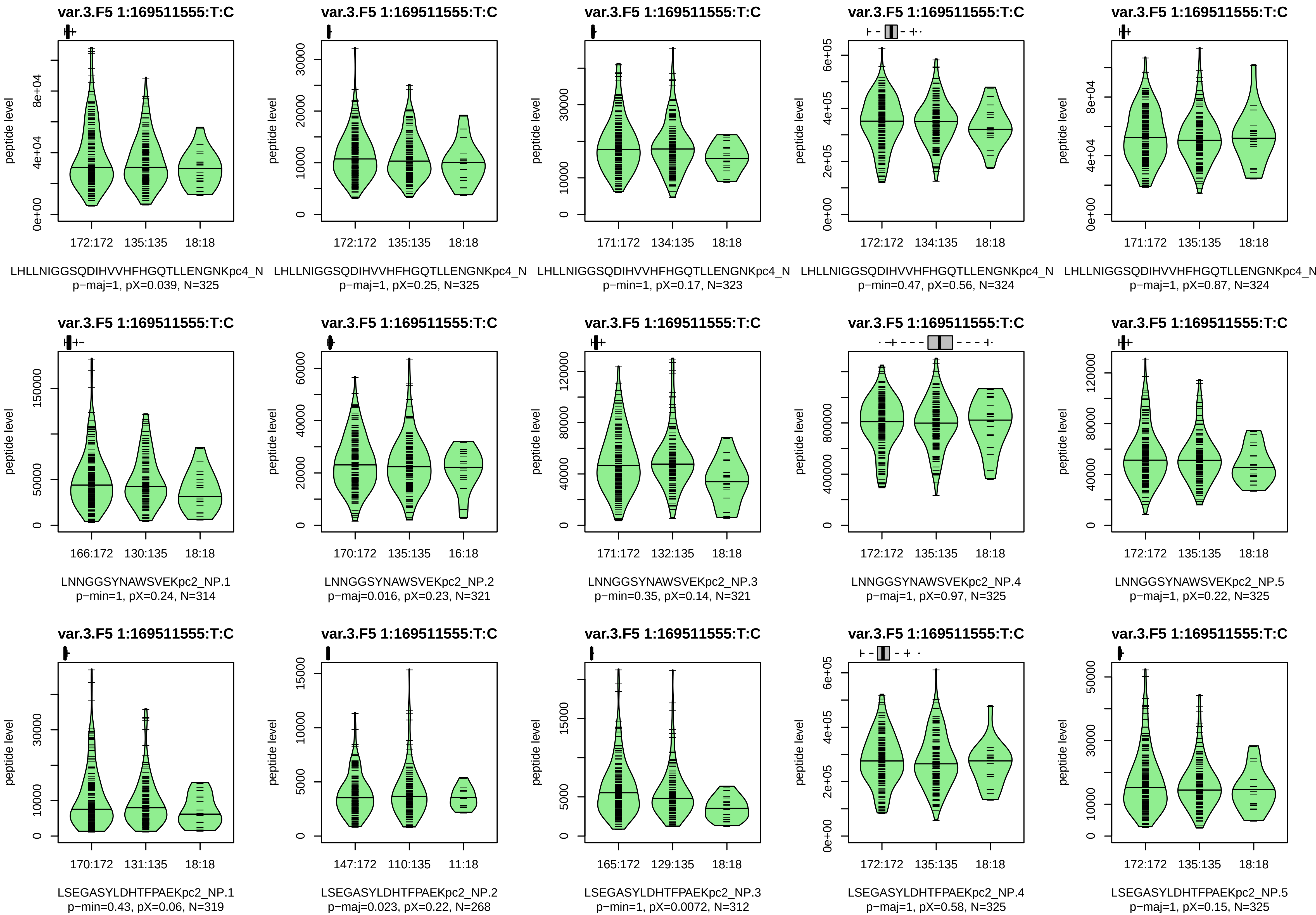
var.3.F5 1:169511555:T:C
peptide level
172:172 135:135 18:18
LHLLNIGGSQDIHVVHFHGQTLLENGNKpc4_N
p−maj=1, pX=0.039, N=325
var.3.F5 1:169511555:T:C
peptide level
172:172 135:135 18:18
LHLLNIGGSQDIHVVHFHGQTLLENGNKpc4_N
p−maj=1, pX=0.25, N=325
var.3.F5 1:169511555:T:C
peptide level
171:172 134:135 18:18
LHLLNIGGSQDIHVVHFHGQTLLENGNKpc4_N
p−min=1, pX=0.17, N=323
var.3.F5 1:169511555:T:C
peptide level
172:172 134:135 18:18
LHLLNIGGSQDIHVVHFHGQTLLENGNKpc4_N
p−min=0.47, pX=0.56, N=324
var.3.F5 1:169511555:T:C
peptide level
171:172 135:135 18:18
LHLLNIGGSQDIHVVHFHGQTLLENGNKpc4_N
p−maj=1, pX=0.87, N=324
var.3.F5 1:169511555:T:C
peptide level
166:172 130:135 18:18
LNNGGSYNAWSVEKpc2_NP.1
p−min=1, pX=0.24, N=314
var.3.F5 1:169511555:T:C
peptide level
170:172 135:135 16:18
LNNGGSYNAWSVEKpc2_NP.2
p−maj=0.016, pX=0.23, N=321
var.3.F5 1:169511555:T:C
peptide level
171:172 132:135 18:18
LNNGGSYNAWSVEKpc2_NP.3
p−min=0.35, pX=0.14, N=321
var.3.F5 1:169511555:T:C
peptide level
172:172 135:135 18:18
LNNGGSYNAWSVEKpc2_NP.4
p−maj=1, pX=0.97, N=325
var.3.F5 1:169511555:T:C
peptide level
172:172 135:135 18:18
LNNGGSYNAWSVEKpc2_NP.5
p−maj=1, pX=0.22, N=325
var.3.F5 1:169511555:T:C
peptide level
170:172 131:135 18:18
LSEGASYLDHTFPAEKpc2_NP.1
p−min=0.43, pX=0.06, N=319
var.3.F5 1:169511555:T:C
peptide level
147:172 110:135 11:18
LSEGASYLDHTFPAEKpc2_NP.2
p−maj=0.023, pX=0.22, N=268
var.3.F5 1:169511555:T:C
peptide level
165:172 129:135 18:18
LSEGASYLDHTFPAEKpc2_NP.3
p−min=1, pX=0.0072, N=312
var.3.F5 1:169511555:T:C
peptide level
172:172 135:135 18:18
LSEGASYLDHTFPAEKpc2_NP.4
p−maj=1, pX=0.58, N=325
var.3.F5 1:169511555:T:C
peptide level
172:172 135:135 18:18
LSEGASYLDHTFPAEKpc2_NP.5
p−maj=1, pX=0.15, N=325

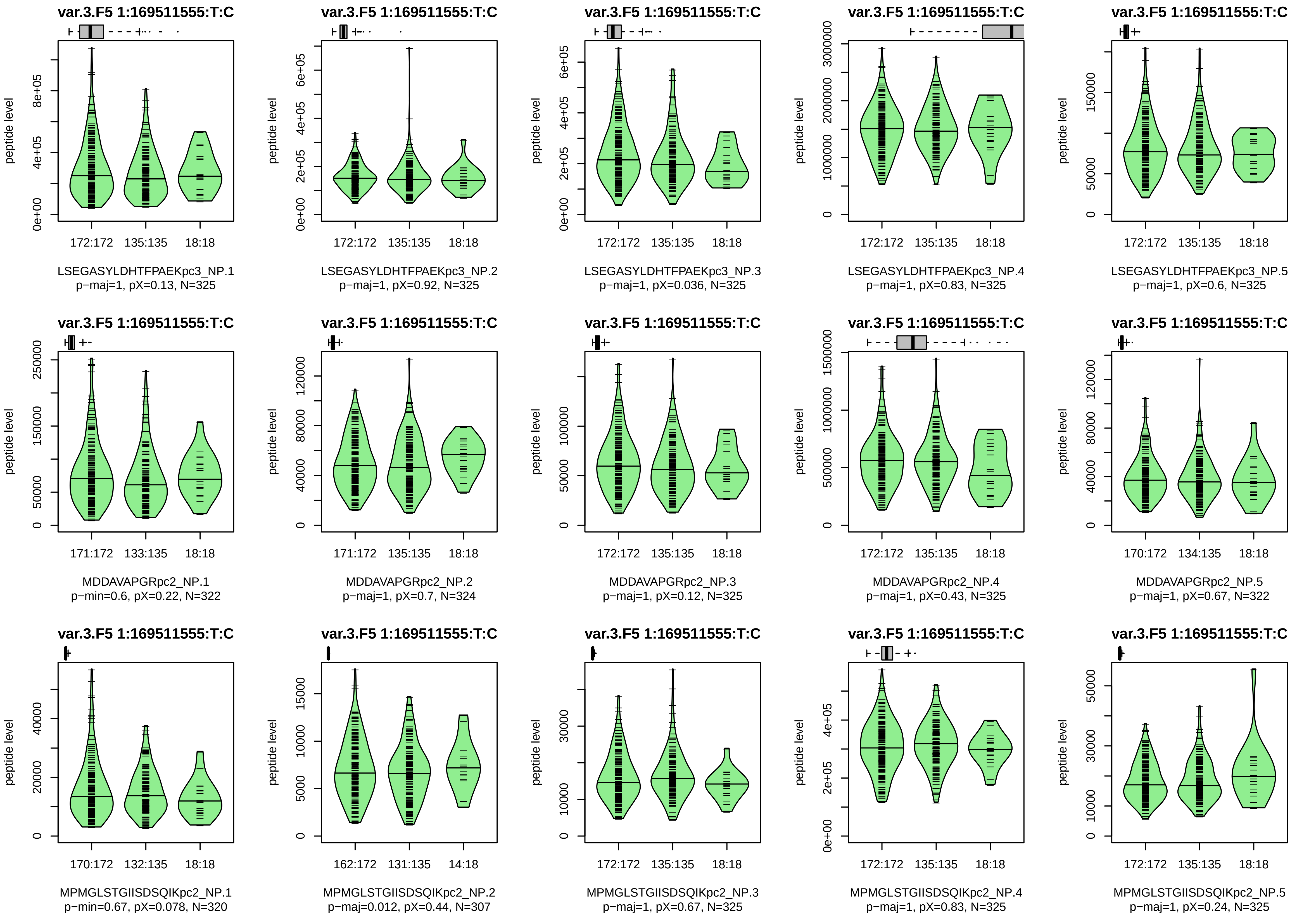

var.3.F5 1:169511555:T:C
peptide level
172:172 135:135 18:18
LSEGASYLDHTFPAEKpc3_NP.1
p−maj=1, pX=0.13, N=325
var.3.F5 1:169511555:T:C
peptide level
172:172 135:135 18:18
LSEGASYLDHTFPAEKpc3_NP.2
p−maj=1, pX=0.92, N=325
var.3.F5 1:169511555:T:C
peptide level
172:172 135:135 18:18
LSEGASYLDHTFPAEKpc3_NP.3
p−maj=1, pX=0.036, N=325
var.3.F5 1:169511555:T:C
peptide level
172:172 135:135 18:18
LSEGASYLDHTFPAEKpc3_NP.4
p−maj=1, pX=0.83, N=325
var.3.F5 1:169511555:T:C
peptide level
172:172 135:135 18:18
LSEGASYLDHTFPAEKpc3_NP.5
p−maj=1, pX=0.6, N=325
var.3.F5 1:169511555:T:C
peptide level
171:172 133:135 18:18
MDDAVAPGRpc2_NP.1
p−min=0.6, pX=0.22, N=322
var.3.F5 1:169511555:T:C
peptide level
171:172 135:135 18:18
MDDAVAPGRpc2_NP.2
p−maj=1, pX=0.7, N=324
var.3.F5 1:169511555:T:C
peptide level
172:172 135:135 18:18
MDDAVAPGRpc2_NP.3
p−maj=1, pX=0.12, N=325
var.3.F5 1:169511555:T:C
peptide level
172:172 135:135 18:18
MDDAVAPGRpc2_NP.4
p−maj=1, pX=0.43, N=325
var.3.F5 1:169511555:T:C
peptide level
170:172 134:135 18:18
MDDAVAPGRpc2_NP.5
p−maj=1, pX=0.67, N=322
var.3.F5 1:169511555:T:C
peptide level
170:172 132:135 18:18
MPMGLSTGIISDSQIKpc2_NP.1
p−min=0.67, pX=0.078, N=320
var.3.F5 1:169511555:T:C
peptide level
162:172 131:135 14:18
MPMGLSTGIISDSQIKpc2_NP.2
p−maj=0.012, pX=0.44, N=307
var.3.F5 1:169511555:T:C
peptide level
172:172 135:135 18:18
MPMGLSTGIISDSQIKpc2_NP.3
p−maj=1, pX=0.67, N=325
var.3.F5 1:169511555:T:C
peptide level
172:172 135:135 18:18
MPMGLSTGIISDSQIKpc2_NP.4
p−maj=1, pX=0.83, N=325
var.3.F5 1:169511555:T:C
peptide level
172:172 135:135 18:18
MPMGLSTGIISDSQIKpc2_NP.5
p−maj=1, pX=0.24, N=325

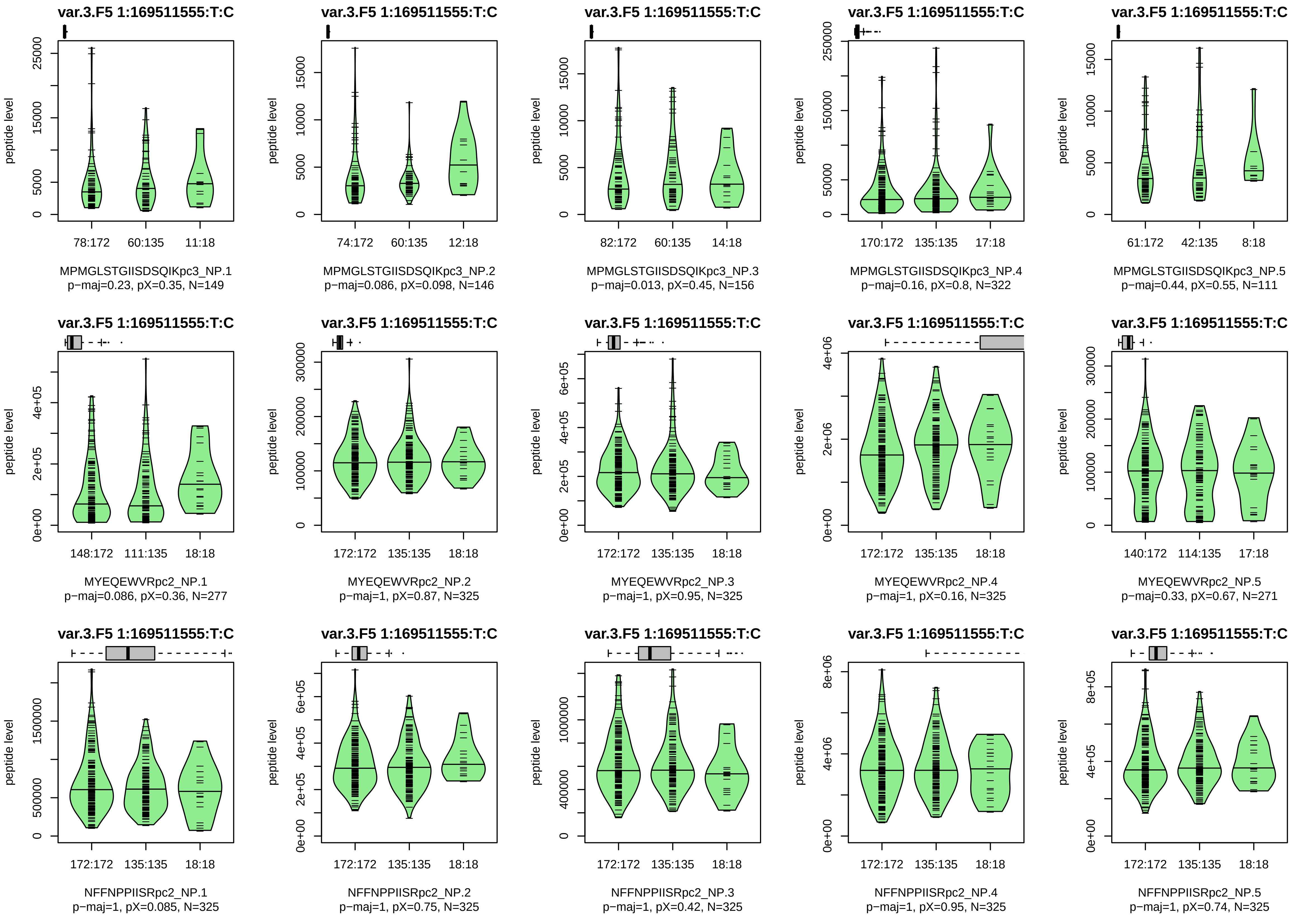
var.3.F5 1:169511555:T:C
peptide level
78:172
60:135
11:18
MPMGLSTGIISDSQIKpc3_NP.1
p−maj=0.23, pX=0.35, N=149
var.3.F5 1:169511555:T:C
peptide level
74:172
60:135
12:18
MPMGLSTGIISDSQIKpc3_NP.2
p−maj=0.086, pX=0.098, N=146
var.3.F5 1:169511555:T:C
peptide level
82:172
60:135
14:18
MPMGLSTGIISDSQIKpc3_NP.3
p−maj=0.013, pX=0.45, N=156
var.3.F5 1:169511555:T:C
peptide level
170:172
135:135
17:18
MPMGLSTGIISDSQIKpc3_NP.4
p−maj=0.16, pX=0.8, N=322
var.3.F5 1:169511555:T:C
peptide level
61:172
42:135
8:18
MPMGLSTGIISDSQIKpc3_NP.5
p−maj=0.44, pX=0.55, N=111
var.3.F5 1:169511555:T:C
peptide level
148:172
111:135
18:18
MYEQEWVRpc2_NP.1
p−maj=0.086, pX=0.36, N=277
var.3.F5 1:169511555:T:C
peptide level
172:172
135:135
18:18
MYEQEWVRpc2_NP.2
p−maj=1, pX=0.87, N=325
var.3.F5 1:169511555:T:C
peptide level
172:172
135:135
18:18
MYEQEWVRpc2_NP.3
p−maj=1, pX=0.95, N=325
var.3.F5 1:169511555:T:C
peptide level
172:172
135:135
18:18
MYEQEWVRpc2_NP.4
p−maj=1, pX=0.16, N=325
var.3.F5 1:169511555:T:C
peptide level
140:172
114:135
17:18
MYEQEWVRpc2_NP.5
p−maj=0.33, pX=0.67, N=271
var.3.F5 1:169511555:T:C
peptide level
172:172
135:135
18:18
NFFNPPIISRpc2_NP.1
p−maj=1, pX=0.085, N=325
var.3.F5 1:169511555:T:C
peptide level
172:172
135:135
18:18
NFFNPPIISRpc2_NP.2
p−maj=1, pX=0.75, N=325
var.3.F5 1:169511555:T:C
peptide level
172:172
135:135
18:18
NFFNPPIISRpc2_NP.3
p−maj=1, pX=0.42, N=325
var.3.F5 1:169511555:T:C
peptide level
172:172
135:135
18:18
NFFNPPIISRpc2_NP.4
p−maj=1, pX=0.95, N=325
var.3.F5 1:169511555:T:C
peptide level
172:172
135:135
18:18
NFFNPPIISRpc2_NP.5
p−maj=1, pX=0.74, N=325

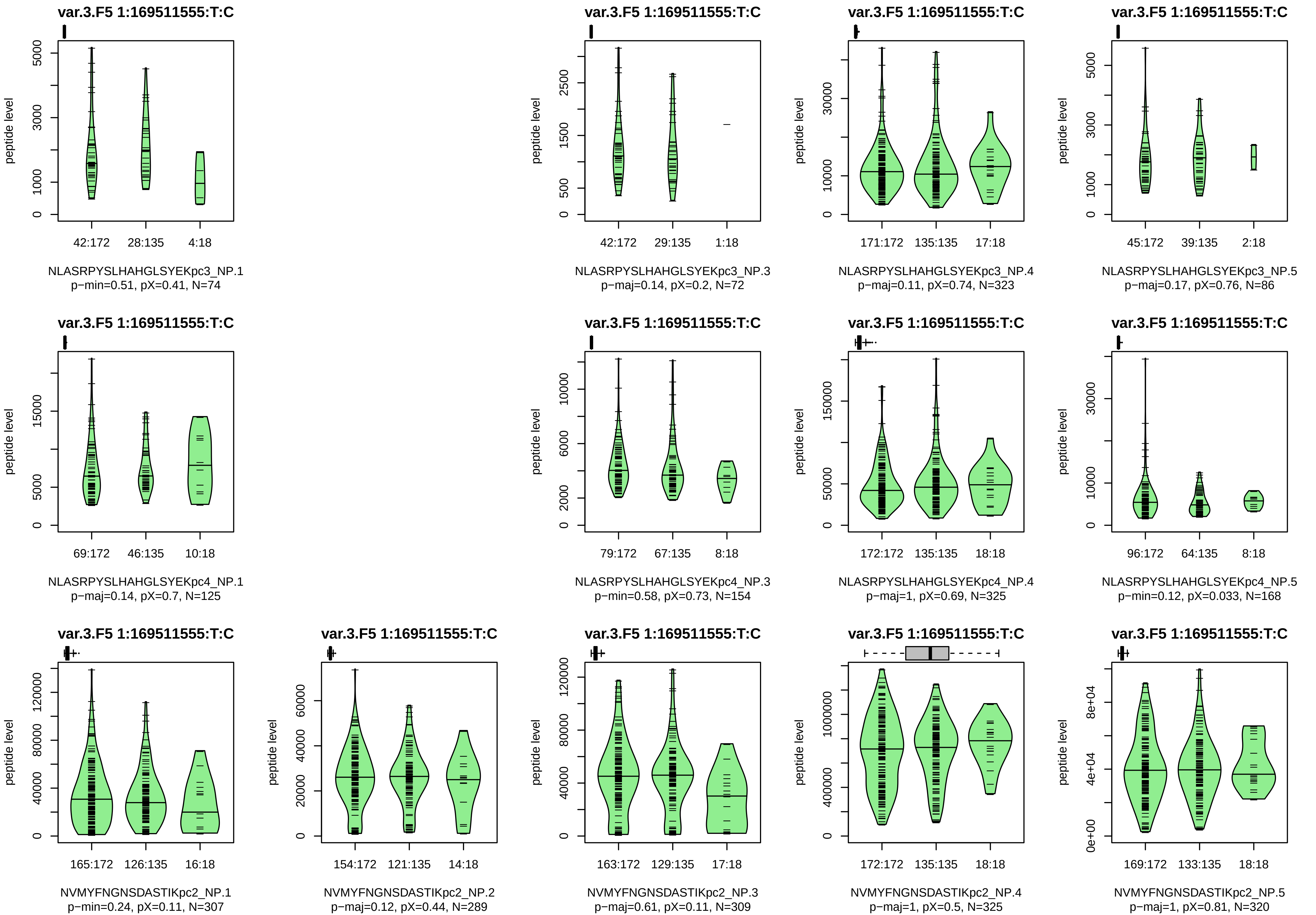

var.3.F5 1:169511555:T:C
peptide level
0
1000
3000
5000
42:172
28:135
4:18
NLASRPYSLHAHGLSYEKpc3_NP.1
p−min=0.51, pX=0.41, N=74
var.3.F5 1:169511555:T:C
peptide level
0
500
1500
2500
42:172
29:135
1:18
NLASRPYSLHAHGLSYEKpc3_NP.3
p−maj=0.14, pX=0.2, N=72
var.3.F5 1:169511555:T:C
peptide level
0
10000
30000
171:172
135:135
17:18
NLASRPYSLHAHGLSYEKpc3_NP.4
p−maj=0.11, pX=0.74, N=323
var.3.F5 1:169511555:T:C
peptide level
0
1000
3000
5000
45:172
39:135
2:18
NLASRPYSLHAHGLSYEKpc3_NP.5
p−maj=0.17, pX=0.76, N=86
var.3.F5 1:169511555:T:C
peptide level
0
5000
15000
69:172
46:135
10:18
NLASRPYSLHAHGLSYEKpc4_NP.1
p−maj=0.14, pX=0.7, N=125
var.3.F5 1:169511555:T:C
peptide level
0
2000
6000
10000
79:172
67:135
8:18
NLASRPYSLHAHGLSYEKpc4_NP.3
p−min=0.58, pX=0.73, N=154
var.3.F5 1:169511555:T:C
peptide level
0
50000
150000
172:172
135:135
18:18
NLASRPYSLHAHGLSYEKpc4_NP.4
p−maj=1, pX=0.69, N=325
var.3.F5 1:169511555:T:C
peptide level
0
10000
30000
96:172
64:135
8:18
NLASRPYSLHAHGLSYEKpc4_NP.5
p−min=0.12, pX=0.033, N=168
var.3.F5 1:169511555:T:C
peptide level
0
40000
80000
120000
165:172
126:135
16:18
NVMYFNGNSDASTIKpc2_NP.1
p−min=0.24, pX=0.11, N=307
var.3.F5 1:169511555:T:C
peptide level
0
20000
40000
60000
154:172
121:135
14:18
NVMYFNGNSDASTIKpc2_NP.2
p−maj=0.12, pX=0.44, N=289
var.3.F5 1:169511555:T:C
peptide level
0
40000
80000
120000
163:172
129:135
17:18
NVMYFNGNSDASTIKpc2_NP.3
p−maj=0.61, pX=0.11, N=309
var.3.F5 1:169511555:T:C
peptide level
0
400000
1000000
172:172
135:135
18:18
NVMYFNGNSDASTIKpc2_NP.4
p−maj=1, pX=0.5, N=325
var.3.F5 1:169511555:T:C
peptide level
0e+00
4e+04
8e+04
169:172
133:135
18:18
NVMYFNGNSDASTIKpc2_NP.5
p−maj=1, pX=0.81, N=320

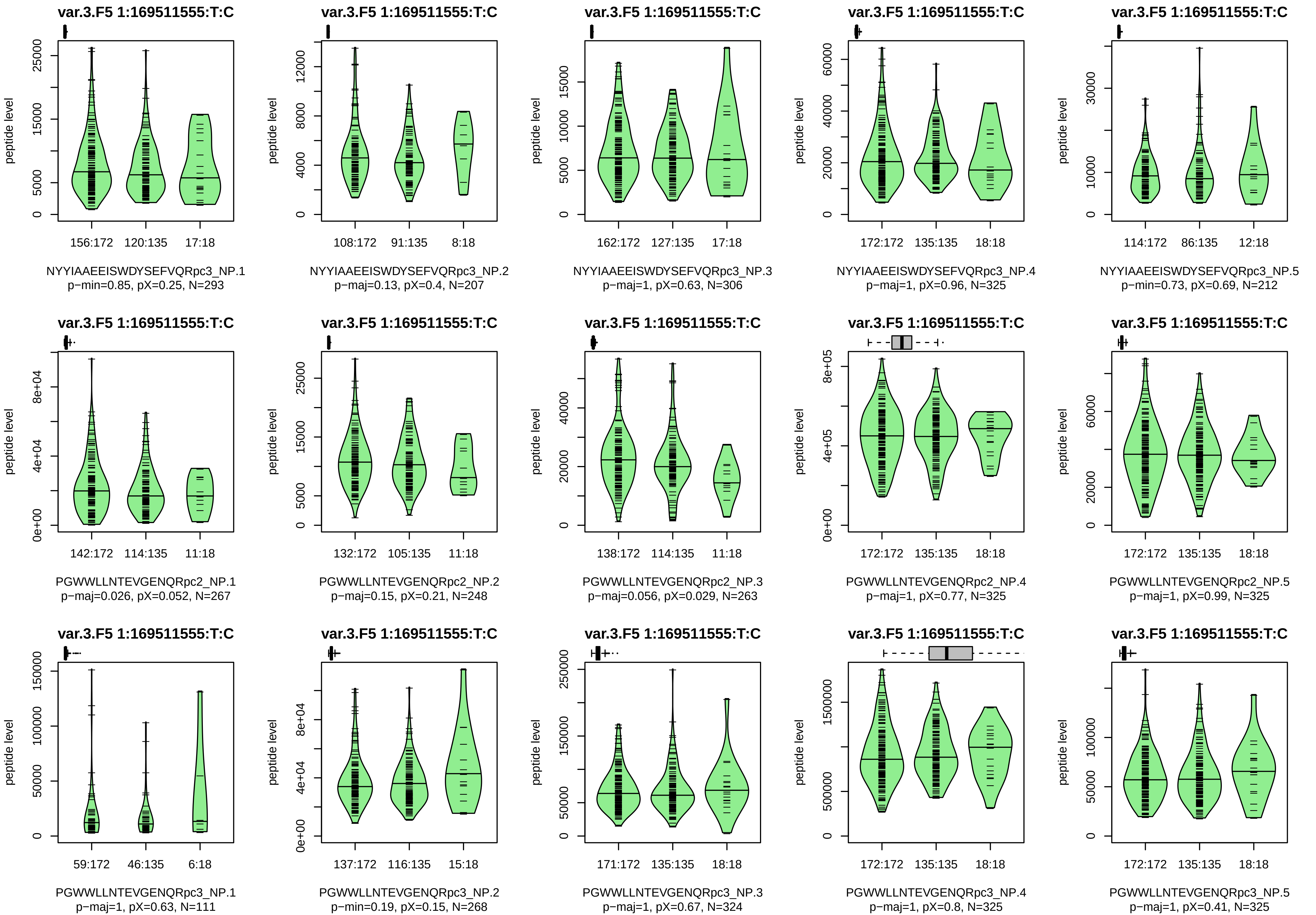
var.3.F5 1:169511555:T:C
peptide level
156:172
120:135
17:18
NYYIAAEEISWDYSEFVQRpc3_NP.1
p−min=0.85, pX=0.25, N=293
var.3.F5 1:169511555:T:C
peptide level
108:172
91:135
8:18
NYYIAAEEISWDYSEFVQRpc3_NP.2
p−maj=0.13, pX=0.4, N=207
var.3.F5 1:169511555:T:C
peptide level
162:172
127:135
17:18
NYYIAAEEISWDYSEFVQRpc3_NP.3
p−maj=1, pX=0.63, N=306
var.3.F5 1:169511555:T:C
peptide level
172:172
135:135
18:18
NYYIAAEEISWDYSEFVQRpc3_NP.4
p−maj=1, pX=0.96, N=325
var.3.F5 1:169511555:T:C
peptide level
114:172
86:135
12:18
NYYIAAEEISWDYSEFVQRpc3_NP.5
p−min=0.73, pX=0.69, N=212
var.3.F5 1:169511555:T:C
peptide level
142:172
114:135
11:18
PGWWLLNTEVGENQRpc2_NP.1
p−maj=0.026, pX=0.052, N=267
var.3.F5 1:169511555:T:C
peptide level
132:172
105:135
11:18
PGWWLLNTEVGENQRpc2_NP.2
p−maj=0.15, pX=0.21, N=248
var.3.F5 1:169511555:T:C
peptide level
138:172
114:135
11:18
PGWWLLNTEVGENQRpc2_NP.3
p−maj=0.056, pX=0.029, N=263
var.3.F5 1:169511555:T:C
peptide level
172:172
135:135
18:18
PGWWLLNTEVGENQRpc2_NP.4
p−maj=1, pX=0.77, N=325
var.3.F5 1:169511555:T:C
peptide level
172:172
135:135
18:18
PGWWLLNTEVGENQRpc2_NP.5
p−maj=1, pX=0.99, N=325
var.3.F5 1:169511555:T:C
peptide level
59:172
46:135
6:18
PGWWLLNTEVGENQRpc3_NP.1
p−maj=1, pX=0.63, N=111
var.3.F5 1:169511555:T:C
peptide level
137:172
116:135
15:18
PGWWLLNTEVGENQRpc3_NP.2
p−min=0.19, pX=0.15, N=268
var.3.F5 1:169511555:T:C
peptide level
171:172
135:135
18:18
PGWWLLNTEVGENQRpc3_NP.3
p−maj=1, pX=0.67, N=324
var.3.F5 1:169511555:T:C
peptide level
172:172
135:135
18:18
PGWWLLNTEVGENQRpc3_NP.4
p−maj=1, pX=0.8, N=325
var.3.F5 1:169511555:T:C
peptide level
172:172
135:135
18:18
PGWWLLNTEVGENQRpc3_NP.5
p−maj=1, pX=0.41, N=325

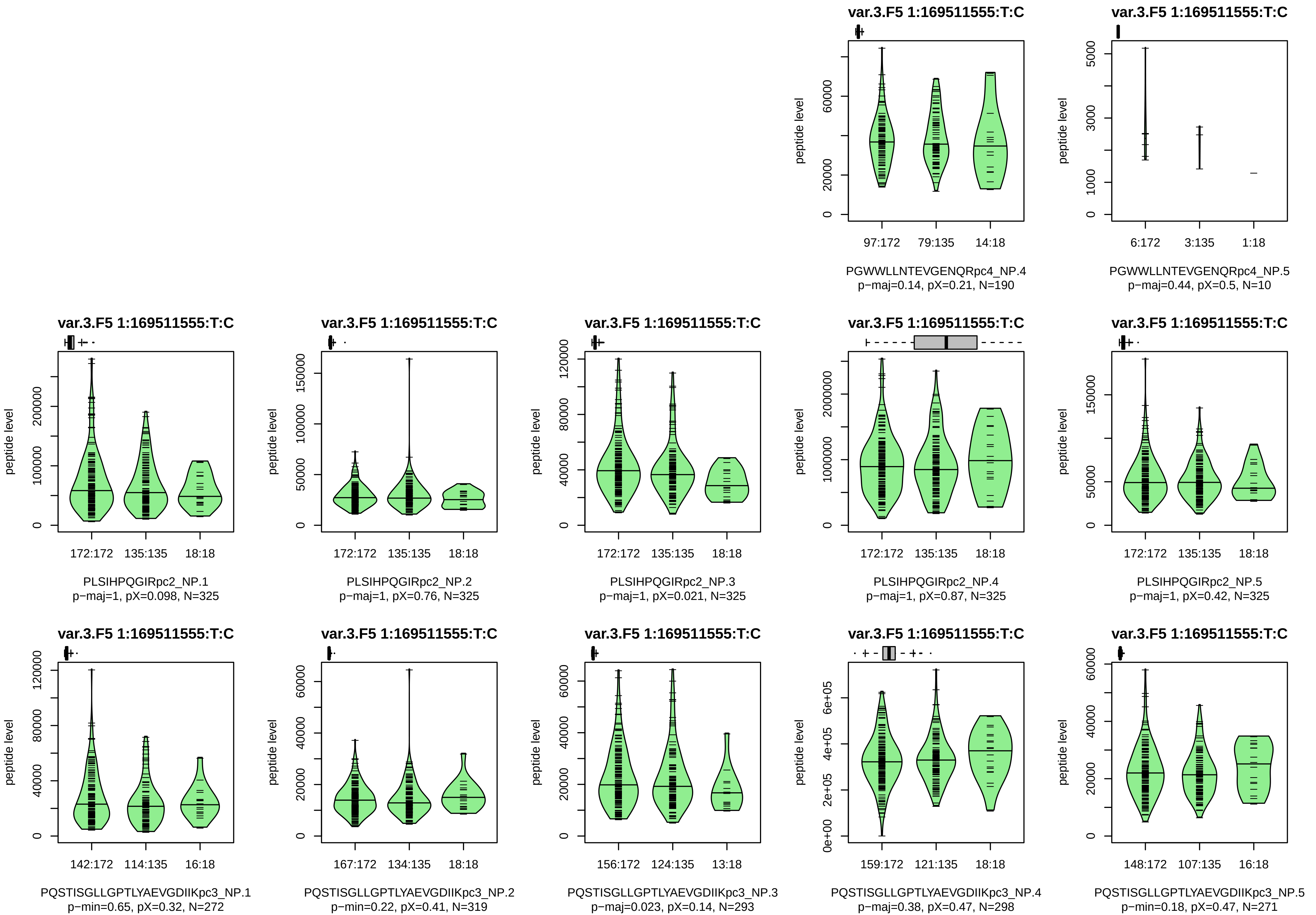

var.3.F5 1:169511555:T:C
peptide level
0
20000
60000
97:172
79:135
14:18
PGWWLLNTEVGENQRpc4_NP.4
p−maj=0.14, pX=0.21, N=190
var.3.F5 1:169511555:T:C
peptide level
0
1000
3000
5000
6:172
3:135
1:18
PGWWLLNTEVGENQRpc4_NP.5
p−maj=0.44, pX=0.5, N=10
var.3.F5 1:169511555:T:C
peptide level
0
100000
200000
172:172
135:135
18:18
PLSIHPQGIRpc2_NP.1
p−maj=1, pX=0.098, N=325
var.3.F5 1:169511555:T:C
peptide level
0
50000
100000
150000
172:172
135:135
18:18
PLSIHPQGIRpc2_NP.2
p−maj=1, pX=0.76, N=325
var.3.F5 1:169511555:T:C
peptide level
0
40000
80000
120000
172:172
135:135
18:18
PLSIHPQGIRpc2_NP.3
p−maj=1, pX=0.021, N=325
var.3.F5 1:169511555:T:C
peptide level
0
1000000
2000000
172:172
135:135
18:18
PLSIHPQGIRpc2_NP.4
p−maj=1, pX=0.87, N=325
var.3.F5 1:169511555:T:C
peptide level
0
50000
150000
172:172
135:135
18:18
PLSIHPQGIRpc2_NP.5
p−maj=1, pX=0.42, N=325
var.3.F5 1:169511555:T:C
peptide level
0
40000
80000
120000
142:172
114:135
16:18
PQSTISGLLGPTLYAEVGDIIKpc3_NP.1
p−min=0.65, pX=0.32, N=272
var.3.F5 1:169511555:T:C
peptide level
0
20000
40000
60000
167:172
134:135
18:18
PQSTISGLLGPTLYAEVGDIIKpc3_NP.2
p−min=0.22, pX=0.41, N=319
var.3.F5 1:169511555:T:C
peptide level
0
20000
40000
60000
156:172
124:135
13:18
PQSTISGLLGPTLYAEVGDIIKpc3_NP.3
p−maj=0.023, pX=0.14, N=293
var.3.F5 1:169511555:T:C
peptide level
0e+00
2e+05
4e+05
6e+05
159:172
121:135
18:18
PQSTISGLLGPTLYAEVGDIIKpc3_NP.4
p−maj=0.38, pX=0.47, N=298
var.3.F5 1:169511555:T:C
peptide level
0
20000
40000
60000
148:172
107:135
16:18
PQSTISGLLGPTLYAEVGDIIKpc3_NP.5
p−min=0.18, pX=0.47, N=271

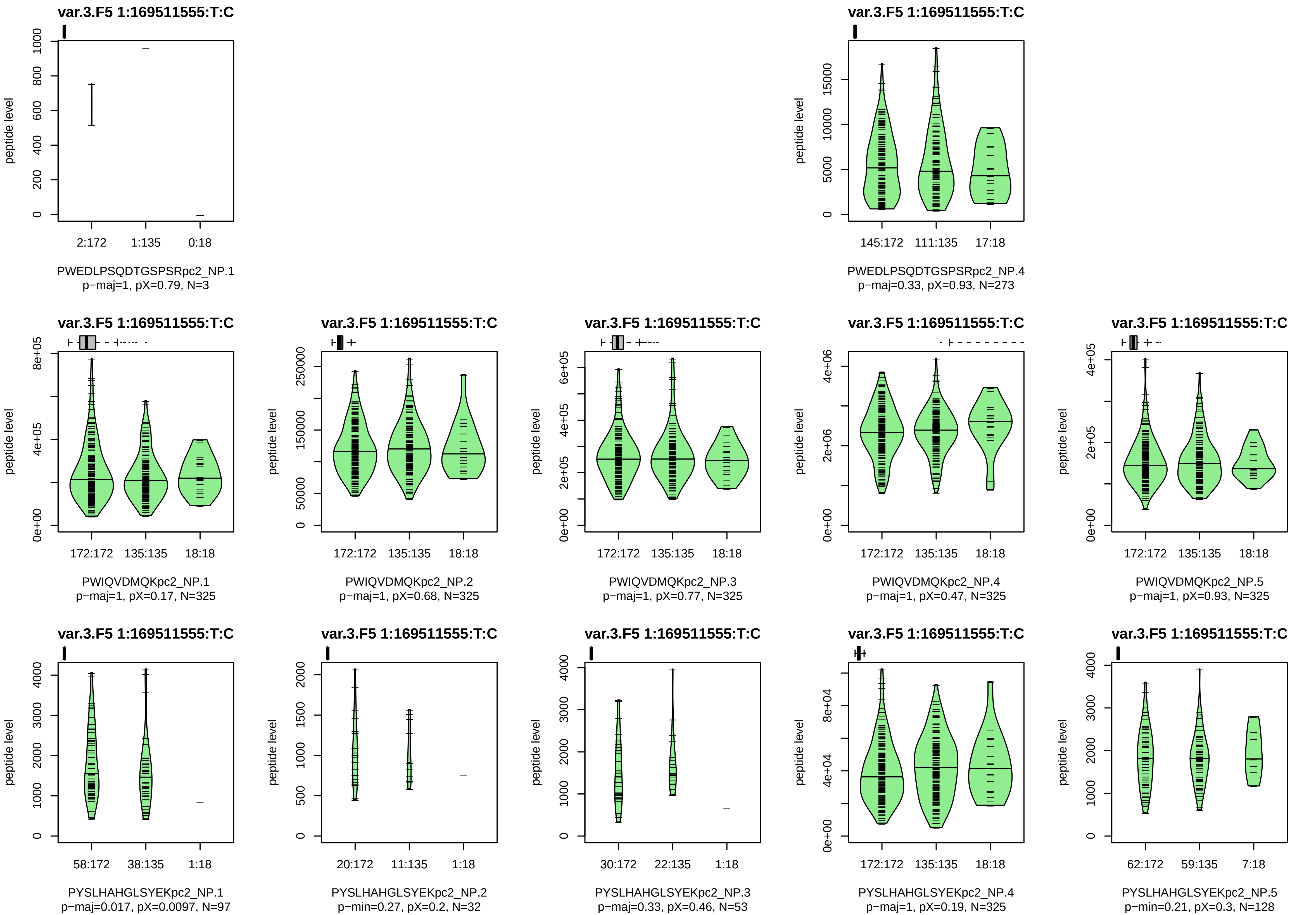
var.3.F5 1:169511555:T:C
peptide level
2:172
1:135
0:18
PWEDLPSQDTGSPSRpc2_NP.1
p−maj=1, pX=0.79, N=3
var.3.F5 1:169511555:T:C
peptide level
145:172
111:135
17:18
PWEDLPSQDTGSPSRpc2_NP.4
p−maj=0.33, pX=0.93, N=273
var.3.F5 1:169511555:T:C
peptide level
172:172
135:135
18:18
PWIQVDMQKpc2_NP.1
p−maj=1, pX=0.17, N=325
var.3.F5 1:169511555:T:C
peptide level
172:172
135:135
18:18
PWIQVDMQKpc2_NP.2
p−maj=1, pX=0.68, N=325
var.3.F5 1:169511555:T:C
peptide level
172:172
135:135
18:18
PWIQVDMQKpc2_NP.3
p−maj=1, pX=0.77, N=325
var.3.F5 1:169511555:T:C
peptide level
172:172
135:135
18:18
PWIQVDMQKpc2_NP.4
p−maj=1, pX=0.47, N=325
var.3.F5 1:169511555:T:C
peptide level
172:172
135:135
18:18
PWIQVDMQKpc2_NP.5
p−maj=1, pX=0.93, N=325
var.3.F5 1:169511555:T:C
peptide level
58:172
38:135
1:18
PYSLHAHGLSYEKpc2_NP.1
p−maj=0.017, pX=0.0097, N=97
var.3.F5 1:169511555:T:C
peptide level
20:172
11:135
1:18
PYSLHAHGLSYEKpc2_NP.2
p−min=0.27, pX=0.2, N=32
var.3.F5 1:169511555:T:C
peptide level
30:172
22:135
1:18
PYSLHAHGLSYEKpc2_NP.3
p−maj=0.33, pX=0.46, N=53
var.3.F5 1:169511555:T:C
peptide level
172:172
135:135
18:18
PYSLHAHGLSYEKpc2_NP.4
p−maj=1, pX=0.19, N=325
var.3.F5 1:169511555:T:C
peptide level
62:172
59:135
7:18
PYSLHAHGLSYEKpc2_NP.5
p−min=0.21, pX=0.3, N=128

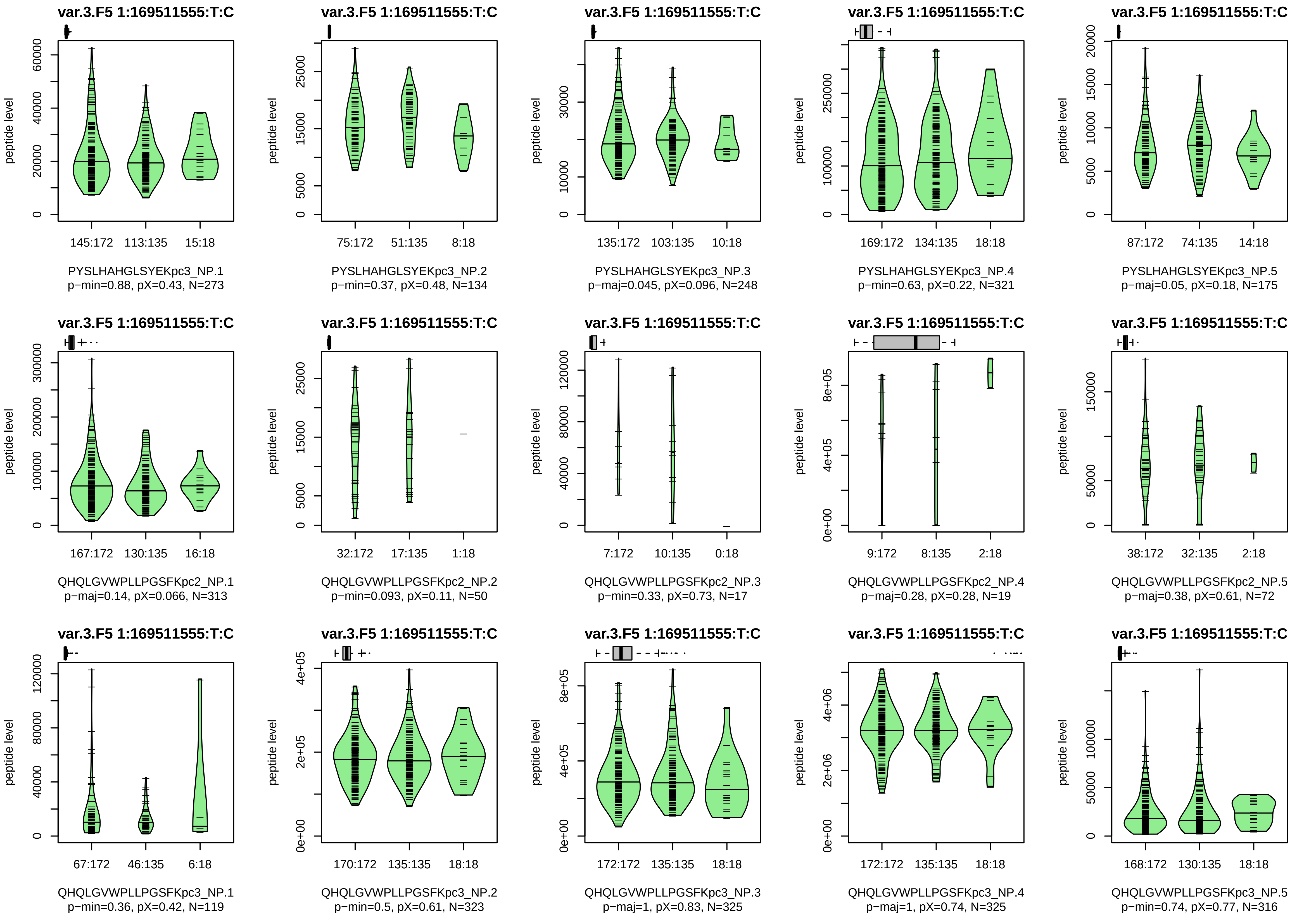
var.3.F5 1:169511555:T:C
peptide level
145:172
113:135
15:18
PYSLHAHGLSYEKpc3_NP.1
p−min=0.88, pX=0.43, N=273
var.3.F5 1:169511555:T:C
peptide level
75:172
51:135
8:18
PYSLHAHGLSYEKpc3_NP.2
p−min=0.37, pX=0.48, N=134
var.3.F5 1:169511555:T:C
peptide level
135:172
103:135
10:18
PYSLHAHGLSYEKpc3_NP.3
p−maj=0.045, pX=0.096, N=248
var.3.F5 1:169511555:T:C
peptide level
169:172
134:135
18:18
PYSLHAHGLSYEKpc3_NP.4
p−min=0.63, pX=0.22, N=321
var.3.F5 1:169511555:T:C
peptide level
87:172
74:135
14:18
PYSLHAHGLSYEKpc3_NP.5
p−maj=0.05, pX=0.18, N=175
var.3.F5 1:169511555:T:C
peptide level
167:172
130:135
16:18
QHQLGVWPLLPGSFKpc2_NP.1
p−maj=0.14, pX=0.066, N=313
var.3.F5 1:169511555:T:C
peptide level
32:172
17:135
1:18
QHQLGVWPLLPGSFKpc2_NP.2
p−min=0.093, pX=0.11, N=50
var.3.F5 1:169511555:T:C
peptide level
7:172
10:135
0:18
QHQLGVWPLLPGSFKpc2_NP.3
p−min=0.33, pX=0.73, N=17
var.3.F5 1:169511555:T:C
peptide level
9:172
8:135
2:18
QHQLGVWPLLPGSFKpc2_NP.4
p−maj=0.28, pX=0.28, N=19
var.3.F5 1:169511555:T:C
peptide level
38:172
32:135
2:18
QHQLGVWPLLPGSFKpc2_NP.5
p−maj=0.38, pX=0.61, N=72
var.3.F5 1:169511555:T:C
peptide level
67:172
46:135
6:18
QHQLGVWPLLPGSFKpc3_NP.1
p−min=0.36, pX=0.42, N=119
var.3.F5 1:169511555:T:C
peptide level
170:172
135:135
18:18
QHQLGVWPLLPGSFKpc3_NP.2
p−min=0.5, pX=0.61, N=323
var.3.F5 1:169511555:T:C
peptide level
172:172
135:135
18:18
QHQLGVWPLLPGSFKpc3_NP.3
p−maj=1, pX=0.83, N=325
var.3.F5 1:169511555:T:C
peptide level
172:172
135:135
18:18
QHQLGVWPLLPGSFKpc3_NP.4
p−maj=1, pX=0.74, N=325
var.3.F5 1:169511555:T:C
peptide level
168:172
130:135
18:18
QHQLGVWPLLPGSFKpc3_NP.5
p−min=0.74, pX=0.77, N=316

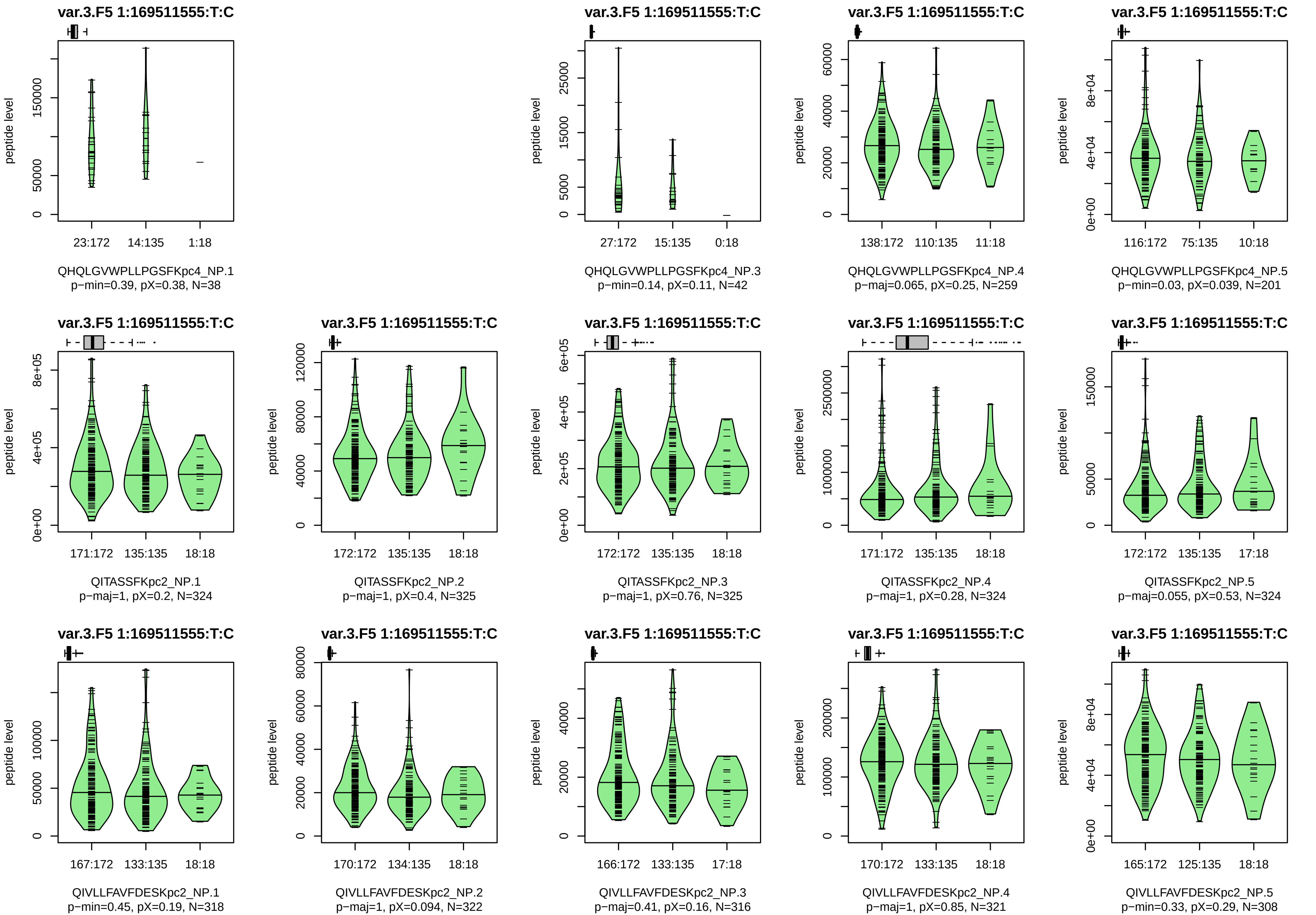
var.3.F5 1:169511555:T:C
peptide level
23:172
14:135
1:18
QHQLGVWPLLPGSFKpc4_NP.1
p−min=0.39, pX=0.38, N=38
var.3.F5 1:169511555:T:C
peptide level
27:172
15:135
0:18
QHQLGVWPLLPGSFKpc4_NP.3
p−min=0.14, pX=0.11, N=42
var.3.F5 1:169511555:T:C
peptide level
138:172
110:135
11:18
QHQLGVWPLLPGSFKpc4_NP.4
p−maj=0.065, pX=0.25, N=259
var.3.F5 1:169511555:T:C
peptide level
116:172
75:135
10:18
QHQLGVWPLLPGSFKpc4_NP.5
p−min=0.03, pX=0.039, N=201
var.3.F5 1:169511555:T:C
peptide level
171:172
135:135
18:18
QITASSFKpc2_NP.1
p−maj=1, pX=0.2, N=324
var.3.F5 1:169511555:T:C
peptide level
172:172
135:135
18:18
QITASSFKpc2_NP.2
p−maj=1, pX=0.4, N=325
var.3.F5 1:169511555:T:C
peptide level
172:172
135:135
18:18
QITASSFKpc2_NP.3
p−maj=1, pX=0.76, N=325
var.3.F5 1:169511555:T:C
peptide level
171:172
135:135
18:18
QITASSFKpc2_NP.4
p−maj=1, pX=0.28, N=324
var.3.F5 1:169511555:T:C
peptide level
172:172
135:135
17:18
QITASSFKpc2_NP.5
p−maj=0.055, pX=0.53, N=324
var.3.F5 1:169511555:T:C
peptide level
167:172
133:135
18:18
QIVLLFAVFDESKpc2_NP.1
p−min=0.45, pX=0.19, N=318
var.3.F5 1:169511555:T:C
peptide level
170:172
134:135
18:18
QIVLLFAVFDESKpc2_NP.2
p−maj=1, pX=0.094, N=322
var.3.F5 1:169511555:T:C
peptide level
166:172
133:135
17:18
QIVLLFAVFDESKpc2_NP.3
p−maj=0.41, pX=0.16, N=316
var.3.F5 1:169511555:T:C
peptide level
170:172
133:135
18:18
QIVLLFAVFDESKpc2_NP.4
p−maj=1, pX=0.85, N=321
var.3.F5 1:169511555:T:C
peptide level
165:172
125:135
18:18
QIVLLFAVFDESKpc2_NP.5
p−min=0.33, pX=0.29, N=308

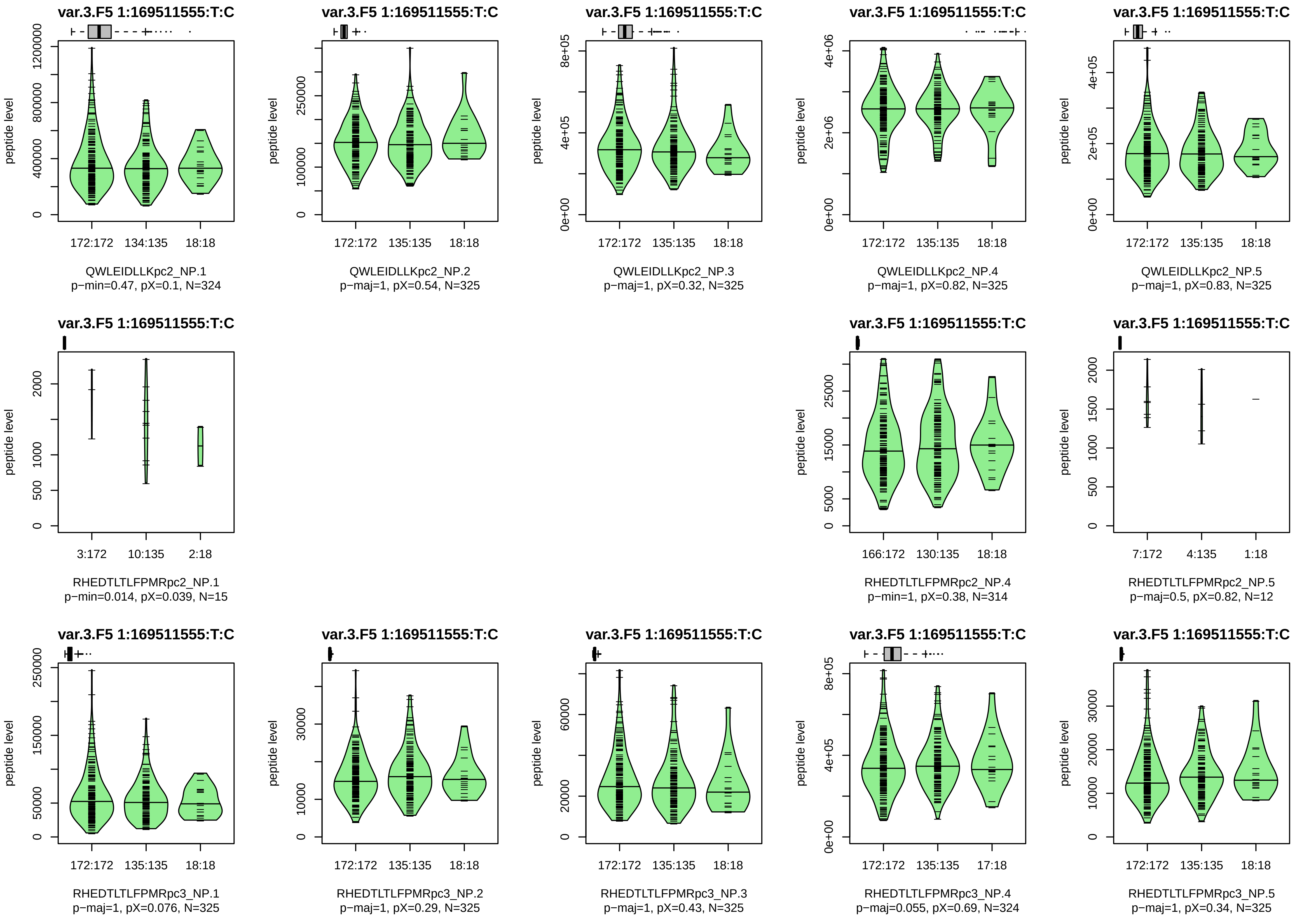
var.3.F5 1:169511555:T:C
peptide level
172:172
134:135
18:18
QWLEIDLLKpc2_NP.1
p−min=0.47, pX=0.1, N=324
var.3.F5 1:169511555:T:C
peptide level
172:172
135:135
18:18
QWLEIDLLKpc2_NP.2
p−maj=1, pX=0.54, N=325
var.3.F5 1:169511555:T:C
peptide level
172:172
135:135
18:18
QWLEIDLLKpc2_NP.3
p−maj=1, pX=0.32, N=325
var.3.F5 1:169511555:T:C
peptide level
172:172
135:135
18:18
QWLEIDLLKpc2_NP.4
p−maj=1, pX=0.82, N=325
var.3.F5 1:169511555:T:C
peptide level
172:172
135:135
18:18
QWLEIDLLKpc2_NP.5
p−maj=1, pX=0.83, N=325
var.3.F5 1:169511555:T:C
peptide level
3:172
10:135
2:18
RHEDTLTLFPMRpc2_NP.1
p−min=0.014, pX=0.039, N=15
var.3.F5 1:169511555:T:C
peptide level
166:172
130:135
18:18
RHEDTLTLFPMRpc2_NP.4
p−min=1, pX=0.38, N=314
var.3.F5 1:169511555:T:C
peptide level
7:172
4:135
1:18
RHEDTLTLFPMRpc2_NP.5
p−maj=0.5, pX=0.82, N=12
var.3.F5 1:169511555:T:C
peptide level
172:172
135:135
18:18
RHEDTLTLFPMRpc3_NP.1
p−maj=1, pX=0.076, N=325
var.3.F5 1:169511555:T:C
peptide level
172:172
135:135
18:18
RHEDTLTLFPMRpc3_NP.2
p−maj=1, pX=0.29, N=325
var.3.F5 1:169511555:T:C
peptide level
172:172
135:135
18:18
RHEDTLTLFPMRpc3_NP.3
p−maj=1, pX=0.43, N=325
var.3.F5 1:169511555:T:C
peptide level
172:172
135:135
17:18
RHEDTLTLFPMRpc3_NP.4
p−maj=0.055, pX=0.69, N=324
var.3.F5 1:169511555:T:C
peptide level
172:172
135:135
18:18
RHEDTLTLFPMRpc3_NP.5
p−maj=1, pX=0.34, N=325

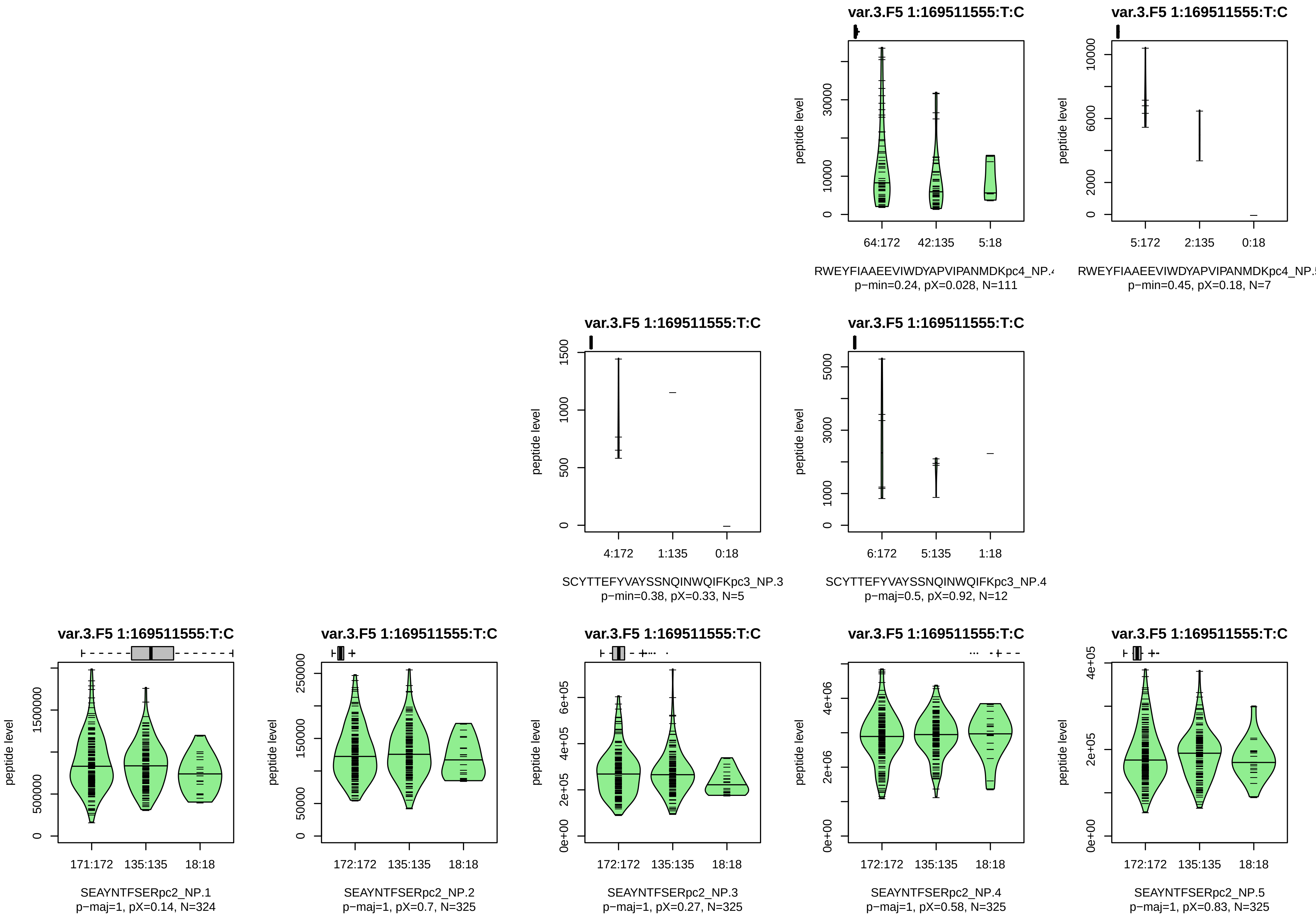
var.3.F5 1:169511555:T:C
peptide level
64:172
42:135
5:18
RWEYFIAAEEVIWDYAPVIPANMDKpc4_NP.4
p−min=0.24, pX=0.028, N=111
var.3.F5 1:169511555:T:C
peptide level
5:172
2:135
0:18
RWEYFIAAEEVIWDYAPVIPANMDKpc4_NP.5
p−min=0.45, pX=0.18, N=7
var.3.F5 1:169511555:T:C
peptide level
4:172
1:135
0:18
SCYTTEFYVAYSSNQINWQIFKpc3_NP.3
p−min=0.38, pX=0.33, N=5
var.3.F5 1:169511555:T:C
peptide level
6:172
5:135
1:18
SCYTTEFYVAYSSNQINWQIFKpc3_NP.4
p−maj=0.5, pX=0.92, N=12
var.3.F5 1:169511555:T:C
peptide level
171:172
135:135
18:18
SEAYNTFSERpc2_NP.1
p−maj=1, pX=0.14, N=324
var.3.F5 1:169511555:T:C
peptide level
172:172
135:135
18:18
SEAYNTFSERpc2_NP.2
p−maj=1, pX=0.7, N=325
var.3.F5 1:169511555:T:C
peptide level
172:172
135:135
18:18
SEAYNTFSERpc2_NP.3
p−maj=1, pX=0.27, N=325
var.3.F5 1:169511555:T:C
peptide level
172:172
135:135
18:18
SEAYNTFSERpc2_NP.4
p−maj=1, pX=0.58, N=325
var.3.F5 1:169511555:T:C
peptide level
172:172
135:135
18:18
SEAYNTFSERpc2_NP.5
p−maj=1, pX=0.83, N=325

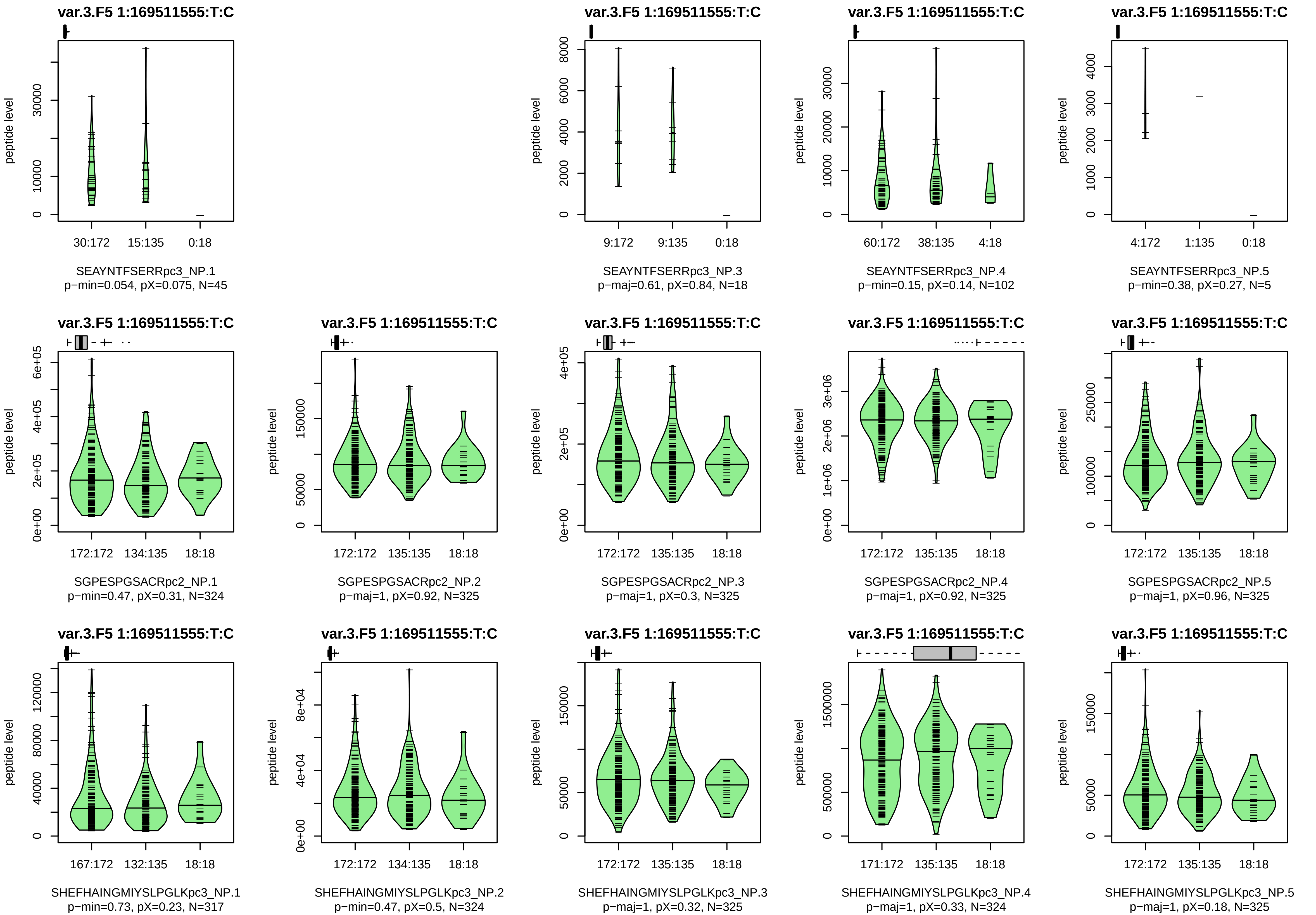
var.3.F5 1:169511555:T:C
peptide level
30:172
15:135
0:18
SEAYNTFSERRpc3_NP.1
p−min=0.054, pX=0.075, N=45
var.3.F5 1:169511555:T:C
peptide level
9:172
9:135
0:18
SEAYNTFSERRpc3_NP.3
p−maj=0.61, pX=0.84, N=18
var.3.F5 1:169511555:T:C
peptide level
60:172
38:135
4:18
SEAYNTFSERRpc3_NP.4
p−min=0.15, pX=0.14, N=102
var.3.F5 1:169511555:T:C
peptide level
4:172
1:135
0:18
SEAYNTFSERRpc3_NP.5
p−min=0.38, pX=0.27, N=5
var.3.F5 1:169511555:T:C
peptide level
172:172
134:135
18:18
SGPESPGSACRpc2_NP.1
p−min=0.47, pX=0.31, N=324
var.3.F5 1:169511555:T:C
peptide level
172:172
135:135
18:18
SGPESPGSACRpc2_NP.2
p−maj=1, pX=0.92, N=325
var.3.F5 1:169511555:T:C
peptide level
172:172
135:135
18:18
SGPESPGSACRpc2_NP.3
p−maj=1, pX=0.3, N=325
var.3.F5 1:169511555:T:C
peptide level
172:172
135:135
18:18
SGPESPGSACRpc2_NP.4
p−maj=1, pX=0.92, N=325
var.3.F5 1:169511555:T:C
peptide level
172:172
135:135
18:18
SGPESPGSACRpc2_NP.5
p−maj=1, pX=0.96, N=325
var.3.F5 1:169511555:T:C
peptide level
167:172
132:135
18:18
SHEFHAINGMIYSLPGLKpc3_NP.1
p−min=0.73, pX=0.23, N=317
var.3.F5 1:169511555:T:C
peptide level
172:172
134:135
18:18
SHEFHAINGMIYSLPGLKpc3_NP.2
p−min=0.47, pX=0.5, N=324
var.3.F5 1:169511555:T:C
peptide level
172:172
135:135
18:18
SHEFHAINGMIYSLPGLKpc3_NP.3
p−maj=1, pX=0.32, N=325
var.3.F5 1:169511555:T:C
peptide level
171:172
135:135
18:18
SHEFHAINGMIYSLPGLKpc3_NP.4
p−maj=1, pX=0.33, N=324
var.3.F5 1:169511555:T:C
peptide level
172:172
135:135
18:18
SHEFHAINGMIYSLPGLKpc3_NP.5
p−maj=1, pX=0.18, N=325

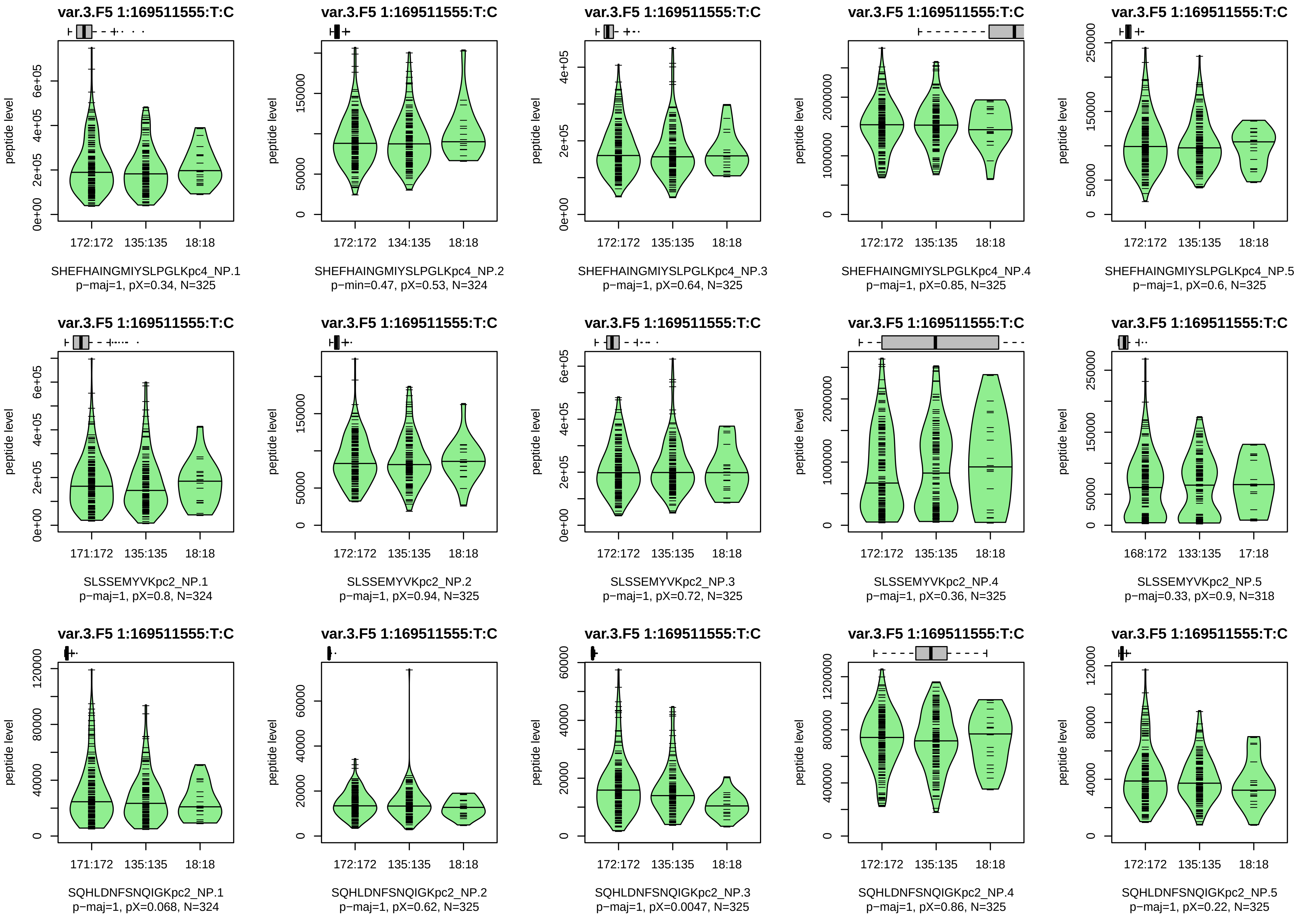
var.3.F5 1:169511555:T:C
peptide level
172:172 135:135 18:18
SHEFHAINGMIYSLPGLKpc4_NP.1
p−maj=1, pX=0.34, N=325
var.3.F5 1:169511555:T:C
peptide level
172:172 134:135 18:18
SHEFHAINGMIYSLPGLKpc4_NP.2
p−min=0.47, pX=0.53, N=324
var.3.F5 1:169511555:T:C
peptide level
172:172 135:135 18:18
SHEFHAINGMIYSLPGLKpc4_NP.3
p−maj=1, pX=0.64, N=325
var.3.F5 1:169511555:T:C
peptide level
172:172 135:135 18:18
SHEFHAINGMIYSLPGLKpc4_NP.4
p−maj=1, pX=0.85, N=325
var.3.F5 1:169511555:T:C
peptide level
172:172 135:135 18:18
SHEFHAINGMIYSLPGLKpc4_NP.5
p−maj=1, pX=0.6, N=325
var.3.F5 1:169511555:T:C
peptide level
171:172 135:135 18:18
SLSSEMYVKpc2_NP.1
p−maj=1, pX=0.8, N=324
var.3.F5 1:169511555:T:C
peptide level
172:172 135:135 18:18
SLSSEMYVKpc2_NP.2
p−maj=1, pX=0.94, N=325
var.3.F5 1:169511555:T:C
peptide level
172:172 135:135 18:18
SLSSEMYVKpc2_NP.3
p−maj=1, pX=0.72, N=325
var.3.F5 1:169511555:T:C
peptide level
172:172 135:135 18:18
SLSSEMYVKpc2_NP.4
p−maj=1, pX=0.36, N=325
var.3.F5 1:169511555:T:C
peptide level
168:172 133:135 17:18
SLSSEMYVKpc2_NP.5
p−maj=0.33, pX=0.9, N=318
var.3.F5 1:169511555:T:C
peptide level
171:172 135:135 18:18
SQHLDNFSNQIGKpc2_NP.1
p−maj=1, pX=0.068, N=324
var.3.F5 1:169511555:T:C
peptide level
172:172 135:135 18:18
SQHLDNFSNQIGKpc2_NP.2
p−maj=1, pX=0.62, N=325
var.3.F5 1:169511555:T:C
peptide level
172:172 135:135 18:18
SQHLDNFSNQIGKpc2_NP.3
p−maj=1, pX=0.0047, N=325
var.3.F5 1:169511555:T:C
peptide level
172:172 135:135 18:18
SQHLDNFSNQIGKpc2_NP.4
p−maj=1, pX=0.86, N=325
var.3.F5 1:169511555:T:C
peptide level
172:172 135:135 18:18
SQHLDNFSNQIGKpc2_NP.5
p−maj=1, pX=0.22, N=325

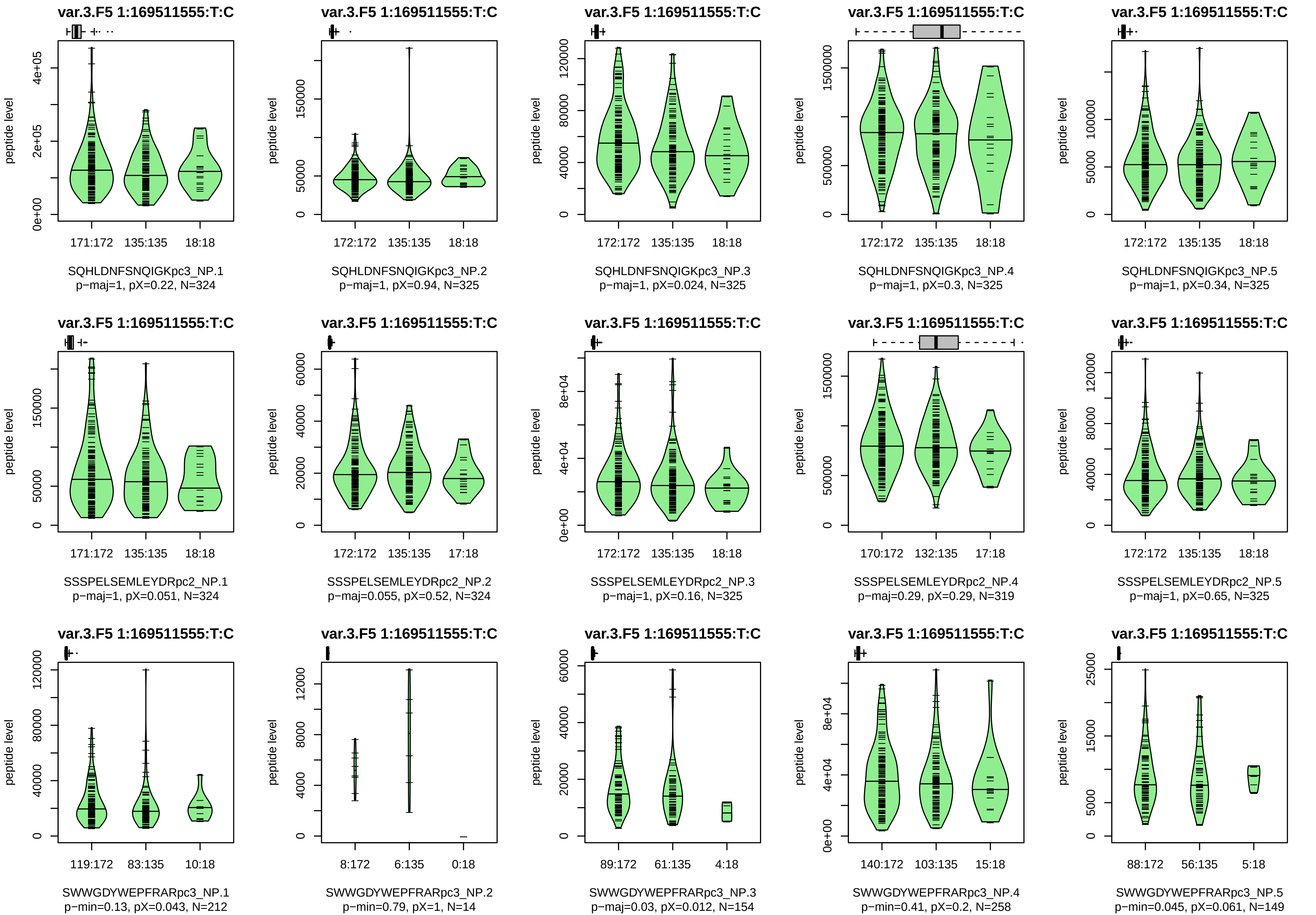

var.3.F5 1:169511555:T:C
peptide level
171:172
135:135
18:18
SQHLDNFSNQIGKpc3_NP.1
p−maj=1, pX=0.22, N=324
var.3.F5 1:169511555:T:C
peptide level
172:172
135:135
18:18
SQHLDNFSNQIGKpc3_NP.2
p−maj=1, pX=0.94, N=325
var.3.F5 1:169511555:T:C
peptide level
172:172
135:135
18:18
SQHLDNFSNQIGKpc3_NP.3
p−maj=1, pX=0.024, N=325
var.3.F5 1:169511555:T:C
peptide level
172:172
135:135
18:18
SQHLDNFSNQIGKpc3_NP.4
p−maj=1, pX=0.3, N=325
var.3.F5 1:169511555:T:C
peptide level
172:172
135:135
18:18
SQHLDNFSNQIGKpc3_NP.5
p−maj=1, pX=0.34, N=325
var.3.F5 1:169511555:T:C
peptide level
171:172
135:135
18:18
SSSPELSEMLEYDRpc2_NP.1
p−maj=1, pX=0.051, N=324
var.3.F5 1:169511555:T:C
peptide level
172:172
135:135
17:18
SSSPELSEMLEYDRpc2_NP.2
p−maj=0.055, pX=0.52, N=324
var.3.F5 1:169511555:T:C
peptide level
172:172
135:135
18:18
SSSPELSEMLEYDRpc2_NP.3
p−maj=1, pX=0.16, N=325
var.3.F5 1:169511555:T:C
peptide level
170:172
132:135
17:18
SSSPELSEMLEYDRpc2_NP.4
p−maj=0.29, pX=0.29, N=319
var.3.F5 1:169511555:T:C
peptide level
172:172
135:135
18:18
SSSPELSEMLEYDRpc2_NP.5
p−maj=1, pX=0.65, N=325
var.3.F5 1:169511555:T:C
peptide level
119:172
83:135
10:18
SWWGDYWEPFRARpc3_NP.1
p−min=0.13, pX=0.043, N=212
var.3.F5 1:169511555:T:C
peptide level
8:172
6:135
0:18
SWWGDYWEPFRARpc3_NP.2
p−min=0.79, pX=1, N=14
var.3.F5 1:169511555:T:C
peptide level
89:172
61:135
4:18
SWWGDYWEPFRARpc3_NP.3
p−maj=0.03, pX=0.012, N=154
var.3.F5 1:169511555:T:C
peptide level
140:172
103:135
15:18
SWWGDYWEPFRARpc3_NP.4
p−min=0.41, pX=0.2, N=258
var.3.F5 1:169511555:T:C
peptide level
88:172
56:135
5:18
SWWGDYWEPFRARpc3_NP.5
p−min=0.045, pX=0.061, N=149

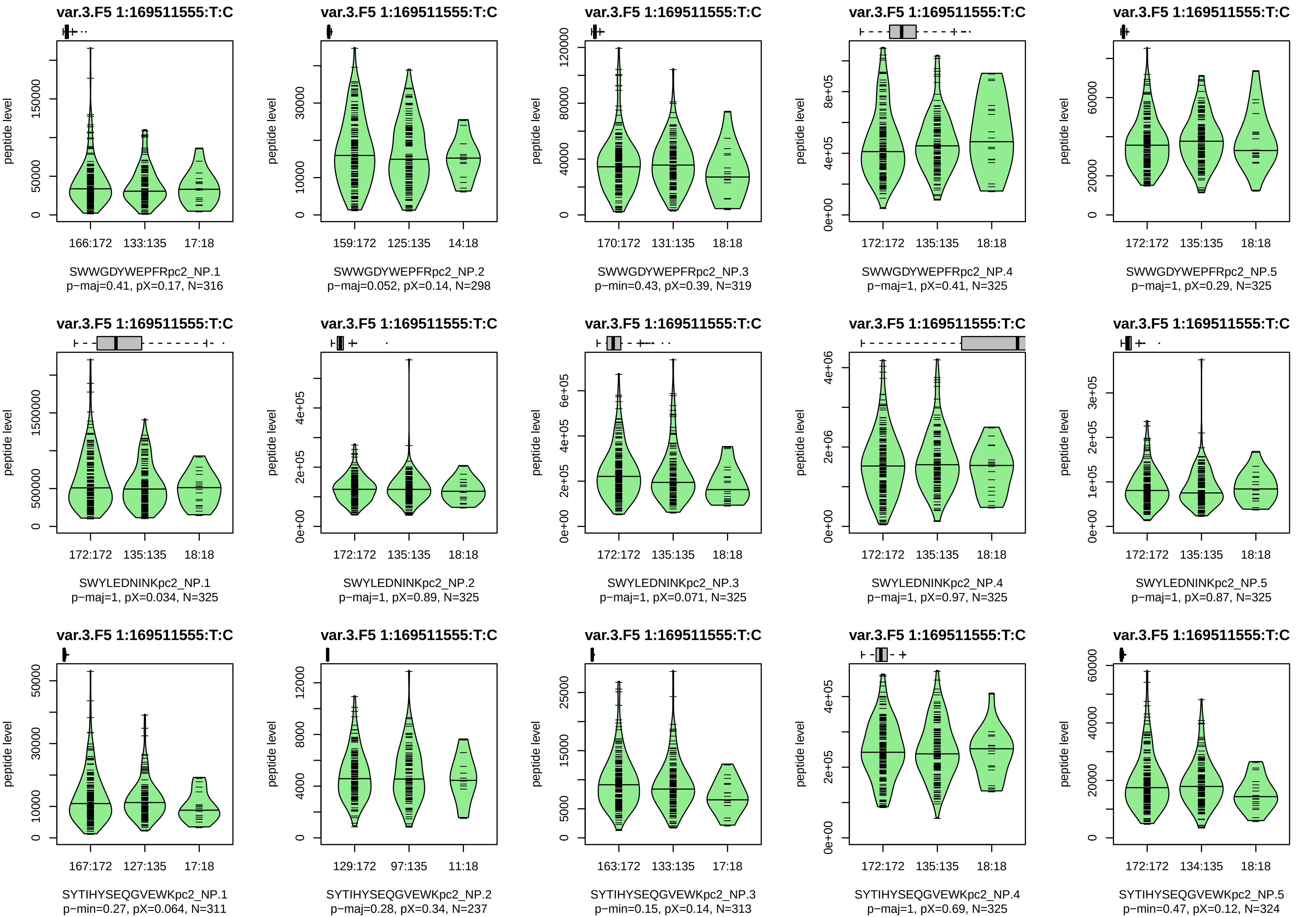
var.3.F5 1:169511555:T:C
peptide level
166:172 133:135 17:18
SWWGDYWEPFRpc2_NP.1
p−maj=0.41, pX=0.17, N=316
var.3.F5 1:169511555:T:C
peptide level
159:172 125:135 14:18
SWWGDYWEPFRpc2_NP.2
p−maj=0.052, pX=0.14, N=298
var.3.F5 1:169511555:T:C
peptide level
170:172 131:135 18:18
SWWGDYWEPFRpc2_NP.3
p−min=0.43, pX=0.39, N=319
var.3.F5 1:169511555:T:C
peptide level
172:172 135:135 18:18
SWWGDYWEPFRpc2_NP.4
p−maj=1, pX=0.41, N=325
var.3.F5 1:169511555:T:C
peptide level
172:172 135:135 18:18
SWWGDYWEPFRpc2_NP.5
p−maj=1, pX=0.29, N=325
var.3.F5 1:169511555:T:C
peptide level
172:172 135:135 18:18
SWYLEDNINKpc2_NP.1
p−maj=1, pX=0.034, N=325
var.3.F5 1:169511555:T:C
peptide level
172:172 135:135 18:18
SWYLEDNINKpc2_NP.2
p−maj=1, pX=0.89, N=325
var.3.F5 1:169511555:T:C
peptide level
172:172 135:135 18:18
SWYLEDNINKpc2_NP.3
p−maj=1, pX=0.071, N=325
var.3.F5 1:169511555:T:C
peptide level
172:172 135:135 18:18
SWYLEDNINKpc2_NP.4
p−maj=1, pX=0.97, N=325
var.3.F5 1:169511555:T:C
peptide level
172:172 135:135 18:18
SWYLEDNINKpc2_NP.5
p−maj=1, pX=0.87, N=325
var.3.F5 1:169511555:T:C
peptide level
167:172 127:135 17:18
SYTIHYSEQGVEWKpc2_NP.1
p−min=0.27, pX=0.064, N=311
var.3.F5 1:169511555:T:C
peptide level
129:172 97:135 11:18
SYTIHYSEQGVEWKpc2_NP.2
p−maj=0.28, pX=0.34, N=237
var.3.F5 1:169511555:T:C
peptide level
163:172 133:135 17:18
SYTIHYSEQGVEWKpc2_NP.3
p−min=0.15, pX=0.14, N=313
var.3.F5 1:169511555:T:C
peptide level
172:172 135:135 18:18
SYTIHYSEQGVEWKpc2_NP.4
p−maj=1, pX=0.69, N=325
var.3.F5 1:169511555:T:C
peptide level
172:172 134:135 18:18
SYTIHYSEQGVEWKpc2_NP.5
p−min=0.47, pX=0.12, N=324

var.3.F5 1:169511555:T:C

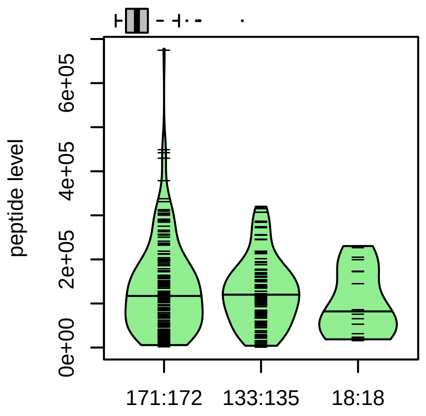

SYTIHYSEQGVEWKpc3_NP.1
p−min=0.6, pX=0.094, N=322

var.3.F5 1:169511555:T:C

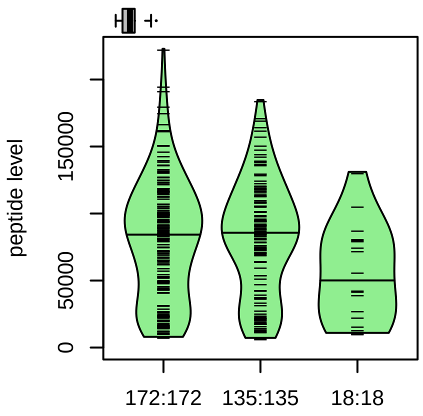

SYTIHYSEQGVEWKpc3_NP.2
p−maj=1, pX=0.29, N=325

var.3.F5 1:169511555:T:C

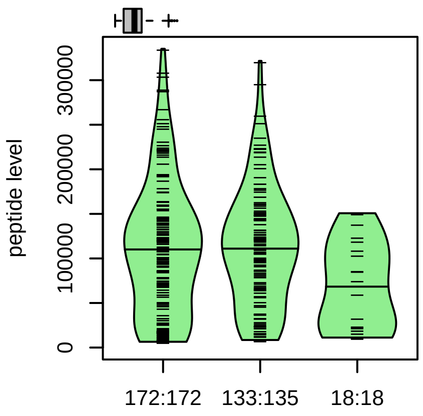

SYTIHYSEQGVEWKpc3_NP.3
p−min=0.22, pX=0.11, N=323

var.3.F5 1:169511555:T:C

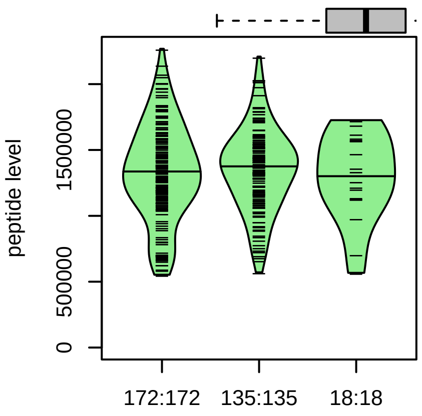

SYTIHYSEQGVEWKpc3_NP.4
p−maj=1, pX=0.96, N=325

var.3.F5 1:169511555:T:C

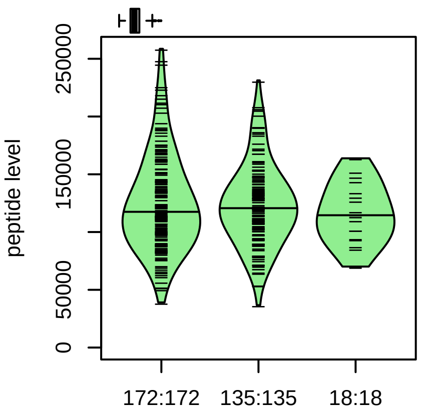

SYTIHYSEQGVEWKpc3_NP.5
p−maj=1, pX=0.37, N=325

var.3.F5 1:169511555:T:C

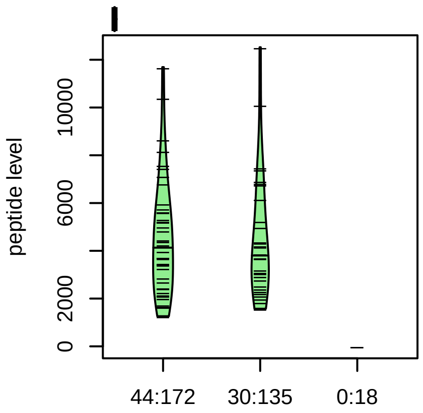

SYTIHYSEQGVEWKPYRpc3_NP.3
p−maj=0.017, pX=0.092, N=74

var.3.F5 1:169511555:T:C

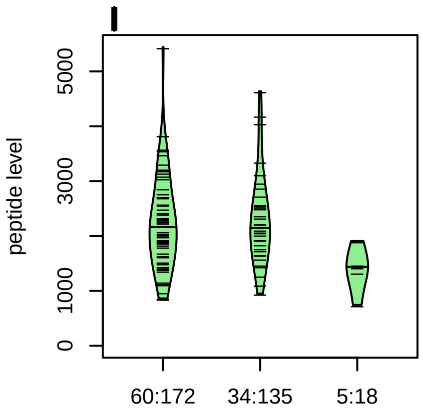

SYTIHYSEQGVEWKPYRpc4_NP.3
p−min=0.071, pX=0.053, N=99

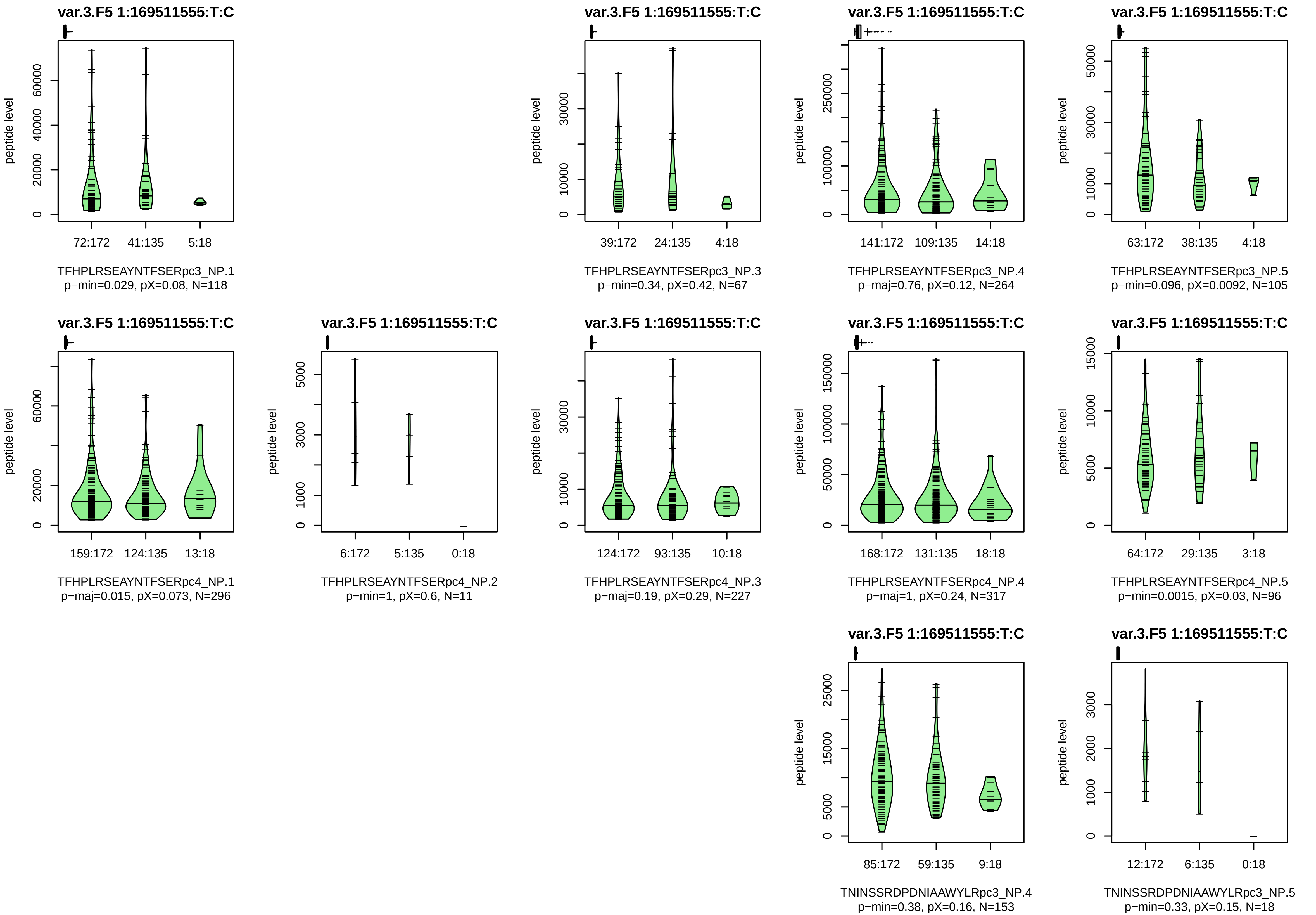

var.3.F5 1:169511555:T:C
peptide level
72:172
41:135
5:18
TFHPLRSEAYNTFSERpc3_NP.1
p−min=0.029, pX=0.08, N=118
var.3.F5 1:169511555:T:C
peptide level
39:172
24:135
4:18
TFHPLRSEAYNTFSERpc3_NP.3
p−min=0.34, pX=0.42, N=67
var.3.F5 1:169511555:T:C
peptide level
141:172
109:135
14:18
TFHPLRSEAYNTFSERpc3_NP.4
p−maj=0.76, pX=0.12, N=264
var.3.F5 1:169511555:T:C
peptide level
63:172
38:135
4:18
TFHPLRSEAYNTFSERpc3_NP.5
p−min=0.096, pX=0.0092, N=105
var.3.F5 1:169511555:T:C
peptide level
159:172
124:135
13:18
TFHPLRSEAYNTFSERpc4_NP.1
p−maj=0.015, pX=0.073, N=296
var.3.F5 1:169511555:T:C
peptide level
6:172
5:135
0:18
TFHPLRSEAYNTFSERpc4_NP.2
p−min=1, pX=0.6, N=11
var.3.F5 1:169511555:T:C
peptide level
124:172
93:135
10:18
TFHPLRSEAYNTFSERpc4_NP.3
p−maj=0.19, pX=0.29, N=227
var.3.F5 1:169511555:T:C
peptide level
168:172
131:135
18:18
TFHPLRSEAYNTFSERpc4_NP.4
p−maj=1, pX=0.24, N=317
var.3.F5 1:169511555:T:C
peptide level
64:172
29:135
3:18
TFHPLRSEAYNTFSERpc4_NP.5
p−min=0.0015, pX=0.03, N=96
var.3.F5 1:169511555:T:C
peptide level
85:172
59:135
9:18
TNINSSRDPDNIAAWYLRpc3_NP.4
p−min=0.38, pX=0.16, N=153
var.3.F5 1:169511555:T:C
peptide level
12:172
6:135
0:18
TNINSSRDPDNIAAWYLRpc3_NP.5
p−min=0.33, pX=0.15, N=18

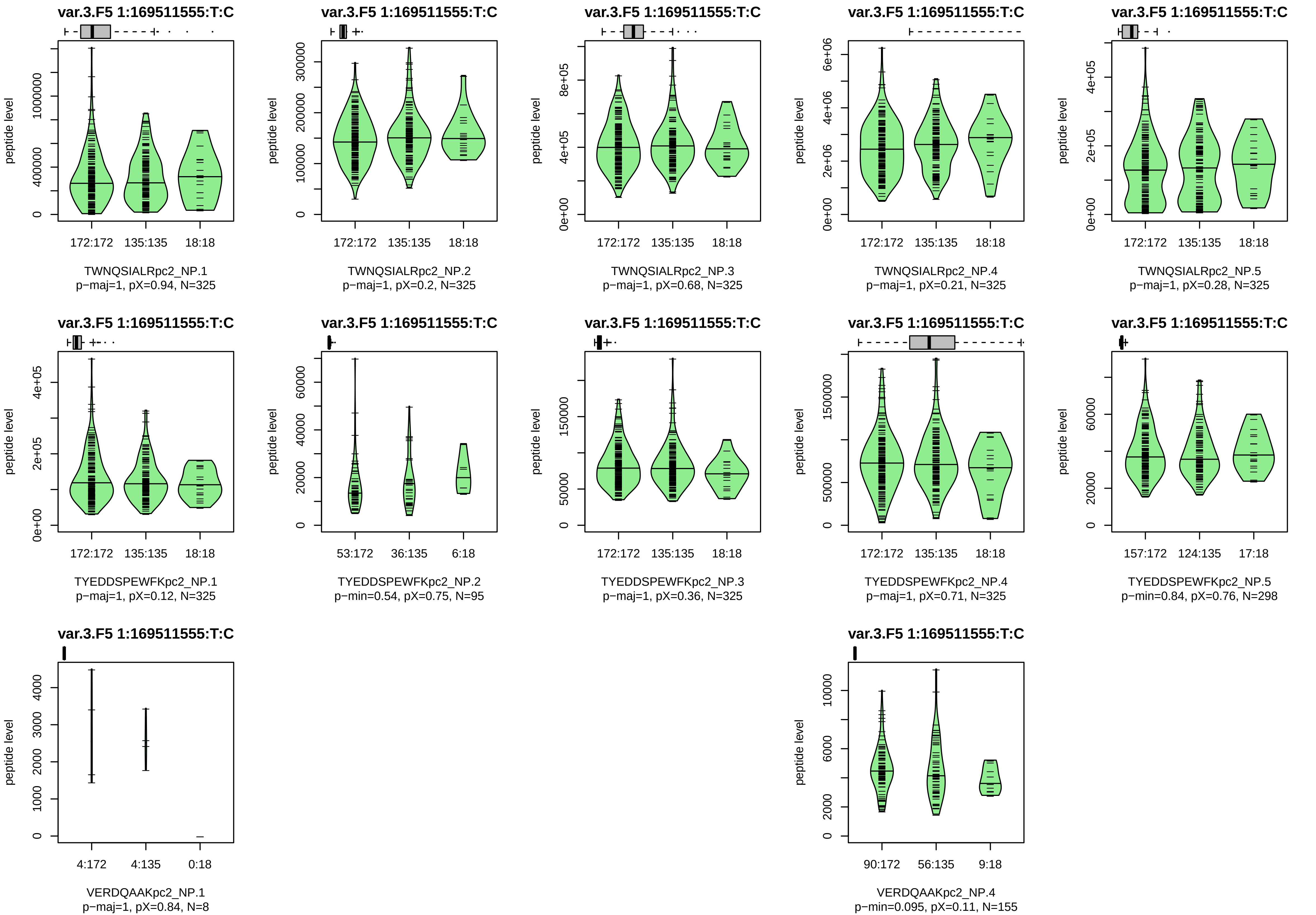
var.3.F5 1:169511555:T:C
peptide level
172:172 135:135 18:18
TWNQSIALRpc2_NP.1
p−maj=1, pX=0.94, N=325
var.3.F5 1:169511555:T:C
172:172 135:135 18:18
TWNQSIALRpc2_NP.2
p−maj=1, pX=0.2, N=325
var.3.F5 1:169511555:T:C
172:172 135:135 18:18
TWNQSIALRpc2_NP.3
p−maj=1, pX=0.68, N=325
var.3.F5 1:169511555:T:C
172:172 135:135 18:18
TWNQSIALRpc2_NP.4
p−maj=1, pX=0.21, N=325
var.3.F5 1:169511555:T:C
172:172 135:135 18:18
TWNQSIALRpc2_NP.5
p−maj=1, pX=0.28, N=325
var.3.F5 1:169511555:T:C
172:172 135:135 18:18
TYEDDSPEWFKpc2_NP.1
p−maj=1, pX=0.12, N=325
var.3.F5 1:169511555:T:C
53:172 36:135 6:18
TYEDDSPEWFKpc2_NP.2
p−min=0.54, pX=0.75, N=95
var.3.F5 1:169511555:T:C
172:172 135:135 18:18
TYEDDSPEWFKpc2_NP.3
p−maj=1, pX=0.36, N=325
var.3.F5 1:169511555:T:C
172:172 135:135 18:18
TYEDDSPEWFKpc2_NP.4
p−maj=1, pX=0.71, N=325
var.3.F5 1:169511555:T:C
157:172 124:135 17:18
TYEDDSPEWFKpc2_NP.5
p−min=0.84, pX=0.76, N=298
var.3.F5 1:169511555:T:C
4:172 4:135 0:18
VERDQAAKpc2_NP.1
p−maj=1, pX=0.84, N=8
var.3.F5 1:169511555:T:C
90:172 56:135 9:18
VERDQAAKpc2_NP.4
p−min=0.095, pX=0.11, N=155

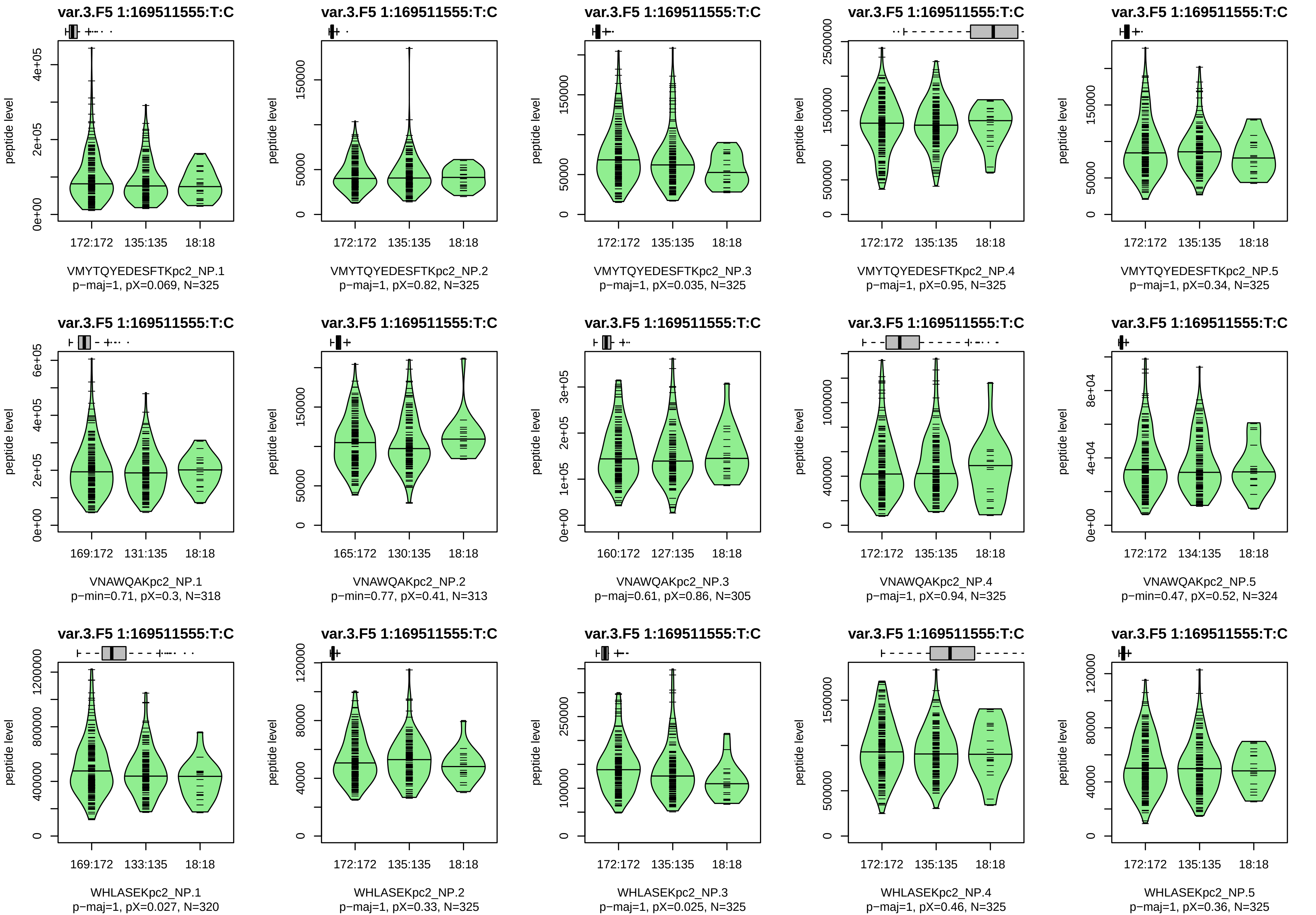

var.3.F5 1:169511555:T:C
peptide level
172:172
135:135
18:18
VMYTQYEDESFTKpc2_NP.1
p−maj=1, pX=0.069, N=325
var.3.F5 1:169511555:T:C
172:172
135:135
18:18
VMYTQYEDESFTKpc2_NP.2
p−maj=1, pX=0.82, N=325
var.3.F5 1:169511555:T:C
172:172
135:135
18:18
VMYTQYEDESFTKpc2_NP.3
p−maj=1, pX=0.035, N=325
var.3.F5 1:169511555:T:C
172:172
135:135
18:18
VMYTQYEDESFTKpc2_NP.4
p−maj=1, pX=0.95, N=325
var.3.F5 1:169511555:T:C
172:172
135:135
18:18
VMYTQYEDESFTKpc2_NP.5
p−maj=1, pX=0.34, N=325
var.3.F5 1:169511555:T:C
169:172
131:135
18:18
VNAWQAKpc2_NP.1
p−min=0.71, pX=0.3, N=318
var.3.F5 1:169511555:T:C
165:172
130:135
18:18
VNAWQAKpc2_NP.2
p−min=0.77, pX=0.41, N=313
var.3.F5 1:169511555:T:C
160:172
127:135
18:18
VNAWQAKpc2_NP.3
p−maj=0.61, pX=0.86, N=305
var.3.F5 1:169511555:T:C
172:172
135:135
18:18
VNAWQAKpc2_NP.4
p−maj=1, pX=0.94, N=325
var.3.F5 1:169511555:T:C
172:172
134:135
18:18
VNAWQAKpc2_NP.5
p−min=0.47, pX=0.52, N=324
var.3.F5 1:169511555:T:C
169:172
133:135
18:18
WHLASEKpc2_NP.1
p−maj=1, pX=0.027, N=320
var.3.F5 1:169511555:T:C
172:172
135:135
18:18
WHLASEKpc2_NP.2
p−maj=1, pX=0.33, N=325
var.3.F5 1:169511555:T:C
172:172
135:135
18:18
WHLASEKpc2_NP.3
p−maj=1, pX=0.025, N=325
var.3.F5 1:169511555:T:C
172:172
135:135
18:18
WHLASEKpc2_NP.4
p−maj=1, pX=0.46, N=325
var.3.F5 1:169511555:T:C
172:172
135:135
18:18
WHLASEKpc2_NP.5
p−maj=1, pX=0.36, N=325

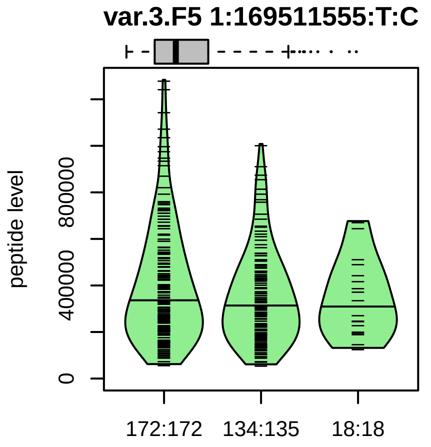

WIISSLTPKpc2_NP.1
p−min=0.47, pX=0.055, N=324

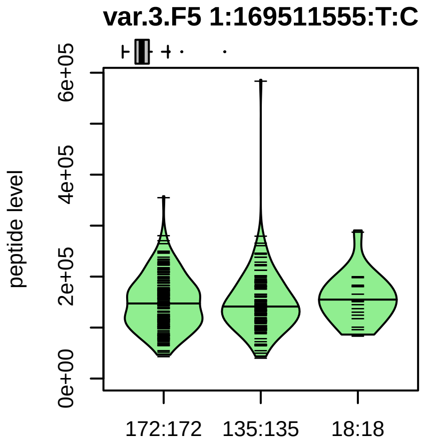

WIISSLTPKpc2_NP.2
p−maj=1, pX=0.69, N=325

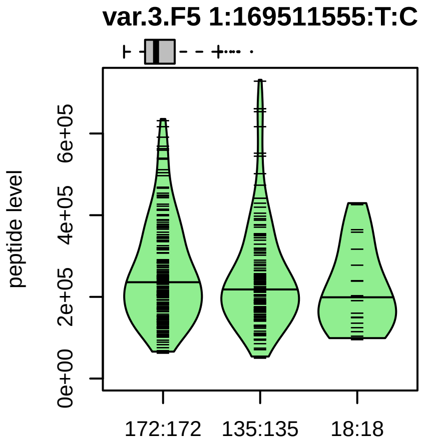

WIISSLTPKpc2_NP.3
p−maj=1, pX=0.15, N=325

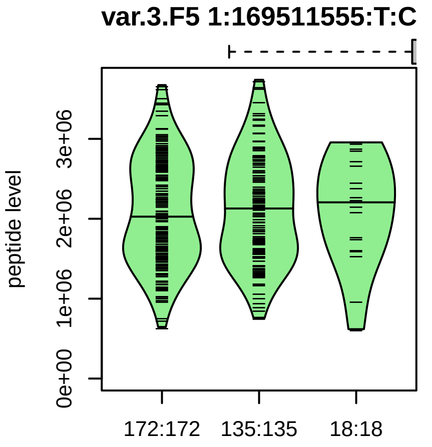

WIISSLTPKpc2_NP.4
p−maj=1, pX=0.76, N=325

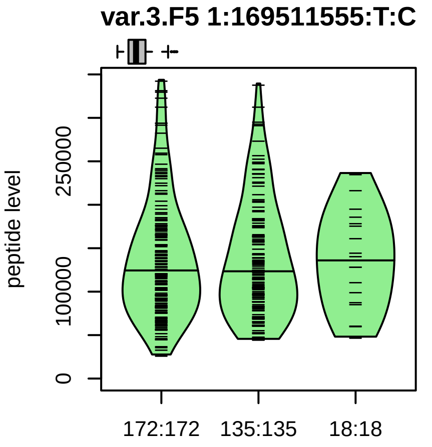

WIISSLTPKpc2_NP.5
p−maj=1, pX=0.99, N=325

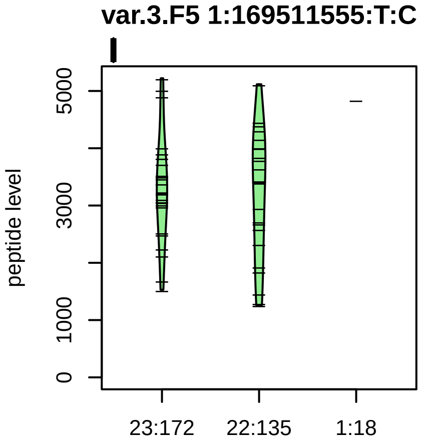

YLDSTFTKpc1_NP.4
p−maj=0.49, pX=0.98, N=46

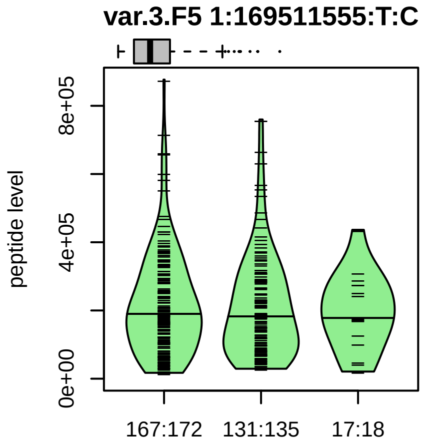

YLDSTFTKpc2_NP.1
p−maj=0.44, pX=0.47, N=315

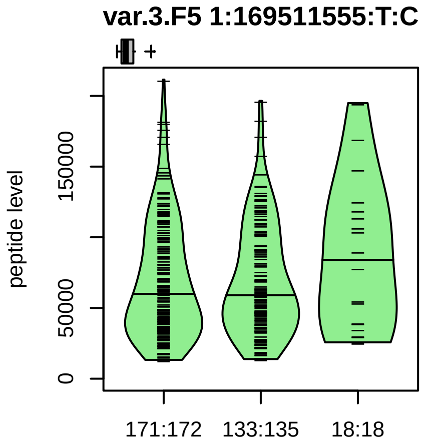

YLDSTFTKpc2_NP.2
p−min=0.6, pX=0.29, N=322

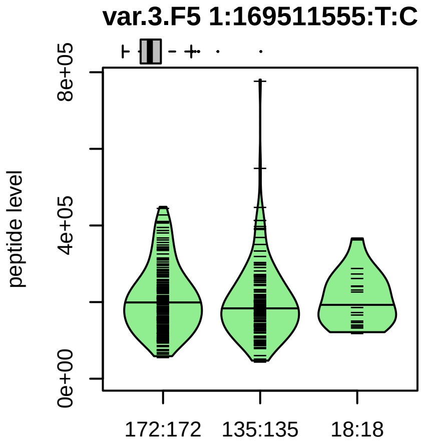

YLDSTFTKpc2_NP.3
p−maj=1, pX=0.57, N=325

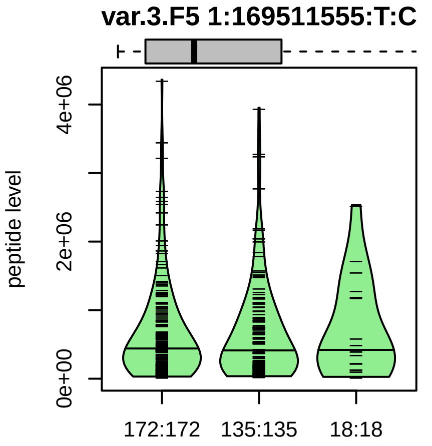

YLDSTFTKpc2_NP.4
p−maj=1, pX=0.89, N=325

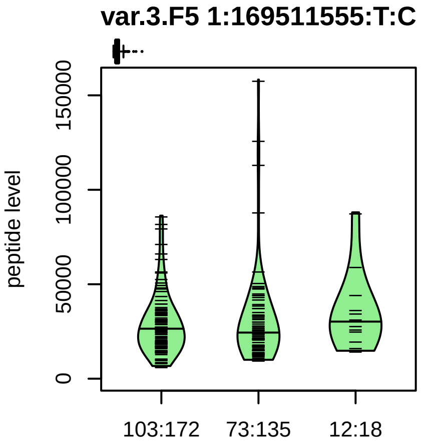

YLDSTFTKpc2_NP.5
p−min=0.43, pX=0.5, N=188

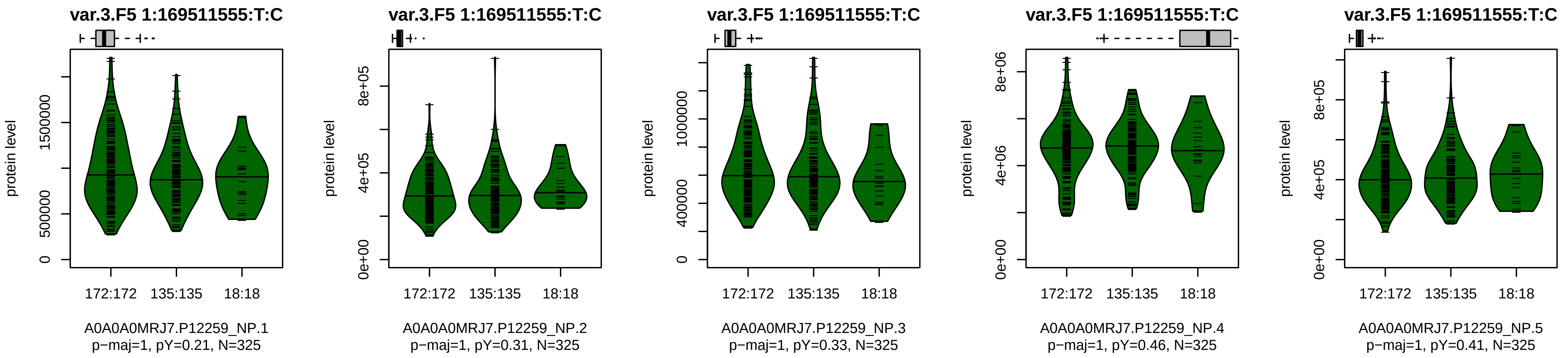
var.3.F5 1:169511555:T:C
protein level
172:172
135:135
18:18
A0A0A0MRJ7.P12259_NP.1
p−maj=1, pY=0.21, N=325
A0A0A0MRJ7.P12259_NP.2
p−maj=1, pY=0.31, N=325
A0A0A0MRJ7.P12259_NP.3
p−maj=1, pY=0.33, N=325
A0A0A0MRJ7.P12259_NP.4
p−maj=1, pY=0.46, N=325
A0A0A0MRJ7.P12259_NP.5
p−maj=1, pY=0.41, N=325

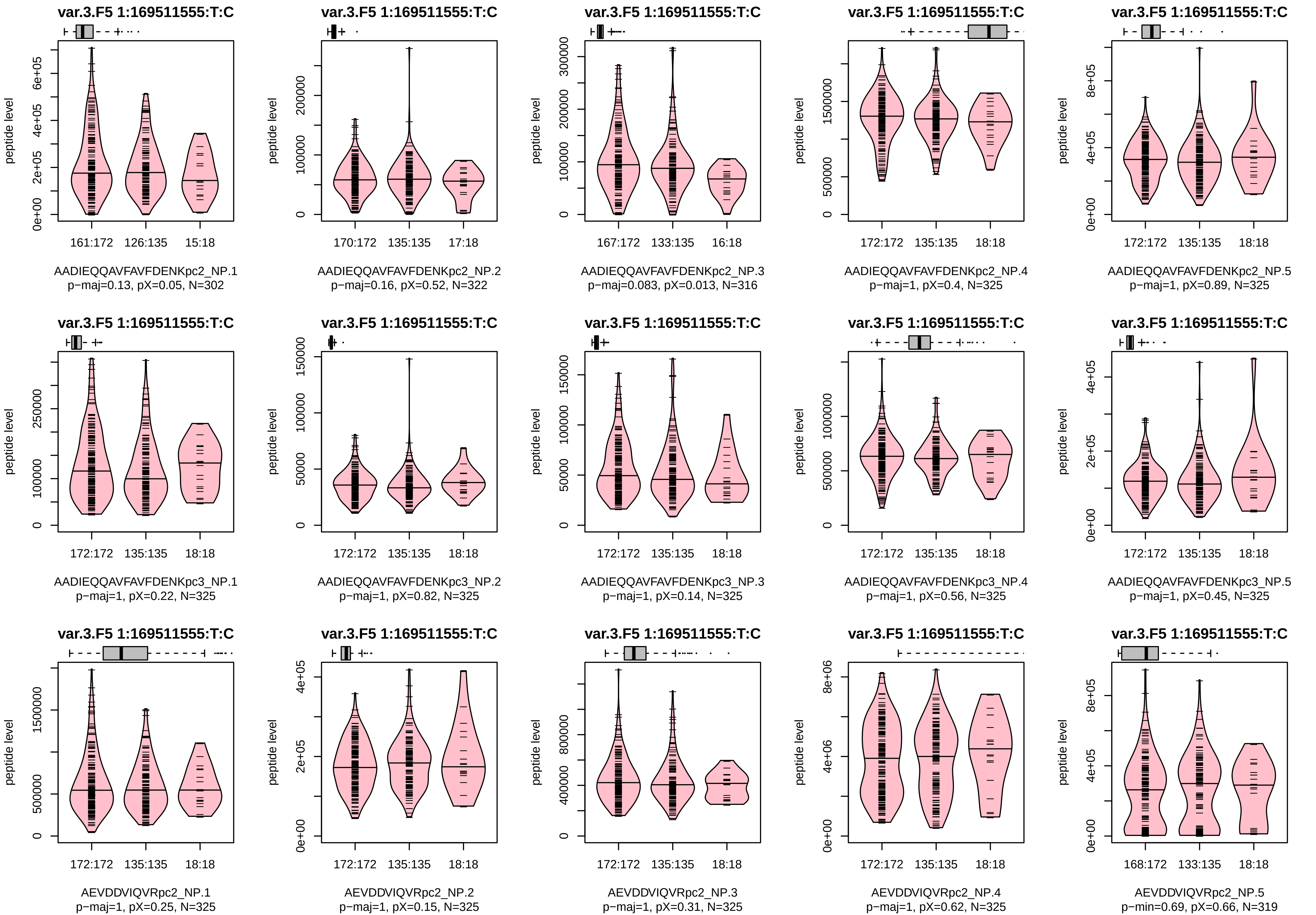
var.3.F5 1:169511555:T:C
peptide level
161:172
126:135
15:18
AADIEQQAVFAVFDENKpc2_NP.1
p−maj=0.13, pX=0.05, N=302
var.3.F5 1:169511555:T:C
peptide level
170:172
135:135
17:18
AADIEQQAVFAVFDENKpc2_NP.2
p−maj=0.16, pX=0.52, N=322
var.3.F5 1:169511555:T:C
peptide level
167:172
133:135
16:18
AADIEQQAVFAVFDENKpc2_NP.3
p−maj=0.083, pX=0.013, N=316
var.3.F5 1:169511555:T:C
peptide level
172:172
135:135
18:18
AADIEQQAVFAVFDENKpc2_NP.4
p−maj=1, pX=0.4, N=325
var.3.F5 1:169511555:T:C
peptide level
172:172
135:135
18:18
AADIEQQAVFAVFDENKpc2_NP.5
p−maj=1, pX=0.89, N=325
var.3.F5 1:169511555:T:C
peptide level
172:172
135:135
18:18
AADIEQQAVFAVFDENKpc3_NP.1
p−maj=1, pX=0.22, N=325
var.3.F5 1:169511555:T:C
peptide level
172:172
135:135
18:18
AADIEQQAVFAVFDENKpc3_NP.2
p−maj=1, pX=0.82, N=325
var.3.F5 1:169511555:T:C
peptide level
172:172
135:135
18:18
AADIEQQAVFAVFDENKpc3_NP.3
p−maj=1, pX=0.14, N=325
var.3.F5 1:169511555:T:C
peptide level
172:172
135:135
18:18
AADIEQQAVFAVFDENKpc3_NP.4
p−maj=1, pX=0.56, N=325
var.3.F5 1:169511555:T:C
peptide level
172:172
135:135
18:18
AADIEQQAVFAVFDENKpc3_NP.5
p−maj=1, pX=0.45, N=325
var.3.F5 1:169511555:T:C
peptide level
172:172
135:135
18:18
AEVDDVIQVRpc2_NP.1
p−maj=1, pX=0.25, N=325
var.3.F5 1:169511555:T:C
peptide level
172:172
135:135
18:18
AEVDDVIQVRpc2_NP.2
p−maj=1, pX=0.15, N=325
var.3.F5 1:169511555:T:C
peptide level
172:172
135:135
18:18
AEVDDVIQVRpc2_NP.3
p−maj=1, pX=0.31, N=325
var.3.F5 1:169511555:T:C
peptide level
172:172
135:135
18:18
AEVDDVIQVRpc2_NP.4
p−maj=1, pX=0.62, N=325
var.3.F5 1:169511555:T:C
peptide level
168:172
133:135
18:18
AEVDDVIQVRpc2_NP.5
p−min=0.69, pX=0.66, N=319

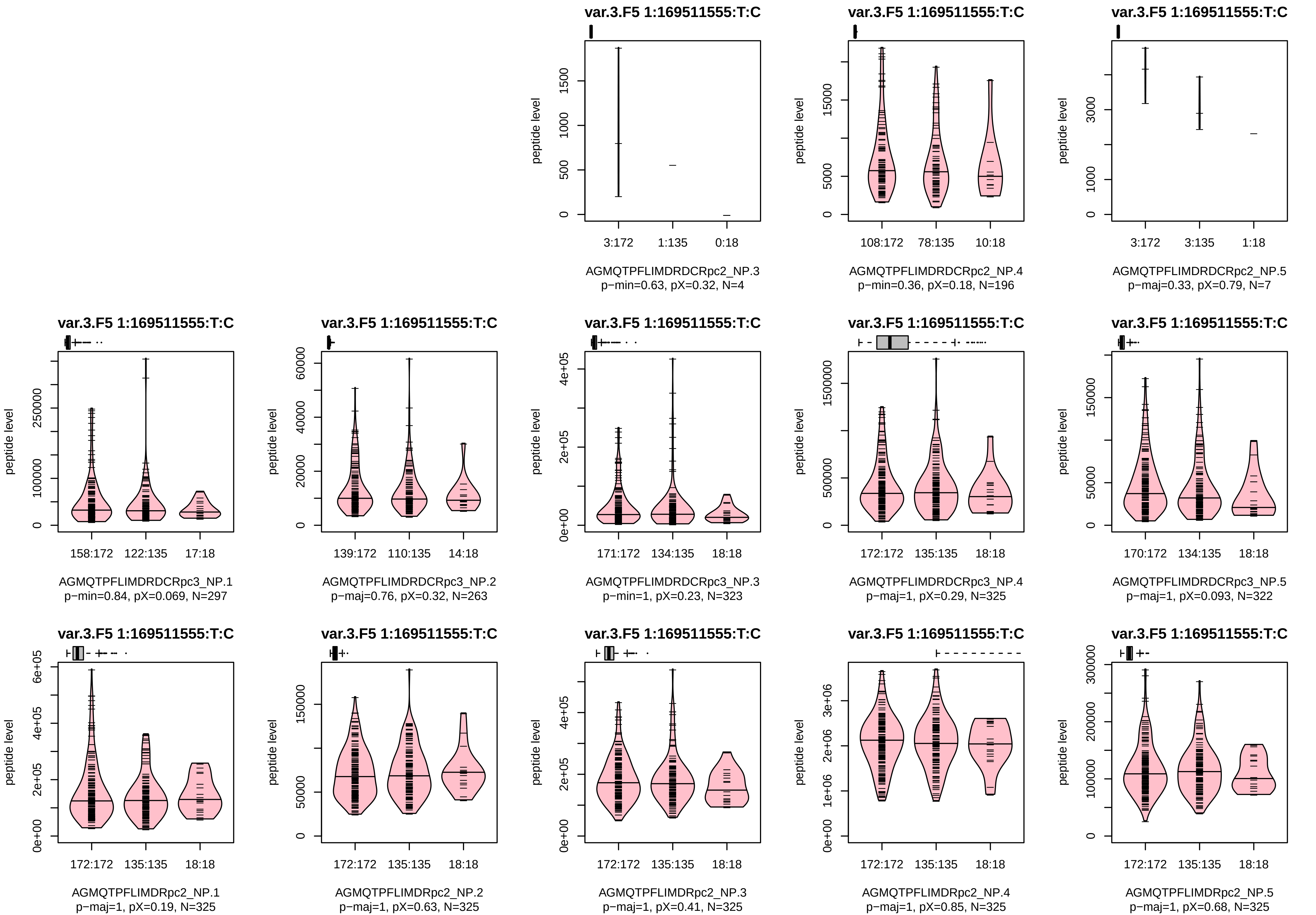
var.3.F5 1:169511555:T:C
peptide level
3:172
1:135
0:18
AGMQTPFLIMDRDCRpc2_NP.3
p−min=0.63, pX=0.32, N=4
var.3.F5 1:169511555:T:C
peptide level
108:172
78:135
10:18
AGMQTPFLIMDRDCRpc2_NP.4
p−min=0.36, pX=0.18, N=196
var.3.F5 1:169511555:T:C
peptide level
3:172
3:135
1:18
AGMQTPFLIMDRDCRpc2_NP.5
p−maj=0.33, pX=0.79, N=7
var.3.F5 1:169511555:T:C
peptide level
158:172
122:135
17:18
AGMQTPFLIMDRDCRpc3_NP.1
p−min=0.84, pX=0.069, N=297
var.3.F5 1:169511555:T:C
peptide level
139:172
110:135
14:18
AGMQTPFLIMDRDCRpc3_NP.2
p−maj=0.76, pX=0.32, N=263
var.3.F5 1:169511555:T:C
peptide level
171:172
134:135
18:18
AGMQTPFLIMDRDCRpc3_NP.3
p−min=1, pX=0.23, N=323
var.3.F5 1:169511555:T:C
peptide level
172:172
135:135
18:18
AGMQTPFLIMDRDCRpc3_NP.4
p−maj=1, pX=0.29, N=325
var.3.F5 1:169511555:T:C
peptide level
170:172
134:135
18:18
AGMQTPFLIMDRDCRpc3_NP.5
p−maj=1, pX=0.093, N=322
var.3.F5 1:169511555:T:C
peptide level
172:172
135:135
18:18
AGMQTPFLIMDRpc2_NP.1
p−maj=1, pX=0.19, N=325
var.3.F5 1:169511555:T:C
peptide level
172:172
135:135
18:18
AGMQTPFLIMDRpc2_NP.2
p−maj=1, pX=0.63, N=325
var.3.F5 1:169511555:T:C
peptide level
172:172
135:135
18:18
AGMQTPFLIMDRpc2_NP.3
p−maj=1, pX=0.41, N=325
var.3.F5 1:169511555:T:C
peptide level
172:172
135:135
18:18
AGMQTPFLIMDRpc2_NP.4
p−maj=1, pX=0.85, N=325
var.3.F5 1:169511555:T:C
peptide level
172:172
135:135
18:18
AGMQTPFLIMDRpc2_NP.5
p−maj=1, pX=0.68, N=325

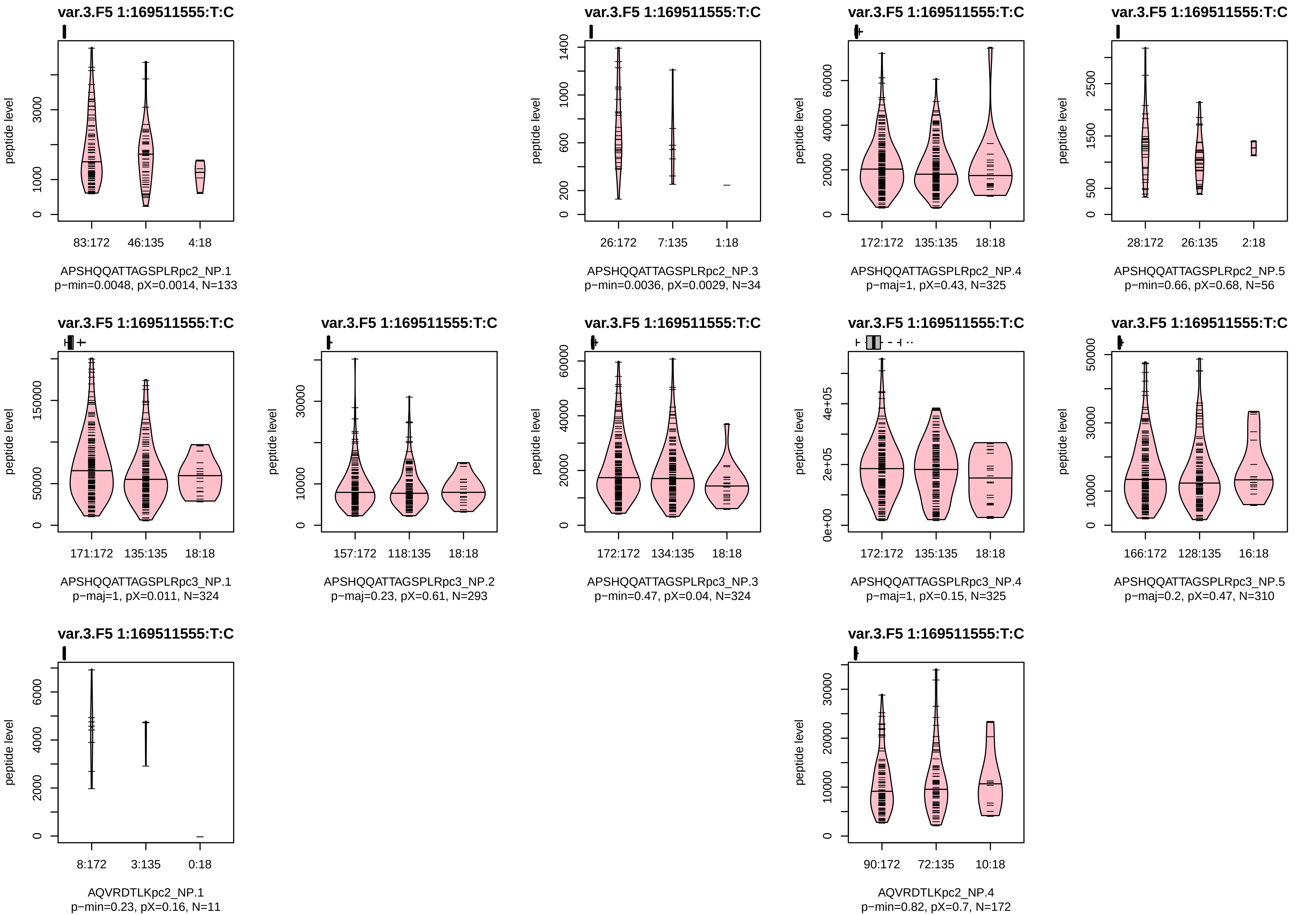
var.3.F5 1:169511555:T:C
peptide level
0
1000
3000
83:172
46:135
4:18
APSHQQATTAGSPLRpc2_NP.1
p−min=0.0048, pX=0.0014, N=133
var.3.F5 1:169511555:T:C
peptide level
0
200
600
1000
1400
26:172
7:135
1:18
APSHQQATTAGSPLRpc2_NP.3
p−min=0.0036, pX=0.0029, N=34
var.3.F5 1:169511555:T:C
peptide level
0
20000
40000
60000
172:172
135:135
18:18
APSHQQATTAGSPLRpc2_NP.4
p−maj=1, pX=0.43, N=325
var.3.F5 1:169511555:T:C
peptide level
0
500
1500
2500
28:172
26:135
2:18
APSHQQATTAGSPLRpc2_NP.5
p−min=0.66, pX=0.68, N=56
var.3.F5 1:169511555:T:C
peptide level
0
50000
150000
171:172
135:135
18:18
APSHQQATTAGSPLRpc3_NP.1
p−maj=1, pX=0.011, N=324
var.3.F5 1:169511555:T:C
peptide level
0
10000
30000
157:172
118:135
18:18
APSHQQATTAGSPLRpc3_NP.2
p−maj=0.23, pX=0.61, N=293
var.3.F5 1:169511555:T:C
peptide level
0
20000
40000
60000
172:172
134:135
18:18
APSHQQATTAGSPLRpc3_NP.3
p−min=0.47, pX=0.04, N=324
var.3.F5 1:169511555:T:C
peptide level
0e+00
2e+05
4e+05
172:172
135:135
18:18
APSHQQATTAGSPLRpc3_NP.4
p−maj=1, pX=0.15, N=325
var.3.F5 1:169511555:T:C
peptide level
0
10000
30000
50000
166:172
128:135
16:18
APSHQQATTAGSPLRpc3_NP.5
p−maj=0.2, pX=0.47, N=310
var.3.F5 1:169511555:T:C
peptide level
0
2000
4000
6000
8:172
3:135
0:18
AQVRDTLKpc2_NP.1
p−min=0.23, pX=0.16, N=11
var.3.F5 1:169511555:T:C
peptide level
0
10000
20000
30000
90:172
72:135
10:18
AQVRDTLKpc2_NP.4
p−min=0.82, pX=0.7, N=172

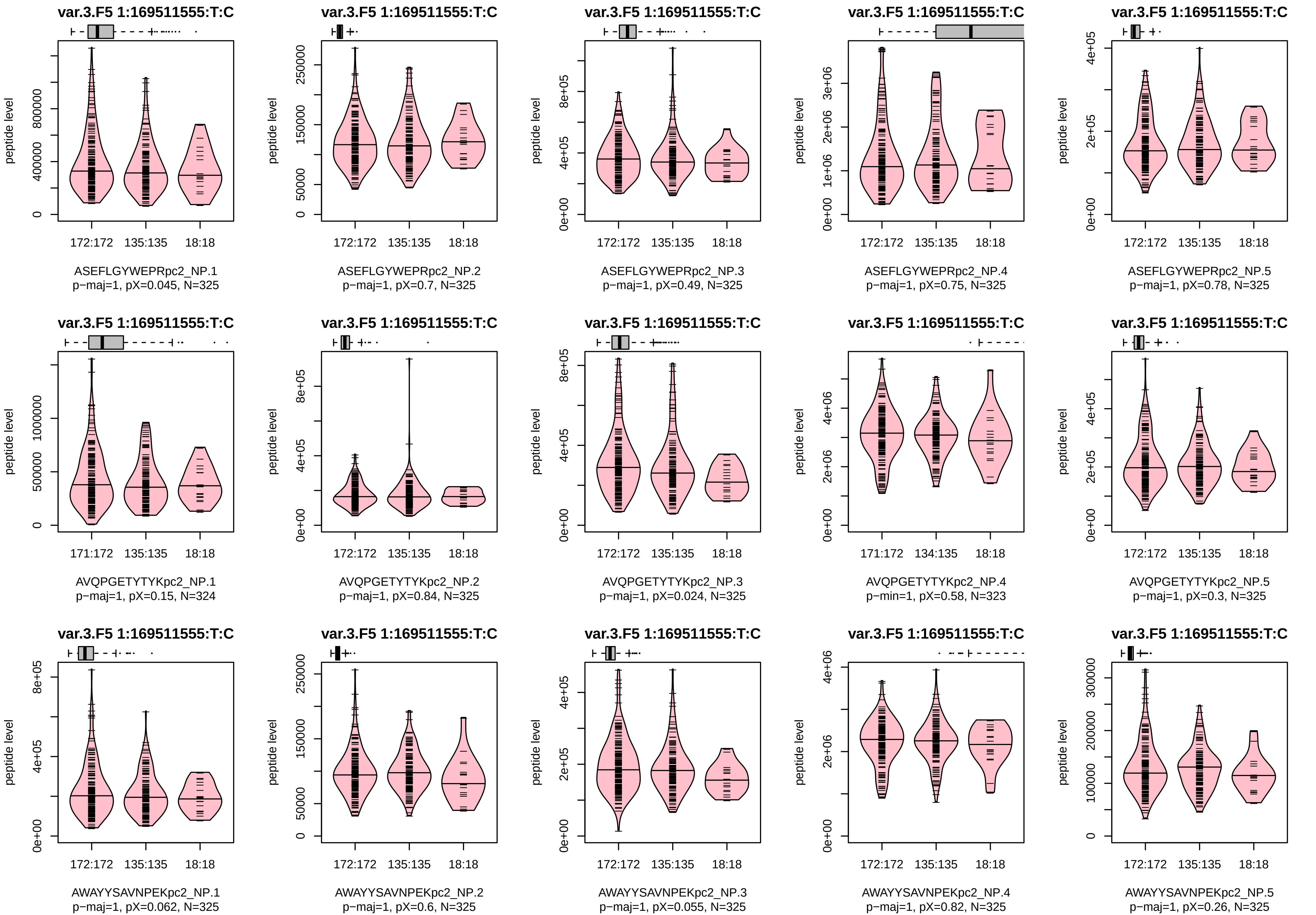
var.3.F5 1:169511555:T:C
peptide level
0
400000
800000
172:172
135:135
18:18
ASEFLGYWEPRpc2_NP.1
p−maj=1, pX=0.045, N=325
var.3.F5 1:169511555:T:C
peptide level
0
50000
150000
250000
172:172
135:135
18:18
ASEFLGYWEPRpc2_NP.2
p−maj=1, pX=0.7, N=325
var.3.F5 1:169511555:T:C
peptide level
0e+00
4e+05
8e+05
172:172
135:135
18:18
ASEFLGYWEPRpc2_NP.3
p−maj=1, pX=0.49, N=325
var.3.F5 1:169511555:T:C
peptide level
0e+00
1e+06
2e+06
3e+06
172:172
135:135
18:18
ASEFLGYWEPRpc2_NP.4
p−maj=1, pX=0.75, N=325
var.3.F5 1:169511555:T:C
peptide level
0e+00
2e+05
4e+05
172:172
135:135
18:18
ASEFLGYWEPRpc2_NP.5
p−maj=1, pX=0.78, N=325
var.3.F5 1:169511555:T:C
peptide level
0
500000
1000000
171:172
135:135
18:18
AVQPGETYTYKpc2_NP.1
p−maj=1, pX=0.15, N=324
var.3.F5 1:169511555:T:C
peptide level
0e+00
4e+05
8e+05
172:172
135:135
18:18
AVQPGETYTYKpc2_NP.2
p−maj=1, pX=0.84, N=325
var.3.F5 1:169511555:T:C
peptide level
0e+00
4e+05
8e+05
172:172
135:135
18:18
AVQPGETYTYKpc2_NP.3
p−maj=1, pX=0.024, N=325
var.3.F5 1:169511555:T:C
peptide level
0e+00
2e+06
4e+06
171:172
134:135
18:18
AVQPGETYTYKpc2_NP.4
p−min=1, pX=0.58, N=323
var.3.F5 1:169511555:T:C
peptide level
0e+00
2e+05
4e+05
172:172
135:135
18:18
AVQPGETYTYKpc2_NP.5
p−maj=1, pX=0.3, N=325
var.3.F5 1:169511555:T:C
peptide level
0e+00
4e+05
8e+05
172:172
135:135
18:18
AWAYYSAVNPEKpc2_NP.1
p−maj=1, pX=0.062, N=325
var.3.F5 1:169511555:T:C
peptide level
0
50000
150000
250000
172:172
135:135
18:18
AWAYYSAVNPEKpc2_NP.2
p−maj=1, pX=0.6, N=325
var.3.F5 1:169511555:T:C
peptide level
0e+00
2e+05
4e+05
172:172
135:135
18:18
AWAYYSAVNPEKpc2_NP.3
p−maj=1, pX=0.055, N=325
var.3.F5 1:169511555:T:C
peptide level
0e+00
2e+06
4e+06
172:172
135:135
18:18
AWAYYSAVNPEKpc2_NP.4
p−maj=1, pX=0.82, N=325
var.3.F5 1:169511555:T:C
peptide level
0
100000
200000
300000
172:172
135:135
18:18
AWAYYSAVNPEKpc2_NP.5
p−maj=1, pX=0.26, N=325

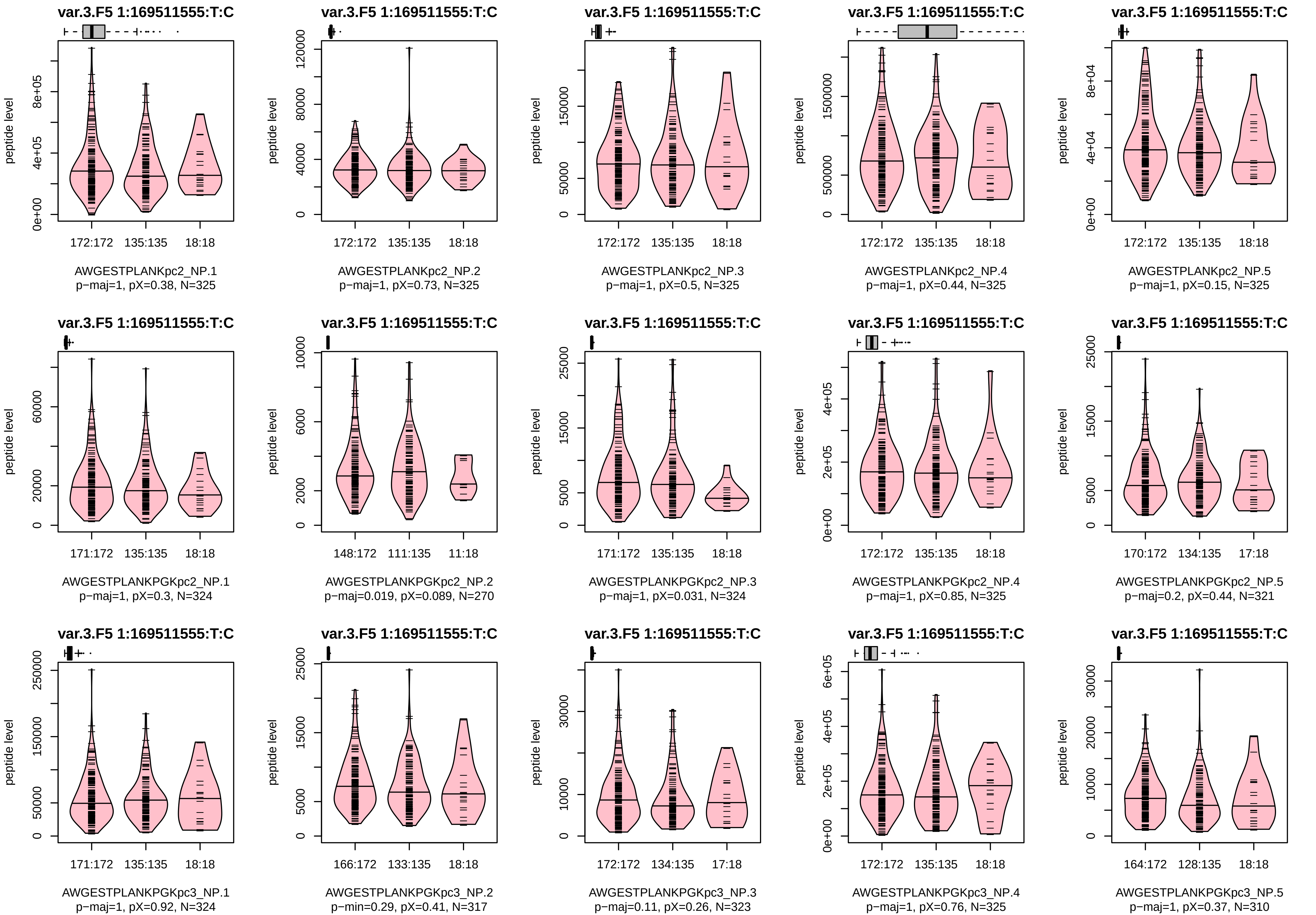
var.3.F5 1:169511555:T:C
peptide level
172:172
135:135
18:18
AWGESTPLANKpc2_NP.1
p−maj=1, pX=0.38, N=325
var.3.F5 1:169511555:T:C
peptide level
172:172
135:135
18:18
AWGESTPLANKpc2_NP.2
p−maj=1, pX=0.73, N=325
var.3.F5 1:169511555:T:C
peptide level
172:172
135:135
18:18
AWGESTPLANKpc2_NP.3
p−maj=1, pX=0.5, N=325
var.3.F5 1:169511555:T:C
peptide level
172:172
135:135
18:18
AWGESTPLANKpc2_NP.4
p−maj=1, pX=0.44, N=325
var.3.F5 1:169511555:T:C
peptide level
172:172
135:135
18:18
AWGESTPLANKpc2_NP.5
p−maj=1, pX=0.15, N=325
var.3.F5 1:169511555:T:C
peptide level
171:172
135:135
18:18
AWGESTPLANKPGKpc2_NP.1
p−maj=1, pX=0.3, N=324
var.3.F5 1:169511555:T:C
peptide level
148:172
111:135
11:18
AWGESTPLANKPGKpc2_NP.2
p−maj=0.019, pX=0.089, N=270
var.3.F5 1:169511555:T:C
peptide level
171:172
135:135
18:18
AWGESTPLANKPGKpc2_NP.3
p−maj=1, pX=0.031, N=324
var.3.F5 1:169511555:T:C
peptide level
172:172
135:135
18:18
AWGESTPLANKPGKpc2_NP.4
p−maj=1, pX=0.85, N=325
var.3.F5 1:169511555:T:C
peptide level
170:172
134:135
17:18
AWGESTPLANKPGKpc2_NP.5
p−maj=0.2, pX=0.44, N=321
var.3.F5 1:169511555:T:C
peptide level
171:172
135:135
18:18
AWGESTPLANKPGKpc3_NP.1
p−maj=1, pX=0.92, N=324
var.3.F5 1:169511555:T:C
peptide level
166:172
133:135
18:18
AWGESTPLANKPGKpc3_NP.2
p−min=0.29, pX=0.41, N=317
var.3.F5 1:169511555:T:C
peptide level
172:172
134:135
17:18
AWGESTPLANKPGKpc3_NP.3
p−maj=0.11, pX=0.26, N=323
var.3.F5 1:169511555:T:C
peptide level
172:172
135:135
18:18
AWGESTPLANKPGKpc3_NP.4
p−maj=1, pX=0.76, N=325
var.3.F5 1:169511555:T:C
peptide level
164:172
128:135
18:18
AWGESTPLANKPGKpc3_NP.5
p−maj=1, pX=0.37, N=310

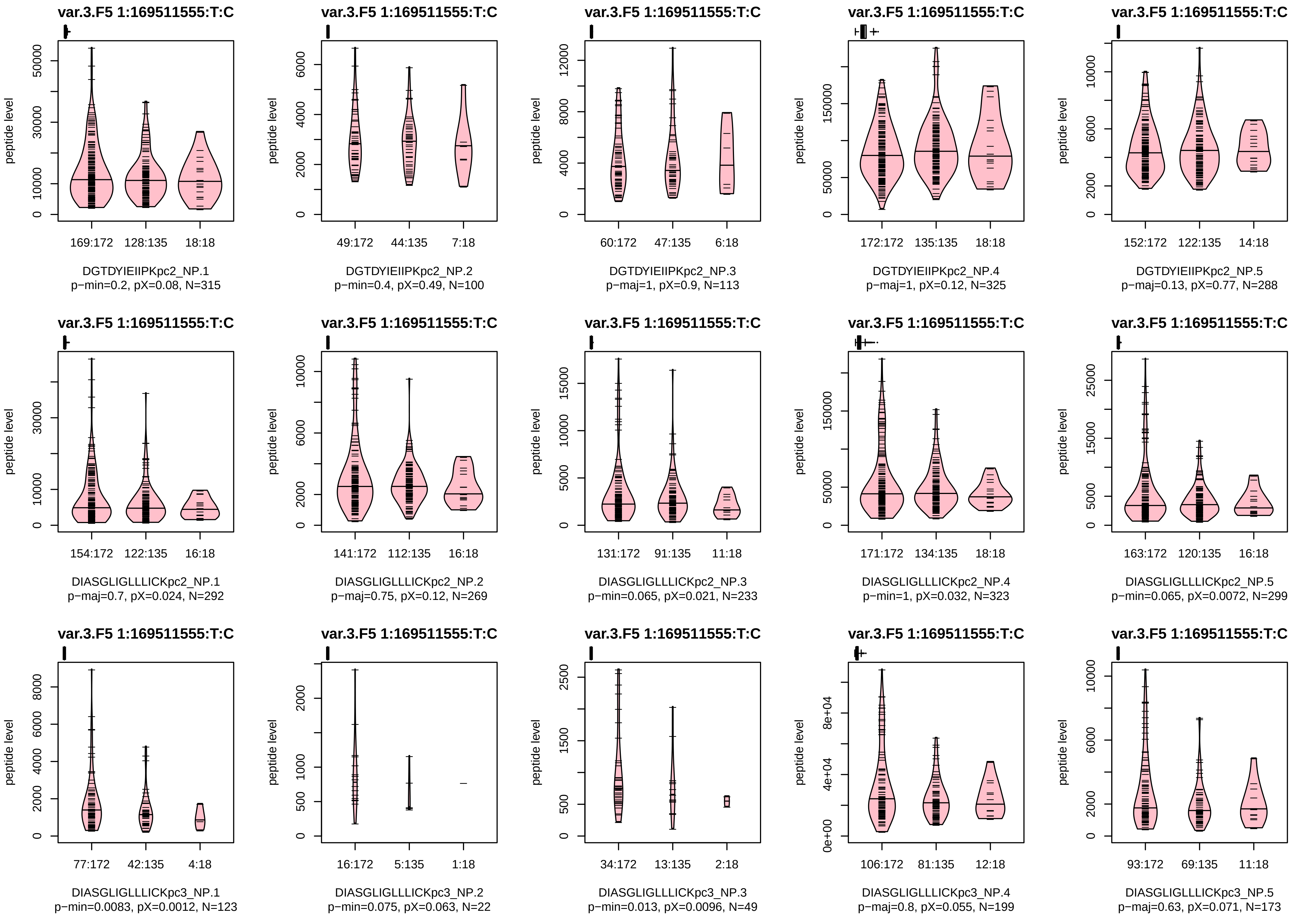
var.3.F5 1:169511555:T:C
peptide level
169:172
128:135
18:18
DGTDYIEIIPKpc2_NP.1
p−min=0.2, pX=0.08, N=315
var.3.F5 1:169511555:T:C
peptide level
49:172
44:135
7:18
DGTDYIEIIPKpc2_NP.2
p−min=0.4, pX=0.49, N=100
var.3.F5 1:169511555:T:C
peptide level
60:172
47:135
6:18
DGTDYIEIIPKpc2_NP.3
p−maj=1, pX=0.9, N=113
var.3.F5 1:169511555:T:C
peptide level
172:172
135:135
18:18
DGTDYIEIIPKpc2_NP.4
p−maj=1, pX=0.12, N=325
var.3.F5 1:169511555:T:C
peptide level
152:172
122:135
14:18
DGTDYIEIIPKpc2_NP.5
p−maj=0.13, pX=0.77, N=288
var.3.F5 1:169511555:T:C
peptide level
154:172
122:135
16:18
DIASGLIGLLLICKpc2_NP.1
p−maj=0.7, pX=0.024, N=292
var.3.F5 1:169511555:T:C
peptide level
141:172
112:135
16:18
DIASGLIGLLLICKpc2_NP.2
p−maj=0.75, pX=0.12, N=269
var.3.F5 1:169511555:T:C
peptide level
131:172
91:135
11:18
DIASGLIGLLLICKpc2_NP.3
p−min=0.065, pX=0.021, N=233
var.3.F5 1:169511555:T:C
peptide level
171:172
134:135
18:18
DIASGLIGLLLICKpc2_NP.4
p−min=1, pX=0.032, N=323
var.3.F5 1:169511555:T:C
peptide level
163:172
120:135
16:18
DIASGLIGLLLICKpc2_NP.5
p−min=0.065, pX=0.0072, N=299
var.3.F5 1:169511555:T:C
peptide level
77:172
42:135
4:18
DIASGLIGLLLICKpc3_NP.1
p−min=0.0083, pX=0.0012, N=123
var.3.F5 1:169511555:T:C
peptide level
16:172
5:135
1:18
DIASGLIGLLLICKpc3_NP.2
p−min=0.075, pX=0.063, N=22
var.3.F5 1:169511555:T:C
peptide level
34:172
13:135
2:18
DIASGLIGLLLICKpc3_NP.3
p−min=0.013, pX=0.0096, N=49
var.3.F5 1:169511555:T:C
peptide level
106:172
81:135
12:18
DIASGLIGLLLICKpc3_NP.4
p−maj=0.8, pX=0.055, N=199
var.3.F5 1:169511555:T:C
peptide level
93:172
69:135
11:18
DIASGLIGLLLICKpc3_NP.5
p−maj=0.63, pX=0.071, N=173

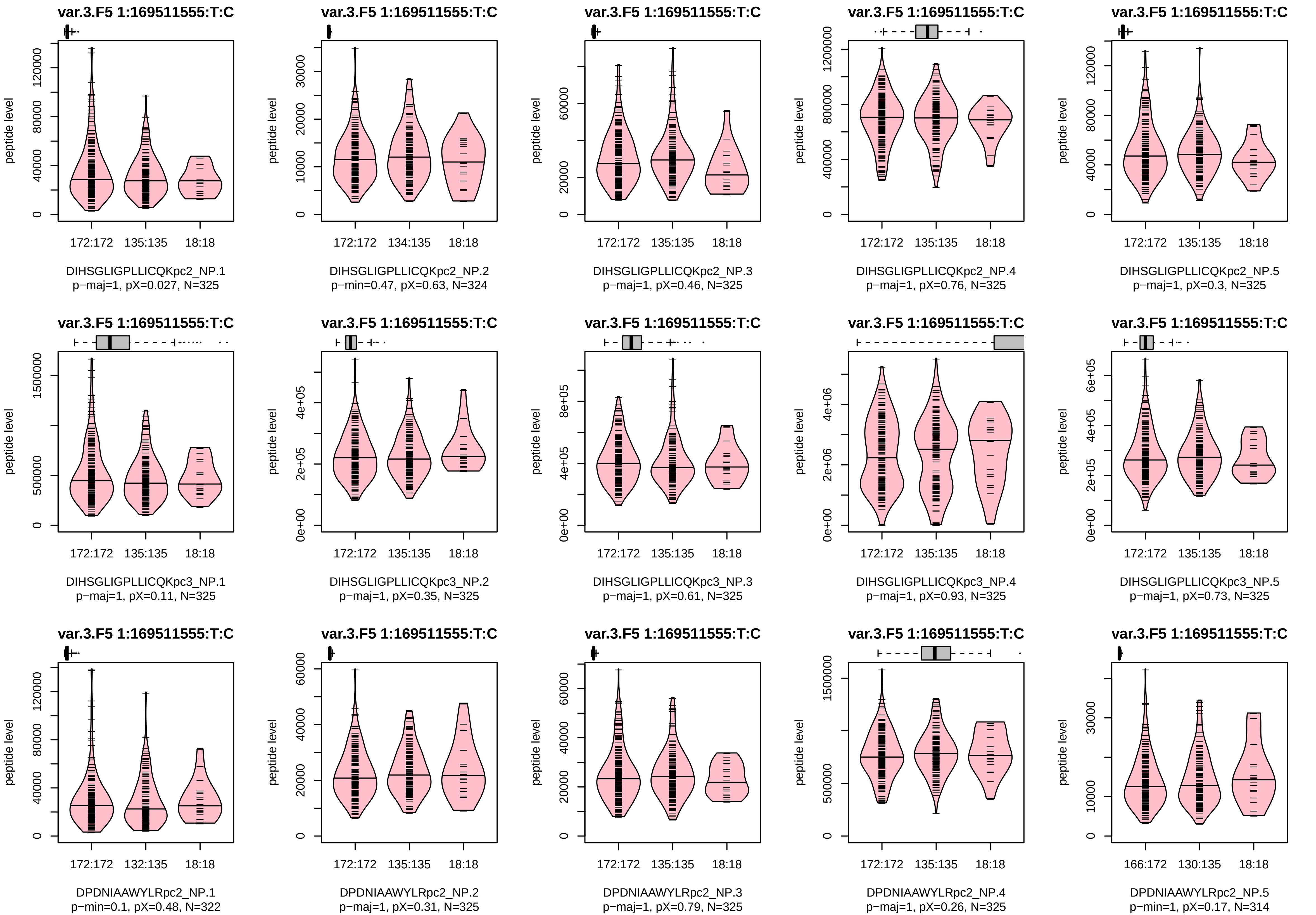
var.3.F5 1:169511555:T:C
peptide level
172:172
135:135
18:18
DIHSGLIGPLLICQKpc2_NP.1
p−maj=1, pX=0.027, N=325
var.3.F5 1:169511555:T:C
peptide level
172:172
134:135
18:18
DIHSGLIGPLLICQKpc2_NP.2
p−min=0.47, pX=0.63, N=324
var.3.F5 1:169511555:T:C
peptide level
172:172
135:135
18:18
DIHSGLIGPLLICQKpc2_NP.3
p−maj=1, pX=0.46, N=325
var.3.F5 1:169511555:T:C
peptide level
172:172
135:135
18:18
DIHSGLIGPLLICQKpc2_NP.4
p−maj=1, pX=0.76, N=325
var.3.F5 1:169511555:T:C
peptide level
172:172
135:135
18:18
DIHSGLIGPLLICQKpc2_NP.5
p−maj=1, pX=0.3, N=325
var.3.F5 1:169511555:T:C
peptide level
172:172
135:135
18:18
DIHSGLIGPLLICQKpc3_NP.1
p−maj=1, pX=0.11, N=325
var.3.F5 1:169511555:T:C
peptide level
172:172
135:135
18:18
DIHSGLIGPLLICQKpc3_NP.2
p−maj=1, pX=0.35, N=325
var.3.F5 1:169511555:T:C
peptide level
172:172
135:135
18:18
DIHSGLIGPLLICQKpc3_NP.3
p−maj=1, pX=0.61, N=325
var.3.F5 1:169511555:T:C
peptide level
172:172
135:135
18:18
DIHSGLIGPLLICQKpc3_NP.4
p−maj=1, pX=0.93, N=325
var.3.F5 1:169511555:T:C
peptide level
172:172
135:135
18:18
DIHSGLIGPLLICQKpc3_NP.5
p−maj=1, pX=0.73, N=325
var.3.F5 1:169511555:T:C
peptide level
172:172
132:135
18:18
DPDNIAAWYLRpc2_NP.1
p−min=0.1, pX=0.48, N=322
var.3.F5 1:169511555:T:C
peptide level
172:172
135:135
18:18
DPDNIAAWYLRpc2_NP.2
p−maj=1, pX=0.31, N=325
var.3.F5 1:169511555:T:C
peptide level
172:172
135:135
18:18
DPDNIAAWYLRpc2_NP.3
p−maj=1, pX=0.79, N=325
var.3.F5 1:169511555:T:C
peptide level
172:172
135:135
18:18
DPDNIAAWYLRpc2_NP.4
p−maj=1, pX=0.26, N=325
var.3.F5 1:169511555:T:C
peptide level
166:172
130:135
18:18
DPDNIAAWYLRpc2_NP.5
p−min=1, pX=0.17, N=314

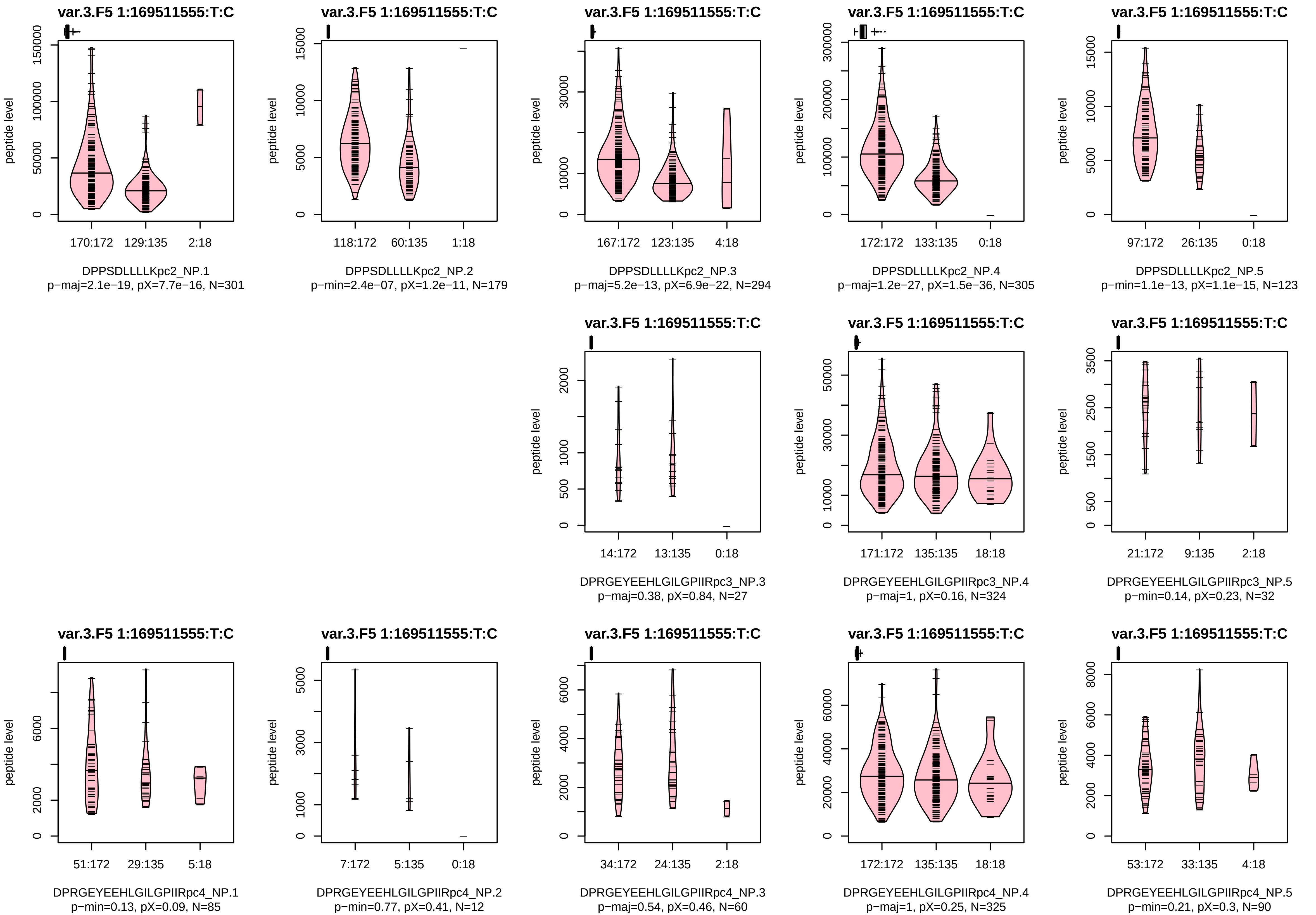
var.3.F5 1:169511555:T:C
peptide level
170:172
129:135
2:18
DPPSDLLLLKpc2_NP.1
p−maj=2.1e−19, pX=7.7e−16, N=301
var.3.F5 1:169511555:T:C
peptide level
118:172
60:135
1:18
DPPSDLLLLKpc2_NP.2
p−min=2.4e−07, pX=1.2e−11, N=179
var.3.F5 1:169511555:T:C
peptide level
167:172
123:135
4:18
DPPSDLLLLKpc2_NP.3
p−maj=5.2e−13, pX=6.9e−22, N=294
var.3.F5 1:169511555:T:C
peptide level
172:172
133:135
0:18
DPPSDLLLLKpc2_NP.4
p−maj=1.2e−27, pX=1.5e−36, N=305
var.3.F5 1:169511555:T:C
peptide level
97:172
26:135
0:18
DPPSDLLLLKpc2_NP.5
p−min=1.1e−13, pX=1.1e−15, N=123
var.3.F5 1:169511555:T:C
peptide level
14:172
13:135
0:18
DPRGEYEEHLGILGPIIRpc3_NP.3
p−maj=0.38, pX=0.84, N=27
var.3.F5 1:169511555:T:C
peptide level
171:172
135:135
18:18
DPRGEYEEHLGILGPIIRpc3_NP.4
p−maj=1, pX=0.16, N=324
var.3.F5 1:169511555:T:C
peptide level
21:172
9:135
2:18
DPRGEYEEHLGILGPIIRpc3_NP.5
p−min=0.14, pX=0.23, N=32
var.3.F5 1:169511555:T:C
peptide level
51:172
29:135
5:18
DPRGEYEEHLGILGPIIRpc4_NP.1
p−min=0.13, pX=0.09, N=85
var.3.F5 1:169511555:T:C
peptide level
7:172
5:135
0:18
DPRGEYEEHLGILGPIIRpc4_NP.2
p−min=0.77, pX=0.41, N=12
var.3.F5 1:169511555:T:C
peptide level
34:172
24:135
2:18
DPRGEYEEHLGILGPIIRpc4_NP.3
p−maj=0.54, pX=0.46, N=60
var.3.F5 1:169511555:T:C
peptide level
172:172
135:135
18:18
DPRGEYEEHLGILGPIIRpc4_NP.4
p−maj=1, pX=0.25, N=325
var.3.F5 1:169511555:T:C
peptide level
53:172
33:135
4:18
DPRGEYEEHLGILGPIIRpc4_NP.5
p−min=0.21, pX=0.3, N=90

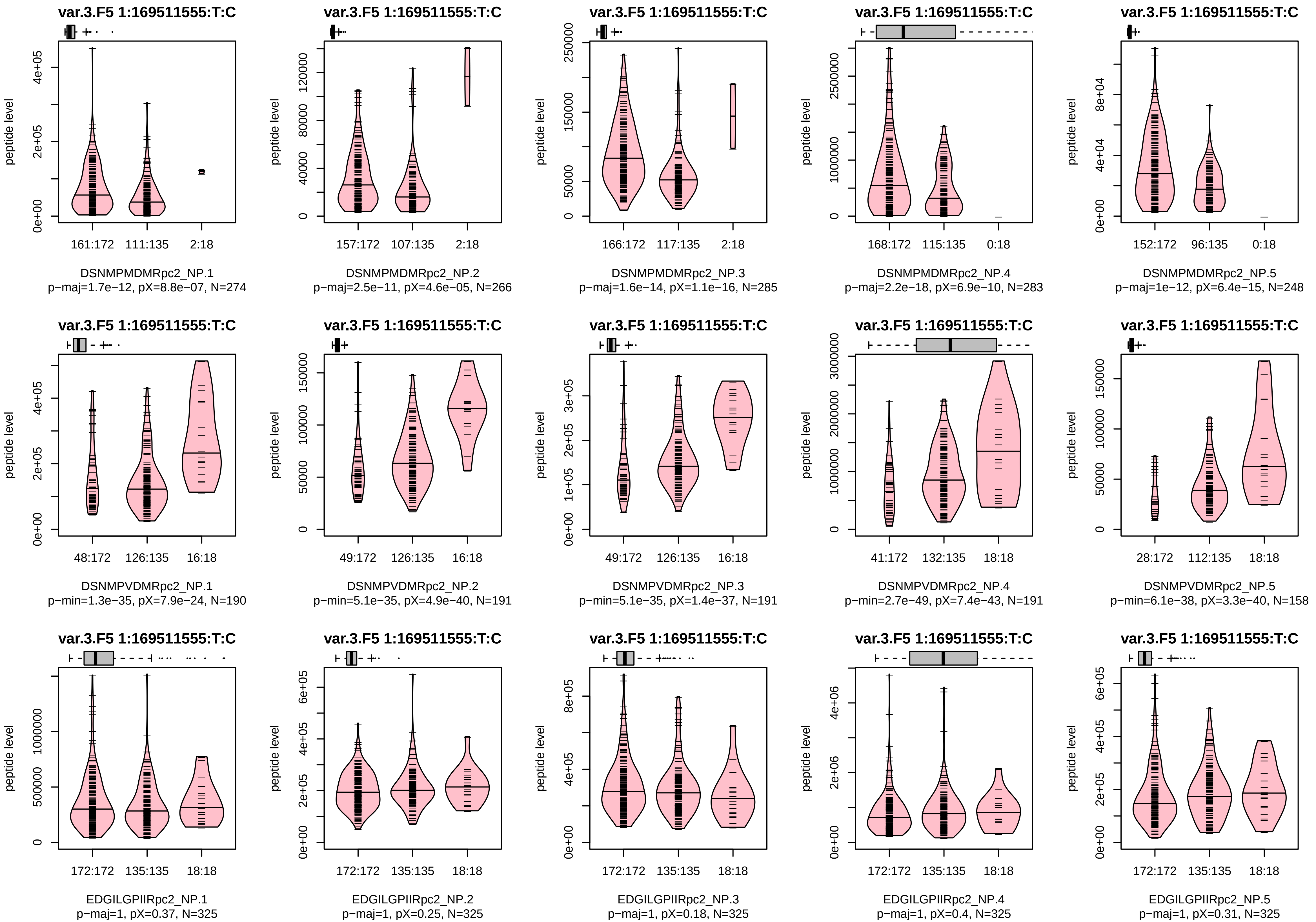
var.3.F5 1:169511555:T:C
peptide level
161:172
111:135
2:18
DSNMPMDMRpc2_NP.1
p−maj=1.7e−12, pX=8.8e−07, N=274
157:172
107:135
2:18
DSNMPMDMRpc2_NP.2
p−maj=2.5e−11, pX=4.6e−05, N=266
166:172
117:135
2:18
DSNMPMDMRpc2_NP.3
p−maj=1.6e−14, pX=1.1e−16, N=285
168:172
115:135
0:18
DSNMPMDMRpc2_NP.4
p−maj=2.2e−18, pX=6.9e−10, N=283
152:172
96:135
0:18
DSNMPMDMRpc2_NP.5
p−maj=1e−12, pX=6.4e−15, N=248
48:172
126:135
16:18
DSNMPVDMRpc2_NP.1
p−min=1.3e−35, pX=7.9e−24, N=190
49:172
126:135
16:18
DSNMPVDMRpc2_NP.2
p−min=5.1e−35, pX=4.9e−40, N=191
49:172
126:135
16:18
DSNMPVDMRpc2_NP.3
p−min=5.1e−35, pX=1.4e−37, N=191
41:172
132:135
18:18
DSNMPVDMRpc2_NP.4
p−min=2.7e−49, pX=7.4e−43, N=191
28:172
112:135
18:18
DSNMPVDMRpc2_NP.5
p−min=6.1e−38, pX=3.3e−40, N=158
172:172
135:135
18:18
EDGILGPIIRpc2_NP.1
p−maj=1, pX=0.37, N=325
EDGILGPIIRpc2_NP.2
p−maj=1, pX=0.25, N=325
EDGILGPIIRpc2_NP.3
p−maj=1, pX=0.18, N=325
EDGILGPIIRpc2_NP.4
p−maj=1, pX=0.4, N=325
EDGILGPIIRpc2_NP.5
p−maj=1, pX=0.31, N=325

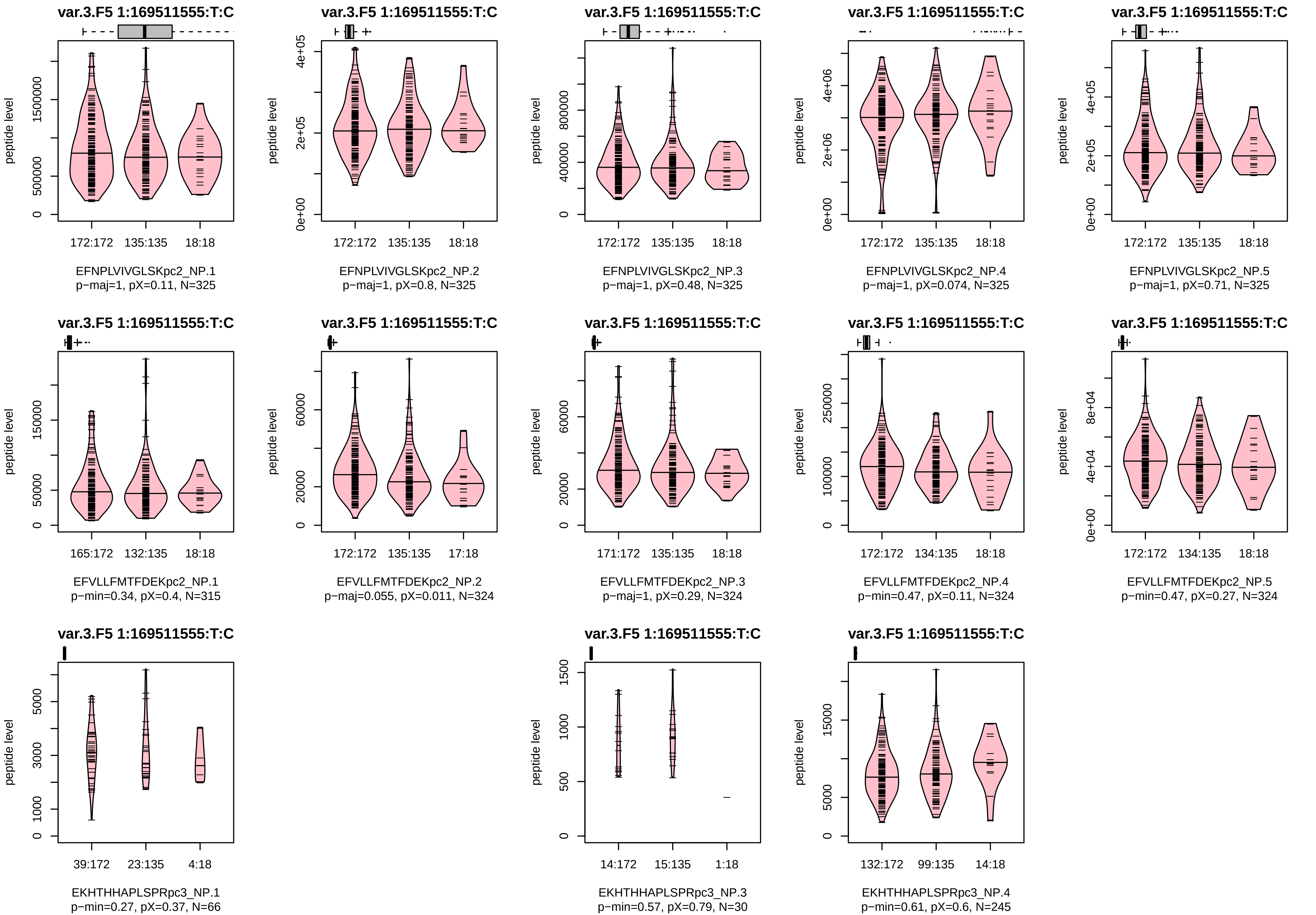
var.3.F5 1:169511555:T:C
peptide level
172:172
135:135
18:18
EFNPLVIVGLSKpc2_NP.1
p−maj=1, pX=0.11, N=325
var.3.F5 1:169511555:T:C
peptide level
172:172
135:135
18:18
EFNPLVIVGLSKpc2_NP.2
p−maj=1, pX=0.8, N=325
var.3.F5 1:169511555:T:C
peptide level
172:172
135:135
18:18
EFNPLVIVGLSKpc2_NP.3
p−maj=1, pX=0.48, N=325
var.3.F5 1:169511555:T:C
peptide level
172:172
135:135
18:18
EFNPLVIVGLSKpc2_NP.4
p−maj=1, pX=0.074, N=325
var.3.F5 1:169511555:T:C
peptide level
172:172
135:135
18:18
EFNPLVIVGLSKpc2_NP.5
p−maj=1, pX=0.71, N=325
var.3.F5 1:169511555:T:C
peptide level
165:172
132:135
18:18
EFVLLFMTFDEKpc2_NP.1
p−min=0.34, pX=0.4, N=315
var.3.F5 1:169511555:T:C
peptide level
172:172
135:135
17:18
EFVLLFMTFDEKpc2_NP.2
p−maj=0.055, pX=0.011, N=324
var.3.F5 1:169511555:T:C
peptide level
171:172
135:135
18:18
EFVLLFMTFDEKpc2_NP.3
p−maj=1, pX=0.29, N=324
var.3.F5 1:169511555:T:C
peptide level
172:172
134:135
18:18
EFVLLFMTFDEKpc2_NP.4
p−min=0.47, pX=0.11, N=324
var.3.F5 1:169511555:T:C
peptide level
172:172
134:135
18:18
EFVLLFMTFDEKpc2_NP.5
p−min=0.47, pX=0.27, N=324
var.3.F5 1:169511555:T:C
peptide level
39:172
23:135
4:18
EKHTHHAPLSPRpc3_NP.1
p−min=0.27, pX=0.37, N=66
var.3.F5 1:169511555:T:C
peptide level
14:172
15:135
1:18
EKHTHHAPLSPRpc3_NP.3
p−min=0.57, pX=0.79, N=30
var.3.F5 1:169511555:T:C
peptide level
132:172
99:135
14:18
EKHTHHAPLSPRpc3_NP.4
p−min=0.61, pX=0.6, N=245

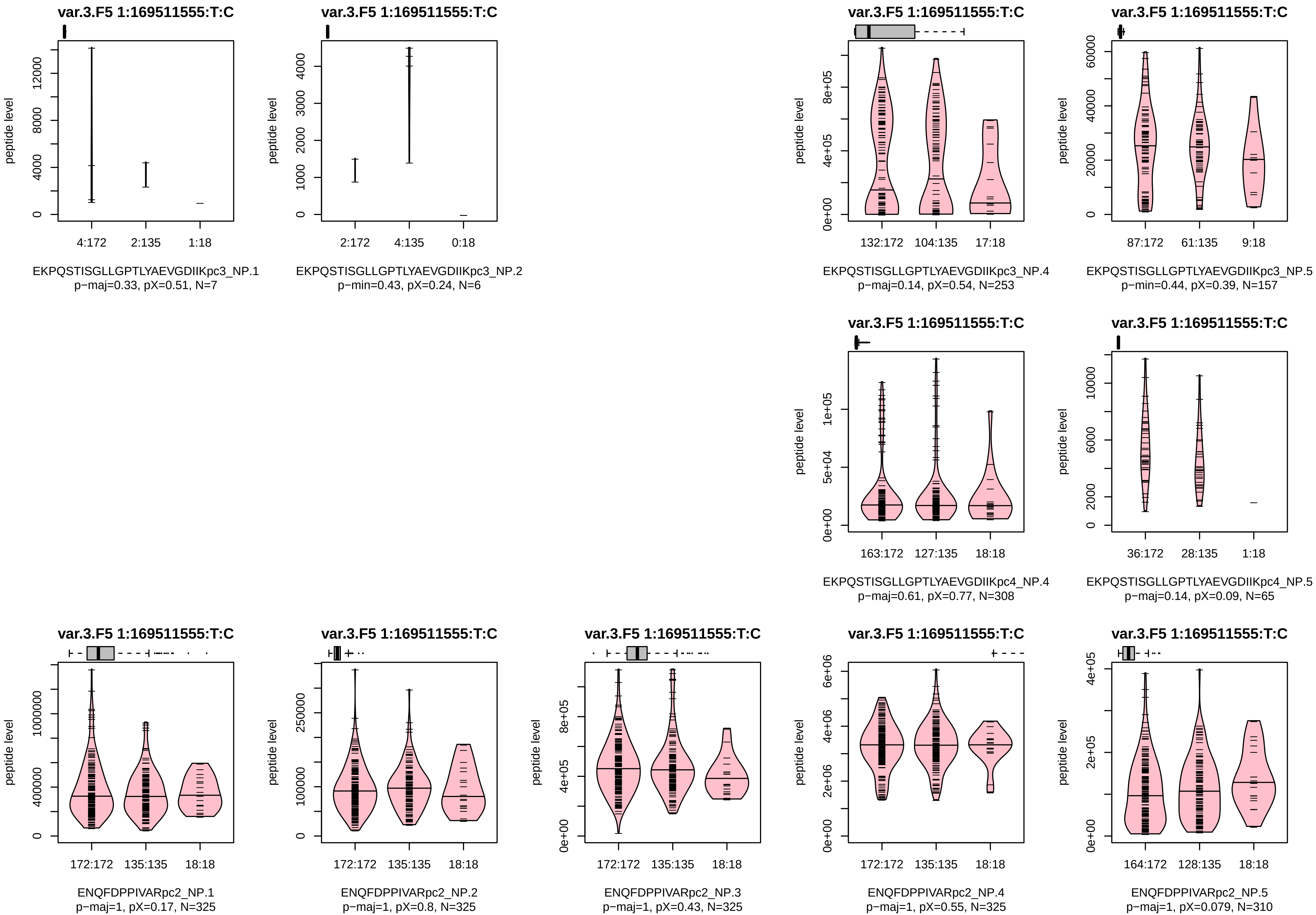
var.3.F5 1:169511555:T:C
peptide level
4:172
2:135
1:18
EKPQSTISGLLGPTLYAEVGDIIKpc3_NP.1
p−maj=0.33, pX=0.51, N=7
var.3.F5 1:169511555:T:C
peptide level
2:172
4:135
0:18
EKPQSTISGLLGPTLYAEVGDIIKpc3_NP.2
p−min=0.43, pX=0.24, N=6
var.3.F5 1:169511555:T:C
peptide level
132:172
104:135
17:18
EKPQSTISGLLGPTLYAEVGDIIKpc3_NP.4
p−maj=0.14, pX=0.54, N=253
var.3.F5 1:169511555:T:C
peptide level
87:172
61:135
9:18
EKPQSTISGLLGPTLYAEVGDIIKpc3_NP.5
p−min=0.44, pX=0.39, N=157
var.3.F5 1:169511555:T:C
peptide level
163:172
127:135
18:18
EKPQSTISGLLGPTLYAEVGDIIKpc4_NP.4
p−maj=0.61, pX=0.77, N=308
var.3.F5 1:169511555:T:C
peptide level
36:172
28:135
1:18
EKPQSTISGLLGPTLYAEVGDIIKpc4_NP.5
p−maj=0.14, pX=0.09, N=65
var.3.F5 1:169511555:T:C
peptide level
172:172
135:135
18:18
ENQFDPPIVARpc2_NP.1
p−maj=1, pX=0.17, N=325
var.3.F5 1:169511555:T:C
peptide level
172:172
135:135
18:18
ENQFDPPIVARpc2_NP.2
p−maj=1, pX=0.8, N=325
var.3.F5 1:169511555:T:C
peptide level
172:172
135:135
18:18
ENQFDPPIVARpc2_NP.3
p−maj=1, pX=0.43, N=325
var.3.F5 1:169511555:T:C
peptide level
172:172
135:135
18:18
ENQFDPPIVARpc2_NP.4
p−maj=1, pX=0.55, N=325
var.3.F5 1:169511555:T:C
peptide level
164:172
128:135
18:18
ENQFDPPIVARpc2_NP.5
p−maj=1, pX=0.079, N=310

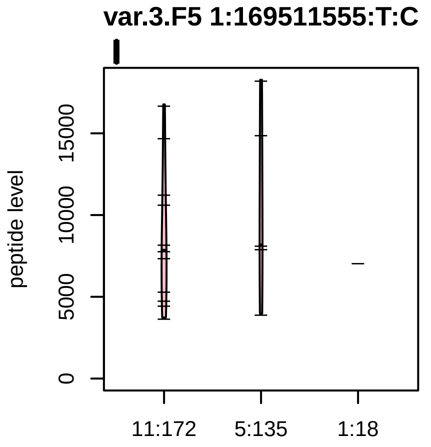

ENQFDPPIVARpc3_NP.1
p−min=0.46, pX=0.56, N=17

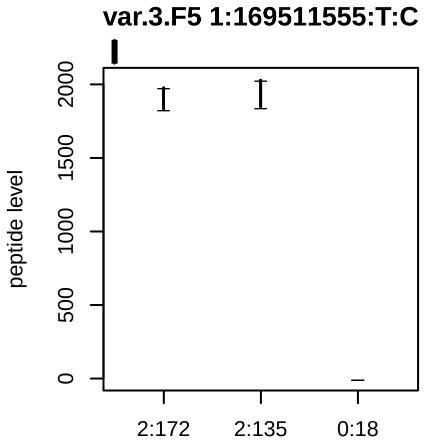

ENQFDPPIVARpc3_NP.2
p−maj=1, pX=0.94, N=4

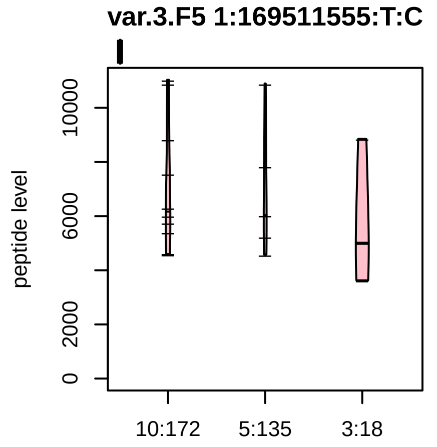

ENQFDPPIVARpc3_NP.3
p−maj=0.069, pX=0.79, N=18

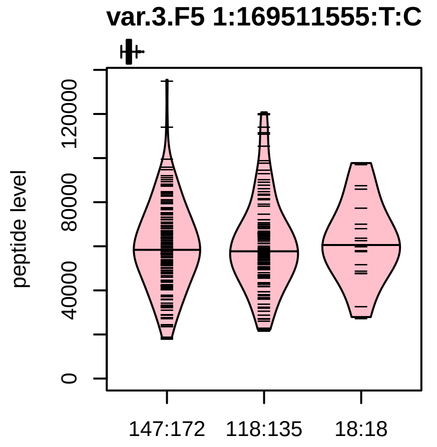

ENQFDPPIVARpc3_NP.4
p−maj=0.14, pX=0.15, N=283

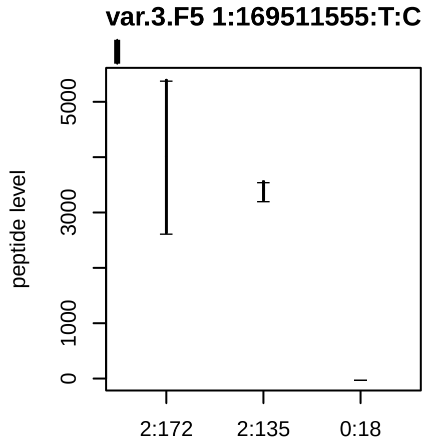

ENQFDPPIVARpc3_NP.5
p−maj=1, pX=0.83, N=4

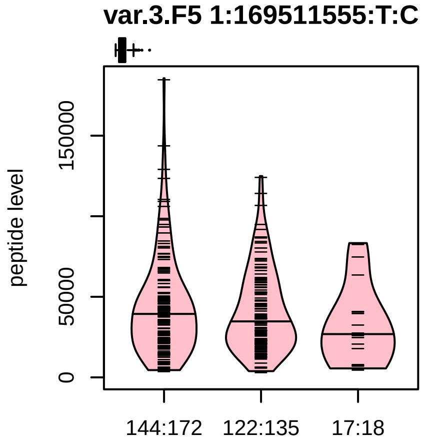

ETDIEDSDDIPEDTTYKKpc3_NP.1
p−min=0.068, pX=0.71, N=283

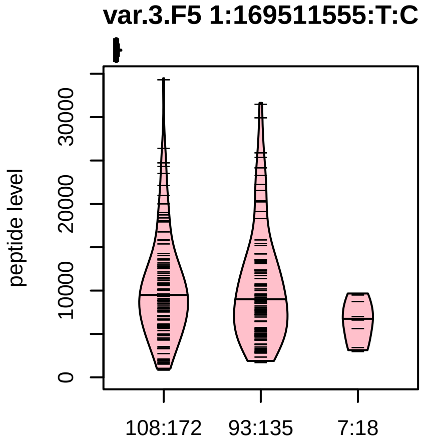

ETDIEDSDDIPEDTTYKKpc3_NP.2
p−maj=0.04, pX=0.46, N=208

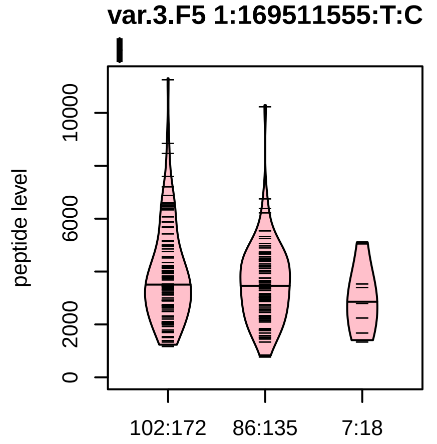

ETDIEDSDDIPEDTTYKKpc3_NP.3
p−maj=0.082, pX=0.18, N=195

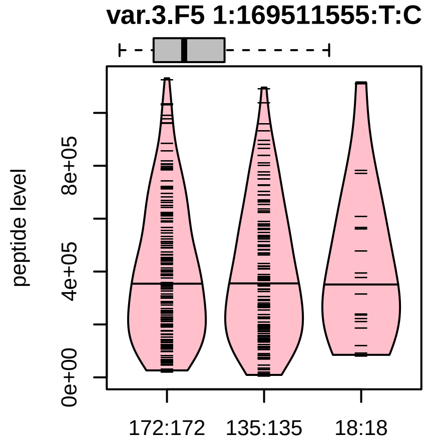

ETDIEDSDDIPEDTTYKKpc3_NP.4
p−maj=1, pX=0.86, N=325

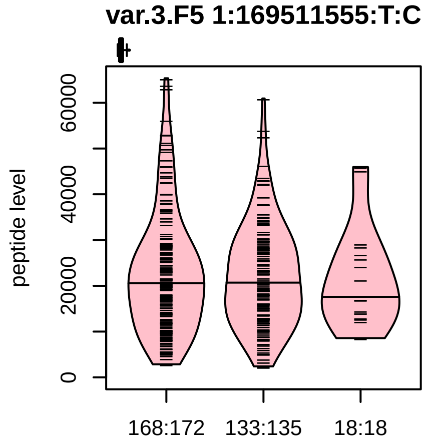

ETDIEDSDDIPEDTTYKKpc3_NP.5
p−min=0.69, pX=0.77, N=319

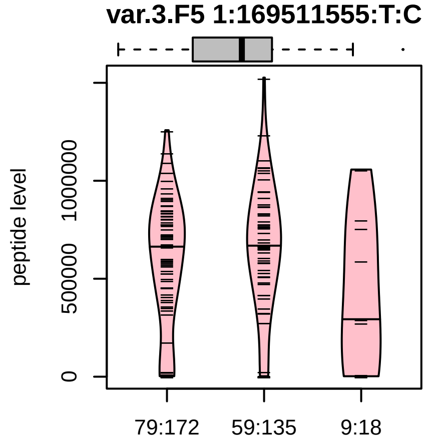

ETDIEDSDDIPEDTTYKpc2_NP.4
p−maj=0.81, pX=1, N=147

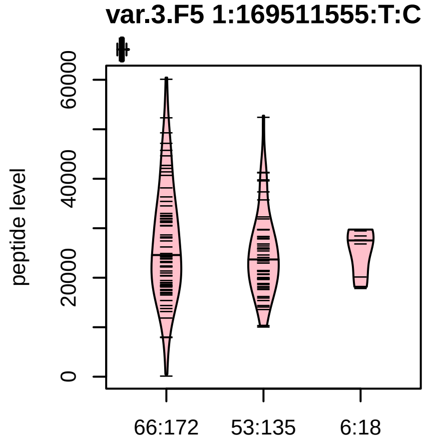

ETDIEDSDDIPEDTTYKpc2_NP.5
p−maj=0.8, pX=0.53, N=125

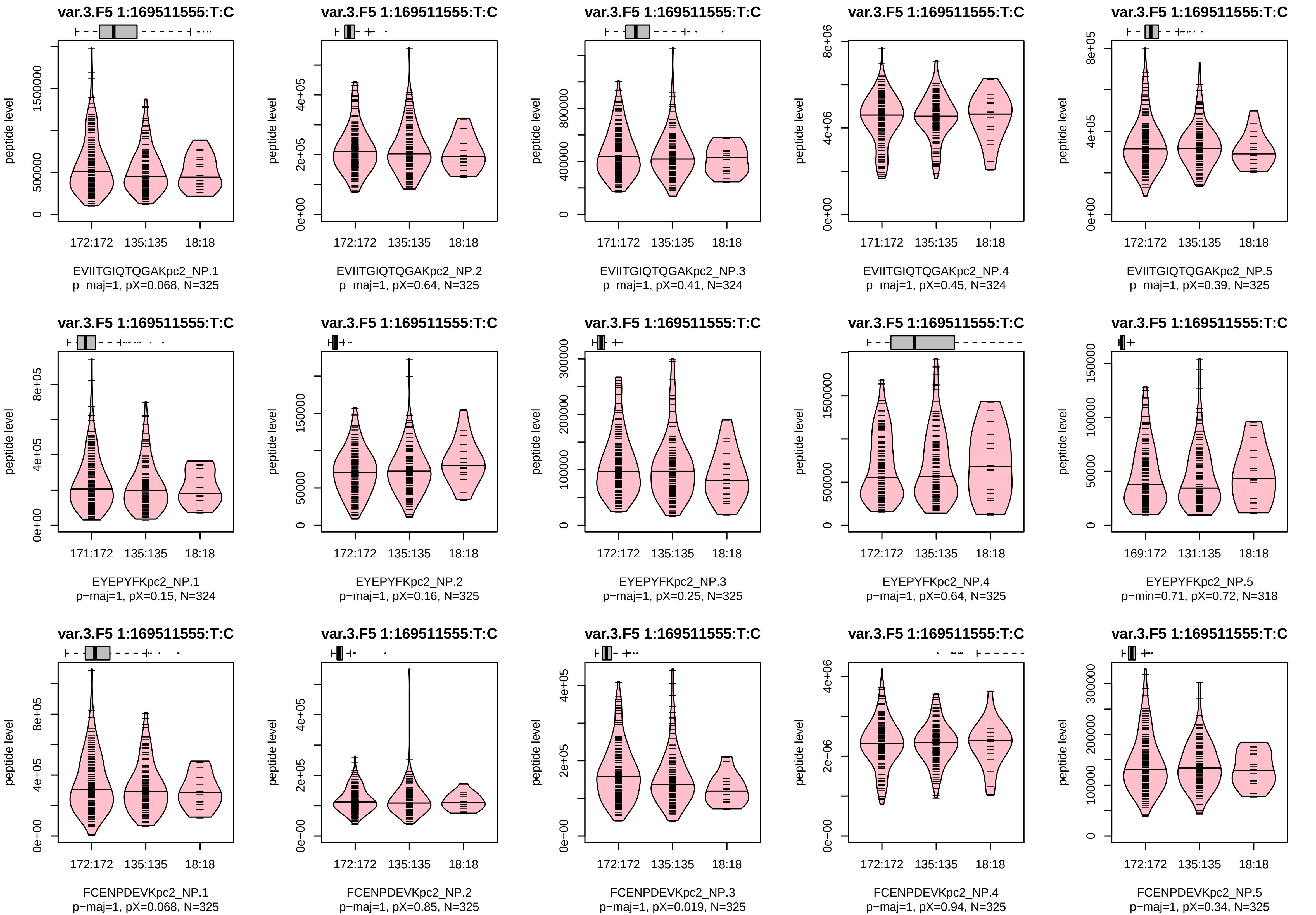
var.3.F5 1:169511555:T:C
peptide level
172:172
135:135
18:18
EVIITGIQTQGAKpc2_NP.1
p−maj=1, pX=0.068, N=325
EVIITGIQTQGAKpc2_NP.2
p−maj=1, pX=0.64, N=325
171:172
EVIITGIQTQGAKpc2_NP.3
p−maj=1, pX=0.41, N=324
EVIITGIQTQGAKpc2_NP.4
p−maj=1, pX=0.45, N=324
EVIITGIQTQGAKpc2_NP.5
p−maj=1, pX=0.39, N=325
EYEPYFKpc2_NP.1
p−maj=1, pX=0.15, N=324
EYEPYFKpc2_NP.2
p−maj=1, pX=0.16, N=325
EYEPYFKpc2_NP.3
p−maj=1, pX=0.25, N=325
EYEPYFKpc2_NP.4
p−maj=1, pX=0.64, N=325
169:172
131:135
EYEPYFKpc2_NP.5
p−min=0.71, pX=0.72, N=318
FCENPDEVKpc2_NP.1
p−maj=1, pX=0.068, N=325
FCENPDEVKpc2_NP.2
p−maj=1, pX=0.85, N=325
FCENPDEVKpc2_NP.3
p−maj=1, pX=0.019, N=325
FCENPDEVKpc2_NP.4
p−maj=1, pX=0.94, N=325
FCENPDEVKpc2_NP.5
p−maj=1, pX=0.34, N=325

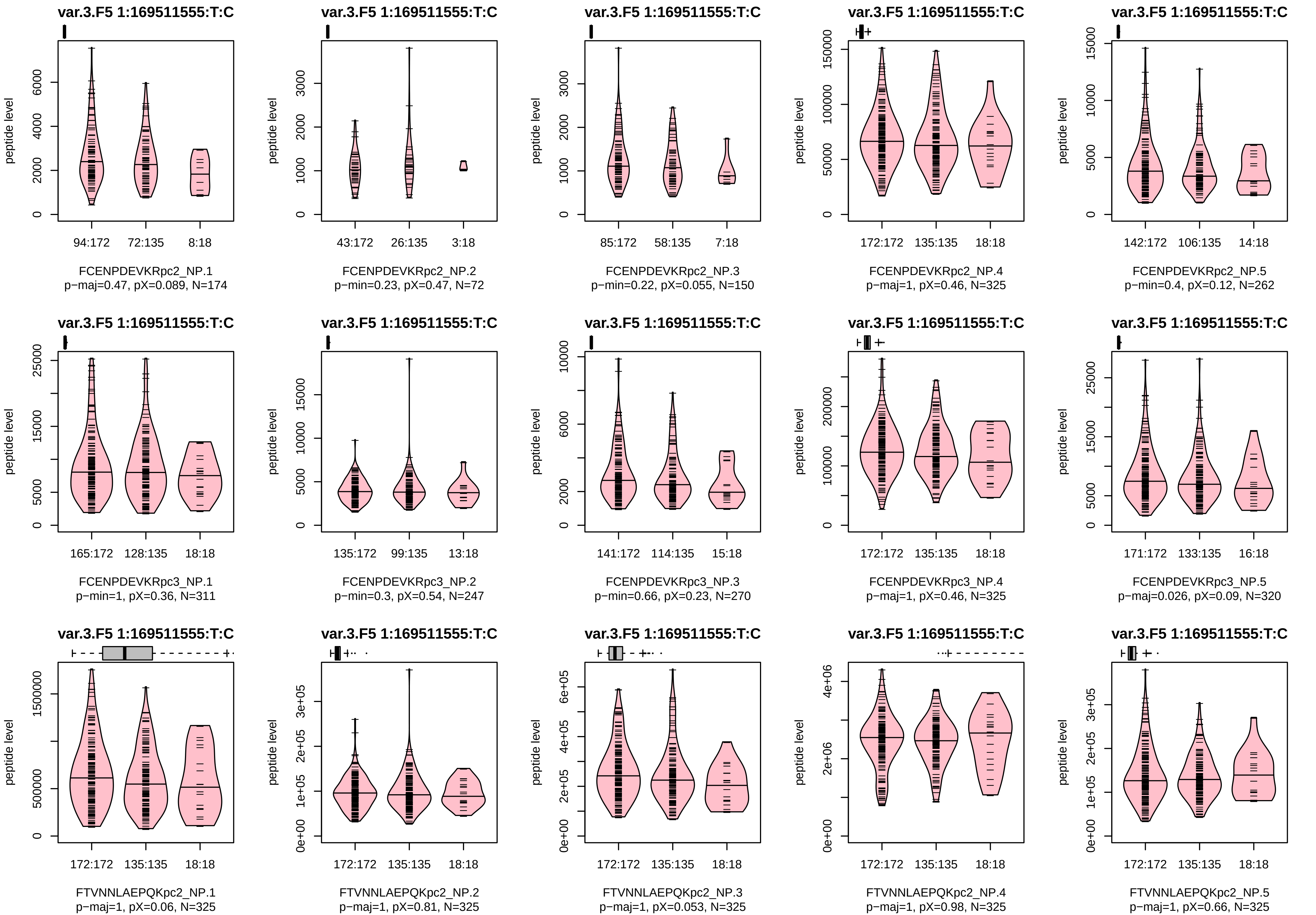

var.3.F5 1:169511555:T:C
peptide level
94:172
72:135
8:18
FCENPDEVKRpc2_NP.1
p−maj=0.47, pX=0.089, N=174
var.3.F5 1:169511555:T:C
peptide level
43:172
26:135
3:18
FCENPDEVKRpc2_NP.2
p−min=0.23, pX=0.47, N=72
var.3.F5 1:169511555:T:C
peptide level
85:172
58:135
7:18
FCENPDEVKRpc2_NP.3
p−min=0.22, pX=0.055, N=150
var.3.F5 1:169511555:T:C
peptide level
172:172
135:135
18:18
FCENPDEVKRpc2_NP.4
p−maj=1, pX=0.46, N=325
var.3.F5 1:169511555:T:C
peptide level
142:172
106:135
14:18
FCENPDEVKRpc2_NP.5
p−min=0.4, pX=0.12, N=262
var.3.F5 1:169511555:T:C
peptide level
165:172
128:135
18:18
FCENPDEVKRpc3_NP.1
p−min=1, pX=0.36, N=311
var.3.F5 1:169511555:T:C
peptide level
135:172
99:135
13:18
FCENPDEVKRpc3_NP.2
p−min=0.3, pX=0.54, N=247
var.3.F5 1:169511555:T:C
peptide level
141:172
114:135
15:18
FCENPDEVKRpc3_NP.3
p−min=0.66, pX=0.23, N=270
var.3.F5 1:169511555:T:C
peptide level
172:172
135:135
18:18
FCENPDEVKRpc3_NP.4
p−maj=1, pX=0.46, N=325
var.3.F5 1:169511555:T:C
peptide level
171:172
133:135
16:18
FCENPDEVKRpc3_NP.5
p−maj=0.026, pX=0.09, N=320
var.3.F5 1:169511555:T:C
peptide level
172:172
135:135
18:18
FTVNNLAEPQKpc2_NP.1
p−maj=1, pX=0.06, N=325
var.3.F5 1:169511555:T:C
peptide level
172:172
135:135
18:18
FTVNNLAEPQKpc2_NP.2
p−maj=1, pX=0.81, N=325
var.3.F5 1:169511555:T:C
peptide level
172:172
135:135
18:18
FTVNNLAEPQKpc2_NP.3
p−maj=1, pX=0.053, N=325
var.3.F5 1:169511555:T:C
peptide level
172:172
135:135
18:18
FTVNNLAEPQKpc2_NP.4
p−maj=1, pX=0.98, N=325
var.3.F5 1:169511555:T:C
peptide level
172:172
135:135
18:18
FTVNNLAEPQKpc2_NP.5
p−maj=1, pX=0.66, N=325

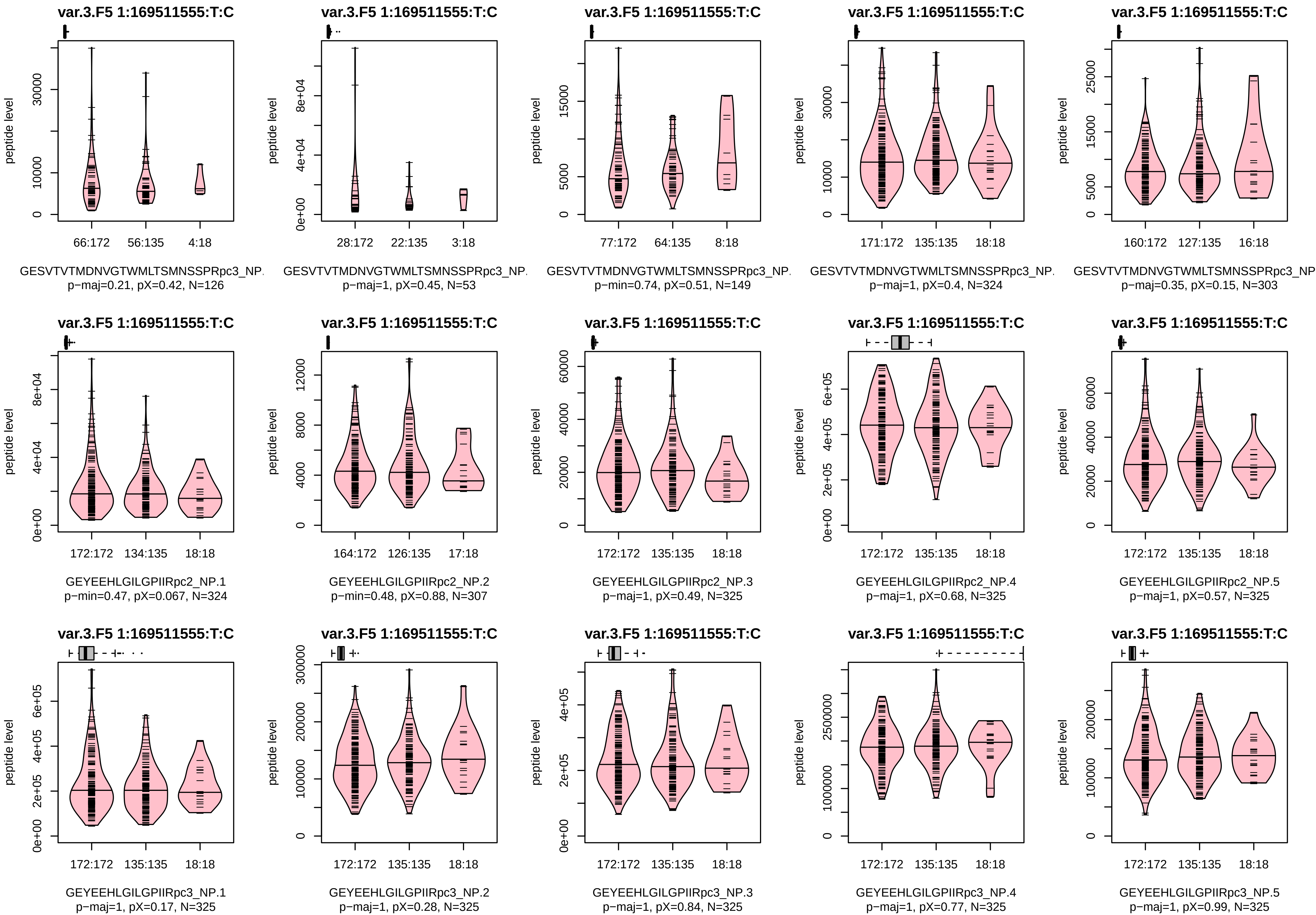
var.3.F5 1:169511555:T:C
peptide level
66:172 56:135 4:18
GESVTVTMDNVGTWMLTSMNSSPRpc3_NP.
p−maj=0.21, pX=0.42, N=126
var.3.F5 1:169511555:T:C
peptide level
28:172 22:135 3:18
GESVTVTMDNVGTWMLTSMNSSPRpc3_NP.
p−maj=1, pX=0.45, N=53
var.3.F5 1:169511555:T:C
peptide level
77:172 64:135 8:18
GESVTVTMDNVGTWMLTSMNSSPRpc3_NP.
p−min=0.74, pX=0.51, N=149
var.3.F5 1:169511555:T:C
peptide level
171:172 135:135 18:18
GESVTVTMDNVGTWMLTSMNSSPRpc3_NP.
p−maj=1, pX=0.4, N=324
var.3.F5 1:169511555:T:C
peptide level
160:172 127:135 16:18
GESVTVTMDNVGTWMLTSMNSSPRpc3_NP
p−maj=0.35, pX=0.15, N=303
var.3.F5 1:169511555:T:C
peptide level
172:172 134:135 18:18
GEYEEHLGILGPIIRpc2_NP.1
p−min=0.47, pX=0.067, N=324
var.3.F5 1:169511555:T:C
peptide level
164:172 126:135 17:18
GEYEEHLGILGPIIRpc2_NP.2
p−min=0.48, pX=0.88, N=307
var.3.F5 1:169511555:T:C
peptide level
172:172 135:135 18:18
GEYEEHLGILGPIIRpc2_NP.3
p−maj=1, pX=0.49, N=325
var.3.F5 1:169511555:T:C
peptide level
172:172 135:135 18:18
GEYEEHLGILGPIIRpc2_NP.4
p−maj=1, pX=0.68, N=325
var.3.F5 1:169511555:T:C
peptide level
172:172 135:135 18:18
GEYEEHLGILGPIIRpc2_NP.5
p−maj=1, pX=0.57, N=325
var.3.F5 1:169511555:T:C
peptide level
172:172 135:135 18:18
GEYEEHLGILGPIIRpc3_NP.1
p−maj=1, pX=0.17, N=325
var.3.F5 1:169511555:T:C
peptide level
172:172 135:135 18:18
GEYEEHLGILGPIIRpc3_NP.2
p−maj=1, pX=0.28, N=325
var.3.F5 1:169511555:T:C
peptide level
172:172 135:135 18:18
GEYEEHLGILGPIIRpc3_NP.3
p−maj=1, pX=0.84, N=325
var.3.F5 1:169511555:T:C
peptide level
172:172 135:135 18:18
GEYEEHLGILGPIIRpc3_NP.4
p−maj=1, pX=0.77, N=325
var.3.F5 1:169511555:T:C
peptide level
172:172 135:135 18:18
GEYEEHLGILGPIIRpc3_NP.5
p−maj=1, pX=0.99, N=325

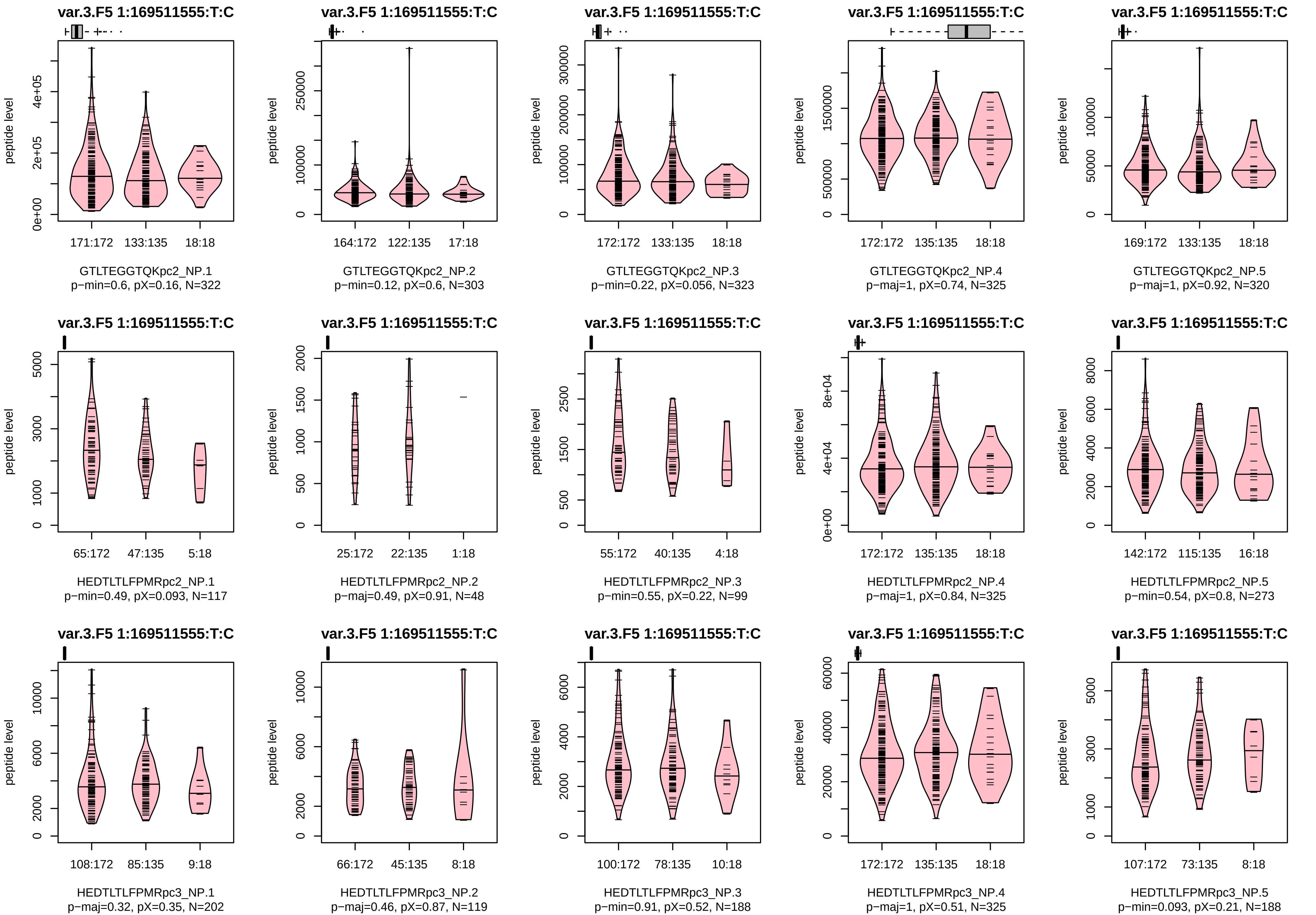
var.3.F5 1:169511555:T:C
peptide level
171:172
133:135
18:18
GTLTEGGTQKpc2_NP.1
p−min=0.6, pX=0.16, N=322
var.3.F5 1:169511555:T:C
peptide level
164:172
122:135
17:18
GTLTEGGTQKpc2_NP.2
p−min=0.12, pX=0.6, N=303
var.3.F5 1:169511555:T:C
peptide level
172:172
133:135
18:18
GTLTEGGTQKpc2_NP.3
p−min=0.22, pX=0.056, N=323
var.3.F5 1:169511555:T:C
peptide level
172:172
135:135
18:18
GTLTEGGTQKpc2_NP.4
p−maj=1, pX=0.74, N=325
var.3.F5 1:169511555:T:C
peptide level
169:172
133:135
18:18
GTLTEGGTQKpc2_NP.5
p−maj=1, pX=0.92, N=320
var.3.F5 1:169511555:T:C
peptide level
65:172
47:135
5:18
HEDTLTLFPMRpc2_NP.1
p−min=0.49, pX=0.093, N=117
var.3.F5 1:169511555:T:C
peptide level
25:172
22:135
1:18
HEDTLTLFPMRpc2_NP.2
p−maj=0.49, pX=0.91, N=48
var.3.F5 1:169511555:T:C
peptide level
55:172
40:135
4:18
HEDTLTLFPMRpc2_NP.3
p−min=0.55, pX=0.22, N=99
var.3.F5 1:169511555:T:C
peptide level
172:172
135:135
18:18
HEDTLTLFPMRpc2_NP.4
p−maj=1, pX=0.84, N=325
var.3.F5 1:169511555:T:C
peptide level
142:172
115:135
16:18
HEDTLTLFPMRpc2_NP.5
p−min=0.54, pX=0.8, N=273
var.3.F5 1:169511555:T:C
peptide level
108:172
85:135
9:18
HEDTLTLFPMRpc3_NP.1
p−maj=0.32, pX=0.35, N=202
var.3.F5 1:169511555:T:C
peptide level
66:172
45:135
8:18
HEDTLTLFPMRpc3_NP.2
p−maj=0.46, pX=0.87, N=119
var.3.F5 1:169511555:T:C
peptide level
100:172
78:135
10:18
HEDTLTLFPMRpc3_NP.3
p−min=0.91, pX=0.52, N=188
var.3.F5 1:169511555:T:C
peptide level
172:172
135:135
18:18
HEDTLTLFPMRpc3_NP.4
p−maj=1, pX=0.51, N=325
var.3.F5 1:169511555:T:C
peptide level
107:172
73:135
8:18
HEDTLTLFPMRpc3_NP.5
p−min=0.093, pX=0.21, N=188

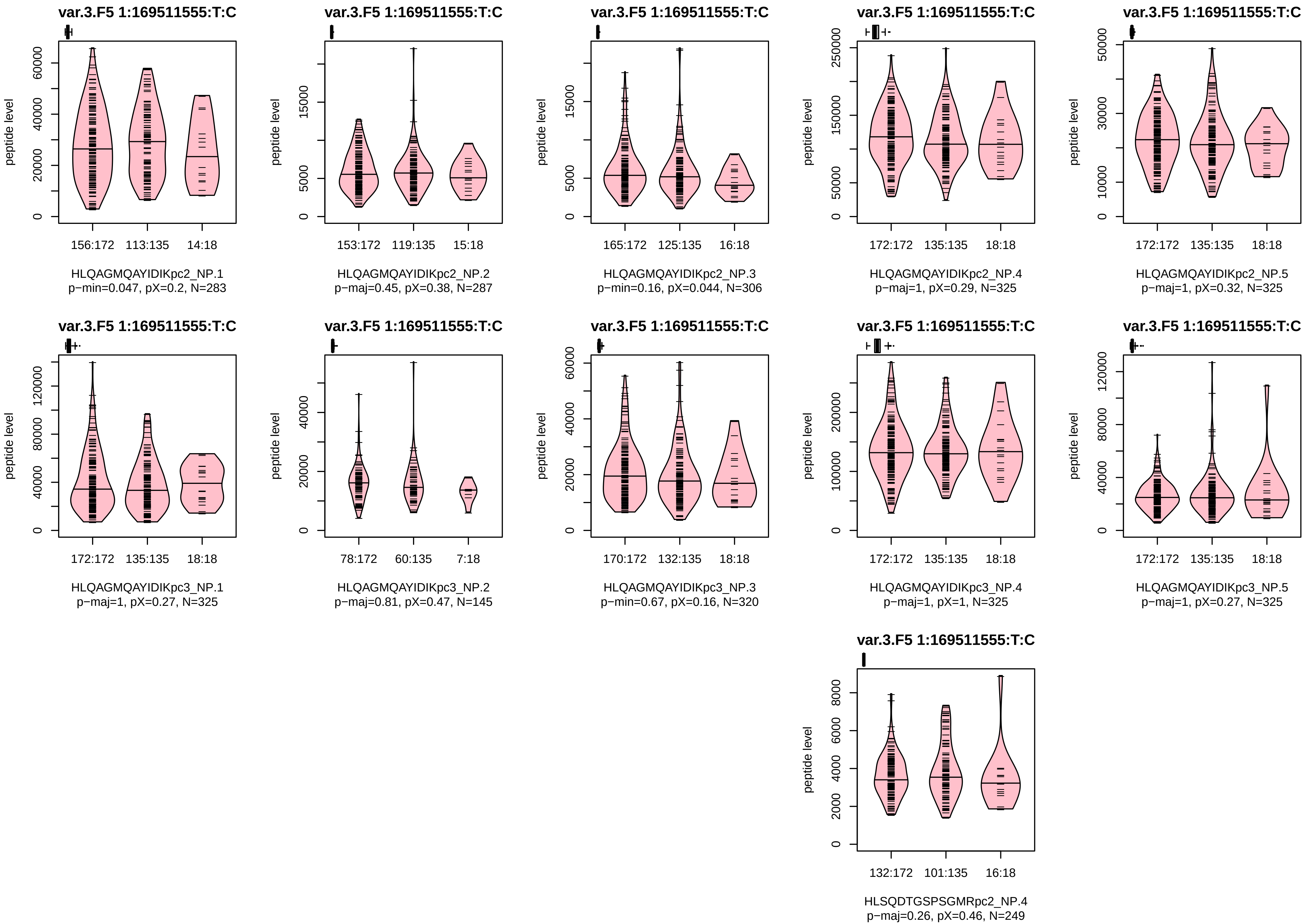
var.3.F5 1:169511555:T:C
peptide level
156:172
113:135
14:18
HLQAGMQAYIDIKpc2_NP.1
p−min=0.047, pX=0.2, N=283
var.3.F5 1:169511555:T:C
peptide level
153:172
119:135
15:18
HLQAGMQAYIDIKpc2_NP.2
p−maj=0.45, pX=0.38, N=287
var.3.F5 1:169511555:T:C
peptide level
165:172
125:135
16:18
HLQAGMQAYIDIKpc2_NP.3
p−min=0.16, pX=0.044, N=306
var.3.F5 1:169511555:T:C
peptide level
172:172
135:135
18:18
HLQAGMQAYIDIKpc2_NP.4
p−maj=1, pX=0.29, N=325
var.3.F5 1:169511555:T:C
peptide level
172:172
135:135
18:18
HLQAGMQAYIDIKpc2_NP.5
p−maj=1, pX=0.32, N=325
var.3.F5 1:169511555:T:C
peptide level
172:172
135:135
18:18
HLQAGMQAYIDIKpc3_NP.1
p−maj=1, pX=0.27, N=325
var.3.F5 1:169511555:T:C
peptide level
78:172
60:135
7:18
HLQAGMQAYIDIKpc3_NP.2
p−maj=0.81, pX=0.47, N=145
var.3.F5 1:169511555:T:C
peptide level
170:172
132:135
18:18
HLQAGMQAYIDIKpc3_NP.3
p−min=0.67, pX=0.16, N=320
var.3.F5 1:169511555:T:C
peptide level
172:172
135:135
18:18
HLQAGMQAYIDIKpc3_NP.4
p−maj=1, pX=1, N=325
var.3.F5 1:169511555:T:C
peptide level
172:172
135:135
18:18
HLQAGMQAYIDIKpc3_NP.5
p−maj=1, pX=0.27, N=325
var.3.F5 1:169511555:T:C
peptide level
132:172
101:135
16:18
HLSQDTGSPSGMRpc2_NP.4
p−maj=0.26, pX=0.46, N=249

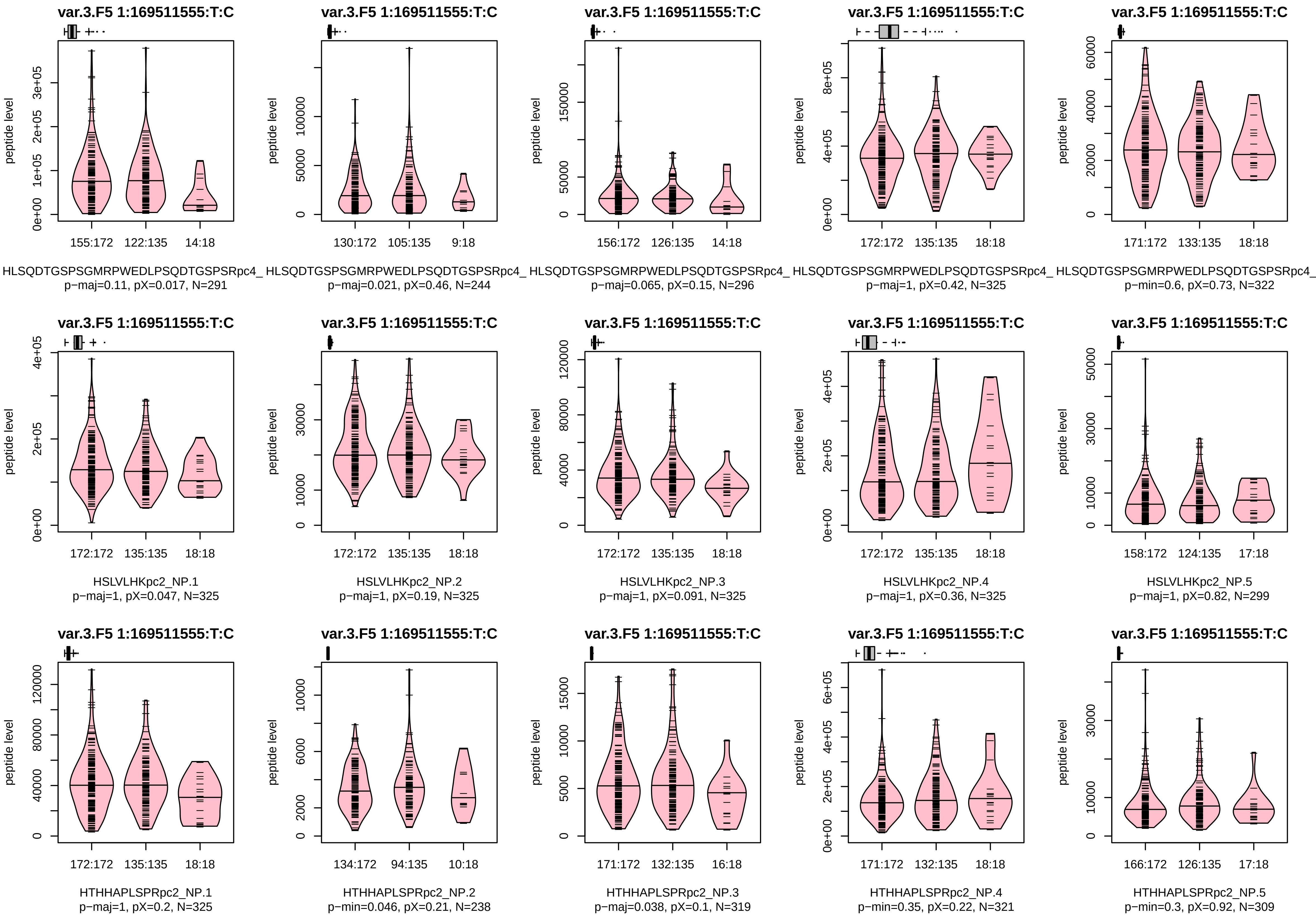
var.3.F5 1:169511555:T:C
peptide level
155:172
122:135
14:18
HLSQDTGSPSGMRPWEDLPSQDTGSPSRpc4_
p−maj=0.11, pX=0.017, N=291
var.3.F5 1:169511555:T:C
peptide level
130:172
105:135
9:18
HLSQDTGSPSGMRPWEDLPSQDTGSPSRpc4_
p−maj=0.021, pX=0.46, N=244
var.3.F5 1:169511555:T:C
peptide level
156:172
126:135
14:18
HLSQDTGSPSGMRPWEDLPSQDTGSPSRpc4_
p−maj=0.065, pX=0.15, N=296
var.3.F5 1:169511555:T:C
peptide level
172:172
135:135
18:18
HLSQDTGSPSGMRPWEDLPSQDTGSPSRpc4_
p−maj=1, pX=0.42, N=325
var.3.F5 1:169511555:T:C
peptide level
171:172
133:135
18:18
HLSQDTGSPSGMRPWEDLPSQDTGSPSRpc4_
p−min=0.6, pX=0.73, N=322
var.3.F5 1:169511555:T:C
peptide level
172:172
135:135
18:18
HSLVLHKpc2_NP.1
p−maj=1, pX=0.047, N=325
var.3.F5 1:169511555:T:C
peptide level
172:172
135:135
18:18
HSLVLHKpc2_NP.2
p−maj=1, pX=0.19, N=325
var.3.F5 1:169511555:T:C
peptide level
172:172
135:135
18:18
HSLVLHKpc2_NP.3
p−maj=1, pX=0.091, N=325
var.3.F5 1:169511555:T:C
peptide level
172:172
135:135
18:18
HSLVLHKpc2_NP.4
p−maj=1, pX=0.36, N=325
var.3.F5 1:169511555:T:C
peptide level
158:172
124:135
17:18
HSLVLHKpc2_NP.5
p−maj=1, pX=0.82, N=299
var.3.F5 1:169511555:T:C
peptide level
172:172
135:135
18:18
HTHHAPLSPRpc2_NP.1
p−maj=1, pX=0.2, N=325
var.3.F5 1:169511555:T:C
peptide level
134:172
94:135
10:18
HTHHAPLSPRpc2_NP.2
p−min=0.046, pX=0.21, N=238
var.3.F5 1:169511555:T:C
peptide level
171:172
132:135
16:18
HTHHAPLSPRpc2_NP.3
p−maj=0.038, pX=0.1, N=319
var.3.F5 1:169511555:T:C
peptide level
171:172
132:135
18:18
HTHHAPLSPRpc2_NP.4
p−min=0.35, pX=0.22, N=321
var.3.F5 1:169511555:T:C
peptide level
166:172
126:135
17:18
HTHHAPLSPRpc2_NP.5
p−min=0.3, pX=0.92, N=309

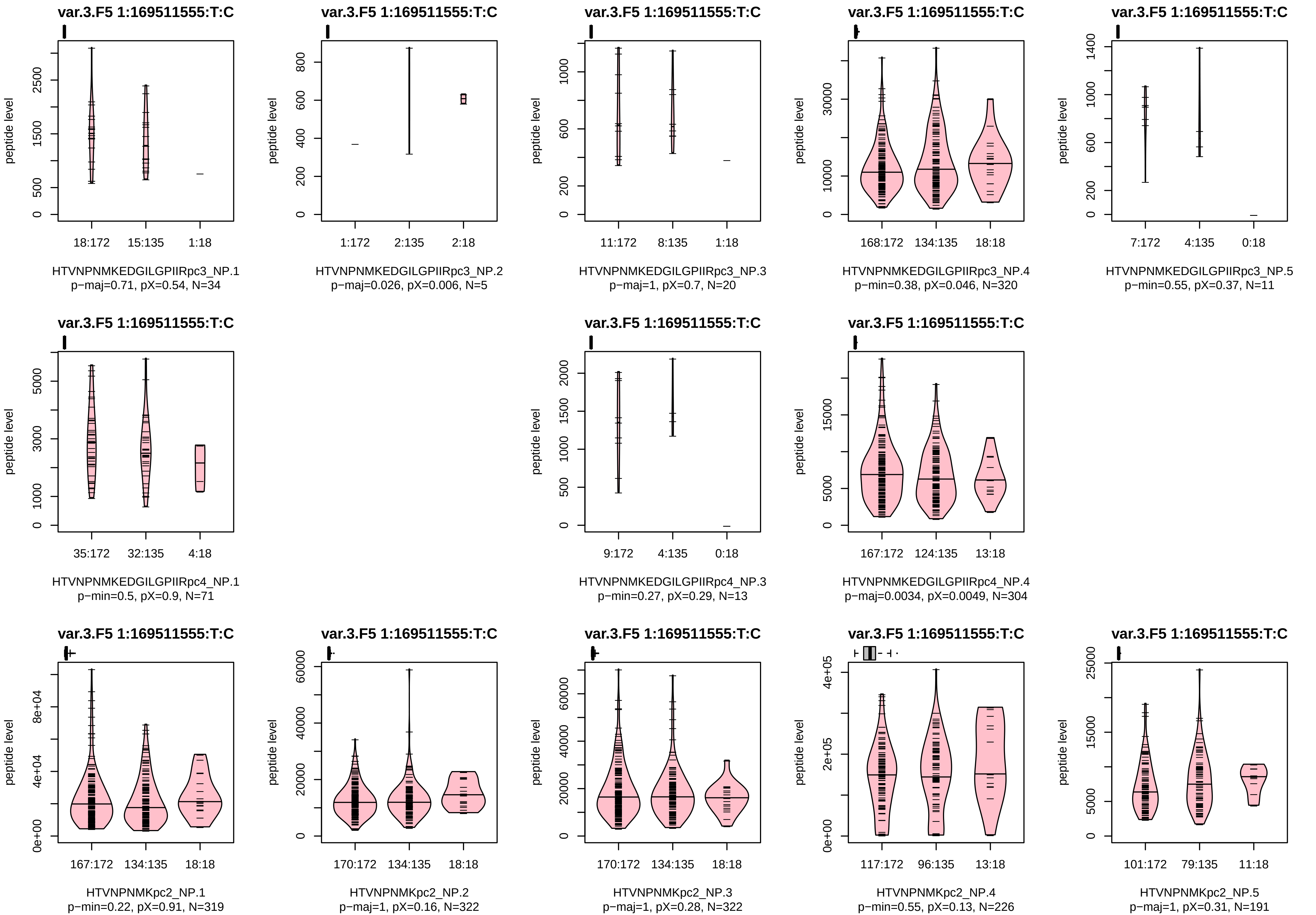
var.3.F5 1:169511555:T:C
peptide level
18:172
15:135
1:18
HTVNPNMKEDGILGPIIRpc3_NP.1
p−maj=0.71, pX=0.54, N=34
var.3.F5 1:169511555:T:C
peptide level
1:172
2:135
2:18
HTVNPNMKEDGILGPIIRpc3_NP.2
p−maj=0.026, pX=0.006, N=5
var.3.F5 1:169511555:T:C
peptide level
11:172
8:135
1:18
HTVNPNMKEDGILGPIIRpc3_NP.3
p−maj=1, pX=0.7, N=20
var.3.F5 1:169511555:T:C
peptide level
168:172
134:135
18:18
HTVNPNMKEDGILGPIIRpc3_NP.4
p−min=0.38, pX=0.046, N=320
var.3.F5 1:169511555:T:C
peptide level
7:172
4:135
0:18
HTVNPNMKEDGILGPIIRpc3_NP.5
p−min=0.55, pX=0.37, N=11
var.3.F5 1:169511555:T:C
peptide level
35:172
32:135
4:18
HTVNPNMKEDGILGPIIRpc4_NP.1
p−min=0.5, pX=0.9, N=71
var.3.F5 1:169511555:T:C
peptide level
9:172
4:135
0:18
HTVNPNMKEDGILGPIIRpc4_NP.3
p−min=0.27, pX=0.29, N=13
var.3.F5 1:169511555:T:C
peptide level
167:172
124:135
13:18
HTVNPNMKEDGILGPIIRpc4_NP.4
p−maj=0.0034, pX=0.0049, N=304
var.3.F5 1:169511555:T:C
peptide level
167:172
134:135
18:18
HTVNPNMKpc2_NP.1
p−min=0.22, pX=0.91, N=319
var.3.F5 1:169511555:T:C
peptide level
170:172
134:135
18:18
HTVNPNMKpc2_NP.2
p−maj=1, pX=0.16, N=322
var.3.F5 1:169511555:T:C
peptide level
170:172
134:135
18:18
HTVNPNMKpc2_NP.3
p−maj=1, pX=0.28, N=322
var.3.F5 1:169511555:T:C
peptide level
117:172
96:135
13:18
HTVNPNMKpc2_NP.4
p−min=0.55, pX=0.13, N=226
var.3.F5 1:169511555:T:C
peptide level
101:172
79:135
11:18
HTVNPNMKpc2_NP.5
p−maj=1, pX=0.31, N=191

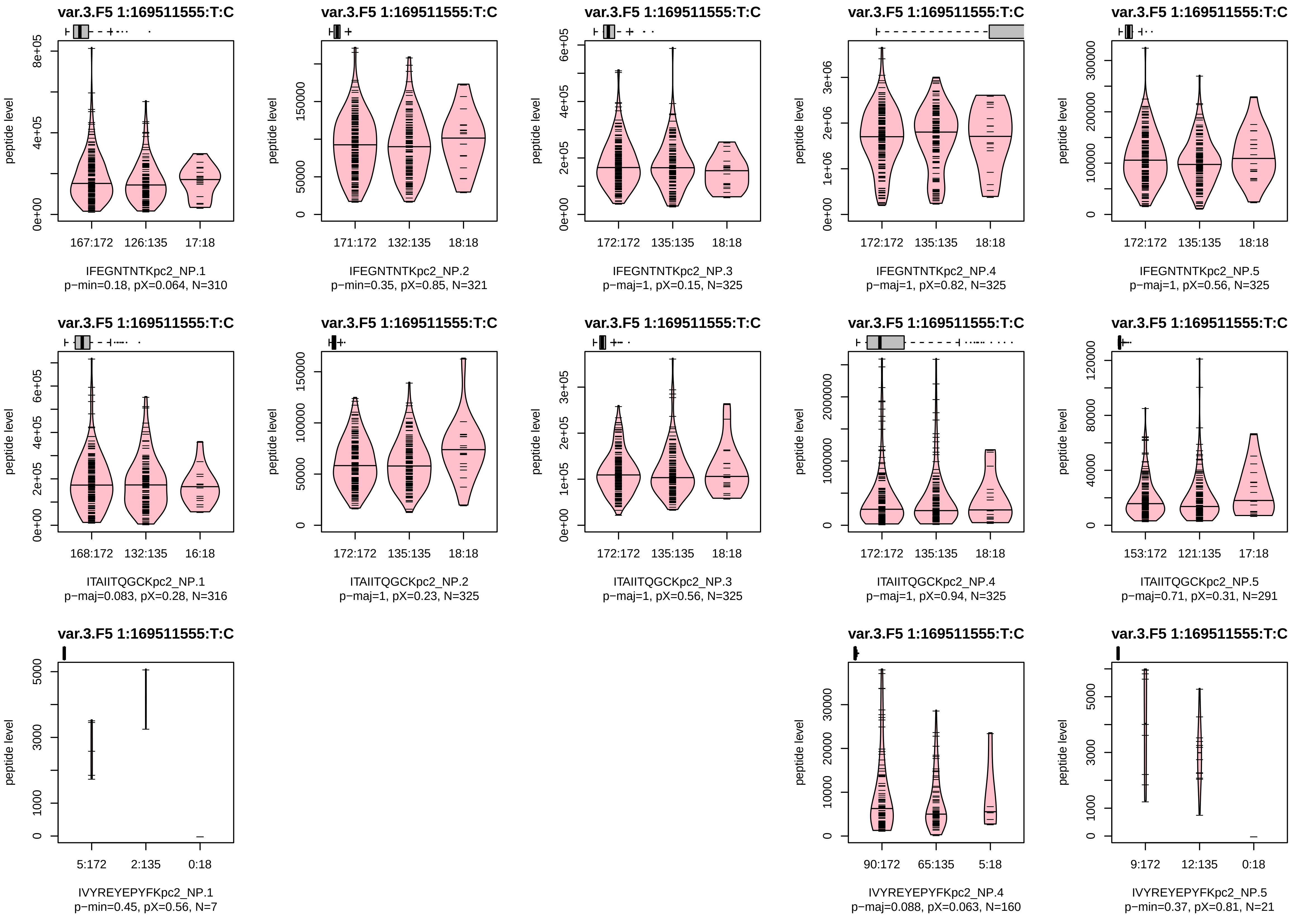
var.3.F5 1:169511555:T:C
peptide level
167:172
126:135
17:18
IFEGNTNTKpc2_NP.1
p−min=0.18, pX=0.064, N=310
var.3.F5 1:169511555:T:C
peptide level
171:172
132:135
18:18
IFEGNTNTKpc2_NP.2
p−min=0.35, pX=0.85, N=321
var.3.F5 1:169511555:T:C
peptide level
172:172
135:135
18:18
IFEGNTNTKpc2_NP.3
p−maj=1, pX=0.15, N=325
var.3.F5 1:169511555:T:C
peptide level
172:172
135:135
18:18
IFEGNTNTKpc2_NP.4
p−maj=1, pX=0.82, N=325
var.3.F5 1:169511555:T:C
peptide level
172:172
135:135
18:18
IFEGNTNTKpc2_NP.5
p−maj=1, pX=0.56, N=325
var.3.F5 1:169511555:T:C
peptide level
168:172
132:135
16:18
ITAIITQGCKpc2_NP.1
p−maj=0.083, pX=0.28, N=316
var.3.F5 1:169511555:T:C
peptide level
172:172
135:135
18:18
ITAIITQGCKpc2_NP.2
p−maj=1, pX=0.23, N=325
var.3.F5 1:169511555:T:C
peptide level
172:172
135:135
18:18
ITAIITQGCKpc2_NP.3
p−maj=1, pX=0.56, N=325
var.3.F5 1:169511555:T:C
peptide level
172:172
135:135
18:18
ITAIITQGCKpc2_NP.4
p−maj=1, pX=0.94, N=325
var.3.F5 1:169511555:T:C
peptide level
153:172
121:135
17:18
ITAIITQGCKpc2_NP.5
p−maj=0.71, pX=0.31, N=291
var.3.F5 1:169511555:T:C
peptide level
5:172
2:135
0:18
IVYREYEPYFKpc2_NP.1
p−min=0.45, pX=0.56, N=7
var.3.F5 1:169511555:T:C
peptide level
90:172
65:135
5:18
IVYREYEPYFKpc2_NP.4
p−maj=0.088, pX=0.063, N=160
var.3.F5 1:169511555:T:C
peptide level
9:172
12:135
0:18
IVYREYEPYFKpc2_NP.5
p−min=0.37, pX=0.81, N=21

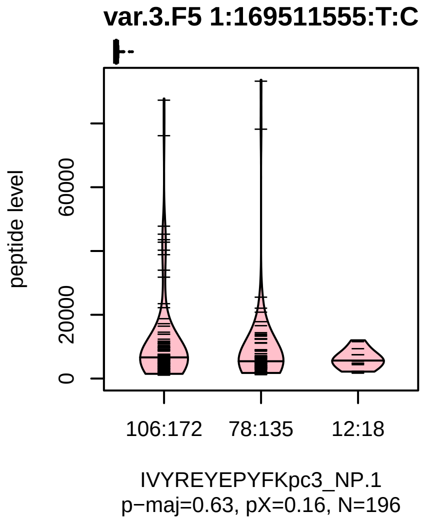
var.3.F5 1:169511555:T:C
peptide level
0
20000
60000
106:172
78:135
12:18
IVYREYEPYFKpc3_NP.1
p−maj=0.63, pX=0.16, N=196

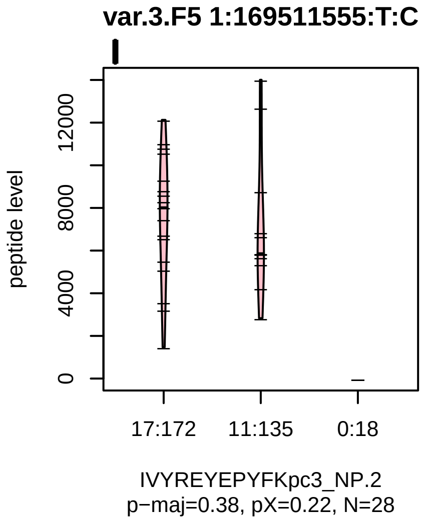
var.3.F5 1:169511555:T:C
peptide level
0
4000
8000
12000
17:172
11:135
0:18
IVYREYEPYFKpc3_NP.2
p−maj=0.38, pX=0.22, N=28

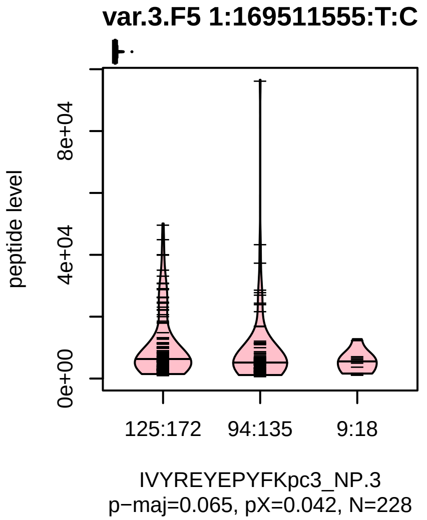
var.3.F5 1:169511555:T:C
peptide level
0e+00
4e+04
8e+04
125:172
94:135
9:18
IVYREYEPYFKpc3_NP.3
p−maj=0.065, pX=0.042, N=228

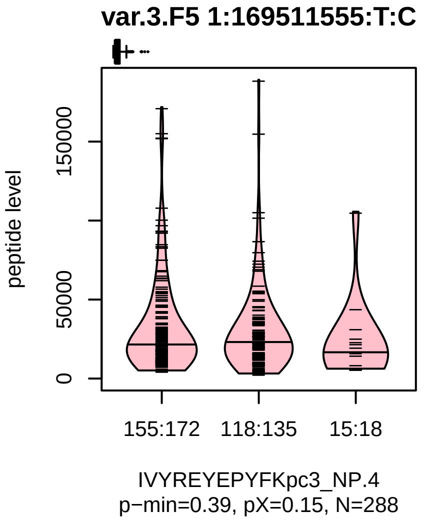
var.3.F5 1:169511555:T:C
peptide level
0
50000
150000
155:172
118:135
15:18
IVYREYEPYFKpc3_NP.4
p−min=0.39, pX=0.15, N=288

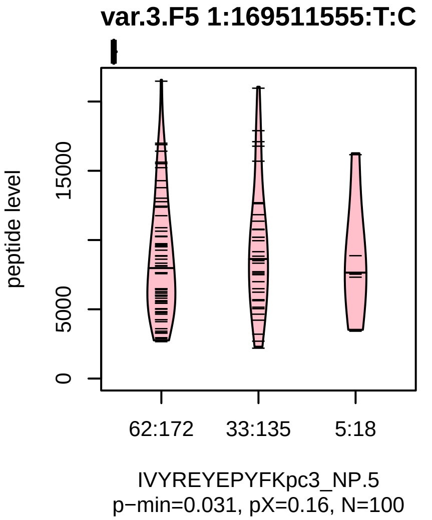
var.3.F5 1:169511555:T:C
peptide level
0
5000
15000
62:172
33:135
5:18
IVYREYEPYFKpc3_NP.5
p−min=0.031, pX=0.16, N=100

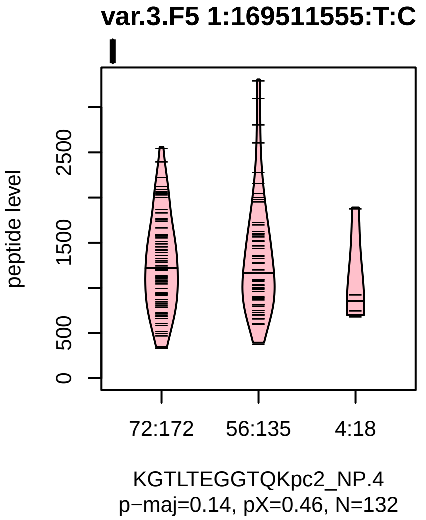
var.3.F5 1:169511555:T:C
peptide level
0
500
1500
2500
72:172
56:135
4:18
KGTLTEGGTQKpc2_NP.4
p−maj=0.14, pX=0.46, N=132

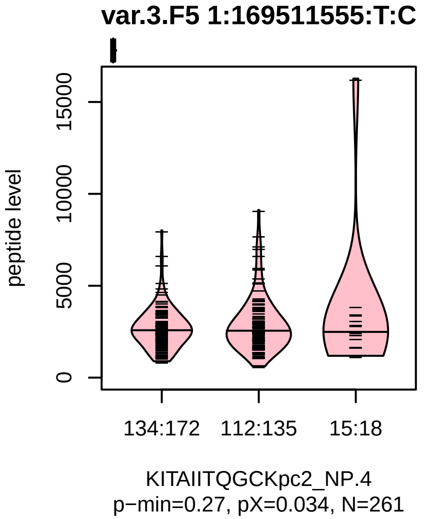
var.3.F5 1:169511555:T:C
peptide level
0
5000
10000
15000
134:172
112:135
15:18
KITAIITQGCKpc2_NP.4
p−min=0.27, pX=0.034, N=261

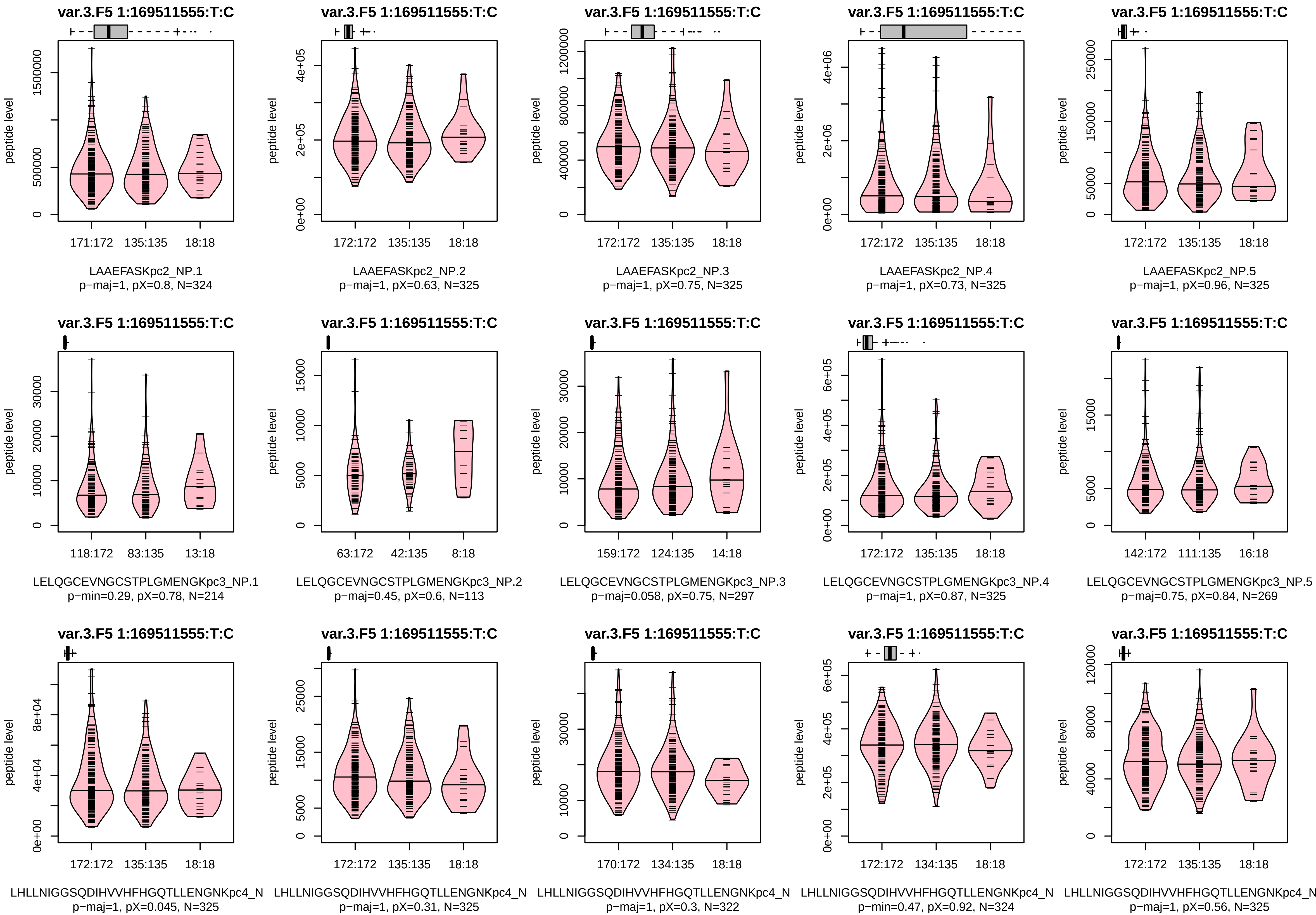
var.3.F5 1:169511555:T:C
peptide level
171:172
135:135
18:18
LAAEFASKpc2_NP.1
p−maj=1, pX=0.8, N=324
var.3.F5 1:169511555:T:C
peptide level
172:172
135:135
18:18
LAAEFASKpc2_NP.2
p−maj=1, pX=0.63, N=325
var.3.F5 1:169511555:T:C
peptide level
172:172
135:135
18:18
LAAEFASKpc2_NP.3
p−maj=1, pX=0.75, N=325
var.3.F5 1:169511555:T:C
peptide level
172:172
135:135
18:18
LAAEFASKpc2_NP.4
p−maj=1, pX=0.73, N=325
var.3.F5 1:169511555:T:C
peptide level
172:172
135:135
18:18
LAAEFASKpc2_NP.5
p−maj=1, pX=0.96, N=325
var.3.F5 1:169511555:T:C
peptide level
118:172
83:135
13:18
LELQGCEVNGCSTPLGMENGKpc3_NP.1
p−min=0.29, pX=0.78, N=214
var.3.F5 1:169511555:T:C
peptide level
63:172
42:135
8:18
LELQGCEVNGCSTPLGMENGKpc3_NP.2
p−maj=0.45, pX=0.6, N=113
var.3.F5 1:169511555:T:C
peptide level
159:172
124:135
14:18
LELQGCEVNGCSTPLGMENGKpc3_NP.3
p−maj=0.058, pX=0.75, N=297
var.3.F5 1:169511555:T:C
peptide level
172:172
135:135
18:18
LELQGCEVNGCSTPLGMENGKpc3_NP.4
p−maj=1, pX=0.87, N=325
var.3.F5 1:169511555:T:C
peptide level
142:172
111:135
16:18
LELQGCEVNGCSTPLGMENGKpc3_NP.5
p−maj=0.75, pX=0.84, N=269
var.3.F5 1:169511555:T:C
peptide level
172:172
135:135
18:18
LHLLNIGGSQDIHVVHFHGQTLLENGNKpc4_N
p−maj=1, pX=0.045, N=325
var.3.F5 1:169511555:T:C
peptide level
172:172
135:135
18:18
LHLLNIGGSQDIHVVHFHGQTLLENGNKpc4_N
p−maj=1, pX=0.31, N=325
var.3.F5 1:169511555:T:C
peptide level
170:172
134:135
18:18
LHLLNIGGSQDIHVVHFHGQTLLENGNKpc4_N
p−maj=1, pX=0.3, N=322
var.3.F5 1:169511555:T:C
peptide level
172:172
134:135
18:18
LHLLNIGGSQDIHVVHFHGQTLLENGNKpc4_N
p−min=0.47, pX=0.92, N=324
var.3.F5 1:169511555:T:C
peptide level
172:172
135:135
18:18
LHLLNIGGSQDIHVVHFHGQTLLENGNKpc4_N
p−maj=1, pX=0.56, N=325

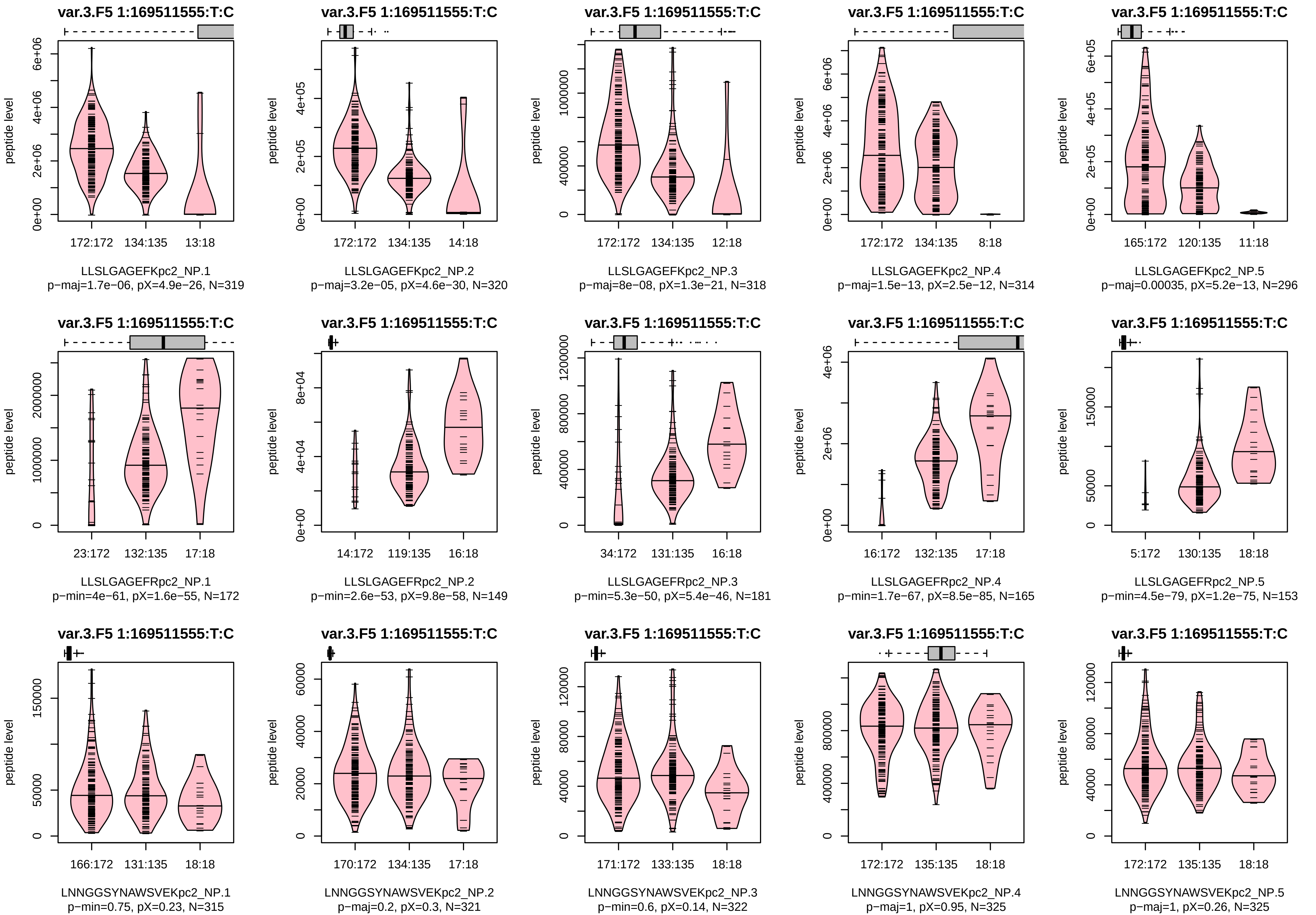
var.3.F5 1:169511555:T:C
peptide level
172:172
134:135
13:18
LLSLGAGEFKpc2_NP.1
p−maj=1.7e−06, pX=4.9e−26, N=319
14:18
LLSLGAGEFKpc2_NP.2
p−maj=3.2e−05, pX=4.6e−30, N=320
12:18
LLSLGAGEFKpc2_NP.3
p−maj=8e−08, pX=1.3e−21, N=318
8:18
LLSLGAGEFKpc2_NP.4
p−maj=1.5e−13, pX=2.5e−12, N=314
165:172
120:135
11:18
LLSLGAGEFKpc2_NP.5
p−maj=0.00035, pX=5.2e−13, N=296
23:172
132:135
17:18
LLSLGAGEFRpc2_NP.1
p−min=4e−61, pX=1.6e−55, N=172
14:172
119:135
16:18
LLSLGAGEFRpc2_NP.2
p−min=2.6e−53, pX=9.8e−58, N=149
34:172
131:135
LLSLGAGEFRpc2_NP.3
p−min=5.3e−50, pX=5.4e−46, N=181
16:172
LLSLGAGEFRpc2_NP.4
p−min=1.7e−67, pX=8.5e−85, N=165
5:172
130:135
18:18
LLSLGAGEFRpc2_NP.5
p−min=4.5e−79, pX=1.2e−75, N=153
166:172
131:135
LNNGGSYNAWSVEKpc2_NP.1
p−min=0.75, pX=0.23, N=315
170:172
134:135
LNNGGSYNAWSVEKpc2_NP.2
p−maj=0.2, pX=0.3, N=321
171:172
133:135
LNNGGSYNAWSVEKpc2_NP.3
p−min=0.6, pX=0.14, N=322
135:135
LNNGGSYNAWSVEKpc2_NP.4
p−maj=1, pX=0.95, N=325
LNNGGSYNAWSVEKpc2_NP.5
p−maj=1, pX=0.26, N=325

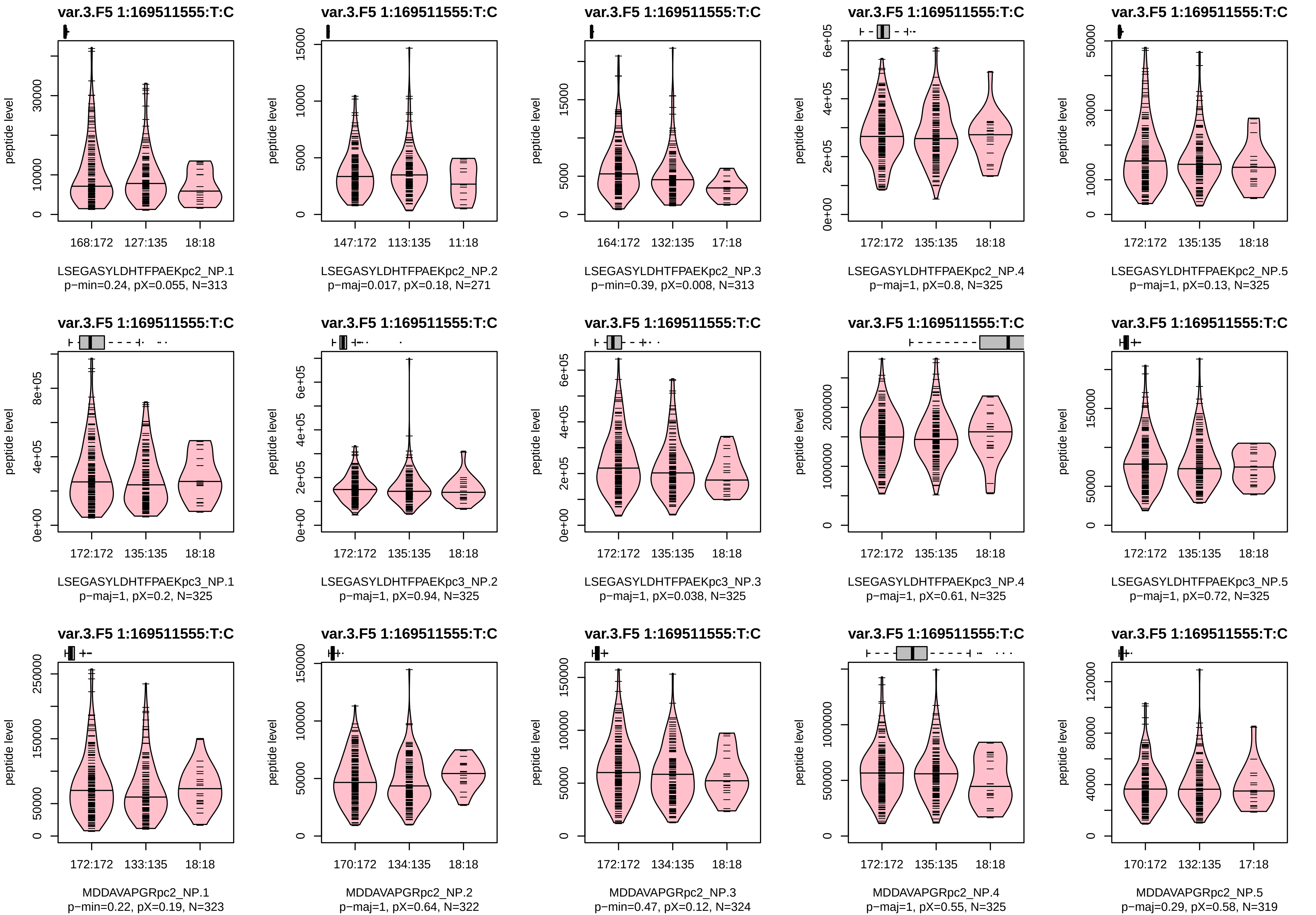
var.3.F5 1:169511555:T:C
peptide level
168:172
127:135
18:18
LSEGASYLDHTFPAEKpc2_NP.1
p−min=0.24, pX=0.055, N=313
var.3.F5 1:169511555:T:C
peptide level
147:172
113:135
11:18
LSEGASYLDHTFPAEKpc2_NP.2
p−maj=0.017, pX=0.18, N=271
var.3.F5 1:169511555:T:C
peptide level
164:172
132:135
17:18
LSEGASYLDHTFPAEKpc2_NP.3
p−min=0.39, pX=0.008, N=313
var.3.F5 1:169511555:T:C
peptide level
172:172
135:135
18:18
LSEGASYLDHTFPAEKpc2_NP.4
p−maj=1, pX=0.8, N=325
var.3.F5 1:169511555:T:C
peptide level
172:172
135:135
18:18
LSEGASYLDHTFPAEKpc2_NP.5
p−maj=1, pX=0.13, N=325
var.3.F5 1:169511555:T:C
peptide level
172:172
135:135
18:18
LSEGASYLDHTFPAEKpc3_NP.1
p−maj=1, pX=0.2, N=325
var.3.F5 1:169511555:T:C
peptide level
172:172
135:135
18:18
LSEGASYLDHTFPAEKpc3_NP.2
p−maj=1, pX=0.94, N=325
var.3.F5 1:169511555:T:C
peptide level
172:172
135:135
18:18
LSEGASYLDHTFPAEKpc3_NP.3
p−maj=1, pX=0.038, N=325
var.3.F5 1:169511555:T:C
peptide level
172:172
135:135
18:18
LSEGASYLDHTFPAEKpc3_NP.4
p−maj=1, pX=0.61, N=325
var.3.F5 1:169511555:T:C
peptide level
172:172
135:135
18:18
LSEGASYLDHTFPAEKpc3_NP.5
p−maj=1, pX=0.72, N=325
var.3.F5 1:169511555:T:C
peptide level
172:172
133:135
18:18
MDDAVAPGRpc2_NP.1
p−min=0.22, pX=0.19, N=323
var.3.F5 1:169511555:T:C
peptide level
170:172
134:135
18:18
MDDAVAPGRpc2_NP.2
p−maj=1, pX=0.64, N=322
var.3.F5 1:169511555:T:C
peptide level
172:172
134:135
18:18
MDDAVAPGRpc2_NP.3
p−min=0.47, pX=0.12, N=324
var.3.F5 1:169511555:T:C
peptide level
172:172
135:135
18:18
MDDAVAPGRpc2_NP.4
p−maj=1, pX=0.55, N=325
var.3.F5 1:169511555:T:C
peptide level
170:172
132:135
17:18
MDDAVAPGRpc2_NP.5
p−maj=0.29, pX=0.58, N=319

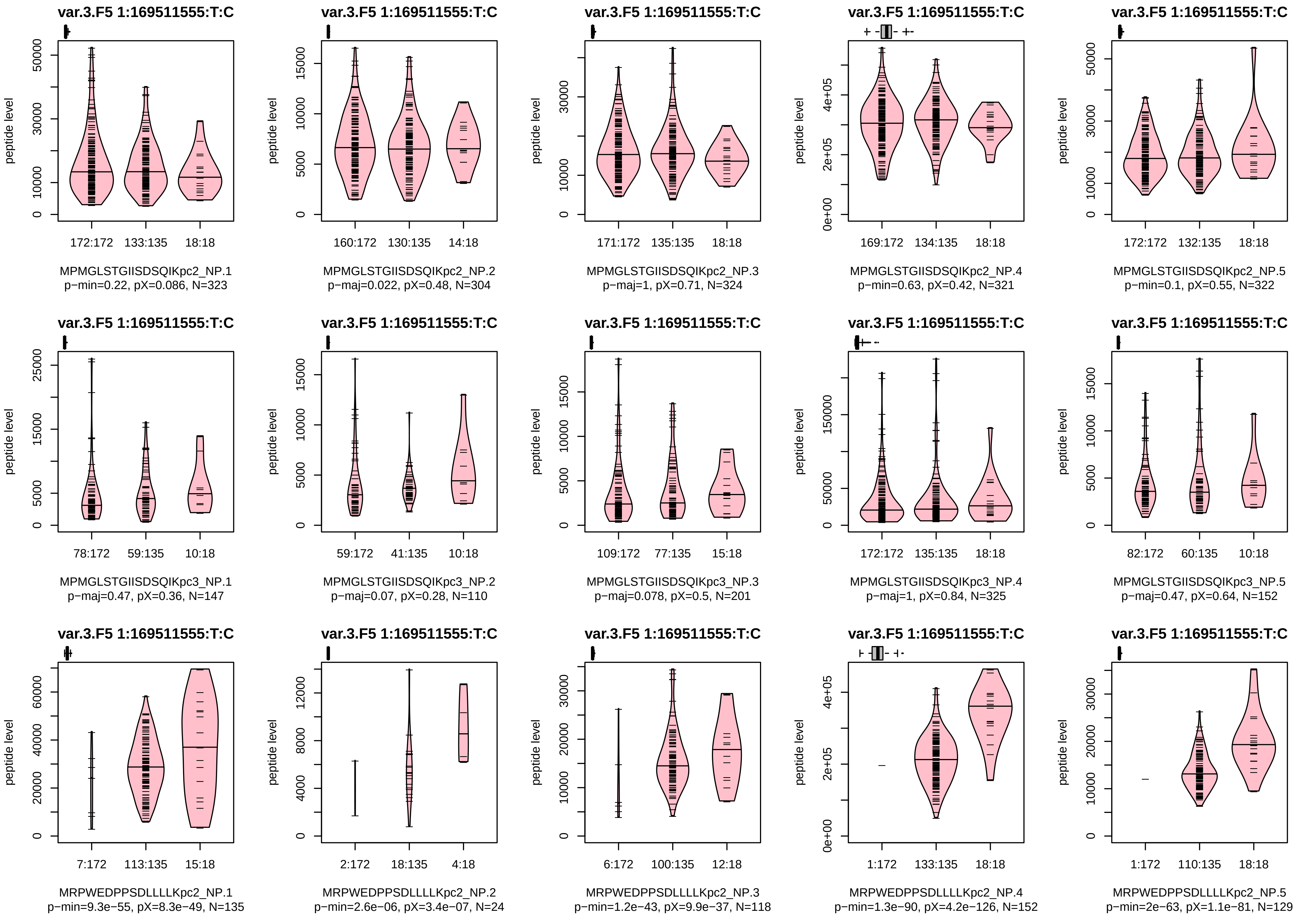

var.3.F5 1:169511555:T:C
peptide level
172:172
133:135
18:18
MPMGLSTGIISDSQIKpc2_NP.1
p−min=0.22, pX=0.086, N=323
var.3.F5 1:169511555:T:C
peptide level
160:172
130:135
14:18
MPMGLSTGIISDSQIKpc2_NP.2
p−maj=0.022, pX=0.48, N=304
var.3.F5 1:169511555:T:C
peptide level
171:172
135:135
18:18
MPMGLSTGIISDSQIKpc2_NP.3
p−maj=1, pX=0.71, N=324
var.3.F5 1:169511555:T:C
peptide level
169:172
134:135
18:18
MPMGLSTGIISDSQIKpc2_NP.4
p−min=0.63, pX=0.42, N=321
var.3.F5 1:169511555:T:C
peptide level
172:172
132:135
18:18
MPMGLSTGIISDSQIKpc2_NP.5
p−min=0.1, pX=0.55, N=322
var.3.F5 1:169511555:T:C
peptide level
78:172
59:135
10:18
MPMGLSTGIISDSQIKpc3_NP.1
p−maj=0.47, pX=0.36, N=147
var.3.F5 1:169511555:T:C
peptide level
59:172
41:135
10:18
MPMGLSTGIISDSQIKpc3_NP.2
p−maj=0.07, pX=0.28, N=110
var.3.F5 1:169511555:T:C
peptide level
109:172
77:135
15:18
MPMGLSTGIISDSQIKpc3_NP.3
p−maj=0.078, pX=0.5, N=201
var.3.F5 1:169511555:T:C
peptide level
172:172
135:135
18:18
MPMGLSTGIISDSQIKpc3_NP.4
p−maj=1, pX=0.84, N=325
var.3.F5 1:169511555:T:C
peptide level
82:172
60:135
10:18
MPMGLSTGIISDSQIKpc3_NP.5
p−maj=0.47, pX=0.64, N=152
var.3.F5 1:169511555:T:C
peptide level
7:172
113:135
15:18
MRPWEDPPSDLLLLKpc2_NP.1
p−min=9.3e−55, pX=8.3e−49, N=135
var.3.F5 1:169511555:T:C
peptide level
2:172
18:135
4:18
MRPWEDPPSDLLLLKpc2_NP.2
p−min=2.6e−06, pX=3.4e−07, N=24
var.3.F5 1:169511555:T:C
peptide level
6:172
100:135
12:18
MRPWEDPPSDLLLLKpc2_NP.3
p−min=1.2e−43, pX=9.9e−37, N=118
var.3.F5 1:169511555:T:C
peptide level
1:172
133:135
18:18
MRPWEDPPSDLLLLKpc2_NP.4
p−min=1.3e−90, pX=4.2e−126, N=152
var.3.F5 1:169511555:T:C
peptide level
1:172
110:135
18:18
MRPWEDPPSDLLLLKpc2_NP.5
p−min=2e−63, pX=1.1e−81, N=129

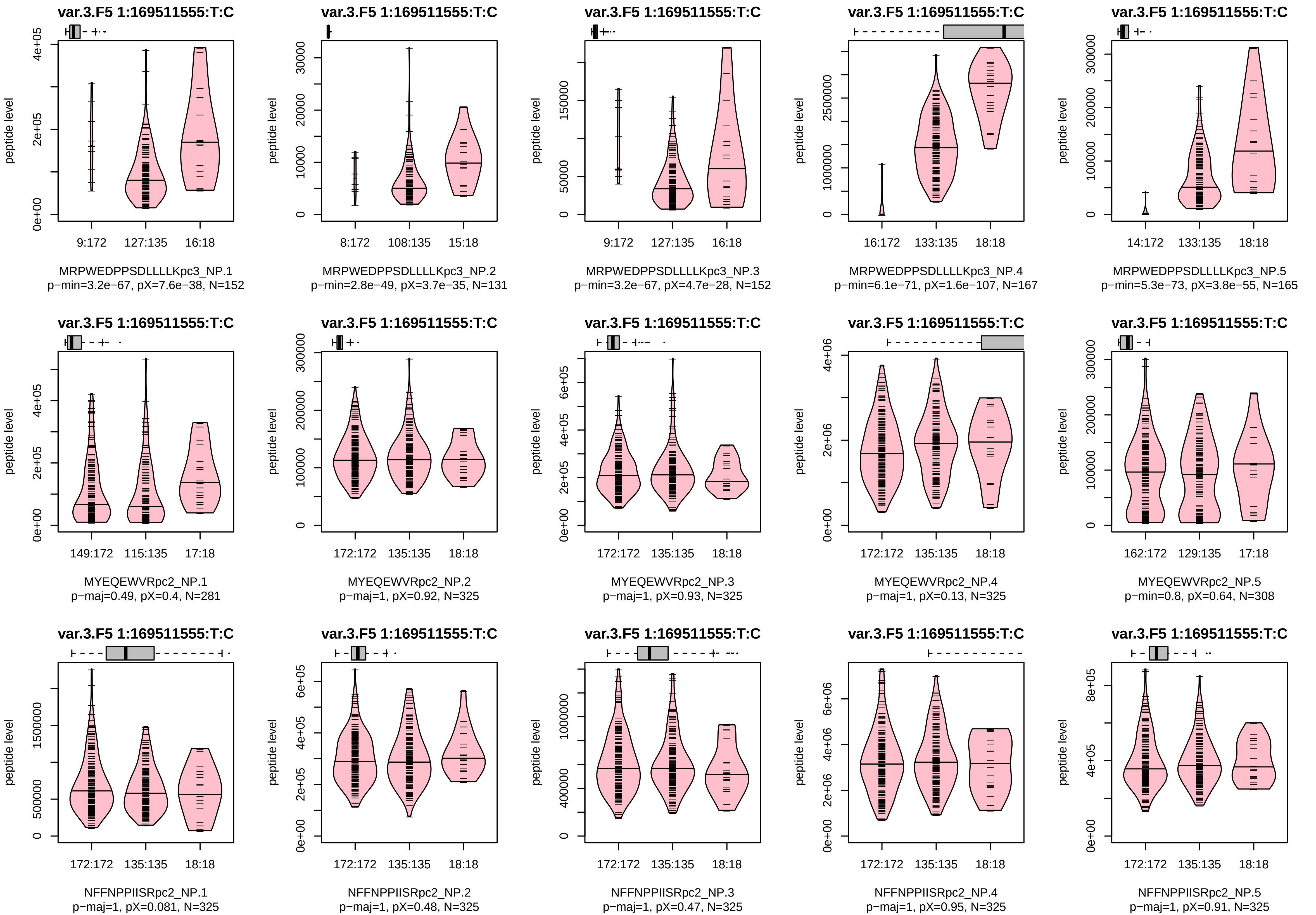
var.3.F5 1:169511555:T:C
peptide level
9:172 127:135 16:18
MRPWEDPPSDLLLLKpc3_NP.1
p−min=3.2e−67, pX=7.6e−38, N=152
var.3.F5 1:169511555:T:C
8:172 108:135 15:18
MRPWEDPPSDLLLLKpc3_NP.2
p−min=2.8e−49, pX=3.7e−35, N=131
var.3.F5 1:169511555:T:C
9:172 127:135 16:18
MRPWEDPPSDLLLLKpc3_NP.3
p−min=3.2e−67, pX=4.7e−28, N=152
var.3.F5 1:169511555:T:C
16:172 133:135 18:18
MRPWEDPPSDLLLLKpc3_NP.4
p−min=6.1e−71, pX=1.6e−107, N=167
var.3.F5 1:169511555:T:C
14:172 133:135 18:18
MRPWEDPPSDLLLLKpc3_NP.5
p−min=5.3e−73, pX=3.8e−55, N=165
var.3.F5 1:169511555:T:C
149:172 115:135 17:18
MYEQEWVRpc2_NP.1
p−maj=0.49, pX=0.4, N=281
var.3.F5 1:169511555:T:C
172:172 135:135 18:18
MYEQEWVRpc2_NP.2
p−maj=1, pX=0.92, N=325
var.3.F5 1:169511555:T:C
172:172 135:135 18:18
MYEQEWVRpc2_NP.3
p−maj=1, pX=0.93, N=325
var.3.F5 1:169511555:T:C
172:172 135:135 18:18
MYEQEWVRpc2_NP.4
p−maj=1, pX=0.13, N=325
var.3.F5 1:169511555:T:C
162:172 129:135 17:18
MYEQEWVRpc2_NP.5
p−min=0.8, pX=0.64, N=308
var.3.F5 1:169511555:T:C
172:172 135:135 18:18
NFFNPPIISRpc2_NP.1
p−maj=1, pX=0.081, N=325
var.3.F5 1:169511555:T:C
172:172 135:135 18:18
NFFNPPIISRpc2_NP.2
p−maj=1, pX=0.48, N=325
var.3.F5 1:169511555:T:C
172:172 135:135 18:18
NFFNPPIISRpc2_NP.3
p−maj=1, pX=0.47, N=325
var.3.F5 1:169511555:T:C
172:172 135:135 18:18
NFFNPPIISRpc2_NP.4
p−maj=1, pX=0.95, N=325
var.3.F5 1:169511555:T:C
172:172 135:135 18:18
NFFNPPIISRpc2_NP.5
p−maj=1, pX=0.91, N=325

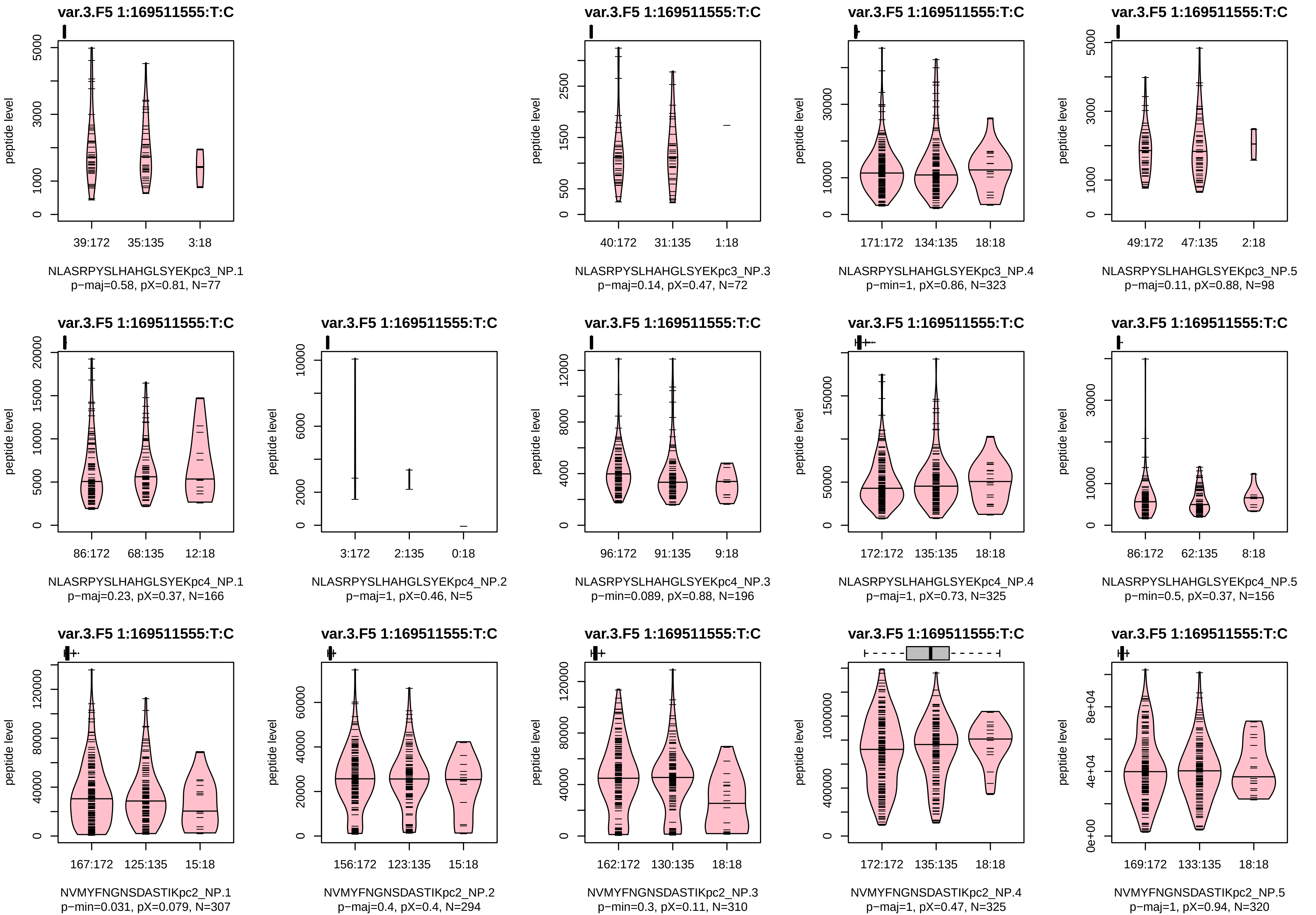

var.3.F5 1:169511555:T:C
peptide level
39:172
35:135
3:18
NLASRPYSLHAHGLSYEKpc3_NP.1
p−maj=0.58, pX=0.81, N=77
var.3.F5 1:169511555:T:C
peptide level
40:172
31:135
1:18
NLASRPYSLHAHGLSYEKpc3_NP.3
p−maj=0.14, pX=0.47, N=72
var.3.F5 1:169511555:T:C
peptide level
171:172
134:135
18:18
NLASRPYSLHAHGLSYEKpc3_NP.4
p−min=1, pX=0.86, N=323
var.3.F5 1:169511555:T:C
peptide level
49:172
47:135
2:18
NLASRPYSLHAHGLSYEKpc3_NP.5
p−maj=0.11, pX=0.88, N=98
var.3.F5 1:169511555:T:C
peptide level
86:172
68:135
12:18
NLASRPYSLHAHGLSYEKpc4_NP.1
p−maj=0.23, pX=0.37, N=166
var.3.F5 1:169511555:T:C
peptide level
3:172
2:135
0:18
NLASRPYSLHAHGLSYEKpc4_NP.2
p−maj=1, pX=0.46, N=5
var.3.F5 1:169511555:T:C
peptide level
96:172
91:135
9:18
NLASRPYSLHAHGLSYEKpc4_NP.3
p−min=0.089, pX=0.88, N=196
var.3.F5 1:169511555:T:C
peptide level
172:172
135:135
18:18
NLASRPYSLHAHGLSYEKpc4_NP.4
p−maj=1, pX=0.73, N=325
var.3.F5 1:169511555:T:C
peptide level
86:172
62:135
8:18
NLASRPYSLHAHGLSYEKpc4_NP.5
p−min=0.5, pX=0.37, N=156
var.3.F5 1:169511555:T:C
peptide level
167:172
125:135
15:18
NVMYFNGNSDASTIKpc2_NP.1
p−min=0.031, pX=0.079, N=307
var.3.F5 1:169511555:T:C
peptide level
156:172
123:135
15:18
NVMYFNGNSDASTIKpc2_NP.2
p−maj=0.4, pX=0.4, N=294
var.3.F5 1:169511555:T:C
peptide level
162:172
130:135
18:18
NVMYFNGNSDASTIKpc2_NP.3
p−min=0.3, pX=0.11, N=310
var.3.F5 1:169511555:T:C
peptide level
172:172
135:135
18:18
NVMYFNGNSDASTIKpc2_NP.4
p−maj=1, pX=0.47, N=325
var.3.F5 1:169511555:T:C
peptide level
169:172
133:135
18:18
NVMYFNGNSDASTIKpc2_NP.5
p−maj=1, pX=0.94, N=320

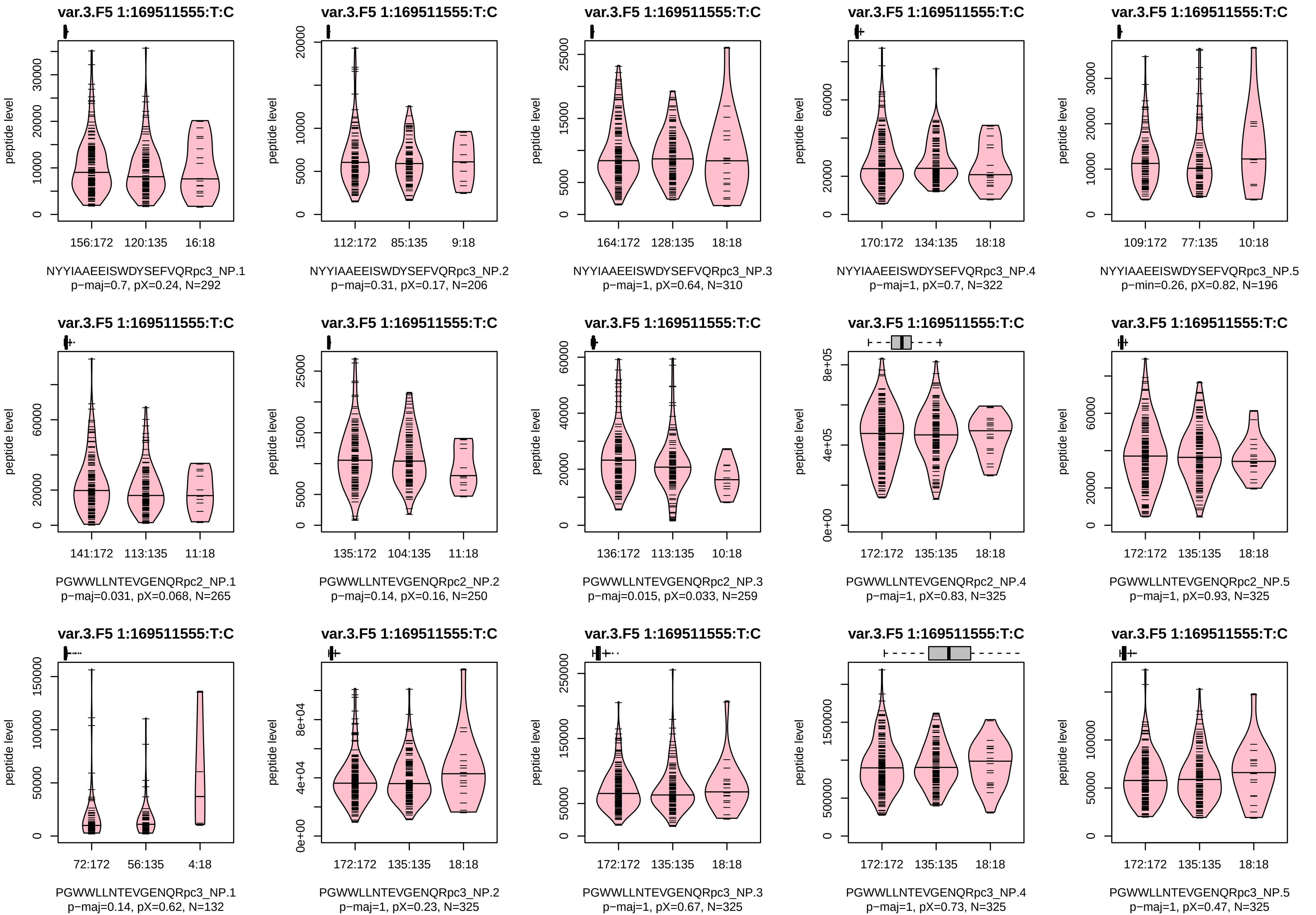
var.3.F5 1:169511555:T:C
peptide level
156:172 120:135 16:18
NYYIAAEEISWDYSEFVQRpc3_NP.1
p−maj=0.7, pX=0.24, N=292
var.3.F5 1:169511555:T:C
peptide level
112:172 85:135 9:18
NYYIAAEEISWDYSEFVQRpc3_NP.2
p−maj=0.31, pX=0.17, N=206
var.3.F5 1:169511555:T:C
peptide level
164:172 128:135 18:18
NYYIAAEEISWDYSEFVQRpc3_NP.3
p−maj=1, pX=0.64, N=310
var.3.F5 1:169511555:T:C
peptide level
170:172 134:135 18:18
NYYIAAEEISWDYSEFVQRpc3_NP.4
p−maj=1, pX=0.7, N=322
var.3.F5 1:169511555:T:C
peptide level
109:172 77:135 10:18
NYYIAAEEISWDYSEFVQRpc3_NP.5
p−min=0.26, pX=0.82, N=196
var.3.F5 1:169511555:T:C
peptide level
141:172 113:135 11:18
PGWWLLNTEVGENQRpc2_NP.1
p−maj=0.031, pX=0.068, N=265
var.3.F5 1:169511555:T:C
peptide level
135:172 104:135 11:18
PGWWLLNTEVGENQRpc2_NP.2
p−maj=0.14, pX=0.16, N=250
var.3.F5 1:169511555:T:C
peptide level
136:172 113:135 10:18
PGWWLLNTEVGENQRpc2_NP.3
p−maj=0.015, pX=0.033, N=259
var.3.F5 1:169511555:T:C
peptide level
172:172 135:135 18:18
PGWWLLNTEVGENQRpc2_NP.4
p−maj=1, pX=0.83, N=325
var.3.F5 1:169511555:T:C
peptide level
172:172 135:135 18:18
PGWWLLNTEVGENQRpc2_NP.5
p−maj=1, pX=0.93, N=325
var.3.F5 1:169511555:T:C
peptide level
72:172 56:135 4:18
PGWWLLNTEVGENQRpc3_NP.1
p−maj=0.14, pX=0.62, N=132
var.3.F5 1:169511555:T:C
peptide level
172:172 135:135 18:18
PGWWLLNTEVGENQRpc3_NP.2
p−maj=1, pX=0.23, N=325
var.3.F5 1:169511555:T:C
peptide level
172:172 135:135 18:18
PGWWLLNTEVGENQRpc3_NP.3
p−maj=1, pX=0.67, N=325
var.3.F5 1:169511555:T:C
peptide level
172:172 135:135 18:18
PGWWLLNTEVGENQRpc3_NP.4
p−maj=1, pX=0.73, N=325
var.3.F5 1:169511555:T:C
peptide level
172:172 135:135 18:18
PGWWLLNTEVGENQRpc3_NP.5
p−maj=1, pX=0.47, N=325

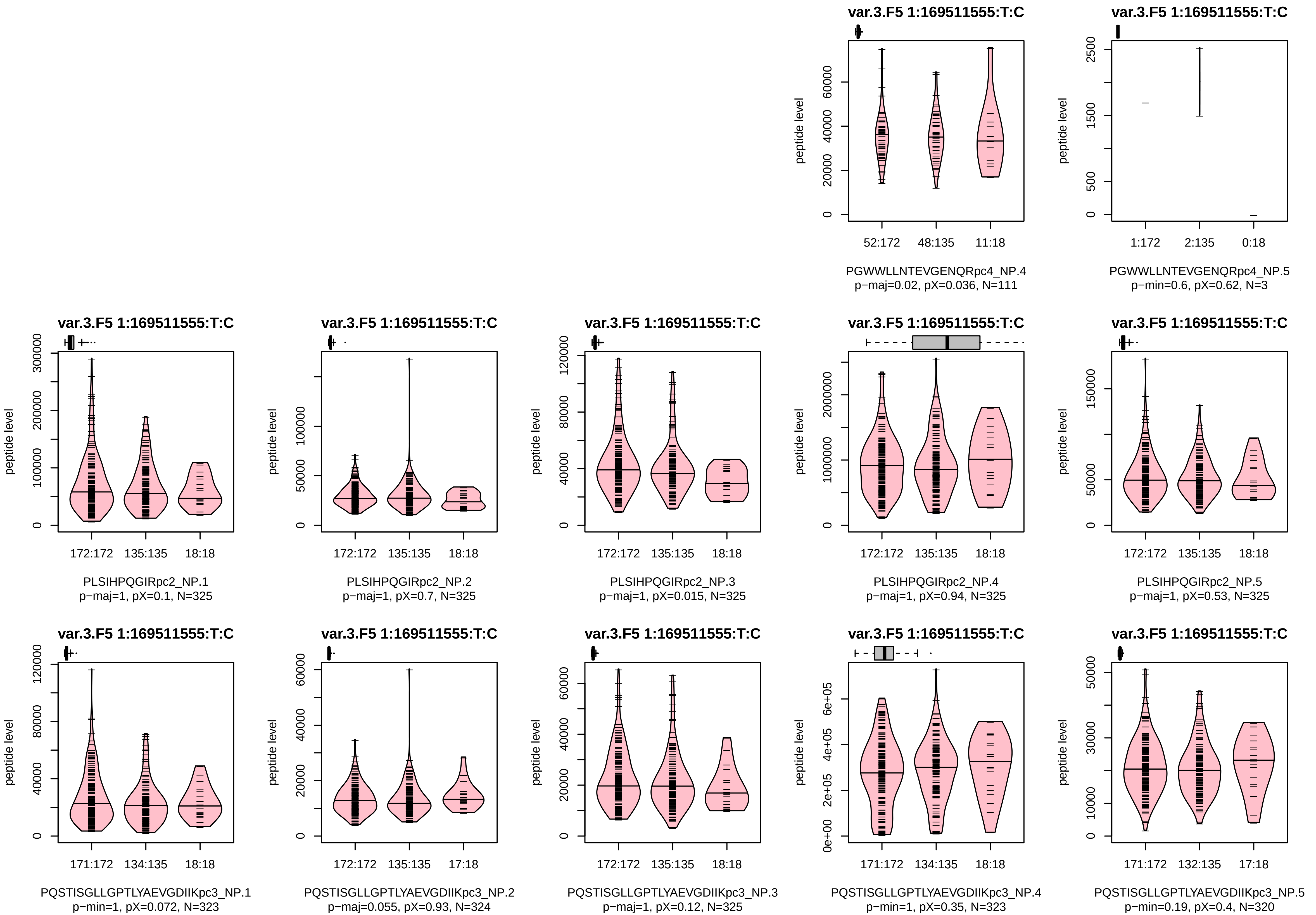
var.3.F5 1:169511555:T:C
peptide level
52:172
48:135
11:18
PGWWLLNTEVGENQRpc4_NP.4
p−maj=0.02, pX=0.036, N=111
var.3.F5 1:169511555:T:C
peptide level
1:172
2:135
0:18
PGWWLLNTEVGENQRpc4_NP.5
p−min=0.6, pX=0.62, N=3
var.3.F5 1:169511555:T:C
peptide level
172:172
135:135
18:18
PLSIHPQGIRpc2_NP.1
p−maj=1, pX=0.1, N=325
var.3.F5 1:169511555:T:C
peptide level
172:172
135:135
18:18
PLSIHPQGIRpc2_NP.2
p−maj=1, pX=0.7, N=325
var.3.F5 1:169511555:T:C
peptide level
172:172
135:135
18:18
PLSIHPQGIRpc2_NP.3
p−maj=1, pX=0.015, N=325
var.3.F5 1:169511555:T:C
peptide level
172:172
135:135
18:18
PLSIHPQGIRpc2_NP.4
p−maj=1, pX=0.94, N=325
var.3.F5 1:169511555:T:C
peptide level
172:172
135:135
18:18
PLSIHPQGIRpc2_NP.5
p−maj=1, pX=0.53, N=325
var.3.F5 1:169511555:T:C
peptide level
171:172
134:135
18:18
PQSTISGLLGPTLYAEVGDIIKpc3_NP.1
p−min=1, pX=0.072, N=323
var.3.F5 1:169511555:T:C
peptide level
172:172
135:135
17:18
PQSTISGLLGPTLYAEVGDIIKpc3_NP.2
p−maj=0.055, pX=0.93, N=324
var.3.F5 1:169511555:T:C
peptide level
172:172
135:135
18:18
PQSTISGLLGPTLYAEVGDIIKpc3_NP.3
p−maj=1, pX=0.12, N=325
var.3.F5 1:169511555:T:C
peptide level
171:172
134:135
18:18
PQSTISGLLGPTLYAEVGDIIKpc3_NP.4
p−min=1, pX=0.35, N=323
var.3.F5 1:169511555:T:C
peptide level
171:172
132:135
17:18
PQSTISGLLGPTLYAEVGDIIKpc3_NP.5
p−min=0.19, pX=0.4, N=320

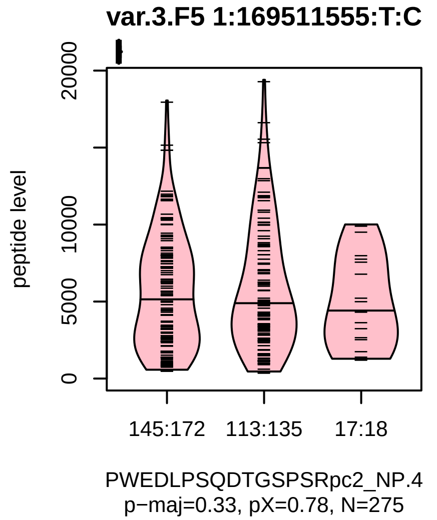
var.3.F5 1:169511555:T:C
peptide level
0
5000
10000
20000
145:172
113:135
17:18
PWEDLPSQDTGSPSRpc2_NP.4
p−maj=0.33, pX=0.78, N=275

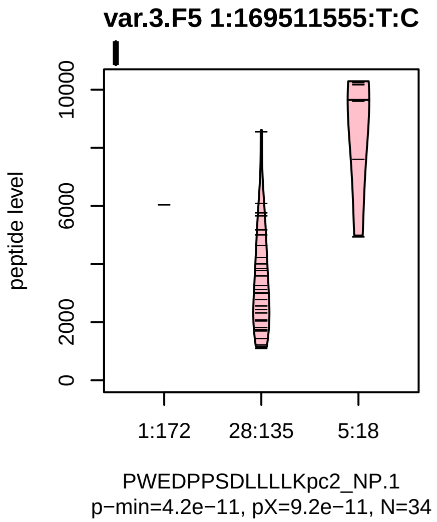
var.3.F5 1:169511555:T:C
peptide level
0
2000
6000
10000
1:172
28:135
5:18
PWEDPPSDLLLLKpc2_NP.1
p−min=4.2e−11, pX=9.2e−11, N=34

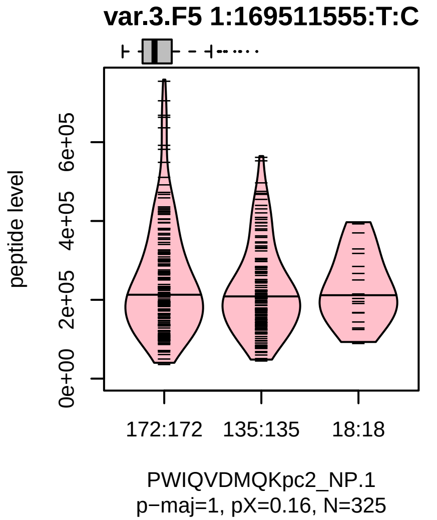
var.3.F5 1:169511555:T:C
peptide level
0e+00
2e+05
4e+05
6e+05
172:172
135:135
18:18
PWIQVDMQKpc2_NP.1
p−maj=1, pX=0.16, N=325

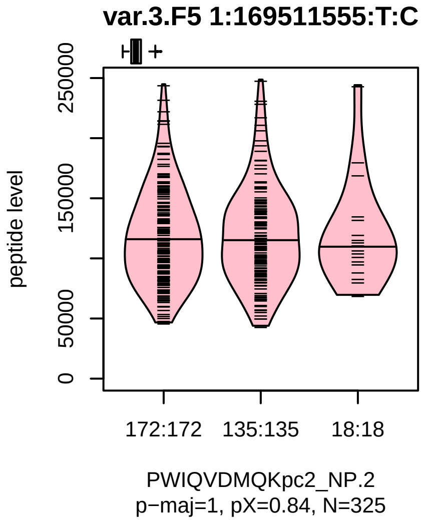
var.3.F5 1:169511555:T:C
peptide level
0
50000
150000
250000
172:172
135:135
18:18
PWIQVDMQKpc2_NP.2
p−maj=1, pX=0.84, N=325

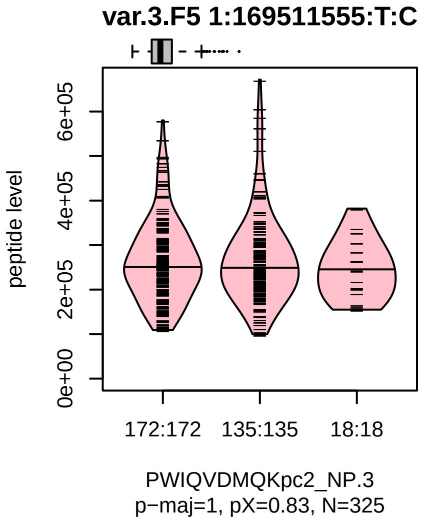
var.3.F5 1:169511555:T:C
peptide level
0e+00
2e+05
4e+05
6e+05
172:172
135:135
18:18
PWIQVDMQKpc2_NP.3
p−maj=1, pX=0.83, N=325

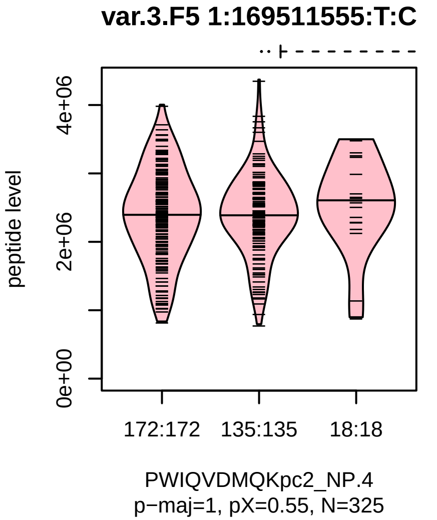
var.3.F5 1:169511555:T:C
peptide level
0e+00
2e+06
4e+06
172:172
135:135
18:18
PWIQVDMQKpc2_NP.4
p−maj=1, pX=0.55, N=325

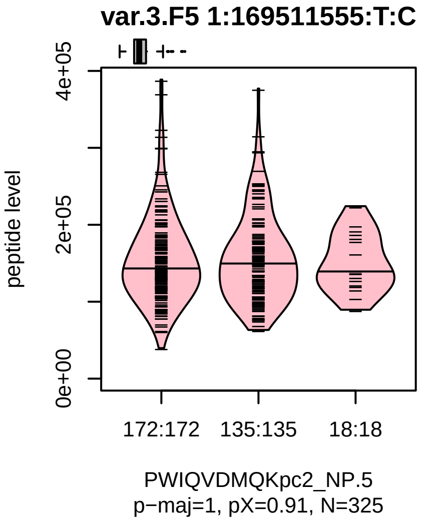
var.3.F5 1:169511555:T:C
peptide level
0e+00
2e+05
4e+05
172:172
135:135
18:18
PWIQVDMQKpc2_NP.5
p−maj=1, pX=0.91, N=325

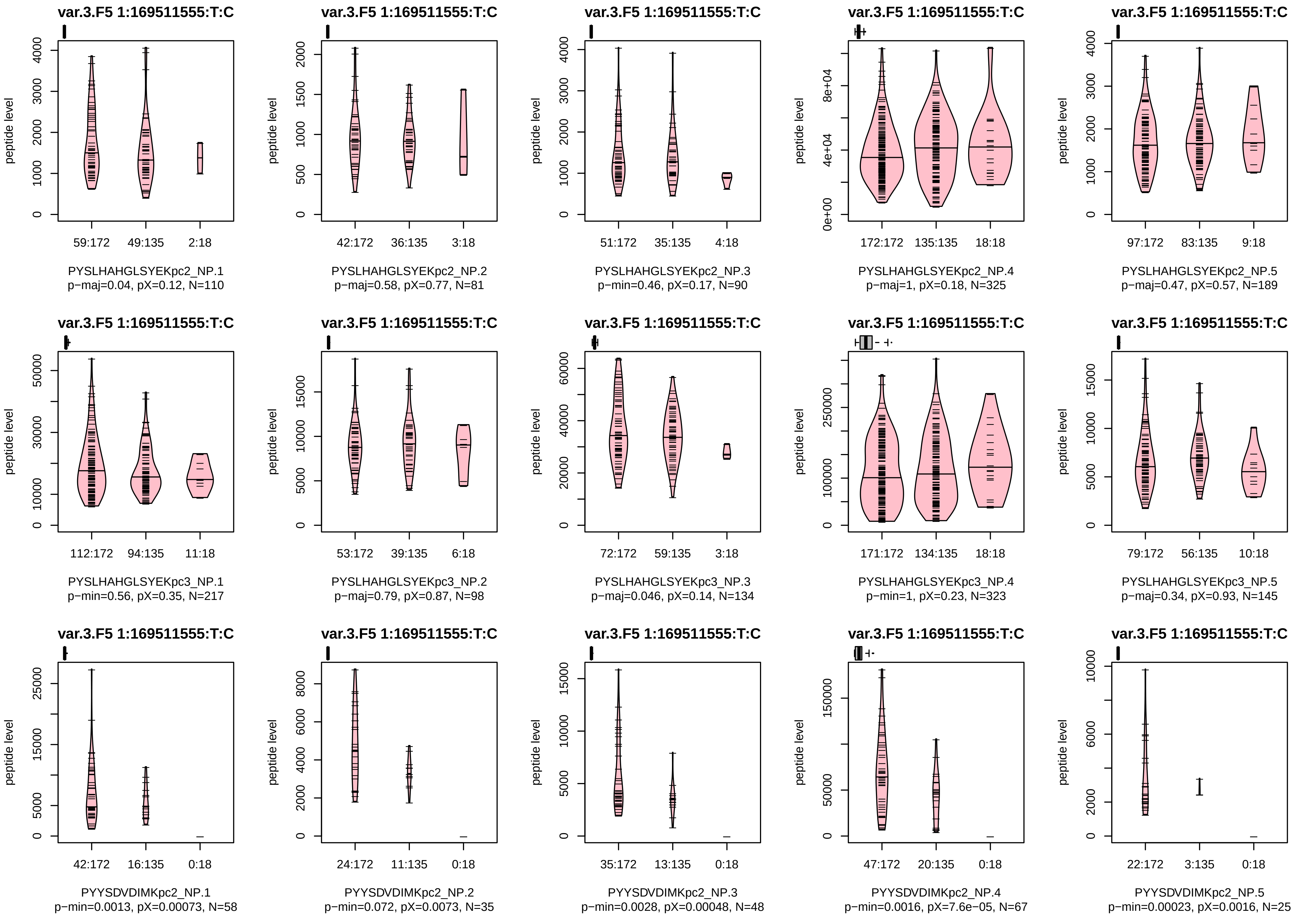
var.3.F5 1:169511555:T:C
peptide level
59:172
49:135
2:18
PYSLHAHGLSYEKpc2_NP.1
p−maj=0.04, pX=0.12, N=110
var.3.F5 1:169511555:T:C
peptide level
42:172
36:135
3:18
PYSLHAHGLSYEKpc2_NP.2
p−maj=0.58, pX=0.77, N=81
var.3.F5 1:169511555:T:C
peptide level
51:172
35:135
4:18
PYSLHAHGLSYEKpc2_NP.3
p−min=0.46, pX=0.17, N=90
var.3.F5 1:169511555:T:C
peptide level
172:172
135:135
18:18
PYSLHAHGLSYEKpc2_NP.4
p−maj=1, pX=0.18, N=325
var.3.F5 1:169511555:T:C
peptide level
97:172
83:135
9:18
PYSLHAHGLSYEKpc2_NP.5
p−maj=0.47, pX=0.57, N=189
var.3.F5 1:169511555:T:C
peptide level
112:172
94:135
11:18
PYSLHAHGLSYEKpc3_NP.1
p−min=0.56, pX=0.35, N=217
var.3.F5 1:169511555:T:C
peptide level
53:172
39:135
6:18
PYSLHAHGLSYEKpc3_NP.2
p−maj=0.79, pX=0.87, N=98
var.3.F5 1:169511555:T:C
peptide level
72:172
59:135
3:18
PYSLHAHGLSYEKpc3_NP.3
p−maj=0.046, pX=0.14, N=134
var.3.F5 1:169511555:T:C
peptide level
171:172
134:135
18:18
PYSLHAHGLSYEKpc3_NP.4
p−min=1, pX=0.23, N=323
var.3.F5 1:169511555:T:C
peptide level
79:172
56:135
10:18
PYSLHAHGLSYEKpc3_NP.5
p−maj=0.34, pX=0.93, N=145
var.3.F5 1:169511555:T:C
peptide level
42:172
16:135
0:18
PYYSDVDIMKpc2_NP.1
p−min=0.0013, pX=0.00073, N=58
var.3.F5 1:169511555:T:C
peptide level
24:172
11:135
0:18
PYYSDVDIMKpc2_NP.2
p−min=0.072, pX=0.0073, N=35
var.3.F5 1:169511555:T:C
peptide level
35:172
13:135
0:18
PYYSDVDIMKpc2_NP.3
p−min=0.0028, pX=0.00048, N=48
var.3.F5 1:169511555:T:C
peptide level
47:172
20:135
0:18
PYYSDVDIMKpc2_NP.4
p−min=0.0016, pX=7.6e−05, N=67
var.3.F5 1:169511555:T:C
peptide level
22:172
3:135
0:18
PYYSDVDIMKpc2_NP.5
p−min=0.00023, pX=0.0016, N=25

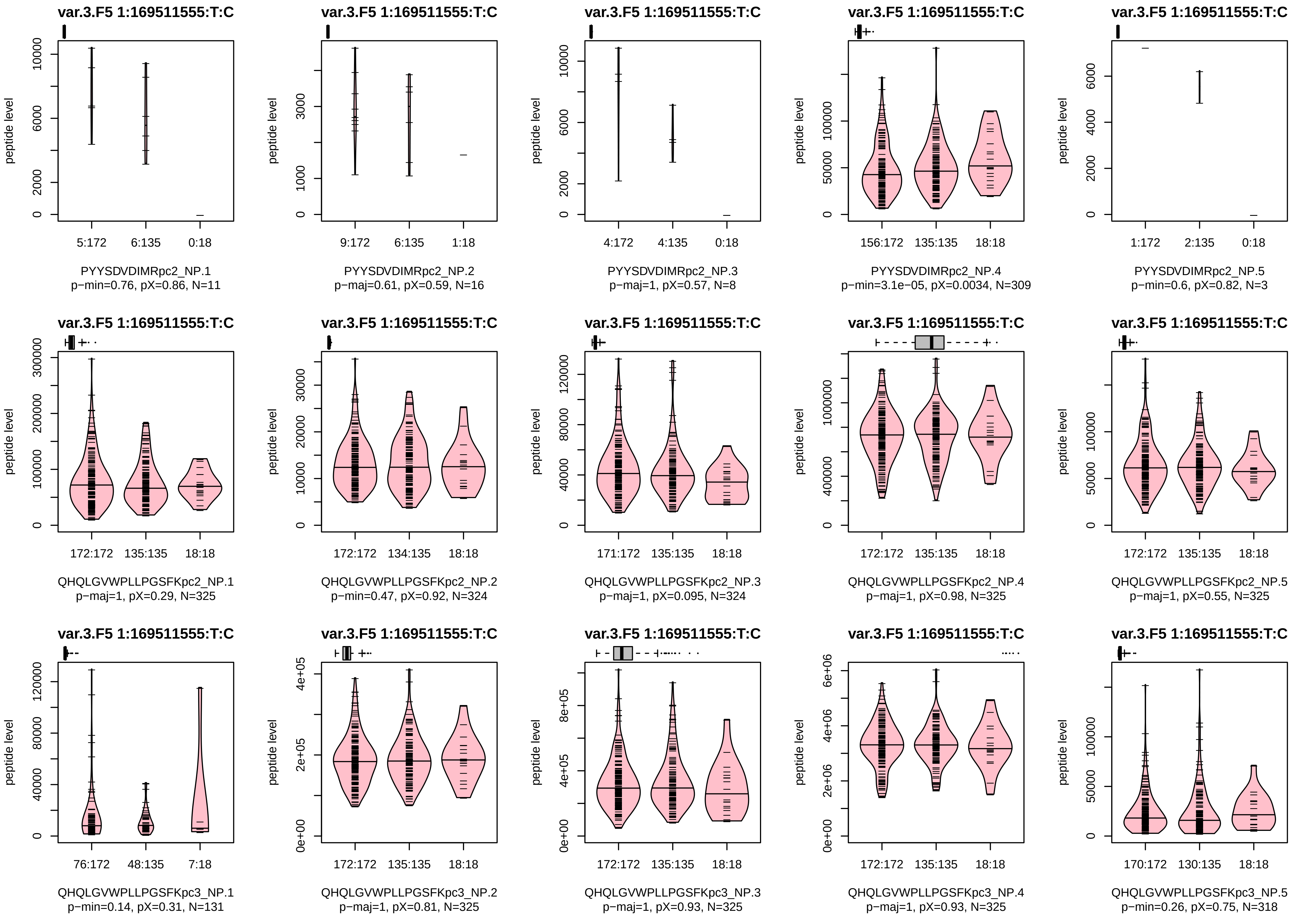
var.3.F5 1:169511555:T:C
peptide level
5:172
6:135
0:18
PYYSDVDIMRpc2_NP.1
p−min=0.76, pX=0.86, N=11
var.3.F5 1:169511555:T:C
peptide level
9:172
6:135
1:18
PYYSDVDIMRpc2_NP.2
p−maj=0.61, pX=0.59, N=16
var.3.F5 1:169511555:T:C
peptide level
4:172
4:135
0:18
PYYSDVDIMRpc2_NP.3
p−maj=1, pX=0.57, N=8
var.3.F5 1:169511555:T:C
peptide level
156:172
135:135
18:18
PYYSDVDIMRpc2_NP.4
p−min=3.1e−05, pX=0.0034, N=309
var.3.F5 1:169511555:T:C
peptide level
1:172
2:135
0:18
PYYSDVDIMRpc2_NP.5
p−min=0.6, pX=0.82, N=3
var.3.F5 1:169511555:T:C
peptide level
172:172
135:135
18:18
QHQLGVWPLLPGSFKpc2_NP.1
p−maj=1, pX=0.29, N=325
var.3.F5 1:169511555:T:C
peptide level
172:172
134:135
18:18
QHQLGVWPLLPGSFKpc2_NP.2
p−min=0.47, pX=0.92, N=324
var.3.F5 1:169511555:T:C
peptide level
171:172
135:135
18:18
QHQLGVWPLLPGSFKpc2_NP.3
p−maj=1, pX=0.095, N=324
var.3.F5 1:169511555:T:C
peptide level
172:172
135:135
18:18
QHQLGVWPLLPGSFKpc2_NP.4
p−maj=1, pX=0.98, N=325
var.3.F5 1:169511555:T:C
peptide level
172:172
135:135
18:18
QHQLGVWPLLPGSFKpc2_NP.5
p−maj=1, pX=0.55, N=325
var.3.F5 1:169511555:T:C
peptide level
76:172
48:135
7:18
QHQLGVWPLLPGSFKpc3_NP.1
p−min=0.14, pX=0.31, N=131
var.3.F5 1:169511555:T:C
peptide level
172:172
135:135
18:18
QHQLGVWPLLPGSFKpc3_NP.2
p−maj=1, pX=0.81, N=325
var.3.F5 1:169511555:T:C
peptide level
172:172
135:135
18:18
QHQLGVWPLLPGSFKpc3_NP.3
p−maj=1, pX=0.93, N=325
var.3.F5 1:169511555:T:C
peptide level
172:172
135:135
18:18
QHQLGVWPLLPGSFKpc3_NP.4
p−maj=1, pX=0.93, N=325
var.3.F5 1:169511555:T:C
peptide level
170:172
130:135
18:18
QHQLGVWPLLPGSFKpc3_NP.5
p−min=0.26, pX=0.75, N=318

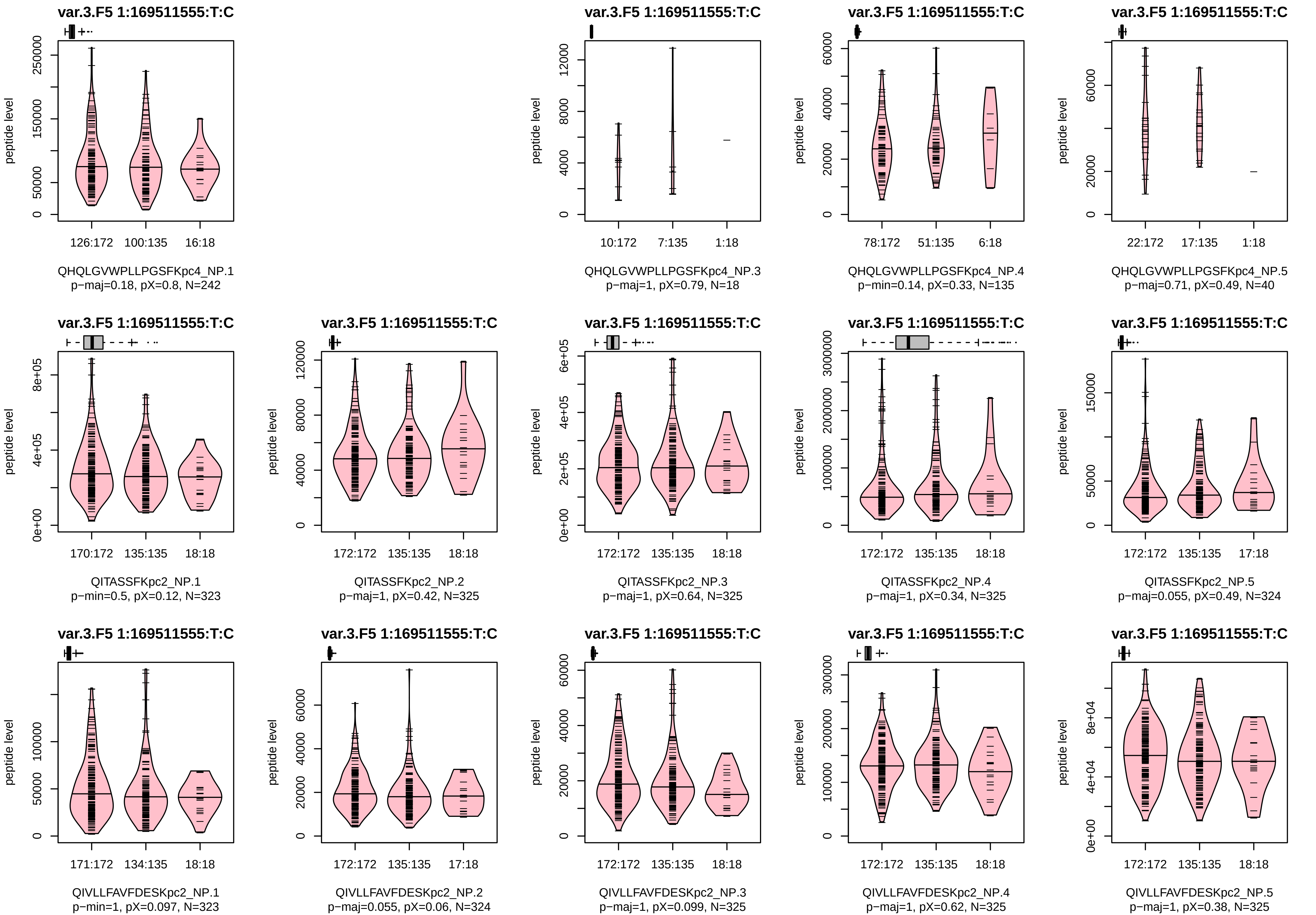
var.3.F5 1:169511555:T:C
peptide level
126:172
100:135
16:18
QHQLGVWPLLPGSFKpc4_NP.1
p−maj=0.18, pX=0.8, N=242
var.3.F5 1:169511555:T:C
peptide level
10:172
7:135
1:18
QHQLGVWPLLPGSFKpc4_NP.3
p−maj=1, pX=0.79, N=18
var.3.F5 1:169511555:T:C
peptide level
78:172
51:135
6:18
QHQLGVWPLLPGSFKpc4_NP.4
p−min=0.14, pX=0.33, N=135
var.3.F5 1:169511555:T:C
peptide level
22:172
17:135
1:18
QHQLGVWPLLPGSFKpc4_NP.5
p−maj=0.71, pX=0.49, N=40
var.3.F5 1:169511555:T:C
peptide level
170:172
135:135
18:18
QITASSFKpc2_NP.1
p−min=0.5, pX=0.12, N=323
var.3.F5 1:169511555:T:C
peptide level
172:172
135:135
18:18
QITASSFKpc2_NP.2
p−maj=1, pX=0.42, N=325
var.3.F5 1:169511555:T:C
peptide level
172:172
135:135
18:18
QITASSFKpc2_NP.3
p−maj=1, pX=0.64, N=325
var.3.F5 1:169511555:T:C
peptide level
172:172
135:135
18:18
QITASSFKpc2_NP.4
p−maj=1, pX=0.34, N=325
var.3.F5 1:169511555:T:C
peptide level
172:172
135:135
17:18
QITASSFKpc2_NP.5
p−maj=0.055, pX=0.49, N=324
var.3.F5 1:169511555:T:C
peptide level
171:172
134:135
18:18
QIVLLFAVFDESKpc2_NP.1
p−min=1, pX=0.097, N=323
var.3.F5 1:169511555:T:C
peptide level
172:172
135:135
17:18
QIVLLFAVFDESKpc2_NP.2
p−maj=0.055, pX=0.06, N=324
var.3.F5 1:169511555:T:C
peptide level
172:172
135:135
18:18
QIVLLFAVFDESKpc2_NP.3
p−maj=1, pX=0.099, N=325
var.3.F5 1:169511555:T:C
peptide level
172:172
135:135
18:18
QIVLLFAVFDESKpc2_NP.4
p−maj=1, pX=0.62, N=325
var.3.F5 1:169511555:T:C
peptide level
172:172
135:135
18:18
QIVLLFAVFDESKpc2_NP.5
p−maj=1, pX=0.38, N=325

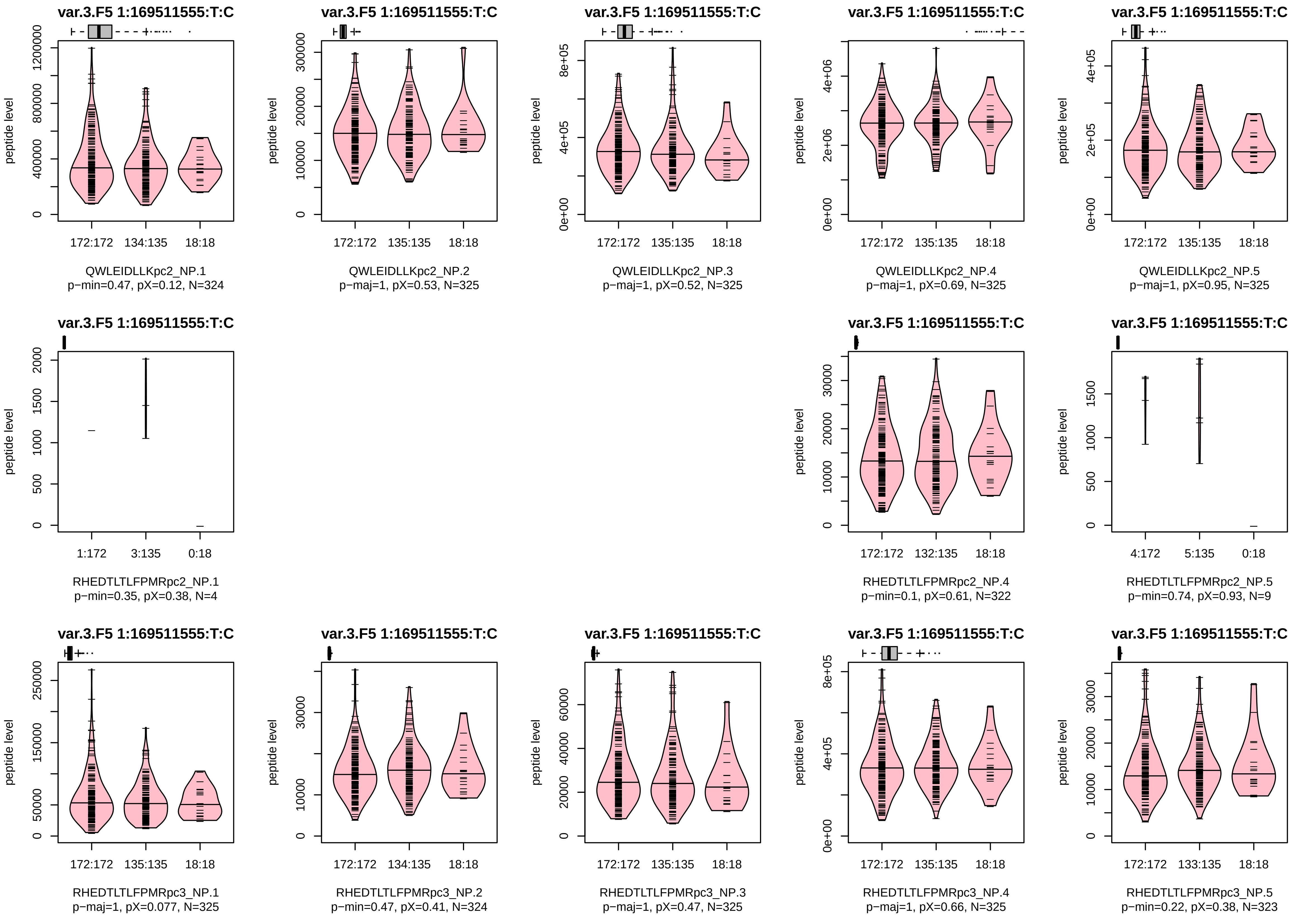

var.3.F5 1:169511555:T:C
peptide level
172:172
134:135
18:18
QWLEIDLLKpc2_NP.1
p−min=0.47, pX=0.12, N=324
var.3.F5 1:169511555:T:C
peptide level
172:172
135:135
18:18
QWLEIDLLKpc2_NP.2
p−maj=1, pX=0.53, N=325
var.3.F5 1:169511555:T:C
peptide level
172:172
135:135
18:18
QWLEIDLLKpc2_NP.3
p−maj=1, pX=0.52, N=325
var.3.F5 1:169511555:T:C
peptide level
172:172
135:135
18:18
QWLEIDLLKpc2_NP.4
p−maj=1, pX=0.69, N=325
var.3.F5 1:169511555:T:C
peptide level
172:172
135:135
18:18
QWLEIDLLKpc2_NP.5
p−maj=1, pX=0.95, N=325
var.3.F5 1:169511555:T:C
peptide level
1:172
3:135
0:18
RHEDTLTLFPMRpc2_NP.1
p−min=0.35, pX=0.38, N=4
var.3.F5 1:169511555:T:C
peptide level
172:172
132:135
18:18
RHEDTLTLFPMRpc2_NP.4
p−min=0.1, pX=0.61, N=322
var.3.F5 1:169511555:T:C
peptide level
4:172
5:135
0:18
RHEDTLTLFPMRpc2_NP.5
p−min=0.74, pX=0.93, N=9
var.3.F5 1:169511555:T:C
peptide level
172:172
135:135
18:18
RHEDTLTLFPMRpc3_NP.1
p−maj=1, pX=0.077, N=325
var.3.F5 1:169511555:T:C
peptide level
172:172
134:135
18:18
RHEDTLTLFPMRpc3_NP.2
p−min=0.47, pX=0.41, N=324
var.3.F5 1:169511555:T:C
peptide level
172:172
135:135
18:18
RHEDTLTLFPMRpc3_NP.3
p−maj=1, pX=0.47, N=325
var.3.F5 1:169511555:T:C
peptide level
172:172
135:135
18:18
RHEDTLTLFPMRpc3_NP.4
p−maj=1, pX=0.66, N=325
var.3.F5 1:169511555:T:C
peptide level
172:172
133:135
18:18
RHEDTLTLFPMRpc3_NP.5
p−min=0.22, pX=0.38, N=323

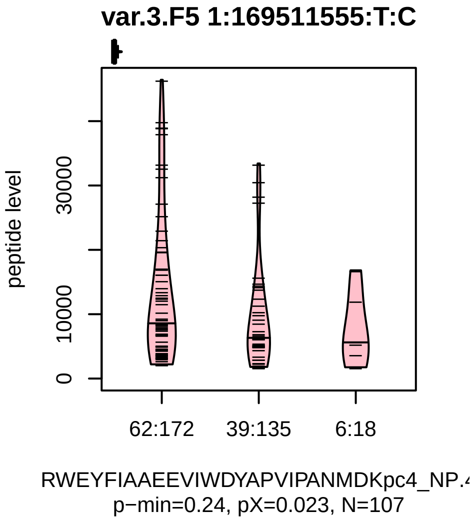
var.3.F5 1:169511555:T:C
peptide level
0
10000
30000
62:172
39:135
6:18
RWEYFIAAEEVIWDYAPVIPANMDKpc4_NP.4
p−min=0.24, pX=0.023, N=107

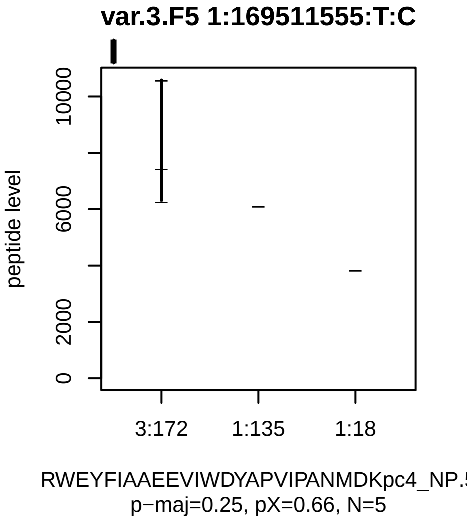
var.3.F5 1:169511555:T:C
peptide level
0
2000
6000
10000
3:172
1:135
1:18
RWEYFIAAEEVIWDYAPVIPANMDKpc4_NP.5
p−maj=0.25, pX=0.66, N=5

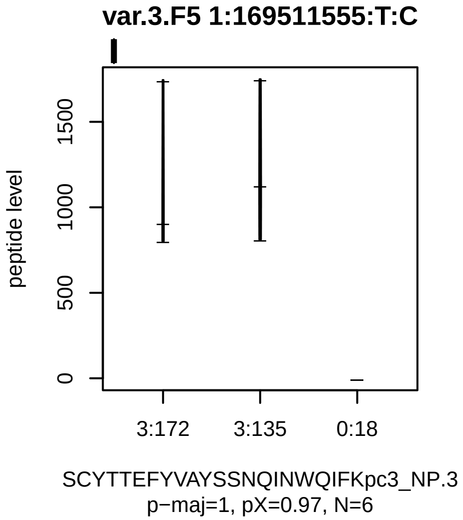
var.3.F5 1:169511555:T:C
peptide level
0
500
1000
1500
3:172
3:135
0:18
SCYTTEFYVAYSSNQINWQIFKpc3_NP.3
p−maj=1, pX=0.97, N=6

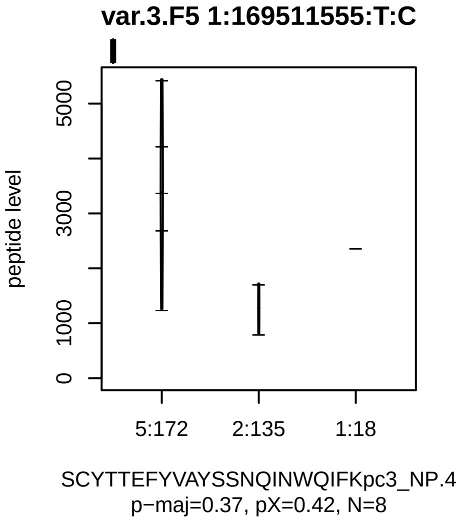
var.3.F5 1:169511555:T:C
peptide level
0
1000
3000
5000
5:172
2:135
1:18
SCYTTEFYVAYSSNQINWQIFKpc3_NP.4
p−maj=0.37, pX=0.42, N=8

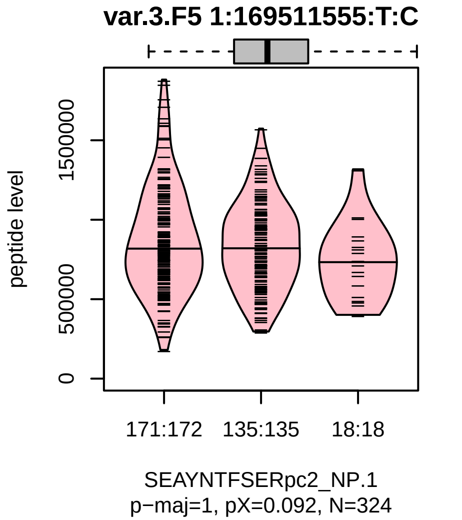
var.3.F5 1:169511555:T:C
peptide level
0
500000
1500000
171:172
135:135
18:18
SEAYNTFSERpc2_NP.1
p−maj=1, pX=0.092, N=324

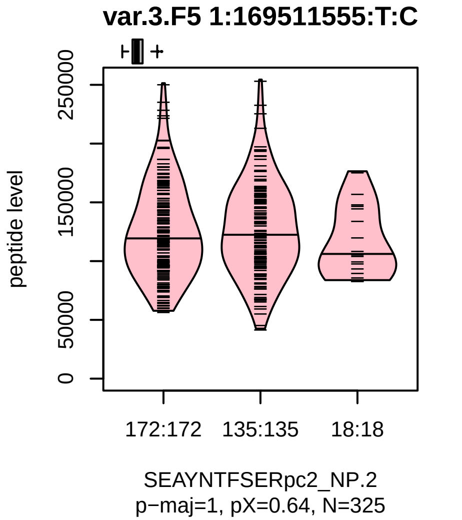
var.3.F5 1:169511555:T:C
peptide level
0
50000
150000
250000
172:172
135:135
18:18
SEAYNTFSERpc2_NP.2
p−maj=1, pX=0.64, N=325

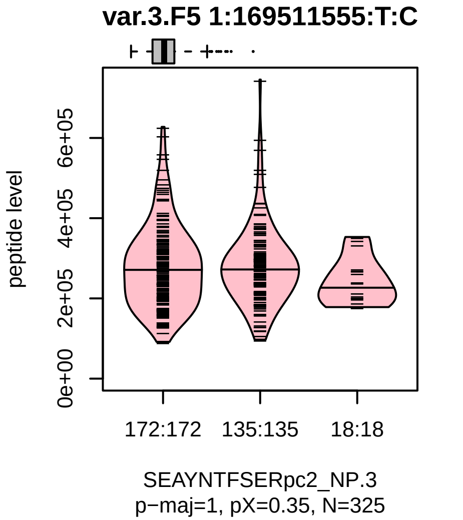
var.3.F5 1:169511555:T:C
peptide level
0e+00
2e+05
4e+05
6e+05
172:172
135:135
18:18
SEAYNTFSERpc2_NP.3
p−maj=1, pX=0.35, N=325

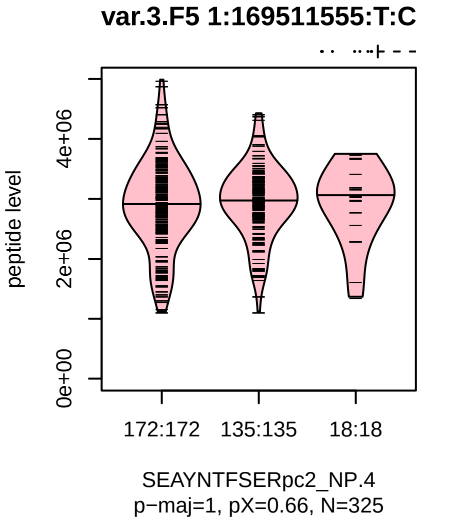
var.3.F5 1:169511555:T:C
peptide level
0e+00
2e+06
4e+06
172:172
135:135
18:18
SEAYNTFSERpc2_NP.4
p−maj=1, pX=0.66, N=325

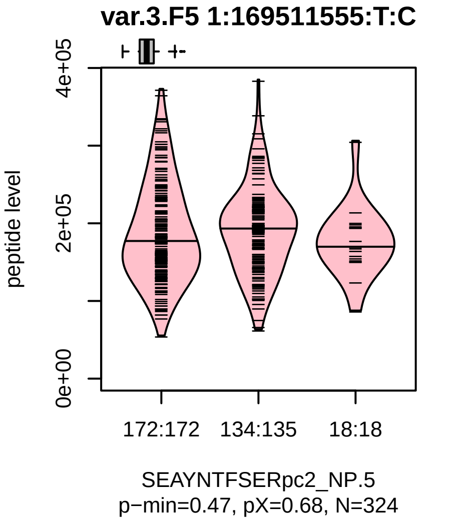
var.3.F5 1:169511555:T:C
peptide level
0e+00
2e+05
4e+05
172:172
134:135
18:18
SEAYNTFSERpc2_NP.5
p−min=0.47, pX=0.68, N=324

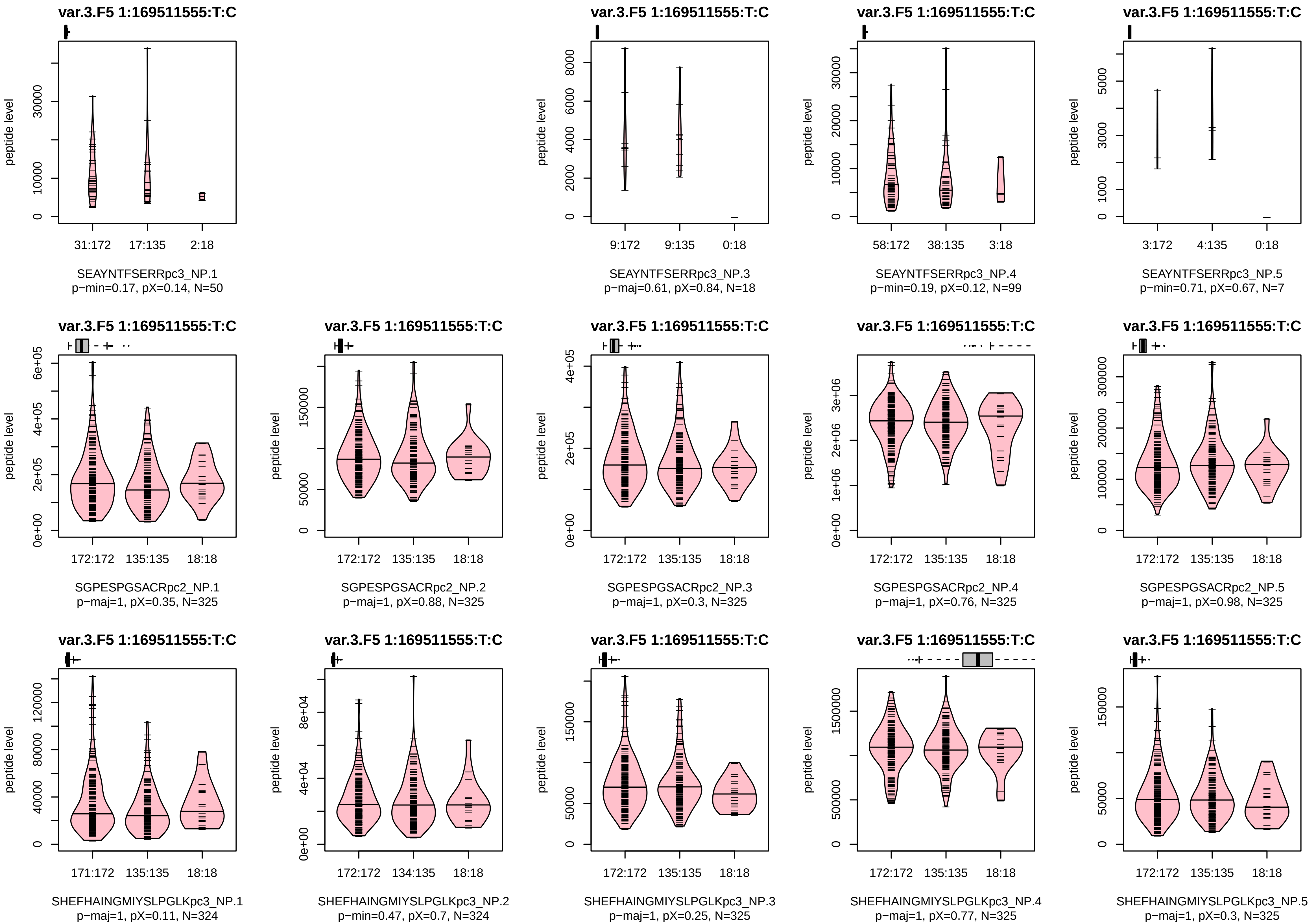
var.3.F5 1:169511555:T:C
peptide level
31:172
17:135
2:18
SEAYNTFSERRpc3_NP.1
p−min=0.17, pX=0.14, N=50
var.3.F5 1:169511555:T:C
peptide level
9:172
9:135
0:18
SEAYNTFSERRpc3_NP.3
p−maj=0.61, pX=0.84, N=18
var.3.F5 1:169511555:T:C
peptide level
58:172
38:135
3:18
SEAYNTFSERRpc3_NP.4
p−min=0.19, pX=0.12, N=99
var.3.F5 1:169511555:T:C
peptide level
3:172
4:135
0:18
SEAYNTFSERRpc3_NP.5
p−min=0.71, pX=0.67, N=7
var.3.F5 1:169511555:T:C
peptide level
172:172
135:135
18:18
SGPESPGSACRpc2_NP.1
p−maj=1, pX=0.35, N=325
var.3.F5 1:169511555:T:C
peptide level
172:172
135:135
18:18
SGPESPGSACRpc2_NP.2
p−maj=1, pX=0.88, N=325
var.3.F5 1:169511555:T:C
peptide level
172:172
135:135
18:18
SGPESPGSACRpc2_NP.3
p−maj=1, pX=0.3, N=325
var.3.F5 1:169511555:T:C
peptide level
172:172
135:135
18:18
SGPESPGSACRpc2_NP.4
p−maj=1, pX=0.76, N=325
var.3.F5 1:169511555:T:C
peptide level
172:172
135:135
18:18
SGPESPGSACRpc2_NP.5
p−maj=1, pX=0.98, N=325
var.3.F5 1:169511555:T:C
peptide level
171:172
135:135
18:18
SHEFHAINGMIYSLPGLKpc3_NP.1
p−maj=1, pX=0.11, N=324
var.3.F5 1:169511555:T:C
peptide level
172:172
134:135
18:18
SHEFHAINGMIYSLPGLKpc3_NP.2
p−min=0.47, pX=0.7, N=324
var.3.F5 1:169511555:T:C
peptide level
172:172
135:135
18:18
SHEFHAINGMIYSLPGLKpc3_NP.3
p−maj=1, pX=0.25, N=325
var.3.F5 1:169511555:T:C
peptide level
172:172
135:135
18:18
SHEFHAINGMIYSLPGLKpc3_NP.4
p−maj=1, pX=0.77, N=325
var.3.F5 1:169511555:T:C
peptide level
172:172
135:135
18:18
SHEFHAINGMIYSLPGLKpc3_NP.5
p−maj=1, pX=0.3, N=325

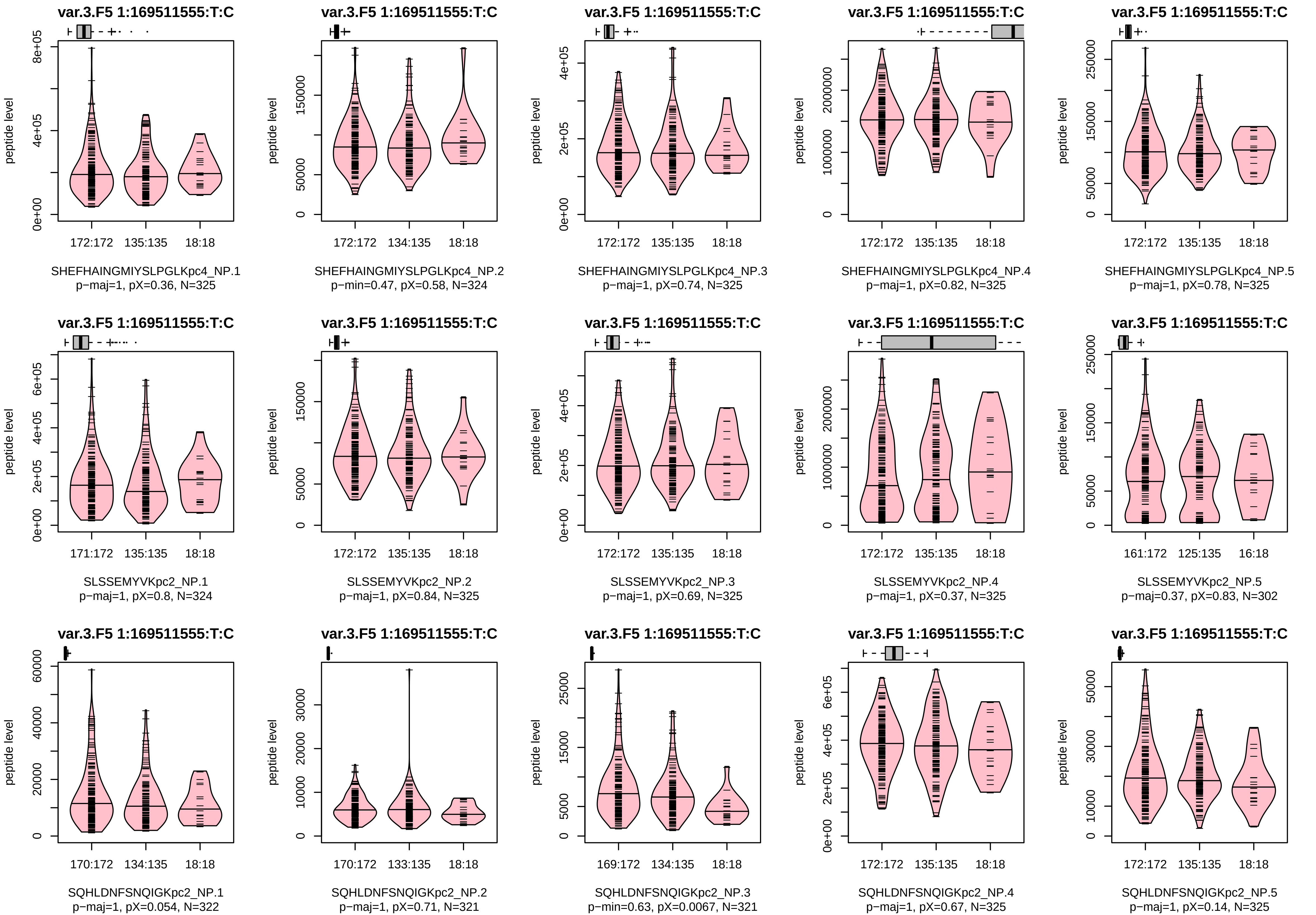
var.3.F5 1:169511555:T:C
peptide level
172:172
135:135
18:18
SHEFHAINGMIYSLPGLKpc4_NP.1
p−maj=1, pX=0.36, N=325
var.3.F5 1:169511555:T:C
peptide level
172:172
134:135
18:18
SHEFHAINGMIYSLPGLKpc4_NP.2
p−min=0.47, pX=0.58, N=324
var.3.F5 1:169511555:T:C
peptide level
172:172
135:135
18:18
SHEFHAINGMIYSLPGLKpc4_NP.3
p−maj=1, pX=0.74, N=325
var.3.F5 1:169511555:T:C
peptide level
172:172
135:135
18:18
SHEFHAINGMIYSLPGLKpc4_NP.4
p−maj=1, pX=0.82, N=325
var.3.F5 1:169511555:T:C
peptide level
172:172
135:135
18:18
SHEFHAINGMIYSLPGLKpc4_NP.5
p−maj=1, pX=0.78, N=325
var.3.F5 1:169511555:T:C
peptide level
171:172
135:135
18:18
SLSSEMYVKpc2_NP.1
p−maj=1, pX=0.8, N=324
var.3.F5 1:169511555:T:C
peptide level
172:172
135:135
18:18
SLSSEMYVKpc2_NP.2
p−maj=1, pX=0.84, N=325
var.3.F5 1:169511555:T:C
peptide level
172:172
135:135
18:18
SLSSEMYVKpc2_NP.3
p−maj=1, pX=0.69, N=325
var.3.F5 1:169511555:T:C
peptide level
172:172
135:135
18:18
SLSSEMYVKpc2_NP.4
p−maj=1, pX=0.37, N=325
var.3.F5 1:169511555:T:C
peptide level
161:172
125:135
16:18
SLSSEMYVKpc2_NP.5
p−maj=0.37, pX=0.83, N=302
var.3.F5 1:169511555:T:C
peptide level
170:172
134:135
18:18
SQHLDNFSNQIGKpc2_NP.1
p−maj=1, pX=0.054, N=322
var.3.F5 1:169511555:T:C
peptide level
170:172
133:135
18:18
SQHLDNFSNQIGKpc2_NP.2
p−maj=1, pX=0.71, N=321
var.3.F5 1:169511555:T:C
peptide level
169:172
134:135
18:18
SQHLDNFSNQIGKpc2_NP.3
p−min=0.63, pX=0.0067, N=321
var.3.F5 1:169511555:T:C
peptide level
172:172
135:135
18:18
SQHLDNFSNQIGKpc2_NP.4
p−maj=1, pX=0.67, N=325
var.3.F5 1:169511555:T:C
peptide level
172:172
135:135
18:18
SQHLDNFSNQIGKpc2_NP.5
p−maj=1, pX=0.14, N=325

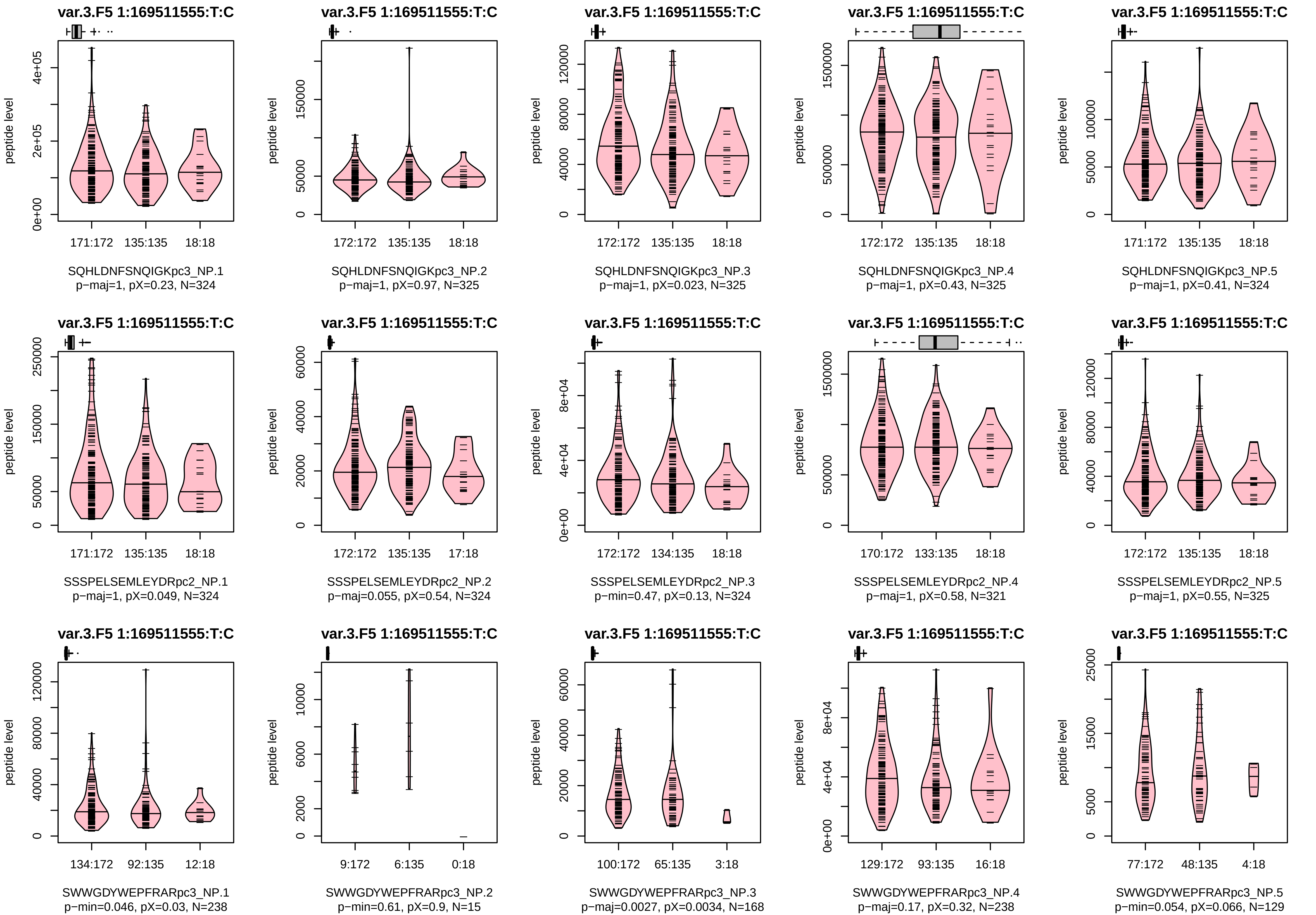
var.3.F5 1:169511555:T:C
peptide level
171:172 135:135 18:18
SQHLDNFSNQIGKpc3_NP.1
p−maj=1, pX=0.23, N=324
var.3.F5 1:169511555:T:C
172:172 135:135 18:18
SQHLDNFSNQIGKpc3_NP.2
p−maj=1, pX=0.97, N=325
var.3.F5 1:169511555:T:C
172:172 135:135 18:18
SQHLDNFSNQIGKpc3_NP.3
p−maj=1, pX=0.023, N=325
var.3.F5 1:169511555:T:C
172:172 135:135 18:18
SQHLDNFSNQIGKpc3_NP.4
p−maj=1, pX=0.43, N=325
var.3.F5 1:169511555:T:C
171:172 135:135 18:18
SQHLDNFSNQIGKpc3_NP.5
p−maj=1, pX=0.41, N=324
var.3.F5 1:169511555:T:C
171:172 135:135 18:18
SSSPELSEMLEYDRpc2_NP.1
p−maj=1, pX=0.049, N=324
var.3.F5 1:169511555:T:C
172:172 135:135 17:18
SSSPELSEMLEYDRpc2_NP.2
p−maj=0.055, pX=0.54, N=324
var.3.F5 1:169511555:T:C
172:172 134:135 18:18
SSSPELSEMLEYDRpc2_NP.3
p−min=0.47, pX=0.13, N=324
var.3.F5 1:169511555:T:C
170:172 133:135 18:18
SSSPELSEMLEYDRpc2_NP.4
p−maj=1, pX=0.58, N=321
var.3.F5 1:169511555:T:C
172:172 135:135 18:18
SSSPELSEMLEYDRpc2_NP.5
p−maj=1, pX=0.55, N=325
var.3.F5 1:169511555:T:C
134:172 92:135 12:18
SWWGDYWEPFRARpc3_NP.1
p−min=0.046, pX=0.03, N=238
var.3.F5 1:169511555:T:C
9:172 6:135 0:18
SWWGDYWEPFRARpc3_NP.2
p−min=0.61, pX=0.9, N=15
var.3.F5 1:169511555:T:C
100:172 65:135 3:18
SWWGDYWEPFRARpc3_NP.3
p−maj=0.0027, pX=0.0034, N=168
var.3.F5 1:169511555:T:C
129:172 93:135 16:18
SWWGDYWEPFRARpc3_NP.4
p−maj=0.17, pX=0.32, N=238
var.3.F5 1:169511555:T:C
77:172 48:135 4:18
SWWGDYWEPFRARpc3_NP.5
p−min=0.054, pX=0.066, N=129

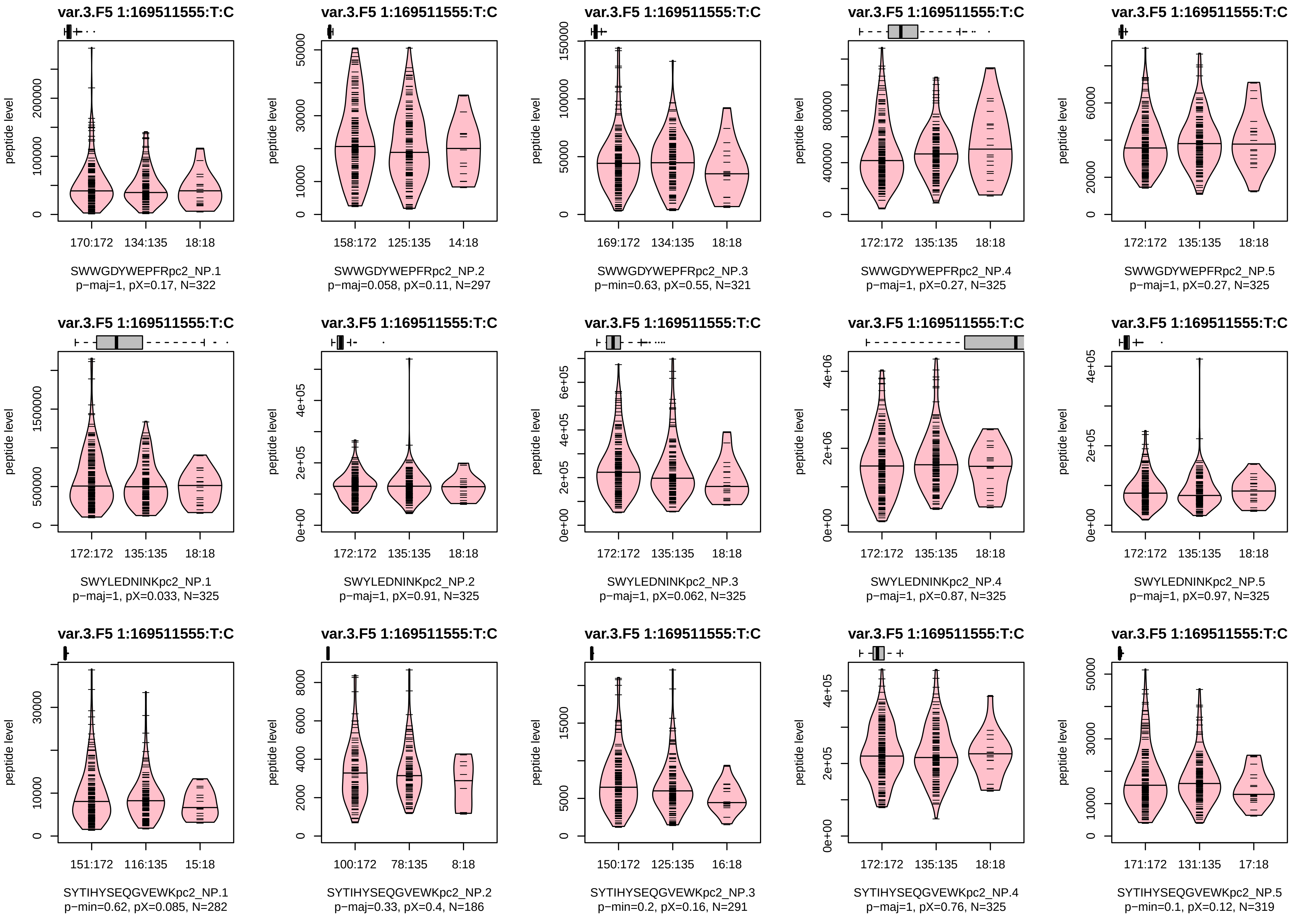

var.3.F5 1:169511555:T:C
peptide level
0
100000
200000
170:172
134:135
18:18
SWWGDYWEPFRpc2_NP.1
p−maj=1, pX=0.17, N=322
var.3.F5 1:169511555:T:C
peptide level
0
10000
30000
50000
158:172
125:135
14:18
SWWGDYWEPFRpc2_NP.2
p−maj=0.058, pX=0.11, N=297
var.3.F5 1:169511555:T:C
peptide level
0
50000
100000
150000
169:172
134:135
18:18
SWWGDYWEPFRpc2_NP.3
p−min=0.63, pX=0.55, N=321
var.3.F5 1:169511555:T:C
peptide level
0
400000
800000
172:172
135:135
18:18
SWWGDYWEPFRpc2_NP.4
p−maj=1, pX=0.27, N=325
var.3.F5 1:169511555:T:C
peptide level
0
20000
60000
172:172
135:135
18:18
SWWGDYWEPFRpc2_NP.5
p−maj=1, pX=0.27, N=325
var.3.F5 1:169511555:T:C
peptide level
0
500000
1500000
172:172
135:135
18:18
SWYLEDNINKpc2_NP.1
p−maj=1, pX=0.033, N=325
var.3.F5 1:169511555:T:C
peptide level
0e+00
2e+05
4e+05
172:172
135:135
18:18
SWYLEDNINKpc2_NP.2
p−maj=1, pX=0.91, N=325
var.3.F5 1:169511555:T:C
peptide level
0e+00
2e+05
4e+05
6e+05
172:172
135:135
18:18
SWYLEDNINKpc2_NP.3
p−maj=1, pX=0.062, N=325
var.3.F5 1:169511555:T:C
peptide level
0e+00
2e+06
4e+06
172:172
135:135
18:18
SWYLEDNINKpc2_NP.4
p−maj=1, pX=0.87, N=325
var.3.F5 1:169511555:T:C
peptide level
0e+00
2e+05
4e+05
172:172
135:135
18:18
SWYLEDNINKpc2_NP.5
p−maj=1, pX=0.97, N=325
var.3.F5 1:169511555:T:C
peptide level
0
10000
30000
151:172
116:135
15:18
SYTIHYSEQGVEWKpc2_NP.1
p−min=0.62, pX=0.085, N=282
var.3.F5 1:169511555:T:C
peptide level
0
2000
4000
6000
8000
100:172
78:135
8:18
SYTIHYSEQGVEWKpc2_NP.2
p−maj=0.33, pX=0.4, N=186
var.3.F5 1:169511555:T:C
peptide level
0
5000
15000
150:172
125:135
16:18
SYTIHYSEQGVEWKpc2_NP.3
p−min=0.2, pX=0.16, N=291
var.3.F5 1:169511555:T:C
peptide level
0e+00
2e+05
4e+05
172:172
135:135
18:18
SYTIHYSEQGVEWKpc2_NP.4
p−maj=1, pX=0.76, N=325
var.3.F5 1:169511555:T:C
peptide level
0
10000
30000
50000
171:172
131:135
17:18
SYTIHYSEQGVEWKpc2_NP.5
p−min=0.1, pX=0.12, N=319

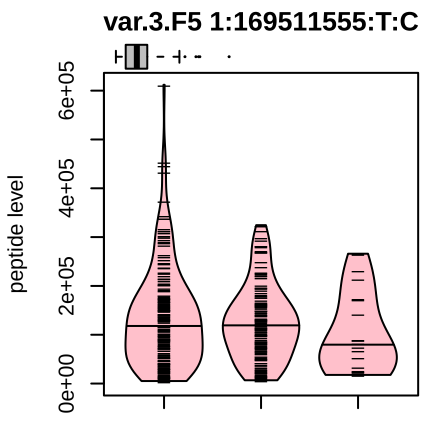

SYTIHYSEQGVEWKpc3_NP.1
p−min=0.6, pX=0.13, N=322

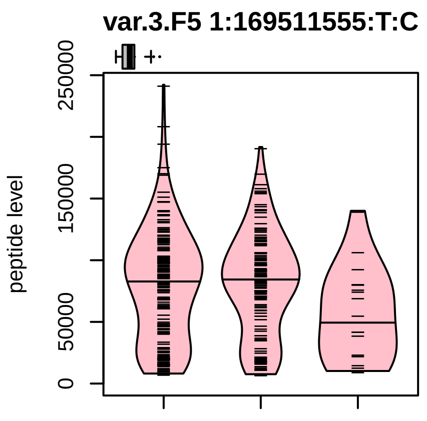

SYTIHYSEQGVEWKpc3_NP.2
p−maj=1, pX=0.23, N=325

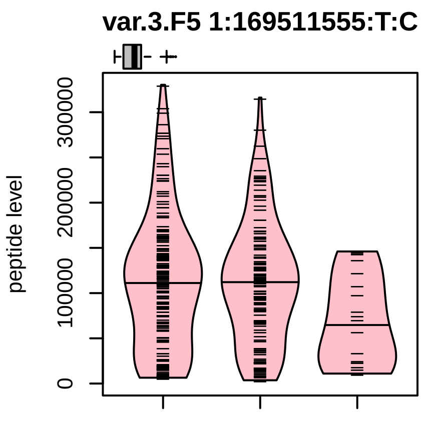

SYTIHYSEQGVEWKpc3_NP.3
p−min=0.47, pX=0.1, N=324

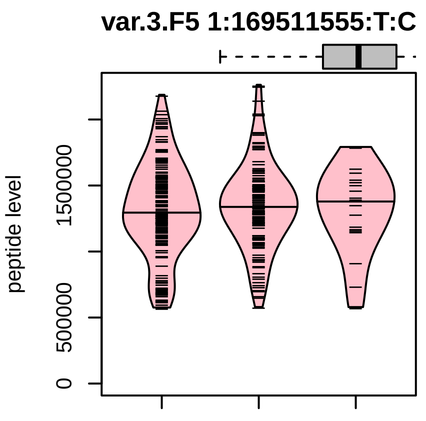

SYTIHYSEQGVEWKpc3_NP.4
p−maj=1, pX=0.7, N=325

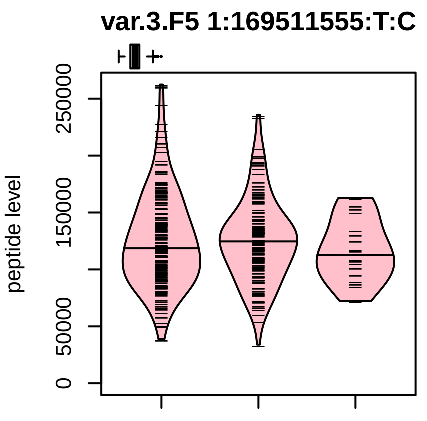

SYTIHYSEQGVEWKpc3_NP.5
p−maj=1, pX=0.5, N=325

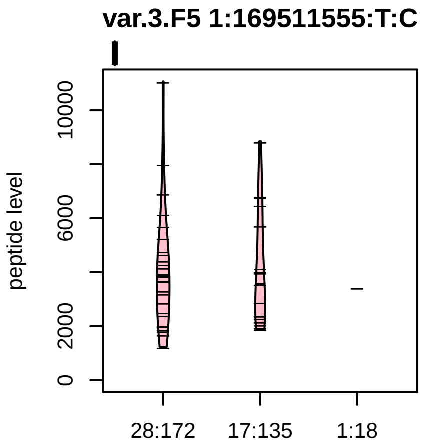

SYTIHYSEQGVEWKPYRpc3_NP.3
p−min=0.27, pX=0.22, N=46

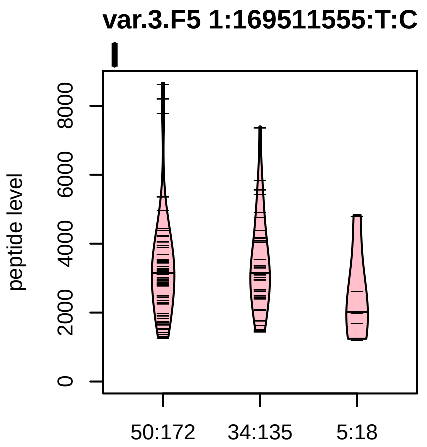

SYTIHYSEQGVEWKPYRpc4_NP.3
p−min=0.53, pX=0.41, N=89

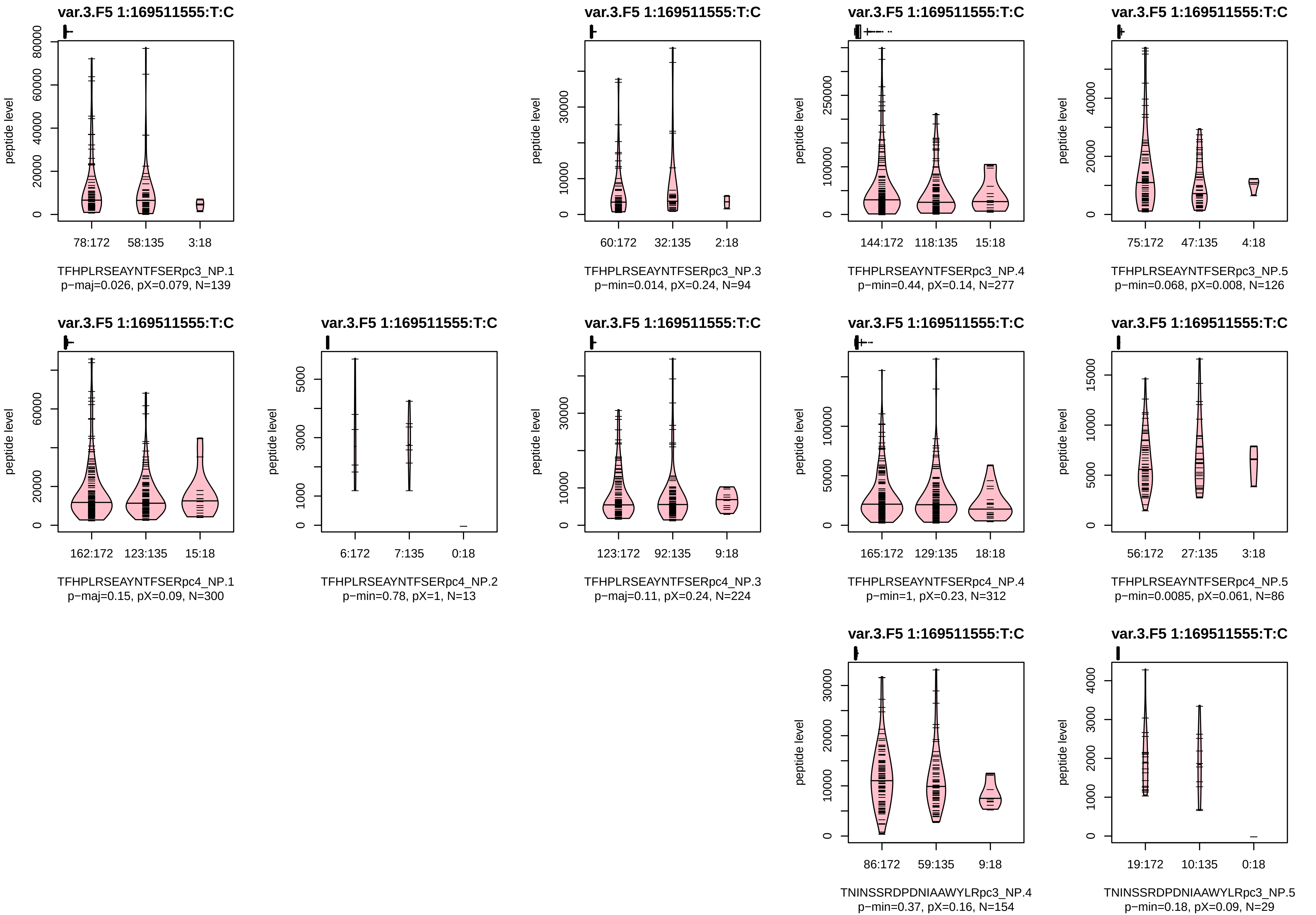

var.3.F5 1:169511555:T:C
peptide level
78:172
58:135
3:18
TFHPLRSEAYNTFSERpc3_NP.1
p−maj=0.026, pX=0.079, N=139
var.3.F5 1:169511555:T:C
peptide level
60:172
32:135
2:18
TFHPLRSEAYNTFSERpc3_NP.3
p−min=0.014, pX=0.24, N=94
var.3.F5 1:169511555:T:C
peptide level
144:172
118:135
15:18
TFHPLRSEAYNTFSERpc3_NP.4
p−min=0.44, pX=0.14, N=277
var.3.F5 1:169511555:T:C
peptide level
75:172
47:135
4:18
TFHPLRSEAYNTFSERpc3_NP.5
p−min=0.068, pX=0.008, N=126
var.3.F5 1:169511555:T:C
peptide level
162:172
123:135
15:18
TFHPLRSEAYNTFSERpc4_NP.1
p−maj=0.15, pX=0.09, N=300
var.3.F5 1:169511555:T:C
peptide level
6:172
7:135
0:18
TFHPLRSEAYNTFSERpc4_NP.2
p−min=0.78, pX=1, N=13
var.3.F5 1:169511555:T:C
peptide level
123:172
92:135
9:18
TFHPLRSEAYNTFSERpc4_NP.3
p−maj=0.11, pX=0.24, N=224
var.3.F5 1:169511555:T:C
peptide level
165:172
129:135
18:18
TFHPLRSEAYNTFSERpc4_NP.4
p−min=1, pX=0.23, N=312
var.3.F5 1:169511555:T:C
peptide level
56:172
27:135
3:18
TFHPLRSEAYNTFSERpc4_NP.5
p−min=0.0085, pX=0.061, N=86
var.3.F5 1:169511555:T:C
peptide level
86:172
59:135
9:18
TNINSSRDPDNIAAWYLRpc3_NP.4
p−min=0.37, pX=0.16, N=154
var.3.F5 1:169511555:T:C
peptide level
19:172
10:135
0:18
TNINSSRDPDNIAAWYLRpc3_NP.5
p−min=0.18, pX=0.09, N=29

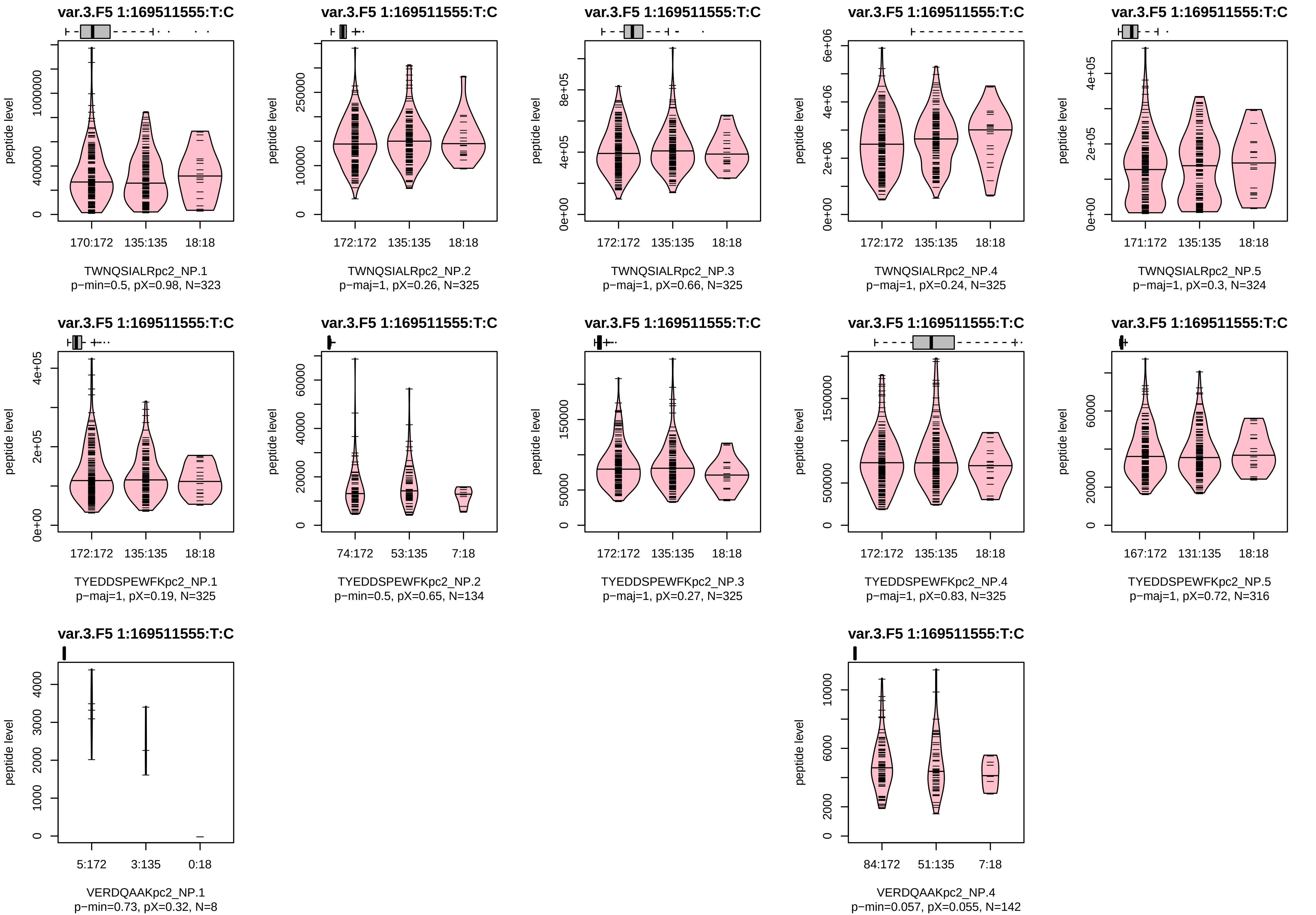
var.3.F5 1:169511555:T:C
peptide level
170:172
135:135
18:18
TWNQSIALRpc2_NP.1
p−min=0.5, pX=0.98, N=323
var.3.F5 1:169511555:T:C
peptide level
172:172
135:135
18:18
TWNQSIALRpc2_NP.2
p−maj=1, pX=0.26, N=325
var.3.F5 1:169511555:T:C
peptide level
172:172
135:135
18:18
TWNQSIALRpc2_NP.3
p−maj=1, pX=0.66, N=325
var.3.F5 1:169511555:T:C
peptide level
172:172
135:135
18:18
TWNQSIALRpc2_NP.4
p−maj=1, pX=0.24, N=325
var.3.F5 1:169511555:T:C
peptide level
171:172
135:135
18:18
TWNQSIALRpc2_NP.5
p−maj=1, pX=0.3, N=324
var.3.F5 1:169511555:T:C
peptide level
172:172
135:135
18:18
TYEDDSPEWFKpc2_NP.1
p−maj=1, pX=0.19, N=325
var.3.F5 1:169511555:T:C
peptide level
74:172
53:135
7:18
TYEDDSPEWFKpc2_NP.2
p−min=0.5, pX=0.65, N=134
var.3.F5 1:169511555:T:C
peptide level
172:172
135:135
18:18
TYEDDSPEWFKpc2_NP.3
p−maj=1, pX=0.27, N=325
var.3.F5 1:169511555:T:C
peptide level
172:172
135:135
18:18
TYEDDSPEWFKpc2_NP.4
p−maj=1, pX=0.83, N=325
var.3.F5 1:169511555:T:C
peptide level
167:172
131:135
18:18
TYEDDSPEWFKpc2_NP.5
p−maj=1, pX=0.72, N=316
var.3.F5 1:169511555:T:C
peptide level
5:172
3:135
0:18
VERDQAAKpc2_NP.1
p−min=0.73, pX=0.32, N=8
var.3.F5 1:169511555:T:C
peptide level
84:172
51:135
7:18
VERDQAAKpc2_NP.4
p−min=0.057, pX=0.055, N=142

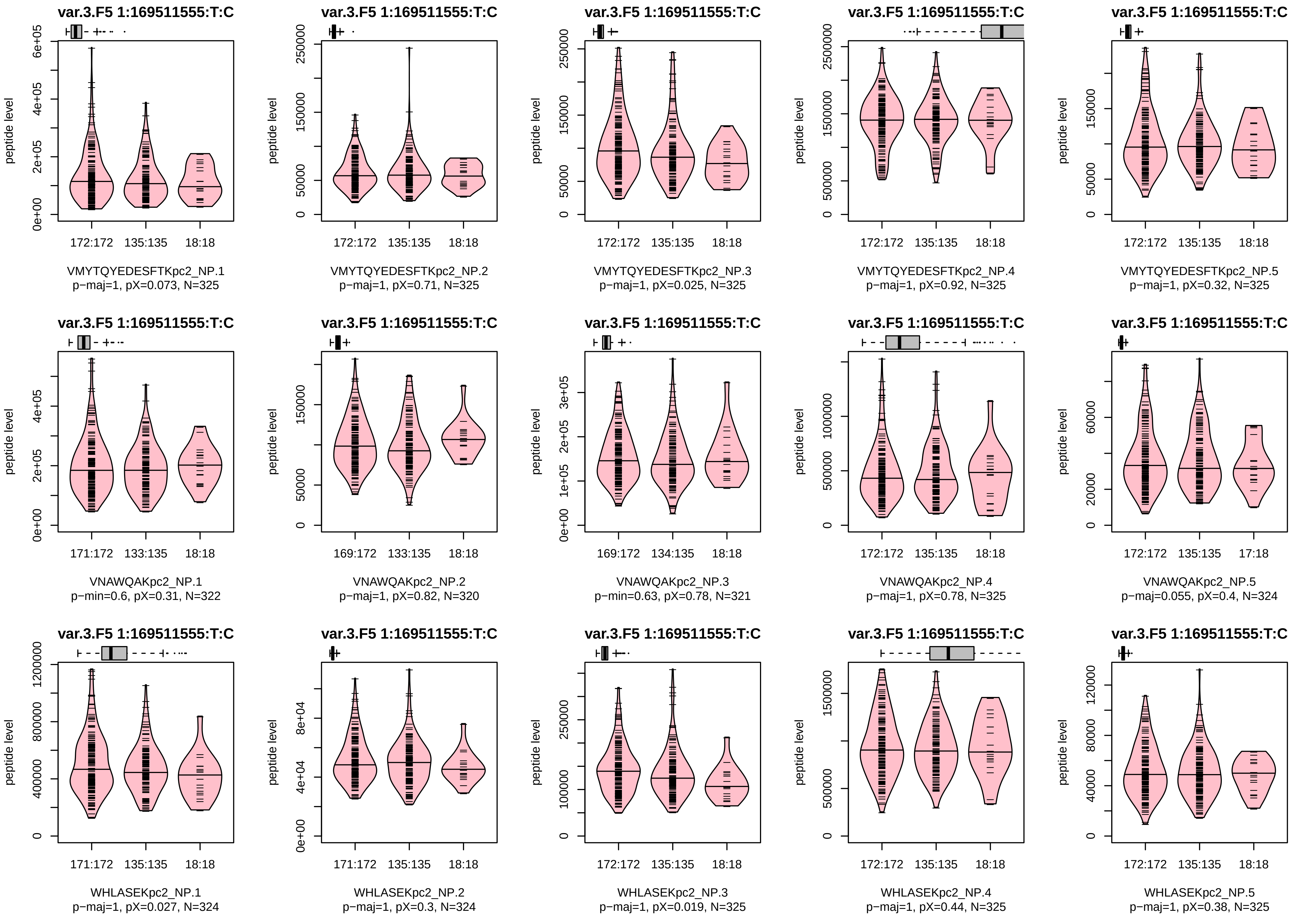
var.3.F5 1:169511555:T:C
peptide level
172:172
135:135
18:18
VMYTQYEDESFTKpc2_NP.1
p−maj=1, pX=0.073, N=325
VMYTQYEDESFTKpc2_NP.2
p−maj=1, pX=0.71, N=325
VMYTQYEDESFTKpc2_NP.3
p−maj=1, pX=0.025, N=325
VMYTQYEDESFTKpc2_NP.4
p−maj=1, pX=0.92, N=325
VMYTQYEDESFTKpc2_NP.5
p−maj=1, pX=0.32, N=325
171:172
133:135
169:172
134:135
17:18
VNAWQAKpc2_NP.1
p−min=0.6, pX=0.31, N=322
VNAWQAKpc2_NP.2
p−maj=1, pX=0.82, N=320
VNAWQAKpc2_NP.3
p−min=0.63, pX=0.78, N=321
VNAWQAKpc2_NP.4
p−maj=1, pX=0.78, N=325
VNAWQAKpc2_NP.5
p−maj=0.055, pX=0.4, N=324
WHLASEKpc2_NP.1
p−maj=1, pX=0.027, N=324
WHLASEKpc2_NP.2
p−maj=1, pX=0.3, N=324
WHLASEKpc2_NP.3
p−maj=1, pX=0.019, N=325
WHLASEKpc2_NP.4
p−maj=1, pX=0.44, N=325
WHLASEKpc2_NP.5
p−maj=1, pX=0.38, N=325

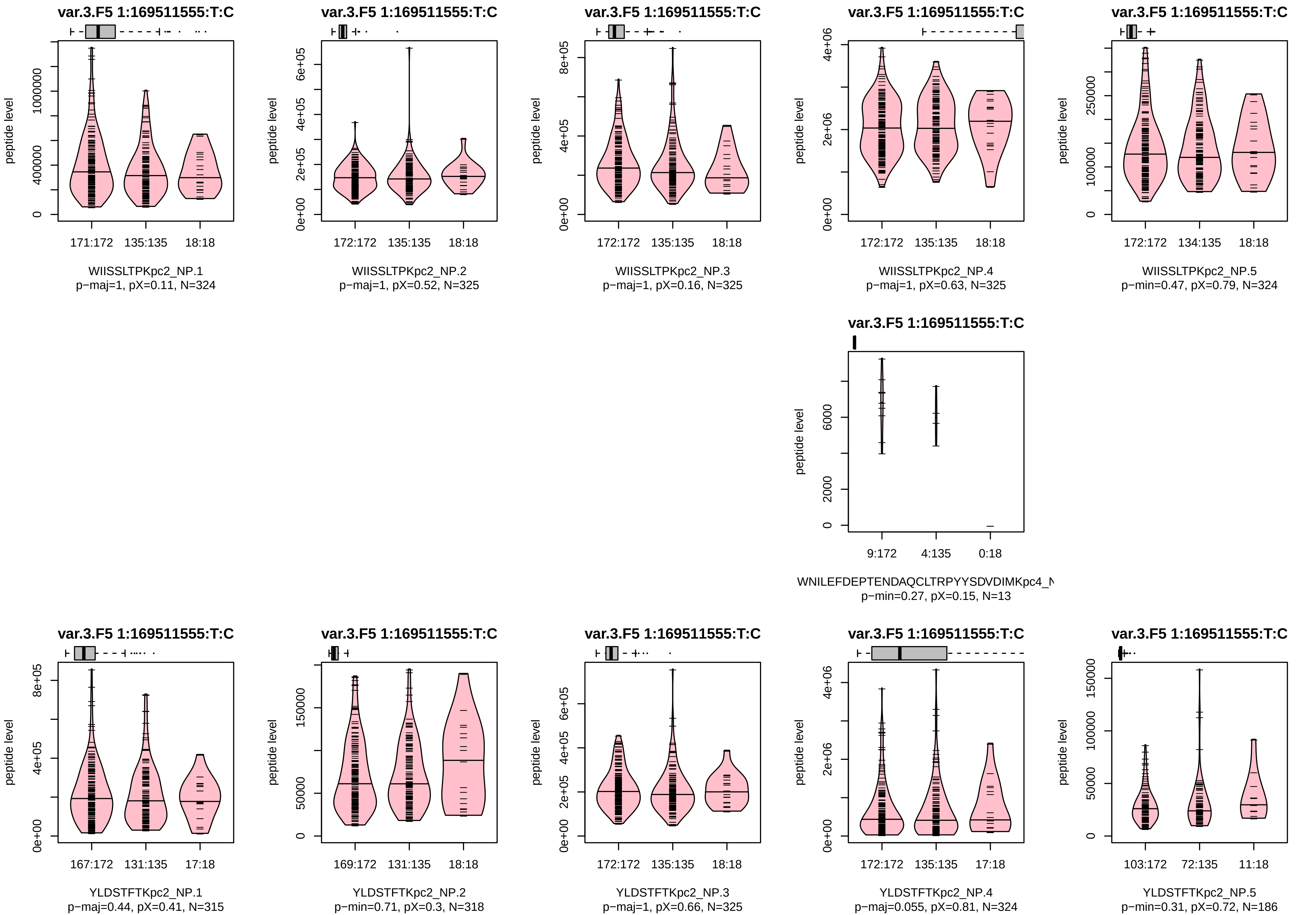

var.3.F5 1:169511555:T:C
peptide level
171:172
135:135
18:18
WIISSLTPKpc2_NP.1
p−maj=1, pX=0.11, N=324
var.3.F5 1:169511555:T:C
172:172
135:135
18:18
WIISSLTPKpc2_NP.2
p−maj=1, pX=0.52, N=325
var.3.F5 1:169511555:T:C
172:172
135:135
18:18
WIISSLTPKpc2_NP.3
p−maj=1, pX=0.16, N=325
var.3.F5 1:169511555:T:C
172:172
135:135
18:18
WIISSLTPKpc2_NP.4
p−maj=1, pX=0.63, N=325
var.3.F5 1:169511555:T:C
172:172
134:135
18:18
WIISSLTPKpc2_NP.5
p−min=0.47, pX=0.79, N=324
var.3.F5 1:169511555:T:C
9:172
4:135
0:18
WNILEFDEPTENDAQCLTRPYYSDVDIMKpc4_N
p−min=0.27, pX=0.15, N=13
var.3.F5 1:169511555:T:C
167:172
131:135
17:18
YLDSTFTKpc2_NP.1
p−maj=0.44, pX=0.41, N=315
var.3.F5 1:169511555:T:C
169:172
131:135
18:18
YLDSTFTKpc2_NP.2
p−min=0.71, pX=0.3, N=318
var.3.F5 1:169511555:T:C
172:172
135:135
18:18
YLDSTFTKpc2_NP.3
p−maj=1, pX=0.66, N=325
var.3.F5 1:169511555:T:C
172:172
135:135
17:18
YLDSTFTKpc2_NP.4
p−maj=0.055, pX=0.81, N=324
var.3.F5 1:169511555:T:C
103:172
72:135
11:18
YLDSTFTKpc2_NP.5
p−min=0.31, pX=0.72, N=186

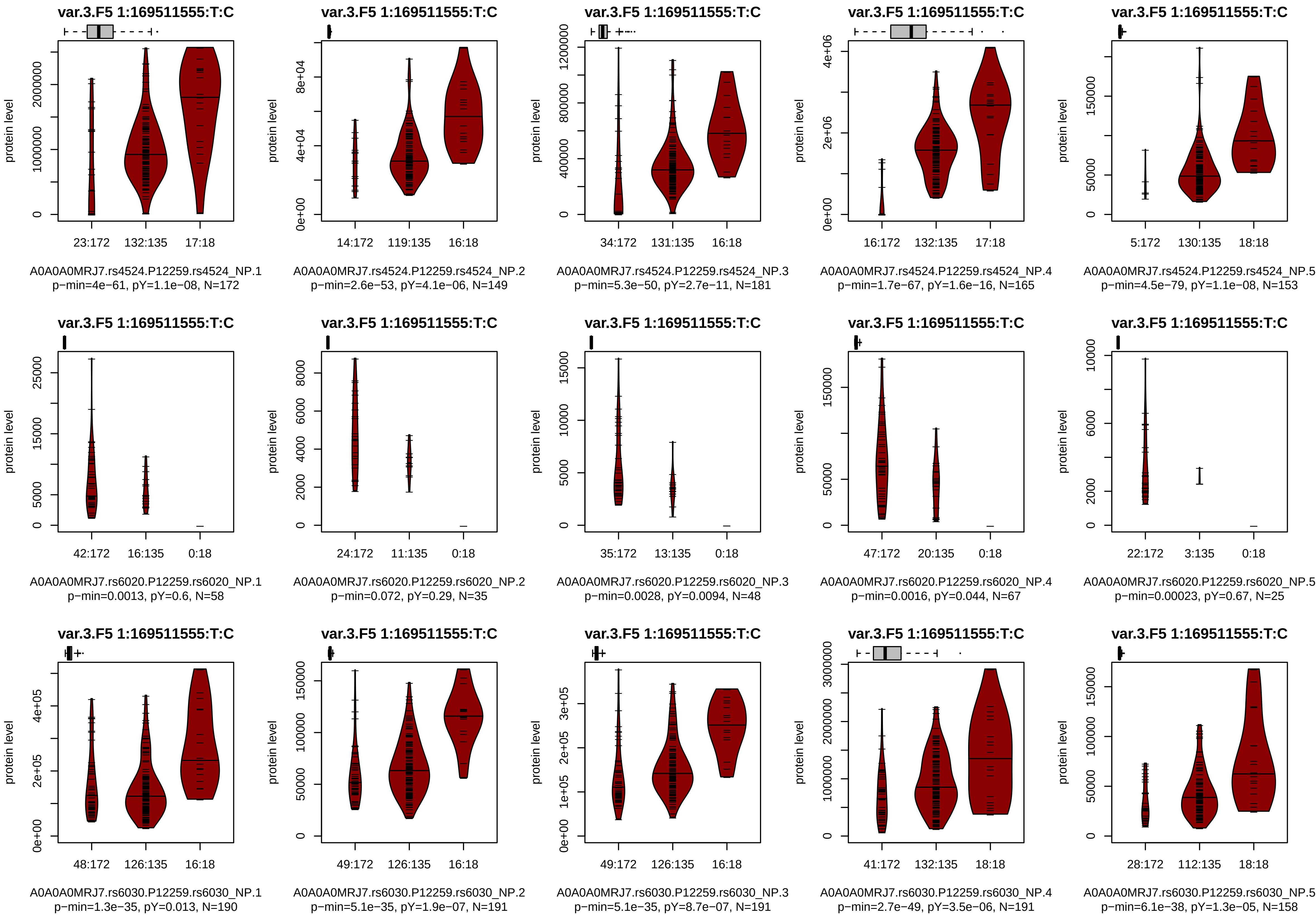
var.3.F5 1:169511555:T:C
protein level
23:172
132:135
17:18
A0A0A0MRJ7.rs4524.P12259.rs4524_NP.1
p−min=4e−61, pY=1.1e−08, N=172
var.3.F5 1:169511555:T:C
protein level
14:172
119:135
16:18
A0A0A0MRJ7.rs4524.P12259.rs4524_NP.2
p−min=2.6e−53, pY=4.1e−06, N=149
var.3.F5 1:169511555:T:C
protein level
34:172
131:135
16:18
A0A0A0MRJ7.rs4524.P12259.rs4524_NP.3
p−min=5.3e−50, pY=2.7e−11, N=181
var.3.F5 1:169511555:T:C
protein level
16:172
132:135
17:18
A0A0A0MRJ7.rs4524.P12259.rs4524_NP.4
p−min=1.7e−67, pY=1.6e−16, N=165
var.3.F5 1:169511555:T:C
protein level
5:172
130:135
18:18
A0A0A0MRJ7.rs4524.P12259.rs4524_NP.5
p−min=4.5e−79, pY=1.1e−08, N=153
var.3.F5 1:169511555:T:C
protein level
42:172
16:135
0:18
A0A0A0MRJ7.rs6020.P12259.rs6020_NP.1
p−min=0.0013, pY=0.6, N=58
var.3.F5 1:169511555:T:C
protein level
24:172
11:135
0:18
A0A0A0MRJ7.rs6020.P12259.rs6020_NP.2
p−min=0.072, pY=0.29, N=35
var.3.F5 1:169511555:T:C
protein level
35:172
13:135
0:18
A0A0A0MRJ7.rs6020.P12259.rs6020_NP.3
p−min=0.0028, pY=0.0094, N=48
var.3.F5 1:169511555:T:C
protein level
47:172
20:135
0:18
A0A0A0MRJ7.rs6020.P12259.rs6020_NP.4
p−min=0.0016, pY=0.044, N=67
var.3.F5 1:169511555:T:C
protein level
22:172
3:135
0:18
A0A0A0MRJ7.rs6020.P12259.rs6020_NP.5
p−min=0.00023, pY=0.67, N=25
var.3.F5 1:169511555:T:C
protein level
48:172
126:135
16:18
A0A0A0MRJ7.rs6030.P12259.rs6030_NP.1
p−min=1.3e−35, pY=0.013, N=190
var.3.F5 1:169511555:T:C
protein level
49:172
126:135
16:18
A0A0A0MRJ7.rs6030.P12259.rs6030_NP.2
p−min=5.1e−35, pY=1.9e−07, N=191
var.3.F5 1:169511555:T:C
protein level
49:172
126:135
16:18
A0A0A0MRJ7.rs6030.P12259.rs6030_NP.3
p−min=5.1e−35, pY=8.7e−07, N=191
var.3.F5 1:169511555:T:C
protein level
41:172
132:135
18:18
A0A0A0MRJ7.rs6030.P12259.rs6030_NP.4
p−min=2.7e−49, pY=3.5e−06, N=191
var.3.F5 1:169511555:T:C
protein level
28:172
112:135
18:18
A0A0A0MRJ7.rs6030.P12259.rs6030_NP.5
p−min=6.1e−38, pY=1.3e−05, N=158

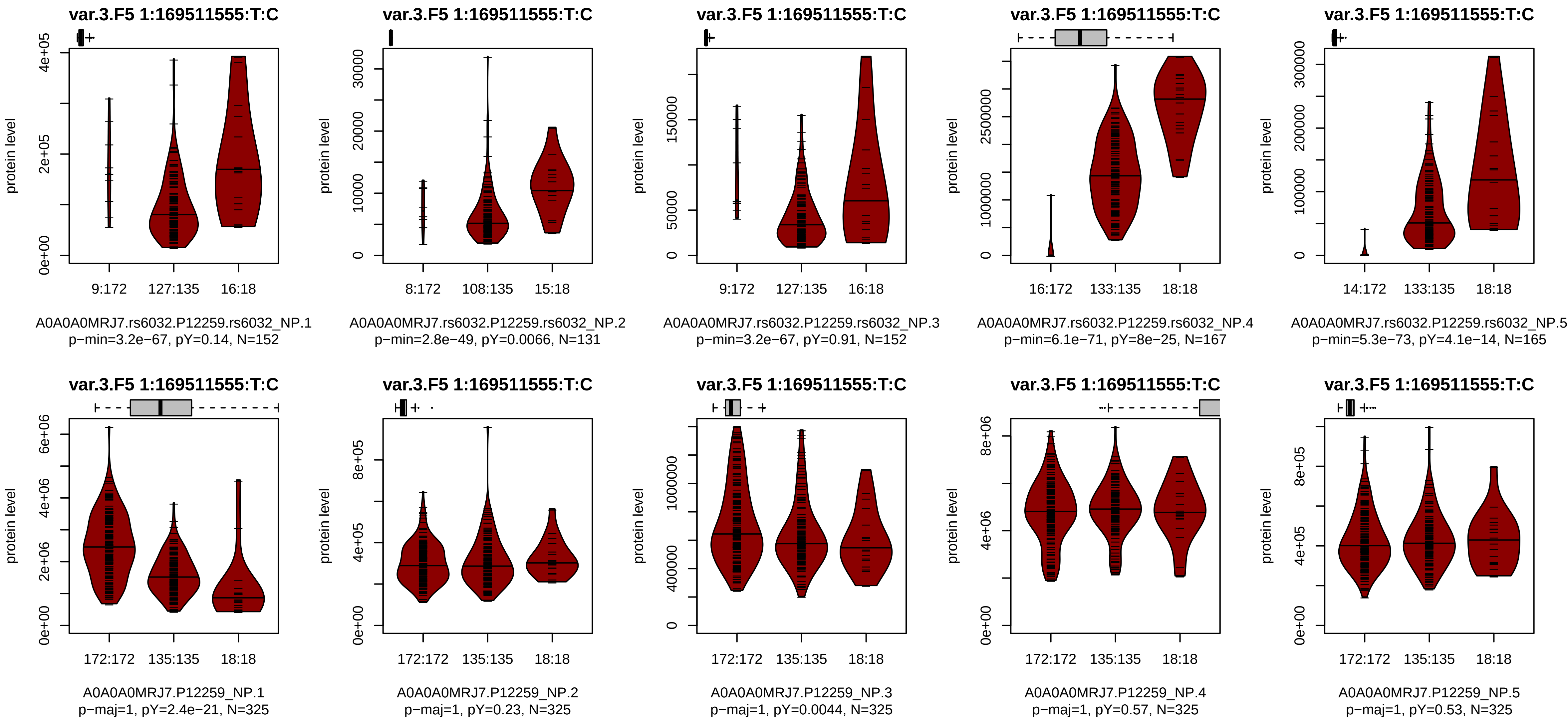
var.3.F5 1:169511555:T:C
protein level
9:172
127:135
16:18
A0A0A0MRJ7.rs6032.P12259.rs6032_NP.1
p−min=3.2e−67, pY=0.14, N=152
var.3.F5 1:169511555:T:C
protein level
8:172
108:135
15:18
A0A0A0MRJ7.rs6032.P12259.rs6032_NP.2
p−min=2.8e−49, pY=0.0066, N=131
var.3.F5 1:169511555:T:C
protein level
9:172
127:135
16:18
A0A0A0MRJ7.rs6032.P12259.rs6032_NP.3
p−min=3.2e−67, pY=0.91, N=152
var.3.F5 1:169511555:T:C
protein level
16:172
133:135
18:18
A0A0A0MRJ7.rs6032.P12259.rs6032_NP.4
p−min=6.1e−71, pY=8e−25, N=167
var.3.F5 1:169511555:T:C
protein level
14:172
133:135
18:18
A0A0A0MRJ7.rs6032.P12259.rs6032_NP.5
p−min=5.3e−73, pY=4.1e−14, N=165
var.3.F5 1:169511555:T:C
protein level
172:172
135:135
18:18
A0A0A0MRJ7.P12259_NP.1
p−maj=1, pY=2.4e−21, N=325
var.3.F5 1:169511555:T:C
protein level
172:172
135:135
18:18
A0A0A0MRJ7.P12259_NP.2
p−maj=1, pY=0.23, N=325
var.3.F5 1:169511555:T:C
protein level
172:172
135:135
18:18
A0A0A0MRJ7.P12259_NP.3
p−maj=1, pY=0.0044, N=325
var.3.F5 1:169511555:T:C
protein level
172:172
135:135
18:18
A0A0A0MRJ7.P12259_NP.4
p−maj=1, pY=0.57, N=325
var.3.F5 1:169511555:T:C
protein level
172:172
135:135
18:18
A0A0A0MRJ7.P12259_NP.5
p−maj=1, pY=0.53, N=325

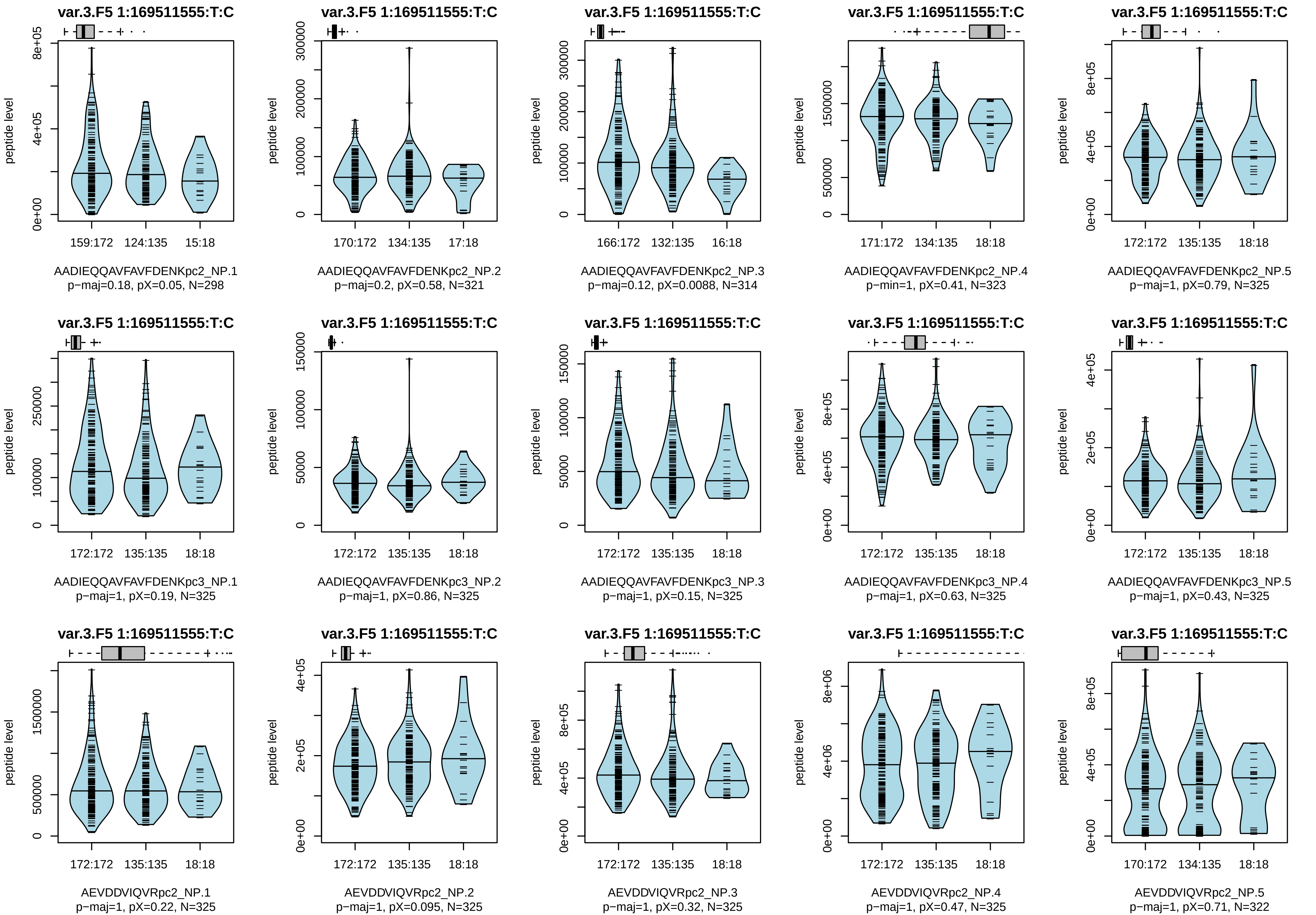
var.3.F5 1:169511555:T:C
peptide level
159:172
124:135
15:18
AADIEQQAVFAVFDENKpc2_NP.1
p−maj=0.18, pX=0.05, N=298
var.3.F5 1:169511555:T:C
peptide level
170:172
134:135
17:18
AADIEQQAVFAVFDENKpc2_NP.2
p−maj=0.2, pX=0.58, N=321
var.3.F5 1:169511555:T:C
peptide level
166:172
132:135
16:18
AADIEQQAVFAVFDENKpc2_NP.3
p−maj=0.12, pX=0.0088, N=314
var.3.F5 1:169511555:T:C
peptide level
171:172
134:135
18:18
AADIEQQAVFAVFDENKpc2_NP.4
p−min=1, pX=0.41, N=323
var.3.F5 1:169511555:T:C
peptide level
172:172
135:135
18:18
AADIEQQAVFAVFDENKpc2_NP.5
p−maj=1, pX=0.79, N=325
var.3.F5 1:169511555:T:C
peptide level
172:172
135:135
18:18
AADIEQQAVFAVFDENKpc3_NP.1
p−maj=1, pX=0.19, N=325
var.3.F5 1:169511555:T:C
peptide level
172:172
135:135
18:18
AADIEQQAVFAVFDENKpc3_NP.2
p−maj=1, pX=0.86, N=325
var.3.F5 1:169511555:T:C
peptide level
172:172
135:135
18:18
AADIEQQAVFAVFDENKpc3_NP.3
p−maj=1, pX=0.15, N=325
var.3.F5 1:169511555:T:C
peptide level
172:172
135:135
18:18
AADIEQQAVFAVFDENKpc3_NP.4
p−maj=1, pX=0.63, N=325
var.3.F5 1:169511555:T:C
peptide level
172:172
135:135
18:18
AADIEQQAVFAVFDENKpc3_NP.5
p−maj=1, pX=0.43, N=325
var.3.F5 1:169511555:T:C
peptide level
172:172
135:135
18:18
AEVDDVIQVRpc2_NP.1
p−maj=1, pX=0.22, N=325
var.3.F5 1:169511555:T:C
peptide level
172:172
135:135
18:18
AEVDDVIQVRpc2_NP.2
p−maj=1, pX=0.095, N=325
var.3.F5 1:169511555:T:C
peptide level
172:172
135:135
18:18
AEVDDVIQVRpc2_NP.3
p−maj=1, pX=0.32, N=325
var.3.F5 1:169511555:T:C
peptide level
172:172
135:135
18:18
AEVDDVIQVRpc2_NP.4
p−maj=1, pX=0.47, N=325
var.3.F5 1:169511555:T:C
peptide level
170:172
134:135
18:18
AEVDDVIQVRpc2_NP.5
p−maj=1, pX=0.71, N=322

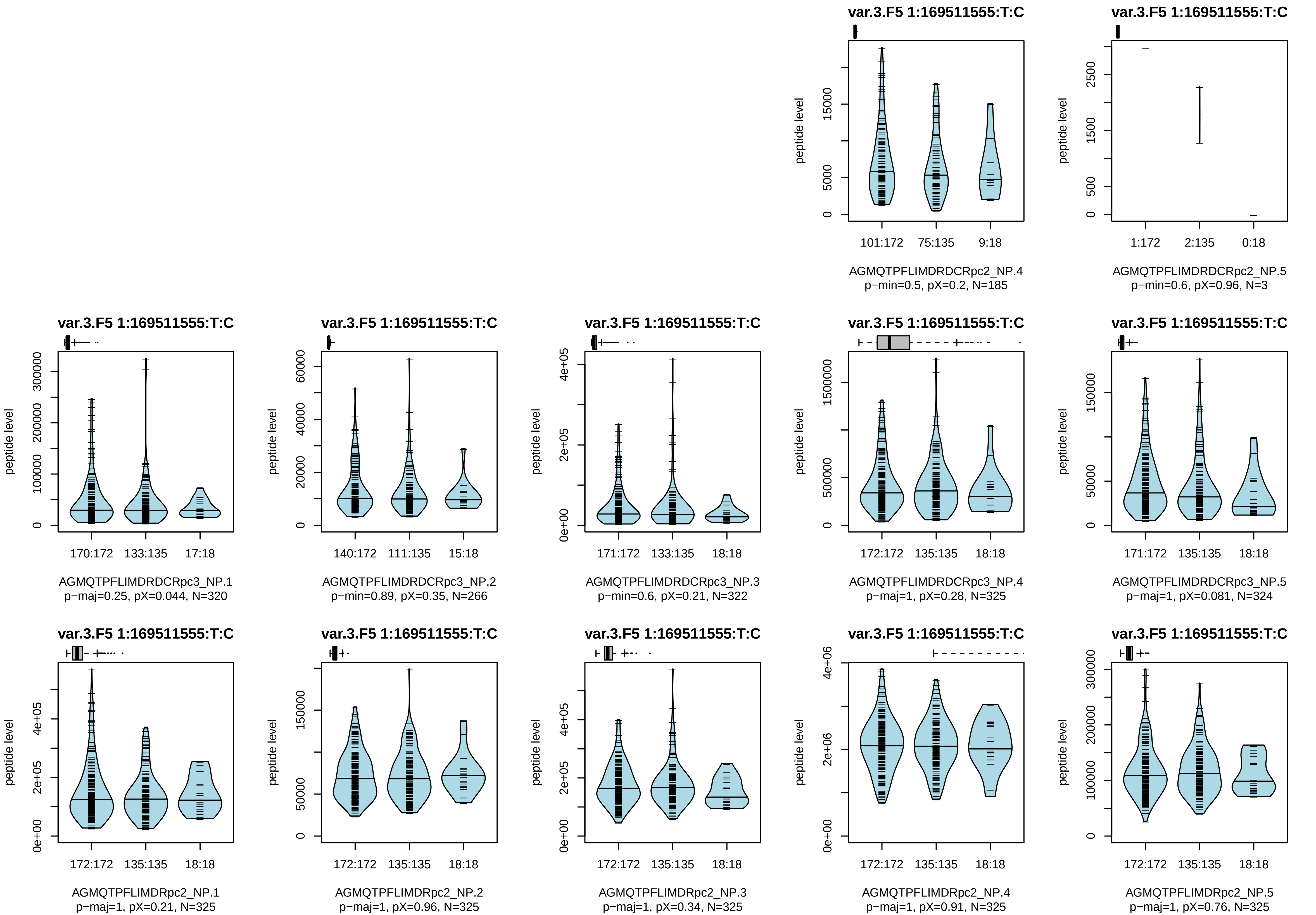
var.3.F5 1:169511555:T:C
peptide level
101:172
75:135
9:18
AGMQTPFLIMDRDCRpc2_NP.4
p−min=0.5, pX=0.2, N=185
var.3.F5 1:169511555:T:C
peptide level
1:172
2:135
0:18
AGMQTPFLIMDRDCRpc2_NP.5
p−min=0.6, pX=0.96, N=3
var.3.F5 1:169511555:T:C
peptide level
170:172
133:135
17:18
AGMQTPFLIMDRDCRpc3_NP.1
p−maj=0.25, pX=0.044, N=320
var.3.F5 1:169511555:T:C
peptide level
140:172
111:135
15:18
AGMQTPFLIMDRDCRpc3_NP.2
p−min=0.89, pX=0.35, N=266
var.3.F5 1:169511555:T:C
peptide level
171:172
133:135
18:18
AGMQTPFLIMDRDCRpc3_NP.3
p−min=0.6, pX=0.21, N=322
var.3.F5 1:169511555:T:C
peptide level
172:172
135:135
18:18
AGMQTPFLIMDRDCRpc3_NP.4
p−maj=1, pX=0.28, N=325
var.3.F5 1:169511555:T:C
peptide level
171:172
135:135
18:18
AGMQTPFLIMDRDCRpc3_NP.5
p−maj=1, pX=0.081, N=324
var.3.F5 1:169511555:T:C
peptide level
172:172
135:135
18:18
AGMQTPFLIMDRpc2_NP.1
p−maj=1, pX=0.21, N=325
var.3.F5 1:169511555:T:C
peptide level
172:172
135:135
18:18
AGMQTPFLIMDRpc2_NP.2
p−maj=1, pX=0.96, N=325
var.3.F5 1:169511555:T:C
peptide level
172:172
135:135
18:18
AGMQTPFLIMDRpc2_NP.3
p−maj=1, pX=0.34, N=325
var.3.F5 1:169511555:T:C
peptide level
172:172
135:135
18:18
AGMQTPFLIMDRpc2_NP.4
p−maj=1, pX=0.91, N=325
var.3.F5 1:169511555:T:C
peptide level
172:172
135:135
18:18
AGMQTPFLIMDRpc2_NP.5
p−maj=1, pX=0.76, N=325

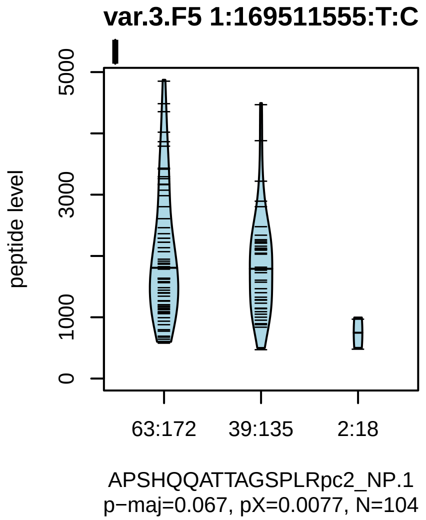
var.3.F5 1:169511555:T:C
peptide level
63:172
39:135
2:18
APSHQQATTAGSPLRpc2_NP.1
p−maj=0.067, pX=0.0077, N=104

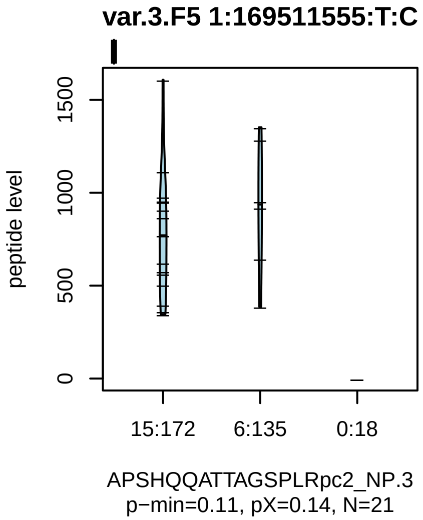
var.3.F5 1:169511555:T:C
peptide level
15:172
6:135
0:18
APSHQQATTAGSPLRpc2_NP.3
p−min=0.11, pX=0.14, N=21

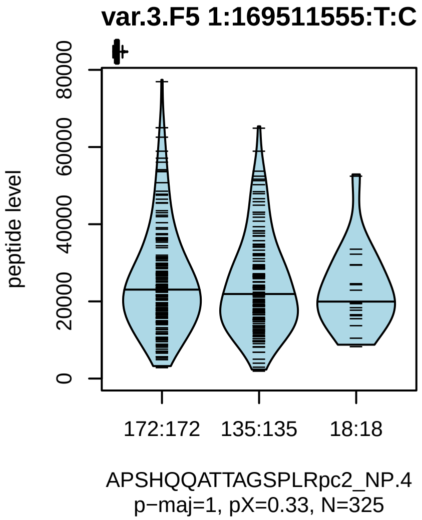
var.3.F5 1:169511555:T:C
peptide level
172:172
135:135
18:18
APSHQQATTAGSPLRpc2_NP.4
p−maj=1, pX=0.33, N=325

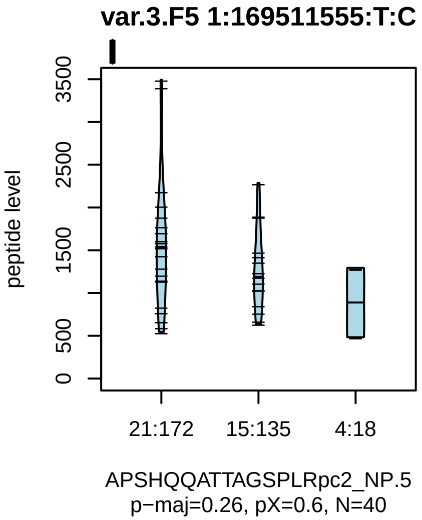
var.3.F5 1:169511555:T:C
peptide level
21:172
15:135
4:18
APSHQQATTAGSPLRpc2_NP.5
p−maj=0.26, pX=0.6, N=40

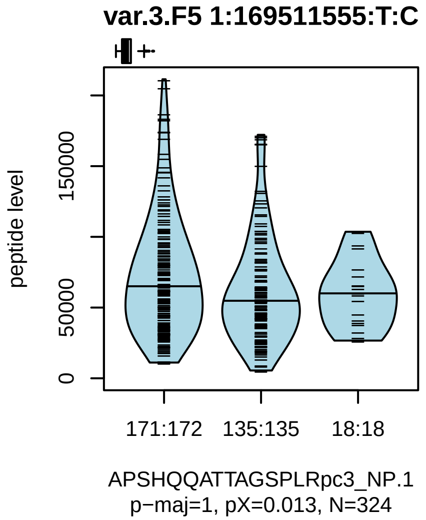
var.3.F5 1:169511555:T:C
peptide level
171:172
135:135
18:18
APSHQQATTAGSPLRpc3_NP.1
p−maj=1, pX=0.013, N=324

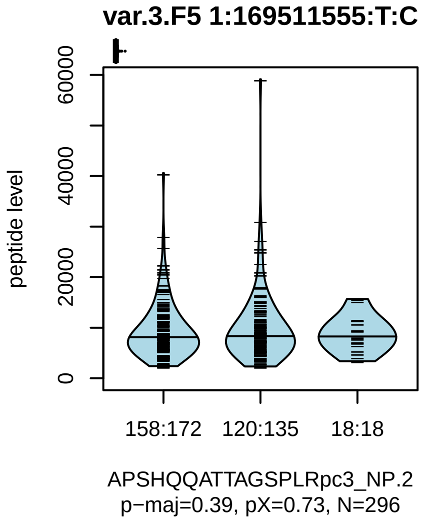
var.3.F5 1:169511555:T:C
peptide level
158:172
120:135
18:18
APSHQQATTAGSPLRpc3_NP.2
p−maj=0.39, pX=0.73, N=296

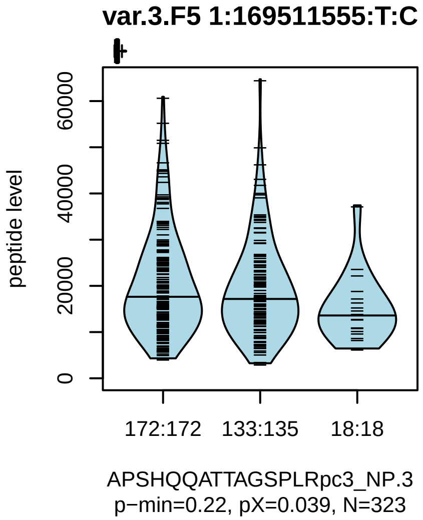
var.3.F5 1:169511555:T:C
peptide level
172:172
133:135
18:18
APSHQQATTAGSPLRpc3_NP.3
p−min=0.22, pX=0.039, N=323

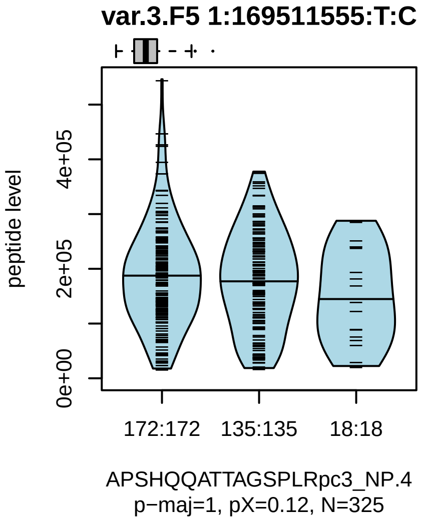
var.3.F5 1:169511555:T:C
peptide level
172:172
135:135
18:18
APSHQQATTAGSPLRpc3_NP.4
p−maj=1, pX=0.12, N=325

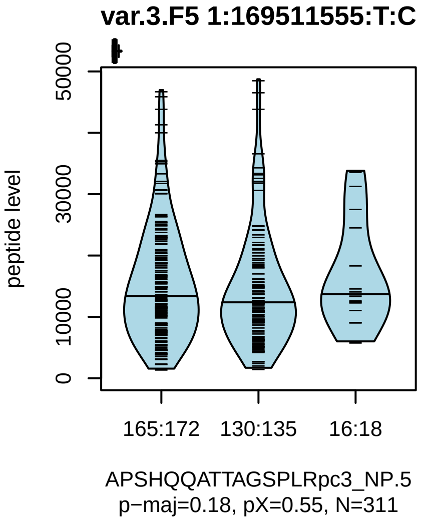
var.3.F5 1:169511555:T:C
peptide level
165:172
130:135
16:18
APSHQQATTAGSPLRpc3_NP.5
p−maj=0.18, pX=0.55, N=311

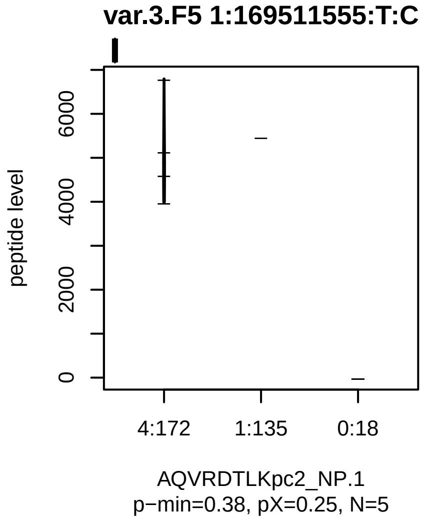
var.3.F5 1:169511555:T:C
peptide level
4:172
1:135
0:18
AQVRDTLKpc2_NP.1
p−min=0.38, pX=0.25, N=5

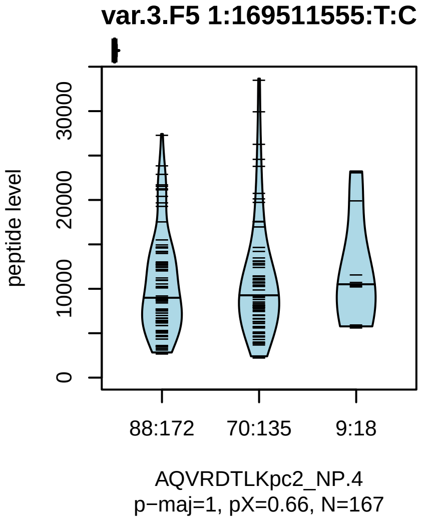
var.3.F5 1:169511555:T:C
peptide level
88:172
70:135
9:18
AQVRDTLKpc2_NP.4
p−maj=1, pX=0.66, N=167

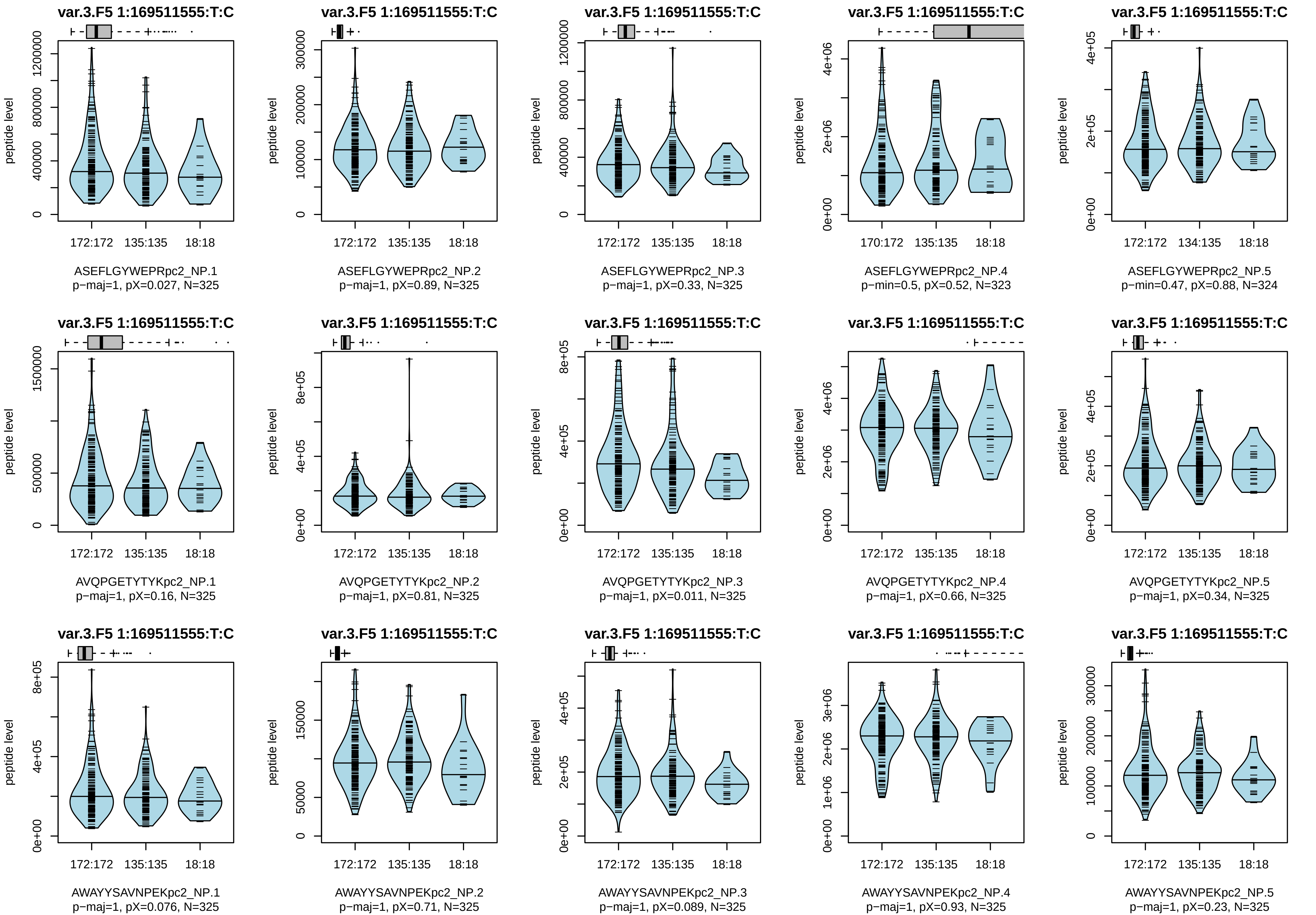

var.3.F5 1:169511555:T:C
peptide level
0
400000
800000
1200000
172:172
135:135
18:18
ASEFLGYWEPRpc2_NP.1
p−maj=1, pX=0.027, N=325
var.3.F5 1:169511555:T:C
peptide level
0
100000
200000
300000
172:172
135:135
18:18
ASEFLGYWEPRpc2_NP.2
p−maj=1, pX=0.89, N=325
var.3.F5 1:169511555:T:C
peptide level
0
400000
800000
1200000
172:172
135:135
18:18
ASEFLGYWEPRpc2_NP.3
p−maj=1, pX=0.33, N=325
var.3.F5 1:169511555:T:C
peptide level
0e+00
2e+06
4e+06
170:172
135:135
18:18
ASEFLGYWEPRpc2_NP.4
p−min=0.5, pX=0.52, N=323
var.3.F5 1:169511555:T:C
peptide level
0e+00
2e+05
4e+05
172:172
134:135
18:18
ASEFLGYWEPRpc2_NP.5
p−min=0.47, pX=0.88, N=324
var.3.F5 1:169511555:T:C
peptide level
0
500000
1500000
172:172
135:135
18:18
AVQPGETYTYKpc2_NP.1
p−maj=1, pX=0.16, N=325
var.3.F5 1:169511555:T:C
peptide level
0e+00
4e+05
8e+05
172:172
135:135
18:18
AVQPGETYTYKpc2_NP.2
p−maj=1, pX=0.81, N=325
var.3.F5 1:169511555:T:C
peptide level
0e+00
4e+05
8e+05
172:172
135:135
18:18
AVQPGETYTYKpc2_NP.3
p−maj=1, pX=0.011, N=325
var.3.F5 1:169511555:T:C
peptide level
0e+00
2e+06
4e+06
172:172
135:135
18:18
AVQPGETYTYKpc2_NP.4
p−maj=1, pX=0.66, N=325
var.3.F5 1:169511555:T:C
peptide level
0e+00
2e+05
4e+05
172:172
135:135
18:18
AVQPGETYTYKpc2_NP.5
p−maj=1, pX=0.34, N=325
var.3.F5 1:169511555:T:C
peptide level
0e+00
4e+05
8e+05
172:172
135:135
18:18
AWAYYSAVNPEKpc2_NP.1
p−maj=1, pX=0.076, N=325
var.3.F5 1:169511555:T:C
peptide level
0
50000
150000
172:172
135:135
18:18
AWAYYSAVNPEKpc2_NP.2
p−maj=1, pX=0.71, N=325
var.3.F5 1:169511555:T:C
peptide level
0e+00
2e+05
4e+05
172:172
135:135
18:18
AWAYYSAVNPEKpc2_NP.3
p−maj=1, pX=0.089, N=325
var.3.F5 1:169511555:T:C
peptide level
0e+00
1e+06
2e+06
3e+06
172:172
135:135
18:18
AWAYYSAVNPEKpc2_NP.4
p−maj=1, pX=0.93, N=325
var.3.F5 1:169511555:T:C
peptide level
0
100000
200000
300000
172:172
135:135
18:18
AWAYYSAVNPEKpc2_NP.5
p−maj=1, pX=0.23, N=325

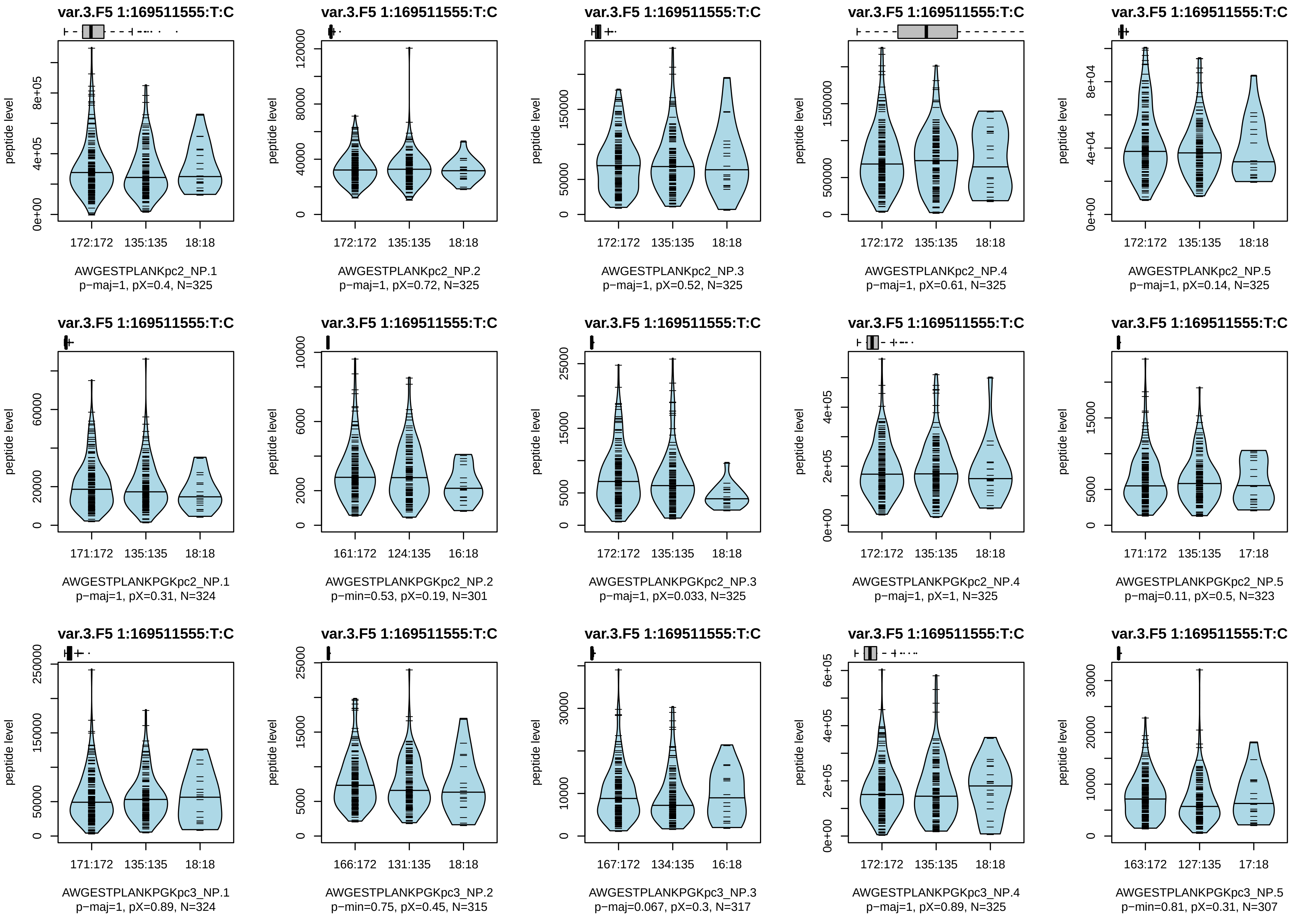
var.3.F5 1:169511555:T:C
172:172 135:135 18:18
AWGESTPLANKpc2_NP.1
p−maj=1, pX=0.4, N=325
var.3.F5 1:169511555:T:C
172:172 135:135 18:18
AWGESTPLANKpc2_NP.2
p−maj=1, pX=0.72, N=325
var.3.F5 1:169511555:T:C
172:172 135:135 18:18
AWGESTPLANKpc2_NP.3
p−maj=1, pX=0.52, N=325
var.3.F5 1:169511555:T:C
172:172 135:135 18:18
AWGESTPLANKpc2_NP.4
p−maj=1, pX=0.61, N=325
var.3.F5 1:169511555:T:C
172:172 135:135 18:18
AWGESTPLANKpc2_NP.5
p−maj=1, pX=0.14, N=325
var.3.F5 1:169511555:T:C
171:172 135:135 18:18
AWGESTPLANKPGKpc2_NP.1
p−maj=1, pX=0.31, N=324
var.3.F5 1:169511555:T:C
161:172 124:135 16:18
AWGESTPLANKPGKpc2_NP.2
p−min=0.53, pX=0.19, N=301
var.3.F5 1:169511555:T:C
172:172 135:135 18:18
AWGESTPLANKPGKpc2_NP.3
p−maj=1, pX=0.033, N=325
var.3.F5 1:169511555:T:C
172:172 135:135 18:18
AWGESTPLANKPGKpc2_NP.4
p−maj=1, pX=1, N=325
var.3.F5 1:169511555:T:C
171:172 135:135 17:18
AWGESTPLANKPGKpc2_NP.5
p−maj=0.11, pX=0.5, N=323
var.3.F5 1:169511555:T:C
171:172 135:135 18:18
AWGESTPLANKPGKpc3_NP.1
p−maj=1, pX=0.89, N=324
var.3.F5 1:169511555:T:C
166:172 131:135 18:18
AWGESTPLANKPGKpc3_NP.2
p−min=0.75, pX=0.45, N=315
var.3.F5 1:169511555:T:C
167:172 134:135 16:18
AWGESTPLANKPGKpc3_NP.3
p−maj=0.067, pX=0.3, N=317
var.3.F5 1:169511555:T:C
172:172 135:135 18:18
AWGESTPLANKPGKpc3_NP.4
p−maj=1, pX=0.89, N=325
var.3.F5 1:169511555:T:C
163:172 127:135 17:18
AWGESTPLANKPGKpc3_NP.5
p−min=0.81, pX=0.31, N=307

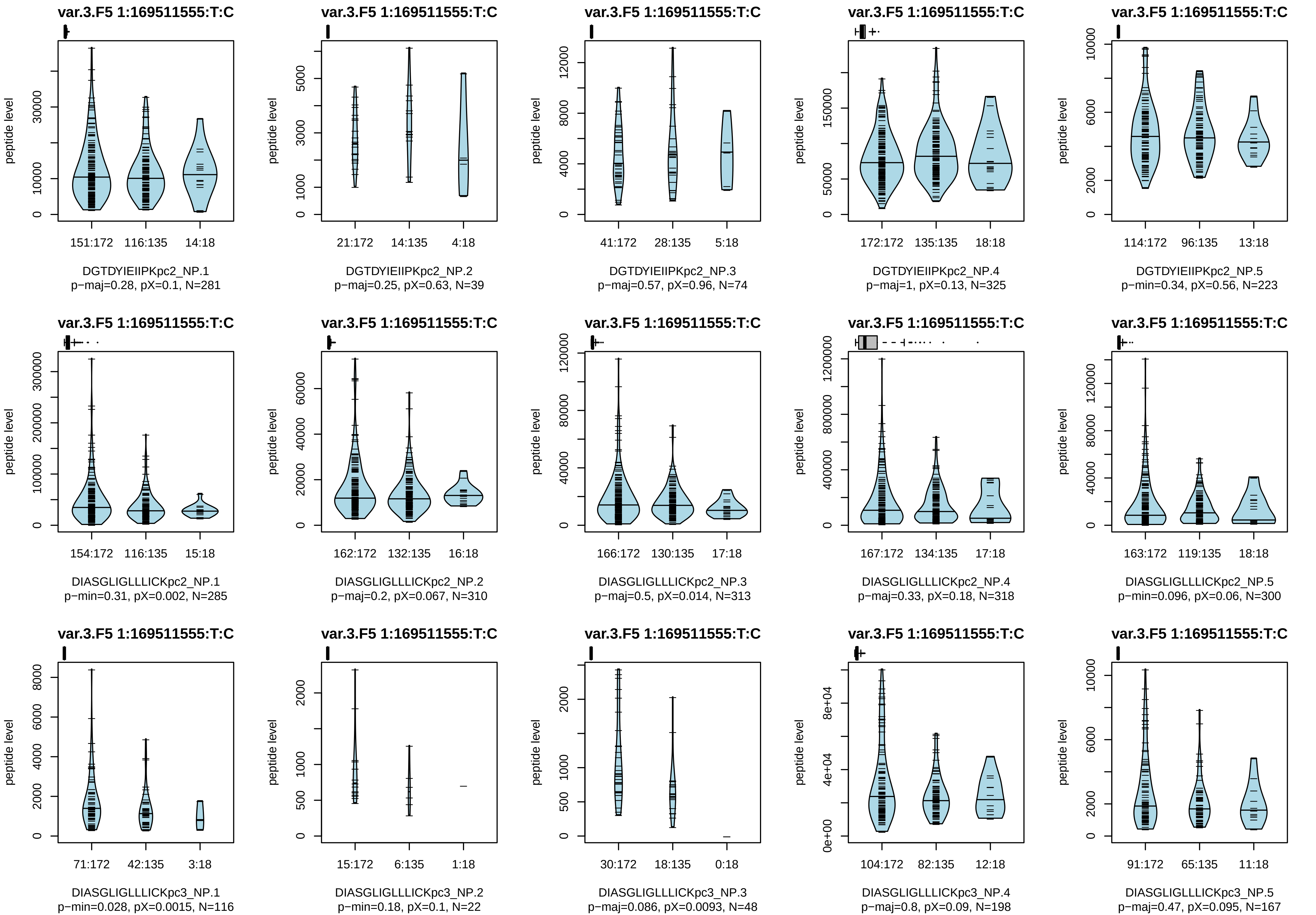
var.3.F5 1:169511555:T:C
peptide level
151:172
116:135
14:18
DGTDYIEIIPKpc2_NP.1
p−maj=0.28, pX=0.1, N=281
var.3.F5 1:169511555:T:C
peptide level
21:172
14:135
4:18
DGTDYIEIIPKpc2_NP.2
p−maj=0.25, pX=0.63, N=39
var.3.F5 1:169511555:T:C
peptide level
41:172
28:135
5:18
DGTDYIEIIPKpc2_NP.3
p−maj=0.57, pX=0.96, N=74
var.3.F5 1:169511555:T:C
peptide level
172:172
135:135
18:18
DGTDYIEIIPKpc2_NP.4
p−maj=1, pX=0.13, N=325
var.3.F5 1:169511555:T:C
peptide level
114:172
96:135
13:18
DGTDYIEIIPKpc2_NP.5
p−min=0.34, pX=0.56, N=223
var.3.F5 1:169511555:T:C
peptide level
154:172
116:135
15:18
DIASGLIGLLLICKpc2_NP.1
p−min=0.31, pX=0.002, N=285
var.3.F5 1:169511555:T:C
peptide level
162:172
132:135
16:18
DIASGLIGLLLICKpc2_NP.2
p−maj=0.2, pX=0.067, N=310
var.3.F5 1:169511555:T:C
peptide level
166:172
130:135
17:18
DIASGLIGLLLICKpc2_NP.3
p−maj=0.5, pX=0.014, N=313
var.3.F5 1:169511555:T:C
peptide level
167:172
134:135
17:18
DIASGLIGLLLICKpc2_NP.4
p−maj=0.33, pX=0.18, N=318
var.3.F5 1:169511555:T:C
peptide level
163:172
119:135
18:18
DIASGLIGLLLICKpc2_NP.5
p−min=0.096, pX=0.06, N=300
var.3.F5 1:169511555:T:C
peptide level
71:172
42:135
3:18
DIASGLIGLLLICKpc3_NP.1
p−min=0.028, pX=0.0015, N=116
var.3.F5 1:169511555:T:C
peptide level
15:172
6:135
1:18
DIASGLIGLLLICKpc3_NP.2
p−min=0.18, pX=0.1, N=22
var.3.F5 1:169511555:T:C
peptide level
30:172
18:135
0:18
DIASGLIGLLLICKpc3_NP.3
p−maj=0.086, pX=0.0093, N=48
var.3.F5 1:169511555:T:C
peptide level
104:172
82:135
12:18
DIASGLIGLLLICKpc3_NP.4
p−maj=0.8, pX=0.09, N=198
var.3.F5 1:169511555:T:C
peptide level
91:172
65:135
11:18
DIASGLIGLLLICKpc3_NP.5
p−maj=0.47, pX=0.095, N=167

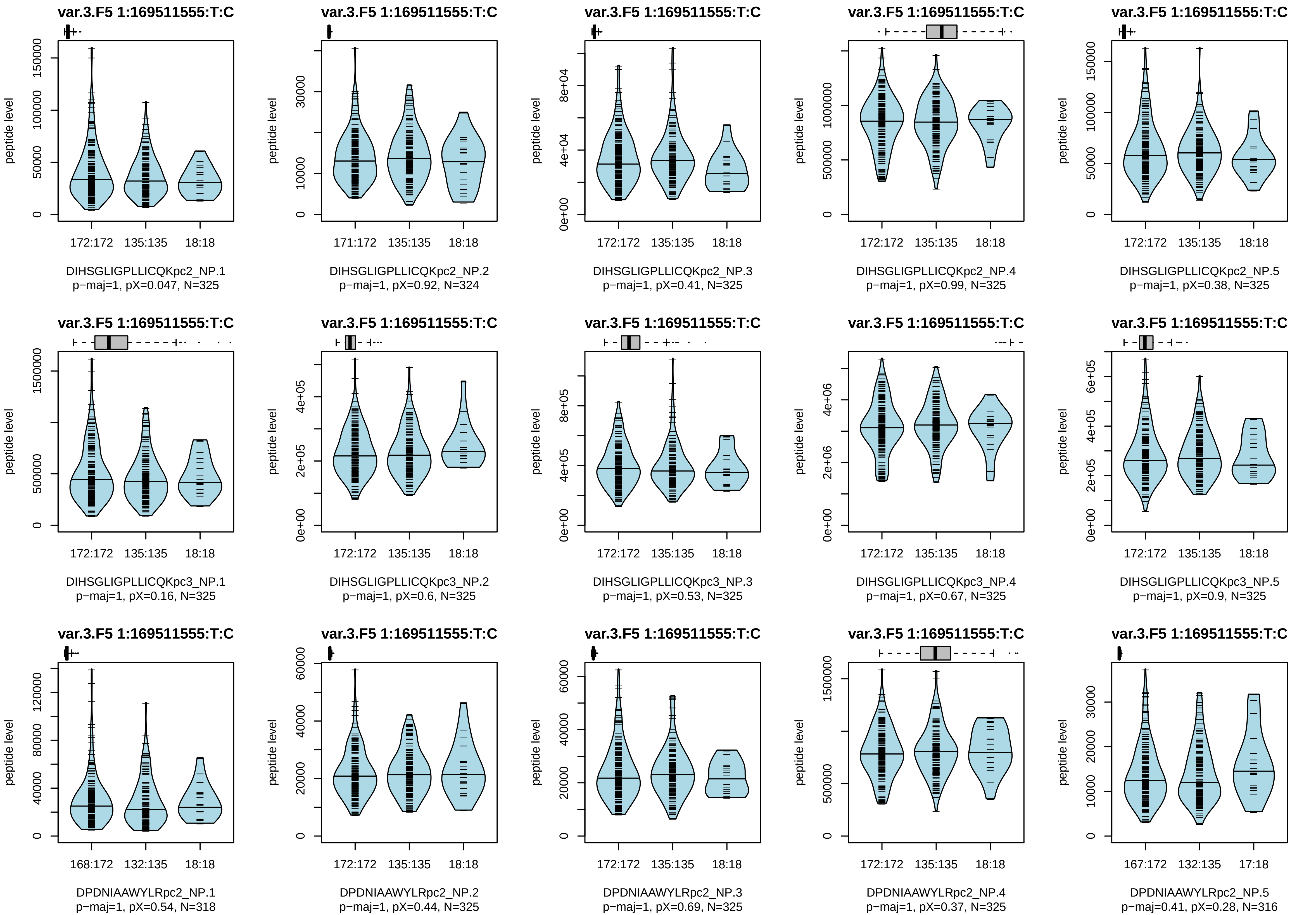
var.3.F5 1:169511555:T:C
peptide level
172:172
135:135
18:18
DIHSGLIGPLLICQKpc2_NP.1
p−maj=1, pX=0.047, N=325
var.3.F5 1:169511555:T:C
peptide level
171:172
135:135
18:18
DIHSGLIGPLLICQKpc2_NP.2
p−maj=1, pX=0.92, N=324
var.3.F5 1:169511555:T:C
peptide level
172:172
135:135
18:18
DIHSGLIGPLLICQKpc2_NP.3
p−maj=1, pX=0.41, N=325
var.3.F5 1:169511555:T:C
peptide level
172:172
135:135
18:18
DIHSGLIGPLLICQKpc2_NP.4
p−maj=1, pX=0.99, N=325
var.3.F5 1:169511555:T:C
peptide level
172:172
135:135
18:18
DIHSGLIGPLLICQKpc2_NP.5
p−maj=1, pX=0.38, N=325
var.3.F5 1:169511555:T:C
peptide level
172:172
135:135
18:18
DIHSGLIGPLLICQKpc3_NP.1
p−maj=1, pX=0.16, N=325
var.3.F5 1:169511555:T:C
peptide level
172:172
135:135
18:18
DIHSGLIGPLLICQKpc3_NP.2
p−maj=1, pX=0.6, N=325
var.3.F5 1:169511555:T:C
peptide level
172:172
135:135
18:18
DIHSGLIGPLLICQKpc3_NP.3
p−maj=1, pX=0.53, N=325
var.3.F5 1:169511555:T:C
peptide level
172:172
135:135
18:18
DIHSGLIGPLLICQKpc3_NP.4
p−maj=1, pX=0.67, N=325
var.3.F5 1:169511555:T:C
peptide level
172:172
135:135
18:18
DIHSGLIGPLLICQKpc3_NP.5
p−maj=1, pX=0.9, N=325
var.3.F5 1:169511555:T:C
peptide level
168:172
132:135
18:18
DPDNIAAWYLRpc2_NP.1
p−maj=1, pX=0.54, N=318
var.3.F5 1:169511555:T:C
peptide level
172:172
135:135
18:18
DPDNIAAWYLRpc2_NP.2
p−maj=1, pX=0.44, N=325
var.3.F5 1:169511555:T:C
peptide level
172:172
135:135
18:18
DPDNIAAWYLRpc2_NP.3
p−maj=1, pX=0.69, N=325
var.3.F5 1:169511555:T:C
peptide level
172:172
135:135
18:18
DPDNIAAWYLRpc2_NP.4
p−maj=1, pX=0.37, N=325
var.3.F5 1:169511555:T:C
peptide level
167:172
132:135
17:18
DPDNIAAWYLRpc2_NP.5
p−maj=0.41, pX=0.28, N=316

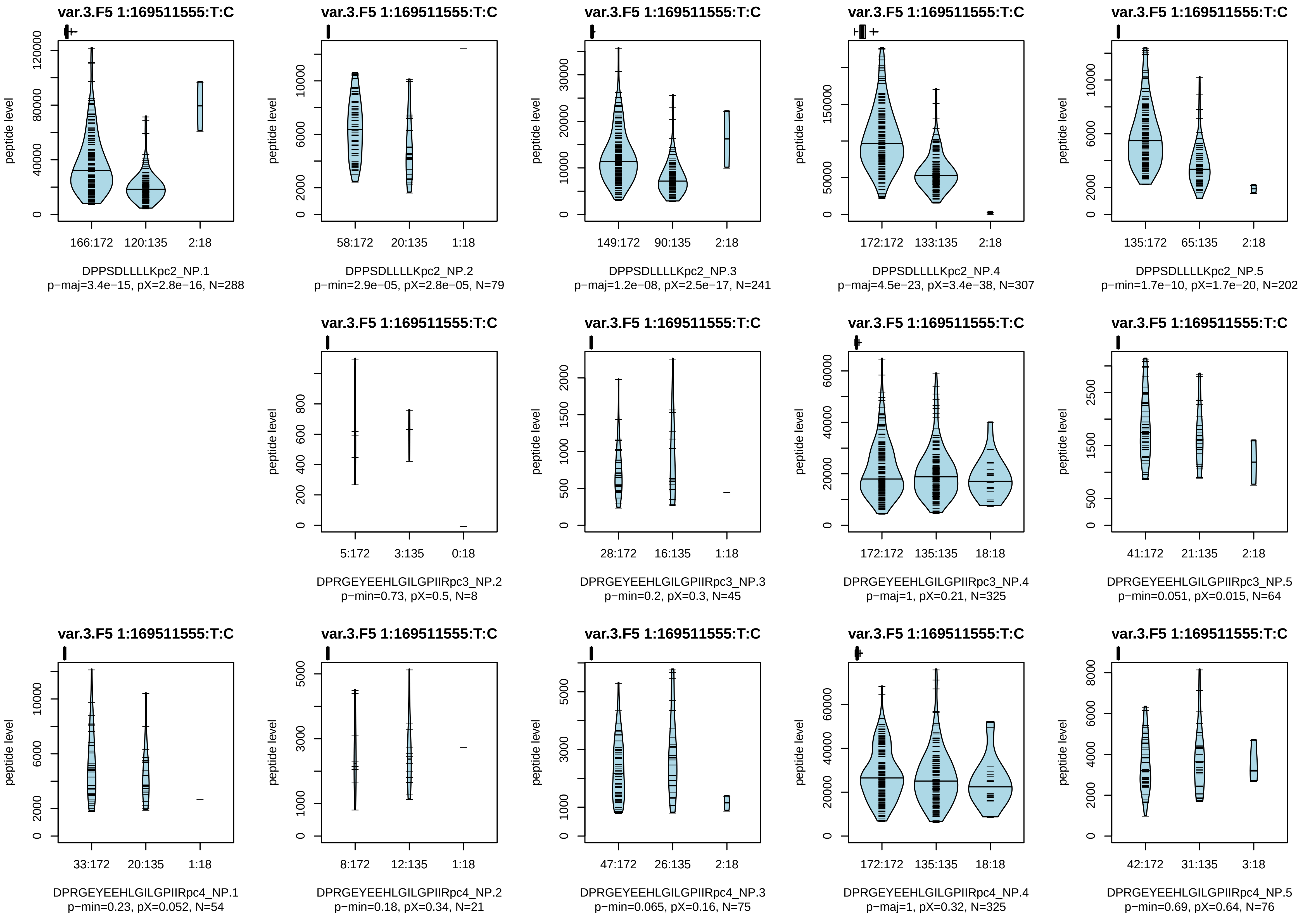
var.3.F5 1:169511555:T:C
166:172
120:135
2:18
DPPSDLLLLKpc2_NP.1
p−maj=3.4e−15, pX=2.8e−16, N=288
var.3.F5 1:169511555:T:C
58:172
20:135
1:18
DPPSDLLLLKpc2_NP.2
p−min=2.9e−05, pX=2.8e−05, N=79
var.3.F5 1:169511555:T:C
149:172
90:135
2:18
DPPSDLLLLKpc2_NP.3
p−maj=1.2e−08, pX=2.5e−17, N=241
var.3.F5 1:169511555:T:C
172:172
133:135
2:18
DPPSDLLLLKpc2_NP.4
p−maj=4.5e−23, pX=3.4e−38, N=307
var.3.F5 1:169511555:T:C
135:172
65:135
2:18
DPPSDLLLLKpc2_NP.5
p−min=1.7e−10, pX=1.7e−20, N=202
var.3.F5 1:169511555:T:C
5:172
3:135
0:18
DPRGEYEEHLGILGPIIRpc3_NP.2
p−min=0.73, pX=0.5, N=8
var.3.F5 1:169511555:T:C
28:172
16:135
1:18
DPRGEYEEHLGILGPIIRpc3_NP.3
p−min=0.2, pX=0.3, N=45
var.3.F5 1:169511555:T:C
172:172
135:135
18:18
DPRGEYEEHLGILGPIIRpc3_NP.4
p−maj=1, pX=0.21, N=325
var.3.F5 1:169511555:T:C
41:172
21:135
2:18
DPRGEYEEHLGILGPIIRpc3_NP.5
p−min=0.051, pX=0.015, N=64
var.3.F5 1:169511555:T:C
33:172
20:135
1:18
DPRGEYEEHLGILGPIIRpc4_NP.1
p−min=0.23, pX=0.052, N=54
var.3.F5 1:169511555:T:C
8:172
12:135
1:18
DPRGEYEEHLGILGPIIRpc4_NP.2
p−min=0.18, pX=0.34, N=21
var.3.F5 1:169511555:T:C
47:172
26:135
2:18
DPRGEYEEHLGILGPIIRpc4_NP.3
p−min=0.065, pX=0.16, N=75
var.3.F5 1:169511555:T:C
172:172
135:135
18:18
DPRGEYEEHLGILGPIIRpc4_NP.4
p−maj=1, pX=0.32, N=325
var.3.F5 1:169511555:T:C
42:172
31:135
3:18
DPRGEYEEHLGILGPIIRpc4_NP.5
p−min=0.69, pX=0.64, N=76
peptide level

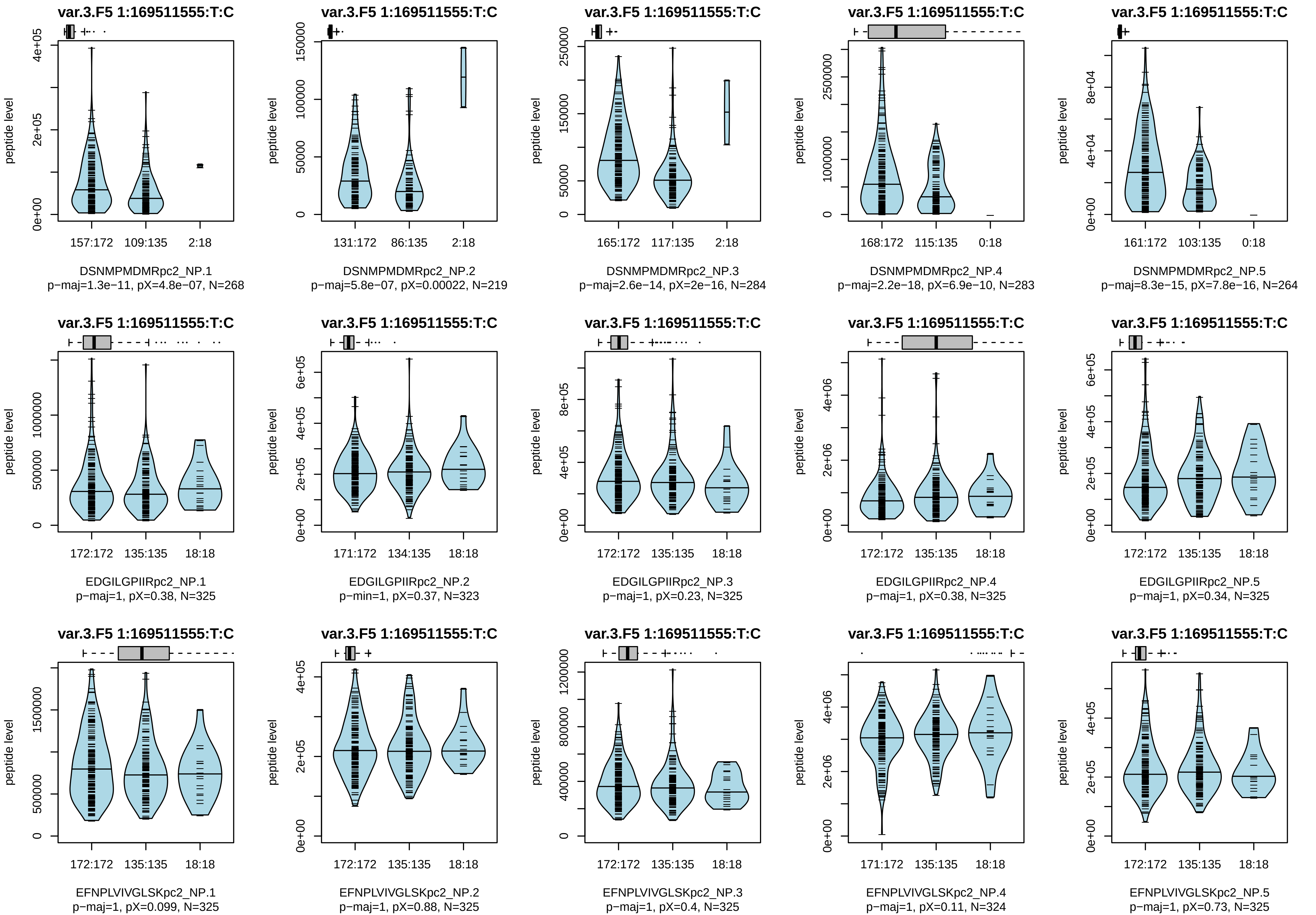

var.3.F5 1:169511555:T:C
peptide level
157:172
109:135
2:18
DSNMPMDMRpc2_NP.1
p−maj=1.3e−11, pX=4.8e−07, N=268
var.3.F5 1:169511555:T:C
peptide level
131:172
86:135
2:18
DSNMPMDMRpc2_NP.2
p−maj=5.8e−07, pX=0.00022, N=219
var.3.F5 1:169511555:T:C
peptide level
165:172
117:135
2:18
DSNMPMDMRpc2_NP.3
p−maj=2.6e−14, pX=2e−16, N=284
var.3.F5 1:169511555:T:C
peptide level
168:172
115:135
0:18
DSNMPMDMRpc2_NP.4
p−maj=2.2e−18, pX=6.9e−10, N=283
var.3.F5 1:169511555:T:C
peptide level
161:172
103:135
0:18
DSNMPMDMRpc2_NP.5
p−maj=8.3e−15, pX=7.8e−16, N=264
var.3.F5 1:169511555:T:C
peptide level
172:172
135:135
18:18
EDGILGPIIRpc2_NP.1
p−maj=1, pX=0.38, N=325
var.3.F5 1:169511555:T:C
peptide level
171:172
134:135
18:18
EDGILGPIIRpc2_NP.2
p−min=1, pX=0.37, N=323
var.3.F5 1:169511555:T:C
peptide level
172:172
135:135
18:18
EDGILGPIIRpc2_NP.3
p−maj=1, pX=0.23, N=325
var.3.F5 1:169511555:T:C
peptide level
172:172
135:135
18:18
EDGILGPIIRpc2_NP.4
p−maj=1, pX=0.38, N=325
var.3.F5 1:169511555:T:C
peptide level
172:172
135:135
18:18
EDGILGPIIRpc2_NP.5
p−maj=1, pX=0.34, N=325
var.3.F5 1:169511555:T:C
peptide level
172:172
135:135
18:18
EFNPLVIVGLSKpc2_NP.1
p−maj=1, pX=0.099, N=325
var.3.F5 1:169511555:T:C
peptide level
172:172
135:135
18:18
EFNPLVIVGLSKpc2_NP.2
p−maj=1, pX=0.88, N=325
var.3.F5 1:169511555:T:C
peptide level
172:172
135:135
18:18
EFNPLVIVGLSKpc2_NP.3
p−maj=1, pX=0.4, N=325
var.3.F5 1:169511555:T:C
peptide level
171:172
135:135
18:18
EFNPLVIVGLSKpc2_NP.4
p−maj=1, pX=0.11, N=324
var.3.F5 1:169511555:T:C
peptide level
172:172
135:135
18:18
EFNPLVIVGLSKpc2_NP.5
p−maj=1, pX=0.73, N=325

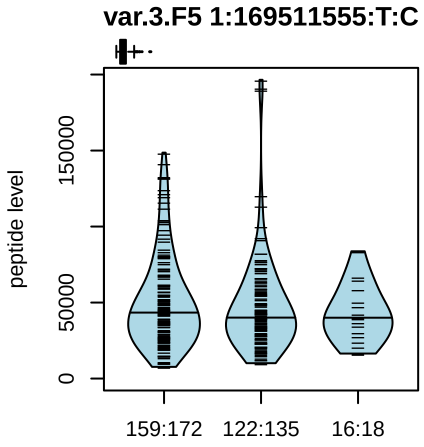

EFVLLFMTFDEKpc2_NP.1
p−min=0.55, pX=0.19, N=297

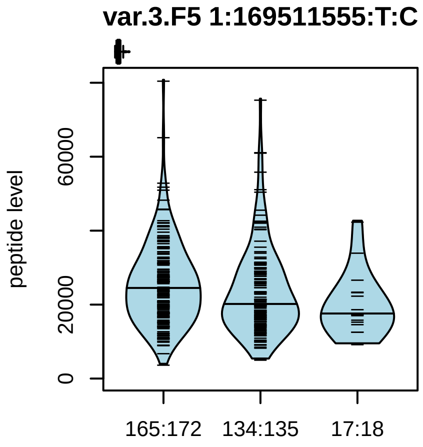

EFVLLFMTFDEKpc2_NP.2
p−min=0.18, pX=0.081, N=316

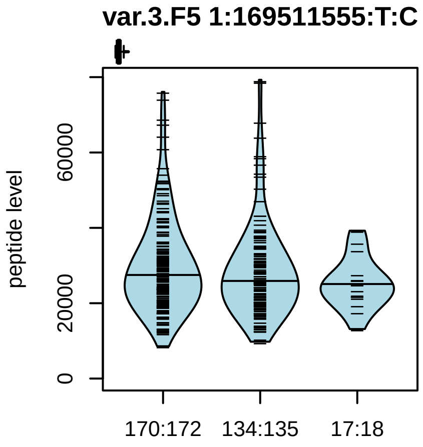

EFVLLFMTFDEKpc2_NP.3
p−maj=0.2, pX=0.092, N=321

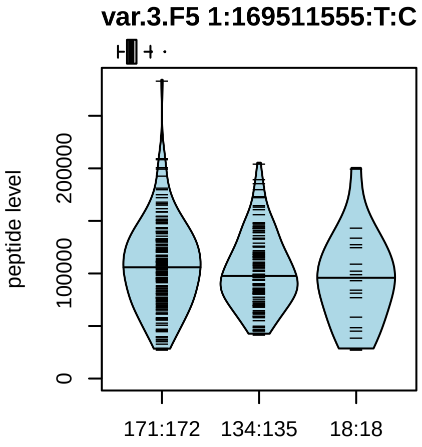

EFVLLFMTFDEKpc2_NP.4
p−min=1, pX=0.14, N=323

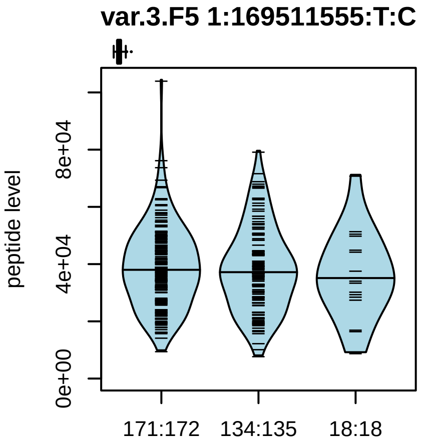

EFVLLFMTFDEKpc2_NP.5
p−min=1, pX=0.46, N=323

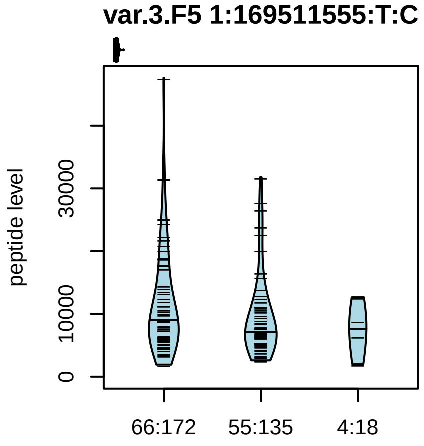

EKPQSTISGLLGPTLYAEVGDIIKpc3_NP.1
p−maj=0.21, pX=0.13, N=125

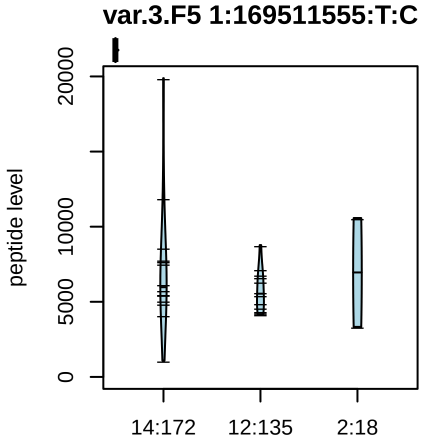

EKPQSTISGLLGPTLYAEVGDIIKpc3_NP.2
p−maj=0.66, pX=1, N=28

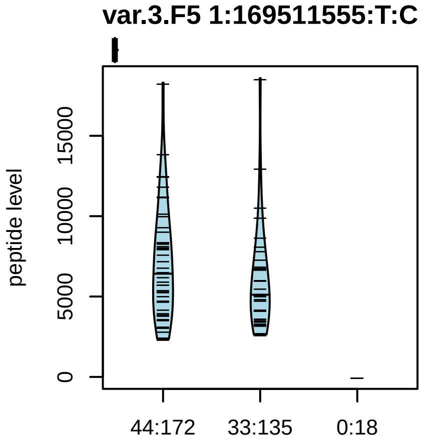

EKPQSTISGLLGPTLYAEVGDIIKpc3_NP.3
p−maj=0.0093, pX=0.053, N=77

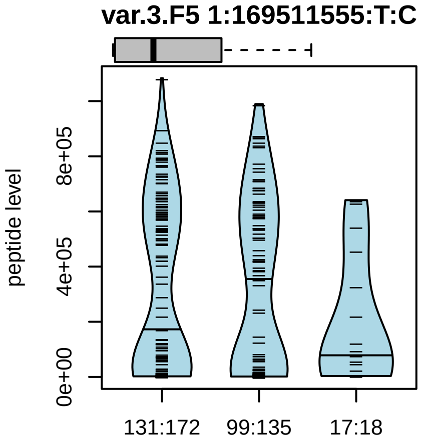

EKPQSTISGLLGPTLYAEVGDIIKpc3_NP.4
p−maj=0.085, pX=0.54, N=247

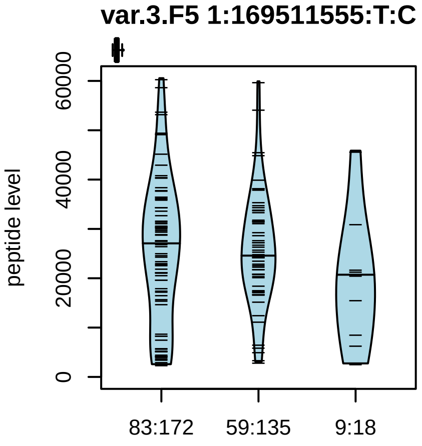

EKPQSTISGLLGPTLYAEVGDIIKpc3_NP.5
p−min=0.51, pX=0.41, N=151

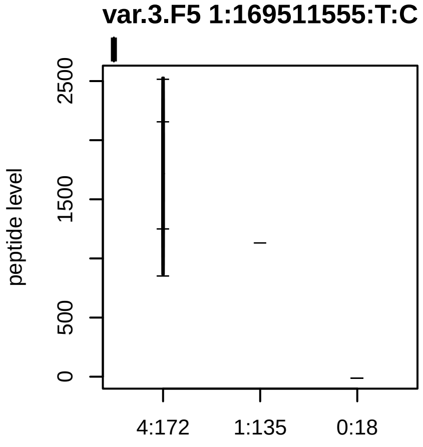

EKPQSTISGLLGPTLYAEVGDIIKpc4_NP.3
p−min=0.38, pX=0.18, N=5

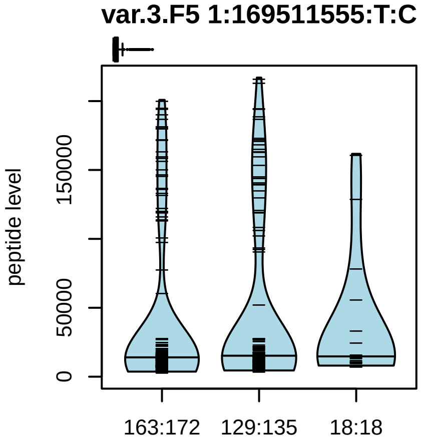

EKPQSTISGLLGPTLYAEVGDIIKpc4_NP.4
p−min=0.61, pX=0.5, N=310

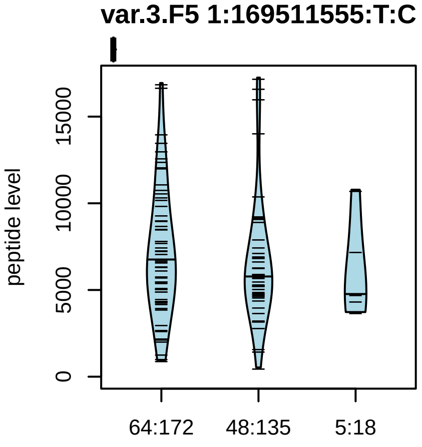

EKPQSTISGLLGPTLYAEVGDIIKpc4_NP.5
p−maj=0.62, pX=0.26, N=117

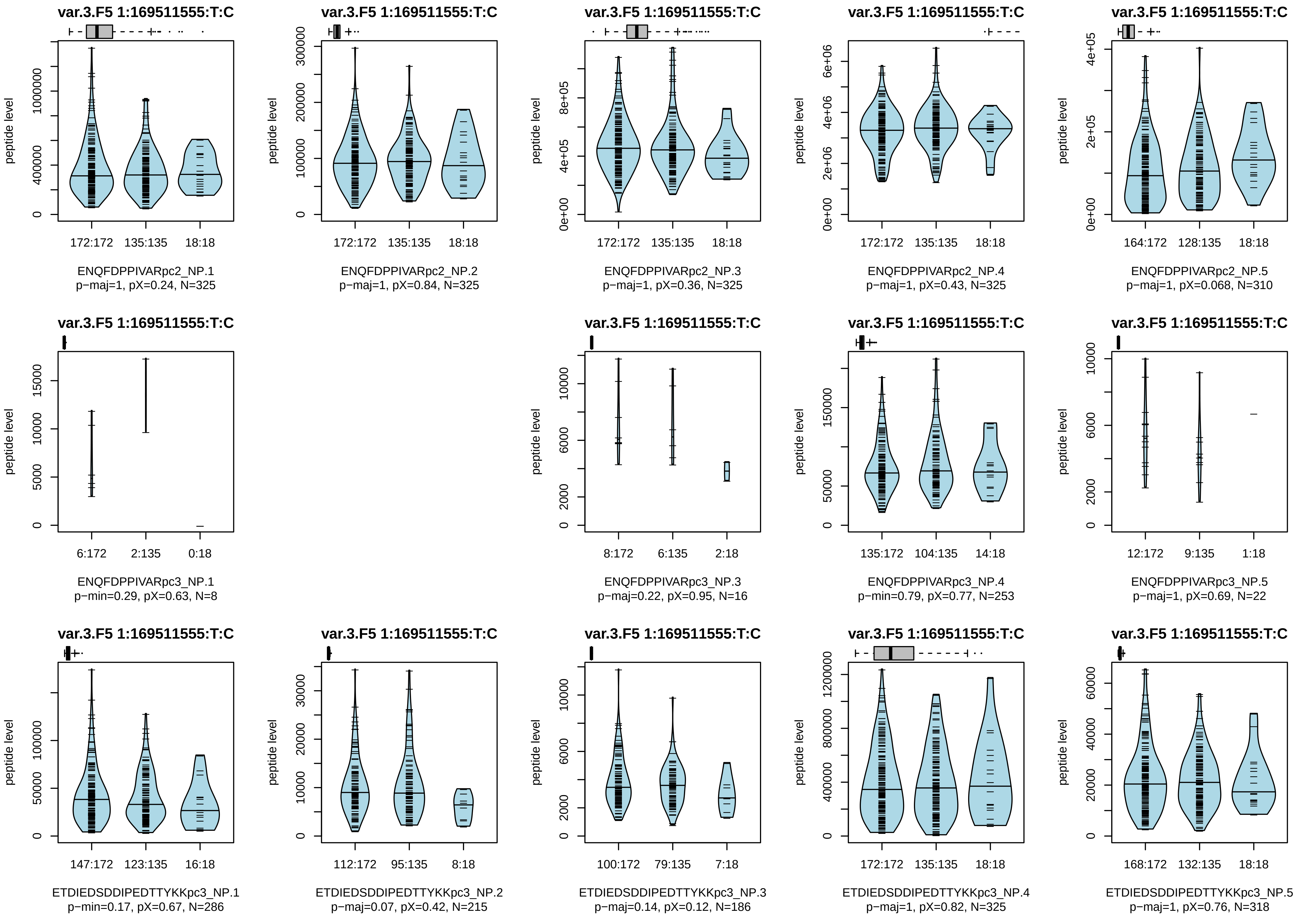
var.3.F5 1:169511555:T:C
peptide level
172:172
135:135
18:18
ENQFDPPIVARpc2_NP.1
p−maj=1, pX=0.24, N=325
var.3.F5 1:169511555:T:C
172:172
135:135
18:18
ENQFDPPIVARpc2_NP.2
p−maj=1, pX=0.84, N=325
var.3.F5 1:169511555:T:C
172:172
135:135
18:18
ENQFDPPIVARpc2_NP.3
p−maj=1, pX=0.36, N=325
var.3.F5 1:169511555:T:C
172:172
135:135
18:18
ENQFDPPIVARpc2_NP.4
p−maj=1, pX=0.43, N=325
var.3.F5 1:169511555:T:C
164:172
128:135
18:18
ENQFDPPIVARpc2_NP.5
p−maj=1, pX=0.068, N=310
var.3.F5 1:169511555:T:C
6:172
2:135
0:18
ENQFDPPIVARpc3_NP.1
p−min=0.29, pX=0.63, N=8
var.3.F5 1:169511555:T:C
8:172
6:135
2:18
ENQFDPPIVARpc3_NP.3
p−maj=0.22, pX=0.95, N=16
var.3.F5 1:169511555:T:C
135:172
104:135
14:18
ENQFDPPIVARpc3_NP.4
p−min=0.79, pX=0.77, N=253
var.3.F5 1:169511555:T:C
12:172
9:135
1:18
ENQFDPPIVARpc3_NP.5
p−maj=1, pX=0.69, N=22
var.3.F5 1:169511555:T:C
147:172
123:135
16:18
ETDIEDSDDIPEDTTYKKpc3_NP.1
p−min=0.17, pX=0.67, N=286
var.3.F5 1:169511555:T:C
112:172
95:135
8:18
ETDIEDSDDIPEDTTYKKpc3_NP.2
p−maj=0.07, pX=0.42, N=215
var.3.F5 1:169511555:T:C
100:172
79:135
7:18
ETDIEDSDDIPEDTTYKKpc3_NP.3
p−maj=0.14, pX=0.12, N=186
var.3.F5 1:169511555:T:C
172:172
135:135
18:18
ETDIEDSDDIPEDTTYKKpc3_NP.4
p−maj=1, pX=0.82, N=325
var.3.F5 1:169511555:T:C
168:172
132:135
18:18
ETDIEDSDDIPEDTTYKKpc3_NP.5
p−maj=1, pX=0.76, N=318

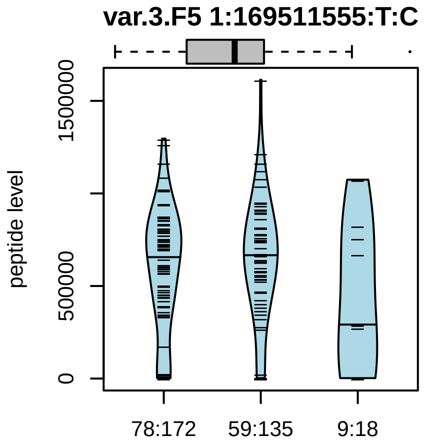

ETDIEDSDDIPEDTTYKpc2_NP.4
p−maj=0.81, pX=0.95, N=146

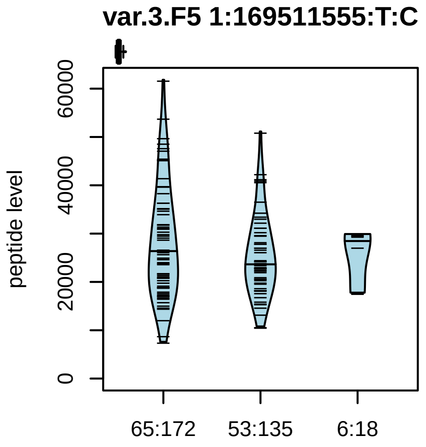

ETDIEDSDDIPEDTTYKpc2_NP.5
p−maj=0.8, pX=0.5, N=124

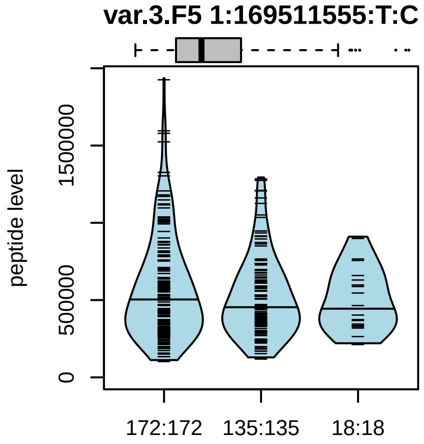

EVIITGIQTQGAKpc2_NP.1
p−maj=1, pX=0.08, N=325

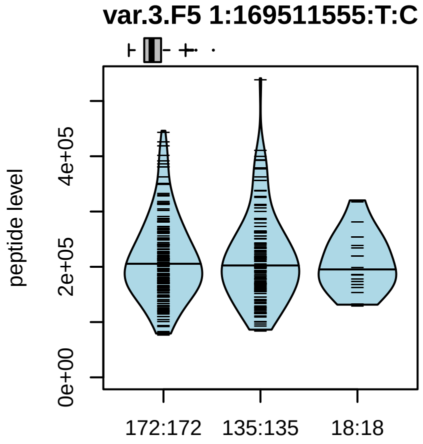

EVIITGIQTQGAKpc2_NP.2
p−maj=1, pX=0.81, N=325

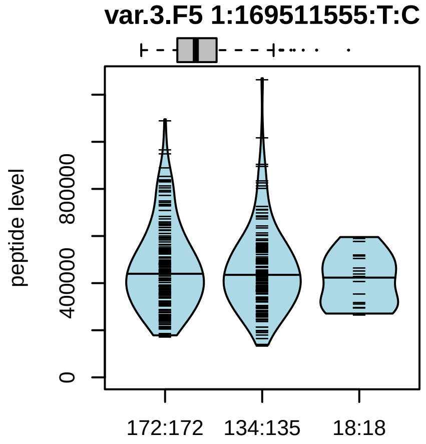

EVIITGIQTQGAKpc2_NP.3
p−min=0.47, pX=0.21, N=324

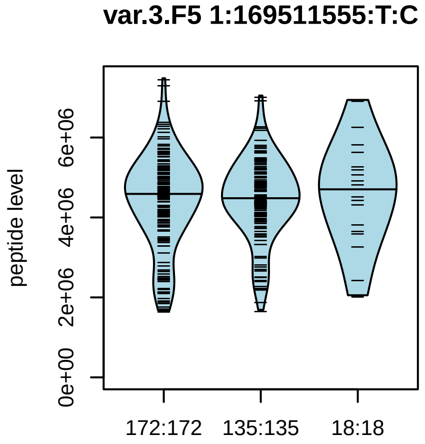

EVIITGIQTQGAKpc2_NP.4
p−maj=1, pX=0.48, N=325

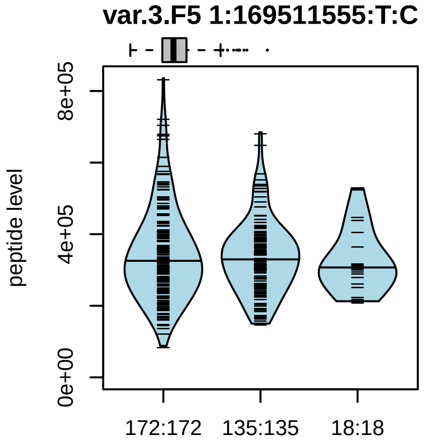

EVIITGIQTQGAKpc2_NP.5
p−maj=1, pX=0.37, N=325

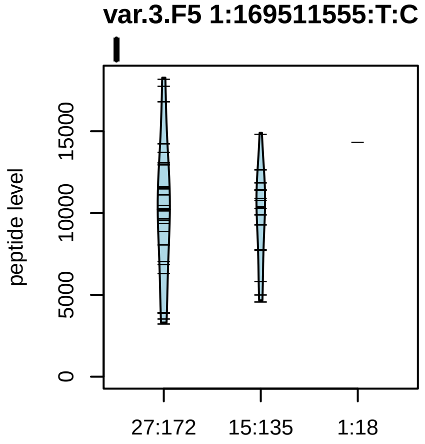

EVIITGIQTQGAKpc3_NP.4
p−min=0.19, pX=0.19, N=43

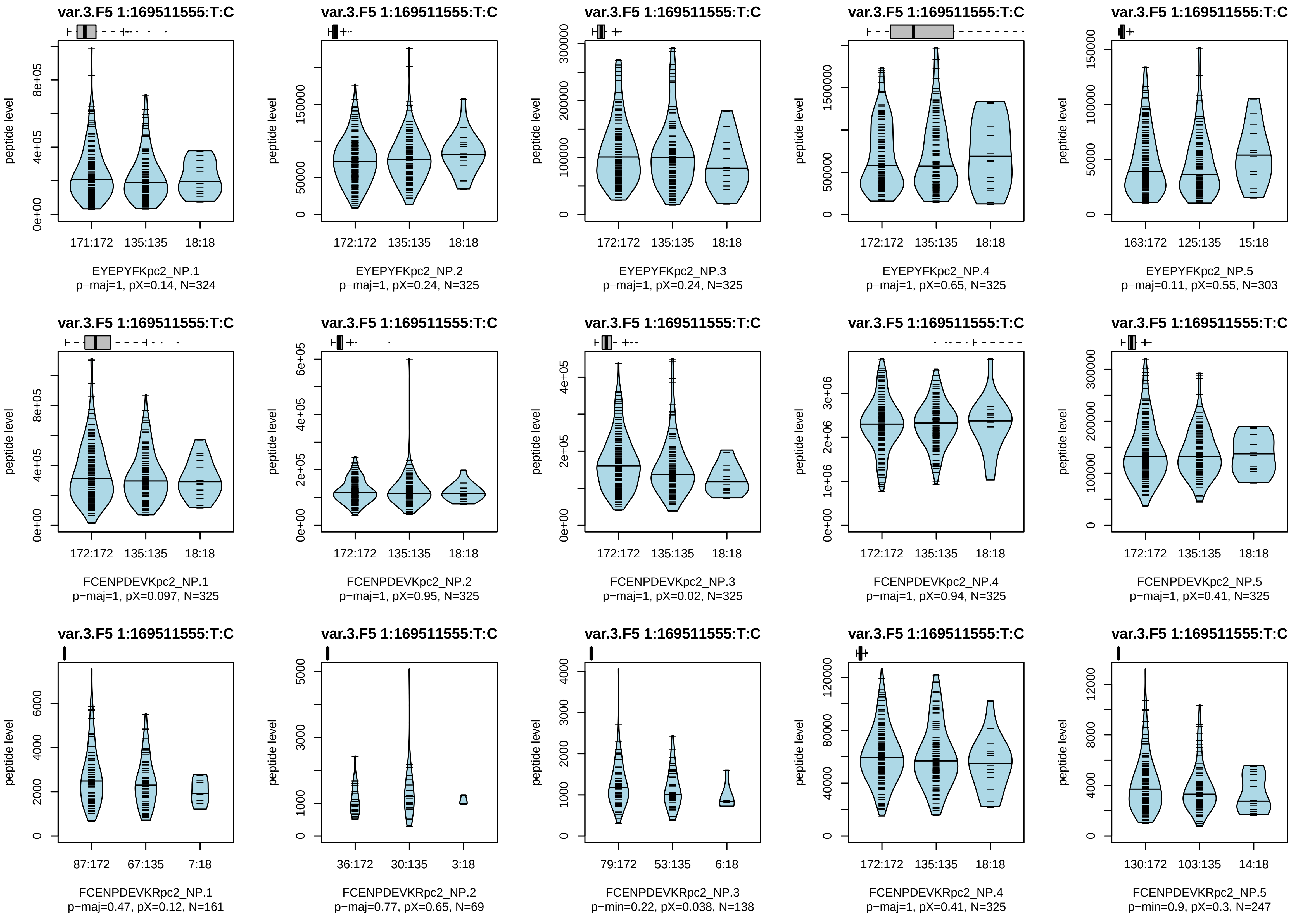
var.3.F5 1:169511555:T:C
peptide level
0e+00
4e+05
8e+05
171:172
135:135
18:18
EYEPYFKpc2_NP.1
p−maj=1, pX=0.14, N=324
var.3.F5 1:169511555:T:C
peptide level
0
50000
150000
172:172
135:135
18:18
EYEPYFKpc2_NP.2
p−maj=1, pX=0.24, N=325
var.3.F5 1:169511555:T:C
peptide level
0
100000
200000
300000
172:172
135:135
18:18
EYEPYFKpc2_NP.3
p−maj=1, pX=0.24, N=325
var.3.F5 1:169511555:T:C
peptide level
0
500000
1500000
172:172
135:135
18:18
EYEPYFKpc2_NP.4
p−maj=1, pX=0.65, N=325
var.3.F5 1:169511555:T:C
peptide level
0
50000
100000
150000
163:172
125:135
15:18
EYEPYFKpc2_NP.5
p−maj=0.11, pX=0.55, N=303
var.3.F5 1:169511555:T:C
peptide level
0e+00
4e+05
8e+05
172:172
135:135
18:18
FCENPDEVKpc2_NP.1
p−maj=1, pX=0.097, N=325
var.3.F5 1:169511555:T:C
peptide level
0e+00
2e+05
4e+05
6e+05
172:172
135:135
18:18
FCENPDEVKpc2_NP.2
p−maj=1, pX=0.95, N=325
var.3.F5 1:169511555:T:C
peptide level
0e+00
2e+05
4e+05
172:172
135:135
18:18
FCENPDEVKpc2_NP.3
p−maj=1, pX=0.02, N=325
var.3.F5 1:169511555:T:C
peptide level
0e+00
1e+06
2e+06
3e+06
172:172
135:135
18:18
FCENPDEVKpc2_NP.4
p−maj=1, pX=0.94, N=325
var.3.F5 1:169511555:T:C
peptide level
0
100000
200000
300000
172:172
135:135
18:18
FCENPDEVKpc2_NP.5
p−maj=1, pX=0.41, N=325
var.3.F5 1:169511555:T:C
peptide level
0
2000
4000
6000
87:172
67:135
7:18
FCENPDEVKRpc2_NP.1
p−maj=0.47, pX=0.12, N=161
var.3.F5 1:169511555:T:C
peptide level
0
1000
3000
5000
36:172
30:135
3:18
FCENPDEVKRpc2_NP.2
p−maj=0.77, pX=0.65, N=69
var.3.F5 1:169511555:T:C
peptide level
0
1000
2000
3000
4000
79:172
53:135
6:18
FCENPDEVKRpc2_NP.3
p−min=0.22, pX=0.038, N=138
var.3.F5 1:169511555:T:C
peptide level
0
40000
80000
120000
172:172
135:135
18:18
FCENPDEVKRpc2_NP.4
p−maj=1, pX=0.41, N=325
var.3.F5 1:169511555:T:C
peptide level
0
4000
8000
12000
130:172
103:135
14:18
FCENPDEVKRpc2_NP.5
p−min=0.9, pX=0.3, N=247

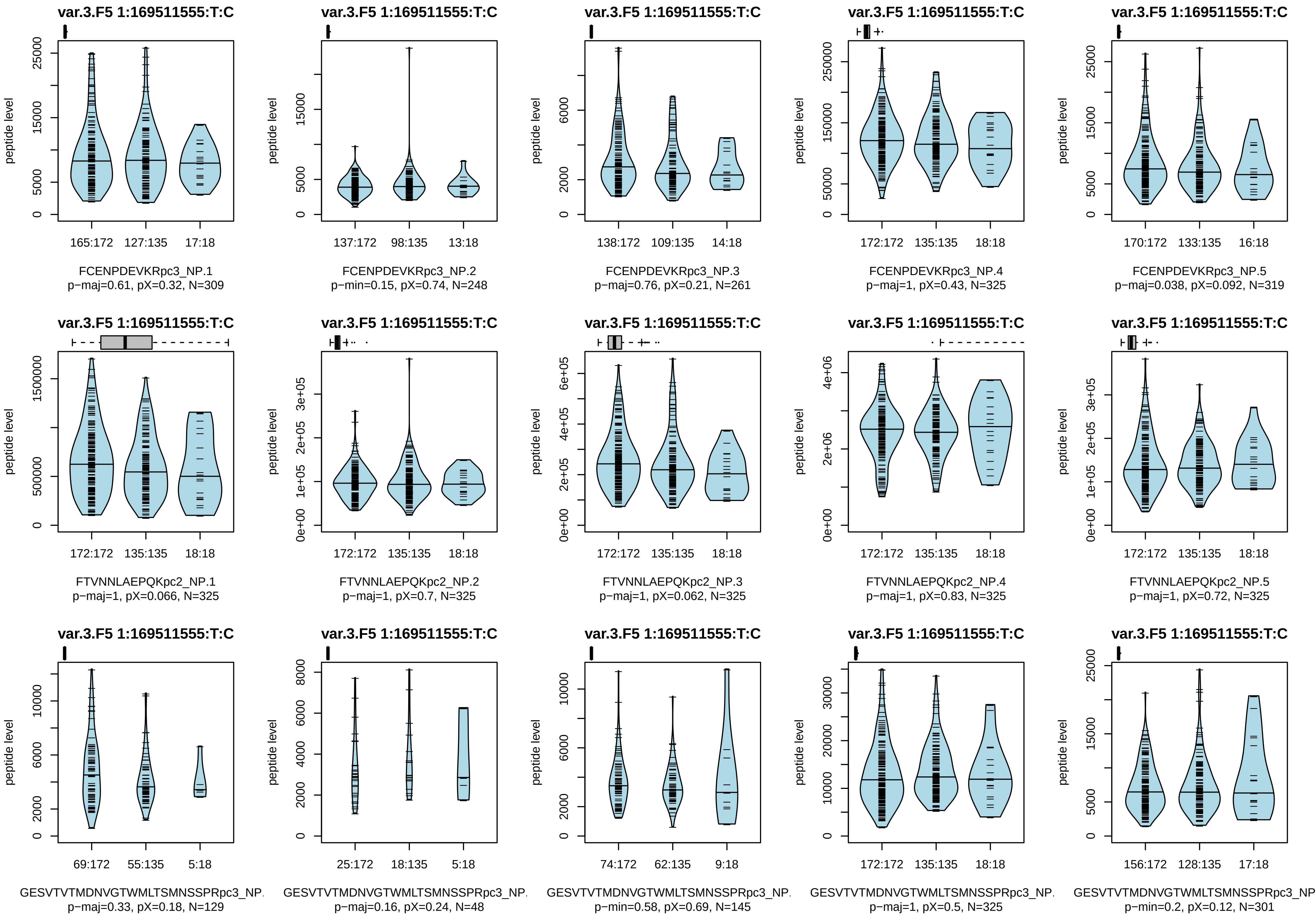
var.3.F5 1:169511555:T:C
peptide level
165:172 127:135 17:18
FCENPDEVKRpc3_NP.1
p−maj=0.61, pX=0.32, N=309
var.3.F5 1:169511555:T:C
peptide level
137:172 98:135 13:18
FCENPDEVKRpc3_NP.2
p−min=0.15, pX=0.74, N=248
var.3.F5 1:169511555:T:C
peptide level
138:172 109:135 14:18
FCENPDEVKRpc3_NP.3
p−maj=0.76, pX=0.21, N=261
var.3.F5 1:169511555:T:C
peptide level
172:172 135:135 18:18
FCENPDEVKRpc3_NP.4
p−maj=1, pX=0.43, N=325
var.3.F5 1:169511555:T:C
peptide level
170:172 133:135 16:18
FCENPDEVKRpc3_NP.5
p−maj=0.038, pX=0.092, N=319
var.3.F5 1:169511555:T:C
peptide level
172:172 135:135 18:18
FTVNNLAEPQKpc2_NP.1
p−maj=1, pX=0.066, N=325
var.3.F5 1:169511555:T:C
peptide level
172:172 135:135 18:18
FTVNNLAEPQKpc2_NP.2
p−maj=1, pX=0.7, N=325
var.3.F5 1:169511555:T:C
peptide level
172:172 135:135 18:18
FTVNNLAEPQKpc2_NP.3
p−maj=1, pX=0.062, N=325
var.3.F5 1:169511555:T:C
peptide level
172:172 135:135 18:18
FTVNNLAEPQKpc2_NP.4
p−maj=1, pX=0.83, N=325
var.3.F5 1:169511555:T:C
peptide level
172:172 135:135 18:18
FTVNNLAEPQKpc2_NP.5
p−maj=1, pX=0.72, N=325
var.3.F5 1:169511555:T:C
peptide level
69:172 55:135 5:18
GESVTVTMDNVGTWMLTSMNSSPRpc3_NP.
p−maj=0.33, pX=0.18, N=129
var.3.F5 1:169511555:T:C
peptide level
25:172 18:135 5:18
GESVTVTMDNVGTWMLTSMNSSPRpc3_NP.
p−maj=0.16, pX=0.24, N=48
var.3.F5 1:169511555:T:C
peptide level
74:172 62:135 9:18
GESVTVTMDNVGTWMLTSMNSSPRpc3_NP.
p−min=0.58, pX=0.69, N=145
var.3.F5 1:169511555:T:C
peptide level
172:172 135:135 18:18
GESVTVTMDNVGTWMLTSMNSSPRpc3_NP.
p−maj=1, pX=0.5, N=325
var.3.F5 1:169511555:T:C
peptide level
156:172 128:135 17:18
GESVTVTMDNVGTWMLTSMNSSPRpc3_NP
p−min=0.2, pX=0.12, N=301

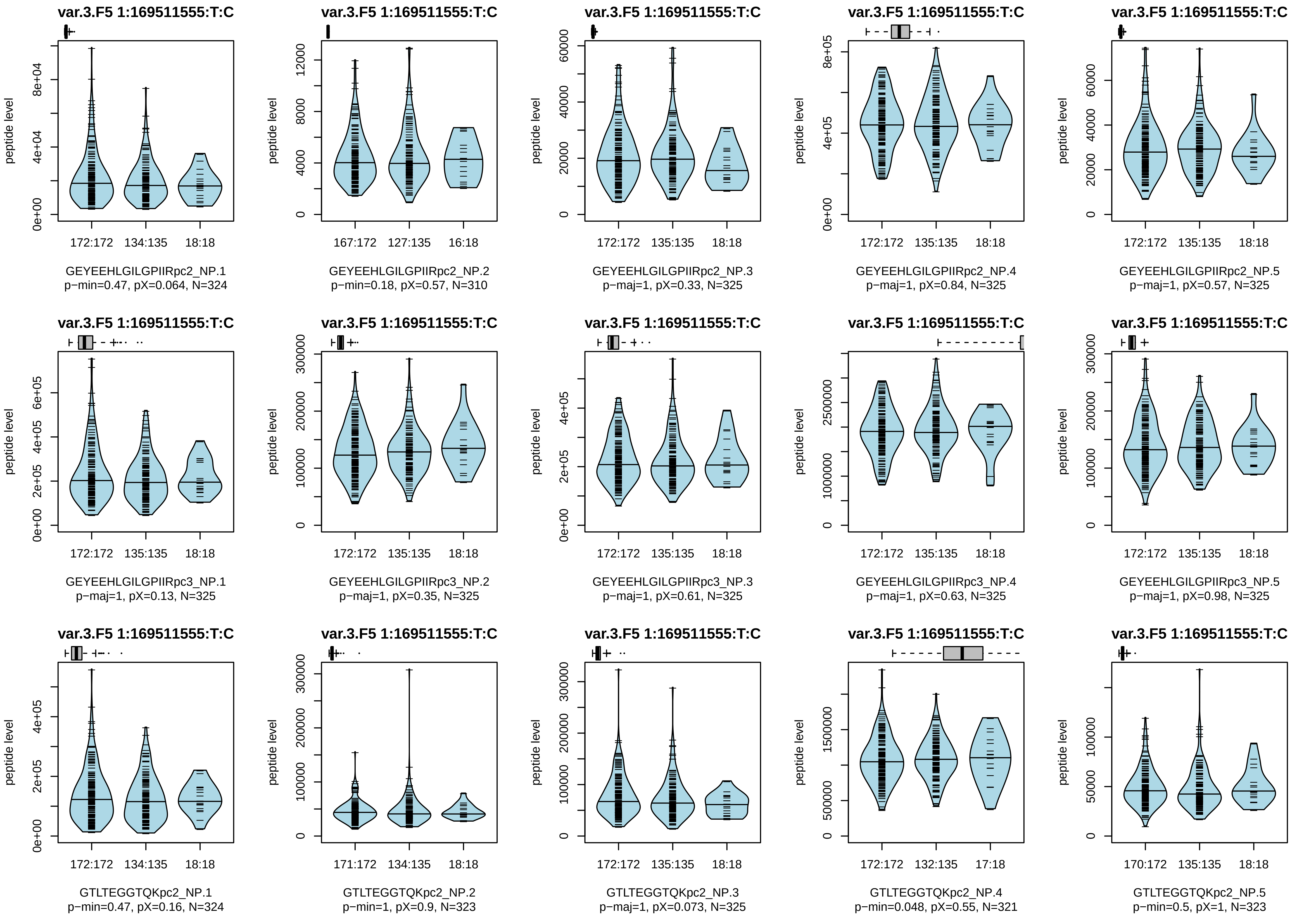

var.3.F5 1:169511555:T:C
peptide level
0e+00 4e+04 8e+04
172:172 134:135 18:18
GEYEEHLGILGPIIRpc2_NP.1
p−min=0.47, pX=0.064, N=324
var.3.F5 1:169511555:T:C
peptide level
0 4000 8000 12000
167:172 127:135 16:18
GEYEEHLGILGPIIRpc2_NP.2
p−min=0.18, pX=0.57, N=310
var.3.F5 1:169511555:T:C
peptide level
0 20000 40000 60000
172:172 135:135 18:18
GEYEEHLGILGPIIRpc2_NP.3
p−maj=1, pX=0.33, N=325
var.3.F5 1:169511555:T:C
peptide level
0e+00 4e+05 8e+05
172:172 135:135 18:18
GEYEEHLGILGPIIRpc2_NP.4
p−maj=1, pX=0.84, N=325
var.3.F5 1:169511555:T:C
peptide level
0 20000 40000 60000
172:172 135:135 18:18
GEYEEHLGILGPIIRpc2_NP.5
p−maj=1, pX=0.57, N=325
var.3.F5 1:169511555:T:C
peptide level
0e+00 2e+05 4e+05 6e+05
172:172 135:135 18:18
GEYEEHLGILGPIIRpc3_NP.1
p−maj=1, pX=0.13, N=325
var.3.F5 1:169511555:T:C
peptide level
0 100000 200000 300000
172:172 135:135 18:18
GEYEEHLGILGPIIRpc3_NP.2
p−maj=1, pX=0.35, N=325
var.3.F5 1:169511555:T:C
peptide level
0e+00 2e+05 4e+05
172:172 135:135 18:18
GEYEEHLGILGPIIRpc3_NP.3
p−maj=1, pX=0.61, N=325
var.3.F5 1:169511555:T:C
peptide level
0 1000000 2500000
172:172 135:135 18:18
GEYEEHLGILGPIIRpc3_NP.4
p−maj=1, pX=0.63, N=325
var.3.F5 1:169511555:T:C
peptide level
0 100000 200000 300000
172:172 135:135 18:18
GEYEEHLGILGPIIRpc3_NP.5
p−maj=1, pX=0.98, N=325
var.3.F5 1:169511555:T:C
peptide level
0e+00 2e+05 4e+05
172:172 134:135 18:18
GTLTEGGTQKpc2_NP.1
p−min=0.47, pX=0.16, N=324
var.3.F5 1:169511555:T:C
peptide level
0 100000 200000 300000
171:172 134:135 18:18
GTLTEGGTQKpc2_NP.2
p−min=1, pX=0.9, N=323
var.3.F5 1:169511555:T:C
peptide level
0 100000 200000 300000
172:172 135:135 18:18
GTLTEGGTQKpc2_NP.3
p−maj=1, pX=0.073, N=325
var.3.F5 1:169511555:T:C
peptide level
0 500000 1500000
172:172 132:135 17:18
GTLTEGGTQKpc2_NP.4
p−min=0.048, pX=0.55, N=321
var.3.F5 1:169511555:T:C
peptide level
0 50000 100000
170:172 135:135 18:18
GTLTEGGTQKpc2_NP.5
p−min=0.5, pX=1, N=323

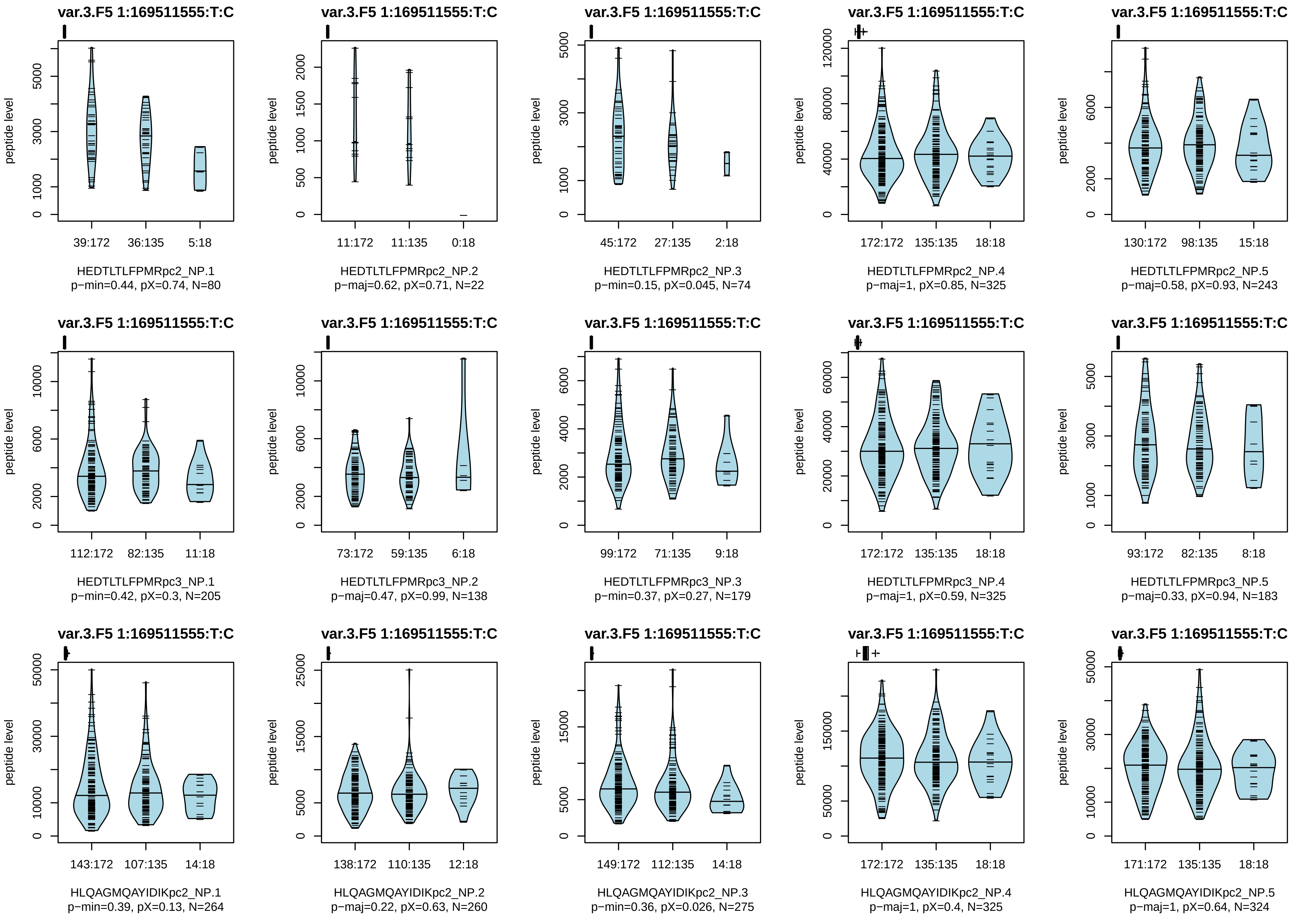
var.3.F5 1:169511555:T:C
peptide level
39:172
36:135
5:18
HEDTLTLFPMRpc2_NP.1
p−min=0.44, pX=0.74, N=80
11:172
11:135
0:18
HEDTLTLFPMRpc2_NP.2
p−maj=0.62, pX=0.71, N=22
45:172
27:135
2:18
HEDTLTLFPMRpc2_NP.3
p−min=0.15, pX=0.045, N=74
172:172
135:135
18:18
HEDTLTLFPMRpc2_NP.4
p−maj=1, pX=0.85, N=325
130:172
98:135
15:18
HEDTLTLFPMRpc2_NP.5
p−maj=0.58, pX=0.93, N=243
112:172
82:135
11:18
HEDTLTLFPMRpc3_NP.1
p−min=0.42, pX=0.3, N=205
73:172
59:135
6:18
HEDTLTLFPMRpc3_NP.2
p−maj=0.47, pX=0.99, N=138
99:172
71:135
9:18
HEDTLTLFPMRpc3_NP.3
p−min=0.37, pX=0.27, N=179
172:172
135:135
18:18
HEDTLTLFPMRpc3_NP.4
p−maj=1, pX=0.59, N=325
93:172
82:135
8:18
HEDTLTLFPMRpc3_NP.5
p−maj=0.33, pX=0.94, N=183
143:172
107:135
14:18
HLQAGMQAYIDIKpc2_NP.1
p−min=0.39, pX=0.13, N=264
138:172
110:135
12:18
HLQAGMQAYIDIKpc2_NP.2
p−maj=0.22, pX=0.63, N=260
149:172
112:135
14:18
HLQAGMQAYIDIKpc2_NP.3
p−min=0.36, pX=0.026, N=275
172:172
135:135
18:18
HLQAGMQAYIDIKpc2_NP.4
p−maj=1, pX=0.4, N=325
171:172
135:135
18:18
HLQAGMQAYIDIKpc2_NP.5
p−maj=1, pX=0.64, N=324

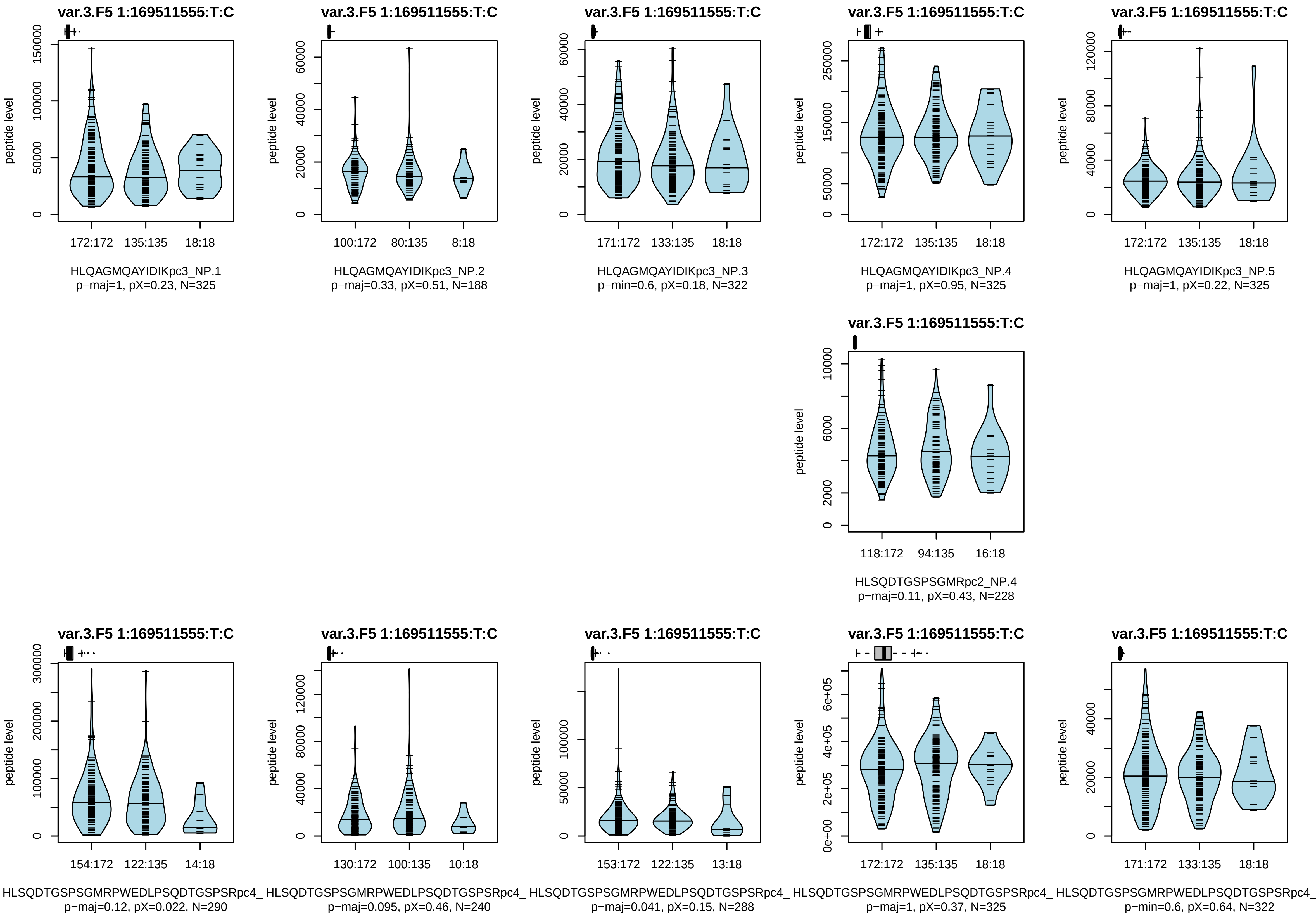
var.3.F5 1:169511555:T:C
peptide level
172:172
135:135
18:18
HLQAGMQAYIDIKpc3_NP.1
p−maj=1, pX=0.23, N=325
var.3.F5 1:169511555:T:C
peptide level
100:172
80:135
8:18
HLQAGMQAYIDIKpc3_NP.2
p−maj=0.33, pX=0.51, N=188
var.3.F5 1:169511555:T:C
peptide level
171:172
133:135
18:18
HLQAGMQAYIDIKpc3_NP.3
p−min=0.6, pX=0.18, N=322
var.3.F5 1:169511555:T:C
peptide level
172:172
135:135
18:18
HLQAGMQAYIDIKpc3_NP.4
p−maj=1, pX=0.95, N=325
var.3.F5 1:169511555:T:C
peptide level
172:172
135:135
18:18
HLQAGMQAYIDIKpc3_NP.5
p−maj=1, pX=0.22, N=325
var.3.F5 1:169511555:T:C
peptide level
118:172
94:135
16:18
HLSQDTGSPSGMRpc2_NP.4
p−maj=0.11, pX=0.43, N=228
var.3.F5 1:169511555:T:C
peptide level
154:172
122:135
14:18
HLSQDTGSPSGMRPWEDLPSQDTGSPSRpc4_
p−maj=0.12, pX=0.022, N=290
var.3.F5 1:169511555:T:C
peptide level
130:172
100:135
10:18
HLSQDTGSPSGMRPWEDLPSQDTGSPSRpc4_
p−maj=0.095, pX=0.46, N=240
var.3.F5 1:169511555:T:C
peptide level
153:172
122:135
13:18
HLSQDTGSPSGMRPWEDLPSQDTGSPSRpc4_
p−maj=0.041, pX=0.15, N=288
var.3.F5 1:169511555:T:C
peptide level
172:172
135:135
18:18
HLSQDTGSPSGMRPWEDLPSQDTGSPSRpc4_
p−maj=1, pX=0.37, N=325
var.3.F5 1:169511555:T:C
peptide level
171:172
133:135
18:18
HLSQDTGSPSGMRPWEDLPSQDTGSPSRpc4_
p−min=0.6, pX=0.64, N=322

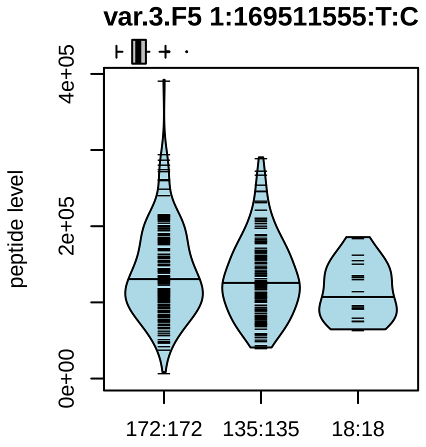

HSLVLHKpc2_NP.1
p−maj=1, pX=0.052, N=325

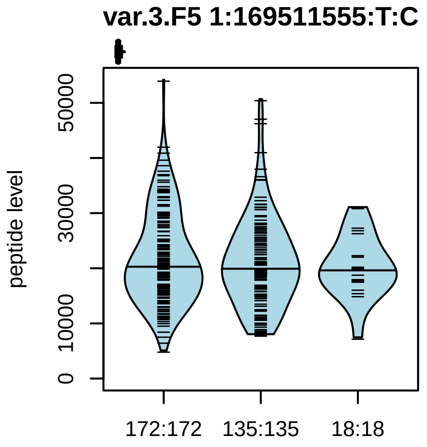

HSLVLHKpc2_NP.2
p−maj=1, pX=0.31, N=325

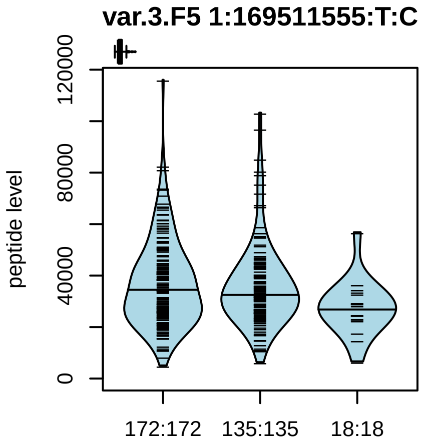

HSLVLHKpc2_NP.3
p−maj=1, pX=0.11, N=325

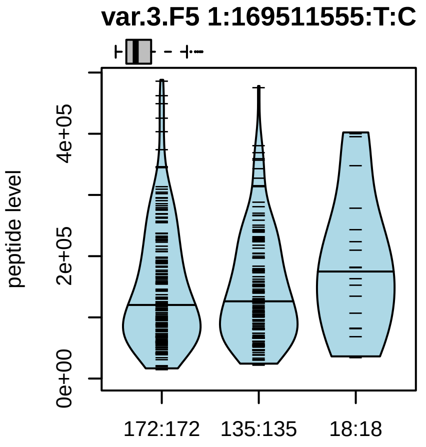

HSLVLHKpc2_NP.4
p−maj=1, pX=0.39, N=325

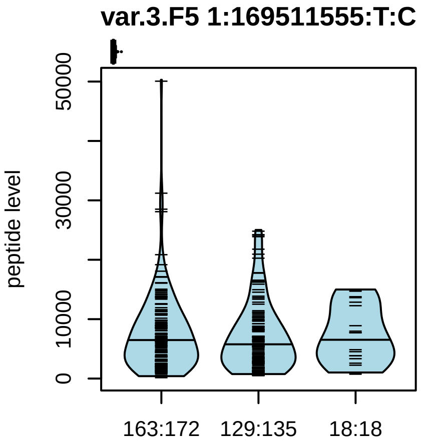

HSLVLHKpc2_NP.5
p−min=0.61, pX=0.86, N=310

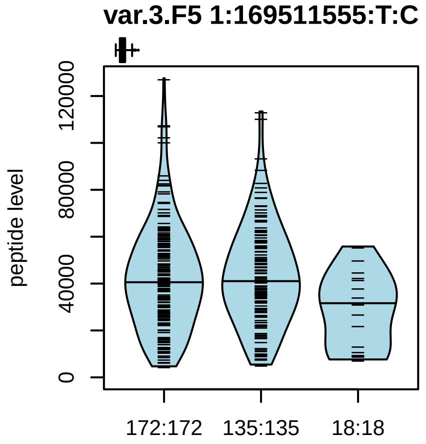

HTHHAPLSPRpc2_NP.1
p−maj=1, pX=0.22, N=325

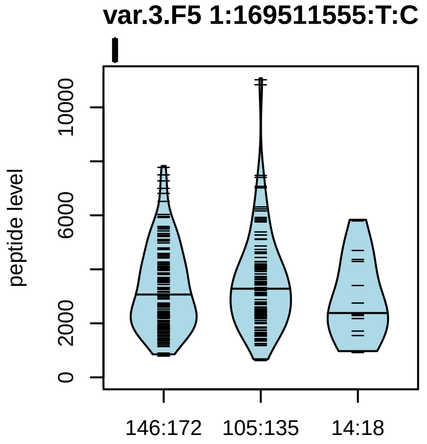

HTHHAPLSPRpc2_NP.2
p−min=0.12, pX=0.41, N=265

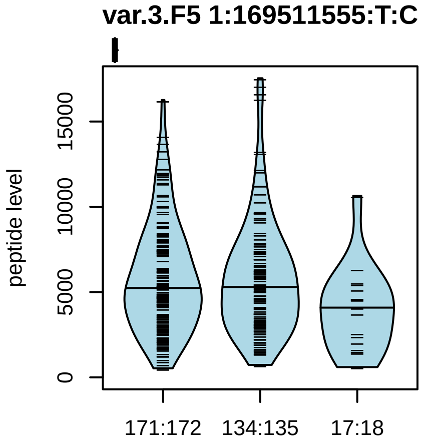

HTHHAPLSPRpc2_NP.3
p−maj=0.16, pX=0.11, N=322

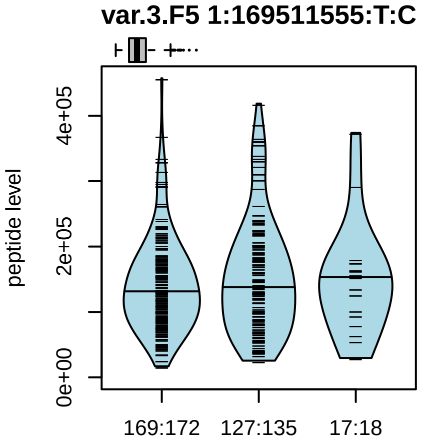

HTHHAPLSPRpc2_NP.4
p−min=0.074, pX=0.69, N=313

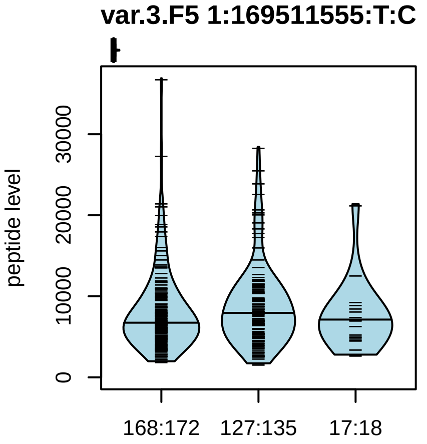

HTHHAPLSPRpc2_NP.5
p−min=0.15, pX=0.91, N=312

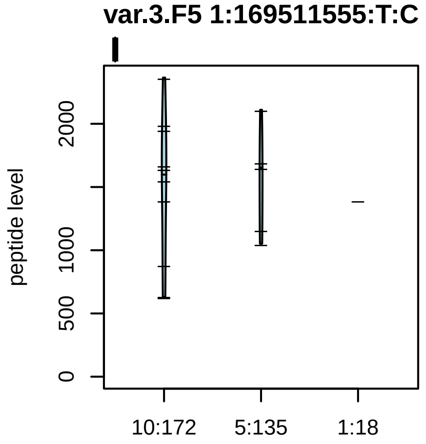

HTVNPNMKEDGILGPIIRpc3_NP.1
p−min=0.46, pX=0.58, N=16

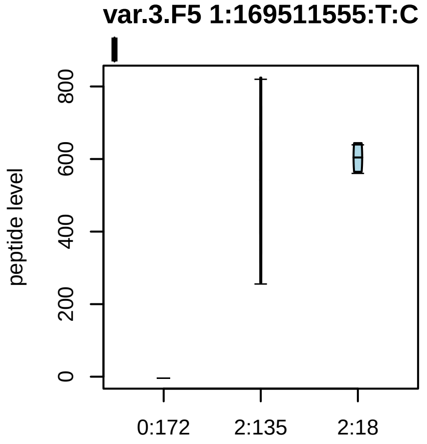

HTVNPNMKEDGILGPIIRpc3_NP.2
p−maj=0.016, pX=0.0015, N=4

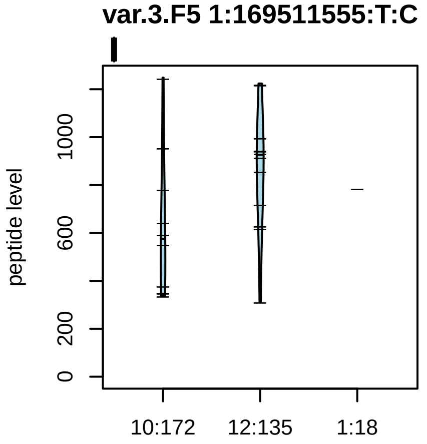

HTVNPNMKEDGILGPIIRpc3_NP.3
p−min=0.39, pX=0.21, N=23

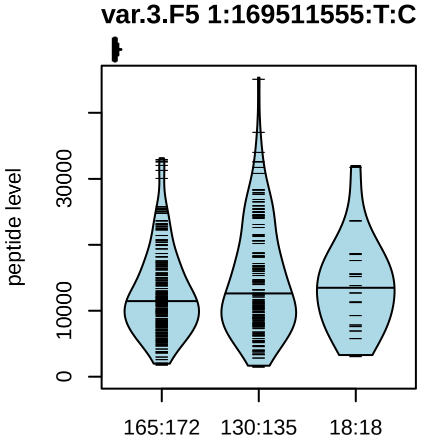

HTVNPNMKEDGILGPIIRpc3_NP.4
p−min=0.77, pX=0.032, N=313

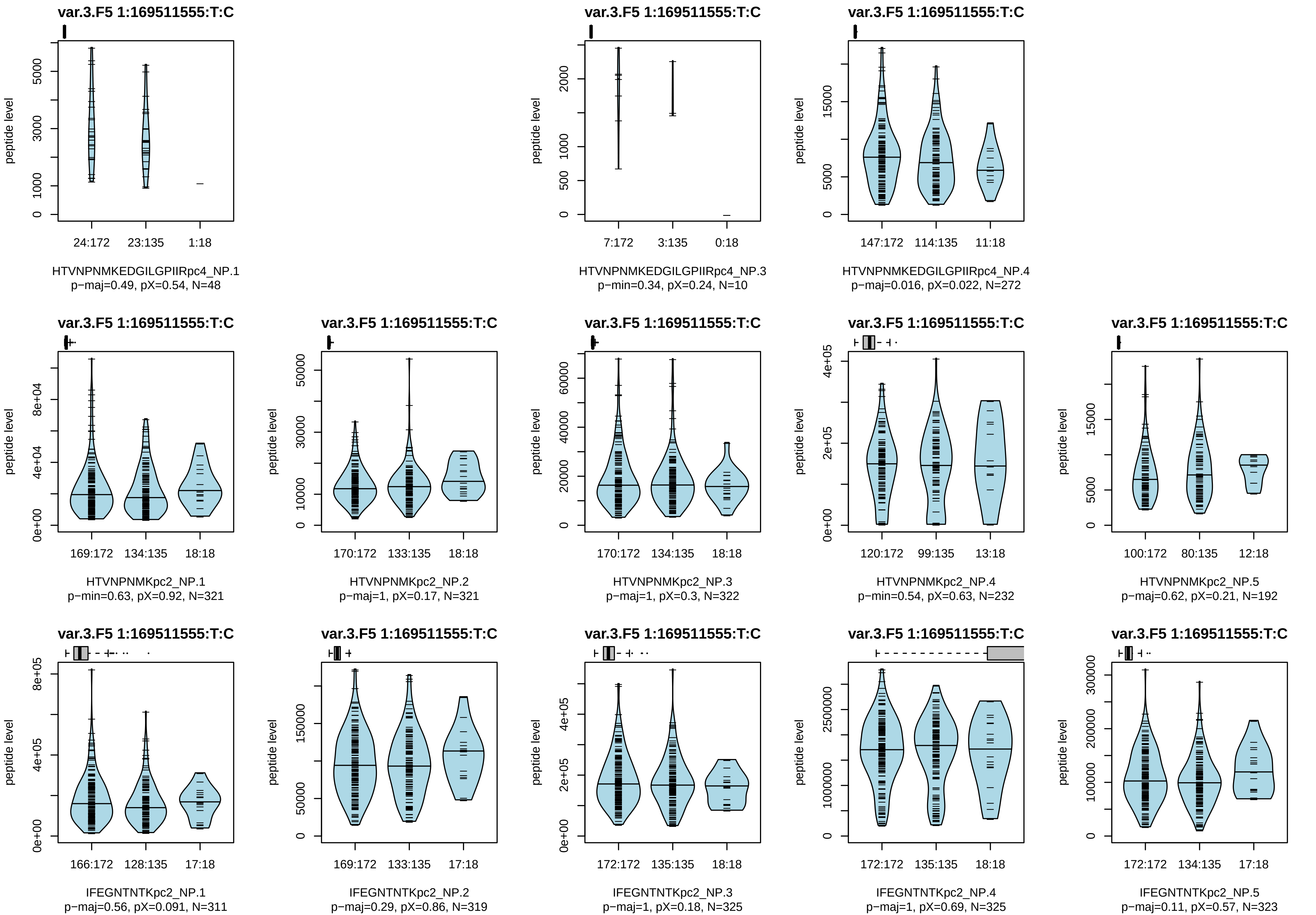
var.3.F5 1:169511555:T:C
peptide level
24:172
23:135
1:18
HTVNPNMKEDGILGPIIRpc4_NP.1
p−maj=0.49, pX=0.54, N=48
var.3.F5 1:169511555:T:C
peptide level
7:172
3:135
0:18
HTVNPNMKEDGILGPIIRpc4_NP.3
p−min=0.34, pX=0.24, N=10
var.3.F5 1:169511555:T:C
peptide level
147:172
114:135
11:18
HTVNPNMKEDGILGPIIRpc4_NP.4
p−maj=0.016, pX=0.022, N=272
var.3.F5 1:169511555:T:C
peptide level
169:172
134:135
18:18
HTVNPNMKpc2_NP.1
p−min=0.63, pX=0.92, N=321
var.3.F5 1:169511555:T:C
peptide level
170:172
133:135
18:18
HTVNPNMKpc2_NP.2
p−maj=1, pX=0.17, N=321
var.3.F5 1:169511555:T:C
peptide level
170:172
134:135
18:18
HTVNPNMKpc2_NP.3
p−maj=1, pX=0.3, N=322
var.3.F5 1:169511555:T:C
peptide level
120:172
99:135
13:18
HTVNPNMKpc2_NP.4
p−min=0.54, pX=0.63, N=232
var.3.F5 1:169511555:T:C
peptide level
100:172
80:135
12:18
HTVNPNMKpc2_NP.5
p−maj=0.62, pX=0.21, N=192
var.3.F5 1:169511555:T:C
peptide level
166:172
128:135
17:18
IFEGNTNTKpc2_NP.1
p−maj=0.56, pX=0.091, N=311
var.3.F5 1:169511555:T:C
peptide level
169:172
133:135
17:18
IFEGNTNTKpc2_NP.2
p−maj=0.29, pX=0.86, N=319
var.3.F5 1:169511555:T:C
peptide level
172:172
135:135
18:18
IFEGNTNTKpc2_NP.3
p−maj=1, pX=0.18, N=325
var.3.F5 1:169511555:T:C
peptide level
172:172
135:135
18:18
IFEGNTNTKpc2_NP.4
p−maj=1, pX=0.69, N=325
var.3.F5 1:169511555:T:C
peptide level
172:172
134:135
17:18
IFEGNTNTKpc2_NP.5
p−maj=0.11, pX=0.57, N=323

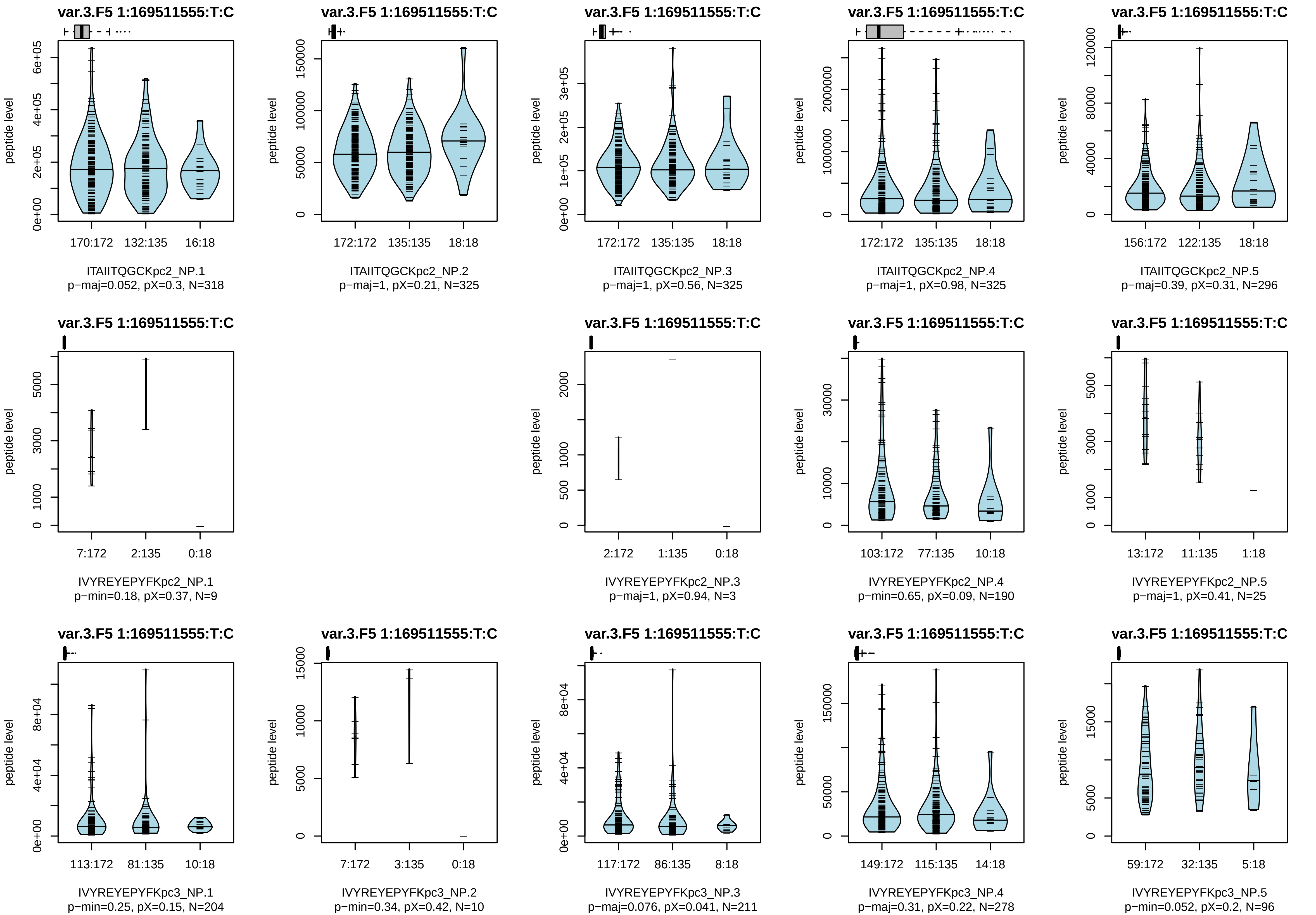
var.3.F5 1:169511555:T:C
peptide level
170:172
132:135
16:18
ITAIITQGCKpc2_NP.1
p−maj=0.052, pX=0.3, N=318
var.3.F5 1:169511555:T:C
peptide level
172:172
135:135
18:18
ITAIITQGCKpc2_NP.2
p−maj=1, pX=0.21, N=325
var.3.F5 1:169511555:T:C
peptide level
172:172
135:135
18:18
ITAIITQGCKpc2_NP.3
p−maj=1, pX=0.56, N=325
var.3.F5 1:169511555:T:C
peptide level
172:172
135:135
18:18
ITAIITQGCKpc2_NP.4
p−maj=1, pX=0.98, N=325
var.3.F5 1:169511555:T:C
peptide level
156:172
122:135
18:18
ITAIITQGCKpc2_NP.5
p−maj=0.39, pX=0.31, N=296
var.3.F5 1:169511555:T:C
peptide level
7:172
2:135
0:18
IVYREYEPYFKpc2_NP.1
p−min=0.18, pX=0.37, N=9
var.3.F5 1:169511555:T:C
peptide level
2:172
1:135
0:18
IVYREYEPYFKpc2_NP.3
p−maj=1, pX=0.94, N=3
var.3.F5 1:169511555:T:C
peptide level
103:172
77:135
10:18
IVYREYEPYFKpc2_NP.4
p−min=0.65, pX=0.09, N=190
var.3.F5 1:169511555:T:C
peptide level
13:172
11:135
1:18
IVYREYEPYFKpc2_NP.5
p−maj=1, pX=0.41, N=25
var.3.F5 1:169511555:T:C
peptide level
113:172
81:135
10:18
IVYREYEPYFKpc3_NP.1
p−min=0.25, pX=0.15, N=204
var.3.F5 1:169511555:T:C
peptide level
7:172
3:135
0:18
IVYREYEPYFKpc3_NP.2
p−min=0.34, pX=0.42, N=10
var.3.F5 1:169511555:T:C
peptide level
117:172
86:135
8:18
IVYREYEPYFKpc3_NP.3
p−maj=0.076, pX=0.041, N=211
var.3.F5 1:169511555:T:C
peptide level
149:172
115:135
14:18
IVYREYEPYFKpc3_NP.4
p−maj=0.31, pX=0.22, N=278
var.3.F5 1:169511555:T:C
peptide level
59:172
32:135
5:18
IVYREYEPYFKpc3_NP.5
p−min=0.052, pX=0.2, N=96

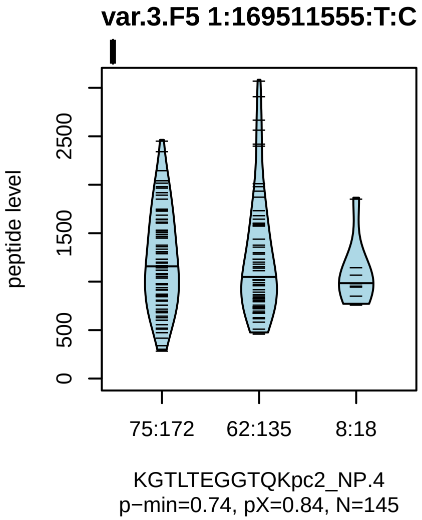
var.3.F5 1:169511555:T:C
peptide level
0
500
1500
2500
75:172
62:135
8:18
KGTLTEGGTQKpc2_NP.4
p−min=0.74, pX=0.84, N=145

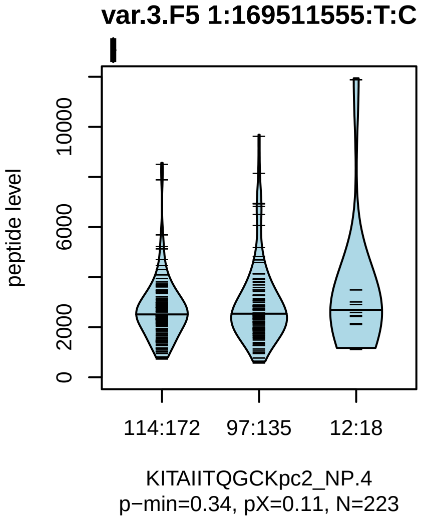
var.3.F5 1:169511555:T:C
peptide level
0
2000
6000
10000
114:172
97:135
12:18
KITAIITQGCKpc2_NP.4
p−min=0.34, pX=0.11, N=223

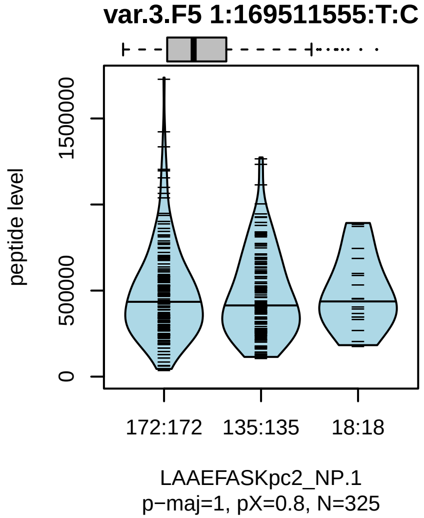
var.3.F5 1:169511555:T:C
peptide level
0
500000
1500000
172:172
135:135
18:18
LAAEFASKpc2_NP.1
p−maj=1, pX=0.8, N=325

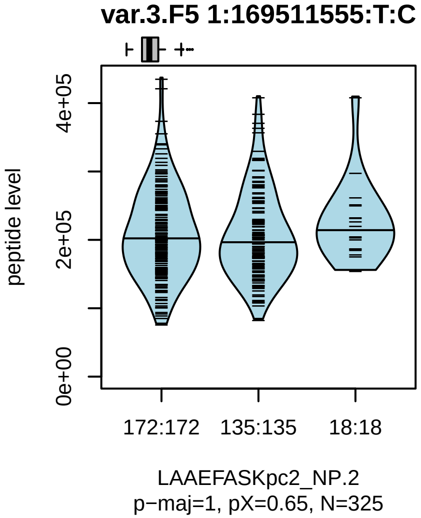
var.3.F5 1:169511555:T:C
peptide level
0e+00
2e+05
4e+05
172:172
135:135
18:18
LAAEFASKpc2_NP.2
p−maj=1, pX=0.65, N=325

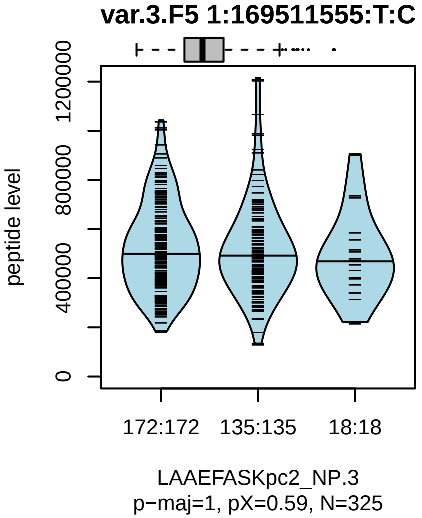
var.3.F5 1:169511555:T:C
peptide level
0
400000
800000
1200000
172:172
135:135
18:18
LAAEFASKpc2_NP.3
p−maj=1, pX=0.59, N=325

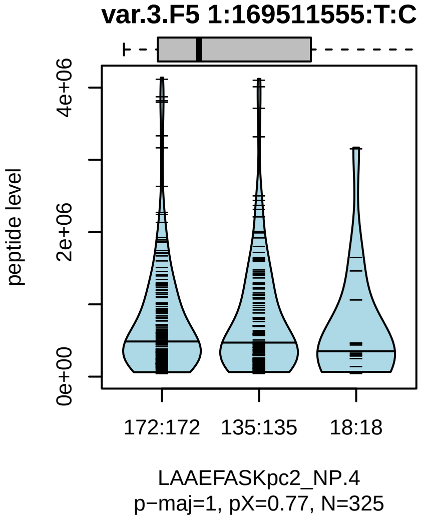
var.3.F5 1:169511555:T:C
peptide level
0e+00
2e+06
4e+06
172:172
135:135
18:18
LAAEFASKpc2_NP.4
p−maj=1, pX=0.77, N=325

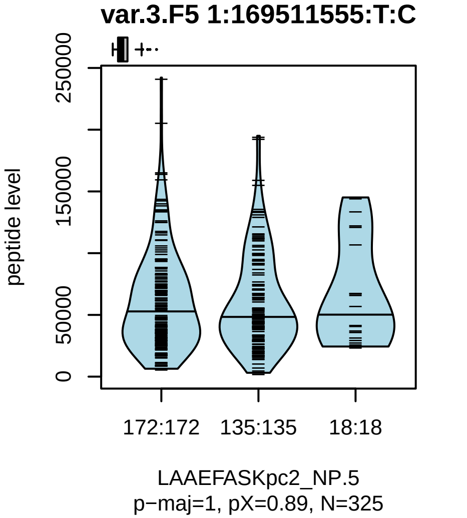
var.3.F5 1:169511555:T:C
peptide level
0
50000
150000
250000
172:172
135:135
18:18
LAAEFASKpc2_NP.5
p−maj=1, pX=0.89, N=325

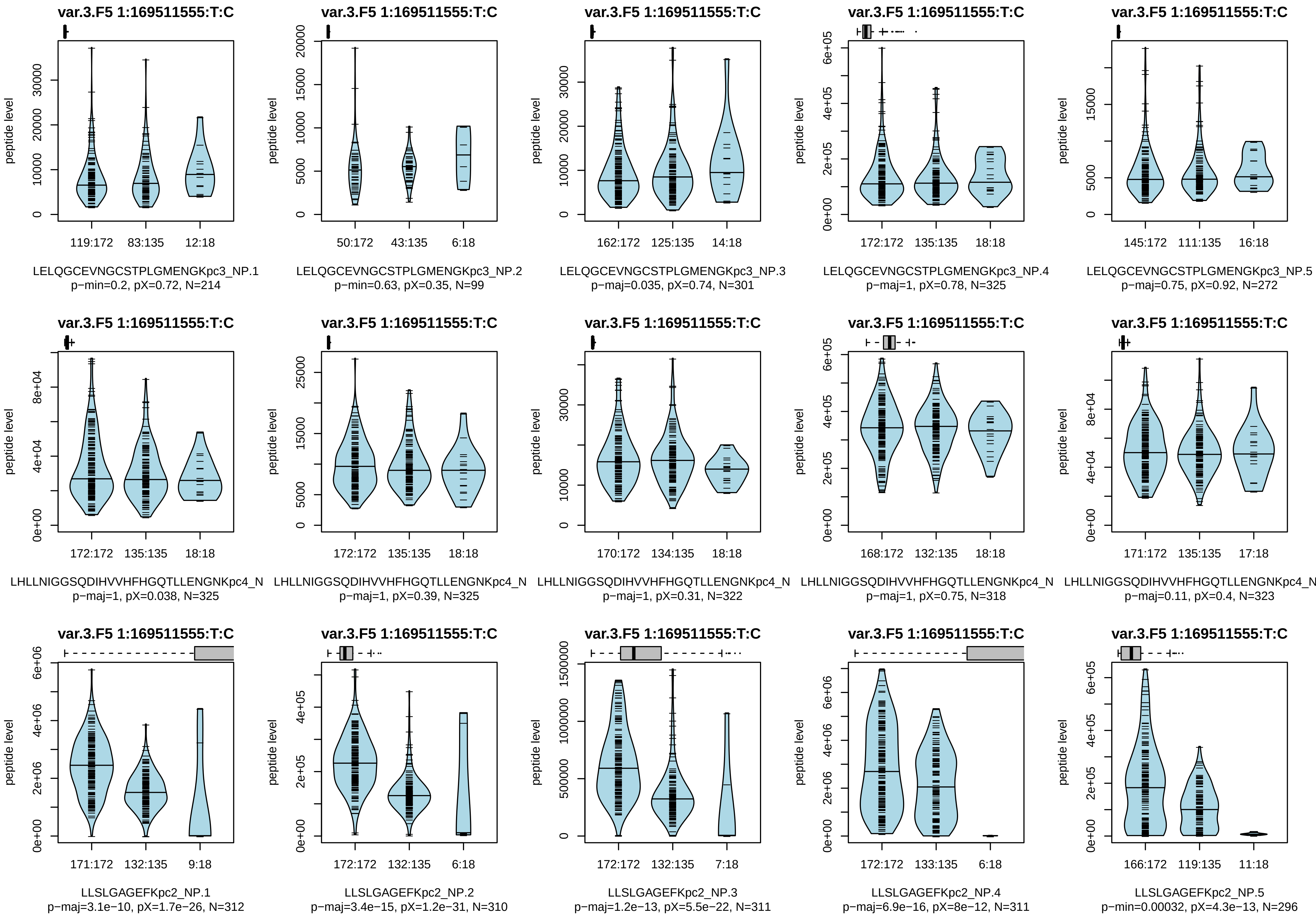
var.3.F5 1:169511555:T:C
peptide level
119:172
83:135
12:18
LELQGCEVNGCSTPLGMENGKpc3_NP.1
p−min=0.2, pX=0.72, N=214
var.3.F5 1:169511555:T:C
peptide level
50:172
43:135
6:18
LELQGCEVNGCSTPLGMENGKpc3_NP.2
p−min=0.63, pX=0.35, N=99
var.3.F5 1:169511555:T:C
peptide level
162:172
125:135
14:18
LELQGCEVNGCSTPLGMENGKpc3_NP.3
p−maj=0.035, pX=0.74, N=301
var.3.F5 1:169511555:T:C
peptide level
172:172
135:135
18:18
LELQGCEVNGCSTPLGMENGKpc3_NP.4
p−maj=1, pX=0.78, N=325
var.3.F5 1:169511555:T:C
peptide level
145:172
111:135
16:18
LELQGCEVNGCSTPLGMENGKpc3_NP.5
p−maj=0.75, pX=0.92, N=272
var.3.F5 1:169511555:T:C
peptide level
172:172
135:135
18:18
LHLLNIGGSQDIHVVHFHGQTLLENGNKpc4_N
p−maj=1, pX=0.038, N=325
var.3.F5 1:169511555:T:C
peptide level
172:172
135:135
18:18
LHLLNIGGSQDIHVVHFHGQTLLENGNKpc4_N
p−maj=1, pX=0.39, N=325
var.3.F5 1:169511555:T:C
peptide level
170:172
134:135
18:18
LHLLNIGGSQDIHVVHFHGQTLLENGNKpc4_N
p−maj=1, pX=0.31, N=322
var.3.F5 1:169511555:T:C
peptide level
168:172
132:135
18:18
LHLLNIGGSQDIHVVHFHGQTLLENGNKpc4_N
p−maj=1, pX=0.75, N=318
var.3.F5 1:169511555:T:C
peptide level
171:172
135:135
17:18
LHLLNIGGSQDIHVVHFHGQTLLENGNKpc4_N
p−maj=0.11, pX=0.4, N=323
var.3.F5 1:169511555:T:C
peptide level
171:172
132:135
9:18
LLSLGAGEFKpc2_NP.1
p−maj=3.1e−10, pX=1.7e−26, N=312
var.3.F5 1:169511555:T:C
peptide level
172:172
132:135
6:18
LLSLGAGEFKpc2_NP.2
p−maj=3.4e−15, pX=1.2e−31, N=310
var.3.F5 1:169511555:T:C
peptide level
172:172
132:135
7:18
LLSLGAGEFKpc2_NP.3
p−maj=1.2e−13, pX=5.5e−22, N=311
var.3.F5 1:169511555:T:C
peptide level
172:172
133:135
6:18
LLSLGAGEFKpc2_NP.4
p−maj=6.9e−16, pX=8e−12, N=311
var.3.F5 1:169511555:T:C
peptide level
166:172
119:135
11:18
LLSLGAGEFKpc2_NP.5
p−min=0.00032, pX=4.3e−13, N=296

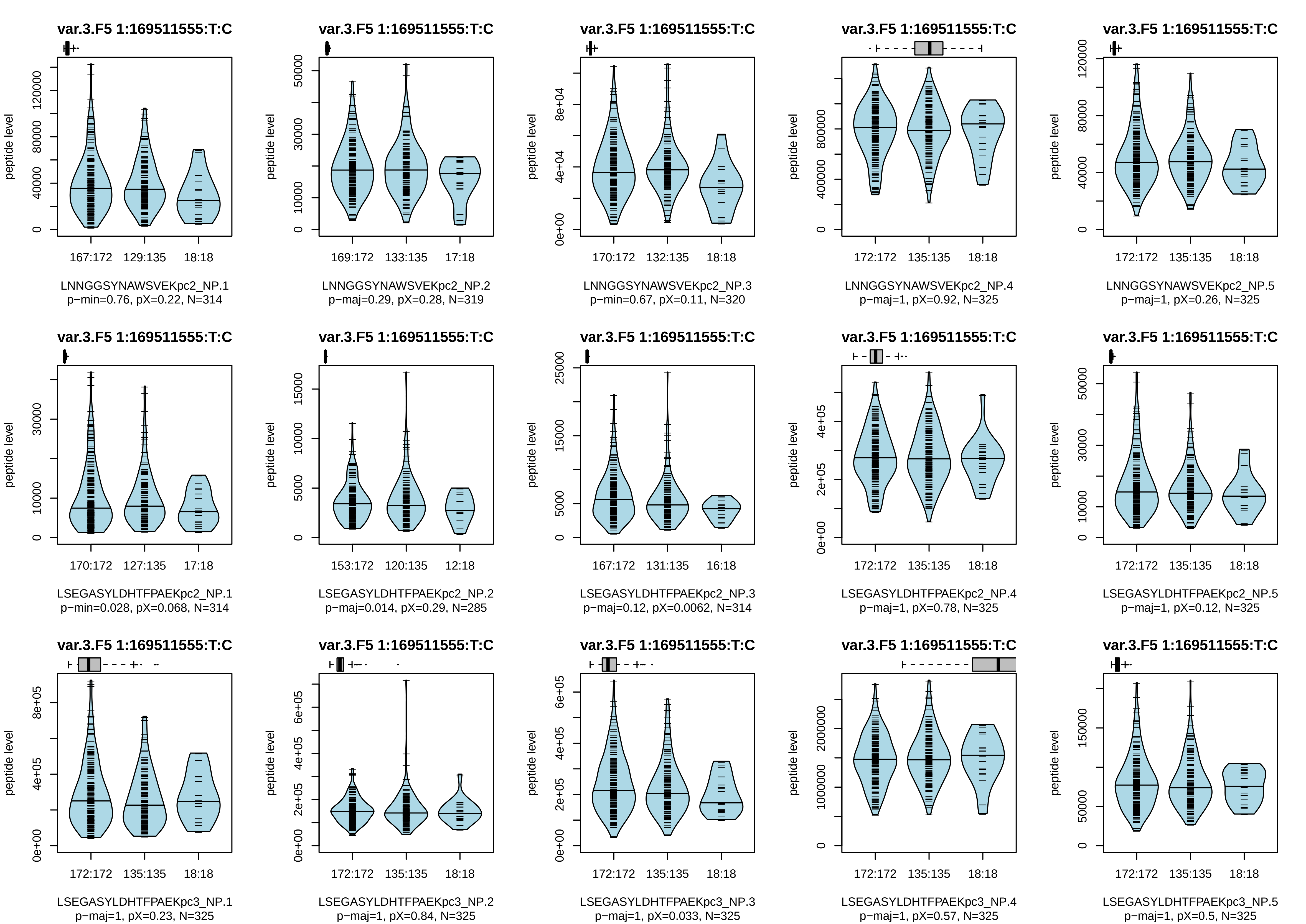

var.3.F5 1:169511555:T:C
peptide level
167:172 129:135 18:18
LNNGGSYNAWSVEKpc2_NP.1
p−min=0.76, pX=0.22, N=314
var.3.F5 1:169511555:T:C
169:172 133:135 17:18
LNNGGSYNAWSVEKpc2_NP.2
p−maj=0.29, pX=0.28, N=319
var.3.F5 1:169511555:T:C
170:172 132:135 18:18
LNNGGSYNAWSVEKpc2_NP.3
p−min=0.67, pX=0.11, N=320
var.3.F5 1:169511555:T:C
172:172 135:135 18:18
LNNGGSYNAWSVEKpc2_NP.4
p−maj=1, pX=0.92, N=325
var.3.F5 1:169511555:T:C
172:172 135:135 18:18
LNNGGSYNAWSVEKpc2_NP.5
p−maj=1, pX=0.26, N=325
var.3.F5 1:169511555:T:C
170:172 127:135 17:18
LSEGASYLDHTFPAEKpc2_NP.1
p−min=0.028, pX=0.068, N=314
var.3.F5 1:169511555:T:C
153:172 120:135 12:18
LSEGASYLDHTFPAEKpc2_NP.2
p−maj=0.014, pX=0.29, N=285
var.3.F5 1:169511555:T:C
167:172 131:135 16:18
LSEGASYLDHTFPAEKpc2_NP.3
p−maj=0.12, pX=0.0062, N=314
var.3.F5 1:169511555:T:C
172:172 135:135 18:18
LSEGASYLDHTFPAEKpc2_NP.4
p−maj=1, pX=0.78, N=325
var.3.F5 1:169511555:T:C
172:172 135:135 18:18
LSEGASYLDHTFPAEKpc2_NP.5
p−maj=1, pX=0.12, N=325
var.3.F5 1:169511555:T:C
172:172 135:135 18:18
LSEGASYLDHTFPAEKpc3_NP.1
p−maj=1, pX=0.23, N=325
var.3.F5 1:169511555:T:C
172:172 135:135 18:18
LSEGASYLDHTFPAEKpc3_NP.2
p−maj=1, pX=0.84, N=325
var.3.F5 1:169511555:T:C
172:172 135:135 18:18
LSEGASYLDHTFPAEKpc3_NP.3
p−maj=1, pX=0.033, N=325
var.3.F5 1:169511555:T:C
172:172 135:135 18:18
LSEGASYLDHTFPAEKpc3_NP.4
p−maj=1, pX=0.57, N=325
var.3.F5 1:169511555:T:C
172:172 135:135 18:18
LSEGASYLDHTFPAEKpc3_NP.5
p−maj=1, pX=0.5, N=325

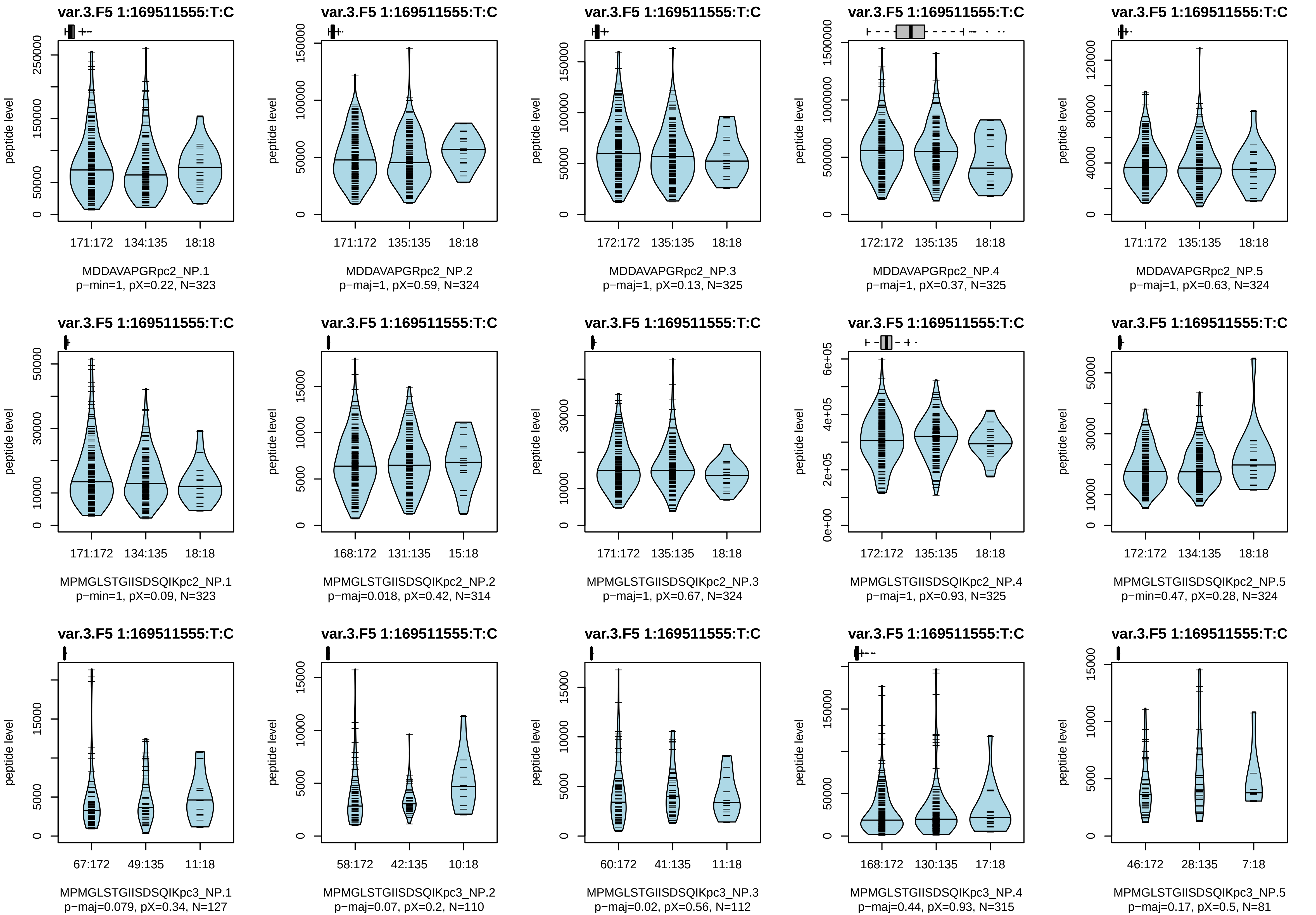

var.3.F5 1:169511555:T:C
peptide level
171:172
134:135
18:18
MDDAVAPGRpc2_NP.1
p−min=1, pX=0.22, N=323
var.3.F5 1:169511555:T:C
171:172
135:135
18:18
MDDAVAPGRpc2_NP.2
p−maj=1, pX=0.59, N=324
var.3.F5 1:169511555:T:C
172:172
135:135
18:18
MDDAVAPGRpc2_NP.3
p−maj=1, pX=0.13, N=325
var.3.F5 1:169511555:T:C
172:172
135:135
18:18
MDDAVAPGRpc2_NP.4
p−maj=1, pX=0.37, N=325
var.3.F5 1:169511555:T:C
171:172
135:135
18:18
MDDAVAPGRpc2_NP.5
p−maj=1, pX=0.63, N=324
var.3.F5 1:169511555:T:C
171:172
134:135
18:18
MPMGLSTGIISDSQIKpc2_NP.1
p−min=1, pX=0.09, N=323
var.3.F5 1:169511555:T:C
168:172
131:135
15:18
MPMGLSTGIISDSQIKpc2_NP.2
p−maj=0.018, pX=0.42, N=314
var.3.F5 1:169511555:T:C
171:172
135:135
18:18
MPMGLSTGIISDSQIKpc2_NP.3
p−maj=1, pX=0.67, N=324
var.3.F5 1:169511555:T:C
172:172
135:135
18:18
MPMGLSTGIISDSQIKpc2_NP.4
p−maj=1, pX=0.93, N=325
var.3.F5 1:169511555:T:C
172:172
134:135
18:18
MPMGLSTGIISDSQIKpc2_NP.5
p−min=0.47, pX=0.28, N=324
var.3.F5 1:169511555:T:C
67:172
49:135
11:18
MPMGLSTGIISDSQIKpc3_NP.1
p−maj=0.079, pX=0.34, N=127
var.3.F5 1:169511555:T:C
58:172
42:135
10:18
MPMGLSTGIISDSQIKpc3_NP.2
p−maj=0.07, pX=0.2, N=110
var.3.F5 1:169511555:T:C
60:172
41:135
11:18
MPMGLSTGIISDSQIKpc3_NP.3
p−maj=0.02, pX=0.56, N=112
var.3.F5 1:169511555:T:C
168:172
130:135
17:18
MPMGLSTGIISDSQIKpc3_NP.4
p−maj=0.44, pX=0.93, N=315
var.3.F5 1:169511555:T:C
46:172
28:135
7:18
MPMGLSTGIISDSQIKpc3_NP.5
p−maj=0.17, pX=0.5, N=81

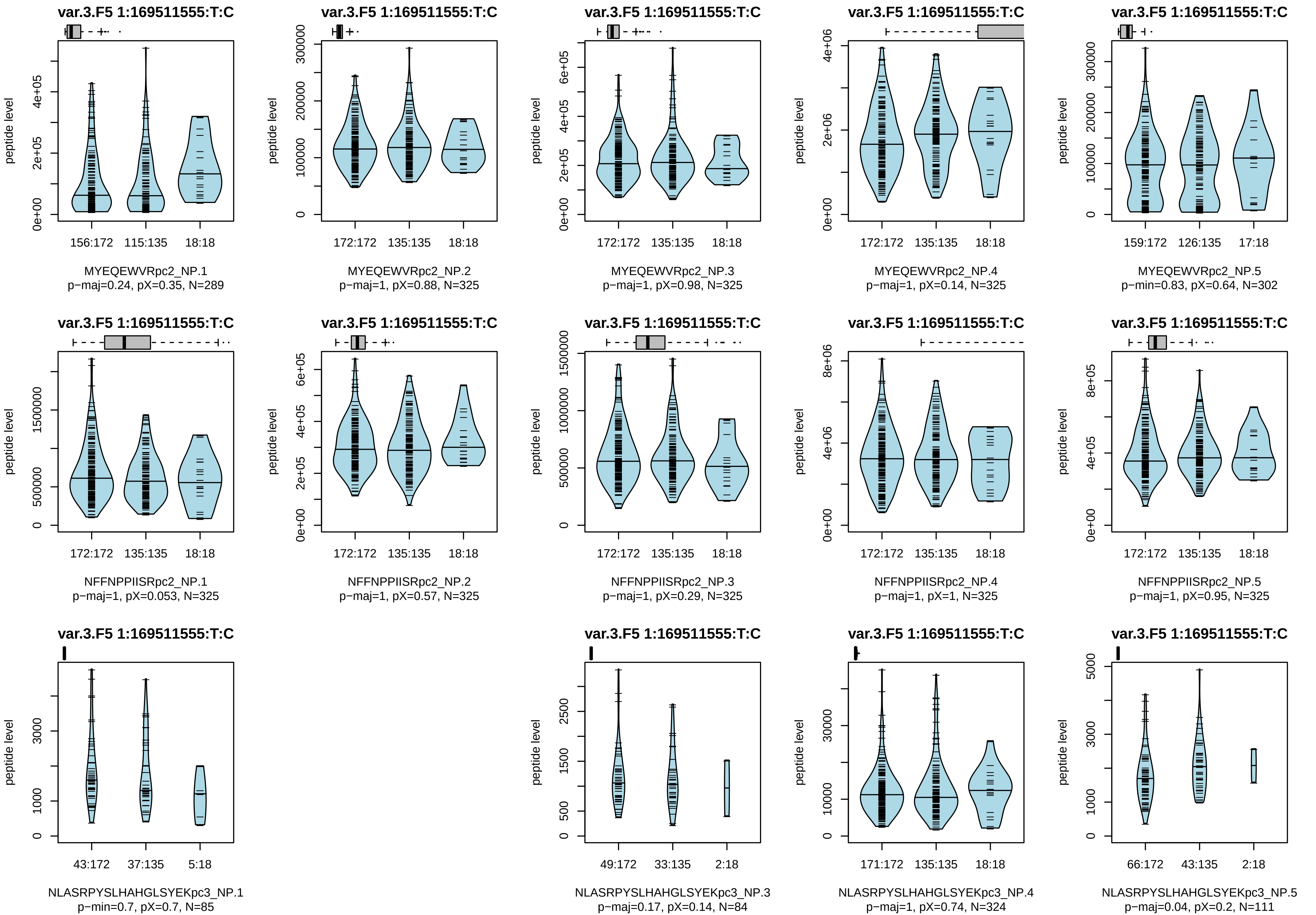

var.3.F5 1:169511555:T:C
peptide level
156:172
115:135
18:18
MYEQEWVRpc2_NP.1
p−maj=0.24, pX=0.35, N=289
var.3.F5 1:169511555:T:C
peptide level
172:172
135:135
18:18
MYEQEWVRpc2_NP.2
p−maj=1, pX=0.88, N=325
var.3.F5 1:169511555:T:C
peptide level
172:172
135:135
18:18
MYEQEWVRpc2_NP.3
p−maj=1, pX=0.98, N=325
var.3.F5 1:169511555:T:C
peptide level
172:172
135:135
18:18
MYEQEWVRpc2_NP.4
p−maj=1, pX=0.14, N=325
var.3.F5 1:169511555:T:C
peptide level
159:172
126:135
17:18
MYEQEWVRpc2_NP.5
p−min=0.83, pX=0.64, N=302
var.3.F5 1:169511555:T:C
peptide level
172:172
135:135
18:18
NFFNPPIISRpc2_NP.1
p−maj=1, pX=0.053, N=325
var.3.F5 1:169511555:T:C
peptide level
172:172
135:135
18:18
NFFNPPIISRpc2_NP.2
p−maj=1, pX=0.57, N=325
var.3.F5 1:169511555:T:C
peptide level
172:172
135:135
18:18
NFFNPPIISRpc2_NP.3
p−maj=1, pX=0.29, N=325
var.3.F5 1:169511555:T:C
peptide level
172:172
135:135
18:18
NFFNPPIISRpc2_NP.4
p−maj=1, pX=1, N=325
var.3.F5 1:169511555:T:C
peptide level
172:172
135:135
18:18
NFFNPPIISRpc2_NP.5
p−maj=1, pX=0.95, N=325
var.3.F5 1:169511555:T:C
peptide level
43:172
37:135
5:18
NLASRPYSLHAHGLSYEKpc3_NP.1
p−min=0.7, pX=0.7, N=85
var.3.F5 1:169511555:T:C
peptide level
49:172
33:135
2:18
NLASRPYSLHAHGLSYEKpc3_NP.3
p−maj=0.17, pX=0.14, N=84
var.3.F5 1:169511555:T:C
peptide level
171:172
135:135
18:18
NLASRPYSLHAHGLSYEKpc3_NP.4
p−maj=1, pX=0.74, N=324
var.3.F5 1:169511555:T:C
peptide level
66:172
43:135
2:18
NLASRPYSLHAHGLSYEKpc3_NP.5
p−maj=0.04, pX=0.2, N=111

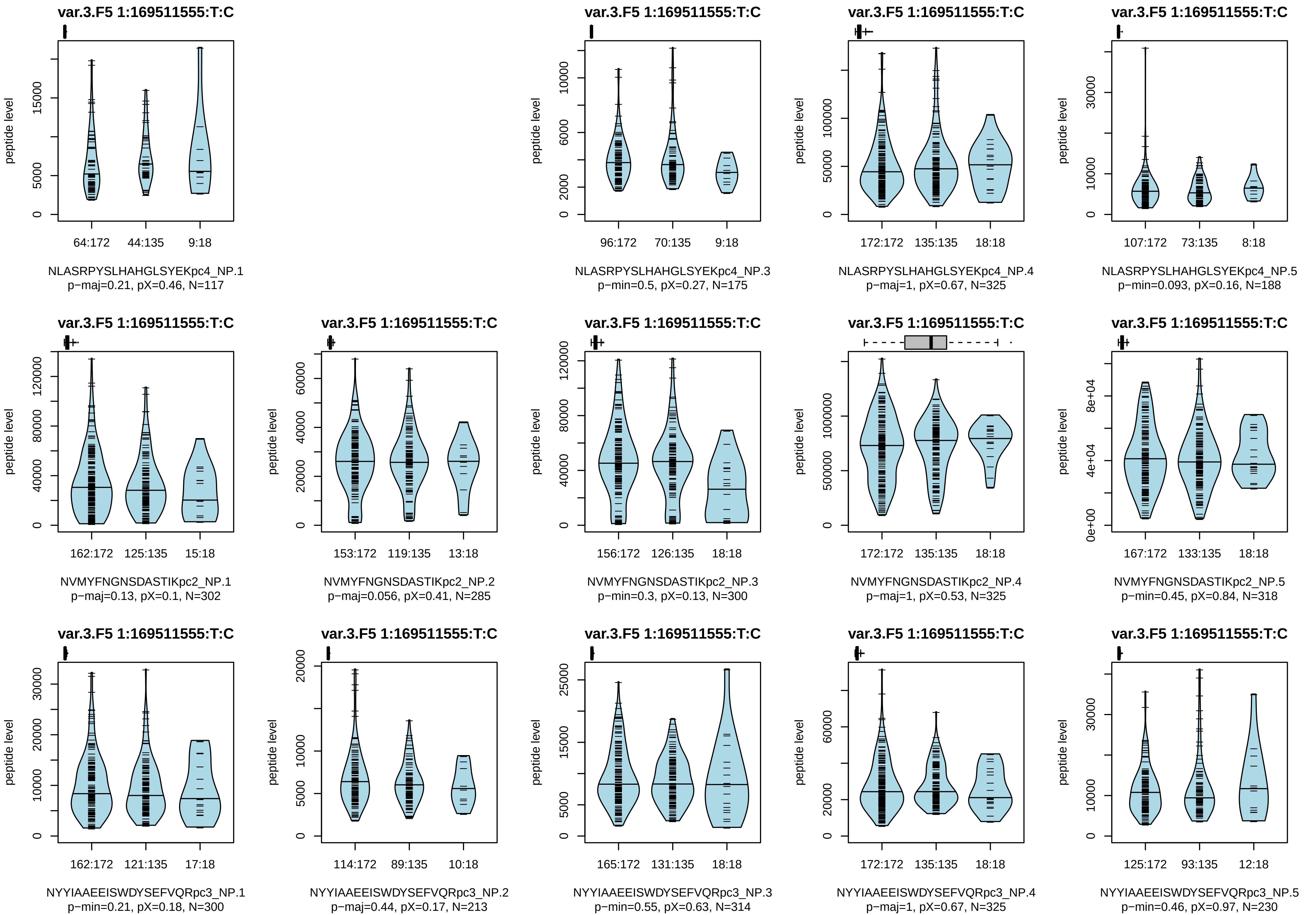

var.3.F5 1:169511555:T:C
peptide level
64:172
44:135
9:18
NLASRPYSLHAHGLSYEKpc4_NP.1
p−maj=0.21, pX=0.46, N=117
96:172
70:135
9:18
NLASRPYSLHAHGLSYEKpc4_NP.3
p−min=0.5, pX=0.27, N=175
172:172
135:135
18:18
NLASRPYSLHAHGLSYEKpc4_NP.4
p−maj=1, pX=0.67, N=325
107:172
73:135
8:18
NLASRPYSLHAHGLSYEKpc4_NP.5
p−min=0.093, pX=0.16, N=188
162:172
125:135
15:18
NVMYFNGNSDASTIKpc2_NP.1
p−maj=0.13, pX=0.1, N=302
153:172
119:135
13:18
NVMYFNGNSDASTIKpc2_NP.2
p−maj=0.056, pX=0.41, N=285
156:172
126:135
18:18
NVMYFNGNSDASTIKpc2_NP.3
p−min=0.3, pX=0.13, N=300
172:172
135:135
18:18
NVMYFNGNSDASTIKpc2_NP.4
p−maj=1, pX=0.53, N=325
167:172
133:135
18:18
NVMYFNGNSDASTIKpc2_NP.5
p−min=0.45, pX=0.84, N=318
162:172
121:135
17:18
NYYIAAEEISWDYSEFVQRpc3_NP.1
p−min=0.21, pX=0.18, N=300
114:172
89:135
10:18
NYYIAAEEISWDYSEFVQRpc3_NP.2
p−maj=0.44, pX=0.17, N=213
165:172
131:135
18:18
NYYIAAEEISWDYSEFVQRpc3_NP.3
p−min=0.55, pX=0.63, N=314
172:172
135:135
18:18
NYYIAAEEISWDYSEFVQRpc3_NP.4
p−maj=1, pX=0.67, N=325
125:172
93:135
12:18
NYYIAAEEISWDYSEFVQRpc3_NP.5
p−min=0.46, pX=0.97, N=230

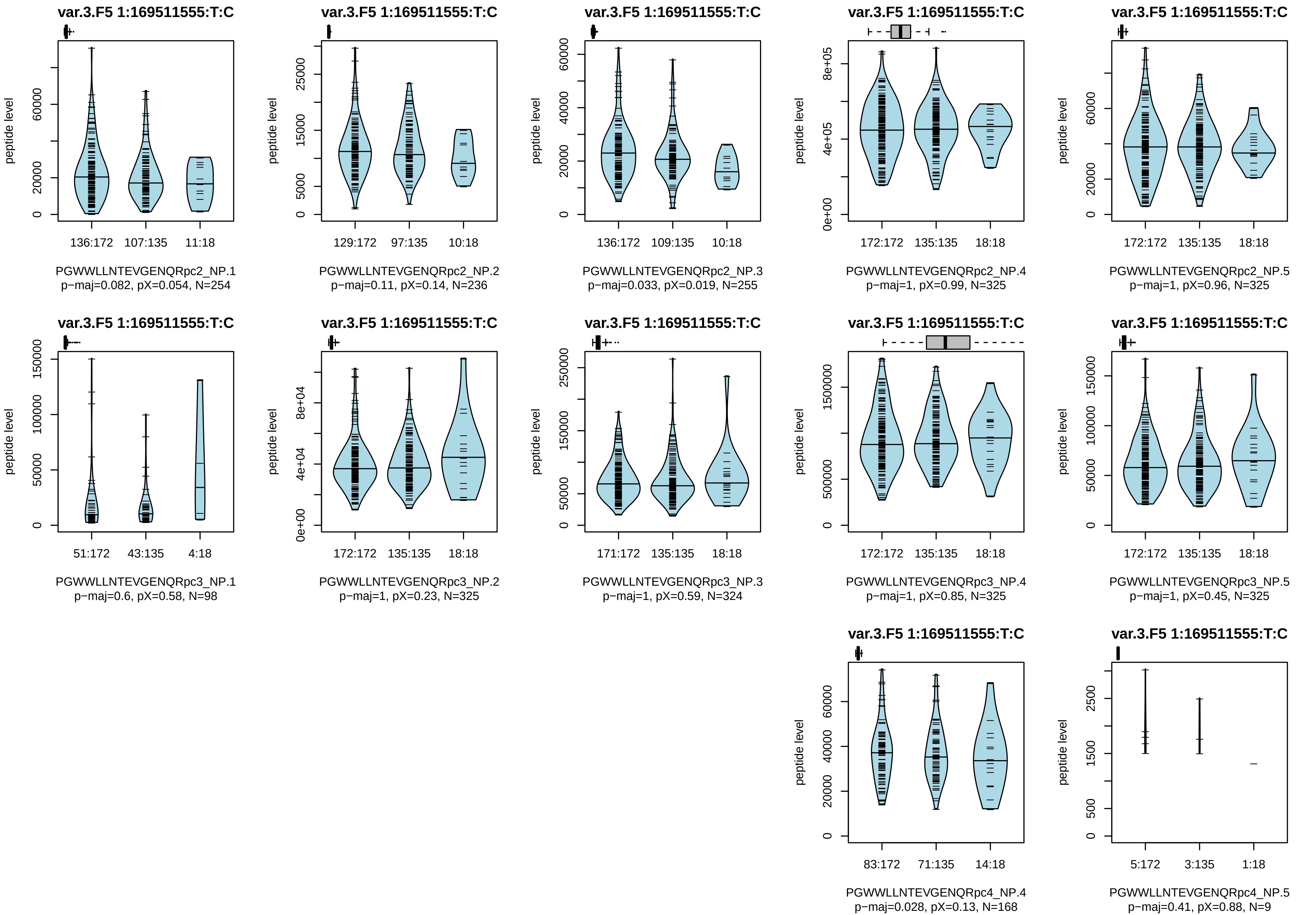
var.3.F5 1:169511555:T:C
peptide level
136:172
107:135
11:18
PGWWLLNTEVGENQRpc2_NP.1
p−maj=0.082, pX=0.054, N=254
var.3.F5 1:169511555:T:C
peptide level
129:172
97:135
10:18
PGWWLLNTEVGENQRpc2_NP.2
p−maj=0.11, pX=0.14, N=236
var.3.F5 1:169511555:T:C
peptide level
136:172
109:135
10:18
PGWWLLNTEVGENQRpc2_NP.3
p−maj=0.033, pX=0.019, N=255
var.3.F5 1:169511555:T:C
peptide level
172:172
135:135
18:18
PGWWLLNTEVGENQRpc2_NP.4
p−maj=1, pX=0.99, N=325
var.3.F5 1:169511555:T:C
peptide level
172:172
135:135
18:18
PGWWLLNTEVGENQRpc2_NP.5
p−maj=1, pX=0.96, N=325
var.3.F5 1:169511555:T:C
peptide level
51:172
43:135
4:18
PGWWLLNTEVGENQRpc3_NP.1
p−maj=0.6, pX=0.58, N=98
var.3.F5 1:169511555:T:C
peptide level
172:172
135:135
18:18
PGWWLLNTEVGENQRpc3_NP.2
p−maj=1, pX=0.23, N=325
var.3.F5 1:169511555:T:C
peptide level
171:172
135:135
18:18
PGWWLLNTEVGENQRpc3_NP.3
p−maj=1, pX=0.59, N=324
var.3.F5 1:169511555:T:C
peptide level
172:172
135:135
18:18
PGWWLLNTEVGENQRpc3_NP.4
p−maj=1, pX=0.85, N=325
var.3.F5 1:169511555:T:C
peptide level
172:172
135:135
18:18
PGWWLLNTEVGENQRpc3_NP.5
p−maj=1, pX=0.45, N=325
var.3.F5 1:169511555:T:C
peptide level
83:172
71:135
14:18
PGWWLLNTEVGENQRpc4_NP.4
p−maj=0.028, pX=0.13, N=168
var.3.F5 1:169511555:T:C
peptide level
5:172
3:135
1:18
PGWWLLNTEVGENQRpc4_NP.5
p−maj=0.41, pX=0.88, N=9

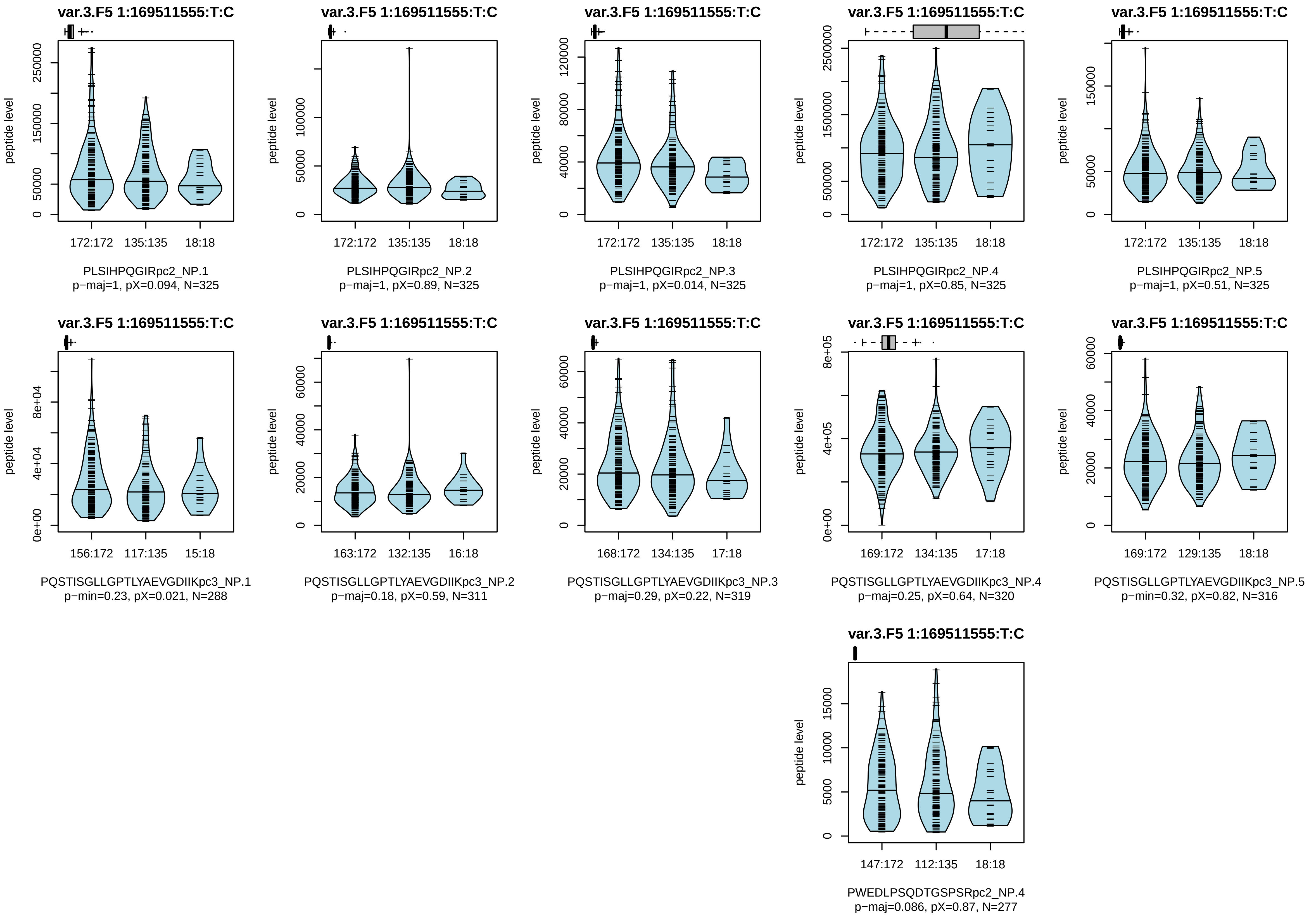
var.3.F5 1:169511555:T:C
peptide level
172:172
135:135
18:18
PLSIHPQGIRpc2_NP.1
p−maj=1, pX=0.094, N=325
PLSIHPQGIRpc2_NP.2
p−maj=1, pX=0.89, N=325
PLSIHPQGIRpc2_NP.3
p−maj=1, pX=0.014, N=325
PLSIHPQGIRpc2_NP.4
p−maj=1, pX=0.85, N=325
PLSIHPQGIRpc2_NP.5
p−maj=1, pX=0.51, N=325
156:172 117:135 15:18
PQSTISGLLGPTLYAEVGDIIKpc3_NP.1
p−min=0.23, pX=0.021, N=288
163:172 132:135 16:18
PQSTISGLLGPTLYAEVGDIIKpc3_NP.2
p−maj=0.18, pX=0.59, N=311
168:172 134:135 17:18
PQSTISGLLGPTLYAEVGDIIKpc3_NP.3
p−maj=0.29, pX=0.22, N=319
169:172 134:135 17:18
PQSTISGLLGPTLYAEVGDIIKpc3_NP.4
p−maj=0.25, pX=0.64, N=320
169:172 129:135 18:18
PQSTISGLLGPTLYAEVGDIIKpc3_NP.5
p−min=0.32, pX=0.82, N=316
147:172 112:135 18:18
PWEDLPSQDTGSPSRpc2_NP.4
p−maj=0.086, pX=0.87, N=277

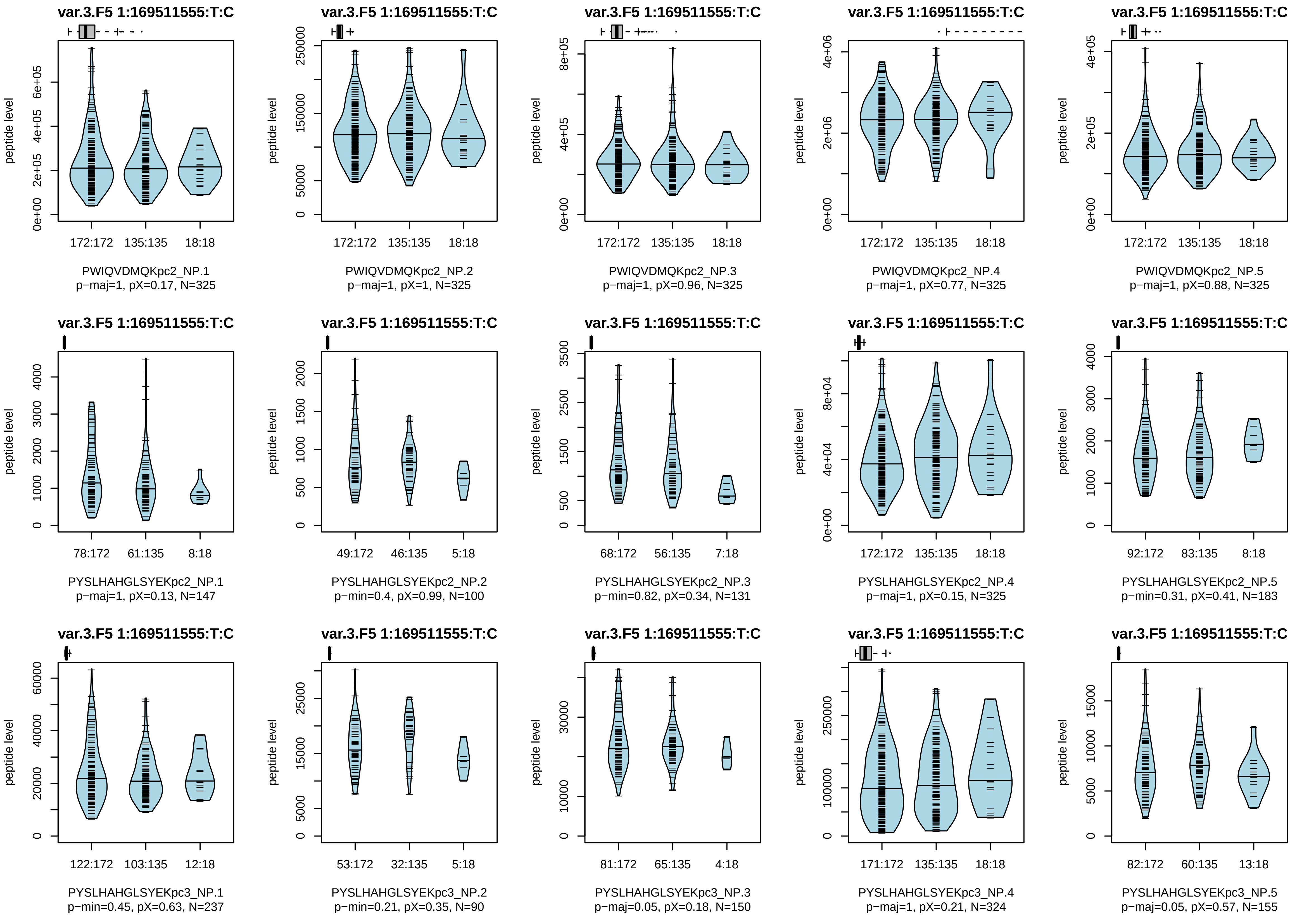
var.3.F5 1:169511555:T:C
peptide level
172:172
135:135
18:18
PWIQVDMQKpc2_NP.1
p−maj=1, pX=0.17, N=325
var.3.F5 1:169511555:T:C
peptide level
172:172
135:135
18:18
PWIQVDMQKpc2_NP.2
p−maj=1, pX=1, N=325
var.3.F5 1:169511555:T:C
peptide level
172:172
135:135
18:18
PWIQVDMQKpc2_NP.3
p−maj=1, pX=0.96, N=325
var.3.F5 1:169511555:T:C
peptide level
172:172
135:135
18:18
PWIQVDMQKpc2_NP.4
p−maj=1, pX=0.77, N=325
var.3.F5 1:169511555:T:C
peptide level
172:172
135:135
18:18
PWIQVDMQKpc2_NP.5
p−maj=1, pX=0.88, N=325
var.3.F5 1:169511555:T:C
peptide level
78:172
61:135
8:18
PYSLHAHGLSYEKpc2_NP.1
p−maj=1, pX=0.13, N=147
var.3.F5 1:169511555:T:C
peptide level
49:172
46:135
5:18
PYSLHAHGLSYEKpc2_NP.2
p−min=0.4, pX=0.99, N=100
var.3.F5 1:169511555:T:C
peptide level
68:172
56:135
7:18
PYSLHAHGLSYEKpc2_NP.3
p−min=0.82, pX=0.34, N=131
var.3.F5 1:169511555:T:C
peptide level
172:172
135:135
18:18
PYSLHAHGLSYEKpc2_NP.4
p−maj=1, pX=0.15, N=325
var.3.F5 1:169511555:T:C
peptide level
92:172
83:135
8:18
PYSLHAHGLSYEKpc2_NP.5
p−min=0.31, pX=0.41, N=183
var.3.F5 1:169511555:T:C
peptide level
122:172
103:135
12:18
PYSLHAHGLSYEKpc3_NP.1
p−min=0.45, pX=0.63, N=237
var.3.F5 1:169511555:T:C
peptide level
53:172
32:135
5:18
PYSLHAHGLSYEKpc3_NP.2
p−min=0.21, pX=0.35, N=90
var.3.F5 1:169511555:T:C
peptide level
81:172
65:135
4:18
PYSLHAHGLSYEKpc3_NP.3
p−maj=0.05, pX=0.18, N=150
var.3.F5 1:169511555:T:C
peptide level
171:172
135:135
18:18
PYSLHAHGLSYEKpc3_NP.4
p−maj=1, pX=0.21, N=324
var.3.F5 1:169511555:T:C
peptide level
82:172
60:135
13:18
PYSLHAHGLSYEKpc3_NP.5
p−maj=0.05, pX=0.57, N=155

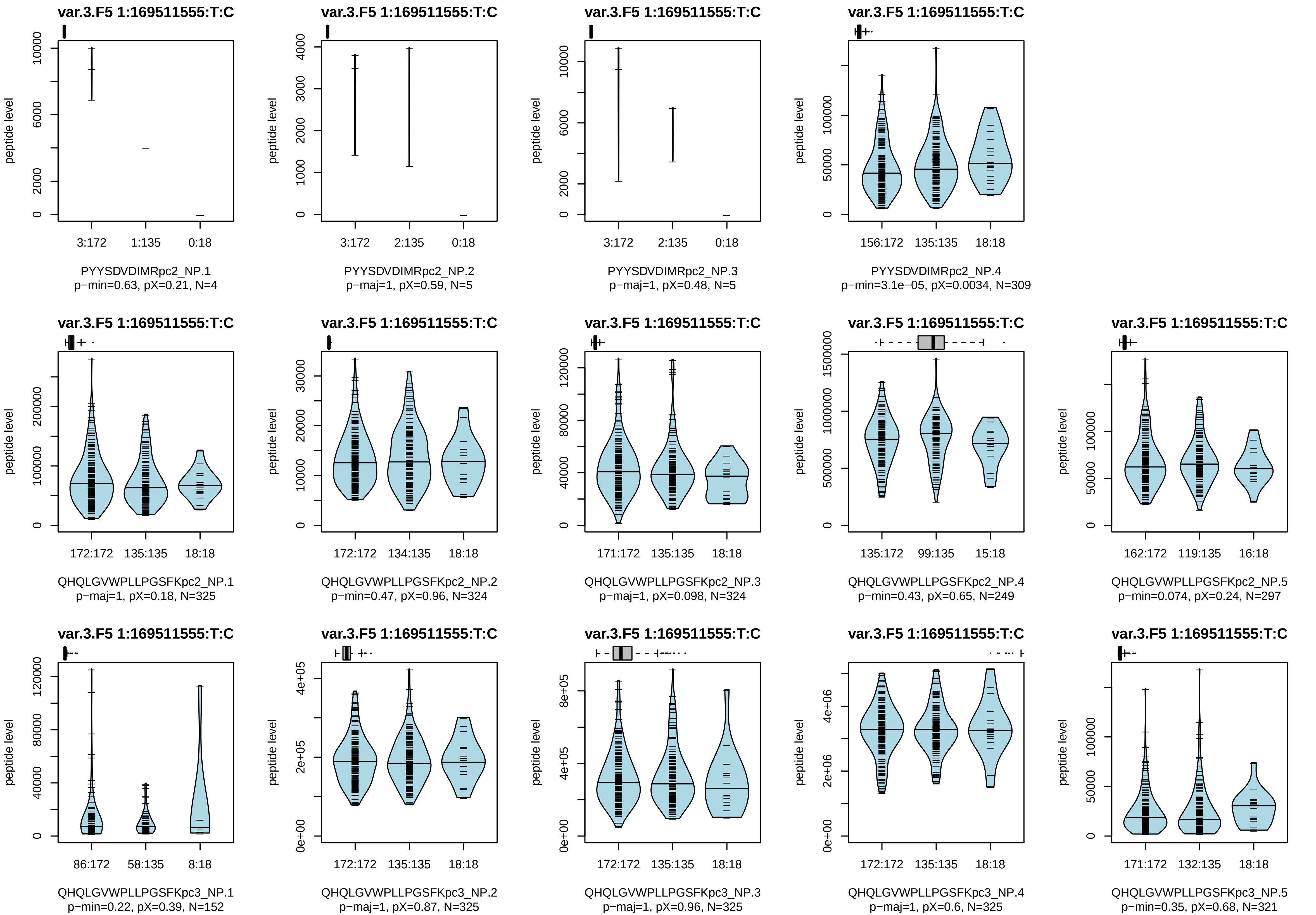
var.3.F5 1:169511555:T:C
peptide level
0
2000
6000
10000
3:172
1:135
0:18
PYYSDVDIMRpc2_NP.1
p−min=0.63, pX=0.21, N=4
var.3.F5 1:169511555:T:C
peptide level
0
1000
2000
3000
4000
3:172
2:135
0:18
PYYSDVDIMRpc2_NP.2
p−maj=1, pX=0.59, N=5
var.3.F5 1:169511555:T:C
peptide level
0
2000
6000
10000
3:172
2:135
0:18
PYYSDVDIMRpc2_NP.3
p−maj=1, pX=0.48, N=5
var.3.F5 1:169511555:T:C
peptide level
0
50000
100000
156:172
135:135
18:18
PYYSDVDIMRpc2_NP.4
p−min=3.1e−05, pX=0.0034, N=309
var.3.F5 1:169511555:T:C
peptide level
0
100000
200000
172:172
135:135
18:18
QHQLGVWPLLPGSFKpc2_NP.1
p−maj=1, pX=0.18, N=325
var.3.F5 1:169511555:T:C
peptide level
0
10000
20000
30000
172:172
134:135
18:18
QHQLGVWPLLPGSFKpc2_NP.2
p−min=0.47, pX=0.96, N=324
var.3.F5 1:169511555:T:C
peptide level
0
40000
80000
120000
171:172
135:135
18:18
QHQLGVWPLLPGSFKpc2_NP.3
p−maj=1, pX=0.098, N=324
var.3.F5 1:169511555:T:C
peptide level
0
500000
1000000
1500000
135:172
99:135
15:18
QHQLGVWPLLPGSFKpc2_NP.4
p−min=0.43, pX=0.65, N=249
var.3.F5 1:169511555:T:C
peptide level
0
50000
100000
162:172
119:135
16:18
QHQLGVWPLLPGSFKpc2_NP.5
p−min=0.074, pX=0.24, N=297
var.3.F5 1:169511555:T:C
peptide level
0
40000
80000
120000
86:172
58:135
8:18
QHQLGVWPLLPGSFKpc3_NP.1
p−min=0.22, pX=0.39, N=152
var.3.F5 1:169511555:T:C
peptide level
0e+00
2e+05
4e+05
172:172
135:135
18:18
QHQLGVWPLLPGSFKpc3_NP.2
p−maj=1, pX=0.87, N=325
var.3.F5 1:169511555:T:C
peptide level
0e+00
4e+05
8e+05
172:172
135:135
18:18
QHQLGVWPLLPGSFKpc3_NP.3
p−maj=1, pX=0.96, N=325
var.3.F5 1:169511555:T:C
peptide level
0e+00
2e+06
4e+06
172:172
135:135
18:18
QHQLGVWPLLPGSFKpc3_NP.4
p−maj=1, pX=0.6, N=325
var.3.F5 1:169511555:T:C
peptide level
0
50000
100000
171:172
132:135
18:18
QHQLGVWPLLPGSFKpc3_NP.5
p−min=0.35, pX=0.68, N=321

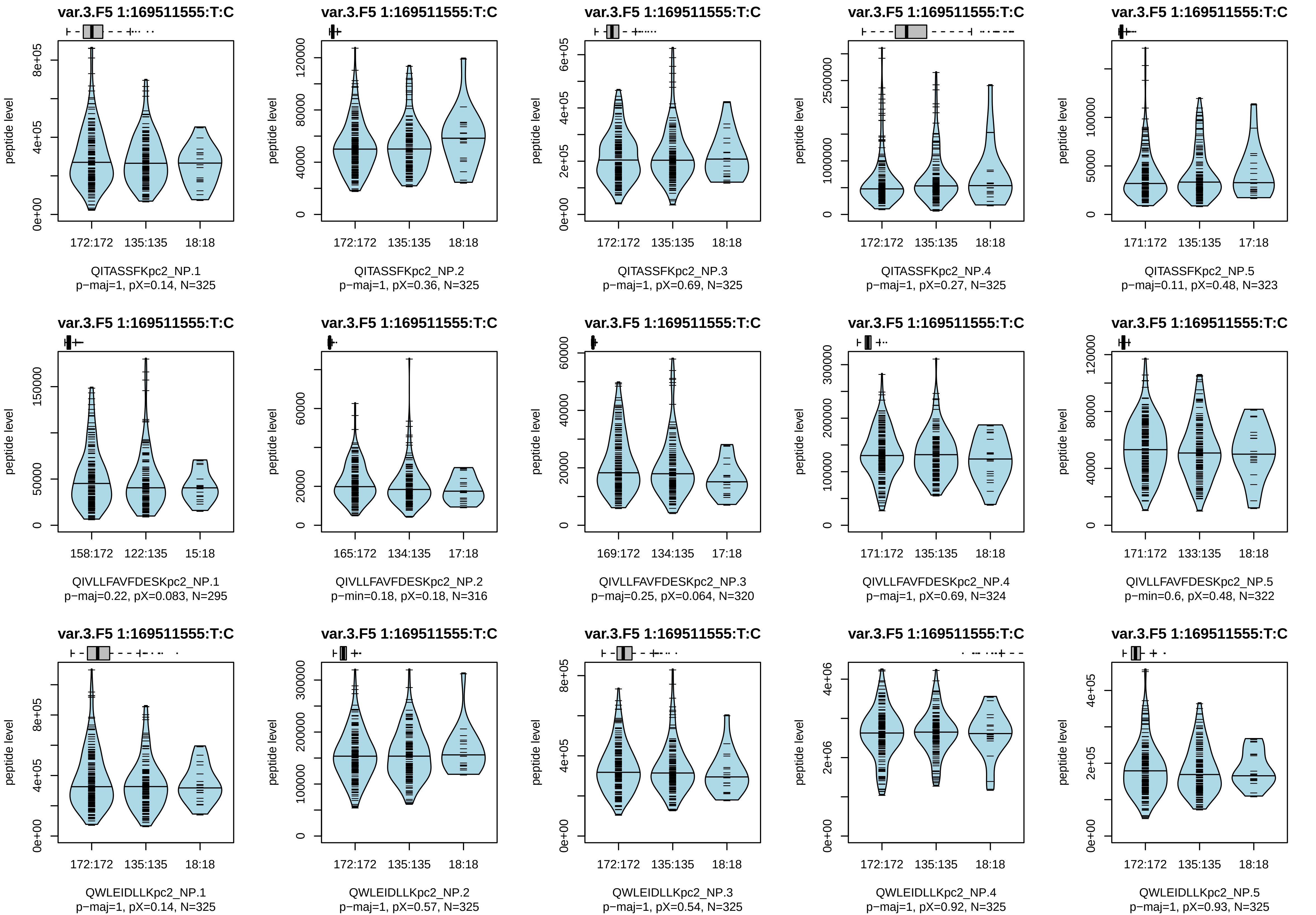
var.3.F5 1:169511555:T:C
peptide level
0e+00
4e+05
8e+05
172:172
135:135
18:18
QITASSFKpc2_NP.1
p−maj=1, pX=0.14, N=325
var.3.F5 1:169511555:T:C
peptide level
0
40000
80000
120000
172:172
135:135
18:18
QITASSFKpc2_NP.2
p−maj=1, pX=0.36, N=325
var.3.F5 1:169511555:T:C
peptide level
0e+00
2e+05
4e+05
6e+05
172:172
135:135
18:18
QITASSFKpc2_NP.3
p−maj=1, pX=0.69, N=325
var.3.F5 1:169511555:T:C
peptide level
0
1000000
2500000
172:172
135:135
18:18
QITASSFKpc2_NP.4
p−maj=1, pX=0.27, N=325
var.3.F5 1:169511555:T:C
peptide level
0
50000
100000
171:172
135:135
17:18
QITASSFKpc2_NP.5
p−maj=0.11, pX=0.48, N=323
var.3.F5 1:169511555:T:C
peptide level
0
50000
150000
158:172
122:135
15:18
QIVLLFAVFDESKpc2_NP.1
p−maj=0.22, pX=0.083, N=295
var.3.F5 1:169511555:T:C
peptide level
0
20000
60000
165:172
134:135
17:18
QIVLLFAVFDESKpc2_NP.2
p−min=0.18, pX=0.18, N=316
var.3.F5 1:169511555:T:C
peptide level
0
20000
40000
60000
169:172
134:135
17:18
QIVLLFAVFDESKpc2_NP.3
p−maj=0.25, pX=0.064, N=320
var.3.F5 1:169511555:T:C
peptide level
0
100000
200000
300000
171:172
135:135
18:18
QIVLLFAVFDESKpc2_NP.4
p−maj=1, pX=0.69, N=324
var.3.F5 1:169511555:T:C
peptide level
0
40000
80000
120000
171:172
133:135
18:18
QIVLLFAVFDESKpc2_NP.5
p−min=0.6, pX=0.48, N=322
var.3.F5 1:169511555:T:C
peptide level
0e+00
4e+05
8e+05
172:172
135:135
18:18
QWLEIDLLKpc2_NP.1
p−maj=1, pX=0.14, N=325
var.3.F5 1:169511555:T:C
peptide level
0
100000
200000
300000
172:172
135:135
18:18
QWLEIDLLKpc2_NP.2
p−maj=1, pX=0.57, N=325
var.3.F5 1:169511555:T:C
peptide level
0e+00
4e+05
8e+05
172:172
135:135
18:18
QWLEIDLLKpc2_NP.3
p−maj=1, pX=0.54, N=325
var.3.F5 1:169511555:T:C
peptide level
0e+00
2e+06
4e+06
172:172
135:135
18:18
QWLEIDLLKpc2_NP.4
p−maj=1, pX=0.92, N=325
var.3.F5 1:169511555:T:C
peptide level
0e+00
2e+05
4e+05
172:172
135:135
18:18
QWLEIDLLKpc2_NP.5
p−maj=1, pX=0.93, N=325

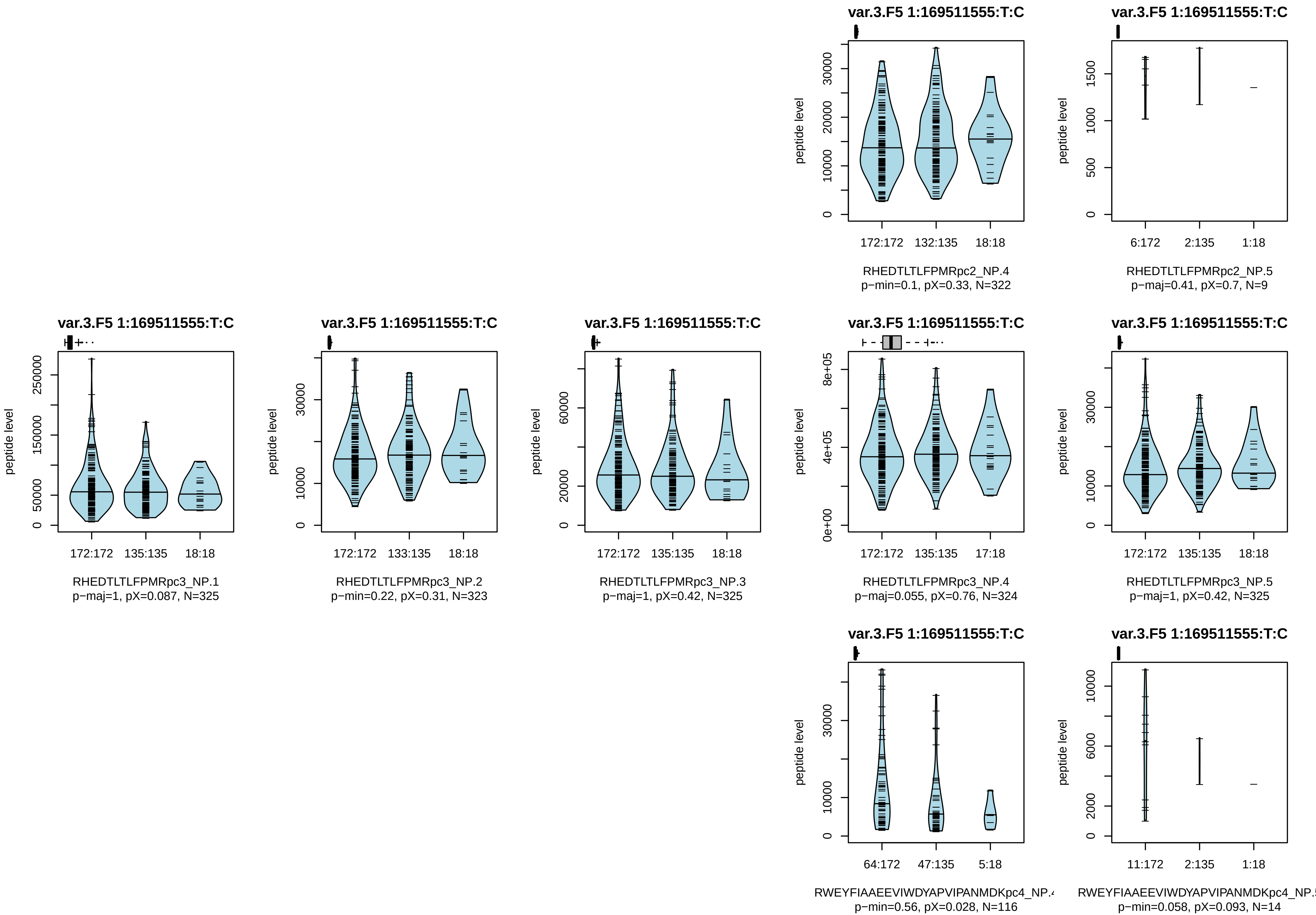

var.3.F5 1:169511555:T:C
peptide level
172:172
132:135
18:18
RHEDTLTLFPMRpc2_NP.4
p−min=0.1, pX=0.33, N=322
var.3.F5 1:169511555:T:C
peptide level
6:172
2:135
1:18
RHEDTLTLFPMRpc2_NP.5
p−maj=0.41, pX=0.7, N=9
var.3.F5 1:169511555:T:C
peptide level
172:172
135:135
18:18
RHEDTLTLFPMRpc3_NP.1
p−maj=1, pX=0.087, N=325
var.3.F5 1:169511555:T:C
peptide level
172:172
133:135
18:18
RHEDTLTLFPMRpc3_NP.2
p−min=0.22, pX=0.31, N=323
var.3.F5 1:169511555:T:C
peptide level
172:172
135:135
18:18
RHEDTLTLFPMRpc3_NP.3
p−maj=1, pX=0.42, N=325
var.3.F5 1:169511555:T:C
peptide level
172:172
135:135
17:18
RHEDTLTLFPMRpc3_NP.4
p−maj=0.055, pX=0.76, N=324
var.3.F5 1:169511555:T:C
peptide level
172:172
135:135
18:18
RHEDTLTLFPMRpc3_NP.5
p−maj=1, pX=0.42, N=325
var.3.F5 1:169511555:T:C
peptide level
64:172
47:135
5:18
RWEYFIAAEEVIWDYAPVIPANMDKpc4_NP.
p−min=0.56, pX=0.028, N=116
var.3.F5 1:169511555:T:C
peptide level
11:172
2:135
1:18
RWEYFIAAEEVIWDYAPVIPANMDKpc4_NP.
p−min=0.058, pX=0.093, N=14

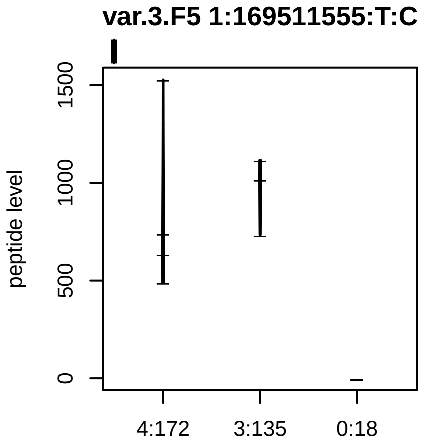

SCYTTEFYVAYSSNQINWQIFKpc3_NP.3
p−maj=1, pX=0.78, N=7

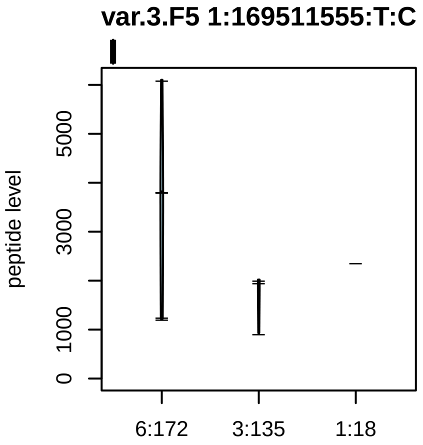

SCYTTEFYVAYSSNQINWQIFKpc3_NP.4
p−maj=0.44, pX=0.42, N=10

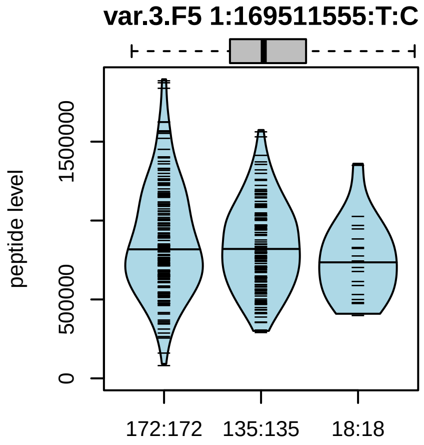

SEAYNTFSERpc2_NP.1
p−maj=1, pX=0.11, N=325

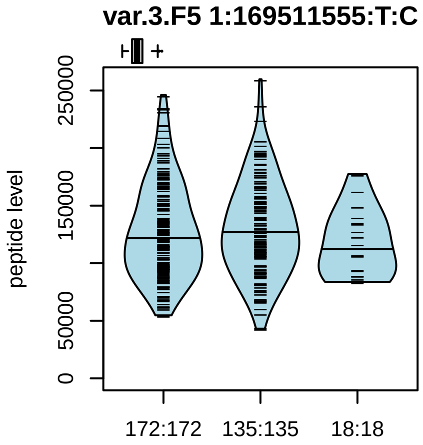

SEAYNTFSERpc2_NP.2
p−maj=1, pX=0.66, N=325

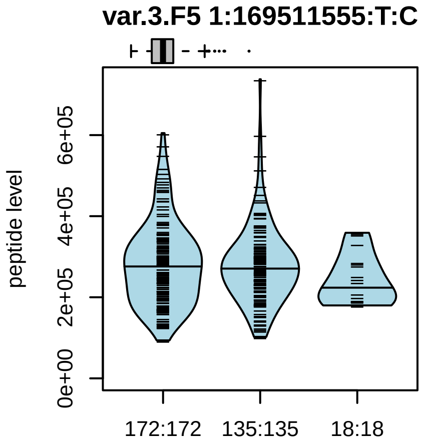

SEAYNTFSERpc2_NP.3
p−maj=1, pX=0.25, N=325

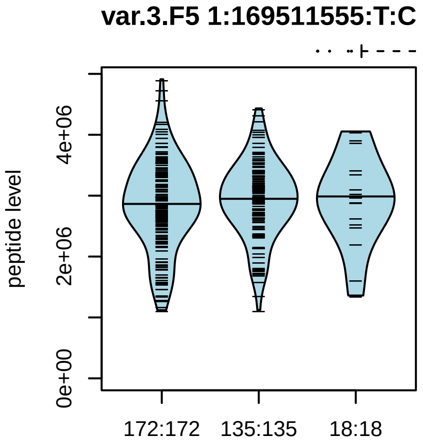

SEAYNTFSERpc2_NP.4
p−maj=1, pX=0.4, N=325

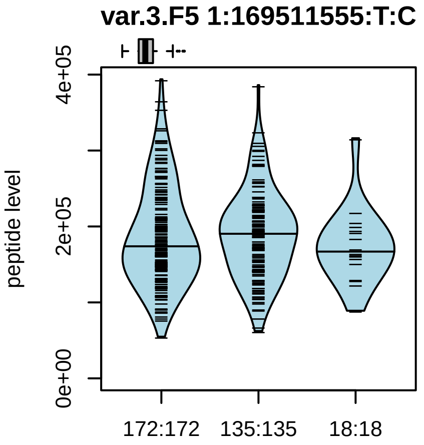

SEAYNTFSERpc2_NP.5
p−maj=1, pX=0.71, N=325

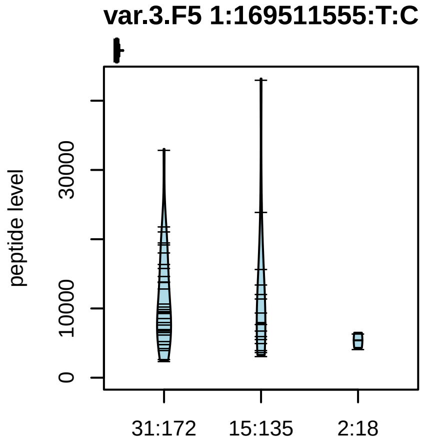

SEAYNTFSERRpc3_NP.1
p−min=0.087, pX=0.11, N=48

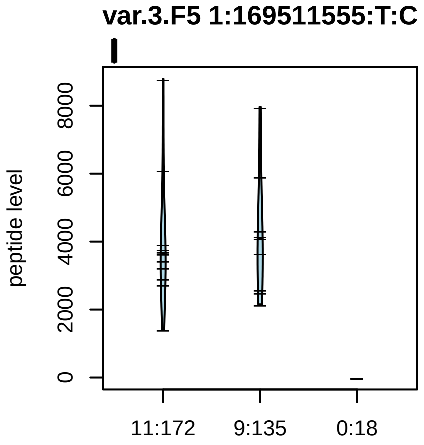

SEAYNTFSERRpc3_NP.3
p−maj=0.61, pX=0.65, N=20

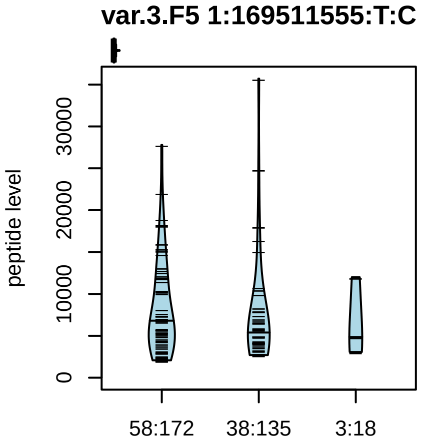

SEAYNTFSERRpc3_NP.4
p−min=0.19, pX=0.11, N=99

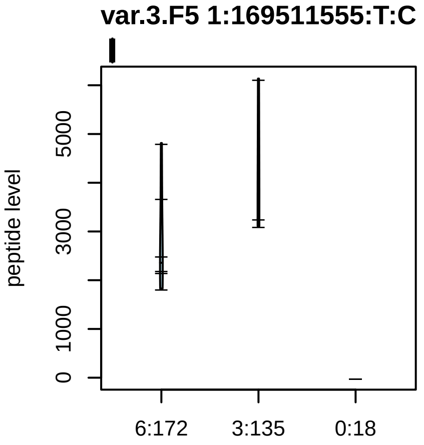

SEAYNTFSERRpc3_NP.5
p−min=0.51, pX=0.62, N=9

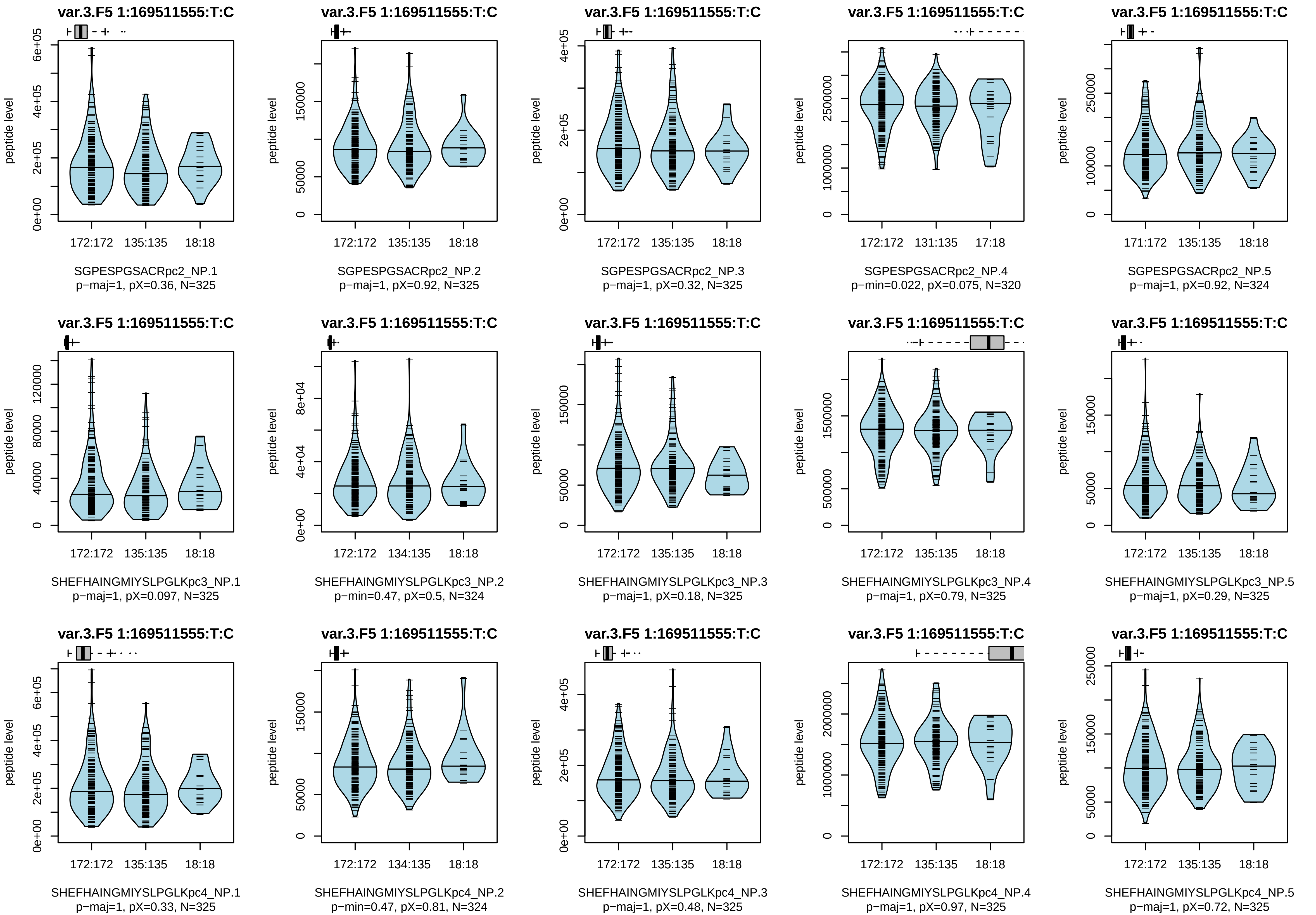
var.3.F5 1:169511555:T:C
peptide level
172:172 135:135 18:18
SGPESPGSACRpc2_NP.1
p−maj=1, pX=0.36, N=325
var.3.F5 1:169511555:T:C
peptide level
172:172 135:135 18:18
SGPESPGSACRpc2_NP.2
p−maj=1, pX=0.92, N=325
var.3.F5 1:169511555:T:C
peptide level
172:172 135:135 18:18
SGPESPGSACRpc2_NP.3
p−maj=1, pX=0.32, N=325
var.3.F5 1:169511555:T:C
peptide level
172:172 131:135 17:18
SGPESPGSACRpc2_NP.4
p−min=0.022, pX=0.075, N=320
var.3.F5 1:169511555:T:C
peptide level
171:172 135:135 18:18
SGPESPGSACRpc2_NP.5
p−maj=1, pX=0.92, N=324
var.3.F5 1:169511555:T:C
peptide level
172:172 135:135 18:18
SHEFHAINGMIYSLPGLKpc3_NP.1
p−maj=1, pX=0.097, N=325
var.3.F5 1:169511555:T:C
peptide level
172:172 134:135 18:18
SHEFHAINGMIYSLPGLKpc3_NP.2
p−min=0.47, pX=0.5, N=324
var.3.F5 1:169511555:T:C
peptide level
172:172 135:135 18:18
SHEFHAINGMIYSLPGLKpc3_NP.3
p−maj=1, pX=0.18, N=325
var.3.F5 1:169511555:T:C
peptide level
172:172 135:135 18:18
SHEFHAINGMIYSLPGLKpc3_NP.4
p−maj=1, pX=0.79, N=325
var.3.F5 1:169511555:T:C
peptide level
172:172 135:135 18:18
SHEFHAINGMIYSLPGLKpc3_NP.5
p−maj=1, pX=0.29, N=325
var.3.F5 1:169511555:T:C
peptide level
172:172 135:135 18:18
SHEFHAINGMIYSLPGLKpc4_NP.1
p−maj=1, pX=0.33, N=325
var.3.F5 1:169511555:T:C
peptide level
172:172 134:135 18:18
SHEFHAINGMIYSLPGLKpc4_NP.2
p−min=0.47, pX=0.81, N=324
var.3.F5 1:169511555:T:C
peptide level
172:172 135:135 18:18
SHEFHAINGMIYSLPGLKpc4_NP.3
p−maj=1, pX=0.48, N=325
var.3.F5 1:169511555:T:C
peptide level
172:172 135:135 18:18
SHEFHAINGMIYSLPGLKpc4_NP.4
p−maj=1, pX=0.97, N=325
var.3.F5 1:169511555:T:C
peptide level
172:172 135:135 18:18
SHEFHAINGMIYSLPGLKpc4_NP.5
p−maj=1, pX=0.72, N=325

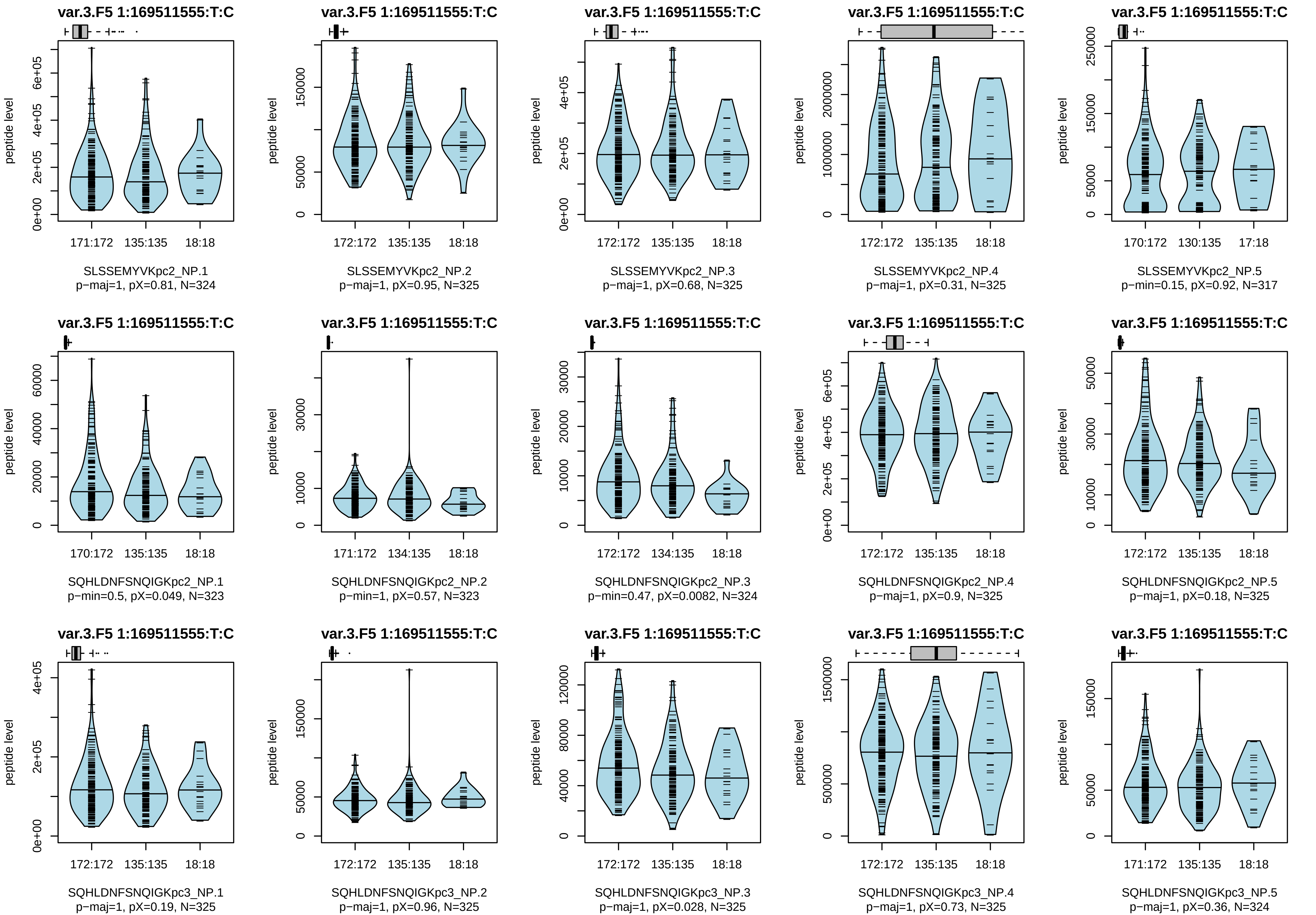
var.3.F5 1:169511555:T:C
peptide level
171:172 135:135 18:18
SLSSEMYVKpc2_NP.1
p−maj=1, pX=0.81, N=324
var.3.F5 1:169511555:T:C
peptide level
172:172 135:135 18:18
SLSSEMYVKpc2_NP.2
p−maj=1, pX=0.95, N=325
var.3.F5 1:169511555:T:C
peptide level
172:172 135:135 18:18
SLSSEMYVKpc2_NP.3
p−maj=1, pX=0.68, N=325
var.3.F5 1:169511555:T:C
peptide level
172:172 135:135 18:18
SLSSEMYVKpc2_NP.4
p−maj=1, pX=0.31, N=325
var.3.F5 1:169511555:T:C
peptide level
170:172 130:135 17:18
SLSSEMYVKpc2_NP.5
p−min=0.15, pX=0.92, N=317
var.3.F5 1:169511555:T:C
peptide level
170:172 135:135 18:18
SQHLDNFSNQIGKpc2_NP.1
p−min=0.5, pX=0.049, N=323
var.3.F5 1:169511555:T:C
peptide level
171:172 134:135 18:18
SQHLDNFSNQIGKpc2_NP.2
p−min=1, pX=0.57, N=323
var.3.F5 1:169511555:T:C
peptide level
172:172 134:135 18:18
SQHLDNFSNQIGKpc2_NP.3
p−min=0.47, pX=0.0082, N=324
var.3.F5 1:169511555:T:C
peptide level
172:172 135:135 18:18
SQHLDNFSNQIGKpc2_NP.4
p−maj=1, pX=0.9, N=325
var.3.F5 1:169511555:T:C
peptide level
172:172 135:135 18:18
SQHLDNFSNQIGKpc2_NP.5
p−maj=1, pX=0.18, N=325
var.3.F5 1:169511555:T:C
peptide level
172:172 135:135 18:18
SQHLDNFSNQIGKpc3_NP.1
p−maj=1, pX=0.19, N=325
var.3.F5 1:169511555:T:C
peptide level
172:172 135:135 18:18
SQHLDNFSNQIGKpc3_NP.2
p−maj=1, pX=0.96, N=325
var.3.F5 1:169511555:T:C
peptide level
172:172 135:135 18:18
SQHLDNFSNQIGKpc3_NP.3
p−maj=1, pX=0.028, N=325
var.3.F5 1:169511555:T:C
peptide level
172:172 135:135 18:18
SQHLDNFSNQIGKpc3_NP.4
p−maj=1, pX=0.73, N=325
var.3.F5 1:169511555:T:C
peptide level
171:172 135:135 18:18
SQHLDNFSNQIGKpc3_NP.5
p−maj=1, pX=0.36, N=324

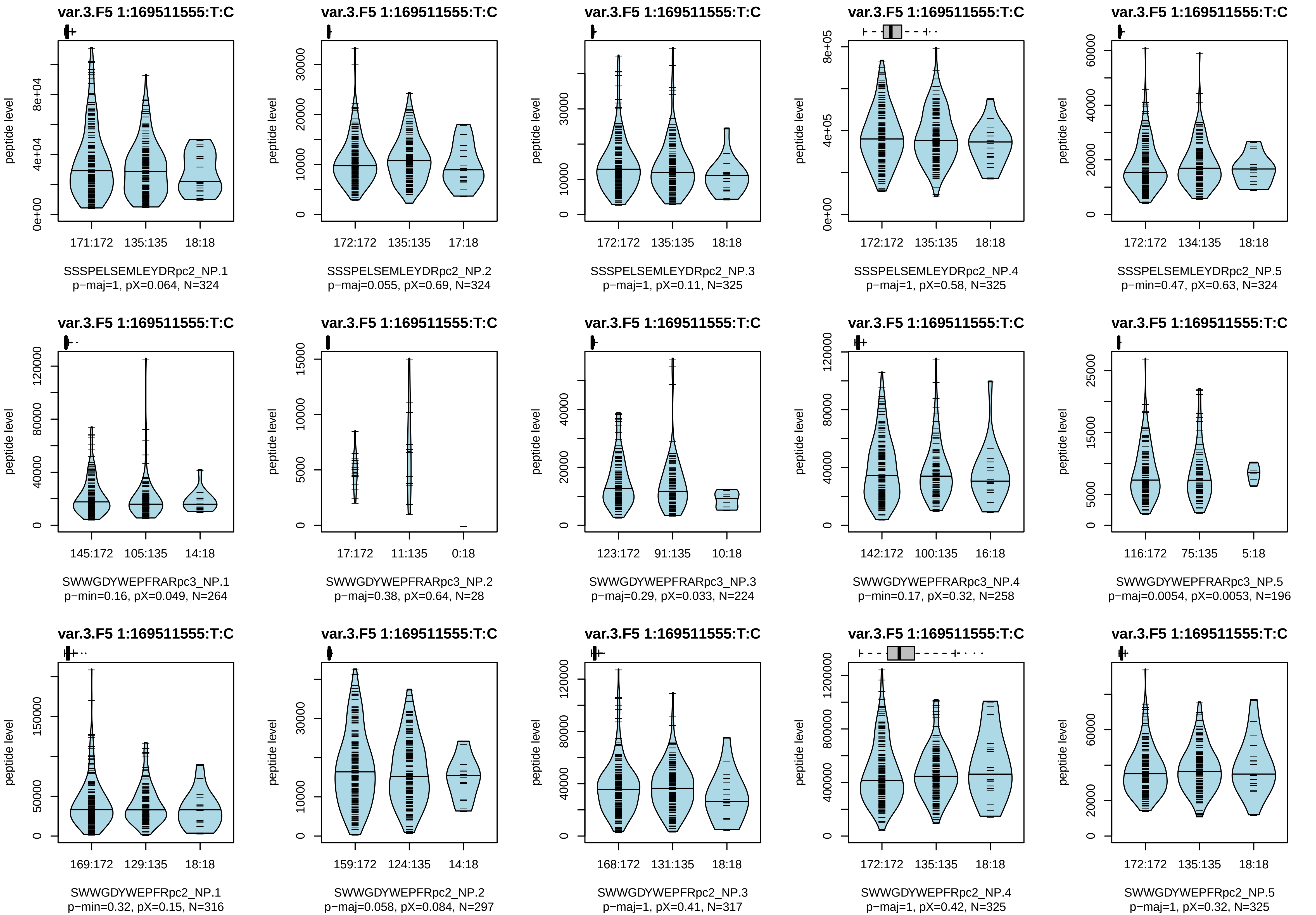
var.3.F5 1:169511555:T:C
peptide level
171:172 135:135 18:18
SSSPELSEMLEYDRpc2_NP.1
p−maj=1, pX=0.064, N=324
var.3.F5 1:169511555:T:C
peptide level
172:172 135:135 17:18
SSSPELSEMLEYDRpc2_NP.2
p−maj=0.055, pX=0.69, N=324
var.3.F5 1:169511555:T:C
peptide level
172:172 135:135 18:18
SSSPELSEMLEYDRpc2_NP.3
p−maj=1, pX=0.11, N=325
var.3.F5 1:169511555:T:C
peptide level
172:172 135:135 18:18
SSSPELSEMLEYDRpc2_NP.4
p−maj=1, pX=0.58, N=325
var.3.F5 1:169511555:T:C
peptide level
172:172 134:135 18:18
SSSPELSEMLEYDRpc2_NP.5
p−min=0.47, pX=0.63, N=324
var.3.F5 1:169511555:T:C
peptide level
145:172 105:135 14:18
SWWGDYWEPFRARpc3_NP.1
p−min=0.16, pX=0.049, N=264
var.3.F5 1:169511555:T:C
peptide level
17:172 11:135 0:18
SWWGDYWEPFRARpc3_NP.2
p−maj=0.38, pX=0.64, N=28
var.3.F5 1:169511555:T:C
peptide level
123:172 91:135 10:18
SWWGDYWEPFRARpc3_NP.3
p−maj=0.29, pX=0.033, N=224
var.3.F5 1:169511555:T:C
peptide level
142:172 100:135 16:18
SWWGDYWEPFRARpc3_NP.4
p−min=0.17, pX=0.32, N=258
var.3.F5 1:169511555:T:C
peptide level
116:172 75:135 5:18
SWWGDYWEPFRARpc3_NP.5
p−maj=0.0054, pX=0.0053, N=196
var.3.F5 1:169511555:T:C
peptide level
169:172 129:135 18:18
SWWGDYWEPFRpc2_NP.1
p−min=0.32, pX=0.15, N=316
var.3.F5 1:169511555:T:C
peptide level
159:172 124:135 14:18
SWWGDYWEPFRpc2_NP.2
p−maj=0.058, pX=0.084, N=297
var.3.F5 1:169511555:T:C
peptide level
168:172 131:135 18:18
SWWGDYWEPFRpc2_NP.3
p−maj=1, pX=0.41, N=317
var.3.F5 1:169511555:T:C
peptide level
172:172 135:135 18:18
SWWGDYWEPFRpc2_NP.4
p−maj=1, pX=0.42, N=325
var.3.F5 1:169511555:T:C
peptide level
172:172 135:135 18:18
SWWGDYWEPFRpc2_NP.5
p−maj=1, pX=0.32, N=325

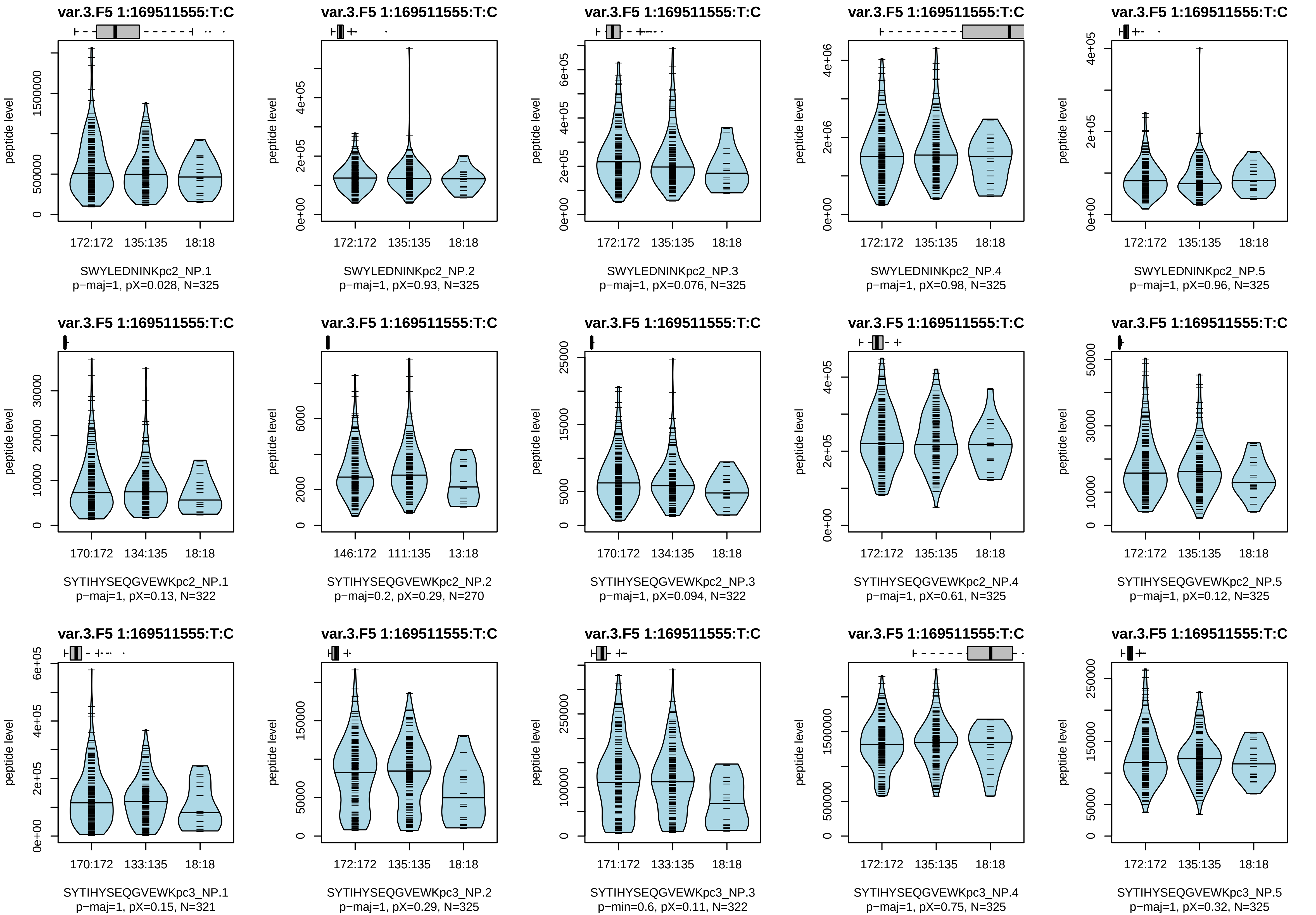
var.3.F5 1:169511555:T:C
peptide level
172:172
135:135
18:18
SWYLEDNINKpc2_NP.1
p−maj=1, pX=0.028, N=325
var.3.F5 1:169511555:T:C
peptide level
172:172
135:135
18:18
SWYLEDNINKpc2_NP.2
p−maj=1, pX=0.93, N=325
var.3.F5 1:169511555:T:C
peptide level
172:172
135:135
18:18
SWYLEDNINKpc2_NP.3
p−maj=1, pX=0.076, N=325
var.3.F5 1:169511555:T:C
peptide level
172:172
135:135
18:18
SWYLEDNINKpc2_NP.4
p−maj=1, pX=0.98, N=325
var.3.F5 1:169511555:T:C
peptide level
172:172
135:135
18:18
SWYLEDNINKpc2_NP.5
p−maj=1, pX=0.96, N=325
var.3.F5 1:169511555:T:C
peptide level
170:172
134:135
18:18
SYTIHYSEQGVEWKpc2_NP.1
p−maj=1, pX=0.13, N=322
var.3.F5 1:169511555:T:C
peptide level
146:172
111:135
13:18
SYTIHYSEQGVEWKpc2_NP.2
p−maj=0.2, pX=0.29, N=270
var.3.F5 1:169511555:T:C
peptide level
170:172
134:135
18:18
SYTIHYSEQGVEWKpc2_NP.3
p−maj=1, pX=0.094, N=322
var.3.F5 1:169511555:T:C
peptide level
172:172
135:135
18:18
SYTIHYSEQGVEWKpc2_NP.4
p−maj=1, pX=0.61, N=325
var.3.F5 1:169511555:T:C
peptide level
172:172
135:135
18:18
SYTIHYSEQGVEWKpc2_NP.5
p−maj=1, pX=0.12, N=325
var.3.F5 1:169511555:T:C
peptide level
170:172
133:135
18:18
SYTIHYSEQGVEWKpc3_NP.1
p−maj=1, pX=0.15, N=321
var.3.F5 1:169511555:T:C
peptide level
172:172
135:135
18:18
SYTIHYSEQGVEWKpc3_NP.2
p−maj=1, pX=0.29, N=325
var.3.F5 1:169511555:T:C
peptide level
171:172
133:135
18:18
SYTIHYSEQGVEWKpc3_NP.3
p−min=0.6, pX=0.11, N=322
var.3.F5 1:169511555:T:C
peptide level
172:172
135:135
18:18
SYTIHYSEQGVEWKpc3_NP.4
p−maj=1, pX=0.75, N=325
var.3.F5 1:169511555:T:C
peptide level
172:172
135:135
18:18
SYTIHYSEQGVEWKpc3_NP.5
p−maj=1, pX=0.32, N=325

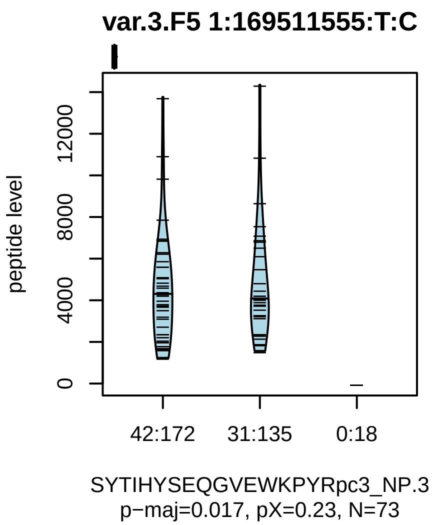
var.3.F5 1:169511555:T:C
peptide level
0
4000
8000
12000
42:172
31:135
0:18
SYTIHYSEQGVEWKPYRpc3_NP.3
p−maj=0.017, pX=0.23, N=73

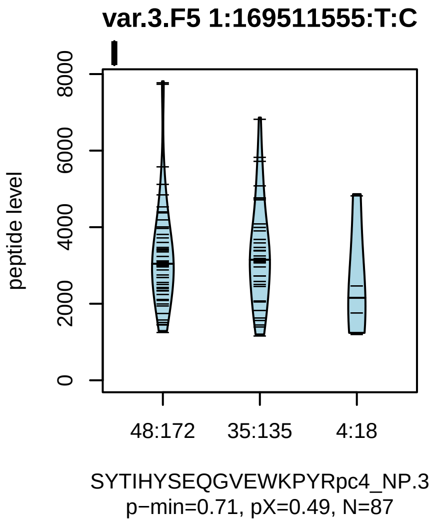
var.3.F5 1:169511555:T:C
peptide level
0
2000
4000
6000
8000
48:172
35:135
4:18
SYTIHYSEQGVEWKPYRpc4_NP.3
p−min=0.71, pX=0.49, N=87

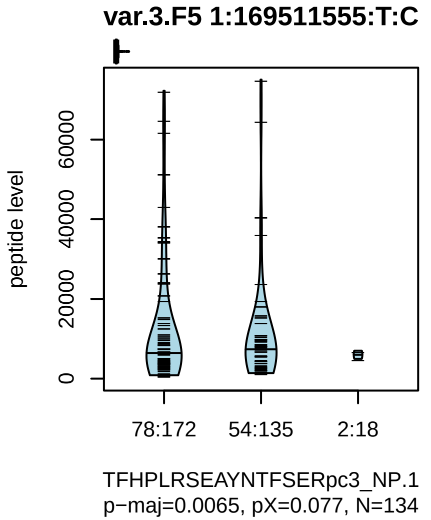
var.3.F5 1:169511555:T:C
peptide level
0
20000
40000
60000
78:172
54:135
2:18
TFHPLRSEAYNTFSERpc3_NP.1
p−maj=0.0065, pX=0.077, N=134

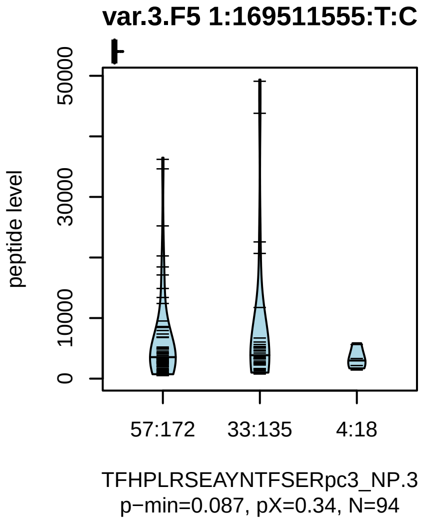
var.3.F5 1:169511555:T:C
peptide level
0
10000
30000
50000
57:172
33:135
4:18
TFHPLRSEAYNTFSERpc3_NP.3
p−min=0.087, pX=0.34, N=94

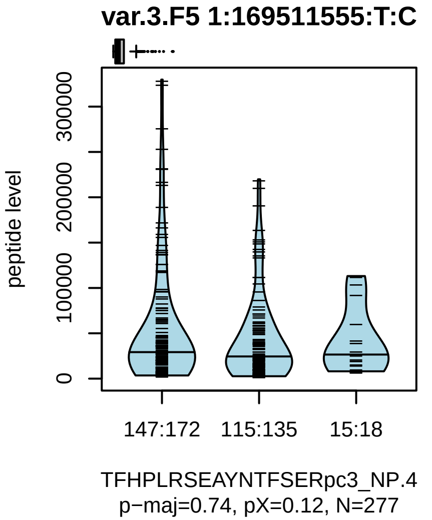
var.3.F5 1:169511555:T:C
peptide level
0
100000
200000
300000
147:172
115:135
15:18
TFHPLRSEAYNTFSERpc3_NP.4
p−maj=0.74, pX=0.12, N=277

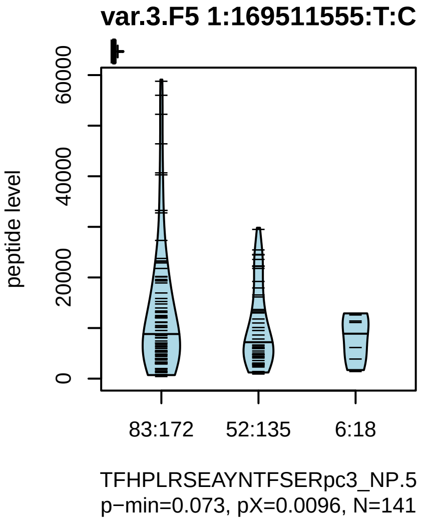
var.3.F5 1:169511555:T:C
peptide level
0
20000
40000
60000
83:172
52:135
6:18
TFHPLRSEAYNTFSERpc3_NP.5
p−min=0.073, pX=0.0096, N=141

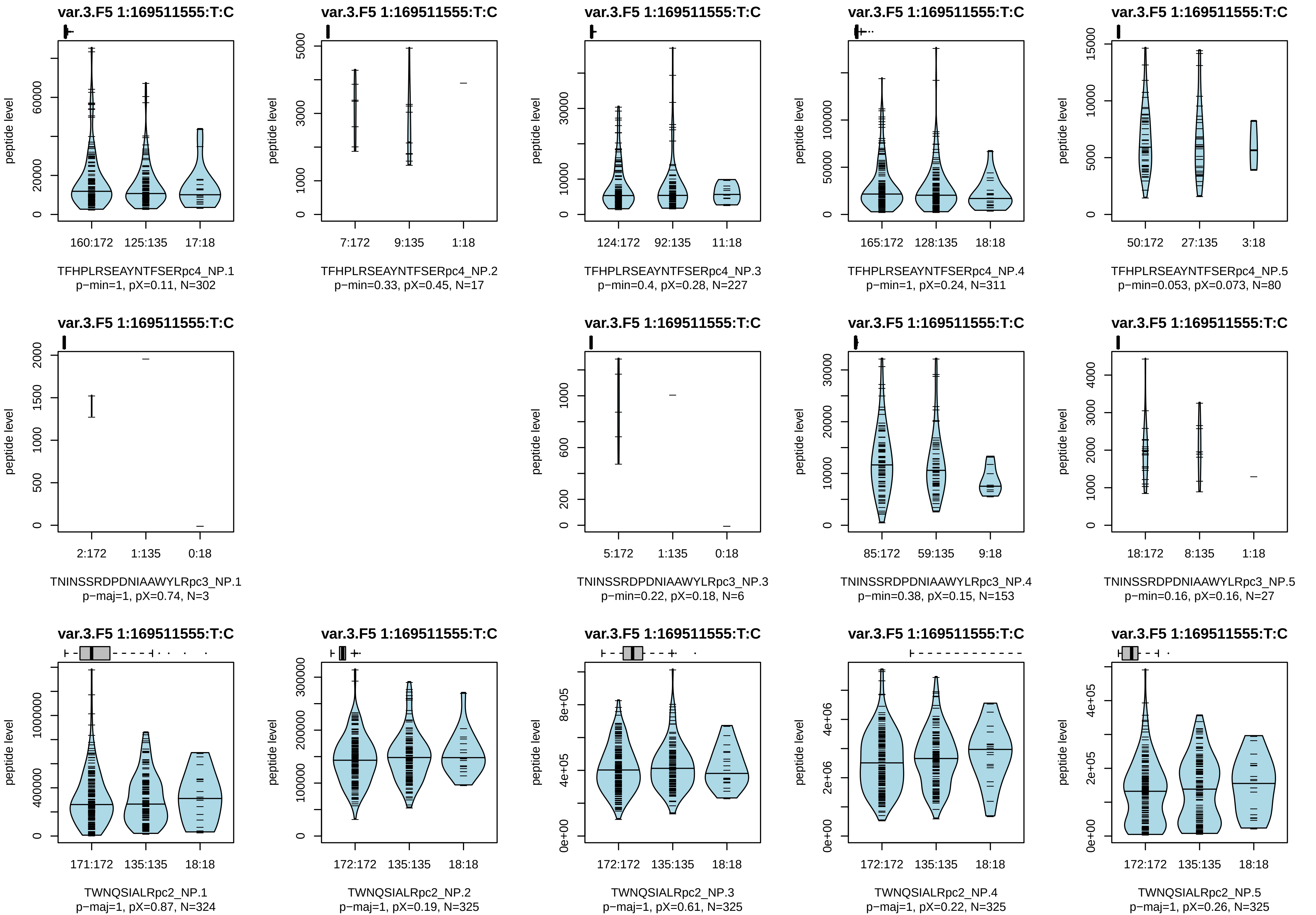
var.3.F5 1:169511555:T:C
peptide level
160:172
125:135
17:18
TFHPLRSEAYNTFSERpc4_NP.1
p−min=1, pX=0.11, N=302
var.3.F5 1:169511555:T:C
peptide level
7:172
9:135
1:18
TFHPLRSEAYNTFSERpc4_NP.2
p−min=0.33, pX=0.45, N=17
var.3.F5 1:169511555:T:C
peptide level
124:172
92:135
11:18
TFHPLRSEAYNTFSERpc4_NP.3
p−min=0.4, pX=0.28, N=227
var.3.F5 1:169511555:T:C
peptide level
165:172
128:135
18:18
TFHPLRSEAYNTFSERpc4_NP.4
p−min=1, pX=0.24, N=311
var.3.F5 1:169511555:T:C
peptide level
50:172
27:135
3:18
TFHPLRSEAYNTFSERpc4_NP.5
p−min=0.053, pX=0.073, N=80
var.3.F5 1:169511555:T:C
peptide level
2:172
1:135
0:18
TNINSSRDPDNIAAWYLRpc3_NP.1
p−maj=1, pX=0.74, N=3
var.3.F5 1:169511555:T:C
peptide level
5:172
1:135
0:18
TNINSSRDPDNIAAWYLRpc3_NP.3
p−min=0.22, pX=0.18, N=6
var.3.F5 1:169511555:T:C
peptide level
85:172
59:135
9:18
TNINSSRDPDNIAAWYLRpc3_NP.4
p−min=0.38, pX=0.15, N=153
var.3.F5 1:169511555:T:C
peptide level
18:172
8:135
1:18
TNINSSRDPDNIAAWYLRpc3_NP.5
p−min=0.16, pX=0.16, N=27
var.3.F5 1:169511555:T:C
peptide level
171:172
135:135
18:18
TWNQSIALRpc2_NP.1
p−maj=1, pX=0.87, N=324
var.3.F5 1:169511555:T:C
peptide level
172:172
135:135
18:18
TWNQSIALRpc2_NP.2
p−maj=1, pX=0.19, N=325
var.3.F5 1:169511555:T:C
peptide level
172:172
135:135
18:18
TWNQSIALRpc2_NP.3
p−maj=1, pX=0.61, N=325
var.3.F5 1:169511555:T:C
peptide level
172:172
135:135
18:18
TWNQSIALRpc2_NP.4
p−maj=1, pX=0.22, N=325
var.3.F5 1:169511555:T:C
peptide level
172:172
135:135
18:18
TWNQSIALRpc2_NP.5
p−maj=1, pX=0.26, N=325

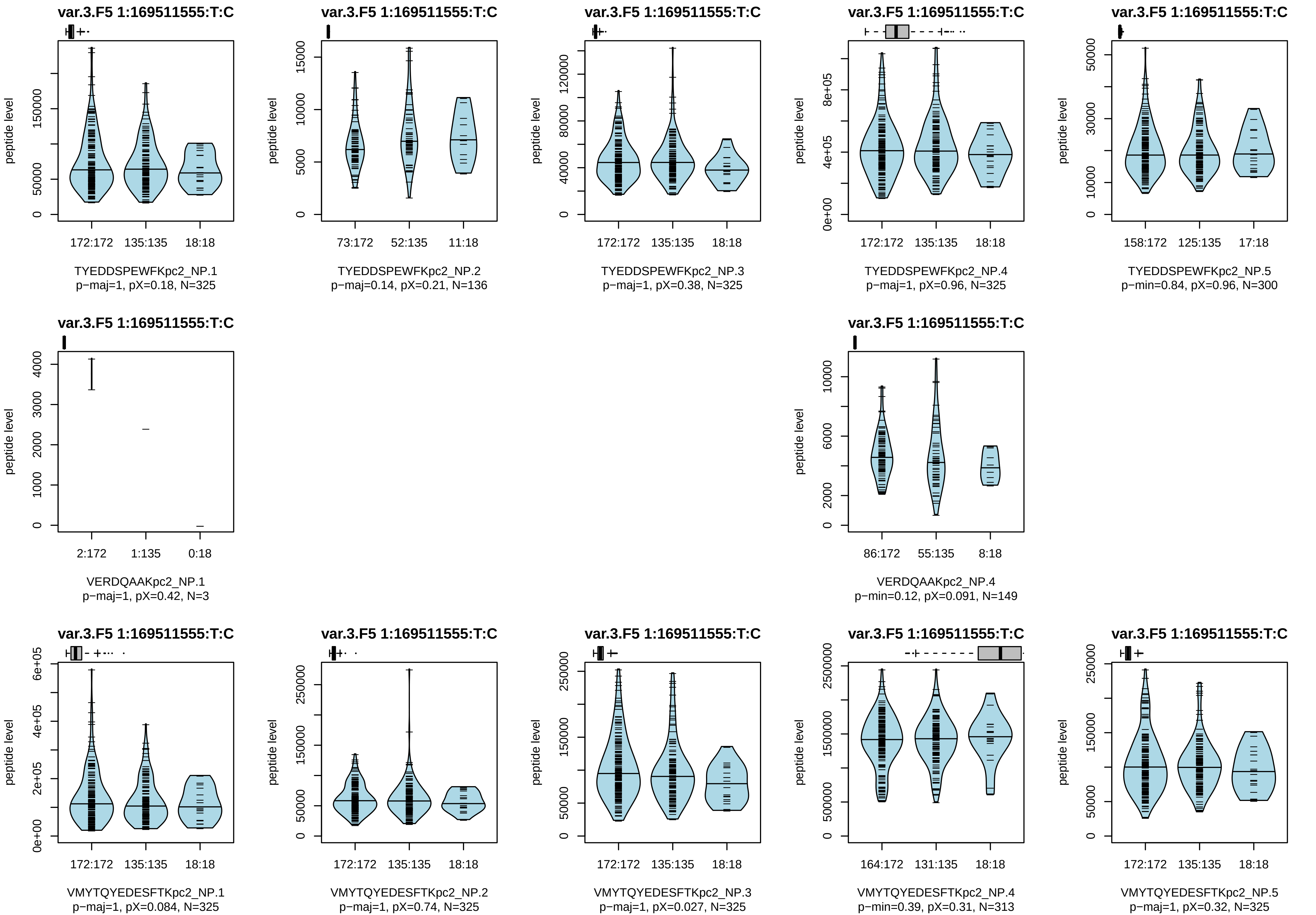
var.3.F5 1:169511555:T:C
peptide level
172:172
135:135
18:18
TYEDDSPEWFKpc2_NP.1
p−maj=1, pX=0.18, N=325
var.3.F5 1:169511555:T:C
73:172
52:135
11:18
TYEDDSPEWFKpc2_NP.2
p−maj=0.14, pX=0.21, N=136
var.3.F5 1:169511555:T:C
172:172
135:135
18:18
TYEDDSPEWFKpc2_NP.3
p−maj=1, pX=0.38, N=325
var.3.F5 1:169511555:T:C
172:172
135:135
18:18
TYEDDSPEWFKpc2_NP.4
p−maj=1, pX=0.96, N=325
var.3.F5 1:169511555:T:C
158:172
125:135
17:18
TYEDDSPEWFKpc2_NP.5
p−min=0.84, pX=0.96, N=300
var.3.F5 1:169511555:T:C
2:172
1:135
0:18
VERDQAAKpc2_NP.1
p−maj=1, pX=0.42, N=3
var.3.F5 1:169511555:T:C
86:172
55:135
8:18
VERDQAAKpc2_NP.4
p−min=0.12, pX=0.091, N=149
var.3.F5 1:169511555:T:C
172:172
135:135
18:18
VMYTQYEDESFTKpc2_NP.1
p−maj=1, pX=0.084, N=325
var.3.F5 1:169511555:T:C
172:172
135:135
18:18
VMYTQYEDESFTKpc2_NP.2
p−maj=1, pX=0.74, N=325
var.3.F5 1:169511555:T:C
172:172
135:135
18:18
VMYTQYEDESFTKpc2_NP.3
p−maj=1, pX=0.027, N=325
var.3.F5 1:169511555:T:C
164:172
131:135
18:18
VMYTQYEDESFTKpc2_NP.4
p−min=0.39, pX=0.31, N=313
var.3.F5 1:169511555:T:C
172:172
135:135
18:18
VMYTQYEDESFTKpc2_NP.5
p−maj=1, pX=0.32, N=325

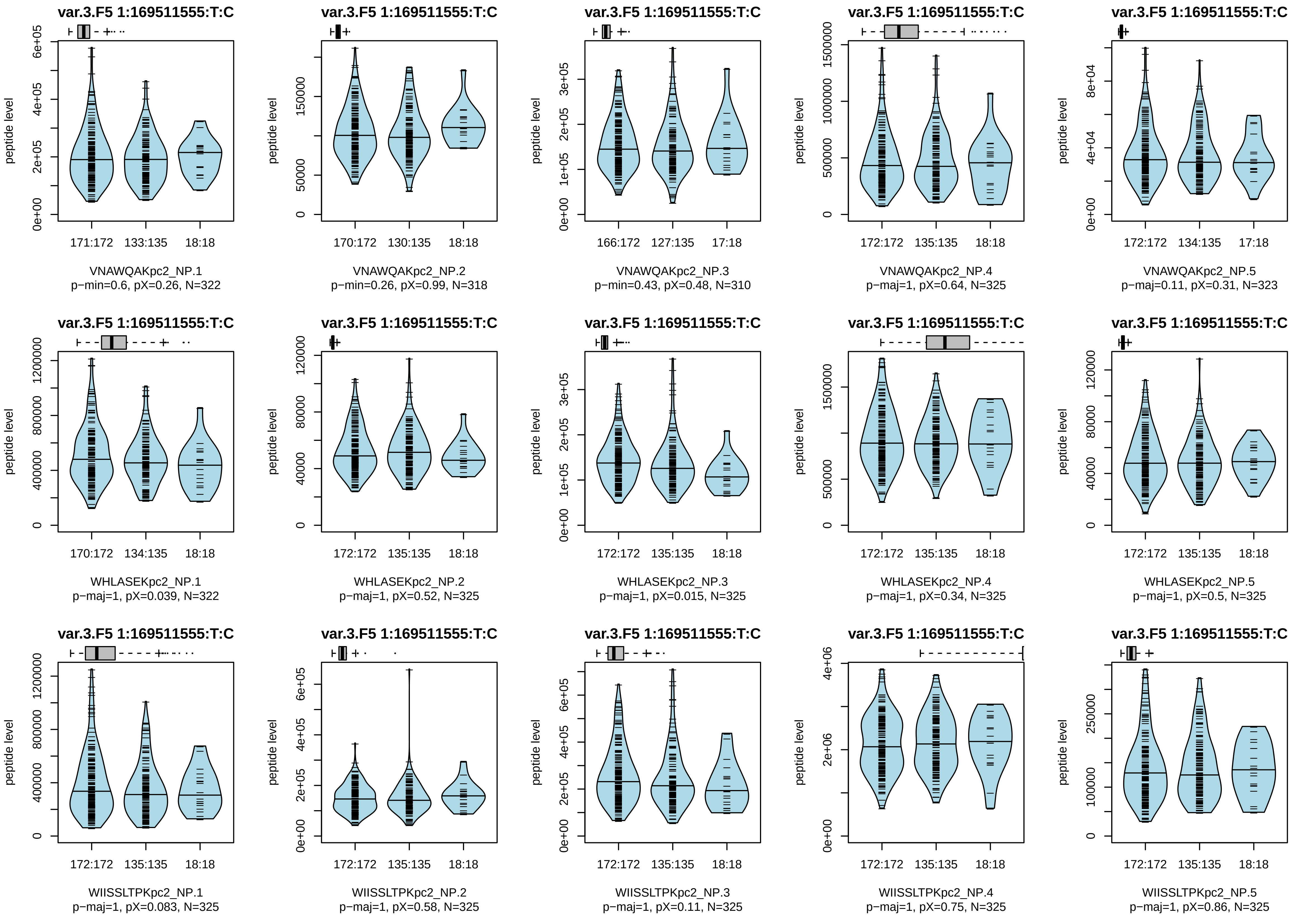
var.3.F5 1:169511555:T:C
peptide level
171:172
133:135
18:18
VNAWQAKpc2_NP.1
p−min=0.6, pX=0.26, N=322
var.3.F5 1:169511555:T:C
peptide level
170:172
130:135
18:18
VNAWQAKpc2_NP.2
p−min=0.26, pX=0.99, N=318
var.3.F5 1:169511555:T:C
peptide level
166:172
127:135
17:18
VNAWQAKpc2_NP.3
p−min=0.43, pX=0.48, N=310
var.3.F5 1:169511555:T:C
peptide level
172:172
135:135
18:18
VNAWQAKpc2_NP.4
p−maj=1, pX=0.64, N=325
var.3.F5 1:169511555:T:C
peptide level
172:172
134:135
17:18
VNAWQAKpc2_NP.5
p−maj=0.11, pX=0.31, N=323
var.3.F5 1:169511555:T:C
peptide level
170:172
134:135
18:18
WHLASEKpc2_NP.1
p−maj=1, pX=0.039, N=322
var.3.F5 1:169511555:T:C
peptide level
172:172
135:135
18:18
WHLASEKpc2_NP.2
p−maj=1, pX=0.52, N=325
var.3.F5 1:169511555:T:C
peptide level
172:172
135:135
18:18
WHLASEKpc2_NP.3
p−maj=1, pX=0.015, N=325
var.3.F5 1:169511555:T:C
peptide level
172:172
135:135
18:18
WHLASEKpc2_NP.4
p−maj=1, pX=0.34, N=325
var.3.F5 1:169511555:T:C
peptide level
172:172
135:135
18:18
WHLASEKpc2_NP.5
p−maj=1, pX=0.5, N=325
var.3.F5 1:169511555:T:C
peptide level
172:172
135:135
18:18
WIISSLTPKpc2_NP.1
p−maj=1, pX=0.083, N=325
var.3.F5 1:169511555:T:C
peptide level
172:172
135:135
18:18
WIISSLTPKpc2_NP.2
p−maj=1, pX=0.58, N=325
var.3.F5 1:169511555:T:C
peptide level
172:172
135:135
18:18
WIISSLTPKpc2_NP.3
p−maj=1, pX=0.11, N=325
var.3.F5 1:169511555:T:C
peptide level
172:172
135:135
18:18
WIISSLTPKpc2_NP.4
p−maj=1, pX=0.75, N=325
var.3.F5 1:169511555:T:C
peptide level
172:172
135:135
18:18
WIISSLTPKpc2_NP.5
p−maj=1, pX=0.86, N=325

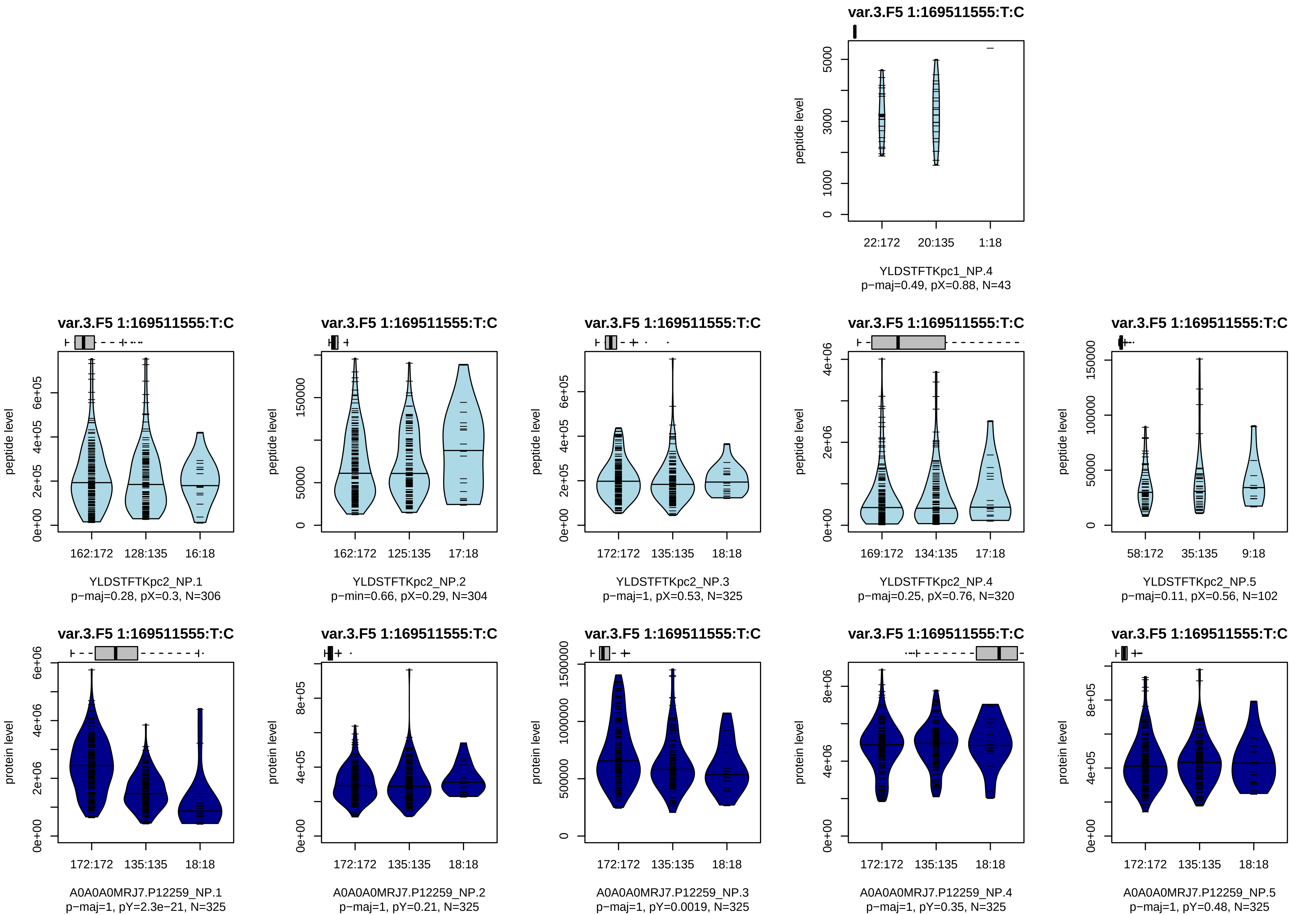
var.3.F5 1:169511555:T:C
peptide level
22:172
20:135
1:18
YLDSTFTKpc1_NP.4
p−maj=0.49, pX=0.88, N=43
var.3.F5 1:169511555:T:C
peptide level
162:172
128:135
16:18
YLDSTFTKpc2_NP.1
p−maj=0.28, pX=0.3, N=306
var.3.F5 1:169511555:T:C
peptide level
162:172
125:135
17:18
YLDSTFTKpc2_NP.2
p−min=0.66, pX=0.29, N=304
var.3.F5 1:169511555:T:C
peptide level
172:172
135:135
18:18
YLDSTFTKpc2_NP.3
p−maj=1, pX=0.53, N=325
var.3.F5 1:169511555:T:C
peptide level
169:172
134:135
17:18
YLDSTFTKpc2_NP.4
p−maj=0.25, pX=0.76, N=320
var.3.F5 1:169511555:T:C
peptide level
58:172
35:135
9:18
YLDSTFTKpc2_NP.5
p−maj=0.11, pX=0.56, N=102
var.3.F5 1:169511555:T:C
protein level
172:172
135:135
18:18
A0A0A0MRJ7.P12259_NP.1
p−maj=1, pY=2.3e−21, N=325
var.3.F5 1:169511555:T:C
protein level
172:172
135:135
18:18
A0A0A0MRJ7.P12259_NP.2
p−maj=1, pY=0.21, N=325
var.3.F5 1:169511555:T:C
protein level
172:172
135:135
18:18
A0A0A0MRJ7.P12259_NP.3
p−maj=1, pY=0.0019, N=325
var.3.F5 1:169511555:T:C
protein level
172:172
135:135
18:18
A0A0A0MRJ7.P12259_NP.4
p−maj=1, pY=0.35, N=325
var.3.F5 1:169511555:T:C
protein level
172:172
135:135
18:18
A0A0A0MRJ7.P12259_NP.5
p−maj=1, pY=0.48, N=325

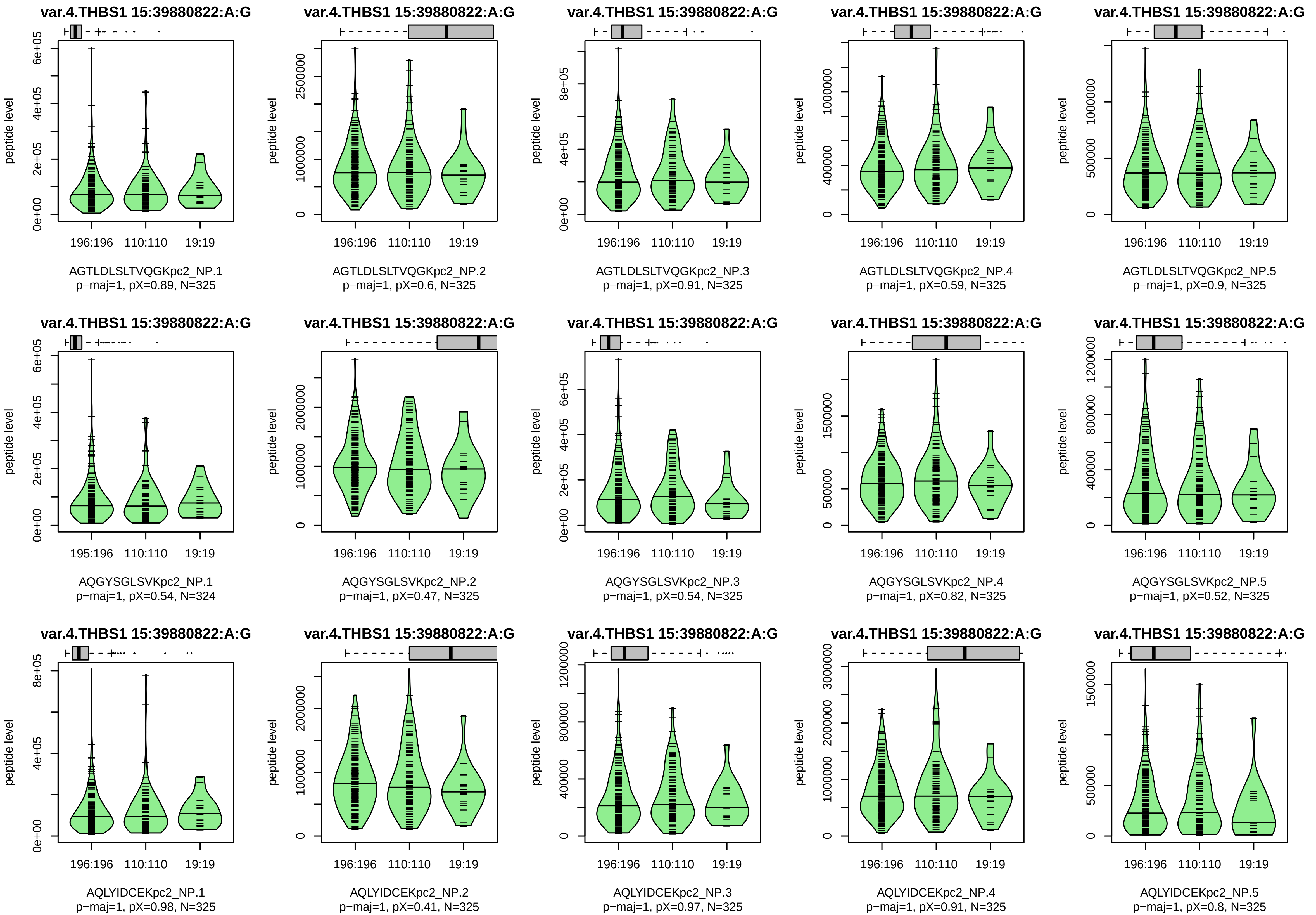
var.4.THBS1 15:39880822:A:G
peptide level
196:196 110:110 19:19
AGTLDLSLTVQGKpc2_NP.1
p−maj=1, pX=0.89, N=325
AGTLDLSLTVQGKpc2_NP.2
p−maj=1, pX=0.6, N=325
AGTLDLSLTVQGKpc2_NP.3
p−maj=1, pX=0.91, N=325
AGTLDLSLTVQGKpc2_NP.4
p−maj=1, pX=0.59, N=325
AGTLDLSLTVQGKpc2_NP.5
p−maj=1, pX=0.9, N=325
195:196 110:110 19:19
AQGYSGLSVKpc2_NP.1
p−maj=1, pX=0.54, N=324
AQGYSGLSVKpc2_NP.2
p−maj=1, pX=0.47, N=325
AQGYSGLSVKpc2_NP.3
p−maj=1, pX=0.54, N=325
AQGYSGLSVKpc2_NP.4
p−maj=1, pX=0.82, N=325
AQGYSGLSVKpc2_NP.5
p−maj=1, pX=0.52, N=325
AQLYIDCEKpc2_NP.1
p−maj=1, pX=0.98, N=325
AQLYIDCEKpc2_NP.2
p−maj=1, pX=0.41, N=325
AQLYIDCEKpc2_NP.3
p−maj=1, pX=0.97, N=325
AQLYIDCEKpc2_NP.4
p−maj=1, pX=0.91, N=325
AQLYIDCEKpc2_NP.5
p−maj=1, pX=0.8, N=325

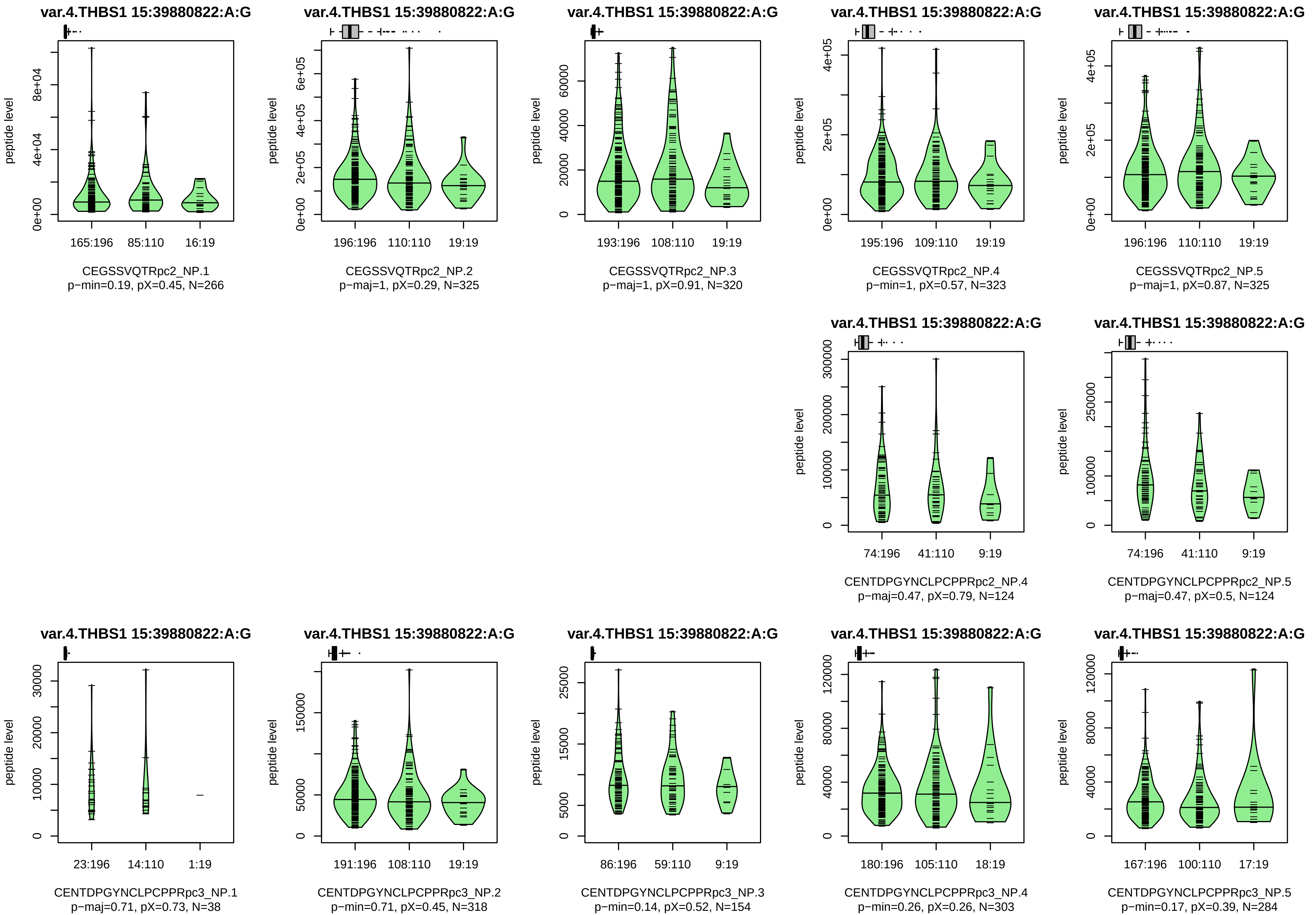
var.4.THBS1 15:39880822:A:G
peptide level
0e+00 4e+04 8e+04
165:196 85:110 16:19
CEGSSVQTRpc2_NP.1
p−min=0.19, pX=0.45, N=266
var.4.THBS1 15:39880822:A:G
peptide level
0e+00 2e+05 4e+05 6e+05
196:196 110:110 19:19
CEGSSVQTRpc2_NP.2
p−maj=1, pX=0.29, N=325
var.4.THBS1 15:39880822:A:G
peptide level
0 20000 40000 60000
193:196 108:110 19:19
CEGSSVQTRpc2_NP.3
p−maj=1, pX=0.91, N=320
var.4.THBS1 15:39880822:A:G
peptide level
0e+00 2e+05 4e+05
195:196 109:110 19:19
CEGSSVQTRpc2_NP.4
p−min=1, pX=0.57, N=323
var.4.THBS1 15:39880822:A:G
peptide level
0e+00 2e+05 4e+05
196:196 110:110 19:19
CEGSSVQTRpc2_NP.5
p−maj=1, pX=0.87, N=325
var.4.THBS1 15:39880822:A:G
peptide level
0 100000 200000 300000
74:196 41:110 9:19
CENTDPGYNCLPCPPRpc2_NP.4
p−maj=0.47, pX=0.79, N=124
var.4.THBS1 15:39880822:A:G
peptide level
0 100000 250000
74:196 41:110 9:19
CENTDPGYNCLPCPPRpc2_NP.5
p−maj=0.47, pX=0.5, N=124
var.4.THBS1 15:39880822:A:G
peptide level
0 10000 20000 30000
23:196 14:110 1:19
CENTDPGYNCLPCPPRpc3_NP.1
p−maj=0.71, pX=0.73, N=38
var.4.THBS1 15:39880822:A:G
peptide level
0 50000 150000
191:196 108:110 19:19
CENTDPGYNCLPCPPRpc3_NP.2
p−min=0.71, pX=0.45, N=318
var.4.THBS1 15:39880822:A:G
peptide level
0 5000 15000 25000
86:196 59:110 9:19
CENTDPGYNCLPCPPRpc3_NP.3
p−min=0.14, pX=0.52, N=154
var.4.THBS1 15:39880822:A:G
peptide level
0 40000 80000 120000
180:196 105:110 18:19
CENTDPGYNCLPCPPRpc3_NP.4
p−min=0.26, pX=0.26, N=303
var.4.THBS1 15:39880822:A:G
peptide level
0 40000 80000 120000
167:196 100:110 17:19
CENTDPGYNCLPCPPRpc3_NP.5
p−min=0.17, pX=0.39, N=284

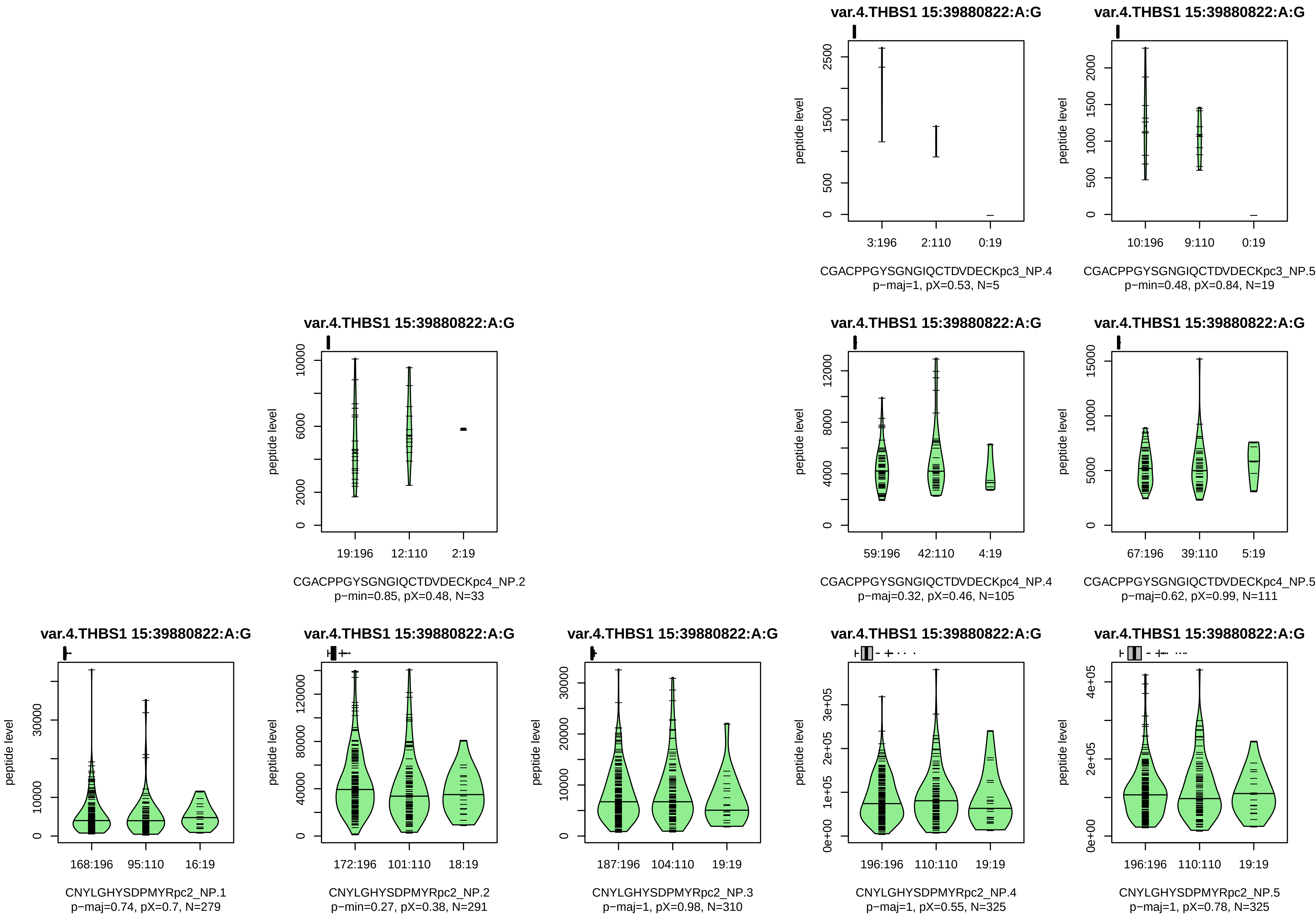

var.4.THBS1 15:39880822:A:G
peptide level
3:196
2:110
0:19
CGACPPGYSGNGIQCTDVDECKpc3_NP.4
p−maj=1, pX=0.53, N=5
var.4.THBS1 15:39880822:A:G
peptide level
10:196
9:110
0:19
CGACPPGYSGNGIQCTDVDECKpc3_NP.5
p−min=0.48, pX=0.84, N=19
var.4.THBS1 15:39880822:A:G
peptide level
19:196
12:110
2:19
CGACPPGYSGNGIQCTDVDECKpc4_NP.2
p−min=0.85, pX=0.48, N=33
var.4.THBS1 15:39880822:A:G
peptide level
59:196
42:110
4:19
CGACPPGYSGNGIQCTDVDECKpc4_NP.4
p−maj=0.32, pX=0.46, N=105
var.4.THBS1 15:39880822:A:G
peptide level
67:196
39:110
5:19
CGACPPGYSGNGIQCTDVDECKpc4_NP.5
p−maj=0.62, pX=0.99, N=111
var.4.THBS1 15:39880822:A:G
peptide level
168:196
95:110
16:19
CNYLGHYSDPMYRpc2_NP.1
p−maj=0.74, pX=0.7, N=279
var.4.THBS1 15:39880822:A:G
peptide level
172:196
101:110
18:19
CNYLGHYSDPMYRpc2_NP.2
p−min=0.27, pX=0.38, N=291
var.4.THBS1 15:39880822:A:G
peptide level
187:196
104:110
19:19
CNYLGHYSDPMYRpc2_NP.3
p−maj=1, pX=0.98, N=310
var.4.THBS1 15:39880822:A:G
peptide level
196:196
110:110
19:19
CNYLGHYSDPMYRpc2_NP.4
p−maj=1, pX=0.55, N=325
var.4.THBS1 15:39880822:A:G
peptide level
196:196
110:110
19:19
CNYLGHYSDPMYRpc2_NP.5
p−maj=1, pX=0.78, N=325

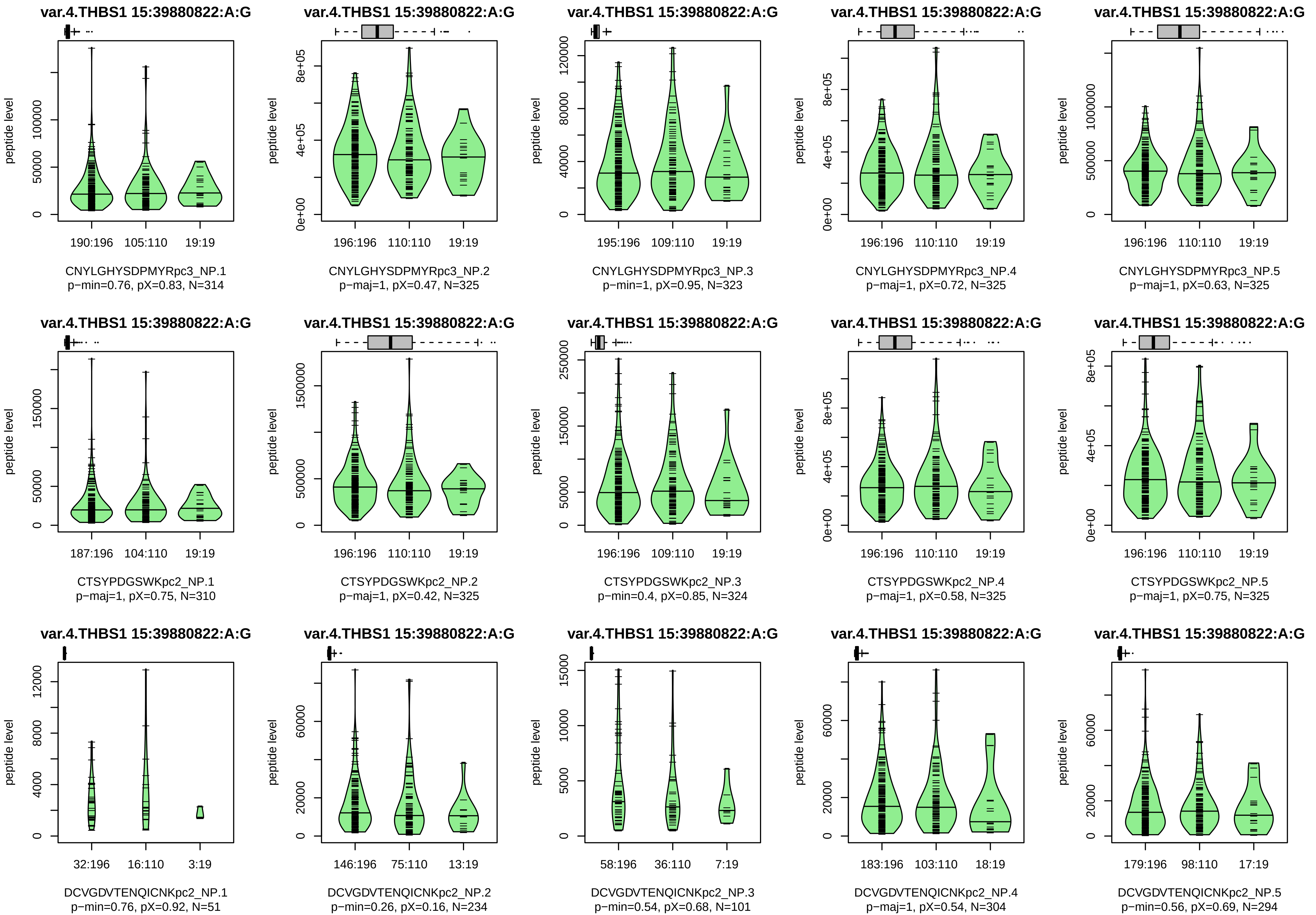
var.4.THBS1 15:39880822:A:G
peptide level
190:196
105:110
19:19
CNYLGHYSDPMYRpc3_NP.1
p−min=0.76, pX=0.83, N=314
var.4.THBS1 15:39880822:A:G
196:196
110:110
19:19
CNYLGHYSDPMYRpc3_NP.2
p−maj=1, pX=0.47, N=325
var.4.THBS1 15:39880822:A:G
195:196
109:110
19:19
CNYLGHYSDPMYRpc3_NP.3
p−min=1, pX=0.95, N=323
var.4.THBS1 15:39880822:A:G
196:196
110:110
19:19
CNYLGHYSDPMYRpc3_NP.4
p−maj=1, pX=0.72, N=325
var.4.THBS1 15:39880822:A:G
196:196
110:110
19:19
CNYLGHYSDPMYRpc3_NP.5
p−maj=1, pX=0.63, N=325
var.4.THBS1 15:39880822:A:G
187:196
104:110
19:19
CTSYPDGSWKpc2_NP.1
p−maj=1, pX=0.75, N=310
var.4.THBS1 15:39880822:A:G
196:196
110:110
19:19
CTSYPDGSWKpc2_NP.2
p−maj=1, pX=0.42, N=325
var.4.THBS1 15:39880822:A:G
196:196
109:110
19:19
CTSYPDGSWKpc2_NP.3
p−min=0.4, pX=0.85, N=324
var.4.THBS1 15:39880822:A:G
196:196
110:110
19:19
CTSYPDGSWKpc2_NP.4
p−maj=1, pX=0.58, N=325
var.4.THBS1 15:39880822:A:G
196:196
110:110
19:19
CTSYPDGSWKpc2_NP.5
p−maj=1, pX=0.75, N=325
var.4.THBS1 15:39880822:A:G
32:196
16:110
3:19
DCVGDVTENQICNKpc2_NP.1
p−min=0.76, pX=0.92, N=51
var.4.THBS1 15:39880822:A:G
146:196
75:110
13:19
DCVGDVTENQICNKpc2_NP.2
p−min=0.26, pX=0.16, N=234
var.4.THBS1 15:39880822:A:G
58:196
36:110
7:19
DCVGDVTENQICNKpc2_NP.3
p−min=0.54, pX=0.68, N=101
var.4.THBS1 15:39880822:A:G
183:196
103:110
18:19
DCVGDVTENQICNKpc2_NP.4
p−maj=1, pX=0.54, N=304
var.4.THBS1 15:39880822:A:G
179:196
98:110
17:19
DCVGDVTENQICNKpc2_NP.5
p−min=0.56, pX=0.69, N=294

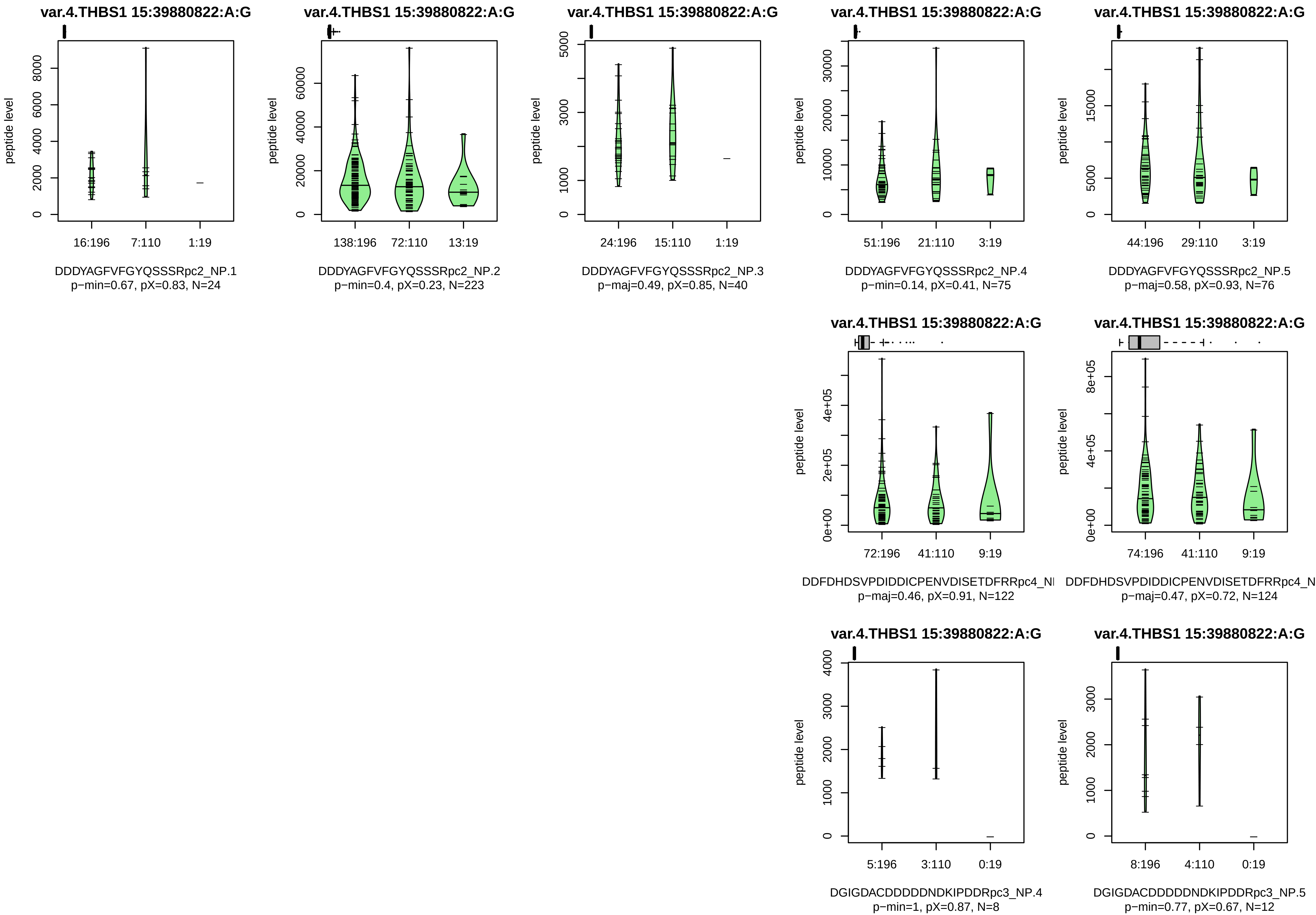

var.4.THBS1 15:39880822:A:G
peptide level
16:196
7:110
1:19
DDDYAGFVFGYQSSSRpc2_NP.1
p−min=0.67, pX=0.83, N=24
var.4.THBS1 15:39880822:A:G
peptide level
138:196
72:110
13:19
DDDYAGFVFGYQSSSRpc2_NP.2
p−min=0.4, pX=0.23, N=223
var.4.THBS1 15:39880822:A:G
peptide level
24:196
15:110
1:19
DDDYAGFVFGYQSSSRpc2_NP.3
p−maj=0.49, pX=0.85, N=40
var.4.THBS1 15:39880822:A:G
peptide level
51:196
21:110
3:19
DDDYAGFVFGYQSSSRpc2_NP.4
p−min=0.14, pX=0.41, N=75
var.4.THBS1 15:39880822:A:G
peptide level
44:196
29:110
3:19
DDDYAGFVFGYQSSSRpc2_NP.5
p−maj=0.58, pX=0.93, N=76
var.4.THBS1 15:39880822:A:G
peptide level
72:196
41:110
9:19
DDFDHDSVPDIDDICPENVDISETDFRRpc4_NI
p−maj=0.46, pX=0.91, N=122
var.4.THBS1 15:39880822:A:G
peptide level
74:196
41:110
9:19
DDFDHDSVPDIDDICPENVDISETDFRRpc4_N
p−maj=0.47, pX=0.72, N=124
var.4.THBS1 15:39880822:A:G
peptide level
5:196
3:110
0:19
DGIGDACDDDDDNDKIPDDRpc3_NP.4
p−min=1, pX=0.87, N=8
var.4.THBS1 15:39880822:A:G
peptide level
8:196
4:110
0:19
DGIGDACDDDDDNDKIPDDRpc3_NP.5
p−min=0.77, pX=0.67, N=12

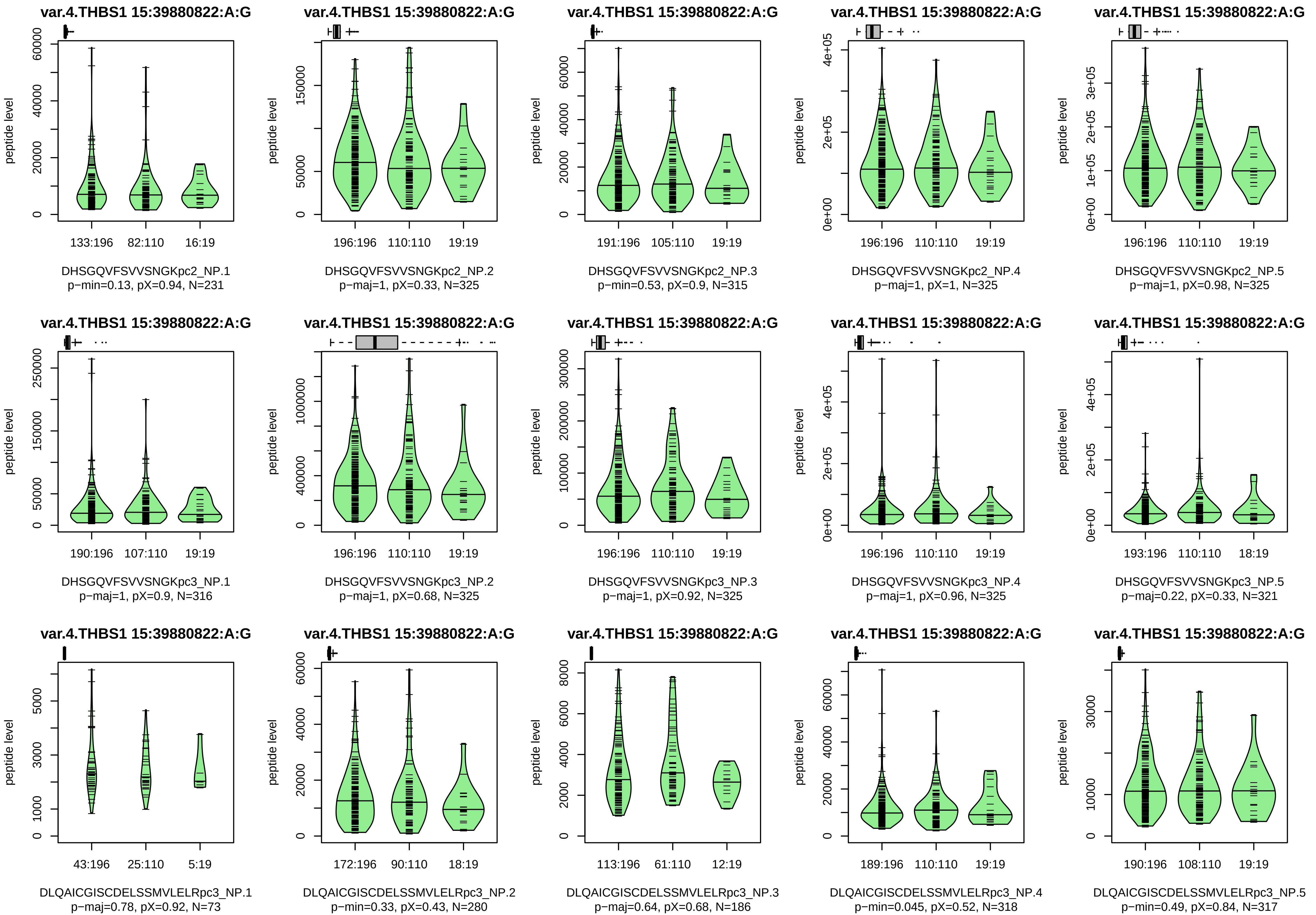
var.4.THBS1 15:39880822:A:G
peptide level
133:196
82:110
16:19
DHSGQVFSVVSNGKpc2_NP.1
p−min=0.13, pX=0.94, N=231
var.4.THBS1 15:39880822:A:G
peptide level
196:196
110:110
19:19
DHSGQVFSVVSNGKpc2_NP.2
p−maj=1, pX=0.33, N=325
var.4.THBS1 15:39880822:A:G
peptide level
191:196
105:110
19:19
DHSGQVFSVVSNGKpc2_NP.3
p−min=0.53, pX=0.9, N=315
var.4.THBS1 15:39880822:A:G
peptide level
196:196
110:110
19:19
DHSGQVFSVVSNGKpc2_NP.4
p−maj=1, pX=1, N=325
var.4.THBS1 15:39880822:A:G
peptide level
196:196
110:110
19:19
DHSGQVFSVVSNGKpc2_NP.5
p−maj=1, pX=0.98, N=325
var.4.THBS1 15:39880822:A:G
peptide level
190:196
107:110
19:19
DHSGQVFSVVSNGKpc3_NP.1
p−maj=1, pX=0.9, N=316
var.4.THBS1 15:39880822:A:G
peptide level
196:196
110:110
19:19
DHSGQVFSVVSNGKpc3_NP.2
p−maj=1, pX=0.68, N=325
var.4.THBS1 15:39880822:A:G
peptide level
196:196
110:110
19:19
DHSGQVFSVVSNGKpc3_NP.3
p−maj=1, pX=0.92, N=325
var.4.THBS1 15:39880822:A:G
peptide level
196:196
110:110
19:19
DHSGQVFSVVSNGKpc3_NP.4
p−maj=1, pX=0.96, N=325
var.4.THBS1 15:39880822:A:G
peptide level
193:196
110:110
18:19
DHSGQVFSVVSNGKpc3_NP.5
p−maj=0.22, pX=0.33, N=321
var.4.THBS1 15:39880822:A:G
peptide level
43:196
25:110
5:19
DLQAICGISCDELSSMVLELRpc3_NP.1
p−maj=0.78, pX=0.92, N=73
var.4.THBS1 15:39880822:A:G
peptide level
172:196
90:110
18:19
DLQAICGISCDELSSMVLELRpc3_NP.2
p−min=0.33, pX=0.43, N=280
var.4.THBS1 15:39880822:A:G
peptide level
113:196
61:110
12:19
DLQAICGISCDELSSMVLELRpc3_NP.3
p−maj=0.64, pX=0.68, N=186
var.4.THBS1 15:39880822:A:G
peptide level
189:196
110:110
19:19
DLQAICGISCDELSSMVLELRpc3_NP.4
p−min=0.045, pX=0.52, N=318
var.4.THBS1 15:39880822:A:G
peptide level
190:196
108:110
19:19
DLQAICGISCDELSSMVLELRpc3_NP.5
p−min=0.49, pX=0.84, N=317

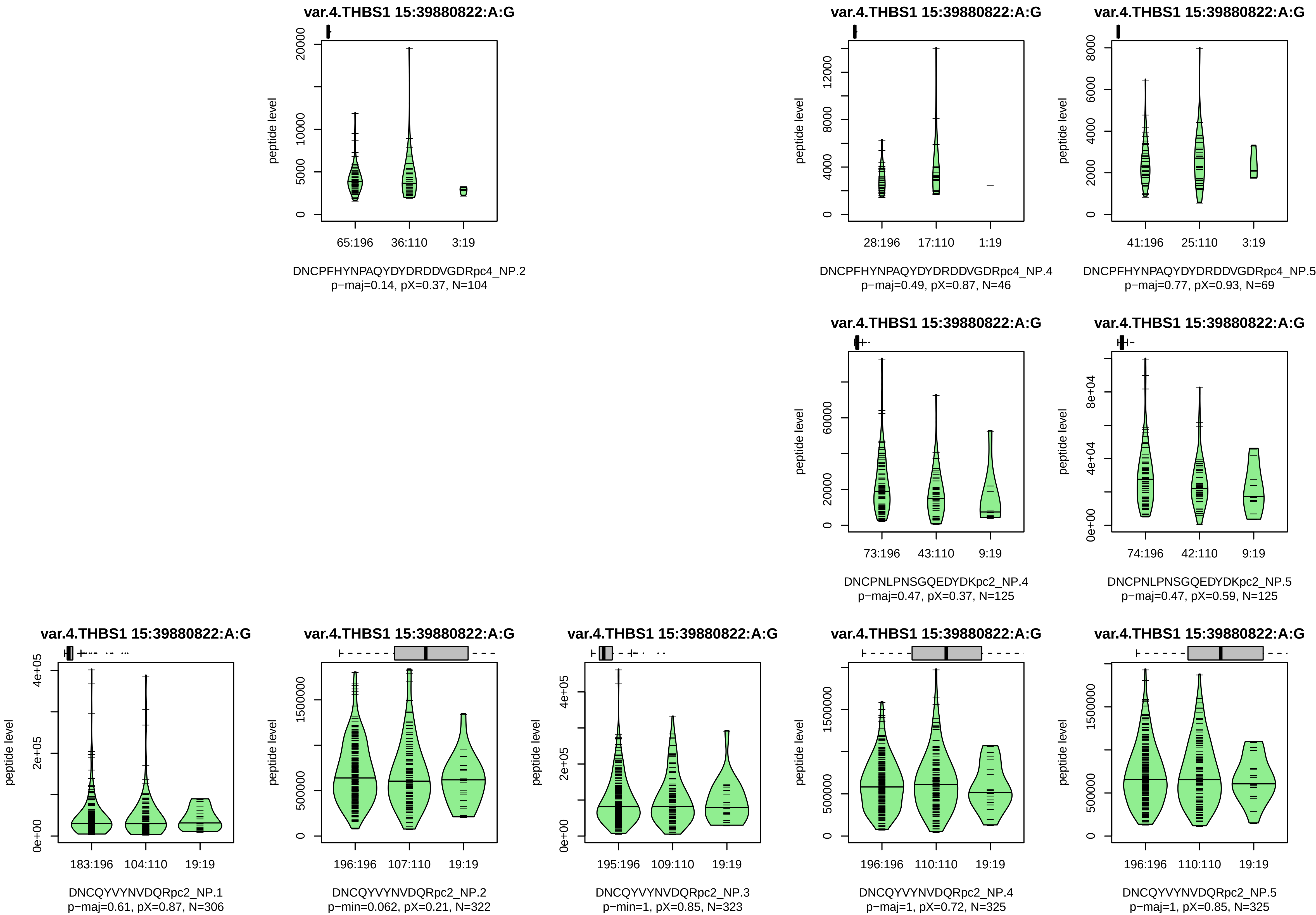

var.4.THBS1 15:39880822:A:G
peptide level
0
5000
10000
20000
65:196
36:110
3:19
DNCPFHYNPAQYDYDRDDVGDRpc4_NP.2
p−maj=0.14, pX=0.37, N=104
var.4.THBS1 15:39880822:A:G
peptide level
0
4000
8000
12000
28:196
17:110
1:19
DNCPFHYNPAQYDYDRDDVGDRpc4_NP.4
p−maj=0.49, pX=0.87, N=46
var.4.THBS1 15:39880822:A:G
peptide level
0
2000
4000
6000
8000
41:196
25:110
3:19
DNCPFHYNPAQYDYDRDDVGDRpc4_NP.5
p−maj=0.77, pX=0.93, N=69
var.4.THBS1 15:39880822:A:G
peptide level
0
20000
60000
73:196
43:110
9:19
DNCPNLPNSGQEDYDKpc2_NP.4
p−maj=0.47, pX=0.37, N=125
var.4.THBS1 15:39880822:A:G
peptide level
0e+00
4e+04
8e+04
74:196
42:110
9:19
DNCPNLPNSGQEDYDKpc2_NP.5
p−maj=0.47, pX=0.59, N=125
var.4.THBS1 15:39880822:A:G
peptide level
0e+00
2e+05
4e+05
183:196
104:110
19:19
DNCQYVYNVDQRpc2_NP.1
p−maj=0.61, pX=0.87, N=306
var.4.THBS1 15:39880822:A:G
peptide level
0
500000
1500000
196:196
107:110
19:19
DNCQYVYNVDQRpc2_NP.2
p−min=0.062, pX=0.21, N=322
var.4.THBS1 15:39880822:A:G
peptide level
0e+00
2e+05
4e+05
195:196
109:110
19:19
DNCQYVYNVDQRpc2_NP.3
p−min=1, pX=0.85, N=323
var.4.THBS1 15:39880822:A:G
peptide level
0
500000
1500000
196:196
110:110
19:19
DNCQYVYNVDQRpc2_NP.4
p−maj=1, pX=0.72, N=325
var.4.THBS1 15:39880822:A:G
peptide level
0
500000
1500000
196:196
110:110
19:19
DNCQYVYNVDQRpc2_NP.5
p−maj=1, pX=0.85, N=325

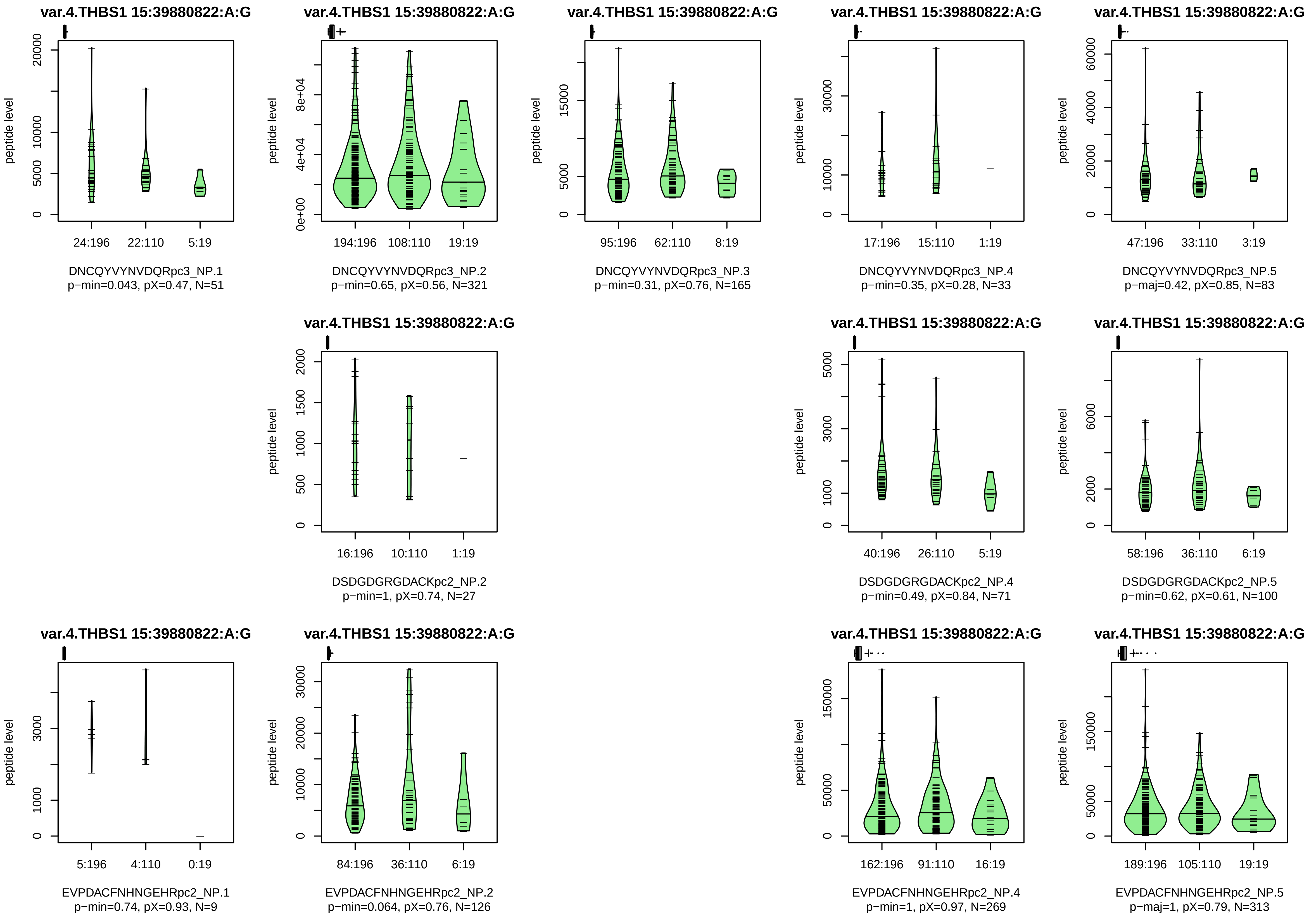
var.4.THBS1 15:39880822:A:G
peptide level
24:196
22:110
5:19
DNCQYVYNVDQRpc3_NP.1
p−min=0.043, pX=0.47, N=51
var.4.THBS1 15:39880822:A:G
peptide level
194:196
108:110
19:19
DNCQYVYNVDQRpc3_NP.2
p−min=0.65, pX=0.56, N=321
var.4.THBS1 15:39880822:A:G
peptide level
95:196
62:110
8:19
DNCQYVYNVDQRpc3_NP.3
p−min=0.31, pX=0.76, N=165
var.4.THBS1 15:39880822:A:G
peptide level
17:196
15:110
1:19
DNCQYVYNVDQRpc3_NP.4
p−min=0.35, pX=0.28, N=33
var.4.THBS1 15:39880822:A:G
peptide level
47:196
33:110
3:19
DNCQYVYNVDQRpc3_NP.5
p−maj=0.42, pX=0.85, N=83
var.4.THBS1 15:39880822:A:G
peptide level
16:196
10:110
1:19
DSDGDGRGDACKpc2_NP.2
p−min=1, pX=0.74, N=27
var.4.THBS1 15:39880822:A:G
peptide level
40:196
26:110
5:19
DSDGDGRGDACKpc2_NP.4
p−min=0.49, pX=0.84, N=71
var.4.THBS1 15:39880822:A:G
peptide level
58:196
36:110
6:19
DSDGDGRGDACKpc2_NP.5
p−min=0.62, pX=0.61, N=100
var.4.THBS1 15:39880822:A:G
peptide level
5:196
4:110
0:19
EVPDACFNHNGEHRpc2_NP.1
p−min=0.74, pX=0.93, N=9
var.4.THBS1 15:39880822:A:G
peptide level
84:196
36:110
6:19
EVPDACFNHNGEHRpc2_NP.2
p−min=0.064, pX=0.76, N=126
var.4.THBS1 15:39880822:A:G
peptide level
162:196
91:110
16:19
EVPDACFNHNGEHRpc2_NP.4
p−min=1, pX=0.97, N=269
var.4.THBS1 15:39880822:A:G
peptide level
189:196
105:110
19:19
EVPDACFNHNGEHRpc2_NP.5
p−maj=1, pX=0.79, N=313

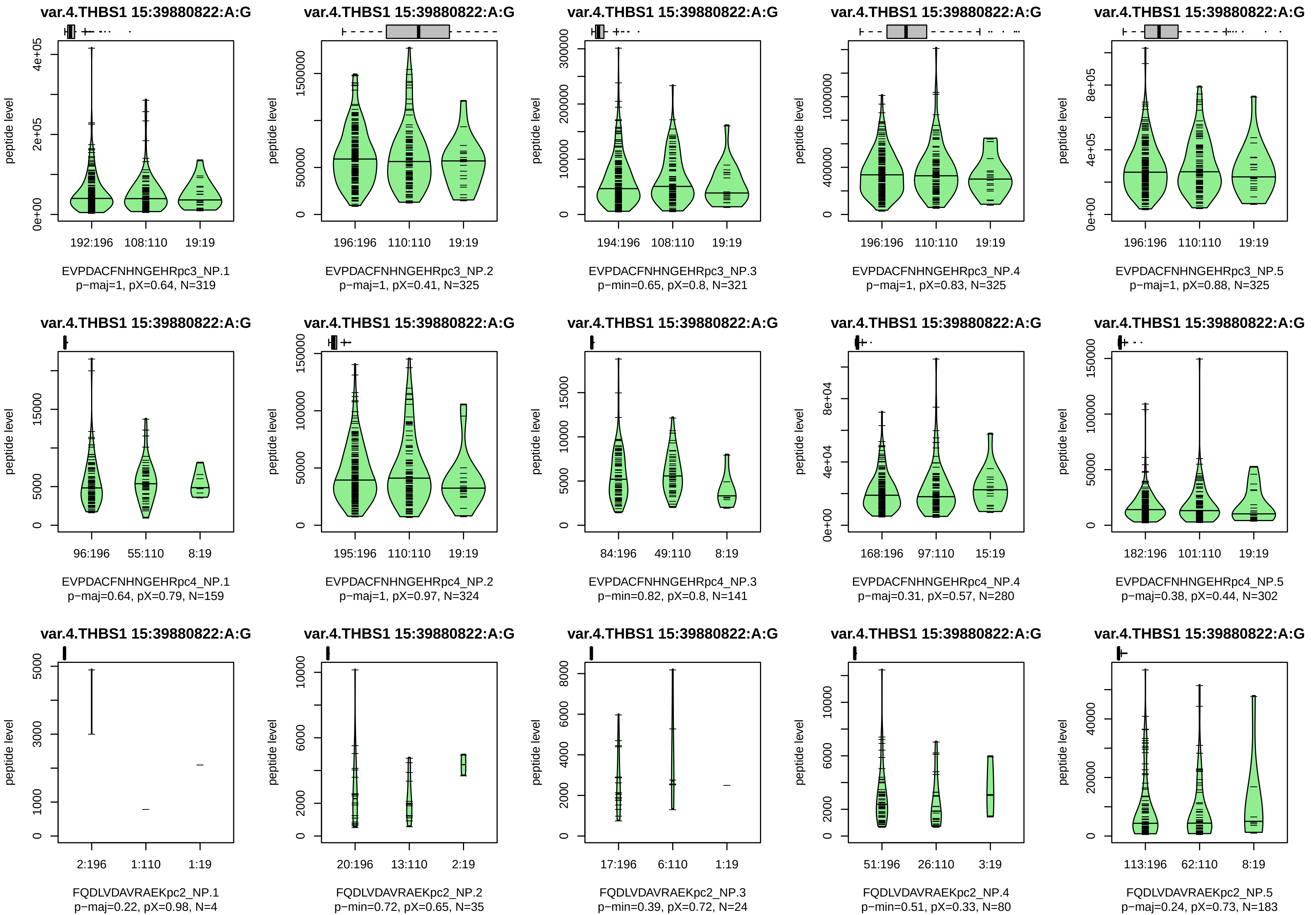

var.4.THBS1 15:39880822:A:G
peptide level
192:196
108:110
19:19
EVPDACFNHNGEHRpc3_NP.1
p−maj=1, pX=0.64, N=319
var.4.THBS1 15:39880822:A:G
peptide level
196:196
110:110
19:19
EVPDACFNHNGEHRpc3_NP.2
p−maj=1, pX=0.41, N=325
var.4.THBS1 15:39880822:A:G
peptide level
194:196
108:110
19:19
EVPDACFNHNGEHRpc3_NP.3
p−min=0.65, pX=0.8, N=321
var.4.THBS1 15:39880822:A:G
peptide level
196:196
110:110
19:19
EVPDACFNHNGEHRpc3_NP.4
p−maj=1, pX=0.83, N=325
var.4.THBS1 15:39880822:A:G
peptide level
196:196
110:110
19:19
EVPDACFNHNGEHRpc3_NP.5
p−maj=1, pX=0.88, N=325
var.4.THBS1 15:39880822:A:G
peptide level
96:196
55:110
8:19
EVPDACFNHNGEHRpc4_NP.1
p−maj=0.64, pX=0.79, N=159
var.4.THBS1 15:39880822:A:G
peptide level
195:196
110:110
19:19
EVPDACFNHNGEHRpc4_NP.2
p−maj=1, pX=0.97, N=324
var.4.THBS1 15:39880822:A:G
peptide level
84:196
49:110
8:19
EVPDACFNHNGEHRpc4_NP.3
p−min=0.82, pX=0.8, N=141
var.4.THBS1 15:39880822:A:G
peptide level
168:196
97:110
15:19
EVPDACFNHNGEHRpc4_NP.4
p−maj=0.31, pX=0.57, N=280
var.4.THBS1 15:39880822:A:G
peptide level
182:196
101:110
19:19
EVPDACFNHNGEHRpc4_NP.5
p−maj=0.38, pX=0.44, N=302
var.4.THBS1 15:39880822:A:G
peptide level
2:196
1:110
1:19
FQDLVDAVRAEKpc2_NP.1
p−maj=0.22, pX=0.98, N=4
var.4.THBS1 15:39880822:A:G
peptide level
20:196
13:110
2:19
FQDLVDAVRAEKpc2_NP.2
p−min=0.72, pX=0.65, N=35
var.4.THBS1 15:39880822:A:G
peptide level
17:196
6:110
1:19
FQDLVDAVRAEKpc2_NP.3
p−min=0.39, pX=0.72, N=24
var.4.THBS1 15:39880822:A:G
peptide level
51:196
26:110
3:19
FQDLVDAVRAEKpc2_NP.4
p−min=0.51, pX=0.33, N=80
var.4.THBS1 15:39880822:A:G
peptide level
113:196
62:110
8:19
FQDLVDAVRAEKpc2_NP.5
p−maj=0.24, pX=0.73, N=183

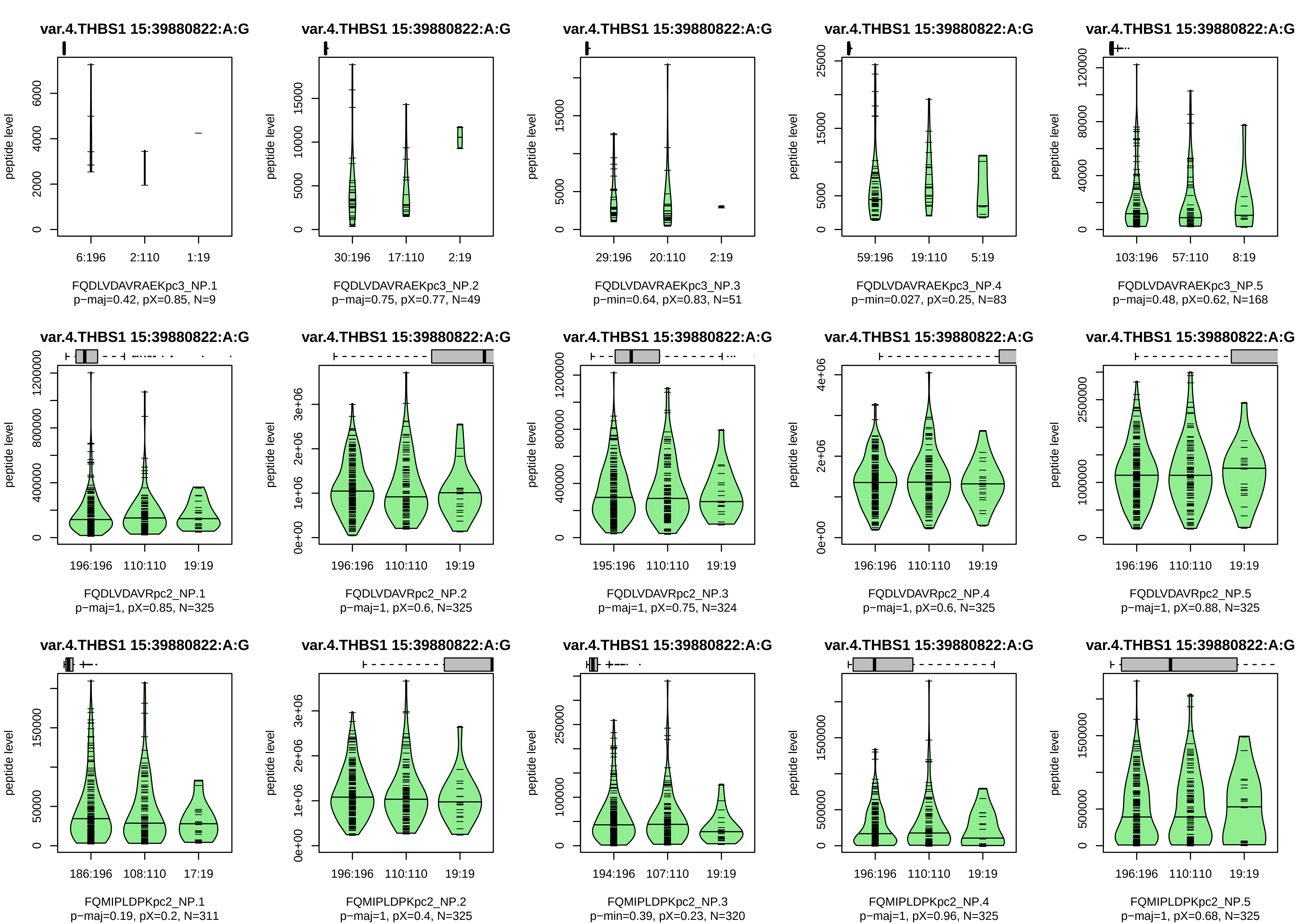
var.4.THBS1 15:39880822:A:G
peptide level
6:196
2:110
1:19
FQDLVDAVRAEKpc3_NP.1
p−maj=0.42, pX=0.85, N=9
var.4.THBS1 15:39880822:A:G
peptide level
30:196
17:110
2:19
FQDLVDAVRAEKpc3_NP.2
p−maj=0.75, pX=0.77, N=49
var.4.THBS1 15:39880822:A:G
peptide level
29:196
20:110
2:19
FQDLVDAVRAEKpc3_NP.3
p−min=0.64, pX=0.83, N=51
var.4.THBS1 15:39880822:A:G
peptide level
59:196
19:110
5:19
FQDLVDAVRAEKpc3_NP.4
p−min=0.027, pX=0.25, N=83
var.4.THBS1 15:39880822:A:G
peptide level
103:196
57:110
8:19
FQDLVDAVRAEKpc3_NP.5
p−maj=0.48, pX=0.62, N=168
var.4.THBS1 15:39880822:A:G
peptide level
196:196
110:110
19:19
FQDLVDAVRpc2_NP.1
p−maj=1, pX=0.85, N=325
var.4.THBS1 15:39880822:A:G
peptide level
196:196
110:110
19:19
FQDLVDAVRpc2_NP.2
p−maj=1, pX=0.6, N=325
var.4.THBS1 15:39880822:A:G
peptide level
195:196
110:110
19:19
FQDLVDAVRpc2_NP.3
p−maj=1, pX=0.75, N=324
var.4.THBS1 15:39880822:A:G
peptide level
196:196
110:110
19:19
FQDLVDAVRpc2_NP.4
p−maj=1, pX=0.6, N=325
var.4.THBS1 15:39880822:A:G
peptide level
196:196
110:110
19:19
FQDLVDAVRpc2_NP.5
p−maj=1, pX=0.88, N=325
var.4.THBS1 15:39880822:A:G
peptide level
186:196
108:110
17:19
FQMIPLDPKpc2_NP.1
p−maj=0.19, pX=0.2, N=311
var.4.THBS1 15:39880822:A:G
peptide level
196:196
110:110
19:19
FQMIPLDPKpc2_NP.2
p−maj=1, pX=0.4, N=325
var.4.THBS1 15:39880822:A:G
peptide level
194:196
107:110
19:19
FQMIPLDPKpc2_NP.3
p−min=0.39, pX=0.23, N=320
var.4.THBS1 15:39880822:A:G
peptide level
196:196
110:110
19:19
FQMIPLDPKpc2_NP.4
p−maj=1, pX=0.96, N=325
var.4.THBS1 15:39880822:A:G
peptide level
196:196
110:110
19:19
FQMIPLDPKpc2_NP.5
p−maj=1, pX=0.68, N=325

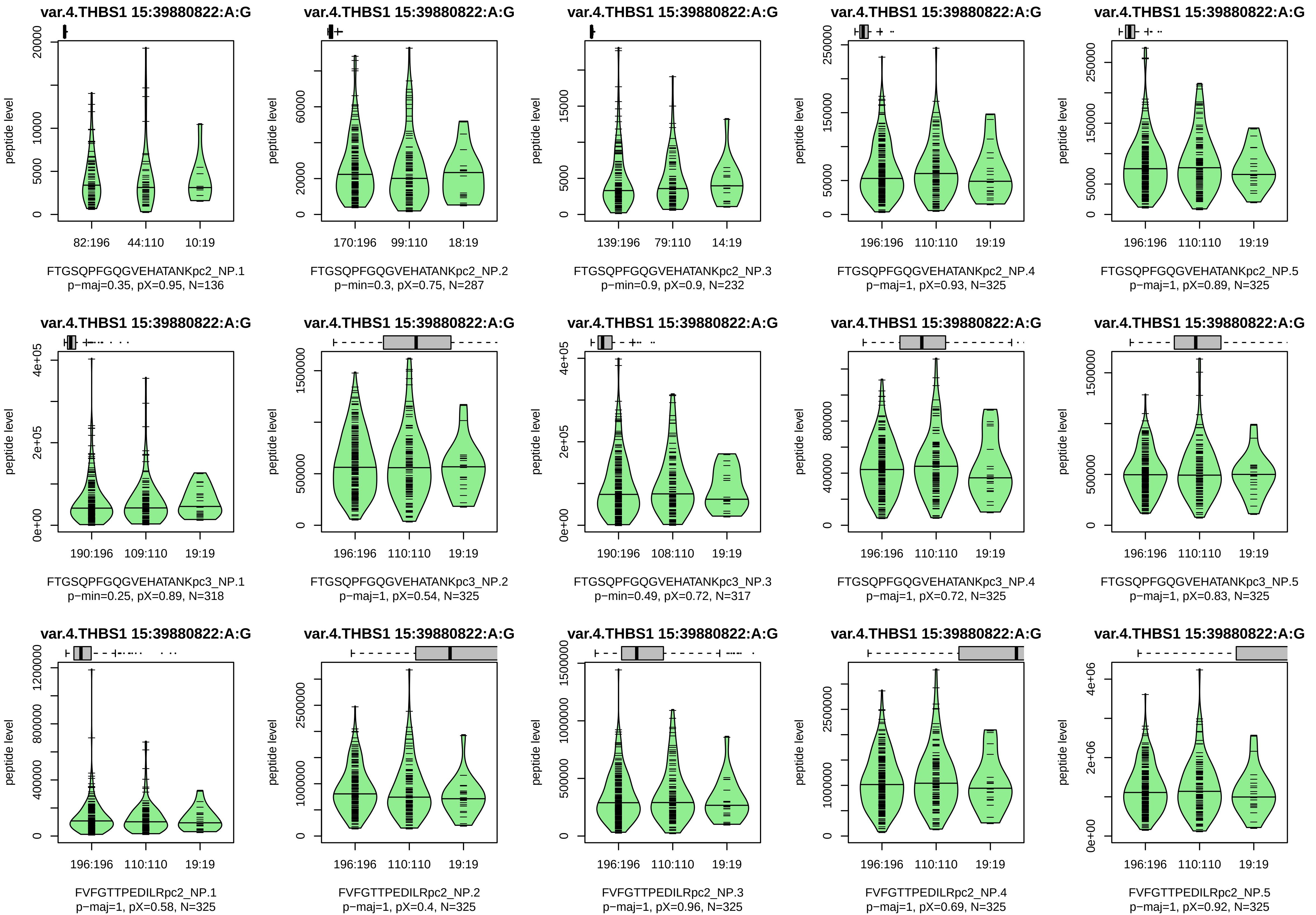
var.4.THBS1 15:39880822:A:G
peptide level
82:196
44:110
10:19
FTGSQPFGQGVEHATANKpc2_NP.1
p−maj=0.35, pX=0.95, N=136
var.4.THBS1 15:39880822:A:G
peptide level
170:196
99:110
18:19
FTGSQPFGQGVEHATANKpc2_NP.2
p−min=0.3, pX=0.75, N=287
var.4.THBS1 15:39880822:A:G
peptide level
139:196
79:110
14:19
FTGSQPFGQGVEHATANKpc2_NP.3
p−min=0.9, pX=0.9, N=232
var.4.THBS1 15:39880822:A:G
peptide level
196:196
110:110
19:19
FTGSQPFGQGVEHATANKpc2_NP.4
p−maj=1, pX=0.93, N=325
var.4.THBS1 15:39880822:A:G
peptide level
196:196
110:110
19:19
FTGSQPFGQGVEHATANKpc2_NP.5
p−maj=1, pX=0.89, N=325
var.4.THBS1 15:39880822:A:G
peptide level
190:196
109:110
19:19
FTGSQPFGQGVEHATANKpc3_NP.1
p−min=0.25, pX=0.89, N=318
var.4.THBS1 15:39880822:A:G
peptide level
196:196
110:110
19:19
FTGSQPFGQGVEHATANKpc3_NP.2
p−maj=1, pX=0.54, N=325
var.4.THBS1 15:39880822:A:G
peptide level
190:196
108:110
19:19
FTGSQPFGQGVEHATANKpc3_NP.3
p−min=0.49, pX=0.72, N=317
var.4.THBS1 15:39880822:A:G
peptide level
196:196
110:110
19:19
FTGSQPFGQGVEHATANKpc3_NP.4
p−maj=1, pX=0.72, N=325
var.4.THBS1 15:39880822:A:G
peptide level
196:196
110:110
19:19
FTGSQPFGQGVEHATANKpc3_NP.5
p−maj=1, pX=0.83, N=325
var.4.THBS1 15:39880822:A:G
peptide level
196:196
110:110
19:19
FVFGTTPEDILRpc2_NP.1
p−maj=1, pX=0.58, N=325
var.4.THBS1 15:39880822:A:G
peptide level
196:196
110:110
19:19
FVFGTTPEDILRpc2_NP.2
p−maj=1, pX=0.4, N=325
var.4.THBS1 15:39880822:A:G
peptide level
196:196
110:110
19:19
FVFGTTPEDILRpc2_NP.3
p−maj=1, pX=0.96, N=325
var.4.THBS1 15:39880822:A:G
peptide level
196:196
110:110
19:19
FVFGTTPEDILRpc2_NP.4
p−maj=1, pX=0.69, N=325
var.4.THBS1 15:39880822:A:G
peptide level
196:196
110:110
19:19
FVFGTTPEDILRpc2_NP.5
p−maj=1, pX=0.92, N=325

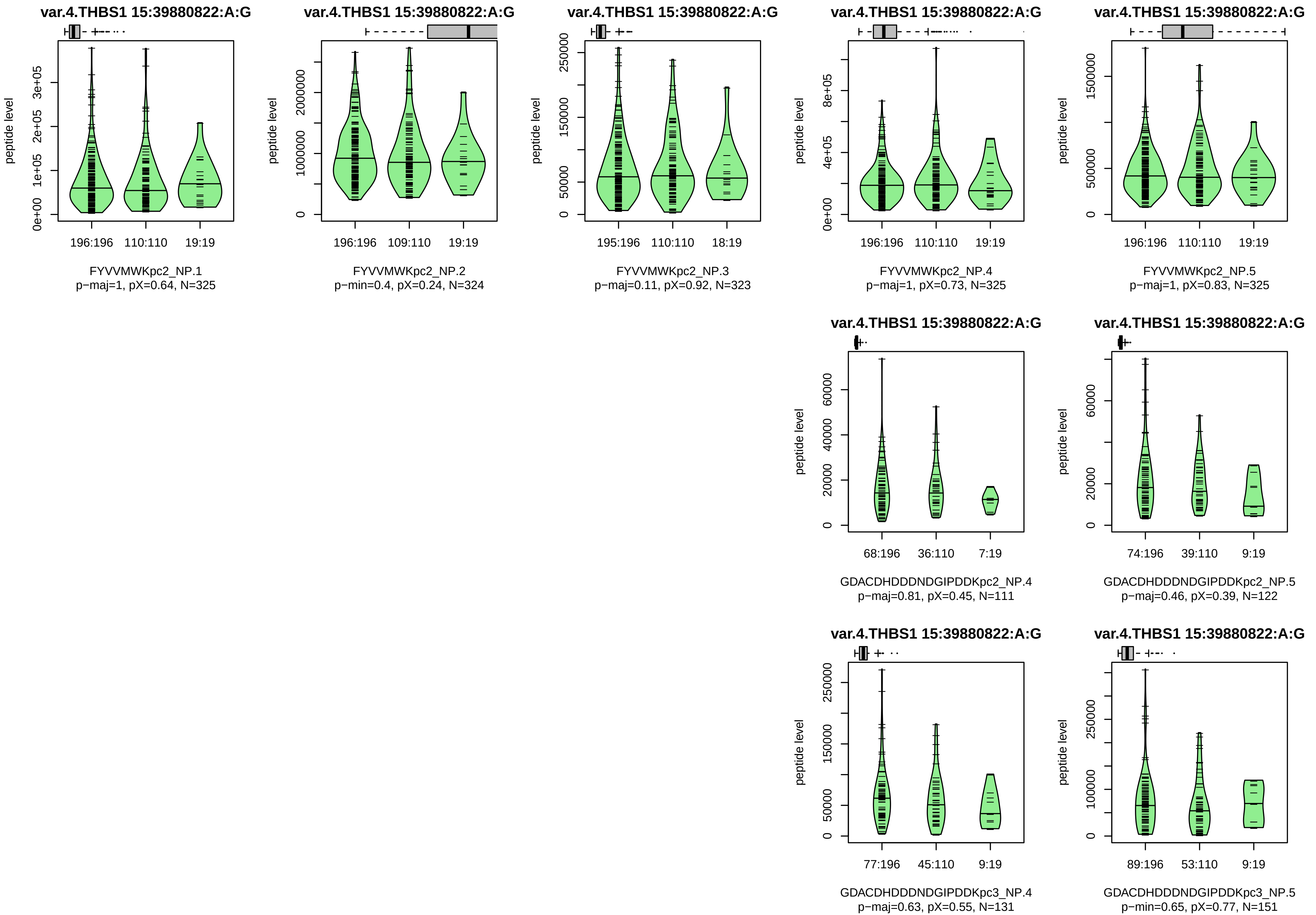

var.4.THBS1 15:39880822:A:G
peptide level
0e+00
1e+05
2e+05
3e+05
196:196
110:110
19:19
FYVVMWKpc2_NP.1
p−maj=1, pX=0.64, N=325
var.4.THBS1 15:39880822:A:G
peptide level
0
1000000
2000000
196:196
109:110
19:19
FYVVMWKpc2_NP.2
p−min=0.4, pX=0.24, N=324
var.4.THBS1 15:39880822:A:G
peptide level
0
50000
150000
250000
195:196
110:110
18:19
FYVVMWKpc2_NP.3
p−maj=0.11, pX=0.92, N=323
var.4.THBS1 15:39880822:A:G
peptide level
0e+00
4e+05
8e+05
196:196
110:110
19:19
FYVVMWKpc2_NP.4
p−maj=1, pX=0.73, N=325
var.4.THBS1 15:39880822:A:G
peptide level
0
500000
1500000
196:196
110:110
19:19
FYVVMWKpc2_NP.5
p−maj=1, pX=0.83, N=325
var.4.THBS1 15:39880822:A:G
peptide level
0
20000
40000
60000
68:196
36:110
7:19
GDACDHDDDNDGIPDDKpc2_NP.4
p−maj=0.81, pX=0.45, N=111
var.4.THBS1 15:39880822:A:G
peptide level
0
20000
60000
74:196
39:110
9:19
GDACDHDDDNDGIPDDKpc2_NP.5
p−maj=0.46, pX=0.39, N=122
var.4.THBS1 15:39880822:A:G
peptide level
0
50000
150000
250000
77:196
45:110
9:19
GDACDHDDDNDGIPDDKpc3_NP.4
p−maj=0.63, pX=0.55, N=131
var.4.THBS1 15:39880822:A:G
peptide level
0
100000
250000
89:196
53:110
9:19
GDACDHDDDNDGIPDDKpc3_NP.5
p−min=0.65, pX=0.77, N=151

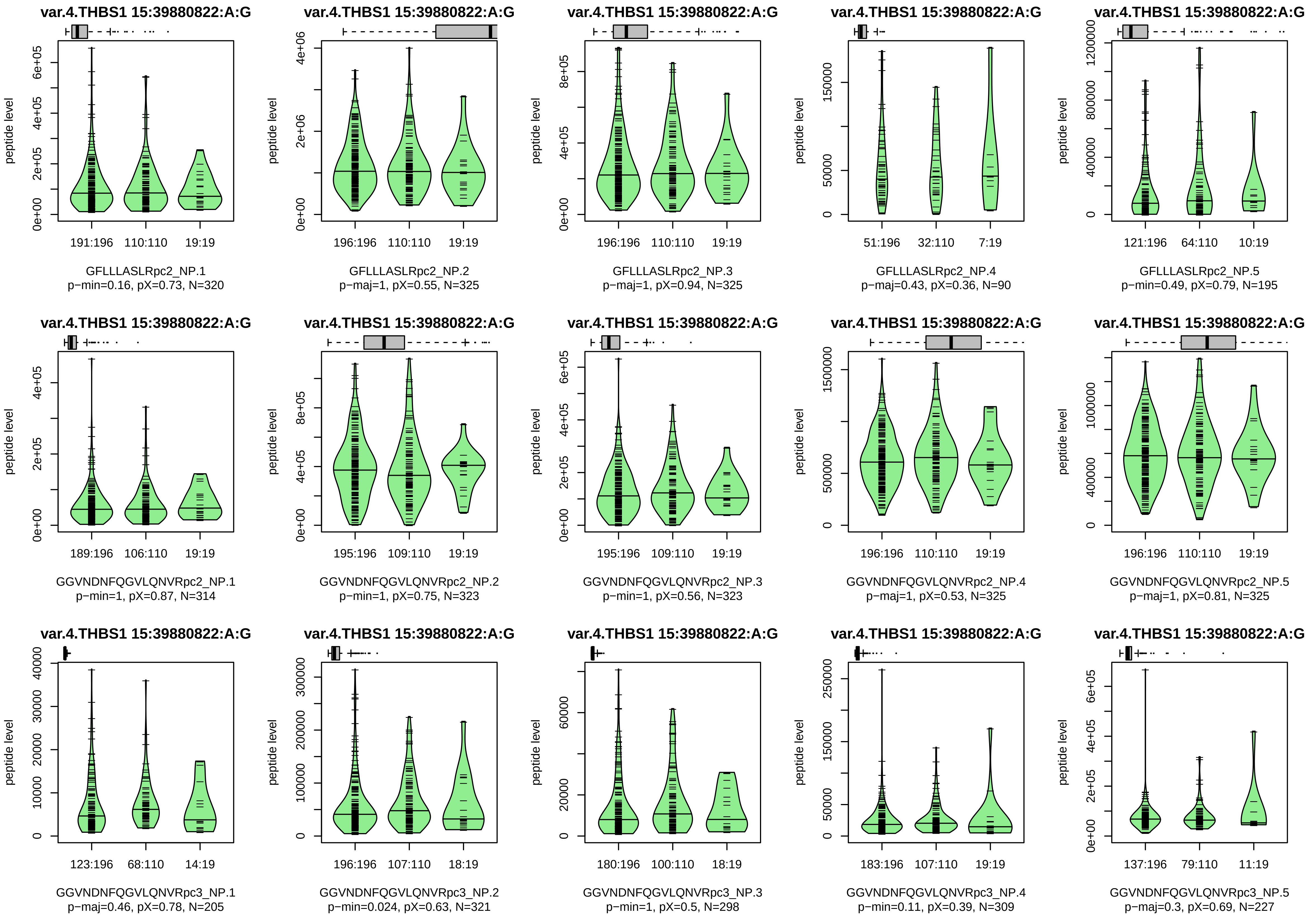

var.4.THBS1 15:39880822:A:G
peptide level
191:196
110:110
19:19
GFLLLASLRpc2_NP.1
p−min=0.16, pX=0.73, N=320
var.4.THBS1 15:39880822:A:G
196:196
110:110
19:19
GFLLLASLRpc2_NP.2
p−maj=1, pX=0.55, N=325
var.4.THBS1 15:39880822:A:G
196:196
110:110
19:19
GFLLLASLRpc2_NP.3
p−maj=1, pX=0.94, N=325
var.4.THBS1 15:39880822:A:G
51:196
32:110
7:19
GFLLLASLRpc2_NP.4
p−maj=0.43, pX=0.36, N=90
var.4.THBS1 15:39880822:A:G
121:196
64:110
10:19
GFLLLASLRpc2_NP.5
p−min=0.49, pX=0.79, N=195
var.4.THBS1 15:39880822:A:G
189:196
106:110
19:19
GGVNDNFQGVLQNVRpc2_NP.1
p−min=1, pX=0.87, N=314
var.4.THBS1 15:39880822:A:G
195:196
109:110
19:19
GGVNDNFQGVLQNVRpc2_NP.2
p−min=1, pX=0.75, N=323
var.4.THBS1 15:39880822:A:G
195:196
109:110
19:19
GGVNDNFQGVLQNVRpc2_NP.3
p−min=1, pX=0.56, N=323
var.4.THBS1 15:39880822:A:G
196:196
110:110
19:19
GGVNDNFQGVLQNVRpc2_NP.4
p−maj=1, pX=0.53, N=325
var.4.THBS1 15:39880822:A:G
196:196
110:110
19:19
GGVNDNFQGVLQNVRpc2_NP.5
p−maj=1, pX=0.81, N=325
var.4.THBS1 15:39880822:A:G
123:196
68:110
14:19
GGVNDNFQGVLQNVRpc3_NP.1
p−maj=0.46, pX=0.78, N=205
var.4.THBS1 15:39880822:A:G
196:196
107:110
18:19
GGVNDNFQGVLQNVRpc3_NP.2
p−min=0.024, pX=0.63, N=321
var.4.THBS1 15:39880822:A:G
180:196
100:110
18:19
GGVNDNFQGVLQNVRpc3_NP.3
p−min=1, pX=0.5, N=298
var.4.THBS1 15:39880822:A:G
183:196
107:110
19:19
GGVNDNFQGVLQNVRpc3_NP.4
p−min=0.11, pX=0.39, N=309
var.4.THBS1 15:39880822:A:G
137:196
79:110
11:19
GGVNDNFQGVLQNVRpc3_NP.5
p−maj=0.3, pX=0.69, N=227

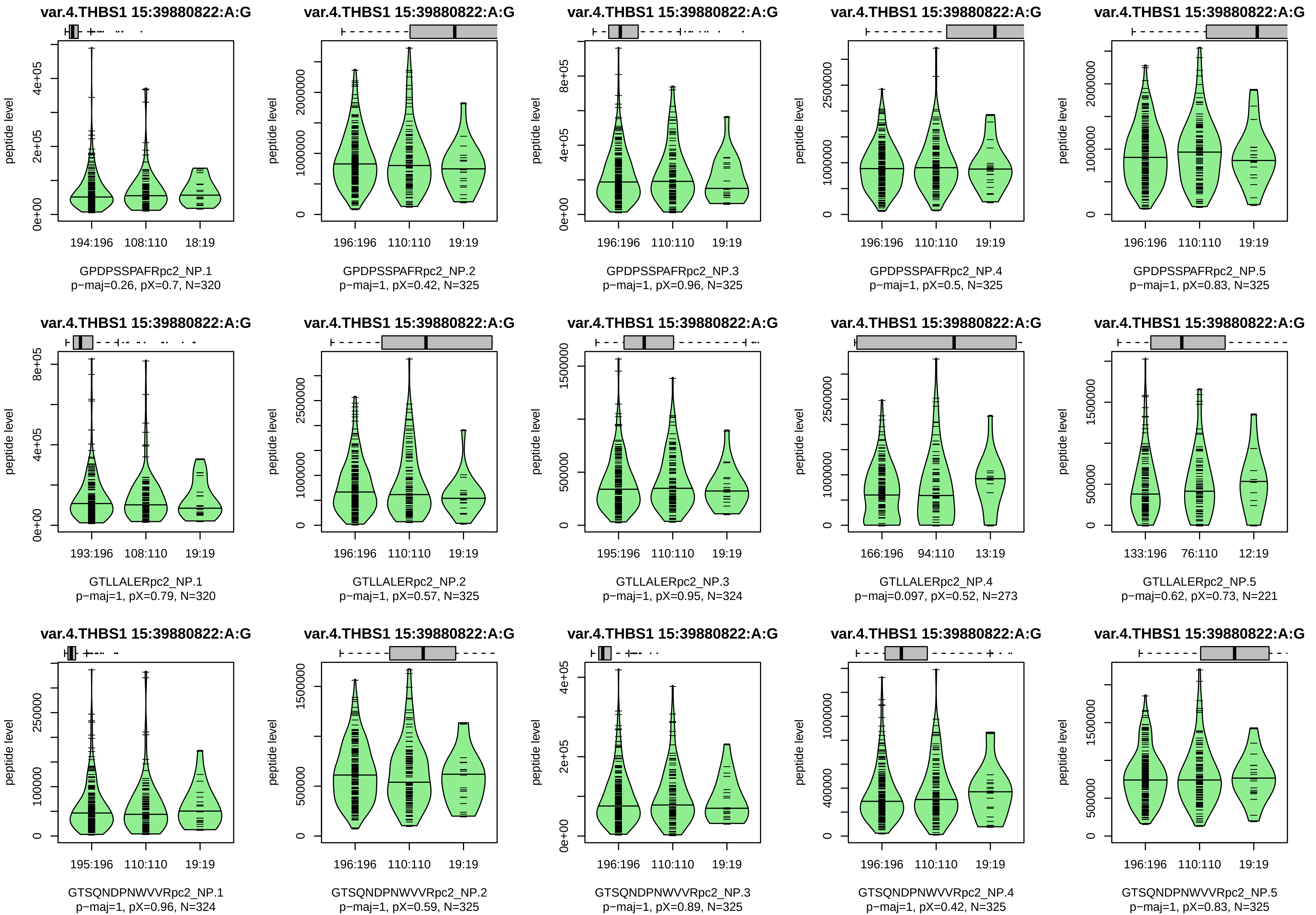
var.4.THBS1 15:39880822:A:G
peptide level
194:196
108:110
18:19
GPDPSSPAFRpc2_NP.1
p−maj=0.26, pX=0.7, N=320
var.4.THBS1 15:39880822:A:G
peptide level
196:196
110:110
19:19
GPDPSSPAFRpc2_NP.2
p−maj=1, pX=0.42, N=325
var.4.THBS1 15:39880822:A:G
peptide level
196:196
110:110
19:19
GPDPSSPAFRpc2_NP.3
p−maj=1, pX=0.96, N=325
var.4.THBS1 15:39880822:A:G
peptide level
196:196
110:110
19:19
GPDPSSPAFRpc2_NP.4
p−maj=1, pX=0.5, N=325
var.4.THBS1 15:39880822:A:G
peptide level
196:196
110:110
19:19
GPDPSSPAFRpc2_NP.5
p−maj=1, pX=0.83, N=325
var.4.THBS1 15:39880822:A:G
peptide level
193:196
108:110
19:19
GTLLALERpc2_NP.1
p−maj=1, pX=0.79, N=320
var.4.THBS1 15:39880822:A:G
peptide level
196:196
110:110
19:19
GTLLALERpc2_NP.2
p−maj=1, pX=0.57, N=325
var.4.THBS1 15:39880822:A:G
peptide level
195:196
110:110
19:19
GTLLALERpc2_NP.3
p−maj=1, pX=0.95, N=324
var.4.THBS1 15:39880822:A:G
peptide level
166:196
94:110
13:19
GTLLALERpc2_NP.4
p−maj=0.097, pX=0.52, N=273
var.4.THBS1 15:39880822:A:G
peptide level
133:196
76:110
12:19
GTLLALERpc2_NP.5
p−maj=0.62, pX=0.73, N=221
var.4.THBS1 15:39880822:A:G
peptide level
195:196
110:110
19:19
GTSQNDPNWVVRpc2_NP.1
p−maj=1, pX=0.96, N=324
var.4.THBS1 15:39880822:A:G
peptide level
196:196
110:110
19:19
GTSQNDPNWVVRpc2_NP.2
p−maj=1, pX=0.59, N=325
var.4.THBS1 15:39880822:A:G
peptide level
196:196
110:110
19:19
GTSQNDPNWVVRpc2_NP.3
p−maj=1, pX=0.89, N=325
var.4.THBS1 15:39880822:A:G
peptide level
196:196
110:110
19:19
GTSQNDPNWVVRpc2_NP.4
p−maj=1, pX=0.42, N=325
var.4.THBS1 15:39880822:A:G
peptide level
196:196
110:110
19:19
GTSQNDPNWVVRpc2_NP.5
p−maj=1, pX=0.83, N=325

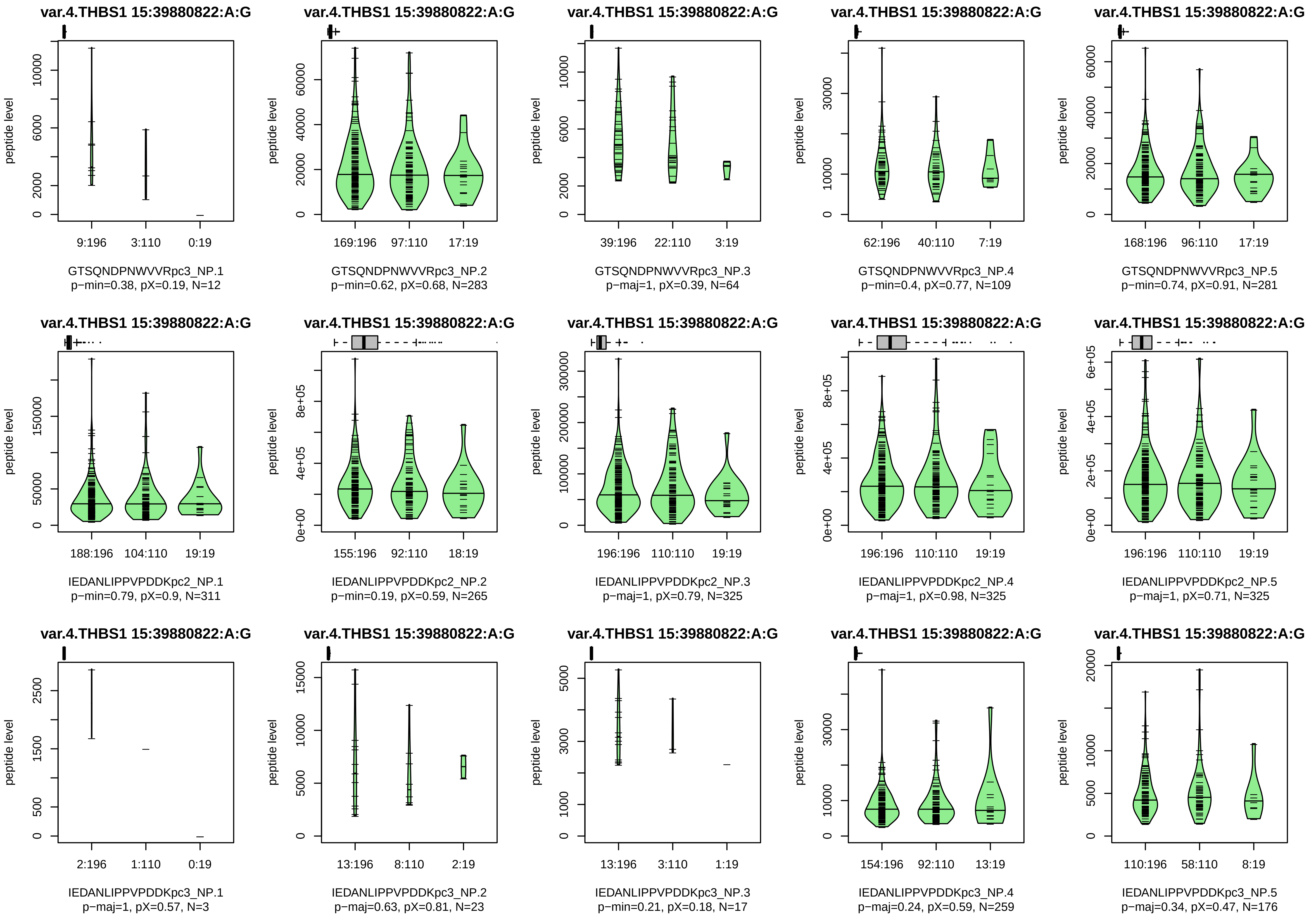
var.4.THBS1 15:39880822:A:G
peptide level
9:196
3:110
0:19
GTSQNDPNWVVRpc3_NP.1
p−min=0.38, pX=0.19, N=12
var.4.THBS1 15:39880822:A:G
peptide level
169:196
97:110
17:19
GTSQNDPNWVVRpc3_NP.2
p−min=0.62, pX=0.68, N=283
var.4.THBS1 15:39880822:A:G
peptide level
39:196
22:110
3:19
GTSQNDPNWVVRpc3_NP.3
p−maj=1, pX=0.39, N=64
var.4.THBS1 15:39880822:A:G
peptide level
62:196
40:110
7:19
GTSQNDPNWVVRpc3_NP.4
p−min=0.4, pX=0.77, N=109
var.4.THBS1 15:39880822:A:G
peptide level
168:196
96:110
17:19
GTSQNDPNWVVRpc3_NP.5
p−min=0.74, pX=0.91, N=281
var.4.THBS1 15:39880822:A:G
peptide level
188:196
104:110
19:19
IEDANLIPPVPDDKpc2_NP.1
p−min=0.79, pX=0.9, N=311
var.4.THBS1 15:39880822:A:G
peptide level
155:196
92:110
18:19
IEDANLIPPVPDDKpc2_NP.2
p−min=0.19, pX=0.59, N=265
var.4.THBS1 15:39880822:A:G
peptide level
196:196
110:110
19:19
IEDANLIPPVPDDKpc2_NP.3
p−maj=1, pX=0.79, N=325
var.4.THBS1 15:39880822:A:G
peptide level
196:196
110:110
19:19
IEDANLIPPVPDDKpc2_NP.4
p−maj=1, pX=0.98, N=325
var.4.THBS1 15:39880822:A:G
peptide level
196:196
110:110
19:19
IEDANLIPPVPDDKpc2_NP.5
p−maj=1, pX=0.71, N=325
var.4.THBS1 15:39880822:A:G
peptide level
2:196
1:110
0:19
IEDANLIPPVPDDKpc3_NP.1
p−maj=1, pX=0.57, N=3
var.4.THBS1 15:39880822:A:G
peptide level
13:196
8:110
2:19
IEDANLIPPVPDDKpc3_NP.2
p−maj=0.63, pX=0.81, N=23
var.4.THBS1 15:39880822:A:G
peptide level
13:196
3:110
1:19
IEDANLIPPVPDDKpc3_NP.3
p−min=0.21, pX=0.18, N=17
var.4.THBS1 15:39880822:A:G
peptide level
154:196
92:110
13:19
IEDANLIPPVPDDKpc3_NP.4
p−maj=0.24, pX=0.59, N=259
var.4.THBS1 15:39880822:A:G
peptide level
110:196
58:110
8:19
IEDANLIPPVPDDKpc3_NP.5
p−maj=0.34, pX=0.47, N=176

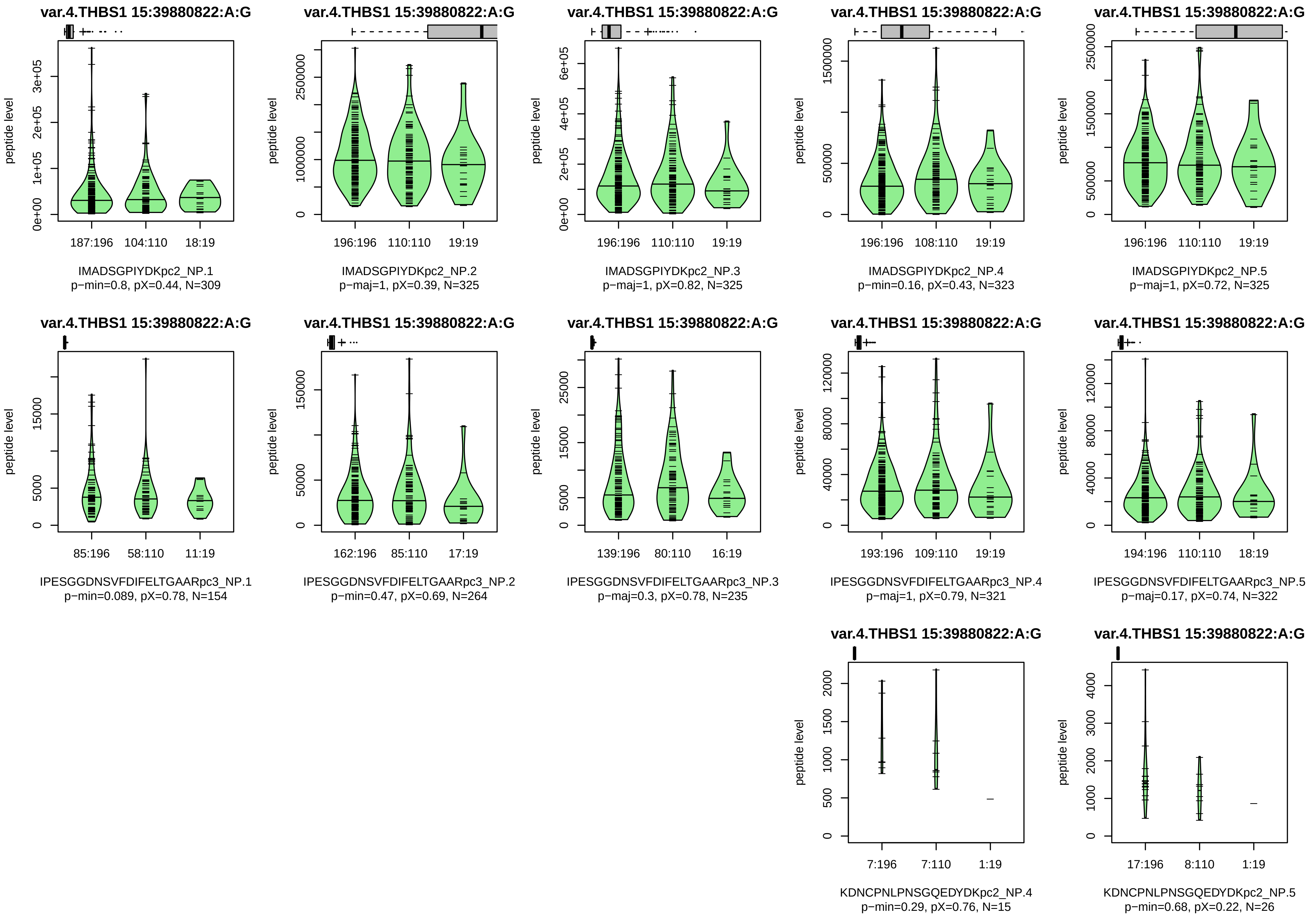
var.4.THBS1 15:39880822:A:G
peptide level
187:196
104:110
18:19
IMADSGPIYDKpc2_NP.1
p−min=0.8, pX=0.44, N=309
var.4.THBS1 15:39880822:A:G
196:196
110:110
19:19
IMADSGPIYDKpc2_NP.2
p−maj=1, pX=0.39, N=325
var.4.THBS1 15:39880822:A:G
196:196
110:110
19:19
IMADSGPIYDKpc2_NP.3
p−maj=1, pX=0.82, N=325
var.4.THBS1 15:39880822:A:G
196:196
108:110
19:19
IMADSGPIYDKpc2_NP.4
p−min=0.16, pX=0.43, N=323
var.4.THBS1 15:39880822:A:G
196:196
110:110
19:19
IMADSGPIYDKpc2_NP.5
p−maj=1, pX=0.72, N=325
var.4.THBS1 15:39880822:A:G
85:196
58:110
11:19
IPESGGDNSVFDIFELTGAARpc3_NP.1
p−min=0.089, pX=0.78, N=154
var.4.THBS1 15:39880822:A:G
162:196
85:110
17:19
IPESGGDNSVFDIFELTGAARpc3_NP.2
p−min=0.47, pX=0.69, N=264
var.4.THBS1 15:39880822:A:G
139:196
80:110
16:19
IPESGGDNSVFDIFELTGAARpc3_NP.3
p−maj=0.3, pX=0.78, N=235
var.4.THBS1 15:39880822:A:G
193:196
109:110
19:19
IPESGGDNSVFDIFELTGAARpc3_NP.4
p−maj=1, pX=0.79, N=321
var.4.THBS1 15:39880822:A:G
194:196
110:110
18:19
IPESGGDNSVFDIFELTGAARpc3_NP.5
p−maj=0.17, pX=0.74, N=322
var.4.THBS1 15:39880822:A:G
7:196
7:110
1:19
KDNCPNLPNSGQEDYDKpc2_NP.4
p−min=0.29, pX=0.76, N=15
var.4.THBS1 15:39880822:A:G
17:196
8:110
1:19
KDNCPNLPNSGQEDYDKpc2_NP.5
p−min=0.68, pX=0.22, N=26

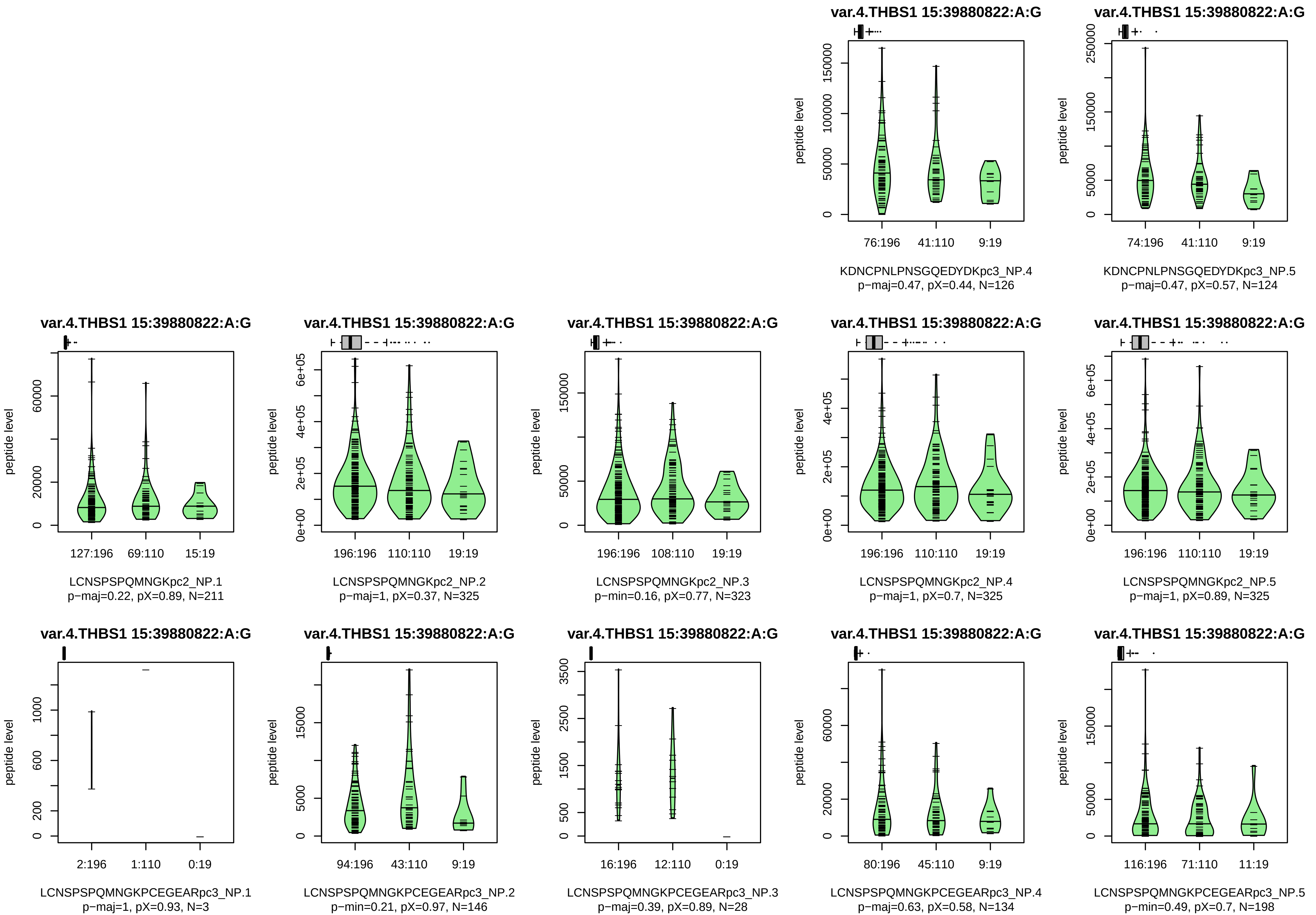

var.4.THBS1 15:39880822:A:G
peptide level
0
50000
100000
150000
76:196
41:110
9:19
KDNCPNLPNSGQEDYDKpc3_NP.4
p−maj=0.47, pX=0.44, N=126
var.4.THBS1 15:39880822:A:G
peptide level
0
50000
150000
250000
74:196
41:110
9:19
KDNCPNLPNSGQEDYDKpc3_NP.5
p−maj=0.47, pX=0.57, N=124
var.4.THBS1 15:39880822:A:G
peptide level
0
20000
60000
127:196
69:110
15:19
LCNSPSPQMNGKpc2_NP.1
p−maj=0.22, pX=0.89, N=211
var.4.THBS1 15:39880822:A:G
peptide level
0e+00
2e+05
4e+05
6e+05
196:196
110:110
19:19
LCNSPSPQMNGKpc2_NP.2
p−maj=1, pX=0.37, N=325
var.4.THBS1 15:39880822:A:G
peptide level
0
50000
150000
196:196
108:110
19:19
LCNSPSPQMNGKpc2_NP.3
p−min=0.16, pX=0.77, N=323
var.4.THBS1 15:39880822:A:G
peptide level
0e+00
2e+05
4e+05
196:196
110:110
19:19
LCNSPSPQMNGKpc2_NP.4
p−maj=1, pX=0.7, N=325
var.4.THBS1 15:39880822:A:G
peptide level
0e+00
2e+05
4e+05
6e+05
196:196
110:110
19:19
LCNSPSPQMNGKpc2_NP.5
p−maj=1, pX=0.89, N=325
var.4.THBS1 15:39880822:A:G
peptide level
0
200
600
1000
2:196
1:110
0:19
LCNSPSPQMNGKPCEGEARpc3_NP.1
p−maj=1, pX=0.93, N=3
var.4.THBS1 15:39880822:A:G
peptide level
0
5000
15000
94:196
43:110
9:19
LCNSPSPQMNGKPCEGEARpc3_NP.2
p−min=0.21, pX=0.97, N=146
var.4.THBS1 15:39880822:A:G
peptide level
0
500
1500
2500
3500
16:196
12:110
0:19
LCNSPSPQMNGKPCEGEARpc3_NP.3
p−maj=0.39, pX=0.89, N=28
var.4.THBS1 15:39880822:A:G
peptide level
0
20000
60000
80:196
45:110
9:19
LCNSPSPQMNGKPCEGEARpc3_NP.4
p−maj=0.63, pX=0.58, N=134
var.4.THBS1 15:39880822:A:G
peptide level
0
50000
150000
116:196
71:110
11:19
LCNSPSPQMNGKPCEGEARpc3_NP.5
p−min=0.49, pX=0.7, N=198

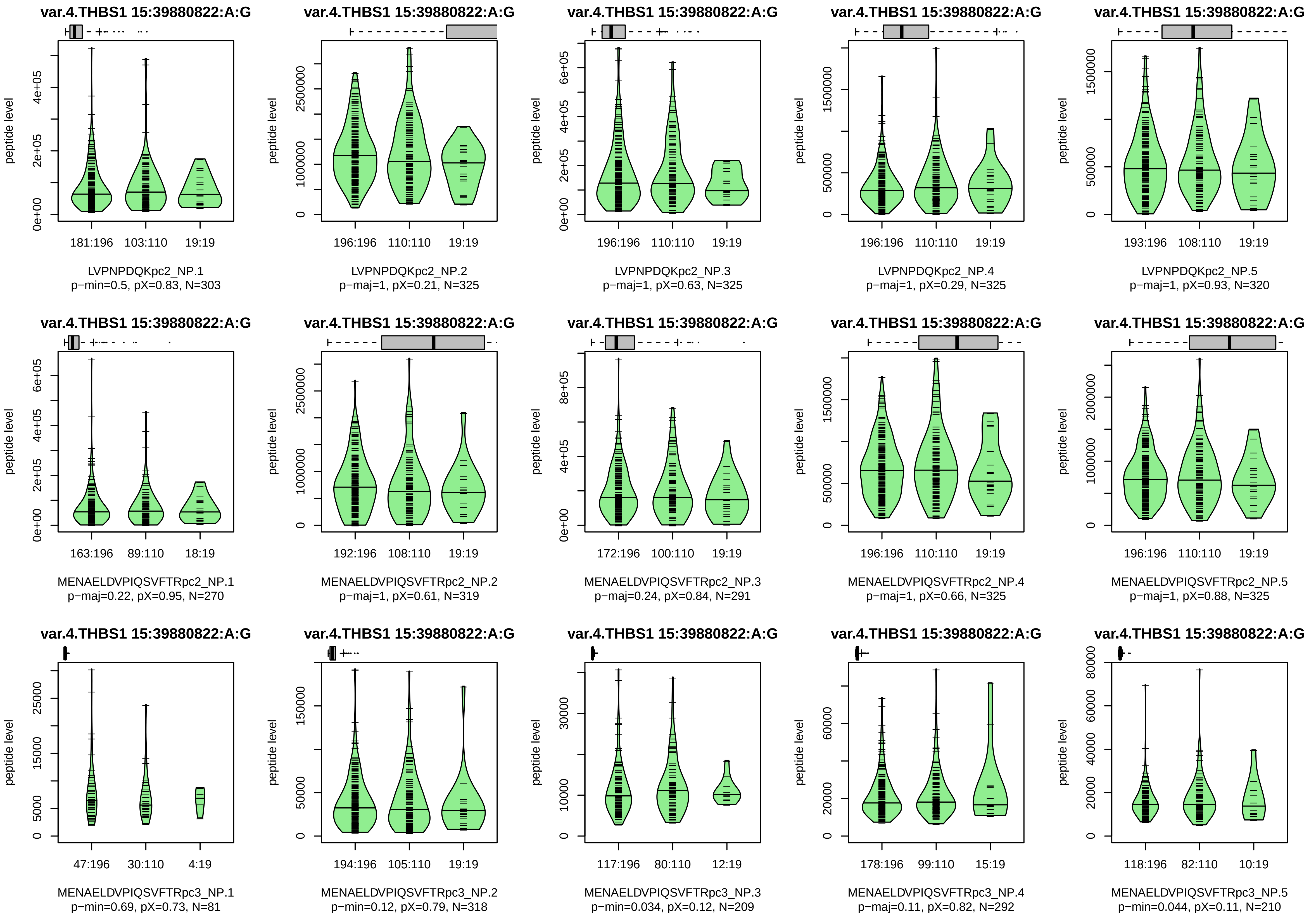
var.4.THBS1 15:39880822:A:G
peptide level
0e+00
2e+05
4e+05
181:196
103:110
19:19
LVPNPDQKpc2_NP.1
p−min=0.5, pX=0.83, N=303
var.4.THBS1 15:39880822:A:G
peptide level
0
1000000
2500000
196:196
110:110
19:19
LVPNPDQKpc2_NP.2
p−maj=1, pX=0.21, N=325
var.4.THBS1 15:39880822:A:G
peptide level
0e+00
2e+05
4e+05
6e+05
196:196
110:110
19:19
LVPNPDQKpc2_NP.3
p−maj=1, pX=0.63, N=325
var.4.THBS1 15:39880822:A:G
peptide level
0
500000
1500000
196:196
110:110
19:19
LVPNPDQKpc2_NP.4
p−maj=1, pX=0.29, N=325
var.4.THBS1 15:39880822:A:G
peptide level
0
500000
1500000
193:196
108:110
19:19
LVPNPDQKpc2_NP.5
p−maj=1, pX=0.93, N=320
var.4.THBS1 15:39880822:A:G
peptide level
0e+00
2e+05
4e+05
6e+05
163:196
89:110
18:19
MENAELDVPIQSVFTRpc2_NP.1
p−maj=0.22, pX=0.95, N=270
var.4.THBS1 15:39880822:A:G
peptide level
0
1000000
2500000
192:196
108:110
19:19
MENAELDVPIQSVFTRpc2_NP.2
p−maj=1, pX=0.61, N=319
var.4.THBS1 15:39880822:A:G
peptide level
0e+00
4e+05
8e+05
172:196
100:110
19:19
MENAELDVPIQSVFTRpc2_NP.3
p−maj=0.24, pX=0.84, N=291
var.4.THBS1 15:39880822:A:G
peptide level
0
500000
1500000
196:196
110:110
19:19
MENAELDVPIQSVFTRpc2_NP.4
p−maj=1, pX=0.66, N=325
var.4.THBS1 15:39880822:A:G
peptide level
0
1000000
2000000
196:196
110:110
19:19
MENAELDVPIQSVFTRpc2_NP.5
p−maj=1, pX=0.88, N=325
var.4.THBS1 15:39880822:A:G
peptide level
0
5000
15000
25000
47:196
30:110
4:19
MENAELDVPIQSVFTRpc3_NP.1
p−min=0.69, pX=0.73, N=81
var.4.THBS1 15:39880822:A:G
peptide level
0
50000
150000
194:196
105:110
19:19
MENAELDVPIQSVFTRpc3_NP.2
p−min=0.12, pX=0.79, N=318
var.4.THBS1 15:39880822:A:G
peptide level
0
10000
30000
117:196
80:110
12:19
MENAELDVPIQSVFTRpc3_NP.3
p−min=0.034, pX=0.12, N=209
var.4.THBS1 15:39880822:A:G
peptide level
0
20000
60000
178:196
99:110
15:19
MENAELDVPIQSVFTRpc3_NP.4
p−maj=0.11, pX=0.82, N=292
var.4.THBS1 15:39880822:A:G
peptide level
0
20000
40000
60000
80000
118:196
82:110
10:19
MENAELDVPIQSVFTRpc3_NP.5
p−min=0.044, pX=0.11, N=210

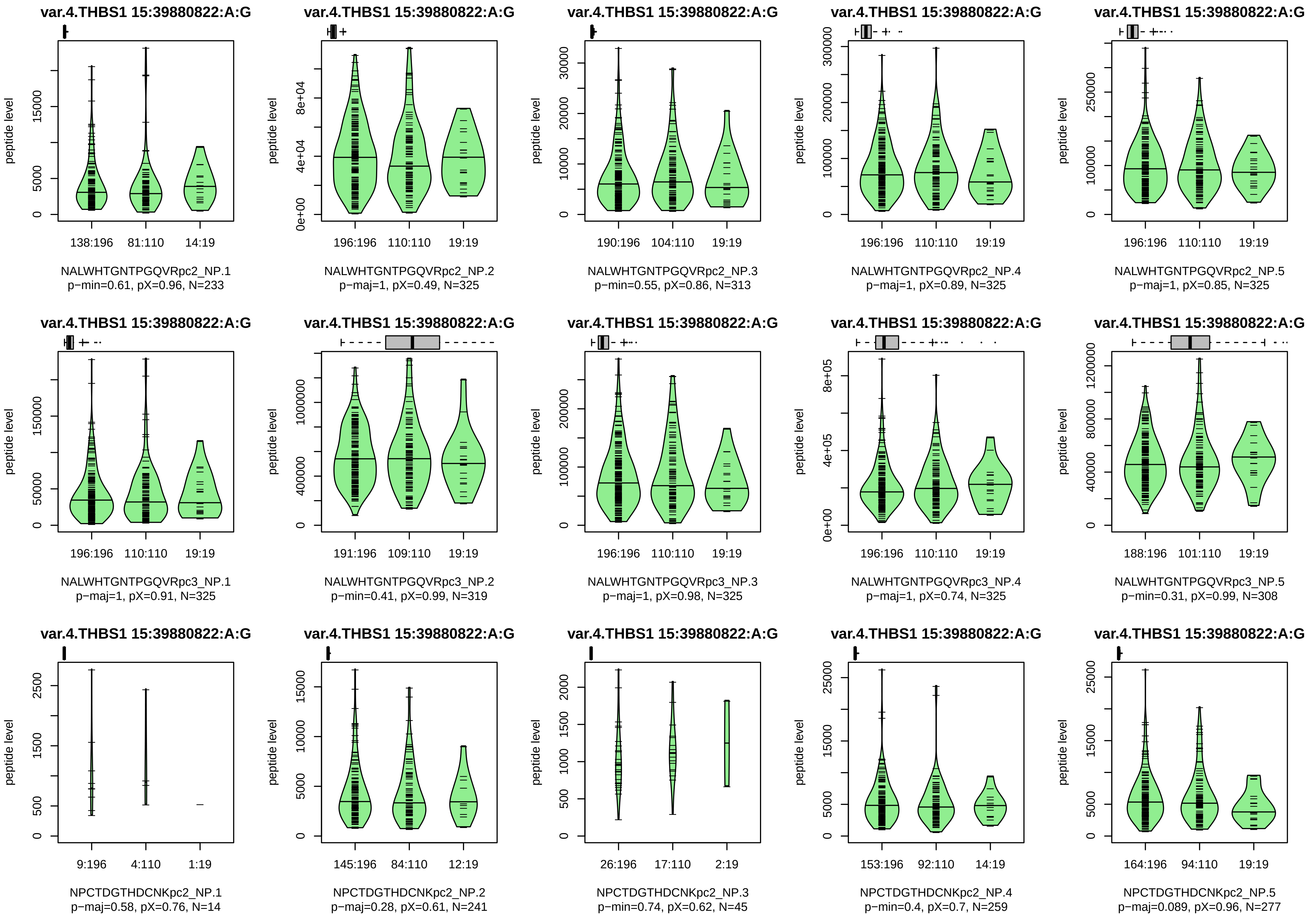
var.4.THBS1 15:39880822:A:G
peptide level
138:196
81:110
14:19
NALWHTGNTPGQVRpc2_NP.1
p−min=0.61, pX=0.96, N=233
var.4.THBS1 15:39880822:A:G
peptide level
196:196
110:110
19:19
NALWHTGNTPGQVRpc2_NP.2
p−maj=1, pX=0.49, N=325
var.4.THBS1 15:39880822:A:G
peptide level
190:196
104:110
19:19
NALWHTGNTPGQVRpc2_NP.3
p−min=0.55, pX=0.86, N=313
var.4.THBS1 15:39880822:A:G
peptide level
196:196
110:110
19:19
NALWHTGNTPGQVRpc2_NP.4
p−maj=1, pX=0.89, N=325
var.4.THBS1 15:39880822:A:G
peptide level
196:196
110:110
19:19
NALWHTGNTPGQVRpc2_NP.5
p−maj=1, pX=0.85, N=325
var.4.THBS1 15:39880822:A:G
peptide level
196:196
110:110
19:19
NALWHTGNTPGQVRpc3_NP.1
p−maj=1, pX=0.91, N=325
var.4.THBS1 15:39880822:A:G
peptide level
191:196
109:110
19:19
NALWHTGNTPGQVRpc3_NP.2
p−min=0.41, pX=0.99, N=319
var.4.THBS1 15:39880822:A:G
peptide level
196:196
110:110
19:19
NALWHTGNTPGQVRpc3_NP.3
p−maj=1, pX=0.98, N=325
var.4.THBS1 15:39880822:A:G
peptide level
196:196
110:110
19:19
NALWHTGNTPGQVRpc3_NP.4
p−maj=1, pX=0.74, N=325
var.4.THBS1 15:39880822:A:G
peptide level
188:196
101:110
19:19
NALWHTGNTPGQVRpc3_NP.5
p−min=0.31, pX=0.99, N=308
var.4.THBS1 15:39880822:A:G
peptide level
9:196
4:110
1:19
NPCTDGTHDCNKpc2_NP.1
p−maj=0.58, pX=0.76, N=14
var.4.THBS1 15:39880822:A:G
peptide level
145:196
84:110
12:19
NPCTDGTHDCNKpc2_NP.2
p−maj=0.28, pX=0.61, N=241
var.4.THBS1 15:39880822:A:G
peptide level
26:196
17:110
2:19
NPCTDGTHDCNKpc2_NP.3
p−min=0.74, pX=0.62, N=45
var.4.THBS1 15:39880822:A:G
peptide level
153:196
92:110
14:19
NPCTDGTHDCNKpc2_NP.4
p−min=0.4, pX=0.7, N=259
var.4.THBS1 15:39880822:A:G
peptide level
164:196
94:110
19:19
NPCTDGTHDCNKpc2_NP.5
p−maj=0.089, pX=0.96, N=277

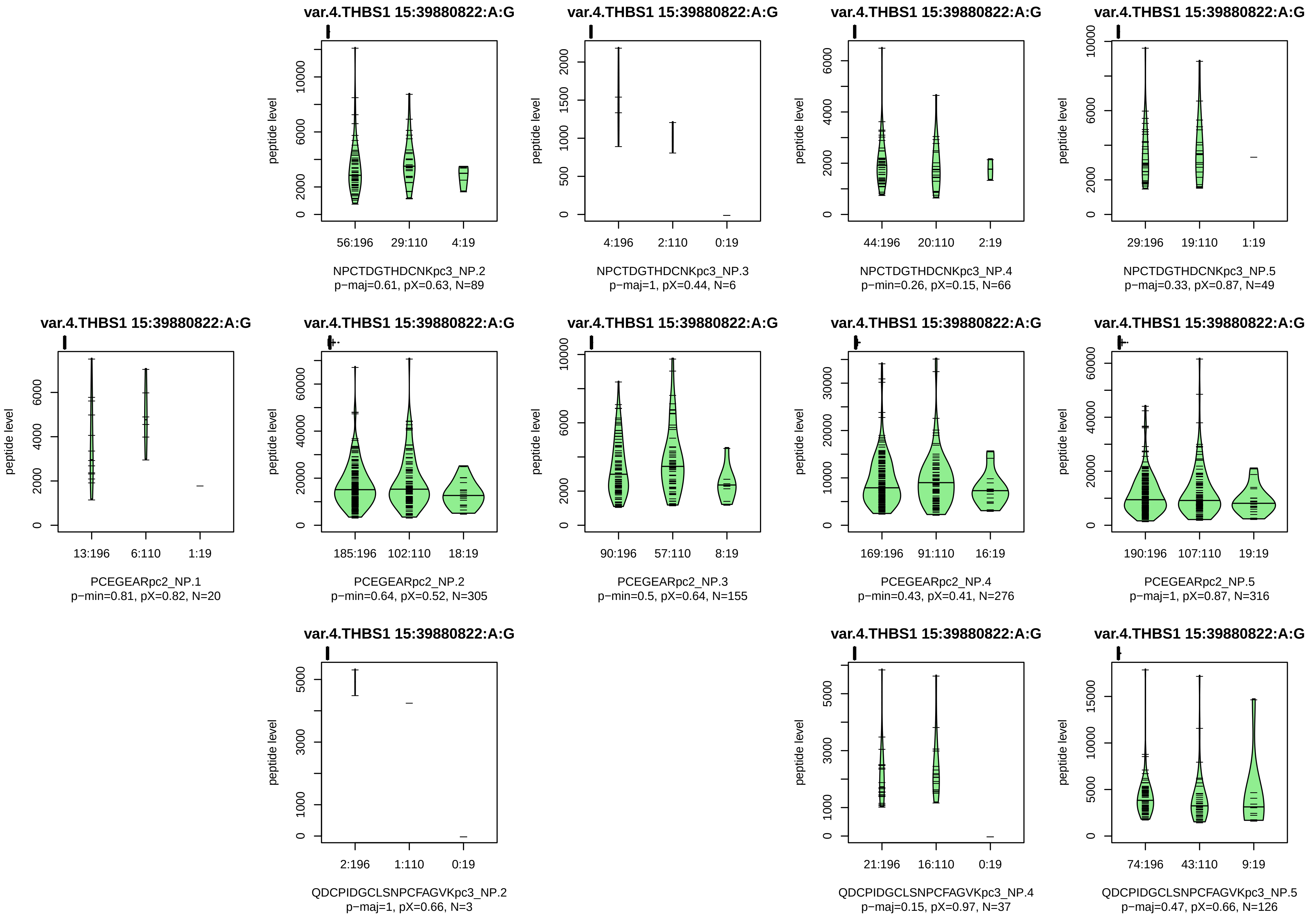
var.4.THBS1 15:39880822:A:G
56:196
29:110
4:19
NPCTDGTHDCNKpc3_NP.2
p−maj=0.61, pX=0.63, N=89
var.4.THBS1 15:39880822:A:G
4:196
2:110
0:19
NPCTDGTHDCNKpc3_NP.3
p−maj=1, pX=0.44, N=6
var.4.THBS1 15:39880822:A:G
44:196
20:110
2:19
NPCTDGTHDCNKpc3_NP.4
p−min=0.26, pX=0.15, N=66
var.4.THBS1 15:39880822:A:G
29:196
19:110
1:19
NPCTDGTHDCNKpc3_NP.5
p−maj=0.33, pX=0.87, N=49
var.4.THBS1 15:39880822:A:G
13:196
6:110
1:19
PCEGEARpc2_NP.1
p−min=0.81, pX=0.82, N=20
var.4.THBS1 15:39880822:A:G
185:196
102:110
18:19
PCEGEARpc2_NP.2
p−min=0.64, pX=0.52, N=305
var.4.THBS1 15:39880822:A:G
90:196
57:110
8:19
PCEGEARpc2_NP.3
p−min=0.5, pX=0.64, N=155
var.4.THBS1 15:39880822:A:G
169:196
91:110
16:19
PCEGEARpc2_NP.4
p−min=0.43, pX=0.41, N=276
var.4.THBS1 15:39880822:A:G
190:196
107:110
19:19
PCEGEARpc2_NP.5
p−maj=1, pX=0.87, N=316
var.4.THBS1 15:39880822:A:G
2:196
1:110
0:19
QDCPIDGCLSNPCFAGVKpc3_NP.2
p−maj=1, pX=0.66, N=3
var.4.THBS1 15:39880822:A:G
21:196
16:110
0:19
QDCPIDGCLSNPCFAGVKpc3_NP.4
p−maj=0.15, pX=0.97, N=37
var.4.THBS1 15:39880822:A:G
74:196
43:110
9:19
QDCPIDGCLSNPCFAGVKpc3_NP.5
p−maj=0.47, pX=0.66, N=126
peptide level

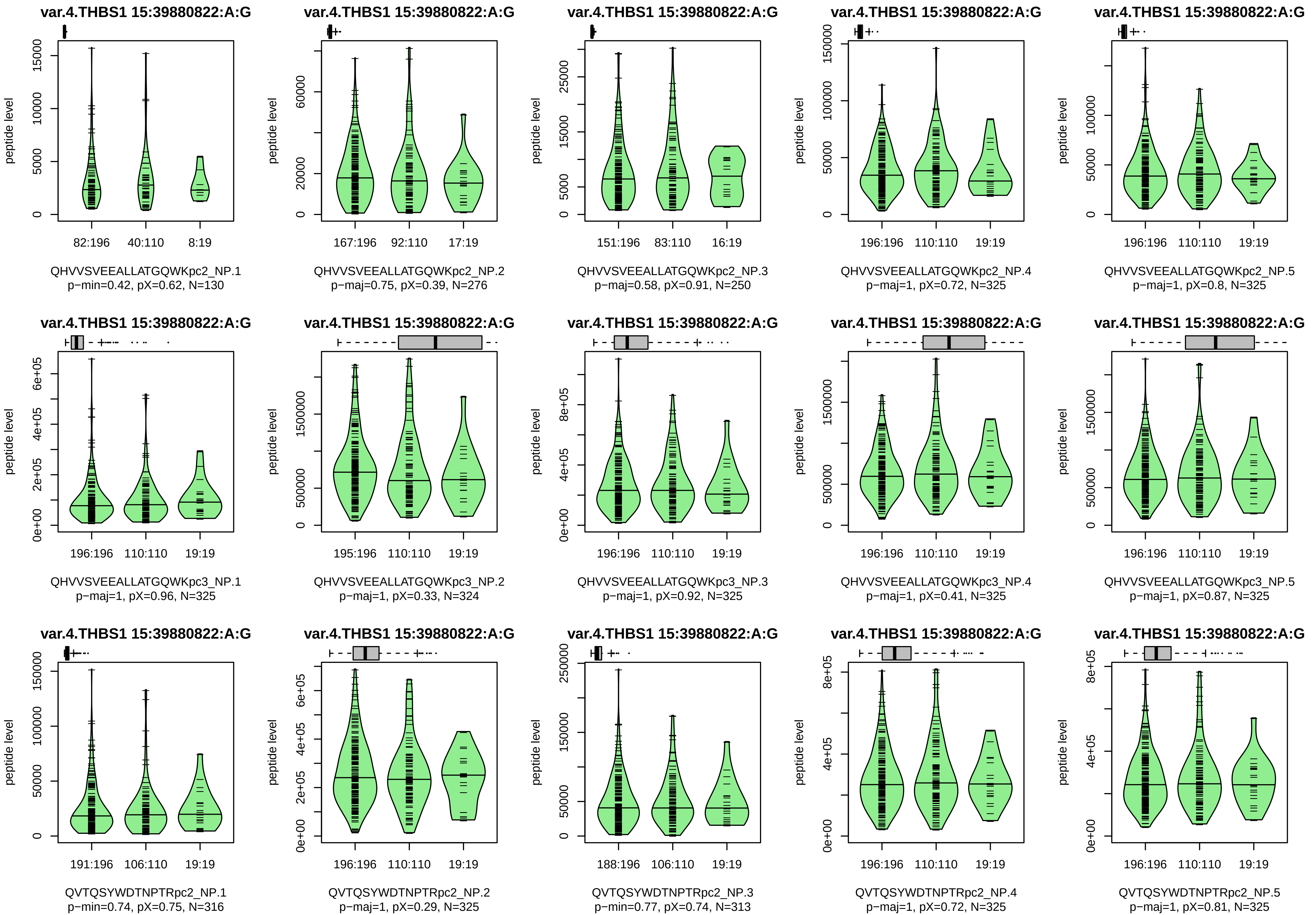
var.4.THBS1 15:39880822:A:G
peptide level
82:196 40:110 8:19
QHVVSVEEALLATGQWKpc2_NP.1
p−min=0.42, pX=0.62, N=130
var.4.THBS1 15:39880822:A:G
peptide level
167:196 92:110 17:19
QHVVSVEEALLATGQWKpc2_NP.2
p−maj=0.75, pX=0.39, N=276
var.4.THBS1 15:39880822:A:G
peptide level
151:196 83:110 16:19
QHVVSVEEALLATGQWKpc2_NP.3
p−maj=0.58, pX=0.91, N=250
var.4.THBS1 15:39880822:A:G
peptide level
196:196 110:110 19:19
QHVVSVEEALLATGQWKpc2_NP.4
p−maj=1, pX=0.72, N=325
var.4.THBS1 15:39880822:A:G
peptide level
196:196 110:110 19:19
QHVVSVEEALLATGQWKpc2_NP.5
p−maj=1, pX=0.8, N=325
var.4.THBS1 15:39880822:A:G
peptide level
196:196 110:110 19:19
QHVVSVEEALLATGQWKpc3_NP.1
p−maj=1, pX=0.96, N=325
var.4.THBS1 15:39880822:A:G
peptide level
195:196 110:110 19:19
QHVVSVEEALLATGQWKpc3_NP.2
p−maj=1, pX=0.33, N=324
var.4.THBS1 15:39880822:A:G
peptide level
196:196 110:110 19:19
QHVVSVEEALLATGQWKpc3_NP.3
p−maj=1, pX=0.92, N=325
var.4.THBS1 15:39880822:A:G
peptide level
196:196 110:110 19:19
QHVVSVEEALLATGQWKpc3_NP.4
p−maj=1, pX=0.41, N=325
var.4.THBS1 15:39880822:A:G
peptide level
196:196 110:110 19:19
QHVVSVEEALLATGQWKpc3_NP.5
p−maj=1, pX=0.87, N=325
var.4.THBS1 15:39880822:A:G
peptide level
191:196 106:110 19:19
QVTQSYWDTNPTRpc2_NP.1
p−min=0.74, pX=0.75, N=316
var.4.THBS1 15:39880822:A:G
peptide level
196:196 110:110 19:19
QVTQSYWDTNPTRpc2_NP.2
p−maj=1, pX=0.29, N=325
var.4.THBS1 15:39880822:A:G
peptide level
188:196 106:110 19:19
QVTQSYWDTNPTRpc2_NP.3
p−min=0.77, pX=0.74, N=313
var.4.THBS1 15:39880822:A:G
peptide level
196:196 110:110 19:19
QVTQSYWDTNPTRpc2_NP.4
p−maj=1, pX=0.72, N=325
var.4.THBS1 15:39880822:A:G
peptide level
196:196 110:110 19:19
QVTQSYWDTNPTRpc2_NP.5
p−maj=1, pX=0.81, N=325

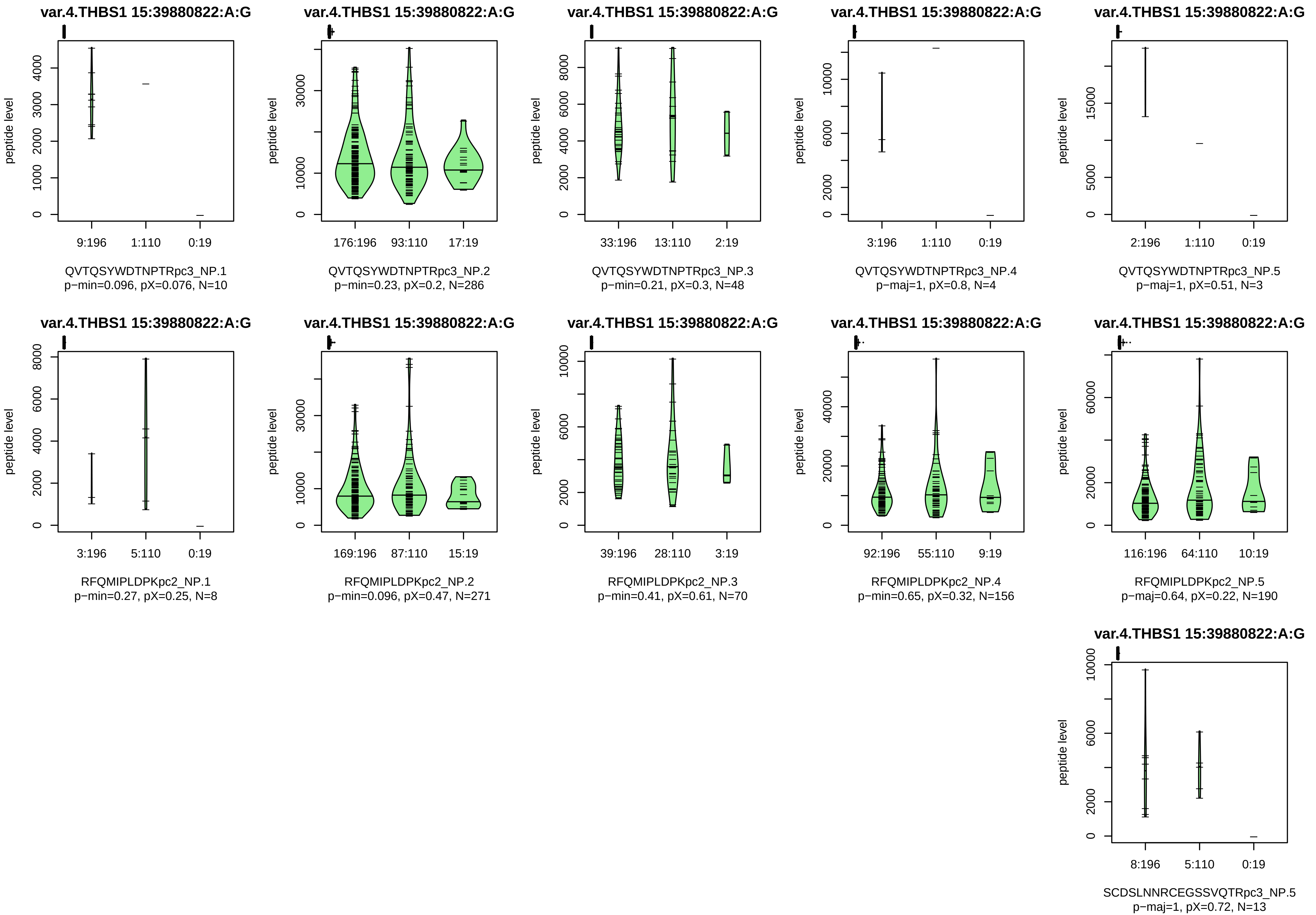

var.4.THBS1 15:39880822:A:G
peptide level
9:196
1:110
0:19
QVTQSYWDTNPTRpc3_NP.1
p−min=0.096, pX=0.076, N=10
var.4.THBS1 15:39880822:A:G
peptide level
176:196
93:110
17:19
QVTQSYWDTNPTRpc3_NP.2
p−min=0.23, pX=0.2, N=286
var.4.THBS1 15:39880822:A:G
peptide level
33:196
13:110
2:19
QVTQSYWDTNPTRpc3_NP.3
p−min=0.21, pX=0.3, N=48
var.4.THBS1 15:39880822:A:G
peptide level
3:196
1:110
0:19
QVTQSYWDTNPTRpc3_NP.4
p−maj=1, pX=0.8, N=4
var.4.THBS1 15:39880822:A:G
peptide level
2:196
1:110
0:19
QVTQSYWDTNPTRpc3_NP.5
p−maj=1, pX=0.51, N=3
var.4.THBS1 15:39880822:A:G
peptide level
3:196
5:110
0:19
RFQMIPLDPKpc2_NP.1
p−min=0.27, pX=0.25, N=8
var.4.THBS1 15:39880822:A:G
peptide level
169:196
87:110
15:19
RFQMIPLDPKpc2_NP.2
p−min=0.096, pX=0.47, N=271
var.4.THBS1 15:39880822:A:G
peptide level
39:196
28:110
3:19
RFQMIPLDPKpc2_NP.3
p−min=0.41, pX=0.61, N=70
var.4.THBS1 15:39880822:A:G
peptide level
92:196
55:110
9:19
RFQMIPLDPKpc2_NP.4
p−min=0.65, pX=0.32, N=156
var.4.THBS1 15:39880822:A:G
peptide level
116:196
64:110
10:19
RFQMIPLDPKpc2_NP.5
p−maj=0.64, pX=0.22, N=190
var.4.THBS1 15:39880822:A:G
peptide level
8:196
5:110
0:19
SCDSLNNRCEGSSVQTRpc3_NP.5
p−maj=1, pX=0.72, N=13

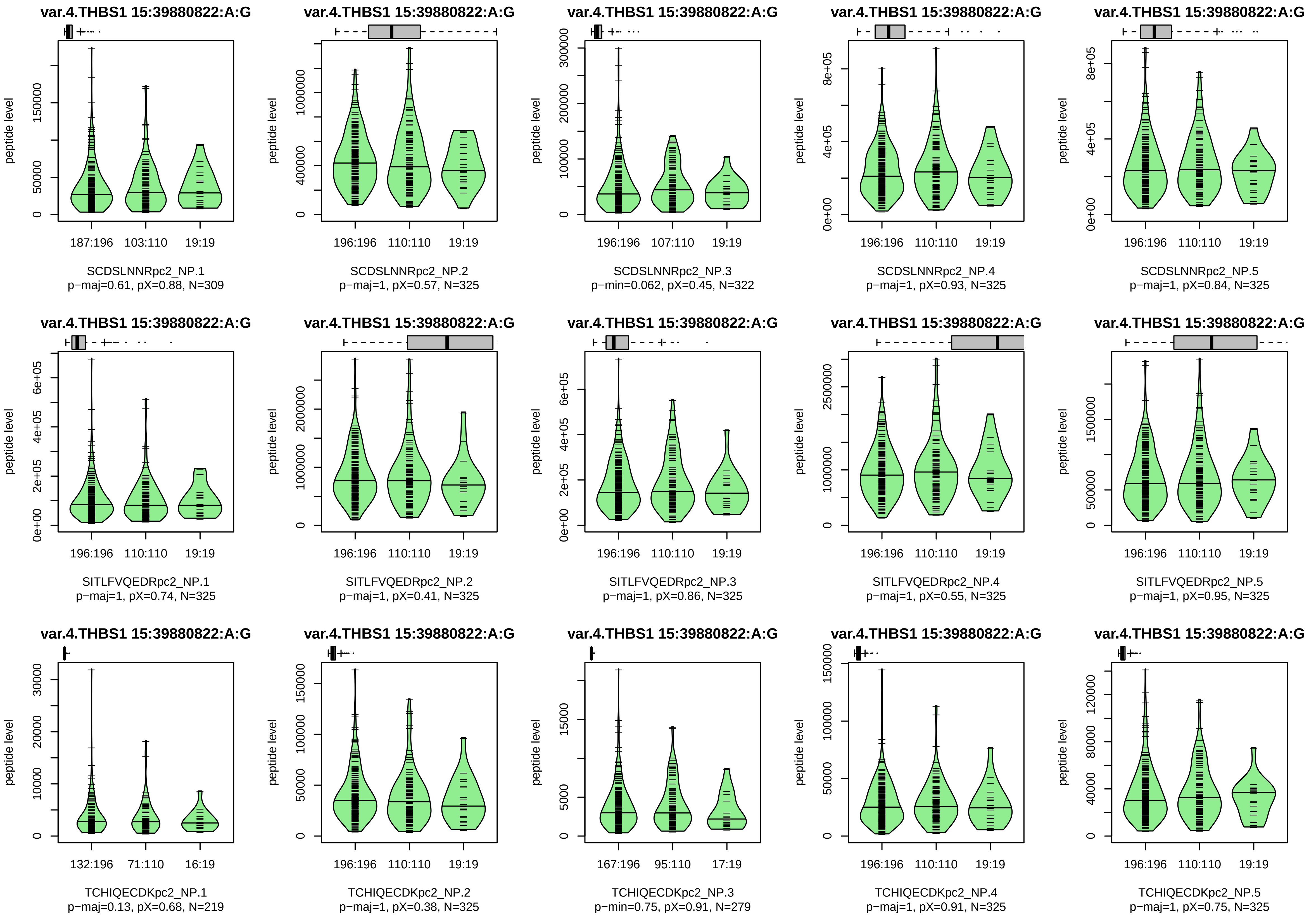
var.4.THBS1 15:39880822:A:G
peptide level
187:196
103:110
19:19
SCDSLNNRpc2_NP.1
p−maj=0.61, pX=0.88, N=309
var.4.THBS1 15:39880822:A:G
peptide level
196:196
110:110
19:19
SCDSLNNRpc2_NP.2
p−maj=1, pX=0.57, N=325
var.4.THBS1 15:39880822:A:G
peptide level
196:196
107:110
19:19
SCDSLNNRpc2_NP.3
p−min=0.062, pX=0.45, N=322
var.4.THBS1 15:39880822:A:G
peptide level
196:196
110:110
19:19
SCDSLNNRpc2_NP.4
p−maj=1, pX=0.93, N=325
var.4.THBS1 15:39880822:A:G
peptide level
196:196
110:110
19:19
SCDSLNNRpc2_NP.5
p−maj=1, pX=0.84, N=325
var.4.THBS1 15:39880822:A:G
peptide level
196:196
110:110
19:19
SITLFVQEDRpc2_NP.1
p−maj=1, pX=0.74, N=325
var.4.THBS1 15:39880822:A:G
peptide level
196:196
110:110
19:19
SITLFVQEDRpc2_NP.2
p−maj=1, pX=0.41, N=325
var.4.THBS1 15:39880822:A:G
peptide level
196:196
110:110
19:19
SITLFVQEDRpc2_NP.3
p−maj=1, pX=0.86, N=325
var.4.THBS1 15:39880822:A:G
peptide level
196:196
110:110
19:19
SITLFVQEDRpc2_NP.4
p−maj=1, pX=0.55, N=325
var.4.THBS1 15:39880822:A:G
peptide level
196:196
110:110
19:19
SITLFVQEDRpc2_NP.5
p−maj=1, pX=0.95, N=325
var.4.THBS1 15:39880822:A:G
peptide level
132:196
71:110
16:19
TCHIQECDKpc2_NP.1
p−maj=0.13, pX=0.68, N=219
var.4.THBS1 15:39880822:A:G
peptide level
196:196
110:110
19:19
TCHIQECDKpc2_NP.2
p−maj=1, pX=0.38, N=325
var.4.THBS1 15:39880822:A:G
peptide level
167:196
95:110
17:19
TCHIQECDKpc2_NP.3
p−min=0.75, pX=0.91, N=279
var.4.THBS1 15:39880822:A:G
peptide level
196:196
110:110
19:19
TCHIQECDKpc2_NP.4
p−maj=1, pX=0.91, N=325
var.4.THBS1 15:39880822:A:G
peptide level
196:196
110:110
19:19
TCHIQECDKpc2_NP.5
p−maj=1, pX=0.75, N=325

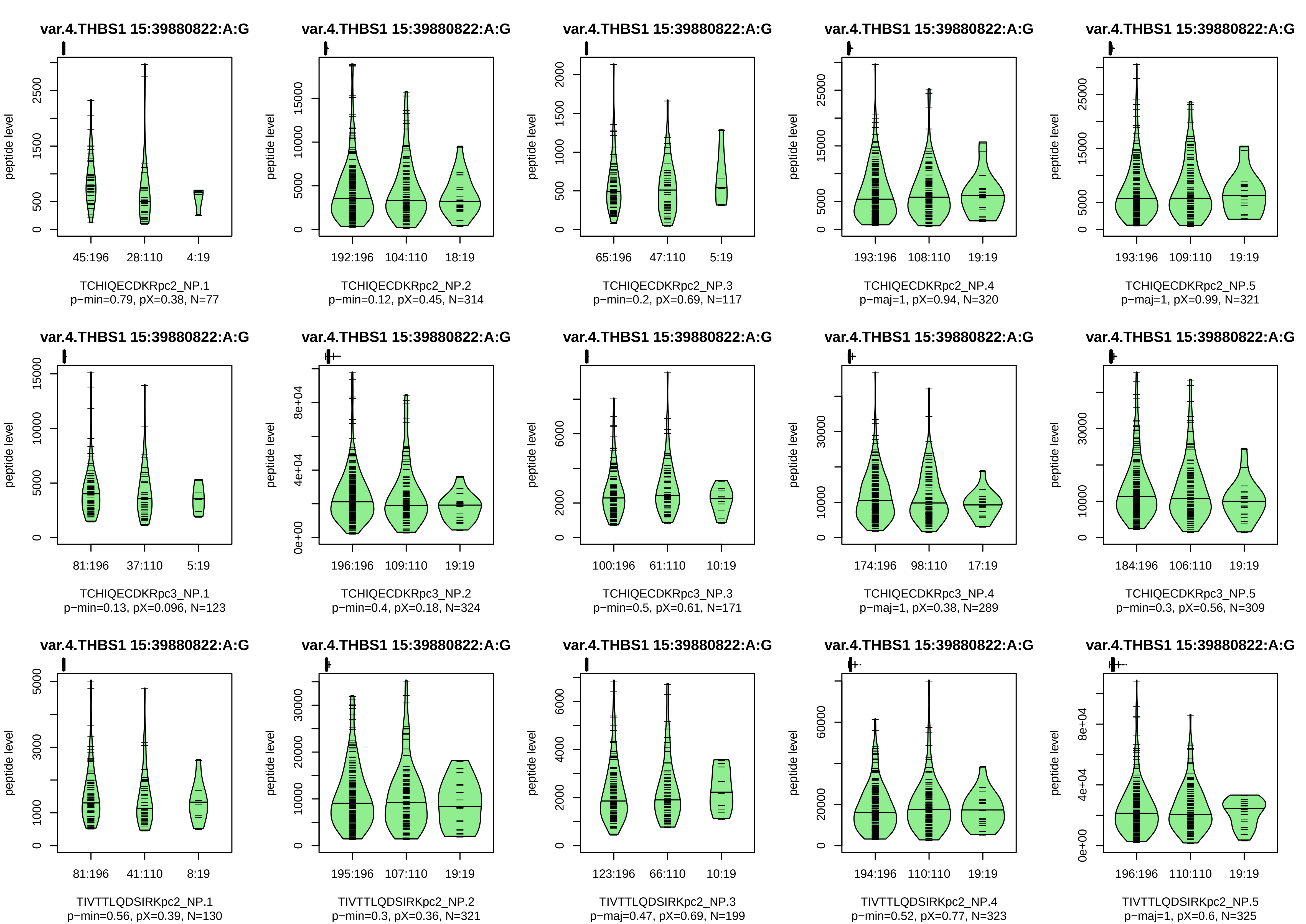
var.4.THBS1 15:39880822:A:G
peptide level
45:196
28:110
4:19
TCHIQECDKRpc2_NP.1
p−min=0.79, pX=0.38, N=77
var.4.THBS1 15:39880822:A:G
peptide level
192:196
104:110
18:19
TCHIQECDKRpc2_NP.2
p−min=0.12, pX=0.45, N=314
var.4.THBS1 15:39880822:A:G
peptide level
65:196
47:110
5:19
TCHIQECDKRpc2_NP.3
p−min=0.2, pX=0.69, N=117
var.4.THBS1 15:39880822:A:G
peptide level
193:196
108:110
19:19
TCHIQECDKRpc2_NP.4
p−maj=1, pX=0.94, N=320
var.4.THBS1 15:39880822:A:G
peptide level
193:196
109:110
19:19
TCHIQECDKRpc2_NP.5
p−maj=1, pX=0.99, N=321
var.4.THBS1 15:39880822:A:G
peptide level
81:196
37:110
5:19
TCHIQECDKRpc3_NP.1
p−min=0.13, pX=0.096, N=123
var.4.THBS1 15:39880822:A:G
peptide level
196:196
109:110
19:19
TCHIQECDKRpc3_NP.2
p−min=0.4, pX=0.18, N=324
var.4.THBS1 15:39880822:A:G
peptide level
100:196
61:110
10:19
TCHIQECDKRpc3_NP.3
p−min=0.5, pX=0.61, N=171
var.4.THBS1 15:39880822:A:G
peptide level
174:196
98:110
17:19
TCHIQECDKRpc3_NP.4
p−maj=1, pX=0.38, N=289
var.4.THBS1 15:39880822:A:G
peptide level
184:196
106:110
19:19
TCHIQECDKRpc3_NP.5
p−min=0.3, pX=0.56, N=309
var.4.THBS1 15:39880822:A:G
peptide level
81:196
41:110
8:19
TIVTTLQDSIRKpc2_NP.1
p−min=0.56, pX=0.39, N=130
var.4.THBS1 15:39880822:A:G
peptide level
195:196
107:110
19:19
TIVTTLQDSIRKpc2_NP.2
p−min=0.3, pX=0.36, N=321
var.4.THBS1 15:39880822:A:G
peptide level
123:196
66:110
10:19
TIVTTLQDSIRKpc2_NP.3
p−maj=0.47, pX=0.69, N=199
var.4.THBS1 15:39880822:A:G
peptide level
194:196
110:110
19:19
TIVTTLQDSIRKpc2_NP.4
p−min=0.52, pX=0.77, N=323
var.4.THBS1 15:39880822:A:G
peptide level
196:196
110:110
19:19
TIVTTLQDSIRKpc2_NP.5
p−maj=1, pX=0.6, N=325

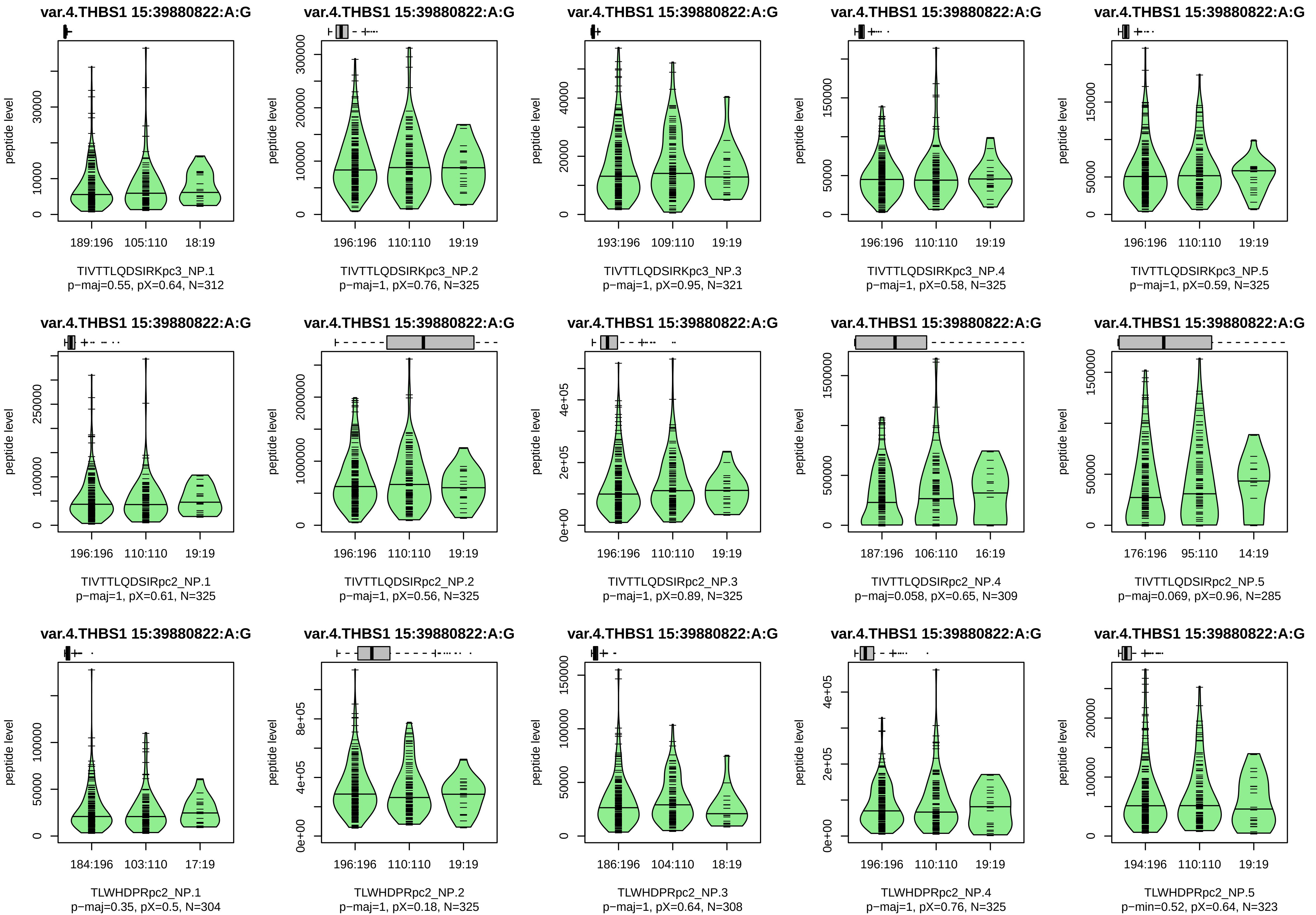
var.4.THBS1 15:39880822:A:G
peptide level
189:196
105:110
18:19
TIVTTLQDSIRKpc3_NP.1
p−maj=0.55, pX=0.64, N=312
var.4.THBS1 15:39880822:A:G
peptide level
196:196
110:110
19:19
TIVTTLQDSIRKpc3_NP.2
p−maj=1, pX=0.76, N=325
var.4.THBS1 15:39880822:A:G
peptide level
193:196
109:110
19:19
TIVTTLQDSIRKpc3_NP.3
p−maj=1, pX=0.95, N=321
var.4.THBS1 15:39880822:A:G
peptide level
196:196
110:110
19:19
TIVTTLQDSIRKpc3_NP.4
p−maj=1, pX=0.58, N=325
var.4.THBS1 15:39880822:A:G
peptide level
196:196
110:110
19:19
TIVTTLQDSIRKpc3_NP.5
p−maj=1, pX=0.59, N=325
var.4.THBS1 15:39880822:A:G
peptide level
196:196
110:110
19:19
TIVTTLQDSIRpc2_NP.1
p−maj=1, pX=0.61, N=325
var.4.THBS1 15:39880822:A:G
peptide level
196:196
110:110
19:19
TIVTTLQDSIRpc2_NP.2
p−maj=1, pX=0.56, N=325
var.4.THBS1 15:39880822:A:G
peptide level
196:196
110:110
19:19
TIVTTLQDSIRpc2_NP.3
p−maj=1, pX=0.89, N=325
var.4.THBS1 15:39880822:A:G
peptide level
187:196
106:110
16:19
TIVTTLQDSIRpc2_NP.4
p−maj=0.058, pX=0.65, N=309
var.4.THBS1 15:39880822:A:G
peptide level
176:196
95:110
14:19
TIVTTLQDSIRpc2_NP.5
p−maj=0.069, pX=0.96, N=285
var.4.THBS1 15:39880822:A:G
peptide level
184:196
103:110
17:19
TLWHDPRpc2_NP.1
p−maj=0.35, pX=0.5, N=304
var.4.THBS1 15:39880822:A:G
peptide level
196:196
110:110
19:19
TLWHDPRpc2_NP.2
p−maj=1, pX=0.18, N=325
var.4.THBS1 15:39880822:A:G
peptide level
186:196
104:110
18:19
TLWHDPRpc2_NP.3
p−maj=1, pX=0.64, N=308
var.4.THBS1 15:39880822:A:G
peptide level
196:196
110:110
19:19
TLWHDPRpc2_NP.4
p−maj=1, pX=0.76, N=325
var.4.THBS1 15:39880822:A:G
peptide level
194:196
110:110
19:19
TLWHDPRpc2_NP.5
p−min=0.52, pX=0.64, N=323

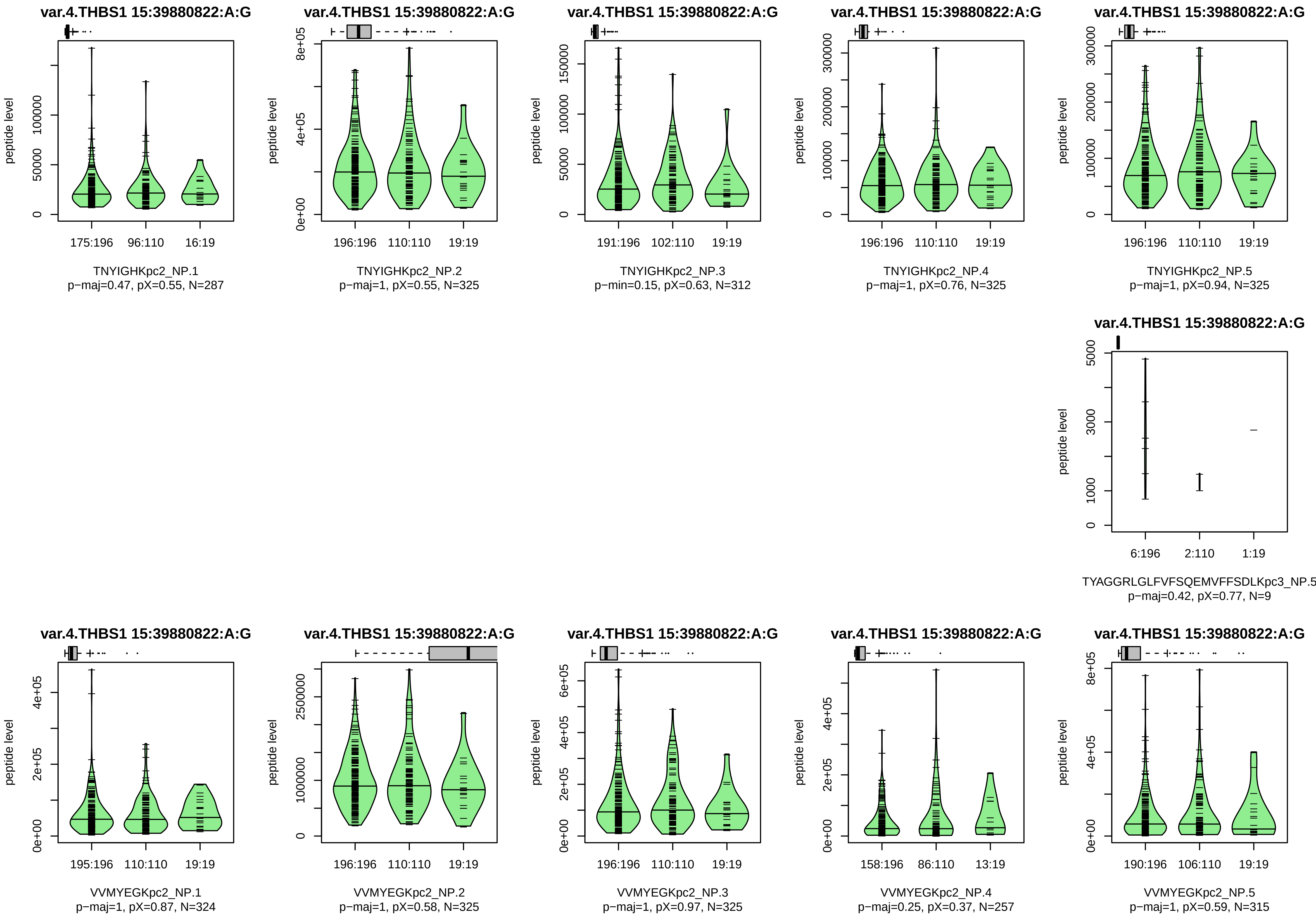
var.4.THBS1 15:39880822:A:G
peptide level
175:196
96:110
16:19
TNYIGHKpc2_NP.1
p−maj=0.47, pX=0.55, N=287
var.4.THBS1 15:39880822:A:G
196:196
110:110
19:19
TNYIGHKpc2_NP.2
p−maj=1, pX=0.55, N=325
var.4.THBS1 15:39880822:A:G
191:196
102:110
19:19
TNYIGHKpc2_NP.3
p−min=0.15, pX=0.63, N=312
var.4.THBS1 15:39880822:A:G
196:196
110:110
19:19
TNYIGHKpc2_NP.4
p−maj=1, pX=0.76, N=325
var.4.THBS1 15:39880822:A:G
196:196
110:110
19:19
TNYIGHKpc2_NP.5
p−maj=1, pX=0.94, N=325
var.4.THBS1 15:39880822:A:G
6:196
2:110
1:19
TYAGGRLGLFVFSQEMVFFSDLKpc3_NP.5
p−maj=0.42, pX=0.77, N=9
var.4.THBS1 15:39880822:A:G
195:196
110:110
19:19
VVMYEGKpc2_NP.1
p−maj=1, pX=0.87, N=324
var.4.THBS1 15:39880822:A:G
196:196
110:110
19:19
VVMYEGKpc2_NP.2
p−maj=1, pX=0.58, N=325
var.4.THBS1 15:39880822:A:G
196:196
110:110
19:19
VVMYEGKpc2_NP.3
p−maj=1, pX=0.97, N=325
var.4.THBS1 15:39880822:A:G
158:196
86:110
13:19
VVMYEGKpc2_NP.4
p−maj=0.25, pX=0.37, N=257
var.4.THBS1 15:39880822:A:G
190:196
106:110
19:19
VVMYEGKpc2_NP.5
p−maj=1, pX=0.59, N=315

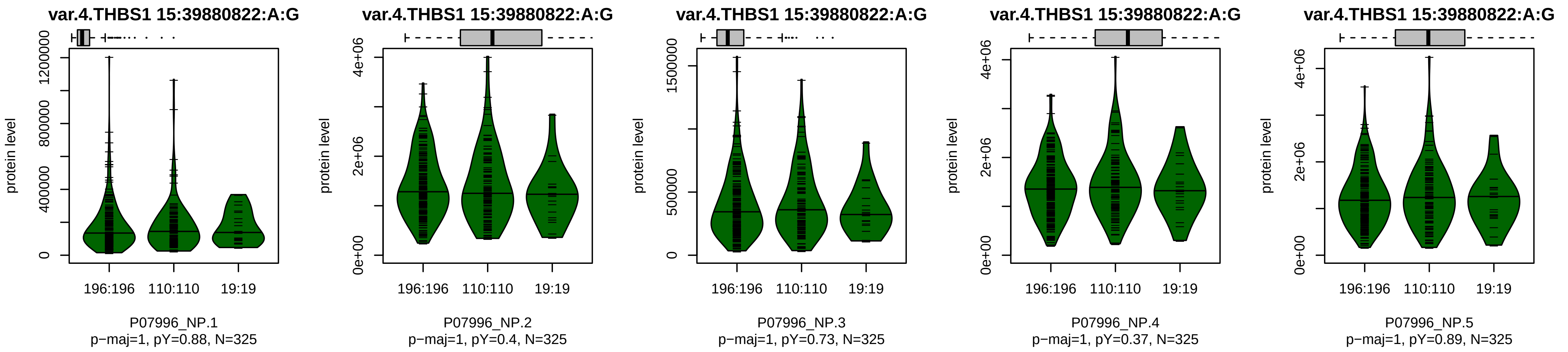

var.4.THBS1 15:39880822:A:G
protein level
0
400000
800000
1200000
196:196
110:110
19:19
P07996_NP.1
p−maj=1, pY=0.88, N=325
var.4.THBS1 15:39880822:A:G
protein level
0e+00
2e+06
4e+06
196:196
110:110
19:19
P07996_NP.2
p−maj=1, pY=0.4, N=325
var.4.THBS1 15:39880822:A:G
protein level
0
500000
1500000
196:196
110:110
19:19
P07996_NP.3
p−maj=1, pY=0.73, N=325
var.4.THBS1 15:39880822:A:G
protein level
0e+00
2e+06
4e+06
196:196
110:110
19:19
P07996_NP.4
p−maj=1, pY=0.37, N=325
var.4.THBS1 15:39880822:A:G
protein level
0e+00
2e+06
4e+06
196:196
110:110
19:19
P07996_NP.5
p−maj=1, pY=0.89, N=325

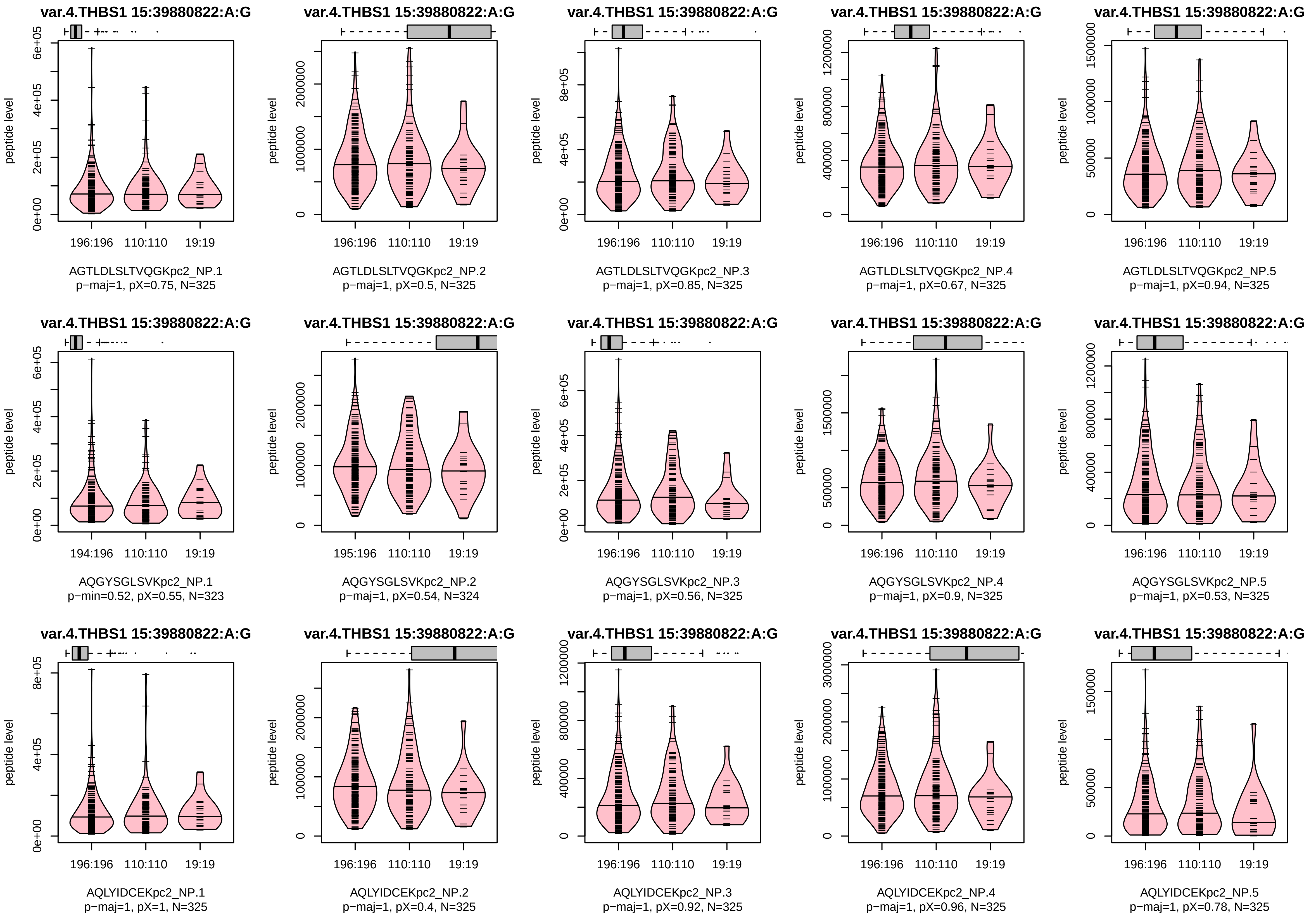
var.4.THBS1 15:39880822:A:G
peptide level
196:196
110:110
19:19
AGTLDLSLTVQGKpc2_NP.1
p−maj=1, pX=0.75, N=325
var.4.THBS1 15:39880822:A:G
peptide level
196:196
110:110
19:19
AGTLDLSLTVQGKpc2_NP.2
p−maj=1, pX=0.5, N=325
var.4.THBS1 15:39880822:A:G
peptide level
196:196
110:110
19:19
AGTLDLSLTVQGKpc2_NP.3
p−maj=1, pX=0.85, N=325
var.4.THBS1 15:39880822:A:G
peptide level
196:196
110:110
19:19
AGTLDLSLTVQGKpc2_NP.4
p−maj=1, pX=0.67, N=325
var.4.THBS1 15:39880822:A:G
peptide level
196:196
110:110
19:19
AGTLDLSLTVQGKpc2_NP.5
p−maj=1, pX=0.94, N=325
var.4.THBS1 15:39880822:A:G
peptide level
194:196
110:110
19:19
AQGYSGLSVKpc2_NP.1
p−min=0.52, pX=0.55, N=323
var.4.THBS1 15:39880822:A:G
peptide level
195:196
110:110
19:19
AQGYSGLSVKpc2_NP.2
p−maj=1, pX=0.54, N=324
var.4.THBS1 15:39880822:A:G
peptide level
196:196
110:110
19:19
AQGYSGLSVKpc2_NP.3
p−maj=1, pX=0.56, N=325
var.4.THBS1 15:39880822:A:G
peptide level
196:196
110:110
19:19
AQGYSGLSVKpc2_NP.4
p−maj=1, pX=0.9, N=325
var.4.THBS1 15:39880822:A:G
peptide level
196:196
110:110
19:19
AQGYSGLSVKpc2_NP.5
p−maj=1, pX=0.53, N=325
var.4.THBS1 15:39880822:A:G
peptide level
196:196
110:110
19:19
AQLYIDCEKpc2_NP.1
p−maj=1, pX=1, N=325
var.4.THBS1 15:39880822:A:G
peptide level
196:196
110:110
19:19
AQLYIDCEKpc2_NP.2
p−maj=1, pX=0.4, N=325
var.4.THBS1 15:39880822:A:G
peptide level
196:196
110:110
19:19
AQLYIDCEKpc2_NP.3
p−maj=1, pX=0.92, N=325
var.4.THBS1 15:39880822:A:G
peptide level
196:196
110:110
19:19
AQLYIDCEKpc2_NP.4
p−maj=1, pX=0.96, N=325
var.4.THBS1 15:39880822:A:G
peptide level
196:196
110:110
19:19
AQLYIDCEKpc2_NP.5
p−maj=1, pX=0.78, N=325

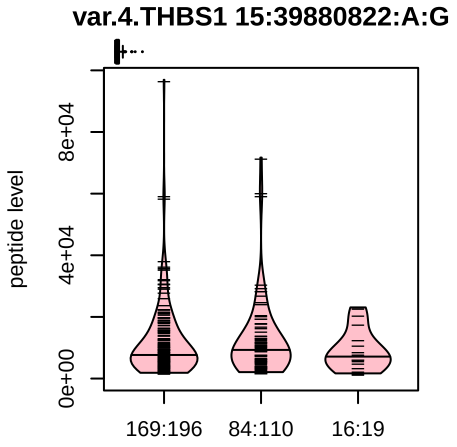

CEGSSVQTRpc2_NP.1
p−min=0.051, pX=0.36, N=269

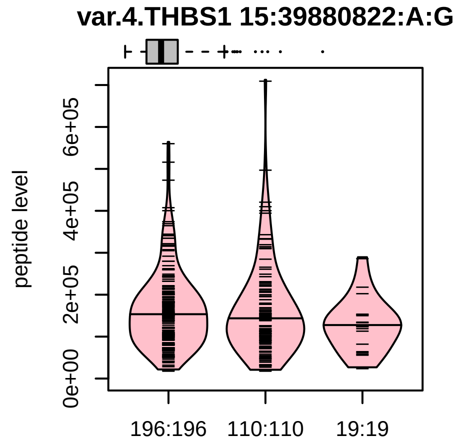

CEGSSVQTRpc2_NP.2
p−maj=1, pX=0.3, N=325

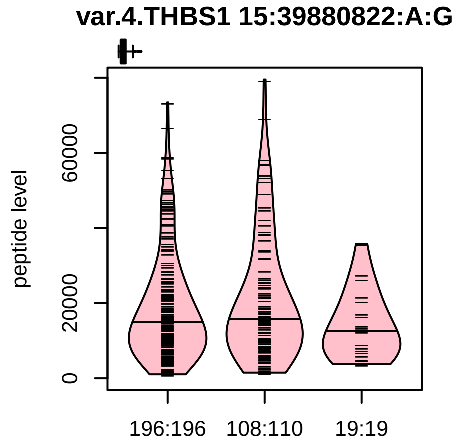

CEGSSVQTRpc2_NP.3
p−min=0.16, pX=0.88, N=323

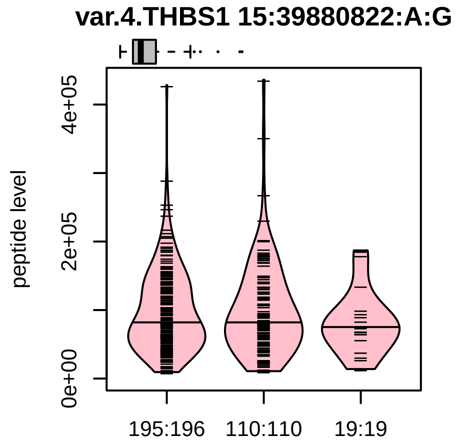

CEGSSVQTRpc2_NP.4
p−maj=1, pX=0.57, N=324

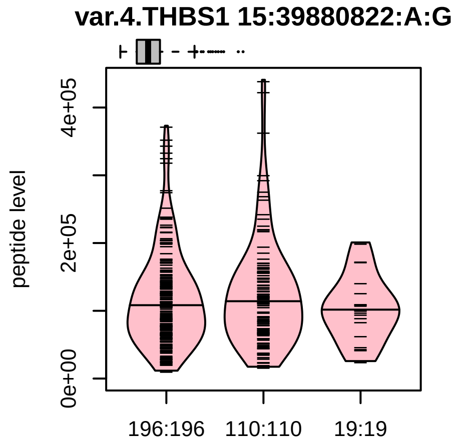

CEGSSVQTRpc2_NP.5
p−maj=1, pX=0.92, N=325

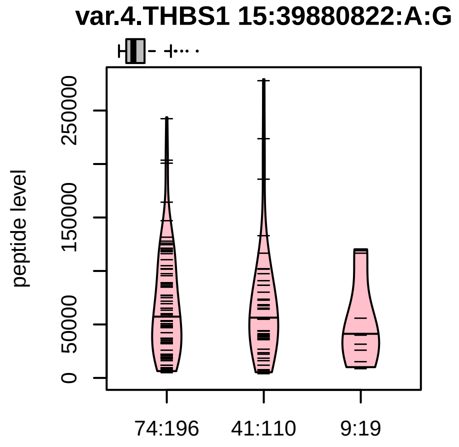

CENTDPGYNCLPCPPRpc2_NP.4
p−maj=0.47, pX=0.85, N=124

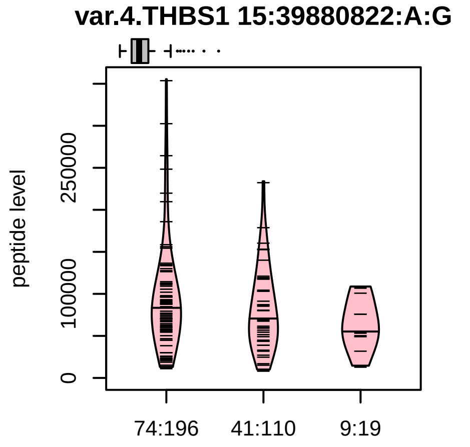

CENTDPGYNCLPCPPRpc2_NP.5
p−maj=0.47, pX=0.43, N=124

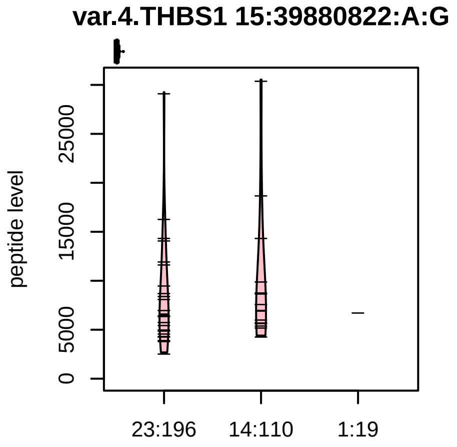

CENTDPGYNCLPCPPRpc3_NP.1
p−maj=0.71, pX=0.9, N=38

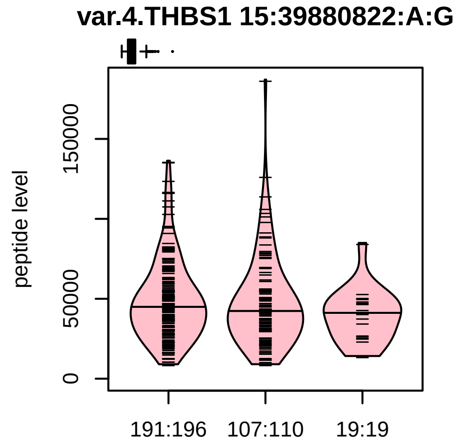

CENTDPGYNCLPCPPRpc3_NP.2
p−min=1, pX=0.28, N=317

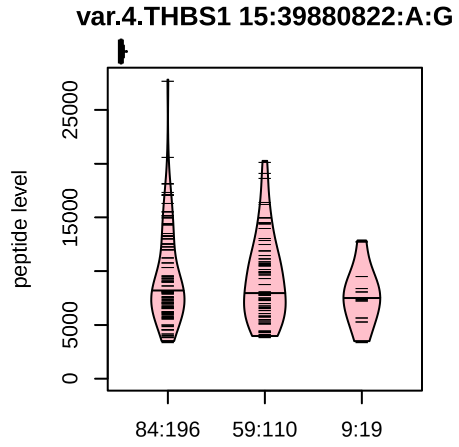

CENTDPGYNCLPCPPRpc3_NP.3
p−min=0.089, pX=0.46, N=152

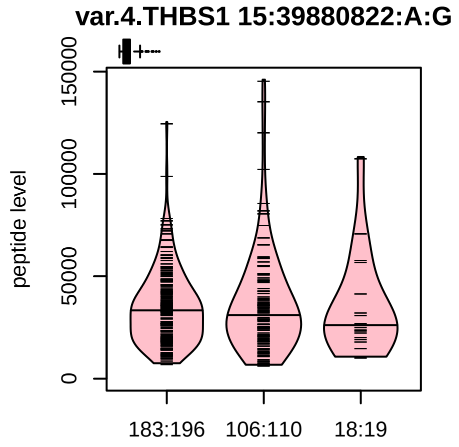

CENTDPGYNCLPCPPRpc3_NP.4
p−min=0.33, pX=0.29, N=307

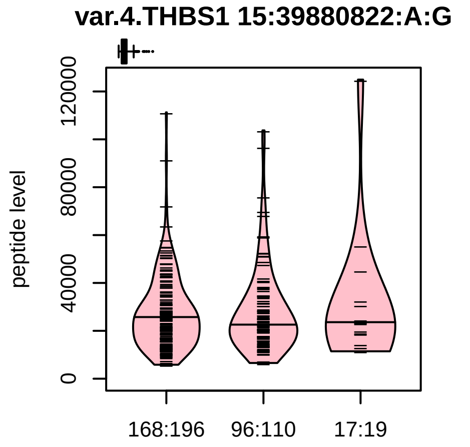

CENTDPGYNCLPCPPRpc3_NP.5
p−min=0.74, pX=0.39, N=281

var.4.THBS1 15:39880822:A:G

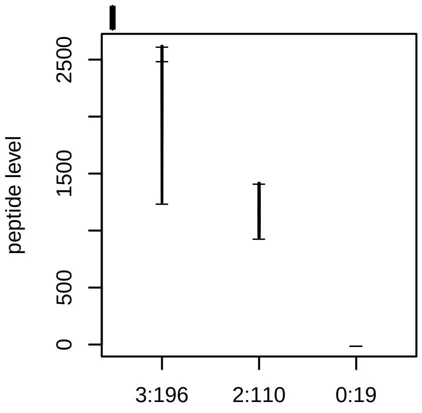

CGACPPGYSGNGIQCTDVDECKpc3_NP.4
p−maj=1, pX=0.52, N=5

var.4.THBS1 15:39880822:A:G

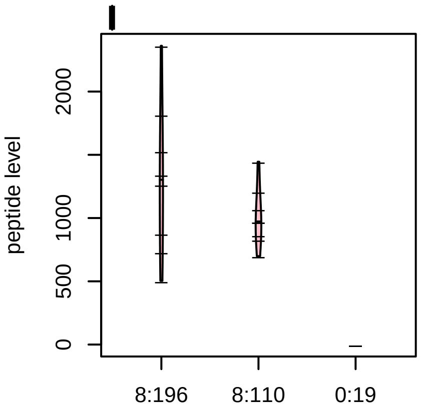

CGACPPGYSGNGIQCTDVDECKpc3_NP.5
p−min=0.44, pX=0.9, N=16

var.4.THBS1 15:39880822:A:G

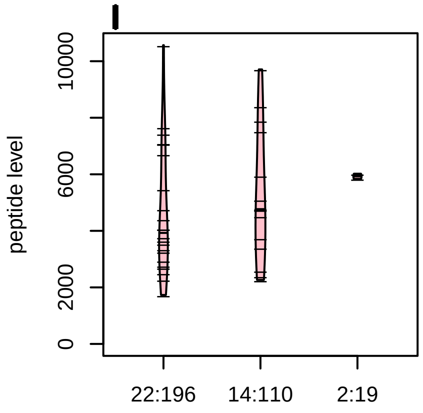

CGACPPGYSGNGIQCTDVDECKpc4_NP.2
p−min=0.86, pX=0.54, N=38

var.4.THBS1 15:39880822:A:G

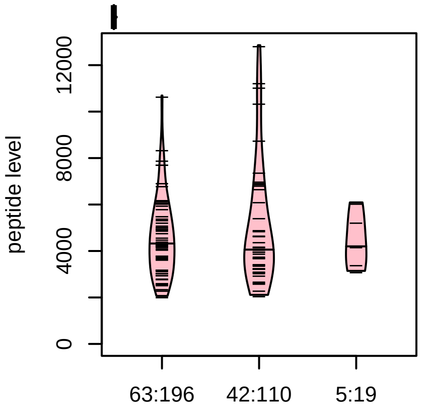

CGACPPGYSGNGIQCTDVDECKpc4_NP.4
p−min=0.47, pX=0.52, N=110

var.4.THBS1 15:39880822:A:G

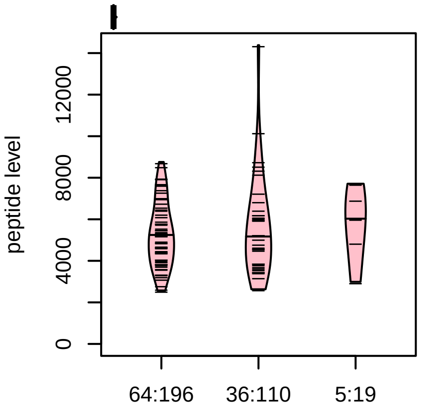

CGACPPGYSGNGIQCTDVDECKpc4_NP.5
p−maj=0.8, pX=0.98, N=105

var.4.THBS1 15:39880822:A:G

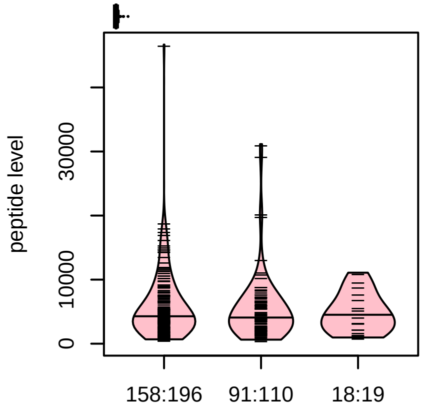

CNYLGHYSDPMYRpc2_NP.1
p−maj=0.22, pX=0.75, N=267

var.4.THBS1 15:39880822:A:G

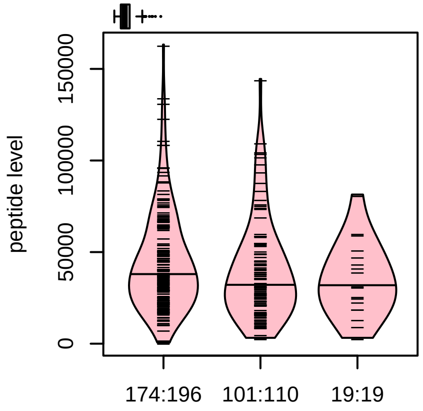

CNYLGHYSDPMYRpc2_NP.2
p−maj=0.24, pX=0.32, N=294

var.4.THBS1 15:39880822:A:G

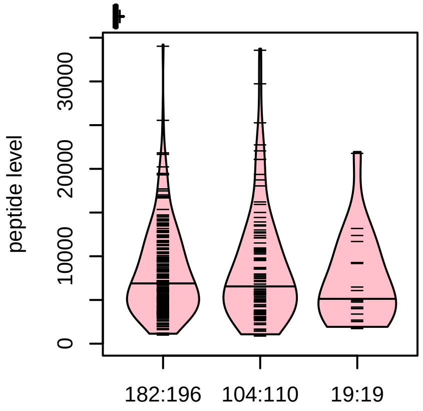

CNYLGHYSDPMYRpc2_NP.3
p−min=0.48, pX=0.85, N=305

var.4.THBS1 15:39880822:A:G

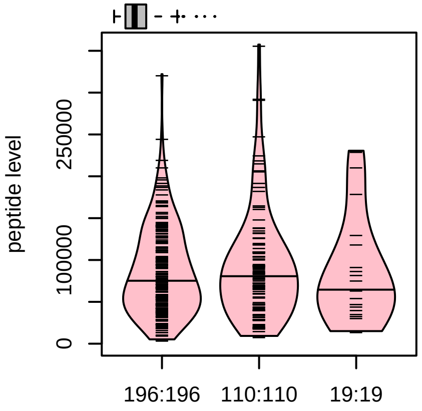

CNYLGHYSDPMYRpc2_NP.4
p−maj=1, pX=0.58, N=325

var.4.THBS1 15:39880822:A:G

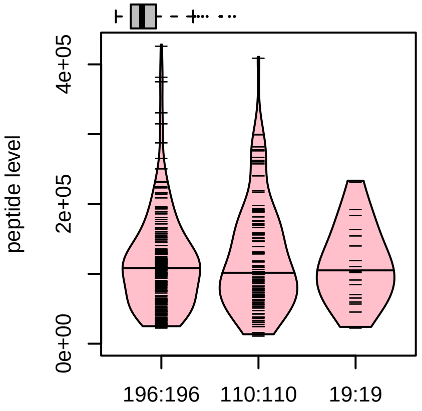

CNYLGHYSDPMYRpc2_NP.5
p−maj=1, pX=0.71, N=325

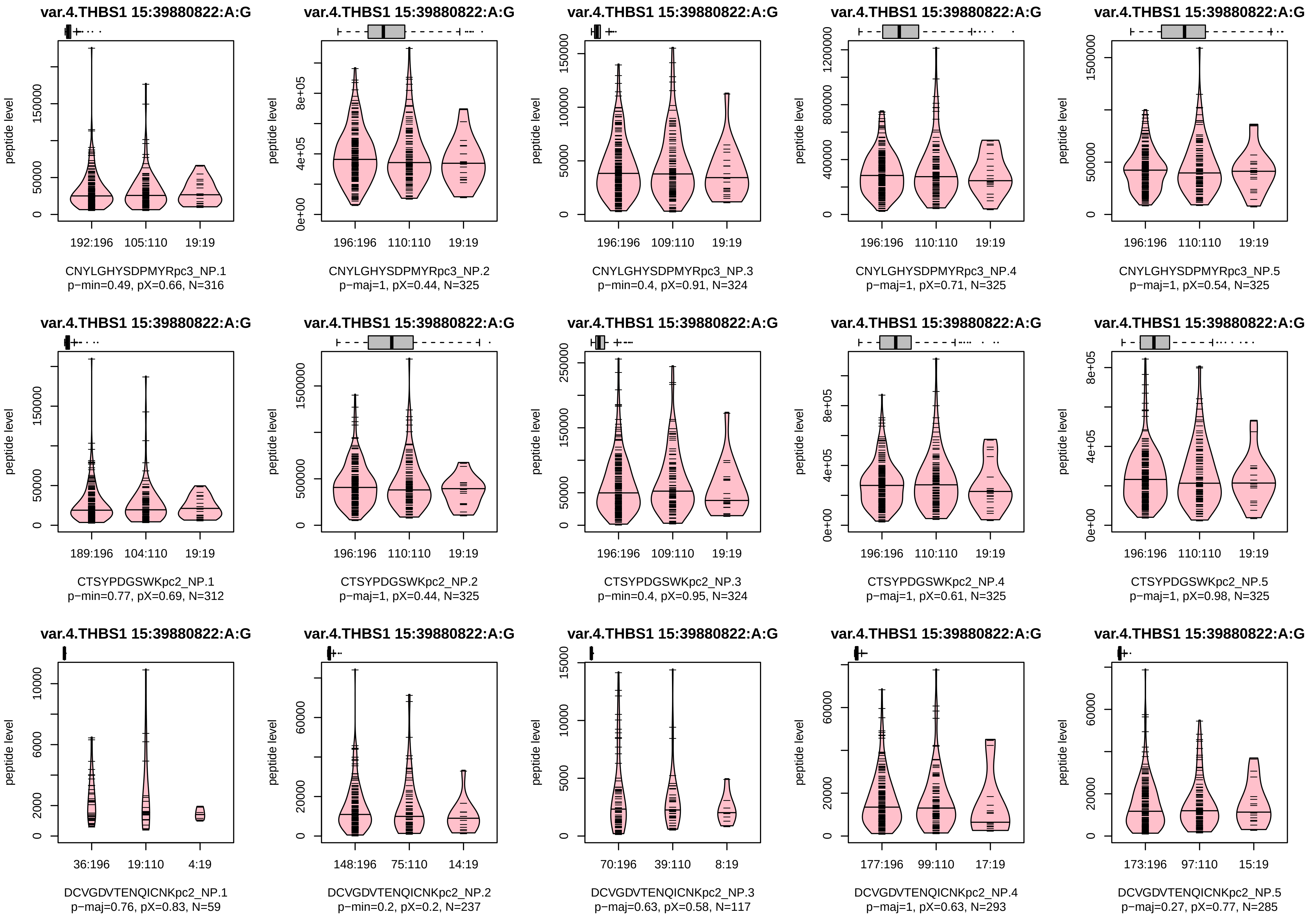

var.4.THBS1 15:39880822:A:G
peptide level
192:196
105:110
19:19
CNYLGHYSDPMYRpc3_NP.1
p−min=0.49, pX=0.66, N=316
var.4.THBS1 15:39880822:A:G
peptide level
196:196
110:110
19:19
CNYLGHYSDPMYRpc3_NP.2
p−maj=1, pX=0.44, N=325
var.4.THBS1 15:39880822:A:G
peptide level
196:196
109:110
19:19
CNYLGHYSDPMYRpc3_NP.3
p−min=0.4, pX=0.91, N=324
var.4.THBS1 15:39880822:A:G
peptide level
196:196
110:110
19:19
CNYLGHYSDPMYRpc3_NP.4
p−maj=1, pX=0.71, N=325
var.4.THBS1 15:39880822:A:G
peptide level
196:196
110:110
19:19
CNYLGHYSDPMYRpc3_NP.5
p−maj=1, pX=0.54, N=325
var.4.THBS1 15:39880822:A:G
peptide level
189:196
104:110
19:19
CTSYPDGSWKpc2_NP.1
p−min=0.77, pX=0.69, N=312
var.4.THBS1 15:39880822:A:G
peptide level
196:196
110:110
19:19
CTSYPDGSWKpc2_NP.2
p−maj=1, pX=0.44, N=325
var.4.THBS1 15:39880822:A:G
peptide level
196:196
109:110
19:19
CTSYPDGSWKpc2_NP.3
p−min=0.4, pX=0.95, N=324
var.4.THBS1 15:39880822:A:G
peptide level
196:196
110:110
19:19
CTSYPDGSWKpc2_NP.4
p−maj=1, pX=0.61, N=325
var.4.THBS1 15:39880822:A:G
peptide level
196:196
110:110
19:19
CTSYPDGSWKpc2_NP.5
p−maj=1, pX=0.98, N=325
var.4.THBS1 15:39880822:A:G
peptide level
36:196
19:110
4:19
DCVGDVTENQICNKpc2_NP.1
p−maj=0.76, pX=0.83, N=59
var.4.THBS1 15:39880822:A:G
peptide level
148:196
75:110
14:19
DCVGDVTENQICNKpc2_NP.2
p−min=0.2, pX=0.2, N=237
var.4.THBS1 15:39880822:A:G
peptide level
70:196
39:110
8:19
DCVGDVTENQICNKpc2_NP.3
p−maj=0.63, pX=0.58, N=117
var.4.THBS1 15:39880822:A:G
peptide level
177:196
99:110
17:19
DCVGDVTENQICNKpc2_NP.4
p−maj=1, pX=0.63, N=293
var.4.THBS1 15:39880822:A:G
peptide level
173:196
97:110
15:19
DCVGDVTENQICNKpc2_NP.5
p−maj=0.27, pX=0.77, N=285

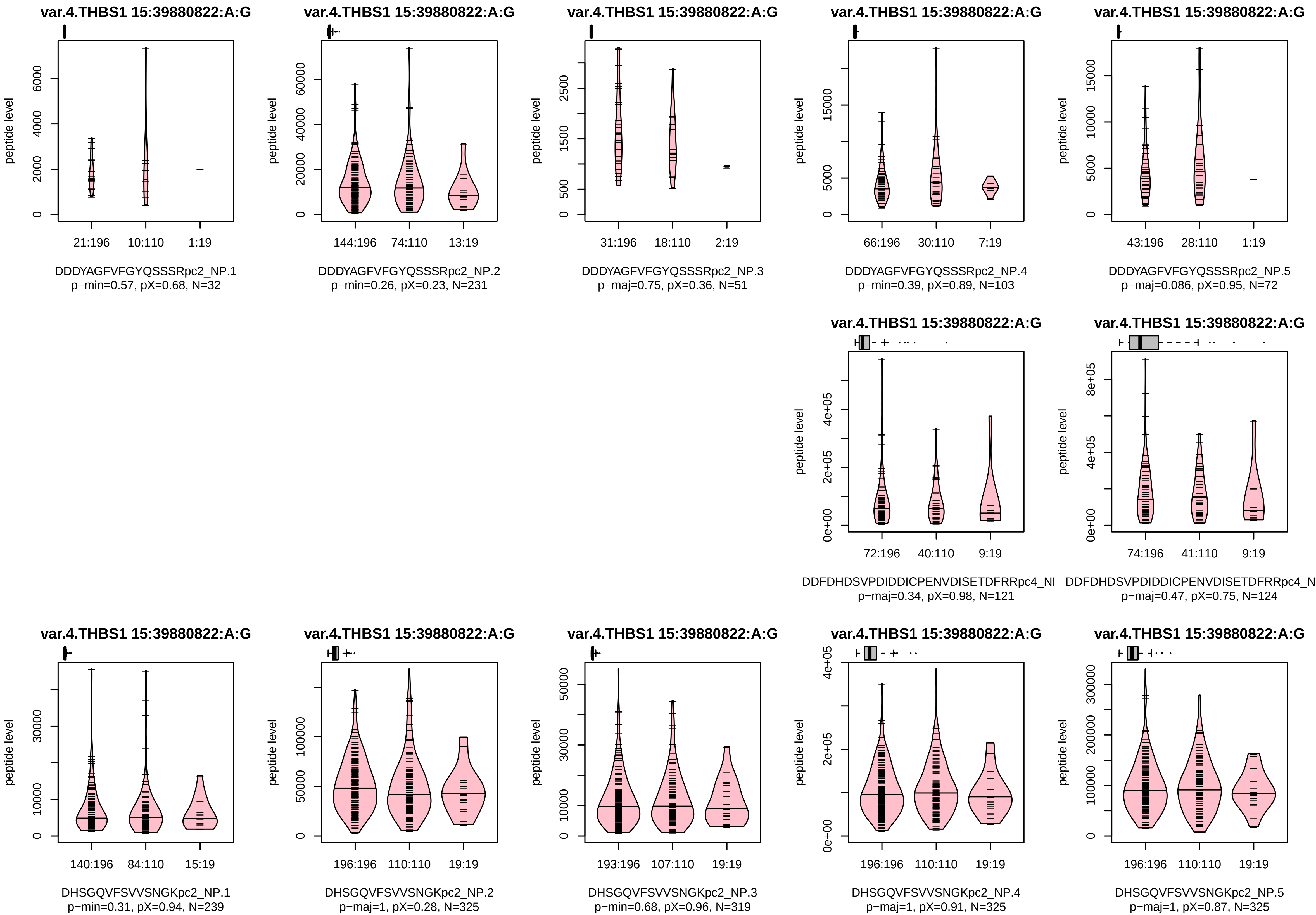
var.4.THBS1 15:39880822:A:G
peptide level
21:196
10:110
1:19
DDDYAGFVFGYQSSSRpc2_NP.1
p−min=0.57, pX=0.68, N=32
var.4.THBS1 15:39880822:A:G
peptide level
144:196
74:110
13:19
DDDYAGFVFGYQSSSRpc2_NP.2
p−min=0.26, pX=0.23, N=231
var.4.THBS1 15:39880822:A:G
peptide level
31:196
18:110
2:19
DDDYAGFVFGYQSSSRpc2_NP.3
p−maj=0.75, pX=0.36, N=51
var.4.THBS1 15:39880822:A:G
peptide level
66:196
30:110
7:19
DDDYAGFVFGYQSSSRpc2_NP.4
p−min=0.39, pX=0.89, N=103
var.4.THBS1 15:39880822:A:G
peptide level
43:196
28:110
1:19
DDDYAGFVFGYQSSSRpc2_NP.5
p−maj=0.086, pX=0.95, N=72
var.4.THBS1 15:39880822:A:G
peptide level
72:196
40:110
9:19
DDFDHDSVPDIDDICPENVDISETDFRRpc4_NI
p−maj=0.34, pX=0.98, N=121
var.4.THBS1 15:39880822:A:G
peptide level
74:196
41:110
9:19
DDFDHDSVPDIDDICPENVDISETDFRRpc4_N
p−maj=0.47, pX=0.75, N=124
var.4.THBS1 15:39880822:A:G
peptide level
140:196
84:110
15:19
DHSGQVFSVVSNGKpc2_NP.1
p−min=0.31, pX=0.94, N=239
var.4.THBS1 15:39880822:A:G
peptide level
196:196
110:110
19:19
DHSGQVFSVVSNGKpc2_NP.2
p−maj=1, pX=0.28, N=325
var.4.THBS1 15:39880822:A:G
peptide level
193:196
107:110
19:19
DHSGQVFSVVSNGKpc2_NP.3
p−min=0.68, pX=0.96, N=319
var.4.THBS1 15:39880822:A:G
peptide level
196:196
110:110
19:19
DHSGQVFSVVSNGKpc2_NP.4
p−maj=1, pX=0.91, N=325
var.4.THBS1 15:39880822:A:G
peptide level
196:196
110:110
19:19
DHSGQVFSVVSNGKpc2_NP.5
p−maj=1, pX=0.87, N=325

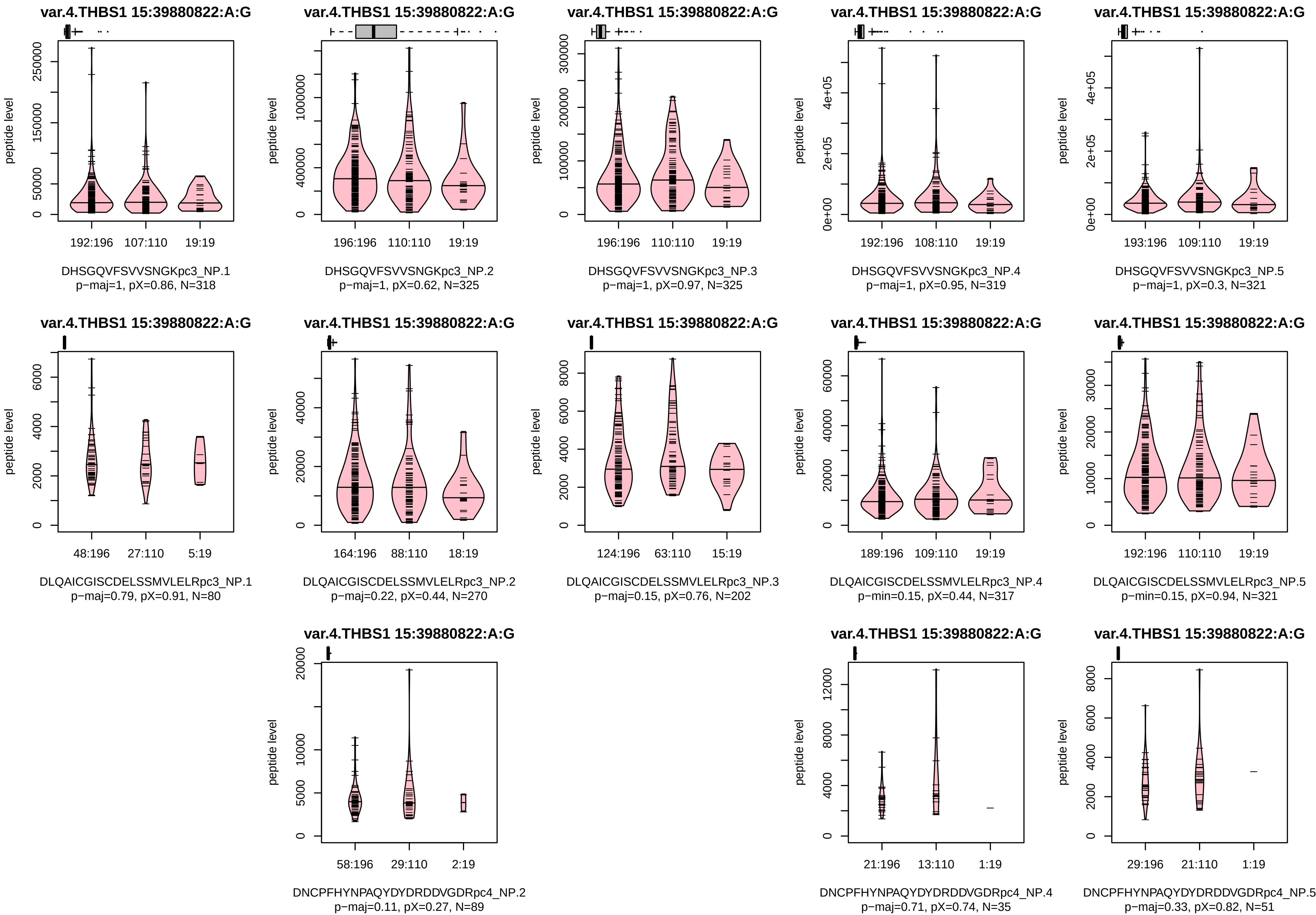
var.4.THBS1 15:39880822:A:G
peptide level
192:196 107:110 19:19
DHSGQVFSVVSNGKpc3_NP.1
p−maj=1, pX=0.86, N=318
var.4.THBS1 15:39880822:A:G
196:196 110:110 19:19
DHSGQVFSVVSNGKpc3_NP.2
p−maj=1, pX=0.62, N=325
var.4.THBS1 15:39880822:A:G
196:196 110:110 19:19
DHSGQVFSVVSNGKpc3_NP.3
p−maj=1, pX=0.97, N=325
var.4.THBS1 15:39880822:A:G
192:196 108:110 19:19
DHSGQVFSVVSNGKpc3_NP.4
p−maj=1, pX=0.95, N=319
var.4.THBS1 15:39880822:A:G
193:196 109:110 19:19
DHSGQVFSVVSNGKpc3_NP.5
p−maj=1, pX=0.3, N=321
var.4.THBS1 15:39880822:A:G
48:196 27:110 5:19
DLQAICGISCDELSSMVLELRpc3_NP.1
p−maj=0.79, pX=0.91, N=80
var.4.THBS1 15:39880822:A:G
164:196 88:110 18:19
DLQAICGISCDELSSMVLELRpc3_NP.2
p−maj=0.22, pX=0.44, N=270
var.4.THBS1 15:39880822:A:G
124:196 63:110 15:19
DLQAICGISCDELSSMVLELRpc3_NP.3
p−maj=0.15, pX=0.76, N=202
var.4.THBS1 15:39880822:A:G
189:196 109:110 19:19
DLQAICGISCDELSSMVLELRpc3_NP.4
p−min=0.15, pX=0.44, N=317
var.4.THBS1 15:39880822:A:G
192:196 110:110 19:19
DLQAICGISCDELSSMVLELRpc3_NP.5
p−min=0.15, pX=0.94, N=321
var.4.THBS1 15:39880822:A:G
58:196 29:110 2:19
DNCPFHYNPAQYDYDRDDVGDRpc4_NP.2
p−maj=0.11, pX=0.27, N=89
var.4.THBS1 15:39880822:A:G
21:196 13:110 1:19
DNCPFHYNPAQYDYDRDDVGDRpc4_NP.4
p−maj=0.71, pX=0.74, N=35
var.4.THBS1 15:39880822:A:G
29:196 21:110 1:19
DNCPFHYNPAQYDYDRDDVGDRpc4_NP.5
p−maj=0.33, pX=0.82, N=51

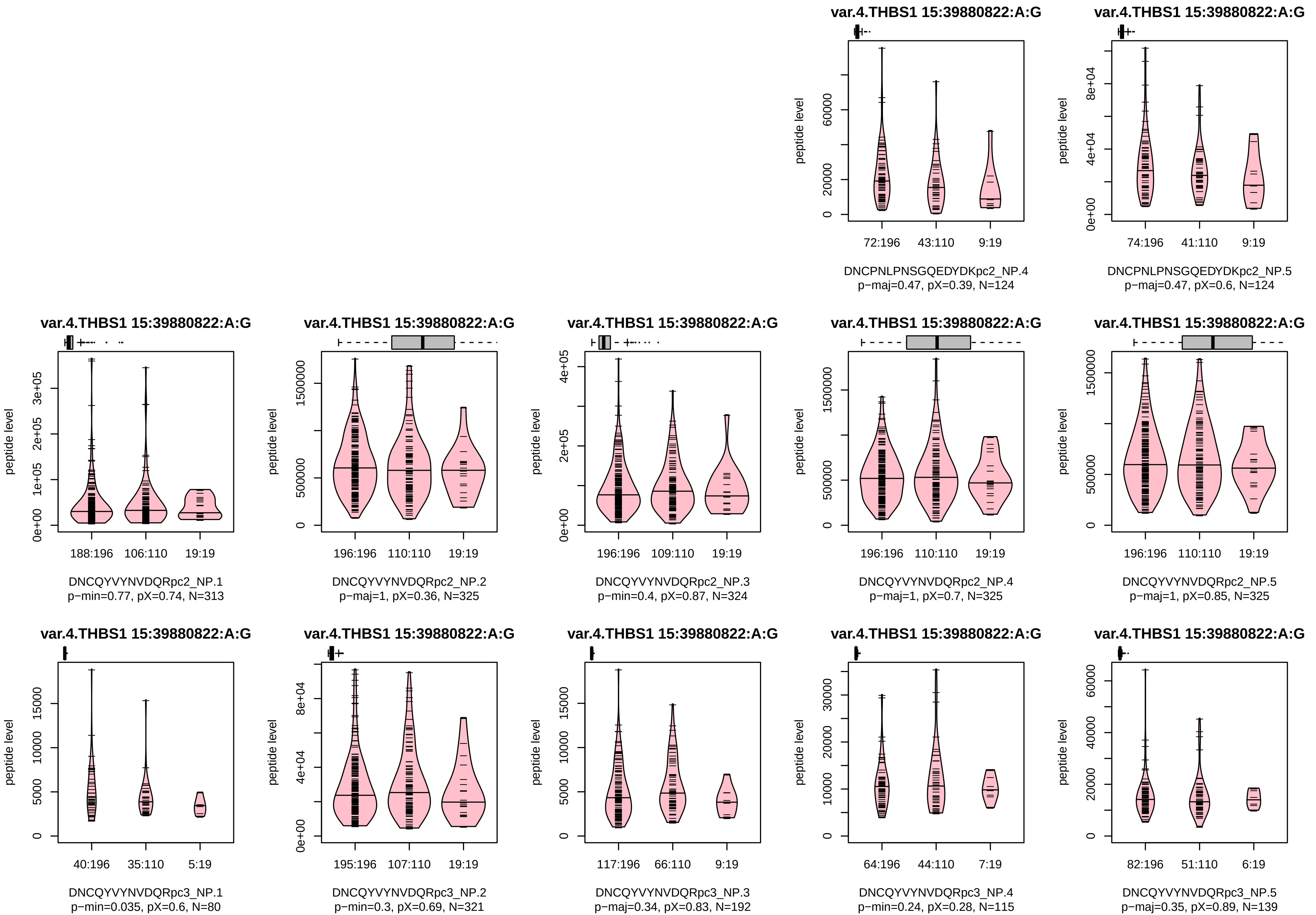
var.4.THBS1 15:39880822:A:G
peptide level
72:196
43:110
9:19
DNCPNLPNSGQEDYDKpc2_NP.4
p−maj=0.47, pX=0.39, N=124
var.4.THBS1 15:39880822:A:G
peptide level
74:196
41:110
9:19
DNCPNLPNSGQEDYDKpc2_NP.5
p−maj=0.47, pX=0.6, N=124
var.4.THBS1 15:39880822:A:G
peptide level
188:196
106:110
19:19
DNCQYVYNVDQRpc2_NP.1
p−min=0.77, pX=0.74, N=313
var.4.THBS1 15:39880822:A:G
peptide level
196:196
110:110
19:19
DNCQYVYNVDQRpc2_NP.2
p−maj=1, pX=0.36, N=325
var.4.THBS1 15:39880822:A:G
peptide level
196:196
109:110
19:19
DNCQYVYNVDQRpc2_NP.3
p−min=0.4, pX=0.87, N=324
var.4.THBS1 15:39880822:A:G
peptide level
196:196
110:110
19:19
DNCQYVYNVDQRpc2_NP.4
p−maj=1, pX=0.7, N=325
var.4.THBS1 15:39880822:A:G
peptide level
196:196
110:110
19:19
DNCQYVYNVDQRpc2_NP.5
p−maj=1, pX=0.85, N=325
var.4.THBS1 15:39880822:A:G
peptide level
40:196
35:110
5:19
DNCQYVYNVDQRpc3_NP.1
p−min=0.035, pX=0.6, N=80
var.4.THBS1 15:39880822:A:G
peptide level
195:196
107:110
19:19
DNCQYVYNVDQRpc3_NP.2
p−min=0.3, pX=0.69, N=321
var.4.THBS1 15:39880822:A:G
peptide level
117:196
66:110
9:19
DNCQYVYNVDQRpc3_NP.3
p−maj=0.34, pX=0.83, N=192
var.4.THBS1 15:39880822:A:G
peptide level
64:196
44:110
7:19
DNCQYVYNVDQRpc3_NP.4
p−min=0.24, pX=0.28, N=115
var.4.THBS1 15:39880822:A:G
peptide level
82:196
51:110
6:19
DNCQYVYNVDQRpc3_NP.5
p−maj=0.35, pX=0.89, N=139

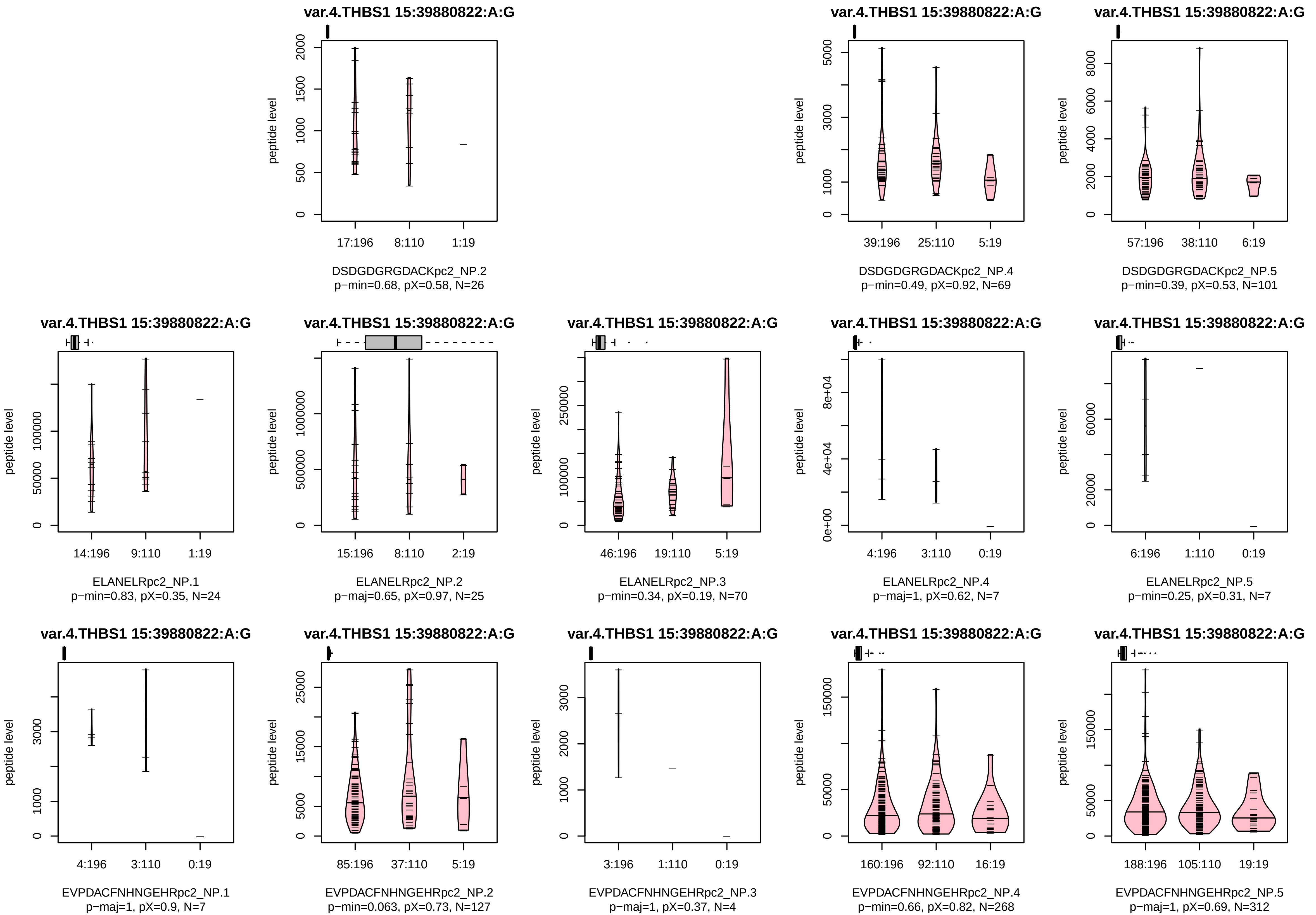
var.4.THBS1 15:39880822:A:G
peptide level
17:196
8:110
1:19
DSDGDGRGDACKpc2_NP.2
p−min=0.68, pX=0.58, N=26
var.4.THBS1 15:39880822:A:G
peptide level
39:196
25:110
5:19
DSDGDGRGDACKpc2_NP.4
p−min=0.49, pX=0.92, N=69
var.4.THBS1 15:39880822:A:G
peptide level
57:196
38:110
6:19
DSDGDGRGDACKpc2_NP.5
p−min=0.39, pX=0.53, N=101
var.4.THBS1 15:39880822:A:G
peptide level
14:196
9:110
1:19
ELANELRpc2_NP.1
p−min=0.83, pX=0.35, N=24
var.4.THBS1 15:39880822:A:G
peptide level
15:196
8:110
2:19
ELANELRpc2_NP.2
p−maj=0.65, pX=0.97, N=25
var.4.THBS1 15:39880822:A:G
peptide level
46:196
19:110
5:19
ELANELRpc2_NP.3
p−min=0.34, pX=0.19, N=70
var.4.THBS1 15:39880822:A:G
peptide level
4:196
3:110
0:19
ELANELRpc2_NP.4
p−maj=1, pX=0.62, N=7
var.4.THBS1 15:39880822:A:G
peptide level
6:196
1:110
0:19
ELANELRpc2_NP.5
p−min=0.25, pX=0.31, N=7
var.4.THBS1 15:39880822:A:G
peptide level
4:196
3:110
0:19
EVPDACFNHNGEHRpc2_NP.1
p−maj=1, pX=0.9, N=7
var.4.THBS1 15:39880822:A:G
peptide level
85:196
37:110
5:19
EVPDACFNHNGEHRpc2_NP.2
p−min=0.063, pX=0.73, N=127
var.4.THBS1 15:39880822:A:G
peptide level
3:196
1:110
0:19
EVPDACFNHNGEHRpc2_NP.3
p−maj=1, pX=0.37, N=4
var.4.THBS1 15:39880822:A:G
peptide level
160:196
92:110
16:19
EVPDACFNHNGEHRpc2_NP.4
p−min=0.66, pX=0.82, N=268
var.4.THBS1 15:39880822:A:G
peptide level
188:196
105:110
19:19
EVPDACFNHNGEHRpc2_NP.5
p−maj=1, pX=0.69, N=312

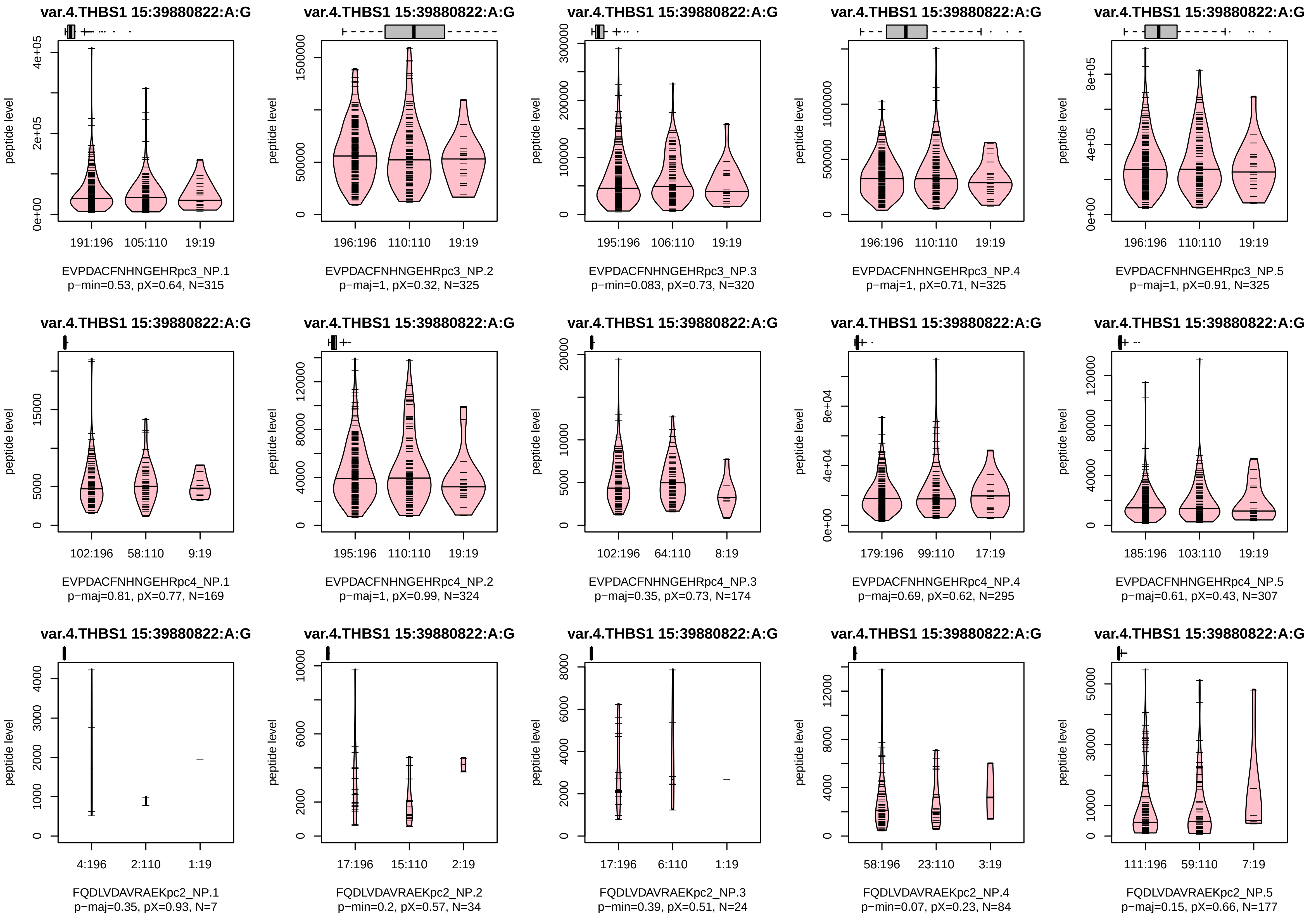
var.4.THBS1 15:39880822:A:G
peptide level
191:196
105:110
19:19
EVPDACFNHNGEHRpc3_NP.1
p−min=0.53, pX=0.64, N=315
var.4.THBS1 15:39880822:A:G
peptide level
196:196
110:110
19:19
EVPDACFNHNGEHRpc3_NP.2
p−maj=1, pX=0.32, N=325
var.4.THBS1 15:39880822:A:G
peptide level
195:196
106:110
19:19
EVPDACFNHNGEHRpc3_NP.3
p−min=0.083, pX=0.73, N=320
var.4.THBS1 15:39880822:A:G
peptide level
196:196
110:110
19:19
EVPDACFNHNGEHRpc3_NP.4
p−maj=1, pX=0.71, N=325
var.4.THBS1 15:39880822:A:G
peptide level
196:196
110:110
19:19
EVPDACFNHNGEHRpc3_NP.5
p−maj=1, pX=0.91, N=325
var.4.THBS1 15:39880822:A:G
peptide level
102:196
58:110
9:19
EVPDACFNHNGEHRpc4_NP.1
p−maj=0.81, pX=0.77, N=169
var.4.THBS1 15:39880822:A:G
peptide level
195:196
110:110
19:19
EVPDACFNHNGEHRpc4_NP.2
p−maj=1, pX=0.99, N=324
var.4.THBS1 15:39880822:A:G
peptide level
102:196
64:110
8:19
EVPDACFNHNGEHRpc4_NP.3
p−maj=0.35, pX=0.73, N=174
var.4.THBS1 15:39880822:A:G
peptide level
179:196
99:110
17:19
EVPDACFNHNGEHRpc4_NP.4
p−maj=0.69, pX=0.62, N=295
var.4.THBS1 15:39880822:A:G
peptide level
185:196
103:110
19:19
EVPDACFNHNGEHRpc4_NP.5
p−maj=0.61, pX=0.43, N=307
var.4.THBS1 15:39880822:A:G
peptide level
4:196
2:110
1:19
FQDLVDAVRAEKpc2_NP.1
p−maj=0.35, pX=0.93, N=7
var.4.THBS1 15:39880822:A:G
peptide level
17:196
15:110
2:19
FQDLVDAVRAEKpc2_NP.2
p−min=0.2, pX=0.57, N=34
var.4.THBS1 15:39880822:A:G
peptide level
17:196
6:110
1:19
FQDLVDAVRAEKpc2_NP.3
p−min=0.39, pX=0.51, N=24
var.4.THBS1 15:39880822:A:G
peptide level
58:196
23:110
3:19
FQDLVDAVRAEKpc2_NP.4
p−min=0.07, pX=0.23, N=84
var.4.THBS1 15:39880822:A:G
peptide level
111:196
59:110
7:19
FQDLVDAVRAEKpc2_NP.5
p−maj=0.15, pX=0.66, N=177

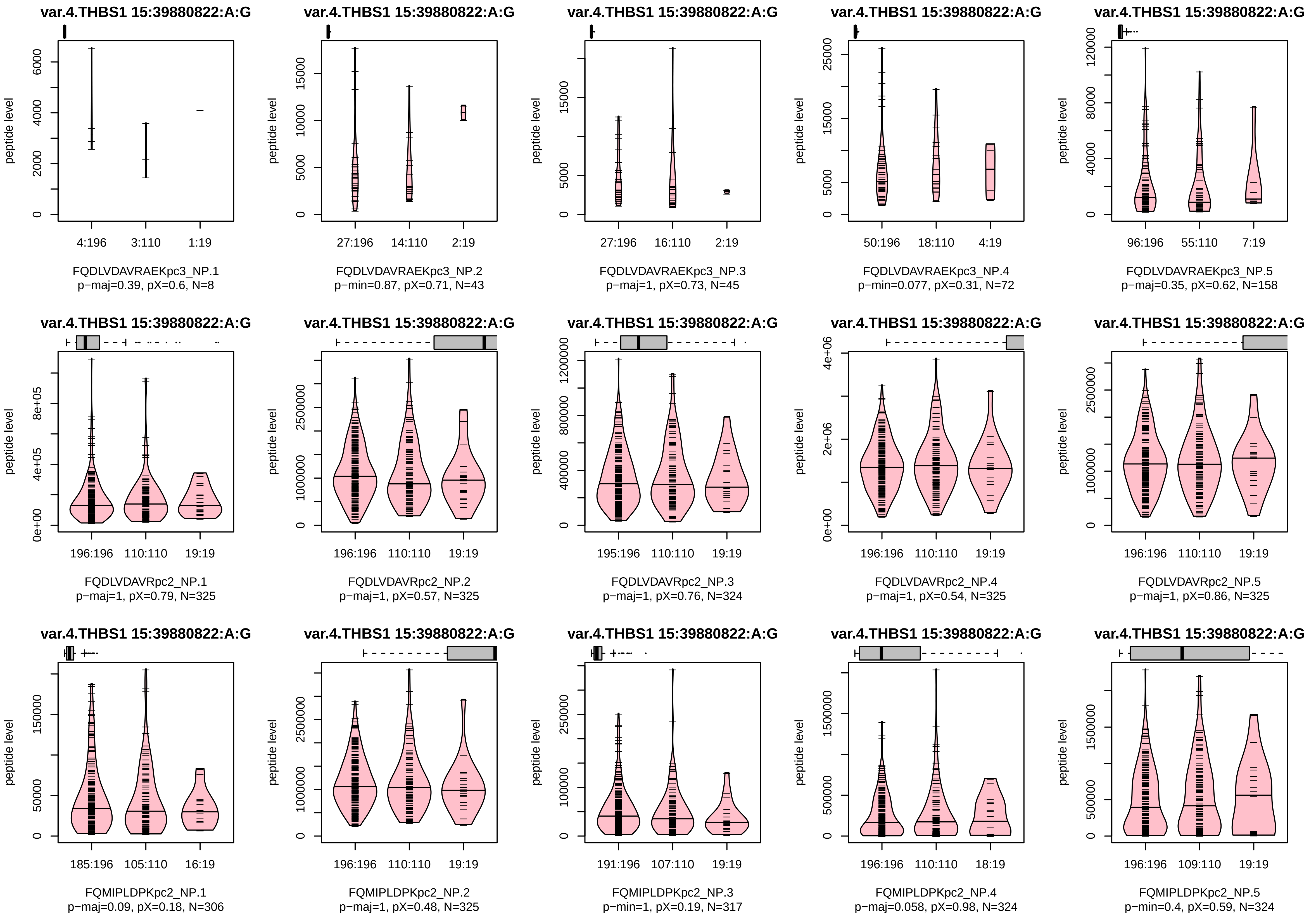
var.4.THBS1 15:39880822:A:G
peptide level
0
2000
4000
6000
4:196
3:110
1:19
FQDLVDAVRAEKpc3_NP.1
p−maj=0.39, pX=0.6, N=8
var.4.THBS1 15:39880822:A:G
peptide level
0
5000
10000
15000
27:196
14:110
2:19
FQDLVDAVRAEKpc3_NP.2
p−min=0.87, pX=0.71, N=43
var.4.THBS1 15:39880822:A:G
peptide level
0
5000
15000
27:196
16:110
2:19
FQDLVDAVRAEKpc3_NP.3
p−maj=1, pX=0.73, N=45
var.4.THBS1 15:39880822:A:G
peptide level
0
5000
15000
25000
50:196
18:110
4:19
FQDLVDAVRAEKpc3_NP.4
p−min=0.077, pX=0.31, N=72
var.4.THBS1 15:39880822:A:G
peptide level
0
40000
80000
120000
96:196
55:110
7:19
FQDLVDAVRAEKpc3_NP.5
p−maj=0.35, pX=0.62, N=158
var.4.THBS1 15:39880822:A:G
peptide level
0e+00
4e+05
8e+05
196:196
110:110
19:19
FQDLVDAVRpc2_NP.1
p−maj=1, pX=0.79, N=325
var.4.THBS1 15:39880822:A:G
peptide level
0
1000000
2500000
196:196
110:110
19:19
FQDLVDAVRpc2_NP.2
p−maj=1, pX=0.57, N=325
var.4.THBS1 15:39880822:A:G
peptide level
0
400000
800000
1200000
195:196
110:110
19:19
FQDLVDAVRpc2_NP.3
p−maj=1, pX=0.76, N=324
var.4.THBS1 15:39880822:A:G
peptide level
0e+00
2e+06
4e+06
196:196
110:110
19:19
FQDLVDAVRpc2_NP.4
p−maj=1, pX=0.54, N=325
var.4.THBS1 15:39880822:A:G
peptide level
0
1000000
2500000
196:196
110:110
19:19
FQDLVDAVRpc2_NP.5
p−maj=1, pX=0.86, N=325
var.4.THBS1 15:39880822:A:G
peptide level
0
50000
150000
185:196
105:110
16:19
FQMIPLDPKpc2_NP.1
p−maj=0.09, pX=0.18, N=306
var.4.THBS1 15:39880822:A:G
peptide level
0
1000000
2500000
196:196
110:110
19:19
FQMIPLDPKpc2_NP.2
p−maj=1, pX=0.48, N=325
var.4.THBS1 15:39880822:A:G
peptide level
0
100000
250000
191:196
107:110
19:19
FQMIPLDPKpc2_NP.3
p−min=1, pX=0.19, N=317
var.4.THBS1 15:39880822:A:G
peptide level
0
500000
1500000
196:196
110:110
18:19
FQMIPLDPKpc2_NP.4
p−maj=0.058, pX=0.98, N=324
var.4.THBS1 15:39880822:A:G
peptide level
0
500000
1500000
196:196
109:110
19:19
FQMIPLDPKpc2_NP.5
p−min=0.4, pX=0.59, N=324

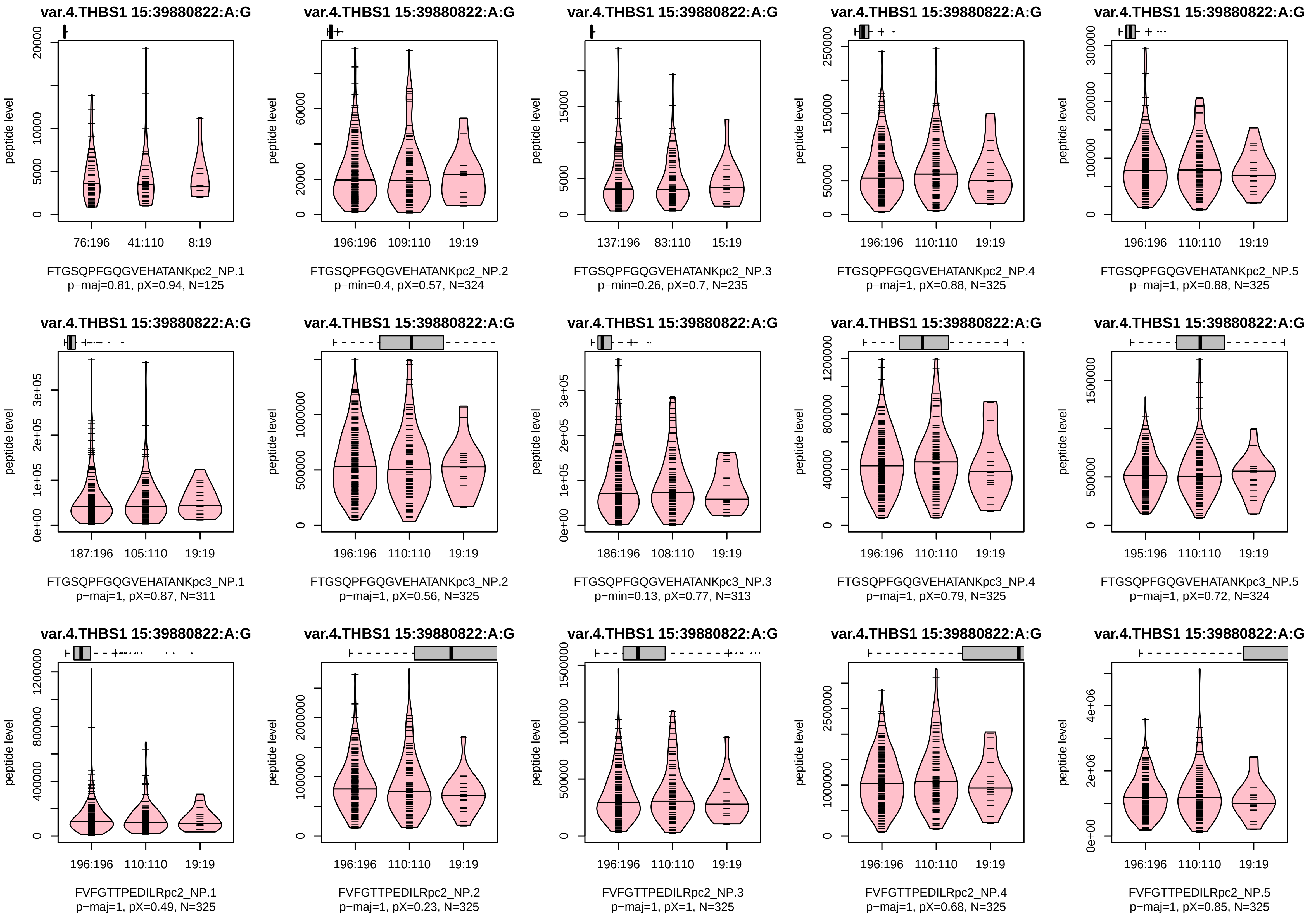

var.4.THBS1 15:39880822:A:G
peptide level
0
5000
10000
20000
76:196
41:110
8:19
FTGSQPFGQGVEHATANKpc2_NP.1
p−maj=0.81, pX=0.94, N=125
var.4.THBS1 15:39880822:A:G
peptide level
0
20000
60000
196:196
109:110
19:19
FTGSQPFGQGVEHATANKpc2_NP.2
p−min=0.4, pX=0.57, N=324
var.4.THBS1 15:39880822:A:G
peptide level
0
5000
15000
137:196
83:110
15:19
FTGSQPFGQGVEHATANKpc2_NP.3
p−min=0.26, pX=0.7, N=235
var.4.THBS1 15:39880822:A:G
peptide level
0
50000
150000
250000
196:196
110:110
19:19
FTGSQPFGQGVEHATANKpc2_NP.4
p−maj=1, pX=0.88, N=325
var.4.THBS1 15:39880822:A:G
peptide level
0
100000
200000
300000
196:196
110:110
19:19
FTGSQPFGQGVEHATANKpc2_NP.5
p−maj=1, pX=0.88, N=325
var.4.THBS1 15:39880822:A:G
peptide level
0e+00
1e+05
2e+05
3e+05
187:196
105:110
19:19
FTGSQPFGQGVEHATANKpc3_NP.1
p−maj=1, pX=0.87, N=311
var.4.THBS1 15:39880822:A:G
peptide level
0
500000
1000000
196:196
110:110
19:19
FTGSQPFGQGVEHATANKpc3_NP.2
p−maj=1, pX=0.56, N=325
var.4.THBS1 15:39880822:A:G
peptide level
0e+00
1e+05
2e+05
3e+05
186:196
108:110
19:19
FTGSQPFGQGVEHATANKpc3_NP.3
p−min=0.13, pX=0.77, N=313
var.4.THBS1 15:39880822:A:G
peptide level
0
400000
800000
1200000
196:196
110:110
19:19
FTGSQPFGQGVEHATANKpc3_NP.4
p−maj=1, pX=0.79, N=325
var.4.THBS1 15:39880822:A:G
peptide level
0
500000
1500000
195:196
110:110
19:19
FTGSQPFGQGVEHATANKpc3_NP.5
p−maj=1, pX=0.72, N=324
var.4.THBS1 15:39880822:A:G
peptide level
0
400000
800000
1200000
196:196
110:110
19:19
FVFGTTPEDILRpc2_NP.1
p−maj=1, pX=0.49, N=325
var.4.THBS1 15:39880822:A:G
peptide level
0
1000000
2000000
196:196
110:110
19:19
FVFGTTPEDILRpc2_NP.2
p−maj=1, pX=0.23, N=325
var.4.THBS1 15:39880822:A:G
peptide level
0
500000
1000000
1500000
196:196
110:110
19:19
FVFGTTPEDILRpc2_NP.3
p−maj=1, pX=1, N=325
var.4.THBS1 15:39880822:A:G
peptide level
0
1000000
2500000
196:196
110:110
19:19
FVFGTTPEDILRpc2_NP.4
p−maj=1, pX=0.68, N=325
var.4.THBS1 15:39880822:A:G
peptide level
0e+00
2e+06
4e+06
196:196
110:110
19:19
FVFGTTPEDILRpc2_NP.5
p−maj=1, pX=0.85, N=325

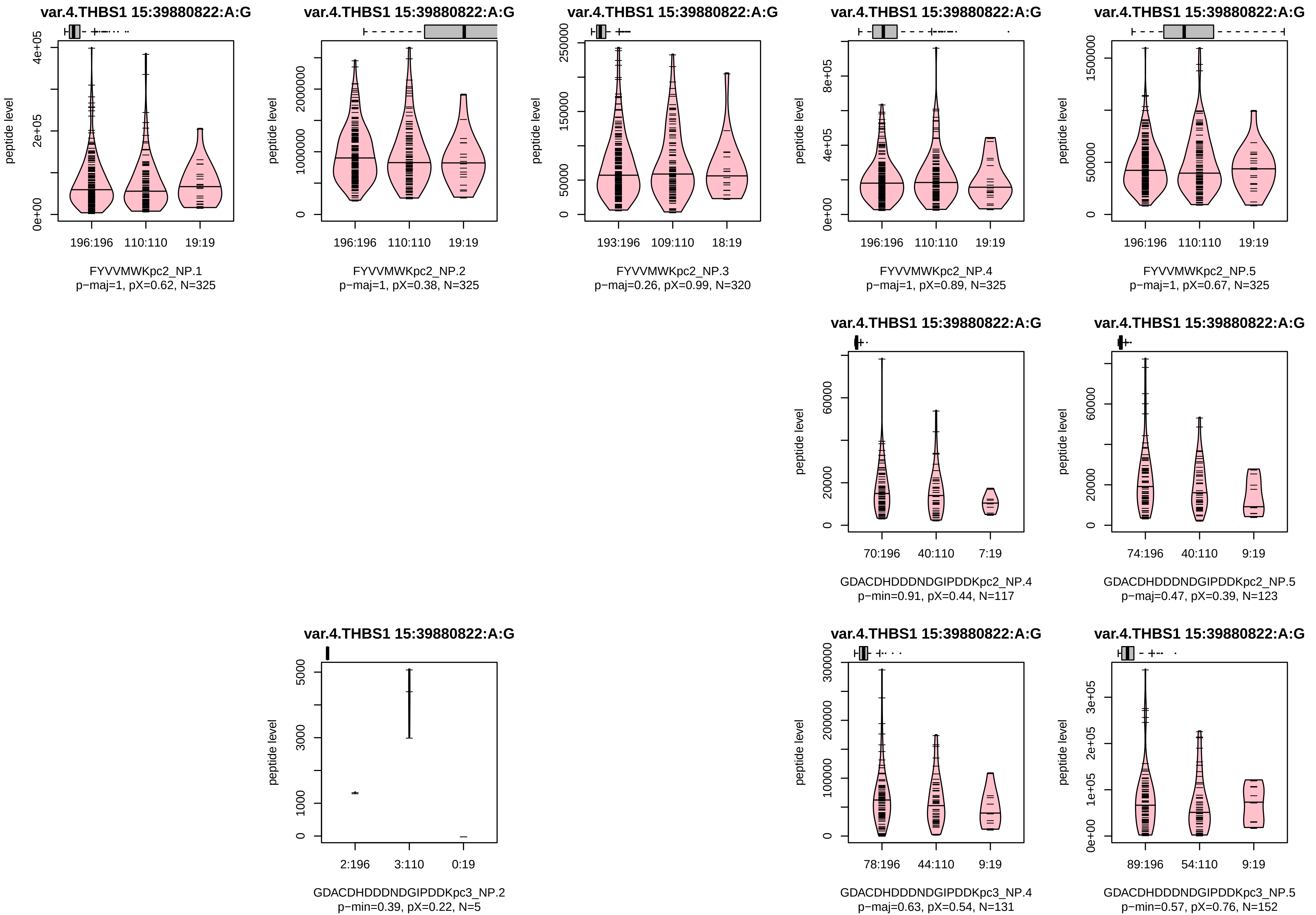

var.4.THBS1 15:39880822:A:G
peptide level
0e+00
2e+05
4e+05
196:196
110:110
19:19
FYVVMWKpc2_NP.1
p−maj=1, pX=0.62, N=325
var.4.THBS1 15:39880822:A:G
peptide level
0
1000000
2000000
196:196
110:110
19:19
FYVVMWKpc2_NP.2
p−maj=1, pX=0.38, N=325
var.4.THBS1 15:39880822:A:G
peptide level
0
50000
150000
250000
193:196
109:110
18:19
FYVVMWKpc2_NP.3
p−maj=0.26, pX=0.99, N=320
var.4.THBS1 15:39880822:A:G
peptide level
0e+00
4e+05
8e+05
196:196
110:110
19:19
FYVVMWKpc2_NP.4
p−maj=1, pX=0.89, N=325
var.4.THBS1 15:39880822:A:G
peptide level
0
500000
1500000
196:196
110:110
19:19
FYVVMWKpc2_NP.5
p−maj=1, pX=0.67, N=325
var.4.THBS1 15:39880822:A:G
peptide level
0
20000
60000
70:196
40:110
7:19
GDACDHDDDNDGIPDDKpc2_NP.4
p−min=0.91, pX=0.44, N=117
var.4.THBS1 15:39880822:A:G
peptide level
0
20000
60000
74:196
40:110
9:19
GDACDHDDDNDGIPDDKpc2_NP.5
p−maj=0.47, pX=0.39, N=123
var.4.THBS1 15:39880822:A:G
peptide level
0
1000
3000
5000
2:196
3:110
0:19
GDACDHDDDNDGIPDDKpc3_NP.2
p−min=0.39, pX=0.22, N=5
var.4.THBS1 15:39880822:A:G
peptide level
0
100000
200000
300000
78:196
44:110
9:19
GDACDHDDDNDGIPDDKpc3_NP.4
p−maj=0.63, pX=0.54, N=131
var.4.THBS1 15:39880822:A:G
peptide level
0e+00
1e+05
2e+05
3e+05
89:196
54:110
9:19
GDACDHDDDNDGIPDDKpc3_NP.5
p−min=0.57, pX=0.76, N=152

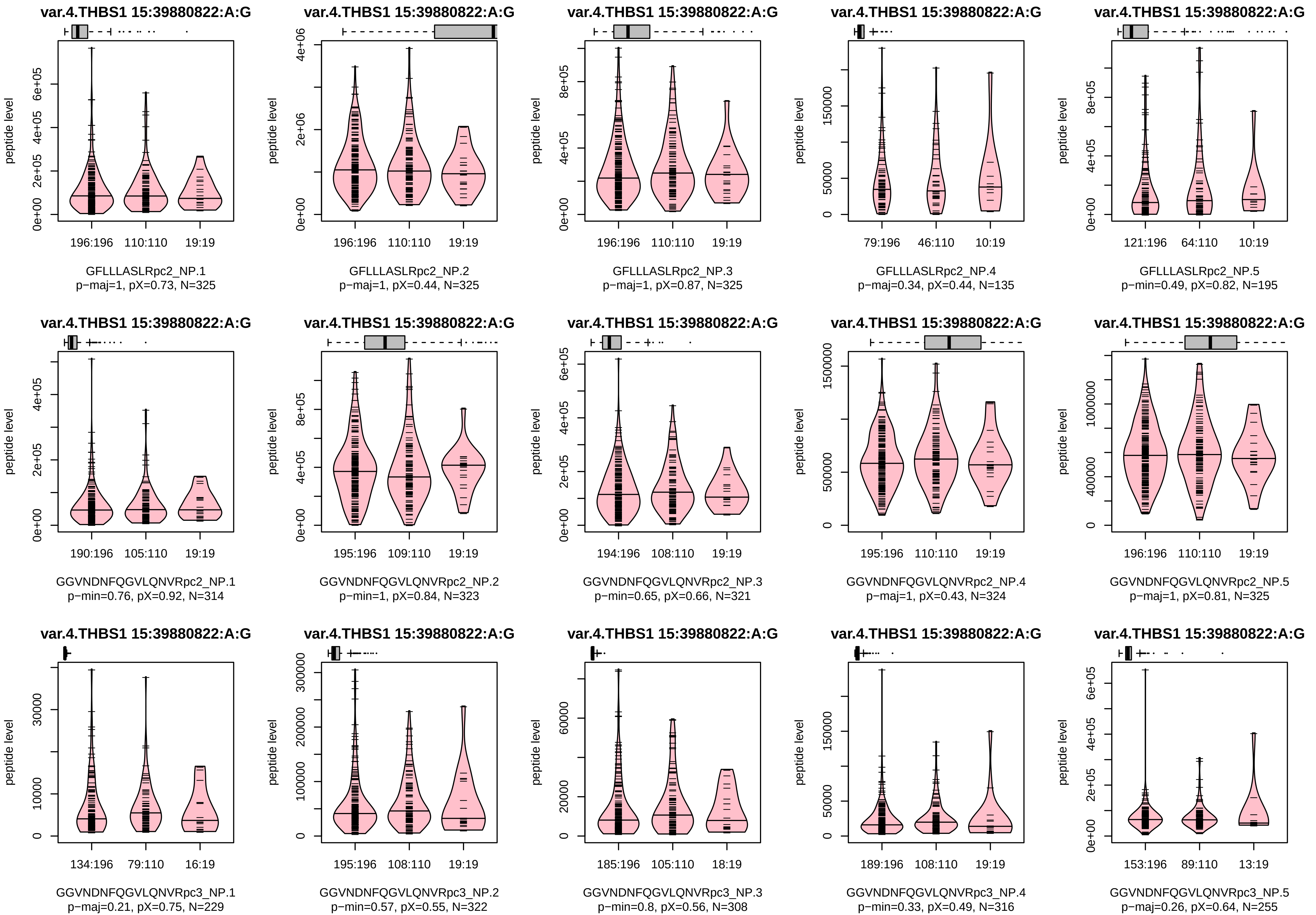
var.4.THBS1 15:39880822:A:G
peptide level
196:196 110:110 19:19
GFLLLASLRpc2_NP.1
p−maj=1, pX=0.73, N=325
var.4.THBS1 15:39880822:A:G
peptide level
196:196 110:110 19:19
GFLLLASLRpc2_NP.2
p−maj=1, pX=0.44, N=325
var.4.THBS1 15:39880822:A:G
peptide level
196:196 110:110 19:19
GFLLLASLRpc2_NP.3
p−maj=1, pX=0.87, N=325
var.4.THBS1 15:39880822:A:G
peptide level
79:196 46:110 10:19
GFLLLASLRpc2_NP.4
p−maj=0.34, pX=0.44, N=135
var.4.THBS1 15:39880822:A:G
peptide level
121:196 64:110 10:19
GFLLLASLRpc2_NP.5
p−min=0.49, pX=0.82, N=195
var.4.THBS1 15:39880822:A:G
peptide level
190:196 105:110 19:19
GGVNDNFQGVLQNVRpc2_NP.1
p−min=0.76, pX=0.92, N=314
var.4.THBS1 15:39880822:A:G
peptide level
195:196 109:110 19:19
GGVNDNFQGVLQNVRpc2_NP.2
p−min=1, pX=0.84, N=323
var.4.THBS1 15:39880822:A:G
peptide level
194:196 108:110 19:19
GGVNDNFQGVLQNVRpc2_NP.3
p−min=0.65, pX=0.66, N=321
var.4.THBS1 15:39880822:A:G
peptide level
195:196 110:110 19:19
GGVNDNFQGVLQNVRpc2_NP.4
p−maj=1, pX=0.43, N=324
var.4.THBS1 15:39880822:A:G
peptide level
196:196 110:110 19:19
GGVNDNFQGVLQNVRpc2_NP.5
p−maj=1, pX=0.81, N=325
var.4.THBS1 15:39880822:A:G
peptide level
134:196 79:110 16:19
GGVNDNFQGVLQNVRpc3_NP.1
p−maj=0.21, pX=0.75, N=229
var.4.THBS1 15:39880822:A:G
peptide level
195:196 108:110 19:19
GGVNDNFQGVLQNVRpc3_NP.2
p−min=0.57, pX=0.55, N=322
var.4.THBS1 15:39880822:A:G
peptide level
185:196 105:110 18:19
GGVNDNFQGVLQNVRpc3_NP.3
p−min=0.8, pX=0.56, N=308
var.4.THBS1 15:39880822:A:G
peptide level
189:196 108:110 19:19
GGVNDNFQGVLQNVRpc3_NP.4
p−min=0.33, pX=0.49, N=316
var.4.THBS1 15:39880822:A:G
peptide level
153:196 89:110 13:19
GGVNDNFQGVLQNVRpc3_NP.5
p−maj=0.26, pX=0.64, N=255

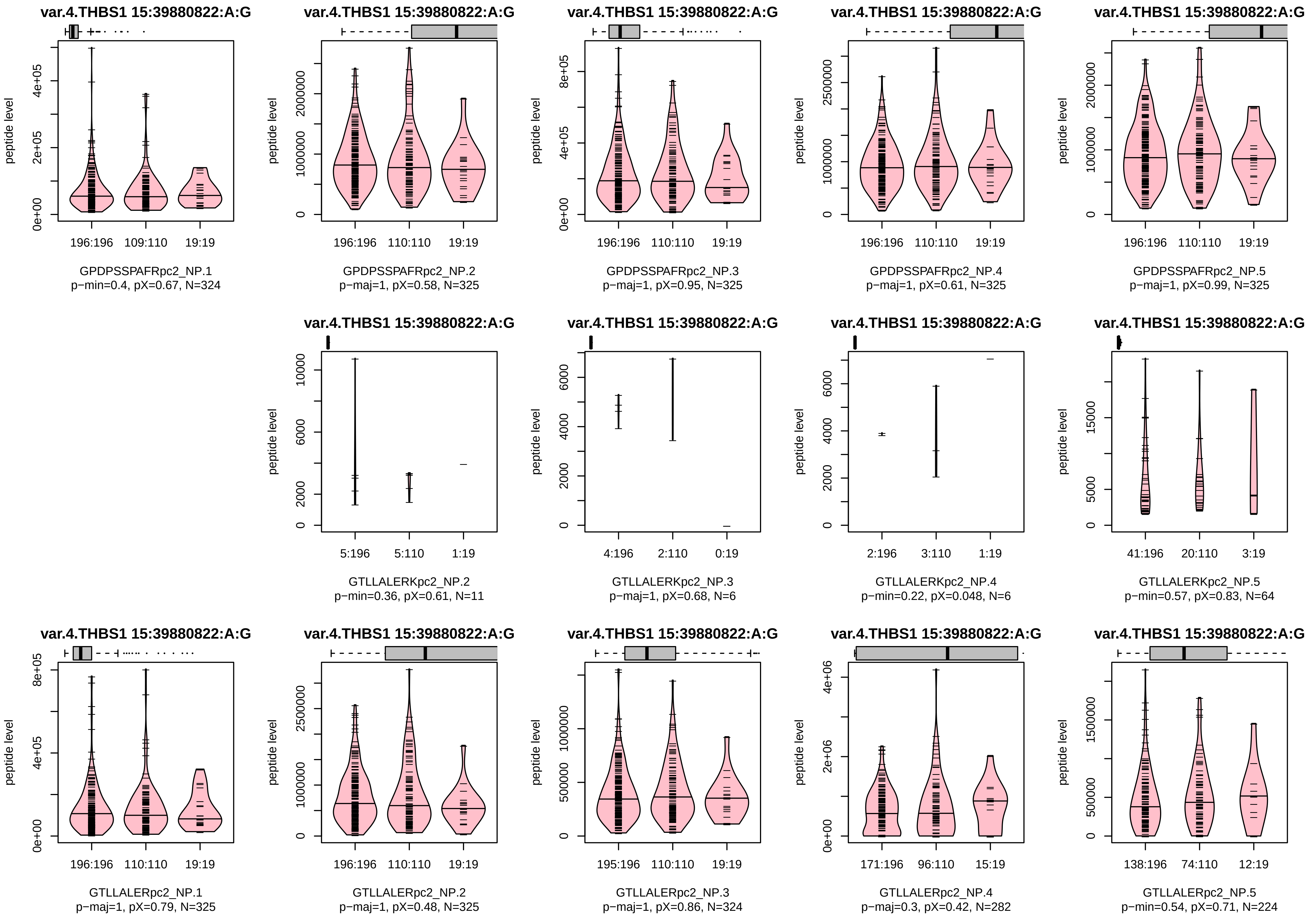
var.4.THBS1 15:39880822:A:G
peptide level
196:196 109:110 19:19
GPDPSSPAFRpc2_NP.1
p−min=0.4, pX=0.67, N=324
var.4.THBS1 15:39880822:A:G
peptide level
196:196 110:110 19:19
GPDPSSPAFRpc2_NP.2
p−maj=1, pX=0.58, N=325
var.4.THBS1 15:39880822:A:G
peptide level
196:196 110:110 19:19
GPDPSSPAFRpc2_NP.3
p−maj=1, pX=0.95, N=325
var.4.THBS1 15:39880822:A:G
peptide level
196:196 110:110 19:19
GPDPSSPAFRpc2_NP.4
p−maj=1, pX=0.61, N=325
var.4.THBS1 15:39880822:A:G
peptide level
196:196 110:110 19:19
GPDPSSPAFRpc2_NP.5
p−maj=1, pX=0.99, N=325
var.4.THBS1 15:39880822:A:G
peptide level
5:196 5:110 1:19
GTLLALERKpc2_NP.2
p−min=0.36, pX=0.61, N=11
var.4.THBS1 15:39880822:A:G
peptide level
4:196 2:110 0:19
GTLLALERKpc2_NP.3
p−maj=1, pX=0.68, N=6
var.4.THBS1 15:39880822:A:G
peptide level
2:196 3:110 1:19
GTLLALERKpc2_NP.4
p−min=0.22, pX=0.048, N=6
var.4.THBS1 15:39880822:A:G
peptide level
41:196 20:110 3:19
GTLLALERKpc2_NP.5
p−min=0.57, pX=0.83, N=64
var.4.THBS1 15:39880822:A:G
peptide level
196:196 110:110 19:19
GTLLALERpc2_NP.1
p−maj=1, pX=0.79, N=325
var.4.THBS1 15:39880822:A:G
peptide level
196:196 110:110 19:19
GTLLALERpc2_NP.2
p−maj=1, pX=0.48, N=325
var.4.THBS1 15:39880822:A:G
peptide level
195:196 110:110 19:19
GTLLALERpc2_NP.3
p−maj=1, pX=0.86, N=324
var.4.THBS1 15:39880822:A:G
peptide level
171:196 96:110 15:19
GTLLALERpc2_NP.4
p−maj=0.3, pX=0.42, N=282
var.4.THBS1 15:39880822:A:G
peptide level
138:196 74:110 12:19
GTLLALERpc2_NP.5
p−min=0.54, pX=0.71, N=224

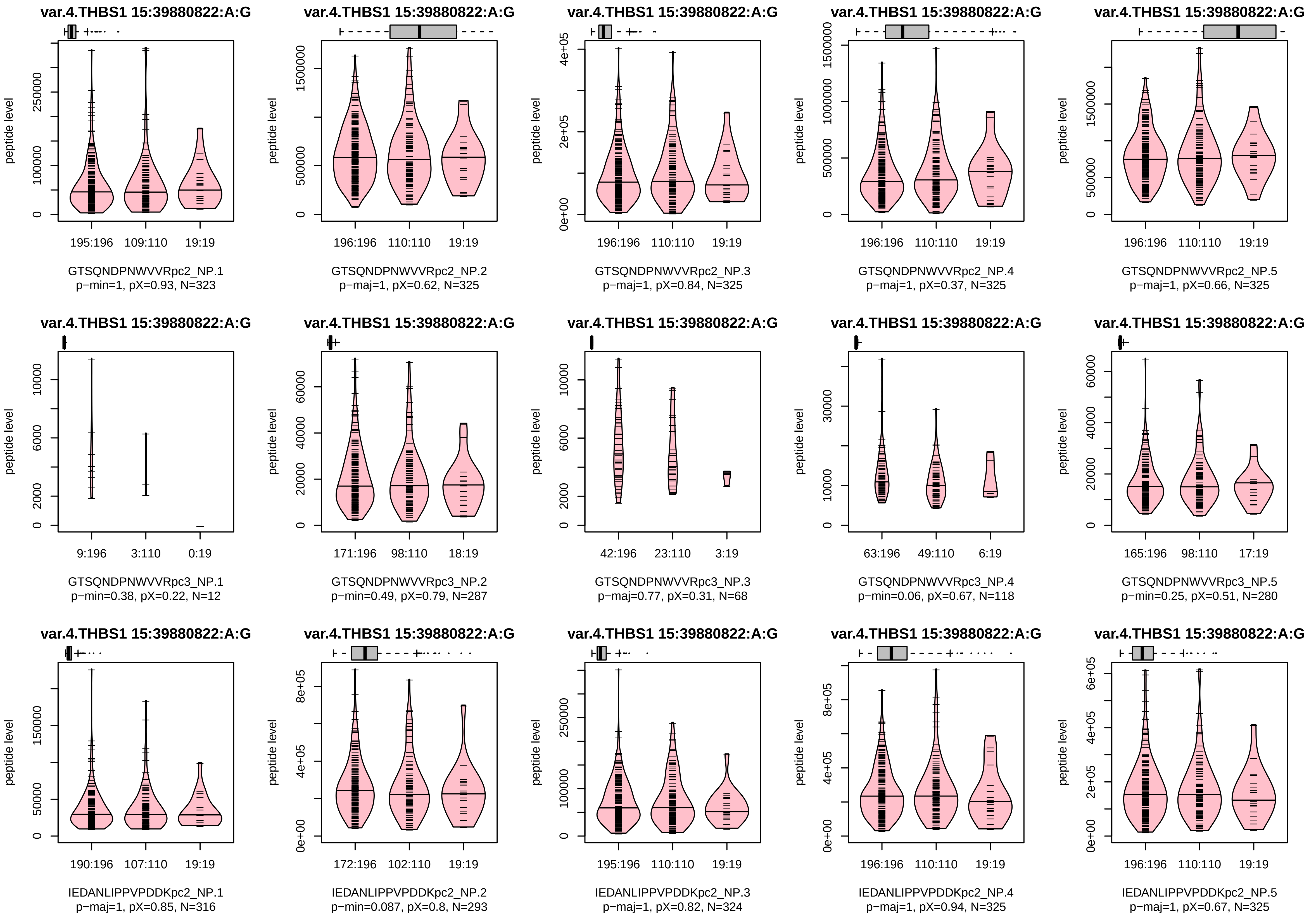
var.4.THBS1 15:39880822:A:G
peptide level
195:196
109:110
19:19
GTSQNDPNWVVRpc2_NP.1
p−min=1, pX=0.93, N=323
var.4.THBS1 15:39880822:A:G
peptide level
196:196
110:110
19:19
GTSQNDPNWVVRpc2_NP.2
p−maj=1, pX=0.62, N=325
var.4.THBS1 15:39880822:A:G
peptide level
196:196
110:110
19:19
GTSQNDPNWVVRpc2_NP.3
p−maj=1, pX=0.84, N=325
var.4.THBS1 15:39880822:A:G
peptide level
196:196
110:110
19:19
GTSQNDPNWVVRpc2_NP.4
p−maj=1, pX=0.37, N=325
var.4.THBS1 15:39880822:A:G
peptide level
196:196
110:110
19:19
GTSQNDPNWVVRpc2_NP.5
p−maj=1, pX=0.66, N=325
var.4.THBS1 15:39880822:A:G
peptide level
9:196
3:110
0:19
GTSQNDPNWVVRpc3_NP.1
p−min=0.38, pX=0.22, N=12
var.4.THBS1 15:39880822:A:G
peptide level
171:196
98:110
18:19
GTSQNDPNWVVRpc3_NP.2
p−min=0.49, pX=0.79, N=287
var.4.THBS1 15:39880822:A:G
peptide level
42:196
23:110
3:19
GTSQNDPNWVVRpc3_NP.3
p−maj=0.77, pX=0.31, N=68
var.4.THBS1 15:39880822:A:G
peptide level
63:196
49:110
6:19
GTSQNDPNWVVRpc3_NP.4
p−min=0.06, pX=0.67, N=118
var.4.THBS1 15:39880822:A:G
peptide level
165:196
98:110
17:19
GTSQNDPNWVVRpc3_NP.5
p−min=0.25, pX=0.51, N=280
var.4.THBS1 15:39880822:A:G
peptide level
190:196
107:110
19:19
IEDANLIPPVPDDKpc2_NP.1
p−maj=1, pX=0.85, N=316
var.4.THBS1 15:39880822:A:G
peptide level
172:196
102:110
19:19
IEDANLIPPVPDDKpc2_NP.2
p−min=0.087, pX=0.8, N=293
var.4.THBS1 15:39880822:A:G
peptide level
195:196
110:110
19:19
IEDANLIPPVPDDKpc2_NP.3
p−maj=1, pX=0.82, N=324
var.4.THBS1 15:39880822:A:G
peptide level
196:196
110:110
19:19
IEDANLIPPVPDDKpc2_NP.4
p−maj=1, pX=0.94, N=325
var.4.THBS1 15:39880822:A:G
peptide level
196:196
110:110
19:19
IEDANLIPPVPDDKpc2_NP.5
p−maj=1, pX=0.67, N=325

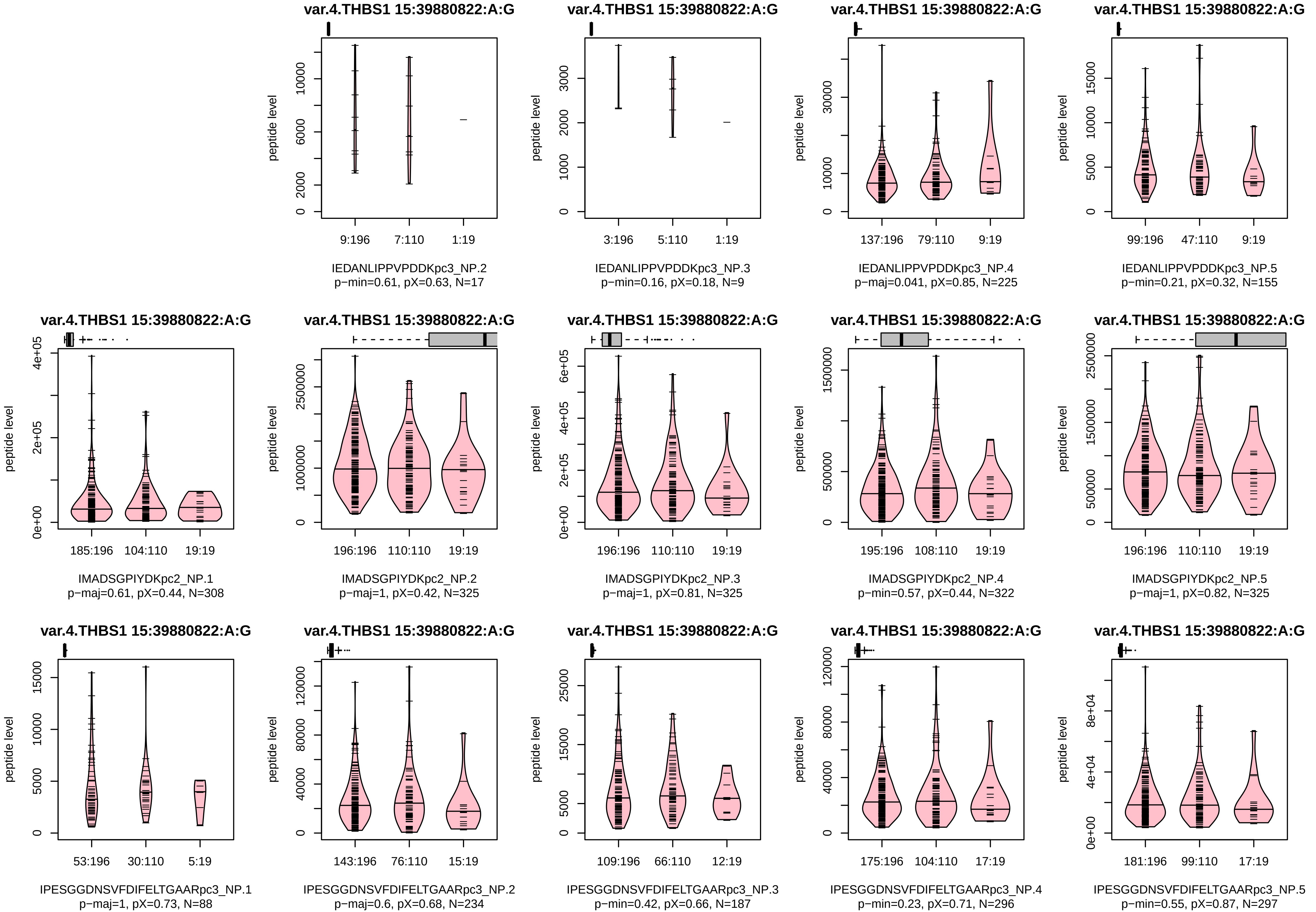
var.4.THBS1 15:39880822:A:G
peptide level
9:196
7:110
1:19
IEDANLIPPVPDDKpc3_NP.2
p−min=0.61, pX=0.63, N=17
var.4.THBS1 15:39880822:A:G
peptide level
3:196
5:110
1:19
IEDANLIPPVPDDKpc3_NP.3
p−min=0.16, pX=0.18, N=9
var.4.THBS1 15:39880822:A:G
peptide level
137:196
79:110
9:19
IEDANLIPPVPDDKpc3_NP.4
p−maj=0.041, pX=0.85, N=225
var.4.THBS1 15:39880822:A:G
peptide level
99:196
47:110
9:19
IEDANLIPPVPDDKpc3_NP.5
p−min=0.21, pX=0.32, N=155
var.4.THBS1 15:39880822:A:G
peptide level
185:196
104:110
19:19
IMADSGPIYDKpc2_NP.1
p−maj=0.61, pX=0.44, N=308
var.4.THBS1 15:39880822:A:G
peptide level
196:196
110:110
19:19
IMADSGPIYDKpc2_NP.2
p−maj=1, pX=0.42, N=325
var.4.THBS1 15:39880822:A:G
peptide level
196:196
110:110
19:19
IMADSGPIYDKpc2_NP.3
p−maj=1, pX=0.81, N=325
var.4.THBS1 15:39880822:A:G
peptide level
195:196
108:110
19:19
IMADSGPIYDKpc2_NP.4
p−min=0.57, pX=0.44, N=322
var.4.THBS1 15:39880822:A:G
peptide level
196:196
110:110
19:19
IMADSGPIYDKpc2_NP.5
p−maj=1, pX=0.82, N=325
var.4.THBS1 15:39880822:A:G
peptide level
53:196
30:110
5:19
IPESGGDNSVFDIFELTGAARpc3_NP.1
p−maj=1, pX=0.73, N=88
var.4.THBS1 15:39880822:A:G
peptide level
143:196
76:110
15:19
IPESGGDNSVFDIFELTGAARpc3_NP.2
p−maj=0.6, pX=0.68, N=234
var.4.THBS1 15:39880822:A:G
peptide level
109:196
66:110
12:19
IPESGGDNSVFDIFELTGAARpc3_NP.3
p−min=0.42, pX=0.66, N=187
var.4.THBS1 15:39880822:A:G
peptide level
175:196
104:110
17:19
IPESGGDNSVFDIFELTGAARpc3_NP.4
p−min=0.23, pX=0.71, N=296
var.4.THBS1 15:39880822:A:G
peptide level
181:196
99:110
17:19
IPESGGDNSVFDIFELTGAARpc3_NP.5
p−min=0.55, pX=0.87, N=297

var.4.THBS1 15:39880822:A:G

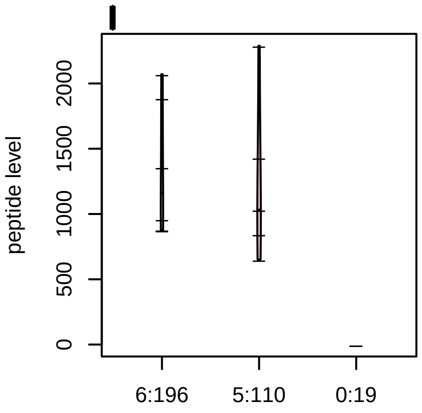

KDNCPNLPNSGQEDYDKpc2_NP.4
p−min=0.76, pX=0.93, N=11

var.4.THBS1 15:39880822:A:G

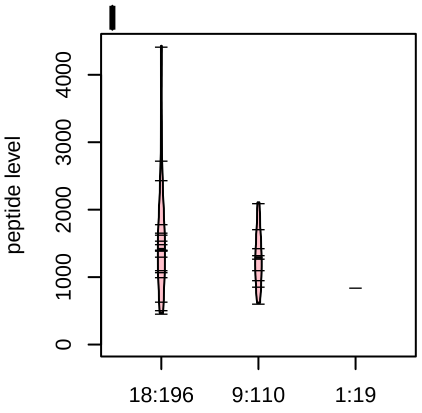

KDNCPNLPNSGQEDYDKpc2_NP.5
p−min=0.69, pX=0.3, N=28

var.4.THBS1 15:39880822:A:G

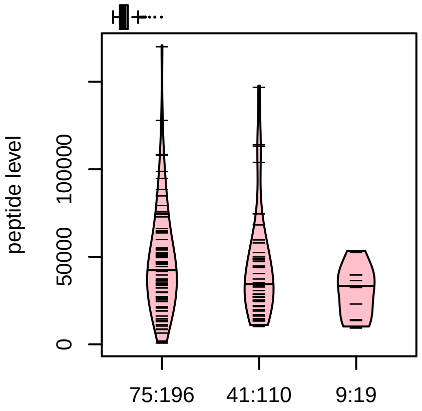

KDNCPNLPNSGQEDYDKpc3_NP.4
p−maj=0.47, pX=0.45, N=125

var.4.THBS1 15:39880822:A:G

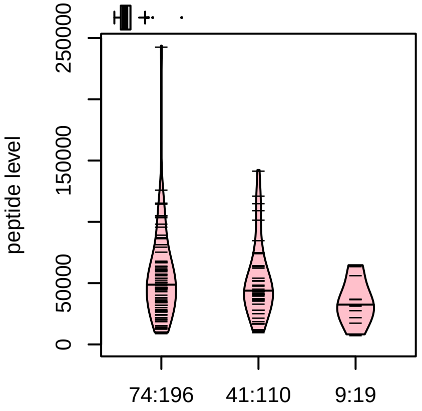

KDNCPNLPNSGQEDYDKpc3_NP.5
p−maj=0.47, pX=0.54, N=124

var.4.THBS1 15:39880822:A:G

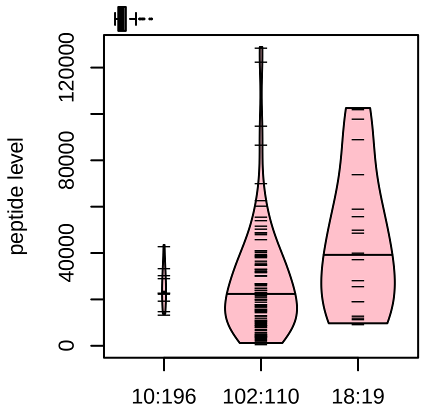

LCNNPAPQFGGKpc2_NP.1
p−min=8.5e−65, pX=9.8e−39, N=130

var.4.THBS1 15:39880822:A:G

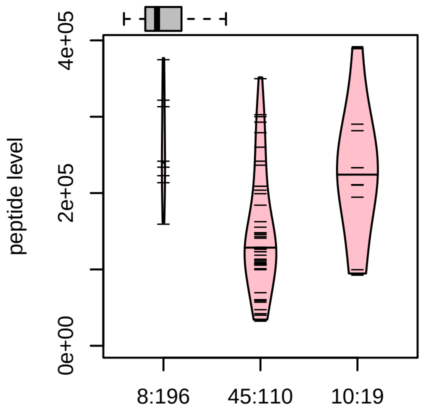

LCNNPAPQFGGKpc2_NP.2
p−min=4.1e−18, pX=1.5e−13, N=63

var.4.THBS1 15:39880822:A:G

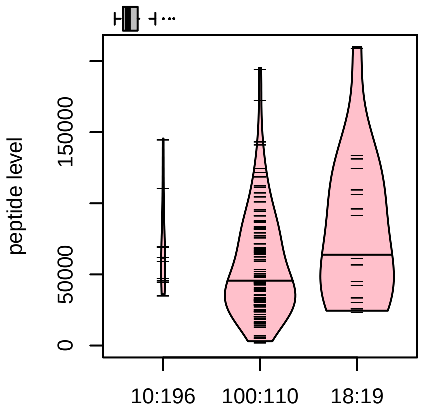

LCNNPAPQFGGKpc2_NP.3
p−min=2.5e−62, pX=2e−41, N=128

var.4.THBS1 15:39880822:A:G

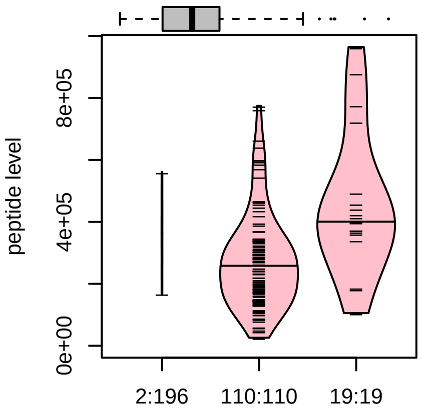

LCNNPAPQFGGKpc2_NP.4
p−min=2.9e−90, pX=1.1e−75, N=131

var.4.THBS1 15:39880822:A:G

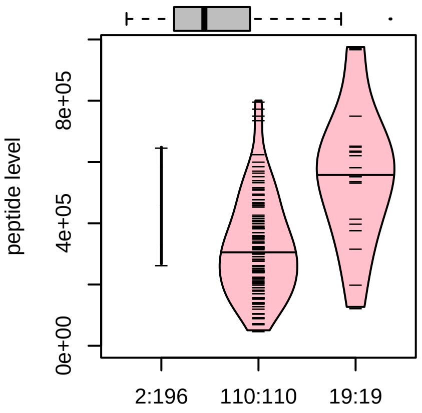

LCNNPAPQFGGKpc2_NP.5
p−min=2.9e−90, pX=1.9e−88, N=131

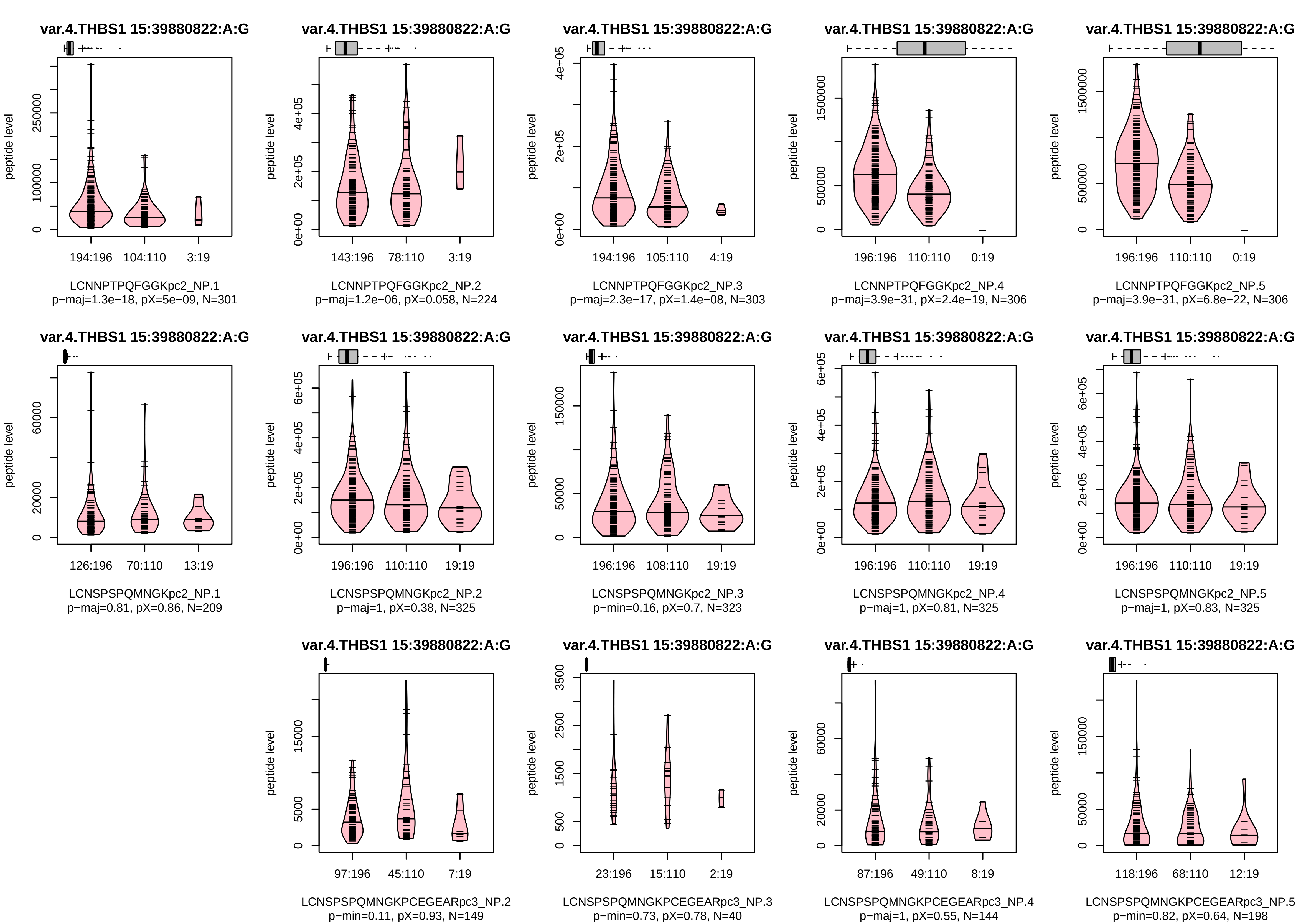
var.4.THBS1 15:39880822:A:G
peptide level
194:196 104:110 3:19
LCNNPTPQFGGKpc2_NP.1
p−maj=1.3e−18, pX=5e−09, N=301
var.4.THBS1 15:39880822:A:G
143:196 78:110 3:19
LCNNPTPQFGGKpc2_NP.2
p−maj=1.2e−06, pX=0.058, N=224
var.4.THBS1 15:39880822:A:G
194:196 105:110 4:19
LCNNPTPQFGGKpc2_NP.3
p−maj=2.3e−17, pX=1.4e−08, N=303
var.4.THBS1 15:39880822:A:G
196:196 110:110 0:19
LCNNPTPQFGGKpc2_NP.4
p−maj=3.9e−31, pX=2.4e−19, N=306
var.4.THBS1 15:39880822:A:G
196:196 110:110 0:19
LCNNPTPQFGGKpc2_NP.5
p−maj=3.9e−31, pX=6.8e−22, N=306
var.4.THBS1 15:39880822:A:G
126:196 70:110 13:19
LCNSPSPQMNGKpc2_NP.1
p−maj=0.81, pX=0.86, N=209
var.4.THBS1 15:39880822:A:G
196:196 110:110 19:19
LCNSPSPQMNGKpc2_NP.2
p−maj=1, pX=0.38, N=325
var.4.THBS1 15:39880822:A:G
196:196 108:110 19:19
LCNSPSPQMNGKpc2_NP.3
p−min=0.16, pX=0.7, N=323
var.4.THBS1 15:39880822:A:G
196:196 110:110 19:19
LCNSPSPQMNGKpc2_NP.4
p−maj=1, pX=0.81, N=325
var.4.THBS1 15:39880822:A:G
196:196 110:110 19:19
LCNSPSPQMNGKpc2_NP.5
p−maj=1, pX=0.83, N=325
var.4.THBS1 15:39880822:A:G
97:196 45:110 7:19
LCNSPSPQMNGKPCEGEARpc3_NP.2
p−min=0.11, pX=0.93, N=149
var.4.THBS1 15:39880822:A:G
23:196 15:110 2:19
LCNSPSPQMNGKPCEGEARpc3_NP.3
p−min=0.73, pX=0.78, N=40
var.4.THBS1 15:39880822:A:G
87:196 49:110 8:19
LCNSPSPQMNGKPCEGEARpc3_NP.4
p−maj=1, pX=0.55, N=144
var.4.THBS1 15:39880822:A:G
118:196 68:110 12:19
LCNSPSPQMNGKPCEGEARpc3_NP.5
p−min=0.82, pX=0.64, N=198

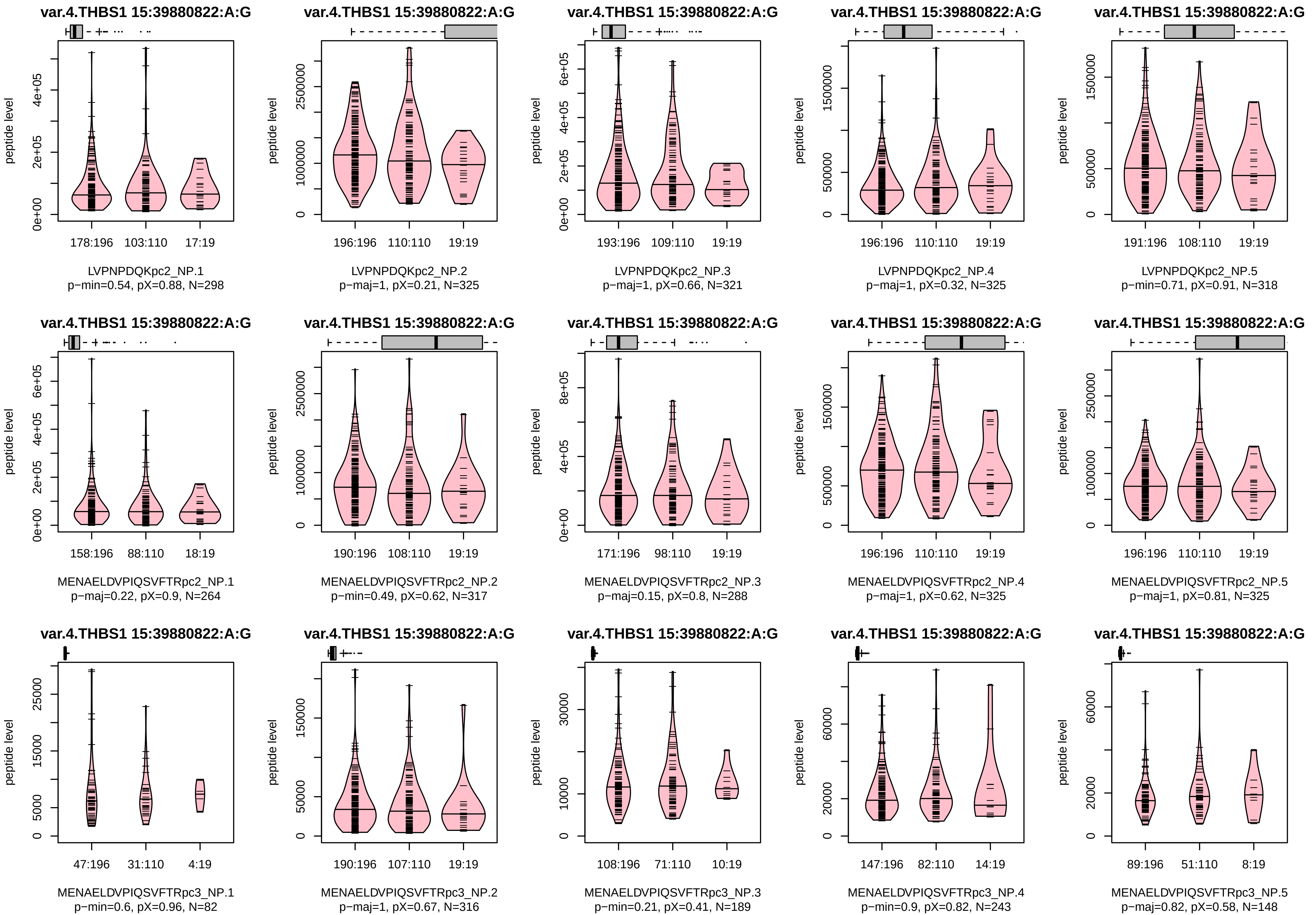
var.4.THBS1 15:39880822:A:G
peptide level
178:196 103:110 17:19
LVPNPDQKpc2_NP.1
p−min=0.54, pX=0.88, N=298
var.4.THBS1 15:39880822:A:G
peptide level
196:196 110:110 19:19
LVPNPDQKpc2_NP.2
p−maj=1, pX=0.21, N=325
var.4.THBS1 15:39880822:A:G
peptide level
193:196 109:110 19:19
LVPNPDQKpc2_NP.3
p−maj=1, pX=0.66, N=321
var.4.THBS1 15:39880822:A:G
peptide level
196:196 110:110 19:19
LVPNPDQKpc2_NP.4
p−maj=1, pX=0.32, N=325
var.4.THBS1 15:39880822:A:G
peptide level
191:196 108:110 19:19
LVPNPDQKpc2_NP.5
p−min=0.71, pX=0.91, N=318
var.4.THBS1 15:39880822:A:G
peptide level
158:196 88:110 18:19
MENAELDVPIQSVFTRpc2_NP.1
p−maj=0.22, pX=0.9, N=264
var.4.THBS1 15:39880822:A:G
peptide level
190:196 108:110 19:19
MENAELDVPIQSVFTRpc2_NP.2
p−min=0.49, pX=0.62, N=317
var.4.THBS1 15:39880822:A:G
peptide level
171:196 98:110 19:19
MENAELDVPIQSVFTRpc2_NP.3
p−maj=0.15, pX=0.8, N=288
var.4.THBS1 15:39880822:A:G
peptide level
196:196 110:110 19:19
MENAELDVPIQSVFTRpc2_NP.4
p−maj=1, pX=0.62, N=325
var.4.THBS1 15:39880822:A:G
peptide level
196:196 110:110 19:19
MENAELDVPIQSVFTRpc2_NP.5
p−maj=1, pX=0.81, N=325
var.4.THBS1 15:39880822:A:G
peptide level
47:196 31:110 4:19
MENAELDVPIQSVFTRpc3_NP.1
p−min=0.6, pX=0.96, N=82
var.4.THBS1 15:39880822:A:G
peptide level
190:196 107:110 19:19
MENAELDVPIQSVFTRpc3_NP.2
p−maj=1, pX=0.67, N=316
var.4.THBS1 15:39880822:A:G
peptide level
108:196 71:110 10:19
MENAELDVPIQSVFTRpc3_NP.3
p−min=0.21, pX=0.41, N=189
var.4.THBS1 15:39880822:A:G
peptide level
147:196 82:110 14:19
MENAELDVPIQSVFTRpc3_NP.4
p−min=0.9, pX=0.82, N=243
var.4.THBS1 15:39880822:A:G
peptide level
89:196 51:110 8:19
MENAELDVPIQSVFTRpc3_NP.5
p−maj=0.82, pX=0.58, N=148

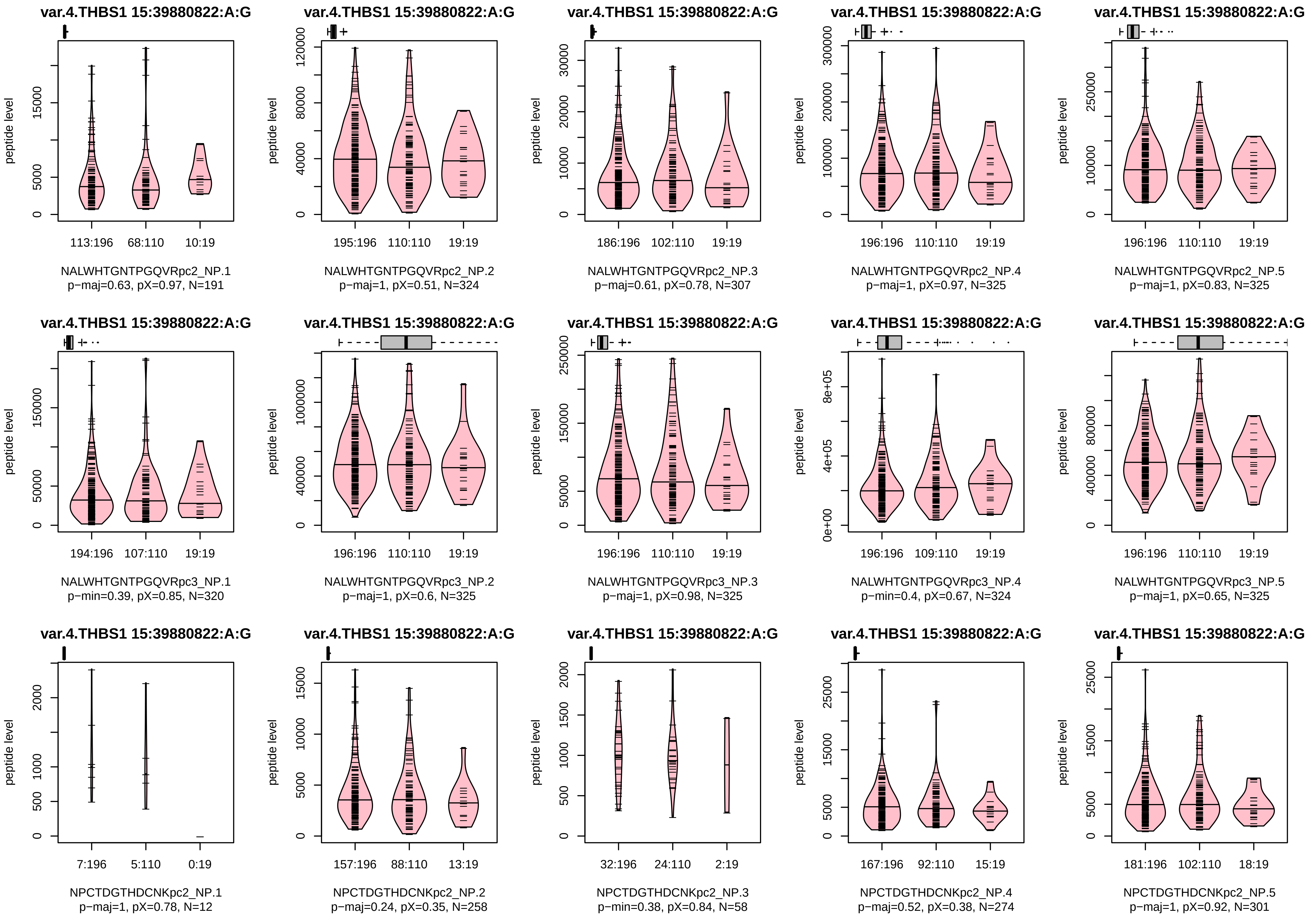
var.4.THBS1 15:39880822:A:G
peptide level
113:196
68:110
10:19
NALWHTGNTPGQVRpc2_NP.1
p−maj=0.63, pX=0.97, N=191
var.4.THBS1 15:39880822:A:G
peptide level
195:196
110:110
19:19
NALWHTGNTPGQVRpc2_NP.2
p−maj=1, pX=0.51, N=324
var.4.THBS1 15:39880822:A:G
peptide level
186:196
102:110
19:19
NALWHTGNTPGQVRpc2_NP.3
p−maj=0.61, pX=0.78, N=307
var.4.THBS1 15:39880822:A:G
peptide level
196:196
110:110
19:19
NALWHTGNTPGQVRpc2_NP.4
p−maj=1, pX=0.97, N=325
var.4.THBS1 15:39880822:A:G
peptide level
196:196
110:110
19:19
NALWHTGNTPGQVRpc2_NP.5
p−maj=1, pX=0.83, N=325
var.4.THBS1 15:39880822:A:G
peptide level
194:196
107:110
19:19
NALWHTGNTPGQVRpc3_NP.1
p−min=0.39, pX=0.85, N=320
var.4.THBS1 15:39880822:A:G
peptide level
196:196
110:110
19:19
NALWHTGNTPGQVRpc3_NP.2
p−maj=1, pX=0.6, N=325
var.4.THBS1 15:39880822:A:G
peptide level
196:196
110:110
19:19
NALWHTGNTPGQVRpc3_NP.3
p−maj=1, pX=0.98, N=325
var.4.THBS1 15:39880822:A:G
peptide level
196:196
109:110
19:19
NALWHTGNTPGQVRpc3_NP.4
p−min=0.4, pX=0.67, N=324
var.4.THBS1 15:39880822:A:G
peptide level
196:196
110:110
19:19
NALWHTGNTPGQVRpc3_NP.5
p−maj=1, pX=0.65, N=325
var.4.THBS1 15:39880822:A:G
peptide level
7:196
5:110
0:19
NPCTDGTHDCNKpc2_NP.1
p−maj=1, pX=0.78, N=12
var.4.THBS1 15:39880822:A:G
peptide level
157:196
88:110
13:19
NPCTDGTHDCNKpc2_NP.2
p−maj=0.24, pX=0.35, N=258
var.4.THBS1 15:39880822:A:G
peptide level
32:196
24:110
2:19
NPCTDGTHDCNKpc2_NP.3
p−min=0.38, pX=0.84, N=58
var.4.THBS1 15:39880822:A:G
peptide level
167:196
92:110
15:19
NPCTDGTHDCNKpc2_NP.4
p−maj=0.52, pX=0.38, N=274
var.4.THBS1 15:39880822:A:G
peptide level
181:196
102:110
18:19
NPCTDGTHDCNKpc2_NP.5
p−maj=1, pX=0.92, N=301

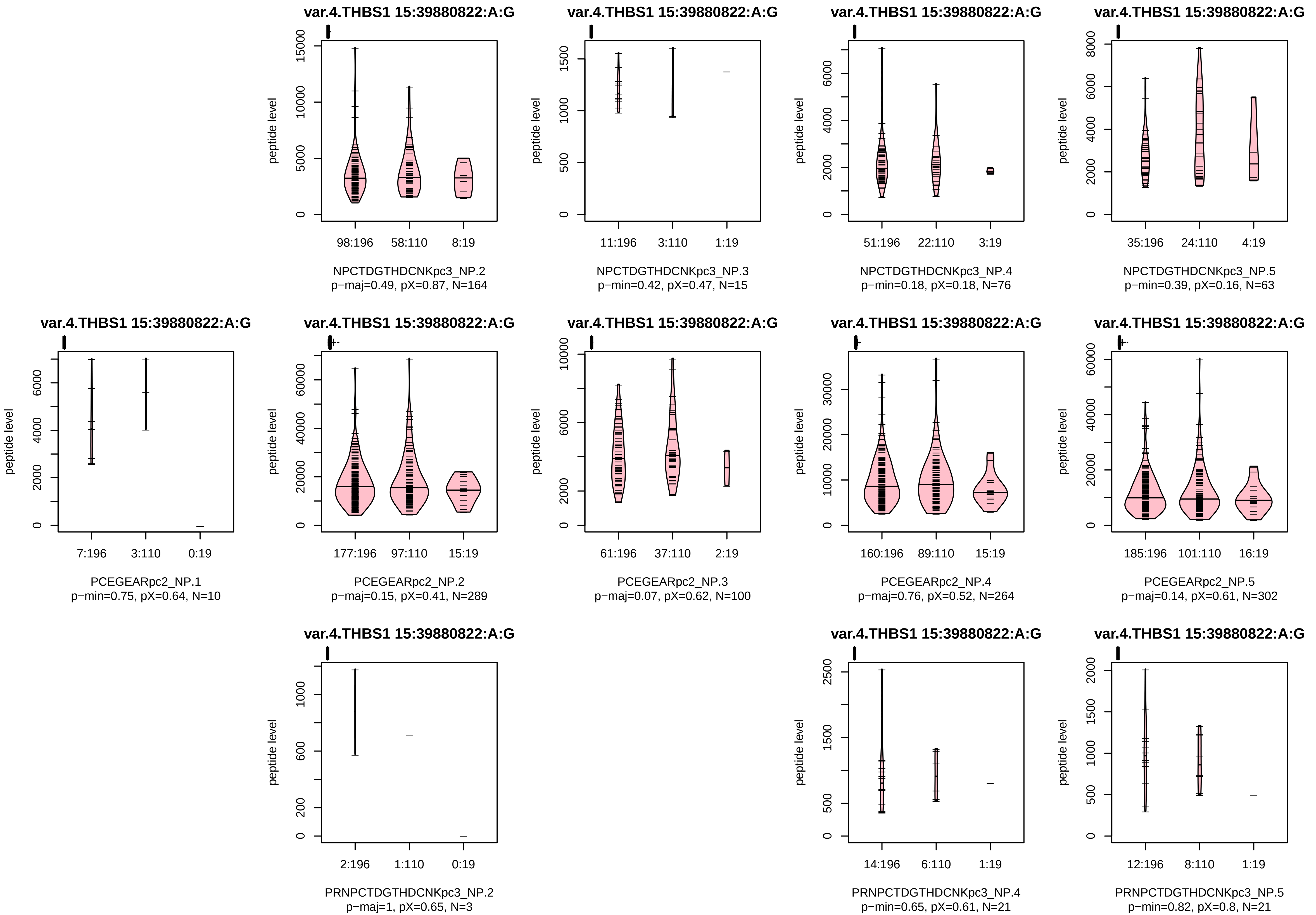
var.4.THBS1 15:39880822:A:G
peptide level
98:196
58:110
8:19
NPCTDGTHDCNKpc3_NP.2
p−maj=0.49, pX=0.87, N=164
var.4.THBS1 15:39880822:A:G
peptide level
11:196
3:110
1:19
NPCTDGTHDCNKpc3_NP.3
p−min=0.42, pX=0.47, N=15
var.4.THBS1 15:39880822:A:G
peptide level
51:196
22:110
3:19
NPCTDGTHDCNKpc3_NP.4
p−min=0.18, pX=0.18, N=76
var.4.THBS1 15:39880822:A:G
peptide level
35:196
24:110
4:19
NPCTDGTHDCNKpc3_NP.5
p−min=0.39, pX=0.16, N=63
var.4.THBS1 15:39880822:A:G
peptide level
7:196
3:110
0:19
PCEGEARpc2_NP.1
p−min=0.75, pX=0.64, N=10
var.4.THBS1 15:39880822:A:G
peptide level
177:196
97:110
15:19
PCEGEARpc2_NP.2
p−maj=0.15, pX=0.41, N=289
var.4.THBS1 15:39880822:A:G
peptide level
61:196
37:110
2:19
PCEGEARpc2_NP.3
p−maj=0.07, pX=0.62, N=100
var.4.THBS1 15:39880822:A:G
peptide level
160:196
89:110
15:19
PCEGEARpc2_NP.4
p−maj=0.76, pX=0.52, N=264
var.4.THBS1 15:39880822:A:G
peptide level
185:196
101:110
16:19
PCEGEARpc2_NP.5
p−maj=0.14, pX=0.61, N=302
var.4.THBS1 15:39880822:A:G
peptide level
2:196
1:110
0:19
PRNPCTDGTHDCNKpc3_NP.2
p−maj=1, pX=0.65, N=3
var.4.THBS1 15:39880822:A:G
peptide level
14:196
6:110
1:19
PRNPCTDGTHDCNKpc3_NP.4
p−min=0.65, pX=0.61, N=21
var.4.THBS1 15:39880822:A:G
peptide level
12:196
8:110
1:19
PRNPCTDGTHDCNKpc3_NP.5
p−min=0.82, pX=0.8, N=21

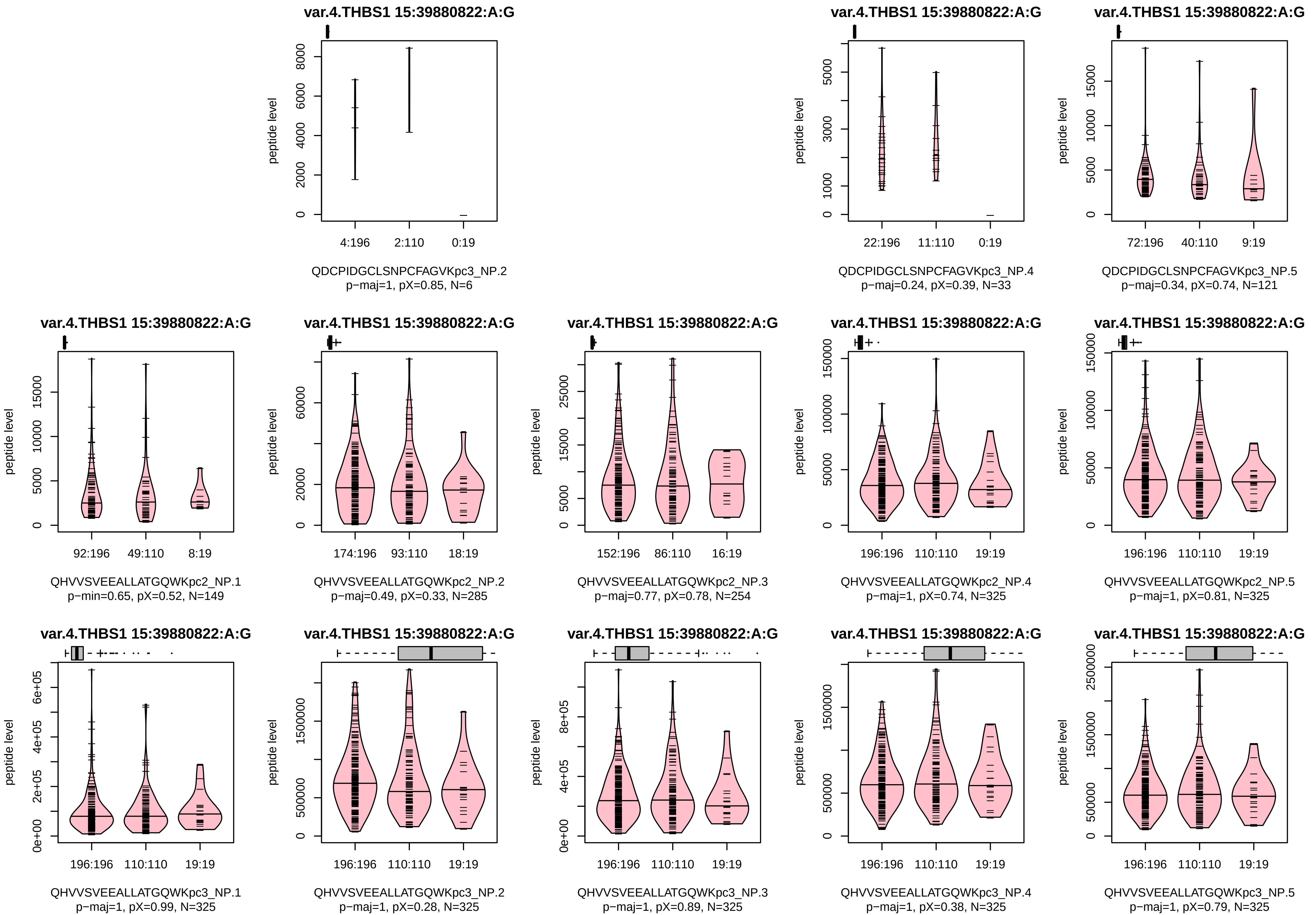
var.4.THBS1 15:39880822:A:G
peptide level
4:196
2:110
0:19
QDCPIDGCLSNPCFAGVKpc3_NP.2
p−maj=1, pX=0.85, N=6
var.4.THBS1 15:39880822:A:G
peptide level
22:196
11:110
0:19
QDCPIDGCLSNPCFAGVKpc3_NP.4
p−maj=0.24, pX=0.39, N=33
var.4.THBS1 15:39880822:A:G
peptide level
72:196
40:110
9:19
QDCPIDGCLSNPCFAGVKpc3_NP.5
p−maj=0.34, pX=0.74, N=121
var.4.THBS1 15:39880822:A:G
peptide level
92:196
49:110
8:19
QHVVSVEEALLATGQWKpc2_NP.1
p−min=0.65, pX=0.52, N=149
var.4.THBS1 15:39880822:A:G
peptide level
174:196
93:110
18:19
QHVVSVEEALLATGQWKpc2_NP.2
p−maj=0.49, pX=0.33, N=285
var.4.THBS1 15:39880822:A:G
peptide level
152:196
86:110
16:19
QHVVSVEEALLATGQWKpc2_NP.3
p−maj=0.77, pX=0.78, N=254
var.4.THBS1 15:39880822:A:G
peptide level
196:196
110:110
19:19
QHVVSVEEALLATGQWKpc2_NP.4
p−maj=1, pX=0.74, N=325
var.4.THBS1 15:39880822:A:G
peptide level
196:196
110:110
19:19
QHVVSVEEALLATGQWKpc2_NP.5
p−maj=1, pX=0.81, N=325
var.4.THBS1 15:39880822:A:G
peptide level
196:196
110:110
19:19
QHVVSVEEALLATGQWKpc3_NP.1
p−maj=1, pX=0.99, N=325
var.4.THBS1 15:39880822:A:G
peptide level
196:196
110:110
19:19
QHVVSVEEALLATGQWKpc3_NP.2
p−maj=1, pX=0.28, N=325
var.4.THBS1 15:39880822:A:G
peptide level
196:196
110:110
19:19
QHVVSVEEALLATGQWKpc3_NP.3
p−maj=1, pX=0.89, N=325
var.4.THBS1 15:39880822:A:G
peptide level
196:196
110:110
19:19
QHVVSVEEALLATGQWKpc3_NP.4
p−maj=1, pX=0.38, N=325
var.4.THBS1 15:39880822:A:G
peptide level
196:196
110:110
19:19
QHVVSVEEALLATGQWKpc3_NP.5
p−maj=1, pX=0.79, N=325

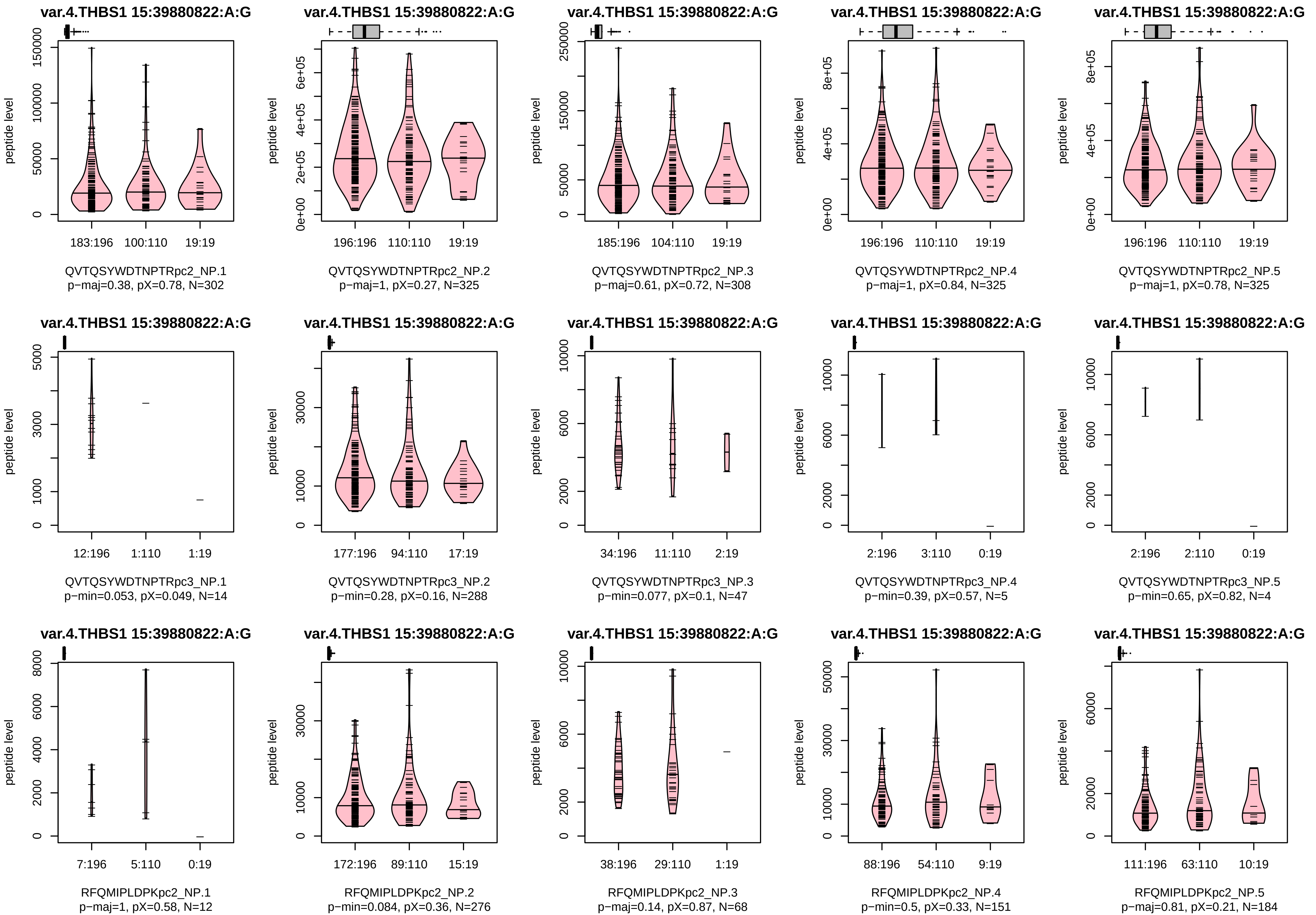
var.4.THBS1 15:39880822:A:G
peptide level
183:196
100:110
19:19
QVTQSYWDTNPTRpc2_NP.1
p−maj=0.38, pX=0.78, N=302
var.4.THBS1 15:39880822:A:G
196:196
110:110
19:19
QVTQSYWDTNPTRpc2_NP.2
p−maj=1, pX=0.27, N=325
var.4.THBS1 15:39880822:A:G
185:196
104:110
19:19
QVTQSYWDTNPTRpc2_NP.3
p−maj=0.61, pX=0.72, N=308
var.4.THBS1 15:39880822:A:G
196:196
110:110
19:19
QVTQSYWDTNPTRpc2_NP.4
p−maj=1, pX=0.84, N=325
var.4.THBS1 15:39880822:A:G
196:196
110:110
19:19
QVTQSYWDTNPTRpc2_NP.5
p−maj=1, pX=0.78, N=325
var.4.THBS1 15:39880822:A:G
12:196
1:110
1:19
QVTQSYWDTNPTRpc3_NP.1
p−min=0.053, pX=0.049, N=14
var.4.THBS1 15:39880822:A:G
177:196
94:110
17:19
QVTQSYWDTNPTRpc3_NP.2
p−min=0.28, pX=0.16, N=288
var.4.THBS1 15:39880822:A:G
34:196
11:110
2:19
QVTQSYWDTNPTRpc3_NP.3
p−min=0.077, pX=0.1, N=47
var.4.THBS1 15:39880822:A:G
2:196
3:110
0:19
QVTQSYWDTNPTRpc3_NP.4
p−min=0.39, pX=0.57, N=5
var.4.THBS1 15:39880822:A:G
2:196
2:110
0:19
QVTQSYWDTNPTRpc3_NP.5
p−min=0.65, pX=0.82, N=4
var.4.THBS1 15:39880822:A:G
7:196
5:110
0:19
RFQMIPLDPKpc2_NP.1
p−maj=1, pX=0.58, N=12
var.4.THBS1 15:39880822:A:G
172:196
89:110
15:19
RFQMIPLDPKpc2_NP.2
p−min=0.084, pX=0.36, N=276
var.4.THBS1 15:39880822:A:G
38:196
29:110
1:19
RFQMIPLDPKpc2_NP.3
p−maj=0.14, pX=0.87, N=68
var.4.THBS1 15:39880822:A:G
88:196
54:110
9:19
RFQMIPLDPKpc2_NP.4
p−min=0.5, pX=0.33, N=151
var.4.THBS1 15:39880822:A:G
111:196
63:110
10:19
RFQMIPLDPKpc2_NP.5
p−maj=0.81, pX=0.21, N=184

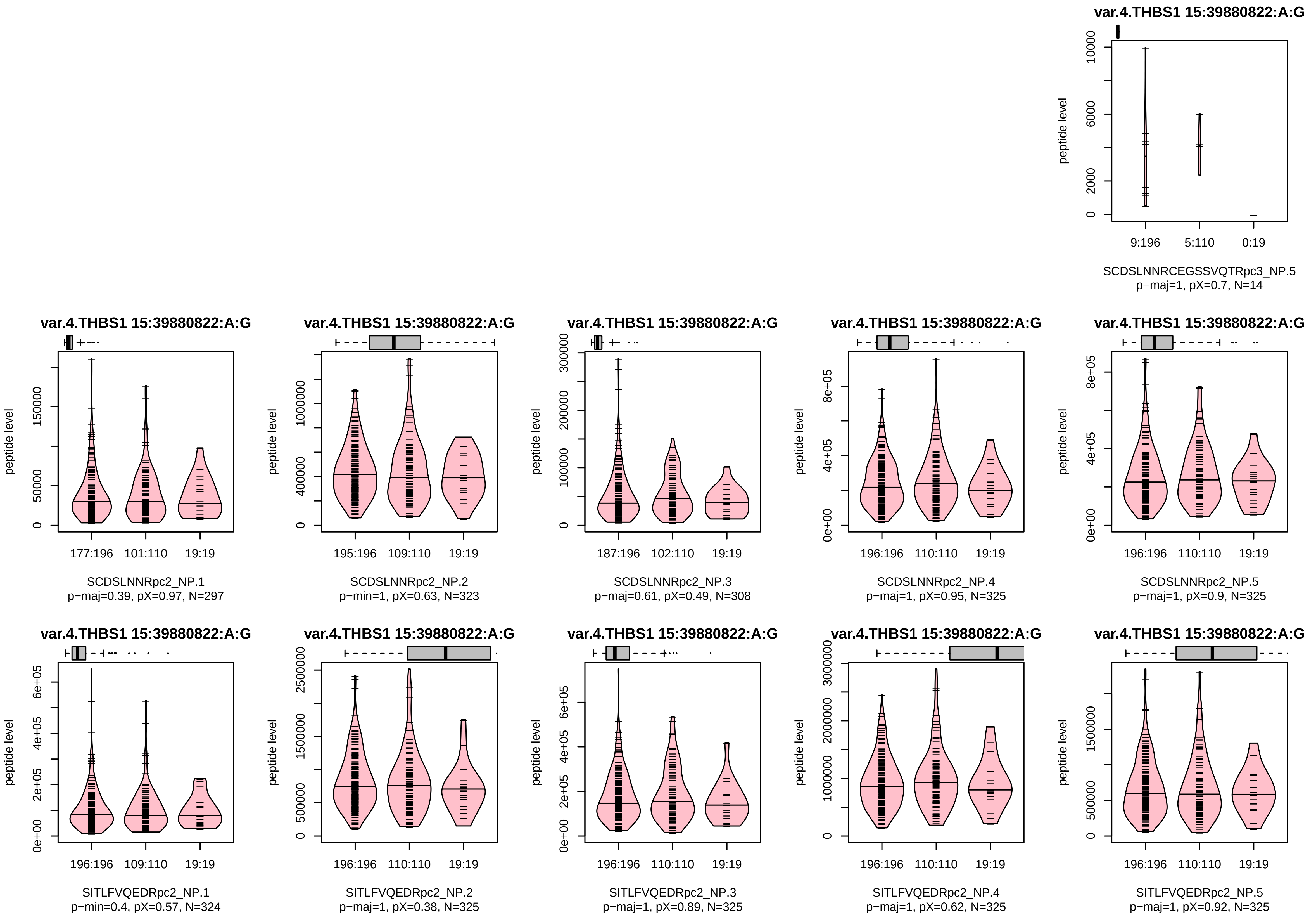
var.4.THBS1 15:39880822:A:G
peptide level
9:196 5:110 0:19
SCDSLNNRCEGSSVQTRpc3_NP.5
p−maj=1, pX=0.7, N=14
var.4.THBS1 15:39880822:A:G
177:196 101:110 19:19
SCDSLNNRpc2_NP.1
p−maj=0.39, pX=0.97, N=297
var.4.THBS1 15:39880822:A:G
195:196 109:110 19:19
SCDSLNNRpc2_NP.2
p−min=1, pX=0.63, N=323
var.4.THBS1 15:39880822:A:G
187:196 102:110 19:19
SCDSLNNRpc2_NP.3
p−maj=0.61, pX=0.49, N=308
var.4.THBS1 15:39880822:A:G
196:196 110:110 19:19
SCDSLNNRpc2_NP.4
p−maj=1, pX=0.95, N=325
var.4.THBS1 15:39880822:A:G
196:196 110:110 19:19
SCDSLNNRpc2_NP.5
p−maj=1, pX=0.9, N=325
var.4.THBS1 15:39880822:A:G
196:196 109:110 19:19
SITLFVQEDRpc2_NP.1
p−min=0.4, pX=0.57, N=324
var.4.THBS1 15:39880822:A:G
196:196 110:110 19:19
SITLFVQEDRpc2_NP.2
p−maj=1, pX=0.38, N=325
var.4.THBS1 15:39880822:A:G
196:196 110:110 19:19
SITLFVQEDRpc2_NP.3
p−maj=1, pX=0.89, N=325
var.4.THBS1 15:39880822:A:G
196:196 110:110 19:19
SITLFVQEDRpc2_NP.4
p−maj=1, pX=0.62, N=325
var.4.THBS1 15:39880822:A:G
196:196 110:110 19:19
SITLFVQEDRpc2_NP.5
p−maj=1, pX=0.92, N=325

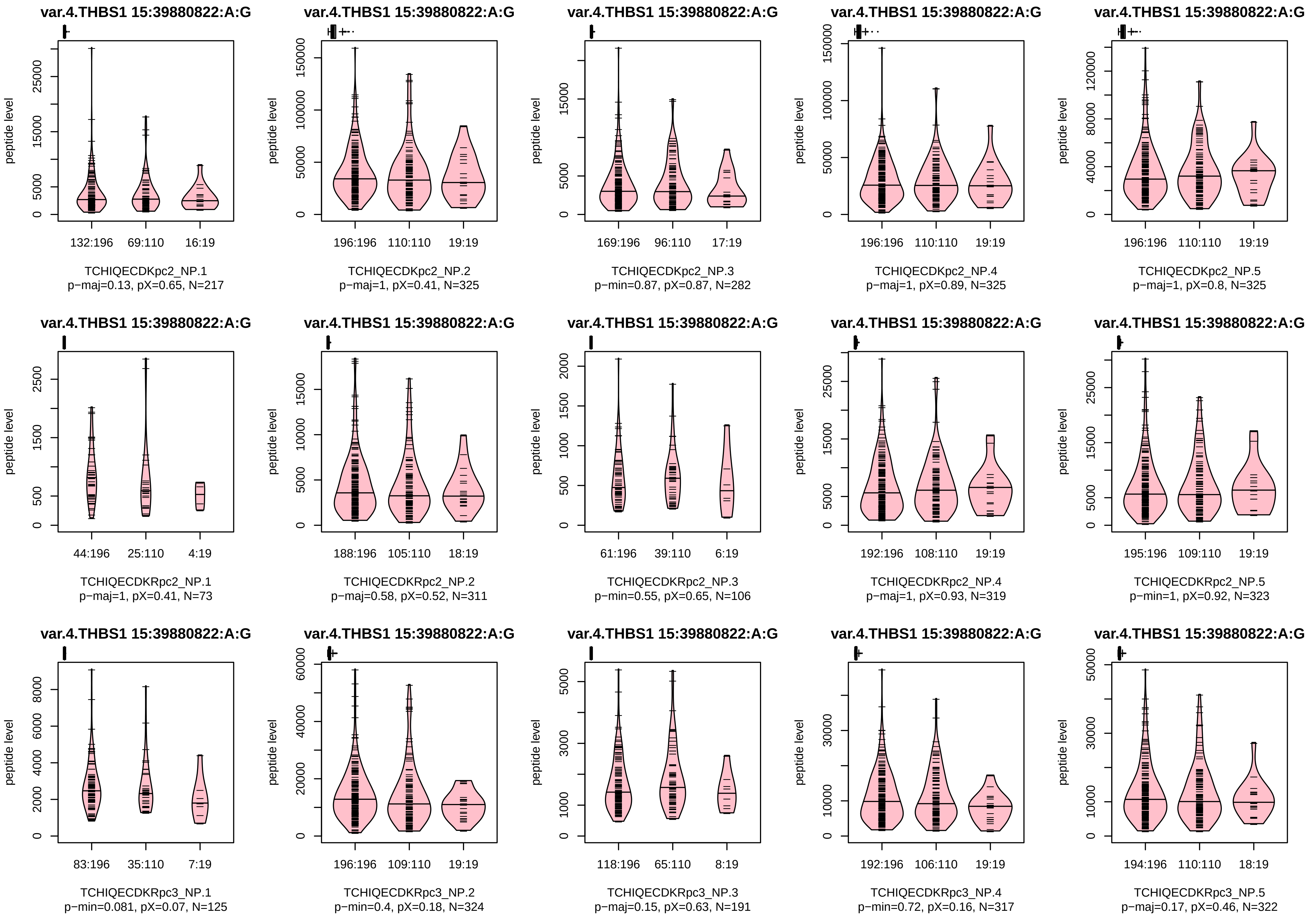

var.4.THBS1 15:39880822:A:G
peptide level
132:196
69:110
16:19
TCHIQECDKpc2_NP.1
p−maj=0.13, pX=0.65, N=217
var.4.THBS1 15:39880822:A:G
peptide level
196:196
110:110
19:19
TCHIQECDKpc2_NP.2
p−maj=1, pX=0.41, N=325
var.4.THBS1 15:39880822:A:G
peptide level
169:196
96:110
17:19
TCHIQECDKpc2_NP.3
p−min=0.87, pX=0.87, N=282
var.4.THBS1 15:39880822:A:G
peptide level
196:196
110:110
19:19
TCHIQECDKpc2_NP.4
p−maj=1, pX=0.89, N=325
var.4.THBS1 15:39880822:A:G
peptide level
196:196
110:110
19:19
TCHIQECDKpc2_NP.5
p−maj=1, pX=0.8, N=325
var.4.THBS1 15:39880822:A:G
peptide level
44:196
25:110
4:19
TCHIQECDKRpc2_NP.1
p−maj=1, pX=0.41, N=73
var.4.THBS1 15:39880822:A:G
peptide level
188:196
105:110
18:19
TCHIQECDKRpc2_NP.2
p−maj=0.58, pX=0.52, N=311
var.4.THBS1 15:39880822:A:G
peptide level
61:196
39:110
6:19
TCHIQECDKRpc2_NP.3
p−min=0.55, pX=0.65, N=106
var.4.THBS1 15:39880822:A:G
peptide level
192:196
108:110
19:19
TCHIQECDKRpc2_NP.4
p−maj=1, pX=0.93, N=319
var.4.THBS1 15:39880822:A:G
peptide level
195:196
109:110
19:19
TCHIQECDKRpc2_NP.5
p−min=1, pX=0.92, N=323
var.4.THBS1 15:39880822:A:G
peptide level
83:196
35:110
7:19
TCHIQECDKRpc3_NP.1
p−min=0.081, pX=0.07, N=125
var.4.THBS1 15:39880822:A:G
peptide level
196:196
109:110
19:19
TCHIQECDKRpc3_NP.2
p−min=0.4, pX=0.18, N=324
var.4.THBS1 15:39880822:A:G
peptide level
118:196
65:110
8:19
TCHIQECDKRpc3_NP.3
p−maj=0.15, pX=0.63, N=191
var.4.THBS1 15:39880822:A:G
peptide level
192:196
106:110
19:19
TCHIQECDKRpc3_NP.4
p−min=0.72, pX=0.16, N=317
var.4.THBS1 15:39880822:A:G
peptide level
194:196
110:110
18:19
TCHIQECDKRpc3_NP.5
p−maj=0.17, pX=0.46, N=322

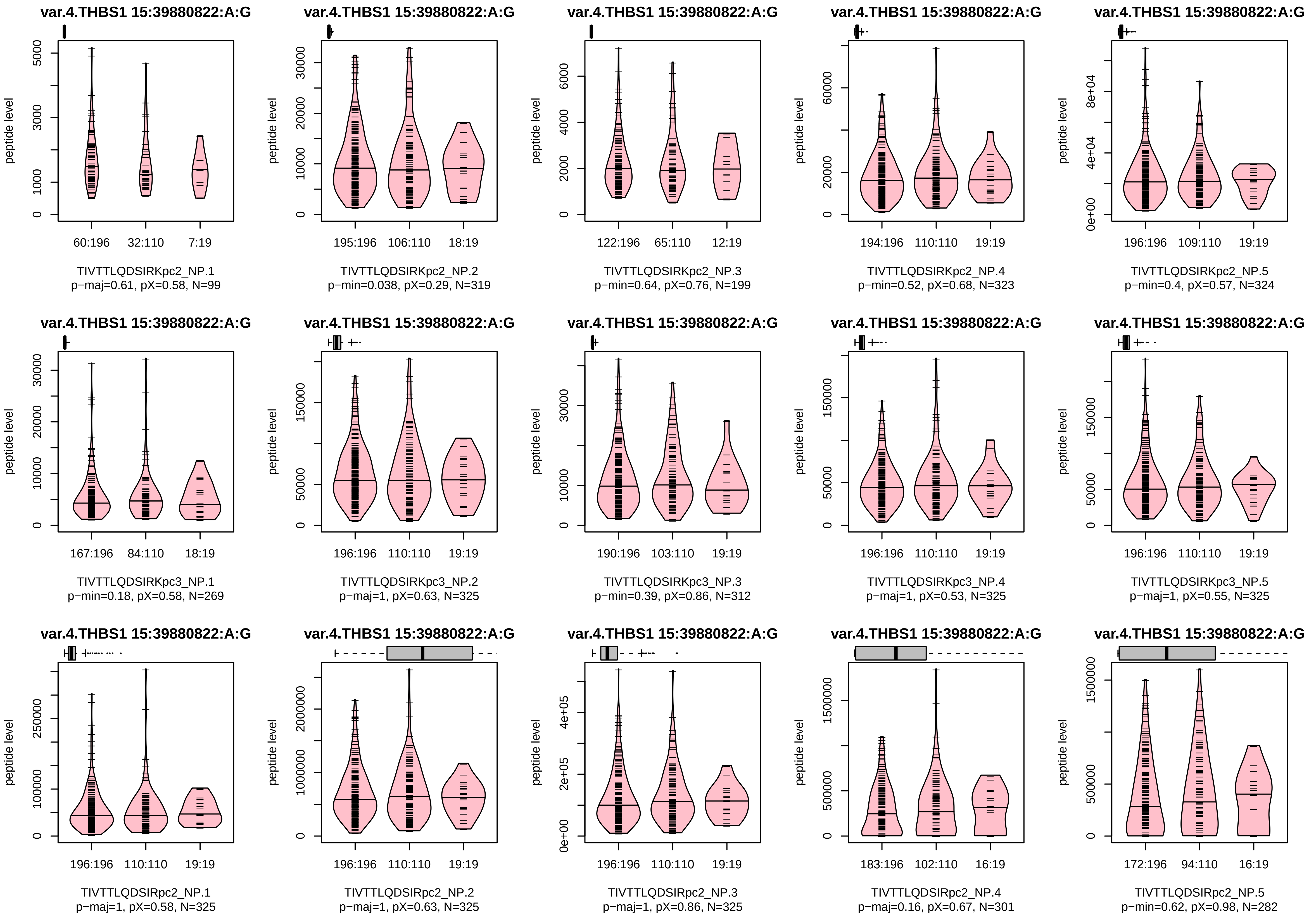
var.4.THBS1 15:39880822:A:G
peptide level
60:196
32:110
7:19
TIVTTLQDSIRKpc2_NP.1
p−maj=0.61, pX=0.58, N=99
var.4.THBS1 15:39880822:A:G
peptide level
195:196
106:110
18:19
TIVTTLQDSIRKpc2_NP.2
p−min=0.038, pX=0.29, N=319
var.4.THBS1 15:39880822:A:G
peptide level
122:196
65:110
12:19
TIVTTLQDSIRKpc2_NP.3
p−min=0.64, pX=0.76, N=199
var.4.THBS1 15:39880822:A:G
peptide level
194:196
110:110
19:19
TIVTTLQDSIRKpc2_NP.4
p−min=0.52, pX=0.68, N=323
var.4.THBS1 15:39880822:A:G
peptide level
196:196
109:110
19:19
TIVTTLQDSIRKpc2_NP.5
p−min=0.4, pX=0.57, N=324
var.4.THBS1 15:39880822:A:G
peptide level
167:196
84:110
18:19
TIVTTLQDSIRKpc3_NP.1
p−min=0.18, pX=0.58, N=269
var.4.THBS1 15:39880822:A:G
peptide level
196:196
110:110
19:19
TIVTTLQDSIRKpc3_NP.2
p−maj=1, pX=0.63, N=325
var.4.THBS1 15:39880822:A:G
peptide level
190:196
103:110
19:19
TIVTTLQDSIRKpc3_NP.3
p−min=0.39, pX=0.86, N=312
var.4.THBS1 15:39880822:A:G
peptide level
196:196
110:110
19:19
TIVTTLQDSIRKpc3_NP.4
p−maj=1, pX=0.53, N=325
var.4.THBS1 15:39880822:A:G
peptide level
196:196
110:110
19:19
TIVTTLQDSIRKpc3_NP.5
p−maj=1, pX=0.55, N=325
var.4.THBS1 15:39880822:A:G
peptide level
196:196
110:110
19:19
TIVTTLQDSIRpc2_NP.1
p−maj=1, pX=0.58, N=325
var.4.THBS1 15:39880822:A:G
peptide level
196:196
110:110
19:19
TIVTTLQDSIRpc2_NP.2
p−maj=1, pX=0.63, N=325
var.4.THBS1 15:39880822:A:G
peptide level
196:196
110:110
19:19
TIVTTLQDSIRpc2_NP.3
p−maj=1, pX=0.86, N=325
var.4.THBS1 15:39880822:A:G
peptide level
183:196
102:110
16:19
TIVTTLQDSIRpc2_NP.4
p−maj=0.16, pX=0.67, N=301
var.4.THBS1 15:39880822:A:G
peptide level
172:196
94:110
16:19
TIVTTLQDSIRpc2_NP.5
p−min=0.62, pX=0.98, N=282

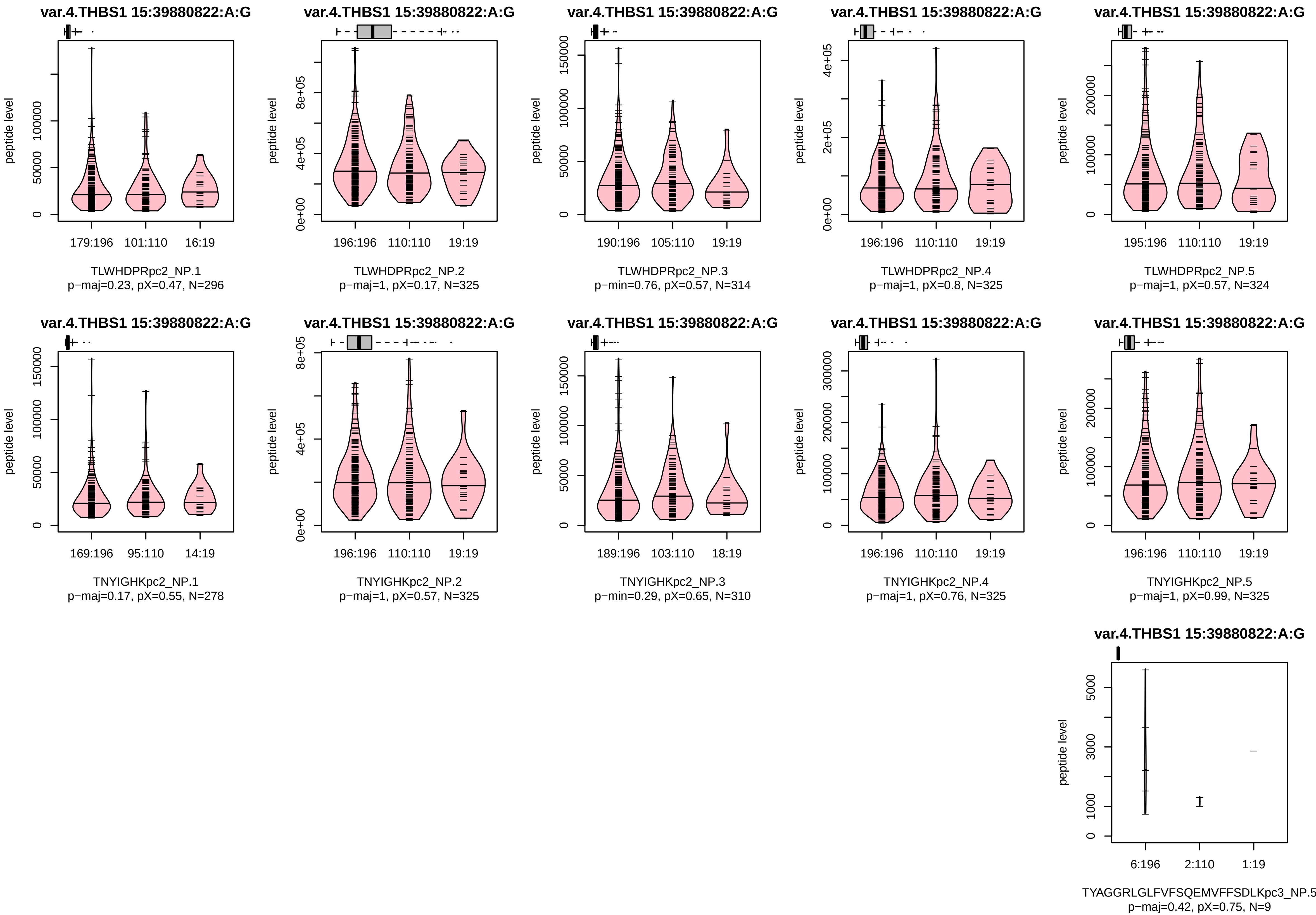
var.4.THBS1 15:39880822:A:G
peptide level
179:196
101:110
16:19
TLWHDPRpc2_NP.1
p−maj=0.23, pX=0.47, N=296
var.4.THBS1 15:39880822:A:G
peptide level
196:196
110:110
19:19
TLWHDPRpc2_NP.2
p−maj=1, pX=0.17, N=325
var.4.THBS1 15:39880822:A:G
peptide level
190:196
105:110
19:19
TLWHDPRpc2_NP.3
p−min=0.76, pX=0.57, N=314
var.4.THBS1 15:39880822:A:G
peptide level
196:196
110:110
19:19
TLWHDPRpc2_NP.4
p−maj=1, pX=0.8, N=325
var.4.THBS1 15:39880822:A:G
peptide level
195:196
110:110
19:19
TLWHDPRpc2_NP.5
p−maj=1, pX=0.57, N=324
var.4.THBS1 15:39880822:A:G
peptide level
169:196
95:110
14:19
TNYIGHKpc2_NP.1
p−maj=0.17, pX=0.55, N=278
var.4.THBS1 15:39880822:A:G
peptide level
196:196
110:110
19:19
TNYIGHKpc2_NP.2
p−maj=1, pX=0.57, N=325
var.4.THBS1 15:39880822:A:G
peptide level
189:196
103:110
18:19
TNYIGHKpc2_NP.3
p−min=0.29, pX=0.65, N=310
var.4.THBS1 15:39880822:A:G
peptide level
196:196
110:110
19:19
TNYIGHKpc2_NP.4
p−maj=1, pX=0.76, N=325
var.4.THBS1 15:39880822:A:G
peptide level
196:196
110:110
19:19
TNYIGHKpc2_NP.5
p−maj=1, pX=0.99, N=325
var.4.THBS1 15:39880822:A:G
peptide level
6:196
2:110
1:19
TYAGGRLGLFVFSQEMVFFSDLKpc3_NP.5
p−maj=0.42, pX=0.75, N=9

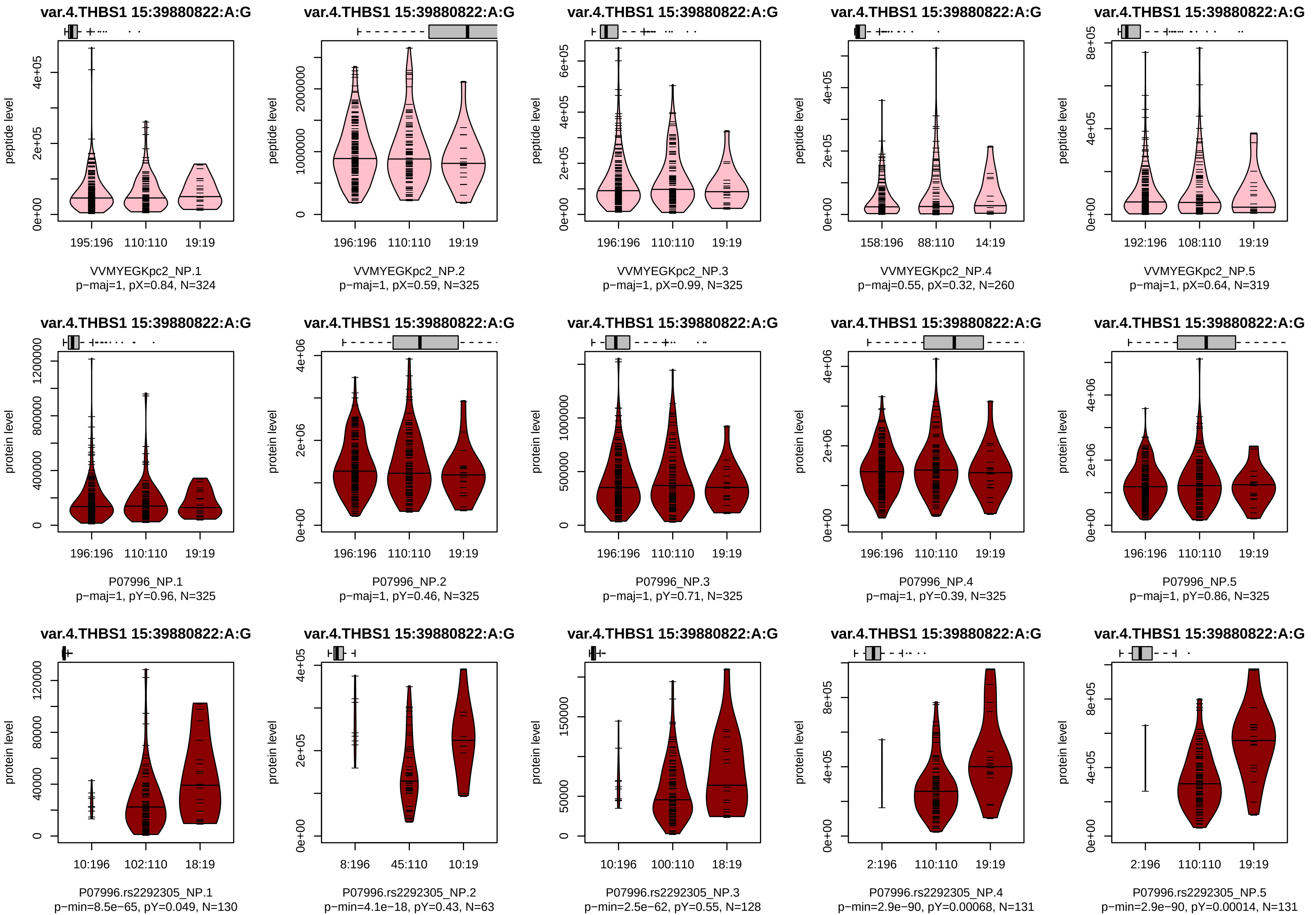
var.4.THBS1 15:39880822:A:G
peptide level
195:196
110:110
19:19
VVMYEGKpc2_NP.1
p−maj=1, pX=0.84, N=324
var.4.THBS1 15:39880822:A:G
peptide level
196:196
110:110
19:19
VVMYEGKpc2_NP.2
p−maj=1, pX=0.59, N=325
var.4.THBS1 15:39880822:A:G
peptide level
196:196
110:110
19:19
VVMYEGKpc2_NP.3
p−maj=1, pX=0.99, N=325
var.4.THBS1 15:39880822:A:G
peptide level
158:196
88:110
14:19
VVMYEGKpc2_NP.4
p−maj=0.55, pX=0.32, N=260
var.4.THBS1 15:39880822:A:G
peptide level
192:196
108:110
19:19
VVMYEGKpc2_NP.5
p−maj=1, pX=0.64, N=319
var.4.THBS1 15:39880822:A:G
protein level
196:196
110:110
19:19
P07996_NP.1
p−maj=1, pY=0.96, N=325
var.4.THBS1 15:39880822:A:G
protein level
196:196
110:110
19:19
P07996_NP.2
p−maj=1, pY=0.46, N=325
var.4.THBS1 15:39880822:A:G
protein level
196:196
110:110
19:19
P07996_NP.3
p−maj=1, pY=0.71, N=325
var.4.THBS1 15:39880822:A:G
protein level
196:196
110:110
19:19
P07996_NP.4
p−maj=1, pY=0.39, N=325
var.4.THBS1 15:39880822:A:G
protein level
196:196
110:110
19:19
P07996_NP.5
p−maj=1, pY=0.86, N=325
var.4.THBS1 15:39880822:A:G
protein level
10:196
102:110
18:19
P07996.rs2292305_NP.1
p−min=8.5e−65, pY=0.049, N=130
var.4.THBS1 15:39880822:A:G
protein level
8:196
45:110
10:19
P07996.rs2292305_NP.2
p−min=4.1e−18, pY=0.43, N=63
var.4.THBS1 15:39880822:A:G
protein level
10:196
100:110
18:19
P07996.rs2292305_NP.3
p−min=2.5e−62, pY=0.55, N=128
var.4.THBS1 15:39880822:A:G
protein level
2:196
110:110
19:19
P07996.rs2292305_NP.4
p−min=2.9e−90, pY=0.00068, N=131
var.4.THBS1 15:39880822:A:G
protein level
2:196
110:110
19:19
P07996.rs2292305_NP.5
p−min=2.9e−90, pY=0.00014, N=131

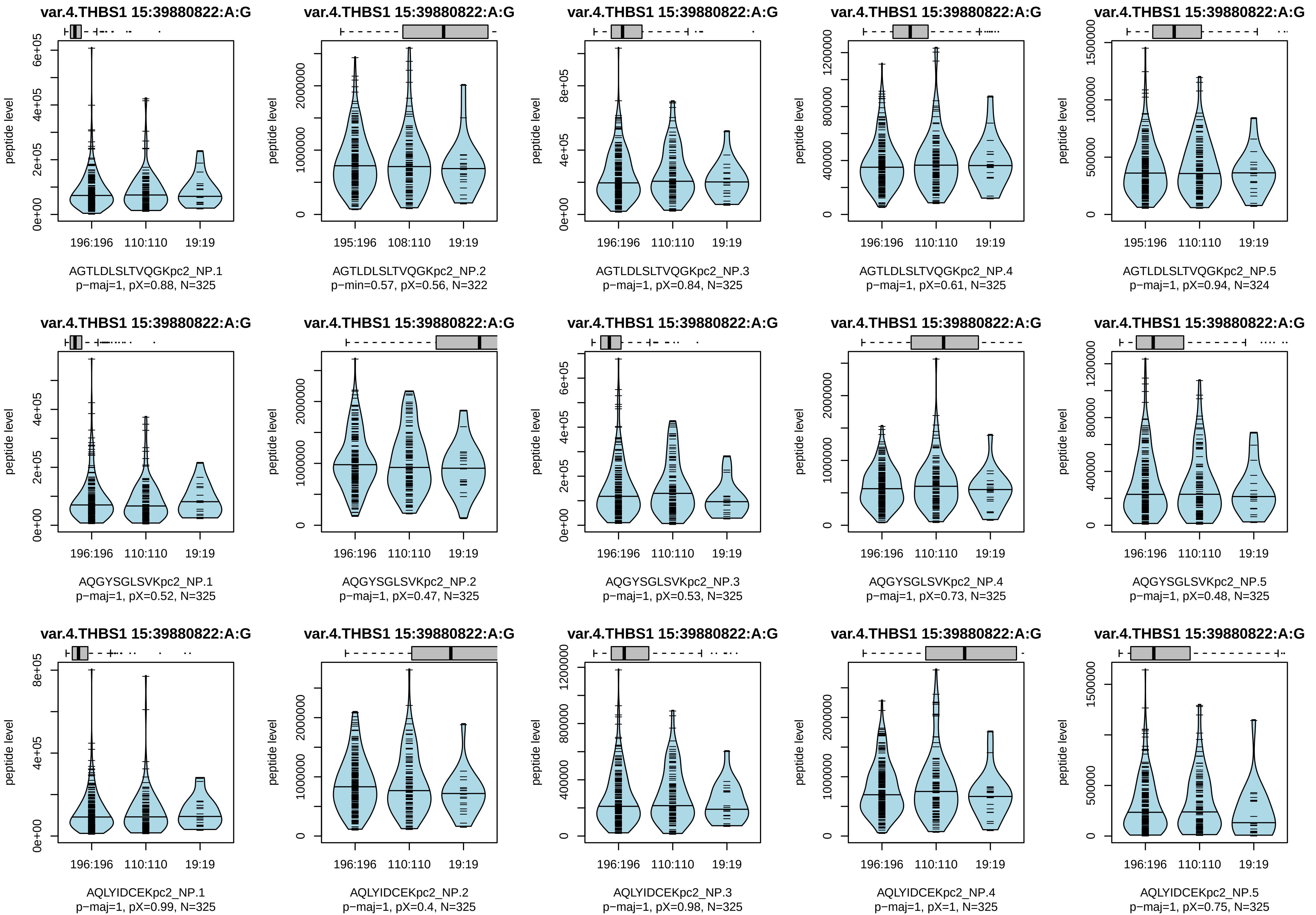
var.4.THBS1 15:39880822:A:G
peptide level
196:196
110:110
19:19
AGTLDLSLTVQGKpc2_NP.1
p−maj=1, pX=0.88, N=325
var.4.THBS1 15:39880822:A:G
peptide level
195:196
108:110
19:19
AGTLDLSLTVQGKpc2_NP.2
p−min=0.57, pX=0.56, N=322
var.4.THBS1 15:39880822:A:G
peptide level
196:196
110:110
19:19
AGTLDLSLTVQGKpc2_NP.3
p−maj=1, pX=0.84, N=325
var.4.THBS1 15:39880822:A:G
peptide level
196:196
110:110
19:19
AGTLDLSLTVQGKpc2_NP.4
p−maj=1, pX=0.61, N=325
var.4.THBS1 15:39880822:A:G
peptide level
195:196
110:110
19:19
AGTLDLSLTVQGKpc2_NP.5
p−maj=1, pX=0.94, N=324
var.4.THBS1 15:39880822:A:G
peptide level
196:196
110:110
19:19
AQGYSGLSVKpc2_NP.1
p−maj=1, pX=0.52, N=325
var.4.THBS1 15:39880822:A:G
peptide level
196:196
110:110
19:19
AQGYSGLSVKpc2_NP.2
p−maj=1, pX=0.47, N=325
var.4.THBS1 15:39880822:A:G
peptide level
196:196
110:110
19:19
AQGYSGLSVKpc2_NP.3
p−maj=1, pX=0.53, N=325
var.4.THBS1 15:39880822:A:G
peptide level
196:196
110:110
19:19
AQGYSGLSVKpc2_NP.4
p−maj=1, pX=0.73, N=325
var.4.THBS1 15:39880822:A:G
peptide level
196:196
110:110
19:19
AQGYSGLSVKpc2_NP.5
p−maj=1, pX=0.48, N=325
var.4.THBS1 15:39880822:A:G
peptide level
196:196
110:110
19:19
AQLYIDCEKpc2_NP.1
p−maj=1, pX=0.99, N=325
var.4.THBS1 15:39880822:A:G
peptide level
196:196
110:110
19:19
AQLYIDCEKpc2_NP.2
p−maj=1, pX=0.4, N=325
var.4.THBS1 15:39880822:A:G
peptide level
196:196
110:110
19:19
AQLYIDCEKpc2_NP.3
p−maj=1, pX=0.98, N=325
var.4.THBS1 15:39880822:A:G
peptide level
196:196
110:110
19:19
AQLYIDCEKpc2_NP.4
p−maj=1, pX=1, N=325
var.4.THBS1 15:39880822:A:G
peptide level
196:196
110:110
19:19
AQLYIDCEKpc2_NP.5
p−maj=1, pX=0.75, N=325

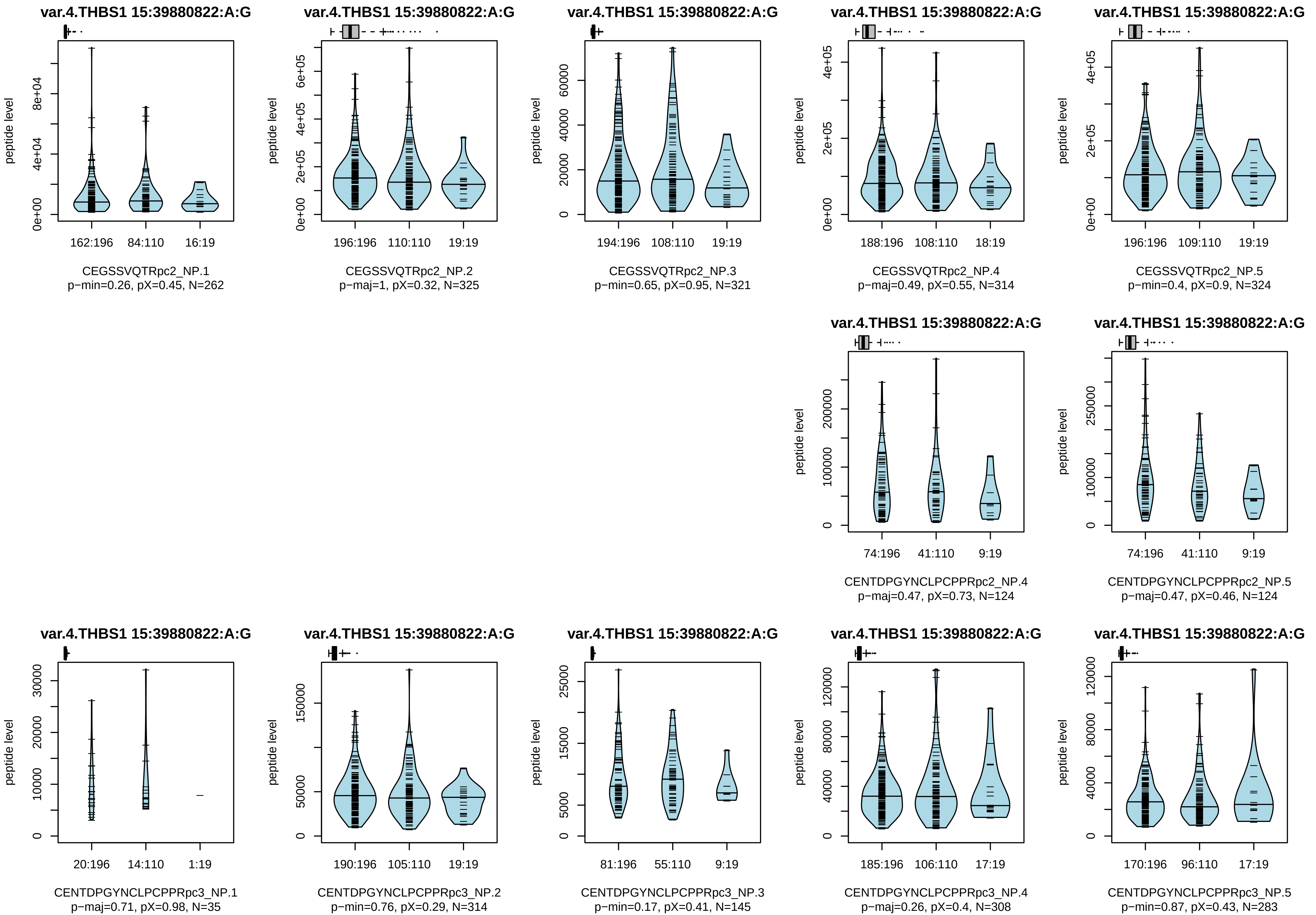
var.4.THBS1 15:39880822:A:G
peptide level
162:196
84:110
16:19
CEGSSVQTRpc2_NP.1
p−min=0.26, pX=0.45, N=262
var.4.THBS1 15:39880822:A:G
196:196
110:110
19:19
CEGSSVQTRpc2_NP.2
p−maj=1, pX=0.32, N=325
var.4.THBS1 15:39880822:A:G
194:196
108:110
19:19
CEGSSVQTRpc2_NP.3
p−min=0.65, pX=0.95, N=321
var.4.THBS1 15:39880822:A:G
188:196
108:110
18:19
CEGSSVQTRpc2_NP.4
p−maj=0.49, pX=0.55, N=314
var.4.THBS1 15:39880822:A:G
196:196
109:110
19:19
CEGSSVQTRpc2_NP.5
p−min=0.4, pX=0.9, N=324
var.4.THBS1 15:39880822:A:G
74:196
41:110
9:19
CENTDPGYNCLPCPPRpc2_NP.4
p−maj=0.47, pX=0.73, N=124
var.4.THBS1 15:39880822:A:G
74:196
41:110
9:19
CENTDPGYNCLPCPPRpc2_NP.5
p−maj=0.47, pX=0.46, N=124
var.4.THBS1 15:39880822:A:G
20:196
14:110
1:19
CENTDPGYNCLPCPPRpc3_NP.1
p−maj=0.71, pX=0.98, N=35
var.4.THBS1 15:39880822:A:G
190:196
105:110
19:19
CENTDPGYNCLPCPPRpc3_NP.2
p−min=0.76, pX=0.29, N=314
var.4.THBS1 15:39880822:A:G
81:196
55:110
9:19
CENTDPGYNCLPCPPRpc3_NP.3
p−min=0.17, pX=0.41, N=145
var.4.THBS1 15:39880822:A:G
185:196
106:110
17:19
CENTDPGYNCLPCPPRpc3_NP.4
p−maj=0.26, pX=0.4, N=308
var.4.THBS1 15:39880822:A:G
170:196
96:110
17:19
CENTDPGYNCLPCPPRpc3_NP.5
p−min=0.87, pX=0.43, N=283

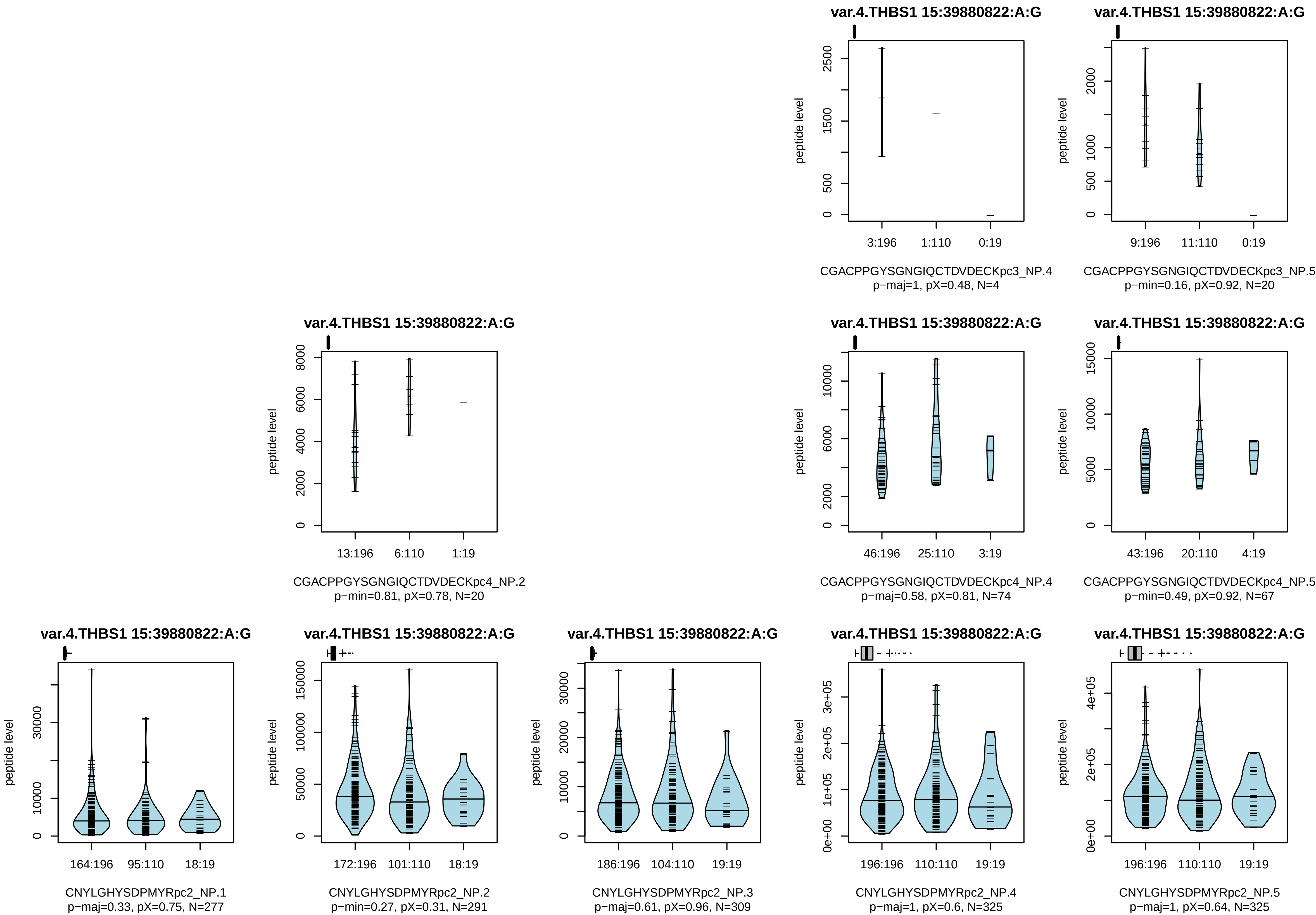
var.4.THBS1 15:39880822:A:G
peptide level
3:196
1:110
0:19
CGACPPGYSGNGIQCTDVDECKpc3_NP.4
p−maj=1, pX=0.48, N=4
var.4.THBS1 15:39880822:A:G
peptide level
9:196
11:110
0:19
CGACPPGYSGNGIQCTDVDECKpc3_NP.5
p−min=0.16, pX=0.92, N=20
var.4.THBS1 15:39880822:A:G
peptide level
13:196
6:110
1:19
CGACPPGYSGNGIQCTDVDECKpc4_NP.2
p−min=0.81, pX=0.78, N=20
var.4.THBS1 15:39880822:A:G
peptide level
46:196
25:110
3:19
CGACPPGYSGNGIQCTDVDECKpc4_NP.4
p−maj=0.58, pX=0.81, N=74
var.4.THBS1 15:39880822:A:G
peptide level
43:196
20:110
4:19
CGACPPGYSGNGIQCTDVDECKpc4_NP.5
p−min=0.49, pX=0.92, N=67
var.4.THBS1 15:39880822:A:G
peptide level
164:196
95:110
18:19
CNYLGHYSDPMYRpc2_NP.1
p−maj=0.33, pX=0.75, N=277
var.4.THBS1 15:39880822:A:G
peptide level
172:196
101:110
18:19
CNYLGHYSDPMYRpc2_NP.2
p−min=0.27, pX=0.31, N=291
var.4.THBS1 15:39880822:A:G
peptide level
186:196
104:110
19:19
CNYLGHYSDPMYRpc2_NP.3
p−maj=0.61, pX=0.96, N=309
var.4.THBS1 15:39880822:A:G
peptide level
196:196
110:110
19:19
CNYLGHYSDPMYRpc2_NP.4
p−maj=1, pX=0.6, N=325
var.4.THBS1 15:39880822:A:G
peptide level
196:196
110:110
19:19
CNYLGHYSDPMYRpc2_NP.5
p−maj=1, pX=0.64, N=325

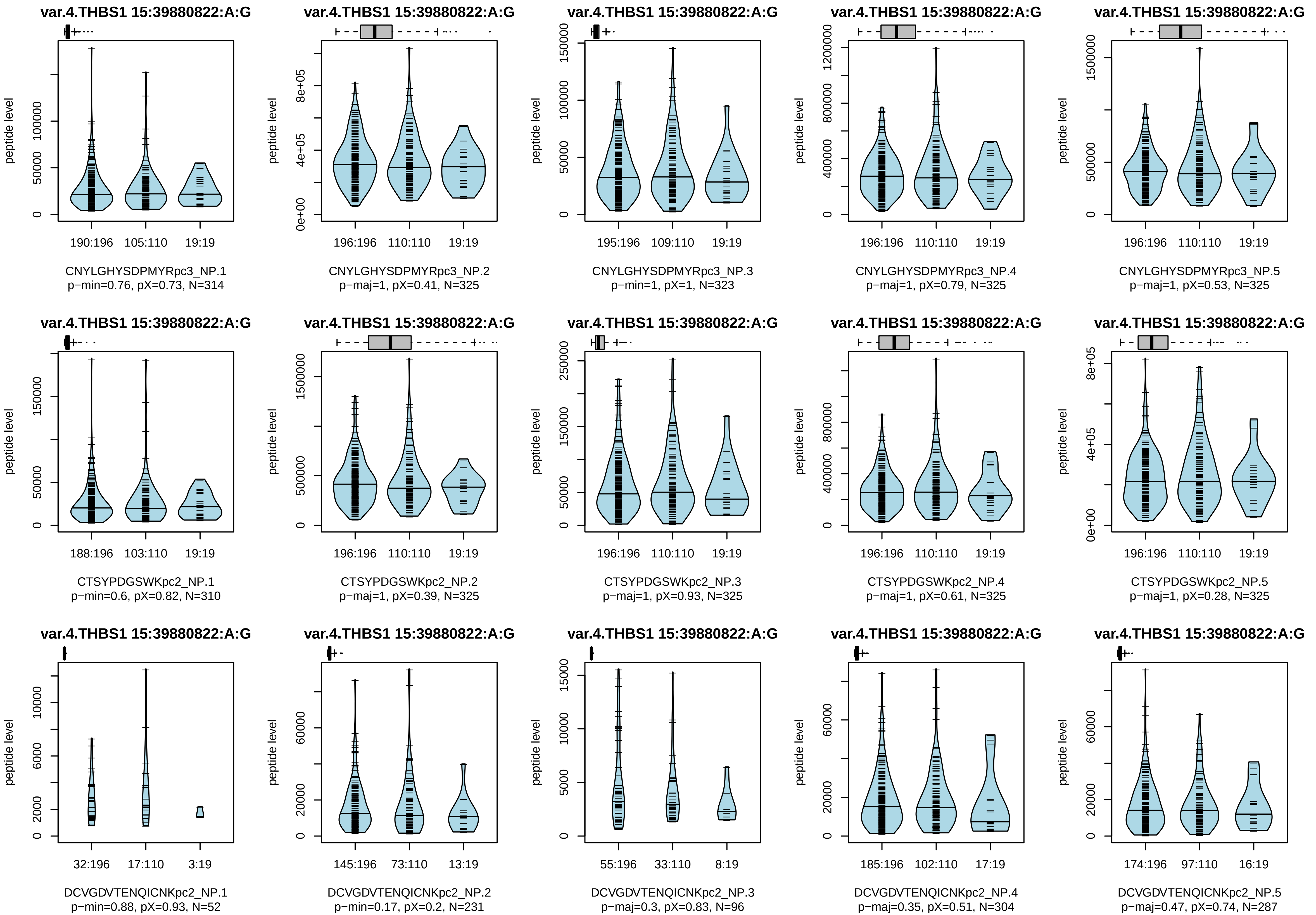
var.4.THBS1 15:39880822:A:G
peptide level
190:196
105:110
19:19
CNYLGHYSDPMYRpc3_NP.1
p−min=0.76, pX=0.73, N=314
var.4.THBS1 15:39880822:A:G
196:196
110:110
19:19
CNYLGHYSDPMYRpc3_NP.2
p−maj=1, pX=0.41, N=325
var.4.THBS1 15:39880822:A:G
195:196
109:110
19:19
CNYLGHYSDPMYRpc3_NP.3
p−min=1, pX=1, N=323
var.4.THBS1 15:39880822:A:G
196:196
110:110
19:19
CNYLGHYSDPMYRpc3_NP.4
p−maj=1, pX=0.79, N=325
var.4.THBS1 15:39880822:A:G
196:196
110:110
19:19
CNYLGHYSDPMYRpc3_NP.5
p−maj=1, pX=0.53, N=325
var.4.THBS1 15:39880822:A:G
188:196
103:110
19:19
CTSYPDGSWKpc2_NP.1
p−min=0.6, pX=0.82, N=310
var.4.THBS1 15:39880822:A:G
196:196
110:110
19:19
CTSYPDGSWKpc2_NP.2
p−maj=1, pX=0.39, N=325
var.4.THBS1 15:39880822:A:G
196:196
110:110
19:19
CTSYPDGSWKpc2_NP.3
p−maj=1, pX=0.93, N=325
var.4.THBS1 15:39880822:A:G
196:196
110:110
19:19
CTSYPDGSWKpc2_NP.4
p−maj=1, pX=0.61, N=325
var.4.THBS1 15:39880822:A:G
196:196
110:110
19:19
CTSYPDGSWKpc2_NP.5
p−maj=1, pX=0.28, N=325
var.4.THBS1 15:39880822:A:G
32:196
17:110
3:19
DCVGDVTENQICNKpc2_NP.1
p−min=0.88, pX=0.93, N=52
var.4.THBS1 15:39880822:A:G
145:196
73:110
13:19
DCVGDVTENQICNKpc2_NP.2
p−min=0.17, pX=0.2, N=231
var.4.THBS1 15:39880822:A:G
55:196
33:110
8:19
DCVGDVTENQICNKpc2_NP.3
p−maj=0.3, pX=0.83, N=96
var.4.THBS1 15:39880822:A:G
185:196
102:110
17:19
DCVGDVTENQICNKpc2_NP.4
p−maj=0.35, pX=0.51, N=304
var.4.THBS1 15:39880822:A:G
174:196
97:110
16:19
DCVGDVTENQICNKpc2_NP.5
p−maj=0.47, pX=0.74, N=287

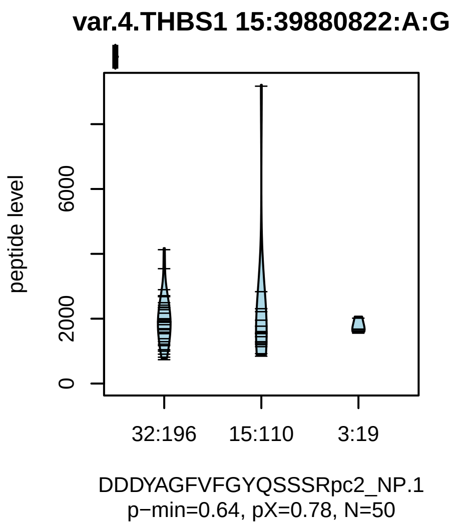
var.4.THBS1 15:39880822:A:G
peptide level
0
2000
6000
32:196
15:110
3:19
DDDYAGFVFGYQSSSRpc2_NP.1
p−min=0.64, pX=0.78, N=50

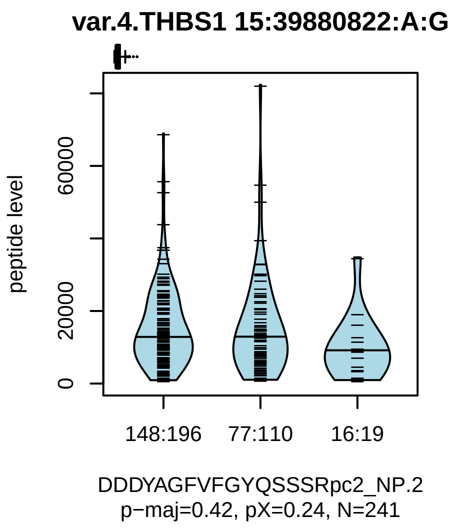
var.4.THBS1 15:39880822:A:G
peptide level
0
20000
60000
148:196
77:110
16:19
DDDYAGFVFGYQSSSRpc2_NP.2
p−maj=0.42, pX=0.24, N=241

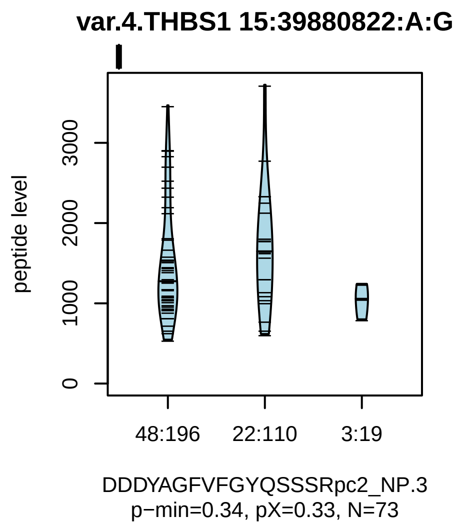
var.4.THBS1 15:39880822:A:G
peptide level
0
1000
2000
3000
48:196
22:110
3:19
DDDYAGFVFGYQSSSRpc2_NP.3
p−min=0.34, pX=0.33, N=73

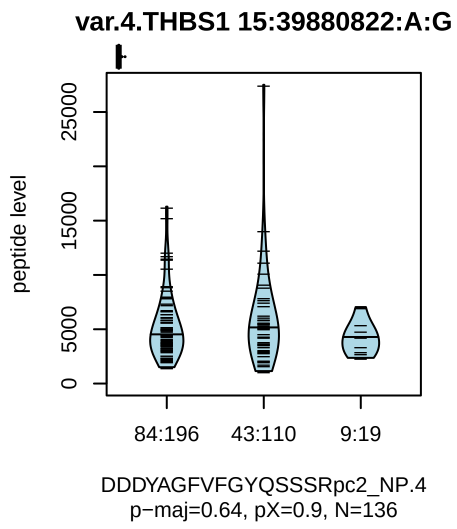
var.4.THBS1 15:39880822:A:G
peptide level
0
5000
15000
25000
84:196
43:110
9:19
DDDYAGFVFGYQSSSRpc2_NP.4
p−maj=0.64, pX=0.9, N=136

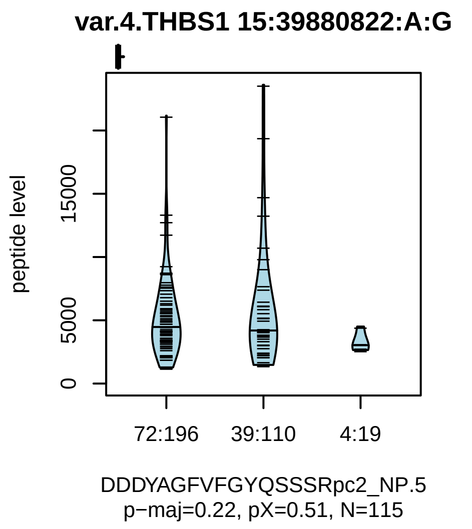
var.4.THBS1 15:39880822:A:G
peptide level
0
5000
15000
72:196
39:110
4:19
DDDYAGFVFGYQSSSRpc2_NP.5
p−maj=0.22, pX=0.51, N=115

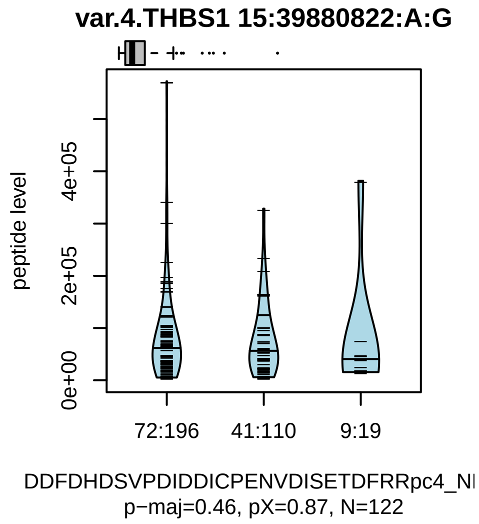
var.4.THBS1 15:39880822:A:G
peptide level
0e+00
2e+05
4e+05
72:196
41:110
9:19
DDFDHDSVPDIDDICPENVDISETDFRRpc4_NI
p−maj=0.46, pX=0.87, N=122

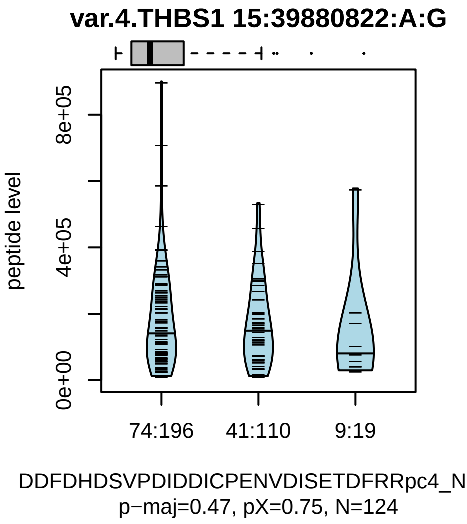
var.4.THBS1 15:39880822:A:G
peptide level
0e+00
4e+05
8e+05
74:196
41:110
9:19
DDFDHDSVPDIDDICPENVDISETDFRRpc4_N
p−maj=0.47, pX=0.75, N=124

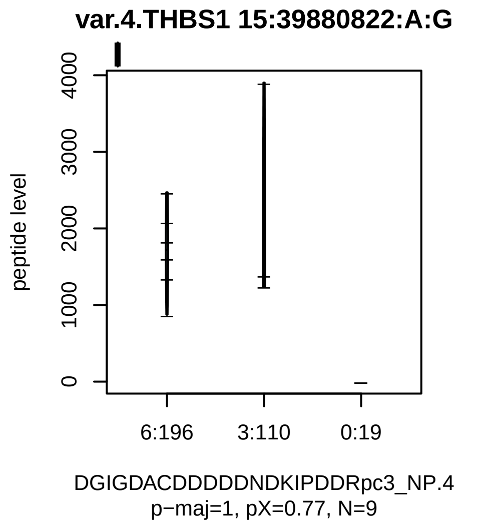
var.4.THBS1 15:39880822:A:G
peptide level
0
1000
2000
3000
4000
6:196
3:110
0:19
DGIGDACDDDDDNDKIPDDRpc3_NP.4
p−maj=1, pX=0.77, N=9

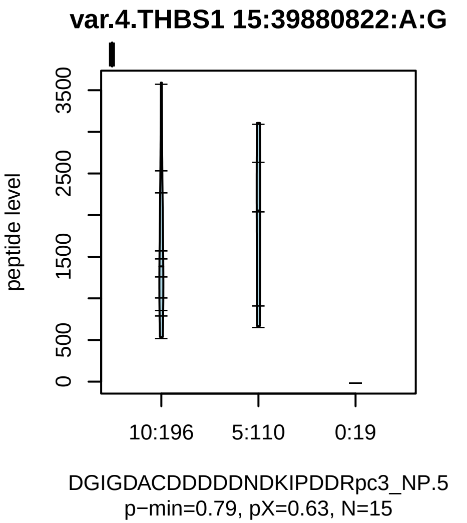
var.4.THBS1 15:39880822:A:G
peptide level
0
500
1500
2500
3500
10:196
5:110
0:19
DGIGDACDDDDDNDKIPDDRpc3_NP.5
p−min=0.79, pX=0.63, N=15

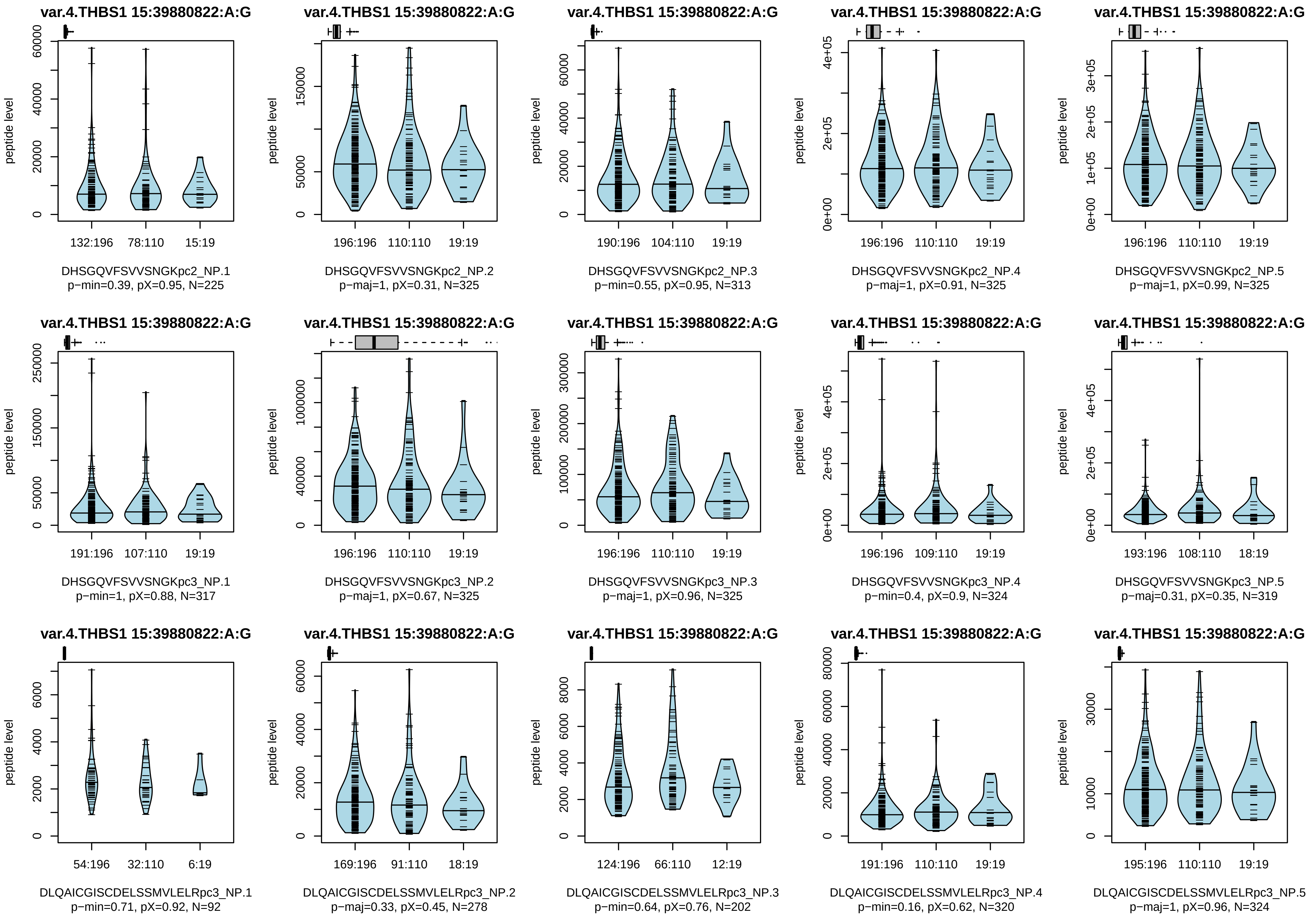
var.4.THBS1 15:39880822:A:G
peptide level
132:196
78:110
15:19
DHSGQVFSVVSNGKpc2_NP.1
p−min=0.39, pX=0.95, N=225
var.4.THBS1 15:39880822:A:G
196:196
110:110
19:19
DHSGQVFSVVSNGKpc2_NP.2
p−maj=1, pX=0.31, N=325
var.4.THBS1 15:39880822:A:G
190:196
104:110
19:19
DHSGQVFSVVSNGKpc2_NP.3
p−min=0.55, pX=0.95, N=313
var.4.THBS1 15:39880822:A:G
196:196
110:110
19:19
DHSGQVFSVVSNGKpc2_NP.4
p−maj=1, pX=0.91, N=325
var.4.THBS1 15:39880822:A:G
196:196
110:110
19:19
DHSGQVFSVVSNGKpc2_NP.5
p−maj=1, pX=0.99, N=325
var.4.THBS1 15:39880822:A:G
191:196
107:110
19:19
DHSGQVFSVVSNGKpc3_NP.1
p−min=1, pX=0.88, N=317
var.4.THBS1 15:39880822:A:G
196:196
110:110
19:19
DHSGQVFSVVSNGKpc3_NP.2
p−maj=1, pX=0.67, N=325
var.4.THBS1 15:39880822:A:G
196:196
110:110
19:19
DHSGQVFSVVSNGKpc3_NP.3
p−maj=1, pX=0.96, N=325
var.4.THBS1 15:39880822:A:G
196:196
109:110
19:19
DHSGQVFSVVSNGKpc3_NP.4
p−min=0.4, pX=0.9, N=324
var.4.THBS1 15:39880822:A:G
193:196
108:110
18:19
DHSGQVFSVVSNGKpc3_NP.5
p−maj=0.31, pX=0.35, N=319
var.4.THBS1 15:39880822:A:G
54:196
32:110
6:19
DLQAICGISCDELSSMVLELRpc3_NP.1
p−min=0.71, pX=0.92, N=92
var.4.THBS1 15:39880822:A:G
169:196
91:110
18:19
DLQAICGISCDELSSMVLELRpc3_NP.2
p−maj=0.33, pX=0.45, N=278
var.4.THBS1 15:39880822:A:G
124:196
66:110
12:19
DLQAICGISCDELSSMVLELRpc3_NP.3
p−min=0.64, pX=0.76, N=202
var.4.THBS1 15:39880822:A:G
191:196
110:110
19:19
DLQAICGISCDELSSMVLELRpc3_NP.4
p−min=0.16, pX=0.62, N=320
var.4.THBS1 15:39880822:A:G
195:196
110:110
19:19
DLQAICGISCDELSSMVLELRpc3_NP.5
p−maj=1, pX=0.96, N=324

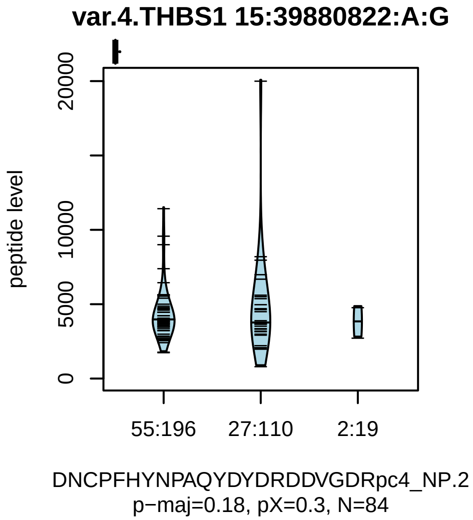
var.4.THBS1 15:39880822:A:G
peptide level
0
5000
10000
20000
55:196
27:110
2:19
DNCPFHYNPAQYDYDRDDVGDRpc4_NP.2
p−maj=0.18, pX=0.3, N=84

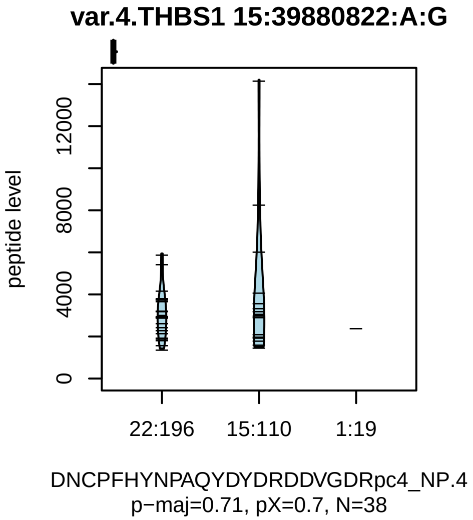
var.4.THBS1 15:39880822:A:G
peptide level
0
4000
8000
12000
22:196
15:110
1:19
DNCPFHYNPAQYDYDRDDVGDRpc4_NP.4
p−maj=0.71, pX=0.7, N=38

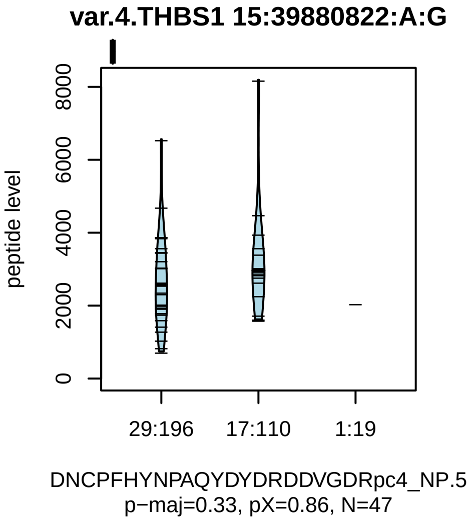
var.4.THBS1 15:39880822:A:G
peptide level
0
2000
4000
6000
8000
29:196
17:110
1:19
DNCPFHYNPAQYDYDRDDVGDRpc4_NP.5
p−maj=0.33, pX=0.86, N=47

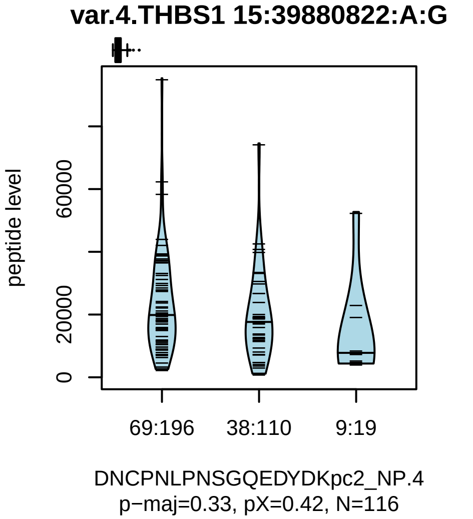
var.4.THBS1 15:39880822:A:G
peptide level
0
20000
40000
60000
69:196
38:110
9:19
DNCPNLPNSGQEDYDKpc2_NP.4
p−maj=0.33, pX=0.42, N=116

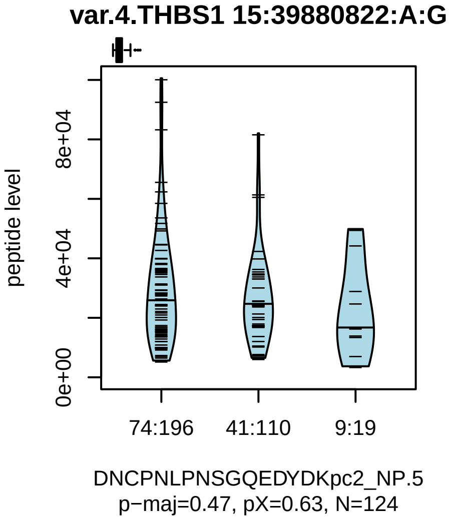
var.4.THBS1 15:39880822:A:G
peptide level
0e+00
4e+04
8e+04
74:196
41:110
9:19
DNCPNLPNSGQEDYDKpc2_NP.5
p−maj=0.47, pX=0.63, N=124

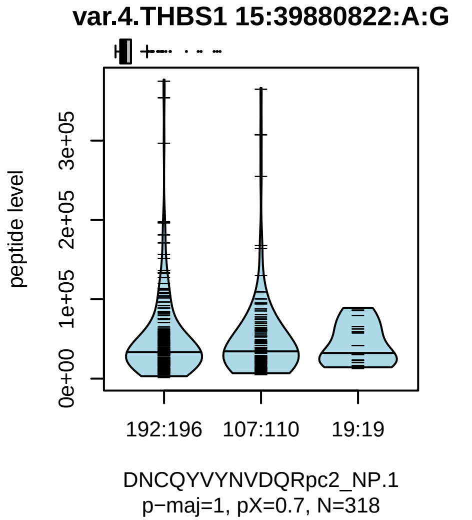
var.4.THBS1 15:39880822:A:G
peptide level
0e+00
1e+05
2e+05
3e+05
192:196
107:110
19:19
DNCQYVYNVDQRpc2_NP.1
p−maj=1, pX=0.7, N=318

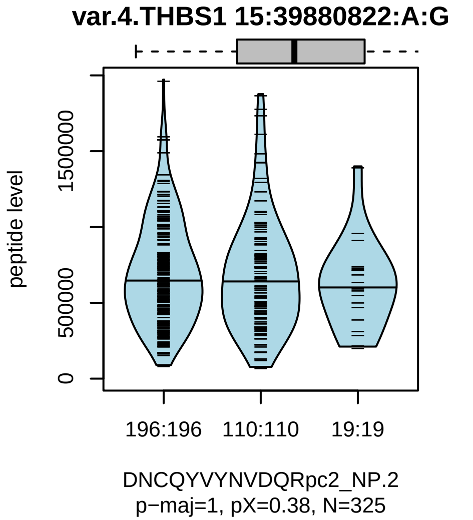
var.4.THBS1 15:39880822:A:G
peptide level
0
500000
1500000
196:196
110:110
19:19
DNCQYVYNVDQRpc2_NP.2
p−maj=1, pX=0.38, N=325

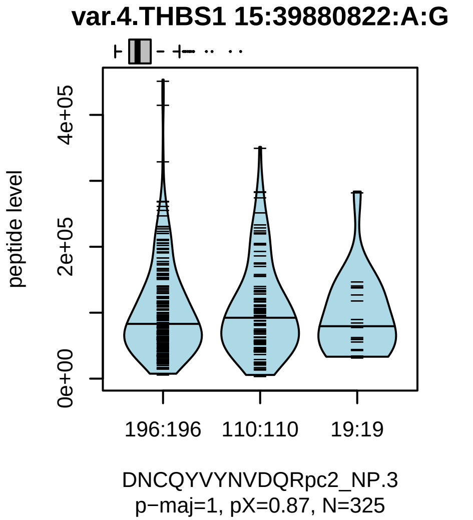
var.4.THBS1 15:39880822:A:G
peptide level
0e+00
2e+05
4e+05
196:196
110:110
19:19
DNCQYVYNVDQRpc2_NP.3
p−maj=1, pX=0.87, N=325

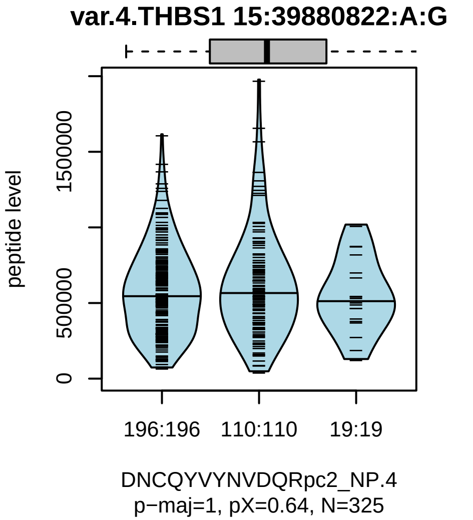
var.4.THBS1 15:39880822:A:G
peptide level
0
500000
1500000
196:196
110:110
19:19
DNCQYVYNVDQRpc2_NP.4
p−maj=1, pX=0.64, N=325

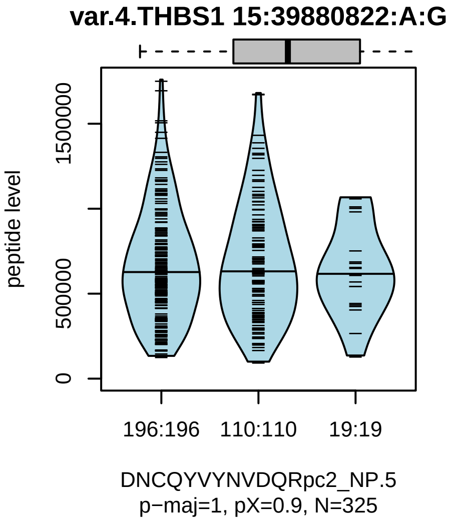
var.4.THBS1 15:39880822:A:G
peptide level
0
500000
1500000
196:196
110:110
19:19
DNCQYVYNVDQRpc2_NP.5
p−maj=1, pX=0.9, N=325

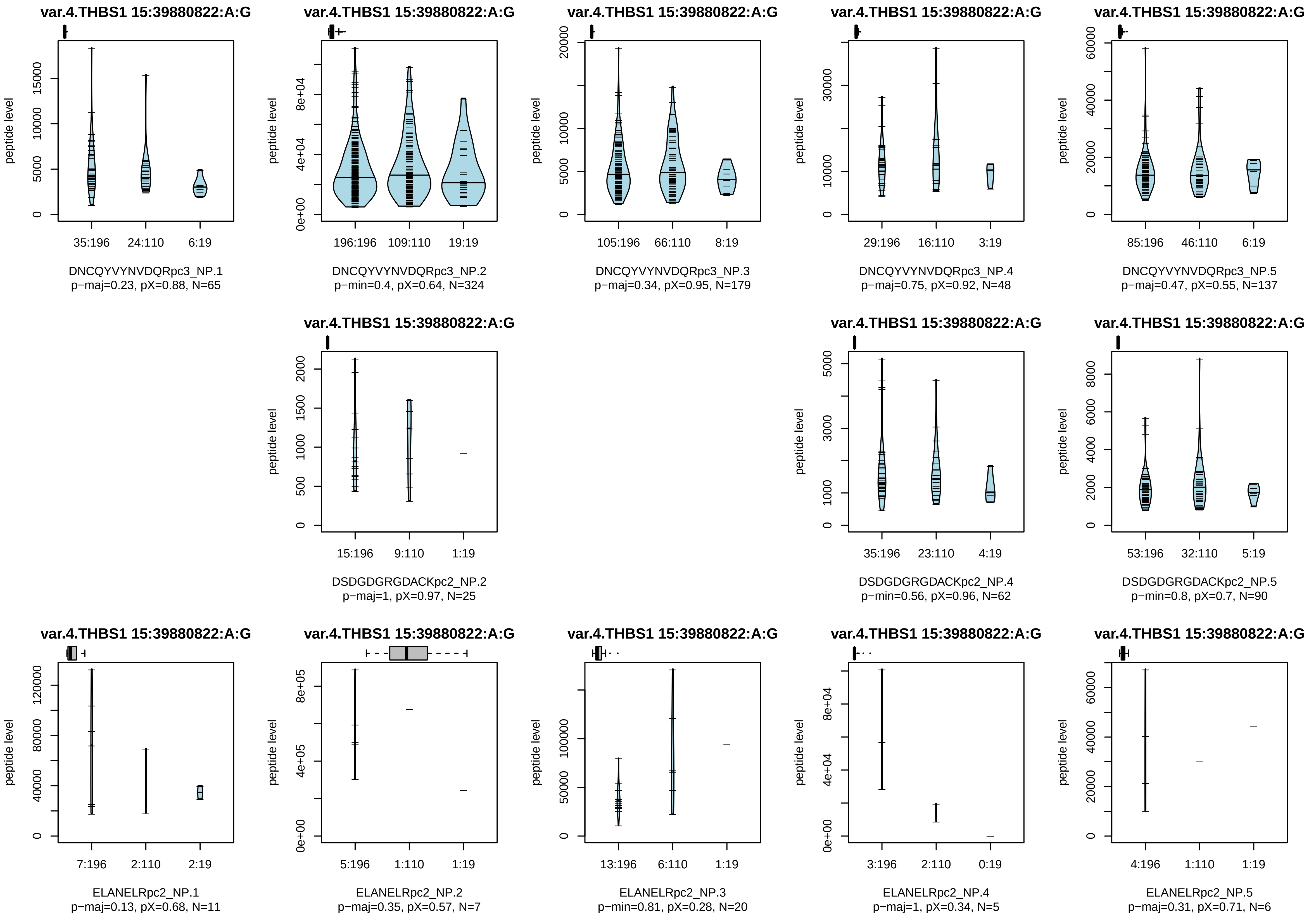
var.4.THBS1 15:39880822:A:G
peptide level
0
5000
10000
15000
35:196
24:110
6:19
DNCQYVYNVDQRpc3_NP.1
p−maj=0.23, pX=0.88, N=65
var.4.THBS1 15:39880822:A:G
peptide level
0e+00
4e+04
8e+04
196:196
109:110
19:19
DNCQYVYNVDQRpc3_NP.2
p−min=0.4, pX=0.64, N=324
var.4.THBS1 15:39880822:A:G
peptide level
0
5000
10000
20000
105:196
66:110
8:19
DNCQYVYNVDQRpc3_NP.3
p−maj=0.34, pX=0.95, N=179
var.4.THBS1 15:39880822:A:G
peptide level
0
10000
30000
29:196
16:110
3:19
DNCQYVYNVDQRpc3_NP.4
p−maj=0.75, pX=0.92, N=48
var.4.THBS1 15:39880822:A:G
peptide level
0
20000
40000
60000
85:196
46:110
6:19
DNCQYVYNVDQRpc3_NP.5
p−maj=0.47, pX=0.55, N=137
var.4.THBS1 15:39880822:A:G
peptide level
0
500
1000
1500
2000
15:196
9:110
1:19
DSDGDGRGDACKpc2_NP.2
p−maj=1, pX=0.97, N=25
var.4.THBS1 15:39880822:A:G
peptide level
0
1000
3000
5000
35:196
23:110
4:19
DSDGDGRGDACKpc2_NP.4
p−min=0.56, pX=0.96, N=62
var.4.THBS1 15:39880822:A:G
peptide level
0
2000
4000
6000
8000
53:196
32:110
5:19
DSDGDGRGDACKpc2_NP.5
p−min=0.8, pX=0.7, N=90
var.4.THBS1 15:39880822:A:G
peptide level
0
40000
80000
120000
7:196
2:110
2:19
ELANELRpc2_NP.1
p−maj=0.13, pX=0.68, N=11
var.4.THBS1 15:39880822:A:G
peptide level
0e+00
4e+05
8e+05
5:196
1:110
1:19
ELANELRpc2_NP.2
p−maj=0.35, pX=0.57, N=7
var.4.THBS1 15:39880822:A:G
peptide level
0
50000
100000
13:196
6:110
1:19
ELANELRpc2_NP.3
p−min=0.81, pX=0.28, N=20
var.4.THBS1 15:39880822:A:G
peptide level
0e+00
4e+04
8e+04
3:196
2:110
0:19
ELANELRpc2_NP.4
p−maj=1, pX=0.34, N=5
var.4.THBS1 15:39880822:A:G
peptide level
0
20000
40000
60000
4:196
1:110
1:19
ELANELRpc2_NP.5
p−maj=0.31, pX=0.71, N=6

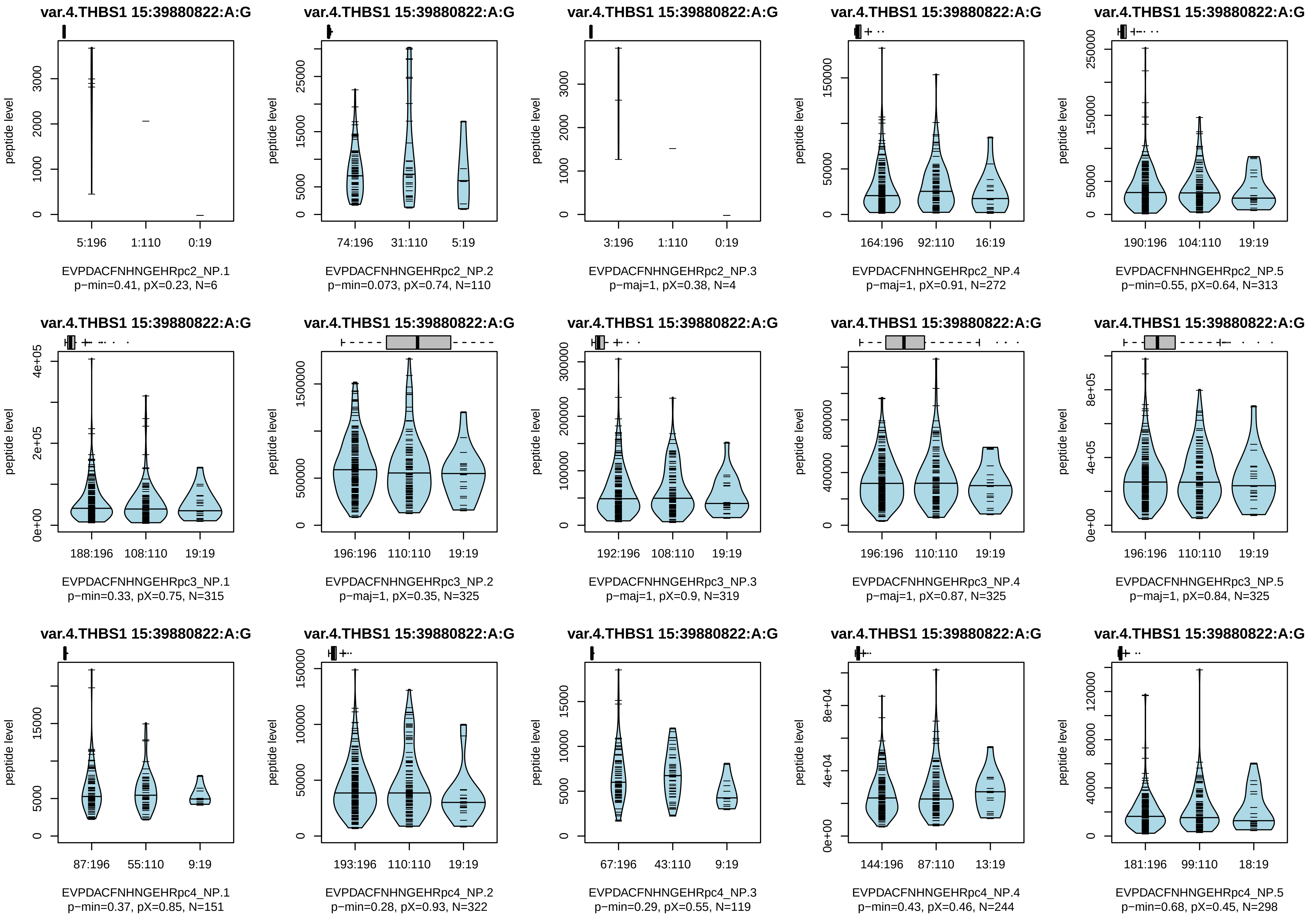
var.4.THBS1 15:39880822:A:G
peptide level
5:196
1:110
0:19
EVPDACFNHNGEHRpc2_NP.1
p−min=0.41, pX=0.23, N=6
var.4.THBS1 15:39880822:A:G
peptide level
74:196
31:110
5:19
EVPDACFNHNGEHRpc2_NP.2
p−min=0.073, pX=0.74, N=110
var.4.THBS1 15:39880822:A:G
peptide level
3:196
1:110
0:19
EVPDACFNHNGEHRpc2_NP.3
p−maj=1, pX=0.38, N=4
var.4.THBS1 15:39880822:A:G
peptide level
164:196
92:110
16:19
EVPDACFNHNGEHRpc2_NP.4
p−maj=1, pX=0.91, N=272
var.4.THBS1 15:39880822:A:G
peptide level
190:196
104:110
19:19
EVPDACFNHNGEHRpc2_NP.5
p−min=0.55, pX=0.64, N=313
var.4.THBS1 15:39880822:A:G
peptide level
188:196
108:110
19:19
EVPDACFNHNGEHRpc3_NP.1
p−min=0.33, pX=0.75, N=315
var.4.THBS1 15:39880822:A:G
peptide level
196:196
110:110
19:19
EVPDACFNHNGEHRpc3_NP.2
p−maj=1, pX=0.35, N=325
var.4.THBS1 15:39880822:A:G
peptide level
192:196
108:110
19:19
EVPDACFNHNGEHRpc3_NP.3
p−maj=1, pX=0.9, N=319
var.4.THBS1 15:39880822:A:G
peptide level
196:196
110:110
19:19
EVPDACFNHNGEHRpc3_NP.4
p−maj=1, pX=0.87, N=325
var.4.THBS1 15:39880822:A:G
peptide level
196:196
110:110
19:19
EVPDACFNHNGEHRpc3_NP.5
p−maj=1, pX=0.84, N=325
var.4.THBS1 15:39880822:A:G
peptide level
87:196
55:110
9:19
EVPDACFNHNGEHRpc4_NP.1
p−min=0.37, pX=0.85, N=151
var.4.THBS1 15:39880822:A:G
peptide level
193:196
110:110
19:19
EVPDACFNHNGEHRpc4_NP.2
p−min=0.28, pX=0.93, N=322
var.4.THBS1 15:39880822:A:G
peptide level
67:196
43:110
9:19
EVPDACFNHNGEHRpc4_NP.3
p−min=0.29, pX=0.55, N=119
var.4.THBS1 15:39880822:A:G
peptide level
144:196
87:110
13:19
EVPDACFNHNGEHRpc4_NP.4
p−min=0.43, pX=0.46, N=244
var.4.THBS1 15:39880822:A:G
peptide level
181:196
99:110
18:19
EVPDACFNHNGEHRpc4_NP.5
p−min=0.68, pX=0.45, N=298

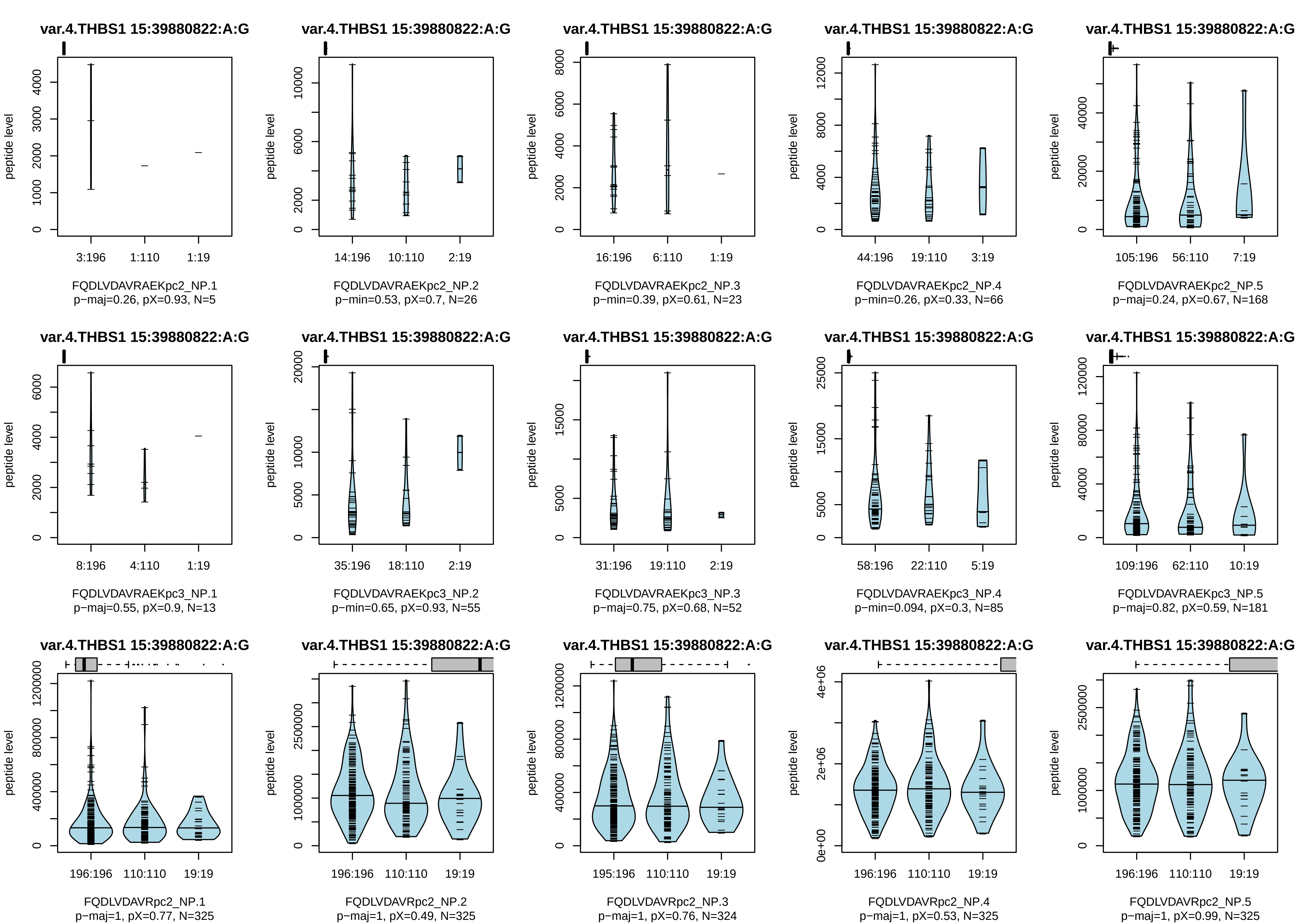
var.4.THBS1 15:39880822:A:G
peptide level
3:196
1:110
1:19
FQDLVDAVRAEKpc2_NP.1
p−maj=0.26, pX=0.93, N=5
14:196
10:110
2:19
FQDLVDAVRAEKpc2_NP.2
p−min=0.53, pX=0.7, N=26
16:196
6:110
1:19
FQDLVDAVRAEKpc2_NP.3
p−min=0.39, pX=0.61, N=23
44:196
19:110
3:19
FQDLVDAVRAEKpc2_NP.4
p−min=0.26, pX=0.33, N=66
105:196
56:110
7:19
FQDLVDAVRAEKpc2_NP.5
p−maj=0.24, pX=0.67, N=168
8:196
4:110
1:19
FQDLVDAVRAEKpc3_NP.1
p−maj=0.55, pX=0.9, N=13
35:196
18:110
2:19
FQDLVDAVRAEKpc3_NP.2
p−min=0.65, pX=0.93, N=55
31:196
19:110
2:19
FQDLVDAVRAEKpc3_NP.3
p−maj=0.75, pX=0.68, N=52
58:196
22:110
5:19
FQDLVDAVRAEKpc3_NP.4
p−min=0.094, pX=0.3, N=85
109:196
62:110
10:19
FQDLVDAVRAEKpc3_NP.5
p−maj=0.82, pX=0.59, N=181
196:196
110:110
19:19
FQDLVDAVRpc2_NP.1
p−maj=1, pX=0.77, N=325
196:196
110:110
19:19
FQDLVDAVRpc2_NP.2
p−maj=1, pX=0.49, N=325
195:196
110:110
19:19
FQDLVDAVRpc2_NP.3
p−maj=1, pX=0.76, N=324
196:196
110:110
19:19
FQDLVDAVRpc2_NP.4
p−maj=1, pX=0.53, N=325
196:196
110:110
19:19
FQDLVDAVRpc2_NP.5
p−maj=1, pX=0.99, N=325

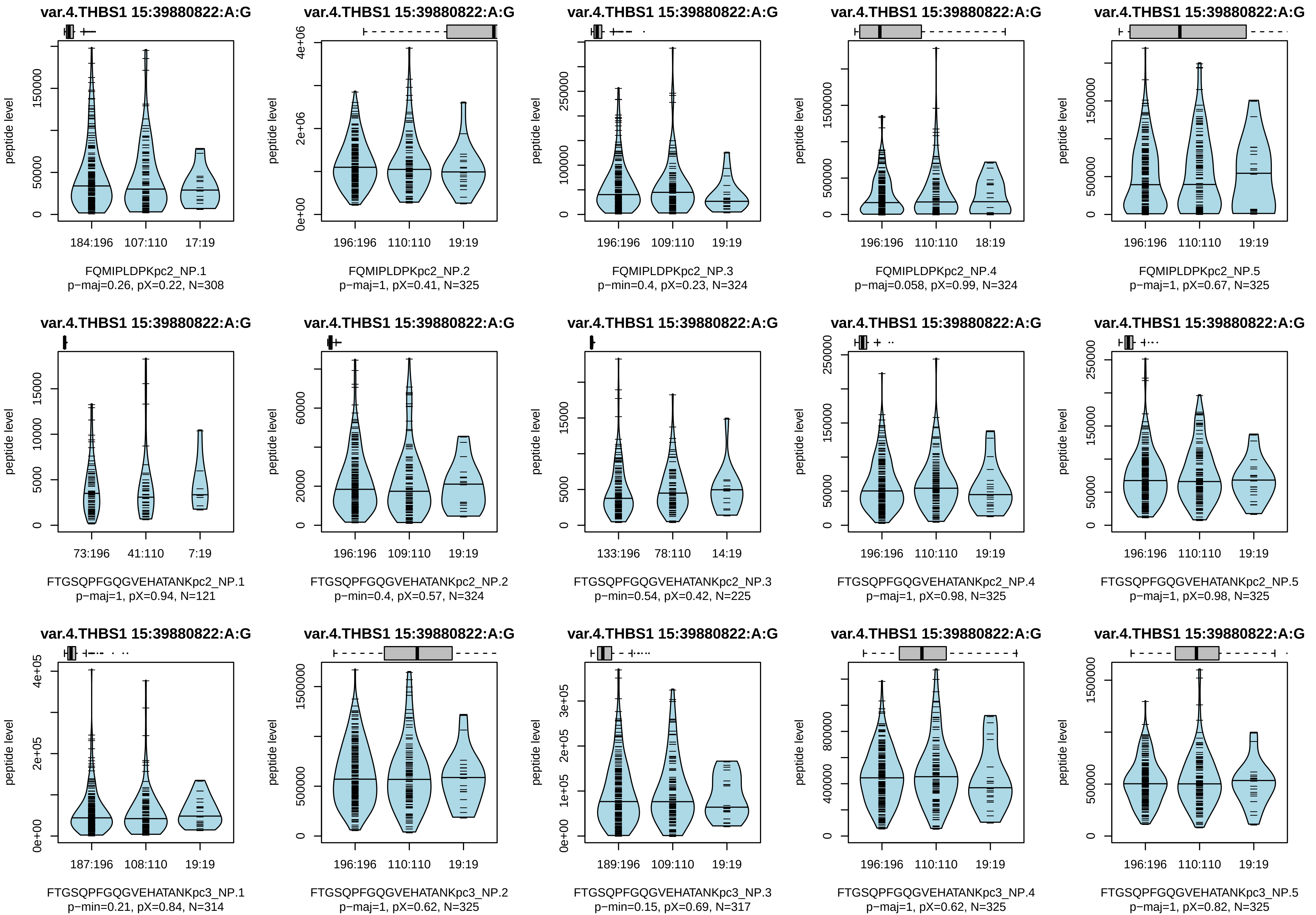
var.4.THBS1 15:39880822:A:G
peptide level
184:196
107:110
17:19
FQMIPLDPKpc2_NP.1
p−maj=0.26, pX=0.22, N=308
var.4.THBS1 15:39880822:A:G
peptide level
196:196
110:110
19:19
FQMIPLDPKpc2_NP.2
p−maj=1, pX=0.41, N=325
var.4.THBS1 15:39880822:A:G
peptide level
196:196
109:110
19:19
FQMIPLDPKpc2_NP.3
p−min=0.4, pX=0.23, N=324
var.4.THBS1 15:39880822:A:G
peptide level
196:196
110:110
18:19
FQMIPLDPKpc2_NP.4
p−maj=0.058, pX=0.99, N=324
var.4.THBS1 15:39880822:A:G
peptide level
196:196
110:110
19:19
FQMIPLDPKpc2_NP.5
p−maj=1, pX=0.67, N=325
var.4.THBS1 15:39880822:A:G
peptide level
73:196
41:110
7:19
FTGSQPFGQGVEHATANKpc2_NP.1
p−maj=1, pX=0.94, N=121
var.4.THBS1 15:39880822:A:G
peptide level
196:196
109:110
19:19
FTGSQPFGQGVEHATANKpc2_NP.2
p−min=0.4, pX=0.57, N=324
var.4.THBS1 15:39880822:A:G
peptide level
133:196
78:110
14:19
FTGSQPFGQGVEHATANKpc2_NP.3
p−min=0.54, pX=0.42, N=225
var.4.THBS1 15:39880822:A:G
peptide level
196:196
110:110
19:19
FTGSQPFGQGVEHATANKpc2_NP.4
p−maj=1, pX=0.98, N=325
var.4.THBS1 15:39880822:A:G
peptide level
196:196
110:110
19:19
FTGSQPFGQGVEHATANKpc2_NP.5
p−maj=1, pX=0.98, N=325
var.4.THBS1 15:39880822:A:G
peptide level
187:196
108:110
19:19
FTGSQPFGQGVEHATANKpc3_NP.1
p−min=0.21, pX=0.84, N=314
var.4.THBS1 15:39880822:A:G
peptide level
196:196
110:110
19:19
FTGSQPFGQGVEHATANKpc3_NP.2
p−maj=1, pX=0.62, N=325
var.4.THBS1 15:39880822:A:G
peptide level
189:196
109:110
19:19
FTGSQPFGQGVEHATANKpc3_NP.3
p−min=0.15, pX=0.69, N=317
var.4.THBS1 15:39880822:A:G
peptide level
196:196
110:110
19:19
FTGSQPFGQGVEHATANKpc3_NP.4
p−maj=1, pX=0.62, N=325
var.4.THBS1 15:39880822:A:G
peptide level
196:196
110:110
19:19
FTGSQPFGQGVEHATANKpc3_NP.5
p−maj=1, pX=0.82, N=325

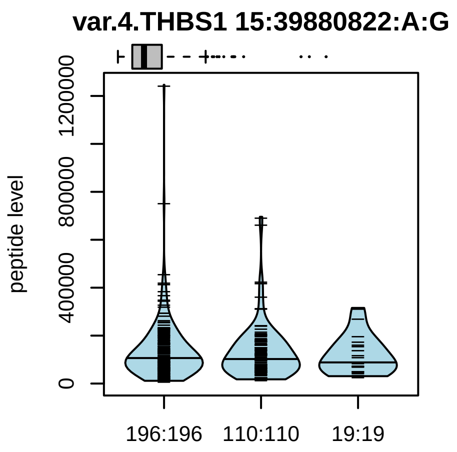

FVFGTTPEDILRpc2_NP.1
p−maj=1, pX=0.56, N=325

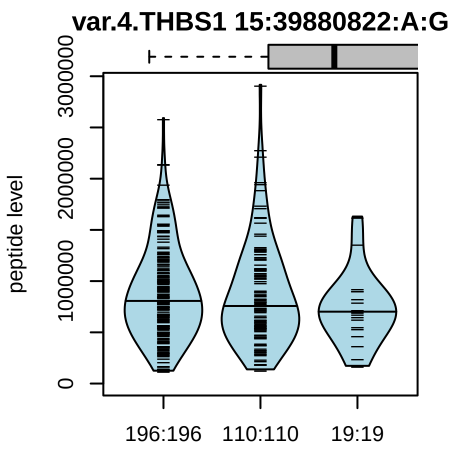

FVFGTTPEDILRpc2_NP.2
p−maj=1, pX=0.31, N=325

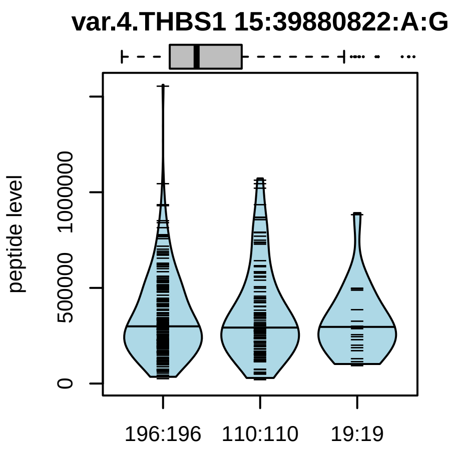

FVFGTTPEDILRpc2_NP.3
p−maj=1, pX=0.95, N=325

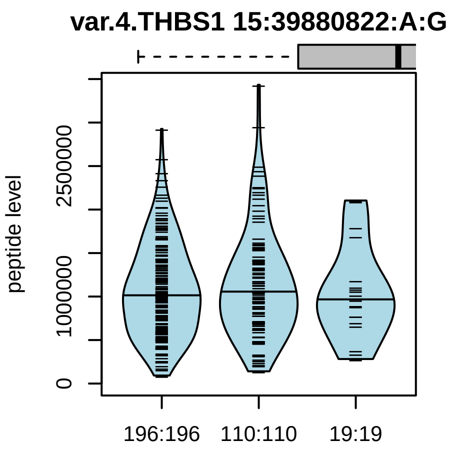

FVFGTTPEDILRpc2_NP.4
p−maj=1, pX=0.69, N=325

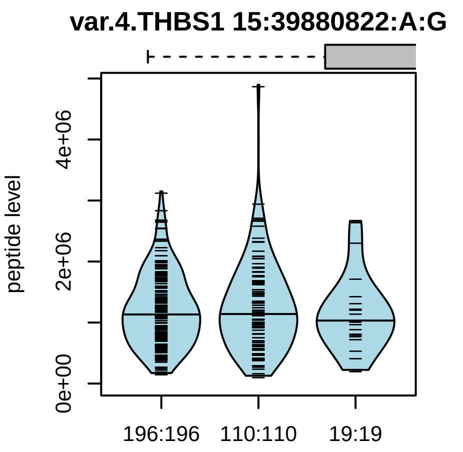

FVFGTTPEDILRpc2_NP.5
p−maj=1, pX=0.87, N=325

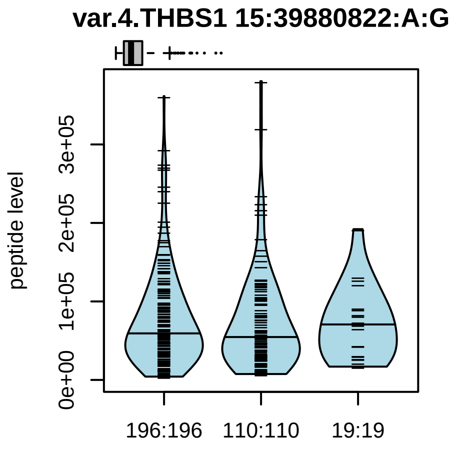

FYVVMWKpc2_NP.1
p−maj=1, pX=0.64, N=325

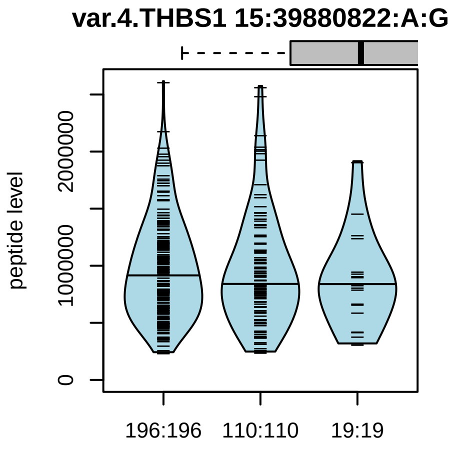

FYVVMWKpc2_NP.2
p−maj=1, pX=0.36, N=325

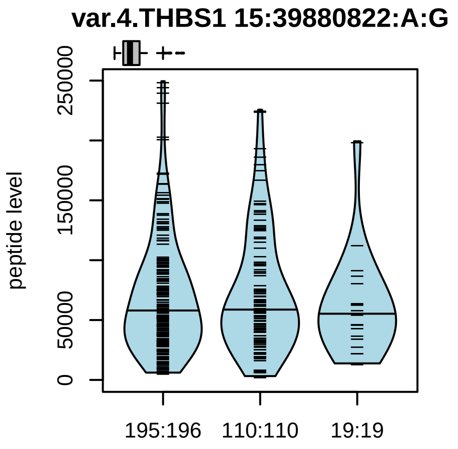

FYVVMWKpc2_NP.3
p−maj=1, pX=0.89, N=324

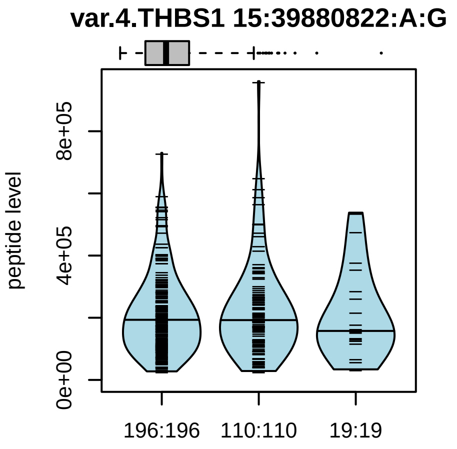

FYVVMWKpc2_NP.4
p−maj=1, pX=0.7, N=325

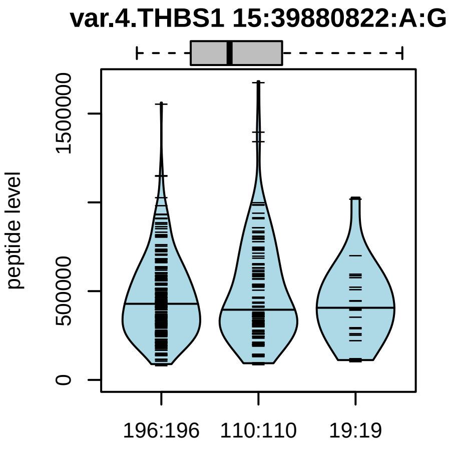

FYVVMWKpc2_NP.5
p−maj=1, pX=0.8, N=325

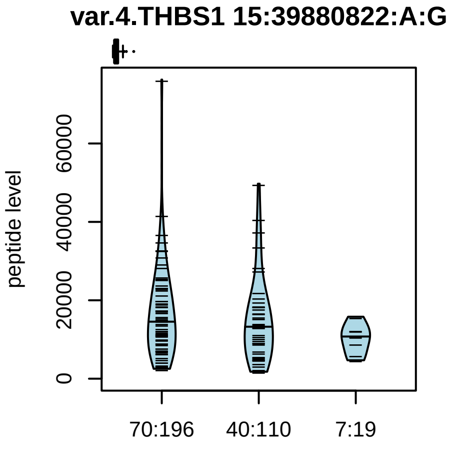

GDACDHDDDNDGIPDDKpc2_NP.4
p−min=0.91, pX=0.42, N=117

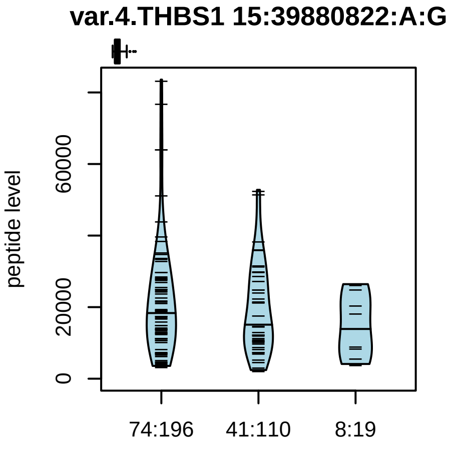

GDACDHDDDNDGIPDDKpc2_NP.5
p−maj=0.81, pX=0.4, N=123

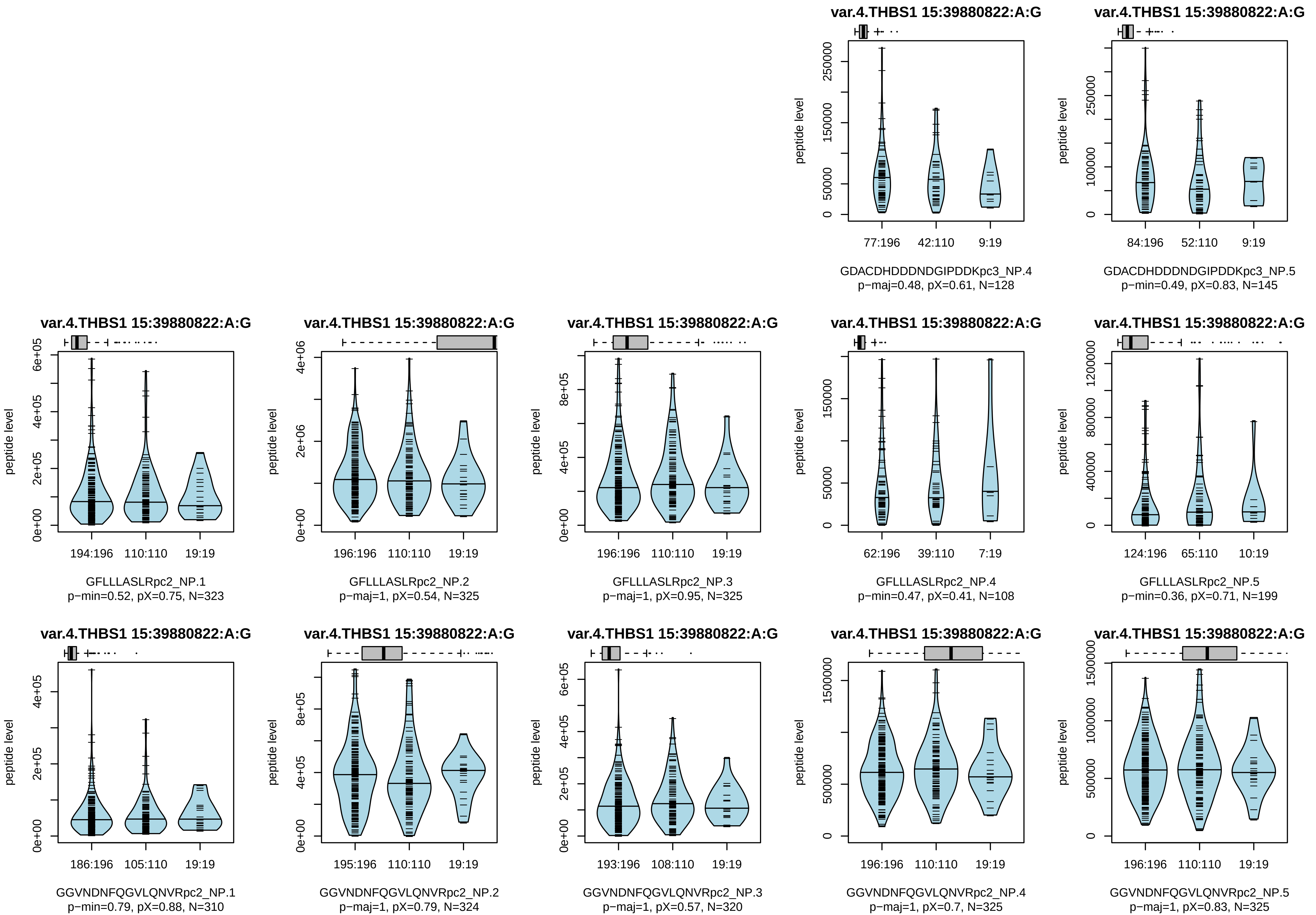

var.4.THBS1 15:39880822:A:G
peptide level
77:196
42:110
9:19
GDACDHDDDNDGIPDDKpc3_NP.4
p−maj=0.48, pX=0.61, N=128
var.4.THBS1 15:39880822:A:G
peptide level
84:196
52:110
9:19
GDACDHDDDNDGIPDDKpc3_NP.5
p−min=0.49, pX=0.83, N=145
var.4.THBS1 15:39880822:A:G
peptide level
194:196
110:110
19:19
GFLLLASLRpc2_NP.1
p−min=0.52, pX=0.75, N=323
var.4.THBS1 15:39880822:A:G
peptide level
196:196
110:110
19:19
GFLLLASLRpc2_NP.2
p−maj=1, pX=0.54, N=325
var.4.THBS1 15:39880822:A:G
peptide level
196:196
110:110
19:19
GFLLLASLRpc2_NP.3
p−maj=1, pX=0.95, N=325
var.4.THBS1 15:39880822:A:G
peptide level
62:196
39:110
7:19
GFLLLASLRpc2_NP.4
p−min=0.47, pX=0.41, N=108
var.4.THBS1 15:39880822:A:G
peptide level
124:196
65:110
10:19
GFLLLASLRpc2_NP.5
p−min=0.36, pX=0.71, N=199
var.4.THBS1 15:39880822:A:G
peptide level
186:196
105:110
19:19
GGVNDNFQGVLQNVRpc2_NP.1
p−min=0.79, pX=0.88, N=310
var.4.THBS1 15:39880822:A:G
peptide level
195:196
110:110
19:19
GGVNDNFQGVLQNVRpc2_NP.2
p−maj=1, pX=0.79, N=324
var.4.THBS1 15:39880822:A:G
peptide level
193:196
108:110
19:19
GGVNDNFQGVLQNVRpc2_NP.3
p−maj=1, pX=0.57, N=320
var.4.THBS1 15:39880822:A:G
peptide level
196:196
110:110
19:19
GGVNDNFQGVLQNVRpc2_NP.4
p−maj=1, pX=0.7, N=325
var.4.THBS1 15:39880822:A:G
peptide level
196:196
110:110
19:19
GGVNDNFQGVLQNVRpc2_NP.5
p−maj=1, pX=0.83, N=325

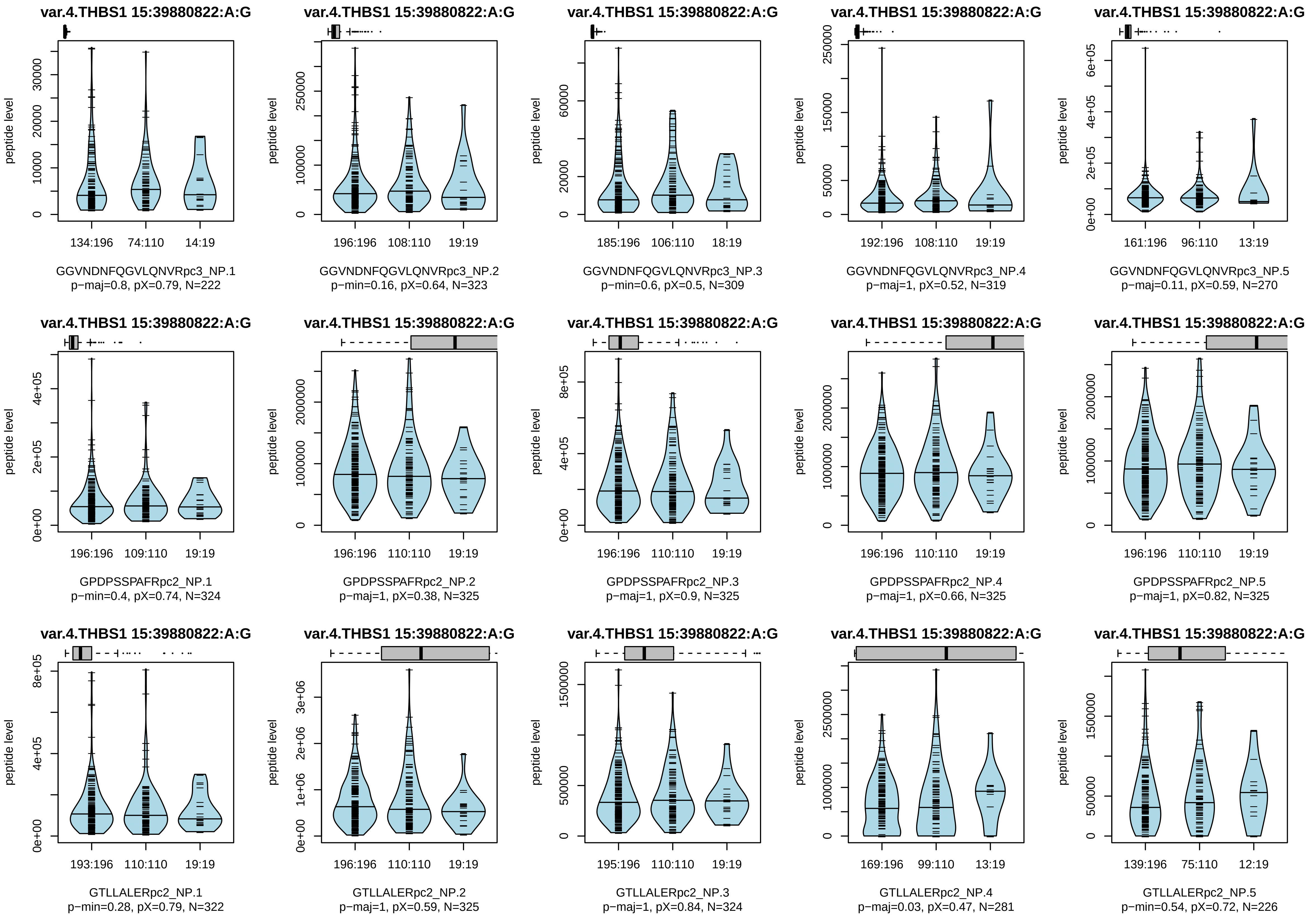

var.4.THBS1 15:39880822:A:G
peptide level
134:196
74:110
14:19
GGVNDNFQGVLQNVRpc3_NP.1
p−maj=0.8, pX=0.79, N=222
var.4.THBS1 15:39880822:A:G
peptide level
196:196
108:110
19:19
GGVNDNFQGVLQNVRpc3_NP.2
p−min=0.16, pX=0.64, N=323
var.4.THBS1 15:39880822:A:G
peptide level
185:196
106:110
18:19
GGVNDNFQGVLQNVRpc3_NP.3
p−min=0.6, pX=0.5, N=309
var.4.THBS1 15:39880822:A:G
peptide level
192:196
108:110
19:19
GGVNDNFQGVLQNVRpc3_NP.4
p−maj=1, pX=0.52, N=319
var.4.THBS1 15:39880822:A:G
peptide level
161:196
96:110
13:19
GGVNDNFQGVLQNVRpc3_NP.5
p−maj=0.11, pX=0.59, N=270
var.4.THBS1 15:39880822:A:G
peptide level
196:196
109:110
19:19
GPDPSSPAFRpc2_NP.1
p−min=0.4, pX=0.74, N=324
var.4.THBS1 15:39880822:A:G
peptide level
196:196
110:110
19:19
GPDPSSPAFRpc2_NP.2
p−maj=1, pX=0.38, N=325
var.4.THBS1 15:39880822:A:G
peptide level
196:196
110:110
19:19
GPDPSSPAFRpc2_NP.3
p−maj=1, pX=0.9, N=325
var.4.THBS1 15:39880822:A:G
peptide level
196:196
110:110
19:19
GPDPSSPAFRpc2_NP.4
p−maj=1, pX=0.66, N=325
var.4.THBS1 15:39880822:A:G
peptide level
196:196
110:110
19:19
GPDPSSPAFRpc2_NP.5
p−maj=1, pX=0.82, N=325
var.4.THBS1 15:39880822:A:G
peptide level
193:196
110:110
19:19
GTLLALERpc2_NP.1
p−min=0.28, pX=0.79, N=322
var.4.THBS1 15:39880822:A:G
peptide level
196:196
110:110
19:19
GTLLALERpc2_NP.2
p−maj=1, pX=0.59, N=325
var.4.THBS1 15:39880822:A:G
peptide level
195:196
110:110
19:19
GTLLALERpc2_NP.3
p−maj=1, pX=0.84, N=324
var.4.THBS1 15:39880822:A:G
peptide level
169:196
99:110
13:19
GTLLALERpc2_NP.4
p−maj=0.03, pX=0.47, N=281
var.4.THBS1 15:39880822:A:G
peptide level
139:196
75:110
12:19
GTLLALERpc2_NP.5
p−min=0.54, pX=0.72, N=226

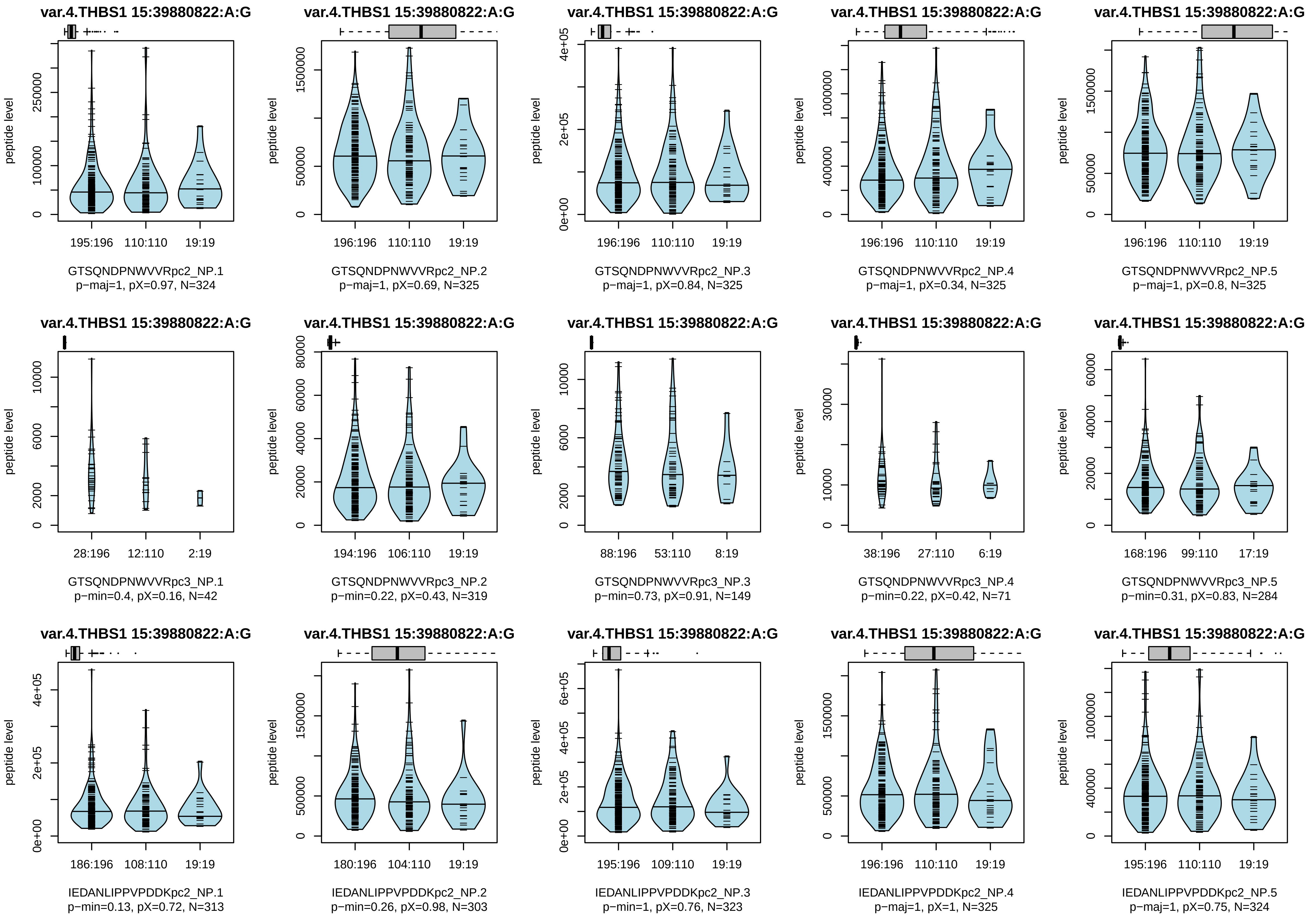

var.4.THBS1 15:39880822:A:G
peptide level
195:196 110:110 19:19
GTSQNDPNWVVRpc2_NP.1
p−maj=1, pX=0.97, N=324
var.4.THBS1 15:39880822:A:G
peptide level
196:196 110:110 19:19
GTSQNDPNWVVRpc2_NP.2
p−maj=1, pX=0.69, N=325
var.4.THBS1 15:39880822:A:G
peptide level
196:196 110:110 19:19
GTSQNDPNWVVRpc2_NP.3
p−maj=1, pX=0.84, N=325
var.4.THBS1 15:39880822:A:G
peptide level
196:196 110:110 19:19
GTSQNDPNWVVRpc2_NP.4
p−maj=1, pX=0.34, N=325
var.4.THBS1 15:39880822:A:G
peptide level
196:196 110:110 19:19
GTSQNDPNWVVRpc2_NP.5
p−maj=1, pX=0.8, N=325
var.4.THBS1 15:39880822:A:G
peptide level
28:196 12:110 2:19
GTSQNDPNWVVRpc3_NP.1
p−min=0.4, pX=0.16, N=42
var.4.THBS1 15:39880822:A:G
peptide level
194:196 106:110 19:19
GTSQNDPNWVVRpc3_NP.2
p−min=0.22, pX=0.43, N=319
var.4.THBS1 15:39880822:A:G
peptide level
88:196 53:110 8:19
GTSQNDPNWVVRpc3_NP.3
p−min=0.73, pX=0.91, N=149
var.4.THBS1 15:39880822:A:G
peptide level
38:196 27:110 6:19
GTSQNDPNWVVRpc3_NP.4
p−min=0.22, pX=0.42, N=71
var.4.THBS1 15:39880822:A:G
peptide level
168:196 99:110 17:19
GTSQNDPNWVVRpc3_NP.5
p−min=0.31, pX=0.83, N=284
var.4.THBS1 15:39880822:A:G
peptide level
186:196 108:110 19:19
IEDANLIPPVPDDKpc2_NP.1
p−min=0.13, pX=0.72, N=313
var.4.THBS1 15:39880822:A:G
peptide level
180:196 104:110 19:19
IEDANLIPPVPDDKpc2_NP.2
p−min=0.26, pX=0.98, N=303
var.4.THBS1 15:39880822:A:G
peptide level
195:196 109:110 19:19
IEDANLIPPVPDDKpc2_NP.3
p−min=1, pX=0.76, N=323
var.4.THBS1 15:39880822:A:G
peptide level
196:196 110:110 19:19
IEDANLIPPVPDDKpc2_NP.4
p−maj=1, pX=1, N=325
var.4.THBS1 15:39880822:A:G
peptide level
195:196 110:110 19:19
IEDANLIPPVPDDKpc2_NP.5
p−maj=1, pX=0.75, N=324

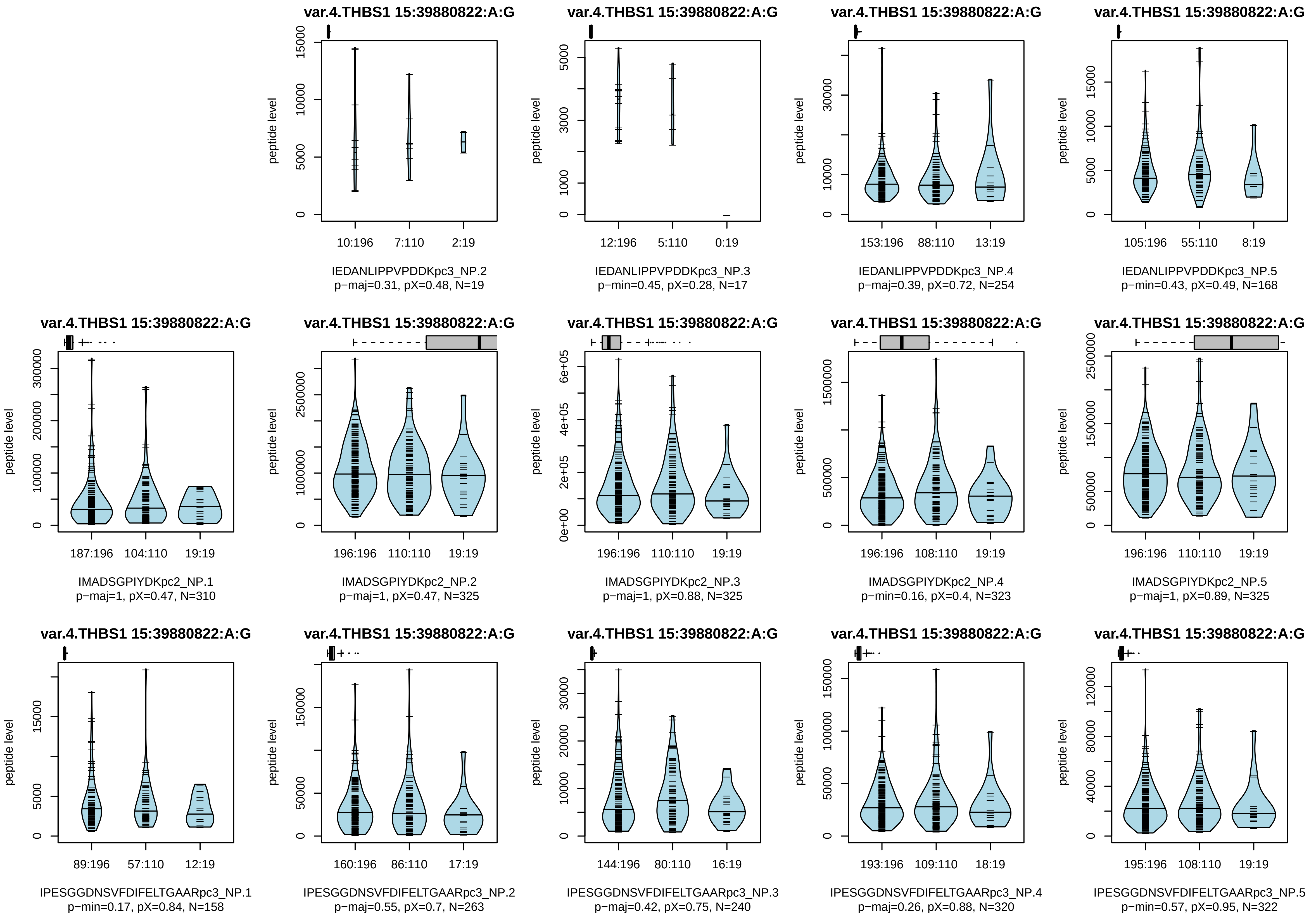
var.4.THBS1 15:39880822:A:G
peptide level
10:196
7:110
2:19
IEDANLIPPVPDDKpc3_NP.2
p−maj=0.31, pX=0.48, N=19
var.4.THBS1 15:39880822:A:G
peptide level
12:196
5:110
0:19
IEDANLIPPVPDDKpc3_NP.3
p−min=0.45, pX=0.28, N=17
var.4.THBS1 15:39880822:A:G
peptide level
153:196
88:110
13:19
IEDANLIPPVPDDKpc3_NP.4
p−maj=0.39, pX=0.72, N=254
var.4.THBS1 15:39880822:A:G
peptide level
105:196
55:110
8:19
IEDANLIPPVPDDKpc3_NP.5
p−min=0.43, pX=0.49, N=168
var.4.THBS1 15:39880822:A:G
peptide level
187:196
104:110
19:19
IMADSGPIYDKpc2_NP.1
p−maj=1, pX=0.47, N=310
var.4.THBS1 15:39880822:A:G
peptide level
196:196
110:110
19:19
IMADSGPIYDKpc2_NP.2
p−maj=1, pX=0.47, N=325
var.4.THBS1 15:39880822:A:G
peptide level
196:196
110:110
19:19
IMADSGPIYDKpc2_NP.3
p−maj=1, pX=0.88, N=325
var.4.THBS1 15:39880822:A:G
peptide level
196:196
108:110
19:19
IMADSGPIYDKpc2_NP.4
p−min=0.16, pX=0.4, N=323
var.4.THBS1 15:39880822:A:G
peptide level
196:196
110:110
19:19
IMADSGPIYDKpc2_NP.5
p−maj=1, pX=0.89, N=325
var.4.THBS1 15:39880822:A:G
peptide level
89:196
57:110
12:19
IPESGGDNSVFDIFELTGAARpc3_NP.1
p−min=0.17, pX=0.84, N=158
var.4.THBS1 15:39880822:A:G
peptide level
160:196
86:110
17:19
IPESGGDNSVFDIFELTGAARpc3_NP.2
p−maj=0.55, pX=0.7, N=263
var.4.THBS1 15:39880822:A:G
peptide level
144:196
80:110
16:19
IPESGGDNSVFDIFELTGAARpc3_NP.3
p−maj=0.42, pX=0.75, N=240
var.4.THBS1 15:39880822:A:G
peptide level
193:196
109:110
18:19
IPESGGDNSVFDIFELTGAARpc3_NP.4
p−maj=0.26, pX=0.88, N=320
var.4.THBS1 15:39880822:A:G
peptide level
195:196
108:110
19:19
IPESGGDNSVFDIFELTGAARpc3_NP.5
p−min=0.57, pX=0.95, N=322

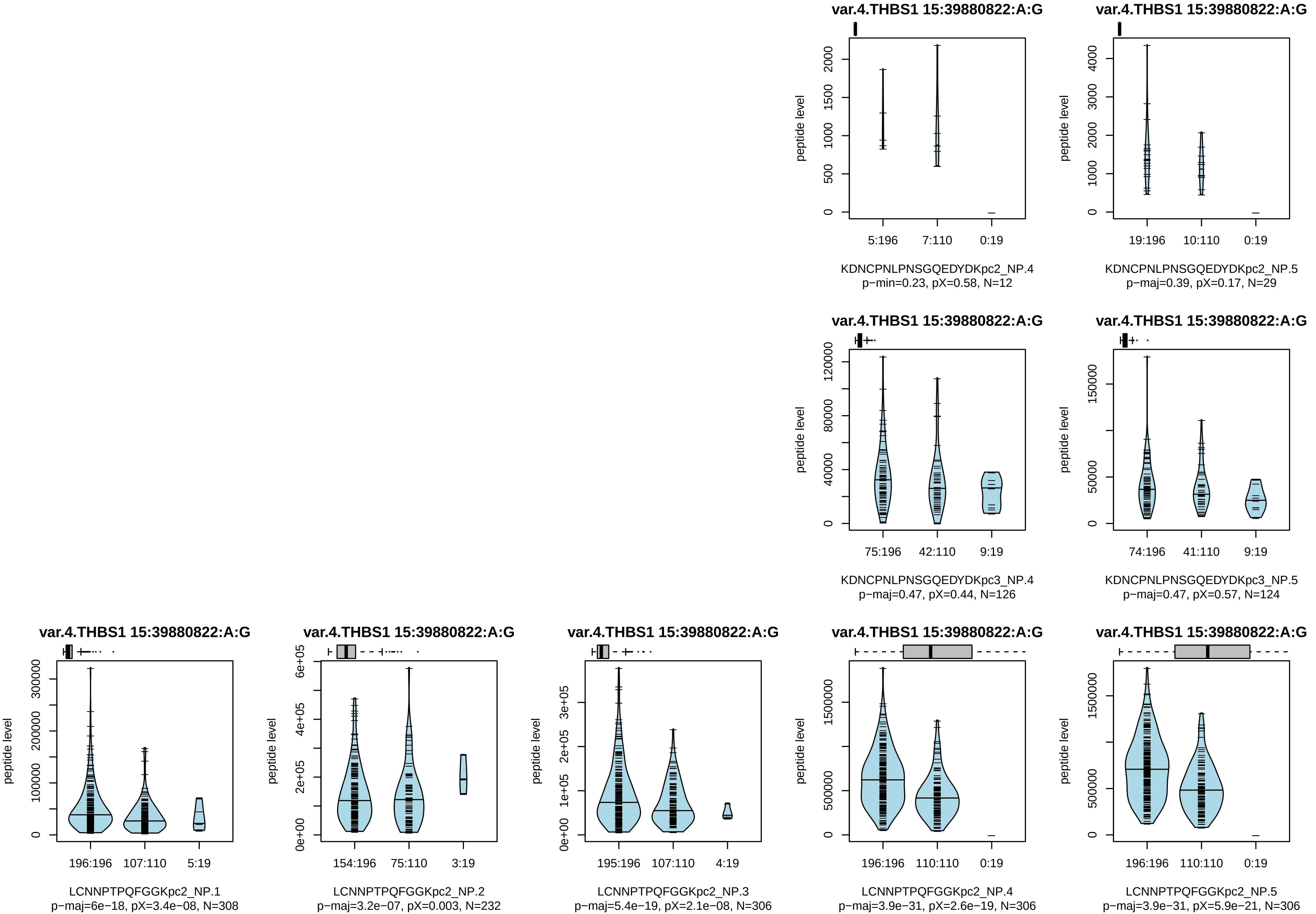
var.4.THBS1 15:39880822:A:G
peptide level
5:196
7:110
0:19
KDNCPNLPNSGQEDYDKpc2_NP.4
p−min=0.23, pX=0.58, N=12
var.4.THBS1 15:39880822:A:G
peptide level
19:196
10:110
0:19
KDNCPNLPNSGQEDYDKpc2_NP.5
p−maj=0.39, pX=0.17, N=29
var.4.THBS1 15:39880822:A:G
peptide level
75:196
42:110
9:19
KDNCPNLPNSGQEDYDKpc3_NP.4
p−maj=0.47, pX=0.44, N=126
var.4.THBS1 15:39880822:A:G
peptide level
74:196
41:110
9:19
KDNCPNLPNSGQEDYDKpc3_NP.5
p−maj=0.47, pX=0.57, N=124
var.4.THBS1 15:39880822:A:G
peptide level
196:196
107:110
5:19
LCNNPTPQFGGKpc2_NP.1
p−maj=6e−18, pX=3.4e−08, N=308
var.4.THBS1 15:39880822:A:G
peptide level
154:196
75:110
3:19
LCNNPTPQFGGKpc2_NP.2
p−maj=3.2e−07, pX=0.003, N=232
var.4.THBS1 15:39880822:A:G
peptide level
195:196
107:110
4:19
LCNNPTPQFGGKpc2_NP.3
p−maj=5.4e−19, pX=2.1e−08, N=306
var.4.THBS1 15:39880822:A:G
peptide level
196:196
110:110
0:19
LCNNPTPQFGGKpc2_NP.4
p−maj=3.9e−31, pX=2.6e−19, N=306
var.4.THBS1 15:39880822:A:G
peptide level
196:196
110:110
0:19
LCNNPTPQFGGKpc2_NP.5
p−maj=3.9e−31, pX=5.9e−21, N=306

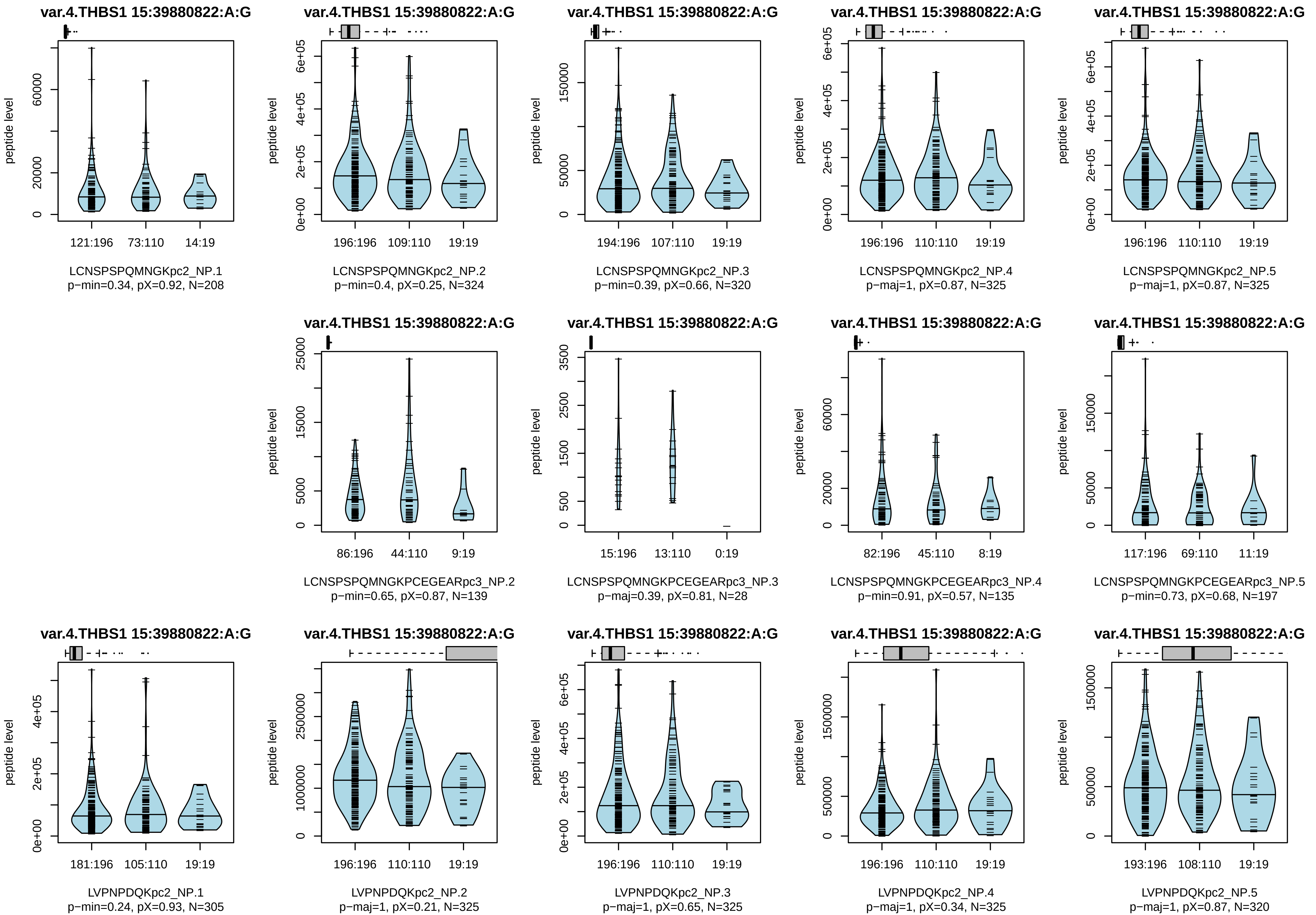
var.4.THBS1 15:39880822:A:G
peptide level
121:196
73:110
14:19
LCNSPSPQMNGKpc2_NP.1
p−min=0.34, pX=0.92, N=208
var.4.THBS1 15:39880822:A:G
peptide level
196:196
109:110
19:19
LCNSPSPQMNGKpc2_NP.2
p−min=0.4, pX=0.25, N=324
var.4.THBS1 15:39880822:A:G
peptide level
194:196
107:110
19:19
LCNSPSPQMNGKpc2_NP.3
p−min=0.39, pX=0.66, N=320
var.4.THBS1 15:39880822:A:G
peptide level
196:196
110:110
19:19
LCNSPSPQMNGKpc2_NP.4
p−maj=1, pX=0.87, N=325
var.4.THBS1 15:39880822:A:G
peptide level
196:196
110:110
19:19
LCNSPSPQMNGKpc2_NP.5
p−maj=1, pX=0.87, N=325
var.4.THBS1 15:39880822:A:G
peptide level
86:196
44:110
9:19
LCNSPSPQMNGKPCEGEARpc3_NP.2
p−min=0.65, pX=0.87, N=139
var.4.THBS1 15:39880822:A:G
peptide level
15:196
13:110
0:19
LCNSPSPQMNGKPCEGEARpc3_NP.3
p−maj=0.39, pX=0.81, N=28
var.4.THBS1 15:39880822:A:G
peptide level
82:196
45:110
8:19
LCNSPSPQMNGKPCEGEARpc3_NP.4
p−min=0.91, pX=0.57, N=135
var.4.THBS1 15:39880822:A:G
peptide level
117:196
69:110
11:19
LCNSPSPQMNGKPCEGEARpc3_NP.5
p−min=0.73, pX=0.68, N=197
var.4.THBS1 15:39880822:A:G
peptide level
181:196
105:110
19:19
LVPNPDQKpc2_NP.1
p−min=0.24, pX=0.93, N=305
var.4.THBS1 15:39880822:A:G
peptide level
196:196
110:110
19:19
LVPNPDQKpc2_NP.2
p−maj=1, pX=0.21, N=325
var.4.THBS1 15:39880822:A:G
peptide level
196:196
110:110
19:19
LVPNPDQKpc2_NP.3
p−maj=1, pX=0.65, N=325
var.4.THBS1 15:39880822:A:G
peptide level
196:196
110:110
19:19
LVPNPDQKpc2_NP.4
p−maj=1, pX=0.34, N=325
var.4.THBS1 15:39880822:A:G
peptide level
193:196
108:110
19:19
LVPNPDQKpc2_NP.5
p−maj=1, pX=0.87, N=320

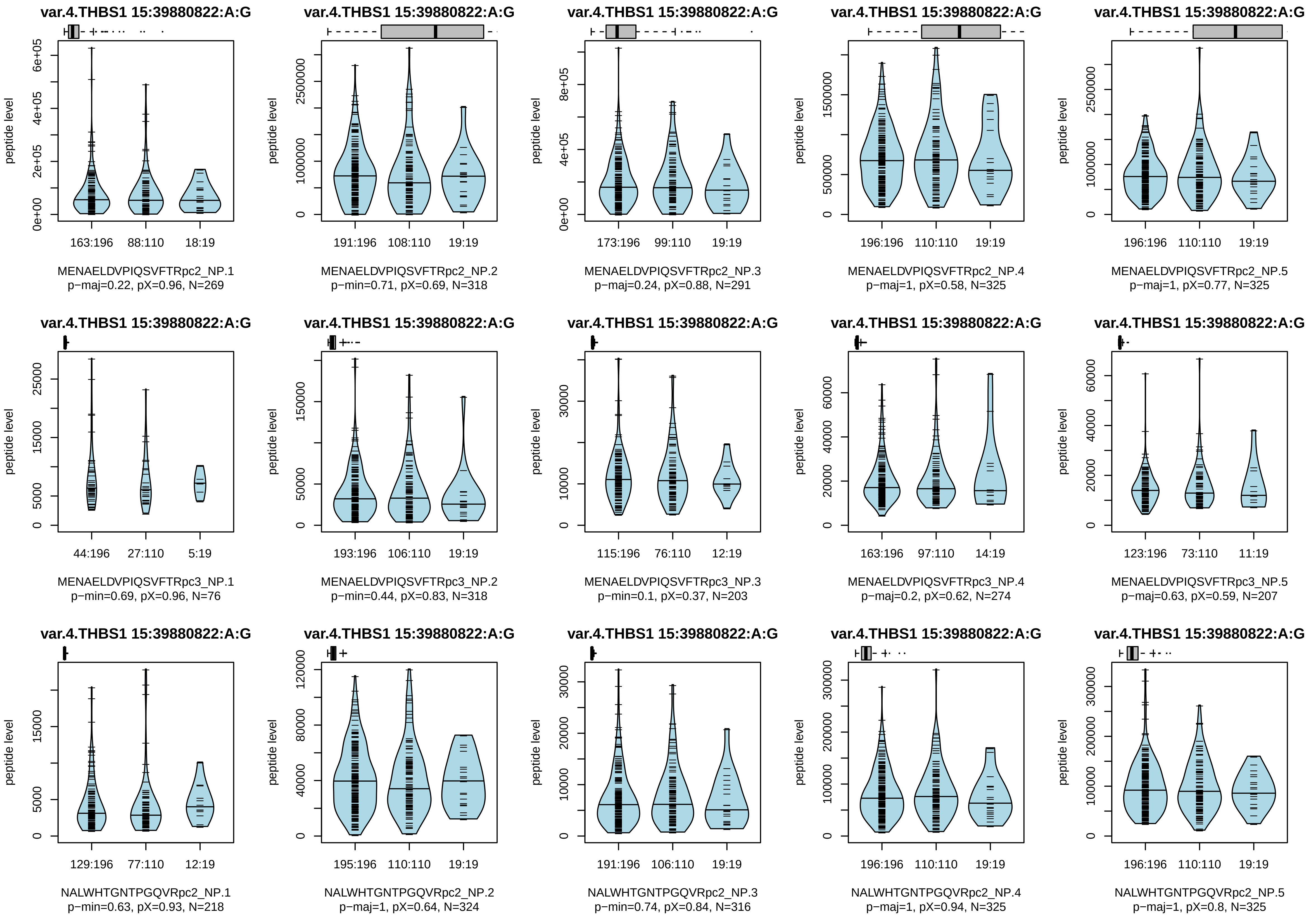
var.4.THBS1 15:39880822:A:G
peptide level
163:196
88:110
18:19
MENAELDVPIQSVFTRpc2_NP.1
p−maj=0.22, pX=0.96, N=269
var.4.THBS1 15:39880822:A:G
peptide level
191:196
108:110
19:19
MENAELDVPIQSVFTRpc2_NP.2
p−min=0.71, pX=0.69, N=318
var.4.THBS1 15:39880822:A:G
peptide level
173:196
99:110
19:19
MENAELDVPIQSVFTRpc2_NP.3
p−maj=0.24, pX=0.88, N=291
var.4.THBS1 15:39880822:A:G
peptide level
196:196
110:110
19:19
MENAELDVPIQSVFTRpc2_NP.4
p−maj=1, pX=0.58, N=325
var.4.THBS1 15:39880822:A:G
peptide level
196:196
110:110
19:19
MENAELDVPIQSVFTRpc2_NP.5
p−maj=1, pX=0.77, N=325
var.4.THBS1 15:39880822:A:G
peptide level
44:196
27:110
5:19
MENAELDVPIQSVFTRpc3_NP.1
p−min=0.69, pX=0.96, N=76
var.4.THBS1 15:39880822:A:G
peptide level
193:196
106:110
19:19
MENAELDVPIQSVFTRpc3_NP.2
p−min=0.44, pX=0.83, N=318
var.4.THBS1 15:39880822:A:G
peptide level
115:196
76:110
12:19
MENAELDVPIQSVFTRpc3_NP.3
p−min=0.1, pX=0.37, N=203
var.4.THBS1 15:39880822:A:G
peptide level
163:196
97:110
14:19
MENAELDVPIQSVFTRpc3_NP.4
p−maj=0.2, pX=0.62, N=274
var.4.THBS1 15:39880822:A:G
peptide level
123:196
73:110
11:19
MENAELDVPIQSVFTRpc3_NP.5
p−maj=0.63, pX=0.59, N=207
var.4.THBS1 15:39880822:A:G
peptide level
129:196
77:110
12:19
NALWHTGNTPGQVRpc2_NP.1
p−min=0.63, pX=0.93, N=218
var.4.THBS1 15:39880822:A:G
peptide level
195:196
110:110
19:19
NALWHTGNTPGQVRpc2_NP.2
p−maj=1, pX=0.64, N=324
var.4.THBS1 15:39880822:A:G
peptide level
191:196
106:110
19:19
NALWHTGNTPGQVRpc2_NP.3
p−min=0.74, pX=0.84, N=316
var.4.THBS1 15:39880822:A:G
peptide level
196:196
110:110
19:19
NALWHTGNTPGQVRpc2_NP.4
p−maj=1, pX=0.94, N=325
var.4.THBS1 15:39880822:A:G
peptide level
196:196
110:110
19:19
NALWHTGNTPGQVRpc2_NP.5
p−maj=1, pX=0.8, N=325

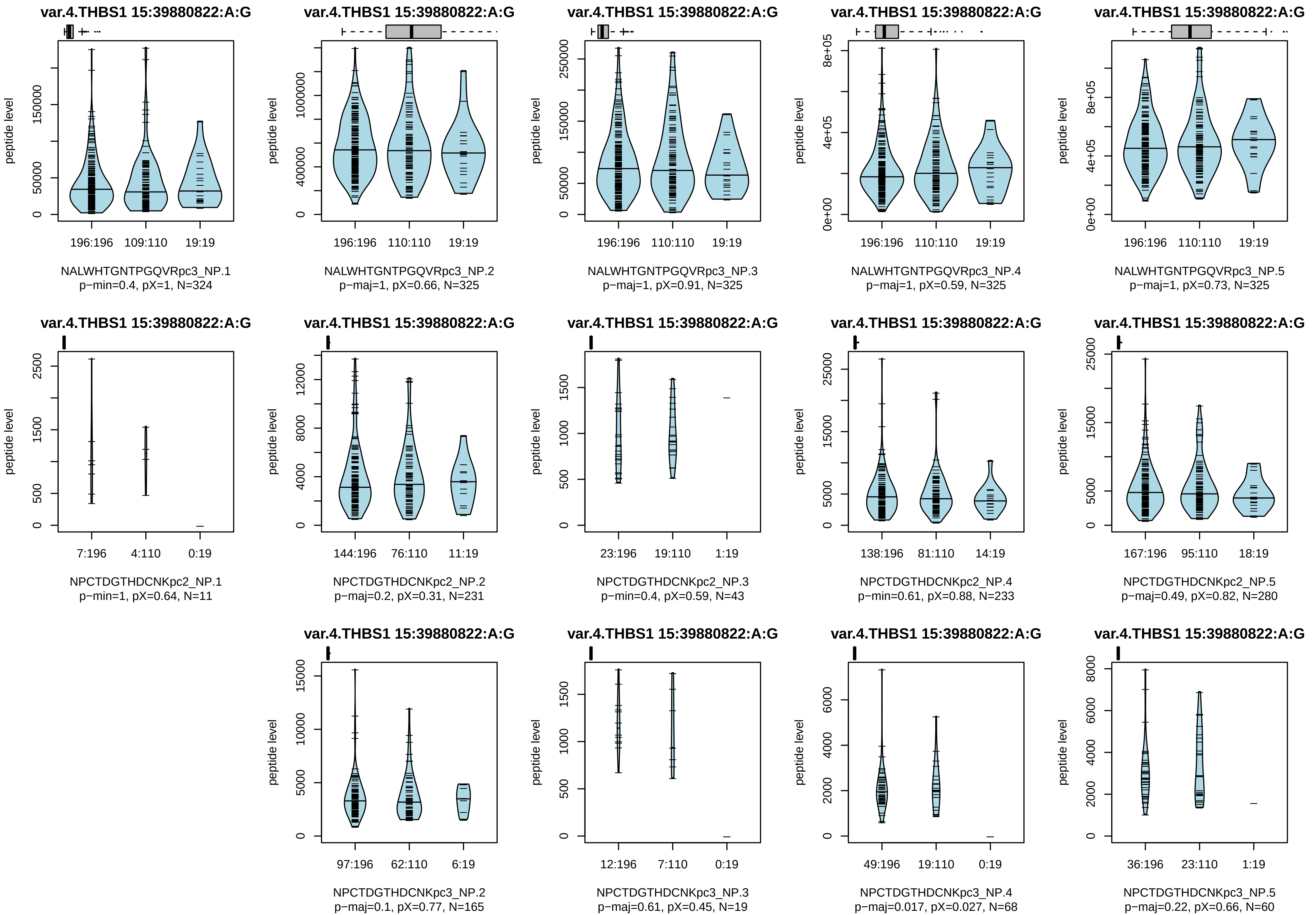
var.4.THBS1 15:39880822:A:G
peptide level
196:196
109:110
19:19
NALWHTGNTPGQVRpc3_NP.1
p−min=0.4, pX=1, N=324
var.4.THBS1 15:39880822:A:G
peptide level
196:196
110:110
19:19
NALWHTGNTPGQVRpc3_NP.2
p−maj=1, pX=0.66, N=325
var.4.THBS1 15:39880822:A:G
peptide level
196:196
110:110
19:19
NALWHTGNTPGQVRpc3_NP.3
p−maj=1, pX=0.91, N=325
var.4.THBS1 15:39880822:A:G
peptide level
196:196
110:110
19:19
NALWHTGNTPGQVRpc3_NP.4
p−maj=1, pX=0.59, N=325
var.4.THBS1 15:39880822:A:G
peptide level
196:196
110:110
19:19
NALWHTGNTPGQVRpc3_NP.5
p−maj=1, pX=0.73, N=325
var.4.THBS1 15:39880822:A:G
peptide level
7:196
4:110
0:19
NPCTDGTHDCNKpc2_NP.1
p−min=1, pX=0.64, N=11
var.4.THBS1 15:39880822:A:G
peptide level
144:196
76:110
11:19
NPCTDGTHDCNKpc2_NP.2
p−maj=0.2, pX=0.31, N=231
var.4.THBS1 15:39880822:A:G
peptide level
23:196
19:110
1:19
NPCTDGTHDCNKpc2_NP.3
p−min=0.4, pX=0.59, N=43
var.4.THBS1 15:39880822:A:G
peptide level
138:196
81:110
14:19
NPCTDGTHDCNKpc2_NP.4
p−min=0.61, pX=0.88, N=233
var.4.THBS1 15:39880822:A:G
peptide level
167:196
95:110
18:19
NPCTDGTHDCNKpc2_NP.5
p−maj=0.49, pX=0.82, N=280
var.4.THBS1 15:39880822:A:G
peptide level
97:196
62:110
6:19
NPCTDGTHDCNKpc3_NP.2
p−maj=0.1, pX=0.77, N=165
var.4.THBS1 15:39880822:A:G
peptide level
12:196
7:110
0:19
NPCTDGTHDCNKpc3_NP.3
p−maj=0.61, pX=0.45, N=19
var.4.THBS1 15:39880822:A:G
peptide level
49:196
19:110
0:19
NPCTDGTHDCNKpc3_NP.4
p−maj=0.017, pX=0.027, N=68
var.4.THBS1 15:39880822:A:G
peptide level
36:196
23:110
1:19
NPCTDGTHDCNKpc3_NP.5
p−maj=0.22, pX=0.66, N=60

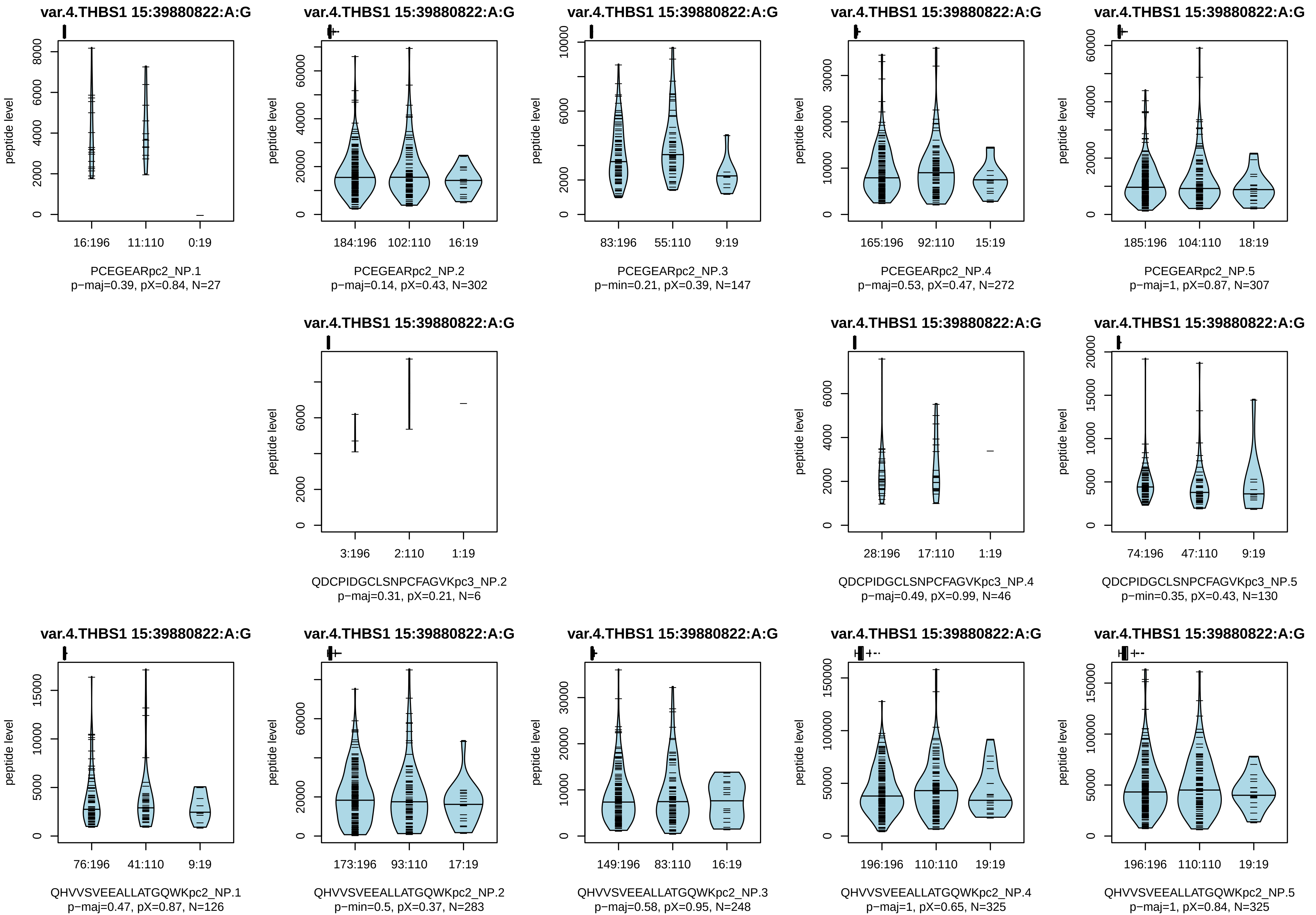
var.4.THBS1 15:39880822:A:G
peptide level
16:196
11:110
0:19
PCEGEARpc2_NP.1
p−maj=0.39, pX=0.84, N=27
var.4.THBS1 15:39880822:A:G
peptide level
184:196
102:110
16:19
PCEGEARpc2_NP.2
p−maj=0.14, pX=0.43, N=302
var.4.THBS1 15:39880822:A:G
peptide level
83:196
55:110
9:19
PCEGEARpc2_NP.3
p−min=0.21, pX=0.39, N=147
var.4.THBS1 15:39880822:A:G
peptide level
165:196
92:110
15:19
PCEGEARpc2_NP.4
p−maj=0.53, pX=0.47, N=272
var.4.THBS1 15:39880822:A:G
peptide level
185:196
104:110
18:19
PCEGEARpc2_NP.5
p−maj=1, pX=0.87, N=307
var.4.THBS1 15:39880822:A:G
peptide level
3:196
2:110
1:19
QDCPIDGCLSNPCFAGVKpc3_NP.2
p−maj=0.31, pX=0.21, N=6
var.4.THBS1 15:39880822:A:G
peptide level
28:196
17:110
1:19
QDCPIDGCLSNPCFAGVKpc3_NP.4
p−maj=0.49, pX=0.99, N=46
var.4.THBS1 15:39880822:A:G
peptide level
74:196
47:110
9:19
QDCPIDGCLSNPCFAGVKpc3_NP.5
p−min=0.35, pX=0.43, N=130
var.4.THBS1 15:39880822:A:G
peptide level
76:196
41:110
9:19
QHVVSVEEALLATGQWKpc2_NP.1
p−maj=0.47, pX=0.87, N=126
var.4.THBS1 15:39880822:A:G
peptide level
173:196
93:110
17:19
QHVVSVEEALLATGQWKpc2_NP.2
p−min=0.5, pX=0.37, N=283
var.4.THBS1 15:39880822:A:G
peptide level
149:196
83:110
16:19
QHVVSVEEALLATGQWKpc2_NP.3
p−maj=0.58, pX=0.95, N=248
var.4.THBS1 15:39880822:A:G
peptide level
196:196
110:110
19:19
QHVVSVEEALLATGQWKpc2_NP.4
p−maj=1, pX=0.65, N=325
var.4.THBS1 15:39880822:A:G
peptide level
196:196
110:110
19:19
QHVVSVEEALLATGQWKpc2_NP.5
p−maj=1, pX=0.84, N=325

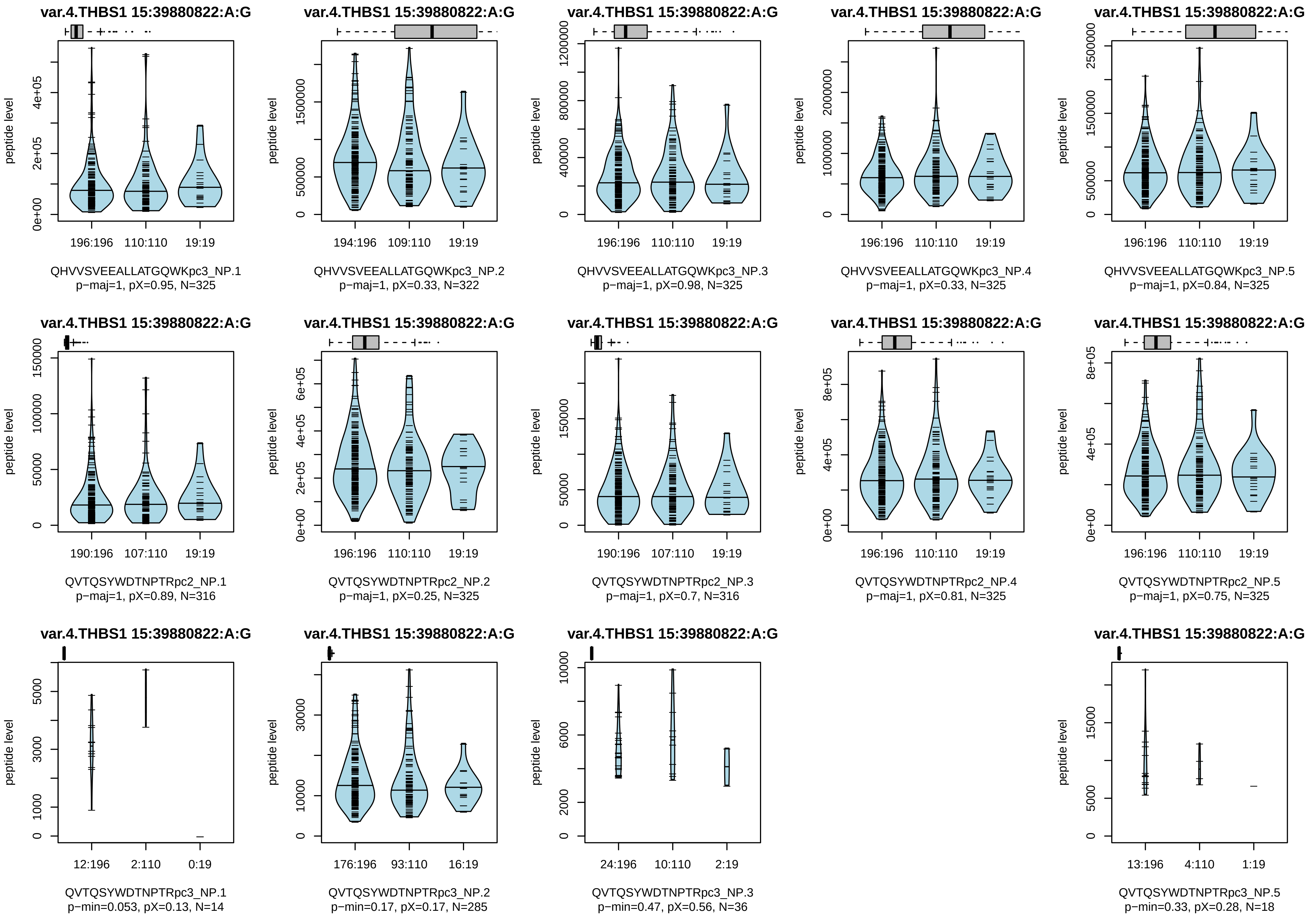
var.4.THBS1 15:39880822:A:G
peptide level
0e+00
2e+05
4e+05
196:196
110:110
19:19
QHVVSVEEALLATGQWKpc3_NP.1
p−maj=1, pX=0.95, N=325
var.4.THBS1 15:39880822:A:G
peptide level
0
500000
1500000
194:196
109:110
19:19
QHVVSVEEALLATGQWKpc3_NP.2
p−maj=1, pX=0.33, N=322
var.4.THBS1 15:39880822:A:G
peptide level
0
400000
800000
1200000
196:196
110:110
19:19
QHVVSVEEALLATGQWKpc3_NP.3
p−maj=1, pX=0.98, N=325
var.4.THBS1 15:39880822:A:G
peptide level
0
1000000
2000000
196:196
110:110
19:19
QHVVSVEEALLATGQWKpc3_NP.4
p−maj=1, pX=0.33, N=325
var.4.THBS1 15:39880822:A:G
peptide level
0
500000
1500000
2500000
196:196
110:110
19:19
QHVVSVEEALLATGQWKpc3_NP.5
p−maj=1, pX=0.84, N=325
var.4.THBS1 15:39880822:A:G
peptide level
0
50000
100000
150000
190:196
107:110
19:19
QVTQSYWDTNPTRpc2_NP.1
p−maj=1, pX=0.89, N=316
var.4.THBS1 15:39880822:A:G
peptide level
0e+00
2e+05
4e+05
6e+05
196:196
110:110
19:19
QVTQSYWDTNPTRpc2_NP.2
p−maj=1, pX=0.25, N=325
var.4.THBS1 15:39880822:A:G
peptide level
0
50000
150000
190:196
107:110
19:19
QVTQSYWDTNPTRpc2_NP.3
p−maj=1, pX=0.7, N=316
var.4.THBS1 15:39880822:A:G
peptide level
0e+00
4e+05
8e+05
196:196
110:110
19:19
QVTQSYWDTNPTRpc2_NP.4
p−maj=1, pX=0.81, N=325
var.4.THBS1 15:39880822:A:G
peptide level
0e+00
4e+05
8e+05
196:196
110:110
19:19
QVTQSYWDTNPTRpc2_NP.5
p−maj=1, pX=0.75, N=325
var.4.THBS1 15:39880822:A:G
peptide level
0
1000
3000
5000
12:196
2:110
0:19
QVTQSYWDTNPTRpc3_NP.1
p−min=0.053, pX=0.13, N=14
var.4.THBS1 15:39880822:A:G
peptide level
0
10000
30000
176:196
93:110
16:19
QVTQSYWDTNPTRpc3_NP.2
p−min=0.17, pX=0.17, N=285
var.4.THBS1 15:39880822:A:G
peptide level
0
2000
6000
10000
24:196
10:110
2:19
QVTQSYWDTNPTRpc3_NP.3
p−min=0.47, pX=0.56, N=36
var.4.THBS1 15:39880822:A:G
peptide level
0
5000
15000
13:196
4:110
1:19
QVTQSYWDTNPTRpc3_NP.5
p−min=0.33, pX=0.28, N=18

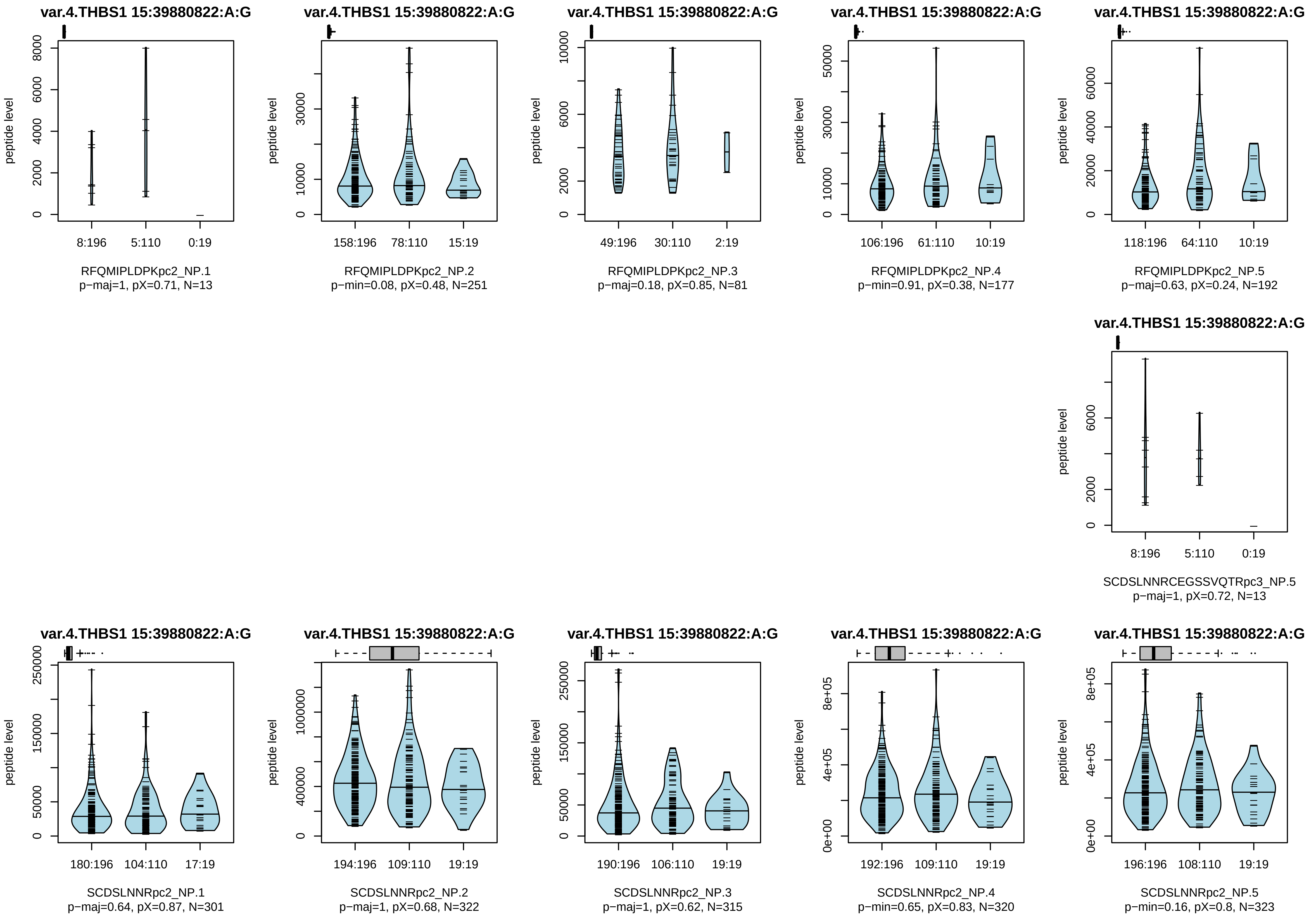
var.4.THBS1 15:39880822:A:G
peptide level
8:196
5:110
0:19
RFQMIPLDPKpc2_NP.1
p−maj=1, pX=0.71, N=13
var.4.THBS1 15:39880822:A:G
peptide level
158:196
78:110
15:19
RFQMIPLDPKpc2_NP.2
p−min=0.08, pX=0.48, N=251
var.4.THBS1 15:39880822:A:G
peptide level
49:196
30:110
2:19
RFQMIPLDPKpc2_NP.3
p−maj=0.18, pX=0.85, N=81
var.4.THBS1 15:39880822:A:G
peptide level
106:196
61:110
10:19
RFQMIPLDPKpc2_NP.4
p−min=0.91, pX=0.38, N=177
var.4.THBS1 15:39880822:A:G
peptide level
118:196
64:110
10:19
RFQMIPLDPKpc2_NP.5
p−maj=0.63, pX=0.24, N=192
var.4.THBS1 15:39880822:A:G
peptide level
8:196
5:110
0:19
SCDSLNNRCEGSSVQTRpc3_NP.5
p−maj=1, pX=0.72, N=13
var.4.THBS1 15:39880822:A:G
peptide level
180:196
104:110
17:19
SCDSLNNRpc2_NP.1
p−maj=0.64, pX=0.87, N=301
var.4.THBS1 15:39880822:A:G
peptide level
194:196
109:110
19:19
SCDSLNNRpc2_NP.2
p−maj=1, pX=0.68, N=322
var.4.THBS1 15:39880822:A:G
peptide level
190:196
106:110
19:19
SCDSLNNRpc2_NP.3
p−maj=1, pX=0.62, N=315
var.4.THBS1 15:39880822:A:G
peptide level
192:196
109:110
19:19
SCDSLNNRpc2_NP.4
p−min=0.65, pX=0.83, N=320
var.4.THBS1 15:39880822:A:G
peptide level
196:196
108:110
19:19
SCDSLNNRpc2_NP.5
p−min=0.16, pX=0.8, N=323

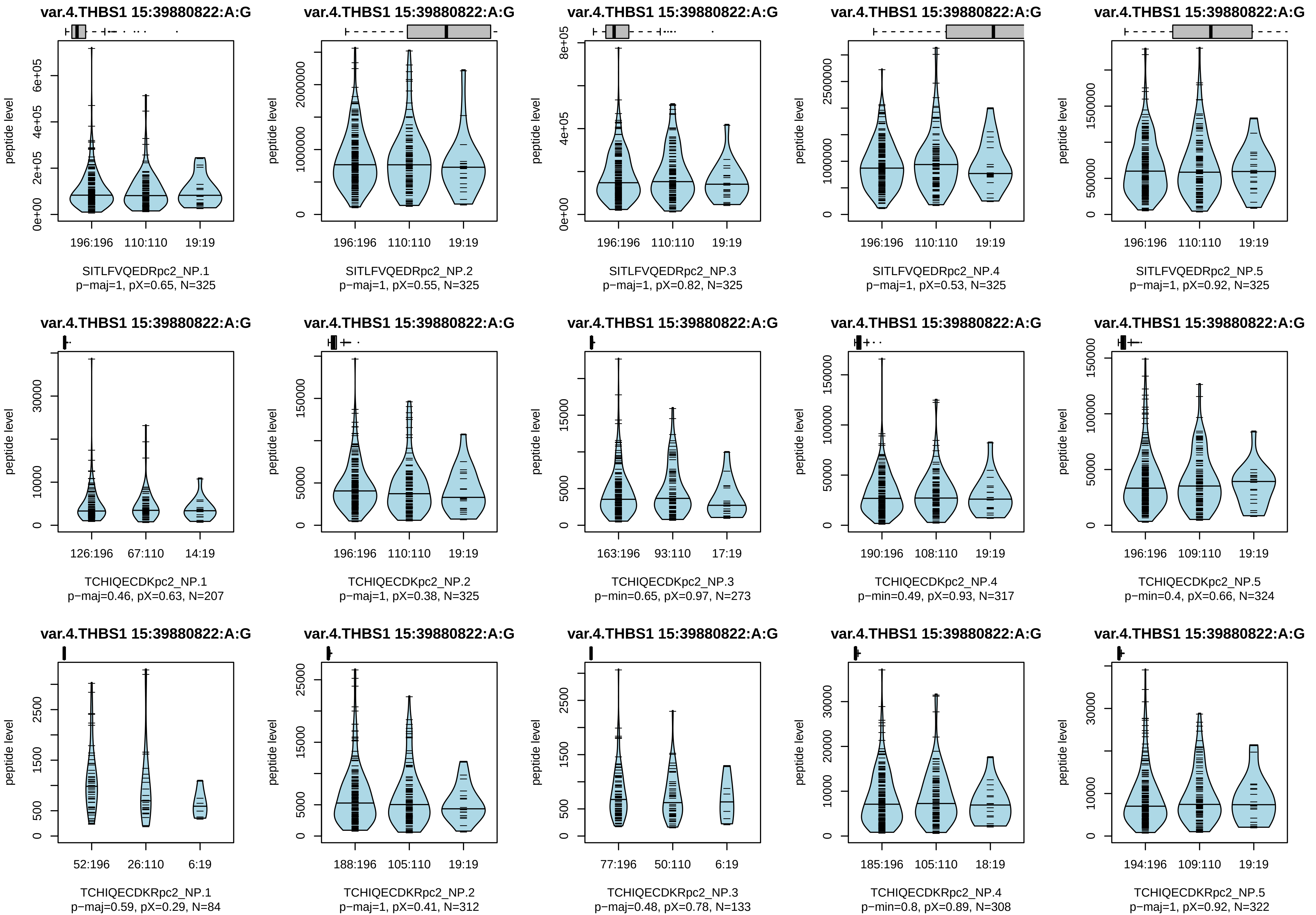
var.4.THBS1 15:39880822:A:G
peptide level
196:196
110:110
19:19
SITLFVQEDRpc2_NP.1
p−maj=1, pX=0.65, N=325
var.4.THBS1 15:39880822:A:G
peptide level
196:196
110:110
19:19
SITLFVQEDRpc2_NP.2
p−maj=1, pX=0.55, N=325
var.4.THBS1 15:39880822:A:G
peptide level
196:196
110:110
19:19
SITLFVQEDRpc2_NP.3
p−maj=1, pX=0.82, N=325
var.4.THBS1 15:39880822:A:G
peptide level
196:196
110:110
19:19
SITLFVQEDRpc2_NP.4
p−maj=1, pX=0.53, N=325
var.4.THBS1 15:39880822:A:G
peptide level
196:196
110:110
19:19
SITLFVQEDRpc2_NP.5
p−maj=1, pX=0.92, N=325
var.4.THBS1 15:39880822:A:G
peptide level
126:196
67:110
14:19
TCHIQECDKpc2_NP.1
p−maj=0.46, pX=0.63, N=207
var.4.THBS1 15:39880822:A:G
peptide level
196:196
110:110
19:19
TCHIQECDKpc2_NP.2
p−maj=1, pX=0.38, N=325
var.4.THBS1 15:39880822:A:G
peptide level
163:196
93:110
17:19
TCHIQECDKpc2_NP.3
p−min=0.65, pX=0.97, N=273
var.4.THBS1 15:39880822:A:G
peptide level
190:196
108:110
19:19
TCHIQECDKpc2_NP.4
p−min=0.49, pX=0.93, N=317
var.4.THBS1 15:39880822:A:G
peptide level
196:196
109:110
19:19
TCHIQECDKpc2_NP.5
p−min=0.4, pX=0.66, N=324
var.4.THBS1 15:39880822:A:G
peptide level
52:196
26:110
6:19
TCHIQECDKRpc2_NP.1
p−maj=0.59, pX=0.29, N=84
var.4.THBS1 15:39880822:A:G
peptide level
188:196
105:110
19:19
TCHIQECDKRpc2_NP.2
p−maj=1, pX=0.41, N=312
var.4.THBS1 15:39880822:A:G
peptide level
77:196
50:110
6:19
TCHIQECDKRpc2_NP.3
p−maj=0.48, pX=0.78, N=133
var.4.THBS1 15:39880822:A:G
peptide level
185:196
105:110
18:19
TCHIQECDKRpc2_NP.4
p−min=0.8, pX=0.89, N=308
var.4.THBS1 15:39880822:A:G
peptide level
194:196
109:110
19:19
TCHIQECDKRpc2_NP.5
p−maj=1, pX=0.92, N=322

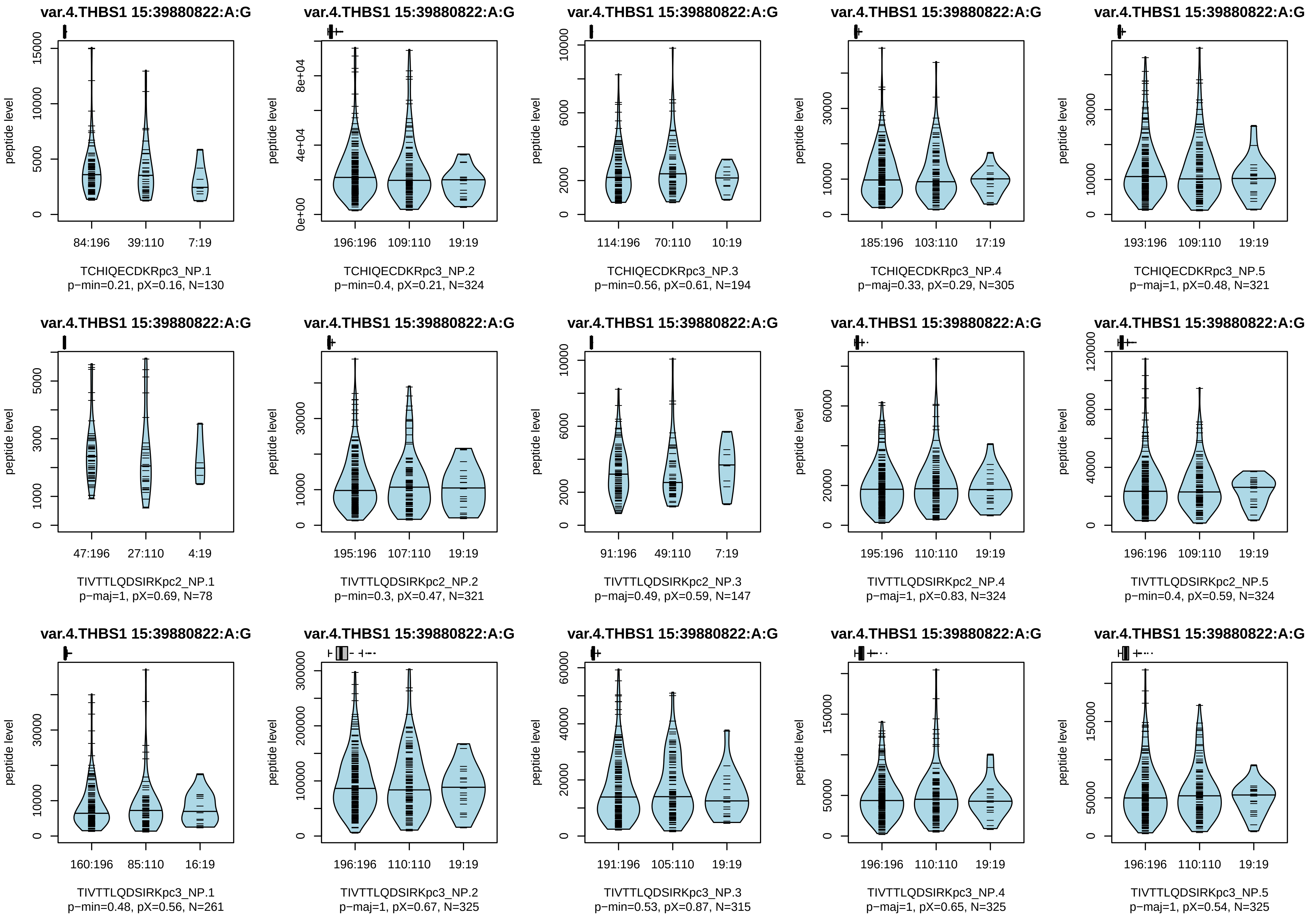
var.4.THBS1 15:39880822:A:G
peptide level
84:196
39:110
7:19
TCHIQECDKRpc3_NP.1
p−min=0.21, pX=0.16, N=130
var.4.THBS1 15:39880822:A:G
peptide level
196:196
109:110
19:19
TCHIQECDKRpc3_NP.2
p−min=0.4, pX=0.21, N=324
var.4.THBS1 15:39880822:A:G
peptide level
114:196
70:110
10:19
TCHIQECDKRpc3_NP.3
p−min=0.56, pX=0.61, N=194
var.4.THBS1 15:39880822:A:G
peptide level
185:196
103:110
17:19
TCHIQECDKRpc3_NP.4
p−maj=0.33, pX=0.29, N=305
var.4.THBS1 15:39880822:A:G
peptide level
193:196
109:110
19:19
TCHIQECDKRpc3_NP.5
p−maj=1, pX=0.48, N=321
var.4.THBS1 15:39880822:A:G
peptide level
47:196
27:110
4:19
TIVTTLQDSIRKpc2_NP.1
p−maj=1, pX=0.69, N=78
var.4.THBS1 15:39880822:A:G
peptide level
195:196
107:110
19:19
TIVTTLQDSIRKpc2_NP.2
p−min=0.3, pX=0.47, N=321
var.4.THBS1 15:39880822:A:G
peptide level
91:196
49:110
7:19
TIVTTLQDSIRKpc2_NP.3
p−maj=0.49, pX=0.59, N=147
var.4.THBS1 15:39880822:A:G
peptide level
195:196
110:110
19:19
TIVTTLQDSIRKpc2_NP.4
p−maj=1, pX=0.83, N=324
var.4.THBS1 15:39880822:A:G
peptide level
196:196
109:110
19:19
TIVTTLQDSIRKpc2_NP.5
p−min=0.4, pX=0.59, N=324
var.4.THBS1 15:39880822:A:G
peptide level
160:196
85:110
16:19
TIVTTLQDSIRKpc3_NP.1
p−min=0.48, pX=0.56, N=261
var.4.THBS1 15:39880822:A:G
peptide level
196:196
110:110
19:19
TIVTTLQDSIRKpc3_NP.2
p−maj=1, pX=0.67, N=325
var.4.THBS1 15:39880822:A:G
peptide level
191:196
105:110
19:19
TIVTTLQDSIRKpc3_NP.3
p−min=0.53, pX=0.87, N=315
var.4.THBS1 15:39880822:A:G
peptide level
196:196
110:110
19:19
TIVTTLQDSIRKpc3_NP.4
p−maj=1, pX=0.65, N=325
var.4.THBS1 15:39880822:A:G
peptide level
196:196
110:110
19:19
TIVTTLQDSIRKpc3_NP.5
p−maj=1, pX=0.54, N=325

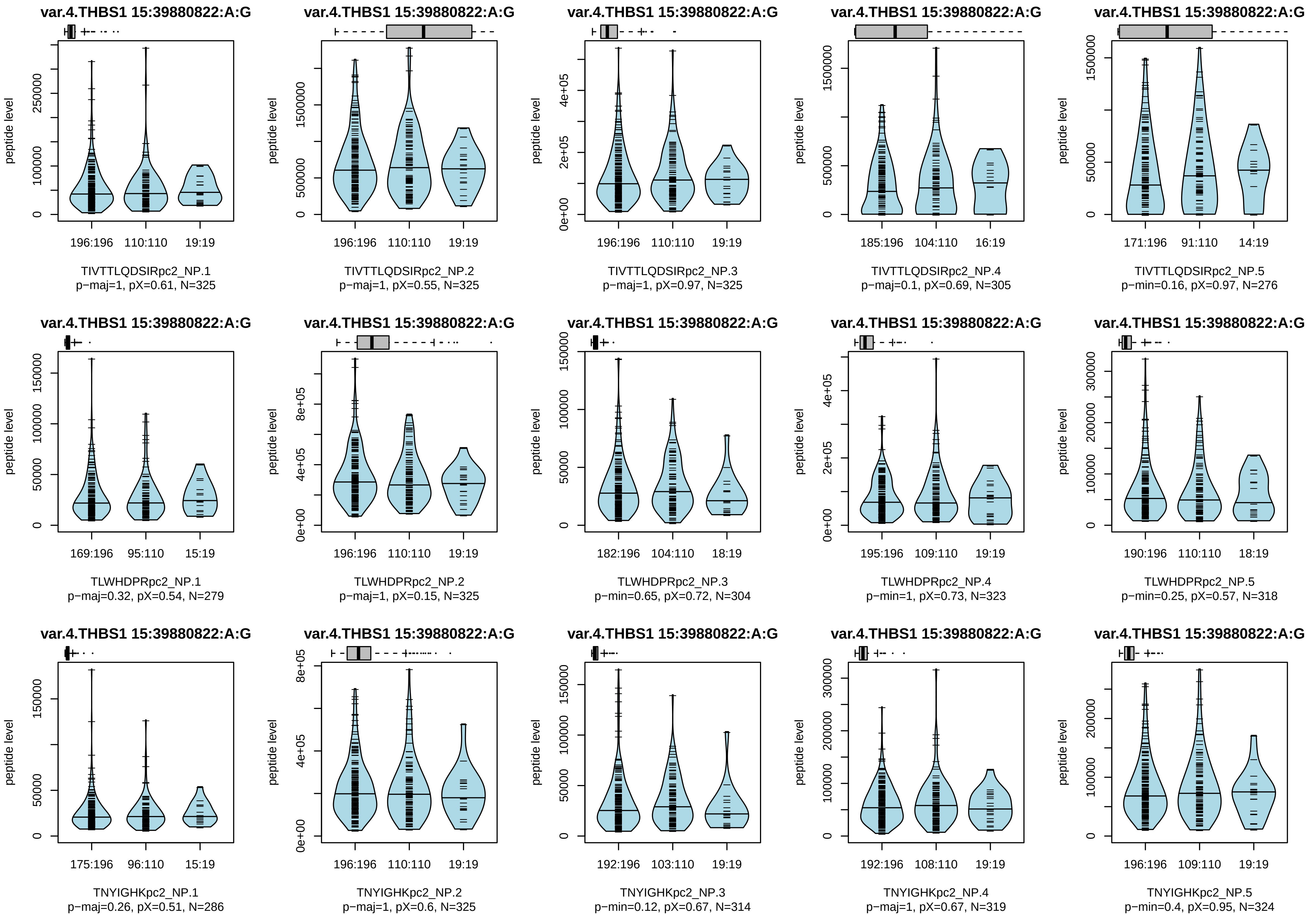
var.4.THBS1 15:39880822:A:G
peptide level
196:196
110:110
19:19
TIVTTLQDSIRpc2_NP.1
p−maj=1, pX=0.61, N=325
var.4.THBS1 15:39880822:A:G
196:196
110:110
19:19
TIVTTLQDSIRpc2_NP.2
p−maj=1, pX=0.55, N=325
var.4.THBS1 15:39880822:A:G
196:196
110:110
19:19
TIVTTLQDSIRpc2_NP.3
p−maj=1, pX=0.97, N=325
var.4.THBS1 15:39880822:A:G
185:196
104:110
16:19
TIVTTLQDSIRpc2_NP.4
p−maj=0.1, pX=0.69, N=305
var.4.THBS1 15:39880822:A:G
171:196
91:110
14:19
TIVTTLQDSIRpc2_NP.5
p−min=0.16, pX=0.97, N=276
var.4.THBS1 15:39880822:A:G
169:196
95:110
15:19
TLWHDPRpc2_NP.1
p−maj=0.32, pX=0.54, N=279
var.4.THBS1 15:39880822:A:G
196:196
110:110
19:19
TLWHDPRpc2_NP.2
p−maj=1, pX=0.15, N=325
var.4.THBS1 15:39880822:A:G
182:196
104:110
18:19
TLWHDPRpc2_NP.3
p−min=0.65, pX=0.72, N=304
var.4.THBS1 15:39880822:A:G
195:196
109:110
19:19
TLWHDPRpc2_NP.4
p−min=1, pX=0.73, N=323
var.4.THBS1 15:39880822:A:G
190:196
110:110
18:19
TLWHDPRpc2_NP.5
p−min=0.25, pX=0.57, N=318
var.4.THBS1 15:39880822:A:G
175:196
96:110
15:19
TNYIGHKpc2_NP.1
p−maj=0.26, pX=0.51, N=286
var.4.THBS1 15:39880822:A:G
196:196
110:110
19:19
TNYIGHKpc2_NP.2
p−maj=1, pX=0.6, N=325
var.4.THBS1 15:39880822:A:G
192:196
103:110
19:19
TNYIGHKpc2_NP.3
p−min=0.12, pX=0.67, N=314
var.4.THBS1 15:39880822:A:G
192:196
108:110
19:19
TNYIGHKpc2_NP.4
p−maj=1, pX=0.67, N=319
var.4.THBS1 15:39880822:A:G
196:196
109:110
19:19
TNYIGHKpc2_NP.5
p−min=0.4, pX=0.95, N=324

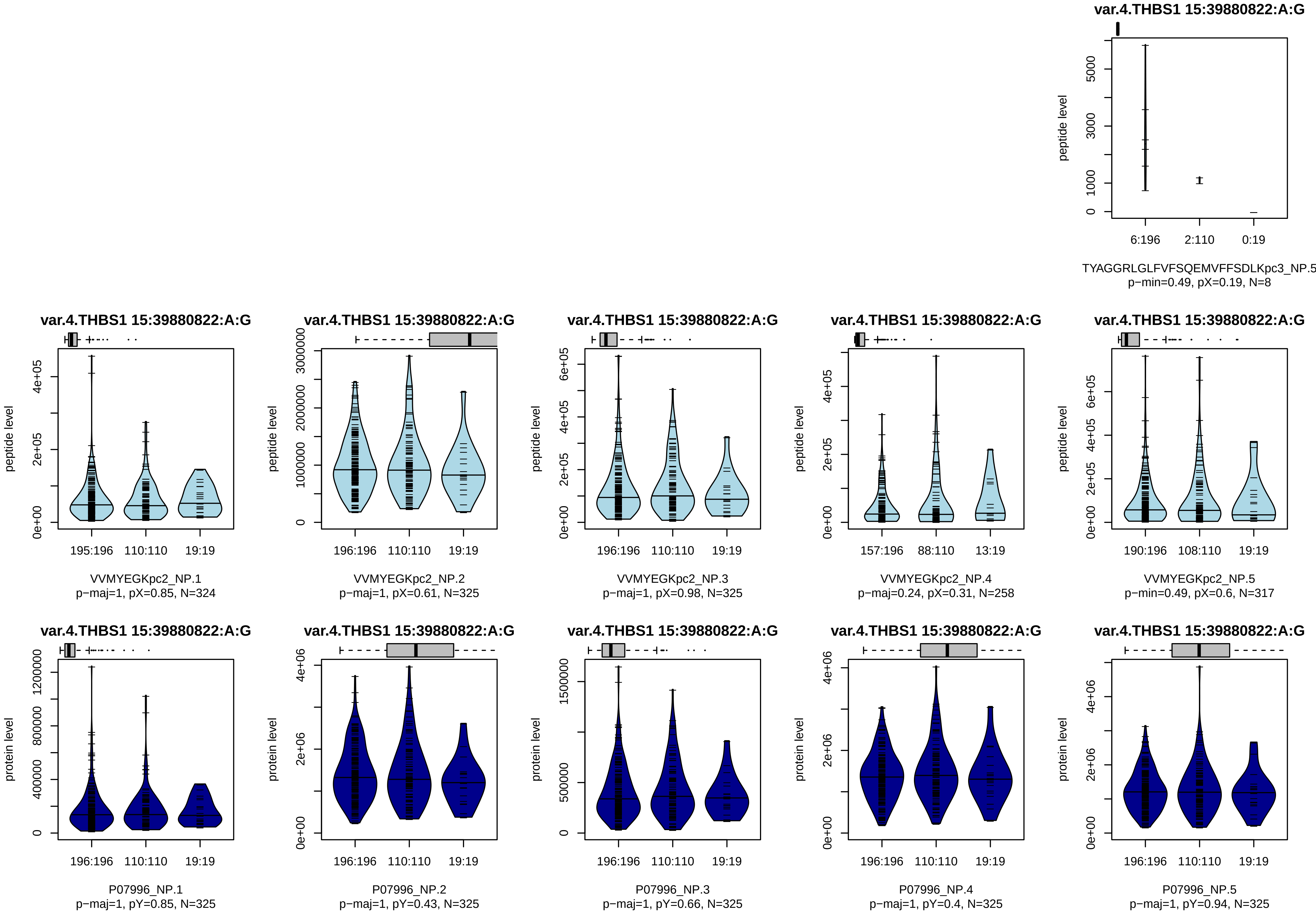

var.4.THBS1 15:39880822:A:G
peptide level
6:196
2:110
0:19
TYAGGRLGLFVFSQEMVFFSDLKpc3_NP.5
p−min=0.49, pX=0.19, N=8
var.4.THBS1 15:39880822:A:G
peptide level
195:196
110:110
19:19
VVMYEGKpc2_NP.1
p−maj=1, pX=0.85, N=324
var.4.THBS1 15:39880822:A:G
peptide level
196:196
110:110
19:19
VVMYEGKpc2_NP.2
p−maj=1, pX=0.61, N=325
var.4.THBS1 15:39880822:A:G
peptide level
196:196
110:110
19:19
VVMYEGKpc2_NP.3
p−maj=1, pX=0.98, N=325
var.4.THBS1 15:39880822:A:G
peptide level
157:196
88:110
13:19
VVMYEGKpc2_NP.4
p−maj=0.24, pX=0.31, N=258
var.4.THBS1 15:39880822:A:G
peptide level
190:196
108:110
19:19
VVMYEGKpc2_NP.5
p−min=0.49, pX=0.6, N=317
var.4.THBS1 15:39880822:A:G
protein level
196:196
110:110
19:19
P07996_NP.1
p−maj=1, pY=0.85, N=325
var.4.THBS1 15:39880822:A:G
protein level
196:196
110:110
19:19
P07996_NP.2
p−maj=1, pY=0.43, N=325
var.4.THBS1 15:39880822:A:G
protein level
196:196
110:110
19:19
P07996_NP.3
p−maj=1, pY=0.66, N=325
var.4.THBS1 15:39880822:A:G
protein level
196:196
110:110
19:19
P07996_NP.4
p−maj=1, pY=0.4, N=325
var.4.THBS1 15:39880822:A:G
protein level
196:196
110:110
19:19
P07996_NP.5
p−maj=1, pY=0.94, N=325

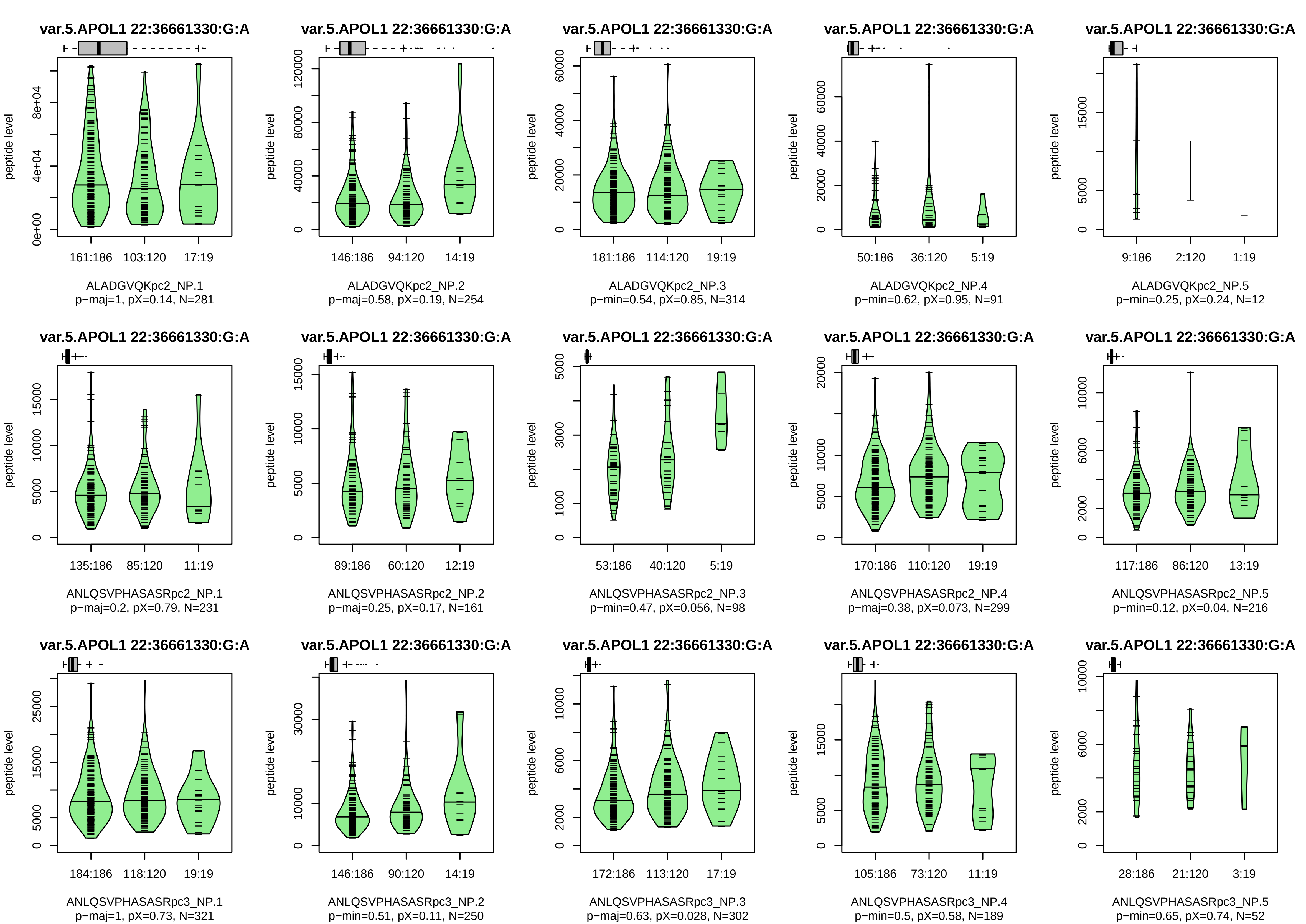
var.5.APOL1 22:36661330:G:A
peptide level
161:186 103:120 17:19
ALADGVQKpc2_NP.1
p−maj=1, pX=0.14, N=281
var.5.APOL1 22:36661330:G:A
peptide level
146:186 94:120 14:19
ALADGVQKpc2_NP.2
p−maj=0.58, pX=0.19, N=254
var.5.APOL1 22:36661330:G:A
peptide level
181:186 114:120 19:19
ALADGVQKpc2_NP.3
p−min=0.54, pX=0.85, N=314
var.5.APOL1 22:36661330:G:A
peptide level
50:186 36:120 5:19
ALADGVQKpc2_NP.4
p−min=0.62, pX=0.95, N=91
var.5.APOL1 22:36661330:G:A
peptide level
9:186 2:120 1:19
ALADGVQKpc2_NP.5
p−min=0.25, pX=0.24, N=12
var.5.APOL1 22:36661330:G:A
peptide level
135:186 85:120 11:19
ANLQSVPHASASRpc2_NP.1
p−maj=0.2, pX=0.79, N=231
var.5.APOL1 22:36661330:G:A
peptide level
89:186 60:120 12:19
ANLQSVPHASASRpc2_NP.2
p−maj=0.25, pX=0.17, N=161
var.5.APOL1 22:36661330:G:A
peptide level
53:186 40:120 5:19
ANLQSVPHASASRpc2_NP.3
p−min=0.47, pX=0.056, N=98
var.5.APOL1 22:36661330:G:A
peptide level
170:186 110:120 19:19
ANLQSVPHASASRpc2_NP.4
p−maj=0.38, pX=0.073, N=299
var.5.APOL1 22:36661330:G:A
peptide level
117:186 86:120 13:19
ANLQSVPHASASRpc2_NP.5
p−min=0.12, pX=0.04, N=216
var.5.APOL1 22:36661330:G:A
peptide level
184:186 118:120 19:19
ANLQSVPHASASRpc3_NP.1
p−maj=1, pX=0.73, N=321
var.5.APOL1 22:36661330:G:A
peptide level
146:186 90:120 14:19
ANLQSVPHASASRpc3_NP.2
p−min=0.51, pX=0.11, N=250
var.5.APOL1 22:36661330:G:A
peptide level
172:186 113:120 17:19
ANLQSVPHASASRpc3_NP.3
p−maj=0.63, pX=0.028, N=302
var.5.APOL1 22:36661330:G:A
peptide level
105:186 73:120 11:19
ANLQSVPHASASRpc3_NP.4
p−min=0.5, pX=0.58, N=189
var.5.APOL1 22:36661330:G:A
peptide level
28:186 21:120 3:19
ANLQSVPHASASRpc3_NP.5
p−min=0.65, pX=0.74, N=52

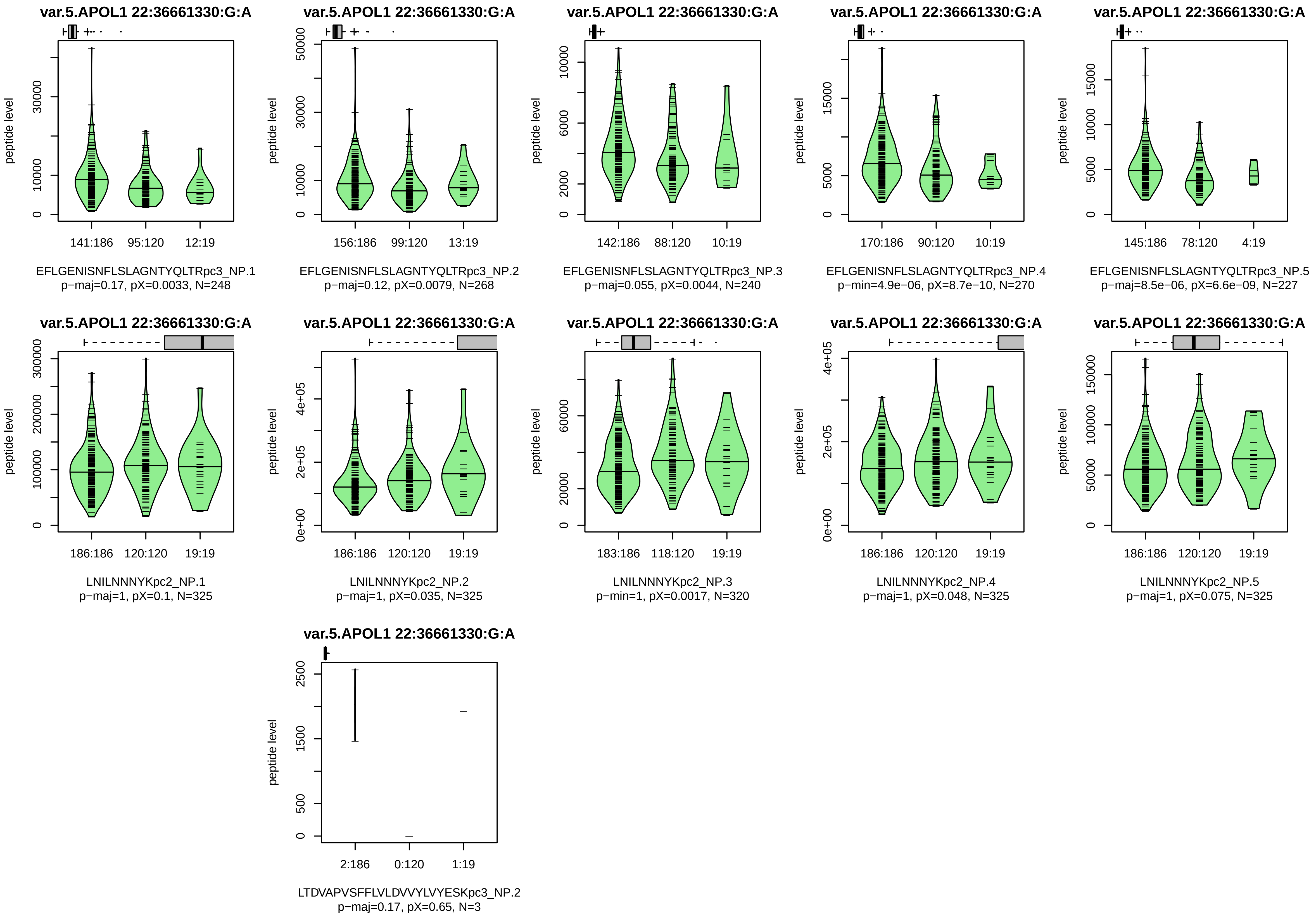

var.5.APOL1 22:36661330:G:A
peptide level
141:186
95:120
12:19
EFLGENISNFLSLAGNTYQLTRpc3_NP.1
p−maj=0.17, pX=0.0033, N=248
var.5.APOL1 22:36661330:G:A
peptide level
156:186
99:120
13:19
EFLGENISNFLSLAGNTYQLTRpc3_NP.2
p−maj=0.12, pX=0.0079, N=268
var.5.APOL1 22:36661330:G:A
peptide level
142:186
88:120
10:19
EFLGENISNFLSLAGNTYQLTRpc3_NP.3
p−maj=0.055, pX=0.0044, N=240
var.5.APOL1 22:36661330:G:A
peptide level
170:186
90:120
10:19
EFLGENISNFLSLAGNTYQLTRpc3_NP.4
p−min=4.9e−06, pX=8.7e−10, N=270
var.5.APOL1 22:36661330:G:A
peptide level
145:186
78:120
4:19
EFLGENISNFLSLAGNTYQLTRpc3_NP.5
p−maj=8.5e−06, pX=6.6e−09, N=227
var.5.APOL1 22:36661330:G:A
peptide level
186:186
120:120
19:19
LNILNNNYKpc2_NP.1
p−maj=1, pX=0.1, N=325
var.5.APOL1 22:36661330:G:A
peptide level
186:186
120:120
19:19
LNILNNNYKpc2_NP.2
p−maj=1, pX=0.035, N=325
var.5.APOL1 22:36661330:G:A
peptide level
183:186
118:120
19:19
LNILNNNYKpc2_NP.3
p−min=1, pX=0.0017, N=320
var.5.APOL1 22:36661330:G:A
peptide level
186:186
120:120
19:19
LNILNNNYKpc2_NP.4
p−maj=1, pX=0.048, N=325
var.5.APOL1 22:36661330:G:A
peptide level
186:186
120:120
19:19
LNILNNNYKpc2_NP.5
p−maj=1, pX=0.075, N=325
var.5.APOL1 22:36661330:G:A
peptide level
2:186
0:120
1:19
LTDVAPVSFFLVLDVVYLVYESKpc3_NP.2
p−maj=0.17, pX=0.65, N=3

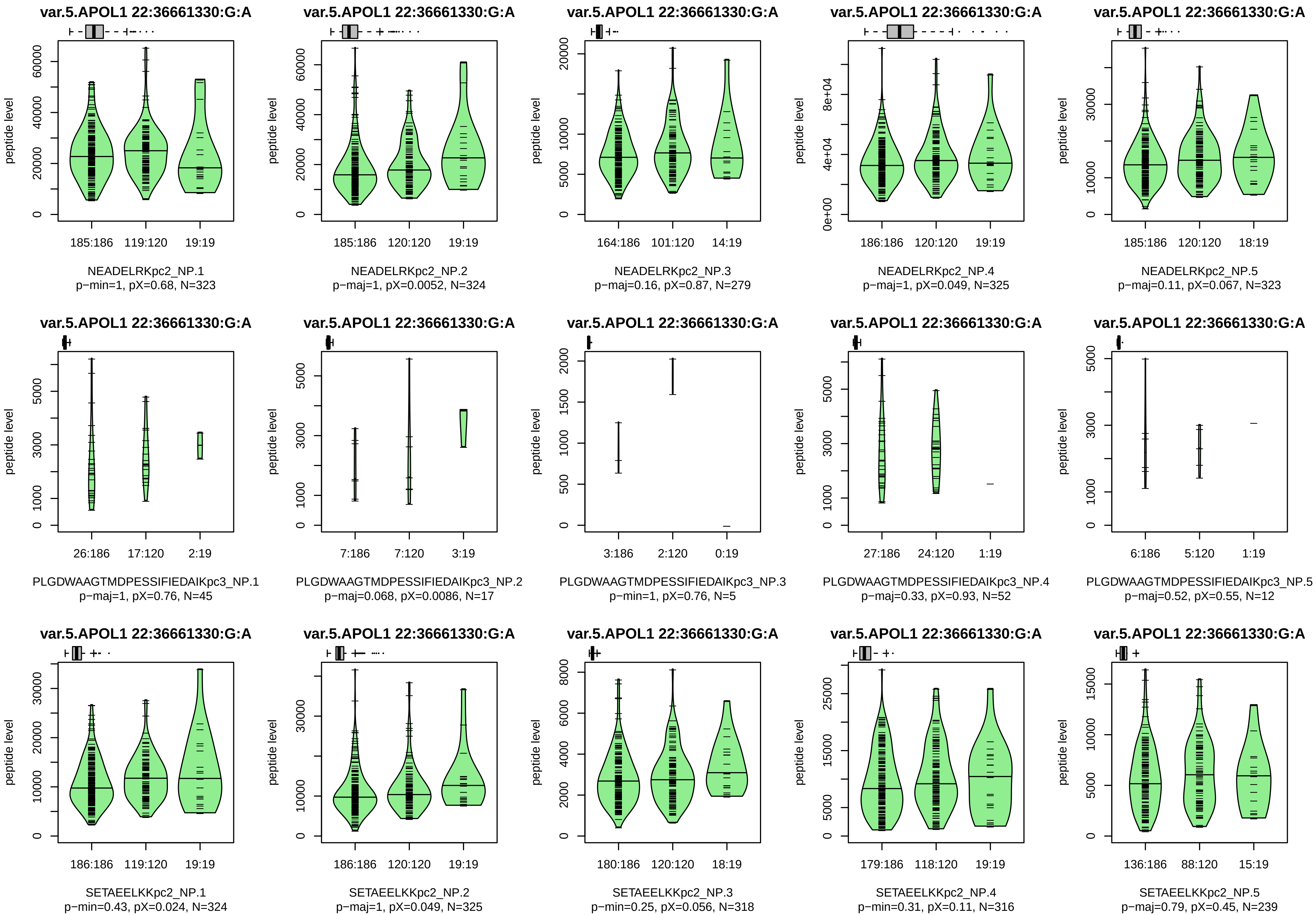
var.5.APOL1 22:36661330:G:A
peptide level
185:186
119:120
19:19
NEADELRKpc2_NP.1
p−min=1, pX=0.68, N=323
var.5.APOL1 22:36661330:G:A
185:186
120:120
19:19
NEADELRKpc2_NP.2
p−maj=1, pX=0.0052, N=324
var.5.APOL1 22:36661330:G:A
164:186
101:120
14:19
NEADELRKpc2_NP.3
p−maj=0.16, pX=0.87, N=279
var.5.APOL1 22:36661330:G:A
186:186
120:120
19:19
NEADELRKpc2_NP.4
p−maj=1, pX=0.049, N=325
var.5.APOL1 22:36661330:G:A
185:186
120:120
18:19
NEADELRKpc2_NP.5
p−maj=0.11, pX=0.067, N=323
var.5.APOL1 22:36661330:G:A
26:186
17:120
2:19
PLGDWAAGTMDPESSIFIEDAIKpc3_NP.1
p−maj=1, pX=0.76, N=45
var.5.APOL1 22:36661330:G:A
7:186
7:120
3:19
PLGDWAAGTMDPESSIFIEDAIKpc3_NP.2
p−maj=0.068, pX=0.0086, N=17
var.5.APOL1 22:36661330:G:A
3:186
2:120
0:19
PLGDWAAGTMDPESSIFIEDAIKpc3_NP.3
p−min=1, pX=0.76, N=5
var.5.APOL1 22:36661330:G:A
27:186
24:120
1:19
PLGDWAAGTMDPESSIFIEDAIKpc3_NP.4
p−maj=0.33, pX=0.93, N=52
var.5.APOL1 22:36661330:G:A
6:186
5:120
1:19
PLGDWAAGTMDPESSIFIEDAIKpc3_NP.5
p−maj=0.52, pX=0.55, N=12
var.5.APOL1 22:36661330:G:A
186:186
119:120
19:19
SETAEELKKpc2_NP.1
p−min=0.43, pX=0.024, N=324
var.5.APOL1 22:36661330:G:A
186:186
120:120
19:19
SETAEELKKpc2_NP.2
p−maj=1, pX=0.049, N=325
var.5.APOL1 22:36661330:G:A
180:186
120:120
18:19
SETAEELKKpc2_NP.3
p−min=0.25, pX=0.056, N=318
var.5.APOL1 22:36661330:G:A
179:186
118:120
19:19
SETAEELKKpc2_NP.4
p−min=0.31, pX=0.11, N=316
var.5.APOL1 22:36661330:G:A
136:186
88:120
15:19
SETAEELKKpc2_NP.5
p−maj=0.79, pX=0.45, N=239

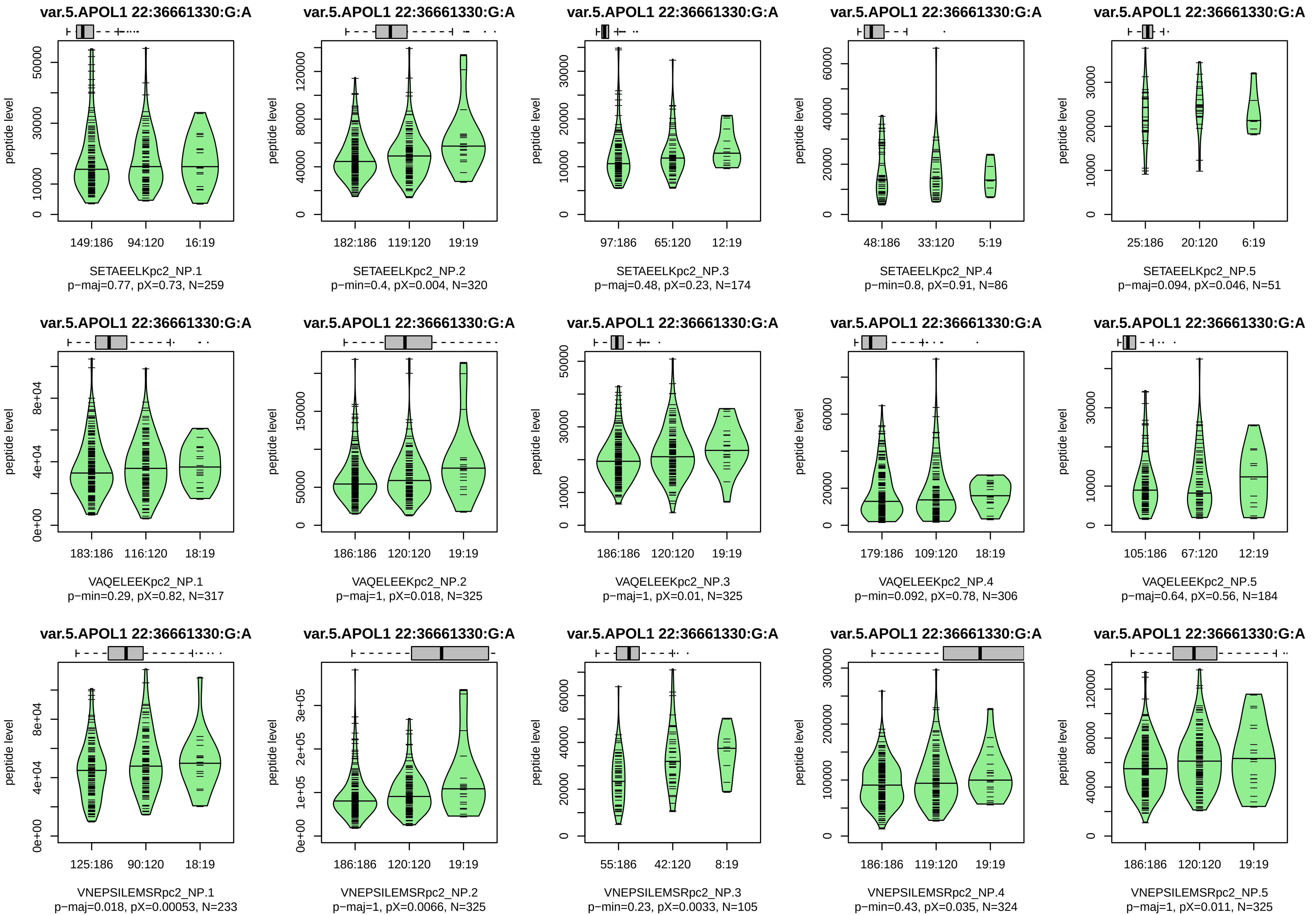
var.5.APOL1 22:36661330:G:A
peptide level
149:186
94:120
16:19
SETAEELKpc2_NP.1
p−maj=0.77, pX=0.73, N=259
var.5.APOL1 22:36661330:G:A
peptide level
182:186
119:120
19:19
SETAEELKpc2_NP.2
p−min=0.4, pX=0.004, N=320
var.5.APOL1 22:36661330:G:A
peptide level
97:186
65:120
12:19
SETAEELKpc2_NP.3
p−maj=0.48, pX=0.23, N=174
var.5.APOL1 22:36661330:G:A
peptide level
48:186
33:120
5:19
SETAEELKpc2_NP.4
p−min=0.8, pX=0.91, N=86
var.5.APOL1 22:36661330:G:A
peptide level
25:186
20:120
6:19
SETAEELKpc2_NP.5
p−maj=0.094, pX=0.046, N=51
var.5.APOL1 22:36661330:G:A
peptide level
183:186
116:120
18:19
VAQELEEKpc2_NP.1
p−min=0.29, pX=0.82, N=317
var.5.APOL1 22:36661330:G:A
peptide level
186:186
120:120
19:19
VAQELEEKpc2_NP.2
p−maj=1, pX=0.018, N=325
var.5.APOL1 22:36661330:G:A
peptide level
186:186
120:120
19:19
VAQELEEKpc2_NP.3
p−maj=1, pX=0.01, N=325
var.5.APOL1 22:36661330:G:A
peptide level
179:186
109:120
18:19
VAQELEEKpc2_NP.4
p−min=0.092, pX=0.78, N=306
var.5.APOL1 22:36661330:G:A
peptide level
105:186
67:120
12:19
VAQELEEKpc2_NP.5
p−maj=0.64, pX=0.56, N=184
var.5.APOL1 22:36661330:G:A
peptide level
125:186
90:120
18:19
VNEPSILEMSRpc2_NP.1
p−maj=0.018, pX=0.00053, N=233
var.5.APOL1 22:36661330:G:A
peptide level
186:186
120:120
19:19
VNEPSILEMSRpc2_NP.2
p−maj=1, pX=0.0066, N=325
var.5.APOL1 22:36661330:G:A
peptide level
55:186
42:120
8:19
VNEPSILEMSRpc2_NP.3
p−min=0.23, pX=0.0033, N=105
var.5.APOL1 22:36661330:G:A
peptide level
186:186
119:120
19:19
VNEPSILEMSRpc2_NP.4
p−min=0.43, pX=0.035, N=324
var.5.APOL1 22:36661330:G:A
peptide level
186:186
120:120
19:19
VNEPSILEMSRpc2_NP.5
p−maj=1, pX=0.011, N=325

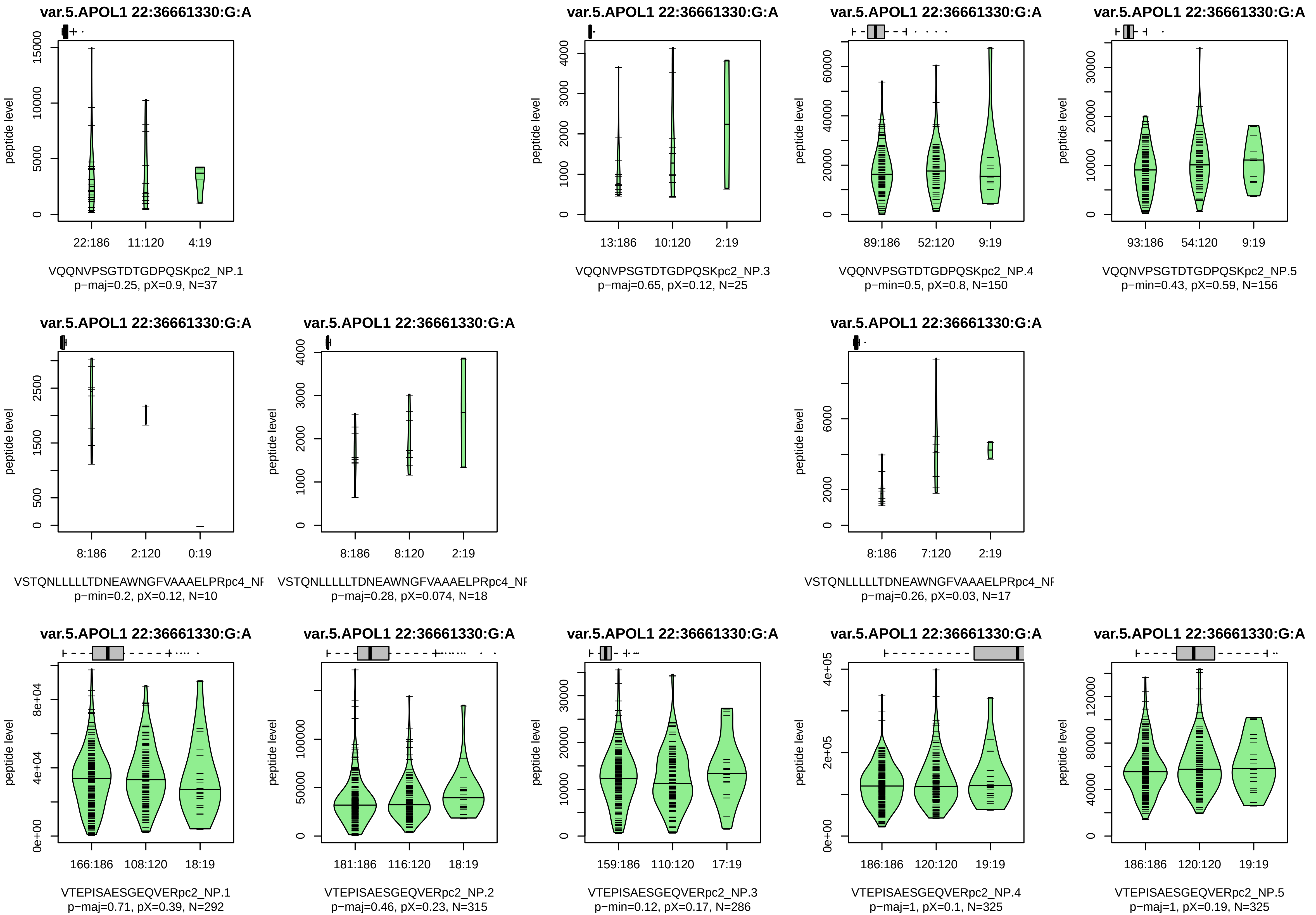
var.5.APOL1 22:36661330:G:A
peptide level
22:186
11:120
4:19
VQQNVPSGTDTGDPQSKpc2_NP.1
p−maj=0.25, pX=0.9, N=37
var.5.APOL1 22:36661330:G:A
peptide level
13:186
10:120
2:19
VQQNVPSGTDTGDPQSKpc2_NP.3
p−maj=0.65, pX=0.12, N=25
var.5.APOL1 22:36661330:G:A
peptide level
89:186
52:120
9:19
VQQNVPSGTDTGDPQSKpc2_NP.4
p−min=0.5, pX=0.8, N=150
var.5.APOL1 22:36661330:G:A
peptide level
93:186
54:120
9:19
VQQNVPSGTDTGDPQSKpc2_NP.5
p−min=0.43, pX=0.59, N=156
var.5.APOL1 22:36661330:G:A
peptide level
8:186
2:120
0:19
VSTQNLLLLLTDNEAWNGFVAAAELPRpc4_NF
p−min=0.2, pX=0.12, N=10
var.5.APOL1 22:36661330:G:A
peptide level
8:186
8:120
2:19
VSTQNLLLLLTDNEAWNGFVAAAELPRpc4_NF
p−maj=0.28, pX=0.074, N=18
var.5.APOL1 22:36661330:G:A
peptide level
8:186
7:120
2:19
VSTQNLLLLLTDNEAWNGFVAAAELPRpc4_NF
p−maj=0.26, pX=0.03, N=17
var.5.APOL1 22:36661330:G:A
peptide level
166:186
108:120
18:19
VTEPISAESGEQVERpc2_NP.1
p−maj=0.71, pX=0.39, N=292
var.5.APOL1 22:36661330:G:A
peptide level
181:186
116:120
18:19
VTEPISAESGEQVERpc2_NP.2
p−maj=0.46, pX=0.23, N=315
var.5.APOL1 22:36661330:G:A
peptide level
159:186
110:120
17:19
VTEPISAESGEQVERpc2_NP.3
p−min=0.12, pX=0.17, N=286
var.5.APOL1 22:36661330:G:A
peptide level
186:186
120:120
19:19
VTEPISAESGEQVERpc2_NP.4
p−maj=1, pX=0.1, N=325
var.5.APOL1 22:36661330:G:A
peptide level
186:186
120:120
19:19
VTEPISAESGEQVERpc2_NP.5
p−maj=1, pX=0.19, N=325

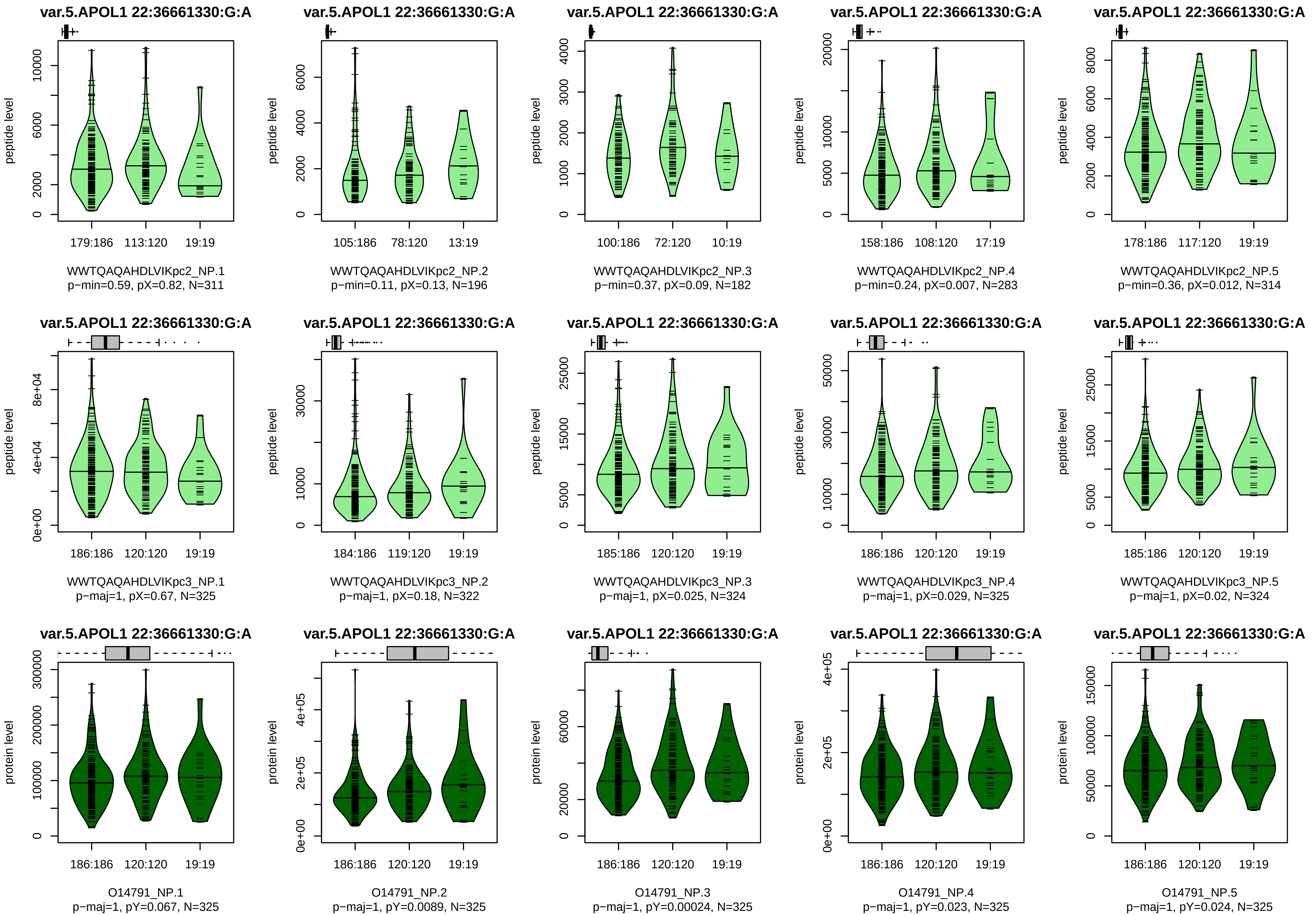

var.5.APOL1 22:36661330:G:A
peptide level
0
2000
6000
10000
179:186
113:120
19:19
WWTQAQAHDLVIKpc2_NP.1
p−min=0.59, pX=0.82, N=311
var.5.APOL1 22:36661330:G:A
peptide level
0
2000
4000
6000
105:186
78:120
13:19
WWTQAQAHDLVIKpc2_NP.2
p−min=0.11, pX=0.13, N=196
var.5.APOL1 22:36661330:G:A
peptide level
0
1000
2000
3000
4000
100:186
72:120
10:19
WWTQAQAHDLVIKpc2_NP.3
p−min=0.37, pX=0.09, N=182
var.5.APOL1 22:36661330:G:A
peptide level
0
5000
10000
20000
158:186
108:120
17:19
WWTQAQAHDLVIKpc2_NP.4
p−min=0.24, pX=0.007, N=283
var.5.APOL1 22:36661330:G:A
peptide level
0
2000
4000
6000
8000
178:186
117:120
19:19
WWTQAQAHDLVIKpc2_NP.5
p−min=0.36, pX=0.012, N=314
var.5.APOL1 22:36661330:G:A
peptide level
0e+00
4e+04
8e+04
186:186
120:120
19:19
WWTQAQAHDLVIKpc3_NP.1
p−maj=1, pX=0.67, N=325
var.5.APOL1 22:36661330:G:A
peptide level
0
10000
30000
184:186
119:120
19:19
WWTQAQAHDLVIKpc3_NP.2
p−maj=1, pX=0.18, N=322
var.5.APOL1 22:36661330:G:A
peptide level
0
5000
15000
25000
185:186
120:120
19:19
WWTQAQAHDLVIKpc3_NP.3
p−maj=1, pX=0.025, N=324
var.5.APOL1 22:36661330:G:A
peptide level
0
10000
30000
50000
186:186
120:120
19:19
WWTQAQAHDLVIKpc3_NP.4
p−maj=1, pX=0.029, N=325
var.5.APOL1 22:36661330:G:A
peptide level
0
5000
15000
25000
185:186
120:120
19:19
WWTQAQAHDLVIKpc3_NP.5
p−maj=1, pX=0.02, N=324
var.5.APOL1 22:36661330:G:A
protein level
0
100000
200000
300000
186:186
120:120
19:19
O14791_NP.1
p−maj=1, pY=0.067, N=325
var.5.APOL1 22:36661330:G:A
protein level
0e+00
2e+05
4e+05
186:186
120:120
19:19
O14791_NP.2
p−maj=1, pY=0.0089, N=325
var.5.APOL1 22:36661330:G:A
protein level
0
20000
60000
186:186
120:120
19:19
O14791_NP.3
p−maj=1, pY=0.00024, N=325
var.5.APOL1 22:36661330:G:A
protein level
0e+00
2e+05
4e+05
186:186
120:120
19:19
O14791_NP.4
p−maj=1, pY=0.023, N=325
var.5.APOL1 22:36661330:G:A
protein level
0
50000
100000
150000
186:186
120:120
19:19
O14791_NP.5
p−maj=1, pY=0.024, N=325

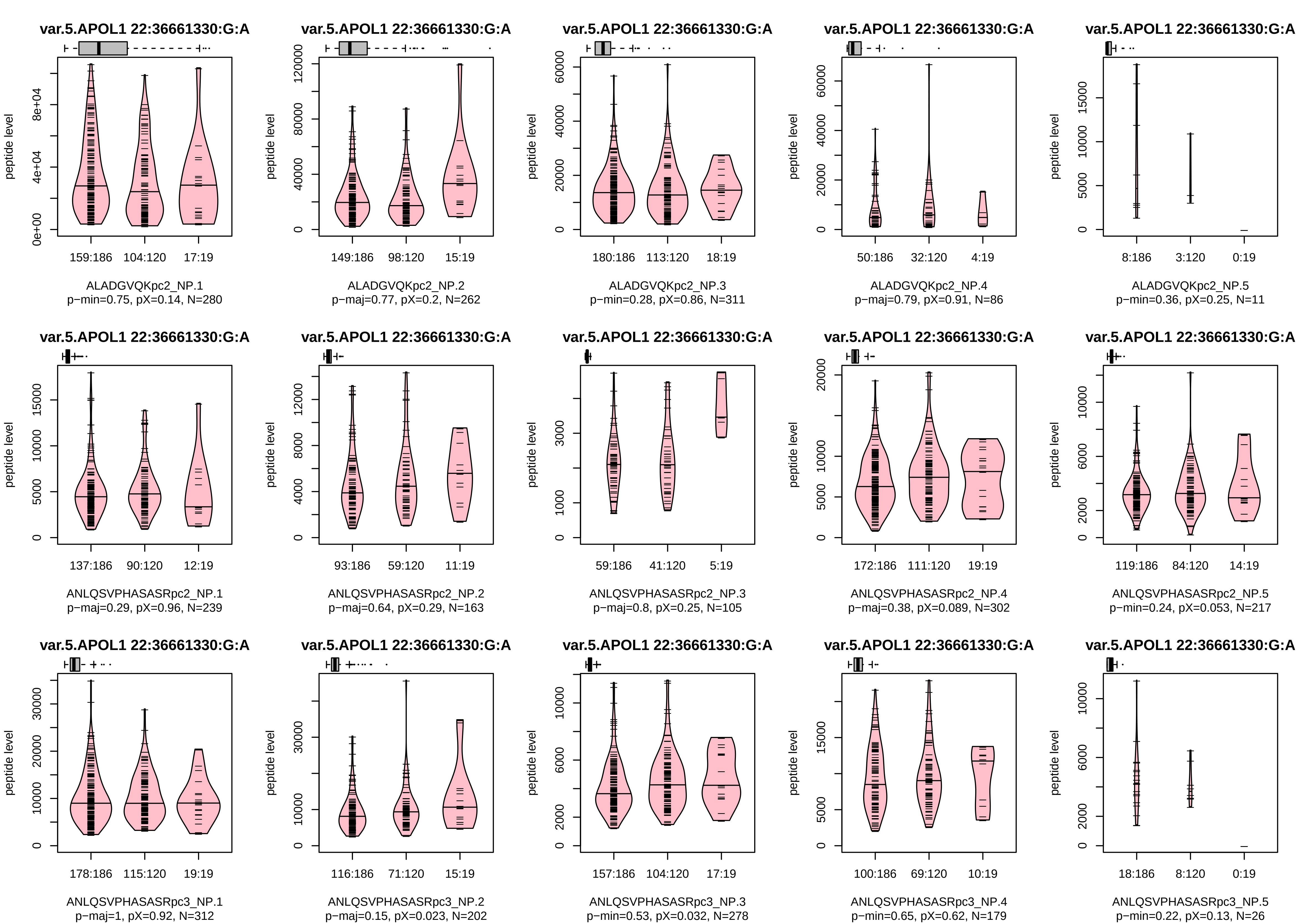
var.5.APOL1 22:36661330:G:A
peptide level
159:186
104:120
17:19
ALADGVQKpc2_NP.1
p−min=0.75, pX=0.14, N=280
var.5.APOL1 22:36661330:G:A
peptide level
149:186
98:120
15:19
ALADGVQKpc2_NP.2
p−maj=0.77, pX=0.2, N=262
var.5.APOL1 22:36661330:G:A
peptide level
180:186
113:120
18:19
ALADGVQKpc2_NP.3
p−min=0.28, pX=0.86, N=311
var.5.APOL1 22:36661330:G:A
peptide level
50:186
32:120
4:19
ALADGVQKpc2_NP.4
p−maj=0.79, pX=0.91, N=86
var.5.APOL1 22:36661330:G:A
peptide level
8:186
3:120
0:19
ALADGVQKpc2_NP.5
p−min=0.36, pX=0.25, N=11
var.5.APOL1 22:36661330:G:A
peptide level
137:186
90:120
12:19
ANLQSVPHASASRpc2_NP.1
p−maj=0.29, pX=0.96, N=239
var.5.APOL1 22:36661330:G:A
peptide level
93:186
59:120
11:19
ANLQSVPHASASRpc2_NP.2
p−maj=0.64, pX=0.29, N=163
var.5.APOL1 22:36661330:G:A
peptide level
59:186
41:120
5:19
ANLQSVPHASASRpc2_NP.3
p−maj=0.8, pX=0.25, N=105
var.5.APOL1 22:36661330:G:A
peptide level
172:186
111:120
19:19
ANLQSVPHASASRpc2_NP.4
p−maj=0.38, pX=0.089, N=302
var.5.APOL1 22:36661330:G:A
peptide level
119:186
84:120
14:19
ANLQSVPHASASRpc2_NP.5
p−min=0.24, pX=0.053, N=217
var.5.APOL1 22:36661330:G:A
peptide level
178:186
115:120
19:19
ANLQSVPHASASRpc3_NP.1
p−maj=1, pX=0.92, N=312
var.5.APOL1 22:36661330:G:A
peptide level
116:186
71:120
15:19
ANLQSVPHASASRpc3_NP.2
p−maj=0.15, pX=0.023, N=202
var.5.APOL1 22:36661330:G:A
peptide level
157:186
104:120
17:19
ANLQSVPHASASRpc3_NP.3
p−min=0.53, pX=0.032, N=278
var.5.APOL1 22:36661330:G:A
peptide level
100:186
69:120
10:19
ANLQSVPHASASRpc3_NP.4
p−min=0.65, pX=0.62, N=179
var.5.APOL1 22:36661330:G:A
peptide level
18:186
8:120
0:19
ANLQSVPHASASRpc3_NP.5
p−min=0.22, pX=0.13, N=26

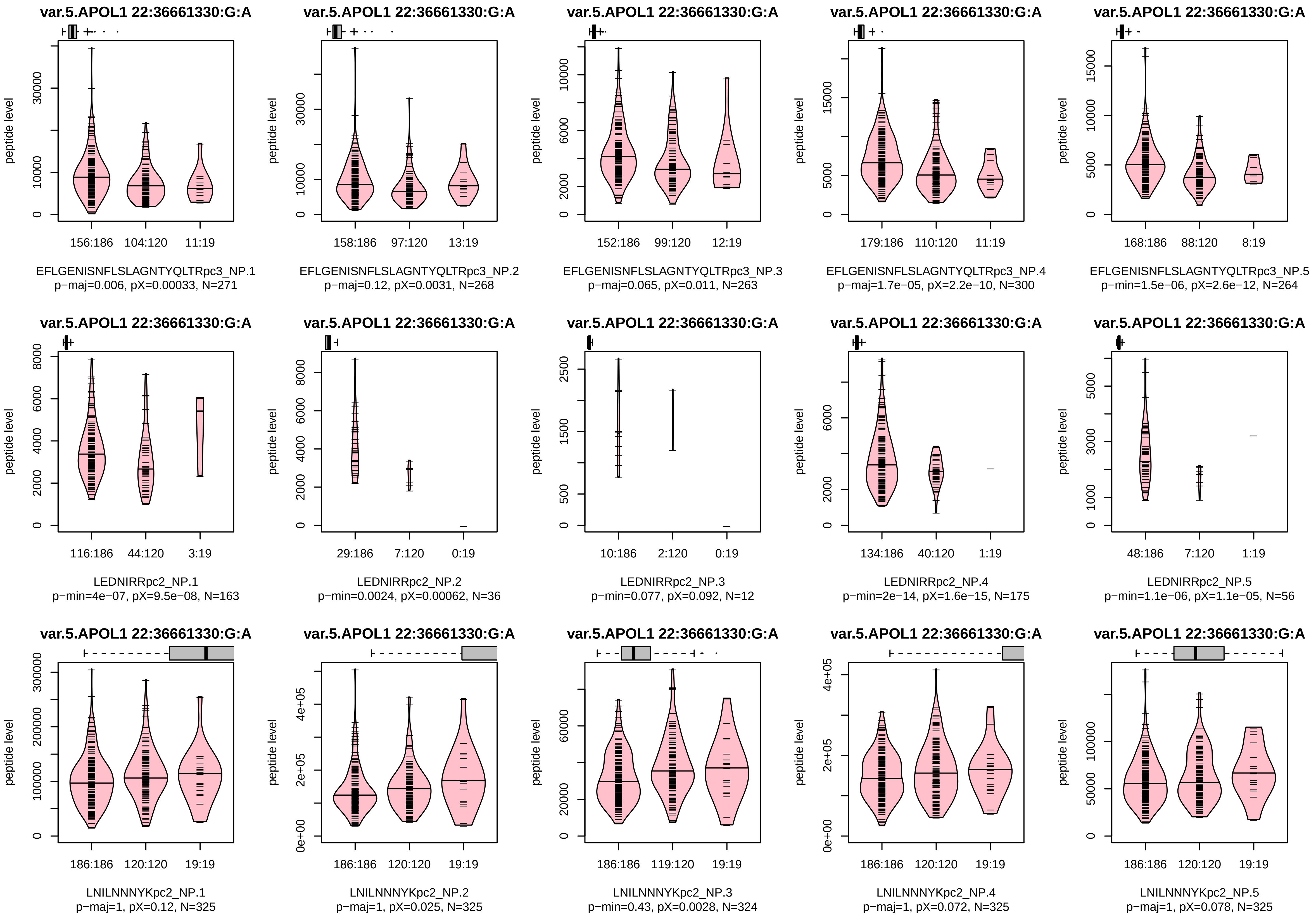
var.5.APOL1 22:36661330:G:A
peptide level
156:186
104:120
11:19
EFLGENISNFLSLAGNTYQLTRpc3_NP.1
p−maj=0.006, pX=0.00033, N=271
var.5.APOL1 22:36661330:G:A
peptide level
158:186
97:120
13:19
EFLGENISNFLSLAGNTYQLTRpc3_NP.2
p−maj=0.12, pX=0.0031, N=268
var.5.APOL1 22:36661330:G:A
peptide level
152:186
99:120
12:19
EFLGENISNFLSLAGNTYQLTRpc3_NP.3
p−maj=0.065, pX=0.011, N=263
var.5.APOL1 22:36661330:G:A
peptide level
179:186
110:120
11:19
EFLGENISNFLSLAGNTYQLTRpc3_NP.4
p−maj=1.7e−05, pX=2.2e−10, N=300
var.5.APOL1 22:36661330:G:A
peptide level
168:186
88:120
8:19
EFLGENISNFLSLAGNTYQLTRpc3_NP.5
p−min=1.5e−06, pX=2.6e−12, N=264
var.5.APOL1 22:36661330:G:A
peptide level
116:186
44:120
3:19
LEDNIRRpc2_NP.1
p−min=4e−07, pX=9.5e−08, N=163
var.5.APOL1 22:36661330:G:A
peptide level
29:186
7:120
0:19
LEDNIRRpc2_NP.2
p−min=0.0024, pX=0.00062, N=36
var.5.APOL1 22:36661330:G:A
peptide level
10:186
2:120
0:19
LEDNIRRpc2_NP.3
p−min=0.077, pX=0.092, N=12
var.5.APOL1 22:36661330:G:A
peptide level
134:186
40:120
1:19
LEDNIRRpc2_NP.4
p−min=2e−14, pX=1.6e−15, N=175
var.5.APOL1 22:36661330:G:A
peptide level
48:186
7:120
1:19
LEDNIRRpc2_NP.5
p−min=1.1e−06, pX=1.1e−05, N=56
var.5.APOL1 22:36661330:G:A
peptide level
186:186
120:120
19:19
LNILNNNYKpc2_NP.1
p−maj=1, pX=0.12, N=325
var.5.APOL1 22:36661330:G:A
peptide level
186:186
120:120
19:19
LNILNNNYKpc2_NP.2
p−maj=1, pX=0.025, N=325
var.5.APOL1 22:36661330:G:A
peptide level
186:186
119:120
19:19
LNILNNNYKpc2_NP.3
p−min=0.43, pX=0.0028, N=324
var.5.APOL1 22:36661330:G:A
peptide level
186:186
120:120
19:19
LNILNNNYKpc2_NP.4
p−maj=1, pX=0.072, N=325
var.5.APOL1 22:36661330:G:A
peptide level
186:186
120:120
19:19
LNILNNNYKpc2_NP.5
p−maj=1, pX=0.078, N=325

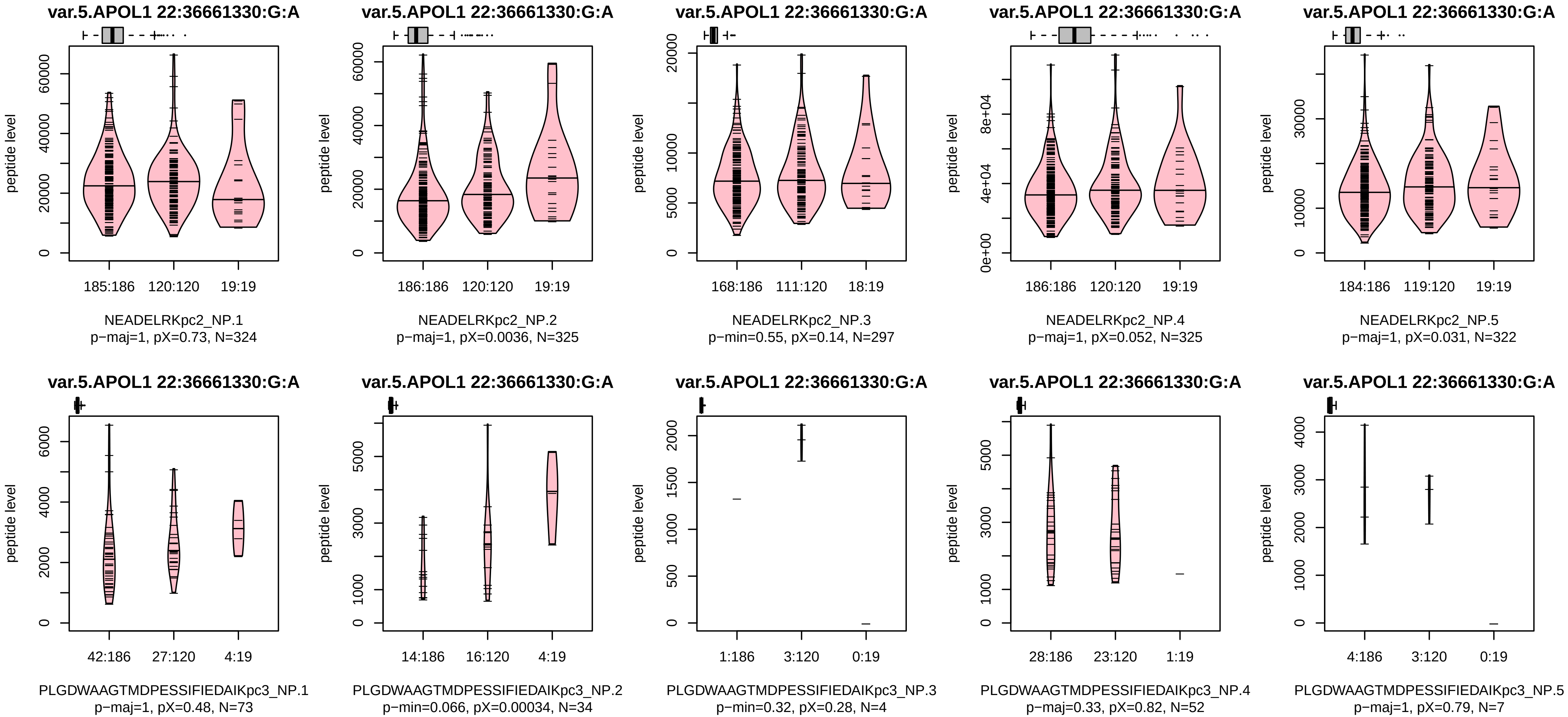
var.5.APOL1 22:36661330:G:A
peptide level
185:186
120:120
19:19
NEADELRKpc2_NP.1
p−maj=1, pX=0.73, N=324
var.5.APOL1 22:36661330:G:A
peptide level
186:186
120:120
19:19
NEADELRKpc2_NP.2
p−maj=1, pX=0.0036, N=325
var.5.APOL1 22:36661330:G:A
peptide level
168:186
111:120
18:19
NEADELRKpc2_NP.3
p−min=0.55, pX=0.14, N=297
var.5.APOL1 22:36661330:G:A
peptide level
186:186
120:120
19:19
NEADELRKpc2_NP.4
p−maj=1, pX=0.052, N=325
var.5.APOL1 22:36661330:G:A
peptide level
184:186
119:120
19:19
NEADELRKpc2_NP.5
p−maj=1, pX=0.031, N=322
var.5.APOL1 22:36661330:G:A
peptide level
42:186
27:120
4:19
PLGDWAAGTMDPESSIFIEDAIKpc3_NP.1
p−maj=1, pX=0.48, N=73
var.5.APOL1 22:36661330:G:A
peptide level
14:186
16:120
4:19
PLGDWAAGTMDPESSIFIEDAIKpc3_NP.2
p−min=0.066, pX=0.00034, N=34
var.5.APOL1 22:36661330:G:A
peptide level
1:186
3:120
0:19
PLGDWAAGTMDPESSIFIEDAIKpc3_NP.3
p−min=0.32, pX=0.28, N=4
var.5.APOL1 22:36661330:G:A
peptide level
28:186
23:120
1:19
PLGDWAAGTMDPESSIFIEDAIKpc3_NP.4
p−maj=0.33, pX=0.82, N=52
var.5.APOL1 22:36661330:G:A
peptide level
4:186
3:120
0:19
PLGDWAAGTMDPESSIFIEDAIKpc3_NP.5
p−maj=1, pX=0.79, N=7

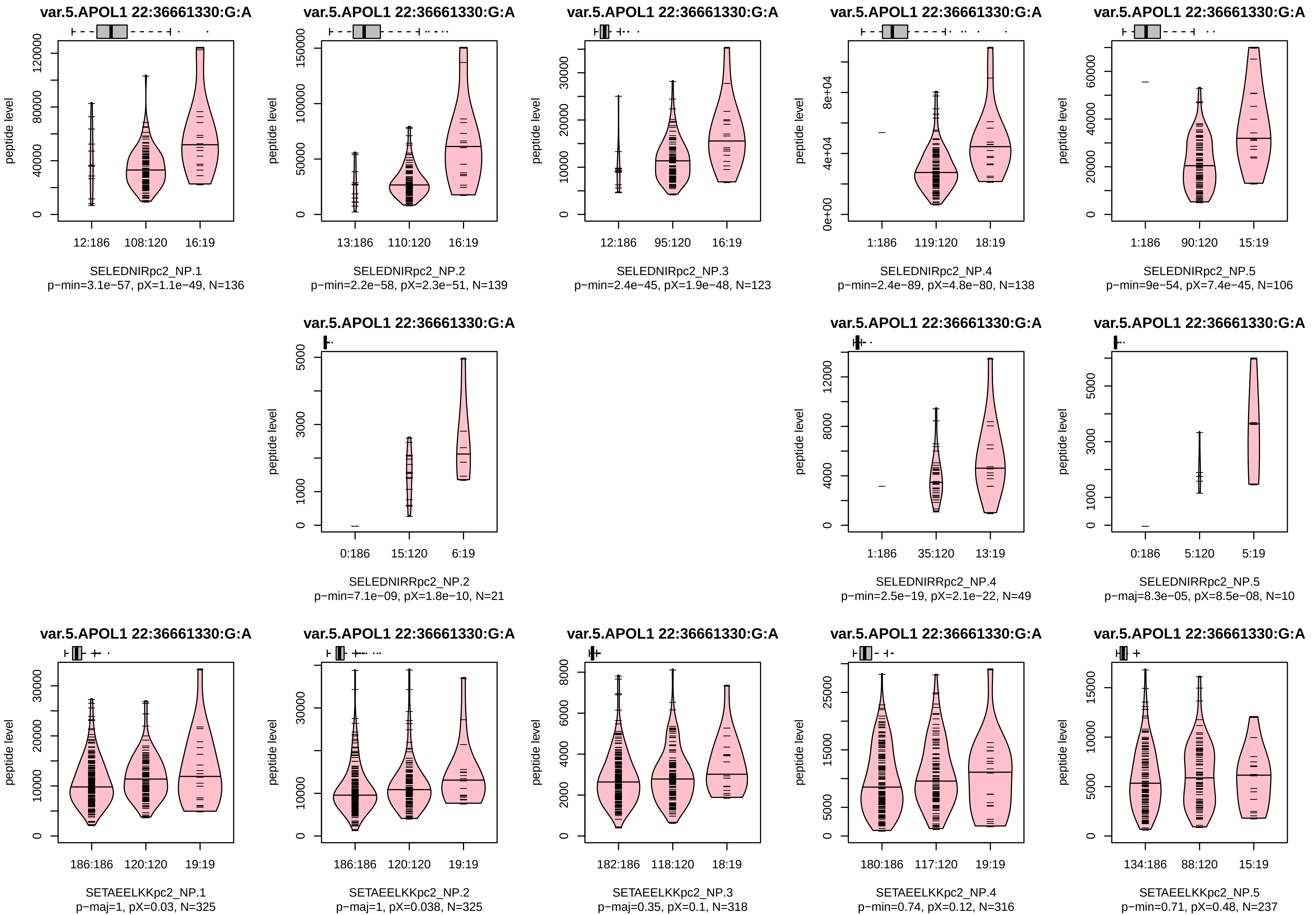

var.5.APOL1 22:36661330:G:A
peptide level
12:186
108:120
16:19
SELEDNIRpc2_NP.1
p−min=3.1e−57, pX=1.1e−49, N=136
var.5.APOL1 22:36661330:G:A
peptide level
13:186
110:120
16:19
SELEDNIRpc2_NP.2
p−min=2.2e−58, pX=2.3e−51, N=139
var.5.APOL1 22:36661330:G:A
peptide level
12:186
95:120
16:19
SELEDNIRpc2_NP.3
p−min=2.4e−45, pX=1.9e−48, N=123
var.5.APOL1 22:36661330:G:A
peptide level
1:186
119:120
18:19
SELEDNIRpc2_NP.4
p−min=2.4e−89, pX=4.8e−80, N=138
var.5.APOL1 22:36661330:G:A
peptide level
1:186
90:120
15:19
SELEDNIRpc2_NP.5
p−min=9e−54, pX=7.4e−45, N=106
var.5.APOL1 22:36661330:G:A
peptide level
0:186
15:120
6:19
SELEDNIRRpc2_NP.2
p−min=7.1e−09, pX=1.8e−10, N=21
var.5.APOL1 22:36661330:G:A
peptide level
1:186
35:120
13:19
SELEDNIRRpc2_NP.4
p−min=2.5e−19, pX=2.1e−22, N=49
var.5.APOL1 22:36661330:G:A
peptide level
0:186
5:120
5:19
SELEDNIRRpc2_NP.5
p−maj=8.3e−05, pX=8.5e−08, N=10
var.5.APOL1 22:36661330:G:A
peptide level
186:186
120:120
19:19
SETAEELKKpc2_NP.1
p−maj=1, pX=0.03, N=325
var.5.APOL1 22:36661330:G:A
peptide level
186:186
120:120
19:19
SETAEELKKpc2_NP.2
p−maj=1, pX=0.038, N=325
var.5.APOL1 22:36661330:G:A
peptide level
182:186
118:120
18:19
SETAEELKKpc2_NP.3
p−maj=0.35, pX=0.1, N=318
var.5.APOL1 22:36661330:G:A
peptide level
180:186
117:120
19:19
SETAEELKKpc2_NP.4
p−min=0.74, pX=0.12, N=316
var.5.APOL1 22:36661330:G:A
peptide level
134:186
88:120
15:19
SETAEELKKpc2_NP.5
p−min=0.71, pX=0.48, N=237

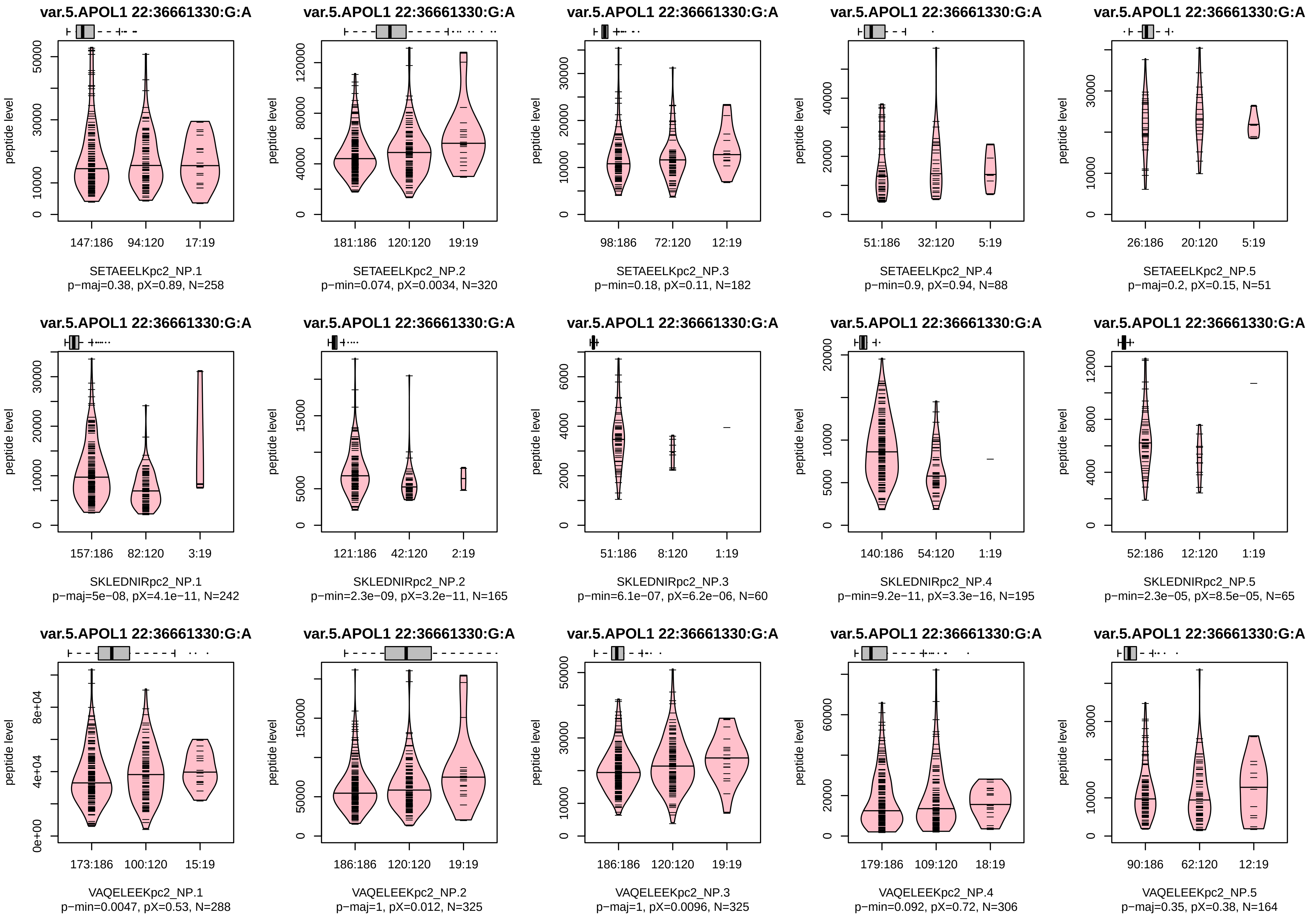
var.5.APOL1 22:36661330:G:A
peptide level
147:186
94:120
17:19
SETAEELKpc2_NP.1
p−maj=0.38, pX=0.89, N=258
var.5.APOL1 22:36661330:G:A
peptide level
181:186
120:120
19:19
SETAEELKpc2_NP.2
p−min=0.074, pX=0.0034, N=320
var.5.APOL1 22:36661330:G:A
peptide level
98:186
72:120
12:19
SETAEELKpc2_NP.3
p−min=0.18, pX=0.11, N=182
var.5.APOL1 22:36661330:G:A
peptide level
51:186
32:120
5:19
SETAEELKpc2_NP.4
p−min=0.9, pX=0.94, N=88
var.5.APOL1 22:36661330:G:A
peptide level
26:186
20:120
5:19
SETAEELKpc2_NP.5
p−maj=0.2, pX=0.15, N=51
var.5.APOL1 22:36661330:G:A
peptide level
157:186
82:120
3:19
SKLEDNIRpc2_NP.1
p−maj=5e−08, pX=4.1e−11, N=242
var.5.APOL1 22:36661330:G:A
peptide level
121:186
42:120
2:19
SKLEDNIRpc2_NP.2
p−min=2.3e−09, pX=3.2e−11, N=165
var.5.APOL1 22:36661330:G:A
peptide level
51:186
8:120
1:19
SKLEDNIRpc2_NP.3
p−min=6.1e−07, pX=6.2e−06, N=60
var.5.APOL1 22:36661330:G:A
peptide level
140:186
54:120
1:19
SKLEDNIRpc2_NP.4
p−min=9.2e−11, pX=3.3e−16, N=195
var.5.APOL1 22:36661330:G:A
peptide level
52:186
12:120
1:19
SKLEDNIRpc2_NP.5
p−min=2.3e−05, pX=8.5e−05, N=65
var.5.APOL1 22:36661330:G:A
peptide level
173:186
100:120
15:19
VAQELEEKpc2_NP.1
p−min=0.0047, pX=0.53, N=288
var.5.APOL1 22:36661330:G:A
peptide level
186:186
120:120
19:19
VAQELEEKpc2_NP.2
p−maj=1, pX=0.012, N=325
var.5.APOL1 22:36661330:G:A
peptide level
186:186
120:120
19:19
VAQELEEKpc2_NP.3
p−maj=1, pX=0.0096, N=325
var.5.APOL1 22:36661330:G:A
peptide level
179:186
109:120
18:19
VAQELEEKpc2_NP.4
p−min=0.092, pX=0.72, N=306
var.5.APOL1 22:36661330:G:A
peptide level
90:186
62:120
12:19
VAQELEEKpc2_NP.5
p−maj=0.35, pX=0.38, N=164

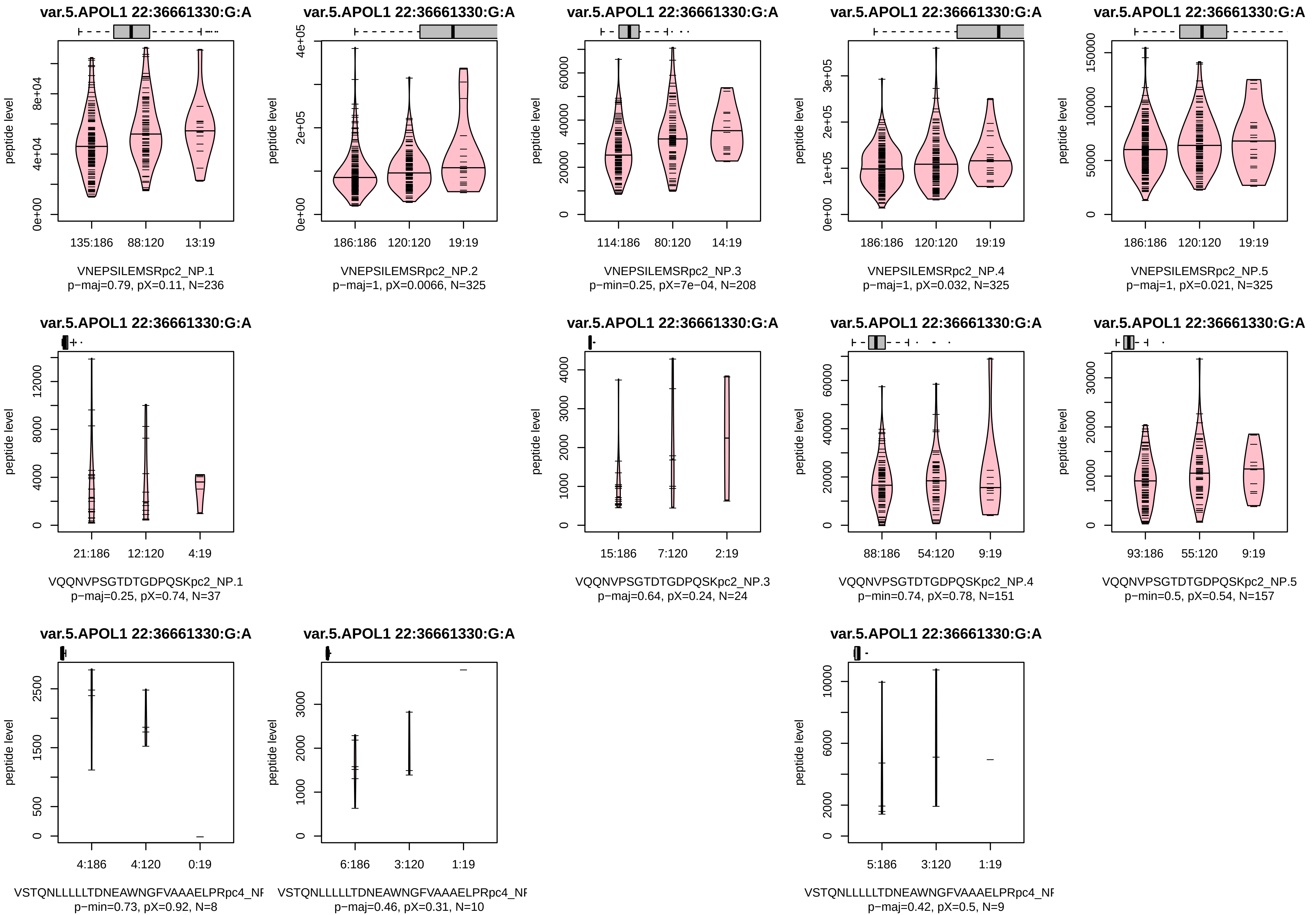

var.5.APOL1 22:36661330:G:A
peptide level
135:186
88:120
13:19
VNEPSILEMSRpc2_NP.1
p−maj=0.79, pX=0.11, N=236
var.5.APOL1 22:36661330:G:A
peptide level
186:186
120:120
19:19
VNEPSILEMSRpc2_NP.2
p−maj=1, pX=0.0066, N=325
var.5.APOL1 22:36661330:G:A
peptide level
114:186
80:120
14:19
VNEPSILEMSRpc2_NP.3
p−min=0.25, pX=7e−04, N=208
var.5.APOL1 22:36661330:G:A
peptide level
186:186
120:120
19:19
VNEPSILEMSRpc2_NP.4
p−maj=1, pX=0.032, N=325
var.5.APOL1 22:36661330:G:A
peptide level
186:186
120:120
19:19
VNEPSILEMSRpc2_NP.5
p−maj=1, pX=0.021, N=325
var.5.APOL1 22:36661330:G:A
peptide level
21:186
12:120
4:19
VQQNVPSGTDTGDPQSKpc2_NP.1
p−maj=0.25, pX=0.74, N=37
var.5.APOL1 22:36661330:G:A
peptide level
15:186
7:120
2:19
VQQNVPSGTDTGDPQSKpc2_NP.3
p−maj=0.64, pX=0.24, N=24
var.5.APOL1 22:36661330:G:A
peptide level
88:186
54:120
9:19
VQQNVPSGTDTGDPQSKpc2_NP.4
p−min=0.74, pX=0.78, N=151
var.5.APOL1 22:36661330:G:A
peptide level
93:186
55:120
9:19
VQQNVPSGTDTGDPQSKpc2_NP.5
p−min=0.5, pX=0.54, N=157
var.5.APOL1 22:36661330:G:A
peptide level
4:186
4:120
0:19
VSTQNLLLLLTDNEAWNGFVAAAELPRpc4_NF
p−min=0.73, pX=0.92, N=8
var.5.APOL1 22:36661330:G:A
peptide level
6:186
3:120
1:19
VSTQNLLLLLTDNEAWNGFVAAAELPRpc4_NF
p−maj=0.46, pX=0.31, N=10
var.5.APOL1 22:36661330:G:A
peptide level
5:186
3:120
1:19
VSTQNLLLLLTDNEAWNGFVAAAELPRpc4_NF
p−maj=0.42, pX=0.5, N=9

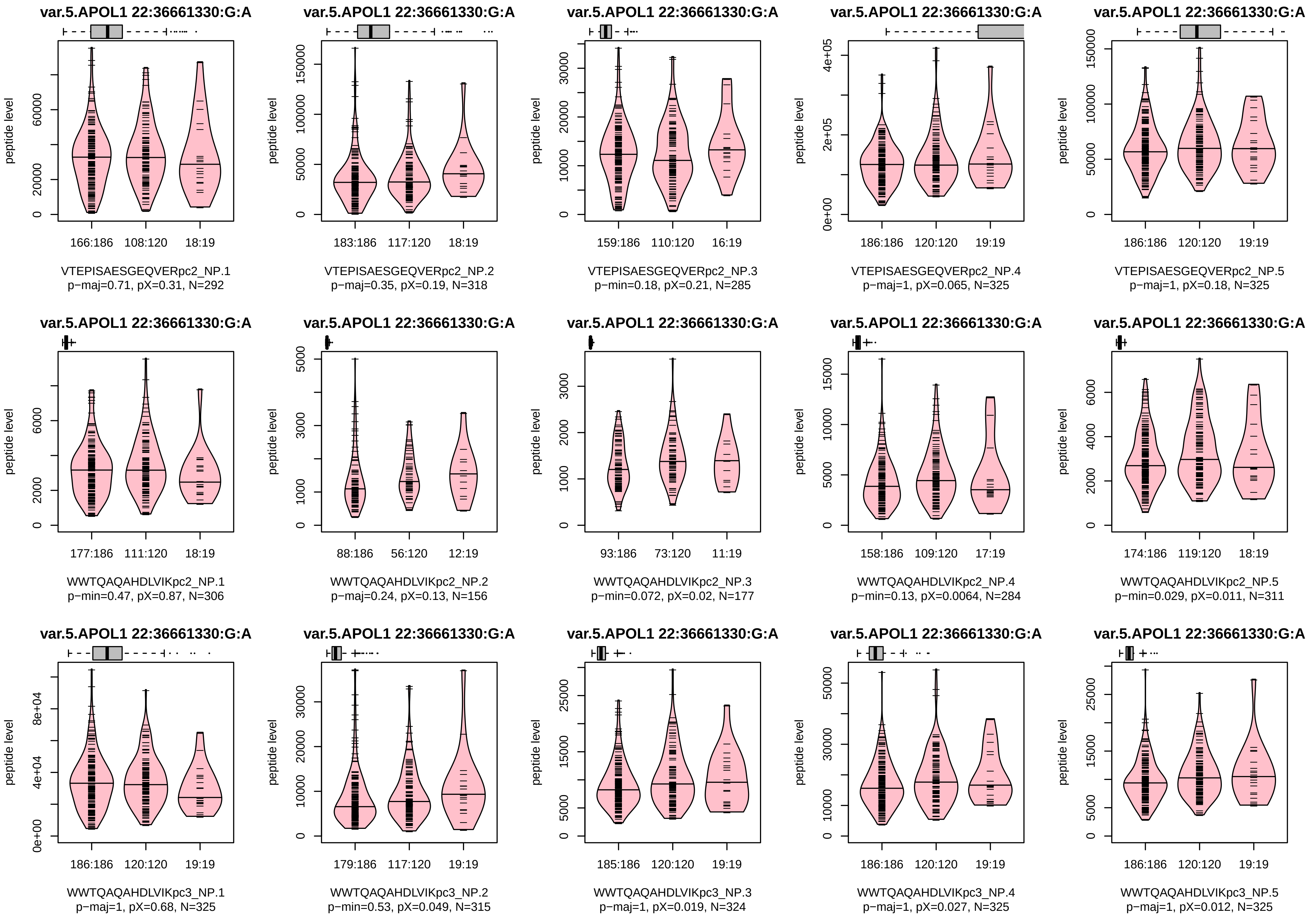
var.5.APOL1 22:36661330:G:A
peptide level
166:186
108:120
18:19
VTEPISAESGEQVERpc2_NP.1
p−maj=0.71, pX=0.31, N=292
var.5.APOL1 22:36661330:G:A
peptide level
183:186
117:120
18:19
VTEPISAESGEQVERpc2_NP.2
p−maj=0.35, pX=0.19, N=318
var.5.APOL1 22:36661330:G:A
peptide level
159:186
110:120
16:19
VTEPISAESGEQVERpc2_NP.3
p−min=0.18, pX=0.21, N=285
var.5.APOL1 22:36661330:G:A
peptide level
186:186
120:120
19:19
VTEPISAESGEQVERpc2_NP.4
p−maj=1, pX=0.065, N=325
var.5.APOL1 22:36661330:G:A
peptide level
186:186
120:120
19:19
VTEPISAESGEQVERpc2_NP.5
p−maj=1, pX=0.18, N=325
var.5.APOL1 22:36661330:G:A
peptide level
177:186
111:120
18:19
WWTQAQAHDLVIKpc2_NP.1
p−min=0.47, pX=0.87, N=306
var.5.APOL1 22:36661330:G:A
peptide level
88:186
56:120
12:19
WWTQAQAHDLVIKpc2_NP.2
p−maj=0.24, pX=0.13, N=156
var.5.APOL1 22:36661330:G:A
peptide level
93:186
73:120
11:19
WWTQAQAHDLVIKpc2_NP.3
p−min=0.072, pX=0.02, N=177
var.5.APOL1 22:36661330:G:A
peptide level
158:186
109:120
17:19
WWTQAQAHDLVIKpc2_NP.4
p−min=0.13, pX=0.0064, N=284
var.5.APOL1 22:36661330:G:A
peptide level
174:186
119:120
18:19
WWTQAQAHDLVIKpc2_NP.5
p−min=0.029, pX=0.011, N=311
var.5.APOL1 22:36661330:G:A
peptide level
186:186
120:120
19:19
WWTQAQAHDLVIKpc3_NP.1
p−maj=1, pX=0.68, N=325
var.5.APOL1 22:36661330:G:A
peptide level
179:186
117:120
19:19
WWTQAQAHDLVIKpc3_NP.2
p−min=0.53, pX=0.049, N=315
var.5.APOL1 22:36661330:G:A
peptide level
185:186
120:120
19:19
WWTQAQAHDLVIKpc3_NP.3
p−maj=1, pX=0.019, N=324
var.5.APOL1 22:36661330:G:A
peptide level
186:186
120:120
19:19
WWTQAQAHDLVIKpc3_NP.4
p−maj=1, pX=0.027, N=325
var.5.APOL1 22:36661330:G:A
peptide level
186:186
120:120
19:19
WWTQAQAHDLVIKpc3_NP.5
p−maj=1, pX=0.012, N=325

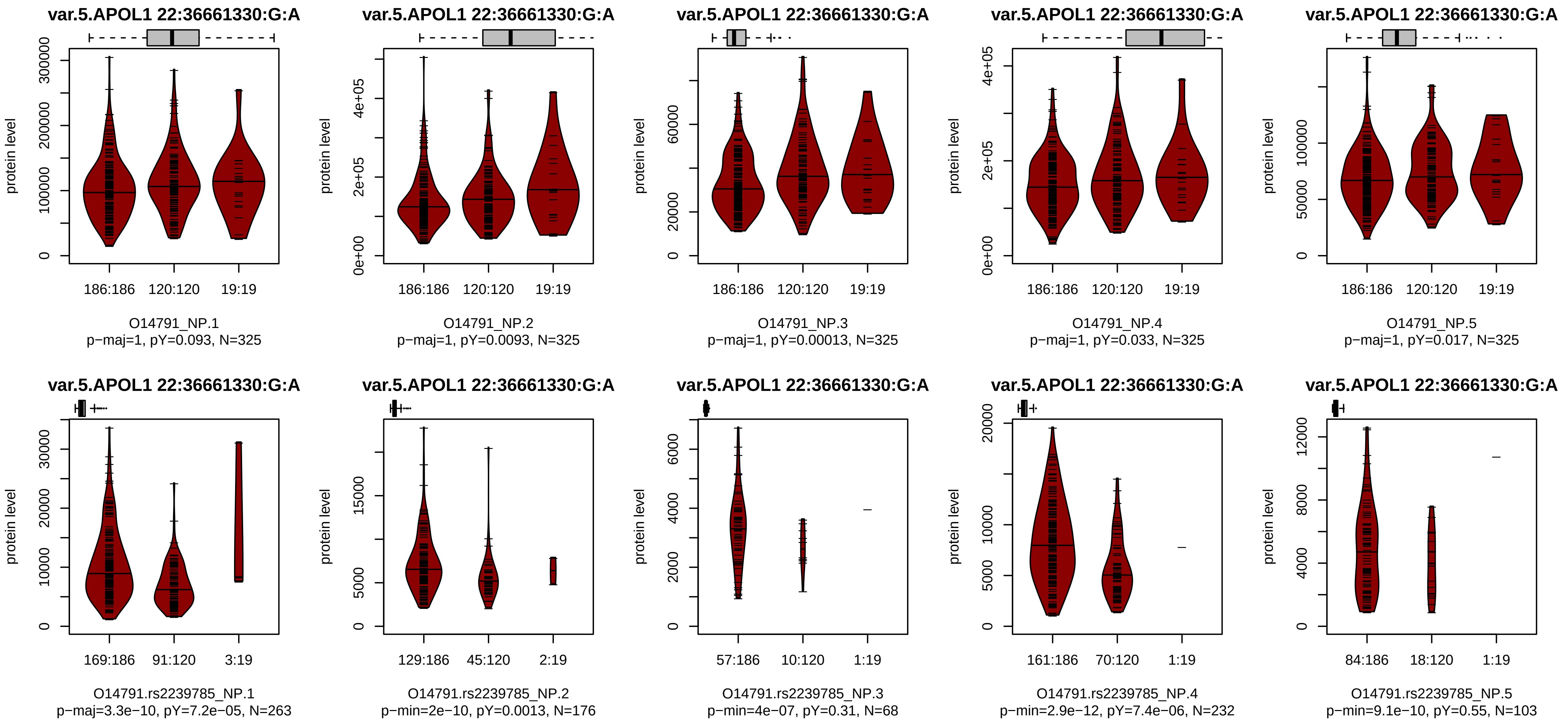
var.5.APOL1 22:36661330:G:A
protein level
186:186
120:120
19:19
O14791_NP.1
p−maj=1, pY=0.093, N=325
var.5.APOL1 22:36661330:G:A
protein level
186:186
120:120
19:19
O14791_NP.2
p−maj=1, pY=0.0093, N=325
var.5.APOL1 22:36661330:G:A
protein level
186:186
120:120
19:19
O14791_NP.3
p−maj=1, pY=0.00013, N=325
var.5.APOL1 22:36661330:G:A
protein level
186:186
120:120
19:19
O14791_NP.4
p−maj=1, pY=0.033, N=325
var.5.APOL1 22:36661330:G:A
protein level
186:186
120:120
19:19
O14791_NP.5
p−maj=1, pY=0.017, N=325
var.5.APOL1 22:36661330:G:A
protein level
169:186
91:120
3:19
O14791.rs2239785_NP.1
p−maj=3.3e−10, pY=7.2e−05, N=263
var.5.APOL1 22:36661330:G:A
protein level
129:186
45:120
2:19
O14791.rs2239785_NP.2
p−min=2e−10, pY=0.0013, N=176
var.5.APOL1 22:36661330:G:A
protein level
57:186
10:120
1:19
O14791.rs2239785_NP.3
p−min=4e−07, pY=0.31, N=68
var.5.APOL1 22:36661330:G:A
protein level
161:186
70:120
1:19
O14791.rs2239785_NP.4
p−min=2.9e−12, pY=7.4e−06, N=232
var.5.APOL1 22:36661330:G:A
protein level
84:186
18:120
1:19
O14791.rs2239785_NP.5
p−min=9.1e−10, pY=0.55, N=103

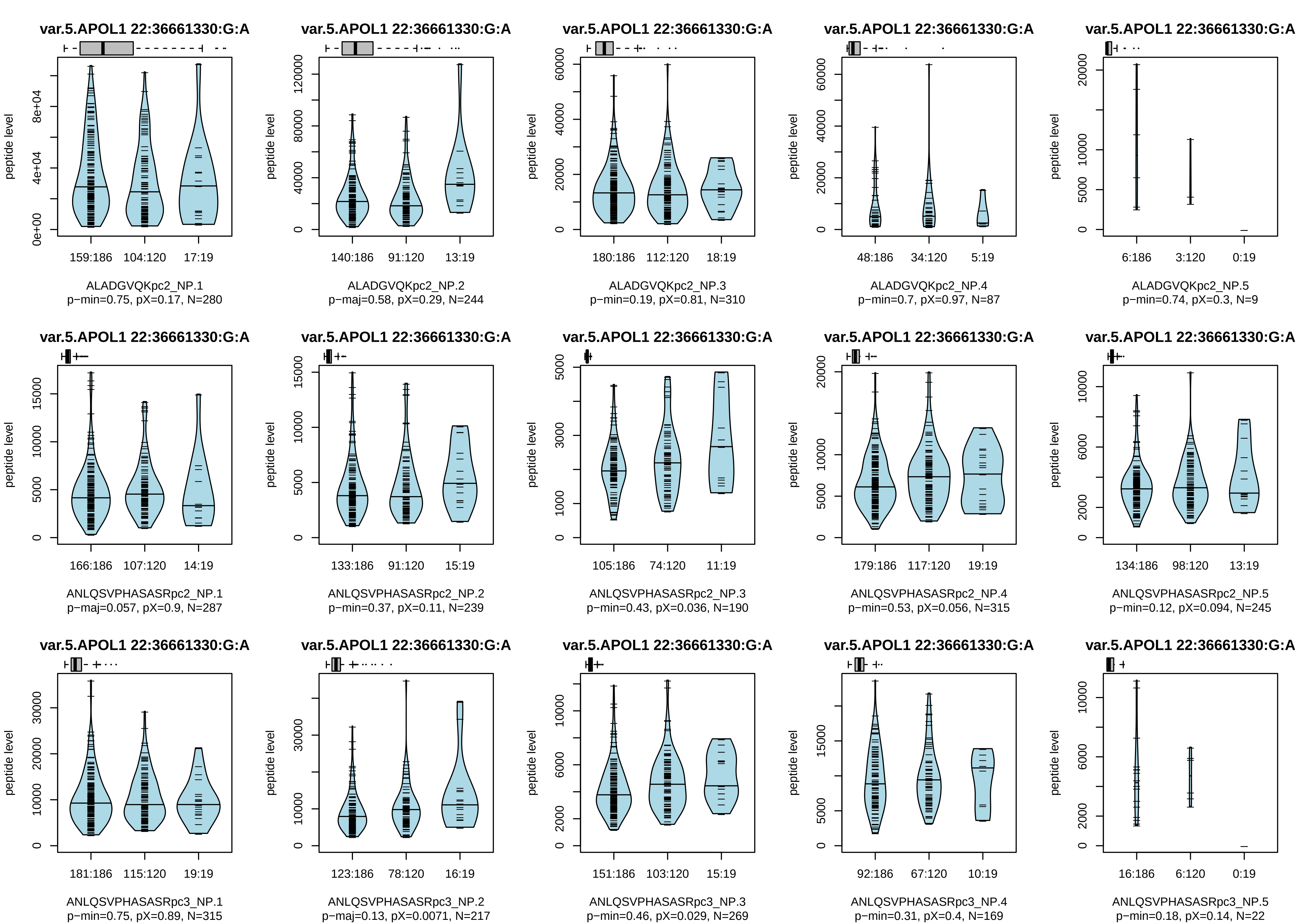
var.5.APOL1 22:36661330:G:A
peptide level
159:186
104:120
17:19
ALADGVQKpc2_NP.1
p−min=0.75, pX=0.17, N=280
var.5.APOL1 22:36661330:G:A
140:186
91:120
13:19
ALADGVQKpc2_NP.2
p−maj=0.58, pX=0.29, N=244
var.5.APOL1 22:36661330:G:A
180:186
112:120
18:19
ALADGVQKpc2_NP.3
p−min=0.19, pX=0.81, N=310
var.5.APOL1 22:36661330:G:A
48:186
34:120
5:19
ALADGVQKpc2_NP.4
p−min=0.7, pX=0.97, N=87
var.5.APOL1 22:36661330:G:A
6:186
3:120
0:19
ALADGVQKpc2_NP.5
p−min=0.74, pX=0.3, N=9
var.5.APOL1 22:36661330:G:A
166:186
107:120
14:19
ANLQSVPHASASRpc2_NP.1
p−maj=0.057, pX=0.9, N=287
var.5.APOL1 22:36661330:G:A
133:186
91:120
15:19
ANLQSVPHASASRpc2_NP.2
p−min=0.37, pX=0.11, N=239
var.5.APOL1 22:36661330:G:A
105:186
74:120
11:19
ANLQSVPHASASRpc2_NP.3
p−min=0.43, pX=0.036, N=190
var.5.APOL1 22:36661330:G:A
179:186
117:120
19:19
ANLQSVPHASASRpc2_NP.4
p−min=0.53, pX=0.056, N=315
var.5.APOL1 22:36661330:G:A
134:186
98:120
13:19
ANLQSVPHASASRpc2_NP.5
p−min=0.12, pX=0.094, N=245
var.5.APOL1 22:36661330:G:A
181:186
115:120
19:19
ANLQSVPHASASRpc3_NP.1
p−min=0.75, pX=0.89, N=315
var.5.APOL1 22:36661330:G:A
123:186
78:120
16:19
ANLQSVPHASASRpc3_NP.2
p−maj=0.13, pX=0.0071, N=217
var.5.APOL1 22:36661330:G:A
151:186
103:120
15:19
ANLQSVPHASASRpc3_NP.3
p−min=0.46, pX=0.029, N=269
var.5.APOL1 22:36661330:G:A
92:186
67:120
10:19
ANLQSVPHASASRpc3_NP.4
p−min=0.31, pX=0.4, N=169
var.5.APOL1 22:36661330:G:A
16:186
6:120
0:19
ANLQSVPHASASRpc3_NP.5
p−min=0.18, pX=0.14, N=22

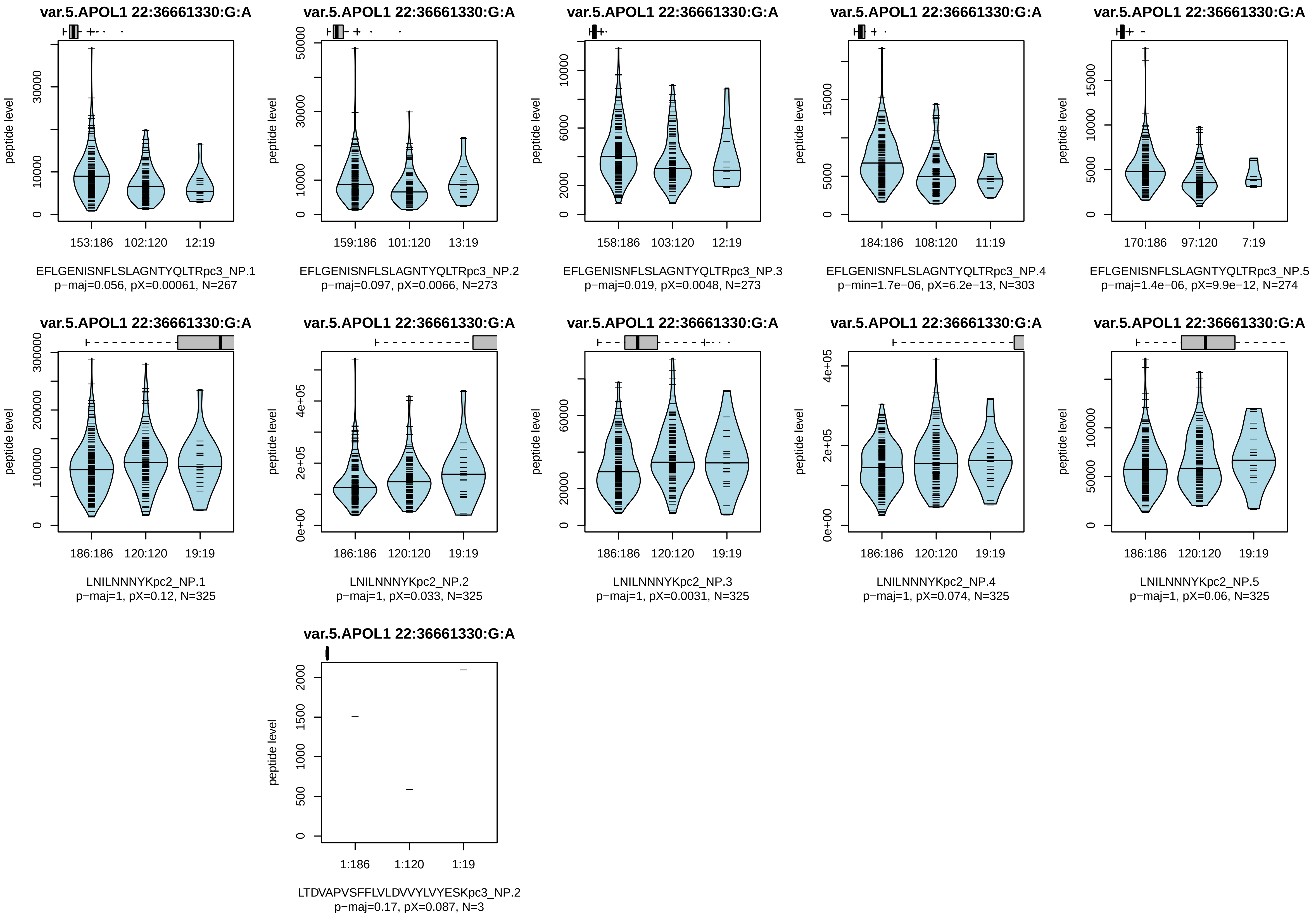
var.5.APOL1 22:36661330:G:A
peptide level
153:186
102:120
12:19
EFLGENISNFLSLAGNTYQLTRpc3_NP.1
p−maj=0.056, pX=0.00061, N=267
var.5.APOL1 22:36661330:G:A
peptide level
159:186
101:120
13:19
EFLGENISNFLSLAGNTYQLTRpc3_NP.2
p−maj=0.097, pX=0.0066, N=273
var.5.APOL1 22:36661330:G:A
peptide level
158:186
103:120
12:19
EFLGENISNFLSLAGNTYQLTRpc3_NP.3
p−maj=0.019, pX=0.0048, N=273
var.5.APOL1 22:36661330:G:A
peptide level
184:186
108:120
11:19
EFLGENISNFLSLAGNTYQLTRpc3_NP.4
p−min=1.7e−06, pX=6.2e−13, N=303
var.5.APOL1 22:36661330:G:A
peptide level
170:186
97:120
7:19
EFLGENISNFLSLAGNTYQLTRpc3_NP.5
p−maj=1.4e−06, pX=9.9e−12, N=274
var.5.APOL1 22:36661330:G:A
peptide level
186:186
120:120
19:19
LNILNNNYKpc2_NP.1
p−maj=1, pX=0.12, N=325
var.5.APOL1 22:36661330:G:A
peptide level
186:186
120:120
19:19
LNILNNNYKpc2_NP.2
p−maj=1, pX=0.033, N=325
var.5.APOL1 22:36661330:G:A
peptide level
186:186
120:120
19:19
LNILNNNYKpc2_NP.3
p−maj=1, pX=0.0031, N=325
var.5.APOL1 22:36661330:G:A
peptide level
186:186
120:120
19:19
LNILNNNYKpc2_NP.4
p−maj=1, pX=0.074, N=325
var.5.APOL1 22:36661330:G:A
peptide level
186:186
120:120
19:19
LNILNNNYKpc2_NP.5
p−maj=1, pX=0.06, N=325
var.5.APOL1 22:36661330:G:A
peptide level
1:186
1:120
1:19
LTDVAPVSFFLVLDVVYLVYESKpc3_NP.2
p−maj=0.17, pX=0.087, N=3

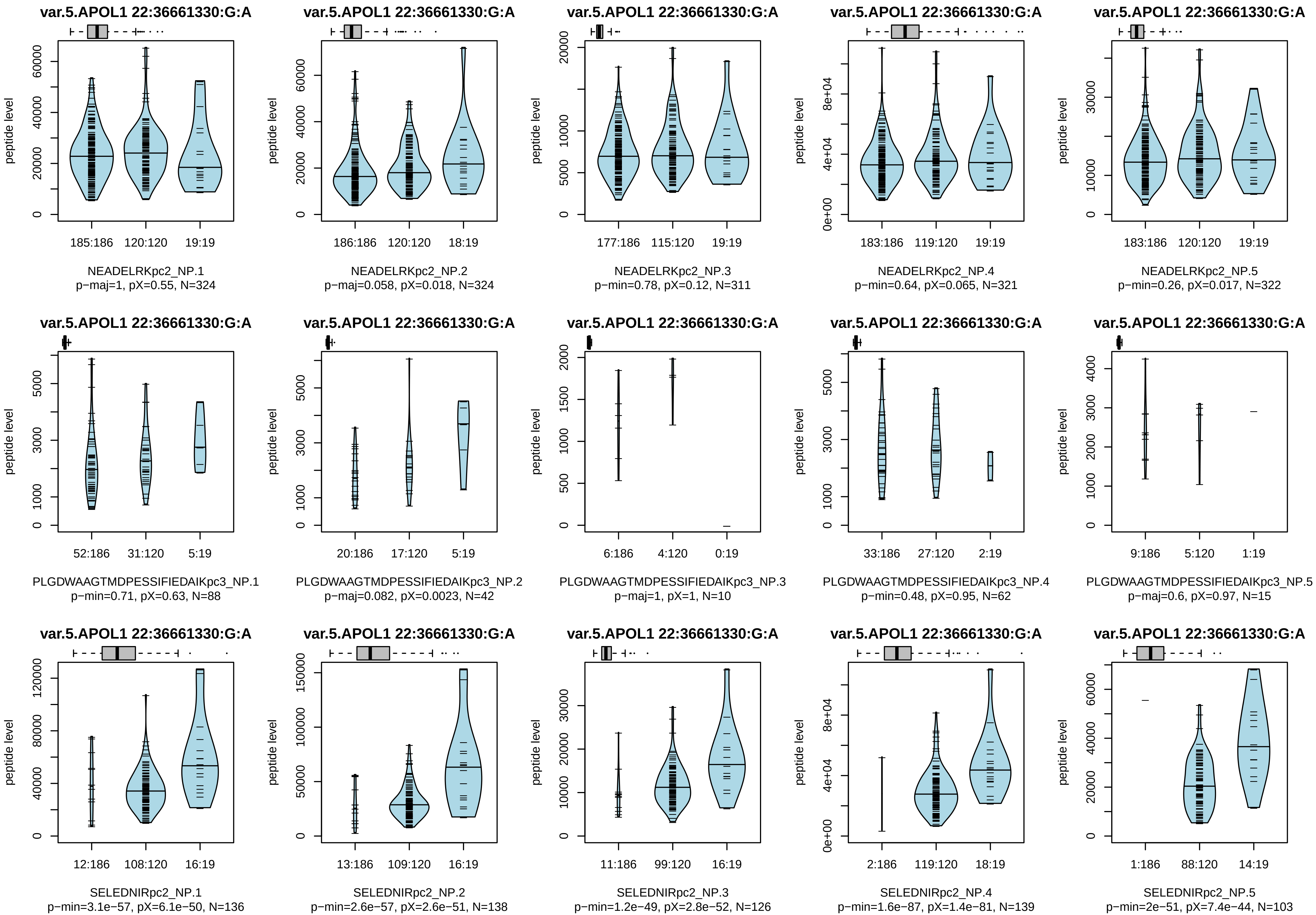
var.5.APOL1 22:36661330:G:A
peptide level
185:186
120:120
19:19
NEADELRKpc2_NP.1
p−maj=1, pX=0.55, N=324
var.5.APOL1 22:36661330:G:A
peptide level
186:186
120:120
18:19
NEADELRKpc2_NP.2
p−maj=0.058, pX=0.018, N=324
var.5.APOL1 22:36661330:G:A
peptide level
177:186
115:120
19:19
NEADELRKpc2_NP.3
p−min=0.78, pX=0.12, N=311
var.5.APOL1 22:36661330:G:A
peptide level
183:186
119:120
19:19
NEADELRKpc2_NP.4
p−min=0.64, pX=0.065, N=321
var.5.APOL1 22:36661330:G:A
peptide level
183:186
120:120
19:19
NEADELRKpc2_NP.5
p−min=0.26, pX=0.017, N=322
var.5.APOL1 22:36661330:G:A
peptide level
52:186
31:120
5:19
PLGDWAAGTMDPESSIFIEDAIKpc3_NP.1
p−min=0.71, pX=0.63, N=88
var.5.APOL1 22:36661330:G:A
peptide level
20:186
17:120
5:19
PLGDWAAGTMDPESSIFIEDAIKpc3_NP.2
p−maj=0.082, pX=0.0023, N=42
var.5.APOL1 22:36661330:G:A
peptide level
6:186
4:120
0:19
PLGDWAAGTMDPESSIFIEDAIKpc3_NP.3
p−maj=1, pX=1, N=10
var.5.APOL1 22:36661330:G:A
peptide level
33:186
27:120
2:19
PLGDWAAGTMDPESSIFIEDAIKpc3_NP.4
p−min=0.48, pX=0.95, N=62
var.5.APOL1 22:36661330:G:A
peptide level
9:186
5:120
1:19
PLGDWAAGTMDPESSIFIEDAIKpc3_NP.5
p−maj=0.6, pX=0.97, N=15
var.5.APOL1 22:36661330:G:A
peptide level
12:186
108:120
16:19
SELEDNIRpc2_NP.1
p−min=3.1e−57, pX=6.1e−50, N=136
var.5.APOL1 22:36661330:G:A
peptide level
13:186
109:120
16:19
SELEDNIRpc2_NP.2
p−min=2.6e−57, pX=2.6e−51, N=138
var.5.APOL1 22:36661330:G:A
peptide level
11:186
99:120
16:19
SELEDNIRpc2_NP.3
p−min=1.2e−49, pX=2.8e−52, N=126
var.5.APOL1 22:36661330:G:A
peptide level
2:186
119:120
18:19
SELEDNIRpc2_NP.4
p−min=1.6e−87, pX=1.4e−81, N=139
var.5.APOL1 22:36661330:G:A
peptide level
1:186
88:120
14:19
SELEDNIRpc2_NP.5
p−min=2e−51, pX=7.4e−44, N=103

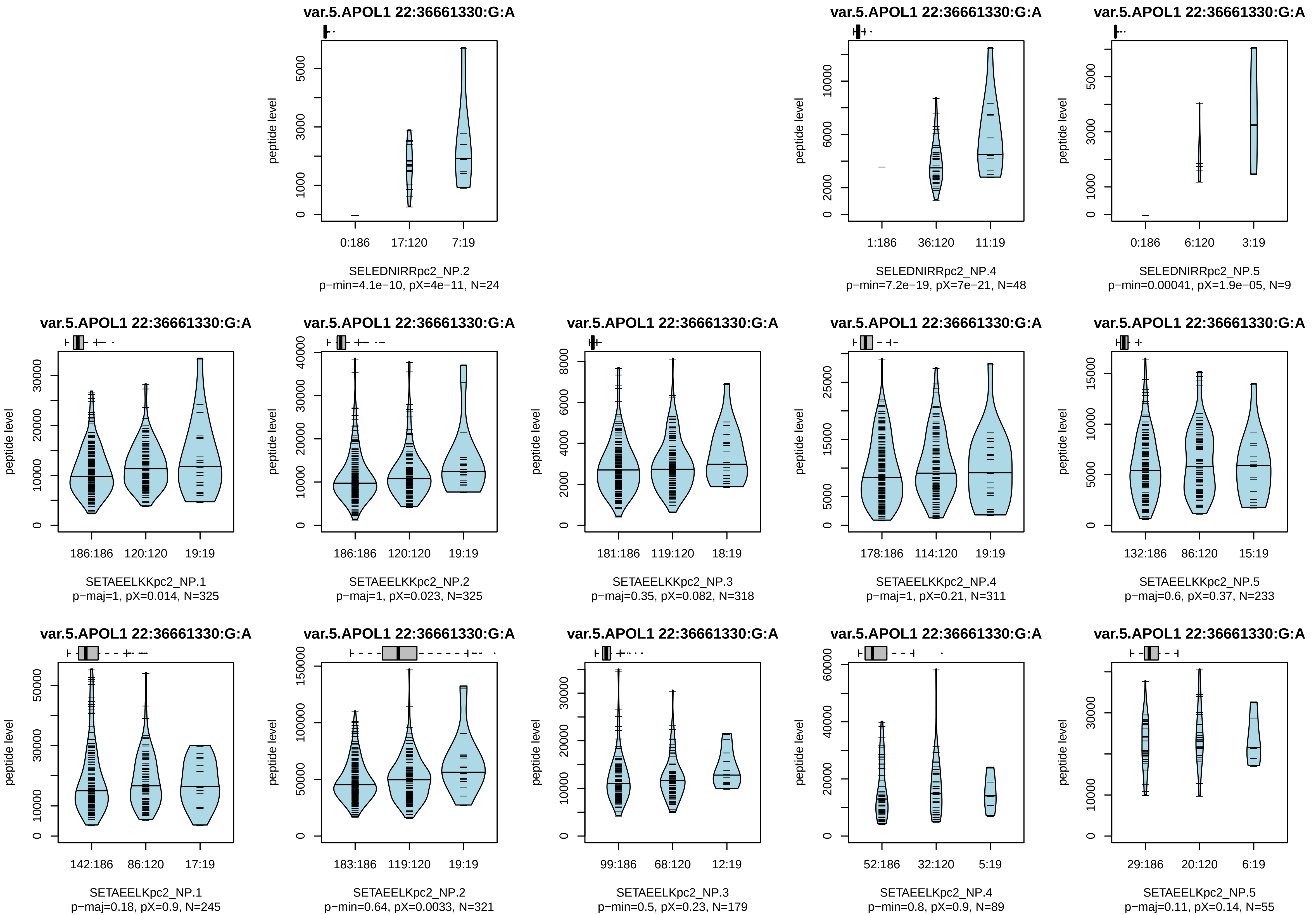
var.5.APOL1 22:36661330:G:A
peptide level
0:186
17:120
7:19
SELEDNIRRpc2_NP.2
p−min=4.1e−10, pX=4e−11, N=24
var.5.APOL1 22:36661330:G:A
peptide level
1:186
36:120
11:19
SELEDNIRRpc2_NP.4
p−min=7.2e−19, pX=7e−21, N=48
var.5.APOL1 22:36661330:G:A
peptide level
0:186
6:120
3:19
SELEDNIRRpc2_NP.5
p−min=0.00041, pX=1.9e−05, N=9
var.5.APOL1 22:36661330:G:A
peptide level
186:186
120:120
19:19
SETAEELKKpc2_NP.1
p−maj=1, pX=0.014, N=325
var.5.APOL1 22:36661330:G:A
peptide level
186:186
120:120
19:19
SETAEELKKpc2_NP.2
p−maj=1, pX=0.023, N=325
var.5.APOL1 22:36661330:G:A
peptide level
181:186
119:120
18:19
SETAEELKKpc2_NP.3
p−maj=0.35, pX=0.082, N=318
var.5.APOL1 22:36661330:G:A
peptide level
178:186
114:120
19:19
SETAEELKKpc2_NP.4
p−maj=1, pX=0.21, N=311
var.5.APOL1 22:36661330:G:A
peptide level
132:186
86:120
15:19
SETAEELKKpc2_NP.5
p−maj=0.6, pX=0.37, N=233
var.5.APOL1 22:36661330:G:A
peptide level
142:186
86:120
17:19
SETAEELKpc2_NP.1
p−maj=0.18, pX=0.9, N=245
var.5.APOL1 22:36661330:G:A
peptide level
183:186
119:120
19:19
SETAEELKpc2_NP.2
p−min=0.64, pX=0.0033, N=321
var.5.APOL1 22:36661330:G:A
peptide level
99:186
68:120
12:19
SETAEELKpc2_NP.3
p−min=0.5, pX=0.23, N=179
var.5.APOL1 22:36661330:G:A
peptide level
52:186
32:120
5:19
SETAEELKpc2_NP.4
p−min=0.8, pX=0.9, N=89
var.5.APOL1 22:36661330:G:A
peptide level
29:186
20:120
6:19
SETAEELKpc2_NP.5
p−maj=0.11, pX=0.14, N=55

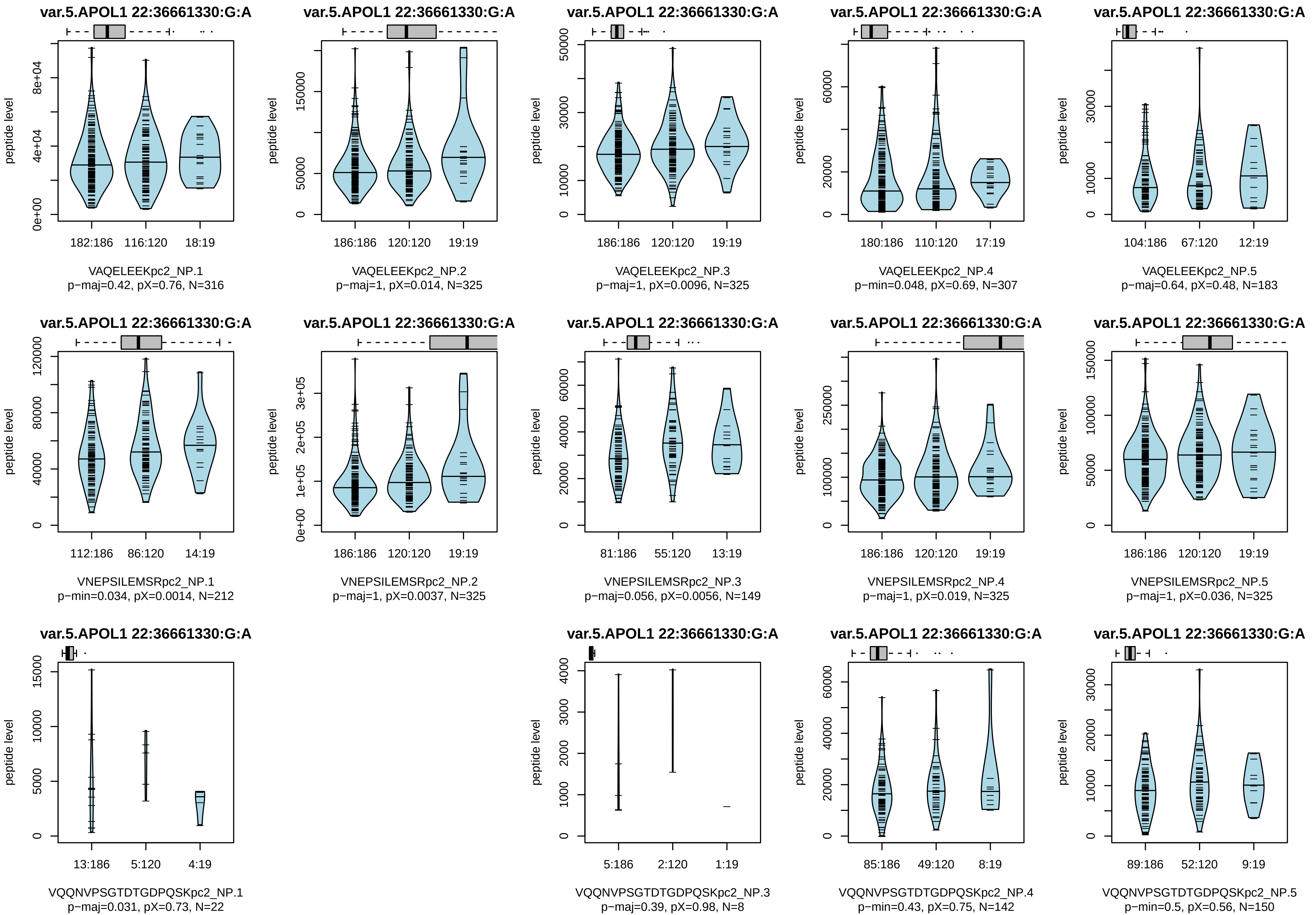
var.5.APOL1 22:36661330:G:A
peptide level
182:186
116:120
18:19
VAQELEEKpc2_NP.1
p−maj=0.42, pX=0.76, N=316
var.5.APOL1 22:36661330:G:A
peptide level
186:186
120:120
19:19
VAQELEEKpc2_NP.2
p−maj=1, pX=0.014, N=325
var.5.APOL1 22:36661330:G:A
peptide level
186:186
120:120
19:19
VAQELEEKpc2_NP.3
p−maj=1, pX=0.0096, N=325
var.5.APOL1 22:36661330:G:A
peptide level
180:186
110:120
17:19
VAQELEEKpc2_NP.4
p−min=0.048, pX=0.69, N=307
var.5.APOL1 22:36661330:G:A
peptide level
104:186
67:120
12:19
VAQELEEKpc2_NP.5
p−maj=0.64, pX=0.48, N=183
var.5.APOL1 22:36661330:G:A
peptide level
112:186
86:120
14:19
VNEPSILEMSRpc2_NP.1
p−min=0.034, pX=0.0014, N=212
var.5.APOL1 22:36661330:G:A
peptide level
186:186
120:120
19:19
VNEPSILEMSRpc2_NP.2
p−maj=1, pX=0.0037, N=325
var.5.APOL1 22:36661330:G:A
peptide level
81:186
55:120
13:19
VNEPSILEMSRpc2_NP.3
p−maj=0.056, pX=0.0056, N=149
var.5.APOL1 22:36661330:G:A
peptide level
186:186
120:120
19:19
VNEPSILEMSRpc2_NP.4
p−maj=1, pX=0.019, N=325
var.5.APOL1 22:36661330:G:A
peptide level
186:186
120:120
19:19
VNEPSILEMSRpc2_NP.5
p−maj=1, pX=0.036, N=325
var.5.APOL1 22:36661330:G:A
peptide level
13:186
5:120
4:19
VQQNVPSGTDTGDPQSKpc2_NP.1
p−maj=0.031, pX=0.73, N=22
var.5.APOL1 22:36661330:G:A
peptide level
5:186
2:120
1:19
VQQNVPSGTDTGDPQSKpc2_NP.3
p−maj=0.39, pX=0.98, N=8
var.5.APOL1 22:36661330:G:A
peptide level
85:186
49:120
8:19
VQQNVPSGTDTGDPQSKpc2_NP.4
p−min=0.43, pX=0.75, N=142
var.5.APOL1 22:36661330:G:A
peptide level
89:186
52:120
9:19
VQQNVPSGTDTGDPQSKpc2_NP.5
p−min=0.5, pX=0.56, N=150

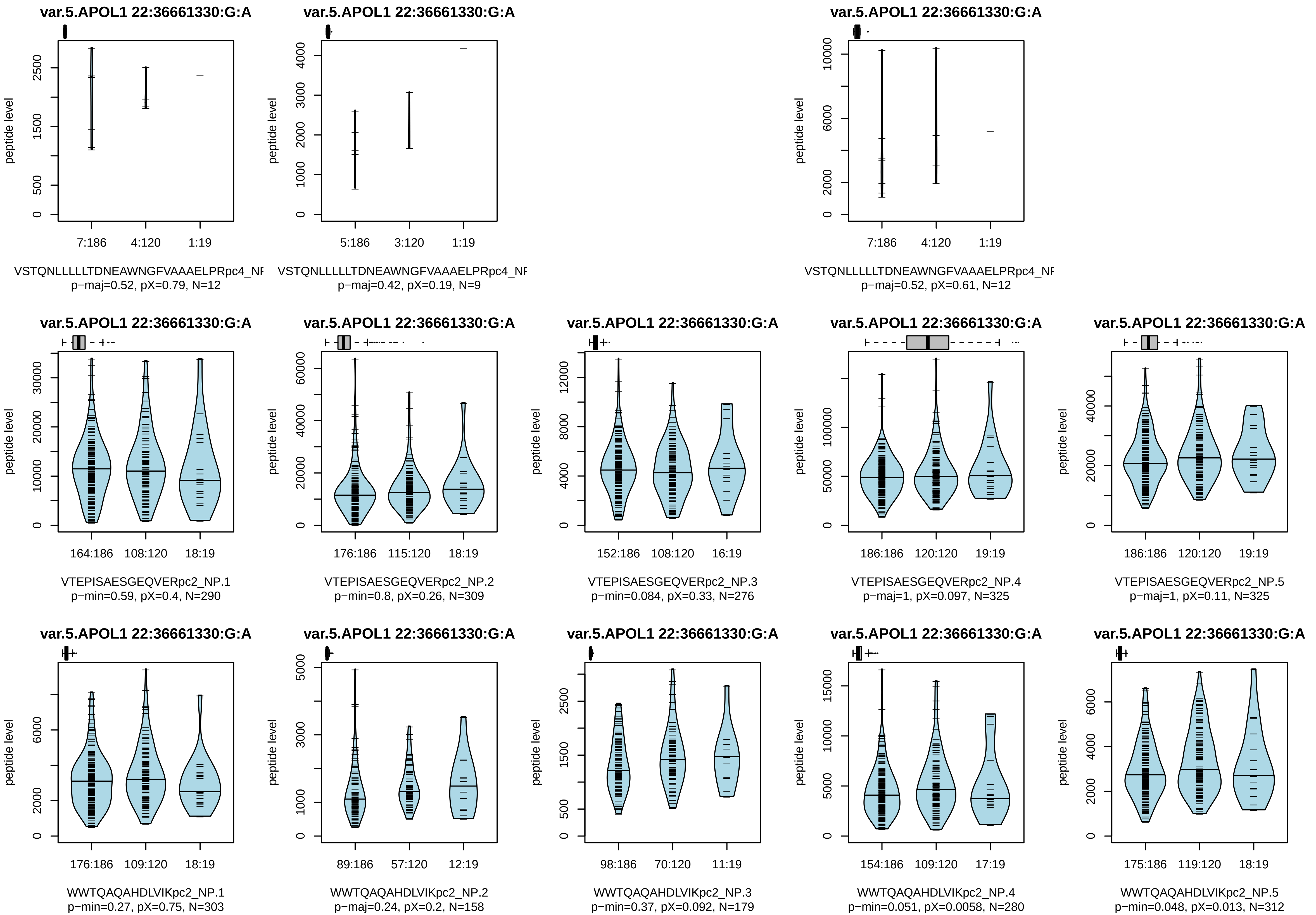
var.5.APOL1 22:36661330:G:A
peptide level
7:186
4:120
1:19
VSTQNLLLLLTDNEAWNGFVAAAELPRpc4_NF
p−maj=0.52, pX=0.79, N=12
var.5.APOL1 22:36661330:G:A
peptide level
5:186
3:120
1:19
VSTQNLLLLLTDNEAWNGFVAAAELPRpc4_NF
p−maj=0.42, pX=0.19, N=9
var.5.APOL1 22:36661330:G:A
peptide level
7:186
4:120
1:19
VSTQNLLLLLTDNEAWNGFVAAAELPRpc4_NF
p−maj=0.52, pX=0.61, N=12
var.5.APOL1 22:36661330:G:A
peptide level
164:186
108:120
18:19
VTEPISAESGEQVERpc2_NP.1
p−min=0.59, pX=0.4, N=290
var.5.APOL1 22:36661330:G:A
peptide level
176:186
115:120
18:19
VTEPISAESGEQVERpc2_NP.2
p−min=0.8, pX=0.26, N=309
var.5.APOL1 22:36661330:G:A
peptide level
152:186
108:120
16:19
VTEPISAESGEQVERpc2_NP.3
p−min=0.084, pX=0.33, N=276
var.5.APOL1 22:36661330:G:A
peptide level
186:186
120:120
19:19
VTEPISAESGEQVERpc2_NP.4
p−maj=1, pX=0.097, N=325
var.5.APOL1 22:36661330:G:A
peptide level
186:186
120:120
19:19
VTEPISAESGEQVERpc2_NP.5
p−maj=1, pX=0.11, N=325
var.5.APOL1 22:36661330:G:A
peptide level
176:186
109:120
18:19
WWTQAQAHDLVIKpc2_NP.1
p−min=0.27, pX=0.75, N=303
var.5.APOL1 22:36661330:G:A
peptide level
89:186
57:120
12:19
WWTQAQAHDLVIKpc2_NP.2
p−maj=0.24, pX=0.2, N=158
var.5.APOL1 22:36661330:G:A
peptide level
98:186
70:120
11:19
WWTQAQAHDLVIKpc2_NP.3
p−min=0.37, pX=0.092, N=179
var.5.APOL1 22:36661330:G:A
peptide level
154:186
109:120
17:19
WWTQAQAHDLVIKpc2_NP.4
p−min=0.051, pX=0.0058, N=280
var.5.APOL1 22:36661330:G:A
peptide level
175:186
119:120
18:19
WWTQAQAHDLVIKpc2_NP.5
p−min=0.048, pX=0.013, N=312

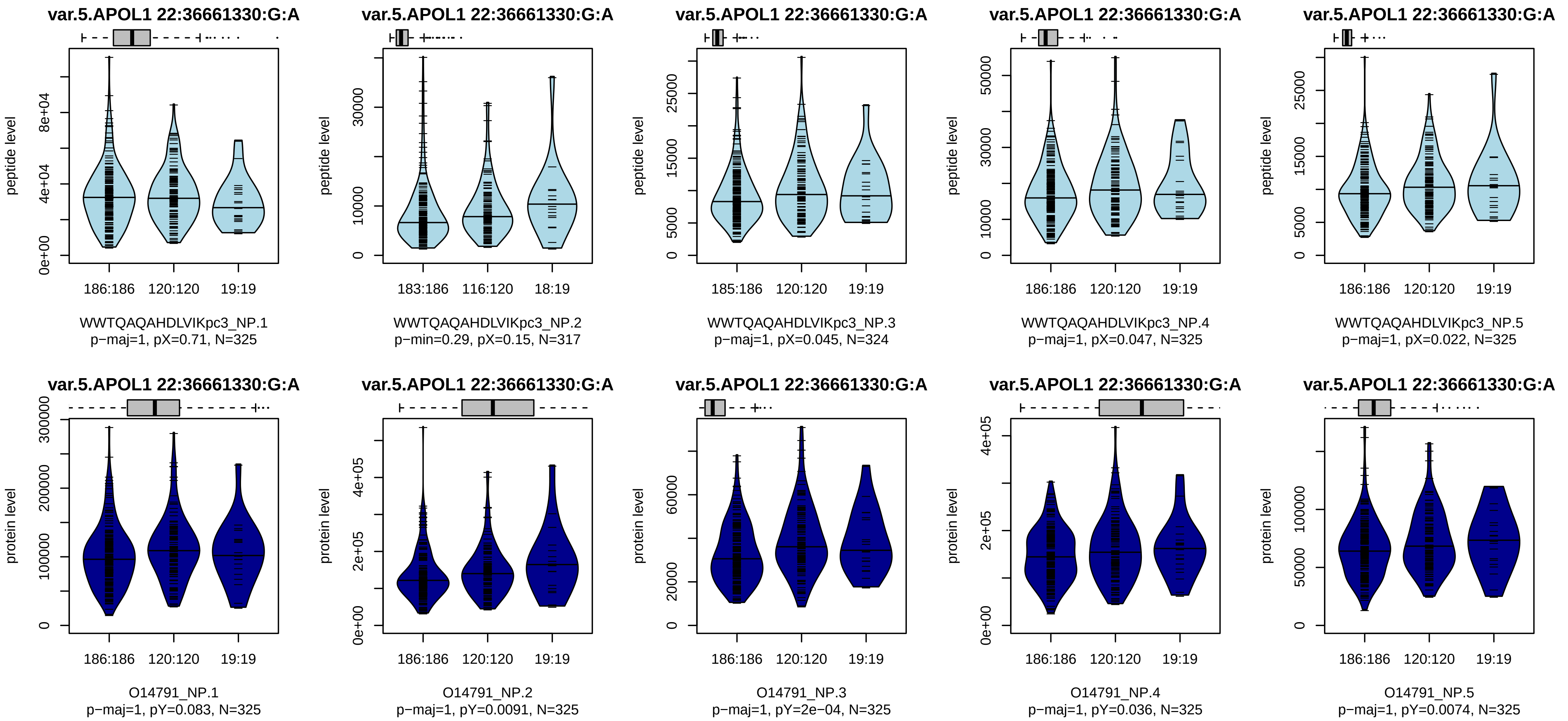
var.5.APOL1 22:36661330:G:A
peptide level
0e+00
4e+04
8e+04
186:186
120:120
19:19
WWTQAQAHDLVIKpc3_NP.1
p−maj=1, pX=0.71, N=325
var.5.APOL1 22:36661330:G:A
peptide level
0
10000
30000
183:186
116:120
18:19
WWTQAQAHDLVIKpc3_NP.2
p−min=0.29, pX=0.15, N=317
var.5.APOL1 22:36661330:G:A
peptide level
0
5000
15000
25000
185:186
120:120
19:19
WWTQAQAHDLVIKpc3_NP.3
p−maj=1, pX=0.045, N=324
var.5.APOL1 22:36661330:G:A
peptide level
0
10000
30000
50000
186:186
120:120
19:19
WWTQAQAHDLVIKpc3_NP.4
p−maj=1, pX=0.047, N=325
var.5.APOL1 22:36661330:G:A
peptide level
0
5000
15000
25000
186:186
120:120
19:19
WWTQAQAHDLVIKpc3_NP.5
p−maj=1, pX=0.022, N=325
var.5.APOL1 22:36661330:G:A
protein level
0
100000
200000
300000
186:186
120:120
19:19
O14791_NP.1
p−maj=1, pY=0.083, N=325
var.5.APOL1 22:36661330:G:A
protein level
0e+00
2e+05
4e+05
186:186
120:120
19:19
O14791_NP.2
p−maj=1, pY=0.0091, N=325
var.5.APOL1 22:36661330:G:A
protein level
0
20000
60000
186:186
120:120
19:19
O14791_NP.3
p−maj=1, pY=2e−04, N=325
var.5.APOL1 22:36661330:G:A
protein level
0e+00
2e+05
4e+05
186:186
120:120
19:19
O14791_NP.4
p−maj=1, pY=0.036, N=325
var.5.APOL1 22:36661330:G:A
protein level
0
50000
100000
186:186
120:120
19:19
O14791_NP.5
p−maj=1, pY=0.0074, N=325

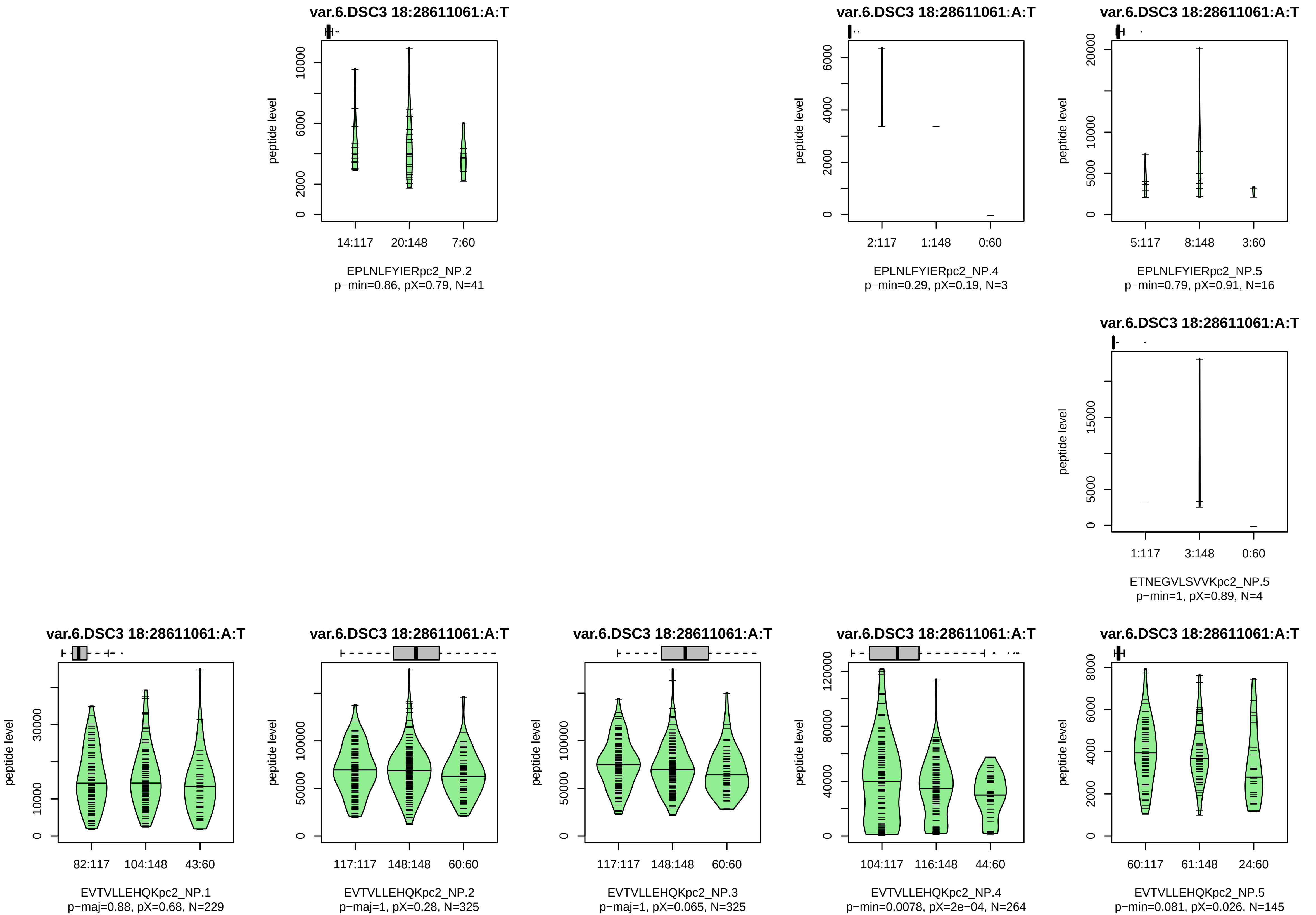
var.6.DSC3 18:28611061:A:T
peptide level
0
2000
6000
10000
14:117
20:148
7:60
EPLNLFYIERpc2_NP.2
p−min=0.86, pX=0.79, N=41
var.6.DSC3 18:28611061:A:T
peptide level
0
2000
4000
6000
2:117
1:148
0:60
EPLNLFYIERpc2_NP.4
p−min=0.29, pX=0.19, N=3
var.6.DSC3 18:28611061:A:T
peptide level
0
5000
10000
20000
5:117
8:148
3:60
EPLNLFYIERpc2_NP.5
p−min=0.79, pX=0.91, N=16
var.6.DSC3 18:28611061:A:T
peptide level
0
5000
15000
1:117
3:148
0:60
ETNEGVLSVVKpc2_NP.5
p−min=1, pX=0.89, N=4
var.6.DSC3 18:28611061:A:T
peptide level
0
10000
30000
82:117
104:148
43:60
EVTVLLEHQKpc2_NP.1
p−maj=0.88, pX=0.68, N=229
var.6.DSC3 18:28611061:A:T
peptide level
0
50000
100000
117:117
148:148
60:60
EVTVLLEHQKpc2_NP.2
p−maj=1, pX=0.28, N=325
var.6.DSC3 18:28611061:A:T
peptide level
0
50000
100000
117:117
148:148
60:60
EVTVLLEHQKpc2_NP.3
p−maj=1, pX=0.065, N=325
var.6.DSC3 18:28611061:A:T
peptide level
0
40000
80000
120000
104:117
116:148
44:60
EVTVLLEHQKpc2_NP.4
p−min=0.0078, pX=2e−04, N=264
var.6.DSC3 18:28611061:A:T
peptide level
0
2000
4000
6000
8000
60:117
61:148
24:60
EVTVLLEHQKpc2_NP.5
p−min=0.081, pX=0.026, N=145

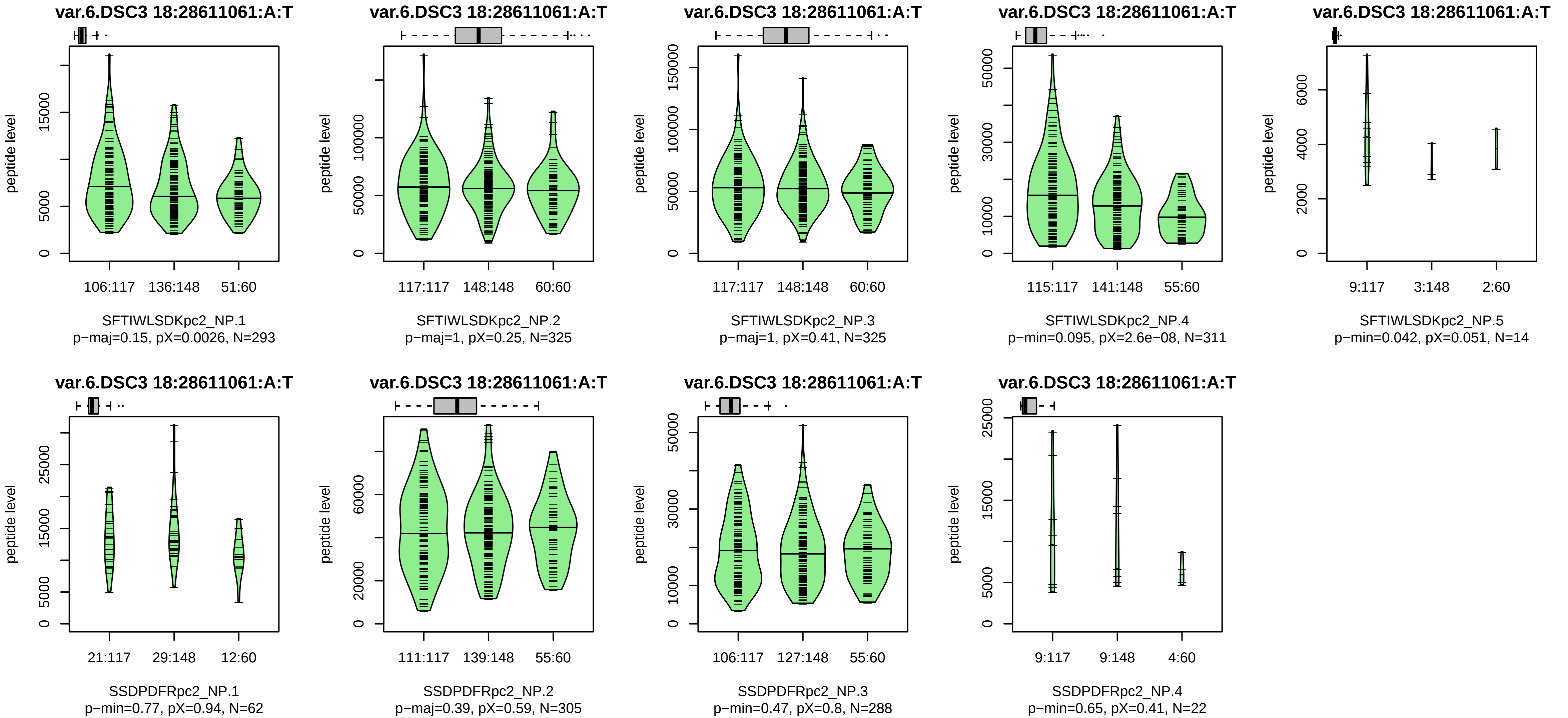
var.6.DSC3 18:28611061:A:T
peptide level
0
5000
15000
106:117
136:148
51:60
SFTIWLSDKpc2_NP.1
p−maj=0.15, pX=0.0026, N=293
var.6.DSC3 18:28611061:A:T
peptide level
0
50000
100000
117:117
148:148
60:60
SFTIWLSDKpc2_NP.2
p−maj=1, pX=0.25, N=325
var.6.DSC3 18:28611061:A:T
peptide level
0
50000
100000
150000
117:117
148:148
60:60
SFTIWLSDKpc2_NP.3
p−maj=1, pX=0.41, N=325
var.6.DSC3 18:28611061:A:T
peptide level
0
10000
30000
50000
115:117
141:148
55:60
SFTIWLSDKpc2_NP.4
p−min=0.095, pX=2.6e−08, N=311
var.6.DSC3 18:28611061:A:T
peptide level
0
2000
4000
6000
9:117
3:148
2:60
SFTIWLSDKpc2_NP.5
p−min=0.042, pX=0.051, N=14
var.6.DSC3 18:28611061:A:T
peptide level
0
5000
15000
25000
21:117
29:148
12:60
SSDPDFRpc2_NP.1
p−min=0.77, pX=0.94, N=62
var.6.DSC3 18:28611061:A:T
peptide level
0
20000
60000
111:117
139:148
55:60
SSDPDFRpc2_NP.2
p−maj=0.39, pX=0.59, N=305
var.6.DSC3 18:28611061:A:T
peptide level
0
10000
30000
50000
106:117
127:148
55:60
SSDPDFRpc2_NP.3
p−min=0.47, pX=0.8, N=288
var.6.DSC3 18:28611061:A:T
peptide level
0
5000
15000
25000
9:117
9:148
4:60
SSDPDFRpc2_NP.4
p−min=0.65, pX=0.41, N=22

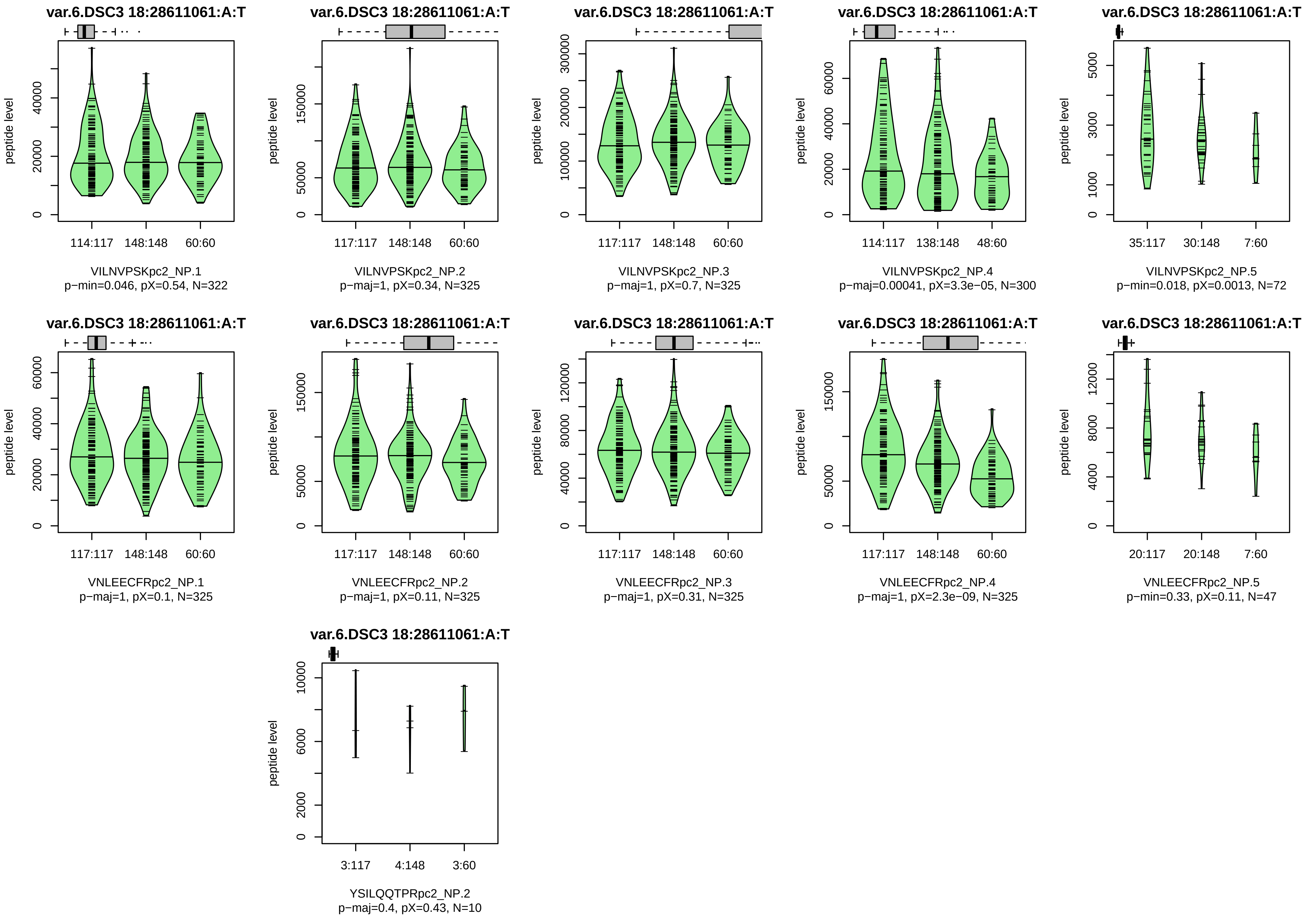
var.6.DSC3 18:28611061:A:T
peptide level
114:117 148:148 60:60
VILNVPSKpc2_NP.1
p−min=0.046, pX=0.54, N=322
var.6.DSC3 18:28611061:A:T
peptide level
117:117 148:148 60:60
VILNVPSKpc2_NP.2
p−maj=1, pX=0.34, N=325
var.6.DSC3 18:28611061:A:T
peptide level
117:117 148:148 60:60
VILNVPSKpc2_NP.3
p−maj=1, pX=0.7, N=325
var.6.DSC3 18:28611061:A:T
peptide level
114:117 138:148 48:60
VILNVPSKpc2_NP.4
p−maj=0.00041, pX=3.3e−05, N=300
var.6.DSC3 18:28611061:A:T
peptide level
35:117 30:148 7:60
VILNVPSKpc2_NP.5
p−min=0.018, pX=0.0013, N=72
var.6.DSC3 18:28611061:A:T
peptide level
117:117 148:148 60:60
VNLEECFRpc2_NP.1
p−maj=1, pX=0.1, N=325
var.6.DSC3 18:28611061:A:T
peptide level
117:117 148:148 60:60
VNLEECFRpc2_NP.2
p−maj=1, pX=0.11, N=325
var.6.DSC3 18:28611061:A:T
peptide level
117:117 148:148 60:60
VNLEECFRpc2_NP.3
p−maj=1, pX=0.31, N=325
var.6.DSC3 18:28611061:A:T
peptide level
117:117 148:148 60:60
VNLEECFRpc2_NP.4
p−maj=1, pX=2.3e−09, N=325
var.6.DSC3 18:28611061:A:T
peptide level
20:117 20:148 7:60
VNLEECFRpc2_NP.5
p−min=0.33, pX=0.11, N=47
var.6.DSC3 18:28611061:A:T
peptide level
3:117 4:148 3:60
YSILQQTPRpc2_NP.2
p−maj=0.4, pX=0.43, N=10

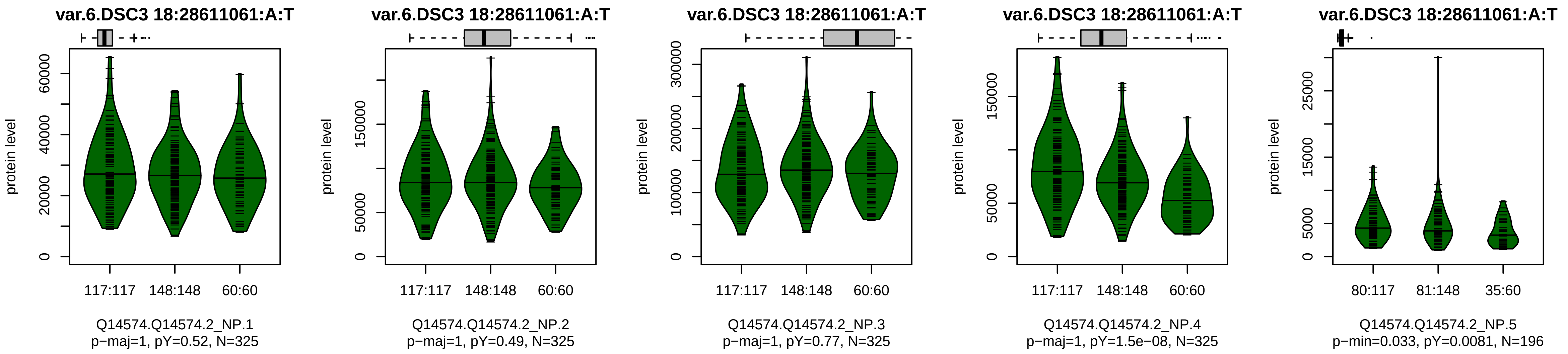

var.6.DSC3 18:28611061:A:T
protein level
117:117
148:148
60:60
Q14574.Q14574.2_NP.1
p−maj=1, pY=0.52, N=325
var.6.DSC3 18:28611061:A:T
protein level
117:117
148:148
60:60
Q14574.Q14574.2_NP.2
p−maj=1, pY=0.49, N=325
var.6.DSC3 18:28611061:A:T
protein level
117:117
148:148
60:60
Q14574.Q14574.2_NP.3
p−maj=1, pY=0.77, N=325
var.6.DSC3 18:28611061:A:T
protein level
117:117
148:148
60:60
Q14574.Q14574.2_NP.4
p−maj=1, pY=1.5e−08, N=325
var.6.DSC3 18:28611061:A:T
protein level
80:117
81:148
35:60
Q14574.Q14574.2_NP.5
p−min=0.033, pY=0.0081, N=196

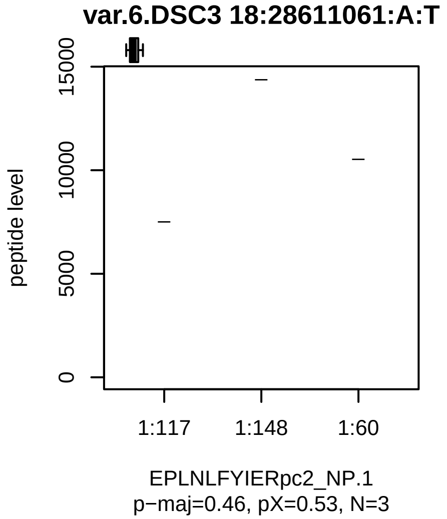
var.6.DSC3 18:28611061:A:T
peptide level
0
5000
10000
15000
1:117
1:148
1:60
EPLNLFYIERpc2_NP.1
p−maj=0.46, pX=0.53, N=3

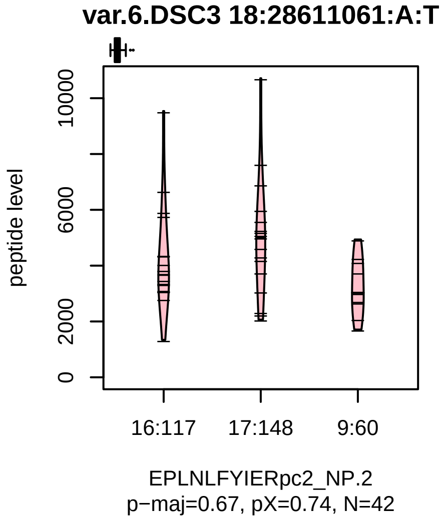
var.6.DSC3 18:28611061:A:T
peptide level
0
2000
6000
10000
16:117
17:148
9:60
EPLNLFYIERpc2_NP.2
p−maj=0.67, pX=0.74, N=42

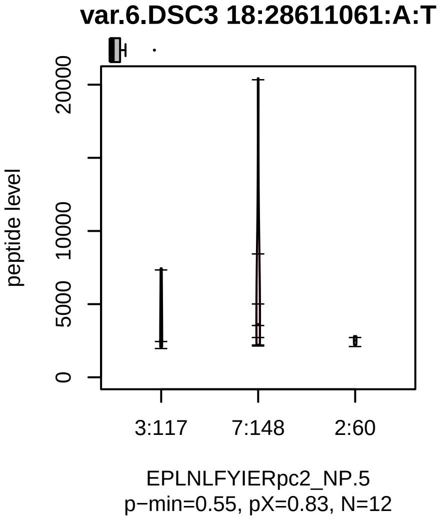
var.6.DSC3 18:28611061:A:T
peptide level
0
5000
10000
20000
3:117
7:148
2:60
EPLNLFYIERpc2_NP.5
p−min=0.55, pX=0.83, N=12

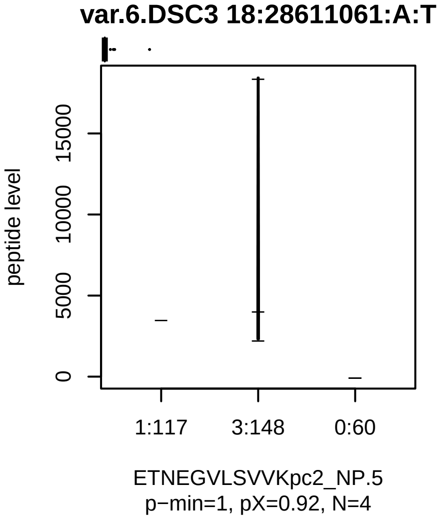
var.6.DSC3 18:28611061:A:T
peptide level
0
5000
10000
15000
1:117
3:148
0:60
ETNEGVLSVVKpc2_NP.5
p−min=1, pX=0.92, N=4

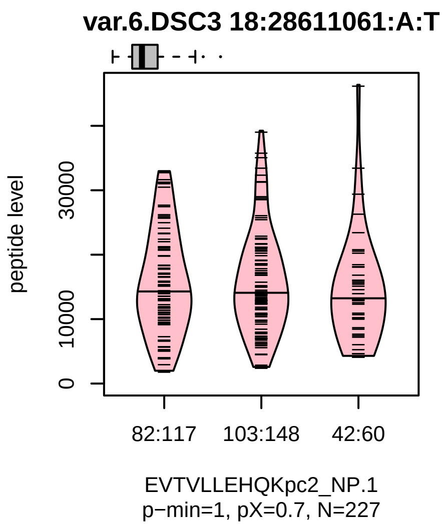
var.6.DSC3 18:28611061:A:T
peptide level
0
10000
30000
82:117
103:148
42:60
EVTVLLEHQKpc2_NP.1
p−min=1, pX=0.7, N=227

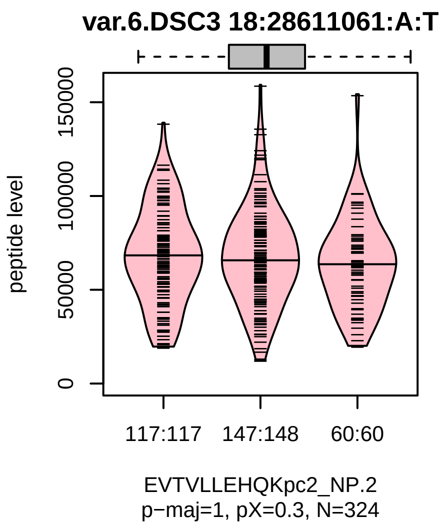
var.6.DSC3 18:28611061:A:T
peptide level
0
50000
100000
150000
117:117
147:148
60:60
EVTVLLEHQKpc2_NP.2
p−maj=1, pX=0.3, N=324

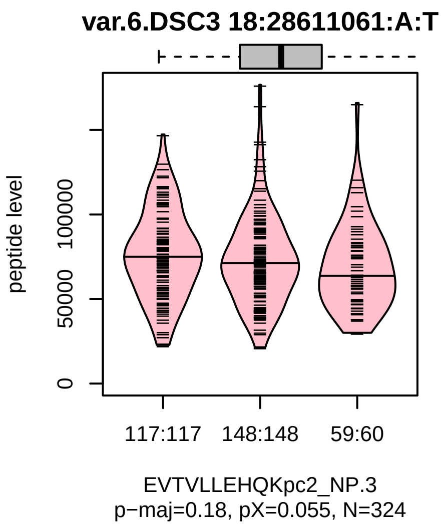
var.6.DSC3 18:28611061:A:T
peptide level
0
50000
100000
117:117
148:148
59:60
EVTVLLEHQKpc2_NP.3
p−maj=0.18, pX=0.055, N=324

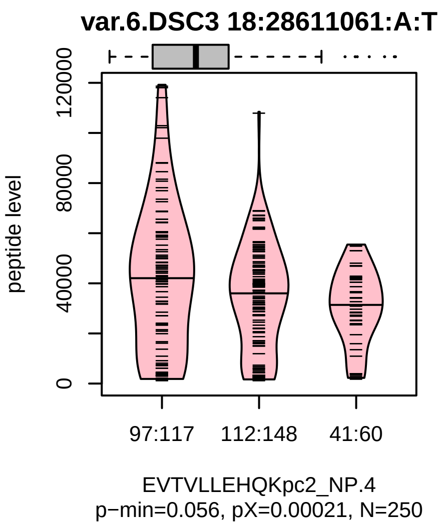
var.6.DSC3 18:28611061:A:T
peptide level
0
40000
80000
120000
97:117
112:148
41:60
EVTVLLEHQKpc2_NP.4
p−min=0.056, pX=0.00021, N=250

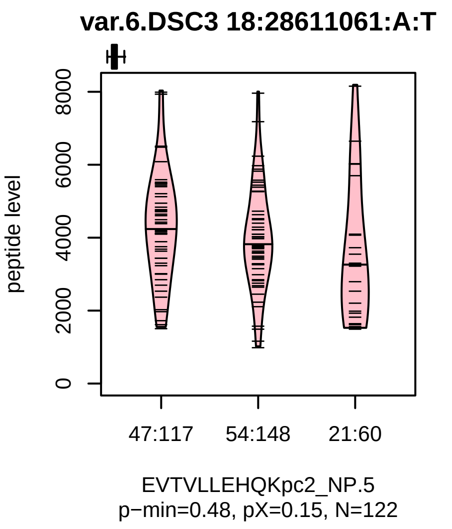
var.6.DSC3 18:28611061:A:T
peptide level
0
2000
4000
6000
8000
47:117
54:148
21:60
EVTVLLEHQKpc2_NP.5
p−min=0.48, pX=0.15, N=122

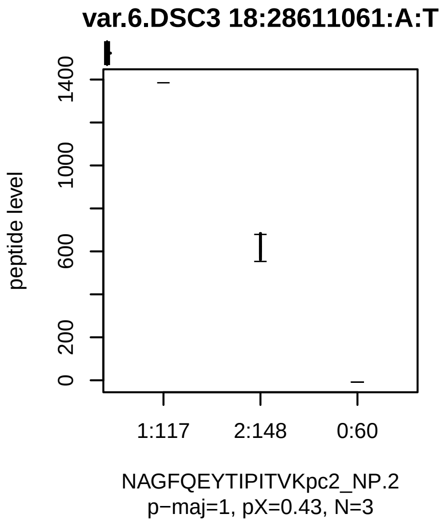
var.6.DSC3 18:28611061:A:T
peptide level
0
200
600
1000
1400
1:117
2:148
0:60
NAGFQEYTIPITVKpc2_NP.2
p−maj=1, pX=0.43, N=3

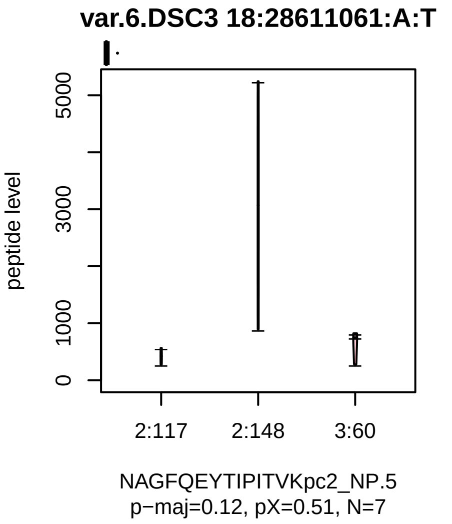
var.6.DSC3 18:28611061:A:T
peptide level
0
1000
3000
5000
2:117
2:148
3:60
NAGFQEYTIPITVKpc2_NP.5
p−maj=0.12, pX=0.51, N=7

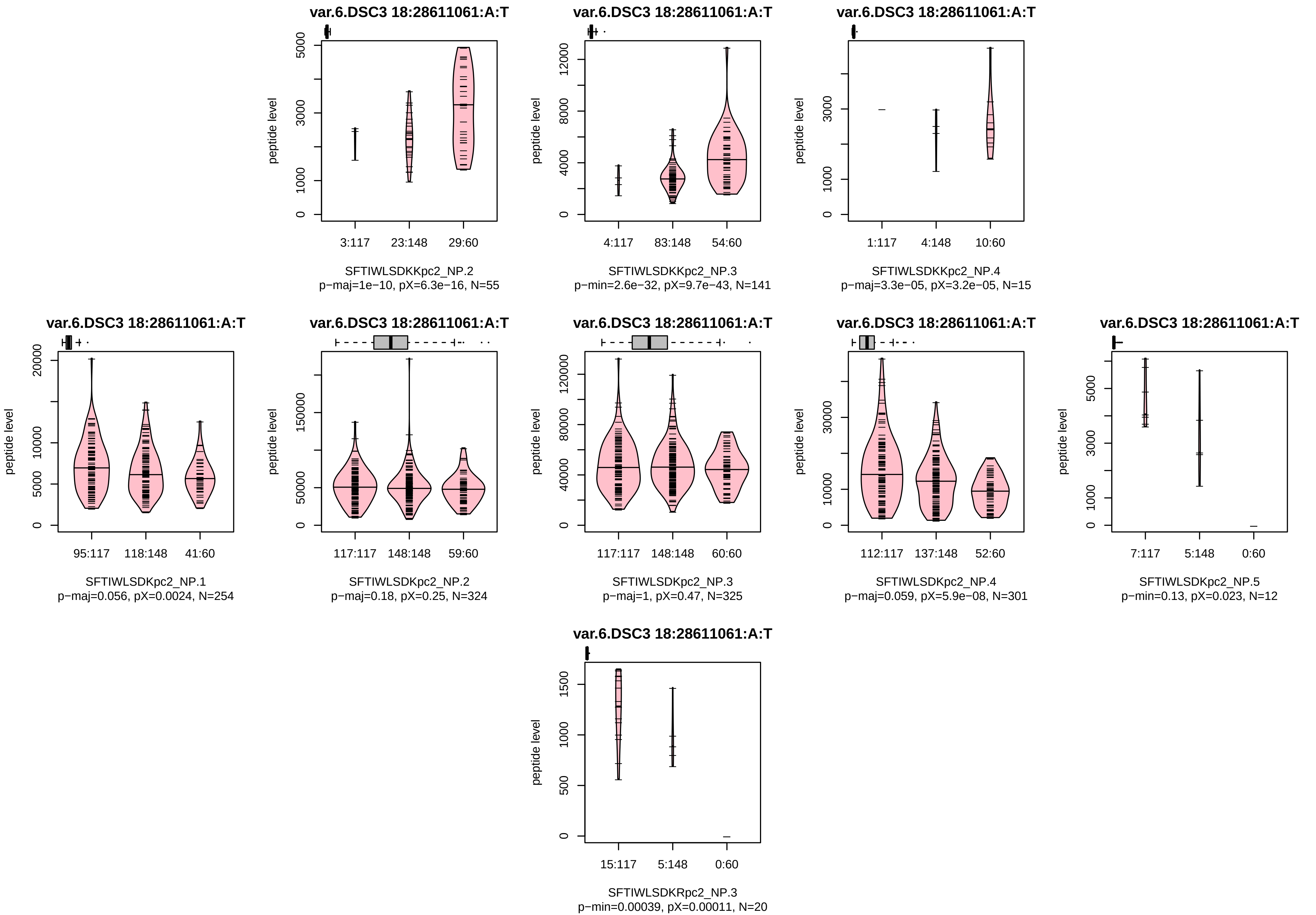

var.6.DSC3 18:28611061:A:T
peptide level
0
1000
3000
5000
3:117
23:148
29:60
SFTIWLSDKKpc2_NP.2
p−maj=1e−10, pX=6.3e−16, N=55
var.6.DSC3 18:28611061:A:T
peptide level
0
4000
8000
12000
4:117
83:148
54:60
SFTIWLSDKKpc2_NP.3
p−min=2.6e−32, pX=9.7e−43, N=141
var.6.DSC3 18:28611061:A:T
peptide level
0
1000
3000
1:117
4:148
10:60
SFTIWLSDKKpc2_NP.4
p−maj=3.3e−05, pX=3.2e−05, N=15
var.6.DSC3 18:28611061:A:T
peptide level
0
5000
10000
20000
95:117
118:148
41:60
SFTIWLSDKpc2_NP.1
p−maj=0.056, pX=0.0024, N=254
var.6.DSC3 18:28611061:A:T
peptide level
0
50000
150000
117:117
148:148
59:60
SFTIWLSDKpc2_NP.2
p−maj=0.18, pX=0.25, N=324
var.6.DSC3 18:28611061:A:T
peptide level
0
40000
80000
120000
117:117
148:148
60:60
SFTIWLSDKpc2_NP.3
p−maj=1, pX=0.47, N=325
var.6.DSC3 18:28611061:A:T
peptide level
0
10000
30000
112:117
137:148
52:60
SFTIWLSDKpc2_NP.4
p−maj=0.059, pX=5.9e−08, N=301
var.6.DSC3 18:28611061:A:T
peptide level
0
1000
3000
5000
7:117
5:148
0:60
SFTIWLSDKpc2_NP.5
p−min=0.13, pX=0.023, N=12
var.6.DSC3 18:28611061:A:T
peptide level
0
500
1000
1500
15:117
5:148
0:60
SFTIWLSDKRpc2_NP.3
p−min=0.00039, pX=0.00011, N=20

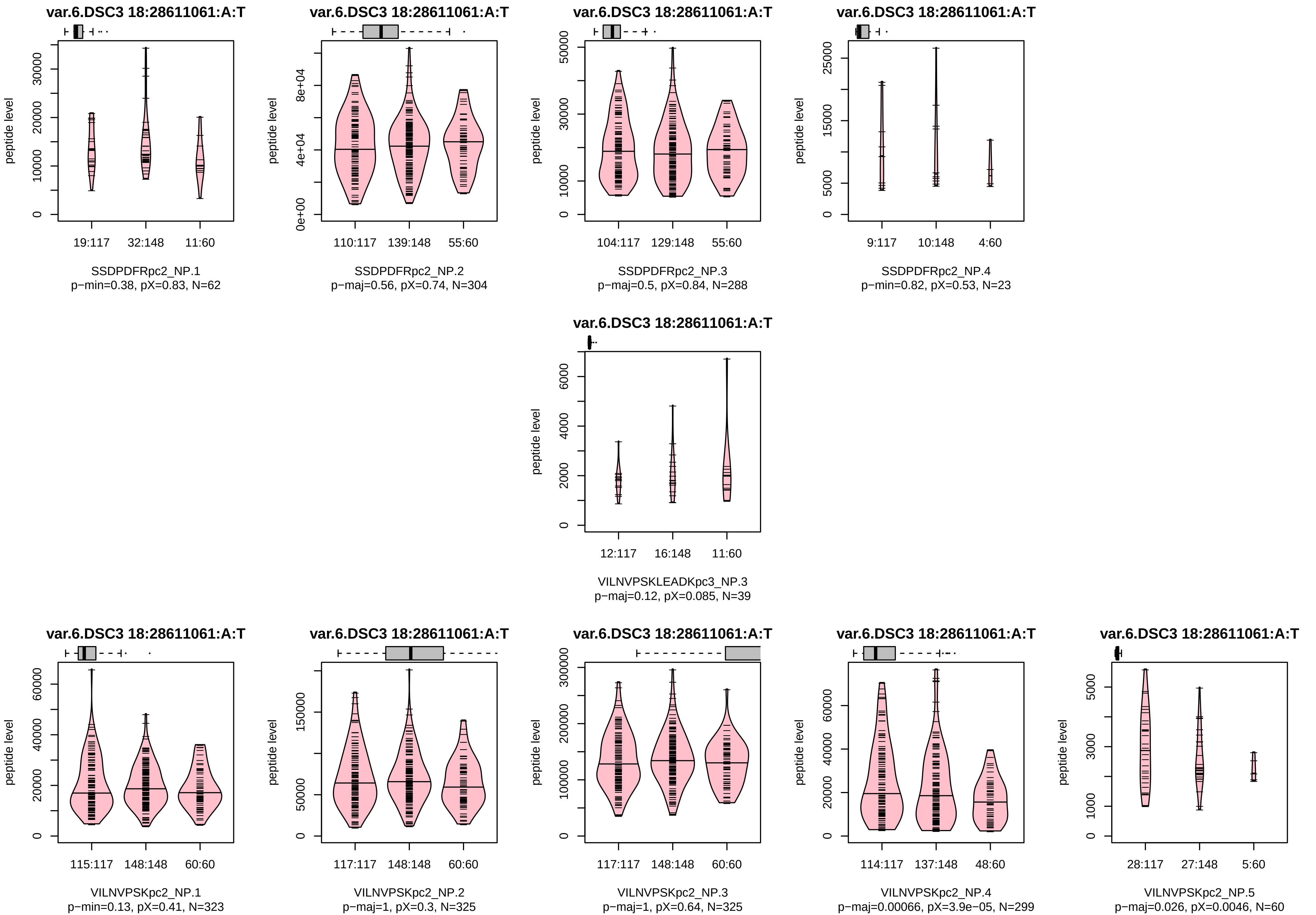
var.6.DSC3 18:28611061:A:T
peptide level
19:117
32:148
11:60
SSDPDFRpc2_NP.1
p−min=0.38, pX=0.83, N=62
var.6.DSC3 18:28611061:A:T
peptide level
110:117
139:148
55:60
SSDPDFRpc2_NP.2
p−maj=0.56, pX=0.74, N=304
var.6.DSC3 18:28611061:A:T
peptide level
104:117
129:148
55:60
SSDPDFRpc2_NP.3
p−maj=0.5, pX=0.84, N=288
var.6.DSC3 18:28611061:A:T
peptide level
9:117
10:148
4:60
SSDPDFRpc2_NP.4
p−min=0.82, pX=0.53, N=23
var.6.DSC3 18:28611061:A:T
peptide level
12:117
16:148
11:60
VILNVPSKLEADKpc3_NP.3
p−maj=0.12, pX=0.085, N=39
var.6.DSC3 18:28611061:A:T
peptide level
115:117
148:148
60:60
VILNVPSKpc2_NP.1
p−min=0.13, pX=0.41, N=323
var.6.DSC3 18:28611061:A:T
peptide level
117:117
148:148
60:60
VILNVPSKpc2_NP.2
p−maj=1, pX=0.3, N=325
var.6.DSC3 18:28611061:A:T
peptide level
117:117
148:148
60:60
VILNVPSKpc2_NP.3
p−maj=1, pX=0.64, N=325
var.6.DSC3 18:28611061:A:T
peptide level
114:117
137:148
48:60
VILNVPSKpc2_NP.4
p−maj=0.00066, pX=3.9e−05, N=299
var.6.DSC3 18:28611061:A:T
peptide level
28:117
27:148
5:60
VILNVPSKpc2_NP.5
p−maj=0.026, pX=0.0046, N=60

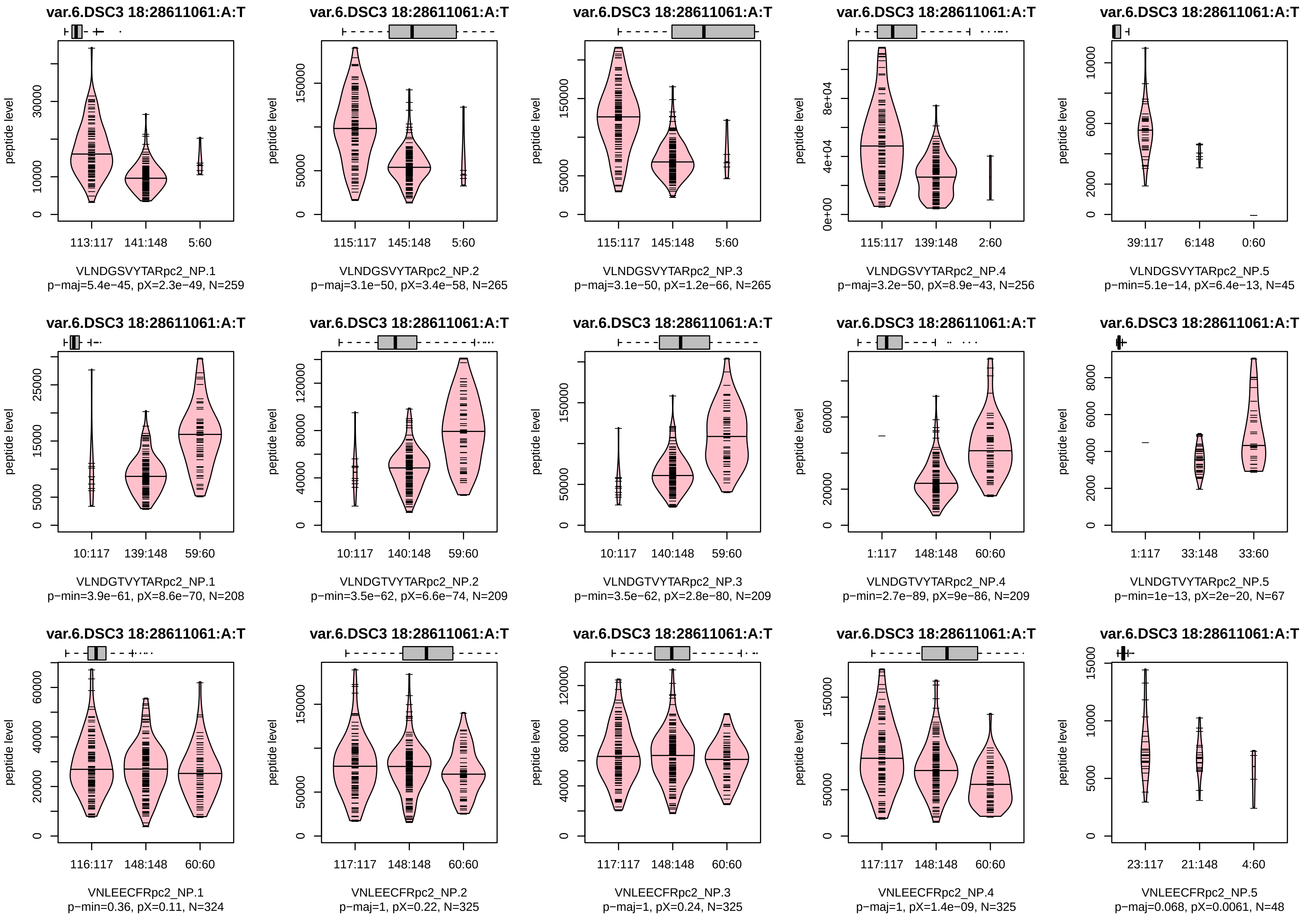
var.6.DSC3 18:28611061:A:T
peptide level
113:117
141:148
5:60
VLNDGSVYTARpc2_NP.1
p−maj=5.4e−45, pX=2.3e−49, N=259
var.6.DSC3 18:28611061:A:T
115:117
145:148
5:60
VLNDGSVYTARpc2_NP.2
p−maj=3.1e−50, pX=3.4e−58, N=265
var.6.DSC3 18:28611061:A:T
115:117
145:148
5:60
VLNDGSVYTARpc2_NP.3
p−maj=3.1e−50, pX=1.2e−66, N=265
var.6.DSC3 18:28611061:A:T
115:117
139:148
2:60
VLNDGSVYTARpc2_NP.4
p−maj=3.2e−50, pX=8.9e−43, N=256
var.6.DSC3 18:28611061:A:T
39:117
6:148
0:60
VLNDGSVYTARpc2_NP.5
p−min=5.1e−14, pX=6.4e−13, N=45
var.6.DSC3 18:28611061:A:T
10:117
139:148
59:60
VLNDGTVYTARpc2_NP.1
p−min=3.9e−61, pX=8.6e−70, N=208
var.6.DSC3 18:28611061:A:T
10:117
140:148
59:60
VLNDGTVYTARpc2_NP.2
p−min=3.5e−62, pX=6.6e−74, N=209
var.6.DSC3 18:28611061:A:T
10:117
140:148
59:60
VLNDGTVYTARpc2_NP.3
p−min=3.5e−62, pX=2.8e−80, N=209
var.6.DSC3 18:28611061:A:T
1:117
148:148
60:60
VLNDGTVYTARpc2_NP.4
p−min=2.7e−89, pX=9e−86, N=209
var.6.DSC3 18:28611061:A:T
1:117
33:148
33:60
VLNDGTVYTARpc2_NP.5
p−min=1e−13, pX=2e−20, N=67
var.6.DSC3 18:28611061:A:T
116:117
148:148
60:60
VNLEECFRpc2_NP.1
p−min=0.36, pX=0.11, N=324
var.6.DSC3 18:28611061:A:T
117:117
148:148
60:60
VNLEECFRpc2_NP.2
p−maj=1, pX=0.22, N=325
var.6.DSC3 18:28611061:A:T
117:117
148:148
60:60
VNLEECFRpc2_NP.3
p−maj=1, pX=0.24, N=325
var.6.DSC3 18:28611061:A:T
117:117
148:148
60:60
VNLEECFRpc2_NP.4
p−maj=1, pX=1.4e−09, N=325
var.6.DSC3 18:28611061:A:T
23:117
21:148
4:60
VNLEECFRpc2_NP.5
p−maj=0.068, pX=0.0061, N=48

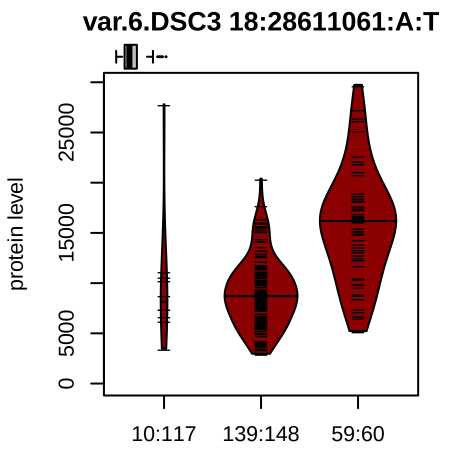

Q14574.2.rs276937.Q14574.rs276937_NP.1
p−min=3.9e−61, pY=3.3e−14, N=208

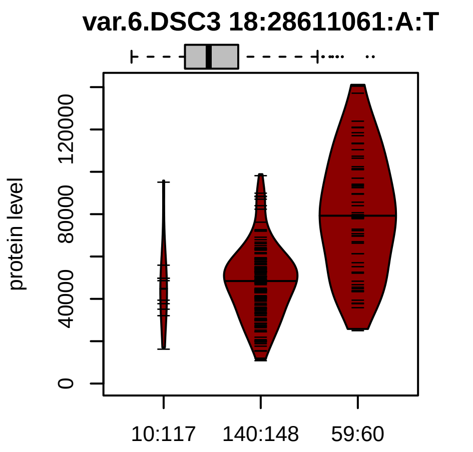

Q14574.2.rs276937.Q14574.rs276937_NP.2
p−min=3.5e−62, pY=4.9e−14, N=209

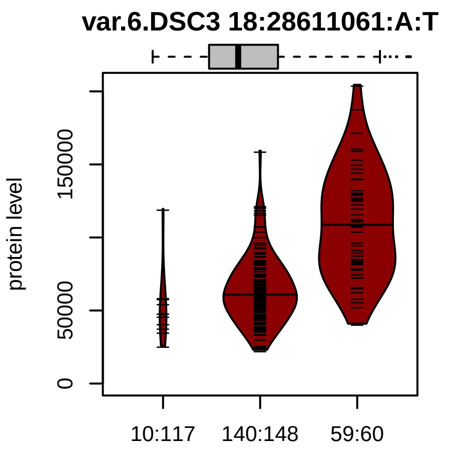

Q14574.2.rs276937.Q14574.rs276937_NP.3
p−min=3.5e−62, pY=1.1e−19, N=209

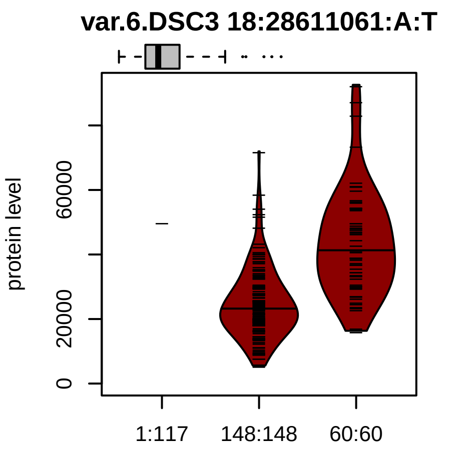

Q14574.2.rs276937.Q14574.rs276937_NP.4
p−min=2.7e−89, pY=4.6e−17, N=209

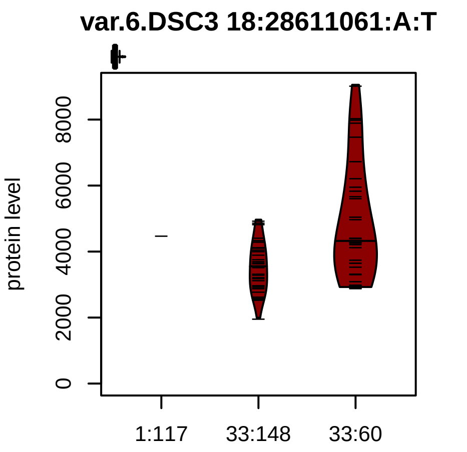

Q14574.2.rs276937.Q14574.rs276937_NP.5
p−min=1e−13, pY=0.0013, N=67

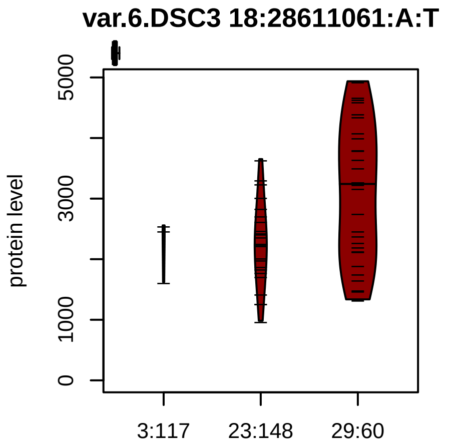

Q14574.2.rs276938.Q14574.rs276938_NP.2
p−maj=1e−10, pY=0.0034, N=55

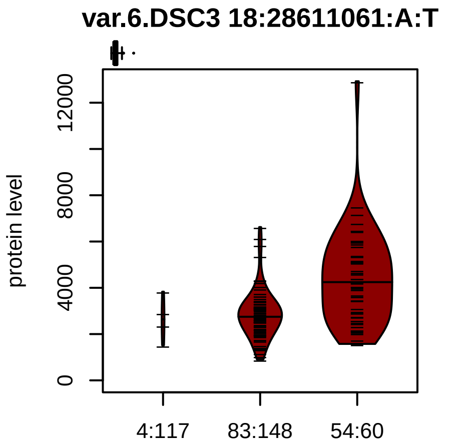

Q14574.2.rs276938.Q14574.rs276938_NP.3
p−min=2.6e−32, pY=1.9e−07, N=141

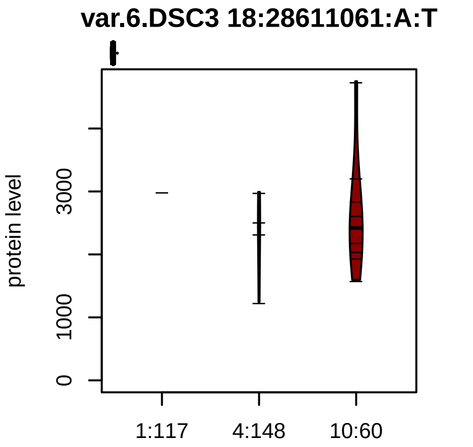

Q14574.2.rs276938.Q14574.rs276938_NP.4
p−maj=3.3e−05, pY=0.85, N=15

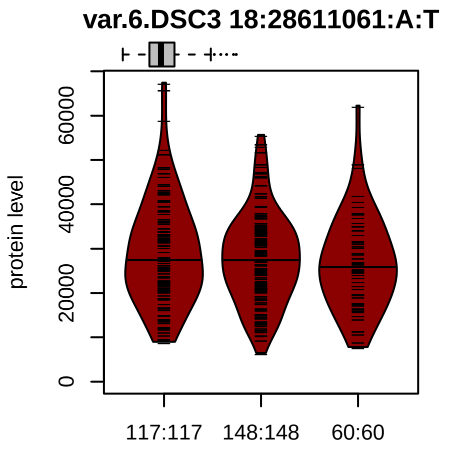

Q14574.Q14574.2_NP.1
p−maj=1, pY=0.51, N=325

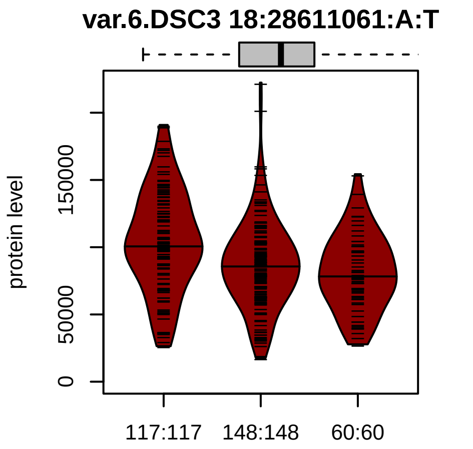

Q14574.Q14574.2_NP.2
p−maj=1, pY=0.00012, N=325

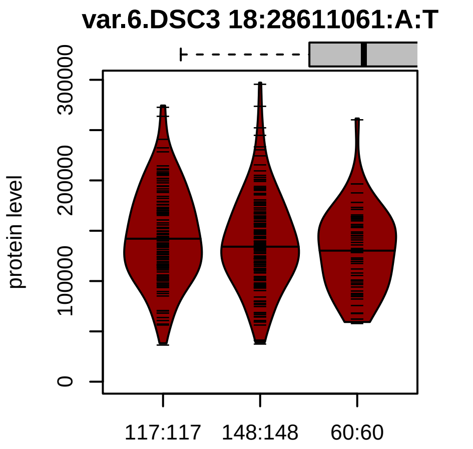

Q14574.Q14574.2_NP.3
p−maj=1, pY=0.048, N=325

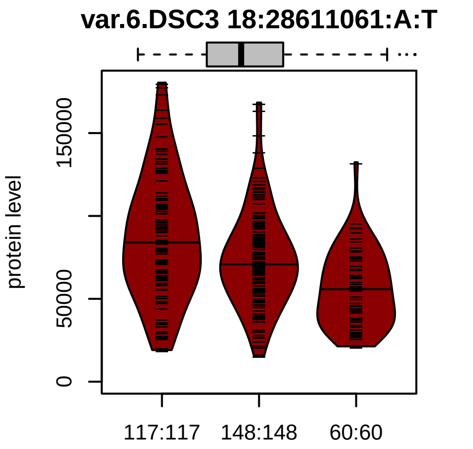

Q14574.Q14574.2_NP.4
p−maj=1, pY=1.6e−08, N=325

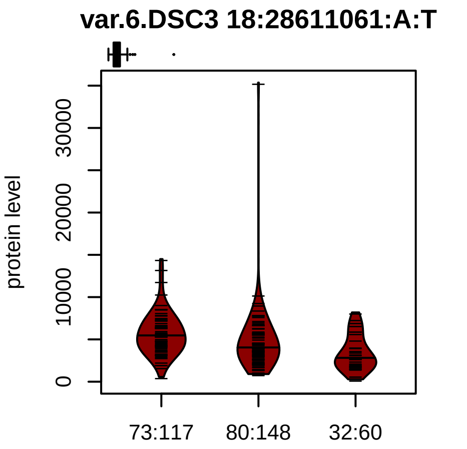

Q14574.Q14574.2_NP.5
p−min=0.16, pY=8.8e−06, N=185

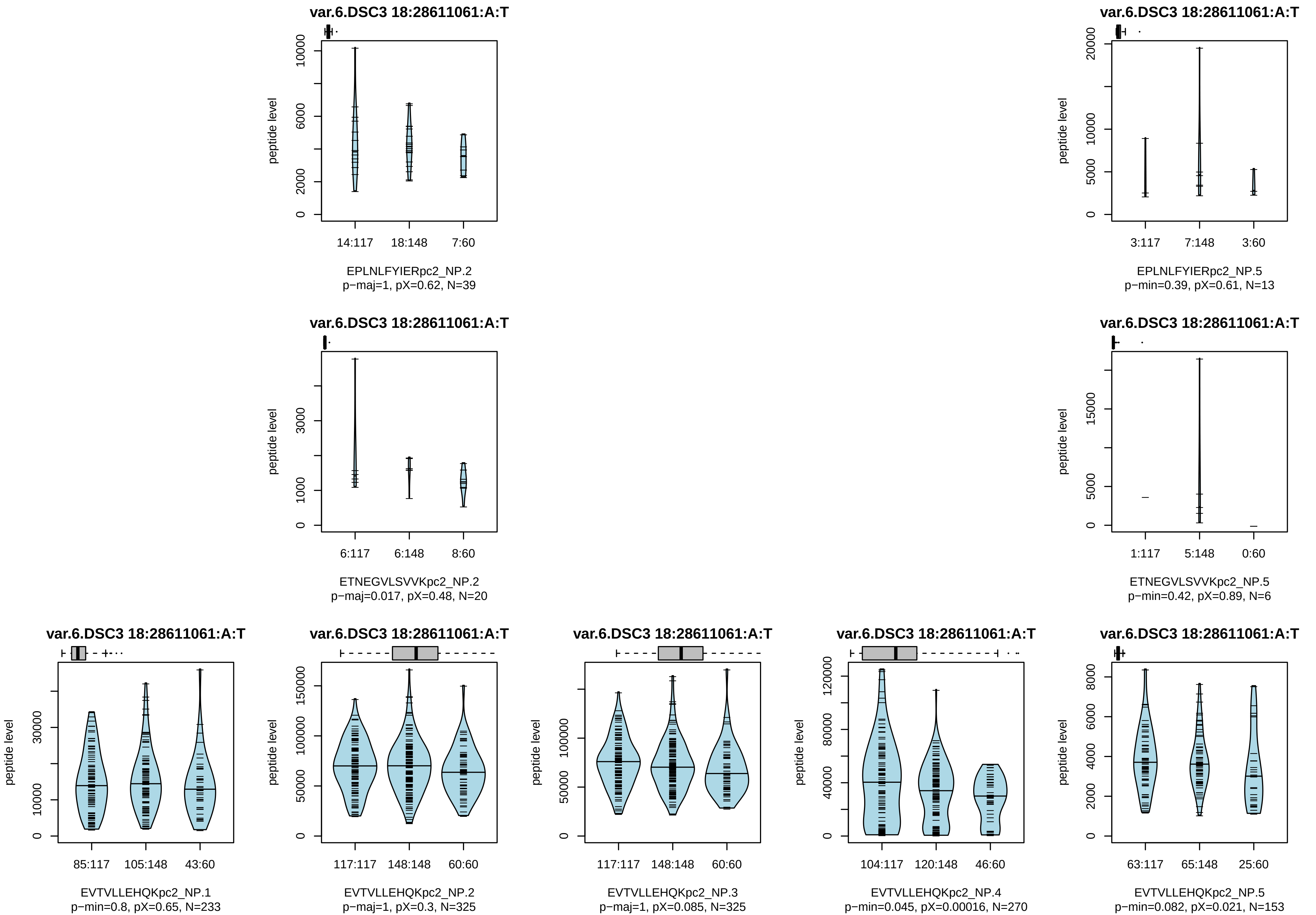
var.6.DSC3 18:28611061:A:T
peptide level
0
2000
6000
10000
14:117
18:148
7:60
EPLNLFYIERpc2_NP.2
p−maj=1, pX=0.62, N=39
var.6.DSC3 18:28611061:A:T
peptide level
0
5000
10000
20000
3:117
7:148
3:60
EPLNLFYIERpc2_NP.5
p−min=0.39, pX=0.61, N=13
var.6.DSC3 18:28611061:A:T
peptide level
0
1000
3000
6:117
6:148
8:60
ETNEGVLSVVKpc2_NP.2
p−maj=0.017, pX=0.48, N=20
var.6.DSC3 18:28611061:A:T
peptide level
0
5000
15000
1:117
5:148
0:60
ETNEGVLSVVKpc2_NP.5
p−min=0.42, pX=0.89, N=6
var.6.DSC3 18:28611061:A:T
peptide level
0
10000
30000
85:117
105:148
43:60
EVTVLLEHQKpc2_NP.1
p−min=0.8, pX=0.65, N=233
var.6.DSC3 18:28611061:A:T
peptide level
0
50000
100000
150000
117:117
148:148
60:60
EVTVLLEHQKpc2_NP.2
p−maj=1, pX=0.3, N=325
var.6.DSC3 18:28611061:A:T
peptide level
0
50000
100000
117:117
148:148
60:60
EVTVLLEHQKpc2_NP.3
p−maj=1, pX=0.085, N=325
var.6.DSC3 18:28611061:A:T
peptide level
0
40000
80000
120000
104:117
120:148
46:60
EVTVLLEHQKpc2_NP.4
p−min=0.045, pX=0.00016, N=270
var.6.DSC3 18:28611061:A:T
peptide level
0
2000
4000
6000
8000
63:117
65:148
25:60
EVTVLLEHQKpc2_NP.5
p−min=0.082, pX=0.021, N=153

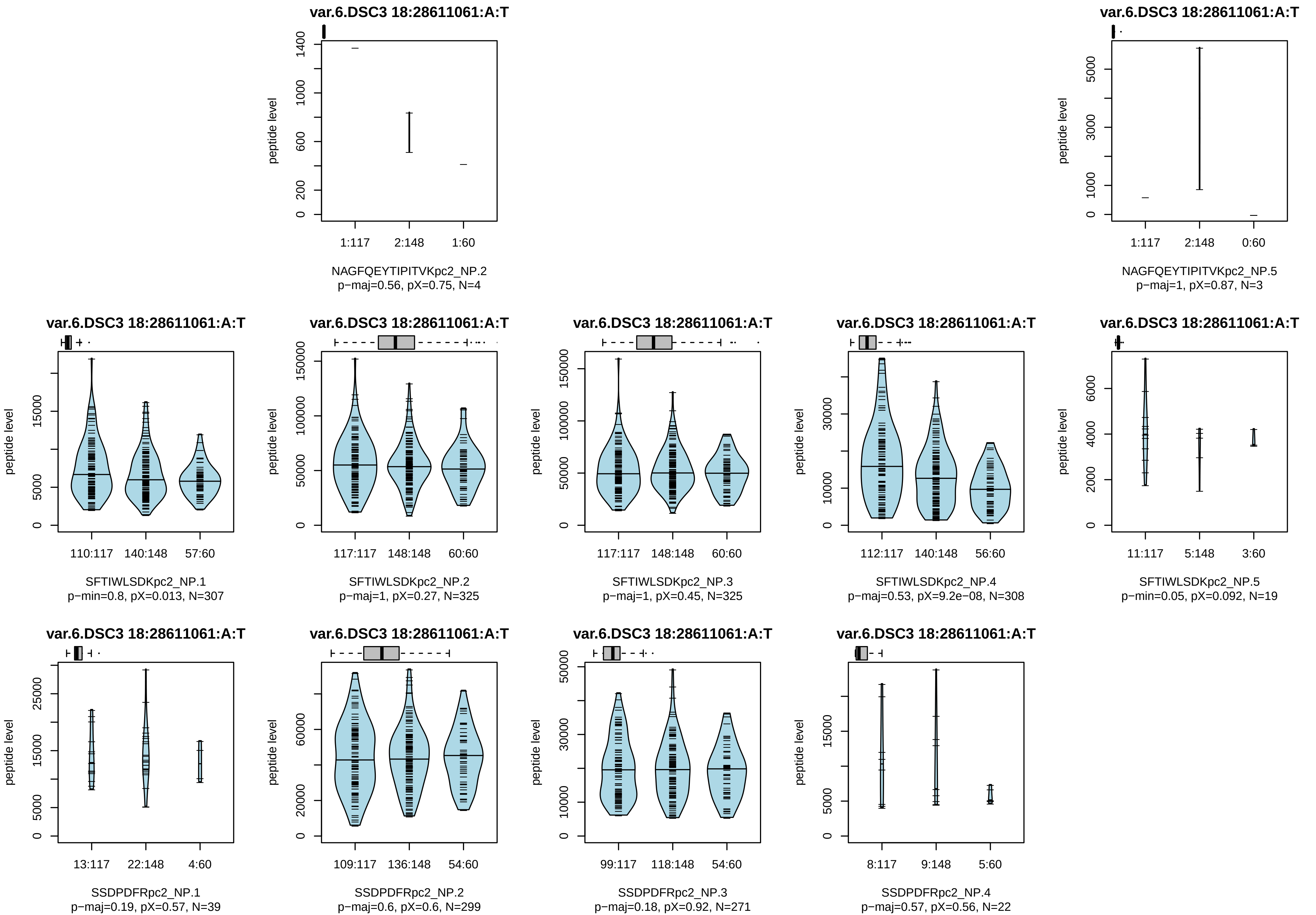
var.6.DSC3 18:28611061:A:T
peptide level
1:117
2:148
1:60
NAGFQEYTIPITVKpc2_NP.2
p−maj=0.56, pX=0.75, N=4
var.6.DSC3 18:28611061:A:T
peptide level
1:117
2:148
0:60
NAGFQEYTIPITVKpc2_NP.5
p−maj=1, pX=0.87, N=3
var.6.DSC3 18:28611061:A:T
peptide level
110:117
140:148
57:60
SFTIWLSDKpc2_NP.1
p−min=0.8, pX=0.013, N=307
var.6.DSC3 18:28611061:A:T
peptide level
117:117
148:148
60:60
SFTIWLSDKpc2_NP.2
p−maj=1, pX=0.27, N=325
var.6.DSC3 18:28611061:A:T
peptide level
117:117
148:148
60:60
SFTIWLSDKpc2_NP.3
p−maj=1, pX=0.45, N=325
var.6.DSC3 18:28611061:A:T
peptide level
112:117
140:148
56:60
SFTIWLSDKpc2_NP.4
p−maj=0.53, pX=9.2e−08, N=308
var.6.DSC3 18:28611061:A:T
peptide level
11:117
5:148
3:60
SFTIWLSDKpc2_NP.5
p−min=0.05, pX=0.092, N=19
var.6.DSC3 18:28611061:A:T
peptide level
13:117
22:148
4:60
SSDPDFRpc2_NP.1
p−maj=0.19, pX=0.57, N=39
var.6.DSC3 18:28611061:A:T
peptide level
109:117
136:148
54:60
SSDPDFRpc2_NP.2
p−maj=0.6, pX=0.6, N=299
var.6.DSC3 18:28611061:A:T
peptide level
99:117
118:148
54:60
SSDPDFRpc2_NP.3
p−maj=0.18, pX=0.92, N=271
var.6.DSC3 18:28611061:A:T
peptide level
8:117
9:148
5:60
SSDPDFRpc2_NP.4
p−maj=0.57, pX=0.56, N=22

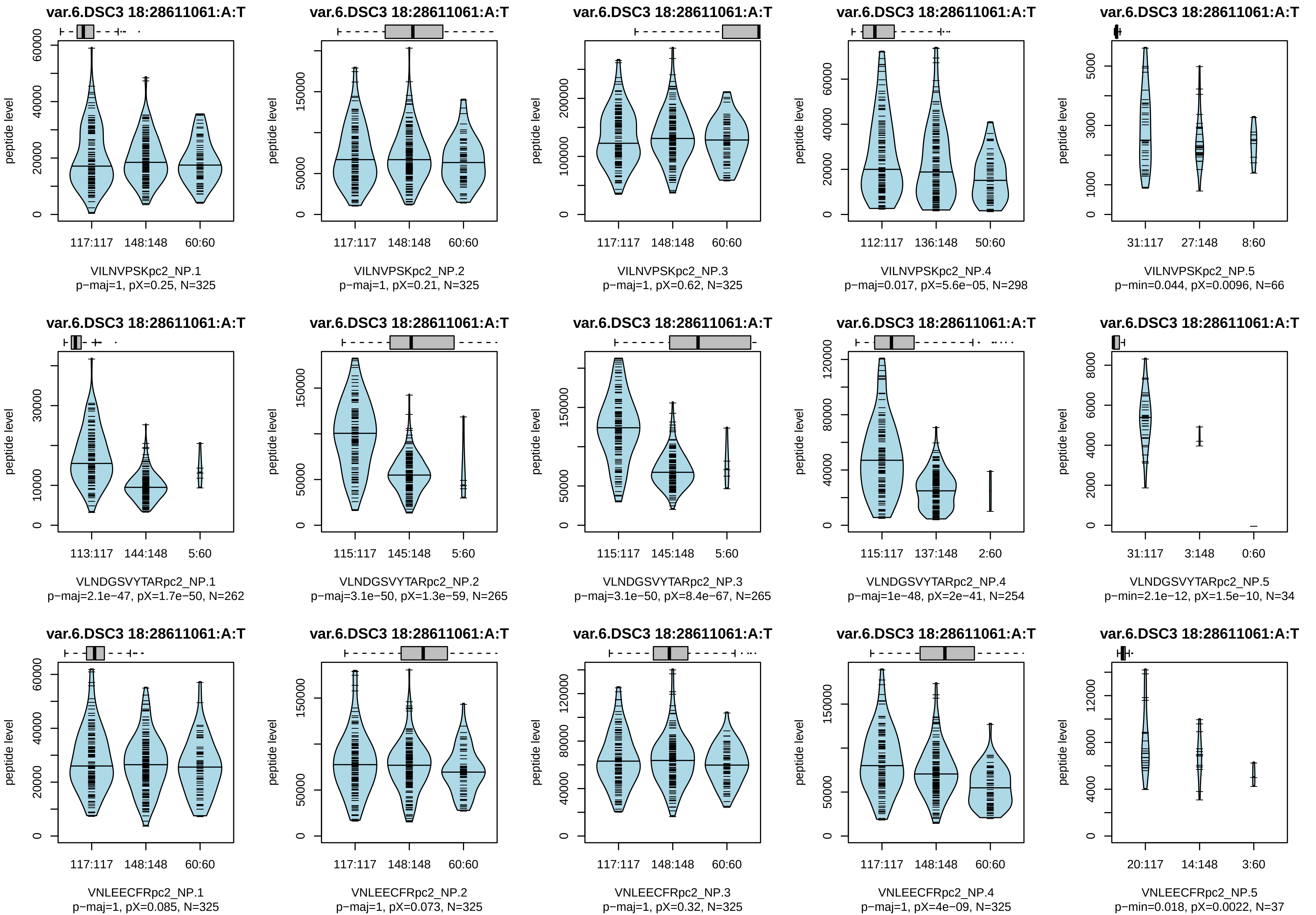
var.6.DSC3 18:28611061:A:T
peptide level
117:117 148:148 60:60
VILNVPSKpc2_NP.1
p−maj=1, pX=0.25, N=325
var.6.DSC3 18:28611061:A:T
peptide level
117:117 148:148 60:60
VILNVPSKpc2_NP.2
p−maj=1, pX=0.21, N=325
var.6.DSC3 18:28611061:A:T
peptide level
117:117 148:148 60:60
VILNVPSKpc2_NP.3
p−maj=1, pX=0.62, N=325
var.6.DSC3 18:28611061:A:T
peptide level
112:117 136:148 50:60
VILNVPSKpc2_NP.4
p−maj=0.017, pX=5.6e−05, N=298
var.6.DSC3 18:28611061:A:T
peptide level
31:117 27:148 8:60
VILNVPSKpc2_NP.5
p−min=0.044, pX=0.0096, N=66
var.6.DSC3 18:28611061:A:T
peptide level
113:117 144:148 5:60
VLNDGSVYTARpc2_NP.1
p−maj=2.1e−47, pX=1.7e−50, N=262
var.6.DSC3 18:28611061:A:T
peptide level
115:117 145:148 5:60
VLNDGSVYTARpc2_NP.2
p−maj=3.1e−50, pX=1.3e−59, N=265
var.6.DSC3 18:28611061:A:T
peptide level
115:117 145:148 5:60
VLNDGSVYTARpc2_NP.3
p−maj=3.1e−50, pX=8.4e−67, N=265
var.6.DSC3 18:28611061:A:T
peptide level
115:117 137:148 2:60
VLNDGSVYTARpc2_NP.4
p−maj=1e−48, pX=2e−41, N=254
var.6.DSC3 18:28611061:A:T
peptide level
31:117 3:148 0:60
VLNDGSVYTARpc2_NP.5
p−min=2.1e−12, pX=1.5e−10, N=34
var.6.DSC3 18:28611061:A:T
peptide level
117:117 148:148 60:60
VNLEECFRpc2_NP.1
p−maj=1, pX=0.085, N=325
var.6.DSC3 18:28611061:A:T
peptide level
117:117 148:148 60:60
VNLEECFRpc2_NP.2
p−maj=1, pX=0.073, N=325
var.6.DSC3 18:28611061:A:T
peptide level
117:117 148:148 60:60
VNLEECFRpc2_NP.3
p−maj=1, pX=0.32, N=325
var.6.DSC3 18:28611061:A:T
peptide level
117:117 148:148 60:60
VNLEECFRpc2_NP.4
p−maj=1, pX=4e−09, N=325
var.6.DSC3 18:28611061:A:T
peptide level
20:117 14:148 3:60
VNLEECFRpc2_NP.5
p−min=0.018, pX=0.0022, N=37

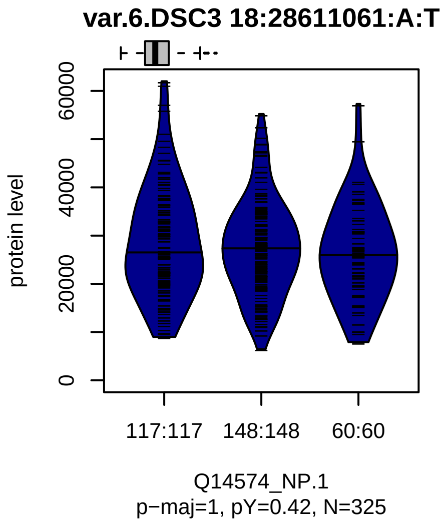
var.6.DSC3 18:28611061:A:T
protein level
0
20000
40000
60000
117:117
148:148
60:60
Q14574_NP.1
p−maj=1, pY=0.42, N=325

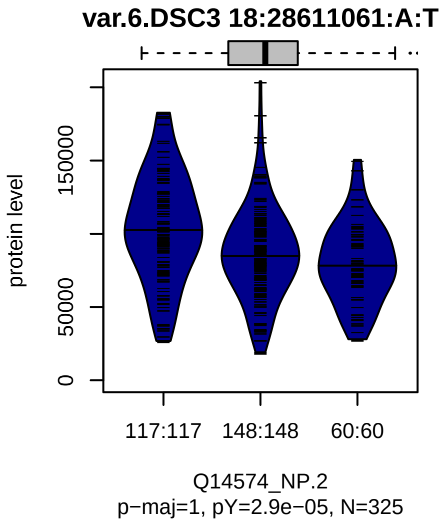
var.6.DSC3 18:28611061:A:T
protein level
0
50000
150000
117:117
148:148
60:60
Q14574_NP.2
p−maj=1, pY=2.9e−05, N=325

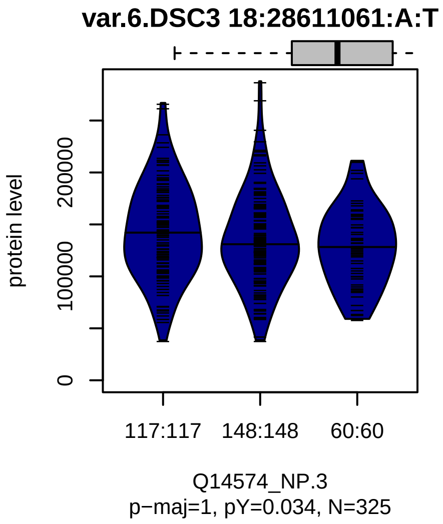
var.6.DSC3 18:28611061:A:T
protein level
0
100000
200000
117:117
148:148
60:60
Q14574_NP.3
p−maj=1, pY=0.034, N=325

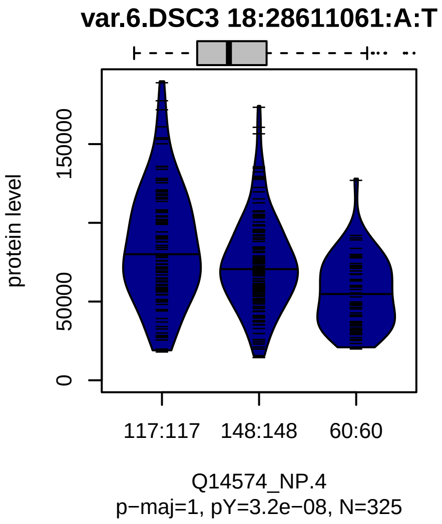
var.6.DSC3 18:28611061:A:T
protein level
0
50000
150000
117:117
148:148
60:60
Q14574_NP.4
p−maj=1, pY=3.2e−08, N=325

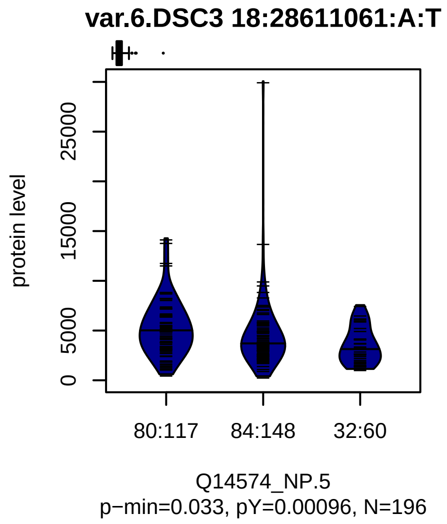
var.6.DSC3 18:28611061:A:T
protein level
0
5000
15000
25000
80:117
84:148
32:60
Q14574_NP.5
p−min=0.033, pY=0.00096, N=196

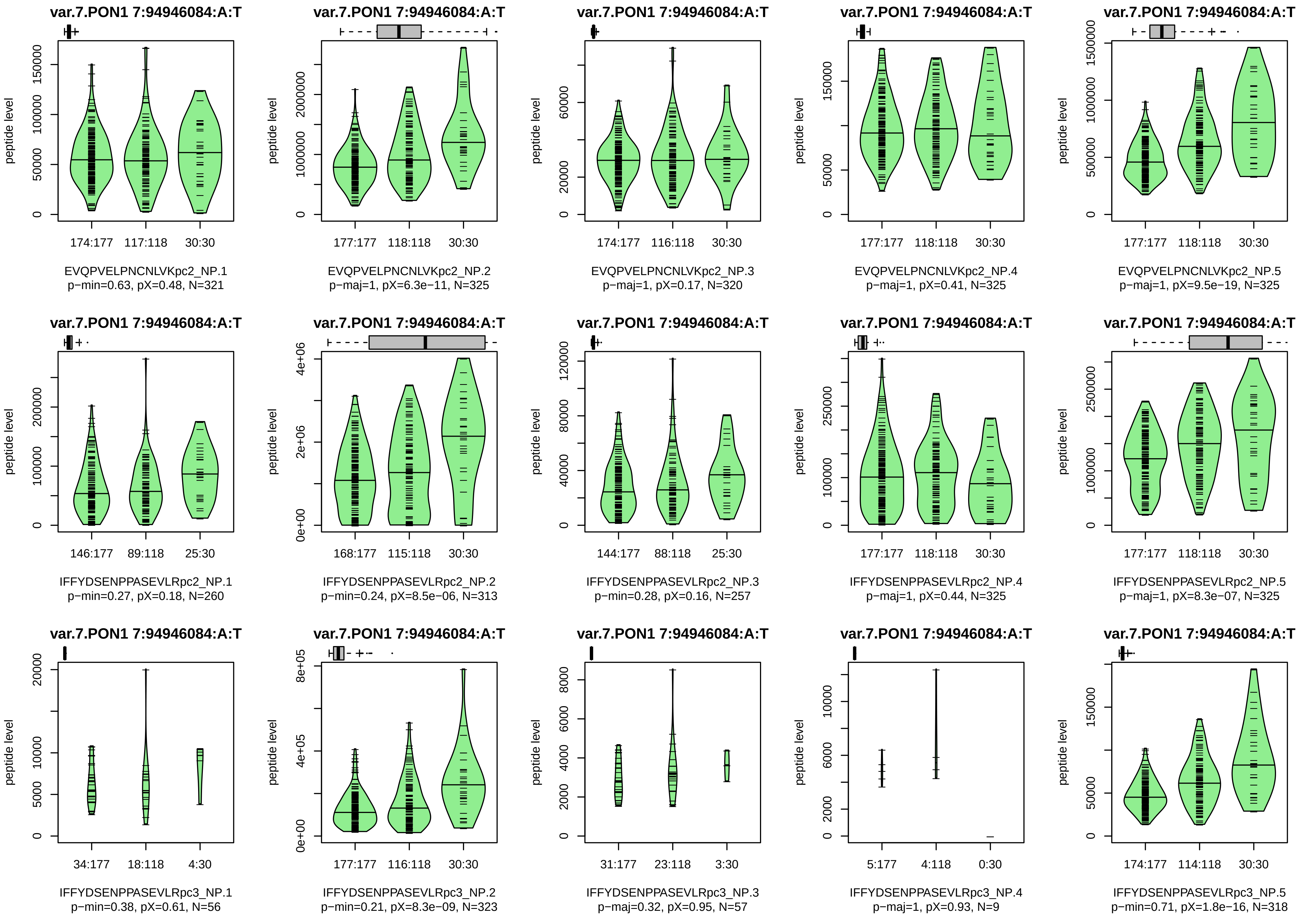
var.7.PON1 7:94946084:A:T
peptide level
174:177 117:118 30:30
EVQPVELPNCNLVKpc2_NP.1
p−min=0.63, pX=0.48, N=321
var.7.PON1 7:94946084:A:T
peptide level
177:177 118:118 30:30
EVQPVELPNCNLVKpc2_NP.2
p−maj=1, pX=6.3e−11, N=325
var.7.PON1 7:94946084:A:T
peptide level
174:177 116:118 30:30
EVQPVELPNCNLVKpc2_NP.3
p−maj=1, pX=0.17, N=320
var.7.PON1 7:94946084:A:T
peptide level
177:177 118:118 30:30
EVQPVELPNCNLVKpc2_NP.4
p−maj=1, pX=0.41, N=325
var.7.PON1 7:94946084:A:T
peptide level
177:177 118:118 30:30
EVQPVELPNCNLVKpc2_NP.5
p−maj=1, pX=9.5e−19, N=325
var.7.PON1 7:94946084:A:T
peptide level
146:177 89:118 25:30
IFFYDSENPPASEVLRpc2_NP.1
p−min=0.27, pX=0.18, N=260
var.7.PON1 7:94946084:A:T
peptide level
168:177 115:118 30:30
IFFYDSENPPASEVLRpc2_NP.2
p−min=0.24, pX=8.5e−06, N=313
var.7.PON1 7:94946084:A:T
peptide level
144:177 88:118 25:30
IFFYDSENPPASEVLRpc2_NP.3
p−min=0.28, pX=0.16, N=257
var.7.PON1 7:94946084:A:T
peptide level
177:177 118:118 30:30
IFFYDSENPPASEVLRpc2_NP.4
p−maj=1, pX=0.44, N=325
var.7.PON1 7:94946084:A:T
peptide level
177:177 118:118 30:30
IFFYDSENPPASEVLRpc2_NP.5
p−maj=1, pX=8.3e−07, N=325
var.7.PON1 7:94946084:A:T
peptide level
34:177 18:118 4:30
IFFYDSENPPASEVLRpc3_NP.1
p−min=0.38, pX=0.61, N=56
var.7.PON1 7:94946084:A:T
peptide level
177:177 116:118 30:30
IFFYDSENPPASEVLRpc3_NP.2
p−min=0.21, pX=8.3e−09, N=323
var.7.PON1 7:94946084:A:T
peptide level
31:177 23:118 3:30
IFFYDSENPPASEVLRpc3_NP.3
p−maj=0.32, pX=0.95, N=57
var.7.PON1 7:94946084:A:T
peptide level
5:177 4:118 0:30
IFFYDSENPPASEVLRpc3_NP.4
p−maj=1, pX=0.93, N=9
var.7.PON1 7:94946084:A:T
peptide level
174:177 114:118 30:30
IFFYDSENPPASEVLRpc3_NP.5
p−min=0.71, pX=1.8e−16, N=318

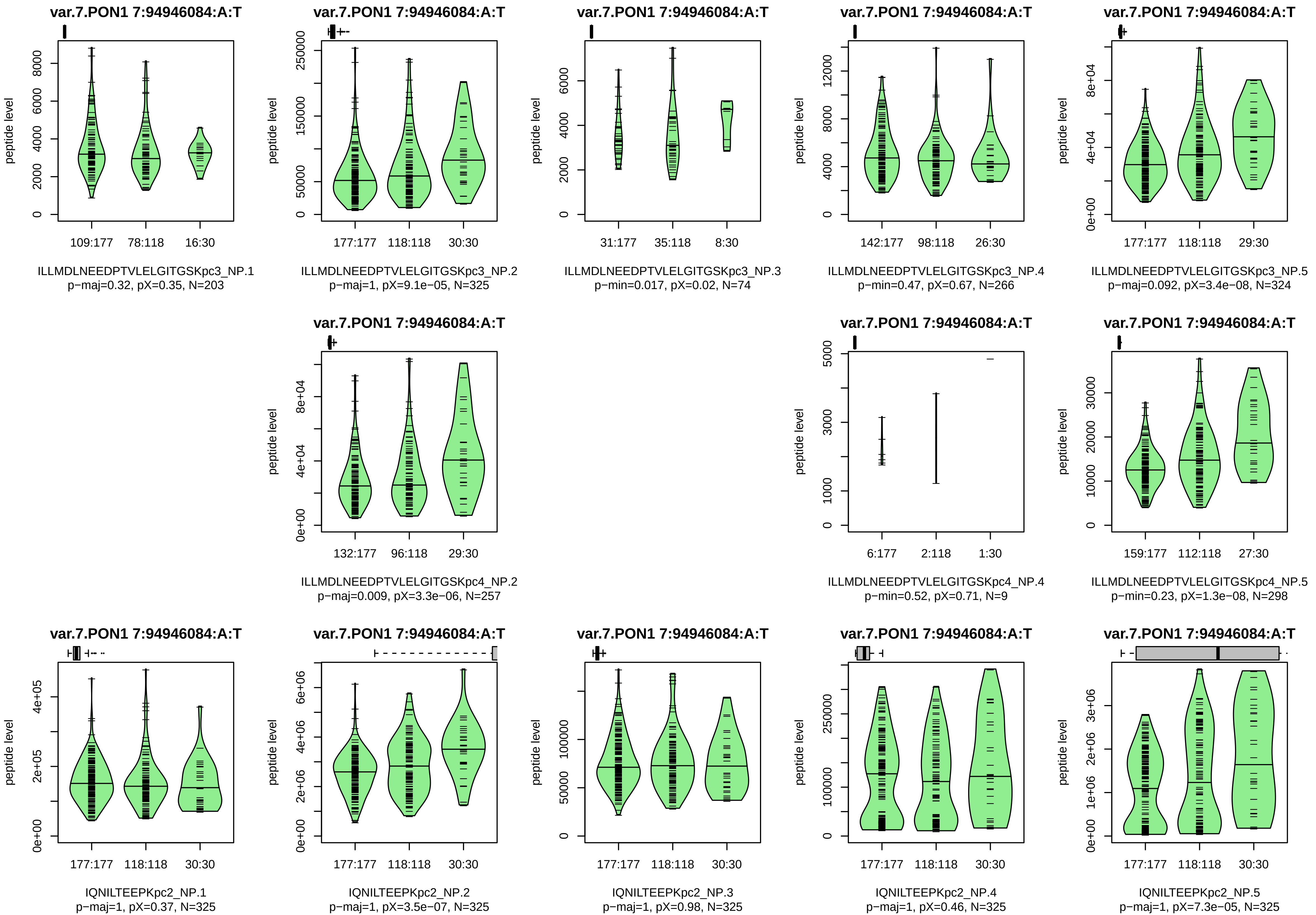

var.7.PON1 7:94946084:A:T
peptide level
109:177
78:118
16:30
ILLMDLNEEDPTVLELGITGSKpc3_NP.1
p−maj=0.32, pX=0.35, N=203
var.7.PON1 7:94946084:A:T
peptide level
177:177
118:118
30:30
ILLMDLNEEDPTVLELGITGSKpc3_NP.2
p−maj=1, pX=9.1e−05, N=325
var.7.PON1 7:94946084:A:T
peptide level
31:177
35:118
8:30
ILLMDLNEEDPTVLELGITGSKpc3_NP.3
p−min=0.017, pX=0.02, N=74
var.7.PON1 7:94946084:A:T
peptide level
142:177
98:118
26:30
ILLMDLNEEDPTVLELGITGSKpc3_NP.4
p−min=0.47, pX=0.67, N=266
var.7.PON1 7:94946084:A:T
peptide level
177:177
118:118
29:30
ILLMDLNEEDPTVLELGITGSKpc3_NP.5
p−maj=0.092, pX=3.4e−08, N=324
var.7.PON1 7:94946084:A:T
peptide level
132:177
96:118
29:30
ILLMDLNEEDPTVLELGITGSKpc4_NP.2
p−maj=0.009, pX=3.3e−06, N=257
var.7.PON1 7:94946084:A:T
peptide level
6:177
2:118
1:30
ILLMDLNEEDPTVLELGITGSKpc4_NP.4
p−min=0.52, pX=0.71, N=9
var.7.PON1 7:94946084:A:T
peptide level
159:177
112:118
27:30
ILLMDLNEEDPTVLELGITGSKpc4_NP.5
p−min=0.23, pX=1.3e−08, N=298
var.7.PON1 7:94946084:A:T
peptide level
177:177
118:118
30:30
IQNILTEEPKpc2_NP.1
p−maj=1, pX=0.37, N=325
var.7.PON1 7:94946084:A:T
peptide level
177:177
118:118
30:30
IQNILTEEPKpc2_NP.2
p−maj=1, pX=3.5e−07, N=325
var.7.PON1 7:94946084:A:T
peptide level
177:177
118:118
30:30
IQNILTEEPKpc2_NP.3
p−maj=1, pX=0.98, N=325
var.7.PON1 7:94946084:A:T
peptide level
177:177
118:118
30:30
IQNILTEEPKpc2_NP.4
p−maj=1, pX=0.46, N=325
var.7.PON1 7:94946084:A:T
peptide level
177:177
118:118
30:30
IQNILTEEPKpc2_NP.5
p−maj=1, pX=7.3e−05, N=325

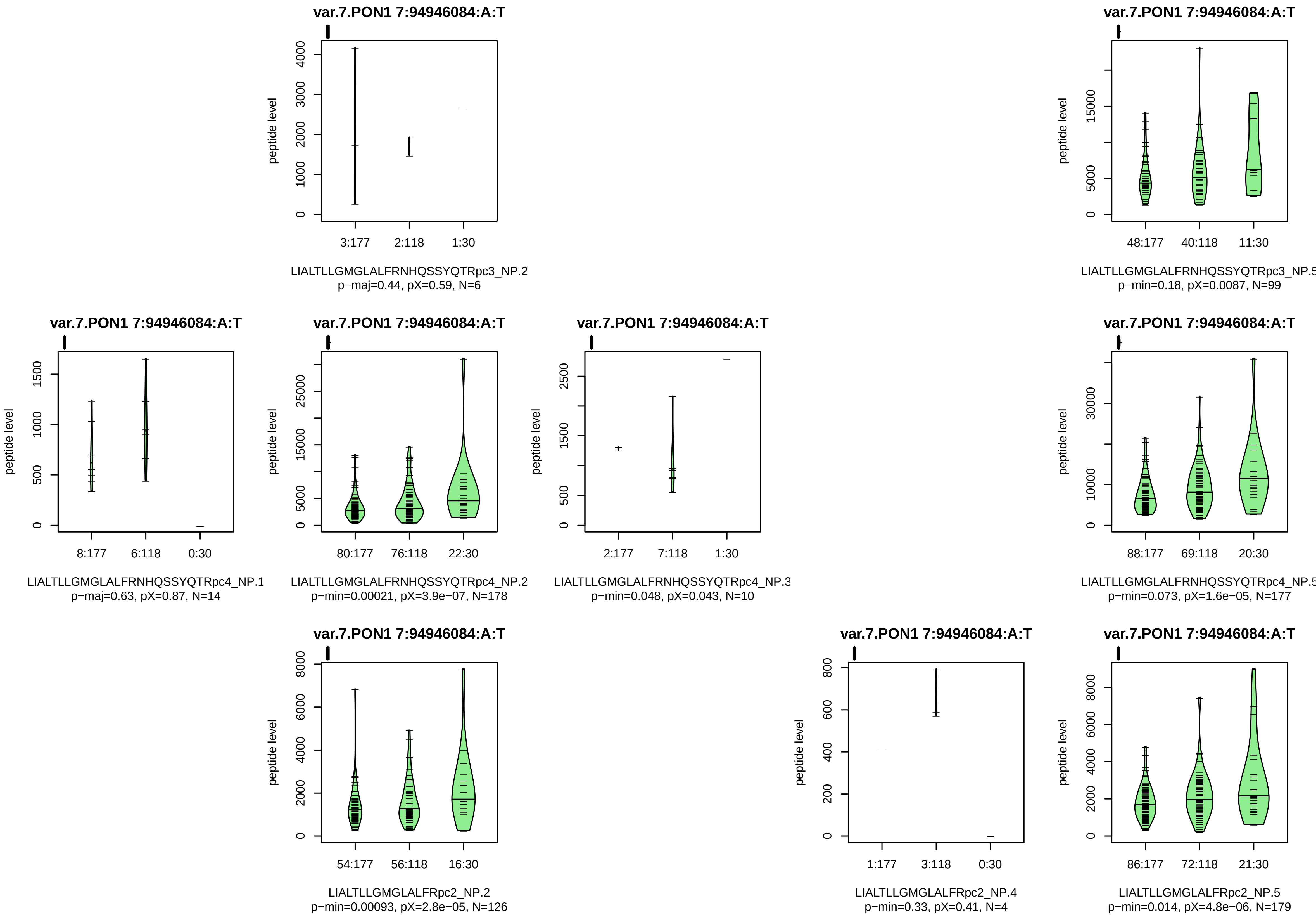
var.7.PON1 7:94946084:A:T
peptide level
3:177
2:118
1:30
LIALTLLGMGLALFRNHQSSYQTRpc3_NP.2
p−maj=0.44, pX=0.59, N=6
var.7.PON1 7:94946084:A:T
peptide level
48:177
40:118
11:30
LIALTLLGMGLALFRNHQSSYQTRpc3_NP.5
p−min=0.18, pX=0.0087, N=99
var.7.PON1 7:94946084:A:T
peptide level
8:177
6:118
0:30
LIALTLLGMGLALFRNHQSSYQTRpc4_NP.1
p−maj=0.63, pX=0.87, N=14
var.7.PON1 7:94946084:A:T
peptide level
80:177
76:118
22:30
LIALTLLGMGLALFRNHQSSYQTRpc4_NP.2
p−min=0.00021, pX=3.9e−07, N=178
var.7.PON1 7:94946084:A:T
peptide level
2:177
7:118
1:30
LIALTLLGMGLALFRNHQSSYQTRpc4_NP.3
p−min=0.048, pX=0.043, N=10
var.7.PON1 7:94946084:A:T
peptide level
88:177
69:118
20:30
LIALTLLGMGLALFRNHQSSYQTRpc4_NP.5
p−min=0.073, pX=1.6e−05, N=177
var.7.PON1 7:94946084:A:T
peptide level
54:177
56:118
16:30
LIALTLLGMGLALFRpc2_NP.2
p−min=0.00093, pX=2.8e−05, N=126
var.7.PON1 7:94946084:A:T
peptide level
1:177
3:118
0:30
LIALTLLGMGLALFRpc2_NP.4
p−min=0.33, pX=0.41, N=4
var.7.PON1 7:94946084:A:T
peptide level
86:177
72:118
21:30
LIALTLLGMGLALFRpc2_NP.5
p−min=0.014, pX=4.8e−06, N=179

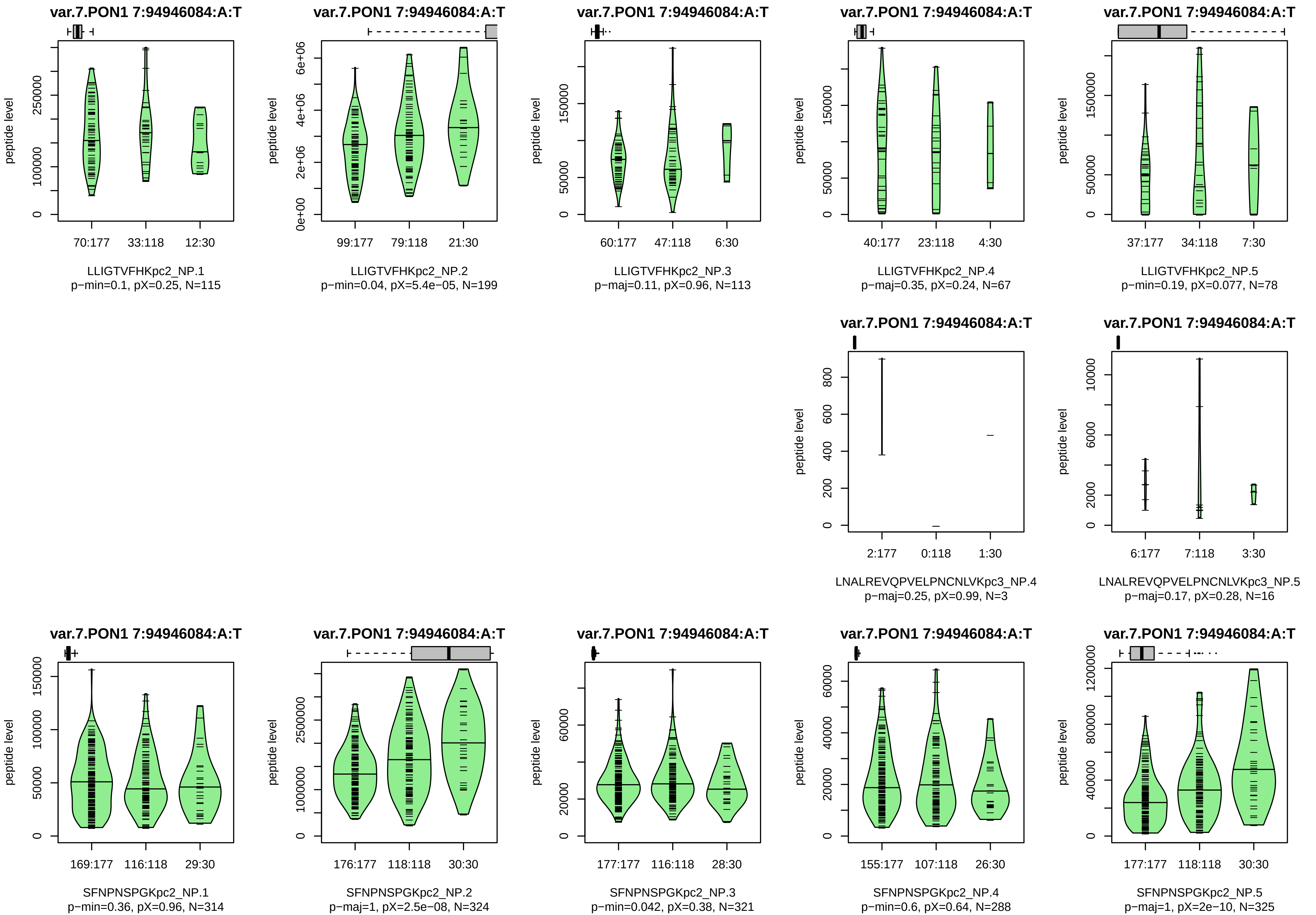
var.7.PON1 7:94946084:A:T
70:177
33:118
12:30
LLIGTVFHKpc2_NP.1
p−min=0.1, pX=0.25, N=115
var.7.PON1 7:94946084:A:T
99:177
79:118
21:30
LLIGTVFHKpc2_NP.2
p−min=0.04, pX=5.4e−05, N=199
var.7.PON1 7:94946084:A:T
60:177
47:118
6:30
LLIGTVFHKpc2_NP.3
p−maj=0.11, pX=0.96, N=113
var.7.PON1 7:94946084:A:T
40:177
23:118
4:30
LLIGTVFHKpc2_NP.4
p−maj=0.35, pX=0.24, N=67
var.7.PON1 7:94946084:A:T
37:177
34:118
7:30
LLIGTVFHKpc2_NP.5
p−min=0.19, pX=0.077, N=78
var.7.PON1 7:94946084:A:T
2:177
0:118
1:30
LNALREVQPVELPNCNLVKpc3_NP.4
p−maj=0.25, pX=0.99, N=3
var.7.PON1 7:94946084:A:T
6:177
7:118
3:30
LNALREVQPVELPNCNLVKpc3_NP.5
p−maj=0.17, pX=0.28, N=16
var.7.PON1 7:94946084:A:T
169:177
116:118
29:30
SFNPNSPGKpc2_NP.1
p−min=0.36, pX=0.96, N=314
var.7.PON1 7:94946084:A:T
176:177
118:118
30:30
SFNPNSPGKpc2_NP.2
p−maj=1, pX=2.5e−08, N=324
var.7.PON1 7:94946084:A:T
177:177
116:118
28:30
SFNPNSPGKpc2_NP.3
p−min=0.042, pX=0.38, N=321
var.7.PON1 7:94946084:A:T
155:177
107:118
26:30
SFNPNSPGKpc2_NP.4
p−min=0.6, pX=0.64, N=288
var.7.PON1 7:94946084:A:T
177:177
118:118
30:30
SFNPNSPGKpc2_NP.5
p−maj=1, pX=2e−10, N=325
peptide level

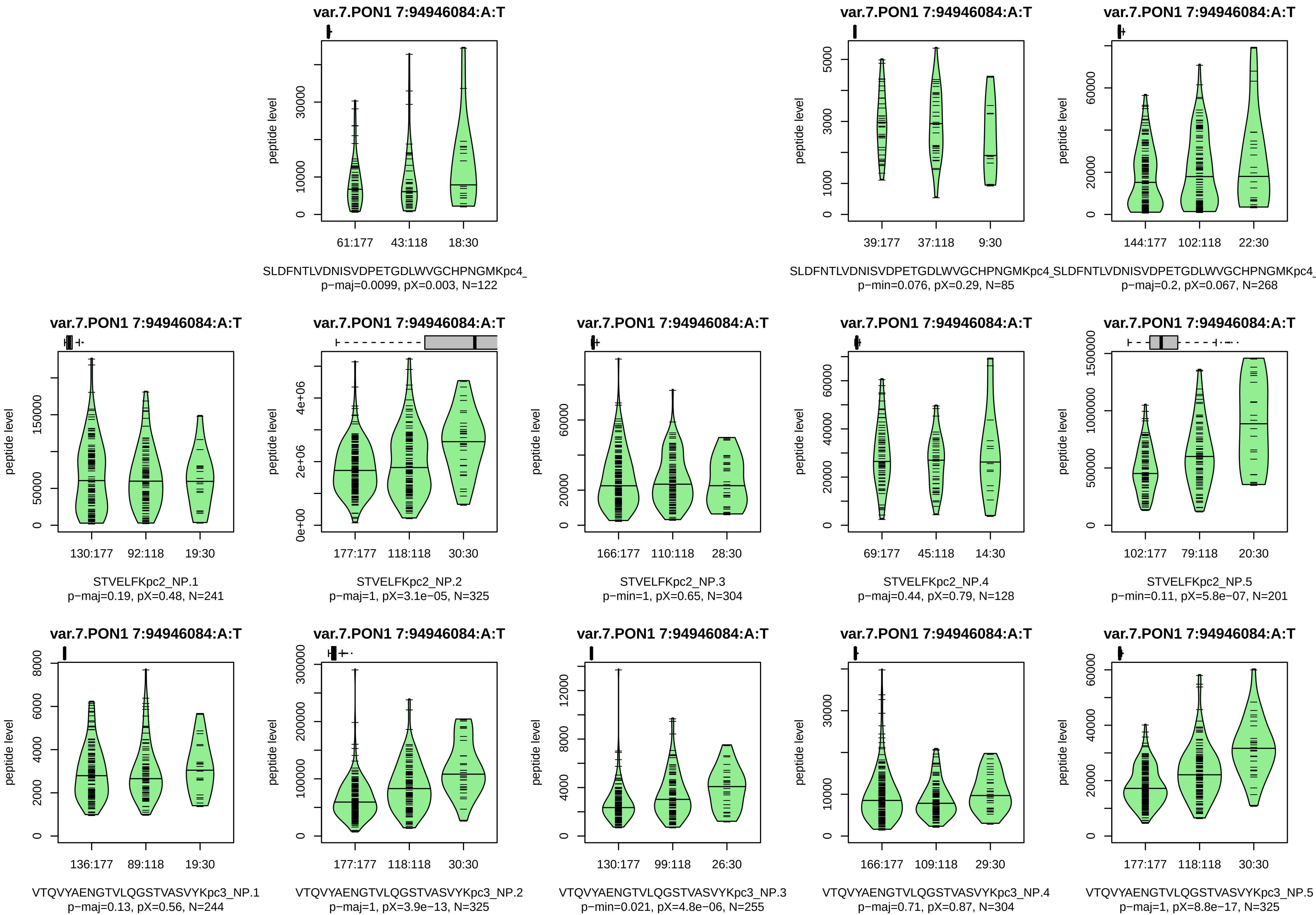
var.7.PON1 7:94946084:A:T
peptide level
61:177
43:118
18:30
SLDFNTLVDNISVDPETGDLWVGCHPNGMKpc4_
p−maj=0.0099, pX=0.003, N=122
var.7.PON1 7:94946084:A:T
peptide level
39:177
37:118
9:30
SLDFNTLVDNISVDPETGDLWVGCHPNGMKpc4_
p−min=0.076, pX=0.29, N=85
var.7.PON1 7:94946084:A:T
peptide level
144:177
102:118
22:30
SLDFNTLVDNISVDPETGDLWVGCHPNGMKpc4_
p−maj=0.2, pX=0.067, N=268
var.7.PON1 7:94946084:A:T
peptide level
130:177
92:118
19:30
STVELFKpc2_NP.1
p−maj=0.19, pX=0.48, N=241
var.7.PON1 7:94946084:A:T
peptide level
177:177
118:118
30:30
STVELFKpc2_NP.2
p−maj=1, pX=3.1e−05, N=325
var.7.PON1 7:94946084:A:T
peptide level
166:177
110:118
28:30
STVELFKpc2_NP.3
p−min=1, pX=0.65, N=304
var.7.PON1 7:94946084:A:T
peptide level
69:177
45:118
14:30
STVELFKpc2_NP.4
p−maj=0.44, pX=0.79, N=128
var.7.PON1 7:94946084:A:T
peptide level
102:177
79:118
20:30
STVELFKpc2_NP.5
p−min=0.11, pX=5.8e−07, N=201
var.7.PON1 7:94946084:A:T
peptide level
136:177
89:118
19:30
VTQVYAENGTVLQGSTVASVYKpc3_NP.1
p−maj=0.13, pX=0.56, N=244
var.7.PON1 7:94946084:A:T
peptide level
177:177
118:118
30:30
VTQVYAENGTVLQGSTVASVYKpc3_NP.2
p−maj=1, pX=3.9e−13, N=325
var.7.PON1 7:94946084:A:T
peptide level
130:177
99:118
26:30
VTQVYAENGTVLQGSTVASVYKpc3_NP.3
p−min=0.021, pX=4.8e−06, N=255
var.7.PON1 7:94946084:A:T
peptide level
166:177
109:118
29:30
VTQVYAENGTVLQGSTVASVYKpc3_NP.4
p−maj=0.71, pX=0.87, N=304
var.7.PON1 7:94946084:A:T
peptide level
177:177
118:118
30:30
VTQVYAENGTVLQGSTVASVYKpc3_NP.5
p−maj=1, pX=8.8e−17, N=325

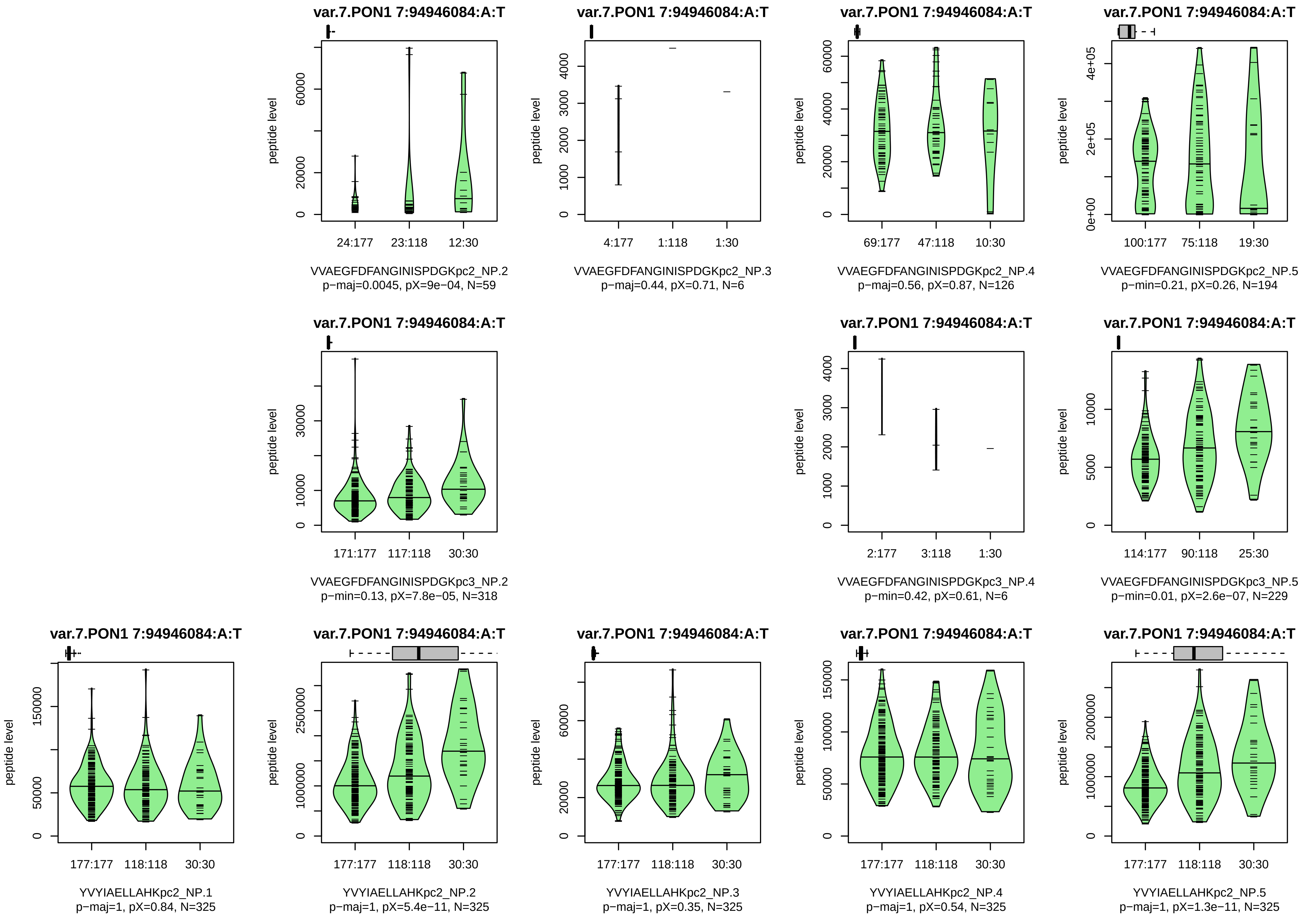
var.7.PON1 7:94946084:A:T
peptide level
24:177
23:118
12:30
VVAEGFDFANGINISPDGKpc2_NP.2
p−maj=0.0045, pX=9e−04, N=59
var.7.PON1 7:94946084:A:T
peptide level
4:177
1:118
1:30
VVAEGFDFANGINISPDGKpc2_NP.3
p−maj=0.44, pX=0.71, N=6
var.7.PON1 7:94946084:A:T
peptide level
69:177
47:118
10:30
VVAEGFDFANGINISPDGKpc2_NP.4
p−maj=0.56, pX=0.87, N=126
var.7.PON1 7:94946084:A:T
peptide level
100:177
75:118
19:30
VVAEGFDFANGINISPDGKpc2_NP.5
p−min=0.21, pX=0.26, N=194
var.7.PON1 7:94946084:A:T
peptide level
171:177
117:118
30:30
VVAEGFDFANGINISPDGKpc3_NP.2
p−min=0.13, pX=7.8e−05, N=318
var.7.PON1 7:94946084:A:T
peptide level
2:177
3:118
1:30
VVAEGFDFANGINISPDGKpc3_NP.4
p−min=0.42, pX=0.61, N=6
var.7.PON1 7:94946084:A:T
peptide level
114:177
90:118
25:30
VVAEGFDFANGINISPDGKpc3_NP.5
p−min=0.01, pX=2.6e−07, N=229
var.7.PON1 7:94946084:A:T
peptide level
177:177
118:118
30:30
YVYIAELLAHKpc2_NP.1
p−maj=1, pX=0.84, N=325
var.7.PON1 7:94946084:A:T
peptide level
177:177
118:118
30:30
YVYIAELLAHKpc2_NP.2
p−maj=1, pX=5.4e−11, N=325
var.7.PON1 7:94946084:A:T
peptide level
177:177
118:118
30:30
YVYIAELLAHKpc2_NP.3
p−maj=1, pX=0.35, N=325
var.7.PON1 7:94946084:A:T
peptide level
177:177
118:118
30:30
YVYIAELLAHKpc2_NP.4
p−maj=1, pX=0.54, N=325
var.7.PON1 7:94946084:A:T
peptide level
177:177
118:118
30:30
YVYIAELLAHKpc2_NP.5
p−maj=1, pX=1.3e−11, N=325

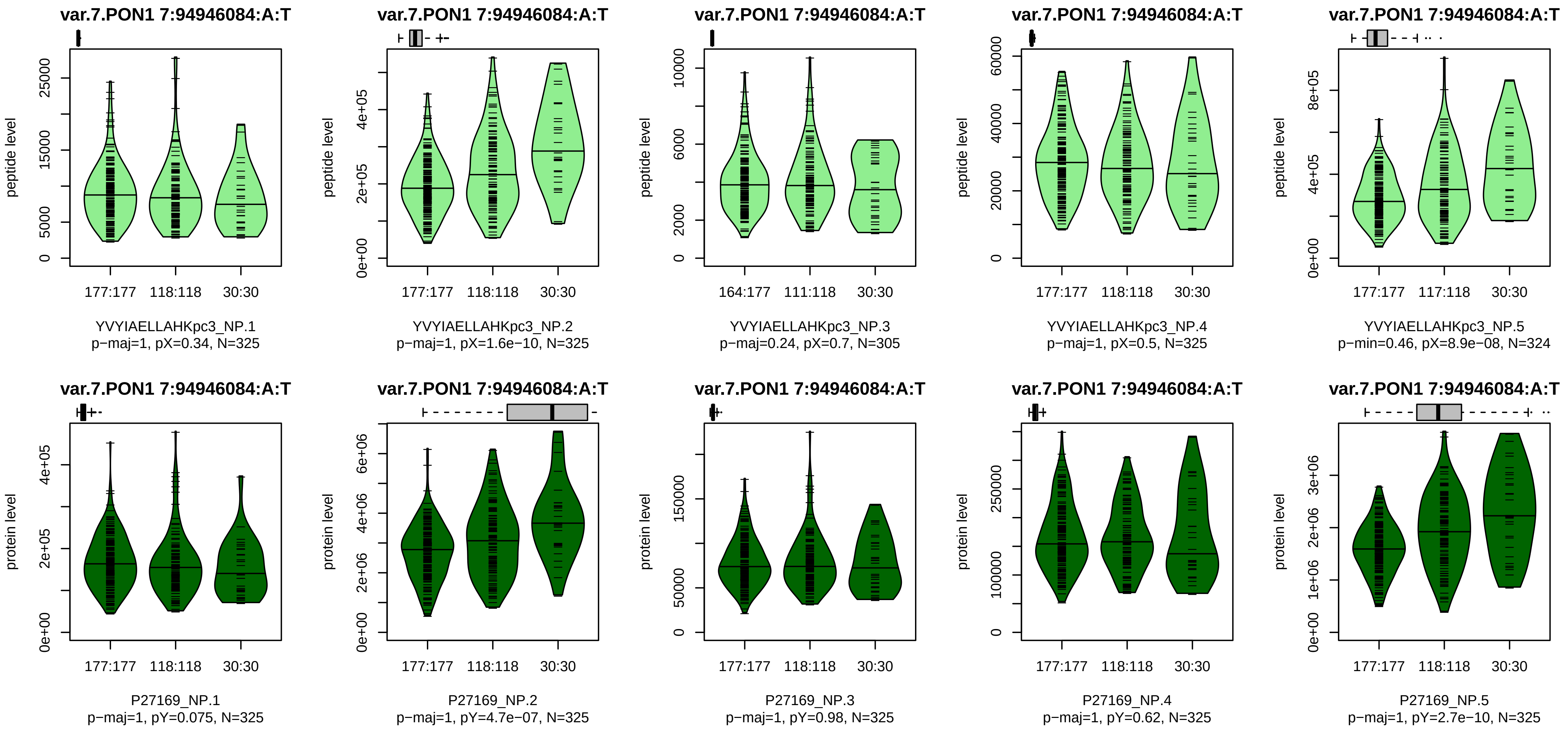
var.7.PON1 7:94946084:A:T
peptide level
0
5000
15000
25000
177:177
118:118
30:30
YVYIAELLAHKpc3_NP.1
p−maj=1, pX=0.34, N=325
var.7.PON1 7:94946084:A:T
peptide level
0e+00
2e+05
4e+05
177:177
118:118
30:30
YVYIAELLAHKpc3_NP.2
p−maj=1, pX=1.6e−10, N=325
var.7.PON1 7:94946084:A:T
peptide level
0
2000
6000
10000
164:177
111:118
30:30
YVYIAELLAHKpc3_NP.3
p−maj=0.24, pX=0.7, N=305
var.7.PON1 7:94946084:A:T
peptide level
0
20000
40000
60000
177:177
118:118
30:30
YVYIAELLAHKpc3_NP.4
p−maj=1, pX=0.5, N=325
var.7.PON1 7:94946084:A:T
peptide level
0e+00
4e+05
8e+05
177:177
117:118
30:30
YVYIAELLAHKpc3_NP.5
p−min=0.46, pX=8.9e−08, N=324
var.7.PON1 7:94946084:A:T
protein level
0e+00
2e+05
4e+05
177:177
118:118
30:30
P27169_NP.1
p−maj=1, pY=0.075, N=325
var.7.PON1 7:94946084:A:T
protein level
0e+00
2e+06
4e+06
6e+06
177:177
118:118
30:30
P27169_NP.2
p−maj=1, pY=4.7e−07, N=325
var.7.PON1 7:94946084:A:T
protein level
0
50000
150000
177:177
118:118
30:30
P27169_NP.3
p−maj=1, pY=0.98, N=325
var.7.PON1 7:94946084:A:T
protein level
0
100000
250000
177:177
118:118
30:30
P27169_NP.4
p−maj=1, pY=0.62, N=325
var.7.PON1 7:94946084:A:T
protein level
0e+00
1e+06
2e+06
3e+06
177:177
118:118
30:30
P27169_NP.5
p−maj=1, pY=2.7e−10, N=325

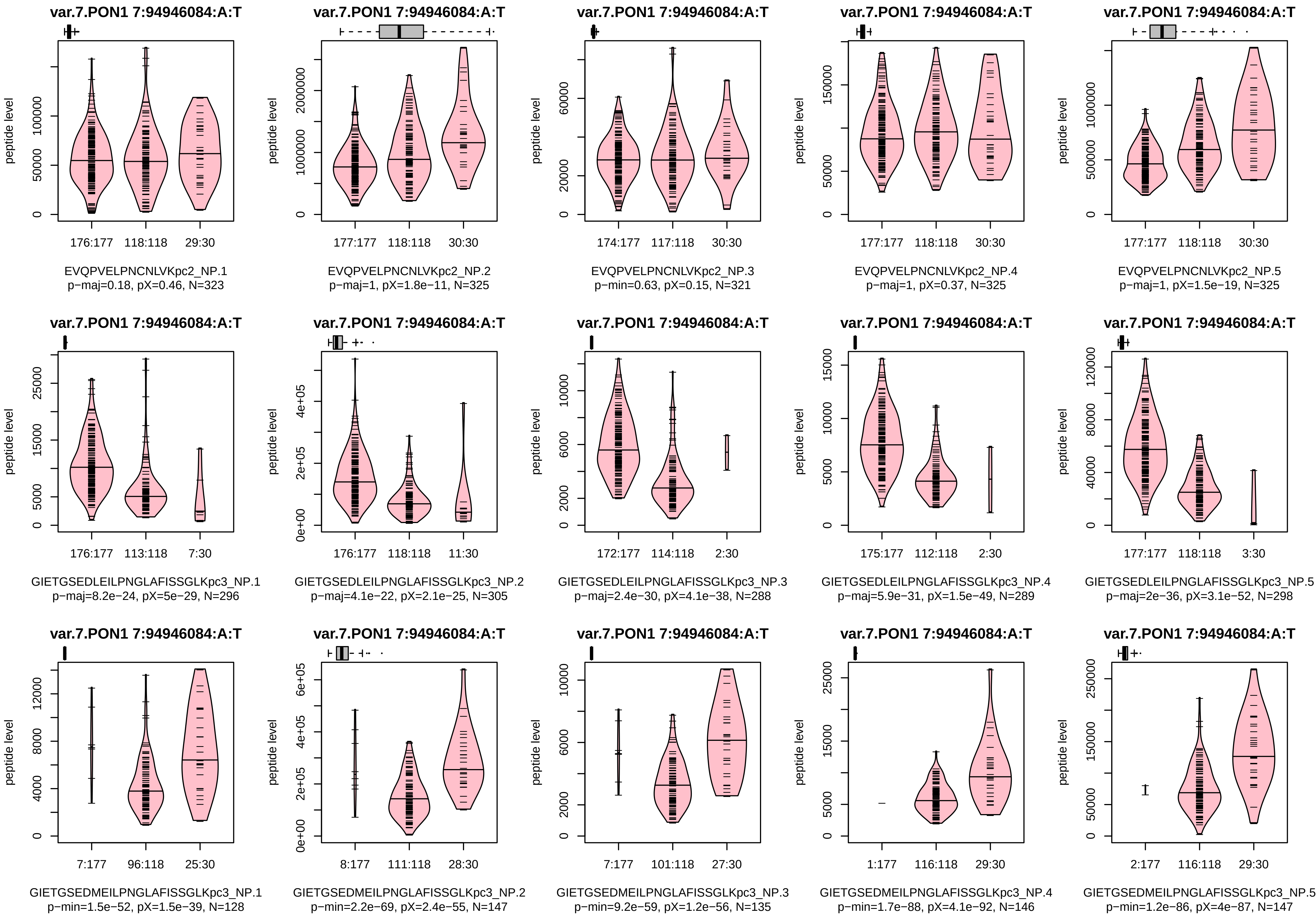
var.7.PON1 7:94946084:A:T
peptide level
176:177
118:118
29:30
EVQPVELPNCNLVKpc2_NP.1
p−maj=0.18, pX=0.46, N=323
var.7.PON1 7:94946084:A:T
peptide level
177:177
118:118
30:30
EVQPVELPNCNLVKpc2_NP.2
p−maj=1, pX=1.8e−11, N=325
var.7.PON1 7:94946084:A:T
peptide level
174:177
117:118
30:30
EVQPVELPNCNLVKpc2_NP.3
p−min=0.63, pX=0.15, N=321
var.7.PON1 7:94946084:A:T
peptide level
177:177
118:118
30:30
EVQPVELPNCNLVKpc2_NP.4
p−maj=1, pX=0.37, N=325
var.7.PON1 7:94946084:A:T
peptide level
177:177
118:118
30:30
EVQPVELPNCNLVKpc2_NP.5
p−maj=1, pX=1.5e−19, N=325
var.7.PON1 7:94946084:A:T
peptide level
176:177
113:118
7:30
GIETGSEDLEILPNGLAFISSGLKpc3_NP.1
p−maj=8.2e−24, pX=5e−29, N=296
var.7.PON1 7:94946084:A:T
peptide level
176:177
118:118
11:30
GIETGSEDLEILPNGLAFISSGLKpc3_NP.2
p−maj=4.1e−22, pX=2.1e−25, N=305
var.7.PON1 7:94946084:A:T
peptide level
172:177
114:118
2:30
GIETGSEDLEILPNGLAFISSGLKpc3_NP.3
p−maj=2.4e−30, pX=4.1e−38, N=288
var.7.PON1 7:94946084:A:T
peptide level
175:177
112:118
2:30
GIETGSEDLEILPNGLAFISSGLKpc3_NP.4
p−maj=5.9e−31, pX=1.5e−49, N=289
var.7.PON1 7:94946084:A:T
peptide level
177:177
118:118
3:30
GIETGSEDLEILPNGLAFISSGLKpc3_NP.5
p−maj=2e−36, pX=3.1e−52, N=298
var.7.PON1 7:94946084:A:T
peptide level
7:177
96:118
25:30
GIETGSEDMEILPNGLAFISSGLKpc3_NP.1
p−min=1.5e−52, pX=1.5e−39, N=128
var.7.PON1 7:94946084:A:T
peptide level
8:177
111:118
28:30
GIETGSEDMEILPNGLAFISSGLKpc3_NP.2
p−min=2.2e−69, pX=2.4e−55, N=147
var.7.PON1 7:94946084:A:T
peptide level
7:177
101:118
27:30
GIETGSEDMEILPNGLAFISSGLKpc3_NP.3
p−min=9.2e−59, pX=1.2e−56, N=135
var.7.PON1 7:94946084:A:T
peptide level
1:177
116:118
29:30
GIETGSEDMEILPNGLAFISSGLKpc3_NP.4
p−min=1.7e−88, pX=4.1e−92, N=146
var.7.PON1 7:94946084:A:T
peptide level
2:177
116:118
29:30
GIETGSEDMEILPNGLAFISSGLKpc3_NP.5
p−min=1.2e−86, pX=4e−87, N=147

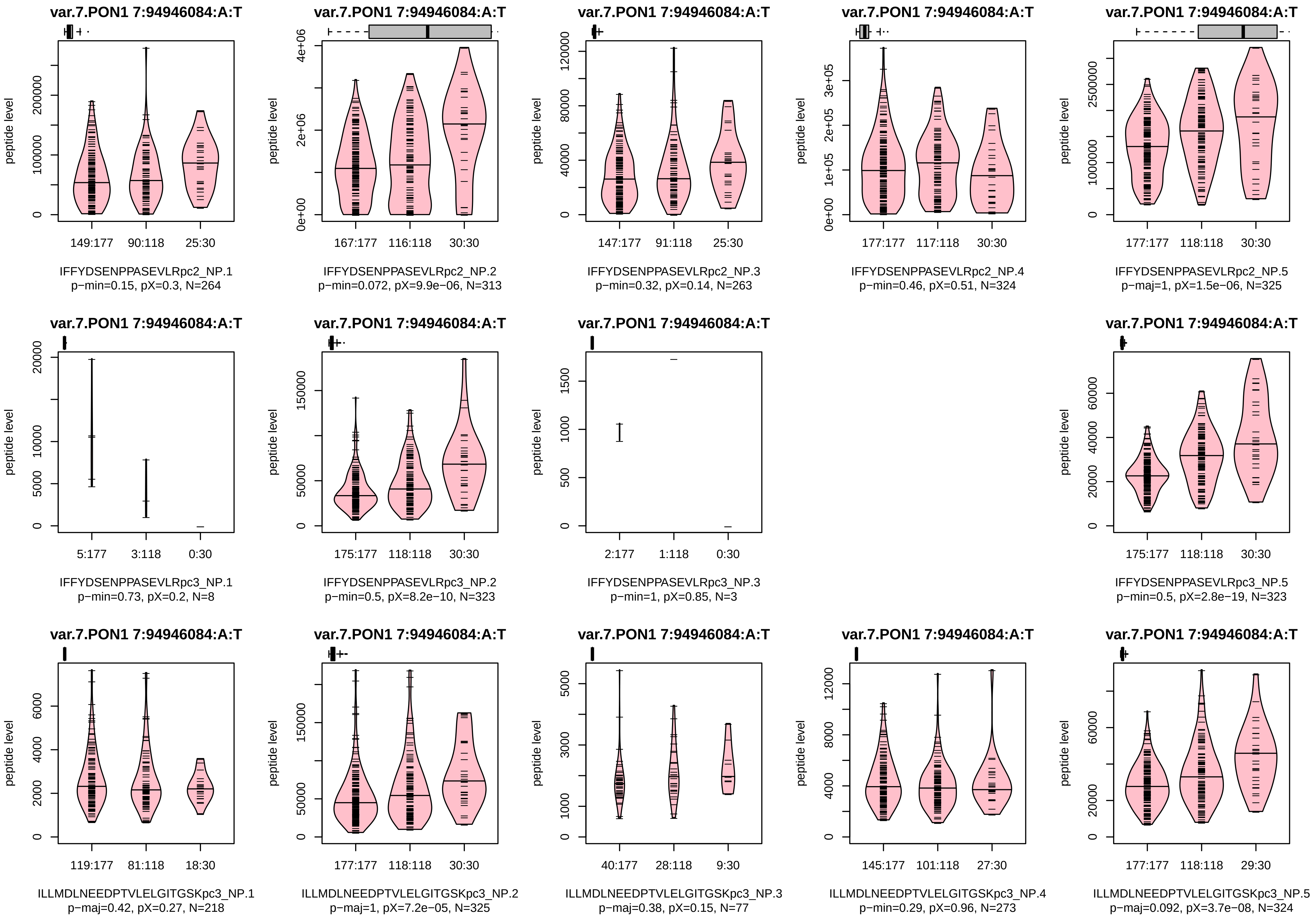

var.7.PON1 7:94946084:A:T
peptide level
0
100000
200000
149:177
90:118
25:30
IFFYDSENPPASEVLRpc2_NP.1
p−min=0.15, pX=0.3, N=264
var.7.PON1 7:94946084:A:T
peptide level
0e+00
2e+06
4e+06
167:177
116:118
30:30
IFFYDSENPPASEVLRpc2_NP.2
p−min=0.072, pX=9.9e−06, N=313
var.7.PON1 7:94946084:A:T
peptide level
0
40000
80000
120000
147:177
91:118
25:30
IFFYDSENPPASEVLRpc2_NP.3
p−min=0.32, pX=0.14, N=263
var.7.PON1 7:94946084:A:T
peptide level
0e+00
1e+05
2e+05
3e+05
177:177
117:118
30:30
IFFYDSENPPASEVLRpc2_NP.4
p−min=0.46, pX=0.51, N=324
var.7.PON1 7:94946084:A:T
peptide level
0
1000000
2500000
177:177
118:118
30:30
IFFYDSENPPASEVLRpc2_NP.5
p−maj=1, pX=1.5e−06, N=325
var.7.PON1 7:94946084:A:T
peptide level
0
5000
10000
20000
5:177
3:118
0:30
IFFYDSENPPASEVLRpc3_NP.1
p−min=0.73, pX=0.2, N=8
var.7.PON1 7:94946084:A:T
peptide level
0
50000
150000
175:177
118:118
30:30
IFFYDSENPPASEVLRpc3_NP.2
p−min=0.5, pX=8.2e−10, N=323
var.7.PON1 7:94946084:A:T
peptide level
0
500
1000
1500
2:177
1:118
0:30
IFFYDSENPPASEVLRpc3_NP.3
p−min=1, pX=0.85, N=3
var.7.PON1 7:94946084:A:T
peptide level
0
20000
40000
60000
175:177
118:118
30:30
IFFYDSENPPASEVLRpc3_NP.5
p−min=0.5, pX=2.8e−19, N=323
var.7.PON1 7:94946084:A:T
peptide level
0
2000
4000
6000
119:177
81:118
18:30
ILLMDLNEEDPTVLELGITGSKpc3_NP.1
p−maj=0.42, pX=0.27, N=218
var.7.PON1 7:94946084:A:T
peptide level
0
50000
150000
177:177
118:118
30:30
ILLMDLNEEDPTVLELGITGSKpc3_NP.2
p−maj=1, pX=7.2e−05, N=325
var.7.PON1 7:94946084:A:T
peptide level
0
1000
3000
5000
40:177
28:118
9:30
ILLMDLNEEDPTVLELGITGSKpc3_NP.3
p−maj=0.38, pX=0.15, N=77
var.7.PON1 7:94946084:A:T
peptide level
0
4000
8000
12000
145:177
101:118
27:30
ILLMDLNEEDPTVLELGITGSKpc3_NP.4
p−min=0.29, pX=0.96, N=273
var.7.PON1 7:94946084:A:T
peptide level
0
20000
60000
177:177
118:118
29:30
ILLMDLNEEDPTVLELGITGSKpc3_NP.5
p−maj=0.092, pX=3.7e−08, N=324

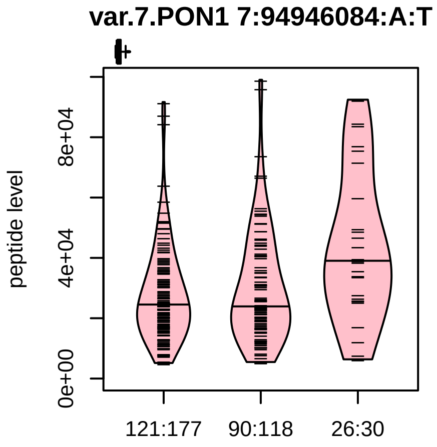

ILLMDLNEEDPTVLELGITGSKpc4_NP.2
p−min=0.046, pX=3.4e−05, N=237

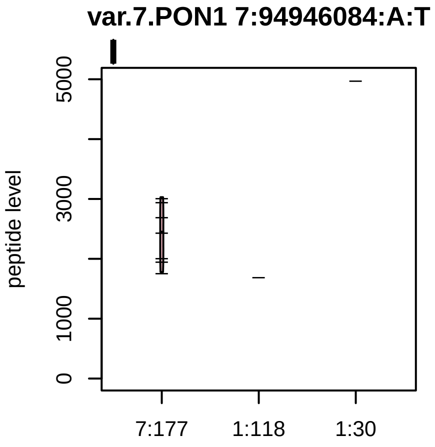

ILLMDLNEEDPTVLELGITGSKpc4_NP.4
p−min=0.19, pX=0.82, N=9

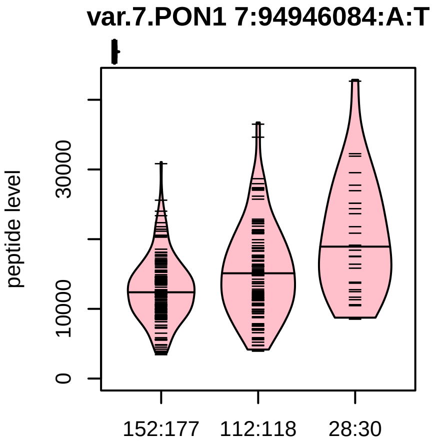

ILLMDLNEEDPTVLELGITGSKpc4_NP.5
p−min=0.0099, pX=2.2e−10, N=292

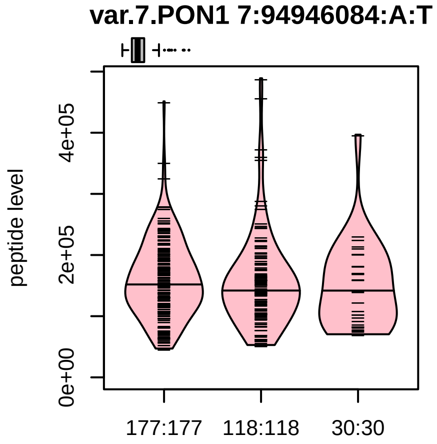

IQNILTEEPKpc2_NP.1
p−maj=1, pX=0.33, N=325

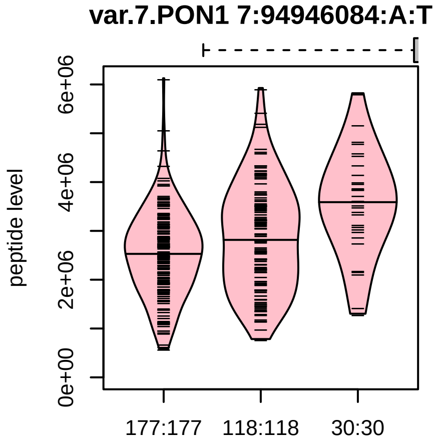

IQNILTEEPKpc2_NP.2
p−maj=1, pX=2e−07, N=325

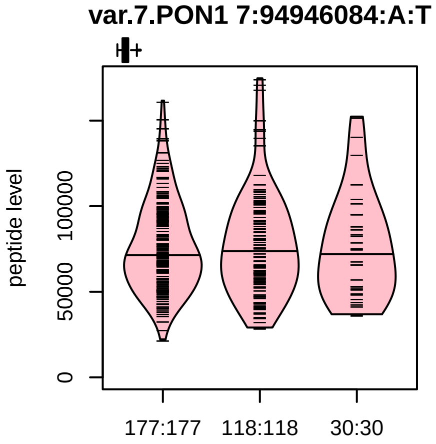

IQNILTEEPKpc2_NP.3
p−maj=1, pX=0.86, N=325

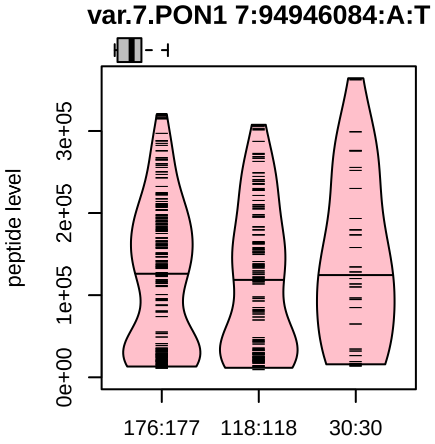

IQNILTEEPKpc2_NP.4
p−maj=1, pX=0.49, N=324

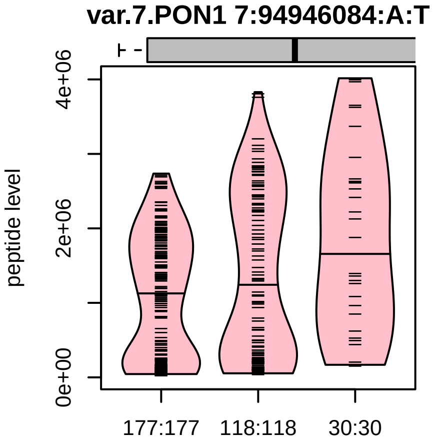

IQNILTEEPKpc2_NP.5
p−maj=1, pX=8e−05, N=325

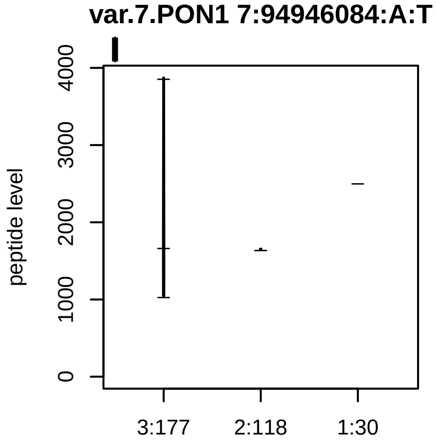

LIALTLLGMGLALFRNHQSSYQTRpc3_NP.2
p−maj=0.44, pX=0.67, N=6

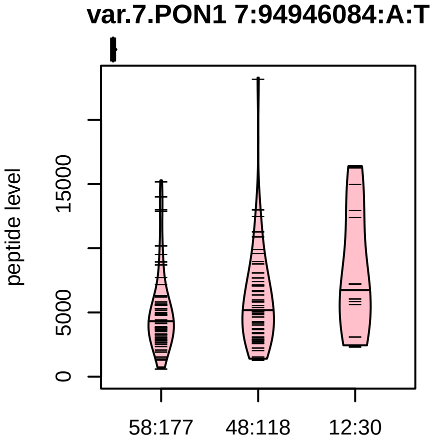

LIALTLLGMGLALFRNHQSSYQTRpc3_NP.5
p−min=0.17, pX=0.0089, N=118

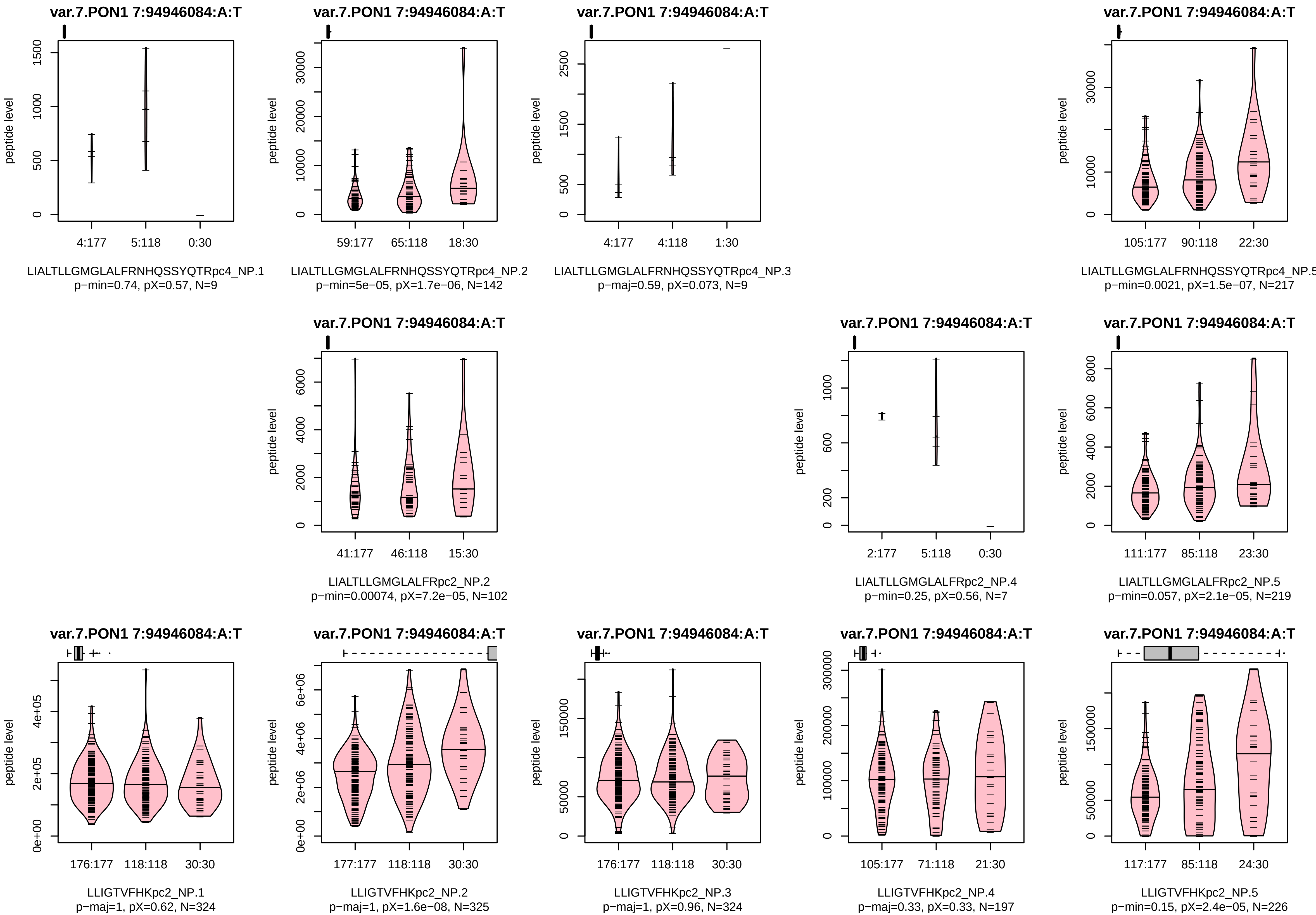
var.7.PON1 7:94946084:A:T
peptide level
4:177
5:118
0:30
LIALTLLGMGLALFRNHQSSYQTRpc4_NP.1
p−min=0.74, pX=0.57, N=9
var.7.PON1 7:94946084:A:T
peptide level
59:177
65:118
18:30
LIALTLLGMGLALFRNHQSSYQTRpc4_NP.2
p−min=5e−05, pX=1.7e−06, N=142
var.7.PON1 7:94946084:A:T
peptide level
4:177
4:118
1:30
LIALTLLGMGLALFRNHQSSYQTRpc4_NP.3
p−maj=0.59, pX=0.073, N=9
var.7.PON1 7:94946084:A:T
peptide level
105:177
90:118
22:30
LIALTLLGMGLALFRNHQSSYQTRpc4_NP.5
p−min=0.0021, pX=1.5e−07, N=217
var.7.PON1 7:94946084:A:T
peptide level
41:177
46:118
15:30
LIALTLLGMGLALFRpc2_NP.2
p−min=0.00074, pX=7.2e−05, N=102
var.7.PON1 7:94946084:A:T
peptide level
2:177
5:118
0:30
LIALTLLGMGLALFRpc2_NP.4
p−min=0.25, pX=0.56, N=7
var.7.PON1 7:94946084:A:T
peptide level
111:177
85:118
23:30
LIALTLLGMGLALFRpc2_NP.5
p−min=0.057, pX=2.1e−05, N=219
var.7.PON1 7:94946084:A:T
peptide level
176:177
118:118
30:30
LLIGTVFHKpc2_NP.1
p−maj=1, pX=0.62, N=324
var.7.PON1 7:94946084:A:T
peptide level
177:177
118:118
30:30
LLIGTVFHKpc2_NP.2
p−maj=1, pX=1.6e−08, N=325
var.7.PON1 7:94946084:A:T
peptide level
176:177
118:118
30:30
LLIGTVFHKpc2_NP.3
p−maj=1, pX=0.96, N=324
var.7.PON1 7:94946084:A:T
peptide level
105:177
71:118
21:30
LLIGTVFHKpc2_NP.4
p−maj=0.33, pX=0.33, N=197
var.7.PON1 7:94946084:A:T
peptide level
117:177
85:118
24:30
LLIGTVFHKpc2_NP.5
p−min=0.15, pX=2.4e−05, N=226

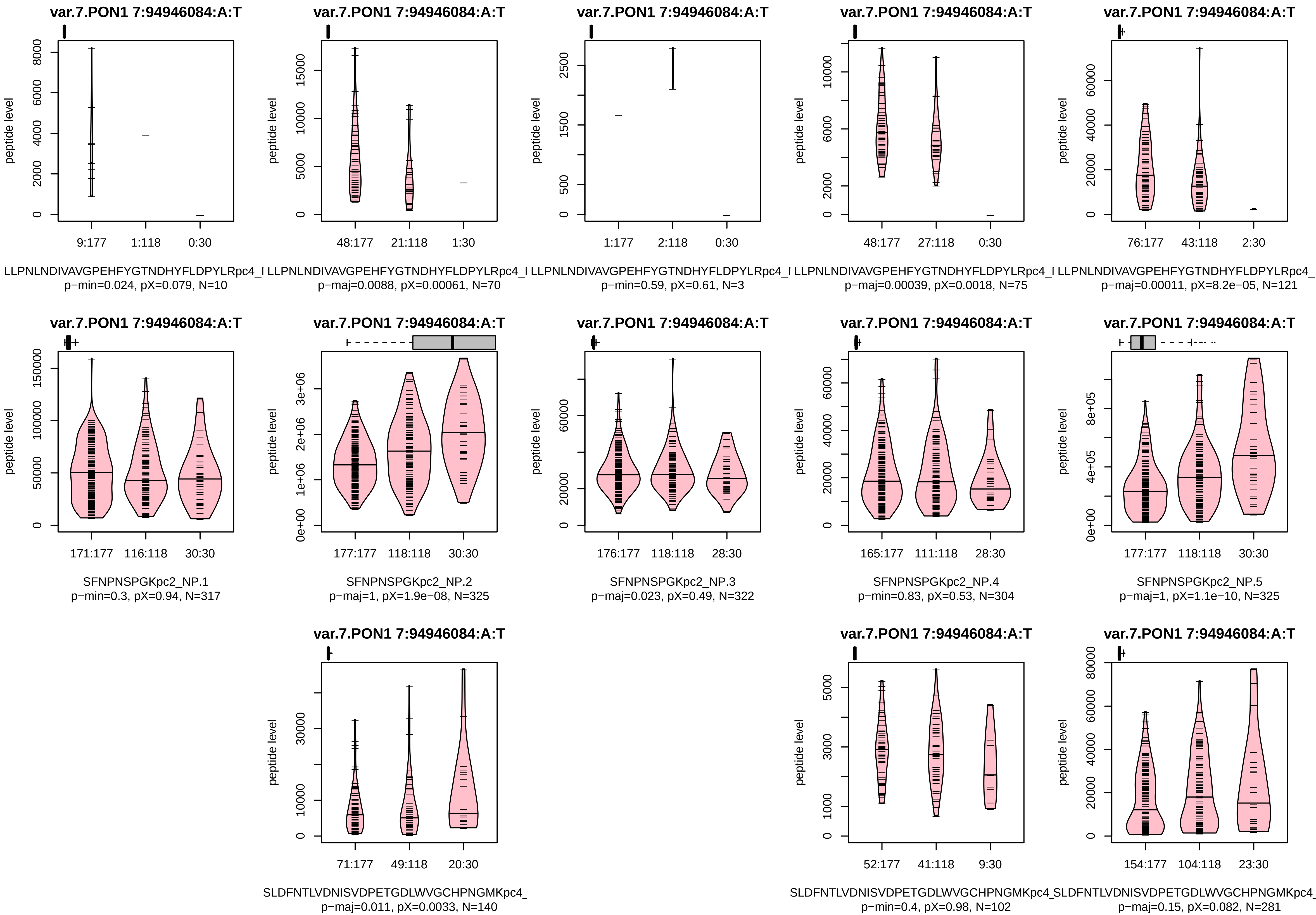
var.7.PON1 7:94946084:A:T
peptide level
9:177
1:118
0:30
LLPNLNDIVAVGPEHFYGTNDHYFLDPYLRpc4_I
p−min=0.024, pX=0.079, N=10
var.7.PON1 7:94946084:A:T
peptide level
48:177
21:118
1:30
LLPNLNDIVAVGPEHFYGTNDHYFLDPYLRpc4_I
p−maj=0.0088, pX=0.00061, N=70
var.7.PON1 7:94946084:A:T
peptide level
1:177
2:118
0:30
LLPNLNDIVAVGPEHFYGTNDHYFLDPYLRpc4_I
p−min=0.59, pX=0.61, N=3
var.7.PON1 7:94946084:A:T
peptide level
48:177
27:118
0:30
LLPNLNDIVAVGPEHFYGTNDHYFLDPYLRpc4_I
p−maj=0.00039, pX=0.0018, N=75
var.7.PON1 7:94946084:A:T
peptide level
76:177
43:118
2:30
LLPNLNDIVAVGPEHFYGTNDHYFLDPYLRpc4_I
p−maj=0.00011, pX=8.2e−05, N=121
var.7.PON1 7:94946084:A:T
peptide level
171:177
116:118
30:30
SFNPNSPGKpc2_NP.1
p−min=0.3, pX=0.94, N=317
var.7.PON1 7:94946084:A:T
peptide level
177:177
118:118
30:30
SFNPNSPGKpc2_NP.2
p−maj=1, pX=1.9e−08, N=325
var.7.PON1 7:94946084:A:T
peptide level
176:177
118:118
28:30
SFNPNSPGKpc2_NP.3
p−maj=0.023, pX=0.49, N=322
var.7.PON1 7:94946084:A:T
peptide level
165:177
111:118
28:30
SFNPNSPGKpc2_NP.4
p−min=0.83, pX=0.53, N=304
var.7.PON1 7:94946084:A:T
peptide level
177:177
118:118
30:30
SFNPNSPGKpc2_NP.5
p−maj=1, pX=1.1e−10, N=325
var.7.PON1 7:94946084:A:T
peptide level
71:177
49:118
20:30
SLDFNTLVDNISVDPETGDLWVGCHPNGMKpc4_
p−maj=0.011, pX=0.0033, N=140
var.7.PON1 7:94946084:A:T
peptide level
52:177
41:118
9:30
SLDFNTLVDNISVDPETGDLWVGCHPNGMKpc4_
p−min=0.4, pX=0.98, N=102
var.7.PON1 7:94946084:A:T
peptide level
154:177
104:118
23:30
SLDFNTLVDNISVDPETGDLWVGCHPNGMKpc4_
p−maj=0.15, pX=0.082, N=281

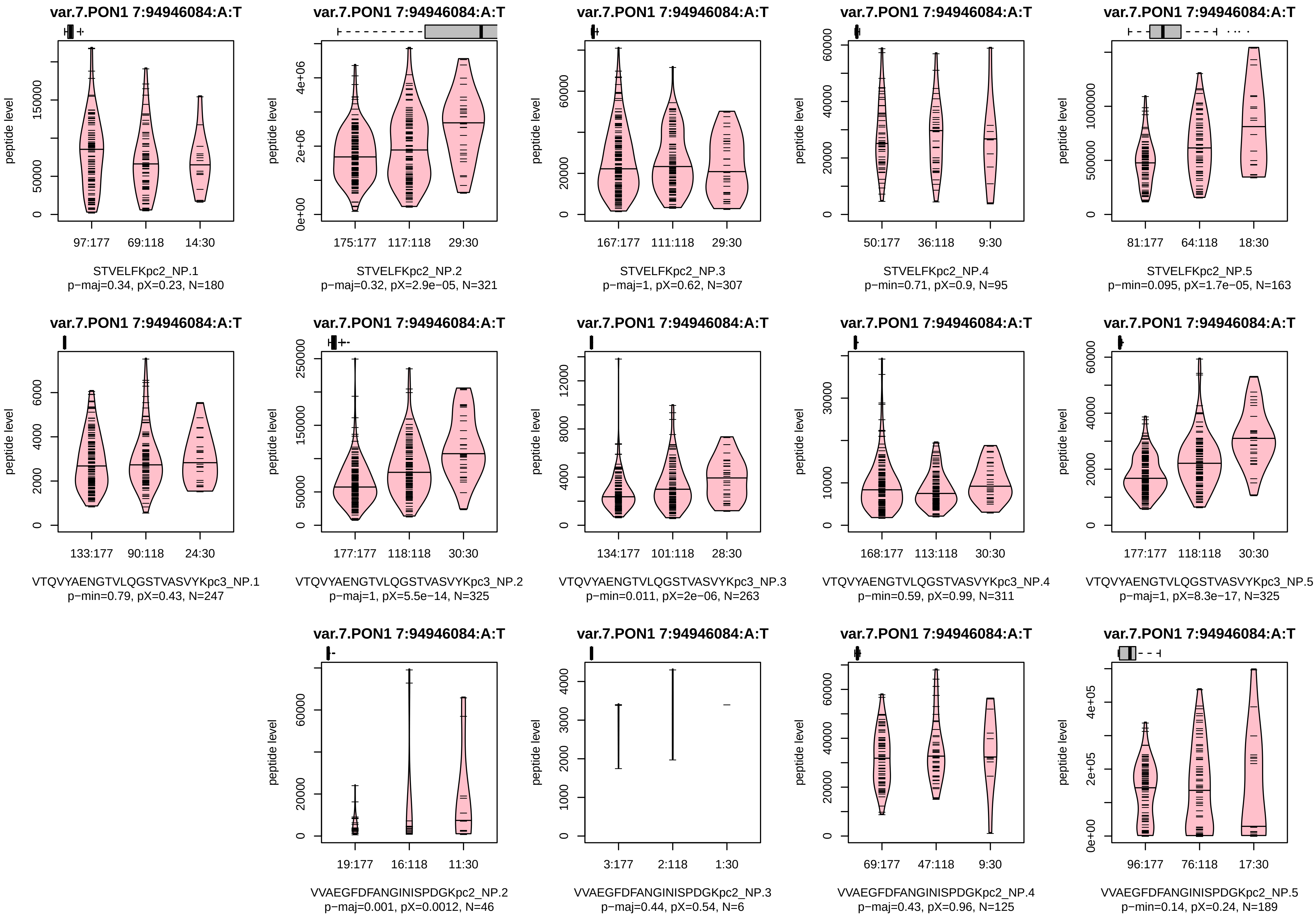
var.7.PON1 7:94946084:A:T
peptide level
97:177
69:118
14:30
STVELFKpc2_NP.1
p−maj=0.34, pX=0.23, N=180
var.7.PON1 7:94946084:A:T
175:177
117:118
29:30
STVELFKpc2_NP.2
p−maj=0.32, pX=2.9e−05, N=321
var.7.PON1 7:94946084:A:T
167:177
111:118
29:30
STVELFKpc2_NP.3
p−maj=1, pX=0.62, N=307
var.7.PON1 7:94946084:A:T
50:177
36:118
9:30
STVELFKpc2_NP.4
p−min=0.71, pX=0.9, N=95
var.7.PON1 7:94946084:A:T
81:177
64:118
18:30
STVELFKpc2_NP.5
p−min=0.095, pX=1.7e−05, N=163
var.7.PON1 7:94946084:A:T
133:177
90:118
24:30
VTQVYAENGTVLQGSTVASVYKpc3_NP.1
p−min=0.79, pX=0.43, N=247
var.7.PON1 7:94946084:A:T
177:177
118:118
30:30
VTQVYAENGTVLQGSTVASVYKpc3_NP.2
p−maj=1, pX=5.5e−14, N=325
var.7.PON1 7:94946084:A:T
134:177
101:118
28:30
VTQVYAENGTVLQGSTVASVYKpc3_NP.3
p−min=0.011, pX=2e−06, N=263
var.7.PON1 7:94946084:A:T
168:177
113:118
30:30
VTQVYAENGTVLQGSTVASVYKpc3_NP.4
p−min=0.59, pX=0.99, N=311
var.7.PON1 7:94946084:A:T
177:177
118:118
30:30
VTQVYAENGTVLQGSTVASVYKpc3_NP.5
p−maj=1, pX=8.3e−17, N=325
var.7.PON1 7:94946084:A:T
19:177
16:118
11:30
VVAEGFDFANGINISPDGKpc2_NP.2
p−maj=0.001, pX=0.0012, N=46
var.7.PON1 7:94946084:A:T
3:177
2:118
1:30
VVAEGFDFANGINISPDGKpc2_NP.3
p−maj=0.44, pX=0.54, N=6
var.7.PON1 7:94946084:A:T
69:177
47:118
9:30
VVAEGFDFANGINISPDGKpc2_NP.4
p−maj=0.43, pX=0.96, N=125
var.7.PON1 7:94946084:A:T
96:177
76:118
17:30
VVAEGFDFANGINISPDGKpc2_NP.5
p−min=0.14, pX=0.24, N=189

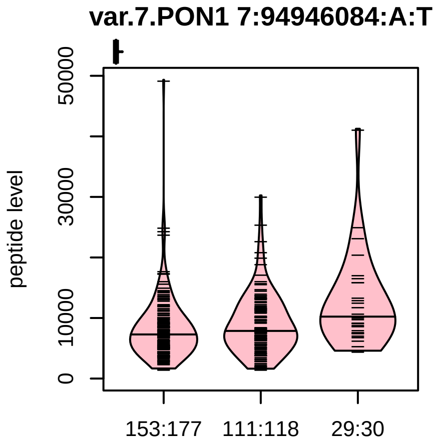

VVAEGFDFANGINISPDGKpc3_NP.2
p−min=0.015, pX=2.5e−05, N=293

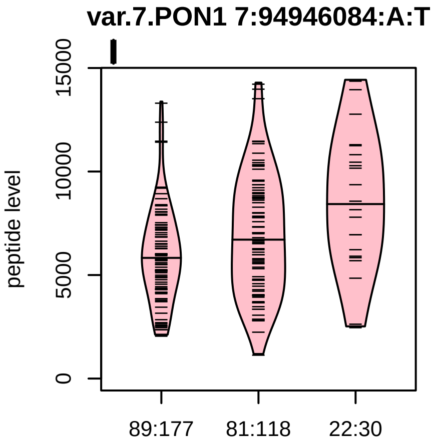

VVAEGFDFANGINISPDGKpc3_NP.5
p−min=0.00045, pX=1.9e−07, N=192

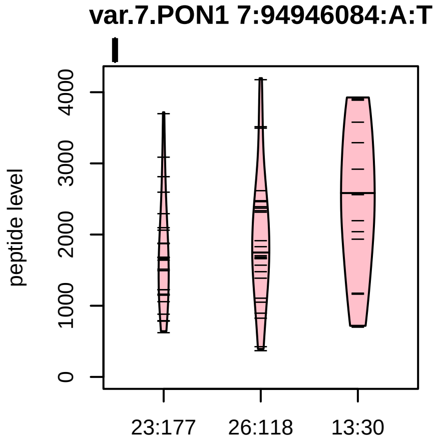

VVAEGFDFANGINISPDGKYVYIAELLAHKpc4_N
p−maj=0.0011, pX=3.1e−06, N=62

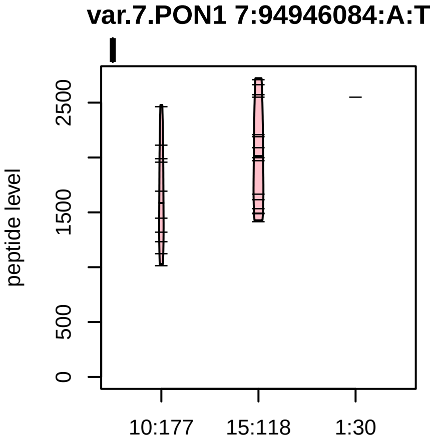

VVAEGFDFANGINISPDGKYVYIAELLAHKpc4_N
p−min=0.1, pX=0.18, N=26

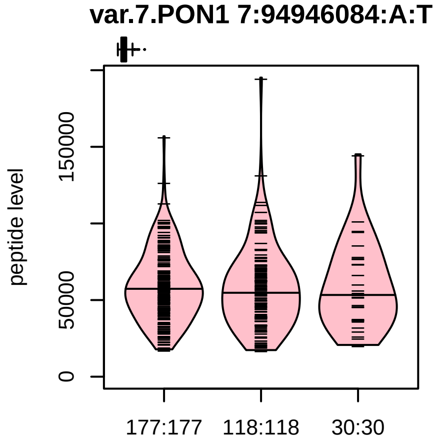

YVYIAELLAHKpc2_NP.1
p−maj=1, pX=0.81, N=325

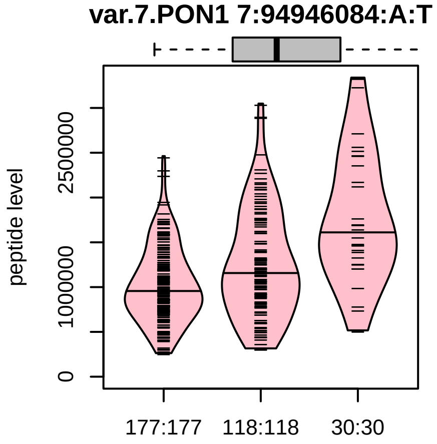

YVYIAELLAHKpc2_NP.2
p−maj=1, pX=6.5e−12, N=325

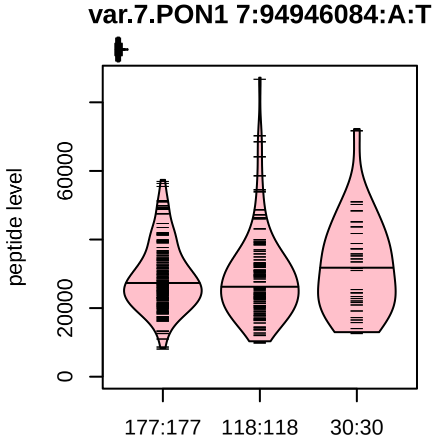

YVYIAELLAHKpc2_NP.3
p−maj=1, pX=0.32, N=325

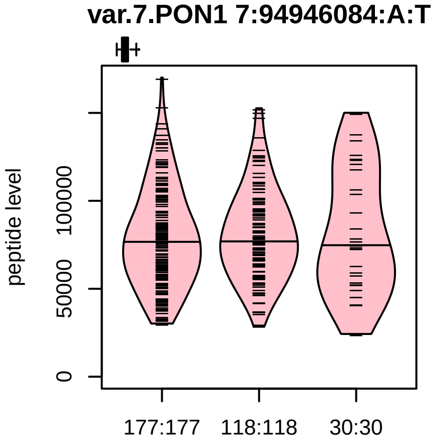

YVYIAELLAHKpc2_NP.4
p−maj=1, pX=0.54, N=325

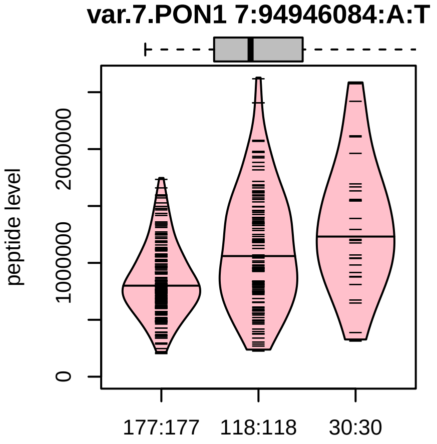

YVYIAELLAHKpc2_NP.5
p−maj=1, pX=4.1e−12, N=325

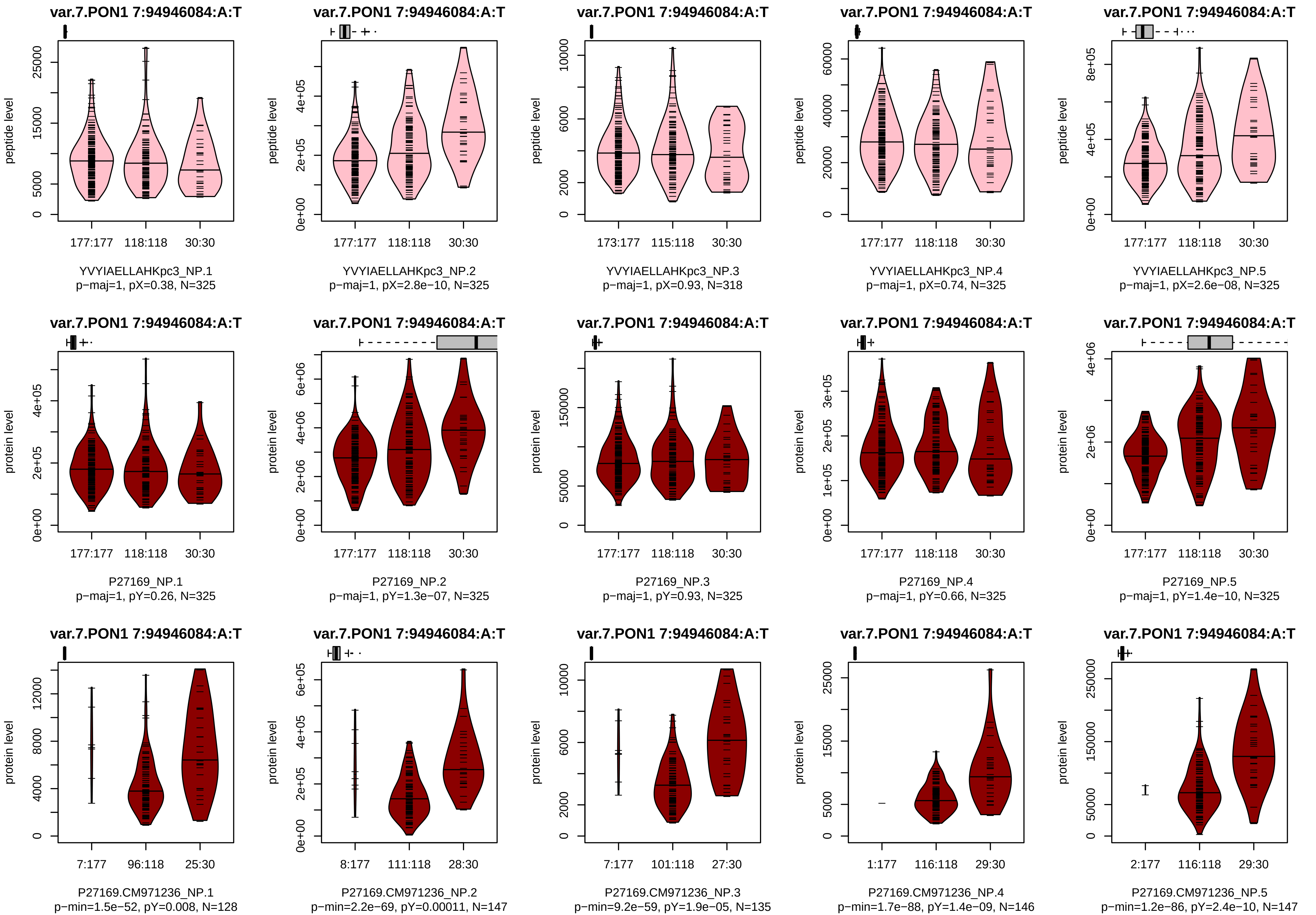
var.7.PON1 7:94946084:A:T
peptide level
177:177
118:118
30:30
YVYIAELLAHKpc3_NP.1
p−maj=1, pX=0.38, N=325
var.7.PON1 7:94946084:A:T
peptide level
177:177
118:118
30:30
YVYIAELLAHKpc3_NP.2
p−maj=1, pX=2.8e−10, N=325
var.7.PON1 7:94946084:A:T
peptide level
173:177
115:118
30:30
YVYIAELLAHKpc3_NP.3
p−maj=1, pX=0.93, N=318
var.7.PON1 7:94946084:A:T
peptide level
177:177
118:118
30:30
YVYIAELLAHKpc3_NP.4
p−maj=1, pX=0.74, N=325
var.7.PON1 7:94946084:A:T
peptide level
177:177
118:118
30:30
YVYIAELLAHKpc3_NP.5
p−maj=1, pX=2.6e−08, N=325
var.7.PON1 7:94946084:A:T
protein level
177:177
118:118
30:30
P27169_NP.1
p−maj=1, pY=0.26, N=325
var.7.PON1 7:94946084:A:T
protein level
177:177
118:118
30:30
P27169_NP.2
p−maj=1, pY=1.3e−07, N=325
var.7.PON1 7:94946084:A:T
protein level
177:177
118:118
30:30
P27169_NP.3
p−maj=1, pY=0.93, N=325
var.7.PON1 7:94946084:A:T
protein level
177:177
118:118
30:30
P27169_NP.4
p−maj=1, pY=0.66, N=325
var.7.PON1 7:94946084:A:T
protein level
177:177
118:118
30:30
P27169_NP.5
p−maj=1, pY=1.4e−10, N=325
var.7.PON1 7:94946084:A:T
protein level
7:177
96:118
25:30
P27169.CM971236_NP.1
p−min=1.5e−52, pY=0.008, N=128
var.7.PON1 7:94946084:A:T
protein level
8:177
111:118
28:30
P27169.CM971236_NP.2
p−min=2.2e−69, pY=0.00011, N=147
var.7.PON1 7:94946084:A:T
protein level
7:177
101:118
27:30
P27169.CM971236_NP.3
p−min=9.2e−59, pY=1.9e−05, N=135
var.7.PON1 7:94946084:A:T
protein level
1:177
116:118
29:30
P27169.CM971236_NP.4
p−min=1.7e−88, pY=1.4e−09, N=146
var.7.PON1 7:94946084:A:T
protein level
2:177
116:118
29:30
P27169.CM971236_NP.5
p−min=1.2e−86, pY=2.4e−10, N=147

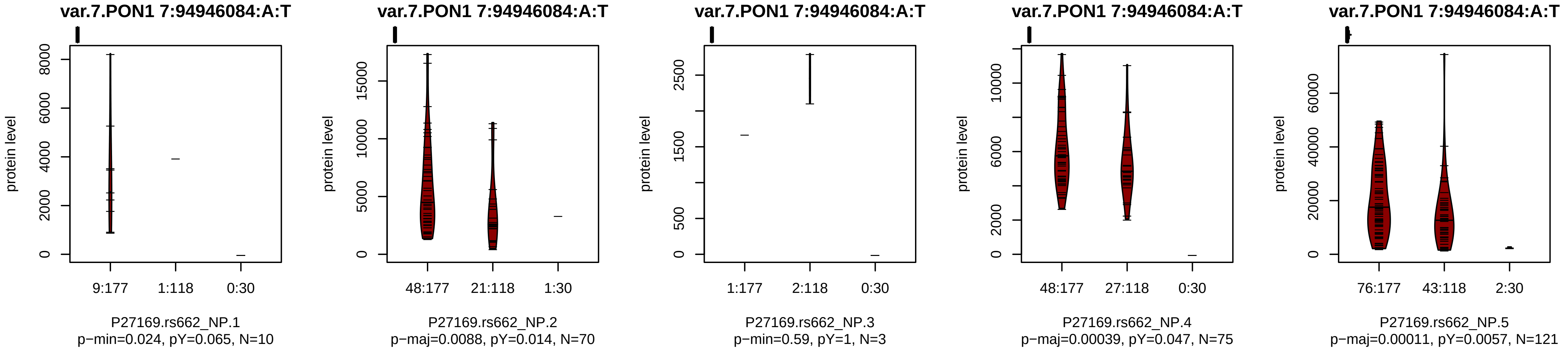
var.7.PON1 7:94946084:A:T
protein level
9:177
1:118
0:30
P27169.rs662_NP.1
p−min=0.024, pY=0.065, N=10
var.7.PON1 7:94946084:A:T
protein level
48:177
21:118
1:30
P27169.rs662_NP.2
p−maj=0.0088, pY=0.014, N=70
var.7.PON1 7:94946084:A:T
protein level
1:177
2:118
0:30
P27169.rs662_NP.3
p−min=0.59, pY=1, N=3
var.7.PON1 7:94946084:A:T
protein level
48:177
27:118
0:30
P27169.rs662_NP.4
p−maj=0.00039, pY=0.047, N=75
var.7.PON1 7:94946084:A:T
protein level
76:177
43:118
2:30
P27169.rs662_NP.5
p−maj=0.00011, pY=0.0057, N=121

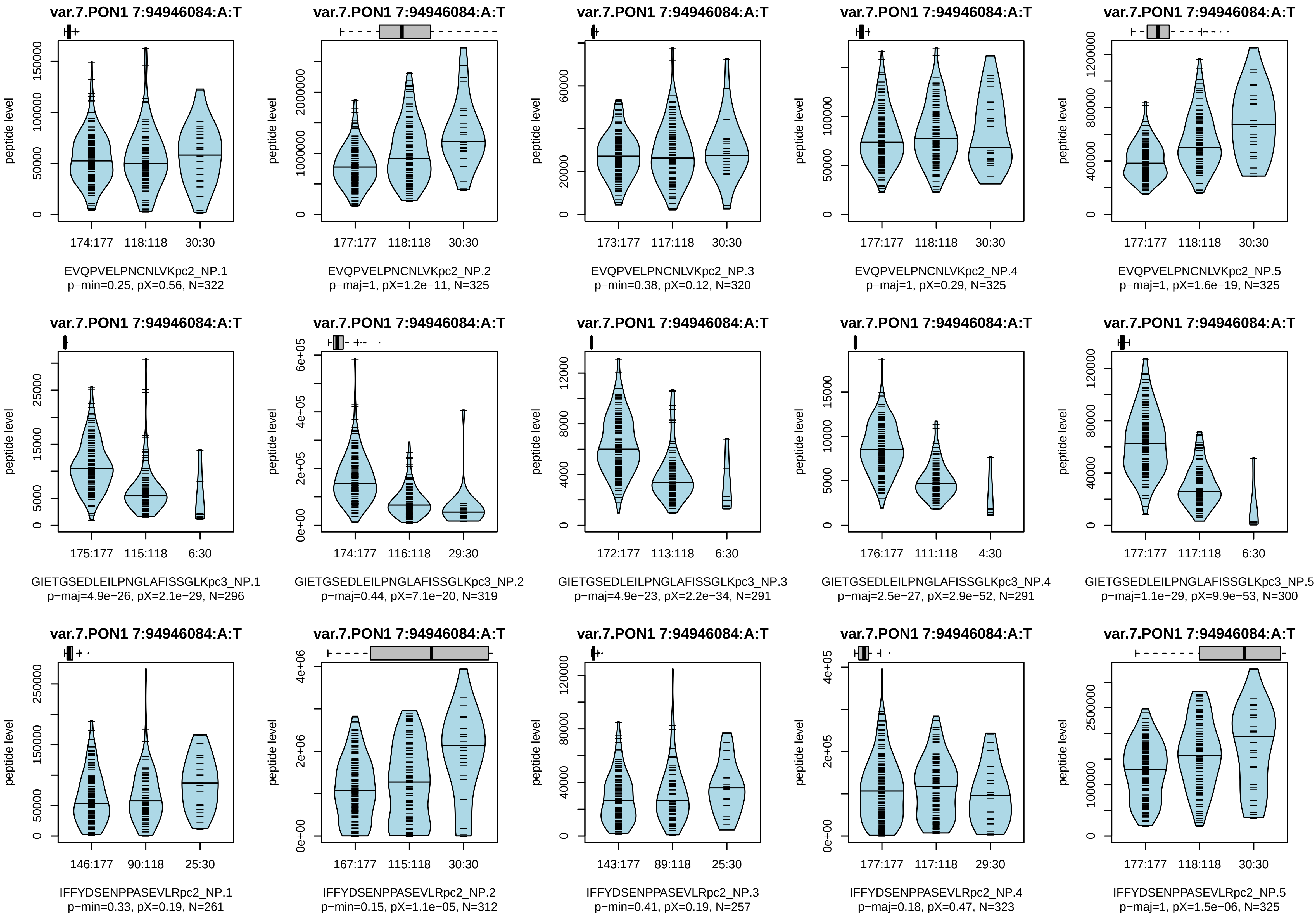

var.7.PON1 7:94946084:A:T
peptide level
174:177 118:118 30:30
EVQPVELPNCNLVKpc2_NP.1
p−min=0.25, pX=0.56, N=322
var.7.PON1 7:94946084:A:T
peptide level
177:177 118:118 30:30
EVQPVELPNCNLVKpc2_NP.2
p−maj=1, pX=1.2e−11, N=325
var.7.PON1 7:94946084:A:T
peptide level
173:177 117:118 30:30
EVQPVELPNCNLVKpc2_NP.3
p−min=0.38, pX=0.12, N=320
var.7.PON1 7:94946084:A:T
peptide level
177:177 118:118 30:30
EVQPVELPNCNLVKpc2_NP.4
p−maj=1, pX=0.29, N=325
var.7.PON1 7:94946084:A:T
peptide level
177:177 118:118 30:30
EVQPVELPNCNLVKpc2_NP.5
p−maj=1, pX=1.6e−19, N=325
var.7.PON1 7:94946084:A:T
peptide level
175:177 115:118 6:30
GIETGSEDLEILPNGLAFISSGLKpc3_NP.1
p−maj=4.9e−26, pX=2.1e−29, N=296
var.7.PON1 7:94946084:A:T
peptide level
174:177 116:118 29:30
GIETGSEDLEILPNGLAFISSGLKpc3_NP.2
p−maj=0.44, pX=7.1e−20, N=319
var.7.PON1 7:94946084:A:T
peptide level
172:177 113:118 6:30
GIETGSEDLEILPNGLAFISSGLKpc3_NP.3
p−maj=4.9e−23, pX=2.2e−34, N=291
var.7.PON1 7:94946084:A:T
peptide level
176:177 111:118 4:30
GIETGSEDLEILPNGLAFISSGLKpc3_NP.4
p−maj=2.5e−27, pX=2.9e−52, N=291
var.7.PON1 7:94946084:A:T
peptide level
177:177 117:118 6:30
GIETGSEDLEILPNGLAFISSGLKpc3_NP.5
p−maj=1.1e−29, pX=9.9e−53, N=300
var.7.PON1 7:94946084:A:T
peptide level
146:177 90:118 25:30
IFFYDSENPPASEVLRpc2_NP.1
p−min=0.33, pX=0.19, N=261
var.7.PON1 7:94946084:A:T
peptide level
167:177 115:118 30:30
IFFYDSENPPASEVLRpc2_NP.2
p−min=0.15, pX=1.1e−05, N=312
var.7.PON1 7:94946084:A:T
peptide level
143:177 89:118 25:30
IFFYDSENPPASEVLRpc2_NP.3
p−min=0.41, pX=0.19, N=257
var.7.PON1 7:94946084:A:T
peptide level
177:177 117:118 29:30
IFFYDSENPPASEVLRpc2_NP.4
p−maj=0.18, pX=0.47, N=323
var.7.PON1 7:94946084:A:T
peptide level
177:177 118:118 30:30
IFFYDSENPPASEVLRpc2_NP.5
p−maj=1, pX=1.5e−06, N=325

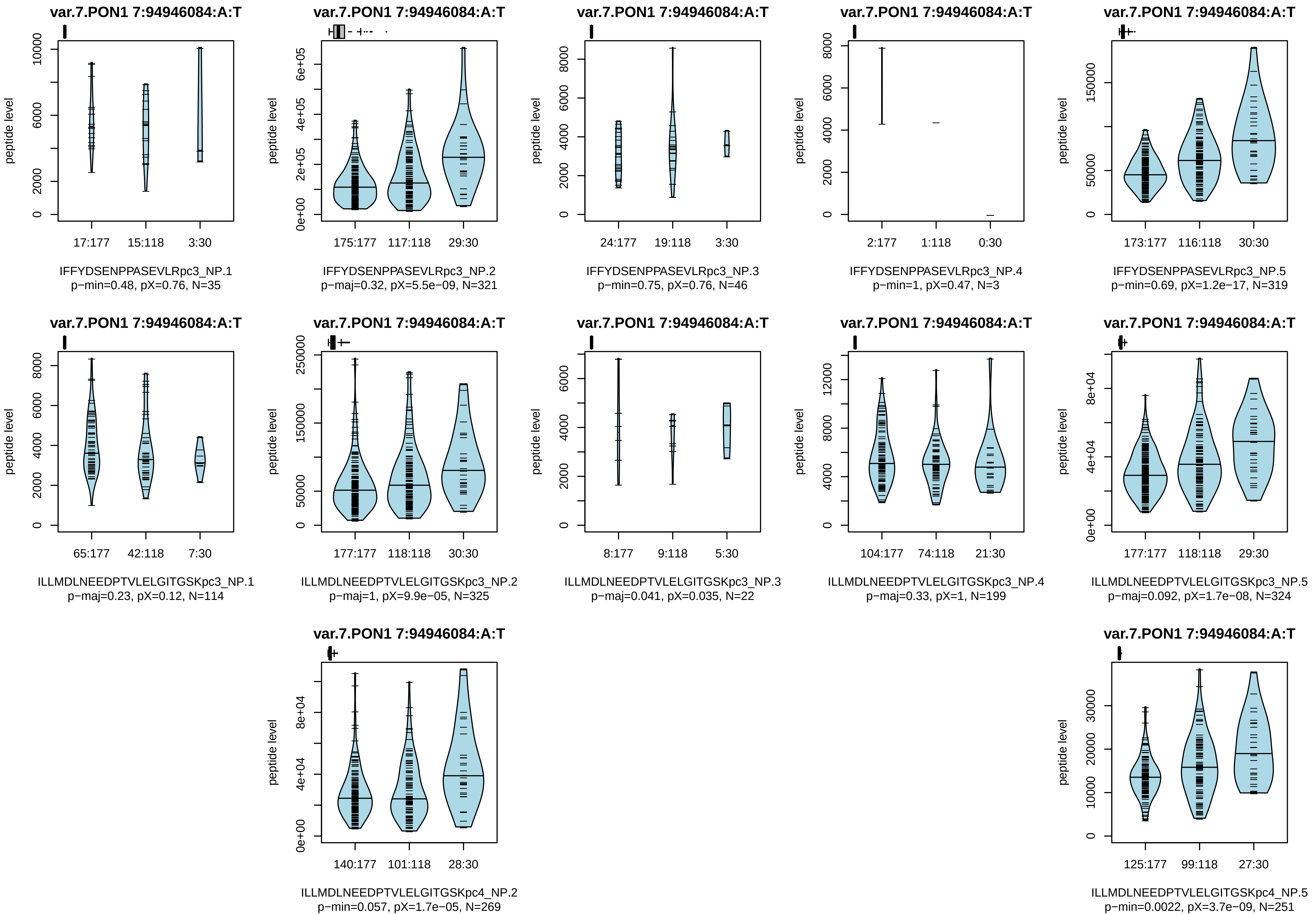
var.7.PON1 7:94946084:A:T
peptide level
17:177
15:118
3:30
IFFYDSENPPASEVLRpc3_NP.1
p−min=0.48, pX=0.76, N=35
var.7.PON1 7:94946084:A:T
peptide level
175:177
117:118
29:30
IFFYDSENPPASEVLRpc3_NP.2
p−maj=0.32, pX=5.5e−09, N=321
var.7.PON1 7:94946084:A:T
peptide level
24:177
19:118
3:30
IFFYDSENPPASEVLRpc3_NP.3
p−min=0.75, pX=0.76, N=46
var.7.PON1 7:94946084:A:T
peptide level
2:177
1:118
0:30
IFFYDSENPPASEVLRpc3_NP.4
p−min=1, pX=0.47, N=3
var.7.PON1 7:94946084:A:T
peptide level
173:177
116:118
30:30
IFFYDSENPPASEVLRpc3_NP.5
p−min=0.69, pX=1.2e−17, N=319
var.7.PON1 7:94946084:A:T
peptide level
65:177
42:118
7:30
ILLMDLNEEDPTVLELGITGSKpc3_NP.1
p−maj=0.23, pX=0.12, N=114
var.7.PON1 7:94946084:A:T
peptide level
177:177
118:118
30:30
ILLMDLNEEDPTVLELGITGSKpc3_NP.2
p−maj=1, pX=9.9e−05, N=325
var.7.PON1 7:94946084:A:T
peptide level
8:177
9:118
5:30
ILLMDLNEEDPTVLELGITGSKpc3_NP.3
p−maj=0.041, pX=0.035, N=22
var.7.PON1 7:94946084:A:T
peptide level
104:177
74:118
21:30
ILLMDLNEEDPTVLELGITGSKpc3_NP.4
p−maj=0.33, pX=1, N=199
var.7.PON1 7:94946084:A:T
peptide level
177:177
118:118
29:30
ILLMDLNEEDPTVLELGITGSKpc3_NP.5
p−maj=0.092, pX=1.7e−08, N=324
var.7.PON1 7:94946084:A:T
peptide level
140:177
101:118
28:30
ILLMDLNEEDPTVLELGITGSKpc4_NP.2
p−min=0.057, pX=1.7e−05, N=269
var.7.PON1 7:94946084:A:T
peptide level
125:177
99:118
27:30
ILLMDLNEEDPTVLELGITGSKpc4_NP.5
p−min=0.0022, pX=3.7e−09, N=251

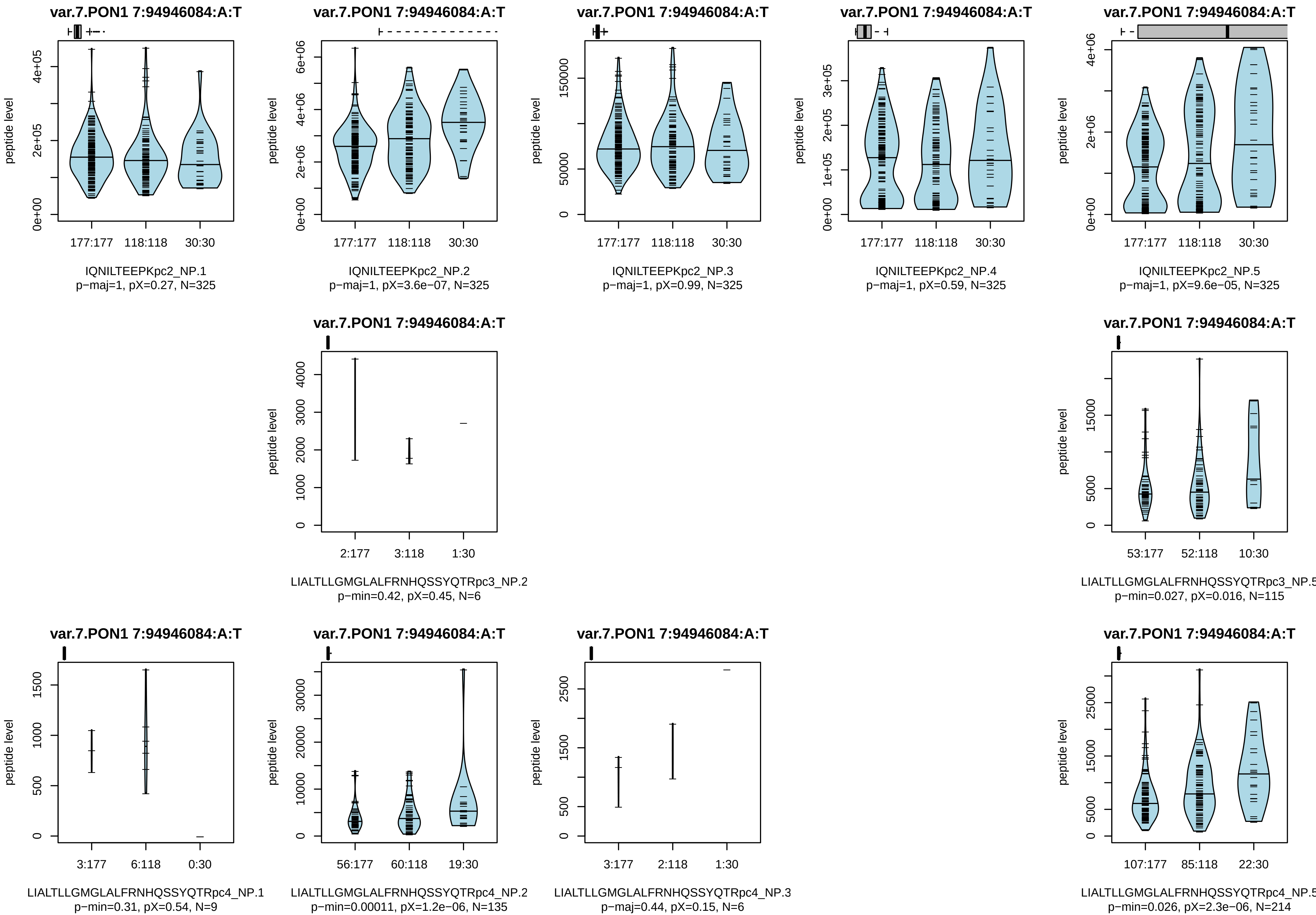

var.7.PON1 7:94946084:A:T
peptide level
177:177
118:118
30:30
IQNILTEEPKpc2_NP.1
p−maj=1, pX=0.27, N=325
var.7.PON1 7:94946084:A:T
peptide level
177:177
118:118
30:30
IQNILTEEPKpc2_NP.2
p−maj=1, pX=3.6e−07, N=325
var.7.PON1 7:94946084:A:T
peptide level
177:177
118:118
30:30
IQNILTEEPKpc2_NP.3
p−maj=1, pX=0.99, N=325
var.7.PON1 7:94946084:A:T
peptide level
177:177
118:118
30:30
IQNILTEEPKpc2_NP.4
p−maj=1, pX=0.59, N=325
var.7.PON1 7:94946084:A:T
peptide level
177:177
118:118
30:30
IQNILTEEPKpc2_NP.5
p−maj=1, pX=9.6e−05, N=325
var.7.PON1 7:94946084:A:T
peptide level
2:177
3:118
1:30
LIALTLLGMGLALFRNHQSSYQTRpc3_NP.2
p−min=0.42, pX=0.45, N=6
var.7.PON1 7:94946084:A:T
peptide level
53:177
52:118
10:30
LIALTLLGMGLALFRNHQSSYQTRpc3_NP.5
p−min=0.027, pX=0.016, N=115
var.7.PON1 7:94946084:A:T
peptide level
3:177
6:118
0:30
LIALTLLGMGLALFRNHQSSYQTRpc4_NP.1
p−min=0.31, pX=0.54, N=9
var.7.PON1 7:94946084:A:T
peptide level
56:177
60:118
19:30
LIALTLLGMGLALFRNHQSSYQTRpc4_NP.2
p−min=0.00011, pX=1.2e−06, N=135
var.7.PON1 7:94946084:A:T
peptide level
3:177
2:118
1:30
LIALTLLGMGLALFRNHQSSYQTRpc4_NP.3
p−maj=0.44, pX=0.15, N=6
var.7.PON1 7:94946084:A:T
peptide level
107:177
85:118
22:30
LIALTLLGMGLALFRNHQSSYQTRpc4_NP.5
p−min=0.026, pX=2.3e−06, N=214

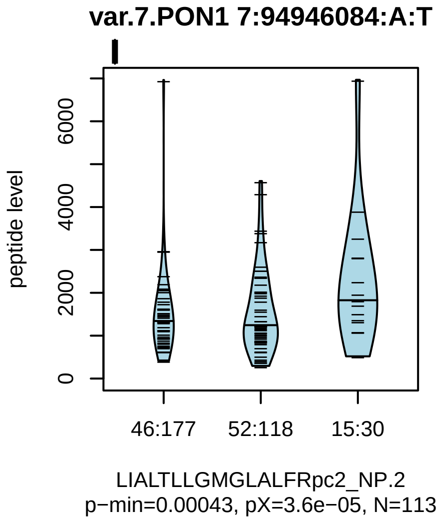
var.7.PON1 7:94946084:A:T
peptide level
0
2000
4000
6000
46:177
52:118
15:30
LIALTLLGMGLALFRpc2_NP.2
p−min=0.00043, pX=3.6e−05, N=113

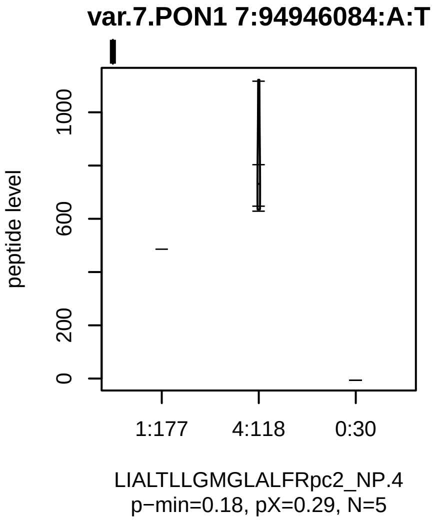
var.7.PON1 7:94946084:A:T
peptide level
0
200
600
1000
1:177
4:118
0:30
LIALTLLGMGLALFRpc2_NP.4
p−min=0.18, pX=0.29, N=5

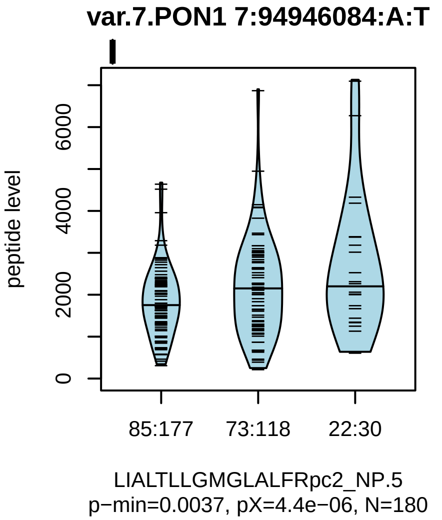
var.7.PON1 7:94946084:A:T
peptide level
0
2000
4000
6000
85:177
73:118
22:30
LIALTLLGMGLALFRpc2_NP.5
p−min=0.0037, pX=4.4e−06, N=180

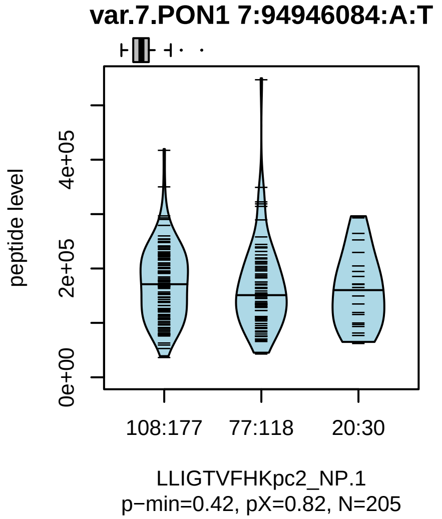
var.7.PON1 7:94946084:A:T
peptide level
0e+00
2e+05
4e+05
108:177
77:118
20:30
LLIGTVFHKpc2_NP.1
p−min=0.42, pX=0.82, N=205

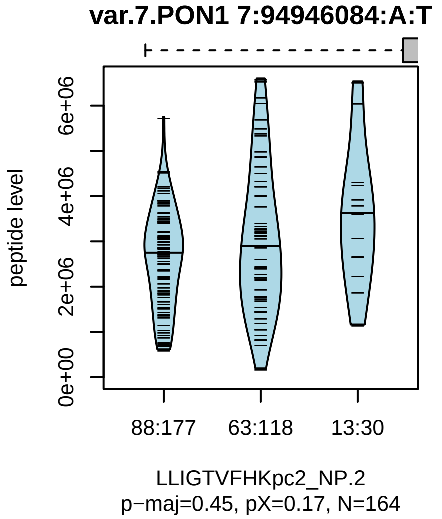
var.7.PON1 7:94946084:A:T
peptide level
0e+00
2e+06
4e+06
6e+06
88:177
63:118
13:30
LLIGTVFHKpc2_NP.2
p−maj=0.45, pX=0.17, N=164

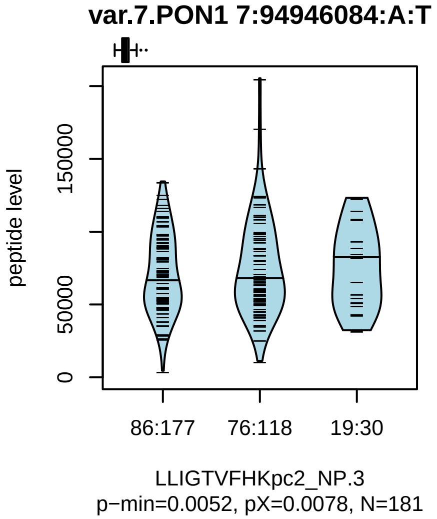
var.7.PON1 7:94946084:A:T
peptide level
0
50000
150000
86:177
76:118
19:30
LLIGTVFHKpc2_NP.3
p−min=0.0052, pX=0.0078, N=181

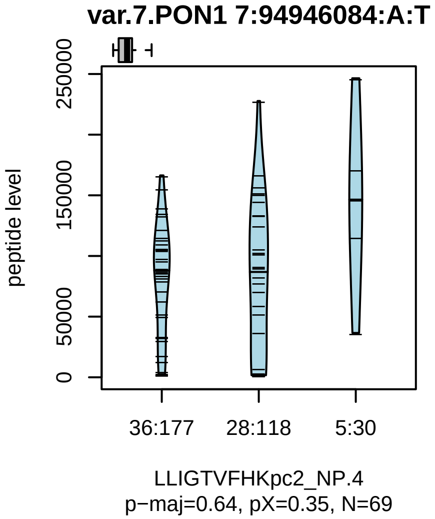
var.7.PON1 7:94946084:A:T
peptide level
0
50000
150000
250000
36:177
28:118
5:30
LLIGTVFHKpc2_NP.4
p−maj=0.64, pX=0.35, N=69

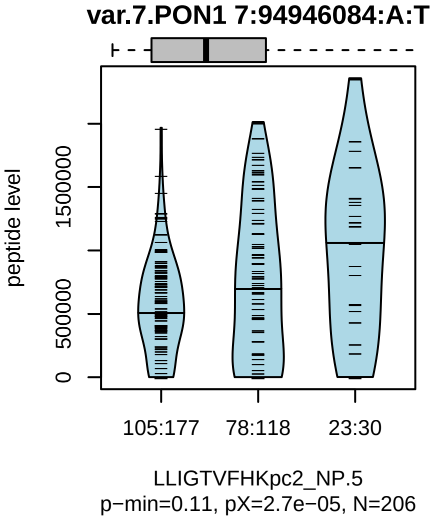
var.7.PON1 7:94946084:A:T
peptide level
0
500000
1500000
105:177
78:118
23:30
LLIGTVFHKpc2_NP.5
p−min=0.11, pX=2.7e−05, N=206

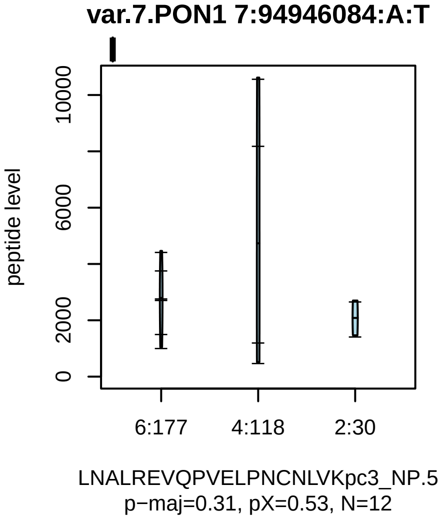
var.7.PON1 7:94946084:A:T
peptide level
0
2000
6000
10000
6:177
4:118
2:30
LNALREVQPVELPNCNLVKpc3_NP.5
p−maj=0.31, pX=0.53, N=12

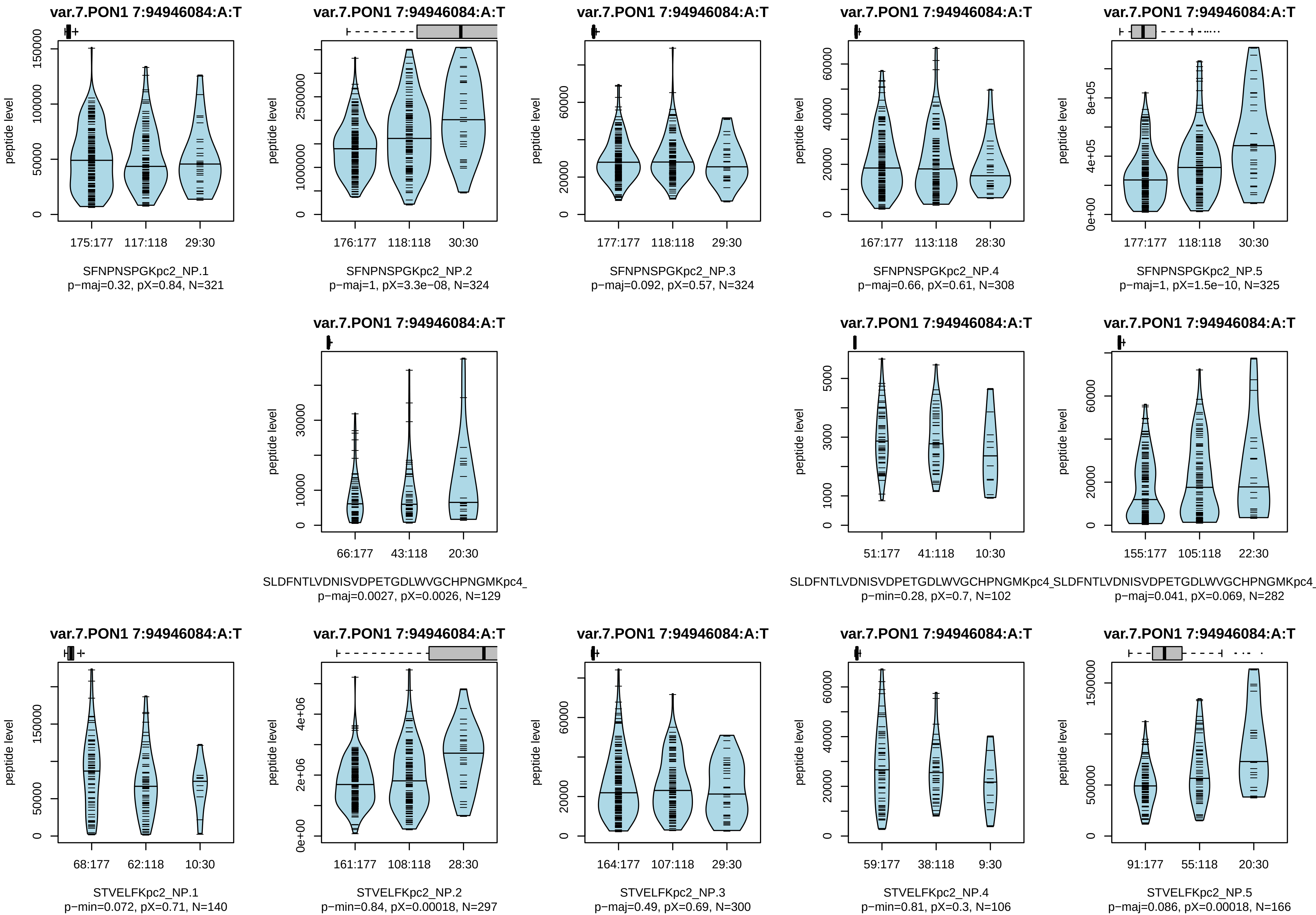

var.7.PON1 7:94946084:A:T
peptide level
0
50000
100000
150000
175:177
117:118
29:30
SFNPNSPGKpc2_NP.1
p−maj=0.32, pX=0.84, N=321
var.7.PON1 7:94946084:A:T
peptide level
0
1000000
2500000
176:177
118:118
30:30
SFNPNSPGKpc2_NP.2
p−maj=1, pX=3.3e−08, N=324
var.7.PON1 7:94946084:A:T
peptide level
0
20000
60000
177:177
118:118
29:30
SFNPNSPGKpc2_NP.3
p−maj=0.092, pX=0.57, N=324
var.7.PON1 7:94946084:A:T
peptide level
0
20000
40000
60000
167:177
113:118
28:30
SFNPNSPGKpc2_NP.4
p−maj=0.66, pX=0.61, N=308
var.7.PON1 7:94946084:A:T
peptide level
0e+00
4e+05
8e+05
177:177
118:118
30:30
SFNPNSPGKpc2_NP.5
p−maj=1, pX=1.5e−10, N=325
var.7.PON1 7:94946084:A:T
peptide level
0
10000
30000
66:177
43:118
20:30
SLDFNTLVDNISVDPETGDLWVGCHPNGMKpc4_
p−maj=0.0027, pX=0.0026, N=129
var.7.PON1 7:94946084:A:T
peptide level
0
1000
3000
5000
51:177
41:118
10:30
SLDFNTLVDNISVDPETGDLWVGCHPNGMKpc4_
p−min=0.28, pX=0.7, N=102
var.7.PON1 7:94946084:A:T
peptide level
0
20000
60000
155:177
105:118
22:30
SLDFNTLVDNISVDPETGDLWVGCHPNGMKpc4_
p−maj=0.041, pX=0.069, N=282
var.7.PON1 7:94946084:A:T
peptide level
0
50000
150000
68:177
62:118
10:30
STVELFKpc2_NP.1
p−min=0.072, pX=0.71, N=140
var.7.PON1 7:94946084:A:T
peptide level
0e+00
2e+06
4e+06
161:177
108:118
28:30
STVELFKpc2_NP.2
p−min=0.84, pX=0.00018, N=297
var.7.PON1 7:94946084:A:T
peptide level
0
20000
60000
164:177
107:118
29:30
STVELFKpc2_NP.3
p−maj=0.49, pX=0.69, N=300
var.7.PON1 7:94946084:A:T
peptide level
0
20000
40000
60000
59:177
38:118
9:30
STVELFKpc2_NP.4
p−min=0.81, pX=0.3, N=106
var.7.PON1 7:94946084:A:T
peptide level
0
500000
1500000
91:177
55:118
20:30
STVELFKpc2_NP.5
p−maj=0.086, pX=0.00018, N=166

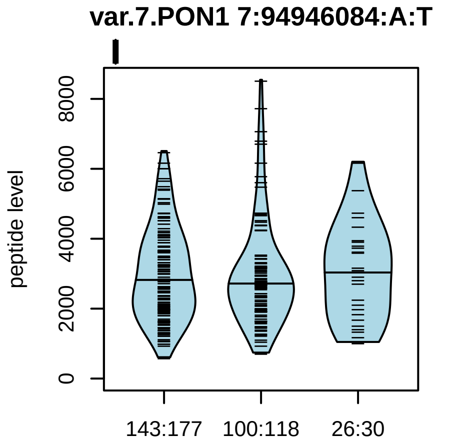

VTQVYAENGTVLQGSTVASVYKpc3_NP.1
p−min=0.38, pX=0.43, N=269

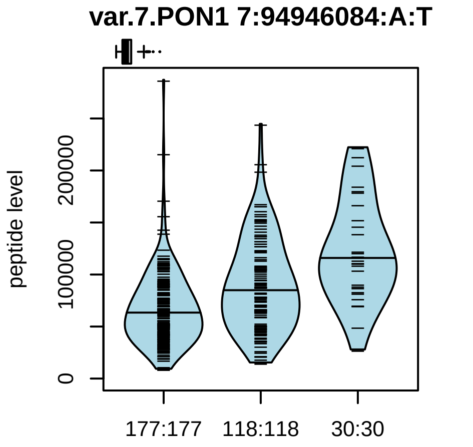

VTQVYAENGTVLQGSTVASVYKpc3_NP.2
p−maj=1, pX=2.3e−13, N=325

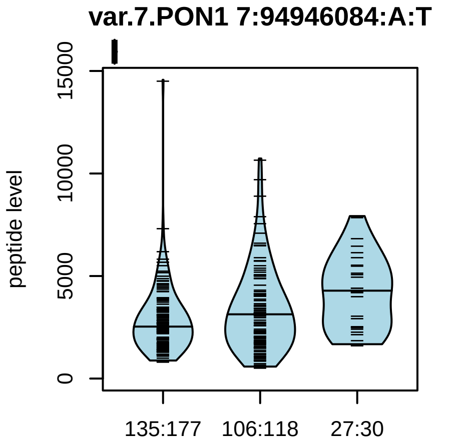

VTQVYAENGTVLQGSTVASVYKpc3_NP.3
p−min=0.0013, pX=2.7e−06, N=268

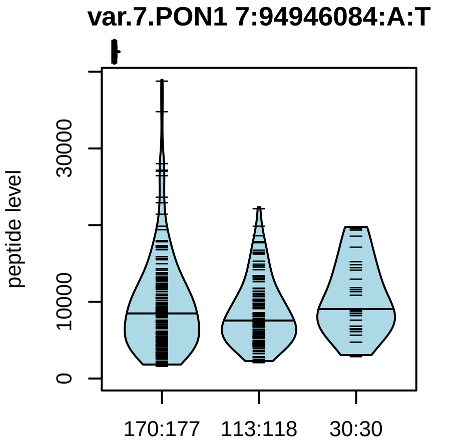

VTQVYAENGTVLQGSTVASVYKpc3_NP.4
p−maj=0.61, pX=0.91, N=313

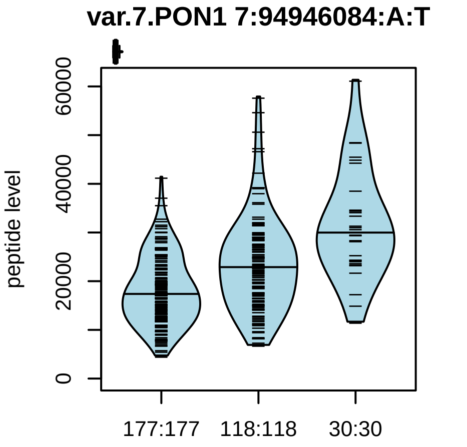

VTQVYAENGTVLQGSTVASVYKpc3_NP.5
p−maj=1, pX=3.2e−16, N=325

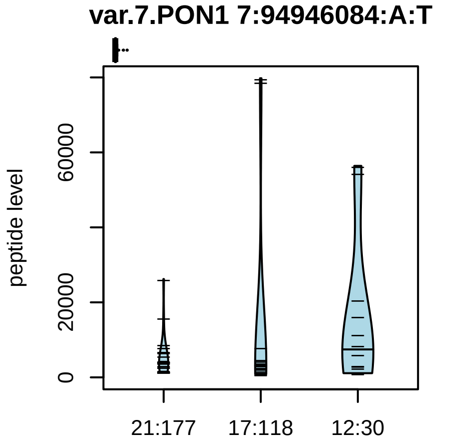

VVAEGFDFANGINISPDGKpc2_NP.2
p−maj=5e−04, pX=0.0015, N=50

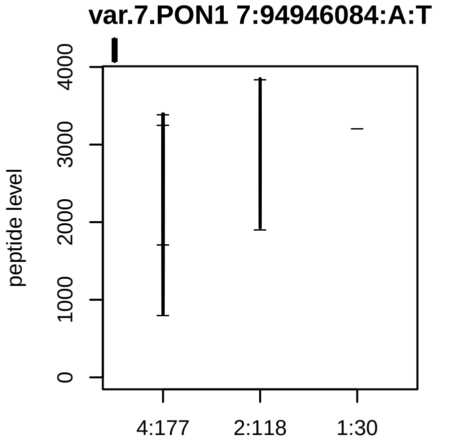

VVAEGFDFANGINISPDGKpc2_NP.3
p−maj=0.5, pX=0.64, N=7

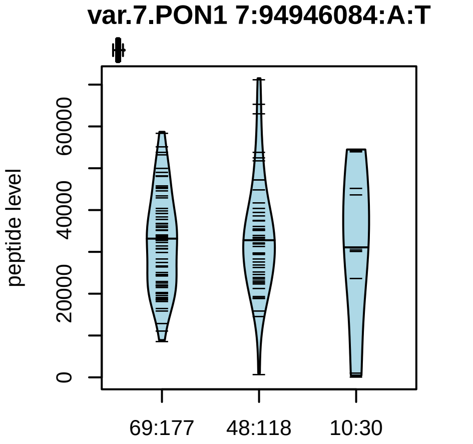

VVAEGFDFANGINISPDGKpc2_NP.4
p−maj=0.56, pX=0.91, N=127

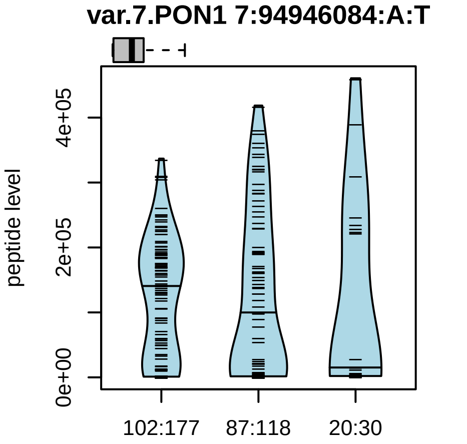

VVAEGFDFANGINISPDGKpc2_NP.5
p−min=0.0074, pX=0.24, N=209

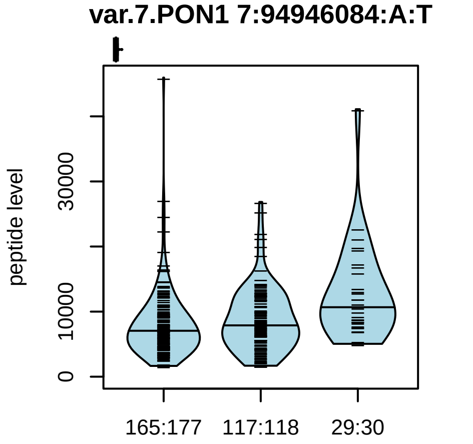

VVAEGFDFANGINISPDGKpc3_NP.2
p−min=0.025, pX=1.4e−05, N=311

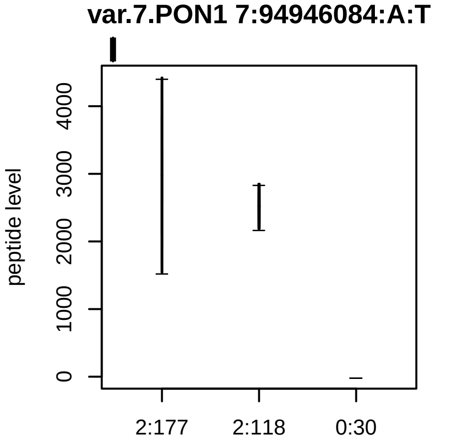

VVAEGFDFANGINISPDGKpc3_NP.4
p−maj=1, pX=0.8, N=4

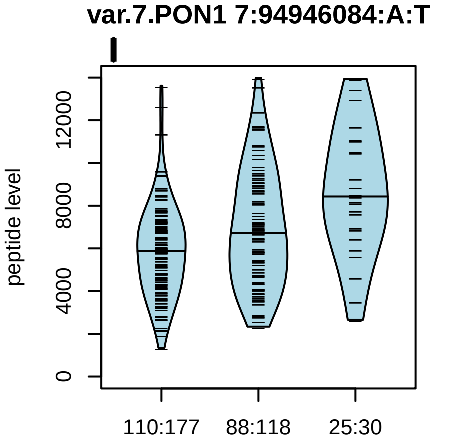

VVAEGFDFANGINISPDGKpc3_NP.5
p−min=0.0081, pX=7.1e−08, N=223

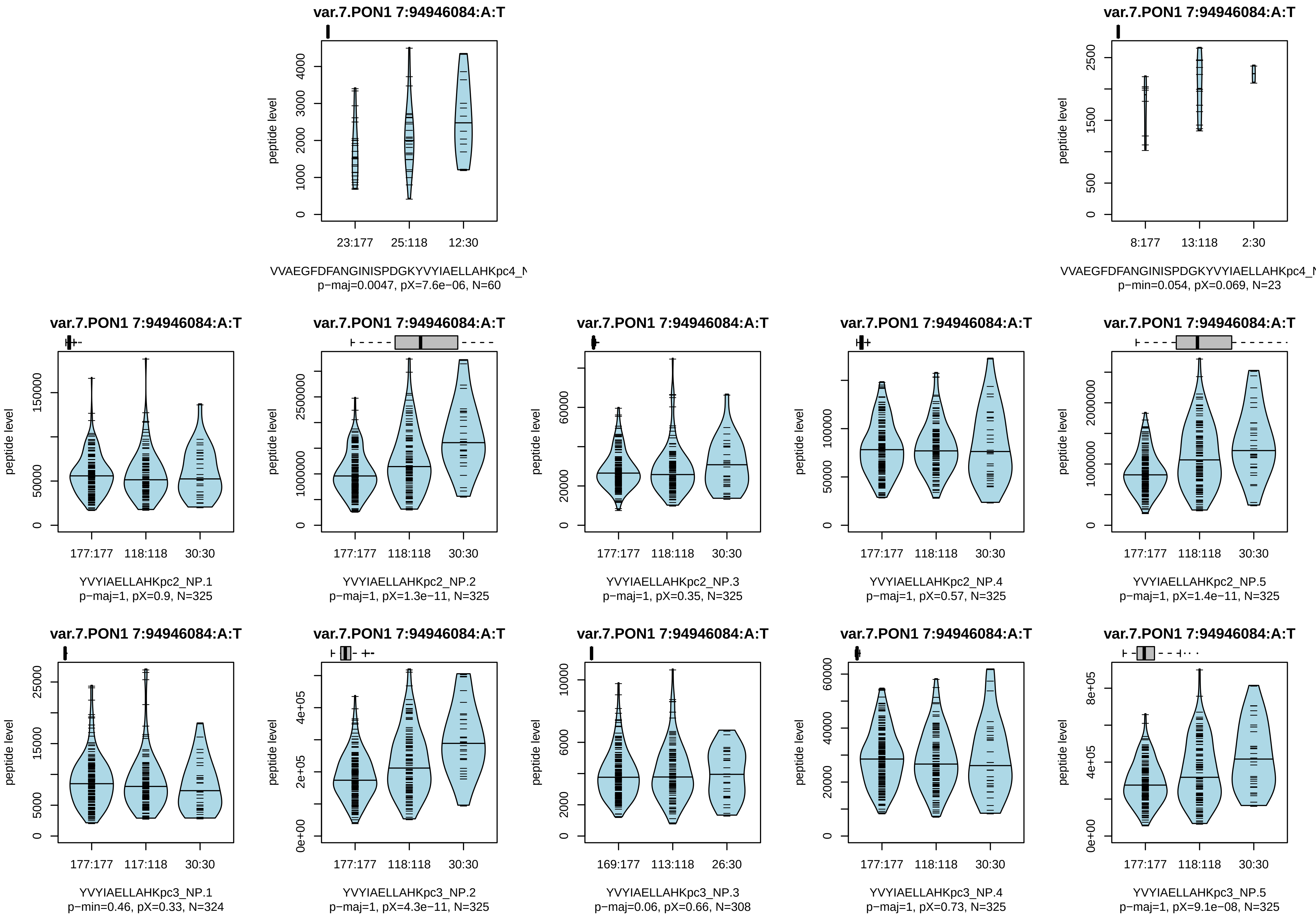
var.7.PON1 7:94946084:A:T
peptide level
23:177 25:118 12:30
VVAEGFDFANGINISPDGKYVYIAELLAHKpc4_N
p−maj=0.0047, pX=7.6e−06, N=60
var.7.PON1 7:94946084:A:T
peptide level
8:177 13:118 2:30
VVAEGFDFANGINISPDGKYVYIAELLAHKpc4_N
p−min=0.054, pX=0.069, N=23
var.7.PON1 7:94946084:A:T
peptide level
177:177 118:118 30:30
YVYIAELLAHKpc2_NP.1
p−maj=1, pX=0.9, N=325
var.7.PON1 7:94946084:A:T
peptide level
177:177 118:118 30:30
YVYIAELLAHKpc2_NP.2
p−maj=1, pX=1.3e−11, N=325
var.7.PON1 7:94946084:A:T
peptide level
177:177 118:118 30:30
YVYIAELLAHKpc2_NP.3
p−maj=1, pX=0.35, N=325
var.7.PON1 7:94946084:A:T
peptide level
177:177 118:118 30:30
YVYIAELLAHKpc2_NP.4
p−maj=1, pX=0.57, N=325
var.7.PON1 7:94946084:A:T
peptide level
177:177 118:118 30:30
YVYIAELLAHKpc2_NP.5
p−maj=1, pX=1.4e−11, N=325
var.7.PON1 7:94946084:A:T
peptide level
177:177 117:118 30:30
YVYIAELLAHKpc3_NP.1
p−min=0.46, pX=0.33, N=324
var.7.PON1 7:94946084:A:T
peptide level
177:177 118:118 30:30
YVYIAELLAHKpc3_NP.2
p−maj=1, pX=4.3e−11, N=325
var.7.PON1 7:94946084:A:T
peptide level
169:177 113:118 26:30
YVYIAELLAHKpc3_NP.3
p−maj=0.06, pX=0.66, N=308
var.7.PON1 7:94946084:A:T
peptide level
177:177 118:118 30:30
YVYIAELLAHKpc3_NP.4
p−maj=1, pX=0.73, N=325
var.7.PON1 7:94946084:A:T
peptide level
177:177 118:118 30:30
YVYIAELLAHKpc3_NP.5
p−maj=1, pX=9.1e−08, N=325

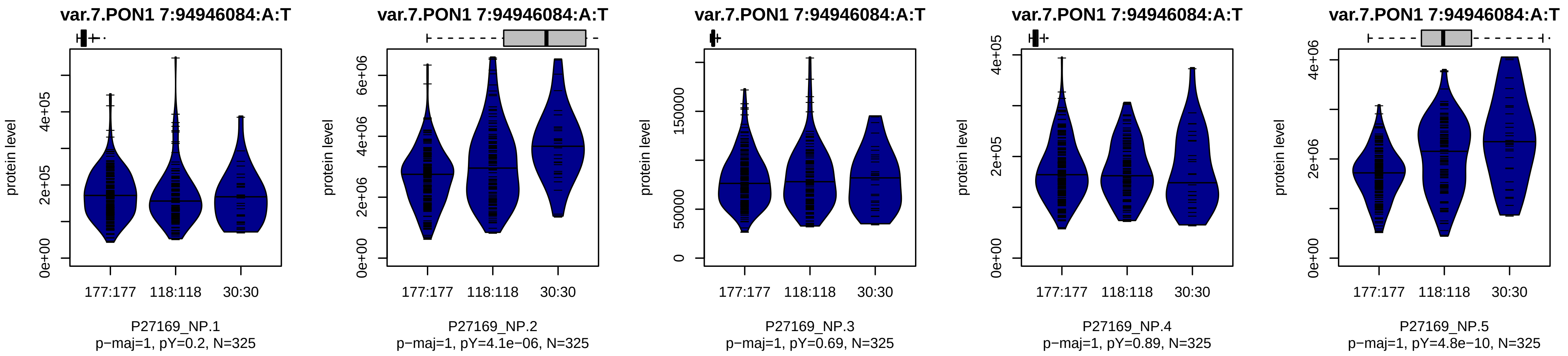
var.7.PON1 7:94946084:A:T
protein level
0e+00
2e+05
4e+05
177:177
118:118
30:30
P27169_NP.1
p−maj=1, pY=0.2, N=325
var.7.PON1 7:94946084:A:T
protein level
0e+00
2e+06
4e+06
6e+06
177:177
118:118
30:30
P27169_NP.2
p−maj=1, pY=4.1e−06, N=325
var.7.PON1 7:94946084:A:T
protein level
0
50000
150000
177:177
118:118
30:30
P27169_NP.3
p−maj=1, pY=0.69, N=325
var.7.PON1 7:94946084:A:T
protein level
0e+00
2e+05
4e+05
177:177
118:118
30:30
P27169_NP.4
p−maj=1, pY=0.89, N=325
var.7.PON1 7:94946084:A:T
protein level
0e+00
2e+06
4e+06
177:177
118:118
30:30
P27169_NP.5
p−maj=1, pY=4.8e−10, N=325

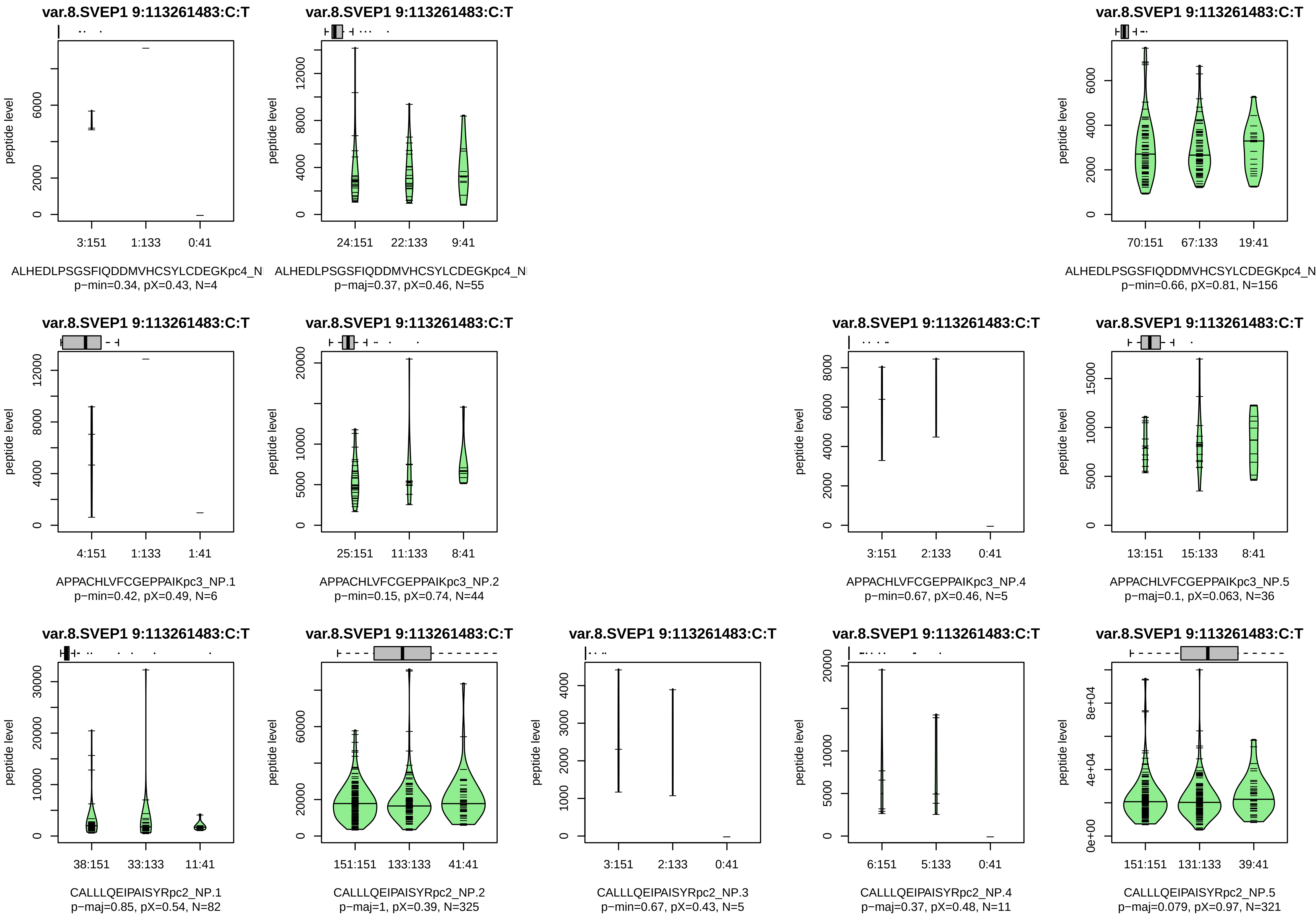
var.8.SVEP1 9:113261483:C:T
peptide level
3:151
1:133
0:41
ALHEDLPSGSFIQDDMVHCSYLCDEGKpc4_N
p−min=0.34, pX=0.43, N=4
var.8.SVEP1 9:113261483:C:T
peptide level
24:151
22:133
9:41
ALHEDLPSGSFIQDDMVHCSYLCDEGKpc4_N
p−maj=0.37, pX=0.46, N=55
var.8.SVEP1 9:113261483:C:T
peptide level
70:151
67:133
19:41
ALHEDLPSGSFIQDDMVHCSYLCDEGKpc4_N
p−min=0.66, pX=0.81, N=156
var.8.SVEP1 9:113261483:C:T
peptide level
4:151
1:133
1:41
APPACHLVFCGEPPAIKpc3_NP.1
p−min=0.42, pX=0.49, N=6
var.8.SVEP1 9:113261483:C:T
peptide level
25:151
11:133
8:41
APPACHLVFCGEPPAIKpc3_NP.2
p−min=0.15, pX=0.74, N=44
var.8.SVEP1 9:113261483:C:T
peptide level
3:151
2:133
0:41
APPACHLVFCGEPPAIKpc3_NP.4
p−min=0.67, pX=0.46, N=5
var.8.SVEP1 9:113261483:C:T
peptide level
13:151
15:133
8:41
APPACHLVFCGEPPAIKpc3_NP.5
p−maj=0.1, pX=0.063, N=36
var.8.SVEP1 9:113261483:C:T
peptide level
38:151
33:133
11:41
CALLLQEIPAISYRpc2_NP.1
p−maj=0.85, pX=0.54, N=82
var.8.SVEP1 9:113261483:C:T
peptide level
151:151
133:133
41:41
CALLLQEIPAISYRpc2_NP.2
p−maj=1, pX=0.39, N=325
var.8.SVEP1 9:113261483:C:T
peptide level
3:151
2:133
0:41
CALLLQEIPAISYRpc2_NP.3
p−min=0.67, pX=0.43, N=5
var.8.SVEP1 9:113261483:C:T
peptide level
6:151
5:133
0:41
CALLLQEIPAISYRpc2_NP.4
p−maj=0.37, pX=0.48, N=11
var.8.SVEP1 9:113261483:C:T
peptide level
151:151
131:133
39:41
CALLLQEIPAISYRpc2_NP.5
p−maj=0.079, pX=0.97, N=321

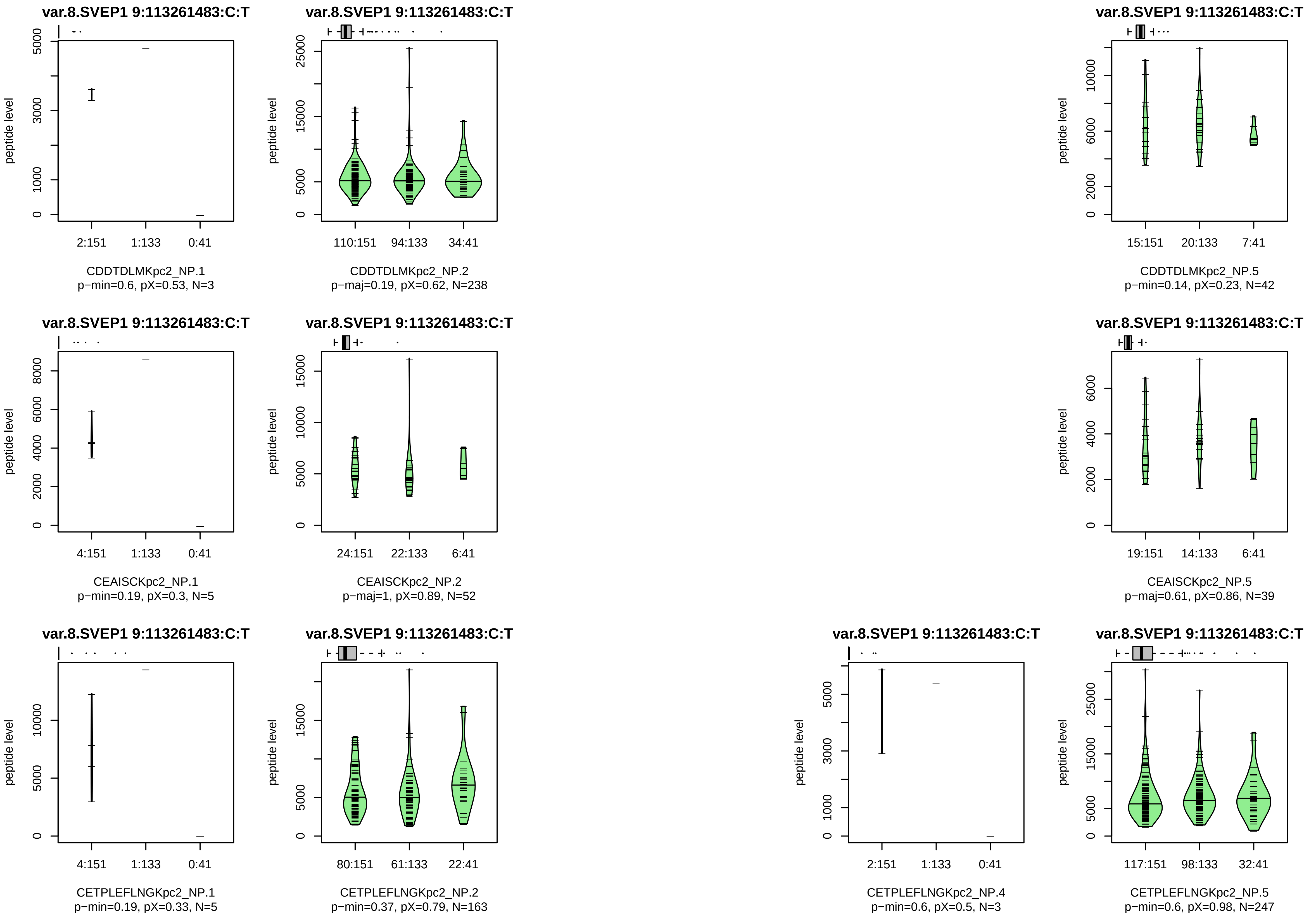
var.8.SVEP1 9:113261483:C:T
peptide level
2:151
1:133
0:41
CDDTDLMKpc2_NP.1
p−min=0.6, pX=0.53, N=3
var.8.SVEP1 9:113261483:C:T
peptide level
110:151
94:133
34:41
CDDTDLMKpc2_NP.2
p−maj=0.19, pX=0.62, N=238
var.8.SVEP1 9:113261483:C:T
peptide level
15:151
20:133
7:41
CDDTDLMKpc2_NP.5
p−min=0.14, pX=0.23, N=42
var.8.SVEP1 9:113261483:C:T
peptide level
4:151
1:133
0:41
CEAISCKpc2_NP.1
p−min=0.19, pX=0.3, N=5
var.8.SVEP1 9:113261483:C:T
peptide level
24:151
22:133
6:41
CEAISCKpc2_NP.2
p−maj=1, pX=0.89, N=52
var.8.SVEP1 9:113261483:C:T
peptide level
19:151
14:133
6:41
CEAISCKpc2_NP.5
p−maj=0.61, pX=0.86, N=39
var.8.SVEP1 9:113261483:C:T
peptide level
4:151
1:133
0:41
CETPLEFLNGKpc2_NP.1
p−min=0.19, pX=0.33, N=5
var.8.SVEP1 9:113261483:C:T
peptide level
80:151
61:133
22:41
CETPLEFLNGKpc2_NP.2
p−min=0.37, pX=0.79, N=163
var.8.SVEP1 9:113261483:C:T
peptide level
2:151
1:133
0:41
CETPLEFLNGKpc2_NP.4
p−min=0.6, pX=0.5, N=3
var.8.SVEP1 9:113261483:C:T
peptide level
117:151
98:133
32:41
CETPLEFLNGKpc2_NP.5
p−min=0.6, pX=0.98, N=247

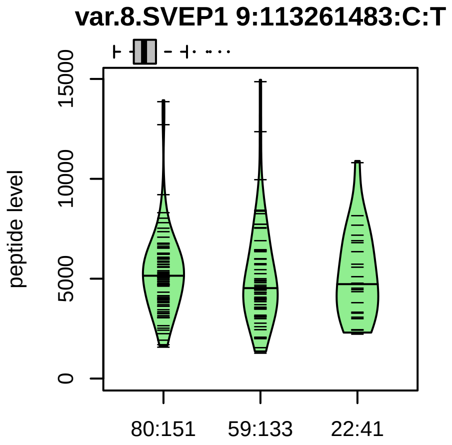

CGTHTGHFECICEKpc3_NP.2
p−min=0.27, pX=0.53, N=161

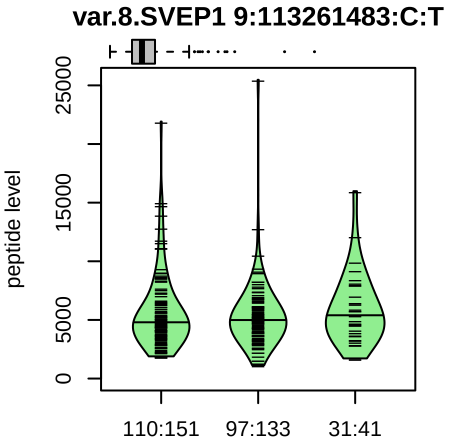

CGTHTGHFECICEKpc3_NP.5
p−maj=0.85, pX=0.74, N=238

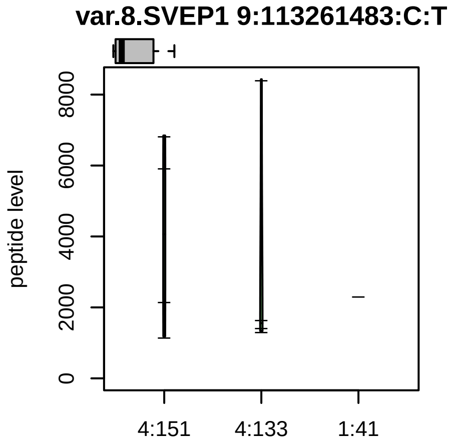

CHTAVCQSPCLNGGKpc3_NP.1
p−maj=1, pX=0.72, N=9

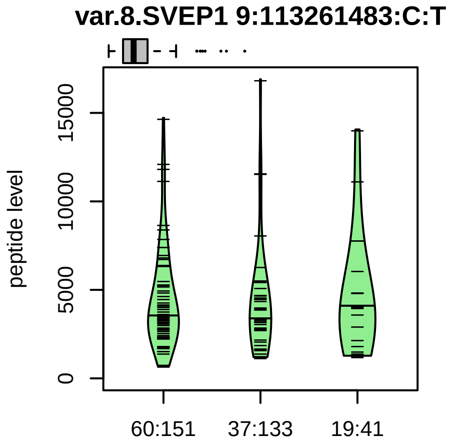

CHTAVCQSPCLNGGKpc3_NP.2
p−maj=0.16, pX=0.86, N=116

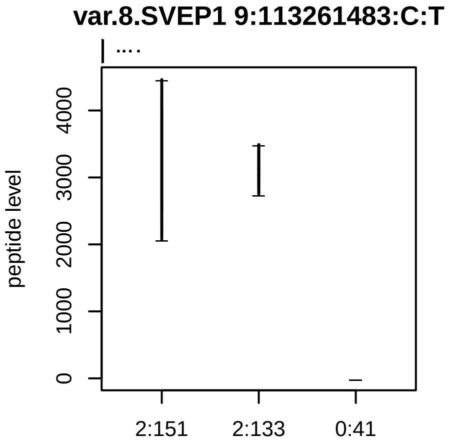

CHTAVCQSPCLNGGKpc3_NP.4
p−maj=1, pX=0.63, N=4

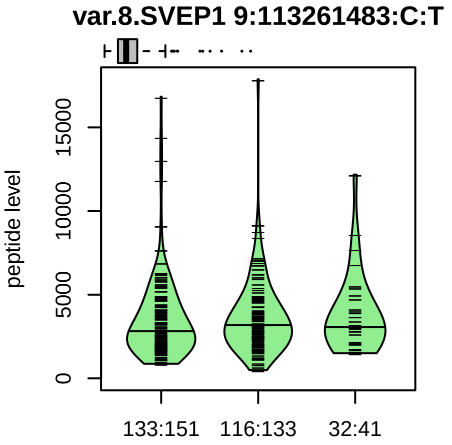

CHTAVCQSPCLNGGKpc3_NP.5
p−maj=0.14, pX=0.87, N=281

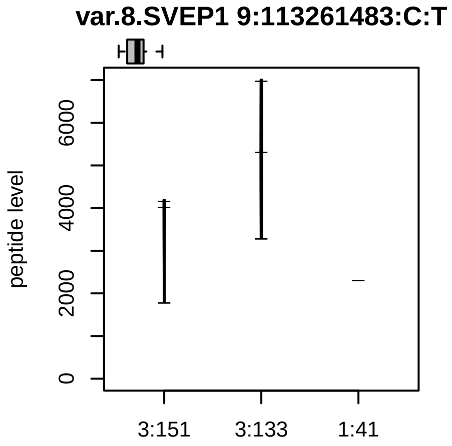

CIAHFCEKPPSVSYSILESVSKpc4_NP.1
p−maj=1, pX=0.82, N=7

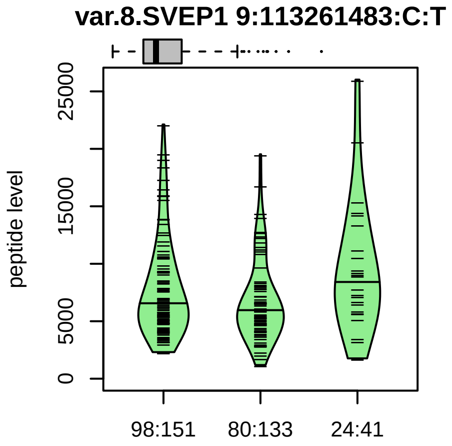

CIAHFCEKPPSVSYSILESVSKpc4_NP.2
p−min=0.36, pX=0.79, N=202

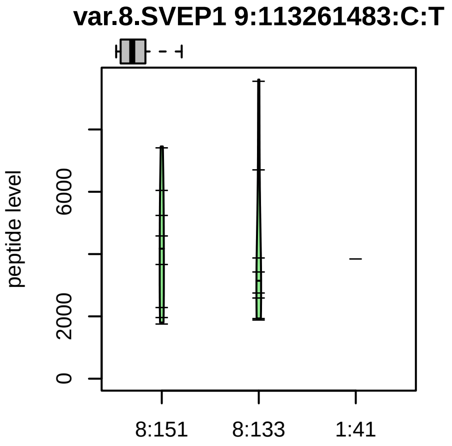

CIAHFCEKPPSVSYSILESVSKpc4_NP.4
p−maj=0.71, pX=0.67, N=17

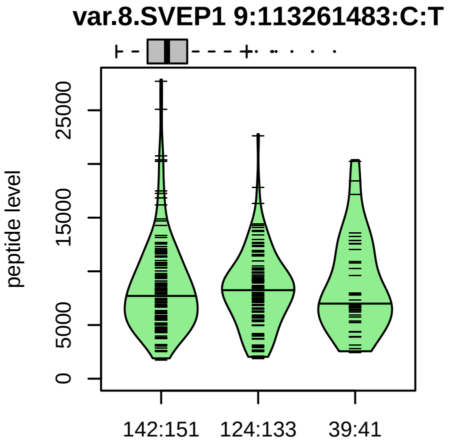

CIAHFCEKPPSVSYSILESVSKpc4_NP.5
p−maj=1, pX=0.99, N=305

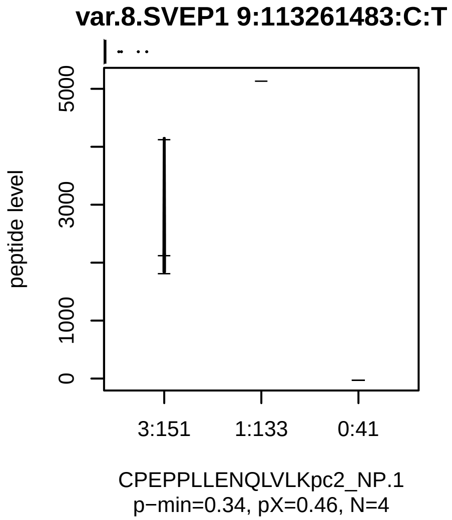
var.8.SVEP1 9:113261483:C:T
peptide level
0
1000
3000
5000
3:151
1:133
0:41
CPEPPLLENQLVLKpc2_NP.1
p−min=0.34, pX=0.46, N=4

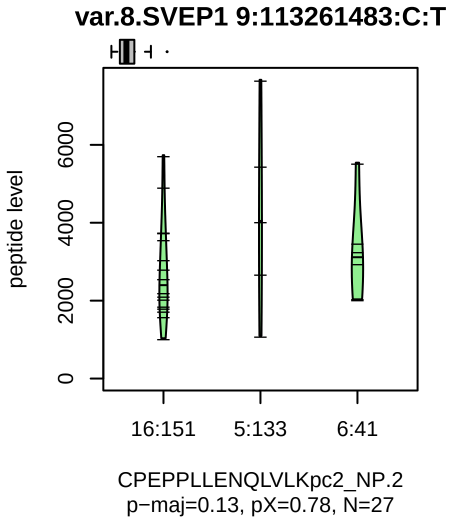
var.8.SVEP1 9:113261483:C:T
peptide level
0
2000
4000
6000
16:151
5:133
6:41
CPEPPLLENQLVLKpc2_NP.2
p−maj=0.13, pX=0.78, N=27

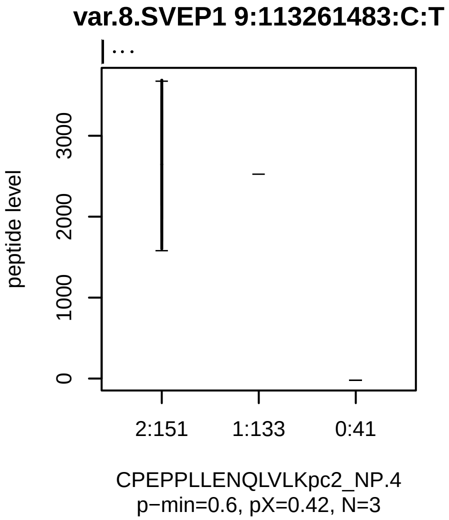
var.8.SVEP1 9:113261483:C:T
peptide level
0
1000
2000
3000
2:151
1:133
0:41
CPEPPLLENQLVLKpc2_NP.4
p−min=0.6, pX=0.42, N=3

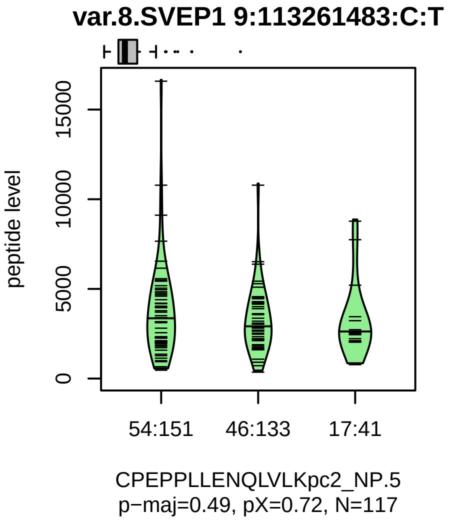
var.8.SVEP1 9:113261483:C:T
peptide level
0
5000
10000
15000
54:151
46:133
17:41
CPEPPLLENQLVLKpc2_NP.5
p−maj=0.49, pX=0.72, N=117

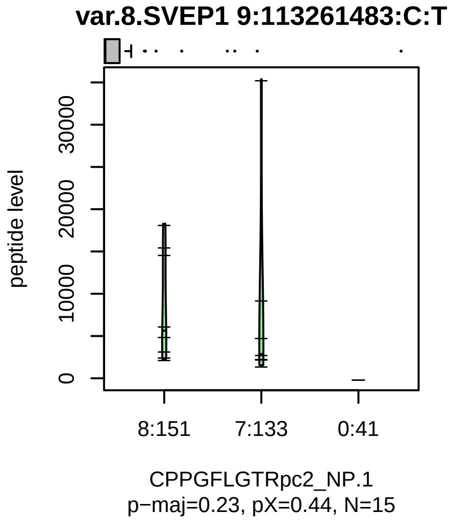
var.8.SVEP1 9:113261483:C:T
peptide level
0
10000
20000
30000
8:151
7:133
0:41
CPPGFLGTRpc2_NP.1
p−maj=0.23, pX=0.44, N=15

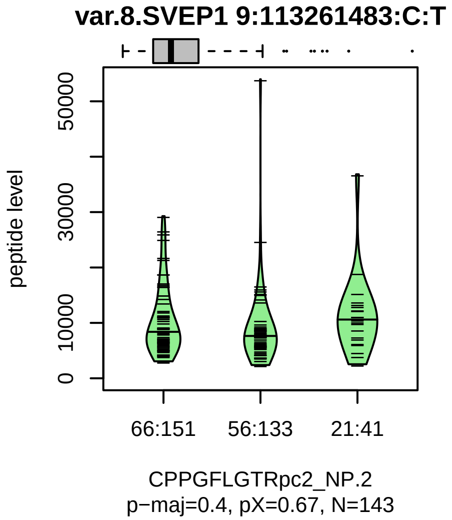
var.8.SVEP1 9:113261483:C:T
peptide level
0
10000
30000
50000
66:151
56:133
21:41
CPPGFLGTRpc2_NP.2
p−maj=0.4, pX=0.67, N=143

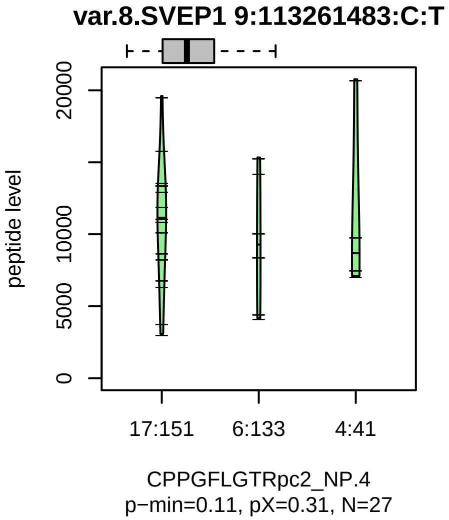
var.8.SVEP1 9:113261483:C:T
peptide level
0
5000
10000
20000
17:151
6:133
4:41
CPPGFLGTRpc2_NP.4
p−min=0.11, pX=0.31, N=27

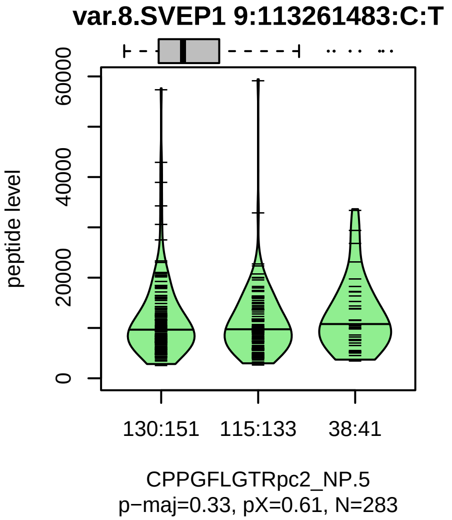
var.8.SVEP1 9:113261483:C:T
peptide level
0
20000
40000
60000
130:151
115:133
38:41
CPPGFLGTRpc2_NP.5
p−maj=0.33, pX=0.61, N=283

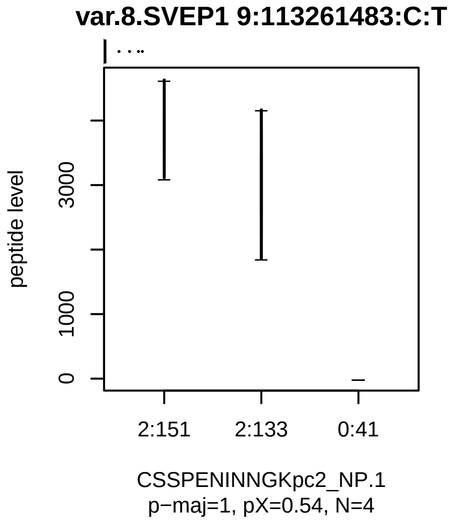
var.8.SVEP1 9:113261483:C:T
peptide level
0
1000
3000
2:151
2:133
0:41
CSSPENINNGKpc2_NP.1
p−maj=1, pX=0.54, N=4

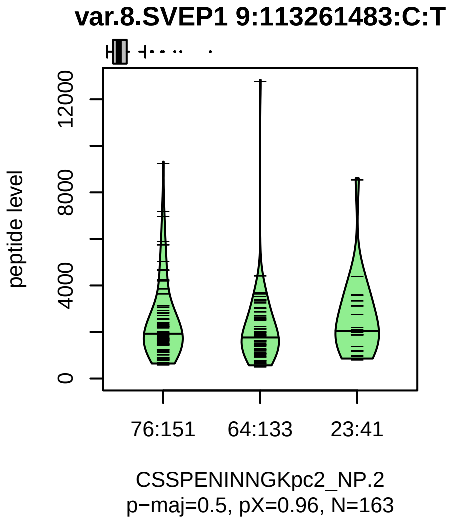
var.8.SVEP1 9:113261483:C:T
peptide level
0
4000
8000
12000
76:151
64:133
23:41
CSSPENINNGKpc2_NP.2
p−maj=0.5, pX=0.96, N=163

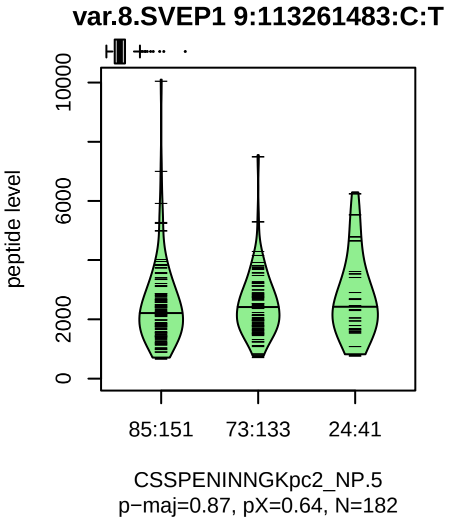
var.8.SVEP1 9:113261483:C:T
peptide level
0
2000
6000
10000
85:151
73:133
24:41
CSSPENINNGKpc2_NP.5
p−maj=0.87, pX=0.64, N=182

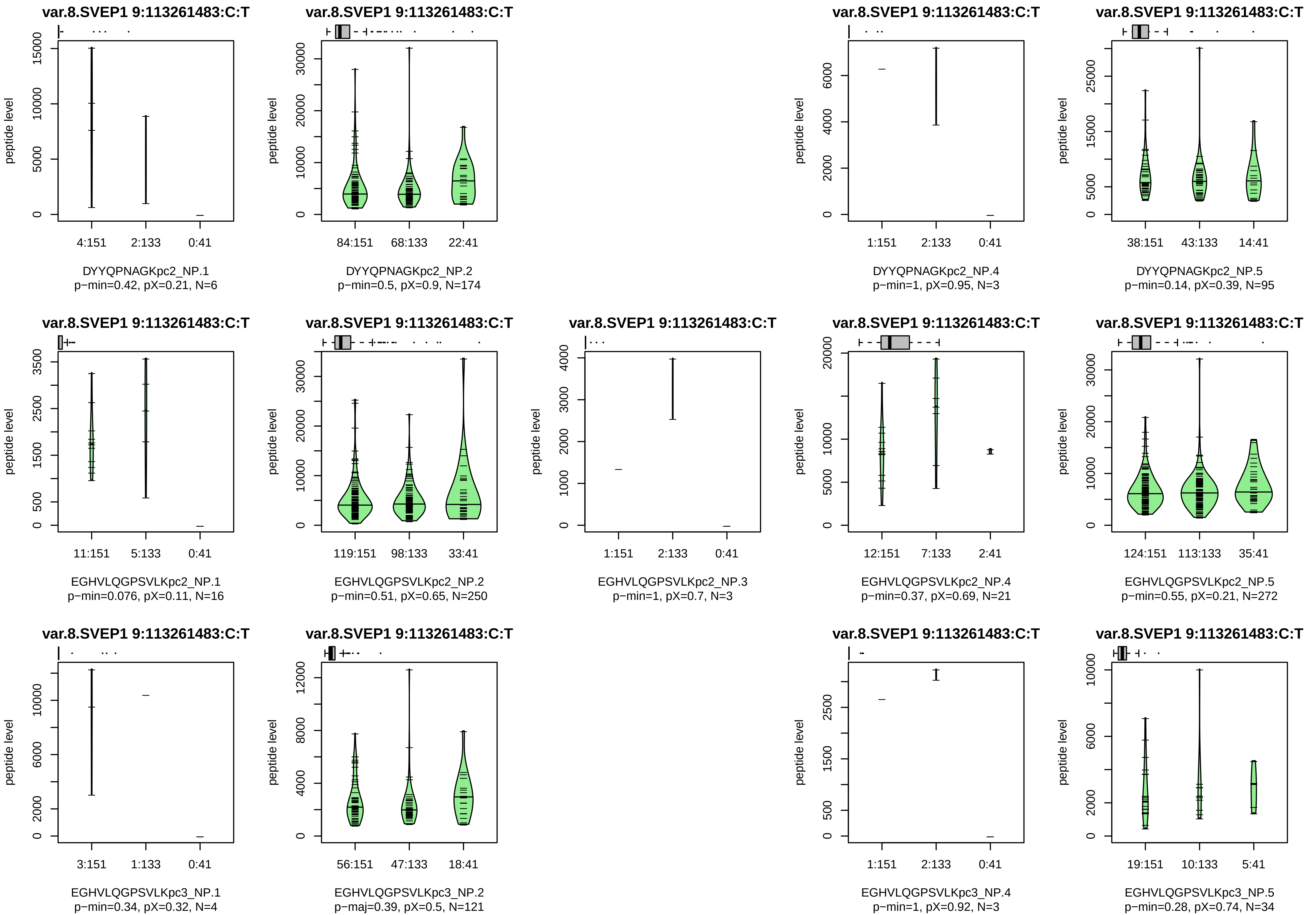
var.8.SVEP1 9:113261483:C:T
peptide level
4:151
2:133
0:41
DYYQPNAGKpc2_NP.1
p−min=0.42, pX=0.21, N=6
var.8.SVEP1 9:113261483:C:T
peptide level
84:151
68:133
22:41
DYYQPNAGKpc2_NP.2
p−min=0.5, pX=0.9, N=174
var.8.SVEP1 9:113261483:C:T
peptide level
1:151
2:133
0:41
DYYQPNAGKpc2_NP.4
p−min=1, pX=0.95, N=3
var.8.SVEP1 9:113261483:C:T
peptide level
38:151
43:133
14:41
DYYQPNAGKpc2_NP.5
p−min=0.14, pX=0.39, N=95
var.8.SVEP1 9:113261483:C:T
peptide level
11:151
5:133
0:41
EGHVLQGPSVLKpc2_NP.1
p−min=0.076, pX=0.11, N=16
var.8.SVEP1 9:113261483:C:T
peptide level
119:151
98:133
33:41
EGHVLQGPSVLKpc2_NP.2
p−min=0.51, pX=0.65, N=250
var.8.SVEP1 9:113261483:C:T
peptide level
1:151
2:133
0:41
EGHVLQGPSVLKpc2_NP.3
p−min=1, pX=0.7, N=3
var.8.SVEP1 9:113261483:C:T
peptide level
12:151
7:133
2:41
EGHVLQGPSVLKpc2_NP.4
p−min=0.37, pX=0.69, N=21
var.8.SVEP1 9:113261483:C:T
peptide level
124:151
113:133
35:41
EGHVLQGPSVLKpc2_NP.5
p−min=0.55, pX=0.21, N=272
var.8.SVEP1 9:113261483:C:T
peptide level
3:151
1:133
0:41
EGHVLQGPSVLKpc3_NP.1
p−min=0.34, pX=0.32, N=4
var.8.SVEP1 9:113261483:C:T
peptide level
56:151
47:133
18:41
EGHVLQGPSVLKpc3_NP.2
p−maj=0.39, pX=0.5, N=121
var.8.SVEP1 9:113261483:C:T
peptide level
1:151
2:133
0:41
EGHVLQGPSVLKpc3_NP.4
p−min=1, pX=0.92, N=3
var.8.SVEP1 9:113261483:C:T
peptide level
19:151
10:133
5:41
EGHVLQGPSVLKpc3_NP.5
p−min=0.28, pX=0.74, N=34

var.8.SVEP1 9:113261483:C:T

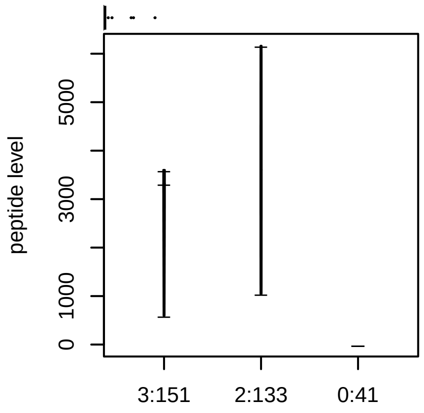

EGYTLAGLDTIECLADGKpc2_NP.1
p−min=0.67, pX=0.64, N=5

var.8.SVEP1 9:113261483:C:T

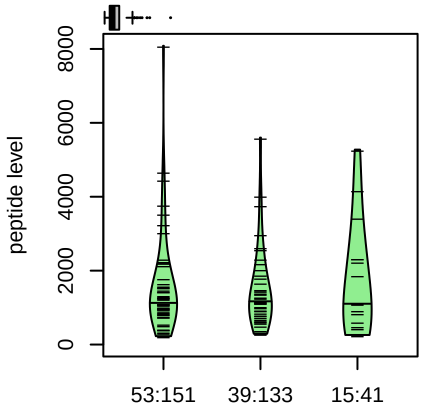

EGYTLAGLDTIECLADGKpc2_NP.2
p−min=0.48, pX=1, N=107

var.8.SVEP1 9:113261483:C:T

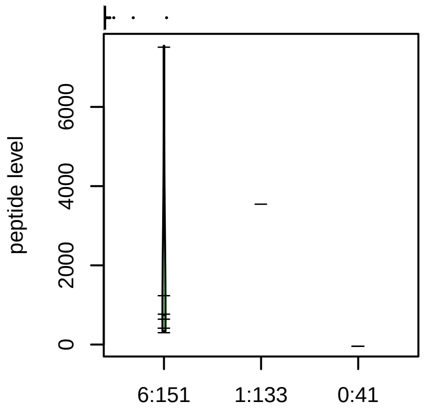

EGYTLAGLDTIECLADGKpc2_NP.4
p−min=0.053, pX=0.3, N=7

var.8.SVEP1 9:113261483:C:T

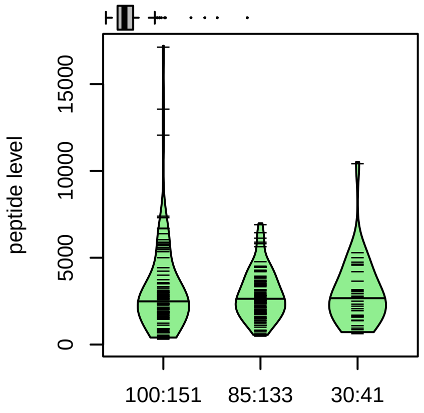

EGYTLAGLDTIECLADGKpc2_NP.5
p−maj=0.38, pX=0.88, N=215

var.8.SVEP1 9:113261483:C:T

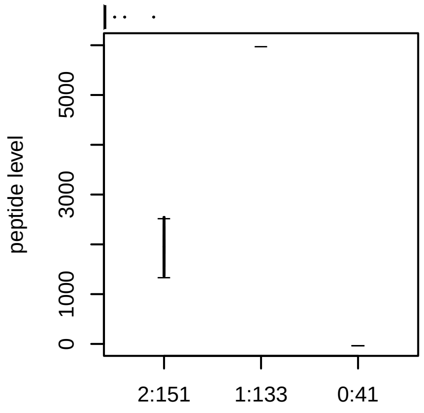

EIEYTCNEGFLLEGARpc2_NP.1
p−min=0.6, pX=0.9, N=3

var.8.SVEP1 9:113261483:C:T

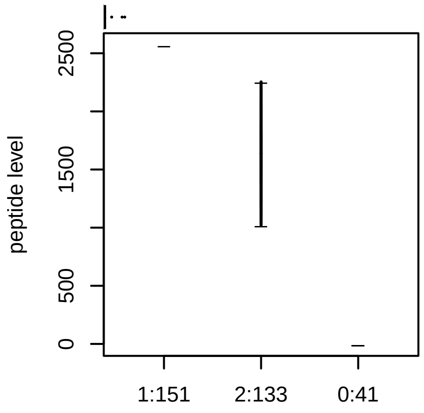

EIEYTCNEGFLLEGARpc2_NP.4
p−min=1, pX=0.81, N=3

var.8.SVEP1 9:113261483:C:T

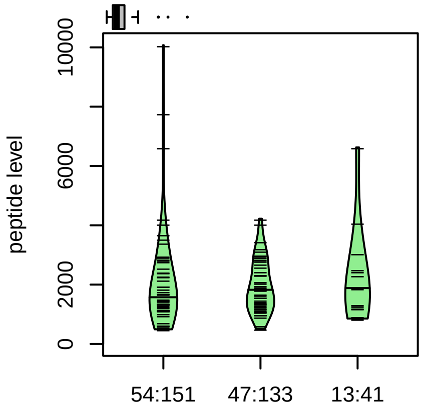

EIEYTCNEGFLLEGARpc2_NP.5
p−maj=0.73, pX=0.7, N=114

var.8.SVEP1 9:113261483:C:T

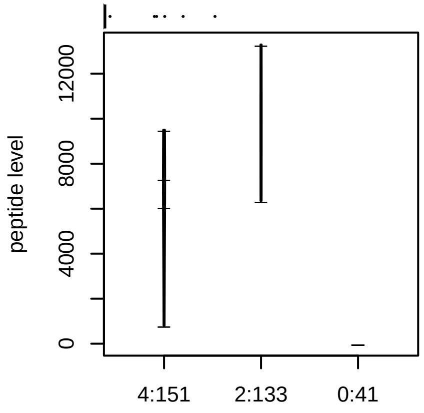

ELNDMASTPKpc2_NP.1
p−min=0.42, pX=0.51, N=6

var.8.SVEP1 9:113261483:C:T

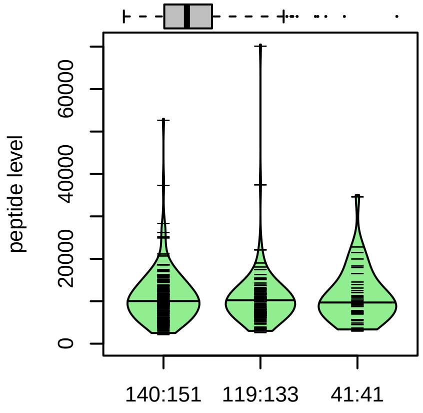

ELNDMASTPKpc2_NP.2
p−maj=0.055, pX=0.93, N=300

var.8.SVEP1 9:113261483:C:T

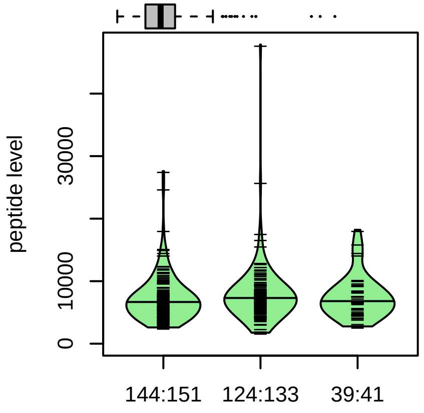

ELNDMASTPKpc2_NP.5
p−min=0.63, pX=0.59, N=307

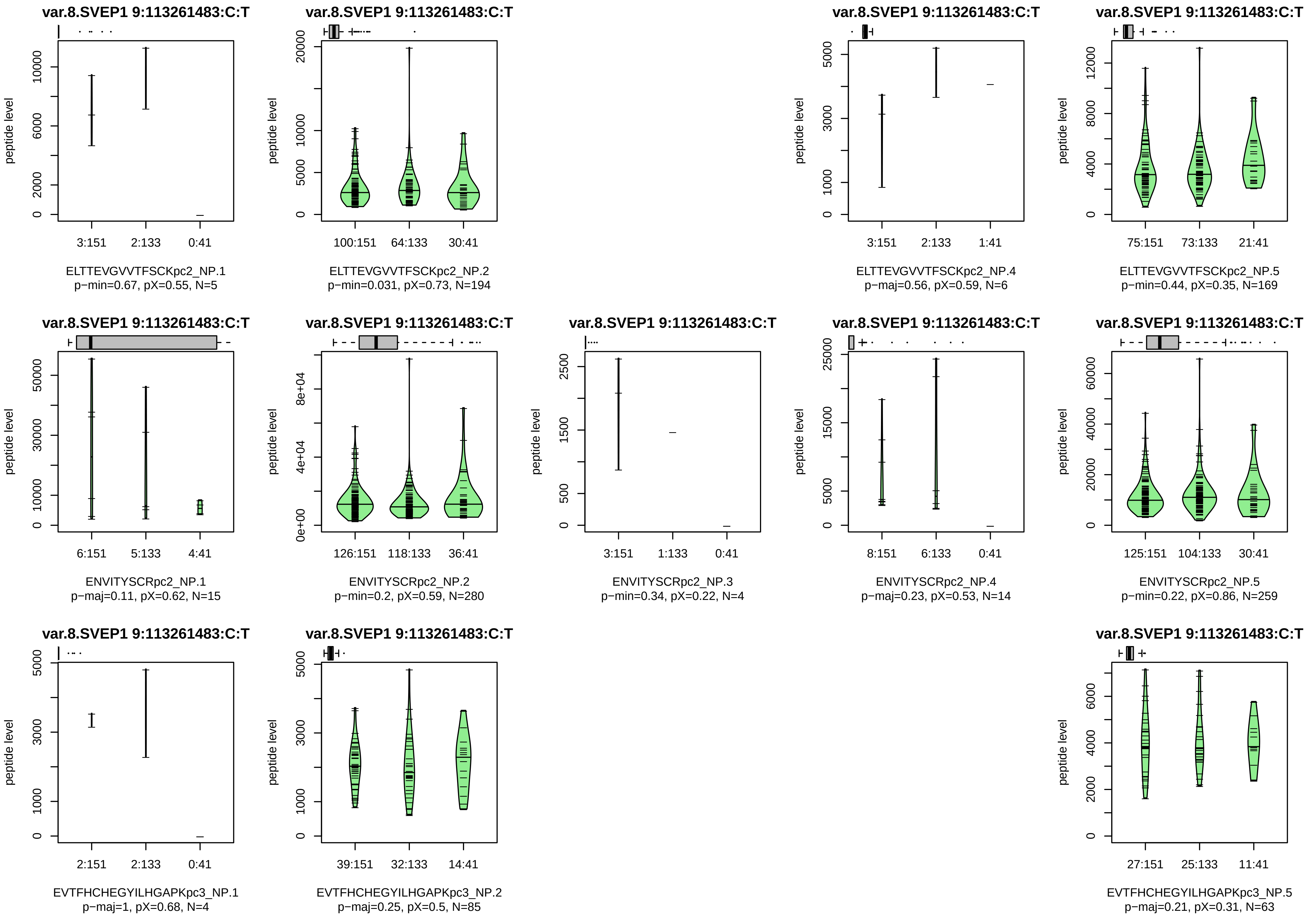

var.8.SVEP1 9:113261483:C:T
peptide level
3:151
2:133
0:41
ELTTEVGVVTFSCKpc2_NP.1
p−min=0.67, pX=0.55, N=5
var.8.SVEP1 9:113261483:C:T
peptide level
100:151
64:133
30:41
ELTTEVGVVTFSCKpc2_NP.2
p−min=0.031, pX=0.73, N=194
var.8.SVEP1 9:113261483:C:T
peptide level
3:151
2:133
1:41
ELTTEVGVVTFSCKpc2_NP.4
p−maj=0.56, pX=0.59, N=6
var.8.SVEP1 9:113261483:C:T
peptide level
75:151
73:133
21:41
ELTTEVGVVTFSCKpc2_NP.5
p−min=0.44, pX=0.35, N=169
var.8.SVEP1 9:113261483:C:T
peptide level
6:151
5:133
4:41
ENVITYSCRpc2_NP.1
p−maj=0.11, pX=0.62, N=15
var.8.SVEP1 9:113261483:C:T
peptide level
126:151
118:133
36:41
ENVITYSCRpc2_NP.2
p−min=0.2, pX=0.59, N=280
var.8.SVEP1 9:113261483:C:T
peptide level
3:151
1:133
0:41
ENVITYSCRpc2_NP.3
p−min=0.34, pX=0.22, N=4
var.8.SVEP1 9:113261483:C:T
peptide level
8:151
6:133
0:41
ENVITYSCRpc2_NP.4
p−maj=0.23, pX=0.53, N=14
var.8.SVEP1 9:113261483:C:T
peptide level
125:151
104:133
30:41
ENVITYSCRpc2_NP.5
p−min=0.22, pX=0.86, N=259
var.8.SVEP1 9:113261483:C:T
peptide level
2:151
2:133
0:41
EVTFHCHEGYILHGAPKpc3_NP.1
p−maj=1, pX=0.68, N=4
var.8.SVEP1 9:113261483:C:T
peptide level
39:151
32:133
14:41
EVTFHCHEGYILHGAPKpc3_NP.2
p−maj=0.25, pX=0.5, N=85
var.8.SVEP1 9:113261483:C:T
peptide level
27:151
25:133
11:41
EVTFHCHEGYILHGAPKpc3_NP.5
p−maj=0.21, pX=0.31, N=63

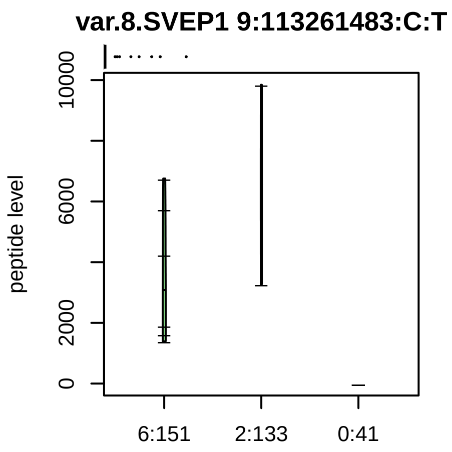

EVTFHCHEGYILHGAPKpc4_NP.1
p−min=0.15, pX=0.32, N=8

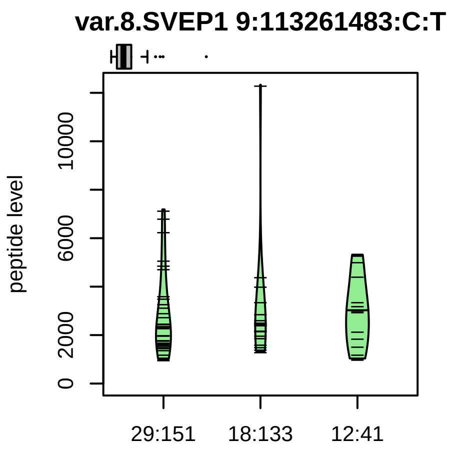

EVTFHCHEGYILHGAPKpc4_NP.2
p−maj=0.08, pX=0.56, N=59

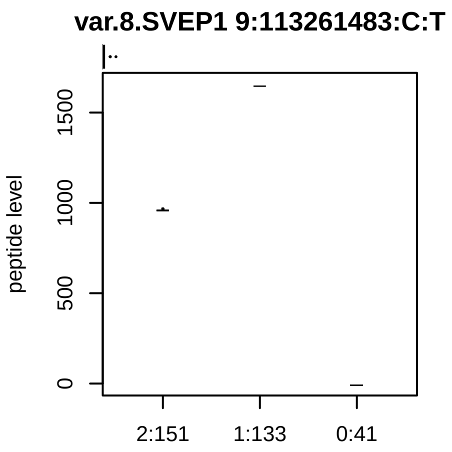

EVTFHCHEGYILHGAPKpc4_NP.3
p−min=0.6, pX=0.63, N=3

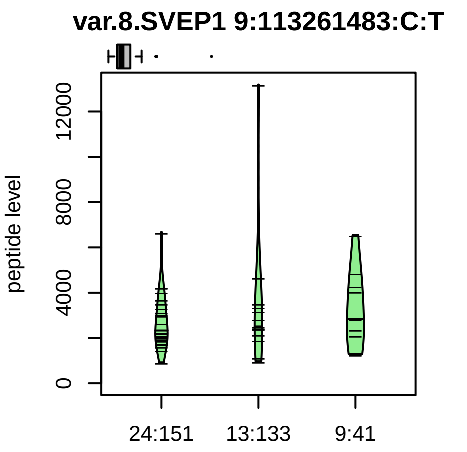

EVTFHCHEGYILHGAPKpc4_NP.5
p−maj=0.15, pX=0.5, N=46

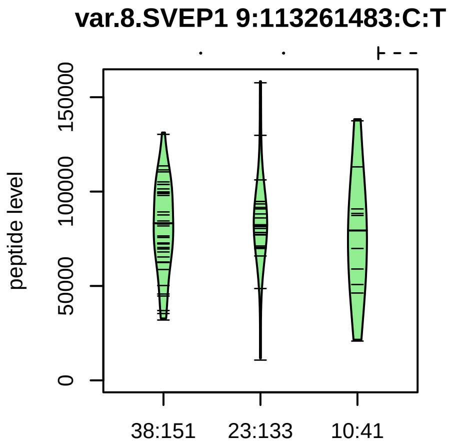

FAAGSVVSFKpc2_NP.2
p−min=0.18, pX=0.45, N=71

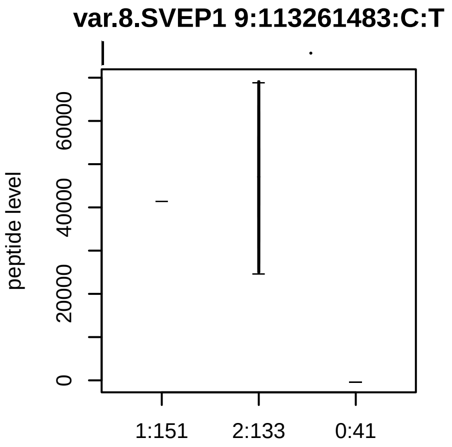

FAAGSVVSFKpc2_NP.4
p−min=1, pX=0.94, N=3

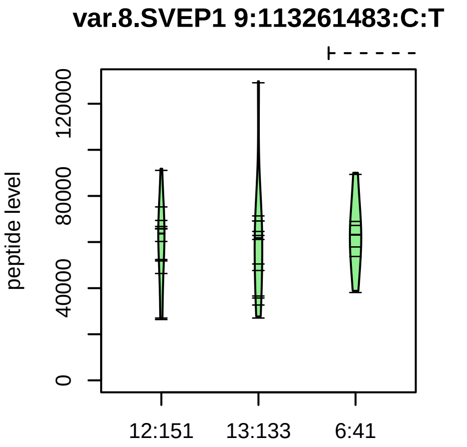

FAAGSVVSFKpc2_NP.5
p−maj=0.25, pX=0.2, N=31

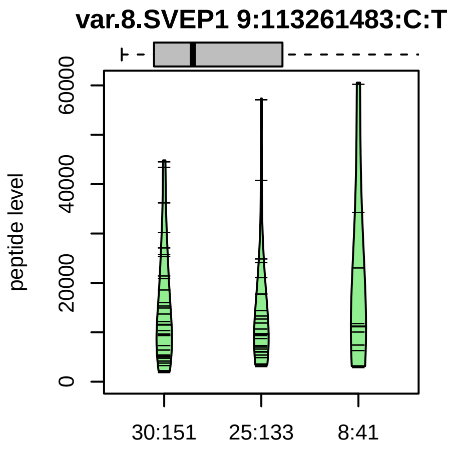

FGTICYVSCRpc2_NP.1
p−min=0.89, pX=0.83, N=63

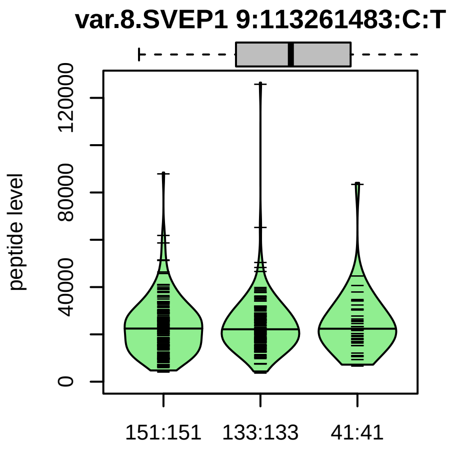

FGTICYVSCRpc2_NP.2
p−maj=1, pX=0.6, N=325

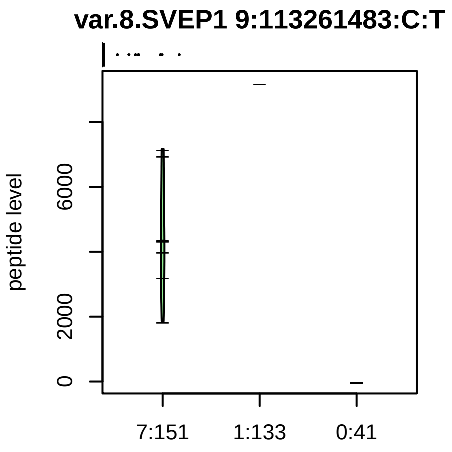

FGTICYVSCRpc2_NP.3
p−min=0.027, pX=0.098, N=8

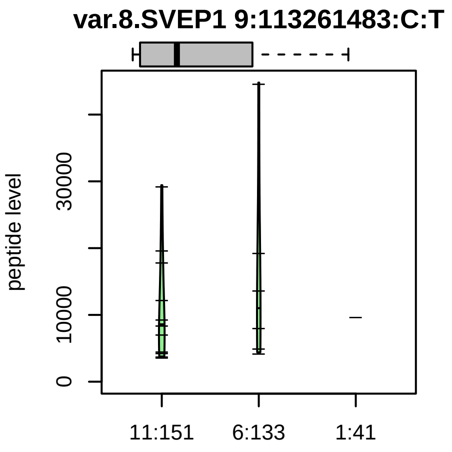

FGTICYVSCRpc2_NP.4
p−min=0.23, pX=0.46, N=18

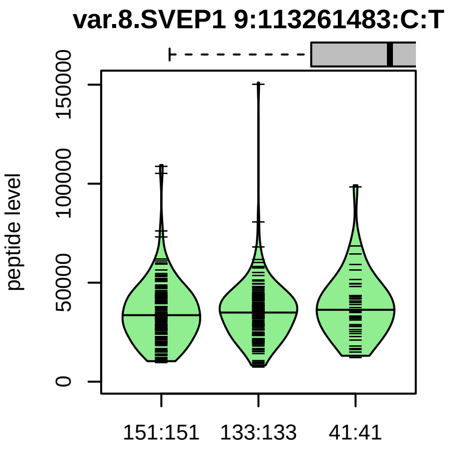

FGTICYVSCRpc2_NP.5
p−maj=1, pX=0.28, N=325

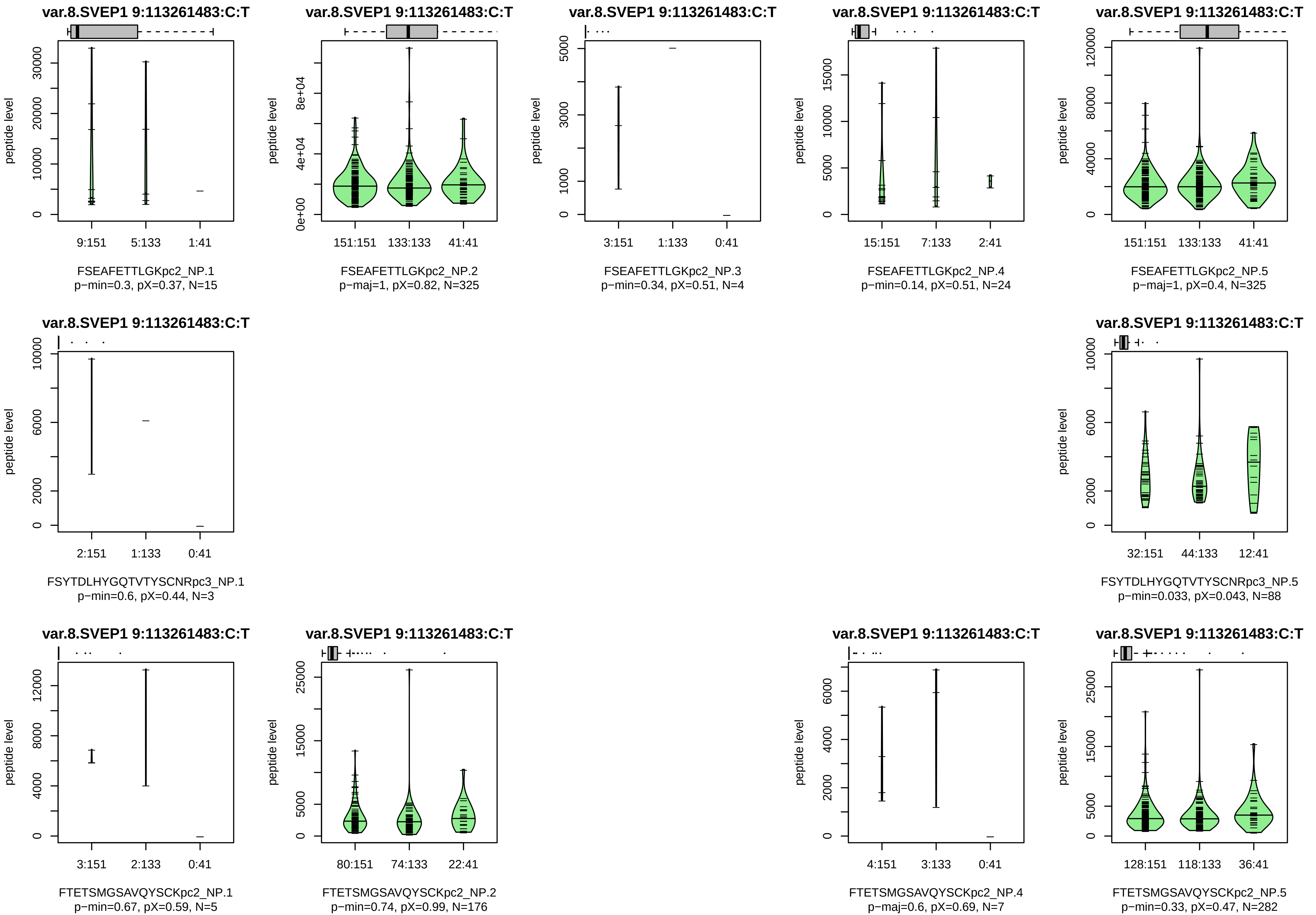

var.8.SVEP1 9:113261483:C:T
peptide level
9:151
5:133
1:41
FSEAFETTLGKpc2_NP.1
p−min=0.3, pX=0.37, N=15
var.8.SVEP1 9:113261483:C:T
peptide level
151:151
133:133
41:41
FSEAFETTLGKpc2_NP.2
p−maj=1, pX=0.82, N=325
var.8.SVEP1 9:113261483:C:T
peptide level
3:151
1:133
0:41
FSEAFETTLGKpc2_NP.3
p−min=0.34, pX=0.51, N=4
var.8.SVEP1 9:113261483:C:T
peptide level
15:151
7:133
2:41
FSEAFETTLGKpc2_NP.4
p−min=0.14, pX=0.51, N=24
var.8.SVEP1 9:113261483:C:T
peptide level
151:151
133:133
41:41
FSEAFETTLGKpc2_NP.5
p−maj=1, pX=0.4, N=325
var.8.SVEP1 9:113261483:C:T
peptide level
2:151
1:133
0:41
FSYTDLHYGQTVTYSCNRpc3_NP.1
p−min=0.6, pX=0.44, N=3
var.8.SVEP1 9:113261483:C:T
peptide level
32:151
44:133
12:41
FSYTDLHYGQTVTYSCNRpc3_NP.5
p−min=0.033, pX=0.043, N=88
var.8.SVEP1 9:113261483:C:T
peptide level
3:151
2:133
0:41
FTETSMGSAVQYSCKpc2_NP.1
p−min=0.67, pX=0.59, N=5
var.8.SVEP1 9:113261483:C:T
peptide level
80:151
74:133
22:41
FTETSMGSAVQYSCKpc2_NP.2
p−min=0.74, pX=0.99, N=176
var.8.SVEP1 9:113261483:C:T
peptide level
4:151
3:133
0:41
FTETSMGSAVQYSCKpc2_NP.4
p−maj=0.6, pX=0.69, N=7
var.8.SVEP1 9:113261483:C:T
peptide level
128:151
118:133
36:41
FTETSMGSAVQYSCKpc2_NP.5
p−min=0.33, pX=0.47, N=282

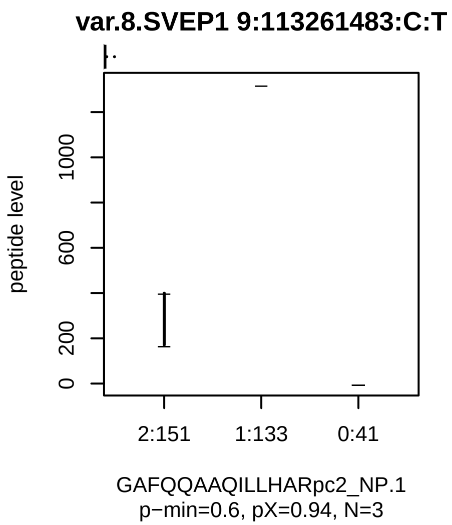
var.8.SVEP1 9:113261483:C:T
peptide level
0
200
600
1000
2:151
1:133
0:41
GAFQQAAQILLHARpc2_NP.1
p−min=0.6, pX=0.94, N=3

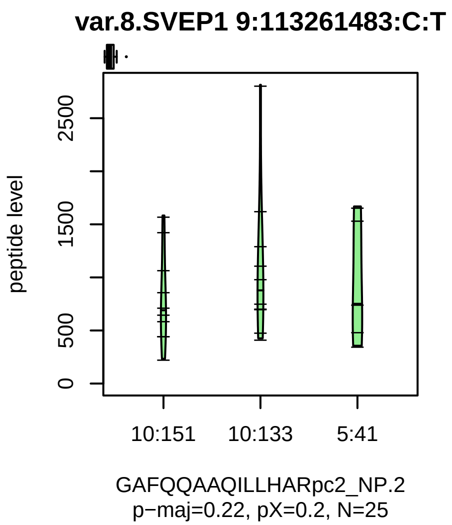
var.8.SVEP1 9:113261483:C:T
peptide level
0
500
1500
2500
10:151
10:133
5:41
GAFQQAAQILLHARpc2_NP.2
p−maj=0.22, pX=0.2, N=25

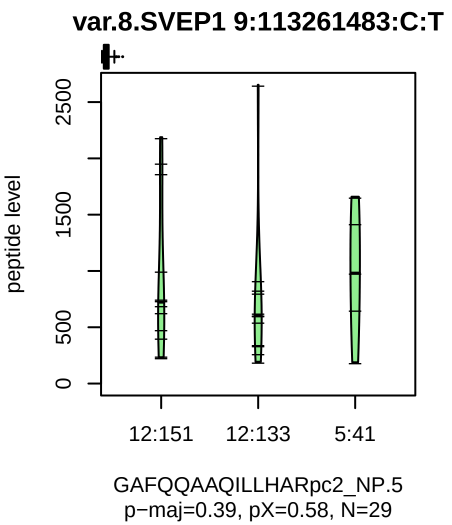
var.8.SVEP1 9:113261483:C:T
peptide level
0
500
1500
2500
12:151
12:133
5:41
GAFQQAAQILLHARpc2_NP.5
p−maj=0.39, pX=0.58, N=29

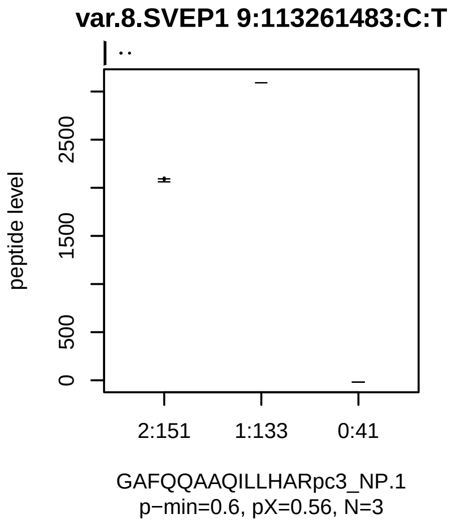
var.8.SVEP1 9:113261483:C:T
peptide level
0
500
1500
2500
2:151
1:133
0:41
GAFQQAAQILLHARpc3_NP.1
p−min=0.6, pX=0.56, N=3

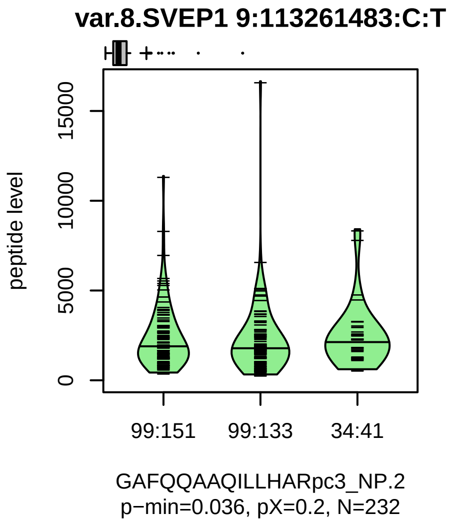
var.8.SVEP1 9:113261483:C:T
peptide level
0
5000
10000
15000
99:151
99:133
34:41
GAFQQAAQILLHARpc3_NP.2
p−min=0.036, pX=0.2, N=232

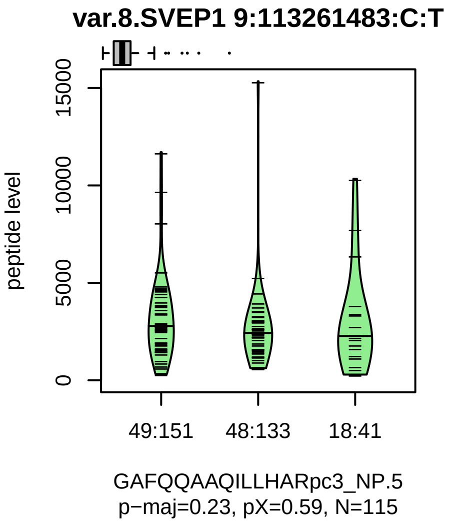
var.8.SVEP1 9:113261483:C:T
peptide level
0
5000
10000
15000
49:151
48:133
18:41
GAFQQAAQILLHARpc3_NP.5
p−maj=0.23, pX=0.59, N=115

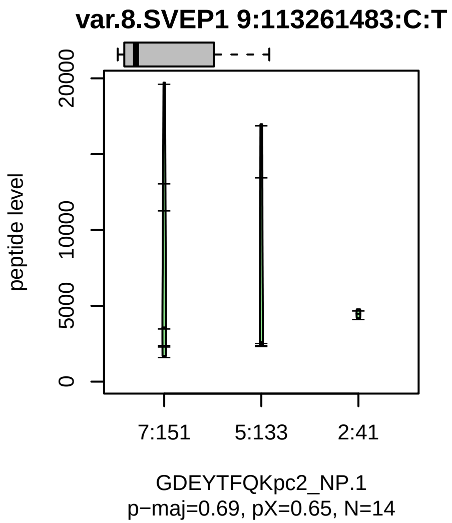
var.8.SVEP1 9:113261483:C:T
peptide level
0
5000
10000
20000
7:151
5:133
2:41
GDEYTFQKpc2_NP.1
p−maj=0.69, pX=0.65, N=14

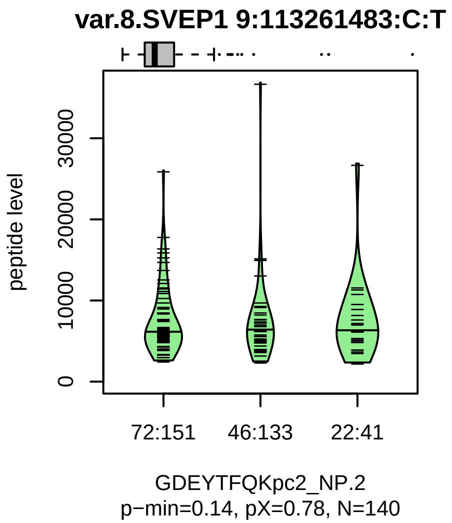
var.8.SVEP1 9:113261483:C:T
peptide level
0
10000
20000
30000
72:151
46:133
22:41
GDEYTFQKpc2_NP.2
p−min=0.14, pX=0.78, N=140

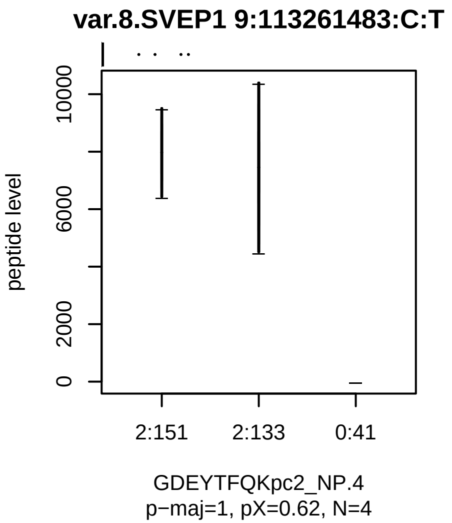
var.8.SVEP1 9:113261483:C:T
peptide level
0
2000
6000
10000
2:151
2:133
0:41
GDEYTFQKpc2_NP.4
p−maj=1, pX=0.62, N=4

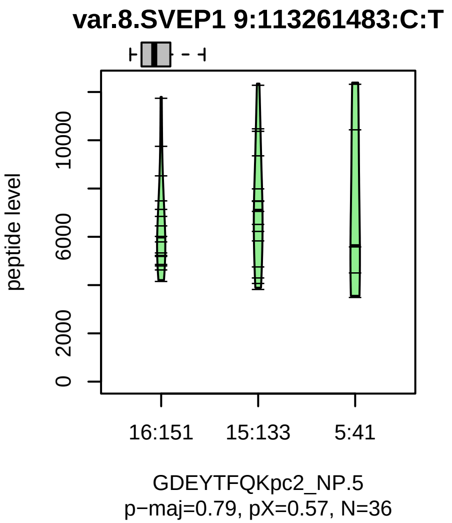
var.8.SVEP1 9:113261483:C:T
peptide level
0
2000
6000
10000
16:151
15:133
5:41
GDEYTFQKpc2_NP.5
p−maj=0.79, pX=0.57, N=36

var.8.SVEP1 9:113261483:C:T

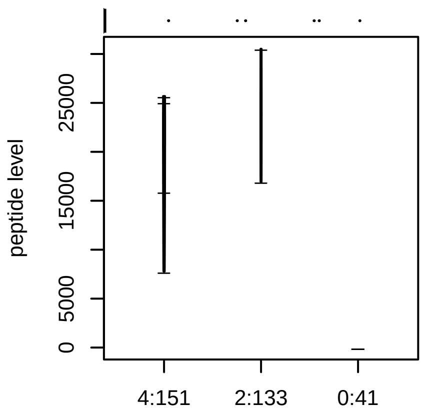

GENFEVGSKpc2_NP.1
p−min=0.42, pX=0.36, N=6

var.8.SVEP1 9:113261483:C:T

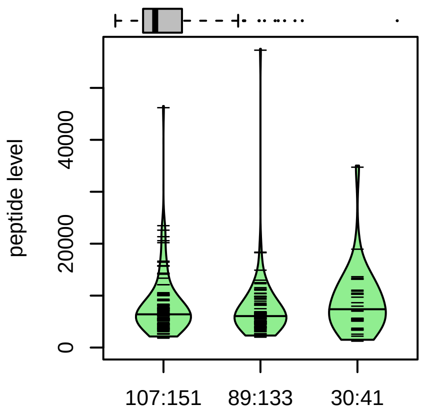

GENFEVGSKpc2_NP.2
p−min=0.72, pX=0.9, N=226

var.8.SVEP1 9:113261483:C:T

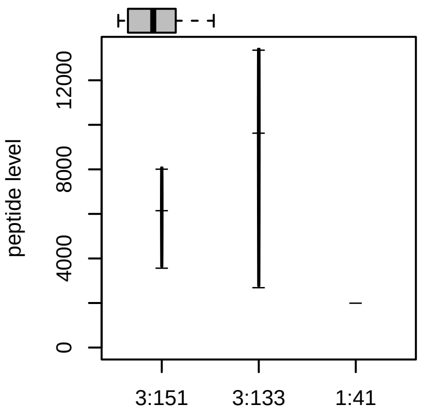

GENFEVGSKpc2_NP.4
p−maj=1, pX=0.98, N=7

var.8.SVEP1 9:113261483:C:T

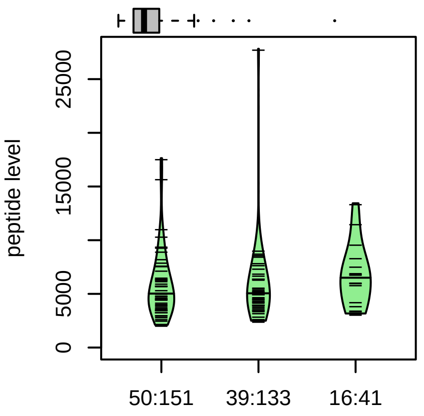

GENFEVGSKpc2_NP.5
p−maj=0.37, pX=0.5, N=105

var.8.SVEP1 9:113261483:C:T

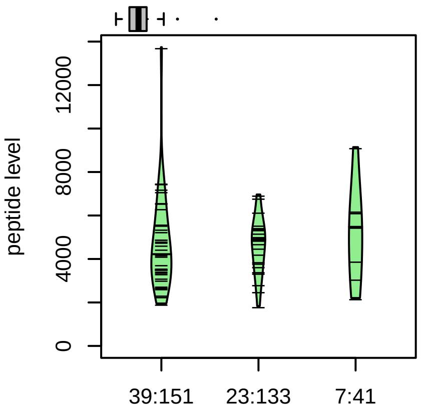

GGQWNPSPMSIQCIPVRpc2_NP.5
p−min=0.077, pX=0.17, N=69

var.8.SVEP1 9:113261483:C:T

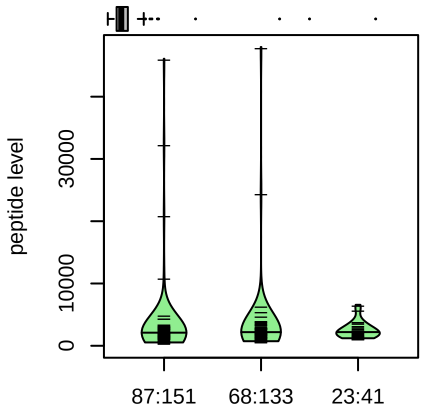

GNVLAWPDFLSGIVGKpc2_NP.1
p−min=0.37, pX=0.54, N=178

var.8.SVEP1 9:113261483:C:T

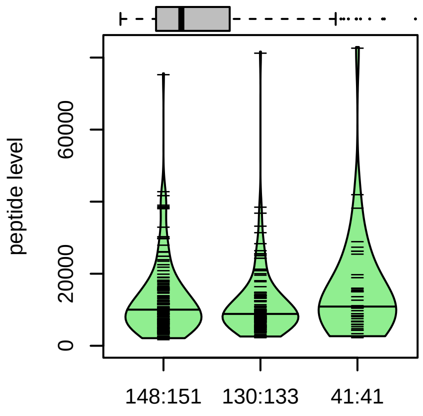

GNVLAWPDFLSGIVGKpc2_NP.2
p−maj=1, pX=0.42, N=319

var.8.SVEP1 9:113261483:C:T

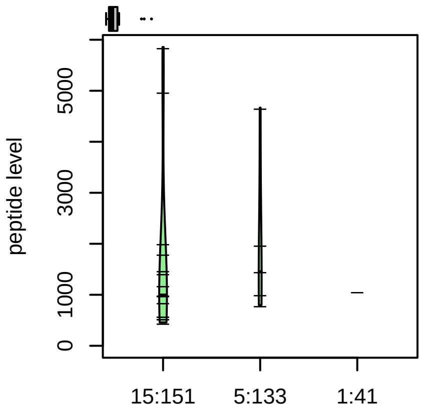

GNVLAWPDFLSGIVGKpc2_NP.3
p−min=0.023, pX=0.11, N=21

var.8.SVEP1 9:113261483:C:T

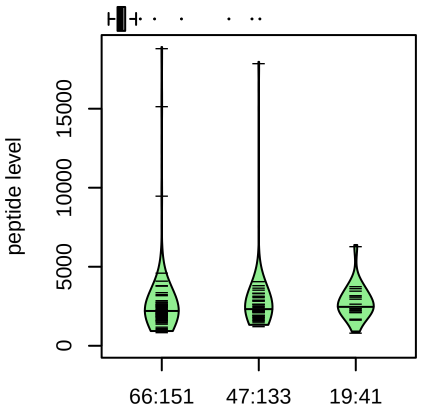

GNVLAWPDFLSGIVGKpc2_NP.4
p−min=0.31, pX=0.73, N=132

var.8.SVEP1 9:113261483:C:T

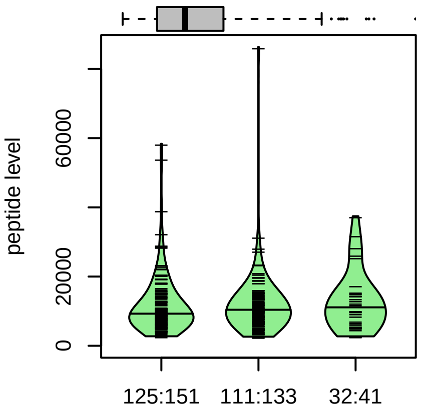

GNVLAWPDFLSGIVGKpc2_NP.5
p−maj=0.51, pX=0.86, N=268

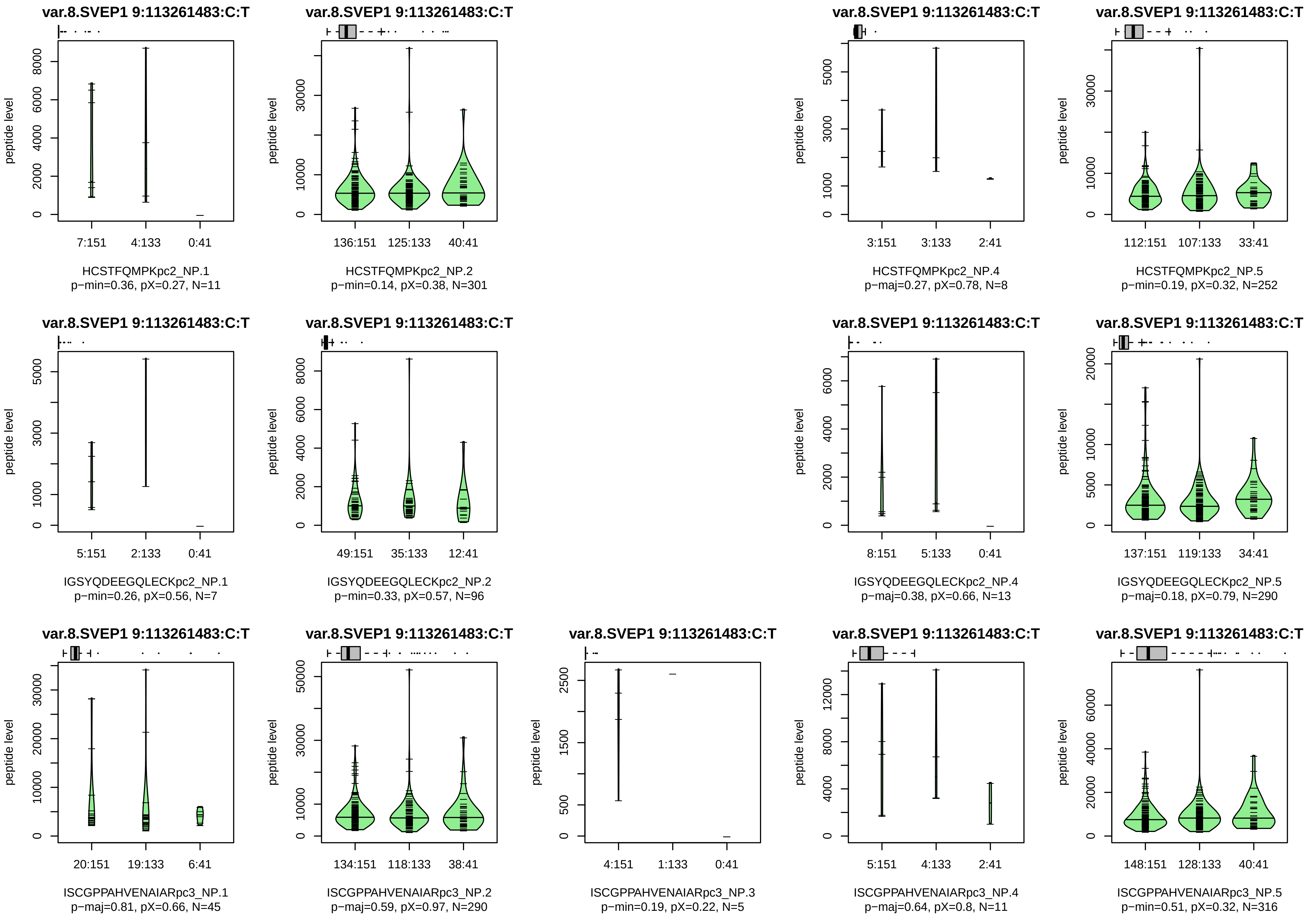

var.8.SVEP1 9:113261483:C:T
peptide level
7:151
4:133
0:41
HCSTFQMPKpc2_NP.1
p−min=0.36, pX=0.27, N=11
var.8.SVEP1 9:113261483:C:T
peptide level
136:151
125:133
40:41
HCSTFQMPKpc2_NP.2
p−min=0.14, pX=0.38, N=301
var.8.SVEP1 9:113261483:C:T
peptide level
3:151
3:133
2:41
HCSTFQMPKpc2_NP.4
p−maj=0.27, pX=0.78, N=8
var.8.SVEP1 9:113261483:C:T
peptide level
112:151
107:133
33:41
HCSTFQMPKpc2_NP.5
p−min=0.19, pX=0.32, N=252
var.8.SVEP1 9:113261483:C:T
peptide level
5:151
2:133
0:41
IGSYQDEEGQLECKpc2_NP.1
p−min=0.26, pX=0.56, N=7
var.8.SVEP1 9:113261483:C:T
peptide level
49:151
35:133
12:41
IGSYQDEEGQLECKpc2_NP.2
p−min=0.33, pX=0.57, N=96
var.8.SVEP1 9:113261483:C:T
peptide level
8:151
5:133
0:41
IGSYQDEEGQLECKpc2_NP.4
p−maj=0.38, pX=0.66, N=13
var.8.SVEP1 9:113261483:C:T
peptide level
137:151
119:133
34:41
IGSYQDEEGQLECKpc2_NP.5
p−maj=0.18, pX=0.79, N=290
var.8.SVEP1 9:113261483:C:T
peptide level
20:151
19:133
6:41
ISCGPPAHVENAIARpc3_NP.1
p−maj=0.81, pX=0.66, N=45
var.8.SVEP1 9:113261483:C:T
peptide level
134:151
118:133
38:41
ISCGPPAHVENAIARpc3_NP.2
p−maj=0.59, pX=0.97, N=290
var.8.SVEP1 9:113261483:C:T
peptide level
4:151
1:133
0:41
ISCGPPAHVENAIARpc3_NP.3
p−min=0.19, pX=0.22, N=5
var.8.SVEP1 9:113261483:C:T
peptide level
5:151
4:133
2:41
ISCGPPAHVENAIARpc3_NP.4
p−maj=0.64, pX=0.8, N=11
var.8.SVEP1 9:113261483:C:T
peptide level
148:151
128:133
40:41
ISCGPPAHVENAIARpc3_NP.5
p−min=0.51, pX=0.32, N=316

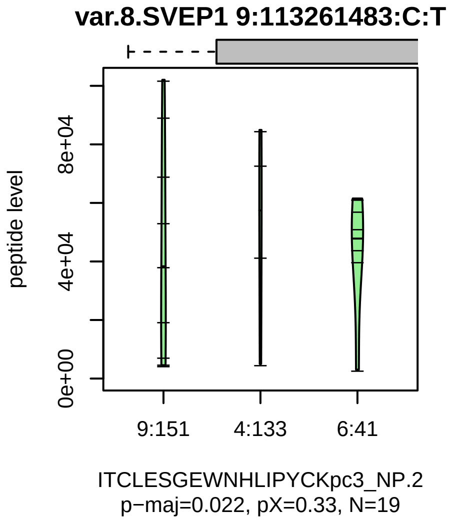
var.8.SVEP1 9:113261483:C:T
peptide level
9:151
4:133
6:41
ITCLESGEWNHLIPYCKpc3_NP.2
p−maj=0.022, pX=0.33, N=19

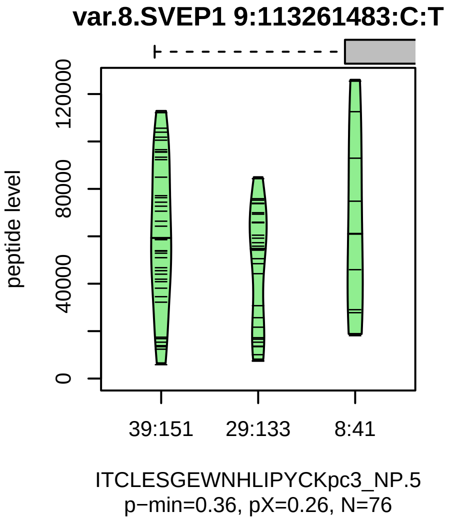
var.8.SVEP1 9:113261483:C:T
peptide level
39:151
29:133
8:41
ITCLESGEWNHLIPYCKpc3_NP.5
p−min=0.36, pX=0.26, N=76

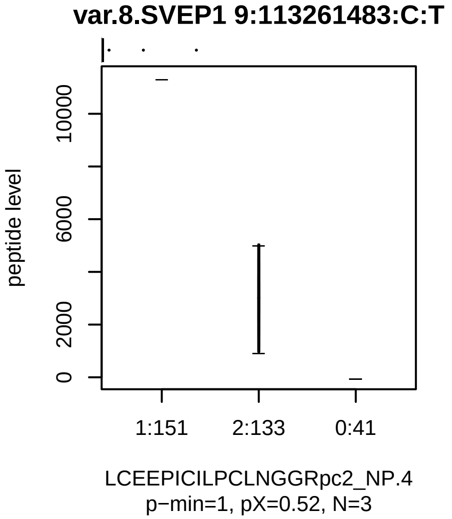
var.8.SVEP1 9:113261483:C:T
peptide level
1:151
2:133
0:41
LCEEPICILPCLNGGRpc2_NP.4
p−min=1, pX=0.52, N=3

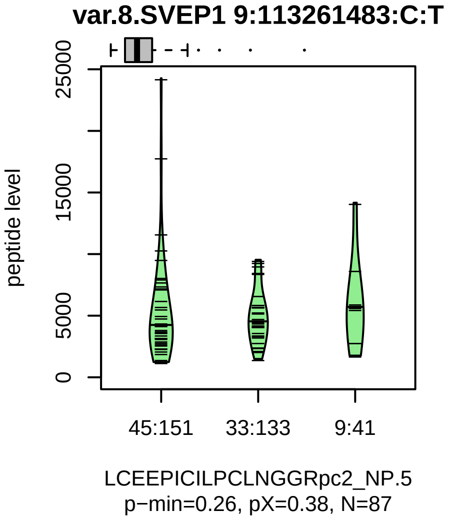
var.8.SVEP1 9:113261483:C:T
peptide level
45:151
33:133
9:41
LCEEPICILPCLNGGRpc2_NP.5
p−min=0.26, pX=0.38, N=87

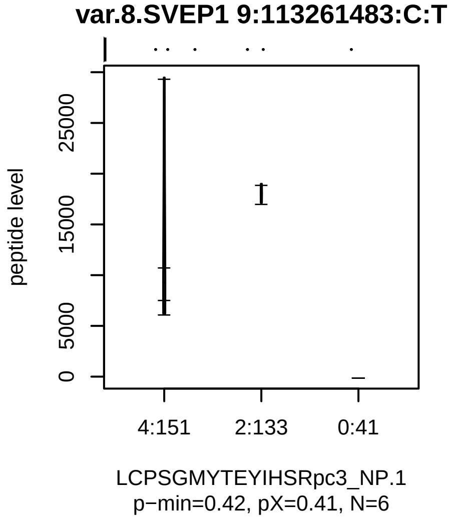
var.8.SVEP1 9:113261483:C:T
peptide level
4:151
2:133
0:41
LCPSGMYTEYIHSRpc3_NP.1
p−min=0.42, pX=0.41, N=6

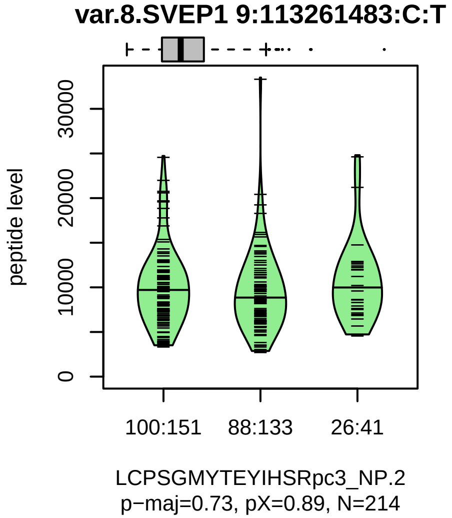
var.8.SVEP1 9:113261483:C:T
peptide level
100:151
88:133
26:41
LCPSGMYTEYIHSRpc3_NP.2
p−maj=0.73, pX=0.89, N=214

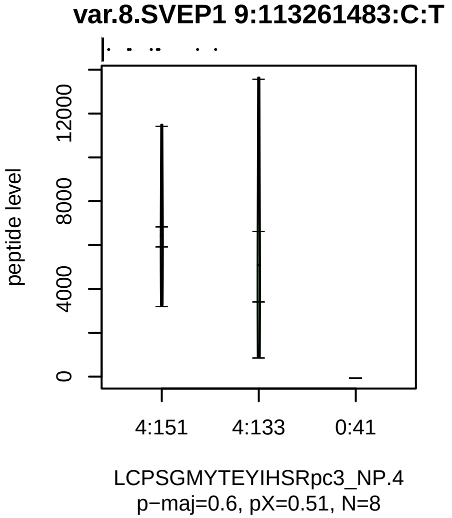
var.8.SVEP1 9:113261483:C:T
peptide level
4:151
4:133
0:41
LCPSGMYTEYIHSRpc3_NP.4
p−maj=0.6, pX=0.51, N=8

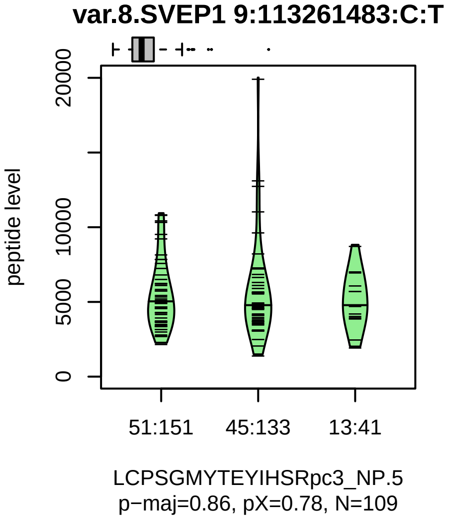
var.8.SVEP1 9:113261483:C:T
peptide level
51:151
45:133
13:41
LCPSGMYTEYIHSRpc3_NP.5
p−maj=0.86, pX=0.78, N=109

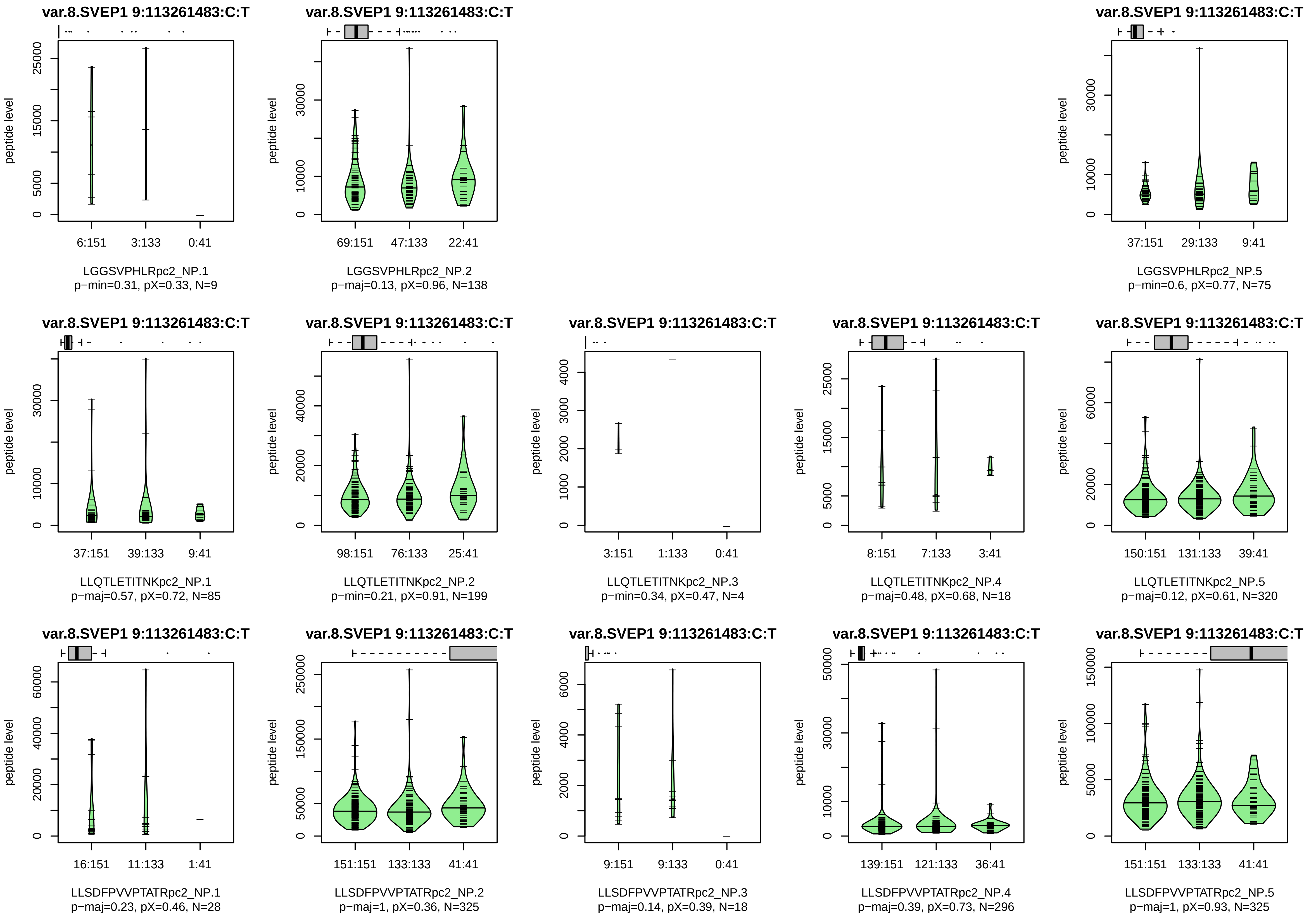
var.8.SVEP1 9:113261483:C:T
peptide level
6:151 3:133 0:41
LGGSVPHLRpc2_NP.1
p−min=0.31, pX=0.33, N=9
var.8.SVEP1 9:113261483:C:T
peptide level
69:151 47:133 22:41
LGGSVPHLRpc2_NP.2
p−maj=0.13, pX=0.96, N=138
var.8.SVEP1 9:113261483:C:T
peptide level
37:151 29:133 9:41
LGGSVPHLRpc2_NP.5
p−min=0.6, pX=0.77, N=75
var.8.SVEP1 9:113261483:C:T
peptide level
37:151 39:133 9:41
LLQTLETITNKpc2_NP.1
p−maj=0.57, pX=0.72, N=85
var.8.SVEP1 9:113261483:C:T
peptide level
98:151 76:133 25:41
LLQTLETITNKpc2_NP.2
p−min=0.21, pX=0.91, N=199
var.8.SVEP1 9:113261483:C:T
peptide level
3:151 1:133 0:41
LLQTLETITNKpc2_NP.3
p−min=0.34, pX=0.47, N=4
var.8.SVEP1 9:113261483:C:T
peptide level
8:151 7:133 3:41
LLQTLETITNKpc2_NP.4
p−maj=0.48, pX=0.68, N=18
var.8.SVEP1 9:113261483:C:T
peptide level
150:151 131:133 39:41
LLQTLETITNKpc2_NP.5
p−maj=0.12, pX=0.61, N=320
var.8.SVEP1 9:113261483:C:T
peptide level
16:151 11:133 1:41
LLSDFPVVPTATRpc2_NP.1
p−maj=0.23, pX=0.46, N=28
var.8.SVEP1 9:113261483:C:T
peptide level
151:151 133:133 41:41
LLSDFPVVPTATRpc2_NP.2
p−maj=1, pX=0.36, N=325
var.8.SVEP1 9:113261483:C:T
peptide level
9:151 9:133 0:41
LLSDFPVVPTATRpc2_NP.3
p−maj=0.14, pX=0.39, N=18
var.8.SVEP1 9:113261483:C:T
peptide level
139:151 121:133 36:41
LLSDFPVVPTATRpc2_NP.4
p−maj=0.39, pX=0.73, N=296
var.8.SVEP1 9:113261483:C:T
peptide level
151:151 133:133 41:41
LLSDFPVVPTATRpc2_NP.5
p−maj=1, pX=0.93, N=325

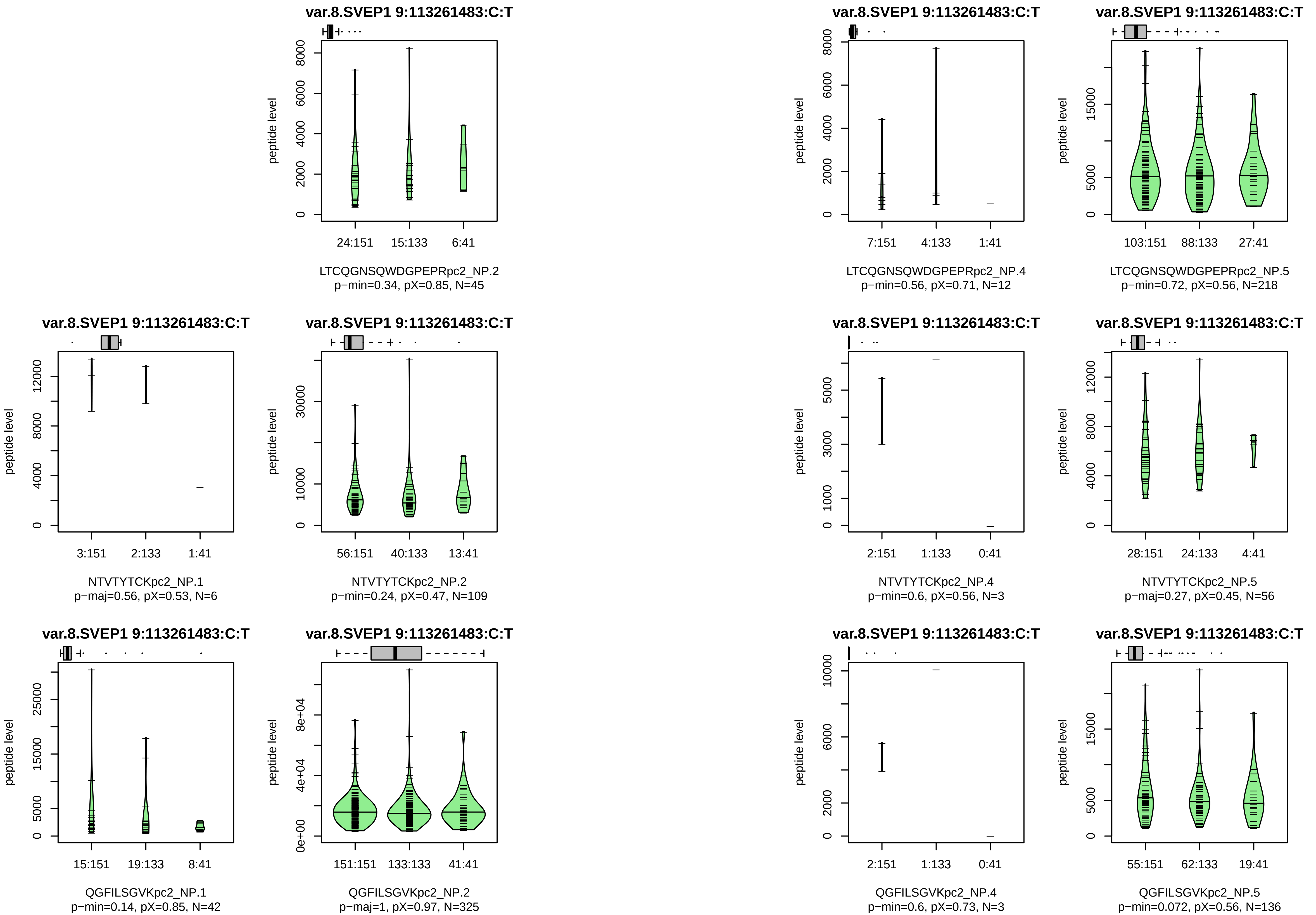
var.8.SVEP1 9:113261483:C:T
peptide level
0
2000
4000
6000
8000
24:151
15:133
6:41
LTCQGNSQWDGPEPRpc2_NP.2
p−min=0.34, pX=0.85, N=45
var.8.SVEP1 9:113261483:C:T
peptide level
0
2000
4000
6000
8000
7:151
4:133
1:41
LTCQGNSQWDGPEPRpc2_NP.4
p−min=0.56, pX=0.71, N=12
var.8.SVEP1 9:113261483:C:T
peptide level
0
5000
15000
103:151
88:133
27:41
LTCQGNSQWDGPEPRpc2_NP.5
p−min=0.72, pX=0.56, N=218
var.8.SVEP1 9:113261483:C:T
peptide level
0
4000
8000
12000
3:151
2:133
1:41
NTVTYTCKpc2_NP.1
p−maj=0.56, pX=0.53, N=6
var.8.SVEP1 9:113261483:C:T
peptide level
0
10000
30000
56:151
40:133
13:41
NTVTYTCKpc2_NP.2
p−min=0.24, pX=0.47, N=109
var.8.SVEP1 9:113261483:C:T
peptide level
0
1000
3000
5000
2:151
1:133
0:41
NTVTYTCKpc2_NP.4
p−min=0.6, pX=0.56, N=3
var.8.SVEP1 9:113261483:C:T
peptide level
0
4000
8000
12000
28:151
24:133
4:41
NTVTYTCKpc2_NP.5
p−maj=0.27, pX=0.45, N=56
var.8.SVEP1 9:113261483:C:T
peptide level
0
5000
15000
25000
15:151
19:133
8:41
QGFILSGVKpc2_NP.1
p−min=0.14, pX=0.85, N=42
var.8.SVEP1 9:113261483:C:T
peptide level
0e+00
4e+04
8e+04
151:151
133:133
41:41
QGFILSGVKpc2_NP.2
p−maj=1, pX=0.97, N=325
var.8.SVEP1 9:113261483:C:T
peptide level
0
2000
6000
10000
2:151
1:133
0:41
QGFILSGVKpc2_NP.4
p−min=0.6, pX=0.73, N=3
var.8.SVEP1 9:113261483:C:T
peptide level
0
5000
15000
55:151
62:133
19:41
QGFILSGVKpc2_NP.5
p−min=0.072, pX=0.56, N=136

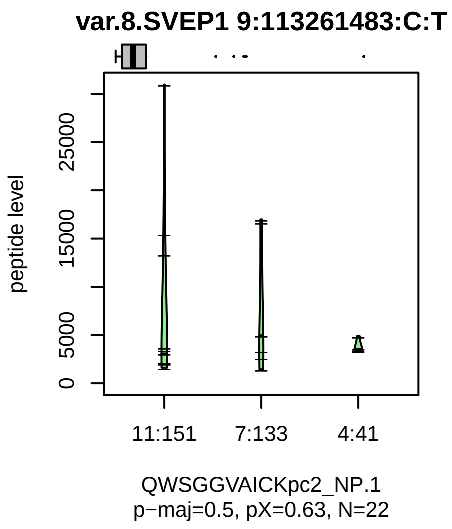
var.8.SVEP1 9:113261483:C:T
peptide level
0
5000
15000
25000
11:151
7:133
4:41
QWSGGVAICKpc2_NP.1
p−maj=0.5, pX=0.63, N=22

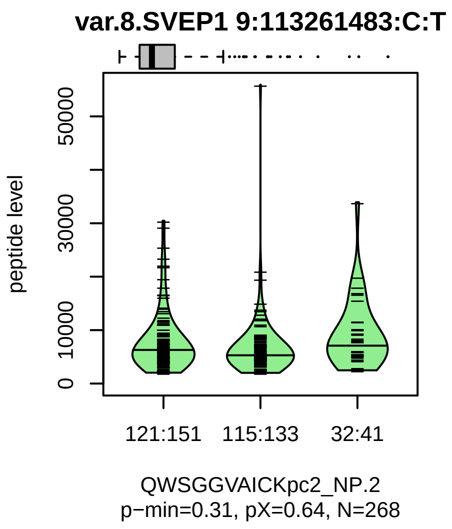
var.8.SVEP1 9:113261483:C:T
peptide level
0
10000
30000
50000
121:151
115:133
32:41
QWSGGVAICKpc2_NP.2
p−min=0.31, pX=0.64, N=268

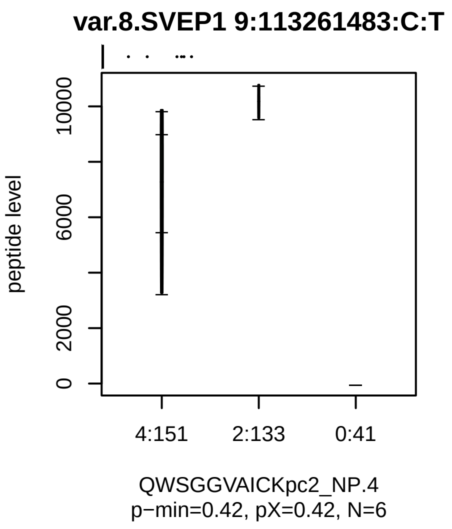
var.8.SVEP1 9:113261483:C:T
peptide level
0
2000
6000
10000
4:151
2:133
0:41
QWSGGVAICKpc2_NP.4
p−min=0.42, pX=0.42, N=6

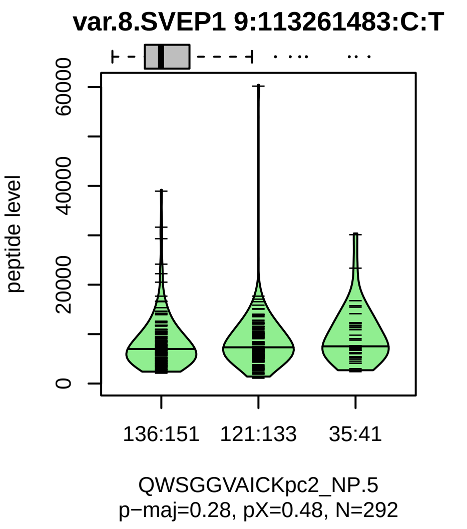
var.8.SVEP1 9:113261483:C:T
peptide level
0
20000
40000
60000
136:151
121:133
35:41
QWSGGVAICKpc2_NP.5
p−maj=0.28, pX=0.48, N=292

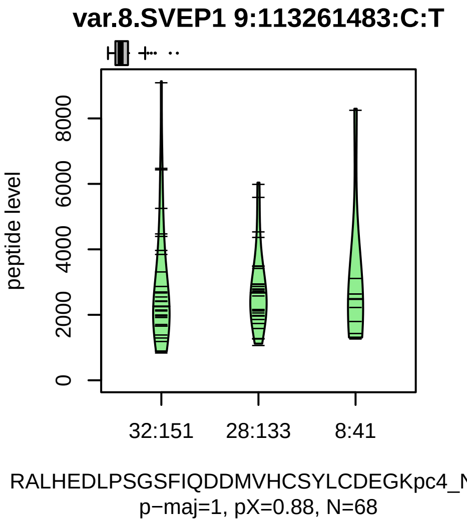
var.8.SVEP1 9:113261483:C:T
peptide level
0
2000
4000
6000
8000
32:151
28:133
8:41
RALHEDLPSGSFIQDDMVHCSYLCDEGKpc4_N
p−maj=1, pX=0.88, N=68

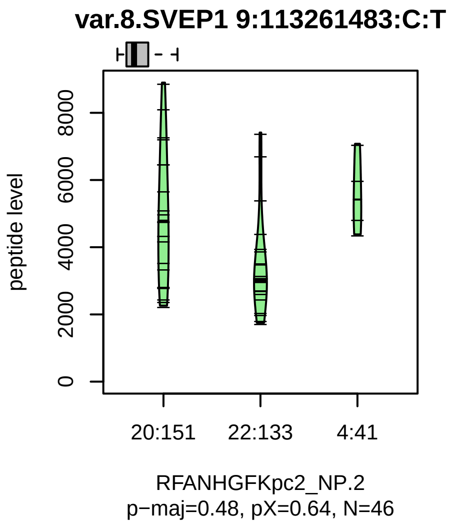
var.8.SVEP1 9:113261483:C:T
peptide level
0
2000
4000
6000
8000
20:151
22:133
4:41
RFANHGFKpc2_NP.2
p−maj=0.48, pX=0.64, N=46

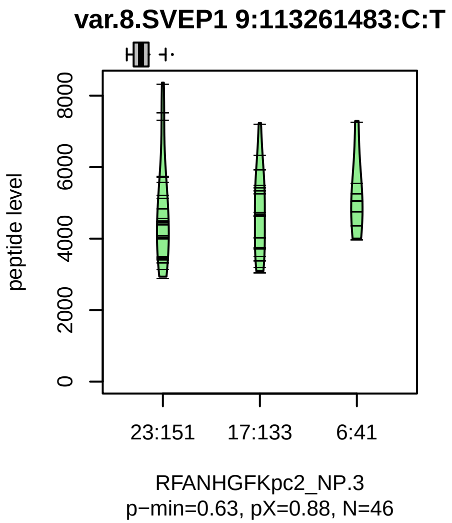
var.8.SVEP1 9:113261483:C:T
peptide level
0
2000
4000
6000
8000
23:151
17:133
6:41
RFANHGFKpc2_NP.3
p−min=0.63, pX=0.88, N=46

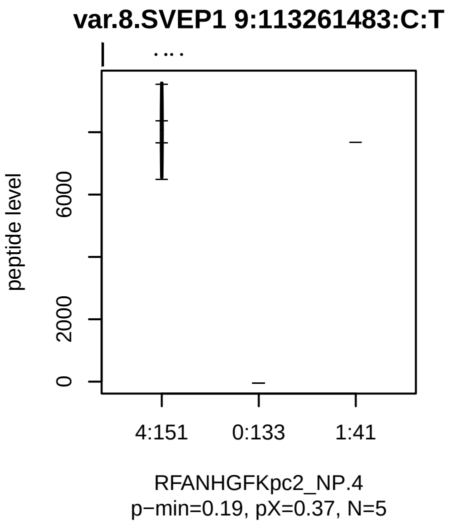
var.8.SVEP1 9:113261483:C:T
peptide level
0
2000
6000
4:151
0:133
1:41
RFANHGFKpc2_NP.4
p−min=0.19, pX=0.37, N=5

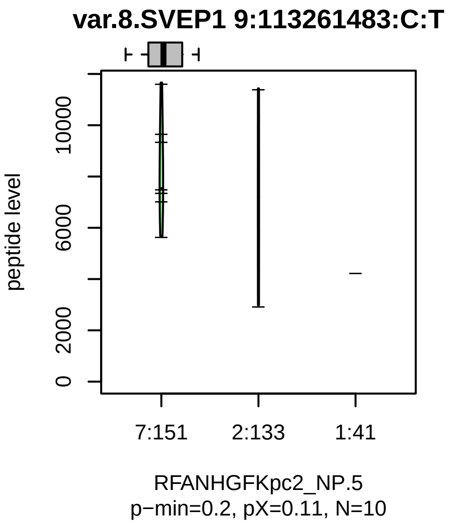
var.8.SVEP1 9:113261483:C:T
peptide level
0
2000
6000
10000
7:151
2:133
1:41
RFANHGFKpc2_NP.5
p−min=0.2, pX=0.11, N=10

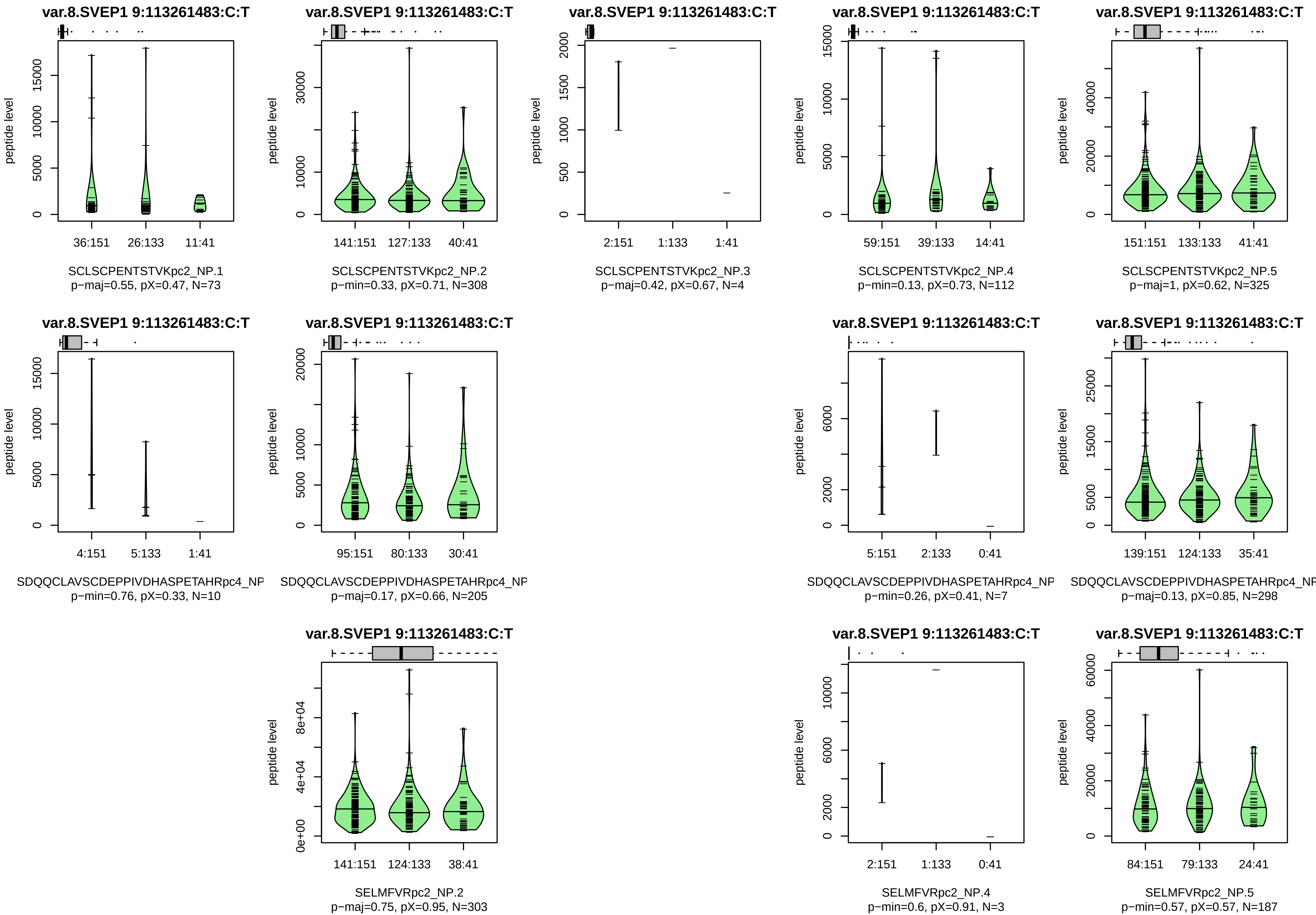
var.8.SVEP1 9:113261483:C:T
peptide level
36:151
26:133
11:41
SCLSCPENTSTVKpc2_NP.1
p−maj=0.55, pX=0.47, N=73
var.8.SVEP1 9:113261483:C:T
peptide level
141:151
127:133
40:41
SCLSCPENTSTVKpc2_NP.2
p−min=0.33, pX=0.71, N=308
var.8.SVEP1 9:113261483:C:T
peptide level
2:151
1:133
1:41
SCLSCPENTSTVKpc2_NP.3
p−maj=0.42, pX=0.67, N=4
var.8.SVEP1 9:113261483:C:T
peptide level
59:151
39:133
14:41
SCLSCPENTSTVKpc2_NP.4
p−min=0.13, pX=0.73, N=112
var.8.SVEP1 9:113261483:C:T
peptide level
151:151
133:133
41:41
SCLSCPENTSTVKpc2_NP.5
p−maj=1, pX=0.62, N=325
var.8.SVEP1 9:113261483:C:T
peptide level
4:151
5:133
1:41
SDQQCLAVSCDEPPIVDHASPETAHRpc4_NP
p−min=0.76, pX=0.33, N=10
var.8.SVEP1 9:113261483:C:T
peptide level
95:151
80:133
30:41
SDQQCLAVSCDEPPIVDHASPETAHRpc4_NP
p−maj=0.17, pX=0.66, N=205
var.8.SVEP1 9:113261483:C:T
peptide level
5:151
2:133
0:41
SDQQCLAVSCDEPPIVDHASPETAHRpc4_NP
p−min=0.26, pX=0.41, N=7
var.8.SVEP1 9:113261483:C:T
peptide level
139:151
124:133
35:41
SDQQCLAVSCDEPPIVDHASPETAHRpc4_NP
p−maj=0.13, pX=0.85, N=298
var.8.SVEP1 9:113261483:C:T
peptide level
141:151
124:133
38:41
SELMFVRpc2_NP.2
p−maj=0.75, pX=0.95, N=303
var.8.SVEP1 9:113261483:C:T
peptide level
2:151
1:133
0:41
SELMFVRpc2_NP.4
p−min=0.6, pX=0.91, N=3
var.8.SVEP1 9:113261483:C:T
peptide level
84:151
79:133
24:41
SELMFVRpc2_NP.5
p−min=0.57, pX=0.57, N=187

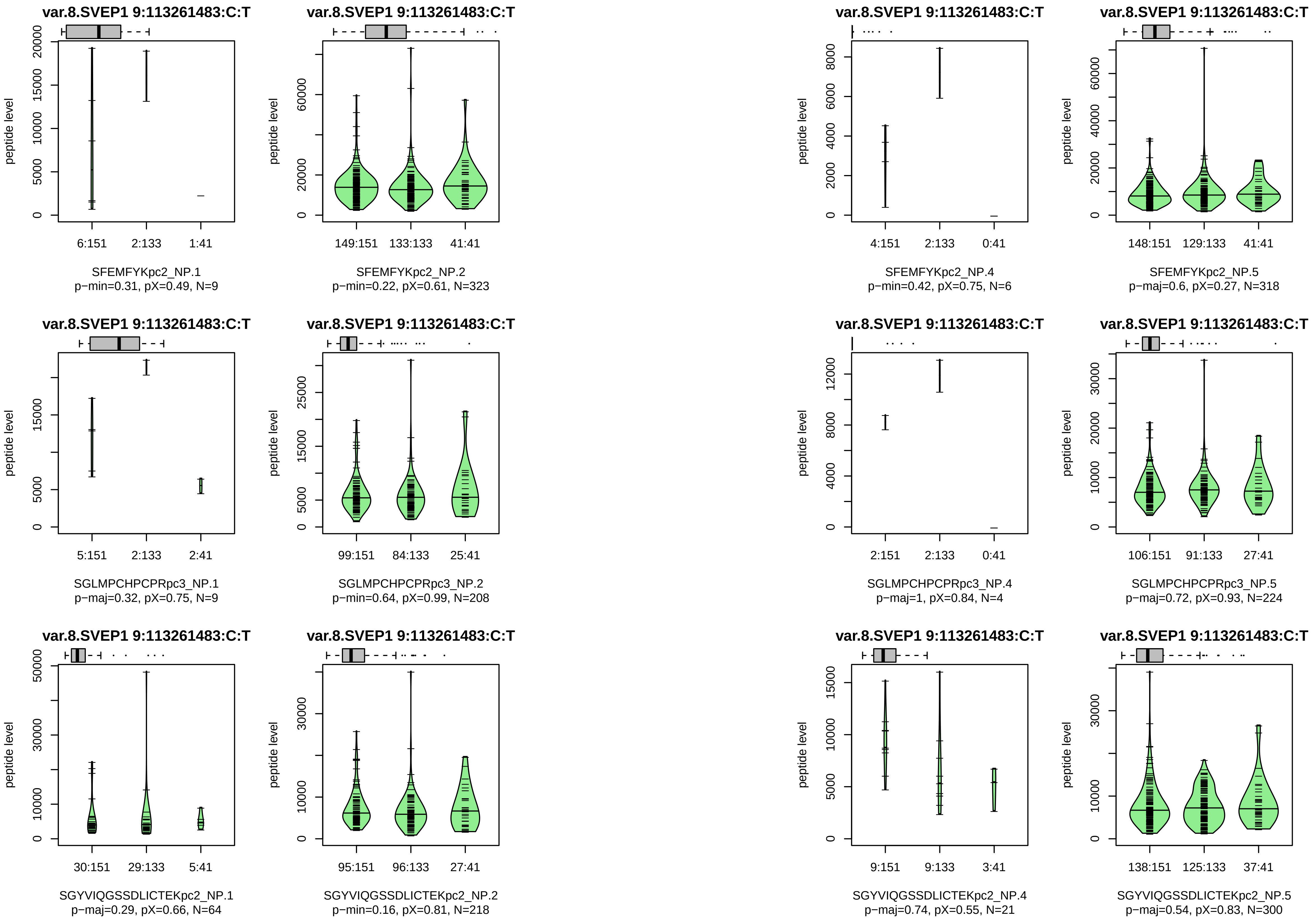
var.8.SVEP1 9:113261483:C:T
peptide level
6:151
2:133
1:41
SFEMFYKpc2_NP.1
p−min=0.31, pX=0.49, N=9
var.8.SVEP1 9:113261483:C:T
peptide level
149:151
133:133
41:41
SFEMFYKpc2_NP.2
p−min=0.22, pX=0.61, N=323
var.8.SVEP1 9:113261483:C:T
peptide level
4:151
2:133
0:41
SFEMFYKpc2_NP.4
p−min=0.42, pX=0.75, N=6
var.8.SVEP1 9:113261483:C:T
peptide level
148:151
129:133
41:41
SFEMFYKpc2_NP.5
p−maj=0.6, pX=0.27, N=318
var.8.SVEP1 9:113261483:C:T
peptide level
5:151
2:133
2:41
SGLMPCHPCPRpc3_NP.1
p−maj=0.32, pX=0.75, N=9
var.8.SVEP1 9:113261483:C:T
peptide level
99:151
84:133
25:41
SGLMPCHPCPRpc3_NP.2
p−min=0.64, pX=0.99, N=208
var.8.SVEP1 9:113261483:C:T
peptide level
2:151
2:133
0:41
SGLMPCHPCPRpc3_NP.4
p−maj=1, pX=0.84, N=4
var.8.SVEP1 9:113261483:C:T
peptide level
106:151
91:133
27:41
SGLMPCHPCPRpc3_NP.5
p−maj=0.72, pX=0.93, N=224
var.8.SVEP1 9:113261483:C:T
peptide level
30:151
29:133
5:41
SGYVIQGSSDLICTEKpc2_NP.1
p−maj=0.29, pX=0.66, N=64
var.8.SVEP1 9:113261483:C:T
peptide level
95:151
96:133
27:41
SGYVIQGSSDLICTEKpc2_NP.2
p−min=0.16, pX=0.81, N=218
var.8.SVEP1 9:113261483:C:T
peptide level
9:151
9:133
3:41
SGYVIQGSSDLICTEKpc2_NP.4
p−maj=0.74, pX=0.55, N=21
var.8.SVEP1 9:113261483:C:T
peptide level
138:151
125:133
37:41
SGYVIQGSSDLICTEKpc2_NP.5
p−maj=0.54, pX=0.83, N=300

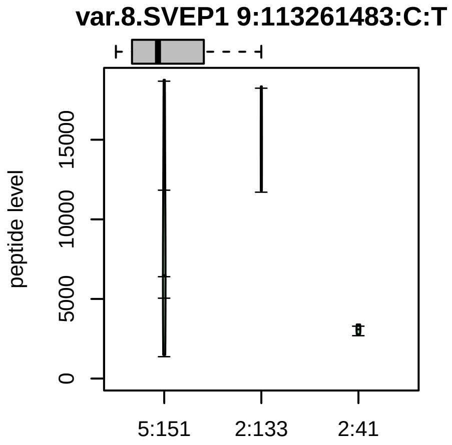

SIFCSDCPRpc2_NP.1
p−maj=0.32, pX=0.64, N=9

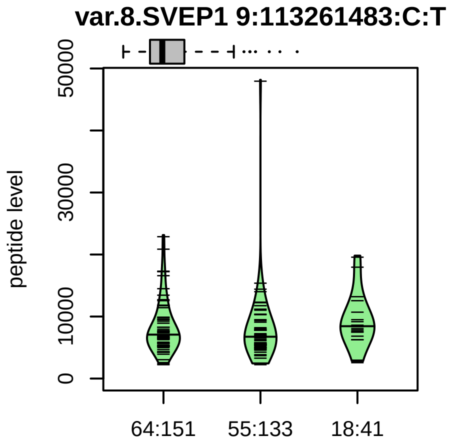

SIFCSDCPRpc2_NP.2
p−maj=0.87, pX=0.68, N=137

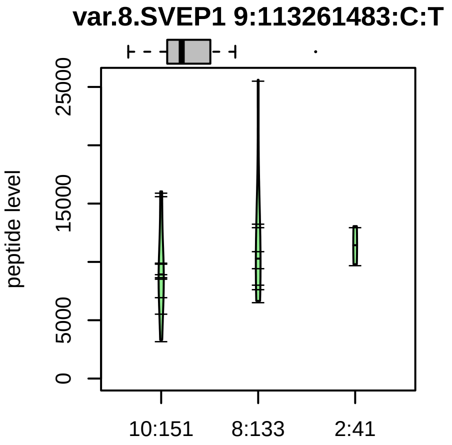

SIFCSDCPRpc2_NP.5
p−min=0.82, pX=0.99, N=20

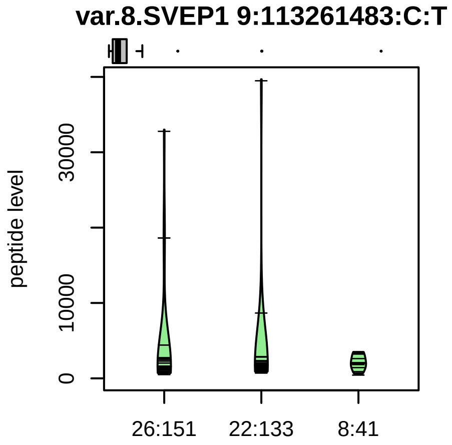

SLATSCPEELSKpc2_NP.1
p−maj=0.66, pX=0.6, N=56

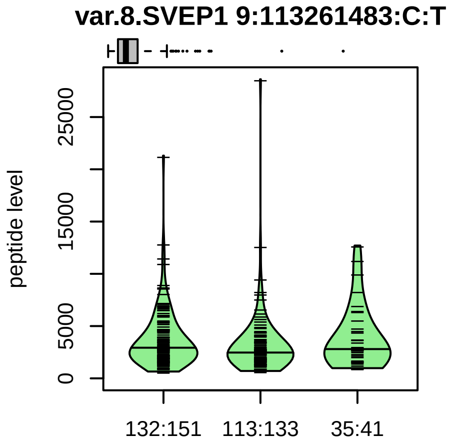

SLATSCPEELSKpc2_NP.2
p−min=0.63, pX=0.59, N=280

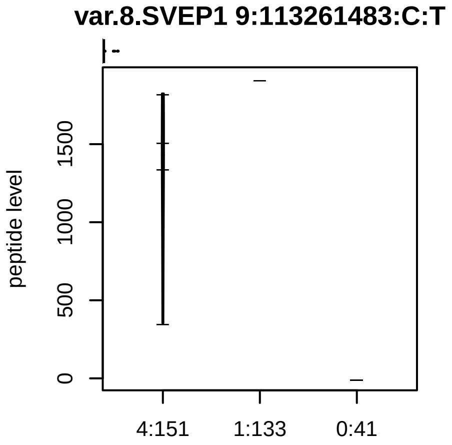

SLATSCPEELSKpc2_NP.3
p−min=0.19, pX=0.24, N=5

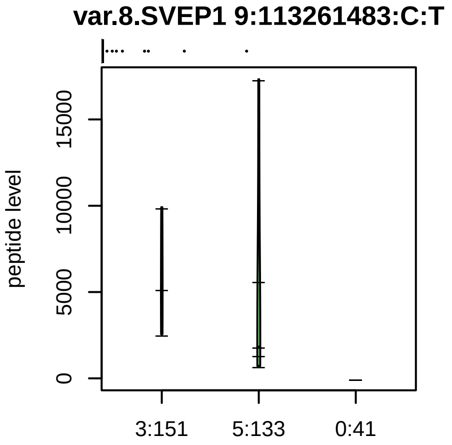

SLATSCPEELSKpc2_NP.4
p−maj=0.6, pX=0.86, N=8

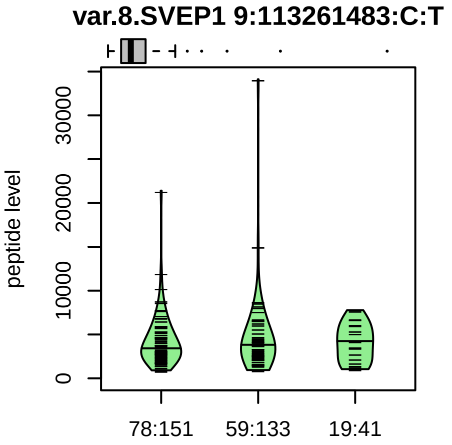

SLATSCPEELSKpc2_NP.5
p−min=0.22, pX=0.51, N=156

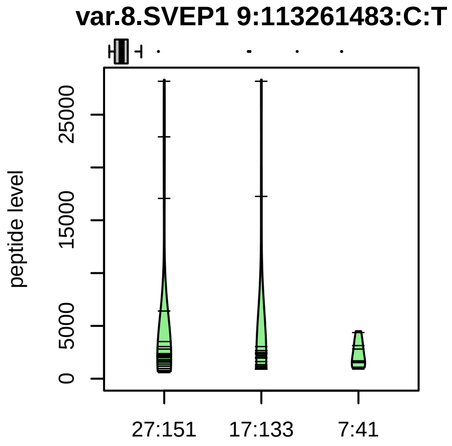

SPPPVQVSEKpc2_NP.1
p−min=0.36, pX=0.39, N=51

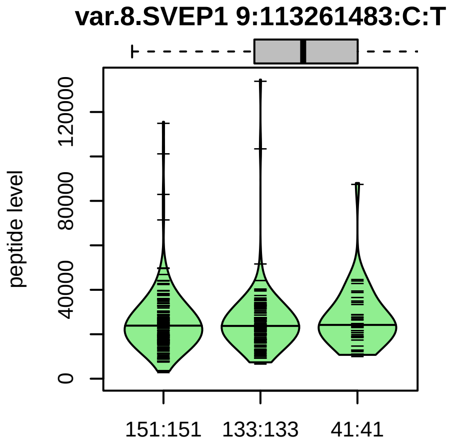

SPPPVQVSEKpc2_NP.2
p−maj=1, pX=0.81, N=325

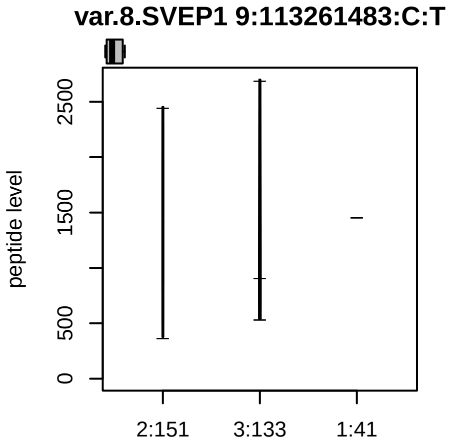

SPPPVQVSEKpc2_NP.3
p−maj=0.56, pX=0.59, N=6

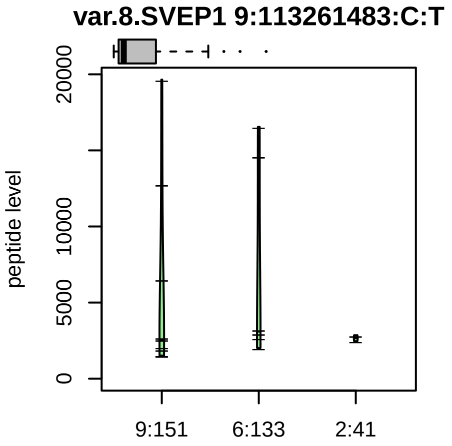

SPPPVQVSEKpc2_NP.4
p−min=0.62, pX=0.59, N=17

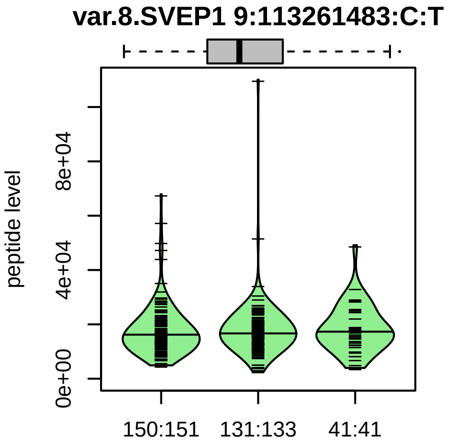

SPPPVQVSEKpc2_NP.5
p−min=1, pX=0.63, N=322

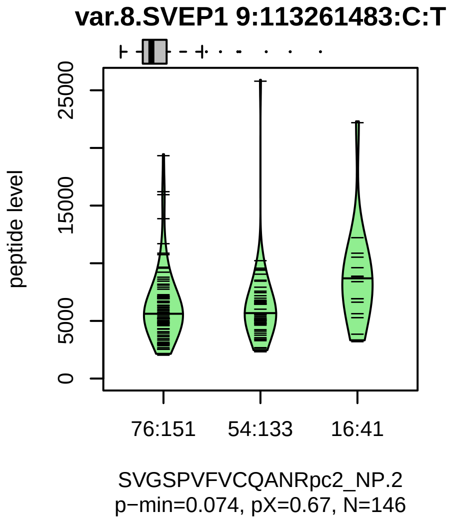
var.8.SVEP1 9:113261483:C:T
peptide level
0
5000
15000
25000
76:151
54:133
16:41
SVGSPVFVCQANRpc2_NP.2
p−min=0.074, pX=0.67, N=146
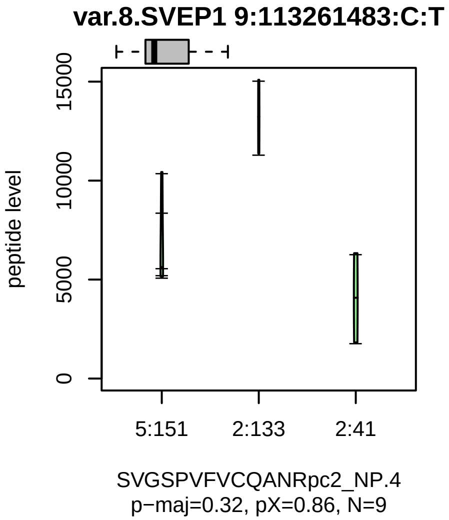
var.8.SVEP1 9:113261483:C:T
peptide level
0
5000
10000
15000
5:151
2:133
2:41
SVGSPVFVCQANRpc2_NP.4
p−maj=0.32, pX=0.86, N=9
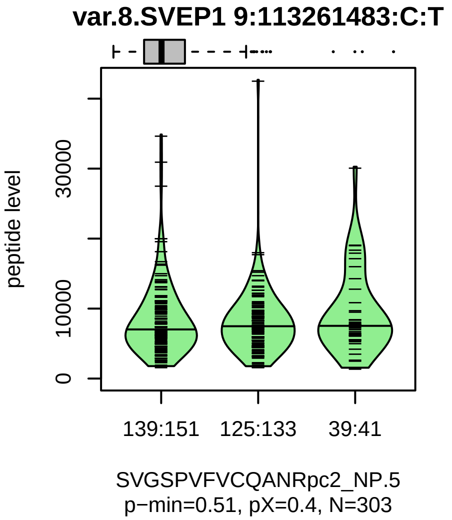
var.8.SVEP1 9:113261483:C:T
peptide level
0
10000
30000
139:151
125:133
39:41
SVGSPVFVCQANRpc2_NP.5
p−min=0.51, pX=0.4, N=303
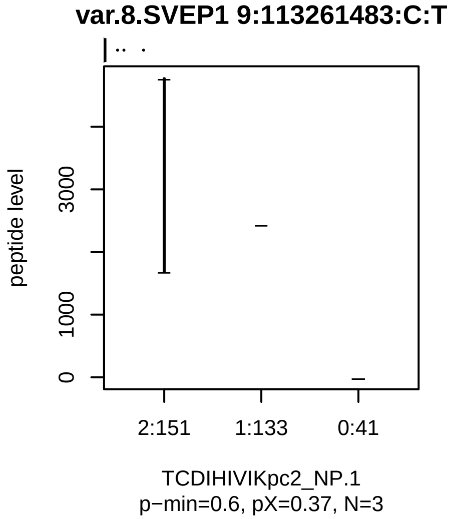
var.8.SVEP1 9:113261483:C:T
peptide level
0
1000
3000
2:151
1:133
0:41
TCDIHIVIKpc2_NP.1
p−min=0.6, pX=0.37, N=3
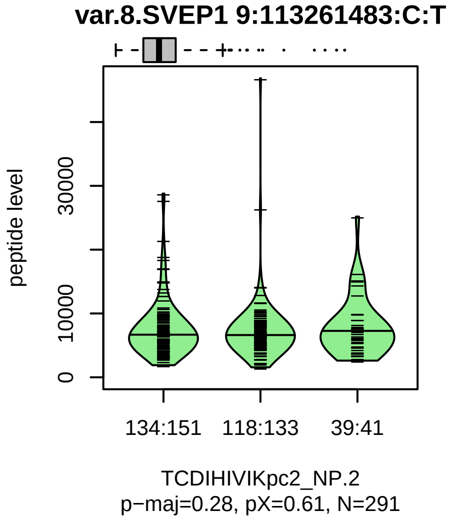
var.8.SVEP1 9:113261483:C:T
peptide level
0
10000
30000
134:151
118:133
39:41
TCDIHIVIKpc2_NP.2
p−maj=0.28, pX=0.61, N=291
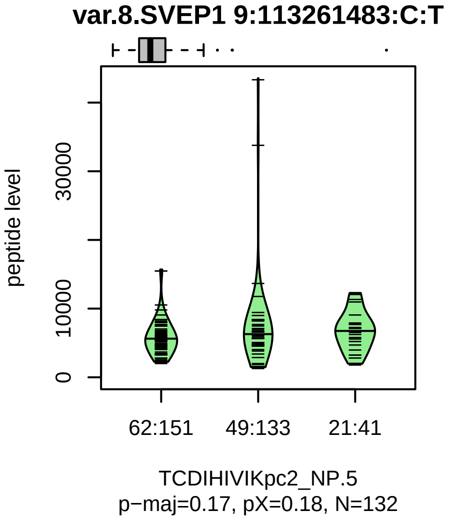
var.8.SVEP1 9:113261483:C:T
peptide level
0
10000
30000
62:151
49:133
21:41
TCDIHIVIKpc2_NP.5
p−maj=0.17, pX=0.18, N=132
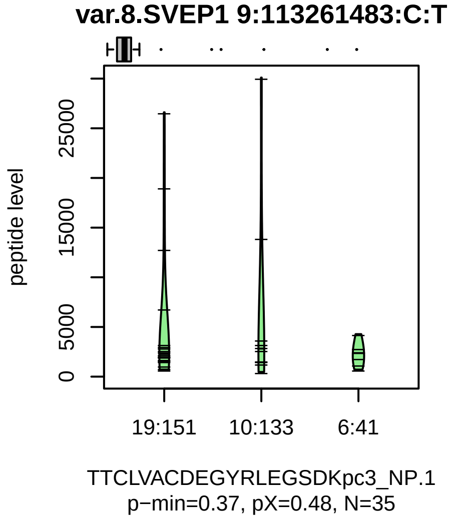
var.8.SVEP1 9:113261483:C:T
peptide level
0
5000
15000
25000
19:151
10:133
6:41
TTCLVACDEGYRLEGSDKpc3_NP.1
p−min=0.37, pX=0.48, N=35
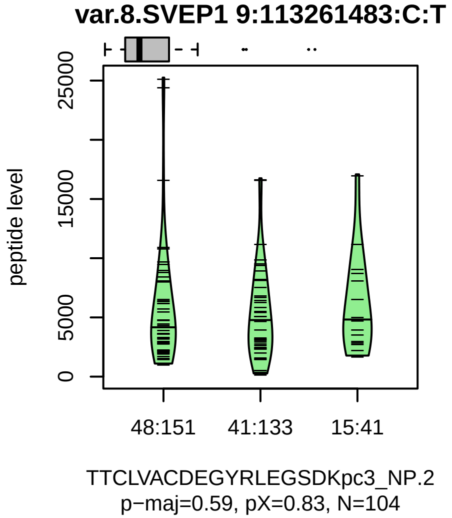
var.8.SVEP1 9:113261483:C:T
peptide level
0
5000
15000
25000
48:151
41:133
15:41
TTCLVACDEGYRLEGSDKpc3_NP.2
p−maj=0.59, pX=0.83, N=104
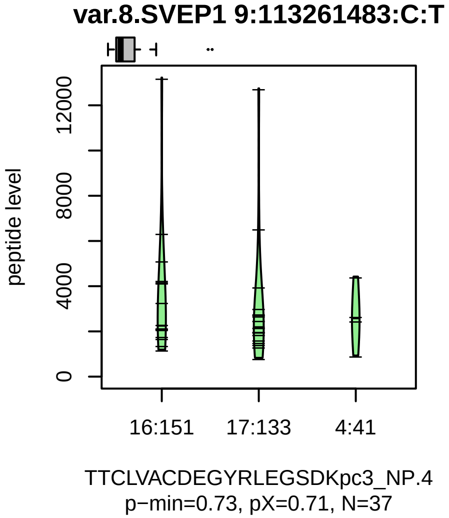
var.8.SVEP1 9:113261483:C:T
peptide level
0
4000
8000
12000
16:151
17:133
4:41
TTCLVACDEGYRLEGSDKpc3_NP.4
p−min=0.73, pX=0.71, N=37
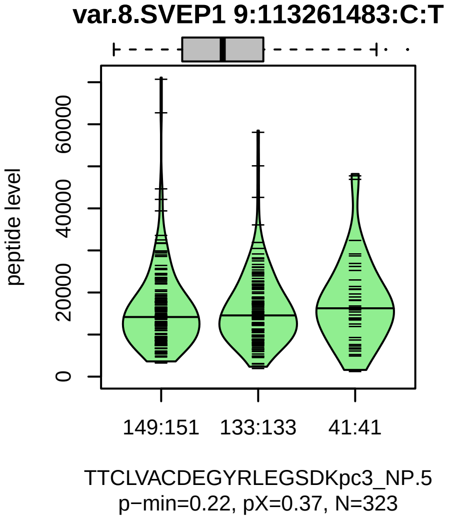
var.8.SVEP1 9:113261483:C:T
peptide level
0
20000
40000
60000
149:151
133:133
41:41
TTCLVACDEGYRLEGSDKpc3_NP.5
p−min=0.22, pX=0.37, N=323

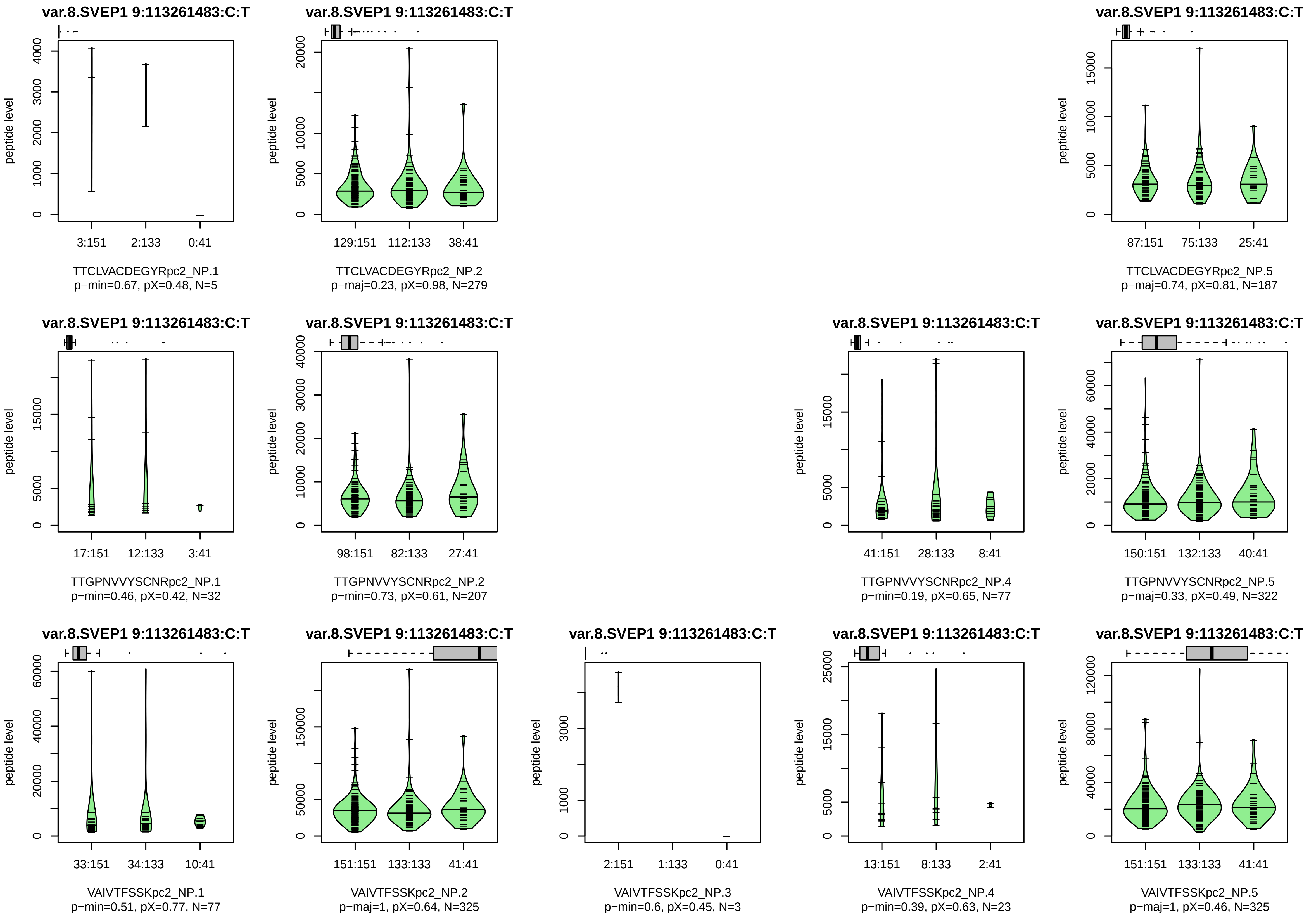

var.8.SVEP1 9:113261483:C:T
peptide level
3:151 2:133 0:41
TTCLVACDEGYRpc2_NP.1
p−min=0.67, pX=0.48, N=5
var.8.SVEP1 9:113261483:C:T
peptide level
129:151 112:133 38:41
TTCLVACDEGYRpc2_NP.2
p−maj=0.23, pX=0.98, N=279
var.8.SVEP1 9:113261483:C:T
peptide level
87:151 75:133 25:41
TTCLVACDEGYRpc2_NP.5
p−maj=0.74, pX=0.81, N=187
var.8.SVEP1 9:113261483:C:T
peptide level
17:151 12:133 3:41
TTGPNVVYSCNRpc2_NP.1
p−min=0.46, pX=0.42, N=32
var.8.SVEP1 9:113261483:C:T
peptide level
98:151 82:133 27:41
TTGPNVVYSCNRpc2_NP.2
p−min=0.73, pX=0.61, N=207
var.8.SVEP1 9:113261483:C:T
peptide level
41:151 28:133 8:41
TTGPNVVYSCNRpc2_NP.4
p−min=0.19, pX=0.65, N=77
var.8.SVEP1 9:113261483:C:T
peptide level
150:151 132:133 40:41
TTGPNVVYSCNRpc2_NP.5
p−maj=0.33, pX=0.49, N=322
var.8.SVEP1 9:113261483:C:T
peptide level
33:151 34:133 10:41
VAIVTFSSKpc2_NP.1
p−min=0.51, pX=0.77, N=77
var.8.SVEP1 9:113261483:C:T
peptide level
151:151 133:133 41:41
VAIVTFSSKpc2_NP.2
p−maj=1, pX=0.64, N=325
var.8.SVEP1 9:113261483:C:T
peptide level
2:151 1:133 0:41
VAIVTFSSKpc2_NP.3
p−min=0.6, pX=0.45, N=3
var.8.SVEP1 9:113261483:C:T
peptide level
13:151 8:133 2:41
VAIVTFSSKpc2_NP.4
p−min=0.39, pX=0.63, N=23
var.8.SVEP1 9:113261483:C:T
peptide level
151:151 133:133 41:41
VAIVTFSSKpc2_NP.5
p−maj=1, pX=0.46, N=325

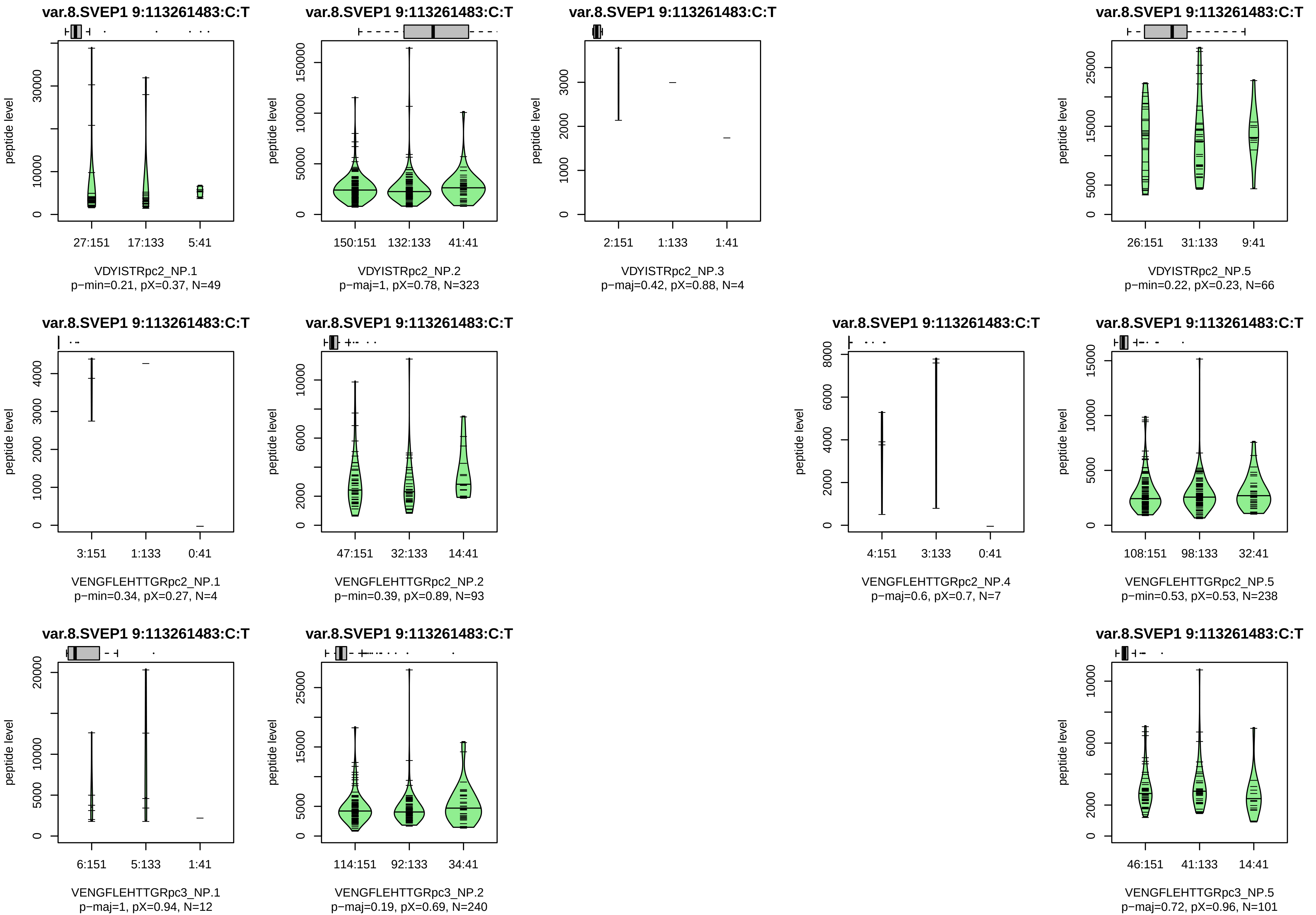
var.8.SVEP1 9:113261483:C:T
peptide level
27:151
17:133
5:41
VDYISTRpc2_NP.1
p−min=0.21, pX=0.37, N=49
var.8.SVEP1 9:113261483:C:T
150:151
132:133
41:41
VDYISTRpc2_NP.2
p−maj=1, pX=0.78, N=323
var.8.SVEP1 9:113261483:C:T
2:151
1:133
1:41
VDYISTRpc2_NP.3
p−maj=0.42, pX=0.88, N=4
var.8.SVEP1 9:113261483:C:T
26:151
31:133
9:41
VDYISTRpc2_NP.5
p−min=0.22, pX=0.23, N=66
var.8.SVEP1 9:113261483:C:T
3:151
1:133
0:41
VENGFLEHTTGRpc2_NP.1
p−min=0.34, pX=0.27, N=4
var.8.SVEP1 9:113261483:C:T
47:151
32:133
14:41
VENGFLEHTTGRpc2_NP.2
p−min=0.39, pX=0.89, N=93
var.8.SVEP1 9:113261483:C:T
4:151
3:133
0:41
VENGFLEHTTGRpc2_NP.4
p−maj=0.6, pX=0.7, N=7
var.8.SVEP1 9:113261483:C:T
108:151
98:133
32:41
VENGFLEHTTGRpc2_NP.5
p−min=0.53, pX=0.53, N=238
var.8.SVEP1 9:113261483:C:T
6:151
5:133
1:41
VENGFLEHTTGRpc3_NP.1
p−maj=1, pX=0.94, N=12
var.8.SVEP1 9:113261483:C:T
114:151
92:133
34:41
VENGFLEHTTGRpc3_NP.2
p−maj=0.19, pX=0.69, N=240
var.8.SVEP1 9:113261483:C:T
46:151
41:133
14:41
VENGFLEHTTGRpc3_NP.5
p−maj=0.72, pX=0.96, N=101

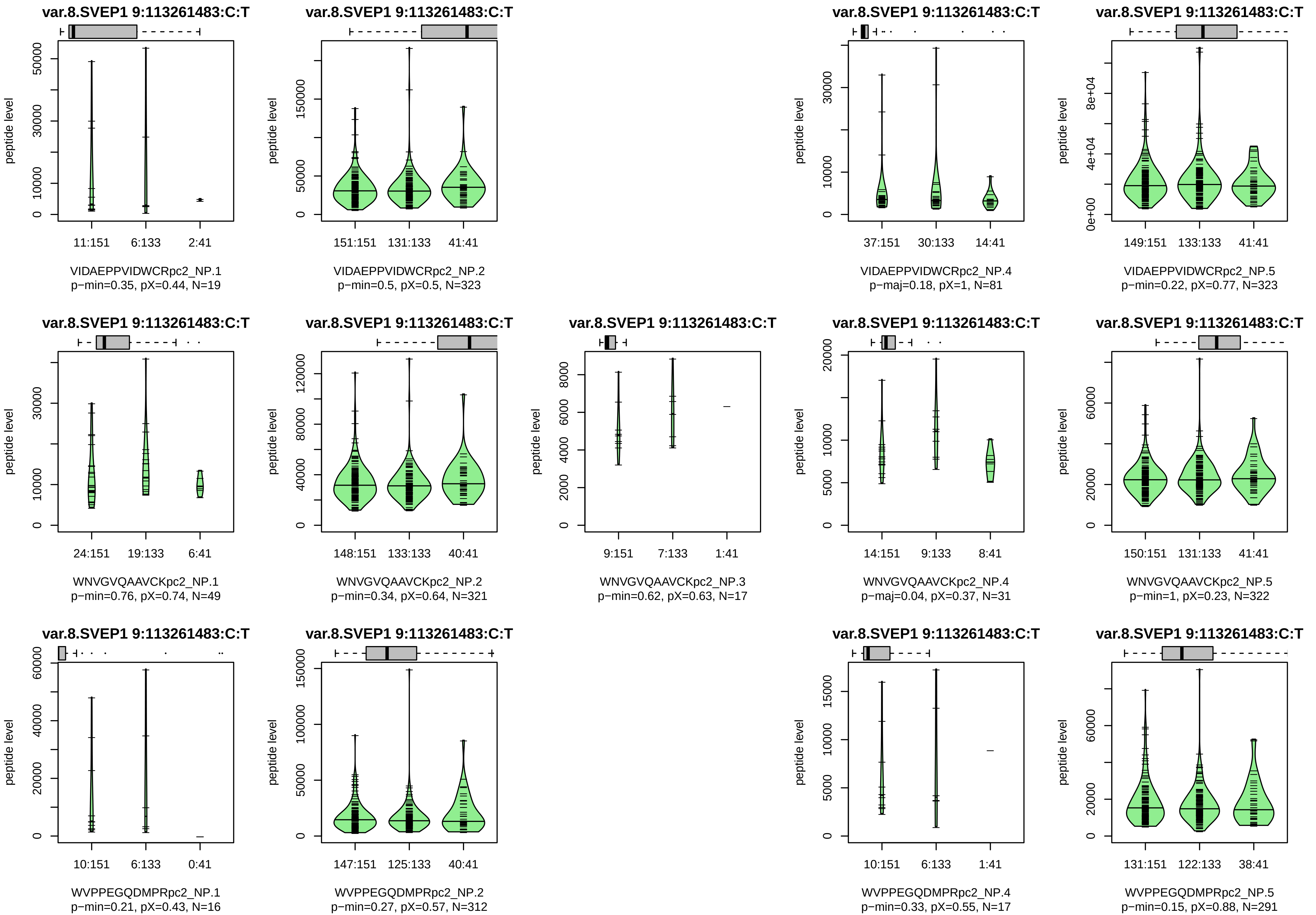
var.8.SVEP1 9:113261483:C:T
peptide level
11:151
6:133
2:41
VIDAEPPVIDWCRpc2_NP.1
p−min=0.35, pX=0.44, N=19
var.8.SVEP1 9:113261483:C:T
peptide level
151:151
131:133
41:41
VIDAEPPVIDWCRpc2_NP.2
p−min=0.5, pX=0.5, N=323
var.8.SVEP1 9:113261483:C:T
peptide level
37:151
30:133
14:41
VIDAEPPVIDWCRpc2_NP.4
p−maj=0.18, pX=1, N=81
var.8.SVEP1 9:113261483:C:T
peptide level
149:151
133:133
41:41
VIDAEPPVIDWCRpc2_NP.5
p−min=0.22, pX=0.77, N=323
var.8.SVEP1 9:113261483:C:T
peptide level
24:151
19:133
6:41
WNVGVQAAVCKpc2_NP.1
p−min=0.76, pX=0.74, N=49
var.8.SVEP1 9:113261483:C:T
peptide level
148:151
133:133
40:41
WNVGVQAAVCKpc2_NP.2
p−min=0.34, pX=0.64, N=321
var.8.SVEP1 9:113261483:C:T
peptide level
9:151
7:133
1:41
WNVGVQAAVCKpc2_NP.3
p−min=0.62, pX=0.63, N=17
var.8.SVEP1 9:113261483:C:T
peptide level
14:151
9:133
8:41
WNVGVQAAVCKpc2_NP.4
p−maj=0.04, pX=0.37, N=31
var.8.SVEP1 9:113261483:C:T
peptide level
150:151
131:133
41:41
WNVGVQAAVCKpc2_NP.5
p−min=1, pX=0.23, N=322
var.8.SVEP1 9:113261483:C:T
peptide level
10:151
6:133
0:41
WVPPEGQDMPRpc2_NP.1
p−min=0.21, pX=0.43, N=16
var.8.SVEP1 9:113261483:C:T
peptide level
147:151
125:133
40:41
WVPPEGQDMPRpc2_NP.2
p−min=0.27, pX=0.57, N=312
var.8.SVEP1 9:113261483:C:T
peptide level
10:151
6:133
1:41
WVPPEGQDMPRpc2_NP.4
p−min=0.33, pX=0.55, N=17
var.8.SVEP1 9:113261483:C:T
peptide level
131:151
122:133
38:41
WVPPEGQDMPRpc2_NP.5
p−min=0.15, pX=0.88, N=291

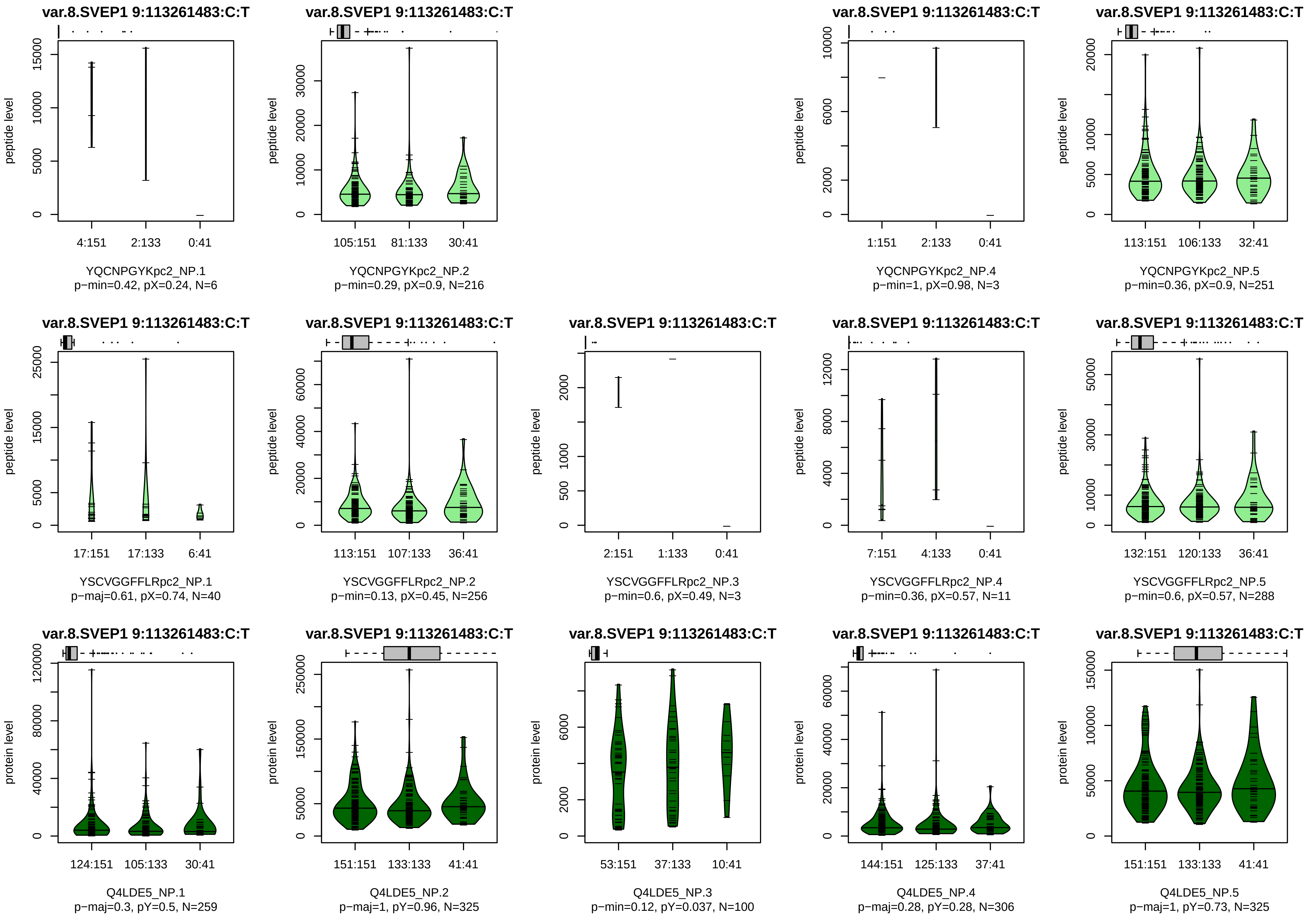

var.8.SVEP1 9:113261483:C:T
peptide level
4:151
2:133
0:41
YQCNPGYKpc2_NP.1
p−min=0.42, pX=0.24, N=6
var.8.SVEP1 9:113261483:C:T
peptide level
105:151
81:133
30:41
YQCNPGYKpc2_NP.2
p−min=0.29, pX=0.9, N=216
var.8.SVEP1 9:113261483:C:T
peptide level
1:151
2:133
0:41
YQCNPGYKpc2_NP.4
p−min=1, pX=0.98, N=3
var.8.SVEP1 9:113261483:C:T
peptide level
113:151
106:133
32:41
YQCNPGYKpc2_NP.5
p−min=0.36, pX=0.9, N=251
var.8.SVEP1 9:113261483:C:T
peptide level
17:151
17:133
6:41
YSCVGGFFLRpc2_NP.1
p−maj=0.61, pX=0.74, N=40
var.8.SVEP1 9:113261483:C:T
peptide level
113:151
107:133
36:41
YSCVGGFFLRpc2_NP.2
p−min=0.13, pX=0.45, N=256
var.8.SVEP1 9:113261483:C:T
peptide level
2:151
1:133
0:41
YSCVGGFFLRpc2_NP.3
p−min=0.6, pX=0.49, N=3
var.8.SVEP1 9:113261483:C:T
peptide level
7:151
4:133
0:41
YSCVGGFFLRpc2_NP.4
p−min=0.36, pX=0.57, N=11
var.8.SVEP1 9:113261483:C:T
peptide level
132:151
120:133
36:41
YSCVGGFFLRpc2_NP.5
p−min=0.6, pX=0.57, N=288
var.8.SVEP1 9:113261483:C:T
protein level
124:151
105:133
30:41
Q4LDE5_NP.1
p−maj=0.3, pY=0.5, N=259
var.8.SVEP1 9:113261483:C:T
protein level
151:151
133:133
41:41
Q4LDE5_NP.2
p−maj=1, pY=0.96, N=325
var.8.SVEP1 9:113261483:C:T
protein level
53:151
37:133
10:41
Q4LDE5_NP.3
p−min=0.12, pY=0.037, N=100
var.8.SVEP1 9:113261483:C:T
protein level
144:151
125:133
37:41
Q4LDE5_NP.4
p−maj=0.28, pY=0.28, N=306
var.8.SVEP1 9:113261483:C:T
protein level
151:151
133:133
41:41
Q4LDE5_NP.5
p−maj=1, pY=0.73, N=325

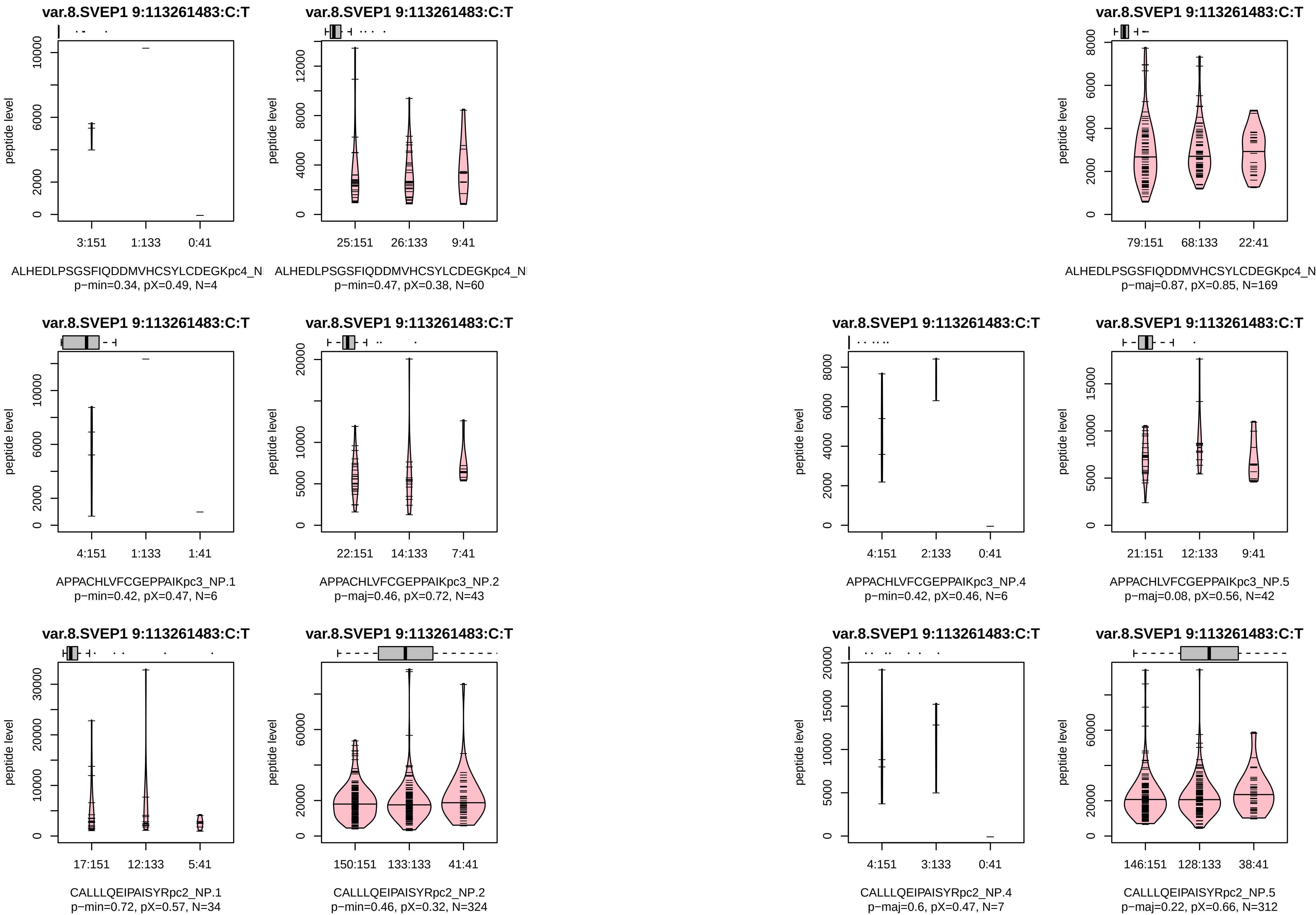
var.8.SVEP1 9:113261483:C:T
peptide level
3:151
1:133
0:41
ALHEDLPSGSFIQDDMVHCSYLCDEGKpc4_N
p−min=0.34, pX=0.49, N=4
var.8.SVEP1 9:113261483:C:T
peptide level
25:151
26:133
9:41
ALHEDLPSGSFIQDDMVHCSYLCDEGKpc4_N
p−min=0.47, pX=0.38, N=60
var.8.SVEP1 9:113261483:C:T
peptide level
79:151
68:133
22:41
ALHEDLPSGSFIQDDMVHCSYLCDEGKpc4_N
p−maj=0.87, pX=0.85, N=169
var.8.SVEP1 9:113261483:C:T
peptide level
4:151
1:133
1:41
APPACHLVFCGEPPAIKpc3_NP.1
p−min=0.42, pX=0.47, N=6
var.8.SVEP1 9:113261483:C:T
peptide level
22:151
14:133
7:41
APPACHLVFCGEPPAIKpc3_NP.2
p−maj=0.46, pX=0.72, N=43
var.8.SVEP1 9:113261483:C:T
peptide level
4:151
2:133
0:41
APPACHLVFCGEPPAIKpc3_NP.4
p−min=0.42, pX=0.46, N=6
var.8.SVEP1 9:113261483:C:T
peptide level
21:151
12:133
9:41
APPACHLVFCGEPPAIKpc3_NP.5
p−maj=0.08, pX=0.56, N=42
var.8.SVEP1 9:113261483:C:T
peptide level
17:151
12:133
5:41
CALLLQEIPAISYRpc2_NP.1
p−min=0.72, pX=0.57, N=34
var.8.SVEP1 9:113261483:C:T
peptide level
150:151
133:133
41:41
CALLLQEIPAISYRpc2_NP.2
p−min=0.46, pX=0.32, N=324
var.8.SVEP1 9:113261483:C:T
peptide level
4:151
3:133
0:41
CALLLQEIPAISYRpc2_NP.4
p−maj=0.6, pX=0.47, N=7
var.8.SVEP1 9:113261483:C:T
peptide level
146:151
128:133
38:41
CALLLQEIPAISYRpc2_NP.5
p−maj=0.22, pX=0.66, N=312

var.8.SVEP1 9:113261483:C:T

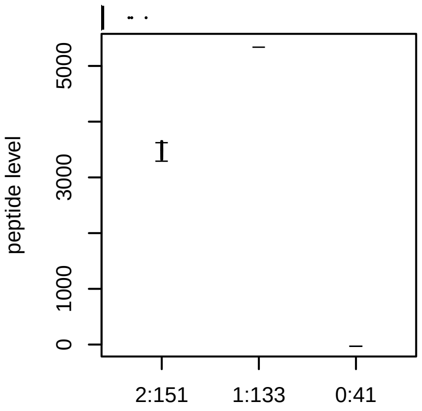

CDDTDLMKpc2_NP.1
p−min=0.6, pX=0.58, N=3

var.8.SVEP1 9:113261483:C:T

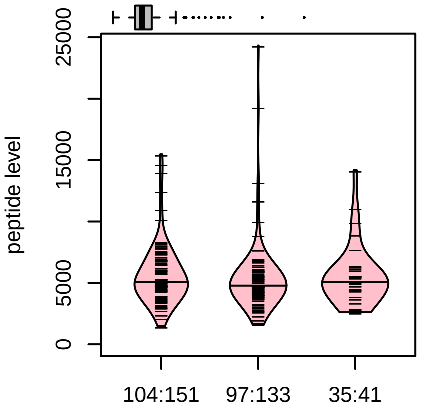

CDDTDLMKpc2_NP.2
p−maj=0.06, pX=0.26, N=236

var.8.SVEP1 9:113261483:C:T

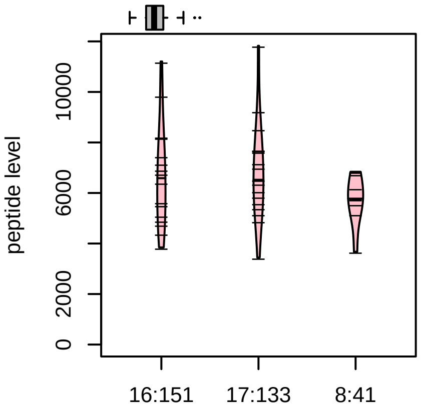

CDDTDLMKpc2_NP.5
p−maj=0.2, pX=0.29, N=41

var.8.SVEP1 9:113261483:C:T

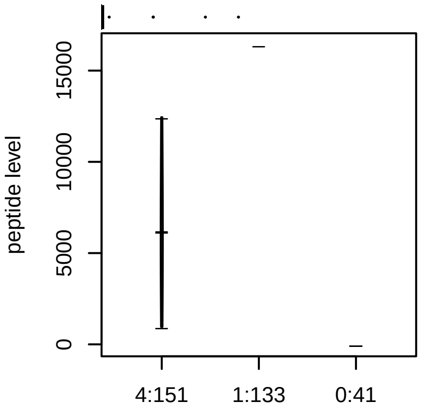

CETPLEFLNGKpc2_NP.1
p−min=0.19, pX=0.45, N=5

var.8.SVEP1 9:113261483:C:T

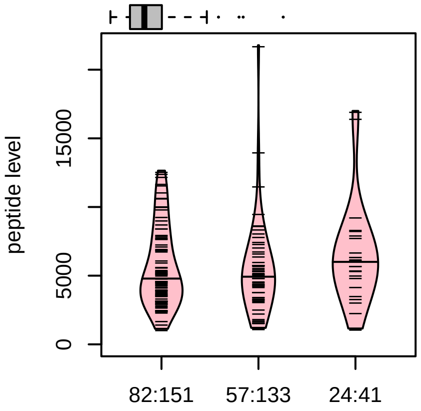

CETPLEFLNGKpc2_NP.2
p−min=0.18, pX=0.93, N=163

var.8.SVEP1 9:113261483:C:T

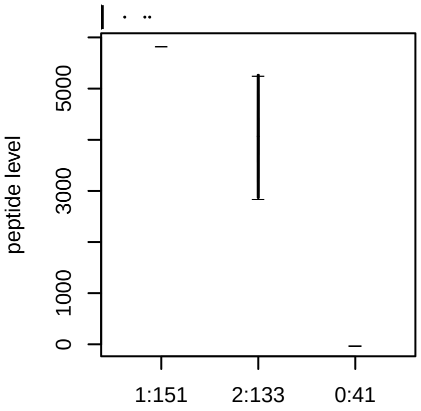

CETPLEFLNGKpc2_NP.4
p−min=1, pX=0.85, N=3

var.8.SVEP1 9:113261483:C:T

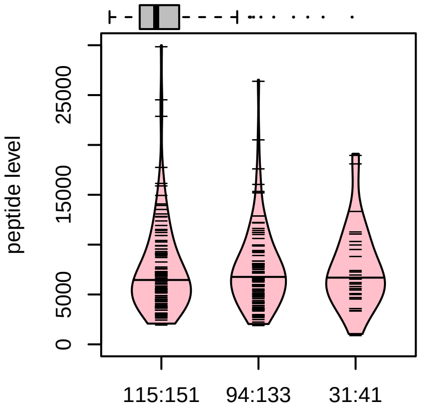

CETPLEFLNGKpc2_NP.5
p−min=0.45, pX=0.89, N=240

var.8.SVEP1 9:113261483:C:T

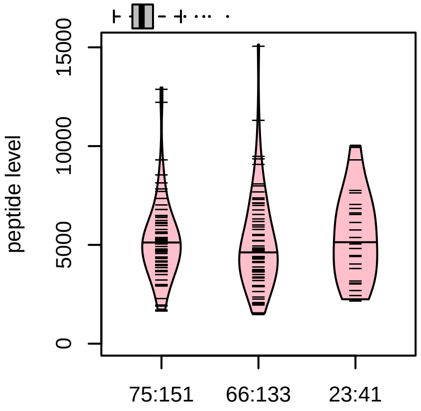

CGTHTGHFECICEKpc3_NP.2
p−maj=0.51, pX=0.52, N=164

var.8.SVEP1 9:113261483:C:T

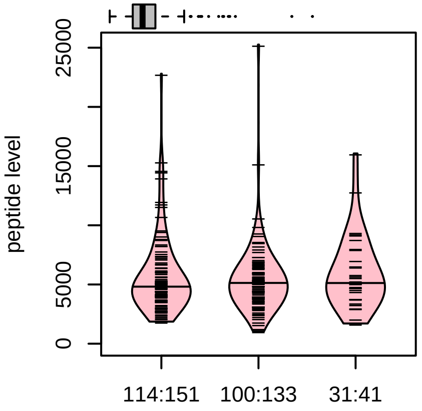

CGTHTGHFECICEKpc3_NP.5
p−maj=1, pX=0.89, N=245

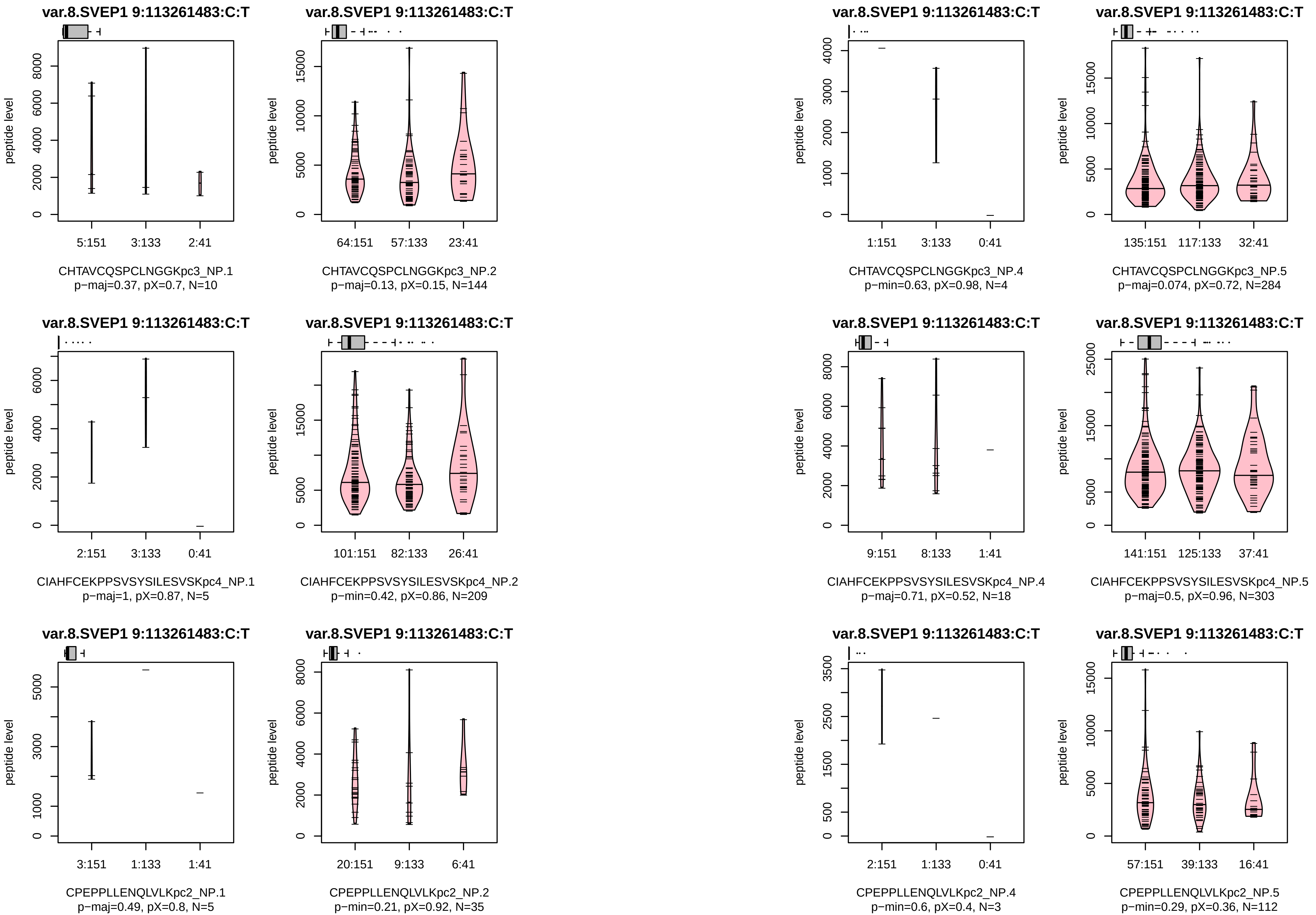

var.8.SVEP1 9:113261483:C:T
peptide level
5:151
3:133
2:41
CHTAVCQSPCLNGGKpc3_NP.1
p−maj=0.37, pX=0.7, N=10
var.8.SVEP1 9:113261483:C:T
peptide level
64:151
57:133
23:41
CHTAVCQSPCLNGGKpc3_NP.2
p−maj=0.13, pX=0.15, N=144
var.8.SVEP1 9:113261483:C:T
peptide level
1:151
3:133
0:41
CHTAVCQSPCLNGGKpc3_NP.4
p−min=0.63, pX=0.98, N=4
var.8.SVEP1 9:113261483:C:T
peptide level
135:151
117:133
32:41
CHTAVCQSPCLNGGKpc3_NP.5
p−maj=0.074, pX=0.72, N=284
var.8.SVEP1 9:113261483:C:T
peptide level
2:151
3:133
0:41
CIAHFCEKPPSVSYSILESVSKpc4_NP.1
p−maj=1, pX=0.87, N=5
var.8.SVEP1 9:113261483:C:T
peptide level
101:151
82:133
26:41
CIAHFCEKPPSVSYSILESVSKpc4_NP.2
p−min=0.42, pX=0.86, N=209
var.8.SVEP1 9:113261483:C:T
peptide level
9:151
8:133
1:41
CIAHFCEKPPSVSYSILESVSKpc4_NP.4
p−maj=0.71, pX=0.52, N=18
var.8.SVEP1 9:113261483:C:T
peptide level
141:151
125:133
37:41
CIAHFCEKPPSVSYSILESVSKpc4_NP.5
p−maj=0.5, pX=0.96, N=303
var.8.SVEP1 9:113261483:C:T
peptide level
3:151
1:133
1:41
CPEPPLLENQLVLKpc2_NP.1
p−maj=0.49, pX=0.8, N=5
var.8.SVEP1 9:113261483:C:T
peptide level
20:151
9:133
6:41
CPEPPLLENQLVLKpc2_NP.2
p−min=0.21, pX=0.92, N=35
var.8.SVEP1 9:113261483:C:T
peptide level
2:151
1:133
0:41
CPEPPLLENQLVLKpc2_NP.4
p−min=0.6, pX=0.4, N=3
var.8.SVEP1 9:113261483:C:T
peptide level
57:151
39:133
16:41
CPEPPLLENQLVLKpc2_NP.5
p−min=0.29, pX=0.36, N=112

var.8.SVEP1 9:113261483:C:T

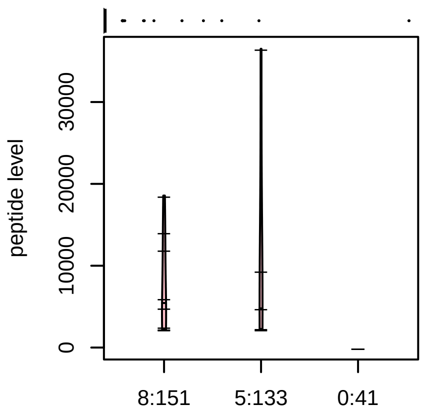

CPPGFLGTRpc2_NP.1
p−maj=0.38, pX=0.48, N=13

var.8.SVEP1 9:113261483:C:T

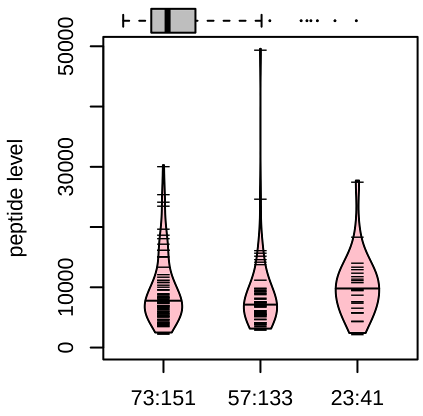

CPPGFLGTRpc2_NP.2
p−maj=0.24, pX=0.8, N=153

var.8.SVEP1 9:113261483:C:T

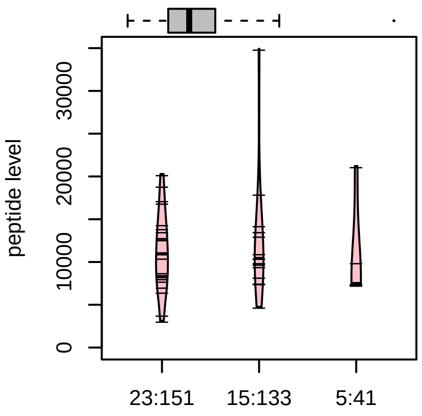

CPPGFLGTRpc2_NP.4
p−min=0.33, pX=0.51, N=43

var.8.SVEP1 9:113261483:C:T

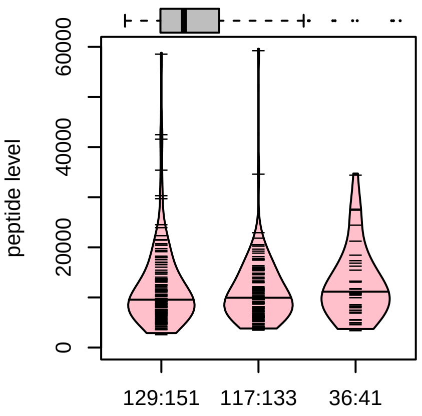

CPPGFLGTRpc2_NP.5
p−min=0.52, pX=0.74, N=282

var.8.SVEP1 9:113261483:C:T

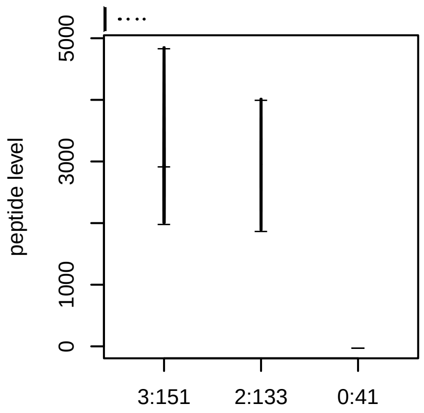

CSSPENINNGKpc2_NP.1
p−min=0.67, pX=0.38, N=5

var.8.SVEP1 9:113261483:C:T

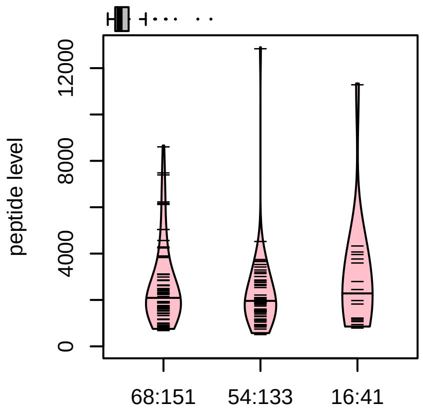

CSSPENINNGKpc2_NP.2
p−min=0.43, pX=0.52, N=138

var.8.SVEP1 9:113261483:C:T

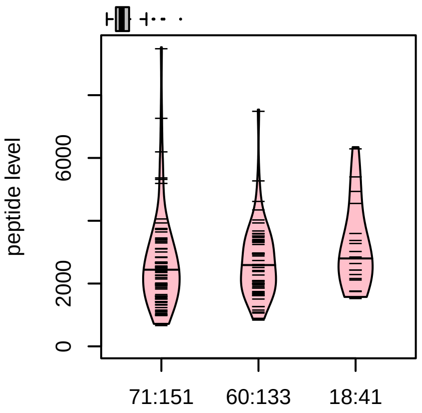

CSSPENINNGKpc2_NP.5
p−min=0.74, pX=0.75, N=149

var.8.SVEP1 9:113261483:C:T

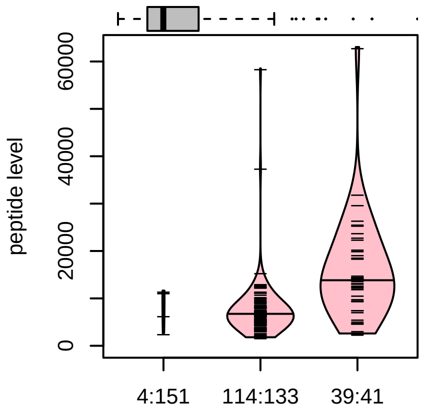

DIIISPHNCGKpc2_NP.2
p−min=5.6e−63, pX=1.3e−40, N=157

var.8.SVEP1 9:113261483:C:T

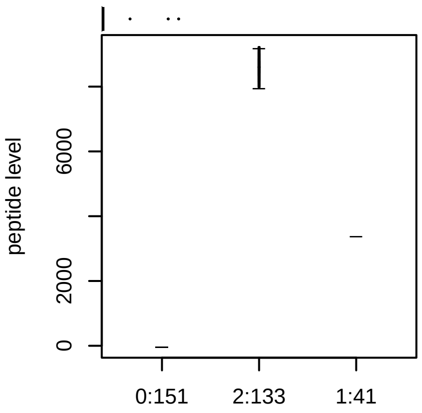

DIIISPHNCGKpc2_NP.4
p−min=0.25, pX=0.23, N=3

var.8.SVEP1 9:113261483:C:T

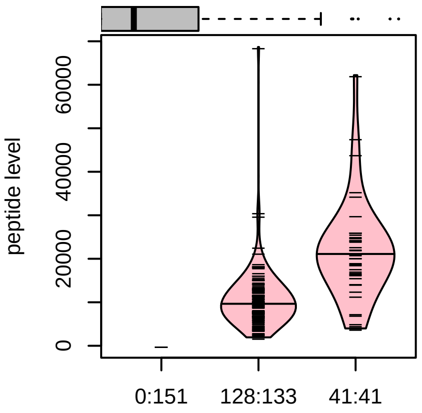

DIIISPHNCGKpc2_NP.5
p−min=5.4e−88, pX=4.2e−65, N=169

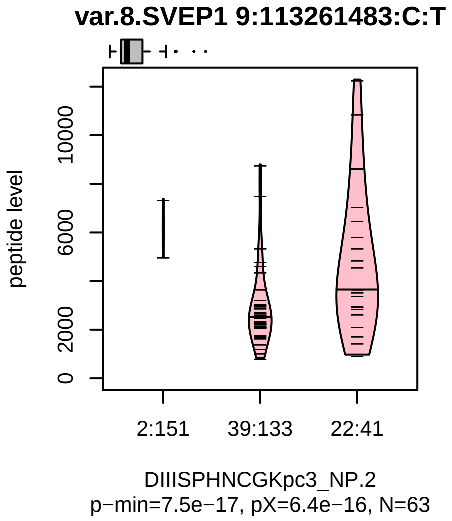
var.8.SVEP1 9:113261483:C:T
peptide level
0
2000
6000
10000
2:151
39:133
22:41
DIIISPHNCGKpc3_NP.2
p−min=7.5e−17, pX=6.4e−16, N=63

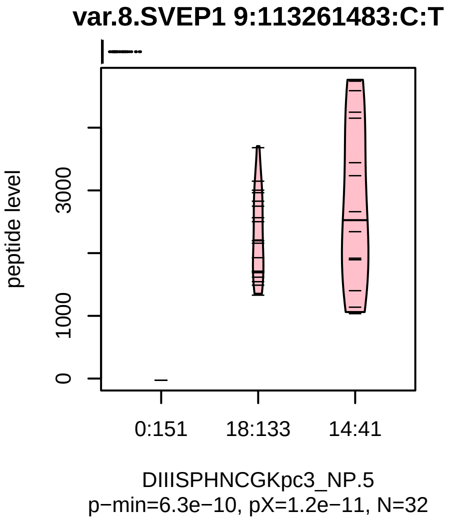
var.8.SVEP1 9:113261483:C:T
peptide level
0
1000
3000
0:151
18:133
14:41
DIIISPHNCGKpc3_NP.5
p−min=6.3e−10, pX=1.2e−11, N=32

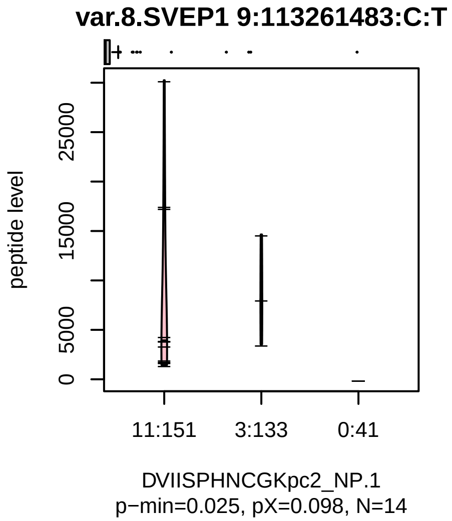
var.8.SVEP1 9:113261483:C:T
peptide level
0
5000
15000
25000
11:151
3:133
0:41
DVIISPHNCGKpc2_NP.1
p−min=0.025, pX=0.098, N=14

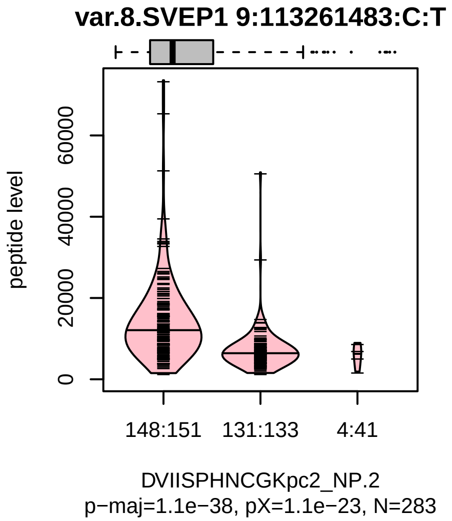
var.8.SVEP1 9:113261483:C:T
peptide level
0
20000
40000
60000
148:151
131:133
4:41
DVIISPHNCGKpc2_NP.2
p−maj=1.1e−38, pX=1.1e−23, N=283

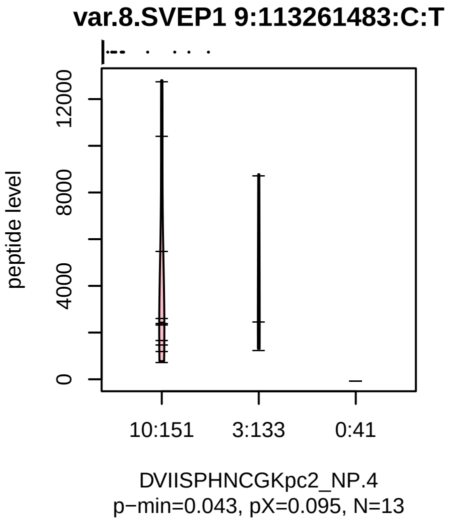
var.8.SVEP1 9:113261483:C:T
peptide level
0
4000
8000
12000
10:151
3:133
0:41
DVIISPHNCGKpc2_NP.4
p−min=0.043, pX=0.095, N=13

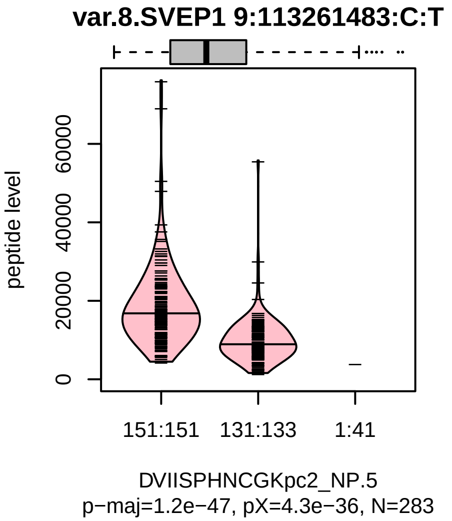
var.8.SVEP1 9:113261483:C:T
peptide level
0
20000
40000
60000
151:151
131:133
1:41
DVIISPHNCGKpc2_NP.5
p−maj=1.2e−47, pX=4.3e−36, N=283

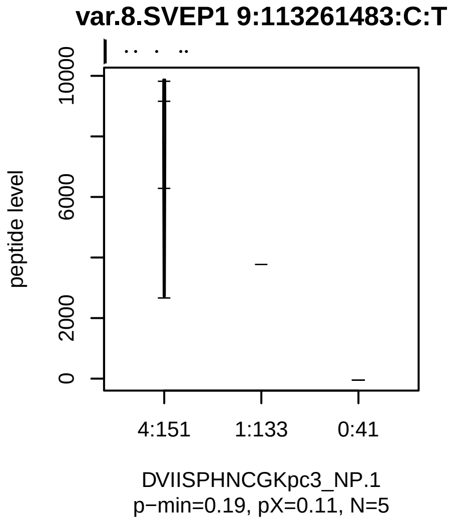
var.8.SVEP1 9:113261483:C:T
peptide level
0
2000
6000
10000
4:151
1:133
0:41
DVIISPHNCGKpc3_NP.1
p−min=0.19, pX=0.11, N=5

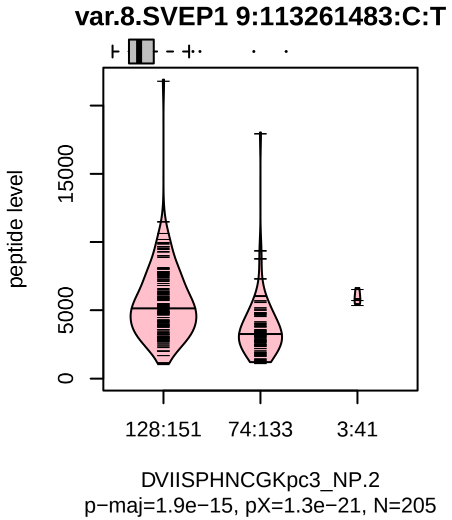
var.8.SVEP1 9:113261483:C:T
peptide level
0
5000
15000
128:151
74:133
3:41
DVIISPHNCGKpc3_NP.2
p−maj=1.9e−15, pX=1.3e−21, N=205

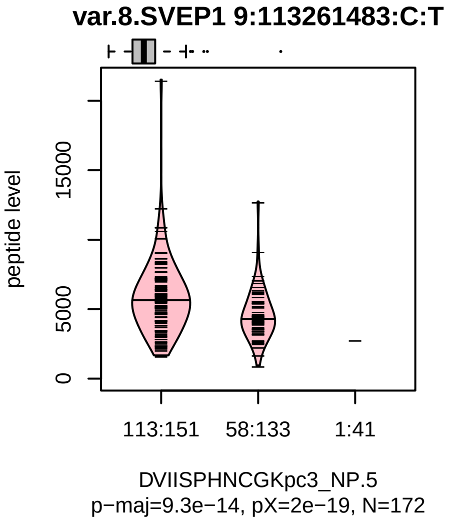
var.8.SVEP1 9:113261483:C:T
peptide level
0
5000
15000
113:151
58:133
1:41
DVIISPHNCGKpc3_NP.5
p−maj=9.3e−14, pX=2e−19, N=172

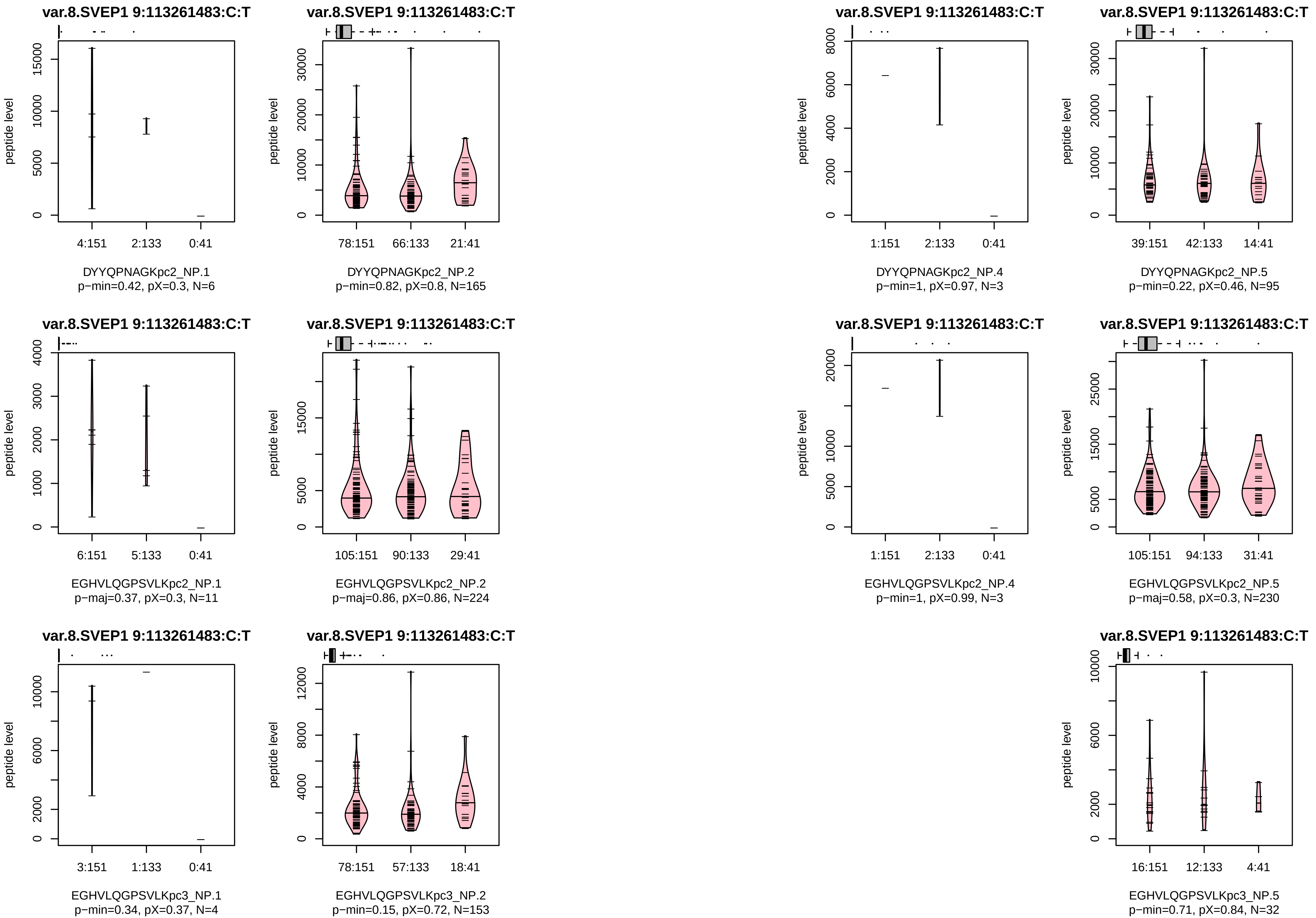
var.8.SVEP1 9:113261483:C:T
peptide level
4:151
2:133
0:41
DYYQPNAGKpc2_NP.1
p−min=0.42, pX=0.3, N=6
var.8.SVEP1 9:113261483:C:T
peptide level
78:151
66:133
21:41
DYYQPNAGKpc2_NP.2
p−min=0.82, pX=0.8, N=165
var.8.SVEP1 9:113261483:C:T
peptide level
1:151
2:133
0:41
DYYQPNAGKpc2_NP.4
p−min=1, pX=0.97, N=3
var.8.SVEP1 9:113261483:C:T
peptide level
39:151
42:133
14:41
DYYQPNAGKpc2_NP.5
p−min=0.22, pX=0.46, N=95
var.8.SVEP1 9:113261483:C:T
peptide level
6:151
5:133
0:41
EGHVLQGPSVLKpc2_NP.1
p−maj=0.37, pX=0.3, N=11
var.8.SVEP1 9:113261483:C:T
peptide level
105:151
90:133
29:41
EGHVLQGPSVLKpc2_NP.2
p−maj=0.86, pX=0.86, N=224
var.8.SVEP1 9:113261483:C:T
peptide level
1:151
2:133
0:41
EGHVLQGPSVLKpc2_NP.4
p−min=1, pX=0.99, N=3
var.8.SVEP1 9:113261483:C:T
peptide level
105:151
94:133
31:41
EGHVLQGPSVLKpc2_NP.5
p−maj=0.58, pX=0.3, N=230
var.8.SVEP1 9:113261483:C:T
peptide level
3:151
1:133
0:41
EGHVLQGPSVLKpc3_NP.1
p−min=0.34, pX=0.37, N=4
var.8.SVEP1 9:113261483:C:T
peptide level
78:151
57:133
18:41
EGHVLQGPSVLKpc3_NP.2
p−min=0.15, pX=0.72, N=153
var.8.SVEP1 9:113261483:C:T
peptide level
16:151
12:133
4:41
EGHVLQGPSVLKpc3_NP.5
p−min=0.71, pX=0.84, N=32

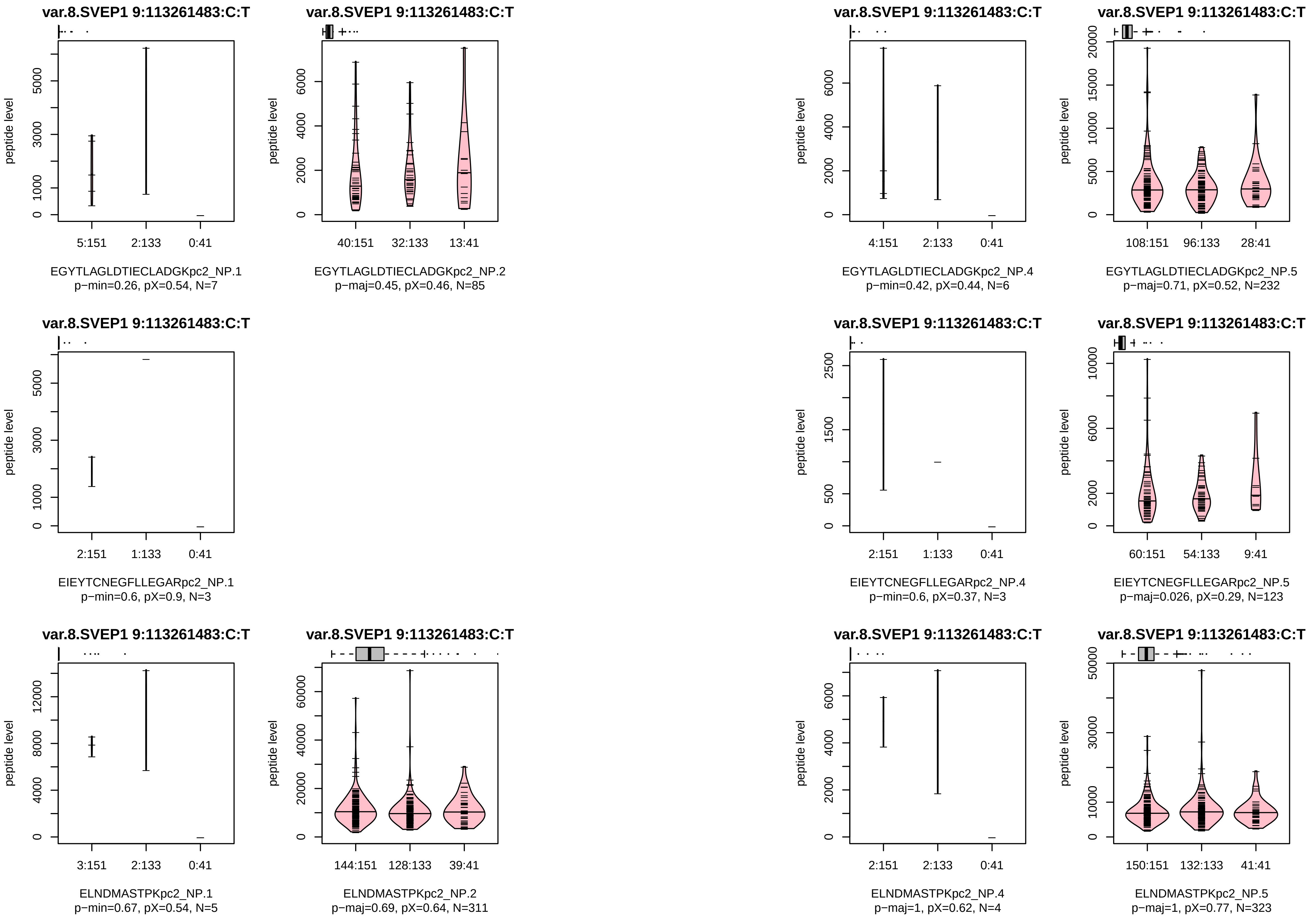

var.8.SVEP1 9:113261483:C:T
peptide level
5:151
2:133
0:41
EGYTLAGLDTIECLADGKpc2_NP.1
p−min=0.26, pX=0.54, N=7
var.8.SVEP1 9:113261483:C:T
peptide level
40:151
32:133
13:41
EGYTLAGLDTIECLADGKpc2_NP.2
p−maj=0.45, pX=0.46, N=85
var.8.SVEP1 9:113261483:C:T
peptide level
4:151
2:133
0:41
EGYTLAGLDTIECLADGKpc2_NP.4
p−min=0.42, pX=0.44, N=6
var.8.SVEP1 9:113261483:C:T
peptide level
108:151
96:133
28:41
EGYTLAGLDTIECLADGKpc2_NP.5
p−maj=0.71, pX=0.52, N=232
var.8.SVEP1 9:113261483:C:T
peptide level
2:151
1:133
0:41
EIEYTCNEGFLLEGARpc2_NP.1
p−min=0.6, pX=0.9, N=3
var.8.SVEP1 9:113261483:C:T
peptide level
2:151
1:133
0:41
EIEYTCNEGFLLEGARpc2_NP.4
p−min=0.6, pX=0.37, N=3
var.8.SVEP1 9:113261483:C:T
peptide level
60:151
54:133
9:41
EIEYTCNEGFLLEGARpc2_NP.5
p−maj=0.026, pX=0.29, N=123
var.8.SVEP1 9:113261483:C:T
peptide level
3:151
2:133
0:41
ELNDMASTPKpc2_NP.1
p−min=0.67, pX=0.54, N=5
var.8.SVEP1 9:113261483:C:T
peptide level
144:151
128:133
39:41
ELNDMASTPKpc2_NP.2
p−maj=0.69, pX=0.64, N=311
var.8.SVEP1 9:113261483:C:T
peptide level
2:151
2:133
0:41
ELNDMASTPKpc2_NP.4
p−maj=1, pX=0.62, N=4
var.8.SVEP1 9:113261483:C:T
peptide level
150:151
132:133
41:41
ELNDMASTPKpc2_NP.5
p−maj=1, pX=0.77, N=323

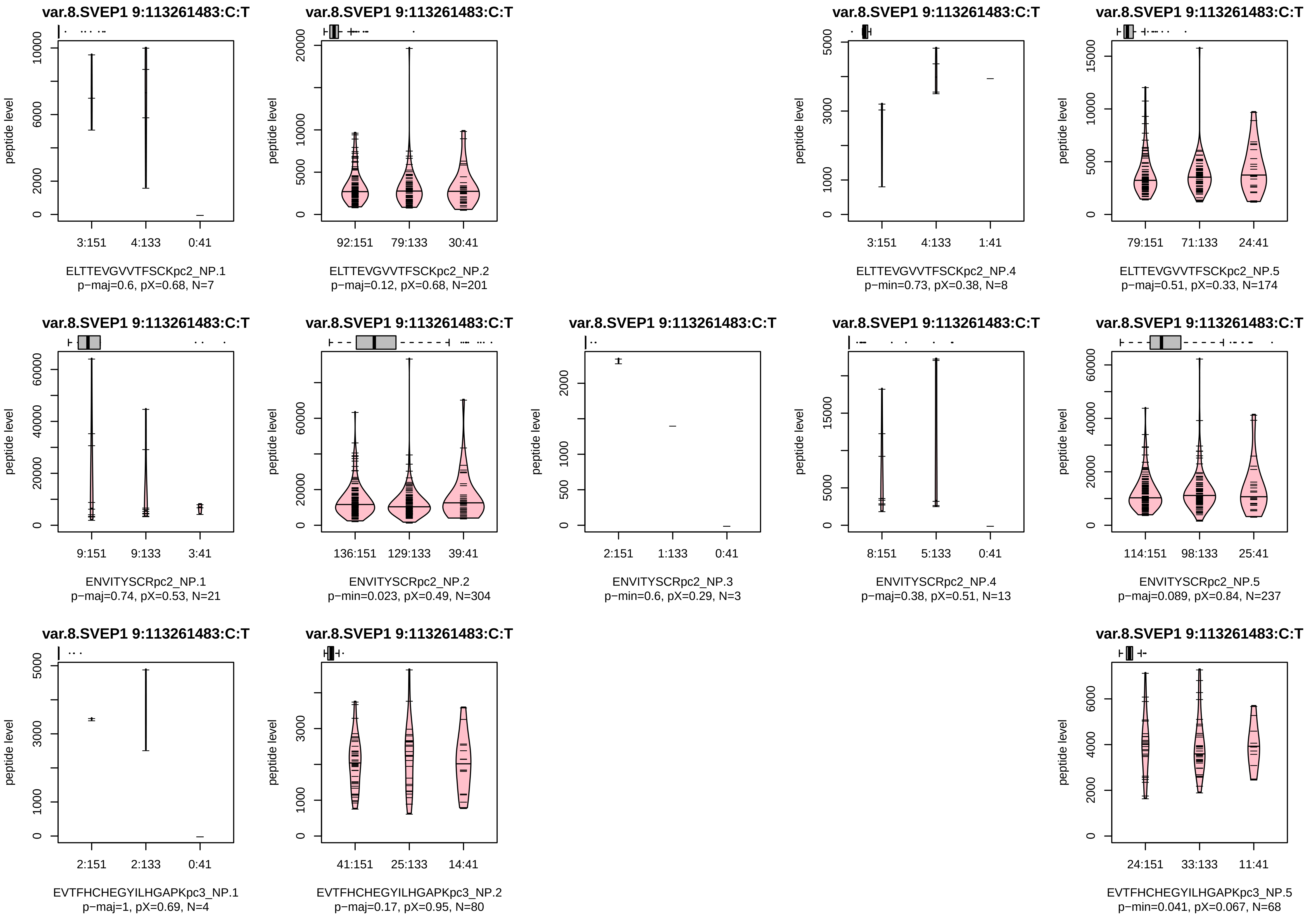
var.8.SVEP1 9:113261483:C:T
peptide level
3:151 4:133 0:41
ELTTEVGVVTFSCKpc2_NP.1
p−maj=0.6, pX=0.68, N=7
var.8.SVEP1 9:113261483:C:T
peptide level
92:151 79:133 30:41
ELTTEVGVVTFSCKpc2_NP.2
p−maj=0.12, pX=0.68, N=201
var.8.SVEP1 9:113261483:C:T
peptide level
3:151 4:133 1:41
ELTTEVGVVTFSCKpc2_NP.4
p−min=0.73, pX=0.38, N=8
var.8.SVEP1 9:113261483:C:T
peptide level
79:151 71:133 24:41
ELTTEVGVVTFSCKpc2_NP.5
p−maj=0.51, pX=0.33, N=174
var.8.SVEP1 9:113261483:C:T
peptide level
9:151 9:133 3:41
ENVITYSCRpc2_NP.1
p−maj=0.74, pX=0.53, N=21
var.8.SVEP1 9:113261483:C:T
peptide level
136:151 129:133 39:41
ENVITYSCRpc2_NP.2
p−min=0.023, pX=0.49, N=304
var.8.SVEP1 9:113261483:C:T
peptide level
2:151 1:133 0:41
ENVITYSCRpc2_NP.3
p−min=0.6, pX=0.29, N=3
var.8.SVEP1 9:113261483:C:T
peptide level
8:151 5:133 0:41
ENVITYSCRpc2_NP.4
p−maj=0.38, pX=0.51, N=13
var.8.SVEP1 9:113261483:C:T
peptide level
114:151 98:133 25:41
ENVITYSCRpc2_NP.5
p−maj=0.089, pX=0.84, N=237
var.8.SVEP1 9:113261483:C:T
peptide level
2:151 2:133 0:41
EVTFHCHEGYILHGAPKpc3_NP.1
p−maj=1, pX=0.69, N=4
var.8.SVEP1 9:113261483:C:T
peptide level
41:151 25:133 14:41
EVTFHCHEGYILHGAPKpc3_NP.2
p−maj=0.17, pX=0.95, N=80
var.8.SVEP1 9:113261483:C:T
peptide level
24:151 33:133 11:41
EVTFHCHEGYILHGAPKpc3_NP.5
p−min=0.041, pX=0.067, N=68

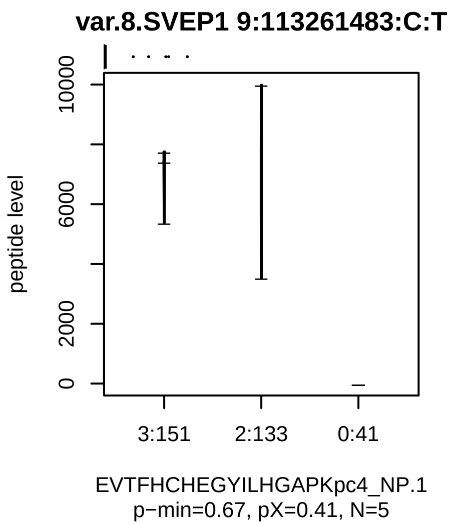
var.8.SVEP1 9:113261483:C:T
peptide level
0
2000
6000
10000
3:151
2:133
0:41
EVTFHCHEGYILHGAPKpc4_NP.1
p−min=0.67, pX=0.41, N=5

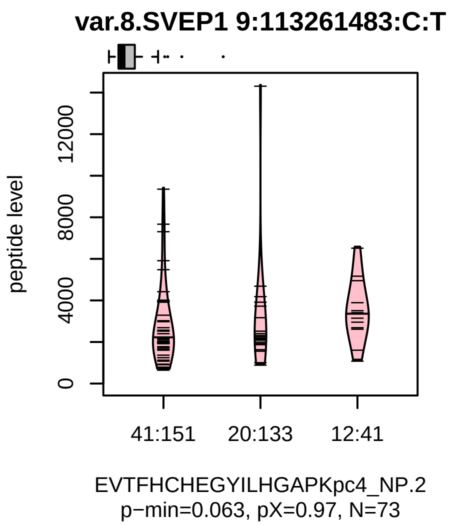
var.8.SVEP1 9:113261483:C:T
peptide level
0
4000
8000
12000
41:151
20:133
12:41
EVTFHCHEGYILHGAPKpc4_NP.2
p−min=0.063, pX=0.97, N=73

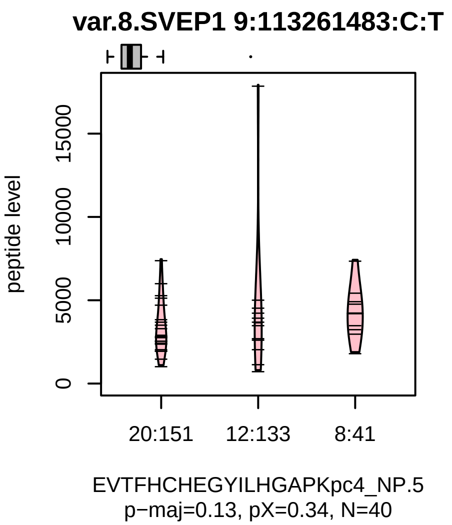
var.8.SVEP1 9:113261483:C:T
peptide level
0
5000
10000
15000
20:151
12:133
8:41
EVTFHCHEGYILHGAPKpc4_NP.5
p−maj=0.13, pX=0.34, N=40

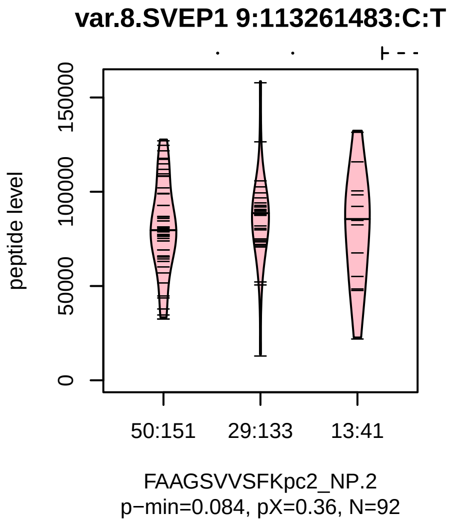
var.8.SVEP1 9:113261483:C:T
peptide level
0
50000
100000
150000
50:151
29:133
13:41
FAAGSVVSFKpc2_NP.2
p−min=0.084, pX=0.36, N=92

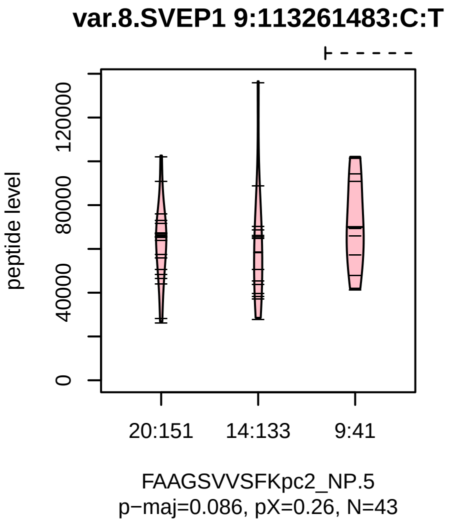
var.8.SVEP1 9:113261483:C:T
peptide level
0
40000
80000
120000
20:151
14:133
9:41
FAAGSVVSFKpc2_NP.5
p−maj=0.086, pX=0.26, N=43

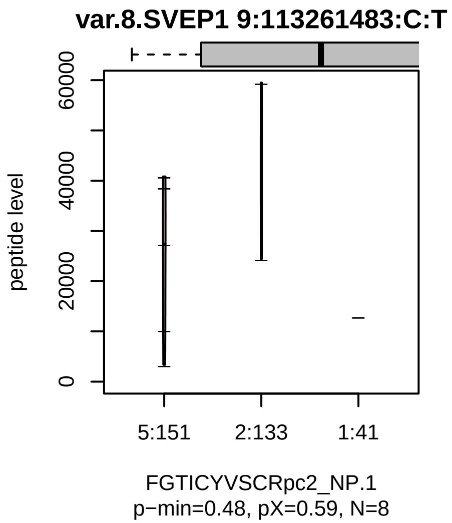
var.8.SVEP1 9:113261483:C:T
peptide level
0
20000
40000
60000
5:151
2:133
1:41
FGTICYVSCRpc2_NP.1
p−min=0.48, pX=0.59, N=8

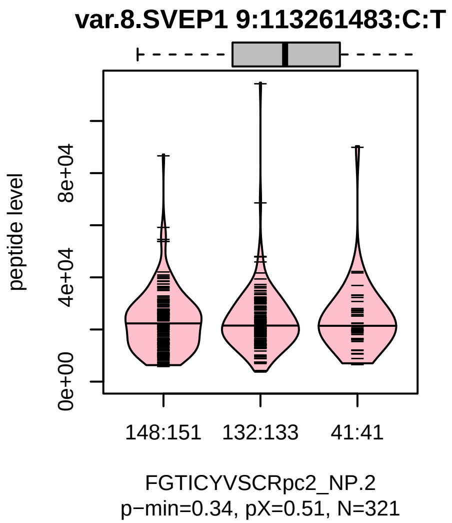
var.8.SVEP1 9:113261483:C:T
peptide level
0e+00
4e+04
8e+04
148:151
132:133
41:41
FGTICYVSCRpc2_NP.2
p−min=0.34, pX=0.51, N=321

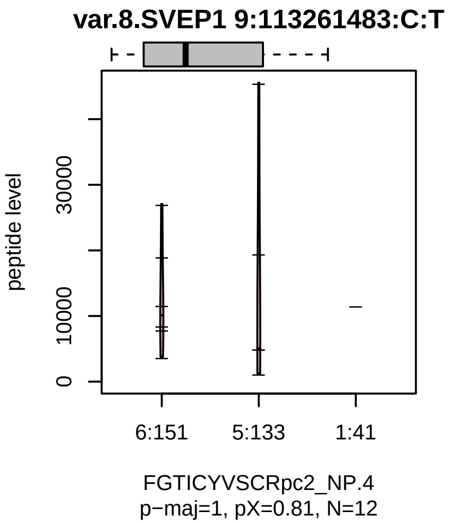
var.8.SVEP1 9:113261483:C:T
peptide level
0
10000
30000
6:151
5:133
1:41
FGTICYVSCRpc2_NP.4
p−maj=1, pX=0.81, N=12

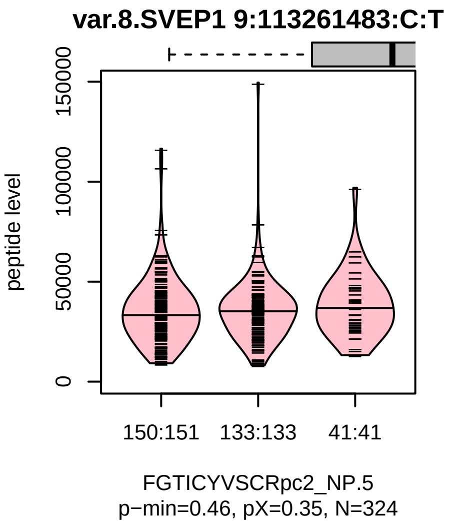
var.8.SVEP1 9:113261483:C:T
peptide level
0
50000
100000
150000
150:151
133:133
41:41
FGTICYVSCRpc2_NP.5
p−min=0.46, pX=0.35, N=324

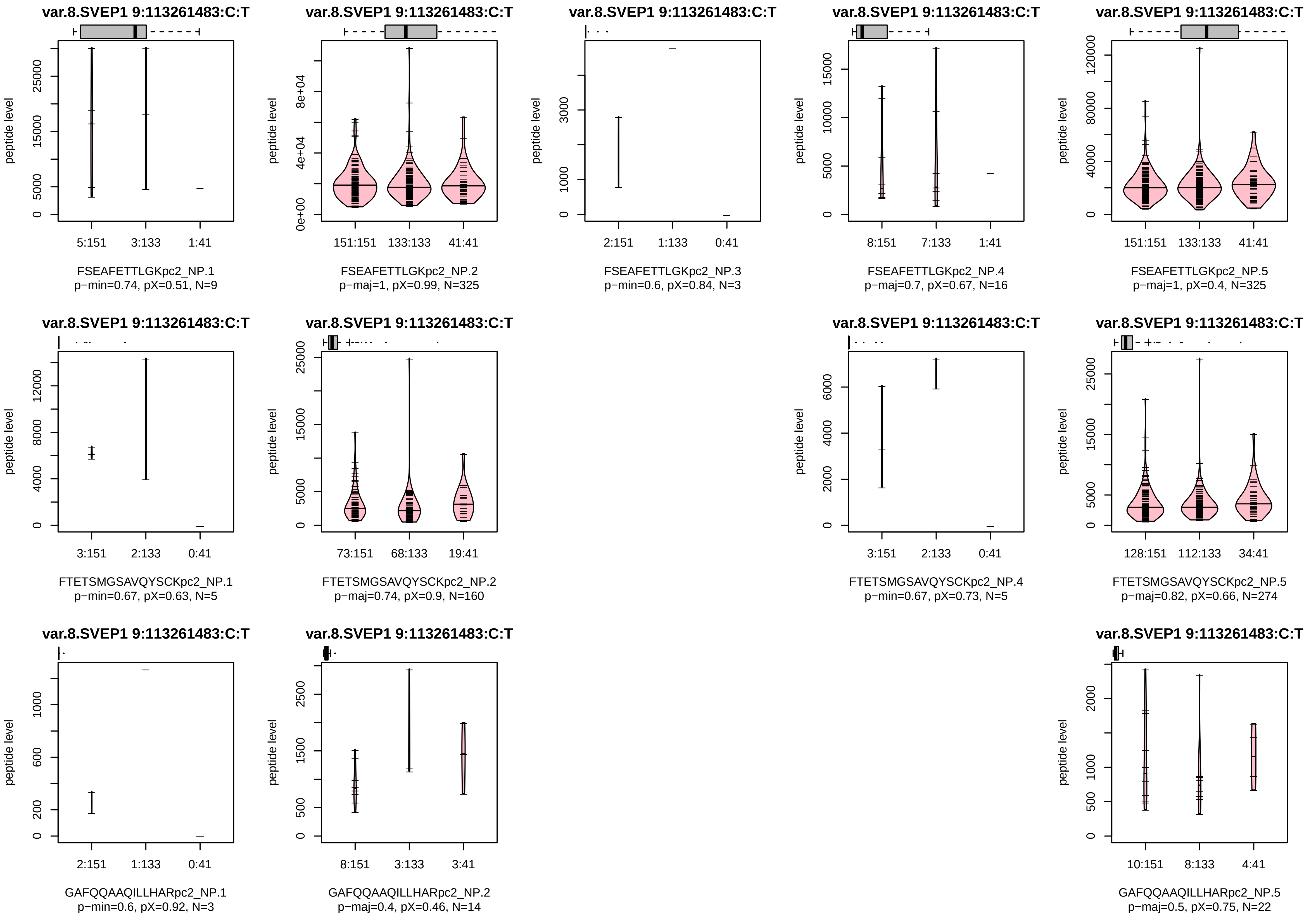
var.8.SVEP1 9:113261483:C:T
peptide level
5:151
3:133
1:41
FSEAFETTLGKpc2_NP.1
p−min=0.74, pX=0.51, N=9
var.8.SVEP1 9:113261483:C:T
peptide level
151:151
133:133
41:41
FSEAFETTLGKpc2_NP.2
p−maj=1, pX=0.99, N=325
var.8.SVEP1 9:113261483:C:T
peptide level
2:151
1:133
0:41
FSEAFETTLGKpc2_NP.3
p−min=0.6, pX=0.84, N=3
var.8.SVEP1 9:113261483:C:T
peptide level
8:151
7:133
1:41
FSEAFETTLGKpc2_NP.4
p−maj=0.7, pX=0.67, N=16
var.8.SVEP1 9:113261483:C:T
peptide level
151:151
133:133
41:41
FSEAFETTLGKpc2_NP.5
p−maj=1, pX=0.4, N=325
var.8.SVEP1 9:113261483:C:T
peptide level
3:151
2:133
0:41
FTETSMGSAVQYSCKpc2_NP.1
p−min=0.67, pX=0.63, N=5
var.8.SVEP1 9:113261483:C:T
peptide level
73:151
68:133
19:41
FTETSMGSAVQYSCKpc2_NP.2
p−maj=0.74, pX=0.9, N=160
var.8.SVEP1 9:113261483:C:T
peptide level
3:151
2:133
0:41
FTETSMGSAVQYSCKpc2_NP.4
p−min=0.67, pX=0.73, N=5
var.8.SVEP1 9:113261483:C:T
peptide level
128:151
112:133
34:41
FTETSMGSAVQYSCKpc2_NP.5
p−maj=0.82, pX=0.66, N=274
var.8.SVEP1 9:113261483:C:T
peptide level
2:151
1:133
0:41
GAFQQAAQILLHARpc2_NP.1
p−min=0.6, pX=0.92, N=3
var.8.SVEP1 9:113261483:C:T
peptide level
8:151
3:133
3:41
GAFQQAAQILLHARpc2_NP.2
p−maj=0.4, pX=0.46, N=14
var.8.SVEP1 9:113261483:C:T
peptide level
10:151
8:133
4:41
GAFQQAAQILLHARpc2_NP.5
p−maj=0.5, pX=0.75, N=22

var.8.SVEP1 9:113261483:C:T

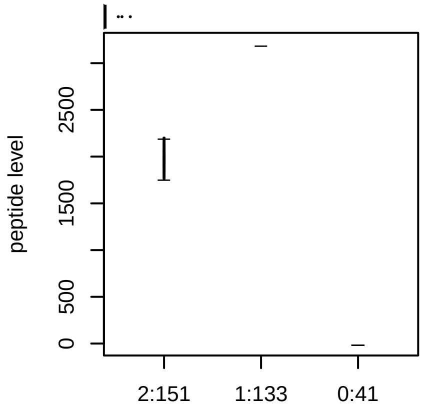

GAFQQAAQILLHARpc3_NP.1
p−min=0.6, pX=0.6, N=3

var.8.SVEP1 9:113261483:C:T

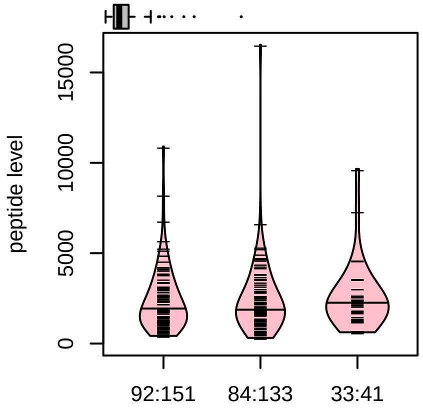

GAFQQAAQILLHARpc3_NP.2
p−maj=0.023, pX=0.11, N=209

var.8.SVEP1 9:113261483:C:T

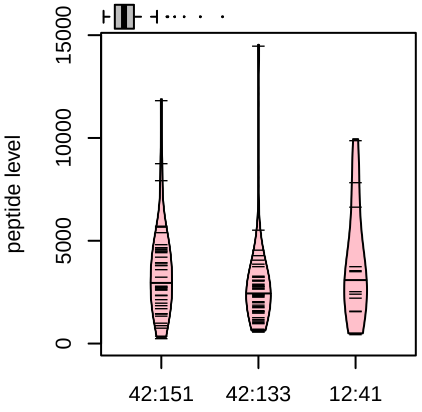

GAFQQAAQILLHARpc3_NP.5
p−min=0.54, pX=0.87, N=96

var.8.SVEP1 9:113261483:C:T

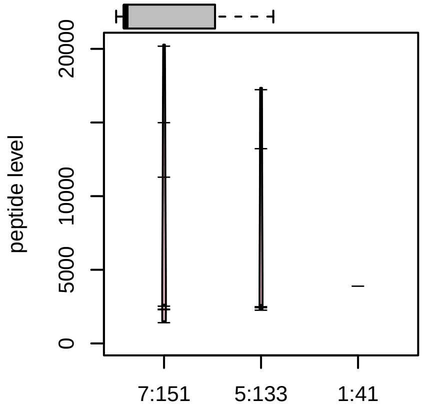

GDEYTFQKpc2_NP.1
p−min=0.78, pX=0.45, N=13

var.8.SVEP1 9:113261483:C:T

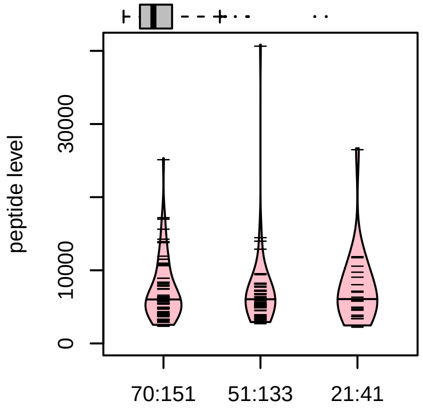

GDEYTFQKpc2_NP.2
p−maj=0.32, pX=0.98, N=142

var.8.SVEP1 9:113261483:C:T

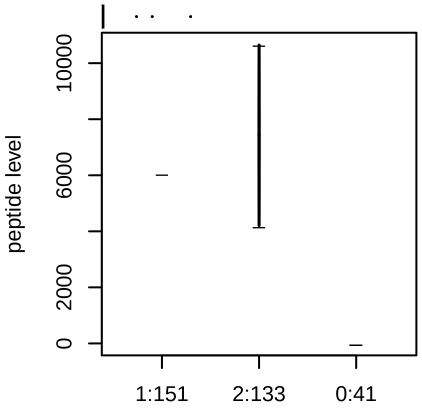

GDEYTFQKpc2_NP.4
p−min=1, pX=0.91, N=3

var.8.SVEP1 9:113261483:C:T

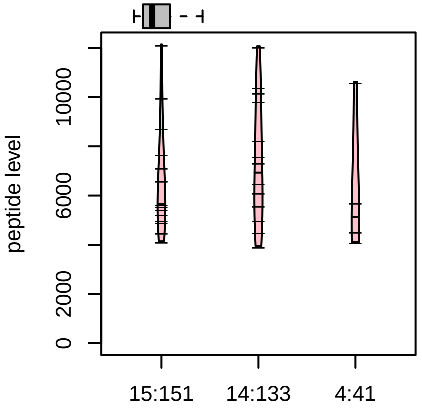

GDEYTFQKpc2_NP.5
p−maj=1, pX=0.94, N=33

var.8.SVEP1 9:113261483:C:T

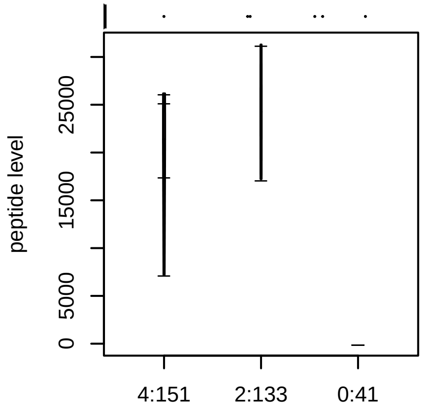

GENFEVGSKpc2_NP.1
p−min=0.42, pX=0.36, N=6

var.8.SVEP1 9:113261483:C:T

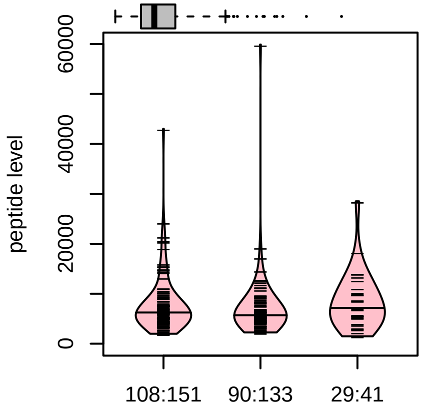

GENFEVGSKpc2_NP.2
p−min=0.55, pX=0.95, N=227

var.8.SVEP1 9:113261483:C:T

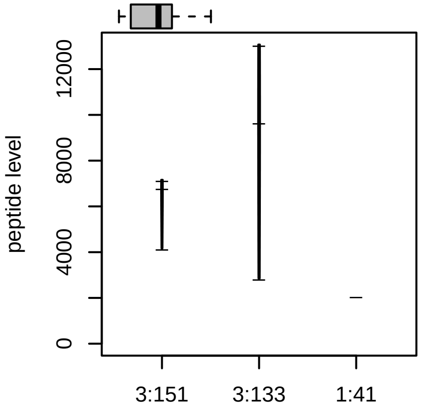

GENFEVGSKpc2_NP.4
p−maj=1, pX=0.97, N=7

var.8.SVEP1 9:113261483:C:T

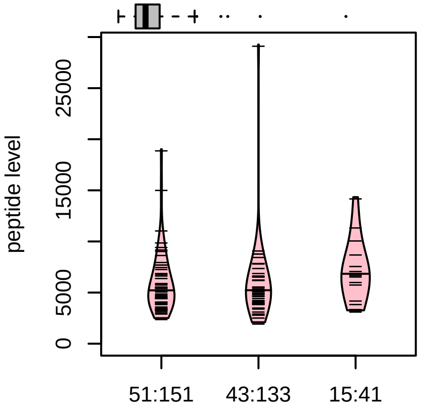

GENFEVGSKpc2_NP.5
p−maj=0.72, pX=0.56, N=109

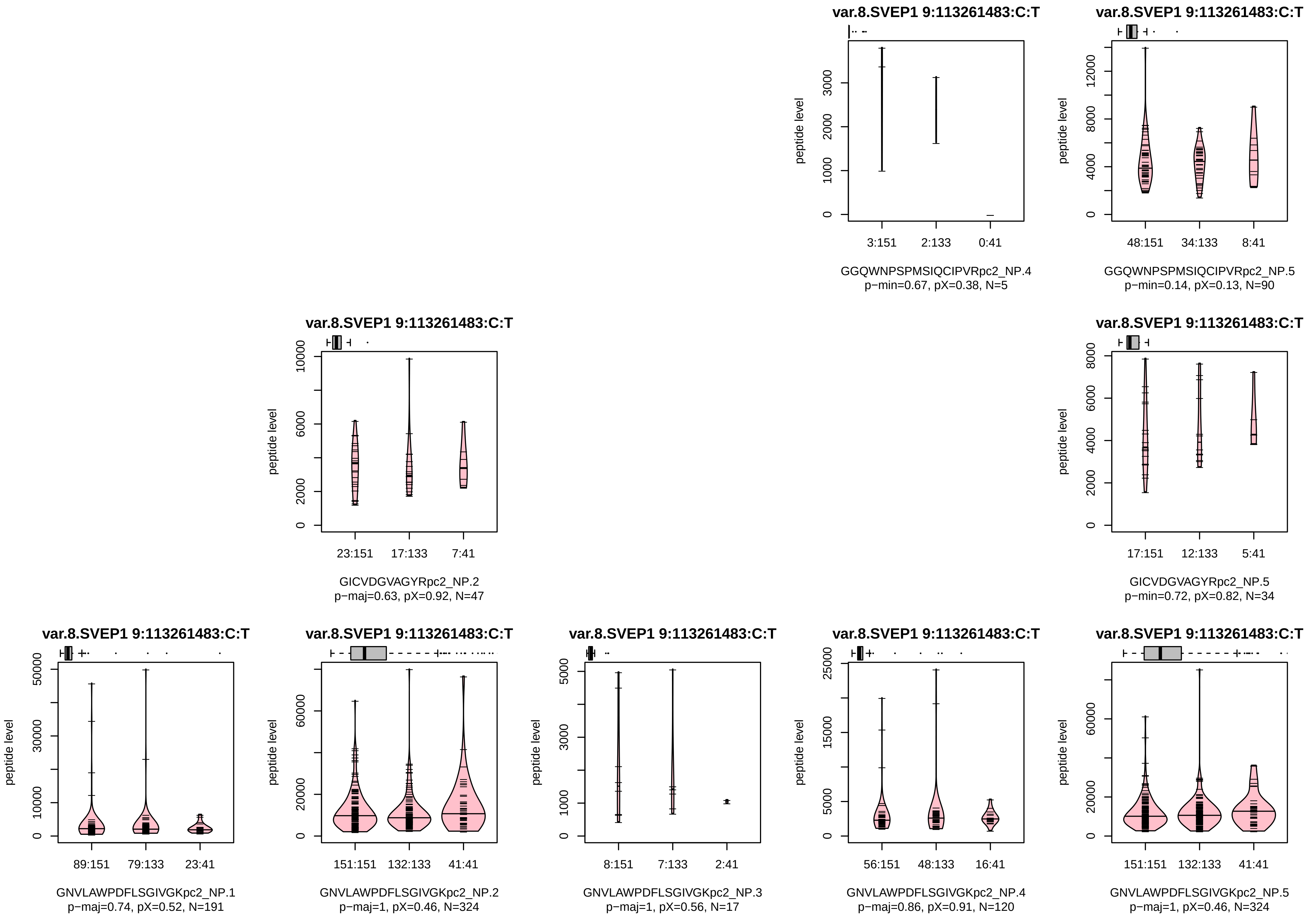
var.8.SVEP1 9:113261483:C:T
peptide level
3:151 2:133 0:41
GGQWNPSPMSIQCIPVRpc2_NP.4
p−min=0.67, pX=0.38, N=5
var.8.SVEP1 9:113261483:C:T
peptide level
48:151 34:133 8:41
GGQWNPSPMSIQCIPVRpc2_NP.5
p−min=0.14, pX=0.13, N=90
var.8.SVEP1 9:113261483:C:T
peptide level
23:151 17:133 7:41
GICVDGVAGYRpc2_NP.2
p−maj=0.63, pX=0.92, N=47
var.8.SVEP1 9:113261483:C:T
peptide level
17:151 12:133 5:41
GICVDGVAGYRpc2_NP.5
p−min=0.72, pX=0.82, N=34
var.8.SVEP1 9:113261483:C:T
peptide level
89:151 79:133 23:41
GNVLAWPDFLSGIVGKpc2_NP.1
p−maj=0.74, pX=0.52, N=191
var.8.SVEP1 9:113261483:C:T
peptide level
151:151 132:133 41:41
GNVLAWPDFLSGIVGKpc2_NP.2
p−maj=1, pX=0.46, N=324
var.8.SVEP1 9:113261483:C:T
peptide level
8:151 7:133 2:41
GNVLAWPDFLSGIVGKpc2_NP.3
p−maj=1, pX=0.56, N=17
var.8.SVEP1 9:113261483:C:T
peptide level
56:151 48:133 16:41
GNVLAWPDFLSGIVGKpc2_NP.4
p−maj=0.86, pX=0.91, N=120
var.8.SVEP1 9:113261483:C:T
peptide level
151:151 132:133 41:41
GNVLAWPDFLSGIVGKpc2_NP.5
p−maj=1, pX=0.46, N=324

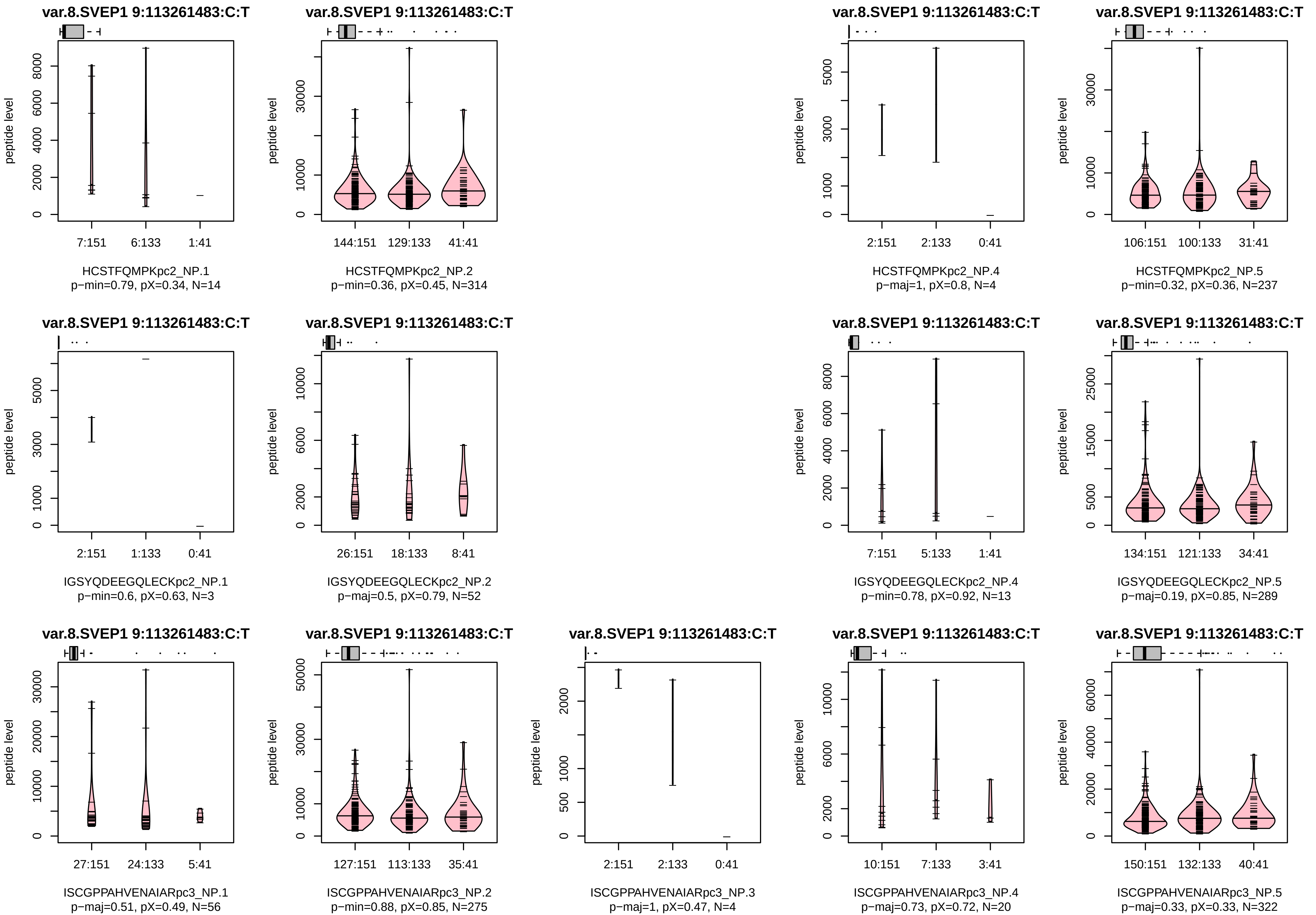
var.8.SVEP1 9:113261483:C:T
peptide level
7:151
6:133
1:41
HCSTFQMPKpc2_NP.1
p−min=0.79, pX=0.34, N=14
var.8.SVEP1 9:113261483:C:T
peptide level
144:151
129:133
41:41
HCSTFQMPKpc2_NP.2
p−min=0.36, pX=0.45, N=314
var.8.SVEP1 9:113261483:C:T
peptide level
2:151
2:133
0:41
HCSTFQMPKpc2_NP.4
p−maj=1, pX=0.8, N=4
var.8.SVEP1 9:113261483:C:T
peptide level
106:151
100:133
31:41
HCSTFQMPKpc2_NP.5
p−min=0.32, pX=0.36, N=237
var.8.SVEP1 9:113261483:C:T
peptide level
2:151
1:133
0:41
IGSYQDEEGQLECKpc2_NP.1
p−min=0.6, pX=0.63, N=3
var.8.SVEP1 9:113261483:C:T
peptide level
26:151
18:133
8:41
IGSYQDEEGQLECKpc2_NP.2
p−maj=0.5, pX=0.79, N=52
var.8.SVEP1 9:113261483:C:T
peptide level
7:151
5:133
1:41
IGSYQDEEGQLECKpc2_NP.4
p−min=0.78, pX=0.92, N=13
var.8.SVEP1 9:113261483:C:T
peptide level
134:151
121:133
34:41
IGSYQDEEGQLECKpc2_NP.5
p−maj=0.19, pX=0.85, N=289
var.8.SVEP1 9:113261483:C:T
peptide level
27:151
24:133
5:41
ISCGPPAHVENAIARpc3_NP.1
p−maj=0.51, pX=0.49, N=56
var.8.SVEP1 9:113261483:C:T
peptide level
127:151
113:133
35:41
ISCGPPAHVENAIARpc3_NP.2
p−min=0.88, pX=0.85, N=275
var.8.SVEP1 9:113261483:C:T
peptide level
2:151
2:133
0:41
ISCGPPAHVENAIARpc3_NP.3
p−maj=1, pX=0.47, N=4
var.8.SVEP1 9:113261483:C:T
peptide level
10:151
7:133
3:41
ISCGPPAHVENAIARpc3_NP.4
p−maj=0.73, pX=0.72, N=20
var.8.SVEP1 9:113261483:C:T
peptide level
150:151
132:133
40:41
ISCGPPAHVENAIARpc3_NP.5
p−maj=0.33, pX=0.33, N=322

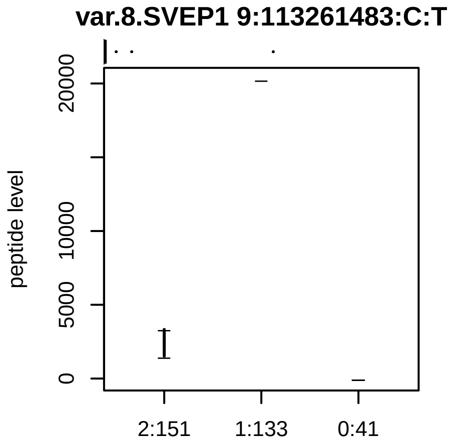

ITCLESGEWNHLIPYCKpc3_NP.1
p−min=0.6, pX=0.8, N=3

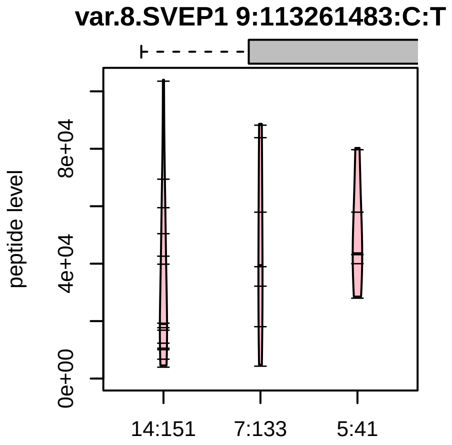

ITCLESGEWNHLIPYCKpc3_NP.2
p−maj=0.35, pX=0.42, N=26

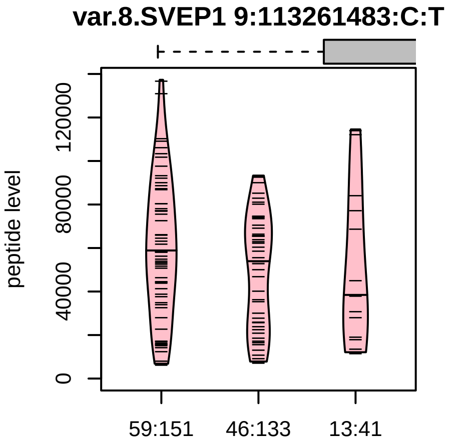

ITCLESGEWNHLIPYCKpc3_NP.5
p−min=0.36, pX=0.079, N=118

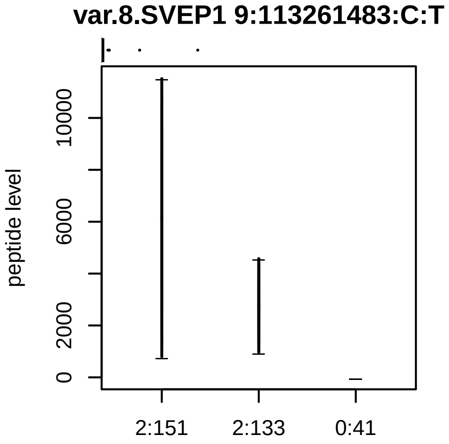

LCEEPICILPCLNGGRpc2_NP.4
p−maj=1, pX=0.47, N=4

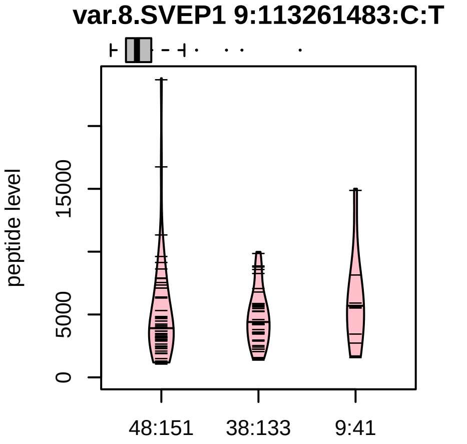

LCEEPICILPCLNGGRpc2_NP.5
p−maj=0.36, pX=0.41, N=95

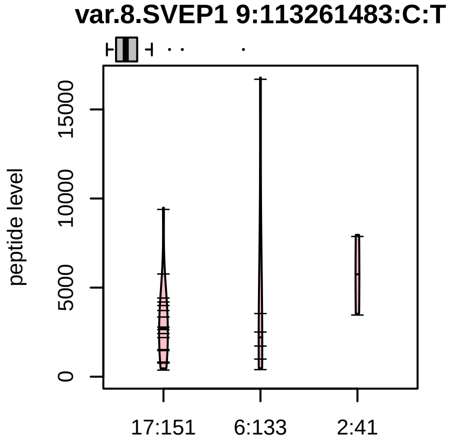

LCPSGMYTEYIHSRpc2_NP.2
p−min=0.035, pX=0.55, N=25

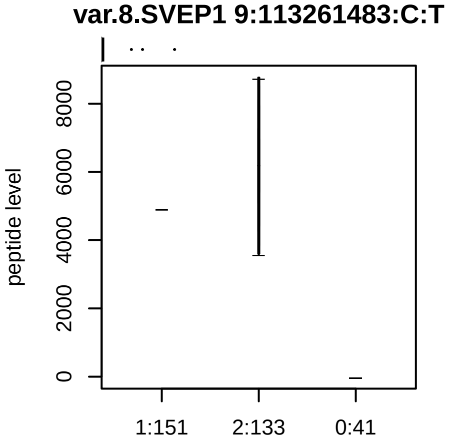

LCPSGMYTEYIHSRpc2_NP.4
p−min=1, pX=0.9, N=3

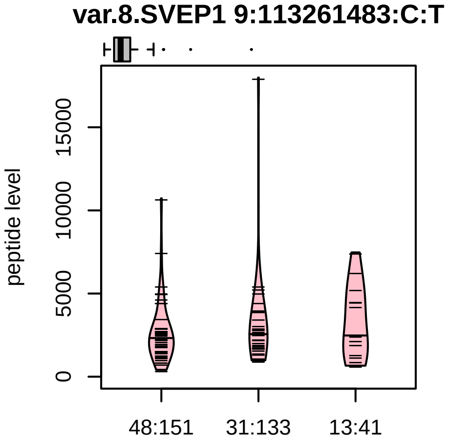

LCPSGMYTEYIHSRpc2_NP.5
p−min=0.22, pX=0.76, N=92

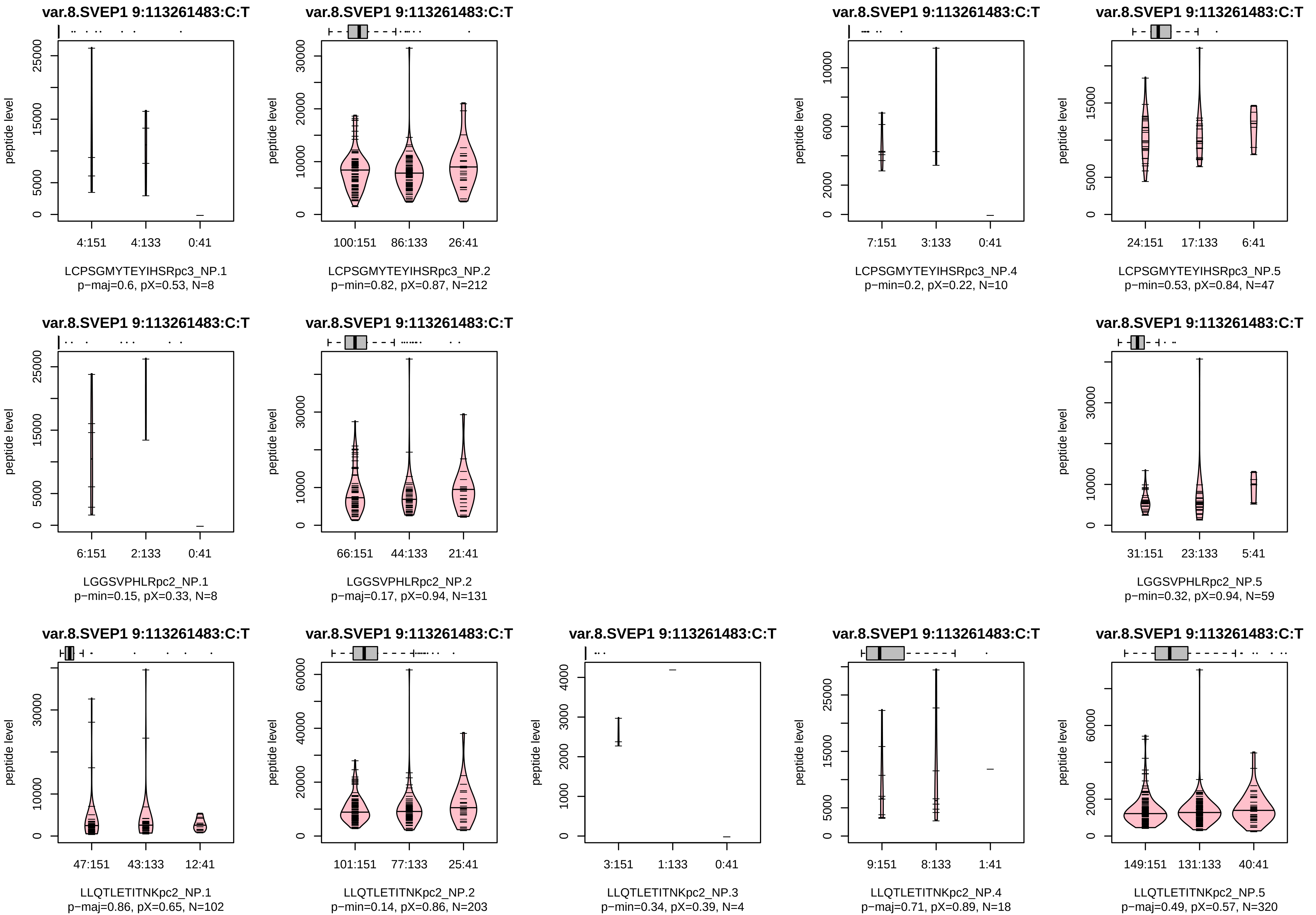
var.8.SVEP1 9:113261483:C:T
peptide level
0
5000
15000
25000
4:151
4:133
0:41
LCPSGMYTEYIHSRpc3_NP.1
p−maj=0.6, pX=0.53, N=8
var.8.SVEP1 9:113261483:C:T
peptide level
0
10000
20000
30000
100:151
86:133
26:41
LCPSGMYTEYIHSRpc3_NP.2
p−min=0.82, pX=0.87, N=212
var.8.SVEP1 9:113261483:C:T
peptide level
0
2000
6000
10000
7:151
3:133
0:41
LCPSGMYTEYIHSRpc3_NP.4
p−min=0.2, pX=0.22, N=10
var.8.SVEP1 9:113261483:C:T
peptide level
0
5000
15000
24:151
17:133
6:41
LCPSGMYTEYIHSRpc3_NP.5
p−min=0.53, pX=0.84, N=47
var.8.SVEP1 9:113261483:C:T
peptide level
0
5000
15000
25000
6:151
2:133
0:41
LGGSVPHLRpc2_NP.1
p−min=0.15, pX=0.33, N=8
var.8.SVEP1 9:113261483:C:T
peptide level
0
10000
30000
66:151
44:133
21:41
LGGSVPHLRpc2_NP.2
p−maj=0.17, pX=0.94, N=131
var.8.SVEP1 9:113261483:C:T
peptide level
0
10000
30000
31:151
23:133
5:41
LGGSVPHLRpc2_NP.5
p−min=0.32, pX=0.94, N=59
var.8.SVEP1 9:113261483:C:T
peptide level
0
10000
30000
47:151
43:133
12:41
LLQTLETITNKpc2_NP.1
p−maj=0.86, pX=0.65, N=102
var.8.SVEP1 9:113261483:C:T
peptide level
0
20000
40000
60000
101:151
77:133
25:41
LLQTLETITNKpc2_NP.2
p−min=0.14, pX=0.86, N=203
var.8.SVEP1 9:113261483:C:T
peptide level
0
1000
2000
3000
4000
3:151
1:133
0:41
LLQTLETITNKpc2_NP.3
p−min=0.34, pX=0.39, N=4
var.8.SVEP1 9:113261483:C:T
peptide level
0
5000
15000
25000
9:151
8:133
1:41
LLQTLETITNKpc2_NP.4
p−maj=0.71, pX=0.89, N=18
var.8.SVEP1 9:113261483:C:T
peptide level
0
20000
60000
149:151
131:133
40:41
LLQTLETITNKpc2_NP.5
p−maj=0.49, pX=0.57, N=320

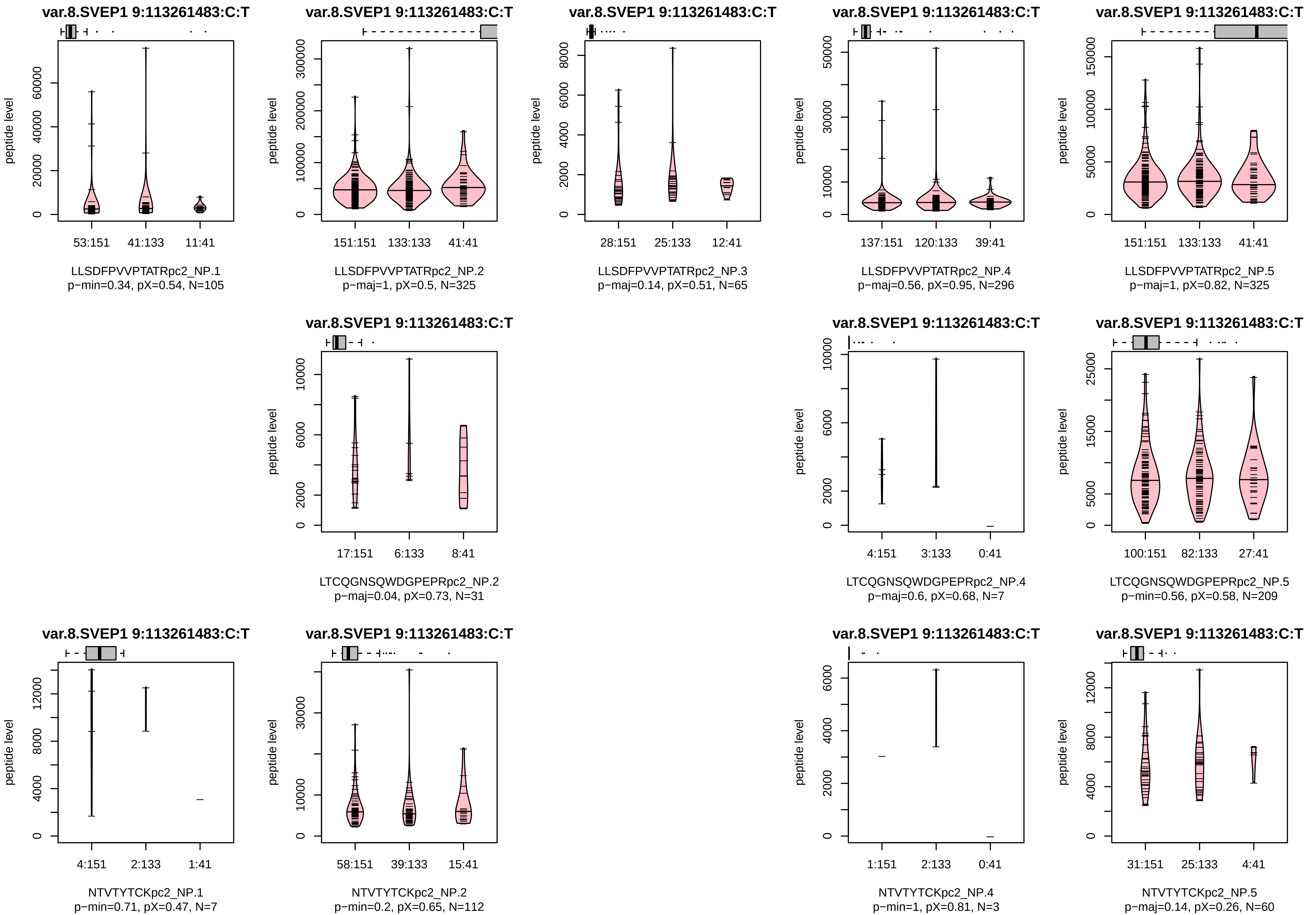
var.8.SVEP1 9:113261483:C:T
peptide level
0
20000
40000
60000
53:151
41:133
11:41
LLSDFPVVPTATRpc2_NP.1
p−min=0.34, pX=0.54, N=105
var.8.SVEP1 9:113261483:C:T
peptide level
0
100000
200000
300000
151:151
133:133
41:41
LLSDFPVVPTATRpc2_NP.2
p−maj=1, pX=0.5, N=325
var.8.SVEP1 9:113261483:C:T
peptide level
0
2000
4000
6000
8000
28:151
25:133
12:41
LLSDFPVVPTATRpc2_NP.3
p−maj=0.14, pX=0.51, N=65
var.8.SVEP1 9:113261483:C:T
peptide level
0
10000
30000
50000
137:151
120:133
39:41
LLSDFPVVPTATRpc2_NP.4
p−maj=0.56, pX=0.95, N=296
var.8.SVEP1 9:113261483:C:T
peptide level
0
50000
100000
150000
151:151
133:133
41:41
LLSDFPVVPTATRpc2_NP.5
p−maj=1, pX=0.82, N=325
var.8.SVEP1 9:113261483:C:T
peptide level
0
2000
6000
10000
17:151
6:133
8:41
LTCQGNSQWDGPEPRpc2_NP.2
p−maj=0.04, pX=0.73, N=31
var.8.SVEP1 9:113261483:C:T
peptide level
0
2000
6000
10000
4:151
3:133
0:41
LTCQGNSQWDGPEPRpc2_NP.4
p−maj=0.6, pX=0.68, N=7
var.8.SVEP1 9:113261483:C:T
peptide level
0
5000
15000
25000
100:151
82:133
27:41
LTCQGNSQWDGPEPRpc2_NP.5
p−min=0.56, pX=0.58, N=209
var.8.SVEP1 9:113261483:C:T
peptide level
0
4000
8000
12000
4:151
2:133
1:41
NTVTYTCKpc2_NP.1
p−min=0.71, pX=0.47, N=7
var.8.SVEP1 9:113261483:C:T
peptide level
0
10000
30000
58:151
39:133
15:41
NTVTYTCKpc2_NP.2
p−min=0.2, pX=0.65, N=112
var.8.SVEP1 9:113261483:C:T
peptide level
0
2000
4000
6000
1:151
2:133
0:41
NTVTYTCKpc2_NP.4
p−min=1, pX=0.81, N=3
var.8.SVEP1 9:113261483:C:T
peptide level
0
4000
8000
12000
31:151
25:133
4:41
NTVTYTCKpc2_NP.5
p−maj=0.14, pX=0.26, N=60

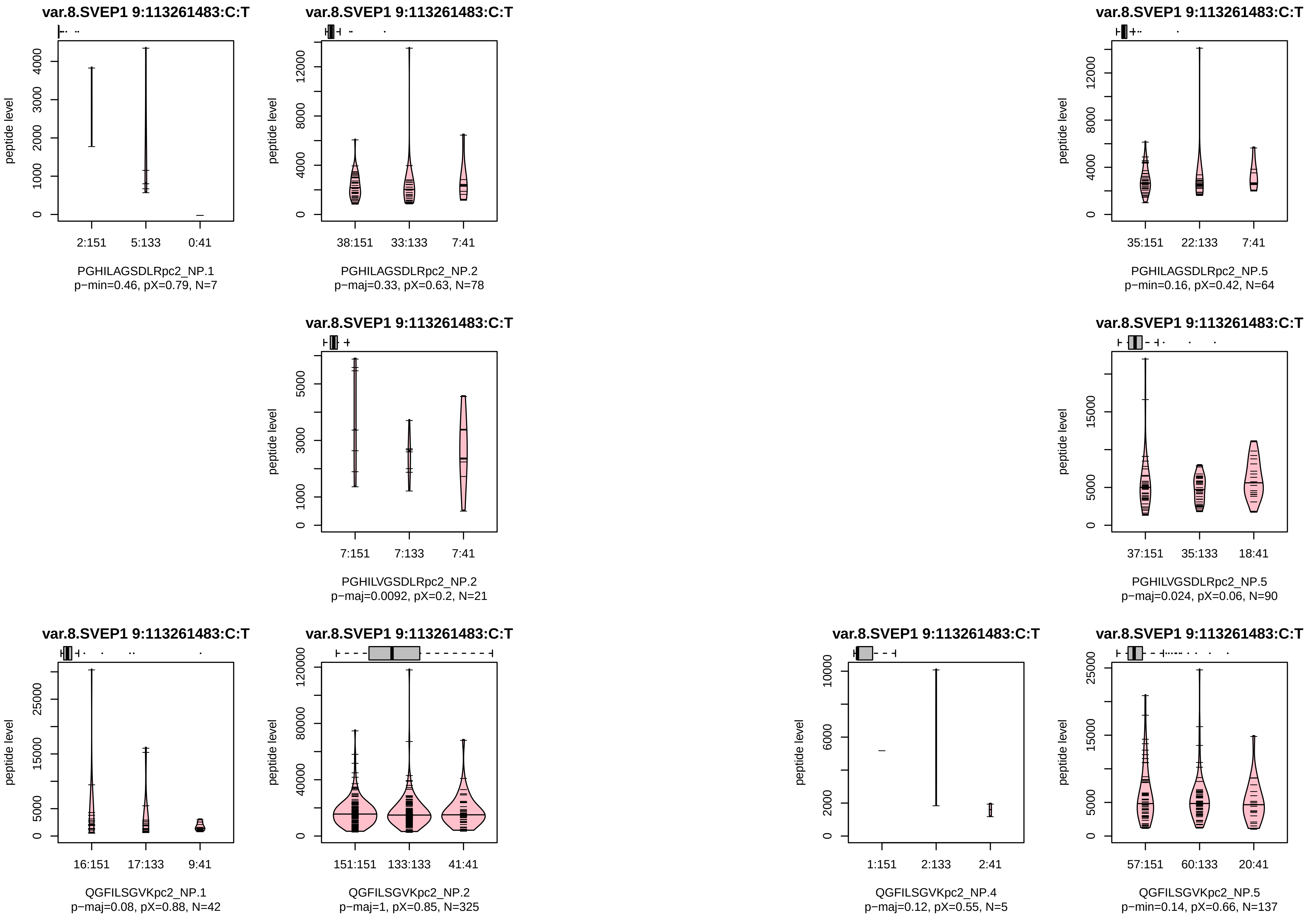
var.8.SVEP1 9:113261483:C:T
peptide level
2:151
5:133
0:41
PGHILAGSDLRpc2_NP.1
p−min=0.46, pX=0.79, N=7
var.8.SVEP1 9:113261483:C:T
peptide level
38:151
33:133
7:41
PGHILAGSDLRpc2_NP.2
p−maj=0.33, pX=0.63, N=78
var.8.SVEP1 9:113261483:C:T
peptide level
35:151
22:133
7:41
PGHILAGSDLRpc2_NP.5
p−min=0.16, pX=0.42, N=64
var.8.SVEP1 9:113261483:C:T
peptide level
7:151
7:133
7:41
PGHILVGSDLRpc2_NP.2
p−maj=0.0092, pX=0.2, N=21
var.8.SVEP1 9:113261483:C:T
peptide level
37:151
35:133
18:41
PGHILVGSDLRpc2_NP.5
p−maj=0.024, pX=0.06, N=90
var.8.SVEP1 9:113261483:C:T
peptide level
16:151
17:133
9:41
QGFILSGVKpc2_NP.1
p−maj=0.08, pX=0.88, N=42
var.8.SVEP1 9:113261483:C:T
peptide level
151:151
133:133
41:41
QGFILSGVKpc2_NP.2
p−maj=1, pX=0.85, N=325
var.8.SVEP1 9:113261483:C:T
peptide level
1:151
2:133
2:41
QGFILSGVKpc2_NP.4
p−maj=0.12, pX=0.55, N=5
var.8.SVEP1 9:113261483:C:T
peptide level
57:151
60:133
20:41
QGFILSGVKpc2_NP.5
p−min=0.14, pX=0.66, N=137

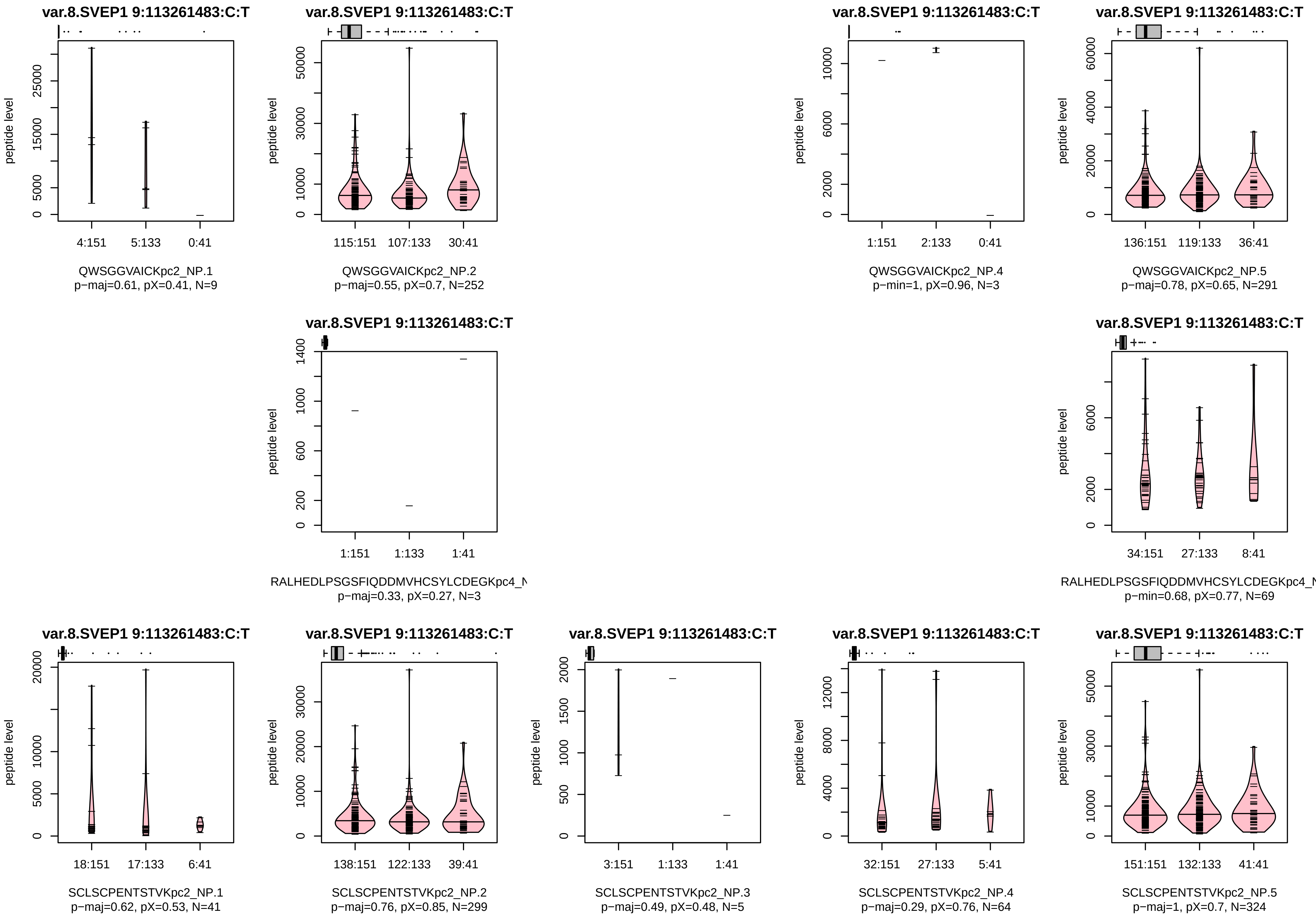
var.8.SVEP1 9:113261483:C:T
peptide level
4:151
5:133
0:41
QWSGGVAICKpc2_NP.1
p−maj=0.61, pX=0.41, N=9
var.8.SVEP1 9:113261483:C:T
peptide level
115:151
107:133
30:41
QWSGGVAICKpc2_NP.2
p−maj=0.55, pX=0.7, N=252
var.8.SVEP1 9:113261483:C:T
peptide level
1:151
2:133
0:41
QWSGGVAICKpc2_NP.4
p−min=1, pX=0.96, N=3
var.8.SVEP1 9:113261483:C:T
peptide level
136:151
119:133
36:41
QWSGGVAICKpc2_NP.5
p−maj=0.78, pX=0.65, N=291
var.8.SVEP1 9:113261483:C:T
peptide level
1:151
1:133
1:41
RALHEDLPSGSFIQDDMVHCSYLCDEGKpc4_N
p−maj=0.33, pX=0.27, N=3
var.8.SVEP1 9:113261483:C:T
peptide level
34:151
27:133
8:41
RALHEDLPSGSFIQDDMVHCSYLCDEGKpc4_N
p−min=0.68, pX=0.77, N=69
var.8.SVEP1 9:113261483:C:T
peptide level
18:151
17:133
6:41
SCLSCPENTSTVKpc2_NP.1
p−maj=0.62, pX=0.53, N=41
var.8.SVEP1 9:113261483:C:T
peptide level
138:151
122:133
39:41
SCLSCPENTSTVKpc2_NP.2
p−maj=0.76, pX=0.85, N=299
var.8.SVEP1 9:113261483:C:T
peptide level
3:151
1:133
1:41
SCLSCPENTSTVKpc2_NP.3
p−maj=0.49, pX=0.48, N=5
var.8.SVEP1 9:113261483:C:T
peptide level
32:151
27:133
5:41
SCLSCPENTSTVKpc2_NP.4
p−maj=0.29, pX=0.76, N=64
var.8.SVEP1 9:113261483:C:T
peptide level
151:151
132:133
41:41
SCLSCPENTSTVKpc2_NP.5
p−maj=1, pX=0.7, N=324

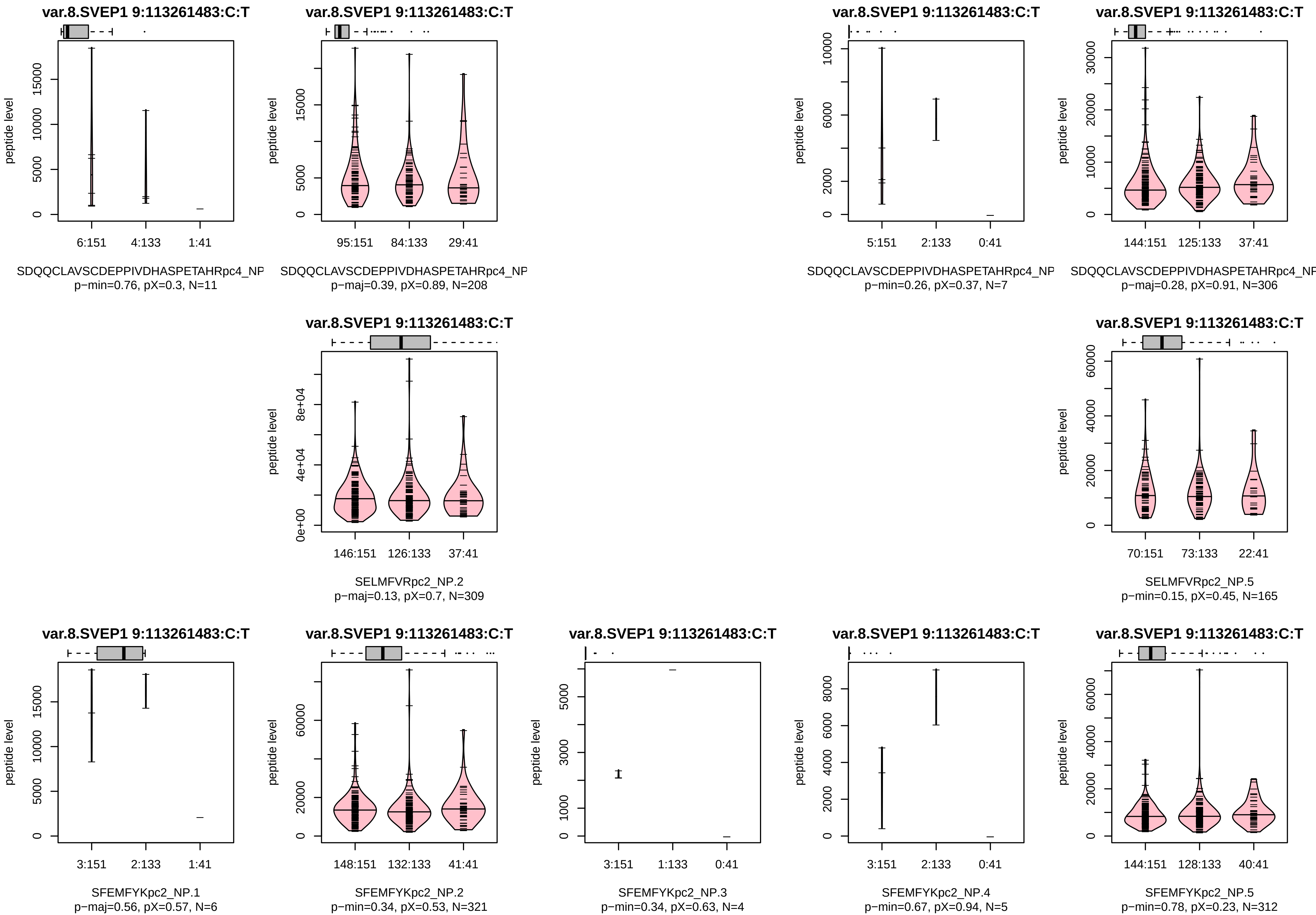
var.8.SVEP1 9:113261483:C:T
peptide level
6:151
4:133
1:41
SDQQCLAVSCDEPPIVDHASPETAHRpc4_NP
p−min=0.76, pX=0.3, N=11
var.8.SVEP1 9:113261483:C:T
peptide level
95:151
84:133
29:41
SDQQCLAVSCDEPPIVDHASPETAHRpc4_NP
p−maj=0.39, pX=0.89, N=208
var.8.SVEP1 9:113261483:C:T
peptide level
5:151
2:133
0:41
SDQQCLAVSCDEPPIVDHASPETAHRpc4_NP
p−min=0.26, pX=0.37, N=7
var.8.SVEP1 9:113261483:C:T
peptide level
144:151
125:133
37:41
SDQQCLAVSCDEPPIVDHASPETAHRpc4_NP
p−maj=0.28, pX=0.91, N=306
var.8.SVEP1 9:113261483:C:T
peptide level
146:151
126:133
37:41
SELMFVRpc2_NP.2
p−maj=0.13, pX=0.7, N=309
var.8.SVEP1 9:113261483:C:T
peptide level
70:151
73:133
22:41
SELMFVRpc2_NP.5
p−min=0.15, pX=0.45, N=165
var.8.SVEP1 9:113261483:C:T
peptide level
3:151
2:133
1:41
SFEMFYKpc2_NP.1
p−maj=0.56, pX=0.57, N=6
var.8.SVEP1 9:113261483:C:T
peptide level
148:151
132:133
41:41
SFEMFYKpc2_NP.2
p−min=0.34, pX=0.53, N=321
var.8.SVEP1 9:113261483:C:T
peptide level
3:151
1:133
0:41
SFEMFYKpc2_NP.3
p−min=0.34, pX=0.63, N=4
var.8.SVEP1 9:113261483:C:T
peptide level
3:151
2:133
0:41
SFEMFYKpc2_NP.4
p−min=0.67, pX=0.94, N=5
var.8.SVEP1 9:113261483:C:T
peptide level
144:151
128:133
40:41
SFEMFYKpc2_NP.5
p−min=0.78, pX=0.23, N=312

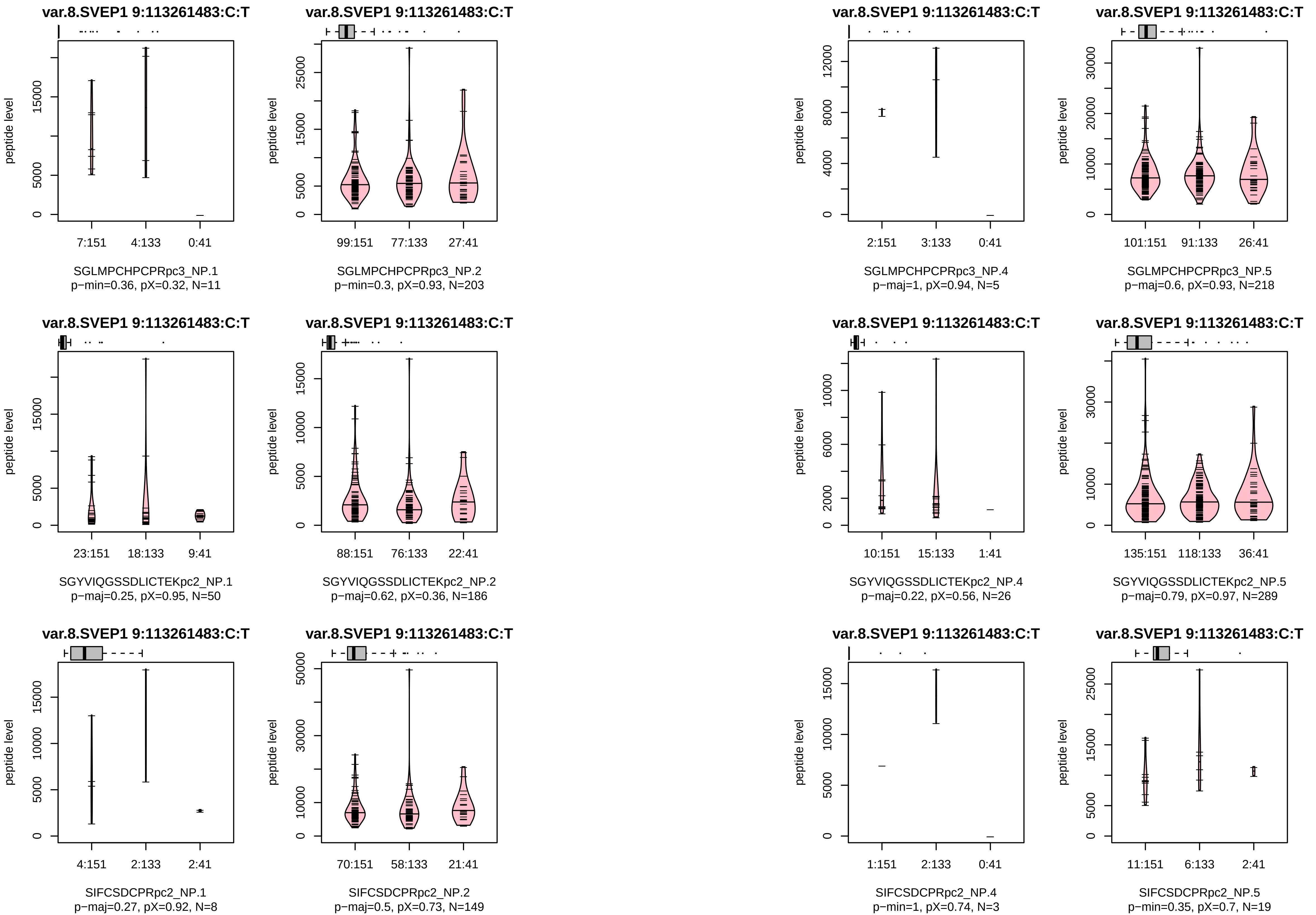

var.8.SVEP1 9:113261483:C:T
peptide level
7:151
4:133
0:41
SGLMPCHPCPRpc3_NP.1
p−min=0.36, pX=0.32, N=11
var.8.SVEP1 9:113261483:C:T
peptide level
99:151
77:133
27:41
SGLMPCHPCPRpc3_NP.2
p−min=0.3, pX=0.93, N=203
var.8.SVEP1 9:113261483:C:T
peptide level
2:151
3:133
0:41
SGLMPCHPCPRpc3_NP.4
p−maj=1, pX=0.94, N=5
var.8.SVEP1 9:113261483:C:T
peptide level
101:151
91:133
26:41
SGLMPCHPCPRpc3_NP.5
p−maj=0.6, pX=0.93, N=218
var.8.SVEP1 9:113261483:C:T
peptide level
23:151
18:133
9:41
SGYVIQGSSDLICTEKpc2_NP.1
p−maj=0.25, pX=0.95, N=50
var.8.SVEP1 9:113261483:C:T
peptide level
88:151
76:133
22:41
SGYVIQGSSDLICTEKpc2_NP.2
p−maj=0.62, pX=0.36, N=186
var.8.SVEP1 9:113261483:C:T
peptide level
10:151
15:133
1:41
SGYVIQGSSDLICTEKpc2_NP.4
p−maj=0.22, pX=0.56, N=26
var.8.SVEP1 9:113261483:C:T
peptide level
135:151
118:133
36:41
SGYVIQGSSDLICTEKpc2_NP.5
p−maj=0.79, pX=0.97, N=289
var.8.SVEP1 9:113261483:C:T
peptide level
4:151
2:133
2:41
SIFCSDCPRpc2_NP.1
p−maj=0.27, pX=0.92, N=8
var.8.SVEP1 9:113261483:C:T
peptide level
70:151
58:133
21:41
SIFCSDCPRpc2_NP.2
p−maj=0.5, pX=0.73, N=149
var.8.SVEP1 9:113261483:C:T
peptide level
1:151
2:133
0:41
SIFCSDCPRpc2_NP.4
p−min=1, pX=0.74, N=3
var.8.SVEP1 9:113261483:C:T
peptide level
11:151
6:133
2:41
SIFCSDCPRpc2_NP.5
p−min=0.35, pX=0.7, N=19

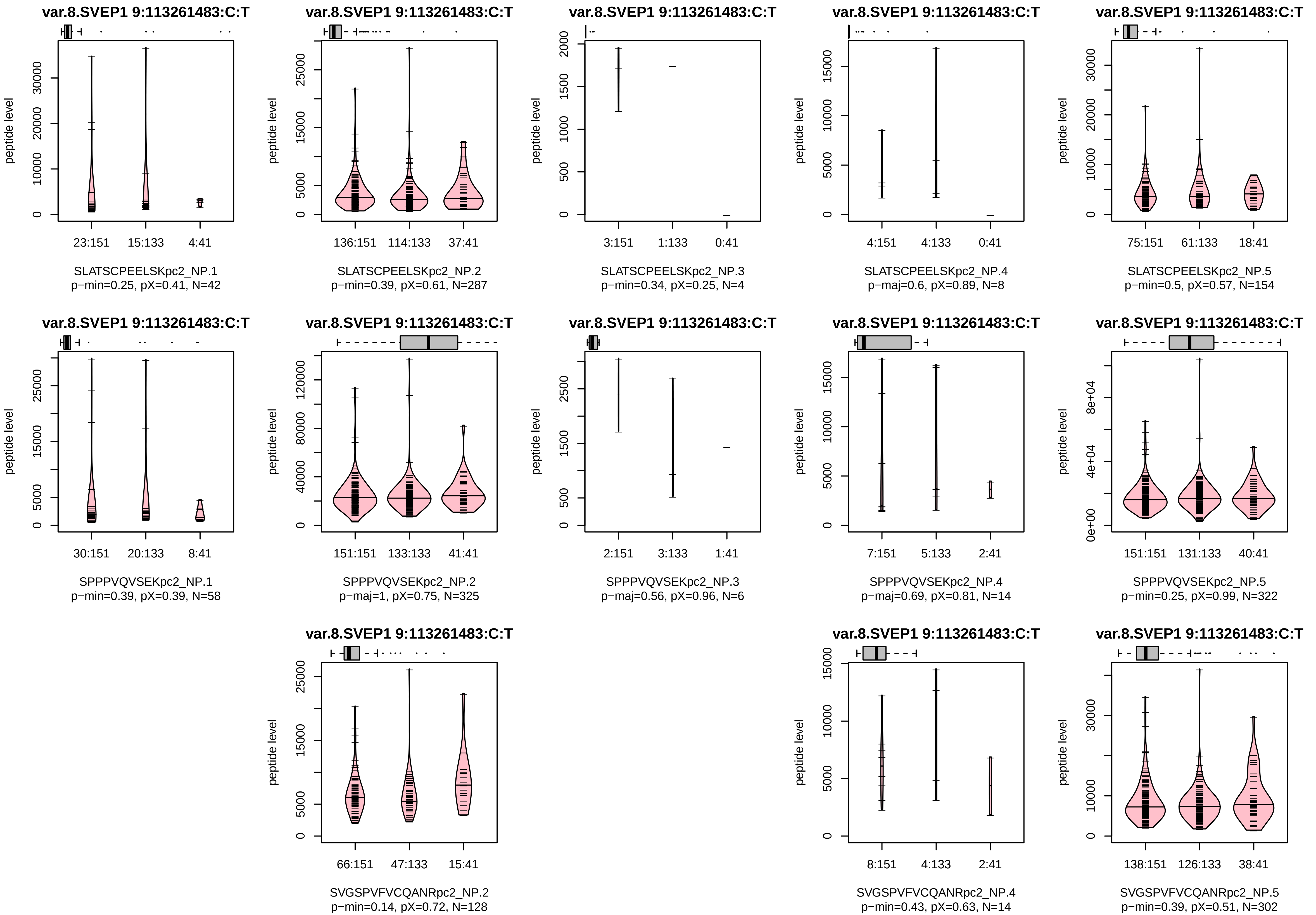

var.8.SVEP1 9:113261483:C:T
peptide level
23:151
15:133
4:41
SLATSCPEELSKpc2_NP.1
p−min=0.25, pX=0.41, N=42
var.8.SVEP1 9:113261483:C:T
peptide level
136:151
114:133
37:41
SLATSCPEELSKpc2_NP.2
p−min=0.39, pX=0.61, N=287
var.8.SVEP1 9:113261483:C:T
peptide level
3:151
1:133
0:41
SLATSCPEELSKpc2_NP.3
p−min=0.34, pX=0.25, N=4
var.8.SVEP1 9:113261483:C:T
peptide level
4:151
4:133
0:41
SLATSCPEELSKpc2_NP.4
p−maj=0.6, pX=0.89, N=8
var.8.SVEP1 9:113261483:C:T
peptide level
75:151
61:133
18:41
SLATSCPEELSKpc2_NP.5
p−min=0.5, pX=0.57, N=154
var.8.SVEP1 9:113261483:C:T
peptide level
30:151
20:133
8:41
SPPPVQVSEKpc2_NP.1
p−min=0.39, pX=0.39, N=58
var.8.SVEP1 9:113261483:C:T
peptide level
151:151
133:133
41:41
SPPPVQVSEKpc2_NP.2
p−maj=1, pX=0.75, N=325
var.8.SVEP1 9:113261483:C:T
peptide level
2:151
3:133
1:41
SPPPVQVSEKpc2_NP.3
p−maj=0.56, pX=0.96, N=6
var.8.SVEP1 9:113261483:C:T
peptide level
7:151
5:133
2:41
SPPPVQVSEKpc2_NP.4
p−maj=0.69, pX=0.81, N=14
var.8.SVEP1 9:113261483:C:T
peptide level
151:151
131:133
40:41
SPPPVQVSEKpc2_NP.5
p−min=0.25, pX=0.99, N=322
var.8.SVEP1 9:113261483:C:T
peptide level
66:151
47:133
15:41
SVGSPVFVCQANRpc2_NP.2
p−min=0.14, pX=0.72, N=128
var.8.SVEP1 9:113261483:C:T
peptide level
8:151
4:133
2:41
SVGSPVFVCQANRpc2_NP.4
p−min=0.43, pX=0.63, N=14
var.8.SVEP1 9:113261483:C:T
peptide level
138:151
126:133
38:41
SVGSPVFVCQANRpc2_NP.5
p−min=0.39, pX=0.51, N=302

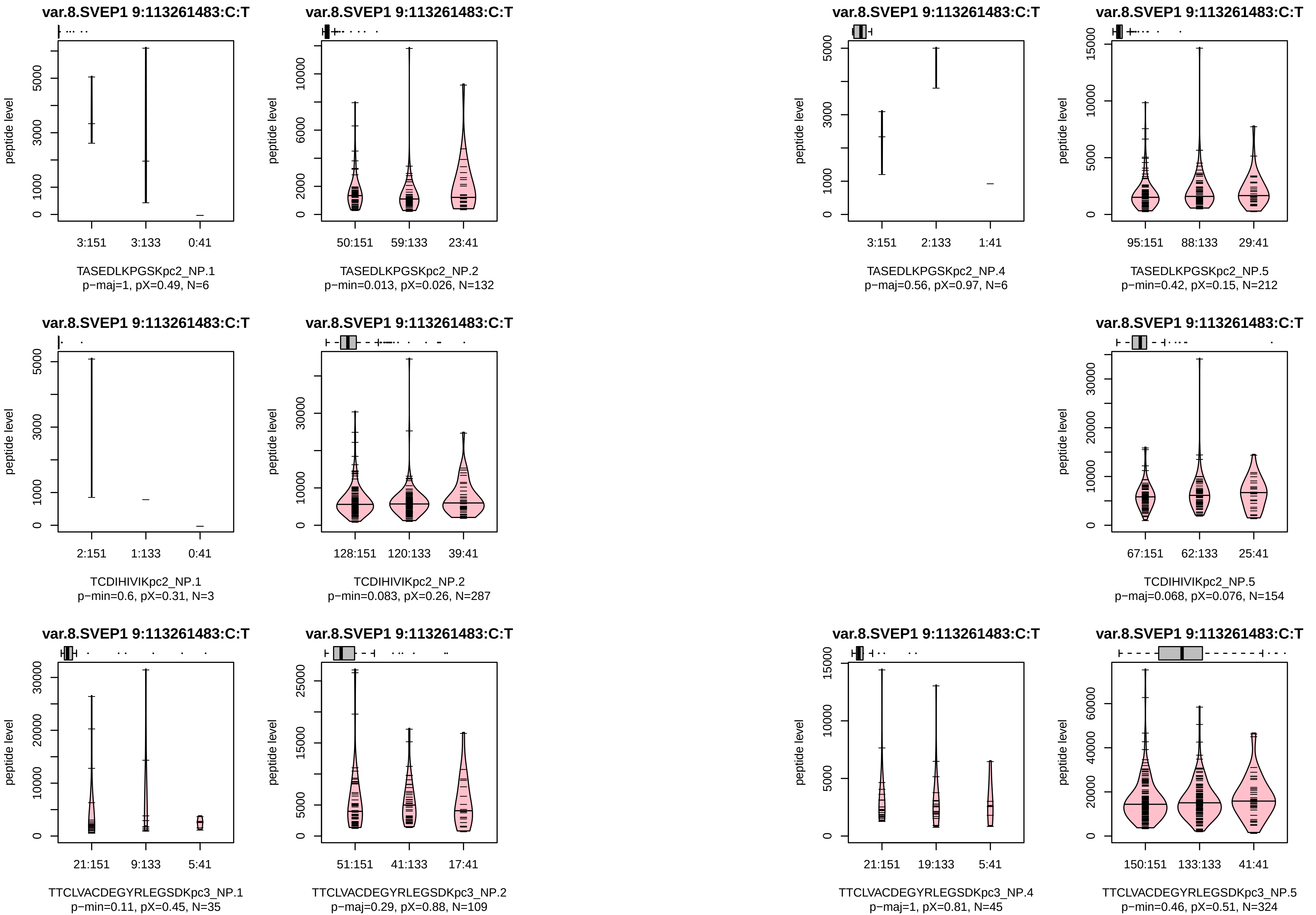

var.8.SVEP1 9:113261483:C:T
peptide level
3:151
3:133
0:41
TASEDLKPGSKpc2_NP.1
p−maj=1, pX=0.49, N=6
var.8.SVEP1 9:113261483:C:T
peptide level
50:151
59:133
23:41
TASEDLKPGSKpc2_NP.2
p−min=0.013, pX=0.026, N=132
var.8.SVEP1 9:113261483:C:T
peptide level
3:151
2:133
1:41
TASEDLKPGSKpc2_NP.4
p−maj=0.56, pX=0.97, N=6
var.8.SVEP1 9:113261483:C:T
peptide level
95:151
88:133
29:41
TASEDLKPGSKpc2_NP.5
p−min=0.42, pX=0.15, N=212
var.8.SVEP1 9:113261483:C:T
peptide level
2:151
1:133
0:41
TCDIHIVIKpc2_NP.1
p−min=0.6, pX=0.31, N=3
var.8.SVEP1 9:113261483:C:T
peptide level
128:151
120:133
39:41
TCDIHIVIKpc2_NP.2
p−min=0.083, pX=0.26, N=287
var.8.SVEP1 9:113261483:C:T
peptide level
67:151
62:133
25:41
TCDIHIVIKpc2_NP.5
p−maj=0.068, pX=0.076, N=154
var.8.SVEP1 9:113261483:C:T
peptide level
21:151
9:133
5:41
TTCLVACDEGYRLEGSDKpc3_NP.1
p−min=0.11, pX=0.45, N=35
var.8.SVEP1 9:113261483:C:T
peptide level
51:151
41:133
17:41
TTCLVACDEGYRLEGSDKpc3_NP.2
p−maj=0.29, pX=0.88, N=109
var.8.SVEP1 9:113261483:C:T
peptide level
21:151
19:133
5:41
TTCLVACDEGYRLEGSDKpc3_NP.4
p−maj=1, pX=0.81, N=45
var.8.SVEP1 9:113261483:C:T
peptide level
150:151
133:133
41:41
TTCLVACDEGYRLEGSDKpc3_NP.5
p−min=0.46, pX=0.51, N=324

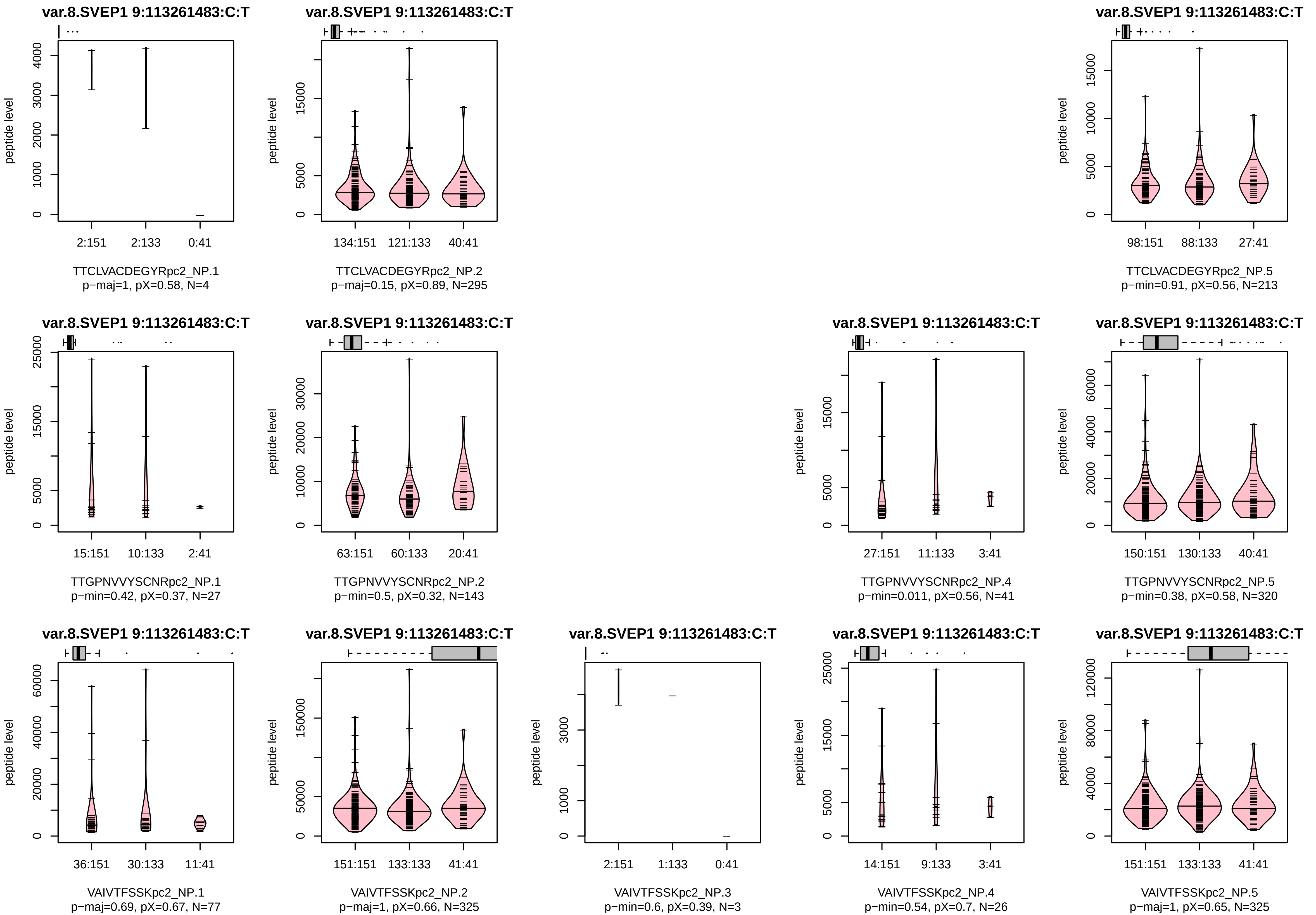
var.8.SVEP1 9:113261483:C:T
peptide level
2:151
2:133
0:41
TTCLVACDEGYRpc2_NP.1
p−maj=1, pX=0.58, N=4
var.8.SVEP1 9:113261483:C:T
peptide level
134:151
121:133
40:41
TTCLVACDEGYRpc2_NP.2
p−maj=0.15, pX=0.89, N=295
var.8.SVEP1 9:113261483:C:T
peptide level
98:151
88:133
27:41
TTCLVACDEGYRpc2_NP.5
p−min=0.91, pX=0.56, N=213
var.8.SVEP1 9:113261483:C:T
peptide level
15:151
10:133
2:41
TTGPNVVYSCNRpc2_NP.1
p−min=0.42, pX=0.37, N=27
var.8.SVEP1 9:113261483:C:T
peptide level
63:151
60:133
20:41
TTGPNVVYSCNRpc2_NP.2
p−min=0.5, pX=0.32, N=143
var.8.SVEP1 9:113261483:C:T
peptide level
27:151
11:133
3:41
TTGPNVVYSCNRpc2_NP.4
p−min=0.011, pX=0.56, N=41
var.8.SVEP1 9:113261483:C:T
peptide level
150:151
130:133
40:41
TTGPNVVYSCNRpc2_NP.5
p−min=0.38, pX=0.58, N=320
var.8.SVEP1 9:113261483:C:T
peptide level
36:151
30:133
11:41
VAIVTFSSKpc2_NP.1
p−maj=0.69, pX=0.67, N=77
var.8.SVEP1 9:113261483:C:T
peptide level
151:151
133:133
41:41
VAIVTFSSKpc2_NP.2
p−maj=1, pX=0.66, N=325
var.8.SVEP1 9:113261483:C:T
peptide level
2:151
1:133
0:41
VAIVTFSSKpc2_NP.3
p−min=0.6, pX=0.39, N=3
var.8.SVEP1 9:113261483:C:T
peptide level
14:151
9:133
3:41
VAIVTFSSKpc2_NP.4
p−min=0.54, pX=0.7, N=26
var.8.SVEP1 9:113261483:C:T
peptide level
151:151
133:133
41:41
VAIVTFSSKpc2_NP.5
p−maj=1, pX=0.65, N=325

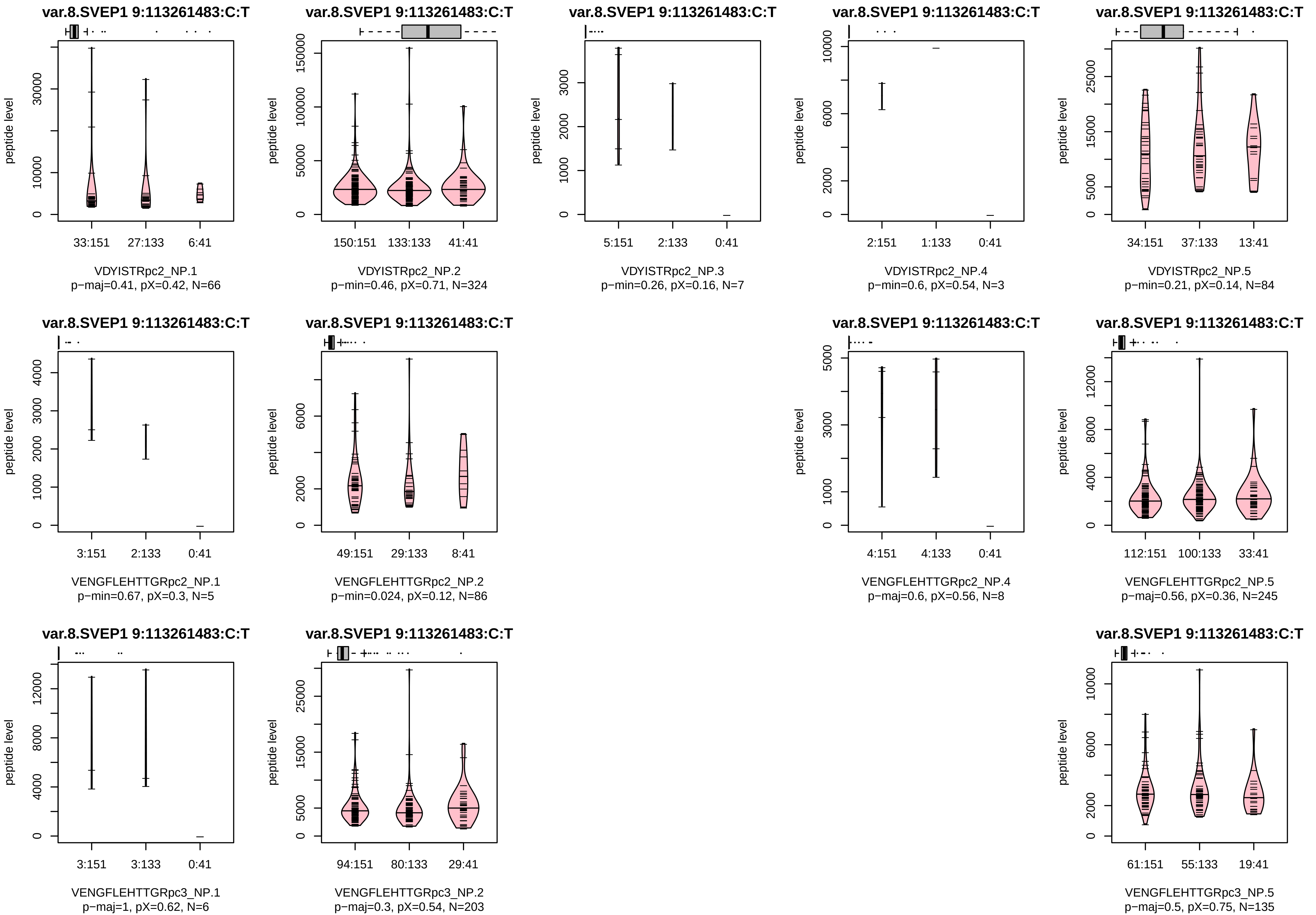

var.8.SVEP1 9:113261483:C:T
peptide level
33:151
27:133
6:41
VDYISTRpc2_NP.1
p−maj=0.41, pX=0.42, N=66
var.8.SVEP1 9:113261483:C:T
peptide level
150:151
133:133
41:41
VDYISTRpc2_NP.2
p−min=0.46, pX=0.71, N=324
var.8.SVEP1 9:113261483:C:T
peptide level
5:151
2:133
0:41
VDYISTRpc2_NP.3
p−min=0.26, pX=0.16, N=7
var.8.SVEP1 9:113261483:C:T
peptide level
2:151
1:133
0:41
VDYISTRpc2_NP.4
p−min=0.6, pX=0.54, N=3
var.8.SVEP1 9:113261483:C:T
peptide level
34:151
37:133
13:41
VDYISTRpc2_NP.5
p−min=0.21, pX=0.14, N=84
var.8.SVEP1 9:113261483:C:T
peptide level
3:151
2:133
0:41
VENGFLEHTTGRpc2_NP.1
p−min=0.67, pX=0.3, N=5
var.8.SVEP1 9:113261483:C:T
peptide level
49:151
29:133
8:41
VENGFLEHTTGRpc2_NP.2
p−min=0.024, pX=0.12, N=86
var.8.SVEP1 9:113261483:C:T
peptide level
4:151
4:133
0:41
VENGFLEHTTGRpc2_NP.4
p−maj=0.6, pX=0.56, N=8
var.8.SVEP1 9:113261483:C:T
peptide level
112:151
100:133
33:41
VENGFLEHTTGRpc2_NP.5
p−maj=0.56, pX=0.36, N=245
var.8.SVEP1 9:113261483:C:T
peptide level
3:151
3:133
0:41
VENGFLEHTTGRpc3_NP.1
p−maj=1, pX=0.62, N=6
var.8.SVEP1 9:113261483:C:T
peptide level
94:151
80:133
29:41
VENGFLEHTTGRpc3_NP.2
p−maj=0.3, pX=0.54, N=203
var.8.SVEP1 9:113261483:C:T
peptide level
61:151
55:133
19:41
VENGFLEHTTGRpc3_NP.5
p−maj=0.5, pX=0.75, N=135

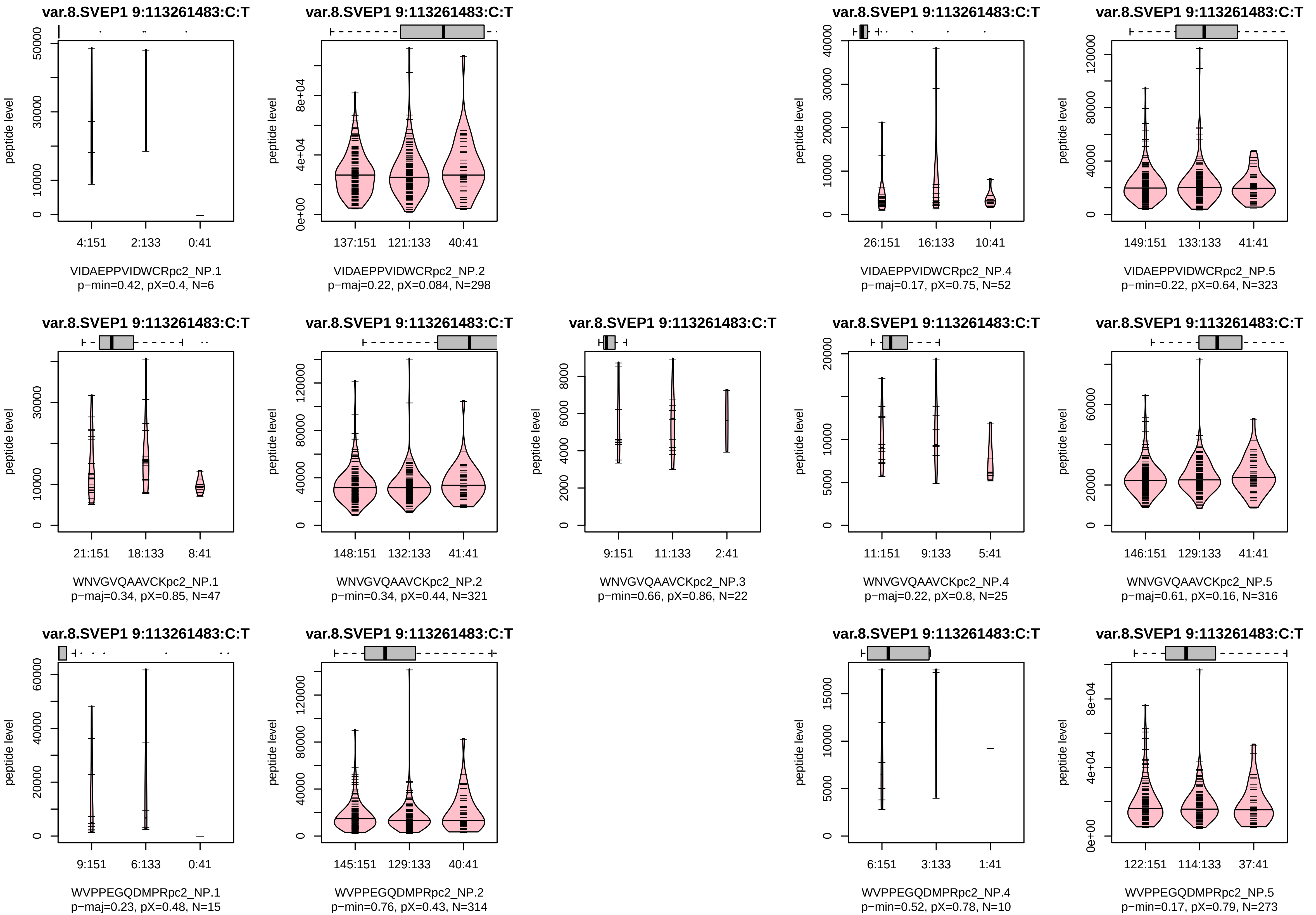

var.8.SVEP1 9:113261483:C:T
peptide level
4:151
2:133
0:41
VIDAEPPVIDWCRpc2_NP.1
p−min=0.42, pX=0.4, N=6
var.8.SVEP1 9:113261483:C:T
peptide level
137:151
121:133
40:41
VIDAEPPVIDWCRpc2_NP.2
p−maj=0.22, pX=0.084, N=298
var.8.SVEP1 9:113261483:C:T
peptide level
26:151
16:133
10:41
VIDAEPPVIDWCRpc2_NP.4
p−maj=0.17, pX=0.75, N=52
var.8.SVEP1 9:113261483:C:T
peptide level
149:151
133:133
41:41
VIDAEPPVIDWCRpc2_NP.5
p−min=0.22, pX=0.64, N=323
var.8.SVEP1 9:113261483:C:T
peptide level
21:151
18:133
8:41
WNVGVQAAVCKpc2_NP.1
p−maj=0.34, pX=0.85, N=47
var.8.SVEP1 9:113261483:C:T
peptide level
148:151
132:133
41:41
WNVGVQAAVCKpc2_NP.2
p−min=0.34, pX=0.44, N=321
var.8.SVEP1 9:113261483:C:T
peptide level
9:151
11:133
2:41
WNVGVQAAVCKpc2_NP.3
p−min=0.66, pX=0.86, N=22
var.8.SVEP1 9:113261483:C:T
peptide level
11:151
9:133
5:41
WNVGVQAAVCKpc2_NP.4
p−maj=0.22, pX=0.8, N=25
var.8.SVEP1 9:113261483:C:T
peptide level
146:151
129:133
41:41
WNVGVQAAVCKpc2_NP.5
p−maj=0.61, pX=0.16, N=316
var.8.SVEP1 9:113261483:C:T
peptide level
9:151
6:133
0:41
WVPPEGQDMPRpc2_NP.1
p−maj=0.23, pX=0.48, N=15
var.8.SVEP1 9:113261483:C:T
peptide level
145:151
129:133
40:41
WVPPEGQDMPRpc2_NP.2
p−min=0.76, pX=0.43, N=314
var.8.SVEP1 9:113261483:C:T
peptide level
6:151
3:133
1:41
WVPPEGQDMPRpc2_NP.4
p−min=0.52, pX=0.78, N=10
var.8.SVEP1 9:113261483:C:T
peptide level
122:151
114:133
37:41
WVPPEGQDMPRpc2_NP.5
p−min=0.17, pX=0.79, N=273

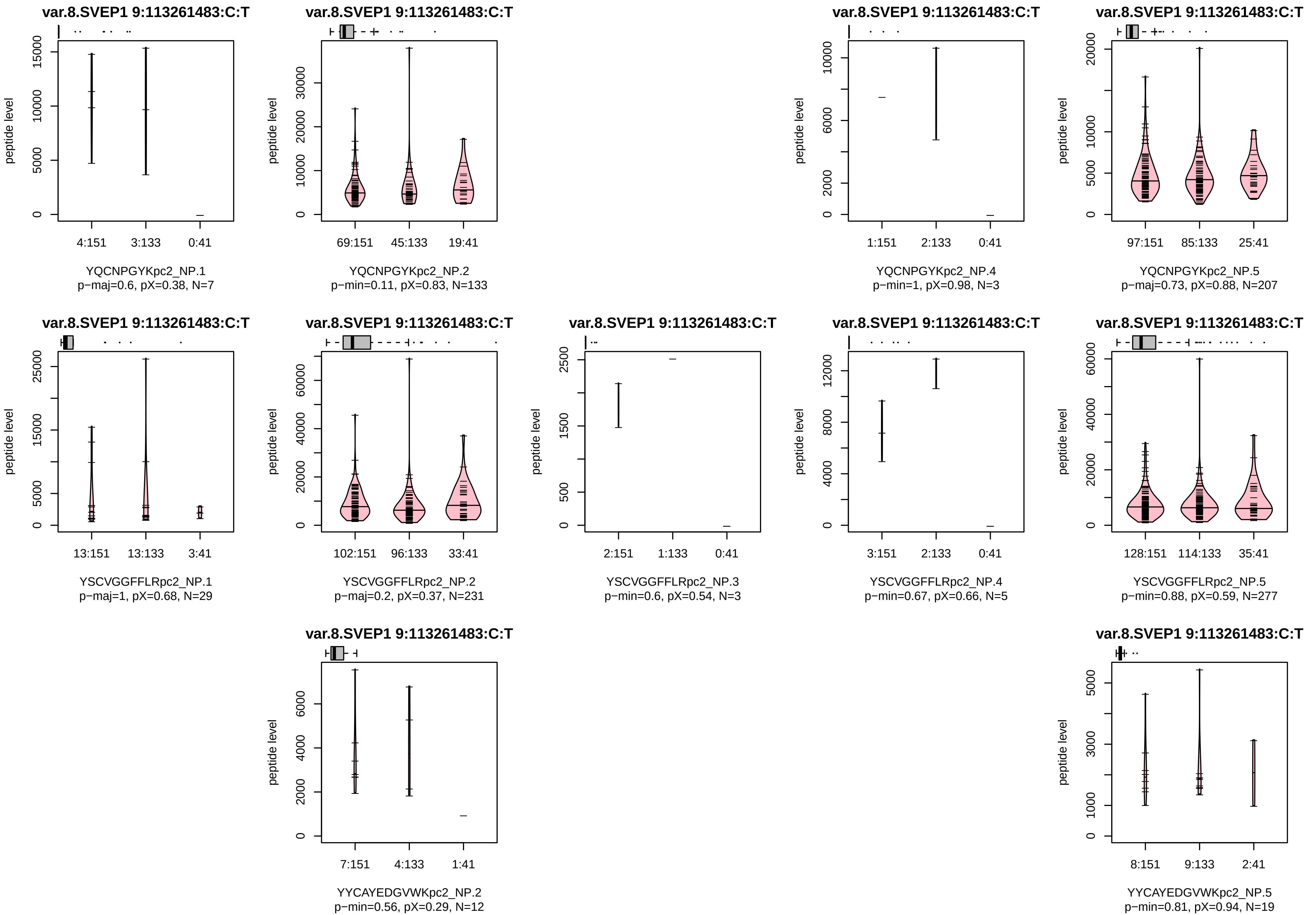
var.8.SVEP1 9:113261483:C:T
peptide level
4:151
3:133
0:41
YQCNPGYKpc2_NP.1
p−maj=0.6, pX=0.38, N=7
var.8.SVEP1 9:113261483:C:T
peptide level
69:151
45:133
19:41
YQCNPGYKpc2_NP.2
p−min=0.11, pX=0.83, N=133
var.8.SVEP1 9:113261483:C:T
peptide level
1:151
2:133
0:41
YQCNPGYKpc2_NP.4
p−min=1, pX=0.98, N=3
var.8.SVEP1 9:113261483:C:T
peptide level
97:151
85:133
25:41
YQCNPGYKpc2_NP.5
p−maj=0.73, pX=0.88, N=207
var.8.SVEP1 9:113261483:C:T
peptide level
13:151
13:133
3:41
YSCVGGFFLRpc2_NP.1
p−maj=1, pX=0.68, N=29
var.8.SVEP1 9:113261483:C:T
peptide level
102:151
96:133
33:41
YSCVGGFFLRpc2_NP.2
p−maj=0.2, pX=0.37, N=231
var.8.SVEP1 9:113261483:C:T
peptide level
2:151
1:133
0:41
YSCVGGFFLRpc2_NP.3
p−min=0.6, pX=0.54, N=3
var.8.SVEP1 9:113261483:C:T
peptide level
3:151
2:133
0:41
YSCVGGFFLRpc2_NP.4
p−min=0.67, pX=0.66, N=5
var.8.SVEP1 9:113261483:C:T
peptide level
128:151
114:133
35:41
YSCVGGFFLRpc2_NP.5
p−min=0.88, pX=0.59, N=277
var.8.SVEP1 9:113261483:C:T
peptide level
7:151
4:133
1:41
YYCAYEDGVWKpc2_NP.2
p−min=0.56, pX=0.29, N=12
var.8.SVEP1 9:113261483:C:T
peptide level
8:151
9:133
2:41
YYCAYEDGVWKpc2_NP.5
p−min=0.81, pX=0.94, N=19

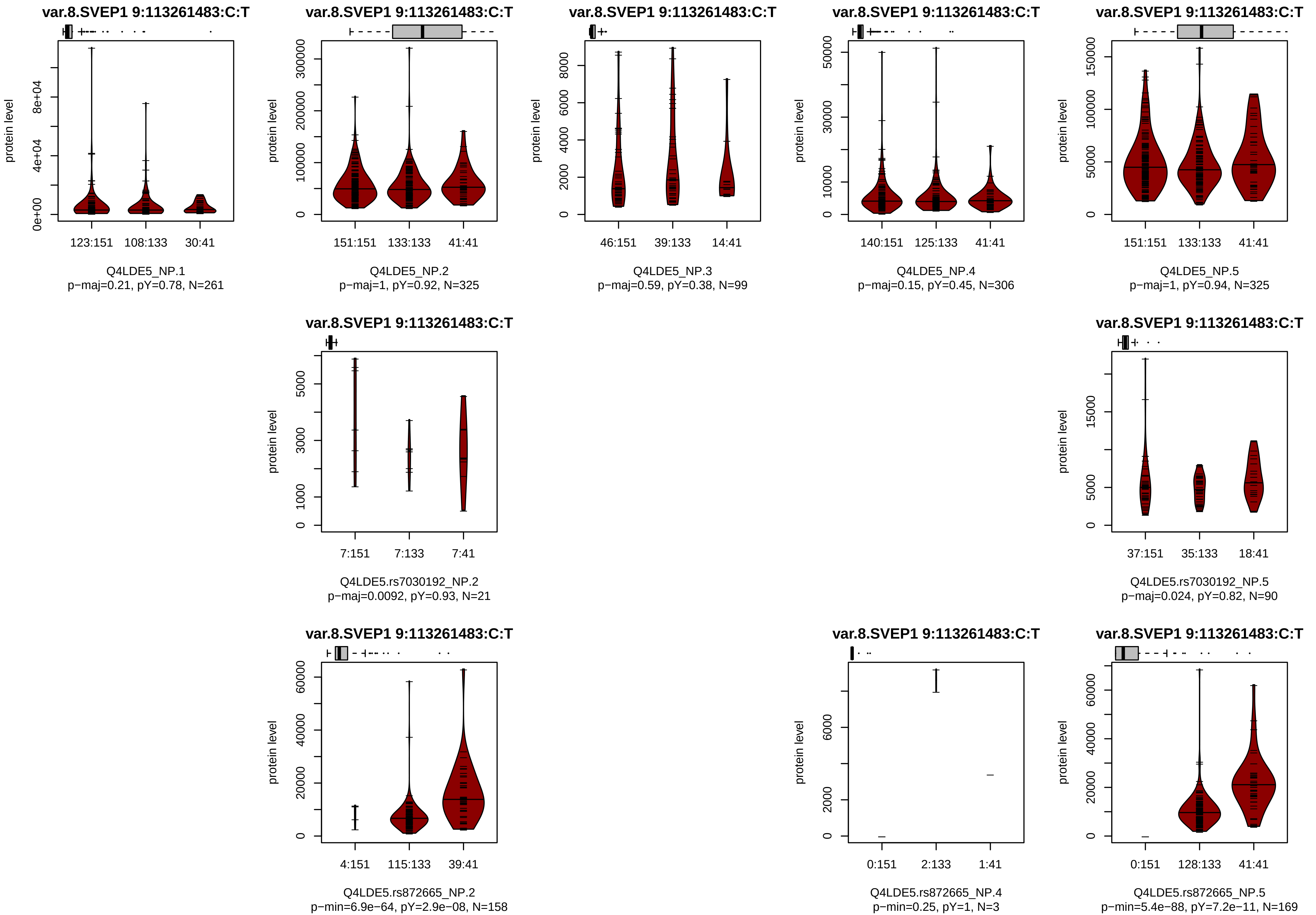
var.8.SVEP1 9:113261483:C:T
protein level
123:151
108:133
30:41
Q4LDE5_NP.1
p−maj=0.21, pY=0.78, N=261
var.8.SVEP1 9:113261483:C:T
151:151
133:133
41:41
Q4LDE5_NP.2
p−maj=1, pY=0.92, N=325
var.8.SVEP1 9:113261483:C:T
46:151
39:133
14:41
Q4LDE5_NP.3
p−maj=0.59, pY=0.38, N=99
var.8.SVEP1 9:113261483:C:T
140:151
125:133
41:41
Q4LDE5_NP.4
p−maj=0.15, pY=0.45, N=306
var.8.SVEP1 9:113261483:C:T
151:151
133:133
41:41
Q4LDE5_NP.5
p−maj=1, pY=0.94, N=325
var.8.SVEP1 9:113261483:C:T
7:151
7:133
7:41
Q4LDE5.rs7030192_NP.2
p−maj=0.0092, pY=0.93, N=21
var.8.SVEP1 9:113261483:C:T
37:151
35:133
18:41
Q4LDE5.rs7030192_NP.5
p−maj=0.024, pY=0.82, N=90
var.8.SVEP1 9:113261483:C:T
4:151
115:133
39:41
Q4LDE5.rs872665_NP.2
p−min=6.9e−64, pY=2.9e−08, N=158
var.8.SVEP1 9:113261483:C:T
0:151
2:133
1:41
Q4LDE5.rs872665_NP.4
p−min=0.25, pY=1, N=3
var.8.SVEP1 9:113261483:C:T
0:151
128:133
41:41
Q4LDE5.rs872665_NP.5
p−min=5.4e−88, pY=7.2e−11, N=169

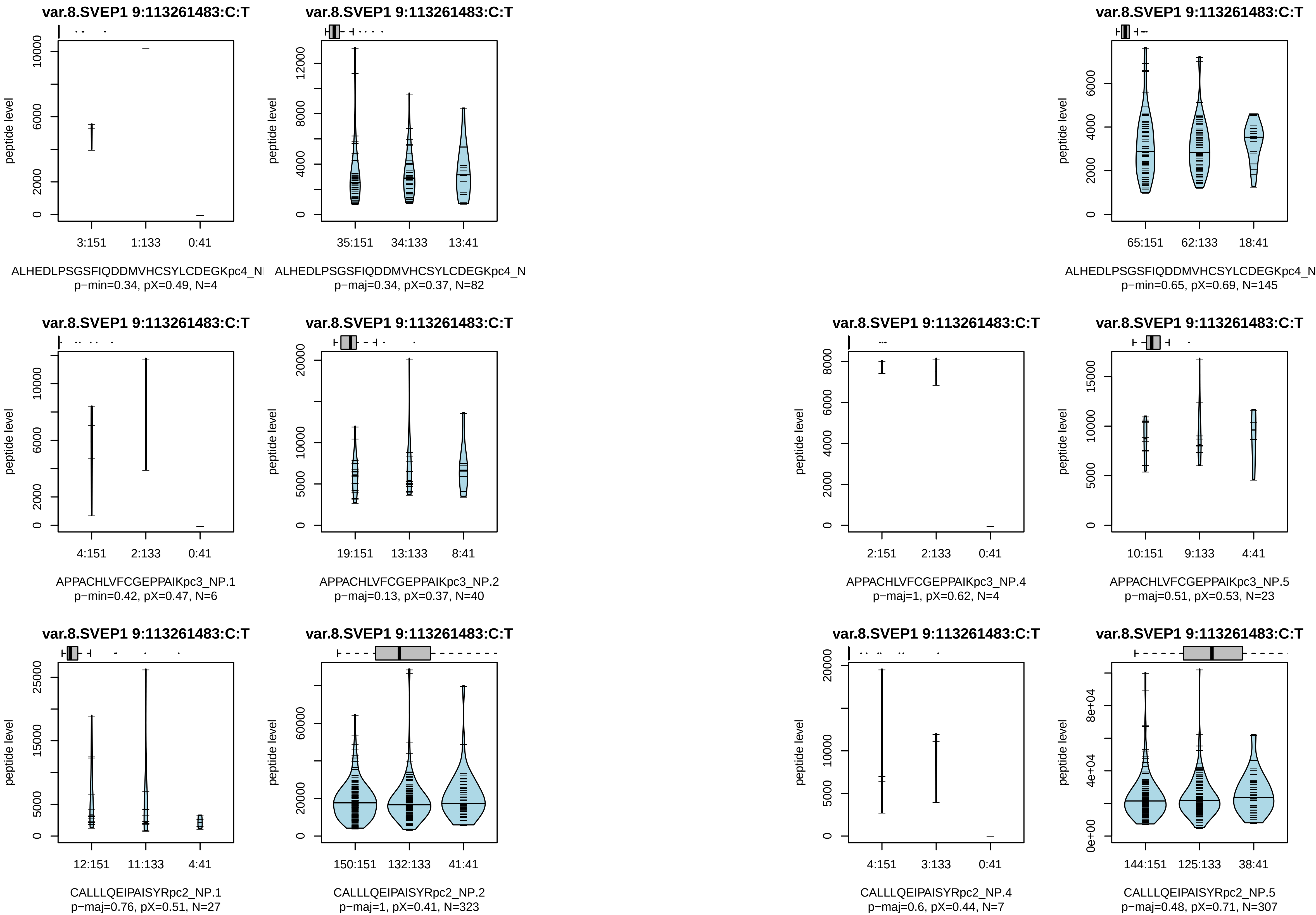
var.8.SVEP1 9:113261483:C:T
peptide level
3:151
1:133
0:41
ALHEDLPSGSFIQDDMVHCSYLCDEGKpc4_N
p−min=0.34, pX=0.49, N=4
var.8.SVEP1 9:113261483:C:T
peptide level
35:151
34:133
13:41
ALHEDLPSGSFIQDDMVHCSYLCDEGKpc4_N
p−maj=0.34, pX=0.37, N=82
var.8.SVEP1 9:113261483:C:T
peptide level
65:151
62:133
18:41
ALHEDLPSGSFIQDDMVHCSYLCDEGKpc4_N
p−min=0.65, pX=0.69, N=145
var.8.SVEP1 9:113261483:C:T
peptide level
4:151
2:133
0:41
APPACHLVFCGEPPAIKpc3_NP.1
p−min=0.42, pX=0.47, N=6
var.8.SVEP1 9:113261483:C:T
peptide level
19:151
13:133
8:41
APPACHLVFCGEPPAIKpc3_NP.2
p−maj=0.13, pX=0.37, N=40
var.8.SVEP1 9:113261483:C:T
peptide level
2:151
2:133
0:41
APPACHLVFCGEPPAIKpc3_NP.4
p−maj=1, pX=0.62, N=4
var.8.SVEP1 9:113261483:C:T
peptide level
10:151
9:133
4:41
APPACHLVFCGEPPAIKpc3_NP.5
p−maj=0.51, pX=0.53, N=23
var.8.SVEP1 9:113261483:C:T
peptide level
12:151
11:133
4:41
CALLLQEIPAISYRpc2_NP.1
p−maj=0.76, pX=0.51, N=27
var.8.SVEP1 9:113261483:C:T
peptide level
150:151
132:133
41:41
CALLLQEIPAISYRpc2_NP.2
p−maj=1, pX=0.41, N=323
var.8.SVEP1 9:113261483:C:T
peptide level
4:151
3:133
0:41
CALLLQEIPAISYRpc2_NP.4
p−maj=0.6, pX=0.44, N=7
var.8.SVEP1 9:113261483:C:T
peptide level
144:151
125:133
38:41
CALLLQEIPAISYRpc2_NP.5
p−maj=0.48, pX=0.71, N=307

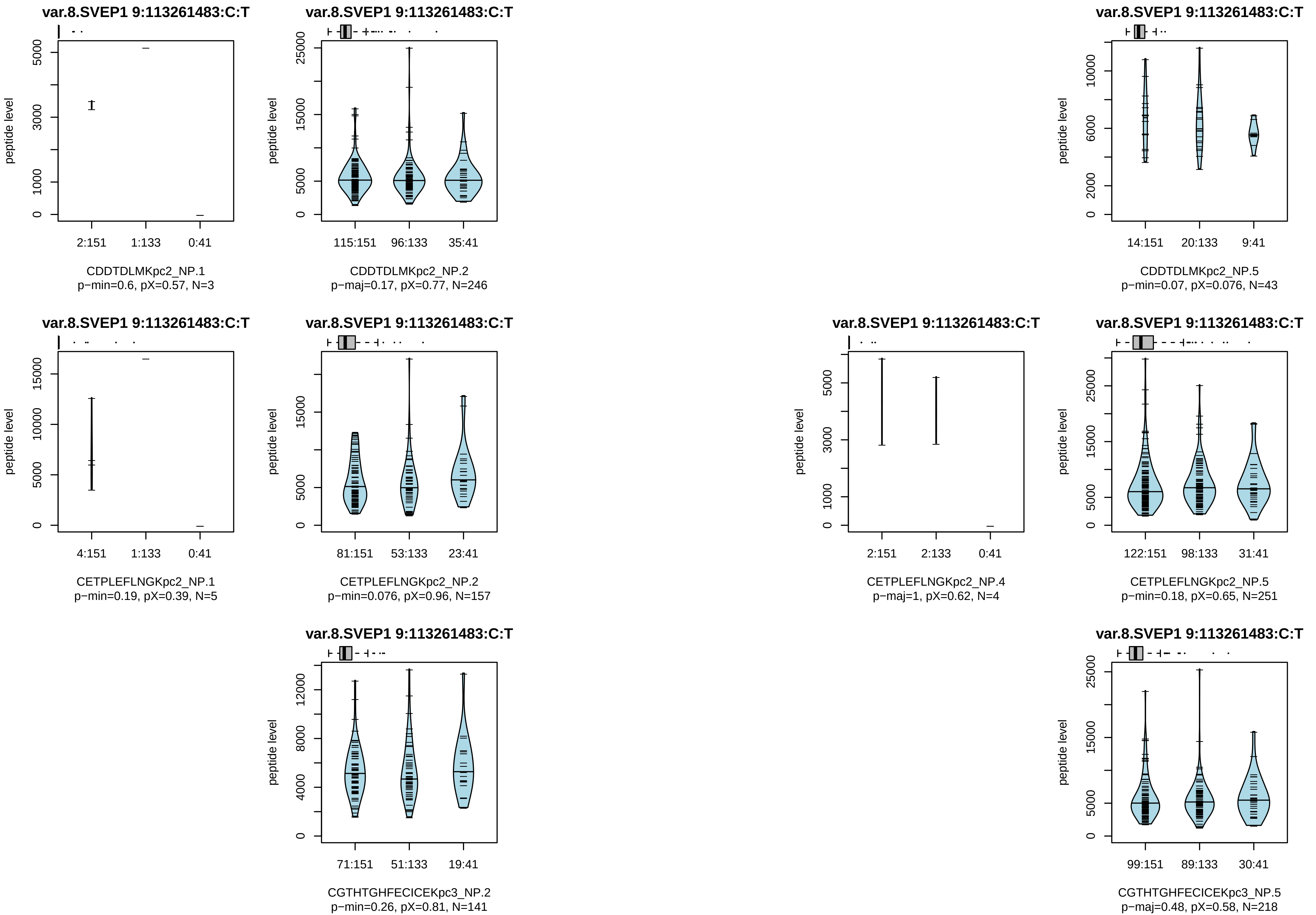
var.8.SVEP1 9:113261483:C:T
peptide level
2:151 1:133 0:41
CDDTDLMKpc2_NP.1
p−min=0.6, pX=0.57, N=3
var.8.SVEP1 9:113261483:C:T
peptide level
115:151 96:133 35:41
CDDTDLMKpc2_NP.2
p−maj=0.17, pX=0.77, N=246
var.8.SVEP1 9:113261483:C:T
peptide level
14:151 20:133 9:41
CDDTDLMKpc2_NP.5
p−min=0.07, pX=0.076, N=43
var.8.SVEP1 9:113261483:C:T
peptide level
4:151 1:133 0:41
CETPLEFLNGKpc2_NP.1
p−min=0.19, pX=0.39, N=5
var.8.SVEP1 9:113261483:C:T
peptide level
81:151 53:133 23:41
CETPLEFLNGKpc2_NP.2
p−min=0.076, pX=0.96, N=157
var.8.SVEP1 9:113261483:C:T
peptide level
2:151 2:133 0:41
CETPLEFLNGKpc2_NP.4
p−maj=1, pX=0.62, N=4
var.8.SVEP1 9:113261483:C:T
peptide level
122:151 98:133 31:41
CETPLEFLNGKpc2_NP.5
p−min=0.18, pX=0.65, N=251
var.8.SVEP1 9:113261483:C:T
peptide level
71:151 51:133 19:41
CGTHTGHFECICEKpc3_NP.2
p−min=0.26, pX=0.81, N=141
var.8.SVEP1 9:113261483:C:T
peptide level
99:151 89:133 30:41
CGTHTGHFECICEKpc3_NP.5
p−maj=0.48, pX=0.58, N=218

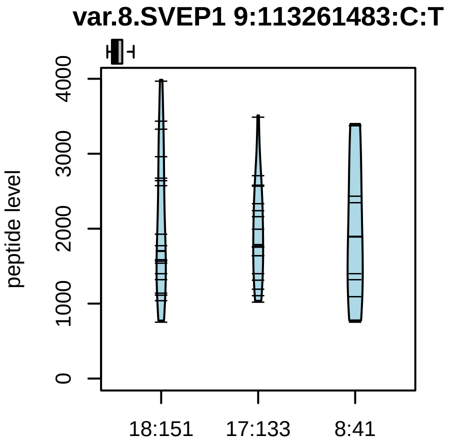

CHCLSSWTGHNCSRpc3_NP.5
p−maj=0.22, pX=0.36, N=43

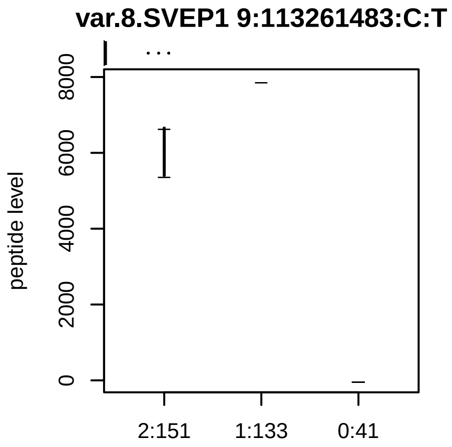

CHTAVCQSPCLNGGKpc3_NP.1
p−min=0.6, pX=0.51, N=3

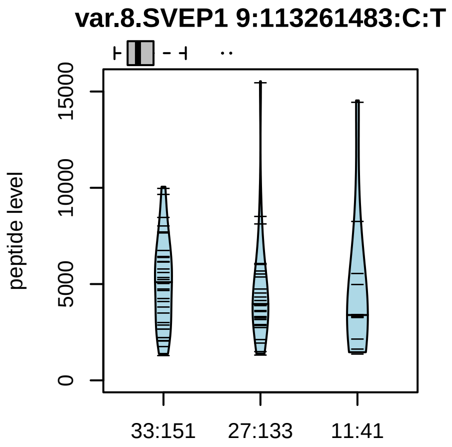

CHTAVCQSPCLNGGKpc3_NP.2
p−maj=0.42, pX=0.95, N=71

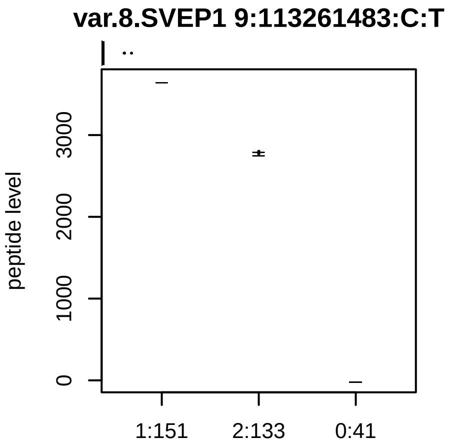

CHTAVCQSPCLNGGKpc3_NP.4
p−min=1, pX=0.89, N=3

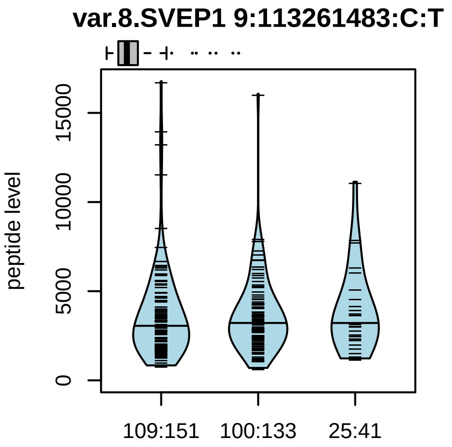

CHTAVCQSPCLNGGKpc3_NP.5
p−maj=0.097, pX=0.76, N=234

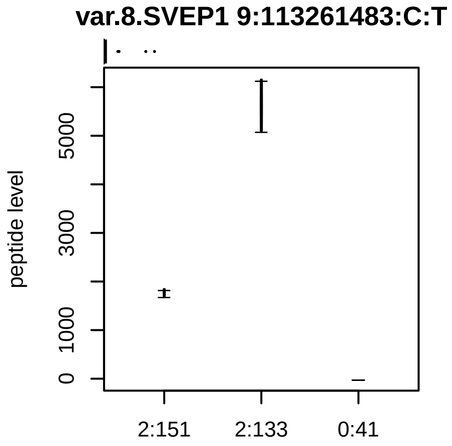

CIAHFCEKPPSVSYSILESVSKpc4_NP.1
p−maj=1, pX=0.8, N=4

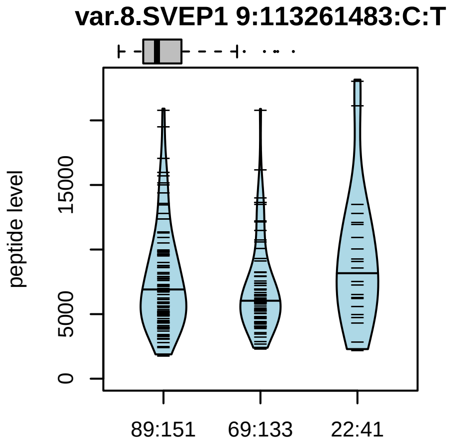

CIAHFCEKPPSVSYSILESVSKpc4_NP.2
p−min=0.26, pX=0.77, N=180

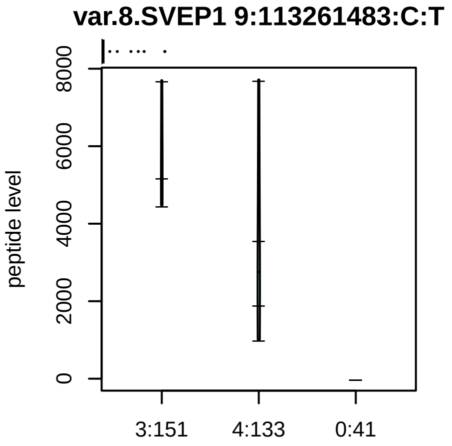

CIAHFCEKPPSVSYSILESVSKpc4_NP.4
p−maj=0.6, pX=0.47, N=7

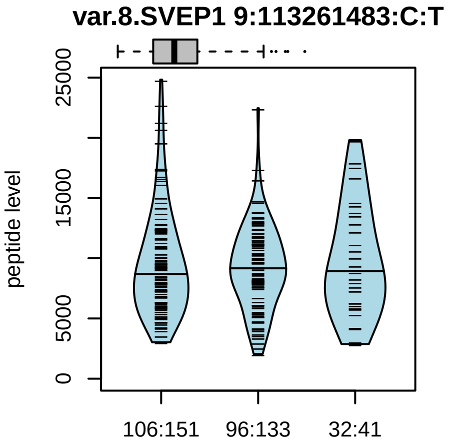

CIAHFCEKPPSVSYSILESVSKpc4_NP.5
p−maj=0.46, pX=0.48, N=234

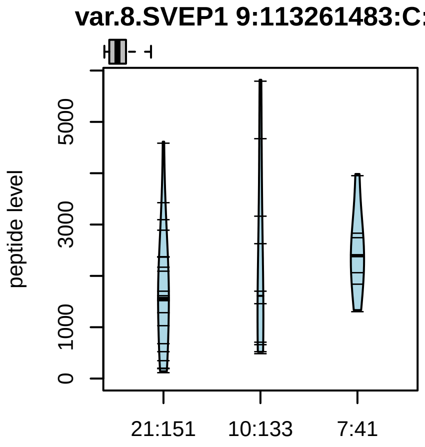

CPEPPLLENQLVLKpc2_NP.2
p−maj=0.29, pX=0.48, N=38

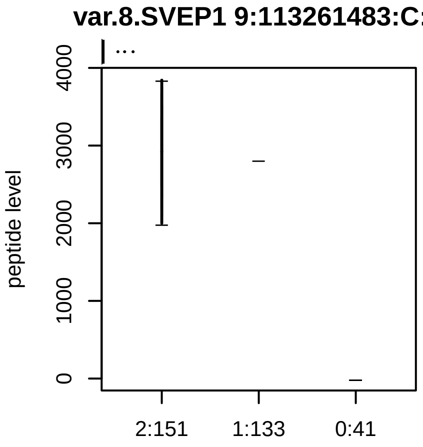

CPEPPLLENQLVLKpc2_NP.4
p−min=0.6, pX=0.41, N=3

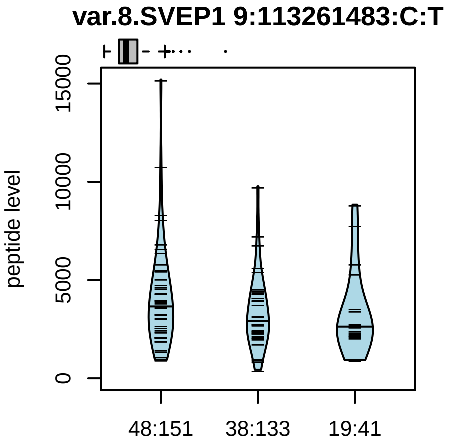

CPEPPLLENQLVLKpc2_NP.5
p−maj=0.049, pX=0.94, N=105

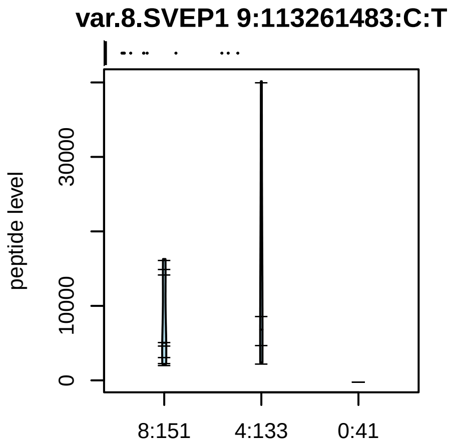

CPPGFLGTRpc2_NP.1
p−min=0.24, pX=0.5, N=12

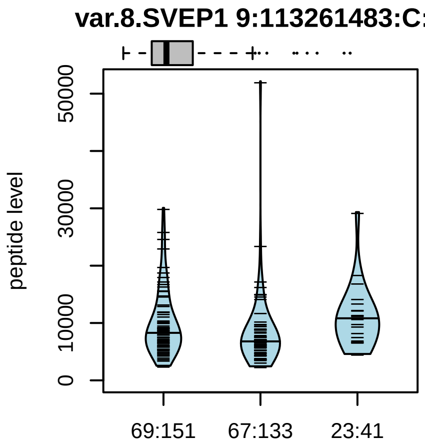

CPPGFLGTRpc2_NP.2
p−min=0.32, pX=0.41, N=159

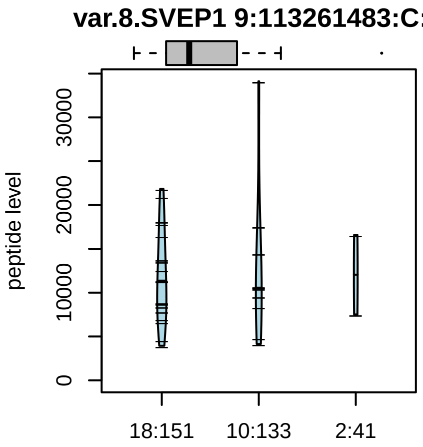

CPPGFLGTRpc2_NP.4
p−min=0.13, pX=0.18, N=30

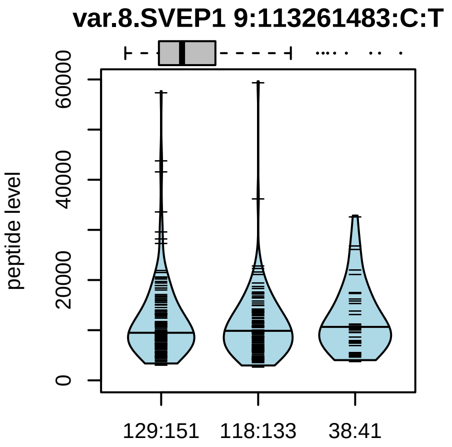

CPPGFLGTRpc2_NP.5
p−min=0.31, pX=0.66, N=285

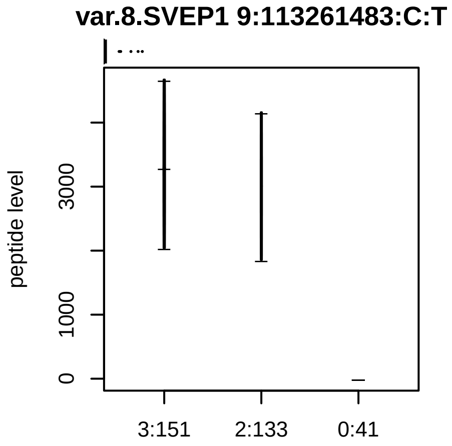

CSSPENINNGKpc2_NP.1
p−min=0.67, pX=0.38, N=5

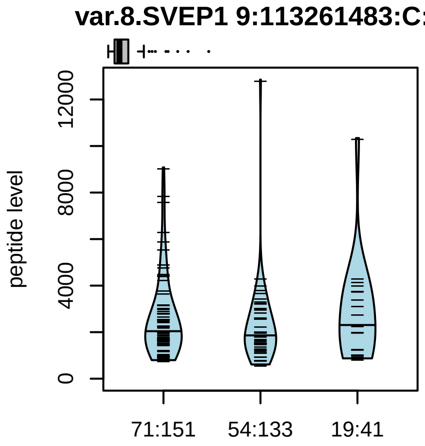

CSSPENINNGKpc2_NP.2
p−min=0.37, pX=0.66, N=144

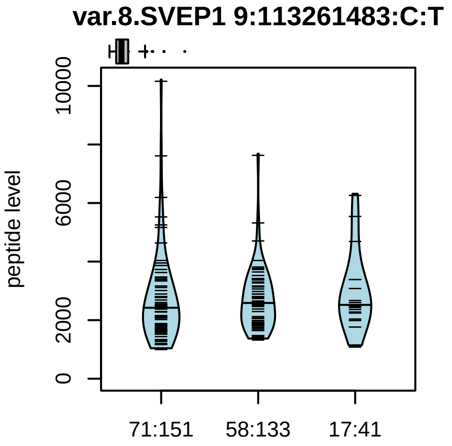

CSSPENINNGKpc2_NP.5
p−min=0.5, pX=0.79, N=146

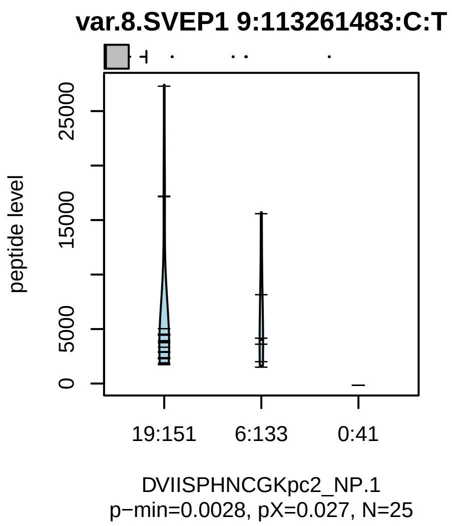
var.8.SVEP1 9:113261483:C:T
peptide level
19:151
6:133
0:41
DVIISPHNCGKpc2_NP.1
p−min=0.0028, pX=0.027, N=25

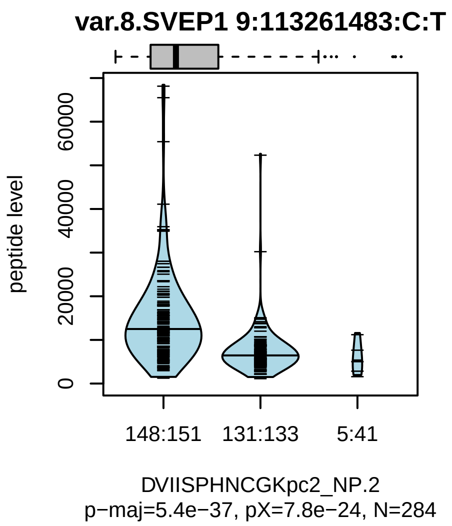
var.8.SVEP1 9:113261483:C:T
peptide level
148:151
131:133
5:41
DVIISPHNCGKpc2_NP.2
p−maj=5.4e−37, pX=7.8e−24, N=284

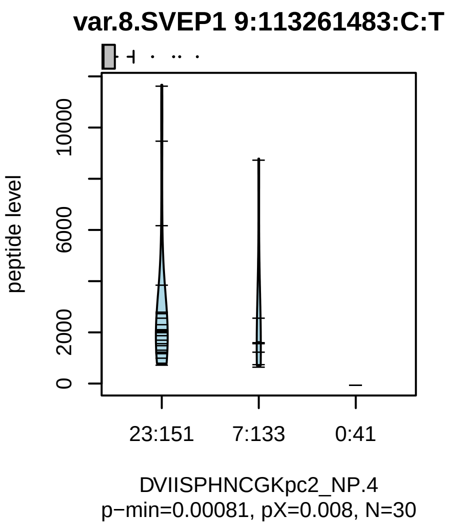
var.8.SVEP1 9:113261483:C:T
peptide level
23:151
7:133
0:41
DVIISPHNCGKpc2_NP.4
p−min=0.00081, pX=0.008, N=30

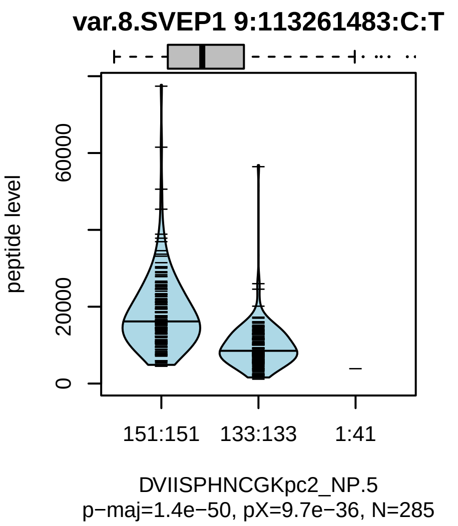
var.8.SVEP1 9:113261483:C:T
peptide level
151:151
133:133
1:41
DVIISPHNCGKpc2_NP.5
p−maj=1.4e−50, pX=9.7e−36, N=285

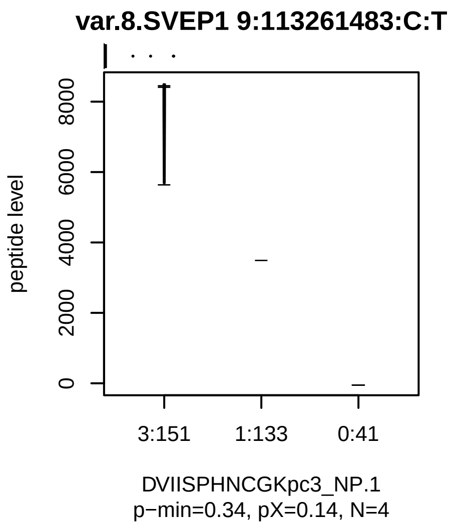
var.8.SVEP1 9:113261483:C:T
peptide level
3:151
1:133
0:41
DVIISPHNCGKpc3_NP.1
p−min=0.34, pX=0.14, N=4

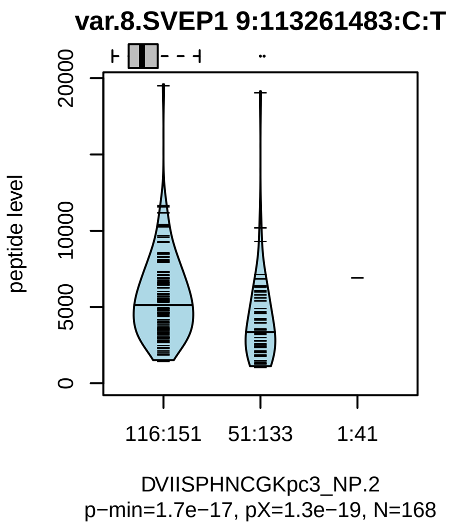
var.8.SVEP1 9:113261483:C:T
peptide level
116:151
51:133
1:41
DVIISPHNCGKpc3_NP.2
p−min=1.7e−17, pX=1.3e−19, N=168

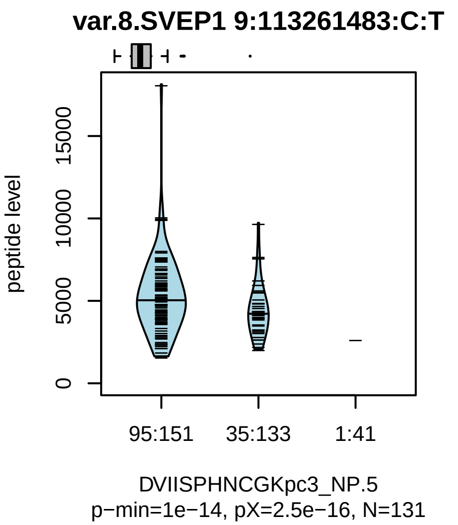
var.8.SVEP1 9:113261483:C:T
peptide level
95:151
35:133
1:41
DVIISPHNCGKpc3_NP.5
p−min=1e−14, pX=2.5e−16, N=131

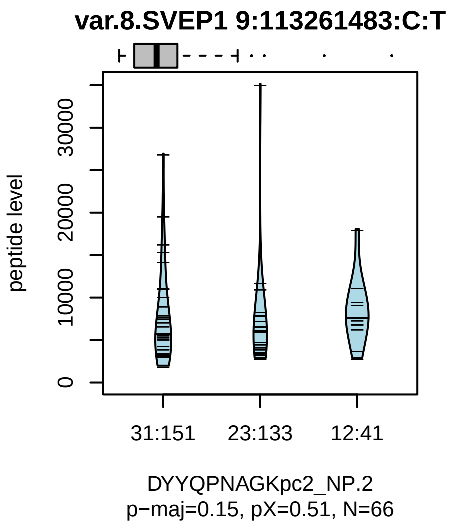
var.8.SVEP1 9:113261483:C:T
peptide level
31:151
23:133
12:41
DYYQPNAGKpc2_NP.2
p−maj=0.15, pX=0.51, N=66

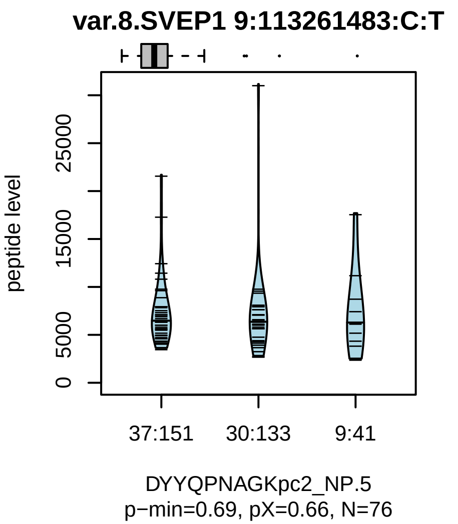
var.8.SVEP1 9:113261483:C:T
peptide level
37:151
30:133
9:41
DYYQPNAGKpc2_NP.5
p−min=0.69, pX=0.66, N=76

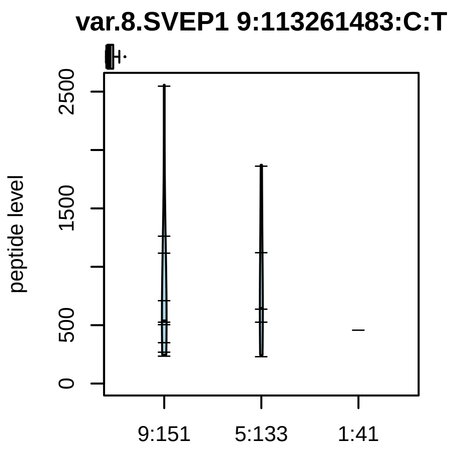

EGHVLQGPSVLKpc2_NP.1
p−min=0.3, pX=0.29, N=15

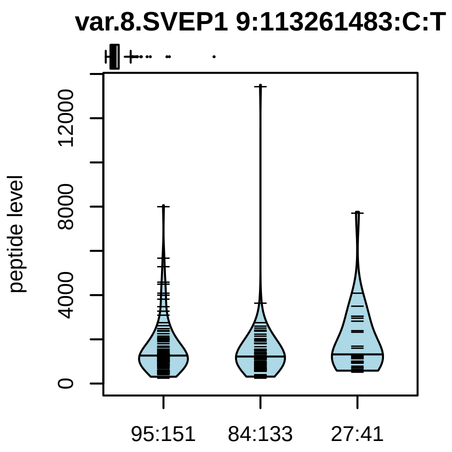

EGHVLQGPSVLKpc2_NP.2
p−maj=0.86, pX=0.65, N=206

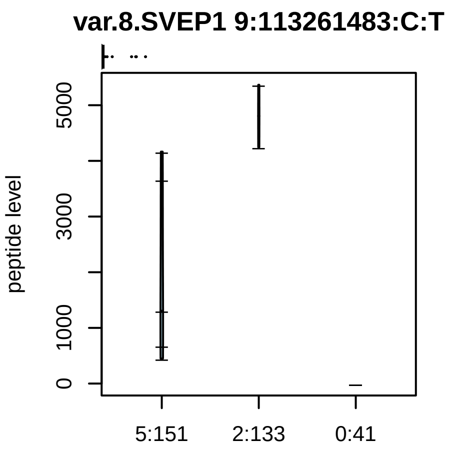

EGHVLQGPSVLKpc2_NP.4
p−min=0.26, pX=0.56, N=7

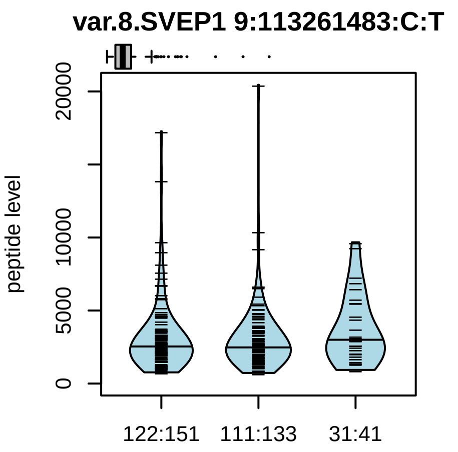

EGHVLQGPSVLKpc2_NP.5
p−maj=0.39, pX=0.56, N=264

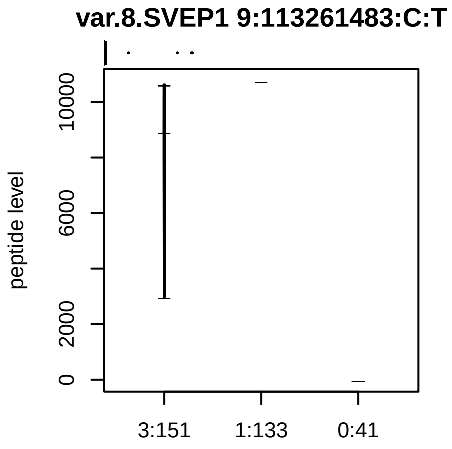

EGHVLQGPSVLKpc3_NP.1
p−min=0.34, pX=0.36, N=4

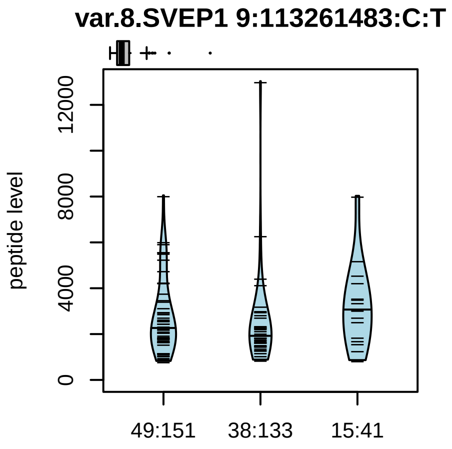

EGHVLQGPSVLKpc3_NP.2
p−maj=0.47, pX=0.74, N=102

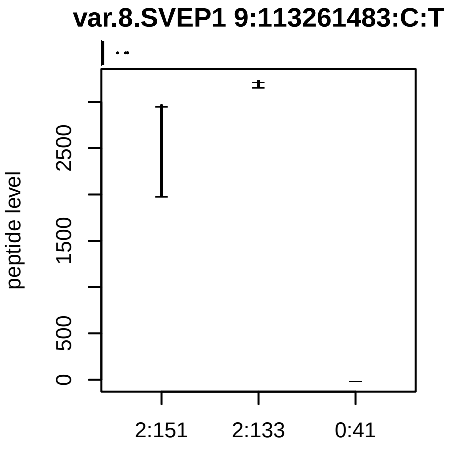

EGHVLQGPSVLKpc3_NP.4
p−maj=1, pX=0.78, N=4

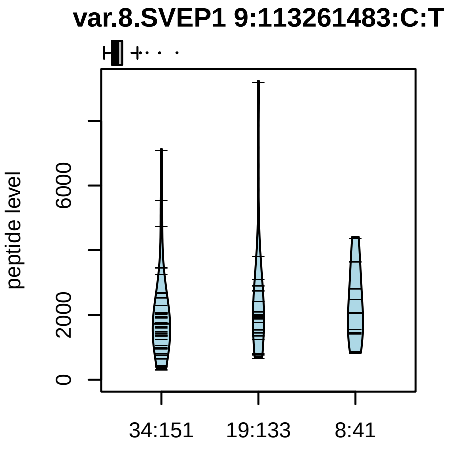

EGHVLQGPSVLKpc3_NP.5
p−min=0.12, pX=0.7, N=61

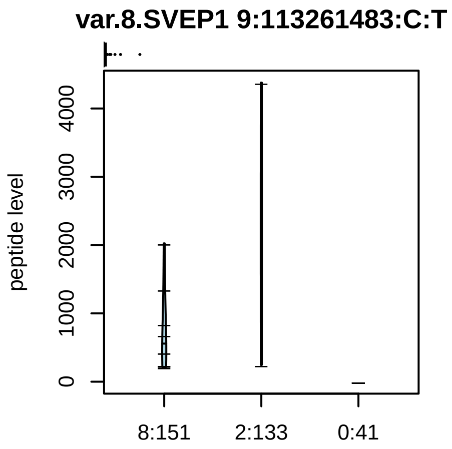

EGYTLAGLDTIECLADGKpc2_NP.1
p−min=0.049, pX=0.5, N=10

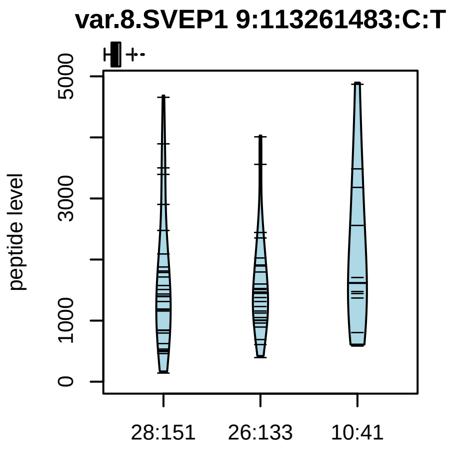

EGYTLAGLDTIECLADGKpc2_NP.2
p−maj=0.41, pX=0.23, N=64

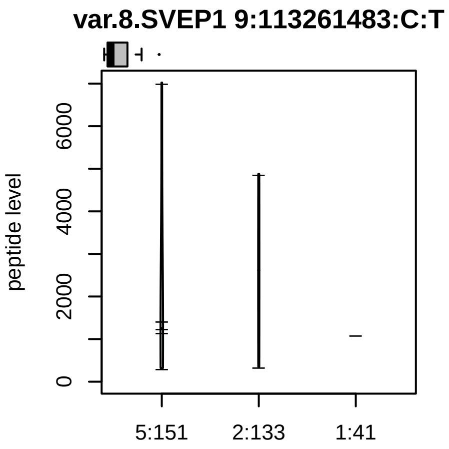

EGYTLAGLDTIECLADGKpc2_NP.4
p−min=0.48, pX=0.5, N=8

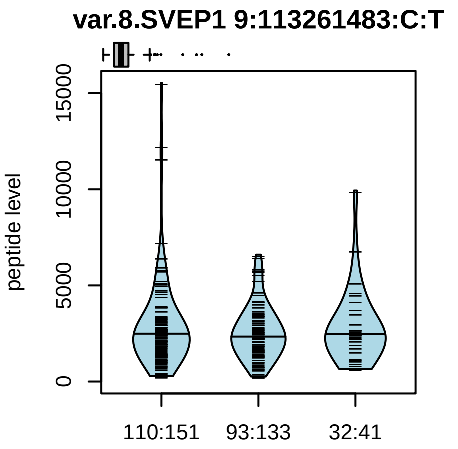

EGYTLAGLDTIECLADGKpc2_NP.5
p−maj=0.46, pX=0.77, N=235

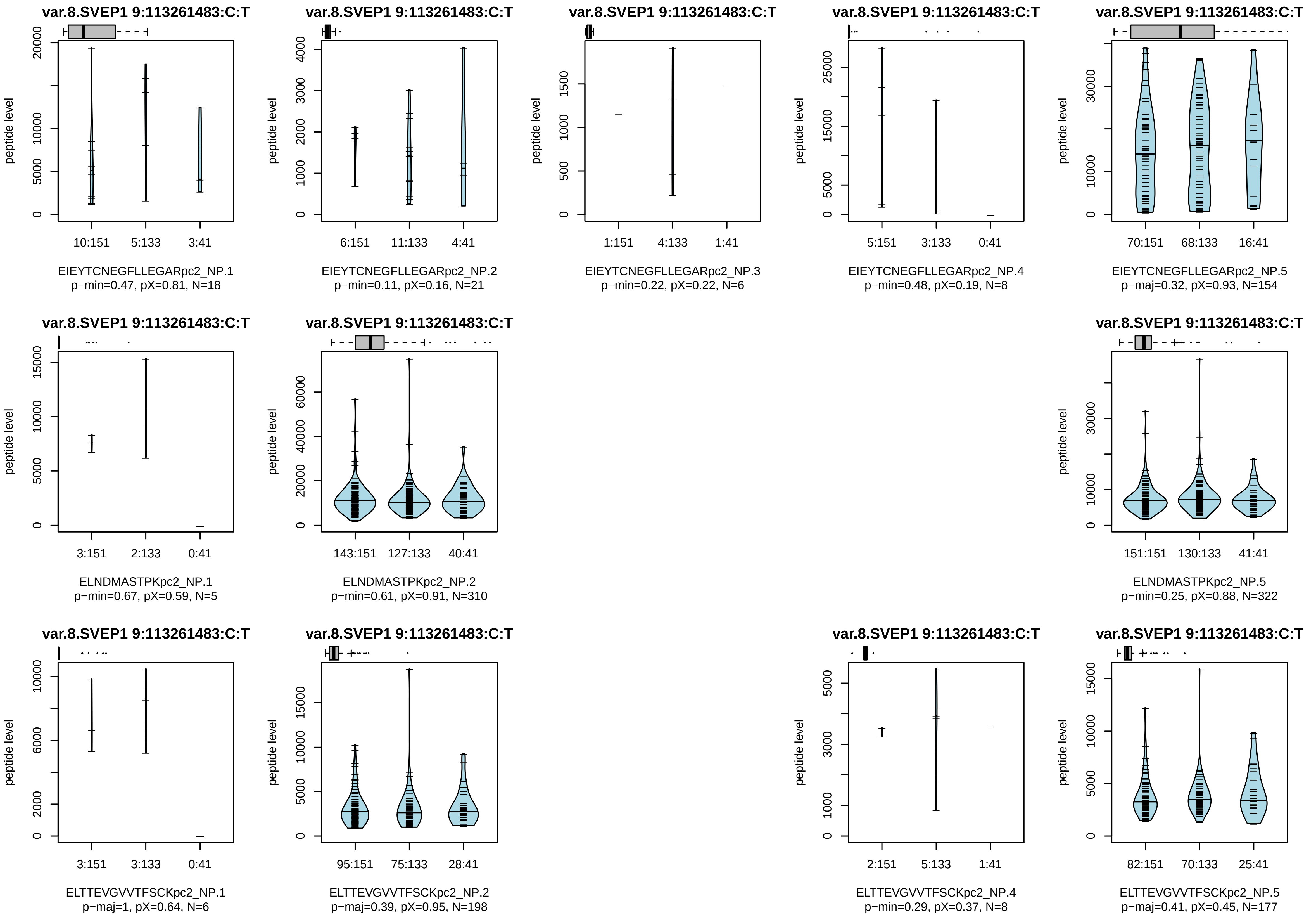
var.8.SVEP1 9:113261483:C:T
peptide level
10:151
5:133
3:41
EIEYTCNEGFLLEGARpc2_NP.1
p−min=0.47, pX=0.81, N=18
var.8.SVEP1 9:113261483:C:T
6:151
11:133
4:41
EIEYTCNEGFLLEGARpc2_NP.2
p−min=0.11, pX=0.16, N=21
var.8.SVEP1 9:113261483:C:T
1:151
4:133
1:41
EIEYTCNEGFLLEGARpc2_NP.3
p−min=0.22, pX=0.22, N=6
var.8.SVEP1 9:113261483:C:T
5:151
3:133
0:41
EIEYTCNEGFLLEGARpc2_NP.4
p−min=0.48, pX=0.19, N=8
var.8.SVEP1 9:113261483:C:T
70:151
68:133
16:41
EIEYTCNEGFLLEGARpc2_NP.5
p−maj=0.32, pX=0.93, N=154
var.8.SVEP1 9:113261483:C:T
3:151
2:133
0:41
ELNDMASTPKpc2_NP.1
p−min=0.67, pX=0.59, N=5
var.8.SVEP1 9:113261483:C:T
143:151
127:133
40:41
ELNDMASTPKpc2_NP.2
p−min=0.61, pX=0.91, N=310
var.8.SVEP1 9:113261483:C:T
151:151
130:133
41:41
ELNDMASTPKpc2_NP.5
p−min=0.25, pX=0.88, N=322
var.8.SVEP1 9:113261483:C:T
3:151
3:133
0:41
ELTTEVGVVTFSCKpc2_NP.1
p−maj=1, pX=0.64, N=6
var.8.SVEP1 9:113261483:C:T
95:151
75:133
28:41
ELTTEVGVVTFSCKpc2_NP.2
p−maj=0.39, pX=0.95, N=198
var.8.SVEP1 9:113261483:C:T
2:151
5:133
1:41
ELTTEVGVVTFSCKpc2_NP.4
p−min=0.29, pX=0.37, N=8
var.8.SVEP1 9:113261483:C:T
82:151
70:133
25:41
ELTTEVGVVTFSCKpc2_NP.5
p−maj=0.41, pX=0.45, N=177

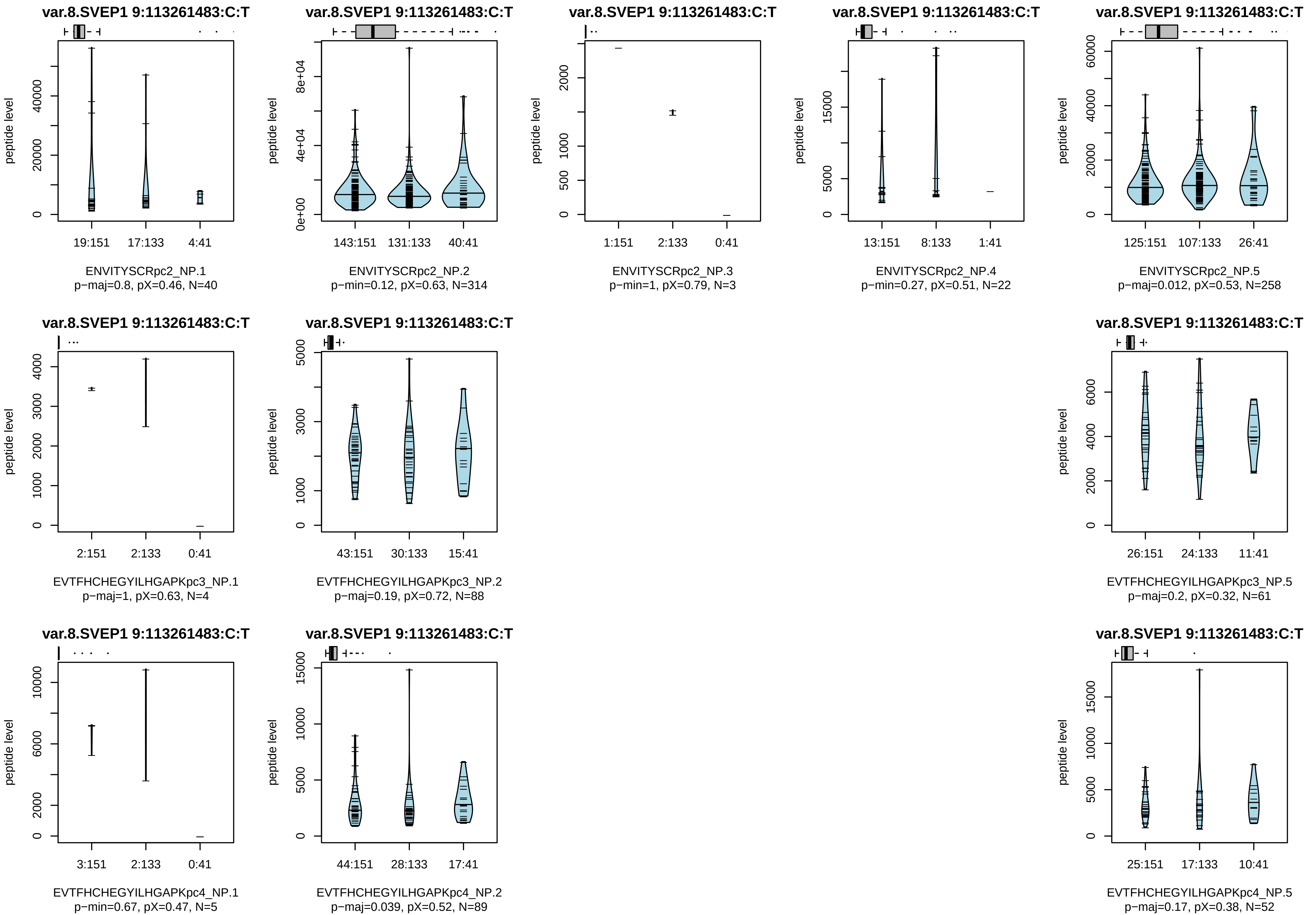
var.8.SVEP1 9:113261483:C:T
peptide level
19:151
17:133
4:41
ENVITYSCRpc2_NP.1
p−maj=0.8, pX=0.46, N=40
var.8.SVEP1 9:113261483:C:T
peptide level
143:151
131:133
40:41
ENVITYSCRpc2_NP.2
p−min=0.12, pX=0.63, N=314
var.8.SVEP1 9:113261483:C:T
peptide level
1:151
2:133
0:41
ENVITYSCRpc2_NP.3
p−min=1, pX=0.79, N=3
var.8.SVEP1 9:113261483:C:T
peptide level
13:151
8:133
1:41
ENVITYSCRpc2_NP.4
p−min=0.27, pX=0.51, N=22
var.8.SVEP1 9:113261483:C:T
peptide level
125:151
107:133
26:41
ENVITYSCRpc2_NP.5
p−maj=0.012, pX=0.53, N=258
var.8.SVEP1 9:113261483:C:T
peptide level
2:151
2:133
0:41
EVTFHCHEGYILHGAPKpc3_NP.1
p−maj=1, pX=0.63, N=4
var.8.SVEP1 9:113261483:C:T
peptide level
43:151
30:133
15:41
EVTFHCHEGYILHGAPKpc3_NP.2
p−maj=0.19, pX=0.72, N=88
var.8.SVEP1 9:113261483:C:T
peptide level
26:151
24:133
11:41
EVTFHCHEGYILHGAPKpc3_NP.5
p−maj=0.2, pX=0.32, N=61
var.8.SVEP1 9:113261483:C:T
peptide level
3:151
2:133
0:41
EVTFHCHEGYILHGAPKpc4_NP.1
p−min=0.67, pX=0.47, N=5
var.8.SVEP1 9:113261483:C:T
peptide level
44:151
28:133
17:41
EVTFHCHEGYILHGAPKpc4_NP.2
p−maj=0.039, pX=0.52, N=89
var.8.SVEP1 9:113261483:C:T
peptide level
25:151
17:133
10:41
EVTFHCHEGYILHGAPKpc4_NP.5
p−maj=0.17, pX=0.38, N=52

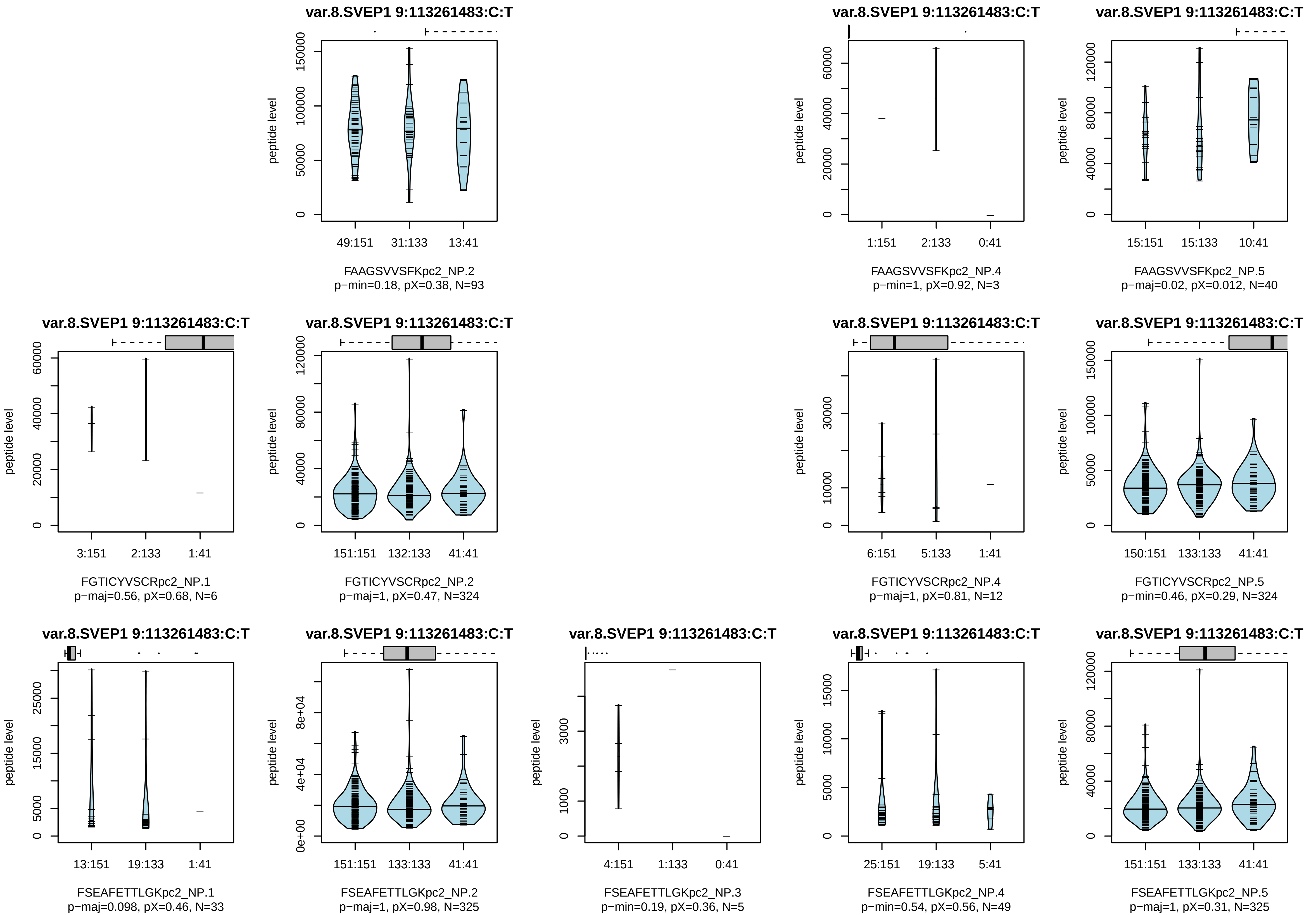
var.8.SVEP1 9:113261483:C:T
peptide level
49:151
31:133
13:41
FAAGSVVSFKpc2_NP.2
p−min=0.18, pX=0.38, N=93
var.8.SVEP1 9:113261483:C:T
peptide level
1:151
2:133
0:41
FAAGSVVSFKpc2_NP.4
p−min=1, pX=0.92, N=3
var.8.SVEP1 9:113261483:C:T
peptide level
15:151
15:133
10:41
FAAGSVVSFKpc2_NP.5
p−maj=0.02, pX=0.012, N=40
var.8.SVEP1 9:113261483:C:T
peptide level
3:151
2:133
1:41
FGTICYVSCRpc2_NP.1
p−maj=0.56, pX=0.68, N=6
var.8.SVEP1 9:113261483:C:T
peptide level
151:151
132:133
41:41
FGTICYVSCRpc2_NP.2
p−maj=1, pX=0.47, N=324
var.8.SVEP1 9:113261483:C:T
peptide level
6:151
5:133
1:41
FGTICYVSCRpc2_NP.4
p−maj=1, pX=0.81, N=12
var.8.SVEP1 9:113261483:C:T
peptide level
150:151
133:133
41:41
FGTICYVSCRpc2_NP.5
p−min=0.46, pX=0.29, N=324
var.8.SVEP1 9:113261483:C:T
peptide level
13:151
19:133
1:41
FSEAFETTLGKpc2_NP.1
p−maj=0.098, pX=0.46, N=33
var.8.SVEP1 9:113261483:C:T
peptide level
151:151
133:133
41:41
FSEAFETTLGKpc2_NP.2
p−maj=1, pX=0.98, N=325
var.8.SVEP1 9:113261483:C:T
peptide level
4:151
1:133
0:41
FSEAFETTLGKpc2_NP.3
p−min=0.19, pX=0.36, N=5
var.8.SVEP1 9:113261483:C:T
peptide level
25:151
19:133
5:41
FSEAFETTLGKpc2_NP.4
p−min=0.54, pX=0.56, N=49
var.8.SVEP1 9:113261483:C:T
peptide level
151:151
133:133
41:41
FSEAFETTLGKpc2_NP.5
p−maj=1, pX=0.31, N=325

var.8.SVEP1 9:113261483:C:T

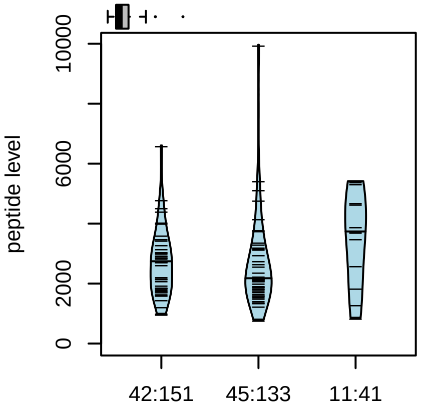

FSYTDLHYGQTVTYSCNRpc3_NP.5
p−min=0.4, pX=0.39, N=98

var.8.SVEP1 9:113261483:C:T

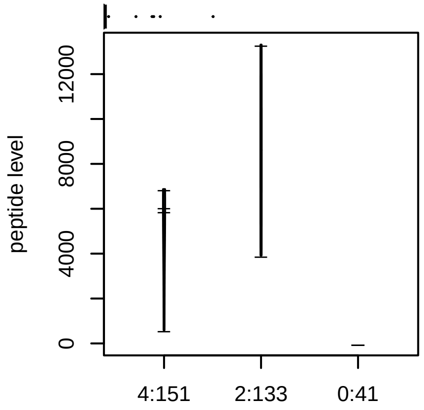

FTETSMGSAVQYSCKpc2_NP.1
p−min=0.42, pX=0.56, N=6

var.8.SVEP1 9:113261483:C:T

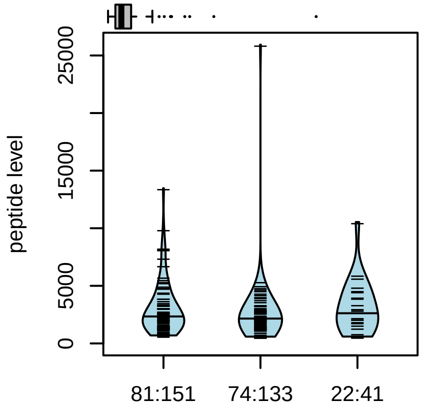

FTETSMGSAVQYSCKpc2_NP.2
p−min=0.82, pX=0.86, N=177

var.8.SVEP1 9:113261483:C:T

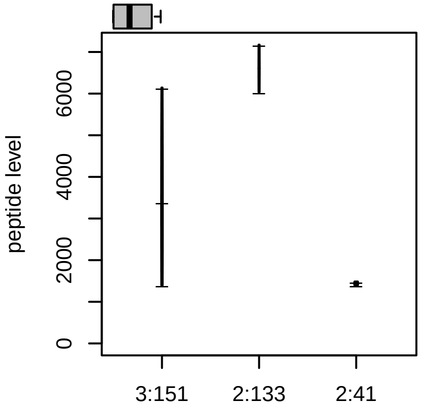

FTETSMGSAVQYSCKpc2_NP.4
p−maj=0.22, pX=0.89, N=7

var.8.SVEP1 9:113261483:C:T

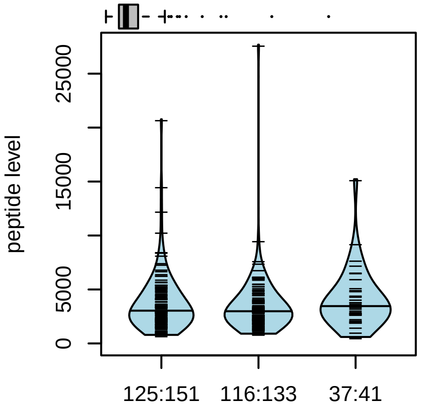

FTETSMGSAVQYSCKpc2_NP.5
p−min=0.21, pX=0.43, N=278

var.8.SVEP1 9:113261483:C:T

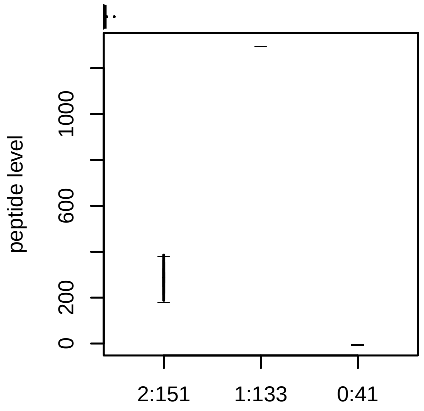

GAFQQAAQILLHARpc2_NP.1
p−min=0.6, pX=0.95, N=3

var.8.SVEP1 9:113261483:C:T

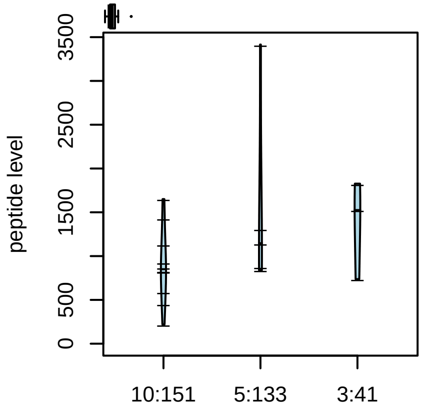

GAFQQAAQILLHARpc2_NP.2
p−min=0.47, pX=0.58, N=18

var.8.SVEP1 9:113261483:C:T

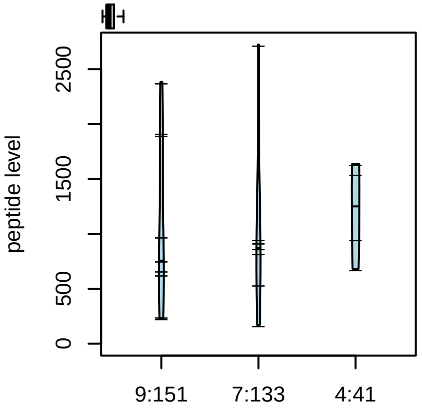

GAFQQAAQILLHARpc2_NP.5
p−maj=0.3, pX=0.54, N=20

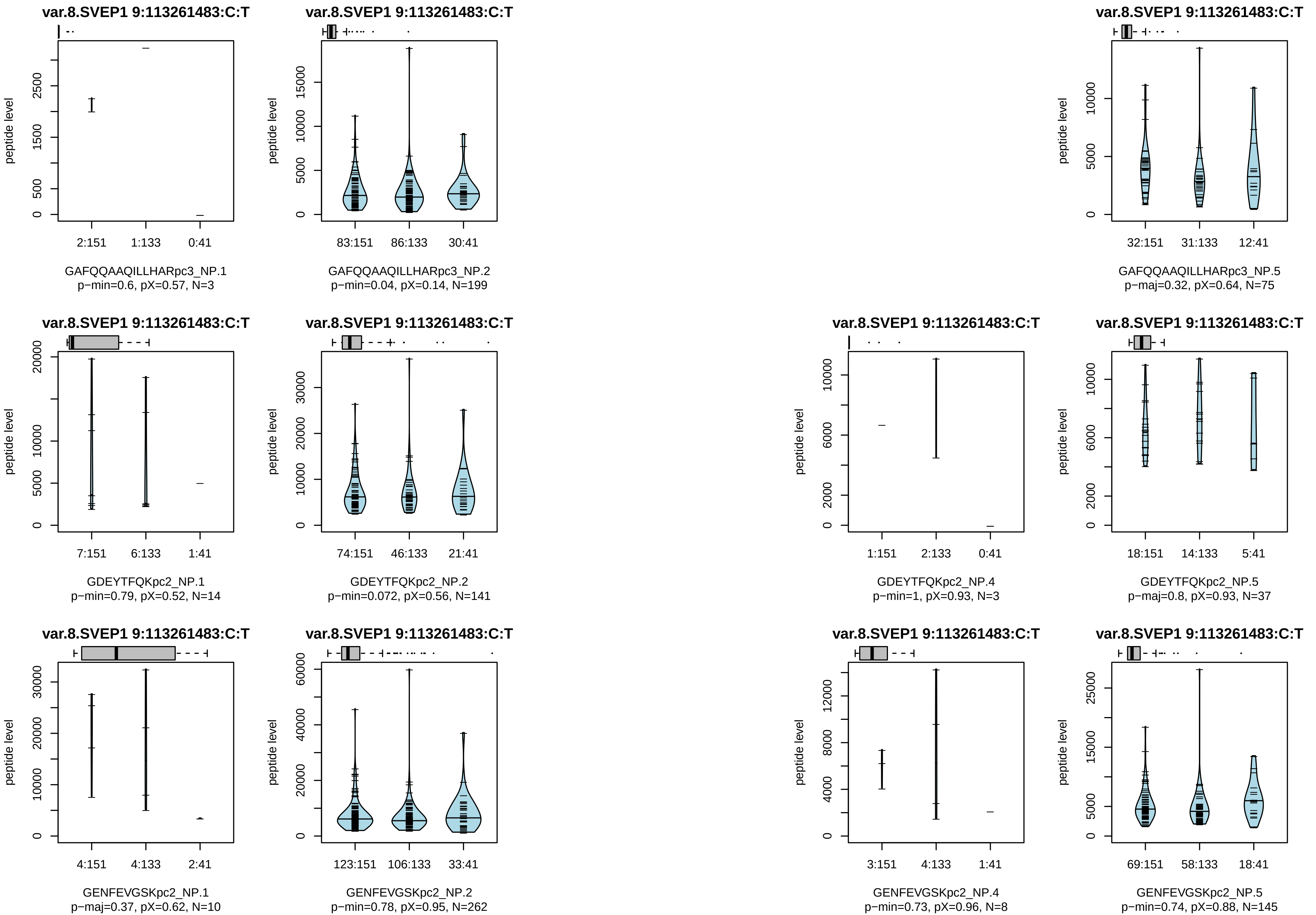

var.8.SVEP1 9:113261483:C:T
peptide level
2:151
1:133
0:41
GAFQQAAQILLHARpc3_NP.1
p−min=0.6, pX=0.57, N=3
var.8.SVEP1 9:113261483:C:T
peptide level
83:151
86:133
30:41
GAFQQAAQILLHARpc3_NP.2
p−min=0.04, pX=0.14, N=199
var.8.SVEP1 9:113261483:C:T
peptide level
32:151
31:133
12:41
GAFQQAAQILLHARpc3_NP.5
p−maj=0.32, pX=0.64, N=75
var.8.SVEP1 9:113261483:C:T
peptide level
7:151
6:133
1:41
GDEYTFQKpc2_NP.1
p−min=0.79, pX=0.52, N=14
var.8.SVEP1 9:113261483:C:T
peptide level
74:151
46:133
21:41
GDEYTFQKpc2_NP.2
p−min=0.072, pX=0.56, N=141
var.8.SVEP1 9:113261483:C:T
peptide level
1:151
2:133
0:41
GDEYTFQKpc2_NP.4
p−min=1, pX=0.93, N=3
var.8.SVEP1 9:113261483:C:T
peptide level
18:151
14:133
5:41
GDEYTFQKpc2_NP.5
p−maj=0.8, pX=0.93, N=37
var.8.SVEP1 9:113261483:C:T
peptide level
4:151
4:133
2:41
GENFEVGSKpc2_NP.1
p−maj=0.37, pX=0.62, N=10
var.8.SVEP1 9:113261483:C:T
peptide level
123:151
106:133
33:41
GENFEVGSKpc2_NP.2
p−min=0.78, pX=0.95, N=262
var.8.SVEP1 9:113261483:C:T
peptide level
3:151
4:133
1:41
GENFEVGSKpc2_NP.4
p−min=0.73, pX=0.96, N=8
var.8.SVEP1 9:113261483:C:T
peptide level
69:151
58:133
18:41
GENFEVGSKpc2_NP.5
p−min=0.74, pX=0.88, N=145

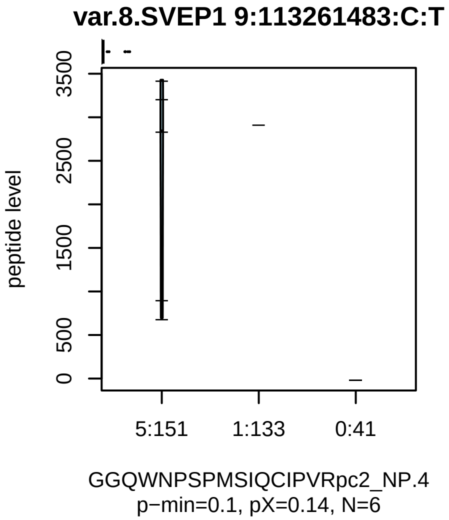
var.8.SVEP1 9:113261483:C:T
peptide level
5:151
1:133
0:41
GGQWNPSPMSIQCIPVRpc2_NP.4
p−min=0.1, pX=0.14, N=6

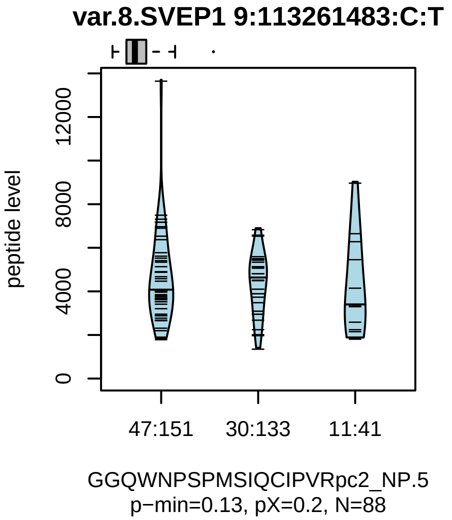
var.8.SVEP1 9:113261483:C:T
peptide level
47:151
30:133
11:41
GGQWNPSPMSIQCIPVRpc2_NP.5
p−min=0.13, pX=0.2, N=88

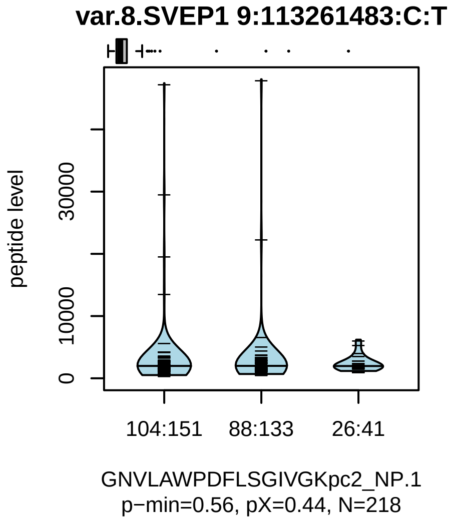
var.8.SVEP1 9:113261483:C:T
peptide level
104:151
88:133
26:41
GNVLAWPDFLSGIVGKpc2_NP.1
p−min=0.56, pX=0.44, N=218

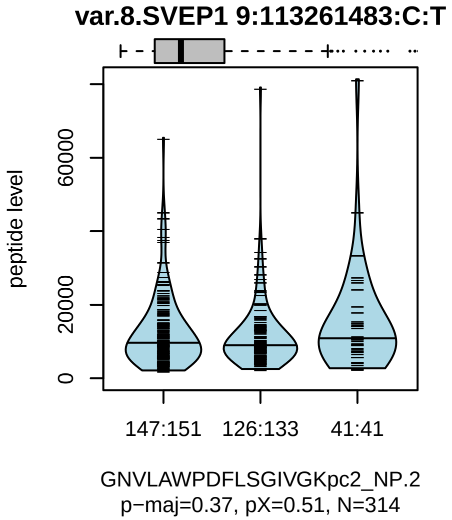
var.8.SVEP1 9:113261483:C:T
peptide level
147:151
126:133
41:41
GNVLAWPDFLSGIVGKpc2_NP.2
p−maj=0.37, pX=0.51, N=314

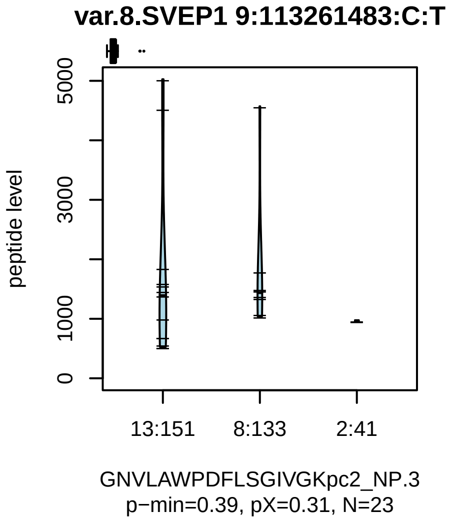
var.8.SVEP1 9:113261483:C:T
peptide level
13:151
8:133
2:41
GNVLAWPDFLSGIVGKpc2_NP.3
p−min=0.39, pX=0.31, N=23

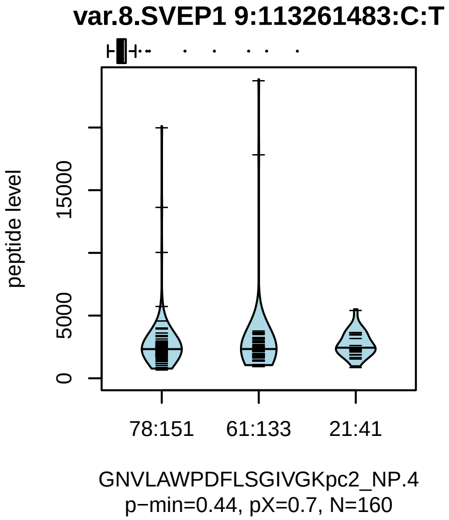
var.8.SVEP1 9:113261483:C:T
peptide level
78:151
61:133
21:41
GNVLAWPDFLSGIVGKpc2_NP.4
p−min=0.44, pX=0.7, N=160

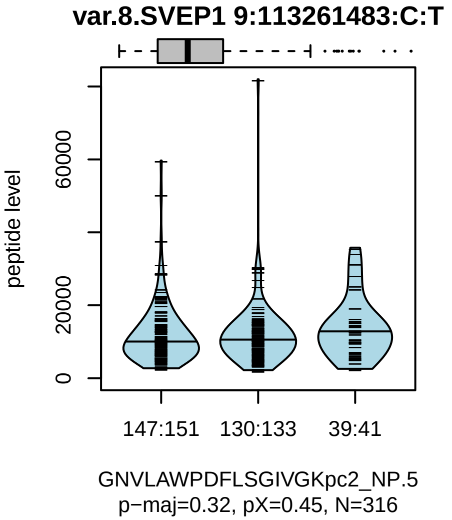
var.8.SVEP1 9:113261483:C:T
peptide level
147:151
130:133
39:41
GNVLAWPDFLSGIVGKpc2_NP.5
p−maj=0.32, pX=0.45, N=316

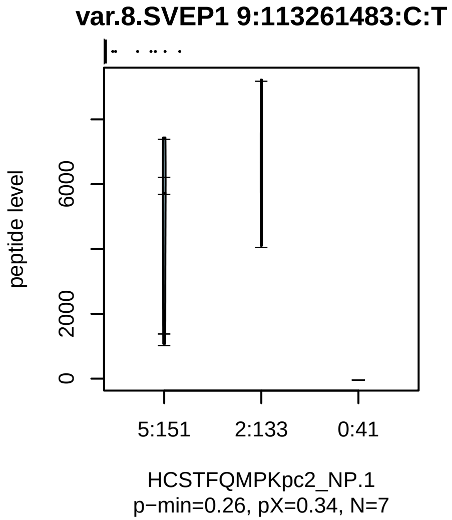
var.8.SVEP1 9:113261483:C:T
peptide level
5:151
2:133
0:41
HCSTFQMPKpc2_NP.1
p−min=0.26, pX=0.34, N=7

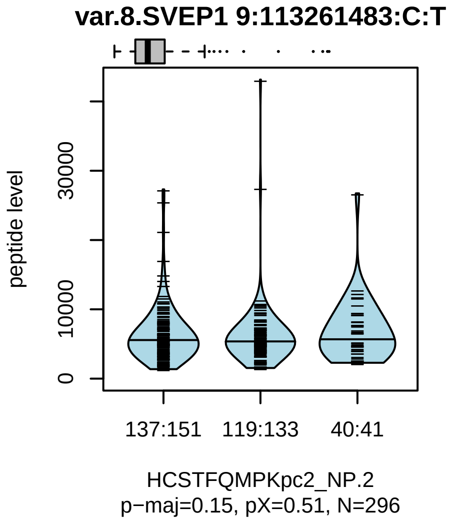
var.8.SVEP1 9:113261483:C:T
peptide level
137:151
119:133
40:41
HCSTFQMPKpc2_NP.2
p−maj=0.15, pX=0.51, N=296

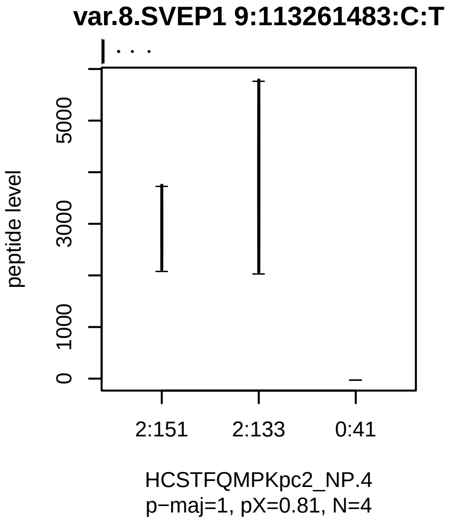
var.8.SVEP1 9:113261483:C:T
peptide level
2:151
2:133
0:41
HCSTFQMPKpc2_NP.4
p−maj=1, pX=0.81, N=4

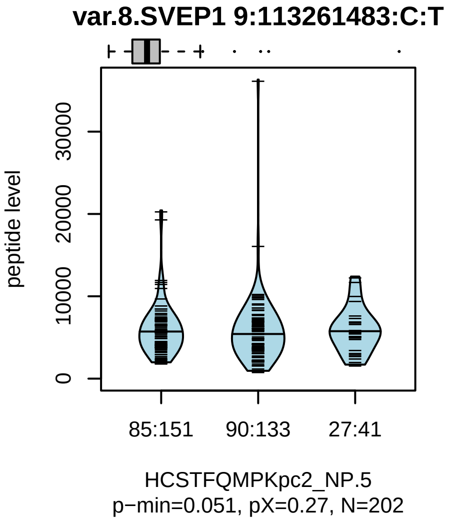
var.8.SVEP1 9:113261483:C:T
peptide level
85:151
90:133
27:41
HCSTFQMPKpc2_NP.5
p−min=0.051, pX=0.27, N=202

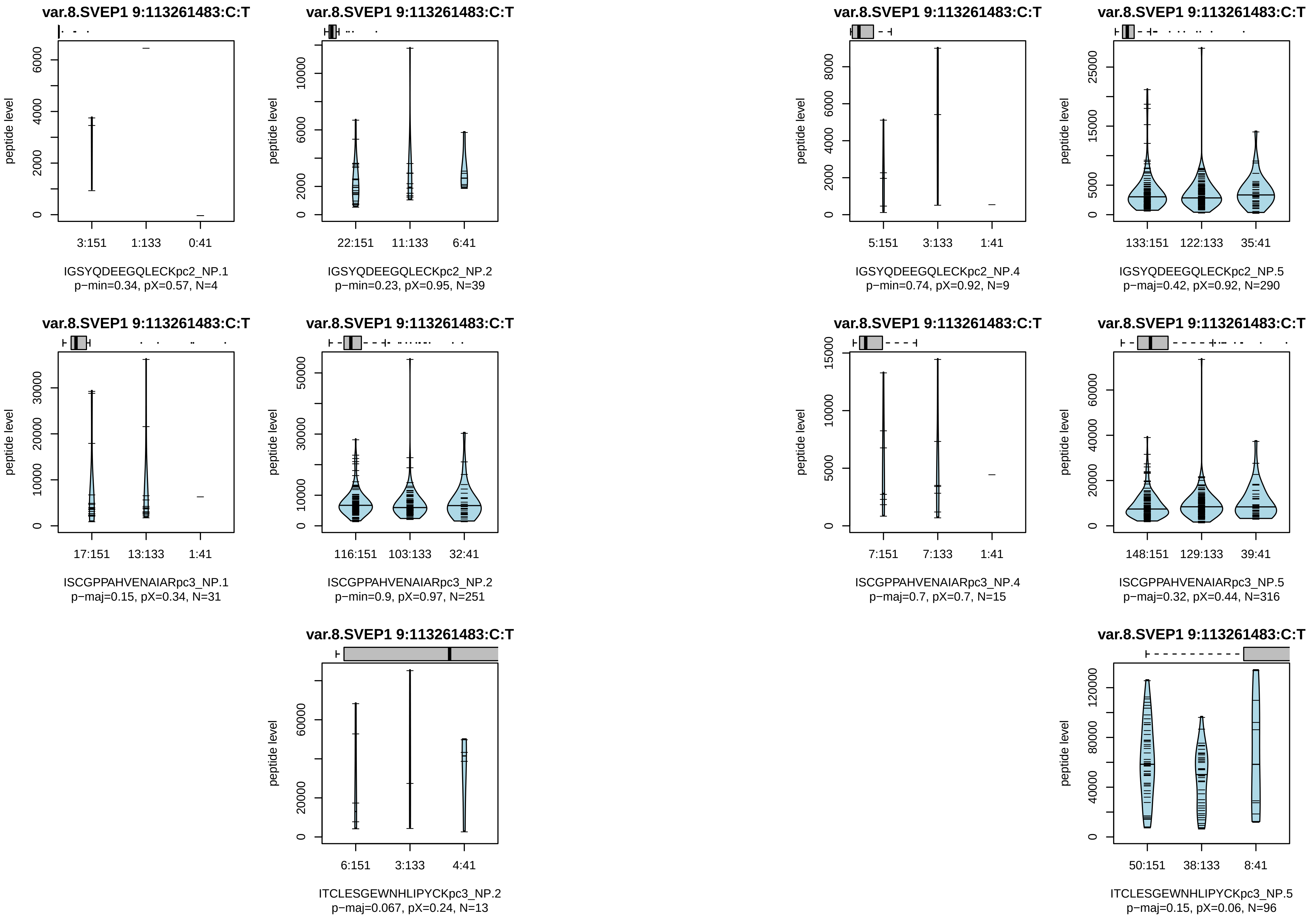
var.8.SVEP1 9:113261483:C:T
peptide level
3:151
1:133
0:41
IGSYQDEEGQLECKpc2_NP.1
p−min=0.34, pX=0.57, N=4
var.8.SVEP1 9:113261483:C:T
peptide level
22:151
11:133
6:41
IGSYQDEEGQLECKpc2_NP.2
p−min=0.23, pX=0.95, N=39
var.8.SVEP1 9:113261483:C:T
peptide level
5:151
3:133
1:41
IGSYQDEEGQLECKpc2_NP.4
p−min=0.74, pX=0.92, N=9
var.8.SVEP1 9:113261483:C:T
peptide level
133:151
122:133
35:41
IGSYQDEEGQLECKpc2_NP.5
p−maj=0.42, pX=0.92, N=290
var.8.SVEP1 9:113261483:C:T
peptide level
17:151
13:133
1:41
ISCGPPAHVENAIARpc3_NP.1
p−maj=0.15, pX=0.34, N=31
var.8.SVEP1 9:113261483:C:T
peptide level
116:151
103:133
32:41
ISCGPPAHVENAIARpc3_NP.2
p−min=0.9, pX=0.97, N=251
var.8.SVEP1 9:113261483:C:T
peptide level
7:151
7:133
1:41
ISCGPPAHVENAIARpc3_NP.4
p−maj=0.7, pX=0.7, N=15
var.8.SVEP1 9:113261483:C:T
peptide level
148:151
129:133
39:41
ISCGPPAHVENAIARpc3_NP.5
p−maj=0.32, pX=0.44, N=316
var.8.SVEP1 9:113261483:C:T
peptide level
6:151
3:133
4:41
ITCLESGEWNHLIPYCKpc3_NP.2
p−maj=0.067, pX=0.24, N=13
var.8.SVEP1 9:113261483:C:T
peptide level
50:151
38:133
8:41
ITCLESGEWNHLIPYCKpc3_NP.5
p−maj=0.15, pX=0.06, N=96

var.8.SVEP1 9:113261483:C:T

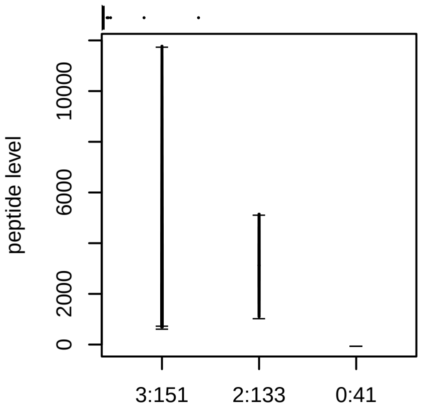

LCEEPICILPCLNGGRpc2_NP.4
p−min=0.67, pX=0.46, N=5

var.8.SVEP1 9:113261483:C:T

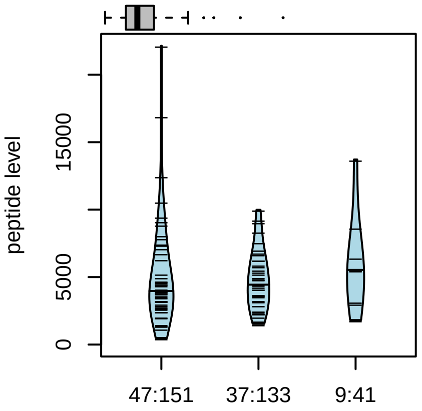

LCEEPICILPCLNGGRpc2_NP.5
p−maj=0.36, pX=0.38, N=93

var.8.SVEP1 9:113261483:C:T

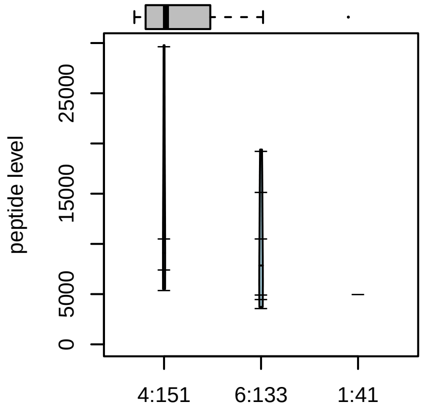

LCPSGMYTEYIHSRpc3_NP.1
p−min=0.55, pX=0.77, N=11

var.8.SVEP1 9:113261483:C:T

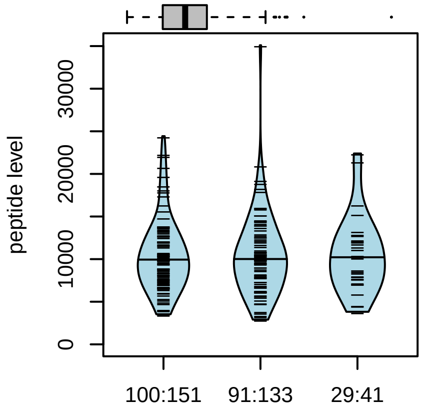

LCPSGMYTEYIHSRpc3_NP.2
p−min=0.64, pX=0.6, N=220

var.8.SVEP1 9:113261483:C:T

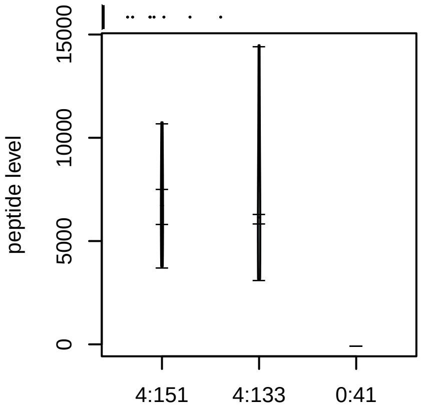

LCPSGMYTEYIHSRpc3_NP.4
p−maj=0.6, pX=0.59, N=8

var.8.SVEP1 9:113261483:C:T

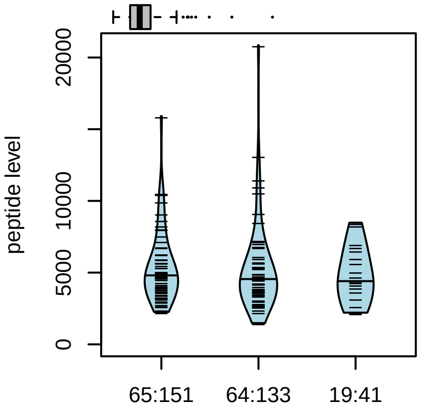

LCPSGMYTEYIHSRpc3_NP.5
p−min=0.44, pX=0.9, N=148

var.8.SVEP1 9:113261483:C:T

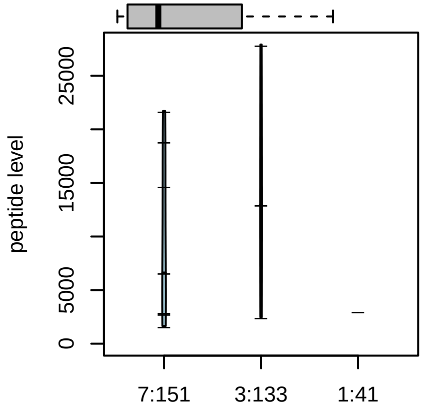

LGGSVPHLRpc2_NP.1
p−min=0.36, pX=0.38, N=11

var.8.SVEP1 9:113261483:C:T

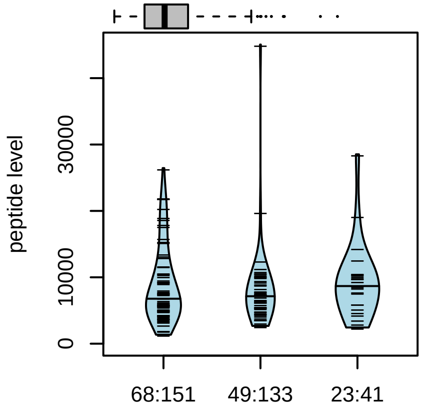

LGGSVPHLRpc2_NP.2
p−maj=0.091, pX=0.78, N=140

var.8.SVEP1 9:113261483:C:T

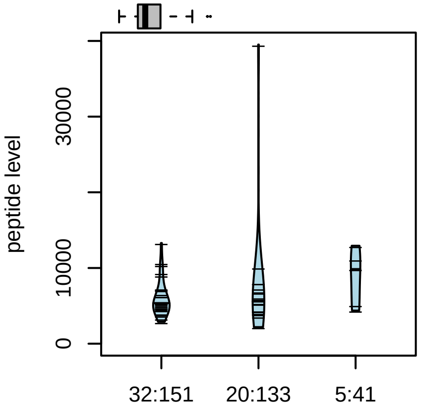

LGGSVPHLRpc2_NP.5
p−min=0.11, pX=0.65, N=57

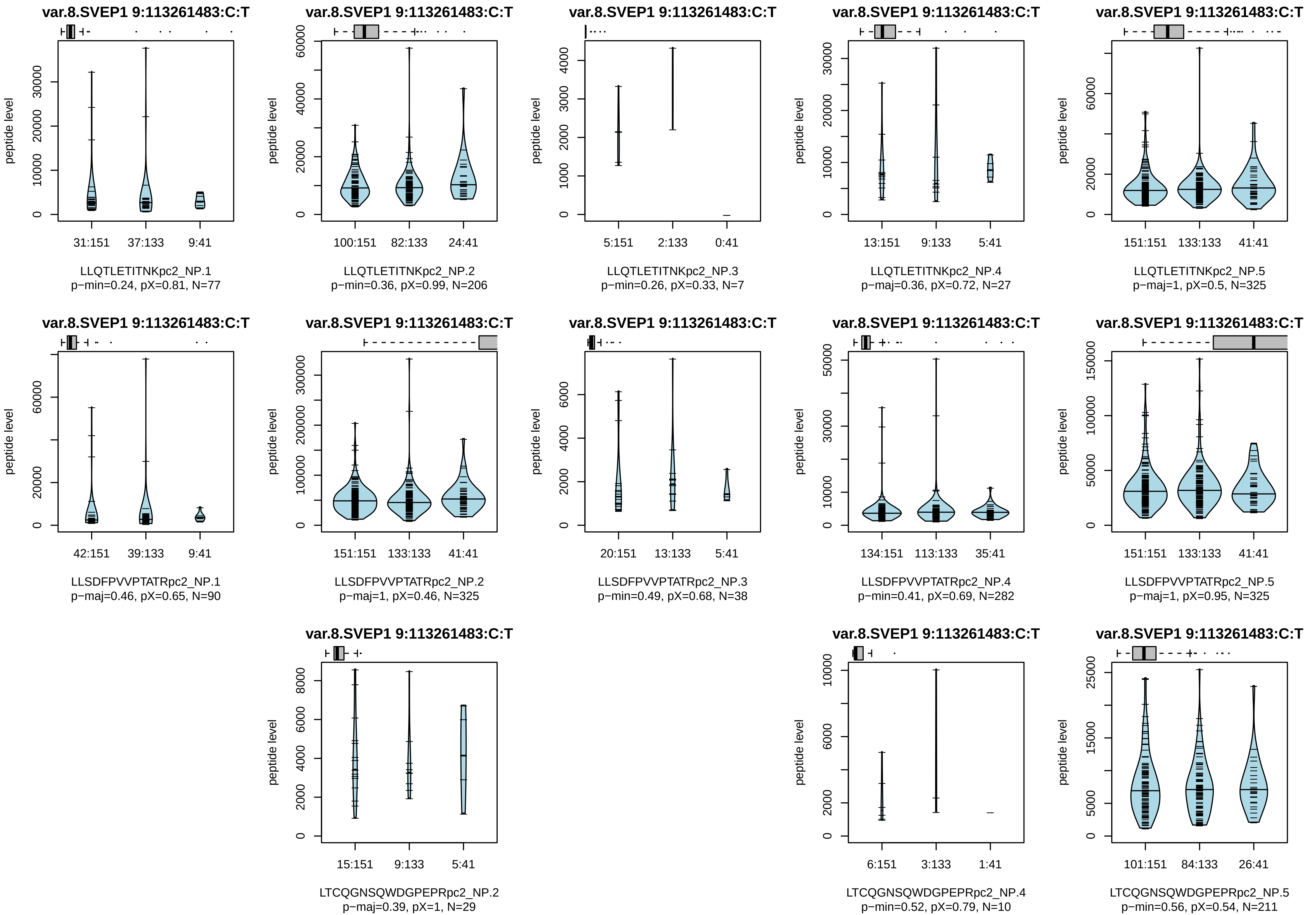

var.8.SVEP1 9:113261483:C:T
peptide level
31:151
37:133
9:41
LLQTLETITNKpc2_NP.1
p−min=0.24, pX=0.81, N=77
var.8.SVEP1 9:113261483:C:T
peptide level
100:151
82:133
24:41
LLQTLETITNKpc2_NP.2
p−min=0.36, pX=0.99, N=206
var.8.SVEP1 9:113261483:C:T
peptide level
5:151
2:133
0:41
LLQTLETITNKpc2_NP.3
p−min=0.26, pX=0.33, N=7
var.8.SVEP1 9:113261483:C:T
peptide level
13:151
9:133
5:41
LLQTLETITNKpc2_NP.4
p−maj=0.36, pX=0.72, N=27
var.8.SVEP1 9:113261483:C:T
peptide level
151:151
133:133
41:41
LLQTLETITNKpc2_NP.5
p−maj=1, pX=0.5, N=325
var.8.SVEP1 9:113261483:C:T
peptide level
42:151
39:133
9:41
LLSDFPVVPTATRpc2_NP.1
p−maj=0.46, pX=0.65, N=90
var.8.SVEP1 9:113261483:C:T
peptide level
151:151
133:133
41:41
LLSDFPVVPTATRpc2_NP.2
p−maj=1, pX=0.46, N=325
var.8.SVEP1 9:113261483:C:T
peptide level
20:151
13:133
5:41
LLSDFPVVPTATRpc2_NP.3
p−min=0.49, pX=0.68, N=38
var.8.SVEP1 9:113261483:C:T
peptide level
134:151
113:133
35:41
LLSDFPVVPTATRpc2_NP.4
p−min=0.41, pX=0.69, N=282
var.8.SVEP1 9:113261483:C:T
peptide level
151:151
133:133
41:41
LLSDFPVVPTATRpc2_NP.5
p−maj=1, pX=0.95, N=325
var.8.SVEP1 9:113261483:C:T
peptide level
15:151
9:133
5:41
LTCQGNSQWDGPEPRpc2_NP.2
p−maj=0.39, pX=1, N=29
var.8.SVEP1 9:113261483:C:T
peptide level
6:151
3:133
1:41
LTCQGNSQWDGPEPRpc2_NP.4
p−min=0.52, pX=0.79, N=10
var.8.SVEP1 9:113261483:C:T
peptide level
101:151
84:133
26:41
LTCQGNSQWDGPEPRpc2_NP.5
p−min=0.56, pX=0.54, N=211

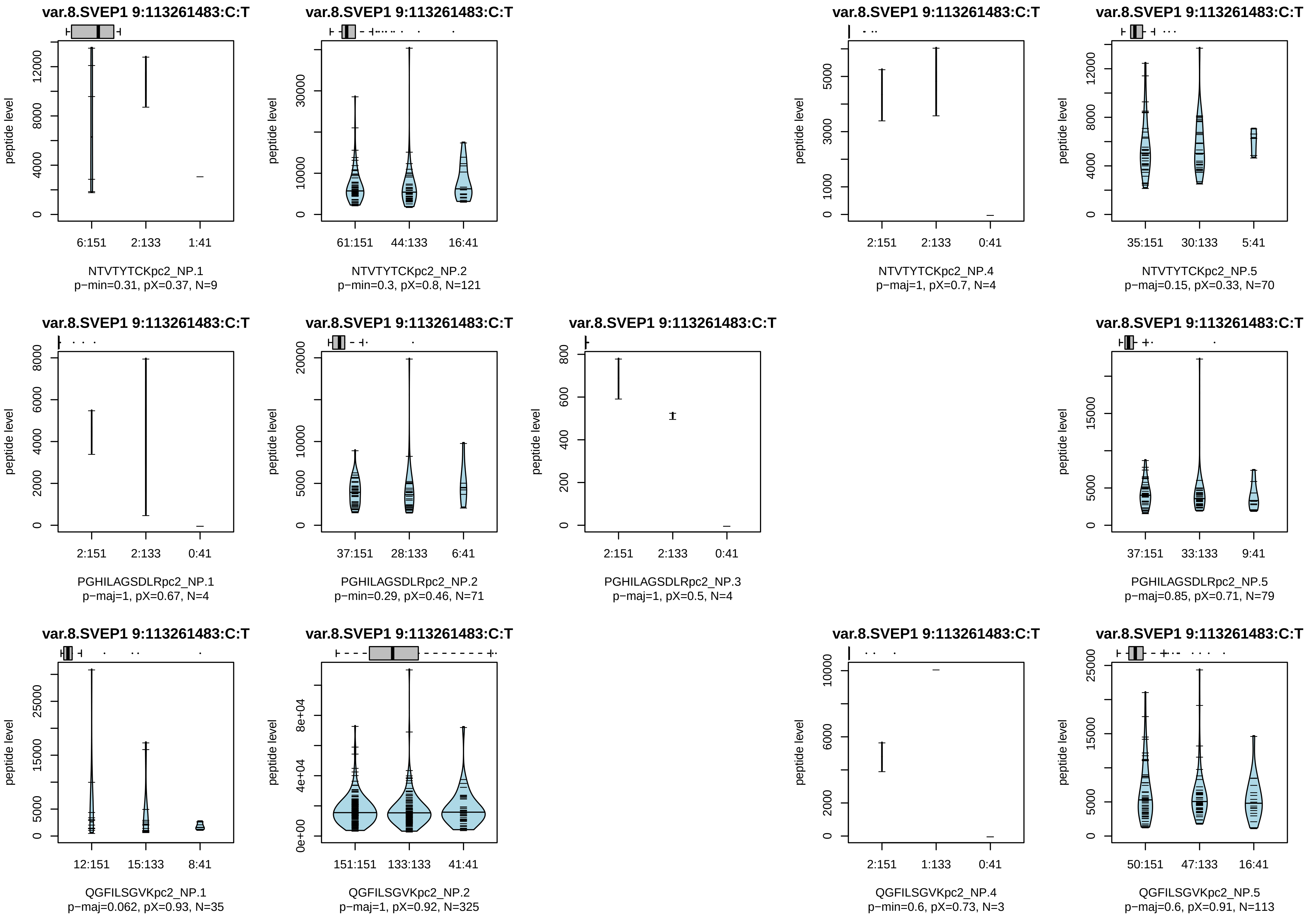

var.8.SVEP1 9:113261483:C:T
peptide level
0
4000
8000
12000
6:151
2:133
1:41
NTVTYTCKpc2_NP.1
p−min=0.31, pX=0.37, N=9
var.8.SVEP1 9:113261483:C:T
peptide level
0
10000
30000
61:151
44:133
16:41
NTVTYTCKpc2_NP.2
p−min=0.3, pX=0.8, N=121
var.8.SVEP1 9:113261483:C:T
peptide level
0
1000
3000
5000
2:151
2:133
0:41
NTVTYTCKpc2_NP.4
p−maj=1, pX=0.7, N=4
var.8.SVEP1 9:113261483:C:T
peptide level
0
4000
8000
12000
35:151
30:133
5:41
NTVTYTCKpc2_NP.5
p−maj=0.15, pX=0.33, N=70
var.8.SVEP1 9:113261483:C:T
peptide level
0
2000
4000
6000
8000
2:151
2:133
0:41
PGHILAGSDLRpc2_NP.1
p−maj=1, pX=0.67, N=4
var.8.SVEP1 9:113261483:C:T
peptide level
0
5000
10000
20000
37:151
28:133
6:41
PGHILAGSDLRpc2_NP.2
p−min=0.29, pX=0.46, N=71
var.8.SVEP1 9:113261483:C:T
peptide level
0
200
400
600
800
2:151
2:133
0:41
PGHILAGSDLRpc2_NP.3
p−maj=1, pX=0.5, N=4
var.8.SVEP1 9:113261483:C:T
peptide level
0
5000
15000
37:151
33:133
9:41
PGHILAGSDLRpc2_NP.5
p−maj=0.85, pX=0.71, N=79
var.8.SVEP1 9:113261483:C:T
peptide level
0
5000
15000
25000
12:151
15:133
8:41
QGFILSGVKpc2_NP.1
p−maj=0.062, pX=0.93, N=35
var.8.SVEP1 9:113261483:C:T
peptide level
0e+00
4e+04
8e+04
151:151
133:133
41:41
QGFILSGVKpc2_NP.2
p−maj=1, pX=0.92, N=325
var.8.SVEP1 9:113261483:C:T
peptide level
0
2000
6000
10000
2:151
1:133
0:41
QGFILSGVKpc2_NP.4
p−min=0.6, pX=0.73, N=3
var.8.SVEP1 9:113261483:C:T
peptide level
0
5000
15000
25000
50:151
47:133
16:41
QGFILSGVKpc2_NP.5
p−maj=0.6, pX=0.91, N=113

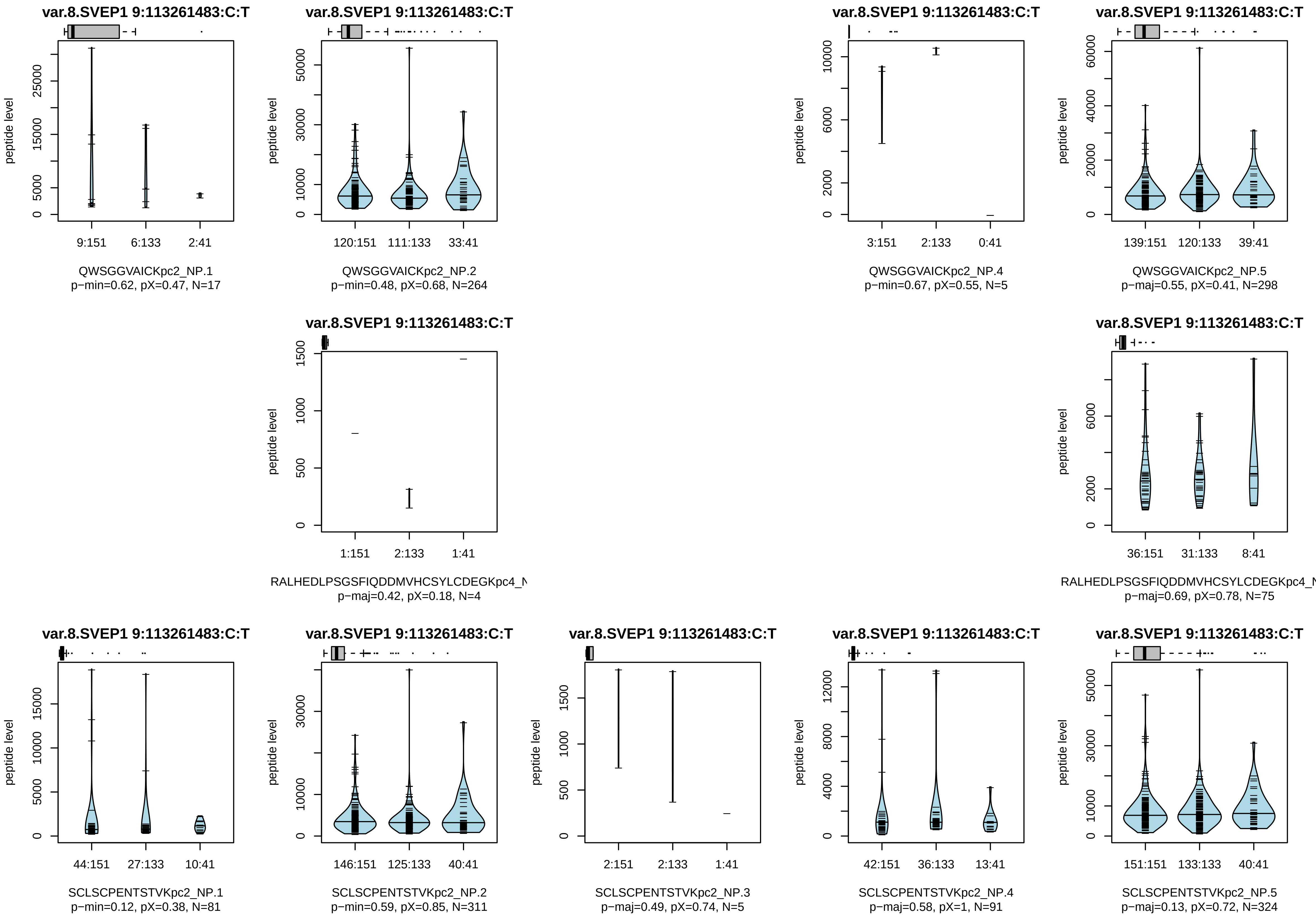
var.8.SVEP1 9:113261483:C:T
peptide level
9:151
6:133
2:41
QWSGGVAICKpc2_NP.1
p−min=0.62, pX=0.47, N=17
var.8.SVEP1 9:113261483:C:T
peptide level
120:151
111:133
33:41
QWSGGVAICKpc2_NP.2
p−min=0.48, pX=0.68, N=264
var.8.SVEP1 9:113261483:C:T
peptide level
3:151
2:133
0:41
QWSGGVAICKpc2_NP.4
p−min=0.67, pX=0.55, N=5
var.8.SVEP1 9:113261483:C:T
peptide level
139:151
120:133
39:41
QWSGGVAICKpc2_NP.5
p−maj=0.55, pX=0.41, N=298
var.8.SVEP1 9:113261483:C:T
peptide level
1:151
2:133
1:41
RALHEDLPSGSFIQDDMVHCSYLCDEGKpc4_N
p−maj=0.42, pX=0.18, N=4
var.8.SVEP1 9:113261483:C:T
peptide level
36:151
31:133
8:41
RALHEDLPSGSFIQDDMVHCSYLCDEGKpc4_N
p−maj=0.69, pX=0.78, N=75
var.8.SVEP1 9:113261483:C:T
peptide level
44:151
27:133
10:41
SCLSCPENTSTVKpc2_NP.1
p−min=0.12, pX=0.38, N=81
var.8.SVEP1 9:113261483:C:T
peptide level
146:151
125:133
40:41
SCLSCPENTSTVKpc2_NP.2
p−min=0.59, pX=0.85, N=311
var.8.SVEP1 9:113261483:C:T
peptide level
2:151
2:133
1:41
SCLSCPENTSTVKpc2_NP.3
p−maj=0.49, pX=0.74, N=5
var.8.SVEP1 9:113261483:C:T
peptide level
42:151
36:133
13:41
SCLSCPENTSTVKpc2_NP.4
p−maj=0.58, pX=1, N=91
var.8.SVEP1 9:113261483:C:T
peptide level
151:151
133:133
40:41
SCLSCPENTSTVKpc2_NP.5
p−maj=0.13, pX=0.72, N=324

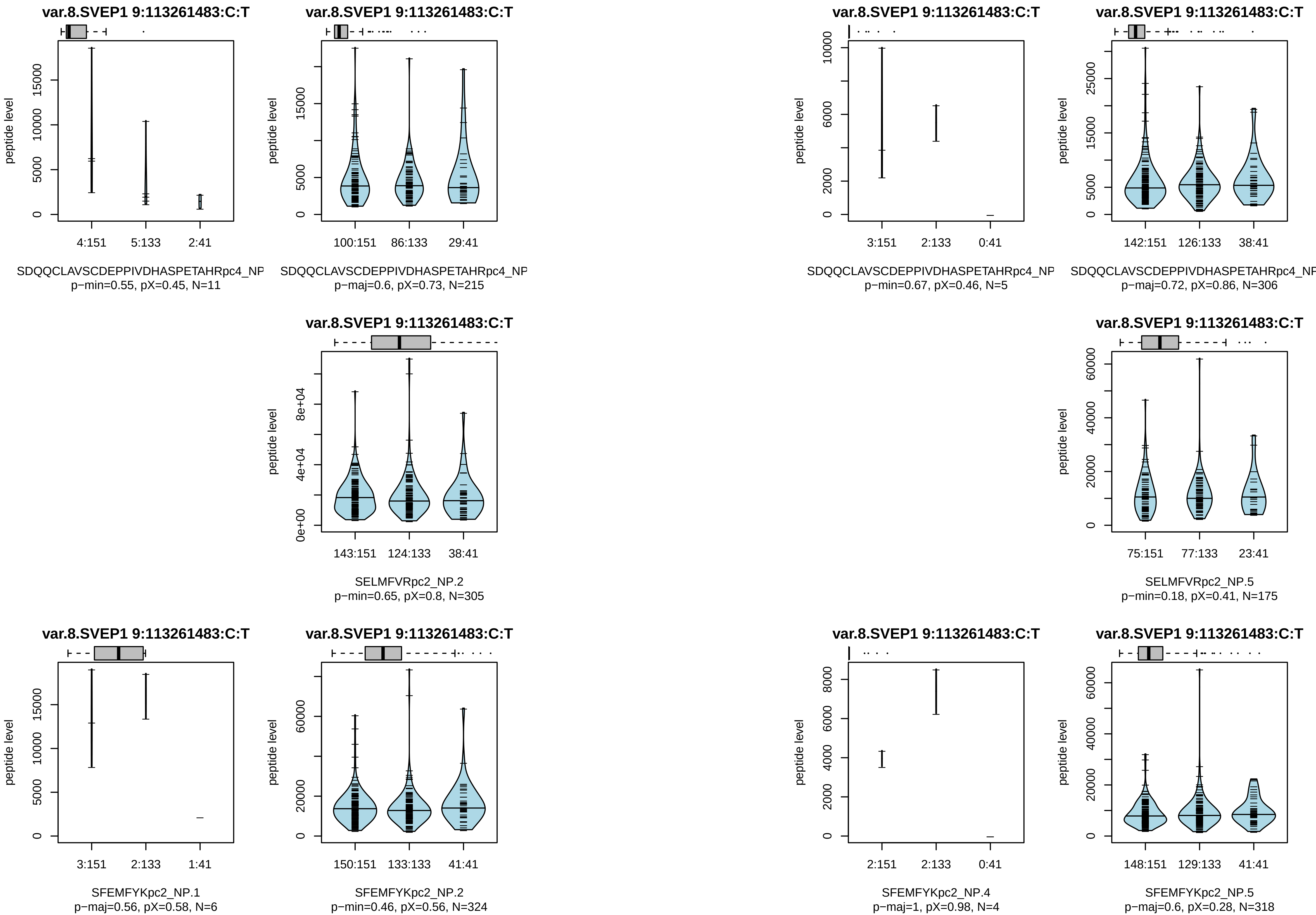
var.8.SVEP1 9:113261483:C:T
peptide level
4:151
5:133
2:41
SDQQCLAVSCDEPPIVDHASPETAHRpc4_NP
p−min=0.55, pX=0.45, N=11
var.8.SVEP1 9:113261483:C:T
peptide level
100:151
86:133
29:41
SDQQCLAVSCDEPPIVDHASPETAHRpc4_NP
p−maj=0.6, pX=0.73, N=215
var.8.SVEP1 9:113261483:C:T
peptide level
3:151
2:133
0:41
SDQQCLAVSCDEPPIVDHASPETAHRpc4_NP
p−min=0.67, pX=0.46, N=5
var.8.SVEP1 9:113261483:C:T
peptide level
142:151
126:133
38:41
SDQQCLAVSCDEPPIVDHASPETAHRpc4_NP
p−maj=0.72, pX=0.86, N=306
var.8.SVEP1 9:113261483:C:T
peptide level
143:151
124:133
38:41
SELMFVRpc2_NP.2
p−min=0.65, pX=0.8, N=305
var.8.SVEP1 9:113261483:C:T
peptide level
75:151
77:133
23:41
SELMFVRpc2_NP.5
p−min=0.18, pX=0.41, N=175
var.8.SVEP1 9:113261483:C:T
peptide level
3:151
2:133
1:41
SFEMFYKpc2_NP.1
p−maj=0.56, pX=0.58, N=6
var.8.SVEP1 9:113261483:C:T
peptide level
150:151
133:133
41:41
SFEMFYKpc2_NP.2
p−min=0.46, pX=0.56, N=324
var.8.SVEP1 9:113261483:C:T
peptide level
2:151
2:133
0:41
SFEMFYKpc2_NP.4
p−maj=1, pX=0.98, N=4
var.8.SVEP1 9:113261483:C:T
peptide level
148:151
129:133
41:41
SFEMFYKpc2_NP.5
p−maj=0.6, pX=0.28, N=318

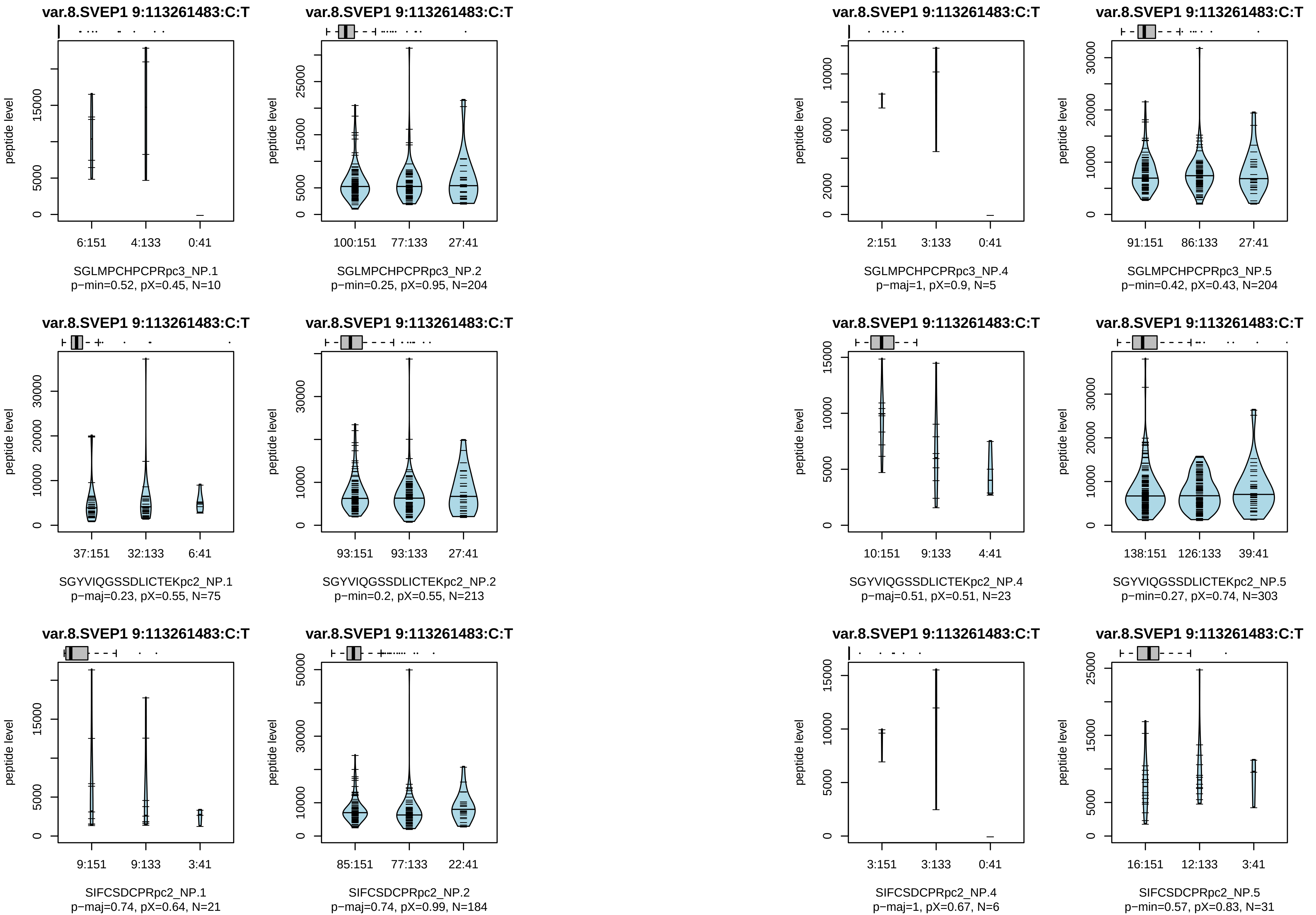
var.8.SVEP1 9:113261483:C:T
peptide level
6:151
4:133
0:41
SGLMPCHPCPRpc3_NP.1
p−min=0.52, pX=0.45, N=10
var.8.SVEP1 9:113261483:C:T
100:151
77:133
27:41
SGLMPCHPCPRpc3_NP.2
p−min=0.25, pX=0.95, N=204
var.8.SVEP1 9:113261483:C:T
2:151
3:133
0:41
SGLMPCHPCPRpc3_NP.4
p−maj=1, pX=0.9, N=5
var.8.SVEP1 9:113261483:C:T
91:151
86:133
27:41
SGLMPCHPCPRpc3_NP.5
p−min=0.42, pX=0.43, N=204
var.8.SVEP1 9:113261483:C:T
37:151
32:133
6:41
SGYVIQGSSDLICTEKpc2_NP.1
p−maj=0.23, pX=0.55, N=75
var.8.SVEP1 9:113261483:C:T
93:151
93:133
27:41
SGYVIQGSSDLICTEKpc2_NP.2
p−min=0.2, pX=0.55, N=213
var.8.SVEP1 9:113261483:C:T
10:151
9:133
4:41
SGYVIQGSSDLICTEKpc2_NP.4
p−maj=0.51, pX=0.51, N=23
var.8.SVEP1 9:113261483:C:T
138:151
126:133
39:41
SGYVIQGSSDLICTEKpc2_NP.5
p−min=0.27, pX=0.74, N=303
var.8.SVEP1 9:113261483:C:T
9:151
9:133
3:41
SIFCSDCPRpc2_NP.1
p−maj=0.74, pX=0.64, N=21
var.8.SVEP1 9:113261483:C:T
85:151
77:133
22:41
SIFCSDCPRpc2_NP.2
p−maj=0.74, pX=0.99, N=184
var.8.SVEP1 9:113261483:C:T
3:151
3:133
0:41
SIFCSDCPRpc2_NP.4
p−maj=1, pX=0.67, N=6
var.8.SVEP1 9:113261483:C:T
16:151
12:133
3:41
SIFCSDCPRpc2_NP.5
p−min=0.57, pX=0.83, N=31

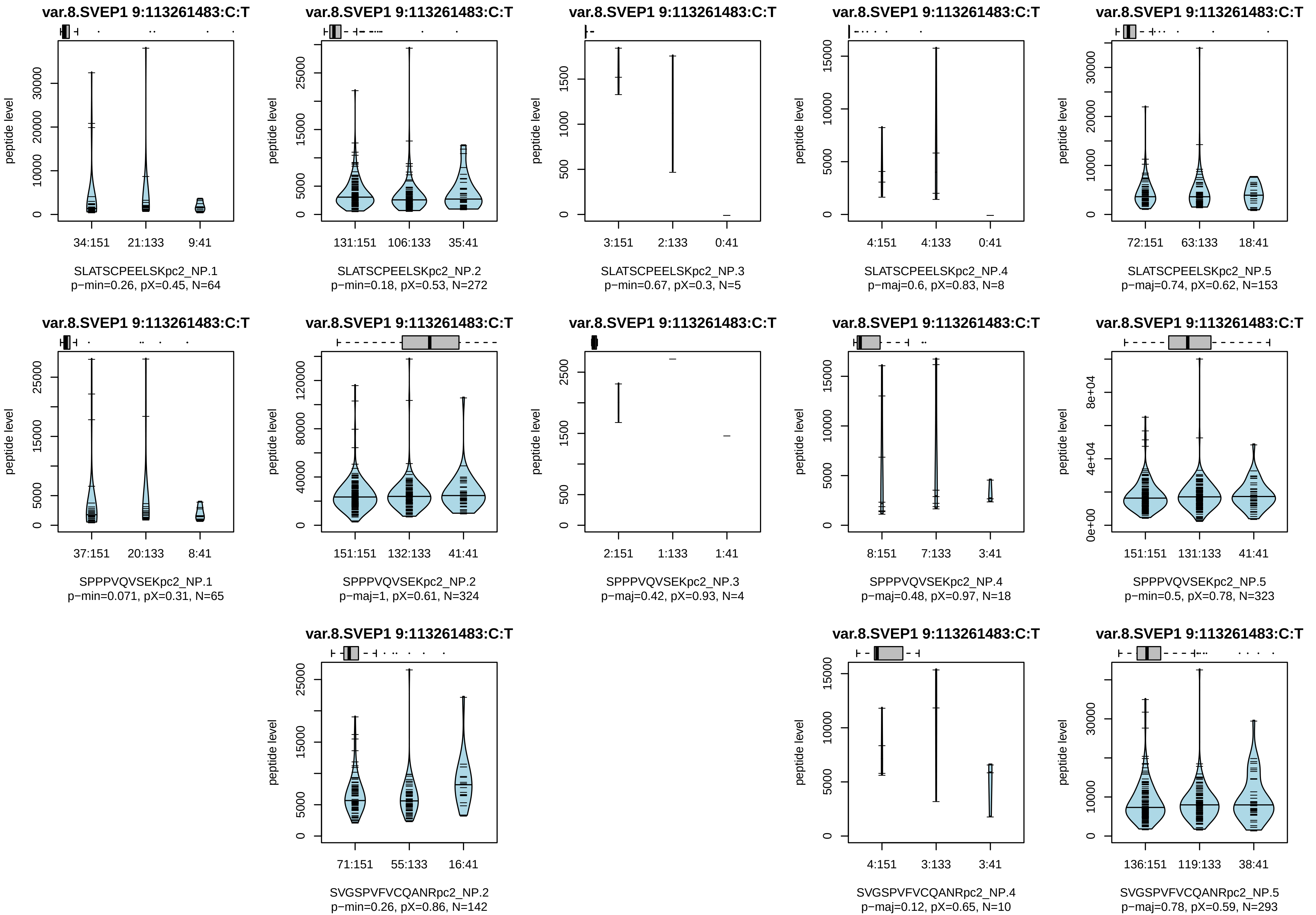

var.8.SVEP1 9:113261483:C:T
peptide level
34:151
21:133
9:41
SLATSCPEELSKpc2_NP.1
p−min=0.26, pX=0.45, N=64
var.8.SVEP1 9:113261483:C:T
peptide level
131:151
106:133
35:41
SLATSCPEELSKpc2_NP.2
p−min=0.18, pX=0.53, N=272
var.8.SVEP1 9:113261483:C:T
peptide level
3:151
2:133
0:41
SLATSCPEELSKpc2_NP.3
p−min=0.67, pX=0.3, N=5
var.8.SVEP1 9:113261483:C:T
peptide level
4:151
4:133
0:41
SLATSCPEELSKpc2_NP.4
p−maj=0.6, pX=0.83, N=8
var.8.SVEP1 9:113261483:C:T
peptide level
72:151
63:133
18:41
SLATSCPEELSKpc2_NP.5
p−maj=0.74, pX=0.62, N=153
var.8.SVEP1 9:113261483:C:T
peptide level
37:151
20:133
8:41
SPPPVQVSEKpc2_NP.1
p−min=0.071, pX=0.31, N=65
var.8.SVEP1 9:113261483:C:T
peptide level
151:151
132:133
41:41
SPPPVQVSEKpc2_NP.2
p−maj=1, pX=0.61, N=324
var.8.SVEP1 9:113261483:C:T
peptide level
2:151
1:133
1:41
SPPPVQVSEKpc2_NP.3
p−maj=0.42, pX=0.93, N=4
var.8.SVEP1 9:113261483:C:T
peptide level
8:151
7:133
3:41
SPPPVQVSEKpc2_NP.4
p−maj=0.48, pX=0.97, N=18
var.8.SVEP1 9:113261483:C:T
peptide level
151:151
131:133
41:41
SPPPVQVSEKpc2_NP.5
p−min=0.5, pX=0.78, N=323
var.8.SVEP1 9:113261483:C:T
peptide level
71:151
55:133
16:41
SVGSPVFVCQANRpc2_NP.2
p−min=0.26, pX=0.86, N=142
var.8.SVEP1 9:113261483:C:T
peptide level
4:151
3:133
3:41
SVGSPVFVCQANRpc2_NP.4
p−maj=0.12, pX=0.65, N=10
var.8.SVEP1 9:113261483:C:T
peptide level
136:151
119:133
38:41
SVGSPVFVCQANRpc2_NP.5
p−maj=0.78, pX=0.59, N=293

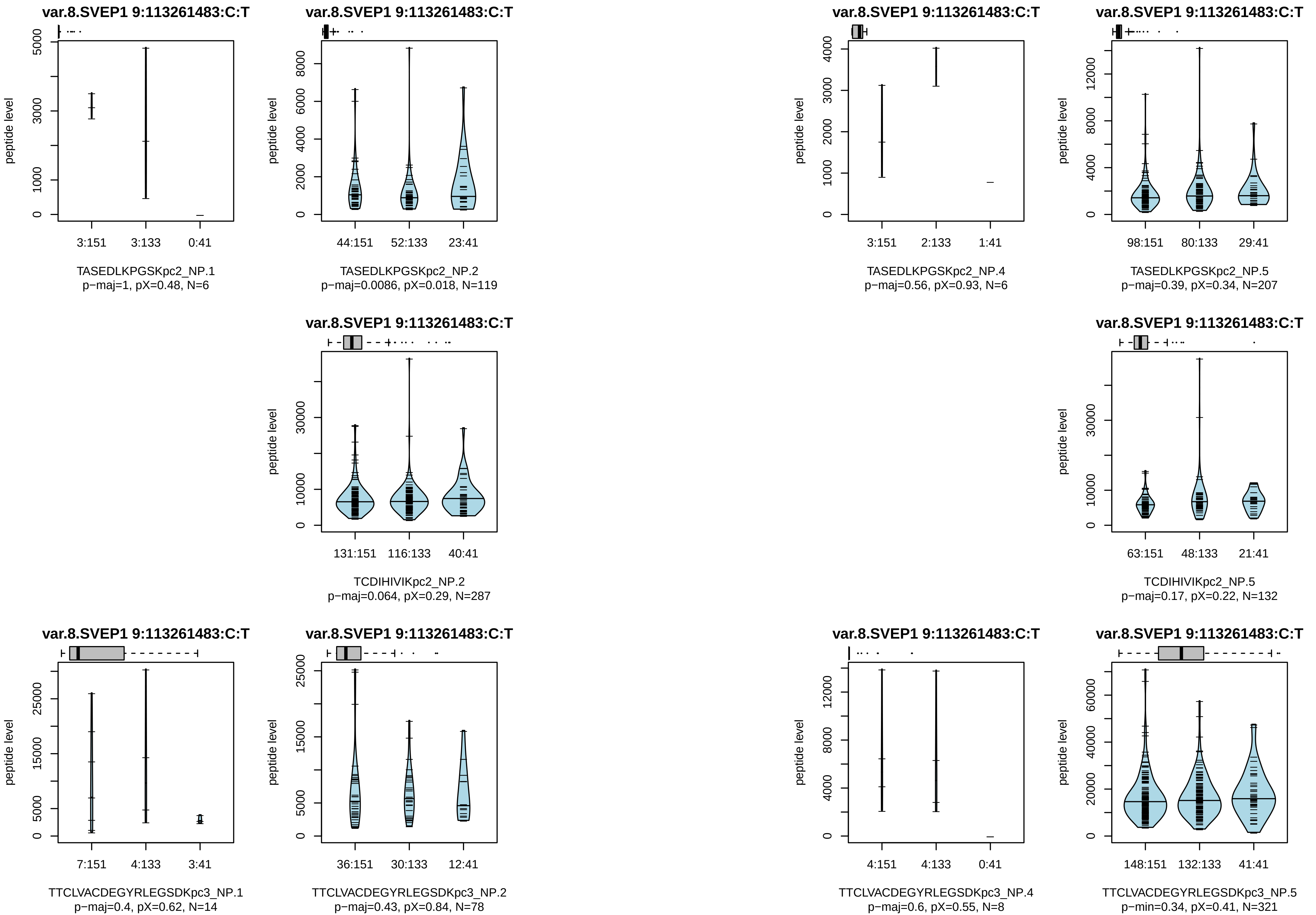
var.8.SVEP1 9:113261483:C:T
peptide level
3:151
3:133
0:41
TASEDLKPGSKpc2_NP.1
p−maj=1, pX=0.48, N=6
var.8.SVEP1 9:113261483:C:T
peptide level
44:151
52:133
23:41
TASEDLKPGSKpc2_NP.2
p−maj=0.0086, pX=0.018, N=119
var.8.SVEP1 9:113261483:C:T
peptide level
3:151
2:133
1:41
TASEDLKPGSKpc2_NP.4
p−maj=0.56, pX=0.93, N=6
var.8.SVEP1 9:113261483:C:T
peptide level
98:151
80:133
29:41
TASEDLKPGSKpc2_NP.5
p−maj=0.39, pX=0.34, N=207
var.8.SVEP1 9:113261483:C:T
peptide level
131:151
116:133
40:41
TCDIHIVIKpc2_NP.2
p−maj=0.064, pX=0.29, N=287
var.8.SVEP1 9:113261483:C:T
peptide level
63:151
48:133
21:41
TCDIHIVIKpc2_NP.5
p−maj=0.17, pX=0.22, N=132
var.8.SVEP1 9:113261483:C:T
peptide level
7:151
4:133
3:41
TTCLVACDEGYRLEGSDKpc3_NP.1
p−maj=0.4, pX=0.62, N=14
var.8.SVEP1 9:113261483:C:T
peptide level
36:151
30:133
12:41
TTCLVACDEGYRLEGSDKpc3_NP.2
p−maj=0.43, pX=0.84, N=78
var.8.SVEP1 9:113261483:C:T
peptide level
4:151
4:133
0:41
TTCLVACDEGYRLEGSDKpc3_NP.4
p−maj=0.6, pX=0.55, N=8
var.8.SVEP1 9:113261483:C:T
peptide level
148:151
132:133
41:41
TTCLVACDEGYRLEGSDKpc3_NP.5
p−min=0.34, pX=0.41, N=321

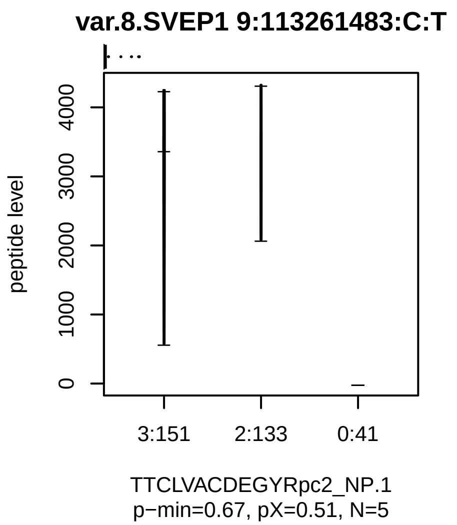
var.8.SVEP1 9:113261483:C:T
peptide level
0
1000
2000
3000
4000
3:151
2:133
0:41
TTCLVACDEGYRpc2_NP.1
p−min=0.67, pX=0.51, N=5

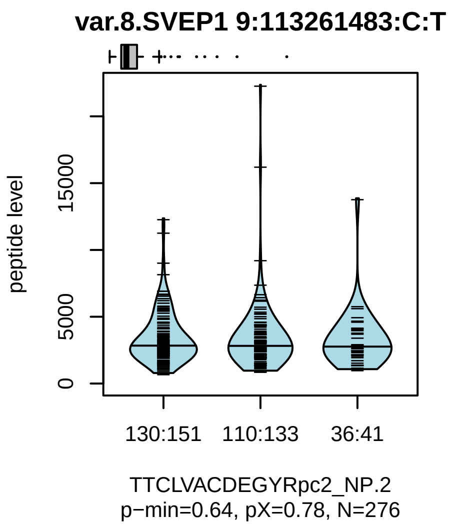
var.8.SVEP1 9:113261483:C:T
peptide level
0
5000
15000
130:151
110:133
36:41
TTCLVACDEGYRpc2_NP.2
p−min=0.64, pX=0.78, N=276

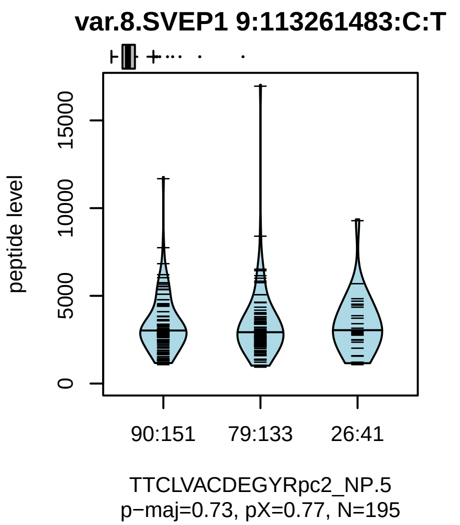
var.8.SVEP1 9:113261483:C:T
peptide level
0
5000
10000
15000
90:151
79:133
26:41
TTCLVACDEGYRpc2_NP.5
p−maj=0.73, pX=0.77, N=195

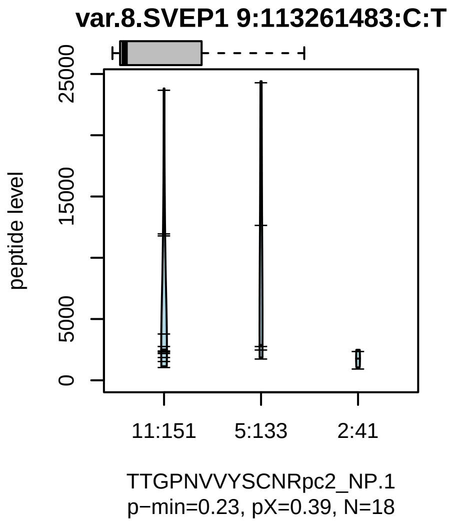
var.8.SVEP1 9:113261483:C:T
peptide level
0
5000
15000
25000
11:151
5:133
2:41
TTGPNVVYSCNRpc2_NP.1
p−min=0.23, pX=0.39, N=18

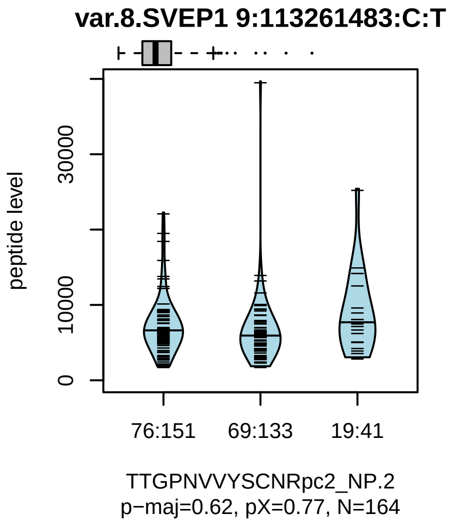
var.8.SVEP1 9:113261483:C:T
peptide level
0
10000
30000
76:151
69:133
19:41
TTGPNVVYSCNRpc2_NP.2
p−maj=0.62, pX=0.77, N=164

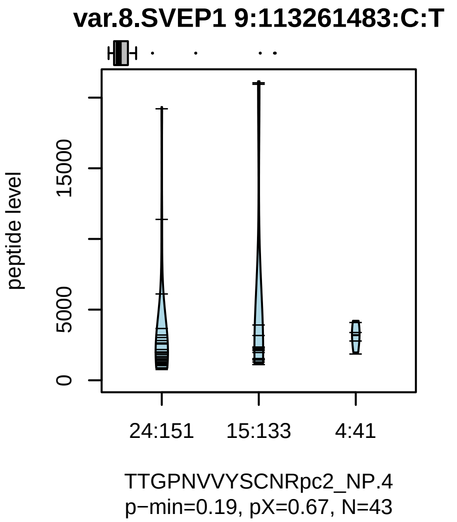
var.8.SVEP1 9:113261483:C:T
peptide level
0
5000
15000
24:151
15:133
4:41
TTGPNVVYSCNRpc2_NP.4
p−min=0.19, pX=0.67, N=43

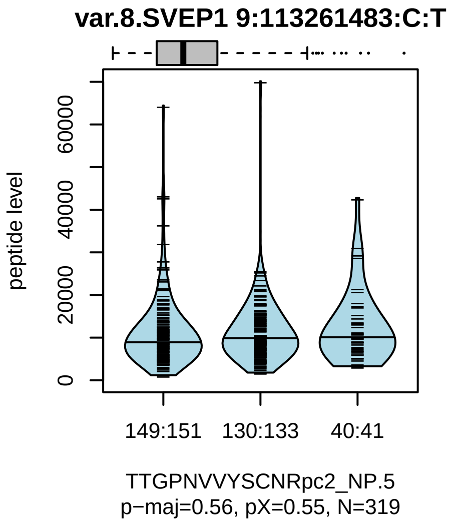
var.8.SVEP1 9:113261483:C:T
peptide level
0
20000
40000
60000
149:151
130:133
40:41
TTGPNVVYSCNRpc2_NP.5
p−maj=0.56, pX=0.55, N=319

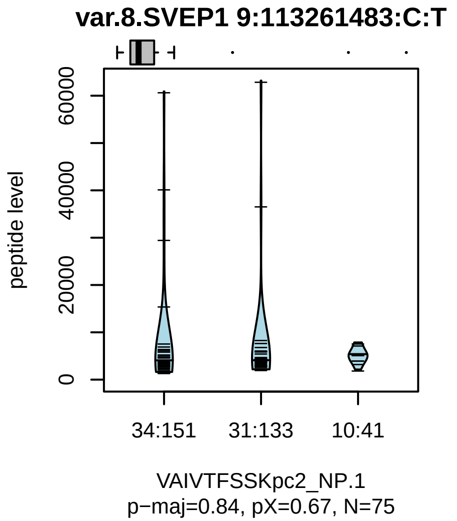
var.8.SVEP1 9:113261483:C:T
peptide level
0
20000
40000
60000
34:151
31:133
10:41
VAIVTFSSKpc2_NP.1
p−maj=0.84, pX=0.67, N=75

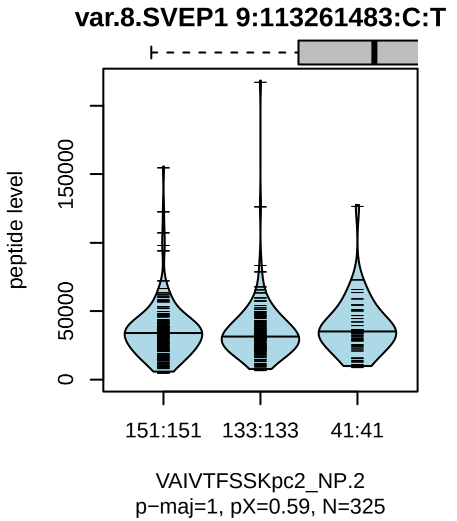
var.8.SVEP1 9:113261483:C:T
peptide level
0
50000
150000
151:151
133:133
41:41
VAIVTFSSKpc2_NP.2
p−maj=1, pX=0.59, N=325

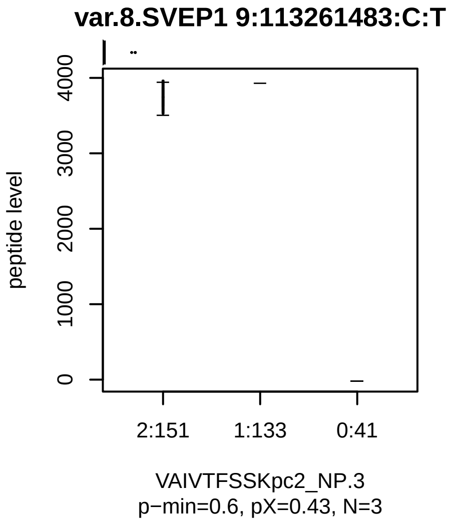
var.8.SVEP1 9:113261483:C:T
peptide level
0
1000
2000
3000
4000
2:151
1:133
0:41
VAIVTFSSKpc2_NP.3
p−min=0.6, pX=0.43, N=3

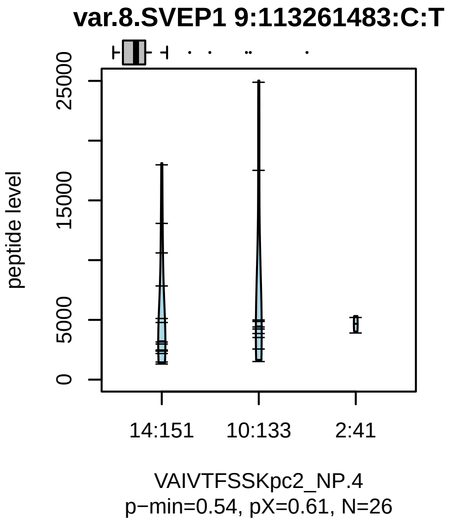
var.8.SVEP1 9:113261483:C:T
peptide level
0
5000
15000
25000
14:151
10:133
2:41
VAIVTFSSKpc2_NP.4
p−min=0.54, pX=0.61, N=26

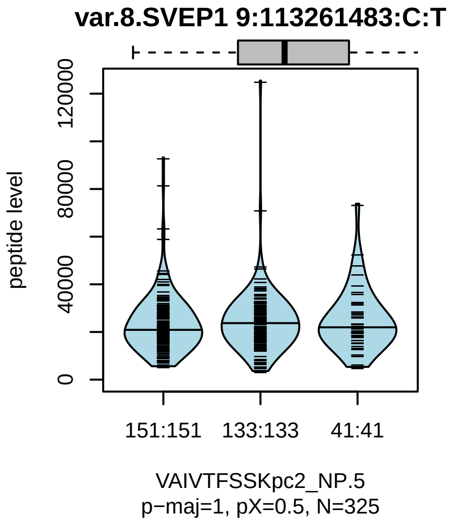
var.8.SVEP1 9:113261483:C:T
peptide level
0
40000
80000
120000
151:151
133:133
41:41
VAIVTFSSKpc2_NP.5
p−maj=1, pX=0.5, N=325

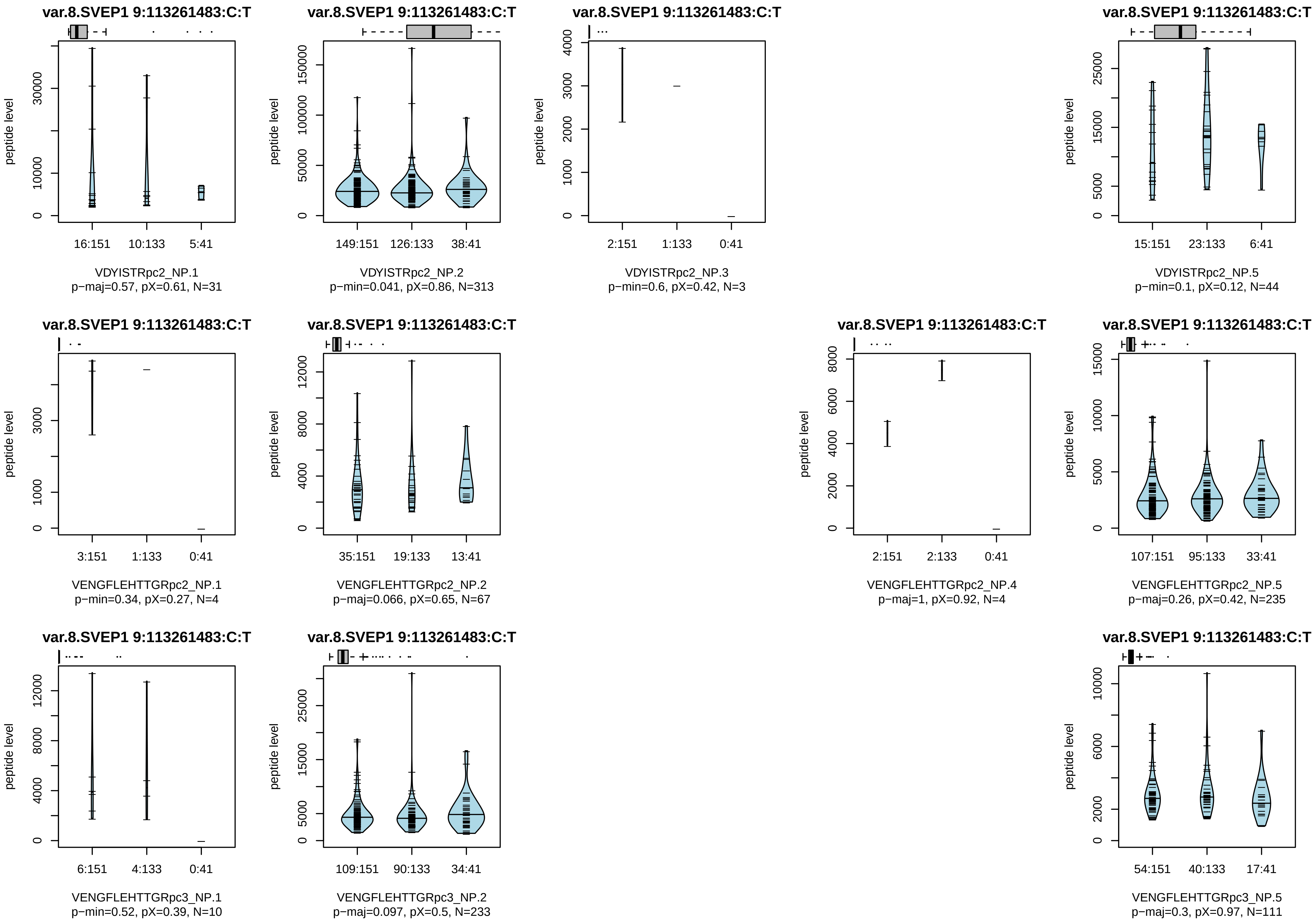
var.8.SVEP1 9:113261483:C:T
peptide level
16:151
10:133
5:41
VDYISTRpc2_NP.1
p−maj=0.57, pX=0.61, N=31
var.8.SVEP1 9:113261483:C:T
peptide level
149:151
126:133
38:41
VDYISTRpc2_NP.2
p−min=0.041, pX=0.86, N=313
var.8.SVEP1 9:113261483:C:T
peptide level
2:151
1:133
0:41
VDYISTRpc2_NP.3
p−min=0.6, pX=0.42, N=3
var.8.SVEP1 9:113261483:C:T
peptide level
15:151
23:133
6:41
VDYISTRpc2_NP.5
p−min=0.1, pX=0.12, N=44
var.8.SVEP1 9:113261483:C:T
peptide level
3:151
1:133
0:41
VENGFLEHTTGRpc2_NP.1
p−min=0.34, pX=0.27, N=4
var.8.SVEP1 9:113261483:C:T
peptide level
35:151
19:133
13:41
VENGFLEHTTGRpc2_NP.2
p−maj=0.066, pX=0.65, N=67
var.8.SVEP1 9:113261483:C:T
peptide level
2:151
2:133
0:41
VENGFLEHTTGRpc2_NP.4
p−maj=1, pX=0.92, N=4
var.8.SVEP1 9:113261483:C:T
peptide level
107:151
95:133
33:41
VENGFLEHTTGRpc2_NP.5
p−maj=0.26, pX=0.42, N=235
var.8.SVEP1 9:113261483:C:T
peptide level
6:151
4:133
0:41
VENGFLEHTTGRpc3_NP.1
p−min=0.52, pX=0.39, N=10
var.8.SVEP1 9:113261483:C:T
peptide level
109:151
90:133
34:41
VENGFLEHTTGRpc3_NP.2
p−maj=0.097, pX=0.5, N=233
var.8.SVEP1 9:113261483:C:T
peptide level
54:151
40:133
17:41
VENGFLEHTTGRpc3_NP.5
p−maj=0.3, pX=0.97, N=111

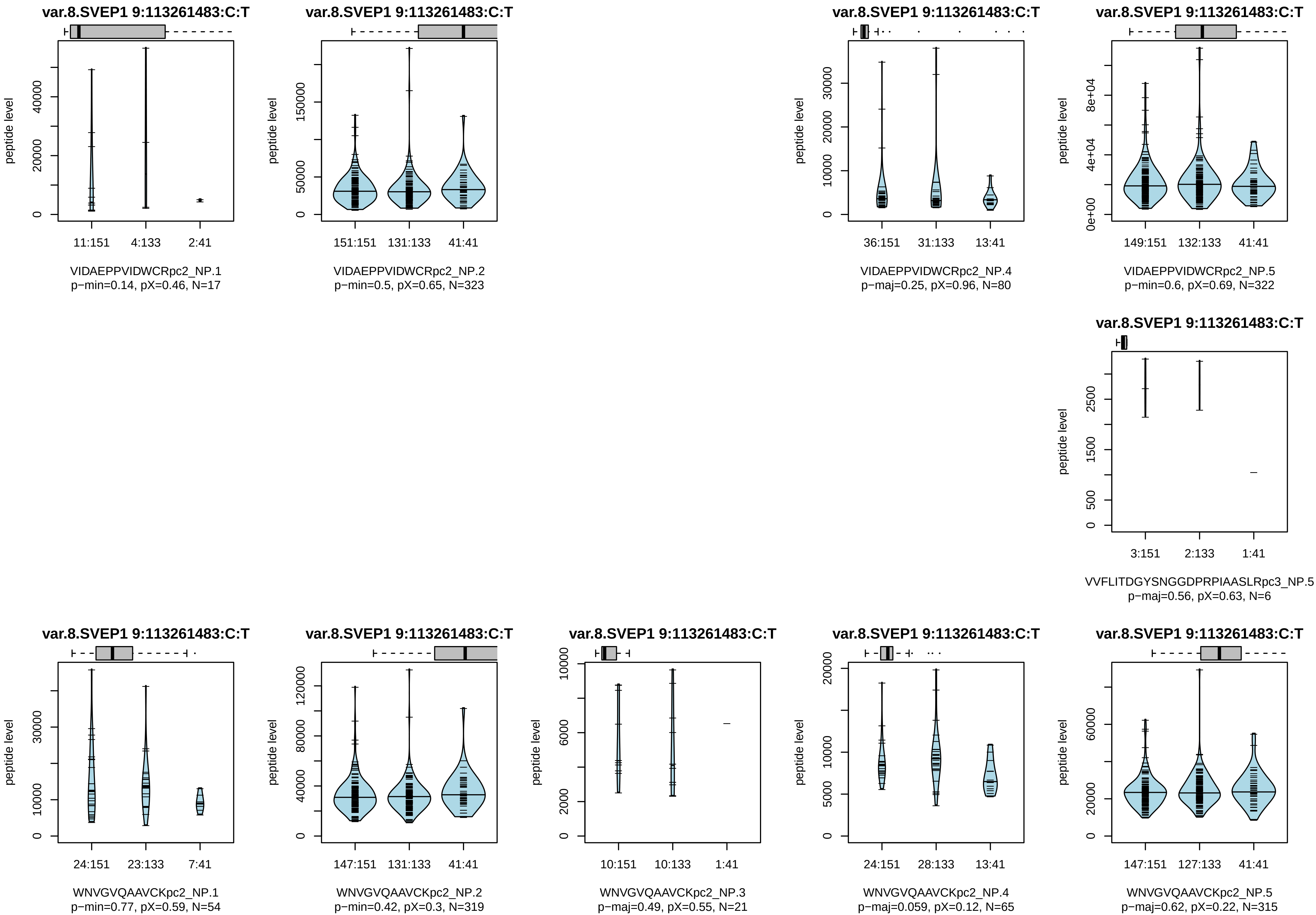
var.8.SVEP1 9:113261483:C:T
peptide level
11:151
4:133
2:41
VIDAEPPVIDWCRpc2_NP.1
p−min=0.14, pX=0.46, N=17
var.8.SVEP1 9:113261483:C:T
peptide level
151:151
131:133
41:41
VIDAEPPVIDWCRpc2_NP.2
p−min=0.5, pX=0.65, N=323
var.8.SVEP1 9:113261483:C:T
peptide level
36:151
31:133
13:41
VIDAEPPVIDWCRpc2_NP.4
p−maj=0.25, pX=0.96, N=80
var.8.SVEP1 9:113261483:C:T
peptide level
149:151
132:133
41:41
VIDAEPPVIDWCRpc2_NP.5
p−min=0.6, pX=0.69, N=322
var.8.SVEP1 9:113261483:C:T
peptide level
3:151
2:133
1:41
VVFLITDGYSNGGDPRPIAASLRpc3_NP.5
p−maj=0.56, pX=0.63, N=6
var.8.SVEP1 9:113261483:C:T
peptide level
24:151
23:133
7:41
WNVGVQAAVCKpc2_NP.1
p−min=0.77, pX=0.59, N=54
var.8.SVEP1 9:113261483:C:T
peptide level
147:151
131:133
41:41
WNVGVQAAVCKpc2_NP.2
p−min=0.42, pX=0.3, N=319
var.8.SVEP1 9:113261483:C:T
peptide level
10:151
10:133
1:41
WNVGVQAAVCKpc2_NP.3
p−maj=0.49, pX=0.55, N=21
var.8.SVEP1 9:113261483:C:T
peptide level
24:151
28:133
13:41
WNVGVQAAVCKpc2_NP.4
p−maj=0.059, pX=0.12, N=65
var.8.SVEP1 9:113261483:C:T
peptide level
147:151
127:133
41:41
WNVGVQAAVCKpc2_NP.5
p−maj=0.62, pX=0.22, N=315

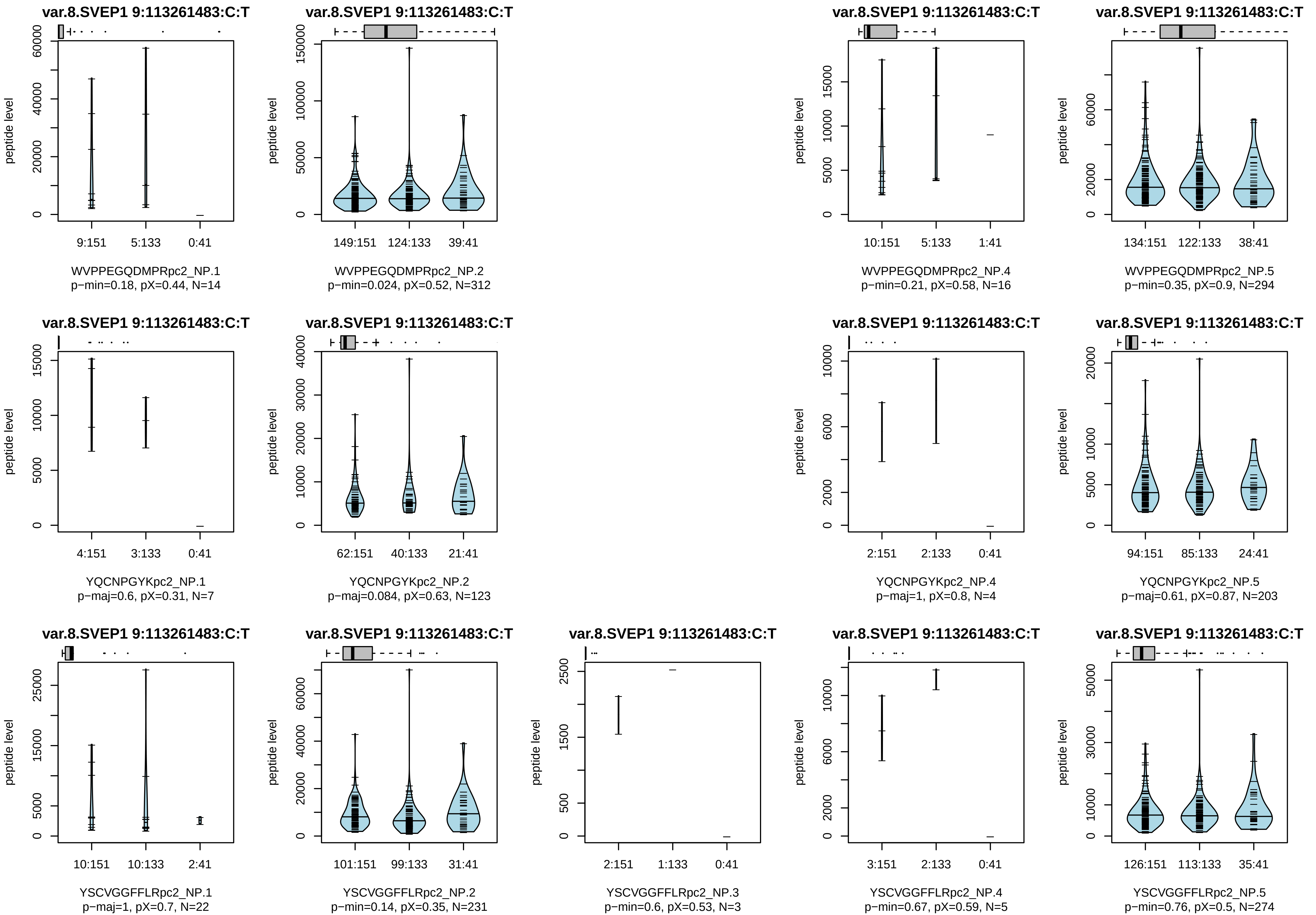
var.8.SVEP1 9:113261483:C:T
peptide level
9:151
5:133
0:41
WVPPEGQDMPRpc2_NP.1
p−min=0.18, pX=0.44, N=14
var.8.SVEP1 9:113261483:C:T
peptide level
149:151
124:133
39:41
WVPPEGQDMPRpc2_NP.2
p−min=0.024, pX=0.52, N=312
var.8.SVEP1 9:113261483:C:T
peptide level
10:151
5:133
1:41
WVPPEGQDMPRpc2_NP.4
p−min=0.21, pX=0.58, N=16
var.8.SVEP1 9:113261483:C:T
peptide level
134:151
122:133
38:41
WVPPEGQDMPRpc2_NP.5
p−min=0.35, pX=0.9, N=294
var.8.SVEP1 9:113261483:C:T
peptide level
4:151
3:133
0:41
YQCNPGYKpc2_NP.1
p−maj=0.6, pX=0.31, N=7
var.8.SVEP1 9:113261483:C:T
peptide level
62:151
40:133
21:41
YQCNPGYKpc2_NP.2
p−maj=0.084, pX=0.63, N=123
var.8.SVEP1 9:113261483:C:T
peptide level
2:151
2:133
0:41
YQCNPGYKpc2_NP.4
p−maj=1, pX=0.8, N=4
var.8.SVEP1 9:113261483:C:T
peptide level
94:151
85:133
24:41
YQCNPGYKpc2_NP.5
p−maj=0.61, pX=0.87, N=203
var.8.SVEP1 9:113261483:C:T
peptide level
10:151
10:133
2:41
YSCVGGFFLRpc2_NP.1
p−maj=1, pX=0.7, N=22
var.8.SVEP1 9:113261483:C:T
peptide level
101:151
99:133
31:41
YSCVGGFFLRpc2_NP.2
p−min=0.14, pX=0.35, N=231
var.8.SVEP1 9:113261483:C:T
peptide level
2:151
1:133
0:41
YSCVGGFFLRpc2_NP.3
p−min=0.6, pX=0.53, N=3
var.8.SVEP1 9:113261483:C:T
peptide level
3:151
2:133
0:41
YSCVGGFFLRpc2_NP.4
p−min=0.67, pX=0.59, N=5
var.8.SVEP1 9:113261483:C:T
peptide level
126:151
113:133
35:41
YSCVGGFFLRpc2_NP.5
p−min=0.76, pX=0.5, N=274

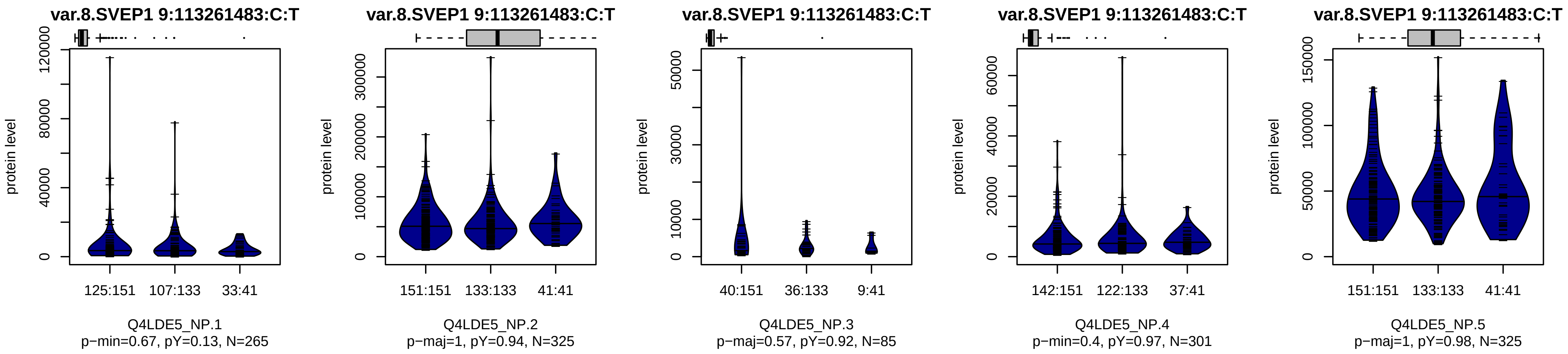

var.8.SVEP1 9:113261483:C:T
protein level
125:151
107:133
33:41
Q4LDE5_NP.1
p−min=0.67, pY=0.13, N=265
var.8.SVEP1 9:113261483:C:T
protein level
151:151
133:133
41:41
Q4LDE5_NP.2
p−maj=1, pY=0.94, N=325
var.8.SVEP1 9:113261483:C:T
protein level
40:151
36:133
9:41
Q4LDE5_NP.3
p−maj=0.57, pY=0.92, N=85
var.8.SVEP1 9:113261483:C:T
protein level
142:151
122:133
37:41
Q4LDE5_NP.4
p−min=0.4, pY=0.97, N=301
var.8.SVEP1 9:113261483:C:T
protein level
151:151
133:133
41:41
Q4LDE5_NP.5
p−maj=1, pY=0.98, N=325

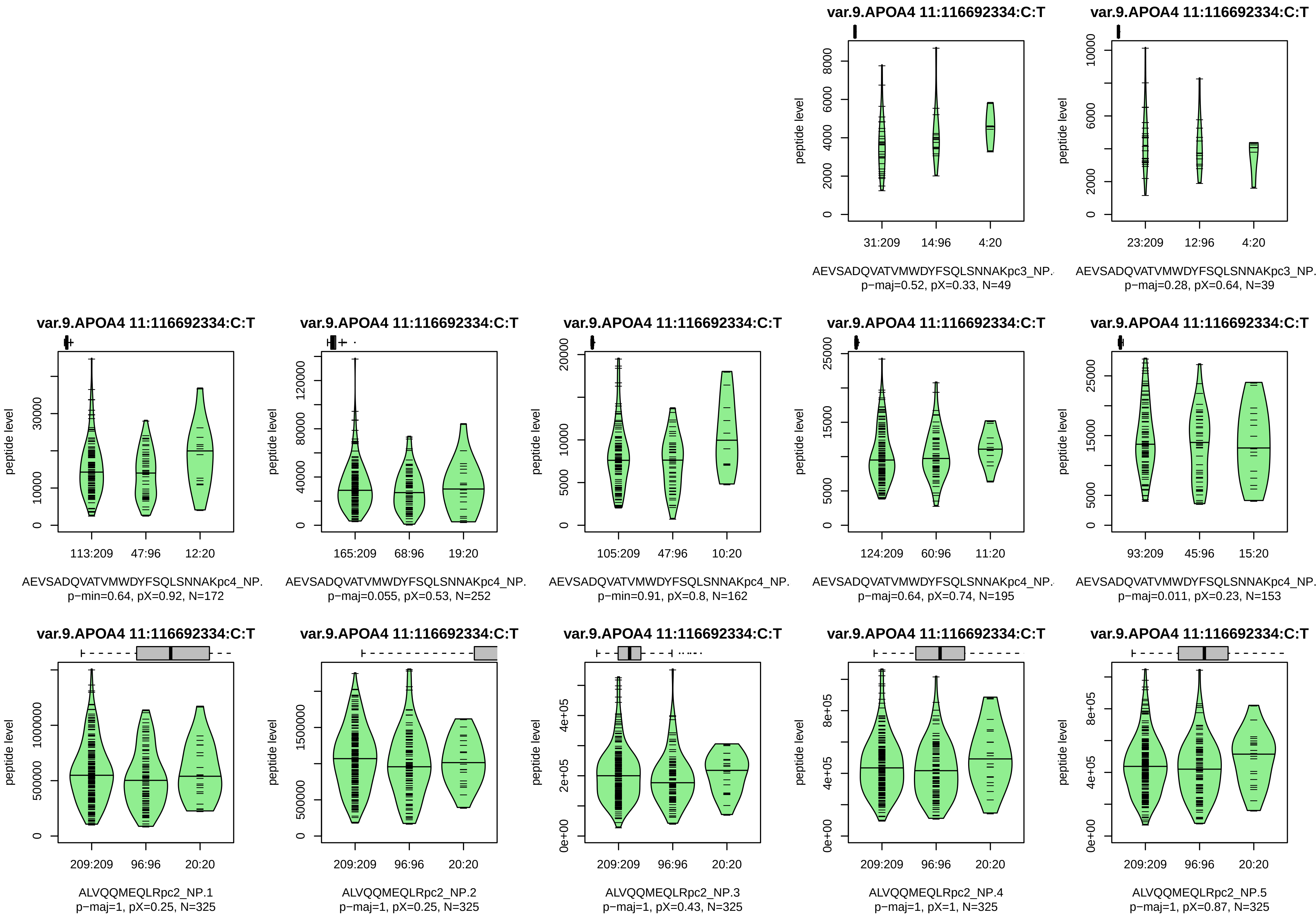
var.9.APOA4 11:116692334:C:T
peptide level
31:209
14:96
4:20
AEVSADQVATVMWDYFSQLSNNAKpc3_NP.
p−maj=0.52, pX=0.33, N=49
var.9.APOA4 11:116692334:C:T
peptide level
23:209
12:96
4:20
AEVSADQVATVMWDYFSQLSNNAKpc3_NP.
p−maj=0.28, pX=0.64, N=39
var.9.APOA4 11:116692334:C:T
peptide level
113:209
47:96
12:20
AEVSADQVATVMWDYFSQLSNNAKpc4_NP.
p−min=0.64, pX=0.92, N=172
var.9.APOA4 11:116692334:C:T
peptide level
165:209
68:96
19:20
AEVSADQVATVMWDYFSQLSNNAKpc4_NP.
p−maj=0.055, pX=0.53, N=252
var.9.APOA4 11:116692334:C:T
peptide level
105:209
47:96
10:20
AEVSADQVATVMWDYFSQLSNNAKpc4_NP.
p−min=0.91, pX=0.8, N=162
var.9.APOA4 11:116692334:C:T
peptide level
124:209
60:96
11:20
AEVSADQVATVMWDYFSQLSNNAKpc4_NP.
p−maj=0.64, pX=0.74, N=195
var.9.APOA4 11:116692334:C:T
peptide level
93:209
45:96
15:20
AEVSADQVATVMWDYFSQLSNNAKpc4_NP.
p−maj=0.011, pX=0.23, N=153
var.9.APOA4 11:116692334:C:T
peptide level
209:209
96:96
20:20
ALVQQMEQLRpc2_NP.1
p−maj=1, pX=0.25, N=325
var.9.APOA4 11:116692334:C:T
peptide level
209:209
96:96
20:20
ALVQQMEQLRpc2_NP.2
p−maj=1, pX=0.25, N=325
var.9.APOA4 11:116692334:C:T
peptide level
209:209
96:96
20:20
ALVQQMEQLRpc2_NP.3
p−maj=1, pX=0.43, N=325
var.9.APOA4 11:116692334:C:T
peptide level
209:209
96:96
20:20
ALVQQMEQLRpc2_NP.4
p−maj=1, pX=1, N=325
var.9.APOA4 11:116692334:C:T
peptide level
209:209
96:96
20:20
ALVQQMEQLRpc2_NP.5
p−maj=1, pX=0.87, N=325

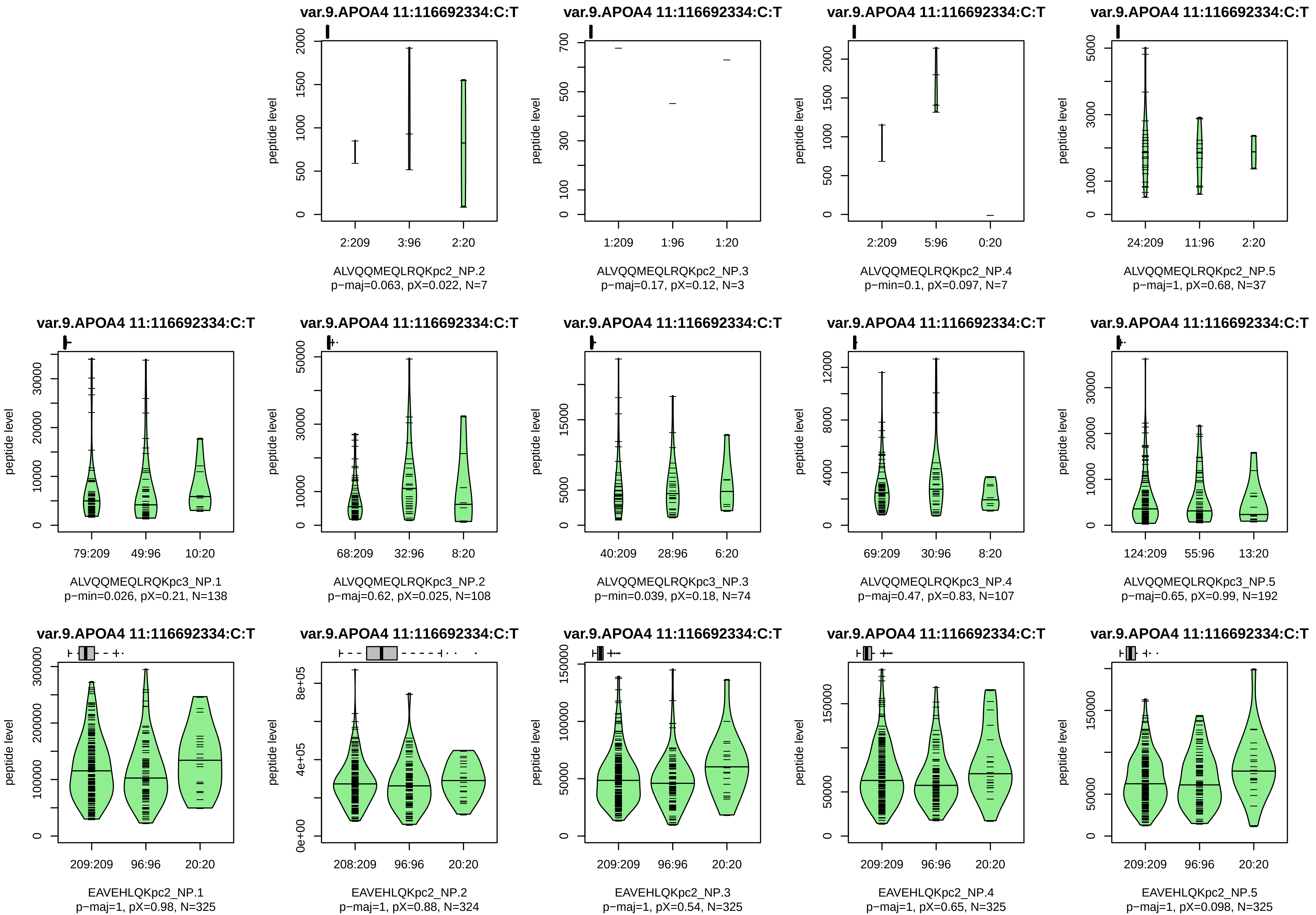
var.9.APOA4 11:116692334:C:T
peptide level
2:209
3:96
2:20
ALVQQMEQLRQKpc2_NP.2
p−maj=0.063, pX=0.022, N=7
var.9.APOA4 11:116692334:C:T
peptide level
1:209
1:96
1:20
ALVQQMEQLRQKpc2_NP.3
p−maj=0.17, pX=0.12, N=3
var.9.APOA4 11:116692334:C:T
peptide level
2:209
5:96
0:20
ALVQQMEQLRQKpc2_NP.4
p−min=0.1, pX=0.097, N=7
var.9.APOA4 11:116692334:C:T
peptide level
24:209
11:96
2:20
ALVQQMEQLRQKpc2_NP.5
p−maj=1, pX=0.68, N=37
var.9.APOA4 11:116692334:C:T
peptide level
79:209
49:96
10:20
ALVQQMEQLRQKpc3_NP.1
p−min=0.026, pX=0.21, N=138
var.9.APOA4 11:116692334:C:T
peptide level
68:209
32:96
8:20
ALVQQMEQLRQKpc3_NP.2
p−maj=0.62, pX=0.025, N=108
var.9.APOA4 11:116692334:C:T
peptide level
40:209
28:96
6:20
ALVQQMEQLRQKpc3_NP.3
p−min=0.039, pX=0.18, N=74
var.9.APOA4 11:116692334:C:T
peptide level
69:209
30:96
8:20
ALVQQMEQLRQKpc3_NP.4
p−maj=0.47, pX=0.83, N=107
var.9.APOA4 11:116692334:C:T
peptide level
124:209
55:96
13:20
ALVQQMEQLRQKpc3_NP.5
p−maj=0.65, pX=0.99, N=192
var.9.APOA4 11:116692334:C:T
peptide level
209:209
96:96
20:20
EAVEHLQKpc2_NP.1
p−maj=1, pX=0.98, N=325
var.9.APOA4 11:116692334:C:T
peptide level
208:209
96:96
20:20
EAVEHLQKpc2_NP.2
p−maj=1, pX=0.88, N=324
var.9.APOA4 11:116692334:C:T
peptide level
209:209
96:96
20:20
EAVEHLQKpc2_NP.3
p−maj=1, pX=0.54, N=325
var.9.APOA4 11:116692334:C:T
peptide level
209:209
96:96
20:20
EAVEHLQKpc2_NP.4
p−maj=1, pX=0.65, N=325
var.9.APOA4 11:116692334:C:T
peptide level
209:209
96:96
20:20
EAVEHLQKpc2_NP.5
p−maj=1, pX=0.098, N=325

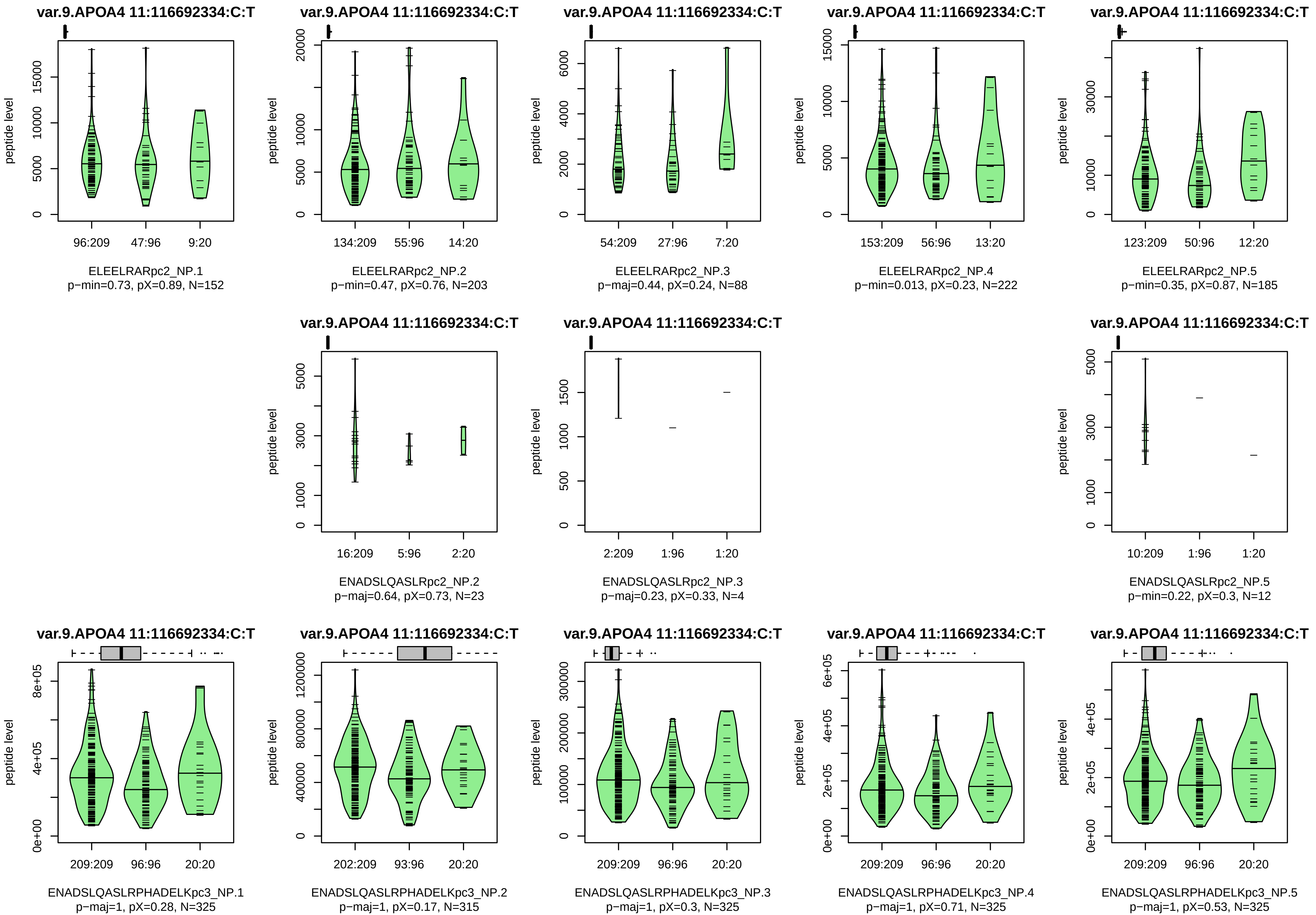

var.9.APOA4 11:116692334:C:T
peptide level
96:209
47:96
9:20
ELEELRARpc2_NP.1
p−min=0.73, pX=0.89, N=152
var.9.APOA4 11:116692334:C:T
peptide level
134:209
55:96
14:20
ELEELRARpc2_NP.2
p−min=0.47, pX=0.76, N=203
var.9.APOA4 11:116692334:C:T
peptide level
54:209
27:96
7:20
ELEELRARpc2_NP.3
p−maj=0.44, pX=0.24, N=88
var.9.APOA4 11:116692334:C:T
peptide level
153:209
56:96
13:20
ELEELRARpc2_NP.4
p−min=0.013, pX=0.23, N=222
var.9.APOA4 11:116692334:C:T
peptide level
123:209
50:96
12:20
ELEELRARpc2_NP.5
p−min=0.35, pX=0.87, N=185
var.9.APOA4 11:116692334:C:T
peptide level
16:209
5:96
2:20
ENADSLQASLRpc2_NP.2
p−maj=0.64, pX=0.73, N=23
var.9.APOA4 11:116692334:C:T
peptide level
2:209
1:96
1:20
ENADSLQASLRpc2_NP.3
p−maj=0.23, pX=0.33, N=4
var.9.APOA4 11:116692334:C:T
peptide level
10:209
1:96
1:20
ENADSLQASLRpc2_NP.5
p−min=0.22, pX=0.3, N=12
var.9.APOA4 11:116692334:C:T
peptide level
209:209
96:96
20:20
ENADSLQASLRPHADELKpc3_NP.1
p−maj=1, pX=0.28, N=325
var.9.APOA4 11:116692334:C:T
peptide level
202:209
93:96
20:20
ENADSLQASLRPHADELKpc3_NP.2
p−maj=1, pX=0.17, N=315
var.9.APOA4 11:116692334:C:T
peptide level
209:209
96:96
20:20
ENADSLQASLRPHADELKpc3_NP.3
p−maj=1, pX=0.3, N=325
var.9.APOA4 11:116692334:C:T
peptide level
209:209
96:96
20:20
ENADSLQASLRPHADELKpc3_NP.4
p−maj=1, pX=0.71, N=325
var.9.APOA4 11:116692334:C:T
peptide level
209:209
96:96
20:20
ENADSLQASLRPHADELKpc3_NP.5
p−maj=1, pX=0.53, N=325

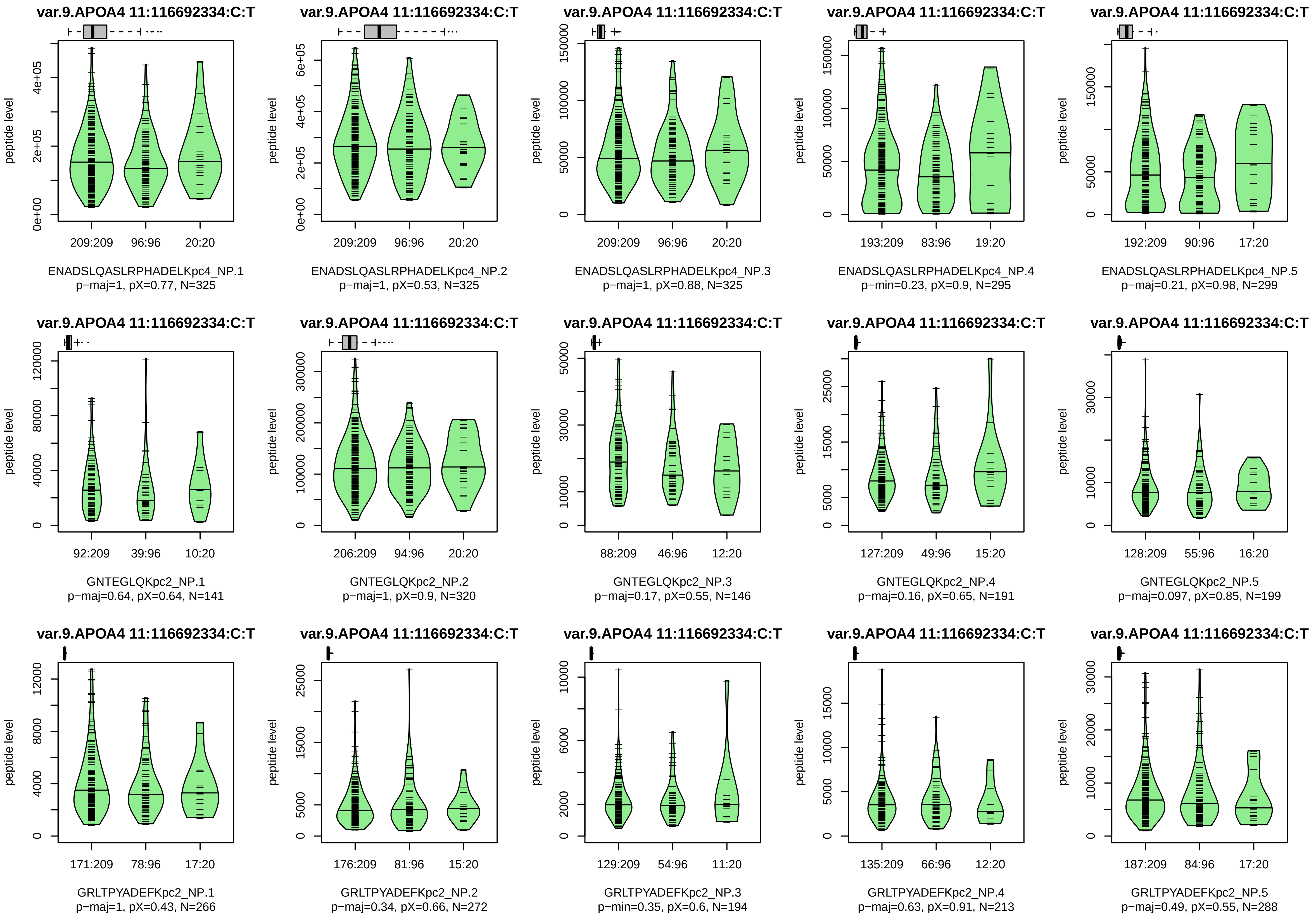
var.9.APOA4 11:116692334:C:T
peptide level
209:209
96:96
20:20
ENADSLQASLRPHADELKpc4_NP.1
p−maj=1, pX=0.77, N=325
var.9.APOA4 11:116692334:C:T
peptide level
209:209
96:96
20:20
ENADSLQASLRPHADELKpc4_NP.2
p−maj=1, pX=0.53, N=325
var.9.APOA4 11:116692334:C:T
peptide level
209:209
96:96
20:20
ENADSLQASLRPHADELKpc4_NP.3
p−maj=1, pX=0.88, N=325
var.9.APOA4 11:116692334:C:T
peptide level
193:209
83:96
19:20
ENADSLQASLRPHADELKpc4_NP.4
p−min=0.23, pX=0.9, N=295
var.9.APOA4 11:116692334:C:T
peptide level
192:209
90:96
17:20
ENADSLQASLRPHADELKpc4_NP.5
p−maj=0.21, pX=0.98, N=299
var.9.APOA4 11:116692334:C:T
peptide level
92:209
39:96
10:20
GNTEGLQKpc2_NP.1
p−maj=0.64, pX=0.64, N=141
var.9.APOA4 11:116692334:C:T
peptide level
206:209
94:96
20:20
GNTEGLQKpc2_NP.2
p−maj=1, pX=0.9, N=320
var.9.APOA4 11:116692334:C:T
peptide level
88:209
46:96
12:20
GNTEGLQKpc2_NP.3
p−maj=0.17, pX=0.55, N=146
var.9.APOA4 11:116692334:C:T
peptide level
127:209
49:96
15:20
GNTEGLQKpc2_NP.4
p−maj=0.16, pX=0.65, N=191
var.9.APOA4 11:116692334:C:T
peptide level
128:209
55:96
16:20
GNTEGLQKpc2_NP.5
p−maj=0.097, pX=0.85, N=199
var.9.APOA4 11:116692334:C:T
peptide level
171:209
78:96
17:20
GRLTPYADEFKpc2_NP.1
p−maj=1, pX=0.43, N=266
var.9.APOA4 11:116692334:C:T
peptide level
176:209
81:96
15:20
GRLTPYADEFKpc2_NP.2
p−maj=0.34, pX=0.66, N=272
var.9.APOA4 11:116692334:C:T
peptide level
129:209
54:96
11:20
GRLTPYADEFKpc2_NP.3
p−min=0.35, pX=0.6, N=194
var.9.APOA4 11:116692334:C:T
peptide level
135:209
66:96
12:20
GRLTPYADEFKpc2_NP.4
p−maj=0.63, pX=0.91, N=213
var.9.APOA4 11:116692334:C:T
peptide level
187:209
84:96
17:20
GRLTPYADEFKpc2_NP.5
p−maj=0.49, pX=0.55, N=288

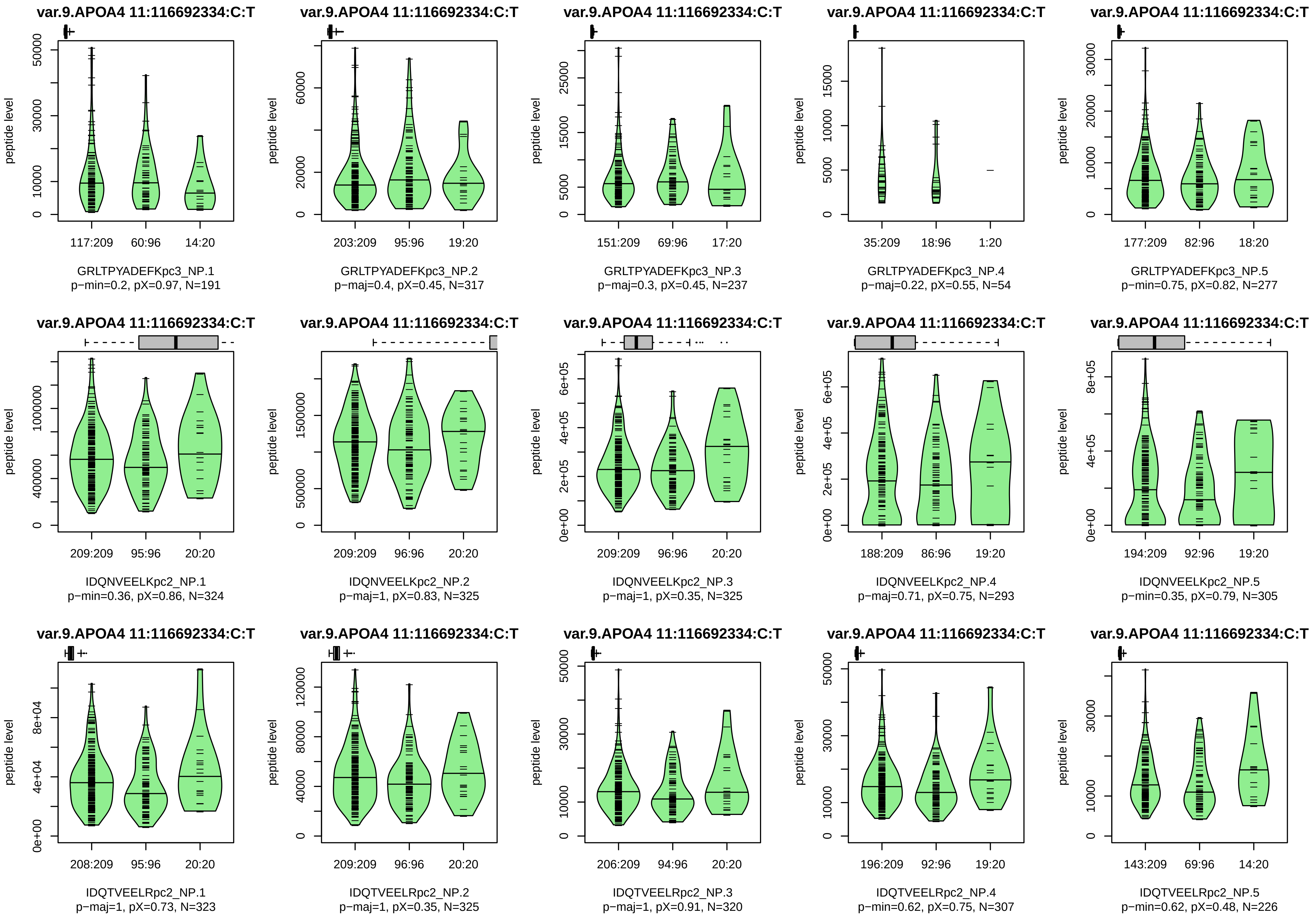
var.9.APOA4 11:116692334:C:T
peptide level
117:209
60:96
14:20
GRLTPYADEFKpc3_NP.1
p−min=0.2, pX=0.97, N=191
var.9.APOA4 11:116692334:C:T
peptide level
203:209
95:96
19:20
GRLTPYADEFKpc3_NP.2
p−maj=0.4, pX=0.45, N=317
var.9.APOA4 11:116692334:C:T
peptide level
151:209
69:96
17:20
GRLTPYADEFKpc3_NP.3
p−maj=0.3, pX=0.45, N=237
var.9.APOA4 11:116692334:C:T
peptide level
35:209
18:96
1:20
GRLTPYADEFKpc3_NP.4
p−maj=0.22, pX=0.55, N=54
var.9.APOA4 11:116692334:C:T
peptide level
177:209
82:96
18:20
GRLTPYADEFKpc3_NP.5
p−min=0.75, pX=0.82, N=277
var.9.APOA4 11:116692334:C:T
peptide level
209:209
95:96
20:20
IDQNVEELKpc2_NP.1
p−min=0.36, pX=0.86, N=324
var.9.APOA4 11:116692334:C:T
peptide level
209:209
96:96
20:20
IDQNVEELKpc2_NP.2
p−maj=1, pX=0.83, N=325
var.9.APOA4 11:116692334:C:T
peptide level
209:209
96:96
20:20
IDQNVEELKpc2_NP.3
p−maj=1, pX=0.35, N=325
var.9.APOA4 11:116692334:C:T
peptide level
188:209
86:96
19:20
IDQNVEELKpc2_NP.4
p−maj=0.71, pX=0.75, N=293
var.9.APOA4 11:116692334:C:T
peptide level
194:209
92:96
19:20
IDQNVEELKpc2_NP.5
p−min=0.35, pX=0.79, N=305
var.9.APOA4 11:116692334:C:T
peptide level
208:209
95:96
20:20
IDQTVEELRpc2_NP.1
p−maj=1, pX=0.73, N=323
var.9.APOA4 11:116692334:C:T
peptide level
209:209
96:96
20:20
IDQTVEELRpc2_NP.2
p−maj=1, pX=0.35, N=325
var.9.APOA4 11:116692334:C:T
peptide level
206:209
94:96
20:20
IDQTVEELRpc2_NP.3
p−maj=1, pX=0.91, N=320
var.9.APOA4 11:116692334:C:T
peptide level
196:209
92:96
19:20
IDQTVEELRpc2_NP.4
p−min=0.62, pX=0.75, N=307
var.9.APOA4 11:116692334:C:T
peptide level
143:209
69:96
14:20
IDQTVEELRpc2_NP.5
p−min=0.62, pX=0.48, N=226

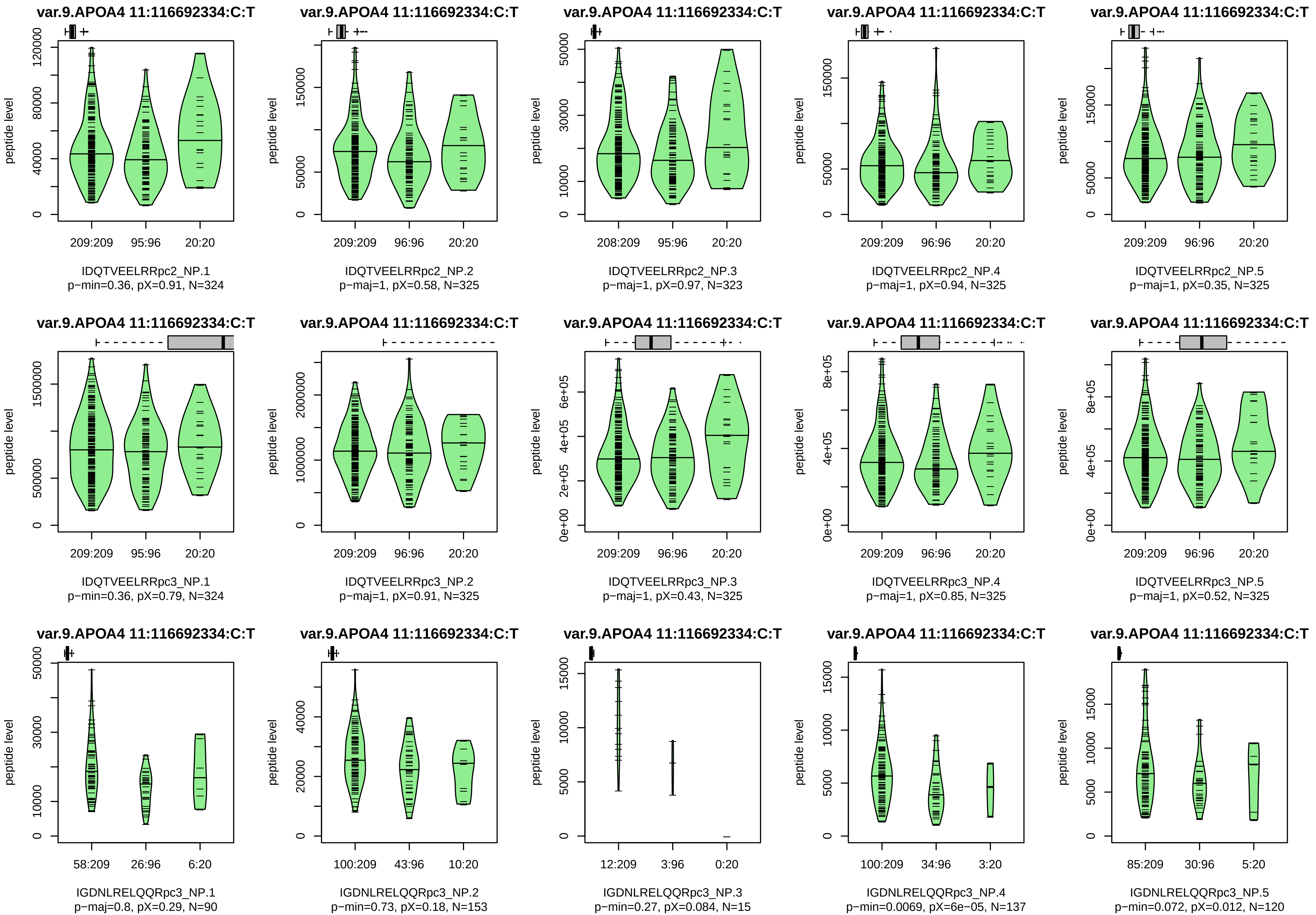

var.9.APOA4 11:116692334:C:T
peptide level
209:209
95:96
20:20
IDQTVEELRRpc2_NP.1
p−min=0.36, pX=0.91, N=324
var.9.APOA4 11:116692334:C:T
peptide level
209:209
96:96
20:20
IDQTVEELRRpc2_NP.2
p−maj=1, pX=0.58, N=325
var.9.APOA4 11:116692334:C:T
peptide level
208:209
95:96
20:20
IDQTVEELRRpc2_NP.3
p−maj=1, pX=0.97, N=323
var.9.APOA4 11:116692334:C:T
peptide level
209:209
96:96
20:20
IDQTVEELRRpc2_NP.4
p−maj=1, pX=0.94, N=325
var.9.APOA4 11:116692334:C:T
peptide level
209:209
96:96
20:20
IDQTVEELRRpc2_NP.5
p−maj=1, pX=0.35, N=325
var.9.APOA4 11:116692334:C:T
peptide level
209:209
95:96
20:20
IDQTVEELRRpc3_NP.1
p−min=0.36, pX=0.79, N=324
var.9.APOA4 11:116692334:C:T
peptide level
209:209
96:96
20:20
IDQTVEELRRpc3_NP.2
p−maj=1, pX=0.91, N=325
var.9.APOA4 11:116692334:C:T
peptide level
209:209
96:96
20:20
IDQTVEELRRpc3_NP.3
p−maj=1, pX=0.43, N=325
var.9.APOA4 11:116692334:C:T
peptide level
209:209
96:96
20:20
IDQTVEELRRpc3_NP.4
p−maj=1, pX=0.85, N=325
var.9.APOA4 11:116692334:C:T
peptide level
209:209
96:96
20:20
IDQTVEELRRpc3_NP.5
p−maj=1, pX=0.52, N=325
var.9.APOA4 11:116692334:C:T
peptide level
58:209
26:96
6:20
IGDNLRELQQRpc3_NP.1
p−maj=0.8, pX=0.29, N=90
var.9.APOA4 11:116692334:C:T
peptide level
100:209
43:96
10:20
IGDNLRELQQRpc3_NP.2
p−min=0.73, pX=0.18, N=153
var.9.APOA4 11:116692334:C:T
peptide level
12:209
3:96
0:20
IGDNLRELQQRpc3_NP.3
p−min=0.27, pX=0.084, N=15
var.9.APOA4 11:116692334:C:T
peptide level
100:209
34:96
3:20
IGDNLRELQQRpc3_NP.4
p−min=0.0069, pX=6e−05, N=137
var.9.APOA4 11:116692334:C:T
peptide level
85:209
30:96
5:20
IGDNLRELQQRpc3_NP.5
p−min=0.072, pX=0.012, N=120

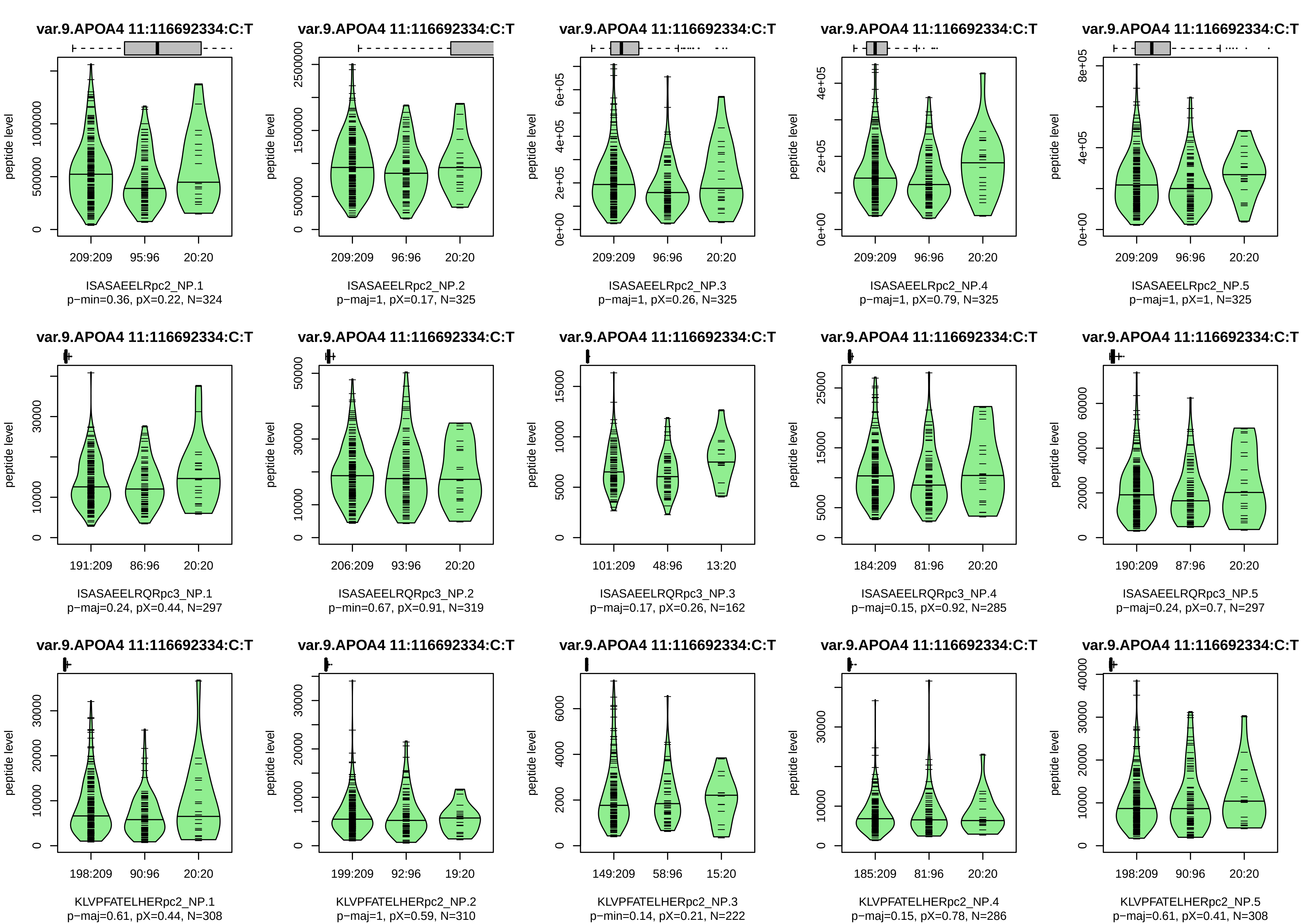
var.9.APOA4 11:116692334:C:T
peptide level
0
500000
1000000
209:209
95:96
20:20
ISASAEELRpc2_NP.1
p−min=0.36, pX=0.22, N=324
var.9.APOA4 11:116692334:C:T
peptide level
0
500000
1500000
2500000
209:209
96:96
20:20
ISASAEELRpc2_NP.2
p−maj=1, pX=0.17, N=325
var.9.APOA4 11:116692334:C:T
peptide level
0e+00
2e+05
4e+05
6e+05
209:209
96:96
20:20
ISASAEELRpc2_NP.3
p−maj=1, pX=0.26, N=325
var.9.APOA4 11:116692334:C:T
peptide level
0e+00
2e+05
4e+05
209:209
96:96
20:20
ISASAEELRpc2_NP.4
p−maj=1, pX=0.79, N=325
var.9.APOA4 11:116692334:C:T
peptide level
0e+00
4e+05
8e+05
209:209
96:96
20:20
ISASAEELRpc2_NP.5
p−maj=1, pX=1, N=325
var.9.APOA4 11:116692334:C:T
peptide level
0
10000
30000
191:209
86:96
20:20
ISASAEELRQRpc3_NP.1
p−maj=0.24, pX=0.44, N=297
var.9.APOA4 11:116692334:C:T
peptide level
0
10000
30000
50000
206:209
93:96
20:20
ISASAEELRQRpc3_NP.2
p−min=0.67, pX=0.91, N=319
var.9.APOA4 11:116692334:C:T
peptide level
0
5000
10000
15000
101:209
48:96
13:20
ISASAEELRQRpc3_NP.3
p−maj=0.17, pX=0.26, N=162
var.9.APOA4 11:116692334:C:T
peptide level
0
5000
15000
25000
184:209
81:96
20:20
ISASAEELRQRpc3_NP.4
p−maj=0.15, pX=0.92, N=285
var.9.APOA4 11:116692334:C:T
peptide level
0
20000
40000
60000
190:209
87:96
20:20
ISASAEELRQRpc3_NP.5
p−maj=0.24, pX=0.7, N=297
var.9.APOA4 11:116692334:C:T
peptide level
0
10000
20000
30000
198:209
90:96
20:20
KLVPFATELHERpc2_NP.1
p−maj=0.61, pX=0.44, N=308
var.9.APOA4 11:116692334:C:T
peptide level
0
10000
20000
30000
199:209
92:96
19:20
KLVPFATELHERpc2_NP.2
p−maj=1, pX=0.59, N=310
var.9.APOA4 11:116692334:C:T
peptide level
0
2000
4000
6000
149:209
58:96
15:20
KLVPFATELHERpc2_NP.3
p−min=0.14, pX=0.21, N=222
var.9.APOA4 11:116692334:C:T
peptide level
0
10000
30000
185:209
81:96
20:20
KLVPFATELHERpc2_NP.4
p−maj=0.15, pX=0.78, N=286
var.9.APOA4 11:116692334:C:T
peptide level
0
10000
20000
30000
40000
198:209
90:96
20:20
KLVPFATELHERpc2_NP.5
p−maj=0.61, pX=0.41, N=308

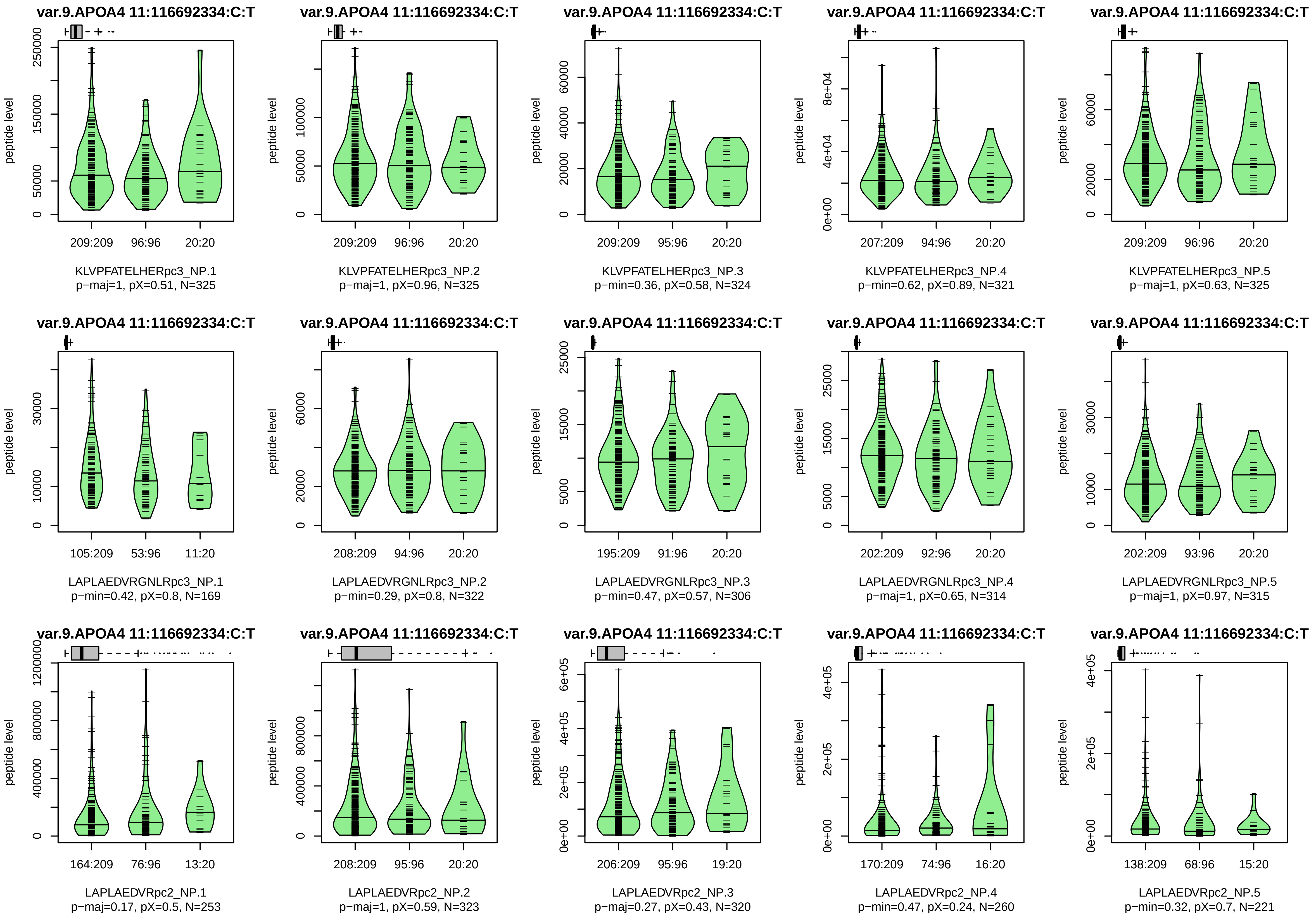
var.9.APOA4 11:116692334:C:T
peptide level
209:209
96:96
20:20
KLVPFATELHERpc3_NP.1
p−maj=1, pX=0.51, N=325
var.9.APOA4 11:116692334:C:T
209:209
96:96
20:20
KLVPFATELHERpc3_NP.2
p−maj=1, pX=0.96, N=325
var.9.APOA4 11:116692334:C:T
209:209
95:96
20:20
KLVPFATELHERpc3_NP.3
p−min=0.36, pX=0.58, N=324
var.9.APOA4 11:116692334:C:T
207:209
94:96
20:20
KLVPFATELHERpc3_NP.4
p−min=0.62, pX=0.89, N=321
var.9.APOA4 11:116692334:C:T
209:209
96:96
20:20
KLVPFATELHERpc3_NP.5
p−maj=1, pX=0.63, N=325
var.9.APOA4 11:116692334:C:T
105:209
53:96
11:20
LAPLAEDVRGNLRpc3_NP.1
p−min=0.42, pX=0.8, N=169
var.9.APOA4 11:116692334:C:T
208:209
94:96
20:20
LAPLAEDVRGNLRpc3_NP.2
p−min=0.29, pX=0.8, N=322
var.9.APOA4 11:116692334:C:T
195:209
91:96
20:20
LAPLAEDVRGNLRpc3_NP.3
p−min=0.47, pX=0.57, N=306
var.9.APOA4 11:116692334:C:T
202:209
92:96
20:20
LAPLAEDVRGNLRpc3_NP.4
p−maj=1, pX=0.65, N=314
var.9.APOA4 11:116692334:C:T
202:209
93:96
20:20
LAPLAEDVRGNLRpc3_NP.5
p−maj=1, pX=0.97, N=315
var.9.APOA4 11:116692334:C:T
164:209
76:96
13:20
LAPLAEDVRpc2_NP.1
p−maj=0.17, pX=0.5, N=253
var.9.APOA4 11:116692334:C:T
208:209
95:96
20:20
LAPLAEDVRpc2_NP.2
p−maj=1, pX=0.59, N=323
var.9.APOA4 11:116692334:C:T
206:209
95:96
19:20
LAPLAEDVRpc2_NP.3
p−maj=0.27, pX=0.43, N=320
var.9.APOA4 11:116692334:C:T
170:209
74:96
16:20
LAPLAEDVRpc2_NP.4
p−min=0.47, pX=0.24, N=260
var.9.APOA4 11:116692334:C:T
138:209
68:96
15:20
LAPLAEDVRpc2_NP.5
p−min=0.32, pX=0.7, N=221

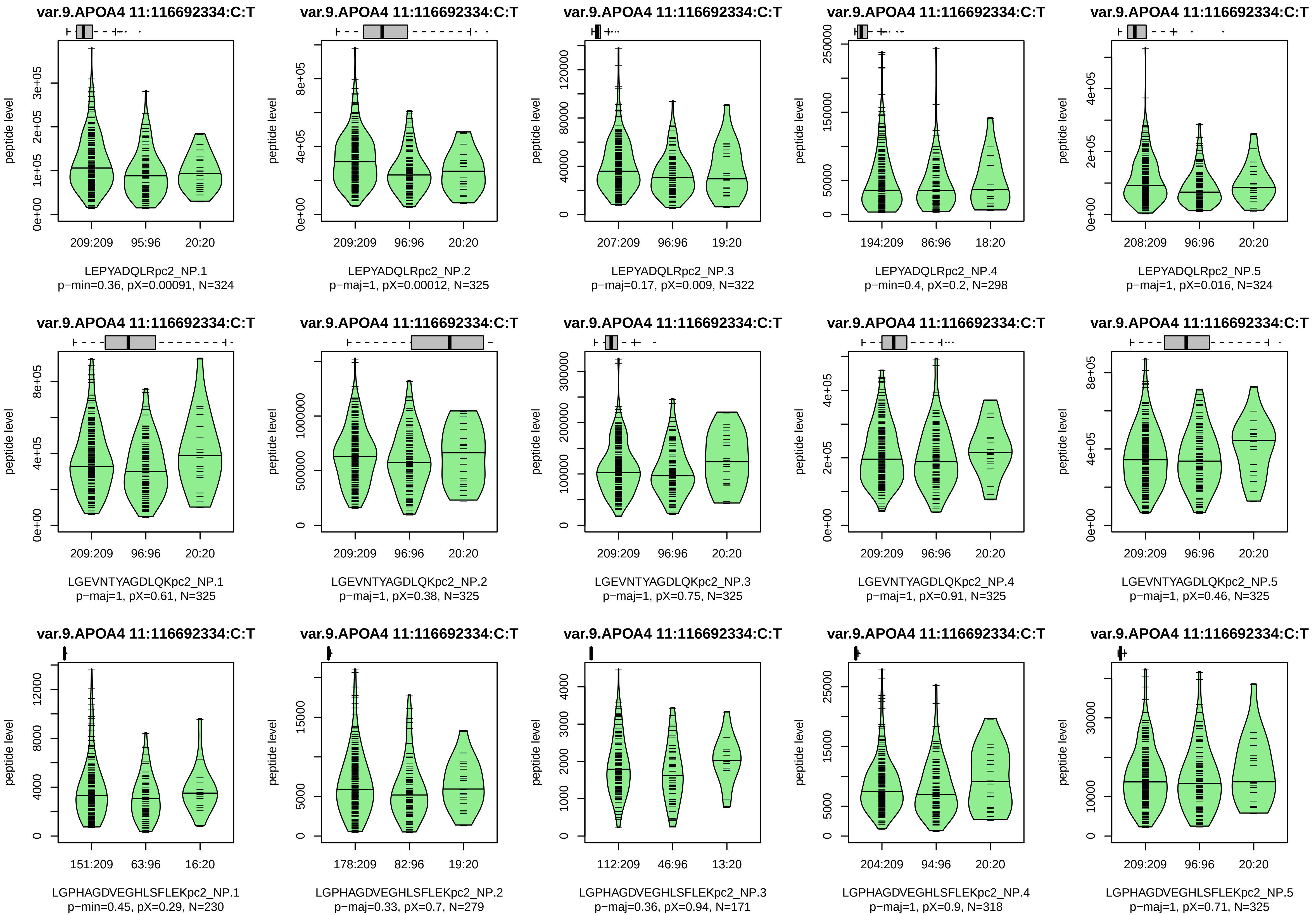
var.9.APOA4 11:116692334:C:T
peptide level
0e+00
1e+05
2e+05
3e+05
209:209
95:96
20:20
LEPYADQLRpc2_NP.1
p−min=0.36, pX=0.00091, N=324
var.9.APOA4 11:116692334:C:T
peptide level
0e+00
4e+05
8e+05
209:209
96:96
20:20
LEPYADQLRpc2_NP.2
p−maj=1, pX=0.00012, N=325
var.9.APOA4 11:116692334:C:T
peptide level
0
40000
80000
120000
207:209
96:96
19:20
LEPYADQLRpc2_NP.3
p−maj=0.17, pX=0.009, N=322
var.9.APOA4 11:116692334:C:T
peptide level
0
50000
150000
250000
194:209
86:96
18:20
LEPYADQLRpc2_NP.4
p−min=0.4, pX=0.2, N=298
var.9.APOA4 11:116692334:C:T
peptide level
0e+00
2e+05
4e+05
208:209
96:96
20:20
LEPYADQLRpc2_NP.5
p−maj=1, pX=0.016, N=324
var.9.APOA4 11:116692334:C:T
peptide level
0e+00
4e+05
8e+05
209:209
96:96
20:20
LGEVNTYAGDLQKpc2_NP.1
p−maj=1, pX=0.61, N=325
var.9.APOA4 11:116692334:C:T
peptide level
0
500000
1000000
209:209
96:96
20:20
LGEVNTYAGDLQKpc2_NP.2
p−maj=1, pX=0.38, N=325
var.9.APOA4 11:116692334:C:T
peptide level
0
100000
200000
300000
209:209
96:96
20:20
LGEVNTYAGDLQKpc2_NP.3
p−maj=1, pX=0.75, N=325
var.9.APOA4 11:116692334:C:T
peptide level
0e+00
2e+05
4e+05
209:209
96:96
20:20
LGEVNTYAGDLQKpc2_NP.4
p−maj=1, pX=0.91, N=325
var.9.APOA4 11:116692334:C:T
peptide level
0e+00
4e+05
8e+05
209:209
96:96
20:20
LGEVNTYAGDLQKpc2_NP.5
p−maj=1, pX=0.46, N=325
var.9.APOA4 11:116692334:C:T
peptide level
0
4000
8000
12000
151:209
63:96
16:20
LGPHAGDVEGHLSFLEKpc2_NP.1
p−min=0.45, pX=0.29, N=230
var.9.APOA4 11:116692334:C:T
peptide level
0
5000
15000
178:209
82:96
19:20
LGPHAGDVEGHLSFLEKpc2_NP.2
p−maj=0.33, pX=0.7, N=279
var.9.APOA4 11:116692334:C:T
peptide level
0
1000
2000
3000
4000
112:209
46:96
13:20
LGPHAGDVEGHLSFLEKpc2_NP.3
p−maj=0.36, pX=0.94, N=171
var.9.APOA4 11:116692334:C:T
peptide level
0
5000
15000
25000
204:209
94:96
20:20
LGPHAGDVEGHLSFLEKpc2_NP.4
p−maj=1, pX=0.9, N=318
var.9.APOA4 11:116692334:C:T
peptide level
0
10000
30000
209:209
96:96
20:20
LGPHAGDVEGHLSFLEKpc2_NP.5
p−maj=1, pX=0.71, N=325

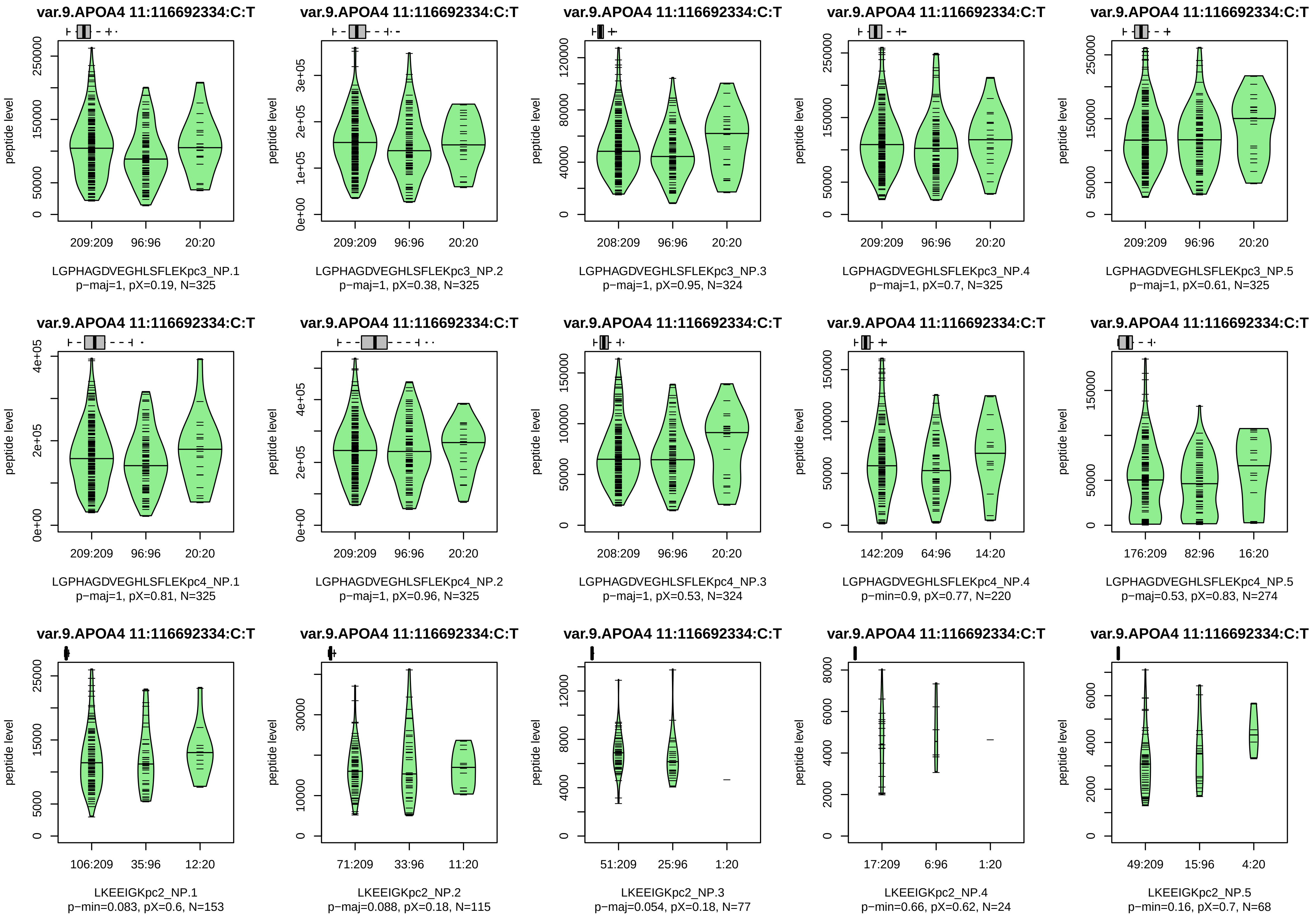
var.9.APOA4 11:116692334:C:T
peptide level
0
50000
150000
250000
209:209
96:96
20:20
LGPHAGDVEGHLSFLEKpc3_NP.1
p−maj=1, pX=0.19, N=325
var.9.APOA4 11:116692334:C:T
peptide level
0e+00
1e+05
2e+05
3e+05
209:209
96:96
20:20
LGPHAGDVEGHLSFLEKpc3_NP.2
p−maj=1, pX=0.38, N=325
var.9.APOA4 11:116692334:C:T
peptide level
0
40000
80000
120000
208:209
96:96
20:20
LGPHAGDVEGHLSFLEKpc3_NP.3
p−maj=1, pX=0.95, N=324
var.9.APOA4 11:116692334:C:T
peptide level
0
50000
150000
250000
209:209
96:96
20:20
LGPHAGDVEGHLSFLEKpc3_NP.4
p−maj=1, pX=0.7, N=325
var.9.APOA4 11:116692334:C:T
peptide level
0
50000
150000
250000
209:209
96:96
20:20
LGPHAGDVEGHLSFLEKpc3_NP.5
p−maj=1, pX=0.61, N=325
var.9.APOA4 11:116692334:C:T
peptide level
0e+00
2e+05
4e+05
209:209
96:96
20:20
LGPHAGDVEGHLSFLEKpc4_NP.1
p−maj=1, pX=0.81, N=325
var.9.APOA4 11:116692334:C:T
peptide level
0e+00
2e+05
4e+05
209:209
96:96
20:20
LGPHAGDVEGHLSFLEKpc4_NP.2
p−maj=1, pX=0.96, N=325
var.9.APOA4 11:116692334:C:T
peptide level
0
50000
100000
150000
208:209
96:96
20:20
LGPHAGDVEGHLSFLEKpc4_NP.3
p−maj=1, pX=0.53, N=324
var.9.APOA4 11:116692334:C:T
peptide level
0
50000
100000
150000
142:209
64:96
14:20
LGPHAGDVEGHLSFLEKpc4_NP.4
p−min=0.9, pX=0.77, N=220
var.9.APOA4 11:116692334:C:T
peptide level
50000
150000
176:209
82:96
16:20
LGPHAGDVEGHLSFLEKpc4_NP.5
p−maj=0.53, pX=0.83, N=274
var.9.APOA4 11:116692334:C:T
peptide level
0
5000
15000
25000
106:209
35:96
12:20
LKEEIGKpc2_NP.1
p−min=0.083, pX=0.6, N=153
var.9.APOA4 11:116692334:C:T
peptide level
0
10000
30000
71:209
33:96
11:20
LKEEIGKpc2_NP.2
p−maj=0.088, pX=0.18, N=115
var.9.APOA4 11:116692334:C:T
peptide level
0
4000
8000
12000
51:209
25:96
1:20
LKEEIGKpc2_NP.3
p−maj=0.054, pX=0.18, N=77
var.9.APOA4 11:116692334:C:T
peptide level
0
2000
4000
6000
8000
17:209
6:96
1:20
LKEEIGKpc2_NP.4
p−min=0.66, pX=0.62, N=24
var.9.APOA4 11:116692334:C:T
peptide level
0
2000
4000
6000
49:209
15:96
4:20
LKEEIGKpc2_NP.5
p−min=0.16, pX=0.7, N=68

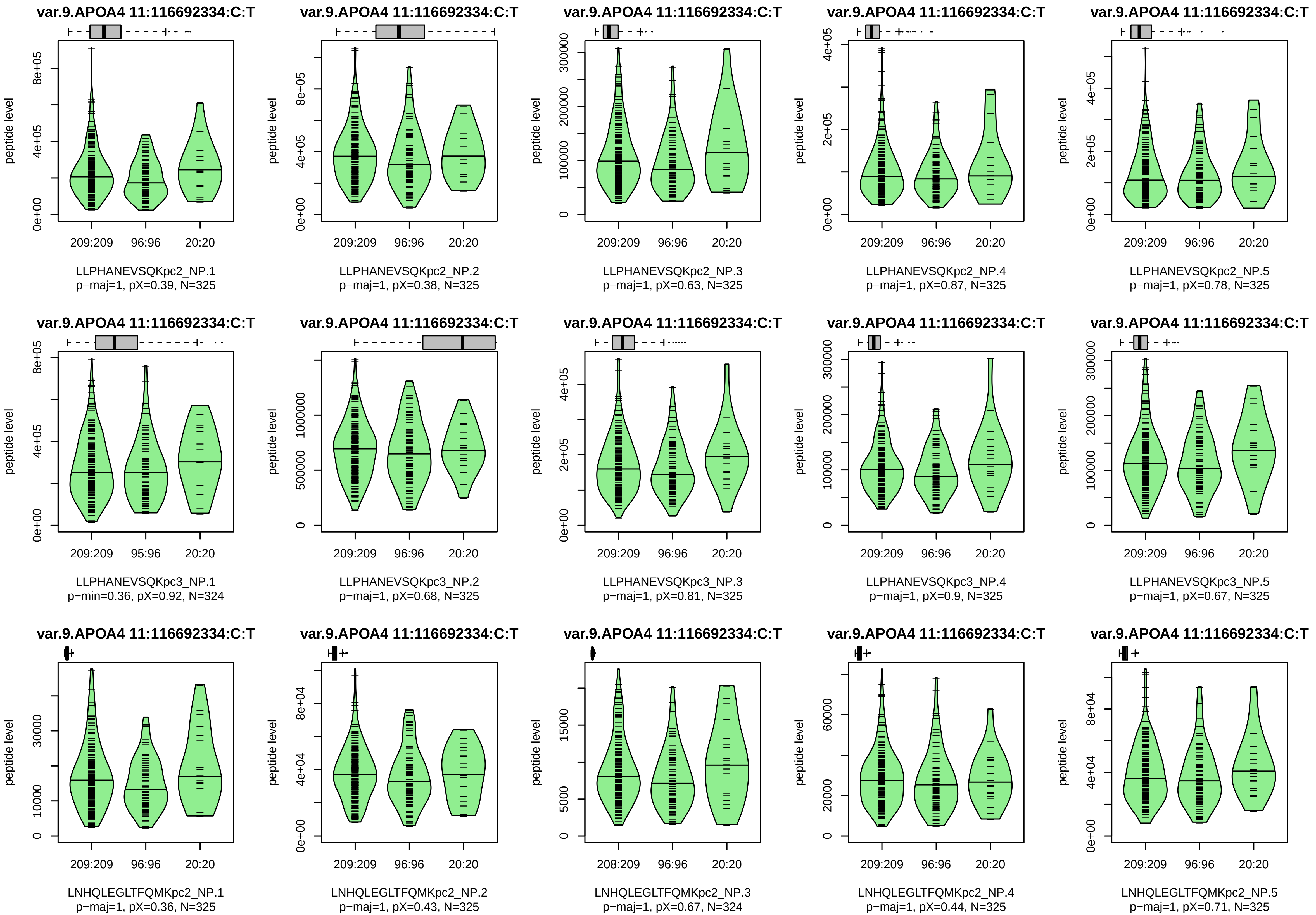
var.9.APOA4 11:116692334:C:T
peptide level
209:209
96:96
20:20
LLPHANEVSQKpc2_NP.1
p−maj=1, pX=0.39, N=325
var.9.APOA4 11:116692334:C:T
209:209
96:96
20:20
LLPHANEVSQKpc2_NP.2
p−maj=1, pX=0.38, N=325
var.9.APOA4 11:116692334:C:T
209:209
96:96
20:20
LLPHANEVSQKpc2_NP.3
p−maj=1, pX=0.63, N=325
var.9.APOA4 11:116692334:C:T
209:209
96:96
20:20
LLPHANEVSQKpc2_NP.4
p−maj=1, pX=0.87, N=325
var.9.APOA4 11:116692334:C:T
209:209
96:96
20:20
LLPHANEVSQKpc2_NP.5
p−maj=1, pX=0.78, N=325
var.9.APOA4 11:116692334:C:T
209:209
95:96
20:20
LLPHANEVSQKpc3_NP.1
p−min=0.36, pX=0.92, N=324
var.9.APOA4 11:116692334:C:T
209:209
96:96
20:20
LLPHANEVSQKpc3_NP.2
p−maj=1, pX=0.68, N=325
var.9.APOA4 11:116692334:C:T
209:209
96:96
20:20
LLPHANEVSQKpc3_NP.3
p−maj=1, pX=0.81, N=325
var.9.APOA4 11:116692334:C:T
209:209
96:96
20:20
LLPHANEVSQKpc3_NP.4
p−maj=1, pX=0.9, N=325
var.9.APOA4 11:116692334:C:T
209:209
96:96
20:20
LLPHANEVSQKpc3_NP.5
p−maj=1, pX=0.67, N=325
var.9.APOA4 11:116692334:C:T
209:209
96:96
20:20
LNHQLEGLTFQMKpc2_NP.1
p−maj=1, pX=0.36, N=325
var.9.APOA4 11:116692334:C:T
209:209
96:96
20:20
LNHQLEGLTFQMKpc2_NP.2
p−maj=1, pX=0.43, N=325
var.9.APOA4 11:116692334:C:T
208:209
96:96
20:20
LNHQLEGLTFQMKpc2_NP.3
p−maj=1, pX=0.67, N=324
var.9.APOA4 11:116692334:C:T
209:209
96:96
20:20
LNHQLEGLTFQMKpc2_NP.4
p−maj=1, pX=0.44, N=325
var.9.APOA4 11:116692334:C:T
209:209
96:96
20:20
LNHQLEGLTFQMKpc2_NP.5
p−maj=1, pX=0.71, N=325

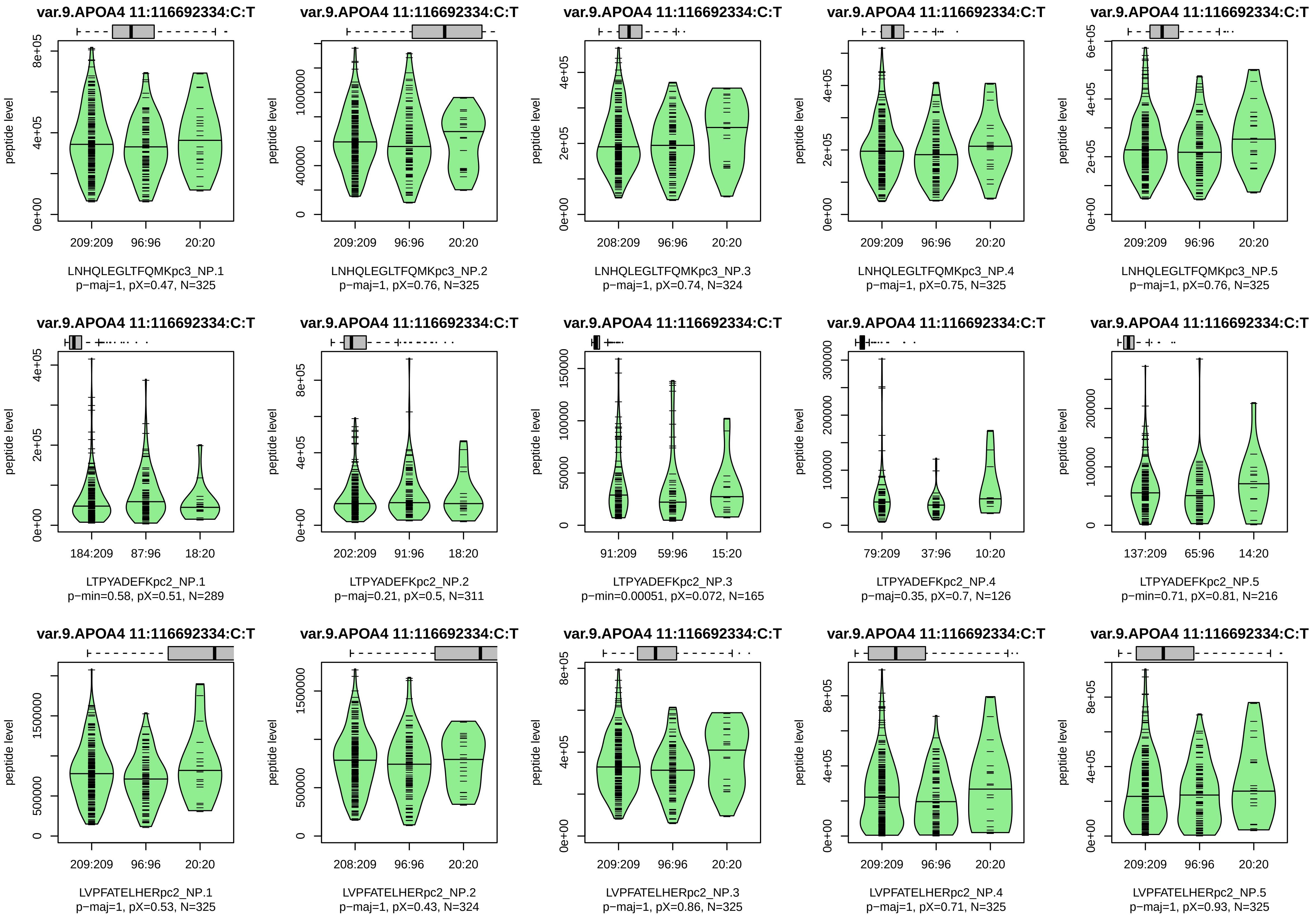

var.9.APOA4 11:116692334:C:T
peptide level
0e+00
4e+05
8e+05
209:209
96:96
20:20
LNHQLEGLTFQMKpc3_NP.1
p−maj=1, pX=0.47, N=325
var.9.APOA4 11:116692334:C:T
peptide level
0
400000
1000000
209:209
96:96
20:20
LNHQLEGLTFQMKpc3_NP.2
p−maj=1, pX=0.76, N=325
var.9.APOA4 11:116692334:C:T
peptide level
0e+00
2e+05
4e+05
208:209
96:96
20:20
LNHQLEGLTFQMKpc3_NP.3
p−maj=1, pX=0.74, N=324
var.9.APOA4 11:116692334:C:T
peptide level
0e+00
2e+05
4e+05
209:209
96:96
20:20
LNHQLEGLTFQMKpc3_NP.4
p−maj=1, pX=0.75, N=325
var.9.APOA4 11:116692334:C:T
peptide level
0e+00
2e+05
4e+05
6e+05
209:209
96:96
20:20
LNHQLEGLTFQMKpc3_NP.5
p−maj=1, pX=0.76, N=325
var.9.APOA4 11:116692334:C:T
peptide level
0e+00
2e+05
4e+05
184:209
87:96
18:20
LTPYADEFKpc2_NP.1
p−min=0.58, pX=0.51, N=289
var.9.APOA4 11:116692334:C:T
peptide level
0e+00
4e+05
8e+05
202:209
91:96
18:20
LTPYADEFKpc2_NP.2
p−maj=0.21, pX=0.5, N=311
var.9.APOA4 11:116692334:C:T
peptide level
0
50000
100000
150000
91:209
59:96
15:20
LTPYADEFKpc2_NP.3
p−min=0.00051, pX=0.072, N=165
var.9.APOA4 11:116692334:C:T
peptide level
0
100000
200000
300000
79:209
37:96
10:20
LTPYADEFKpc2_NP.4
p−maj=0.35, pX=0.7, N=126
var.9.APOA4 11:116692334:C:T
peptide level
0
100000
200000
137:209
65:96
14:20
LTPYADEFKpc2_NP.5
p−min=0.71, pX=0.81, N=216
var.9.APOA4 11:116692334:C:T
peptide level
0
500000
1500000
209:209
96:96
20:20
LVPFATELHERpc2_NP.1
p−maj=1, pX=0.53, N=325
var.9.APOA4 11:116692334:C:T
peptide level
0
500000
1500000
208:209
96:96
20:20
LVPFATELHERpc2_NP.2
p−maj=1, pX=0.43, N=324
var.9.APOA4 11:116692334:C:T
peptide level
0e+00
4e+05
8e+05
209:209
96:96
20:20
LVPFATELHERpc2_NP.3
p−maj=1, pX=0.86, N=325
var.9.APOA4 11:116692334:C:T
peptide level
0e+00
4e+05
8e+05
209:209
96:96
20:20
LVPFATELHERpc2_NP.4
p−maj=1, pX=0.71, N=325
var.9.APOA4 11:116692334:C:T
peptide level
0e+00
4e+05
8e+05
209:209
96:96
20:20
LVPFATELHERpc2_NP.5
p−maj=1, pX=0.93, N=325

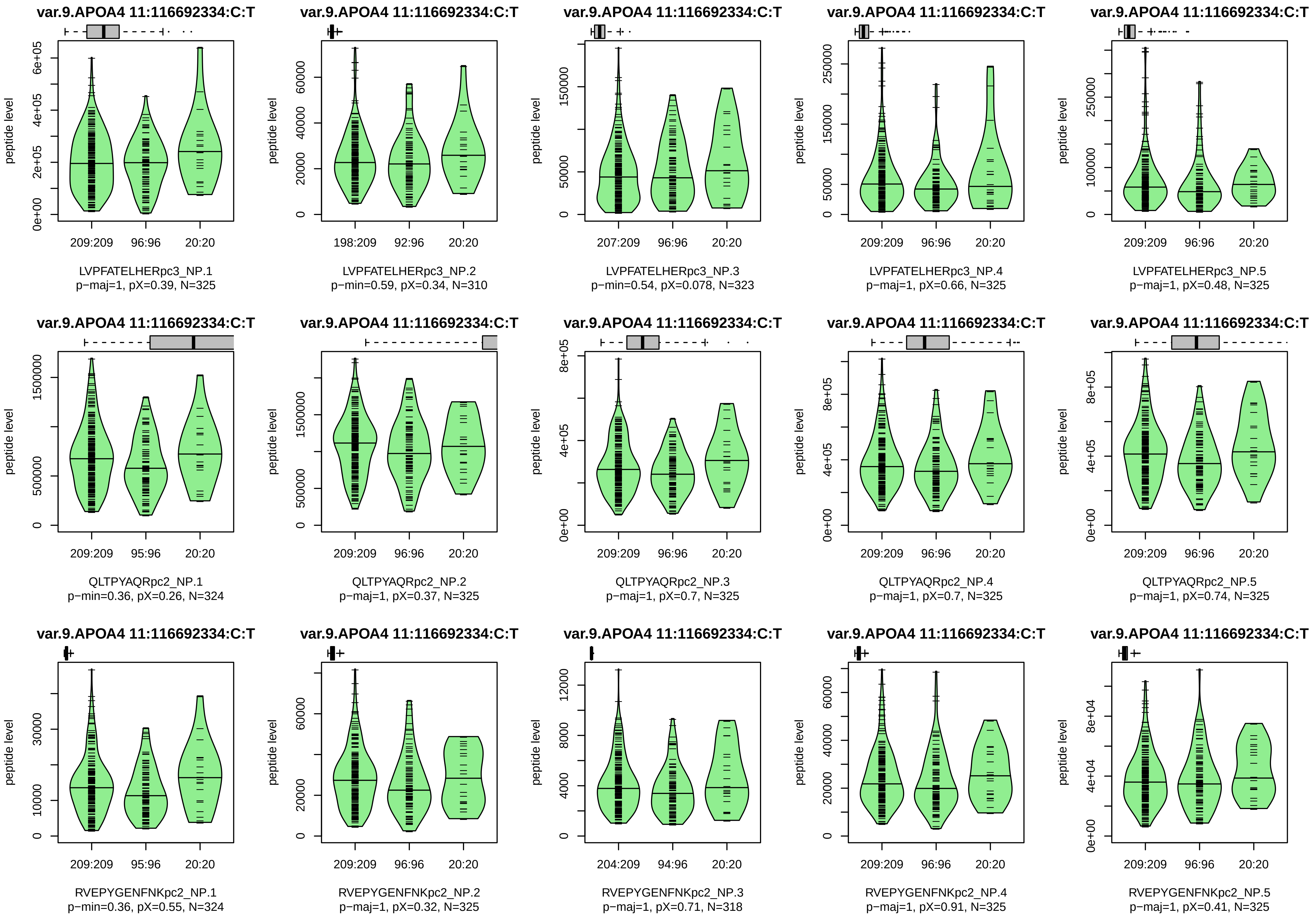
var.9.APOA4 11:116692334:C:T
peptide level
209:209
96:96
20:20
LVPFATELHERpc3_NP.1
p−maj=1, pX=0.39, N=325
var.9.APOA4 11:116692334:C:T
peptide level
198:209
92:96
20:20
LVPFATELHERpc3_NP.2
p−min=0.59, pX=0.34, N=310
var.9.APOA4 11:116692334:C:T
peptide level
207:209
96:96
20:20
LVPFATELHERpc3_NP.3
p−min=0.54, pX=0.078, N=323
var.9.APOA4 11:116692334:C:T
peptide level
209:209
96:96
20:20
LVPFATELHERpc3_NP.4
p−maj=1, pX=0.66, N=325
var.9.APOA4 11:116692334:C:T
peptide level
209:209
96:96
20:20
LVPFATELHERpc3_NP.5
p−maj=1, pX=0.48, N=325
var.9.APOA4 11:116692334:C:T
peptide level
209:209
95:96
20:20
QLTPYAQRpc2_NP.1
p−min=0.36, pX=0.26, N=324
var.9.APOA4 11:116692334:C:T
peptide level
209:209
96:96
20:20
QLTPYAQRpc2_NP.2
p−maj=1, pX=0.37, N=325
var.9.APOA4 11:116692334:C:T
peptide level
209:209
96:96
20:20
QLTPYAQRpc2_NP.3
p−maj=1, pX=0.7, N=325
var.9.APOA4 11:116692334:C:T
peptide level
209:209
96:96
20:20
QLTPYAQRpc2_NP.4
p−maj=1, pX=0.7, N=325
var.9.APOA4 11:116692334:C:T
peptide level
209:209
96:96
20:20
QLTPYAQRpc2_NP.5
p−maj=1, pX=0.74, N=325
var.9.APOA4 11:116692334:C:T
peptide level
209:209
95:96
20:20
RVEPYGENFNKpc2_NP.1
p−min=0.36, pX=0.55, N=324
var.9.APOA4 11:116692334:C:T
peptide level
209:209
96:96
20:20
RVEPYGENFNKpc2_NP.2
p−maj=1, pX=0.32, N=325
var.9.APOA4 11:116692334:C:T
peptide level
204:209
94:96
20:20
RVEPYGENFNKpc2_NP.3
p−maj=1, pX=0.71, N=318
var.9.APOA4 11:116692334:C:T
peptide level
209:209
96:96
20:20
RVEPYGENFNKpc2_NP.4
p−maj=1, pX=0.91, N=325
var.9.APOA4 11:116692334:C:T
peptide level
209:209
96:96
20:20
RVEPYGENFNKpc2_NP.5
p−maj=1, pX=0.41, N=325

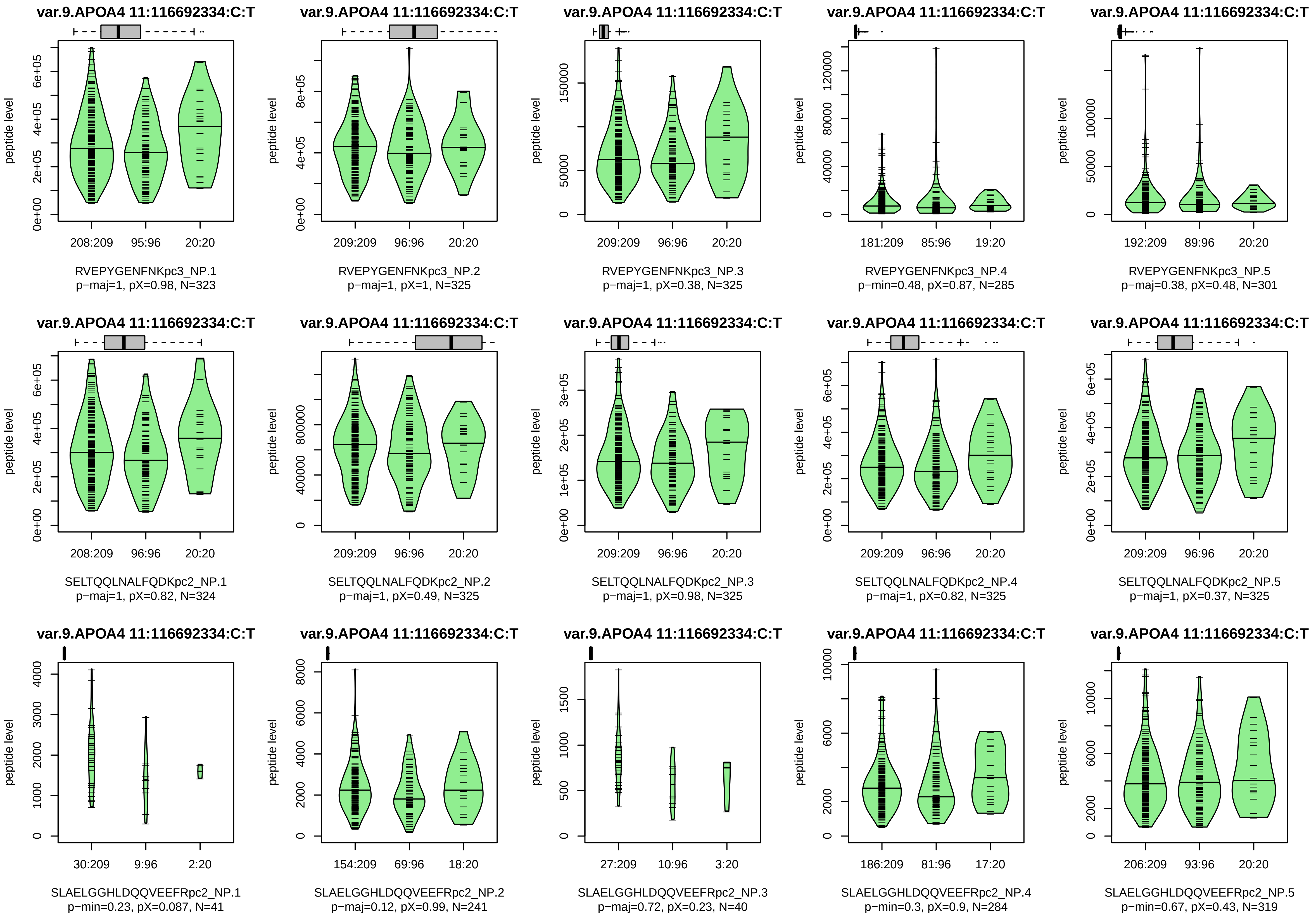
var.9.APOA4 11:116692334:C:T
peptide level
208:209
95:96
20:20
RVEPYGENFNKpc3_NP.1
p−maj=1, pX=0.98, N=323
var.9.APOA4 11:116692334:C:T
peptide level
209:209
96:96
20:20
RVEPYGENFNKpc3_NP.2
p−maj=1, pX=1, N=325
var.9.APOA4 11:116692334:C:T
peptide level
209:209
96:96
20:20
RVEPYGENFNKpc3_NP.3
p−maj=1, pX=0.38, N=325
var.9.APOA4 11:116692334:C:T
peptide level
181:209
85:96
19:20
RVEPYGENFNKpc3_NP.4
p−min=0.48, pX=0.87, N=285
var.9.APOA4 11:116692334:C:T
peptide level
192:209
89:96
20:20
RVEPYGENFNKpc3_NP.5
p−maj=0.38, pX=0.48, N=301
var.9.APOA4 11:116692334:C:T
peptide level
208:209
96:96
20:20
SELTQQLNALFQDKpc2_NP.1
p−maj=1, pX=0.82, N=324
var.9.APOA4 11:116692334:C:T
peptide level
209:209
96:96
20:20
SELTQQLNALFQDKpc2_NP.2
p−maj=1, pX=0.49, N=325
var.9.APOA4 11:116692334:C:T
peptide level
209:209
96:96
20:20
SELTQQLNALFQDKpc2_NP.3
p−maj=1, pX=0.98, N=325
var.9.APOA4 11:116692334:C:T
peptide level
209:209
96:96
20:20
SELTQQLNALFQDKpc2_NP.4
p−maj=1, pX=0.82, N=325
var.9.APOA4 11:116692334:C:T
peptide level
209:209
96:96
20:20
SELTQQLNALFQDKpc2_NP.5
p−maj=1, pX=0.37, N=325
var.9.APOA4 11:116692334:C:T
peptide level
30:209
9:96
2:20
SLAELGGHLDQQVEEFRpc2_NP.1
p−min=0.23, pX=0.087, N=41
var.9.APOA4 11:116692334:C:T
peptide level
154:209
69:96
18:20
SLAELGGHLDQQVEEFRpc2_NP.2
p−maj=0.12, pX=0.99, N=241
var.9.APOA4 11:116692334:C:T
peptide level
27:209
10:96
3:20
SLAELGGHLDQQVEEFRpc2_NP.3
p−maj=0.72, pX=0.23, N=40
var.9.APOA4 11:116692334:C:T
peptide level
186:209
81:96
17:20
SLAELGGHLDQQVEEFRpc2_NP.4
p−min=0.3, pX=0.9, N=284
var.9.APOA4 11:116692334:C:T
peptide level
206:209
93:96
20:20
SLAELGGHLDQQVEEFRpc2_NP.5
p−min=0.67, pX=0.43, N=319

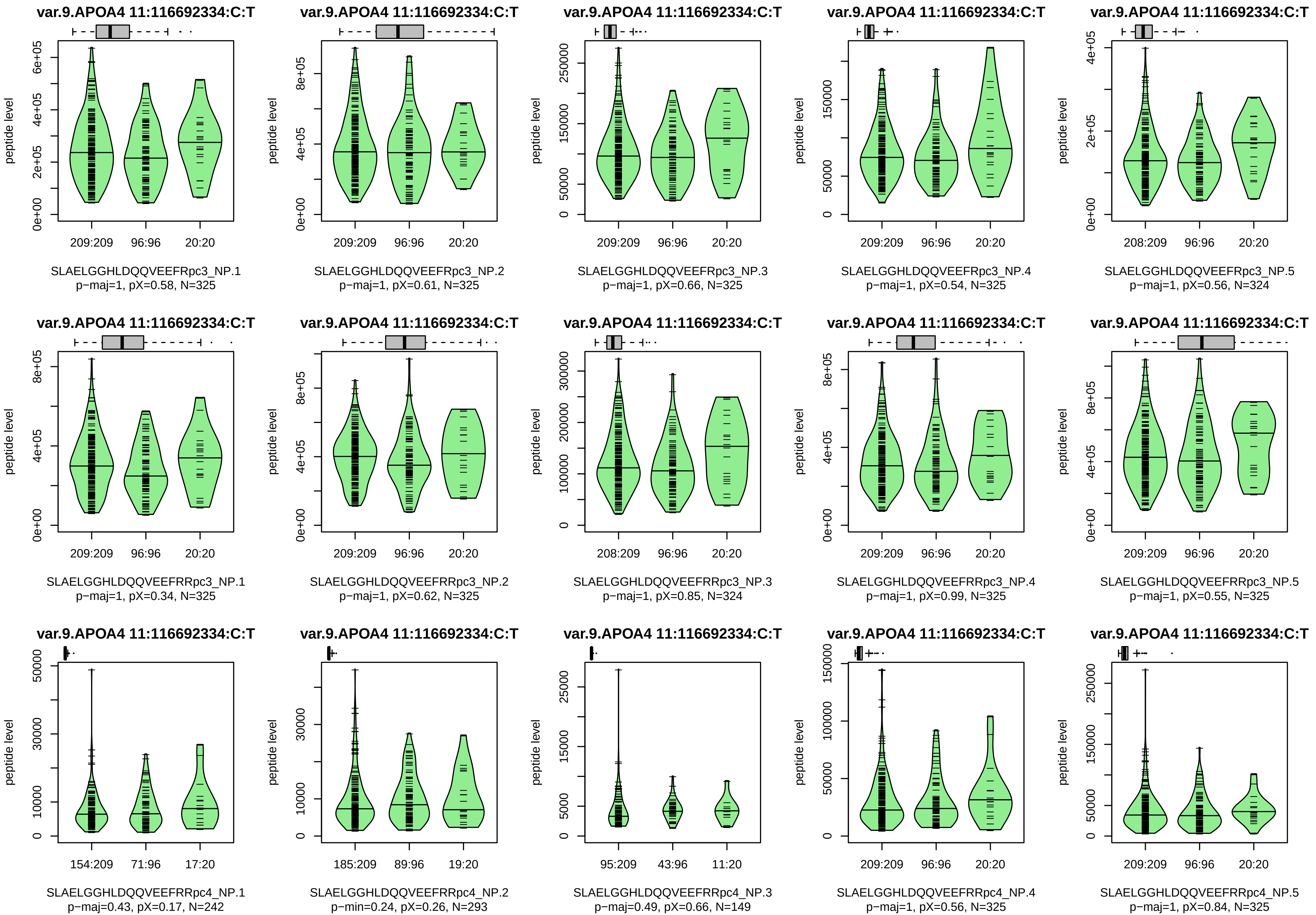
var.9.APOA4 11:116692334:C:T
peptide level
209:209
96:96
20:20
SLAELGGHLDQQVEEFRpc3_NP.1
p−maj=1, pX=0.58, N=325
var.9.APOA4 11:116692334:C:T
peptide level
209:209
96:96
20:20
SLAELGGHLDQQVEEFRpc3_NP.2
p−maj=1, pX=0.61, N=325
var.9.APOA4 11:116692334:C:T
peptide level
209:209
96:96
20:20
SLAELGGHLDQQVEEFRpc3_NP.3
p−maj=1, pX=0.66, N=325
var.9.APOA4 11:116692334:C:T
peptide level
209:209
96:96
20:20
SLAELGGHLDQQVEEFRpc3_NP.4
p−maj=1, pX=0.54, N=325
var.9.APOA4 11:116692334:C:T
peptide level
208:209
96:96
20:20
SLAELGGHLDQQVEEFRpc3_NP.5
p−maj=1, pX=0.56, N=324
var.9.APOA4 11:116692334:C:T
peptide level
209:209
96:96
20:20
SLAELGGHLDQQVEEFRRpc3_NP.1
p−maj=1, pX=0.34, N=325
var.9.APOA4 11:116692334:C:T
peptide level
209:209
96:96
20:20
SLAELGGHLDQQVEEFRRpc3_NP.2
p−maj=1, pX=0.62, N=325
var.9.APOA4 11:116692334:C:T
peptide level
208:209
96:96
20:20
SLAELGGHLDQQVEEFRRpc3_NP.3
p−maj=1, pX=0.85, N=324
var.9.APOA4 11:116692334:C:T
peptide level
209:209
96:96
20:20
SLAELGGHLDQQVEEFRRpc3_NP.4
p−maj=1, pX=0.99, N=325
var.9.APOA4 11:116692334:C:T
peptide level
209:209
96:96
20:20
SLAELGGHLDQQVEEFRRpc3_NP.5
p−maj=1, pX=0.55, N=325
var.9.APOA4 11:116692334:C:T
peptide level
154:209
71:96
17:20
SLAELGGHLDQQVEEFRRpc4_NP.1
p−maj=0.43, pX=0.17, N=242
var.9.APOA4 11:116692334:C:T
peptide level
185:209
89:96
19:20
SLAELGGHLDQQVEEFRRpc4_NP.2
p−min=0.24, pX=0.26, N=293
var.9.APOA4 11:116692334:C:T
peptide level
95:209
43:96
11:20
SLAELGGHLDQQVEEFRRpc4_NP.3
p−maj=0.49, pX=0.66, N=149
var.9.APOA4 11:116692334:C:T
peptide level
209:209
96:96
20:20
SLAELGGHLDQQVEEFRRpc4_NP.4
p−maj=1, pX=0.56, N=325
var.9.APOA4 11:116692334:C:T
peptide level
209:209
96:96
20:20
SLAELGGHLDQQVEEFRRpc4_NP.5
p−maj=1, pX=0.84, N=325

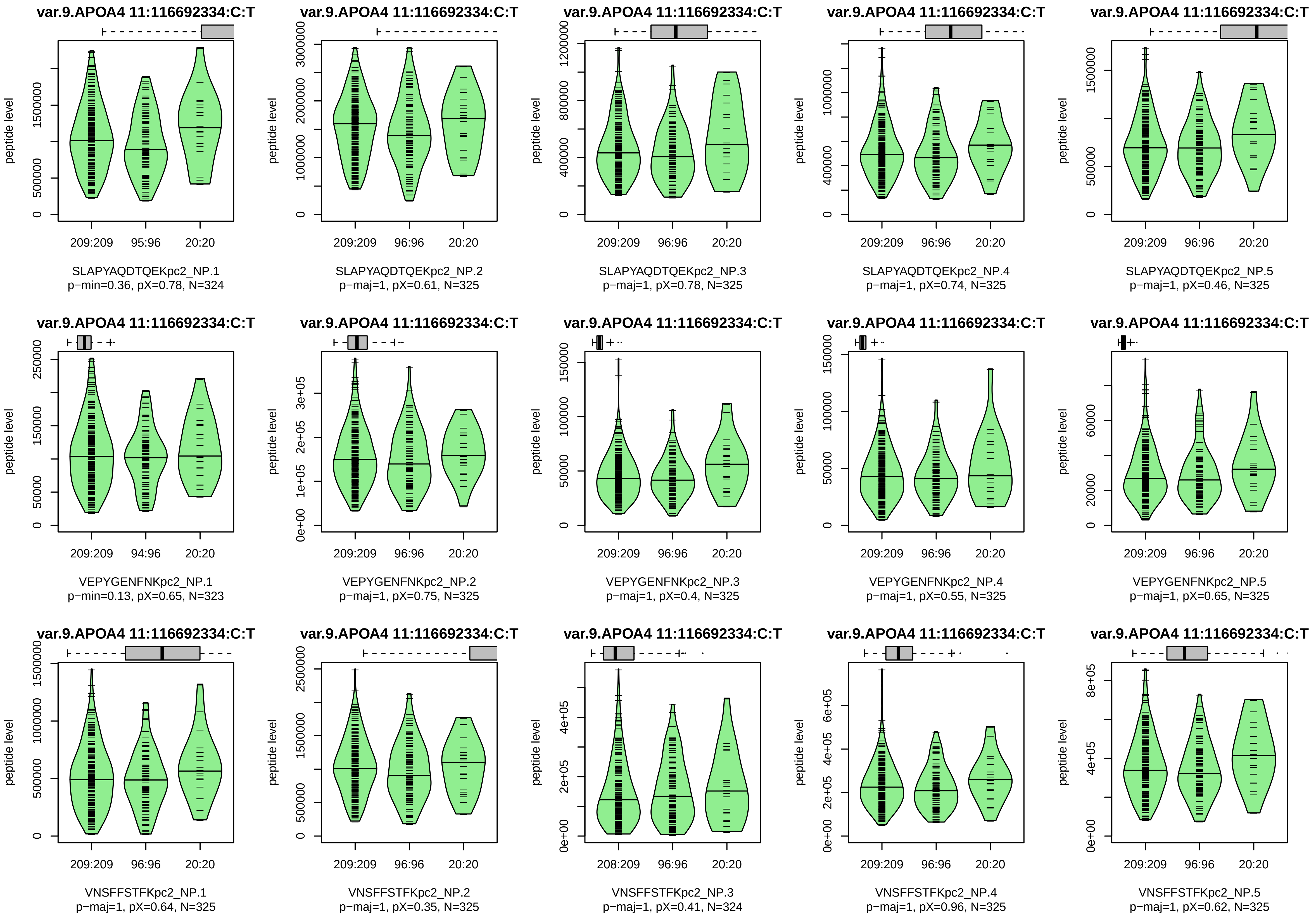
var.9.APOA4 11:116692334:C:T
peptide level
209:209
95:96
20:20
SLAPYAQDTQEKpc2_NP.1
p−min=0.36, pX=0.78, N=324
var.9.APOA4 11:116692334:C:T
peptide level
209:209
96:96
20:20
SLAPYAQDTQEKpc2_NP.2
p−maj=1, pX=0.61, N=325
var.9.APOA4 11:116692334:C:T
peptide level
209:209
96:96
20:20
SLAPYAQDTQEKpc2_NP.3
p−maj=1, pX=0.78, N=325
var.9.APOA4 11:116692334:C:T
peptide level
209:209
96:96
20:20
SLAPYAQDTQEKpc2_NP.4
p−maj=1, pX=0.74, N=325
var.9.APOA4 11:116692334:C:T
peptide level
209:209
96:96
20:20
SLAPYAQDTQEKpc2_NP.5
p−maj=1, pX=0.46, N=325
var.9.APOA4 11:116692334:C:T
peptide level
209:209
94:96
20:20
VEPYGENFNKpc2_NP.1
p−min=0.13, pX=0.65, N=323
var.9.APOA4 11:116692334:C:T
peptide level
209:209
96:96
20:20
VEPYGENFNKpc2_NP.2
p−maj=1, pX=0.75, N=325
var.9.APOA4 11:116692334:C:T
peptide level
209:209
96:96
20:20
VEPYGENFNKpc2_NP.3
p−maj=1, pX=0.4, N=325
var.9.APOA4 11:116692334:C:T
peptide level
209:209
96:96
20:20
VEPYGENFNKpc2_NP.4
p−maj=1, pX=0.55, N=325
var.9.APOA4 11:116692334:C:T
peptide level
209:209
96:96
20:20
VEPYGENFNKpc2_NP.5
p−maj=1, pX=0.65, N=325
var.9.APOA4 11:116692334:C:T
peptide level
209:209
96:96
20:20
VNSFFSTFKpc2_NP.1
p−maj=1, pX=0.64, N=325
var.9.APOA4 11:116692334:C:T
peptide level
209:209
96:96
20:20
VNSFFSTFKpc2_NP.2
p−maj=1, pX=0.35, N=325
var.9.APOA4 11:116692334:C:T
peptide level
208:209
96:96
20:20
VNSFFSTFKpc2_NP.3
p−maj=1, pX=0.41, N=324
var.9.APOA4 11:116692334:C:T
peptide level
209:209
96:96
20:20
VNSFFSTFKpc2_NP.4
p−maj=1, pX=0.96, N=325
var.9.APOA4 11:116692334:C:T
peptide level
209:209
96:96
20:20
VNSFFSTFKpc2_NP.5
p−maj=1, pX=0.62, N=325

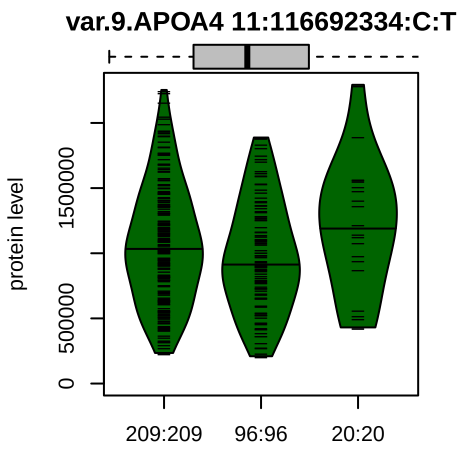

P06727_NP.1
p−maj=1, pY=0.72, N=325

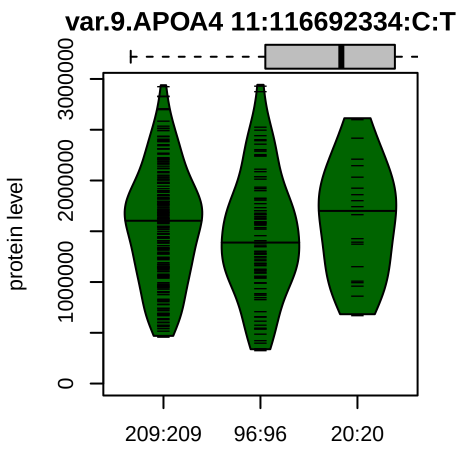

P06727_NP.2
p−maj=1, pY=0.91, N=325

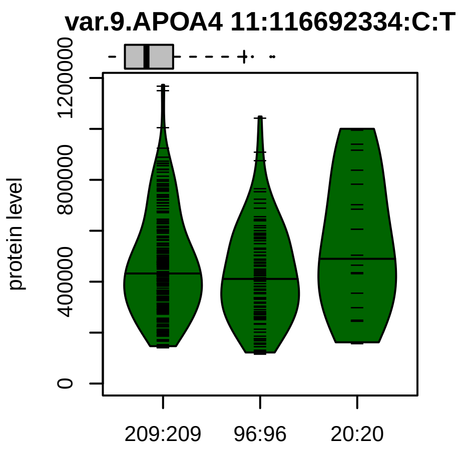

P06727_NP.3
p−maj=1, pY=0.74, N=325

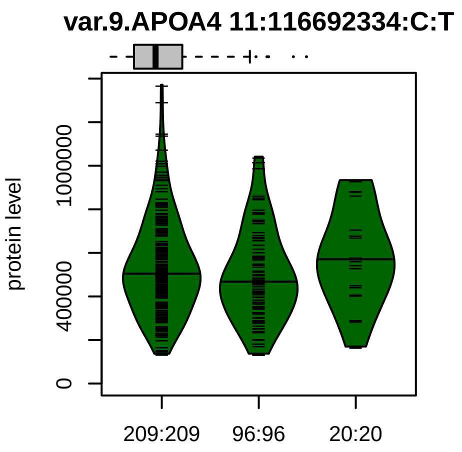

P06727_NP.4
p−maj=1, pY=0.99, N=325

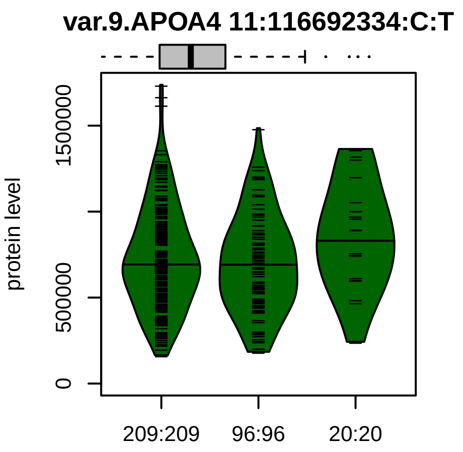

P06727_NP.5
p−maj=1, pY=0.23, N=325

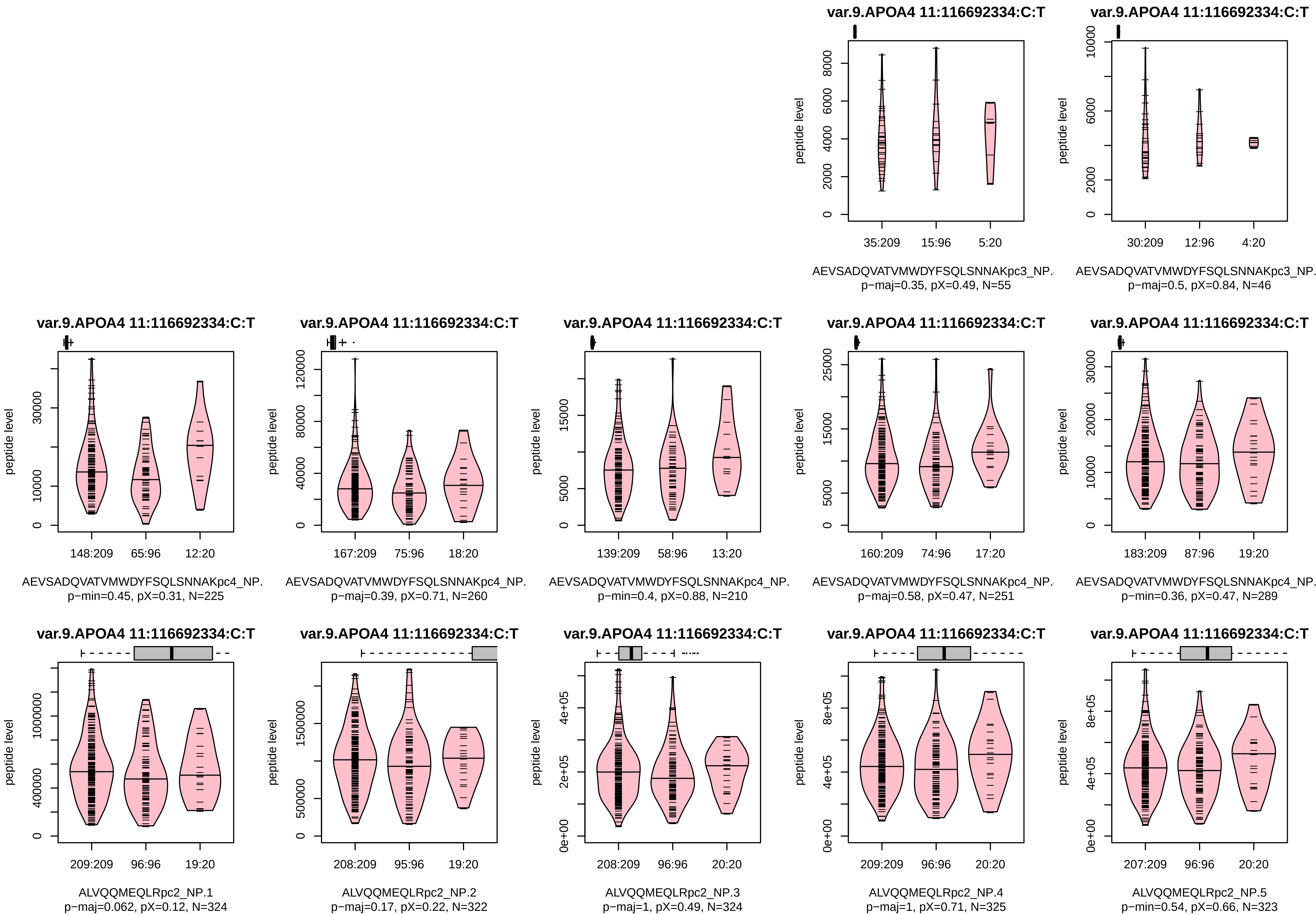

var.9.APOA4 11:116692334:C:T
peptide level
35:209
15:96
5:20
AEVSADQVATVMWDYFSQLSNNAKpc3_NP.
p−maj=0.35, pX=0.49, N=55
var.9.APOA4 11:116692334:C:T
peptide level
30:209
12:96
4:20
AEVSADQVATVMWDYFSQLSNNAKpc3_NP.
p−maj=0.5, pX=0.84, N=46
var.9.APOA4 11:116692334:C:T
peptide level
148:209
65:96
12:20
AEVSADQVATVMWDYFSQLSNNAKpc4_NP.
p−min=0.45, pX=0.31, N=225
var.9.APOA4 11:116692334:C:T
peptide level
167:209
75:96
18:20
AEVSADQVATVMWDYFSQLSNNAKpc4_NP.
p−maj=0.39, pX=0.71, N=260
var.9.APOA4 11:116692334:C:T
peptide level
139:209
58:96
13:20
AEVSADQVATVMWDYFSQLSNNAKpc4_NP.
p−min=0.4, pX=0.88, N=210
var.9.APOA4 11:116692334:C:T
peptide level
160:209
74:96
17:20
AEVSADQVATVMWDYFSQLSNNAKpc4_NP.
p−maj=0.58, pX=0.47, N=251
var.9.APOA4 11:116692334:C:T
peptide level
183:209
87:96
19:20
AEVSADQVATVMWDYFSQLSNNAKpc4_NP.
p−min=0.36, pX=0.47, N=289
var.9.APOA4 11:116692334:C:T
peptide level
209:209
96:96
19:20
ALVQQMEQLRpc2_NP.1
p−maj=0.062, pX=0.12, N=324
var.9.APOA4 11:116692334:C:T
peptide level
208:209
95:96
19:20
ALVQQMEQLRpc2_NP.2
p−maj=0.17, pX=0.22, N=322
var.9.APOA4 11:116692334:C:T
peptide level
208:209
96:96
20:20
ALVQQMEQLRpc2_NP.3
p−maj=1, pX=0.49, N=324
var.9.APOA4 11:116692334:C:T
peptide level
209:209
96:96
20:20
ALVQQMEQLRpc2_NP.4
p−maj=1, pX=0.71, N=325
var.9.APOA4 11:116692334:C:T
peptide level
207:209
96:96
20:20
ALVQQMEQLRpc2_NP.5
p−min=0.54, pX=0.66, N=323

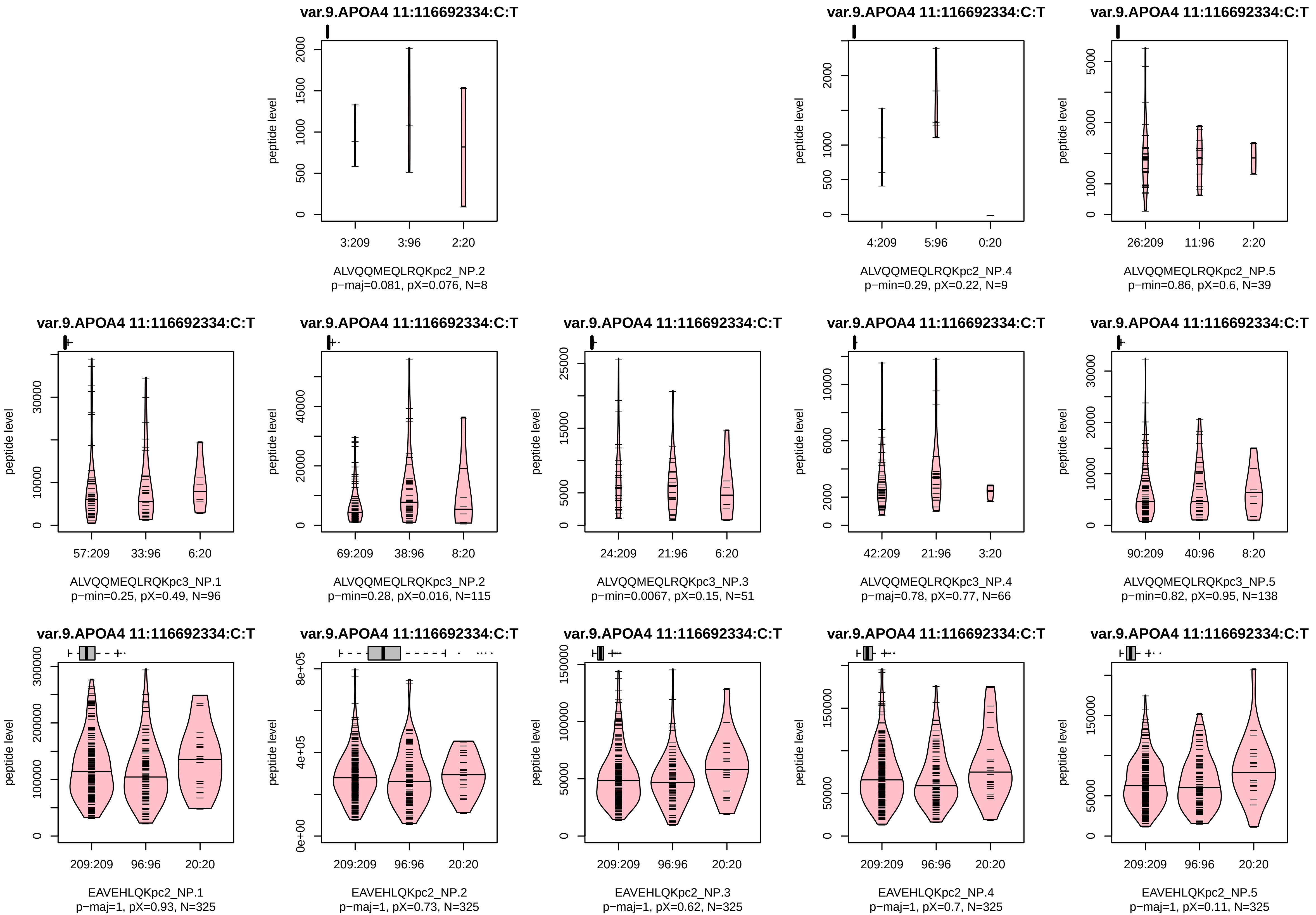
var.9.APOA4 11:116692334:C:T
peptide level
3:209
3:96
2:20
ALVQQMEQLRQKpc2_NP.2
p−maj=0.081, pX=0.076, N=8
var.9.APOA4 11:116692334:C:T
peptide level
4:209
5:96
0:20
ALVQQMEQLRQKpc2_NP.4
p−min=0.29, pX=0.22, N=9
var.9.APOA4 11:116692334:C:T
peptide level
26:209
11:96
2:20
ALVQQMEQLRQKpc2_NP.5
p−min=0.86, pX=0.6, N=39
var.9.APOA4 11:116692334:C:T
peptide level
57:209
33:96
6:20
ALVQQMEQLRQKpc3_NP.1
p−min=0.25, pX=0.49, N=96
var.9.APOA4 11:116692334:C:T
peptide level
69:209
38:96
8:20
ALVQQMEQLRQKpc3_NP.2
p−min=0.28, pX=0.016, N=115
var.9.APOA4 11:116692334:C:T
peptide level
24:209
21:96
6:20
ALVQQMEQLRQKpc3_NP.3
p−min=0.0067, pX=0.15, N=51
var.9.APOA4 11:116692334:C:T
peptide level
42:209
21:96
3:20
ALVQQMEQLRQKpc3_NP.4
p−maj=0.78, pX=0.77, N=66
var.9.APOA4 11:116692334:C:T
peptide level
90:209
40:96
8:20
ALVQQMEQLRQKpc3_NP.5
p−min=0.82, pX=0.95, N=138
var.9.APOA4 11:116692334:C:T
peptide level
209:209
96:96
20:20
EAVEHLQKpc2_NP.1
p−maj=1, pX=0.93, N=325
var.9.APOA4 11:116692334:C:T
peptide level
209:209
96:96
20:20
EAVEHLQKpc2_NP.2
p−maj=1, pX=0.73, N=325
var.9.APOA4 11:116692334:C:T
peptide level
209:209
96:96
20:20
EAVEHLQKpc2_NP.3
p−maj=1, pX=0.62, N=325
var.9.APOA4 11:116692334:C:T
peptide level
209:209
96:96
20:20
EAVEHLQKpc2_NP.4
p−maj=1, pX=0.7, N=325
var.9.APOA4 11:116692334:C:T
peptide level
209:209
96:96
20:20
EAVEHLQKpc2_NP.5
p−maj=1, pX=0.11, N=325

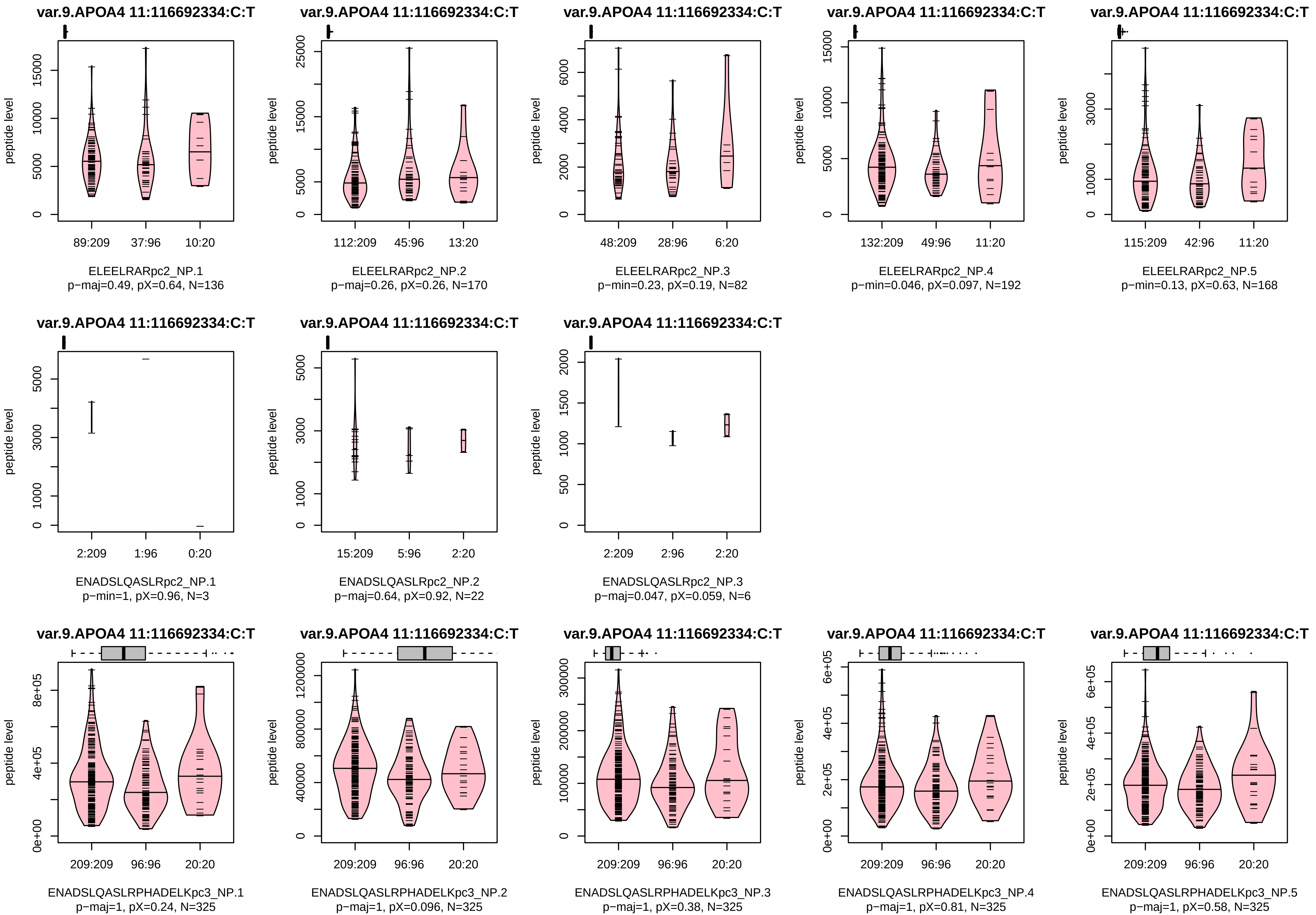
var.9.APOA4 11:116692334:C:T
peptide level
89:209
37:96
10:20
ELEELRARpc2_NP.1
p−maj=0.49, pX=0.64, N=136
var.9.APOA4 11:116692334:C:T
peptide level
112:209
45:96
13:20
ELEELRARpc2_NP.2
p−maj=0.26, pX=0.26, N=170
var.9.APOA4 11:116692334:C:T
peptide level
48:209
28:96
6:20
ELEELRARpc2_NP.3
p−min=0.23, pX=0.19, N=82
var.9.APOA4 11:116692334:C:T
peptide level
132:209
49:96
11:20
ELEELRARpc2_NP.4
p−min=0.046, pX=0.097, N=192
var.9.APOA4 11:116692334:C:T
peptide level
115:209
42:96
11:20
ELEELRARpc2_NP.5
p−min=0.13, pX=0.63, N=168
var.9.APOA4 11:116692334:C:T
peptide level
2:209
1:96
0:20
ENADSLQASLRpc2_NP.1
p−min=1, pX=0.96, N=3
var.9.APOA4 11:116692334:C:T
peptide level
15:209
5:96
2:20
ENADSLQASLRpc2_NP.2
p−maj=0.64, pX=0.92, N=22
var.9.APOA4 11:116692334:C:T
peptide level
2:209
2:96
2:20
ENADSLQASLRpc2_NP.3
p−maj=0.047, pX=0.059, N=6
var.9.APOA4 11:116692334:C:T
peptide level
209:209
96:96
20:20
ENADSLQASLRPHADELKpc3_NP.1
p−maj=1, pX=0.24, N=325
var.9.APOA4 11:116692334:C:T
peptide level
209:209
96:96
20:20
ENADSLQASLRPHADELKpc3_NP.2
p−maj=1, pX=0.096, N=325
var.9.APOA4 11:116692334:C:T
peptide level
209:209
96:96
20:20
ENADSLQASLRPHADELKpc3_NP.3
p−maj=1, pX=0.38, N=325
var.9.APOA4 11:116692334:C:T
peptide level
209:209
96:96
20:20
ENADSLQASLRPHADELKpc3_NP.4
p−maj=1, pX=0.81, N=325
var.9.APOA4 11:116692334:C:T
peptide level
209:209
96:96
20:20
ENADSLQASLRPHADELKpc3_NP.5
p−maj=1, pX=0.58, N=325

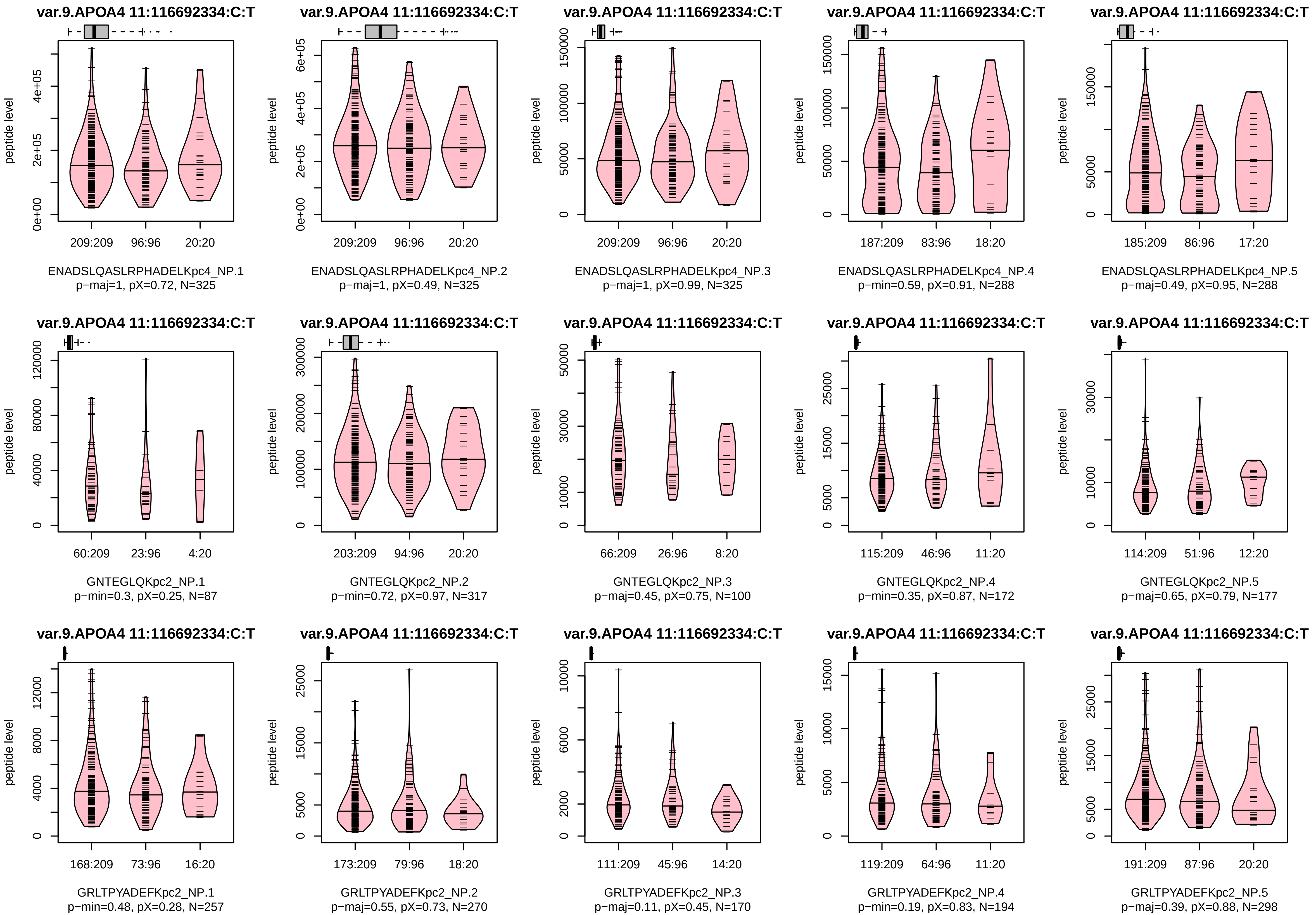
var.9.APOA4 11:116692334:C:T
peptide level
209:209
96:96
20:20
ENADSLQASLRPHADELKpc4_NP.1
p−maj=1, pX=0.72, N=325
var.9.APOA4 11:116692334:C:T
peptide level
209:209
96:96
20:20
ENADSLQASLRPHADELKpc4_NP.2
p−maj=1, pX=0.49, N=325
var.9.APOA4 11:116692334:C:T
peptide level
209:209
96:96
20:20
ENADSLQASLRPHADELKpc4_NP.3
p−maj=1, pX=0.99, N=325
var.9.APOA4 11:116692334:C:T
peptide level
187:209
83:96
18:20
ENADSLQASLRPHADELKpc4_NP.4
p−min=0.59, pX=0.91, N=288
var.9.APOA4 11:116692334:C:T
peptide level
185:209
86:96
17:20
ENADSLQASLRPHADELKpc4_NP.5
p−maj=0.49, pX=0.95, N=288
var.9.APOA4 11:116692334:C:T
peptide level
60:209
23:96
4:20
GNTEGLQKpc2_NP.1
p−min=0.3, pX=0.25, N=87
var.9.APOA4 11:116692334:C:T
peptide level
203:209
94:96
20:20
GNTEGLQKpc2_NP.2
p−min=0.72, pX=0.97, N=317
var.9.APOA4 11:116692334:C:T
peptide level
66:209
26:96
8:20
GNTEGLQKpc2_NP.3
p−maj=0.45, pX=0.75, N=100
var.9.APOA4 11:116692334:C:T
peptide level
115:209
46:96
11:20
GNTEGLQKpc2_NP.4
p−min=0.35, pX=0.87, N=172
var.9.APOA4 11:116692334:C:T
peptide level
114:209
51:96
12:20
GNTEGLQKpc2_NP.5
p−maj=0.65, pX=0.79, N=177
var.9.APOA4 11:116692334:C:T
peptide level
168:209
73:96
16:20
GRLTPYADEFKpc2_NP.1
p−min=0.48, pX=0.28, N=257
var.9.APOA4 11:116692334:C:T
peptide level
173:209
79:96
18:20
GRLTPYADEFKpc2_NP.2
p−maj=0.55, pX=0.73, N=270
var.9.APOA4 11:116692334:C:T
peptide level
111:209
45:96
14:20
GRLTPYADEFKpc2_NP.3
p−maj=0.11, pX=0.45, N=170
var.9.APOA4 11:116692334:C:T
peptide level
119:209
64:96
11:20
GRLTPYADEFKpc2_NP.4
p−min=0.19, pX=0.83, N=194
var.9.APOA4 11:116692334:C:T
peptide level
191:209
87:96
20:20
GRLTPYADEFKpc2_NP.5
p−maj=0.39, pX=0.88, N=298

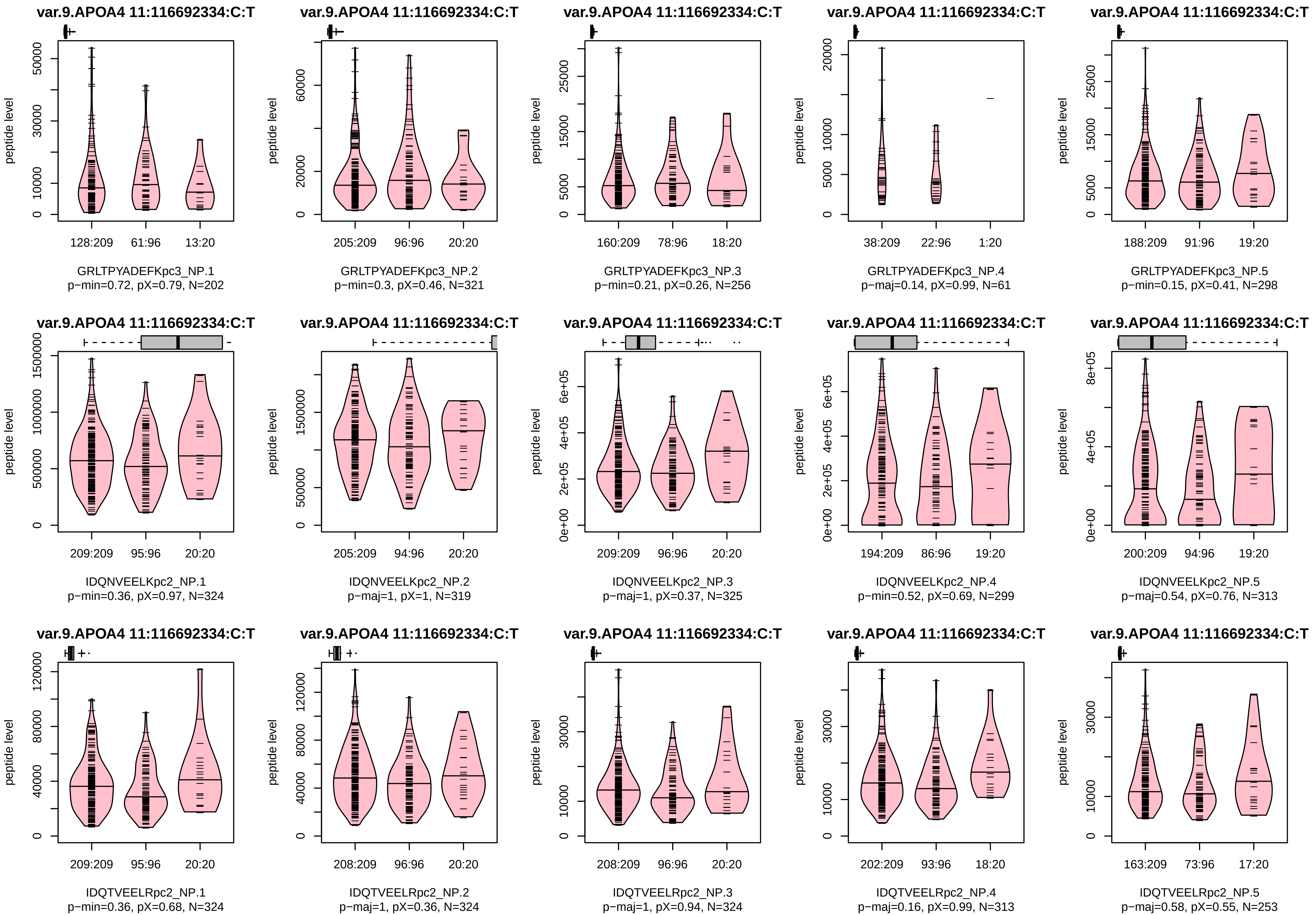

var.9.APOA4 11:116692334:C:T
peptide level
128:209
61:96
13:20
GRLTPYADEFKpc3_NP.1
p−min=0.72, pX=0.79, N=202
var.9.APOA4 11:116692334:C:T
peptide level
205:209
96:96
20:20
GRLTPYADEFKpc3_NP.2
p−min=0.3, pX=0.46, N=321
var.9.APOA4 11:116692334:C:T
peptide level
160:209
78:96
18:20
GRLTPYADEFKpc3_NP.3
p−min=0.21, pX=0.26, N=256
var.9.APOA4 11:116692334:C:T
peptide level
38:209
22:96
1:20
GRLTPYADEFKpc3_NP.4
p−maj=0.14, pX=0.99, N=61
var.9.APOA4 11:116692334:C:T
peptide level
188:209
91:96
19:20
GRLTPYADEFKpc3_NP.5
p−min=0.15, pX=0.41, N=298
var.9.APOA4 11:116692334:C:T
peptide level
209:209
95:96
20:20
IDQNVEELKpc2_NP.1
p−min=0.36, pX=0.97, N=324
var.9.APOA4 11:116692334:C:T
peptide level
205:209
94:96
20:20
IDQNVEELKpc2_NP.2
p−maj=1, pX=1, N=319
var.9.APOA4 11:116692334:C:T
peptide level
209:209
96:96
20:20
IDQNVEELKpc2_NP.3
p−maj=1, pX=0.37, N=325
var.9.APOA4 11:116692334:C:T
peptide level
194:209
86:96
19:20
IDQNVEELKpc2_NP.4
p−min=0.52, pX=0.69, N=299
var.9.APOA4 11:116692334:C:T
peptide level
200:209
94:96
19:20
IDQNVEELKpc2_NP.5
p−maj=0.54, pX=0.76, N=313
var.9.APOA4 11:116692334:C:T
peptide level
209:209
95:96
20:20
IDQTVEELRpc2_NP.1
p−min=0.36, pX=0.68, N=324
var.9.APOA4 11:116692334:C:T
peptide level
208:209
96:96
20:20
IDQTVEELRpc2_NP.2
p−maj=1, pX=0.36, N=324
var.9.APOA4 11:116692334:C:T
peptide level
208:209
96:96
20:20
IDQTVEELRpc2_NP.3
p−maj=1, pX=0.94, N=324
var.9.APOA4 11:116692334:C:T
peptide level
202:209
93:96
18:20
IDQTVEELRpc2_NP.4
p−maj=0.16, pX=0.99, N=313
var.9.APOA4 11:116692334:C:T
peptide level
163:209
73:96
17:20
IDQTVEELRpc2_NP.5
p−maj=0.58, pX=0.55, N=253

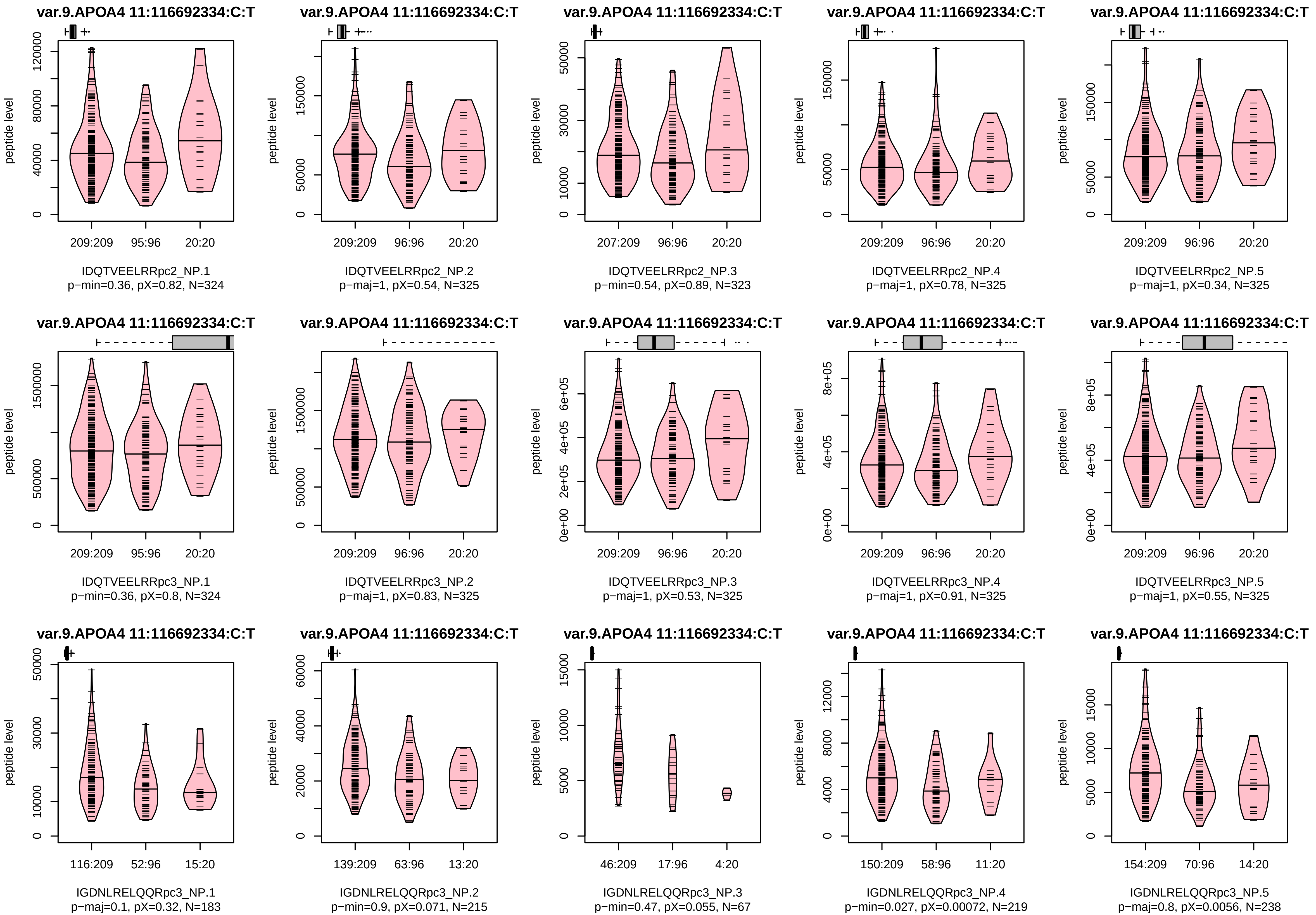
var.9.APOA4 11:116692334:C:T
peptide level
209:209
95:96
20:20
IDQTVEELRRpc2_NP.1
p−min=0.36, pX=0.82, N=324
var.9.APOA4 11:116692334:C:T
209:209
96:96
20:20
IDQTVEELRRpc2_NP.2
p−maj=1, pX=0.54, N=325
var.9.APOA4 11:116692334:C:T
207:209
96:96
20:20
IDQTVEELRRpc2_NP.3
p−min=0.54, pX=0.89, N=323
var.9.APOA4 11:116692334:C:T
209:209
96:96
20:20
IDQTVEELRRpc2_NP.4
p−maj=1, pX=0.78, N=325
var.9.APOA4 11:116692334:C:T
209:209
96:96
20:20
IDQTVEELRRpc2_NP.5
p−maj=1, pX=0.34, N=325
var.9.APOA4 11:116692334:C:T
209:209
95:96
20:20
IDQTVEELRRpc3_NP.1
p−min=0.36, pX=0.8, N=324
var.9.APOA4 11:116692334:C:T
209:209
96:96
20:20
IDQTVEELRRpc3_NP.2
p−maj=1, pX=0.83, N=325
var.9.APOA4 11:116692334:C:T
209:209
96:96
20:20
IDQTVEELRRpc3_NP.3
p−maj=1, pX=0.53, N=325
var.9.APOA4 11:116692334:C:T
209:209
96:96
20:20
IDQTVEELRRpc3_NP.4
p−maj=1, pX=0.91, N=325
var.9.APOA4 11:116692334:C:T
209:209
96:96
20:20
IDQTVEELRRpc3_NP.5
p−maj=1, pX=0.55, N=325
var.9.APOA4 11:116692334:C:T
116:209
52:96
15:20
IGDNLRELQQRpc3_NP.1
p−maj=0.1, pX=0.32, N=183
var.9.APOA4 11:116692334:C:T
139:209
63:96
13:20
IGDNLRELQQRpc3_NP.2
p−min=0.9, pX=0.071, N=215
var.9.APOA4 11:116692334:C:T
46:209
17:96
4:20
IGDNLRELQQRpc3_NP.3
p−min=0.47, pX=0.055, N=67
var.9.APOA4 11:116692334:C:T
150:209
58:96
11:20
IGDNLRELQQRpc3_NP.4
p−min=0.027, pX=0.00072, N=219
var.9.APOA4 11:116692334:C:T
154:209
70:96
14:20
IGDNLRELQQRpc3_NP.5
p−maj=0.8, pX=0.0056, N=238

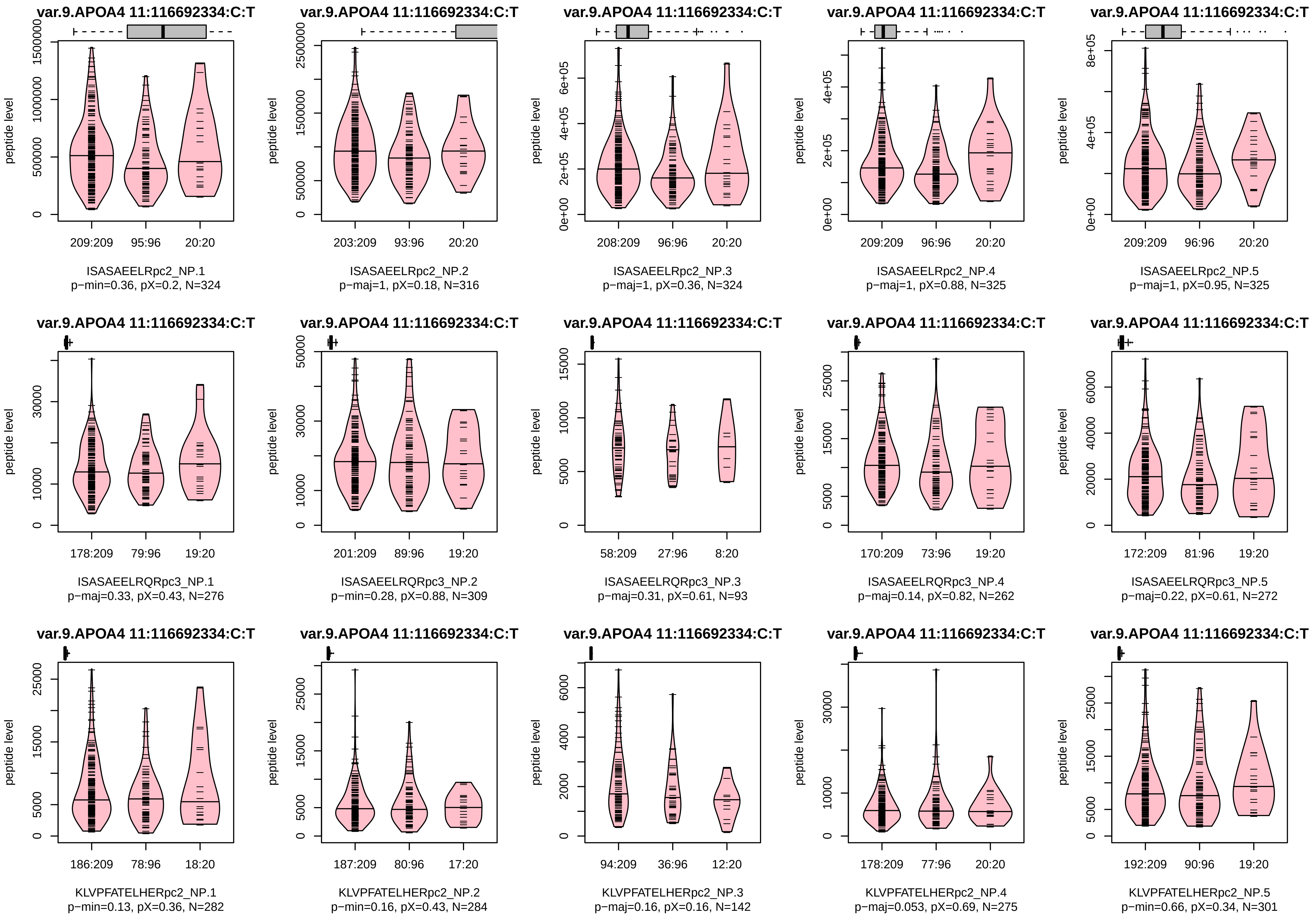
var.9.APOA4 11:116692334:C:T
peptide level
209:209 95:96 20:20
ISASAEELRpc2_NP.1
p−min=0.36, pX=0.2, N=324
var.9.APOA4 11:116692334:C:T
peptide level
203:209 93:96 20:20
ISASAEELRpc2_NP.2
p−maj=1, pX=0.18, N=316
var.9.APOA4 11:116692334:C:T
peptide level
208:209 96:96 20:20
ISASAEELRpc2_NP.3
p−maj=1, pX=0.36, N=324
var.9.APOA4 11:116692334:C:T
peptide level
209:209 96:96 20:20
ISASAEELRpc2_NP.4
p−maj=1, pX=0.88, N=325
var.9.APOA4 11:116692334:C:T
peptide level
209:209 96:96 20:20
ISASAEELRpc2_NP.5
p−maj=1, pX=0.95, N=325
var.9.APOA4 11:116692334:C:T
peptide level
178:209 79:96 19:20
ISASAEELRQRpc3_NP.1
p−maj=0.33, pX=0.43, N=276
var.9.APOA4 11:116692334:C:T
peptide level
201:209 89:96 19:20
ISASAEELRQRpc3_NP.2
p−min=0.28, pX=0.88, N=309
var.9.APOA4 11:116692334:C:T
peptide level
58:209 27:96 8:20
ISASAEELRQRpc3_NP.3
p−maj=0.31, pX=0.61, N=93
var.9.APOA4 11:116692334:C:T
peptide level
170:209 73:96 19:20
ISASAEELRQRpc3_NP.4
p−maj=0.14, pX=0.82, N=262
var.9.APOA4 11:116692334:C:T
peptide level
172:209 81:96 19:20
ISASAEELRQRpc3_NP.5
p−maj=0.22, pX=0.61, N=272
var.9.APOA4 11:116692334:C:T
peptide level
186:209 78:96 18:20
KLVPFATELHERpc2_NP.1
p−min=0.13, pX=0.36, N=282
var.9.APOA4 11:116692334:C:T
peptide level
187:209 80:96 17:20
KLVPFATELHERpc2_NP.2
p−min=0.16, pX=0.43, N=284
var.9.APOA4 11:116692334:C:T
peptide level
94:209 36:96 12:20
KLVPFATELHERpc2_NP.3
p−maj=0.16, pX=0.16, N=142
var.9.APOA4 11:116692334:C:T
peptide level
178:209 77:96 20:20
KLVPFATELHERpc2_NP.4
p−maj=0.053, pX=0.69, N=275
var.9.APOA4 11:116692334:C:T
peptide level
192:209 90:96 19:20
KLVPFATELHERpc2_NP.5
p−min=0.66, pX=0.34, N=301

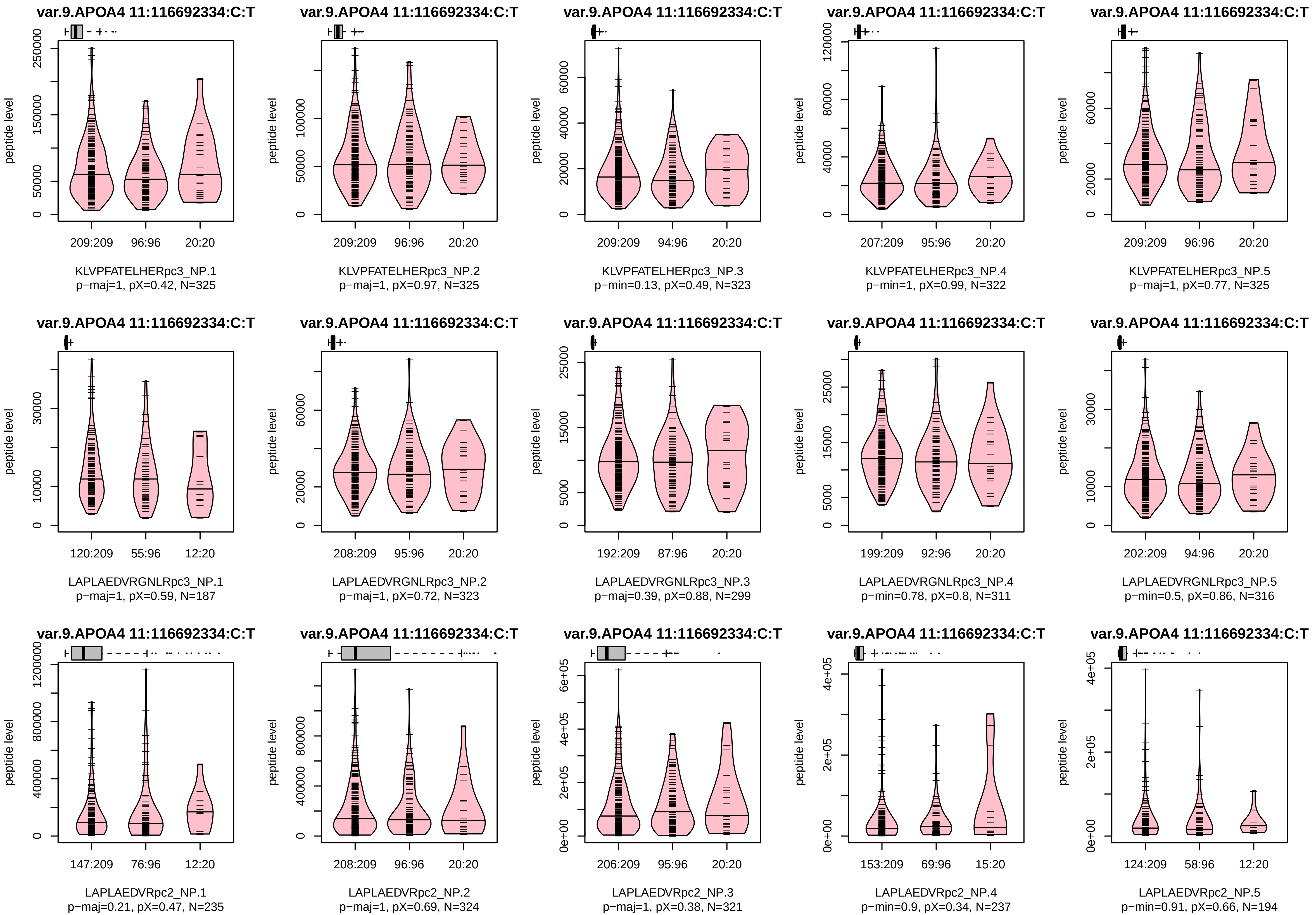
var.9.APOA4 11:116692334:C:T
peptide level
209:209
96:96
20:20
KLVPFATELHERpc3_NP.1
p−maj=1, pX=0.42, N=325
var.9.APOA4 11:116692334:C:T
peptide level
209:209
96:96
20:20
KLVPFATELHERpc3_NP.2
p−maj=1, pX=0.97, N=325
var.9.APOA4 11:116692334:C:T
peptide level
209:209
94:96
20:20
KLVPFATELHERpc3_NP.3
p−min=0.13, pX=0.49, N=323
var.9.APOA4 11:116692334:C:T
peptide level
207:209
95:96
20:20
KLVPFATELHERpc3_NP.4
p−min=1, pX=0.99, N=322
var.9.APOA4 11:116692334:C:T
peptide level
209:209
96:96
20:20
KLVPFATELHERpc3_NP.5
p−maj=1, pX=0.77, N=325
var.9.APOA4 11:116692334:C:T
peptide level
120:209
55:96
12:20
LAPLAEDVRGNLRpc3_NP.1
p−maj=1, pX=0.59, N=187
var.9.APOA4 11:116692334:C:T
peptide level
208:209
95:96
20:20
LAPLAEDVRGNLRpc3_NP.2
p−maj=1, pX=0.72, N=323
var.9.APOA4 11:116692334:C:T
peptide level
192:209
87:96
20:20
LAPLAEDVRGNLRpc3_NP.3
p−maj=0.39, pX=0.88, N=299
var.9.APOA4 11:116692334:C:T
peptide level
199:209
92:96
20:20
LAPLAEDVRGNLRpc3_NP.4
p−min=0.78, pX=0.8, N=311
var.9.APOA4 11:116692334:C:T
peptide level
202:209
94:96
20:20
LAPLAEDVRGNLRpc3_NP.5
p−min=0.5, pX=0.86, N=316
var.9.APOA4 11:116692334:C:T
peptide level
147:209
76:96
12:20
LAPLAEDVRpc2_NP.1
p−maj=0.21, pX=0.47, N=235
var.9.APOA4 11:116692334:C:T
peptide level
208:209
96:96
20:20
LAPLAEDVRpc2_NP.2
p−maj=1, pX=0.69, N=324
var.9.APOA4 11:116692334:C:T
peptide level
206:209
95:96
20:20
LAPLAEDVRpc2_NP.3
p−maj=1, pX=0.38, N=321
var.9.APOA4 11:116692334:C:T
peptide level
153:209
69:96
15:20
LAPLAEDVRpc2_NP.4
p−min=0.9, pX=0.34, N=237
var.9.APOA4 11:116692334:C:T
peptide level
124:209
58:96
12:20
LAPLAEDVRpc2_NP.5
p−min=0.91, pX=0.66, N=194

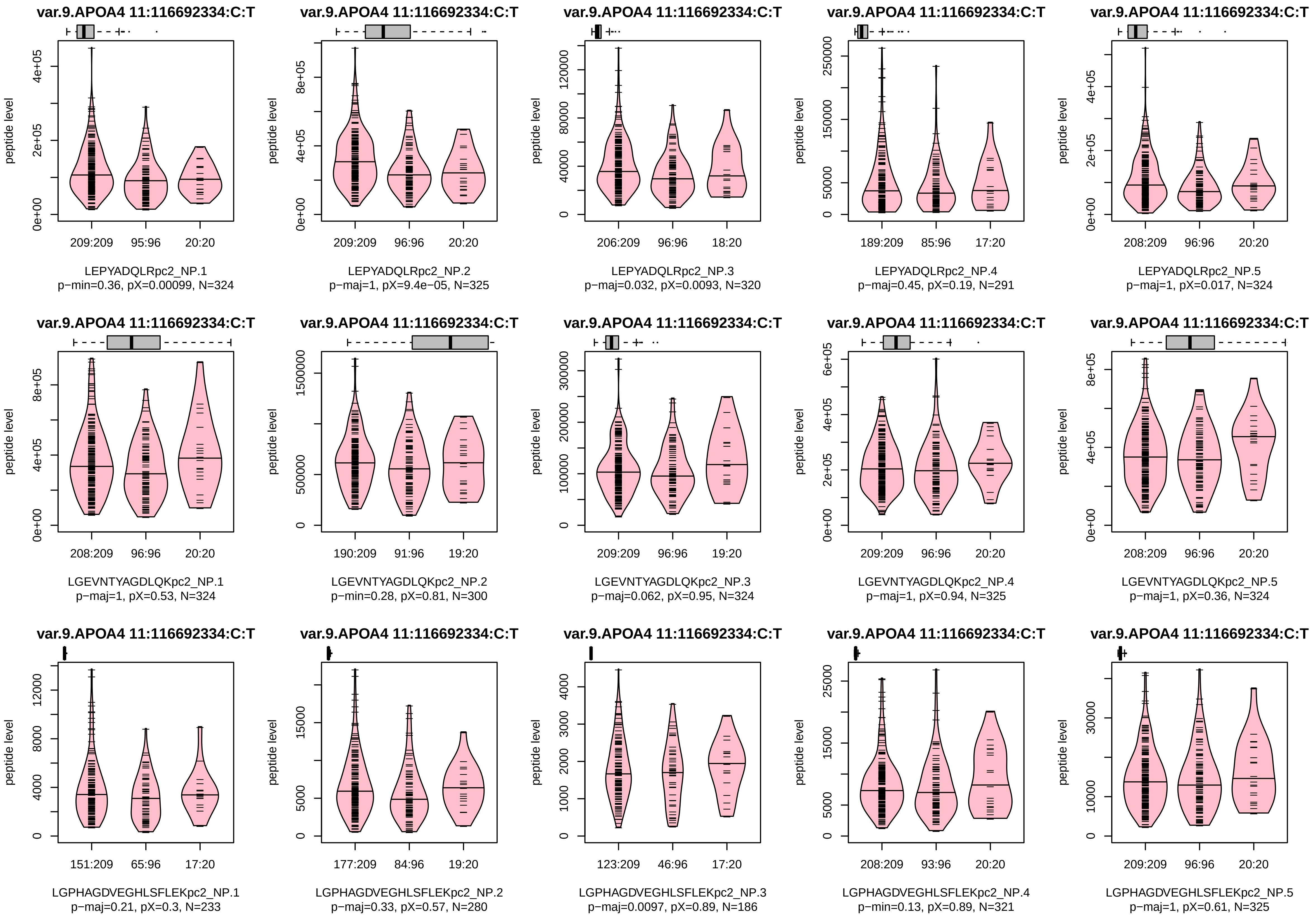
var.9.APOA4 11:116692334:C:T
peptide level
209:209
95:96
20:20
LEPYADQLRpc2_NP.1
p−min=0.36, pX=0.00099, N=324
var.9.APOA4 11:116692334:C:T
peptide level
209:209
96:96
20:20
LEPYADQLRpc2_NP.2
p−maj=1, pX=9.4e−05, N=325
var.9.APOA4 11:116692334:C:T
peptide level
206:209
96:96
18:20
LEPYADQLRpc2_NP.3
p−maj=0.032, pX=0.0093, N=320
var.9.APOA4 11:116692334:C:T
peptide level
189:209
85:96
17:20
LEPYADQLRpc2_NP.4
p−maj=0.45, pX=0.19, N=291
var.9.APOA4 11:116692334:C:T
peptide level
208:209
96:96
20:20
LEPYADQLRpc2_NP.5
p−maj=1, pX=0.017, N=324
var.9.APOA4 11:116692334:C:T
peptide level
208:209
96:96
20:20
LGEVNTYAGDLQKpc2_NP.1
p−maj=1, pX=0.53, N=324
var.9.APOA4 11:116692334:C:T
peptide level
190:209
91:96
19:20
LGEVNTYAGDLQKpc2_NP.2
p−min=0.28, pX=0.81, N=300
var.9.APOA4 11:116692334:C:T
peptide level
209:209
96:96
19:20
LGEVNTYAGDLQKpc2_NP.3
p−maj=0.062, pX=0.95, N=324
var.9.APOA4 11:116692334:C:T
peptide level
209:209
96:96
20:20
LGEVNTYAGDLQKpc2_NP.4
p−maj=1, pX=0.94, N=325
var.9.APOA4 11:116692334:C:T
peptide level
208:209
96:96
20:20
LGEVNTYAGDLQKpc2_NP.5
p−maj=1, pX=0.36, N=324
var.9.APOA4 11:116692334:C:T
peptide level
151:209
65:96
17:20
LGPHAGDVEGHLSFLEKpc2_NP.1
p−maj=0.21, pX=0.3, N=233
var.9.APOA4 11:116692334:C:T
peptide level
177:209
84:96
19:20
LGPHAGDVEGHLSFLEKpc2_NP.2
p−maj=0.33, pX=0.57, N=280
var.9.APOA4 11:116692334:C:T
peptide level
123:209
46:96
17:20
LGPHAGDVEGHLSFLEKpc2_NP.3
p−maj=0.0097, pX=0.89, N=186
var.9.APOA4 11:116692334:C:T
peptide level
208:209
93:96
20:20
LGPHAGDVEGHLSFLEKpc2_NP.4
p−min=0.13, pX=0.89, N=321
var.9.APOA4 11:116692334:C:T
peptide level
209:209
96:96
20:20
LGPHAGDVEGHLSFLEKpc2_NP.5
p−maj=1, pX=0.61, N=325

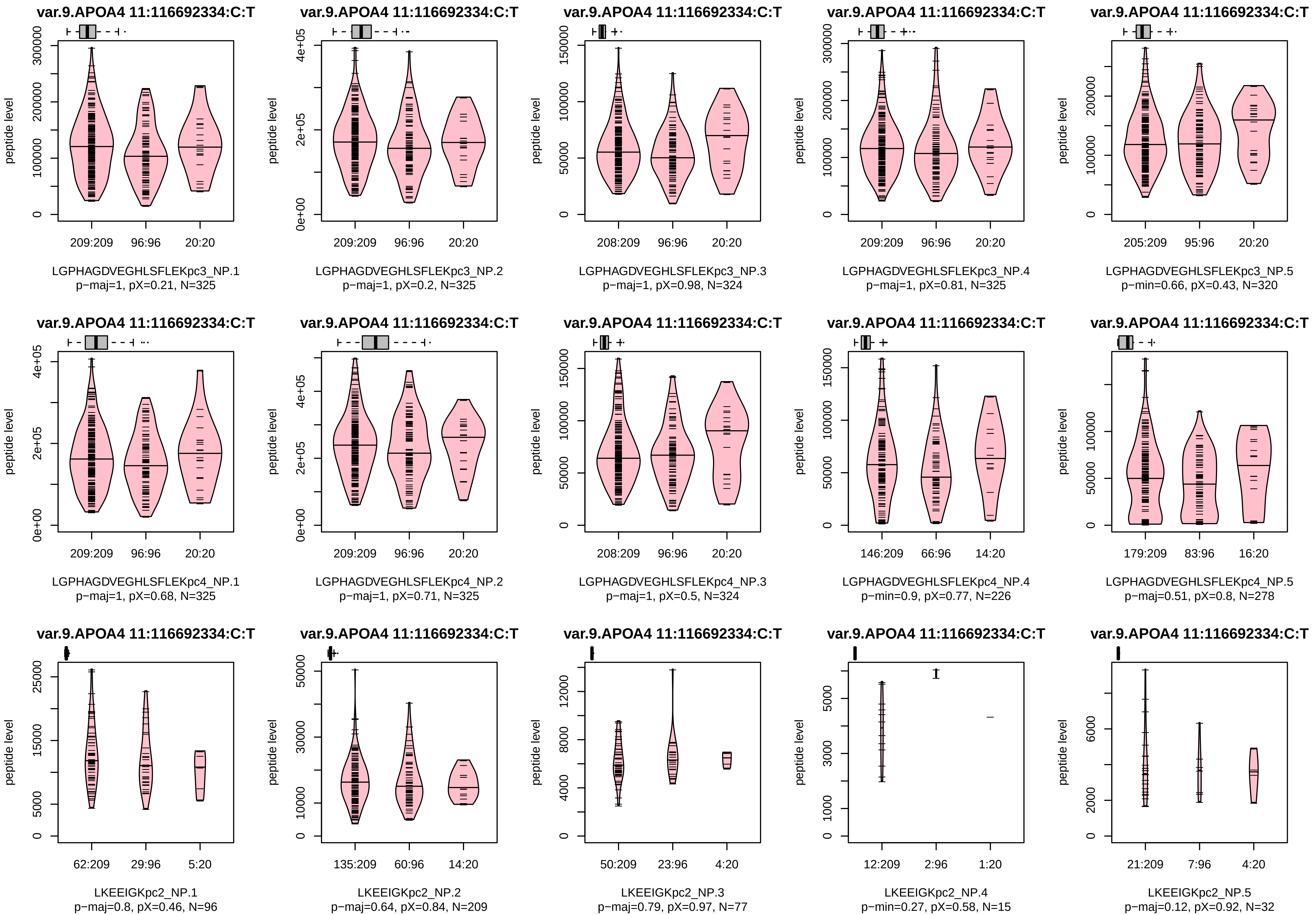

var.9.APOA4 11:116692334:C:T
peptide level
0
100000
200000
300000
209:209
96:96
20:20
LGPHAGDVEGHLSFLEKpc3_NP.1
p−maj=1, pX=0.21, N=325
var.9.APOA4 11:116692334:C:T
peptide level
0e+00
2e+05
4e+05
209:209
96:96
20:20
LGPHAGDVEGHLSFLEKpc3_NP.2
p−maj=1, pX=0.2, N=325
var.9.APOA4 11:116692334:C:T
peptide level
0
50000
100000
150000
208:209
96:96
20:20
LGPHAGDVEGHLSFLEKpc3_NP.3
p−maj=1, pX=0.98, N=324
var.9.APOA4 11:116692334:C:T
peptide level
0
100000
200000
300000
209:209
96:96
20:20
LGPHAGDVEGHLSFLEKpc3_NP.4
p−maj=1, pX=0.81, N=325
var.9.APOA4 11:116692334:C:T
peptide level
0
100000
200000
205:209
95:96
20:20
LGPHAGDVEGHLSFLEKpc3_NP.5
p−min=0.66, pX=0.43, N=320
var.9.APOA4 11:116692334:C:T
peptide level
0e+00
2e+05
4e+05
209:209
96:96
20:20
LGPHAGDVEGHLSFLEKpc4_NP.1
p−maj=1, pX=0.68, N=325
var.9.APOA4 11:116692334:C:T
peptide level
0e+00
2e+05
4e+05
209:209
96:96
20:20
LGPHAGDVEGHLSFLEKpc4_NP.2
p−maj=1, pX=0.71, N=325
var.9.APOA4 11:116692334:C:T
peptide level
0
50000
100000
150000
208:209
96:96
20:20
LGPHAGDVEGHLSFLEKpc4_NP.3
p−maj=1, pX=0.5, N=324
var.9.APOA4 11:116692334:C:T
peptide level
0
50000
100000
150000
146:209
66:96
14:20
LGPHAGDVEGHLSFLEKpc4_NP.4
p−min=0.9, pX=0.77, N=226
var.9.APOA4 11:116692334:C:T
peptide level
0
50000
100000
179:209
83:96
16:20
LGPHAGDVEGHLSFLEKpc4_NP.5
p−maj=0.51, pX=0.8, N=278
var.9.APOA4 11:116692334:C:T
peptide level
0
5000
15000
25000
62:209
29:96
5:20
LKEEIGKpc2_NP.1
p−maj=0.8, pX=0.46, N=96
var.9.APOA4 11:116692334:C:T
peptide level
0
10000
30000
50000
135:209
60:96
14:20
LKEEIGKpc2_NP.2
p−maj=0.64, pX=0.84, N=209
var.9.APOA4 11:116692334:C:T
peptide level
0
4000
8000
12000
50:209
23:96
4:20
LKEEIGKpc2_NP.3
p−maj=0.79, pX=0.97, N=77
var.9.APOA4 11:116692334:C:T
peptide level
1000
3000
5000
12:209
2:96
1:20
LKEEIGKpc2_NP.4
p−min=0.27, pX=0.58, N=15
var.9.APOA4 11:116692334:C:T
peptide level
0
2000
6000
21:209
7:96
4:20
LKEEIGKpc2_NP.5
p−maj=0.12, pX=0.92, N=32

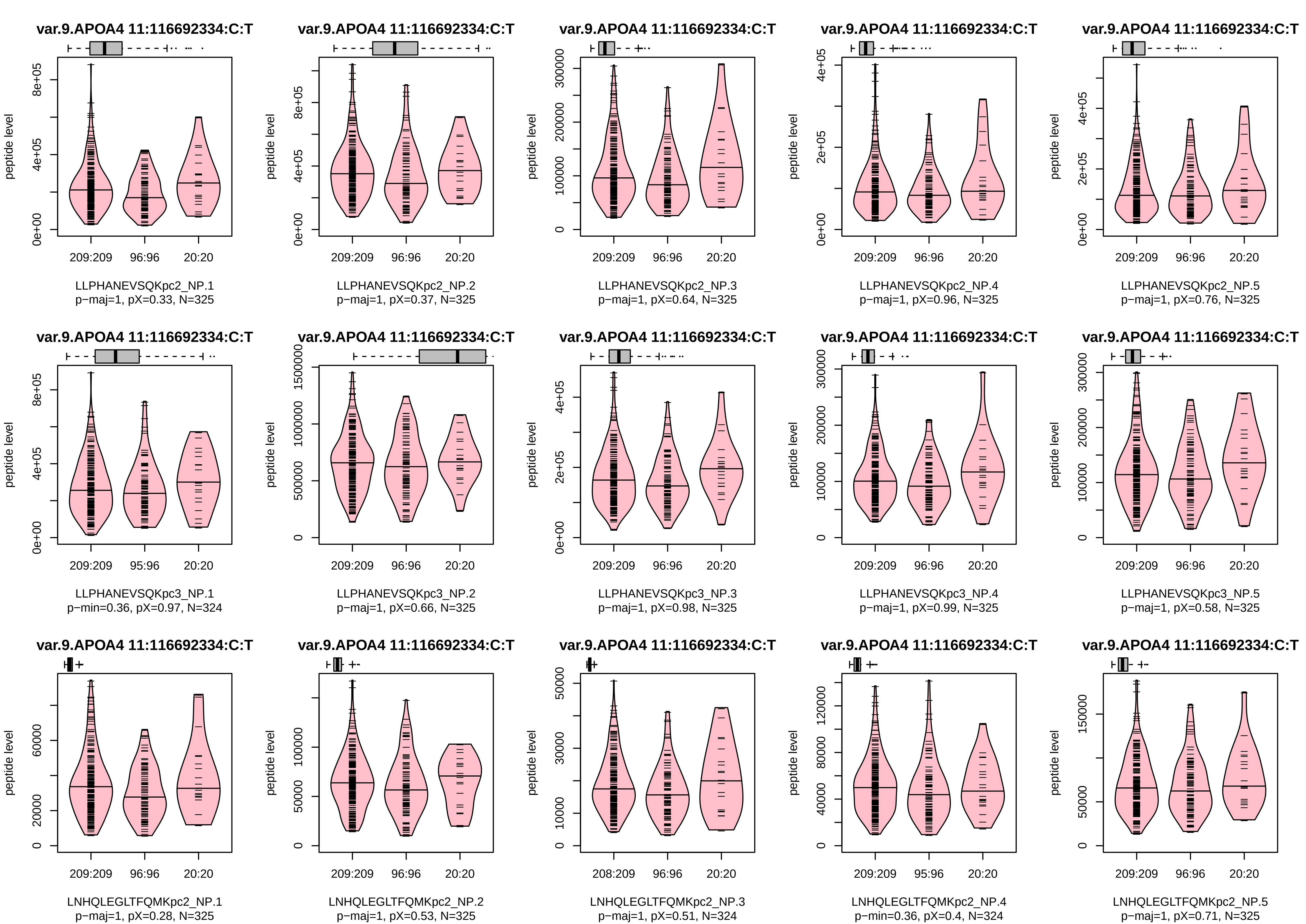

var.9.APOA4 11:116692334:C:T
peptide level
209:209
96:96
20:20
LLPHANEVSQKpc2_NP.1
p−maj=1, pX=0.33, N=325
var.9.APOA4 11:116692334:C:T
peptide level
209:209
96:96
20:20
LLPHANEVSQKpc2_NP.2
p−maj=1, pX=0.37, N=325
var.9.APOA4 11:116692334:C:T
peptide level
209:209
96:96
20:20
LLPHANEVSQKpc2_NP.3
p−maj=1, pX=0.64, N=325
var.9.APOA4 11:116692334:C:T
peptide level
209:209
96:96
20:20
LLPHANEVSQKpc2_NP.4
p−maj=1, pX=0.96, N=325
var.9.APOA4 11:116692334:C:T
peptide level
209:209
96:96
20:20
LLPHANEVSQKpc2_NP.5
p−maj=1, pX=0.76, N=325
var.9.APOA4 11:116692334:C:T
peptide level
209:209
95:96
20:20
LLPHANEVSQKpc3_NP.1
p−min=0.36, pX=0.97, N=324
var.9.APOA4 11:116692334:C:T
peptide level
209:209
96:96
20:20
LLPHANEVSQKpc3_NP.2
p−maj=1, pX=0.66, N=325
var.9.APOA4 11:116692334:C:T
peptide level
209:209
96:96
20:20
LLPHANEVSQKpc3_NP.3
p−maj=1, pX=0.98, N=325
var.9.APOA4 11:116692334:C:T
peptide level
209:209
96:96
20:20
LLPHANEVSQKpc3_NP.4
p−maj=1, pX=0.99, N=325
var.9.APOA4 11:116692334:C:T
peptide level
209:209
96:96
20:20
LLPHANEVSQKpc3_NP.5
p−maj=1, pX=0.58, N=325
var.9.APOA4 11:116692334:C:T
peptide level
209:209
96:96
20:20
LNHQLEGLTFQMKpc2_NP.1
p−maj=1, pX=0.28, N=325
var.9.APOA4 11:116692334:C:T
peptide level
209:209
96:96
20:20
LNHQLEGLTFQMKpc2_NP.2
p−maj=1, pX=0.53, N=325
var.9.APOA4 11:116692334:C:T
peptide level
208:209
96:96
20:20
LNHQLEGLTFQMKpc2_NP.3
p−maj=1, pX=0.51, N=324
var.9.APOA4 11:116692334:C:T
peptide level
209:209
95:96
20:20
LNHQLEGLTFQMKpc2_NP.4
p−min=0.36, pX=0.4, N=324
var.9.APOA4 11:116692334:C:T
peptide level
209:209
96:96
20:20
LNHQLEGLTFQMKpc2_NP.5
p−maj=1, pX=0.71, N=325

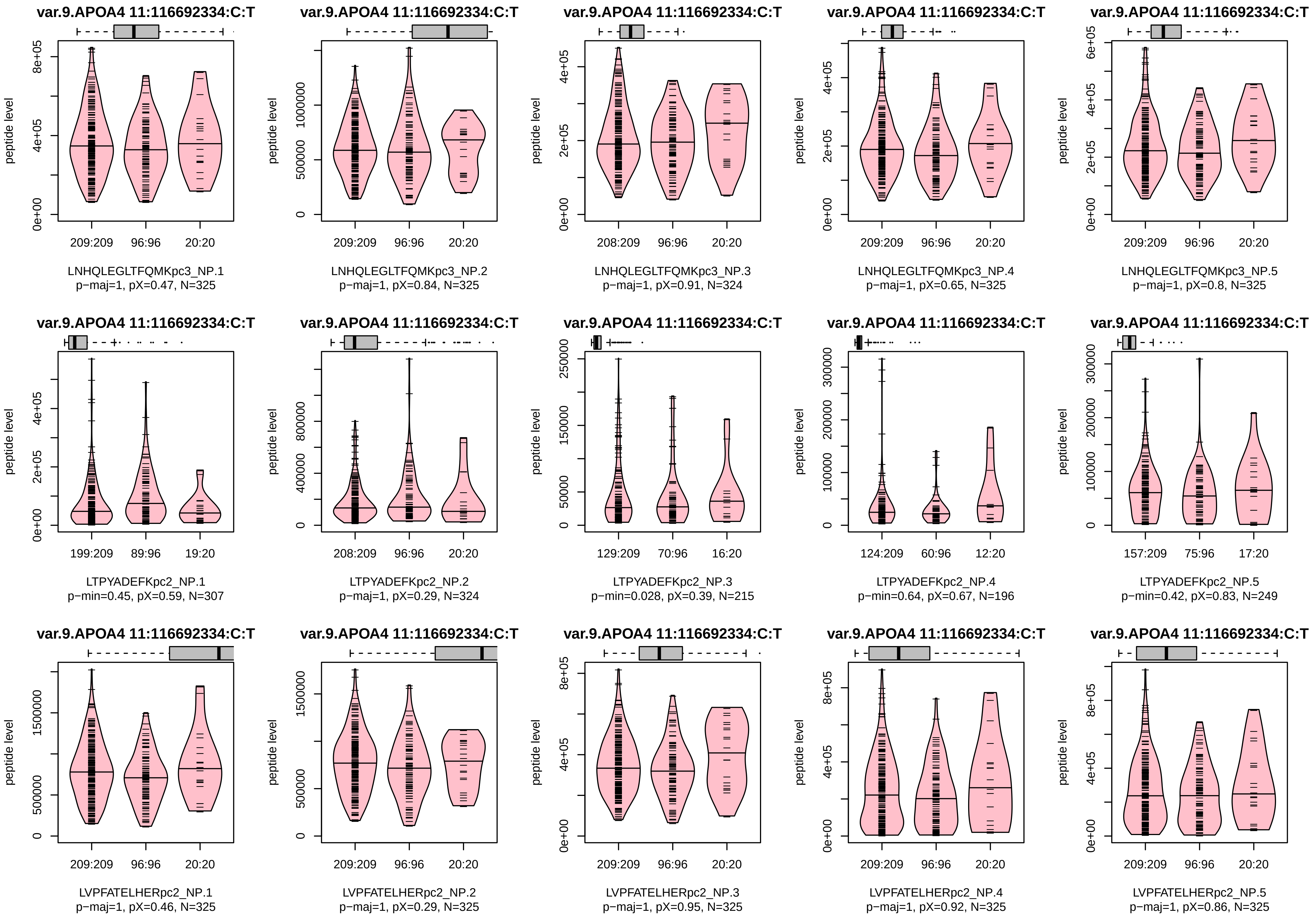
var.9.APOA4 11:116692334:C:T
peptide level
209:209
96:96
20:20
LNHQLEGLTFQMKpc3_NP.1
p−maj=1, pX=0.47, N=325
var.9.APOA4 11:116692334:C:T
peptide level
209:209
96:96
20:20
LNHQLEGLTFQMKpc3_NP.2
p−maj=1, pX=0.84, N=325
var.9.APOA4 11:116692334:C:T
peptide level
208:209
96:96
20:20
LNHQLEGLTFQMKpc3_NP.3
p−maj=1, pX=0.91, N=324
var.9.APOA4 11:116692334:C:T
peptide level
209:209
96:96
20:20
LNHQLEGLTFQMKpc3_NP.4
p−maj=1, pX=0.65, N=325
var.9.APOA4 11:116692334:C:T
peptide level
209:209
96:96
20:20
LNHQLEGLTFQMKpc3_NP.5
p−maj=1, pX=0.8, N=325
var.9.APOA4 11:116692334:C:T
peptide level
199:209
89:96
19:20
LTPYADEFKpc2_NP.1
p−min=0.45, pX=0.59, N=307
var.9.APOA4 11:116692334:C:T
peptide level
208:209
96:96
20:20
LTPYADEFKpc2_NP.2
p−maj=1, pX=0.29, N=324
var.9.APOA4 11:116692334:C:T
peptide level
129:209
70:96
16:20
LTPYADEFKpc2_NP.3
p−min=0.028, pX=0.39, N=215
var.9.APOA4 11:116692334:C:T
peptide level
124:209
60:96
12:20
LTPYADEFKpc2_NP.4
p−min=0.64, pX=0.67, N=196
var.9.APOA4 11:116692334:C:T
peptide level
157:209
75:96
17:20
LTPYADEFKpc2_NP.5
p−min=0.42, pX=0.83, N=249
var.9.APOA4 11:116692334:C:T
peptide level
209:209
96:96
20:20
LVPFATELHERpc2_NP.1
p−maj=1, pX=0.46, N=325
var.9.APOA4 11:116692334:C:T
peptide level
209:209
96:96
20:20
LVPFATELHERpc2_NP.2
p−maj=1, pX=0.29, N=325
var.9.APOA4 11:116692334:C:T
peptide level
209:209
96:96
20:20
LVPFATELHERpc2_NP.3
p−maj=1, pX=0.95, N=325
var.9.APOA4 11:116692334:C:T
peptide level
209:209
96:96
20:20
LVPFATELHERpc2_NP.4
p−maj=1, pX=0.92, N=325
var.9.APOA4 11:116692334:C:T
peptide level
209:209
96:96
20:20
LVPFATELHERpc2_NP.5
p−maj=1, pX=0.86, N=325

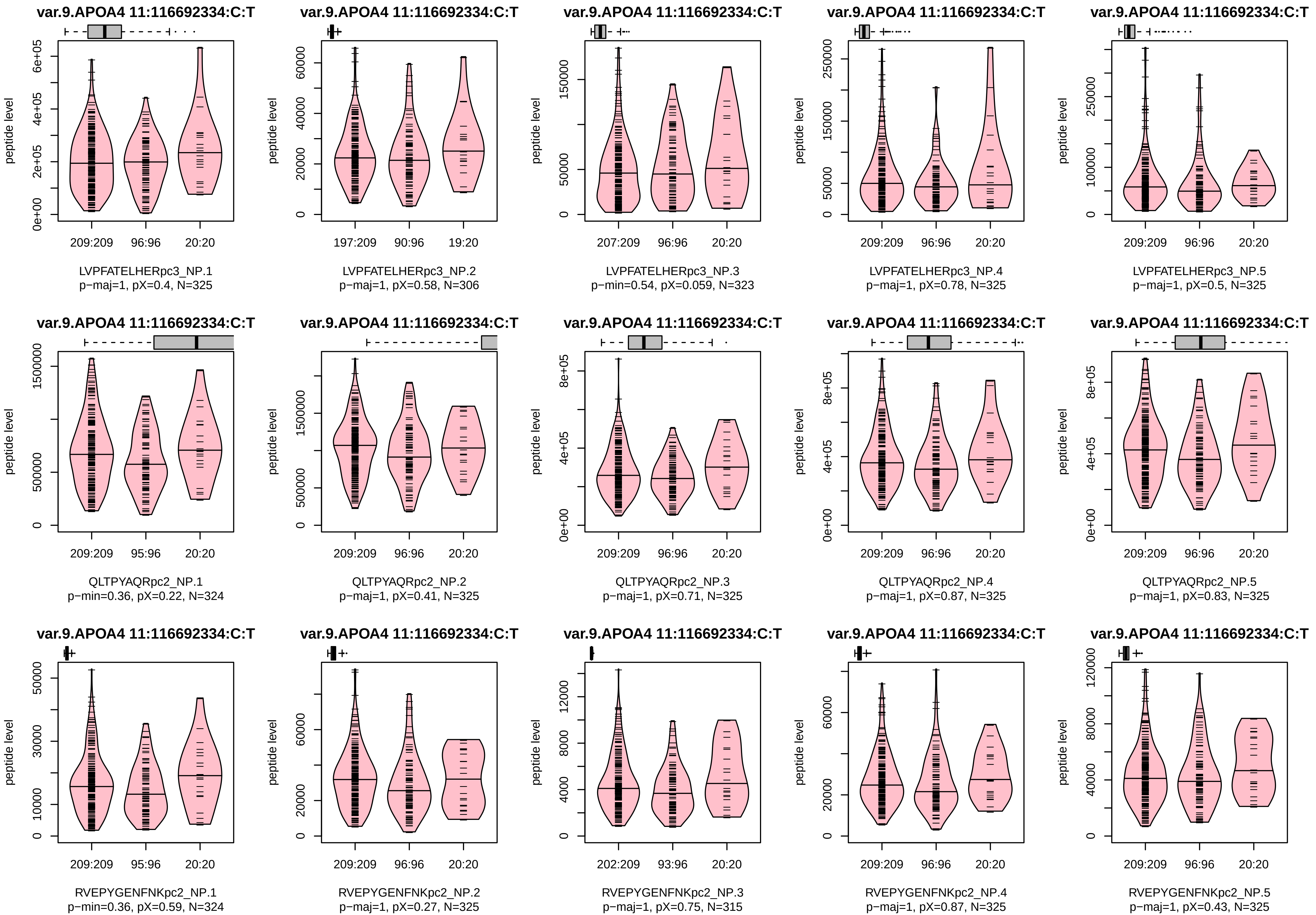

var.9.APOA4 11:116692334:C:T
peptide level
209:209
96:96
20:20
LVPFATELHERpc3_NP.1
p−maj=1, pX=0.4, N=325
var.9.APOA4 11:116692334:C:T
peptide level
197:209
90:96
19:20
LVPFATELHERpc3_NP.2
p−maj=1, pX=0.58, N=306
var.9.APOA4 11:116692334:C:T
peptide level
207:209
96:96
20:20
LVPFATELHERpc3_NP.3
p−min=0.54, pX=0.059, N=323
var.9.APOA4 11:116692334:C:T
peptide level
209:209
96:96
20:20
LVPFATELHERpc3_NP.4
p−maj=1, pX=0.78, N=325
var.9.APOA4 11:116692334:C:T
peptide level
209:209
96:96
20:20
LVPFATELHERpc3_NP.5
p−maj=1, pX=0.5, N=325
var.9.APOA4 11:116692334:C:T
peptide level
209:209
95:96
20:20
QLTPYAQRpc2_NP.1
p−min=0.36, pX=0.22, N=324
var.9.APOA4 11:116692334:C:T
peptide level
209:209
96:96
20:20
QLTPYAQRpc2_NP.2
p−maj=1, pX=0.41, N=325
var.9.APOA4 11:116692334:C:T
peptide level
209:209
96:96
20:20
QLTPYAQRpc2_NP.3
p−maj=1, pX=0.71, N=325
var.9.APOA4 11:116692334:C:T
peptide level
209:209
96:96
20:20
QLTPYAQRpc2_NP.4
p−maj=1, pX=0.87, N=325
var.9.APOA4 11:116692334:C:T
peptide level
209:209
96:96
20:20
QLTPYAQRpc2_NP.5
p−maj=1, pX=0.83, N=325
var.9.APOA4 11:116692334:C:T
peptide level
209:209
95:96
20:20
RVEPYGENFNKpc2_NP.1
p−min=0.36, pX=0.59, N=324
var.9.APOA4 11:116692334:C:T
peptide level
209:209
96:96
20:20
RVEPYGENFNKpc2_NP.2
p−maj=1, pX=0.27, N=325
var.9.APOA4 11:116692334:C:T
peptide level
202:209
93:96
20:20
RVEPYGENFNKpc2_NP.3
p−maj=1, pX=0.75, N=315
var.9.APOA4 11:116692334:C:T
peptide level
209:209
96:96
20:20
RVEPYGENFNKpc2_NP.4
p−maj=1, pX=0.87, N=325
var.9.APOA4 11:116692334:C:T
peptide level
209:209
96:96
20:20
RVEPYGENFNKpc2_NP.5
p−maj=1, pX=0.43, N=325

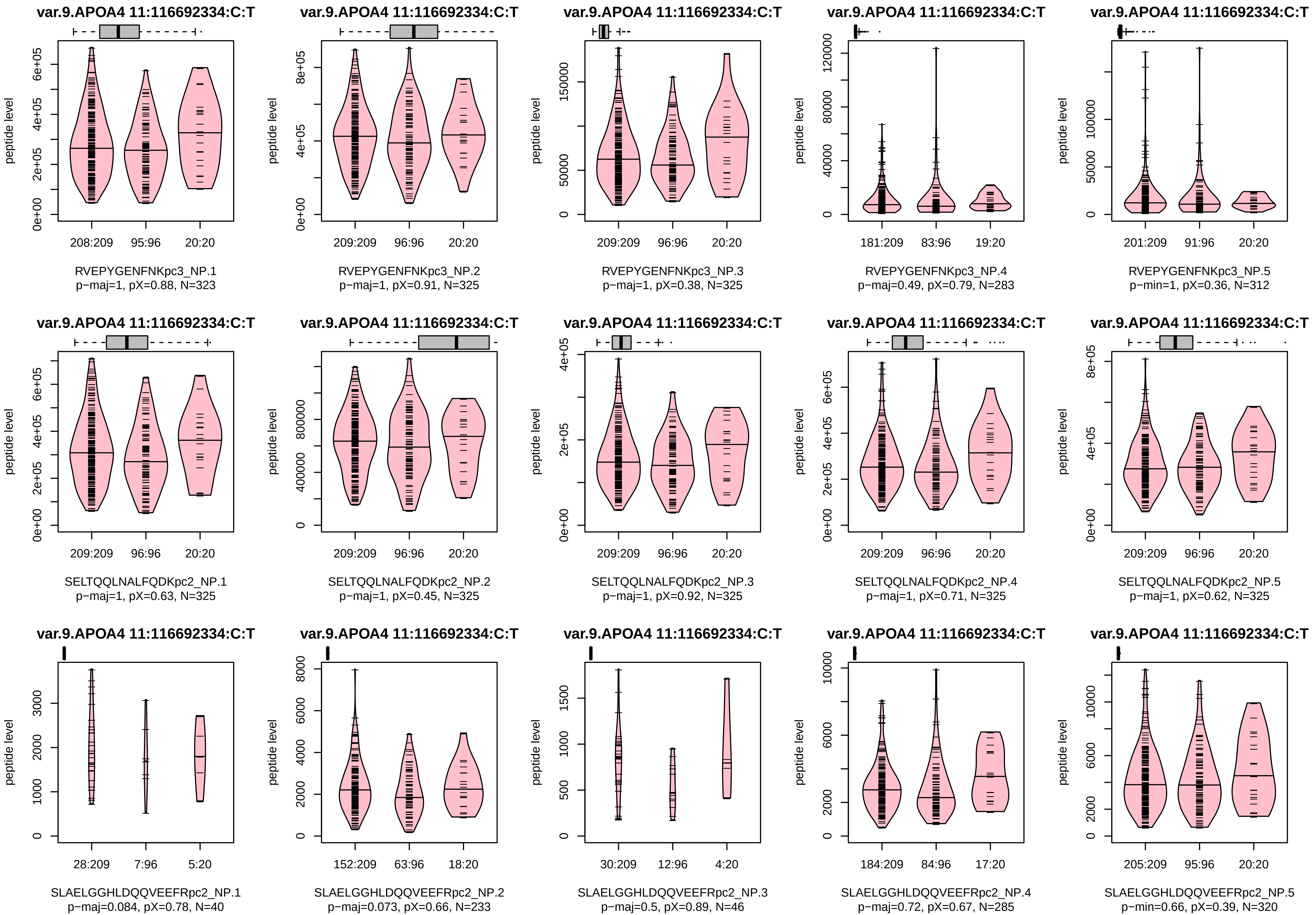
var.9.APOA4 11:116692334:C:T
peptide level
208:209
95:96
20:20
RVEPYGENFNKpc3_NP.1
p−maj=1, pX=0.88, N=323
var.9.APOA4 11:116692334:C:T
peptide level
209:209
96:96
20:20
RVEPYGENFNKpc3_NP.2
p−maj=1, pX=0.91, N=325
var.9.APOA4 11:116692334:C:T
peptide level
209:209
96:96
20:20
RVEPYGENFNKpc3_NP.3
p−maj=1, pX=0.38, N=325
var.9.APOA4 11:116692334:C:T
peptide level
181:209
83:96
19:20
RVEPYGENFNKpc3_NP.4
p−maj=0.49, pX=0.79, N=283
var.9.APOA4 11:116692334:C:T
peptide level
201:209
91:96
20:20
RVEPYGENFNKpc3_NP.5
p−min=1, pX=0.36, N=312
var.9.APOA4 11:116692334:C:T
peptide level
209:209
96:96
20:20
SELTQQLNALFQDKpc2_NP.1
p−maj=1, pX=0.63, N=325
var.9.APOA4 11:116692334:C:T
peptide level
209:209
96:96
20:20
SELTQQLNALFQDKpc2_NP.2
p−maj=1, pX=0.45, N=325
var.9.APOA4 11:116692334:C:T
peptide level
209:209
96:96
20:20
SELTQQLNALFQDKpc2_NP.3
p−maj=1, pX=0.92, N=325
var.9.APOA4 11:116692334:C:T
peptide level
209:209
96:96
20:20
SELTQQLNALFQDKpc2_NP.4
p−maj=1, pX=0.71, N=325
var.9.APOA4 11:116692334:C:T
peptide level
209:209
96:96
20:20
SELTQQLNALFQDKpc2_NP.5
p−maj=1, pX=0.62, N=325
var.9.APOA4 11:116692334:C:T
peptide level
28:209
7:96
5:20
SLAELGGHLDQQVEEFRpc2_NP.1
p−maj=0.084, pX=0.78, N=40
var.9.APOA4 11:116692334:C:T
peptide level
152:209
63:96
18:20
SLAELGGHLDQQVEEFRpc2_NP.2
p−maj=0.073, pX=0.66, N=233
var.9.APOA4 11:116692334:C:T
peptide level
30:209
12:96
4:20
SLAELGGHLDQQVEEFRpc2_NP.3
p−maj=0.5, pX=0.89, N=46
var.9.APOA4 11:116692334:C:T
peptide level
184:209
84:96
17:20
SLAELGGHLDQQVEEFRpc2_NP.4
p−maj=0.72, pX=0.67, N=285
var.9.APOA4 11:116692334:C:T
peptide level
205:209
95:96
20:20
SLAELGGHLDQQVEEFRpc2_NP.5
p−min=0.66, pX=0.39, N=320

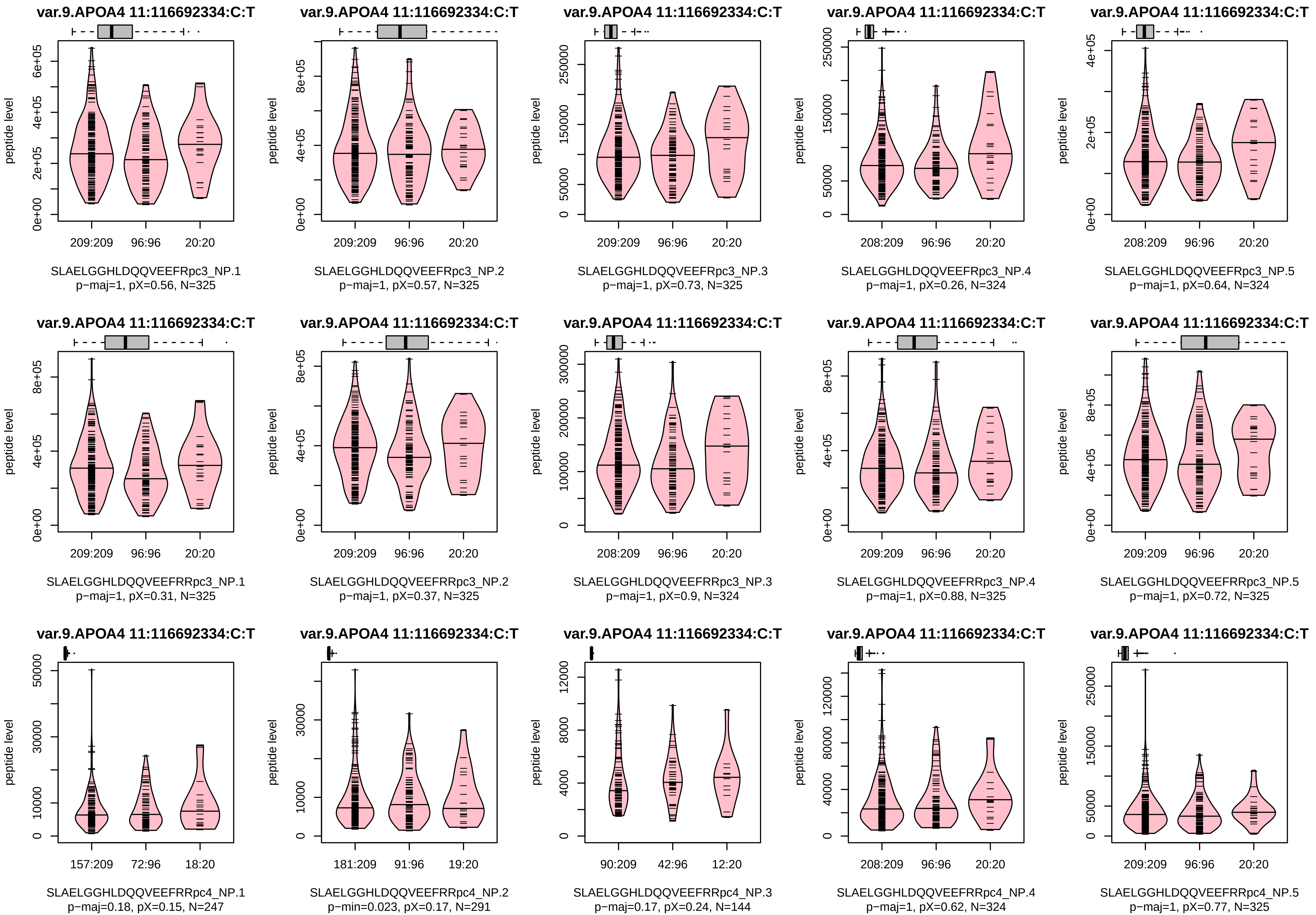
var.9.APOA4 11:116692334:C:T
peptide level
209:209
96:96
20:20
SLAELGGHLDQQVEEFRpc3_NP.1
p−maj=1, pX=0.56, N=325
var.9.APOA4 11:116692334:C:T
peptide level
209:209
96:96
20:20
SLAELGGHLDQQVEEFRpc3_NP.2
p−maj=1, pX=0.57, N=325
var.9.APOA4 11:116692334:C:T
peptide level
209:209
96:96
20:20
SLAELGGHLDQQVEEFRpc3_NP.3
p−maj=1, pX=0.73, N=325
var.9.APOA4 11:116692334:C:T
peptide level
208:209
96:96
20:20
SLAELGGHLDQQVEEFRpc3_NP.4
p−maj=1, pX=0.26, N=324
var.9.APOA4 11:116692334:C:T
peptide level
208:209
96:96
20:20
SLAELGGHLDQQVEEFRpc3_NP.5
p−maj=1, pX=0.64, N=324
var.9.APOA4 11:116692334:C:T
peptide level
209:209
96:96
20:20
SLAELGGHLDQQVEEFRRpc3_NP.1
p−maj=1, pX=0.31, N=325
var.9.APOA4 11:116692334:C:T
peptide level
209:209
96:96
20:20
SLAELGGHLDQQVEEFRRpc3_NP.2
p−maj=1, pX=0.37, N=325
var.9.APOA4 11:116692334:C:T
peptide level
208:209
96:96
20:20
SLAELGGHLDQQVEEFRRpc3_NP.3
p−maj=1, pX=0.9, N=324
var.9.APOA4 11:116692334:C:T
peptide level
209:209
96:96
20:20
SLAELGGHLDQQVEEFRRpc3_NP.4
p−maj=1, pX=0.88, N=325
var.9.APOA4 11:116692334:C:T
peptide level
209:209
96:96
20:20
SLAELGGHLDQQVEEFRRpc3_NP.5
p−maj=1, pX=0.72, N=325
var.9.APOA4 11:116692334:C:T
peptide level
157:209
72:96
18:20
SLAELGGHLDQQVEEFRRpc4_NP.1
p−maj=0.18, pX=0.15, N=247
var.9.APOA4 11:116692334:C:T
peptide level
181:209
91:96
19:20
SLAELGGHLDQQVEEFRRpc4_NP.2
p−min=0.023, pX=0.17, N=291
var.9.APOA4 11:116692334:C:T
peptide level
90:209
42:96
12:20
SLAELGGHLDQQVEEFRRpc4_NP.3
p−maj=0.17, pX=0.24, N=144
var.9.APOA4 11:116692334:C:T
peptide level
208:209
96:96
20:20
SLAELGGHLDQQVEEFRRpc4_NP.4
p−maj=1, pX=0.62, N=324
var.9.APOA4 11:116692334:C:T
peptide level
209:209
96:96
20:20
SLAELGGHLDQQVEEFRRpc4_NP.5
p−maj=1, pX=0.77, N=325

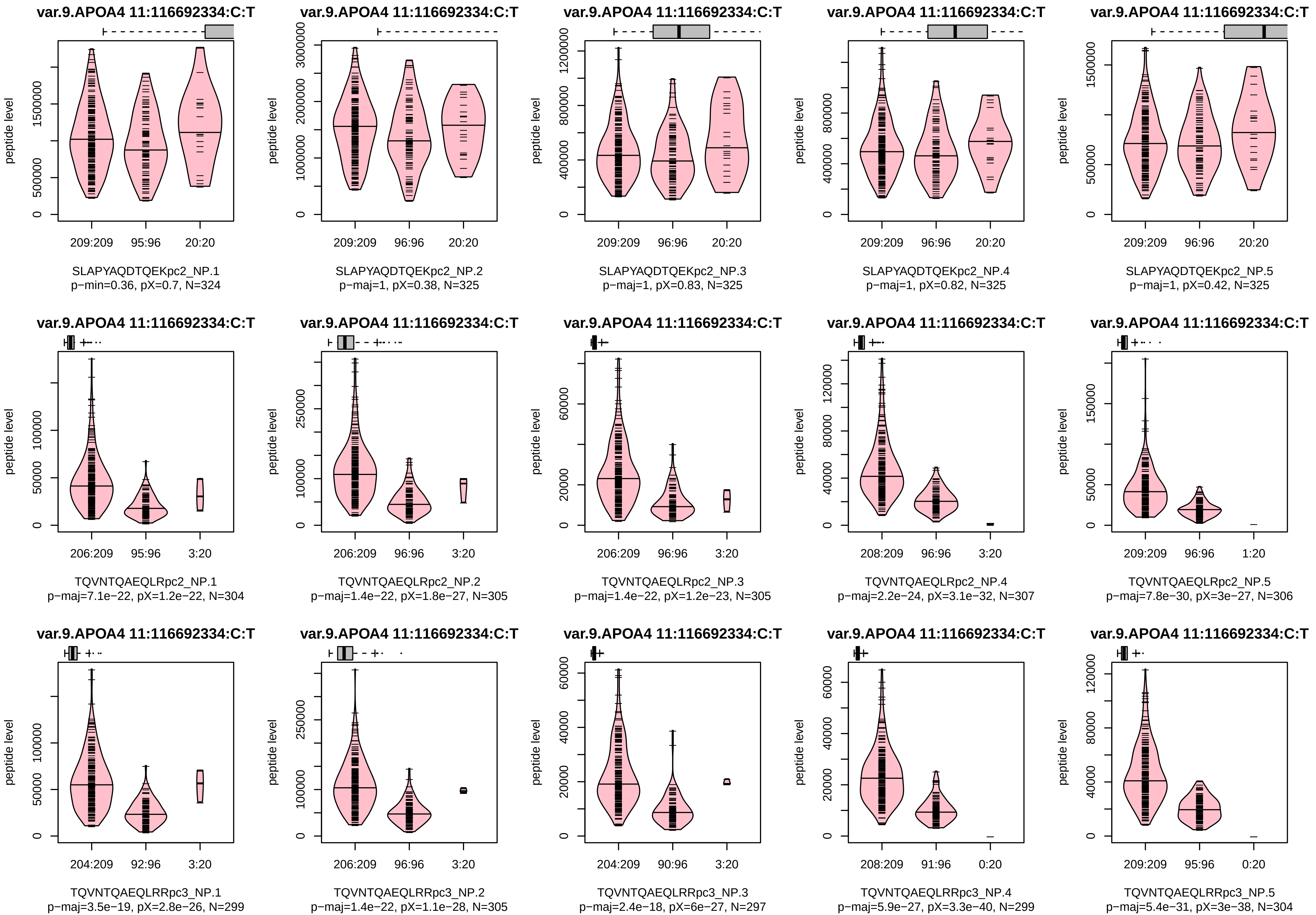
var.9.APOA4 11:116692334:C:T
peptide level
209:209
95:96
20:20
SLAPYAQDTQEKpc2_NP.1
p−min=0.36, pX=0.7, N=324
var.9.APOA4 11:116692334:C:T
209:209
96:96
20:20
SLAPYAQDTQEKpc2_NP.2
p−maj=1, pX=0.38, N=325
var.9.APOA4 11:116692334:C:T
209:209
96:96
20:20
SLAPYAQDTQEKpc2_NP.3
p−maj=1, pX=0.83, N=325
var.9.APOA4 11:116692334:C:T
209:209
96:96
20:20
SLAPYAQDTQEKpc2_NP.4
p−maj=1, pX=0.82, N=325
var.9.APOA4 11:116692334:C:T
209:209
96:96
20:20
SLAPYAQDTQEKpc2_NP.5
p−maj=1, pX=0.42, N=325
var.9.APOA4 11:116692334:C:T
206:209
95:96
3:20
TQVNTQAEQLRpc2_NP.1
p−maj=7.1e−22, pX=1.2e−22, N=304
var.9.APOA4 11:116692334:C:T
206:209
96:96
3:20
TQVNTQAEQLRpc2_NP.2
p−maj=1.4e−22, pX=1.8e−27, N=305
var.9.APOA4 11:116692334:C:T
206:209
96:96
3:20
TQVNTQAEQLRpc2_NP.3
p−maj=1.4e−22, pX=1.2e−23, N=305
var.9.APOA4 11:116692334:C:T
208:209
96:96
3:20
TQVNTQAEQLRpc2_NP.4
p−maj=2.2e−24, pX=3.1e−32, N=307
var.9.APOA4 11:116692334:C:T
209:209
96:96
1:20
TQVNTQAEQLRpc2_NP.5
p−maj=7.8e−30, pX=3e−27, N=306
var.9.APOA4 11:116692334:C:T
204:209
92:96
3:20
TQVNTQAEQLRRpc3_NP.1
p−maj=3.5e−19, pX=2.8e−26, N=299
var.9.APOA4 11:116692334:C:T
206:209
96:96
3:20
TQVNTQAEQLRRpc3_NP.2
p−maj=1.4e−22, pX=1.1e−28, N=305
var.9.APOA4 11:116692334:C:T
204:209
90:96
3:20
TQVNTQAEQLRRpc3_NP.3
p−maj=2.4e−18, pX=6e−27, N=297
var.9.APOA4 11:116692334:C:T
208:209
91:96
0:20
TQVNTQAEQLRRpc3_NP.4
p−maj=5.9e−27, pX=3.3e−40, N=299
var.9.APOA4 11:116692334:C:T
209:209
95:96
0:20
TQVNTQAEQLRRpc3_NP.5
p−maj=5.4e−31, pX=3e−38, N=304

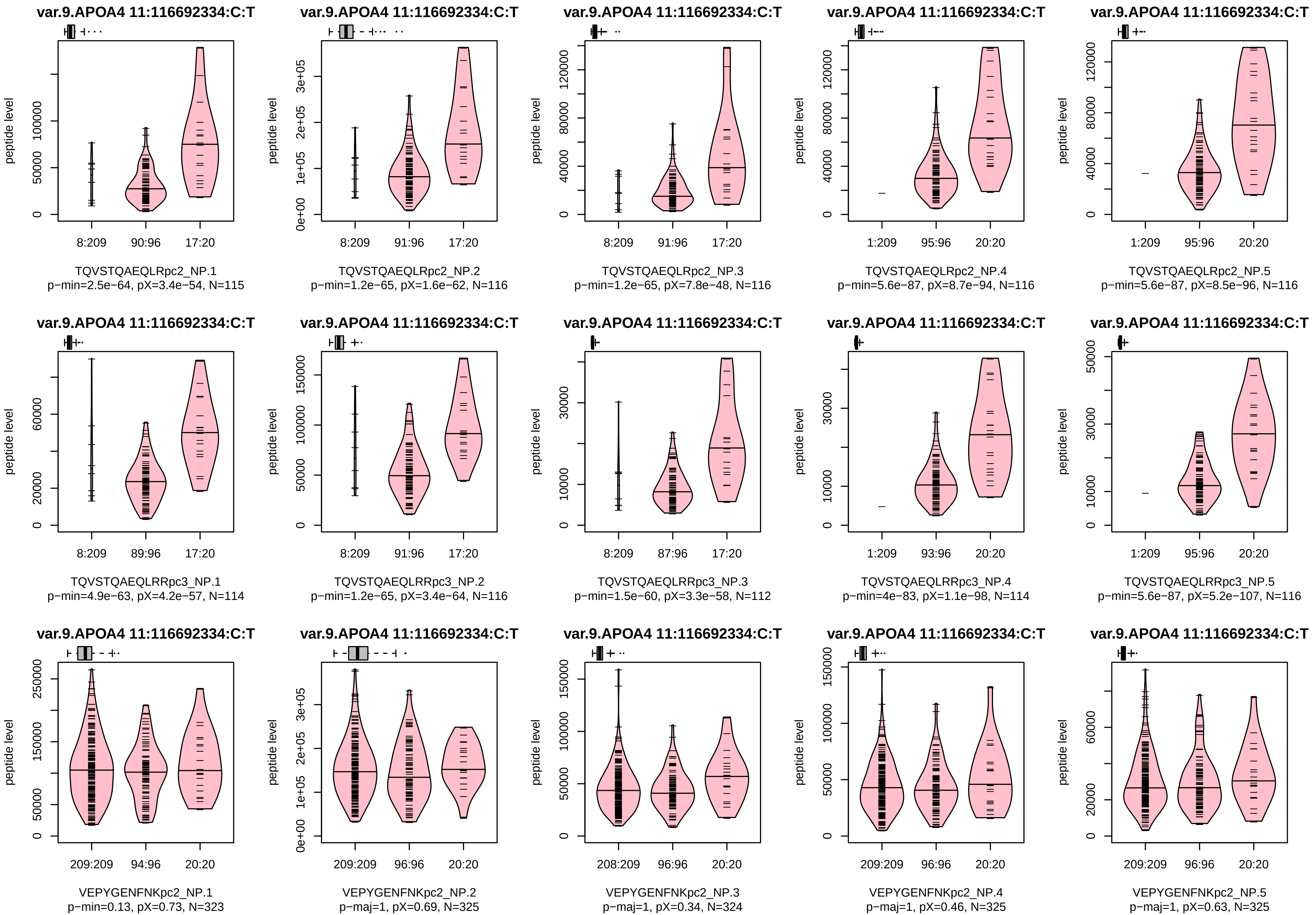
var.9.APOA4 11:116692334:C:T
peptide level
8:209
90:96
17:20
TQVSTQAEQLRpc2_NP.1
p−min=2.5e−64, pX=3.4e−54, N=115
var.9.APOA4 11:116692334:C:T
peptide level
8:209
91:96
17:20
TQVSTQAEQLRpc2_NP.2
p−min=1.2e−65, pX=1.6e−62, N=116
var.9.APOA4 11:116692334:C:T
peptide level
8:209
91:96
17:20
TQVSTQAEQLRpc2_NP.3
p−min=1.2e−65, pX=7.8e−48, N=116
var.9.APOA4 11:116692334:C:T
peptide level
1:209
95:96
20:20
TQVSTQAEQLRpc2_NP.4
p−min=5.6e−87, pX=8.7e−94, N=116
var.9.APOA4 11:116692334:C:T
peptide level
1:209
95:96
20:20
TQVSTQAEQLRpc2_NP.5
p−min=5.6e−87, pX=8.5e−96, N=116
var.9.APOA4 11:116692334:C:T
peptide level
8:209
89:96
17:20
TQVSTQAEQLRRpc3_NP.1
p−min=4.9e−63, pX=4.2e−57, N=114
var.9.APOA4 11:116692334:C:T
peptide level
8:209
91:96
17:20
TQVSTQAEQLRRpc3_NP.2
p−min=1.2e−65, pX=3.4e−64, N=116
var.9.APOA4 11:116692334:C:T
peptide level
8:209
87:96
17:20
TQVSTQAEQLRRpc3_NP.3
p−min=1.5e−60, pX=3.3e−58, N=112
var.9.APOA4 11:116692334:C:T
peptide level
1:209
93:96
20:20
TQVSTQAEQLRRpc3_NP.4
p−min=4e−83, pX=1.1e−98, N=114
var.9.APOA4 11:116692334:C:T
peptide level
1:209
95:96
20:20
TQVSTQAEQLRRpc3_NP.5
p−min=5.6e−87, pX=5.2e−107, N=116
var.9.APOA4 11:116692334:C:T
peptide level
209:209
94:96
20:20
VEPYGENFNKpc2_NP.1
p−min=0.13, pX=0.73, N=323
var.9.APOA4 11:116692334:C:T
peptide level
209:209
96:96
20:20
VEPYGENFNKpc2_NP.2
p−maj=1, pX=0.69, N=325
var.9.APOA4 11:116692334:C:T
peptide level
208:209
96:96
20:20
VEPYGENFNKpc2_NP.3
p−maj=1, pX=0.34, N=324
var.9.APOA4 11:116692334:C:T
peptide level
209:209
96:96
20:20
VEPYGENFNKpc2_NP.4
p−maj=1, pX=0.46, N=325
var.9.APOA4 11:116692334:C:T
peptide level
209:209
96:96
20:20
VEPYGENFNKpc2_NP.5
p−maj=1, pX=0.63, N=325

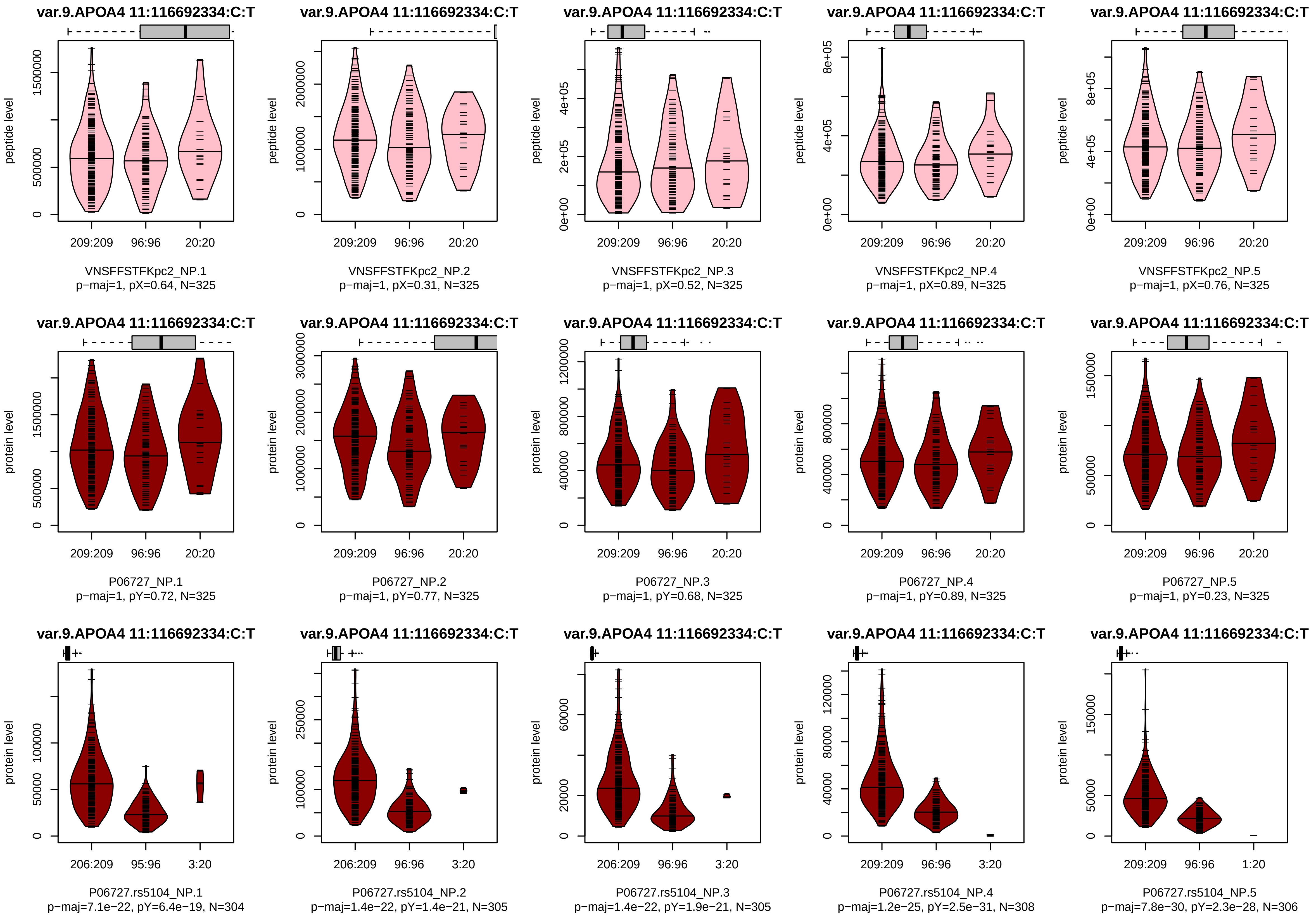
var.9.APOA4 11:116692334:C:T
peptide level
209:209
96:96
20:20
VNSFFSTFKpc2_NP.1
p−maj=1, pX=0.64, N=325
var.9.APOA4 11:116692334:C:T
peptide level
209:209
96:96
20:20
VNSFFSTFKpc2_NP.2
p−maj=1, pX=0.31, N=325
var.9.APOA4 11:116692334:C:T
peptide level
209:209
96:96
20:20
VNSFFSTFKpc2_NP.3
p−maj=1, pX=0.52, N=325
var.9.APOA4 11:116692334:C:T
peptide level
209:209
96:96
20:20
VNSFFSTFKpc2_NP.4
p−maj=1, pX=0.89, N=325
var.9.APOA4 11:116692334:C:T
peptide level
209:209
96:96
20:20
VNSFFSTFKpc2_NP.5
p−maj=1, pX=0.76, N=325
var.9.APOA4 11:116692334:C:T
protein level
209:209
96:96
20:20
P06727_NP.1
p−maj=1, pY=0.72, N=325
var.9.APOA4 11:116692334:C:T
protein level
209:209
96:96
20:20
P06727_NP.2
p−maj=1, pY=0.77, N=325
var.9.APOA4 11:116692334:C:T
protein level
209:209
96:96
20:20
P06727_NP.3
p−maj=1, pY=0.68, N=325
var.9.APOA4 11:116692334:C:T
protein level
209:209
96:96
20:20
P06727_NP.4
p−maj=1, pY=0.89, N=325
var.9.APOA4 11:116692334:C:T
protein level
209:209
96:96
20:20
P06727_NP.5
p−maj=1, pY=0.23, N=325
var.9.APOA4 11:116692334:C:T
protein level
206:209
95:96
3:20
P06727.rs5104_NP.1
p−maj=7.1e−22, pY=6.4e−19, N=304
var.9.APOA4 11:116692334:C:T
protein level
206:209
96:96
3:20
P06727.rs5104_NP.2
p−maj=1.4e−22, pY=1.4e−21, N=305
var.9.APOA4 11:116692334:C:T
protein level
206:209
96:96
3:20
P06727.rs5104_NP.3
p−maj=1.4e−22, pY=1.9e−21, N=305
var.9.APOA4 11:116692334:C:T
protein level
209:209
96:96
3:20
P06727.rs5104_NP.4
p−maj=1.2e−25, pY=2.5e−31, N=308
var.9.APOA4 11:116692334:C:T
protein level
209:209
96:96
1:20
P06727.rs5104_NP.5
p−maj=7.8e−30, pY=2.3e−28, N=306

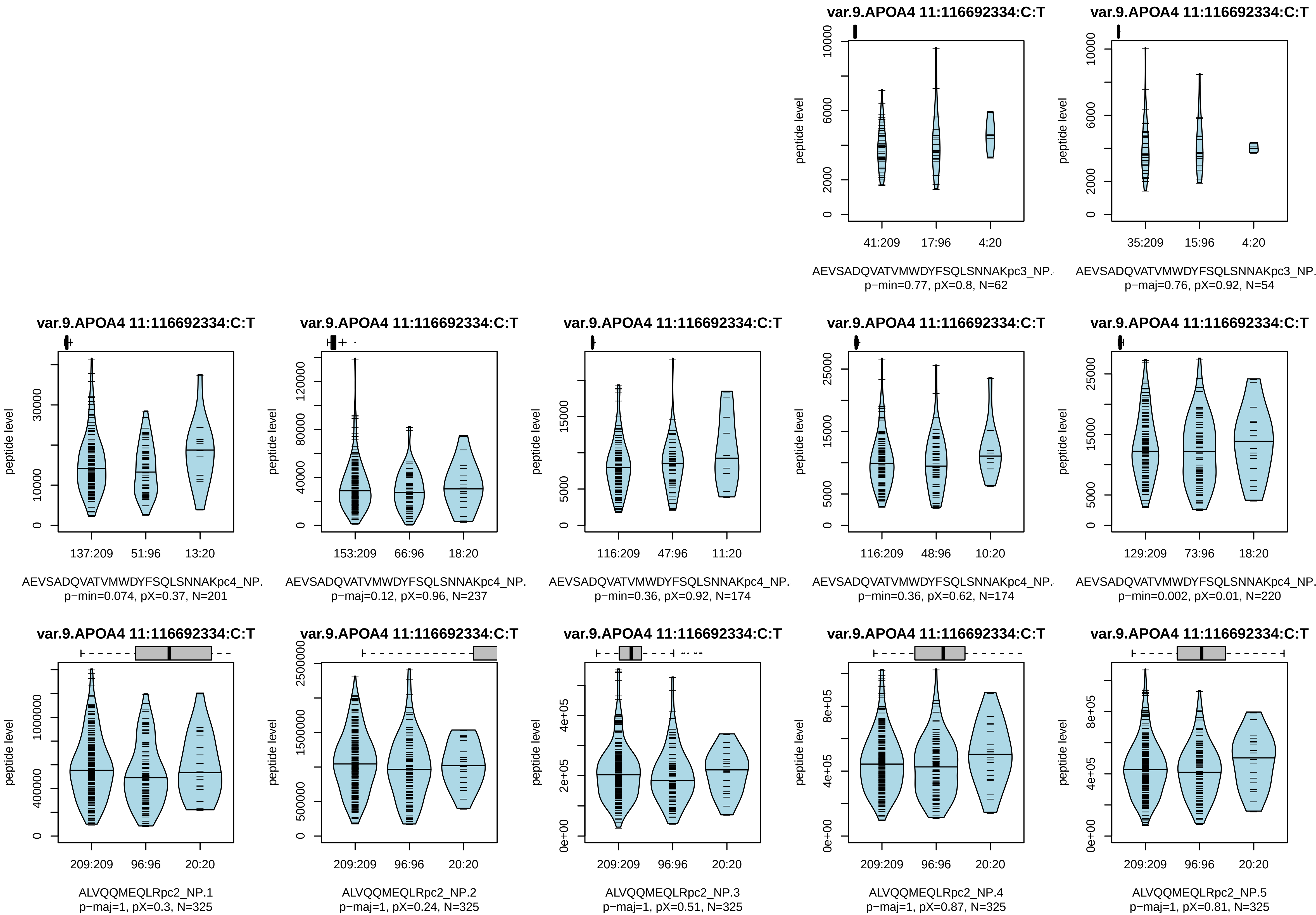
var.9.APOA4 11:116692334:C:T
peptide level
41:209
17:96
4:20
AEVSADQVATVMWDYFSQLSNNAKpc3_NP.
p−min=0.77, pX=0.8, N=62
var.9.APOA4 11:116692334:C:T
peptide level
35:209
15:96
4:20
AEVSADQVATVMWDYFSQLSNNAKpc3_NP.
p−maj=0.76, pX=0.92, N=54
var.9.APOA4 11:116692334:C:T
peptide level
137:209
51:96
13:20
AEVSADQVATVMWDYFSQLSNNAKpc4_NP.
p−min=0.074, pX=0.37, N=201
var.9.APOA4 11:116692334:C:T
peptide level
153:209
66:96
18:20
AEVSADQVATVMWDYFSQLSNNAKpc4_NP.
p−maj=0.12, pX=0.96, N=237
var.9.APOA4 11:116692334:C:T
peptide level
116:209
47:96
11:20
AEVSADQVATVMWDYFSQLSNNAKpc4_NP.
p−min=0.36, pX=0.92, N=174
var.9.APOA4 11:116692334:C:T
peptide level
116:209
48:96
10:20
AEVSADQVATVMWDYFSQLSNNAKpc4_NP.
p−min=0.36, pX=0.62, N=174
var.9.APOA4 11:116692334:C:T
peptide level
129:209
73:96
18:20
AEVSADQVATVMWDYFSQLSNNAKpc4_NP.
p−min=0.002, pX=0.01, N=220
var.9.APOA4 11:116692334:C:T
peptide level
209:209
96:96
20:20
ALVQQMEQLRpc2_NP.1
p−maj=1, pX=0.3, N=325
var.9.APOA4 11:116692334:C:T
peptide level
209:209
96:96
20:20
ALVQQMEQLRpc2_NP.2
p−maj=1, pX=0.24, N=325
var.9.APOA4 11:116692334:C:T
peptide level
209:209
96:96
20:20
ALVQQMEQLRpc2_NP.3
p−maj=1, pX=0.51, N=325
var.9.APOA4 11:116692334:C:T
peptide level
209:209
96:96
20:20
ALVQQMEQLRpc2_NP.4
p−maj=1, pX=0.87, N=325
var.9.APOA4 11:116692334:C:T
peptide level
209:209
96:96
20:20
ALVQQMEQLRpc2_NP.5
p−maj=1, pX=0.81, N=325

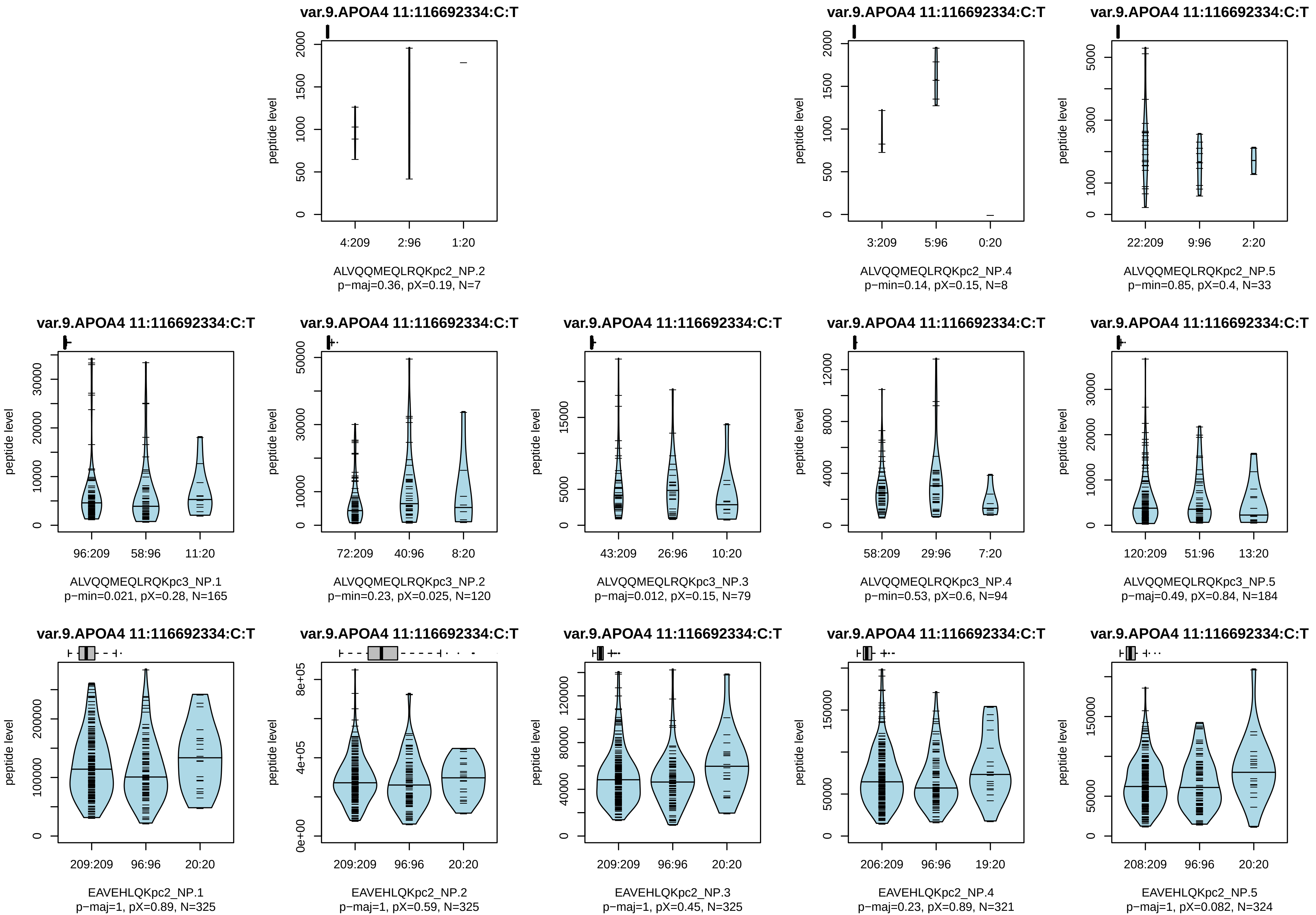
var.9.APOA4 11:116692334:C:T
peptide level
4:209
2:96
1:20
ALVQQMEQLRQKpc2_NP.2
p−maj=0.36, pX=0.19, N=7
var.9.APOA4 11:116692334:C:T
peptide level
3:209
5:96
0:20
ALVQQMEQLRQKpc2_NP.4
p−min=0.14, pX=0.15, N=8
var.9.APOA4 11:116692334:C:T
peptide level
22:209
9:96
2:20
ALVQQMEQLRQKpc2_NP.5
p−min=0.85, pX=0.4, N=33
var.9.APOA4 11:116692334:C:T
peptide level
96:209
58:96
11:20
ALVQQMEQLRQKpc3_NP.1
p−min=0.021, pX=0.28, N=165
var.9.APOA4 11:116692334:C:T
peptide level
72:209
40:96
8:20
ALVQQMEQLRQKpc3_NP.2
p−min=0.23, pX=0.025, N=120
var.9.APOA4 11:116692334:C:T
peptide level
43:209
26:96
10:20
ALVQQMEQLRQKpc3_NP.3
p−maj=0.012, pX=0.15, N=79
var.9.APOA4 11:116692334:C:T
peptide level
58:209
29:96
7:20
ALVQQMEQLRQKpc3_NP.4
p−min=0.53, pX=0.6, N=94
var.9.APOA4 11:116692334:C:T
peptide level
120:209
51:96
13:20
ALVQQMEQLRQKpc3_NP.5
p−maj=0.49, pX=0.84, N=184
var.9.APOA4 11:116692334:C:T
peptide level
209:209
96:96
20:20
EAVEHLQKpc2_NP.1
p−maj=1, pX=0.89, N=325
var.9.APOA4 11:116692334:C:T
peptide level
209:209
96:96
20:20
EAVEHLQKpc2_NP.2
p−maj=1, pX=0.59, N=325
var.9.APOA4 11:116692334:C:T
peptide level
209:209
96:96
20:20
EAVEHLQKpc2_NP.3
p−maj=1, pX=0.45, N=325
var.9.APOA4 11:116692334:C:T
peptide level
206:209
96:96
19:20
EAVEHLQKpc2_NP.4
p−maj=0.23, pX=0.89, N=321
var.9.APOA4 11:116692334:C:T
peptide level
208:209
96:96
20:20
EAVEHLQKpc2_NP.5
p−maj=1, pX=0.082, N=324

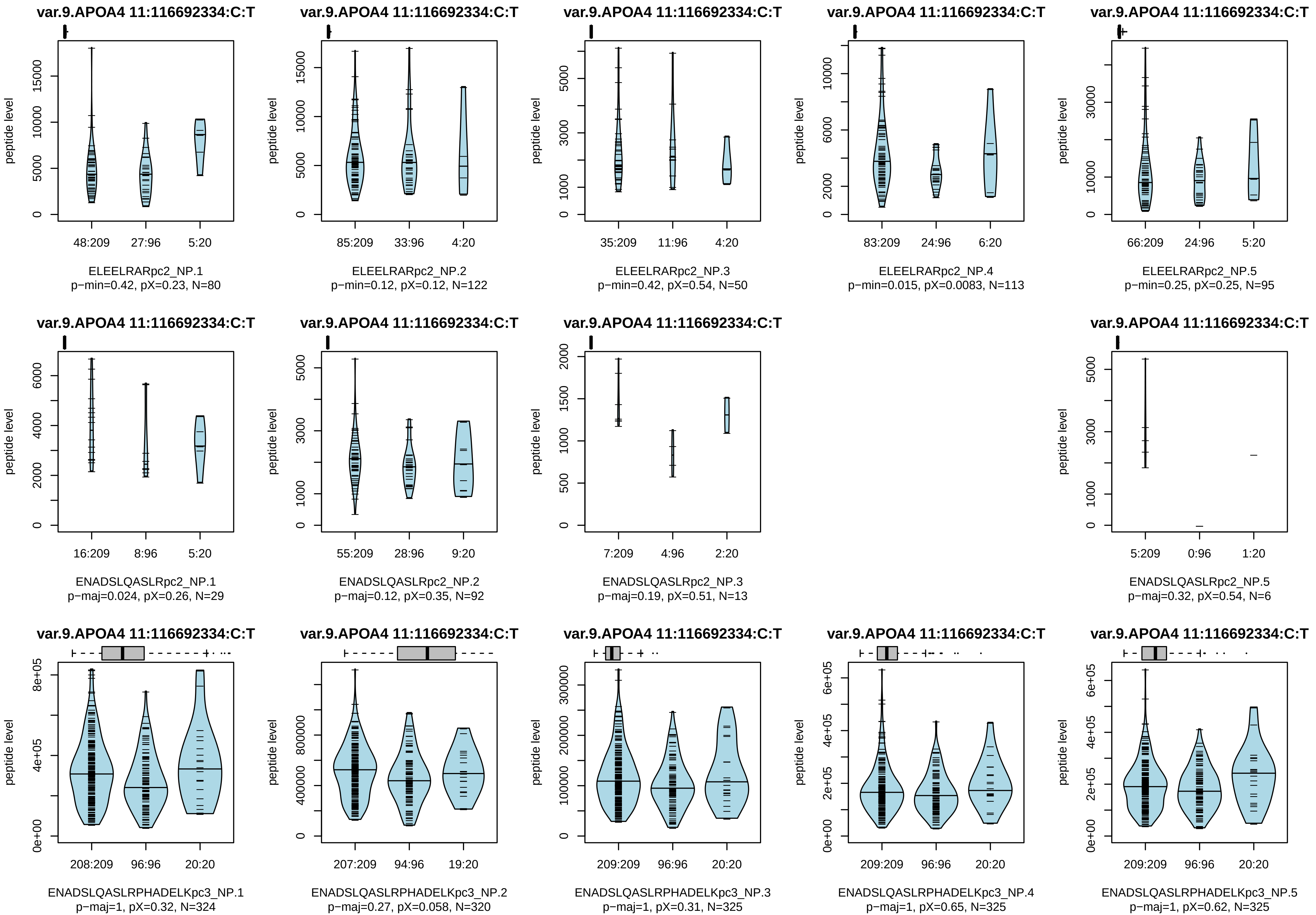

var.9.APOA4 11:116692334:C:T
peptide level
48:209
27:96
5:20
ELEELRARpc2_NP.1
p−min=0.42, pX=0.23, N=80
85:209
33:96
4:20
ELEELRARpc2_NP.2
p−min=0.12, pX=0.12, N=122
35:209
11:96
4:20
ELEELRARpc2_NP.3
p−min=0.42, pX=0.54, N=50
83:209
24:96
6:20
ELEELRARpc2_NP.4
p−min=0.015, pX=0.0083, N=113
66:209
24:96
5:20
ELEELRARpc2_NP.5
p−min=0.25, pX=0.25, N=95
16:209
8:96
5:20
ENADSLQASLRpc2_NP.1
p−maj=0.024, pX=0.26, N=29
55:209
28:96
9:20
ENADSLQASLRpc2_NP.2
p−maj=0.12, pX=0.35, N=92
7:209
4:96
2:20
ENADSLQASLRpc2_NP.3
p−maj=0.19, pX=0.51, N=13
5:209
0:96
1:20
ENADSLQASLRpc2_NP.5
p−maj=0.32, pX=0.54, N=6
208:209
96:96
20:20
ENADSLQASLRPHADELKpc3_NP.1
p−maj=1, pX=0.32, N=324
207:209
94:96
19:20
ENADSLQASLRPHADELKpc3_NP.2
p−maj=0.27, pX=0.058, N=320
209:209
96:96
20:20
ENADSLQASLRPHADELKpc3_NP.3
p−maj=1, pX=0.31, N=325
209:209
96:96
20:20
ENADSLQASLRPHADELKpc3_NP.4
p−maj=1, pX=0.65, N=325
209:209
96:96
20:20
ENADSLQASLRPHADELKpc3_NP.5
p−maj=1, pX=0.62, N=325

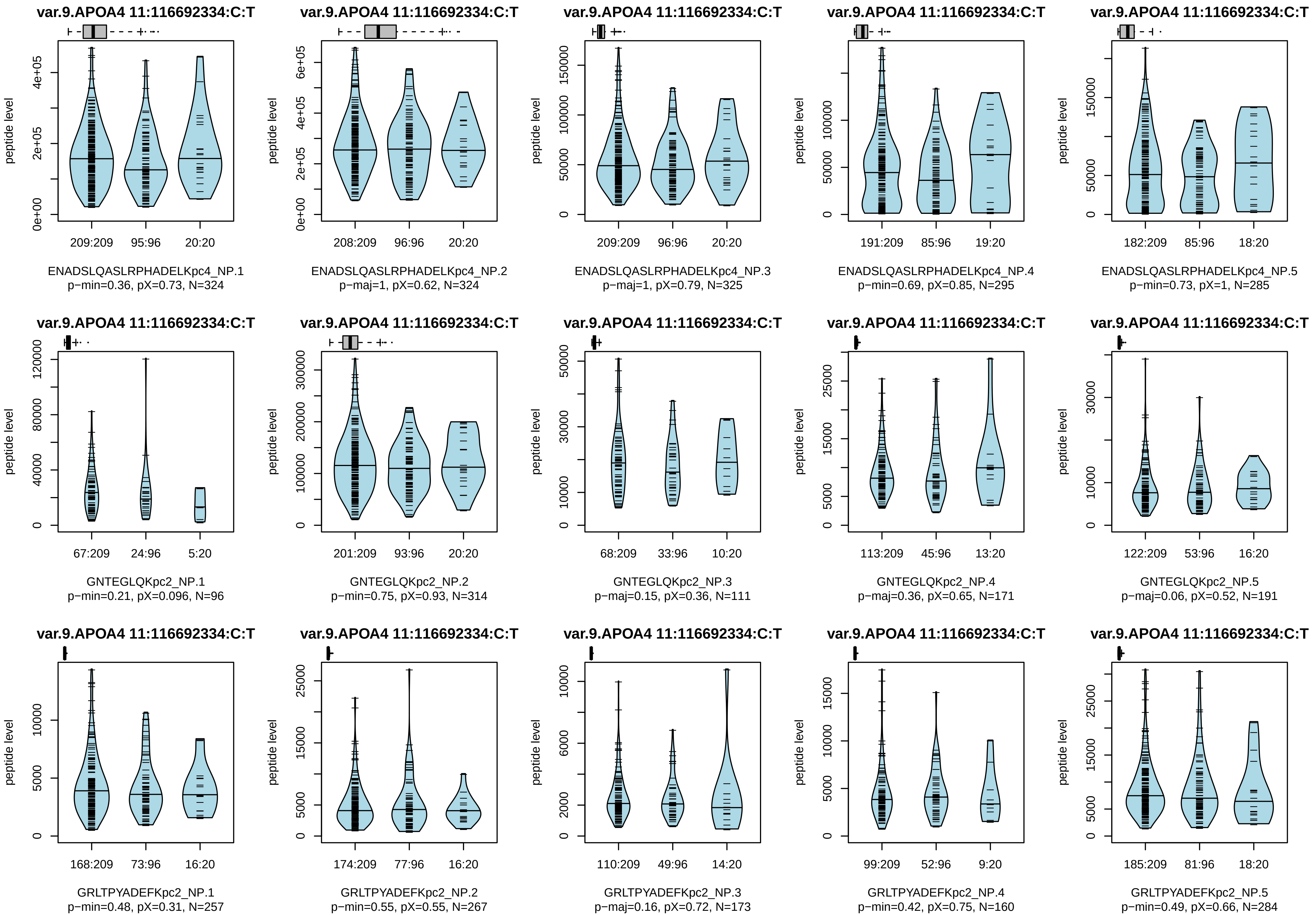

var.9.APOA4 11:116692334:C:T
peptide level
209:209
95:96
20:20
ENADSLQASLRPHADELKpc4_NP.1
p−min=0.36, pX=0.73, N=324
var.9.APOA4 11:116692334:C:T
peptide level
208:209
96:96
20:20
ENADSLQASLRPHADELKpc4_NP.2
p−maj=1, pX=0.62, N=324
var.9.APOA4 11:116692334:C:T
peptide level
209:209
96:96
20:20
ENADSLQASLRPHADELKpc4_NP.3
p−maj=1, pX=0.79, N=325
var.9.APOA4 11:116692334:C:T
peptide level
191:209
85:96
19:20
ENADSLQASLRPHADELKpc4_NP.4
p−min=0.69, pX=0.85, N=295
var.9.APOA4 11:116692334:C:T
peptide level
182:209
85:96
18:20
ENADSLQASLRPHADELKpc4_NP.5
p−min=0.73, pX=1, N=285
var.9.APOA4 11:116692334:C:T
peptide level
67:209
24:96
5:20
GNTEGLQKpc2_NP.1
p−min=0.21, pX=0.096, N=96
var.9.APOA4 11:116692334:C:T
peptide level
201:209
93:96
20:20
GNTEGLQKpc2_NP.2
p−min=0.75, pX=0.93, N=314
var.9.APOA4 11:116692334:C:T
peptide level
68:209
33:96
10:20
GNTEGLQKpc2_NP.3
p−maj=0.15, pX=0.36, N=111
var.9.APOA4 11:116692334:C:T
peptide level
113:209
45:96
13:20
GNTEGLQKpc2_NP.4
p−maj=0.36, pX=0.65, N=171
var.9.APOA4 11:116692334:C:T
peptide level
122:209
53:96
16:20
GNTEGLQKpc2_NP.5
p−maj=0.06, pX=0.52, N=191
var.9.APOA4 11:116692334:C:T
peptide level
168:209
73:96
16:20
GRLTPYADEFKpc2_NP.1
p−min=0.48, pX=0.31, N=257
var.9.APOA4 11:116692334:C:T
peptide level
174:209
77:96
16:20
GRLTPYADEFKpc2_NP.2
p−min=0.55, pX=0.55, N=267
var.9.APOA4 11:116692334:C:T
peptide level
110:209
49:96
14:20
GRLTPYADEFKpc2_NP.3
p−maj=0.16, pX=0.72, N=173
var.9.APOA4 11:116692334:C:T
peptide level
99:209
52:96
9:20
GRLTPYADEFKpc2_NP.4
p−min=0.42, pX=0.75, N=160
var.9.APOA4 11:116692334:C:T
peptide level
185:209
81:96
18:20
GRLTPYADEFKpc2_NP.5
p−min=0.49, pX=0.66, N=284

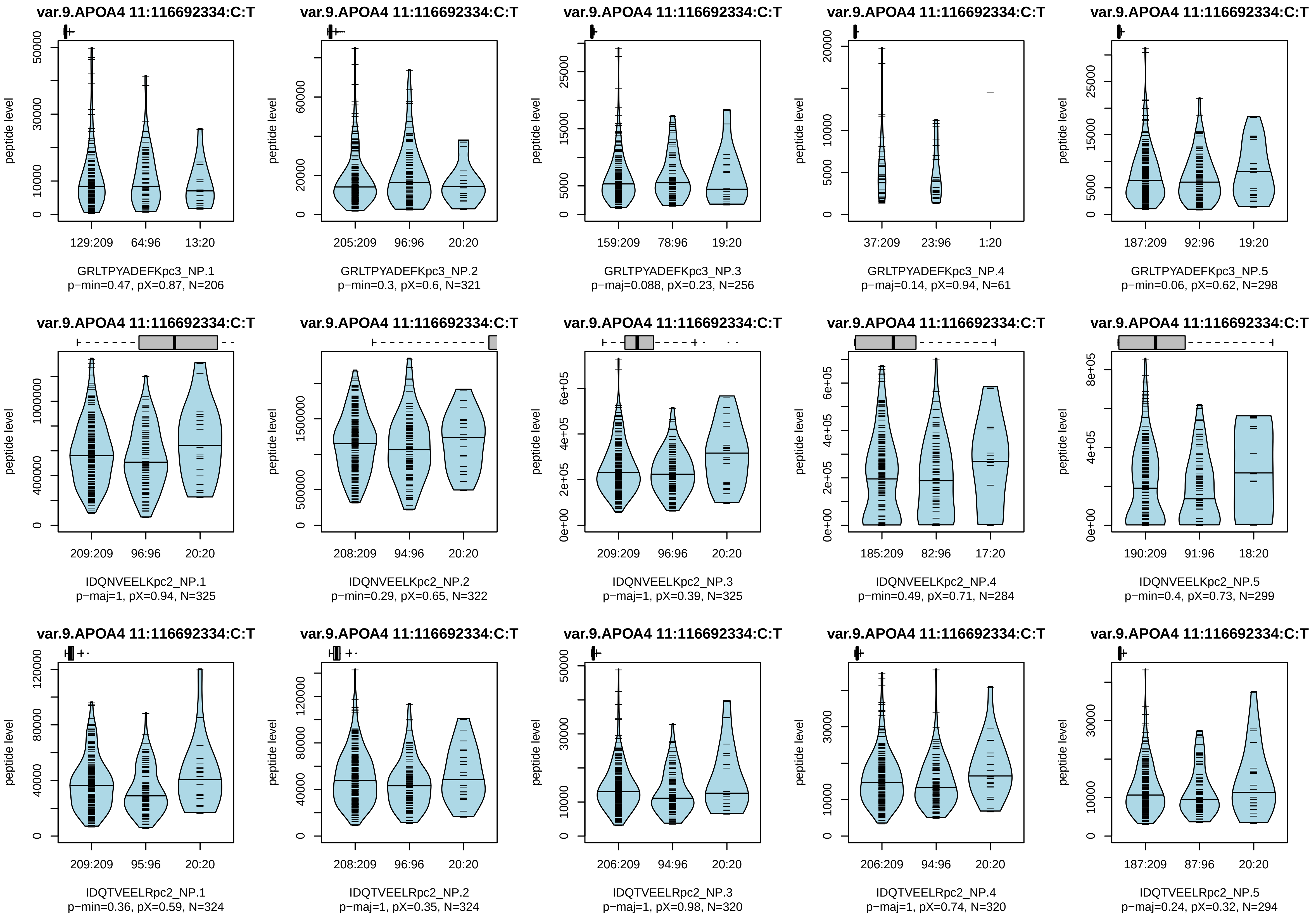
var.9.APOA4 11:116692334:C:T
peptide level
129:209
64:96
13:20
GRLTPYADEFKpc3_NP.1
p−min=0.47, pX=0.87, N=206
var.9.APOA4 11:116692334:C:T
peptide level
205:209
96:96
20:20
GRLTPYADEFKpc3_NP.2
p−min=0.3, pX=0.6, N=321
var.9.APOA4 11:116692334:C:T
peptide level
159:209
78:96
19:20
GRLTPYADEFKpc3_NP.3
p−maj=0.088, pX=0.23, N=256
var.9.APOA4 11:116692334:C:T
peptide level
37:209
23:96
1:20
GRLTPYADEFKpc3_NP.4
p−maj=0.14, pX=0.94, N=61
var.9.APOA4 11:116692334:C:T
peptide level
187:209
92:96
19:20
GRLTPYADEFKpc3_NP.5
p−min=0.06, pX=0.62, N=298
var.9.APOA4 11:116692334:C:T
peptide level
209:209
96:96
20:20
IDQNVEELKpc2_NP.1
p−maj=1, pX=0.94, N=325
var.9.APOA4 11:116692334:C:T
peptide level
208:209
94:96
20:20
IDQNVEELKpc2_NP.2
p−min=0.29, pX=0.65, N=322
var.9.APOA4 11:116692334:C:T
peptide level
209:209
96:96
20:20
IDQNVEELKpc2_NP.3
p−maj=1, pX=0.39, N=325
var.9.APOA4 11:116692334:C:T
peptide level
185:209
82:96
17:20
IDQNVEELKpc2_NP.4
p−min=0.49, pX=0.71, N=284
var.9.APOA4 11:116692334:C:T
peptide level
190:209
91:96
18:20
IDQNVEELKpc2_NP.5
p−min=0.4, pX=0.73, N=299
var.9.APOA4 11:116692334:C:T
peptide level
209:209
95:96
20:20
IDQTVEELRpc2_NP.1
p−min=0.36, pX=0.59, N=324
var.9.APOA4 11:116692334:C:T
peptide level
208:209
96:96
20:20
IDQTVEELRpc2_NP.2
p−maj=1, pX=0.35, N=324
var.9.APOA4 11:116692334:C:T
peptide level
206:209
94:96
20:20
IDQTVEELRpc2_NP.3
p−maj=1, pX=0.98, N=320
var.9.APOA4 11:116692334:C:T
peptide level
206:209
94:96
20:20
IDQTVEELRpc2_NP.4
p−maj=1, pX=0.74, N=320
var.9.APOA4 11:116692334:C:T
peptide level
187:209
87:96
20:20
IDQTVEELRpc2_NP.5
p−maj=0.24, pX=0.32, N=294

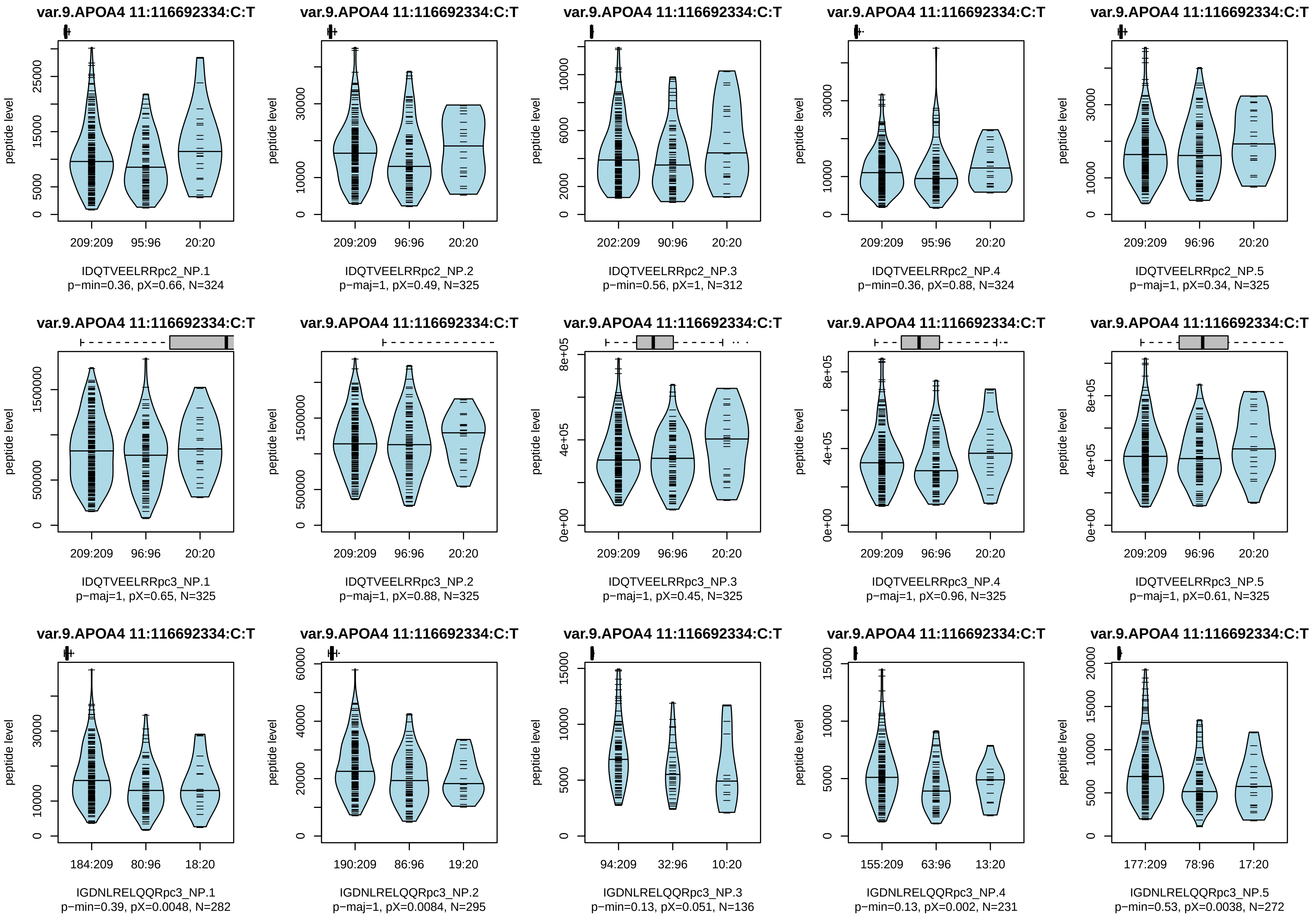
var.9.APOA4 11:116692334:C:T
peptide level
209:209
95:96
20:20
IDQTVEELRRpc2_NP.1
p−min=0.36, pX=0.66, N=324
var.9.APOA4 11:116692334:C:T
peptide level
209:209
96:96
20:20
IDQTVEELRRpc2_NP.2
p−maj=1, pX=0.49, N=325
var.9.APOA4 11:116692334:C:T
peptide level
202:209
90:96
20:20
IDQTVEELRRpc2_NP.3
p−min=0.56, pX=1, N=312
var.9.APOA4 11:116692334:C:T
peptide level
209:209
95:96
20:20
IDQTVEELRRpc2_NP.4
p−min=0.36, pX=0.88, N=324
var.9.APOA4 11:116692334:C:T
peptide level
209:209
96:96
20:20
IDQTVEELRRpc2_NP.5
p−maj=1, pX=0.34, N=325
var.9.APOA4 11:116692334:C:T
peptide level
209:209
96:96
20:20
IDQTVEELRRpc3_NP.1
p−maj=1, pX=0.65, N=325
var.9.APOA4 11:116692334:C:T
peptide level
209:209
96:96
20:20
IDQTVEELRRpc3_NP.2
p−maj=1, pX=0.88, N=325
var.9.APOA4 11:116692334:C:T
peptide level
209:209
96:96
20:20
IDQTVEELRRpc3_NP.3
p−maj=1, pX=0.45, N=325
var.9.APOA4 11:116692334:C:T
peptide level
209:209
96:96
20:20
IDQTVEELRRpc3_NP.4
p−maj=1, pX=0.96, N=325
var.9.APOA4 11:116692334:C:T
peptide level
209:209
96:96
20:20
IDQTVEELRRpc3_NP.5
p−maj=1, pX=0.61, N=325
var.9.APOA4 11:116692334:C:T
peptide level
184:209
80:96
18:20
IGDNLRELQQRpc3_NP.1
p−min=0.39, pX=0.0048, N=282
var.9.APOA4 11:116692334:C:T
peptide level
190:209
86:96
19:20
IGDNLRELQQRpc3_NP.2
p−maj=1, pX=0.0084, N=295
var.9.APOA4 11:116692334:C:T
peptide level
94:209
32:96
10:20
IGDNLRELQQRpc3_NP.3
p−min=0.13, pX=0.051, N=136
var.9.APOA4 11:116692334:C:T
peptide level
155:209
63:96
13:20
IGDNLRELQQRpc3_NP.4
p−min=0.13, pX=0.002, N=231
var.9.APOA4 11:116692334:C:T
peptide level
177:209
78:96
17:20
IGDNLRELQQRpc3_NP.5
p−min=0.53, pX=0.0038, N=272

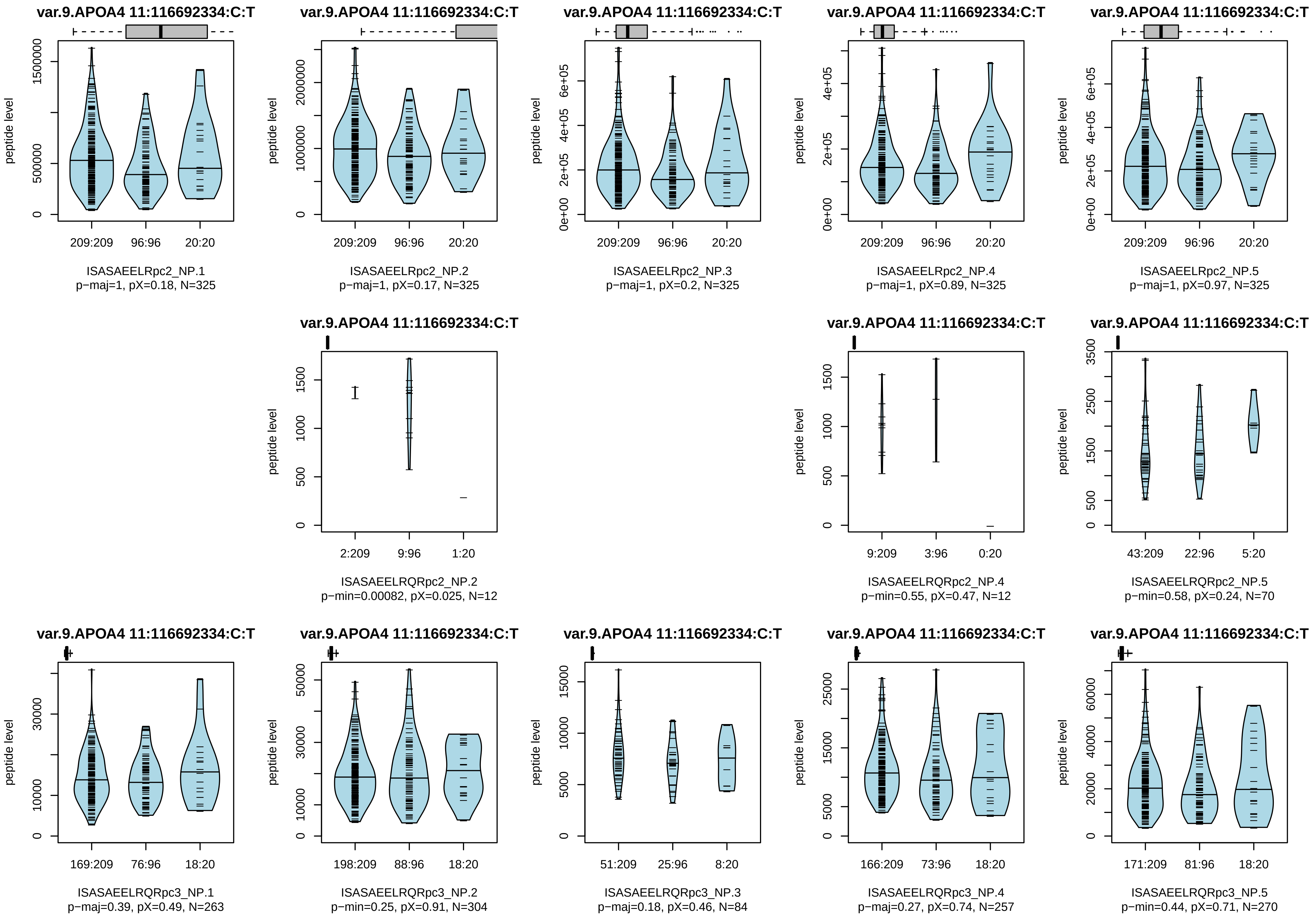
var.9.APOA4 11:116692334:C:T
peptide level
209:209
96:96
20:20
ISASAEELRpc2_NP.1
p−maj=1, pX=0.18, N=325
var.9.APOA4 11:116692334:C:T
209:209
96:96
20:20
ISASAEELRpc2_NP.2
p−maj=1, pX=0.17, N=325
var.9.APOA4 11:116692334:C:T
209:209
96:96
20:20
ISASAEELRpc2_NP.3
p−maj=1, pX=0.2, N=325
var.9.APOA4 11:116692334:C:T
209:209
96:96
20:20
ISASAEELRpc2_NP.4
p−maj=1, pX=0.89, N=325
var.9.APOA4 11:116692334:C:T
209:209
96:96
20:20
ISASAEELRpc2_NP.5
p−maj=1, pX=0.97, N=325
var.9.APOA4 11:116692334:C:T
2:209
9:96
1:20
ISASAEELRQRpc2_NP.2
p−min=0.00082, pX=0.025, N=12
var.9.APOA4 11:116692334:C:T
9:209
3:96
0:20
ISASAEELRQRpc2_NP.4
p−min=0.55, pX=0.47, N=12
var.9.APOA4 11:116692334:C:T
43:209
22:96
5:20
ISASAEELRQRpc2_NP.5
p−min=0.58, pX=0.24, N=70
var.9.APOA4 11:116692334:C:T
169:209
76:96
18:20
ISASAEELRQRpc3_NP.1
p−maj=0.39, pX=0.49, N=263
var.9.APOA4 11:116692334:C:T
198:209
88:96
18:20
ISASAEELRQRpc3_NP.2
p−min=0.25, pX=0.91, N=304
var.9.APOA4 11:116692334:C:T
51:209
25:96
8:20
ISASAEELRQRpc3_NP.3
p−maj=0.18, pX=0.46, N=84
var.9.APOA4 11:116692334:C:T
166:209
73:96
18:20
ISASAEELRQRpc3_NP.4
p−maj=0.27, pX=0.74, N=257
var.9.APOA4 11:116692334:C:T
171:209
81:96
18:20
ISASAEELRQRpc3_NP.5
p−min=0.44, pX=0.71, N=270

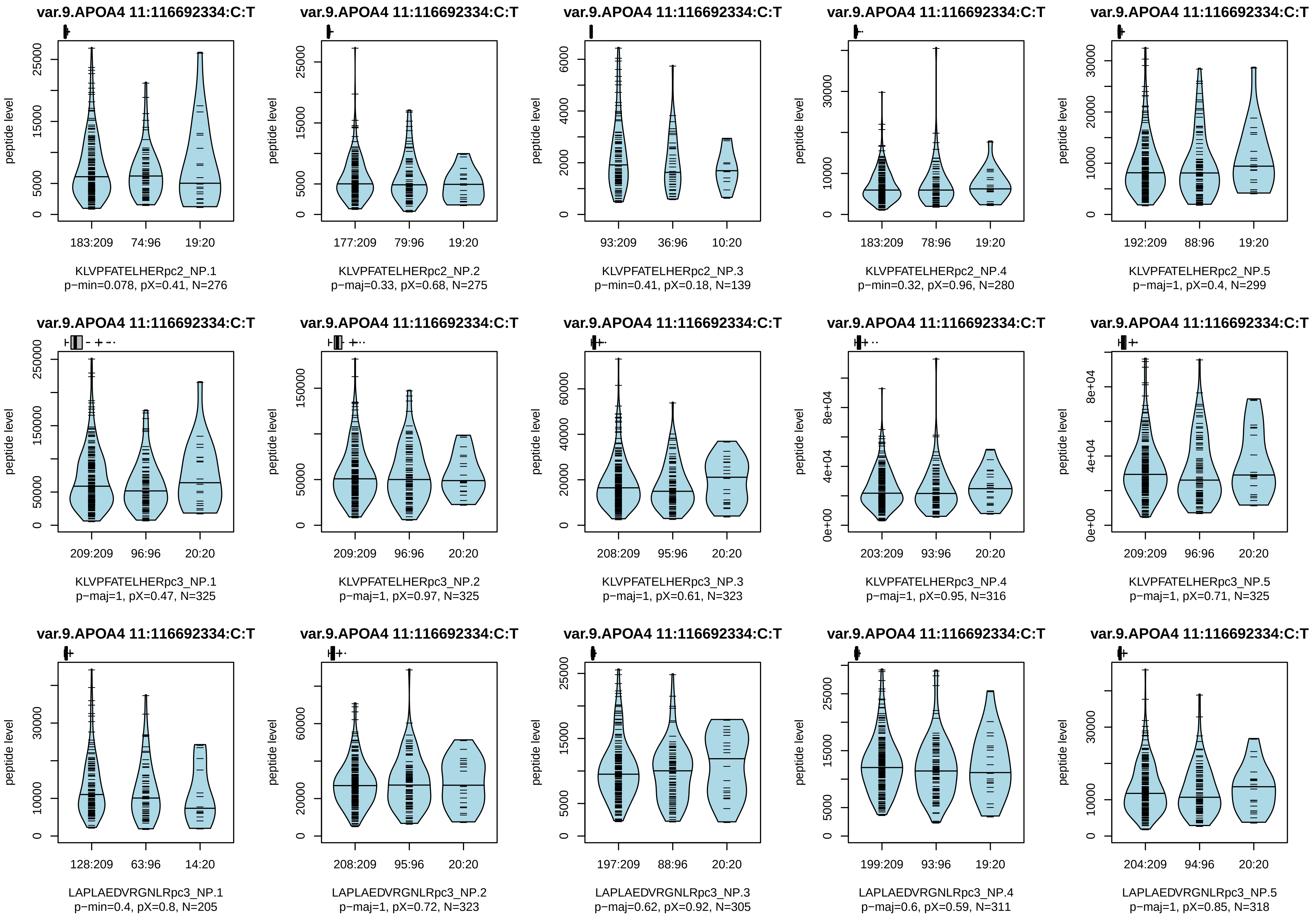
var.9.APOA4 11:116692334:C:T
peptide level
183:209
74:96
19:20
KLVPFATELHERpc2_NP.1
p−min=0.078, pX=0.41, N=276
var.9.APOA4 11:116692334:C:T
peptide level
177:209
79:96
19:20
KLVPFATELHERpc2_NP.2
p−maj=0.33, pX=0.68, N=275
var.9.APOA4 11:116692334:C:T
peptide level
93:209
36:96
10:20
KLVPFATELHERpc2_NP.3
p−min=0.41, pX=0.18, N=139
var.9.APOA4 11:116692334:C:T
peptide level
183:209
78:96
19:20
KLVPFATELHERpc2_NP.4
p−min=0.32, pX=0.96, N=280
var.9.APOA4 11:116692334:C:T
peptide level
192:209
88:96
19:20
KLVPFATELHERpc2_NP.5
p−maj=1, pX=0.4, N=299
var.9.APOA4 11:116692334:C:T
peptide level
209:209
96:96
20:20
KLVPFATELHERpc3_NP.1
p−maj=1, pX=0.47, N=325
var.9.APOA4 11:116692334:C:T
peptide level
209:209
96:96
20:20
KLVPFATELHERpc3_NP.2
p−maj=1, pX=0.97, N=325
var.9.APOA4 11:116692334:C:T
peptide level
208:209
95:96
20:20
KLVPFATELHERpc3_NP.3
p−maj=1, pX=0.61, N=323
var.9.APOA4 11:116692334:C:T
peptide level
203:209
93:96
20:20
KLVPFATELHERpc3_NP.4
p−maj=1, pX=0.95, N=316
var.9.APOA4 11:116692334:C:T
peptide level
209:209
96:96
20:20
KLVPFATELHERpc3_NP.5
p−maj=1, pX=0.71, N=325
var.9.APOA4 11:116692334:C:T
peptide level
128:209
63:96
14:20
LAPLAEDVRGNLRpc3_NP.1
p−min=0.4, pX=0.8, N=205
var.9.APOA4 11:116692334:C:T
peptide level
208:209
95:96
20:20
LAPLAEDVRGNLRpc3_NP.2
p−maj=1, pX=0.72, N=323
var.9.APOA4 11:116692334:C:T
peptide level
197:209
88:96
20:20
LAPLAEDVRGNLRpc3_NP.3
p−maj=0.62, pX=0.92, N=305
var.9.APOA4 11:116692334:C:T
peptide level
199:209
93:96
19:20
LAPLAEDVRGNLRpc3_NP.4
p−maj=0.6, pX=0.59, N=311
var.9.APOA4 11:116692334:C:T
peptide level
204:209
94:96
20:20
LAPLAEDVRGNLRpc3_NP.5
p−maj=1, pX=0.85, N=318

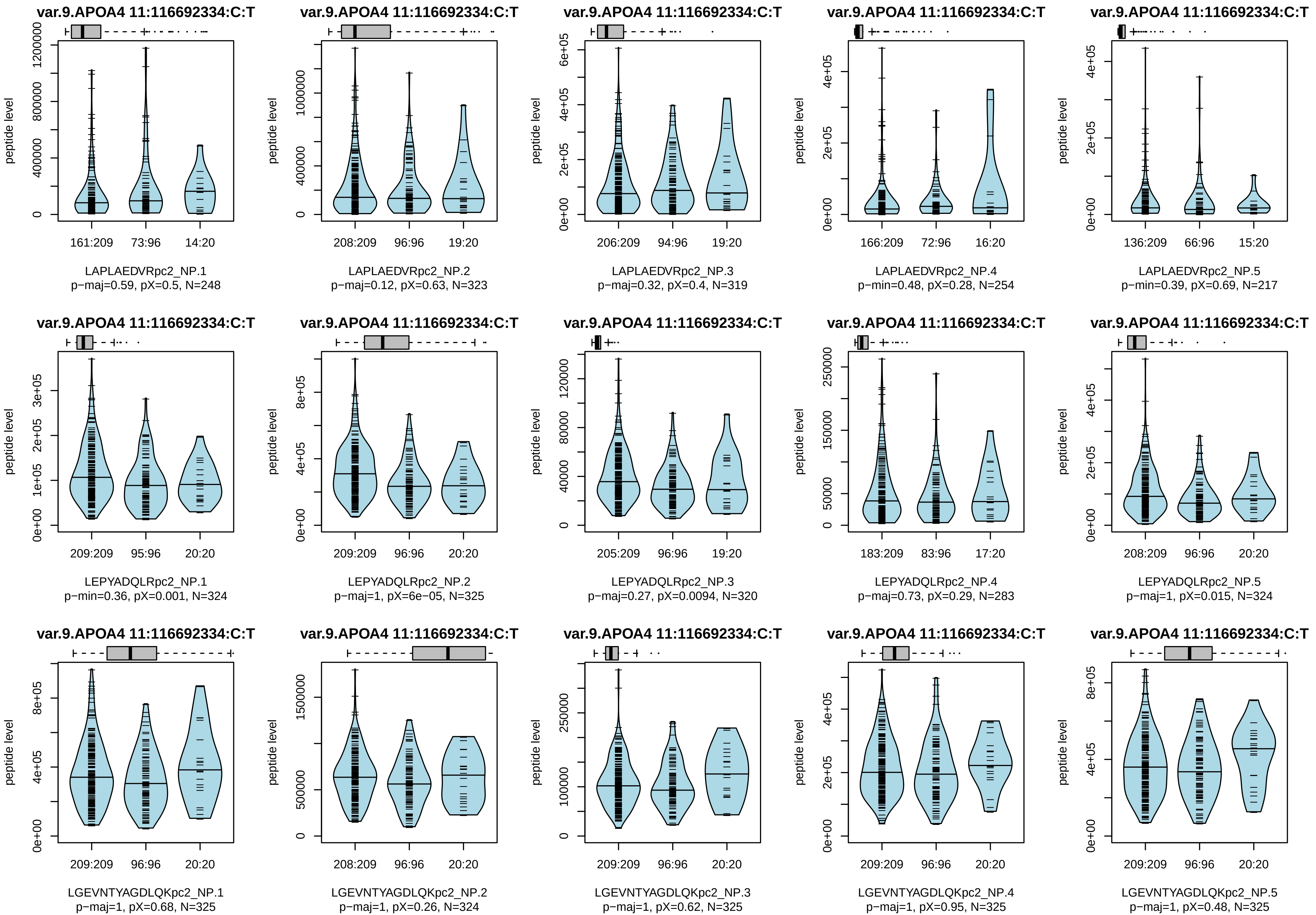

var.9.APOA4 11:116692334:C:T
peptide level
161:209
73:96
14:20
LAPLAEDVRpc2_NP.1
p−maj=0.59, pX=0.5, N=248
var.9.APOA4 11:116692334:C:T
peptide level
208:209
96:96
19:20
LAPLAEDVRpc2_NP.2
p−maj=0.12, pX=0.63, N=323
var.9.APOA4 11:116692334:C:T
peptide level
206:209
94:96
19:20
LAPLAEDVRpc2_NP.3
p−maj=0.32, pX=0.4, N=319
var.9.APOA4 11:116692334:C:T
peptide level
166:209
72:96
16:20
LAPLAEDVRpc2_NP.4
p−min=0.48, pX=0.28, N=254
var.9.APOA4 11:116692334:C:T
peptide level
136:209
66:96
15:20
LAPLAEDVRpc2_NP.5
p−min=0.39, pX=0.69, N=217
var.9.APOA4 11:116692334:C:T
peptide level
209:209
95:96
20:20
LEPYADQLRpc2_NP.1
p−min=0.36, pX=0.001, N=324
var.9.APOA4 11:116692334:C:T
peptide level
209:209
96:96
20:20
LEPYADQLRpc2_NP.2
p−maj=1, pX=6e−05, N=325
var.9.APOA4 11:116692334:C:T
peptide level
205:209
96:96
19:20
LEPYADQLRpc2_NP.3
p−maj=0.27, pX=0.0094, N=320
var.9.APOA4 11:116692334:C:T
peptide level
183:209
83:96
17:20
LEPYADQLRpc2_NP.4
p−maj=0.73, pX=0.29, N=283
var.9.APOA4 11:116692334:C:T
peptide level
208:209
96:96
20:20
LEPYADQLRpc2_NP.5
p−maj=1, pX=0.015, N=324
var.9.APOA4 11:116692334:C:T
peptide level
209:209
96:96
20:20
LGEVNTYAGDLQKpc2_NP.1
p−maj=1, pX=0.68, N=325
var.9.APOA4 11:116692334:C:T
peptide level
208:209
96:96
20:20
LGEVNTYAGDLQKpc2_NP.2
p−maj=1, pX=0.26, N=324
var.9.APOA4 11:116692334:C:T
peptide level
209:209
96:96
20:20
LGEVNTYAGDLQKpc2_NP.3
p−maj=1, pX=0.62, N=325
var.9.APOA4 11:116692334:C:T
peptide level
209:209
96:96
20:20
LGEVNTYAGDLQKpc2_NP.4
p−maj=1, pX=0.95, N=325
var.9.APOA4 11:116692334:C:T
peptide level
209:209
96:96
20:20
LGEVNTYAGDLQKpc2_NP.5
p−maj=1, pX=0.48, N=325

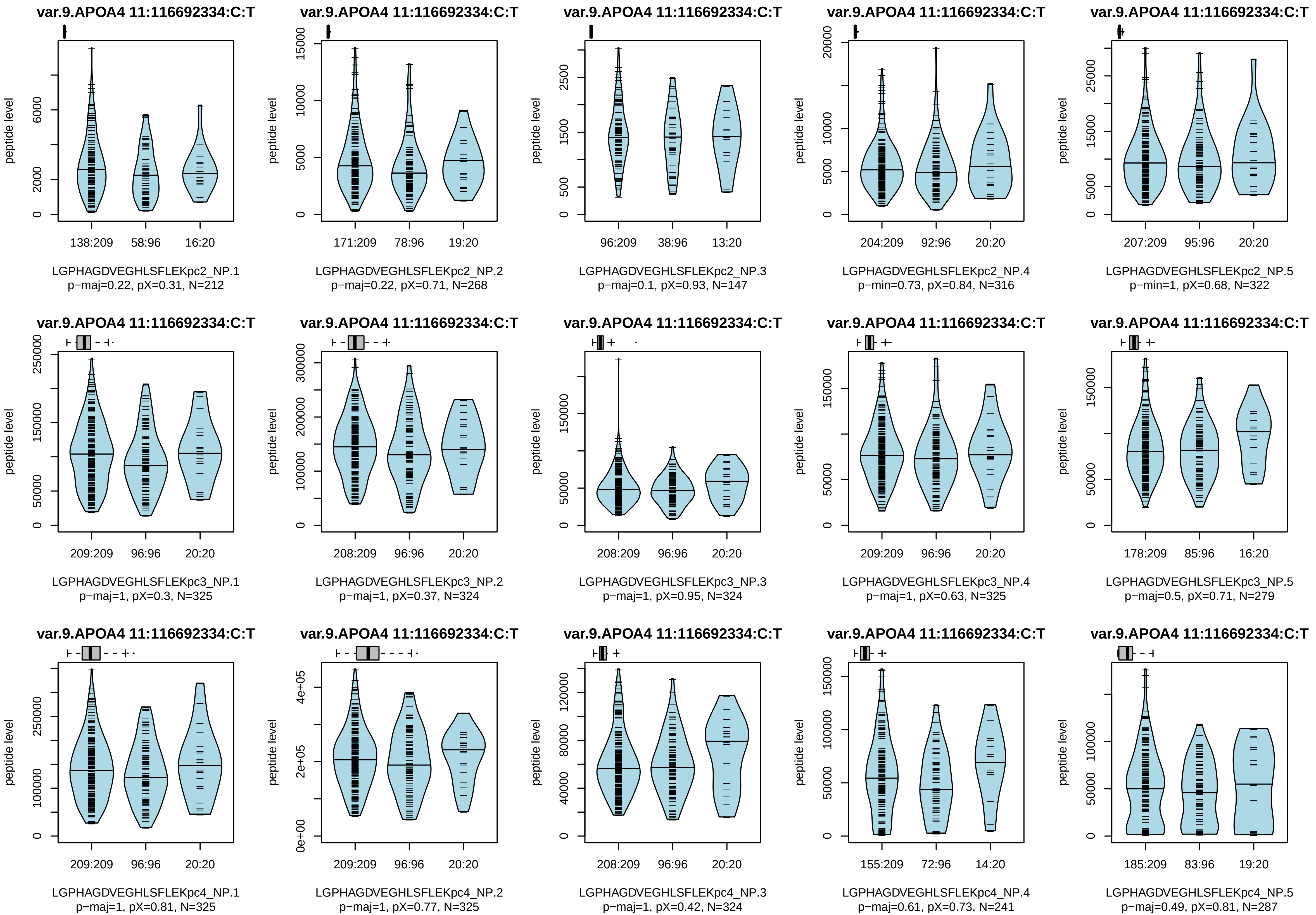
var.9.APOA4 11:116692334:C:T
peptide level
138:209
58:96
16:20
LGPHAGDVEGHLSFLEKpc2_NP.1
p−maj=0.22, pX=0.31, N=212
var.9.APOA4 11:116692334:C:T
peptide level
171:209
78:96
19:20
LGPHAGDVEGHLSFLEKpc2_NP.2
p−maj=0.22, pX=0.71, N=268
var.9.APOA4 11:116692334:C:T
peptide level
96:209
38:96
13:20
LGPHAGDVEGHLSFLEKpc2_NP.3
p−maj=0.1, pX=0.93, N=147
var.9.APOA4 11:116692334:C:T
peptide level
204:209
92:96
20:20
LGPHAGDVEGHLSFLEKpc2_NP.4
p−min=0.73, pX=0.84, N=316
var.9.APOA4 11:116692334:C:T
peptide level
207:209
95:96
20:20
LGPHAGDVEGHLSFLEKpc2_NP.5
p−min=1, pX=0.68, N=322
var.9.APOA4 11:116692334:C:T
peptide level
209:209
96:96
20:20
LGPHAGDVEGHLSFLEKpc3_NP.1
p−maj=1, pX=0.3, N=325
var.9.APOA4 11:116692334:C:T
peptide level
208:209
96:96
20:20
LGPHAGDVEGHLSFLEKpc3_NP.2
p−maj=1, pX=0.37, N=324
var.9.APOA4 11:116692334:C:T
peptide level
208:209
96:96
20:20
LGPHAGDVEGHLSFLEKpc3_NP.3
p−maj=1, pX=0.95, N=324
var.9.APOA4 11:116692334:C:T
peptide level
209:209
96:96
20:20
LGPHAGDVEGHLSFLEKpc3_NP.4
p−maj=1, pX=0.63, N=325
var.9.APOA4 11:116692334:C:T
peptide level
178:209
85:96
16:20
LGPHAGDVEGHLSFLEKpc3_NP.5
p−maj=0.5, pX=0.71, N=279
var.9.APOA4 11:116692334:C:T
peptide level
209:209
96:96
20:20
LGPHAGDVEGHLSFLEKpc4_NP.1
p−maj=1, pX=0.81, N=325
var.9.APOA4 11:116692334:C:T
peptide level
209:209
96:96
20:20
LGPHAGDVEGHLSFLEKpc4_NP.2
p−maj=1, pX=0.77, N=325
var.9.APOA4 11:116692334:C:T
peptide level
208:209
96:96
20:20
LGPHAGDVEGHLSFLEKpc4_NP.3
p−maj=1, pX=0.42, N=324
var.9.APOA4 11:116692334:C:T
peptide level
155:209
72:96
14:20
LGPHAGDVEGHLSFLEKpc4_NP.4
p−maj=0.61, pX=0.73, N=241
var.9.APOA4 11:116692334:C:T
peptide level
185:209
83:96
19:20
LGPHAGDVEGHLSFLEKpc4_NP.5
p−maj=0.49, pX=0.81, N=287

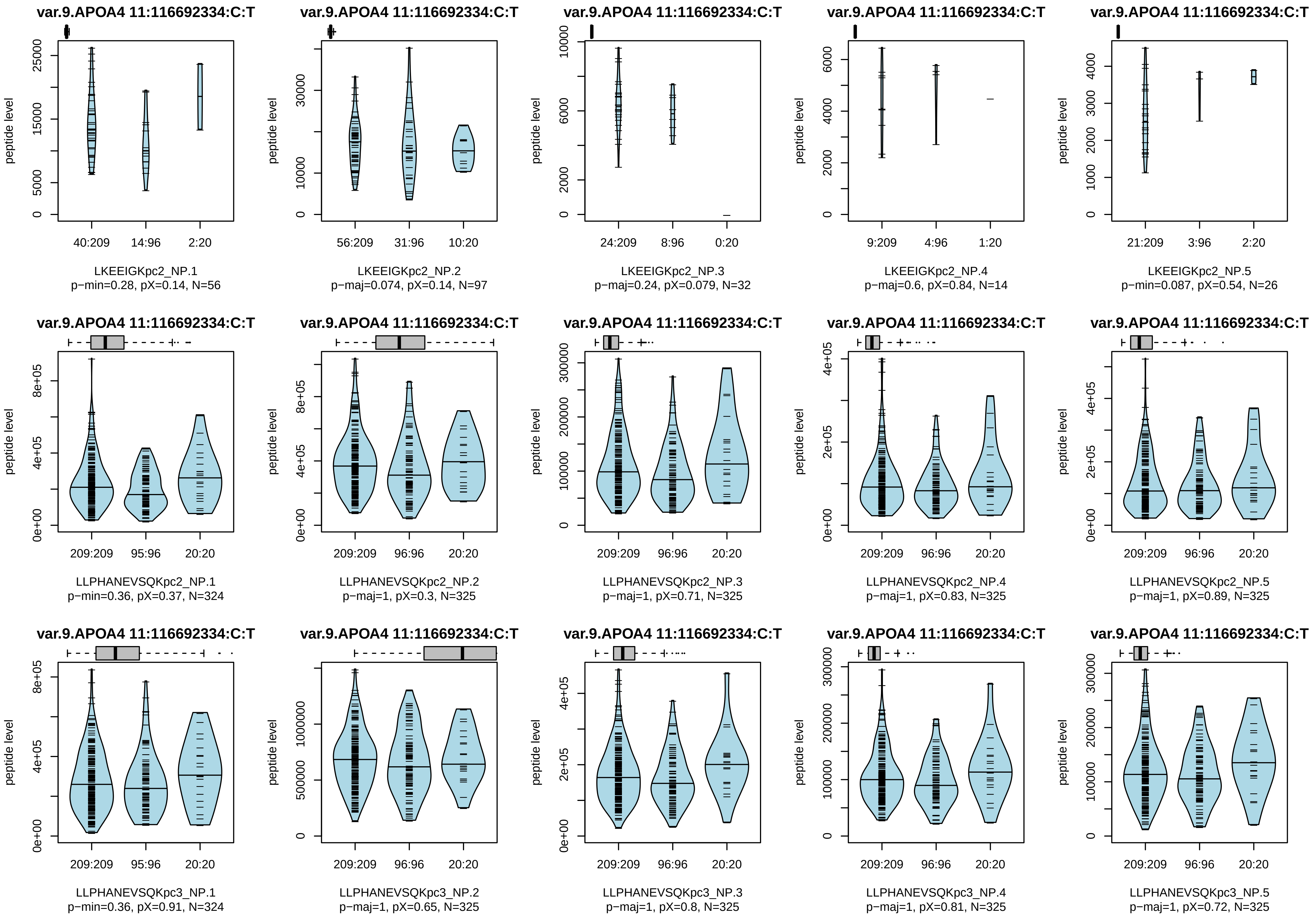
var.9.APOA4 11:116692334:C:T
peptide level
40:209
14:96
2:20
LKEEIGKpc2_NP.1
p−min=0.28, pX=0.14, N=56
var.9.APOA4 11:116692334:C:T
peptide level
56:209
31:96
10:20
LKEEIGKpc2_NP.2
p−maj=0.074, pX=0.14, N=97
var.9.APOA4 11:116692334:C:T
peptide level
24:209
8:96
0:20
LKEEIGKpc2_NP.3
p−maj=0.24, pX=0.079, N=32
var.9.APOA4 11:116692334:C:T
peptide level
9:209
4:96
1:20
LKEEIGKpc2_NP.4
p−maj=0.6, pX=0.84, N=14
var.9.APOA4 11:116692334:C:T
peptide level
21:209
3:96
2:20
LKEEIGKpc2_NP.5
p−min=0.087, pX=0.54, N=26
var.9.APOA4 11:116692334:C:T
peptide level
209:209
95:96
20:20
LLPHANEVSQKpc2_NP.1
p−min=0.36, pX=0.37, N=324
var.9.APOA4 11:116692334:C:T
peptide level
209:209
96:96
20:20
LLPHANEVSQKpc2_NP.2
p−maj=1, pX=0.3, N=325
var.9.APOA4 11:116692334:C:T
peptide level
209:209
96:96
20:20
LLPHANEVSQKpc2_NP.3
p−maj=1, pX=0.71, N=325
var.9.APOA4 11:116692334:C:T
peptide level
209:209
96:96
20:20
LLPHANEVSQKpc2_NP.4
p−maj=1, pX=0.83, N=325
var.9.APOA4 11:116692334:C:T
peptide level
209:209
96:96
20:20
LLPHANEVSQKpc2_NP.5
p−maj=1, pX=0.89, N=325
var.9.APOA4 11:116692334:C:T
peptide level
209:209
95:96
20:20
LLPHANEVSQKpc3_NP.1
p−min=0.36, pX=0.91, N=324
var.9.APOA4 11:116692334:C:T
peptide level
209:209
96:96
20:20
LLPHANEVSQKpc3_NP.2
p−maj=1, pX=0.65, N=325
var.9.APOA4 11:116692334:C:T
peptide level
209:209
96:96
20:20
LLPHANEVSQKpc3_NP.3
p−maj=1, pX=0.8, N=325
var.9.APOA4 11:116692334:C:T
peptide level
209:209
96:96
20:20
LLPHANEVSQKpc3_NP.4
p−maj=1, pX=0.81, N=325
var.9.APOA4 11:116692334:C:T
peptide level
209:209
96:96
20:20
LLPHANEVSQKpc3_NP.5
p−maj=1, pX=0.72, N=325

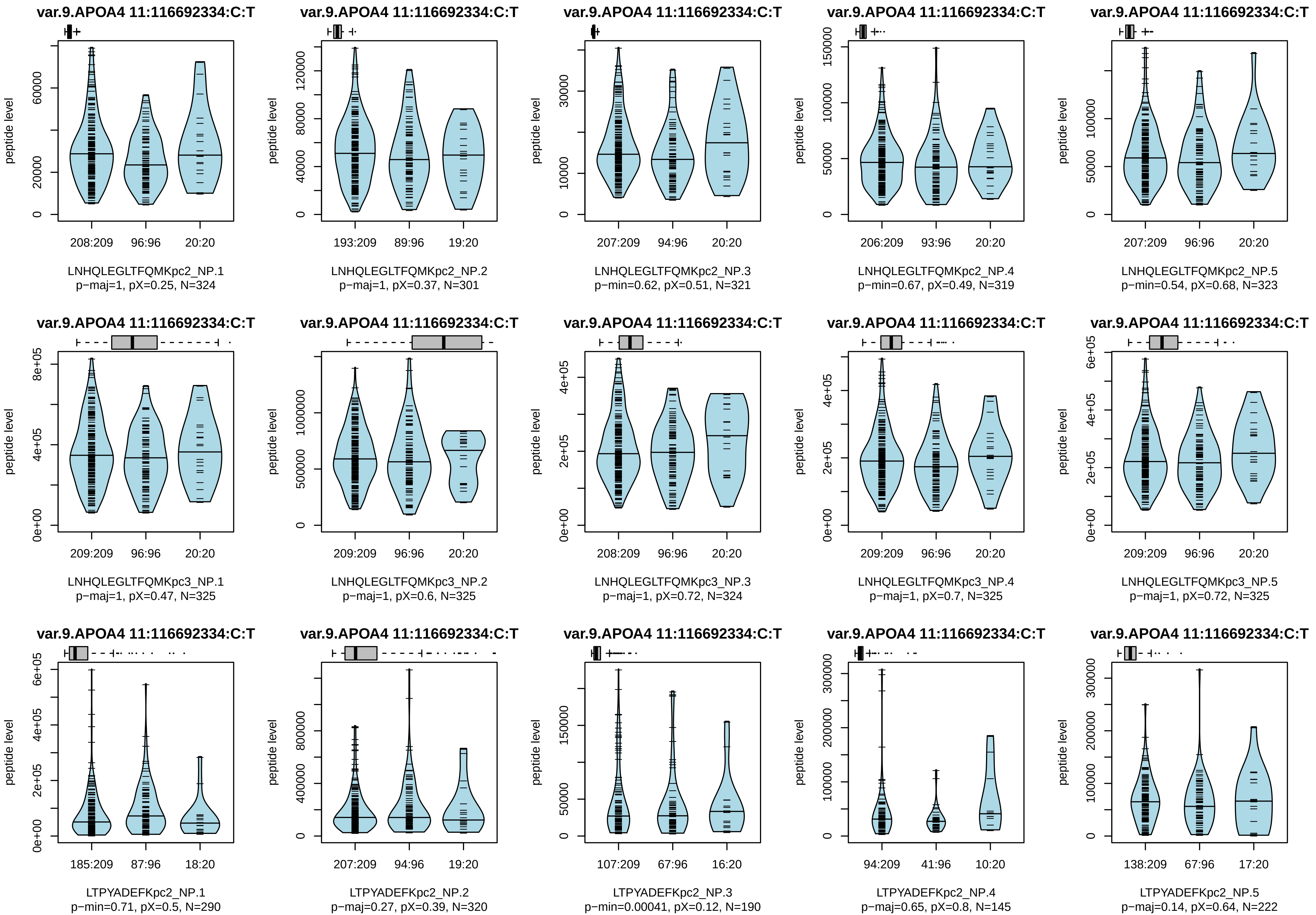
var.9.APOA4 11:116692334:C:T
peptide level
208:209
96:96
20:20
LNHQLEGLTFQMKpc2_NP.1
p−maj=1, pX=0.25, N=324
var.9.APOA4 11:116692334:C:T
peptide level
193:209
89:96
19:20
LNHQLEGLTFQMKpc2_NP.2
p−maj=1, pX=0.37, N=301
var.9.APOA4 11:116692334:C:T
peptide level
207:209
94:96
20:20
LNHQLEGLTFQMKpc2_NP.3
p−min=0.62, pX=0.51, N=321
var.9.APOA4 11:116692334:C:T
peptide level
206:209
93:96
20:20
LNHQLEGLTFQMKpc2_NP.4
p−min=0.67, pX=0.49, N=319
var.9.APOA4 11:116692334:C:T
peptide level
207:209
96:96
20:20
LNHQLEGLTFQMKpc2_NP.5
p−min=0.54, pX=0.68, N=323
var.9.APOA4 11:116692334:C:T
peptide level
209:209
96:96
20:20
LNHQLEGLTFQMKpc3_NP.1
p−maj=1, pX=0.47, N=325
var.9.APOA4 11:116692334:C:T
peptide level
209:209
96:96
20:20
LNHQLEGLTFQMKpc3_NP.2
p−maj=1, pX=0.6, N=325
var.9.APOA4 11:116692334:C:T
peptide level
208:209
96:96
20:20
LNHQLEGLTFQMKpc3_NP.3
p−maj=1, pX=0.72, N=324
var.9.APOA4 11:116692334:C:T
peptide level
209:209
96:96
20:20
LNHQLEGLTFQMKpc3_NP.4
p−maj=1, pX=0.7, N=325
var.9.APOA4 11:116692334:C:T
peptide level
209:209
96:96
20:20
LNHQLEGLTFQMKpc3_NP.5
p−maj=1, pX=0.72, N=325
var.9.APOA4 11:116692334:C:T
peptide level
185:209
87:96
18:20
LTPYADEFKpc2_NP.1
p−min=0.71, pX=0.5, N=290
var.9.APOA4 11:116692334:C:T
peptide level
207:209
94:96
19:20
LTPYADEFKpc2_NP.2
p−maj=0.27, pX=0.39, N=320
var.9.APOA4 11:116692334:C:T
peptide level
107:209
67:96
16:20
LTPYADEFKpc2_NP.3
p−min=0.00041, pX=0.12, N=190
var.9.APOA4 11:116692334:C:T
peptide level
94:209
41:96
10:20
LTPYADEFKpc2_NP.4
p−maj=0.65, pX=0.8, N=145
var.9.APOA4 11:116692334:C:T
peptide level
138:209
67:96
17:20
LTPYADEFKpc2_NP.5
p−maj=0.14, pX=0.64, N=222

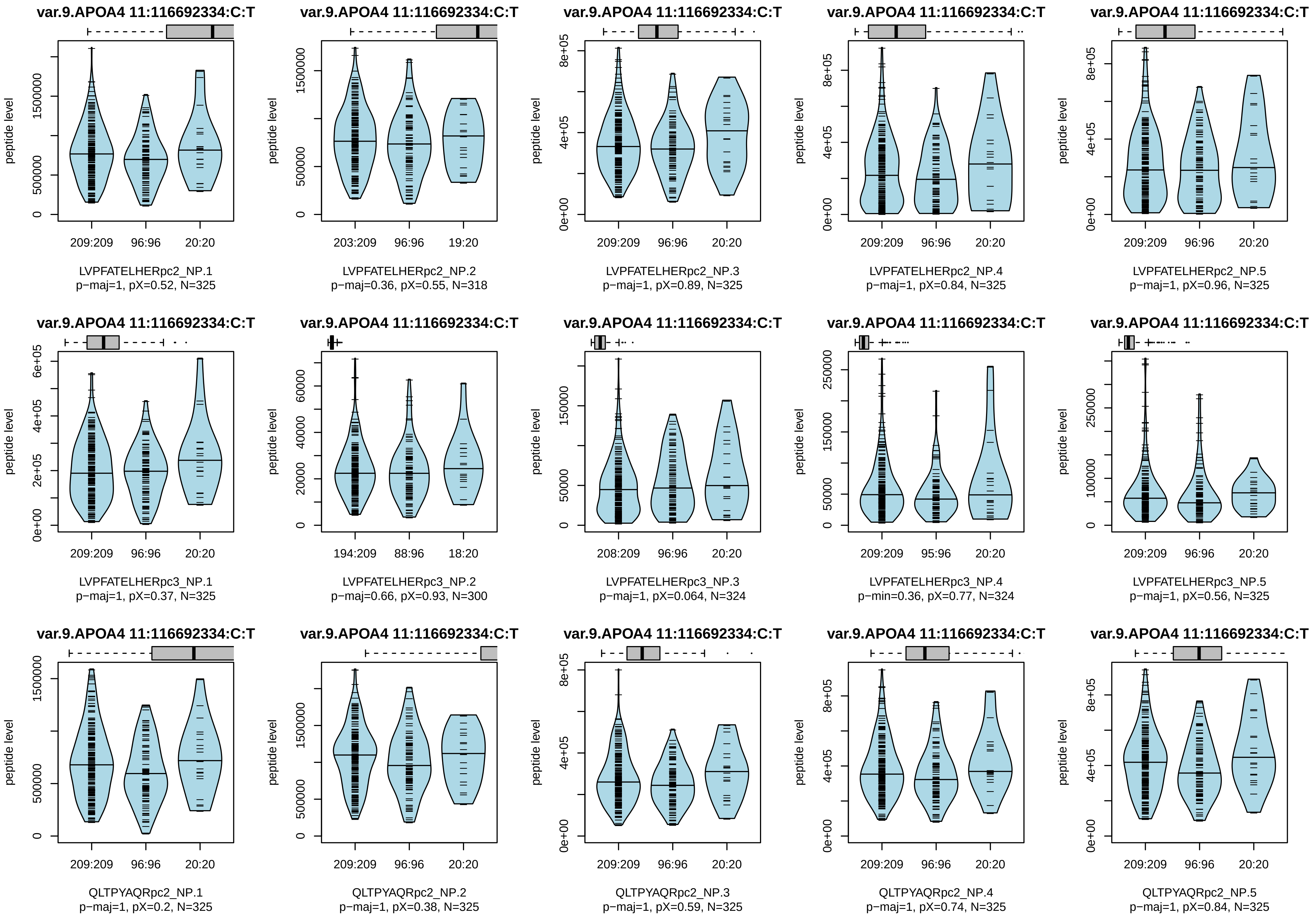
var.9.APOA4 11:116692334:C:T
peptide level
209:209
96:96
20:20
LVPFATELHERpc2_NP.1
p−maj=1, pX=0.52, N=325
var.9.APOA4 11:116692334:C:T
peptide level
203:209
96:96
19:20
LVPFATELHERpc2_NP.2
p−maj=0.36, pX=0.55, N=318
var.9.APOA4 11:116692334:C:T
peptide level
209:209
96:96
20:20
LVPFATELHERpc2_NP.3
p−maj=1, pX=0.89, N=325
var.9.APOA4 11:116692334:C:T
peptide level
209:209
96:96
20:20
LVPFATELHERpc2_NP.4
p−maj=1, pX=0.84, N=325
var.9.APOA4 11:116692334:C:T
peptide level
209:209
96:96
20:20
LVPFATELHERpc2_NP.5
p−maj=1, pX=0.96, N=325
var.9.APOA4 11:116692334:C:T
peptide level
209:209
96:96
20:20
LVPFATELHERpc3_NP.1
p−maj=1, pX=0.37, N=325
var.9.APOA4 11:116692334:C:T
peptide level
194:209
88:96
18:20
LVPFATELHERpc3_NP.2
p−maj=0.66, pX=0.93, N=300
var.9.APOA4 11:116692334:C:T
peptide level
208:209
96:96
20:20
LVPFATELHERpc3_NP.3
p−maj=1, pX=0.064, N=324
var.9.APOA4 11:116692334:C:T
peptide level
209:209
95:96
20:20
LVPFATELHERpc3_NP.4
p−min=0.36, pX=0.77, N=324
var.9.APOA4 11:116692334:C:T
peptide level
209:209
96:96
20:20
LVPFATELHERpc3_NP.5
p−maj=1, pX=0.56, N=325
var.9.APOA4 11:116692334:C:T
peptide level
209:209
96:96
20:20
QLTPYAQRpc2_NP.1
p−maj=1, pX=0.2, N=325
var.9.APOA4 11:116692334:C:T
peptide level
209:209
96:96
20:20
QLTPYAQRpc2_NP.2
p−maj=1, pX=0.38, N=325
var.9.APOA4 11:116692334:C:T
peptide level
209:209
96:96
20:20
QLTPYAQRpc2_NP.3
p−maj=1, pX=0.59, N=325
var.9.APOA4 11:116692334:C:T
peptide level
209:209
96:96
20:20
QLTPYAQRpc2_NP.4
p−maj=1, pX=0.74, N=325
var.9.APOA4 11:116692334:C:T
peptide level
209:209
96:96
20:20
QLTPYAQRpc2_NP.5
p−maj=1, pX=0.84, N=325

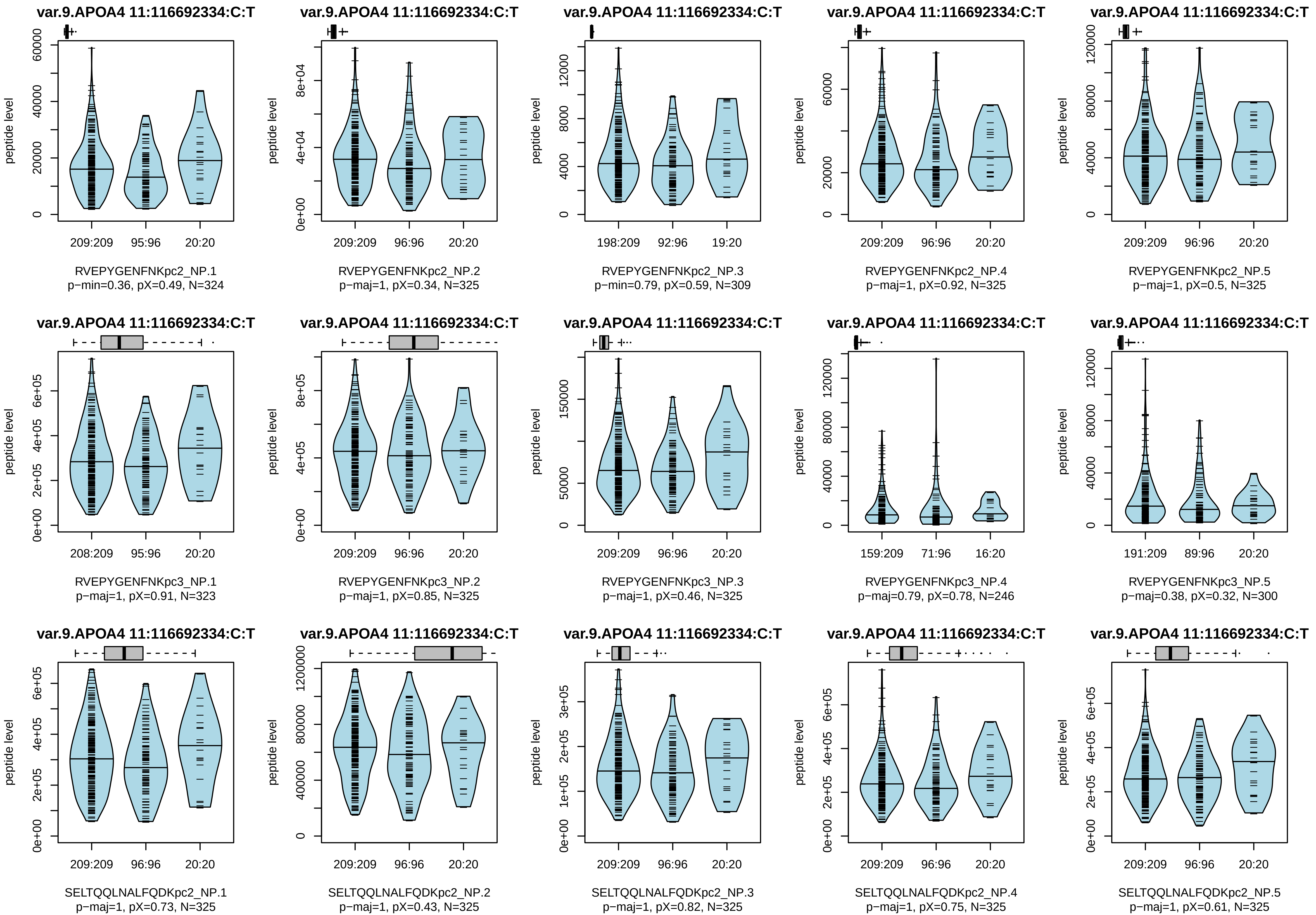
var.9.APOA4 11:116692334:C:T
peptide level
209:209
95:96
20:20
RVEPYGENFNKpc2_NP.1
p−min=0.36, pX=0.49, N=324
var.9.APOA4 11:116692334:C:T
209:209
96:96
20:20
RVEPYGENFNKpc2_NP.2
p−maj=1, pX=0.34, N=325
var.9.APOA4 11:116692334:C:T
198:209
92:96
19:20
RVEPYGENFNKpc2_NP.3
p−min=0.79, pX=0.59, N=309
var.9.APOA4 11:116692334:C:T
209:209
96:96
20:20
RVEPYGENFNKpc2_NP.4
p−maj=1, pX=0.92, N=325
var.9.APOA4 11:116692334:C:T
209:209
96:96
20:20
RVEPYGENFNKpc2_NP.5
p−maj=1, pX=0.5, N=325
var.9.APOA4 11:116692334:C:T
208:209
95:96
20:20
RVEPYGENFNKpc3_NP.1
p−maj=1, pX=0.91, N=323
var.9.APOA4 11:116692334:C:T
209:209
96:96
20:20
RVEPYGENFNKpc3_NP.2
p−maj=1, pX=0.85, N=325
var.9.APOA4 11:116692334:C:T
209:209
96:96
20:20
RVEPYGENFNKpc3_NP.3
p−maj=1, pX=0.46, N=325
var.9.APOA4 11:116692334:C:T
159:209
71:96
16:20
RVEPYGENFNKpc3_NP.4
p−maj=0.79, pX=0.78, N=246
var.9.APOA4 11:116692334:C:T
191:209
89:96
20:20
RVEPYGENFNKpc3_NP.5
p−maj=0.38, pX=0.32, N=300
var.9.APOA4 11:116692334:C:T
209:209
96:96
20:20
SELTQQLNALFQDKpc2_NP.1
p−maj=1, pX=0.73, N=325
var.9.APOA4 11:116692334:C:T
209:209
96:96
20:20
SELTQQLNALFQDKpc2_NP.2
p−maj=1, pX=0.43, N=325
var.9.APOA4 11:116692334:C:T
209:209
96:96
20:20
SELTQQLNALFQDKpc2_NP.3
p−maj=1, pX=0.82, N=325
var.9.APOA4 11:116692334:C:T
209:209
96:96
20:20
SELTQQLNALFQDKpc2_NP.4
p−maj=1, pX=0.75, N=325
var.9.APOA4 11:116692334:C:T
209:209
96:96
20:20
SELTQQLNALFQDKpc2_NP.5
p−maj=1, pX=0.61, N=325

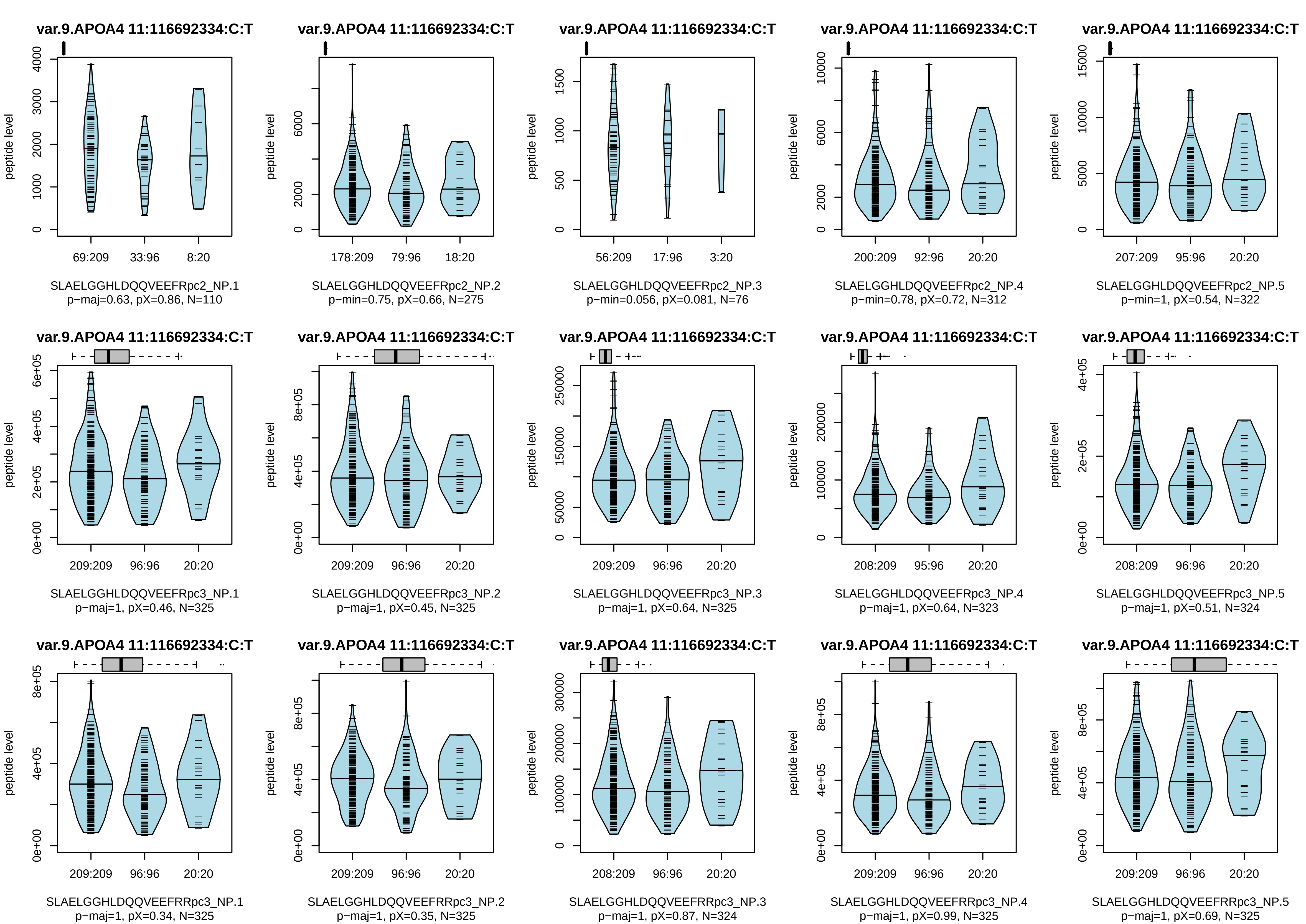
var.9.APOA4 11:116692334:C:T
peptide level
69:209
33:96
8:20
SLAELGGHLDQQVEEFRpc2_NP.1
p−maj=0.63, pX=0.86, N=110
178:209
79:96
18:20
SLAELGGHLDQQVEEFRpc2_NP.2
p−min=0.75, pX=0.66, N=275
56:209
17:96
3:20
SLAELGGHLDQQVEEFRpc2_NP.3
p−min=0.056, pX=0.081, N=76
200:209
92:96
20:20
SLAELGGHLDQQVEEFRpc2_NP.4
p−min=0.78, pX=0.72, N=312
207:209
95:96
20:20
SLAELGGHLDQQVEEFRpc2_NP.5
p−min=1, pX=0.54, N=322
209:209
96:96
20:20
SLAELGGHLDQQVEEFRpc3_NP.1
p−maj=1, pX=0.46, N=325
SLAELGGHLDQQVEEFRpc3_NP.2
p−maj=1, pX=0.45, N=325
SLAELGGHLDQQVEEFRpc3_NP.3
p−maj=1, pX=0.64, N=325
208:209
95:96
SLAELGGHLDQQVEEFRpc3_NP.4
p−maj=1, pX=0.64, N=323
208:209
96:96
SLAELGGHLDQQVEEFRpc3_NP.5
p−maj=1, pX=0.51, N=324
SLAELGGHLDQQVEEFRRpc3_NP.1
p−maj=1, pX=0.34, N=325
SLAELGGHLDQQVEEFRRpc3_NP.2
p−maj=1, pX=0.35, N=325
SLAELGGHLDQQVEEFRRpc3_NP.3
p−maj=1, pX=0.87, N=324
SLAELGGHLDQQVEEFRRpc3_NP.4
p−maj=1, pX=0.99, N=325
SLAELGGHLDQQVEEFRRpc3_NP.5
p−maj=1, pX=0.69, N=325

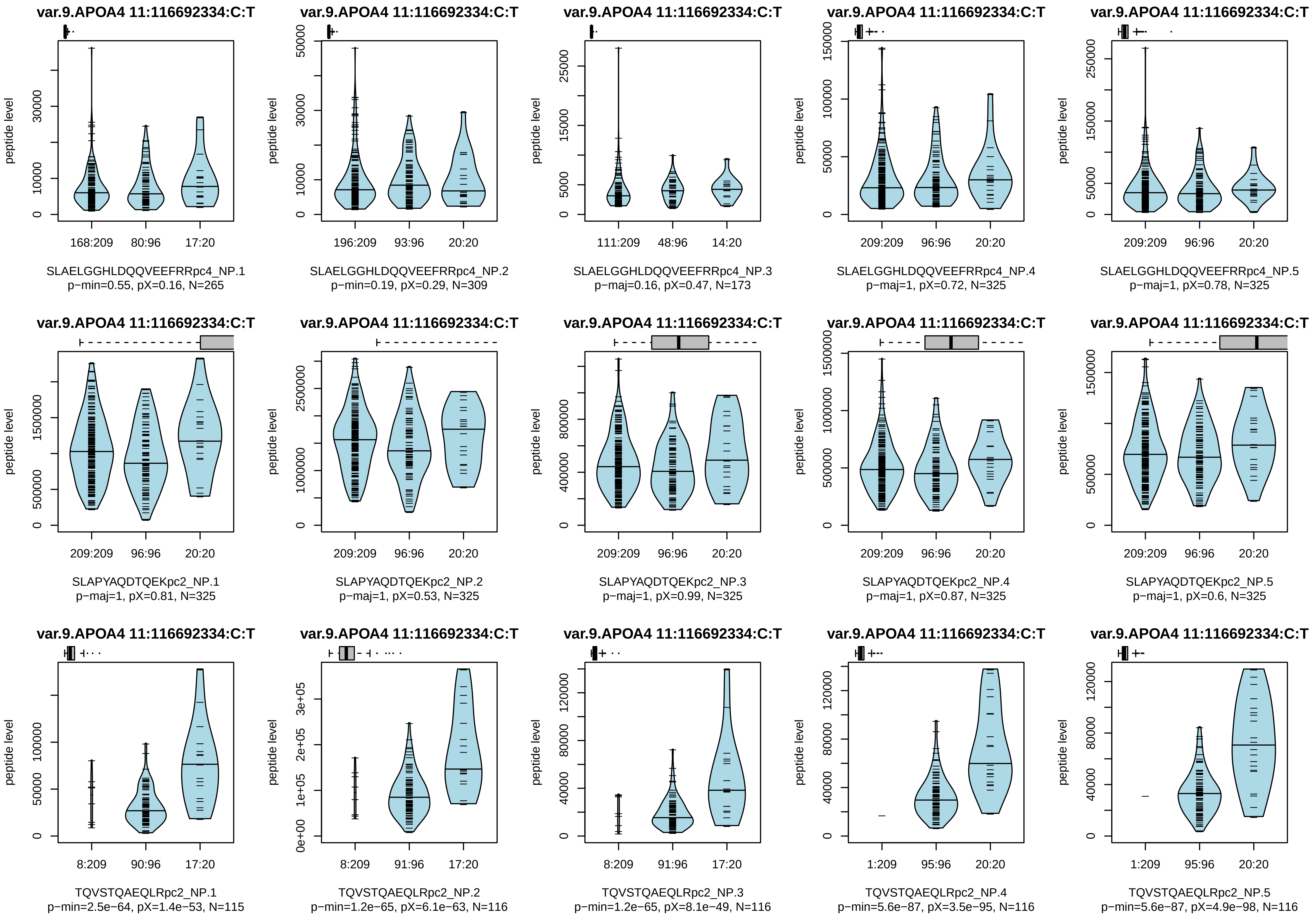

var.9.APOA4 11:116692334:C:T
peptide level
168:209
80:96
17:20
SLAELGGHLDQQVEEFRRpc4_NP.1
p−min=0.55, pX=0.16, N=265
var.9.APOA4 11:116692334:C:T
peptide level
196:209
93:96
20:20
SLAELGGHLDQQVEEFRRpc4_NP.2
p−min=0.19, pX=0.29, N=309
var.9.APOA4 11:116692334:C:T
peptide level
111:209
48:96
14:20
SLAELGGHLDQQVEEFRRpc4_NP.3
p−maj=0.16, pX=0.47, N=173
var.9.APOA4 11:116692334:C:T
peptide level
209:209
96:96
20:20
SLAELGGHLDQQVEEFRRpc4_NP.4
p−maj=1, pX=0.72, N=325
var.9.APOA4 11:116692334:C:T
peptide level
209:209
96:96
20:20
SLAELGGHLDQQVEEFRRpc4_NP.5
p−maj=1, pX=0.78, N=325
var.9.APOA4 11:116692334:C:T
peptide level
209:209
96:96
20:20
SLAPYAQDTQEKpc2_NP.1
p−maj=1, pX=0.81, N=325
var.9.APOA4 11:116692334:C:T
peptide level
209:209
96:96
20:20
SLAPYAQDTQEKpc2_NP.2
p−maj=1, pX=0.53, N=325
var.9.APOA4 11:116692334:C:T
peptide level
209:209
96:96
20:20
SLAPYAQDTQEKpc2_NP.3
p−maj=1, pX=0.99, N=325
var.9.APOA4 11:116692334:C:T
peptide level
209:209
96:96
20:20
SLAPYAQDTQEKpc2_NP.4
p−maj=1, pX=0.87, N=325
var.9.APOA4 11:116692334:C:T
peptide level
209:209
96:96
20:20
SLAPYAQDTQEKpc2_NP.5
p−maj=1, pX=0.6, N=325
var.9.APOA4 11:116692334:C:T
peptide level
8:209
90:96
17:20
TQVSTQAEQLRpc2_NP.1
p−min=2.5e−64, pX=1.4e−53, N=115
var.9.APOA4 11:116692334:C:T
peptide level
8:209
91:96
17:20
TQVSTQAEQLRpc2_NP.2
p−min=1.2e−65, pX=6.1e−63, N=116
var.9.APOA4 11:116692334:C:T
peptide level
8:209
91:96
17:20
TQVSTQAEQLRpc2_NP.3
p−min=1.2e−65, pX=8.1e−49, N=116
var.9.APOA4 11:116692334:C:T
peptide level
1:209
95:96
20:20
TQVSTQAEQLRpc2_NP.4
p−min=5.6e−87, pX=3.5e−95, N=116
var.9.APOA4 11:116692334:C:T
peptide level
1:209
95:96
20:20
TQVSTQAEQLRpc2_NP.5
p−min=5.6e−87, pX=4.9e−98, N=116

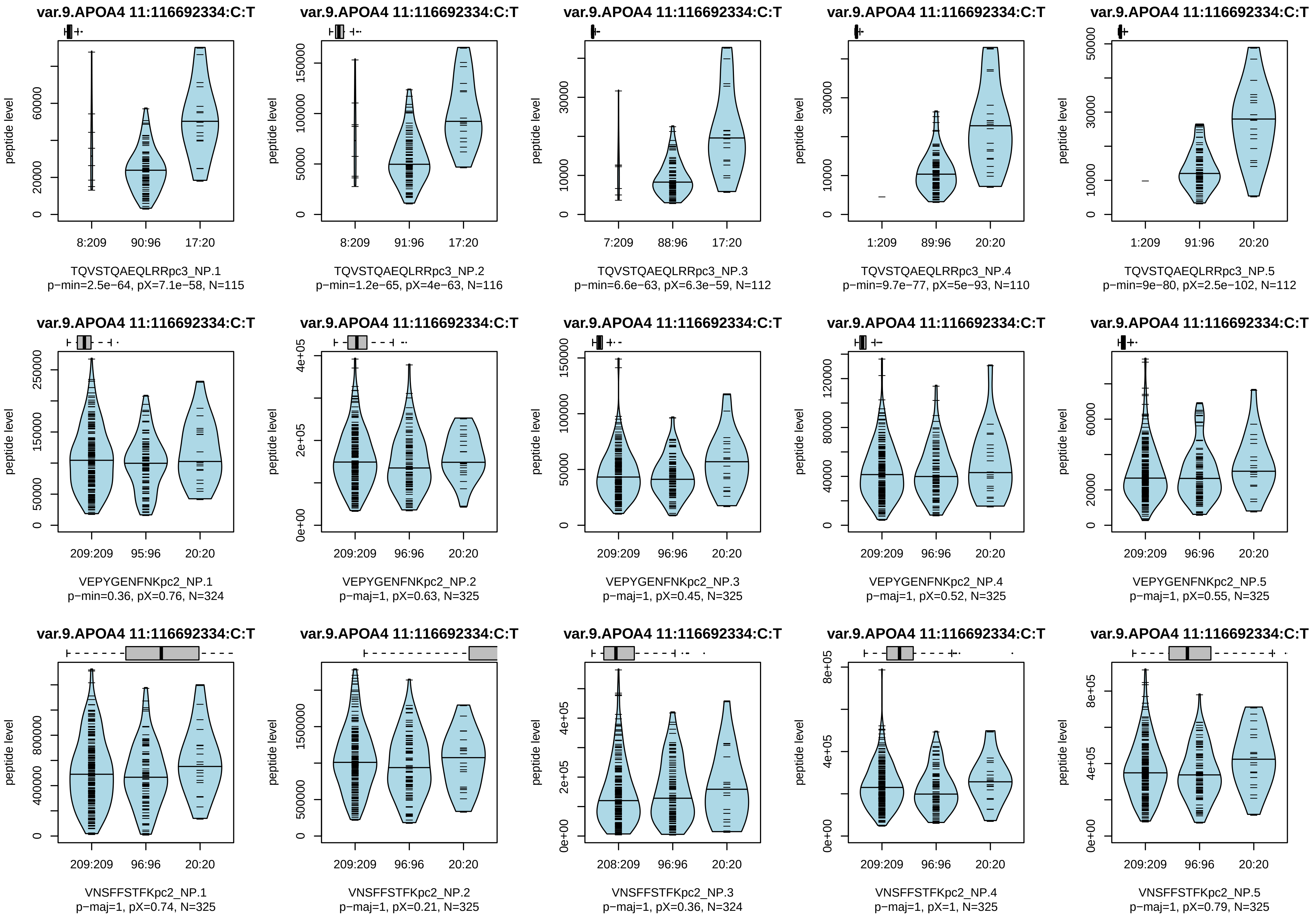
var.9.APOA4 11:116692334:C:T
peptide level
8:209
90:96
17:20
TQVSTQAEQLRRpc3_NP.1
p−min=2.5e−64, pX=7.1e−58, N=115
var.9.APOA4 11:116692334:C:T
peptide level
8:209
91:96
17:20
TQVSTQAEQLRRpc3_NP.2
p−min=1.2e−65, pX=4e−63, N=116
var.9.APOA4 11:116692334:C:T
peptide level
7:209
88:96
17:20
TQVSTQAEQLRRpc3_NP.3
p−min=6.6e−63, pX=6.3e−59, N=112
var.9.APOA4 11:116692334:C:T
peptide level
1:209
89:96
20:20
TQVSTQAEQLRRpc3_NP.4
p−min=9.7e−77, pX=5e−93, N=110
var.9.APOA4 11:116692334:C:T
peptide level
1:209
91:96
20:20
TQVSTQAEQLRRpc3_NP.5
p−min=9e−80, pX=2.5e−102, N=112
var.9.APOA4 11:116692334:C:T
peptide level
209:209
95:96
20:20
VEPYGENFNKpc2_NP.1
p−min=0.36, pX=0.76, N=324
var.9.APOA4 11:116692334:C:T
peptide level
209:209
96:96
20:20
VEPYGENFNKpc2_NP.2
p−maj=1, pX=0.63, N=325
var.9.APOA4 11:116692334:C:T
peptide level
209:209
96:96
20:20
VEPYGENFNKpc2_NP.3
p−maj=1, pX=0.45, N=325
var.9.APOA4 11:116692334:C:T
peptide level
209:209
96:96
20:20
VEPYGENFNKpc2_NP.4
p−maj=1, pX=0.52, N=325
var.9.APOA4 11:116692334:C:T
peptide level
209:209
96:96
20:20
VEPYGENFNKpc2_NP.5
p−maj=1, pX=0.55, N=325
var.9.APOA4 11:116692334:C:T
peptide level
209:209
96:96
20:20
VNSFFSTFKpc2_NP.1
p−maj=1, pX=0.74, N=325
var.9.APOA4 11:116692334:C:T
peptide level
209:209
96:96
20:20
VNSFFSTFKpc2_NP.2
p−maj=1, pX=0.21, N=325
var.9.APOA4 11:116692334:C:T
peptide level
208:209
96:96
20:20
VNSFFSTFKpc2_NP.3
p−maj=1, pX=0.36, N=324
var.9.APOA4 11:116692334:C:T
peptide level
209:209
96:96
20:20
VNSFFSTFKpc2_NP.4
p−maj=1, pX=1, N=325
var.9.APOA4 11:116692334:C:T
peptide level
209:209
96:96
20:20
VNSFFSTFKpc2_NP.5
p−maj=1, pX=0.79, N=325

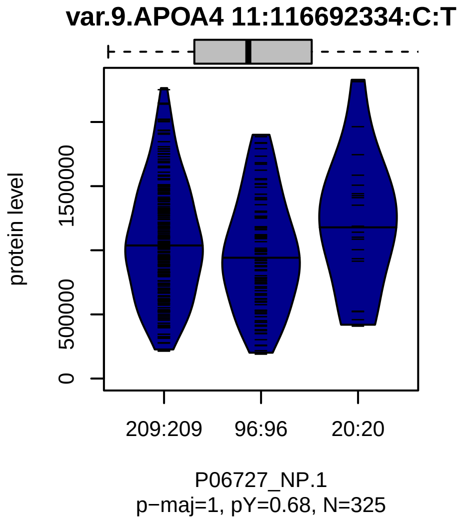
var.9.APOA4 11:116692334:C:T
protein level
0
500000
1500000
209:209
96:96
20:20
P06727_NP.1
p−maj=1, pY=0.68, N=325

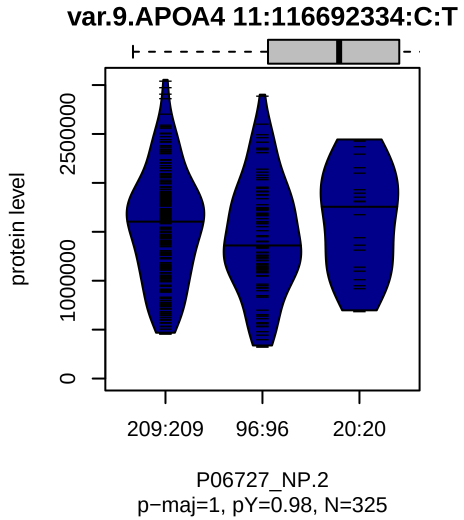
var.9.APOA4 11:116692334:C:T
protein level
0
1000000
2500000
209:209
96:96
20:20
P06727_NP.2
p−maj=1, pY=0.98, N=325

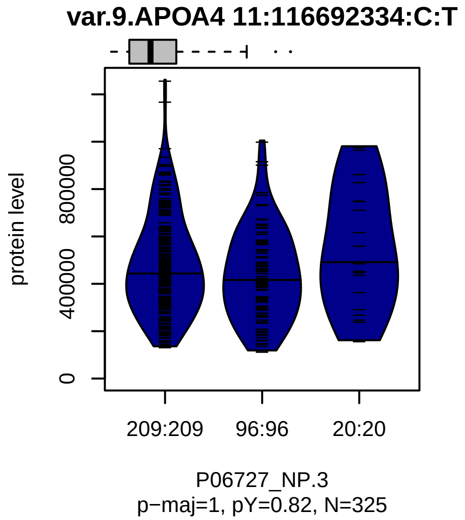
var.9.APOA4 11:116692334:C:T
protein level
0
400000
800000
209:209
96:96
20:20
P06727_NP.3
p−maj=1, pY=0.82, N=325

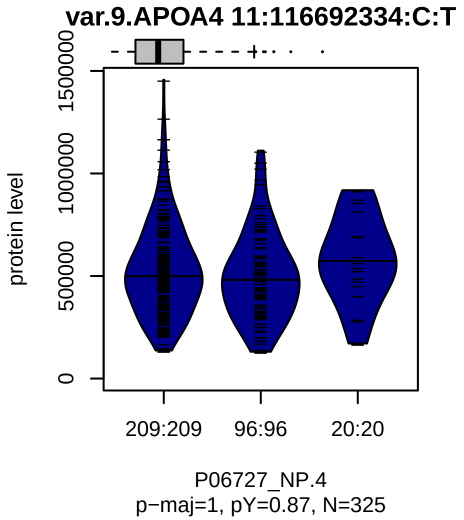
var.9.APOA4 11:116692334:C:T
protein level
0
500000
1000000
1500000
209:209
96:96
20:20
P06727_NP.4
p−maj=1, pY=0.87, N=325

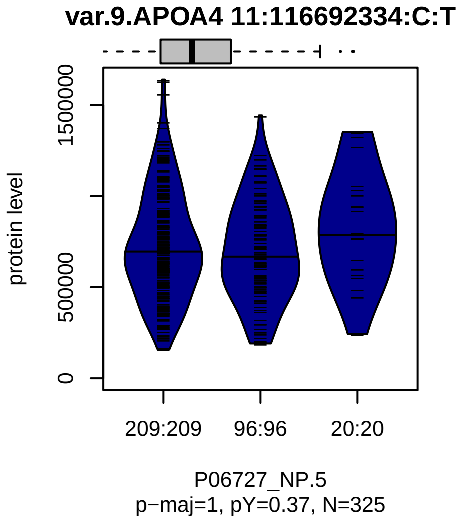
var.9.APOA4 11:116692334:C:T
protein level
0
500000
1500000
209:209
96:96
20:20
P06727_NP.5
p−maj=1, pY=0.37, N=325

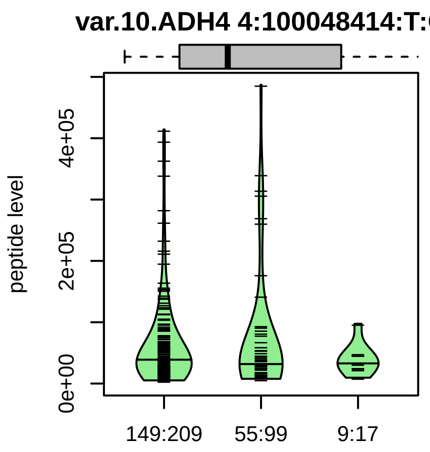

AAIAWEAGKpc2_NP.1
p−min=0.0049, pX=0.15, N=213

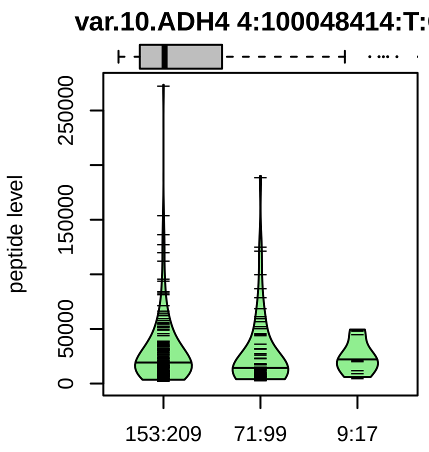

AAIAWEAGKpc2_NP.2
p−maj=0.097, pX=0.2, N=233

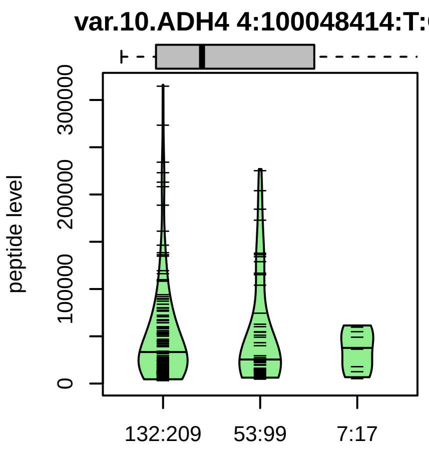

AAIAWEAGKpc2_NP.3
p−min=0.046, pX=0.11, N=192

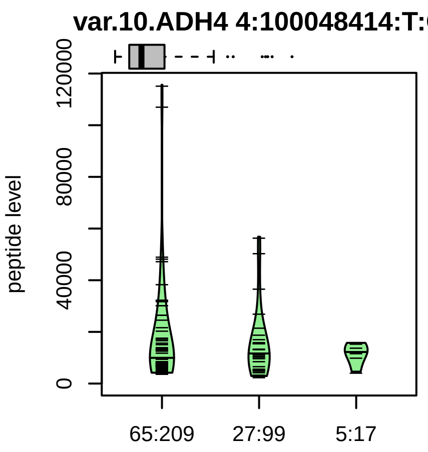

AAIAWEAGKpc2_NP.4
p−min=0.53, pX=0.29, N=97

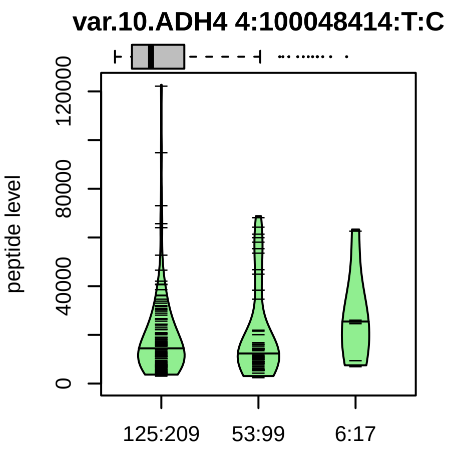

AAIAWEAGKpc2_NP.5
p−maj=0.081, pX=0.65, N=184

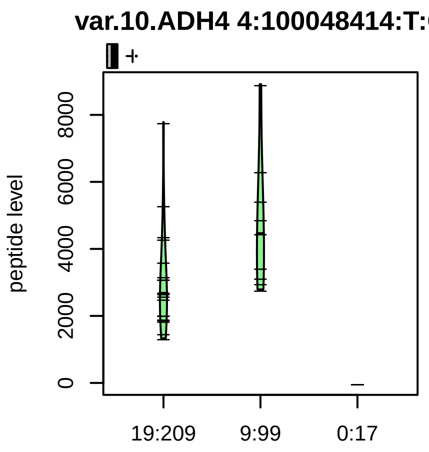

AALDCTTAGWGSCTFIGVAAGSKpc3_NP.2
p−maj=0.38, pX=0.91, N=28

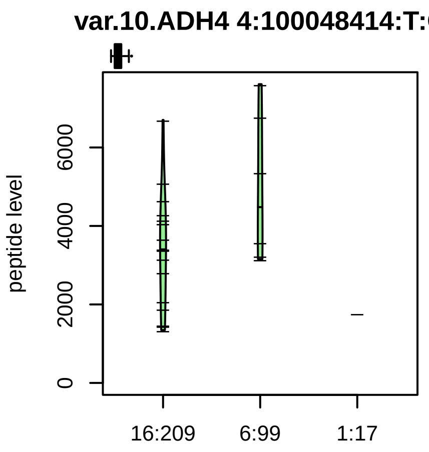

AALDCTTAGWGSCTFIGVAAGSKpc3_NP.3
p−min=0.66, pX=0.89, N=23

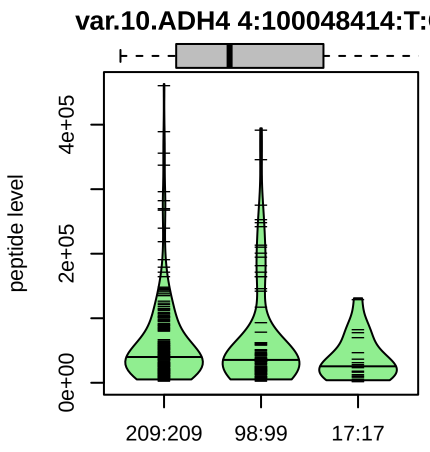

ALGATDCLNPRpc2_NP.1
p−min=0.36, pX=0.33, N=324

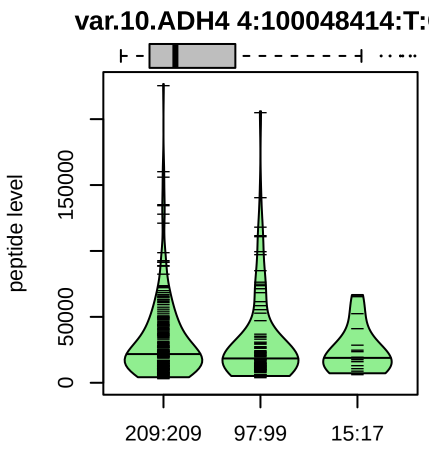

ALGATDCLNPRpc2_NP.2
p−maj=0.015, pX=0.22, N=321

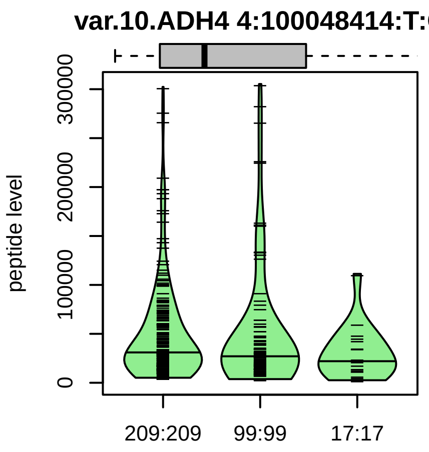

ALGATDCLNPRpc2_NP.3
p−maj=1, pX=0.42, N=325

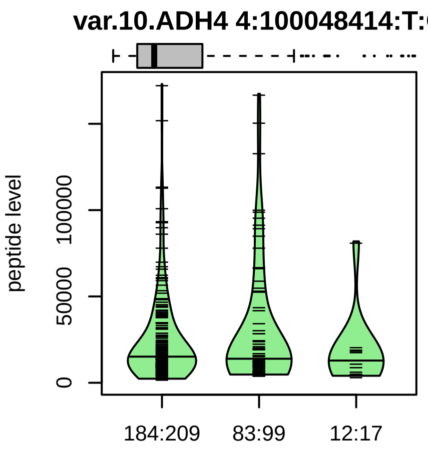

ALGATDCLNPRpc2_NP.4
p−maj=0.075, pX=0.79, N=279

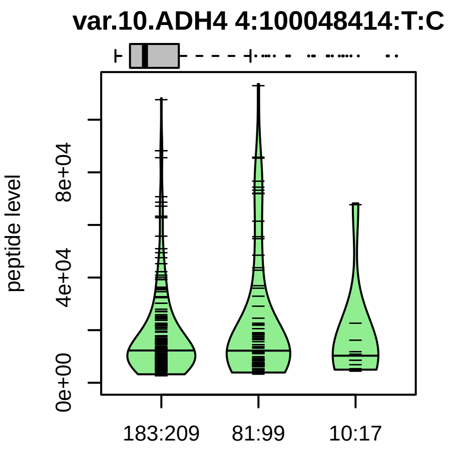

ALGATDCLNPRpc2_NP.5
p−maj=0.0087, pX=0.88, N=274

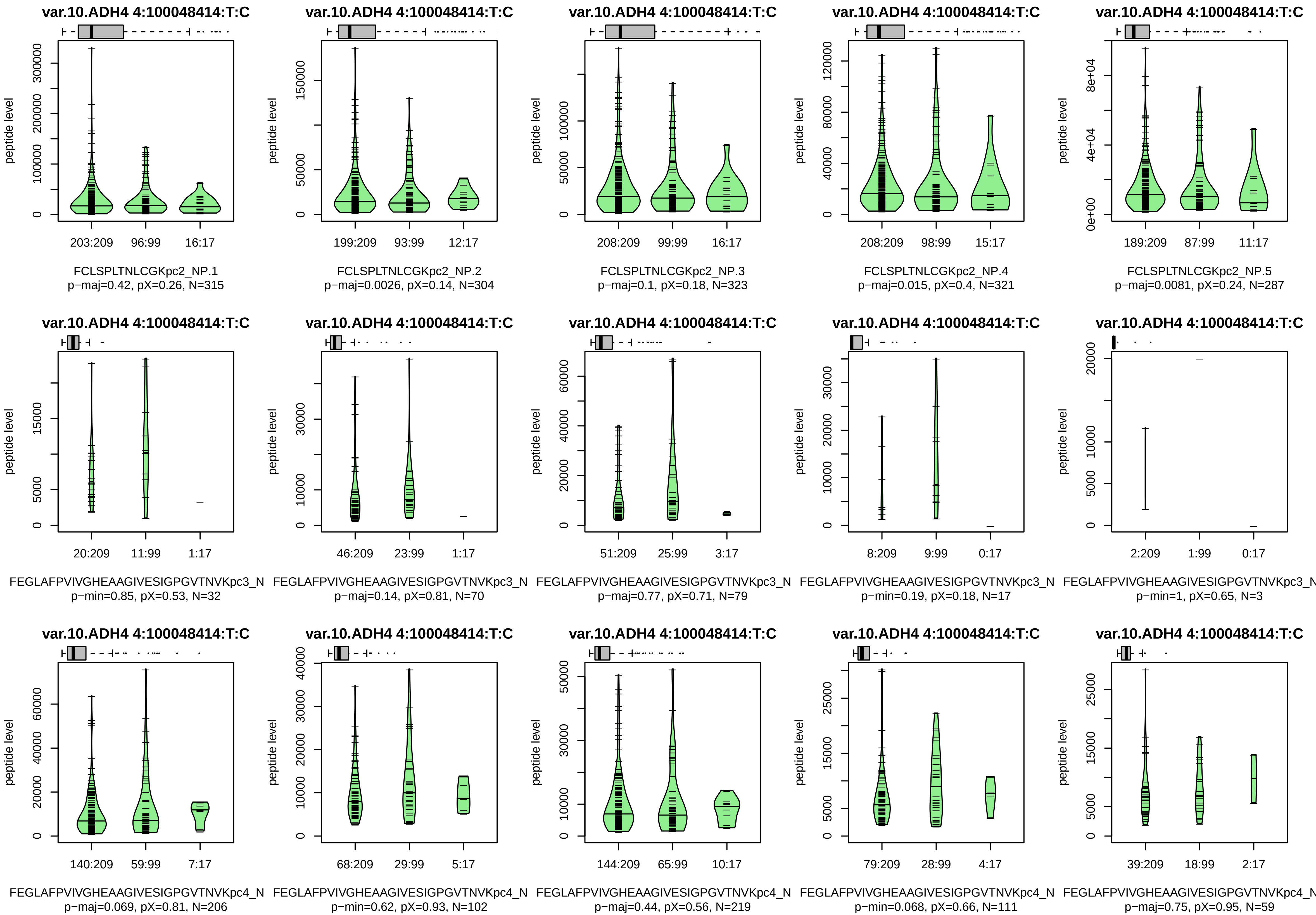
var.10.ADH4 4:100048414:T:C
peptide level
0
100000
200000
300000
203:209
96:99
16:17
FCLSPLTNLCGKpc2_NP.1
p−maj=0.42, pX=0.26, N=315
var.10.ADH4 4:100048414:T:C
peptide level
0
50000
150000
199:209
93:99
12:17
FCLSPLTNLCGKpc2_NP.2
p−maj=0.0026, pX=0.14, N=304
var.10.ADH4 4:100048414:T:C
peptide level
0
50000
100000
208:209
99:99
16:17
FCLSPLTNLCGKpc2_NP.3
p−maj=0.1, pX=0.18, N=323
var.10.ADH4 4:100048414:T:C
peptide level
0
40000
80000
120000
208:209
98:99
15:17
FCLSPLTNLCGKpc2_NP.4
p−maj=0.015, pX=0.4, N=321
var.10.ADH4 4:100048414:T:C
peptide level
0e+00
4e+04
8e+04
189:209
87:99
11:17
FCLSPLTNLCGKpc2_NP.5
p−maj=0.0081, pX=0.24, N=287
var.10.ADH4 4:100048414:T:C
peptide level
0
5000
15000
20:209
11:99
1:17
FEGLAFPVIVGHEAAGIVESIGPGVTNVKpc3_N
p−min=0.85, pX=0.53, N=32
var.10.ADH4 4:100048414:T:C
peptide level
0
10000
30000
46:209
23:99
1:17
FEGLAFPVIVGHEAAGIVESIGPGVTNVKpc3_N
p−maj=0.14, pX=0.81, N=70
var.10.ADH4 4:100048414:T:C
peptide level
0
20000
40000
60000
51:209
25:99
3:17
FEGLAFPVIVGHEAAGIVESIGPGVTNVKpc3_N
p−maj=0.77, pX=0.71, N=79
var.10.ADH4 4:100048414:T:C
peptide level
0
10000
20000
30000
8:209
9:99
0:17
FEGLAFPVIVGHEAAGIVESIGPGVTNVKpc3_N
p−min=0.19, pX=0.18, N=17
var.10.ADH4 4:100048414:T:C
peptide level
0
5000
10000
20000
2:209
1:99
0:17
FEGLAFPVIVGHEAAGIVESIGPGVTNVKpc3_N
p−min=1, pX=0.65, N=3
var.10.ADH4 4:100048414:T:C
peptide level
0
20000
40000
60000
140:209
59:99
7:17
FEGLAFPVIVGHEAAGIVESIGPGVTNVKpc4_N
p−maj=0.069, pX=0.81, N=206
var.10.ADH4 4:100048414:T:C
peptide level
0
10000
20000
30000
40000
68:209
29:99
5:17
FEGLAFPVIVGHEAAGIVESIGPGVTNVKpc4_N
p−min=0.62, pX=0.93, N=102
var.10.ADH4 4:100048414:T:C
peptide level
0
10000
30000
50000
144:209
65:99
10:17
FEGLAFPVIVGHEAAGIVESIGPGVTNVKpc4_N
p−maj=0.44, pX=0.56, N=219
var.10.ADH4 4:100048414:T:C
peptide level
0
5000
15000
25000
79:209
28:99
4:17
FEGLAFPVIVGHEAAGIVESIGPGVTNVKpc4_N
p−min=0.068, pX=0.66, N=111
var.10.ADH4 4:100048414:T:C
peptide level
0
5000
15000
25000
39:209
18:99
2:17
FEGLAFPVIVGHEAAGIVESIGPGVTNVKpc4_N
p−maj=0.75, pX=0.95, N=59

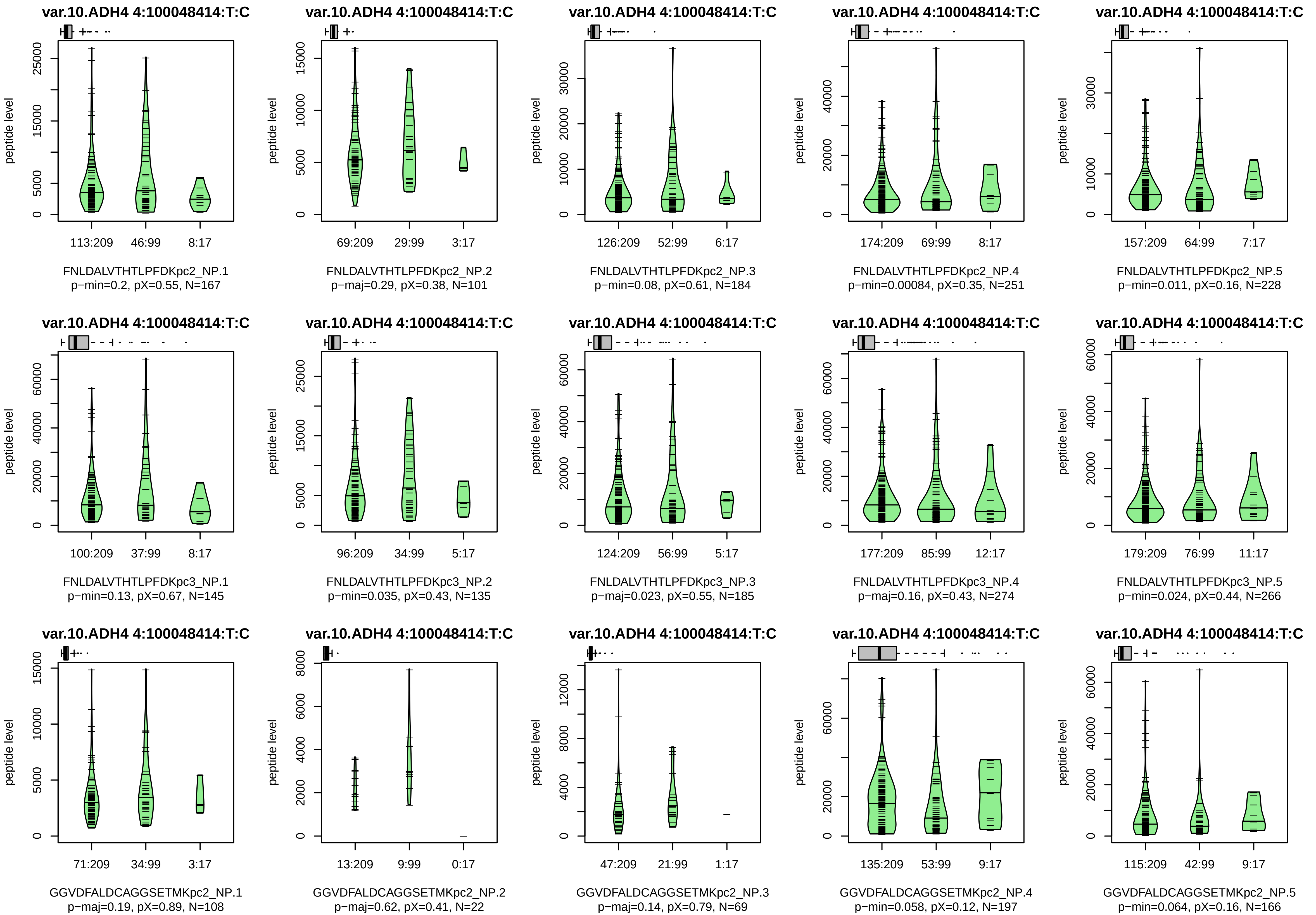

var.10.ADH4 4:100048414:T:C
peptide level
113:209
46:99
8:17
FNLDALVTHTLPFDKpc2_NP.1
p−min=0.2, pX=0.55, N=167
var.10.ADH4 4:100048414:T:C
69:209
29:99
3:17
FNLDALVTHTLPFDKpc2_NP.2
p−maj=0.29, pX=0.38, N=101
var.10.ADH4 4:100048414:T:C
126:209
52:99
6:17
FNLDALVTHTLPFDKpc2_NP.3
p−min=0.08, pX=0.61, N=184
var.10.ADH4 4:100048414:T:C
174:209
69:99
8:17
FNLDALVTHTLPFDKpc2_NP.4
p−min=0.00084, pX=0.35, N=251
var.10.ADH4 4:100048414:T:C
157:209
64:99
7:17
FNLDALVTHTLPFDKpc2_NP.5
p−min=0.011, pX=0.16, N=228
var.10.ADH4 4:100048414:T:C
100:209
37:99
8:17
FNLDALVTHTLPFDKpc3_NP.1
p−min=0.13, pX=0.67, N=145
var.10.ADH4 4:100048414:T:C
96:209
34:99
5:17
FNLDALVTHTLPFDKpc3_NP.2
p−min=0.035, pX=0.43, N=135
var.10.ADH4 4:100048414:T:C
124:209
56:99
5:17
FNLDALVTHTLPFDKpc3_NP.3
p−maj=0.023, pX=0.55, N=185
var.10.ADH4 4:100048414:T:C
177:209
85:99
12:17
FNLDALVTHTLPFDKpc3_NP.4
p−maj=0.16, pX=0.43, N=274
var.10.ADH4 4:100048414:T:C
179:209
76:99
11:17
FNLDALVTHTLPFDKpc3_NP.5
p−min=0.024, pX=0.44, N=266
var.10.ADH4 4:100048414:T:C
71:209
34:99
3:17
GGVDFALDCAGGSETMKpc2_NP.1
p−maj=0.19, pX=0.89, N=108
var.10.ADH4 4:100048414:T:C
13:209
9:99
0:17
GGVDFALDCAGGSETMKpc2_NP.2
p−maj=0.62, pX=0.41, N=22
var.10.ADH4 4:100048414:T:C
47:209
21:99
1:17
GGVDFALDCAGGSETMKpc2_NP.3
p−maj=0.14, pX=0.79, N=69
var.10.ADH4 4:100048414:T:C
135:209
53:99
9:17
GGVDFALDCAGGSETMKpc2_NP.4
p−min=0.058, pX=0.12, N=197
var.10.ADH4 4:100048414:T:C
115:209
42:99
9:17
GGVDFALDCAGGSETMKpc2_NP.5
p−min=0.064, pX=0.16, N=166

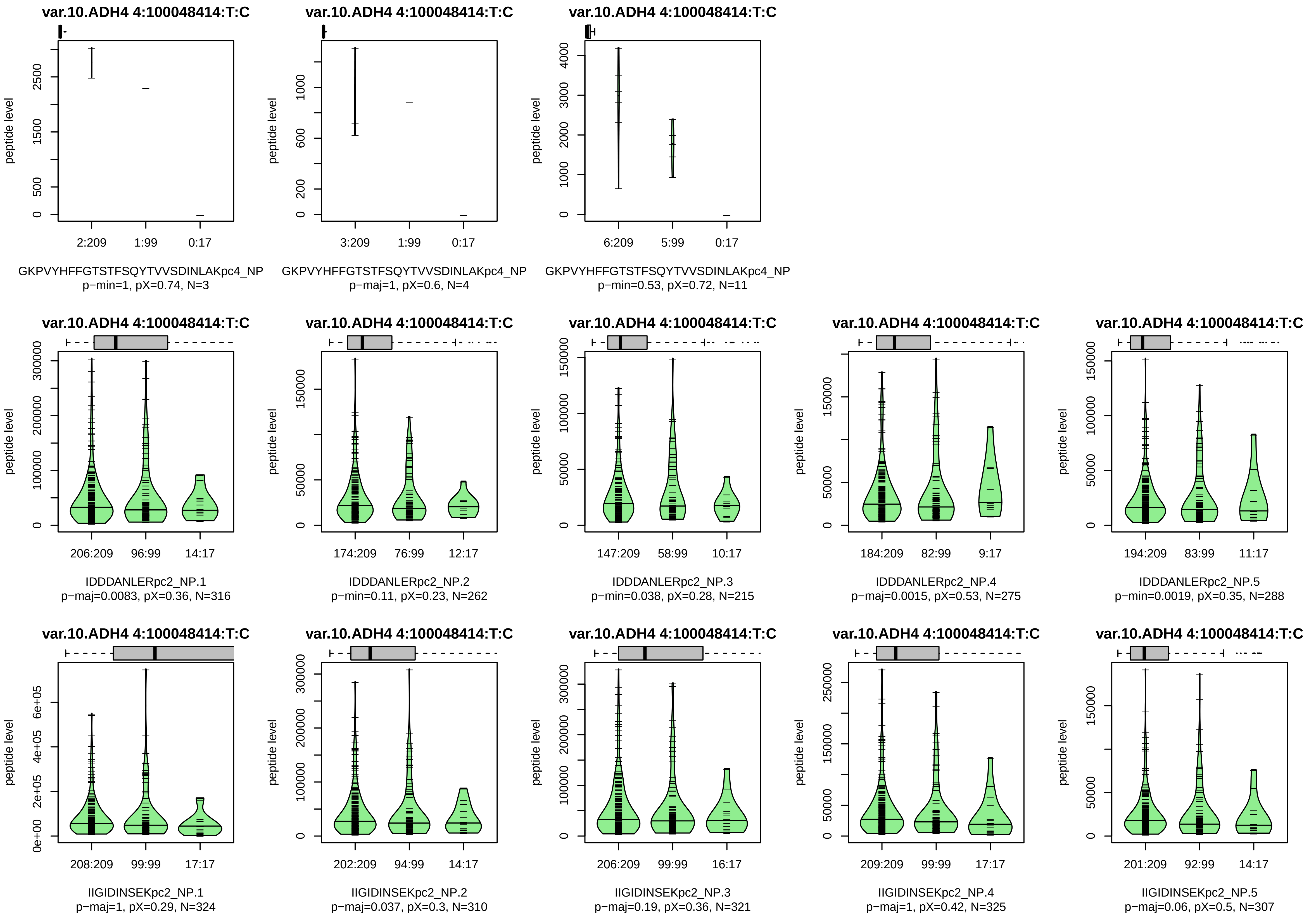

var.10.ADH4 4:100048414:T:C
peptide level
0
500
1500
2500
2:209
1:99
0:17
GKPVYHFFGTSTFSQYTVVSDINLAKpc4_NP
p−min=1, pX=0.74, N=3
var.10.ADH4 4:100048414:T:C
peptide level
0
200
600
1000
3:209
1:99
0:17
GKPVYHFFGTSTFSQYTVVSDINLAKpc4_NP
p−maj=1, pX=0.6, N=4
var.10.ADH4 4:100048414:T:C
peptide level
0
1000
2000
3000
4000
6:209
5:99
0:17
GKPVYHFFGTSTFSQYTVVSDINLAKpc4_NP
p−min=0.53, pX=0.72, N=11
var.10.ADH4 4:100048414:T:C
peptide level
0
100000
200000
300000
206:209
96:99
14:17
IDDDANLERpc2_NP.1
p−maj=0.0083, pX=0.36, N=316
var.10.ADH4 4:100048414:T:C
peptide level
0
50000
150000
174:209
76:99
12:17
IDDDANLERpc2_NP.2
p−min=0.11, pX=0.23, N=262
var.10.ADH4 4:100048414:T:C
peptide level
0
50000
100000
150000
147:209
58:99
10:17
IDDDANLERpc2_NP.3
p−min=0.038, pX=0.28, N=215
var.10.ADH4 4:100048414:T:C
peptide level
0
50000
150000
184:209
82:99
9:17
IDDDANLERpc2_NP.4
p−maj=0.0015, pX=0.53, N=275
var.10.ADH4 4:100048414:T:C
peptide level
0
50000
100000
150000
194:209
83:99
11:17
IDDDANLERpc2_NP.5
p−min=0.0019, pX=0.35, N=288
var.10.ADH4 4:100048414:T:C
peptide level
0e+00
2e+05
4e+05
6e+05
208:209
99:99
17:17
IIGIDINSEKpc2_NP.1
p−maj=1, pX=0.29, N=324
var.10.ADH4 4:100048414:T:C
peptide level
0
100000
200000
300000
202:209
94:99
14:17
IIGIDINSEKpc2_NP.2
p−maj=0.037, pX=0.3, N=310
var.10.ADH4 4:100048414:T:C
peptide level
0
100000
200000
300000
206:209
99:99
16:17
IIGIDINSEKpc2_NP.3
p−maj=0.19, pX=0.36, N=321
var.10.ADH4 4:100048414:T:C
peptide level
0
50000
150000
250000
209:209
99:99
17:17
IIGIDINSEKpc2_NP.4
p−maj=1, pX=0.42, N=325
var.10.ADH4 4:100048414:T:C
peptide level
0
50000
150000
201:209
92:99
14:17
IIGIDINSEKpc2_NP.5
p−maj=0.06, pX=0.5, N=307

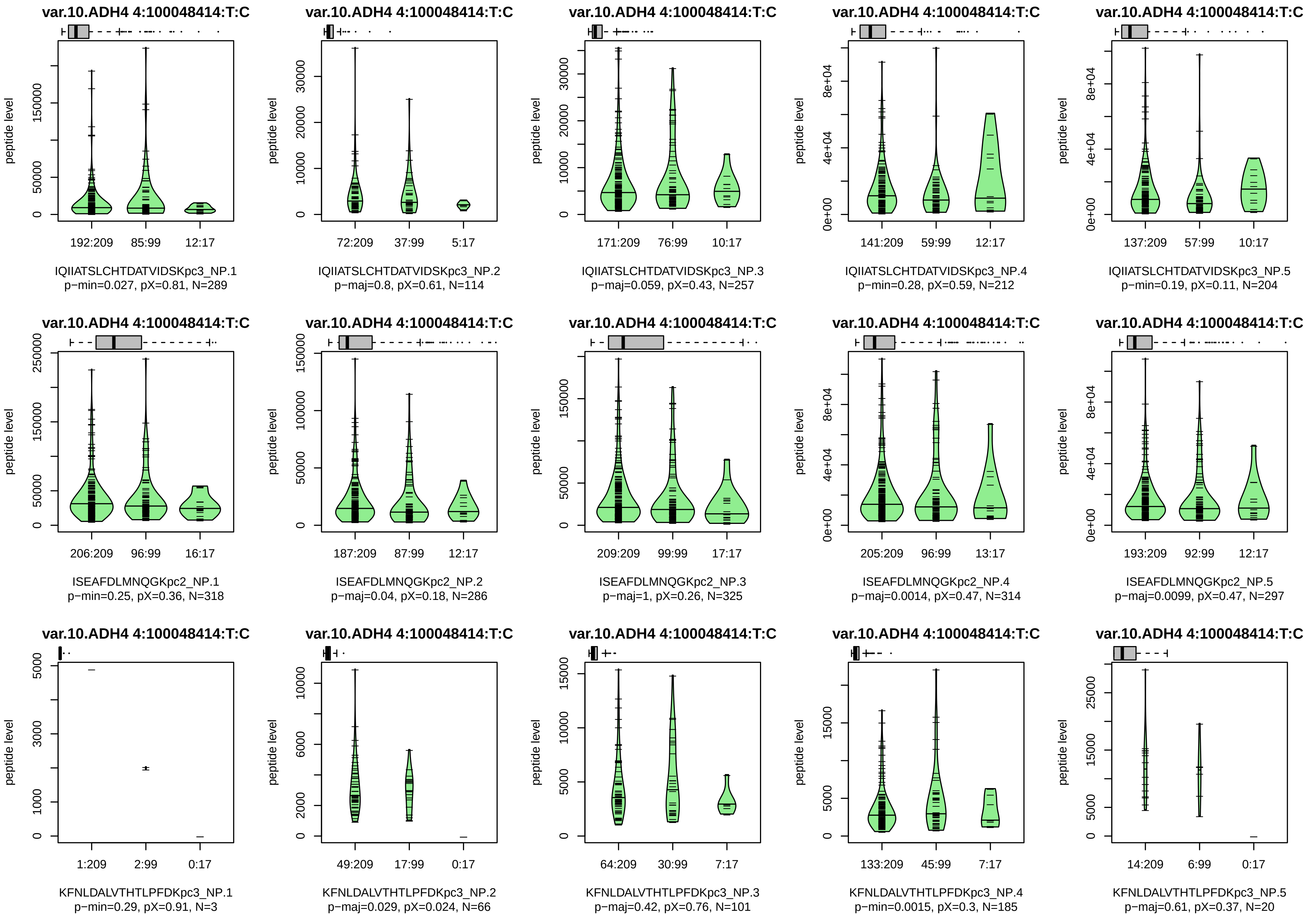
var.10.ADH4 4:100048414:T:C
peptide level
192:209
85:99
12:17
IQIIATSLCHTDATVIDSKpc3_NP.1
p−min=0.027, pX=0.81, N=289
var.10.ADH4 4:100048414:T:C
peptide level
72:209
37:99
5:17
IQIIATSLCHTDATVIDSKpc3_NP.2
p−maj=0.8, pX=0.61, N=114
var.10.ADH4 4:100048414:T:C
peptide level
171:209
76:99
10:17
IQIIATSLCHTDATVIDSKpc3_NP.3
p−maj=0.059, pX=0.43, N=257
var.10.ADH4 4:100048414:T:C
peptide level
141:209
59:99
12:17
IQIIATSLCHTDATVIDSKpc3_NP.4
p−min=0.28, pX=0.59, N=212
var.10.ADH4 4:100048414:T:C
peptide level
137:209
57:99
10:17
IQIIATSLCHTDATVIDSKpc3_NP.5
p−min=0.19, pX=0.11, N=204
var.10.ADH4 4:100048414:T:C
peptide level
206:209
96:99
16:17
ISEAFDLMNQGKpc2_NP.1
p−min=0.25, pX=0.36, N=318
var.10.ADH4 4:100048414:T:C
peptide level
187:209
87:99
12:17
ISEAFDLMNQGKpc2_NP.2
p−maj=0.04, pX=0.18, N=286
var.10.ADH4 4:100048414:T:C
peptide level
209:209
99:99
17:17
ISEAFDLMNQGKpc2_NP.3
p−maj=1, pX=0.26, N=325
var.10.ADH4 4:100048414:T:C
peptide level
205:209
96:99
13:17
ISEAFDLMNQGKpc2_NP.4
p−maj=0.0014, pX=0.47, N=314
var.10.ADH4 4:100048414:T:C
peptide level
193:209
92:99
12:17
ISEAFDLMNQGKpc2_NP.5
p−maj=0.0099, pX=0.47, N=297
var.10.ADH4 4:100048414:T:C
peptide level
1:209
2:99
0:17
KFNLDALVTHTLPFDKpc3_NP.1
p−min=0.29, pX=0.91, N=3
var.10.ADH4 4:100048414:T:C
peptide level
49:209
17:99
0:17
KFNLDALVTHTLPFDKpc3_NP.2
p−maj=0.029, pX=0.024, N=66
var.10.ADH4 4:100048414:T:C
peptide level
64:209
30:99
7:17
KFNLDALVTHTLPFDKpc3_NP.3
p−maj=0.42, pX=0.76, N=101
var.10.ADH4 4:100048414:T:C
peptide level
133:209
45:99
7:17
KFNLDALVTHTLPFDKpc3_NP.4
p−min=0.0015, pX=0.3, N=185
var.10.ADH4 4:100048414:T:C
peptide level
14:209
6:99
0:17
KFNLDALVTHTLPFDKpc3_NP.5
p−maj=0.61, pX=0.37, N=20

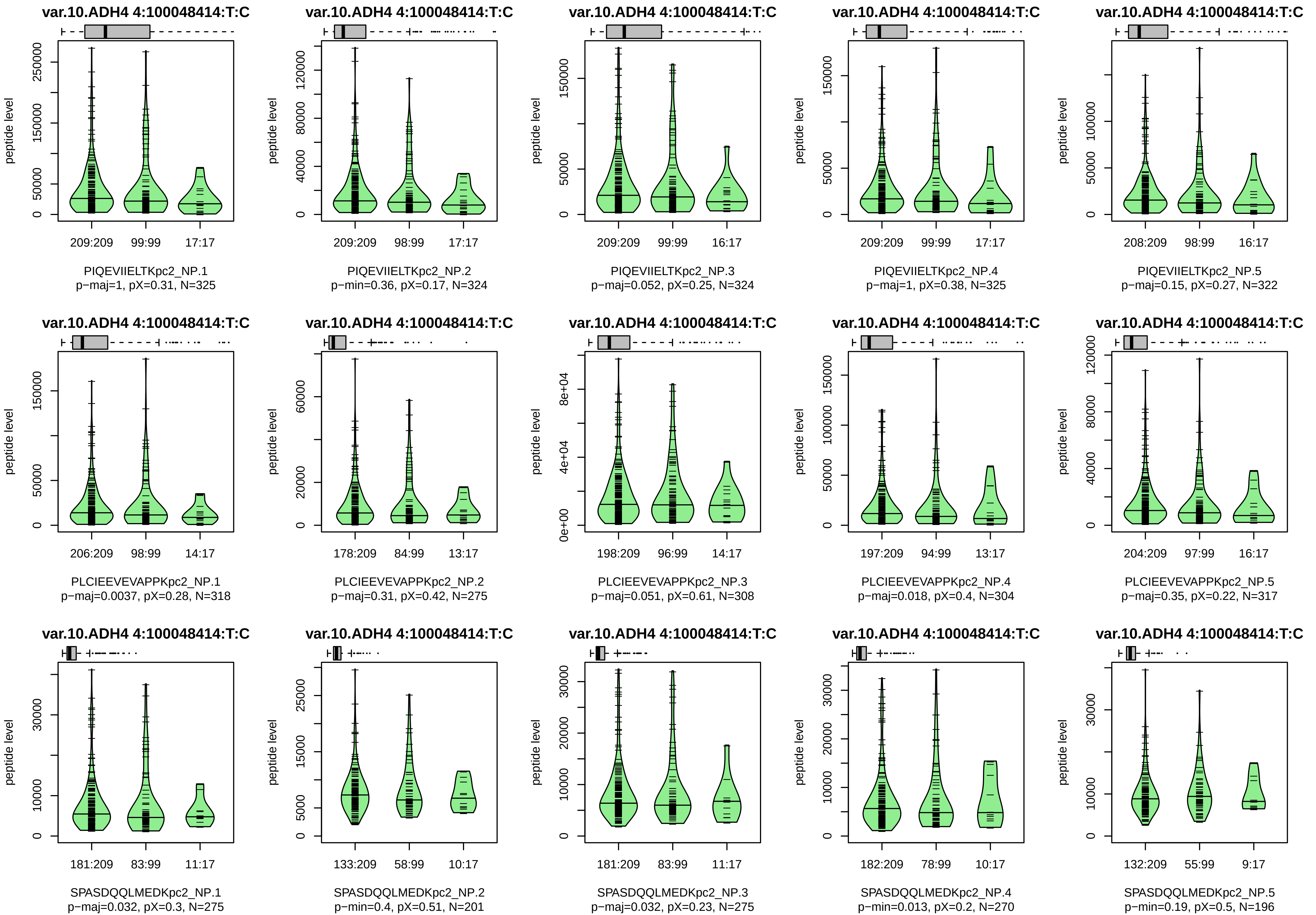
var.10.ADH4 4:100048414:T:C
peptide level
0
50000
150000
250000
209:209
99:99
17:17
PIQEVIIELTKpc2_NP.1
p−maj=1, pX=0.31, N=325
var.10.ADH4 4:100048414:T:C
peptide level
0
40000
80000
120000
209:209
98:99
17:17
PIQEVIIELTKpc2_NP.2
p−min=0.36, pX=0.17, N=324
var.10.ADH4 4:100048414:T:C
peptide level
0
50000
150000
209:209
99:99
16:17
PIQEVIIELTKpc2_NP.3
p−maj=0.052, pX=0.25, N=324
var.10.ADH4 4:100048414:T:C
peptide level
0
50000
150000
209:209
99:99
17:17
PIQEVIIELTKpc2_NP.4
p−maj=1, pX=0.38, N=325
var.10.ADH4 4:100048414:T:C
peptide level
0
50000
100000
208:209
98:99
16:17
PIQEVIIELTKpc2_NP.5
p−maj=0.15, pX=0.27, N=322
var.10.ADH4 4:100048414:T:C
peptide level
0
50000
150000
206:209
98:99
14:17
PLCIEEVEVAPPKpc2_NP.1
p−maj=0.0037, pX=0.28, N=318
var.10.ADH4 4:100048414:T:C
peptide level
0
20000
60000
178:209
84:99
13:17
PLCIEEVEVAPPKpc2_NP.2
p−maj=0.31, pX=0.42, N=275
var.10.ADH4 4:100048414:T:C
peptide level
0e+00
4e+04
8e+04
198:209
96:99
14:17
PLCIEEVEVAPPKpc2_NP.3
p−maj=0.051, pX=0.61, N=308
var.10.ADH4 4:100048414:T:C
peptide level
0
50000
100000
150000
197:209
94:99
13:17
PLCIEEVEVAPPKpc2_NP.4
p−maj=0.018, pX=0.4, N=304
var.10.ADH4 4:100048414:T:C
peptide level
0
40000
80000
120000
204:209
97:99
16:17
PLCIEEVEVAPPKpc2_NP.5
p−maj=0.35, pX=0.22, N=317
var.10.ADH4 4:100048414:T:C
peptide level
0
10000
30000
181:209
83:99
11:17
SPASDQQLMEDKpc2_NP.1
p−maj=0.032, pX=0.3, N=275
var.10.ADH4 4:100048414:T:C
peptide level
0
5000
15000
25000
133:209
58:99
10:17
SPASDQQLMEDKpc2_NP.2
p−min=0.4, pX=0.51, N=201
var.10.ADH4 4:100048414:T:C
peptide level
0
10000
20000
30000
181:209
83:99
11:17
SPASDQQLMEDKpc2_NP.3
p−maj=0.032, pX=0.23, N=275
var.10.ADH4 4:100048414:T:C
peptide level
0
10000
20000
30000
182:209
78:99
10:17
SPASDQQLMEDKpc2_NP.4
p−min=0.013, pX=0.2, N=270
var.10.ADH4 4:100048414:T:C
peptide level
0
10000
30000
132:209
55:99
9:17
SPASDQQLMEDKpc2_NP.5
p−min=0.19, pX=0.5, N=196

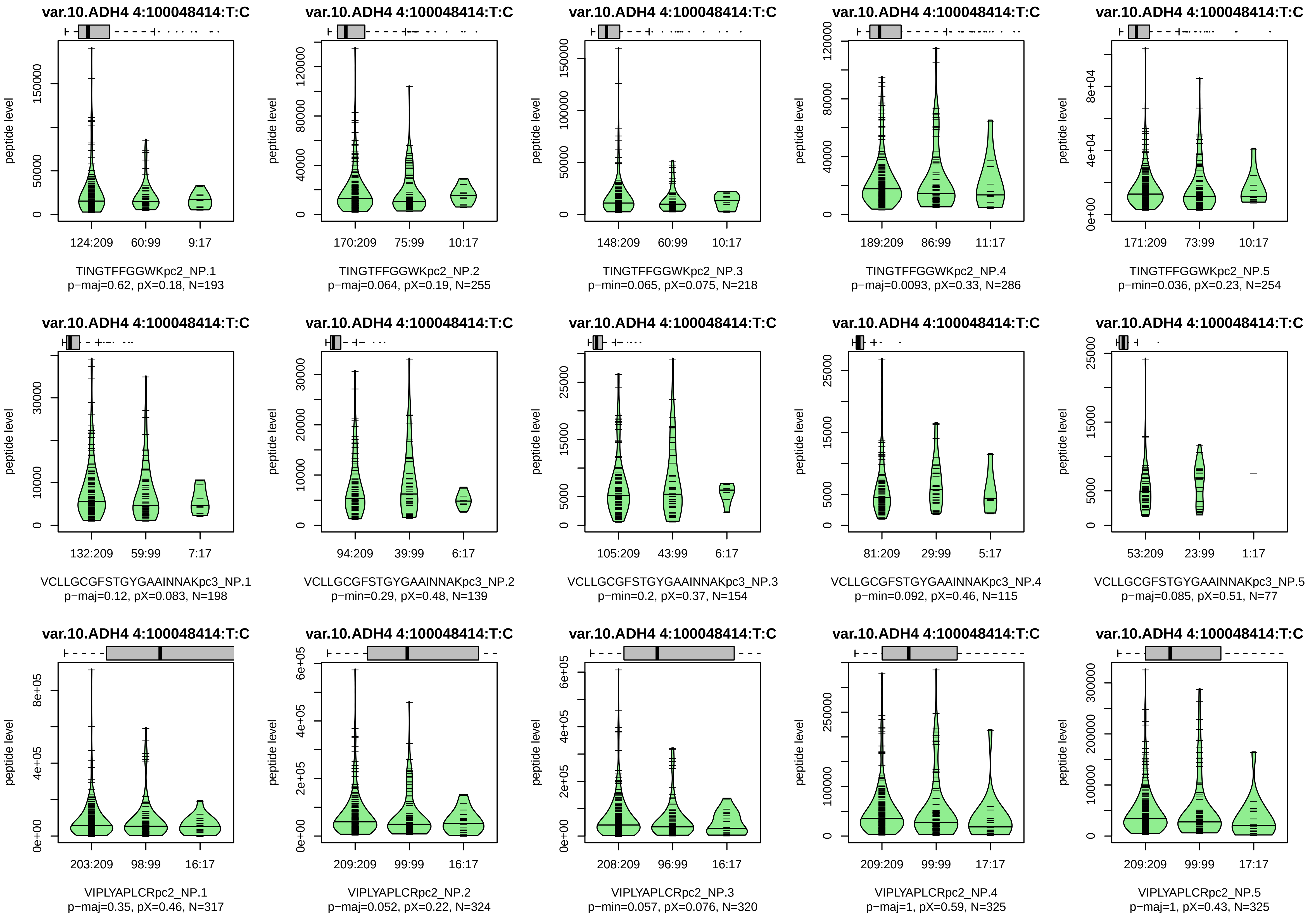
var.10.ADH4 4:100048414:T:C
peptide level
124:209
60:99
9:17
TINGTFFGGWKpc2_NP.1
p−maj=0.62, pX=0.18, N=193
var.10.ADH4 4:100048414:T:C
peptide level
170:209
75:99
10:17
TINGTFFGGWKpc2_NP.2
p−maj=0.064, pX=0.19, N=255
var.10.ADH4 4:100048414:T:C
peptide level
148:209
60:99
10:17
TINGTFFGGWKpc2_NP.3
p−min=0.065, pX=0.075, N=218
var.10.ADH4 4:100048414:T:C
peptide level
189:209
86:99
11:17
TINGTFFGGWKpc2_NP.4
p−maj=0.0093, pX=0.33, N=286
var.10.ADH4 4:100048414:T:C
peptide level
171:209
73:99
10:17
TINGTFFGGWKpc2_NP.5
p−min=0.036, pX=0.23, N=254
var.10.ADH4 4:100048414:T:C
peptide level
132:209
59:99
7:17
VCLLGCGFSTGYGAAINNAKpc3_NP.1
p−maj=0.12, pX=0.083, N=198
var.10.ADH4 4:100048414:T:C
peptide level
94:209
39:99
6:17
VCLLGCGFSTGYGAAINNAKpc3_NP.2
p−min=0.29, pX=0.48, N=139
var.10.ADH4 4:100048414:T:C
peptide level
105:209
43:99
6:17
VCLLGCGFSTGYGAAINNAKpc3_NP.3
p−min=0.2, pX=0.37, N=154
var.10.ADH4 4:100048414:T:C
peptide level
81:209
29:99
5:17
VCLLGCGFSTGYGAAINNAKpc3_NP.4
p−min=0.092, pX=0.46, N=115
var.10.ADH4 4:100048414:T:C
peptide level
53:209
23:99
1:17
VCLLGCGFSTGYGAAINNAKpc3_NP.5
p−maj=0.085, pX=0.51, N=77
var.10.ADH4 4:100048414:T:C
peptide level
203:209
98:99
16:17
VIPLYAPLCRpc2_NP.1
p−maj=0.35, pX=0.46, N=317
var.10.ADH4 4:100048414:T:C
peptide level
209:209
99:99
16:17
VIPLYAPLCRpc2_NP.2
p−maj=0.052, pX=0.22, N=324
var.10.ADH4 4:100048414:T:C
peptide level
208:209
96:99
16:17
VIPLYAPLCRpc2_NP.3
p−min=0.057, pX=0.076, N=320
var.10.ADH4 4:100048414:T:C
peptide level
209:209
99:99
17:17
VIPLYAPLCRpc2_NP.4
p−maj=1, pX=0.59, N=325
var.10.ADH4 4:100048414:T:C
peptide level
209:209
99:99
17:17
VIPLYAPLCRpc2_NP.5
p−maj=1, pX=0.43, N=325

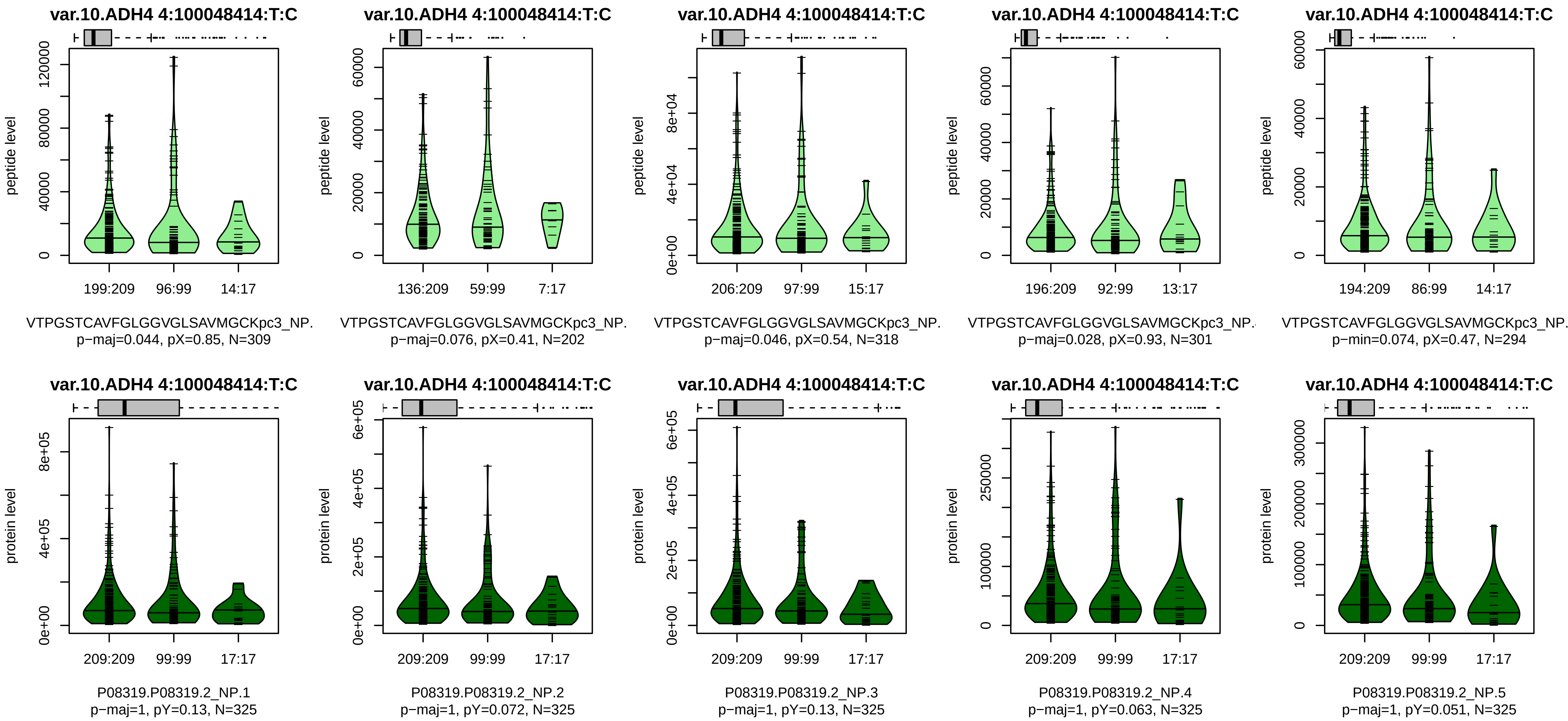

var.10.ADH4 4:100048414:T:C
peptide level
199:209
96:99
14:17
VTPGSTCAVFGLGGVGLSAVMGCKpc3_NP.
p−maj=0.044, pX=0.85, N=309
var.10.ADH4 4:100048414:T:C
peptide level
136:209
59:99
7:17
VTPGSTCAVFGLGGVGLSAVMGCKpc3_NP.
p−maj=0.076, pX=0.41, N=202
var.10.ADH4 4:100048414:T:C
peptide level
206:209
97:99
15:17
VTPGSTCAVFGLGGVGLSAVMGCKpc3_NP.
p−maj=0.046, pX=0.54, N=318
var.10.ADH4 4:100048414:T:C
peptide level
196:209
92:99
13:17
VTPGSTCAVFGLGGVGLSAVMGCKpc3_NP.
p−maj=0.028, pX=0.93, N=301
var.10.ADH4 4:100048414:T:C
peptide level
194:209
86:99
14:17
VTPGSTCAVFGLGGVGLSAVMGCKpc3_NP.
p−min=0.074, pX=0.47, N=294
var.10.ADH4 4:100048414:T:C
protein level
209:209
99:99
17:17
P08319.P08319.2_NP.1
p−maj=1, pY=0.13, N=325
var.10.ADH4 4:100048414:T:C
protein level
209:209
99:99
17:17
P08319.P08319.2_NP.2
p−maj=1, pY=0.072, N=325
var.10.ADH4 4:100048414:T:C
protein level
209:209
99:99
17:17
P08319.P08319.2_NP.3
p−maj=1, pY=0.13, N=325
var.10.ADH4 4:100048414:T:C
protein level
209:209
99:99
17:17
P08319.P08319.2_NP.4
p−maj=1, pY=0.063, N=325
var.10.ADH4 4:100048414:T:C
protein level
209:209
99:99
17:17
P08319.P08319.2_NP.5
p−maj=1, pY=0.051, N=325

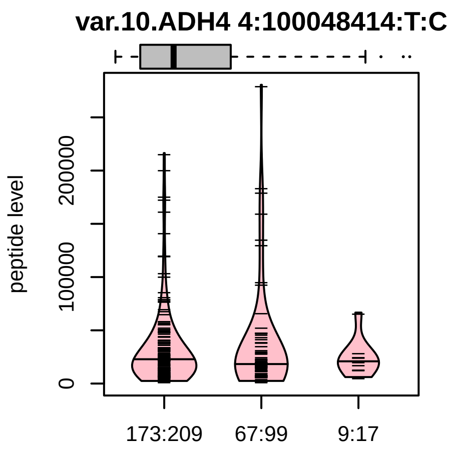

AAIAWEAGKpc2_NP.1
p−min=6e−04, pX=0.25, N=249

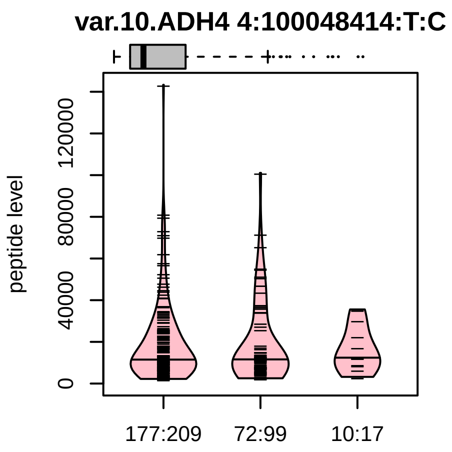

AAIAWEAGKpc2_NP.2
p−min=0.0038, pX=0.12, N=259

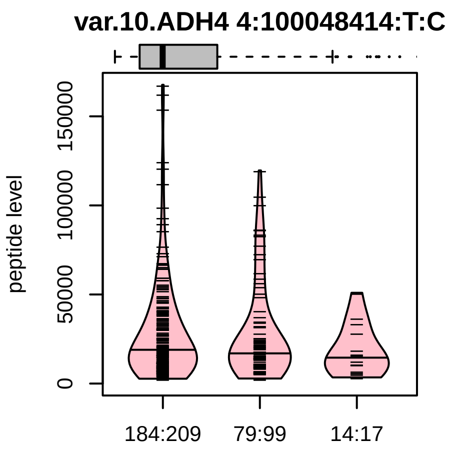

AAIAWEAGKpc2_NP.3
p−min=0.072, pX=0.13, N=277

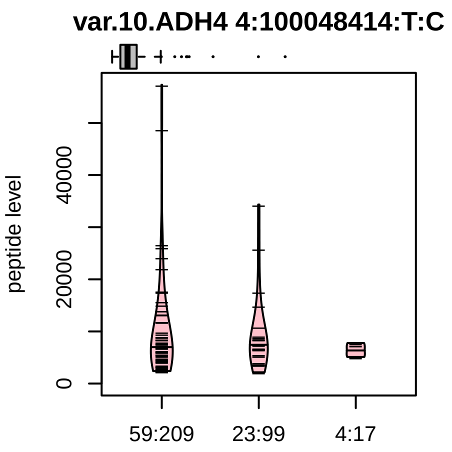

AAIAWEAGKpc2_NP.4
p−min=0.36, pX=0.27, N=86

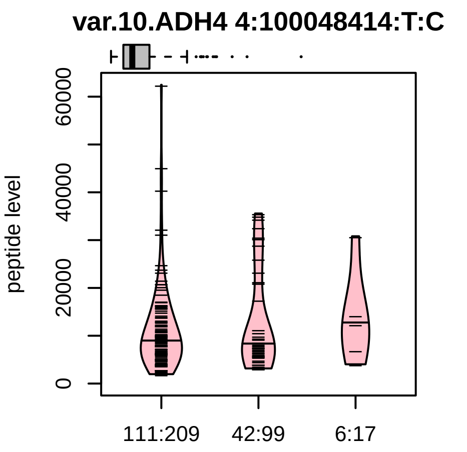

AAIAWEAGKpc2_NP.5
p−min=0.049, pX=0.57, N=159

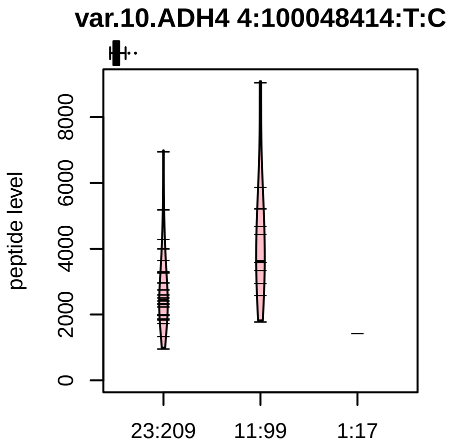

AALDCTTAGWGSCTFIGVAAGSKpc3_NP.2
p−maj=1, pX=0.76, N=35

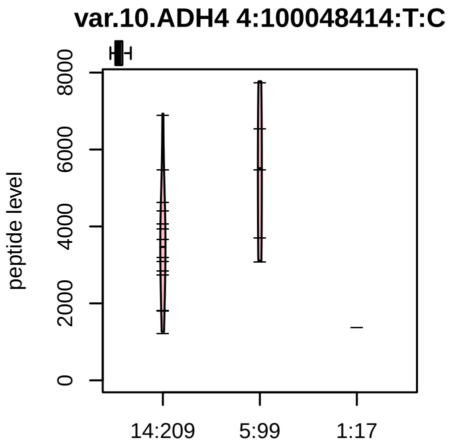

AALDCTTAGWGSCTFIGVAAGSKpc3_NP.3
p−min=0.64, pX=0.82, N=20

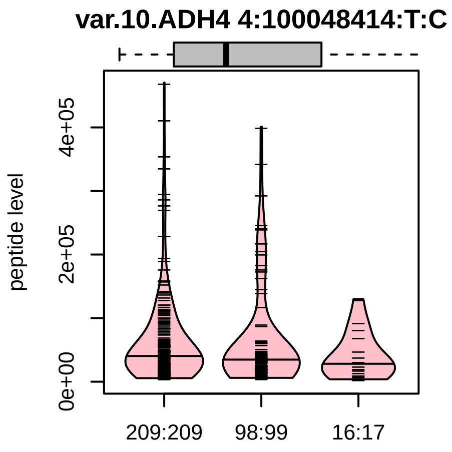

ALGATDCLNPRpc2_NP.1
p−maj=0.1, pX=0.33, N=323

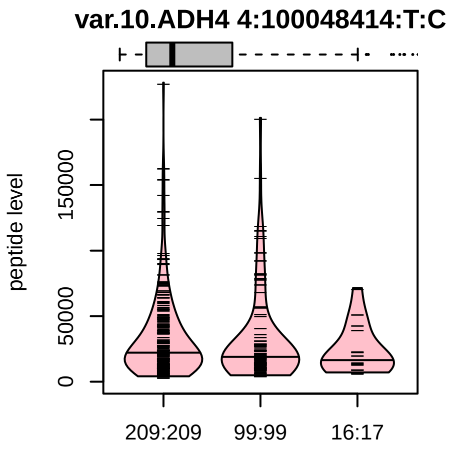

ALGATDCLNPRpc2_NP.2
p−maj=0.052, pX=0.3, N=324

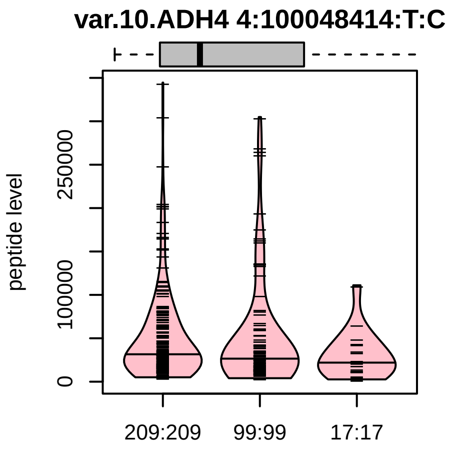

ALGATDCLNPRpc2_NP.3
p−maj=1, pX=0.38, N=325

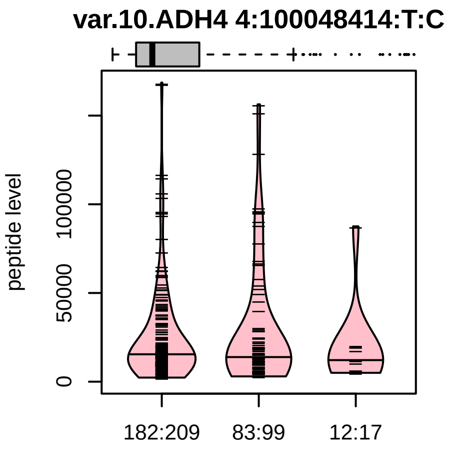

ALGATDCLNPRpc2_NP.4
p−maj=0.15, pX=0.77, N=277

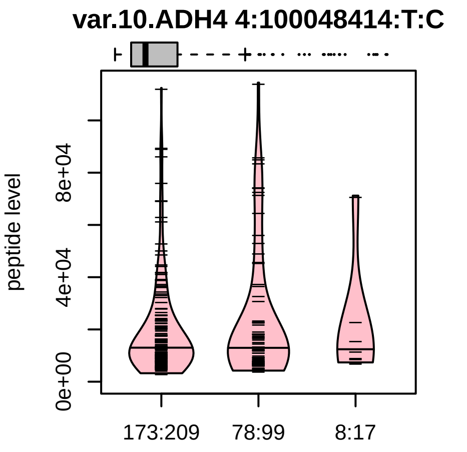

ALGATDCLNPRpc2_NP.5
p−maj=0.0022, pX=0.88, N=259

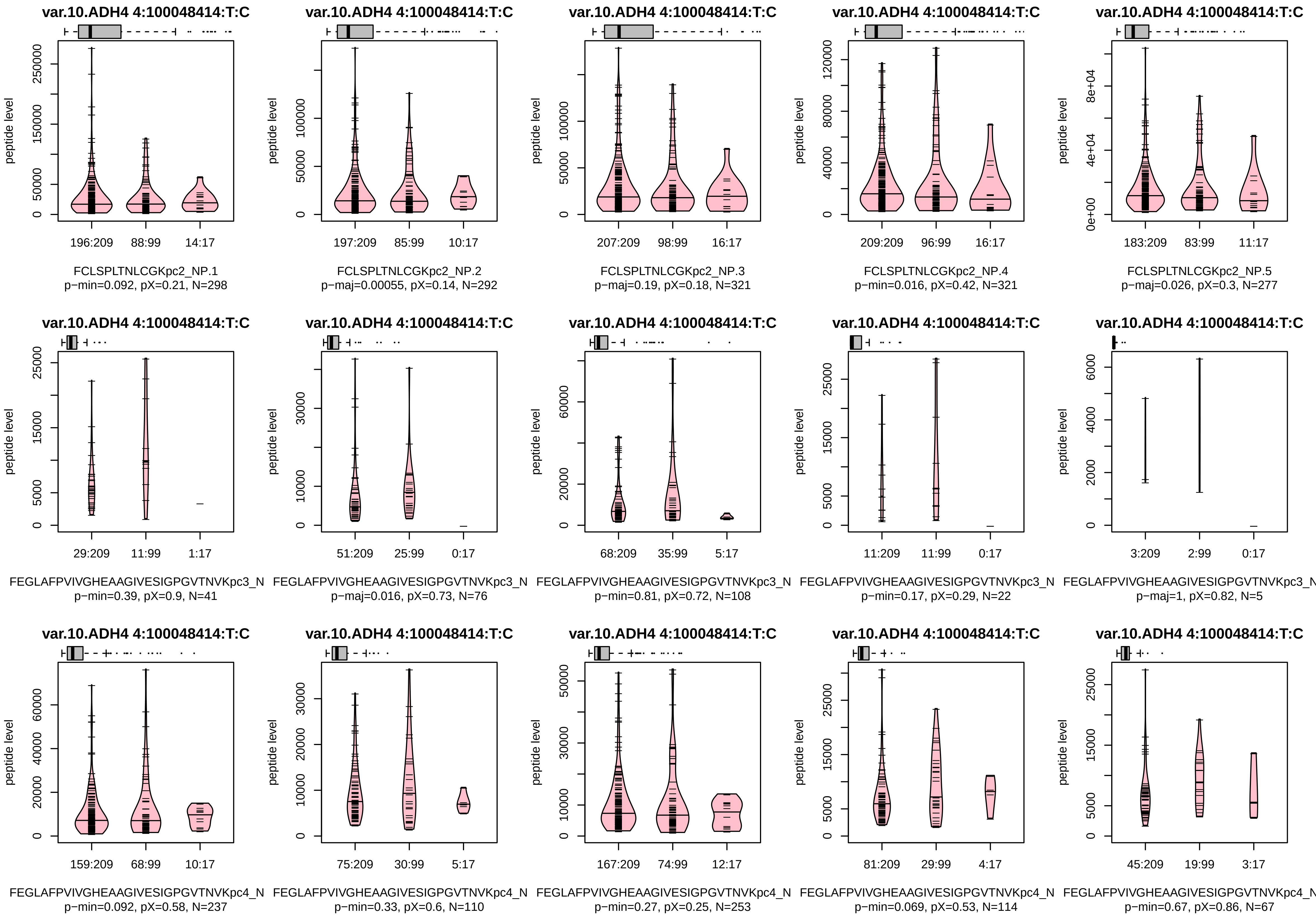
var.10.ADH4 4:100048414:T:C
peptide level
196:209
88:99
14:17
FCLSPLTNLCGKpc2_NP.1
p−min=0.092, pX=0.21, N=298
var.10.ADH4 4:100048414:T:C
peptide level
197:209
85:99
10:17
FCLSPLTNLCGKpc2_NP.2
p−maj=0.00055, pX=0.14, N=292
var.10.ADH4 4:100048414:T:C
peptide level
207:209
98:99
16:17
FCLSPLTNLCGKpc2_NP.3
p−maj=0.19, pX=0.18, N=321
var.10.ADH4 4:100048414:T:C
peptide level
209:209
96:99
16:17
FCLSPLTNLCGKpc2_NP.4
p−min=0.016, pX=0.42, N=321
var.10.ADH4 4:100048414:T:C
peptide level
183:209
83:99
11:17
FCLSPLTNLCGKpc2_NP.5
p−maj=0.026, pX=0.3, N=277
var.10.ADH4 4:100048414:T:C
peptide level
29:209
11:99
1:17
FEGLAFPVIVGHEAAGIVESIGPGVTNVKpc3_N
p−min=0.39, pX=0.9, N=41
var.10.ADH4 4:100048414:T:C
peptide level
51:209
25:99
0:17
FEGLAFPVIVGHEAAGIVESIGPGVTNVKpc3_N
p−maj=0.016, pX=0.73, N=76
var.10.ADH4 4:100048414:T:C
peptide level
68:209
35:99
5:17
FEGLAFPVIVGHEAAGIVESIGPGVTNVKpc3_N
p−min=0.81, pX=0.72, N=108
var.10.ADH4 4:100048414:T:C
peptide level
11:209
11:99
0:17
FEGLAFPVIVGHEAAGIVESIGPGVTNVKpc3_N
p−min=0.17, pX=0.29, N=22
var.10.ADH4 4:100048414:T:C
peptide level
3:209
2:99
0:17
FEGLAFPVIVGHEAAGIVESIGPGVTNVKpc3_N
p−maj=1, pX=0.82, N=5
var.10.ADH4 4:100048414:T:C
peptide level
159:209
68:99
10:17
FEGLAFPVIVGHEAAGIVESIGPGVTNVKpc4_N
p−min=0.092, pX=0.58, N=237
var.10.ADH4 4:100048414:T:C
peptide level
75:209
30:99
5:17
FEGLAFPVIVGHEAAGIVESIGPGVTNVKpc4_N
p−min=0.33, pX=0.6, N=110
var.10.ADH4 4:100048414:T:C
peptide level
167:209
74:99
12:17
FEGLAFPVIVGHEAAGIVESIGPGVTNVKpc4_N
p−min=0.27, pX=0.25, N=253
var.10.ADH4 4:100048414:T:C
peptide level
81:209
29:99
4:17
FEGLAFPVIVGHEAAGIVESIGPGVTNVKpc4_N
p−min=0.069, pX=0.53, N=114
var.10.ADH4 4:100048414:T:C
peptide level
45:209
19:99
3:17
FEGLAFPVIVGHEAAGIVESIGPGVTNVKpc4_N
p−min=0.67, pX=0.86, N=67

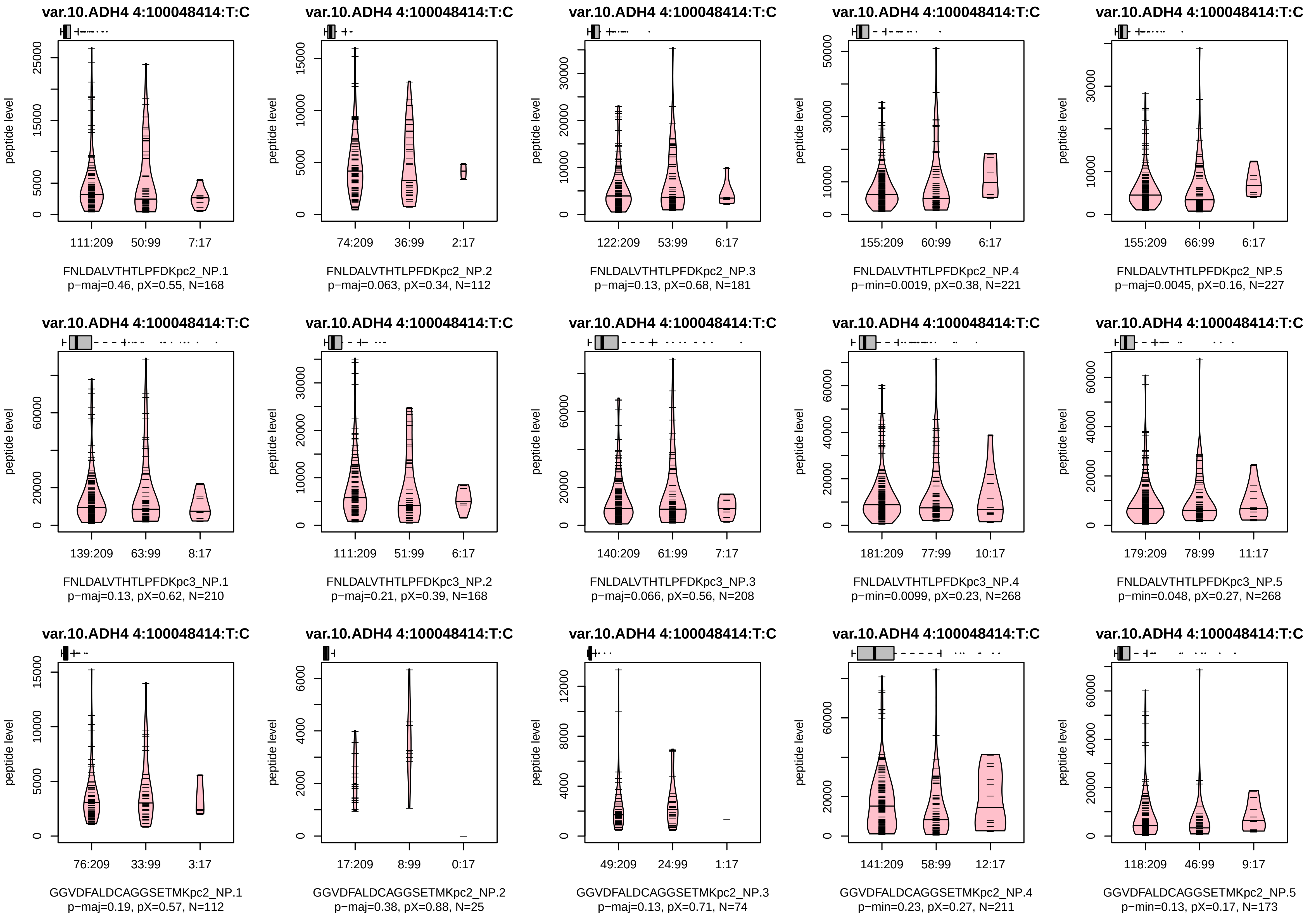
var.10.ADH4 4:100048414:T:C
peptide level
111:209
50:99
7:17
FNLDALVTHTLPFDKpc2_NP.1
p−maj=0.46, pX=0.55, N=168
var.10.ADH4 4:100048414:T:C
peptide level
74:209
36:99
2:17
FNLDALVTHTLPFDKpc2_NP.2
p−maj=0.063, pX=0.34, N=112
var.10.ADH4 4:100048414:T:C
peptide level
122:209
53:99
6:17
FNLDALVTHTLPFDKpc2_NP.3
p−maj=0.13, pX=0.68, N=181
var.10.ADH4 4:100048414:T:C
peptide level
155:209
60:99
6:17
FNLDALVTHTLPFDKpc2_NP.4
p−min=0.0019, pX=0.38, N=221
var.10.ADH4 4:100048414:T:C
peptide level
155:209
66:99
6:17
FNLDALVTHTLPFDKpc2_NP.5
p−maj=0.0045, pX=0.16, N=227
var.10.ADH4 4:100048414:T:C
peptide level
139:209
63:99
8:17
FNLDALVTHTLPFDKpc3_NP.1
p−maj=0.13, pX=0.62, N=210
var.10.ADH4 4:100048414:T:C
peptide level
111:209
51:99
6:17
FNLDALVTHTLPFDKpc3_NP.2
p−maj=0.21, pX=0.39, N=168
var.10.ADH4 4:100048414:T:C
peptide level
140:209
61:99
7:17
FNLDALVTHTLPFDKpc3_NP.3
p−maj=0.066, pX=0.56, N=208
var.10.ADH4 4:100048414:T:C
peptide level
181:209
77:99
10:17
FNLDALVTHTLPFDKpc3_NP.4
p−min=0.0099, pX=0.23, N=268
var.10.ADH4 4:100048414:T:C
peptide level
179:209
78:99
11:17
FNLDALVTHTLPFDKpc3_NP.5
p−min=0.048, pX=0.27, N=268
var.10.ADH4 4:100048414:T:C
peptide level
76:209
33:99
3:17
GGVDFALDCAGGSETMKpc2_NP.1
p−maj=0.19, pX=0.57, N=112
var.10.ADH4 4:100048414:T:C
peptide level
17:209
8:99
0:17
GGVDFALDCAGGSETMKpc2_NP.2
p−maj=0.38, pX=0.88, N=25
var.10.ADH4 4:100048414:T:C
peptide level
49:209
24:99
1:17
GGVDFALDCAGGSETMKpc2_NP.3
p−maj=0.13, pX=0.71, N=74
var.10.ADH4 4:100048414:T:C
peptide level
141:209
58:99
12:17
GGVDFALDCAGGSETMKpc2_NP.4
p−min=0.23, pX=0.27, N=211
var.10.ADH4 4:100048414:T:C
peptide level
118:209
46:99
9:17
GGVDFALDCAGGSETMKpc2_NP.5
p−min=0.13, pX=0.17, N=173

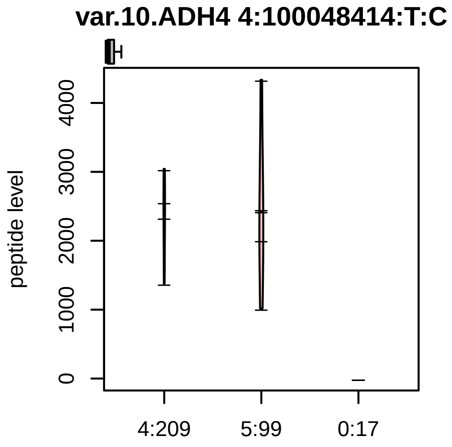

GKPVYHFFGTSTFSQYTVVSDINLAKpc4_NP
p−min=0.29, pX=0.44, N=9

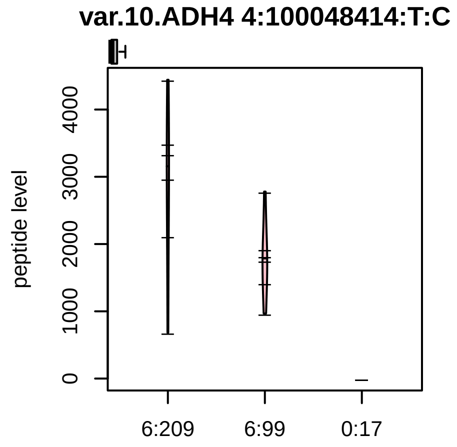

GKPVYHFFGTSTFSQYTVVSDINLAKpc4_NP
p−min=0.36, pX=0.89, N=12

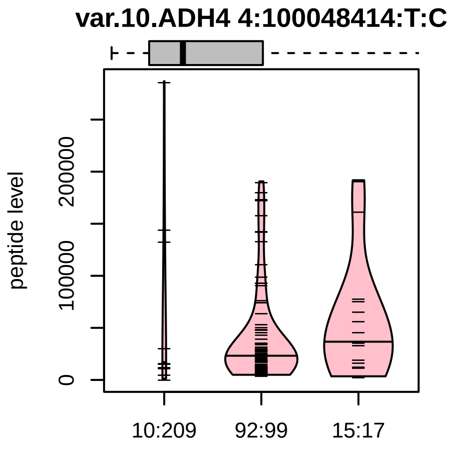

GLTIFPEELIIGRpc2_NP.1
p−min=3.5e−62, pX=1.3e−17, N=117

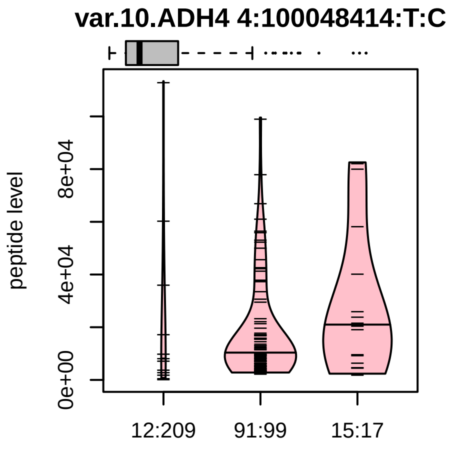

GLTIFPEELIIGRpc2_NP.2
p−min=6.3e−59, pX=1.7e−21, N=118

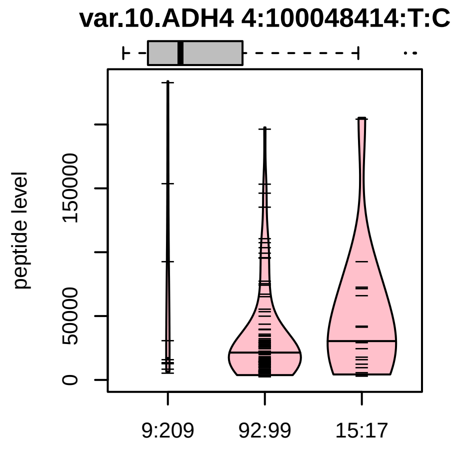

GLTIFPEELIIGRpc2_NP.3
p−min=3.1e−63, pX=9.6e−18, N=116

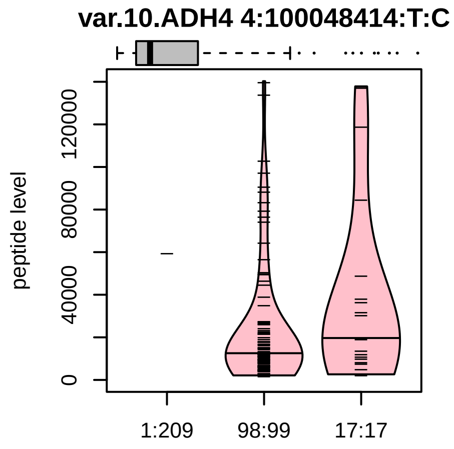

GLTIFPEELIIGRpc2_NP.4
p−min=5.6e−87, pX=8.8e−28, N=116

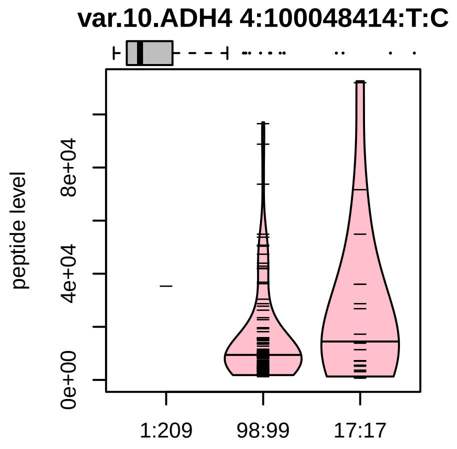

GLTIFPEELIIGRpc2_NP.5
p−min=5.6e−87, pX=6.5e−30, N=116

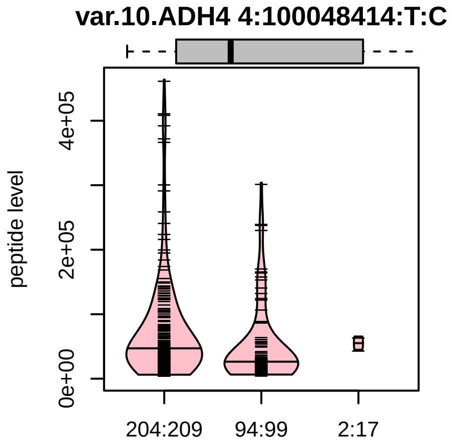

GLTVFPEELIIGRpc2_NP.1
p−maj=1.6e−17, pX=2.8e−05, N=300

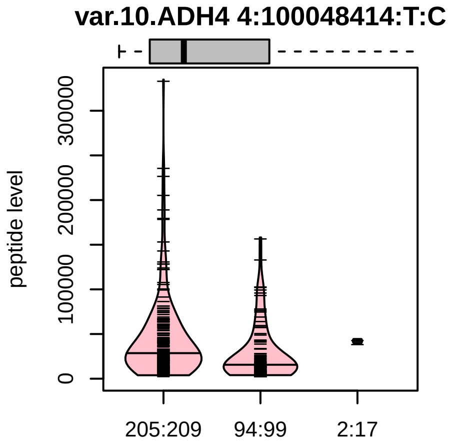

GLTVFPEELIIGRpc2_NP.2
p−maj=6.4e−18, pX=4.5e−06, N=301

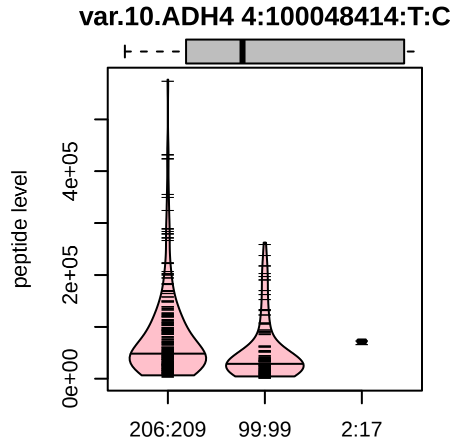

GLTVFPEELIIGRpc2_NP.3
p−maj=4.1e−21, pX=9.6e−06, N=307

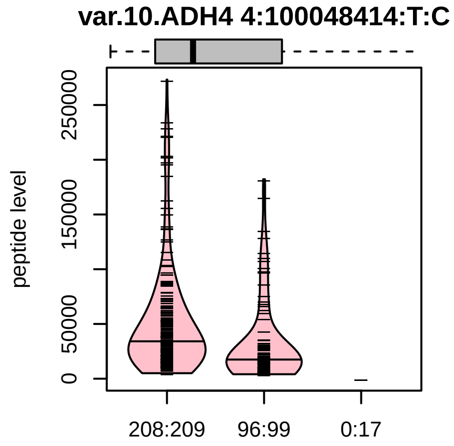

GLTVFPEELIIGRpc2_NP.4
p−maj=6.5e−25, pX=1.2e−07, N=304

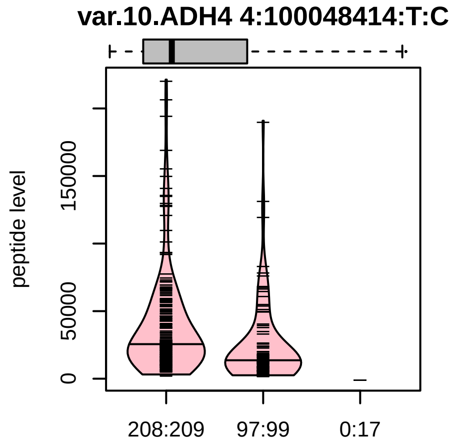

GLTVFPEELIIGRpc2_NP.5
p−maj=1.2e−25, pX=3e−07, N=305

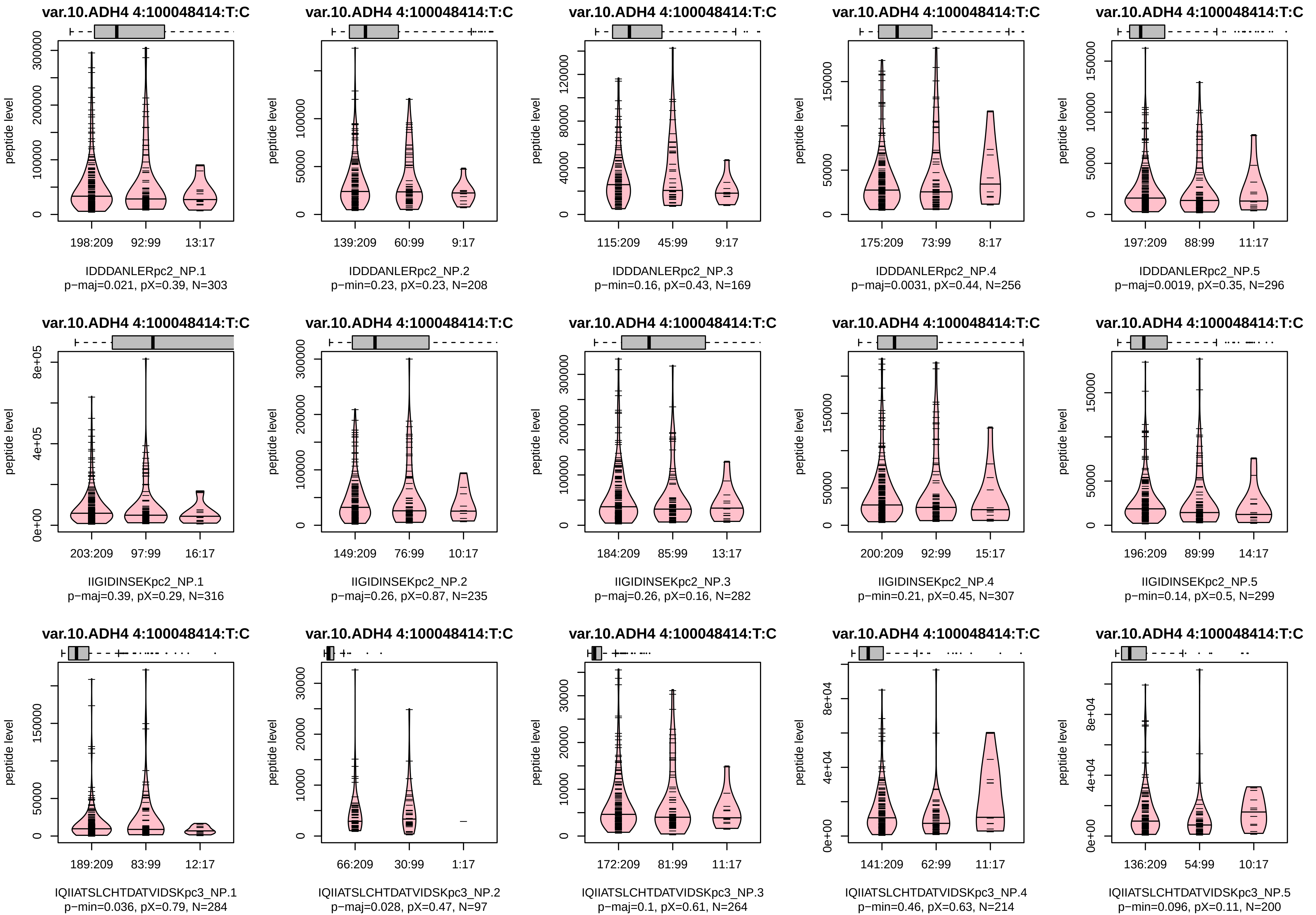
var.10.ADH4 4:100048414:T:C
peptide level
198:209
92:99
13:17
IDDDANLERpc2_NP.1
p−maj=0.021, pX=0.39, N=303
var.10.ADH4 4:100048414:T:C
peptide level
139:209
60:99
9:17
IDDDANLERpc2_NP.2
p−min=0.23, pX=0.23, N=208
var.10.ADH4 4:100048414:T:C
peptide level
115:209
45:99
9:17
IDDDANLERpc2_NP.3
p−min=0.16, pX=0.43, N=169
var.10.ADH4 4:100048414:T:C
peptide level
175:209
73:99
8:17
IDDDANLERpc2_NP.4
p−maj=0.0031, pX=0.44, N=256
var.10.ADH4 4:100048414:T:C
peptide level
197:209
88:99
11:17
IDDDANLERpc2_NP.5
p−maj=0.0019, pX=0.35, N=296
var.10.ADH4 4:100048414:T:C
peptide level
203:209
97:99
16:17
IIGIDINSEKpc2_NP.1
p−maj=0.39, pX=0.29, N=316
var.10.ADH4 4:100048414:T:C
peptide level
149:209
76:99
10:17
IIGIDINSEKpc2_NP.2
p−maj=0.26, pX=0.87, N=235
var.10.ADH4 4:100048414:T:C
peptide level
184:209
85:99
13:17
IIGIDINSEKpc2_NP.3
p−maj=0.26, pX=0.16, N=282
var.10.ADH4 4:100048414:T:C
peptide level
200:209
92:99
15:17
IIGIDINSEKpc2_NP.4
p−min=0.21, pX=0.45, N=307
var.10.ADH4 4:100048414:T:C
peptide level
196:209
89:99
14:17
IIGIDINSEKpc2_NP.5
p−min=0.14, pX=0.5, N=299
var.10.ADH4 4:100048414:T:C
peptide level
189:209
83:99
12:17
IQIIATSLCHTDATVIDSKpc3_NP.1
p−min=0.036, pX=0.79, N=284
var.10.ADH4 4:100048414:T:C
peptide level
66:209
30:99
1:17
IQIIATSLCHTDATVIDSKpc3_NP.2
p−maj=0.028, pX=0.47, N=97
var.10.ADH4 4:100048414:T:C
peptide level
172:209
81:99
11:17
IQIIATSLCHTDATVIDSKpc3_NP.3
p−maj=0.1, pX=0.61, N=264
var.10.ADH4 4:100048414:T:C
peptide level
141:209
62:99
11:17
IQIIATSLCHTDATVIDSKpc3_NP.4
p−min=0.46, pX=0.63, N=214
var.10.ADH4 4:100048414:T:C
peptide level
136:209
54:99
10:17
IQIIATSLCHTDATVIDSKpc3_NP.5
p−min=0.096, pX=0.11, N=200

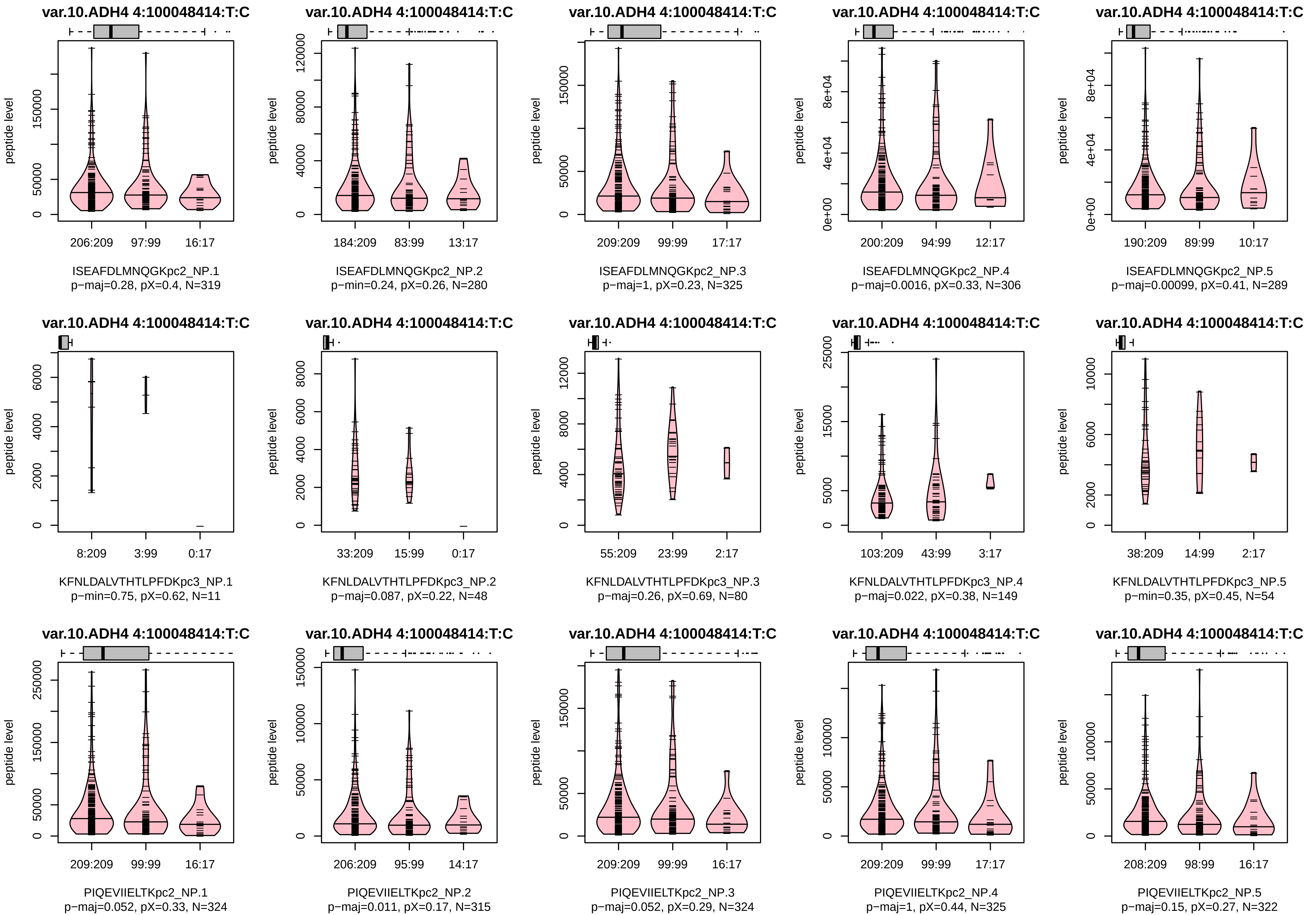
var.10.ADH4 4:100048414:T:C
peptide level
206:209
97:99
16:17
ISEAFDLMNQGKpc2_NP.1
p−maj=0.28, pX=0.4, N=319
var.10.ADH4 4:100048414:T:C
peptide level
184:209
83:99
13:17
ISEAFDLMNQGKpc2_NP.2
p−min=0.24, pX=0.26, N=280
var.10.ADH4 4:100048414:T:C
peptide level
209:209
99:99
17:17
ISEAFDLMNQGKpc2_NP.3
p−maj=1, pX=0.23, N=325
var.10.ADH4 4:100048414:T:C
peptide level
200:209
94:99
12:17
ISEAFDLMNQGKpc2_NP.4
p−maj=0.0016, pX=0.33, N=306
var.10.ADH4 4:100048414:T:C
peptide level
190:209
89:99
10:17
ISEAFDLMNQGKpc2_NP.5
p−maj=0.00099, pX=0.41, N=289
var.10.ADH4 4:100048414:T:C
peptide level
8:209
3:99
0:17
KFNLDALVTHTLPFDKpc3_NP.1
p−min=0.75, pX=0.62, N=11
var.10.ADH4 4:100048414:T:C
peptide level
33:209
15:99
0:17
KFNLDALVTHTLPFDKpc3_NP.2
p−maj=0.087, pX=0.22, N=48
var.10.ADH4 4:100048414:T:C
peptide level
55:209
23:99
2:17
KFNLDALVTHTLPFDKpc3_NP.3
p−maj=0.26, pX=0.69, N=80
var.10.ADH4 4:100048414:T:C
peptide level
103:209
43:99
3:17
KFNLDALVTHTLPFDKpc3_NP.4
p−maj=0.022, pX=0.38, N=149
var.10.ADH4 4:100048414:T:C
peptide level
38:209
14:99
2:17
KFNLDALVTHTLPFDKpc3_NP.5
p−min=0.35, pX=0.45, N=54
var.10.ADH4 4:100048414:T:C
peptide level
209:209
99:99
16:17
PIQEVIIELTKpc2_NP.1
p−maj=0.052, pX=0.33, N=324
var.10.ADH4 4:100048414:T:C
peptide level
206:209
95:99
14:17
PIQEVIIELTKpc2_NP.2
p−maj=0.011, pX=0.17, N=315
var.10.ADH4 4:100048414:T:C
peptide level
209:209
99:99
16:17
PIQEVIIELTKpc2_NP.3
p−maj=0.052, pX=0.29, N=324
var.10.ADH4 4:100048414:T:C
peptide level
209:209
99:99
17:17
PIQEVIIELTKpc2_NP.4
p−maj=1, pX=0.44, N=325
var.10.ADH4 4:100048414:T:C
peptide level
208:209
98:99
16:17
PIQEVIIELTKpc2_NP.5
p−maj=0.15, pX=0.27, N=322

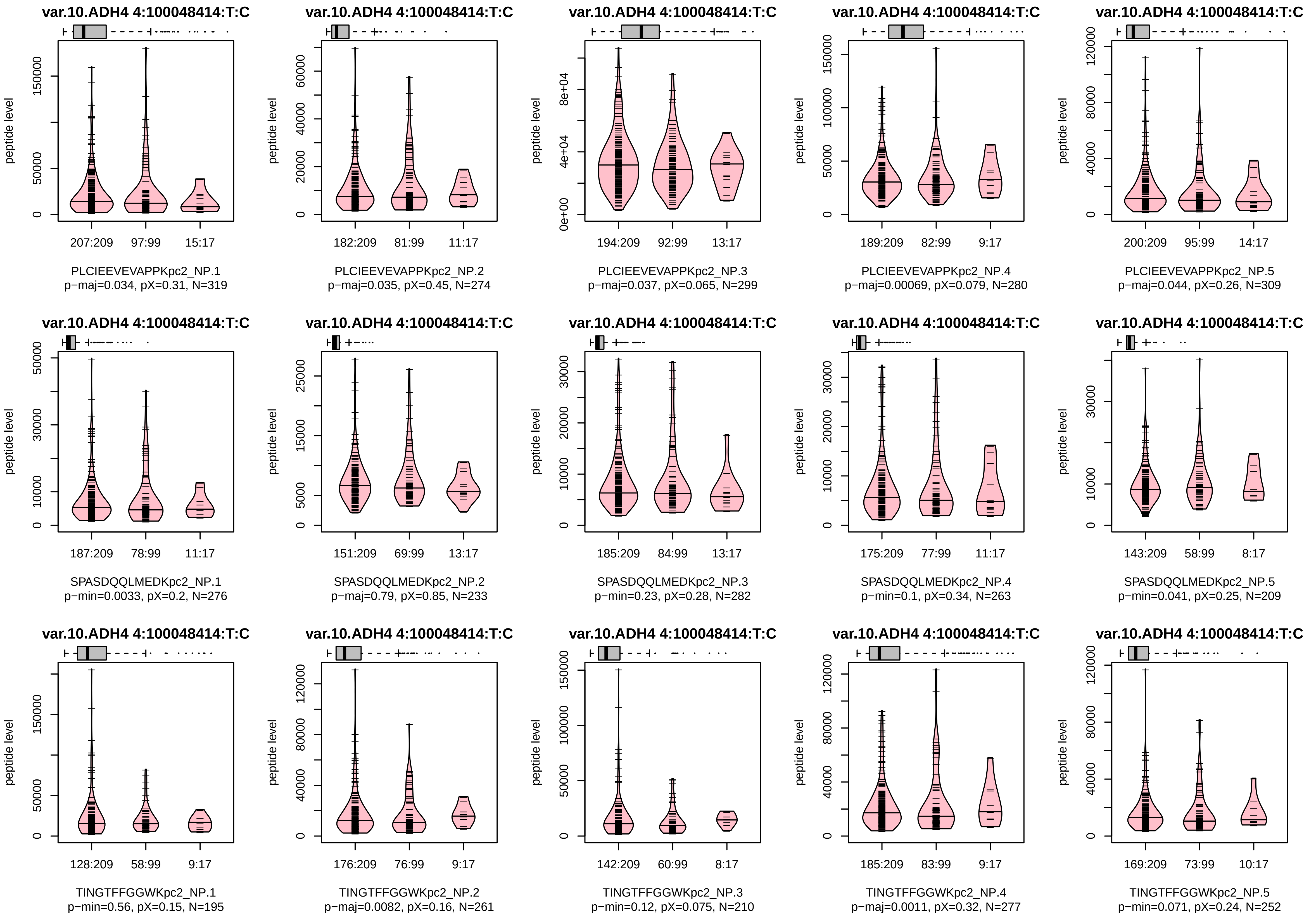

var.10.ADH4 4:100048414:T:C
peptide level
207:209 97:99 15:17
PLCIEEVEVAPPKpc2_NP.1
p−maj=0.034, pX=0.31, N=319
var.10.ADH4 4:100048414:T:C
peptide level
182:209 81:99 11:17
PLCIEEVEVAPPKpc2_NP.2
p−maj=0.035, pX=0.45, N=274
var.10.ADH4 4:100048414:T:C
peptide level
194:209 92:99 13:17
PLCIEEVEVAPPKpc2_NP.3
p−maj=0.037, pX=0.065, N=299
var.10.ADH4 4:100048414:T:C
peptide level
189:209 82:99 9:17
PLCIEEVEVAPPKpc2_NP.4
p−maj=0.00069, pX=0.079, N=280
var.10.ADH4 4:100048414:T:C
peptide level
200:209 95:99 14:17
PLCIEEVEVAPPKpc2_NP.5
p−maj=0.044, pX=0.26, N=309
var.10.ADH4 4:100048414:T:C
peptide level
187:209 78:99 11:17
SPASDQQLMEDKpc2_NP.1
p−min=0.0033, pX=0.2, N=276
var.10.ADH4 4:100048414:T:C
peptide level
151:209 69:99 13:17
SPASDQQLMEDKpc2_NP.2
p−maj=0.79, pX=0.85, N=233
var.10.ADH4 4:100048414:T:C
peptide level
185:209 84:99 13:17
SPASDQQLMEDKpc2_NP.3
p−min=0.23, pX=0.28, N=282
var.10.ADH4 4:100048414:T:C
peptide level
175:209 77:99 11:17
SPASDQQLMEDKpc2_NP.4
p−min=0.1, pX=0.34, N=263
var.10.ADH4 4:100048414:T:C
peptide level
143:209 58:99 8:17
SPASDQQLMEDKpc2_NP.5
p−min=0.041, pX=0.25, N=209
var.10.ADH4 4:100048414:T:C
peptide level
128:209 58:99 9:17
TINGTFFGGWKpc2_NP.1
p−min=0.56, pX=0.15, N=195
var.10.ADH4 4:100048414:T:C
peptide level
176:209 76:99 9:17
TINGTFFGGWKpc2_NP.2
p−maj=0.0082, pX=0.16, N=261
var.10.ADH4 4:100048414:T:C
peptide level
142:209 60:99 8:17
TINGTFFGGWKpc2_NP.3
p−min=0.12, pX=0.075, N=210
var.10.ADH4 4:100048414:T:C
peptide level
185:209 83:99 9:17
TINGTFFGGWKpc2_NP.4
p−maj=0.0011, pX=0.32, N=277
var.10.ADH4 4:100048414:T:C
peptide level
169:209 73:99 10:17
TINGTFFGGWKpc2_NP.5
p−min=0.071, pX=0.24, N=252

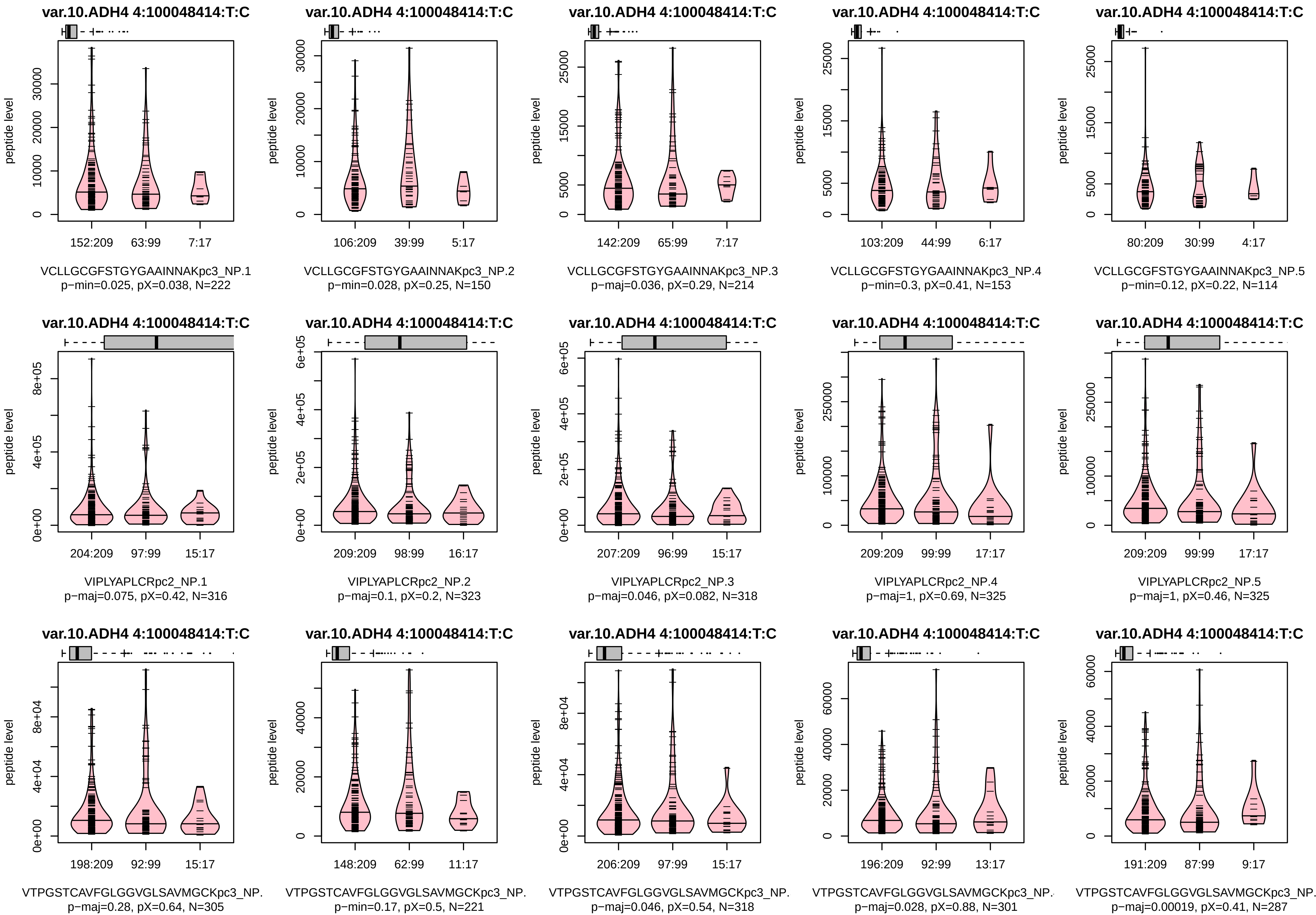
var.10.ADH4 4:100048414:T:C
peptide level
152:209
63:99
7:17
VCLLGCGFSTGYGAAINNAKpc3_NP.1
p−min=0.025, pX=0.038, N=222
var.10.ADH4 4:100048414:T:C
peptide level
106:209
39:99
5:17
VCLLGCGFSTGYGAAINNAKpc3_NP.2
p−min=0.028, pX=0.25, N=150
var.10.ADH4 4:100048414:T:C
peptide level
142:209
65:99
7:17
VCLLGCGFSTGYGAAINNAKpc3_NP.3
p−maj=0.036, pX=0.29, N=214
var.10.ADH4 4:100048414:T:C
peptide level
103:209
44:99
6:17
VCLLGCGFSTGYGAAINNAKpc3_NP.4
p−min=0.3, pX=0.41, N=153
var.10.ADH4 4:100048414:T:C
peptide level
80:209
30:99
4:17
VCLLGCGFSTGYGAAINNAKpc3_NP.5
p−min=0.12, pX=0.22, N=114
var.10.ADH4 4:100048414:T:C
peptide level
204:209
97:99
15:17
VIPLYAPLCRpc2_NP.1
p−maj=0.075, pX=0.42, N=316
var.10.ADH4 4:100048414:T:C
peptide level
209:209
98:99
16:17
VIPLYAPLCRpc2_NP.2
p−maj=0.1, pX=0.2, N=323
var.10.ADH4 4:100048414:T:C
peptide level
207:209
96:99
15:17
VIPLYAPLCRpc2_NP.3
p−maj=0.046, pX=0.082, N=318
var.10.ADH4 4:100048414:T:C
peptide level
209:209
99:99
17:17
VIPLYAPLCRpc2_NP.4
p−maj=1, pX=0.69, N=325
var.10.ADH4 4:100048414:T:C
peptide level
209:209
99:99
17:17
VIPLYAPLCRpc2_NP.5
p−maj=1, pX=0.46, N=325
var.10.ADH4 4:100048414:T:C
peptide level
198:209
92:99
15:17
VTPGSTCAVFGLGGVGLSAVMGCKpc3_NP.
p−maj=0.28, pX=0.64, N=305
var.10.ADH4 4:100048414:T:C
peptide level
148:209
62:99
11:17
VTPGSTCAVFGLGGVGLSAVMGCKpc3_NP.
p−min=0.17, pX=0.5, N=221
var.10.ADH4 4:100048414:T:C
peptide level
206:209
97:99
15:17
VTPGSTCAVFGLGGVGLSAVMGCKpc3_NP.
p−maj=0.046, pX=0.54, N=318
var.10.ADH4 4:100048414:T:C
peptide level
196:209
92:99
13:17
VTPGSTCAVFGLGGVGLSAVMGCKpc3_NP.
p−maj=0.028, pX=0.88, N=301
var.10.ADH4 4:100048414:T:C
peptide level
191:209
87:99
9:17
VTPGSTCAVFGLGGVGLSAVMGCKpc3_NP.
p−maj=0.00019, pX=0.41, N=287

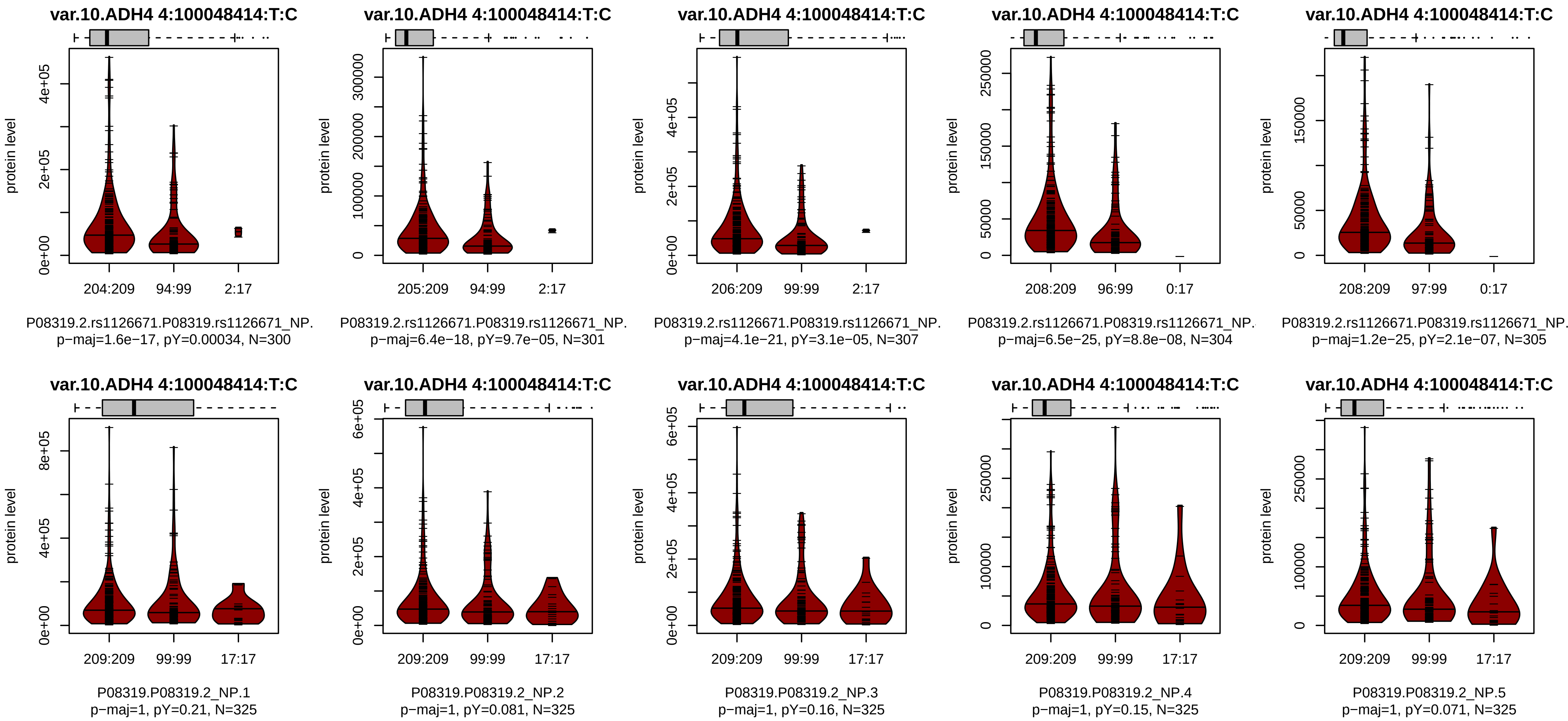
var.10.ADH4 4:100048414:T:C
protein level
204:209
94:99
2:17
P08319.2.rs1126671.P08319.rs1126671_NP.
p−maj=1.6e−17, pY=0.00034, N=300
var.10.ADH4 4:100048414:T:C
205:209
94:99
2:17
P08319.2.rs1126671.P08319.rs1126671_NP.
p−maj=6.4e−18, pY=9.7e−05, N=301
var.10.ADH4 4:100048414:T:C
206:209
99:99
2:17
P08319.2.rs1126671.P08319.rs1126671_NP.
p−maj=4.1e−21, pY=3.1e−05, N=307
var.10.ADH4 4:100048414:T:C
208:209
96:99
0:17
P08319.2.rs1126671.P08319.rs1126671_NP.
p−maj=6.5e−25, pY=8.8e−08, N=304
var.10.ADH4 4:100048414:T:C
208:209
97:99
0:17
P08319.2.rs1126671.P08319.rs1126671_NP.
p−maj=1.2e−25, pY=2.1e−07, N=305
var.10.ADH4 4:100048414:T:C
209:209
99:99
17:17
P08319.P08319.2_NP.1
p−maj=1, pY=0.21, N=325
var.10.ADH4 4:100048414:T:C
209:209
99:99
17:17
P08319.P08319.2_NP.2
p−maj=1, pY=0.081, N=325
var.10.ADH4 4:100048414:T:C
209:209
99:99
17:17
P08319.P08319.2_NP.3
p−maj=1, pY=0.16, N=325
var.10.ADH4 4:100048414:T:C
209:209
99:99
17:17
P08319.P08319.2_NP.4
p−maj=1, pY=0.15, N=325
var.10.ADH4 4:100048414:T:C
209:209
99:99
17:17
P08319.P08319.2_NP.5
p−maj=1, pY=0.071, N=325

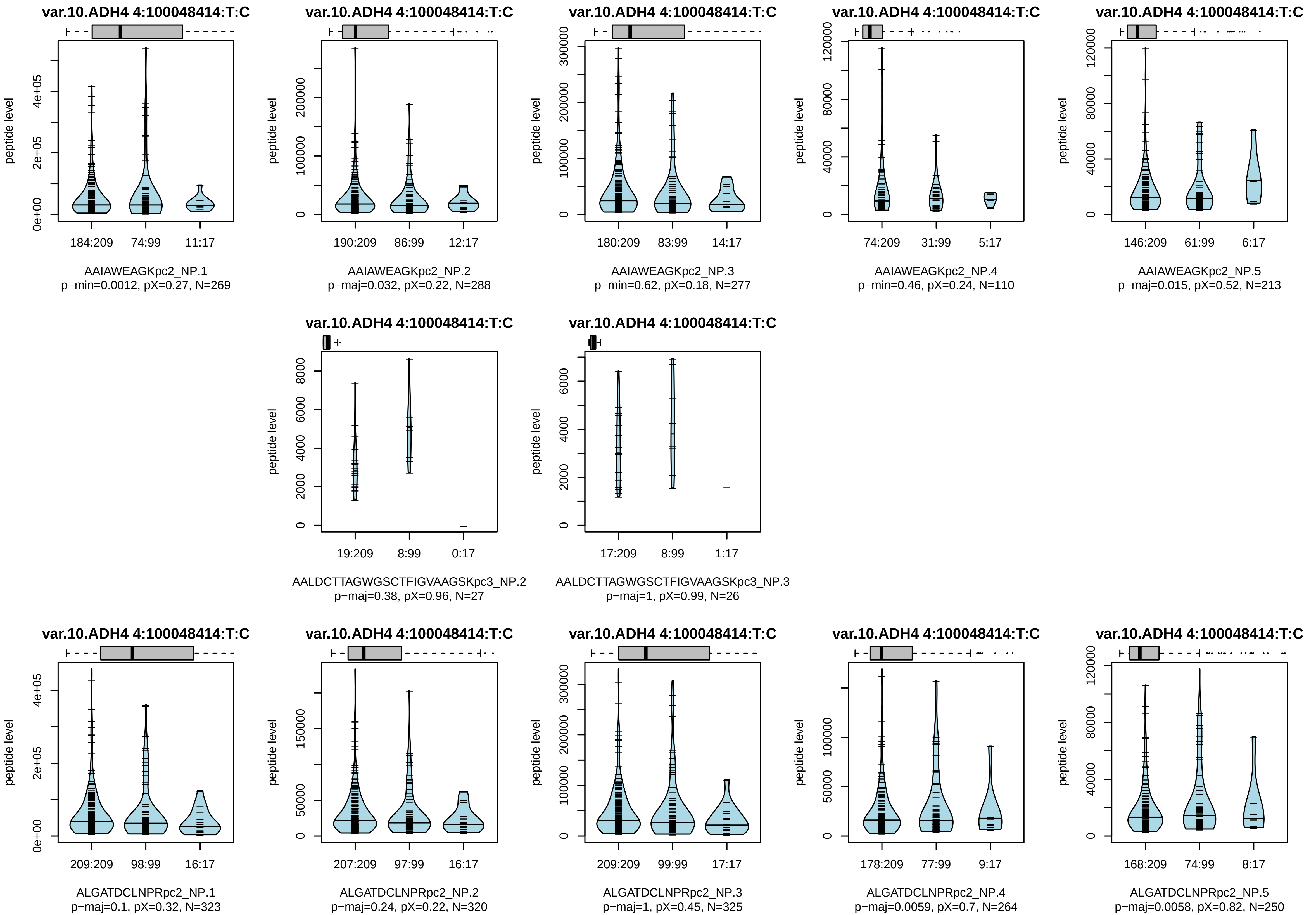

var.10.ADH4 4:100048414:T:C
peptide level
184:209
74:99
11:17
AAIAWEAGKpc2_NP.1
p−min=0.0012, pX=0.27, N=269
var.10.ADH4 4:100048414:T:C
peptide level
190:209
86:99
12:17
AAIAWEAGKpc2_NP.2
p−maj=0.032, pX=0.22, N=288
var.10.ADH4 4:100048414:T:C
peptide level
180:209
83:99
14:17
AAIAWEAGKpc2_NP.3
p−min=0.62, pX=0.18, N=277
var.10.ADH4 4:100048414:T:C
peptide level
74:209
31:99
5:17
AAIAWEAGKpc2_NP.4
p−min=0.46, pX=0.24, N=110
var.10.ADH4 4:100048414:T:C
peptide level
146:209
61:99
6:17
AAIAWEAGKpc2_NP.5
p−maj=0.015, pX=0.52, N=213
var.10.ADH4 4:100048414:T:C
peptide level
19:209
8:99
0:17
AALDCTTAGWGSCTFIGVAAGSKpc3_NP.2
p−maj=0.38, pX=0.96, N=27
var.10.ADH4 4:100048414:T:C
peptide level
17:209
8:99
1:17
AALDCTTAGWGSCTFIGVAAGSKpc3_NP.3
p−maj=1, pX=0.99, N=26
var.10.ADH4 4:100048414:T:C
peptide level
209:209
98:99
16:17
ALGATDCLNPRpc2_NP.1
p−maj=0.1, pX=0.32, N=323
var.10.ADH4 4:100048414:T:C
peptide level
207:209
97:99
16:17
ALGATDCLNPRpc2_NP.2
p−maj=0.24, pX=0.22, N=320
var.10.ADH4 4:100048414:T:C
peptide level
209:209
99:99
17:17
ALGATDCLNPRpc2_NP.3
p−maj=1, pX=0.45, N=325
var.10.ADH4 4:100048414:T:C
peptide level
178:209
77:99
9:17
ALGATDCLNPRpc2_NP.4
p−maj=0.0059, pX=0.7, N=264
var.10.ADH4 4:100048414:T:C
peptide level
168:209
74:99
8:17
ALGATDCLNPRpc2_NP.5
p−maj=0.0058, pX=0.82, N=250

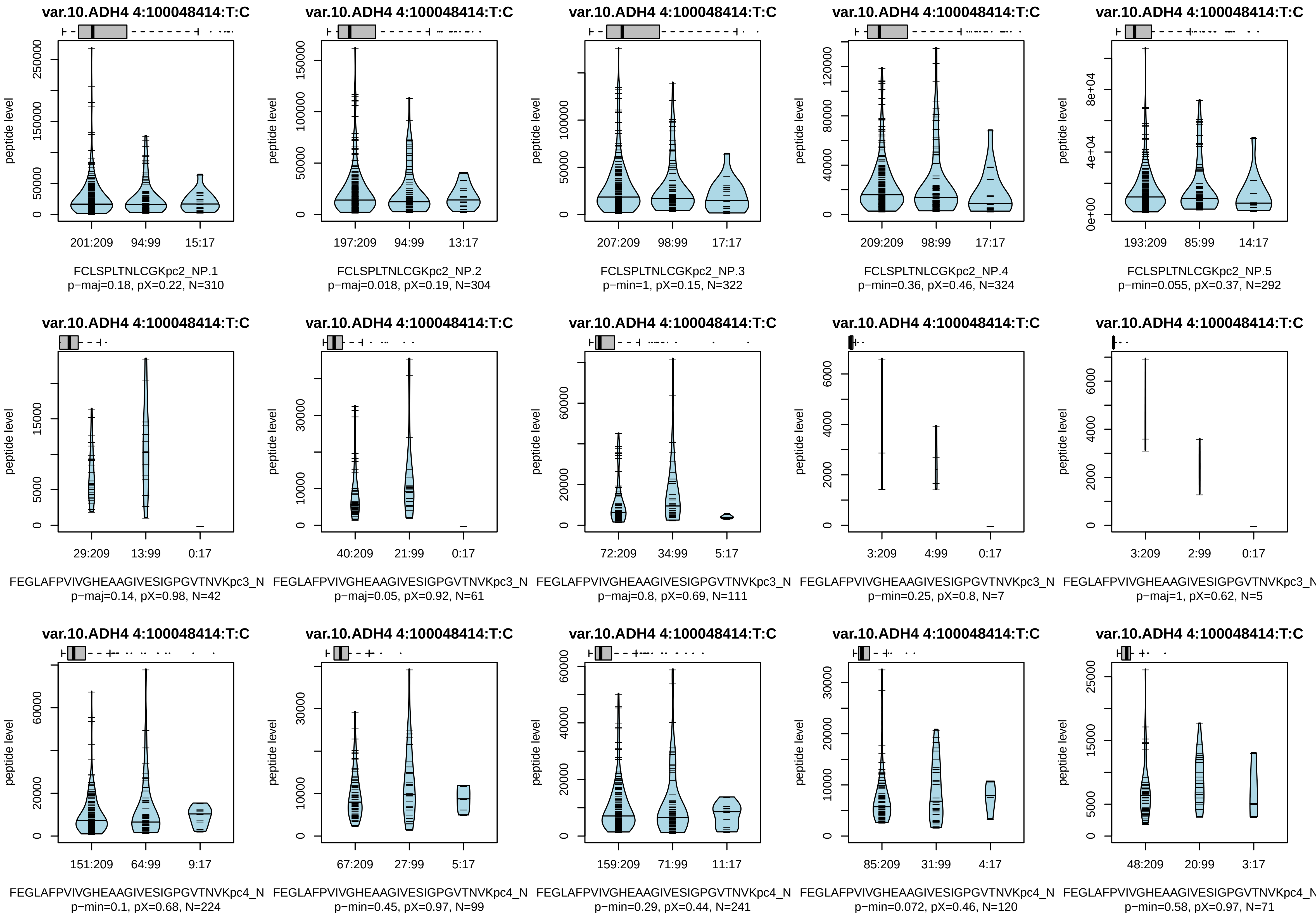

var.10.ADH4 4:100048414:T:C
peptide level
201:209
94:99
15:17
FCLSPLTNLCGKpc2_NP.1
p−maj=0.18, pX=0.22, N=310
var.10.ADH4 4:100048414:T:C
peptide level
197:209
94:99
13:17
FCLSPLTNLCGKpc2_NP.2
p−maj=0.018, pX=0.19, N=304
var.10.ADH4 4:100048414:T:C
peptide level
207:209
98:99
17:17
FCLSPLTNLCGKpc2_NP.3
p−min=1, pX=0.15, N=322
var.10.ADH4 4:100048414:T:C
peptide level
209:209
98:99
17:17
FCLSPLTNLCGKpc2_NP.4
p−min=0.36, pX=0.46, N=324
var.10.ADH4 4:100048414:T:C
peptide level
193:209
85:99
14:17
FCLSPLTNLCGKpc2_NP.5
p−min=0.055, pX=0.37, N=292
var.10.ADH4 4:100048414:T:C
peptide level
29:209
13:99
0:17
FEGLAFPVIVGHEAAGIVESIGPGVTNVKpc3_N
p−maj=0.14, pX=0.98, N=42
var.10.ADH4 4:100048414:T:C
peptide level
40:209
21:99
0:17
FEGLAFPVIVGHEAAGIVESIGPGVTNVKpc3_N
p−maj=0.05, pX=0.92, N=61
var.10.ADH4 4:100048414:T:C
peptide level
72:209
34:99
5:17
FEGLAFPVIVGHEAAGIVESIGPGVTNVKpc3_N
p−maj=0.8, pX=0.69, N=111
var.10.ADH4 4:100048414:T:C
peptide level
3:209
4:99
0:17
FEGLAFPVIVGHEAAGIVESIGPGVTNVKpc3_N
p−min=0.25, pX=0.8, N=7
var.10.ADH4 4:100048414:T:C
peptide level
3:209
2:99
0:17
FEGLAFPVIVGHEAAGIVESIGPGVTNVKpc3_N
p−maj=1, pX=0.62, N=5
var.10.ADH4 4:100048414:T:C
peptide level
151:209
64:99
9:17
FEGLAFPVIVGHEAAGIVESIGPGVTNVKpc4_N
p−min=0.1, pX=0.68, N=224
var.10.ADH4 4:100048414:T:C
peptide level
67:209
27:99
5:17
FEGLAFPVIVGHEAAGIVESIGPGVTNVKpc4_N
p−min=0.45, pX=0.97, N=99
var.10.ADH4 4:100048414:T:C
peptide level
159:209
71:99
11:17
FEGLAFPVIVGHEAAGIVESIGPGVTNVKpc4_N
p−min=0.29, pX=0.44, N=241
var.10.ADH4 4:100048414:T:C
peptide level
85:209
31:99
4:17
FEGLAFPVIVGHEAAGIVESIGPGVTNVKpc4_N
p−min=0.072, pX=0.46, N=120
var.10.ADH4 4:100048414:T:C
peptide level
48:209
20:99
3:17
FEGLAFPVIVGHEAAGIVESIGPGVTNVKpc4_N
p−min=0.58, pX=0.97, N=71

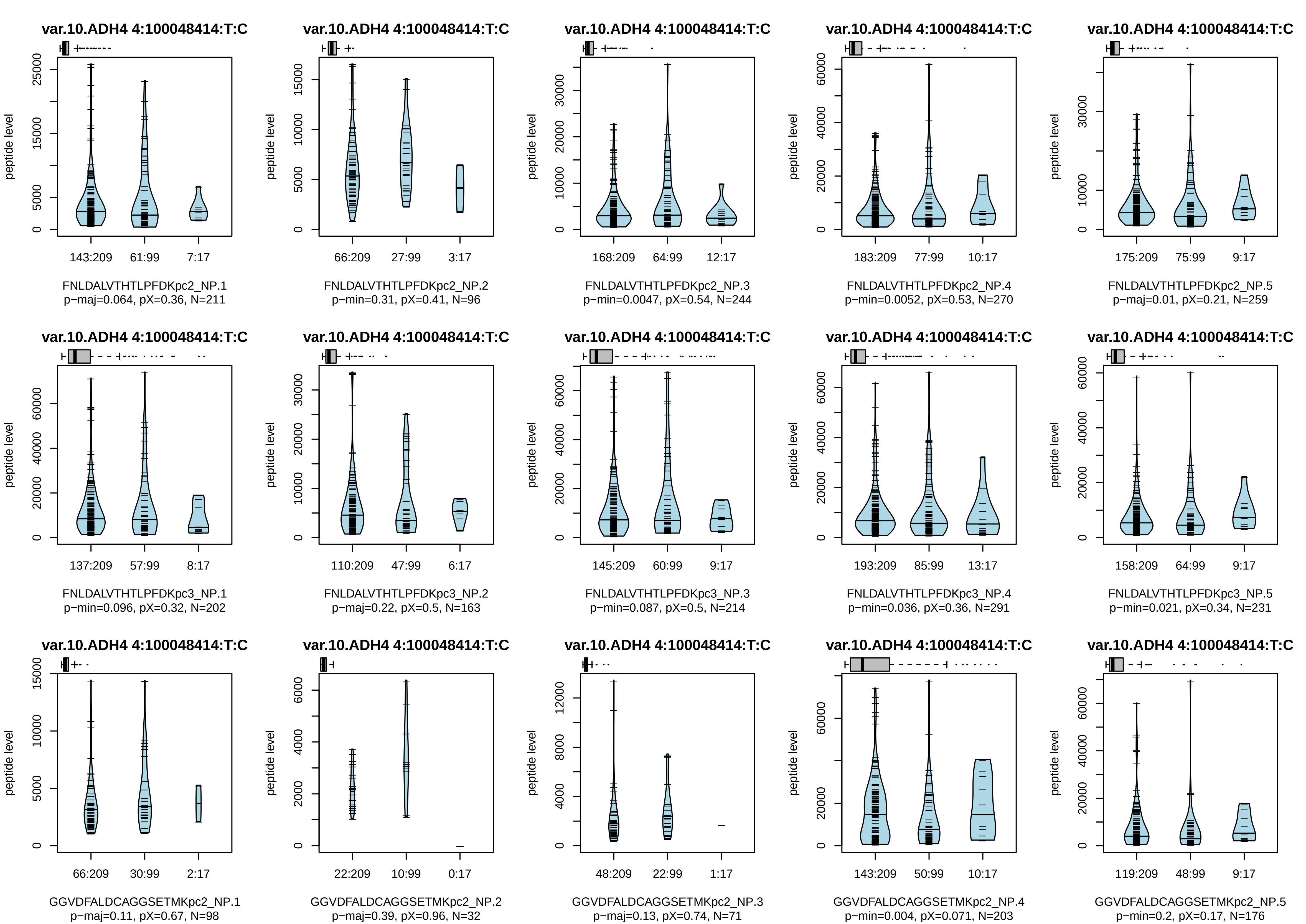
var.10.ADH4 4:100048414:T:C
peptide level
143:209
61:99
7:17
FNLDALVTHTLPFDKpc2_NP.1
p−maj=0.064, pX=0.36, N=211
var.10.ADH4 4:100048414:T:C
peptide level
66:209
27:99
3:17
FNLDALVTHTLPFDKpc2_NP.2
p−min=0.31, pX=0.41, N=96
var.10.ADH4 4:100048414:T:C
peptide level
168:209
64:99
12:17
FNLDALVTHTLPFDKpc2_NP.3
p−min=0.0047, pX=0.54, N=244
var.10.ADH4 4:100048414:T:C
peptide level
183:209
77:99
10:17
FNLDALVTHTLPFDKpc2_NP.4
p−min=0.0052, pX=0.53, N=270
var.10.ADH4 4:100048414:T:C
peptide level
175:209
75:99
9:17
FNLDALVTHTLPFDKpc2_NP.5
p−maj=0.01, pX=0.21, N=259
var.10.ADH4 4:100048414:T:C
peptide level
137:209
57:99
8:17
FNLDALVTHTLPFDKpc3_NP.1
p−min=0.096, pX=0.32, N=202
var.10.ADH4 4:100048414:T:C
peptide level
110:209
47:99
6:17
FNLDALVTHTLPFDKpc3_NP.2
p−maj=0.22, pX=0.5, N=163
var.10.ADH4 4:100048414:T:C
peptide level
145:209
60:99
9:17
FNLDALVTHTLPFDKpc3_NP.3
p−min=0.087, pX=0.5, N=214
var.10.ADH4 4:100048414:T:C
peptide level
193:209
85:99
13:17
FNLDALVTHTLPFDKpc3_NP.4
p−min=0.036, pX=0.36, N=291
var.10.ADH4 4:100048414:T:C
peptide level
158:209
64:99
9:17
FNLDALVTHTLPFDKpc3_NP.5
p−min=0.021, pX=0.34, N=231
var.10.ADH4 4:100048414:T:C
peptide level
66:209
30:99
2:17
GGVDFALDCAGGSETMKpc2_NP.1
p−maj=0.11, pX=0.67, N=98
var.10.ADH4 4:100048414:T:C
peptide level
22:209
10:99
0:17
GGVDFALDCAGGSETMKpc2_NP.2
p−maj=0.39, pX=0.96, N=32
var.10.ADH4 4:100048414:T:C
peptide level
48:209
22:99
1:17
GGVDFALDCAGGSETMKpc2_NP.3
p−maj=0.13, pX=0.74, N=71
var.10.ADH4 4:100048414:T:C
peptide level
143:209
50:99
10:17
GGVDFALDCAGGSETMKpc2_NP.4
p−min=0.004, pX=0.071, N=203
var.10.ADH4 4:100048414:T:C
peptide level
119:209
48:99
9:17
GGVDFALDCAGGSETMKpc2_NP.5
p−min=0.2, pX=0.17, N=176

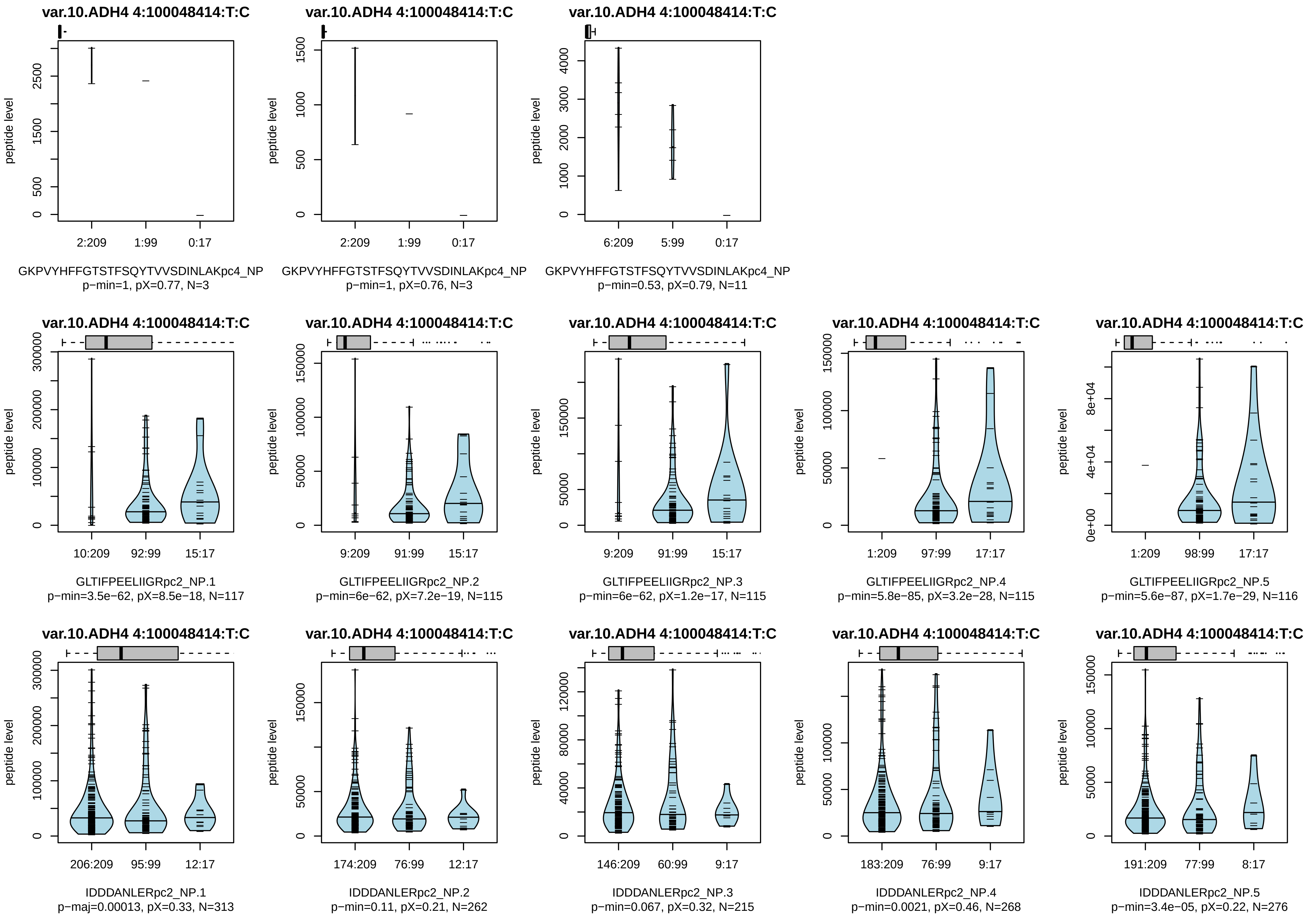
var.10.ADH4 4:100048414:T:C
peptide level
2:209
1:99
0:17
GKPVYHFFGTSTFSQYTVVSDINLAKpc4_NP
p−min=1, pX=0.77, N=3
var.10.ADH4 4:100048414:T:C
peptide level
2:209
1:99
0:17
GKPVYHFFGTSTFSQYTVVSDINLAKpc4_NP
p−min=1, pX=0.76, N=3
var.10.ADH4 4:100048414:T:C
peptide level
6:209
5:99
0:17
GKPVYHFFGTSTFSQYTVVSDINLAKpc4_NP
p−min=0.53, pX=0.79, N=11
var.10.ADH4 4:100048414:T:C
peptide level
10:209
92:99
15:17
GLTIFPEELIIGRpc2_NP.1
p−min=3.5e−62, pX=8.5e−18, N=117
var.10.ADH4 4:100048414:T:C
peptide level
9:209
91:99
15:17
GLTIFPEELIIGRpc2_NP.2
p−min=6e−62, pX=7.2e−19, N=115
var.10.ADH4 4:100048414:T:C
peptide level
9:209
91:99
15:17
GLTIFPEELIIGRpc2_NP.3
p−min=6e−62, pX=1.2e−17, N=115
var.10.ADH4 4:100048414:T:C
peptide level
1:209
97:99
17:17
GLTIFPEELIIGRpc2_NP.4
p−min=5.8e−85, pX=3.2e−28, N=115
var.10.ADH4 4:100048414:T:C
peptide level
1:209
98:99
17:17
GLTIFPEELIIGRpc2_NP.5
p−min=5.6e−87, pX=1.7e−29, N=116
var.10.ADH4 4:100048414:T:C
peptide level
206:209
95:99
12:17
IDDDANLERpc2_NP.1
p−maj=0.00013, pX=0.33, N=313
var.10.ADH4 4:100048414:T:C
peptide level
174:209
76:99
12:17
IDDDANLERpc2_NP.2
p−min=0.11, pX=0.21, N=262
var.10.ADH4 4:100048414:T:C
peptide level
146:209
60:99
9:17
IDDDANLERpc2_NP.3
p−min=0.067, pX=0.32, N=215
var.10.ADH4 4:100048414:T:C
peptide level
183:209
76:99
9:17
IDDDANLERpc2_NP.4
p−min=0.0021, pX=0.46, N=268
var.10.ADH4 4:100048414:T:C
peptide level
191:209
77:99
8:17
IDDDANLERpc2_NP.5
p−min=3.4e−05, pX=0.22, N=276

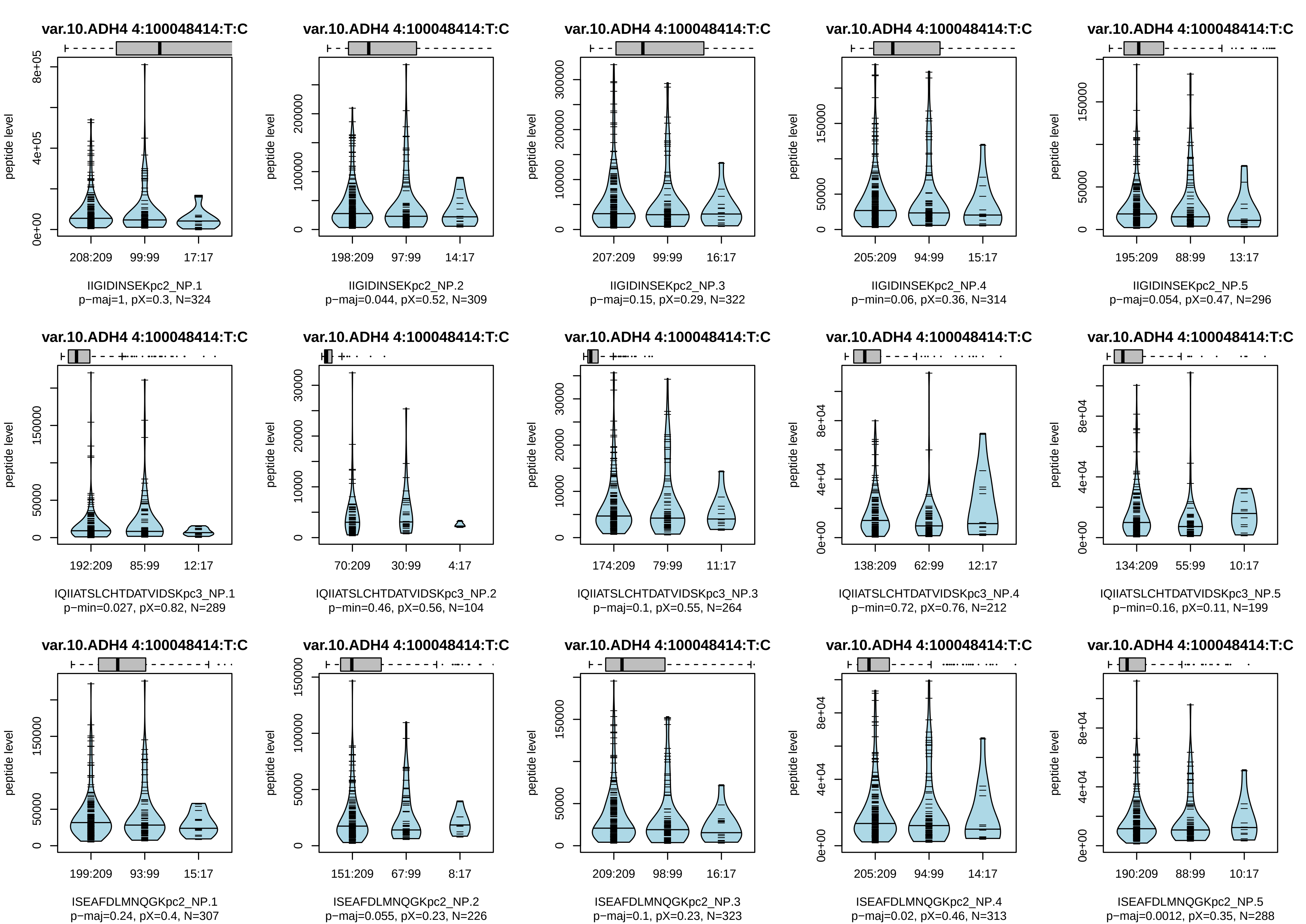
var.10.ADH4 4:100048414:T:C
peptide level
208:209
99:99
17:17
IIGIDINSEKpc2_NP.1
p−maj=1, pX=0.3, N=324
var.10.ADH4 4:100048414:T:C
peptide level
198:209
97:99
14:17
IIGIDINSEKpc2_NP.2
p−maj=0.044, pX=0.52, N=309
var.10.ADH4 4:100048414:T:C
peptide level
207:209
99:99
16:17
IIGIDINSEKpc2_NP.3
p−maj=0.15, pX=0.29, N=322
var.10.ADH4 4:100048414:T:C
peptide level
205:209
94:99
15:17
IIGIDINSEKpc2_NP.4
p−min=0.06, pX=0.36, N=314
var.10.ADH4 4:100048414:T:C
peptide level
195:209
88:99
13:17
IIGIDINSEKpc2_NP.5
p−maj=0.054, pX=0.47, N=296
var.10.ADH4 4:100048414:T:C
peptide level
192:209
85:99
12:17
IQIIATSLCHTDATVIDSKpc3_NP.1
p−min=0.027, pX=0.82, N=289
var.10.ADH4 4:100048414:T:C
peptide level
70:209
30:99
4:17
IQIIATSLCHTDATVIDSKpc3_NP.2
p−min=0.46, pX=0.56, N=104
var.10.ADH4 4:100048414:T:C
peptide level
174:209
79:99
11:17
IQIIATSLCHTDATVIDSKpc3_NP.3
p−maj=0.1, pX=0.55, N=264
var.10.ADH4 4:100048414:T:C
peptide level
138:209
62:99
12:17
IQIIATSLCHTDATVIDSKpc3_NP.4
p−min=0.72, pX=0.76, N=212
var.10.ADH4 4:100048414:T:C
peptide level
134:209
55:99
10:17
IQIIATSLCHTDATVIDSKpc3_NP.5
p−min=0.16, pX=0.11, N=199
var.10.ADH4 4:100048414:T:C
peptide level
199:209
93:99
15:17
ISEAFDLMNQGKpc2_NP.1
p−maj=0.24, pX=0.4, N=307
var.10.ADH4 4:100048414:T:C
peptide level
151:209
67:99
8:17
ISEAFDLMNQGKpc2_NP.2
p−maj=0.055, pX=0.23, N=226
var.10.ADH4 4:100048414:T:C
peptide level
209:209
98:99
16:17
ISEAFDLMNQGKpc2_NP.3
p−maj=0.1, pX=0.23, N=323
var.10.ADH4 4:100048414:T:C
peptide level
205:209
94:99
14:17
ISEAFDLMNQGKpc2_NP.4
p−maj=0.02, pX=0.46, N=313
var.10.ADH4 4:100048414:T:C
peptide level
190:209
88:99
10:17
ISEAFDLMNQGKpc2_NP.5
p−maj=0.0012, pX=0.35, N=288

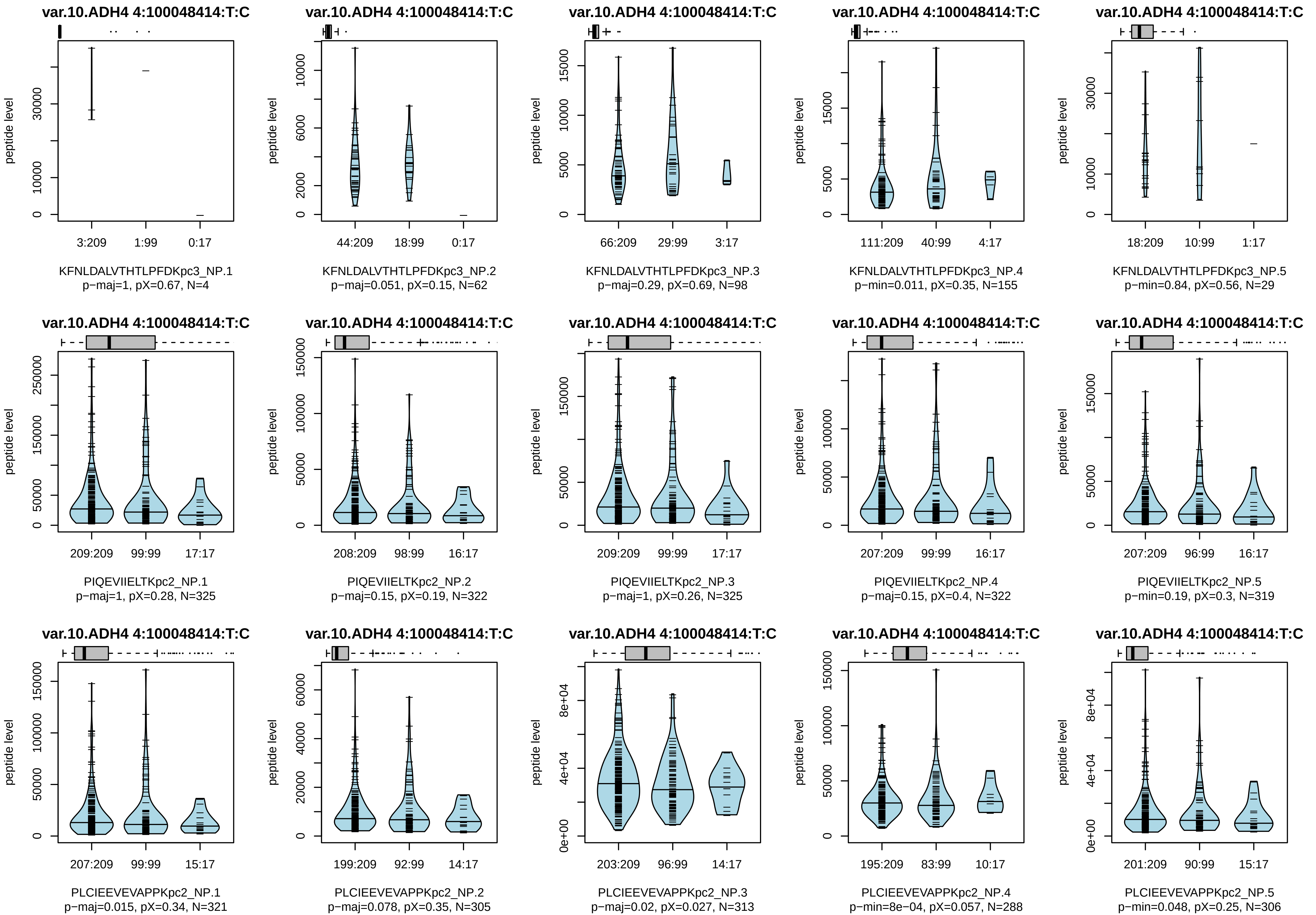
var.10.ADH4 4:100048414:T:C
peptide level
3:209
1:99
0:17
KFNLDALVTHTLPFDKpc3_NP.1
p−maj=1, pX=0.67, N=4
var.10.ADH4 4:100048414:T:C
peptide level
44:209
18:99
0:17
KFNLDALVTHTLPFDKpc3_NP.2
p−maj=0.051, pX=0.15, N=62
var.10.ADH4 4:100048414:T:C
peptide level
66:209
29:99
3:17
KFNLDALVTHTLPFDKpc3_NP.3
p−maj=0.29, pX=0.69, N=98
var.10.ADH4 4:100048414:T:C
peptide level
111:209
40:99
4:17
KFNLDALVTHTLPFDKpc3_NP.4
p−min=0.011, pX=0.35, N=155
var.10.ADH4 4:100048414:T:C
peptide level
18:209
10:99
1:17
KFNLDALVTHTLPFDKpc3_NP.5
p−min=0.84, pX=0.56, N=29
var.10.ADH4 4:100048414:T:C
peptide level
209:209
99:99
17:17
PIQEVIIELTKpc2_NP.1
p−maj=1, pX=0.28, N=325
var.10.ADH4 4:100048414:T:C
peptide level
208:209
98:99
16:17
PIQEVIIELTKpc2_NP.2
p−maj=0.15, pX=0.19, N=322
var.10.ADH4 4:100048414:T:C
peptide level
209:209
99:99
17:17
PIQEVIIELTKpc2_NP.3
p−maj=1, pX=0.26, N=325
var.10.ADH4 4:100048414:T:C
peptide level
207:209
99:99
16:17
PIQEVIIELTKpc2_NP.4
p−maj=0.15, pX=0.4, N=322
var.10.ADH4 4:100048414:T:C
peptide level
207:209
96:99
16:17
PIQEVIIELTKpc2_NP.5
p−min=0.19, pX=0.3, N=319
var.10.ADH4 4:100048414:T:C
peptide level
207:209
99:99
15:17
PLCIEEVEVAPPKpc2_NP.1
p−maj=0.015, pX=0.34, N=321
var.10.ADH4 4:100048414:T:C
peptide level
199:209
92:99
14:17
PLCIEEVEVAPPKpc2_NP.2
p−maj=0.078, pX=0.35, N=305
var.10.ADH4 4:100048414:T:C
peptide level
203:209
96:99
14:17
PLCIEEVEVAPPKpc2_NP.3
p−maj=0.02, pX=0.027, N=313
var.10.ADH4 4:100048414:T:C
peptide level
195:209
83:99
10:17
PLCIEEVEVAPPKpc2_NP.4
p−min=8e−04, pX=0.057, N=288
var.10.ADH4 4:100048414:T:C
peptide level
201:209
90:99
15:17
PLCIEEVEVAPPKpc2_NP.5
p−min=0.048, pX=0.25, N=306

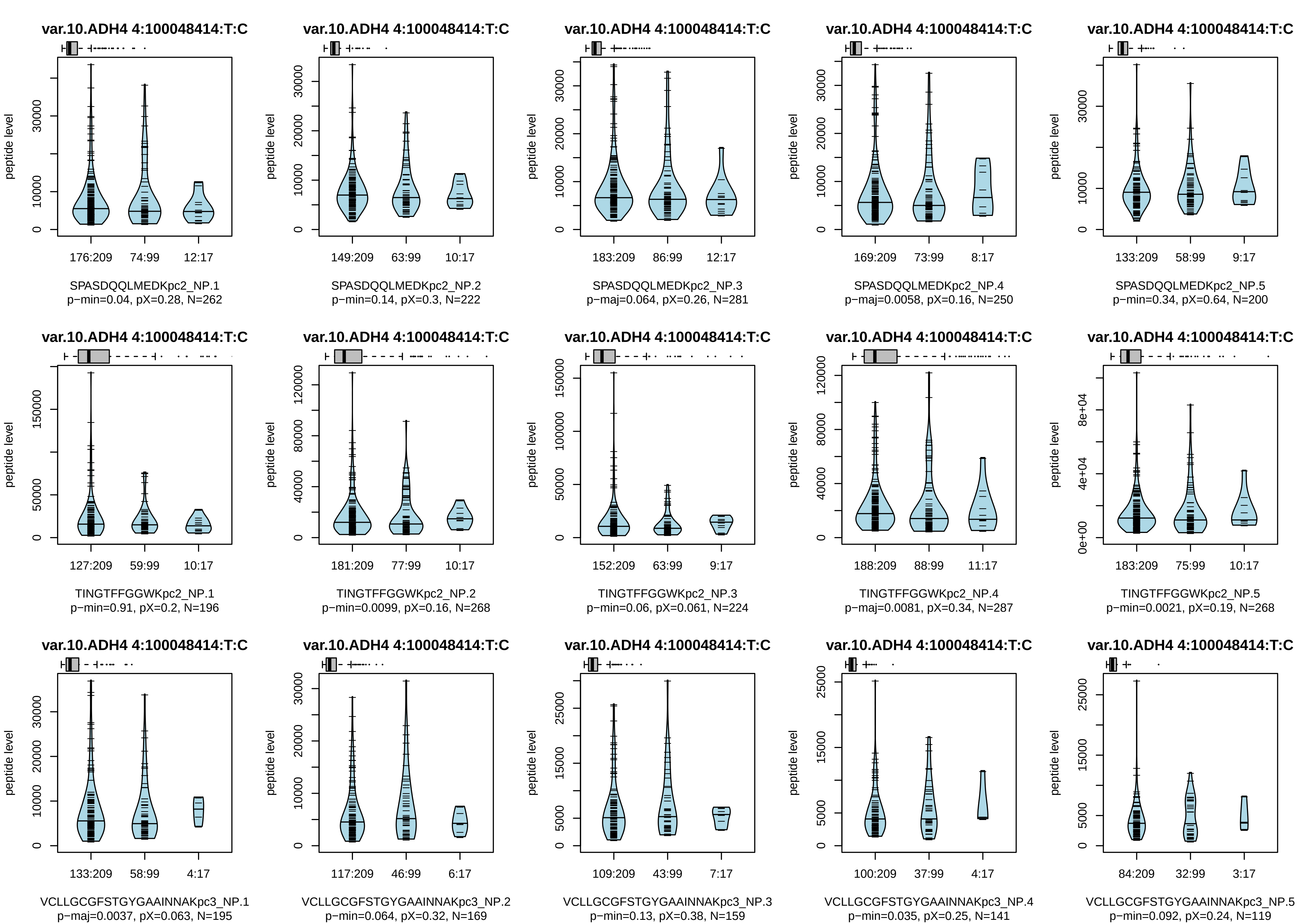
var.10.ADH4 4:100048414:T:C
peptide level
176:209
74:99
12:17
SPASDQQLMEDKpc2_NP.1
p−min=0.04, pX=0.28, N=262
var.10.ADH4 4:100048414:T:C
peptide level
149:209
63:99
10:17
SPASDQQLMEDKpc2_NP.2
p−min=0.14, pX=0.3, N=222
var.10.ADH4 4:100048414:T:C
peptide level
183:209
86:99
12:17
SPASDQQLMEDKpc2_NP.3
p−maj=0.064, pX=0.26, N=281
var.10.ADH4 4:100048414:T:C
peptide level
169:209
73:99
8:17
SPASDQQLMEDKpc2_NP.4
p−maj=0.0058, pX=0.16, N=250
var.10.ADH4 4:100048414:T:C
peptide level
133:209
58:99
9:17
SPASDQQLMEDKpc2_NP.5
p−min=0.34, pX=0.64, N=200
var.10.ADH4 4:100048414:T:C
peptide level
127:209
59:99
10:17
TINGTFFGGWKpc2_NP.1
p−min=0.91, pX=0.2, N=196
var.10.ADH4 4:100048414:T:C
peptide level
181:209
77:99
10:17
TINGTFFGGWKpc2_NP.2
p−min=0.0099, pX=0.16, N=268
var.10.ADH4 4:100048414:T:C
peptide level
152:209
63:99
9:17
TINGTFFGGWKpc2_NP.3
p−min=0.06, pX=0.061, N=224
var.10.ADH4 4:100048414:T:C
peptide level
188:209
88:99
11:17
TINGTFFGGWKpc2_NP.4
p−maj=0.0081, pX=0.34, N=287
var.10.ADH4 4:100048414:T:C
peptide level
183:209
75:99
10:17
TINGTFFGGWKpc2_NP.5
p−min=0.0021, pX=0.19, N=268
var.10.ADH4 4:100048414:T:C
peptide level
133:209
58:99
4:17
VCLLGCGFSTGYGAAINNAKpc3_NP.1
p−maj=0.0037, pX=0.063, N=195
var.10.ADH4 4:100048414:T:C
peptide level
117:209
46:99
6:17
VCLLGCGFSTGYGAAINNAKpc3_NP.2
p−min=0.064, pX=0.32, N=169
var.10.ADH4 4:100048414:T:C
peptide level
109:209
43:99
7:17
VCLLGCGFSTGYGAAINNAKpc3_NP.3
p−min=0.13, pX=0.38, N=159
var.10.ADH4 4:100048414:T:C
peptide level
100:209
37:99
4:17
VCLLGCGFSTGYGAAINNAKpc3_NP.4
p−min=0.035, pX=0.25, N=141
var.10.ADH4 4:100048414:T:C
peptide level
84:209
32:99
3:17
VCLLGCGFSTGYGAAINNAKpc3_NP.5
p−min=0.092, pX=0.24, N=119

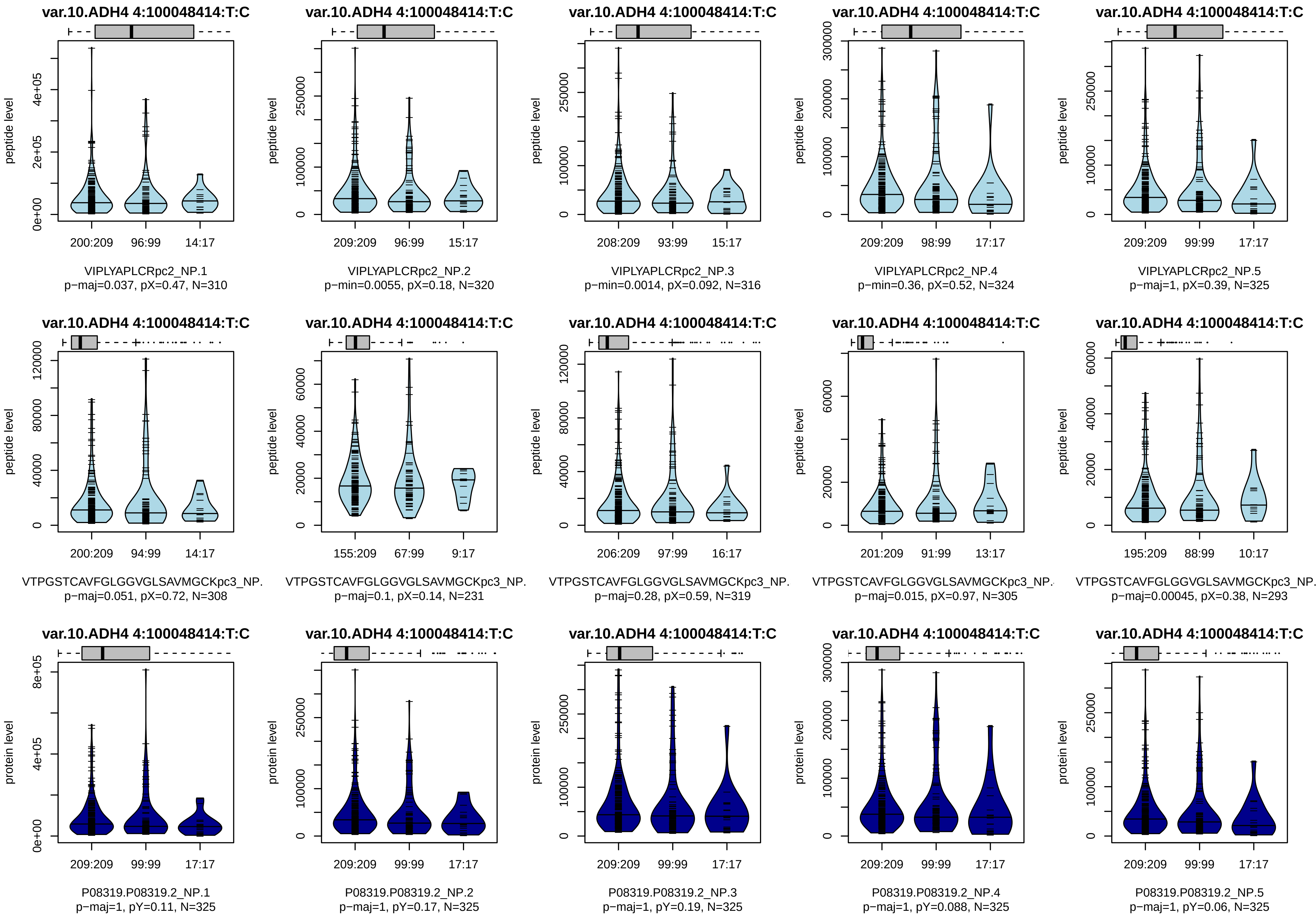
var.10.ADH4 4:100048414:T:C
peptide level
200:209
96:99
14:17
VIPLYAPLCRpc2_NP.1
p−maj=0.037, pX=0.47, N=310
var.10.ADH4 4:100048414:T:C
peptide level
209:209
96:99
15:17
VIPLYAPLCRpc2_NP.2
p−min=0.0055, pX=0.18, N=320
var.10.ADH4 4:100048414:T:C
peptide level
208:209
93:99
15:17
VIPLYAPLCRpc2_NP.3
p−min=0.0014, pX=0.092, N=316
var.10.ADH4 4:100048414:T:C
peptide level
209:209
98:99
17:17
VIPLYAPLCRpc2_NP.4
p−min=0.36, pX=0.52, N=324
var.10.ADH4 4:100048414:T:C
peptide level
209:209
99:99
17:17
VIPLYAPLCRpc2_NP.5
p−maj=1, pX=0.39, N=325
var.10.ADH4 4:100048414:T:C
peptide level
200:209
94:99
14:17
VTPGSTCAVFGLGGVGLSAVMGCKpc3_NP.
p−maj=0.051, pX=0.72, N=308
var.10.ADH4 4:100048414:T:C
peptide level
155:209
67:99
9:17
VTPGSTCAVFGLGGVGLSAVMGCKpc3_NP.
p−maj=0.1, pX=0.14, N=231
var.10.ADH4 4:100048414:T:C
peptide level
206:209
97:99
16:17
VTPGSTCAVFGLGGVGLSAVMGCKpc3_NP.
p−maj=0.28, pX=0.59, N=319
var.10.ADH4 4:100048414:T:C
peptide level
201:209
91:99
13:17
VTPGSTCAVFGLGGVGLSAVMGCKpc3_NP.
p−maj=0.015, pX=0.97, N=305
var.10.ADH4 4:100048414:T:C
peptide level
195:209
88:99
10:17
VTPGSTCAVFGLGGVGLSAVMGCKpc3_NP.
p−maj=0.00045, pX=0.38, N=293
var.10.ADH4 4:100048414:T:C
protein level
209:209
99:99
17:17
P08319.P08319.2_NP.1
p−maj=1, pY=0.11, N=325
var.10.ADH4 4:100048414:T:C
protein level
209:209
99:99
17:17
P08319.P08319.2_NP.2
p−maj=1, pY=0.17, N=325
var.10.ADH4 4:100048414:T:C
protein level
209:209
99:99
17:17
P08319.P08319.2_NP.3
p−maj=1, pY=0.19, N=325
var.10.ADH4 4:100048414:T:C
protein level
209:209
99:99
17:17
P08319.P08319.2_NP.4
p−maj=1, pY=0.088, N=325
var.10.ADH4 4:100048414:T:C
protein level
209:209
99:99
17:17
P08319.P08319.2_NP.5
p−maj=1, pY=0.06, N=325

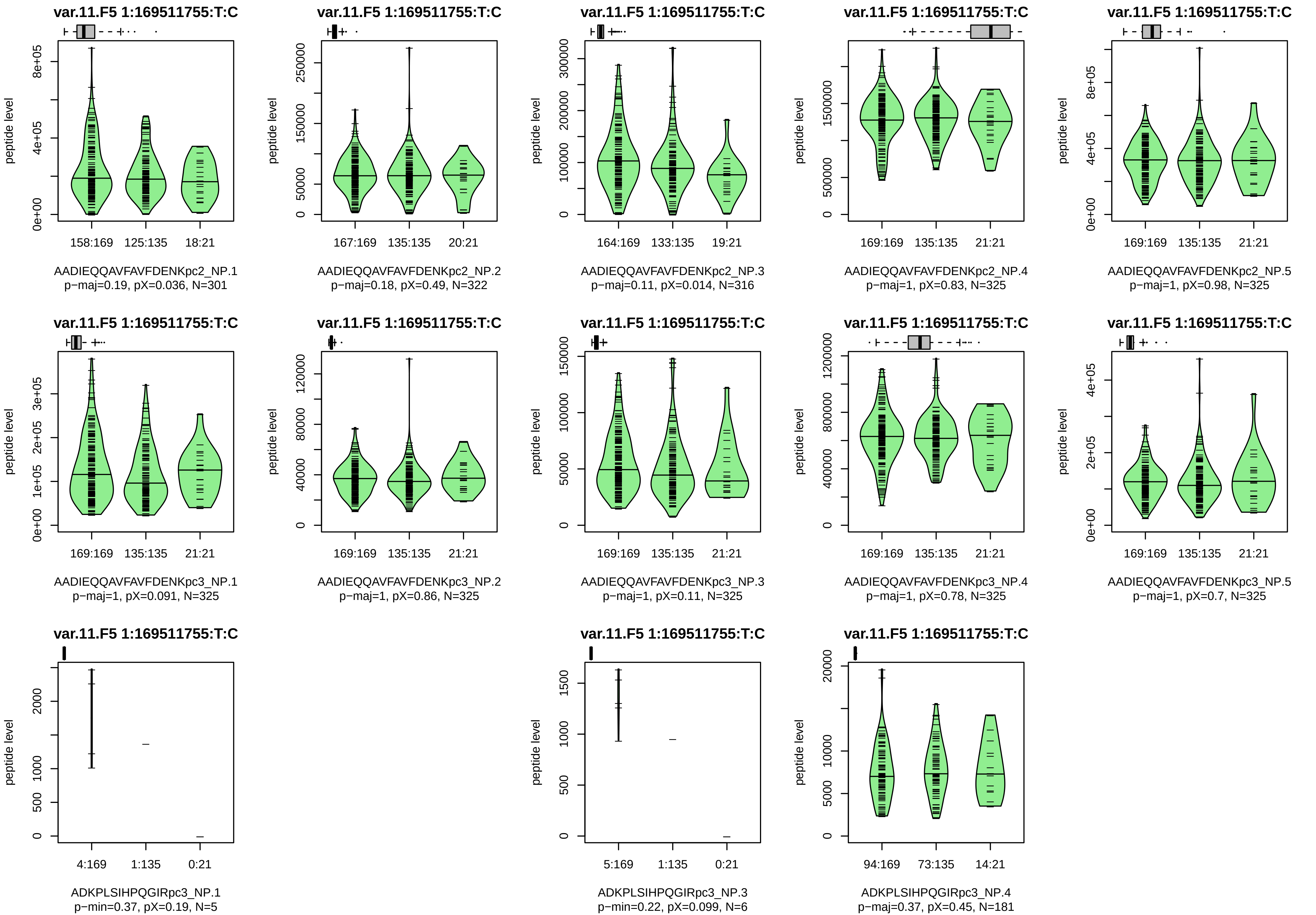
var.11.F5 1:169511755:T:C
peptide level
158:169
125:135
18:21
AADIEQQAVFAVFDENKpc2_NP.1
p−maj=0.19, pX=0.036, N=301
var.11.F5 1:169511755:T:C
peptide level
167:169
135:135
20:21
AADIEQQAVFAVFDENKpc2_NP.2
p−maj=0.18, pX=0.49, N=322
var.11.F5 1:169511755:T:C
peptide level
164:169
133:135
19:21
AADIEQQAVFAVFDENKpc2_NP.3
p−maj=0.11, pX=0.014, N=316
var.11.F5 1:169511755:T:C
peptide level
169:169
135:135
21:21
AADIEQQAVFAVFDENKpc2_NP.4
p−maj=1, pX=0.83, N=325
var.11.F5 1:169511755:T:C
peptide level
169:169
135:135
21:21
AADIEQQAVFAVFDENKpc2_NP.5
p−maj=1, pX=0.98, N=325
var.11.F5 1:169511755:T:C
peptide level
169:169
135:135
21:21
AADIEQQAVFAVFDENKpc3_NP.1
p−maj=1, pX=0.091, N=325
var.11.F5 1:169511755:T:C
peptide level
169:169
135:135
21:21
AADIEQQAVFAVFDENKpc3_NP.2
p−maj=1, pX=0.86, N=325
var.11.F5 1:169511755:T:C
peptide level
169:169
135:135
21:21
AADIEQQAVFAVFDENKpc3_NP.3
p−maj=1, pX=0.11, N=325
var.11.F5 1:169511755:T:C
peptide level
169:169
135:135
21:21
AADIEQQAVFAVFDENKpc3_NP.4
p−maj=1, pX=0.78, N=325
var.11.F5 1:169511755:T:C
peptide level
169:169
135:135
21:21
AADIEQQAVFAVFDENKpc3_NP.5
p−maj=1, pX=0.7, N=325
var.11.F5 1:169511755:T:C
peptide level
4:169
1:135
0:21
ADKPLSIHPQGIRpc3_NP.1
p−min=0.37, pX=0.19, N=5
var.11.F5 1:169511755:T:C
peptide level
5:169
1:135
0:21
ADKPLSIHPQGIRpc3_NP.3
p−min=0.22, pX=0.099, N=6
var.11.F5 1:169511755:T:C
peptide level
94:169
73:135
14:21
ADKPLSIHPQGIRpc3_NP.4
p−maj=0.37, pX=0.45, N=181

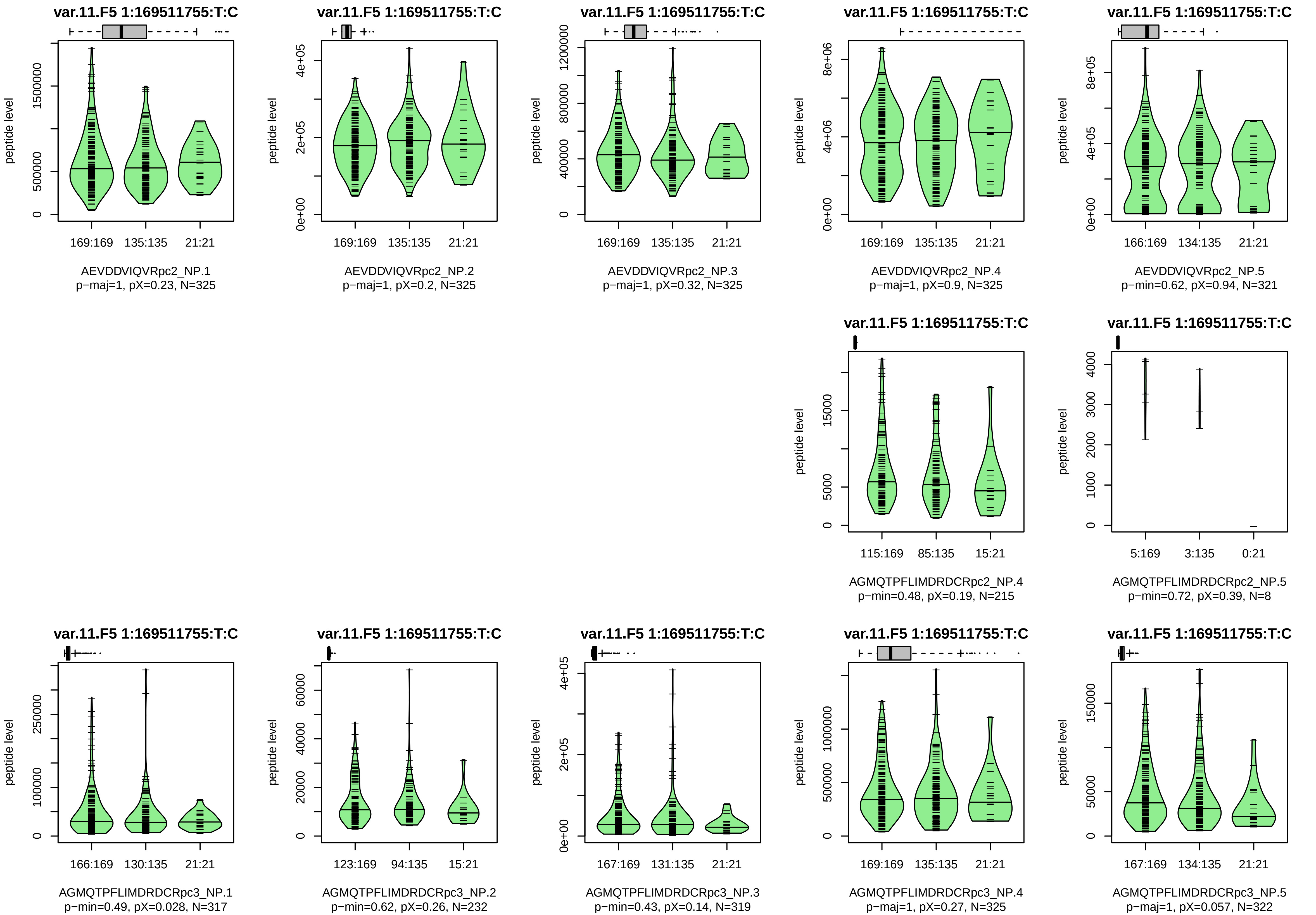

var.11.F5 1:169511755:T:C
peptide level
169:169
135:135
21:21
AEVDDVIQVRpc2_NP.1
p−maj=1, pX=0.23, N=325
var.11.F5 1:169511755:T:C
peptide level
169:169
135:135
21:21
AEVDDVIQVRpc2_NP.2
p−maj=1, pX=0.2, N=325
var.11.F5 1:169511755:T:C
peptide level
169:169
135:135
21:21
AEVDDVIQVRpc2_NP.3
p−maj=1, pX=0.32, N=325
var.11.F5 1:169511755:T:C
peptide level
169:169
135:135
21:21
AEVDDVIQVRpc2_NP.4
p−maj=1, pX=0.9, N=325
var.11.F5 1:169511755:T:C
peptide level
166:169
134:135
21:21
AEVDDVIQVRpc2_NP.5
p−min=0.62, pX=0.94, N=321
var.11.F5 1:169511755:T:C
peptide level
115:169
85:135
15:21
AGMQTPFLIMDRDCRpc2_NP.4
p−min=0.48, pX=0.19, N=215
var.11.F5 1:169511755:T:C
peptide level
5:169
3:135
0:21
AGMQTPFLIMDRDCRpc2_NP.5
p−min=0.72, pX=0.39, N=8
var.11.F5 1:169511755:T:C
peptide level
166:169
130:135
21:21
AGMQTPFLIMDRDCRpc3_NP.1
p−min=0.49, pX=0.028, N=317
var.11.F5 1:169511755:T:C
peptide level
123:169
94:135
15:21
AGMQTPFLIMDRDCRpc3_NP.2
p−min=0.62, pX=0.26, N=232
var.11.F5 1:169511755:T:C
peptide level
167:169
131:135
21:21
AGMQTPFLIMDRDCRpc3_NP.3
p−min=0.43, pX=0.14, N=319
var.11.F5 1:169511755:T:C
peptide level
169:169
135:135
21:21
AGMQTPFLIMDRDCRpc3_NP.4
p−maj=1, pX=0.27, N=325
var.11.F5 1:169511755:T:C
peptide level
167:169
134:135
21:21
AGMQTPFLIMDRDCRpc3_NP.5
p−maj=1, pX=0.057, N=322

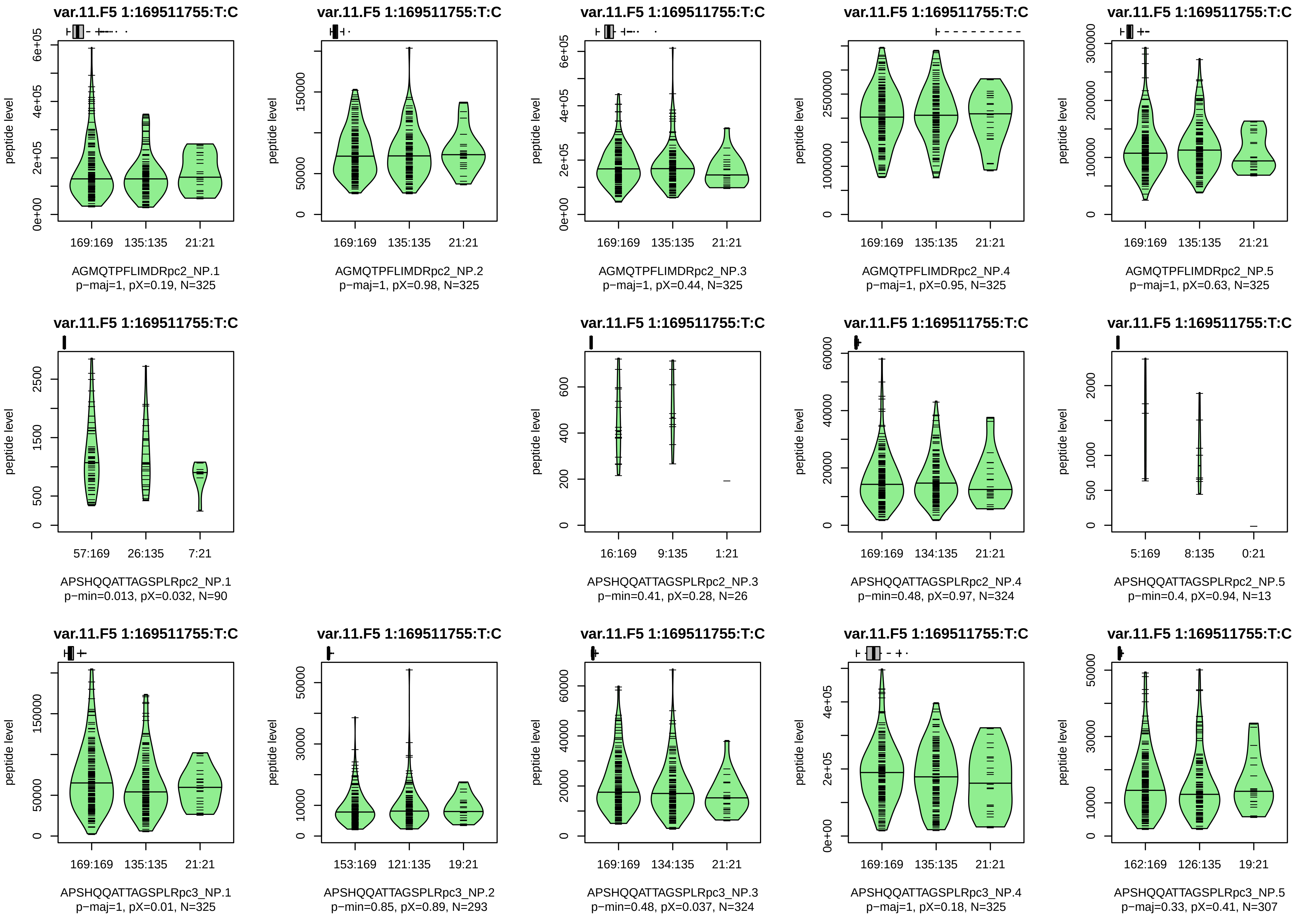
var.11.F5 1:169511755:T:C
peptide level
169:169 135:135 21:21
AGMQTPFLIMDRpc2_NP.1
p−maj=1, pX=0.19, N=325
var.11.F5 1:169511755:T:C
peptide level
169:169 135:135 21:21
AGMQTPFLIMDRpc2_NP.2
p−maj=1, pX=0.98, N=325
var.11.F5 1:169511755:T:C
peptide level
169:169 135:135 21:21
AGMQTPFLIMDRpc2_NP.3
p−maj=1, pX=0.44, N=325
var.11.F5 1:169511755:T:C
peptide level
169:169 135:135 21:21
AGMQTPFLIMDRpc2_NP.4
p−maj=1, pX=0.95, N=325
var.11.F5 1:169511755:T:C
peptide level
169:169 135:135 21:21
AGMQTPFLIMDRpc2_NP.5
p−maj=1, pX=0.63, N=325
var.11.F5 1:169511755:T:C
peptide level
57:169 26:135 7:21
APSHQQATTAGSPLRpc2_NP.1
p−min=0.013, pX=0.032, N=90
var.11.F5 1:169511755:T:C
peptide level
16:169 9:135 1:21
APSHQQATTAGSPLRpc2_NP.3
p−min=0.41, pX=0.28, N=26
var.11.F5 1:169511755:T:C
peptide level
169:169 134:135 21:21
APSHQQATTAGSPLRpc2_NP.4
p−min=0.48, pX=0.97, N=324
var.11.F5 1:169511755:T:C
peptide level
5:169 8:135 0:21
APSHQQATTAGSPLRpc2_NP.5
p−min=0.4, pX=0.94, N=13
var.11.F5 1:169511755:T:C
peptide level
169:169 135:135 21:21
APSHQQATTAGSPLRpc3_NP.1
p−maj=1, pX=0.01, N=325
var.11.F5 1:169511755:T:C
peptide level
153:169 121:135 19:21
APSHQQATTAGSPLRpc3_NP.2
p−min=0.85, pX=0.89, N=293
var.11.F5 1:169511755:T:C
peptide level
169:169 134:135 21:21
APSHQQATTAGSPLRpc3_NP.3
p−min=0.48, pX=0.037, N=324
var.11.F5 1:169511755:T:C
peptide level
169:169 135:135 21:21
APSHQQATTAGSPLRpc3_NP.4
p−maj=1, pX=0.18, N=325
var.11.F5 1:169511755:T:C
peptide level
162:169 126:135 19:21
APSHQQATTAGSPLRpc3_NP.5
p−maj=0.33, pX=0.41, N=307

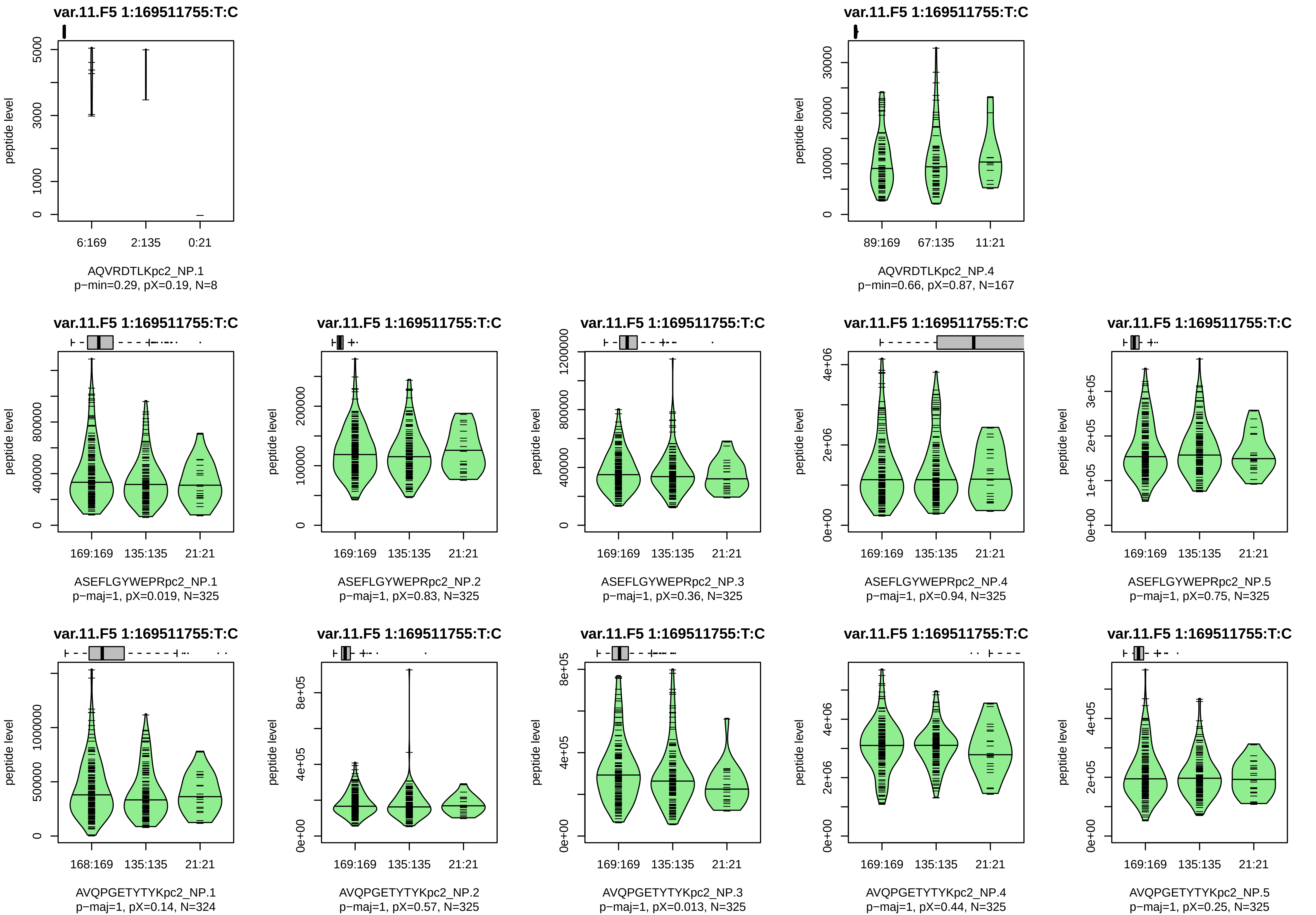
var.11.F5 1:169511755:T:C
peptide level
6:169
2:135
0:21
AQVRDTLKpc2_NP.1
p−min=0.29, pX=0.19, N=8
var.11.F5 1:169511755:T:C
peptide level
89:169
67:135
11:21
AQVRDTLKpc2_NP.4
p−min=0.66, pX=0.87, N=167
var.11.F5 1:169511755:T:C
peptide level
169:169
135:135
21:21
ASEFLGYWEPRpc2_NP.1
p−maj=1, pX=0.019, N=325
var.11.F5 1:169511755:T:C
peptide level
169:169
135:135
21:21
ASEFLGYWEPRpc2_NP.2
p−maj=1, pX=0.83, N=325
var.11.F5 1:169511755:T:C
peptide level
169:169
135:135
21:21
ASEFLGYWEPRpc2_NP.3
p−maj=1, pX=0.36, N=325
var.11.F5 1:169511755:T:C
peptide level
169:169
135:135
21:21
ASEFLGYWEPRpc2_NP.4
p−maj=1, pX=0.94, N=325
var.11.F5 1:169511755:T:C
peptide level
169:169
135:135
21:21
ASEFLGYWEPRpc2_NP.5
p−maj=1, pX=0.75, N=325
var.11.F5 1:169511755:T:C
peptide level
168:169
135:135
21:21
AVQPGETYTYKpc2_NP.1
p−maj=1, pX=0.14, N=324
var.11.F5 1:169511755:T:C
peptide level
169:169
135:135
21:21
AVQPGETYTYKpc2_NP.2
p−maj=1, pX=0.57, N=325
var.11.F5 1:169511755:T:C
peptide level
169:169
135:135
21:21
AVQPGETYTYKpc2_NP.3
p−maj=1, pX=0.013, N=325
var.11.F5 1:169511755:T:C
peptide level
169:169
135:135
21:21
AVQPGETYTYKpc2_NP.4
p−maj=1, pX=0.44, N=325
var.11.F5 1:169511755:T:C
peptide level
169:169
135:135
21:21
AVQPGETYTYKpc2_NP.5
p−maj=1, pX=0.25, N=325

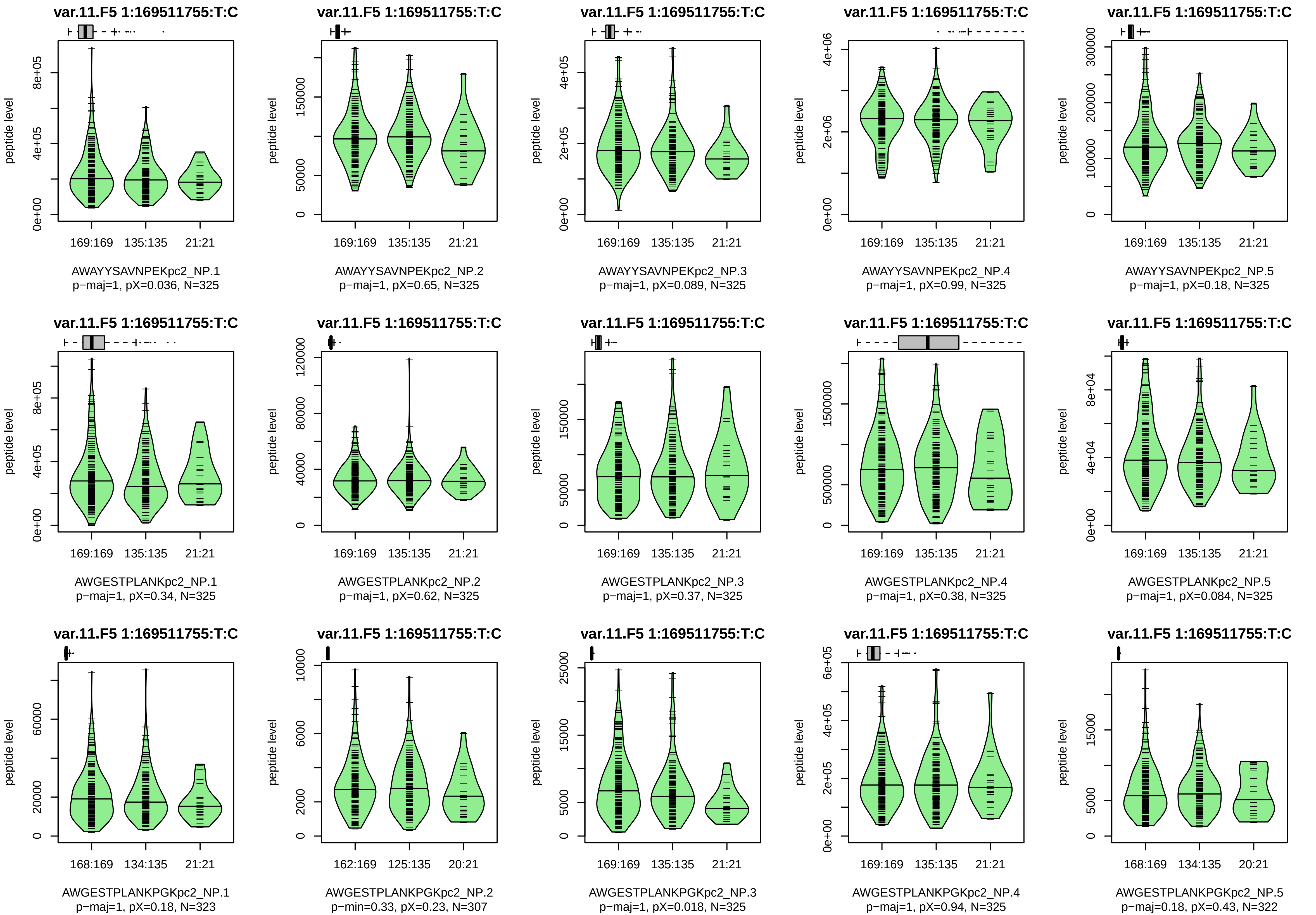
var.11.F5 1:169511755:T:C
peptide level
0e+00
4e+05
8e+05
169:169
135:135
21:21
AWAYYSAVNPEKpc2_NP.1
p−maj=1, pX=0.036, N=325
var.11.F5 1:169511755:T:C
peptide level
0
50000
150000
169:169
135:135
21:21
AWAYYSAVNPEKpc2_NP.2
p−maj=1, pX=0.65, N=325
var.11.F5 1:169511755:T:C
peptide level
0e+00
2e+05
4e+05
169:169
135:135
21:21
AWAYYSAVNPEKpc2_NP.3
p−maj=1, pX=0.089, N=325
var.11.F5 1:169511755:T:C
peptide level
0e+00
2e+06
4e+06
169:169
135:135
21:21
AWAYYSAVNPEKpc2_NP.4
p−maj=1, pX=0.99, N=325
var.11.F5 1:169511755:T:C
peptide level
0
100000
200000
300000
169:169
135:135
21:21
AWAYYSAVNPEKpc2_NP.5
p−maj=1, pX=0.18, N=325
var.11.F5 1:169511755:T:C
peptide level
0e+00
4e+05
8e+05
169:169
135:135
21:21
AWGESTPLANKpc2_NP.1
p−maj=1, pX=0.34, N=325
var.11.F5 1:169511755:T:C
peptide level
0
40000
80000
120000
169:169
135:135
21:21
AWGESTPLANKpc2_NP.2
p−maj=1, pX=0.62, N=325
var.11.F5 1:169511755:T:C
peptide level
0
50000
150000
169:169
135:135
21:21
AWGESTPLANKpc2_NP.3
p−maj=1, pX=0.37, N=325
var.11.F5 1:169511755:T:C
peptide level
0
500000
1500000
169:169
135:135
21:21
AWGESTPLANKpc2_NP.4
p−maj=1, pX=0.38, N=325
var.11.F5 1:169511755:T:C
peptide level
0e+00
4e+04
8e+04
169:169
135:135
21:21
AWGESTPLANKpc2_NP.5
p−maj=1, pX=0.084, N=325
var.11.F5 1:169511755:T:C
peptide level
0
20000
60000
168:169
134:135
21:21
AWGESTPLANKPGKpc2_NP.1
p−maj=1, pX=0.18, N=323
var.11.F5 1:169511755:T:C
peptide level
0
2000
6000
10000
162:169
125:135
20:21
AWGESTPLANKPGKpc2_NP.2
p−min=0.33, pX=0.23, N=307
var.11.F5 1:169511755:T:C
peptide level
0
5000
15000
25000
169:169
135:135
21:21
AWGESTPLANKPGKpc2_NP.3
p−maj=1, pX=0.018, N=325
var.11.F5 1:169511755:T:C
peptide level
0e+00
2e+05
4e+05
6e+05
169:169
135:135
21:21
AWGESTPLANKPGKpc2_NP.4
p−maj=1, pX=0.94, N=325
var.11.F5 1:169511755:T:C
peptide level
0
5000
15000
168:169
134:135
20:21
AWGESTPLANKPGKpc2_NP.5
p−maj=0.18, pX=0.43, N=322

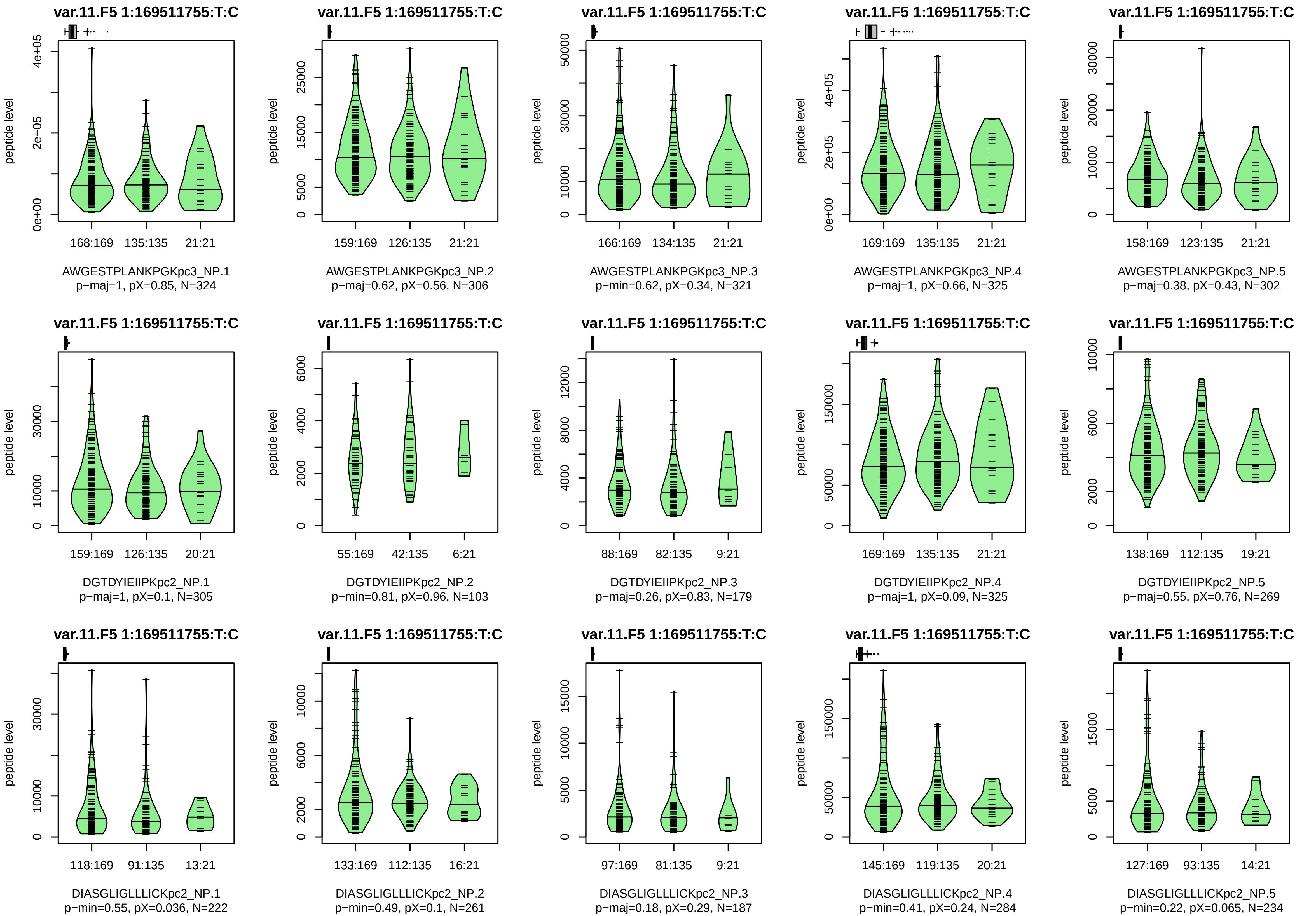
var.11.F5 1:169511755:T:C
peptide level
0e+00
2e+05
4e+05
168:169
135:135
21:21
AWGESTPLANKPGKpc3_NP.1
p−maj=1, pX=0.85, N=324
var.11.F5 1:169511755:T:C
peptide level
0
5000
15000
25000
159:169
126:135
21:21
AWGESTPLANKPGKpc3_NP.2
p−maj=0.62, pX=0.56, N=306
var.11.F5 1:169511755:T:C
peptide level
0
10000
30000
50000
166:169
134:135
21:21
AWGESTPLANKPGKpc3_NP.3
p−min=0.62, pX=0.34, N=321
var.11.F5 1:169511755:T:C
peptide level
0e+00
2e+05
4e+05
169:169
135:135
21:21
AWGESTPLANKPGKpc3_NP.4
p−maj=1, pX=0.66, N=325
var.11.F5 1:169511755:T:C
peptide level
0
10000
20000
30000
158:169
123:135
21:21
AWGESTPLANKPGKpc3_NP.5
p−maj=0.38, pX=0.43, N=302
var.11.F5 1:169511755:T:C
peptide level
0
10000
30000
159:169
126:135
20:21
DGTDYIEIIPKpc2_NP.1
p−maj=1, pX=0.1, N=305
var.11.F5 1:169511755:T:C
peptide level
0
2000
4000
6000
55:169
42:135
6:21
DGTDYIEIIPKpc2_NP.2
p−min=0.81, pX=0.96, N=103
var.11.F5 1:169511755:T:C
peptide level
0
4000
8000
12000
88:169
82:135
9:21
DGTDYIEIIPKpc2_NP.3
p−maj=0.26, pX=0.83, N=179
var.11.F5 1:169511755:T:C
peptide level
0
50000
150000
169:169
135:135
21:21
DGTDYIEIIPKpc2_NP.4
p−maj=1, pX=0.09, N=325
var.11.F5 1:169511755:T:C
peptide level
0
2000
6000
10000
138:169
112:135
19:21
DGTDYIEIIPKpc2_NP.5
p−maj=0.55, pX=0.76, N=269
var.11.F5 1:169511755:T:C
peptide level
0
10000
30000
118:169
91:135
13:21
DIASGLIGLLLICKpc2_NP.1
p−min=0.55, pX=0.036, N=222
var.11.F5 1:169511755:T:C
peptide level
0
2000
6000
10000
133:169
112:135
16:21
DIASGLIGLLLICKpc2_NP.2
p−min=0.49, pX=0.1, N=261
var.11.F5 1:169511755:T:C
peptide level
0
5000
10000
15000
97:169
81:135
9:21
DIASGLIGLLLICKpc2_NP.3
p−maj=0.18, pX=0.29, N=187
var.11.F5 1:169511755:T:C
peptide level
0
50000
150000
145:169
119:135
20:21
DIASGLIGLLLICKpc2_NP.4
p−min=0.41, pX=0.24, N=284
var.11.F5 1:169511755:T:C
peptide level
0
5000
15000
127:169
93:135
14:21
DIASGLIGLLLICKpc2_NP.5
p−min=0.22, pX=0.065, N=234

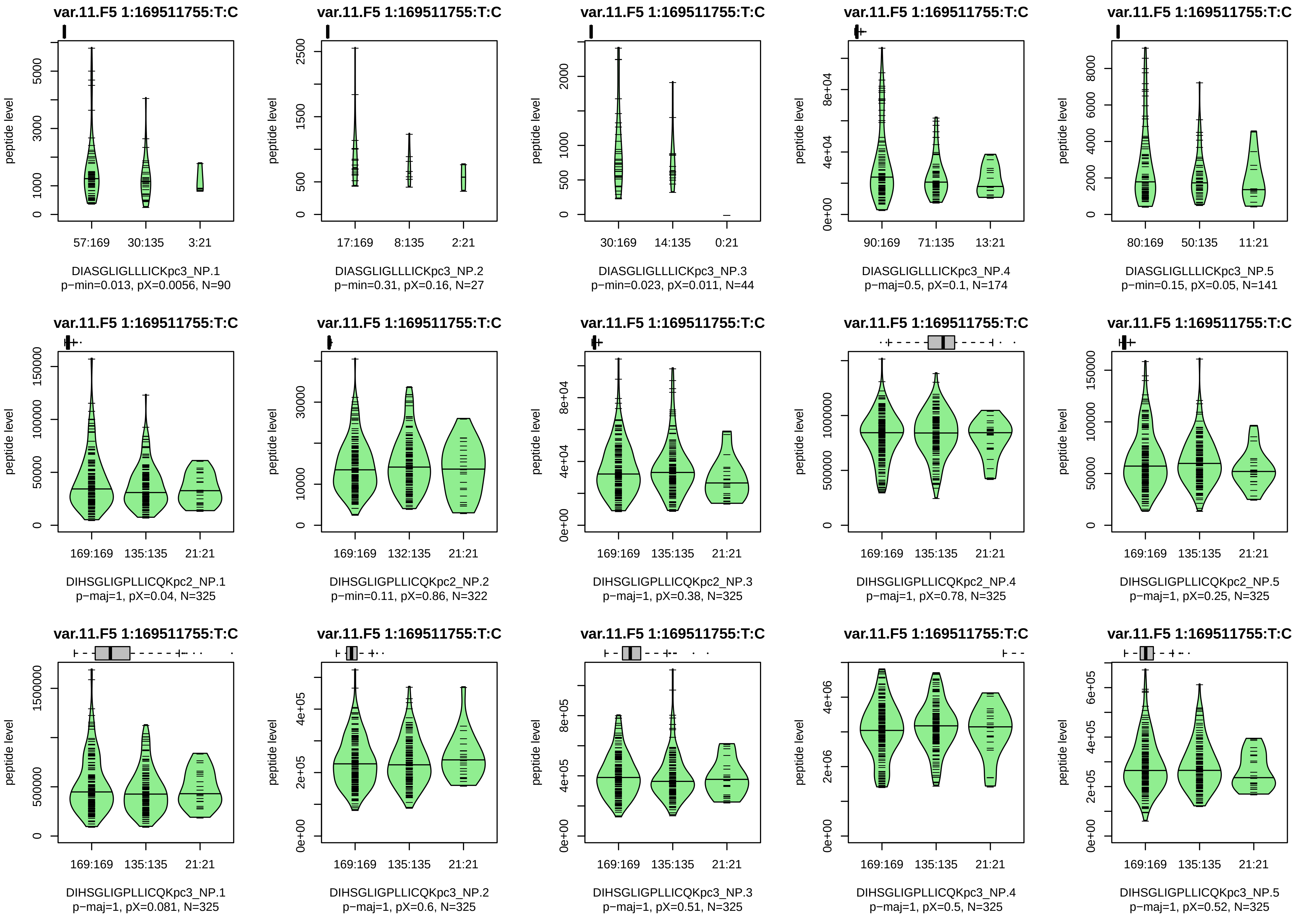
var.11.F5 1:169511755:T:C
peptide level
57:169 30:135 3:21
DIASGLIGLLLICKpc3_NP.1
p−min=0.013, pX=0.0056, N=90
var.11.F5 1:169511755:T:C
peptide level
17:169 8:135 2:21
DIASGLIGLLLICKpc3_NP.2
p−min=0.31, pX=0.16, N=27
var.11.F5 1:169511755:T:C
peptide level
30:169 14:135 0:21
DIASGLIGLLLICKpc3_NP.3
p−min=0.023, pX=0.011, N=44
var.11.F5 1:169511755:T:C
peptide level
90:169 71:135 13:21
DIASGLIGLLLICKpc3_NP.4
p−maj=0.5, pX=0.1, N=174
var.11.F5 1:169511755:T:C
peptide level
80:169 50:135 11:21
DIASGLIGLLLICKpc3_NP.5
p−min=0.15, pX=0.05, N=141
var.11.F5 1:169511755:T:C
peptide level
169:169 135:135 21:21
DIHSGLIGPLLICQKpc2_NP.1
p−maj=1, pX=0.04, N=325
var.11.F5 1:169511755:T:C
peptide level
169:169 132:135 21:21
DIHSGLIGPLLICQKpc2_NP.2
p−min=0.11, pX=0.86, N=322
var.11.F5 1:169511755:T:C
peptide level
169:169 135:135 21:21
DIHSGLIGPLLICQKpc2_NP.3
p−maj=1, pX=0.38, N=325
var.11.F5 1:169511755:T:C
peptide level
169:169 135:135 21:21
DIHSGLIGPLLICQKpc2_NP.4
p−maj=1, pX=0.78, N=325
var.11.F5 1:169511755:T:C
peptide level
169:169 135:135 21:21
DIHSGLIGPLLICQKpc2_NP.5
p−maj=1, pX=0.25, N=325
var.11.F5 1:169511755:T:C
peptide level
169:169 135:135 21:21
DIHSGLIGPLLICQKpc3_NP.1
p−maj=1, pX=0.081, N=325
var.11.F5 1:169511755:T:C
peptide level
169:169 135:135 21:21
DIHSGLIGPLLICQKpc3_NP.2
p−maj=1, pX=0.6, N=325
var.11.F5 1:169511755:T:C
peptide level
169:169 135:135 21:21
DIHSGLIGPLLICQKpc3_NP.3
p−maj=1, pX=0.51, N=325
var.11.F5 1:169511755:T:C
peptide level
169:169 135:135 21:21
DIHSGLIGPLLICQKpc3_NP.4
p−maj=1, pX=0.5, N=325
var.11.F5 1:169511755:T:C
peptide level
169:169 135:135 21:21
DIHSGLIGPLLICQKpc3_NP.5
p−maj=1, pX=0.52, N=325

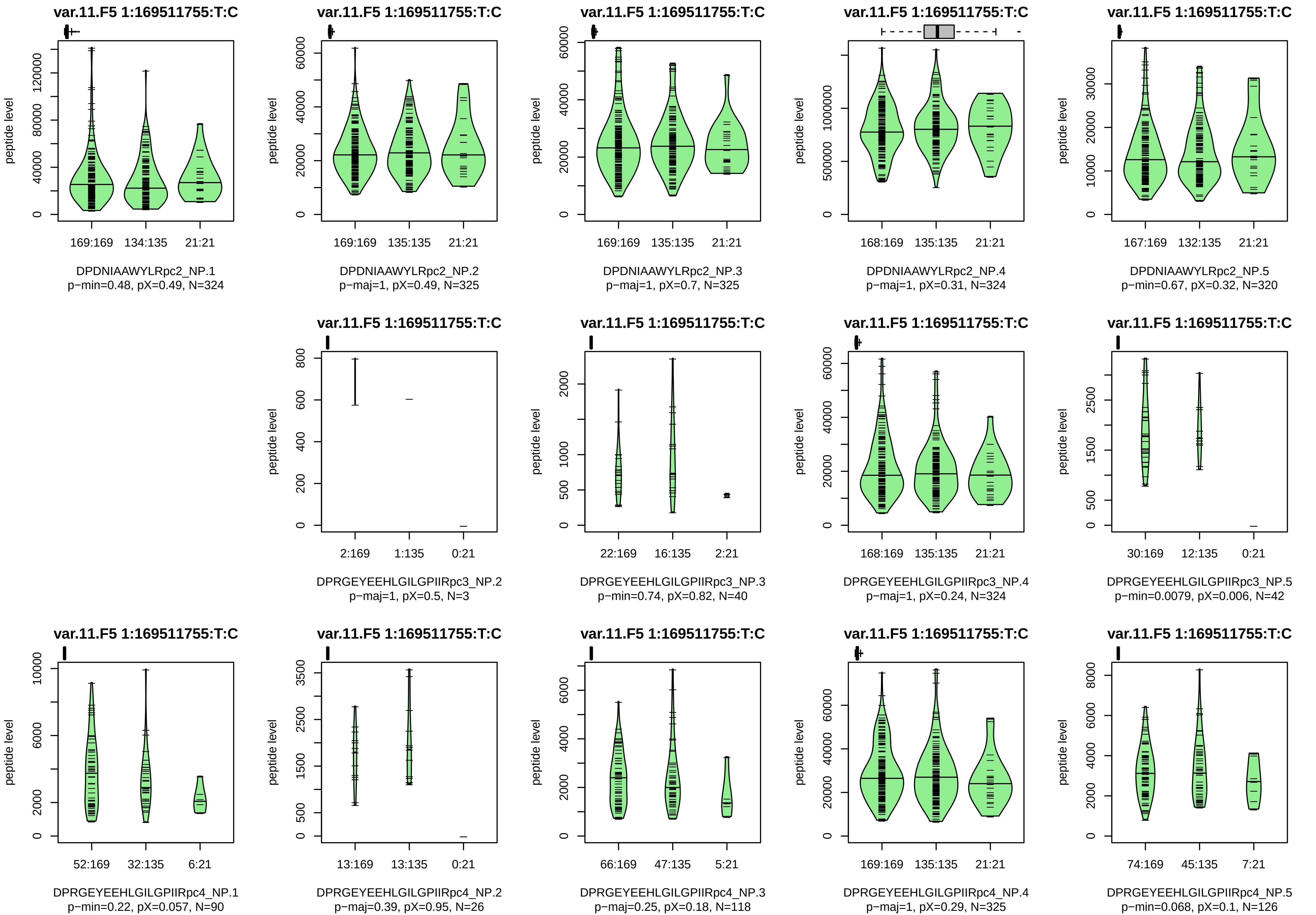
var.11.F5 1:169511755:T:C
peptide level
169:169
134:135
21:21
DPDNIAAWYLRpc2_NP.1
p−min=0.48, pX=0.49, N=324
var.11.F5 1:169511755:T:C
peptide level
169:169
135:135
21:21
DPDNIAAWYLRpc2_NP.2
p−maj=1, pX=0.49, N=325
var.11.F5 1:169511755:T:C
peptide level
169:169
135:135
21:21
DPDNIAAWYLRpc2_NP.3
p−maj=1, pX=0.7, N=325
var.11.F5 1:169511755:T:C
peptide level
168:169
135:135
21:21
DPDNIAAWYLRpc2_NP.4
p−maj=1, pX=0.31, N=324
var.11.F5 1:169511755:T:C
peptide level
167:169
132:135
21:21
DPDNIAAWYLRpc2_NP.5
p−min=0.67, pX=0.32, N=320
var.11.F5 1:169511755:T:C
peptide level
2:169
1:135
0:21
DPRGEYEEHLGILGPIIRpc3_NP.2
p−maj=1, pX=0.5, N=3
var.11.F5 1:169511755:T:C
peptide level
22:169
16:135
2:21
DPRGEYEEHLGILGPIIRpc3_NP.3
p−min=0.74, pX=0.82, N=40
var.11.F5 1:169511755:T:C
peptide level
168:169
135:135
21:21
DPRGEYEEHLGILGPIIRpc3_NP.4
p−maj=1, pX=0.24, N=324
var.11.F5 1:169511755:T:C
peptide level
30:169
12:135
0:21
DPRGEYEEHLGILGPIIRpc3_NP.5
p−min=0.0079, pX=0.006, N=42
var.11.F5 1:169511755:T:C
peptide level
52:169
32:135
6:21
DPRGEYEEHLGILGPIIRpc4_NP.1
p−min=0.22, pX=0.057, N=90
var.11.F5 1:169511755:T:C
peptide level
13:169
13:135
0:21
DPRGEYEEHLGILGPIIRpc4_NP.2
p−maj=0.39, pX=0.95, N=26
var.11.F5 1:169511755:T:C
peptide level
66:169
47:135
5:21
DPRGEYEEHLGILGPIIRpc4_NP.3
p−maj=0.25, pX=0.18, N=118
var.11.F5 1:169511755:T:C
peptide level
169:169
135:135
21:21
DPRGEYEEHLGILGPIIRpc4_NP.4
p−maj=1, pX=0.29, N=325
var.11.F5 1:169511755:T:C
peptide level
74:169
45:135
7:21
DPRGEYEEHLGILGPIIRpc4_NP.5
p−min=0.068, pX=0.1, N=126

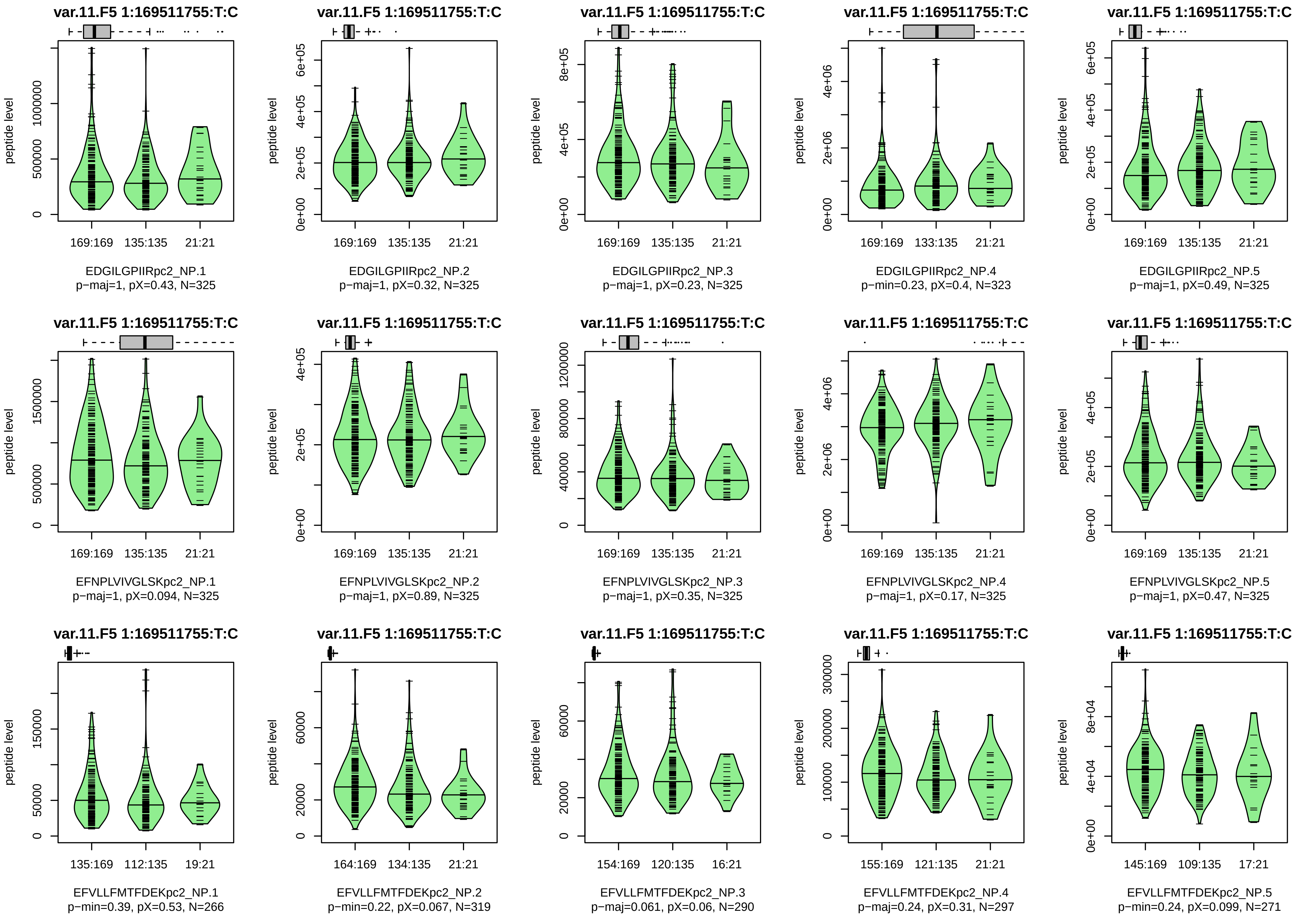
var.11.F5 1:169511755:T:C
peptide level
169:169 135:135 21:21
EDGILGPIIRpc2_NP.1
p−maj=1, pX=0.43, N=325
var.11.F5 1:169511755:T:C
peptide level
169:169 135:135 21:21
EDGILGPIIRpc2_NP.2
p−maj=1, pX=0.32, N=325
var.11.F5 1:169511755:T:C
peptide level
169:169 135:135 21:21
EDGILGPIIRpc2_NP.3
p−maj=1, pX=0.23, N=325
var.11.F5 1:169511755:T:C
peptide level
169:169 133:135 21:21
EDGILGPIIRpc2_NP.4
p−min=0.23, pX=0.4, N=323
var.11.F5 1:169511755:T:C
peptide level
169:169 135:135 21:21
EDGILGPIIRpc2_NP.5
p−maj=1, pX=0.49, N=325
var.11.F5 1:169511755:T:C
peptide level
169:169 135:135 21:21
EFNPLVIVGLSKpc2_NP.1
p−maj=1, pX=0.094, N=325
var.11.F5 1:169511755:T:C
peptide level
169:169 135:135 21:21
EFNPLVIVGLSKpc2_NP.2
p−maj=1, pX=0.89, N=325
var.11.F5 1:169511755:T:C
peptide level
169:169 135:135 21:21
EFNPLVIVGLSKpc2_NP.3
p−maj=1, pX=0.35, N=325
var.11.F5 1:169511755:T:C
peptide level
169:169 135:135 21:21
EFNPLVIVGLSKpc2_NP.4
p−maj=1, pX=0.17, N=325
var.11.F5 1:169511755:T:C
peptide level
169:169 135:135 21:21
EFNPLVIVGLSKpc2_NP.5
p−maj=1, pX=0.47, N=325
var.11.F5 1:169511755:T:C
peptide level
135:169 112:135 19:21
EFVLLFMTFDEKpc2_NP.1
p−min=0.39, pX=0.53, N=266
var.11.F5 1:169511755:T:C
peptide level
164:169 134:135 21:21
EFVLLFMTFDEKpc2_NP.2
p−min=0.22, pX=0.067, N=319
var.11.F5 1:169511755:T:C
peptide level
154:169 120:135 16:21
EFVLLFMTFDEKpc2_NP.3
p−maj=0.061, pX=0.06, N=290
var.11.F5 1:169511755:T:C
peptide level
155:169 121:135 21:21
EFVLLFMTFDEKpc2_NP.4
p−maj=0.24, pX=0.31, N=297
var.11.F5 1:169511755:T:C
peptide level
145:169 109:135 17:21
EFVLLFMTFDEKpc2_NP.5
p−min=0.24, pX=0.099, N=271

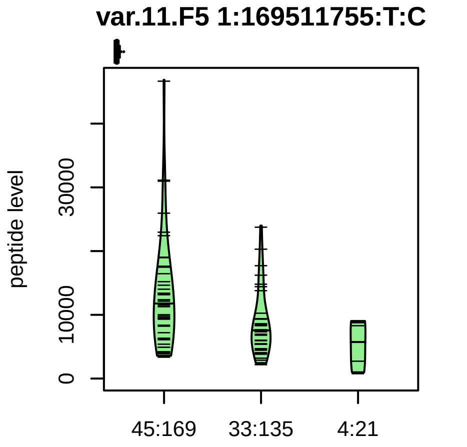

EKPQSTISGLLGPTLYAEVGDIIKpc3_NP.1
p−min=0.61, pX=0.02, N=82

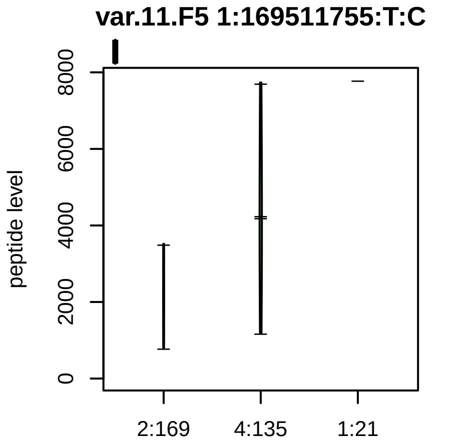

EKPQSTISGLLGPTLYAEVGDIIKpc3_NP.2
p−min=0.27, pX=0.034, N=7

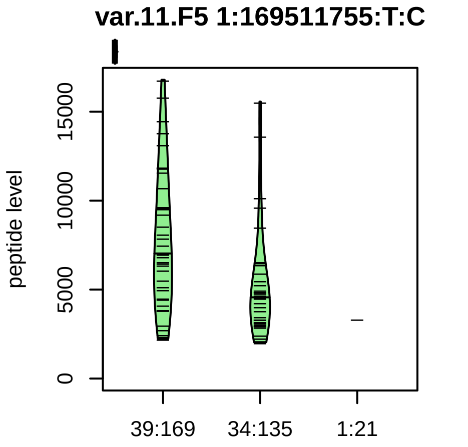

EKPQSTISGLLGPTLYAEVGDIIKpc3_NP.3
p−maj=0.056, pX=0.03, N=74

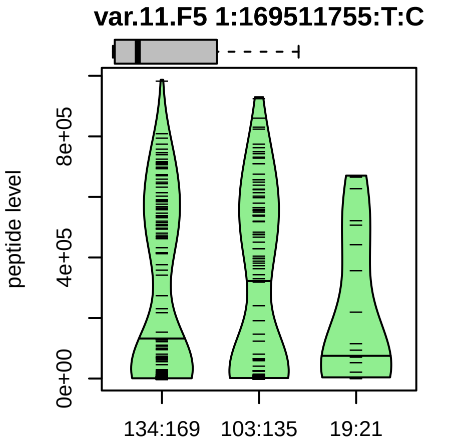

EKPQSTISGLLGPTLYAEVGDIIKpc3_NP.4
p−maj=0.27, pX=0.71, N=256

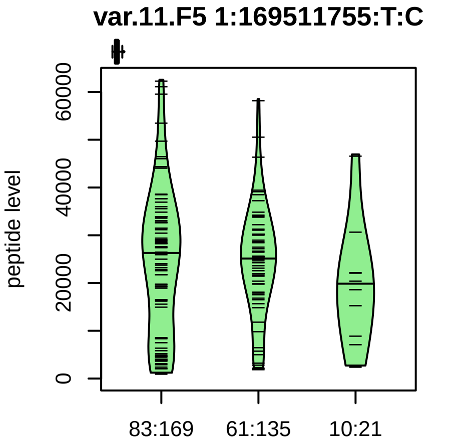

EKPQSTISGLLGPTLYAEVGDIIKpc3_NP.5
p−min=0.58, pX=0.46, N=154

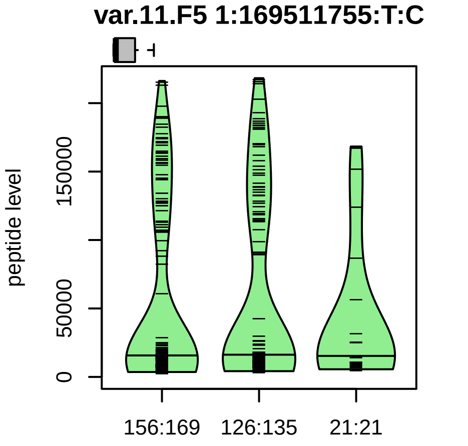

EKPQSTISGLLGPTLYAEVGDIIKpc4_NP.4
p−maj=0.38, pX=1, N=303

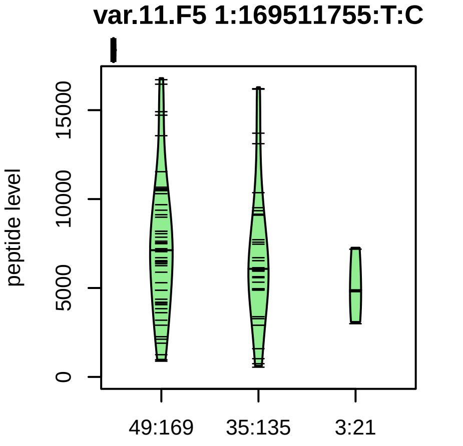

EKPQSTISGLLGPTLYAEVGDIIKpc4_NP.5
p−maj=0.21, pX=0.1, N=87

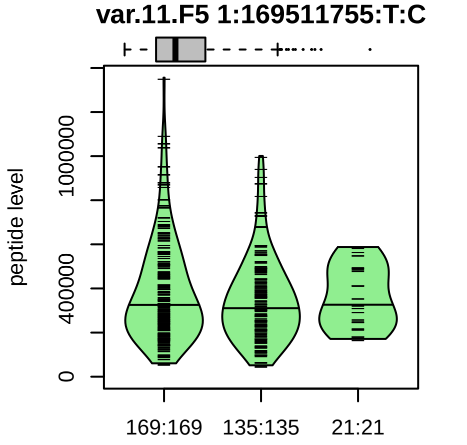

ENQFDPPIVARpc2_NP.1
p−maj=1, pX=0.2, N=325

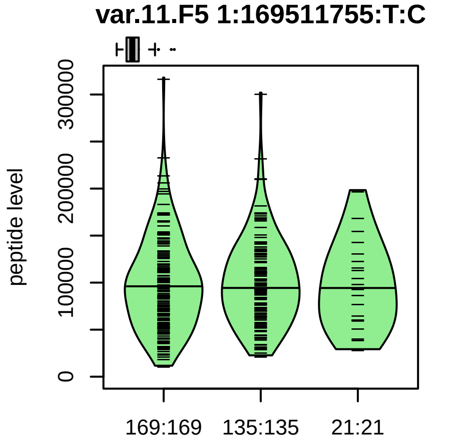

ENQFDPPIVARpc2_NP.2
p−maj=1, pX=0.85, N=325

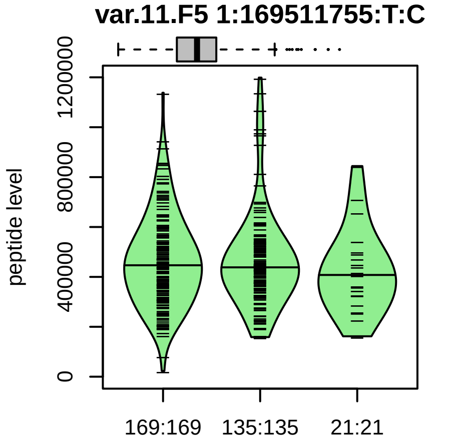

ENQFDPPIVARpc2_NP.3
p−maj=1, pX=0.56, N=325

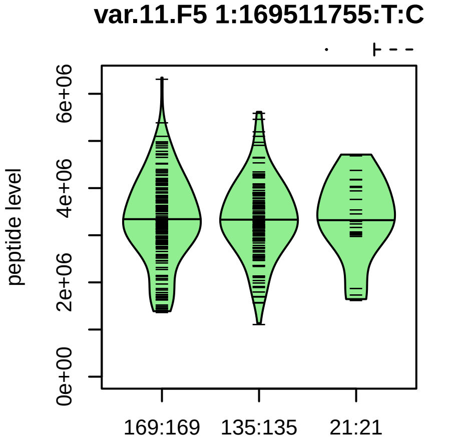

ENQFDPPIVARpc2_NP.4
p−maj=1, pX=0.71, N=325

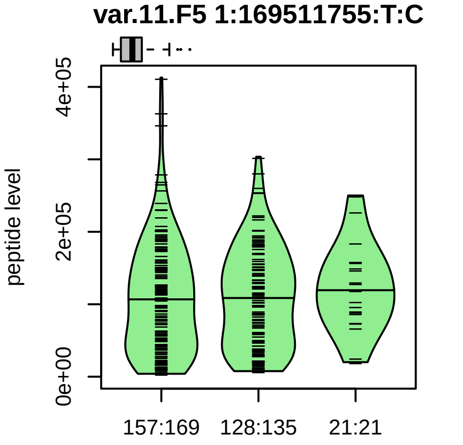

ENQFDPPIVARpc2_NP.5
p−min=0.35, pX=0.35, N=306

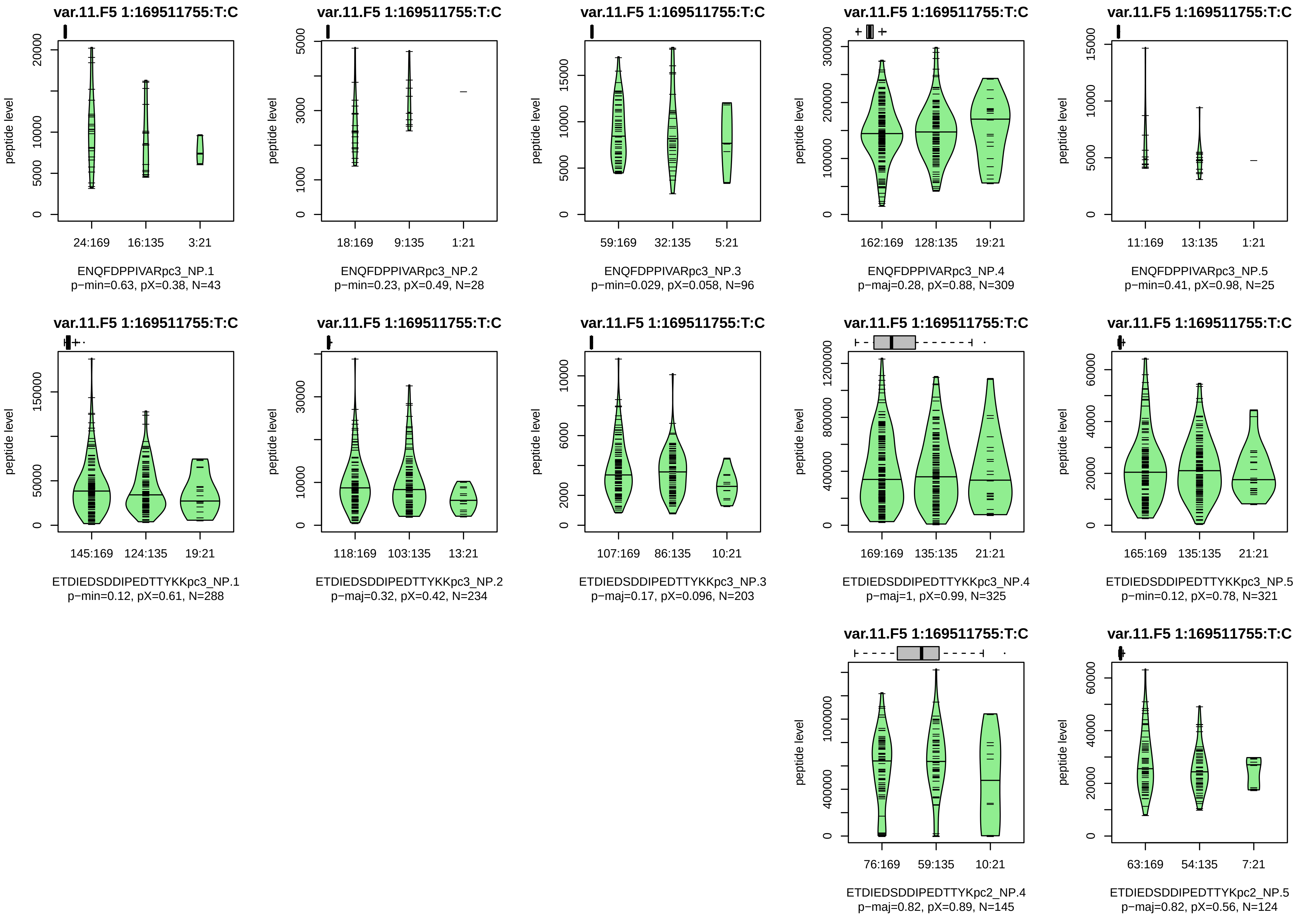
var.11.F5 1:169511755:T:C
peptide level
24:169 16:135 3:21
ENQFDPPIVARpc3_NP.1
p−min=0.63, pX=0.38, N=43
var.11.F5 1:169511755:T:C
peptide level
18:169 9:135 1:21
ENQFDPPIVARpc3_NP.2
p−min=0.23, pX=0.49, N=28
var.11.F5 1:169511755:T:C
peptide level
59:169 32:135 5:21
ENQFDPPIVARpc3_NP.3
p−min=0.029, pX=0.058, N=96
var.11.F5 1:169511755:T:C
peptide level
162:169 128:135 19:21
ENQFDPPIVARpc3_NP.4
p−maj=0.28, pX=0.88, N=309
var.11.F5 1:169511755:T:C
peptide level
11:169 13:135 1:21
ENQFDPPIVARpc3_NP.5
p−min=0.41, pX=0.98, N=25
var.11.F5 1:169511755:T:C
peptide level
145:169 124:135 19:21
ETDIEDSDDIPEDTTYKKpc3_NP.1
p−min=0.12, pX=0.61, N=288
var.11.F5 1:169511755:T:C
peptide level
118:169 103:135 13:21
ETDIEDSDDIPEDTTYKKpc3_NP.2
p−maj=0.32, pX=0.42, N=234
var.11.F5 1:169511755:T:C
peptide level
107:169 86:135 10:21
ETDIEDSDDIPEDTTYKKpc3_NP.3
p−maj=0.17, pX=0.096, N=203
var.11.F5 1:169511755:T:C
peptide level
169:169 135:135 21:21
ETDIEDSDDIPEDTTYKKpc3_NP.4
p−maj=1, pX=0.99, N=325
var.11.F5 1:169511755:T:C
peptide level
165:169 135:135 21:21
ETDIEDSDDIPEDTTYKKpc3_NP.5
p−min=0.12, pX=0.78, N=321
var.11.F5 1:169511755:T:C
peptide level
76:169 59:135 10:21
ETDIEDSDDIPEDTTYKpc2_NP.4
p−maj=0.82, pX=0.89, N=145
var.11.F5 1:169511755:T:C
peptide level
63:169 54:135 7:21
ETDIEDSDDIPEDTTYKpc2_NP.5
p−maj=0.82, pX=0.56, N=124

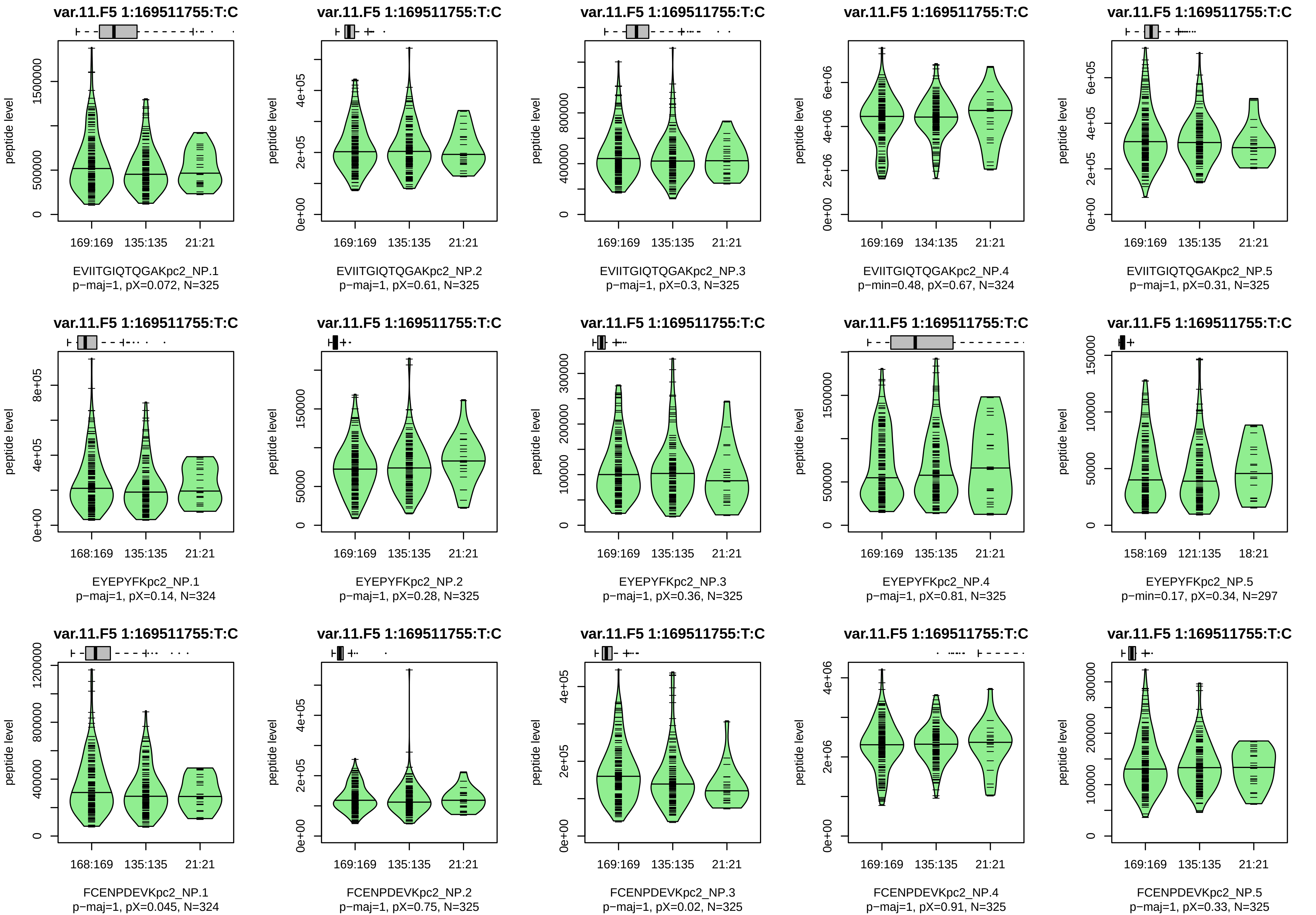
var.11.F5 1:169511755:T:C
peptide level
169:169 135:135 21:21
EVIITGIQTQGAKpc2_NP.1
p−maj=1, pX=0.072, N=325
var.11.F5 1:169511755:T:C
peptide level
169:169 135:135 21:21
EVIITGIQTQGAKpc2_NP.2
p−maj=1, pX=0.61, N=325
var.11.F5 1:169511755:T:C
peptide level
169:169 135:135 21:21
EVIITGIQTQGAKpc2_NP.3
p−maj=1, pX=0.3, N=325
var.11.F5 1:169511755:T:C
peptide level
169:169 134:135 21:21
EVIITGIQTQGAKpc2_NP.4
p−min=0.48, pX=0.67, N=324
var.11.F5 1:169511755:T:C
peptide level
169:169 135:135 21:21
EVIITGIQTQGAKpc2_NP.5
p−maj=1, pX=0.31, N=325
var.11.F5 1:169511755:T:C
peptide level
168:169 135:135 21:21
EYEPYFKpc2_NP.1
p−maj=1, pX=0.14, N=324
var.11.F5 1:169511755:T:C
peptide level
169:169 135:135 21:21
EYEPYFKpc2_NP.2
p−maj=1, pX=0.28, N=325
var.11.F5 1:169511755:T:C
peptide level
169:169 135:135 21:21
EYEPYFKpc2_NP.3
p−maj=1, pX=0.36, N=325
var.11.F5 1:169511755:T:C
peptide level
169:169 135:135 21:21
EYEPYFKpc2_NP.4
p−maj=1, pX=0.81, N=325
var.11.F5 1:169511755:T:C
peptide level
158:169 121:135 18:21
EYEPYFKpc2_NP.5
p−min=0.17, pX=0.34, N=297
var.11.F5 1:169511755:T:C
peptide level
168:169 135:135 21:21
FCENPDEVKpc2_NP.1
p−maj=1, pX=0.045, N=324
var.11.F5 1:169511755:T:C
peptide level
169:169 135:135 21:21
FCENPDEVKpc2_NP.2
p−maj=1, pX=0.75, N=325
var.11.F5 1:169511755:T:C
peptide level
169:169 135:135 21:21
FCENPDEVKpc2_NP.3
p−maj=1, pX=0.02, N=325
var.11.F5 1:169511755:T:C
peptide level
169:169 135:135 21:21
FCENPDEVKpc2_NP.4
p−maj=1, pX=0.91, N=325
var.11.F5 1:169511755:T:C
peptide level
169:169 135:135 21:21
FCENPDEVKpc2_NP.5
p−maj=1, pX=0.33, N=325

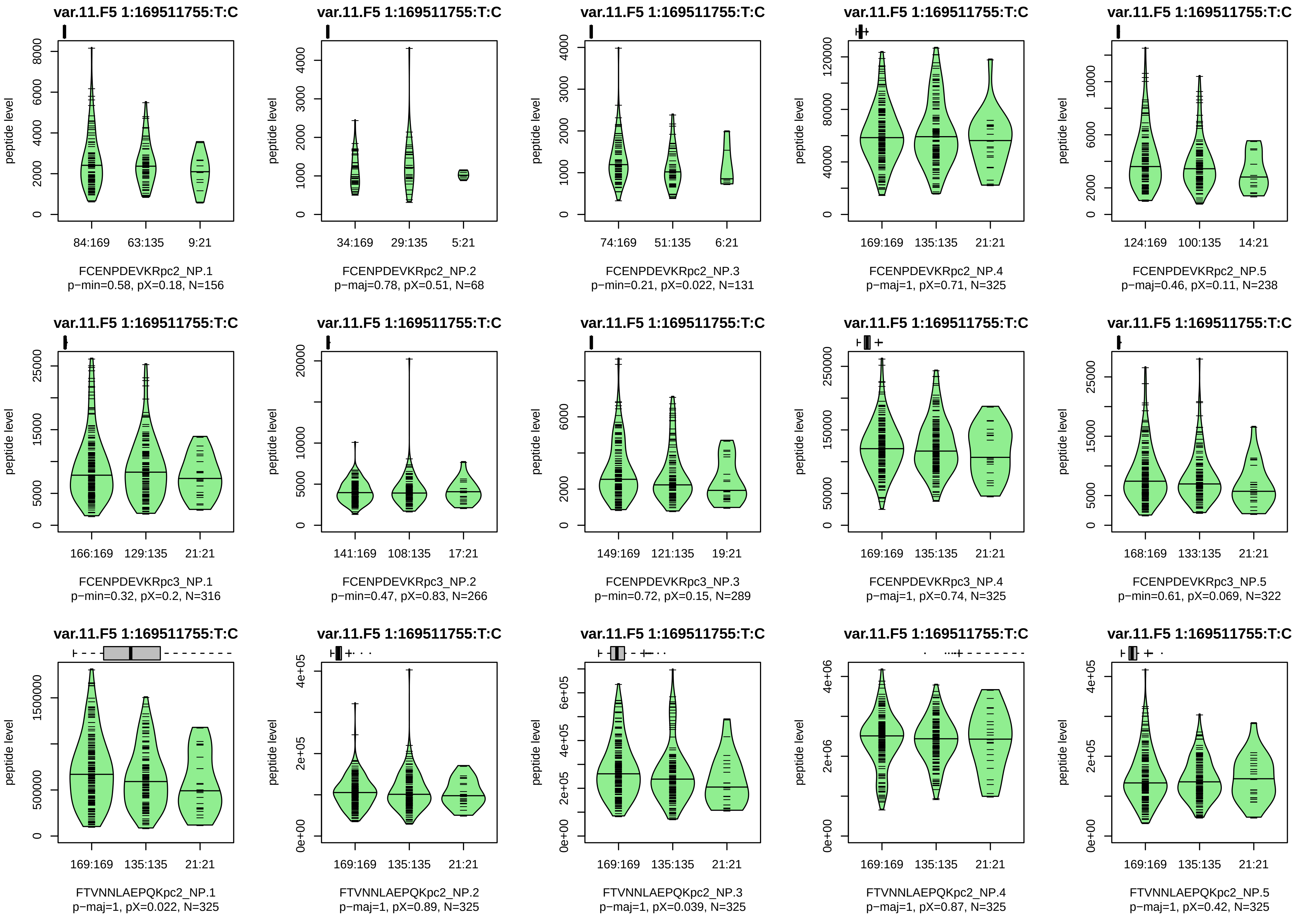
var.11.F5 1:169511755:T:C
peptide level
84:169
63:135
9:21
FCENPDEVKRpc2_NP.1
p−min=0.58, pX=0.18, N=156
var.11.F5 1:169511755:T:C
peptide level
34:169
29:135
5:21
FCENPDEVKRpc2_NP.2
p−maj=0.78, pX=0.51, N=68
var.11.F5 1:169511755:T:C
peptide level
74:169
51:135
6:21
FCENPDEVKRpc2_NP.3
p−min=0.21, pX=0.022, N=131
var.11.F5 1:169511755:T:C
peptide level
169:169
135:135
21:21
FCENPDEVKRpc2_NP.4
p−maj=1, pX=0.71, N=325
var.11.F5 1:169511755:T:C
peptide level
124:169
100:135
14:21
FCENPDEVKRpc2_NP.5
p−maj=0.46, pX=0.11, N=238
var.11.F5 1:169511755:T:C
peptide level
166:169
129:135
21:21
FCENPDEVKRpc3_NP.1
p−min=0.32, pX=0.2, N=316
var.11.F5 1:169511755:T:C
peptide level
141:169
108:135
17:21
FCENPDEVKRpc3_NP.2
p−min=0.47, pX=0.83, N=266
var.11.F5 1:169511755:T:C
peptide level
149:169
121:135
19:21
FCENPDEVKRpc3_NP.3
p−min=0.72, pX=0.15, N=289
var.11.F5 1:169511755:T:C
peptide level
169:169
135:135
21:21
FCENPDEVKRpc3_NP.4
p−maj=1, pX=0.74, N=325
var.11.F5 1:169511755:T:C
peptide level
168:169
133:135
21:21
FCENPDEVKRpc3_NP.5
p−min=0.61, pX=0.069, N=322
var.11.F5 1:169511755:T:C
peptide level
169:169
135:135
21:21
FTVNNLAEPQKpc2_NP.1
p−maj=1, pX=0.022, N=325
var.11.F5 1:169511755:T:C
peptide level
169:169
135:135
21:21
FTVNNLAEPQKpc2_NP.2
p−maj=1, pX=0.89, N=325
var.11.F5 1:169511755:T:C
peptide level
169:169
135:135
21:21
FTVNNLAEPQKpc2_NP.3
p−maj=1, pX=0.039, N=325
var.11.F5 1:169511755:T:C
peptide level
169:169
135:135
21:21
FTVNNLAEPQKpc2_NP.4
p−maj=1, pX=0.87, N=325
var.11.F5 1:169511755:T:C
peptide level
169:169
135:135
21:21
FTVNNLAEPQKpc2_NP.5
p−maj=1, pX=0.42, N=325

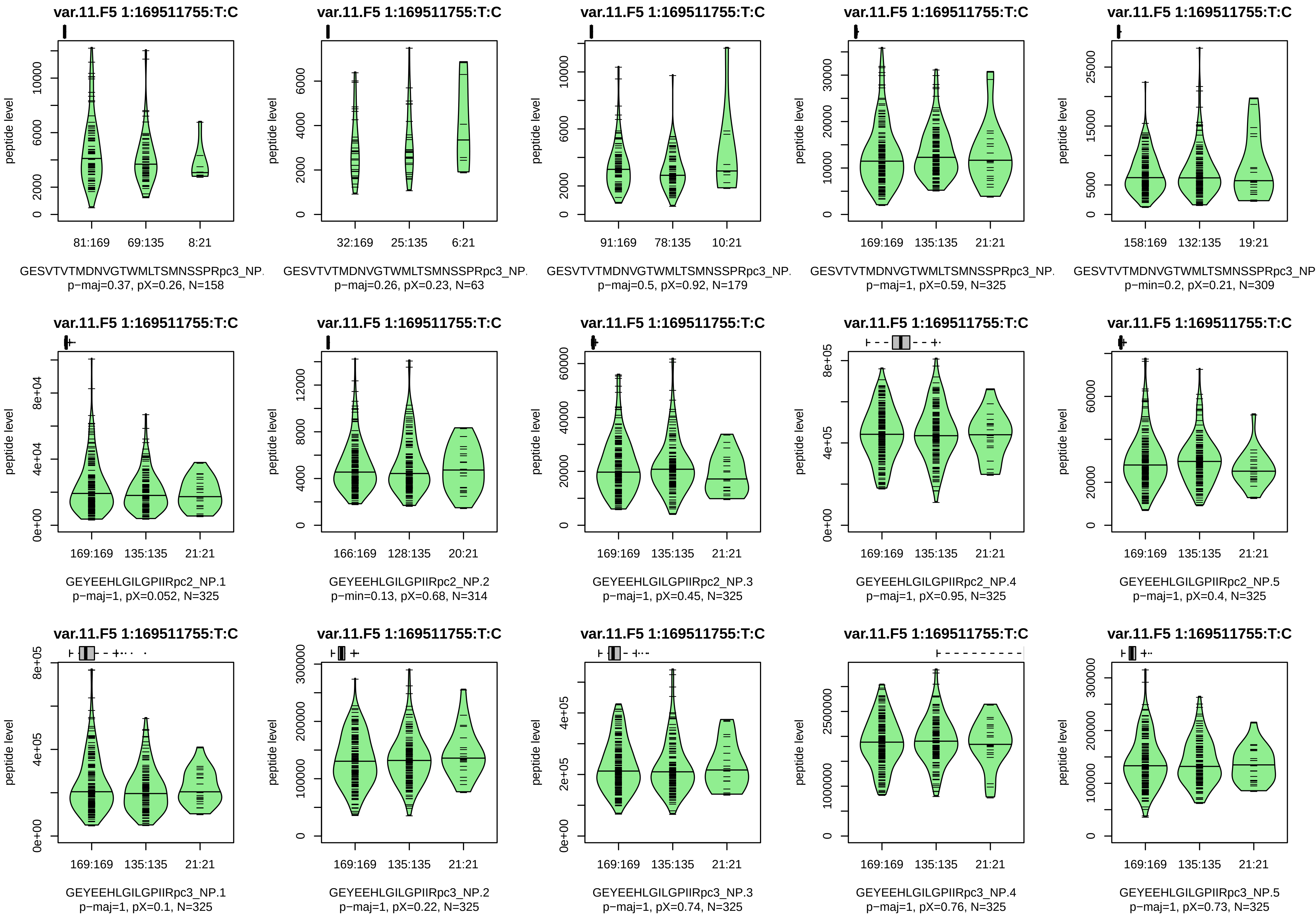
var.11.F5 1:169511755:T:C
peptide level
81:169
69:135
8:21
GESVTVTMDNVGTWMLTSMNSSPRpc3_NP.
p−maj=0.37, pX=0.26, N=158
var.11.F5 1:169511755:T:C
peptide level
32:169
25:135
6:21
GESVTVTMDNVGTWMLTSMNSSPRpc3_NP.
p−maj=0.26, pX=0.23, N=63
var.11.F5 1:169511755:T:C
peptide level
91:169
78:135
10:21
GESVTVTMDNVGTWMLTSMNSSPRpc3_NP.
p−maj=0.5, pX=0.92, N=179
var.11.F5 1:169511755:T:C
peptide level
169:169
135:135
21:21
GESVTVTMDNVGTWMLTSMNSSPRpc3_NP.
p−maj=1, pX=0.59, N=325
var.11.F5 1:169511755:T:C
peptide level
158:169
132:135
19:21
GESVTVTMDNVGTWMLTSMNSSPRpc3_NP
p−min=0.2, pX=0.21, N=309
var.11.F5 1:169511755:T:C
peptide level
169:169
135:135
21:21
GEYEEHLGILGPIIRpc2_NP.1
p−maj=1, pX=0.052, N=325
var.11.F5 1:169511755:T:C
peptide level
166:169
128:135
20:21
GEYEEHLGILGPIIRpc2_NP.2
p−min=0.13, pX=0.68, N=314
var.11.F5 1:169511755:T:C
peptide level
169:169
135:135
21:21
GEYEEHLGILGPIIRpc2_NP.3
p−maj=1, pX=0.45, N=325
var.11.F5 1:169511755:T:C
peptide level
169:169
135:135
21:21
GEYEEHLGILGPIIRpc2_NP.4
p−maj=1, pX=0.95, N=325
var.11.F5 1:169511755:T:C
peptide level
169:169
135:135
21:21
GEYEEHLGILGPIIRpc2_NP.5
p−maj=1, pX=0.4, N=325
var.11.F5 1:169511755:T:C
peptide level
169:169
135:135
21:21
GEYEEHLGILGPIIRpc3_NP.1
p−maj=1, pX=0.1, N=325
var.11.F5 1:169511755:T:C
peptide level
169:169
135:135
21:21
GEYEEHLGILGPIIRpc3_NP.2
p−maj=1, pX=0.22, N=325
var.11.F5 1:169511755:T:C
peptide level
169:169
135:135
21:21
GEYEEHLGILGPIIRpc3_NP.3
p−maj=1, pX=0.74, N=325
var.11.F5 1:169511755:T:C
peptide level
169:169
135:135
21:21
GEYEEHLGILGPIIRpc3_NP.4
p−maj=1, pX=0.76, N=325
var.11.F5 1:169511755:T:C
peptide level
169:169
135:135
21:21
GEYEEHLGILGPIIRpc3_NP.5
p−maj=1, pX=0.73, N=325

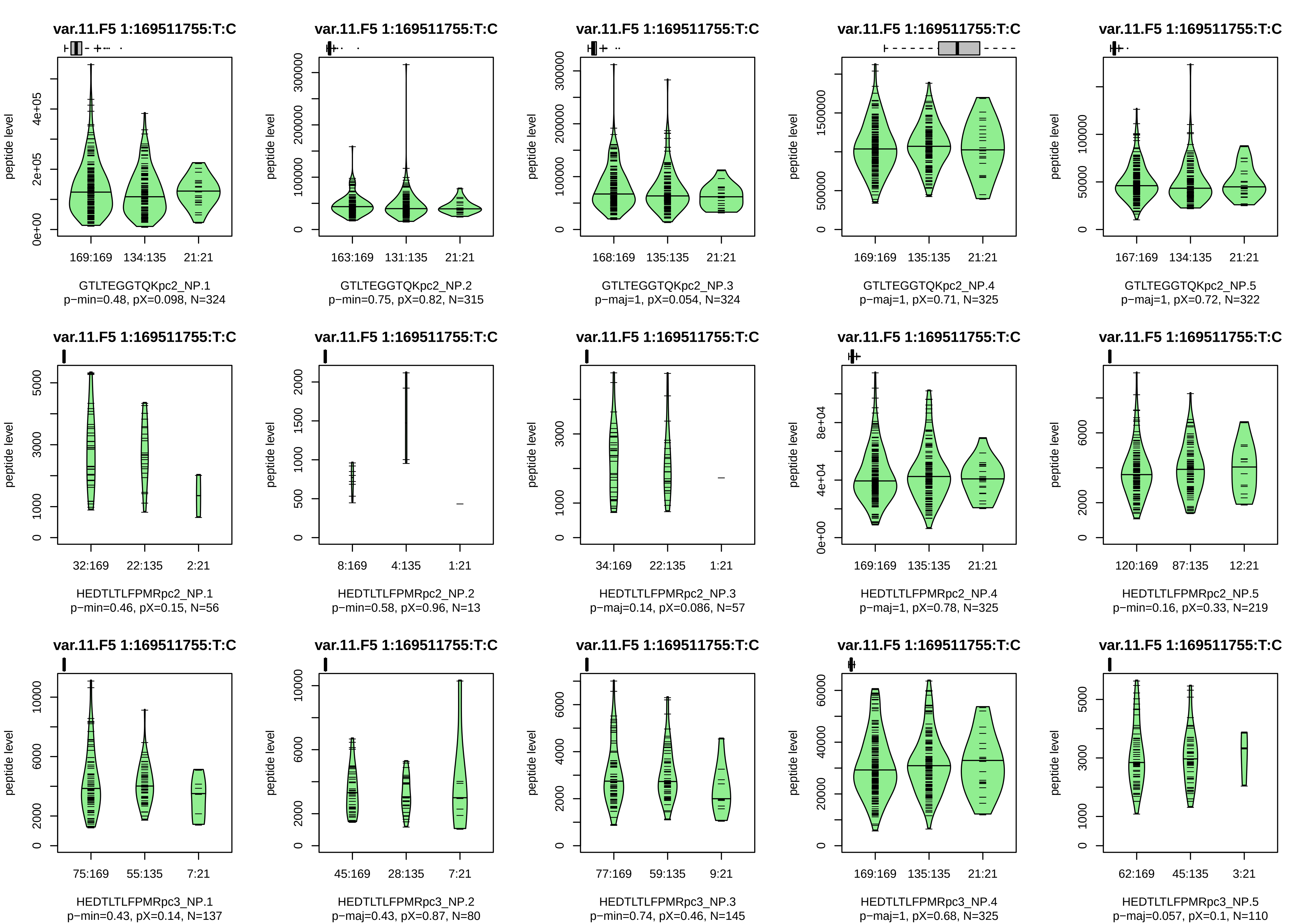
var.11.F5 1:169511755:T:C
peptide level
169:169 134:135 21:21
GTLTEGGTQKpc2_NP.1
p−min=0.48, pX=0.098, N=324
var.11.F5 1:169511755:T:C
peptide level
163:169 131:135 21:21
GTLTEGGTQKpc2_NP.2
p−min=0.75, pX=0.82, N=315
var.11.F5 1:169511755:T:C
peptide level
168:169 135:135 21:21
GTLTEGGTQKpc2_NP.3
p−maj=1, pX=0.054, N=324
var.11.F5 1:169511755:T:C
peptide level
169:169 135:135 21:21
GTLTEGGTQKpc2_NP.4
p−maj=1, pX=0.71, N=325
var.11.F5 1:169511755:T:C
peptide level
167:169 134:135 21:21
GTLTEGGTQKpc2_NP.5
p−maj=1, pX=0.72, N=322
var.11.F5 1:169511755:T:C
peptide level
32:169 22:135 2:21
HEDTLTLFPMRpc2_NP.1
p−min=0.46, pX=0.15, N=56
var.11.F5 1:169511755:T:C
peptide level
8:169 4:135 1:21
HEDTLTLFPMRpc2_NP.2
p−min=0.58, pX=0.96, N=13
var.11.F5 1:169511755:T:C
peptide level
34:169 22:135 1:21
HEDTLTLFPMRpc2_NP.3
p−maj=0.14, pX=0.086, N=57
var.11.F5 1:169511755:T:C
peptide level
169:169 135:135 21:21
HEDTLTLFPMRpc2_NP.4
p−maj=1, pX=0.78, N=325
var.11.F5 1:169511755:T:C
peptide level
120:169 87:135 12:21
HEDTLTLFPMRpc2_NP.5
p−min=0.16, pX=0.33, N=219
var.11.F5 1:169511755:T:C
peptide level
75:169 55:135 7:21
HEDTLTLFPMRpc3_NP.1
p−min=0.43, pX=0.14, N=137
var.11.F5 1:169511755:T:C
peptide level
45:169 28:135 7:21
HEDTLTLFPMRpc3_NP.2
p−maj=0.43, pX=0.87, N=80
var.11.F5 1:169511755:T:C
peptide level
77:169 59:135 9:21
HEDTLTLFPMRpc3_NP.3
p−min=0.74, pX=0.46, N=145
var.11.F5 1:169511755:T:C
peptide level
169:169 135:135 21:21
HEDTLTLFPMRpc3_NP.4
p−maj=1, pX=0.68, N=325
var.11.F5 1:169511755:T:C
peptide level
62:169 45:135 3:21
HEDTLTLFPMRpc3_NP.5
p−maj=0.057, pX=0.1, N=110

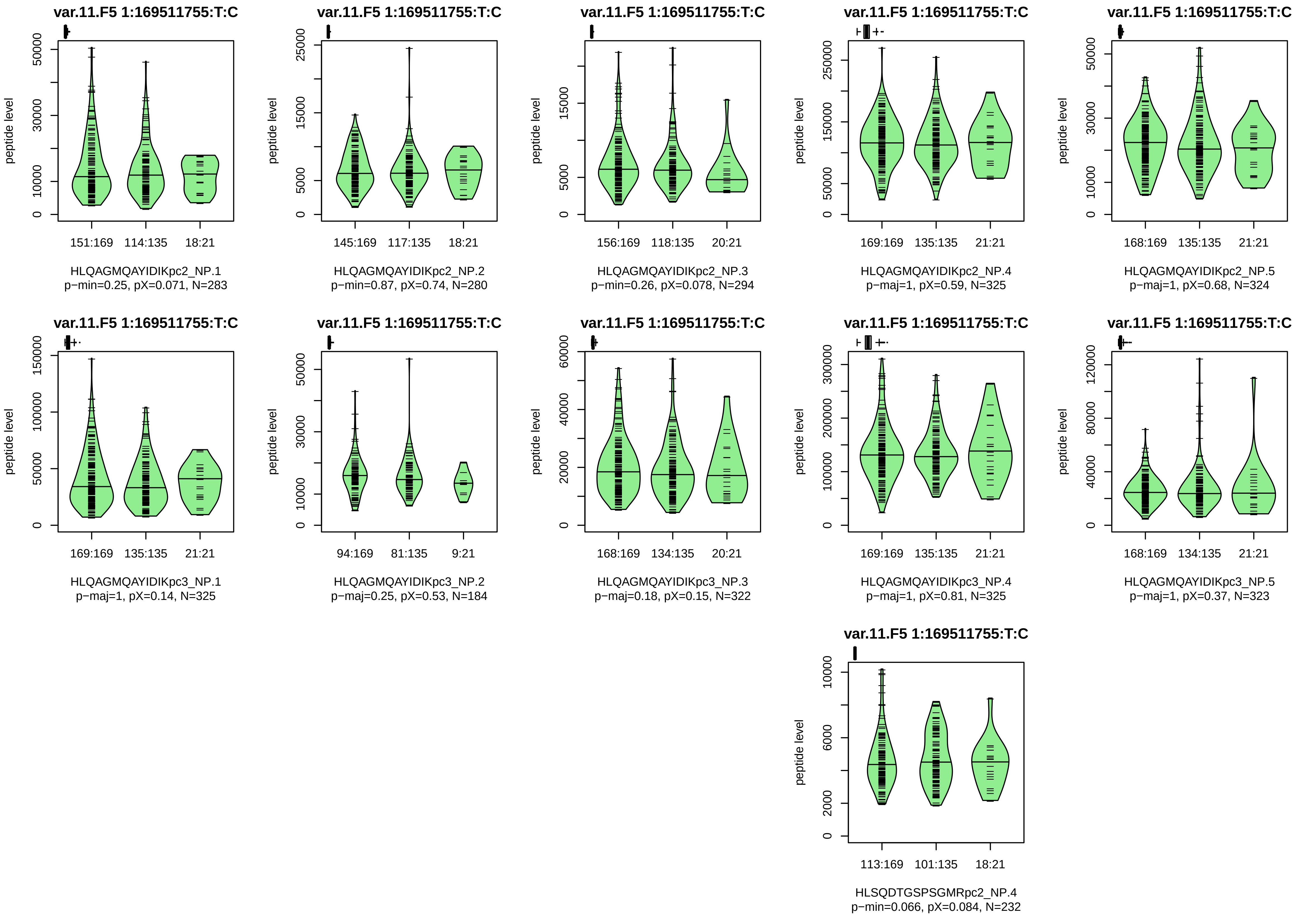
var.11.F5 1:169511755:T:C
peptide level
151:169 114:135 18:21
HLQAGMQAYIDIKpc2_NP.1
p−min=0.25, pX=0.071, N=283
var.11.F5 1:169511755:T:C
peptide level
145:169 117:135 18:21
HLQAGMQAYIDIKpc2_NP.2
p−min=0.87, pX=0.74, N=280
var.11.F5 1:169511755:T:C
peptide level
156:169 118:135 20:21
HLQAGMQAYIDIKpc2_NP.3
p−min=0.26, pX=0.078, N=294
var.11.F5 1:169511755:T:C
peptide level
169:169 135:135 21:21
HLQAGMQAYIDIKpc2_NP.4
p−maj=1, pX=0.59, N=325
var.11.F5 1:169511755:T:C
peptide level
168:169 135:135 21:21
HLQAGMQAYIDIKpc2_NP.5
p−maj=1, pX=0.68, N=324
var.11.F5 1:169511755:T:C
peptide level
169:169 135:135 21:21
HLQAGMQAYIDIKpc3_NP.1
p−maj=1, pX=0.14, N=325
var.11.F5 1:169511755:T:C
peptide level
94:169 81:135 9:21
HLQAGMQAYIDIKpc3_NP.2
p−maj=0.25, pX=0.53, N=184
var.11.F5 1:169511755:T:C
peptide level
168:169 134:135 20:21
HLQAGMQAYIDIKpc3_NP.3
p−maj=0.18, pX=0.15, N=322
var.11.F5 1:169511755:T:C
peptide level
169:169 135:135 21:21
HLQAGMQAYIDIKpc3_NP.4
p−maj=1, pX=0.81, N=325
var.11.F5 1:169511755:T:C
peptide level
168:169 134:135 21:21
HLQAGMQAYIDIKpc3_NP.5
p−maj=1, pX=0.37, N=323
var.11.F5 1:169511755:T:C
peptide level
113:169 101:135 18:21
HLSQDTGSPSGMRpc2_NP.4
p−min=0.066, pX=0.084, N=232

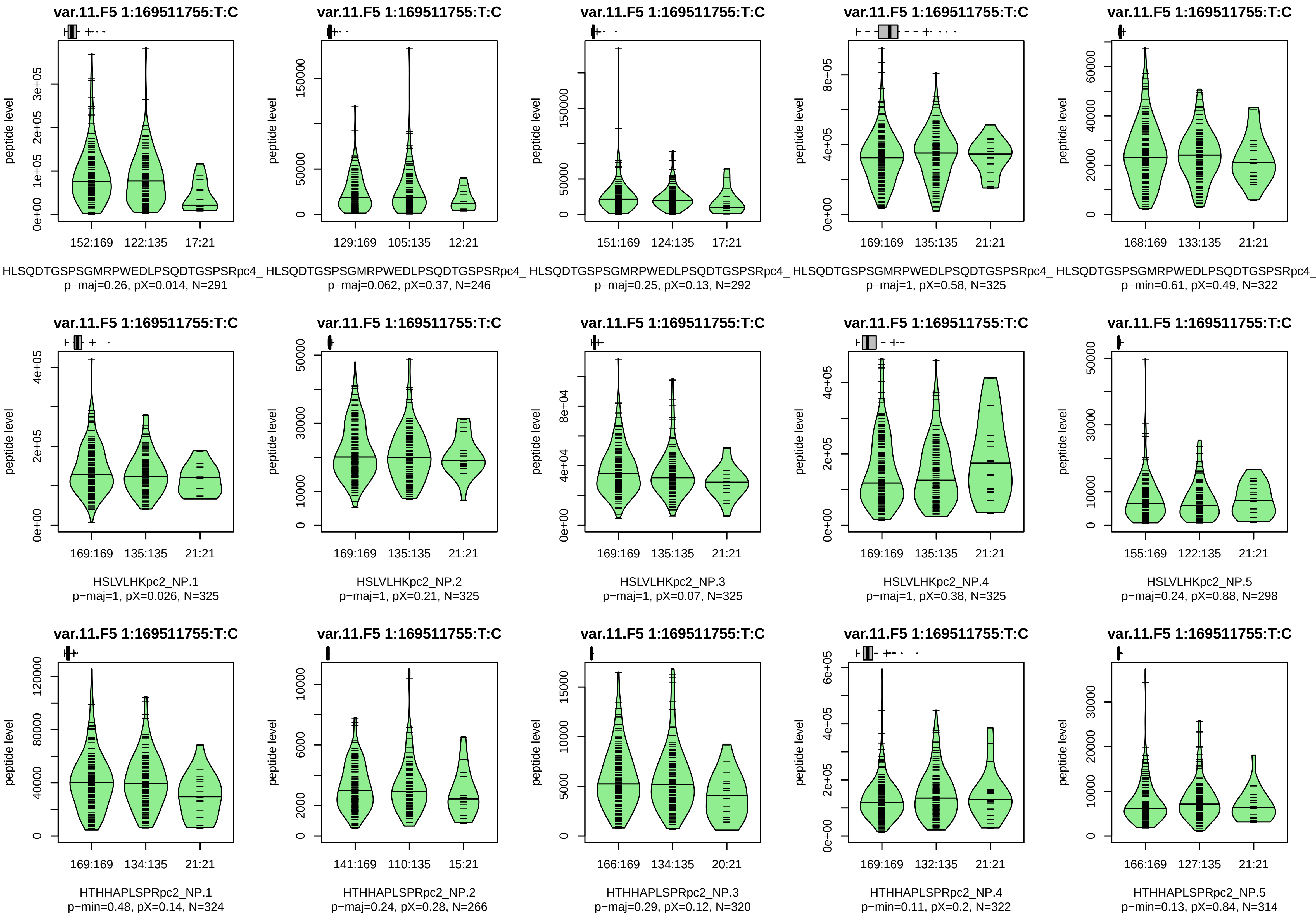
var.11.F5 1:169511755:T:C
peptide level
152:169 122:135 17:21
HLSQDTGSPSGMRPWEDLPSQDTGSPSRpc4_
p−maj=0.26, pX=0.014, N=291
var.11.F5 1:169511755:T:C
peptide level
129:169 105:135 12:21
HLSQDTGSPSGMRPWEDLPSQDTGSPSRpc4_
p−maj=0.062, pX=0.37, N=246
var.11.F5 1:169511755:T:C
peptide level
151:169 124:135 17:21
HLSQDTGSPSGMRPWEDLPSQDTGSPSRpc4_
p−maj=0.25, pX=0.13, N=292
var.11.F5 1:169511755:T:C
peptide level
169:169 135:135 21:21
HLSQDTGSPSGMRPWEDLPSQDTGSPSRpc4_
p−maj=1, pX=0.58, N=325
var.11.F5 1:169511755:T:C
peptide level
168:169 133:135 21:21
HLSQDTGSPSGMRPWEDLPSQDTGSPSRpc4_
p−min=0.61, pX=0.49, N=322
var.11.F5 1:169511755:T:C
peptide level
169:169 135:135 21:21
HSLVLHKpc2_NP.1
p−maj=1, pX=0.026, N=325
var.11.F5 1:169511755:T:C
peptide level
169:169 135:135 21:21
HSLVLHKpc2_NP.2
p−maj=1, pX=0.21, N=325
var.11.F5 1:169511755:T:C
peptide level
169:169 135:135 21:21
HSLVLHKpc2_NP.3
p−maj=1, pX=0.07, N=325
var.11.F5 1:169511755:T:C
peptide level
169:169 135:135 21:21
HSLVLHKpc2_NP.4
p−maj=1, pX=0.38, N=325
var.11.F5 1:169511755:T:C
peptide level
155:169 122:135 21:21
HSLVLHKpc2_NP.5
p−maj=0.24, pX=0.88, N=298
var.11.F5 1:169511755:T:C
peptide level
169:169 134:135 21:21
HTHHAPLSPRpc2_NP.1
p−min=0.48, pX=0.14, N=324
var.11.F5 1:169511755:T:C
peptide level
141:169 110:135 15:21
HTHHAPLSPRpc2_NP.2
p−maj=0.24, pX=0.28, N=266
var.11.F5 1:169511755:T:C
peptide level
166:169 134:135 20:21
HTHHAPLSPRpc2_NP.3
p−maj=0.29, pX=0.12, N=320
var.11.F5 1:169511755:T:C
peptide level
169:169 132:135 21:21
HTHHAPLSPRpc2_NP.4
p−min=0.11, pX=0.2, N=322
var.11.F5 1:169511755:T:C
peptide level
166:169 127:135 21:21
HTHHAPLSPRpc2_NP.5
p−min=0.13, pX=0.84, N=314

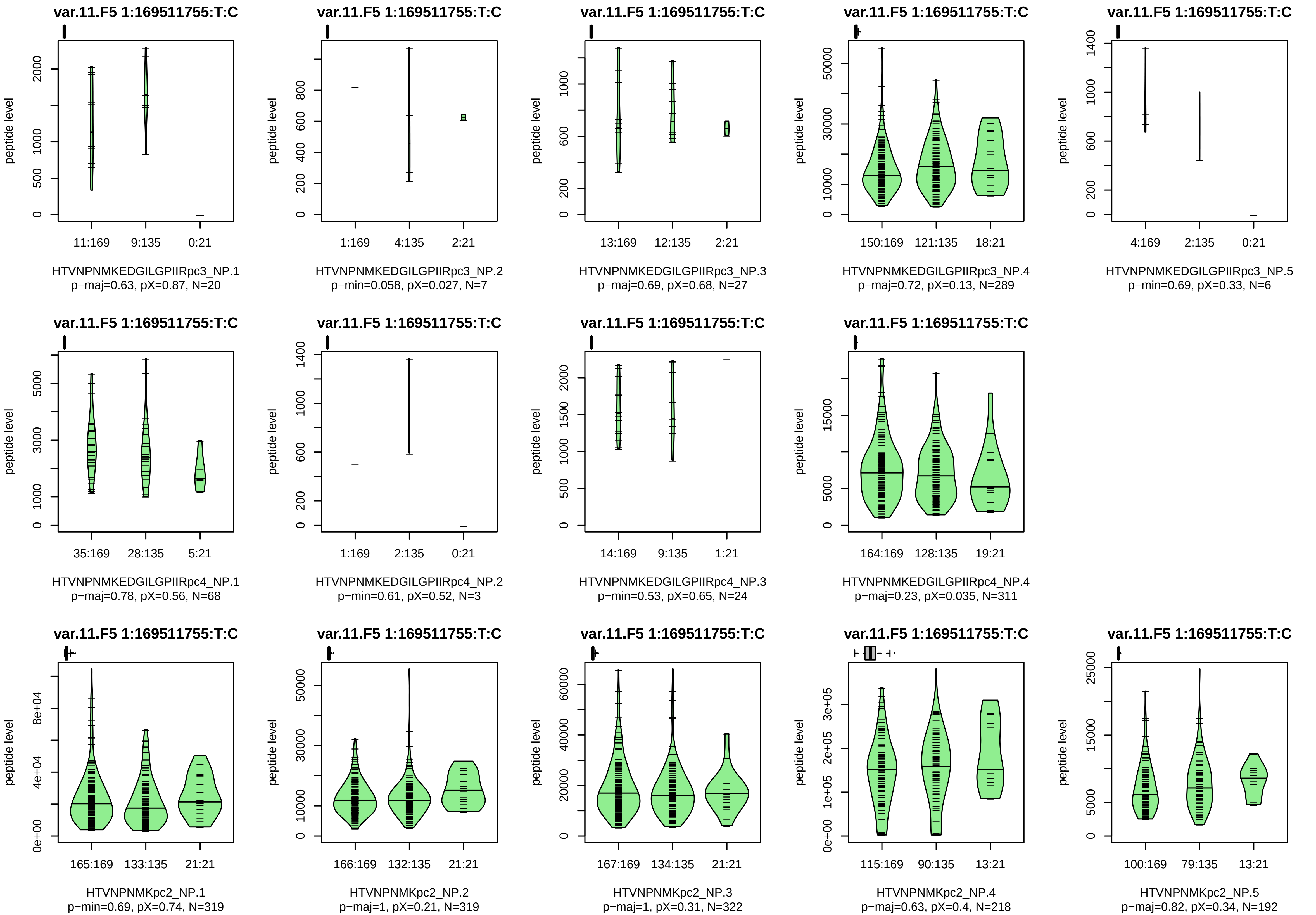
var.11.F5 1:169511755:T:C
peptide level
11:169
9:135
0:21
HTVNPNMKEDGILGPIIRpc3_NP.1
p−maj=0.63, pX=0.87, N=20
var.11.F5 1:169511755:T:C
peptide level
1:169
4:135
2:21
HTVNPNMKEDGILGPIIRpc3_NP.2
p−min=0.058, pX=0.027, N=7
var.11.F5 1:169511755:T:C
peptide level
13:169
12:135
2:21
HTVNPNMKEDGILGPIIRpc3_NP.3
p−maj=0.69, pX=0.68, N=27
var.11.F5 1:169511755:T:C
peptide level
150:169
121:135
18:21
HTVNPNMKEDGILGPIIRpc3_NP.4
p−maj=0.72, pX=0.13, N=289
var.11.F5 1:169511755:T:C
peptide level
4:169
2:135
0:21
HTVNPNMKEDGILGPIIRpc3_NP.5
p−min=0.69, pX=0.33, N=6
var.11.F5 1:169511755:T:C
peptide level
35:169
28:135
5:21
HTVNPNMKEDGILGPIIRpc4_NP.1
p−maj=0.78, pX=0.56, N=68
var.11.F5 1:169511755:T:C
peptide level
1:169
2:135
0:21
HTVNPNMKEDGILGPIIRpc4_NP.2
p−min=0.61, pX=0.52, N=3
var.11.F5 1:169511755:T:C
peptide level
14:169
9:135
1:21
HTVNPNMKEDGILGPIIRpc4_NP.3
p−min=0.53, pX=0.65, N=24
var.11.F5 1:169511755:T:C
peptide level
164:169
128:135
19:21
HTVNPNMKEDGILGPIIRpc4_NP.4
p−maj=0.23, pX=0.035, N=311
var.11.F5 1:169511755:T:C
peptide level
165:169
133:135
21:21
HTVNPNMKpc2_NP.1
p−min=0.69, pX=0.74, N=319
var.11.F5 1:169511755:T:C
peptide level
166:169
132:135
21:21
HTVNPNMKpc2_NP.2
p−maj=1, pX=0.21, N=319
var.11.F5 1:169511755:T:C
peptide level
167:169
134:135
21:21
HTVNPNMKpc2_NP.3
p−maj=1, pX=0.31, N=322
var.11.F5 1:169511755:T:C
peptide level
115:169
90:135
13:21
HTVNPNMKpc2_NP.4
p−maj=0.63, pX=0.4, N=218
var.11.F5 1:169511755:T:C
peptide level
100:169
79:135
13:21
HTVNPNMKpc2_NP.5
p−maj=0.82, pX=0.34, N=192

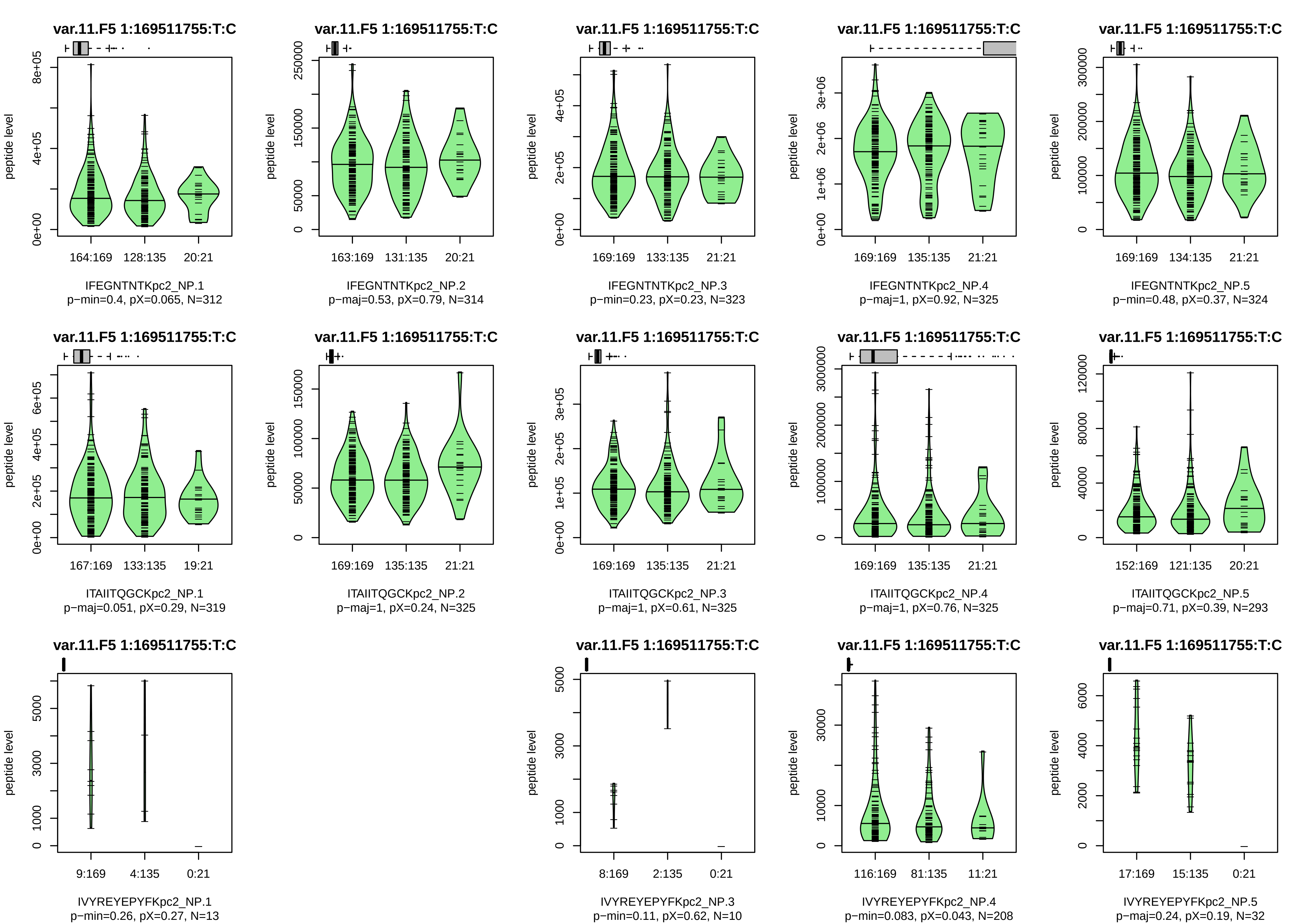

var.11.F5 1:169511755:T:C
peptide level
164:169
128:135
20:21
IFEGNTNTKpc2_NP.1
p−min=0.4, pX=0.065, N=312
var.11.F5 1:169511755:T:C
peptide level
163:169
131:135
20:21
IFEGNTNTKpc2_NP.2
p−maj=0.53, pX=0.79, N=314
var.11.F5 1:169511755:T:C
peptide level
169:169
133:135
21:21
IFEGNTNTKpc2_NP.3
p−min=0.23, pX=0.23, N=323
var.11.F5 1:169511755:T:C
peptide level
169:169
135:135
21:21
IFEGNTNTKpc2_NP.4
p−maj=1, pX=0.92, N=325
var.11.F5 1:169511755:T:C
peptide level
169:169
134:135
21:21
IFEGNTNTKpc2_NP.5
p−min=0.48, pX=0.37, N=324
var.11.F5 1:169511755:T:C
peptide level
167:169
133:135
19:21
ITAIITQGCKpc2_NP.1
p−maj=0.051, pX=0.29, N=319
var.11.F5 1:169511755:T:C
peptide level
169:169
135:135
21:21
ITAIITQGCKpc2_NP.2
p−maj=1, pX=0.24, N=325
var.11.F5 1:169511755:T:C
peptide level
169:169
135:135
21:21
ITAIITQGCKpc2_NP.3
p−maj=1, pX=0.61, N=325
var.11.F5 1:169511755:T:C
peptide level
169:169
135:135
21:21
ITAIITQGCKpc2_NP.4
p−maj=1, pX=0.76, N=325
var.11.F5 1:169511755:T:C
peptide level
152:169
121:135
20:21
ITAIITQGCKpc2_NP.5
p−maj=0.71, pX=0.39, N=293
var.11.F5 1:169511755:T:C
peptide level
9:169
4:135
0:21
IVYREYEPYFKpc2_NP.1
p−min=0.26, pX=0.27, N=13
var.11.F5 1:169511755:T:C
peptide level
8:169
2:135
0:21
IVYREYEPYFKpc2_NP.3
p−min=0.11, pX=0.62, N=10
var.11.F5 1:169511755:T:C
peptide level
116:169
81:135
11:21
IVYREYEPYFKpc2_NP.4
p−min=0.083, pX=0.043, N=208
var.11.F5 1:169511755:T:C
peptide level
17:169
15:135
0:21
IVYREYEPYFKpc2_NP.5
p−maj=0.24, pX=0.19, N=32

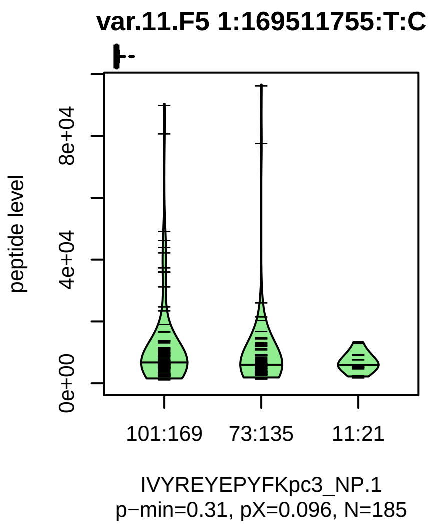
var.11.F5 1:169511755:T:C
peptide level
0e+00
4e+04
8e+04
101:169
73:135
11:21
IVYREYEPYFKpc3_NP.1
p−min=0.31, pX=0.096, N=185

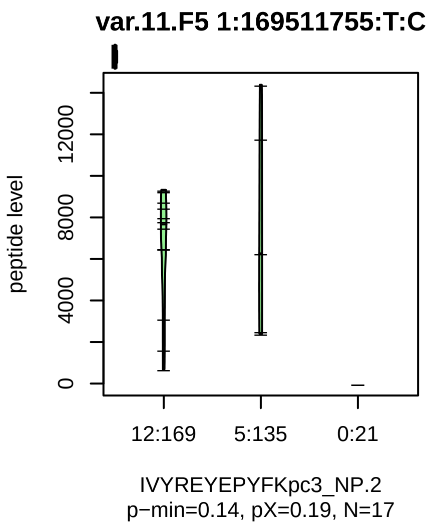
var.11.F5 1:169511755:T:C
peptide level
0
4000
8000
12000
12:169
5:135
0:21
IVYREYEPYFKpc3_NP.2
p−min=0.14, pX=0.19, N=17

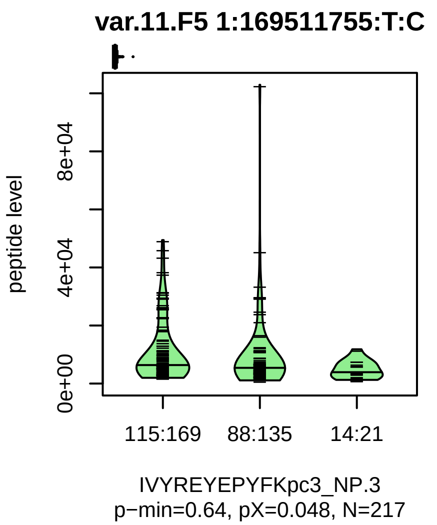
var.11.F5 1:169511755:T:C
peptide level
0e+00
4e+04
8e+04
115:169
88:135
14:21
IVYREYEPYFKpc3_NP.3
p−min=0.64, pX=0.048, N=217

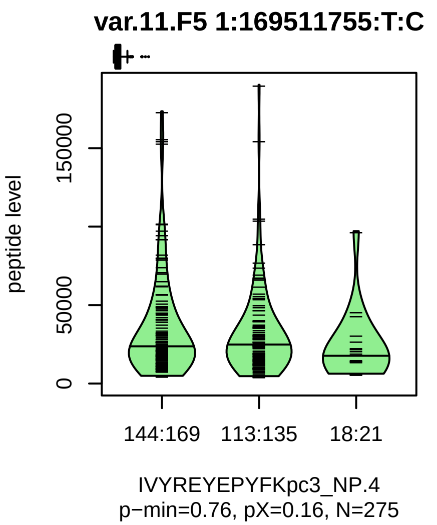
var.11.F5 1:169511755:T:C
peptide level
0
50000
150000
144:169
113:135
18:21
IVYREYEPYFKpc3_NP.4
p−min=0.76, pX=0.16, N=275

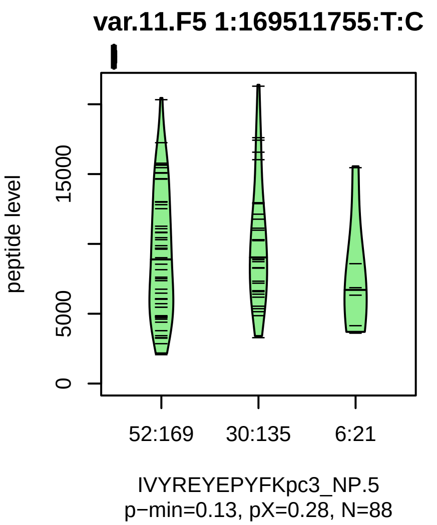
var.11.F5 1:169511755:T:C
peptide level
0
5000
15000
52:169
30:135
6:21
IVYREYEPYFKpc3_NP.5
p−min=0.13, pX=0.28, N=88

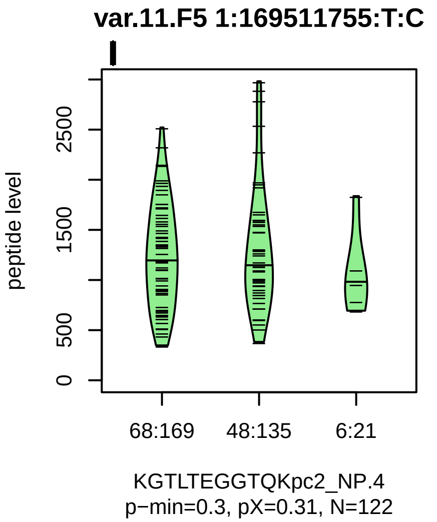
var.11.F5 1:169511755:T:C
peptide level
0
500
1500
2500
68:169
48:135
6:21
KGTLTEGGTQKpc2_NP.4
p−min=0.3, pX=0.31, N=122

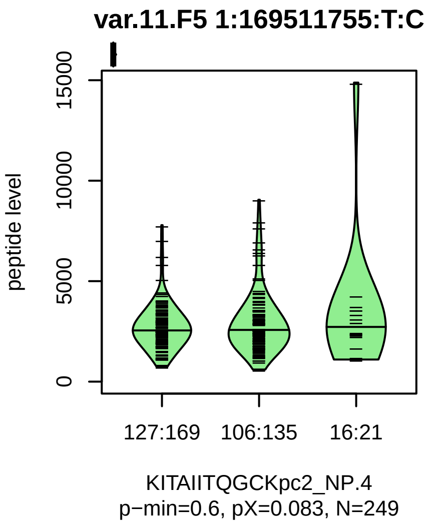
var.11.F5 1:169511755:T:C
peptide level
0
5000
10000
15000
127:169
106:135
16:21
KITAIITQGCKpc2_NP.4
p−min=0.6, pX=0.083, N=249

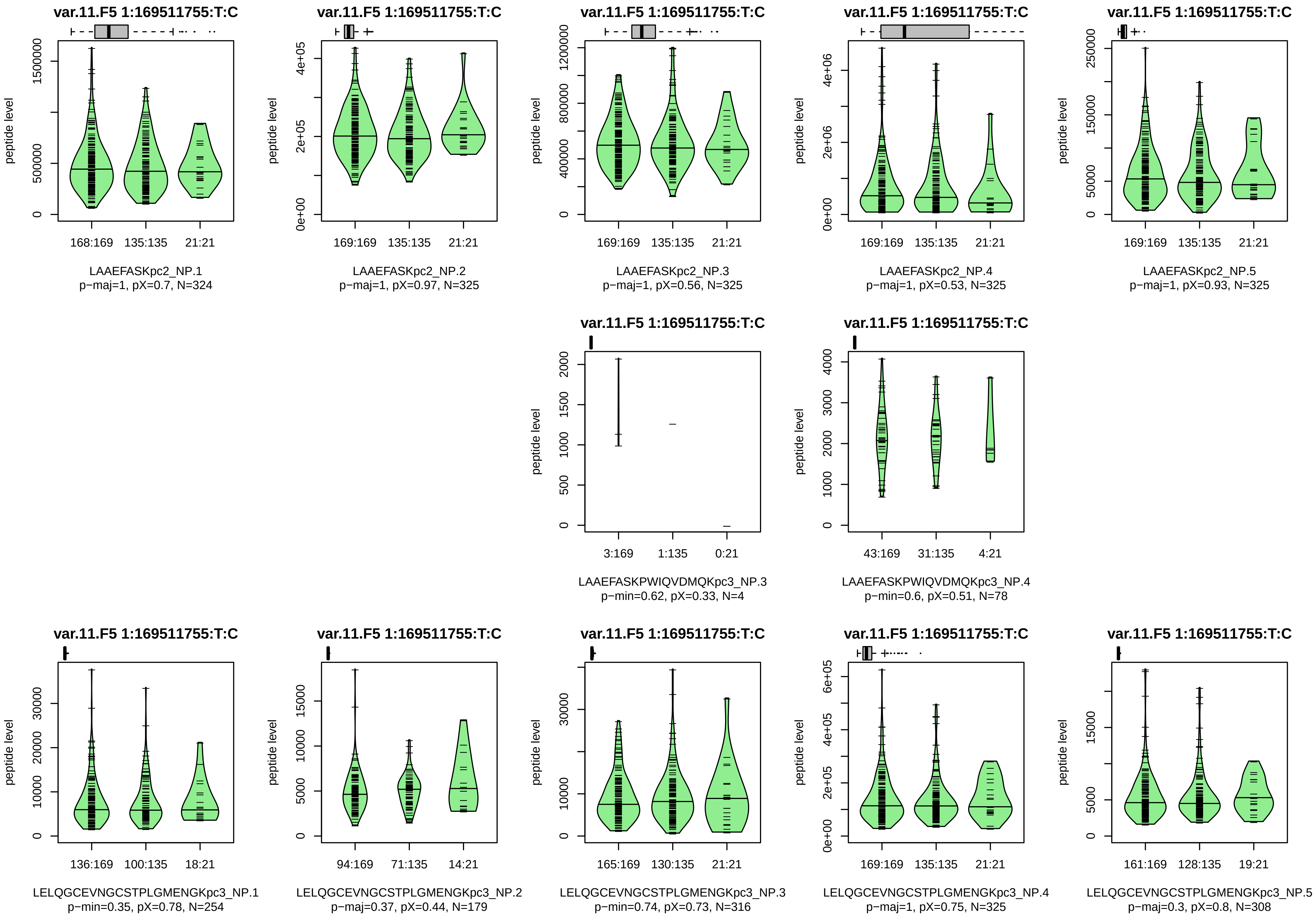
var.11.F5 1:169511755:T:C
peptide level
168:169 135:135 21:21
LAAEFASKpc2_NP.1
p−maj=1, pX=0.7, N=324
var.11.F5 1:169511755:T:C
peptide level
169:169 135:135 21:21
LAAEFASKpc2_NP.2
p−maj=1, pX=0.97, N=325
var.11.F5 1:169511755:T:C
peptide level
169:169 135:135 21:21
LAAEFASKpc2_NP.3
p−maj=1, pX=0.56, N=325
var.11.F5 1:169511755:T:C
peptide level
169:169 135:135 21:21
LAAEFASKpc2_NP.4
p−maj=1, pX=0.53, N=325
var.11.F5 1:169511755:T:C
peptide level
169:169 135:135 21:21
LAAEFASKpc2_NP.5
p−maj=1, pX=0.93, N=325
var.11.F5 1:169511755:T:C
peptide level
3:169 1:135 0:21
LAAEFASKPWIQVDMQKpc3_NP.3
p−min=0.62, pX=0.33, N=4
var.11.F5 1:169511755:T:C
peptide level
43:169 31:135 4:21
LAAEFASKPWIQVDMQKpc3_NP.4
p−min=0.6, pX=0.51, N=78
var.11.F5 1:169511755:T:C
peptide level
136:169 100:135 18:21
LELQGCEVNGCSTPLGMENGKpc3_NP.1
p−min=0.35, pX=0.78, N=254
var.11.F5 1:169511755:T:C
peptide level
94:169 71:135 14:21
LELQGCEVNGCSTPLGMENGKpc3_NP.2
p−maj=0.37, pX=0.44, N=179
var.11.F5 1:169511755:T:C
peptide level
165:169 130:135 21:21
LELQGCEVNGCSTPLGMENGKpc3_NP.3
p−min=0.74, pX=0.73, N=316
var.11.F5 1:169511755:T:C
peptide level
169:169 135:135 21:21
LELQGCEVNGCSTPLGMENGKpc3_NP.4
p−maj=1, pX=0.75, N=325
var.11.F5 1:169511755:T:C
peptide level
161:169 128:135 19:21
LELQGCEVNGCSTPLGMENGKpc3_NP.5
p−maj=0.3, pX=0.8, N=308

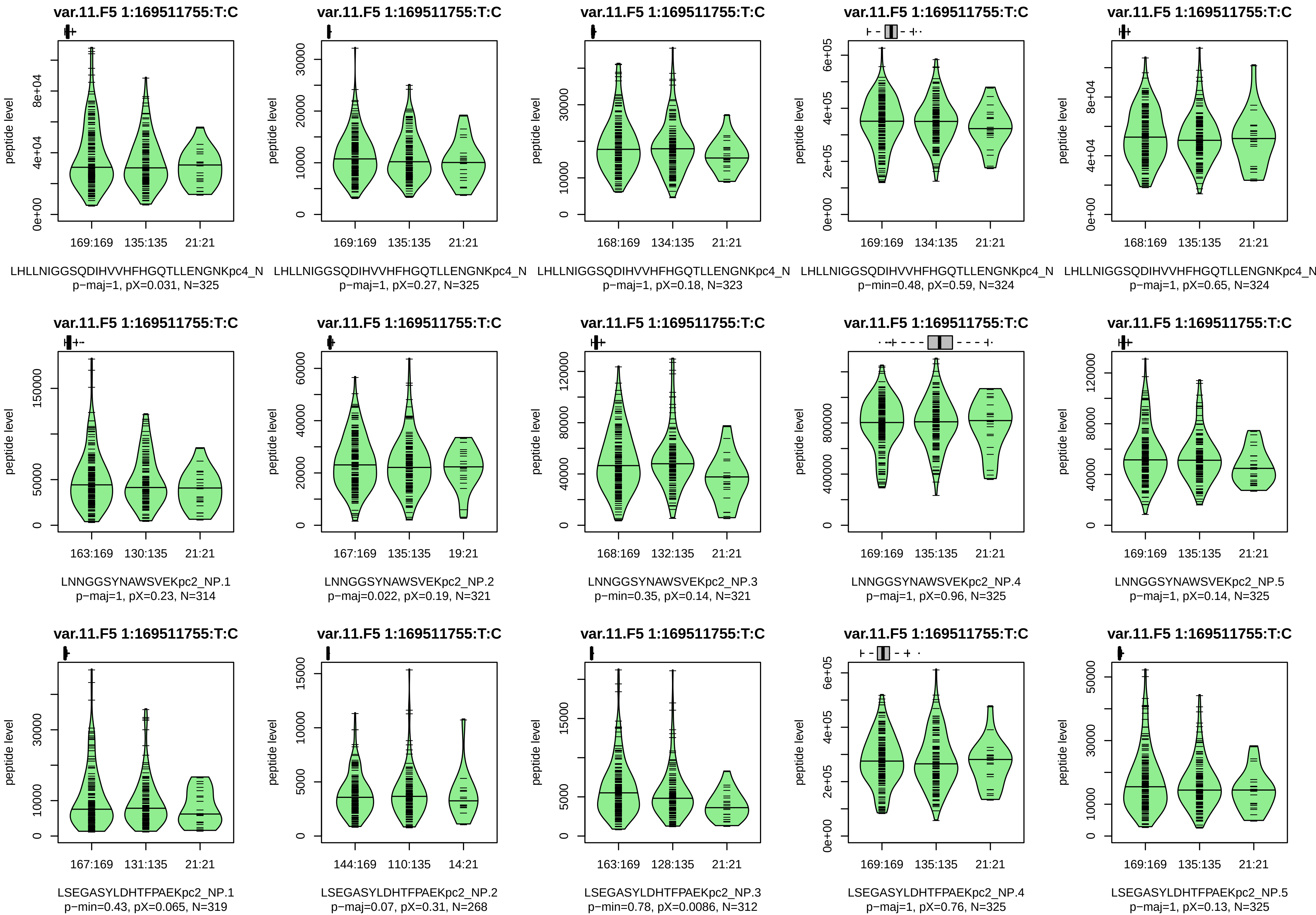
var.11.F5 1:169511755:T:C
peptide level
169:169 135:135 21:21
LHLLNIGGSQDIHVVHFHGQTLLENGNKpc4_N
p−maj=1, pX=0.031, N=325
var.11.F5 1:169511755:T:C
peptide level
169:169 135:135 21:21
LHLLNIGGSQDIHVVHFHGQTLLENGNKpc4_N
p−maj=1, pX=0.27, N=325
var.11.F5 1:169511755:T:C
peptide level
168:169 134:135 21:21
LHLLNIGGSQDIHVVHFHGQTLLENGNKpc4_N
p−maj=1, pX=0.18, N=323
var.11.F5 1:169511755:T:C
peptide level
169:169 134:135 21:21
LHLLNIGGSQDIHVVHFHGQTLLENGNKpc4_N
p−min=0.48, pX=0.59, N=324
var.11.F5 1:169511755:T:C
peptide level
168:169 135:135 21:21
LHLLNIGGSQDIHVVHFHGQTLLENGNKpc4_N
p−maj=1, pX=0.65, N=324
var.11.F5 1:169511755:T:C
peptide level
163:169 130:135 21:21
LNNGGSYNAWSVEKpc2_NP.1
p−maj=1, pX=0.23, N=314
var.11.F5 1:169511755:T:C
peptide level
167:169 135:135 19:21
LNNGGSYNAWSVEKpc2_NP.2
p−maj=0.022, pX=0.19, N=321
var.11.F5 1:169511755:T:C
peptide level
168:169 132:135 21:21
LNNGGSYNAWSVEKpc2_NP.3
p−min=0.35, pX=0.14, N=321
var.11.F5 1:169511755:T:C
peptide level
169:169 135:135 21:21
LNNGGSYNAWSVEKpc2_NP.4
p−maj=1, pX=0.96, N=325
var.11.F5 1:169511755:T:C
peptide level
169:169 135:135 21:21
LNNGGSYNAWSVEKpc2_NP.5
p−maj=1, pX=0.14, N=325
var.11.F5 1:169511755:T:C
peptide level
167:169 131:135 21:21
LSEGASYLDHTFPAEKpc2_NP.1
p−min=0.43, pX=0.065, N=319
var.11.F5 1:169511755:T:C
peptide level
144:169 110:135 14:21
LSEGASYLDHTFPAEKpc2_NP.2
p−maj=0.07, pX=0.31, N=268
var.11.F5 1:169511755:T:C
peptide level
163:169 128:135 21:21
LSEGASYLDHTFPAEKpc2_NP.3
p−min=0.78, pX=0.0086, N=312
var.11.F5 1:169511755:T:C
peptide level
169:169 135:135 21:21
LSEGASYLDHTFPAEKpc2_NP.4
p−maj=1, pX=0.76, N=325
var.11.F5 1:169511755:T:C
peptide level
169:169 135:135 21:21
LSEGASYLDHTFPAEKpc2_NP.5
p−maj=1, pX=0.13, N=325

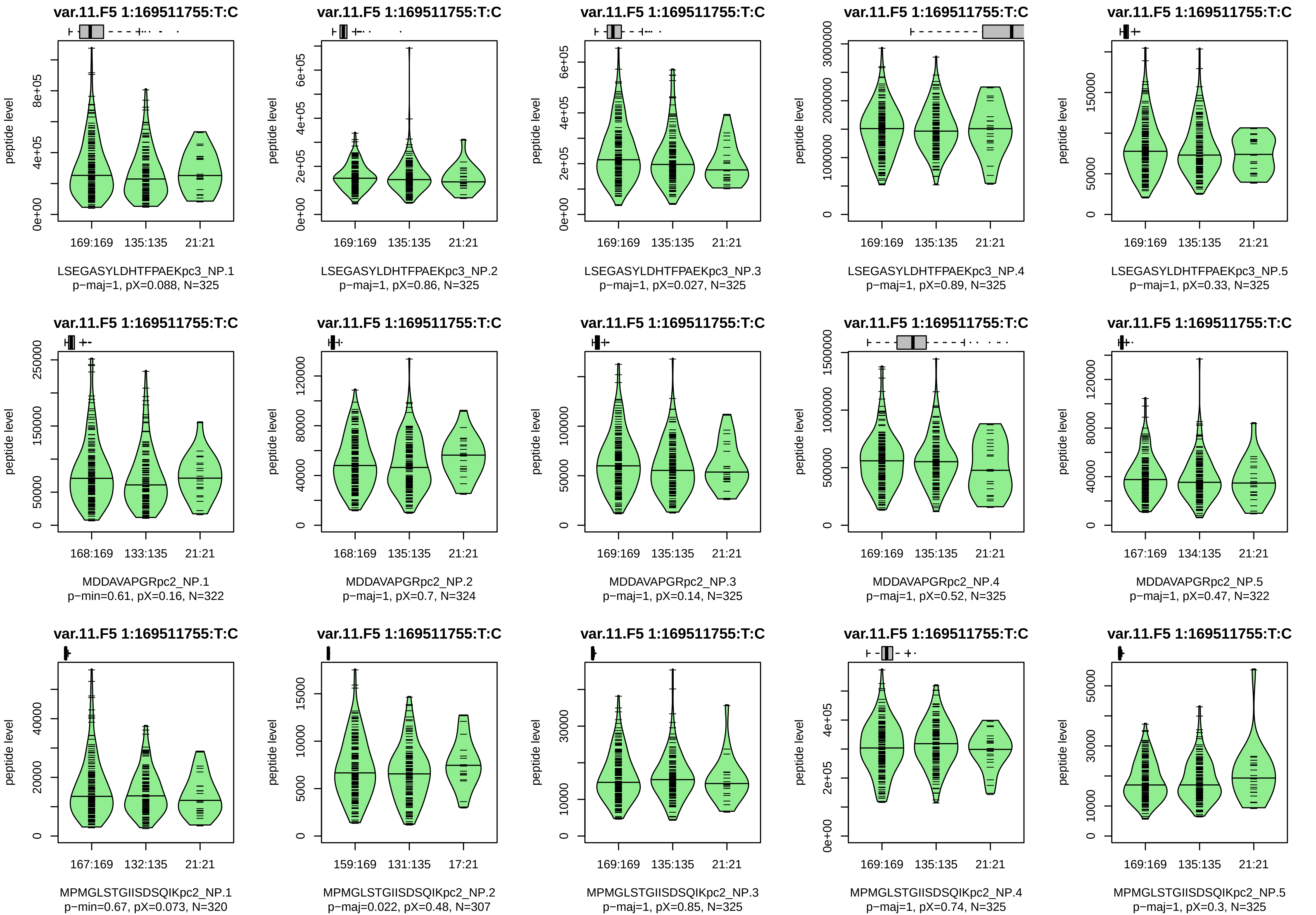
var.11.F5 1:169511755:T:C
peptide level
169:169 135:135 21:21
LSEGASYLDHTFPAEKpc3_NP.1
p−maj=1, pX=0.088, N=325
var.11.F5 1:169511755:T:C
peptide level
169:169 135:135 21:21
LSEGASYLDHTFPAEKpc3_NP.2
p−maj=1, pX=0.86, N=325
var.11.F5 1:169511755:T:C
peptide level
169:169 135:135 21:21
LSEGASYLDHTFPAEKpc3_NP.3
p−maj=1, pX=0.027, N=325
var.11.F5 1:169511755:T:C
peptide level
169:169 135:135 21:21
LSEGASYLDHTFPAEKpc3_NP.4
p−maj=1, pX=0.89, N=325
var.11.F5 1:169511755:T:C
peptide level
169:169 135:135 21:21
LSEGASYLDHTFPAEKpc3_NP.5
p−maj=1, pX=0.33, N=325
var.11.F5 1:169511755:T:C
peptide level
168:169 133:135 21:21
MDDAVAPGRpc2_NP.1
p−min=0.61, pX=0.16, N=322
var.11.F5 1:169511755:T:C
peptide level
168:169 135:135 21:21
MDDAVAPGRpc2_NP.2
p−maj=1, pX=0.7, N=324
var.11.F5 1:169511755:T:C
peptide level
169:169 135:135 21:21
MDDAVAPGRpc2_NP.3
p−maj=1, pX=0.14, N=325
var.11.F5 1:169511755:T:C
peptide level
169:169 135:135 21:21
MDDAVAPGRpc2_NP.4
p−maj=1, pX=0.52, N=325
var.11.F5 1:169511755:T:C
peptide level
167:169 134:135 21:21
MDDAVAPGRpc2_NP.5
p−maj=1, pX=0.47, N=322
var.11.F5 1:169511755:T:C
peptide level
167:169 132:135 21:21
MPMGLSTGIISDSQIKpc2_NP.1
p−min=0.67, pX=0.073, N=320
var.11.F5 1:169511755:T:C
peptide level
159:169 131:135 17:21
MPMGLSTGIISDSQIKpc2_NP.2
p−maj=0.022, pX=0.48, N=307
var.11.F5 1:169511755:T:C
peptide level
169:169 135:135 21:21
MPMGLSTGIISDSQIKpc2_NP.3
p−maj=1, pX=0.85, N=325
var.11.F5 1:169511755:T:C
peptide level
169:169 135:135 21:21
MPMGLSTGIISDSQIKpc2_NP.4
p−maj=1, pX=0.74, N=325
var.11.F5 1:169511755:T:C
peptide level
169:169 135:135 21:21
MPMGLSTGIISDSQIKpc2_NP.5
p−maj=1, pX=0.3, N=325

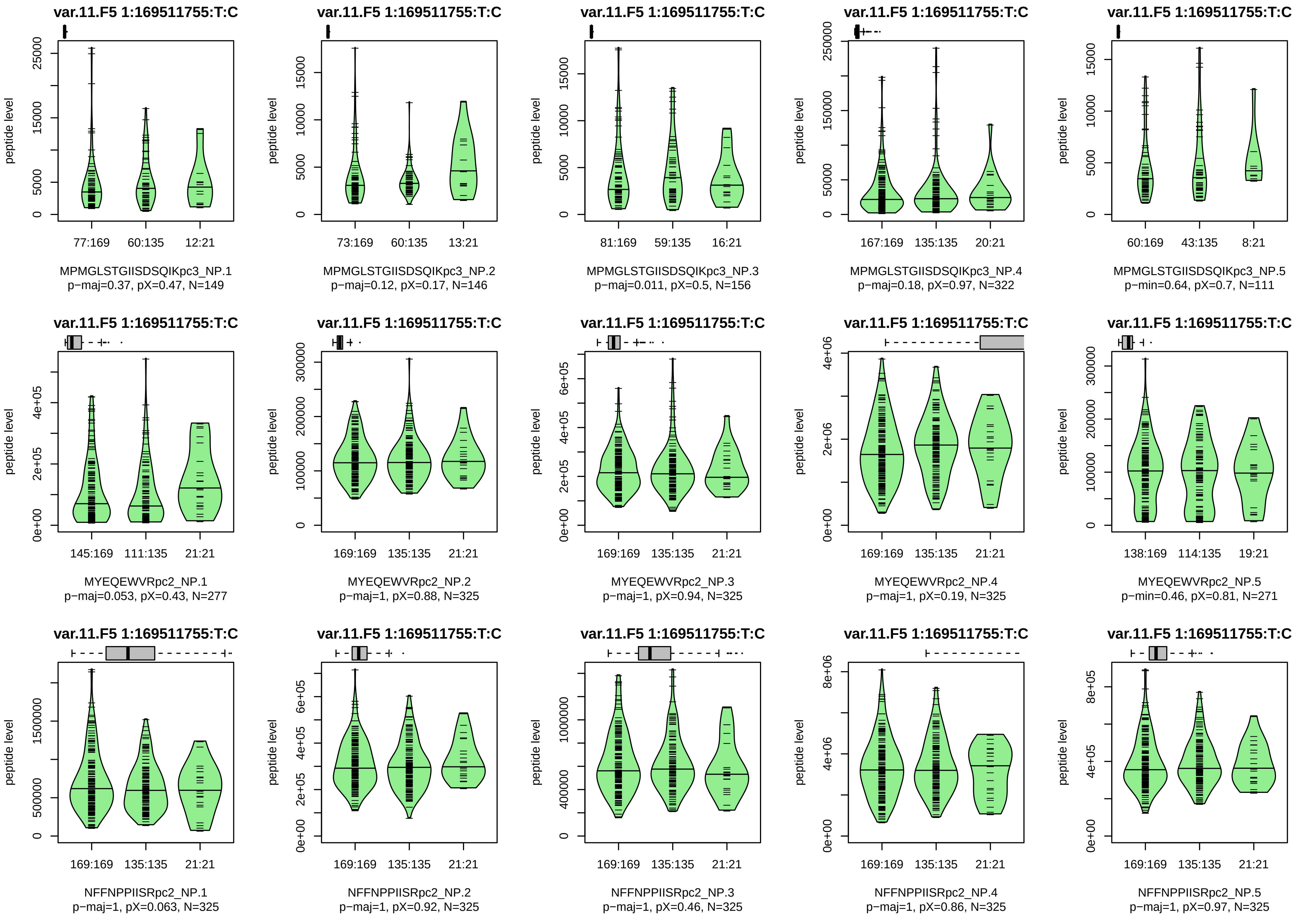

var.11.F5 1:169511755:T:C
peptide level
77:169
60:135
12:21
MPMGLSTGIISDSQIKpc3_NP.1
p−maj=0.37, pX=0.47, N=149
var.11.F5 1:169511755:T:C
peptide level
73:169
60:135
13:21
MPMGLSTGIISDSQIKpc3_NP.2
p−maj=0.12, pX=0.17, N=146
var.11.F5 1:169511755:T:C
peptide level
81:169
59:135
16:21
MPMGLSTGIISDSQIKpc3_NP.3
p−maj=0.011, pX=0.5, N=156
var.11.F5 1:169511755:T:C
peptide level
167:169
135:135
20:21
MPMGLSTGIISDSQIKpc3_NP.4
p−maj=0.18, pX=0.97, N=322
var.11.F5 1:169511755:T:C
peptide level
60:169
43:135
8:21
MPMGLSTGIISDSQIKpc3_NP.5
p−min=0.64, pX=0.7, N=111
var.11.F5 1:169511755:T:C
peptide level
145:169
111:135
21:21
MYEQEWVRpc2_NP.1
p−maj=0.053, pX=0.43, N=277
var.11.F5 1:169511755:T:C
peptide level
169:169
135:135
21:21
MYEQEWVRpc2_NP.2
p−maj=1, pX=0.88, N=325
var.11.F5 1:169511755:T:C
peptide level
169:169
135:135
21:21
MYEQEWVRpc2_NP.3
p−maj=1, pX=0.94, N=325
var.11.F5 1:169511755:T:C
peptide level
169:169
135:135
21:21
MYEQEWVRpc2_NP.4
p−maj=1, pX=0.19, N=325
var.11.F5 1:169511755:T:C
peptide level
138:169
114:135
19:21
MYEQEWVRpc2_NP.5
p−min=0.46, pX=0.81, N=271
var.11.F5 1:169511755:T:C
peptide level
169:169
135:135
21:21
NFFNPPIISRpc2_NP.1
p−maj=1, pX=0.063, N=325
var.11.F5 1:169511755:T:C
peptide level
169:169
135:135
21:21
NFFNPPIISRpc2_NP.2
p−maj=1, pX=0.92, N=325
var.11.F5 1:169511755:T:C
peptide level
169:169
135:135
21:21
NFFNPPIISRpc2_NP.3
p−maj=1, pX=0.46, N=325
var.11.F5 1:169511755:T:C
peptide level
169:169
135:135
21:21
NFFNPPIISRpc2_NP.4
p−maj=1, pX=0.86, N=325
var.11.F5 1:169511755:T:C
peptide level
169:169
135:135
21:21
NFFNPPIISRpc2_NP.5
p−maj=1, pX=0.97, N=325

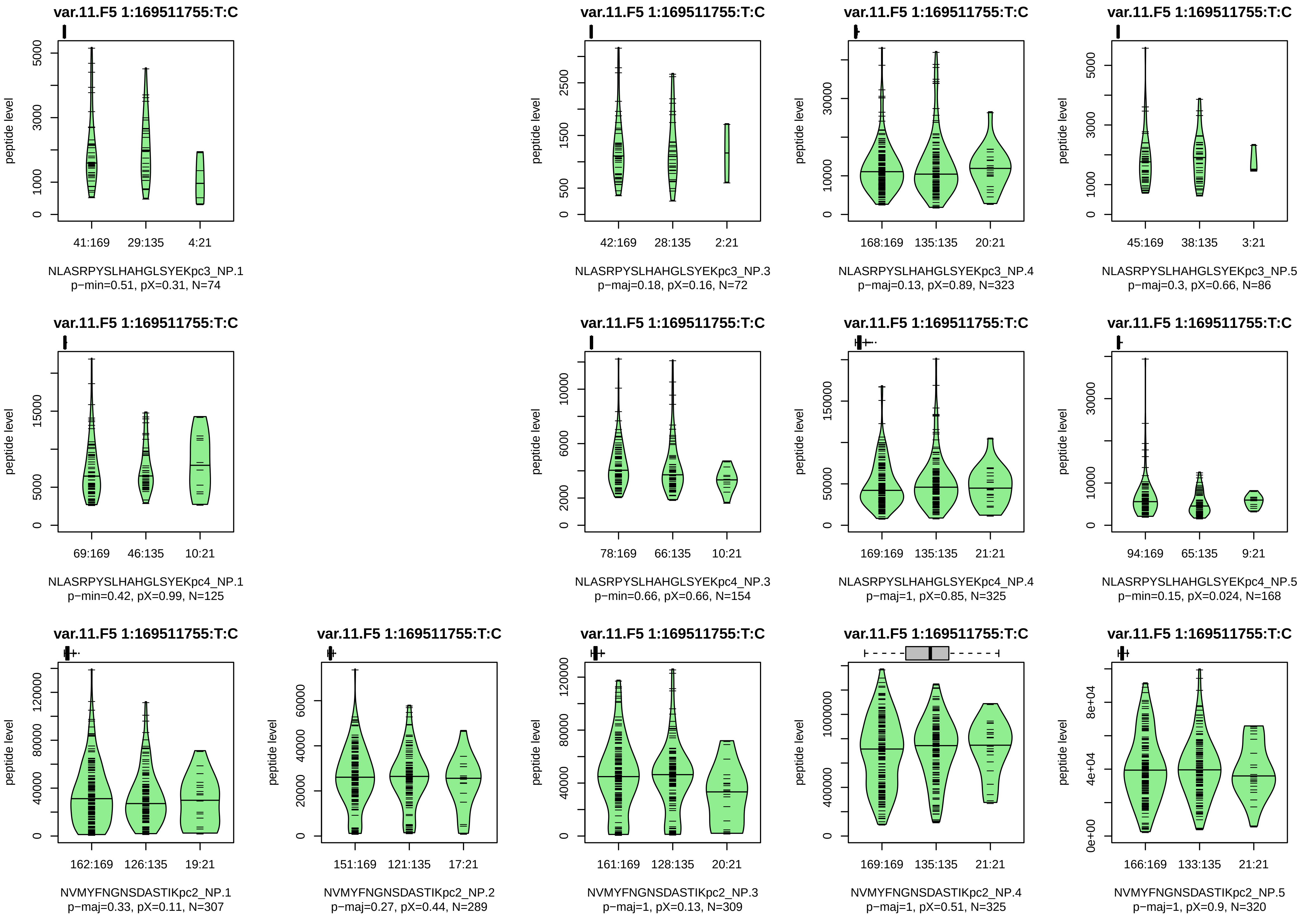
var.11.F5 1:169511755:T:C
peptide level
41:169
29:135
4:21
NLASRPYSLHAHGLSYEKpc3_NP.1
p−min=0.51, pX=0.31, N=74
var.11.F5 1:169511755:T:C
peptide level
42:169
28:135
2:21
NLASRPYSLHAHGLSYEKpc3_NP.3
p−maj=0.18, pX=0.16, N=72
var.11.F5 1:169511755:T:C
peptide level
168:169
135:135
20:21
NLASRPYSLHAHGLSYEKpc3_NP.4
p−maj=0.13, pX=0.89, N=323
var.11.F5 1:169511755:T:C
peptide level
45:169
38:135
3:21
NLASRPYSLHAHGLSYEKpc3_NP.5
p−maj=0.3, pX=0.66, N=86
var.11.F5 1:169511755:T:C
peptide level
69:169
46:135
10:21
NLASRPYSLHAHGLSYEKpc4_NP.1
p−min=0.42, pX=0.99, N=125
var.11.F5 1:169511755:T:C
peptide level
78:169
66:135
10:21
NLASRPYSLHAHGLSYEKpc4_NP.3
p−min=0.66, pX=0.66, N=154
var.11.F5 1:169511755:T:C
peptide level
169:169
135:135
21:21
NLASRPYSLHAHGLSYEKpc4_NP.4
p−maj=1, pX=0.85, N=325
var.11.F5 1:169511755:T:C
peptide level
94:169
65:135
9:21
NLASRPYSLHAHGLSYEKpc4_NP.5
p−min=0.15, pX=0.024, N=168
var.11.F5 1:169511755:T:C
peptide level
162:169
126:135
19:21
NVMYFNGNSDASTIKpc2_NP.1
p−maj=0.33, pX=0.11, N=307
var.11.F5 1:169511755:T:C
peptide level
151:169
121:135
17:21
NVMYFNGNSDASTIKpc2_NP.2
p−maj=0.27, pX=0.44, N=289
var.11.F5 1:169511755:T:C
peptide level
161:169
128:135
20:21
NVMYFNGNSDASTIKpc2_NP.3
p−maj=1, pX=0.13, N=309
var.11.F5 1:169511755:T:C
peptide level
169:169
135:135
21:21
NVMYFNGNSDASTIKpc2_NP.4
p−maj=1, pX=0.51, N=325
var.11.F5 1:169511755:T:C
peptide level
166:169
133:135
21:21
NVMYFNGNSDASTIKpc2_NP.5
p−maj=1, pX=0.9, N=320

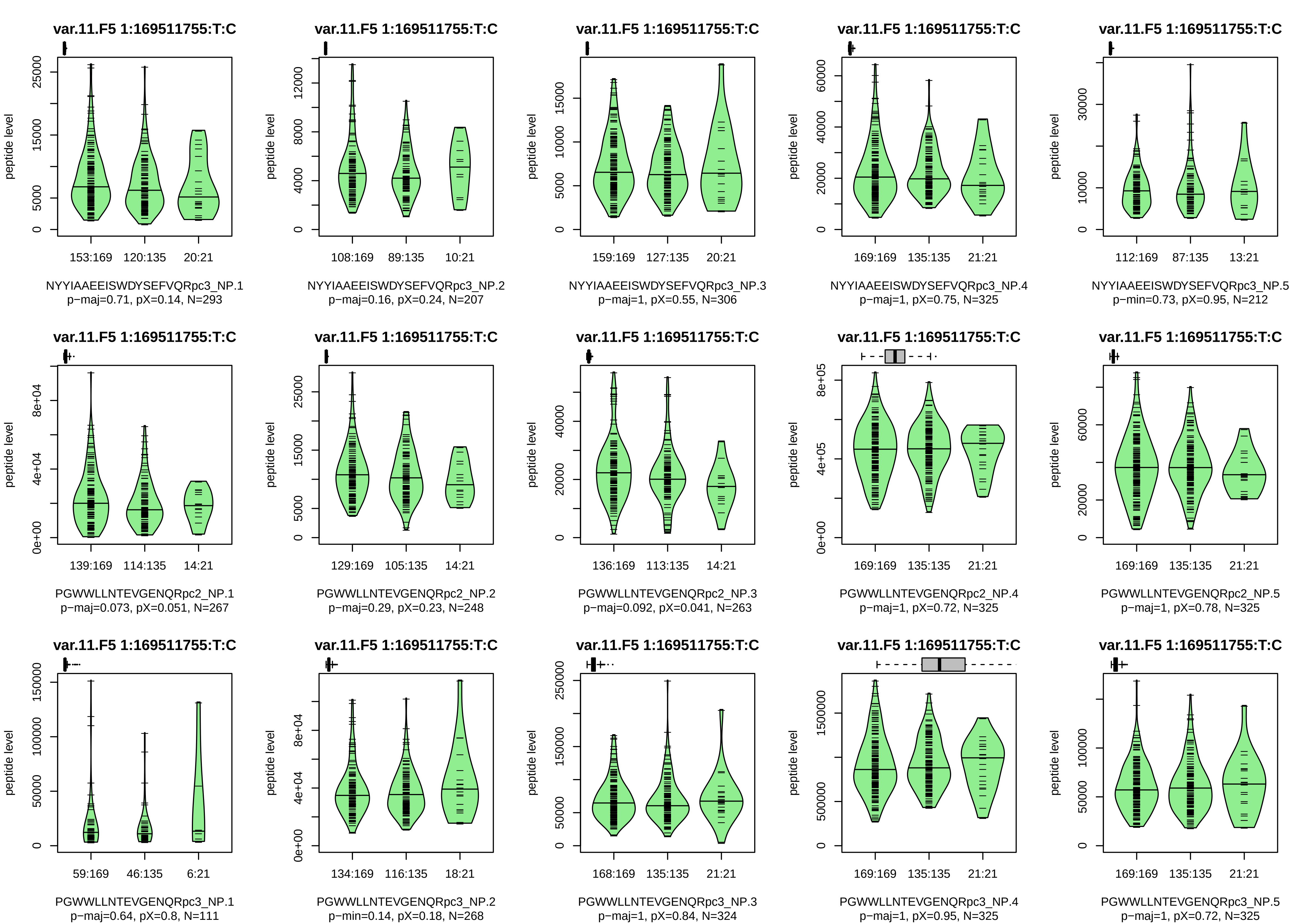
var.11.F5 1:169511755:T:C
peptide level
153:169
120:135
20:21
NYYIAAEEISWDYSEFVQRpc3_NP.1
p−maj=0.71, pX=0.14, N=293
var.11.F5 1:169511755:T:C
peptide level
108:169
89:135
10:21
NYYIAAEEISWDYSEFVQRpc3_NP.2
p−maj=0.16, pX=0.24, N=207
var.11.F5 1:169511755:T:C
peptide level
159:169
127:135
20:21
NYYIAAEEISWDYSEFVQRpc3_NP.3
p−maj=1, pX=0.55, N=306
var.11.F5 1:169511755:T:C
peptide level
169:169
135:135
21:21
NYYIAAEEISWDYSEFVQRpc3_NP.4
p−maj=1, pX=0.75, N=325
var.11.F5 1:169511755:T:C
peptide level
112:169
87:135
13:21
NYYIAAEEISWDYSEFVQRpc3_NP.5
p−min=0.73, pX=0.95, N=212
var.11.F5 1:169511755:T:C
peptide level
139:169
114:135
14:21
PGWWLLNTEVGENQRpc2_NP.1
p−maj=0.073, pX=0.051, N=267
var.11.F5 1:169511755:T:C
peptide level
129:169
105:135
14:21
PGWWLLNTEVGENQRpc2_NP.2
p−maj=0.29, pX=0.23, N=248
var.11.F5 1:169511755:T:C
peptide level
136:169
113:135
14:21
PGWWLLNTEVGENQRpc2_NP.3
p−maj=0.092, pX=0.041, N=263
var.11.F5 1:169511755:T:C
peptide level
169:169
135:135
21:21
PGWWLLNTEVGENQRpc2_NP.4
p−maj=1, pX=0.72, N=325
var.11.F5 1:169511755:T:C
peptide level
169:169
135:135
21:21
PGWWLLNTEVGENQRpc2_NP.5
p−maj=1, pX=0.78, N=325
var.11.F5 1:169511755:T:C
peptide level
59:169
46:135
6:21
PGWWLLNTEVGENQRpc3_NP.1
p−maj=0.64, pX=0.8, N=111
var.11.F5 1:169511755:T:C
peptide level
134:169
116:135
18:21
PGWWLLNTEVGENQRpc3_NP.2
p−min=0.14, pX=0.18, N=268
var.11.F5 1:169511755:T:C
peptide level
168:169
135:135
21:21
PGWWLLNTEVGENQRpc3_NP.3
p−maj=1, pX=0.84, N=324
var.11.F5 1:169511755:T:C
peptide level
169:169
135:135
21:21
PGWWLLNTEVGENQRpc3_NP.4
p−maj=1, pX=0.95, N=325
var.11.F5 1:169511755:T:C
peptide level
169:169
135:135
21:21
PGWWLLNTEVGENQRpc3_NP.5
p−maj=1, pX=0.72, N=325

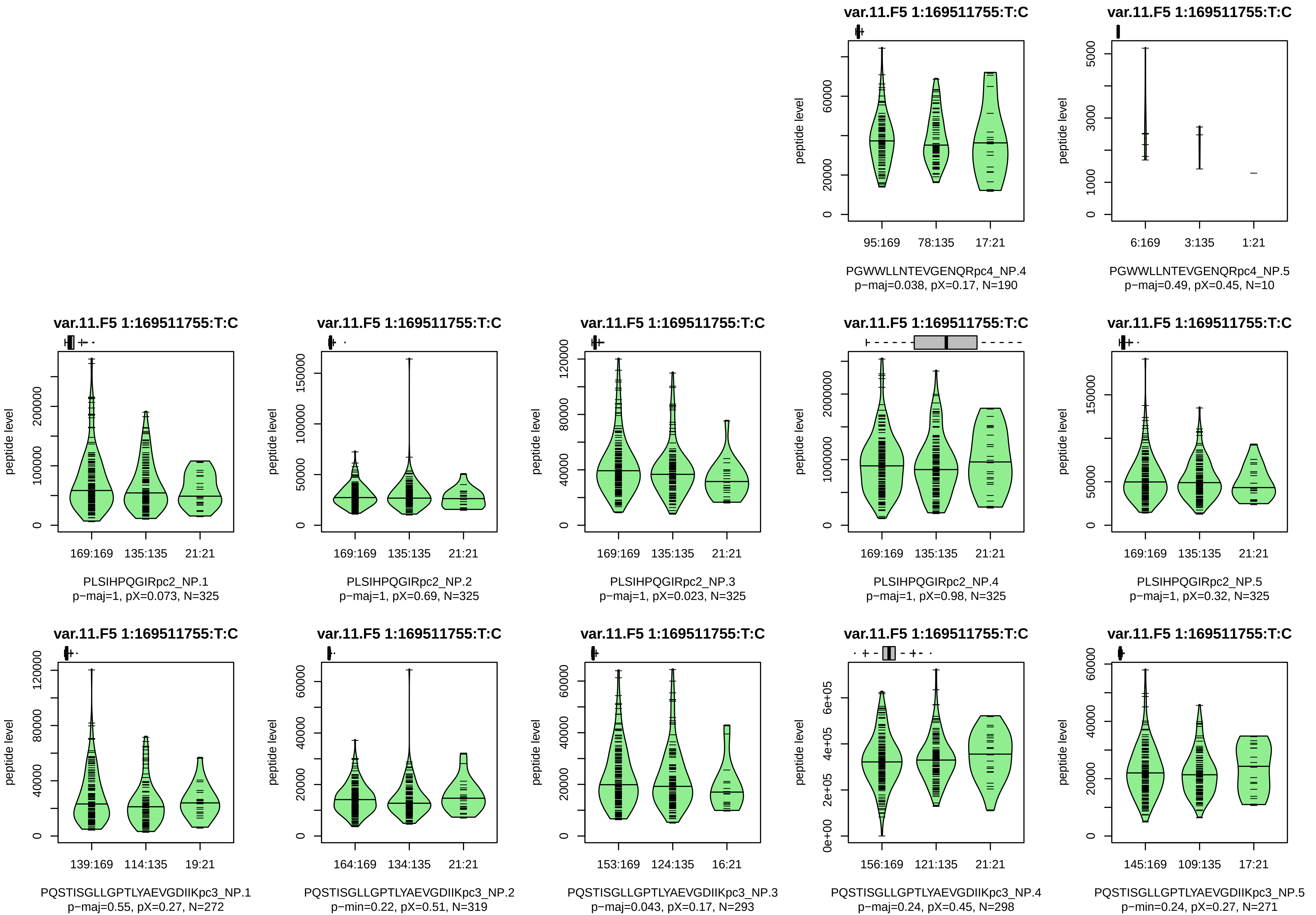

var.11.F5 1:169511755:T:C
peptide level
95:169
78:135
17:21
PGWWLLNTEVGENQRpc4_NP.4
p−maj=0.038, pX=0.17, N=190
var.11.F5 1:169511755:T:C
peptide level
6:169
3:135
1:21
PGWWLLNTEVGENQRpc4_NP.5
p−maj=0.49, pX=0.45, N=10
var.11.F5 1:169511755:T:C
peptide level
169:169
135:135
21:21
PLSIHPQGIRpc2_NP.1
p−maj=1, pX=0.073, N=325
var.11.F5 1:169511755:T:C
peptide level
169:169
135:135
21:21
PLSIHPQGIRpc2_NP.2
p−maj=1, pX=0.69, N=325
var.11.F5 1:169511755:T:C
peptide level
169:169
135:135
21:21
PLSIHPQGIRpc2_NP.3
p−maj=1, pX=0.023, N=325
var.11.F5 1:169511755:T:C
peptide level
169:169
135:135
21:21
PLSIHPQGIRpc2_NP.4
p−maj=1, pX=0.98, N=325
var.11.F5 1:169511755:T:C
peptide level
169:169
135:135
21:21
PLSIHPQGIRpc2_NP.5
p−maj=1, pX=0.32, N=325
var.11.F5 1:169511755:T:C
peptide level
139:169
114:135
19:21
PQSTISGLLGPTLYAEVGDIIKpc3_NP.1
p−maj=0.55, pX=0.27, N=272
var.11.F5 1:169511755:T:C
peptide level
164:169
134:135
21:21
PQSTISGLLGPTLYAEVGDIIKpc3_NP.2
p−min=0.22, pX=0.51, N=319
var.11.F5 1:169511755:T:C
peptide level
153:169
124:135
16:21
PQSTISGLLGPTLYAEVGDIIKpc3_NP.3
p−maj=0.043, pX=0.17, N=293
var.11.F5 1:169511755:T:C
peptide level
156:169
121:135
21:21
PQSTISGLLGPTLYAEVGDIIKpc3_NP.4
p−maj=0.24, pX=0.45, N=298
var.11.F5 1:169511755:T:C
peptide level
145:169
109:135
17:21
PQSTISGLLGPTLYAEVGDIIKpc3_NP.5
p−min=0.24, pX=0.27, N=271

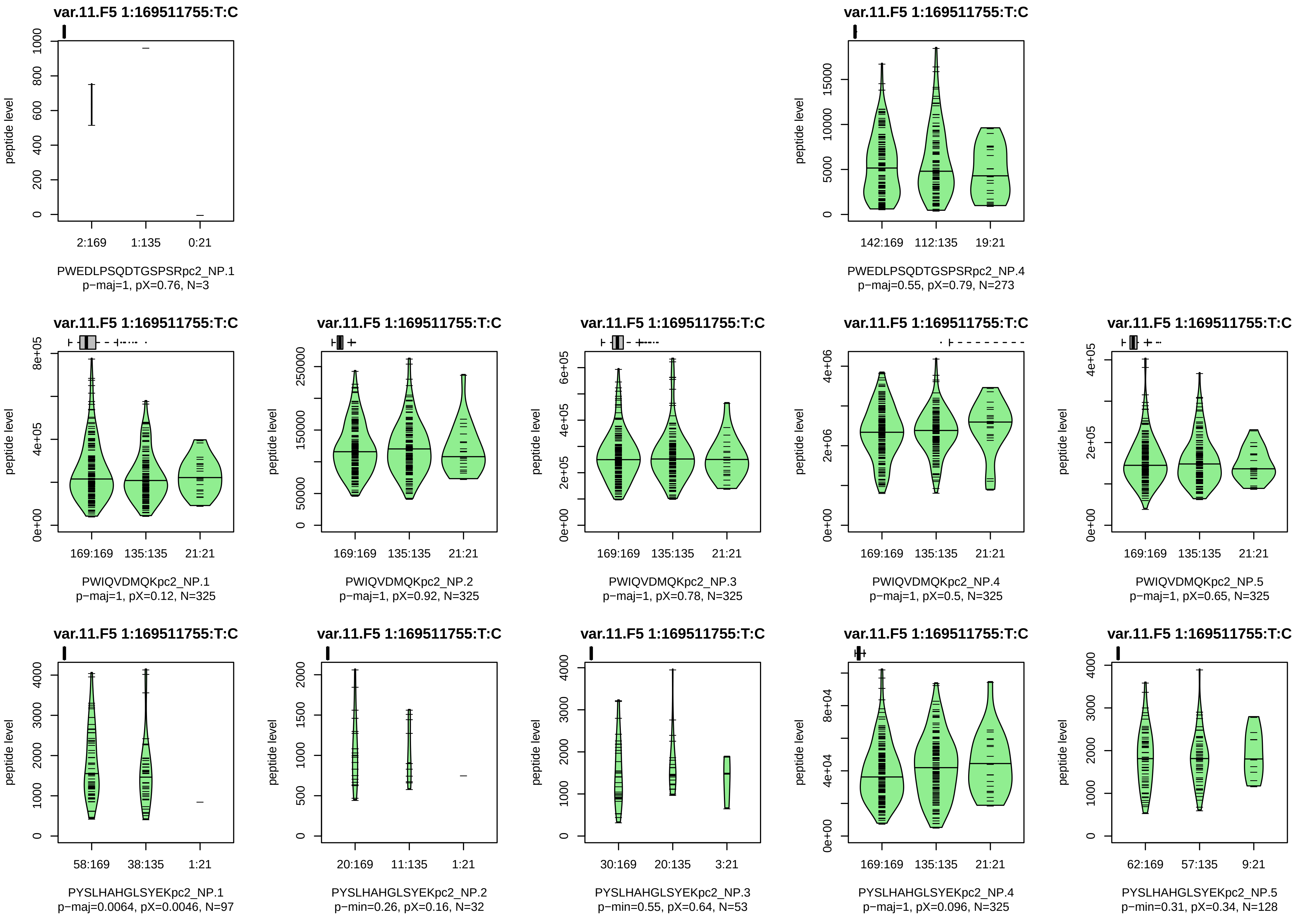
var.11.F5 1:169511755:T:C
peptide level
2:169
1:135
0:21
PWEDLPSQDTGSPSRpc2_NP.1
p−maj=1, pX=0.76, N=3
var.11.F5 1:169511755:T:C
peptide level
142:169
112:135
19:21
PWEDLPSQDTGSPSRpc2_NP.4
p−maj=0.55, pX=0.79, N=273
var.11.F5 1:169511755:T:C
peptide level
169:169
135:135
21:21
PWIQVDMQKpc2_NP.1
p−maj=1, pX=0.12, N=325
var.11.F5 1:169511755:T:C
peptide level
169:169
135:135
21:21
PWIQVDMQKpc2_NP.2
p−maj=1, pX=0.92, N=325
var.11.F5 1:169511755:T:C
peptide level
169:169
135:135
21:21
PWIQVDMQKpc2_NP.3
p−maj=1, pX=0.78, N=325
var.11.F5 1:169511755:T:C
peptide level
169:169
135:135
21:21
PWIQVDMQKpc2_NP.4
p−maj=1, pX=0.5, N=325
var.11.F5 1:169511755:T:C
peptide level
169:169
135:135
21:21
PWIQVDMQKpc2_NP.5
p−maj=1, pX=0.65, N=325
var.11.F5 1:169511755:T:C
peptide level
58:169
38:135
1:21
PYSLHAHGLSYEKpc2_NP.1
p−maj=0.0064, pX=0.0046, N=97
var.11.F5 1:169511755:T:C
peptide level
20:169
11:135
1:21
PYSLHAHGLSYEKpc2_NP.2
p−min=0.26, pX=0.16, N=32
var.11.F5 1:169511755:T:C
peptide level
30:169
20:135
3:21
PYSLHAHGLSYEKpc2_NP.3
p−min=0.55, pX=0.64, N=53
var.11.F5 1:169511755:T:C
peptide level
169:169
135:135
21:21
PYSLHAHGLSYEKpc2_NP.4
p−maj=1, pX=0.096, N=325
var.11.F5 1:169511755:T:C
peptide level
62:169
57:135
9:21
PYSLHAHGLSYEKpc2_NP.5
p−min=0.31, pX=0.34, N=128

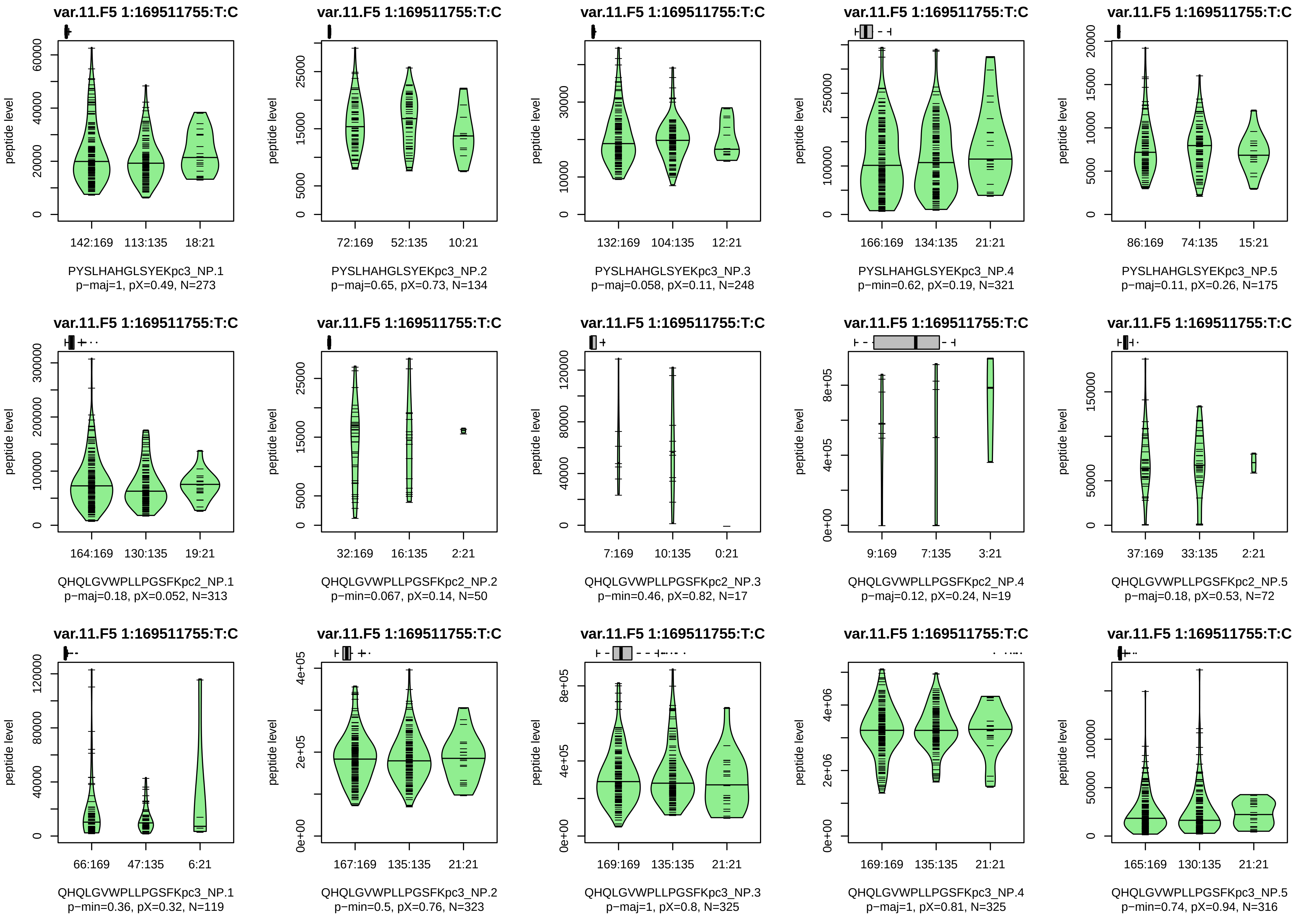
var.11.F5 1:169511755:T:C
peptide level
142:169
113:135
18:21
PYSLHAHGLSYEKpc3_NP.1
p−maj=1, pX=0.49, N=273
var.11.F5 1:169511755:T:C
peptide level
72:169
52:135
10:21
PYSLHAHGLSYEKpc3_NP.2
p−maj=0.65, pX=0.73, N=134
var.11.F5 1:169511755:T:C
peptide level
132:169
104:135
12:21
PYSLHAHGLSYEKpc3_NP.3
p−maj=0.058, pX=0.11, N=248
var.11.F5 1:169511755:T:C
peptide level
166:169
134:135
21:21
PYSLHAHGLSYEKpc3_NP.4
p−min=0.62, pX=0.19, N=321
var.11.F5 1:169511755:T:C
peptide level
86:169
74:135
15:21
PYSLHAHGLSYEKpc3_NP.5
p−maj=0.11, pX=0.26, N=175
var.11.F5 1:169511755:T:C
peptide level
164:169
130:135
19:21
QHQLGVWPLLPGSFKpc2_NP.1
p−maj=0.18, pX=0.052, N=313
var.11.F5 1:169511755:T:C
peptide level
32:169
16:135
2:21
QHQLGVWPLLPGSFKpc2_NP.2
p−min=0.067, pX=0.14, N=50
var.11.F5 1:169511755:T:C
peptide level
7:169
10:135
0:21
QHQLGVWPLLPGSFKpc2_NP.3
p−min=0.46, pX=0.82, N=17
var.11.F5 1:169511755:T:C
peptide level
9:169
7:135
3:21
QHQLGVWPLLPGSFKpc2_NP.4
p−maj=0.12, pX=0.24, N=19
var.11.F5 1:169511755:T:C
peptide level
37:169
33:135
2:21
QHQLGVWPLLPGSFKpc2_NP.5
p−maj=0.18, pX=0.53, N=72
var.11.F5 1:169511755:T:C
peptide level
66:169
47:135
6:21
QHQLGVWPLLPGSFKpc3_NP.1
p−min=0.36, pX=0.32, N=119
var.11.F5 1:169511755:T:C
peptide level
167:169
135:135
21:21
QHQLGVWPLLPGSFKpc3_NP.2
p−min=0.5, pX=0.76, N=323
var.11.F5 1:169511755:T:C
peptide level
169:169
135:135
21:21
QHQLGVWPLLPGSFKpc3_NP.3
p−maj=1, pX=0.8, N=325
var.11.F5 1:169511755:T:C
peptide level
169:169
135:135
21:21
QHQLGVWPLLPGSFKpc3_NP.4
p−maj=1, pX=0.81, N=325
var.11.F5 1:169511755:T:C
peptide level
165:169
130:135
21:21
QHQLGVWPLLPGSFKpc3_NP.5
p−min=0.74, pX=0.94, N=316

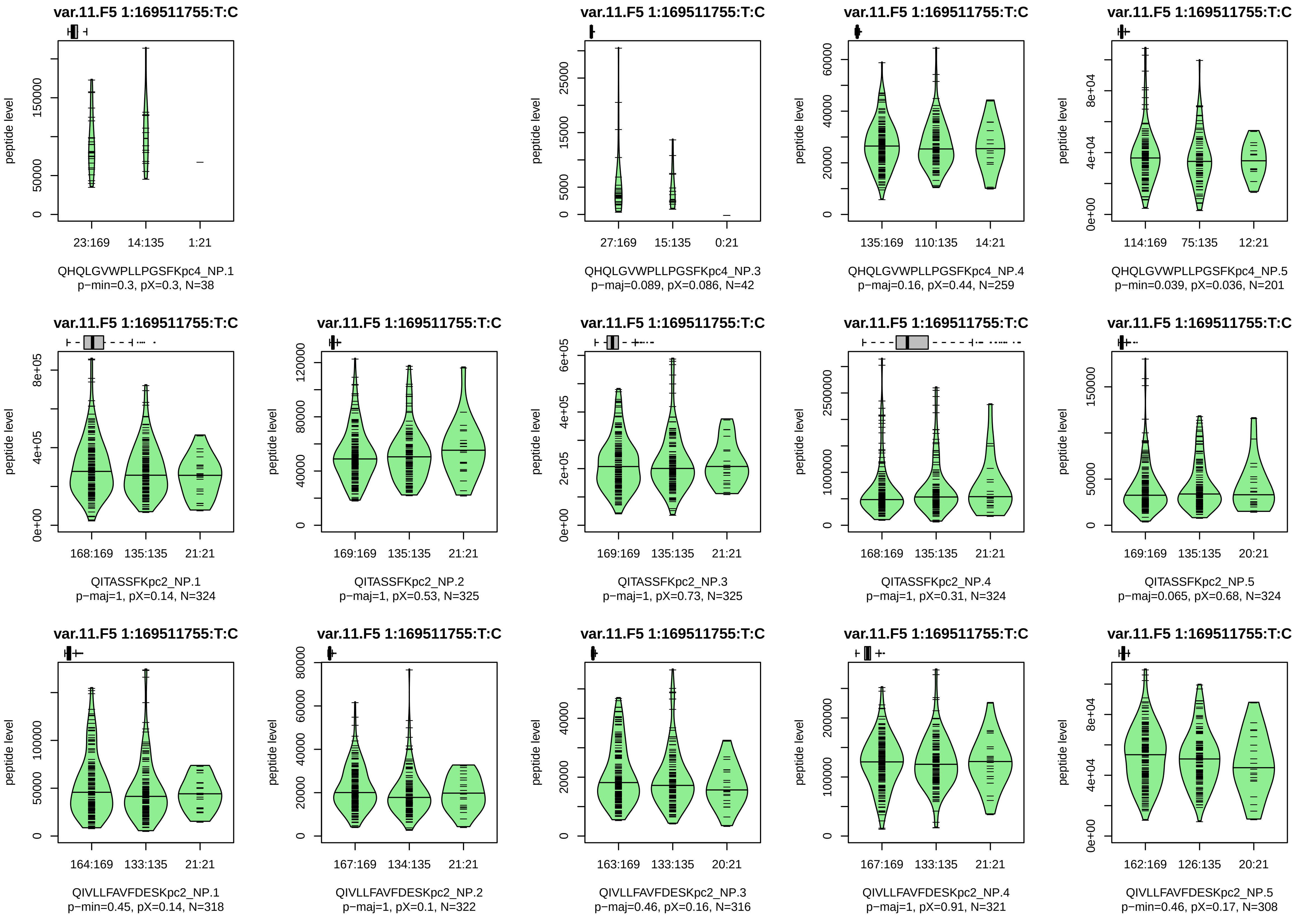
var.11.F5 1:169511755:T:C
peptide level
23:169
14:135
1:21
QHQLGVWPLLPGSFKpc4_NP.1
p−min=0.3, pX=0.3, N=38
var.11.F5 1:169511755:T:C
peptide level
27:169
15:135
0:21
QHQLGVWPLLPGSFKpc4_NP.3
p−maj=0.089, pX=0.086, N=42
var.11.F5 1:169511755:T:C
peptide level
135:169
110:135
14:21
QHQLGVWPLLPGSFKpc4_NP.4
p−maj=0.16, pX=0.44, N=259
var.11.F5 1:169511755:T:C
peptide level
114:169
75:135
12:21
QHQLGVWPLLPGSFKpc4_NP.5
p−min=0.039, pX=0.036, N=201
var.11.F5 1:169511755:T:C
peptide level
168:169
135:135
21:21
QITASSFKpc2_NP.1
p−maj=1, pX=0.14, N=324
var.11.F5 1:169511755:T:C
peptide level
169:169
135:135
21:21
QITASSFKpc2_NP.2
p−maj=1, pX=0.53, N=325
var.11.F5 1:169511755:T:C
peptide level
169:169
135:135
21:21
QITASSFKpc2_NP.3
p−maj=1, pX=0.73, N=325
var.11.F5 1:169511755:T:C
peptide level
168:169
135:135
21:21
QITASSFKpc2_NP.4
p−maj=1, pX=0.31, N=324
var.11.F5 1:169511755:T:C
peptide level
169:169
135:135
20:21
QITASSFKpc2_NP.5
p−maj=0.065, pX=0.68, N=324
var.11.F5 1:169511755:T:C
peptide level
164:169
133:135
21:21
QIVLLFAVFDESKpc2_NP.1
p−min=0.45, pX=0.14, N=318
var.11.F5 1:169511755:T:C
peptide level
167:169
134:135
21:21
QIVLLFAVFDESKpc2_NP.2
p−maj=1, pX=0.1, N=322
var.11.F5 1:169511755:T:C
peptide level
163:169
133:135
20:21
QIVLLFAVFDESKpc2_NP.3
p−maj=0.46, pX=0.16, N=316
var.11.F5 1:169511755:T:C
peptide level
167:169
133:135
21:21
QIVLLFAVFDESKpc2_NP.4
p−maj=1, pX=0.91, N=321
var.11.F5 1:169511755:T:C
peptide level
162:169
126:135
20:21
QIVLLFAVFDESKpc2_NP.5
p−min=0.46, pX=0.17, N=308

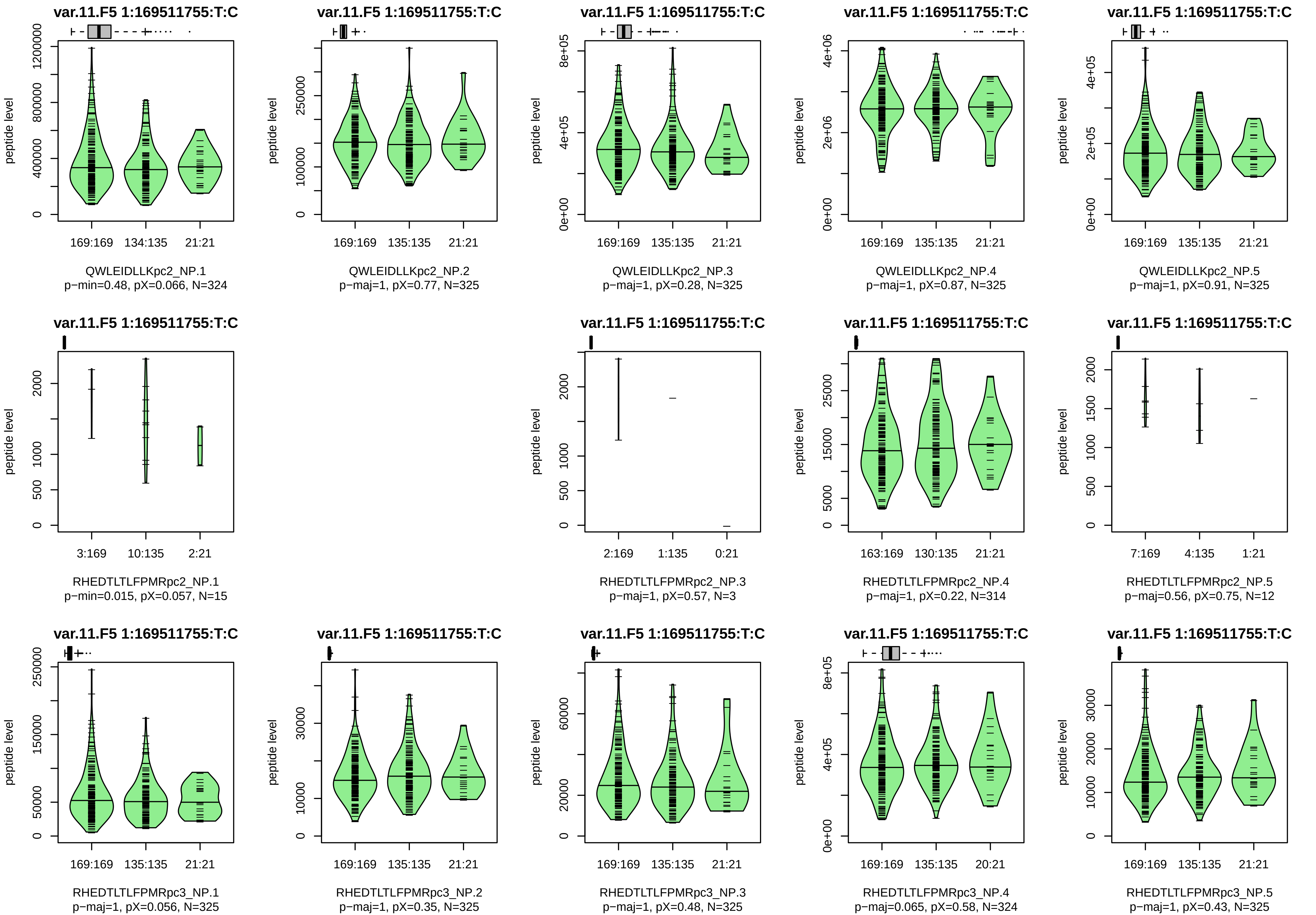
var.11.F5 1:169511755:T:C
peptide level
169:169 134:135 21:21
QWLEIDLLKpc2_NP.1
p−min=0.48, pX=0.066, N=324
var.11.F5 1:169511755:T:C
peptide level
169:169 135:135 21:21
QWLEIDLLKpc2_NP.2
p−maj=1, pX=0.77, N=325
var.11.F5 1:169511755:T:C
peptide level
169:169 135:135 21:21
QWLEIDLLKpc2_NP.3
p−maj=1, pX=0.28, N=325
var.11.F5 1:169511755:T:C
peptide level
169:169 135:135 21:21
QWLEIDLLKpc2_NP.4
p−maj=1, pX=0.87, N=325
var.11.F5 1:169511755:T:C
peptide level
169:169 135:135 21:21
QWLEIDLLKpc2_NP.5
p−maj=1, pX=0.91, N=325
var.11.F5 1:169511755:T:C
peptide level
3:169 10:135 2:21
RHEDTLTLFPMRpc2_NP.1
p−min=0.015, pX=0.057, N=15
var.11.F5 1:169511755:T:C
peptide level
2:169 1:135 0:21
RHEDTLTLFPMRpc2_NP.3
p−maj=1, pX=0.57, N=3
var.11.F5 1:169511755:T:C
peptide level
163:169 130:135 21:21
RHEDTLTLFPMRpc2_NP.4
p−maj=1, pX=0.22, N=314
var.11.F5 1:169511755:T:C
peptide level
7:169 4:135 1:21
RHEDTLTLFPMRpc2_NP.5
p−maj=0.56, pX=0.75, N=12
var.11.F5 1:169511755:T:C
peptide level
169:169 135:135 21:21
RHEDTLTLFPMRpc3_NP.1
p−maj=1, pX=0.056, N=325
var.11.F5 1:169511755:T:C
peptide level
169:169 135:135 21:21
RHEDTLTLFPMRpc3_NP.2
p−maj=1, pX=0.35, N=325
var.11.F5 1:169511755:T:C
peptide level
169:169 135:135 21:21
RHEDTLTLFPMRpc3_NP.3
p−maj=1, pX=0.48, N=325
var.11.F5 1:169511755:T:C
peptide level
169:169 135:135 20:21
RHEDTLTLFPMRpc3_NP.4
p−maj=0.065, pX=0.58, N=324
var.11.F5 1:169511755:T:C
peptide level
169:169 135:135 21:21
RHEDTLTLFPMRpc3_NP.5
p−maj=1, pX=0.43, N=325

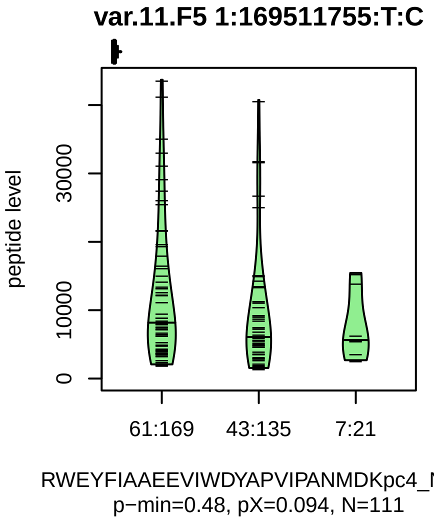
var.11.F5 1:169511755:T:C
peptide level
0
10000
30000
61:169
43:135
7:21
RWEYFIAAEEVIWDYAPVIPANMDKpc4_NP.4
p−min=0.48, pX=0.094, N=111

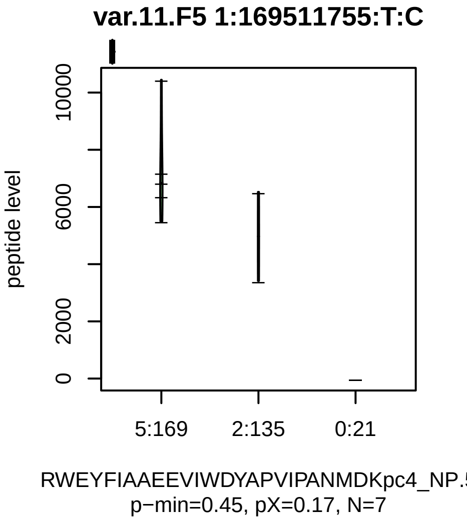
var.11.F5 1:169511755:T:C
peptide level
0
2000
6000
10000
5:169
2:135
0:21
RWEYFIAAEEVIWDYAPVIPANMDKpc4_NP.5
p−min=0.45, pX=0.17, N=7

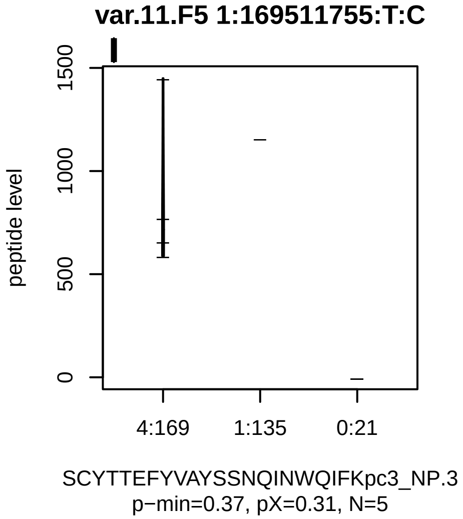
var.11.F5 1:169511755:T:C
peptide level
0
500
1000
1500
4:169
1:135
0:21
SCYTTEFYVAYSSNQINWQIFKpc3_NP.3
p−min=0.37, pX=0.31, N=5

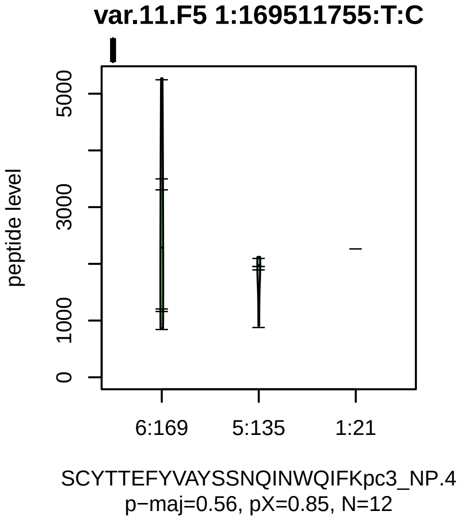
var.11.F5 1:169511755:T:C
peptide level
0
1000
3000
5000
6:169
5:135
1:21
SCYTTEFYVAYSSNQINWQIFKpc3_NP.4
p−maj=0.56, pX=0.85, N=12

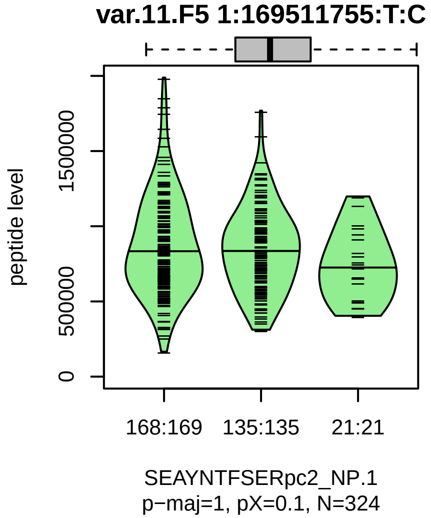
var.11.F5 1:169511755:T:C
peptide level
0
500000
1500000
168:169
135:135
21:21
SEAYNTFSERpc2_NP.1
p−maj=1, pX=0.1, N=324

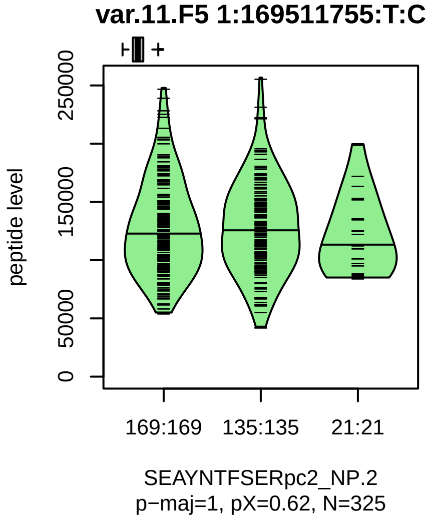
var.11.F5 1:169511755:T:C
peptide level
0
50000
150000
250000
169:169
135:135
21:21
SEAYNTFSERpc2_NP.2
p−maj=1, pX=0.62, N=325

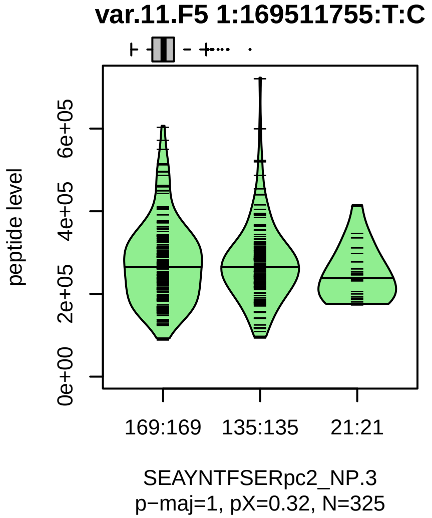
var.11.F5 1:169511755:T:C
peptide level
0e+00
2e+05
4e+05
6e+05
169:169
135:135
21:21
SEAYNTFSERpc2_NP.3
p−maj=1, pX=0.32, N=325

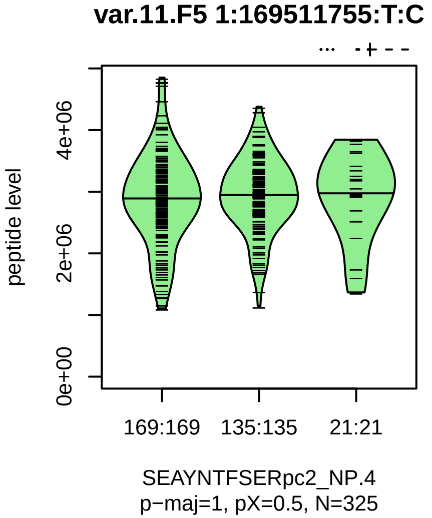
var.11.F5 1:169511755:T:C
peptide level
0e+00
2e+06
4e+06
169:169
135:135
21:21
SEAYNTFSERpc2_NP.4
p−maj=1, pX=0.5, N=325

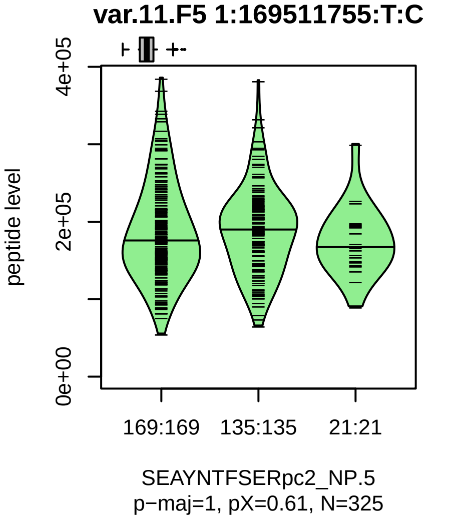
var.11.F5 1:169511755:T:C
peptide level
0e+00
2e+05
4e+05
169:169
135:135
21:21
SEAYNTFSERpc2_NP.5
p−maj=1, pX=0.61, N=325

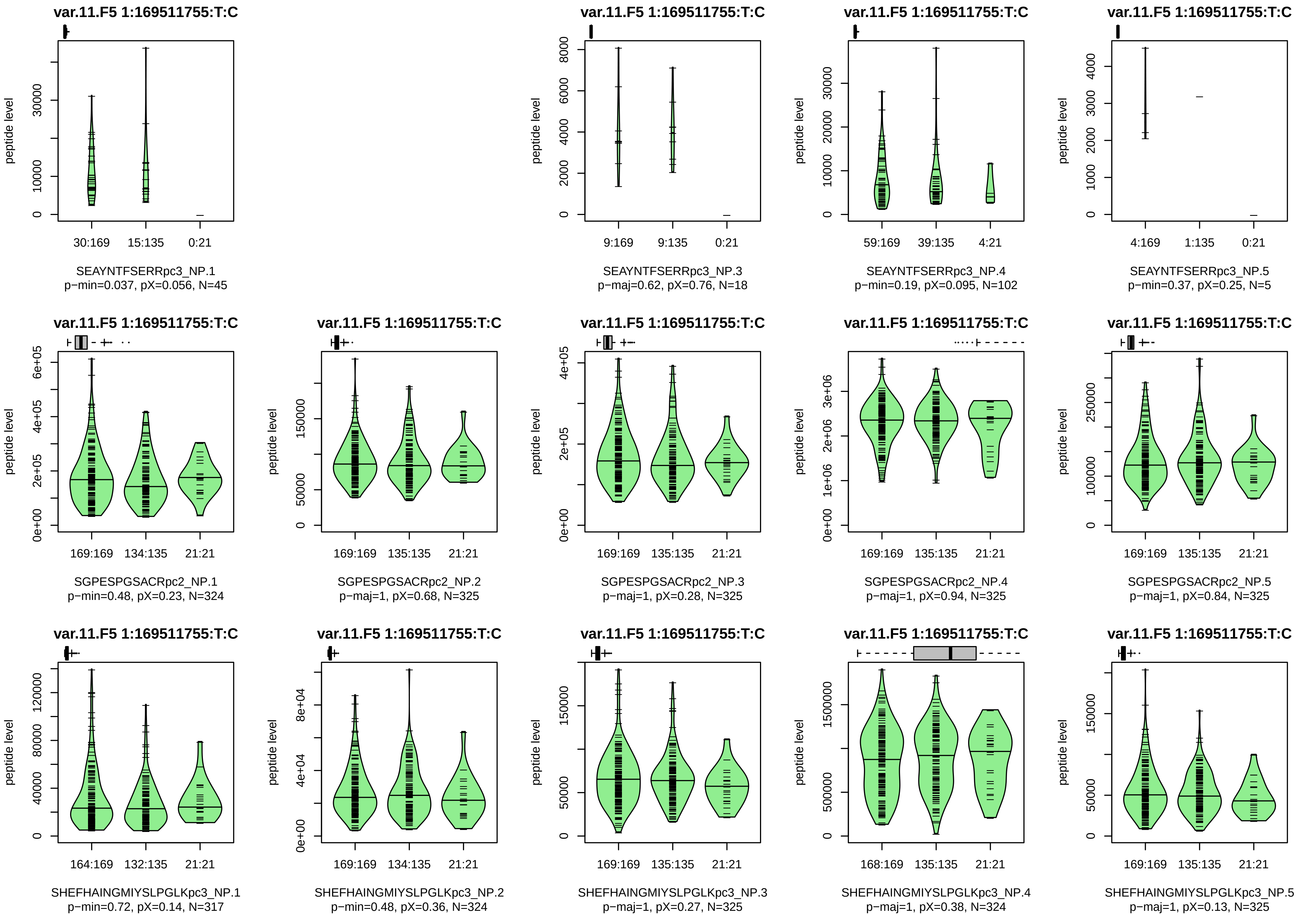
var.11.F5 1:169511755:T:C
peptide level
30:169
15:135
0:21
SEAYNTFSERRpc3_NP.1
p−min=0.037, pX=0.056, N=45
var.11.F5 1:169511755:T:C
peptide level
9:169
9:135
0:21
SEAYNTFSERRpc3_NP.3
p−maj=0.62, pX=0.76, N=18
var.11.F5 1:169511755:T:C
peptide level
59:169
39:135
4:21
SEAYNTFSERRpc3_NP.4
p−min=0.19, pX=0.095, N=102
var.11.F5 1:169511755:T:C
peptide level
4:169
1:135
0:21
SEAYNTFSERRpc3_NP.5
p−min=0.37, pX=0.25, N=5
var.11.F5 1:169511755:T:C
peptide level
169:169
134:135
21:21
SGPESPGSACRpc2_NP.1
p−min=0.48, pX=0.23, N=324
var.11.F5 1:169511755:T:C
peptide level
169:169
135:135
21:21
SGPESPGSACRpc2_NP.2
p−maj=1, pX=0.68, N=325
var.11.F5 1:169511755:T:C
peptide level
169:169
135:135
21:21
SGPESPGSACRpc2_NP.3
p−maj=1, pX=0.28, N=325
var.11.F5 1:169511755:T:C
peptide level
169:169
135:135
21:21
SGPESPGSACRpc2_NP.4
p−maj=1, pX=0.94, N=325
var.11.F5 1:169511755:T:C
peptide level
169:169
135:135
21:21
SGPESPGSACRpc2_NP.5
p−maj=1, pX=0.84, N=325
var.11.F5 1:169511755:T:C
peptide level
164:169
132:135
21:21
SHEFHAINGMIYSLPGLKpc3_NP.1
p−min=0.72, pX=0.14, N=317
var.11.F5 1:169511755:T:C
peptide level
169:169
134:135
21:21
SHEFHAINGMIYSLPGLKpc3_NP.2
p−min=0.48, pX=0.36, N=324
var.11.F5 1:169511755:T:C
peptide level
169:169
135:135
21:21
SHEFHAINGMIYSLPGLKpc3_NP.3
p−maj=1, pX=0.27, N=325
var.11.F5 1:169511755:T:C
peptide level
168:169
135:135
21:21
SHEFHAINGMIYSLPGLKpc3_NP.4
p−maj=1, pX=0.38, N=324
var.11.F5 1:169511755:T:C
peptide level
169:169
135:135
21:21
SHEFHAINGMIYSLPGLKpc3_NP.5
p−maj=1, pX=0.13, N=325

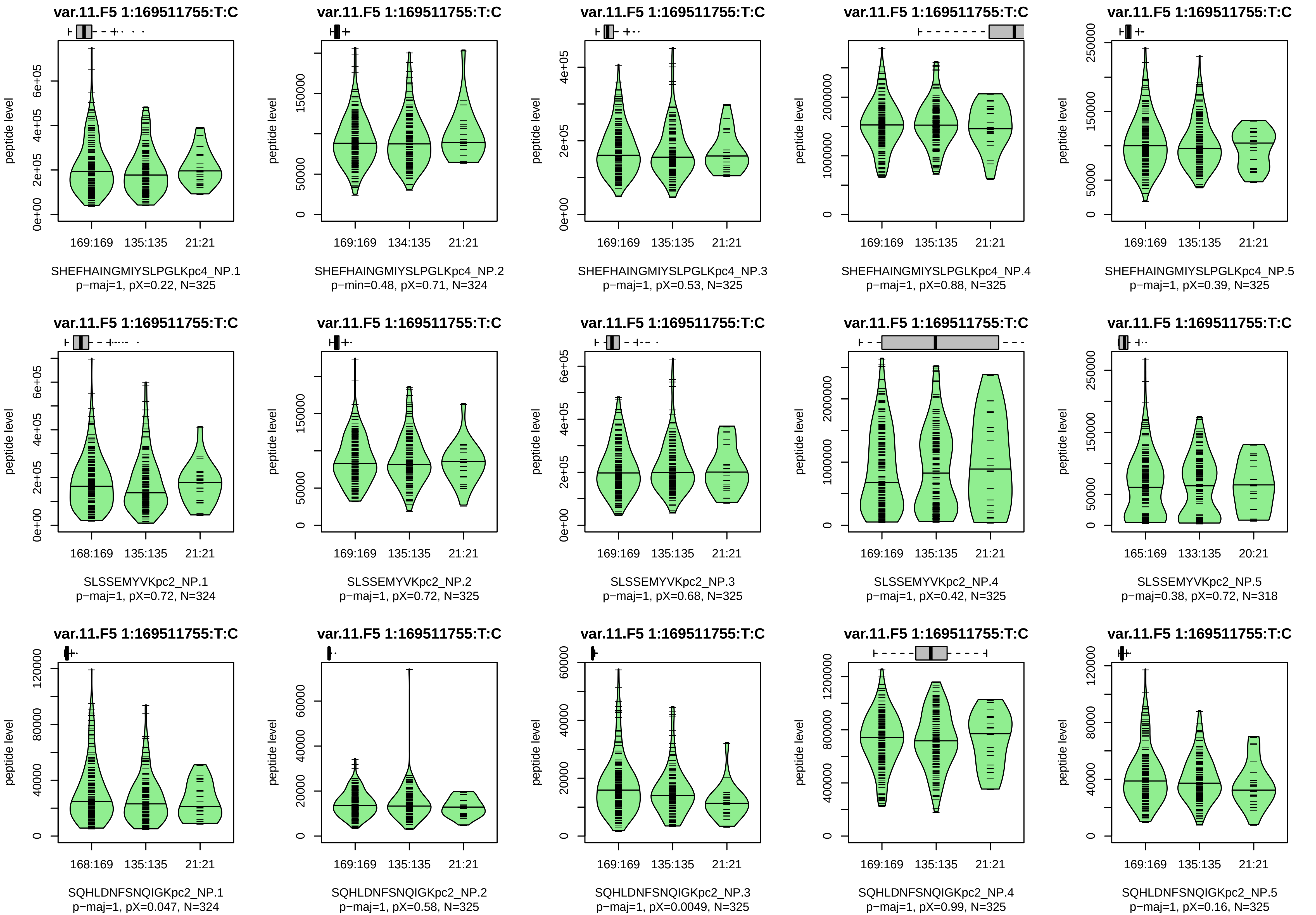
var.11.F5 1:169511755:T:C
peptide level
169:169 135:135 21:21
SHEFHAINGMIYSLPGLKpc4_NP.1
p−maj=1, pX=0.22, N=325
var.11.F5 1:169511755:T:C
peptide level
169:169 134:135 21:21
SHEFHAINGMIYSLPGLKpc4_NP.2
p−min=0.48, pX=0.71, N=324
var.11.F5 1:169511755:T:C
peptide level
169:169 135:135 21:21
SHEFHAINGMIYSLPGLKpc4_NP.3
p−maj=1, pX=0.53, N=325
var.11.F5 1:169511755:T:C
peptide level
169:169 135:135 21:21
SHEFHAINGMIYSLPGLKpc4_NP.4
p−maj=1, pX=0.88, N=325
var.11.F5 1:169511755:T:C
peptide level
169:169 135:135 21:21
SHEFHAINGMIYSLPGLKpc4_NP.5
p−maj=1, pX=0.39, N=325
var.11.F5 1:169511755:T:C
peptide level
168:169 135:135 21:21
SLSSEMYVKpc2_NP.1
p−maj=1, pX=0.72, N=324
var.11.F5 1:169511755:T:C
peptide level
169:169 135:135 21:21
SLSSEMYVKpc2_NP.2
p−maj=1, pX=0.72, N=325
var.11.F5 1:169511755:T:C
peptide level
169:169 135:135 21:21
SLSSEMYVKpc2_NP.3
p−maj=1, pX=0.68, N=325
var.11.F5 1:169511755:T:C
peptide level
169:169 135:135 21:21
SLSSEMYVKpc2_NP.4
p−maj=1, pX=0.42, N=325
var.11.F5 1:169511755:T:C
peptide level
165:169 133:135 20:21
SLSSEMYVKpc2_NP.5
p−maj=0.38, pX=0.72, N=318
var.11.F5 1:169511755:T:C
peptide level
168:169 135:135 21:21
SQHLDNFSNQIGKpc2_NP.1
p−maj=1, pX=0.047, N=324
var.11.F5 1:169511755:T:C
peptide level
169:169 135:135 21:21
SQHLDNFSNQIGKpc2_NP.2
p−maj=1, pX=0.58, N=325
var.11.F5 1:169511755:T:C
peptide level
169:169 135:135 21:21
SQHLDNFSNQIGKpc2_NP.3
p−maj=1, pX=0.0049, N=325
var.11.F5 1:169511755:T:C
peptide level
169:169 135:135 21:21
SQHLDNFSNQIGKpc2_NP.4
p−maj=1, pX=0.99, N=325
var.11.F5 1:169511755:T:C
peptide level
169:169 135:135 21:21
SQHLDNFSNQIGKpc2_NP.5
p−maj=1, pX=0.16, N=325

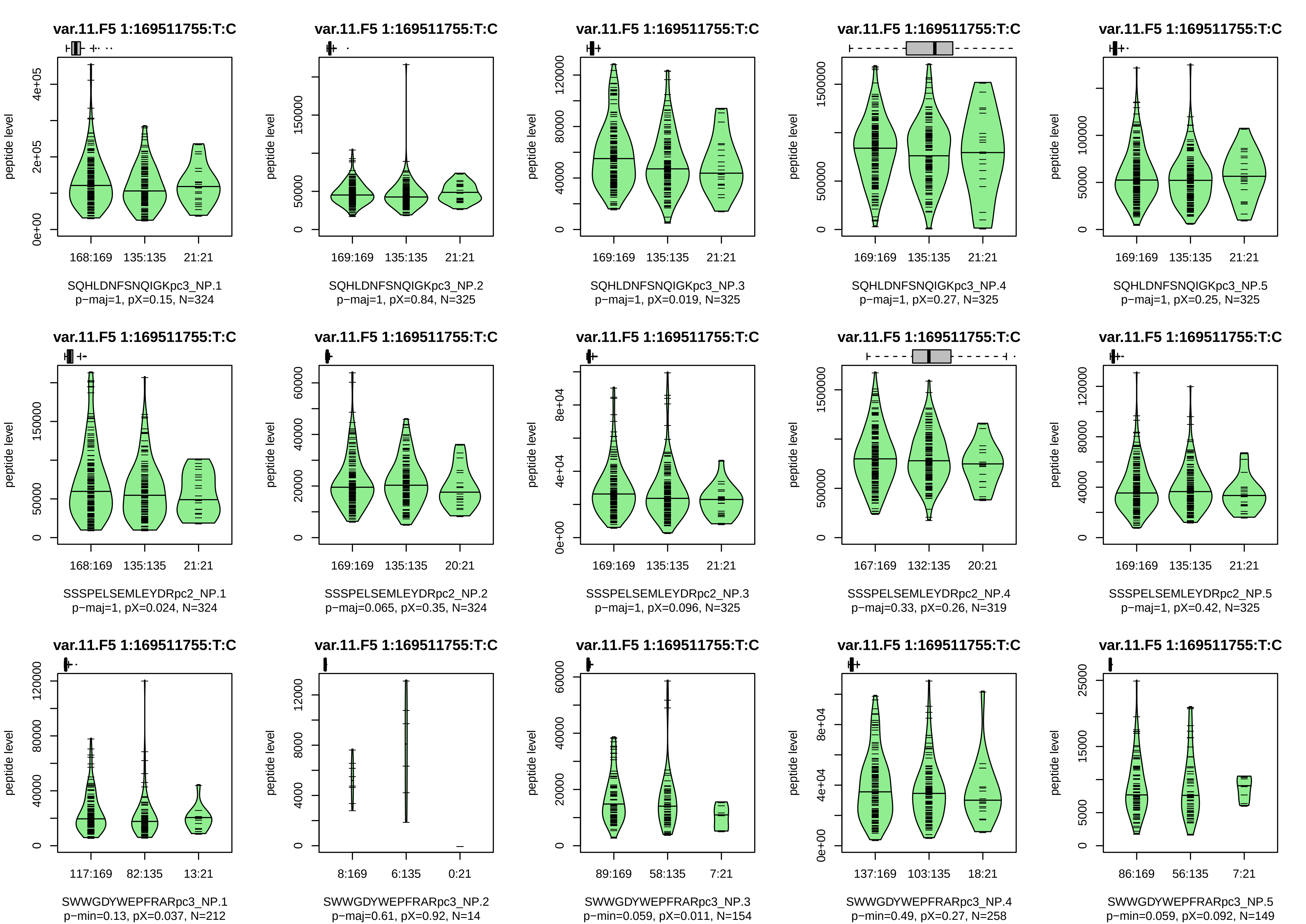
var.11.F5 1:169511755:T:C
peptide level
168:169 135:135 21:21
SQHLDNFSNQIGKpc3_NP.1
p−maj=1, pX=0.15, N=324
var.11.F5 1:169511755:T:C
peptide level
169:169 135:135 21:21
SQHLDNFSNQIGKpc3_NP.2
p−maj=1, pX=0.84, N=325
var.11.F5 1:169511755:T:C
peptide level
169:169 135:135 21:21
SQHLDNFSNQIGKpc3_NP.3
p−maj=1, pX=0.019, N=325
var.11.F5 1:169511755:T:C
peptide level
169:169 135:135 21:21
SQHLDNFSNQIGKpc3_NP.4
p−maj=1, pX=0.27, N=325
var.11.F5 1:169511755:T:C
peptide level
169:169 135:135 21:21
SQHLDNFSNQIGKpc3_NP.5
p−maj=1, pX=0.25, N=325
var.11.F5 1:169511755:T:C
peptide level
168:169 135:135 21:21
SSSPELSEMLEYDRpc2_NP.1
p−maj=1, pX=0.024, N=324
var.11.F5 1:169511755:T:C
peptide level
169:169 135:135 20:21
SSSPELSEMLEYDRpc2_NP.2
p−maj=0.065, pX=0.35, N=324
var.11.F5 1:169511755:T:C
peptide level
169:169 135:135 21:21
SSSPELSEMLEYDRpc2_NP.3
p−maj=1, pX=0.096, N=325
var.11.F5 1:169511755:T:C
peptide level
167:169 132:135 20:21
SSSPELSEMLEYDRpc2_NP.4
p−maj=0.33, pX=0.26, N=319
var.11.F5 1:169511755:T:C
peptide level
169:169 135:135 21:21
SSSPELSEMLEYDRpc2_NP.5
p−maj=1, pX=0.42, N=325
var.11.F5 1:169511755:T:C
peptide level
117:169 82:135 13:21
SWWGDYWEPFRARpc3_NP.1
p−min=0.13, pX=0.037, N=212
var.11.F5 1:169511755:T:C
peptide level
8:169 6:135 0:21
SWWGDYWEPFRARpc3_NP.2
p−maj=0.61, pX=0.92, N=14
var.11.F5 1:169511755:T:C
peptide level
89:169 58:135 7:21
SWWGDYWEPFRARpc3_NP.3
p−min=0.059, pX=0.011, N=154
var.11.F5 1:169511755:T:C
peptide level
137:169 103:135 18:21
SWWGDYWEPFRARpc3_NP.4
p−min=0.49, pX=0.27, N=258
var.11.F5 1:169511755:T:C
peptide level
86:169 56:135 7:21
SWWGDYWEPFRARpc3_NP.5
p−min=0.059, pX=0.092, N=149

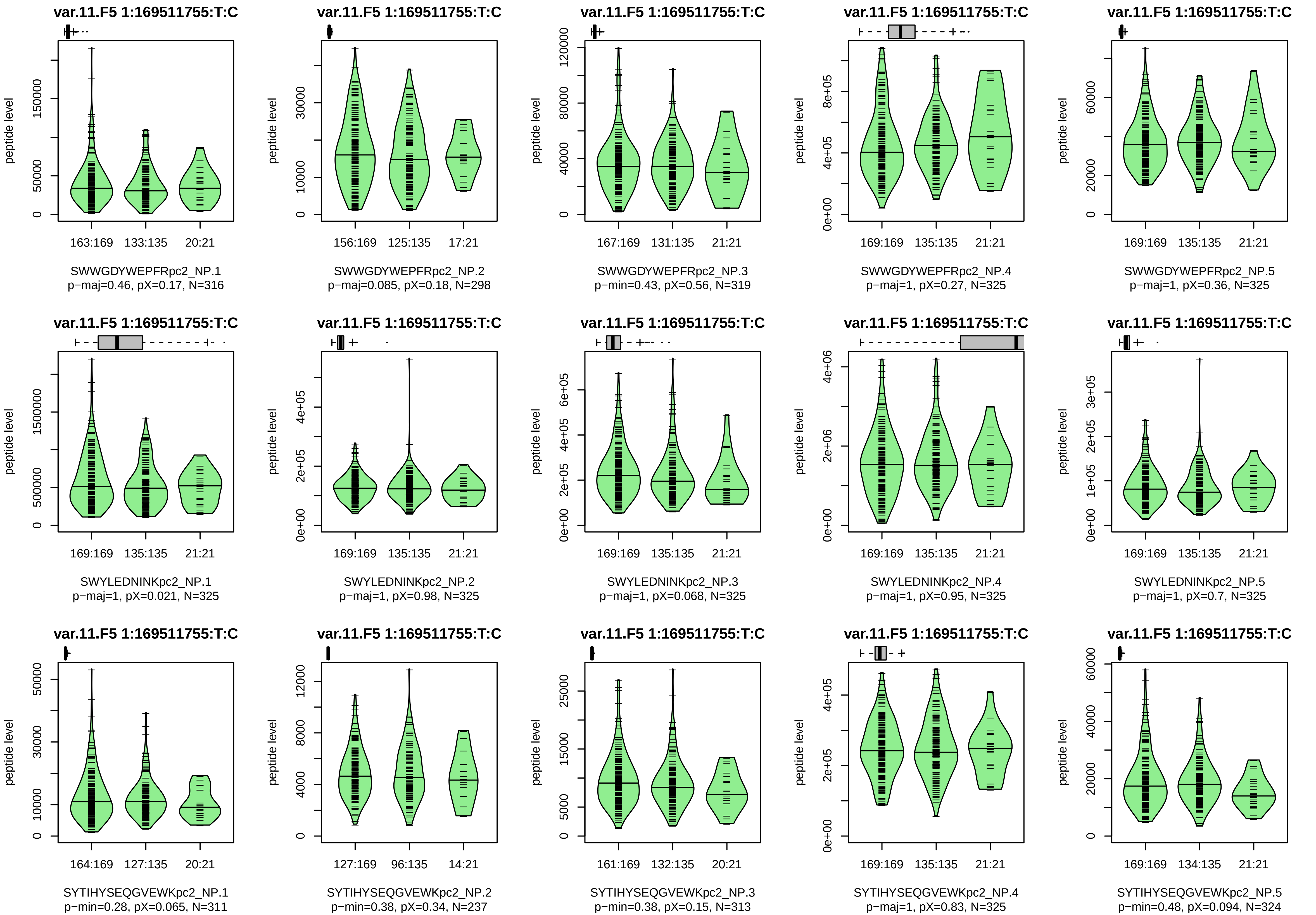
var.11.F5 1:169511755:T:C
163:169 133:135 20:21
SWWGDYWEPFRpc2_NP.1
p−maj=0.46, pX=0.17, N=316
var.11.F5 1:169511755:T:C
156:169 125:135 17:21
SWWGDYWEPFRpc2_NP.2
p−maj=0.085, pX=0.18, N=298
var.11.F5 1:169511755:T:C
167:169 131:135 21:21
SWWGDYWEPFRpc2_NP.3
p−min=0.43, pX=0.56, N=319
var.11.F5 1:169511755:T:C
169:169 135:135 21:21
SWWGDYWEPFRpc2_NP.4
p−maj=1, pX=0.27, N=325
var.11.F5 1:169511755:T:C
169:169 135:135 21:21
SWWGDYWEPFRpc2_NP.5
p−maj=1, pX=0.36, N=325
var.11.F5 1:169511755:T:C
169:169 135:135 21:21
SWYLEDNINKpc2_NP.1
p−maj=1, pX=0.021, N=325
var.11.F5 1:169511755:T:C
169:169 135:135 21:21
SWYLEDNINKpc2_NP.2
p−maj=1, pX=0.98, N=325
var.11.F5 1:169511755:T:C
169:169 135:135 21:21
SWYLEDNINKpc2_NP.3
p−maj=1, pX=0.068, N=325
var.11.F5 1:169511755:T:C
169:169 135:135 21:21
SWYLEDNINKpc2_NP.4
p−maj=1, pX=0.95, N=325
var.11.F5 1:169511755:T:C
169:169 135:135 21:21
SWYLEDNINKpc2_NP.5
p−maj=1, pX=0.7, N=325
var.11.F5 1:169511755:T:C
164:169 127:135 20:21
SYTIHYSEQGVEWKpc2_NP.1
p−min=0.28, pX=0.065, N=311
var.11.F5 1:169511755:T:C
127:169 96:135 14:21
SYTIHYSEQGVEWKpc2_NP.2
p−min=0.38, pX=0.34, N=237
var.11.F5 1:169511755:T:C
161:169 132:135 20:21
SYTIHYSEQGVEWKpc2_NP.3
p−min=0.38, pX=0.15, N=313
var.11.F5 1:169511755:T:C
169:169 135:135 21:21
SYTIHYSEQGVEWKpc2_NP.4
p−maj=1, pX=0.83, N=325
var.11.F5 1:169511755:T:C
169:169 134:135 21:21
SYTIHYSEQGVEWKpc2_NP.5
p−min=0.48, pX=0.094, N=324
peptide level

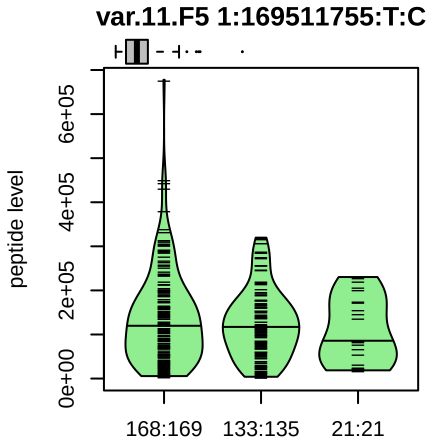

SYTIHYSEQGVEWKpc3_NP.1
p−min=0.61, pX=0.1, N=322

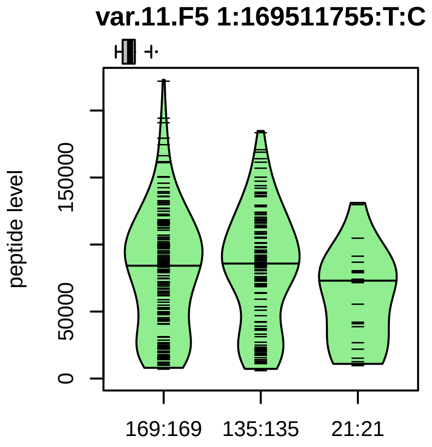

SYTIHYSEQGVEWKpc3_NP.2
p−maj=1, pX=0.3, N=325

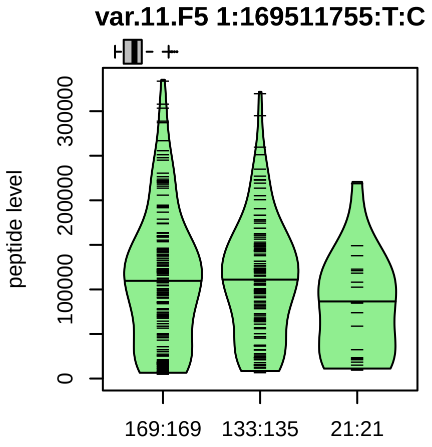

SYTIHYSEQGVEWKpc3_NP.3
p−min=0.23, pX=0.15, N=323

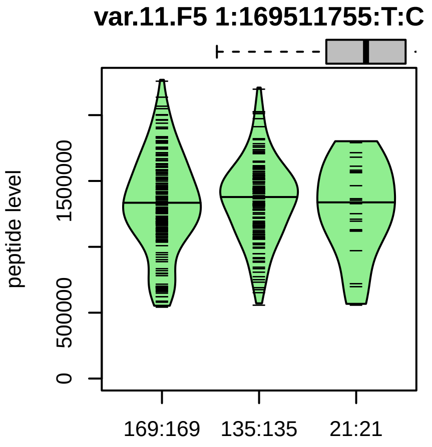

SYTIHYSEQGVEWKpc3_NP.4
p−maj=1, pX=0.92, N=325

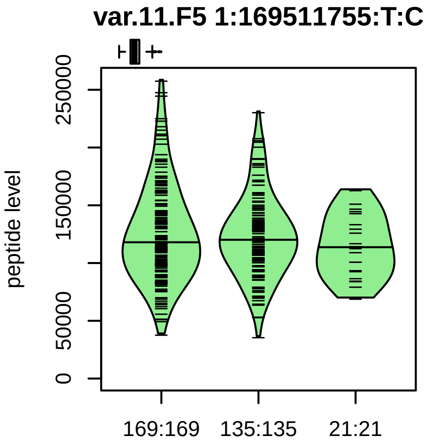

SYTIHYSEQGVEWKpc3_NP.5
p−maj=1, pX=0.23, N=325

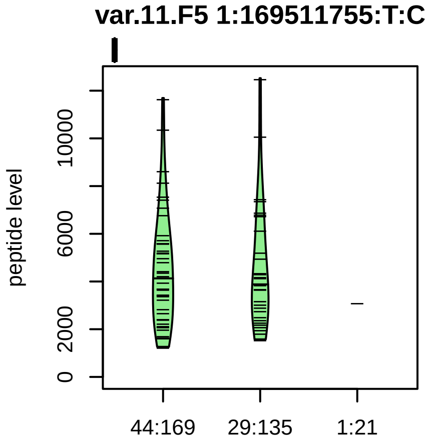

SYTIHYSEQGVEWKPYRpc3_NP.3
p−maj=0.056, pX=0.076, N=74

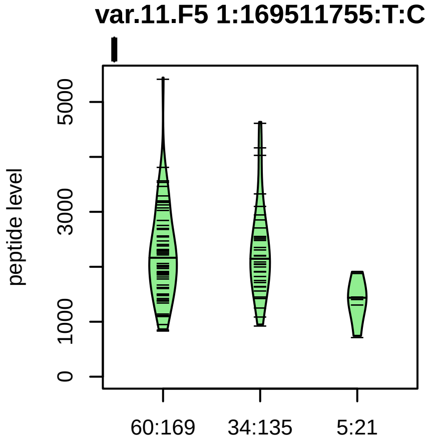

SYTIHYSEQGVEWKPYRpc4_NP.3
p−min=0.041, pX=0.027, N=99

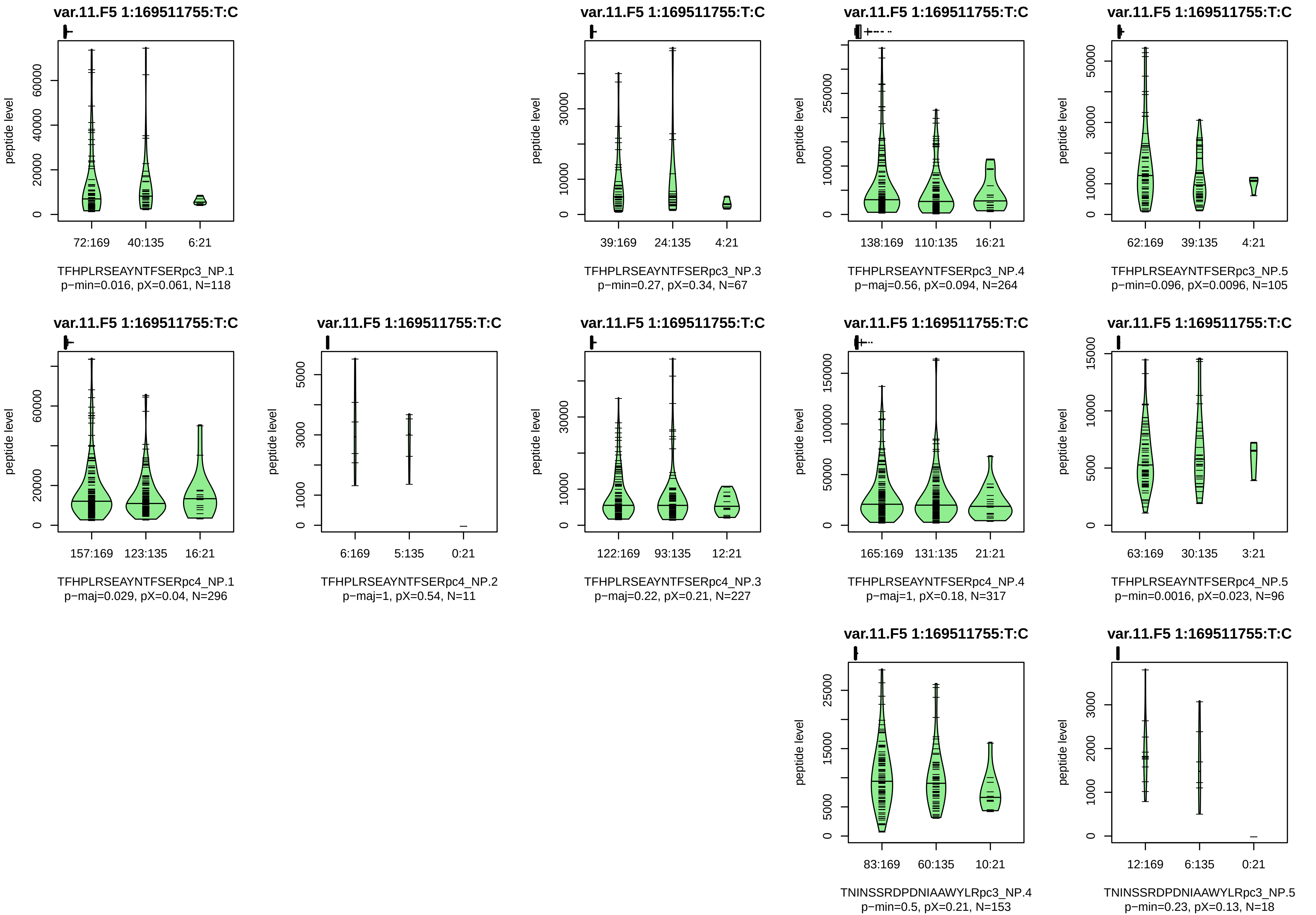
var.11.F5 1:169511755:T:C
peptide level
72:169
40:135
6:21
TFHPLRSEAYNTFSERpc3_NP.1
p−min=0.016, pX=0.061, N=118
var.11.F5 1:169511755:T:C
peptide level
39:169
24:135
4:21
TFHPLRSEAYNTFSERpc3_NP.3
p−min=0.27, pX=0.34, N=67
var.11.F5 1:169511755:T:C
peptide level
138:169
110:135
16:21
TFHPLRSEAYNTFSERpc3_NP.4
p−maj=0.56, pX=0.094, N=264
var.11.F5 1:169511755:T:C
peptide level
62:169
39:135
4:21
TFHPLRSEAYNTFSERpc3_NP.5
p−min=0.096, pX=0.0096, N=105
var.11.F5 1:169511755:T:C
peptide level
157:169
123:135
16:21
TFHPLRSEAYNTFSERpc4_NP.1
p−maj=0.029, pX=0.04, N=296
var.11.F5 1:169511755:T:C
peptide level
6:169
5:135
0:21
TFHPLRSEAYNTFSERpc4_NP.2
p−maj=1, pX=0.54, N=11
var.11.F5 1:169511755:T:C
peptide level
122:169
93:135
12:21
TFHPLRSEAYNTFSERpc4_NP.3
p−maj=0.22, pX=0.21, N=227
var.11.F5 1:169511755:T:C
peptide level
165:169
131:135
21:21
TFHPLRSEAYNTFSERpc4_NP.4
p−maj=1, pX=0.18, N=317
var.11.F5 1:169511755:T:C
peptide level
63:169
30:135
3:21
TFHPLRSEAYNTFSERpc4_NP.5
p−min=0.0016, pX=0.023, N=96
var.11.F5 1:169511755:T:C
peptide level
83:169
60:135
10:21
TNINSSRDPDNIAAWYLRpc3_NP.4
p−min=0.5, pX=0.21, N=153
var.11.F5 1:169511755:T:C
peptide level
12:169
6:135
0:21
TNINSSRDPDNIAAWYLRpc3_NP.5
p−min=0.23, pX=0.13, N=18

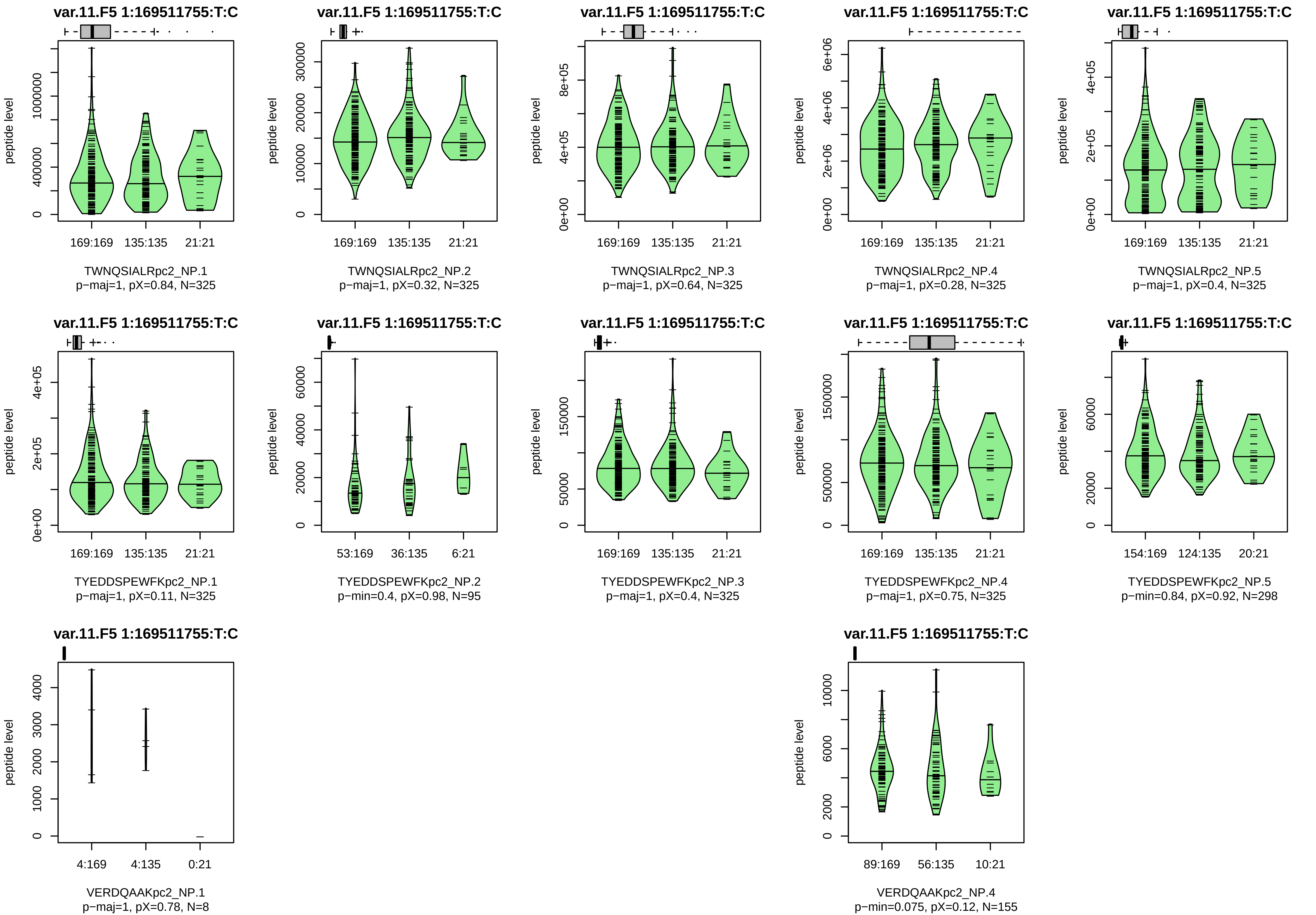
var.11.F5 1:169511755:T:C
peptide level
169:169
135:135
21:21
TWNQSIALRpc2_NP.1
p−maj=1, pX=0.84, N=325
TWNQSIALRpc2_NP.2
p−maj=1, pX=0.32, N=325
TWNQSIALRpc2_NP.3
p−maj=1, pX=0.64, N=325
TWNQSIALRpc2_NP.4
p−maj=1, pX=0.28, N=325
TWNQSIALRpc2_NP.5
p−maj=1, pX=0.4, N=325
TYEDDSPEWFKpc2_NP.1
p−maj=1, pX=0.11, N=325
53:169
36:135
6:21
TYEDDSPEWFKpc2_NP.2
p−min=0.4, pX=0.98, N=95
TYEDDSPEWFKpc2_NP.3
p−maj=1, pX=0.4, N=325
TYEDDSPEWFKpc2_NP.4
p−maj=1, pX=0.75, N=325
154:169
124:135
20:21
TYEDDSPEWFKpc2_NP.5
p−min=0.84, pX=0.92, N=298
4:169
4:135
0:21
VERDQAAKpc2_NP.1
p−maj=1, pX=0.78, N=8
89:169
56:135
10:21
VERDQAAKpc2_NP.4
p−min=0.075, pX=0.12, N=155

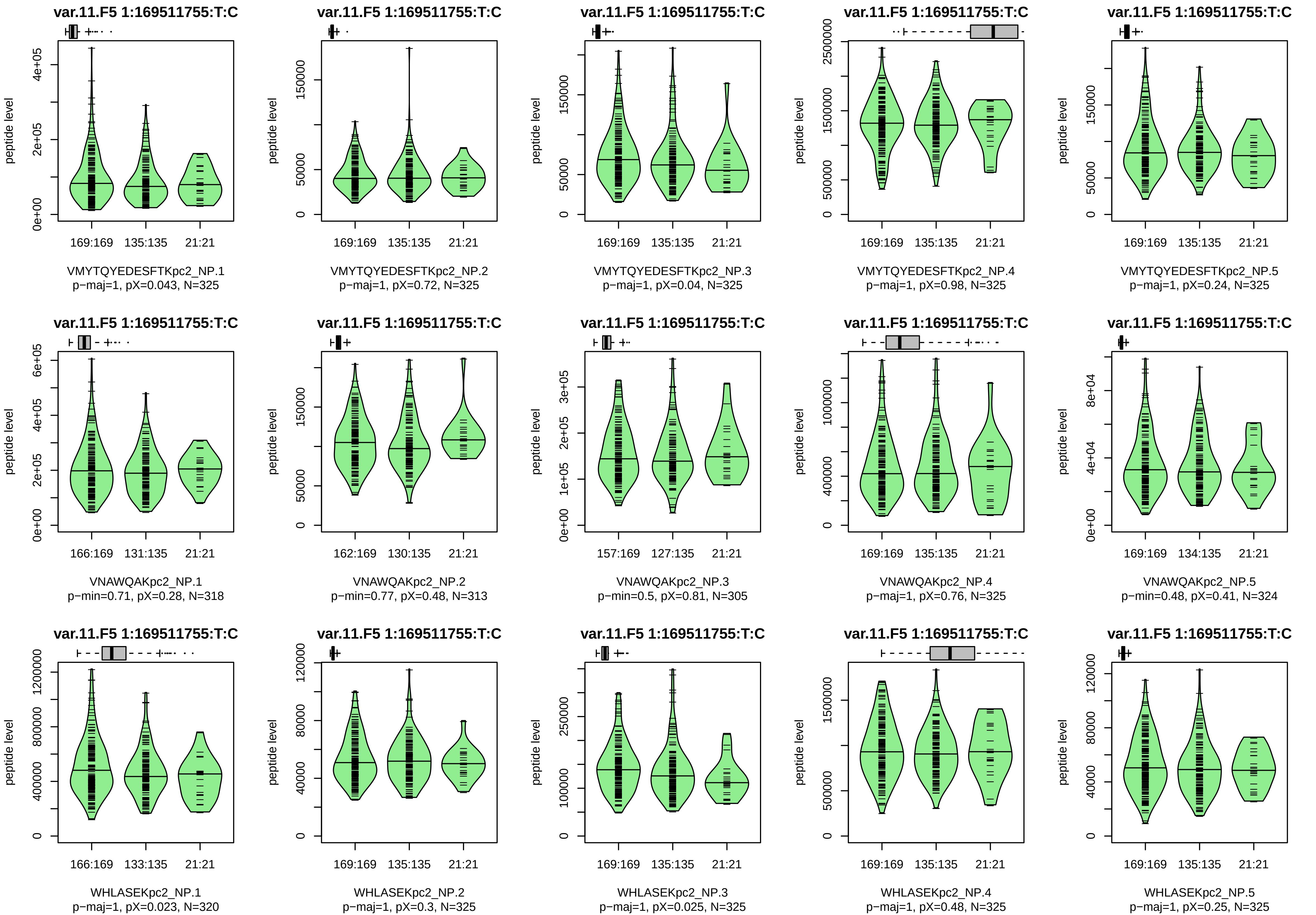
var.11.F5 1:169511755:T:C
peptide level
169:169
135:135
21:21
VMYTQYEDESFTKpc2_NP.1
p−maj=1, pX=0.043, N=325
VMYTQYEDESFTKpc2_NP.2
p−maj=1, pX=0.72, N=325
VMYTQYEDESFTKpc2_NP.3
p−maj=1, pX=0.04, N=325
VMYTQYEDESFTKpc2_NP.4
p−maj=1, pX=0.98, N=325
VMYTQYEDESFTKpc2_NP.5
p−maj=1, pX=0.24, N=325
166:169
131:135
162:169
130:135
157:169
127:135
134:135
VNAWQAKpc2_NP.1
p−min=0.71, pX=0.28, N=318
VNAWQAKpc2_NP.2
p−min=0.77, pX=0.48, N=313
VNAWQAKpc2_NP.3
p−min=0.5, pX=0.81, N=305
VNAWQAKpc2_NP.4
p−maj=1, pX=0.76, N=325
VNAWQAKpc2_NP.5
p−min=0.48, pX=0.41, N=324
133:135
WHLASEKpc2_NP.1
p−maj=1, pX=0.023, N=320
WHLASEKpc2_NP.2
p−maj=1, pX=0.3, N=325
WHLASEKpc2_NP.3
p−maj=1, pX=0.025, N=325
WHLASEKpc2_NP.4
p−maj=1, pX=0.48, N=325
WHLASEKpc2_NP.5
p−maj=1, pX=0.25, N=325

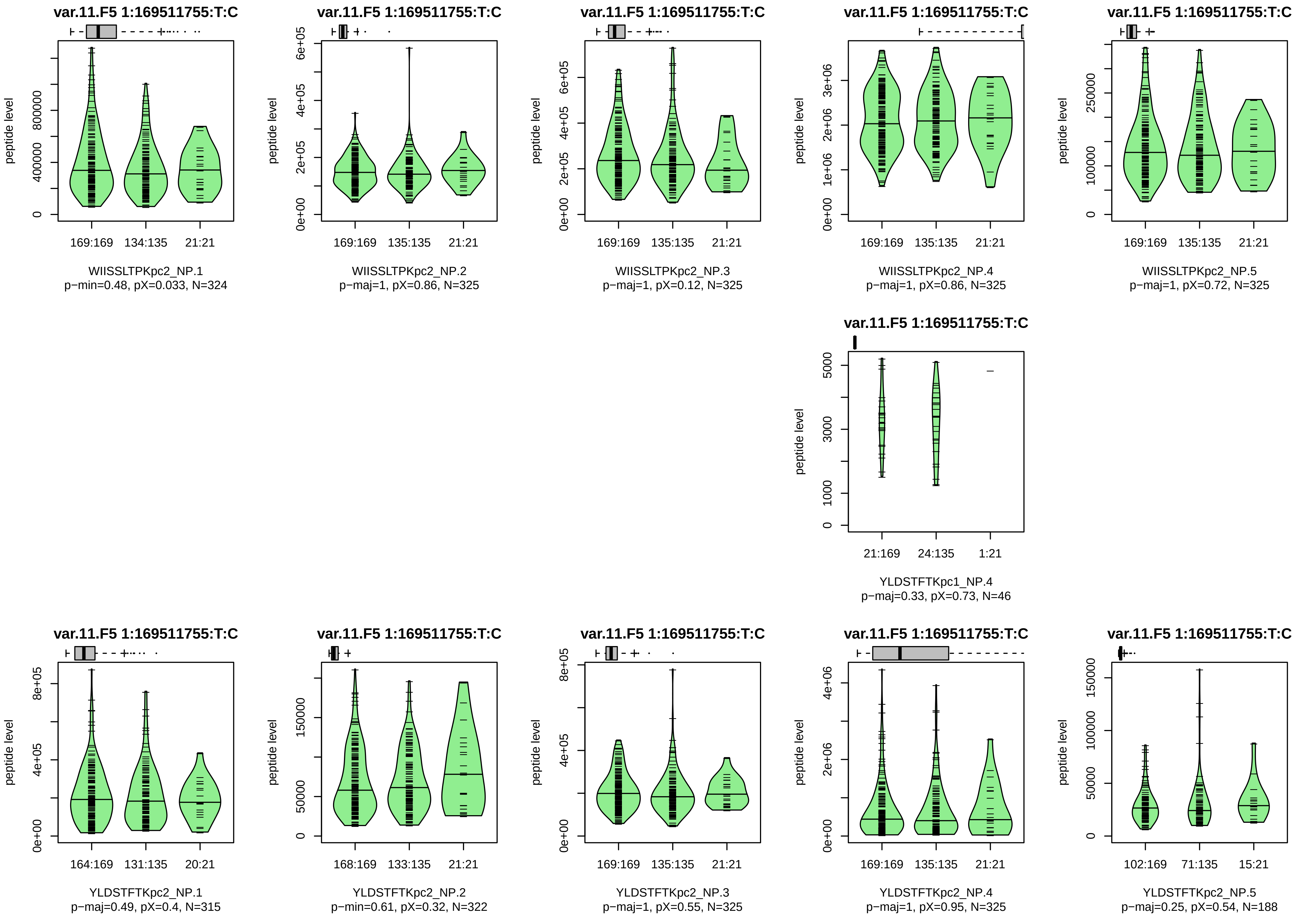
var.11.F5 1:169511755:T:C
peptide level
169:169
134:135
21:21
WIISSLTPKpc2_NP.1
p−min=0.48, pX=0.033, N=324
var.11.F5 1:169511755:T:C
peptide level
169:169
135:135
21:21
WIISSLTPKpc2_NP.2
p−maj=1, pX=0.86, N=325
var.11.F5 1:169511755:T:C
peptide level
169:169
135:135
21:21
WIISSLTPKpc2_NP.3
p−maj=1, pX=0.12, N=325
var.11.F5 1:169511755:T:C
peptide level
169:169
135:135
21:21
WIISSLTPKpc2_NP.4
p−maj=1, pX=0.86, N=325
var.11.F5 1:169511755:T:C
peptide level
169:169
135:135
21:21
WIISSLTPKpc2_NP.5
p−maj=1, pX=0.72, N=325
var.11.F5 1:169511755:T:C
peptide level
21:169
24:135
1:21
YLDSTFTKpc1_NP.4
p−maj=0.33, pX=0.73, N=46
var.11.F5 1:169511755:T:C
peptide level
164:169
131:135
20:21
YLDSTFTKpc2_NP.1
p−maj=0.49, pX=0.4, N=315
var.11.F5 1:169511755:T:C
peptide level
168:169
133:135
21:21
YLDSTFTKpc2_NP.2
p−min=0.61, pX=0.32, N=322
var.11.F5 1:169511755:T:C
peptide level
169:169
135:135
21:21
YLDSTFTKpc2_NP.3
p−maj=1, pX=0.55, N=325
var.11.F5 1:169511755:T:C
peptide level
169:169
135:135
21:21
YLDSTFTKpc2_NP.4
p−maj=1, pX=0.95, N=325
var.11.F5 1:169511755:T:C
peptide level
102:169
71:135
15:21
YLDSTFTKpc2_NP.5
p−maj=0.25, pX=0.54, N=188

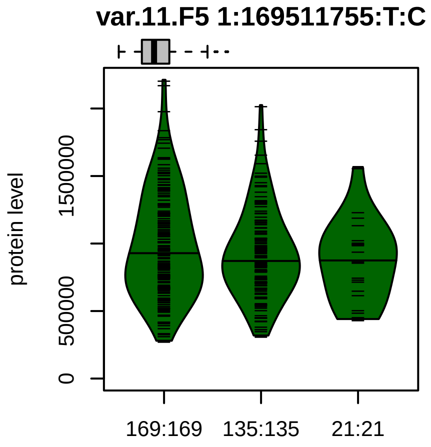

A0A0A0MRJ7.P12259_NP.1
p−maj=1, pY=0.16, N=325

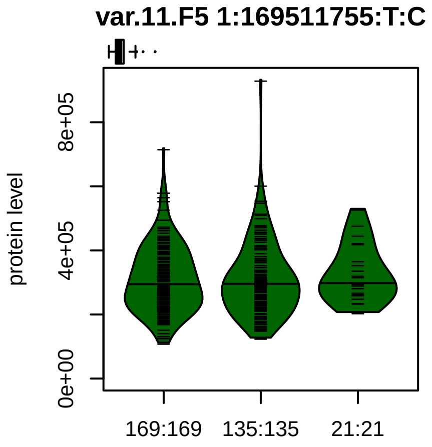

A0A0A0MRJ7.P12259_NP.2
p−maj=1, pY=0.4, N=325

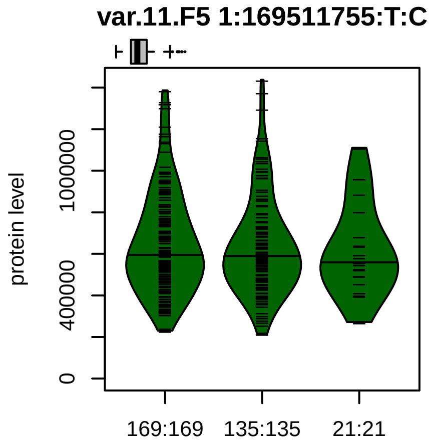

A0A0A0MRJ7.P12259_NP.3
p−maj=1, pY=0.4, N=325

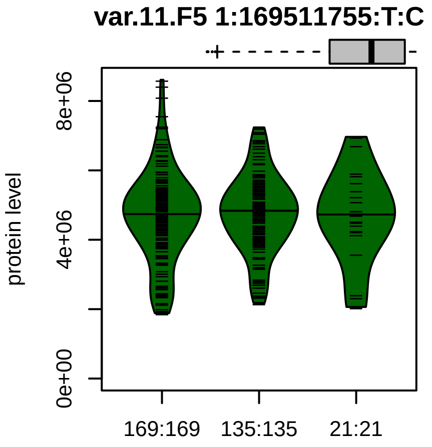

A0A0A0MRJ7.P12259_NP.4
p−maj=1, pY=0.48, N=325

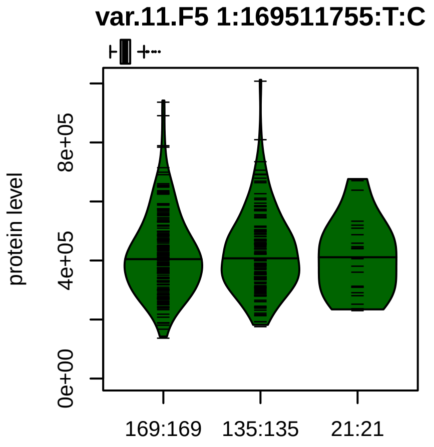

A0A0A0MRJ7.P12259_NP.5
p−maj=1, pY=0.65, N=325

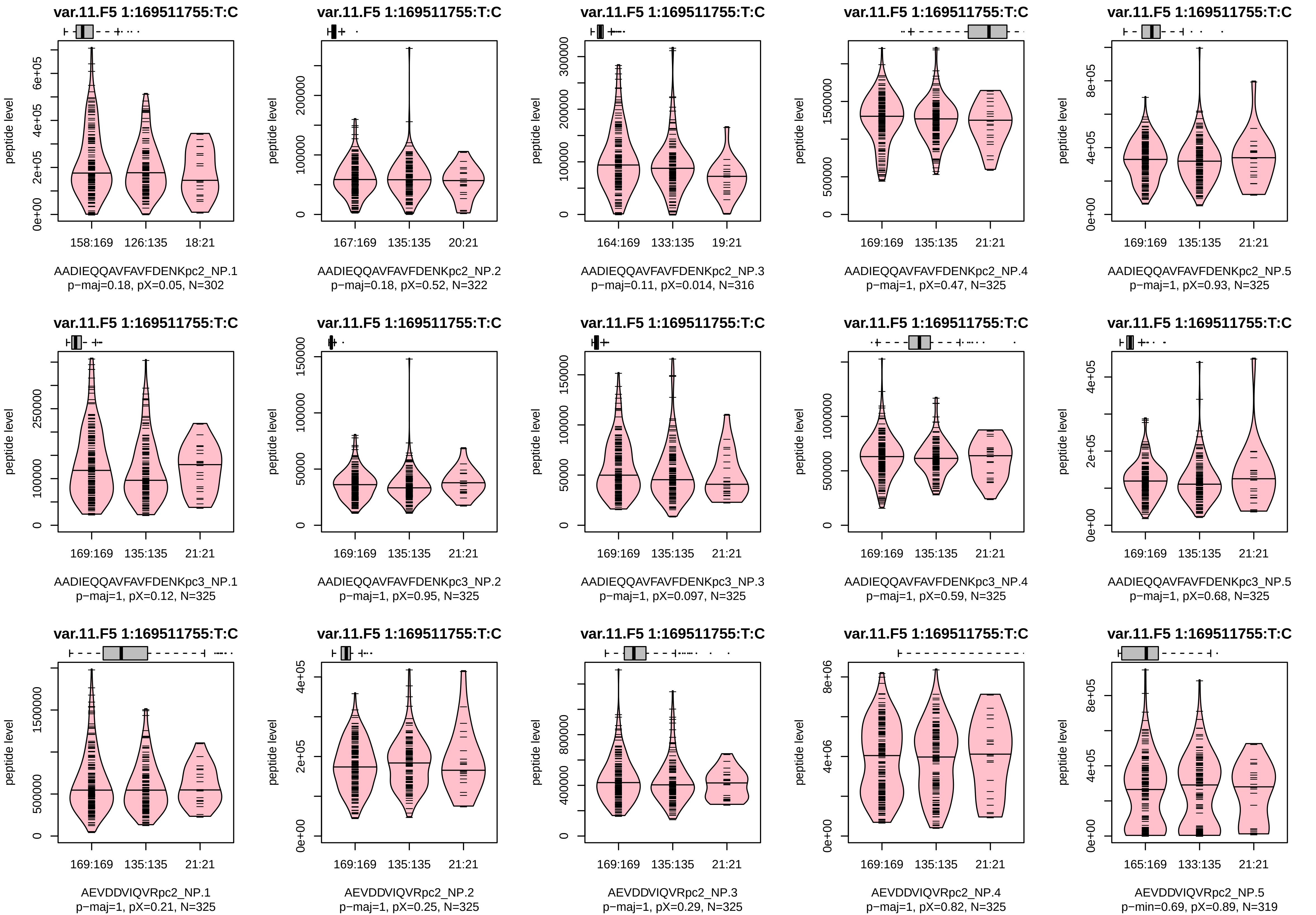

var.11.F5 1:169511755:T:C
peptide level
158:169
126:135
18:21
AADIEQQAVFAVFDENKpc2_NP.1
p−maj=0.18, pX=0.05, N=302
var.11.F5 1:169511755:T:C
peptide level
167:169
135:135
20:21
AADIEQQAVFAVFDENKpc2_NP.2
p−maj=0.18, pX=0.52, N=322
var.11.F5 1:169511755:T:C
peptide level
164:169
133:135
19:21
AADIEQQAVFAVFDENKpc2_NP.3
p−maj=0.11, pX=0.014, N=316
var.11.F5 1:169511755:T:C
peptide level
169:169
135:135
21:21
AADIEQQAVFAVFDENKpc2_NP.4
p−maj=1, pX=0.47, N=325
var.11.F5 1:169511755:T:C
peptide level
169:169
135:135
21:21
AADIEQQAVFAVFDENKpc2_NP.5
p−maj=1, pX=0.93, N=325
var.11.F5 1:169511755:T:C
peptide level
169:169
135:135
21:21
AADIEQQAVFAVFDENKpc3_NP.1
p−maj=1, pX=0.12, N=325
var.11.F5 1:169511755:T:C
peptide level
169:169
135:135
21:21
AADIEQQAVFAVFDENKpc3_NP.2
p−maj=1, pX=0.95, N=325
var.11.F5 1:169511755:T:C
peptide level
169:169
135:135
21:21
AADIEQQAVFAVFDENKpc3_NP.3
p−maj=1, pX=0.097, N=325
var.11.F5 1:169511755:T:C
peptide level
169:169
135:135
21:21
AADIEQQAVFAVFDENKpc3_NP.4
p−maj=1, pX=0.59, N=325
var.11.F5 1:169511755:T:C
peptide level
169:169
135:135
21:21
AADIEQQAVFAVFDENKpc3_NP.5
p−maj=1, pX=0.68, N=325
var.11.F5 1:169511755:T:C
peptide level
169:169
135:135
21:21
AEVDDVIQVRpc2_NP.1
p−maj=1, pX=0.21, N=325
var.11.F5 1:169511755:T:C
peptide level
169:169
135:135
21:21
AEVDDVIQVRpc2_NP.2
p−maj=1, pX=0.25, N=325
var.11.F5 1:169511755:T:C
peptide level
169:169
135:135
21:21
AEVDDVIQVRpc2_NP.3
p−maj=1, pX=0.29, N=325
var.11.F5 1:169511755:T:C
peptide level
169:169
135:135
21:21
AEVDDVIQVRpc2_NP.4
p−maj=1, pX=0.82, N=325
var.11.F5 1:169511755:T:C
peptide level
165:169
133:135
21:21
AEVDDVIQVRpc2_NP.5
p−min=0.69, pX=0.89, N=319

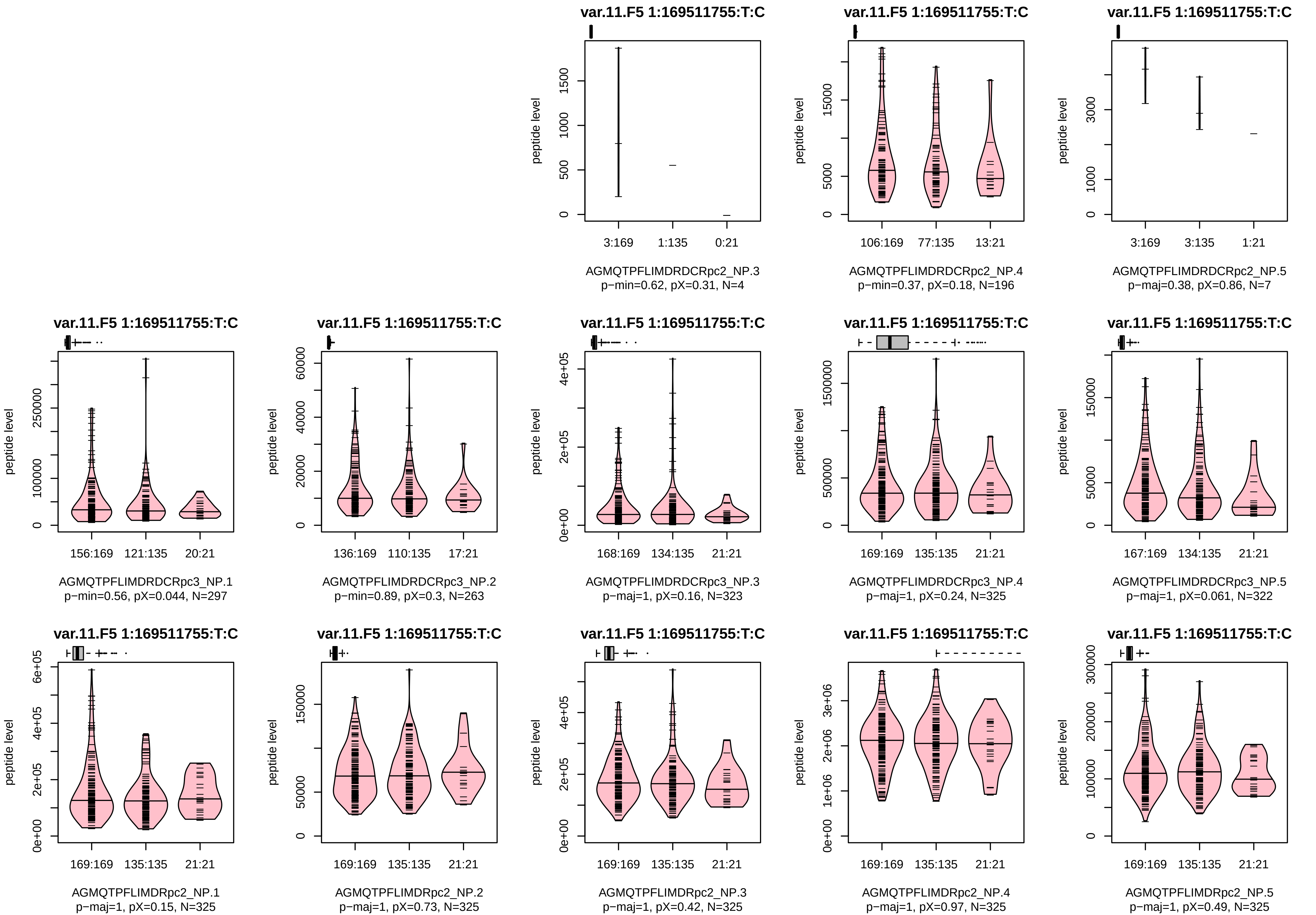
var.11.F5 1:169511755:T:C
peptide level
3:169
1:135
0:21
AGMQTPFLIMDRDCRpc2_NP.3
p−min=0.62, pX=0.31, N=4
var.11.F5 1:169511755:T:C
peptide level
106:169
77:135
13:21
AGMQTPFLIMDRDCRpc2_NP.4
p−min=0.37, pX=0.18, N=196
var.11.F5 1:169511755:T:C
peptide level
3:169
3:135
1:21
AGMQTPFLIMDRDCRpc2_NP.5
p−maj=0.38, pX=0.86, N=7
var.11.F5 1:169511755:T:C
peptide level
156:169
121:135
20:21
AGMQTPFLIMDRDCRpc3_NP.1
p−min=0.56, pX=0.044, N=297
var.11.F5 1:169511755:T:C
peptide level
136:169
110:135
17:21
AGMQTPFLIMDRDCRpc3_NP.2
p−min=0.89, pX=0.3, N=263
var.11.F5 1:169511755:T:C
peptide level
168:169
134:135
21:21
AGMQTPFLIMDRDCRpc3_NP.3
p−maj=1, pX=0.16, N=323
var.11.F5 1:169511755:T:C
peptide level
169:169
135:135
21:21
AGMQTPFLIMDRDCRpc3_NP.4
p−maj=1, pX=0.24, N=325
var.11.F5 1:169511755:T:C
peptide level
167:169
134:135
21:21
AGMQTPFLIMDRDCRpc3_NP.5
p−maj=1, pX=0.061, N=322
var.11.F5 1:169511755:T:C
peptide level
169:169
135:135
21:21
AGMQTPFLIMDRpc2_NP.1
p−maj=1, pX=0.15, N=325
var.11.F5 1:169511755:T:C
peptide level
169:169
135:135
21:21
AGMQTPFLIMDRpc2_NP.2
p−maj=1, pX=0.73, N=325
var.11.F5 1:169511755:T:C
peptide level
169:169
135:135
21:21
AGMQTPFLIMDRpc2_NP.3
p−maj=1, pX=0.42, N=325
var.11.F5 1:169511755:T:C
peptide level
169:169
135:135
21:21
AGMQTPFLIMDRpc2_NP.4
p−maj=1, pX=0.97, N=325
var.11.F5 1:169511755:T:C
peptide level
169:169
135:135
21:21
AGMQTPFLIMDRpc2_NP.5
p−maj=1, pX=0.49, N=325

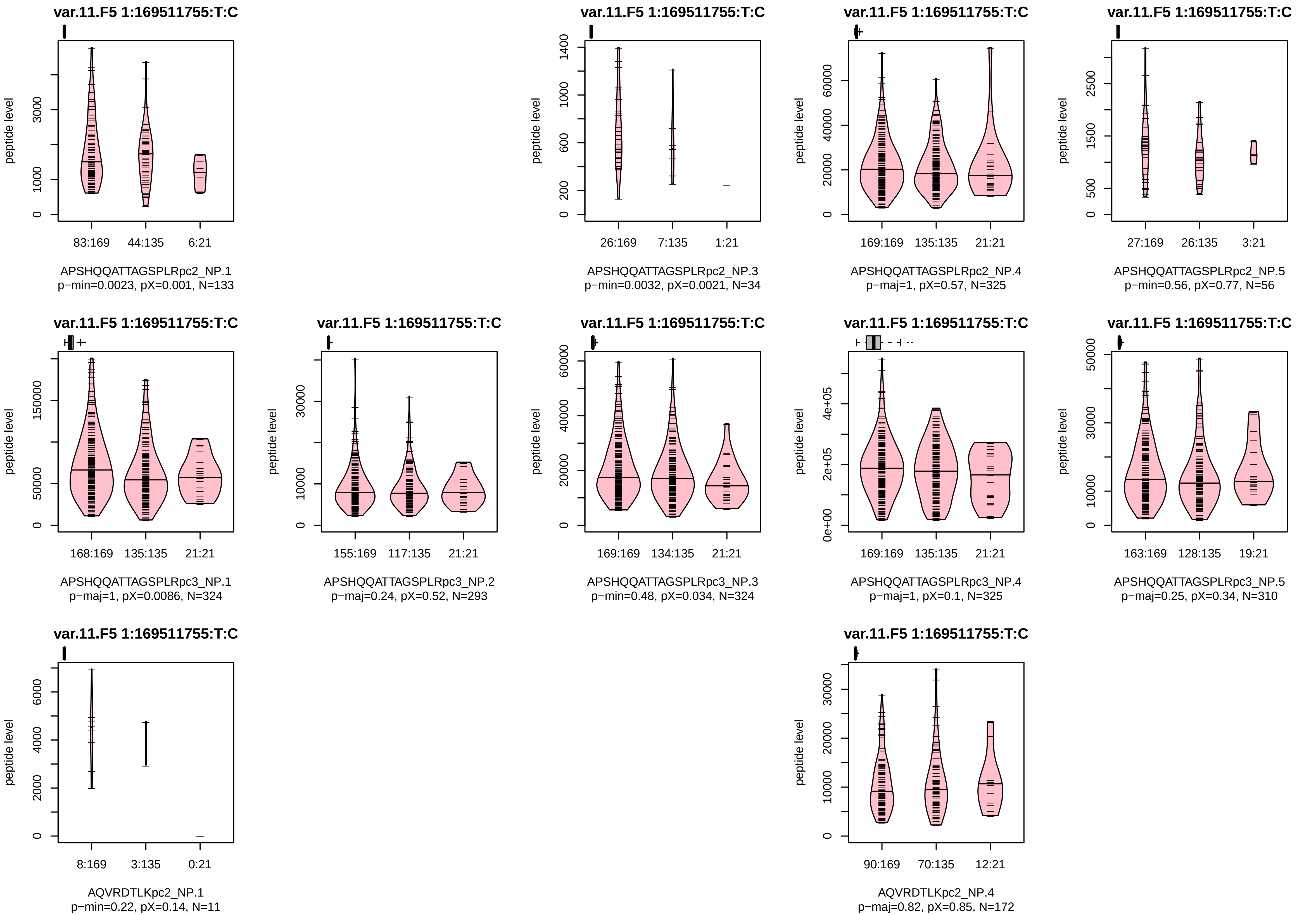
var.11.F5 1:169511755:T:C
peptide level
83:169
44:135
6:21
APSHQQATTAGSPLRpc2_NP.1
p−min=0.0023, pX=0.001, N=133
var.11.F5 1:169511755:T:C
peptide level
26:169
7:135
1:21
APSHQQATTAGSPLRpc2_NP.3
p−min=0.0032, pX=0.0021, N=34
var.11.F5 1:169511755:T:C
peptide level
169:169
135:135
21:21
APSHQQATTAGSPLRpc2_NP.4
p−maj=1, pX=0.57, N=325
var.11.F5 1:169511755:T:C
peptide level
27:169
26:135
3:21
APSHQQATTAGSPLRpc2_NP.5
p−min=0.56, pX=0.77, N=56
var.11.F5 1:169511755:T:C
peptide level
168:169
135:135
21:21
APSHQQATTAGSPLRpc3_NP.1
p−maj=1, pX=0.0086, N=324
var.11.F5 1:169511755:T:C
peptide level
155:169
117:135
21:21
APSHQQATTAGSPLRpc3_NP.2
p−maj=0.24, pX=0.52, N=293
var.11.F5 1:169511755:T:C
peptide level
169:169
134:135
21:21
APSHQQATTAGSPLRpc3_NP.3
p−min=0.48, pX=0.034, N=324
var.11.F5 1:169511755:T:C
peptide level
169:169
135:135
21:21
APSHQQATTAGSPLRpc3_NP.4
p−maj=1, pX=0.1, N=325
var.11.F5 1:169511755:T:C
peptide level
163:169
128:135
19:21
APSHQQATTAGSPLRpc3_NP.5
p−maj=0.25, pX=0.34, N=310
var.11.F5 1:169511755:T:C
peptide level
8:169
3:135
0:21
AQVRDTLKpc2_NP.1
p−min=0.22, pX=0.14, N=11
var.11.F5 1:169511755:T:C
peptide level
90:169
70:135
12:21
AQVRDTLKpc2_NP.4
p−maj=0.82, pX=0.85, N=172

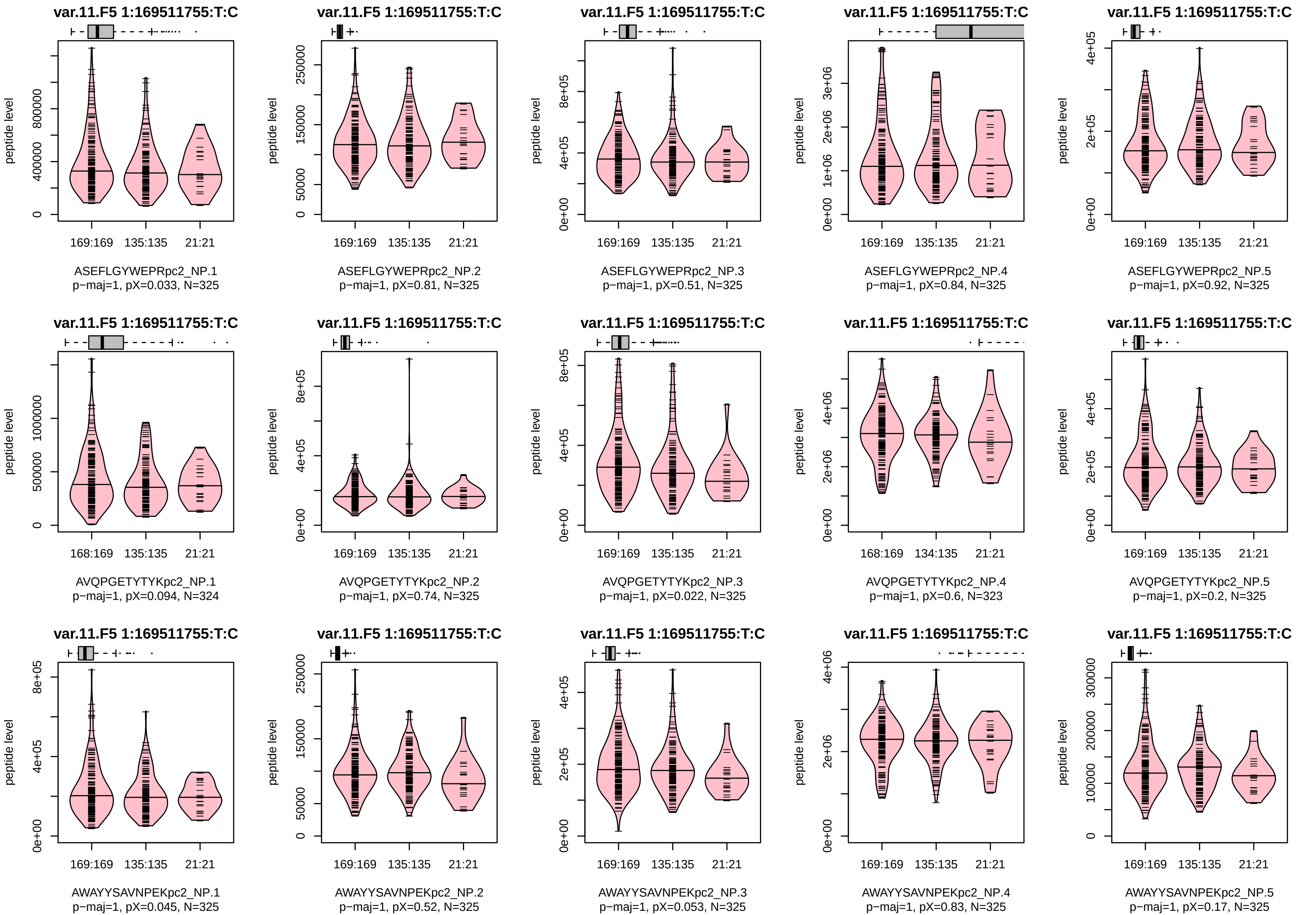
var.11.F5 1:169511755:T:C
peptide level
169:169
135:135
21:21
ASEFLGYWEPRpc2_NP.1
p−maj=1, pX=0.033, N=325
ASEFLGYWEPRpc2_NP.2
p−maj=1, pX=0.81, N=325
ASEFLGYWEPRpc2_NP.3
p−maj=1, pX=0.51, N=325
ASEFLGYWEPRpc2_NP.4
p−maj=1, pX=0.84, N=325
ASEFLGYWEPRpc2_NP.5
p−maj=1, pX=0.92, N=325
168:169
134:135
AVQPGETYTYKpc2_NP.1
p−maj=1, pX=0.094, N=324
AVQPGETYTYKpc2_NP.2
p−maj=1, pX=0.74, N=325
AVQPGETYTYKpc2_NP.3
p−maj=1, pX=0.022, N=325
AVQPGETYTYKpc2_NP.4
p−maj=1, pX=0.6, N=323
AVQPGETYTYKpc2_NP.5
p−maj=1, pX=0.2, N=325
AWAYYSAVNPEKpc2_NP.1
p−maj=1, pX=0.045, N=325
AWAYYSAVNPEKpc2_NP.2
p−maj=1, pX=0.52, N=325
AWAYYSAVNPEKpc2_NP.3
p−maj=1, pX=0.053, N=325
AWAYYSAVNPEKpc2_NP.4
p−maj=1, pX=0.83, N=325
AWAYYSAVNPEKpc2_NP.5
p−maj=1, pX=0.17, N=325

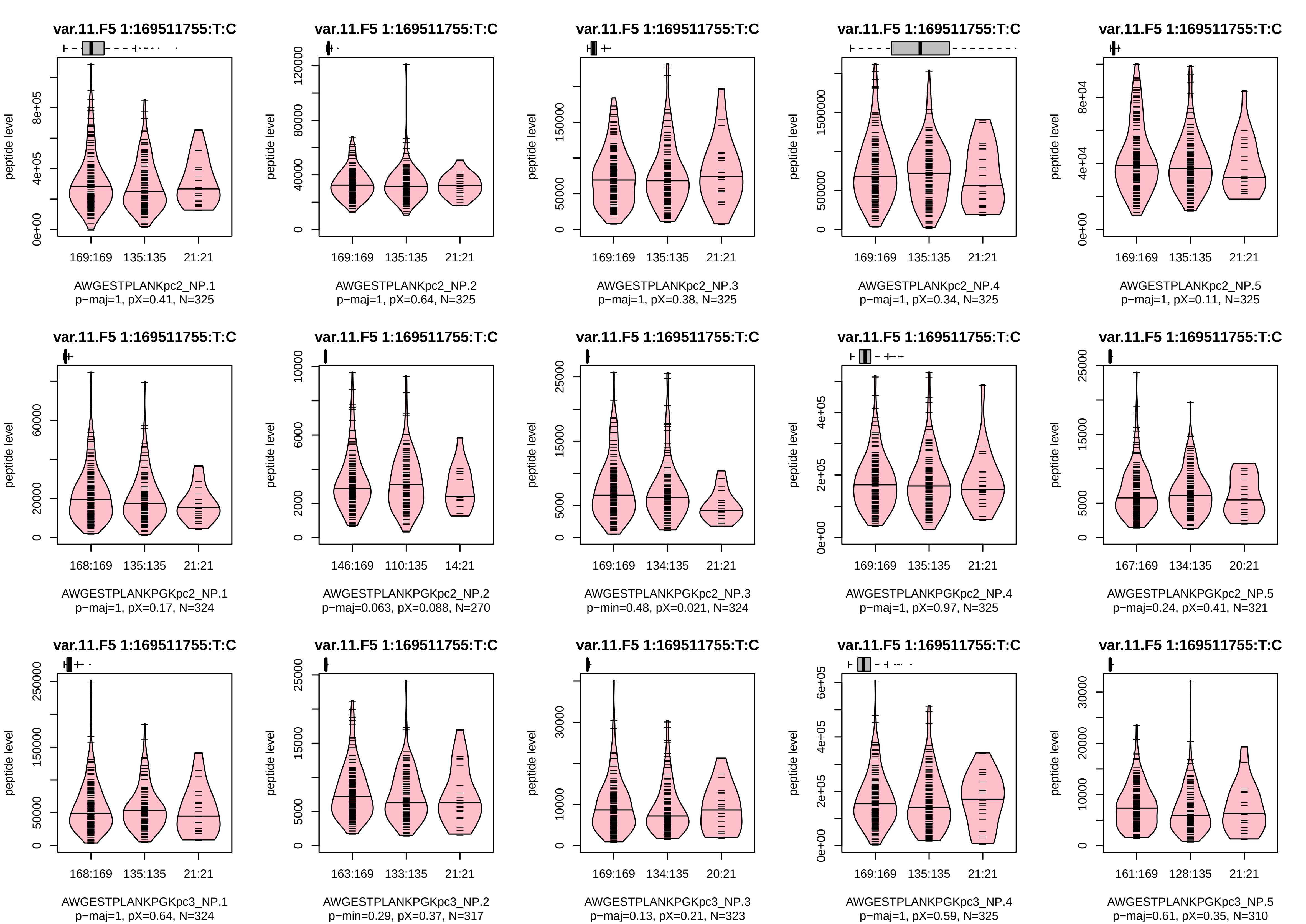
var.11.F5 1:169511755:T:C
peptide level
169:169
135:135
21:21
AWGESTPLANKpc2_NP.1
p−maj=1, pX=0.41, N=325
var.11.F5 1:169511755:T:C
peptide level
169:169
135:135
21:21
AWGESTPLANKpc2_NP.2
p−maj=1, pX=0.64, N=325
var.11.F5 1:169511755:T:C
peptide level
169:169
135:135
21:21
AWGESTPLANKpc2_NP.3
p−maj=1, pX=0.38, N=325
var.11.F5 1:169511755:T:C
peptide level
169:169
135:135
21:21
AWGESTPLANKpc2_NP.4
p−maj=1, pX=0.34, N=325
var.11.F5 1:169511755:T:C
peptide level
169:169
135:135
21:21
AWGESTPLANKpc2_NP.5
p−maj=1, pX=0.11, N=325
var.11.F5 1:169511755:T:C
peptide level
168:169
135:135
21:21
AWGESTPLANKPGKpc2_NP.1
p−maj=1, pX=0.17, N=324
var.11.F5 1:169511755:T:C
peptide level
146:169
110:135
14:21
AWGESTPLANKPGKpc2_NP.2
p−maj=0.063, pX=0.088, N=270
var.11.F5 1:169511755:T:C
peptide level
169:169
134:135
21:21
AWGESTPLANKPGKpc2_NP.3
p−min=0.48, pX=0.021, N=324
var.11.F5 1:169511755:T:C
peptide level
169:169
135:135
21:21
AWGESTPLANKPGKpc2_NP.4
p−maj=1, pX=0.97, N=325
var.11.F5 1:169511755:T:C
peptide level
167:169
134:135
20:21
AWGESTPLANKPGKpc2_NP.5
p−maj=0.24, pX=0.41, N=321
var.11.F5 1:169511755:T:C
peptide level
168:169
135:135
21:21
AWGESTPLANKPGKpc3_NP.1
p−maj=1, pX=0.64, N=324
var.11.F5 1:169511755:T:C
peptide level
163:169
133:135
21:21
AWGESTPLANKPGKpc3_NP.2
p−min=0.29, pX=0.37, N=317
var.11.F5 1:169511755:T:C
peptide level
169:169
134:135
20:21
AWGESTPLANKPGKpc3_NP.3
p−maj=0.13, pX=0.21, N=323
var.11.F5 1:169511755:T:C
peptide level
169:169
135:135
21:21
AWGESTPLANKPGKpc3_NP.4
p−maj=1, pX=0.59, N=325
var.11.F5 1:169511755:T:C
peptide level
161:169
128:135
21:21
AWGESTPLANKPGKpc3_NP.5
p−maj=0.61, pX=0.35, N=310

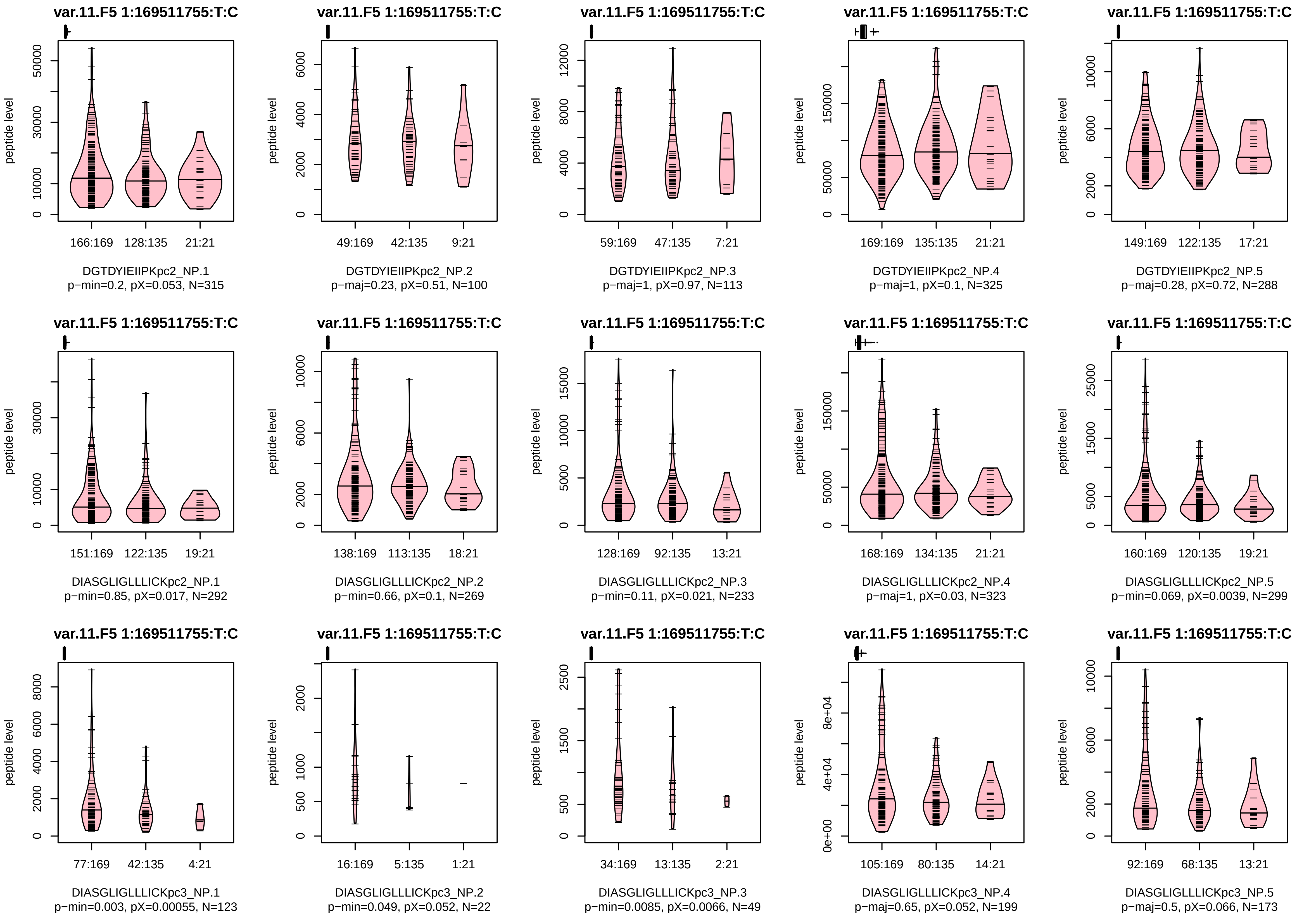
var.11.F5 1:169511755:T:C
peptide level
166:169
128:135
21:21
DGTDYIEIIPKpc2_NP.1
p−min=0.2, pX=0.053, N=315
var.11.F5 1:169511755:T:C
peptide level
49:169
42:135
9:21
DGTDYIEIIPKpc2_NP.2
p−maj=0.23, pX=0.51, N=100
var.11.F5 1:169511755:T:C
peptide level
59:169
47:135
7:21
DGTDYIEIIPKpc2_NP.3
p−maj=1, pX=0.97, N=113
var.11.F5 1:169511755:T:C
peptide level
169:169
135:135
21:21
DGTDYIEIIPKpc2_NP.4
p−maj=1, pX=0.1, N=325
var.11.F5 1:169511755:T:C
peptide level
149:169
122:135
17:21
DGTDYIEIIPKpc2_NP.5
p−maj=0.28, pX=0.72, N=288
var.11.F5 1:169511755:T:C
peptide level
151:169
122:135
19:21
DIASGLIGLLLICKpc2_NP.1
p−min=0.85, pX=0.017, N=292
var.11.F5 1:169511755:T:C
peptide level
138:169
113:135
18:21
DIASGLIGLLLICKpc2_NP.2
p−min=0.66, pX=0.1, N=269
var.11.F5 1:169511755:T:C
peptide level
128:169
92:135
13:21
DIASGLIGLLLICKpc2_NP.3
p−min=0.11, pX=0.021, N=233
var.11.F5 1:169511755:T:C
peptide level
168:169
134:135
21:21
DIASGLIGLLLICKpc2_NP.4
p−maj=1, pX=0.03, N=323
var.11.F5 1:169511755:T:C
peptide level
160:169
120:135
19:21
DIASGLIGLLLICKpc2_NP.5
p−min=0.069, pX=0.0039, N=299
var.11.F5 1:169511755:T:C
peptide level
77:169
42:135
4:21
DIASGLIGLLLICKpc3_NP.1
p−min=0.003, pX=0.00055, N=123
var.11.F5 1:169511755:T:C
peptide level
16:169
5:135
1:21
DIASGLIGLLLICKpc3_NP.2
p−min=0.049, pX=0.052, N=22
var.11.F5 1:169511755:T:C
peptide level
34:169
13:135
2:21
DIASGLIGLLLICKpc3_NP.3
p−min=0.0085, pX=0.0066, N=49
var.11.F5 1:169511755:T:C
peptide level
105:169
80:135
14:21
DIASGLIGLLLICKpc3_NP.4
p−maj=0.65, pX=0.052, N=199
var.11.F5 1:169511755:T:C
peptide level
92:169
68:135
13:21
DIASGLIGLLLICKpc3_NP.5
p−maj=0.5, pX=0.066, N=173

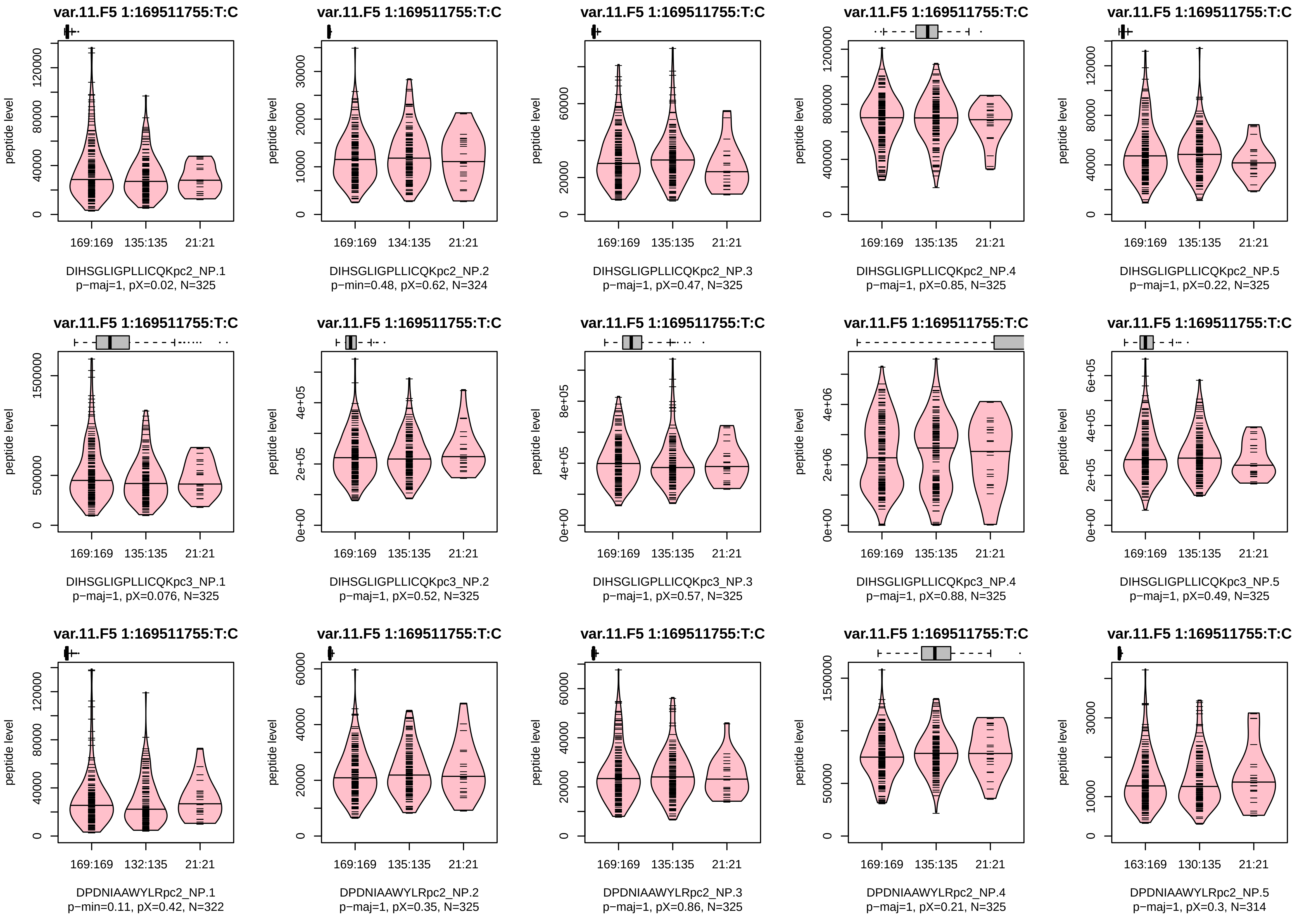

var.11.F5 1:169511755:T:C
peptide level
0
40000
80000
120000
169:169
135:135
21:21
DIHSGLIGPLLICQKpc2_NP.1
p−maj=1, pX=0.02, N=325
var.11.F5 1:169511755:T:C
peptide level
0
10000
20000
30000
169:169
134:135
21:21
DIHSGLIGPLLICQKpc2_NP.2
p−min=0.48, pX=0.62, N=324
var.11.F5 1:169511755:T:C
peptide level
0
20000
60000
169:169
135:135
21:21
DIHSGLIGPLLICQKpc2_NP.3
p−maj=1, pX=0.47, N=325
var.11.F5 1:169511755:T:C
peptide level
0
400000
800000
1200000
169:169
135:135
21:21
DIHSGLIGPLLICQKpc2_NP.4
p−maj=1, pX=0.85, N=325
var.11.F5 1:169511755:T:C
peptide level
0
40000
80000
120000
169:169
135:135
21:21
DIHSGLIGPLLICQKpc2_NP.5
p−maj=1, pX=0.22, N=325
var.11.F5 1:169511755:T:C
peptide level
0
500000
1500000
169:169
135:135
21:21
DIHSGLIGPLLICQKpc3_NP.1
p−maj=1, pX=0.076, N=325
var.11.F5 1:169511755:T:C
peptide level
0e+00
2e+05
4e+05
169:169
135:135
21:21
DIHSGLIGPLLICQKpc3_NP.2
p−maj=1, pX=0.52, N=325
var.11.F5 1:169511755:T:C
peptide level
0e+00
4e+05
8e+05
169:169
135:135
21:21
DIHSGLIGPLLICQKpc3_NP.3
p−maj=1, pX=0.57, N=325
var.11.F5 1:169511755:T:C
peptide level
0e+00
2e+06
4e+06
169:169
135:135
21:21
DIHSGLIGPLLICQKpc3_NP.4
p−maj=1, pX=0.88, N=325
var.11.F5 1:169511755:T:C
peptide level
0e+00
2e+05
4e+05
6e+05
169:169
135:135
21:21
DIHSGLIGPLLICQKpc3_NP.5
p−maj=1, pX=0.49, N=325
var.11.F5 1:169511755:T:C
peptide level
0
40000
80000
120000
169:169
132:135
21:21
DPDNIAAWYLRpc2_NP.1
p−min=0.11, pX=0.42, N=322
var.11.F5 1:169511755:T:C
peptide level
0
20000
40000
60000
169:169
135:135
21:21
DPDNIAAWYLRpc2_NP.2
p−maj=1, pX=0.35, N=325
var.11.F5 1:169511755:T:C
peptide level
0
20000
40000
60000
169:169
135:135
21:21
DPDNIAAWYLRpc2_NP.3
p−maj=1, pX=0.86, N=325
var.11.F5 1:169511755:T:C
peptide level
0
500000
1500000
169:169
135:135
21:21
DPDNIAAWYLRpc2_NP.4
p−maj=1, pX=0.21, N=325
var.11.F5 1:169511755:T:C
peptide level
0
10000
30000
163:169
130:135
21:21
DPDNIAAWYLRpc2_NP.5
p−maj=1, pX=0.3, N=314

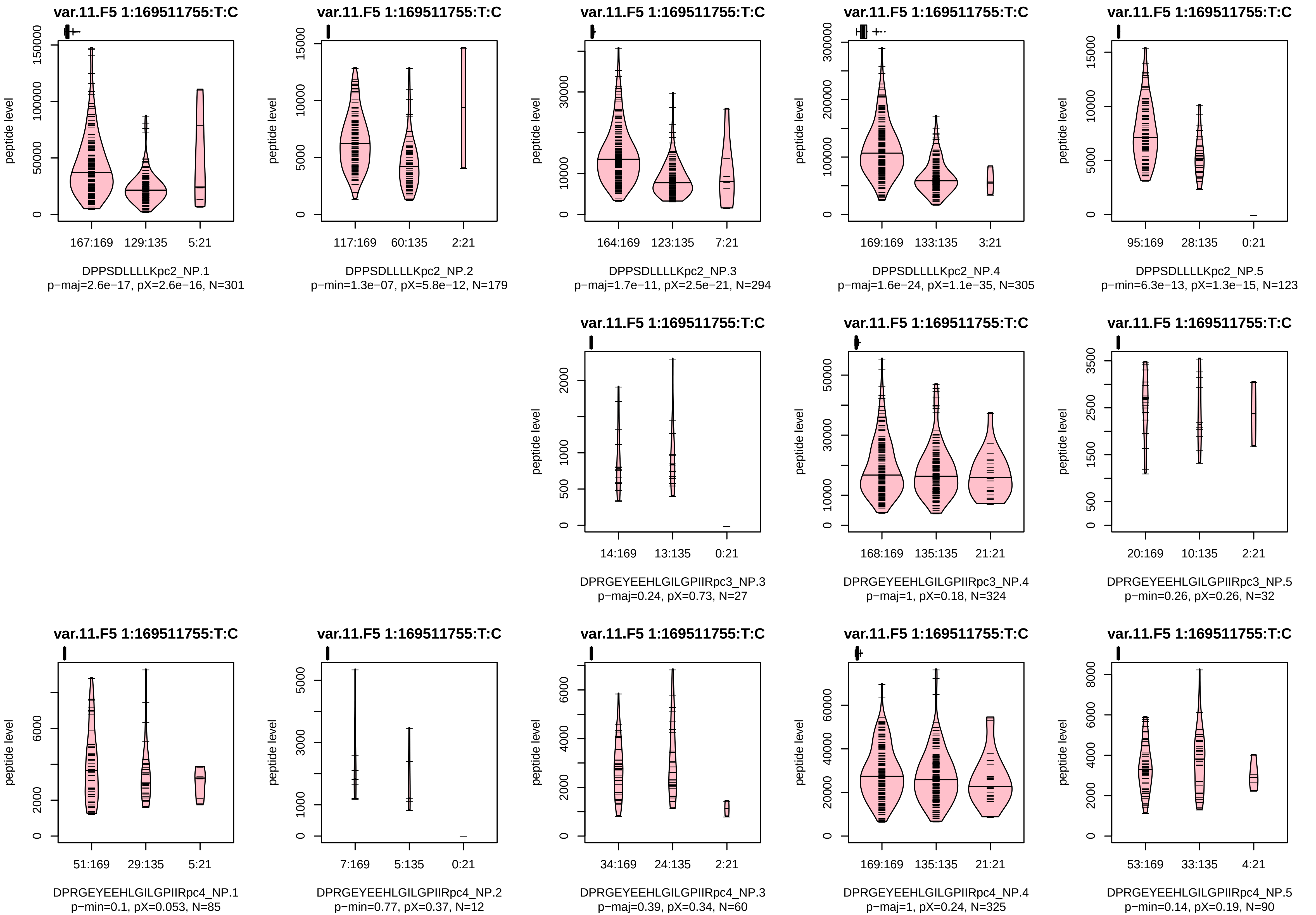

var.11.F5 1:169511755:T:C
peptide level
167:169
129:135
5:21
DPPSDLLLLKpc2_NP.1
p−maj=2.6e−17, pX=2.6e−16, N=301
var.11.F5 1:169511755:T:C
peptide level
117:169
60:135
2:21
DPPSDLLLLKpc2_NP.2
p−min=1.3e−07, pX=5.8e−12, N=179
var.11.F5 1:169511755:T:C
peptide level
164:169
123:135
7:21
DPPSDLLLLKpc2_NP.3
p−maj=1.7e−11, pX=2.5e−21, N=294
var.11.F5 1:169511755:T:C
peptide level
169:169
133:135
3:21
DPPSDLLLLKpc2_NP.4
p−maj=1.6e−24, pX=1.1e−35, N=305
var.11.F5 1:169511755:T:C
peptide level
95:169
28:135
0:21
DPPSDLLLLKpc2_NP.5
p−min=6.3e−13, pX=1.3e−15, N=123
var.11.F5 1:169511755:T:C
peptide level
14:169
13:135
0:21
DPRGEYEEHLGILGPIIRpc3_NP.3
p−maj=0.24, pX=0.73, N=27
var.11.F5 1:169511755:T:C
peptide level
168:169
135:135
21:21
DPRGEYEEHLGILGPIIRpc3_NP.4
p−maj=1, pX=0.18, N=324
var.11.F5 1:169511755:T:C
peptide level
20:169
10:135
2:21
DPRGEYEEHLGILGPIIRpc3_NP.5
p−min=0.26, pX=0.26, N=32
var.11.F5 1:169511755:T:C
peptide level
51:169
29:135
5:21
DPRGEYEEHLGILGPIIRpc4_NP.1
p−min=0.1, pX=0.053, N=85
var.11.F5 1:169511755:T:C
peptide level
7:169
5:135
0:21
DPRGEYEEHLGILGPIIRpc4_NP.2
p−min=0.77, pX=0.37, N=12
var.11.F5 1:169511755:T:C
peptide level
34:169
24:135
2:21
DPRGEYEEHLGILGPIIRpc4_NP.3
p−maj=0.39, pX=0.34, N=60
var.11.F5 1:169511755:T:C
peptide level
169:169
135:135
21:21
DPRGEYEEHLGILGPIIRpc4_NP.4
p−maj=1, pX=0.24, N=325
var.11.F5 1:169511755:T:C
peptide level
53:169
33:135
4:21
DPRGEYEEHLGILGPIIRpc4_NP.5
p−min=0.14, pX=0.19, N=90

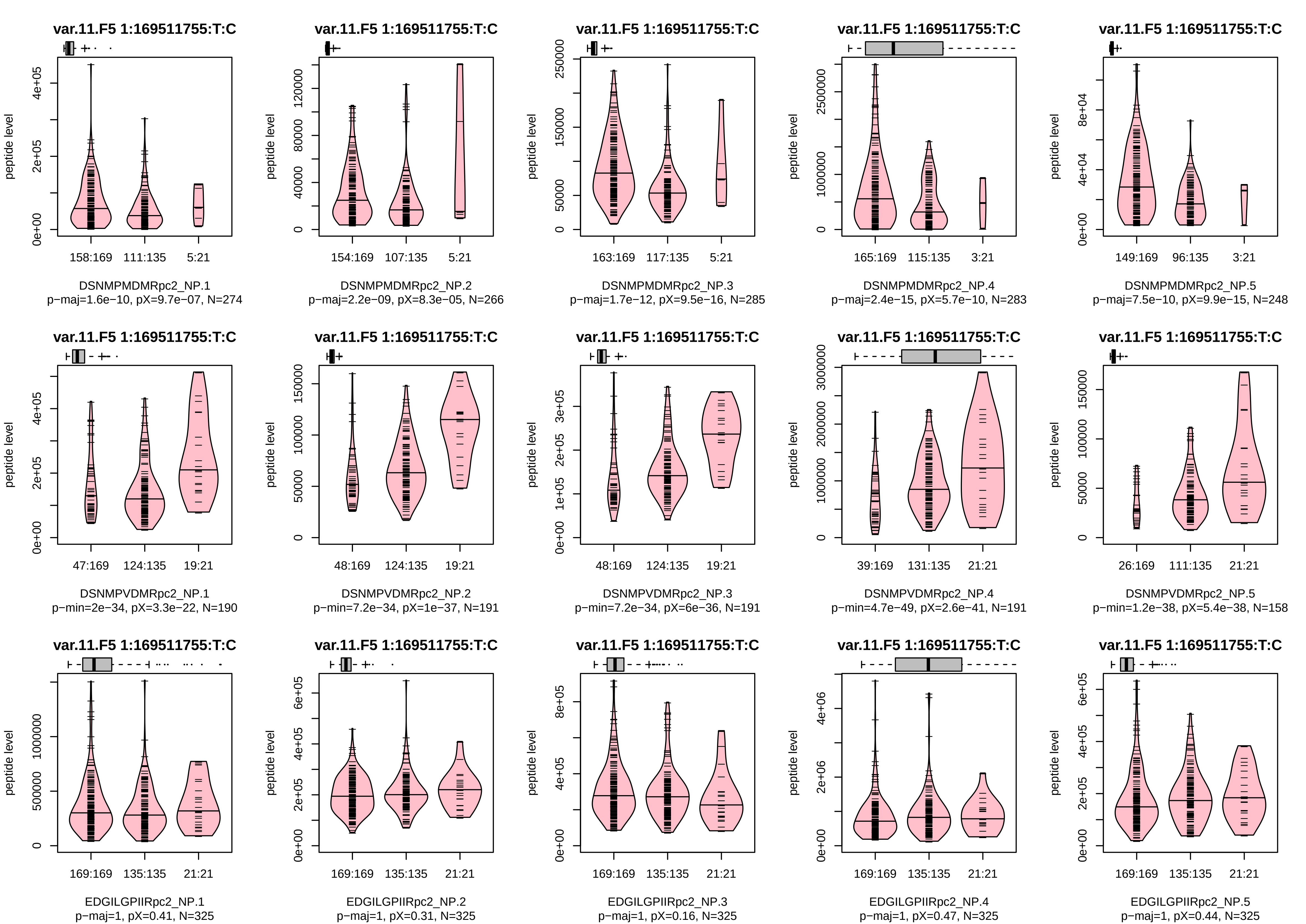
var.11.F5 1:169511755:T:C
peptide level
158:169
111:135
5:21
DSNMPMDMRpc2_NP.1
p−maj=1.6e−10, pX=9.7e−07, N=274
154:169
107:135
5:21
DSNMPMDMRpc2_NP.2
p−maj=2.2e−09, pX=8.3e−05, N=266
163:169
117:135
5:21
DSNMPMDMRpc2_NP.3
p−maj=1.7e−12, pX=9.5e−16, N=285
165:169
115:135
3:21
DSNMPMDMRpc2_NP.4
p−maj=2.4e−15, pX=5.7e−10, N=283
149:169
96:135
3:21
DSNMPMDMRpc2_NP.5
p−maj=7.5e−10, pX=9.9e−15, N=248
47:169
124:135
19:21
DSNMPVDMRpc2_NP.1
p−min=2e−34, pX=3.3e−22, N=190
48:169
124:135
19:21
DSNMPVDMRpc2_NP.2
p−min=7.2e−34, pX=1e−37, N=191
48:169
124:135
19:21
DSNMPVDMRpc2_NP.3
p−min=7.2e−34, pX=6e−36, N=191
39:169
131:135
21:21
DSNMPVDMRpc2_NP.4
p−min=4.7e−49, pX=2.6e−41, N=191
26:169
111:135
21:21
DSNMPVDMRpc2_NP.5
p−min=1.2e−38, pX=5.4e−38, N=158
169:169
135:135
21:21
EDGILGPIIRpc2_NP.1
p−maj=1, pX=0.41, N=325
EDGILGPIIRpc2_NP.2
p−maj=1, pX=0.31, N=325
EDGILGPIIRpc2_NP.3
p−maj=1, pX=0.16, N=325
EDGILGPIIRpc2_NP.4
p−maj=1, pX=0.47, N=325
EDGILGPIIRpc2_NP.5
p−maj=1, pX=0.44, N=325

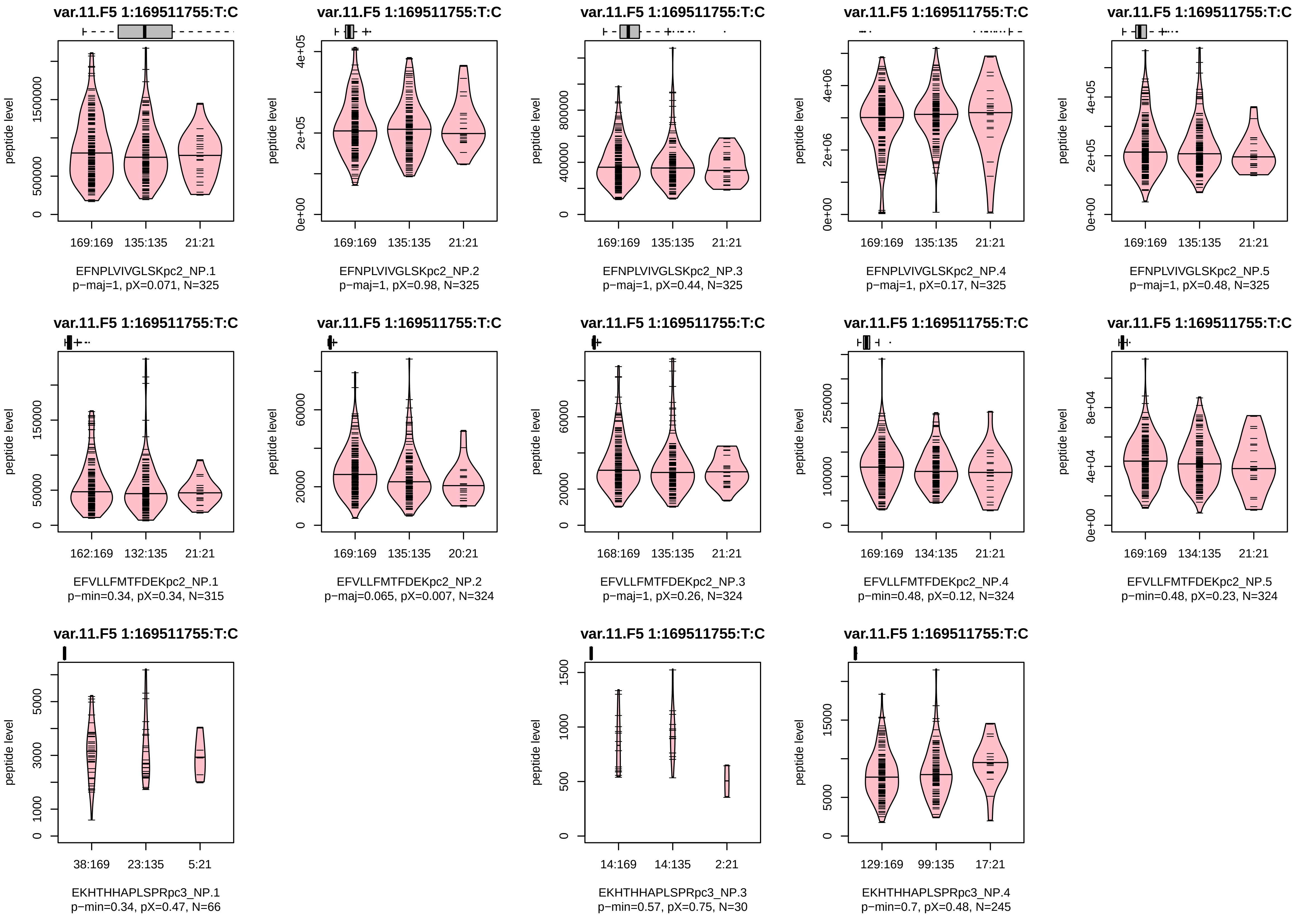
var.11.F5 1:169511755:T:C
peptide level
169:169
135:135
21:21
EFNPLVIVGLSKpc2_NP.1
p−maj=1, pX=0.071, N=325
var.11.F5 1:169511755:T:C
169:169
135:135
21:21
EFNPLVIVGLSKpc2_NP.2
p−maj=1, pX=0.98, N=325
var.11.F5 1:169511755:T:C
169:169
135:135
21:21
EFNPLVIVGLSKpc2_NP.3
p−maj=1, pX=0.44, N=325
var.11.F5 1:169511755:T:C
169:169
135:135
21:21
EFNPLVIVGLSKpc2_NP.4
p−maj=1, pX=0.17, N=325
var.11.F5 1:169511755:T:C
169:169
135:135
21:21
EFNPLVIVGLSKpc2_NP.5
p−maj=1, pX=0.48, N=325
var.11.F5 1:169511755:T:C
162:169
132:135
21:21
EFVLLFMTFDEKpc2_NP.1
p−min=0.34, pX=0.34, N=315
var.11.F5 1:169511755:T:C
169:169
135:135
20:21
EFVLLFMTFDEKpc2_NP.2
p−maj=0.065, pX=0.007, N=324
var.11.F5 1:169511755:T:C
168:169
135:135
21:21
EFVLLFMTFDEKpc2_NP.3
p−maj=1, pX=0.26, N=324
var.11.F5 1:169511755:T:C
169:169
134:135
21:21
EFVLLFMTFDEKpc2_NP.4
p−min=0.48, pX=0.12, N=324
var.11.F5 1:169511755:T:C
169:169
134:135
21:21
EFVLLFMTFDEKpc2_NP.5
p−min=0.48, pX=0.23, N=324
var.11.F5 1:169511755:T:C
38:169
23:135
5:21
EKHTHHAPLSPRpc3_NP.1
p−min=0.34, pX=0.47, N=66
var.11.F5 1:169511755:T:C
14:169
14:135
2:21
EKHTHHAPLSPRpc3_NP.3
p−min=0.57, pX=0.75, N=30
var.11.F5 1:169511755:T:C
129:169
99:135
17:21
EKHTHHAPLSPRpc3_NP.4
p−min=0.7, pX=0.48, N=245

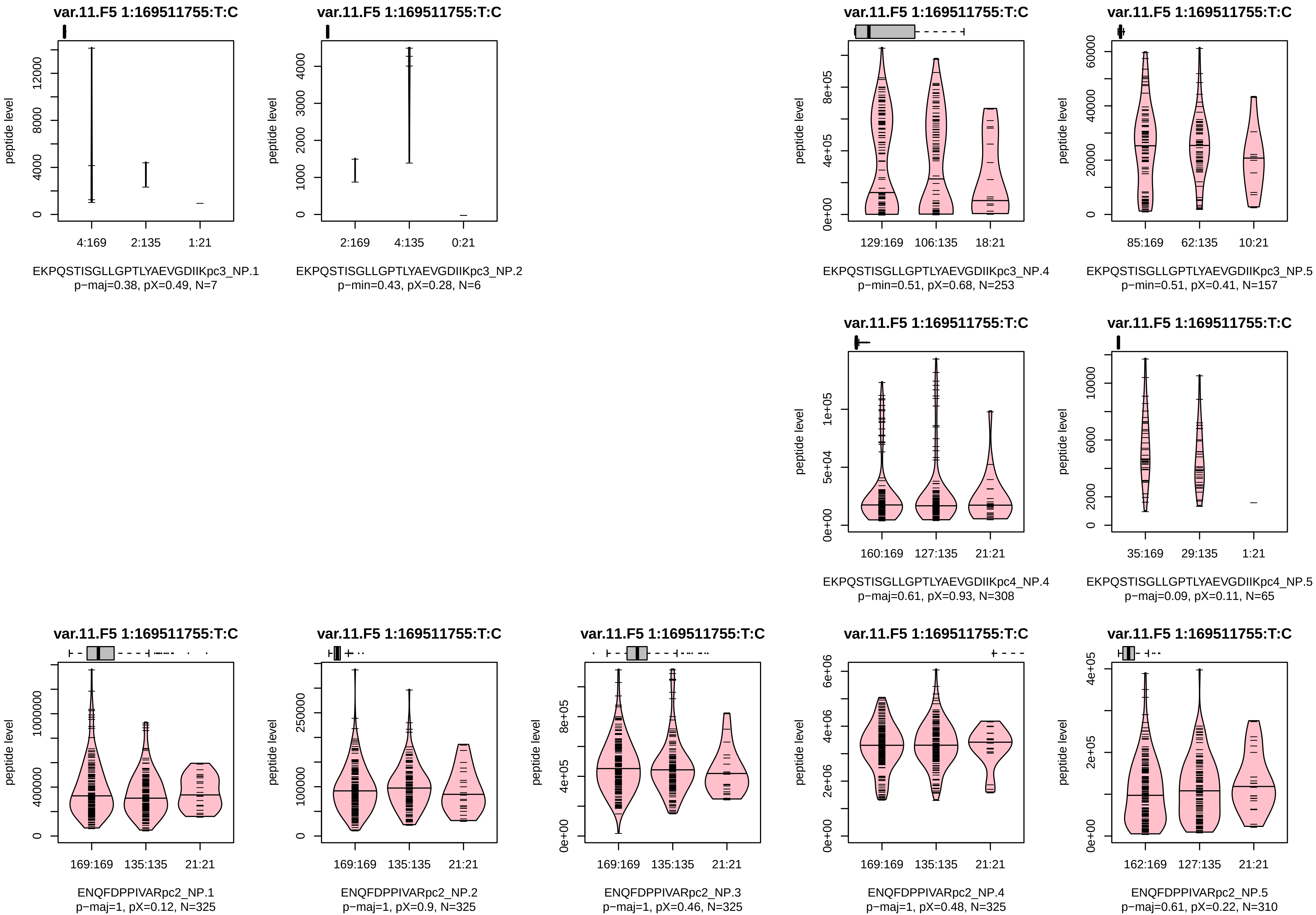
var.11.F5 1:169511755:T:C
peptide level
4:169
2:135
1:21
EKPQSTISGLLGPTLYAEVGDIIKpc3_NP.1
p−maj=0.38, pX=0.49, N=7
var.11.F5 1:169511755:T:C
peptide level
2:169
4:135
0:21
EKPQSTISGLLGPTLYAEVGDIIKpc3_NP.2
p−min=0.43, pX=0.28, N=6
var.11.F5 1:169511755:T:C
peptide level
129:169
106:135
18:21
EKPQSTISGLLGPTLYAEVGDIIKpc3_NP.4
p−min=0.51, pX=0.68, N=253
var.11.F5 1:169511755:T:C
peptide level
85:169
62:135
10:21
EKPQSTISGLLGPTLYAEVGDIIKpc3_NP.5
p−min=0.51, pX=0.41, N=157
var.11.F5 1:169511755:T:C
peptide level
160:169
127:135
21:21
EKPQSTISGLLGPTLYAEVGDIIKpc4_NP.4
p−maj=0.61, pX=0.93, N=308
var.11.F5 1:169511755:T:C
peptide level
35:169
29:135
1:21
EKPQSTISGLLGPTLYAEVGDIIKpc4_NP.5
p−maj=0.09, pX=0.11, N=65
var.11.F5 1:169511755:T:C
peptide level
169:169
135:135
21:21
ENQFDPPIVARpc2_NP.1
p−maj=1, pX=0.12, N=325
var.11.F5 1:169511755:T:C
peptide level
169:169
135:135
21:21
ENQFDPPIVARpc2_NP.2
p−maj=1, pX=0.9, N=325
var.11.F5 1:169511755:T:C
peptide level
169:169
135:135
21:21
ENQFDPPIVARpc2_NP.3
p−maj=1, pX=0.46, N=325
var.11.F5 1:169511755:T:C
peptide level
169:169
135:135
21:21
ENQFDPPIVARpc2_NP.4
p−maj=1, pX=0.48, N=325
var.11.F5 1:169511755:T:C
peptide level
162:169
127:135
21:21
ENQFDPPIVARpc2_NP.5
p−maj=0.61, pX=0.22, N=310

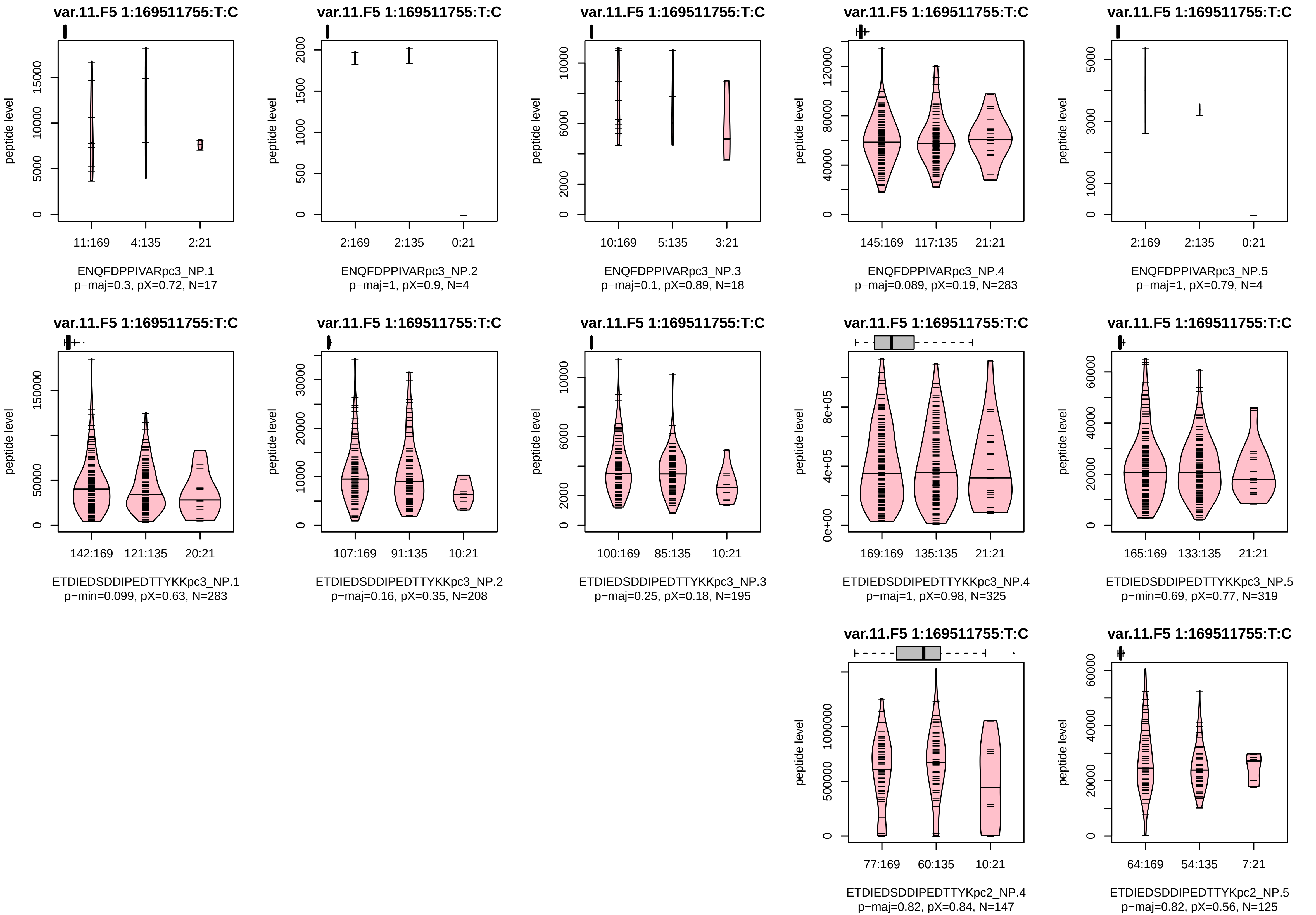
var.11.F5 1:169511755:T:C
peptide level
11:169
4:135
2:21
ENQFDPPIVARpc3_NP.1
p−maj=0.3, pX=0.72, N=17
var.11.F5 1:169511755:T:C
peptide level
2:169
2:135
0:21
ENQFDPPIVARpc3_NP.2
p−maj=1, pX=0.9, N=4
var.11.F5 1:169511755:T:C
peptide level
10:169
5:135
3:21
ENQFDPPIVARpc3_NP.3
p−maj=0.1, pX=0.89, N=18
var.11.F5 1:169511755:T:C
peptide level
145:169
117:135
21:21
ENQFDPPIVARpc3_NP.4
p−maj=0.089, pX=0.19, N=283
var.11.F5 1:169511755:T:C
peptide level
2:169
2:135
0:21
ENQFDPPIVARpc3_NP.5
p−maj=1, pX=0.79, N=4
var.11.F5 1:169511755:T:C
peptide level
142:169
121:135
20:21
ETDIEDSDDIPEDTTYKKpc3_NP.1
p−min=0.099, pX=0.63, N=283
var.11.F5 1:169511755:T:C
peptide level
107:169
91:135
10:21
ETDIEDSDDIPEDTTYKKpc3_NP.2
p−maj=0.16, pX=0.35, N=208
var.11.F5 1:169511755:T:C
peptide level
100:169
85:135
10:21
ETDIEDSDDIPEDTTYKKpc3_NP.3
p−maj=0.25, pX=0.18, N=195
var.11.F5 1:169511755:T:C
peptide level
169:169
135:135
21:21
ETDIEDSDDIPEDTTYKKpc3_NP.4
p−maj=1, pX=0.98, N=325
var.11.F5 1:169511755:T:C
peptide level
165:169
133:135
21:21
ETDIEDSDDIPEDTTYKKpc3_NP.5
p−min=0.69, pX=0.77, N=319
var.11.F5 1:169511755:T:C
peptide level
77:169
60:135
10:21
ETDIEDSDDIPEDTTYKpc2_NP.4
p−maj=0.82, pX=0.84, N=147
var.11.F5 1:169511755:T:C
peptide level
64:169
54:135
7:21
ETDIEDSDDIPEDTTYKpc2_NP.5
p−maj=0.82, pX=0.56, N=125

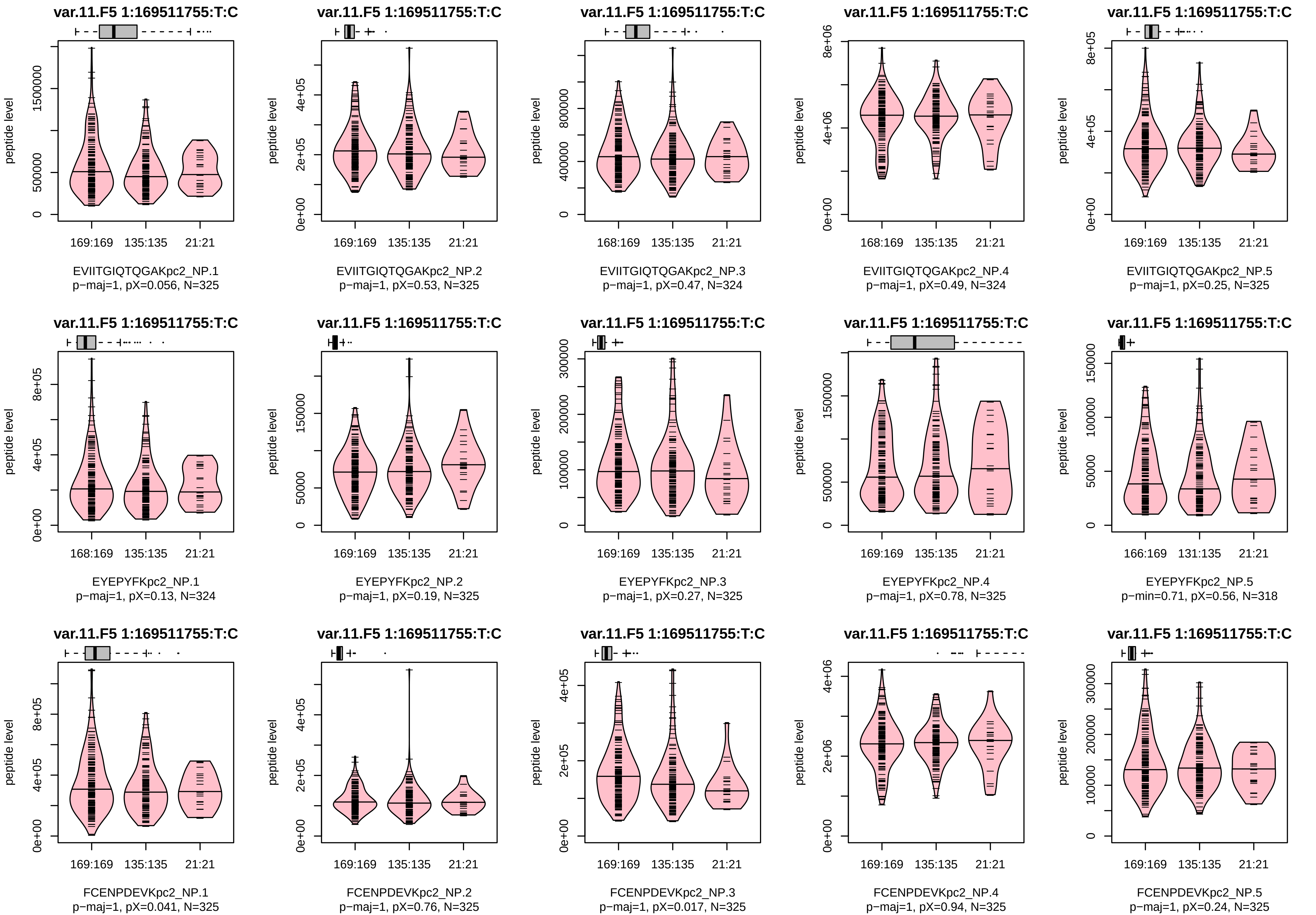
var.11.F5 1:169511755:T:C
peptide level
169:169
135:135
21:21
EVIITGIQTQGAKpc2_NP.1
p−maj=1, pX=0.056, N=325
var.11.F5 1:169511755:T:C
peptide level
169:169
135:135
21:21
EVIITGIQTQGAKpc2_NP.2
p−maj=1, pX=0.53, N=325
var.11.F5 1:169511755:T:C
peptide level
168:169
135:135
21:21
EVIITGIQTQGAKpc2_NP.3
p−maj=1, pX=0.47, N=324
var.11.F5 1:169511755:T:C
peptide level
168:169
135:135
21:21
EVIITGIQTQGAKpc2_NP.4
p−maj=1, pX=0.49, N=324
var.11.F5 1:169511755:T:C
peptide level
169:169
135:135
21:21
EVIITGIQTQGAKpc2_NP.5
p−maj=1, pX=0.25, N=325
var.11.F5 1:169511755:T:C
peptide level
168:169
135:135
21:21
EYEPYFKpc2_NP.1
p−maj=1, pX=0.13, N=324
var.11.F5 1:169511755:T:C
peptide level
169:169
135:135
21:21
EYEPYFKpc2_NP.2
p−maj=1, pX=0.19, N=325
var.11.F5 1:169511755:T:C
peptide level
169:169
135:135
21:21
EYEPYFKpc2_NP.3
p−maj=1, pX=0.27, N=325
var.11.F5 1:169511755:T:C
peptide level
169:169
135:135
21:21
EYEPYFKpc2_NP.4
p−maj=1, pX=0.78, N=325
var.11.F5 1:169511755:T:C
peptide level
166:169
131:135
21:21
EYEPYFKpc2_NP.5
p−min=0.71, pX=0.56, N=318
var.11.F5 1:169511755:T:C
peptide level
169:169
135:135
21:21
FCENPDEVKpc2_NP.1
p−maj=1, pX=0.041, N=325
var.11.F5 1:169511755:T:C
peptide level
169:169
135:135
21:21
FCENPDEVKpc2_NP.2
p−maj=1, pX=0.76, N=325
var.11.F5 1:169511755:T:C
peptide level
169:169
135:135
21:21
FCENPDEVKpc2_NP.3
p−maj=1, pX=0.017, N=325
var.11.F5 1:169511755:T:C
peptide level
169:169
135:135
21:21
FCENPDEVKpc2_NP.4
p−maj=1, pX=0.94, N=325
var.11.F5 1:169511755:T:C
peptide level
169:169
135:135
21:21
FCENPDEVKpc2_NP.5
p−maj=1, pX=0.24, N=325

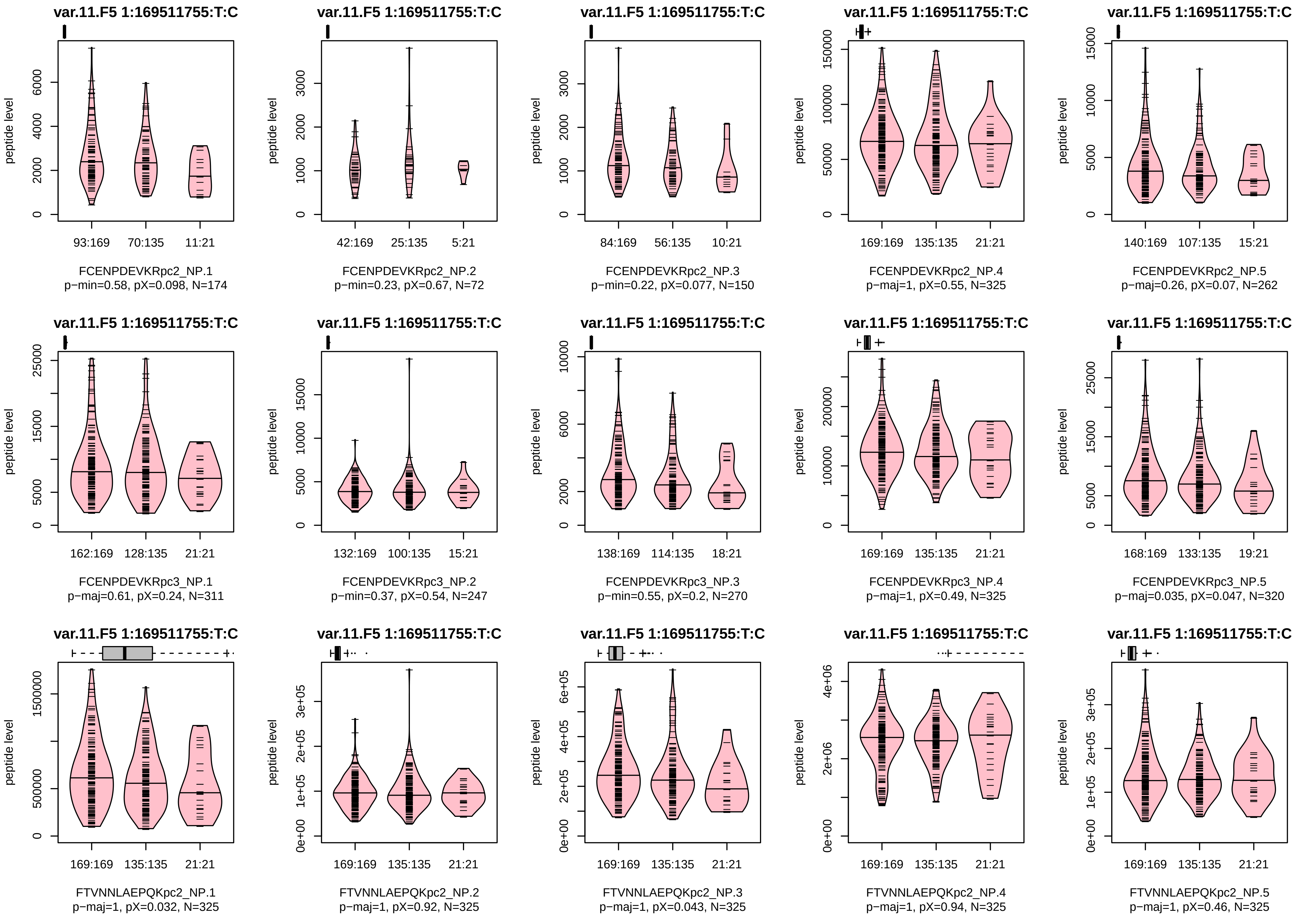
var.11.F5 1:169511755:T:C
peptide level
0
2000
4000
6000
93:169
70:135
11:21
FCENPDEVKRpc2_NP.1
p−min=0.58, pX=0.098, N=174
var.11.F5 1:169511755:T:C
peptide level
0
1000
2000
3000
42:169
25:135
5:21
FCENPDEVKRpc2_NP.2
p−min=0.23, pX=0.67, N=72
var.11.F5 1:169511755:T:C
peptide level
0
1000
2000
3000
84:169
56:135
10:21
FCENPDEVKRpc2_NP.3
p−min=0.22, pX=0.077, N=150
var.11.F5 1:169511755:T:C
peptide level
0
50000
100000
150000
169:169
135:135
21:21
FCENPDEVKRpc2_NP.4
p−maj=1, pX=0.55, N=325
var.11.F5 1:169511755:T:C
peptide level
0
5000
10000
15000
140:169
107:135
15:21
FCENPDEVKRpc2_NP.5
p−maj=0.26, pX=0.07, N=262
var.11.F5 1:169511755:T:C
peptide level
0
5000
15000
25000
162:169
128:135
21:21
FCENPDEVKRpc3_NP.1
p−maj=0.61, pX=0.24, N=311
var.11.F5 1:169511755:T:C
peptide level
0
5000
10000
15000
132:169
100:135
15:21
FCENPDEVKRpc3_NP.2
p−min=0.37, pX=0.54, N=247
var.11.F5 1:169511755:T:C
peptide level
0
2000
6000
10000
138:169
114:135
18:21
FCENPDEVKRpc3_NP.3
p−min=0.55, pX=0.2, N=270
var.11.F5 1:169511755:T:C
peptide level
0
100000
200000
169:169
135:135
21:21
FCENPDEVKRpc3_NP.4
p−maj=1, pX=0.49, N=325
var.11.F5 1:169511755:T:C
peptide level
0
5000
15000
25000
168:169
133:135
19:21
FCENPDEVKRpc3_NP.5
p−maj=0.035, pX=0.047, N=320
var.11.F5 1:169511755:T:C
peptide level
0
500000
1500000
169:169
135:135
21:21
FTVNNLAEPQKpc2_NP.1
p−maj=1, pX=0.032, N=325
var.11.F5 1:169511755:T:C
peptide level
0e+00
1e+05
2e+05
3e+05
169:169
135:135
21:21
FTVNNLAEPQKpc2_NP.2
p−maj=1, pX=0.92, N=325
var.11.F5 1:169511755:T:C
peptide level
0e+00
2e+05
4e+05
6e+05
169:169
135:135
21:21
FTVNNLAEPQKpc2_NP.3
p−maj=1, pX=0.043, N=325
var.11.F5 1:169511755:T:C
peptide level
0e+00
2e+06
4e+06
169:169
135:135
21:21
FTVNNLAEPQKpc2_NP.4
p−maj=1, pX=0.94, N=325
var.11.F5 1:169511755:T:C
peptide level
0e+00
1e+05
2e+05
3e+05
169:169
135:135
21:21
FTVNNLAEPQKpc2_NP.5
p−maj=1, pX=0.46, N=325

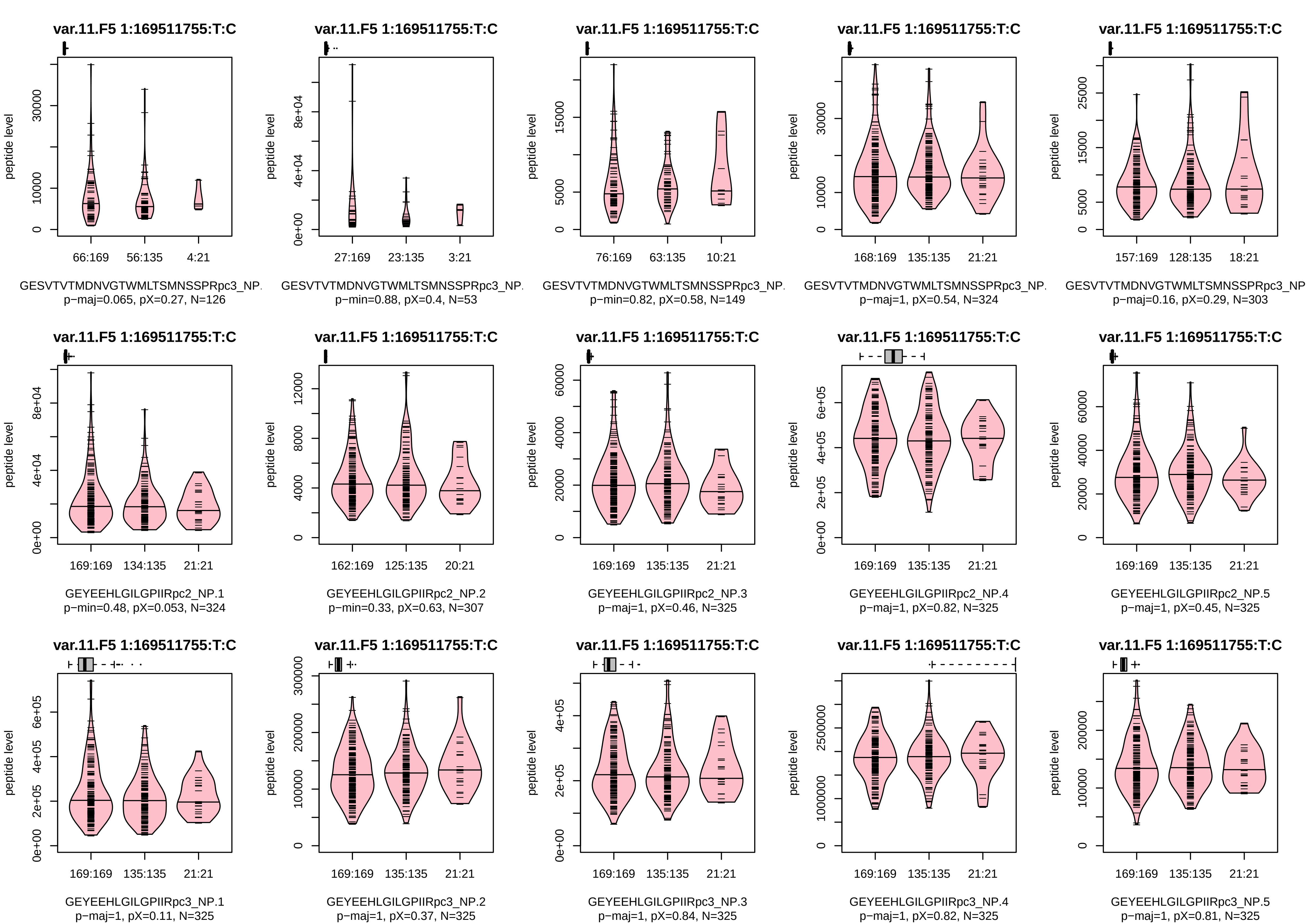
var.11.F5 1:169511755:T:C
peptide level
66:169 56:135 4:21
GESVTVTMDNVGTWMLTSMNSSPRpc3_NP.
p−maj=0.065, pX=0.27, N=126
var.11.F5 1:169511755:T:C
peptide level
27:169 23:135 3:21
GESVTVTMDNVGTWMLTSMNSSPRpc3_NP.
p−min=0.88, pX=0.4, N=53
var.11.F5 1:169511755:T:C
peptide level
76:169 63:135 10:21
GESVTVTMDNVGTWMLTSMNSSPRpc3_NP.
p−min=0.82, pX=0.58, N=149
var.11.F5 1:169511755:T:C
peptide level
168:169 135:135 21:21
GESVTVTMDNVGTWMLTSMNSSPRpc3_NP.
p−maj=1, pX=0.54, N=324
var.11.F5 1:169511755:T:C
peptide level
157:169 128:135 18:21
GESVTVTMDNVGTWMLTSMNSSPRpc3_NP
p−maj=0.16, pX=0.29, N=303
var.11.F5 1:169511755:T:C
peptide level
169:169 134:135 21:21
GEYEEHLGILGPIIRpc2_NP.1
p−min=0.48, pX=0.053, N=324
var.11.F5 1:169511755:T:C
peptide level
162:169 125:135 20:21
GEYEEHLGILGPIIRpc2_NP.2
p−min=0.33, pX=0.63, N=307
var.11.F5 1:169511755:T:C
peptide level
169:169 135:135 21:21
GEYEEHLGILGPIIRpc2_NP.3
p−maj=1, pX=0.46, N=325
var.11.F5 1:169511755:T:C
peptide level
169:169 135:135 21:21
GEYEEHLGILGPIIRpc2_NP.4
p−maj=1, pX=0.82, N=325
var.11.F5 1:169511755:T:C
peptide level
169:169 135:135 21:21
GEYEEHLGILGPIIRpc2_NP.5
p−maj=1, pX=0.45, N=325
var.11.F5 1:169511755:T:C
peptide level
169:169 135:135 21:21
GEYEEHLGILGPIIRpc3_NP.1
p−maj=1, pX=0.11, N=325
var.11.F5 1:169511755:T:C
peptide level
169:169 135:135 21:21
GEYEEHLGILGPIIRpc3_NP.2
p−maj=1, pX=0.37, N=325
var.11.F5 1:169511755:T:C
peptide level
169:169 135:135 21:21
GEYEEHLGILGPIIRpc3_NP.3
p−maj=1, pX=0.84, N=325
var.11.F5 1:169511755:T:C
peptide level
169:169 135:135 21:21
GEYEEHLGILGPIIRpc3_NP.4
p−maj=1, pX=0.82, N=325
var.11.F5 1:169511755:T:C
peptide level
169:169 135:135 21:21
GEYEEHLGILGPIIRpc3_NP.5
p−maj=1, pX=0.81, N=325

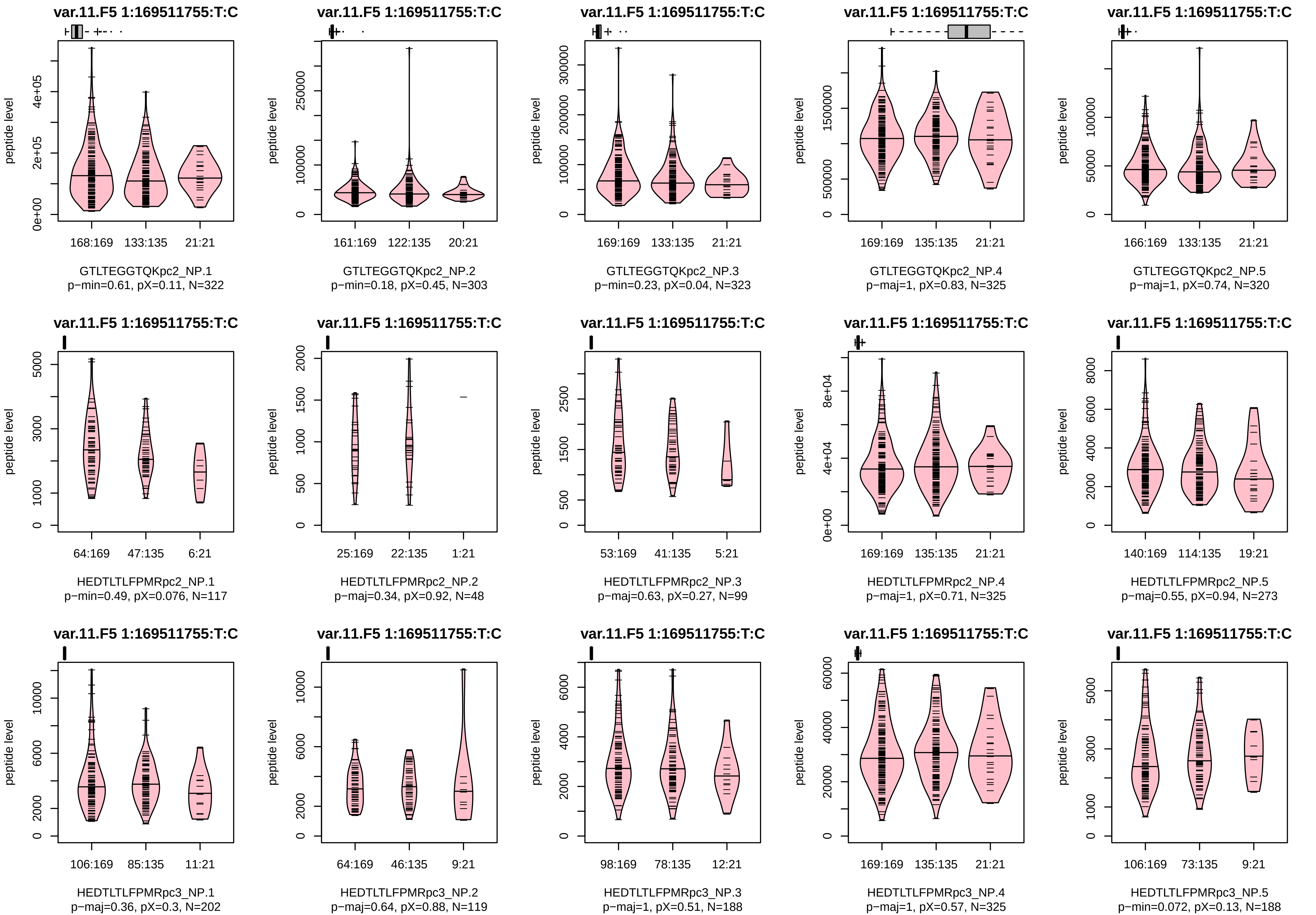
var.11.F5 1:169511755:T:C
peptide level
168:169
133:135
21:21
GTLTEGGTQKpc2_NP.1
p−min=0.61, pX=0.11, N=322
var.11.F5 1:169511755:T:C
peptide level
161:169
122:135
20:21
GTLTEGGTQKpc2_NP.2
p−min=0.18, pX=0.45, N=303
var.11.F5 1:169511755:T:C
peptide level
169:169
133:135
21:21
GTLTEGGTQKpc2_NP.3
p−min=0.23, pX=0.04, N=323
var.11.F5 1:169511755:T:C
peptide level
169:169
135:135
21:21
GTLTEGGTQKpc2_NP.4
p−maj=1, pX=0.83, N=325
var.11.F5 1:169511755:T:C
peptide level
166:169
133:135
21:21
GTLTEGGTQKpc2_NP.5
p−maj=1, pX=0.74, N=320
var.11.F5 1:169511755:T:C
peptide level
64:169
47:135
6:21
HEDTLTLFPMRpc2_NP.1
p−min=0.49, pX=0.076, N=117
var.11.F5 1:169511755:T:C
peptide level
25:169
22:135
1:21
HEDTLTLFPMRpc2_NP.2
p−maj=0.34, pX=0.92, N=48
var.11.F5 1:169511755:T:C
peptide level
53:169
41:135
5:21
HEDTLTLFPMRpc2_NP.3
p−maj=0.63, pX=0.27, N=99
var.11.F5 1:169511755:T:C
peptide level
169:169
135:135
21:21
HEDTLTLFPMRpc2_NP.4
p−maj=1, pX=0.71, N=325
var.11.F5 1:169511755:T:C
peptide level
140:169
114:135
19:21
HEDTLTLFPMRpc2_NP.5
p−maj=0.55, pX=0.94, N=273
var.11.F5 1:169511755:T:C
peptide level
106:169
85:135
11:21
HEDTLTLFPMRpc3_NP.1
p−maj=0.36, pX=0.3, N=202
var.11.F5 1:169511755:T:C
peptide level
64:169
46:135
9:21
HEDTLTLFPMRpc3_NP.2
p−maj=0.64, pX=0.88, N=119
var.11.F5 1:169511755:T:C
peptide level
98:169
78:135
12:21
HEDTLTLFPMRpc3_NP.3
p−maj=1, pX=0.51, N=188
var.11.F5 1:169511755:T:C
peptide level
169:169
135:135
21:21
HEDTLTLFPMRpc3_NP.4
p−maj=1, pX=0.57, N=325
var.11.F5 1:169511755:T:C
peptide level
106:169
73:135
9:21
HEDTLTLFPMRpc3_NP.5
p−min=0.072, pX=0.13, N=188

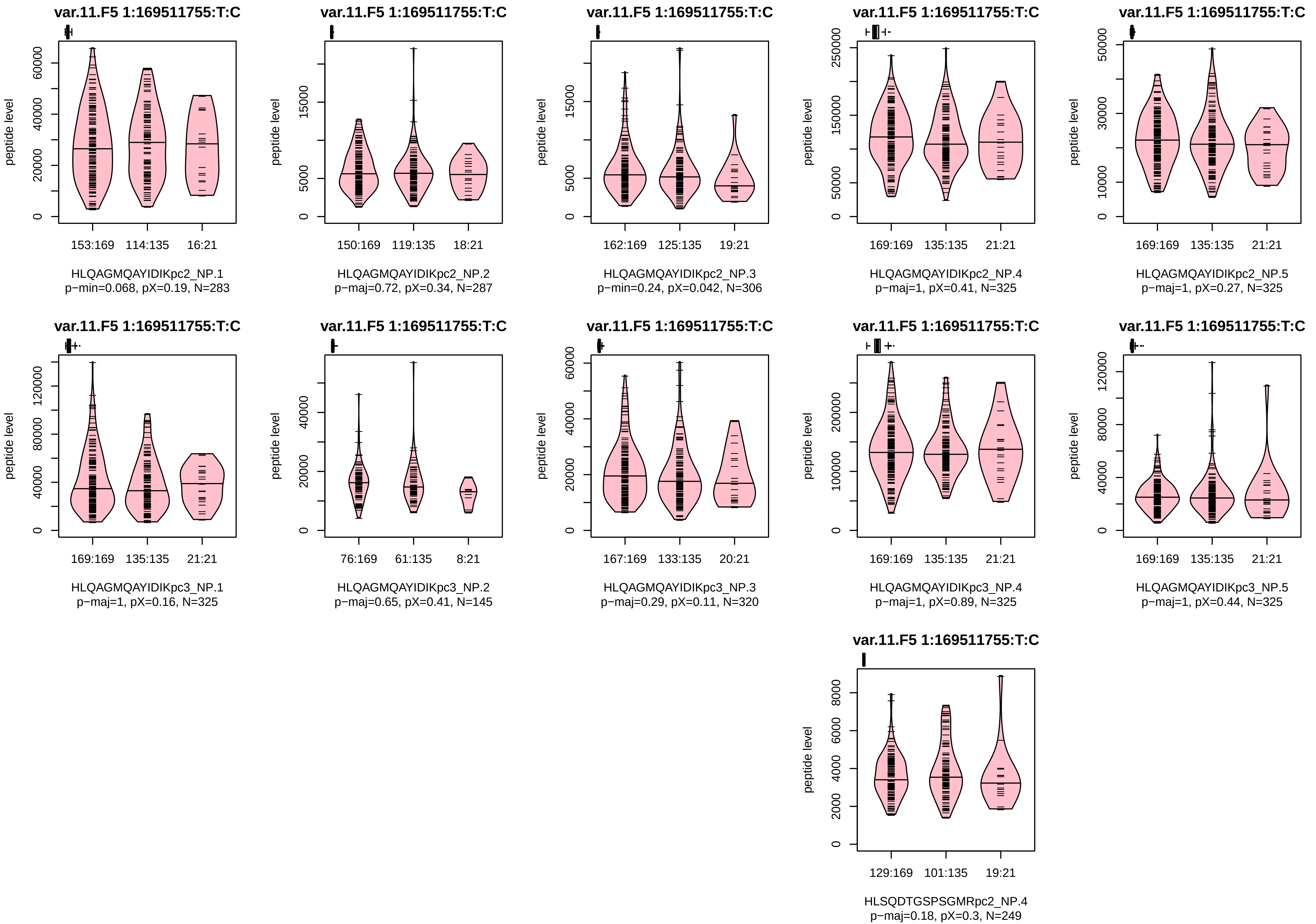
var.11.F5 1:169511755:T:C
peptide level
153:169
114:135
16:21
HLQAGMQAYIDIKpc2_NP.1
p−min=0.068, pX=0.19, N=283
var.11.F5 1:169511755:T:C
150:169
119:135
18:21
HLQAGMQAYIDIKpc2_NP.2
p−maj=0.72, pX=0.34, N=287
var.11.F5 1:169511755:T:C
162:169
125:135
19:21
HLQAGMQAYIDIKpc2_NP.3
p−min=0.24, pX=0.042, N=306
var.11.F5 1:169511755:T:C
169:169
135:135
21:21
HLQAGMQAYIDIKpc2_NP.4
p−maj=1, pX=0.41, N=325
var.11.F5 1:169511755:T:C
169:169
135:135
21:21
HLQAGMQAYIDIKpc2_NP.5
p−maj=1, pX=0.27, N=325
var.11.F5 1:169511755:T:C
169:169
135:135
21:21
HLQAGMQAYIDIKpc3_NP.1
p−maj=1, pX=0.16, N=325
var.11.F5 1:169511755:T:C
76:169
61:135
8:21
HLQAGMQAYIDIKpc3_NP.2
p−maj=0.65, pX=0.41, N=145
var.11.F5 1:169511755:T:C
167:169
133:135
20:21
HLQAGMQAYIDIKpc3_NP.3
p−maj=0.29, pX=0.11, N=320
var.11.F5 1:169511755:T:C
169:169
135:135
21:21
HLQAGMQAYIDIKpc3_NP.4
p−maj=1, pX=0.89, N=325
var.11.F5 1:169511755:T:C
169:169
135:135
21:21
HLQAGMQAYIDIKpc3_NP.5
p−maj=1, pX=0.44, N=325
var.11.F5 1:169511755:T:C
129:169
101:135
19:21
HLSQDTGSPSGMRpc2_NP.4
p−maj=0.18, pX=0.3, N=249

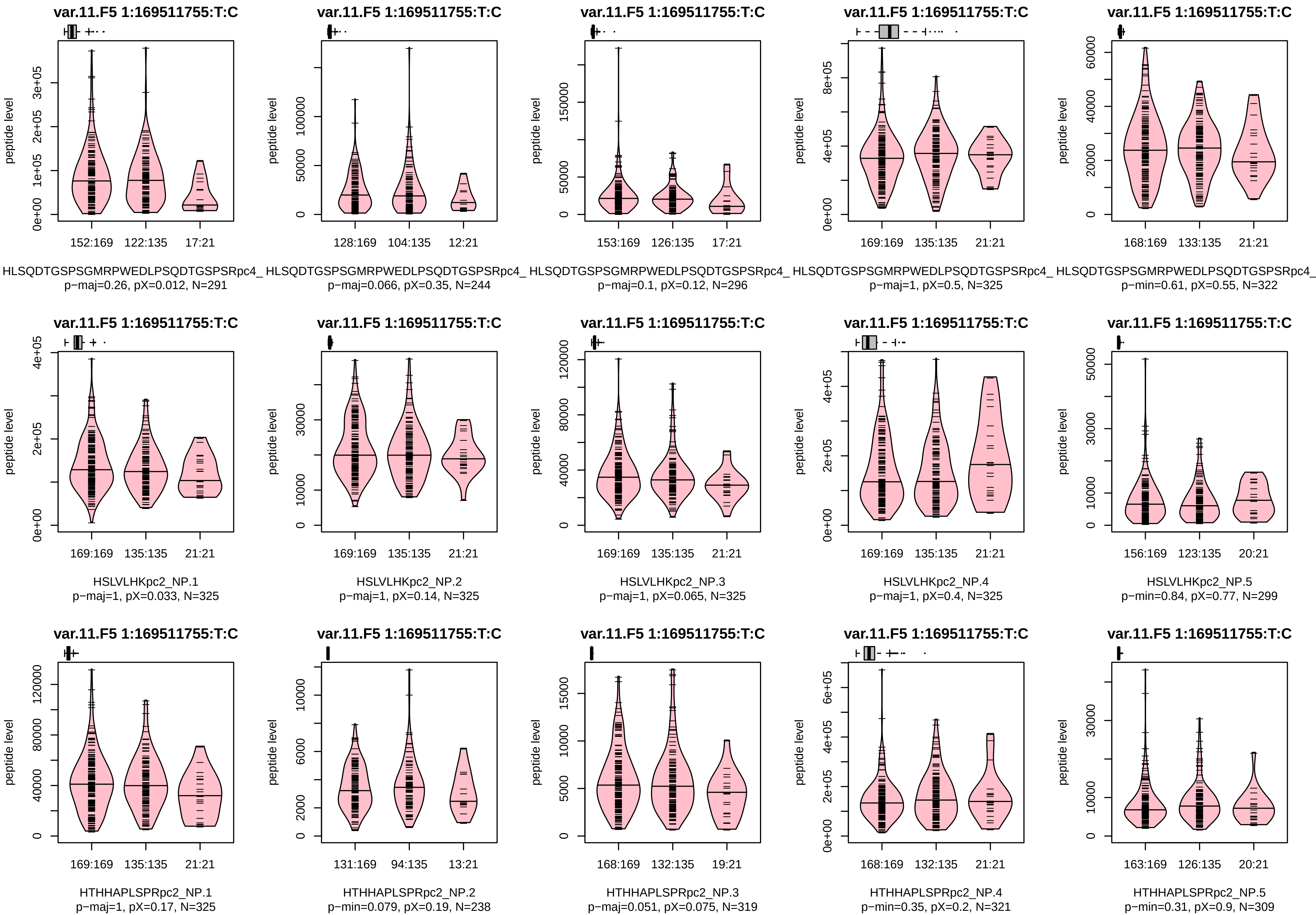
var.11.F5 1:169511755:T:C
peptide level
152:169 122:135 17:21
HLSQDTGSPSGMRPWEDLPSQDTGSPSRpc4_
p−maj=0.26, pX=0.012, N=291
var.11.F5 1:169511755:T:C
peptide level
128:169 104:135 12:21
HLSQDTGSPSGMRPWEDLPSQDTGSPSRpc4_
p−maj=0.066, pX=0.35, N=244
var.11.F5 1:169511755:T:C
peptide level
153:169 126:135 17:21
HLSQDTGSPSGMRPWEDLPSQDTGSPSRpc4_
p−maj=0.1, pX=0.12, N=296
var.11.F5 1:169511755:T:C
peptide level
169:169 135:135 21:21
HLSQDTGSPSGMRPWEDLPSQDTGSPSRpc4_
p−maj=1, pX=0.5, N=325
var.11.F5 1:169511755:T:C
peptide level
168:169 133:135 21:21
HLSQDTGSPSGMRPWEDLPSQDTGSPSRpc4_
p−min=0.61, pX=0.55, N=322
var.11.F5 1:169511755:T:C
peptide level
169:169 135:135 21:21
HSLVLHKpc2_NP.1
p−maj=1, pX=0.033, N=325
var.11.F5 1:169511755:T:C
peptide level
169:169 135:135 21:21
HSLVLHKpc2_NP.2
p−maj=1, pX=0.14, N=325
var.11.F5 1:169511755:T:C
peptide level
169:169 135:135 21:21
HSLVLHKpc2_NP.3
p−maj=1, pX=0.065, N=325
var.11.F5 1:169511755:T:C
peptide level
169:169 135:135 21:21
HSLVLHKpc2_NP.4
p−maj=1, pX=0.4, N=325
var.11.F5 1:169511755:T:C
peptide level
156:169 123:135 20:21
HSLVLHKpc2_NP.5
p−min=0.84, pX=0.77, N=299
var.11.F5 1:169511755:T:C
peptide level
169:169 135:135 21:21
HTHHAPLSPRpc2_NP.1
p−maj=1, pX=0.17, N=325
var.11.F5 1:169511755:T:C
peptide level
131:169 94:135 13:21
HTHHAPLSPRpc2_NP.2
p−min=0.079, pX=0.19, N=238
var.11.F5 1:169511755:T:C
peptide level
168:169 132:135 19:21
HTHHAPLSPRpc2_NP.3
p−maj=0.051, pX=0.075, N=319
var.11.F5 1:169511755:T:C
peptide level
168:169 132:135 21:21
HTHHAPLSPRpc2_NP.4
p−min=0.35, pX=0.2, N=321
var.11.F5 1:169511755:T:C
peptide level
163:169 126:135 20:21
HTHHAPLSPRpc2_NP.5
p−min=0.31, pX=0.9, N=309

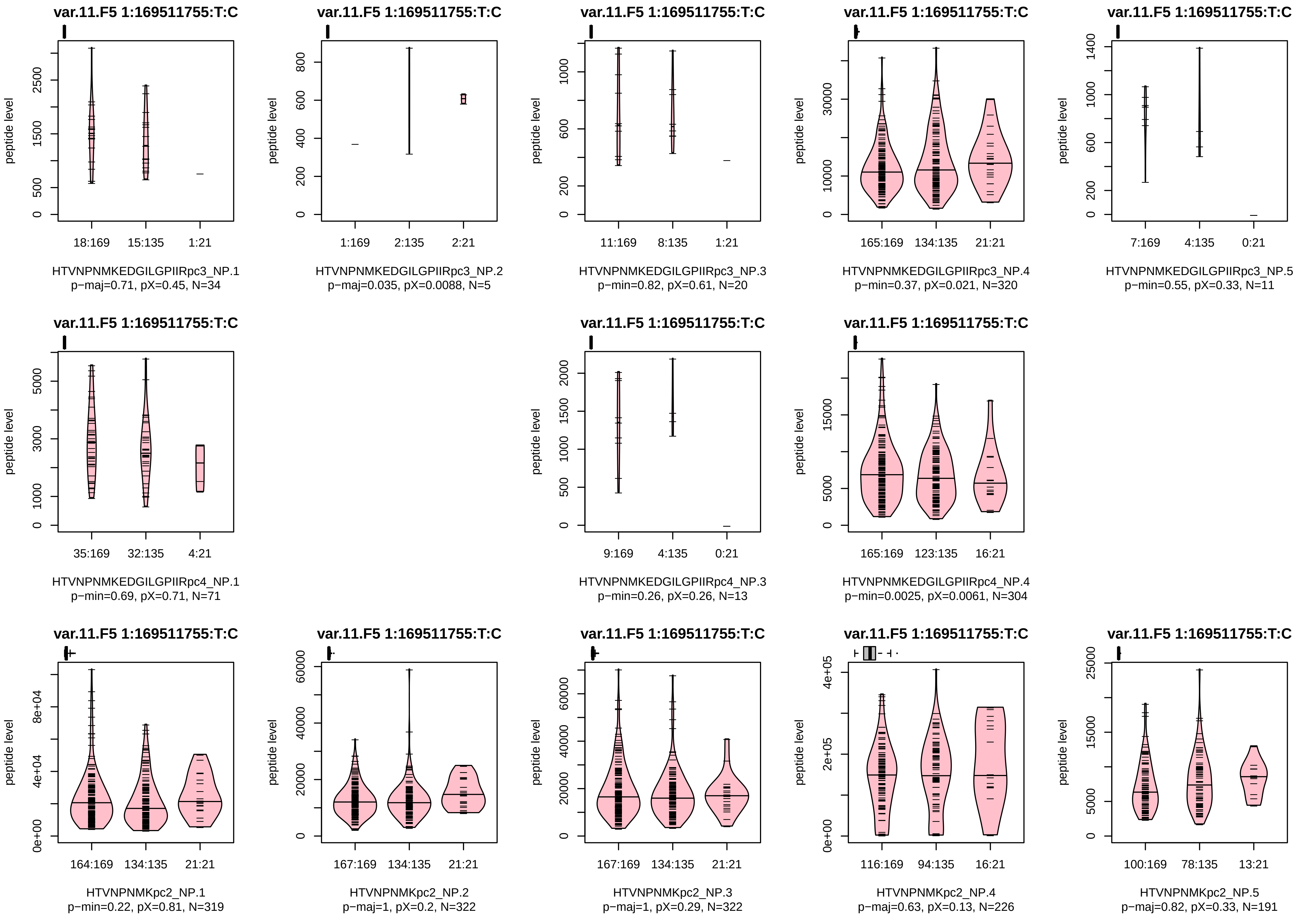
var.11.F5 1:169511755:T:C
peptide level
18:169
15:135
1:21
HTVNPNMKEDGILGPIIRpc3_NP.1
p−maj=0.71, pX=0.45, N=34
var.11.F5 1:169511755:T:C
peptide level
1:169
2:135
2:21
HTVNPNMKEDGILGPIIRpc3_NP.2
p−maj=0.035, pX=0.0088, N=5
var.11.F5 1:169511755:T:C
peptide level
11:169
8:135
1:21
HTVNPNMKEDGILGPIIRpc3_NP.3
p−min=0.82, pX=0.61, N=20
var.11.F5 1:169511755:T:C
peptide level
165:169
134:135
21:21
HTVNPNMKEDGILGPIIRpc3_NP.4
p−min=0.37, pX=0.021, N=320
var.11.F5 1:169511755:T:C
peptide level
7:169
4:135
0:21
HTVNPNMKEDGILGPIIRpc3_NP.5
p−min=0.55, pX=0.33, N=11
var.11.F5 1:169511755:T:C
peptide level
35:169
32:135
4:21
HTVNPNMKEDGILGPIIRpc4_NP.1
p−min=0.69, pX=0.71, N=71
var.11.F5 1:169511755:T:C
peptide level
9:169
4:135
0:21
HTVNPNMKEDGILGPIIRpc4_NP.3
p−min=0.26, pX=0.26, N=13
var.11.F5 1:169511755:T:C
peptide level
165:169
123:135
16:21
HTVNPNMKEDGILGPIIRpc4_NP.4
p−min=0.0025, pX=0.0061, N=304
var.11.F5 1:169511755:T:C
peptide level
164:169
134:135
21:21
HTVNPNMKpc2_NP.1
p−min=0.22, pX=0.81, N=319
var.11.F5 1:169511755:T:C
peptide level
167:169
134:135
21:21
HTVNPNMKpc2_NP.2
p−maj=1, pX=0.2, N=322
var.11.F5 1:169511755:T:C
peptide level
167:169
134:135
21:21
HTVNPNMKpc2_NP.3
p−maj=1, pX=0.29, N=322
var.11.F5 1:169511755:T:C
peptide level
116:169
94:135
16:21
HTVNPNMKpc2_NP.4
p−maj=0.63, pX=0.13, N=226
var.11.F5 1:169511755:T:C
peptide level
100:169
78:135
13:21
HTVNPNMKpc2_NP.5
p−maj=0.82, pX=0.33, N=191

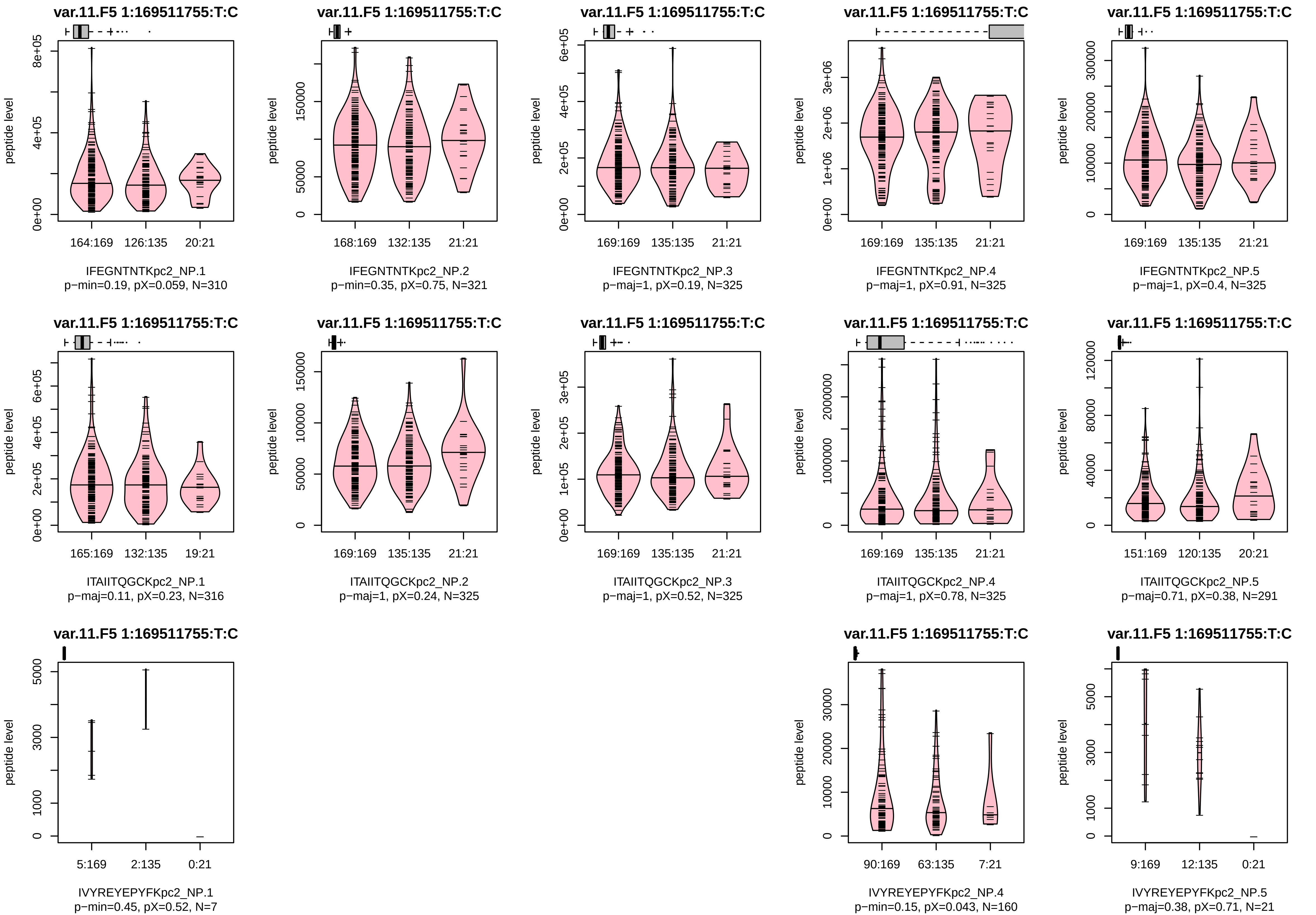

var.11.F5 1:169511755:T:C
peptide level
164:169
126:135
20:21
IFEGNTNTKpc2_NP.1
p−min=0.19, pX=0.059, N=310
var.11.F5 1:169511755:T:C
peptide level
168:169
132:135
21:21
IFEGNTNTKpc2_NP.2
p−min=0.35, pX=0.75, N=321
var.11.F5 1:169511755:T:C
peptide level
169:169
135:135
21:21
IFEGNTNTKpc2_NP.3
p−maj=1, pX=0.19, N=325
var.11.F5 1:169511755:T:C
peptide level
169:169
135:135
21:21
IFEGNTNTKpc2_NP.4
p−maj=1, pX=0.91, N=325
var.11.F5 1:169511755:T:C
peptide level
169:169
135:135
21:21
IFEGNTNTKpc2_NP.5
p−maj=1, pX=0.4, N=325
var.11.F5 1:169511755:T:C
peptide level
165:169
132:135
19:21
ITAIITQGCKpc2_NP.1
p−maj=0.11, pX=0.23, N=316
var.11.F5 1:169511755:T:C
peptide level
169:169
135:135
21:21
ITAIITQGCKpc2_NP.2
p−maj=1, pX=0.24, N=325
var.11.F5 1:169511755:T:C
peptide level
169:169
135:135
21:21
ITAIITQGCKpc2_NP.3
p−maj=1, pX=0.52, N=325
var.11.F5 1:169511755:T:C
peptide level
169:169
135:135
21:21
ITAIITQGCKpc2_NP.4
p−maj=1, pX=0.78, N=325
var.11.F5 1:169511755:T:C
peptide level
151:169
120:135
20:21
ITAIITQGCKpc2_NP.5
p−maj=0.71, pX=0.38, N=291
var.11.F5 1:169511755:T:C
peptide level
5:169
2:135
0:21
IVYREYEPYFKpc2_NP.1
p−min=0.45, pX=0.52, N=7
var.11.F5 1:169511755:T:C
peptide level
90:169
63:135
7:21
IVYREYEPYFKpc2_NP.4
p−min=0.15, pX=0.043, N=160
var.11.F5 1:169511755:T:C
peptide level
9:169
12:135
0:21
IVYREYEPYFKpc2_NP.5
p−maj=0.38, pX=0.71, N=21

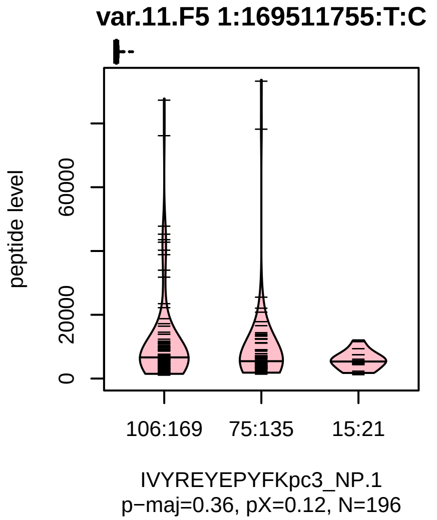
var.11.F5 1:169511755:T:C
peptide level
0
20000
60000
106:169
75:135
15:21
IVYREYEPYFKpc3_NP.1
p−maj=0.36, pX=0.12, N=196
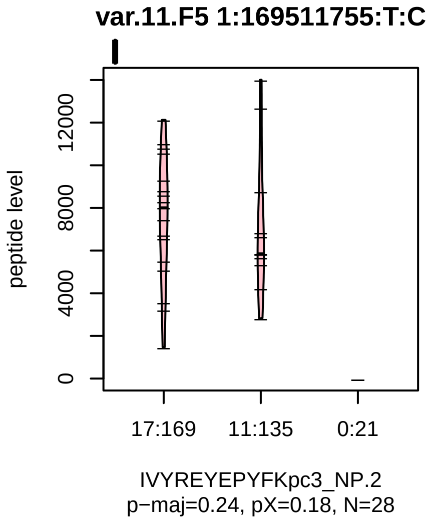
var.11.F5 1:169511755:T:C
peptide level
0
4000
8000
12000
17:169
11:135
0:21
IVYREYEPYFKpc3_NP.2
p−maj=0.24, pX=0.18, N=28
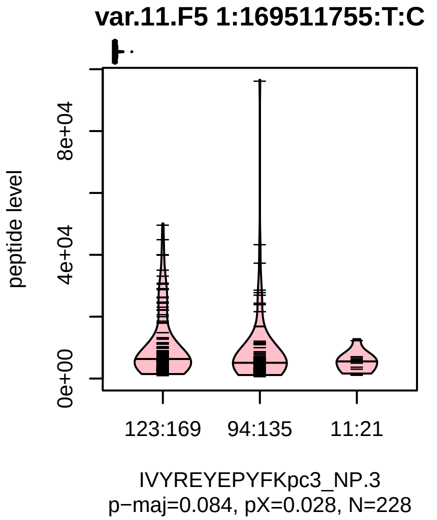
var.11.F5 1:169511755:T:C
peptide level
0e+00
4e+04
8e+04
123:169
94:135
11:21
IVYREYEPYFKpc3_NP.3
p−maj=0.084, pX=0.028, N=228
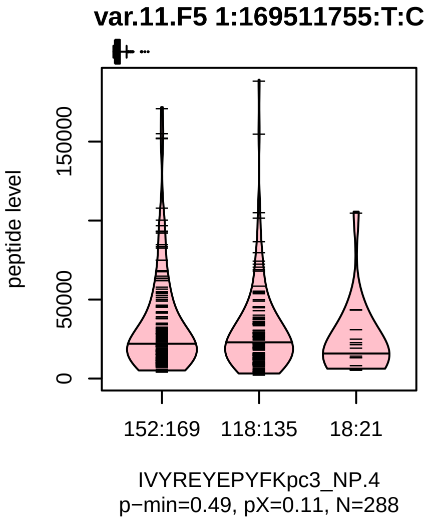
var.11.F5 1:169511755:T:C
peptide level
0
50000
150000
152:169
118:135
18:21
IVYREYEPYFKpc3_NP.4
p−min=0.49, pX=0.11, N=288
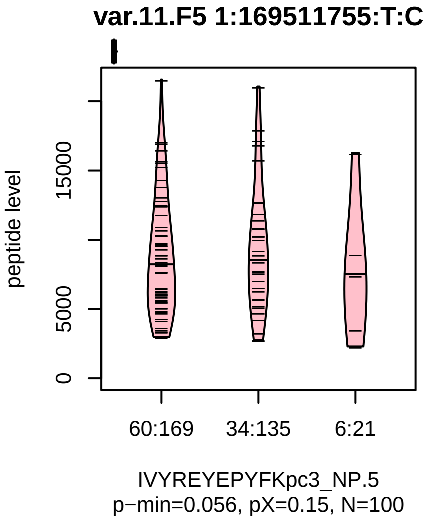
var.11.F5 1:169511755:T:C
peptide level
0
5000
15000
60:169
34:135
6:21
IVYREYEPYFKpc3_NP.5
p−min=0.056, pX=0.15, N=100
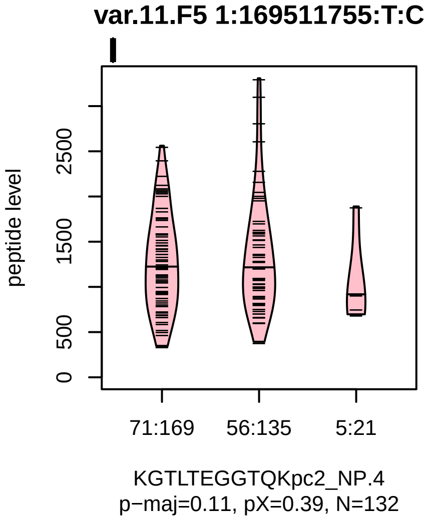
var.11.F5 1:169511755:T:C
peptide level
0
500
1500
2500
71:169
56:135
5:21
KGTLTEGGTQKpc2_NP.4
p−maj=0.11, pX=0.39, N=132
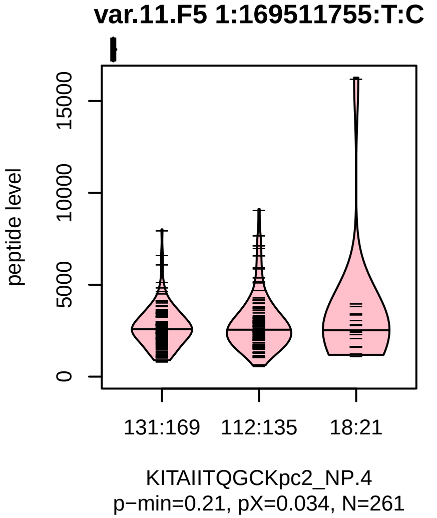
var.11.F5 1:169511755:T:C
peptide level
0
5000
10000
15000
131:169
112:135
18:21
KITAIITQGCKpc2_NP.4
p−min=0.21, pX=0.034, N=261

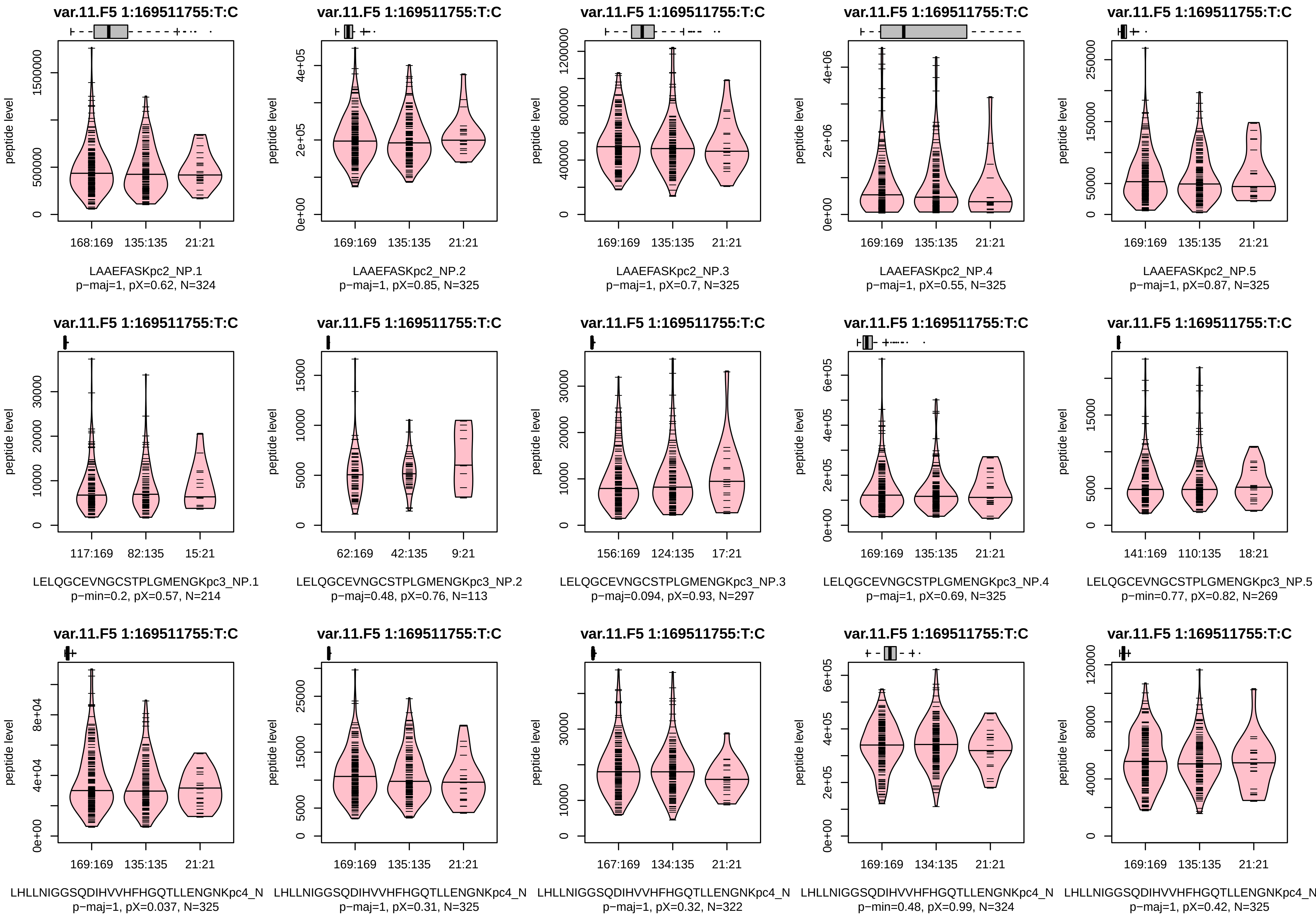
var.11.F5 1:169511755:T:C
peptide level
168:169 135:135 21:21
LAAEFASKpc2_NP.1
p−maj=1, pX=0.62, N=324
var.11.F5 1:169511755:T:C
peptide level
169:169 135:135 21:21
LAAEFASKpc2_NP.2
p−maj=1, pX=0.85, N=325
var.11.F5 1:169511755:T:C
peptide level
169:169 135:135 21:21
LAAEFASKpc2_NP.3
p−maj=1, pX=0.7, N=325
var.11.F5 1:169511755:T:C
peptide level
169:169 135:135 21:21
LAAEFASKpc2_NP.4
p−maj=1, pX=0.55, N=325
var.11.F5 1:169511755:T:C
peptide level
169:169 135:135 21:21
LAAEFASKpc2_NP.5
p−maj=1, pX=0.87, N=325
var.11.F5 1:169511755:T:C
peptide level
117:169 82:135 15:21
LELQGCEVNGCSTPLGMENGKpc3_NP.1
p−min=0.2, pX=0.57, N=214
var.11.F5 1:169511755:T:C
peptide level
62:169 42:135 9:21
LELQGCEVNGCSTPLGMENGKpc3_NP.2
p−maj=0.48, pX=0.76, N=113
var.11.F5 1:169511755:T:C
peptide level
156:169 124:135 17:21
LELQGCEVNGCSTPLGMENGKpc3_NP.3
p−maj=0.094, pX=0.93, N=297
var.11.F5 1:169511755:T:C
peptide level
169:169 135:135 21:21
LELQGCEVNGCSTPLGMENGKpc3_NP.4
p−maj=1, pX=0.69, N=325
var.11.F5 1:169511755:T:C
peptide level
141:169 110:135 18:21
LELQGCEVNGCSTPLGMENGKpc3_NP.5
p−min=0.77, pX=0.82, N=269
var.11.F5 1:169511755:T:C
peptide level
169:169 135:135 21:21
LHLLNIGGSQDIHVVHFHGQTLLENGNKpc4_N
p−maj=1, pX=0.037, N=325
var.11.F5 1:169511755:T:C
peptide level
169:169 135:135 21:21
LHLLNIGGSQDIHVVHFHGQTLLENGNKpc4_N
p−maj=1, pX=0.31, N=325
var.11.F5 1:169511755:T:C
peptide level
167:169 134:135 21:21
LHLLNIGGSQDIHVVHFHGQTLLENGNKpc4_N
p−maj=1, pX=0.32, N=322
var.11.F5 1:169511755:T:C
peptide level
169:169 134:135 21:21
LHLLNIGGSQDIHVVHFHGQTLLENGNKpc4_N
p−min=0.48, pX=0.99, N=324
var.11.F5 1:169511755:T:C
peptide level
169:169 135:135 21:21
LHLLNIGGSQDIHVVHFHGQTLLENGNKpc4_N
p−maj=1, pX=0.42, N=325

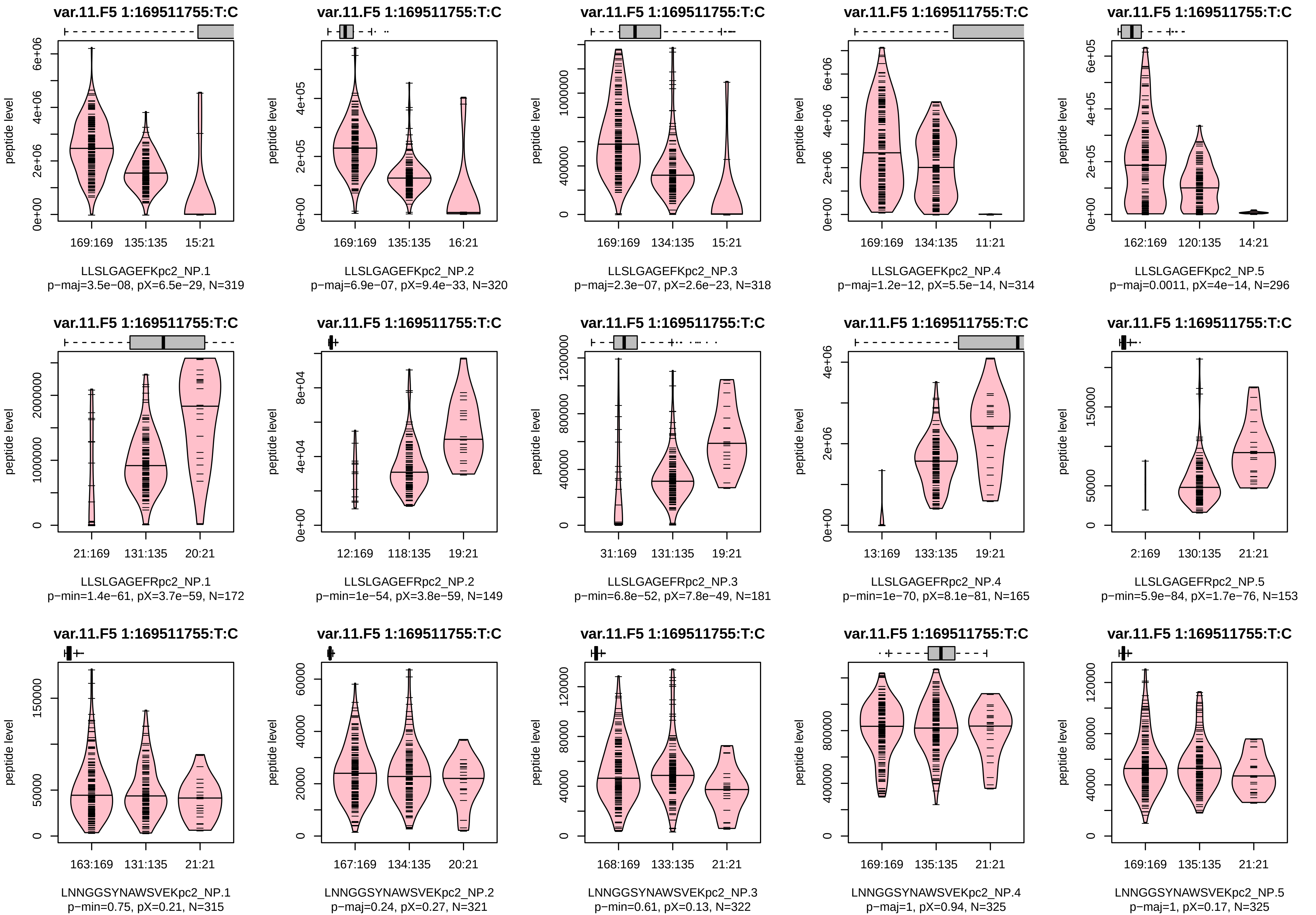

var.11.F5 1:169511755:T:C
peptide level
169:169
135:135
15:21
LLSLGAGEFKpc2_NP.1
p−maj=3.5e−08, pX=6.5e−29, N=319
var.11.F5 1:169511755:T:C
169:169
135:135
16:21
LLSLGAGEFKpc2_NP.2
p−maj=6.9e−07, pX=9.4e−33, N=320
var.11.F5 1:169511755:T:C
169:169
134:135
15:21
LLSLGAGEFKpc2_NP.3
p−maj=2.3e−07, pX=2.6e−23, N=318
var.11.F5 1:169511755:T:C
169:169
134:135
11:21
LLSLGAGEFKpc2_NP.4
p−maj=1.2e−12, pX=5.5e−14, N=314
var.11.F5 1:169511755:T:C
162:169
120:135
14:21
LLSLGAGEFKpc2_NP.5
p−maj=0.0011, pX=4e−14, N=296
var.11.F5 1:169511755:T:C
21:169
131:135
20:21
LLSLGAGEFRpc2_NP.1
p−min=1.4e−61, pX=3.7e−59, N=172
var.11.F5 1:169511755:T:C
12:169
118:135
19:21
LLSLGAGEFRpc2_NP.2
p−min=1e−54, pX=3.8e−59, N=149
var.11.F5 1:169511755:T:C
31:169
131:135
19:21
LLSLGAGEFRpc2_NP.3
p−min=6.8e−52, pX=7.8e−49, N=181
var.11.F5 1:169511755:T:C
13:169
133:135
19:21
LLSLGAGEFRpc2_NP.4
p−min=1e−70, pX=8.1e−81, N=165
var.11.F5 1:169511755:T:C
2:169
130:135
21:21
LLSLGAGEFRpc2_NP.5
p−min=5.9e−84, pX=1.7e−76, N=153
var.11.F5 1:169511755:T:C
163:169
131:135
21:21
LNNGGSYNAWSVEKpc2_NP.1
p−min=0.75, pX=0.21, N=315
var.11.F5 1:169511755:T:C
167:169
134:135
20:21
LNNGGSYNAWSVEKpc2_NP.2
p−maj=0.24, pX=0.27, N=321
var.11.F5 1:169511755:T:C
168:169
133:135
21:21
LNNGGSYNAWSVEKpc2_NP.3
p−min=0.61, pX=0.13, N=322
var.11.F5 1:169511755:T:C
169:169
135:135
21:21
LNNGGSYNAWSVEKpc2_NP.4
p−maj=1, pX=0.94, N=325
var.11.F5 1:169511755:T:C
169:169
135:135
21:21
LNNGGSYNAWSVEKpc2_NP.5
p−maj=1, pX=0.17, N=325

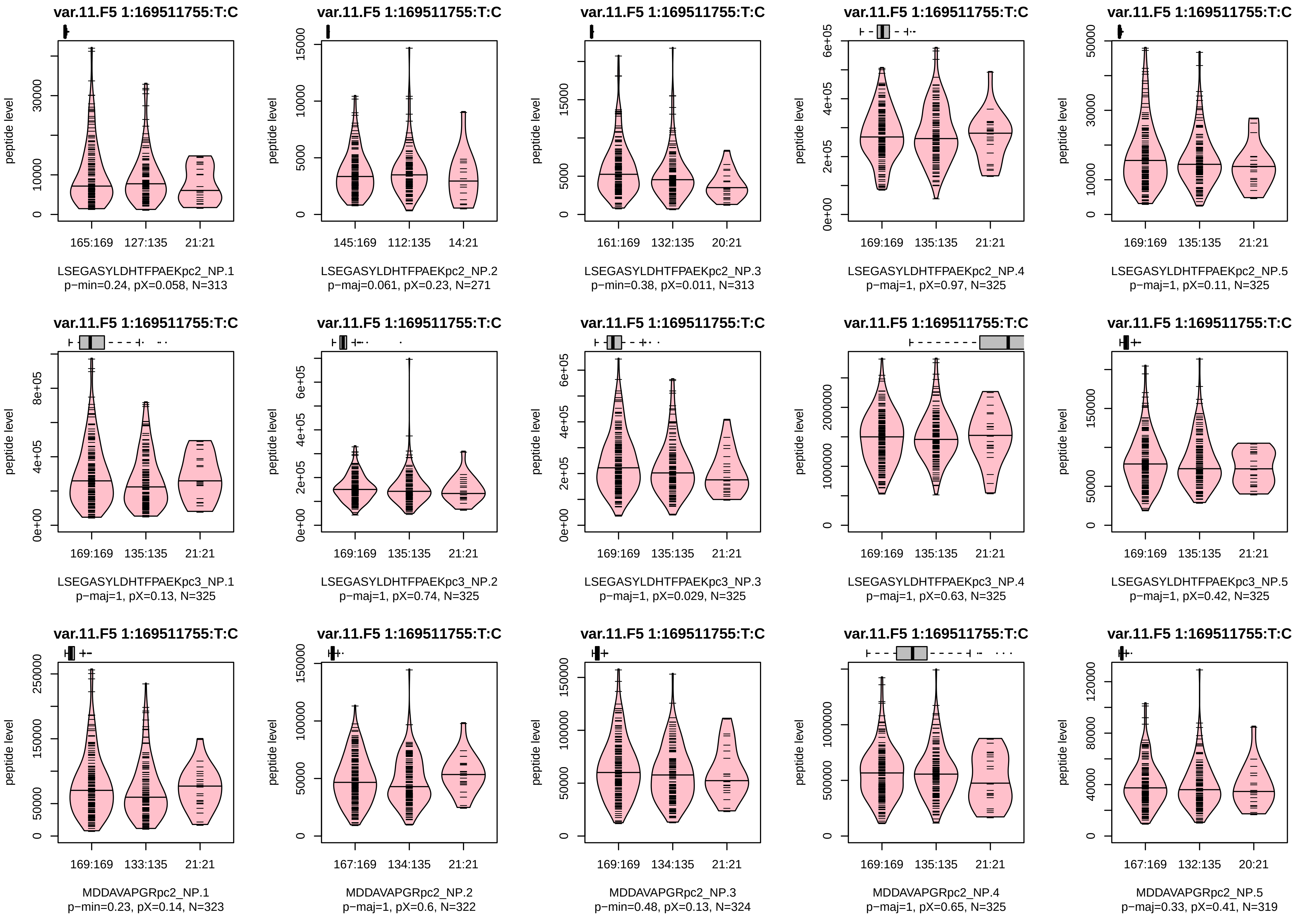
var.11.F5 1:169511755:T:C
peptide level
165:169 127:135 21:21
LSEGASYLDHTFPAEKpc2_NP.1
p−min=0.24, pX=0.058, N=313
var.11.F5 1:169511755:T:C
peptide level
145:169 112:135 14:21
LSEGASYLDHTFPAEKpc2_NP.2
p−maj=0.061, pX=0.23, N=271
var.11.F5 1:169511755:T:C
peptide level
161:169 132:135 20:21
LSEGASYLDHTFPAEKpc2_NP.3
p−min=0.38, pX=0.011, N=313
var.11.F5 1:169511755:T:C
peptide level
169:169 135:135 21:21
LSEGASYLDHTFPAEKpc2_NP.4
p−maj=1, pX=0.97, N=325
var.11.F5 1:169511755:T:C
peptide level
169:169 135:135 21:21
LSEGASYLDHTFPAEKpc2_NP.5
p−maj=1, pX=0.11, N=325
var.11.F5 1:169511755:T:C
peptide level
169:169 135:135 21:21
LSEGASYLDHTFPAEKpc3_NP.1
p−maj=1, pX=0.13, N=325
var.11.F5 1:169511755:T:C
peptide level
169:169 135:135 21:21
LSEGASYLDHTFPAEKpc3_NP.2
p−maj=1, pX=0.74, N=325
var.11.F5 1:169511755:T:C
peptide level
169:169 135:135 21:21
LSEGASYLDHTFPAEKpc3_NP.3
p−maj=1, pX=0.029, N=325
var.11.F5 1:169511755:T:C
peptide level
169:169 135:135 21:21
LSEGASYLDHTFPAEKpc3_NP.4
p−maj=1, pX=0.63, N=325
var.11.F5 1:169511755:T:C
peptide level
169:169 135:135 21:21
LSEGASYLDHTFPAEKpc3_NP.5
p−maj=1, pX=0.42, N=325
var.11.F5 1:169511755:T:C
peptide level
169:169 133:135 21:21
MDDAVAPGRpc2_NP.1
p−min=0.23, pX=0.14, N=323
var.11.F5 1:169511755:T:C
peptide level
167:169 134:135 21:21
MDDAVAPGRpc2_NP.2
p−maj=1, pX=0.6, N=322
var.11.F5 1:169511755:T:C
peptide level
169:169 134:135 21:21
MDDAVAPGRpc2_NP.3
p−min=0.48, pX=0.13, N=324
var.11.F5 1:169511755:T:C
peptide level
169:169 135:135 21:21
MDDAVAPGRpc2_NP.4
p−maj=1, pX=0.65, N=325
var.11.F5 1:169511755:T:C
peptide level
167:169 132:135 20:21
MDDAVAPGRpc2_NP.5
p−maj=0.33, pX=0.41, N=319

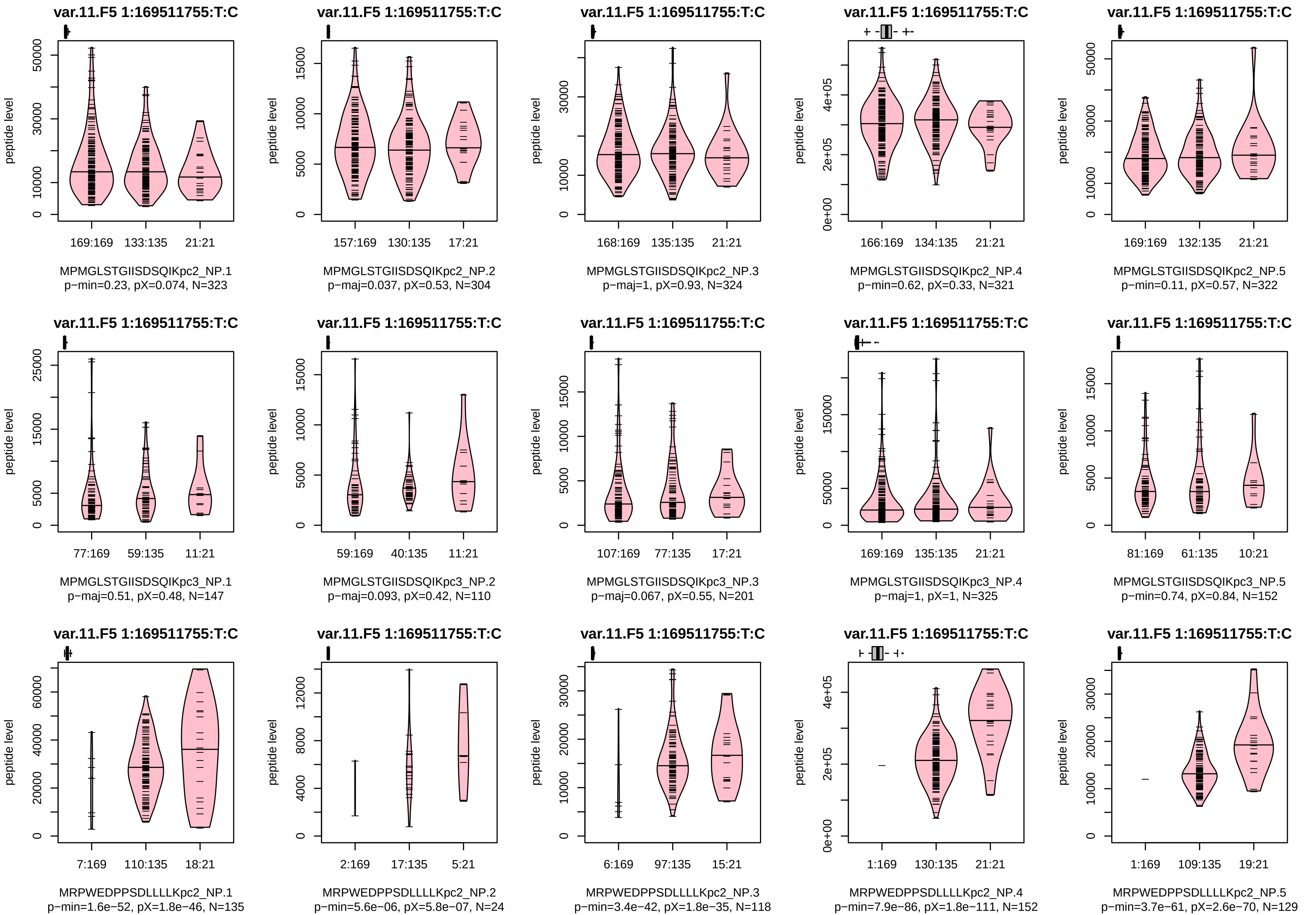

var.11.F5 1:169511755:T:C
peptide level
169:169 133:135 21:21
MPMGLSTGIISDSQIKpc2_NP.1
p−min=0.23, pX=0.074, N=323
var.11.F5 1:169511755:T:C
peptide level
157:169 130:135 17:21
MPMGLSTGIISDSQIKpc2_NP.2
p−maj=0.037, pX=0.53, N=304
var.11.F5 1:169511755:T:C
peptide level
168:169 135:135 21:21
MPMGLSTGIISDSQIKpc2_NP.3
p−maj=1, pX=0.93, N=324
var.11.F5 1:169511755:T:C
peptide level
166:169 134:135 21:21
MPMGLSTGIISDSQIKpc2_NP.4
p−min=0.62, pX=0.33, N=321
var.11.F5 1:169511755:T:C
peptide level
169:169 132:135 21:21
MPMGLSTGIISDSQIKpc2_NP.5
p−min=0.11, pX=0.57, N=322
var.11.F5 1:169511755:T:C
peptide level
77:169 59:135 11:21
MPMGLSTGIISDSQIKpc3_NP.1
p−maj=0.51, pX=0.48, N=147
var.11.F5 1:169511755:T:C
peptide level
59:169 40:135 11:21
MPMGLSTGIISDSQIKpc3_NP.2
p−maj=0.093, pX=0.42, N=110
var.11.F5 1:169511755:T:C
peptide level
107:169 77:135 17:21
MPMGLSTGIISDSQIKpc3_NP.3
p−maj=0.067, pX=0.55, N=201
var.11.F5 1:169511755:T:C
peptide level
169:169 135:135 21:21
MPMGLSTGIISDSQIKpc3_NP.4
p−maj=1, pX=1, N=325
var.11.F5 1:169511755:T:C
peptide level
81:169 61:135 10:21
MPMGLSTGIISDSQIKpc3_NP.5
p−min=0.74, pX=0.84, N=152
var.11.F5 1:169511755:T:C
peptide level
7:169 110:135 18:21
MRPWEDPPSDLLLLKpc2_NP.1
p−min=1.6e−52, pX=1.8e−46, N=135
var.11.F5 1:169511755:T:C
peptide level
2:169 17:135 5:21
MRPWEDPPSDLLLLKpc2_NP.2
p−min=5.6e−06, pX=5.8e−07, N=24
var.11.F5 1:169511755:T:C
peptide level
6:169 97:135 15:21
MRPWEDPPSDLLLLKpc2_NP.3
p−min=3.4e−42, pX=1.8e−35, N=118
var.11.F5 1:169511755:T:C
peptide level
1:169 130:135 21:21
MRPWEDPPSDLLLLKpc2_NP.4
p−min=7.9e−86, pX=1.8e−111, N=152
var.11.F5 1:169511755:T:C
peptide level
1:169 109:135 19:21
MRPWEDPPSDLLLLKpc2_NP.5
p−min=3.7e−61, pX=2.6e−70, N=129

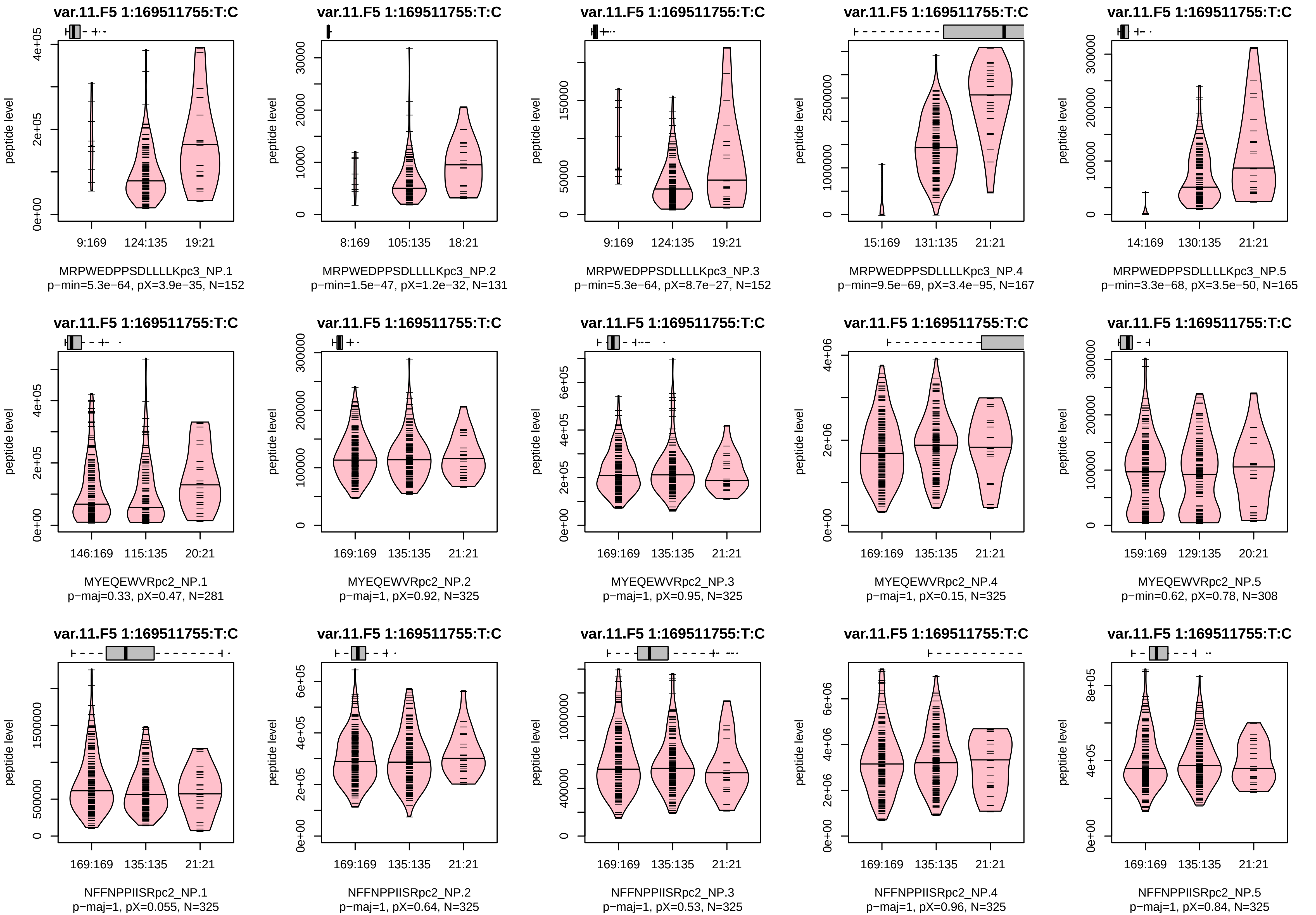
var.11.F5 1:169511755:T:C
peptide level
9:169
124:135
19:21
MRPWEDPPSDLLLLKpc3_NP.1
p−min=5.3e−64, pX=3.9e−35, N=152
var.11.F5 1:169511755:T:C
peptide level
8:169
105:135
18:21
MRPWEDPPSDLLLLKpc3_NP.2
p−min=1.5e−47, pX=1.2e−32, N=131
var.11.F5 1:169511755:T:C
peptide level
9:169
124:135
19:21
MRPWEDPPSDLLLLKpc3_NP.3
p−min=5.3e−64, pX=8.7e−27, N=152
var.11.F5 1:169511755:T:C
peptide level
15:169
131:135
21:21
MRPWEDPPSDLLLLKpc3_NP.4
p−min=9.5e−69, pX=3.4e−95, N=167
var.11.F5 1:169511755:T:C
peptide level
14:169
130:135
21:21
MRPWEDPPSDLLLLKpc3_NP.5
p−min=3.3e−68, pX=3.5e−50, N=165
var.11.F5 1:169511755:T:C
peptide level
146:169
115:135
20:21
MYEQEWVRpc2_NP.1
p−maj=0.33, pX=0.47, N=281
var.11.F5 1:169511755:T:C
peptide level
169:169
135:135
21:21
MYEQEWVRpc2_NP.2
p−maj=1, pX=0.92, N=325
var.11.F5 1:169511755:T:C
peptide level
169:169
135:135
21:21
MYEQEWVRpc2_NP.3
p−maj=1, pX=0.95, N=325
var.11.F5 1:169511755:T:C
peptide level
169:169
135:135
21:21
MYEQEWVRpc2_NP.4
p−maj=1, pX=0.15, N=325
var.11.F5 1:169511755:T:C
peptide level
159:169
129:135
20:21
MYEQEWVRpc2_NP.5
p−min=0.62, pX=0.78, N=308
var.11.F5 1:169511755:T:C
peptide level
169:169
135:135
21:21
NFFNPPIISRpc2_NP.1
p−maj=1, pX=0.055, N=325
var.11.F5 1:169511755:T:C
peptide level
169:169
135:135
21:21
NFFNPPIISRpc2_NP.2
p−maj=1, pX=0.64, N=325
var.11.F5 1:169511755:T:C
peptide level
169:169
135:135
21:21
NFFNPPIISRpc2_NP.3
p−maj=1, pX=0.53, N=325
var.11.F5 1:169511755:T:C
peptide level
169:169
135:135
21:21
NFFNPPIISRpc2_NP.4
p−maj=1, pX=0.96, N=325
var.11.F5 1:169511755:T:C
peptide level
169:169
135:135
21:21
NFFNPPIISRpc2_NP.5
p−maj=1, pX=0.84, N=325

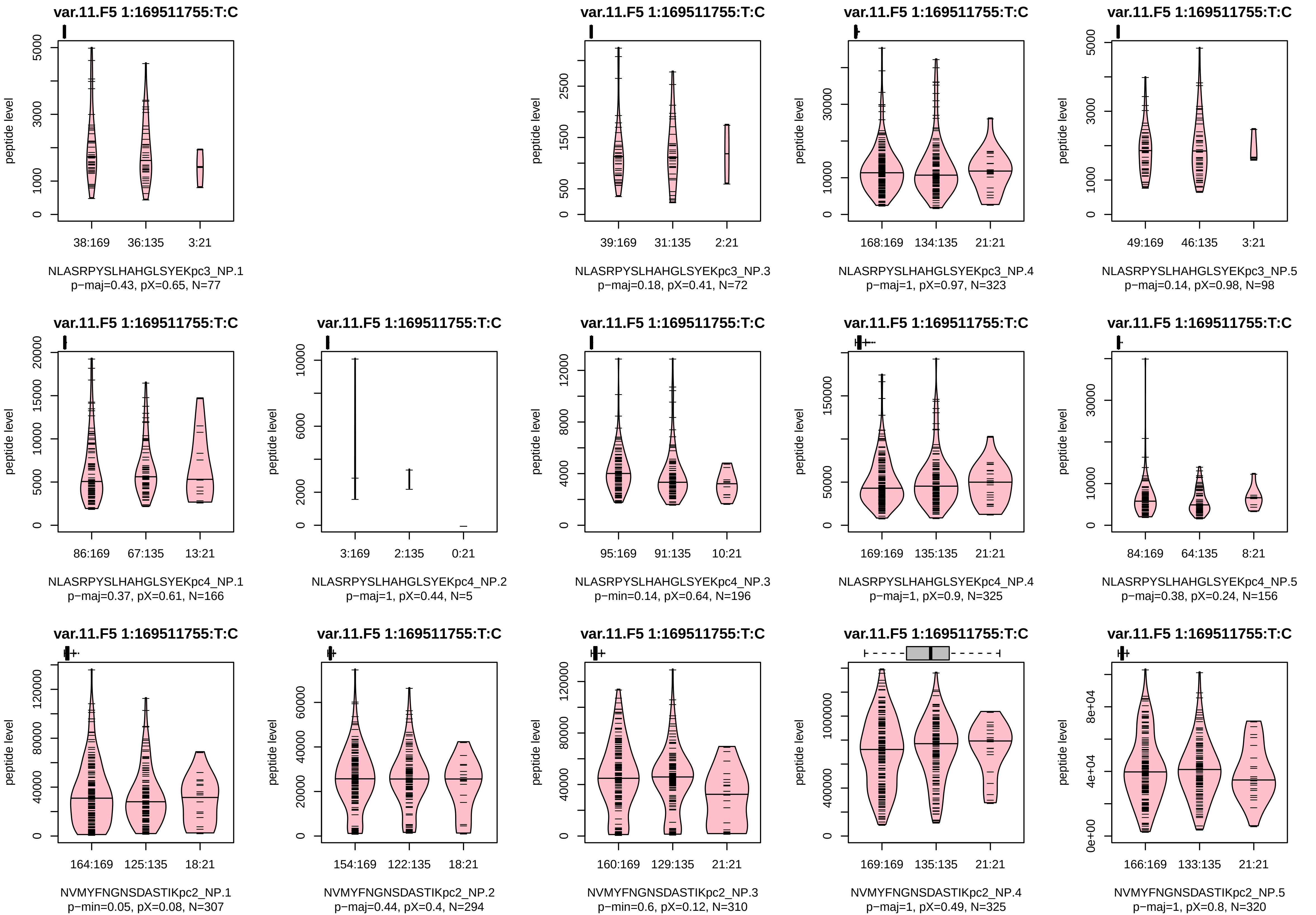

var.11.F5 1:169511755:T:C
peptide level
38:169
36:135
3:21
NLASRPYSLHAHGLSYEKpc3_NP.1
p−maj=0.43, pX=0.65, N=77
var.11.F5 1:169511755:T:C
peptide level
39:169
31:135
2:21
NLASRPYSLHAHGLSYEKpc3_NP.3
p−maj=0.18, pX=0.41, N=72
var.11.F5 1:169511755:T:C
peptide level
168:169
134:135
21:21
NLASRPYSLHAHGLSYEKpc3_NP.4
p−maj=1, pX=0.97, N=323
var.11.F5 1:169511755:T:C
peptide level
49:169
46:135
3:21
NLASRPYSLHAHGLSYEKpc3_NP.5
p−maj=0.14, pX=0.98, N=98
var.11.F5 1:169511755:T:C
peptide level
86:169
67:135
13:21
NLASRPYSLHAHGLSYEKpc4_NP.1
p−maj=0.37, pX=0.61, N=166
var.11.F5 1:169511755:T:C
peptide level
3:169
2:135
0:21
NLASRPYSLHAHGLSYEKpc4_NP.2
p−maj=1, pX=0.44, N=5
var.11.F5 1:169511755:T:C
peptide level
95:169
91:135
10:21
NLASRPYSLHAHGLSYEKpc4_NP.3
p−min=0.14, pX=0.64, N=196
var.11.F5 1:169511755:T:C
peptide level
169:169
135:135
21:21
NLASRPYSLHAHGLSYEKpc4_NP.4
p−maj=1, pX=0.9, N=325
var.11.F5 1:169511755:T:C
peptide level
84:169
64:135
8:21
NLASRPYSLHAHGLSYEKpc4_NP.5
p−maj=0.38, pX=0.24, N=156
var.11.F5 1:169511755:T:C
peptide level
164:169
125:135
18:21
NVMYFNGNSDASTIKpc2_NP.1
p−min=0.05, pX=0.08, N=307
var.11.F5 1:169511755:T:C
peptide level
154:169
122:135
18:21
NVMYFNGNSDASTIKpc2_NP.2
p−maj=0.44, pX=0.4, N=294
var.11.F5 1:169511755:T:C
peptide level
160:169
129:135
21:21
NVMYFNGNSDASTIKpc2_NP.3
p−min=0.6, pX=0.12, N=310
var.11.F5 1:169511755:T:C
peptide level
169:169
135:135
21:21
NVMYFNGNSDASTIKpc2_NP.4
p−maj=1, pX=0.49, N=325
var.11.F5 1:169511755:T:C
peptide level
166:169
133:135
21:21
NVMYFNGNSDASTIKpc2_NP.5
p−maj=1, pX=0.8, N=320

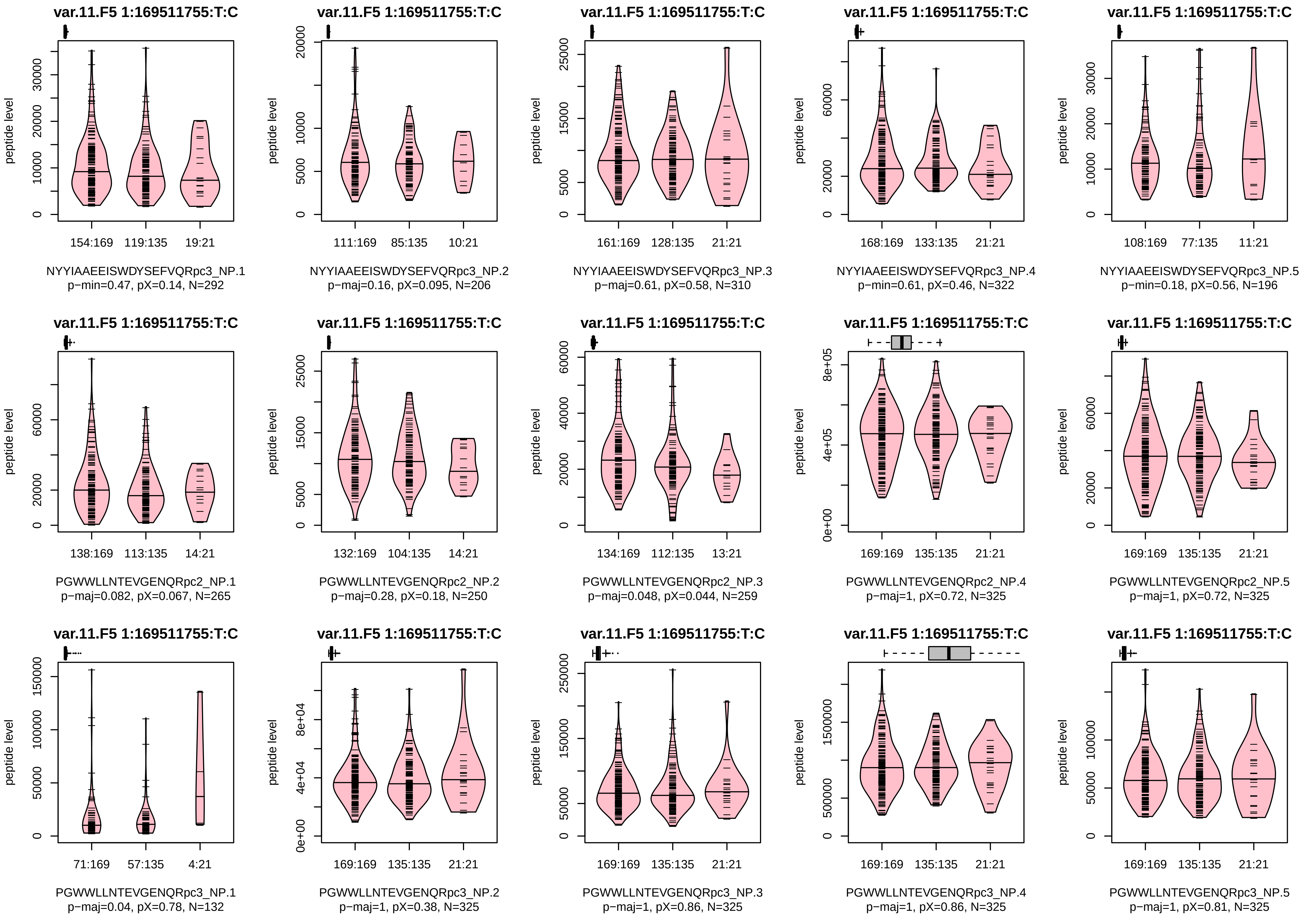
var.11.F5 1:169511755:T:C
peptide level
154:169
119:135
19:21
NYYIAAAEEISWDYSEFVQRpc3_NP.1
p−min=0.47, pX=0.14, N=292
var.11.F5 1:169511755:T:C
peptide level
111:169
85:135
10:21
NYYIAAAEEISWDYSEFVQRpc3_NP.2
p−maj=0.16, pX=0.095, N=206
var.11.F5 1:169511755:T:C
peptide level
161:169
128:135
21:21
NYYIAAAEEISWDYSEFVQRpc3_NP.3
p−maj=0.61, pX=0.58, N=310
var.11.F5 1:169511755:T:C
peptide level
168:169
133:135
21:21
NYYIAAAEEISWDYSEFVQRpc3_NP.4
p−min=0.61, pX=0.46, N=322
var.11.F5 1:169511755:T:C
peptide level
108:169
77:135
11:21
NYYIAAAEEISWDYSEFVQRpc3_NP.5
p−min=0.18, pX=0.56, N=196
var.11.F5 1:169511755:T:C
peptide level
138:169
113:135
14:21
PGWWLLNTEVGENQRpc2_NP.1
p−maj=0.082, pX=0.067, N=265
var.11.F5 1:169511755:T:C
peptide level
132:169
104:135
14:21
PGWWLLNTEVGENQRpc2_NP.2
p−maj=0.28, pX=0.18, N=250
var.11.F5 1:169511755:T:C
peptide level
134:169
112:135
13:21
PGWWLLNTEVGENQRpc2_NP.3
p−maj=0.048, pX=0.044, N=259
var.11.F5 1:169511755:T:C
peptide level
169:169
135:135
21:21
PGWWLLNTEVGENQRpc2_NP.4
p−maj=1, pX=0.72, N=325
var.11.F5 1:169511755:T:C
peptide level
169:169
135:135
21:21
PGWWLLNTEVGENQRpc2_NP.5
p−maj=1, pX=0.72, N=325
var.11.F5 1:169511755:T:C
peptide level
71:169
57:135
4:21
PGWWLLNTEVGENQRpc3_NP.1
p−maj=0.04, pX=0.78, N=132
var.11.F5 1:169511755:T:C
peptide level
169:169
135:135
21:21
PGWWLLNTEVGENQRpc3_NP.2
p−maj=1, pX=0.38, N=325
var.11.F5 1:169511755:T:C
peptide level
169:169
135:135
21:21
PGWWLLNTEVGENQRpc3_NP.3
p−maj=1, pX=0.86, N=325
var.11.F5 1:169511755:T:C
peptide level
169:169
135:135
21:21
PGWWLLNTEVGENQRpc3_NP.4
p−maj=1, pX=0.86, N=325
var.11.F5 1:169511755:T:C
peptide level
169:169
135:135
21:21
PGWWLLNTEVGENQRpc3_NP.5
p−maj=1, pX=0.81, N=325

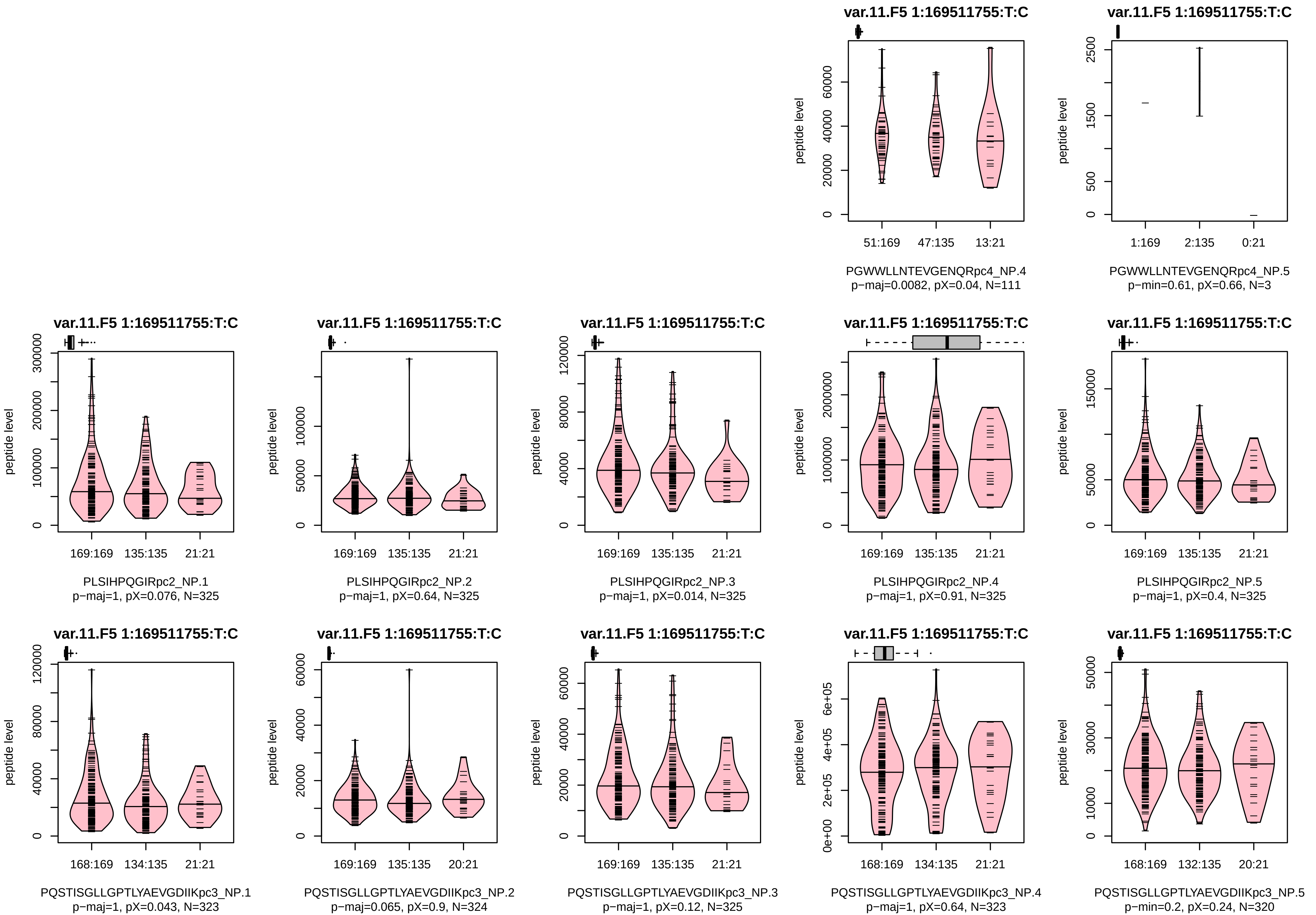

var.11.F5 1:169511755:T:C
peptide level
51:169
47:135
13:21
PGWWLLNTEVGENQRpc4_NP.4
p−maj=0.0082, pX=0.04, N=111
var.11.F5 1:169511755:T:C
peptide level
1:169
2:135
0:21
PGWWLLNTEVGENQRpc4_NP.5
p−min=0.61, pX=0.66, N=3
var.11.F5 1:169511755:T:C
peptide level
169:169
135:135
21:21
PLSIHPQGIRpc2_NP.1
p−maj=1, pX=0.076, N=325
var.11.F5 1:169511755:T:C
peptide level
169:169
135:135
21:21
PLSIHPQGIRpc2_NP.2
p−maj=1, pX=0.64, N=325
var.11.F5 1:169511755:T:C
peptide level
169:169
135:135
21:21
PLSIHPQGIRpc2_NP.3
p−maj=1, pX=0.014, N=325
var.11.F5 1:169511755:T:C
peptide level
169:169
135:135
21:21
PLSIHPQGIRpc2_NP.4
p−maj=1, pX=0.91, N=325
var.11.F5 1:169511755:T:C
peptide level
169:169
135:135
21:21
PLSIHPQGIRpc2_NP.5
p−maj=1, pX=0.4, N=325
var.11.F5 1:169511755:T:C
peptide level
168:169
134:135
21:21
PQSTISGLLGPTLYAEVGDIIKpc3_NP.1
p−maj=1, pX=0.043, N=323
var.11.F5 1:169511755:T:C
peptide level
169:169
135:135
20:21
PQSTISGLLGPTLYAEVGDIIKpc3_NP.2
p−maj=0.065, pX=0.9, N=324
var.11.F5 1:169511755:T:C
peptide level
169:169
135:135
21:21
PQSTISGLLGPTLYAEVGDIIKpc3_NP.3
p−maj=1, pX=0.12, N=325
var.11.F5 1:169511755:T:C
peptide level
168:169
134:135
21:21
PQSTISGLLGPTLYAEVGDIIKpc3_NP.4
p−maj=1, pX=0.64, N=323
var.11.F5 1:169511755:T:C
peptide level
168:169
132:135
20:21
PQSTISGLLGPTLYAEVGDIIKpc3_NP.5
p−min=0.2, pX=0.24, N=320

var.11.F5 1:169511755:T:C

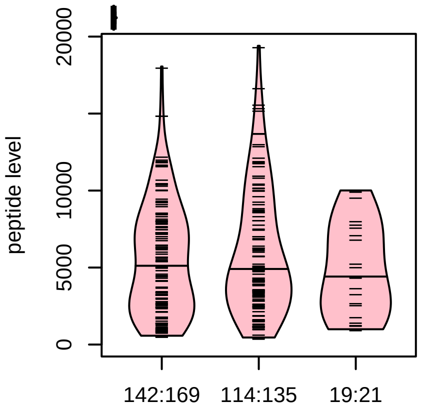

PWEDLPSQDTGSPSRpc2_NP.4
p−maj=0.75, pX=0.65, N=275

var.11.F5 1:169511755:T:C

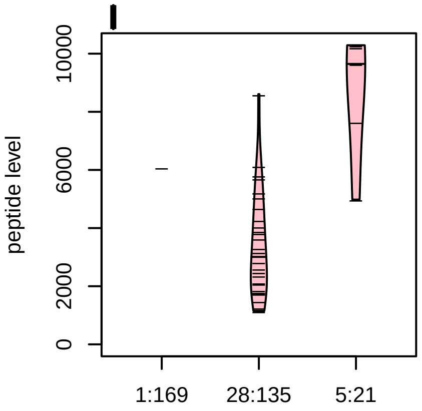

PWEDPPSDLLLLKpc2_NP.1
p−min=1.2e−10, pX=7.7e−10, N=34

var.11.F5 1:169511755:T:C

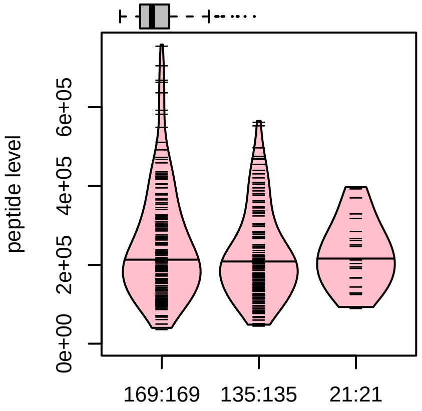

PWIQVDMQKpc2_NP.1
p−maj=1, pX=0.1, N=325

var.11.F5 1:169511755:T:C

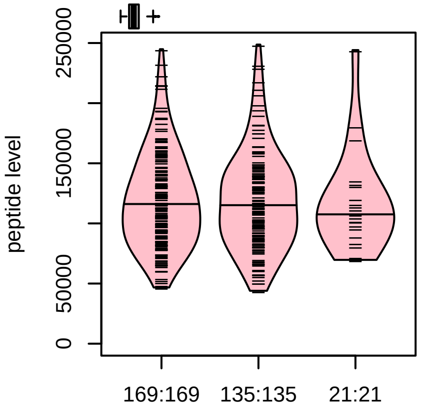

PWIQVDMQKpc2_NP.2
p−maj=1, pX=0.89, N=325

var.11.F5 1:169511755:T:C

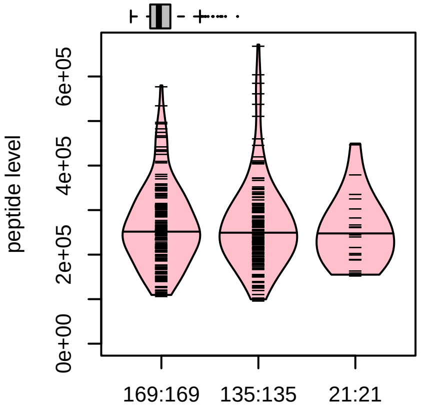

PWIQVDMQKpc2_NP.3
p−maj=1, pX=0.81, N=325

var.11.F5 1:169511755:T:C

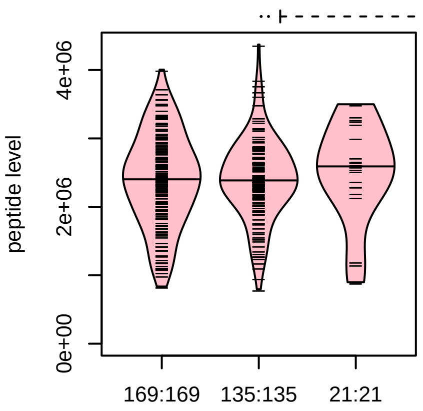

PWIQVDMQKpc2_NP.4
p−maj=1, pX=0.56, N=325

var.11.F5 1:169511755:T:C

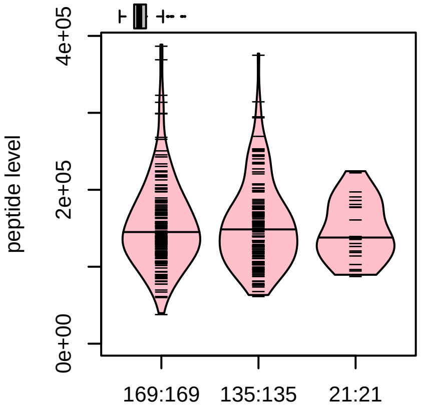

PWIQVDMQKpc2_NP.5
p−maj=1, pX=0.65, N=325

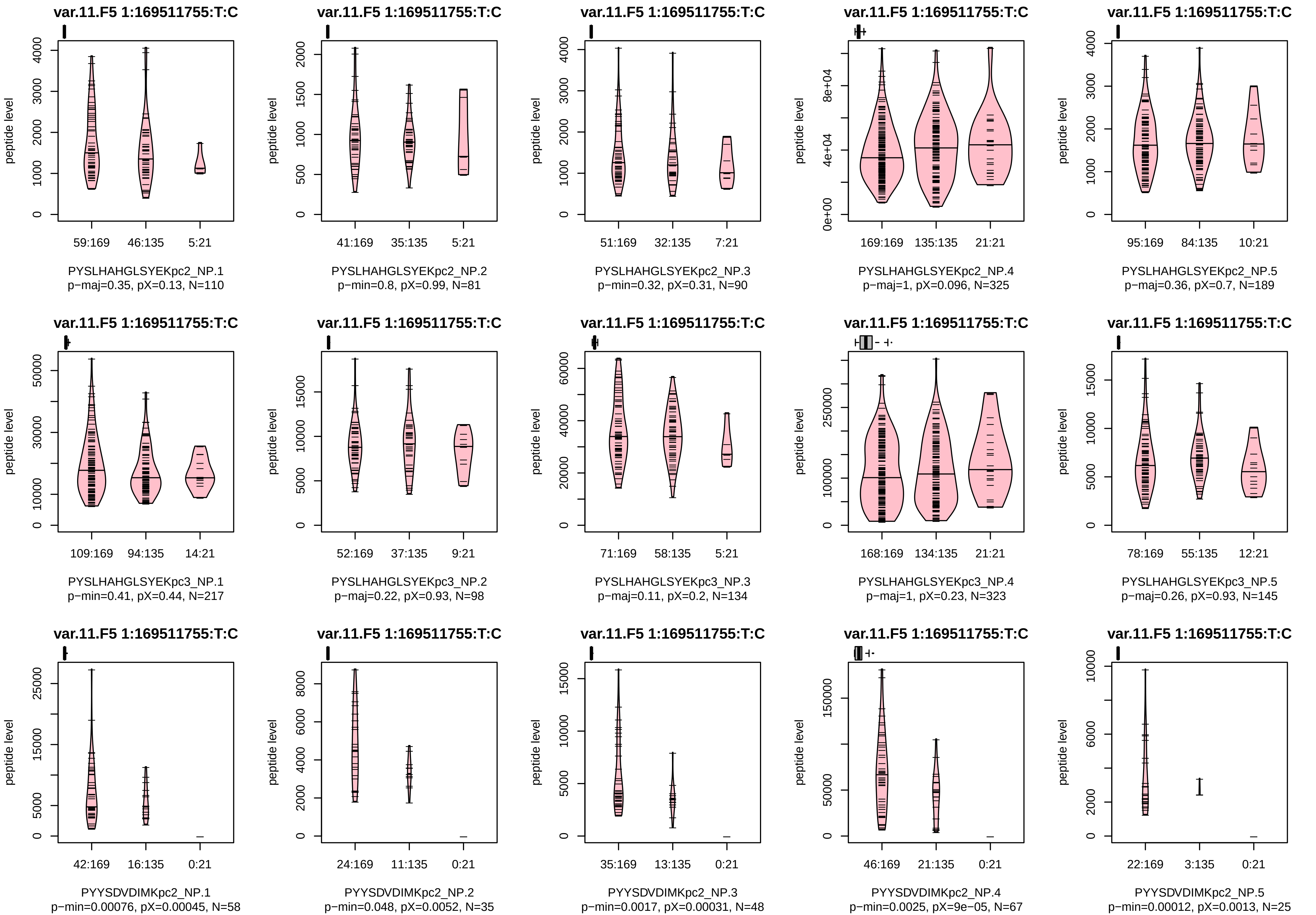

var.11.F5 1:169511755:T:C
peptide level
59:169
46:135
5:21
PYSLHAHGLSYEKpc2_NP.1
p−maj=0.35, pX=0.13, N=110
var.11.F5 1:169511755:T:C
peptide level
41:169
35:135
5:21
PYSLHAHGLSYEKpc2_NP.2
p−min=0.8, pX=0.99, N=81
var.11.F5 1:169511755:T:C
peptide level
51:169
32:135
7:21
PYSLHAHGLSYEKpc2_NP.3
p−min=0.32, pX=0.31, N=90
var.11.F5 1:169511755:T:C
peptide level
169:169
135:135
21:21
PYSLHAHGLSYEKpc2_NP.4
p−maj=1, pX=0.096, N=325
var.11.F5 1:169511755:T:C
peptide level
95:169
84:135
10:21
PYSLHAHGLSYEKpc2_NP.5
p−maj=0.36, pX=0.7, N=189
var.11.F5 1:169511755:T:C
peptide level
109:169
94:135
14:21
PYSLHAHGLSYEKpc3_NP.1
p−min=0.41, pX=0.44, N=217
var.11.F5 1:169511755:T:C
peptide level
52:169
37:135
9:21
PYSLHAHGLSYEKpc3_NP.2
p−maj=0.22, pX=0.93, N=98
var.11.F5 1:169511755:T:C
peptide level
71:169
58:135
5:21
PYSLHAHGLSYEKpc3_NP.3
p−maj=0.11, pX=0.2, N=134
var.11.F5 1:169511755:T:C
peptide level
168:169
134:135
21:21
PYSLHAHGLSYEKpc3_NP.4
p−maj=1, pX=0.23, N=323
var.11.F5 1:169511755:T:C
peptide level
78:169
55:135
12:21
PYSLHAHGLSYEKpc3_NP.5
p−maj=0.26, pX=0.93, N=145
var.11.F5 1:169511755:T:C
peptide level
42:169
16:135
0:21
PYYSDVDIMKpc2_NP.1
p−min=0.00076, pX=0.00045, N=58
var.11.F5 1:169511755:T:C
peptide level
24:169
11:135
0:21
PYYSDVDIMKpc2_NP.2
p−min=0.048, pX=0.0052, N=35
var.11.F5 1:169511755:T:C
peptide level
35:169
13:135
0:21
PYYSDVDIMKpc2_NP.3
p−min=0.0017, pX=0.00031, N=48
var.11.F5 1:169511755:T:C
peptide level
46:169
21:135
0:21
PYYSDVDIMKpc2_NP.4
p−min=0.0025, pX=9e−05, N=67
var.11.F5 1:169511755:T:C
peptide level
22:169
3:135
0:21
PYYSDVDIMKpc2_NP.5
p−min=0.00012, pX=0.0013, N=25

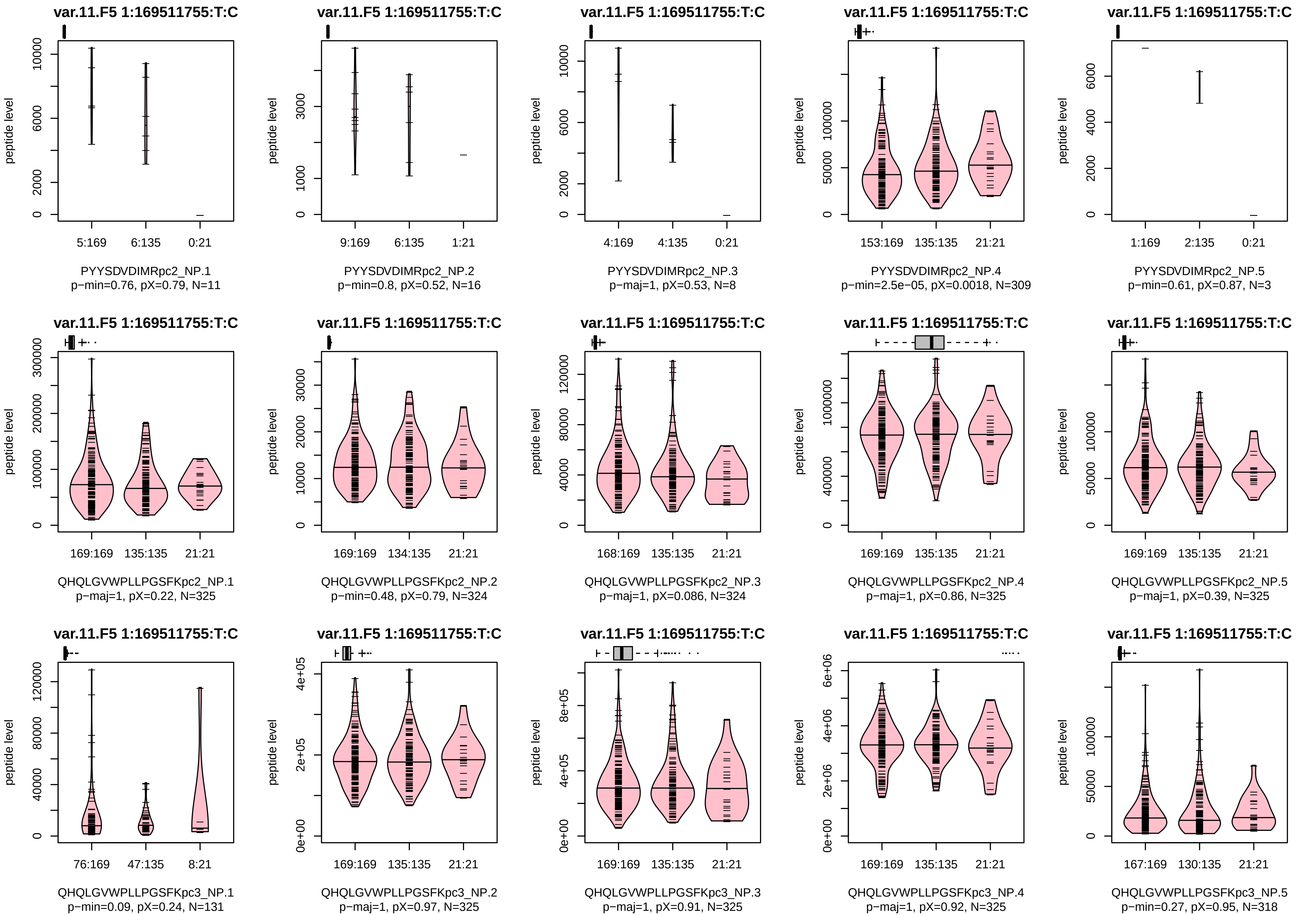

var.11.F5 1:169511755:T:C
5:169 6:135 0:21
PYYSDVDIMRpc2_NP.1
p−min=0.76, pX=0.79, N=11
var.11.F5 1:169511755:T:C
9:169 6:135 1:21
PYYSDVDIMRpc2_NP.2
p−min=0.8, pX=0.52, N=16
var.11.F5 1:169511755:T:C
4:169 4:135 0:21
PYYSDVDIMRpc2_NP.3
p−maj=1, pX=0.53, N=8
var.11.F5 1:169511755:T:C
153:169 135:135 21:21
PYYSDVDIMRpc2_NP.4
p−min=2.5e−05, pX=0.0018, N=309
var.11.F5 1:169511755:T:C
1:169 2:135 0:21
PYYSDVDIMRpc2_NP.5
p−min=0.61, pX=0.87, N=3
var.11.F5 1:169511755:T:C
169:169 135:135 21:21
QHQLGVWPLLPGSFKpc2_NP.1
p−maj=1, pX=0.22, N=325
var.11.F5 1:169511755:T:C
169:169 134:135 21:21
QHQLGVWPLLPGSFKpc2_NP.2
p−min=0.48, pX=0.79, N=324
var.11.F5 1:169511755:T:C
168:169 135:135 21:21
QHQLGVWPLLPGSFKpc2_NP.3
p−maj=1, pX=0.086, N=324
var.11.F5 1:169511755:T:C
169:169 135:135 21:21
QHQLGVWPLLPGSFKpc2_NP.4
p−maj=1, pX=0.86, N=325
var.11.F5 1:169511755:T:C
169:169 135:135 21:21
QHQLGVWPLLPGSFKpc2_NP.5
p−maj=1, pX=0.39, N=325
var.11.F5 1:169511755:T:C
76:169 47:135 8:21
QHQLGVWPLLPGSFKpc3_NP.1
p−min=0.09, pX=0.24, N=131
var.11.F5 1:169511755:T:C
169:169 135:135 21:21
QHQLGVWPLLPGSFKpc3_NP.2
p−maj=1, pX=0.97, N=325
var.11.F5 1:169511755:T:C
169:169 135:135 21:21
QHQLGVWPLLPGSFKpc3_NP.3
p−maj=1, pX=0.91, N=325
var.11.F5 1:169511755:T:C
169:169 135:135 21:21
QHQLGVWPLLPGSFKpc3_NP.4
p−maj=1, pX=0.92, N=325
var.11.F5 1:169511755:T:C
167:169 130:135 21:21
QHQLGVWPLLPGSFKpc3_NP.5
p−min=0.27, pX=0.95, N=318
peptide level

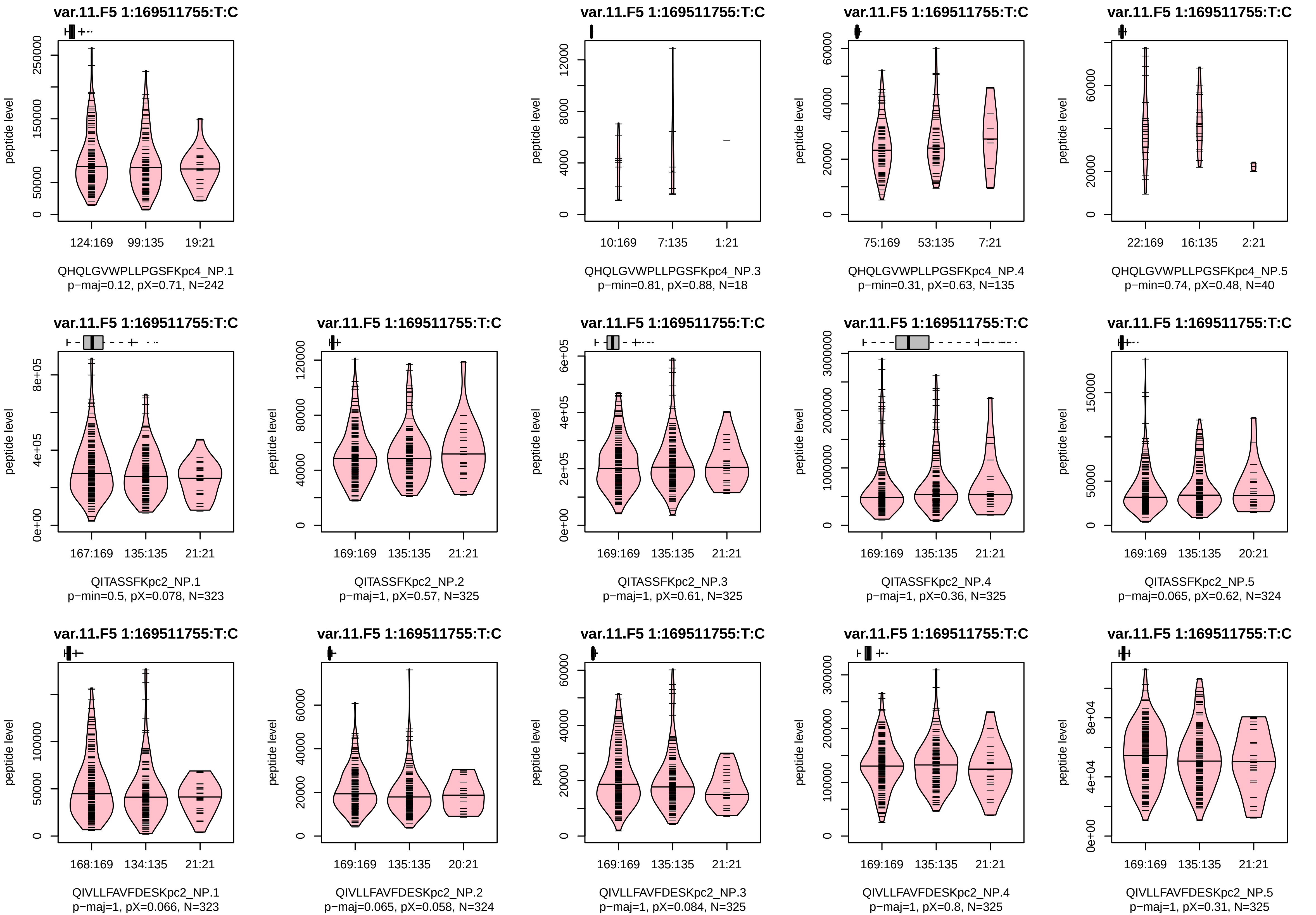

var.11.F5 1:169511755:T:C
peptide level
124:169
99:135
19:21
QHQLGVWPLLPGSFKpc4_NP.1
p−maj=0.12, pX=0.71, N=242
var.11.F5 1:169511755:T:C
peptide level
10:169
7:135
1:21
QHQLGVWPLLPGSFKpc4_NP.3
p−min=0.81, pX=0.88, N=18
var.11.F5 1:169511755:T:C
peptide level
75:169
53:135
7:21
QHQLGVWPLLPGSFKpc4_NP.4
p−min=0.31, pX=0.63, N=135
var.11.F5 1:169511755:T:C
peptide level
22:169
16:135
2:21
QHQLGVWPLLPGSFKpc4_NP.5
p−min=0.74, pX=0.48, N=40
var.11.F5 1:169511755:T:C
peptide level
167:169
135:135
21:21
QITASSFKpc2_NP.1
p−min=0.5, pX=0.078, N=323
var.11.F5 1:169511755:T:C
peptide level
169:169
135:135
21:21
QITASSFKpc2_NP.2
p−maj=1, pX=0.57, N=325
var.11.F5 1:169511755:T:C
peptide level
169:169
135:135
21:21
QITASSFKpc2_NP.3
p−maj=1, pX=0.61, N=325
var.11.F5 1:169511755:T:C
peptide level
169:169
135:135
21:21
QITASSFKpc2_NP.4
p−maj=1, pX=0.36, N=325
var.11.F5 1:169511755:T:C
peptide level
169:169
135:135
20:21
QITASSFKpc2_NP.5
p−maj=0.065, pX=0.62, N=324
var.11.F5 1:169511755:T:C
peptide level
168:169
134:135
21:21
QIVLLFAVFDESKpc2_NP.1
p−maj=1, pX=0.066, N=323
var.11.F5 1:169511755:T:C
peptide level
169:169
135:135
20:21
QIVLLFAVFDESKpc2_NP.2
p−maj=0.065, pX=0.058, N=324
var.11.F5 1:169511755:T:C
peptide level
169:169
135:135
21:21
QIVLLFAVFDESKpc2_NP.3
p−maj=1, pX=0.084, N=325
var.11.F5 1:169511755:T:C
peptide level
169:169
135:135
21:21
QIVLLFAVFDESKpc2_NP.4
p−maj=1, pX=0.8, N=325
var.11.F5 1:169511755:T:C
peptide level
169:169
135:135
21:21
QIVLLFAVFDESKpc2_NP.5
p−maj=1, pX=0.31, N=325

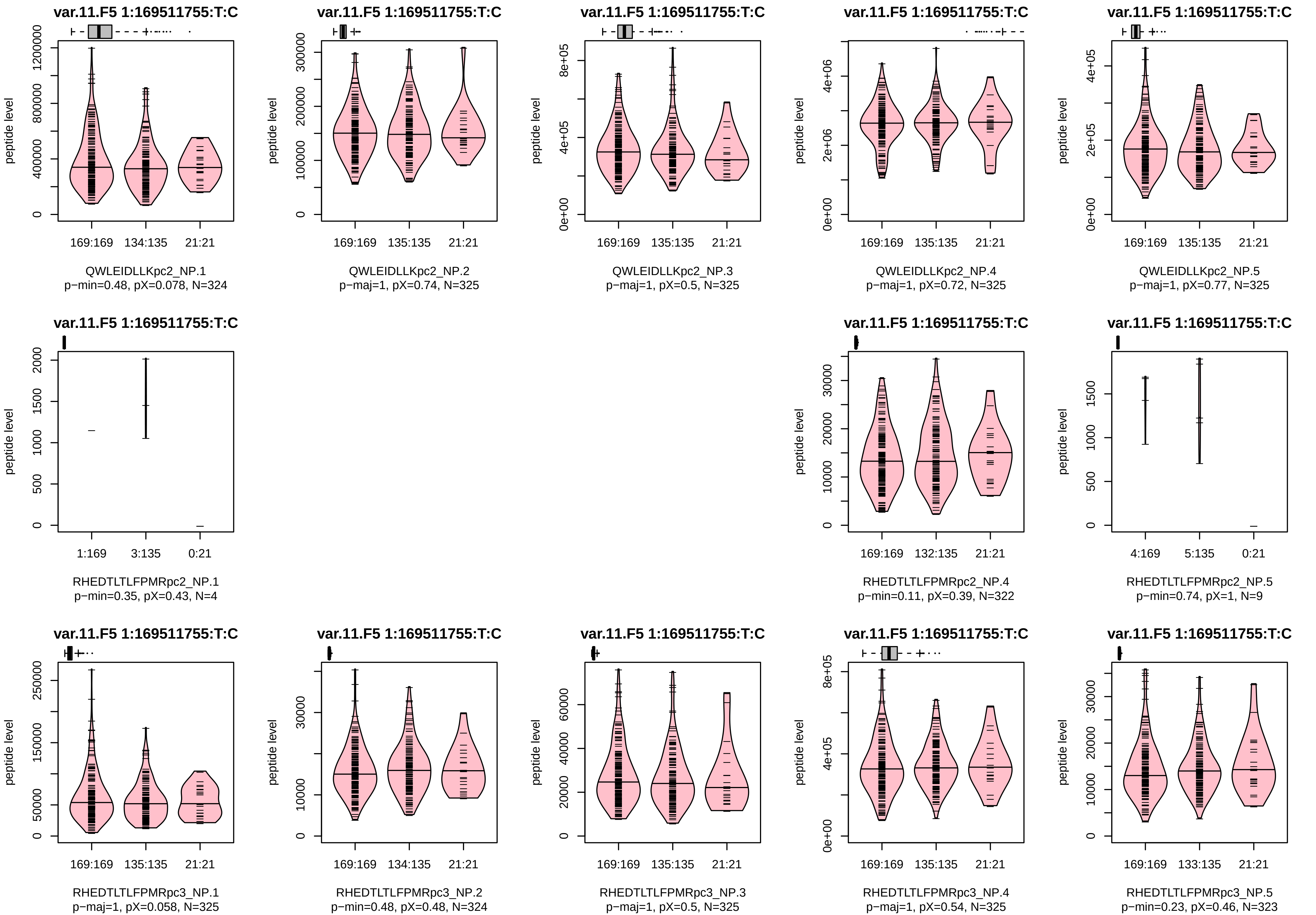

var.11.F5 1:169511755:T:C
peptide level
169:169
134:135
21:21
QWLEIDLLKpc2_NP.1
p−min=0.48, pX=0.078, N=324
var.11.F5 1:169511755:T:C
169:169
135:135
21:21
QWLEIDLLKpc2_NP.2
p−maj=1, pX=0.74, N=325
var.11.F5 1:169511755:T:C
169:169
135:135
21:21
QWLEIDLLKpc2_NP.3
p−maj=1, pX=0.5, N=325
var.11.F5 1:169511755:T:C
169:169
135:135
21:21
QWLEIDLLKpc2_NP.4
p−maj=1, pX=0.72, N=325
var.11.F5 1:169511755:T:C
169:169
135:135
21:21
QWLEIDLLKpc2_NP.5
p−maj=1, pX=0.77, N=325
var.11.F5 1:169511755:T:C
1:169
3:135
0:21
RHEDTLTLFPMRpc2_NP.1
p−min=0.35, pX=0.43, N=4
var.11.F5 1:169511755:T:C
169:169
132:135
21:21
RHEDTLTLFPMRpc2_NP.4
p−min=0.11, pX=0.39, N=322
var.11.F5 1:169511755:T:C
4:169
5:135
0:21
RHEDTLTLFPMRpc2_NP.5
p−min=0.74, pX=1, N=9
var.11.F5 1:169511755:T:C
169:169
135:135
21:21
RHEDTLTLFPMRpc3_NP.1
p−maj=1, pX=0.058, N=325
var.11.F5 1:169511755:T:C
169:169
134:135
21:21
RHEDTLTLFPMRpc3_NP.2
p−min=0.48, pX=0.48, N=324
var.11.F5 1:169511755:T:C
169:169
135:135
21:21
RHEDTLTLFPMRpc3_NP.3
p−maj=1, pX=0.5, N=325
var.11.F5 1:169511755:T:C
169:169
135:135
21:21
RHEDTLTLFPMRpc3_NP.4
p−maj=1, pX=0.54, N=325
var.11.F5 1:169511755:T:C
169:169
133:135
21:21
RHEDTLTLFPMRpc3_NP.5
p−min=0.23, pX=0.46, N=323

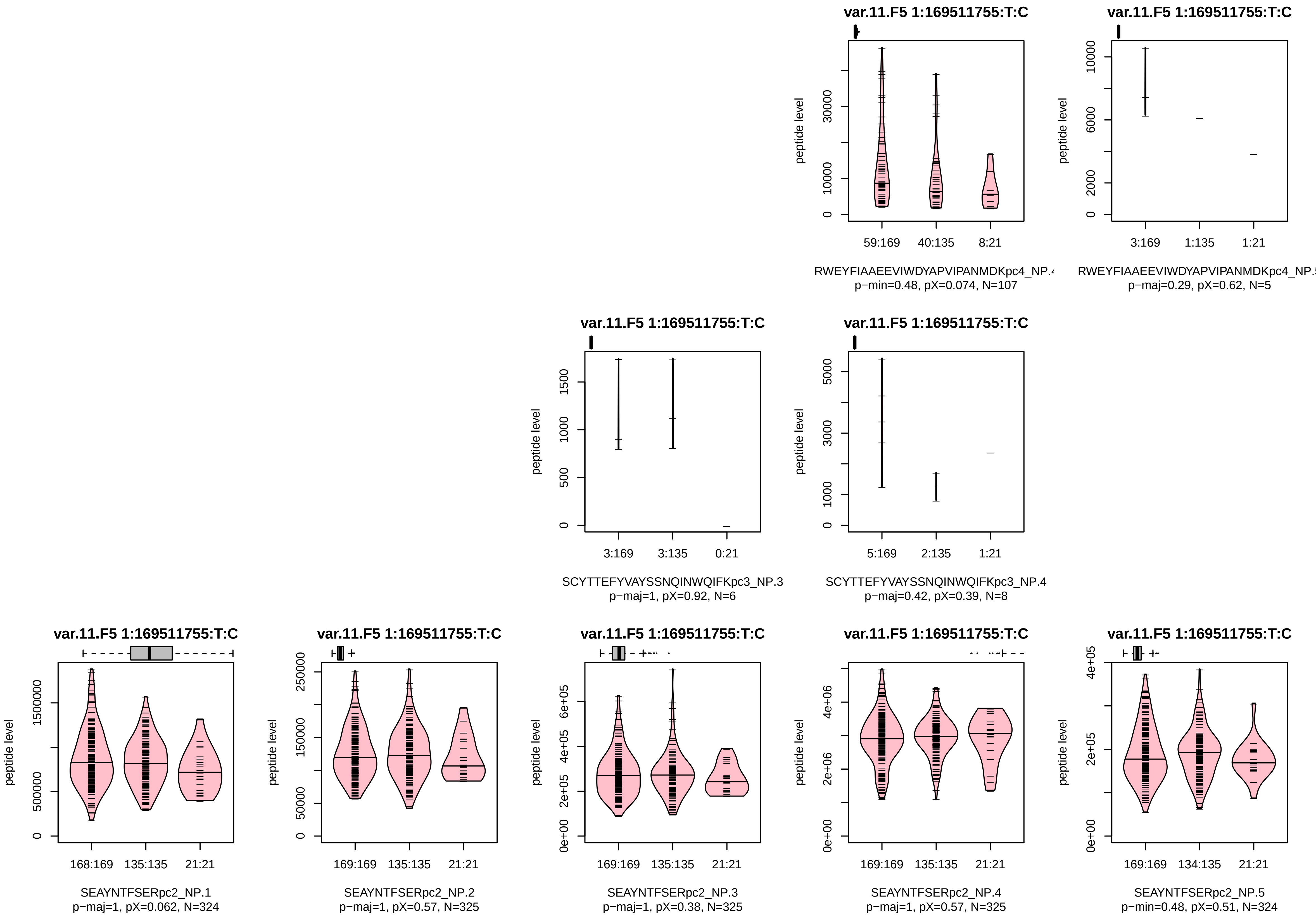

var.11.F5 1:169511755:T:C
peptide level
59:169
40:135
8:21
RWEYFIAAEEVIWDYAPVIPANMDKpc4_NP.4
p−min=0.48, pX=0.074, N=107
var.11.F5 1:169511755:T:C
peptide level
3:169
1:135
1:21
RWEYFIAAEEVIWDYAPVIPANMDKpc4_NP.5
p−maj=0.29, pX=0.62, N=5
var.11.F5 1:169511755:T:C
peptide level
3:169
3:135
0:21
SCYTTEFYVAYSSNQINWQIFKpc3_NP.3
p−maj=1, pX=0.92, N=6
var.11.F5 1:169511755:T:C
peptide level
5:169
2:135
1:21
SCYTTEFYVAYSSNQINWQIFKpc3_NP.4
p−maj=0.42, pX=0.39, N=8
var.11.F5 1:169511755:T:C
peptide level
168:169
135:135
21:21
SEAYNTFSERpc2_NP.1
p−maj=1, pX=0.062, N=324
var.11.F5 1:169511755:T:C
peptide level
169:169
135:135
21:21
SEAYNTFSERpc2_NP.2
p−maj=1, pX=0.57, N=325
var.11.F5 1:169511755:T:C
peptide level
169:169
135:135
21:21
SEAYNTFSERpc2_NP.3
p−maj=1, pX=0.38, N=325
var.11.F5 1:169511755:T:C
peptide level
169:169
135:135
21:21
SEAYNTFSERpc2_NP.4
p−maj=1, pX=0.57, N=325
var.11.F5 1:169511755:T:C
peptide level
169:169
134:135
21:21
SEAYNTFSERpc2_NP.5
p−min=0.48, pX=0.51, N=324

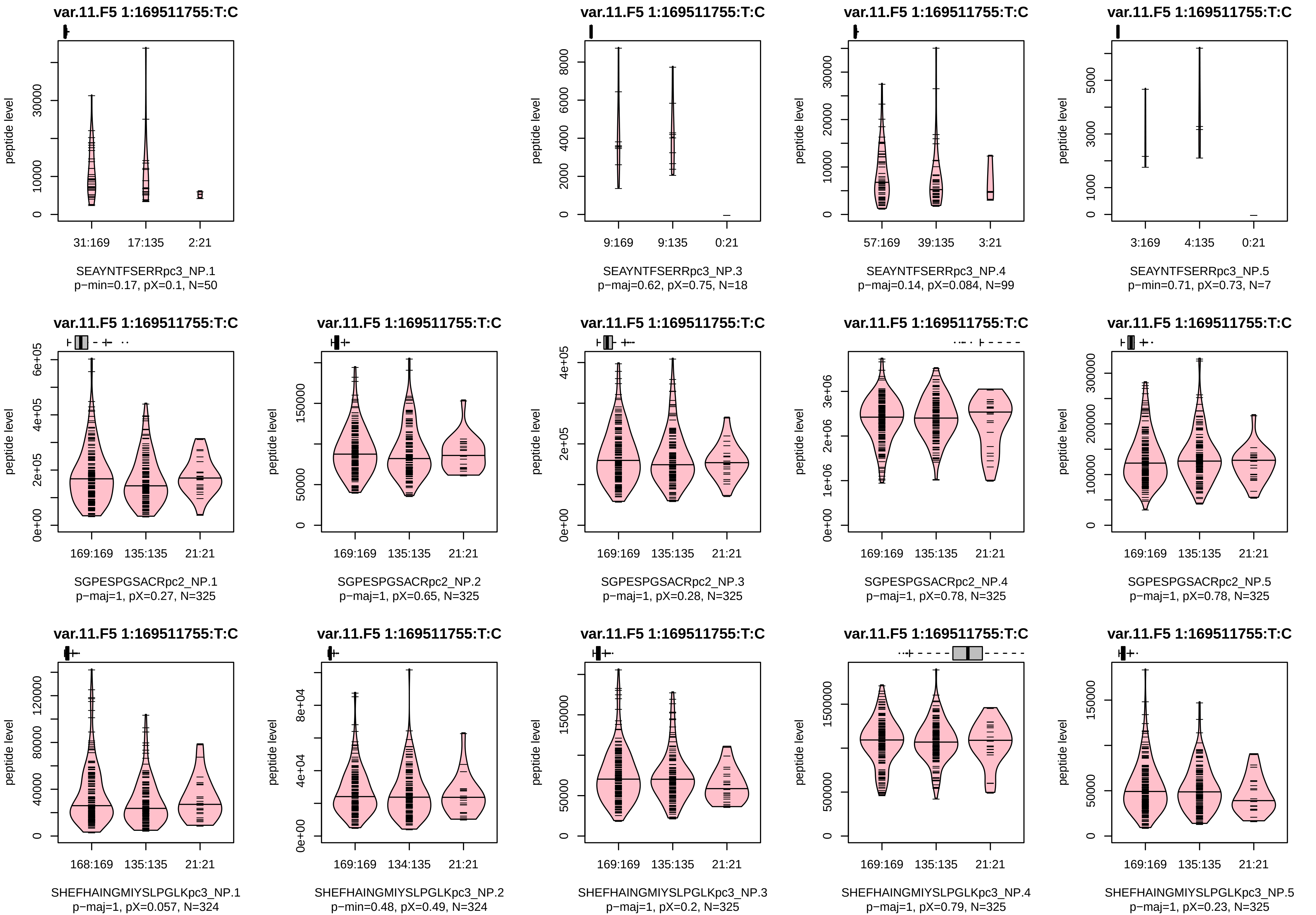
var.11.F5 1:169511755:T:C
peptide level
31:169
17:135
2:21
SEAYNTFSERRpc3_NP.1
p−min=0.17, pX=0.1, N=50
var.11.F5 1:169511755:T:C
peptide level
9:169
9:135
0:21
SEAYNTFSERRpc3_NP.3
p−maj=0.62, pX=0.75, N=18
var.11.F5 1:169511755:T:C
peptide level
57:169
39:135
3:21
SEAYNTFSERRpc3_NP.4
p−maj=0.14, pX=0.084, N=99
var.11.F5 1:169511755:T:C
peptide level
3:169
4:135
0:21
SEAYNTFSERRpc3_NP.5
p−min=0.71, pX=0.73, N=7
var.11.F5 1:169511755:T:C
peptide level
169:169
135:135
21:21
SGPESPGSACRpc2_NP.1
p−maj=1, pX=0.27, N=325
var.11.F5 1:169511755:T:C
peptide level
169:169
135:135
21:21
SGPESPGSACRpc2_NP.2
p−maj=1, pX=0.65, N=325
var.11.F5 1:169511755:T:C
peptide level
169:169
135:135
21:21
SGPESPGSACRpc2_NP.3
p−maj=1, pX=0.28, N=325
var.11.F5 1:169511755:T:C
peptide level
169:169
135:135
21:21
SGPESPGSACRpc2_NP.4
p−maj=1, pX=0.78, N=325
var.11.F5 1:169511755:T:C
peptide level
169:169
135:135
21:21
SGPESPGSACRpc2_NP.5
p−maj=1, pX=0.78, N=325
var.11.F5 1:169511755:T:C
peptide level
168:169
135:135
21:21
SHEFHAINGMIYSLPGLKpc3_NP.1
p−maj=1, pX=0.057, N=324
var.11.F5 1:169511755:T:C
peptide level
169:169
134:135
21:21
SHEFHAINGMIYSLPGLKpc3_NP.2
p−min=0.48, pX=0.49, N=324
var.11.F5 1:169511755:T:C
peptide level
169:169
135:135
21:21
SHEFHAINGMIYSLPGLKpc3_NP.3
p−maj=1, pX=0.2, N=325
var.11.F5 1:169511755:T:C
peptide level
169:169
135:135
21:21
SHEFHAINGMIYSLPGLKpc3_NP.4
p−maj=1, pX=0.79, N=325
var.11.F5 1:169511755:T:C
peptide level
169:169
135:135
21:21
SHEFHAINGMIYSLPGLKpc3_NP.5
p−maj=1, pX=0.23, N=325

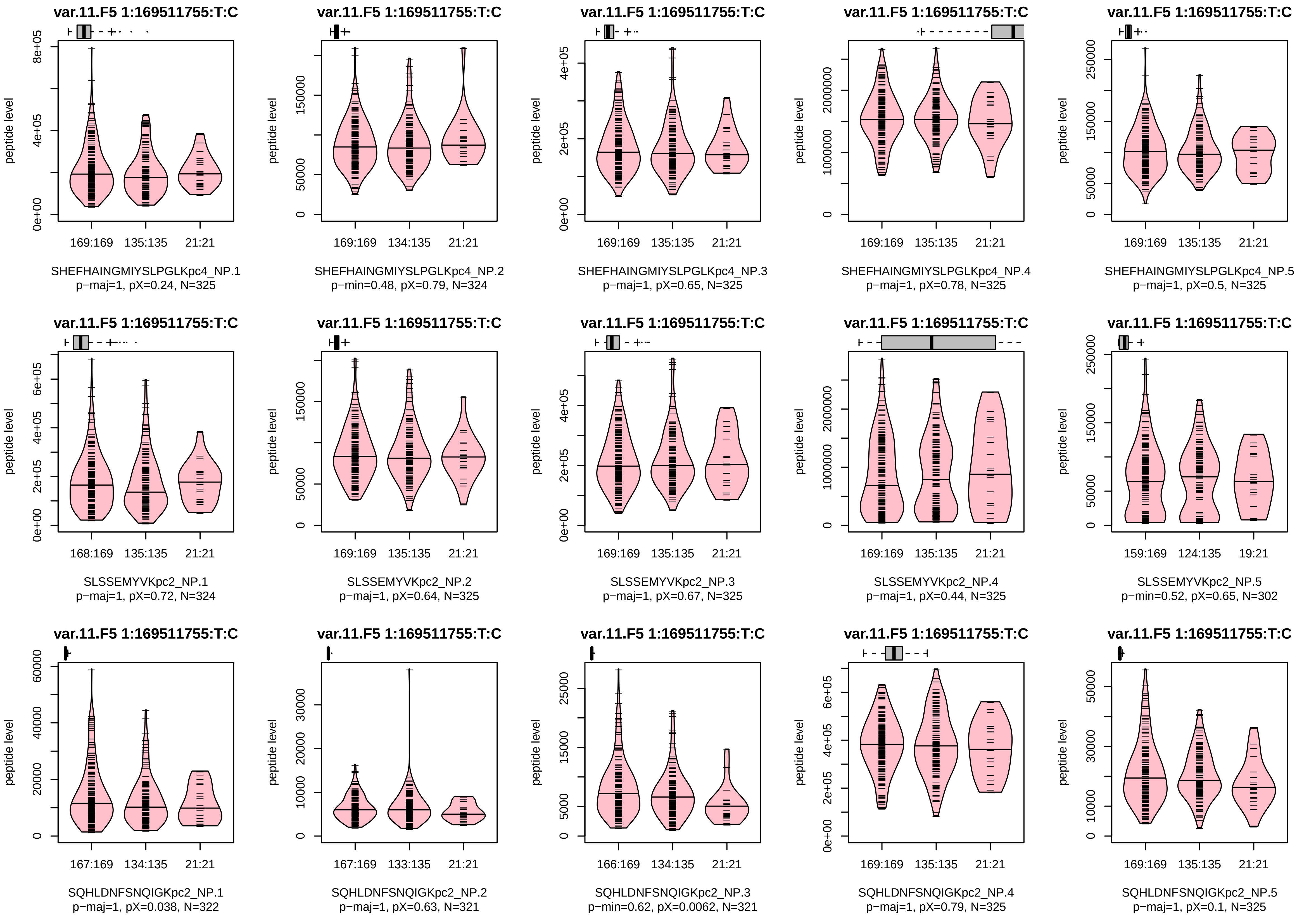

var.11.F5 1:169511755:T:C
peptide level
169:169
135:135
21:21
SHEFHAINGMIYSLPGLKpc4_NP.1
p−maj=1, pX=0.24, N=325
var.11.F5 1:169511755:T:C
peptide level
169:169
134:135
21:21
SHEFHAINGMIYSLPGLKpc4_NP.2
p−min=0.48, pX=0.79, N=324
var.11.F5 1:169511755:T:C
peptide level
169:169
135:135
21:21
SHEFHAINGMIYSLPGLKpc4_NP.3
p−maj=1, pX=0.65, N=325
var.11.F5 1:169511755:T:C
peptide level
169:169
135:135
21:21
SHEFHAINGMIYSLPGLKpc4_NP.4
p−maj=1, pX=0.78, N=325
var.11.F5 1:169511755:T:C
peptide level
169:169
135:135
21:21
SHEFHAINGMIYSLPGLKpc4_NP.5
p−maj=1, pX=0.5, N=325
var.11.F5 1:169511755:T:C
peptide level
168:169
135:135
21:21
SLSSEMYVKpc2_NP.1
p−maj=1, pX=0.72, N=324
var.11.F5 1:169511755:T:C
peptide level
169:169
135:135
21:21
SLSSEMYVKpc2_NP.2
p−maj=1, pX=0.64, N=325
var.11.F5 1:169511755:T:C
peptide level
169:169
135:135
21:21
SLSSEMYVKpc2_NP.3
p−maj=1, pX=0.67, N=325
var.11.F5 1:169511755:T:C
peptide level
169:169
135:135
21:21
SLSSEMYVKpc2_NP.4
p−maj=1, pX=0.44, N=325
var.11.F5 1:169511755:T:C
peptide level
159:169
124:135
19:21
SLSSEMYVKpc2_NP.5
p−min=0.52, pX=0.65, N=302
var.11.F5 1:169511755:T:C
peptide level
167:169
134:135
21:21
SQHLDNFSNQIGKpc2_NP.1
p−maj=1, pX=0.038, N=322
var.11.F5 1:169511755:T:C
peptide level
167:169
133:135
21:21
SQHLDNFSNQIGKpc2_NP.2
p−maj=1, pX=0.63, N=321
var.11.F5 1:169511755:T:C
peptide level
166:169
134:135
21:21
SQHLDNFSNQIGKpc2_NP.3
p−min=0.62, pX=0.0062, N=321
var.11.F5 1:169511755:T:C
peptide level
169:169
135:135
21:21
SQHLDNFSNQIGKpc2_NP.4
p−maj=1, pX=0.79, N=325
var.11.F5 1:169511755:T:C
peptide level
169:169
135:135
21:21
SQHLDNFSNQIGKpc2_NP.5
p−maj=1, pX=0.1, N=325

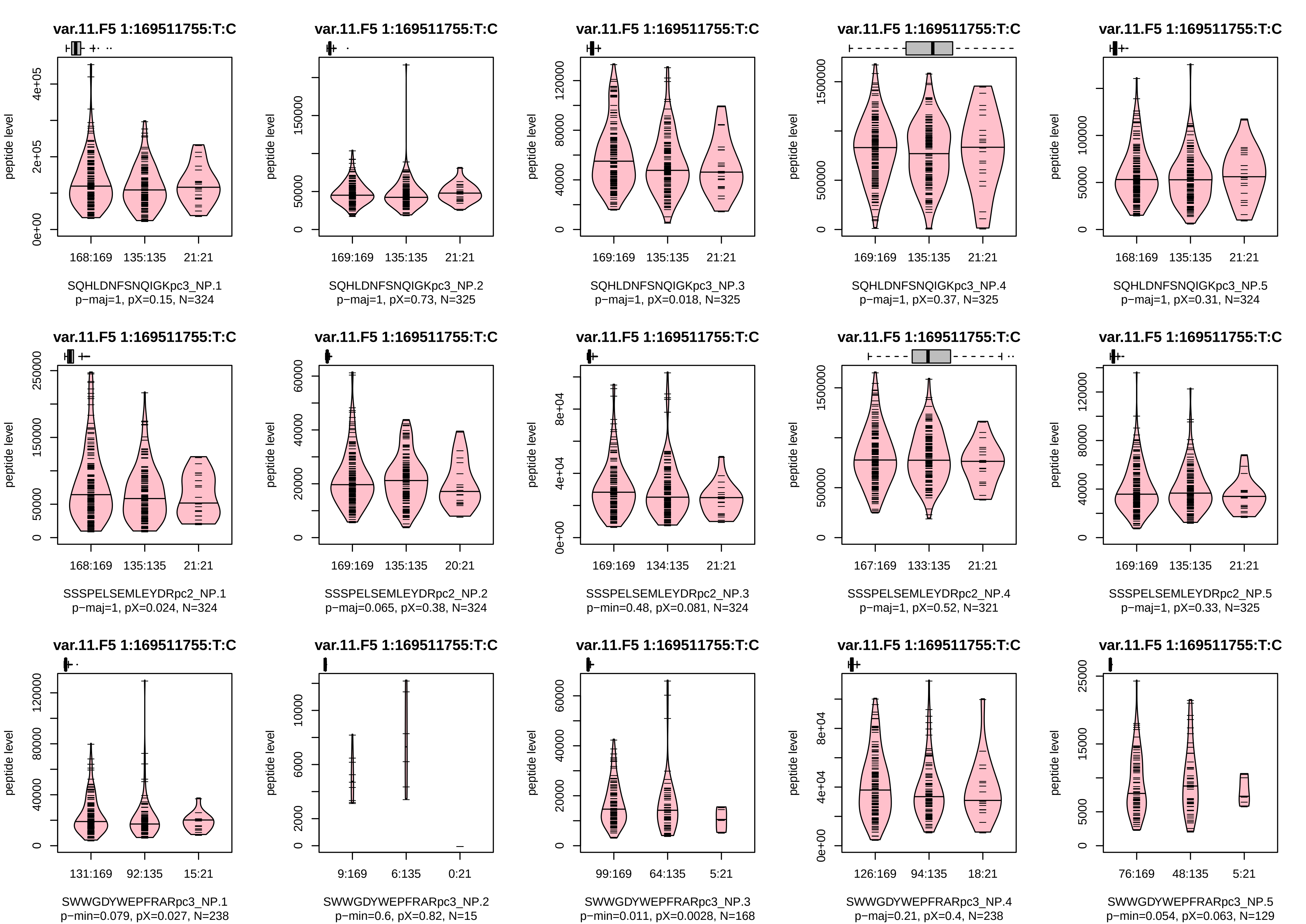
var.11.F5 1:169511755:T:C
peptide level
168:169
135:135
21:21
SQHLDNFSNQIGKpc3_NP.1
p−maj=1, pX=0.15, N=324
var.11.F5 1:169511755:T:C
peptide level
169:169
135:135
21:21
SQHLDNFSNQIGKpc3_NP.2
p−maj=1, pX=0.73, N=325
var.11.F5 1:169511755:T:C
peptide level
169:169
135:135
21:21
SQHLDNFSNQIGKpc3_NP.3
p−maj=1, pX=0.018, N=325
var.11.F5 1:169511755:T:C
peptide level
169:169
135:135
21:21
SQHLDNFSNQIGKpc3_NP.4
p−maj=1, pX=0.37, N=325
var.11.F5 1:169511755:T:C
peptide level
168:169
135:135
21:21
SQHLDNFSNQIGKpc3_NP.5
p−maj=1, pX=0.31, N=324
var.11.F5 1:169511755:T:C
peptide level
168:169
135:135
21:21
SSSPELSEMLEYDRpc2_NP.1
p−maj=1, pX=0.024, N=324
var.11.F5 1:169511755:T:C
peptide level
169:169
135:135
20:21
SSSPELSEMLEYDRpc2_NP.2
p−maj=0.065, pX=0.38, N=324
var.11.F5 1:169511755:T:C
peptide level
169:169
134:135
21:21
SSSPELSEMLEYDRpc2_NP.3
p−min=0.48, pX=0.081, N=324
var.11.F5 1:169511755:T:C
peptide level
167:169
133:135
21:21
SSSPELSEMLEYDRpc2_NP.4
p−maj=1, pX=0.52, N=321
var.11.F5 1:169511755:T:C
peptide level
169:169
135:135
21:21
SSSPELSEMLEYDRpc2_NP.5
p−maj=1, pX=0.33, N=325
var.11.F5 1:169511755:T:C
peptide level
131:169
92:135
15:21
SWWGDYWEPFRARpc3_NP.1
p−min=0.079, pX=0.027, N=238
var.11.F5 1:169511755:T:C
peptide level
9:169
6:135
0:21
SWWGDYWEPFRARpc3_NP.2
p−min=0.6, pX=0.82, N=15
var.11.F5 1:169511755:T:C
peptide level
99:169
64:135
5:21
SWWGDYWEPFRARpc3_NP.3
p−min=0.011, pX=0.0028, N=168
var.11.F5 1:169511755:T:C
peptide level
126:169
94:135
18:21
SWWGDYWEPFRARpc3_NP.4
p−maj=0.21, pX=0.4, N=238
var.11.F5 1:169511755:T:C
peptide level
76:169
48:135
5:21
SWWGDYWEPFRARpc3_NP.5
p−min=0.054, pX=0.063, N=129

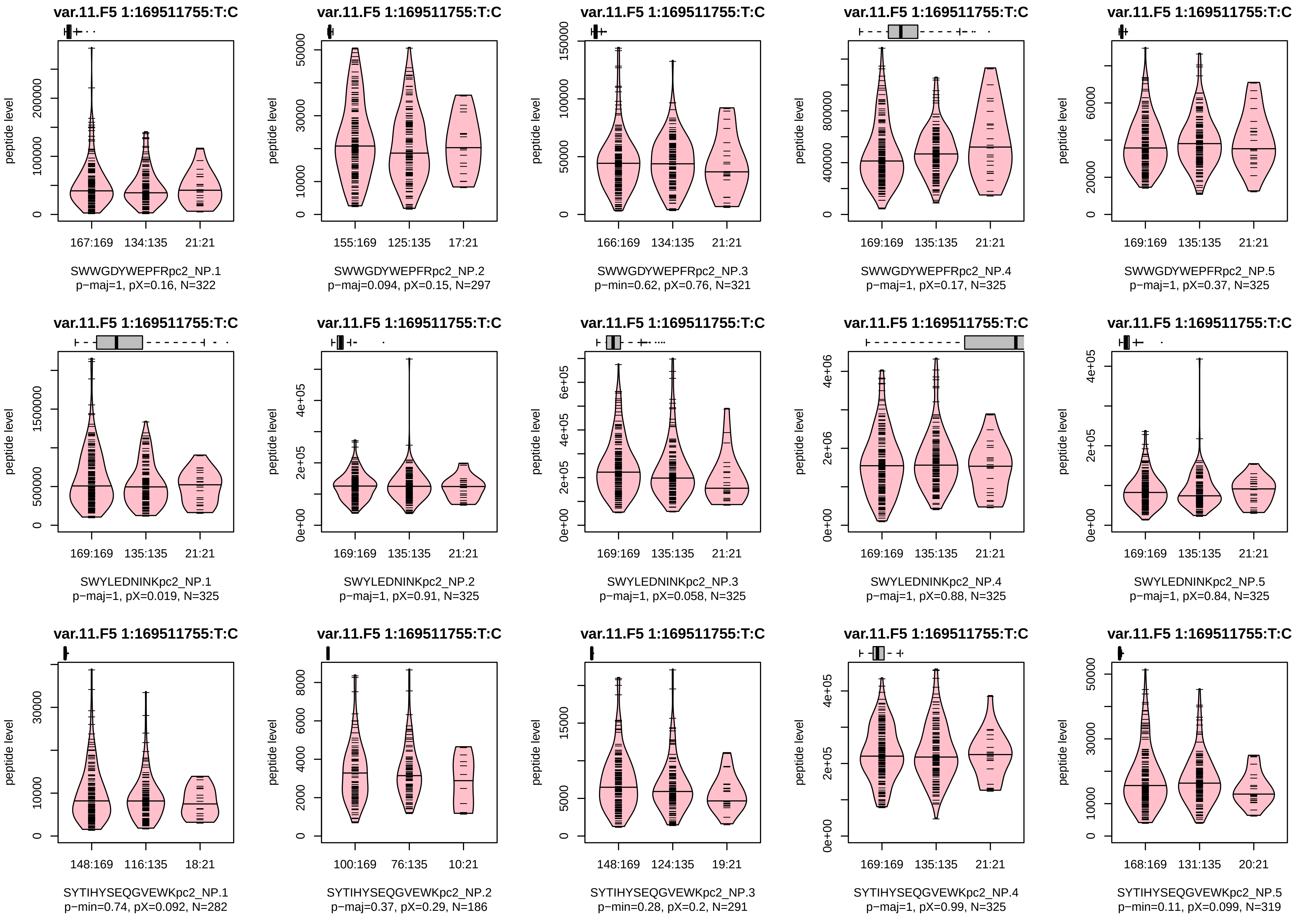
var.11.F5 1:169511755:T:C
peptide level
167:169
134:135
21:21
SWWGDYWEPFRpc2_NP.1
p−maj=1, pX=0.16, N=322
var.11.F5 1:169511755:T:C
peptide level
155:169
125:135
17:21
SWWGDYWEPFRpc2_NP.2
p−maj=0.094, pX=0.15, N=297
var.11.F5 1:169511755:T:C
peptide level
166:169
134:135
21:21
SWWGDYWEPFRpc2_NP.3
p−min=0.62, pX=0.76, N=321
var.11.F5 1:169511755:T:C
peptide level
169:169
135:135
21:21
SWWGDYWEPFRpc2_NP.4
p−maj=1, pX=0.17, N=325
var.11.F5 1:169511755:T:C
peptide level
169:169
135:135
21:21
SWWGDYWEPFRpc2_NP.5
p−maj=1, pX=0.37, N=325
var.11.F5 1:169511755:T:C
peptide level
169:169
135:135
21:21
SWYLEDNINKpc2_NP.1
p−maj=1, pX=0.019, N=325
var.11.F5 1:169511755:T:C
peptide level
169:169
135:135
21:21
SWYLEDNINKpc2_NP.2
p−maj=1, pX=0.91, N=325
var.11.F5 1:169511755:T:C
peptide level
169:169
135:135
21:21
SWYLEDNINKpc2_NP.3
p−maj=1, pX=0.058, N=325
var.11.F5 1:169511755:T:C
peptide level
169:169
135:135
21:21
SWYLEDNINKpc2_NP.4
p−maj=1, pX=0.88, N=325
var.11.F5 1:169511755:T:C
peptide level
169:169
135:135
21:21
SWYLEDNINKpc2_NP.5
p−maj=1, pX=0.84, N=325
var.11.F5 1:169511755:T:C
peptide level
148:169
116:135
18:21
SYTIHYSEQGVEWKpc2_NP.1
p−min=0.74, pX=0.092, N=282
var.11.F5 1:169511755:T:C
peptide level
100:169
76:135
10:21
SYTIHYSEQGVEWKpc2_NP.2
p−maj=0.37, pX=0.29, N=186
var.11.F5 1:169511755:T:C
peptide level
148:169
124:135
19:21
SYTIHYSEQGVEWKpc2_NP.3
p−min=0.28, pX=0.2, N=291
var.11.F5 1:169511755:T:C
peptide level
169:169
135:135
21:21
SYTIHYSEQGVEWKpc2_NP.4
p−maj=1, pX=0.99, N=325
var.11.F5 1:169511755:T:C
peptide level
168:169
131:135
20:21
SYTIHYSEQGVEWKpc2_NP.5
p−min=0.11, pX=0.099, N=319

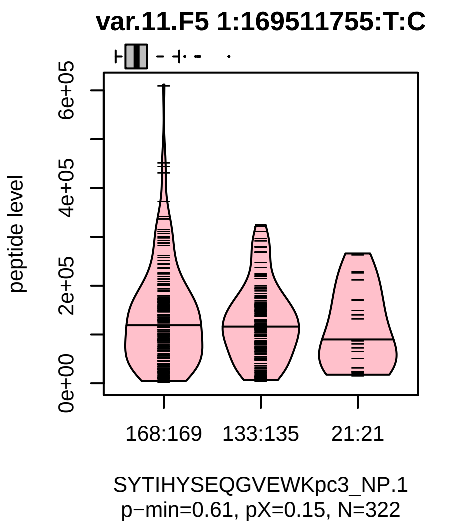
var.11.F5 1:169511755:T:C
peptide level
0e+00 2e+05 4e+05 6e+05
168:169 133:135 21:21
SYTIHYSEQGVEWKpc3_NP.1
p−min=0.61, pX=0.15, N=322

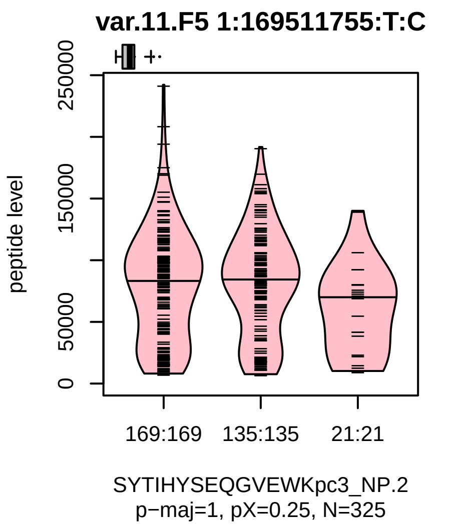
var.11.F5 1:169511755:T:C
peptide level
0 50000 150000 250000
169:169 135:135 21:21
SYTIHYSEQGVEWKpc3_NP.2
p−maj=1, pX=0.25, N=325

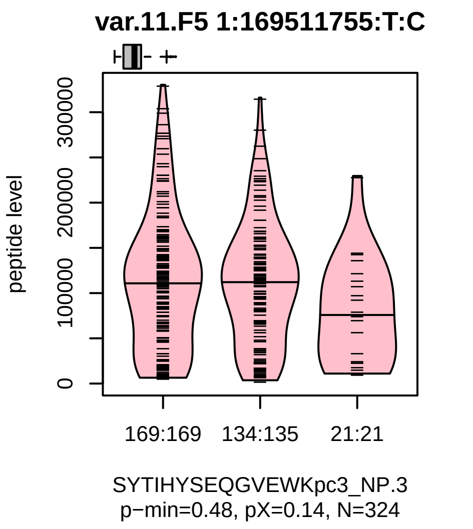
var.11.F5 1:169511755:T:C
peptide level
0 100000 200000 300000
169:169 134:135 21:21
SYTIHYSEQGVEWKpc3_NP.3
p−min=0.48, pX=0.14, N=324

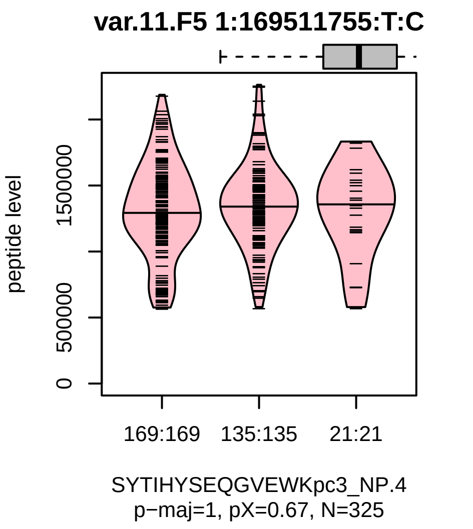
var.11.F5 1:169511755:T:C
peptide level
0 500000 1500000
169:169 135:135 21:21
SYTIHYSEQGVEWKpc3_NP.4
p−maj=1, pX=0.67, N=325

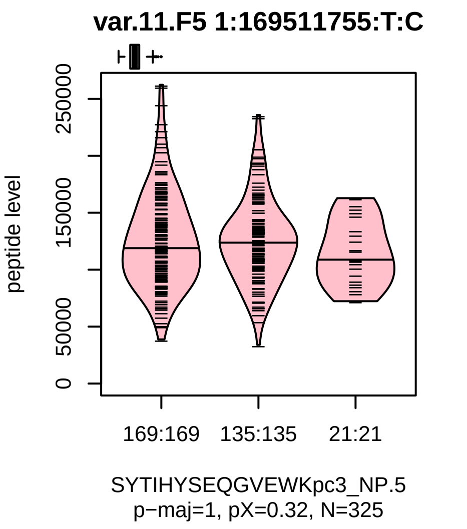
var.11.F5 1:169511755:T:C
peptide level
0 50000 150000 250000
169:169 135:135 21:21
SYTIHYSEQGVEWKpc3_NP.5
p−maj=1, pX=0.32, N=325

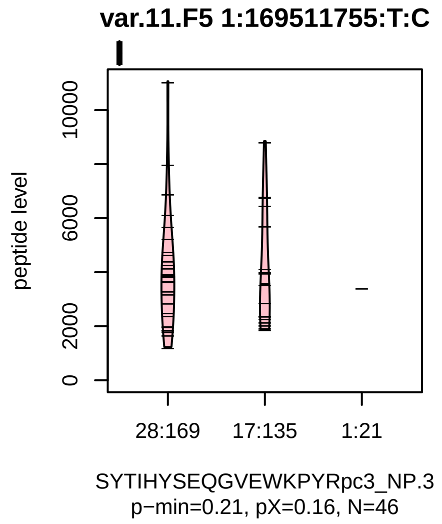
var.11.F5 1:169511755:T:C
peptide level
0 2000 6000 10000
28:169 17:135 1:21
SYTIHYSEQGVEWKPYRpc3_NP.3
p−min=0.21, pX=0.16, N=46

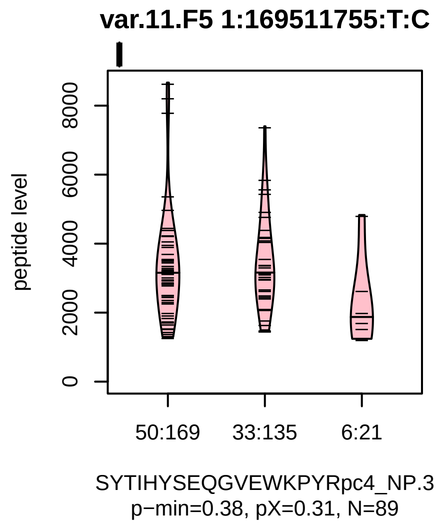
var.11.F5 1:169511755:T:C
peptide level
0 2000 4000 6000 8000
50:169 33:135 6:21
SYTIHYSEQGVEWKPYRpc4_NP.3
p−min=0.38, pX=0.31, N=89

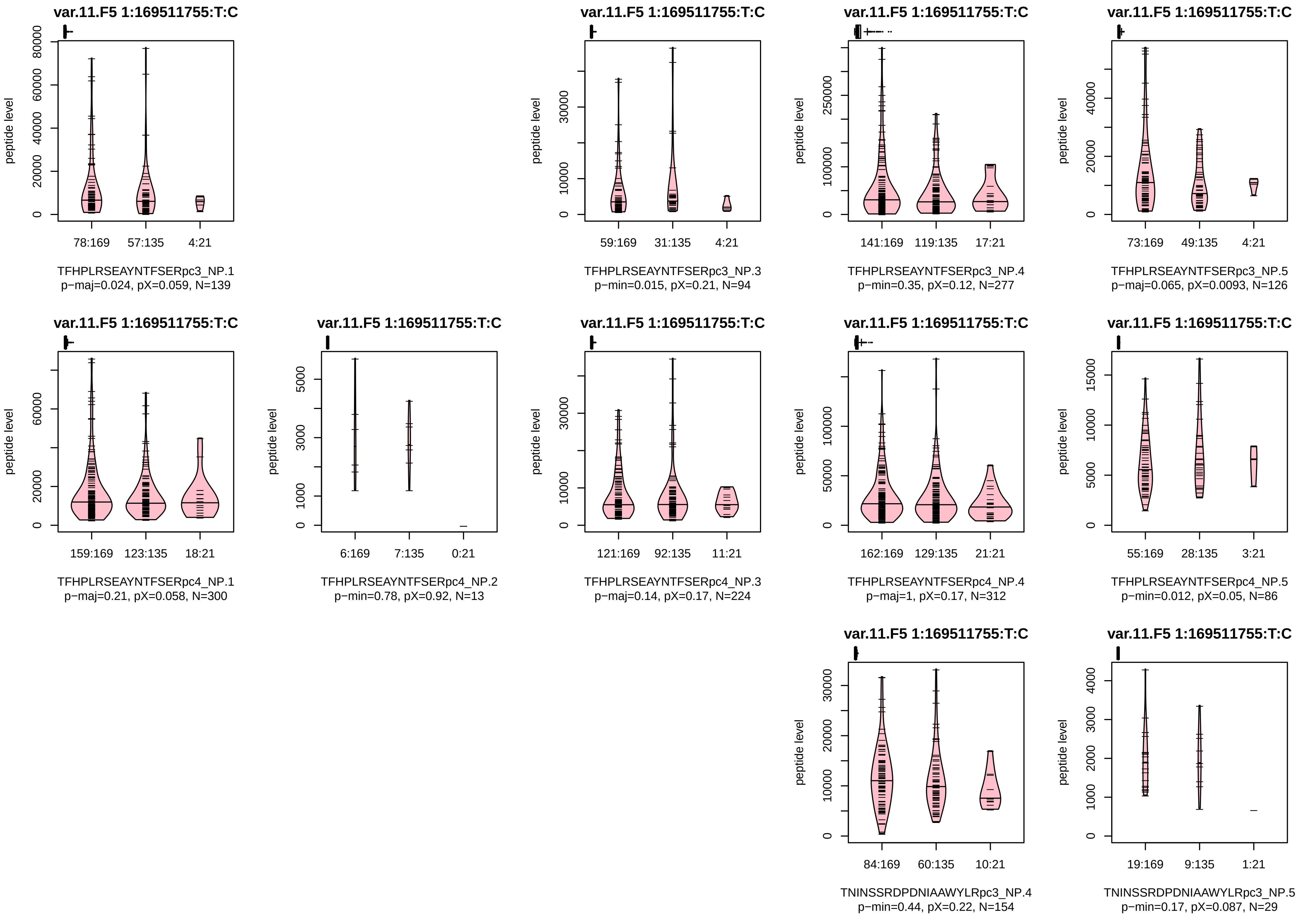
var.11.F5 1:169511755:T:C
78:169
57:135
4:21
TFHPLRSEAYNTFSERpc3_NP.1
p−maj=0.024, pX=0.059, N=139
var.11.F5 1:169511755:T:C
59:169
31:135
4:21
TFHPLRSEAYNTFSERpc3_NP.3
p−min=0.015, pX=0.21, N=94
var.11.F5 1:169511755:T:C
141:169
119:135
17:21
TFHPLRSEAYNTFSERpc3_NP.4
p−min=0.35, pX=0.12, N=277
var.11.F5 1:169511755:T:C
73:169
49:135
4:21
TFHPLRSEAYNTFSERpc3_NP.5
p−maj=0.065, pX=0.0093, N=126
var.11.F5 1:169511755:T:C
159:169
123:135
18:21
TFHPLRSEAYNTFSERpc4_NP.1
p−maj=0.21, pX=0.058, N=300
var.11.F5 1:169511755:T:C
6:169
7:135
0:21
TFHPLRSEAYNTFSERpc4_NP.2
p−min=0.78, pX=0.92, N=13
var.11.F5 1:169511755:T:C
121:169
92:135
11:21
TFHPLRSEAYNTFSERpc4_NP.3
p−maj=0.14, pX=0.17, N=224
var.11.F5 1:169511755:T:C
162:169
129:135
21:21
TFHPLRSEAYNTFSERpc4_NP.4
p−maj=1, pX=0.17, N=312
var.11.F5 1:169511755:T:C
55:169
28:135
3:21
TFHPLRSEAYNTFSERpc4_NP.5
p−min=0.012, pX=0.05, N=86
var.11.F5 1:169511755:T:C
84:169
60:135
10:21
TNINSSRDPDNIAAWYLRpc3_NP.4
p−min=0.44, pX=0.22, N=154
var.11.F5 1:169511755:T:C
19:169
9:135
1:21
TNINSSRDPDNIAAWYLRpc3_NP.5
p−min=0.17, pX=0.087, N=29

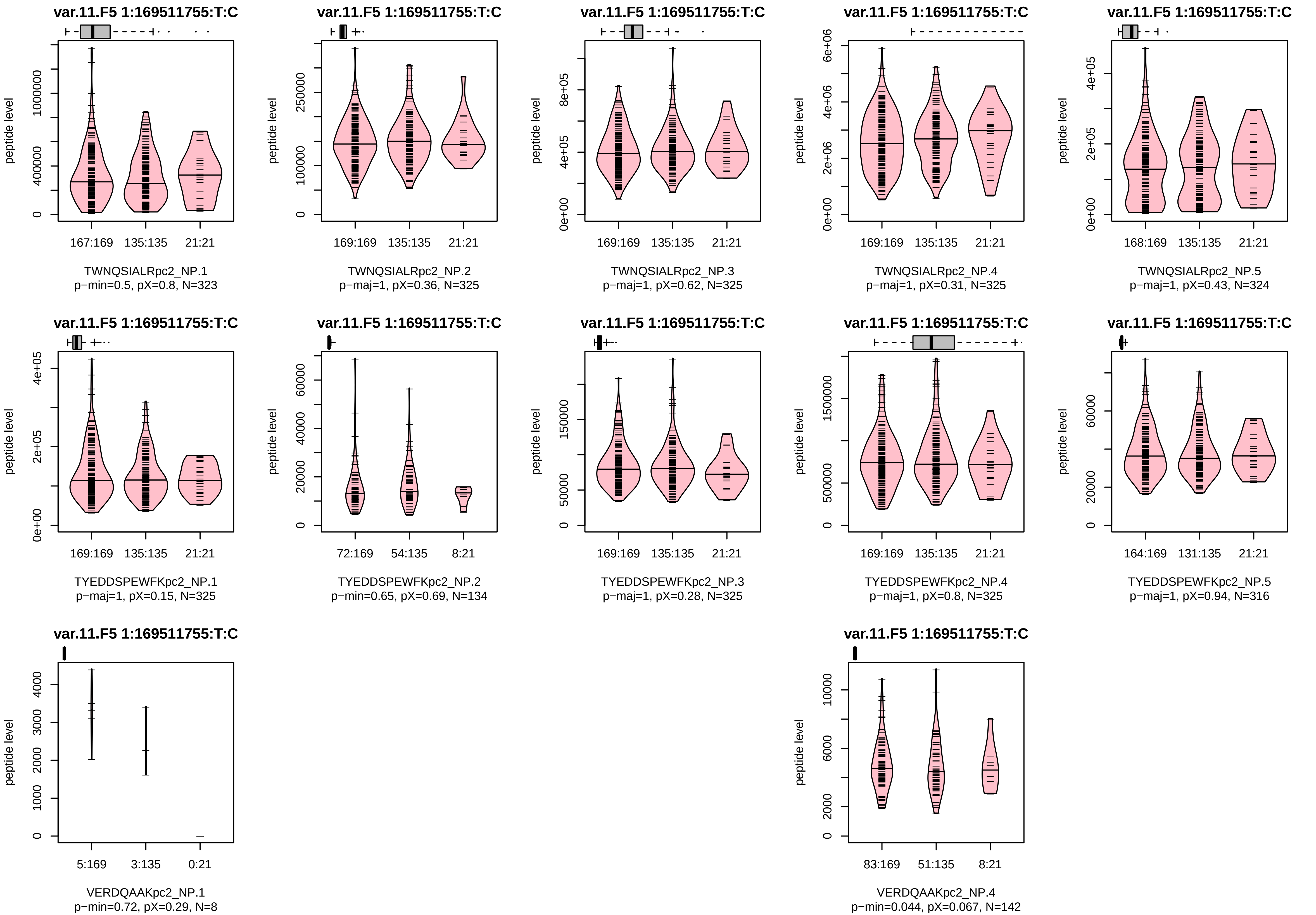
var.11.F5 1:169511755:T:C
peptide level
167:169
135:135
21:21
TWNQSIALRpc2_NP.1
p−min=0.5, pX=0.8, N=323
169:169
TWNQSIALRpc2_NP.2
p−maj=1, pX=0.36, N=325
TWNQSIALRpc2_NP.3
p−maj=1, pX=0.62, N=325
TWNQSIALRpc2_NP.4
p−maj=1, pX=0.31, N=325
168:169
TWNQSIALRpc2_NP.5
p−maj=1, pX=0.43, N=324
TYEDDSPEWFKpc2_NP.1
p−maj=1, pX=0.15, N=325
72:169
54:135
8:21
TYEDDSPEWFKpc2_NP.2
p−min=0.65, pX=0.69, N=134
TYEDDSPEWFKpc2_NP.3
p−maj=1, pX=0.28, N=325
TYEDDSPEWFKpc2_NP.4
p−maj=1, pX=0.8, N=325
164:169
131:135
TYEDDSPEWFKpc2_NP.5
p−maj=1, pX=0.94, N=316
5:169
3:135
0:21
VERDQAAKpc2_NP.1
p−min=0.72, pX=0.29, N=8
83:169
51:135
VERDQAAKpc2_NP.4
p−min=0.044, pX=0.067, N=142

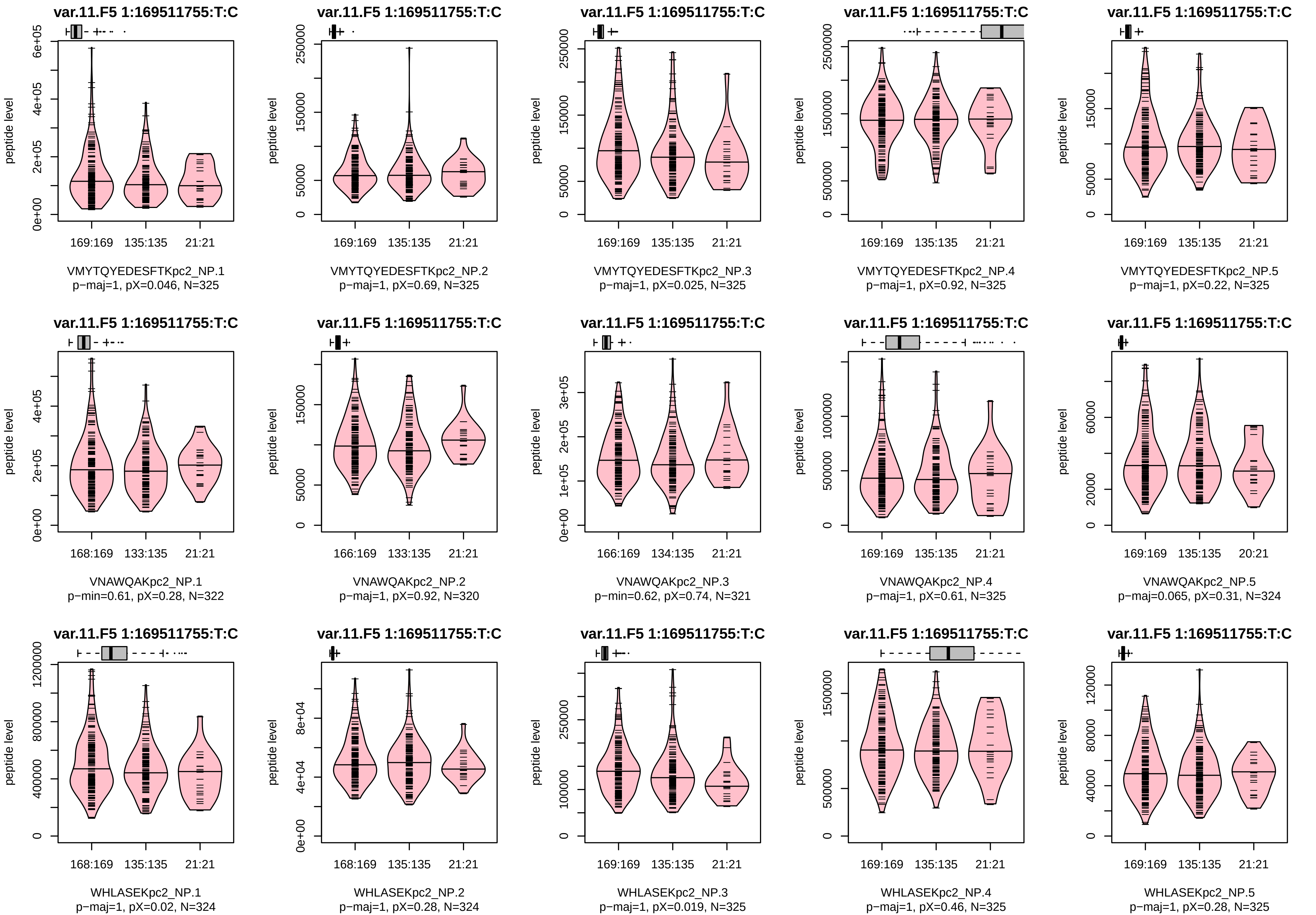

var.11.F5 1:169511755:T:C
peptide level
169:169
135:135
21:21
VMYTQYEDESFTKpc2_NP.1
p−maj=1, pX=0.046, N=325
var.11.F5 1:169511755:T:C
VMYTQYEDESFTKpc2_NP.2
p−maj=1, pX=0.69, N=325
var.11.F5 1:169511755:T:C
VMYTQYEDESFTKpc2_NP.3
p−maj=1, pX=0.025, N=325
var.11.F5 1:169511755:T:C
VMYTQYEDESFTKpc2_NP.4
p−maj=1, pX=0.92, N=325
var.11.F5 1:169511755:T:C
VMYTQYEDESFTKpc2_NP.5
p−maj=1, pX=0.22, N=325
var.11.F5 1:169511755:T:C
168:169
133:135
21:21
VNAWQAKpc2_NP.1
p−min=0.61, pX=0.28, N=322
var.11.F5 1:169511755:T:C
166:169
133:135
21:21
VNAWQAKpc2_NP.2
p−maj=1, pX=0.92, N=320
var.11.F5 1:169511755:T:C
166:169
134:135
21:21
VNAWQAKpc2_NP.3
p−min=0.62, pX=0.74, N=321
var.11.F5 1:169511755:T:C
169:169
135:135
21:21
VNAWQAKpc2_NP.4
p−maj=1, pX=0.61, N=325
var.11.F5 1:169511755:T:C
169:169
135:135
20:21
VNAWQAKpc2_NP.5
p−maj=0.065, pX=0.31, N=324
var.11.F5 1:169511755:T:C
168:169
135:135
21:21
WHLASEKpc2_NP.1
p−maj=1, pX=0.02, N=324
var.11.F5 1:169511755:T:C
168:169
135:135
21:21
WHLASEKpc2_NP.2
p−maj=1, pX=0.28, N=324
var.11.F5 1:169511755:T:C
169:169
135:135
21:21
WHLASEKpc2_NP.3
p−maj=1, pX=0.019, N=325
var.11.F5 1:169511755:T:C
169:169
135:135
21:21
WHLASEKpc2_NP.4
p−maj=1, pX=0.46, N=325
var.11.F5 1:169511755:T:C
169:169
135:135
21:21
WHLASEKpc2_NP.5
p−maj=1, pX=0.28, N=325

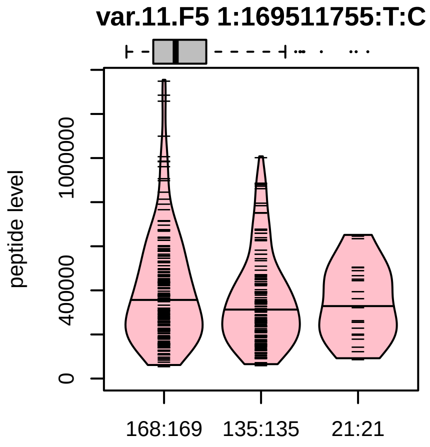

WIISSLTPKpc2_NP.1
p−maj=1, pX=0.073, N=324

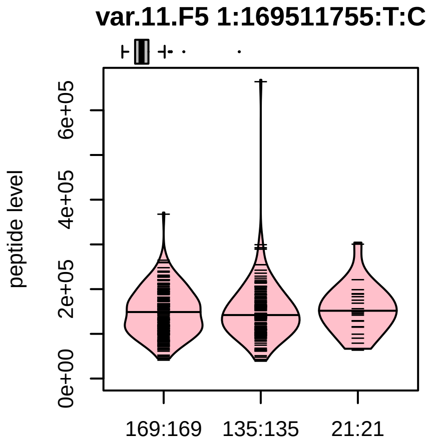

WIISSLTPKpc2_NP.2
p−maj=1, pX=0.67, N=325

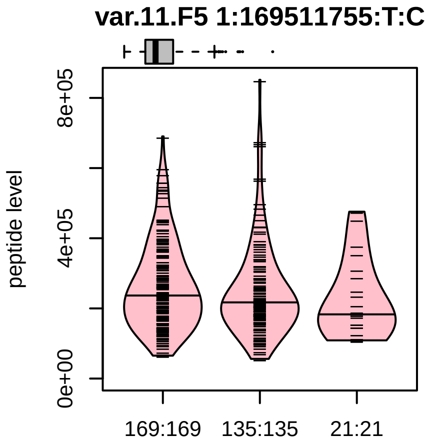

WIISSLTPKpc2_NP.3
p−maj=1, pX=0.14, N=325

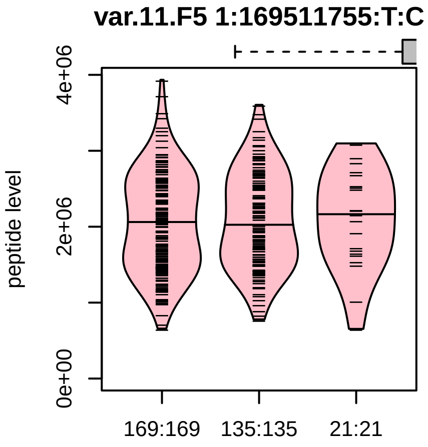

WIISSLTPKpc2_NP.4
p−maj=1, pX=0.71, N=325

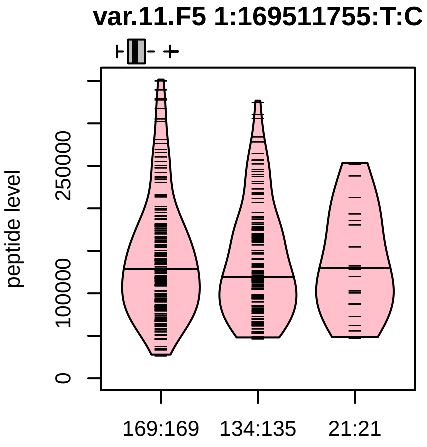

WIISSLTPKpc2_NP.5
p−min=0.48, pX=0.57, N=324

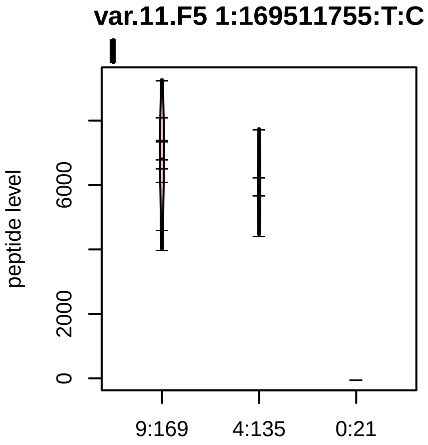

WNILEFDEPTENDAQCLTRPYYSDVDIMKpc4_N
p−min=0.26, pX=0.13, N=13

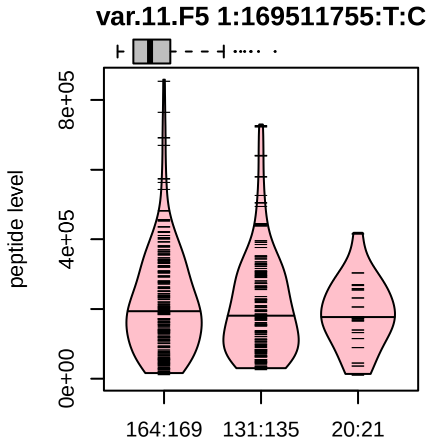

YLDSTFTKpc2_NP.1
p−maj=0.49, pX=0.34, N=315

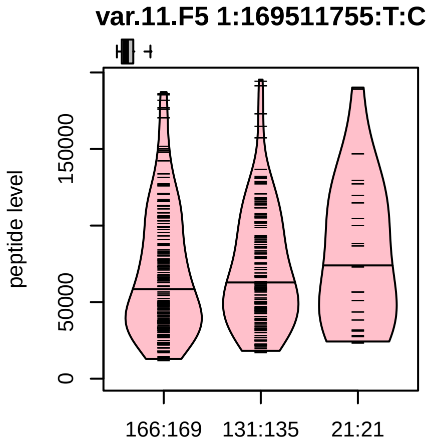

YLDSTFTKpc2_NP.2
p−min=0.71, pX=0.34, N=318

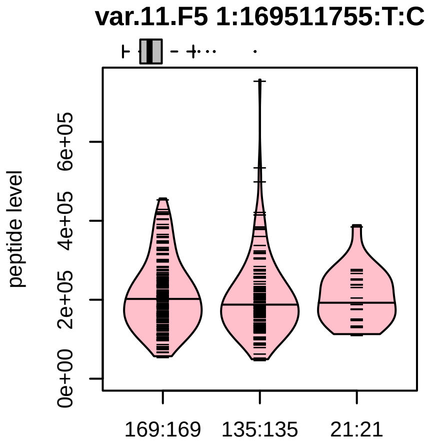

YLDSTFTKpc2_NP.3
p−maj=1, pX=0.61, N=325

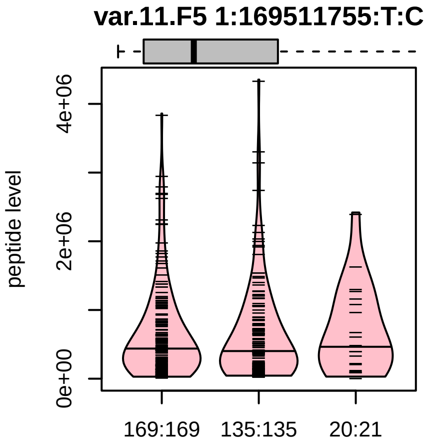

YLDSTFTKpc2_NP.4
p−maj=0.065, pX=0.98, N=324

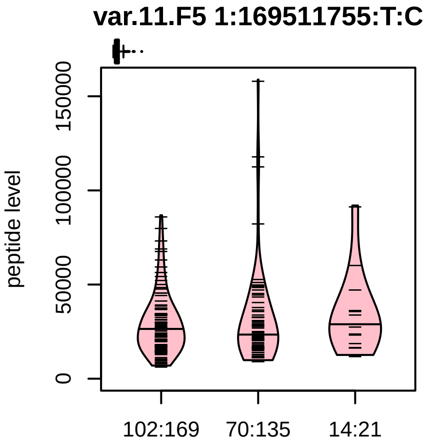

YLDSTFTKpc2_NP.5
p−min=0.26, pX=0.77, N=186

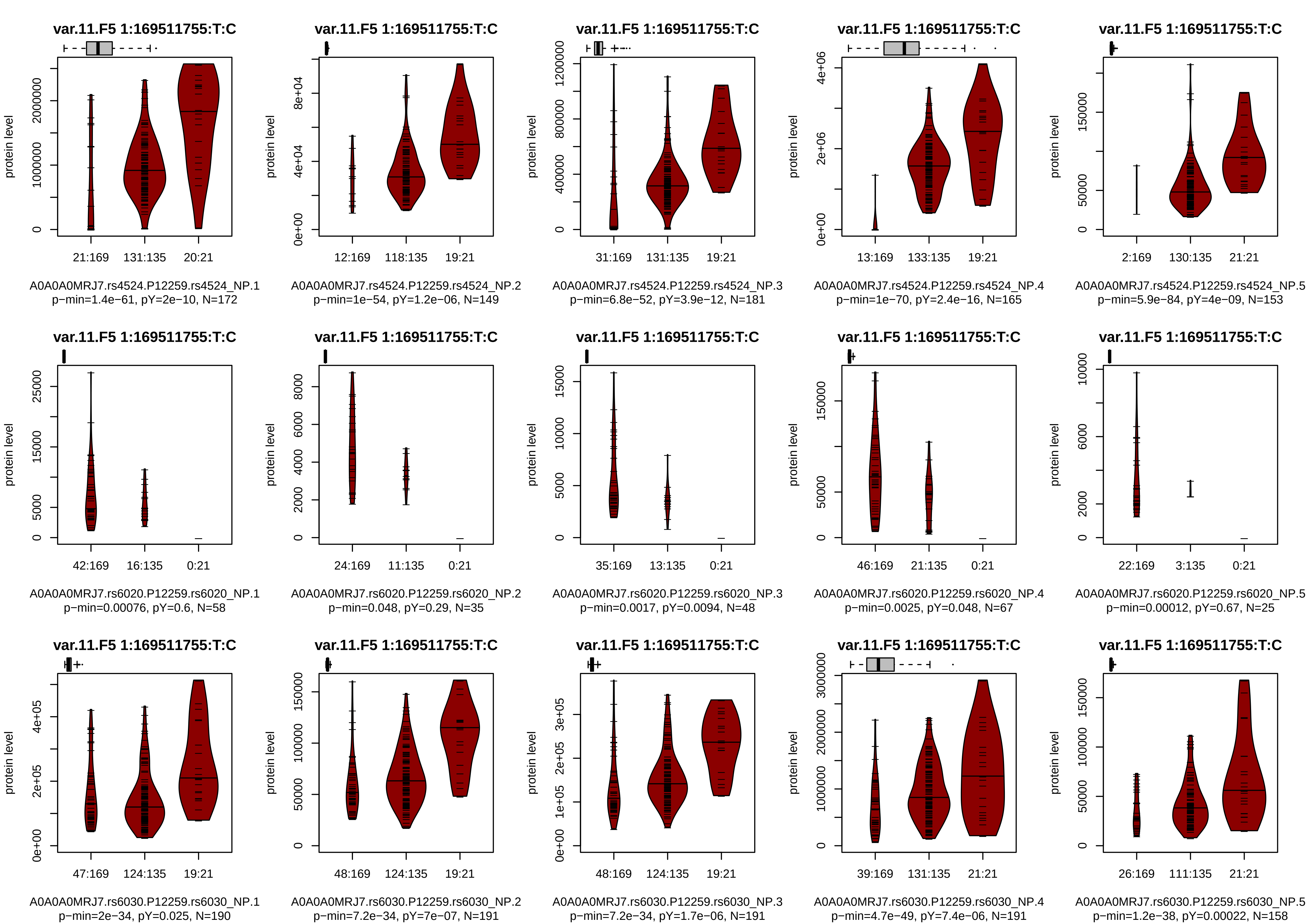
var.11.F5 1:169511755:T:C
protein level
21:169 131:135 20:21
A0A0A0MRJ7.rs4524.P12259.rs4524_NP.1
p−min=1.4e−61, pY=2e−10, N=172
var.11.F5 1:169511755:T:C
protein level
12:169 118:135 19:21
A0A0A0MRJ7.rs4524.P12259.rs4524_NP.2
p−min=1e−54, pY=1.2e−06, N=149
var.11.F5 1:169511755:T:C
protein level
31:169 131:135 19:21
A0A0A0MRJ7.rs4524.P12259.rs4524_NP.3
p−min=6.8e−52, pY=3.9e−12, N=181
var.11.F5 1:169511755:T:C
protein level
13:169 133:135 19:21
A0A0A0MRJ7.rs4524.P12259.rs4524_NP.4
p−min=1e−70, pY=2.4e−16, N=165
var.11.F5 1:169511755:T:C
protein level
2:169 130:135 21:21
A0A0A0MRJ7.rs4524.P12259.rs4524_NP.5
p−min=5.9e−84, pY=4e−09, N=153
var.11.F5 1:169511755:T:C
protein level
42:169 16:135 0:21
A0A0A0MRJ7.rs6020.P12259.rs6020_NP.1
p−min=0.00076, pY=0.6, N=58
var.11.F5 1:169511755:T:C
protein level
24:169 11:135 0:21
A0A0A0MRJ7.rs6020.P12259.rs6020_NP.2
p−min=0.048, pY=0.29, N=35
var.11.F5 1:169511755:T:C
protein level
35:169 13:135 0:21
A0A0A0MRJ7.rs6020.P12259.rs6020_NP.3
p−min=0.0017, pY=0.0094, N=48
var.11.F5 1:169511755:T:C
protein level
46:169 21:135 0:21
A0A0A0MRJ7.rs6020.P12259.rs6020_NP.4
p−min=0.0025, pY=0.048, N=67
var.11.F5 1:169511755:T:C
protein level
22:169 3:135 0:21
A0A0A0MRJ7.rs6020.P12259.rs6020_NP.5
p−min=0.00012, pY=0.67, N=25
var.11.F5 1:169511755:T:C
protein level
47:169 124:135 19:21
A0A0A0MRJ7.rs6030.P12259.rs6030_NP.1
p−min=2e−34, pY=0.025, N=190
var.11.F5 1:169511755:T:C
protein level
48:169 124:135 19:21
A0A0A0MRJ7.rs6030.P12259.rs6030_NP.2
p−min=7.2e−34, pY=7e−07, N=191
var.11.F5 1:169511755:T:C
protein level
48:169 124:135 19:21
A0A0A0MRJ7.rs6030.P12259.rs6030_NP.3
p−min=7.2e−34, pY=1.7e−06, N=191
var.11.F5 1:169511755:T:C
protein level
39:169 131:135 21:21
A0A0A0MRJ7.rs6030.P12259.rs6030_NP.4
p−min=4.7e−49, pY=7.4e−06, N=191
var.11.F5 1:169511755:T:C
protein level
26:169 111:135 21:21
A0A0A0MRJ7.rs6030.P12259.rs6030_NP.5
p−min=1.2e−38, pY=0.00022, N=158

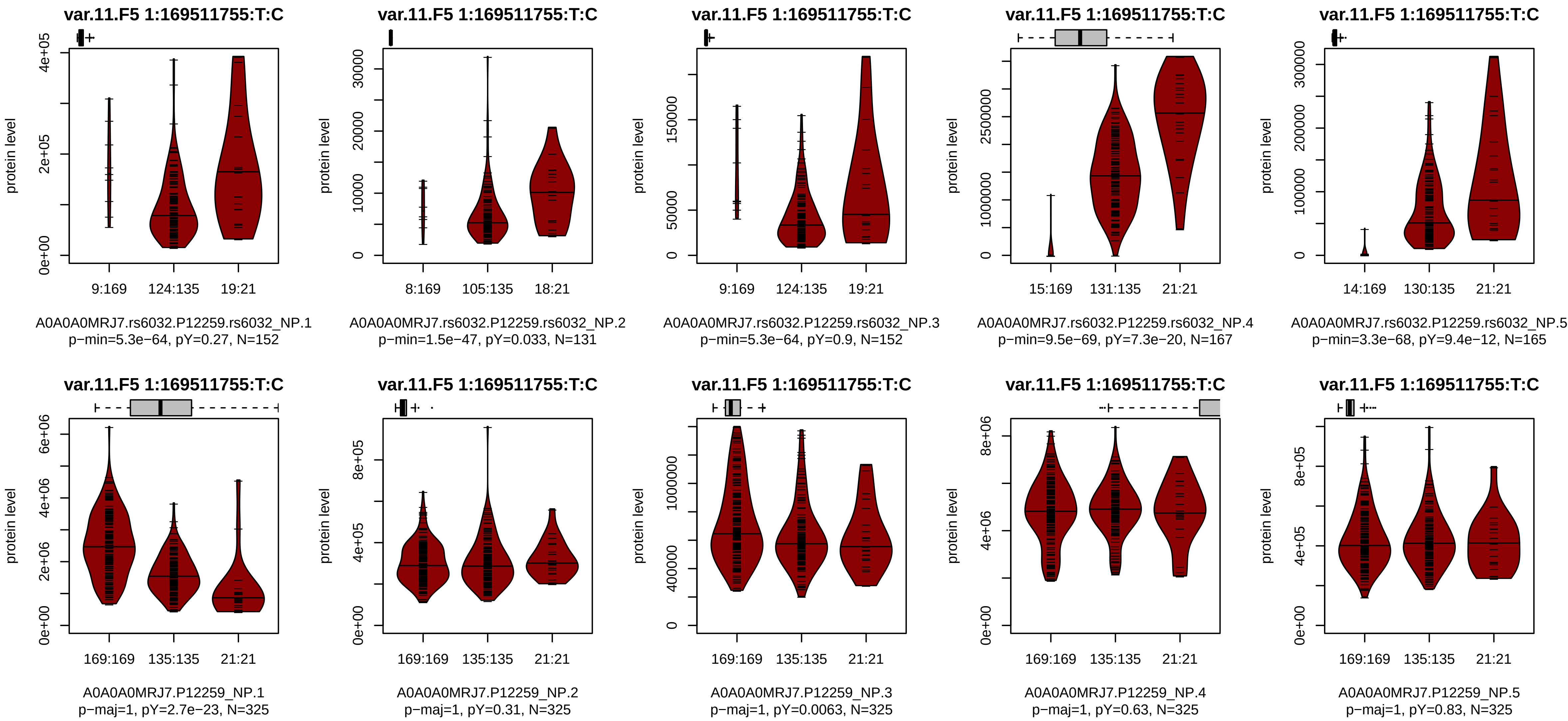
var.11.F5 1:169511755:T:C
protein level
0e+00
2e+05
4e+05
9:169
124:135
19:21
A0A0A0MRJ7.rs6032.P12259.rs6032_NP.1
p−min=5.3e−64, pY=0.27, N=152
var.11.F5 1:169511755:T:C
protein level
0
10000
20000
30000
8:169
105:135
18:21
A0A0A0MRJ7.rs6032.P12259.rs6032_NP.2
p−min=1.5e−47, pY=0.033, N=131
var.11.F5 1:169511755:T:C
protein level
0
50000
150000
9:169
124:135
19:21
A0A0A0MRJ7.rs6032.P12259.rs6032_NP.3
p−min=5.3e−64, pY=0.9, N=152
var.11.F5 1:169511755:T:C
protein level
0
1000000
2500000
15:169
131:135
21:21
A0A0A0MRJ7.rs6032.P12259.rs6032_NP.4
p−min=9.5e−69, pY=7.3e−20, N=167
var.11.F5 1:169511755:T:C
protein level
0
100000
200000
300000
14:169
130:135
21:21
A0A0A0MRJ7.rs6032.P12259.rs6032_NP.5
p−min=3.3e−68, pY=9.4e−12, N=165
var.11.F5 1:169511755:T:C
protein level
0e+00
2e+06
4e+06
6e+06
169:169
135:135
21:21
A0A0A0MRJ7.P12259_NP.1
p−maj=1, pY=2.7e−23, N=325
var.11.F5 1:169511755:T:C
protein level
0e+00
4e+05
8e+05
169:169
135:135
21:21
A0A0A0MRJ7.P12259_NP.2
p−maj=1, pY=0.31, N=325
var.11.F5 1:169511755:T:C
protein level
0
400000
1000000
169:169
135:135
21:21
A0A0A0MRJ7.P12259_NP.3
p−maj=1, pY=0.0063, N=325
var.11.F5 1:169511755:T:C
protein level
0e+00
4e+06
8e+06
169:169
135:135
21:21
A0A0A0MRJ7.P12259_NP.4
p−maj=1, pY=0.63, N=325
var.11.F5 1:169511755:T:C
protein level
0e+00
4e+05
8e+05
169:169
135:135
21:21
A0A0A0MRJ7.P12259_NP.5
p−maj=1, pY=0.83, N=325

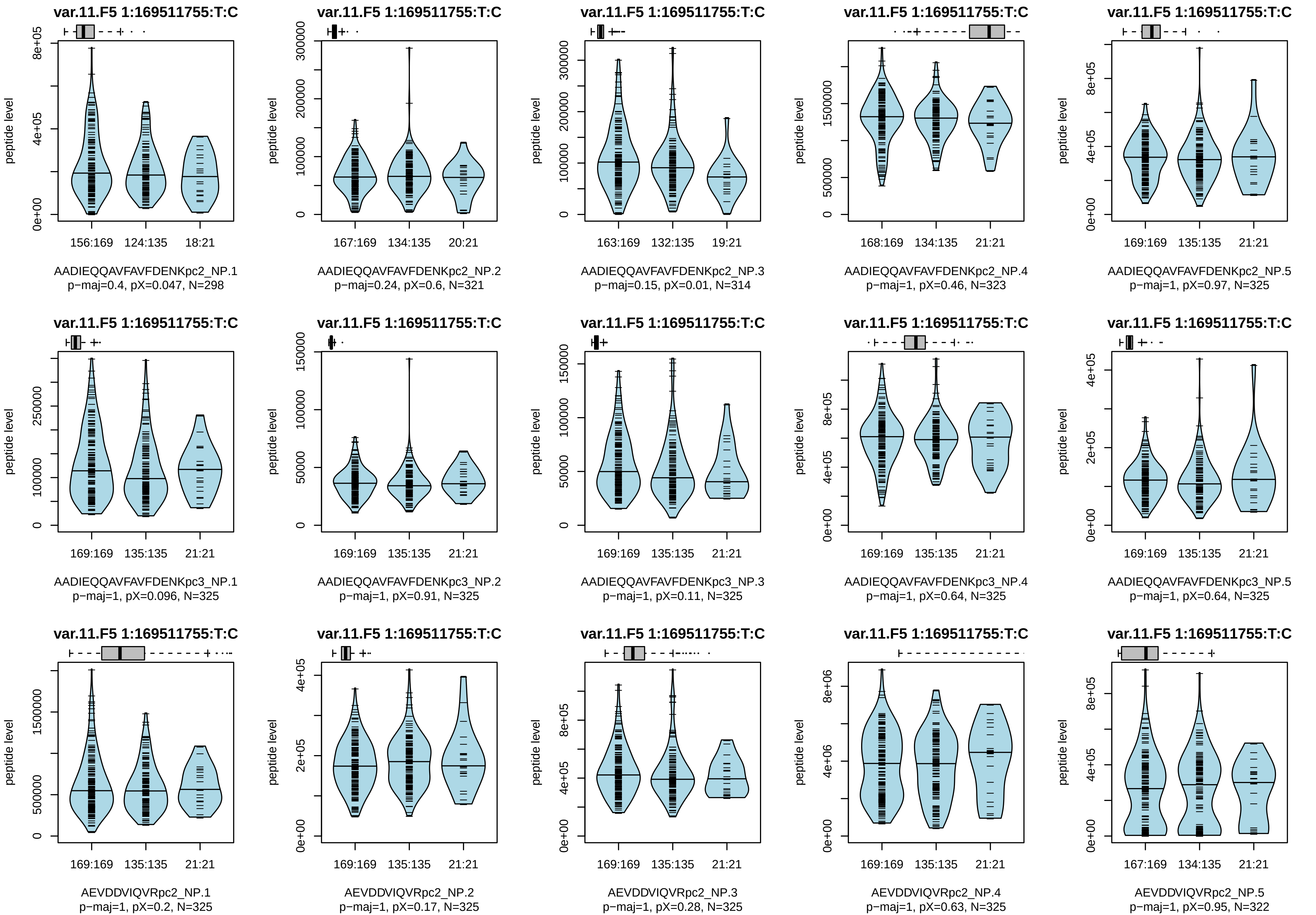
var.11.F5 1:169511755:T:C
peptide level
156:169
124:135
18:21
AADIEQQAVFAVFDENKpc2_NP.1
p−maj=0.4, pX=0.047, N=298
var.11.F5 1:169511755:T:C
peptide level
167:169
134:135
20:21
AADIEQQAVFAVFDENKpc2_NP.2
p−maj=0.24, pX=0.6, N=321
var.11.F5 1:169511755:T:C
peptide level
163:169
132:135
19:21
AADIEQQAVFAVFDENKpc2_NP.3
p−maj=0.15, pX=0.01, N=314
var.11.F5 1:169511755:T:C
peptide level
168:169
134:135
21:21
AADIEQQAVFAVFDENKpc2_NP.4
p−maj=1, pX=0.46, N=323
var.11.F5 1:169511755:T:C
peptide level
169:169
135:135
21:21
AADIEQQAVFAVFDENKpc2_NP.5
p−maj=1, pX=0.97, N=325
var.11.F5 1:169511755:T:C
peptide level
169:169
135:135
21:21
AADIEQQAVFAVFDENKpc3_NP.1
p−maj=1, pX=0.096, N=325
var.11.F5 1:169511755:T:C
peptide level
169:169
135:135
21:21
AADIEQQAVFAVFDENKpc3_NP.2
p−maj=1, pX=0.91, N=325
var.11.F5 1:169511755:T:C
peptide level
169:169
135:135
21:21
AADIEQQAVFAVFDENKpc3_NP.3
p−maj=1, pX=0.11, N=325
var.11.F5 1:169511755:T:C
peptide level
169:169
135:135
21:21
AADIEQQAVFAVFDENKpc3_NP.4
p−maj=1, pX=0.64, N=325
var.11.F5 1:169511755:T:C
peptide level
169:169
135:135
21:21
AADIEQQAVFAVFDENKpc3_NP.5
p−maj=1, pX=0.64, N=325
var.11.F5 1:169511755:T:C
peptide level
169:169
135:135
21:21
AEVDDVIQVRpc2_NP.1
p−maj=1, pX=0.2, N=325
var.11.F5 1:169511755:T:C
peptide level
169:169
135:135
21:21
AEVDDVIQVRpc2_NP.2
p−maj=1, pX=0.17, N=325
var.11.F5 1:169511755:T:C
peptide level
169:169
135:135
21:21
AEVDDVIQVRpc2_NP.3
p−maj=1, pX=0.28, N=325
var.11.F5 1:169511755:T:C
peptide level
169:169
135:135
21:21
AEVDDVIQVRpc2_NP.4
p−maj=1, pX=0.63, N=325
var.11.F5 1:169511755:T:C
peptide level
167:169
134:135
21:21
AEVDDVIQVRpc2_NP.5
p−maj=1, pX=0.95, N=322

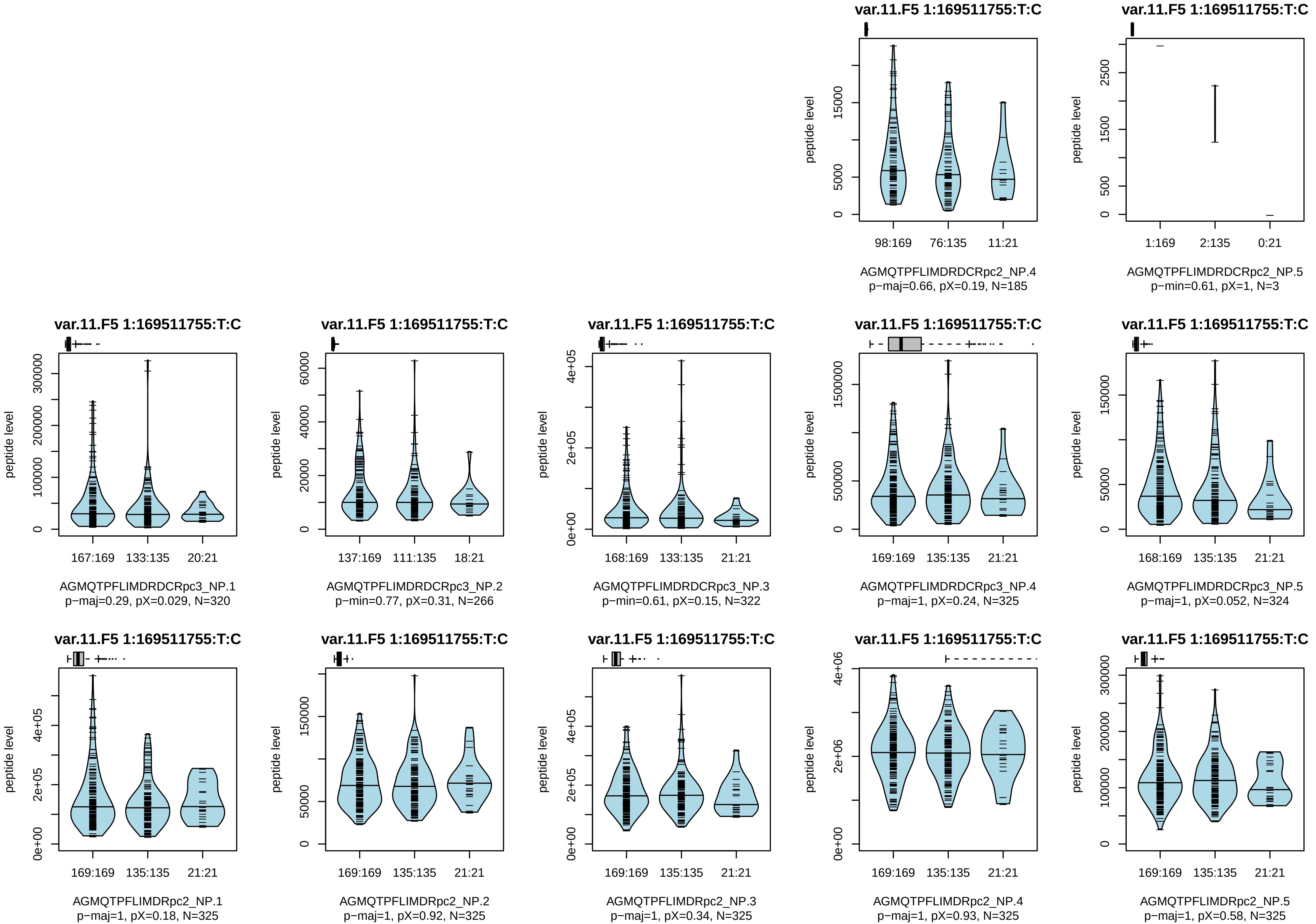

var.11.F5 1:169511755:T:C
peptide level
98:169
76:135
11:21
AGMQTPFLIMDRDCRpc2_NP.4
p−maj=0.66, pX=0.19, N=185
var.11.F5 1:169511755:T:C
peptide level
1:169
2:135
0:21
AGMQTPFLIMDRDCRpc2_NP.5
p−min=0.61, pX=1, N=3
var.11.F5 1:169511755:T:C
peptide level
167:169
133:135
20:21
AGMQTPFLIMDRDCRpc3_NP.1
p−maj=0.29, pX=0.029, N=320
var.11.F5 1:169511755:T:C
peptide level
137:169
111:135
18:21
AGMQTPFLIMDRDCRpc3_NP.2
p−min=0.77, pX=0.31, N=266
var.11.F5 1:169511755:T:C
peptide level
168:169
133:135
21:21
AGMQTPFLIMDRDCRpc3_NP.3
p−min=0.61, pX=0.15, N=322
var.11.F5 1:169511755:T:C
peptide level
169:169
135:135
21:21
AGMQTPFLIMDRDCRpc3_NP.4
p−maj=1, pX=0.24, N=325
var.11.F5 1:169511755:T:C
peptide level
168:169
135:135
21:21
AGMQTPFLIMDRDCRpc3_NP.5
p−maj=1, pX=0.052, N=324
var.11.F5 1:169511755:T:C
peptide level
169:169
135:135
21:21
AGMQTPFLIMDRpc2_NP.1
p−maj=1, pX=0.18, N=325
var.11.F5 1:169511755:T:C
peptide level
169:169
135:135
21:21
AGMQTPFLIMDRpc2_NP.2
p−maj=1, pX=0.92, N=325
var.11.F5 1:169511755:T:C
peptide level
169:169
135:135
21:21
AGMQTPFLIMDRpc2_NP.3
p−maj=1, pX=0.34, N=325
var.11.F5 1:169511755:T:C
peptide level
169:169
135:135
21:21
AGMQTPFLIMDRpc2_NP.4
p−maj=1, pX=0.93, N=325
var.11.F5 1:169511755:T:C
peptide level
169:169
135:135
21:21
AGMQTPFLIMDRpc2_NP.5
p−maj=1, pX=0.58, N=325

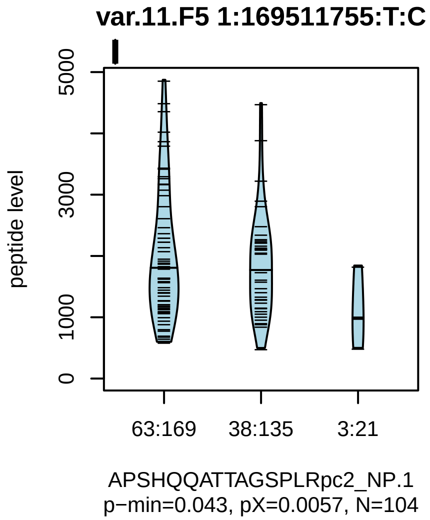
var.11.F5 1:169511755:T:C
peptide level
0
1000
3000
5000
63:169
38:135
3:21
APSHQQATTAGSPLRpc2_NP.1
p−min=0.043, pX=0.0057, N=104

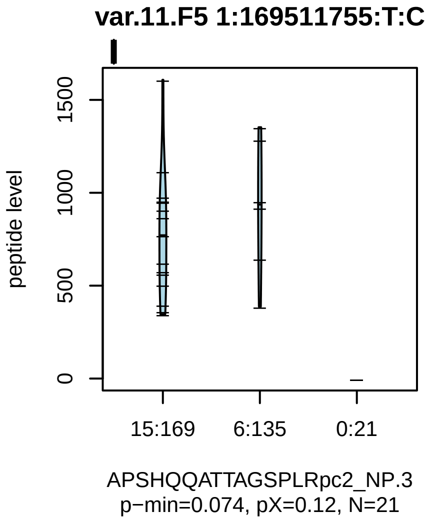
var.11.F5 1:169511755:T:C
peptide level
0
500
1000
1500
15:169
6:135
0:21
APSHQQATTAGSPLRpc2_NP.3
p−min=0.074, pX=0.12, N=21

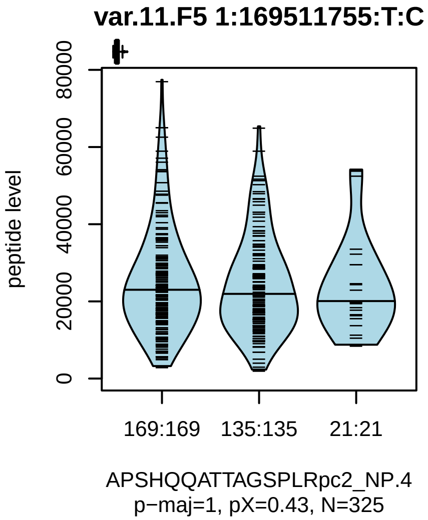
var.11.F5 1:169511755:T:C
peptide level
0
20000
40000
60000
80000
169:169
135:135
21:21
APSHQQATTAGSPLRpc2_NP.4
p−maj=1, pX=0.43, N=325

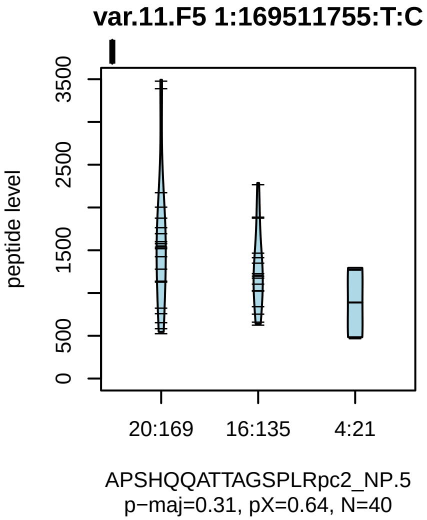
var.11.F5 1:169511755:T:C
peptide level
0
500
1500
2500
3500
20:169
16:135
4:21
APSHQQATTAGSPLRpc2_NP.5
p−maj=0.31, pX=0.64, N=40

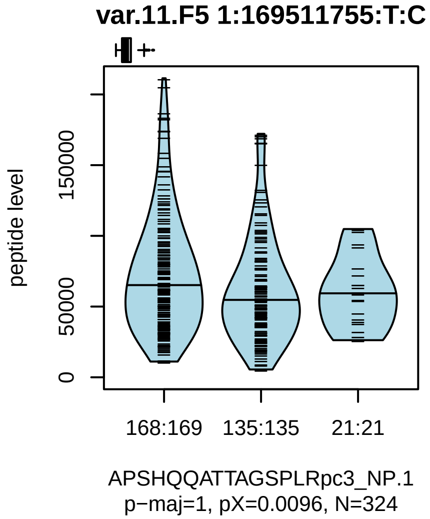
var.11.F5 1:169511755:T:C
peptide level
0
50000
150000
168:169
135:135
21:21
APSHQQATTAGSPLRpc3_NP.1
p−maj=1, pX=0.0096, N=324

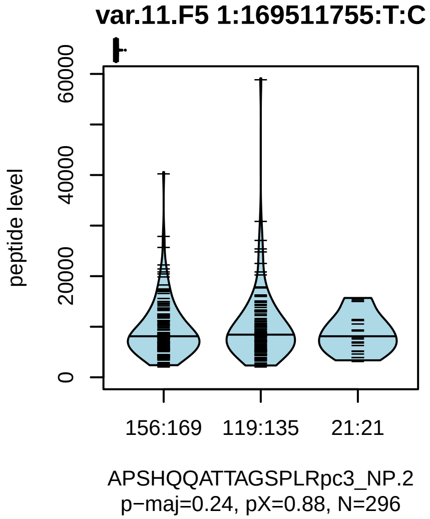
var.11.F5 1:169511755:T:C
peptide level
0
20000
40000
60000
156:169
119:135
21:21
APSHQQATTAGSPLRpc3_NP.2
p−maj=0.24, pX=0.88, N=296

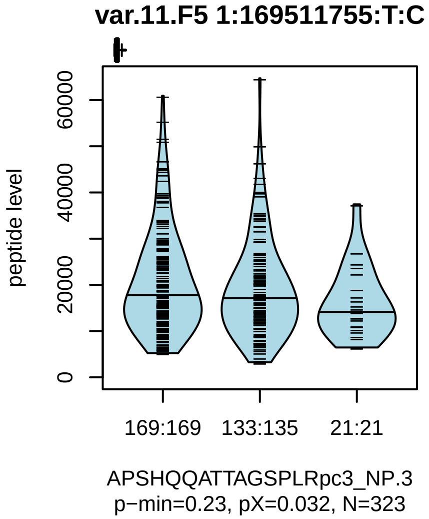
var.11.F5 1:169511755:T:C
peptide level
0
20000
40000
60000
169:169
133:135
21:21
APSHQQATTAGSPLRpc3_NP.3
p−min=0.23, pX=0.032, N=323

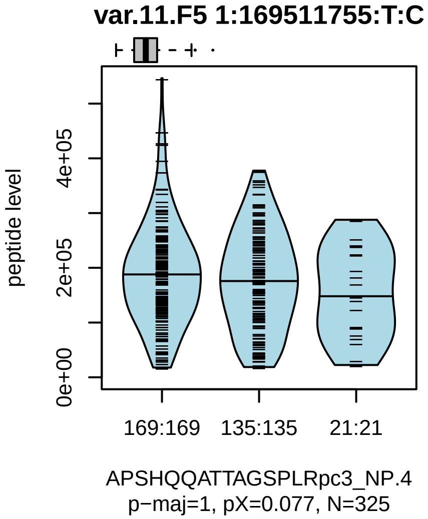
var.11.F5 1:169511755:T:C
peptide level
0e+00
2e+05
4e+05
169:169
135:135
21:21
APSHQQATTAGSPLRpc3_NP.4
p−maj=1, pX=0.077, N=325

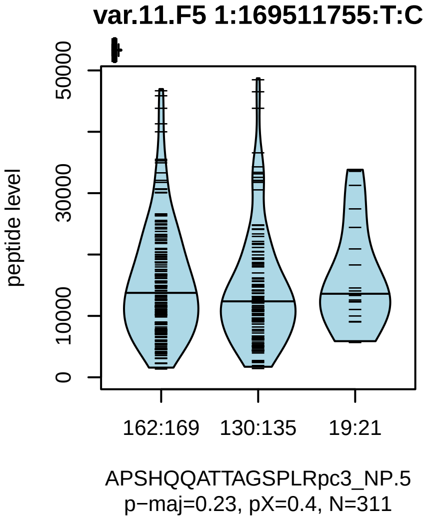
var.11.F5 1:169511755:T:C
peptide level
0
10000
30000
50000
162:169
130:135
19:21
APSHQQATTAGSPLRpc3_NP.5
p−maj=0.23, pX=0.4, N=311

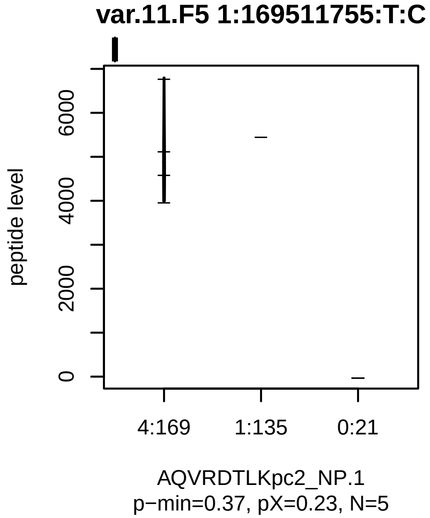
var.11.F5 1:169511755:T:C
peptide level
0
2000
4000
6000
4:169
1:135
0:21
AQVRDTLKpc2_NP.1
p−min=0.37, pX=0.23, N=5

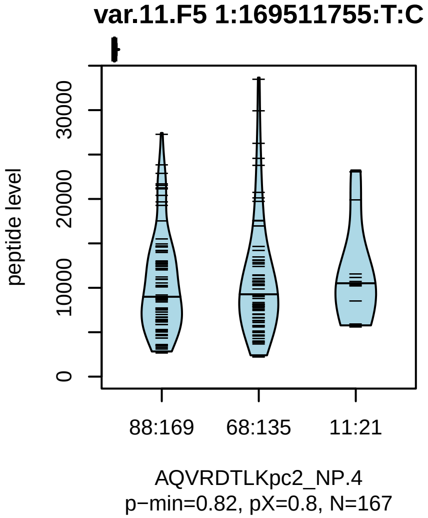
var.11.F5 1:169511755:T:C
peptide level
0
10000
20000
30000
88:169
68:135
11:21
AQVRDTLKpc2_NP.4
p−min=0.82, pX=0.8, N=167

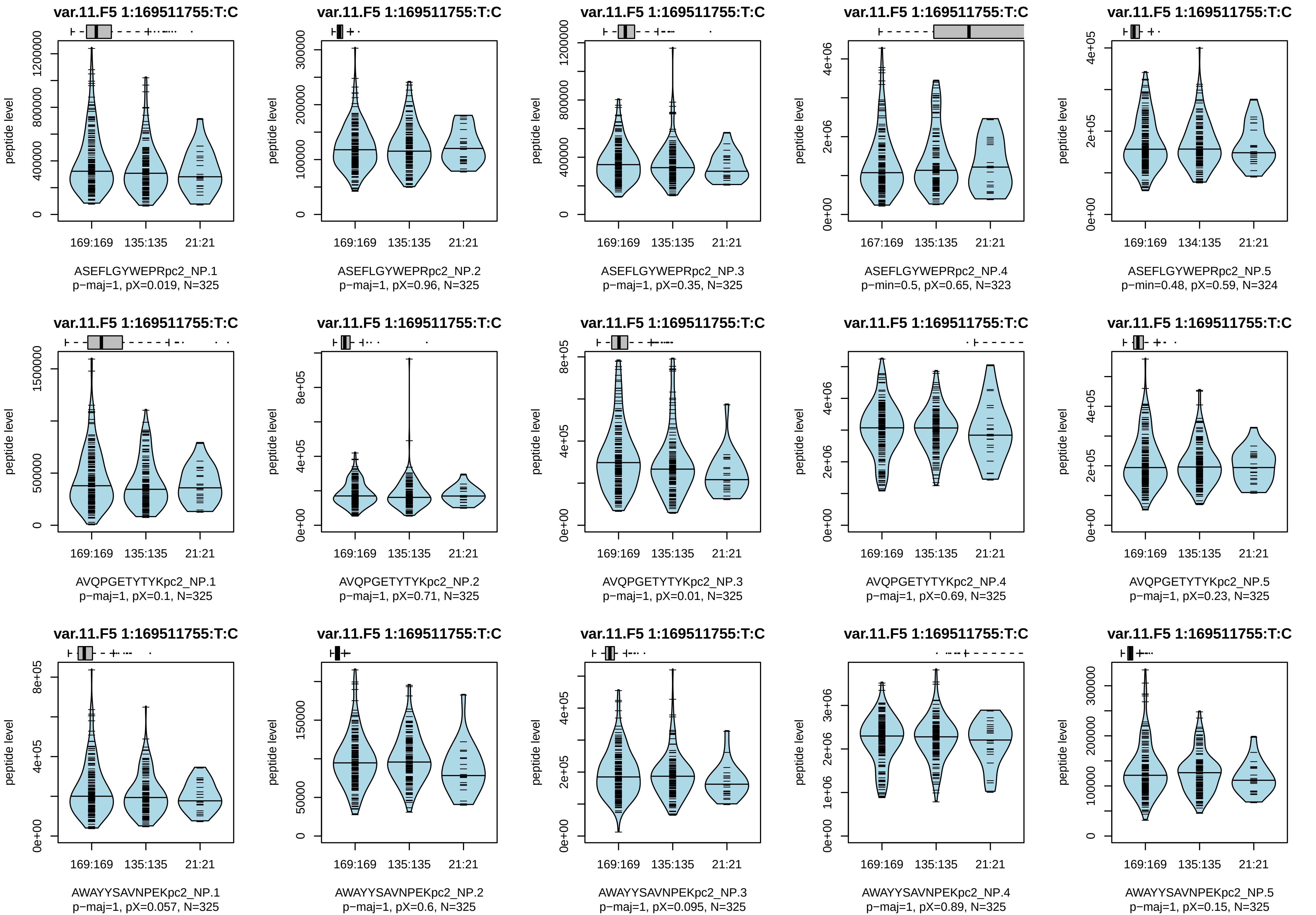
var.11.F5 1:169511755:T:C
peptide level
169:169 135:135 21:21
ASEFLGYWEPRpc2_NP.1
p−maj=1, pX=0.019, N=325
var.11.F5 1:169511755:T:C
peptide level
169:169 135:135 21:21
ASEFLGYWEPRpc2_NP.2
p−maj=1, pX=0.96, N=325
var.11.F5 1:169511755:T:C
peptide level
169:169 135:135 21:21
ASEFLGYWEPRpc2_NP.3
p−maj=1, pX=0.35, N=325
var.11.F5 1:169511755:T:C
peptide level
167:169 135:135 21:21
ASEFLGYWEPRpc2_NP.4
p−min=0.5, pX=0.65, N=323
var.11.F5 1:169511755:T:C
peptide level
169:169 134:135 21:21
ASEFLGYWEPRpc2_NP.5
p−min=0.48, pX=0.59, N=324
var.11.F5 1:169511755:T:C
peptide level
169:169 135:135 21:21
AVQPGETYTYKpc2_NP.1
p−maj=1, pX=0.1, N=325
var.11.F5 1:169511755:T:C
peptide level
169:169 135:135 21:21
AVQPGETYTYKpc2_NP.2
p−maj=1, pX=0.71, N=325
var.11.F5 1:169511755:T:C
peptide level
169:169 135:135 21:21
AVQPGETYTYKpc2_NP.3
p−maj=1, pX=0.01, N=325
var.11.F5 1:169511755:T:C
peptide level
169:169 135:135 21:21
AVQPGETYTYKpc2_NP.4
p−maj=1, pX=0.69, N=325
var.11.F5 1:169511755:T:C
peptide level
169:169 135:135 21:21
AVQPGETYTYKpc2_NP.5
p−maj=1, pX=0.23, N=325
var.11.F5 1:169511755:T:C
peptide level
169:169 135:135 21:21
AWAYYSAVNPEKpc2_NP.1
p−maj=1, pX=0.057, N=325
var.11.F5 1:169511755:T:C
peptide level
169:169 135:135 21:21
AWAYYSAVNPEKpc2_NP.2
p−maj=1, pX=0.6, N=325
var.11.F5 1:169511755:T:C
peptide level
169:169 135:135 21:21
AWAYYSAVNPEKpc2_NP.3
p−maj=1, pX=0.095, N=325
var.11.F5 1:169511755:T:C
peptide level
169:169 135:135 21:21
AWAYYSAVNPEKpc2_NP.4
p−maj=1, pX=0.89, N=325
var.11.F5 1:169511755:T:C
peptide level
169:169 135:135 21:21
AWAYYSAVNPEKpc2_NP.5
p−maj=1, pX=0.15, N=325

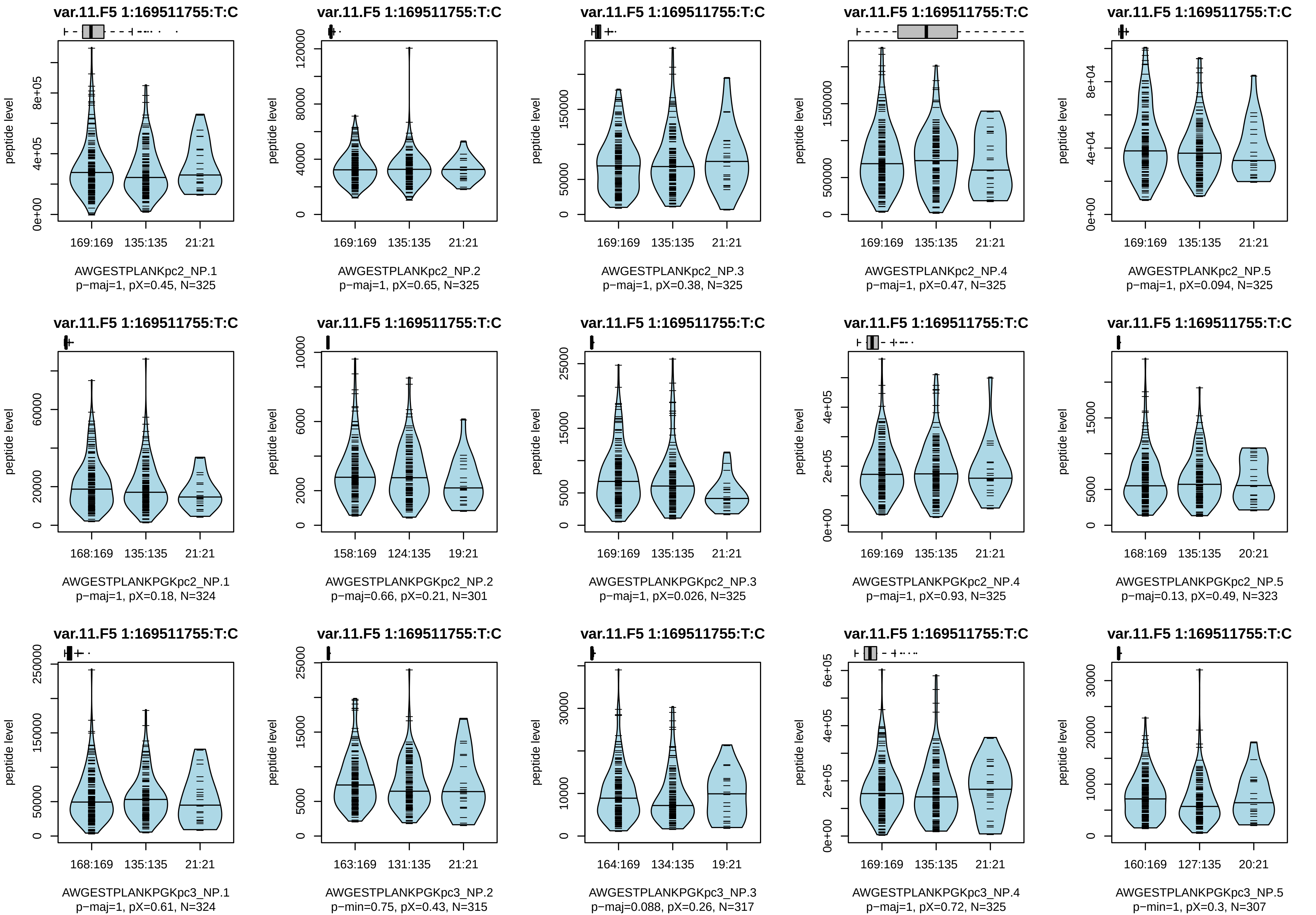

var.11.F5 1:169511755:T:C
peptide level
169:169 135:135 21:21
AWGESTPLANKpc2_NP.1
p−maj=1, pX=0.45, N=325
var.11.F5 1:169511755:T:C
peptide level
169:169 135:135 21:21
AWGESTPLANKpc2_NP.2
p−maj=1, pX=0.65, N=325
var.11.F5 1:169511755:T:C
peptide level
169:169 135:135 21:21
AWGESTPLANKpc2_NP.3
p−maj=1, pX=0.38, N=325
var.11.F5 1:169511755:T:C
peptide level
169:169 135:135 21:21
AWGESTPLANKpc2_NP.4
p−maj=1, pX=0.47, N=325
var.11.F5 1:169511755:T:C
peptide level
169:169 135:135 21:21
AWGESTPLANKpc2_NP.5
p−maj=1, pX=0.094, N=325
var.11.F5 1:169511755:T:C
peptide level
168:169 135:135 21:21
AWGESTPLANKPGKpc2_NP.1
p−maj=1, pX=0.18, N=324
var.11.F5 1:169511755:T:C
peptide level
158:169 124:135 19:21
AWGESTPLANKPGKpc2_NP.2
p−maj=0.66, pX=0.21, N=301
var.11.F5 1:169511755:T:C
peptide level
169:169 135:135 21:21
AWGESTPLANKPGKpc2_NP.3
p−maj=1, pX=0.026, N=325
var.11.F5 1:169511755:T:C
peptide level
169:169 135:135 21:21
AWGESTPLANKPGKpc2_NP.4
p−maj=1, pX=0.93, N=325
var.11.F5 1:169511755:T:C
peptide level
168:169 135:135 20:21
AWGESTPLANKPGKpc2_NP.5
p−maj=0.13, pX=0.49, N=323
var.11.F5 1:169511755:T:C
peptide level
168:169 135:135 21:21
AWGESTPLANKPGKpc3_NP.1
p−maj=1, pX=0.61, N=324
var.11.F5 1:169511755:T:C
peptide level
163:169 131:135 21:21
AWGESTPLANKPGKpc3_NP.2
p−min=0.75, pX=0.43, N=315
var.11.F5 1:169511755:T:C
peptide level
164:169 134:135 19:21
AWGESTPLANKPGKpc3_NP.3
p−maj=0.088, pX=0.26, N=317
var.11.F5 1:169511755:T:C
peptide level
169:169 135:135 21:21
AWGESTPLANKPGKpc3_NP.4
p−maj=1, pX=0.72, N=325
var.11.F5 1:169511755:T:C
peptide level
160:169 127:135 20:21
AWGESTPLANKPGKpc3_NP.5
p−min=1, pX=0.3, N=307

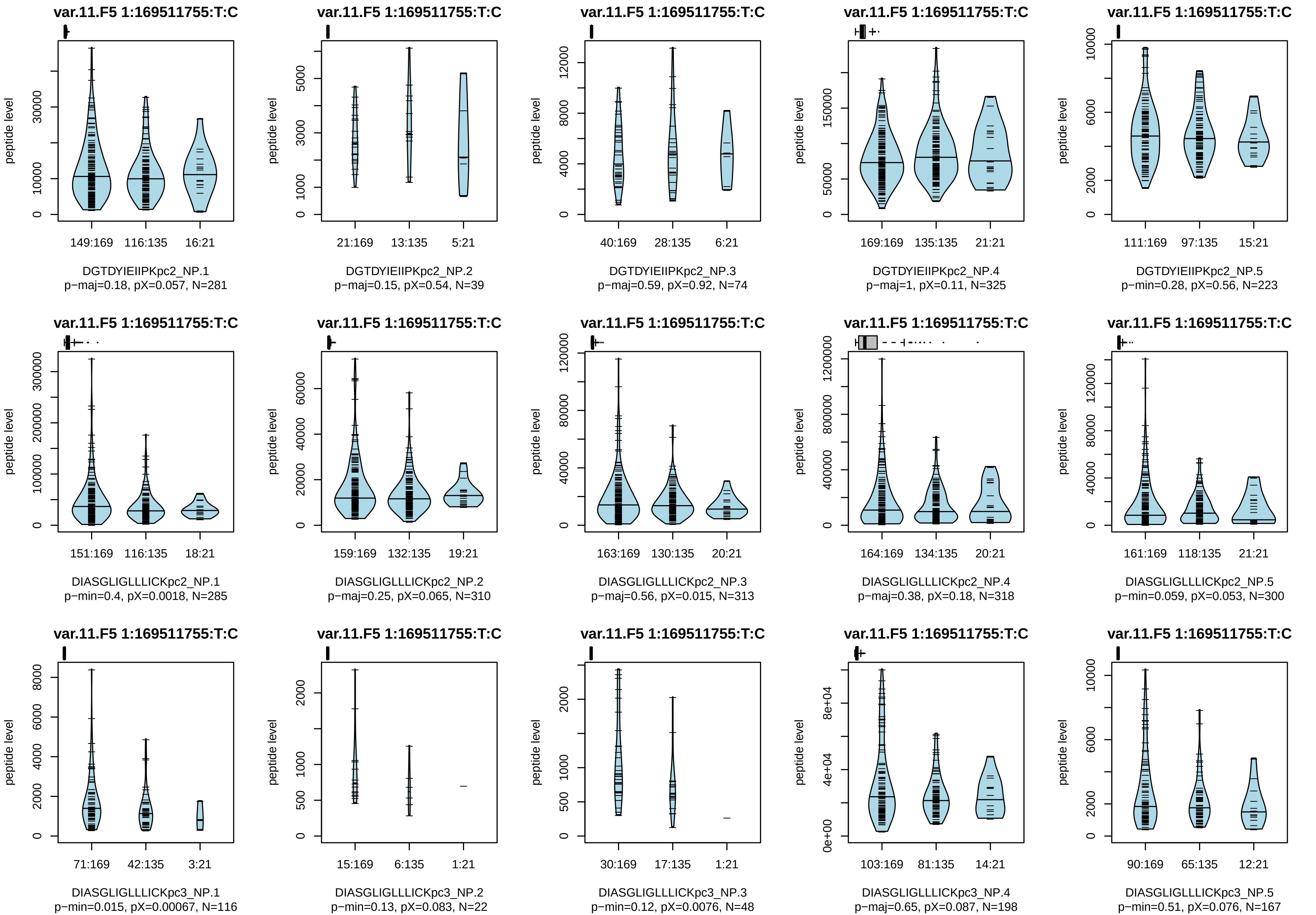

var.11.F5 1:169511755:T:C
peptide level
149:169
116:135
16:21
DGTDYIEIIPKpc2_NP.1
p−maj=0.18, pX=0.057, N=281
var.11.F5 1:169511755:T:C
peptide level
21:169
13:135
5:21
DGTDYIEIIPKpc2_NP.2
p−maj=0.15, pX=0.54, N=39
var.11.F5 1:169511755:T:C
peptide level
40:169
28:135
6:21
DGTDYIEIIPKpc2_NP.3
p−maj=0.59, pX=0.92, N=74
var.11.F5 1:169511755:T:C
peptide level
169:169
135:135
21:21
DGTDYIEIIPKpc2_NP.4
p−maj=1, pX=0.11, N=325
var.11.F5 1:169511755:T:C
peptide level
111:169
97:135
15:21
DGTDYIEIIPKpc2_NP.5
p−min=0.28, pX=0.56, N=223
var.11.F5 1:169511755:T:C
peptide level
151:169
116:135
18:21
DIASGLIGLLLICKpc2_NP.1
p−min=0.4, pX=0.0018, N=285
var.11.F5 1:169511755:T:C
peptide level
159:169
132:135
19:21
DIASGLIGLLLICKpc2_NP.2
p−maj=0.25, pX=0.065, N=310
var.11.F5 1:169511755:T:C
peptide level
163:169
130:135
20:21
DIASGLIGLLLICKpc2_NP.3
p−maj=0.56, pX=0.015, N=313
var.11.F5 1:169511755:T:C
peptide level
164:169
134:135
20:21
DIASGLIGLLLICKpc2_NP.4
p−maj=0.38, pX=0.18, N=318
var.11.F5 1:169511755:T:C
peptide level
161:169
118:135
21:21
DIASGLIGLLLICKpc2_NP.5
p−min=0.059, pX=0.053, N=300
var.11.F5 1:169511755:T:C
peptide level
71:169
42:135
3:21
DIASGLIGLLLICKpc3_NP.1
p−min=0.015, pX=0.00067, N=116
var.11.F5 1:169511755:T:C
peptide level
15:169
6:135
1:21
DIASGLIGLLLICKpc3_NP.2
p−min=0.13, pX=0.083, N=22
var.11.F5 1:169511755:T:C
peptide level
30:169
17:135
1:21
DIASGLIGLLLICKpc3_NP.3
p−min=0.12, pX=0.0076, N=48
var.11.F5 1:169511755:T:C
peptide level
103:169
81:135
14:21
DIASGLIGLLLICKpc3_NP.4
p−maj=0.65, pX=0.087, N=198
var.11.F5 1:169511755:T:C
peptide level
90:169
65:135
12:21
DIASGLIGLLLICKpc3_NP.5
p−min=0.51, pX=0.076, N=167

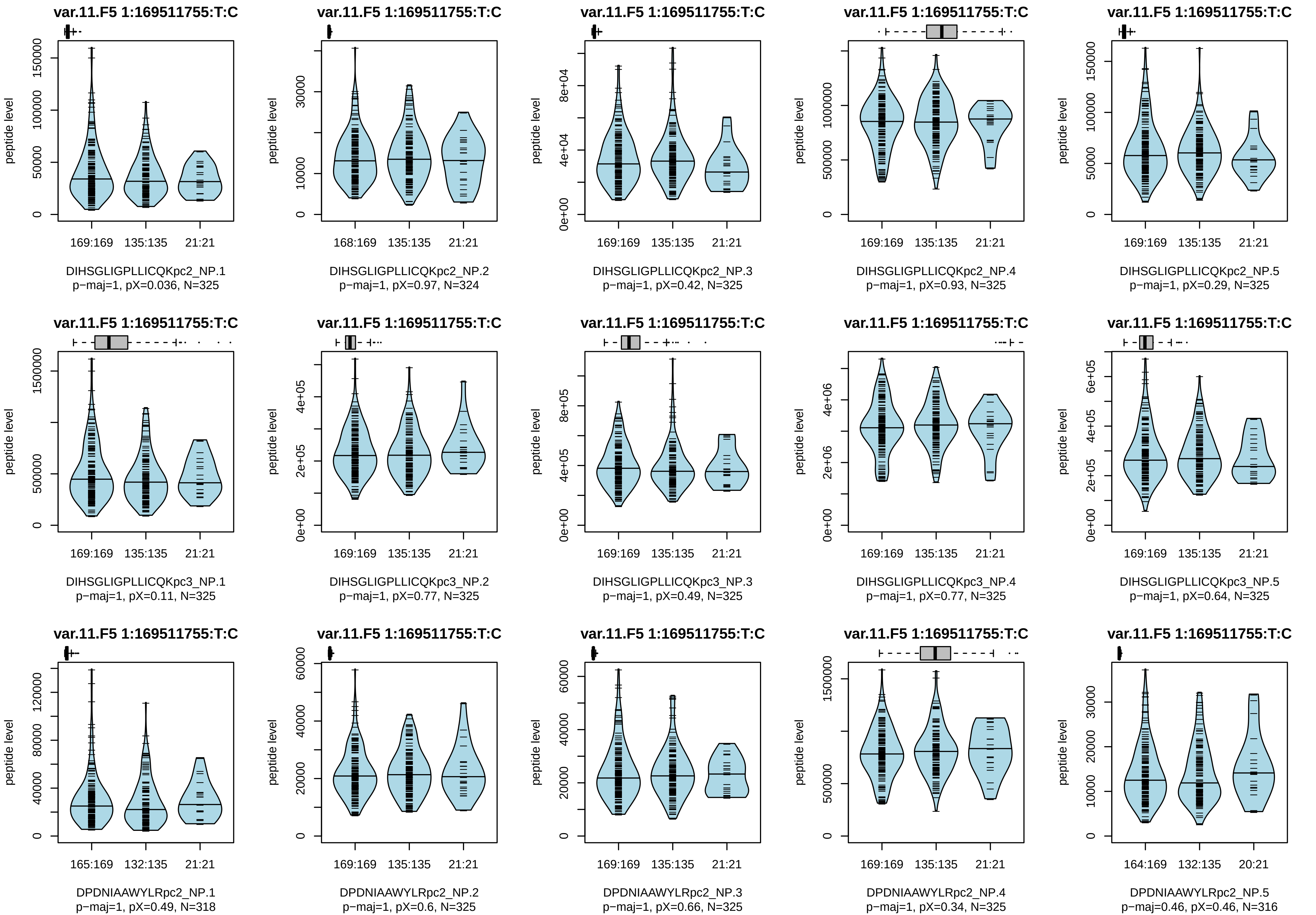
var.11.F5 1:169511755:T:C
peptide level
169:169 135:135 21:21
DIHSGLIGPLLICQKpc2_NP.1
p−maj=1, pX=0.036, N=325
var.11.F5 1:169511755:T:C
peptide level
168:169 135:135 21:21
DIHSGLIGPLLICQKpc2_NP.2
p−maj=1, pX=0.97, N=324
var.11.F5 1:169511755:T:C
peptide level
169:169 135:135 21:21
DIHSGLIGPLLICQKpc2_NP.3
p−maj=1, pX=0.42, N=325
var.11.F5 1:169511755:T:C
peptide level
169:169 135:135 21:21
DIHSGLIGPLLICQKpc2_NP.4
p−maj=1, pX=0.93, N=325
var.11.F5 1:169511755:T:C
peptide level
169:169 135:135 21:21
DIHSGLIGPLLICQKpc2_NP.5
p−maj=1, pX=0.29, N=325
var.11.F5 1:169511755:T:C
peptide level
169:169 135:135 21:21
DIHSGLIGPLLICQKpc3_NP.1
p−maj=1, pX=0.11, N=325
var.11.F5 1:169511755:T:C
peptide level
169:169 135:135 21:21
DIHSGLIGPLLICQKpc3_NP.2
p−maj=1, pX=0.77, N=325
var.11.F5 1:169511755:T:C
peptide level
169:169 135:135 21:21
DIHSGLIGPLLICQKpc3_NP.3
p−maj=1, pX=0.49, N=325
var.11.F5 1:169511755:T:C
peptide level
169:169 135:135 21:21
DIHSGLIGPLLICQKpc3_NP.4
p−maj=1, pX=0.77, N=325
var.11.F5 1:169511755:T:C
peptide level
169:169 135:135 21:21
DIHSGLIGPLLICQKpc3_NP.5
p−maj=1, pX=0.64, N=325
var.11.F5 1:169511755:T:C
peptide level
165:169 132:135 21:21
DPDNIAAWYLRpc2_NP.1
p−maj=1, pX=0.49, N=318
var.11.F5 1:169511755:T:C
peptide level
169:169 135:135 21:21
DPDNIAAWYLRpc2_NP.2
p−maj=1, pX=0.6, N=325
var.11.F5 1:169511755:T:C
peptide level
169:169 135:135 21:21
DPDNIAAWYLRpc2_NP.3
p−maj=1, pX=0.66, N=325
var.11.F5 1:169511755:T:C
peptide level
169:169 135:135 21:21
DPDNIAAWYLRpc2_NP.4
p−maj=1, pX=0.34, N=325
var.11.F5 1:169511755:T:C
peptide level
164:169 132:135 20:21
DPDNIAAWYLRpc2_NP.5
p−maj=0.46, pX=0.46, N=316

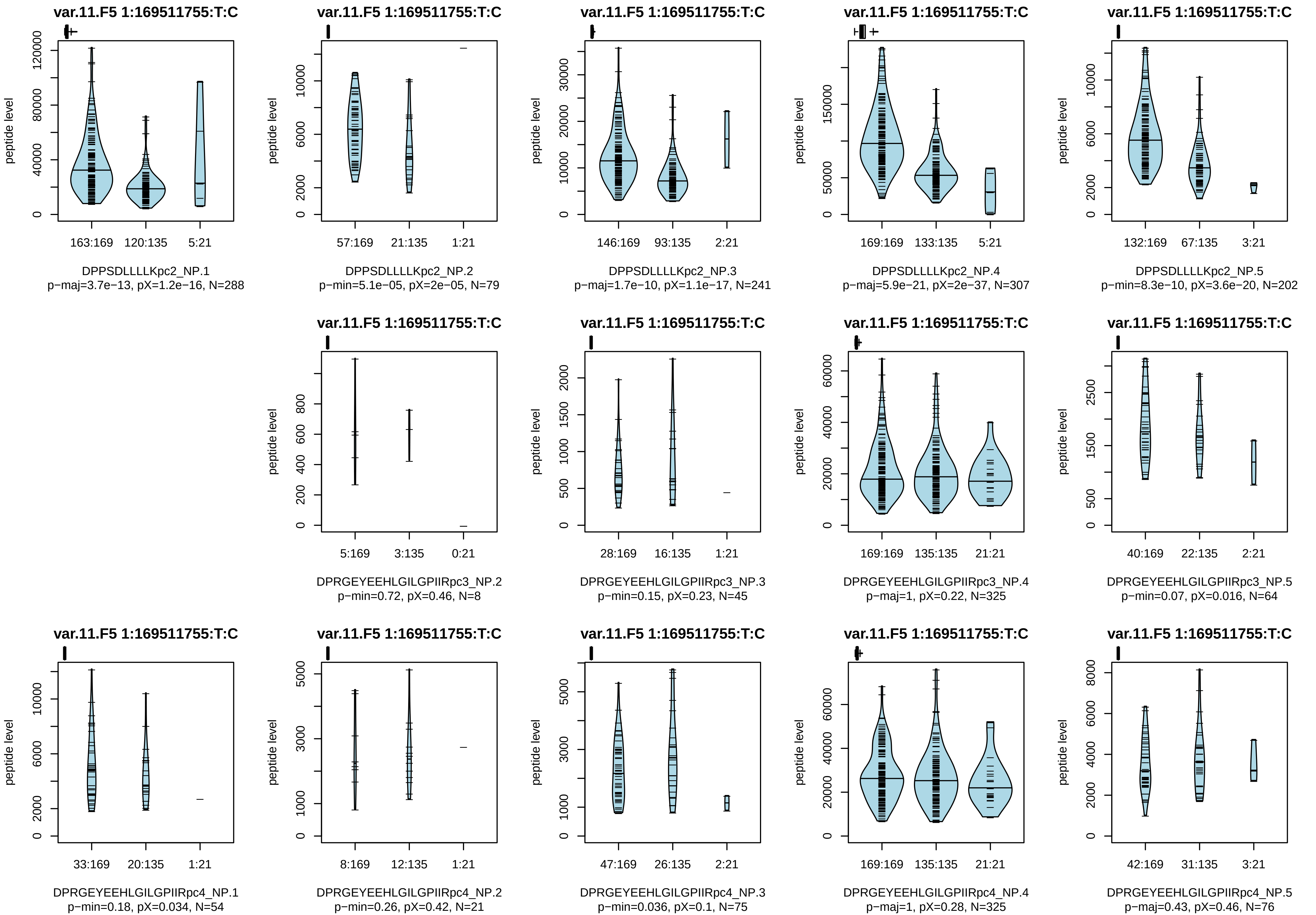
var.11.F5 1:169511755:T:C
peptide level
163:169 120:135 5:21
DPPSDLLLLKpc2_NP.1
p−maj=3.7e−13, pX=1.2e−16, N=288
var.11.F5 1:169511755:T:C
peptide level
57:169 21:135 1:21
DPPSDLLLLKpc2_NP.2
p−min=5.1e−05, pX=2e−05, N=79
var.11.F5 1:169511755:T:C
peptide level
146:169 93:135 2:21
DPPSDLLLLKpc2_NP.3
p−maj=1.7e−10, pX=1.1e−17, N=241
var.11.F5 1:169511755:T:C
peptide level
169:169 133:135 5:21
DPPSDLLLLKpc2_NP.4
p−maj=5.9e−21, pX=2e−37, N=307
var.11.F5 1:169511755:T:C
peptide level
132:169 67:135 3:21
DPPSDLLLLKpc2_NP.5
p−min=8.3e−10, pX=3.6e−20, N=202
var.11.F5 1:169511755:T:C
peptide level
5:169 3:135 0:21
DPRGEYEEHLGILGPIIRpc3_NP.2
p−min=0.72, pX=0.46, N=8
var.11.F5 1:169511755:T:C
peptide level
28:169 16:135 1:21
DPRGEYEEHLGILGPIIRpc3_NP.3
p−min=0.15, pX=0.23, N=45
var.11.F5 1:169511755:T:C
peptide level
169:169 135:135 21:21
DPRGEYEEHLGILGPIIRpc3_NP.4
p−maj=1, pX=0.22, N=325
var.11.F5 1:169511755:T:C
peptide level
40:169 22:135 2:21
DPRGEYEEHLGILGPIIRpc3_NP.5
p−min=0.07, pX=0.016, N=64
var.11.F5 1:169511755:T:C
peptide level
33:169 20:135 1:21
DPRGEYEEHLGILGPIIRpc4_NP.1
p−min=0.18, pX=0.034, N=54
var.11.F5 1:169511755:T:C
peptide level
8:169 12:135 1:21
DPRGEYEEHLGILGPIIRpc4_NP.2
p−min=0.26, pX=0.42, N=21
var.11.F5 1:169511755:T:C
peptide level
47:169 26:135 2:21
DPRGEYEEHLGILGPIIRpc4_NP.3
p−min=0.036, pX=0.1, N=75
var.11.F5 1:169511755:T:C
peptide level
169:169 135:135 21:21
DPRGEYEEHLGILGPIIRpc4_NP.4
p−maj=1, pX=0.28, N=325
var.11.F5 1:169511755:T:C
peptide level
42:169 31:135 3:21
DPRGEYEEHLGILGPIIRpc4_NP.5
p−maj=0.43, pX=0.46, N=76

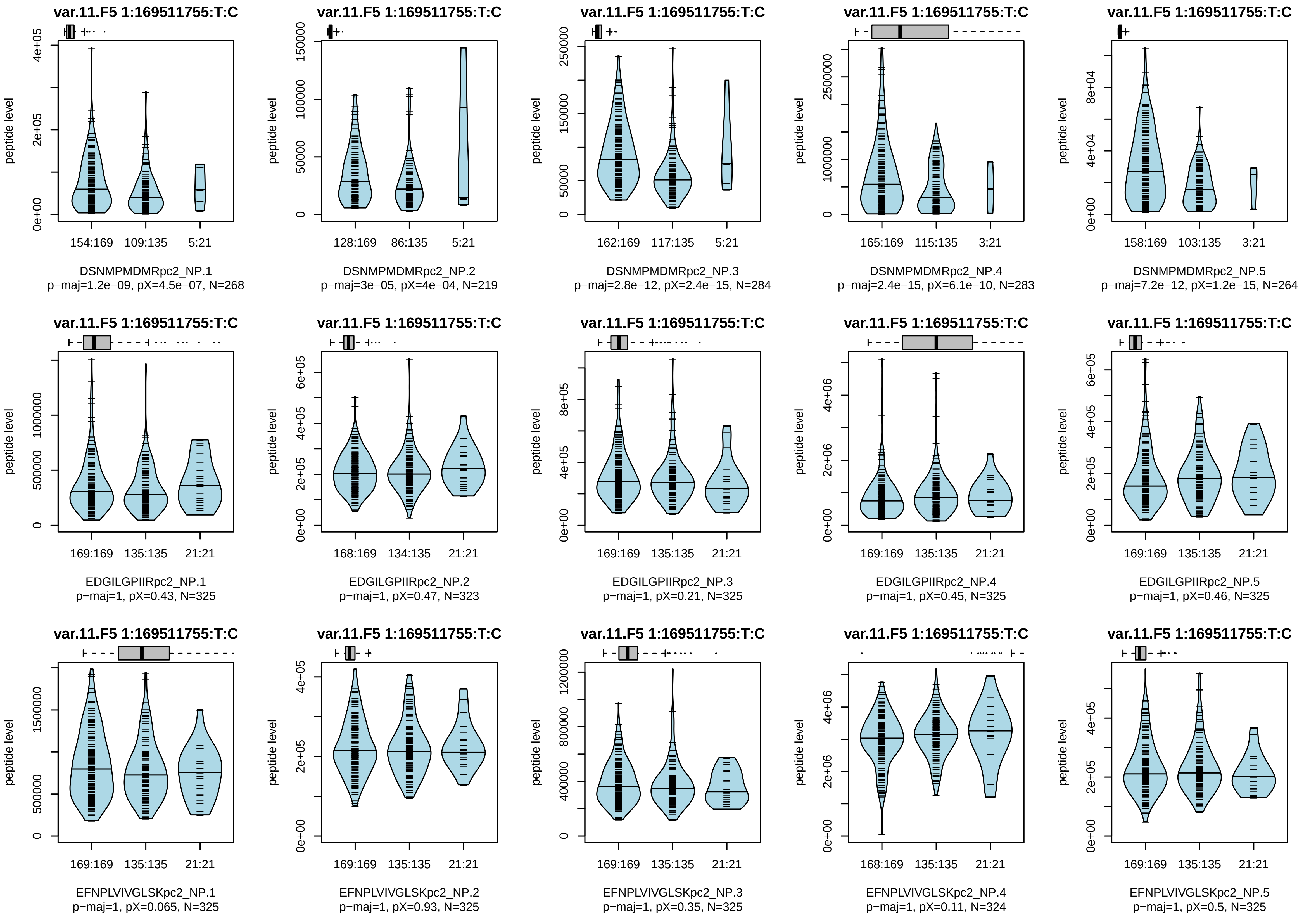
var.11.F5 1:169511755:T:C
peptide level
154:169
109:135
5:21
DSNMPMDMRpc2_NP.1
p−maj=1.2e−09, pX=4.5e−07, N=268
128:169
86:135
5:21
DSNMPMDMRpc2_NP.2
p−maj=3e−05, pX=4e−04, N=219
162:169
117:135
5:21
DSNMPMDMRpc2_NP.3
p−maj=2.8e−12, pX=2.4e−15, N=284
165:169
115:135
3:21
DSNMPMDMRpc2_NP.4
p−maj=2.4e−15, pX=6.1e−10, N=283
158:169
103:135
3:21
DSNMPMDMRpc2_NP.5
p−maj=7.2e−12, pX=1.2e−15, N=264
169:169
135:135
21:21
EDGILGPIIRpc2_NP.1
p−maj=1, pX=0.43, N=325
168:169
134:135
21:21
EDGILGPIIRpc2_NP.2
p−maj=1, pX=0.47, N=323
169:169
135:135
21:21
EDGILGPIIRpc2_NP.3
p−maj=1, pX=0.21, N=325
169:169
135:135
21:21
EDGILGPIIRpc2_NP.4
p−maj=1, pX=0.45, N=325
169:169
135:135
21:21
EDGILGPIIRpc2_NP.5
p−maj=1, pX=0.46, N=325
169:169
135:135
21:21
EFNPLVIVGLSKpc2_NP.1
p−maj=1, pX=0.065, N=325
169:169
135:135
21:21
EFNPLVIVGLSKpc2_NP.2
p−maj=1, pX=0.93, N=325
169:169
135:135
21:21
EFNPLVIVGLSKpc2_NP.3
p−maj=1, pX=0.35, N=325
168:169
135:135
21:21
EFNPLVIVGLSKpc2_NP.4
p−maj=1, pX=0.11, N=324
169:169
135:135
21:21
EFNPLVIVGLSKpc2_NP.5
p−maj=1, pX=0.5, N=325

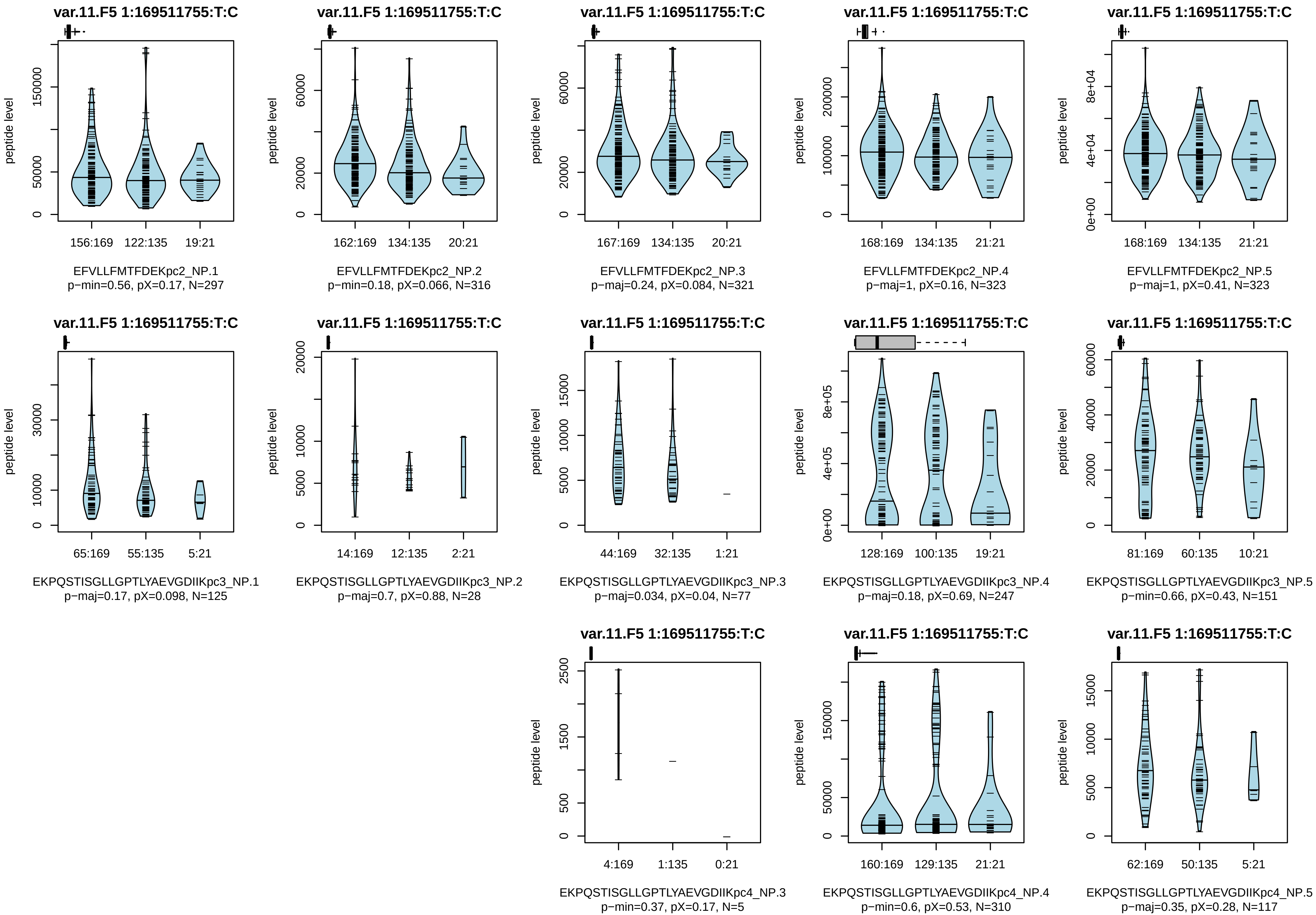
var.11.F5 1:169511755:T:C
peptide level
156:169
122:135
19:21
EFVLLFMTFDEKpc2_NP.1
p−min=0.56, pX=0.17, N=297
var.11.F5 1:169511755:T:C
peptide level
162:169
134:135
20:21
EFVLLFMTFDEKpc2_NP.2
p−min=0.18, pX=0.066, N=316
var.11.F5 1:169511755:T:C
peptide level
167:169
134:135
20:21
EFVLLFMTFDEKpc2_NP.3
p−maj=0.24, pX=0.084, N=321
var.11.F5 1:169511755:T:C
peptide level
168:169
134:135
21:21
EFVLLFMTFDEKpc2_NP.4
p−maj=1, pX=0.16, N=323
var.11.F5 1:169511755:T:C
peptide level
168:169
134:135
21:21
EFVLLFMTFDEKpc2_NP.5
p−maj=1, pX=0.41, N=323
var.11.F5 1:169511755:T:C
peptide level
65:169
55:135
5:21
EKPQSTISGLLGPTLYAEVGDIIKpc3_NP.1
p−maj=0.17, pX=0.098, N=125
var.11.F5 1:169511755:T:C
peptide level
14:169
12:135
2:21
EKPQSTISGLLGPTLYAEVGDIIKpc3_NP.2
p−maj=0.7, pX=0.88, N=28
var.11.F5 1:169511755:T:C
peptide level
44:169
32:135
1:21
EKPQSTISGLLGPTLYAEVGDIIKpc3_NP.3
p−maj=0.034, pX=0.04, N=77
var.11.F5 1:169511755:T:C
peptide level
128:169
100:135
19:21
EKPQSTISGLLGPTLYAEVGDIIKpc3_NP.4
p−maj=0.18, pX=0.69, N=247
var.11.F5 1:169511755:T:C
peptide level
81:169
60:135
10:21
EKPQSTISGLLGPTLYAEVGDIIKpc3_NP.5
p−min=0.66, pX=0.43, N=151
var.11.F5 1:169511755:T:C
peptide level
4:169
1:135
0:21
EKPQSTISGLLGPTLYAEVGDIIKpc4_NP.3
p−min=0.37, pX=0.17, N=5
var.11.F5 1:169511755:T:C
peptide level
160:169
129:135
21:21
EKPQSTISGLLGPTLYAEVGDIIKpc4_NP.4
p−min=0.6, pX=0.53, N=310
var.11.F5 1:169511755:T:C
peptide level
62:169
50:135
5:21
EKPQSTISGLLGPTLYAEVGDIIKpc4_NP.5
p−maj=0.35, pX=0.28, N=117

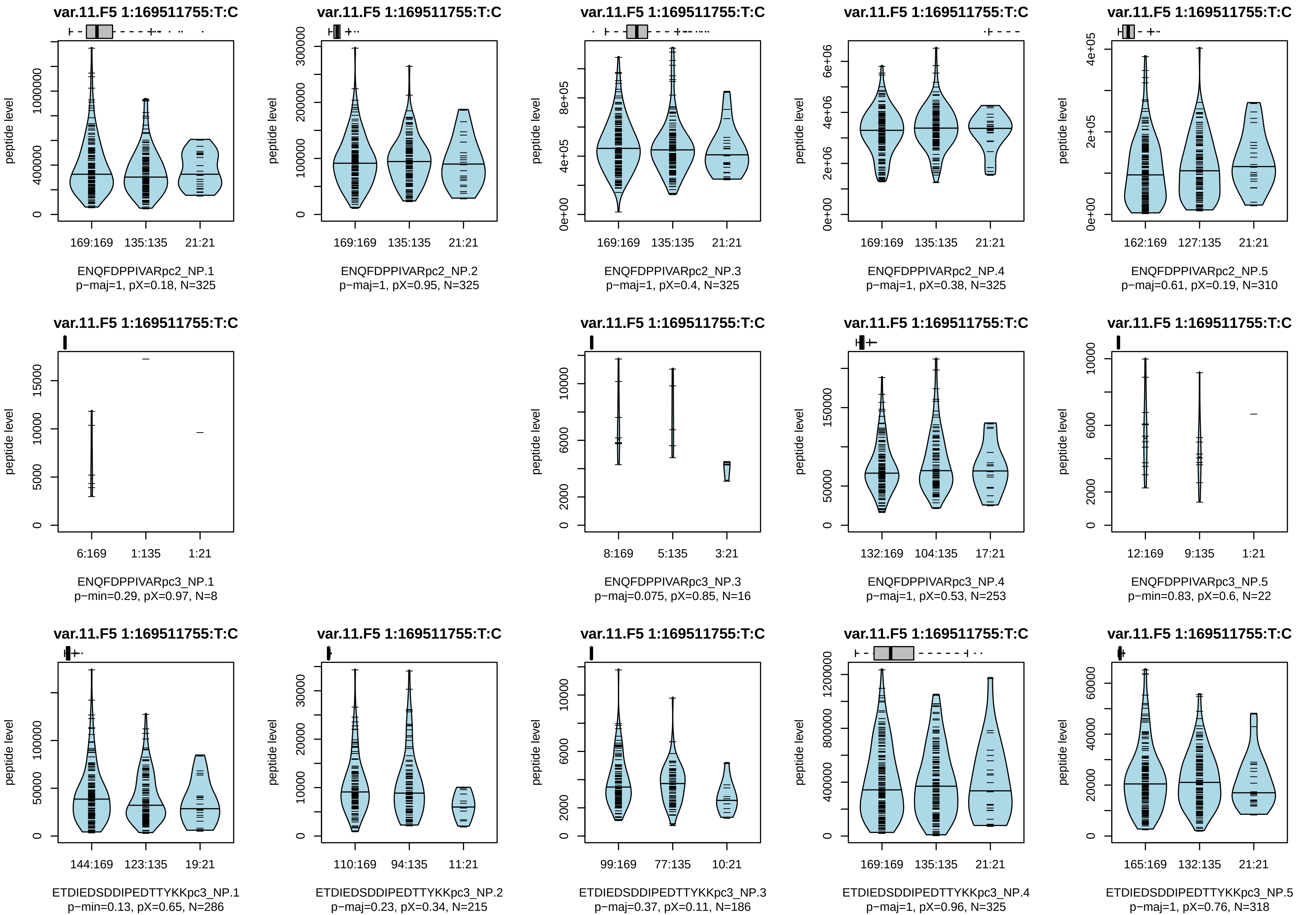
var.11.F5 1:169511755:T:C
peptide level
169:169 135:135 21:21
ENQFDPPIVARpc2_NP.1
p−maj=1, pX=0.18, N=325
var.11.F5 1:169511755:T:C
169:169 135:135 21:21
ENQFDPPIVARpc2_NP.2
p−maj=1, pX=0.95, N=325
var.11.F5 1:169511755:T:C
169:169 135:135 21:21
ENQFDPPIVARpc2_NP.3
p−maj=1, pX=0.4, N=325
var.11.F5 1:169511755:T:C
169:169 135:135 21:21
ENQFDPPIVARpc2_NP.4
p−maj=1, pX=0.38, N=325
var.11.F5 1:169511755:T:C
162:169 127:135 21:21
ENQFDPPIVARpc2_NP.5
p−maj=0.61, pX=0.19, N=310
var.11.F5 1:169511755:T:C
6:169 1:135 1:21
ENQFDPPIVARpc3_NP.1
p−min=0.29, pX=0.97, N=8
var.11.F5 1:169511755:T:C
8:169 5:135 3:21
ENQFDPPIVARpc3_NP.3
p−maj=0.075, pX=0.85, N=16
var.11.F5 1:169511755:T:C
132:169 104:135 17:21
ENQFDPPIVARpc3_NP.4
p−maj=1, pX=0.53, N=253
var.11.F5 1:169511755:T:C
12:169 9:135 1:21
ENQFDPPIVARpc3_NP.5
p−min=0.83, pX=0.6, N=22
var.11.F5 1:169511755:T:C
144:169 123:135 19:21
ETDIEDSDDIPEDTTYKKpc3_NP.1
p−min=0.13, pX=0.65, N=286
var.11.F5 1:169511755:T:C
110:169 94:135 11:21
ETDIEDSDDIPEDTTYKKpc3_NP.2
p−maj=0.23, pX=0.34, N=215
var.11.F5 1:169511755:T:C
99:169 77:135 10:21
ETDIEDSDDIPEDTTYKKpc3_NP.3
p−maj=0.37, pX=0.11, N=186
var.11.F5 1:169511755:T:C
169:169 135:135 21:21
ETDIEDSDDIPEDTTYKKpc3_NP.4
p−maj=1, pX=0.96, N=325
var.11.F5 1:169511755:T:C
165:169 132:135 21:21
ETDIEDSDDIPEDTTYKKpc3_NP.5
p−maj=1, pX=0.76, N=318

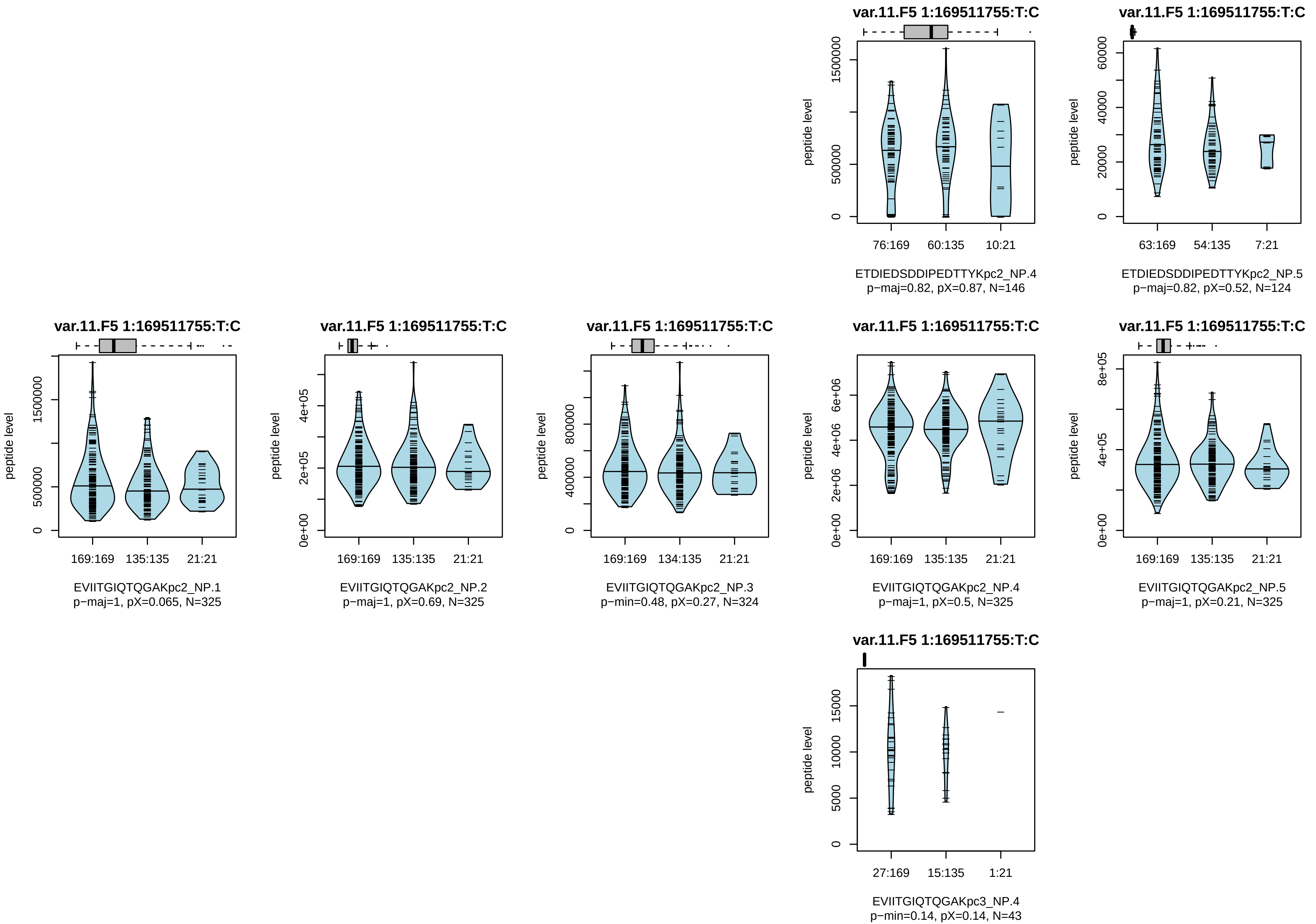
var.11.F5 1:169511755:T:C
peptide level
0
500000
1500000
76:169
60:135
10:21
ETDIEDSDDIPEDTTYKpc2_NP.4
p−maj=0.82, pX=0.87, N=146
var.11.F5 1:169511755:T:C
peptide level
0
20000
40000
60000
63:169
54:135
7:21
ETDIEDSDDIPEDTTYKpc2_NP.5
p−maj=0.82, pX=0.52, N=124
var.11.F5 1:169511755:T:C
peptide level
0
500000
1500000
169:169
135:135
21:21
EVIITGIQTQGAKpc2_NP.1
p−maj=1, pX=0.065, N=325
var.11.F5 1:169511755:T:C
peptide level
0e+00
2e+05
4e+05
169:169
135:135
21:21
EVIITGIQTQGAKpc2_NP.2
p−maj=1, pX=0.69, N=325
var.11.F5 1:169511755:T:C
peptide level
0
400000
800000
169:169
134:135
21:21
EVIITGIQTQGAKpc2_NP.3
p−min=0.48, pX=0.27, N=324
var.11.F5 1:169511755:T:C
peptide level
0e+00
2e+06
4e+06
6e+06
169:169
135:135
21:21
EVIITGIQTQGAKpc2_NP.4
p−maj=1, pX=0.5, N=325
var.11.F5 1:169511755:T:C
peptide level
0e+00
4e+05
8e+05
169:169
135:135
21:21
EVIITGIQTQGAKpc2_NP.5
p−maj=1, pX=0.21, N=325
var.11.F5 1:169511755:T:C
peptide level
0
5000
10000
15000
27:169
15:135
1:21
EVIITGIQTQGAKpc3_NP.4
p−min=0.14, pX=0.14, N=43

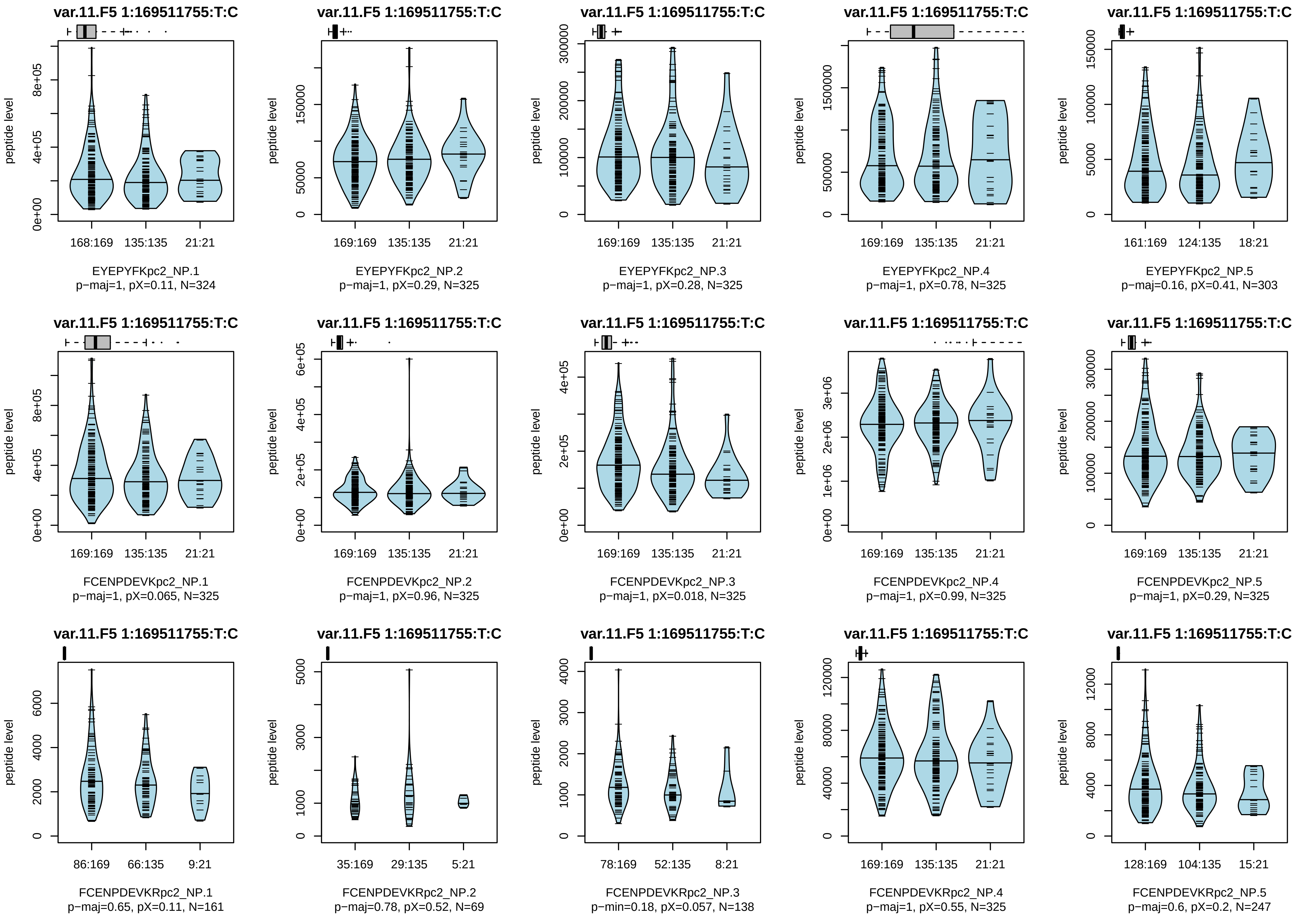
var.11.F5 1:169511755:T:C
peptide level
168:169 135:135 21:21
EYEPYFKpc2_NP.1
p−maj=1, pX=0.11, N=324
var.11.F5 1:169511755:T:C
peptide level
169:169 135:135 21:21
EYEPYFKpc2_NP.2
p−maj=1, pX=0.29, N=325
var.11.F5 1:169511755:T:C
peptide level
169:169 135:135 21:21
EYEPYFKpc2_NP.3
p−maj=1, pX=0.28, N=325
var.11.F5 1:169511755:T:C
peptide level
169:169 135:135 21:21
EYEPYFKpc2_NP.4
p−maj=1, pX=0.78, N=325
var.11.F5 1:169511755:T:C
peptide level
161:169 124:135 18:21
EYEPYFKpc2_NP.5
p−maj=0.16, pX=0.41, N=303
var.11.F5 1:169511755:T:C
peptide level
169:169 135:135 21:21
FCENPDEVKpc2_NP.1
p−maj=1, pX=0.065, N=325
var.11.F5 1:169511755:T:C
peptide level
169:169 135:135 21:21
FCENPDEVKpc2_NP.2
p−maj=1, pX=0.96, N=325
var.11.F5 1:169511755:T:C
peptide level
169:169 135:135 21:21
FCENPDEVKpc2_NP.3
p−maj=1, pX=0.018, N=325
var.11.F5 1:169511755:T:C
peptide level
169:169 135:135 21:21
FCENPDEVKpc2_NP.4
p−maj=1, pX=0.99, N=325
var.11.F5 1:169511755:T:C
peptide level
169:169 135:135 21:21
FCENPDEVKpc2_NP.5
p−maj=1, pX=0.29, N=325
var.11.F5 1:169511755:T:C
peptide level
86:169 66:135 9:21
FCENPDEVKRpc2_NP.1
p−maj=0.65, pX=0.11, N=161
var.11.F5 1:169511755:T:C
peptide level
35:169 29:135 5:21
FCENPDEVKRpc2_NP.2
p−maj=0.78, pX=0.52, N=69
var.11.F5 1:169511755:T:C
peptide level
78:169 52:135 8:21
FCENPDEVKRpc2_NP.3
p−min=0.18, pX=0.057, N=138
var.11.F5 1:169511755:T:C
peptide level
169:169 135:135 21:21
FCENPDEVKRpc2_NP.4
p−maj=1, pX=0.55, N=325
var.11.F5 1:169511755:T:C
peptide level
128:169 104:135 15:21
FCENPDEVKRpc2_NP.5
p−maj=0.6, pX=0.2, N=247

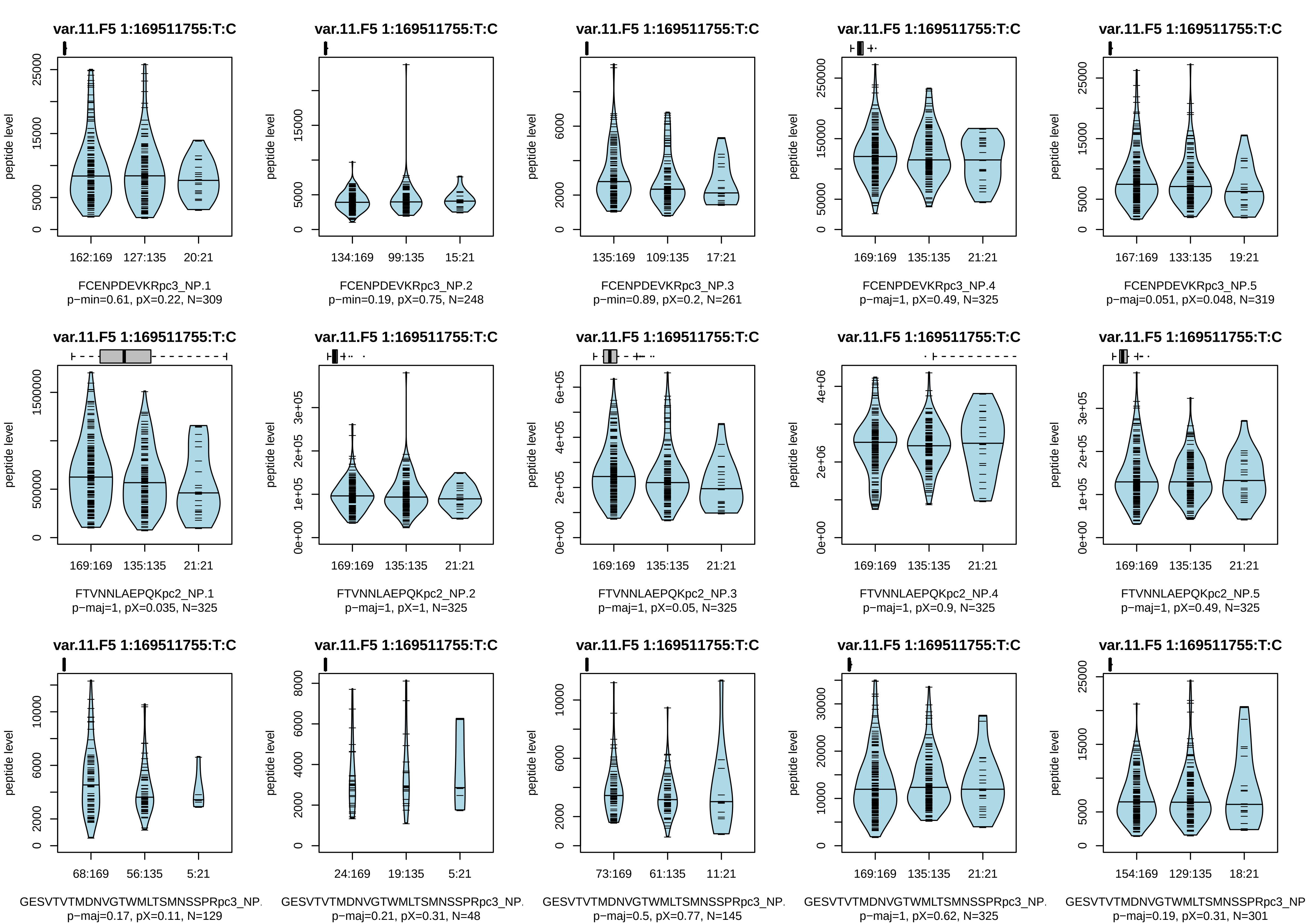
var.11.F5 1:169511755:T:C
peptide level
162:169
127:135
20:21
FCENPDEVKRpc3_NP.1
p−min=0.61, pX=0.22, N=309
134:169
99:135
15:21
FCENPDEVKRpc3_NP.2
p−min=0.19, pX=0.75, N=248
135:169
109:135
17:21
FCENPDEVKRpc3_NP.3
p−min=0.89, pX=0.2, N=261
169:169
135:135
21:21
FCENPDEVKRpc3_NP.4
p−maj=1, pX=0.49, N=325
167:169
133:135
19:21
FCENPDEVKRpc3_NP.5
p−maj=0.051, pX=0.048, N=319
169:169
135:135
21:21
FTVNNLAEPQKpc2_NP.1
p−maj=1, pX=0.035, N=325
FTVNNLAEPQKpc2_NP.2
p−maj=1, pX=1, N=325
FTVNNLAEPQKpc2_NP.3
p−maj=1, pX=0.05, N=325
FTVNNLAEPQKpc2_NP.4
p−maj=1, pX=0.9, N=325
FTVNNLAEPQKpc2_NP.5
p−maj=1, pX=0.49, N=325
68:169
56:135
5:21
GESVTVTMDNVGTWMLTSMNSSPRpc3_NP.
p−maj=0.17, pX=0.11, N=129
24:169
19:135
5:21
GESVTVTMDNVGTWMLTSMNSSPRpc3_NP.
p−maj=0.21, pX=0.31, N=48
73:169
61:135
11:21
GESVTVTMDNVGTWMLTSMNSSPRpc3_NP.
p−maj=0.5, pX=0.77, N=145
169:169
135:135
21:21
GESVTVTMDNVGTWMLTSMNSSPRpc3_NP.
p−maj=1, pX=0.62, N=325
154:169
129:135
18:21
GESVTVTMDNVGTWMLTSMNSSPRpc3_NP
p−maj=0.19, pX=0.31, N=301

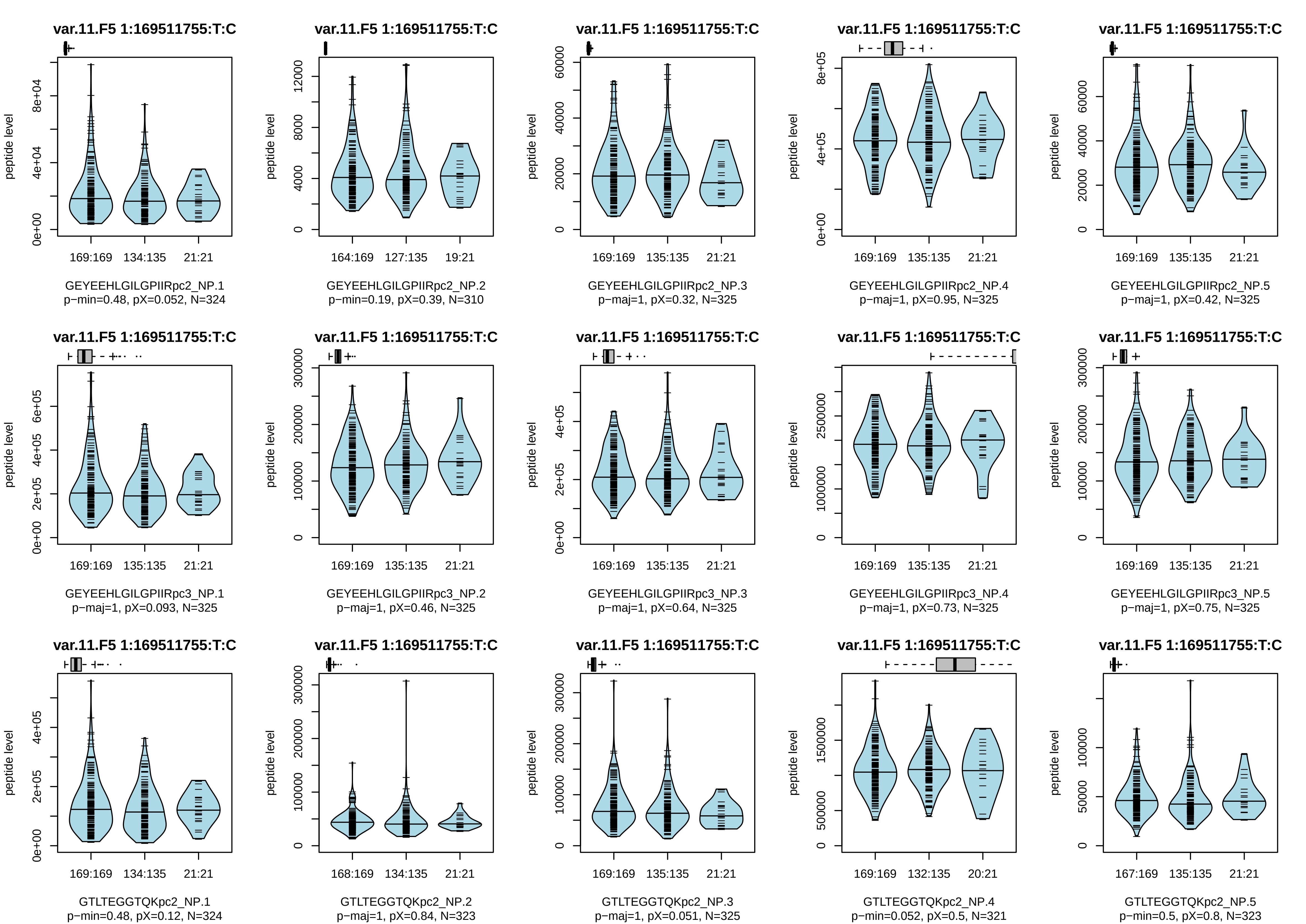
var.11.F5 1:169511755:T:C
peptide level
169:169 134:135 21:21
GEYEEHLGILGPIIRpc2_NP.1
p−min=0.48, pX=0.052, N=324
var.11.F5 1:169511755:T:C
peptide level
164:169 127:135 19:21
GEYEEHLGILGPIIRpc2_NP.2
p−min=0.19, pX=0.39, N=310
var.11.F5 1:169511755:T:C
peptide level
169:169 135:135 21:21
GEYEEHLGILGPIIRpc2_NP.3
p−maj=1, pX=0.32, N=325
var.11.F5 1:169511755:T:C
peptide level
169:169 135:135 21:21
GEYEEHLGILGPIIRpc2_NP.4
p−maj=1, pX=0.95, N=325
var.11.F5 1:169511755:T:C
peptide level
169:169 135:135 21:21
GEYEEHLGILGPIIRpc2_NP.5
p−maj=1, pX=0.42, N=325
var.11.F5 1:169511755:T:C
peptide level
169:169 135:135 21:21
GEYEEHLGILGPIIRpc3_NP.1
p−maj=1, pX=0.093, N=325
var.11.F5 1:169511755:T:C
peptide level
169:169 135:135 21:21
GEYEEHLGILGPIIRpc3_NP.2
p−maj=1, pX=0.46, N=325
var.11.F5 1:169511755:T:C
peptide level
169:169 135:135 21:21
GEYEEHLGILGPIIRpc3_NP.3
p−maj=1, pX=0.64, N=325
var.11.F5 1:169511755:T:C
peptide level
169:169 135:135 21:21
GEYEEHLGILGPIIRpc3_NP.4
p−maj=1, pX=0.73, N=325
var.11.F5 1:169511755:T:C
peptide level
169:169 135:135 21:21
GEYEEHLGILGPIIRpc3_NP.5
p−maj=1, pX=0.75, N=325
var.11.F5 1:169511755:T:C
peptide level
169:169 134:135 21:21
GTLTEGGTQKpc2_NP.1
p−min=0.48, pX=0.12, N=324
var.11.F5 1:169511755:T:C
peptide level
168:169 134:135 21:21
GTLTEGGTQKpc2_NP.2
p−maj=1, pX=0.84, N=323
var.11.F5 1:169511755:T:C
peptide level
169:169 135:135 21:21
GTLTEGGTQKpc2_NP.3
p−maj=1, pX=0.051, N=325
var.11.F5 1:169511755:T:C
peptide level
169:169 132:135 20:21
GTLTEGGTQKpc2_NP.4
p−min=0.052, pX=0.5, N=321
var.11.F5 1:169511755:T:C
peptide level
167:169 135:135 21:21
GTLTEGGTQKpc2_NP.5
p−min=0.5, pX=0.8, N=323

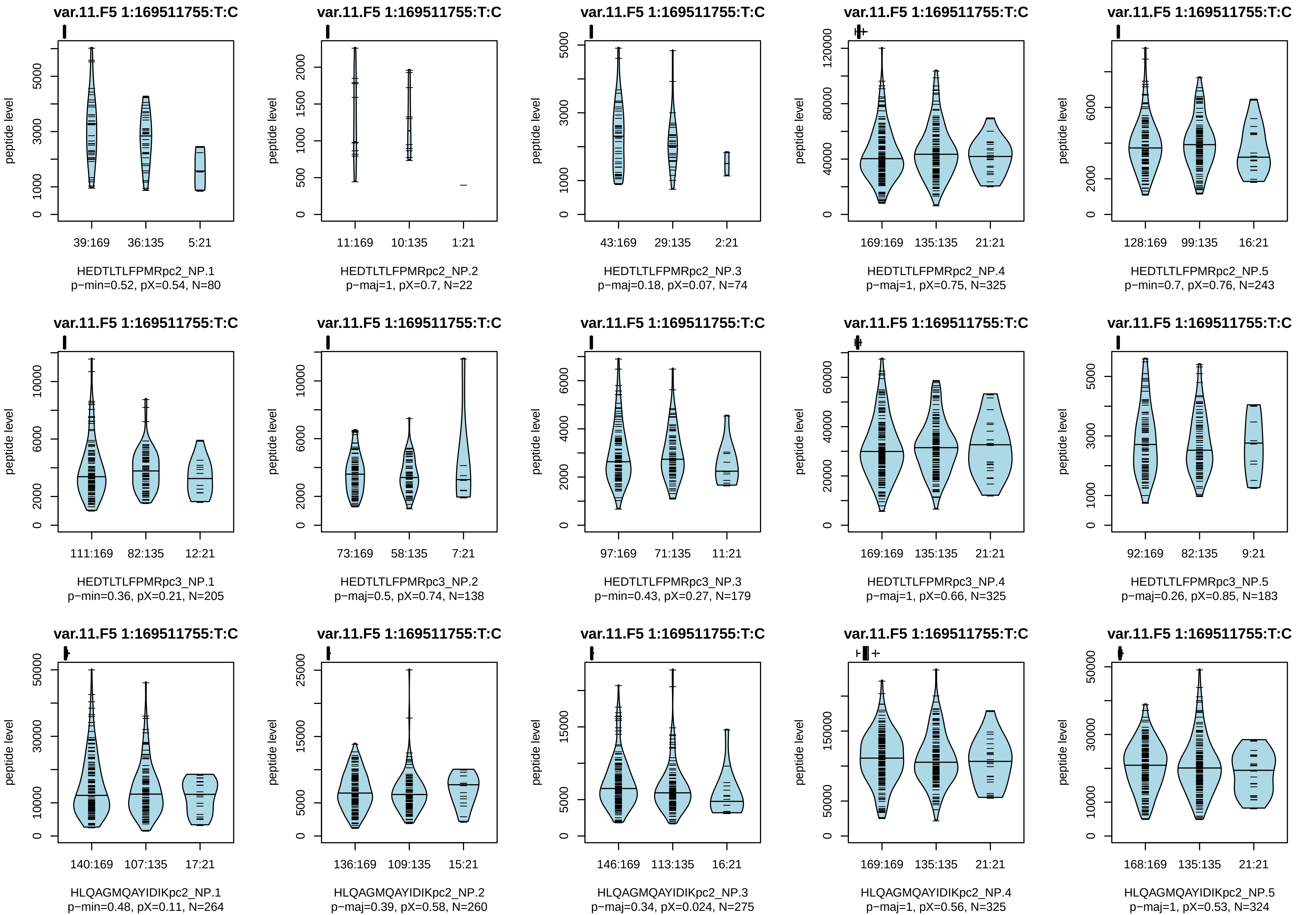
var.11.F5 1:169511755:T:C
peptide level
39:169
36:135
5:21
HEDTLTLFPMRpc2_NP.1
p−min=0.52, pX=0.54, N=80
var.11.F5 1:169511755:T:C
11:169
10:135
1:21
HEDTLTLFPMRpc2_NP.2
p−maj=1, pX=0.7, N=22
var.11.F5 1:169511755:T:C
43:169
29:135
2:21
HEDTLTLFPMRpc2_NP.3
p−maj=0.18, pX=0.07, N=74
var.11.F5 1:169511755:T:C
169:169
135:135
21:21
HEDTLTLFPMRpc2_NP.4
p−maj=1, pX=0.75, N=325
var.11.F5 1:169511755:T:C
128:169
99:135
16:21
HEDTLTLFPMRpc2_NP.5
p−min=0.7, pX=0.76, N=243
var.11.F5 1:169511755:T:C
111:169
82:135
12:21
HEDTLTLFPMRpc3_NP.1
p−min=0.36, pX=0.21, N=205
var.11.F5 1:169511755:T:C
73:169
58:135
7:21
HEDTLTLFPMRpc3_NP.2
p−maj=0.5, pX=0.74, N=138
var.11.F5 1:169511755:T:C
97:169
71:135
11:21
HEDTLTLFPMRpc3_NP.3
p−min=0.43, pX=0.27, N=179
var.11.F5 1:169511755:T:C
169:169
135:135
21:21
HEDTLTLFPMRpc3_NP.4
p−maj=1, pX=0.66, N=325
var.11.F5 1:169511755:T:C
92:169
82:135
9:21
HEDTLTLFPMRpc3_NP.5
p−maj=0.26, pX=0.85, N=183
var.11.F5 1:169511755:T:C
140:169
107:135
17:21
HLQAGMQAYIDIKpc2_NP.1
p−min=0.48, pX=0.11, N=264
var.11.F5 1:169511755:T:C
136:169
109:135
15:21
HLQAGMQAYIDIKpc2_NP.2
p−maj=0.39, pX=0.58, N=260
var.11.F5 1:169511755:T:C
146:169
113:135
16:21
HLQAGMQAYIDIKpc2_NP.3
p−maj=0.34, pX=0.024, N=275
var.11.F5 1:169511755:T:C
169:169
135:135
21:21
HLQAGMQAYIDIKpc2_NP.4
p−maj=1, pX=0.56, N=325
var.11.F5 1:169511755:T:C
168:169
135:135
21:21
HLQAGMQAYIDIKpc2_NP.5
p−maj=1, pX=0.53, N=324

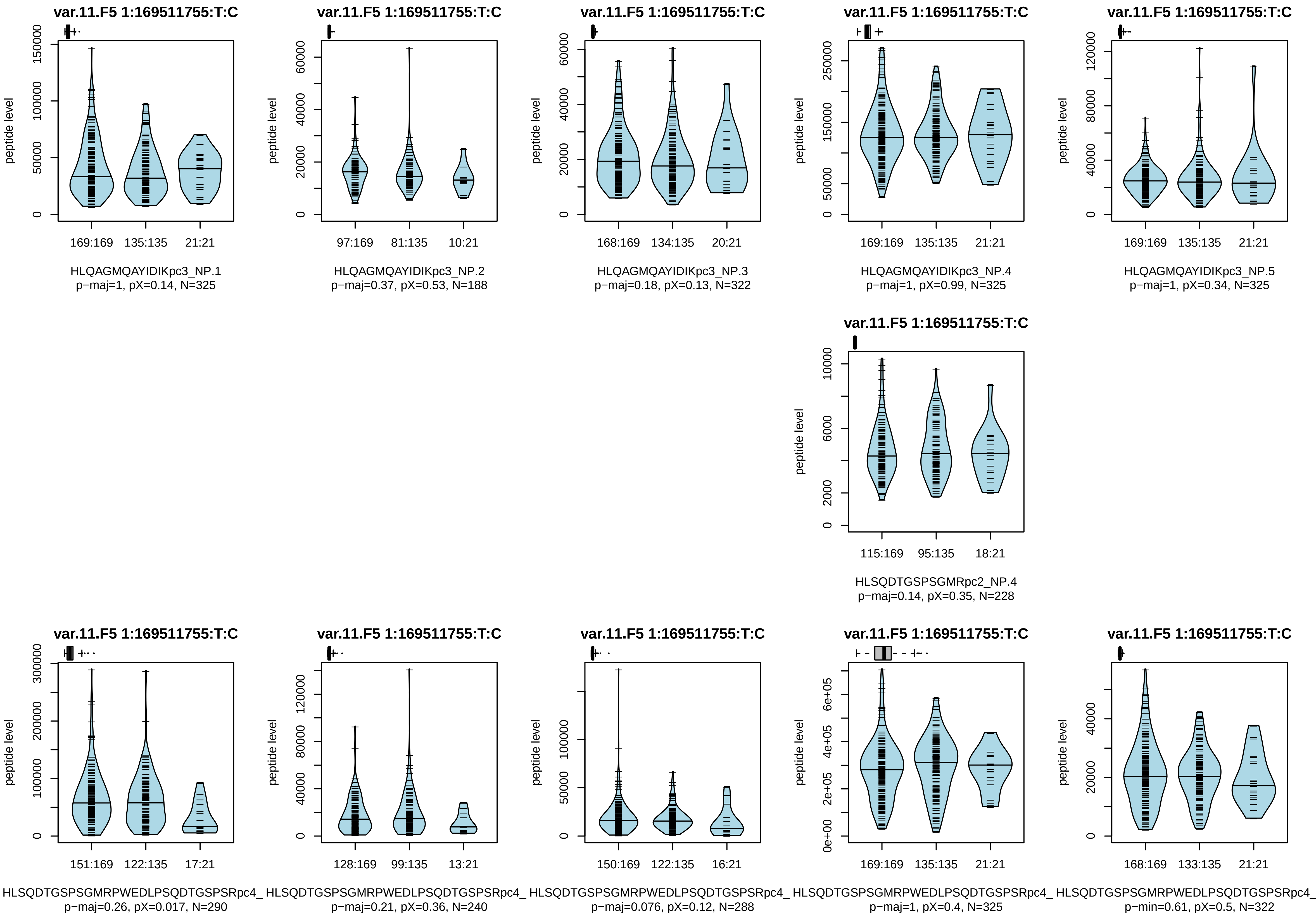
var.11.F5 1:169511755:T:C
peptide level
169:169
135:135
21:21
HLQAGMQAYIDIKpc3_NP.1
p−maj=1, pX=0.14, N=325
var.11.F5 1:169511755:T:C
peptide level
97:169
81:135
10:21
HLQAGMQAYIDIKpc3_NP.2
p−maj=0.37, pX=0.53, N=188
var.11.F5 1:169511755:T:C
peptide level
168:169
134:135
20:21
HLQAGMQAYIDIKpc3_NP.3
p−maj=0.18, pX=0.13, N=322
var.11.F5 1:169511755:T:C
peptide level
169:169
135:135
21:21
HLQAGMQAYIDIKpc3_NP.4
p−maj=1, pX=0.99, N=325
var.11.F5 1:169511755:T:C
peptide level
169:169
135:135
21:21
HLQAGMQAYIDIKpc3_NP.5
p−maj=1, pX=0.34, N=325
var.11.F5 1:169511755:T:C
peptide level
115:169
95:135
18:21
HLSQDTGSPSGMRpc2_NP.4
p−maj=0.14, pX=0.35, N=228
var.11.F5 1:169511755:T:C
peptide level
151:169
122:135
17:21
HLSQDTGSPSGMRPWEDLPSQDTGSPSRpc4_
p−maj=0.26, pX=0.017, N=290
var.11.F5 1:169511755:T:C
peptide level
128:169
99:135
13:21
HLSQDTGSPSGMRPWEDLPSQDTGSPSRpc4_
p−maj=0.21, pX=0.36, N=240
var.11.F5 1:169511755:T:C
peptide level
150:169
122:135
16:21
HLSQDTGSPSGMRPWEDLPSQDTGSPSRpc4_
p−maj=0.076, pX=0.12, N=288
var.11.F5 1:169511755:T:C
peptide level
169:169
135:135
21:21
HLSQDTGSPSGMRPWEDLPSQDTGSPSRpc4_
p−maj=1, pX=0.4, N=325
var.11.F5 1:169511755:T:C
peptide level
168:169
133:135
21:21
HLSQDTGSPSGMRPWEDLPSQDTGSPSRpc4_
p−min=0.61, pX=0.5, N=322

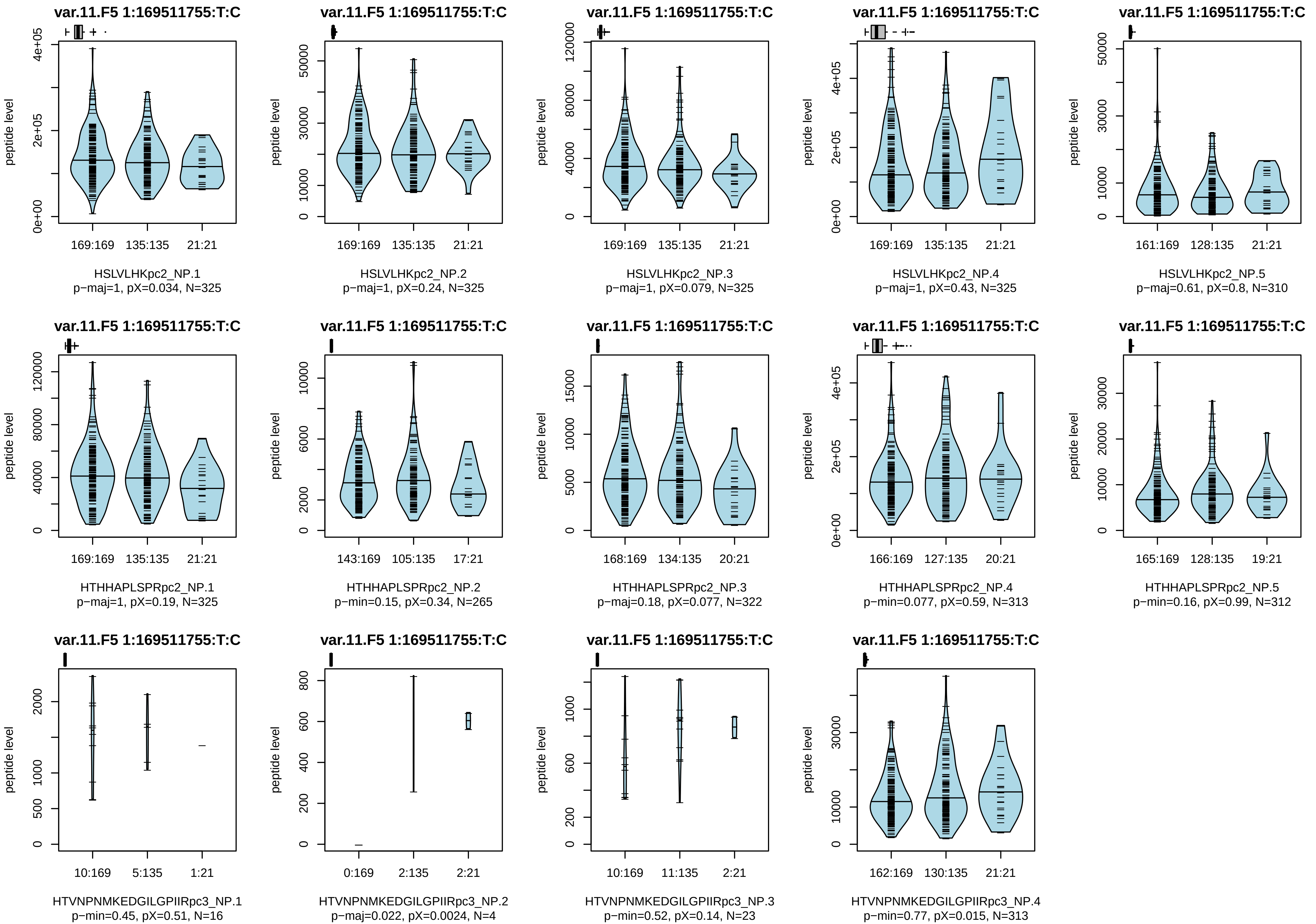
var.11.F5 1:169511755:T:C
peptide level
169:169
135:135
21:21
HSLVLHKpc2_NP.1
p−maj=1, pX=0.034, N=325
var.11.F5 1:169511755:T:C
peptide level
169:169
135:135
21:21
HSLVLHKpc2_NP.2
p−maj=1, pX=0.24, N=325
var.11.F5 1:169511755:T:C
peptide level
169:169
135:135
21:21
HSLVLHKpc2_NP.3
p−maj=1, pX=0.079, N=325
var.11.F5 1:169511755:T:C
peptide level
169:169
135:135
21:21
HSLVLHKpc2_NP.4
p−maj=1, pX=0.43, N=325
var.11.F5 1:169511755:T:C
peptide level
161:169
128:135
21:21
HSLVLHKpc2_NP.5
p−maj=0.61, pX=0.8, N=310
var.11.F5 1:169511755:T:C
peptide level
169:169
135:135
21:21
HTHHAPLSPRpc2_NP.1
p−maj=1, pX=0.19, N=325
var.11.F5 1:169511755:T:C
peptide level
143:169
105:135
17:21
HTHHAPLSPRpc2_NP.2
p−min=0.15, pX=0.34, N=265
var.11.F5 1:169511755:T:C
peptide level
168:169
134:135
20:21
HTHHAPLSPRpc2_NP.3
p−maj=0.18, pX=0.077, N=322
var.11.F5 1:169511755:T:C
peptide level
166:169
127:135
20:21
HTHHAPLSPRpc2_NP.4
p−min=0.077, pX=0.59, N=313
var.11.F5 1:169511755:T:C
peptide level
165:169
128:135
19:21
HTHHAPLSPRpc2_NP.5
p−min=0.16, pX=0.99, N=312
var.11.F5 1:169511755:T:C
peptide level
10:169
5:135
1:21
HTVNPNMKEDGILGPIIRpc3_NP.1
p−min=0.45, pX=0.51, N=16
var.11.F5 1:169511755:T:C
peptide level
0:169
2:135
2:21
HTVNPNMKEDGILGPIIRpc3_NP.2
p−maj=0.022, pX=0.0024, N=4
var.11.F5 1:169511755:T:C
peptide level
10:169
11:135
2:21
HTVNPNMKEDGILGPIIRpc3_NP.3
p−min=0.52, pX=0.14, N=23
var.11.F5 1:169511755:T:C
peptide level
162:169
130:135
21:21
HTVNPNMKEDGILGPIIRpc3_NP.4
p−min=0.77, pX=0.015, N=313

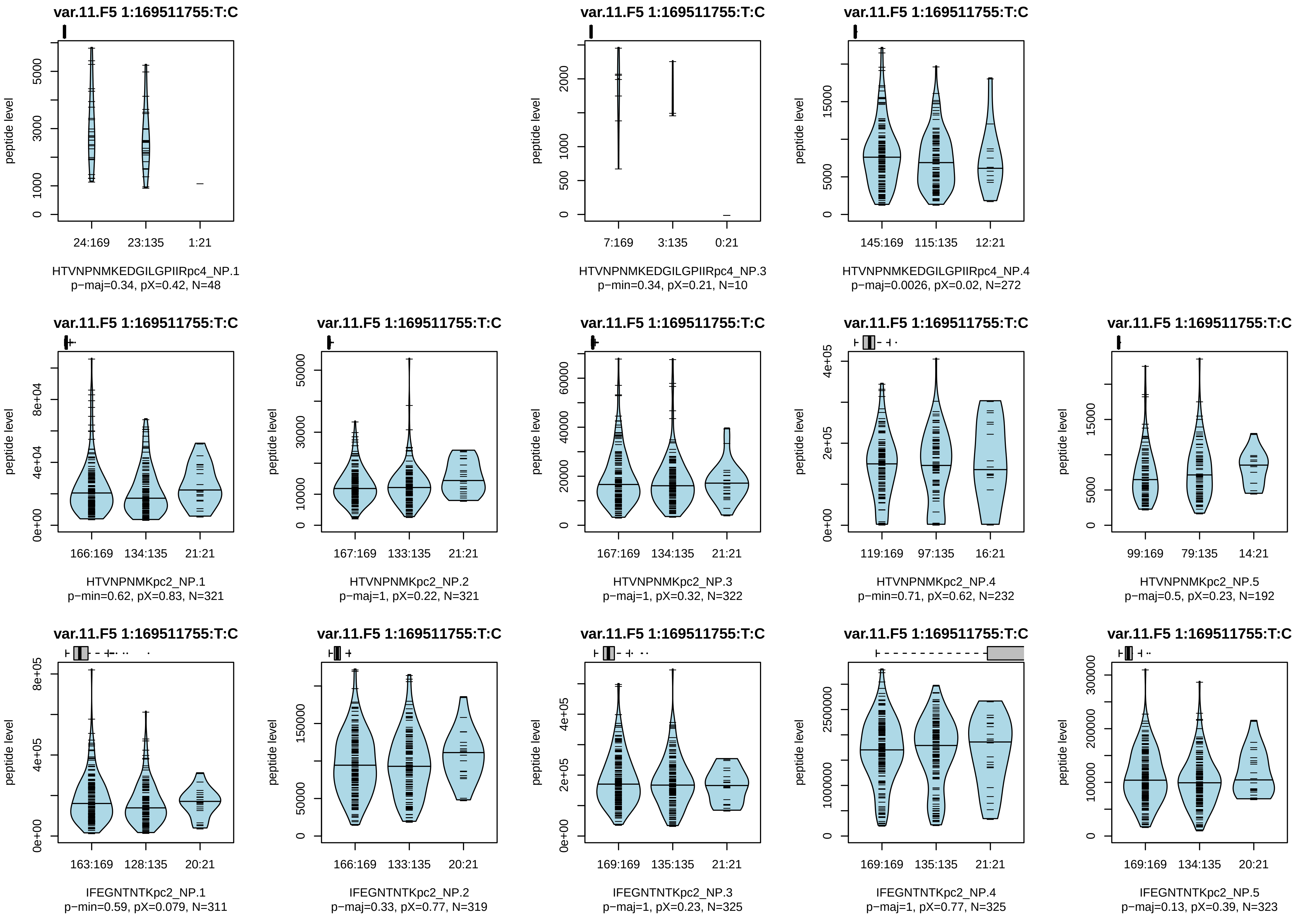
var.11.F5 1:169511755:T:C
peptide level
0
1000
3000
5000
24:169
23:135
1:21
HTVNPNMKEDGILGPIIRpc4_NP.1
p−maj=0.34, pX=0.42, N=48
var.11.F5 1:169511755:T:C
peptide level
0
500
1000
2000
7:169
3:135
0:21
HTVNPNMKEDGILGPIIRpc4_NP.3
p−min=0.34, pX=0.21, N=10
var.11.F5 1:169511755:T:C
peptide level
0
5000
15000
145:169
115:135
12:21
HTVNPNMKEDGILGPIIRpc4_NP.4
p−maj=0.0026, pX=0.02, N=272
var.11.F5 1:169511755:T:C
peptide level
0e+00
4e+04
8e+04
166:169
134:135
21:21
HTVNPNMKpc2_NP.1
p−min=0.62, pX=0.83, N=321
var.11.F5 1:169511755:T:C
peptide level
0
10000
30000
50000
167:169
133:135
21:21
HTVNPNMKpc2_NP.2
p−maj=1, pX=0.22, N=321
var.11.F5 1:169511755:T:C
peptide level
0
20000
40000
60000
167:169
134:135
21:21
HTVNPNMKpc2_NP.3
p−maj=1, pX=0.32, N=322
var.11.F5 1:169511755:T:C
peptide level
0e+00
2e+05
4e+05
119:169
97:135
16:21
HTVNPNMKpc2_NP.4
p−min=0.71, pX=0.62, N=232
var.11.F5 1:169511755:T:C
peptide level
0
5000
15000
99:169
79:135
14:21
HTVNPNMKpc2_NP.5
p−maj=0.5, pX=0.23, N=192
var.11.F5 1:169511755:T:C
peptide level
0e+00
4e+05
8e+05
163:169
128:135
20:21
IFEGNTNTKpc2_NP.1
p−min=0.59, pX=0.079, N=311
var.11.F5 1:169511755:T:C
peptide level
0
50000
150000
166:169
133:135
20:21
IFEGNTNTKpc2_NP.2
p−maj=0.33, pX=0.77, N=319
var.11.F5 1:169511755:T:C
peptide level
0e+00
2e+05
4e+05
169:169
135:135
21:21
IFEGNTNTKpc2_NP.3
p−maj=1, pX=0.23, N=325
var.11.F5 1:169511755:T:C
peptide level
0
1000000
2500000
169:169
135:135
21:21
IFEGNTNTKpc2_NP.4
p−maj=1, pX=0.77, N=325
var.11.F5 1:169511755:T:C
peptide level
0
100000
200000
300000
169:169
134:135
20:21
IFEGNTNTKpc2_NP.5
p−maj=0.13, pX=0.39, N=323

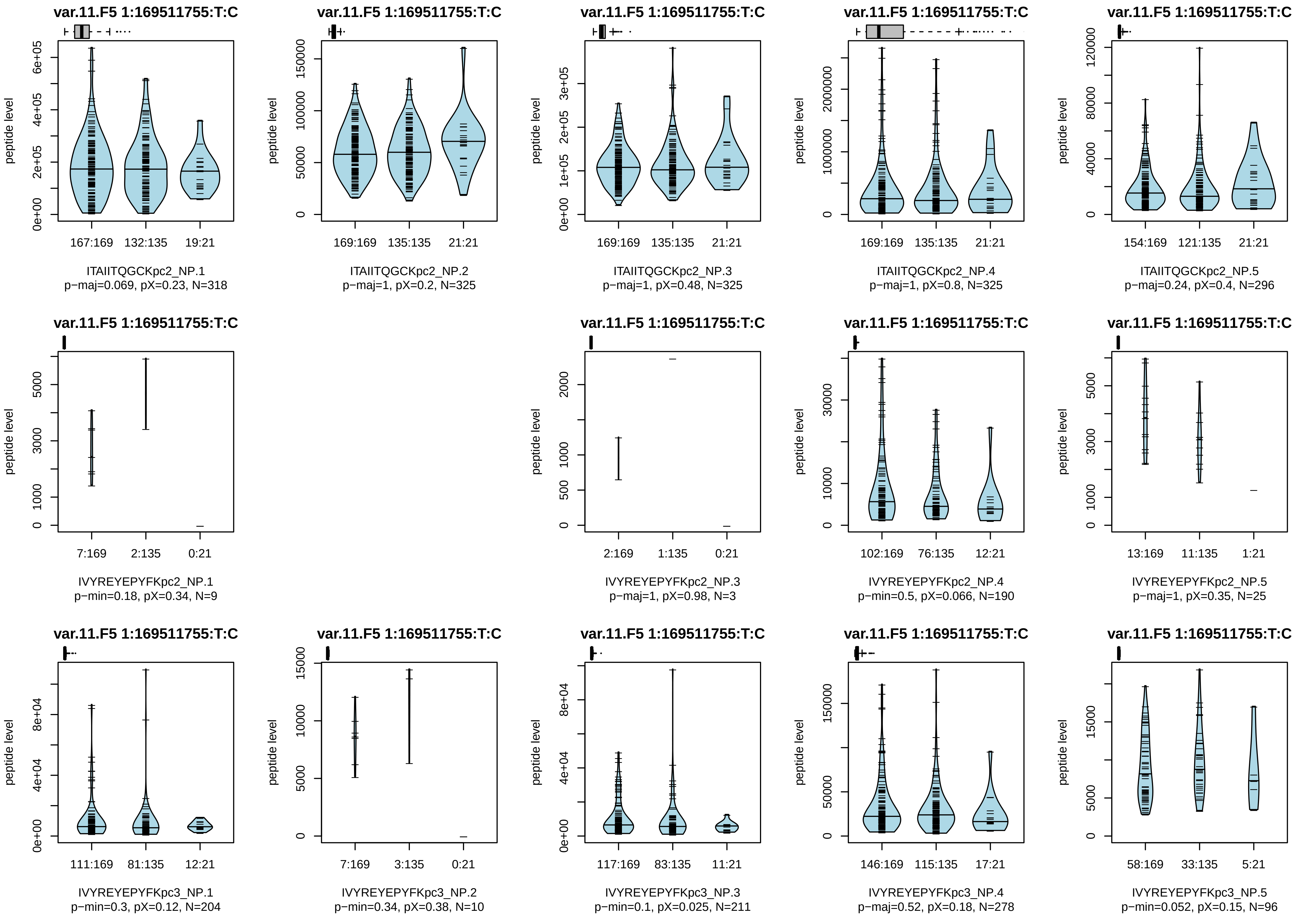
var.11.F5 1:169511755:T:C
peptide level
167:169
132:135
19:21
ITAIITQGCKpc2_NP.1
p−maj=0.069, pX=0.23, N=318
var.11.F5 1:169511755:T:C
peptide level
169:169
135:135
21:21
ITAIITQGCKpc2_NP.2
p−maj=1, pX=0.2, N=325
var.11.F5 1:169511755:T:C
peptide level
169:169
135:135
21:21
ITAIITQGCKpc2_NP.3
p−maj=1, pX=0.48, N=325
var.11.F5 1:169511755:T:C
peptide level
169:169
135:135
21:21
ITAIITQGCKpc2_NP.4
p−maj=1, pX=0.8, N=325
var.11.F5 1:169511755:T:C
peptide level
154:169
121:135
21:21
ITAIITQGCKpc2_NP.5
p−maj=0.24, pX=0.4, N=296
var.11.F5 1:169511755:T:C
peptide level
7:169
2:135
0:21
IVYREYEPYFKpc2_NP.1
p−min=0.18, pX=0.34, N=9
var.11.F5 1:169511755:T:C
peptide level
2:169
1:135
0:21
IVYREYEPYFKpc2_NP.3
p−maj=1, pX=0.98, N=3
var.11.F5 1:169511755:T:C
peptide level
102:169
76:135
12:21
IVYREYEPYFKpc2_NP.4
p−min=0.5, pX=0.066, N=190
var.11.F5 1:169511755:T:C
peptide level
13:169
11:135
1:21
IVYREYEPYFKpc2_NP.5
p−maj=1, pX=0.35, N=25
var.11.F5 1:169511755:T:C
peptide level
111:169
81:135
12:21
IVYREYEPYFKpc3_NP.1
p−min=0.3, pX=0.12, N=204
var.11.F5 1:169511755:T:C
peptide level
7:169
3:135
0:21
IVYREYEPYFKpc3_NP.2
p−min=0.34, pX=0.38, N=10
var.11.F5 1:169511755:T:C
peptide level
117:169
83:135
11:21
IVYREYEPYFKpc3_NP.3
p−min=0.1, pX=0.025, N=211
var.11.F5 1:169511755:T:C
peptide level
146:169
115:135
17:21
IVYREYEPYFKpc3_NP.4
p−maj=0.52, pX=0.18, N=278
var.11.F5 1:169511755:T:C
peptide level
58:169
33:135
5:21
IVYREYEPYFKpc3_NP.5
p−min=0.052, pX=0.15, N=96

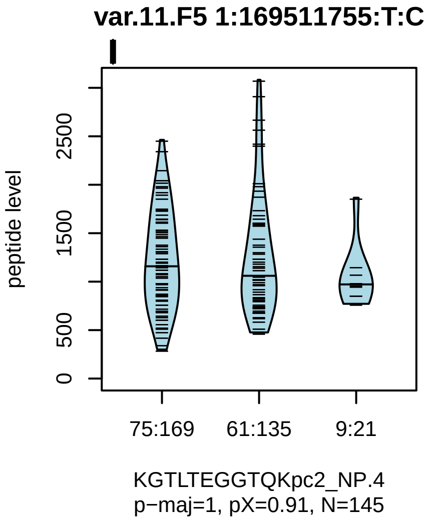
var.11.F5 1:169511755:T:C
peptide level
0
500
1500
2500
75:169
61:135
9:21
KGTLTEGGTQKpc2_NP.4
p−maj=1, pX=0.91, N=145

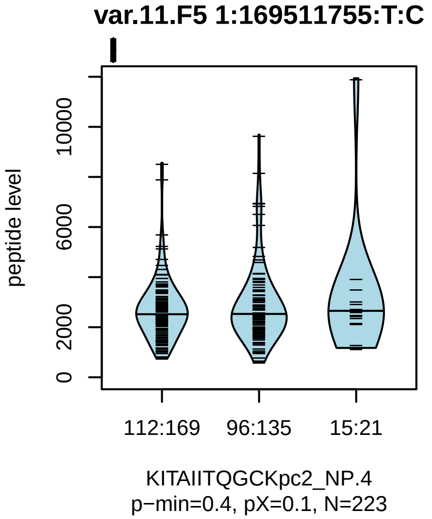
var.11.F5 1:169511755:T:C
peptide level
0
2000
6000
10000
112:169
96:135
15:21
KITAIITQGCKpc2_NP.4
p−min=0.4, pX=0.1, N=223

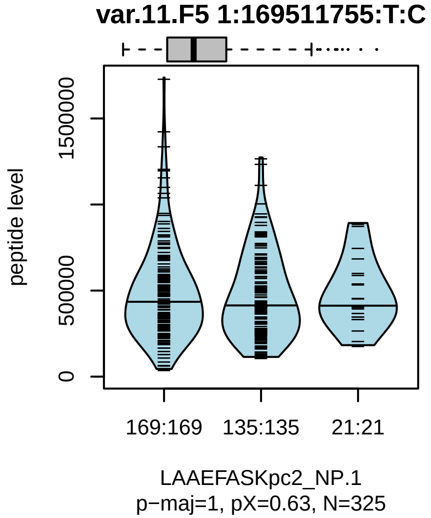
var.11.F5 1:169511755:T:C
peptide level
0
500000
1500000
169:169
135:135
21:21
LAAEFASKpc2_NP.1
p−maj=1, pX=0.63, N=325

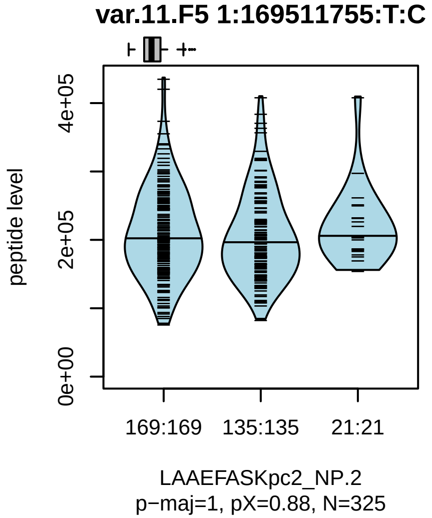
var.11.F5 1:169511755:T:C
peptide level
0e+00
2e+05
4e+05
169:169
135:135
21:21
LAAEFASKpc2_NP.2
p−maj=1, pX=0.88, N=325

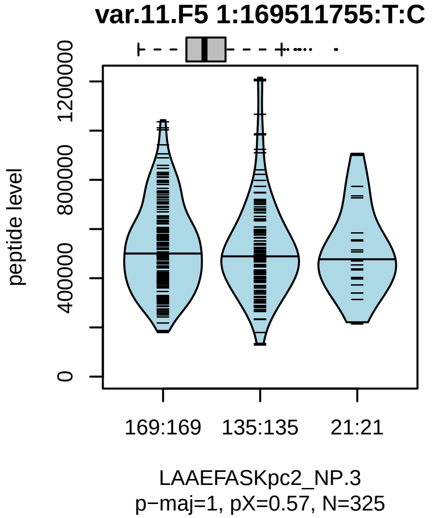
var.11.F5 1:169511755:T:C
peptide level
0
400000
800000
1200000
169:169
135:135
21:21
LAAEFASKpc2_NP.3
p−maj=1, pX=0.57, N=325

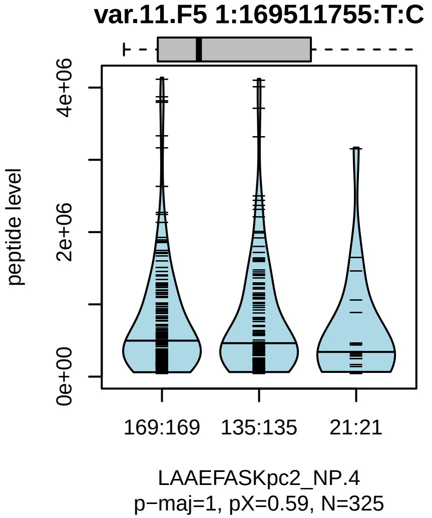
var.11.F5 1:169511755:T:C
peptide level
0e+00
2e+06
4e+06
169:169
135:135
21:21
LAAEFASKpc2_NP.4
p−maj=1, pX=0.59, N=325

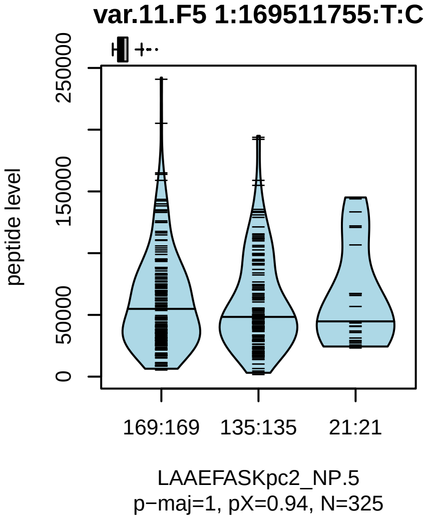
var.11.F5 1:169511755:T:C
peptide level
0
50000
150000
250000
169:169
135:135
21:21
LAAEFASKpc2_NP.5
p−maj=1, pX=0.94, N=325

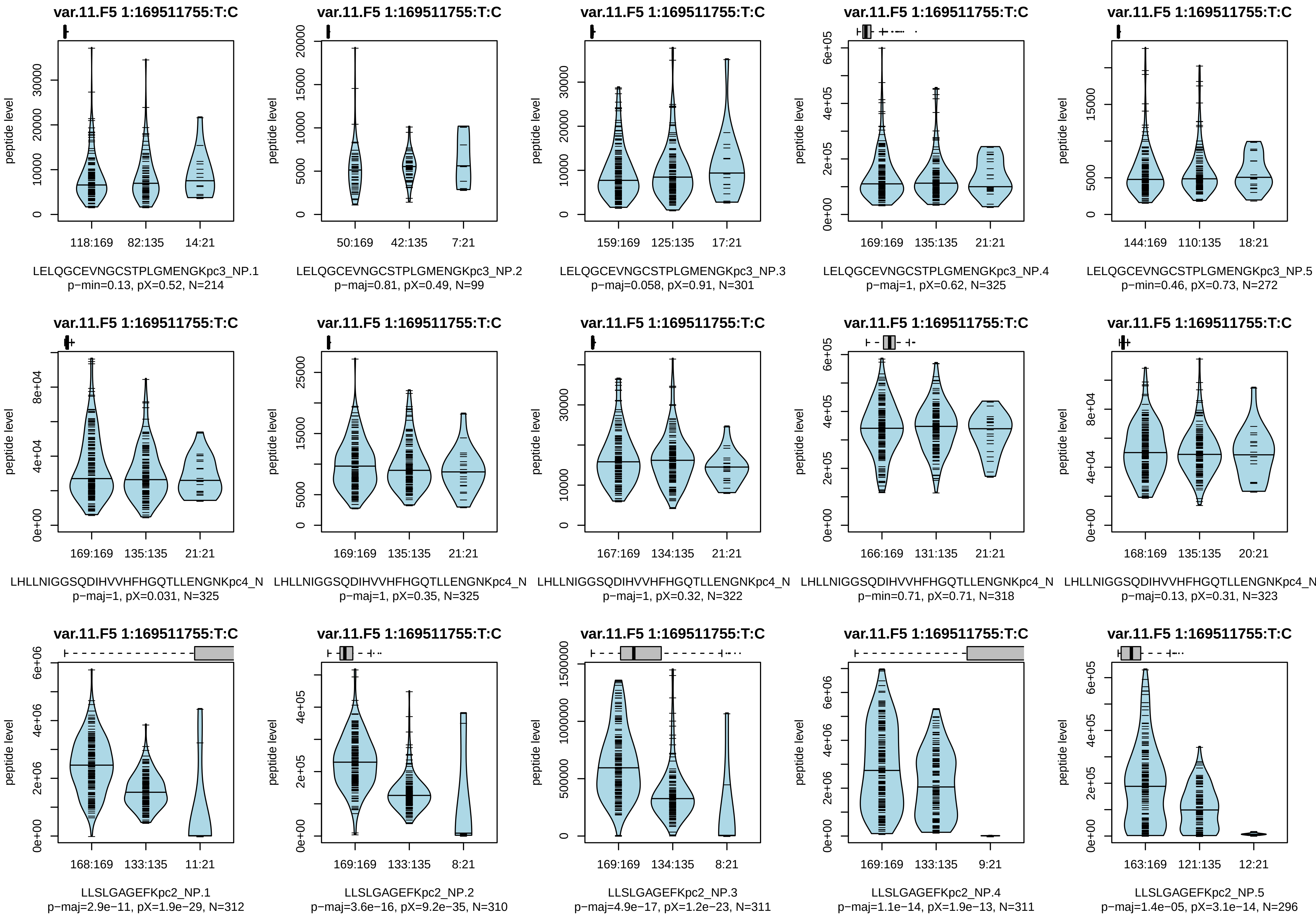
var.11.F5 1:169511755:T:C
peptide level
118:169
82:135
14:21
LELQGCEVNGCSTPLGMENGKpc3_NP.1
p−min=0.13, pX=0.52, N=214
var.11.F5 1:169511755:T:C
peptide level
50:169
42:135
7:21
LELQGCEVNGCSTPLGMENGKpc3_NP.2
p−maj=0.81, pX=0.49, N=99
var.11.F5 1:169511755:T:C
peptide level
159:169
125:135
17:21
LELQGCEVNGCSTPLGMENGKpc3_NP.3
p−maj=0.058, pX=0.91, N=301
var.11.F5 1:169511755:T:C
peptide level
169:169
135:135
21:21
LELQGCEVNGCSTPLGMENGKpc3_NP.4
p−maj=1, pX=0.62, N=325
var.11.F5 1:169511755:T:C
peptide level
144:169
110:135
18:21
LELQGCEVNGCSTPLGMENGKpc3_NP.5
p−min=0.46, pX=0.73, N=272
var.11.F5 1:169511755:T:C
peptide level
169:169
135:135
21:21
LHLLNIGGSQDIHVVHFHGQTLLENGNKpc4_N
p−maj=1, pX=0.031, N=325
var.11.F5 1:169511755:T:C
peptide level
169:169
135:135
21:21
LHLLNIGGSQDIHVVHFHGQTLLENGNKpc4_N
p−maj=1, pX=0.35, N=325
var.11.F5 1:169511755:T:C
peptide level
167:169
134:135
21:21
LHLLNIGGSQDIHVVHFHGQTLLENGNKpc4_N
p−maj=1, pX=0.32, N=322
var.11.F5 1:169511755:T:C
peptide level
166:169
131:135
21:21
LHLLNIGGSQDIHVVHFHGQTLLENGNKpc4_N
p−min=0.71, pX=0.71, N=318
var.11.F5 1:169511755:T:C
peptide level
168:169
135:135
20:21
LHLLNIGGSQDIHVVHFHGQTLLENGNKpc4_N
p−maj=0.13, pX=0.31, N=323
var.11.F5 1:169511755:T:C
peptide level
168:169
133:135
11:21
LLSLGAGEFKpc2_NP.1
p−maj=2.9e−11, pX=1.9e−29, N=312
var.11.F5 1:169511755:T:C
peptide level
169:169
133:135
8:21
LLSLGAGEFKpc2_NP.2
p−maj=3.6e−16, pX=9.2e−35, N=310
var.11.F5 1:169511755:T:C
peptide level
169:169
134:135
8:21
LLSLGAGEFKpc2_NP.3
p−maj=4.9e−17, pX=1.2e−23, N=311
var.11.F5 1:169511755:T:C
peptide level
169:169
133:135
9:21
LLSLGAGEFKpc2_NP.4
p−maj=1.1e−14, pX=1.9e−13, N=311
var.11.F5 1:169511755:T:C
peptide level
163:169
121:135
12:21
LLSLGAGEFKpc2_NP.5
p−maj=1.4e−05, pX=3.1e−14, N=296

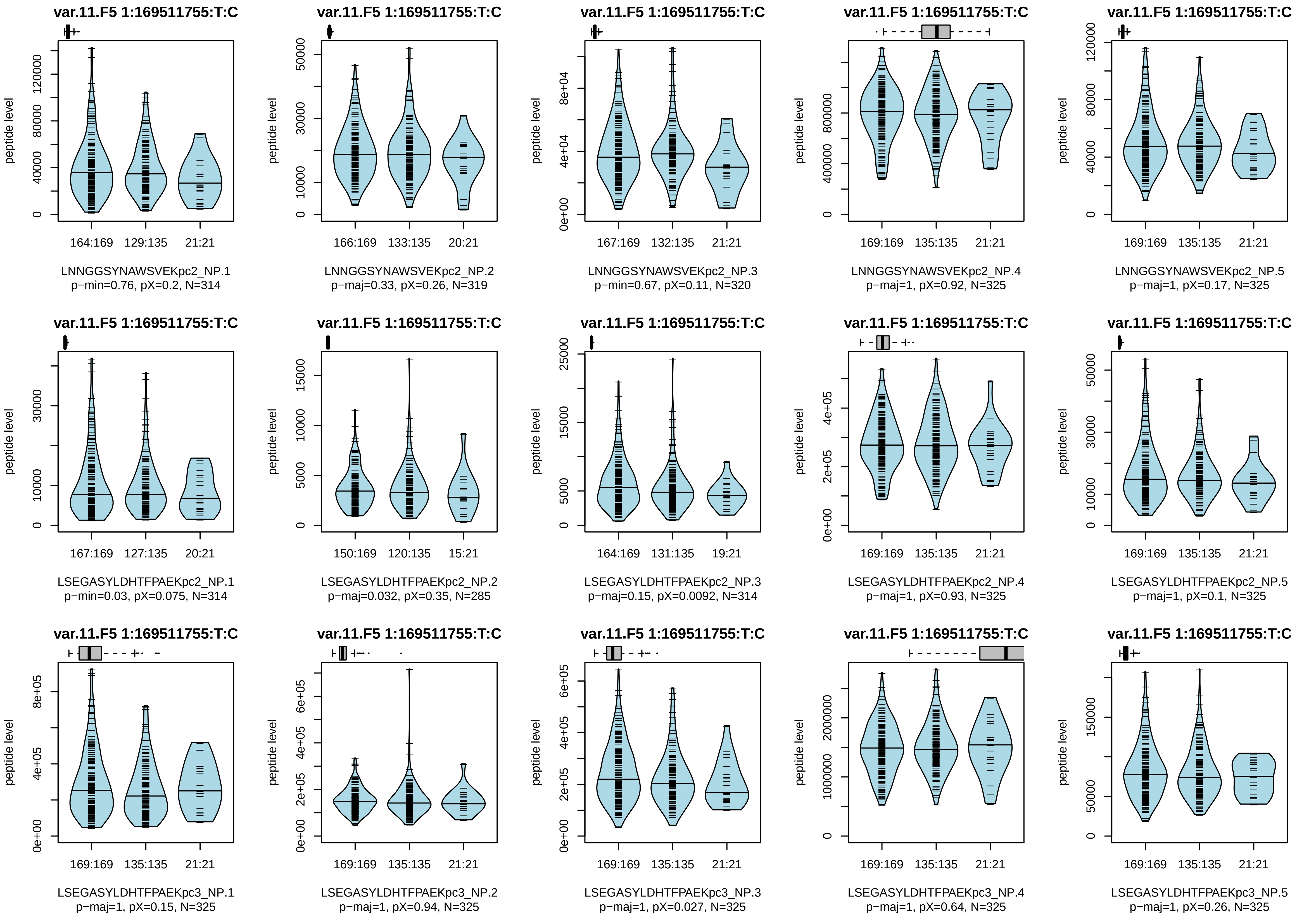
var.11.F5 1:169511755:T:C
peptide level
164:169 129:135 21:21
LNNGGSYNAWSVEKpc2_NP.1
p−min=0.76, pX=0.2, N=314
var.11.F5 1:169511755:T:C
peptide level
166:169 133:135 20:21
LNNGGSYNAWSVEKpc2_NP.2
p−maj=0.33, pX=0.26, N=319
var.11.F5 1:169511755:T:C
peptide level
167:169 132:135 21:21
LNNGGSYNAWSVEKpc2_NP.3
p−min=0.67, pX=0.11, N=320
var.11.F5 1:169511755:T:C
peptide level
169:169 135:135 21:21
LNNGGSYNAWSVEKpc2_NP.4
p−maj=1, pX=0.92, N=325
var.11.F5 1:169511755:T:C
peptide level
169:169 135:135 21:21
LNNGGSYNAWSVEKpc2_NP.5
p−maj=1, pX=0.17, N=325
var.11.F5 1:169511755:T:C
peptide level
167:169 127:135 20:21
LSEGASYLDHTFPAEKpc2_NP.1
p−min=0.03, pX=0.075, N=314
var.11.F5 1:169511755:T:C
peptide level
150:169 120:135 15:21
LSEGASYLDHTFPAEKpc2_NP.2
p−maj=0.032, pX=0.35, N=285
var.11.F5 1:169511755:T:C
peptide level
164:169 131:135 19:21
LSEGASYLDHTFPAEKpc2_NP.3
p−maj=0.15, pX=0.0092, N=314
var.11.F5 1:169511755:T:C
peptide level
169:169 135:135 21:21
LSEGASYLDHTFPAEKpc2_NP.4
p−maj=1, pX=0.93, N=325
var.11.F5 1:169511755:T:C
peptide level
169:169 135:135 21:21
LSEGASYLDHTFPAEKpc2_NP.5
p−maj=1, pX=0.1, N=325
var.11.F5 1:169511755:T:C
peptide level
169:169 135:135 21:21
LSEGASYLDHTFPAEKpc3_NP.1
p−maj=1, pX=0.15, N=325
var.11.F5 1:169511755:T:C
peptide level
169:169 135:135 21:21
LSEGASYLDHTFPAEKpc3_NP.2
p−maj=1, pX=0.94, N=325
var.11.F5 1:169511755:T:C
peptide level
169:169 135:135 21:21
LSEGASYLDHTFPAEKpc3_NP.3
p−maj=1, pX=0.027, N=325
var.11.F5 1:169511755:T:C
peptide level
169:169 135:135 21:21
LSEGASYLDHTFPAEKpc3_NP.4
p−maj=1, pX=0.64, N=325
var.11.F5 1:169511755:T:C
peptide level
169:169 135:135 21:21
LSEGASYLDHTFPAEKpc3_NP.5
p−maj=1, pX=0.26, N=325

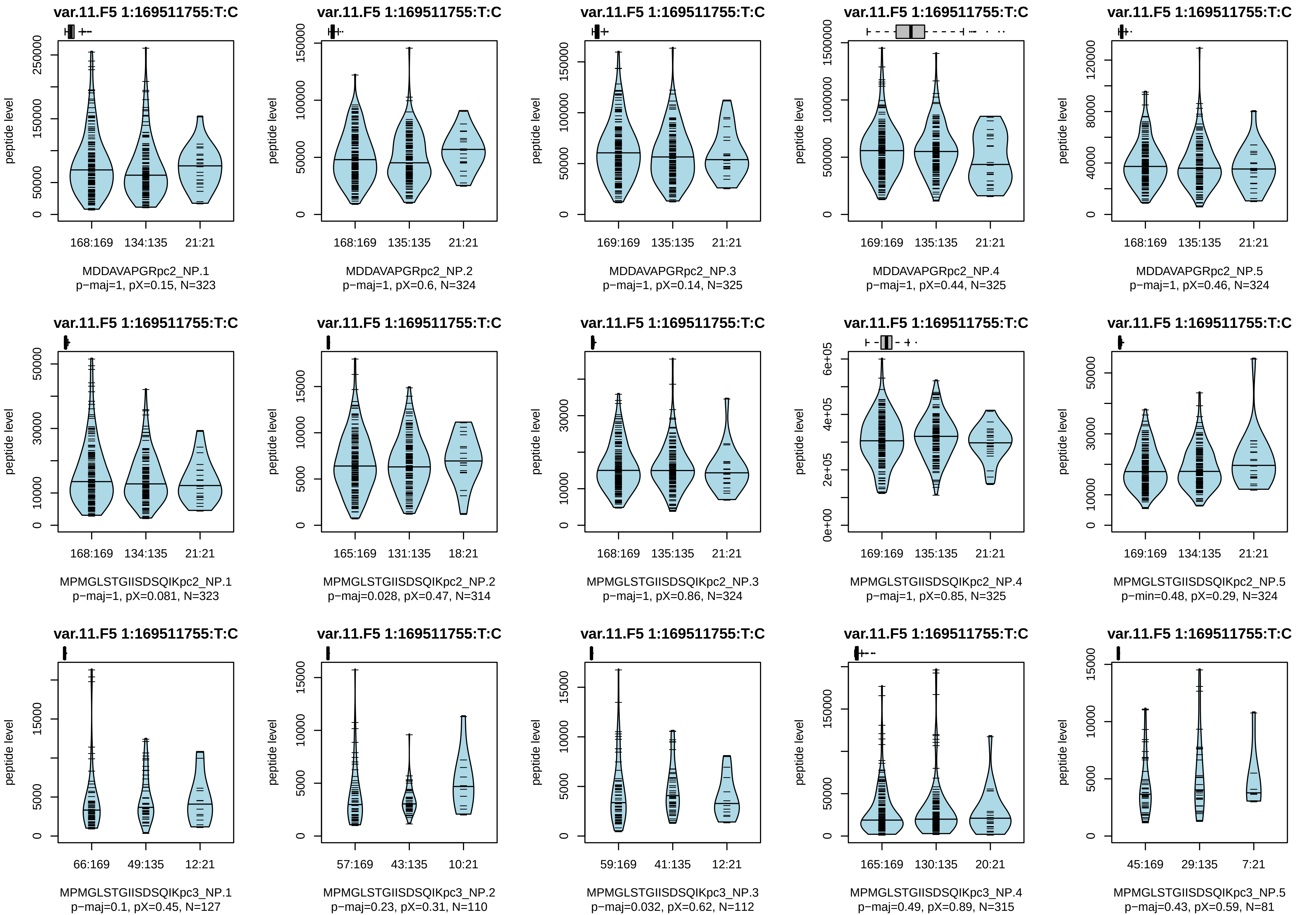

var.11.F5 1:169511755:T:C
peptide level
0
50000
150000
250000
168:169
134:135
21:21
MDDAVAPGRpc2_NP.1
p−maj=1, pX=0.15, N=323
var.11.F5 1:169511755:T:C
peptide level
0
50000
100000
150000
168:169
135:135
21:21
MDDAVAPGRpc2_NP.2
p−maj=1, pX=0.6, N=324
var.11.F5 1:169511755:T:C
peptide level
0
50000
100000
150000
169:169
135:135
21:21
MDDAVAPGRpc2_NP.3
p−maj=1, pX=0.14, N=325
var.11.F5 1:169511755:T:C
peptide level
0
500000
1000000
1500000
169:169
135:135
21:21
MDDAVAPGRpc2_NP.4
p−maj=1, pX=0.44, N=325
var.11.F5 1:169511755:T:C
peptide level
0
40000
80000
120000
168:169
135:135
21:21
MDDAVAPGRpc2_NP.5
p−maj=1, pX=0.46, N=324
var.11.F5 1:169511755:T:C
peptide level
0
10000
30000
50000
168:169
134:135
21:21
MPMGLSTGIISDSQIKpc2_NP.1
p−maj=1, pX=0.081, N=323
var.11.F5 1:169511755:T:C
peptide level
0
5000
10000
15000
165:169
131:135
18:21
MPMGLSTGIISDSQIKpc2_NP.2
p−maj=0.028, pX=0.47, N=314
var.11.F5 1:169511755:T:C
peptide level
0
10000
30000
168:169
135:135
21:21
MPMGLSTGIISDSQIKpc2_NP.3
p−maj=1, pX=0.86, N=324
var.11.F5 1:169511755:T:C
peptide level
0e+00
2e+05
4e+05
6e+05
169:169
135:135
21:21
MPMGLSTGIISDSQIKpc2_NP.4
p−maj=1, pX=0.85, N=325
var.11.F5 1:169511755:T:C
peptide level
0
10000
30000
50000
169:169
134:135
21:21
MPMGLSTGIISDSQIKpc2_NP.5
p−min=0.48, pX=0.29, N=324
var.11.F5 1:169511755:T:C
peptide level
0
5000
15000
66:169
49:135
12:21
MPMGLSTGIISDSQIKpc3_NP.1
p−maj=0.1, pX=0.45, N=127
var.11.F5 1:169511755:T:C
peptide level
0
5000
10000
15000
57:169
43:135
10:21
MPMGLSTGIISDSQIKpc3_NP.2
p−maj=0.23, pX=0.31, N=110
var.11.F5 1:169511755:T:C
peptide level
0
5000
10000
15000
59:169
41:135
12:21
MPMGLSTGIISDSQIKpc3_NP.3
p−maj=0.032, pX=0.62, N=112
var.11.F5 1:169511755:T:C
peptide level
0
50000
150000
165:169
130:135
20:21
MPMGLSTGIISDSQIKpc3_NP.4
p−maj=0.49, pX=0.89, N=315
var.11.F5 1:169511755:T:C
peptide level
0
5000
10000
15000
45:169
29:135
7:21
MPMGLSTGIISDSQIKpc3_NP.5
p−maj=0.43, pX=0.59, N=81

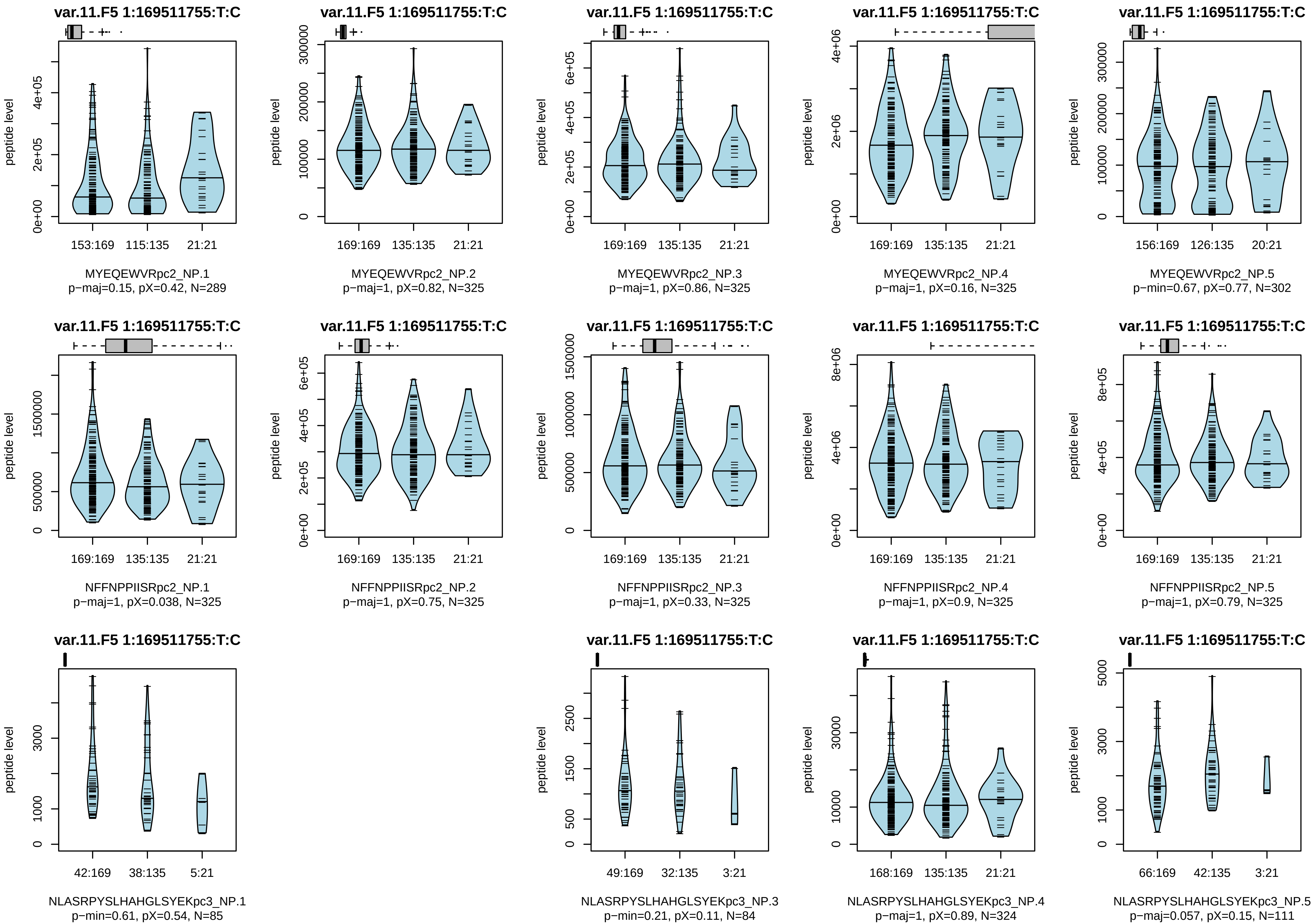
var.11.F5 1:169511755:T:C
peptide level
153:169
115:135
21:21
MYEQEWVRpc2_NP.1
p−maj=0.15, pX=0.42, N=289
var.11.F5 1:169511755:T:C
peptide level
169:169
135:135
21:21
MYEQEWVRpc2_NP.2
p−maj=1, pX=0.82, N=325
var.11.F5 1:169511755:T:C
peptide level
169:169
135:135
21:21
MYEQEWVRpc2_NP.3
p−maj=1, pX=0.86, N=325
var.11.F5 1:169511755:T:C
peptide level
169:169
135:135
21:21
MYEQEWVRpc2_NP.4
p−maj=1, pX=0.16, N=325
var.11.F5 1:169511755:T:C
peptide level
156:169
126:135
20:21
MYEQEWVRpc2_NP.5
p−min=0.67, pX=0.77, N=302
var.11.F5 1:169511755:T:C
peptide level
169:169
135:135
21:21
NFFNPPIISRpc2_NP.1
p−maj=1, pX=0.038, N=325
var.11.F5 1:169511755:T:C
peptide level
169:169
135:135
21:21
NFFNPPIISRpc2_NP.2
p−maj=1, pX=0.75, N=325
var.11.F5 1:169511755:T:C
peptide level
169:169
135:135
21:21
NFFNPPIISRpc2_NP.3
p−maj=1, pX=0.33, N=325
var.11.F5 1:169511755:T:C
peptide level
169:169
135:135
21:21
NFFNPPIISRpc2_NP.4
p−maj=1, pX=0.9, N=325
var.11.F5 1:169511755:T:C
peptide level
169:169
135:135
21:21
NFFNPPIISRpc2_NP.5
p−maj=1, pX=0.79, N=325
var.11.F5 1:169511755:T:C
peptide level
42:169
38:135
5:21
NLASRPYSLHAHGLSYEKpc3_NP.1
p−min=0.61, pX=0.54, N=85
var.11.F5 1:169511755:T:C
peptide level
49:169
32:135
3:21
NLASRPYSLHAHGLSYEKpc3_NP.3
p−min=0.21, pX=0.11, N=84
var.11.F5 1:169511755:T:C
peptide level
168:169
135:135
21:21
NLASRPYSLHAHGLSYEKpc3_NP.4
p−maj=1, pX=0.89, N=324
var.11.F5 1:169511755:T:C
peptide level
66:169
42:135
3:21
NLASRPYSLHAHGLSYEKpc3_NP.5
p−maj=0.057, pX=0.15, N=111

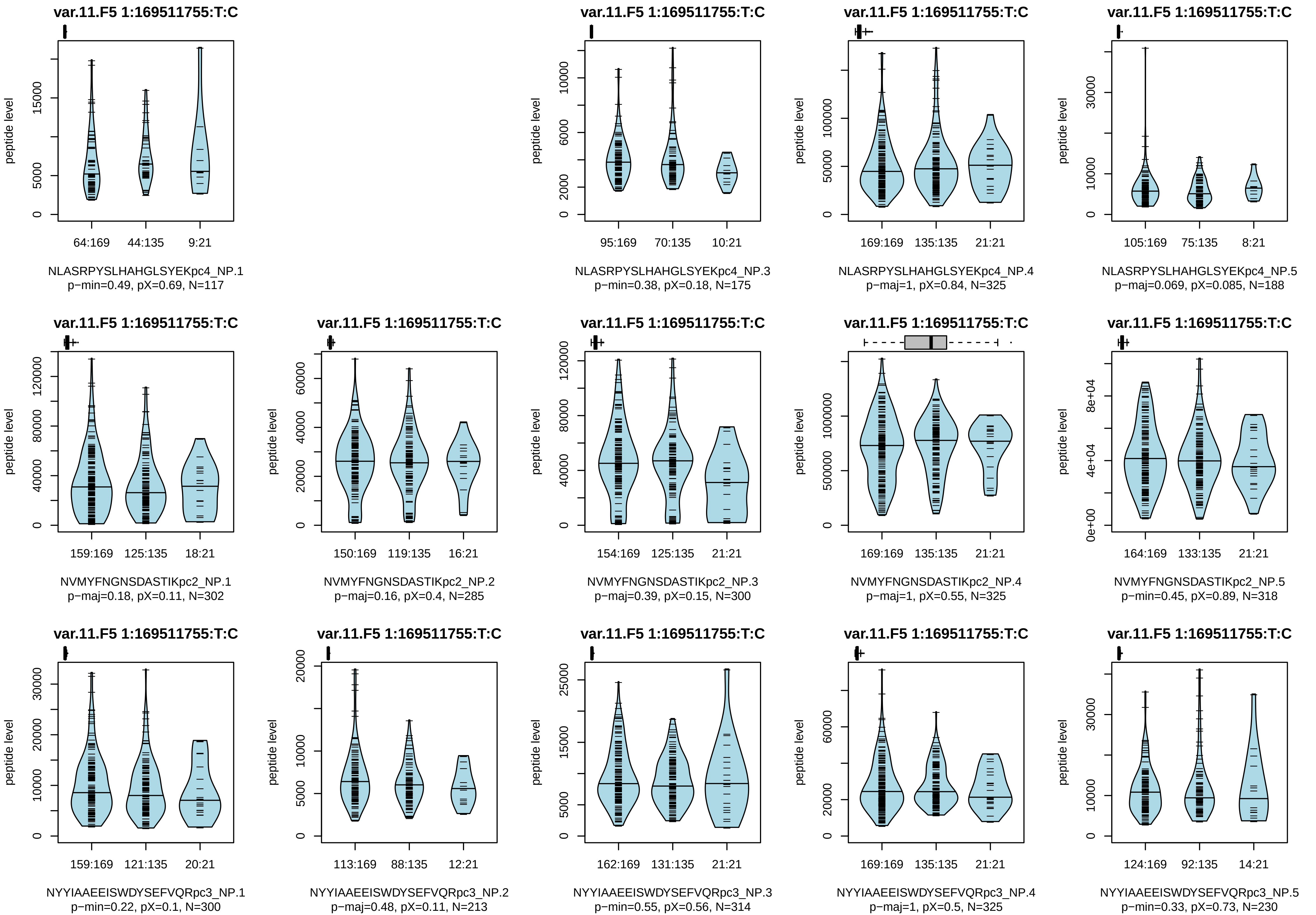
var.11.F5 1:169511755:T:C
peptide level
64:169 44:135 9:21
NLASRPYSLHAHGLSYEKpc4_NP.1
p−min=0.49, pX=0.69, N=117
var.11.F5 1:169511755:T:C
peptide level
95:169 70:135 10:21
NLASRPYSLHAHGLSYEKpc4_NP.3
p−min=0.38, pX=0.18, N=175
var.11.F5 1:169511755:T:C
peptide level
169:169 135:135 21:21
NLASRPYSLHAHGLSYEKpc4_NP.4
p−maj=1, pX=0.84, N=325
var.11.F5 1:169511755:T:C
peptide level
105:169 75:135 8:21
NLASRPYSLHAHGLSYEKpc4_NP.5
p−maj=0.069, pX=0.085, N=188
var.11.F5 1:169511755:T:C
peptide level
159:169 125:135 18:21
NVMYFNGNSDASTIKpc2_NP.1
p−maj=0.18, pX=0.11, N=302
var.11.F5 1:169511755:T:C
peptide level
150:169 119:135 16:21
NVMYFNGNSDASTIKpc2_NP.2
p−maj=0.16, pX=0.4, N=285
var.11.F5 1:169511755:T:C
peptide level
154:169 125:135 21:21
NVMYFNGNSDASTIKpc2_NP.3
p−maj=0.39, pX=0.15, N=300
var.11.F5 1:169511755:T:C
peptide level
169:169 135:135 21:21
NVMYFNGNSDASTIKpc2_NP.4
p−maj=1, pX=0.55, N=325
var.11.F5 1:169511755:T:C
peptide level
164:169 133:135 21:21
NVMYFNGNSDASTIKpc2_NP.5
p−min=0.45, pX=0.89, N=318
var.11.F5 1:169511755:T:C
peptide level
159:169 121:135 20:21
NYYIAAEEISWDYSEFVQRpc3_NP.1
p−min=0.22, pX=0.1, N=300
var.11.F5 1:169511755:T:C
peptide level
113:169 88:135 12:21
NYYIAAEEISWDYSEFVQRpc3_NP.2
p−maj=0.48, pX=0.11, N=213
var.11.F5 1:169511755:T:C
peptide level
162:169 131:135 21:21
NYYIAAEEISWDYSEFVQRpc3_NP.3
p−min=0.55, pX=0.56, N=314
var.11.F5 1:169511755:T:C
peptide level
169:169 135:135 21:21
NYYIAAEEISWDYSEFVQRpc3_NP.4
p−maj=1, pX=0.5, N=325
var.11.F5 1:169511755:T:C
peptide level
124:169 92:135 14:21
NYYIAAEEISWDYSEFVQRpc3_NP.5
p−min=0.33, pX=0.73, N=230

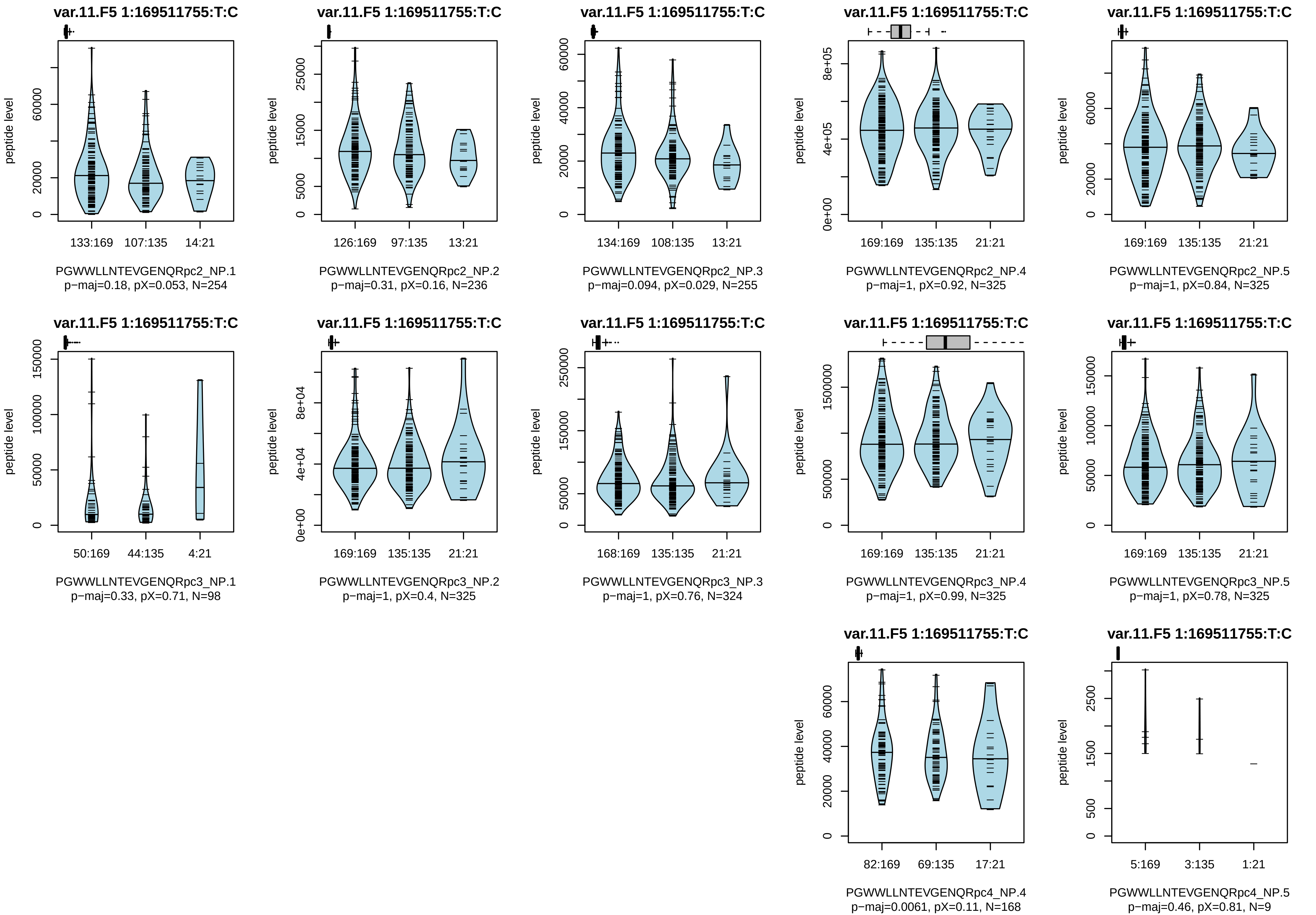

var.11.F5 1:169511755:T:C
peptide level
133:169 107:135 14:21
PGWWLLNTEVGENQRpc2_NP.1
p−maj=0.18, pX=0.053, N=254
var.11.F5 1:169511755:T:C
peptide level
126:169 97:135 13:21
PGWWLLNTEVGENQRpc2_NP.2
p−maj=0.31, pX=0.16, N=236
var.11.F5 1:169511755:T:C
peptide level
134:169 108:135 13:21
PGWWLLNTEVGENQRpc2_NP.3
p−maj=0.094, pX=0.029, N=255
var.11.F5 1:169511755:T:C
peptide level
169:169 135:135 21:21
PGWWLLNTEVGENQRpc2_NP.4
p−maj=1, pX=0.92, N=325
var.11.F5 1:169511755:T:C
peptide level
169:169 135:135 21:21
PGWWLLNTEVGENQRpc2_NP.5
p−maj=1, pX=0.84, N=325
var.11.F5 1:169511755:T:C
peptide level
50:169 44:135 4:21
PGWWLLNTEVGENQRpc3_NP.1
p−maj=0.33, pX=0.71, N=98
var.11.F5 1:169511755:T:C
peptide level
169:169 135:135 21:21
PGWWLLNTEVGENQRpc3_NP.2
p−maj=1, pX=0.4, N=325
var.11.F5 1:169511755:T:C
peptide level
168:169 135:135 21:21
PGWWLLNTEVGENQRpc3_NP.3
p−maj=1, pX=0.76, N=324
var.11.F5 1:169511755:T:C
peptide level
169:169 135:135 21:21
PGWWLLNTEVGENQRpc3_NP.4
p−maj=1, pX=0.99, N=325
var.11.F5 1:169511755:T:C
peptide level
169:169 135:135 21:21
PGWWLLNTEVGENQRpc3_NP.5
p−maj=1, pX=0.78, N=325
var.11.F5 1:169511755:T:C
peptide level
82:169 69:135 17:21
PGWWLLNTEVGENQRpc4_NP.4
p−maj=0.0061, pX=0.11, N=168
var.11.F5 1:169511755:T:C
peptide level
5:169 3:135 1:21
PGWWLLNTEVGENQRpc4_NP.5
p−maj=0.46, pX=0.81, N=9

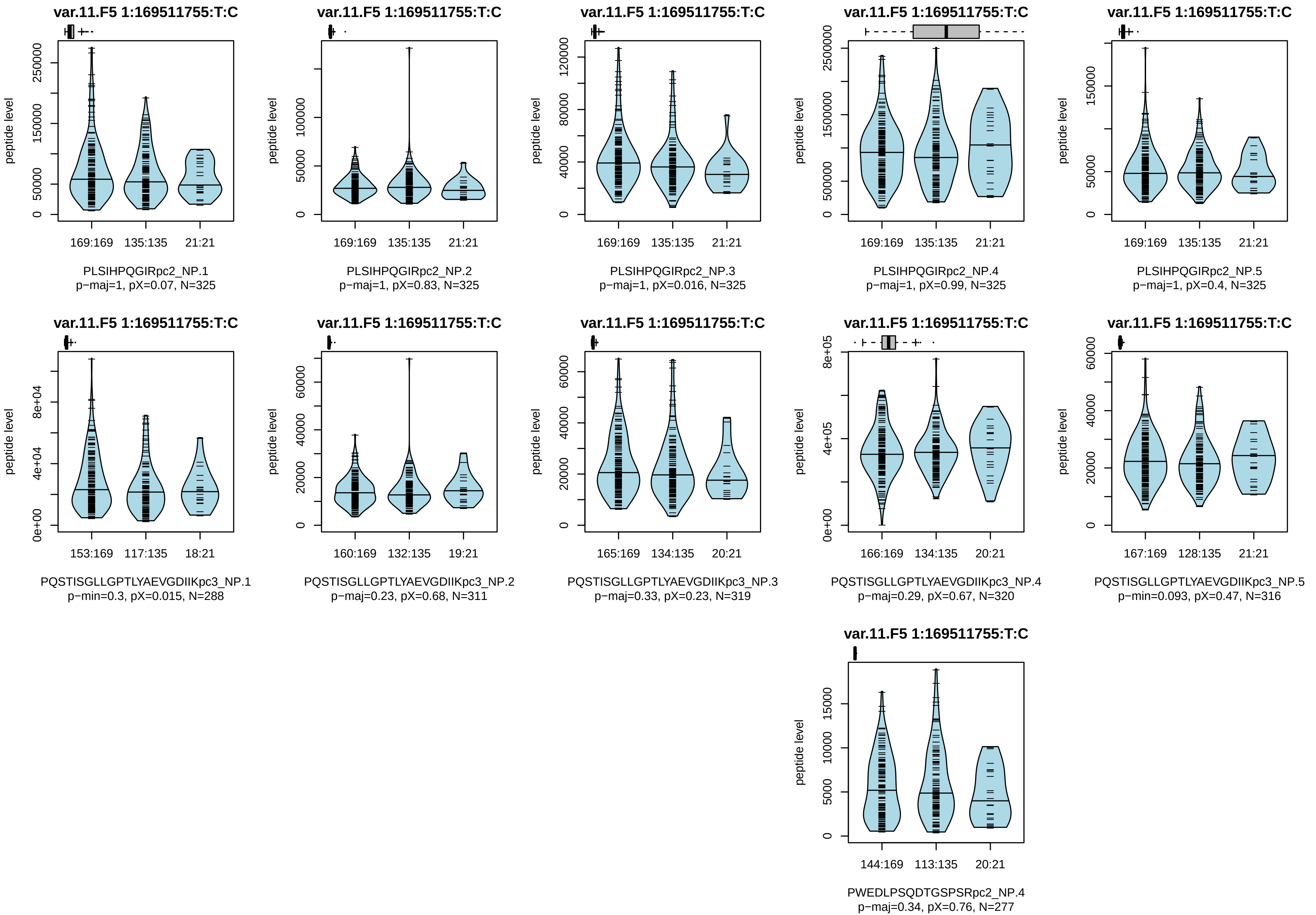

var.11.F5 1:169511755:T:C
peptide level
169:169
135:135
21:21
PLSIHPQGIRpc2_NP.1
p−maj=1, pX=0.07, N=325
var.11.F5 1:169511755:T:C
peptide level
169:169
135:135
21:21
PLSIHPQGIRpc2_NP.2
p−maj=1, pX=0.83, N=325
var.11.F5 1:169511755:T:C
peptide level
169:169
135:135
21:21
PLSIHPQGIRpc2_NP.3
p−maj=1, pX=0.016, N=325
var.11.F5 1:169511755:T:C
peptide level
169:169
135:135
21:21
PLSIHPQGIRpc2_NP.4
p−maj=1, pX=0.99, N=325
var.11.F5 1:169511755:T:C
peptide level
169:169
135:135
21:21
PLSIHPQGIRpc2_NP.5
p−maj=1, pX=0.4, N=325
var.11.F5 1:169511755:T:C
peptide level
153:169
117:135
18:21
PQSTISGLLGPTLYAEVGDIIKpc3_NP.1
p−min=0.3, pX=0.015, N=288
var.11.F5 1:169511755:T:C
peptide level
160:169
132:135
19:21
PQSTISGLLGPTLYAEVGDIIKpc3_NP.2
p−maj=0.23, pX=0.68, N=311
var.11.F5 1:169511755:T:C
peptide level
165:169
134:135
20:21
PQSTISGLLGPTLYAEVGDIIKpc3_NP.3
p−maj=0.33, pX=0.23, N=319
var.11.F5 1:169511755:T:C
peptide level
166:169
134:135
20:21
PQSTISGLLGPTLYAEVGDIIKpc3_NP.4
p−maj=0.29, pX=0.67, N=320
var.11.F5 1:169511755:T:C
peptide level
167:169
128:135
21:21
PQSTISGLLGPTLYAEVGDIIKpc3_NP.5
p−min=0.093, pX=0.47, N=316
var.11.F5 1:169511755:T:C
peptide level
144:169
113:135
20:21
PWEDLPSQDTGSPSRpc2_NP.4
p−maj=0.34, pX=0.76, N=277

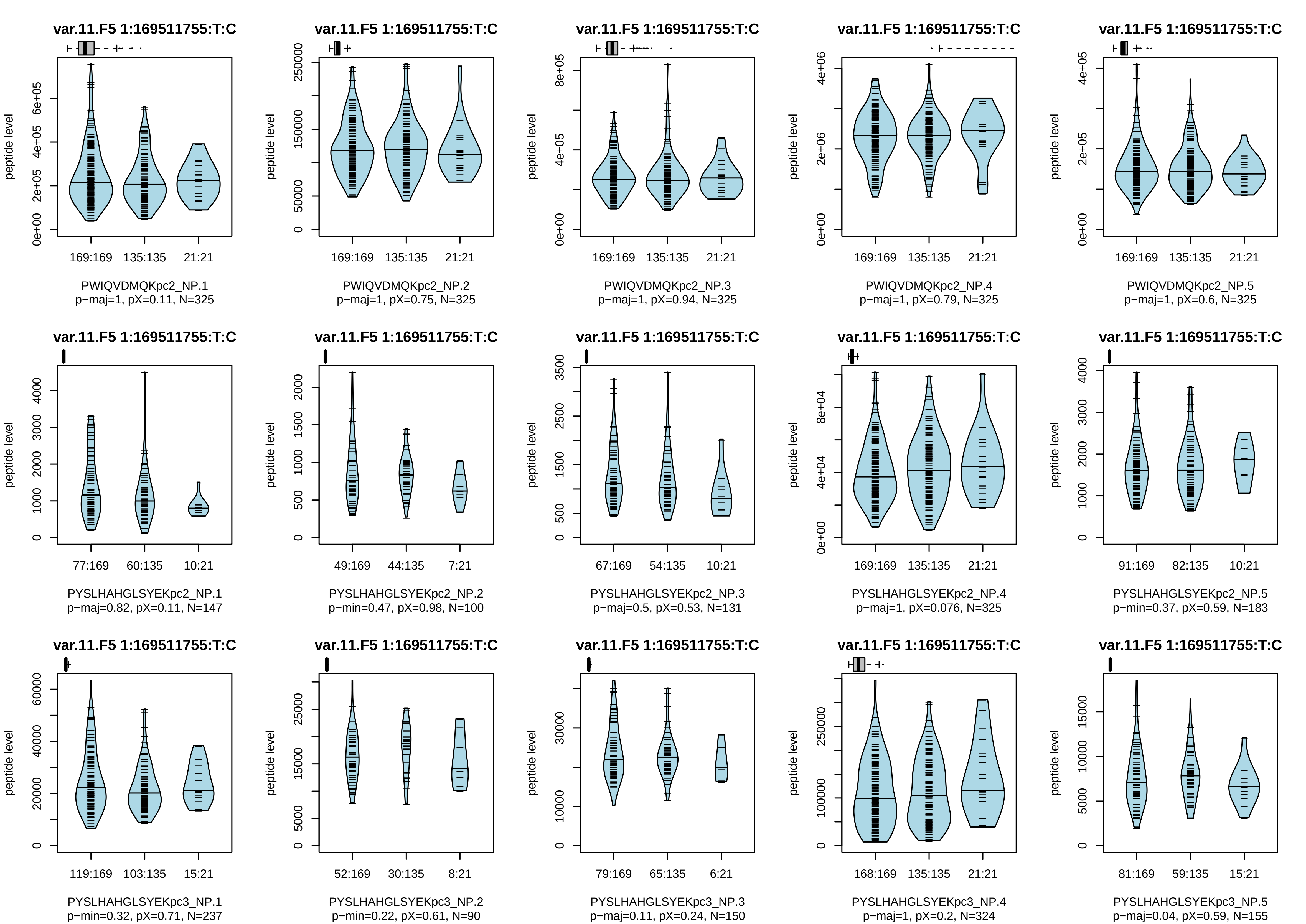
var.11.F5 1:169511755:T:C
peptide level
169:169
135:135
21:21
PWIQVDMQKpc2_NP.1
p−maj=1, pX=0.11, N=325
var.11.F5 1:169511755:T:C
peptide level
169:169
135:135
21:21
PWIQVDMQKpc2_NP.2
p−maj=1, pX=0.75, N=325
var.11.F5 1:169511755:T:C
peptide level
169:169
135:135
21:21
PWIQVDMQKpc2_NP.3
p−maj=1, pX=0.94, N=325
var.11.F5 1:169511755:T:C
peptide level
169:169
135:135
21:21
PWIQVDMQKpc2_NP.4
p−maj=1, pX=0.79, N=325
var.11.F5 1:169511755:T:C
peptide level
169:169
135:135
21:21
PWIQVDMQKpc2_NP.5
p−maj=1, pX=0.6, N=325
var.11.F5 1:169511755:T:C
peptide level
77:169
60:135
10:21
PYSLHAHGLSYEKpc2_NP.1
p−maj=0.82, pX=0.11, N=147
var.11.F5 1:169511755:T:C
peptide level
49:169
44:135
7:21
PYSLHAHGLSYEKpc2_NP.2
p−min=0.47, pX=0.98, N=100
var.11.F5 1:169511755:T:C
peptide level
67:169
54:135
10:21
PYSLHAHGLSYEKpc2_NP.3
p−maj=0.5, pX=0.53, N=131
var.11.F5 1:169511755:T:C
peptide level
169:169
135:135
21:21
PYSLHAHGLSYEKpc2_NP.4
p−maj=1, pX=0.076, N=325
var.11.F5 1:169511755:T:C
peptide level
91:169
82:135
10:21
PYSLHAHGLSYEKpc2_NP.5
p−min=0.37, pX=0.59, N=183
var.11.F5 1:169511755:T:C
peptide level
119:169
103:135
15:21
PYSLHAHGLSYEKpc3_NP.1
p−min=0.32, pX=0.71, N=237
var.11.F5 1:169511755:T:C
peptide level
52:169
30:135
8:21
PYSLHAHGLSYEKpc3_NP.2
p−min=0.22, pX=0.61, N=90
var.11.F5 1:169511755:T:C
peptide level
79:169
65:135
6:21
PYSLHAHGLSYEKpc3_NP.3
p−maj=0.11, pX=0.24, N=150
var.11.F5 1:169511755:T:C
peptide level
168:169
135:135
21:21
PYSLHAHGLSYEKpc3_NP.4
p−maj=1, pX=0.2, N=324
var.11.F5 1:169511755:T:C
peptide level
81:169
59:135
15:21
PYSLHAHGLSYEKpc3_NP.5
p−maj=0.04, pX=0.59, N=155

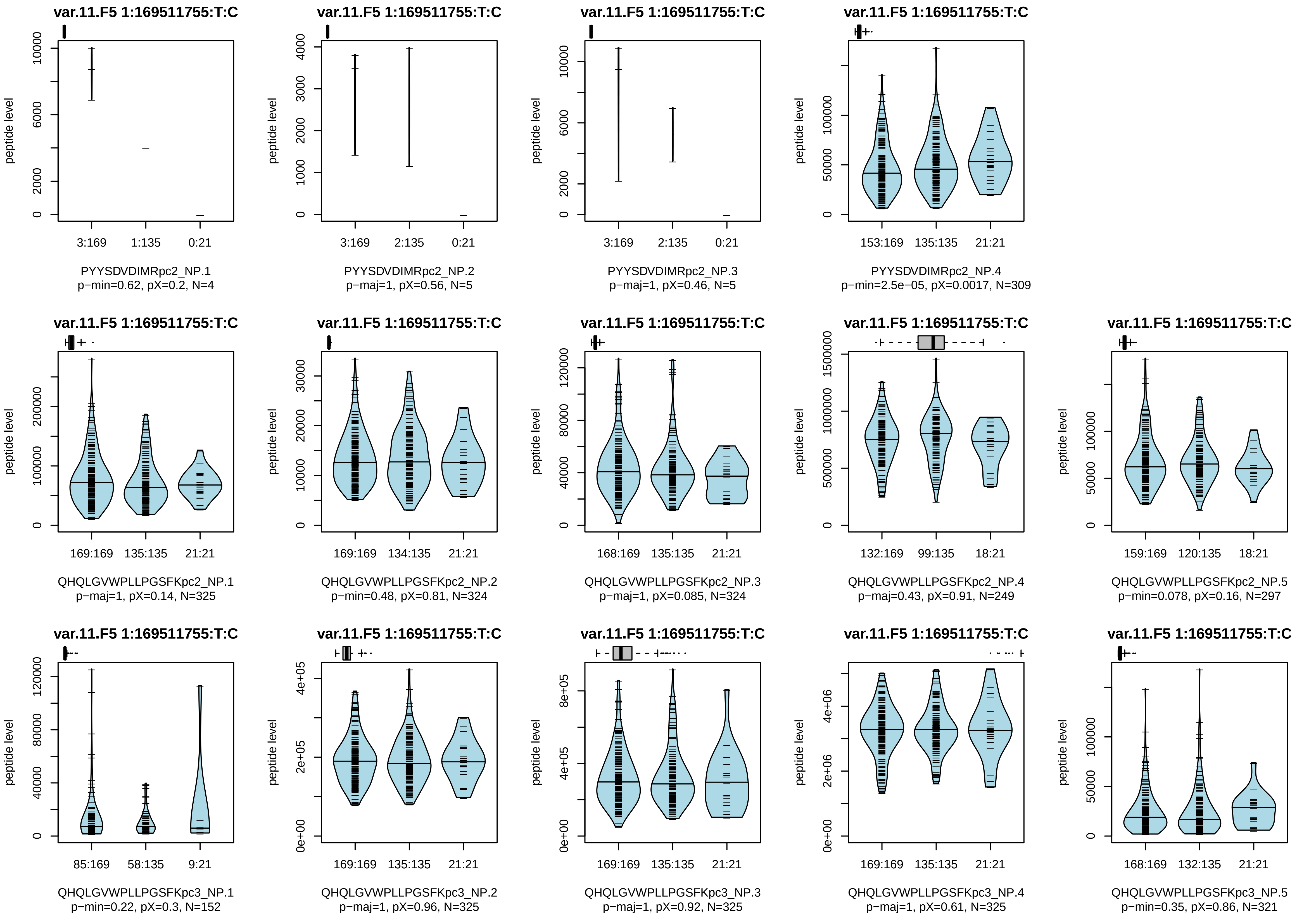
var.11.F5 1:169511755:T:C
peptide level
3:169
1:135
0:21
PYYSDVDIMRpc2_NP.1
p−min=0.62, pX=0.2, N=4
var.11.F5 1:169511755:T:C
peptide level
3:169
2:135
0:21
PYYSDVDIMRpc2_NP.2
p−maj=1, pX=0.56, N=5
var.11.F5 1:169511755:T:C
peptide level
3:169
2:135
0:21
PYYSDVDIMRpc2_NP.3
p−maj=1, pX=0.46, N=5
var.11.F5 1:169511755:T:C
peptide level
153:169
135:135
21:21
PYYSDVDIMRpc2_NP.4
p−min=2.5e−05, pX=0.0017, N=309
var.11.F5 1:169511755:T:C
peptide level
169:169
135:135
21:21
QHQLGVWPLLPGSFKpc2_NP.1
p−maj=1, pX=0.14, N=325
var.11.F5 1:169511755:T:C
peptide level
169:169
134:135
21:21
QHQLGVWPLLPGSFKpc2_NP.2
p−min=0.48, pX=0.81, N=324
var.11.F5 1:169511755:T:C
peptide level
168:169
135:135
21:21
QHQLGVWPLLPGSFKpc2_NP.3
p−maj=1, pX=0.085, N=324
var.11.F5 1:169511755:T:C
peptide level
132:169
99:135
18:21
QHQLGVWPLLPGSFKpc2_NP.4
p−maj=0.43, pX=0.91, N=249
var.11.F5 1:169511755:T:C
peptide level
159:169
120:135
18:21
QHQLGVWPLLPGSFKpc2_NP.5
p−min=0.078, pX=0.16, N=297
var.11.F5 1:169511755:T:C
peptide level
85:169
58:135
9:21
QHQLGVWPLLPGSFKpc3_NP.1
p−min=0.22, pX=0.3, N=152
var.11.F5 1:169511755:T:C
peptide level
169:169
135:135
21:21
QHQLGVWPLLPGSFKpc3_NP.2
p−maj=1, pX=0.96, N=325
var.11.F5 1:169511755:T:C
peptide level
169:169
135:135
21:21
QHQLGVWPLLPGSFKpc3_NP.3
p−maj=1, pX=0.92, N=325
var.11.F5 1:169511755:T:C
peptide level
169:169
135:135
21:21
QHQLGVWPLLPGSFKpc3_NP.4
p−maj=1, pX=0.61, N=325
var.11.F5 1:169511755:T:C
peptide level
168:169
132:135
21:21
QHQLGVWPLLPGSFKpc3_NP.5
p−min=0.35, pX=0.86, N=321

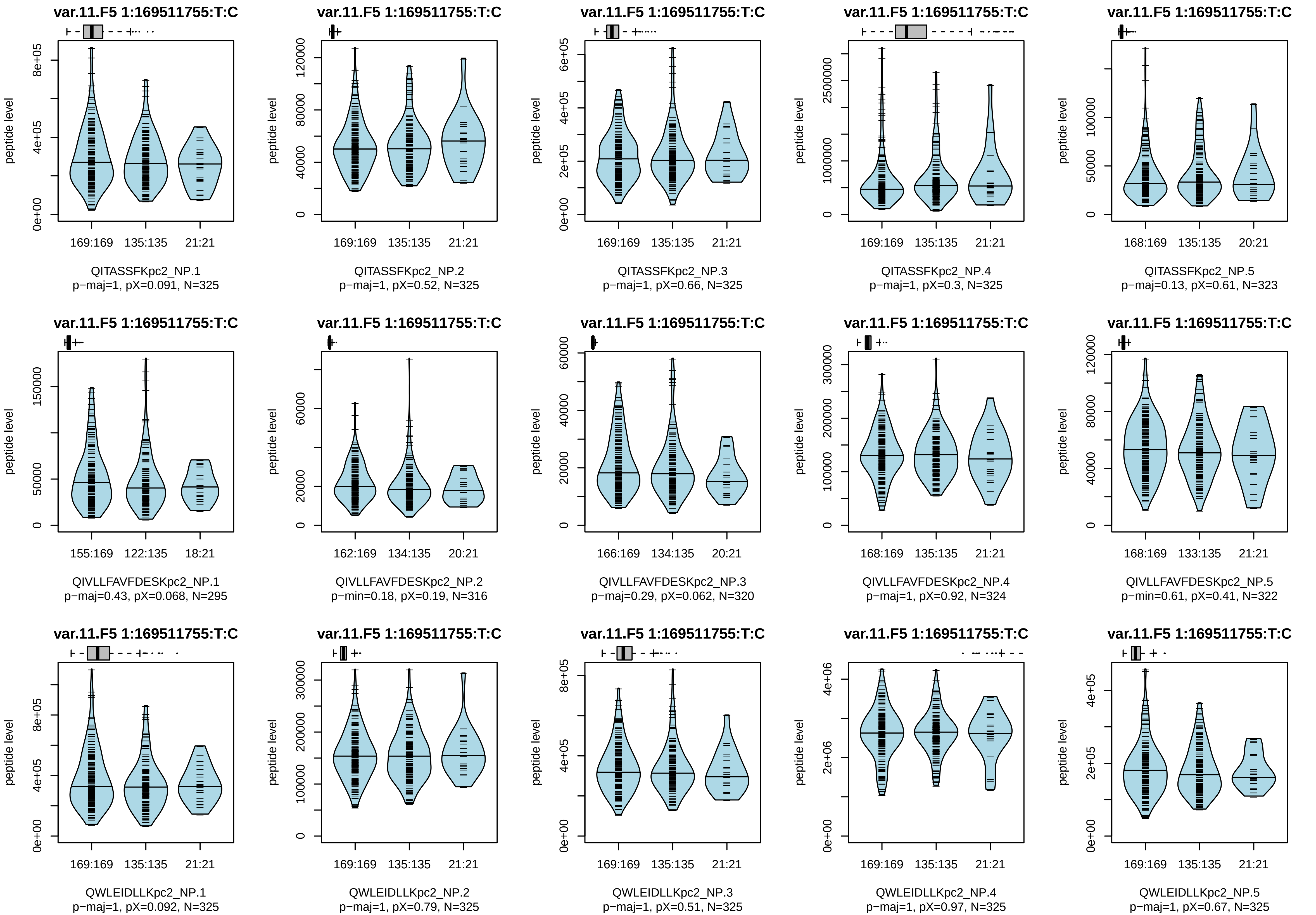

var.11.F5 1:169511755:T:C
peptide level
169:169 135:135 21:21
QITASSFKpc2_NP.1
p−maj=1, pX=0.091, N=325
var.11.F5 1:169511755:T:C
peptide level
169:169 135:135 21:21
QITASSFKpc2_NP.2
p−maj=1, pX=0.52, N=325
var.11.F5 1:169511755:T:C
peptide level
169:169 135:135 21:21
QITASSFKpc2_NP.3
p−maj=1, pX=0.66, N=325
var.11.F5 1:169511755:T:C
peptide level
169:169 135:135 21:21
QITASSFKpc2_NP.4
p−maj=1, pX=0.3, N=325
var.11.F5 1:169511755:T:C
peptide level
168:169 135:135 20:21
QITASSFKpc2_NP.5
p−maj=0.13, pX=0.61, N=323
var.11.F5 1:169511755:T:C
peptide level
155:169 122:135 18:21
QIVLLFAVFDESKpc2_NP.1
p−maj=0.43, pX=0.068, N=295
var.11.F5 1:169511755:T:C
peptide level
162:169 134:135 20:21
QIVLLFAVFDESKpc2_NP.2
p−min=0.18, pX=0.19, N=316
var.11.F5 1:169511755:T:C
peptide level
166:169 134:135 20:21
QIVLLFAVFDESKpc2_NP.3
p−maj=0.29, pX=0.062, N=320
var.11.F5 1:169511755:T:C
peptide level
168:169 135:135 21:21
QIVLLFAVFDESKpc2_NP.4
p−maj=1, pX=0.92, N=324
var.11.F5 1:169511755:T:C
peptide level
168:169 133:135 21:21
QIVLLFAVFDESKpc2_NP.5
p−min=0.61, pX=0.41, N=322
var.11.F5 1:169511755:T:C
peptide level
169:169 135:135 21:21
QWLEIDLLKpc2_NP.1
p−maj=1, pX=0.092, N=325
var.11.F5 1:169511755:T:C
peptide level
169:169 135:135 21:21
QWLEIDLLKpc2_NP.2
p−maj=1, pX=0.79, N=325
var.11.F5 1:169511755:T:C
peptide level
169:169 135:135 21:21
QWLEIDLLKpc2_NP.3
p−maj=1, pX=0.51, N=325
var.11.F5 1:169511755:T:C
peptide level
169:169 135:135 21:21
QWLEIDLLKpc2_NP.4
p−maj=1, pX=0.97, N=325
var.11.F5 1:169511755:T:C
peptide level
169:169 135:135 21:21
QWLEIDLLKpc2_NP.5
p−maj=1, pX=0.67, N=325

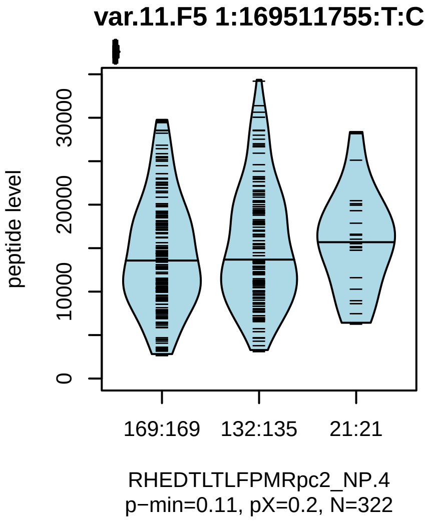
var.11.F5 1:169511755:T:C
peptide level
0
10000
20000
30000
169:169
132:135
21:21
RHEDTLTLFPMRpc2_NP.4
p−min=0.11, pX=0.2, N=322

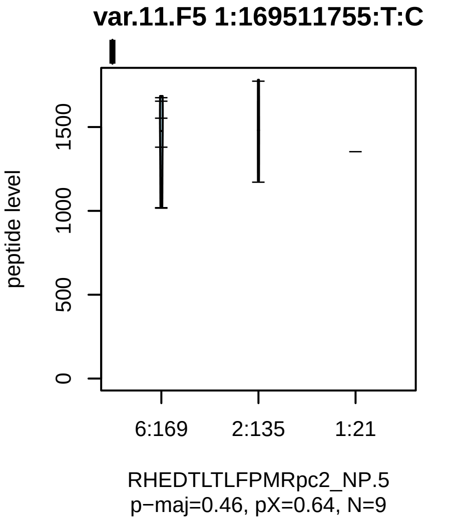
var.11.F5 1:169511755:T:C
peptide level
0
500
1000
1500
6:169
2:135
1:21
RHEDTLTLFPMRpc2_NP.5
p−maj=0.46, pX=0.64, N=9

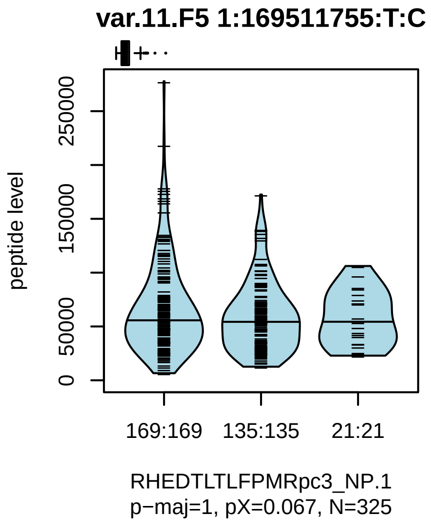
var.11.F5 1:169511755:T:C
peptide level
0
50000
150000
250000
169:169
135:135
21:21
RHEDTLTLFPMRpc3_NP.1
p−maj=1, pX=0.067, N=325

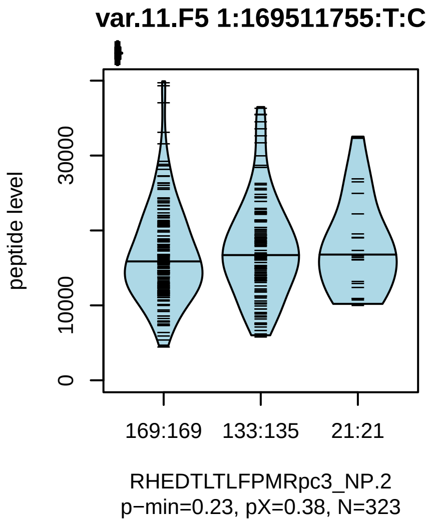
var.11.F5 1:169511755:T:C
peptide level
0
10000
30000
169:169
133:135
21:21
RHEDTLTLFPMRpc3_NP.2
p−min=0.23, pX=0.38, N=323

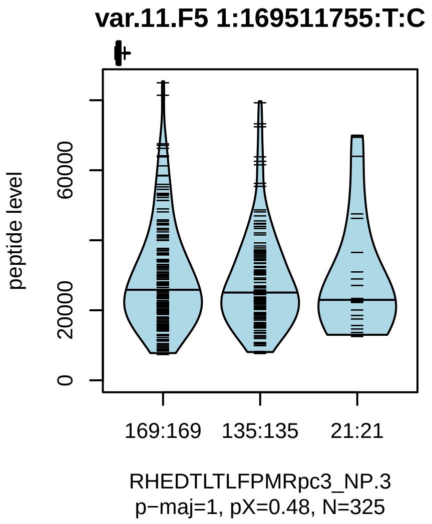
var.11.F5 1:169511755:T:C
peptide level
0
20000
60000
169:169
135:135
21:21
RHEDTLTLFPMRpc3_NP.3
p−maj=1, pX=0.48, N=325

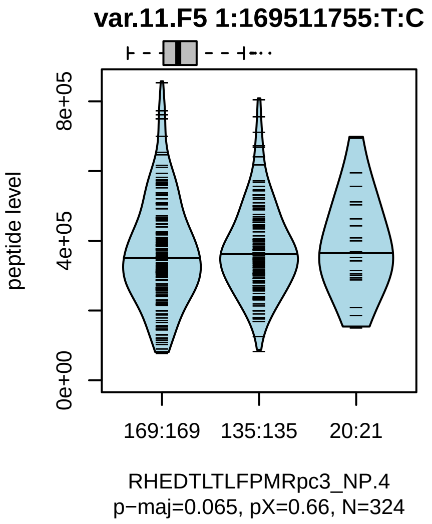
var.11.F5 1:169511755:T:C
peptide level
0e+00
4e+05
8e+05
169:169
135:135
20:21
RHEDTLTLFPMRpc3_NP.4
p−maj=0.065, pX=0.66, N=324

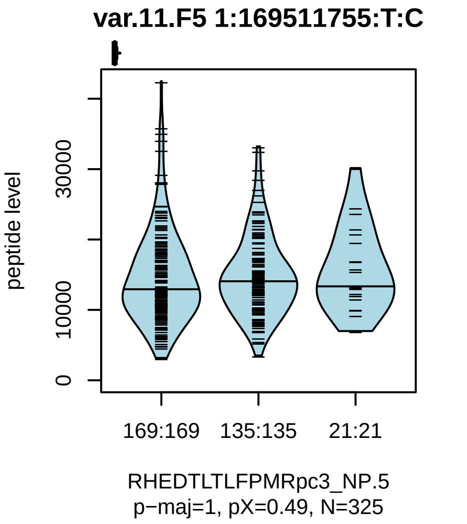
var.11.F5 1:169511755:T:C
peptide level
0
10000
30000
169:169
135:135
21:21
RHEDTLTLFPMRpc3_NP.5
p−maj=1, pX=0.49, N=325

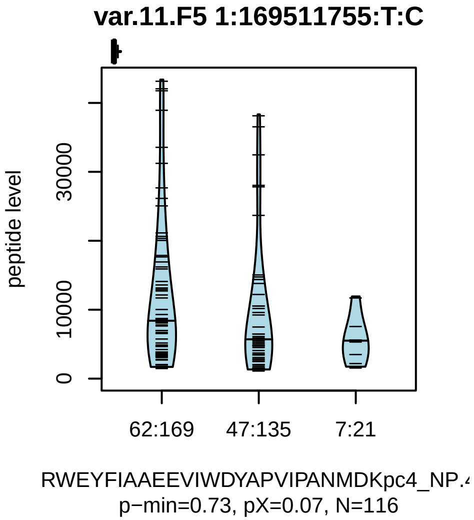
var.11.F5 1:169511755:T:C
peptide level
0
10000
30000
62:169
47:135
7:21
RWEYFIAAEEVIWDYAPVIPANMDKpc4_NP.
p−min=0.73, pX=0.07, N=116

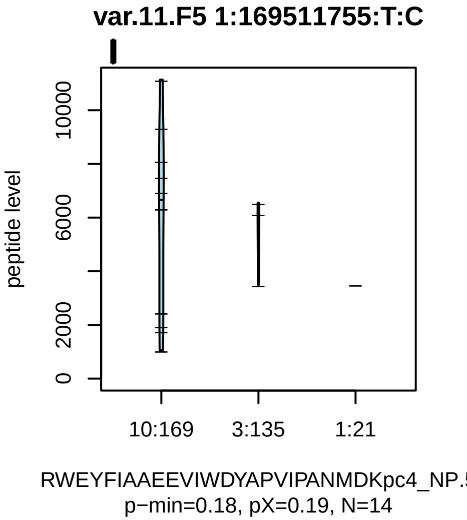
var.11.F5 1:169511755:T:C
peptide level
0
2000
6000
10000
10:169
3:135
1:21
RWEYFIAAEEVIWDYAPVIPANMDKpc4_NP.
p−min=0.18, pX=0.19, N=14

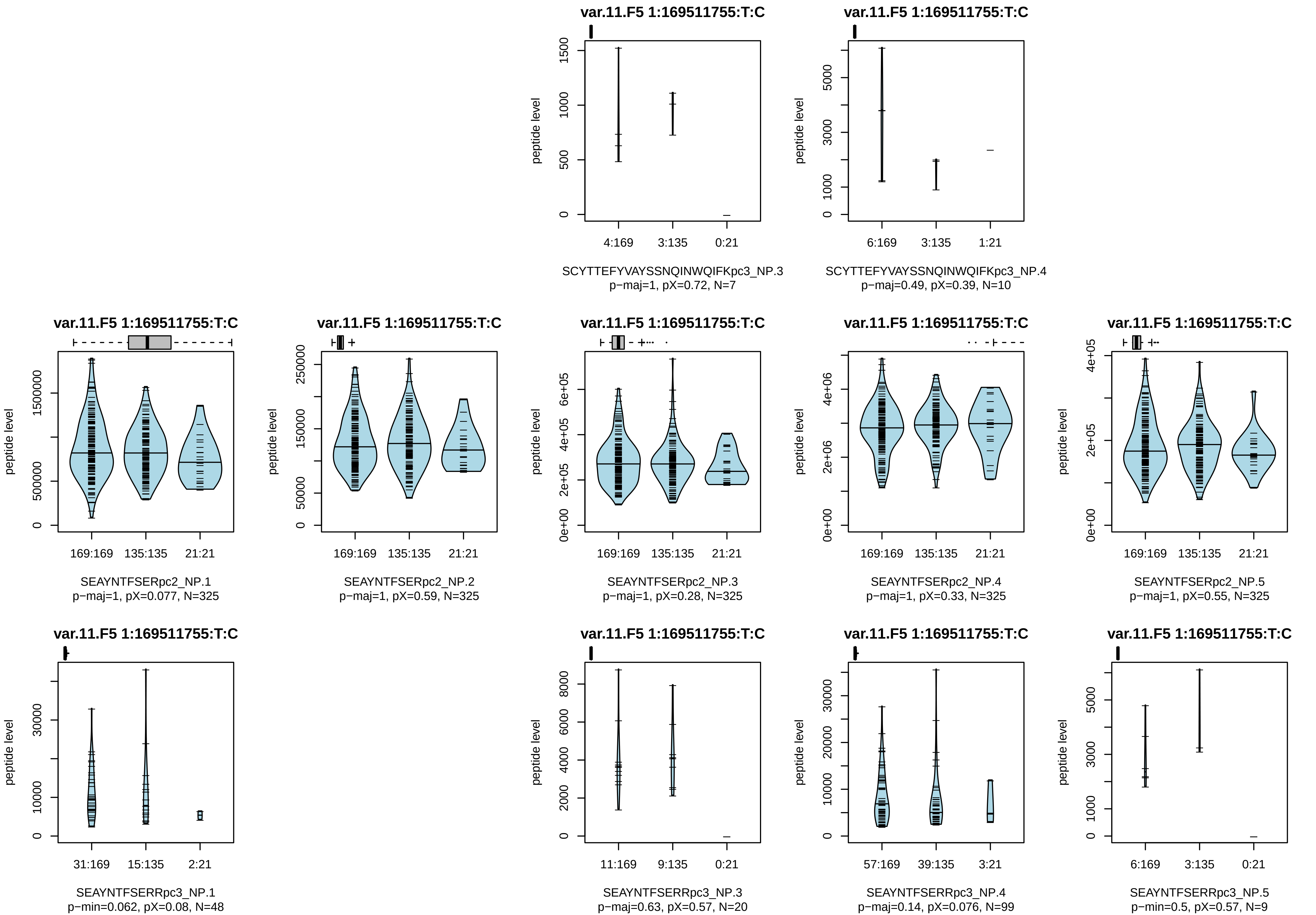
var.11.F5 1:169511755:T:C
peptide level
4:169
3:135
0:21
SCYTTEFYVAYSSNQINWQIFKpc3_NP.3
p−maj=1, pX=0.72, N=7
var.11.F5 1:169511755:T:C
peptide level
6:169
3:135
1:21
SCYTTEFYVAYSSNQINWQIFKpc3_NP.4
p−maj=0.49, pX=0.39, N=10
var.11.F5 1:169511755:T:C
peptide level
169:169
135:135
21:21
SEAYNTFSERpc2_NP.1
p−maj=1, pX=0.077, N=325
var.11.F5 1:169511755:T:C
peptide level
169:169
135:135
21:21
SEAYNTFSERpc2_NP.2
p−maj=1, pX=0.59, N=325
var.11.F5 1:169511755:T:C
peptide level
169:169
135:135
21:21
SEAYNTFSERpc2_NP.3
p−maj=1, pX=0.28, N=325
var.11.F5 1:169511755:T:C
peptide level
169:169
135:135
21:21
SEAYNTFSERpc2_NP.4
p−maj=1, pX=0.33, N=325
var.11.F5 1:169511755:T:C
peptide level
169:169
135:135
21:21
SEAYNTFSERpc2_NP.5
p−maj=1, pX=0.55, N=325
var.11.F5 1:169511755:T:C
peptide level
31:169
15:135
2:21
SEAYNTFSERRpc3_NP.1
p−min=0.062, pX=0.08, N=48
var.11.F5 1:169511755:T:C
peptide level
11:169
9:135
0:21
SEAYNTFSERRpc3_NP.3
p−maj=0.63, pX=0.57, N=20
var.11.F5 1:169511755:T:C
peptide level
57:169
39:135
3:21
SEAYNTFSERRpc3_NP.4
p−maj=0.14, pX=0.076, N=99
var.11.F5 1:169511755:T:C
peptide level
6:169
3:135
0:21
SEAYNTFSERRpc3_NP.5
p−min=0.5, pX=0.57, N=9

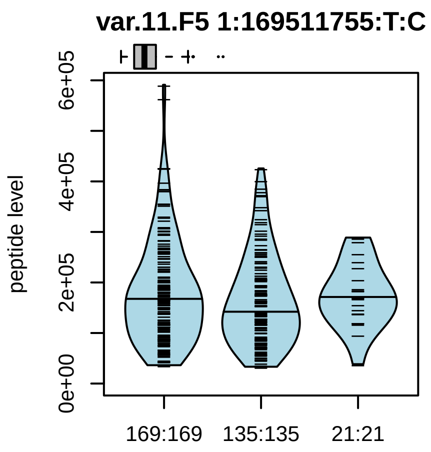

SGPESPGSACRpc2_NP.1
p−maj=1, pX=0.29, N=325

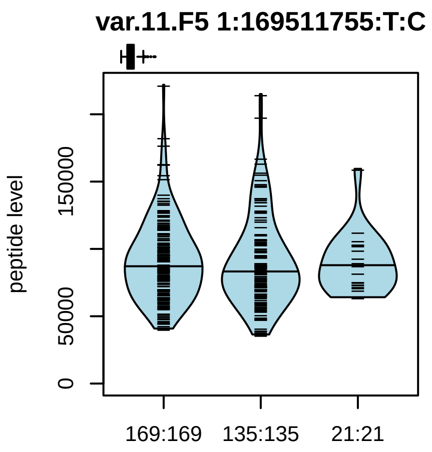

SGPESPGSACRpc2_NP.2
p−maj=1, pX=0.68, N=325

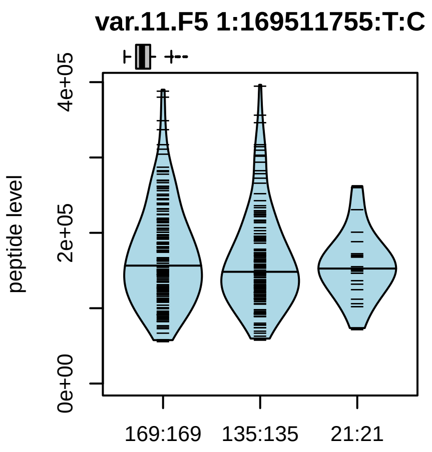

SGPESPGSACRpc2_NP.3
p−maj=1, pX=0.29, N=325

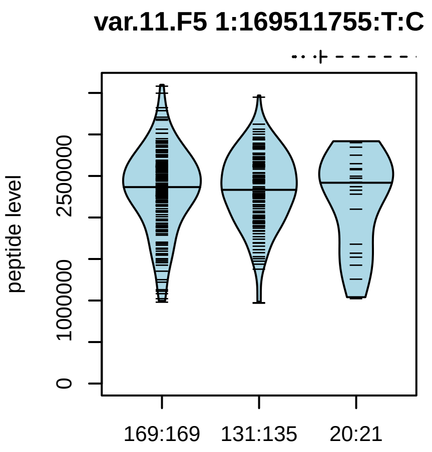

SGPESPGSACRpc2_NP.4
p−min=0.025, pX=0.09, N=320

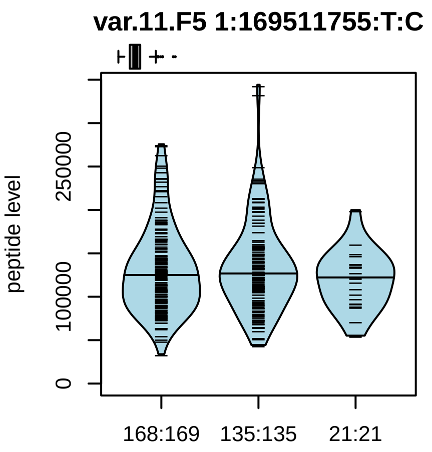

SGPESPGSACRpc2_NP.5
p−maj=1, pX=0.87, N=324

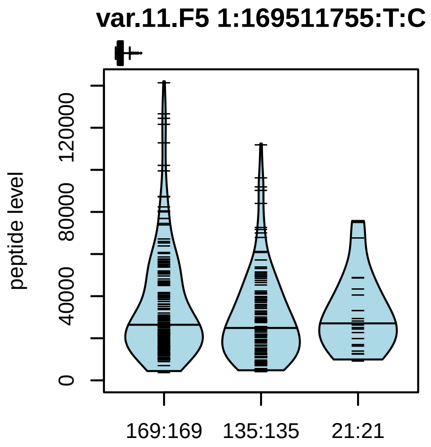

SHEFHAINGMIYSLPGLKpc3_NP.1
p−maj=1, pX=0.047, N=325

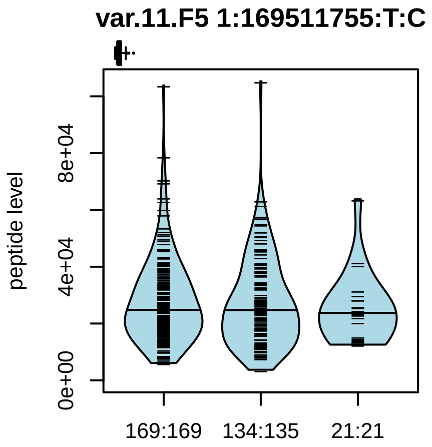

SHEFHAINGMIYSLPGLKpc3_NP.2
p−min=0.48, pX=0.34, N=324

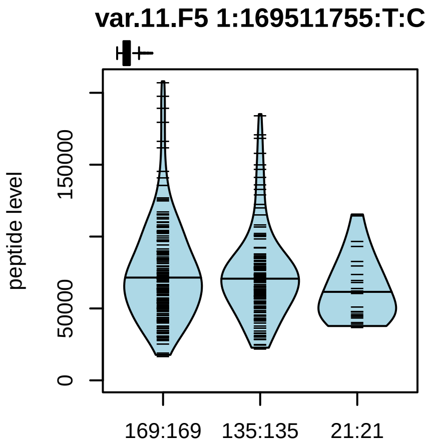

SHEFHAINGMIYSLPGLKpc3_NP.3
p−maj=1, pX=0.14, N=325

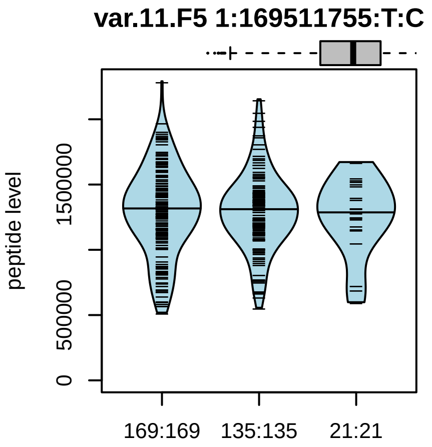

SHEFHAINGMIYSLPGLKpc3_NP.4
p−maj=1, pX=0.75, N=325

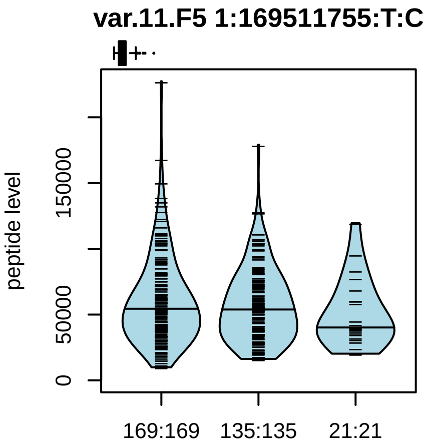

SHEFHAINGMIYSLPGLKpc3_NP.5
p−maj=1, pX=0.21, N=325

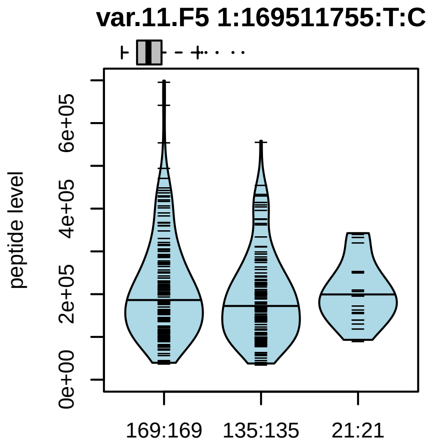

SHEFHAINGMIYSLPGLKpc4_NP.1
p−maj=1, pX=0.21, N=325

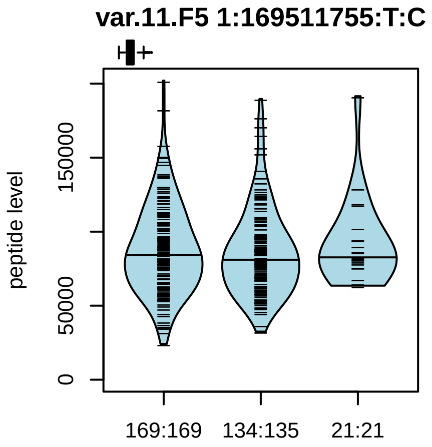

SHEFHAINGMIYSLPGLKpc4_NP.2
p−min=0.48, pX=0.93, N=324

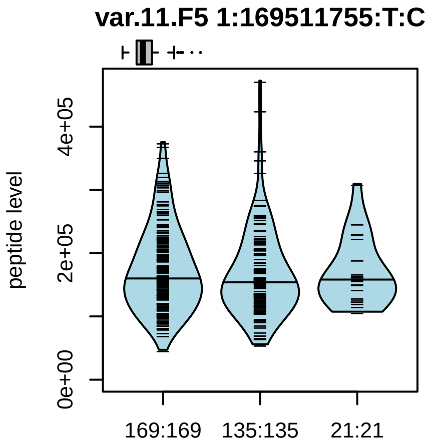

SHEFHAINGMIYSLPGLKpc4_NP.3
p−maj=1, pX=0.41, N=325

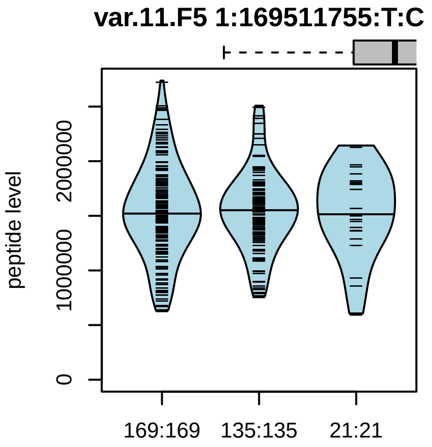

SHEFHAINGMIYSLPGLKpc4_NP.4
p−maj=1, pX=0.97, N=325

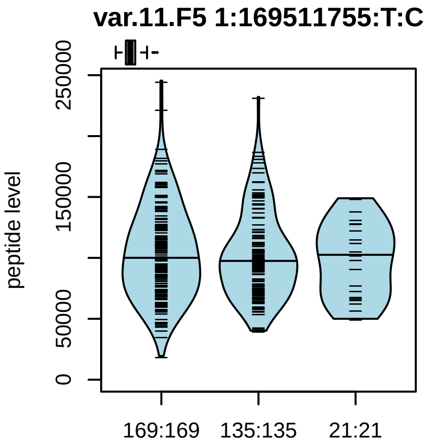

SHEFHAINGMIYSLPGLKpc4_NP.5
p−maj=1, pX=0.43, N=325

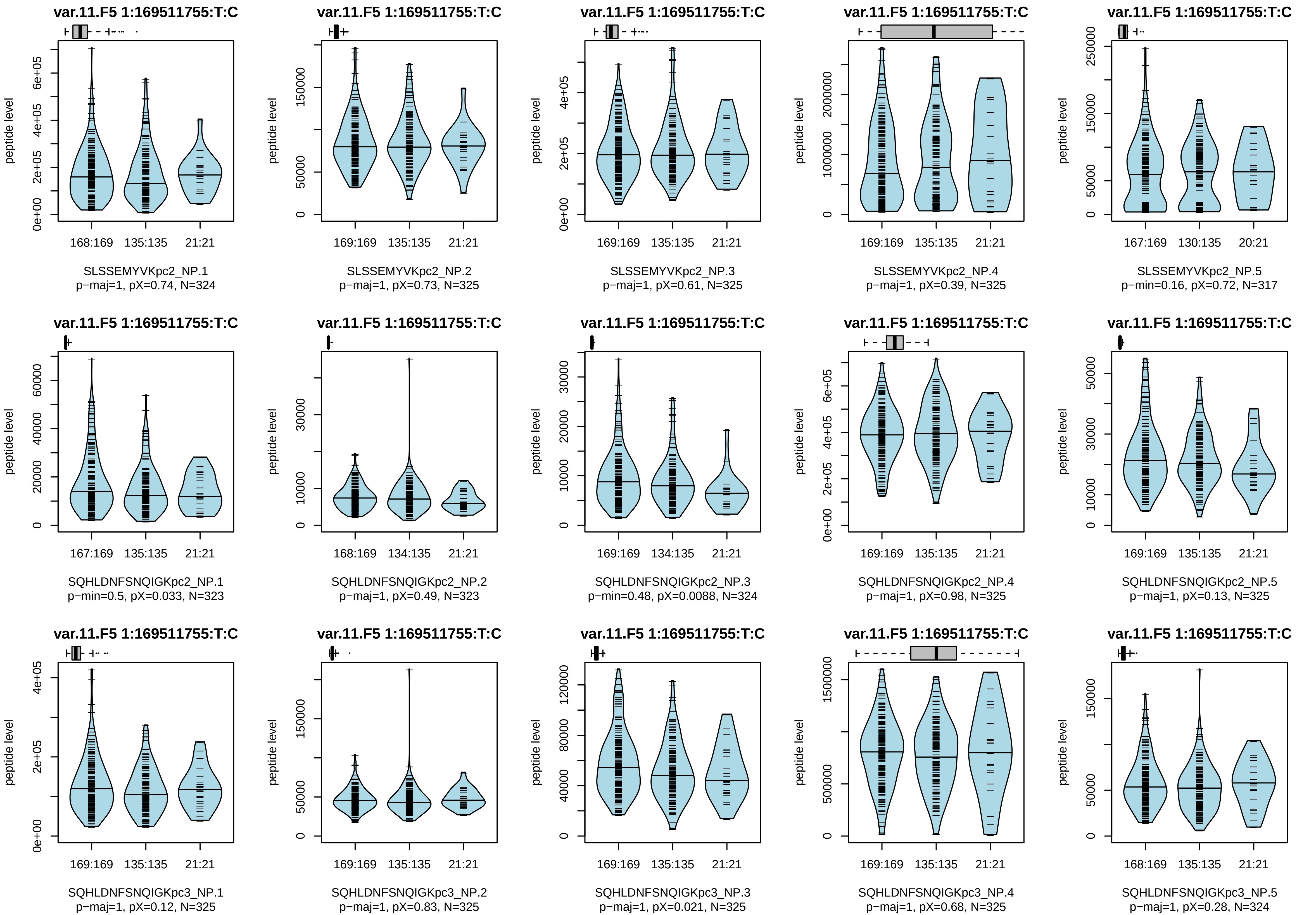

var.11.F5 1:169511755:T:C
peptide level
0e+00
2e+05
4e+05
6e+05
168:169
135:135
21:21
SLSSEMYVKpc2_NP.1
p−maj=1, pX=0.74, N=324
var.11.F5 1:169511755:T:C
peptide level
0
50000
150000
169:169
135:135
21:21
SLSSEMYVKpc2_NP.2
p−maj=1, pX=0.73, N=325
var.11.F5 1:169511755:T:C
peptide level
0e+00
2e+05
4e+05
169:169
135:135
21:21
SLSSEMYVKpc2_NP.3
p−maj=1, pX=0.61, N=325
var.11.F5 1:169511755:T:C
peptide level
0
1000000
2000000
169:169
135:135
21:21
SLSSEMYVKpc2_NP.4
p−maj=1, pX=0.39, N=325
var.11.F5 1:169511755:T:C
peptide level
0
50000
150000
250000
167:169
130:135
20:21
SLSSEMYVKpc2_NP.5
p−min=0.16, pX=0.72, N=317
var.11.F5 1:169511755:T:C
peptide level
0
20000
40000
60000
167:169
135:135
21:21
SQHLDNFSNQIGKpc2_NP.1
p−min=0.5, pX=0.033, N=323
var.11.F5 1:169511755:T:C
peptide level
0
10000
30000
168:169
134:135
21:21
SQHLDNFSNQIGKpc2_NP.2
p−maj=1, pX=0.49, N=323
var.11.F5 1:169511755:T:C
peptide level
0
10000
20000
30000
169:169
134:135
21:21
SQHLDNFSNQIGKpc2_NP.3
p−min=0.48, pX=0.0088, N=324
var.11.F5 1:169511755:T:C
peptide level
0e+00
2e+05
4e+05
6e+05
169:169
135:135
21:21
SQHLDNFSNQIGKpc2_NP.4
p−maj=1, pX=0.98, N=325
var.11.F5 1:169511755:T:C
peptide level
0
10000
30000
50000
169:169
135:135
21:21
SQHLDNFSNQIGKpc2_NP.5
p−maj=1, pX=0.13, N=325
var.11.F5 1:169511755:T:C
peptide level
0e+00
2e+05
4e+05
169:169
135:135
21:21
SQHLDNFSNQIGKpc3_NP.1
p−maj=1, pX=0.12, N=325
var.11.F5 1:169511755:T:C
peptide level
0
50000
150000
169:169
135:135
21:21
SQHLDNFSNQIGKpc3_NP.2
p−maj=1, pX=0.83, N=325
var.11.F5 1:169511755:T:C
peptide level
0
40000
80000
120000
169:169
135:135
21:21
SQHLDNFSNQIGKpc3_NP.3
p−maj=1, pX=0.021, N=325
var.11.F5 1:169511755:T:C
peptide level
0
500000
1500000
169:169
135:135
21:21
SQHLDNFSNQIGKpc3_NP.4
p−maj=1, pX=0.68, N=325
var.11.F5 1:169511755:T:C
peptide level
0
50000
150000
168:169
135:135
21:21
SQHLDNFSNQIGKpc3_NP.5
p−maj=1, pX=0.28, N=324

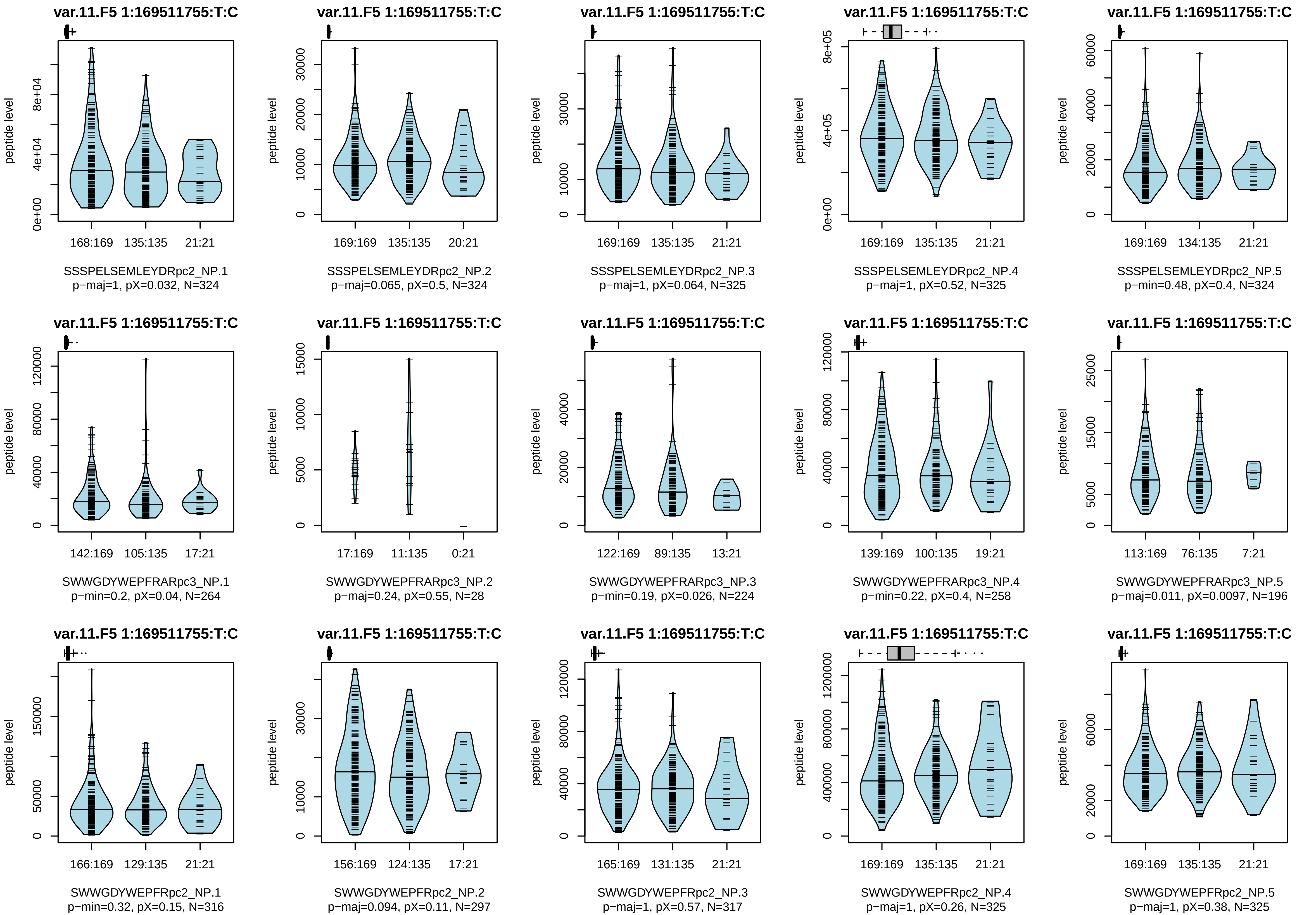
var.11.F5 1:169511755:T:C
peptide level
168:169
135:135
21:21
SSSPELSEMLEYDRpc2_NP.1
p−maj=1, pX=0.032, N=324
var.11.F5 1:169511755:T:C
peptide level
169:169
135:135
20:21
SSSPELSEMLEYDRpc2_NP.2
p−maj=0.065, pX=0.5, N=324
var.11.F5 1:169511755:T:C
peptide level
169:169
135:135
21:21
SSSPELSEMLEYDRpc2_NP.3
p−maj=1, pX=0.064, N=325
var.11.F5 1:169511755:T:C
peptide level
169:169
135:135
21:21
SSSPELSEMLEYDRpc2_NP.4
p−maj=1, pX=0.52, N=325
var.11.F5 1:169511755:T:C
peptide level
169:169
134:135
21:21
SSSPELSEMLEYDRpc2_NP.5
p−min=0.48, pX=0.4, N=324
var.11.F5 1:169511755:T:C
peptide level
142:169
105:135
17:21
SWWGDYWEPFRARpc3_NP.1
p−min=0.2, pX=0.04, N=264
var.11.F5 1:169511755:T:C
peptide level
17:169
11:135
0:21
SWWGDYWEPFRARpc3_NP.2
p−maj=0.24, pX=0.55, N=28
var.11.F5 1:169511755:T:C
peptide level
122:169
89:135
13:21
SWWGDYWEPFRARpc3_NP.3
p−min=0.19, pX=0.026, N=224
var.11.F5 1:169511755:T:C
peptide level
139:169
100:135
19:21
SWWGDYWEPFRARpc3_NP.4
p−min=0.22, pX=0.4, N=258
var.11.F5 1:169511755:T:C
peptide level
113:169
76:135
7:21
SWWGDYWEPFRARpc3_NP.5
p−maj=0.011, pX=0.0097, N=196
var.11.F5 1:169511755:T:C
peptide level
166:169
129:135
21:21
SWWGDYWEPFRpc2_NP.1
p−min=0.32, pX=0.15, N=316
var.11.F5 1:169511755:T:C
peptide level
156:169
124:135
17:21
SWWGDYWEPFRpc2_NP.2
p−maj=0.094, pX=0.11, N=297
var.11.F5 1:169511755:T:C
peptide level
165:169
131:135
21:21
SWWGDYWEPFRpc2_NP.3
p−maj=1, pX=0.57, N=317
var.11.F5 1:169511755:T:C
peptide level
169:169
135:135
21:21
SWWGDYWEPFRpc2_NP.4
p−maj=1, pX=0.26, N=325
var.11.F5 1:169511755:T:C
peptide level
169:169
135:135
21:21
SWWGDYWEPFRpc2_NP.5
p−maj=1, pX=0.38, N=325

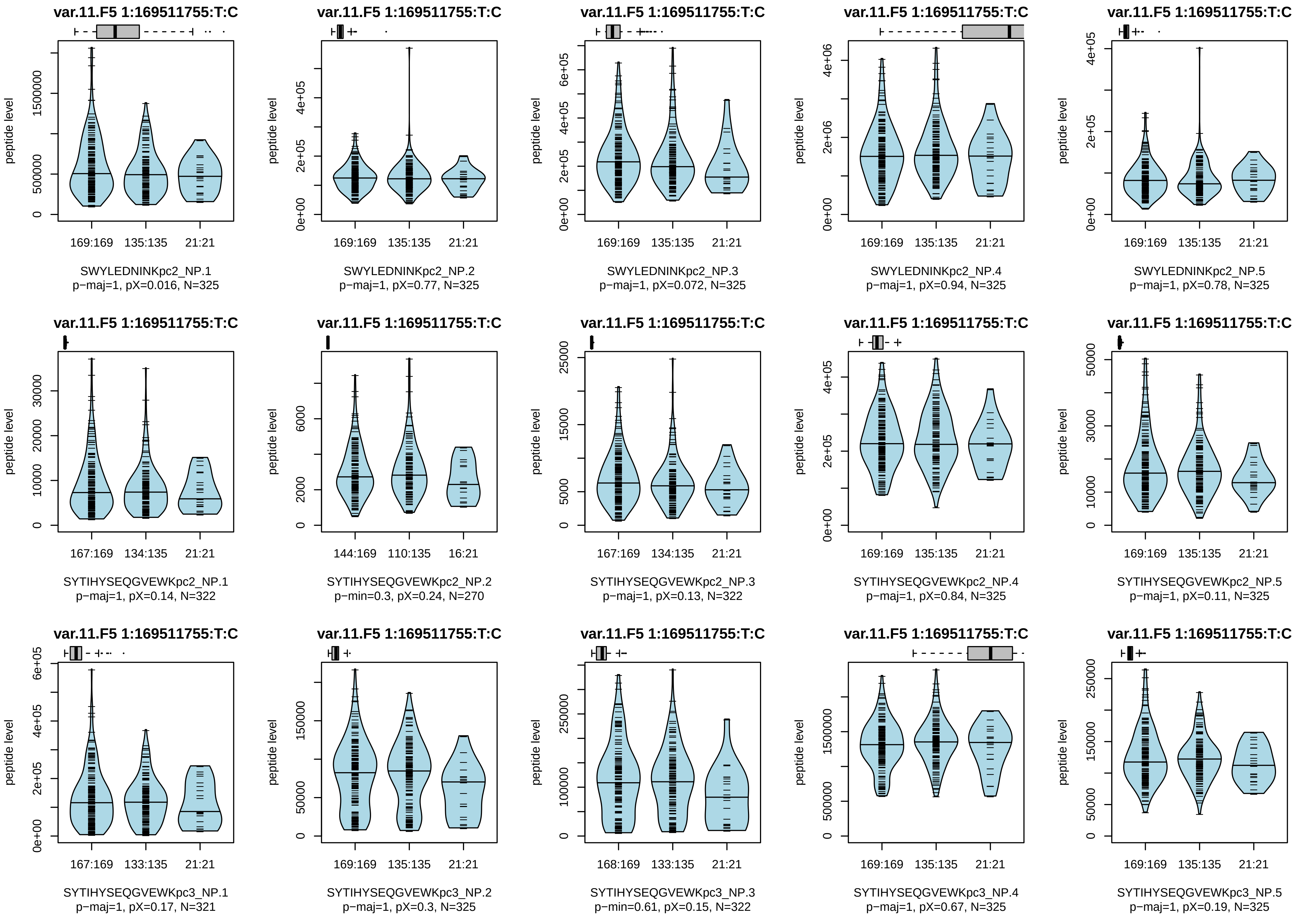
var.11.F5 1:169511755:T:C
peptide level
169:169
135:135
21:21
SWYLEDNINKpc2_NP.1
p−maj=1, pX=0.016, N=325
var.11.F5 1:169511755:T:C
169:169
135:135
21:21
SWYLEDNINKpc2_NP.2
p−maj=1, pX=0.77, N=325
var.11.F5 1:169511755:T:C
169:169
135:135
21:21
SWYLEDNINKpc2_NP.3
p−maj=1, pX=0.072, N=325
var.11.F5 1:169511755:T:C
169:169
135:135
21:21
SWYLEDNINKpc2_NP.4
p−maj=1, pX=0.94, N=325
var.11.F5 1:169511755:T:C
169:169
135:135
21:21
SWYLEDNINKpc2_NP.5
p−maj=1, pX=0.78, N=325
var.11.F5 1:169511755:T:C
167:169
134:135
21:21
SYTIHYSEQGVEWKpc2_NP.1
p−maj=1, pX=0.14, N=322
var.11.F5 1:169511755:T:C
144:169
110:135
16:21
SYTIHYSEQGVEWKpc2_NP.2
p−min=0.3, pX=0.24, N=270
var.11.F5 1:169511755:T:C
167:169
134:135
21:21
SYTIHYSEQGVEWKpc2_NP.3
p−maj=1, pX=0.13, N=322
var.11.F5 1:169511755:T:C
169:169
135:135
21:21
SYTIHYSEQGVEWKpc2_NP.4
p−maj=1, pX=0.84, N=325
var.11.F5 1:169511755:T:C
169:169
135:135
21:21
SYTIHYSEQGVEWKpc2_NP.5
p−maj=1, pX=0.11, N=325
var.11.F5 1:169511755:T:C
167:169
133:135
21:21
SYTIHYSEQGVEWKpc3_NP.1
p−maj=1, pX=0.17, N=321
var.11.F5 1:169511755:T:C
169:169
135:135
21:21
SYTIHYSEQGVEWKpc3_NP.2
p−maj=1, pX=0.3, N=325
var.11.F5 1:169511755:T:C
168:169
133:135
21:21
SYTIHYSEQGVEWKpc3_NP.3
p−min=0.61, pX=0.15, N=322
var.11.F5 1:169511755:T:C
169:169
135:135
21:21
SYTIHYSEQGVEWKpc3_NP.4
p−maj=1, pX=0.67, N=325
var.11.F5 1:169511755:T:C
169:169
135:135
21:21
SYTIHYSEQGVEWKpc3_NP.5
p−maj=1, pX=0.19, N=325

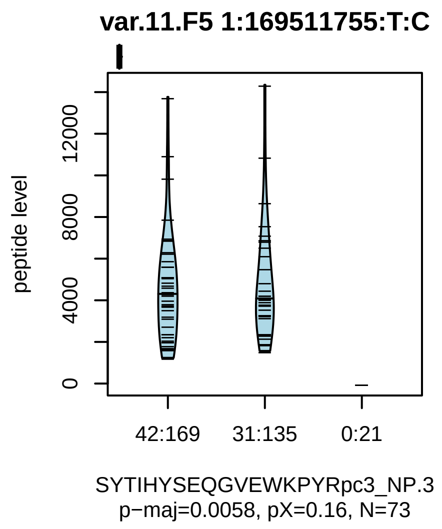
var.11.F5 1:169511755:T:C
peptide level
42:169
31:135
0:21
SYTIHYSEQGVEWKPYRpc3_NP.3
p−maj=0.0058, pX=0.16, N=73

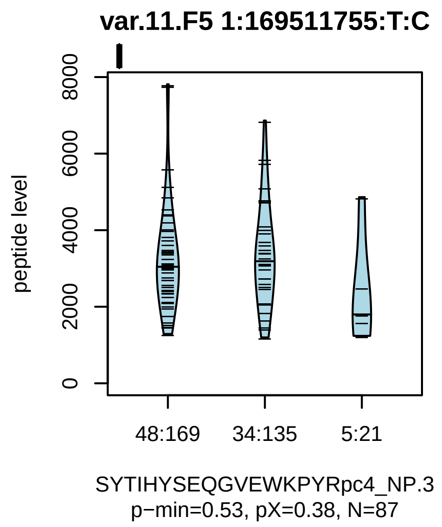
var.11.F5 1:169511755:T:C
peptide level
48:169
34:135
5:21
SYTIHYSEQGVEWKPYRpc4_NP.3
p−min=0.53, pX=0.38, N=87

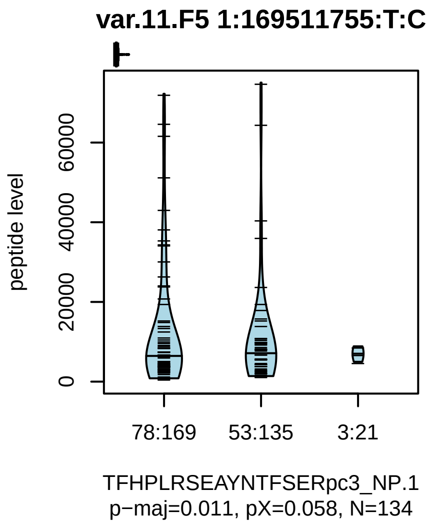
var.11.F5 1:169511755:T:C
peptide level
78:169
53:135
3:21
TFHPLRSEAYNTFSERpc3_NP.1
p−maj=0.011, pX=0.058, N=134

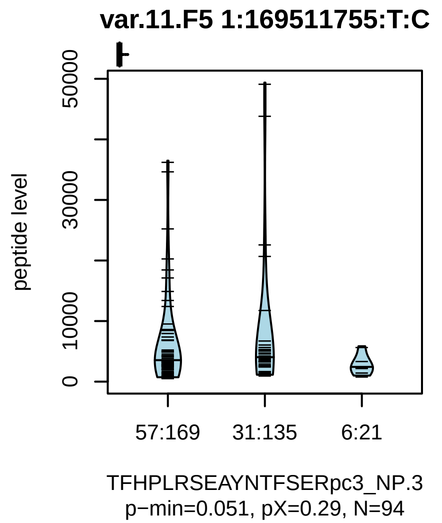
var.11.F5 1:169511755:T:C
peptide level
57:169
31:135
6:21
TFHPLRSEAYNTFSERpc3_NP.3
p−min=0.051, pX=0.29, N=94

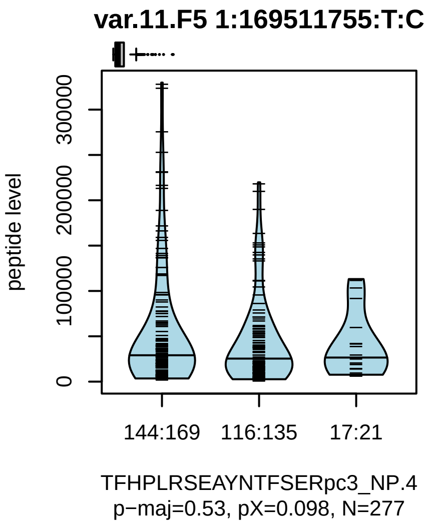
var.11.F5 1:169511755:T:C
peptide level
144:169
116:135
17:21
TFHPLRSEAYNTFSERpc3_NP.4
p−maj=0.53, pX=0.098, N=277

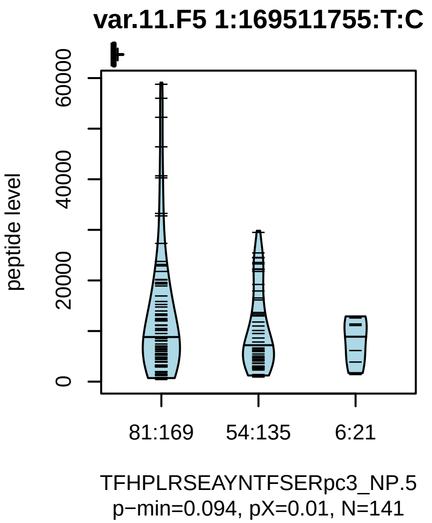
var.11.F5 1:169511755:T:C
peptide level
81:169
54:135
6:21
TFHPLRSEAYNTFSERpc3_NP.5
p−min=0.094, pX=0.01, N=141

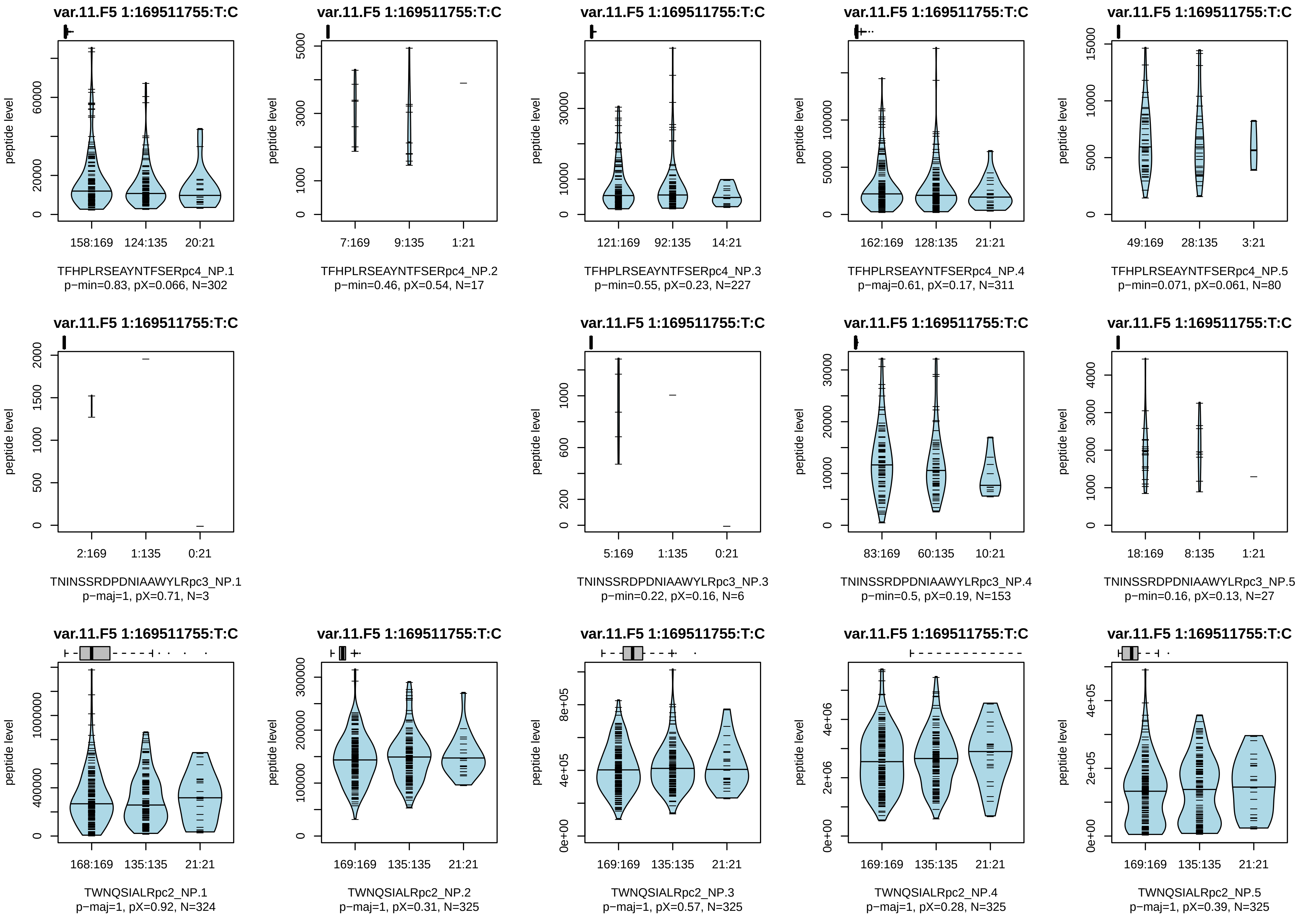
var.11.F5 1:169511755:T:C
peptide level
158:169
124:135
20:21
TFHPLRSEAYNTFSERpc4_NP.1
p−min=0.83, pX=0.066, N=302
var.11.F5 1:169511755:T:C
peptide level
7:169
9:135
1:21
TFHPLRSEAYNTFSERpc4_NP.2
p−min=0.46, pX=0.54, N=17
var.11.F5 1:169511755:T:C
peptide level
121:169
92:135
14:21
TFHPLRSEAYNTFSERpc4_NP.3
p−min=0.55, pX=0.23, N=227
var.11.F5 1:169511755:T:C
peptide level
162:169
128:135
21:21
TFHPLRSEAYNTFSERpc4_NP.4
p−maj=0.61, pX=0.17, N=311
var.11.F5 1:169511755:T:C
peptide level
49:169
28:135
3:21
TFHPLRSEAYNTFSERpc4_NP.5
p−min=0.071, pX=0.061, N=80
var.11.F5 1:169511755:T:C
peptide level
2:169
1:135
0:21
TNINSSRDPDNIAAWYLRpc3_NP.1
p−maj=1, pX=0.71, N=3
var.11.F5 1:169511755:T:C
peptide level
5:169
1:135
0:21
TNINSSRDPDNIAAWYLRpc3_NP.3
p−min=0.22, pX=0.16, N=6
var.11.F5 1:169511755:T:C
peptide level
83:169
60:135
10:21
TNINSSRDPDNIAAWYLRpc3_NP.4
p−min=0.5, pX=0.19, N=153
var.11.F5 1:169511755:T:C
peptide level
18:169
8:135
1:21
TNINSSRDPDNIAAWYLRpc3_NP.5
p−min=0.16, pX=0.13, N=27
var.11.F5 1:169511755:T:C
peptide level
168:169
135:135
21:21
TWNQSIALRpc2_NP.1
p−maj=1, pX=0.92, N=324
var.11.F5 1:169511755:T:C
peptide level
169:169
135:135
21:21
TWNQSIALRpc2_NP.2
p−maj=1, pX=0.31, N=325
var.11.F5 1:169511755:T:C
peptide level
169:169
135:135
21:21
TWNQSIALRpc2_NP.3
p−maj=1, pX=0.57, N=325
var.11.F5 1:169511755:T:C
peptide level
169:169
135:135
21:21
TWNQSIALRpc2_NP.4
p−maj=1, pX=0.28, N=325
var.11.F5 1:169511755:T:C
peptide level
169:169
135:135
21:21
TWNQSIALRpc2_NP.5
p−maj=1, pX=0.39, N=325

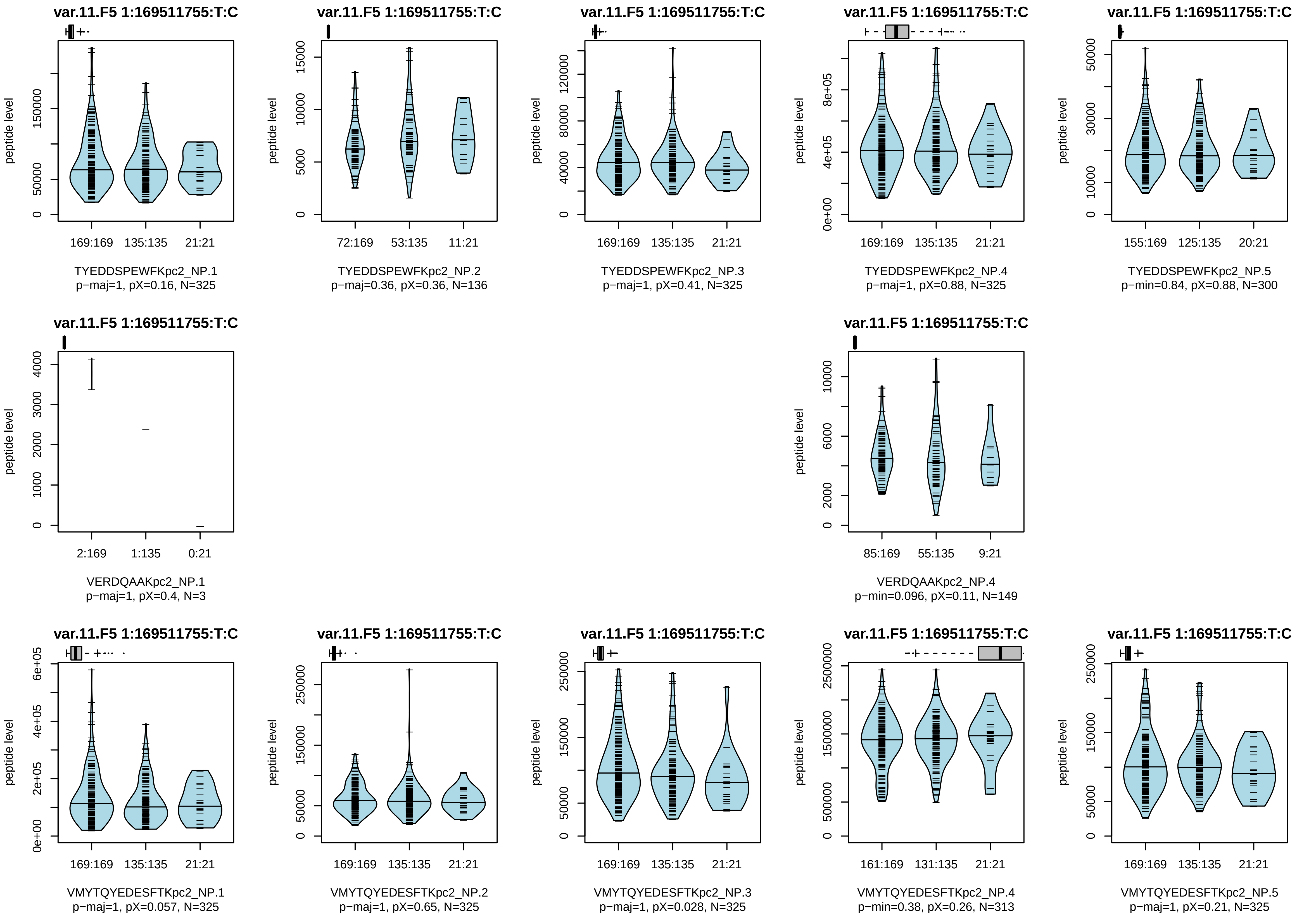

var.11.F5 1:169511755:T:C
peptide level
169:169
135:135
21:21
TYEDDSPEWFKpc2_NP.1
p−maj=1, pX=0.16, N=325
var.11.F5 1:169511755:T:C
peptide level
72:169
53:135
11:21
TYEDDSPEWFKpc2_NP.2
p−maj=0.36, pX=0.36, N=136
var.11.F5 1:169511755:T:C
peptide level
169:169
135:135
21:21
TYEDDSPEWFKpc2_NP.3
p−maj=1, pX=0.41, N=325
var.11.F5 1:169511755:T:C
peptide level
169:169
135:135
21:21
TYEDDSPEWFKpc2_NP.4
p−maj=1, pX=0.88, N=325
var.11.F5 1:169511755:T:C
peptide level
155:169
125:135
20:21
TYEDDSPEWFKpc2_NP.5
p−min=0.84, pX=0.88, N=300
var.11.F5 1:169511755:T:C
peptide level
2:169
1:135
0:21
VERDQAAKpc2_NP.1
p−maj=1, pX=0.4, N=3
var.11.F5 1:169511755:T:C
peptide level
85:169
55:135
9:21
VERDQAAKpc2_NP.4
p−min=0.096, pX=0.11, N=149
var.11.F5 1:169511755:T:C
peptide level
169:169
135:135
21:21
VMYTQYEDESFTKpc2_NP.1
p−maj=1, pX=0.057, N=325
var.11.F5 1:169511755:T:C
peptide level
169:169
135:135
21:21
VMYTQYEDESFTKpc2_NP.2
p−maj=1, pX=0.65, N=325
var.11.F5 1:169511755:T:C
peptide level
169:169
135:135
21:21
VMYTQYEDESFTKpc2_NP.3
p−maj=1, pX=0.028, N=325
var.11.F5 1:169511755:T:C
peptide level
161:169
131:135
21:21
VMYTQYEDESFTKpc2_NP.4
p−min=0.38, pX=0.26, N=313
var.11.F5 1:169511755:T:C
peptide level
169:169
135:135
21:21
VMYTQYEDESFTKpc2_NP.5
p−maj=1, pX=0.21, N=325

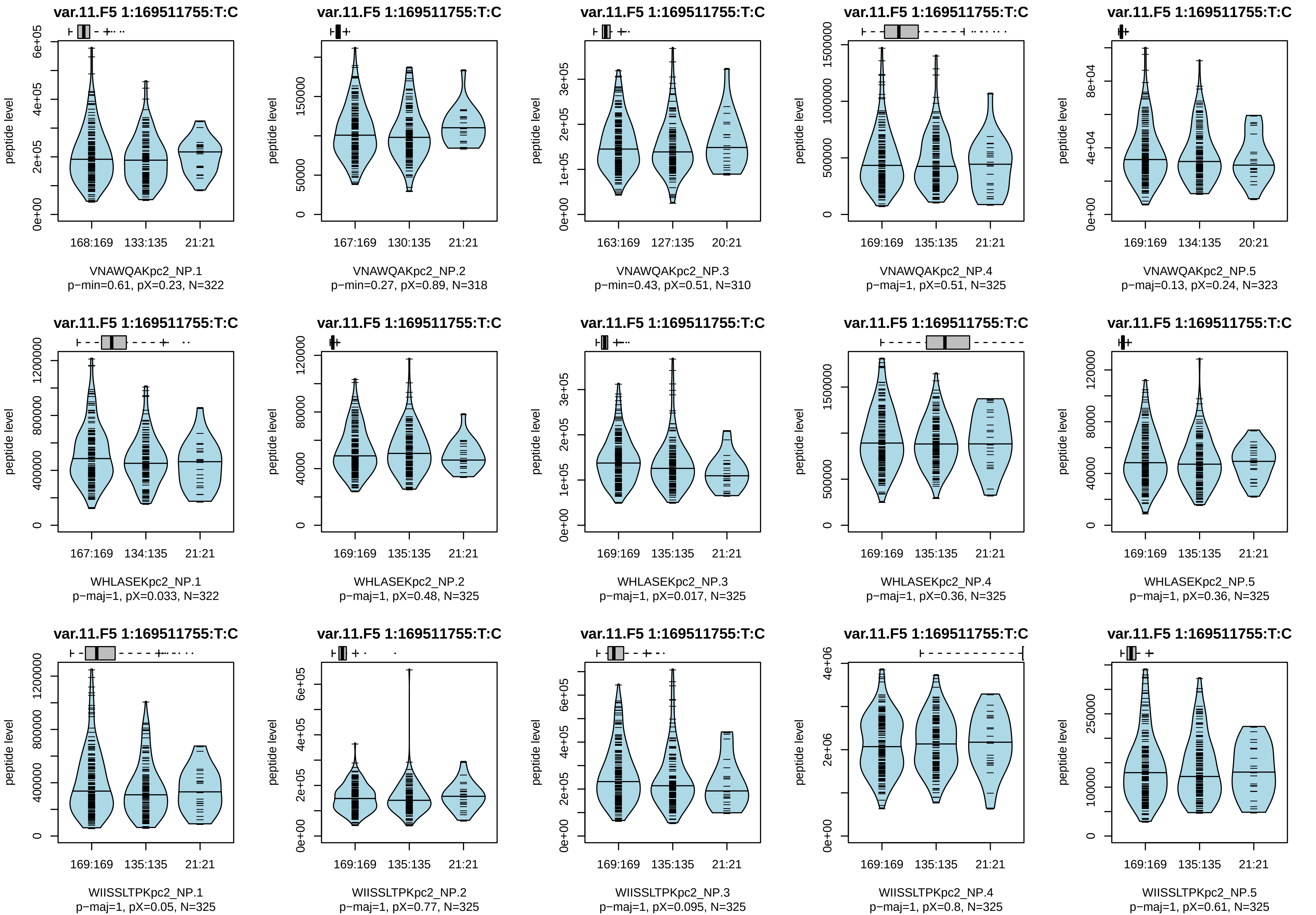
var.11.F5 1:169511755:T:C
peptide level
168:169
133:135
21:21
VNAWQAKpc2_NP.1
p−min=0.61, pX=0.23, N=322
var.11.F5 1:169511755:T:C
167:169
130:135
21:21
VNAWQAKpc2_NP.2
p−min=0.27, pX=0.89, N=318
var.11.F5 1:169511755:T:C
163:169
127:135
20:21
VNAWQAKpc2_NP.3
p−min=0.43, pX=0.51, N=310
var.11.F5 1:169511755:T:C
169:169
135:135
21:21
VNAWQAKpc2_NP.4
p−maj=1, pX=0.51, N=325
var.11.F5 1:169511755:T:C
169:169
134:135
20:21
VNAWQAKpc2_NP.5
p−maj=0.13, pX=0.24, N=323
var.11.F5 1:169511755:T:C
167:169
134:135
21:21
WHLASEKpc2_NP.1
p−maj=1, pX=0.033, N=322
var.11.F5 1:169511755:T:C
169:169
135:135
21:21
WHLASEKpc2_NP.2
p−maj=1, pX=0.48, N=325
var.11.F5 1:169511755:T:C
169:169
135:135
21:21
WHLASEKpc2_NP.3
p−maj=1, pX=0.017, N=325
var.11.F5 1:169511755:T:C
169:169
135:135
21:21
WHLASEKpc2_NP.4
p−maj=1, pX=0.36, N=325
var.11.F5 1:169511755:T:C
169:169
135:135
21:21
WHLASEKpc2_NP.5
p−maj=1, pX=0.36, N=325
var.11.F5 1:169511755:T:C
169:169
135:135
21:21
WIISSLTPKpc2_NP.1
p−maj=1, pX=0.05, N=325
var.11.F5 1:169511755:T:C
169:169
135:135
21:21
WIISSLTPKpc2_NP.2
p−maj=1, pX=0.77, N=325
var.11.F5 1:169511755:T:C
169:169
135:135
21:21
WIISSLTPKpc2_NP.3
p−maj=1, pX=0.095, N=325
var.11.F5 1:169511755:T:C
169:169
135:135
21:21
WIISSLTPKpc2_NP.4
p−maj=1, pX=0.8, N=325
var.11.F5 1:169511755:T:C
169:169
135:135
21:21
WIISSLTPKpc2_NP.5
p−maj=1, pX=0.61, N=325

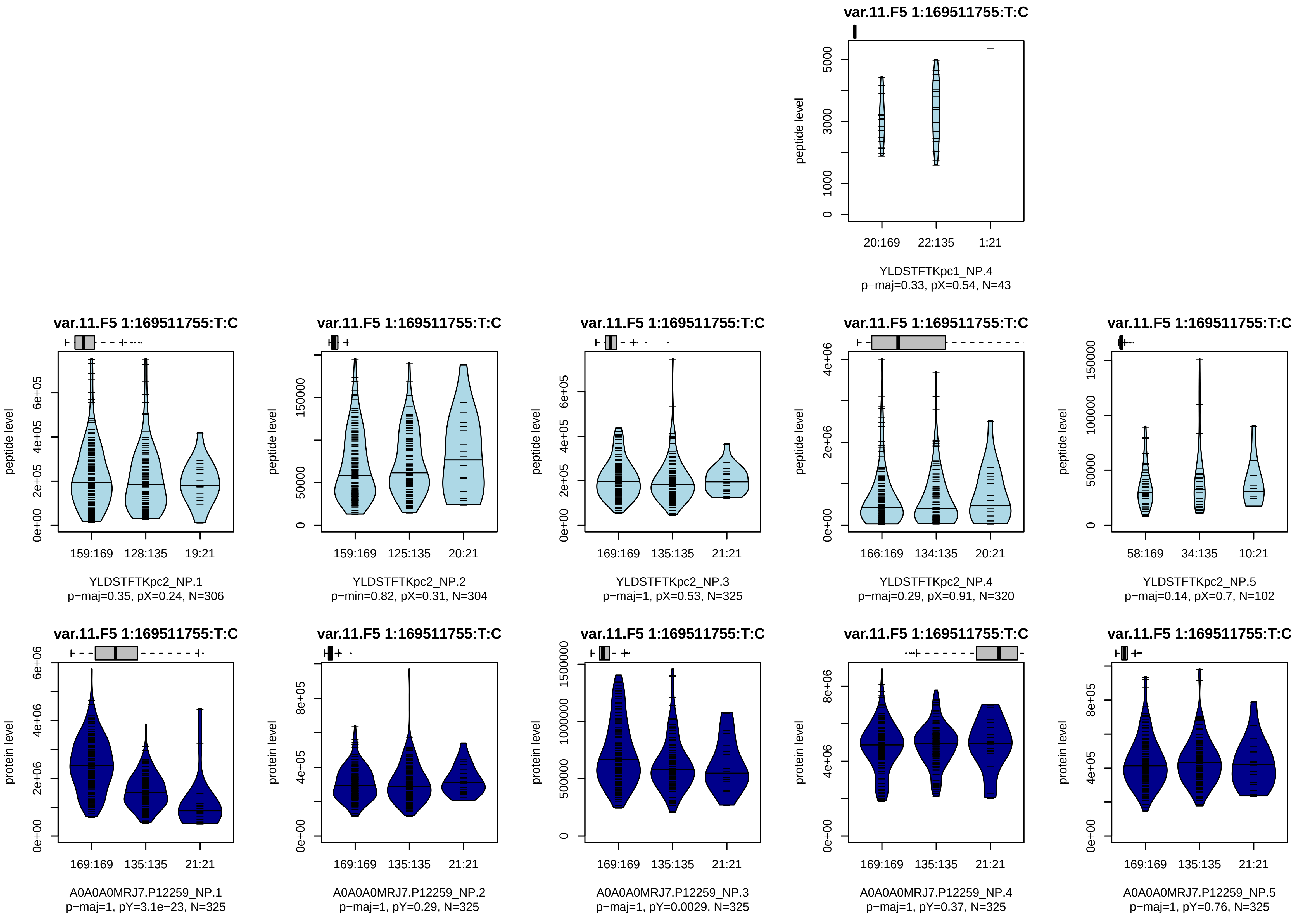

var.11.F5 1:169511755:T:C
peptide level
0
1000
3000
5000
20:169
22:135
1:21
YLDSTFTKpc1_NP.4
p−maj=0.33, pX=0.54, N=43
var.11.F5 1:169511755:T:C
peptide level
0e+00
2e+05
4e+05
6e+05
159:169
128:135
19:21
YLDSTFTKpc2_NP.1
p−maj=0.35, pX=0.24, N=306
var.11.F5 1:169511755:T:C
peptide level
0
50000
150000
159:169
125:135
20:21
YLDSTFTKpc2_NP.2
p−min=0.82, pX=0.31, N=304
var.11.F5 1:169511755:T:C
peptide level
0e+00
2e+05
4e+05
6e+05
169:169
135:135
21:21
YLDSTFTKpc2_NP.3
p−maj=1, pX=0.53, N=325
var.11.F5 1:169511755:T:C
peptide level
0e+00
2e+06
4e+06
166:169
134:135
20:21
YLDSTFTKpc2_NP.4
p−maj=0.29, pX=0.91, N=320
var.11.F5 1:169511755:T:C
peptide level
0
50000
100000
150000
58:169
34:135
10:21
YLDSTFTKpc2_NP.5
p−maj=0.14, pX=0.7, N=102
var.11.F5 1:169511755:T:C
protein level
0e+00
2e+06
4e+06
6e+06
169:169
135:135
21:21
A0A0A0MRJ7.P12259_NP.1
p−maj=1, pY=3.1e−23, N=325
var.11.F5 1:169511755:T:C
protein level
0e+00
4e+05
8e+05
169:169
135:135
21:21
A0A0A0MRJ7.P12259_NP.2
p−maj=1, pY=0.29, N=325
var.11.F5 1:169511755:T:C
protein level
0
500000
1000000
1500000
169:169
135:135
21:21
A0A0A0MRJ7.P12259_NP.3
p−maj=1, pY=0.0029, N=325
var.11.F5 1:169511755:T:C
protein level
0e+00
4e+06
8e+06
169:169
135:135
21:21
A0A0A0MRJ7.P12259_NP.4
p−maj=1, pY=0.37, N=325
var.11.F5 1:169511755:T:C
protein level
0e+00
4e+05
8e+05
169:169
135:135
21:21
A0A0A0MRJ7.P12259_NP.5
p−maj=1, pY=0.76, N=325

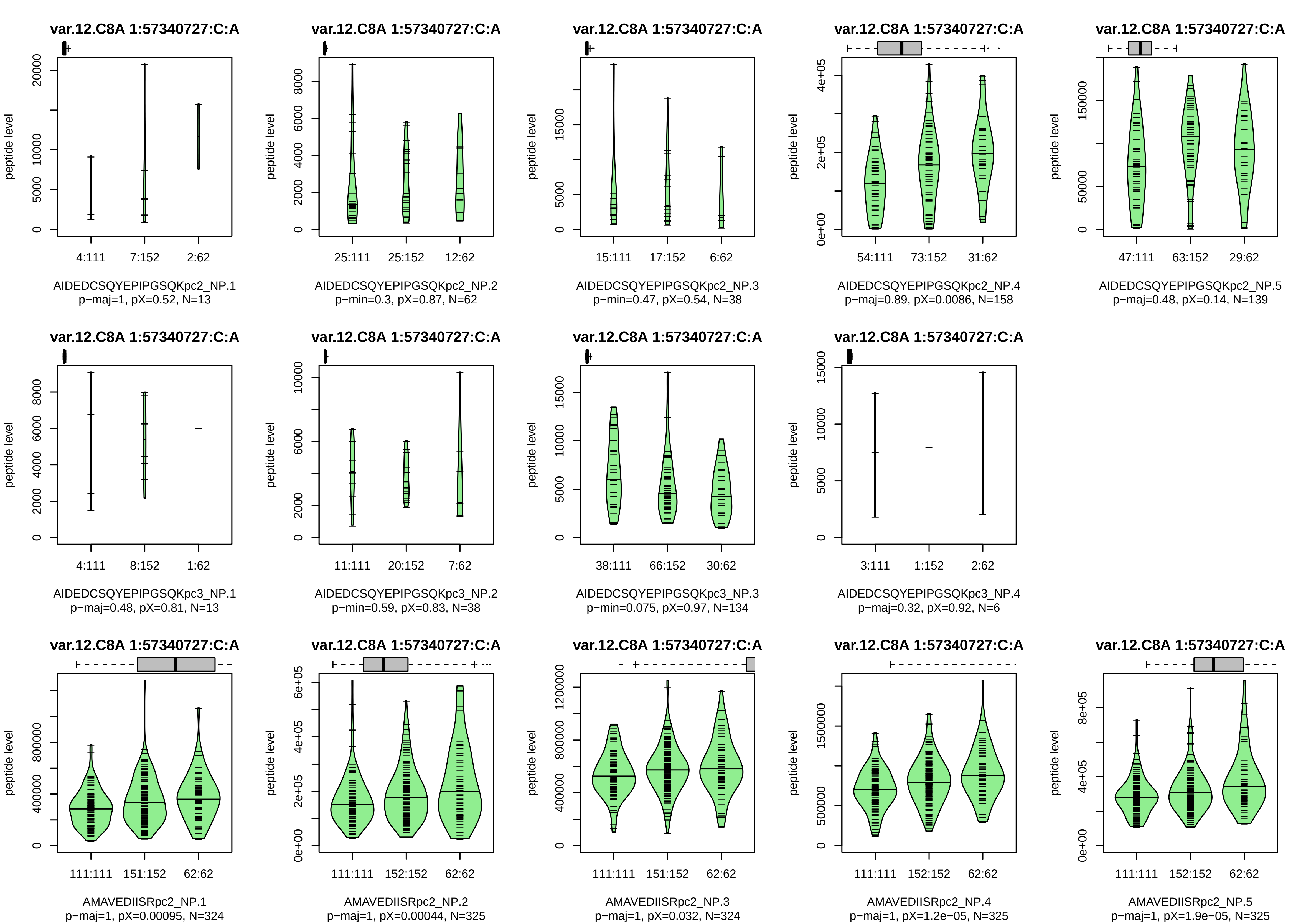

var.12.C8A 1:57340727:C:A
peptide level
4:111
7:152
2:62
AIDEDCSQYEPIPGSQKpc2_NP.1
p−maj=1, pX=0.52, N=13
var.12.C8A 1:57340727:C:A
peptide level
25:111
25:152
12:62
AIDEDCSQYEPIPGSQKpc2_NP.2
p−min=0.3, pX=0.87, N=62
var.12.C8A 1:57340727:C:A
peptide level
15:111
17:152
6:62
AIDEDCSQYEPIPGSQKpc2_NP.3
p−min=0.47, pX=0.54, N=38
var.12.C8A 1:57340727:C:A
peptide level
54:111
73:152
31:62
AIDEDCSQYEPIPGSQKpc2_NP.4
p−maj=0.89, pX=0.0086, N=158
var.12.C8A 1:57340727:C:A
peptide level
47:111
63:152
29:62
AIDEDCSQYEPIPGSQKpc2_NP.5
p−maj=0.48, pX=0.14, N=139
var.12.C8A 1:57340727:C:A
peptide level
4:111
8:152
1:62
AIDEDCSQYEPIPGSQKpc3_NP.1
p−maj=0.48, pX=0.81, N=13
var.12.C8A 1:57340727:C:A
peptide level
11:111
20:152
7:62
AIDEDCSQYEPIPGSQKpc3_NP.2
p−min=0.59, pX=0.83, N=38
var.12.C8A 1:57340727:C:A
peptide level
38:111
66:152
30:62
AIDEDCSQYEPIPGSQKpc3_NP.3
p−min=0.075, pX=0.97, N=134
var.12.C8A 1:57340727:C:A
peptide level
3:111
1:152
2:62
AIDEDCSQYEPIPGSQKpc3_NP.4
p−maj=0.32, pX=0.92, N=6
var.12.C8A 1:57340727:C:A
peptide level
111:111
151:152
62:62
AMAVEDIISRpc2_NP.1
p−maj=1, pX=0.00095, N=324
var.12.C8A 1:57340727:C:A
peptide level
111:111
152:152
62:62
AMAVEDIISRpc2_NP.2
p−maj=1, pX=0.00044, N=325
var.12.C8A 1:57340727:C:A
peptide level
111:111
151:152
62:62
AMAVEDIISRpc2_NP.3
p−maj=1, pX=0.032, N=324
var.12.C8A 1:57340727:C:A
peptide level
111:111
152:152
62:62
AMAVEDIISRpc2_NP.4
p−maj=1, pX=1.2e−05, N=325
var.12.C8A 1:57340727:C:A
peptide level
111:111
152:152
62:62
AMAVEDIISRpc2_NP.5
p−maj=1, pX=1.9e−05, N=325

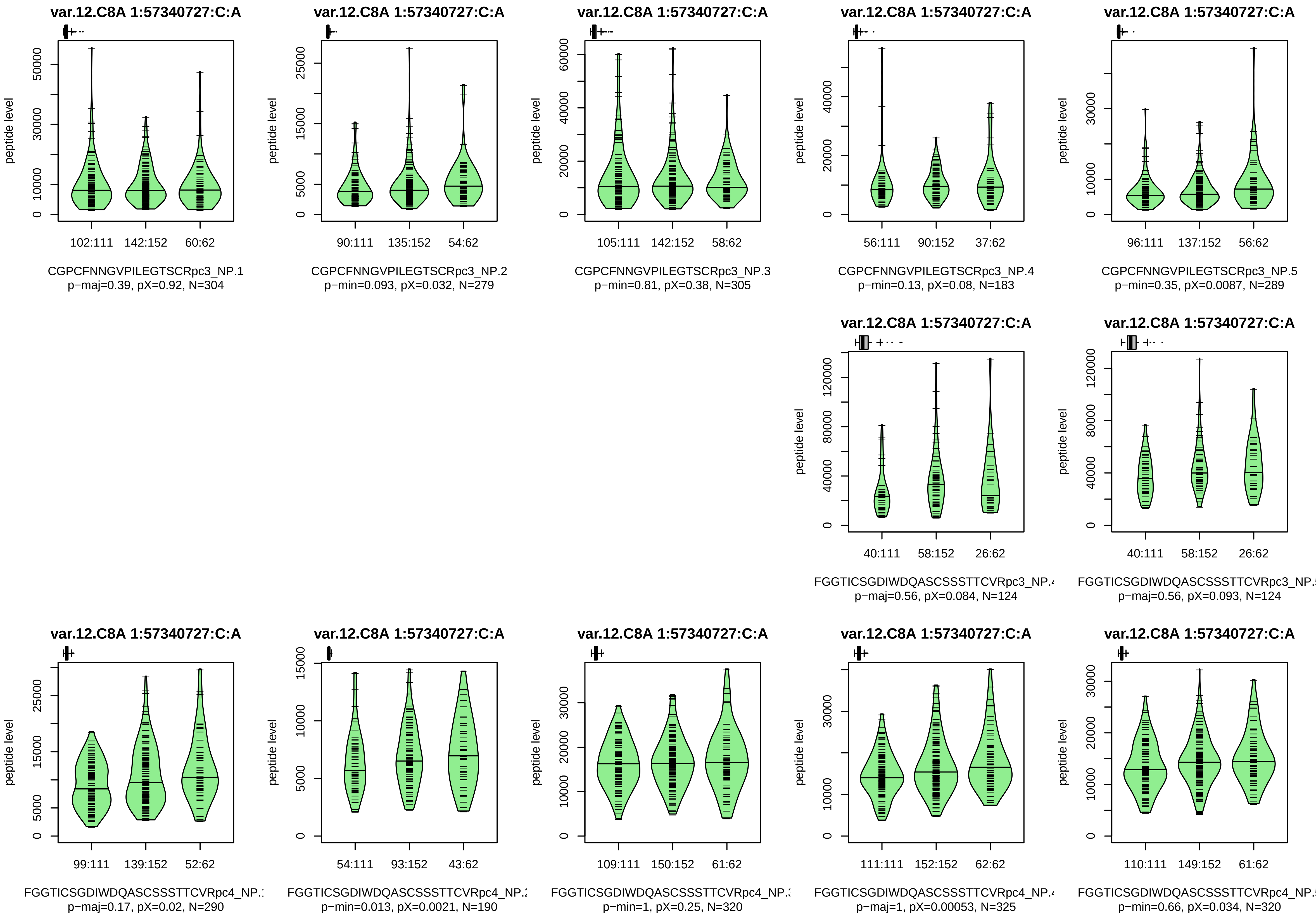

var.12.C8A 1:57340727:C:A
peptide level
102:111
142:152
60:62
CGPCFNNGVPILEGTSCRpc3_NP.1
p−maj=0.39, pX=0.92, N=304
var.12.C8A 1:57340727:C:A
peptide level
90:111
135:152
54:62
CGPCFNNGVPILEGTSCRpc3_NP.2
p−min=0.093, pX=0.032, N=279
var.12.C8A 1:57340727:C:A
peptide level
105:111
142:152
58:62
CGPCFNNGVPILEGTSCRpc3_NP.3
p−min=0.81, pX=0.38, N=305
var.12.C8A 1:57340727:C:A
peptide level
56:111
90:152
37:62
CGPCFNNGVPILEGTSCRpc3_NP.4
p−min=0.13, pX=0.08, N=183
var.12.C8A 1:57340727:C:A
peptide level
96:111
137:152
56:62
CGPCFNNGVPILEGTSCRpc3_NP.5
p−min=0.35, pX=0.0087, N=289
var.12.C8A 1:57340727:C:A
peptide level
40:111
58:152
26:62
p−maj=0.56, pX=0.084, N=124
var.12.C8A 1:57340727:C:A
peptide level
40:111
58:152
26:62
p−maj=0.56, pX=0.093, N=124
var.12.C8A 1:57340727:C:A
peptide level
99:111
139:152
52:62
p−maj=0.17, pX=0.02, N=290
var.12.C8A 1:57340727:C:A
peptide level
54:111
93:152
43:62
p−min=0.013, pX=0.0021, N=190
var.12.C8A 1:57340727:C:A
peptide level
109:111
150:152
61:62
p−min=1, pX=0.25, N=320
var.12.C8A 1:57340727:C:A
peptide level
111:111
152:152
62:62
p−maj=1, pX=0.00053, N=325
var.12.C8A 1:57340727:C:A
peptide level
110:111
149:152
61:62
p−min=0.66, pX=0.034, N=320

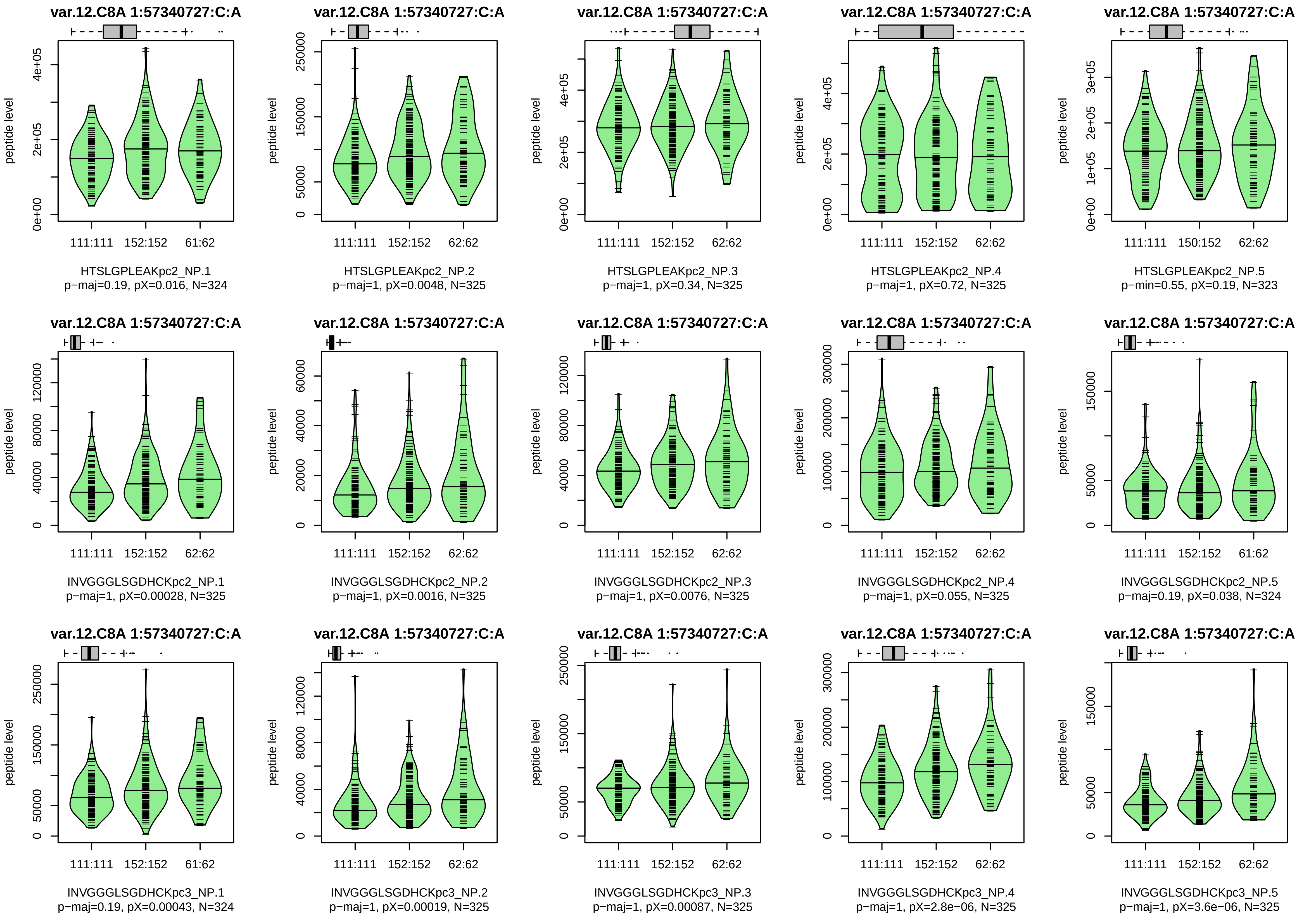
var.12.C8A 1:57340727:C:A
peptide level
0e+00
2e+05
4e+05
111:111
152:152
61:62
HTSLGPLEAKpc2_NP.1
p−maj=0.19, pX=0.016, N=324
var.12.C8A 1:57340727:C:A
peptide level
0
50000
150000
250000
111:111
152:152
62:62
HTSLGPLEAKpc2_NP.2
p−maj=1, pX=0.0048, N=325
var.12.C8A 1:57340727:C:A
peptide level
0e+00
2e+05
4e+05
111:111
152:152
62:62
HTSLGPLEAKpc2_NP.3
p−maj=1, pX=0.34, N=325
var.12.C8A 1:57340727:C:A
peptide level
0e+00
2e+05
4e+05
111:111
152:152
62:62
HTSLGPLEAKpc2_NP.4
p−maj=1, pX=0.72, N=325
var.12.C8A 1:57340727:C:A
peptide level
0e+00
1e+05
2e+05
3e+05
111:111
150:152
62:62
HTSLGPLEAKpc2_NP.5
p−min=0.55, pX=0.19, N=323
var.12.C8A 1:57340727:C:A
peptide level
0
40000
80000
120000
111:111
152:152
62:62
INVGGGLSGDHCKpc2_NP.1
p−maj=1, pX=0.00028, N=325
var.12.C8A 1:57340727:C:A
peptide level
0
20000
40000
60000
111:111
152:152
62:62
INVGGGLSGDHCKpc2_NP.2
p−maj=1, pX=0.0016, N=325
var.12.C8A 1:57340727:C:A
peptide level
0
40000
80000
120000
111:111
152:152
62:62
INVGGGLSGDHCKpc2_NP.3
p−maj=1, pX=0.0076, N=325
var.12.C8A 1:57340727:C:A
peptide level
0
100000
200000
300000
111:111
152:152
62:62
INVGGGLSGDHCKpc2_NP.4
p−maj=1, pX=0.055, N=325
var.12.C8A 1:57340727:C:A
peptide level
0
50000
150000
111:111
152:152
61:62
INVGGGLSGDHCKpc2_NP.5
p−maj=0.19, pX=0.038, N=324
var.12.C8A 1:57340727:C:A
peptide level
0
50000
150000
250000
111:111
152:152
61:62
INVGGGLSGDHCKpc3_NP.1
p−maj=0.19, pX=0.00043, N=324
var.12.C8A 1:57340727:C:A
peptide level
0
40000
80000
120000
111:111
152:152
62:62
INVGGGLSGDHCKpc3_NP.2
p−maj=1, pX=0.00019, N=325
var.12.C8A 1:57340727:C:A
peptide level
0
50000
150000
250000
111:111
152:152
62:62
INVGGGLSGDHCKpc3_NP.3
p−maj=1, pX=0.00087, N=325
var.12.C8A 1:57340727:C:A
peptide level
0
100000
200000
300000
111:111
152:152
62:62
INVGGGLSGDHCKpc3_NP.4
p−maj=1, pX=2.8e−06, N=325
var.12.C8A 1:57340727:C:A
peptide level
0
50000
150000
111:111
152:152
62:62
INVGGGLSGDHCKpc3_NP.5
p−maj=1, pX=3.6e−06, N=325

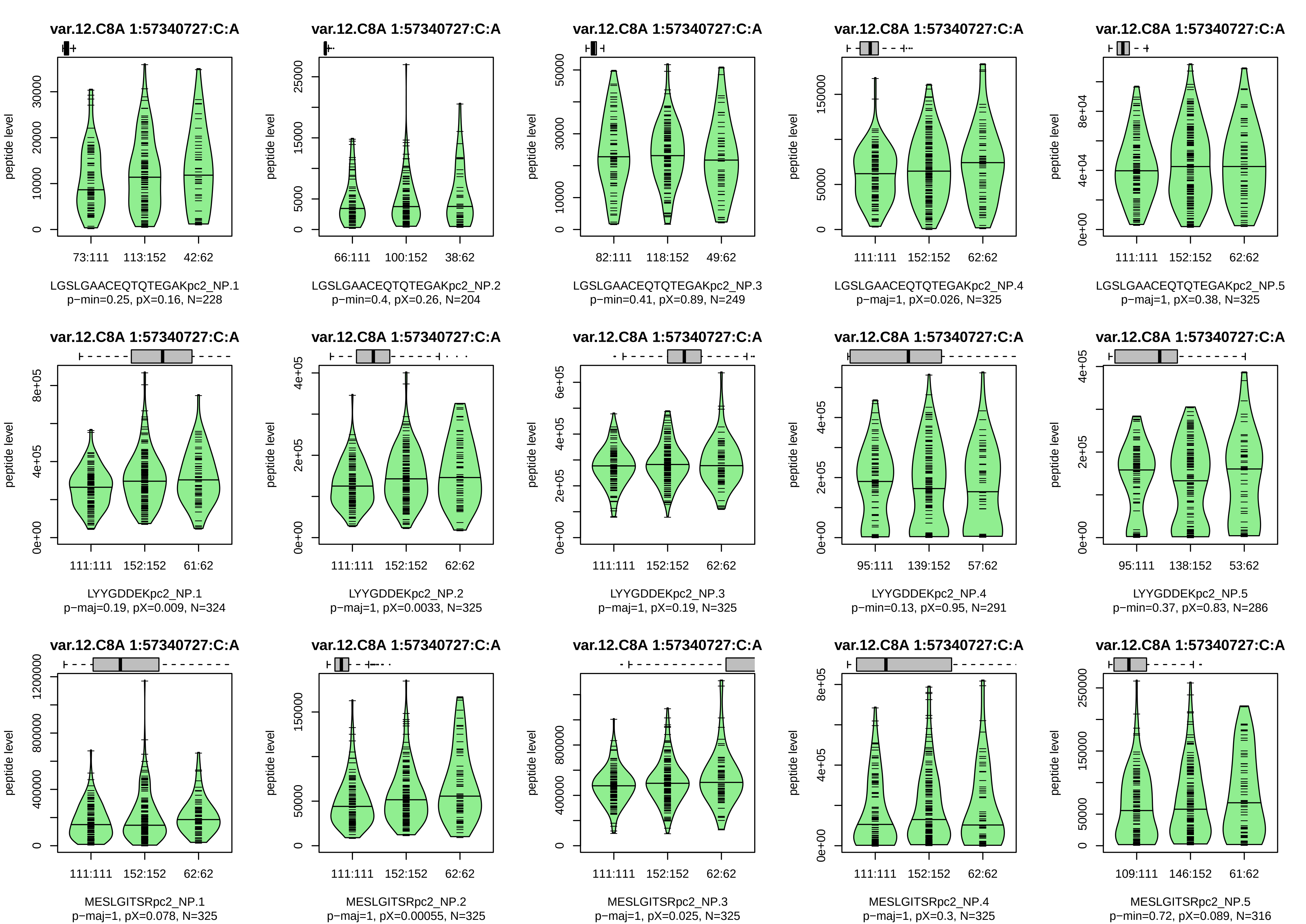
var.12.C8A 1:57340727:C:A
peptide level
73:111
113:152
42:62
LGSLGAACEQTQTEGAKpc2_NP.1
p−min=0.25, pX=0.16, N=228
var.12.C8A 1:57340727:C:A
peptide level
66:111
100:152
38:62
LGSLGAACEQTQTEGAKpc2_NP.2
p−min=0.4, pX=0.26, N=204
var.12.C8A 1:57340727:C:A
peptide level
82:111
118:152
49:62
LGSLGAACEQTQTEGAKpc2_NP.3
p−min=0.41, pX=0.89, N=249
var.12.C8A 1:57340727:C:A
peptide level
111:111
152:152
62:62
LGSLGAACEQTQTEGAKpc2_NP.4
p−maj=1, pX=0.026, N=325
var.12.C8A 1:57340727:C:A
peptide level
111:111
152:152
62:62
LGSLGAACEQTQTEGAKpc2_NP.5
p−maj=1, pX=0.38, N=325
var.12.C8A 1:57340727:C:A
peptide level
111:111
152:152
61:62
LYYGDDEKpc2_NP.1
p−maj=0.19, pX=0.009, N=324
var.12.C8A 1:57340727:C:A
peptide level
111:111
152:152
62:62
LYYGDDEKpc2_NP.2
p−maj=1, pX=0.0033, N=325
var.12.C8A 1:57340727:C:A
peptide level
111:111
152:152
62:62
LYYGDDEKpc2_NP.3
p−maj=1, pX=0.19, N=325
var.12.C8A 1:57340727:C:A
peptide level
95:111
139:152
57:62
LYYGDDEKpc2_NP.4
p−min=0.13, pX=0.95, N=291
var.12.C8A 1:57340727:C:A
peptide level
95:111
138:152
53:62
LYYGDDEKpc2_NP.5
p−min=0.37, pX=0.83, N=286
var.12.C8A 1:57340727:C:A
peptide level
111:111
152:152
62:62
MESLGITSRpc2_NP.1
p−maj=1, pX=0.078, N=325
var.12.C8A 1:57340727:C:A
peptide level
111:111
152:152
62:62
MESLGITSRpc2_NP.2
p−maj=1, pX=0.00055, N=325
var.12.C8A 1:57340727:C:A
peptide level
111:111
152:152
62:62
MESLGITSRpc2_NP.3
p−maj=1, pX=0.025, N=325
var.12.C8A 1:57340727:C:A
peptide level
111:111
152:152
62:62
MESLGITSRpc2_NP.4
p−maj=1, pX=0.3, N=325
var.12.C8A 1:57340727:C:A
peptide level
109:111
146:152
61:62
MESLGITSRpc2_NP.5
p−min=0.72, pX=0.089, N=316

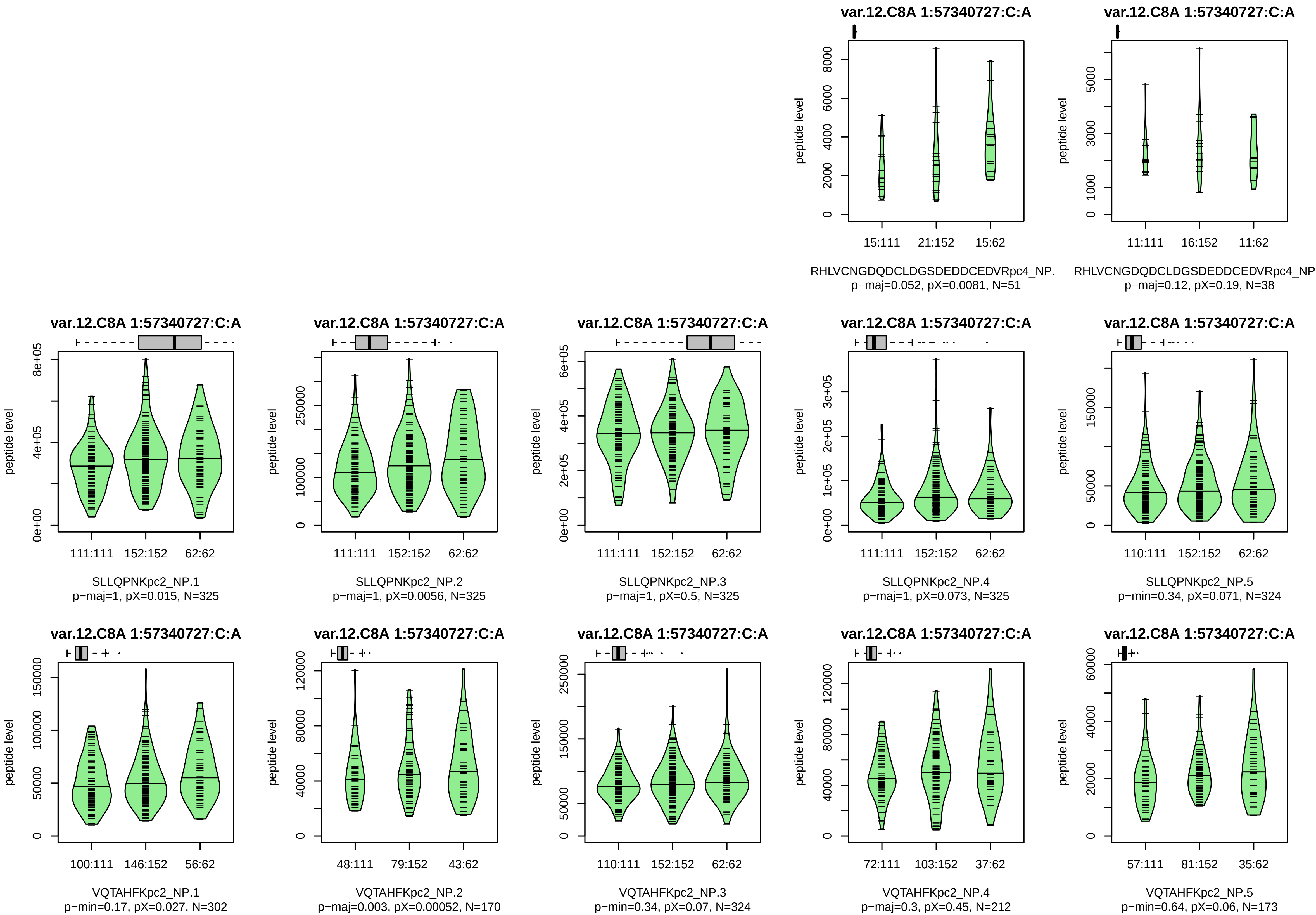
var.12.C8A 1:57340727:C:A
peptide level
15:111
21:152
15:62
RHLVCNGDQDCLDGSDEDDCEDVRpc4_NP.
p−maj=0.052, pX=0.0081, N=51
var.12.C8A 1:57340727:C:A
peptide level
11:111
16:152
11:62
RHLVCNGDQDCLDGSDEDDCEDVRpc4_NP
p−maj=0.12, pX=0.19, N=38
var.12.C8A 1:57340727:C:A
peptide level
111:111
152:152
62:62
SLLQPNKpc2_NP.1
p−maj=1, pX=0.015, N=325
var.12.C8A 1:57340727:C:A
peptide level
111:111
152:152
62:62
SLLQPNKpc2_NP.2
p−maj=1, pX=0.0056, N=325
var.12.C8A 1:57340727:C:A
peptide level
111:111
152:152
62:62
SLLQPNKpc2_NP.3
p−maj=1, pX=0.5, N=325
var.12.C8A 1:57340727:C:A
peptide level
111:111
152:152
62:62
SLLQPNKpc2_NP.4
p−maj=1, pX=0.073, N=325
var.12.C8A 1:57340727:C:A
peptide level
110:111
152:152
62:62
SLLQPNKpc2_NP.5
p−min=0.34, pX=0.071, N=324
var.12.C8A 1:57340727:C:A
peptide level
100:111
146:152
56:62
VQTAHFKpc2_NP.1
p−min=0.17, pX=0.027, N=302
var.12.C8A 1:57340727:C:A
peptide level
48:111
79:152
43:62
VQTAHFKpc2_NP.2
p−maj=0.003, pX=0.00052, N=170
var.12.C8A 1:57340727:C:A
peptide level
110:111
152:152
62:62
VQTAHFKpc2_NP.3
p−min=0.34, pX=0.07, N=324
var.12.C8A 1:57340727:C:A
peptide level
72:111
103:152
37:62
VQTAHFKpc2_NP.4
p−maj=0.3, pX=0.45, N=212
var.12.C8A 1:57340727:C:A
peptide level
57:111
81:152
35:62
VQTAHFKpc2_NP.5
p−min=0.64, pX=0.06, N=173

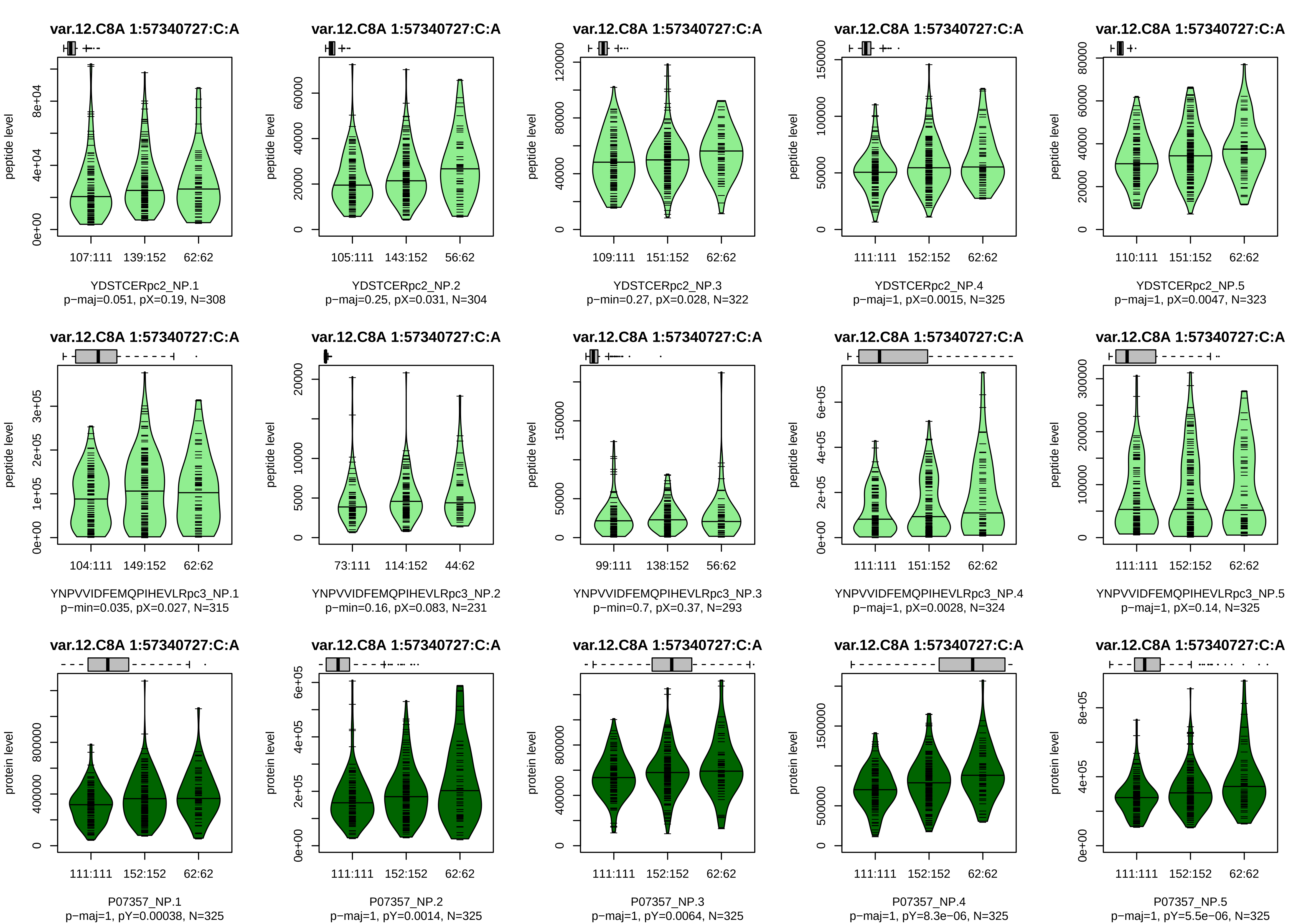
var.12.C8A 1:57340727:C:A
peptide level
107:111
139:152
62:62
YDSTCERpc2_NP.1
p−maj=0.051, pX=0.19, N=308
105:111
143:152
56:62
YDSTCERpc2_NP.2
p−maj=0.25, pX=0.031, N=304
109:111
151:152
62:62
YDSTCERpc2_NP.3
p−min=0.27, pX=0.028, N=322
111:111
152:152
62:62
YDSTCERpc2_NP.4
p−maj=1, pX=0.0015, N=325
110:111
151:152
62:62
YDSTCERpc2_NP.5
p−maj=1, pX=0.0047, N=323
104:111
149:152
62:62
YNPVVIDFEMQPIHEVLRpc3_NP.1
p−min=0.035, pX=0.027, N=315
73:111
114:152
44:62
YNPVVIDFEMQPIHEVLRpc3_NP.2
p−min=0.16, pX=0.083, N=231
99:111
138:152
56:62
YNPVVIDFEMQPIHEVLRpc3_NP.3
p−min=0.7, pX=0.37, N=293
111:111
151:152
62:62
YNPVVIDFEMQPIHEVLRpc3_NP.4
p−maj=1, pX=0.0028, N=324
111:111
152:152
62:62
YNPVVIDFEMQPIHEVLRpc3_NP.5
p−maj=1, pX=0.14, N=325
protein level
111:111
152:152
62:62
P07357_NP.1
p−maj=1, pY=0.00038, N=325
P07357_NP.2
p−maj=1, pY=0.0014, N=325
P07357_NP.3
p−maj=1, pY=0.0064, N=325
P07357_NP.4
p−maj=1, pY=8.3e−06, N=325
P07357_NP.5
p−maj=1, pY=5.5e−06, N=325

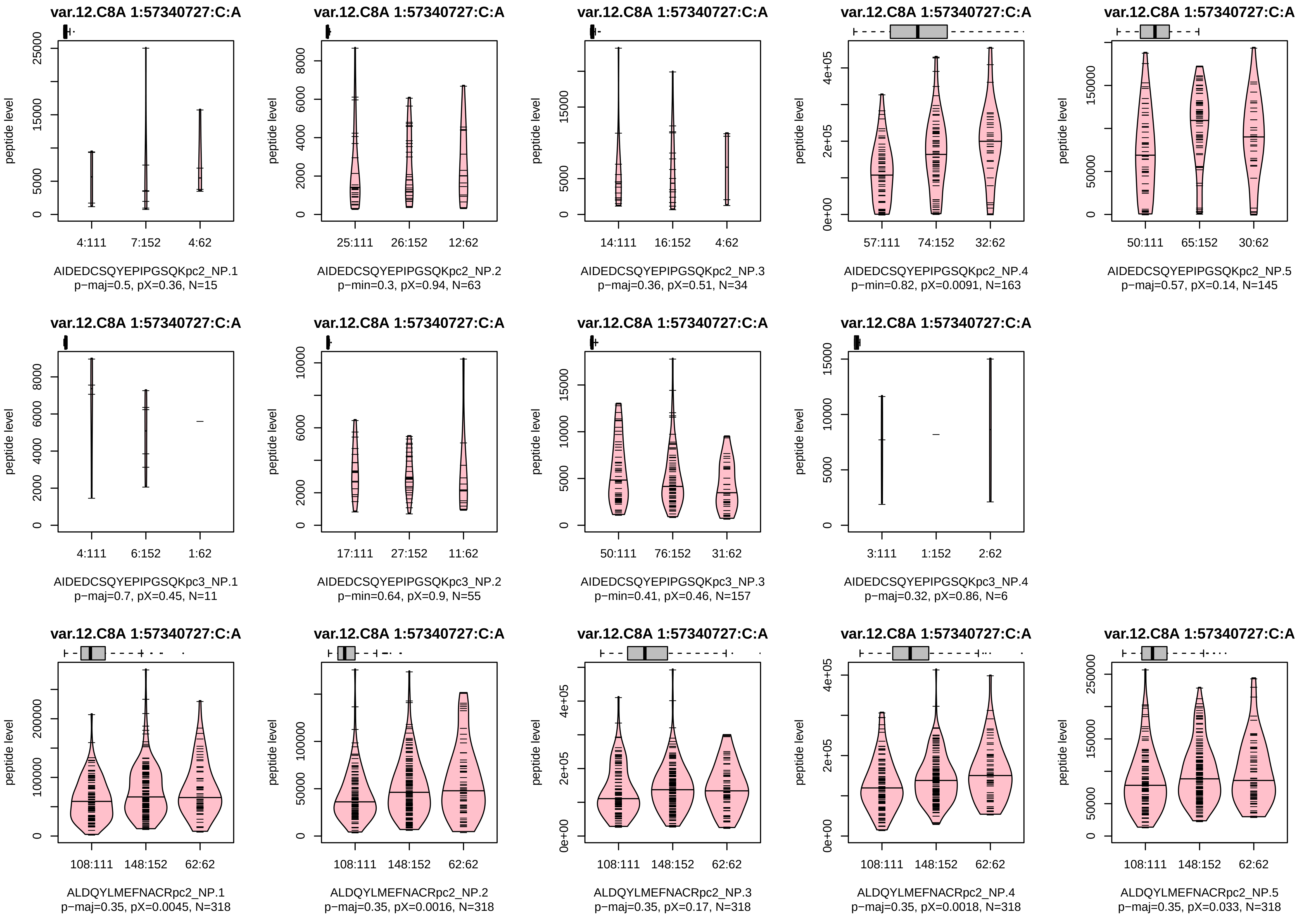
var.12.C8A 1:57340727:C:A
peptide level
4:111 7:152 4:62
AIDEDCSQYEPIPGSQKpc2_NP.1
p−maj=0.5, pX=0.36, N=15
var.12.C8A 1:57340727:C:A
peptide level
25:111 26:152 12:62
AIDEDCSQYEPIPGSQKpc2_NP.2
p−min=0.3, pX=0.94, N=63
var.12.C8A 1:57340727:C:A
peptide level
14:111 16:152 4:62
AIDEDCSQYEPIPGSQKpc2_NP.3
p−maj=0.36, pX=0.51, N=34
var.12.C8A 1:57340727:C:A
peptide level
57:111 74:152 32:62
AIDEDCSQYEPIPGSQKpc2_NP.4
p−min=0.82, pX=0.0091, N=163
var.12.C8A 1:57340727:C:A
peptide level
50:111 65:152 30:62
AIDEDCSQYEPIPGSQKpc2_NP.5
p−maj=0.57, pX=0.14, N=145
var.12.C8A 1:57340727:C:A
peptide level
4:111 6:152 1:62
AIDEDCSQYEPIPGSQKpc3_NP.1
p−maj=0.7, pX=0.45, N=11
var.12.C8A 1:57340727:C:A
peptide level
17:111 27:152 11:62
AIDEDCSQYEPIPGSQKpc3_NP.2
p−min=0.64, pX=0.9, N=55
var.12.C8A 1:57340727:C:A
peptide level
50:111 76:152 31:62
AIDEDCSQYEPIPGSQKpc3_NP.3
p−min=0.41, pX=0.46, N=157
var.12.C8A 1:57340727:C:A
peptide level
3:111 1:152 2:62
AIDEDCSQYEPIPGSQKpc3_NP.4
p−maj=0.32, pX=0.86, N=6
var.12.C8A 1:57340727:C:A
peptide level
108:111 148:152 62:62
ALDQYLMEFNACRpc2_NP.1
p−maj=0.35, pX=0.0045, N=318
var.12.C8A 1:57340727:C:A
peptide level
108:111 148:152 62:62
ALDQYLMEFNACRpc2_NP.2
p−maj=0.35, pX=0.0016, N=318
var.12.C8A 1:57340727:C:A
peptide level
108:111 148:152 62:62
ALDQYLMEFNACRpc2_NP.3
p−maj=0.35, pX=0.17, N=318
var.12.C8A 1:57340727:C:A
peptide level
108:111 148:152 62:62
ALDQYLMEFNACRpc2_NP.4
p−maj=0.35, pX=0.0018, N=318
var.12.C8A 1:57340727:C:A
peptide level
108:111 148:152 62:62
ALDQYLMEFNACRpc2_NP.5
p−maj=0.35, pX=0.033, N=318

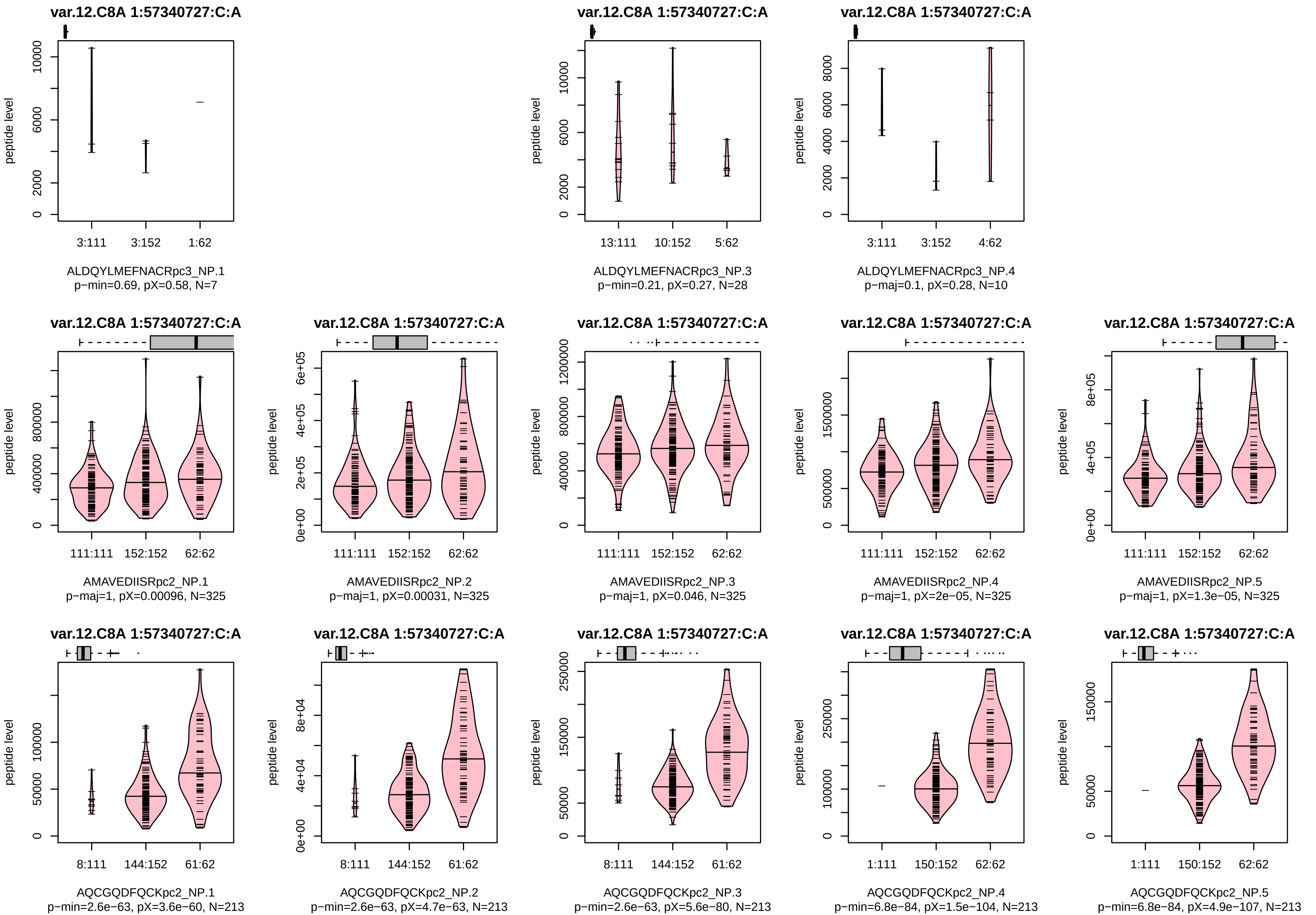
var.12.C8A 1:57340727:C:A
peptide level
3:111
3:152
1:62
ALDQYLMEFNACRpc3_NP.1
p−min=0.69, pX=0.58, N=7
var.12.C8A 1:57340727:C:A
peptide level
13:111
10:152
5:62
ALDQYLMEFNACRpc3_NP.3
p−min=0.21, pX=0.27, N=28
var.12.C8A 1:57340727:C:A
peptide level
3:111
3:152
4:62
ALDQYLMEFNACRpc3_NP.4
p−maj=0.1, pX=0.28, N=10
var.12.C8A 1:57340727:C:A
peptide level
111:111
152:152
62:62
AMAVEDIISRpc2_NP.1
p−maj=1, pX=0.00096, N=325
var.12.C8A 1:57340727:C:A
peptide level
111:111
152:152
62:62
AMAVEDIISRpc2_NP.2
p−maj=1, pX=0.00031, N=325
var.12.C8A 1:57340727:C:A
peptide level
111:111
152:152
62:62
AMAVEDIISRpc2_NP.3
p−maj=1, pX=0.046, N=325
var.12.C8A 1:57340727:C:A
peptide level
111:111
152:152
62:62
AMAVEDIISRpc2_NP.4
p−maj=1, pX=2e−05, N=325
var.12.C8A 1:57340727:C:A
peptide level
111:111
152:152
62:62
AMAVEDIISRpc2_NP.5
p−maj=1, pX=1.3e−05, N=325
var.12.C8A 1:57340727:C:A
peptide level
8:111
144:152
61:62
AQCGQDFQCKpc2_NP.1
p−min=2.6e−63, pX=3.6e−60, N=213
var.12.C8A 1:57340727:C:A
peptide level
8:111
144:152
61:62
AQCGQDFQCKpc2_NP.2
p−min=2.6e−63, pX=4.7e−63, N=213
var.12.C8A 1:57340727:C:A
peptide level
8:111
144:152
61:62
AQCGQDFQCKpc2_NP.3
p−min=2.6e−63, pX=5.6e−80, N=213
var.12.C8A 1:57340727:C:A
peptide level
1:111
150:152
62:62
AQCGQDFQCKpc2_NP.4
p−min=6.8e−84, pX=1.5e−104, N=213
var.12.C8A 1:57340727:C:A
peptide level
1:111
150:152
62:62
AQCGQDFQCKpc2_NP.5
p−min=6.8e−84, pX=4.9e−107, N=213

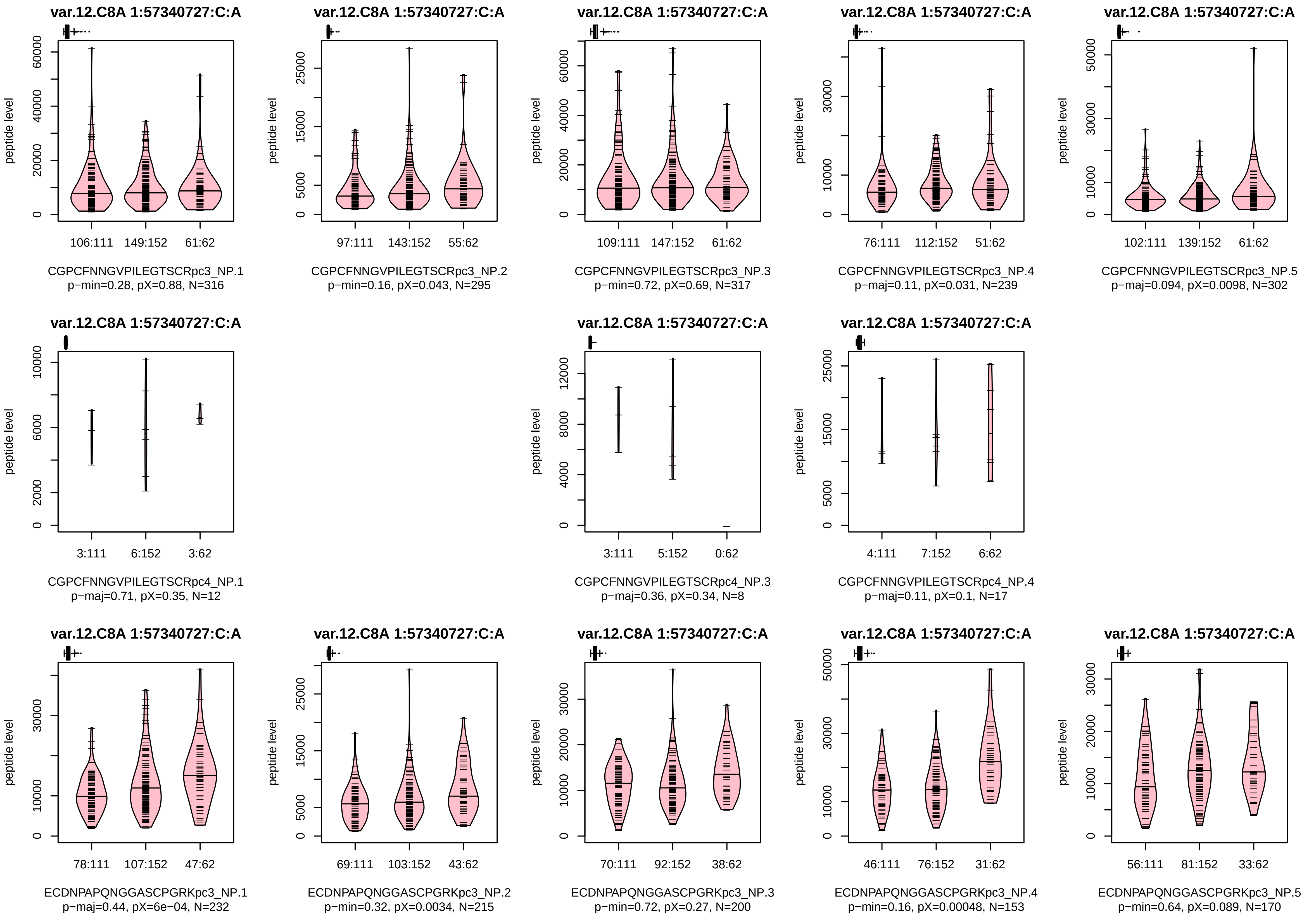
var.12.C8A 1:57340727:C:A
peptide level
106:111
149:152
61:62
CGPCFNNGVPILEGTSCRpc3_NP.1
p−min=0.28, pX=0.88, N=316
var.12.C8A 1:57340727:C:A
peptide level
97:111
143:152
55:62
CGPCFNNGVPILEGTSCRpc3_NP.2
p−min=0.16, pX=0.043, N=295
var.12.C8A 1:57340727:C:A
peptide level
109:111
147:152
61:62
CGPCFNNGVPILEGTSCRpc3_NP.3
p−min=0.72, pX=0.69, N=317
var.12.C8A 1:57340727:C:A
peptide level
76:111
112:152
51:62
CGPCFNNGVPILEGTSCRpc3_NP.4
p−maj=0.11, pX=0.031, N=239
var.12.C8A 1:57340727:C:A
peptide level
102:111
139:152
61:62
CGPCFNNGVPILEGTSCRpc3_NP.5
p−maj=0.094, pX=0.0098, N=302
var.12.C8A 1:57340727:C:A
peptide level
3:111
6:152
3:62
CGPCFNNGVPILEGTSCRpc4_NP.1
p−maj=0.71, pX=0.35, N=12
var.12.C8A 1:57340727:C:A
peptide level
3:111
5:152
0:62
CGPCFNNGVPILEGTSCRpc4_NP.3
p−maj=0.36, pX=0.34, N=8
var.12.C8A 1:57340727:C:A
peptide level
4:111
7:152
6:62
CGPCFNNGVPILEGTSCRpc4_NP.4
p−maj=0.11, pX=0.1, N=17
var.12.C8A 1:57340727:C:A
peptide level
78:111
107:152
47:62
ECDNPAPQNGGASCPGRKpc3_NP.1
p−maj=0.44, pX=6e−04, N=232
var.12.C8A 1:57340727:C:A
peptide level
69:111
103:152
43:62
ECDNPAPQNGGASCPGRKpc3_NP.2
p−min=0.32, pX=0.0034, N=215
var.12.C8A 1:57340727:C:A
peptide level
70:111
92:152
38:62
ECDNPAPQNGGASCPGRKpc3_NP.3
p−min=0.72, pX=0.27, N=200
var.12.C8A 1:57340727:C:A
peptide level
46:111
76:152
31:62
ECDNPAPQNGGASCPGRKpc3_NP.4
p−min=0.16, pX=0.00048, N=153
var.12.C8A 1:57340727:C:A
peptide level
56:111
81:152
33:62
ECDNPAPQNGGASCPGRKpc3_NP.5
p−min=0.64, pX=0.089, N=170

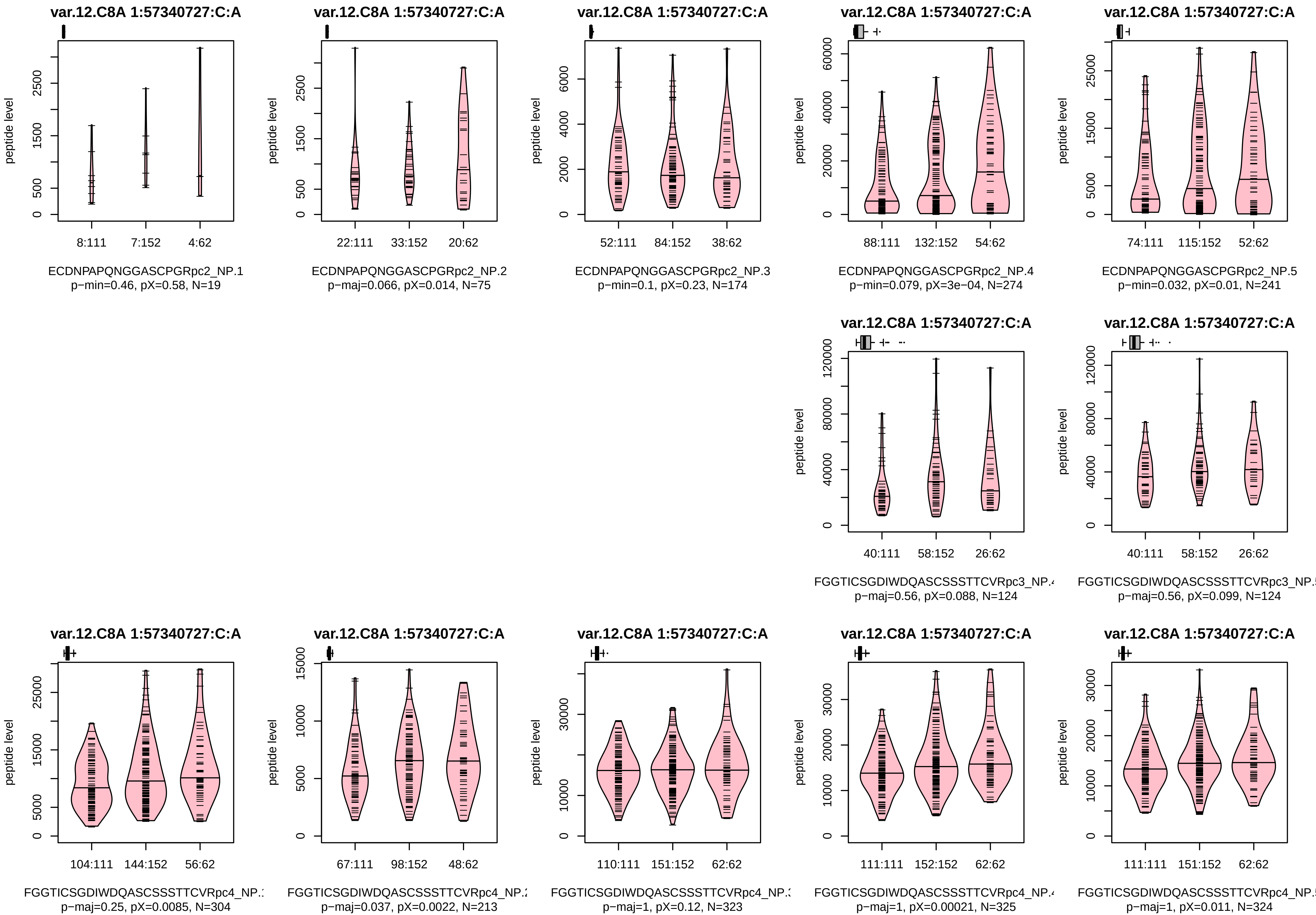
var.12.C8A 1:57340727:C:A
peptide level
8:111
7:152
4:62
ECDNPAPQNGGASCPGRpc2_NP.1
p−min=0.46, pX=0.58, N=19
var.12.C8A 1:57340727:C:A
peptide level
22:111
33:152
20:62
ECDNPAPQNGGASCPGRpc2_NP.2
p−maj=0.066, pX=0.014, N=75
var.12.C8A 1:57340727:C:A
peptide level
52:111
84:152
38:62
ECDNPAPQNGGASCPGRpc2_NP.3
p−min=0.1, pX=0.23, N=174
var.12.C8A 1:57340727:C:A
peptide level
88:111
132:152
54:62
ECDNPAPQNGGASCPGRpc2_NP.4
p−min=0.079, pX=3e−04, N=274
var.12.C8A 1:57340727:C:A
peptide level
74:111
115:152
52:62
ECDNPAPQNGGASCPGRpc2_NP.5
p−min=0.032, pX=0.01, N=241
var.12.C8A 1:57340727:C:A
peptide level
40:111
58:152
26:62
FGGTICSGDIWDQASCSSSTTCVRpc3_NP.
p−maj=0.56, pX=0.088, N=124
var.12.C8A 1:57340727:C:A
peptide level
40:111
58:152
26:62
FGGTICSGDIWDQASCSSSTTCVRpc3_NP.
p−maj=0.56, pX=0.099, N=124
var.12.C8A 1:57340727:C:A
peptide level
104:111
144:152
56:62
FGGTICSGDIWDQASCSSSTTCVRpc4_NP.
p−maj=0.25, pX=0.0085, N=304
var.12.C8A 1:57340727:C:A
peptide level
67:111
98:152
48:62
FGGTICSGDIWDQASCSSSTTCVRpc4_NP.
p−maj=0.037, pX=0.0022, N=213
var.12.C8A 1:57340727:C:A
peptide level
110:111
151:152
62:62
FGGTICSGDIWDQASCSSSTTCVRpc4_NP.
p−maj=1, pX=0.12, N=323
var.12.C8A 1:57340727:C:A
peptide level
111:111
152:152
62:62
FGGTICSGDIWDQASCSSSTTCVRpc4_NP.
p−maj=1, pX=0.00021, N=325
var.12.C8A 1:57340727:C:A
peptide level
111:111
151:152
62:62
FGGTICSGDIWDQASCSSSTTCVRpc4_NP.
p−maj=1, pX=0.011, N=324

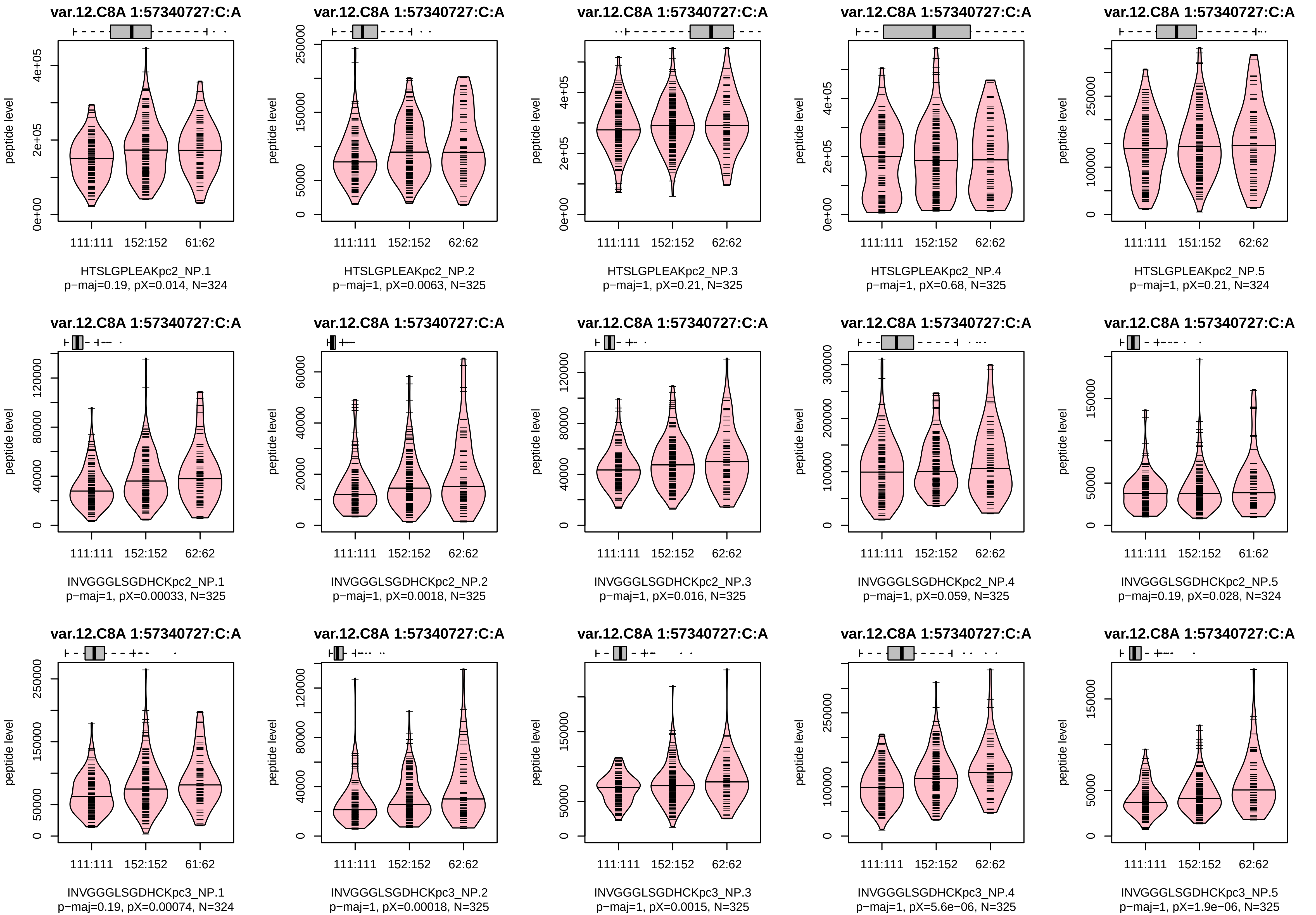

var.12.C8A 1:57340727:C:A
peptide level
111:111 152:152 61:62
HTSLGPLEAKpc2_NP.1
p−maj=0.19, pX=0.014, N=324
var.12.C8A 1:57340727:C:A
peptide level
111:111 152:152 62:62
HTSLGPLEAKpc2_NP.2
p−maj=1, pX=0.0063, N=325
var.12.C8A 1:57340727:C:A
peptide level
111:111 152:152 62:62
HTSLGPLEAKpc2_NP.3
p−maj=1, pX=0.21, N=325
var.12.C8A 1:57340727:C:A
peptide level
111:111 152:152 62:62
HTSLGPLEAKpc2_NP.4
p−maj=1, pX=0.68, N=325
var.12.C8A 1:57340727:C:A
peptide level
111:111 151:152 62:62
HTSLGPLEAKpc2_NP.5
p−maj=1, pX=0.21, N=324
var.12.C8A 1:57340727:C:A
peptide level
111:111 152:152 62:62
INVGGGLSGDHCKpc2_NP.1
p−maj=1, pX=0.00033, N=325
var.12.C8A 1:57340727:C:A
peptide level
111:111 152:152 62:62
INVGGGLSGDHCKpc2_NP.2
p−maj=1, pX=0.0018, N=325
var.12.C8A 1:57340727:C:A
peptide level
111:111 152:152 62:62
INVGGGLSGDHCKpc2_NP.3
p−maj=1, pX=0.016, N=325
var.12.C8A 1:57340727:C:A
peptide level
111:111 152:152 62:62
INVGGGLSGDHCKpc2_NP.4
p−maj=1, pX=0.059, N=325
var.12.C8A 1:57340727:C:A
peptide level
111:111 152:152 61:62
INVGGGLSGDHCKpc2_NP.5
p−maj=0.19, pX=0.028, N=324
var.12.C8A 1:57340727:C:A
peptide level
111:111 152:152 61:62
INVGGGLSGDHCKpc3_NP.1
p−maj=0.19, pX=0.00074, N=324
var.12.C8A 1:57340727:C:A
peptide level
111:111 152:152 62:62
INVGGGLSGDHCKpc3_NP.2
p−maj=1, pX=0.00018, N=325
var.12.C8A 1:57340727:C:A
peptide level
111:111 152:152 62:62
INVGGGLSGDHCKpc3_NP.3
p−maj=1, pX=0.0015, N=325
var.12.C8A 1:57340727:C:A
peptide level
111:111 152:152 62:62
INVGGGLSGDHCKpc3_NP.4
p−maj=1, pX=5.6e−06, N=325
var.12.C8A 1:57340727:C:A
peptide level
111:111 152:152 62:62
INVGGGLSGDHCKpc3_NP.5
p−maj=1, pX=1.9e−06, N=325

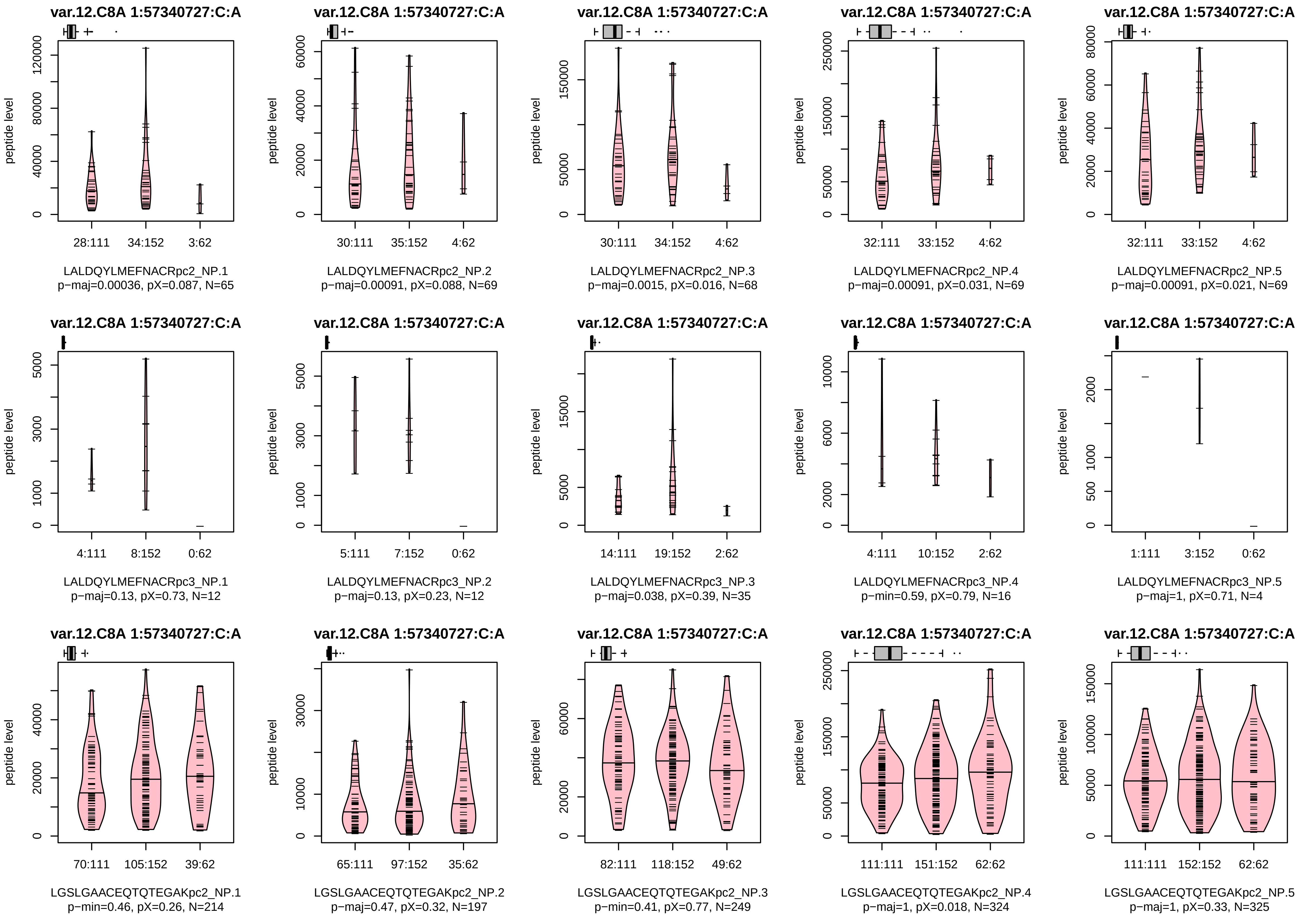

var.12.C8A 1:57340727:C:A
peptide level
28:111
34:152
3:62
LALDQYLMEFNACRpc2_NP.1
p−maj=0.00036, pX=0.087, N=65
var.12.C8A 1:57340727:C:A
peptide level
30:111
35:152
4:62
LALDQYLMEFNACRpc2_NP.2
p−maj=0.00091, pX=0.088, N=69
var.12.C8A 1:57340727:C:A
peptide level
30:111
34:152
4:62
LALDQYLMEFNACRpc2_NP.3
p−maj=0.0015, pX=0.016, N=68
var.12.C8A 1:57340727:C:A
peptide level
32:111
33:152
4:62
LALDQYLMEFNACRpc2_NP.4
p−maj=0.00091, pX=0.031, N=69
var.12.C8A 1:57340727:C:A
peptide level
32:111
33:152
4:62
LALDQYLMEFNACRpc2_NP.5
p−maj=0.00091, pX=0.021, N=69
var.12.C8A 1:57340727:C:A
peptide level
4:111
8:152
0:62
LALDQYLMEFNACRpc3_NP.1
p−maj=0.13, pX=0.73, N=12
var.12.C8A 1:57340727:C:A
peptide level
5:111
7:152
0:62
LALDQYLMEFNACRpc3_NP.2
p−maj=0.13, pX=0.23, N=12
var.12.C8A 1:57340727:C:A
peptide level
14:111
19:152
2:62
LALDQYLMEFNACRpc3_NP.3
p−maj=0.038, pX=0.39, N=35
var.12.C8A 1:57340727:C:A
peptide level
4:111
10:152
2:62
LALDQYLMEFNACRpc3_NP.4
p−min=0.59, pX=0.79, N=16
var.12.C8A 1:57340727:C:A
peptide level
1:111
3:152
0:62
LALDQYLMEFNACRpc3_NP.5
p−maj=1, pX=0.71, N=4
var.12.C8A 1:57340727:C:A
peptide level
70:111
105:152
39:62
LGSLGAACEQTQTEGAKpc2_NP.1
p−min=0.46, pX=0.26, N=214
var.12.C8A 1:57340727:C:A
peptide level
65:111
97:152
35:62
LGSLGAACEQTQTEGAKpc2_NP.2
p−maj=0.47, pX=0.32, N=197
var.12.C8A 1:57340727:C:A
peptide level
82:111
118:152
49:62
LGSLGAACEQTQTEGAKpc2_NP.3
p−min=0.41, pX=0.77, N=249
var.12.C8A 1:57340727:C:A
peptide level
111:111
151:152
62:62
LGSLGAACEQTQTEGAKpc2_NP.4
p−maj=1, pX=0.018, N=324
var.12.C8A 1:57340727:C:A
peptide level
111:111
152:152
62:62
LGSLGAACEQTQTEGAKpc2_NP.5
p−maj=1, pX=0.33, N=325

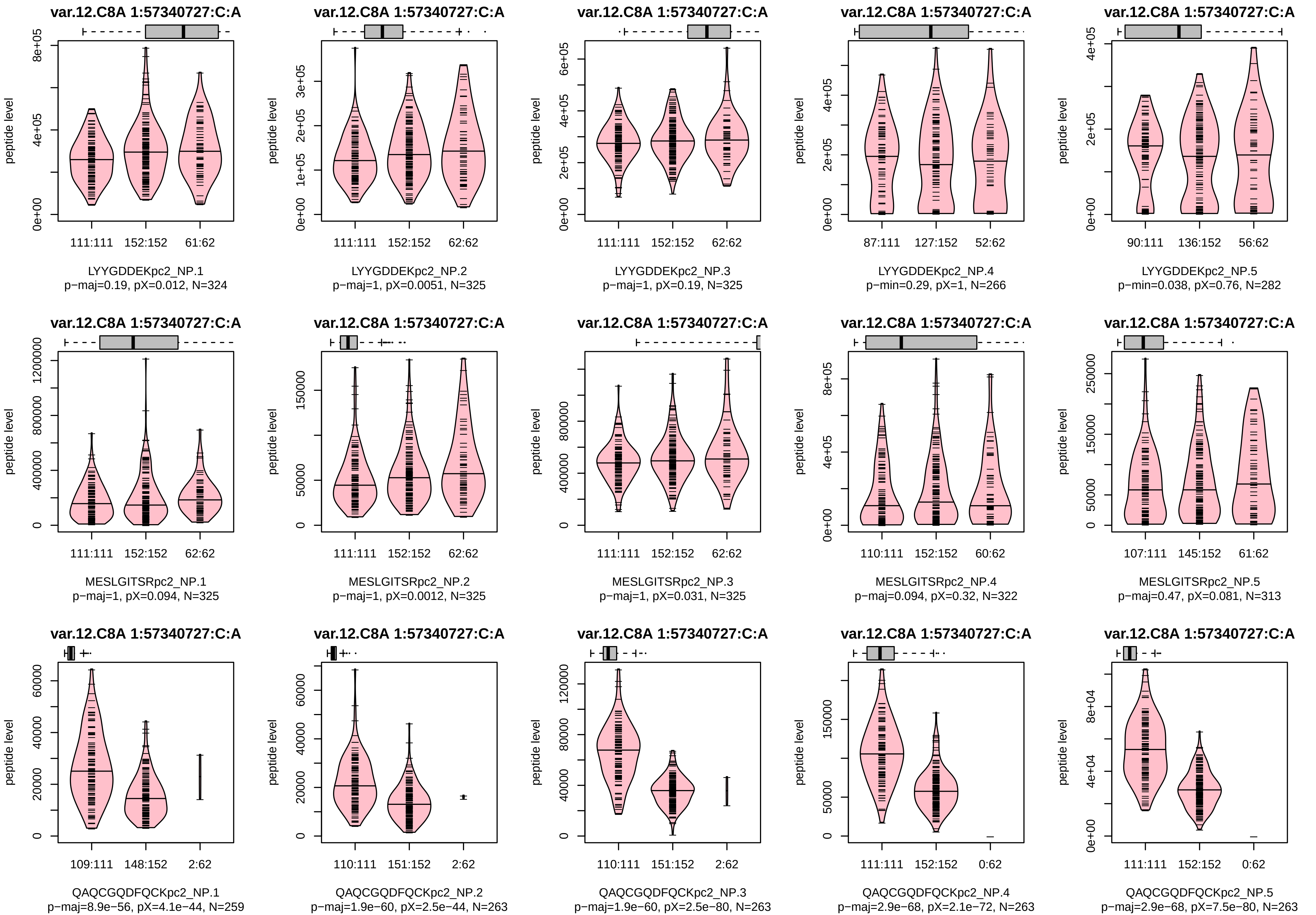
var.12.C8A 1:57340727:C:A
peptide level
111:111 152:152 61:62
LYYGDDEKpc2_NP.1
p−maj=0.19, pX=0.012, N=324
var.12.C8A 1:57340727:C:A
111:111 152:152 62:62
LYYGDDEKpc2_NP.2
p−maj=1, pX=0.0051, N=325
var.12.C8A 1:57340727:C:A
111:111 152:152 62:62
LYYGDDEKpc2_NP.3
p−maj=1, pX=0.19, N=325
var.12.C8A 1:57340727:C:A
87:111 127:152 52:62
LYYGDDEKpc2_NP.4
p−min=0.29, pX=1, N=266
var.12.C8A 1:57340727:C:A
90:111 136:152 56:62
LYYGDDEKpc2_NP.5
p−min=0.038, pX=0.76, N=282
var.12.C8A 1:57340727:C:A
111:111 152:152 62:62
MESLGITSRpc2_NP.1
p−maj=1, pX=0.094, N=325
var.12.C8A 1:57340727:C:A
111:111 152:152 62:62
MESLGITSRpc2_NP.2
p−maj=1, pX=0.0012, N=325
var.12.C8A 1:57340727:C:A
111:111 152:152 62:62
MESLGITSRpc2_NP.3
p−maj=1, pX=0.031, N=325
var.12.C8A 1:57340727:C:A
110:111 152:152 60:62
MESLGITSRpc2_NP.4
p−maj=0.094, pX=0.32, N=322
var.12.C8A 1:57340727:C:A
107:111 145:152 61:62
MESLGITSRpc2_NP.5
p−maj=0.47, pX=0.081, N=313
var.12.C8A 1:57340727:C:A
109:111 148:152 2:62
QAQCGQDFQCKpc2_NP.1
p−maj=8.9e−56, pX=4.1e−44, N=259
var.12.C8A 1:57340727:C:A
110:111 151:152 2:62
QAQCGQDFQCKpc2_NP.2
p−maj=1.9e−60, pX=2.5e−44, N=263
var.12.C8A 1:57340727:C:A
110:111 151:152 2:62
QAQCGQDFQCKpc2_NP.3
p−maj=1.9e−60, pX=2.5e−80, N=263
var.12.C8A 1:57340727:C:A
111:111 152:152 0:62
QAQCGQDFQCKpc2_NP.4
p−maj=2.9e−68, pX=2.1e−72, N=263
var.12.C8A 1:57340727:C:A
111:111 152:152 0:62
QAQCGQDFQCKpc2_NP.5
p−maj=2.9e−68, pX=7.5e−80, N=263

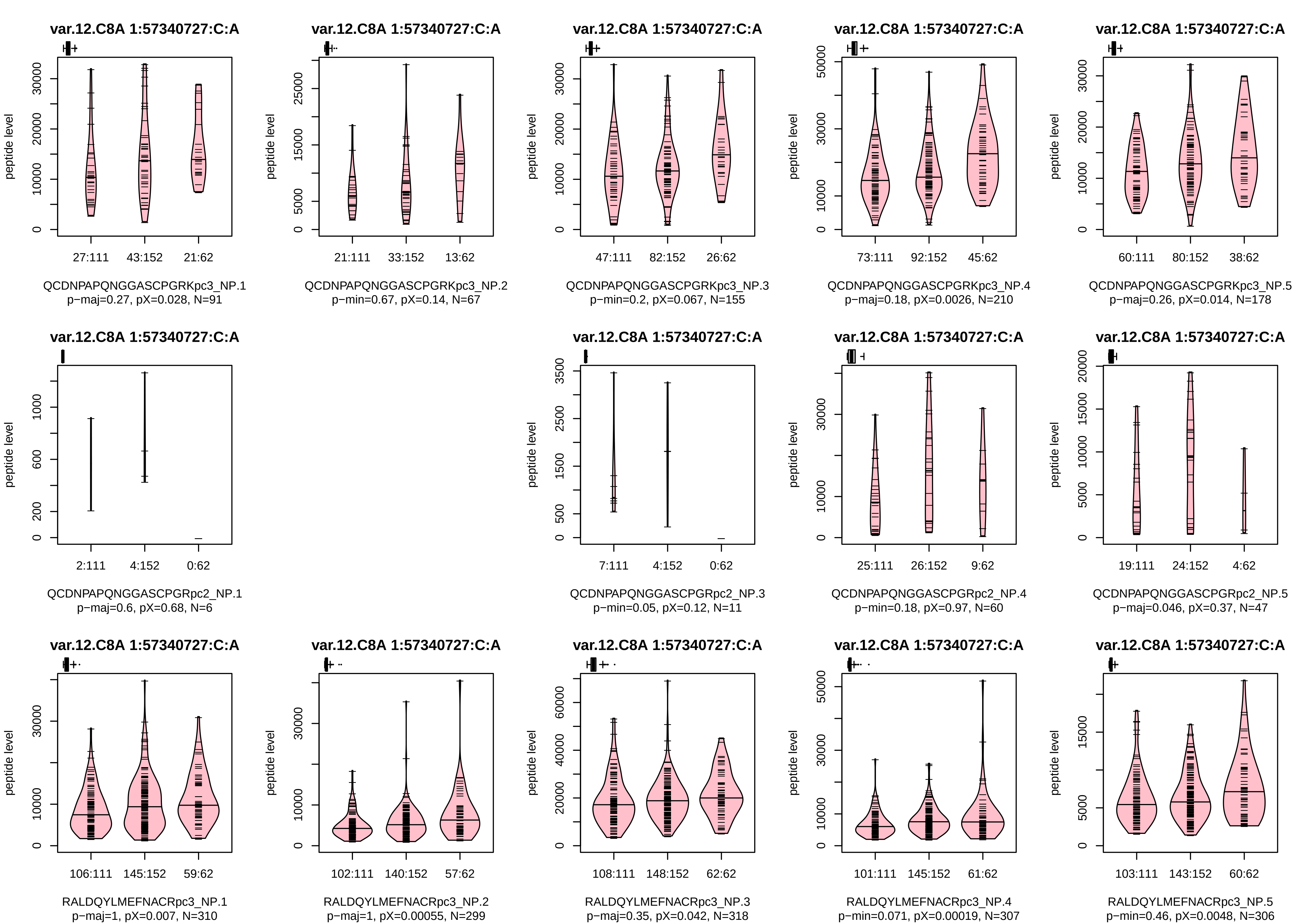
var.12.C8A 1:57340727:C:A
peptide level
27:111
43:152
21:62
QCDNPAPQNGGASCPGRKpc3_NP.1
p−maj=0.27, pX=0.028, N=91
var.12.C8A 1:57340727:C:A
peptide level
21:111
33:152
13:62
QCDNPAPQNGGASCPGRKpc3_NP.2
p−min=0.67, pX=0.14, N=67
var.12.C8A 1:57340727:C:A
peptide level
47:111
82:152
26:62
QCDNPAPQNGGASCPGRKpc3_NP.3
p−min=0.2, pX=0.067, N=155
var.12.C8A 1:57340727:C:A
peptide level
73:111
92:152
45:62
QCDNPAPQNGGASCPGRKpc3_NP.4
p−maj=0.18, pX=0.0026, N=210
var.12.C8A 1:57340727:C:A
peptide level
60:111
80:152
38:62
QCDNPAPQNGGASCPGRKpc3_NP.5
p−maj=0.26, pX=0.014, N=178
var.12.C8A 1:57340727:C:A
peptide level
2:111
4:152
0:62
QCDNPAPQNGGASCPGRpc2_NP.1
p−maj=0.6, pX=0.68, N=6
var.12.C8A 1:57340727:C:A
peptide level
7:111
4:152
0:62
QCDNPAPQNGGASCPGRpc2_NP.3
p−min=0.05, pX=0.12, N=11
var.12.C8A 1:57340727:C:A
peptide level
25:111
26:152
9:62
QCDNPAPQNGGASCPGRpc2_NP.4
p−min=0.18, pX=0.97, N=60
var.12.C8A 1:57340727:C:A
peptide level
19:111
24:152
4:62
QCDNPAPQNGGASCPGRpc2_NP.5
p−maj=0.046, pX=0.37, N=47
var.12.C8A 1:57340727:C:A
peptide level
106:111
145:152
59:62
RALDQYLMEFNACRpc3_NP.1
p−maj=1, pX=0.007, N=310
var.12.C8A 1:57340727:C:A
peptide level
102:111
140:152
57:62
RALDQYLMEFNACRpc3_NP.2
p−maj=1, pX=0.00055, N=299
var.12.C8A 1:57340727:C:A
peptide level
108:111
148:152
62:62
RALDQYLMEFNACRpc3_NP.3
p−maj=0.35, pX=0.042, N=318
var.12.C8A 1:57340727:C:A
peptide level
101:111
145:152
61:62
RALDQYLMEFNACRpc3_NP.4
p−min=0.071, pX=0.00019, N=307
var.12.C8A 1:57340727:C:A
peptide level
103:111
143:152
60:62
RALDQYLMEFNACRpc3_NP.5
p−min=0.46, pX=0.0048, N=306

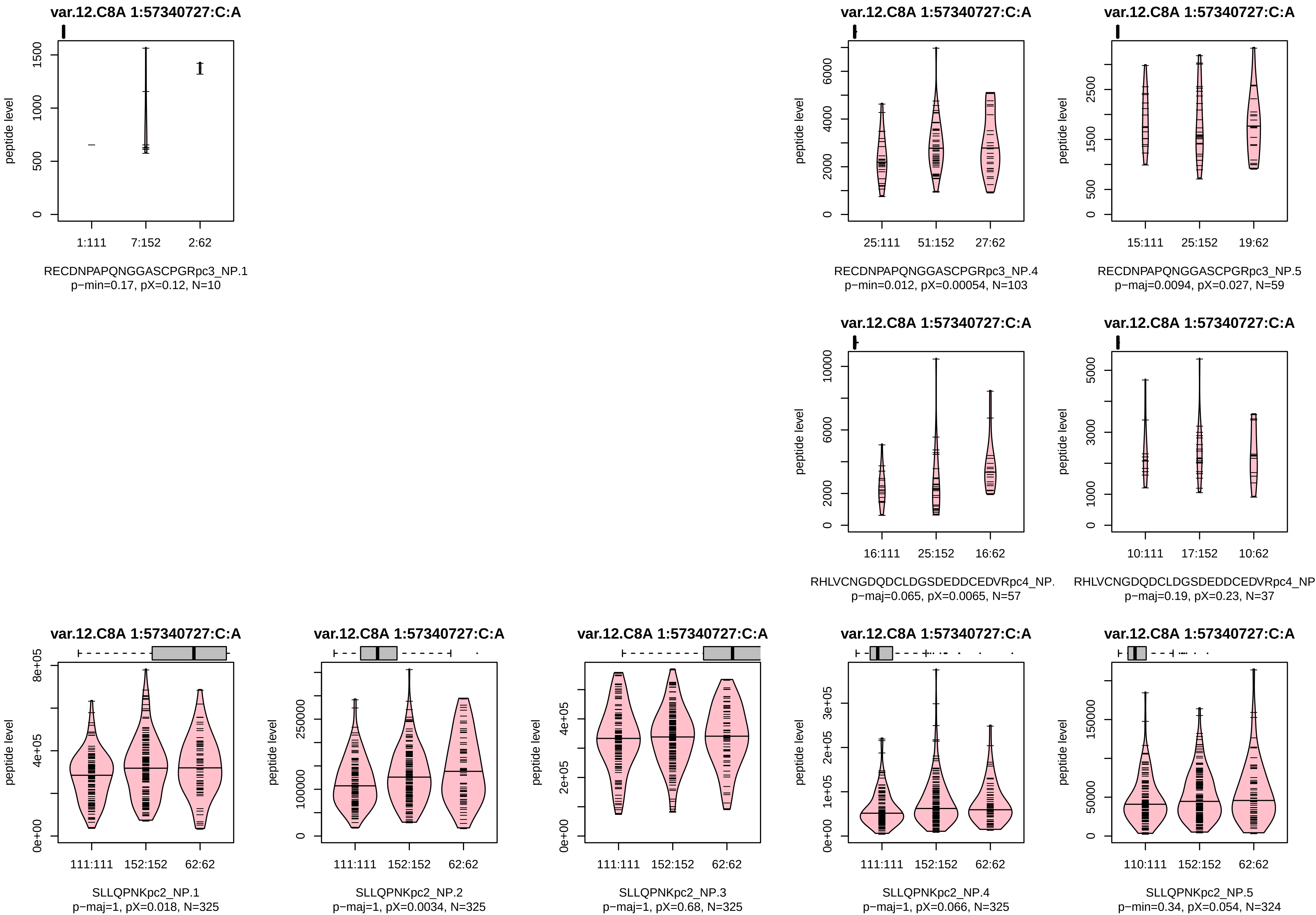
var.12.C8A 1:57340727:C:A
peptide level
1:111
7:152
2:62
RECDNPAPQNGGASCPGRpc3_NP.1
p−min=0.17, pX=0.12, N=10
var.12.C8A 1:57340727:C:A
peptide level
25:111
51:152
27:62
RECDNPAPQNGGASCPGRpc3_NP.4
p−min=0.012, pX=0.00054, N=103
var.12.C8A 1:57340727:C:A
peptide level
15:111
25:152
19:62
RECDNPAPQNGGASCPGRpc3_NP.5
p−maj=0.0094, pX=0.027, N=59
var.12.C8A 1:57340727:C:A
peptide level
16:111
25:152
16:62
RHLVCNGDQDCLDGSDEDDCEDVRpc4_NP.
p−maj=0.065, pX=0.0065, N=57
var.12.C8A 1:57340727:C:A
peptide level
10:111
17:152
10:62
RHLVCNGDQDCLDGSDEDDCEDVRpc4_NP
p−maj=0.19, pX=0.23, N=37
var.12.C8A 1:57340727:C:A
peptide level
111:111
152:152
62:62
SLLQPNKpc2_NP.1
p−maj=1, pX=0.018, N=325
var.12.C8A 1:57340727:C:A
peptide level
111:111
152:152
62:62
SLLQPNKpc2_NP.2
p−maj=1, pX=0.0034, N=325
var.12.C8A 1:57340727:C:A
peptide level
111:111
152:152
62:62
SLLQPNKpc2_NP.3
p−maj=1, pX=0.68, N=325
var.12.C8A 1:57340727:C:A
peptide level
111:111
152:152
62:62
SLLQPNKpc2_NP.4
p−maj=1, pX=0.066, N=325
var.12.C8A 1:57340727:C:A
peptide level
110:111
152:152
62:62
SLLQPNKpc2_NP.5
p−min=0.34, pX=0.054, N=324

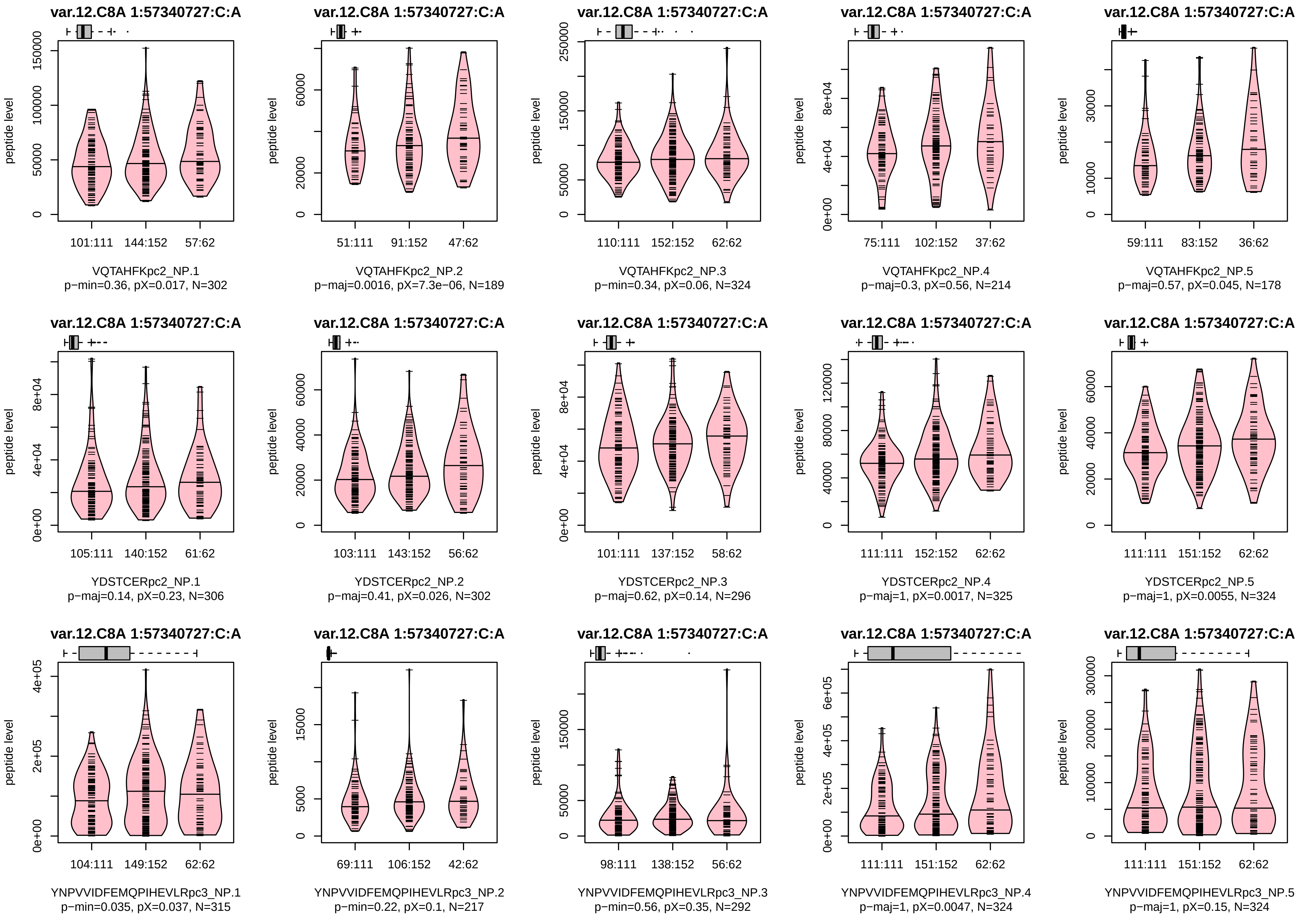
var.12.C8A 1:57340727:C:A
peptide level
101:111
144:152
57:62
VQTAHFKpc2_NP.1
p−min=0.36, pX=0.017, N=302
var.12.C8A 1:57340727:C:A
peptide level
51:111
91:152
47:62
VQTAHFKpc2_NP.2
p−maj=0.0016, pX=7.3e−06, N=189
var.12.C8A 1:57340727:C:A
peptide level
110:111
152:152
62:62
VQTAHFKpc2_NP.3
p−min=0.34, pX=0.06, N=324
var.12.C8A 1:57340727:C:A
peptide level
75:111
102:152
37:62
VQTAHFKpc2_NP.4
p−maj=0.3, pX=0.56, N=214
var.12.C8A 1:57340727:C:A
peptide level
59:111
83:152
36:62
VQTAHFKpc2_NP.5
p−maj=0.57, pX=0.045, N=178
var.12.C8A 1:57340727:C:A
peptide level
105:111
140:152
61:62
YDSTCERpc2_NP.1
p−maj=0.14, pX=0.23, N=306
var.12.C8A 1:57340727:C:A
peptide level
103:111
143:152
56:62
YDSTCERpc2_NP.2
p−maj=0.41, pX=0.026, N=302
var.12.C8A 1:57340727:C:A
peptide level
101:111
137:152
58:62
YDSTCERpc2_NP.3
p−maj=0.62, pX=0.14, N=296
var.12.C8A 1:57340727:C:A
peptide level
111:111
152:152
62:62
YDSTCERpc2_NP.4
p−maj=1, pX=0.0017, N=325
var.12.C8A 1:57340727:C:A
peptide level
111:111
151:152
62:62
YDSTCERpc2_NP.5
p−maj=1, pX=0.0055, N=324
var.12.C8A 1:57340727:C:A
peptide level
104:111
149:152
62:62
YNPVVIDFEMQPIHEVLRpc3_NP.1
p−min=0.035, pX=0.037, N=315
var.12.C8A 1:57340727:C:A
peptide level
69:111
106:152
42:62
YNPVVIDFEMQPIHEVLRpc3_NP.2
p−min=0.22, pX=0.1, N=217
var.12.C8A 1:57340727:C:A
peptide level
98:111
138:152
56:62
YNPVVIDFEMQPIHEVLRpc3_NP.3
p−min=0.56, pX=0.35, N=292
var.12.C8A 1:57340727:C:A
peptide level
111:111
151:152
62:62
YNPVVIDFEMQPIHEVLRpc3_NP.4
p−maj=1, pX=0.0047, N=324
var.12.C8A 1:57340727:C:A
peptide level
111:111
151:152
62:62
YNPVVIDFEMQPIHEVLRpc3_NP.5
p−maj=1, pX=0.15, N=324

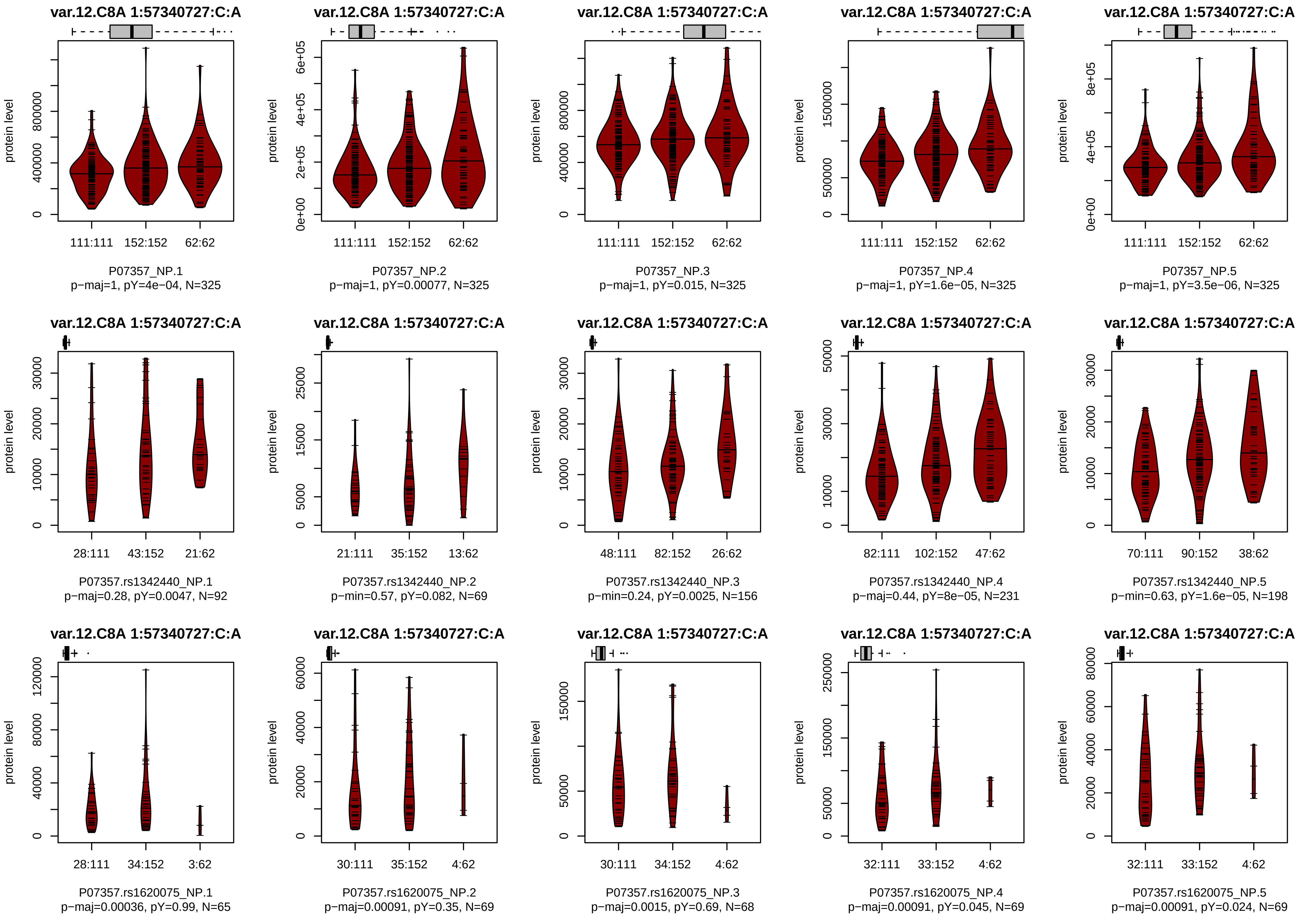

var.12.C8A 1:57340727:C:A
protein level
111:111 152:152 62:62
P07357_NP.1
p−maj=1, pY=4e−04, N=325
var.12.C8A 1:57340727:C:A
protein level
111:111 152:152 62:62
P07357_NP.2
p−maj=1, pY=0.00077, N=325
var.12.C8A 1:57340727:C:A
protein level
111:111 152:152 62:62
P07357_NP.3
p−maj=1, pY=0.015, N=325
var.12.C8A 1:57340727:C:A
protein level
111:111 152:152 62:62
P07357_NP.4
p−maj=1, pY=1.6e−05, N=325
var.12.C8A 1:57340727:C:A
protein level
111:111 152:152 62:62
P07357_NP.5
p−maj=1, pY=3.5e−06, N=325
var.12.C8A 1:57340727:C:A
protein level
28:111 43:152 21:62
P07357.rs1342440_NP.1
p−maj=0.28, pY=0.0047, N=92
var.12.C8A 1:57340727:C:A
protein level
21:111 35:152 13:62
P07357.rs1342440_NP.2
p−min=0.57, pY=0.082, N=69
var.12.C8A 1:57340727:C:A
protein level
48:111 82:152 26:62
P07357.rs1342440_NP.3
p−min=0.24, pY=0.0025, N=156
var.12.C8A 1:57340727:C:A
protein level
82:111 102:152 47:62
P07357.rs1342440_NP.4
p−maj=0.44, pY=8e−05, N=231
var.12.C8A 1:57340727:C:A
protein level
70:111 90:152 38:62
P07357.rs1342440_NP.5
p−min=0.63, pY=1.6e−05, N=198
var.12.C8A 1:57340727:C:A
protein level
28:111 34:152 3:62
P07357.rs1620075_NP.1
p−maj=0.00036, pY=0.99, N=65
var.12.C8A 1:57340727:C:A
protein level
30:111 35:152 4:62
P07357.rs1620075_NP.2
p−maj=0.00091, pY=0.35, N=69
var.12.C8A 1:57340727:C:A
protein level
30:111 34:152 4:62
P07357.rs1620075_NP.3
p−maj=0.0015, pY=0.69, N=68
var.12.C8A 1:57340727:C:A
protein level
32:111 33:152 4:62
P07357.rs1620075_NP.4
p−maj=0.00091, pY=0.045, N=69
var.12.C8A 1:57340727:C:A
protein level
32:111 33:152 4:62
P07357.rs1620075_NP.5
p−maj=0.00091, pY=0.024, N=69

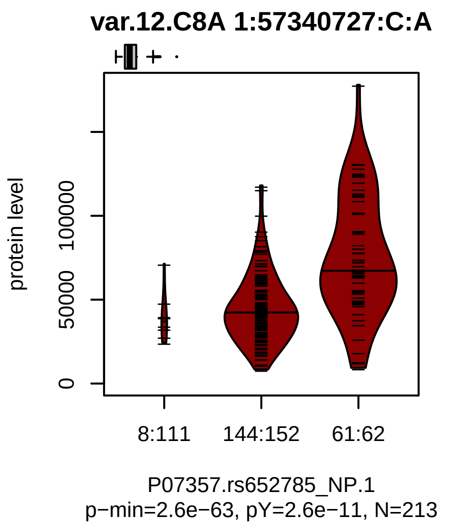
var.12.C8A 1:57340727:C:A
protein level
0
50000
100000
8:111
144:152
61:62
P07357.rs652785_NP.1
p−min=2.6e−63, pY=2.6e−11, N=213

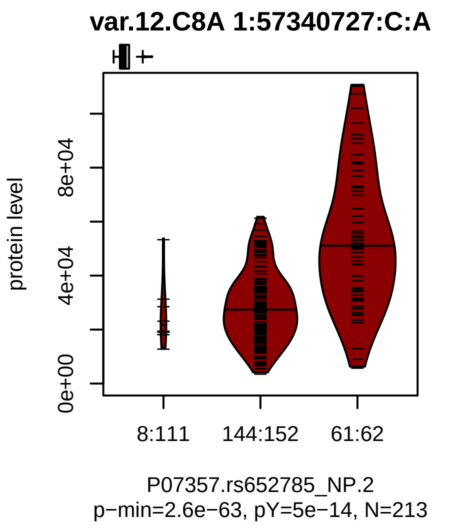
var.12.C8A 1:57340727:C:A
protein level
0e+00
4e+04
8e+04
8:111
144:152
61:62
P07357.rs652785_NP.2
p−min=2.6e−63, pY=5e−14, N=213

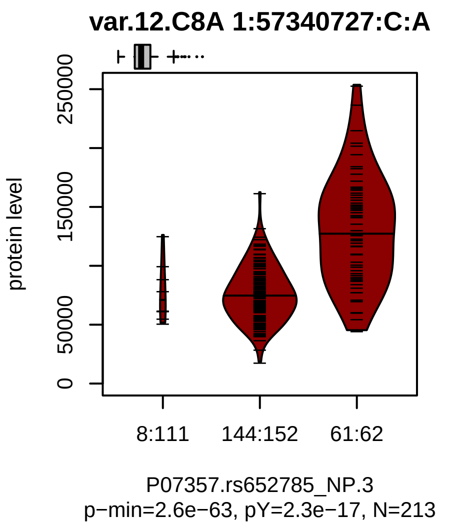
var.12.C8A 1:57340727:C:A
protein level
0
50000
150000
250000
8:111
144:152
61:62
P07357.rs652785_NP.3
p−min=2.6e−63, pY=2.3e−17, N=213

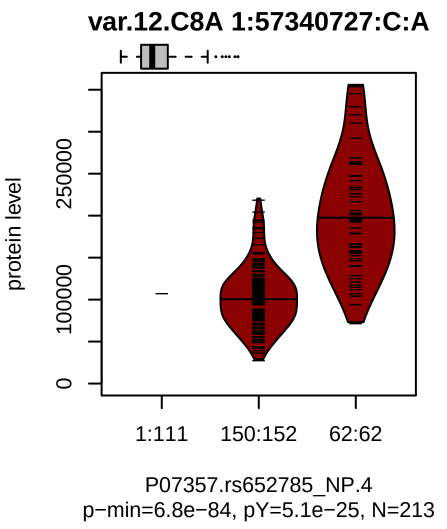
var.12.C8A 1:57340727:C:A
protein level
0
100000
250000
1:111
150:152
62:62
P07357.rs652785_NP.4
p−min=6.8e−84, pY=5.1e−25, N=213

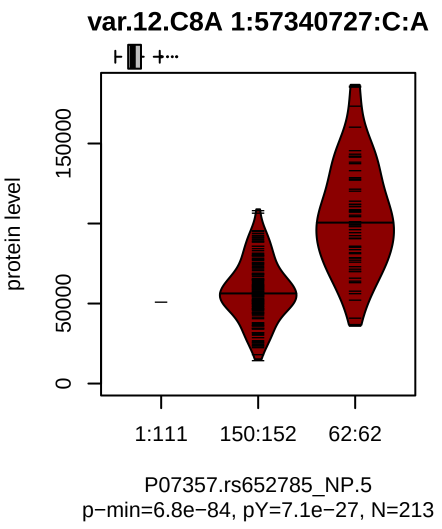
var.12.C8A 1:57340727:C:A
protein level
0
50000
150000
1:111
150:152
62:62
P07357.rs652785_NP.5
p−min=6.8e−84, pY=7.1e−27, N=213

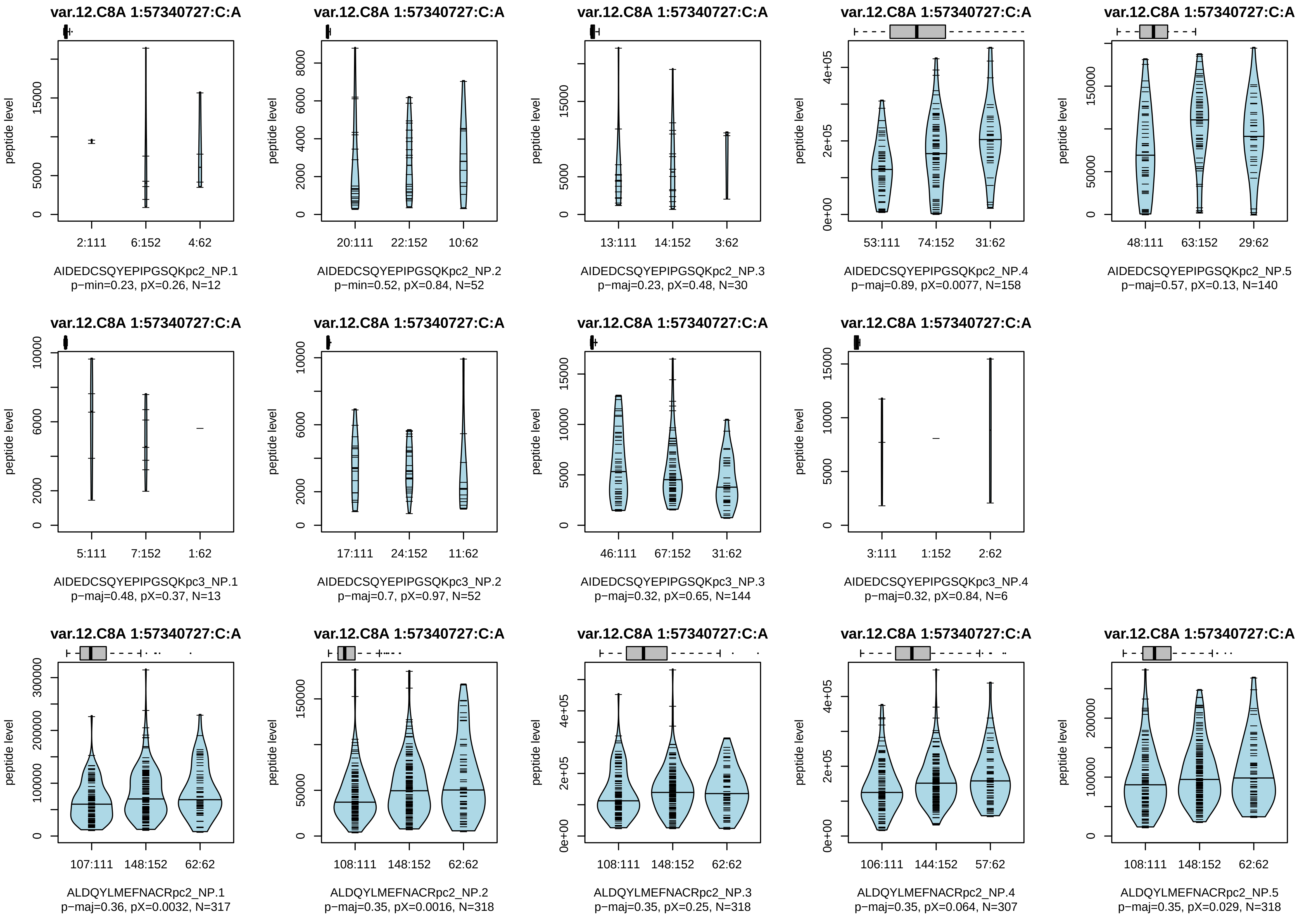
var.12.C8A 1:57340727:C:A
peptide level
2:111
6:152
4:62
AIDEDCSQYEPIPGSQKpc2_NP.1
p−min=0.23, pX=0.26, N=12
var.12.C8A 1:57340727:C:A
peptide level
20:111
22:152
10:62
AIDEDCSQYEPIPGSQKpc2_NP.2
p−min=0.52, pX=0.84, N=52
var.12.C8A 1:57340727:C:A
peptide level
13:111
14:152
3:62
AIDEDCSQYEPIPGSQKpc2_NP.3
p−maj=0.23, pX=0.48, N=30
var.12.C8A 1:57340727:C:A
peptide level
53:111
74:152
31:62
AIDEDCSQYEPIPGSQKpc2_NP.4
p−maj=0.89, pX=0.0077, N=158
var.12.C8A 1:57340727:C:A
peptide level
48:111
63:152
29:62
AIDEDCSQYEPIPGSQKpc2_NP.5
p−maj=0.57, pX=0.13, N=140
var.12.C8A 1:57340727:C:A
peptide level
5:111
7:152
1:62
AIDEDCSQYEPIPGSQKpc3_NP.1
p−maj=0.48, pX=0.37, N=13
var.12.C8A 1:57340727:C:A
peptide level
17:111
24:152
11:62
AIDEDCSQYEPIPGSQKpc3_NP.2
p−maj=0.7, pX=0.97, N=52
var.12.C8A 1:57340727:C:A
peptide level
46:111
67:152
31:62
AIDEDCSQYEPIPGSQKpc3_NP.3
p−maj=0.32, pX=0.65, N=144
var.12.C8A 1:57340727:C:A
peptide level
3:111
1:152
2:62
AIDEDCSQYEPIPGSQKpc3_NP.4
p−maj=0.32, pX=0.84, N=6
var.12.C8A 1:57340727:C:A
peptide level
107:111
148:152
62:62
ALDQYLMEFNACRpc2_NP.1
p−maj=0.36, pX=0.0032, N=317
var.12.C8A 1:57340727:C:A
peptide level
108:111
148:152
62:62
ALDQYLMEFNACRpc2_NP.2
p−maj=0.35, pX=0.0016, N=318
var.12.C8A 1:57340727:C:A
peptide level
108:111
148:152
62:62
ALDQYLMEFNACRpc2_NP.3
p−maj=0.35, pX=0.25, N=318
var.12.C8A 1:57340727:C:A
peptide level
106:111
144:152
57:62
ALDQYLMEFNACRpc2_NP.4
p−maj=0.35, pX=0.064, N=307
var.12.C8A 1:57340727:C:A
peptide level
108:111
148:152
62:62
ALDQYLMEFNACRpc2_NP.5
p−maj=0.35, pX=0.029, N=318

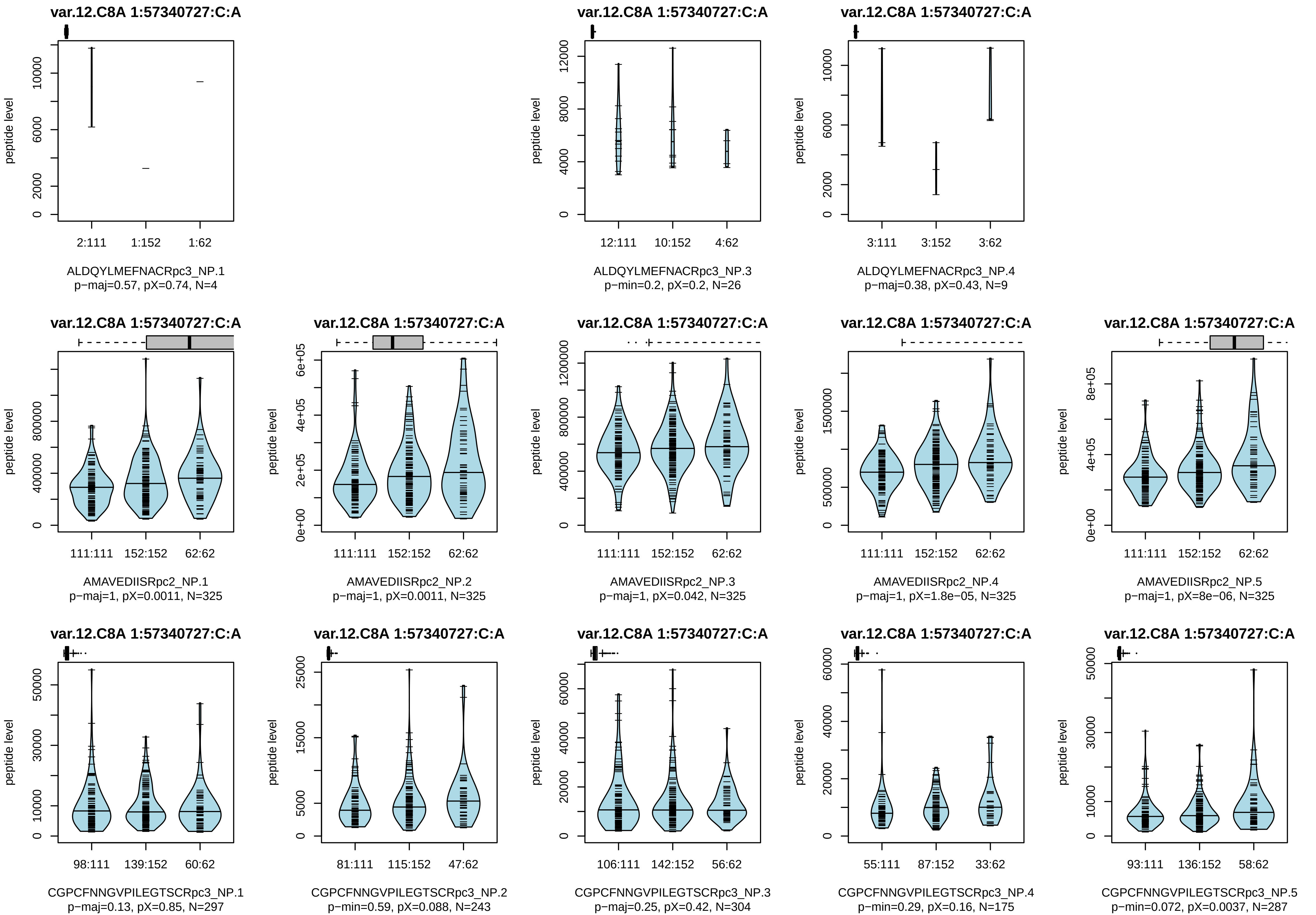
var.12.C8A 1:57340727:C:A
peptide level
2:111
1:152
1:62
ALDQYLMEFNACRpc3_NP.1
p−maj=0.57, pX=0.74, N=4
var.12.C8A 1:57340727:C:A
peptide level
12:111
10:152
4:62
ALDQYLMEFNACRpc3_NP.3
p−min=0.2, pX=0.2, N=26
var.12.C8A 1:57340727:C:A
peptide level
3:111
3:152
3:62
ALDQYLMEFNACRpc3_NP.4
p−maj=0.38, pX=0.43, N=9
var.12.C8A 1:57340727:C:A
peptide level
111:111
152:152
62:62
AMAVEDIISRpc2_NP.1
p−maj=1, pX=0.0011, N=325
var.12.C8A 1:57340727:C:A
peptide level
111:111
152:152
62:62
AMAVEDIISRpc2_NP.2
p−maj=1, pX=0.0011, N=325
var.12.C8A 1:57340727:C:A
peptide level
111:111
152:152
62:62
AMAVEDIISRpc2_NP.3
p−maj=1, pX=0.042, N=325
var.12.C8A 1:57340727:C:A
peptide level
111:111
152:152
62:62
AMAVEDIISRpc2_NP.4
p−maj=1, pX=1.8e−05, N=325
var.12.C8A 1:57340727:C:A
peptide level
111:111
152:152
62:62
AMAVEDIISRpc2_NP.5
p−maj=1, pX=8e−06, N=325
var.12.C8A 1:57340727:C:A
peptide level
98:111
139:152
60:62
CGPCFNNGVPILEGTSCRpc3_NP.1
p−maj=0.13, pX=0.85, N=297
var.12.C8A 1:57340727:C:A
peptide level
81:111
115:152
47:62
CGPCFNNGVPILEGTSCRpc3_NP.2
p−min=0.59, pX=0.088, N=243
var.12.C8A 1:57340727:C:A
peptide level
106:111
142:152
56:62
CGPCFNNGVPILEGTSCRpc3_NP.3
p−maj=0.25, pX=0.42, N=304
var.12.C8A 1:57340727:C:A
peptide level
55:111
87:152
33:62
CGPCFNNGVPILEGTSCRpc3_NP.4
p−min=0.29, pX=0.16, N=175
var.12.C8A 1:57340727:C:A
peptide level
93:111
136:152
58:62
CGPCFNNGVPILEGTSCRpc3_NP.5
p−min=0.072, pX=0.0037, N=287

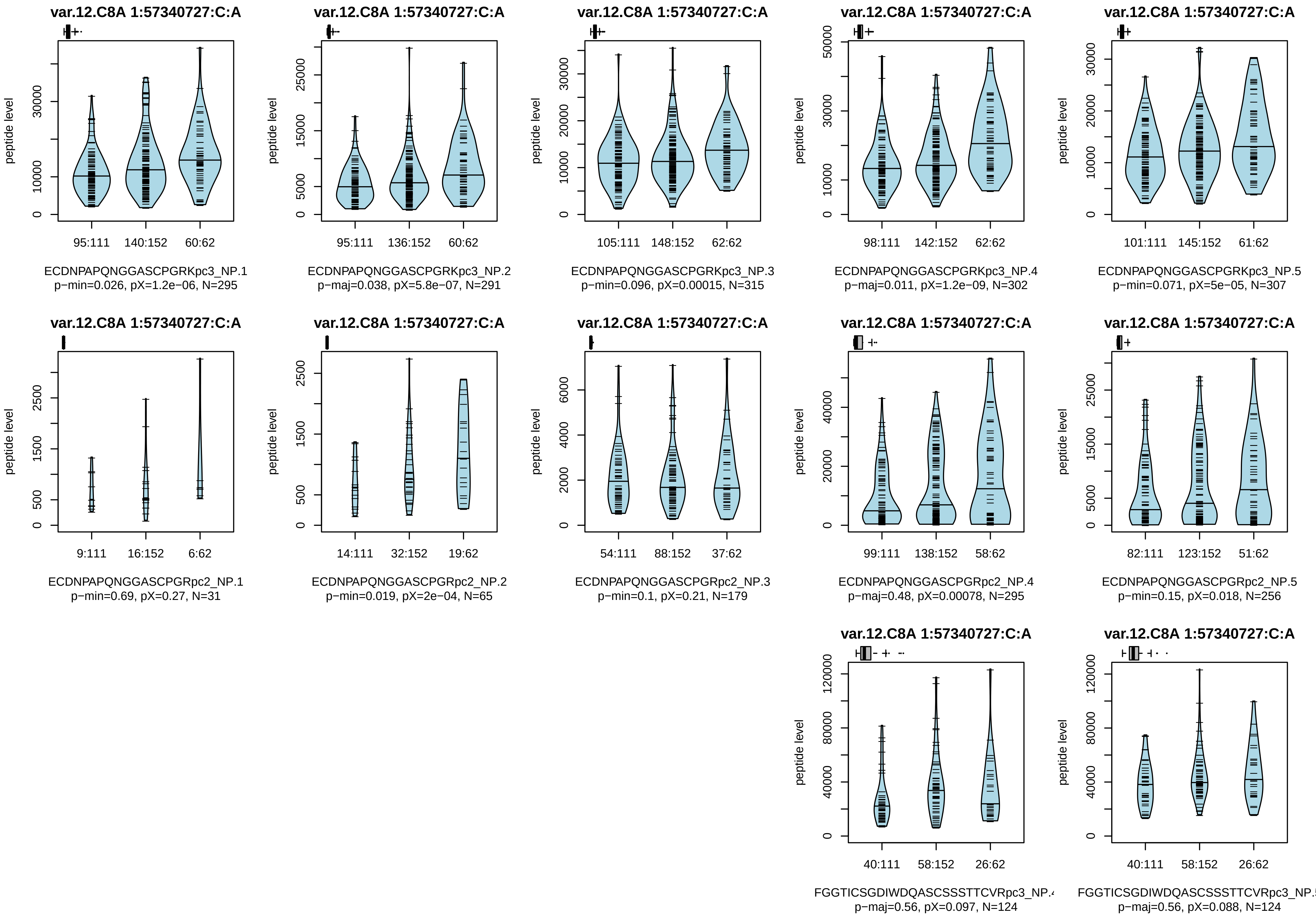

var.12.C8A 1:57340727:C:A
peptide level
95:111
140:152
60:62
ECDNPAPQNGGASCPGRKpc3_NP.1
p−min=0.026, pX=1.2e−06, N=295
var.12.C8A 1:57340727:C:A
peptide level
95:111
136:152
60:62
ECDNPAPQNGGASCPGRKpc3_NP.2
p−maj=0.038, pX=5.8e−07, N=291
var.12.C8A 1:57340727:C:A
peptide level
105:111
148:152
62:62
ECDNPAPQNGGASCPGRKpc3_NP.3
p−min=0.096, pX=0.00015, N=315
var.12.C8A 1:57340727:C:A
peptide level
98:111
142:152
62:62
ECDNPAPQNGGASCPGRKpc3_NP.4
p−maj=0.011, pX=1.2e−09, N=302
var.12.C8A 1:57340727:C:A
peptide level
101:111
145:152
61:62
ECDNPAPQNGGASCPGRKpc3_NP.5
p−min=0.071, pX=5e−05, N=307
var.12.C8A 1:57340727:C:A
peptide level
9:111
16:152
6:62
ECDNPAPQNGGASCPGRpc2_NP.1
p−min=0.69, pX=0.27, N=31
var.12.C8A 1:57340727:C:A
peptide level
14:111
32:152
19:62
ECDNPAPQNGGASCPGRpc2_NP.2
p−min=0.019, pX=2e−04, N=65
var.12.C8A 1:57340727:C:A
peptide level
54:111
88:152
37:62
ECDNPAPQNGGASCPGRpc2_NP.3
p−min=0.1, pX=0.21, N=179
var.12.C8A 1:57340727:C:A
peptide level
99:111
138:152
58:62
ECDNPAPQNGGASCPGRpc2_NP.4
p−maj=0.48, pX=0.00078, N=295
var.12.C8A 1:57340727:C:A
peptide level
82:111
123:152
51:62
ECDNPAPQNGGASCPGRpc2_NP.5
p−min=0.15, pX=0.018, N=256
var.12.C8A 1:57340727:C:A
peptide level
40:111
58:152
26:62
p−maj=0.56, pX=0.097, N=124
var.12.C8A 1:57340727:C:A
peptide level
40:111
58:152
26:62
p−maj=0.56, pX=0.088, N=124

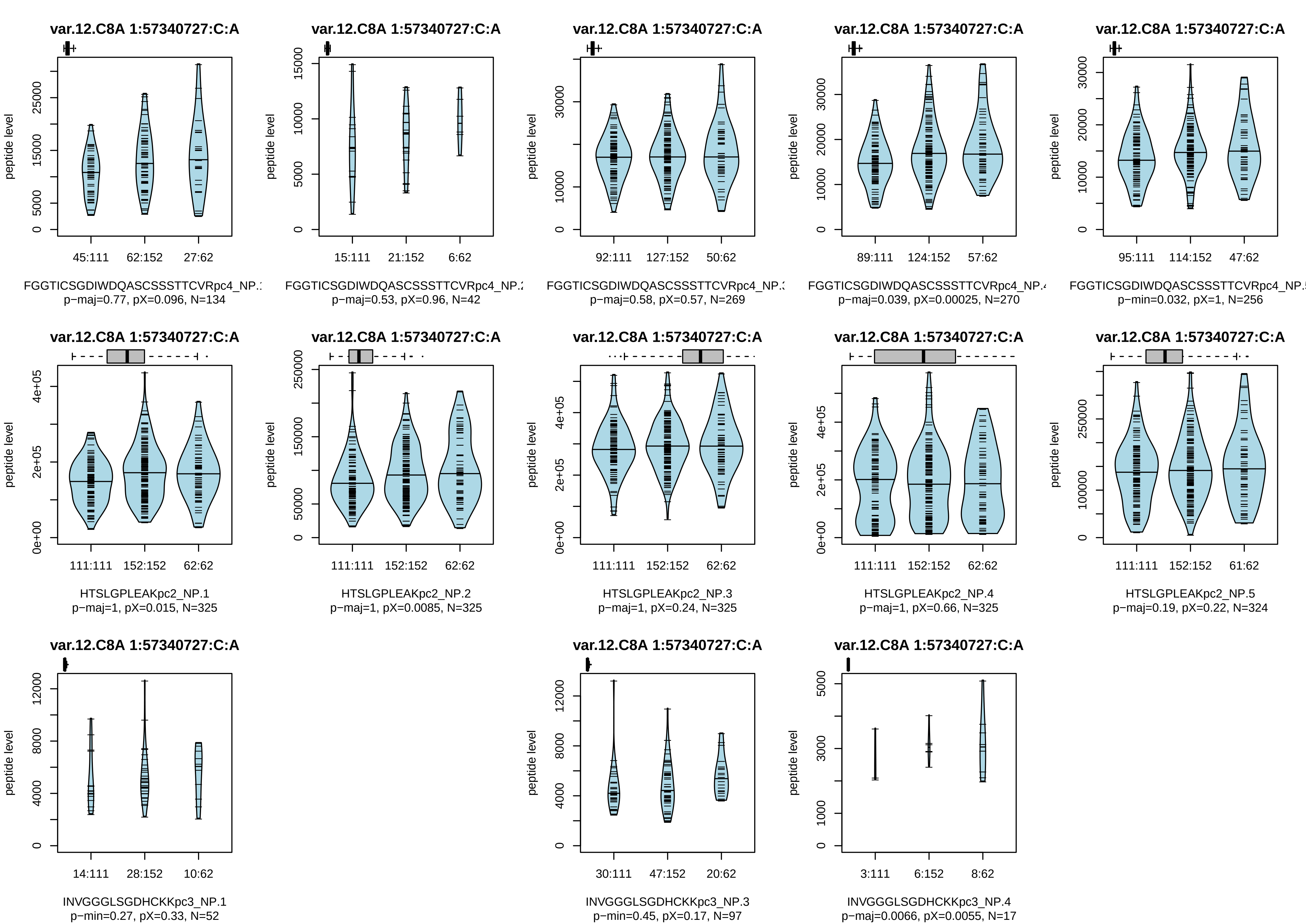
var.12.C8A 1:57340727:C:A
peptide level
45:111
62:152
27:62
FGGTICSGDIWDQASCSSSTTCVRpc4_NP.1
p−maj=0.77, pX=0.096, N=134
var.12.C8A 1:57340727:C:A
peptide level
15:111
21:152
6:62
FGGTICSGDIWDQASCSSSTTCVRpc4_NP.2
p−maj=0.53, pX=0.96, N=42
var.12.C8A 1:57340727:C:A
peptide level
92:111
127:152
50:62
FGGTICSGDIWDQASCSSSTTCVRpc4_NP.3
p−maj=0.58, pX=0.57, N=269
var.12.C8A 1:57340727:C:A
peptide level
89:111
124:152
57:62
FGGTICSGDIWDQASCSSSTTCVRpc4_NP.4
p−maj=0.039, pX=0.00025, N=270
var.12.C8A 1:57340727:C:A
peptide level
95:111
114:152
47:62
FGGTICSGDIWDQASCSSSTTCVRpc4_NP.5
p−min=0.032, pX=1, N=256
var.12.C8A 1:57340727:C:A
peptide level
111:111
152:152
62:62
HTSLGPLEAKpc2_NP.1
p−maj=1, pX=0.015, N=325
var.12.C8A 1:57340727:C:A
peptide level
111:111
152:152
62:62
HTSLGPLEAKpc2_NP.2
p−maj=1, pX=0.0085, N=325
var.12.C8A 1:57340727:C:A
peptide level
111:111
152:152
62:62
HTSLGPLEAKpc2_NP.3
p−maj=1, pX=0.24, N=325
var.12.C8A 1:57340727:C:A
peptide level
111:111
152:152
62:62
HTSLGPLEAKpc2_NP.4
p−maj=1, pX=0.66, N=325
var.12.C8A 1:57340727:C:A
peptide level
111:111
152:152
61:62
HTSLGPLEAKpc2_NP.5
p−maj=0.19, pX=0.22, N=324
var.12.C8A 1:57340727:C:A
peptide level
14:111
28:152
10:62
INVGGGLSGDHCKKpc3_NP.1
p−min=0.27, pX=0.33, N=52
var.12.C8A 1:57340727:C:A
peptide level
30:111
47:152
20:62
INVGGGLSGDHCKKpc3_NP.3
p−min=0.45, pX=0.17, N=97
var.12.C8A 1:57340727:C:A
peptide level
3:111
6:152
8:62
INVGGGLSGDHCKKpc3_NP.4
p−maj=0.0066, pX=0.0055, N=17

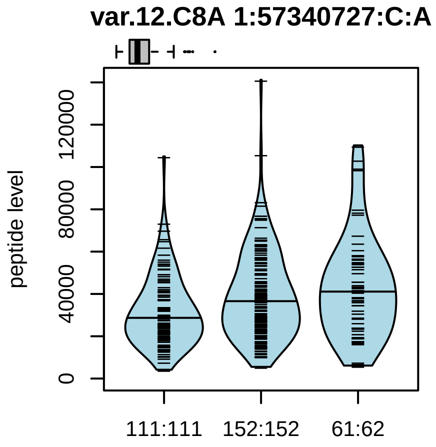

INVGGGLSGDHCKpc2_NP.1
p−maj=0.19, pX=0.00042, N=324

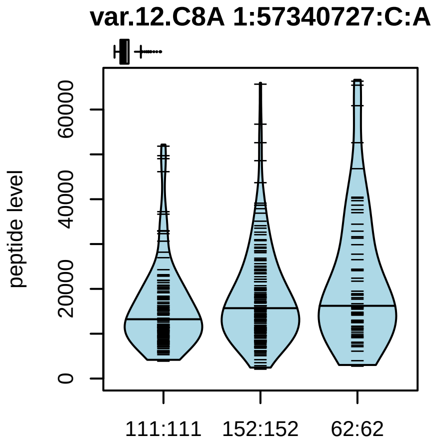

INVGGGLSGDHCKpc2_NP.2
p−maj=1, pX=0.0014, N=325

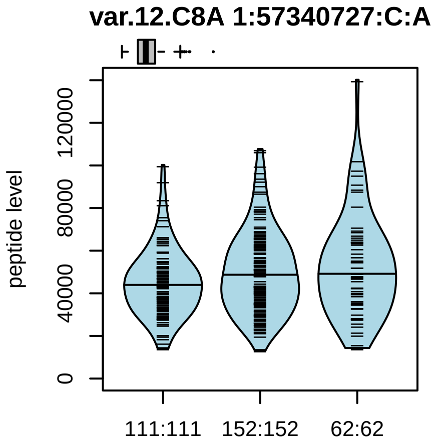

INVGGGLSGDHCKpc2_NP.3
p−maj=1, pX=0.0082, N=325

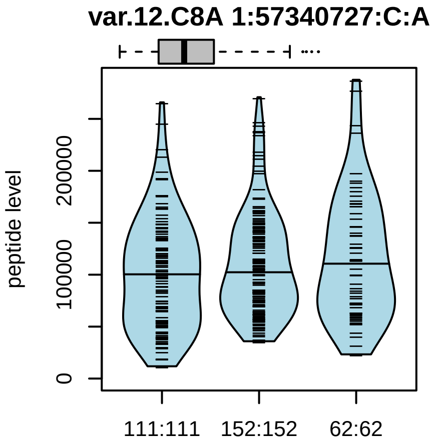

INVGGGLSGDHCKpc2_NP.4
p−maj=1, pX=0.051, N=325

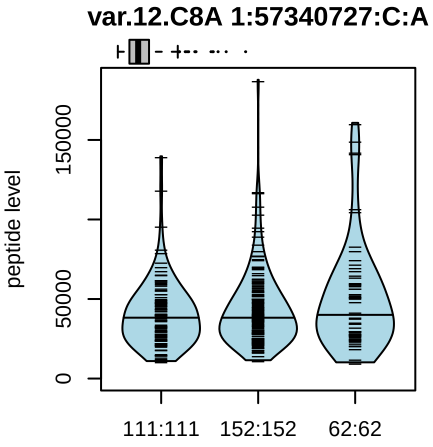

INVGGGLSGDHCKpc2_NP.5
p−maj=1, pX=0.013, N=325

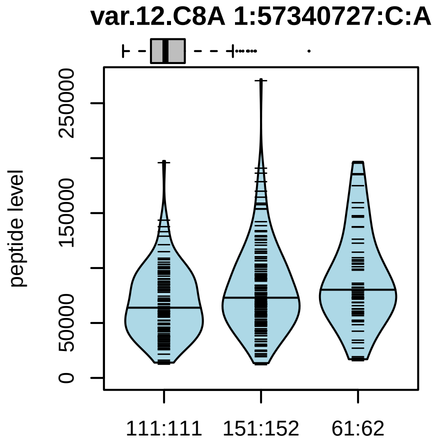

INVGGGLSGDHCKpc3_NP.1
p−maj=0.35, pX=0.00054, N=323

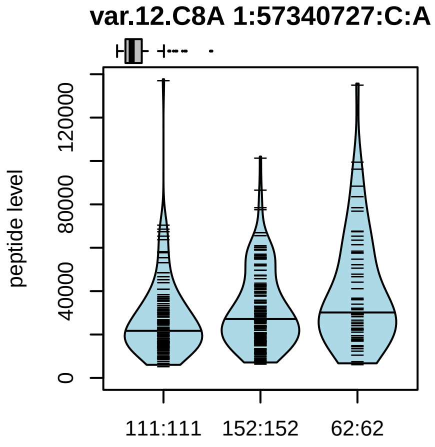

INVGGGLSGDHCKpc3_NP.2
p−maj=1, pX=0.00014, N=325

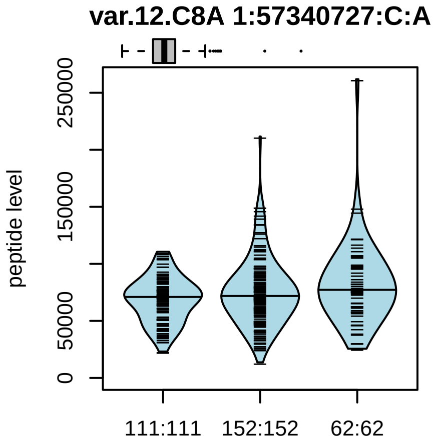

INVGGGLSGDHCKpc3_NP.3
p−maj=1, pX=0.0017, N=325

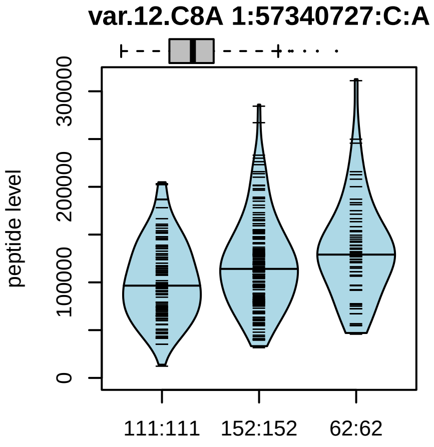

INVGGGLSGDHCKpc3_NP.4
p−maj=1, pX=4.8e−06, N=325

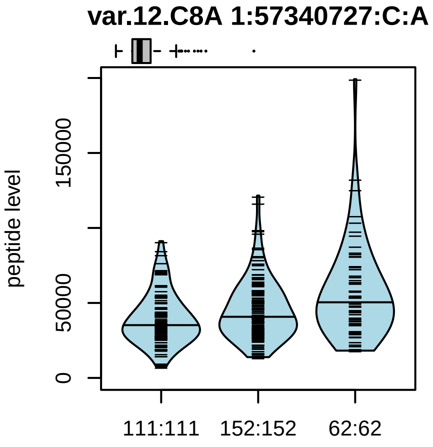

INVGGGLSGDHCKpc3_NP.5
p−maj=1, pX=6.8e−07, N=325

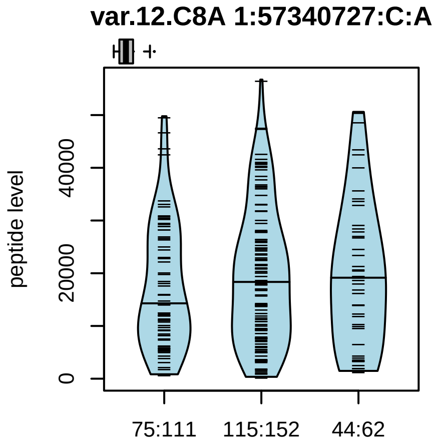

LGSLGAACEQTQTEGAKpc2_NP.1
p−min=0.24, pX=0.19, N=234

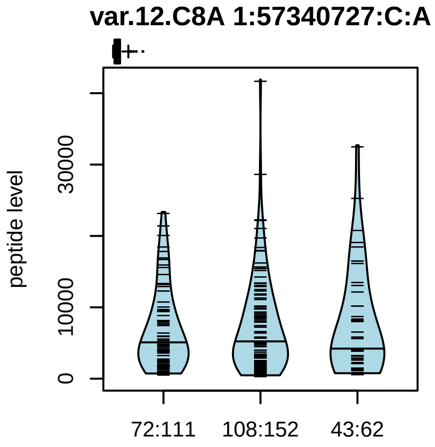

LGSLGAACEQTQTEGAKpc2_NP.2
p−min=0.31, pX=0.29, N=223

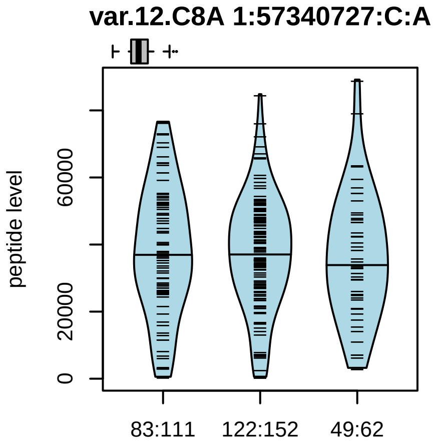

LGSLGAACEQTQTEGAKpc2_NP.3
p−min=0.32, pX=0.79, N=254

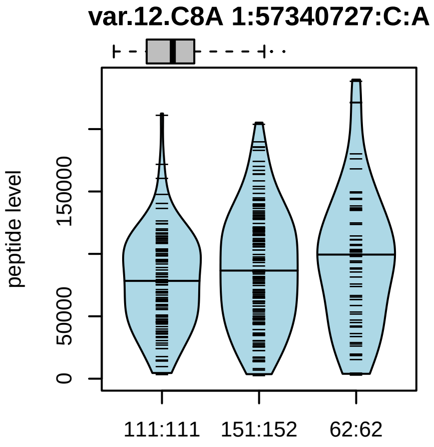

LGSLGAACEQTQTEGAKpc2_NP.4
p−maj=1, pX=0.015, N=324

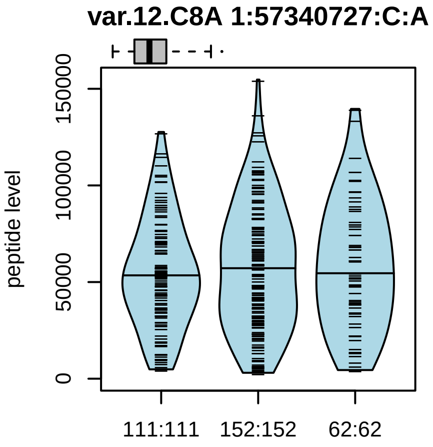

LGSLGAACEQTQTEGAKpc2_NP.5
p−maj=1, pX=0.33, N=325

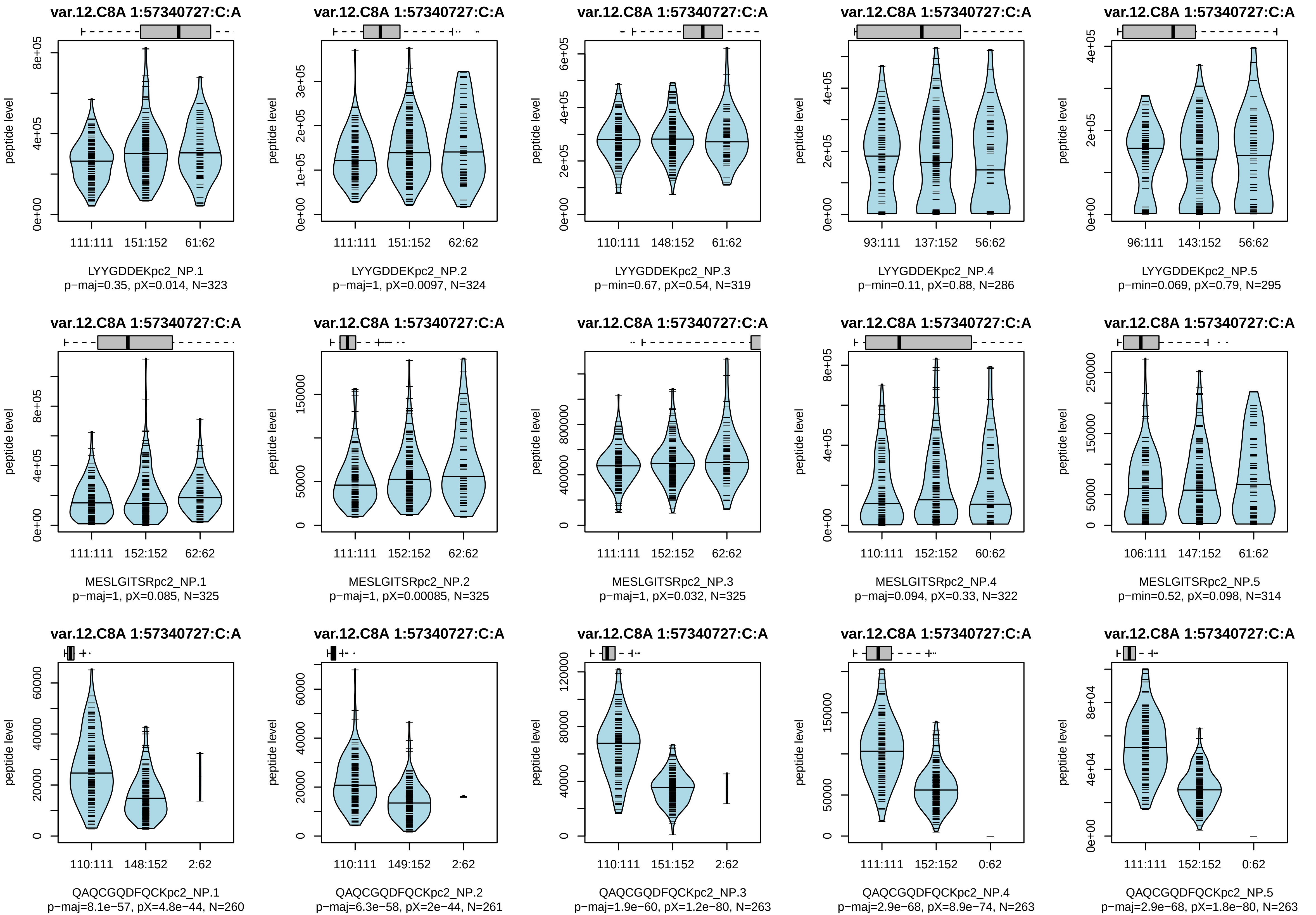
var.12.C8A 1:57340727:C:A
peptide level
111:111
151:152
61:62
LYYGDDEKpc2_NP.1
p−maj=0.35, pX=0.014, N=323
var.12.C8A 1:57340727:C:A
111:111
151:152
62:62
LYYGDDEKpc2_NP.2
p−maj=1, pX=0.0097, N=324
var.12.C8A 1:57340727:C:A
110:111
148:152
61:62
LYYGDDEKpc2_NP.3
p−min=0.67, pX=0.54, N=319
var.12.C8A 1:57340727:C:A
93:111
137:152
56:62
LYYGDDEKpc2_NP.4
p−min=0.11, pX=0.88, N=286
var.12.C8A 1:57340727:C:A
96:111
143:152
56:62
LYYGDDEKpc2_NP.5
p−min=0.069, pX=0.79, N=295
var.12.C8A 1:57340727:C:A
111:111
152:152
62:62
MESLGITSRpc2_NP.1
p−maj=1, pX=0.085, N=325
var.12.C8A 1:57340727:C:A
111:111
152:152
62:62
MESLGITSRpc2_NP.2
p−maj=1, pX=0.00085, N=325
var.12.C8A 1:57340727:C:A
111:111
152:152
62:62
MESLGITSRpc2_NP.3
p−maj=1, pX=0.032, N=325
var.12.C8A 1:57340727:C:A
110:111
152:152
60:62
MESLGITSRpc2_NP.4
p−maj=0.094, pX=0.33, N=322
var.12.C8A 1:57340727:C:A
106:111
147:152
61:62
MESLGITSRpc2_NP.5
p−min=0.52, pX=0.098, N=314
var.12.C8A 1:57340727:C:A
110:111
148:152
2:62
QAQCGQDFQCKpc2_NP.1
p−maj=8.1e−57, pX=4.8e−44, N=260
var.12.C8A 1:57340727:C:A
110:111
149:152
2:62
QAQCGQDFQCKpc2_NP.2
p−maj=6.3e−58, pX=2e−44, N=261
var.12.C8A 1:57340727:C:A
110:111
151:152
2:62
QAQCGQDFQCKpc2_NP.3
p−maj=1.9e−60, pX=1.2e−80, N=263
var.12.C8A 1:57340727:C:A
111:111
152:152
0:62
QAQCGQDFQCKpc2_NP.4
p−maj=2.9e−68, pX=8.9e−74, N=263
var.12.C8A 1:57340727:C:A
111:111
152:152
0:62
QAQCGQDFQCKpc2_NP.5
p−maj=2.9e−68, pX=1.8e−80, N=263

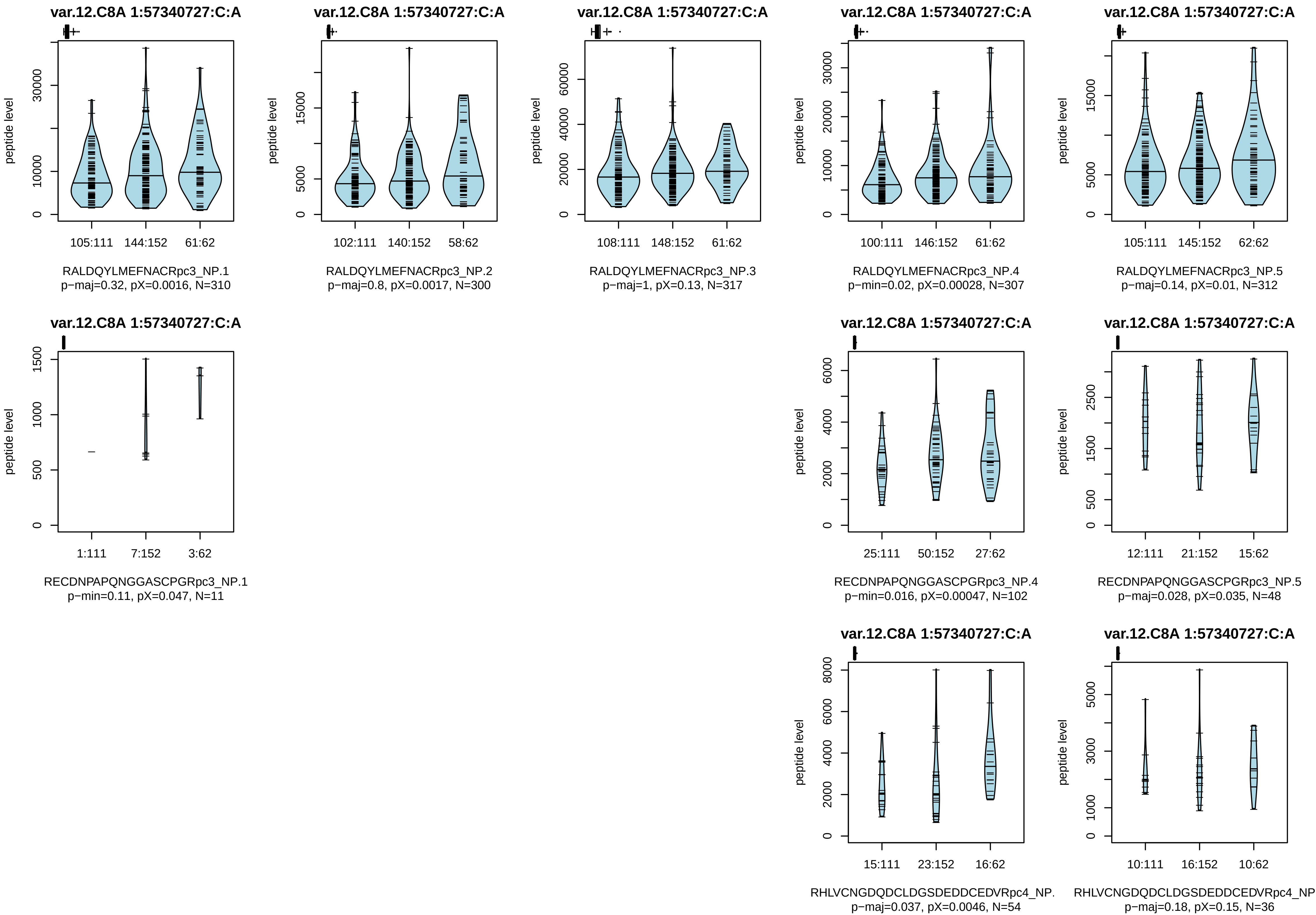
var.12.C8A 1:57340727:C:A
peptide level
105:111
144:152
61:62
RALDQYLMEFNACRpc3_NP.1
p−maj=0.32, pX=0.0016, N=310
var.12.C8A 1:57340727:C:A
peptide level
102:111
140:152
58:62
RALDQYLMEFNACRpc3_NP.2
p−maj=0.8, pX=0.0017, N=300
var.12.C8A 1:57340727:C:A
peptide level
108:111
148:152
61:62
RALDQYLMEFNACRpc3_NP.3
p−maj=1, pX=0.13, N=317
var.12.C8A 1:57340727:C:A
peptide level
100:111
146:152
61:62
RALDQYLMEFNACRpc3_NP.4
p−min=0.02, pX=0.00028, N=307
var.12.C8A 1:57340727:C:A
peptide level
105:111
145:152
62:62
RALDQYLMEFNACRpc3_NP.5
p−maj=0.14, pX=0.01, N=312
var.12.C8A 1:57340727:C:A
peptide level
1:111
7:152
3:62
RECDNPAPQNGGASCPGRpc3_NP.1
p−min=0.11, pX=0.047, N=11
var.12.C8A 1:57340727:C:A
peptide level
25:111
50:152
27:62
RECDNPAPQNGGASCPGRpc3_NP.4
p−min=0.016, pX=0.00047, N=102
var.12.C8A 1:57340727:C:A
peptide level
12:111
21:152
15:62
RECDNPAPQNGGASCPGRpc3_NP.5
p−maj=0.028, pX=0.035, N=48
var.12.C8A 1:57340727:C:A
peptide level
15:111
23:152
16:62
RHLVCNGDQDCLDGSDEDDCEDVRpc4_NP.
p−maj=0.037, pX=0.0046, N=54
var.12.C8A 1:57340727:C:A
peptide level
10:111
16:152
10:62
RHLVCNGDQDCLDGSDEDDCEDVRpc4_NP
p−maj=0.18, pX=0.15, N=36

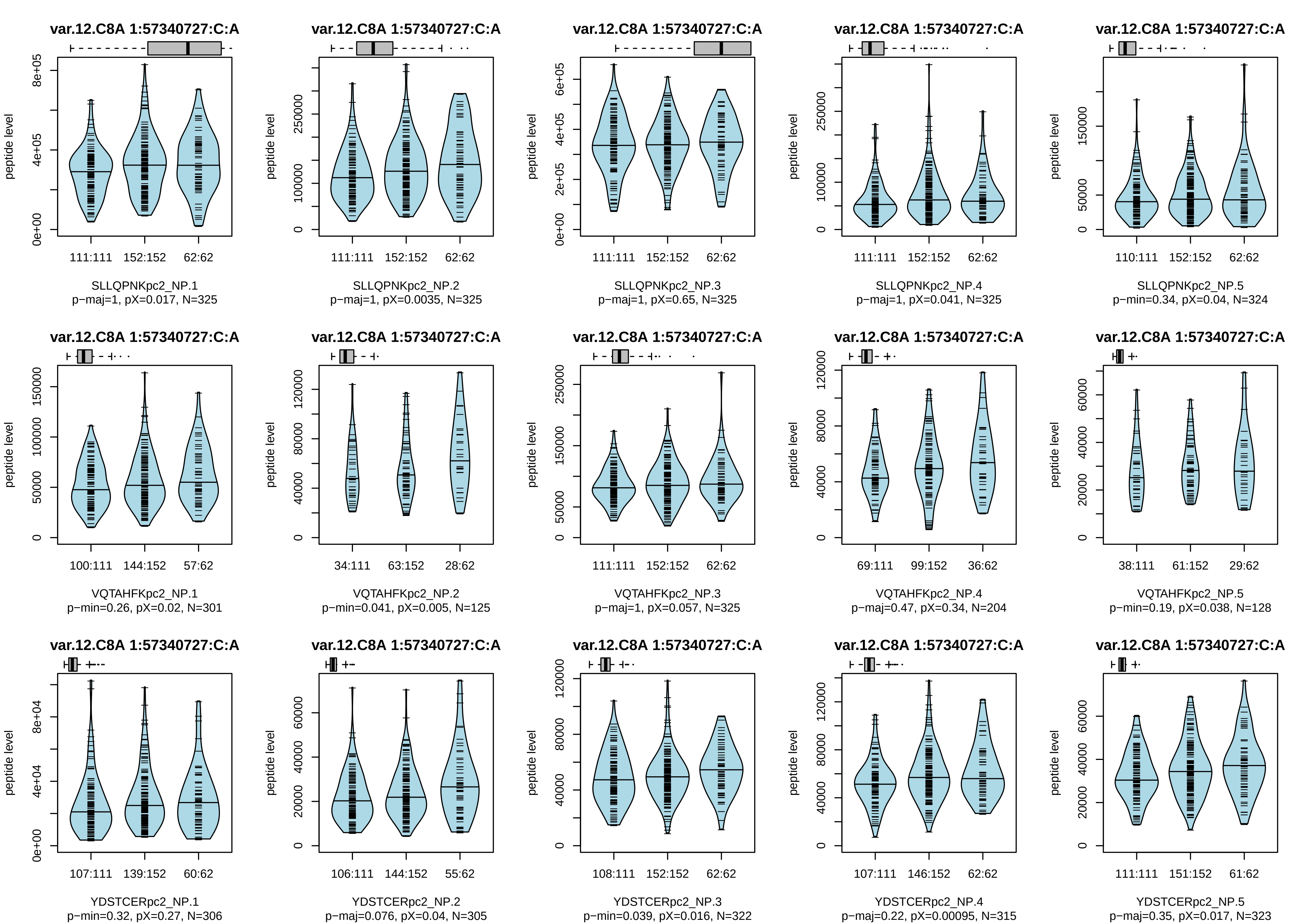

var.12.C8A 1:57340727:C:A
peptide level
111:111 152:152 62:62
SLLQPNKpc2_NP.1
p−maj=1, pX=0.017, N=325
var.12.C8A 1:57340727:C:A
peptide level
111:111 152:152 62:62
SLLQPNKpc2_NP.2
p−maj=1, pX=0.0035, N=325
var.12.C8A 1:57340727:C:A
peptide level
111:111 152:152 62:62
SLLQPNKpc2_NP.3
p−maj=1, pX=0.65, N=325
var.12.C8A 1:57340727:C:A
peptide level
111:111 152:152 62:62
SLLQPNKpc2_NP.4
p−maj=1, pX=0.041, N=325
var.12.C8A 1:57340727:C:A
peptide level
110:111 152:152 62:62
SLLQPNKpc2_NP.5
p−min=0.34, pX=0.04, N=324
var.12.C8A 1:57340727:C:A
peptide level
100:111 144:152 57:62
VQTAHFKpc2_NP.1
p−min=0.26, pX=0.02, N=301
var.12.C8A 1:57340727:C:A
peptide level
34:111 63:152 28:62
VQTAHFKpc2_NP.2
p−min=0.041, pX=0.005, N=125
var.12.C8A 1:57340727:C:A
peptide level
111:111 152:152 62:62
VQTAHFKpc2_NP.3
p−maj=1, pX=0.057, N=325
var.12.C8A 1:57340727:C:A
peptide level
69:111 99:152 36:62
VQTAHFKpc2_NP.4
p−maj=0.47, pX=0.34, N=204
var.12.C8A 1:57340727:C:A
peptide level
38:111 61:152 29:62
VQTAHFKpc2_NP.5
p−min=0.19, pX=0.038, N=128
var.12.C8A 1:57340727:C:A
peptide level
107:111 139:152 60:62
YDSTCERpc2_NP.1
p−min=0.32, pX=0.27, N=306
var.12.C8A 1:57340727:C:A
peptide level
106:111 144:152 55:62
YDSTCERpc2_NP.2
p−maj=0.076, pX=0.04, N=305
var.12.C8A 1:57340727:C:A
peptide level
108:111 152:152 62:62
YDSTCERpc2_NP.3
p−min=0.039, pX=0.016, N=322
var.12.C8A 1:57340727:C:A
peptide level
107:111 146:152 62:62
YDSTCERpc2_NP.4
p−maj=0.22, pX=0.00095, N=315
var.12.C8A 1:57340727:C:A
peptide level
111:111 151:152 61:62
YDSTCERpc2_NP.5
p−maj=0.35, pX=0.017, N=323

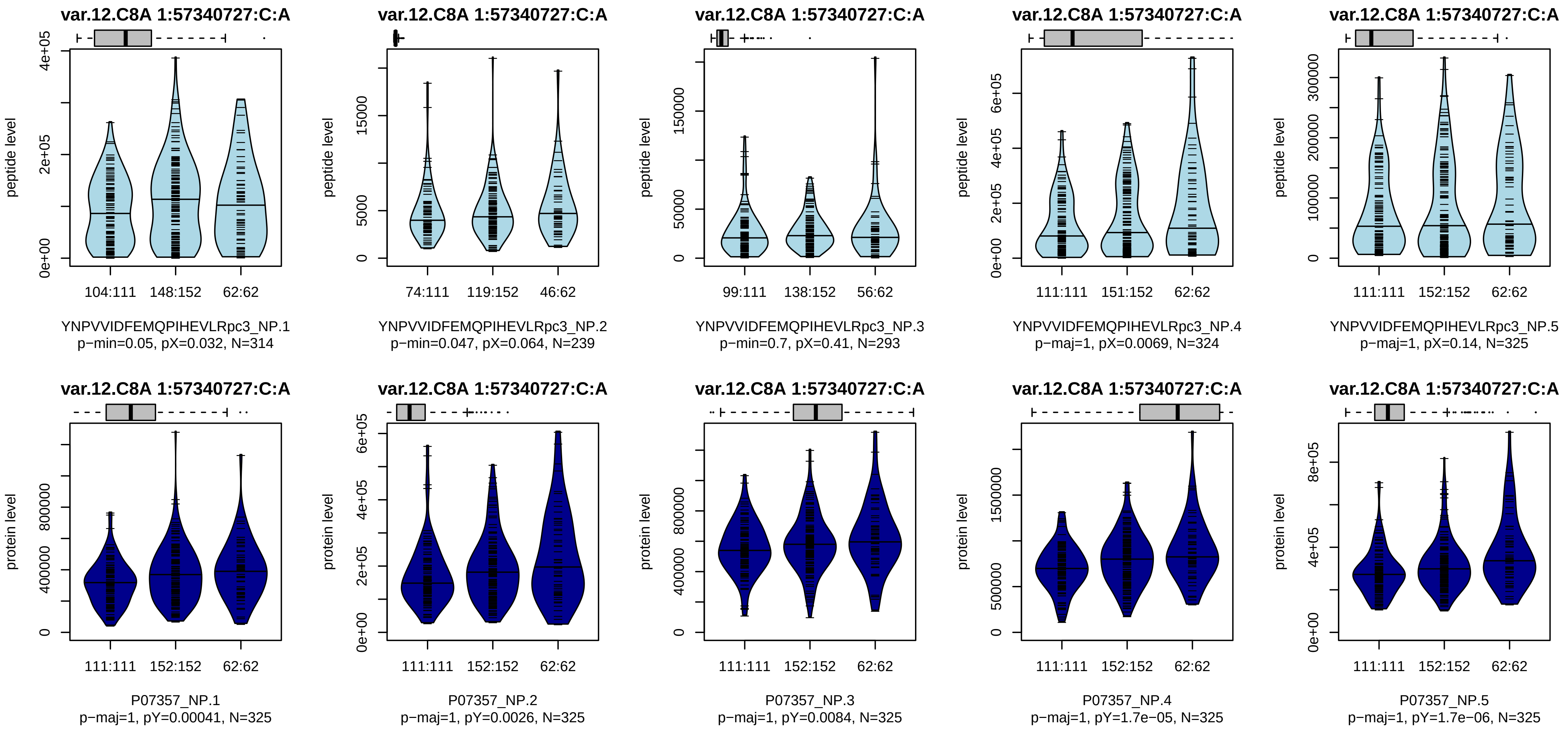

var.12.C8A 1:57340727:C:A
peptide level
0e+00
2e+05
4e+05
104:111
148:152
62:62
YNPVVIDFEMQPIHEVLRpc3_NP.1
p−min=0.05, pX=0.032, N=314
var.12.C8A 1:57340727:C:A
peptide level
0
5000
15000
74:111
119:152
46:62
YNPVVIDFEMQPIHEVLRpc3_NP.2
p−min=0.047, pX=0.064, N=239
var.12.C8A 1:57340727:C:A
peptide level
0
50000
150000
99:111
138:152
56:62
YNPVVIDFEMQPIHEVLRpc3_NP.3
p−min=0.7, pX=0.41, N=293
var.12.C8A 1:57340727:C:A
peptide level
0e+00
2e+05
4e+05
6e+05
111:111
151:152
62:62
YNPVVIDFEMQPIHEVLRpc3_NP.4
p−maj=1, pX=0.0069, N=324
var.12.C8A 1:57340727:C:A
peptide level
0
100000
200000
300000
111:111
152:152
62:62
YNPVVIDFEMQPIHEVLRpc3_NP.5
p−maj=1, pX=0.14, N=325
var.12.C8A 1:57340727:C:A
protein level
0
400000
800000
111:111
152:152
62:62
P07357_NP.1
p−maj=1, pY=0.00041, N=325
var.12.C8A 1:57340727:C:A
protein level
0e+00
2e+05
4e+05
6e+05
111:111
152:152
62:62
P07357_NP.2
p−maj=1, pY=0.0026, N=325
var.12.C8A 1:57340727:C:A
protein level
0
400000
800000
111:111
152:152
62:62
P07357_NP.3
p−maj=1, pY=0.0084, N=325
var.12.C8A 1:57340727:C:A
protein level
0
500000
1500000
111:111
152:152
62:62
P07357_NP.4
p−maj=1, pY=1.7e−05, N=325
var.12.C8A 1:57340727:C:A
protein level
0e+00
4e+05
8e+05
111:111
152:152
62:62
P07357_NP.5
p−maj=1, pY=1.7e−06, N=325

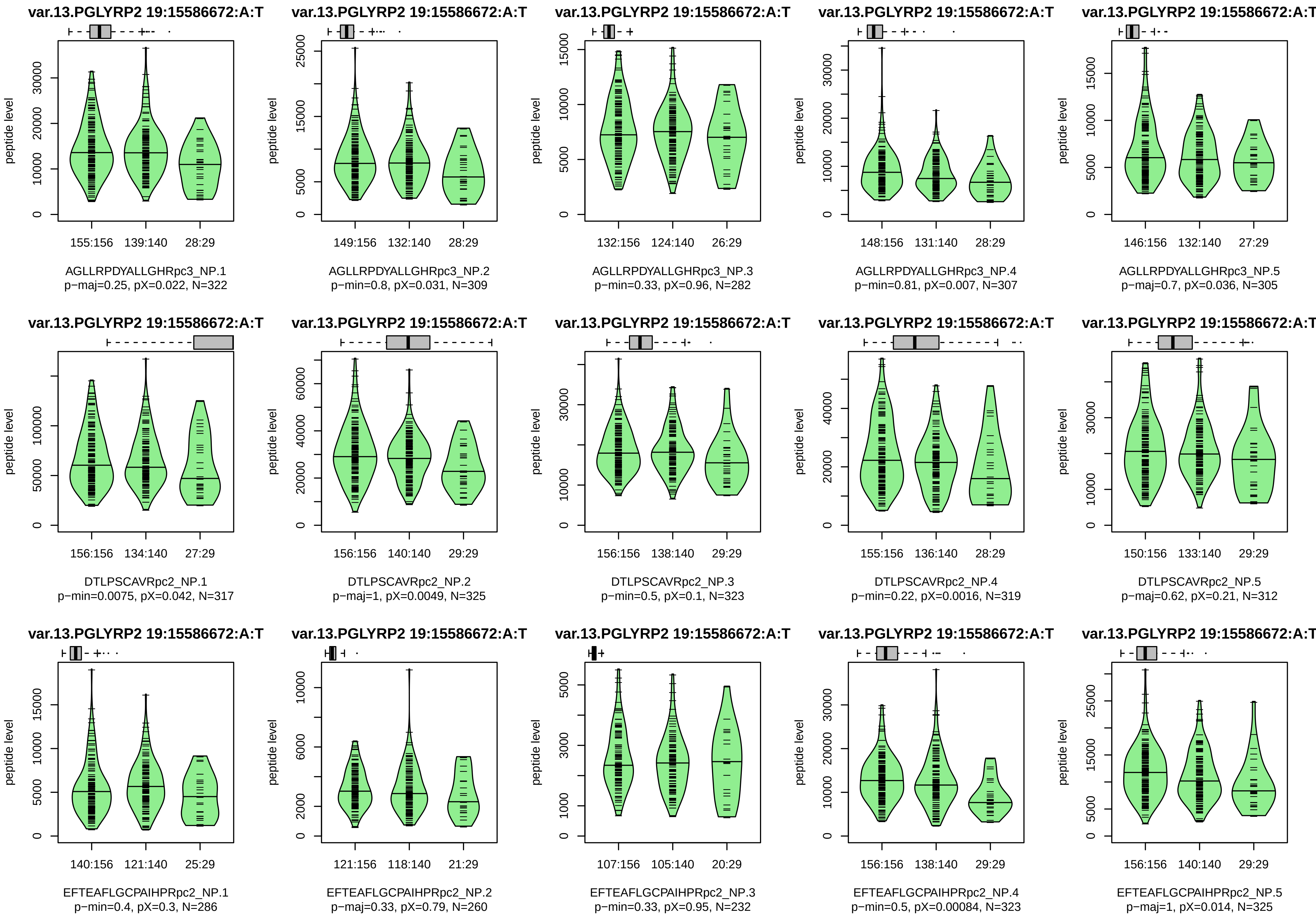

var.13.PGLYRP2 19:15586672:A:T
peptide level
155:156
139:140
28:29
AGLLRPDYALLGHRpc3_NP.1
p−maj=0.25, pX=0.022, N=322
var.13.PGLYRP2 19:15586672:A:T
peptide level
149:156
132:140
28:29
AGLLRPDYALLGHRpc3_NP.2
p−min=0.8, pX=0.031, N=309
var.13.PGLYRP2 19:15586672:A:T
peptide level
132:156
124:140
26:29
AGLLRPDYALLGHRpc3_NP.3
p−min=0.33, pX=0.96, N=282
var.13.PGLYRP2 19:15586672:A:T
peptide level
148:156
131:140
28:29
AGLLRPDYALLGHRpc3_NP.4
p−min=0.81, pX=0.007, N=307
var.13.PGLYRP2 19:15586672:A:T
peptide level
146:156
132:140
27:29
AGLLRPDYALLGHRpc3_NP.5
p−maj=0.7, pX=0.036, N=305
var.13.PGLYRP2 19:15586672:A:T
peptide level
156:156
134:140
27:29
DTLPSCAVRpc2_NP.1
p−min=0.0075, pX=0.042, N=317
var.13.PGLYRP2 19:15586672:A:T
peptide level
156:156
140:140
29:29
DTLPSCAVRpc2_NP.2
p−maj=1, pX=0.0049, N=325
var.13.PGLYRP2 19:15586672:A:T
peptide level
156:156
138:140
29:29
DTLPSCAVRpc2_NP.3
p−min=0.5, pX=0.1, N=323
var.13.PGLYRP2 19:15586672:A:T
peptide level
155:156
136:140
28:29
DTLPSCAVRpc2_NP.4
p−min=0.22, pX=0.0016, N=319
var.13.PGLYRP2 19:15586672:A:T
peptide level
150:156
133:140
29:29
DTLPSCAVRpc2_NP.5
p−maj=0.62, pX=0.21, N=312
var.13.PGLYRP2 19:15586672:A:T
peptide level
140:156
121:140
25:29
EFTEAFLGCPAIHPRpc2_NP.1
p−min=0.4, pX=0.3, N=286
var.13.PGLYRP2 19:15586672:A:T
peptide level
121:156
118:140
21:29
EFTEAFLGCPAIHPRpc2_NP.2
p−maj=0.33, pX=0.79, N=260
var.13.PGLYRP2 19:15586672:A:T
peptide level
107:156
105:140
20:29
EFTEAFLGCPAIHPRpc2_NP.3
p−min=0.33, pX=0.95, N=232
var.13.PGLYRP2 19:15586672:A:T
peptide level
156:156
138:140
29:29
EFTEAFLGCPAIHPRpc2_NP.4
p−min=0.5, pX=0.00084, N=323
var.13.PGLYRP2 19:15586672:A:T
peptide level
156:156
140:140
29:29
EFTEAFLGCPAIHPRpc2_NP.5
p−maj=1, pX=0.014, N=325

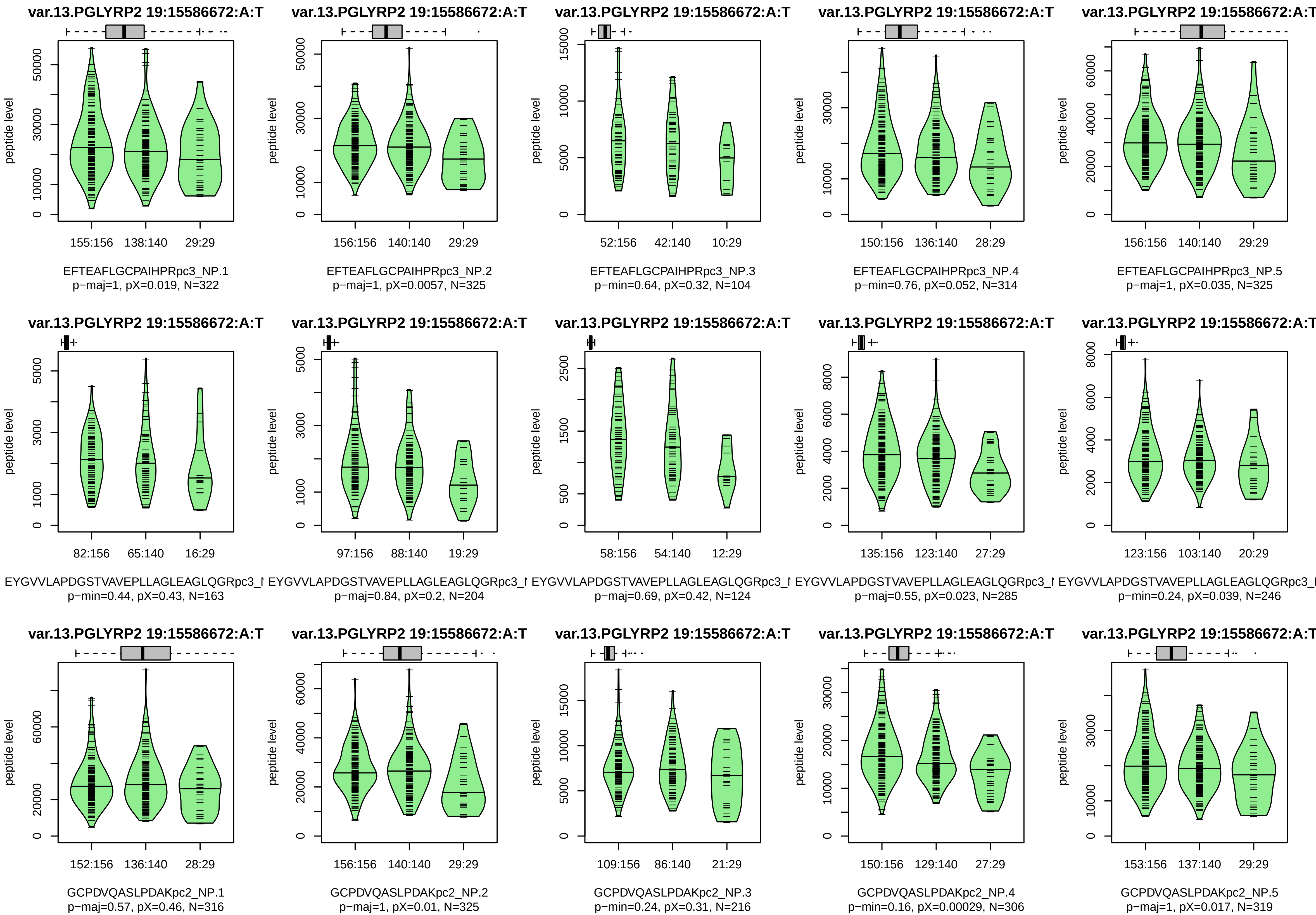

var.13.PGLYRP2 19:15586672:A:T
peptide level
155:156 138:140 29:29
EFTEAFLGCPAIHPRpc3_NP.1
p−maj=1, pX=0.019, N=322
var.13.PGLYRP2 19:15586672:A:T
peptide level
156:156 140:140 29:29
EFTEAFLGCPAIHPRpc3_NP.2
p−maj=1, pX=0.0057, N=325
var.13.PGLYRP2 19:15586672:A:T
peptide level
52:156 42:140 10:29
EFTEAFLGCPAIHPRpc3_NP.3
p−min=0.64, pX=0.32, N=104
var.13.PGLYRP2 19:15586672:A:T
peptide level
150:156 136:140 28:29
EFTEAFLGCPAIHPRpc3_NP.4
p−min=0.76, pX=0.052, N=314
var.13.PGLYRP2 19:15586672:A:T
peptide level
156:156 140:140 29:29
EFTEAFLGCPAIHPRpc3_NP.5
p−maj=1, pX=0.035, N=325
var.13.PGLYRP2 19:15586672:A:T
peptide level
82:156 65:140 16:29
EYGVVLAPDGSTVAVEPLLAGLEAGLQGRpc3_
p−min=0.44, pX=0.43, N=163
var.13.PGLYRP2 19:15586672:A:T
peptide level
97:156 88:140 19:29
EYGVVLAPDGSTVAVEPLLAGLEAGLQGRpc3_
p−maj=0.84, pX=0.2, N=204
var.13.PGLYRP2 19:15586672:A:T
peptide level
58:156 54:140 12:29
EYGVVLAPDGSTVAVEPLLAGLEAGLQGRpc3_
p−maj=0.69, pX=0.42, N=124
var.13.PGLYRP2 19:15586672:A:T
peptide level
135:156 123:140 27:29
EYGVVLAPDGSTVAVEPLLAGLEAGLQGRpc3_
p−maj=0.55, pX=0.023, N=285
var.13.PGLYRP2 19:15586672:A:T
peptide level
123:156 103:140 20:29
EYGVVLAPDGSTVAVEPLLAGLEAGLQGRpc3_
p−min=0.24, pX=0.039, N=246
var.13.PGLYRP2 19:15586672:A:T
peptide level
152:156 136:140 28:29
GCPDVQASLPDAKpc2_NP.1
p−maj=0.57, pX=0.46, N=316
var.13.PGLYRP2 19:15586672:A:T
peptide level
156:156 140:140 29:29
GCPDVQASLPDAKpc2_NP.2
p−maj=1, pX=0.01, N=325
var.13.PGLYRP2 19:15586672:A:T
peptide level
109:156 86:140 21:29
GCPDVQASLPDAKpc2_NP.3
p−min=0.24, pX=0.31, N=216
var.13.PGLYRP2 19:15586672:A:T
peptide level
150:156 129:140 27:29
GCPDVQASLPDAKpc2_NP.4
p−min=0.16, pX=0.00029, N=306
var.13.PGLYRP2 19:15586672:A:T
peptide level
153:156 137:140 29:29
GCPDVQASLPDAKpc2_NP.5
p−maj=1, pX=0.017, N=319

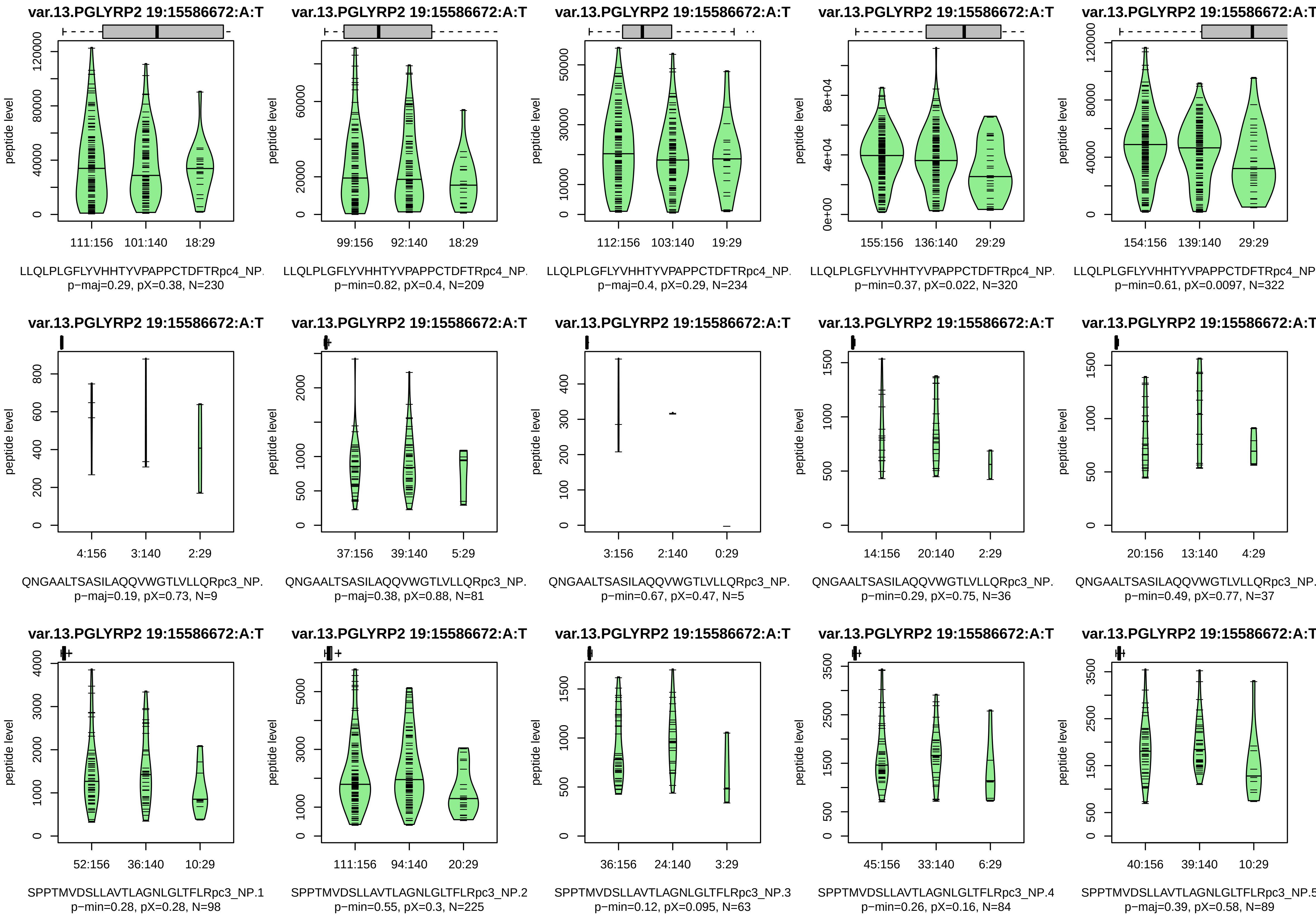
var.13.PGLYRP2 19:15586672:A:T
peptide level
111:156
101:140
18:29
LLQLPLGFLYVHHTYVPAPPCTDFTRpc4_NP.
p−maj=0.29, pX=0.38, N=230
99:156
92:140
18:29
LLQLPLGFLYVHHTYVPAPPCTDFTRpc4_NP.
p−min=0.82, pX=0.4, N=209
112:156
103:140
19:29
LLQLPLGFLYVHHTYVPAPPCTDFTRpc4_NP.
p−maj=0.4, pX=0.29, N=234
155:156
136:140
29:29
LLQLPLGFLYVHHTYVPAPPCTDFTRpc4_NP.
p−min=0.37, pX=0.022, N=320
154:156
139:140
29:29
LLQLPLGFLYVHHTYVPAPPCTDFTRpc4_NP
p−min=0.61, pX=0.0097, N=322
4:156
3:140
2:29
QNGAALTSASILAQQVWGTLVLLQRpc3_NP.
p−maj=0.19, pX=0.73, N=9
37:156
39:140
5:29
QNGAALTSASILAQQVWGTLVLLQRpc3_NP.
p−maj=0.38, pX=0.88, N=81
3:156
2:140
0:29
QNGAALTSASILAQQVWGTLVLLQRpc3_NP.
p−min=0.67, pX=0.47, N=5
14:156
20:140
2:29
QNGAALTSASILAQQVWGTLVLLQRpc3_NP.
p−min=0.29, pX=0.75, N=36
20:156
13:140
4:29
QNGAALTSASILAQQVWGTLVLLQRpc3_NP.
p−min=0.49, pX=0.77, N=37
52:156
36:140
10:29
SPPTMVDSLLAVTLAGNLGLTFLRpc3_NP.1
p−min=0.28, pX=0.28, N=98
111:156
94:140
20:29
SPPTMVDSLLAVTLAGNLGLTFLRpc3_NP.2
p−min=0.55, pX=0.3, N=225
36:156
24:140
3:29
SPPTMVDSLLAVTLAGNLGLTFLRpc3_NP.3
p−min=0.12, pX=0.095, N=63
45:156
33:140
6:29
SPPTMVDSLLAVTLAGNLGLTFLRpc3_NP.4
p−min=0.26, pX=0.16, N=84
40:156
39:140
10:29
SPPTMVDSLLAVTLAGNLGLTFLRpc3_NP.5
p−maj=0.39, pX=0.58, N=89

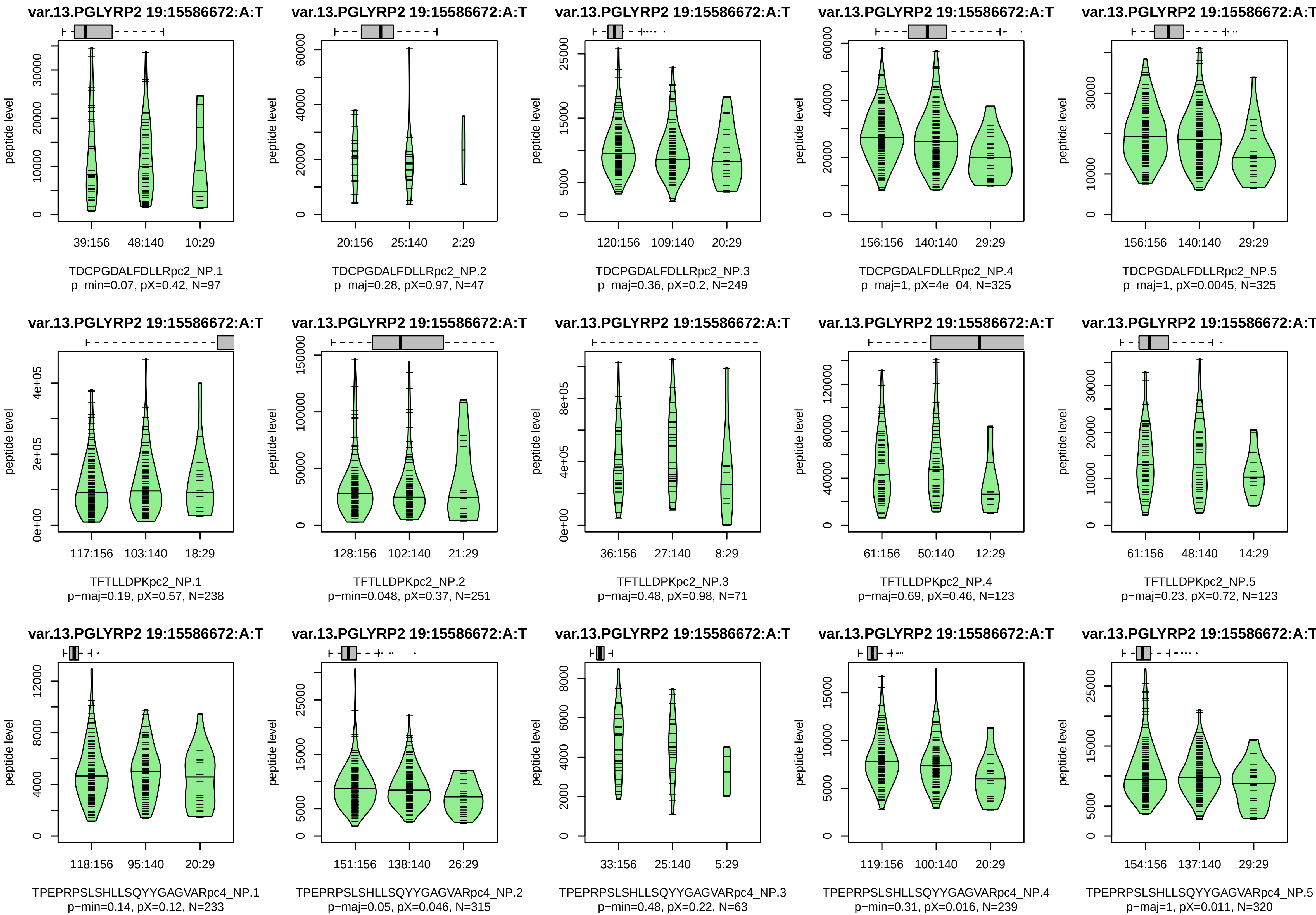
var.13.PGLYRP2 19:15586672:A:T
peptide level
39:156
48:140
10:29
TDCPGDALFDLLRpc2_NP.1
p−min=0.07, pX=0.42, N=97
var.13.PGLYRP2 19:15586672:A:T
peptide level
20:156
25:140
2:29
TDCPGDALFDLLRpc2_NP.2
p−maj=0.28, pX=0.97, N=47
var.13.PGLYRP2 19:15586672:A:T
peptide level
120:156
109:140
20:29
TDCPGDALFDLLRpc2_NP.3
p−maj=0.36, pX=0.2, N=249
var.13.PGLYRP2 19:15586672:A:T
peptide level
156:156
140:140
29:29
TDCPGDALFDLLRpc2_NP.4
p−maj=1, pX=4e−04, N=325
var.13.PGLYRP2 19:15586672:A:T
peptide level
156:156
140:140
29:29
TDCPGDALFDLLRpc2_NP.5
p−maj=1, pX=0.0045, N=325
var.13.PGLYRP2 19:15586672:A:T
peptide level
117:156
103:140
18:29
TFTLLDPKpc2_NP.1
p−maj=0.19, pX=0.57, N=238
var.13.PGLYRP2 19:15586672:A:T
peptide level
128:156
102:140
21:29
TFTLLDPKpc2_NP.2
p−min=0.048, pX=0.37, N=251
var.13.PGLYRP2 19:15586672:A:T
peptide level
36:156
27:140
8:29
TFTLLDPKpc2_NP.3
p−maj=0.48, pX=0.98, N=71
var.13.PGLYRP2 19:15586672:A:T
peptide level
61:156
50:140
12:29
TFTLLDPKpc2_NP.4
p−maj=0.69, pX=0.46, N=123
var.13.PGLYRP2 19:15586672:A:T
peptide level
61:156
48:140
14:29
TFTLLDPKpc2_NP.5
p−maj=0.23, pX=0.72, N=123
var.13.PGLYRP2 19:15586672:A:T
peptide level
118:156
95:140
20:29
TPEPRPSLSHLLSQYYGAGVARpc4_NP.1
p−min=0.14, pX=0.12, N=233
var.13.PGLYRP2 19:15586672:A:T
peptide level
151:156
138:140
26:29
TPEPRPSLSHLLSQYYGAGVARpc4_NP.2
p−maj=0.05, pX=0.046, N=315
var.13.PGLYRP2 19:15586672:A:T
peptide level
33:156
25:140
5:29
TPEPRPSLSHLLSQYYGAGVARpc4_NP.3
p−min=0.48, pX=0.22, N=63
var.13.PGLYRP2 19:15586672:A:T
peptide level
119:156
100:140
20:29
TPEPRPSLSHLLSQYYGAGVARpc4_NP.4
p−min=0.31, pX=0.016, N=239
var.13.PGLYRP2 19:15586672:A:T
peptide level
154:156
137:140
29:29
TPEPRPSLSHLLSQYYGAGVARpc4_NP.5
p−maj=1, pX=0.011, N=320

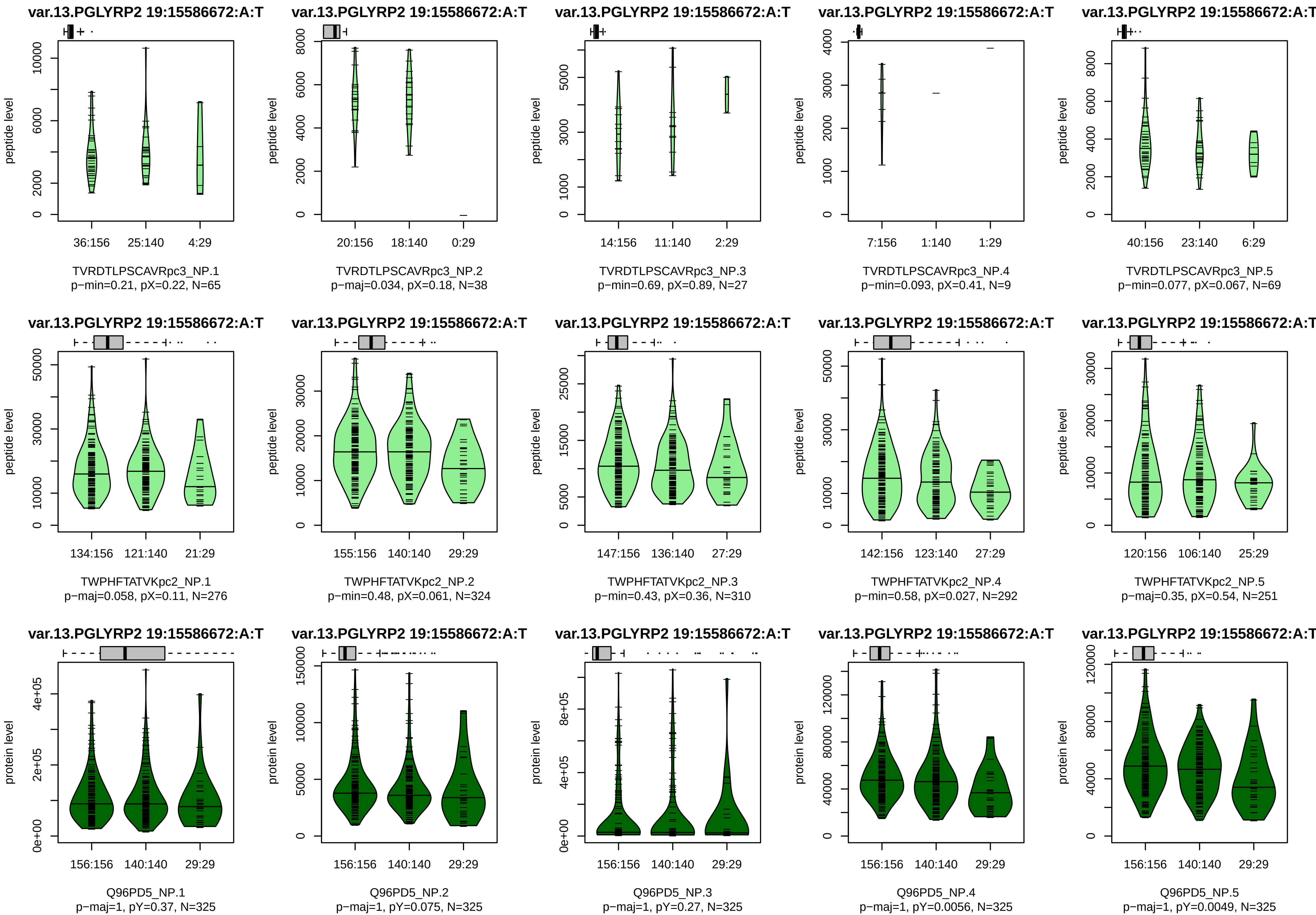

var.13.PGLYRP2 19:15586672:A:T
peptide level
36:156
25:140
4:29
TVRDTLPSCAVRpc3_NP.1
p−min=0.21, pX=0.22, N=65
var.13.PGLYRP2 19:15586672:A:T
peptide level
20:156
18:140
0:29
TVRDTLPSCAVRpc3_NP.2
p−maj=0.034, pX=0.18, N=38
var.13.PGLYRP2 19:15586672:A:T
peptide level
14:156
11:140
2:29
TVRDTLPSCAVRpc3_NP.3
p−min=0.69, pX=0.89, N=27
var.13.PGLYRP2 19:15586672:A:T
peptide level
7:156
1:140
1:29
TVRDTLPSCAVRpc3_NP.4
p−min=0.093, pX=0.41, N=9
var.13.PGLYRP2 19:15586672:A:T
peptide level
40:156
23:140
6:29
TVRDTLPSCAVRpc3_NP.5
p−min=0.077, pX=0.067, N=69
var.13.PGLYRP2 19:15586672:A:T
peptide level
134:156
121:140
21:29
TWPHFTATVKpc2_NP.1
p−maj=0.058, pX=0.11, N=276
var.13.PGLYRP2 19:15586672:A:T
peptide level
155:156
140:140
29:29
TWPHFTATVKpc2_NP.2
p−min=0.48, pX=0.061, N=324
var.13.PGLYRP2 19:15586672:A:T
peptide level
147:156
136:140
27:29
TWPHFTATVKpc2_NP.3
p−min=0.43, pX=0.36, N=310
var.13.PGLYRP2 19:15586672:A:T
peptide level
142:156
123:140
27:29
TWPHFTATVKpc2_NP.4
p−min=0.58, pX=0.027, N=292
var.13.PGLYRP2 19:15586672:A:T
peptide level
120:156
106:140
25:29
TWPHFTATVKpc2_NP.5
p−maj=0.35, pX=0.54, N=251
var.13.PGLYRP2 19:15586672:A:T
protein level
156:156
140:140
29:29
Q96PD5_NP.1
p−maj=1, pY=0.37, N=325
var.13.PGLYRP2 19:15586672:A:T
protein level
156:156
140:140
29:29
Q96PD5_NP.2
p−maj=1, pY=0.075, N=325
var.13.PGLYRP2 19:15586672:A:T
protein level
156:156
140:140
29:29
Q96PD5_NP.3
p−maj=1, pY=0.27, N=325
var.13.PGLYRP2 19:15586672:A:T
protein level
156:156
140:140
29:29
Q96PD5_NP.4
p−maj=1, pY=0.0056, N=325
var.13.PGLYRP2 19:15586672:A:T
protein level
156:156
140:140
29:29
Q96PD5_NP.5
p−maj=1, pY=0.0049, N=325

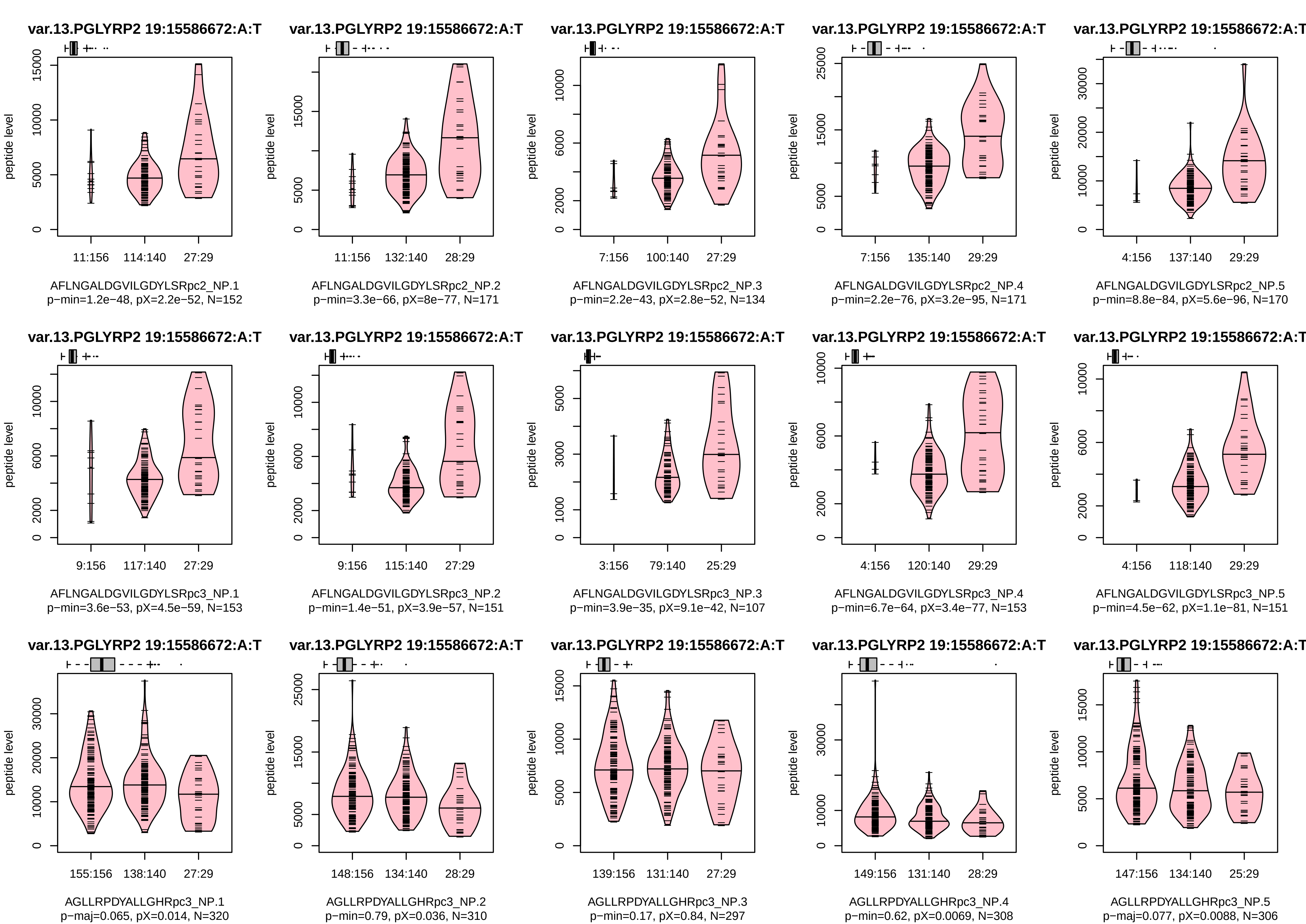
var.13.PGLYRP2 19:15586672:A:T
peptide level
11:156
114:140
27:29
AFLNGALDGVILGDYLSRpc2_NP.1
p−min=1.2e−48, pX=2.2e−52, N=152
var.13.PGLYRP2 19:15586672:A:T
peptide level
11:156
132:140
28:29
AFLNGALDGVILGDYLSRpc2_NP.2
p−min=3.3e−66, pX=8e−77, N=171
var.13.PGLYRP2 19:15586672:A:T
peptide level
7:156
100:140
27:29
AFLNGALDGVILGDYLSRpc2_NP.3
p−min=2.2e−43, pX=2.8e−52, N=134
var.13.PGLYRP2 19:15586672:A:T
peptide level
7:156
135:140
29:29
AFLNGALDGVILGDYLSRpc2_NP.4
p−min=2.2e−76, pX=3.2e−95, N=171
var.13.PGLYRP2 19:15586672:A:T
peptide level
4:156
137:140
29:29
AFLNGALDGVILGDYLSRpc2_NP.5
p−min=8.8e−84, pX=5.6e−96, N=170
var.13.PGLYRP2 19:15586672:A:T
peptide level
9:156
117:140
27:29
AFLNGALDGVILGDYLSRpc3_NP.1
p−min=3.6e−53, pX=4.5e−59, N=153
var.13.PGLYRP2 19:15586672:A:T
peptide level
9:156
115:140
27:29
AFLNGALDGVILGDYLSRpc3_NP.2
p−min=1.4e−51, pX=3.9e−57, N=151
var.13.PGLYRP2 19:15586672:A:T
peptide level
3:156
79:140
25:29
AFLNGALDGVILGDYLSRpc3_NP.3
p−min=3.9e−35, pX=9.1e−42, N=107
var.13.PGLYRP2 19:15586672:A:T
peptide level
4:156
120:140
29:29
AFLNGALDGVILGDYLSRpc3_NP.4
p−min=6.7e−64, pX=3.4e−77, N=153
var.13.PGLYRP2 19:15586672:A:T
peptide level
4:156
118:140
29:29
AFLNGALDGVILGDYLSRpc3_NP.5
p−min=4.5e−62, pX=1.1e−81, N=151
var.13.PGLYRP2 19:15586672:A:T
peptide level
155:156
138:140
27:29
AGLLRPDYALLGHRpc3_NP.1
p−maj=0.065, pX=0.014, N=320
var.13.PGLYRP2 19:15586672:A:T
peptide level
148:156
134:140
28:29
AGLLRPDYALLGHRpc3_NP.2
p−min=0.79, pX=0.036, N=310
var.13.PGLYRP2 19:15586672:A:T
peptide level
139:156
131:140
27:29
AGLLRPDYALLGHRpc3_NP.3
p−min=0.17, pX=0.84, N=297
var.13.PGLYRP2 19:15586672:A:T
peptide level
149:156
131:140
28:29
AGLLRPDYALLGHRpc3_NP.4
p−min=0.62, pX=0.0069, N=308
var.13.PGLYRP2 19:15586672:A:T
peptide level
147:156
134:140
25:29
AGLLRPDYALLGHRpc3_NP.5
p−maj=0.077, pX=0.0088, N=306

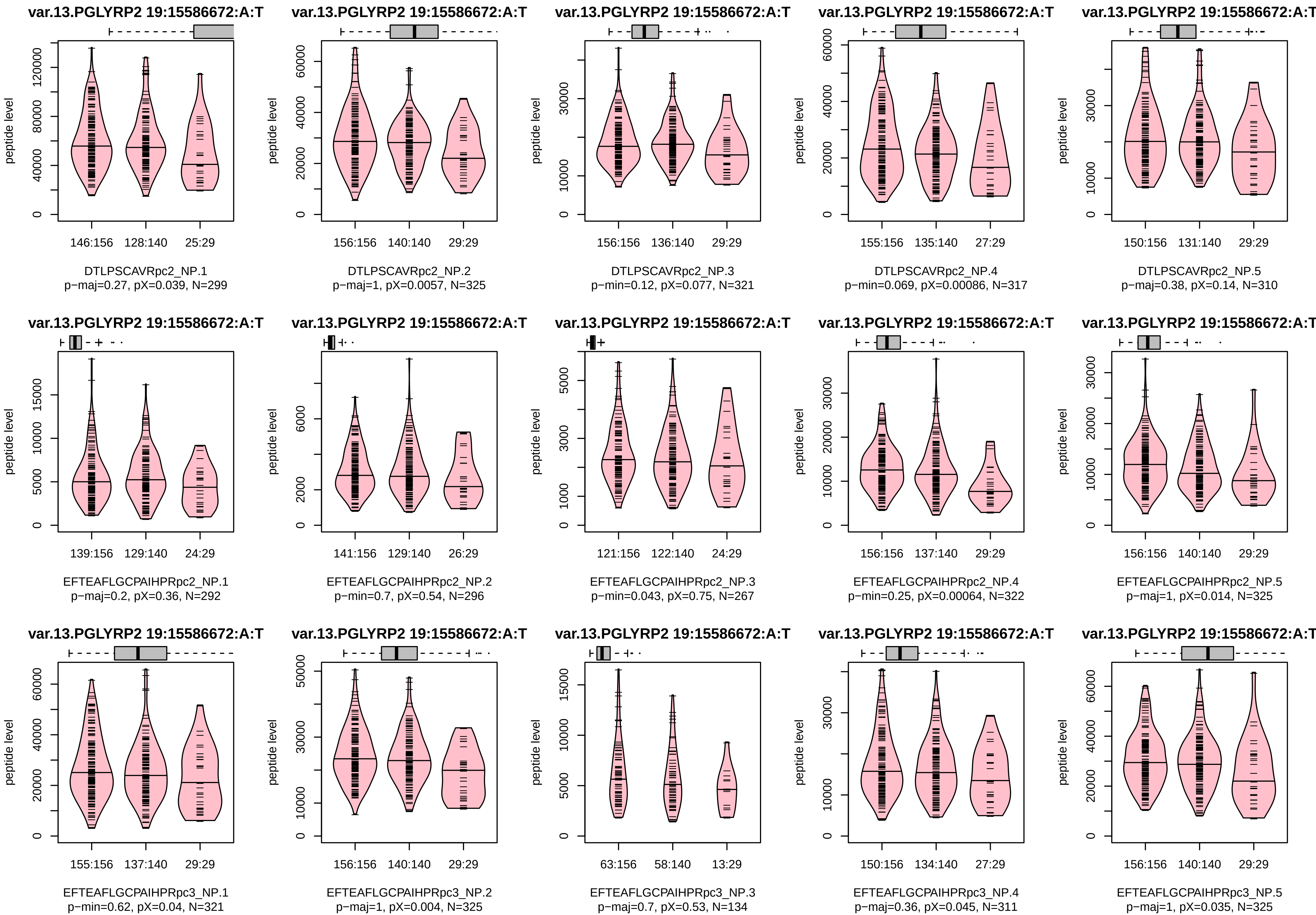
var.13.PGLYRP2 19:15586672:A:T
peptide level
146:156
128:140
25:29
DTLPSCAVRpc2_NP.1
p−maj=0.27, pX=0.039, N=299
var.13.PGLYRP2 19:15586672:A:T
peptide level
156:156
140:140
29:29
DTLPSCAVRpc2_NP.2
p−maj=1, pX=0.0057, N=325
var.13.PGLYRP2 19:15586672:A:T
peptide level
156:156
136:140
29:29
DTLPSCAVRpc2_NP.3
p−min=0.12, pX=0.077, N=321
var.13.PGLYRP2 19:15586672:A:T
peptide level
155:156
135:140
27:29
DTLPSCAVRpc2_NP.4
p−min=0.069, pX=0.00086, N=317
var.13.PGLYRP2 19:15586672:A:T
peptide level
150:156
131:140
29:29
DTLPSCAVRpc2_NP.5
p−maj=0.38, pX=0.14, N=310
var.13.PGLYRP2 19:15586672:A:T
peptide level
139:156
129:140
24:29
EFTEAFLGCPAIHPRpc2_NP.1
p−maj=0.2, pX=0.36, N=292
var.13.PGLYRP2 19:15586672:A:T
peptide level
141:156
129:140
26:29
EFTEAFLGCPAIHPRpc2_NP.2
p−min=0.7, pX=0.54, N=296
var.13.PGLYRP2 19:15586672:A:T
peptide level
121:156
122:140
24:29
EFTEAFLGCPAIHPRpc2_NP.3
p−min=0.043, pX=0.75, N=267
var.13.PGLYRP2 19:15586672:A:T
peptide level
156:156
137:140
29:29
EFTEAFLGCPAIHPRpc2_NP.4
p−min=0.25, pX=0.00064, N=322
var.13.PGLYRP2 19:15586672:A:T
peptide level
156:156
140:140
29:29
EFTEAFLGCPAIHPRpc2_NP.5
p−maj=1, pX=0.014, N=325
var.13.PGLYRP2 19:15586672:A:T
peptide level
155:156
137:140
29:29
EFTEAFLGCPAIHPRpc3_NP.1
p−min=0.62, pX=0.04, N=321
var.13.PGLYRP2 19:15586672:A:T
peptide level
156:156
140:140
29:29
EFTEAFLGCPAIHPRpc3_NP.2
p−maj=1, pX=0.004, N=325
var.13.PGLYRP2 19:15586672:A:T
peptide level
63:156
58:140
13:29
EFTEAFLGCPAIHPRpc3_NP.3
p−maj=0.7, pX=0.53, N=134
var.13.PGLYRP2 19:15586672:A:T
peptide level
150:156
134:140
27:29
EFTEAFLGCPAIHPRpc3_NP.4
p−maj=0.36, pX=0.045, N=311
var.13.PGLYRP2 19:15586672:A:T
peptide level
156:156
140:140
29:29
EFTEAFLGCPAIHPRpc3_NP.5
p−maj=1, pX=0.035, N=325

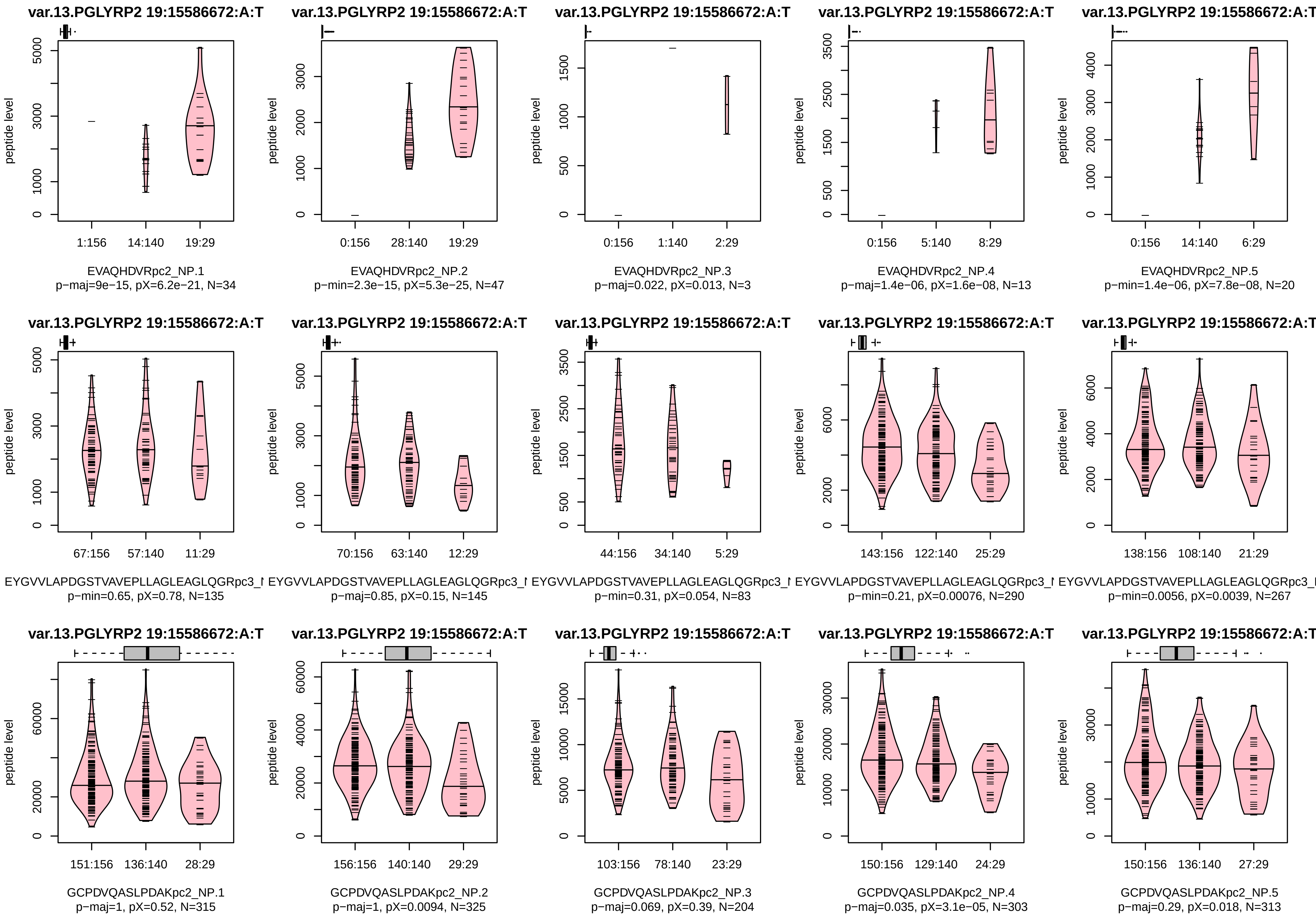
var.13.PGLYRP2 19:15586672:A:T
peptide level
1:156
14:140
19:29
EVAQHDVRpc2_NP.1
p−maj=9e−15, pX=6.2e−21, N=34
var.13.PGLYRP2 19:15586672:A:T
peptide level
0:156
28:140
19:29
EVAQHDVRpc2_NP.2
p−min=2.3e−15, pX=5.3e−25, N=47
var.13.PGLYRP2 19:15586672:A:T
peptide level
0:156
1:140
2:29
EVAQHDVRpc2_NP.3
p−maj=0.022, pX=0.013, N=3
var.13.PGLYRP2 19:15586672:A:T
peptide level
0:156
5:140
8:29
EVAQHDVRpc2_NP.4
p−maj=1.4e−06, pX=1.6e−08, N=13
var.13.PGLYRP2 19:15586672:A:T
peptide level
0:156
14:140
6:29
EVAQHDVRpc2_NP.5
p−min=1.4e−06, pX=7.8e−08, N=20
var.13.PGLYRP2 19:15586672:A:T
peptide level
67:156
57:140
11:29
EYGVVLAPDGSTVAVEPLLAGLEAGLQGRpc3_
p−min=0.65, pX=0.78, N=135
var.13.PGLYRP2 19:15586672:A:T
peptide level
70:156
63:140
12:29
EYGVVLAPDGSTVAVEPLLAGLEAGLQGRpc3_
p−maj=0.85, pX=0.15, N=145
var.13.PGLYRP2 19:15586672:A:T
peptide level
44:156
34:140
5:29
EYGVVLAPDGSTVAVEPLLAGLEAGLQGRpc3_
p−min=0.31, pX=0.054, N=83
var.13.PGLYRP2 19:15586672:A:T
peptide level
143:156
122:140
25:29
EYGVVLAPDGSTVAVEPLLAGLEAGLQGRpc3_
p−min=0.21, pX=0.00076, N=290
var.13.PGLYRP2 19:15586672:A:T
peptide level
138:156
108:140
21:29
EYGVVLAPDGSTVAVEPLLAGLEAGLQGRpc3_
p−min=0.0056, pX=0.0039, N=267
var.13.PGLYRP2 19:15586672:A:T
peptide level
151:156
136:140
28:29
GCPDVQASLPDAKpc2_NP.1
p−maj=1, pX=0.52, N=315
var.13.PGLYRP2 19:15586672:A:T
peptide level
156:156
140:140
29:29
GCPDVQASLPDAKpc2_NP.2
p−maj=1, pX=0.0094, N=325
var.13.PGLYRP2 19:15586672:A:T
peptide level
103:156
78:140
23:29
GCPDVQASLPDAKpc2_NP.3
p−maj=0.069, pX=0.39, N=204
var.13.PGLYRP2 19:15586672:A:T
peptide level
150:156
129:140
24:29
GCPDVQASLPDAKpc2_NP.4
p−maj=0.035, pX=3.1e−05, N=303
var.13.PGLYRP2 19:15586672:A:T
peptide level
150:156
136:140
27:29
GCPDVQASLPDAKpc2_NP.5
p−maj=0.29, pX=0.018, N=313

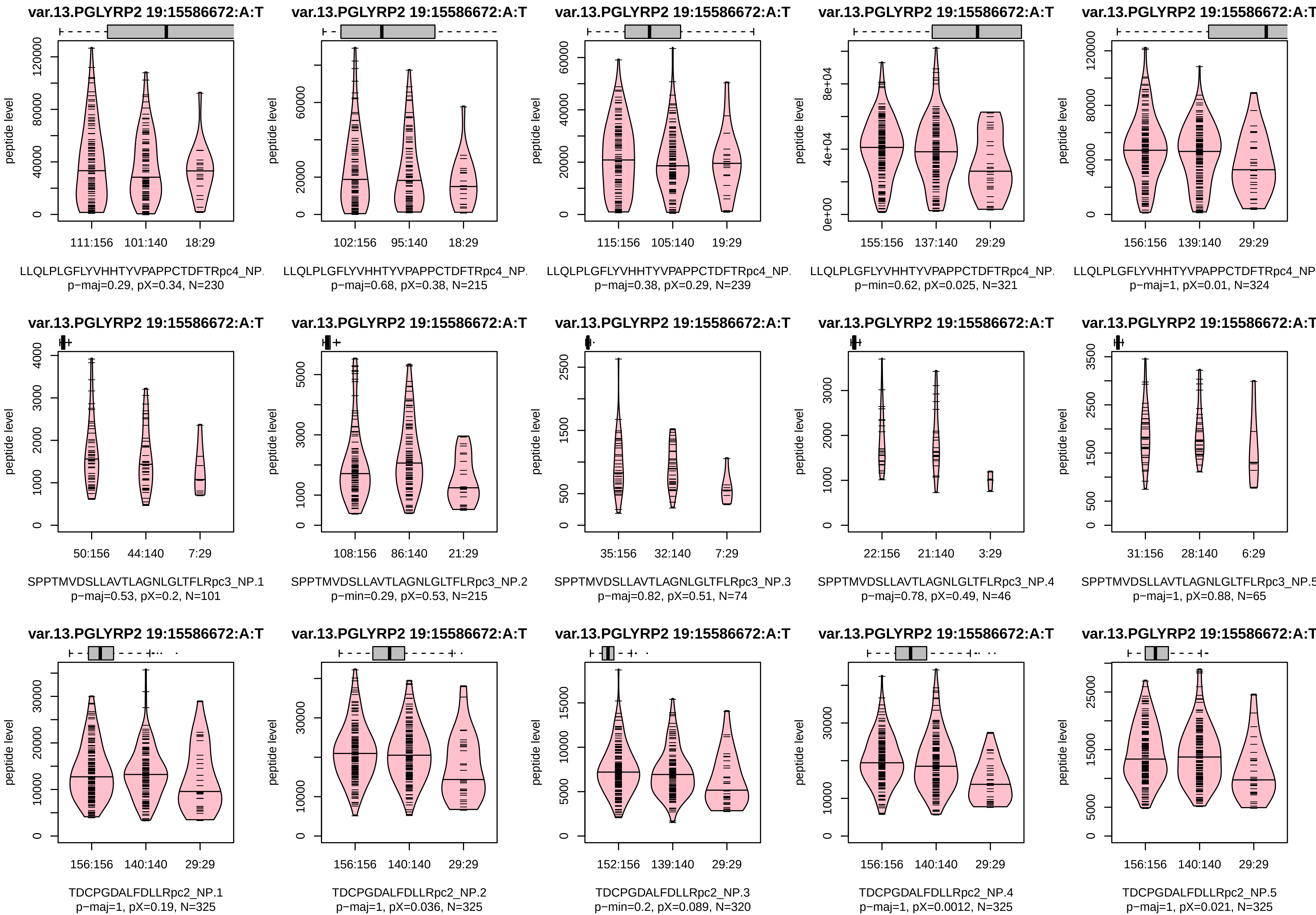
var.13.PGLYRP2 19:15586672:A:T
peptide level
111:156
101:140
18:29
LLQLPLGFLYVHHTYVPAPPCTDFTRpc4_NP.
p−maj=0.29, pX=0.34, N=230
var.13.PGLYRP2 19:15586672:A:T
102:156
95:140
18:29
LLQLPLGFLYVHHTYVPAPPCTDFTRpc4_NP.
p−maj=0.68, pX=0.38, N=215
var.13.PGLYRP2 19:15586672:A:T
115:156
105:140
19:29
LLQLPLGFLYVHHTYVPAPPCTDFTRpc4_NP.
p−maj=0.38, pX=0.29, N=239
var.13.PGLYRP2 19:15586672:A:T
155:156
137:140
29:29
LLQLPLGFLYVHHTYVPAPPCTDFTRpc4_NP.
p−min=0.62, pX=0.025, N=321
var.13.PGLYRP2 19:15586672:A:T
156:156
139:140
29:29
LLQLPLGFLYVHHTYVPAPPCTDFTRpc4_NP
p−maj=1, pX=0.01, N=324
var.13.PGLYRP2 19:15586672:A:T
50:156
44:140
7:29
SPPTMVDSLLAVTLAGNLGLTFLRpc3_NP.1
p−maj=0.53, pX=0.2, N=101
var.13.PGLYRP2 19:15586672:A:T
108:156
86:140
21:29
SPPTMVDSLLAVTLAGNLGLTFLRpc3_NP.2
p−min=0.29, pX=0.53, N=215
var.13.PGLYRP2 19:15586672:A:T
35:156
32:140
7:29
SPPTMVDSLLAVTLAGNLGLTFLRpc3_NP.3
p−maj=0.82, pX=0.51, N=74
var.13.PGLYRP2 19:15586672:A:T
22:156
21:140
3:29
SPPTMVDSLLAVTLAGNLGLTFLRpc3_NP.4
p−maj=0.78, pX=0.49, N=46
var.13.PGLYRP2 19:15586672:A:T
31:156
28:140
6:29
SPPTMVDSLLAVTLAGNLGLTFLRpc3_NP.5
p−maj=1, pX=0.88, N=65
var.13.PGLYRP2 19:15586672:A:T
156:156
140:140
29:29
TDCPGDALFDLLRpc2_NP.1
p−maj=1, pX=0.19, N=325
var.13.PGLYRP2 19:15586672:A:T
156:156
140:140
29:29
TDCPGDALFDLLRpc2_NP.2
p−maj=1, pX=0.036, N=325
var.13.PGLYRP2 19:15586672:A:T
152:156
139:140
29:29
TDCPGDALFDLLRpc2_NP.3
p−min=0.2, pX=0.089, N=320
var.13.PGLYRP2 19:15586672:A:T
156:156
140:140
29:29
TDCPGDALFDLLRpc2_NP.4
p−maj=1, pX=0.0012, N=325
var.13.PGLYRP2 19:15586672:A:T
156:156
140:140
29:29
TDCPGDALFDLLRpc2_NP.5
p−maj=1, pX=0.021, N=325

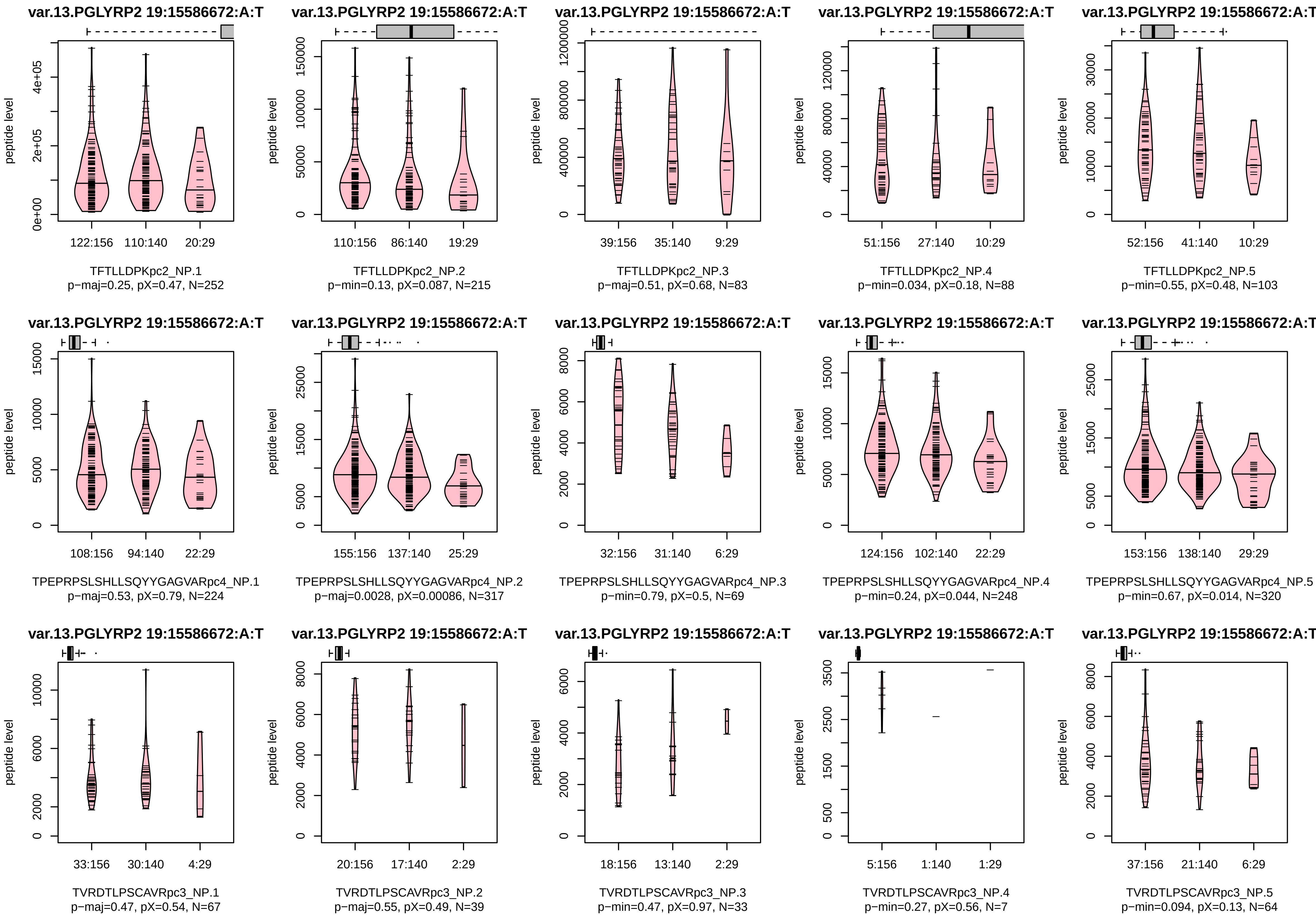
var.13.PGLYRP2 19:15586672:A:T
peptide level
122:156
110:140
20:29
TFTLLDPKpc2_NP.1
p−maj=0.25, pX=0.47, N=252
var.13.PGLYRP2 19:15586672:A:T
peptide level
110:156
86:140
19:29
TFTLLDPKpc2_NP.2
p−min=0.13, pX=0.087, N=215
var.13.PGLYRP2 19:15586672:A:T
peptide level
39:156
35:140
9:29
TFTLLDPKpc2_NP.3
p−maj=0.51, pX=0.68, N=83
var.13.PGLYRP2 19:15586672:A:T
peptide level
51:156
27:140
10:29
TFTLLDPKpc2_NP.4
p−min=0.034, pX=0.18, N=88
var.13.PGLYRP2 19:15586672:A:T
peptide level
52:156
41:140
10:29
TFTLLDPKpc2_NP.5
p−min=0.55, pX=0.48, N=103
var.13.PGLYRP2 19:15586672:A:T
peptide level
108:156
94:140
22:29
TPEPRPSLSHLLSQYYGAGVARpc4_NP.1
p−maj=0.53, pX=0.79, N=224
var.13.PGLYRP2 19:15586672:A:T
peptide level
155:156
137:140
25:29
TPEPRPSLSHLLSQYYGAGVARpc4_NP.2
p−maj=0.0028, pX=0.00086, N=317
var.13.PGLYRP2 19:15586672:A:T
peptide level
32:156
31:140
6:29
TPEPRPSLSHLLSQYYGAGVARpc4_NP.3
p−min=0.79, pX=0.5, N=69
var.13.PGLYRP2 19:15586672:A:T
peptide level
124:156
102:140
22:29
TPEPRPSLSHLLSQYYGAGVARpc4_NP.4
p−min=0.24, pX=0.044, N=248
var.13.PGLYRP2 19:15586672:A:T
peptide level
153:156
138:140
29:29
TPEPRPSLSHLLSQYYGAGVARpc4_NP.5
p−min=0.67, pX=0.014, N=320
var.13.PGLYRP2 19:15586672:A:T
peptide level
33:156
30:140
4:29
TVRDTLPSCAVRpc3_NP.1
p−maj=0.47, pX=0.54, N=67
var.13.PGLYRP2 19:15586672:A:T
peptide level
20:156
17:140
2:29
TVRDTLPSCAVRpc3_NP.2
p−maj=0.55, pX=0.49, N=39
var.13.PGLYRP2 19:15586672:A:T
peptide level
18:156
13:140
2:29
TVRDTLPSCAVRpc3_NP.3
p−min=0.47, pX=0.97, N=33
var.13.PGLYRP2 19:15586672:A:T
peptide level
5:156
1:140
1:29
TVRDTLPSCAVRpc3_NP.4
p−min=0.27, pX=0.56, N=7
var.13.PGLYRP2 19:15586672:A:T
peptide level
37:156
21:140
6:29
TVRDTLPSCAVRpc3_NP.5
p−min=0.094, pX=0.13, N=64

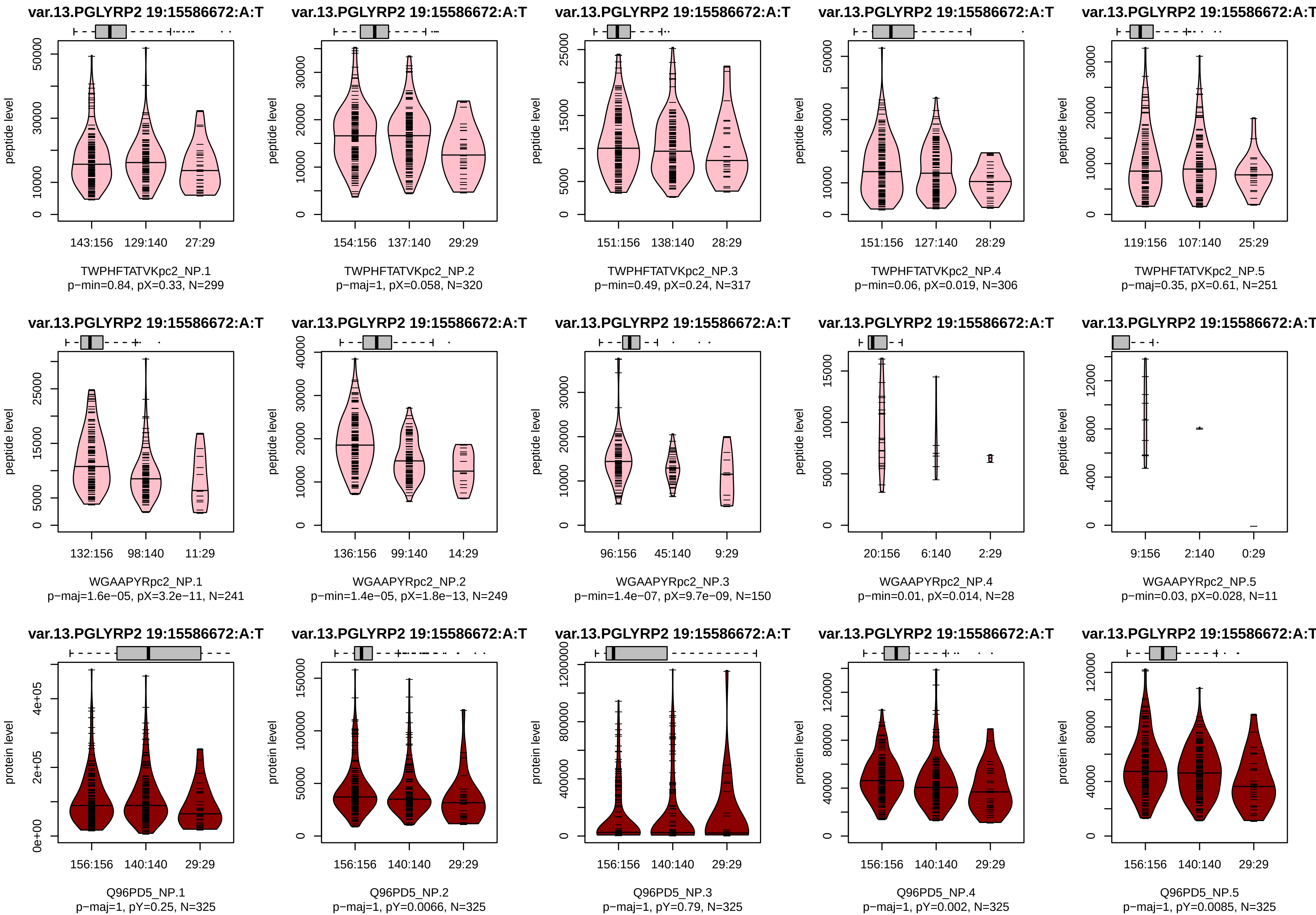
var.13.PGLYRP2 19:15586672:A:T
peptide level
143:156 129:140 27:29
TWPHFTATVKpc2_NP.1
p−min=0.84, pX=0.33, N=299
var.13.PGLYRP2 19:15586672:A:T
peptide level
154:156 137:140 29:29
TWPHFTATVKpc2_NP.2
p−maj=1, pX=0.058, N=320
var.13.PGLYRP2 19:15586672:A:T
peptide level
151:156 138:140 28:29
TWPHFTATVKpc2_NP.3
p−min=0.49, pX=0.24, N=317
var.13.PGLYRP2 19:15586672:A:T
peptide level
151:156 127:140 28:29
TWPHFTATVKpc2_NP.4
p−min=0.06, pX=0.019, N=306
var.13.PGLYRP2 19:15586672:A:T
peptide level
119:156 107:140 25:29
TWPHFTATVKpc2_NP.5
p−maj=0.35, pX=0.61, N=251
var.13.PGLYRP2 19:15586672:A:T
peptide level
132:156 98:140 11:29
WGAAPYRpc2_NP.1
p−maj=1.6e−05, pX=3.2e−11, N=241
var.13.PGLYRP2 19:15586672:A:T
peptide level
136:156 99:140 14:29
WGAAPYRpc2_NP.2
p−min=1.4e−05, pX=1.8e−13, N=249
var.13.PGLYRP2 19:15586672:A:T
peptide level
96:156 45:140 9:29
WGAAPYRpc2_NP.3
p−min=1.4e−07, pX=9.7e−09, N=150
var.13.PGLYRP2 19:15586672:A:T
peptide level
20:156 6:140 2:29
WGAAPYRpc2_NP.4
p−min=0.01, pX=0.014, N=28
var.13.PGLYRP2 19:15586672:A:T
peptide level
9:156 2:140 0:29
WGAAPYRpc2_NP.5
p−min=0.03, pX=0.028, N=11
var.13.PGLYRP2 19:15586672:A:T
protein level
156:156 140:140 29:29
Q96PD5_NP.1
p−maj=1, pY=0.25, N=325
var.13.PGLYRP2 19:15586672:A:T
protein level
156:156 140:140 29:29
Q96PD5_NP.2
p−maj=1, pY=0.0066, N=325
var.13.PGLYRP2 19:15586672:A:T
protein level
156:156 140:140 29:29
Q96PD5_NP.3
p−maj=1, pY=0.79, N=325
var.13.PGLYRP2 19:15586672:A:T
protein level
156:156 140:140 29:29
Q96PD5_NP.4
p−maj=1, pY=0.002, N=325
var.13.PGLYRP2 19:15586672:A:T
protein level
156:156 140:140 29:29
Q96PD5_NP.5
p−maj=1, pY=0.0085, N=325

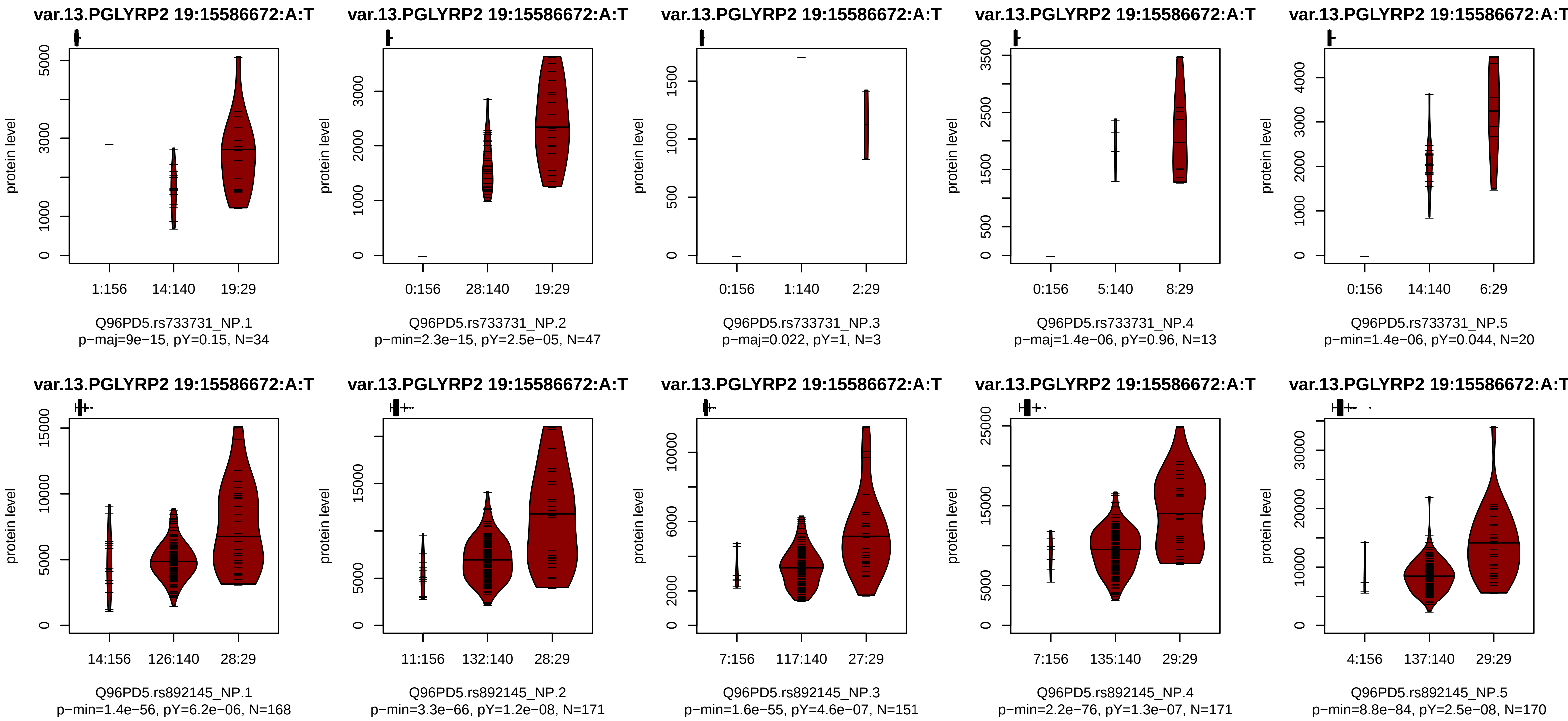

var.13.PGLYRP2 19:15586672:A:T
protein level
1:156
14:140
19:29
Q96PD5.rs733731_NP.1
p−maj=9e−15, pY=0.15, N=34
var.13.PGLYRP2 19:15586672:A:T
0:156
28:140
19:29
Q96PD5.rs733731_NP.2
p−min=2.3e−15, pY=2.5e−05, N=47
var.13.PGLYRP2 19:15586672:A:T
0:156
1:140
2:29
Q96PD5.rs733731_NP.3
p−maj=0.022, pY=1, N=3
var.13.PGLYRP2 19:15586672:A:T
0:156
5:140
8:29
Q96PD5.rs733731_NP.4
p−maj=1.4e−06, pY=0.96, N=13
var.13.PGLYRP2 19:15586672:A:T
0:156
14:140
6:29
Q96PD5.rs733731_NP.5
p−min=1.4e−06, pY=0.044, N=20
var.13.PGLYRP2 19:15586672:A:T
14:156
126:140
28:29
Q96PD5.rs892145_NP.1
p−min=1.4e−56, pY=6.2e−06, N=168
var.13.PGLYRP2 19:15586672:A:T
11:156
132:140
28:29
Q96PD5.rs892145_NP.2
p−min=3.3e−66, pY=1.2e−08, N=171
var.13.PGLYRP2 19:15586672:A:T
7:156
117:140
27:29
Q96PD5.rs892145_NP.3
p−min=1.6e−55, pY=4.6e−07, N=151
var.13.PGLYRP2 19:15586672:A:T
7:156
135:140
29:29
Q96PD5.rs892145_NP.4
p−min=2.2e−76, pY=1.3e−07, N=171
var.13.PGLYRP2 19:15586672:A:T
4:156
137:140
29:29
Q96PD5.rs892145_NP.5
p−min=8.8e−84, pY=2.5e−08, N=170

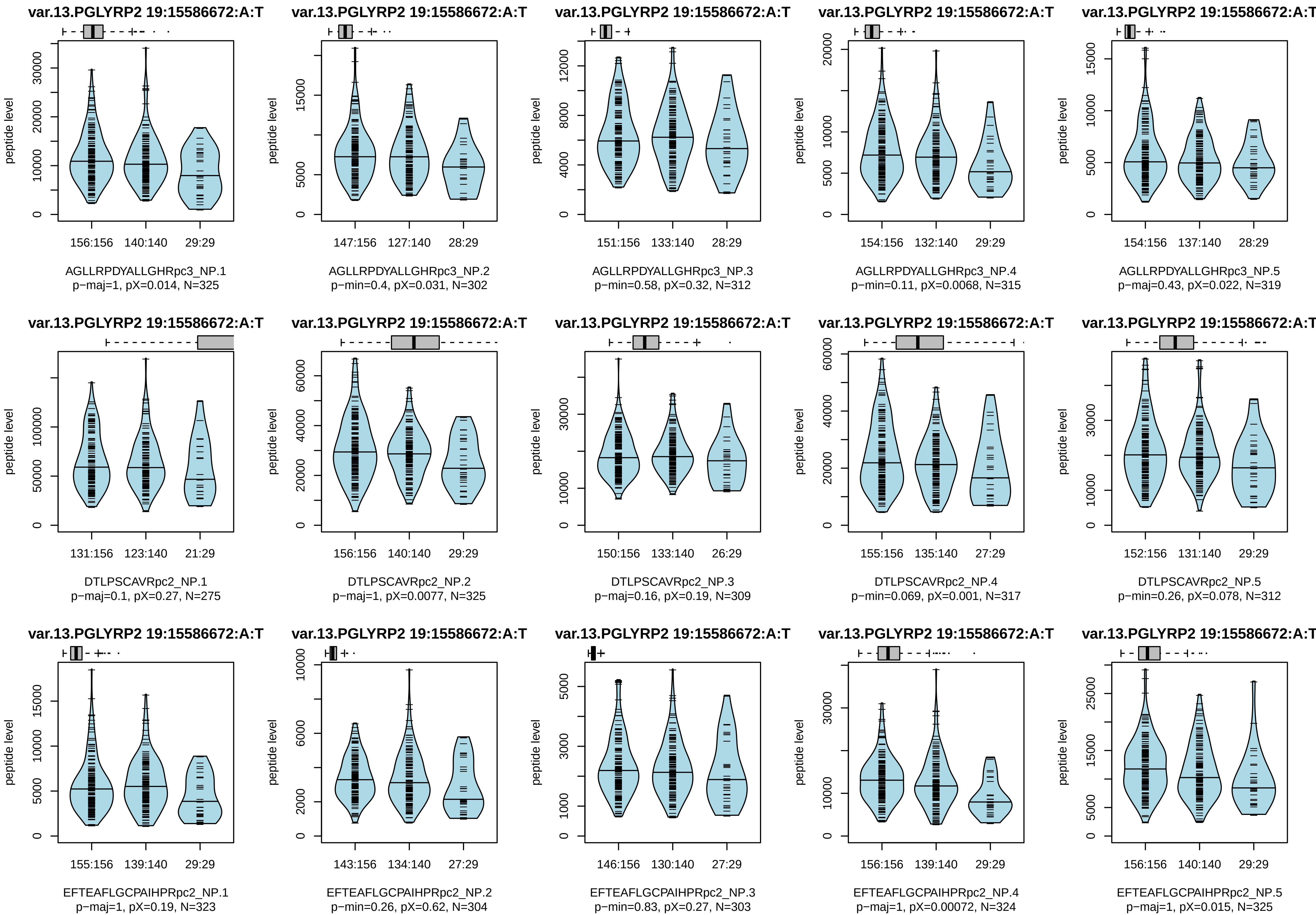
var.13.PGLYRP2 19:15586672:A:T
peptide level
156:156 140:140 29:29
AGLLRPDYALLGHRpc3_NP.1
p−maj=1, pX=0.014, N=325
var.13.PGLYRP2 19:15586672:A:T
peptide level
147:156 127:140 28:29
AGLLRPDYALLGHRpc3_NP.2
p−min=0.4, pX=0.031, N=302
var.13.PGLYRP2 19:15586672:A:T
peptide level
151:156 133:140 28:29
AGLLRPDYALLGHRpc3_NP.3
p−min=0.58, pX=0.32, N=312
var.13.PGLYRP2 19:15586672:A:T
peptide level
154:156 132:140 29:29
AGLLRPDYALLGHRpc3_NP.4
p−min=0.11, pX=0.0068, N=315
var.13.PGLYRP2 19:15586672:A:T
peptide level
154:156 137:140 28:29
AGLLRPDYALLGHRpc3_NP.5
p−maj=0.43, pX=0.022, N=319
var.13.PGLYRP2 19:15586672:A:T
peptide level
131:156 123:140 21:29
DTLPSCAVRpc2_NP.1
p−maj=0.1, pX=0.27, N=275
var.13.PGLYRP2 19:15586672:A:T
peptide level
156:156 140:140 29:29
DTLPSCAVRpc2_NP.2
p−maj=1, pX=0.0077, N=325
var.13.PGLYRP2 19:15586672:A:T
peptide level
150:156 133:140 26:29
DTLPSCAVRpc2_NP.3
p−maj=0.16, pX=0.19, N=309
var.13.PGLYRP2 19:15586672:A:T
peptide level
155:156 135:140 27:29
DTLPSCAVRpc2_NP.4
p−min=0.069, pX=0.001, N=317
var.13.PGLYRP2 19:15586672:A:T
peptide level
152:156 131:140 29:29
DTLPSCAVRpc2_NP.5
p−min=0.26, pX=0.078, N=312
var.13.PGLYRP2 19:15586672:A:T
peptide level
155:156 139:140 29:29
EFTEAFLGCPAIHPRpc2_NP.1
p−maj=1, pX=0.19, N=323
var.13.PGLYRP2 19:15586672:A:T
peptide level
143:156 134:140 27:29
EFTEAFLGCPAIHPRpc2_NP.2
p−min=0.26, pX=0.62, N=304
var.13.PGLYRP2 19:15586672:A:T
peptide level
146:156 130:140 27:29
EFTEAFLGCPAIHPRpc2_NP.3
p−min=0.83, pX=0.27, N=303
var.13.PGLYRP2 19:15586672:A:T
peptide level
156:156 139:140 29:29
EFTEAFLGCPAIHPRpc2_NP.4
p−maj=1, pX=0.00072, N=324
var.13.PGLYRP2 19:15586672:A:T
peptide level
156:156 140:140 29:29
EFTEAFLGCPAIHPRpc2_NP.5
p−maj=1, pX=0.015, N=325

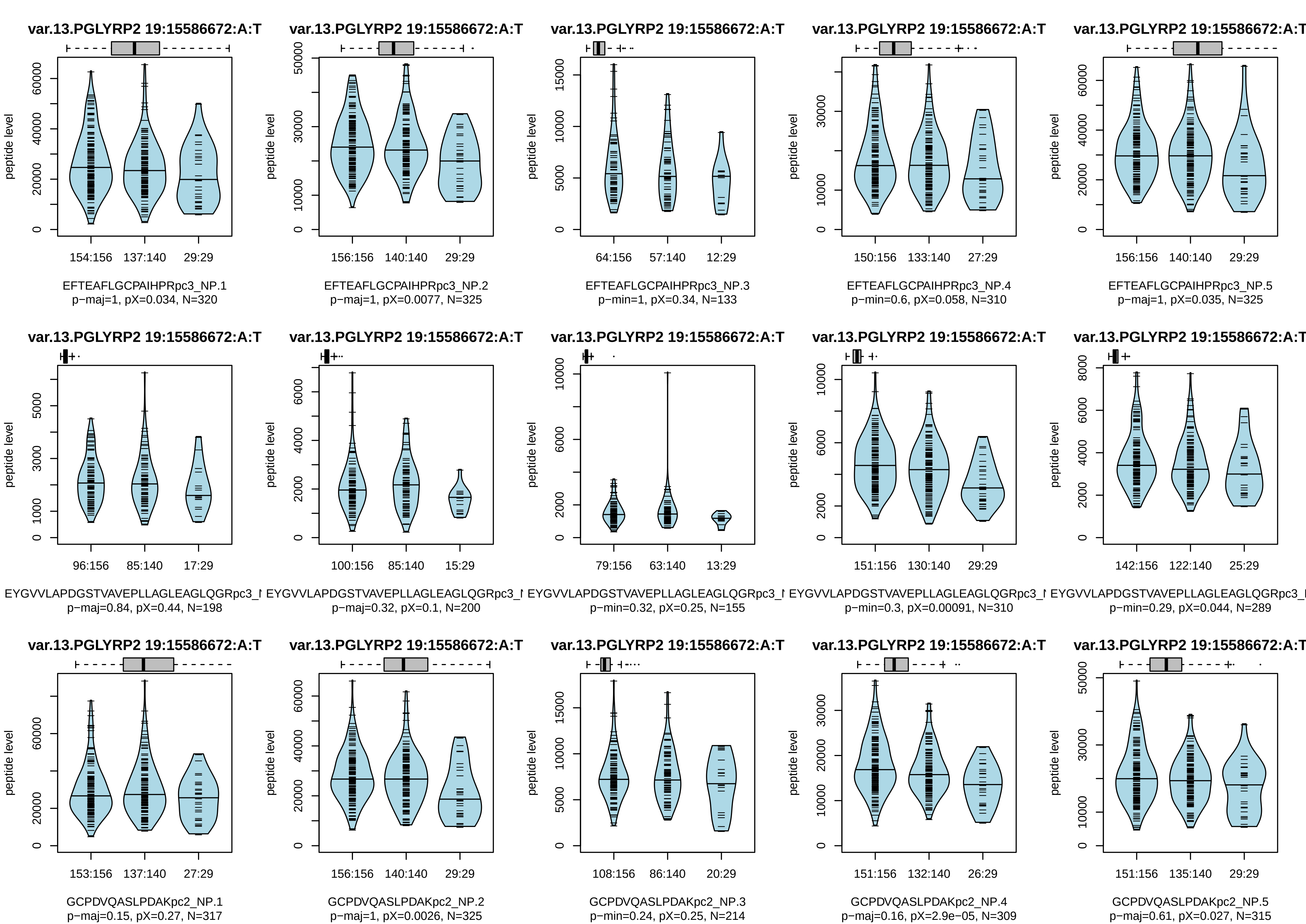
var.13.PGLYRP2 19:15586672:A:T
peptide level
154:156
137:140
29:29
EFTEAFLGCPAIHPRpc3_NP.1
p−maj=1, pX=0.034, N=320
var.13.PGLYRP2 19:15586672:A:T
peptide level
156:156
140:140
29:29
EFTEAFLGCPAIHPRpc3_NP.2
p−maj=1, pX=0.0077, N=325
var.13.PGLYRP2 19:15586672:A:T
peptide level
64:156
57:140
12:29
EFTEAFLGCPAIHPRpc3_NP.3
p−min=1, pX=0.34, N=133
var.13.PGLYRP2 19:15586672:A:T
peptide level
150:156
133:140
27:29
EFTEAFLGCPAIHPRpc3_NP.4
p−min=0.6, pX=0.058, N=310
var.13.PGLYRP2 19:15586672:A:T
peptide level
156:156
140:140
29:29
EFTEAFLGCPAIHPRpc3_NP.5
p−maj=1, pX=0.035, N=325
var.13.PGLYRP2 19:15586672:A:T
peptide level
96:156
85:140
17:29
EYGVVLAPDGSTVAVEPLLAGLEAGLQGRpc3_I
p−maj=0.84, pX=0.44, N=198
var.13.PGLYRP2 19:15586672:A:T
peptide level
100:156
85:140
15:29
EYGVVLAPDGSTVAVEPLLAGLEAGLQGRpc3_I
p−maj=0.32, pX=0.1, N=200
var.13.PGLYRP2 19:15586672:A:T
peptide level
79:156
63:140
13:29
EYGVVLAPDGSTVAVEPLLAGLEAGLQGRpc3_I
p−min=0.32, pX=0.25, N=155
var.13.PGLYRP2 19:15586672:A:T
peptide level
151:156
130:140
29:29
EYGVVLAPDGSTVAVEPLLAGLEAGLQGRpc3_I
p−min=0.3, pX=0.00091, N=310
var.13.PGLYRP2 19:15586672:A:T
peptide level
142:156
122:140
25:29
EYGVVLAPDGSTVAVEPLLAGLEAGLQGRpc3_I
p−min=0.29, pX=0.044, N=289
var.13.PGLYRP2 19:15586672:A:T
peptide level
153:156
137:140
27:29
GCPDVQASLPDAKpc2_NP.1
p−maj=0.15, pX=0.27, N=317
var.13.PGLYRP2 19:15586672:A:T
peptide level
156:156
140:140
29:29
GCPDVQASLPDAKpc2_NP.2
p−maj=1, pX=0.0026, N=325
var.13.PGLYRP2 19:15586672:A:T
peptide level
108:156
86:140
20:29
GCPDVQASLPDAKpc2_NP.3
p−min=0.24, pX=0.25, N=214
var.13.PGLYRP2 19:15586672:A:T
peptide level
151:156
132:140
26:29
GCPDVQASLPDAKpc2_NP.4
p−maj=0.16, pX=2.9e−05, N=309
var.13.PGLYRP2 19:15586672:A:T
peptide level
151:156
135:140
29:29
GCPDVQASLPDAKpc2_NP.5
p−maj=0.61, pX=0.027, N=315

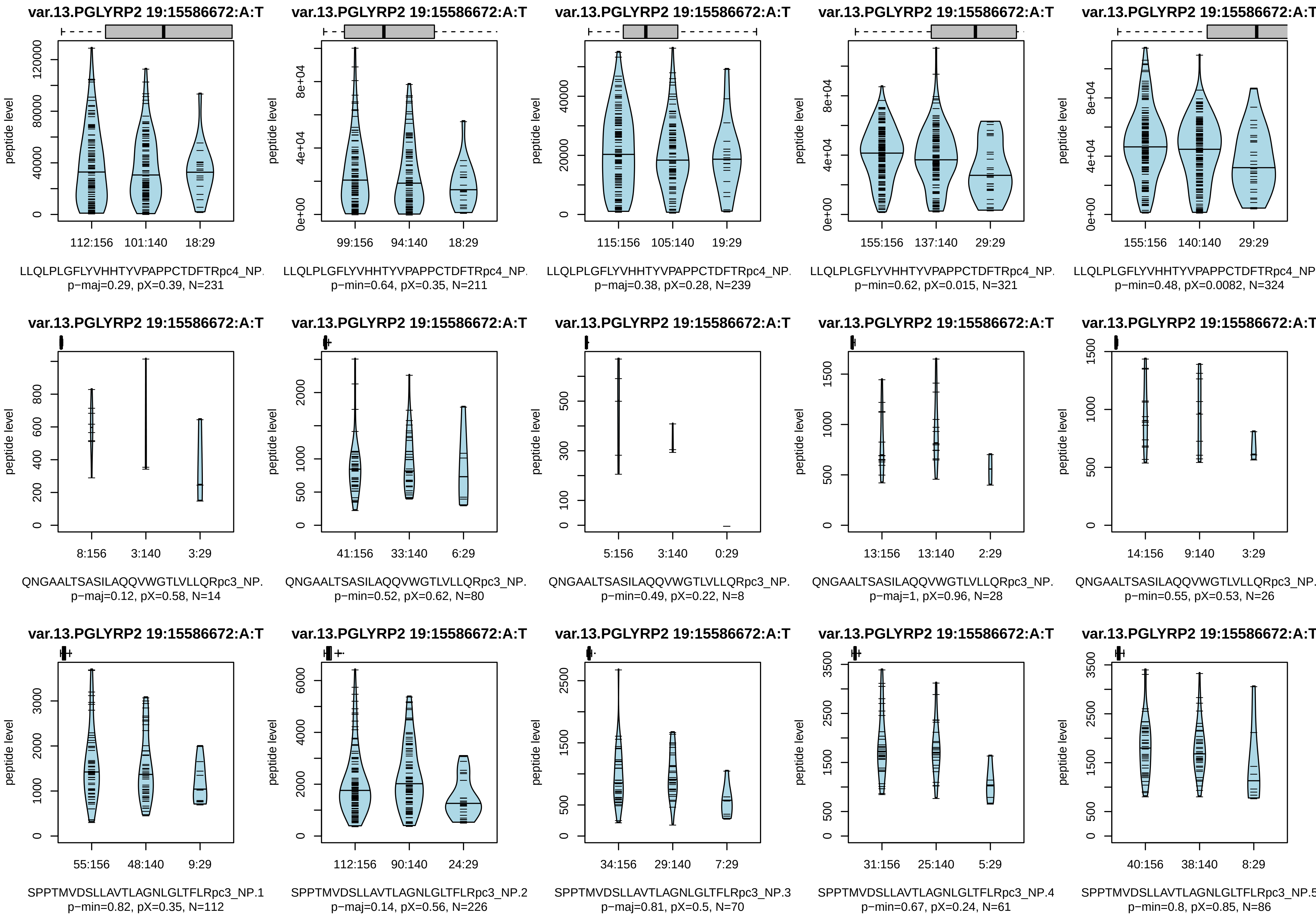

var.13.PGLYRP2 19:15586672:A:T
peptide level
112:156
101:140
18:29
LLQLPLGFLYVHHTYVPAPPCTDFTRpc4_NP.
p−maj=0.29, pX=0.39, N=231
var.13.PGLYRP2 19:15586672:A:T
peptide level
99:156
94:140
18:29
LLQLPLGFLYVHHTYVPAPPCTDFTRpc4_NP.
p−min=0.64, pX=0.35, N=211
var.13.PGLYRP2 19:15586672:A:T
peptide level
115:156
105:140
19:29
LLQLPLGFLYVHHTYVPAPPCTDFTRpc4_NP.
p−maj=0.38, pX=0.28, N=239
var.13.PGLYRP2 19:15586672:A:T
peptide level
155:156
137:140
29:29
LLQLPLGFLYVHHTYVPAPPCTDFTRpc4_NP.
p−min=0.62, pX=0.015, N=321
var.13.PGLYRP2 19:15586672:A:T
peptide level
155:156
140:140
29:29
LLQLPLGFLYVHHTYVPAPPCTDFTRpc4_NP
p−min=0.48, pX=0.0082, N=324
var.13.PGLYRP2 19:15586672:A:T
peptide level
8:156
3:140
3:29
QNGAALTSASILAQQVWGTLVLLQRpc3_NP.
p−maj=0.12, pX=0.58, N=14
var.13.PGLYRP2 19:15586672:A:T
peptide level
41:156
33:140
6:29
QNGAALTSASILAQQVWGTLVLLQRpc3_NP.
p−min=0.52, pX=0.62, N=80
var.13.PGLYRP2 19:15586672:A:T
peptide level
5:156
3:140
0:29
QNGAALTSASILAQQVWGTLVLLQRpc3_NP.
p−min=0.49, pX=0.22, N=8
var.13.PGLYRP2 19:15586672:A:T
peptide level
13:156
13:140
2:29
QNGAALTSASILAQQVWGTLVLLQRpc3_NP.
p−maj=1, pX=0.96, N=28
var.13.PGLYRP2 19:15586672:A:T
peptide level
14:156
9:140
3:29
QNGAALTSASILAQQVWGTLVLLQRpc3_NP.
p−min=0.55, pX=0.53, N=26
var.13.PGLYRP2 19:15586672:A:T
peptide level
55:156
48:140
9:29
SPPTMVDSLLAVTLAGNLGLTFLRpc3_NP.1
p−min=0.82, pX=0.35, N=112
var.13.PGLYRP2 19:15586672:A:T
peptide level
112:156
90:140
24:29
SPPTMVDSLLAVTLAGNLGLTFLRpc3_NP.2
p−maj=0.14, pX=0.56, N=226
var.13.PGLYRP2 19:15586672:A:T
peptide level
34:156
29:140
7:29
SPPTMVDSLLAVTLAGNLGLTFLRpc3_NP.3
p−maj=0.81, pX=0.5, N=70
var.13.PGLYRP2 19:15586672:A:T
peptide level
31:156
25:140
5:29
SPPTMVDSLLAVTLAGNLGLTFLRpc3_NP.4
p−min=0.67, pX=0.24, N=61
var.13.PGLYRP2 19:15586672:A:T
peptide level
40:156
38:140
8:29
SPPTMVDSLLAVTLAGNLGLTFLRpc3_NP.5
p−min=0.8, pX=0.85, N=86

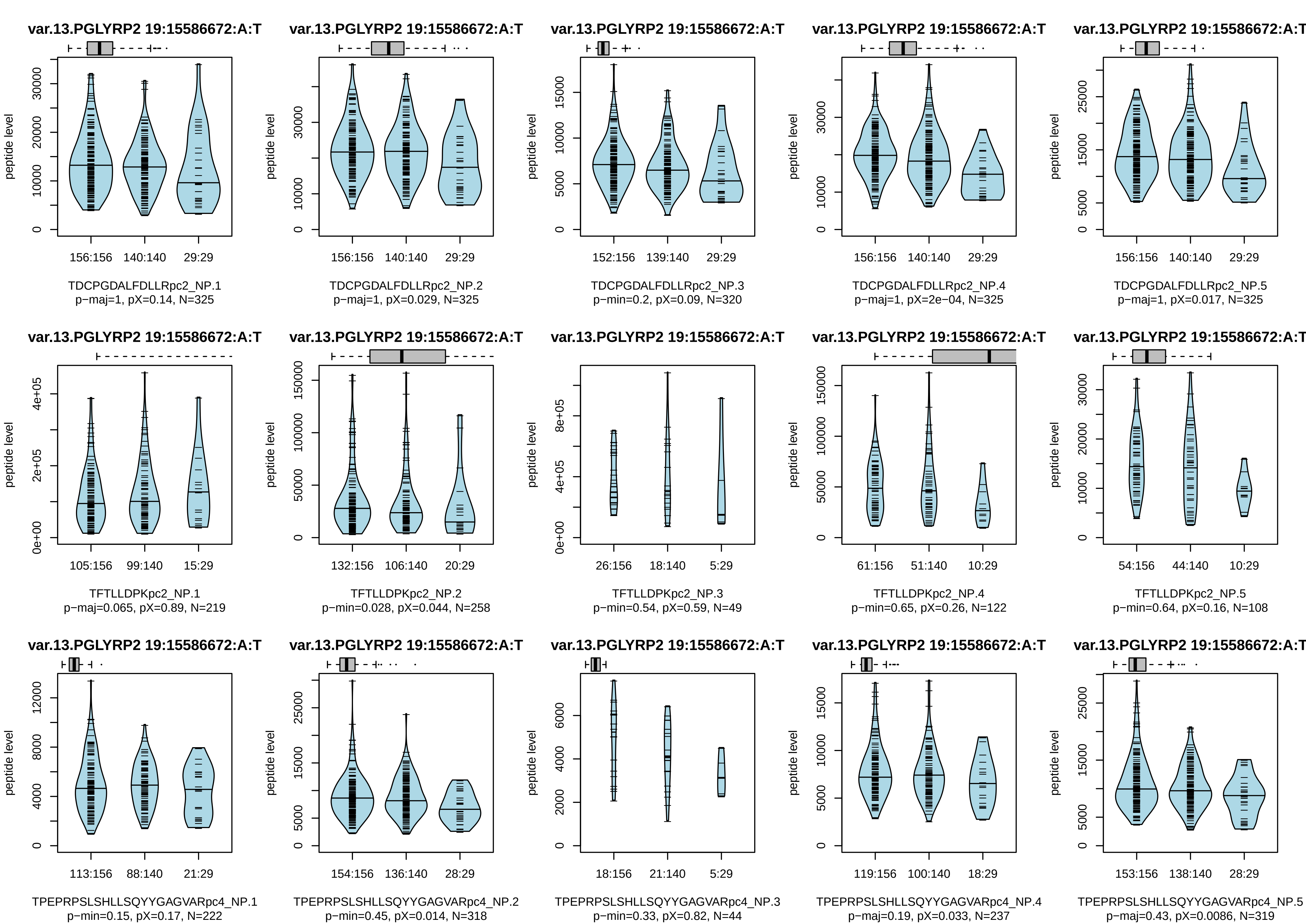
var.13.PGLYRP2 19:15586672:A:T
peptide level
156:156
140:140
29:29
TDCPGDALFDLLRpc2_NP.1
p−maj=1, pX=0.14, N=325
var.13.PGLYRP2 19:15586672:A:T
peptide level
156:156
140:140
29:29
TDCPGDALFDLLRpc2_NP.2
p−maj=1, pX=0.029, N=325
var.13.PGLYRP2 19:15586672:A:T
peptide level
152:156
139:140
29:29
TDCPGDALFDLLRpc2_NP.3
p−min=0.2, pX=0.09, N=320
var.13.PGLYRP2 19:15586672:A:T
peptide level
156:156
140:140
29:29
TDCPGDALFDLLRpc2_NP.4
p−maj=1, pX=2e−04, N=325
var.13.PGLYRP2 19:15586672:A:T
peptide level
156:156
140:140
29:29
TDCPGDALFDLLRpc2_NP.5
p−maj=1, pX=0.017, N=325
var.13.PGLYRP2 19:15586672:A:T
peptide level
105:156
99:140
15:29
TFTLLDPKpc2_NP.1
p−maj=0.065, pX=0.89, N=219
var.13.PGLYRP2 19:15586672:A:T
peptide level
132:156
106:140
20:29
TFTLLDPKpc2_NP.2
p−min=0.028, pX=0.044, N=258
var.13.PGLYRP2 19:15586672:A:T
peptide level
26:156
18:140
5:29
TFTLLDPKpc2_NP.3
p−min=0.54, pX=0.59, N=49
var.13.PGLYRP2 19:15586672:A:T
peptide level
61:156
51:140
10:29
TFTLLDPKpc2_NP.4
p−min=0.65, pX=0.26, N=122
var.13.PGLYRP2 19:15586672:A:T
peptide level
54:156
44:140
10:29
TFTLLDPKpc2_NP.5
p−min=0.64, pX=0.16, N=108
var.13.PGLYRP2 19:15586672:A:T
peptide level
113:156
88:140
21:29
TPEPRPSLSHLLSQYYGAGVARpc4_NP.1
p−min=0.15, pX=0.17, N=222
var.13.PGLYRP2 19:15586672:A:T
peptide level
154:156
136:140
28:29
TPEPRPSLSHLLSQYYGAGVARpc4_NP.2
p−min=0.45, pX=0.014, N=318
var.13.PGLYRP2 19:15586672:A:T
peptide level
18:156
21:140
5:29
TPEPRPSLSHLLSQYYGAGVARpc4_NP.3
p−min=0.33, pX=0.82, N=44
var.13.PGLYRP2 19:15586672:A:T
peptide level
119:156
100:140
18:29
TPEPRPSLSHLLSQYYGAGVARpc4_NP.4
p−maj=0.19, pX=0.033, N=237
var.13.PGLYRP2 19:15586672:A:T
peptide level
153:156
138:140
28:29
TPEPRPSLSHLLSQYYGAGVARpc4_NP.5
p−maj=0.43, pX=0.0086, N=319

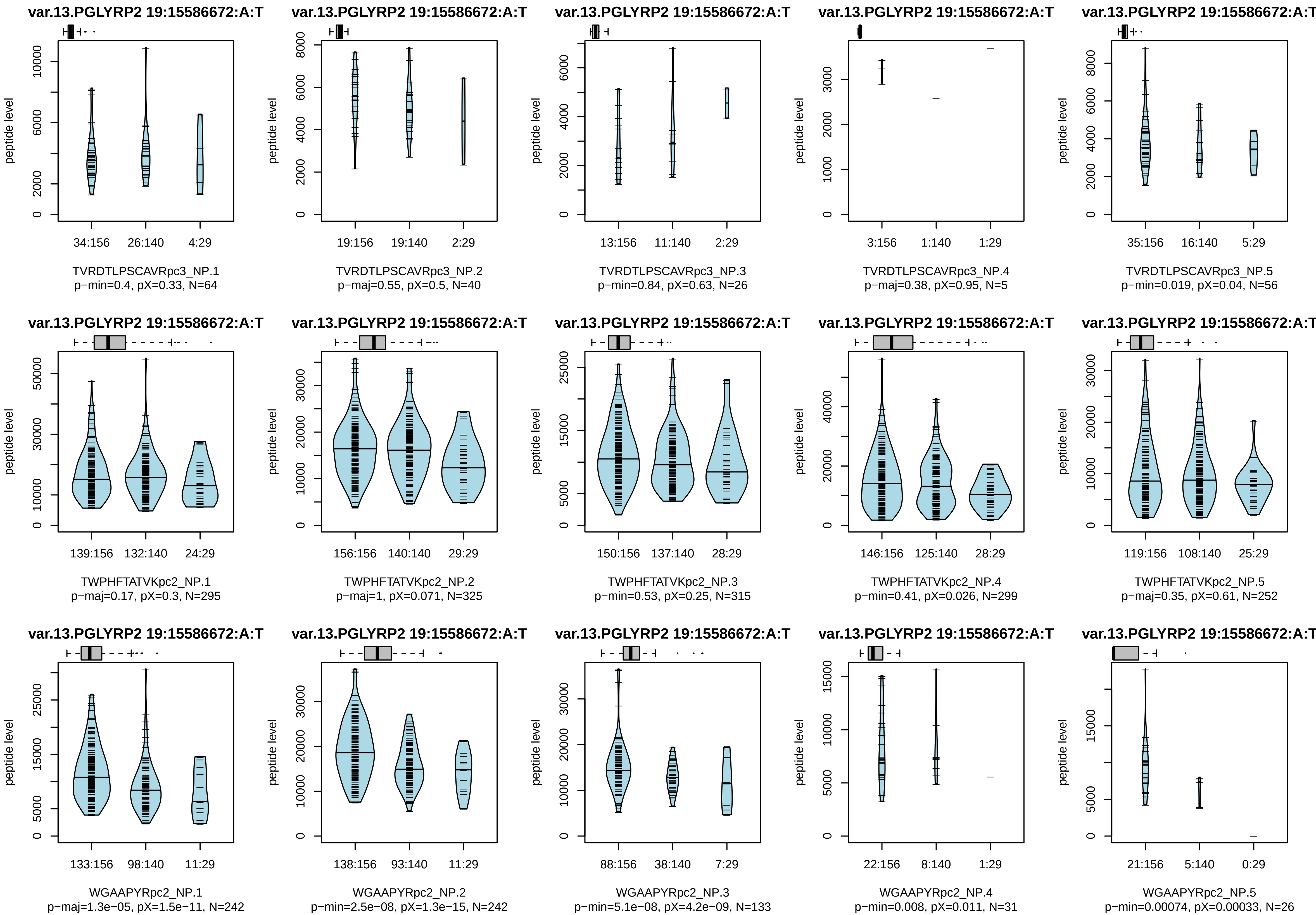
var.13.PGLYRP2 19:15586672:A:T
peptide level
34:156
26:140
4:29
TVRDTLPSCAVRpc3_NP.1
p−min=0.4, pX=0.33, N=64
var.13.PGLYRP2 19:15586672:A:T
peptide level
19:156
19:140
2:29
TVRDTLPSCAVRpc3_NP.2
p−maj=0.55, pX=0.5, N=40
var.13.PGLYRP2 19:15586672:A:T
peptide level
13:156
11:140
2:29
TVRDTLPSCAVRpc3_NP.3
p−min=0.84, pX=0.63, N=26
var.13.PGLYRP2 19:15586672:A:T
peptide level
3:156
1:140
1:29
TVRDTLPSCAVRpc3_NP.4
p−maj=0.38, pX=0.95, N=5
var.13.PGLYRP2 19:15586672:A:T
peptide level
35:156
16:140
5:29
TVRDTLPSCAVRpc3_NP.5
p−min=0.019, pX=0.04, N=56
var.13.PGLYRP2 19:15586672:A:T
peptide level
139:156
132:140
24:29
TWPHFTATVKpc2_NP.1
p−maj=0.17, pX=0.3, N=295
var.13.PGLYRP2 19:15586672:A:T
peptide level
156:156
140:140
29:29
TWPHFTATVKpc2_NP.2
p−maj=1, pX=0.071, N=325
var.13.PGLYRP2 19:15586672:A:T
peptide level
150:156
137:140
28:29
TWPHFTATVKpc2_NP.3
p−min=0.53, pX=0.25, N=315
var.13.PGLYRP2 19:15586672:A:T
peptide level
146:156
125:140
28:29
TWPHFTATVKpc2_NP.4
p−min=0.41, pX=0.026, N=299
var.13.PGLYRP2 19:15586672:A:T
peptide level
119:156
108:140
25:29
TWPHFTATVKpc2_NP.5
p−maj=0.35, pX=0.61, N=252
var.13.PGLYRP2 19:15586672:A:T
peptide level
133:156
98:140
11:29
WGAAPYRpc2_NP.1
p−maj=1.3e−05, pX=1.5e−11, N=242
var.13.PGLYRP2 19:15586672:A:T
peptide level
138:156
93:140
11:29
WGAAPYRpc2_NP.2
p−min=2.5e−08, pX=1.3e−15, N=242
var.13.PGLYRP2 19:15586672:A:T
peptide level
88:156
38:140
7:29
WGAAPYRpc2_NP.3
p−min=5.1e−08, pX=4.2e−09, N=133
var.13.PGLYRP2 19:15586672:A:T
peptide level
22:156
8:140
1:29
WGAAPYRpc2_NP.4
p−min=0.008, pX=0.011, N=31
var.13.PGLYRP2 19:15586672:A:T
peptide level
21:156
5:140
0:29
WGAAPYRpc2_NP.5
p−min=0.00074, pX=0.00033, N=26

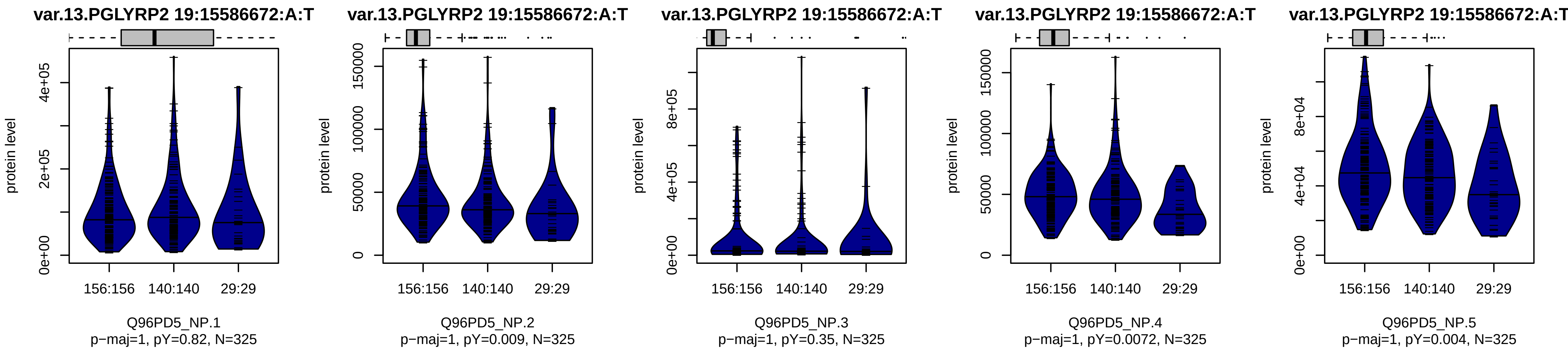

var.13.PGLYRP2 19:15586672:A:T
protein level
156:156
140:140
29:29
Q96PD5_NP.1
p−maj=1, pY=0.82, N=325
var.13.PGLYRP2 19:15586672:A:T
Q96PD5_NP.2
p−maj=1, pY=0.009, N=325
var.13.PGLYRP2 19:15586672:A:T
Q96PD5_NP.3
p−maj=1, pY=0.35, N=325
var.13.PGLYRP2 19:15586672:A:T
Q96PD5_NP.4
p−maj=1, pY=0.0072, N=325
var.13.PGLYRP2 19:15586672:A:T
Q96PD5_NP.5
p−maj=1, pY=0.004, N=325

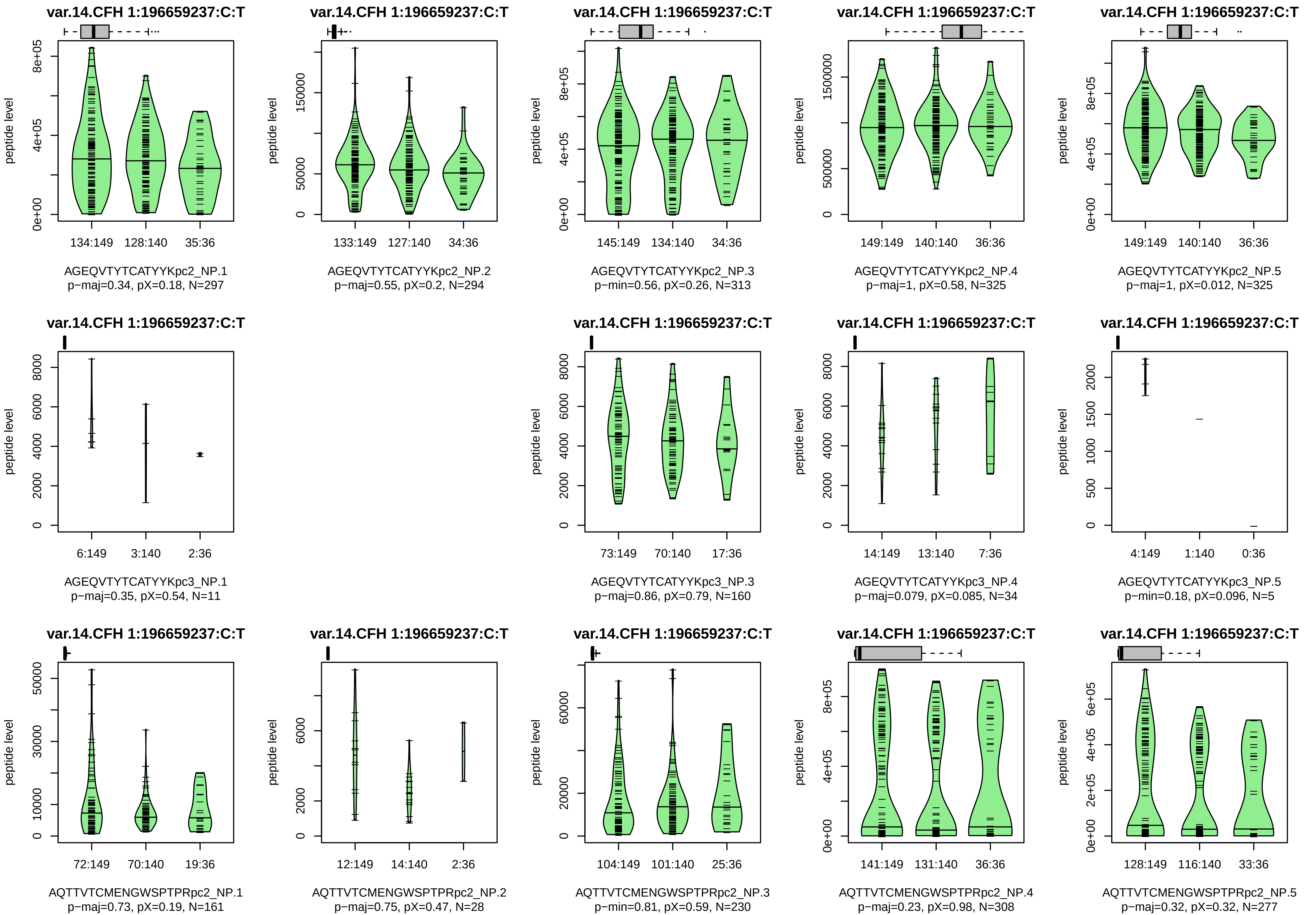
var.14.CFH 1:196659237:C:T
peptide level
134:149
128:140
35:36
AGEQVTYTCATYYKpc2_NP.1
p−maj=0.34, pX=0.18, N=297
var.14.CFH 1:196659237:C:T
peptide level
133:149
127:140
34:36
AGEQVTYTCATYYKpc2_NP.2
p−maj=0.55, pX=0.2, N=294
var.14.CFH 1:196659237:C:T
peptide level
145:149
134:140
34:36
AGEQVTYTCATYYKpc2_NP.3
p−min=0.56, pX=0.26, N=313
var.14.CFH 1:196659237:C:T
peptide level
149:149
140:140
36:36
AGEQVTYTCATYYKpc2_NP.4
p−maj=1, pX=0.58, N=325
var.14.CFH 1:196659237:C:T
peptide level
149:149
140:140
36:36
AGEQVTYTCATYYKpc2_NP.5
p−maj=1, pX=0.012, N=325
var.14.CFH 1:196659237:C:T
peptide level
6:149
3:140
2:36
AGEQVTYTCATYYKpc3_NP.1
p−maj=0.35, pX=0.54, N=11
var.14.CFH 1:196659237:C:T
peptide level
73:149
70:140
17:36
AGEQVTYTCATYYKpc3_NP.3
p−maj=0.86, pX=0.79, N=160
var.14.CFH 1:196659237:C:T
peptide level
14:149
13:140
7:36
AGEQVTYTCATYYKpc3_NP.4
p−maj=0.079, pX=0.085, N=34
var.14.CFH 1:196659237:C:T
peptide level
4:149
1:140
0:36
AGEQVTYTCATYYKpc3_NP.5
p−min=0.18, pX=0.096, N=5
var.14.CFH 1:196659237:C:T
peptide level
72:149
70:140
19:36
AQTTVTCMENGWSPTPRpc2_NP.1
p−maj=0.73, pX=0.19, N=161
var.14.CFH 1:196659237:C:T
peptide level
12:149
14:140
2:36
AQTTVTCMENGWSPTPRpc2_NP.2
p−maj=0.75, pX=0.47, N=28
var.14.CFH 1:196659237:C:T
peptide level
104:149
101:140
25:36
AQTTVTCMENGWSPTPRpc2_NP.3
p−min=0.81, pX=0.59, N=230
var.14.CFH 1:196659237:C:T
peptide level
141:149
131:140
36:36
AQTTVTCMENGWSPTPRpc2_NP.4
p−maj=0.23, pX=0.98, N=308
var.14.CFH 1:196659237:C:T
peptide level
128:149
116:140
33:36
AQTTVTCMENGWSPTPRpc2_NP.5
p−maj=0.32, pX=0.32, N=277

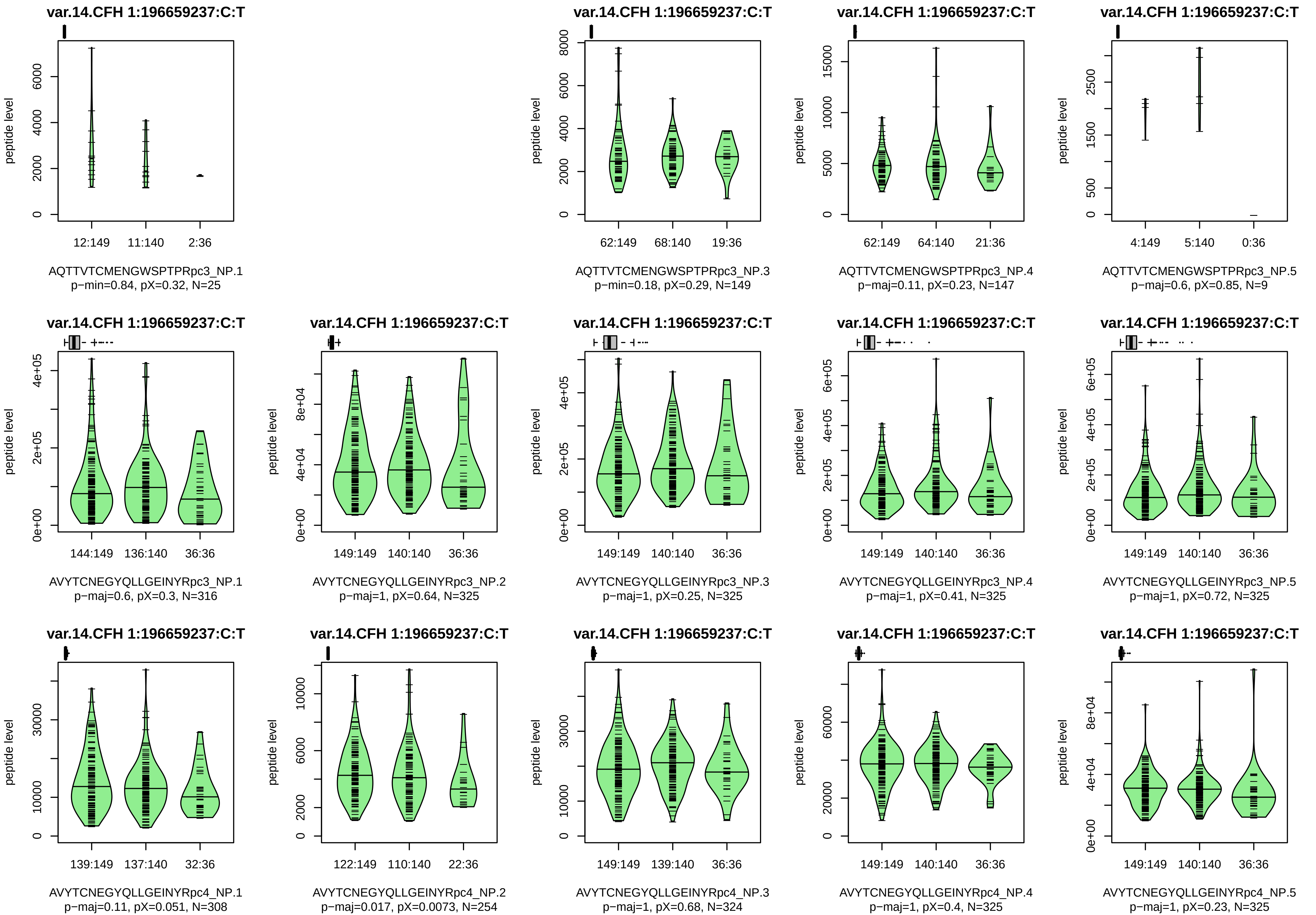
var.14.CFH 1:196659237:C:T
peptide level
12:149
11:140
2:36
AQTTVTCMENGWSPTPRpc3_NP.1
p−min=0.84, pX=0.32, N=25
var.14.CFH 1:196659237:C:T
peptide level
62:149
68:140
19:36
AQTTVTCMENGWSPTPRpc3_NP.3
p−min=0.18, pX=0.29, N=149
var.14.CFH 1:196659237:C:T
peptide level
62:149
64:140
21:36
AQTTVTCMENGWSPTPRpc3_NP.4
p−maj=0.11, pX=0.23, N=147
var.14.CFH 1:196659237:C:T
peptide level
4:149
5:140
0:36
AQTTVTCMENGWSPTPRpc3_NP.5
p−maj=0.6, pX=0.85, N=9
var.14.CFH 1:196659237:C:T
peptide level
144:149
136:140
36:36
AVYTCNEGYQLLGEINYRpc3_NP.1
p−maj=0.6, pX=0.3, N=316
var.14.CFH 1:196659237:C:T
peptide level
149:149
140:140
36:36
AVYTCNEGYQLLGEINYRpc3_NP.2
p−maj=1, pX=0.64, N=325
var.14.CFH 1:196659237:C:T
peptide level
149:149
140:140
36:36
AVYTCNEGYQLLGEINYRpc3_NP.3
p−maj=1, pX=0.25, N=325
var.14.CFH 1:196659237:C:T
peptide level
149:149
140:140
36:36
AVYTCNEGYQLLGEINYRpc3_NP.4
p−maj=1, pX=0.41, N=325
var.14.CFH 1:196659237:C:T
peptide level
149:149
140:140
36:36
AVYTCNEGYQLLGEINYRpc3_NP.5
p−maj=1, pX=0.72, N=325
var.14.CFH 1:196659237:C:T
peptide level
139:149
137:140
32:36
AVYTCNEGYQLLGEINYRpc4_NP.1
p−maj=0.11, pX=0.051, N=308
var.14.CFH 1:196659237:C:T
peptide level
122:149
110:140
22:36
AVYTCNEGYQLLGEINYRpc4_NP.2
p−maj=0.017, pX=0.0073, N=254
var.14.CFH 1:196659237:C:T
peptide level
149:149
139:140
36:36
AVYTCNEGYQLLGEINYRpc4_NP.3
p−maj=1, pX=0.68, N=324
var.14.CFH 1:196659237:C:T
peptide level
149:149
140:140
36:36
AVYTCNEGYQLLGEINYRpc4_NP.4
p−maj=1, pX=0.4, N=325
var.14.CFH 1:196659237:C:T
peptide level
149:149
140:140
36:36
AVYTCNEGYQLLGEINYRpc4_NP.5
p−maj=1, pX=0.23, N=325

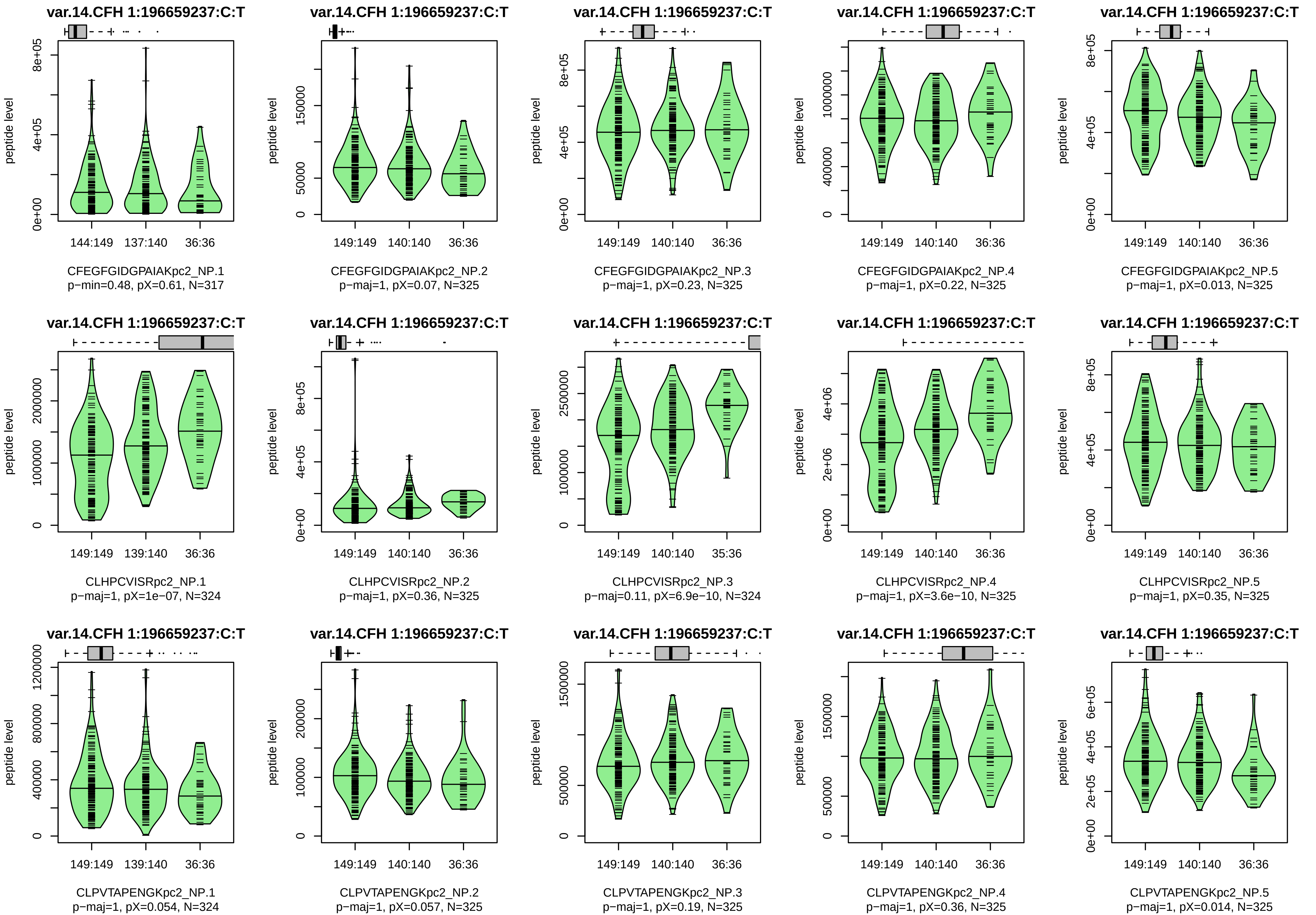
var.14.CFH 1:196659237:C:T
peptide level
144:149 137:140 36:36
CFEGFGIDGPAIAKpc2_NP.1
p−min=0.48, pX=0.61, N=317
var.14.CFH 1:196659237:C:T
peptide level
149:149 140:140 36:36
CFEGFGIDGPAIAKpc2_NP.2
p−maj=1, pX=0.07, N=325
var.14.CFH 1:196659237:C:T
peptide level
149:149 140:140 36:36
CFEGFGIDGPAIAKpc2_NP.3
p−maj=1, pX=0.23, N=325
var.14.CFH 1:196659237:C:T
peptide level
149:149 140:140 36:36
CFEGFGIDGPAIAKpc2_NP.4
p−maj=1, pX=0.22, N=325
var.14.CFH 1:196659237:C:T
peptide level
149:149 140:140 36:36
CFEGFGIDGPAIAKpc2_NP.5
p−maj=1, pX=0.013, N=325
var.14.CFH 1:196659237:C:T
peptide level
149:149 139:140 36:36
CLHPCVISRpc2_NP.1
p−maj=1, pX=1e−07, N=324
var.14.CFH 1:196659237:C:T
peptide level
149:149 140:140 36:36
CLHPCVISRpc2_NP.2
p−maj=1, pX=0.36, N=325
var.14.CFH 1:196659237:C:T
peptide level
149:149 140:140 35:36
CLHPCVISRpc2_NP.3
p−maj=0.11, pX=6.9e−10, N=324
var.14.CFH 1:196659237:C:T
peptide level
149:149 140:140 36:36
CLHPCVISRpc2_NP.4
p−maj=1, pX=3.6e−10, N=325
var.14.CFH 1:196659237:C:T
peptide level
149:149 140:140 36:36
CLHPCVISRpc2_NP.5
p−maj=1, pX=0.35, N=325
var.14.CFH 1:196659237:C:T
peptide level
149:149 139:140 36:36
CLPVTAPENGKpc2_NP.1
p−maj=1, pX=0.054, N=324
var.14.CFH 1:196659237:C:T
peptide level
149:149 140:140 36:36
CLPVTAPENGKpc2_NP.2
p−maj=1, pX=0.057, N=325
var.14.CFH 1:196659237:C:T
peptide level
149:149 140:140 36:36
CLPVTAPENGKpc2_NP.3
p−maj=1, pX=0.19, N=325
var.14.CFH 1:196659237:C:T
peptide level
149:149 140:140 36:36
CLPVTAPENGKpc2_NP.4
p−maj=1, pX=0.36, N=325
var.14.CFH 1:196659237:C:T
peptide level
149:149 140:140 36:36
CLPVTAPENGKpc2_NP.5
p−maj=1, pX=0.014, N=325

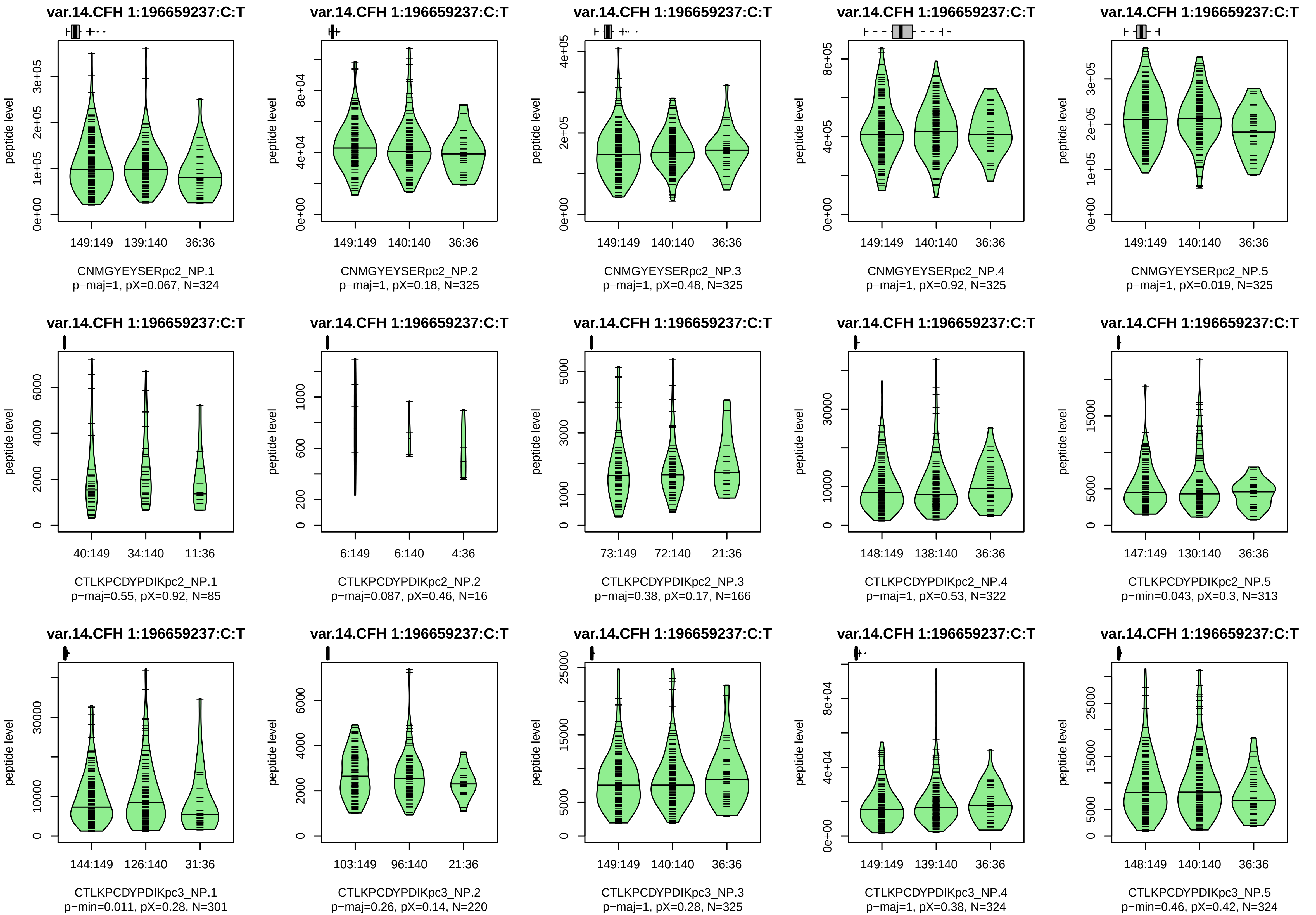
var.14.CFH 1:196659237:C:T
peptide level
149:149 139:140 36:36
CNMGYEYSERpc2_NP.1
p−maj=1, pX=0.067, N=324
var.14.CFH 1:196659237:C:T
peptide level
149:149 140:140 36:36
CNMGYEYSERpc2_NP.2
p−maj=1, pX=0.18, N=325
var.14.CFH 1:196659237:C:T
peptide level
149:149 140:140 36:36
CNMGYEYSERpc2_NP.3
p−maj=1, pX=0.48, N=325
var.14.CFH 1:196659237:C:T
peptide level
149:149 140:140 36:36
CNMGYEYSERpc2_NP.4
p−maj=1, pX=0.92, N=325
var.14.CFH 1:196659237:C:T
peptide level
149:149 140:140 36:36
CNMGYEYSERpc2_NP.5
p−maj=1, pX=0.019, N=325
var.14.CFH 1:196659237:C:T
peptide level
40:149 34:140 11:36
CTLKPCDYPDIKpc2_NP.1
p−maj=0.55, pX=0.92, N=85
var.14.CFH 1:196659237:C:T
peptide level
6:149 6:140 4:36
CTLKPCDYPDIKpc2_NP.2
p−maj=0.087, pX=0.46, N=16
var.14.CFH 1:196659237:C:T
peptide level
73:149 72:140 21:36
CTLKPCDYPDIKpc2_NP.3
p−maj=0.38, pX=0.17, N=166
var.14.CFH 1:196659237:C:T
peptide level
148:149 138:140 36:36
CTLKPCDYPDIKpc2_NP.4
p−maj=1, pX=0.53, N=322
var.14.CFH 1:196659237:C:T
peptide level
147:149 130:140 36:36
CTLKPCDYPDIKpc2_NP.5
p−min=0.043, pX=0.3, N=313
var.14.CFH 1:196659237:C:T
peptide level
144:149 126:140 31:36
CTLKPCDYPDIKpc3_NP.1
p−min=0.011, pX=0.28, N=301
var.14.CFH 1:196659237:C:T
peptide level
103:149 96:140 21:36
CTLKPCDYPDIKpc3_NP.2
p−maj=0.26, pX=0.14, N=220
var.14.CFH 1:196659237:C:T
peptide level
149:149 140:140 36:36
CTLKPCDYPDIKpc3_NP.3
p−maj=1, pX=0.28, N=325
var.14.CFH 1:196659237:C:T
peptide level
149:149 139:140 36:36
CTLKPCDYPDIKpc3_NP.4
p−maj=1, pX=0.38, N=324
var.14.CFH 1:196659237:C:T
peptide level
148:149 140:140 36:36
CTLKPCDYPDIKpc3_NP.5
p−min=0.46, pX=0.42, N=324

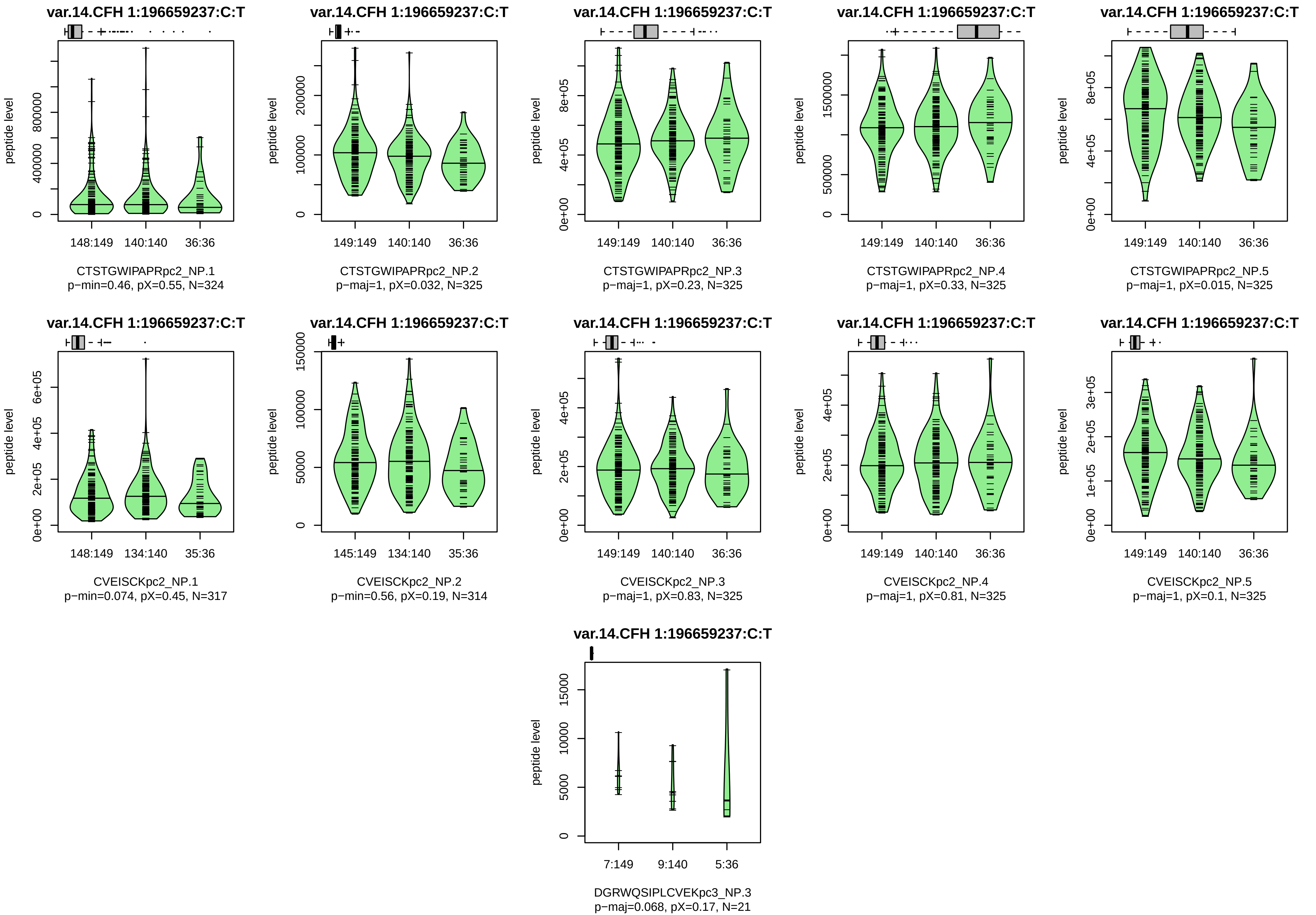

var.14.CFH 1:196659237:C:T
148:149
140:140
36:36
CTSTGWIPAPRpc2_NP.1
p−min=0.46, pX=0.55, N=324
var.14.CFH 1:196659237:C:T
149:149
140:140
36:36
CTSTGWIPAPRpc2_NP.2
p−maj=1, pX=0.032, N=325
var.14.CFH 1:196659237:C:T
149:149
140:140
36:36
CTSTGWIPAPRpc2_NP.3
p−maj=1, pX=0.23, N=325
var.14.CFH 1:196659237:C:T
149:149
140:140
36:36
CTSTGWIPAPRpc2_NP.4
p−maj=1, pX=0.33, N=325
var.14.CFH 1:196659237:C:T
149:149
140:140
36:36
CTSTGWIPAPRpc2_NP.5
p−maj=1, pX=0.015, N=325
var.14.CFH 1:196659237:C:T
148:149
134:140
35:36
CVEISCKpc2_NP.1
p−min=0.074, pX=0.45, N=317
var.14.CFH 1:196659237:C:T
145:149
134:140
35:36
CVEISCKpc2_NP.2
p−min=0.56, pX=0.19, N=314
var.14.CFH 1:196659237:C:T
149:149
140:140
36:36
CVEISCKpc2_NP.3
p−maj=1, pX=0.83, N=325
var.14.CFH 1:196659237:C:T
149:149
140:140
36:36
CVEISCKpc2_NP.4
p−maj=1, pX=0.81, N=325
var.14.CFH 1:196659237:C:T
149:149
140:140
36:36
CVEISCKpc2_NP.5
p−maj=1, pX=0.1, N=325
var.14.CFH 1:196659237:C:T
peptide level
7:149
9:140
5:36
DGRWQSIPLCVEKpc3_NP.3
p−maj=0.068, pX=0.17, N=21

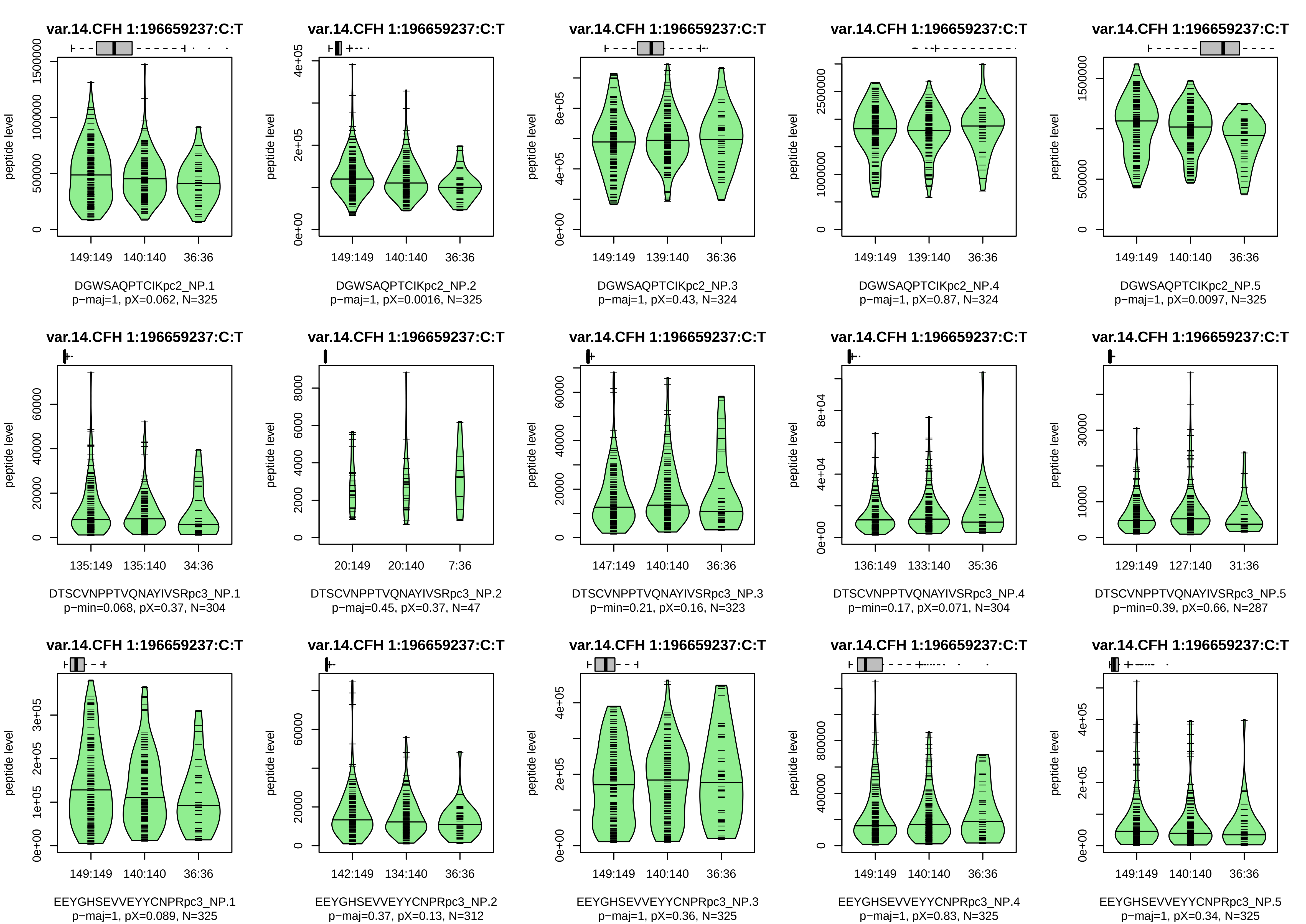
var.14.CFH 1:196659237:C:T
peptide level
149:149 140:140 36:36
DGWSAQPTCIKpc2_NP.1
p−maj=1, pX=0.062, N=325
var.14.CFH 1:196659237:C:T
peptide level
149:149 140:140 36:36
DGWSAQPTCIKpc2_NP.2
p−maj=1, pX=0.0016, N=325
var.14.CFH 1:196659237:C:T
peptide level
149:149 139:140 36:36
DGWSAQPTCIKpc2_NP.3
p−maj=1, pX=0.43, N=324
var.14.CFH 1:196659237:C:T
peptide level
149:149 139:140 36:36
DGWSAQPTCIKpc2_NP.4
p−maj=1, pX=0.87, N=324
var.14.CFH 1:196659237:C:T
peptide level
149:149 140:140 36:36
DGWSAQPTCIKpc2_NP.5
p−maj=1, pX=0.0097, N=325
var.14.CFH 1:196659237:C:T
peptide level
135:149 135:140 34:36
DTSCVNPPTVQNAYIVSRpc3_NP.1
p−min=0.068, pX=0.37, N=304
var.14.CFH 1:196659237:C:T
peptide level
20:149 20:140 7:36
DTSCVNPPTVQNAYIVSRpc3_NP.2
p−maj=0.45, pX=0.37, N=47
var.14.CFH 1:196659237:C:T
peptide level
147:149 140:140 36:36
DTSCVNPPTVQNAYIVSRpc3_NP.3
p−min=0.21, pX=0.16, N=323
var.14.CFH 1:196659237:C:T
peptide level
136:149 133:140 35:36
DTSCVNPPTVQNAYIVSRpc3_NP.4
p−min=0.17, pX=0.071, N=304
var.14.CFH 1:196659237:C:T
peptide level
129:149 127:140 31:36
DTSCVNPPTVQNAYIVSRpc3_NP.5
p−min=0.39, pX=0.66, N=287
var.14.CFH 1:196659237:C:T
peptide level
149:149 140:140 36:36
EEYGHSEVVEYYCNPRpc3_NP.1
p−maj=1, pX=0.089, N=325
var.14.CFH 1:196659237:C:T
peptide level
142:149 134:140 36:36
EEYGHSEVVEYYCNPRpc3_NP.2
p−maj=0.37, pX=0.13, N=312
var.14.CFH 1:196659237:C:T
peptide level
149:149 140:140 36:36
EEYGHSEVVEYYCNPRpc3_NP.3
p−maj=1, pX=0.36, N=325
var.14.CFH 1:196659237:C:T
peptide level
149:149 140:140 36:36
EEYGHSEVVEYYCNPRpc3_NP.4
p−maj=1, pX=0.83, N=325
var.14.CFH 1:196659237:C:T
peptide level
149:149 140:140 36:36
EEYGHSEVVEYYCNPRpc3_NP.5
p−maj=1, pX=0.34, N=325

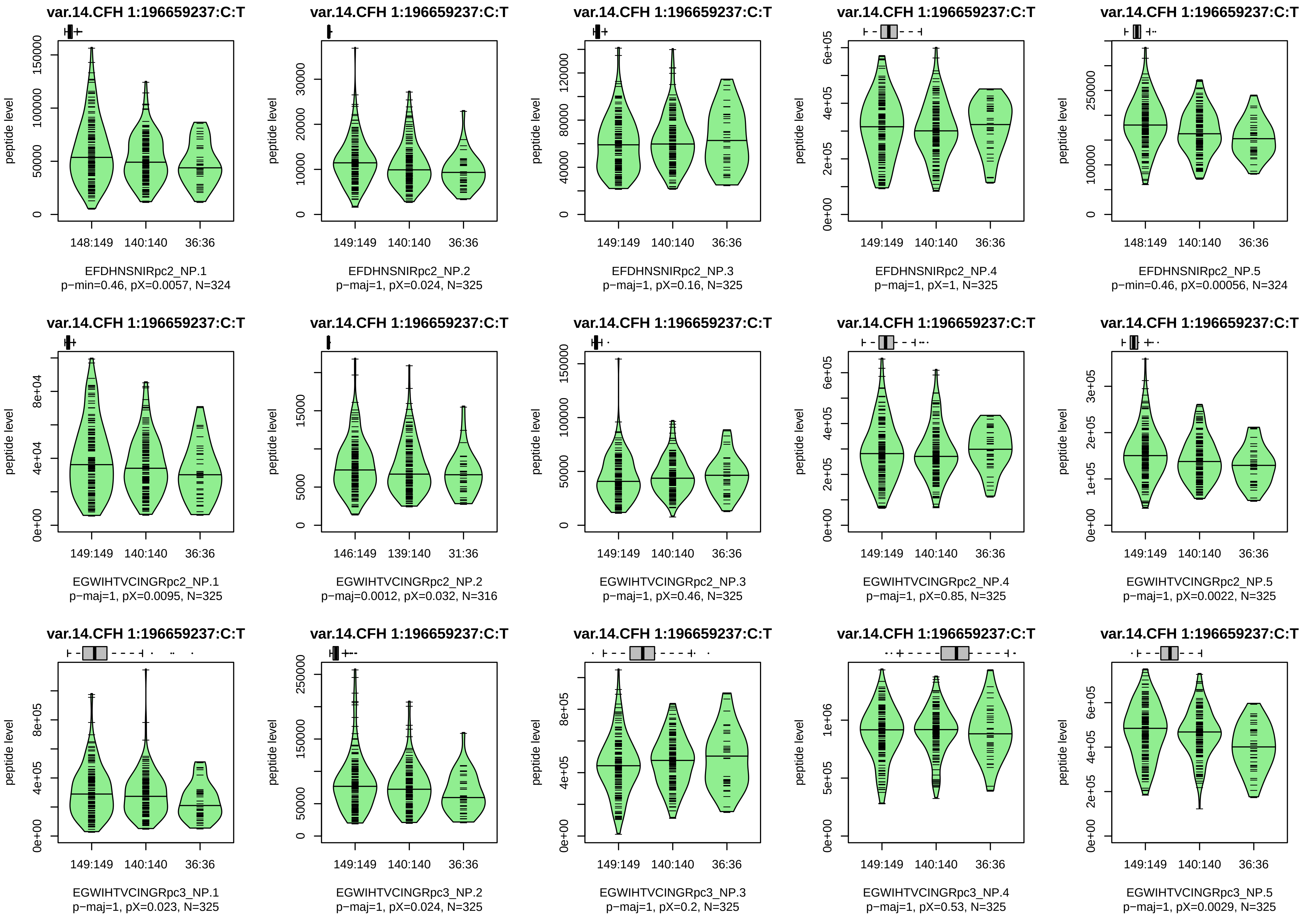

var.14.CFH 1:196659237:C:T
peptide level
148:149
140:140
36:36
EFDHNSNIRpc2_NP.1
p−min=0.46, pX=0.0057, N=324
var.14.CFH 1:196659237:C:T
peptide level
149:149
140:140
36:36
EFDHNSNIRpc2_NP.2
p−maj=1, pX=0.024, N=325
var.14.CFH 1:196659237:C:T
peptide level
149:149
140:140
36:36
EFDHNSNIRpc2_NP.3
p−maj=1, pX=0.16, N=325
var.14.CFH 1:196659237:C:T
peptide level
149:149
140:140
36:36
EFDHNSNIRpc2_NP.4
p−maj=1, pX=1, N=325
var.14.CFH 1:196659237:C:T
peptide level
148:149
140:140
36:36
EFDHNSNIRpc2_NP.5
p−min=0.46, pX=0.00056, N=324
var.14.CFH 1:196659237:C:T
peptide level
149:149
140:140
36:36
EGWIHTVCINGRpc2_NP.1
p−maj=1, pX=0.0095, N=325
var.14.CFH 1:196659237:C:T
peptide level
146:149
139:140
31:36
EGWIHTVCINGRpc2_NP.2
p−maj=0.0012, pX=0.032, N=316
var.14.CFH 1:196659237:C:T
peptide level
149:149
140:140
36:36
EGWIHTVCINGRpc2_NP.3
p−maj=1, pX=0.46, N=325
var.14.CFH 1:196659237:C:T
peptide level
149:149
140:140
36:36
EGWIHTVCINGRpc2_NP.4
p−maj=1, pX=0.85, N=325
var.14.CFH 1:196659237:C:T
peptide level
149:149
140:140
36:36
EGWIHTVCINGRpc2_NP.5
p−maj=1, pX=0.0022, N=325
var.14.CFH 1:196659237:C:T
peptide level
149:149
140:140
36:36
EGWIHTVCINGRpc3_NP.1
p−maj=1, pX=0.023, N=325
var.14.CFH 1:196659237:C:T
peptide level
149:149
140:140
36:36
EGWIHTVCINGRpc3_NP.2
p−maj=1, pX=0.024, N=325
var.14.CFH 1:196659237:C:T
peptide level
149:149
140:140
36:36
EGWIHTVCINGRpc3_NP.3
p−maj=1, pX=0.2, N=325
var.14.CFH 1:196659237:C:T
peptide level
149:149
140:140
36:36
EGWIHTVCINGRpc3_NP.4
p−maj=1, pX=0.53, N=325
var.14.CFH 1:196659237:C:T
peptide level
149:149
140:140
36:36
EGWIHTVCINGRpc3_NP.5
p−maj=1, pX=0.0029, N=325

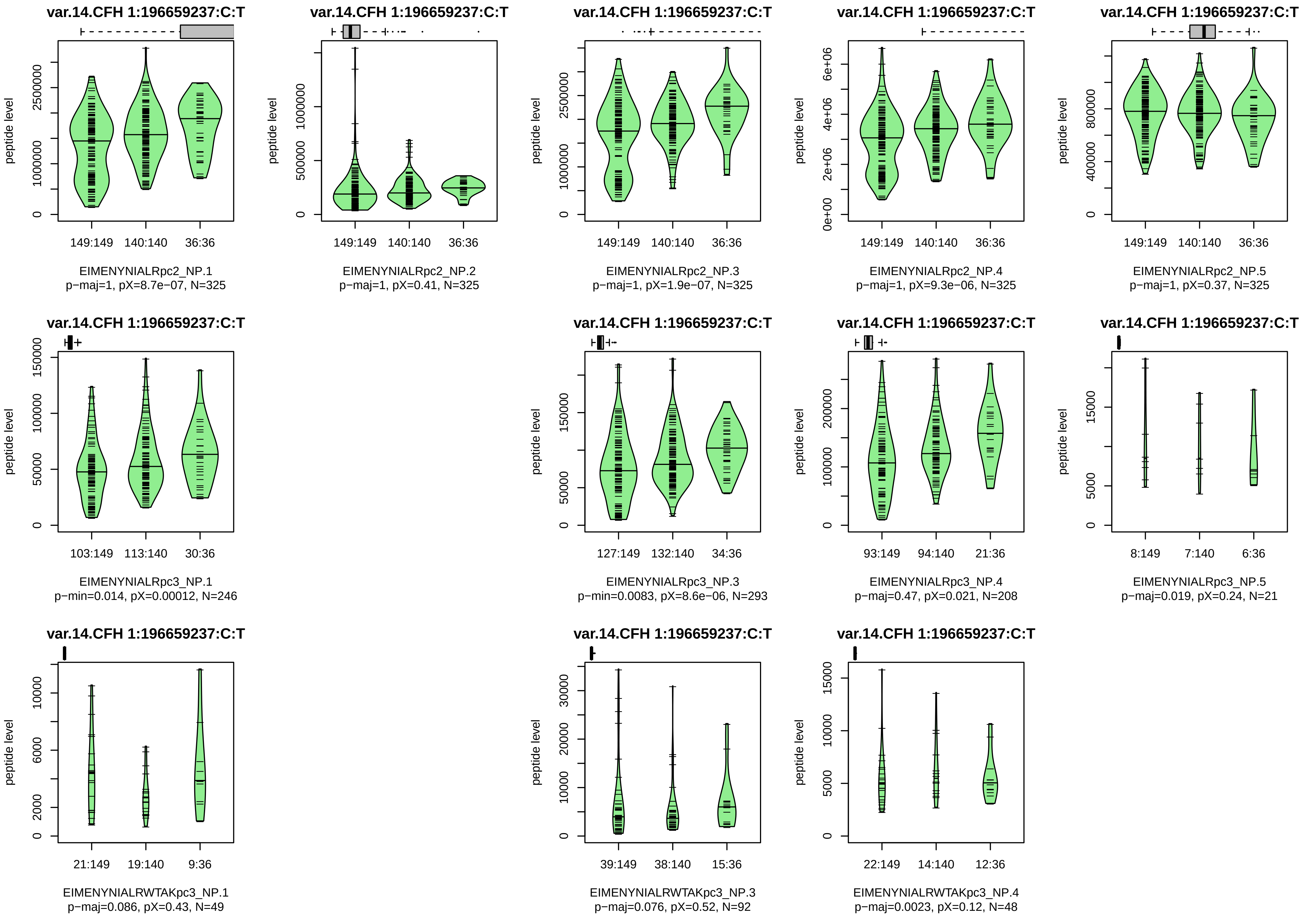
var.14.CFH 1:196659237:C:T
peptide level
149:149
140:140
36:36
EIMENYNIALRpc2_NP.1
p−maj=1, pX=8.7e−07, N=325
EIMENYNIALRpc2_NP.2
p−maj=1, pX=0.41, N=325
EIMENYNIALRpc2_NP.3
p−maj=1, pX=1.9e−07, N=325
EIMENYNIALRpc2_NP.4
p−maj=1, pX=9.3e−06, N=325
EIMENYNIALRpc2_NP.5
p−maj=1, pX=0.37, N=325
103:149
113:140
30:36
EIMENYNIALRpc3_NP.1
p−min=0.014, pX=0.00012, N=246
127:149
132:140
34:36
EIMENYNIALRpc3_NP.3
p−min=0.0083, pX=8.6e−06, N=293
93:149
94:140
21:36
EIMENYNIALRpc3_NP.4
p−maj=0.47, pX=0.021, N=208
8:149
7:140
6:36
EIMENYNIALRpc3_NP.5
p−maj=0.019, pX=0.24, N=21
21:149
19:140
9:36
EIMENYNIALRWTAKpc3_NP.1
p−maj=0.086, pX=0.43, N=49
39:149
38:140
15:36
EIMENYNIALRWTAKpc3_NP.3
p−maj=0.076, pX=0.52, N=92
22:149
14:140
12:36
EIMENYNIALRWTAKpc3_NP.4
p−maj=0.0023, pX=0.12, N=48

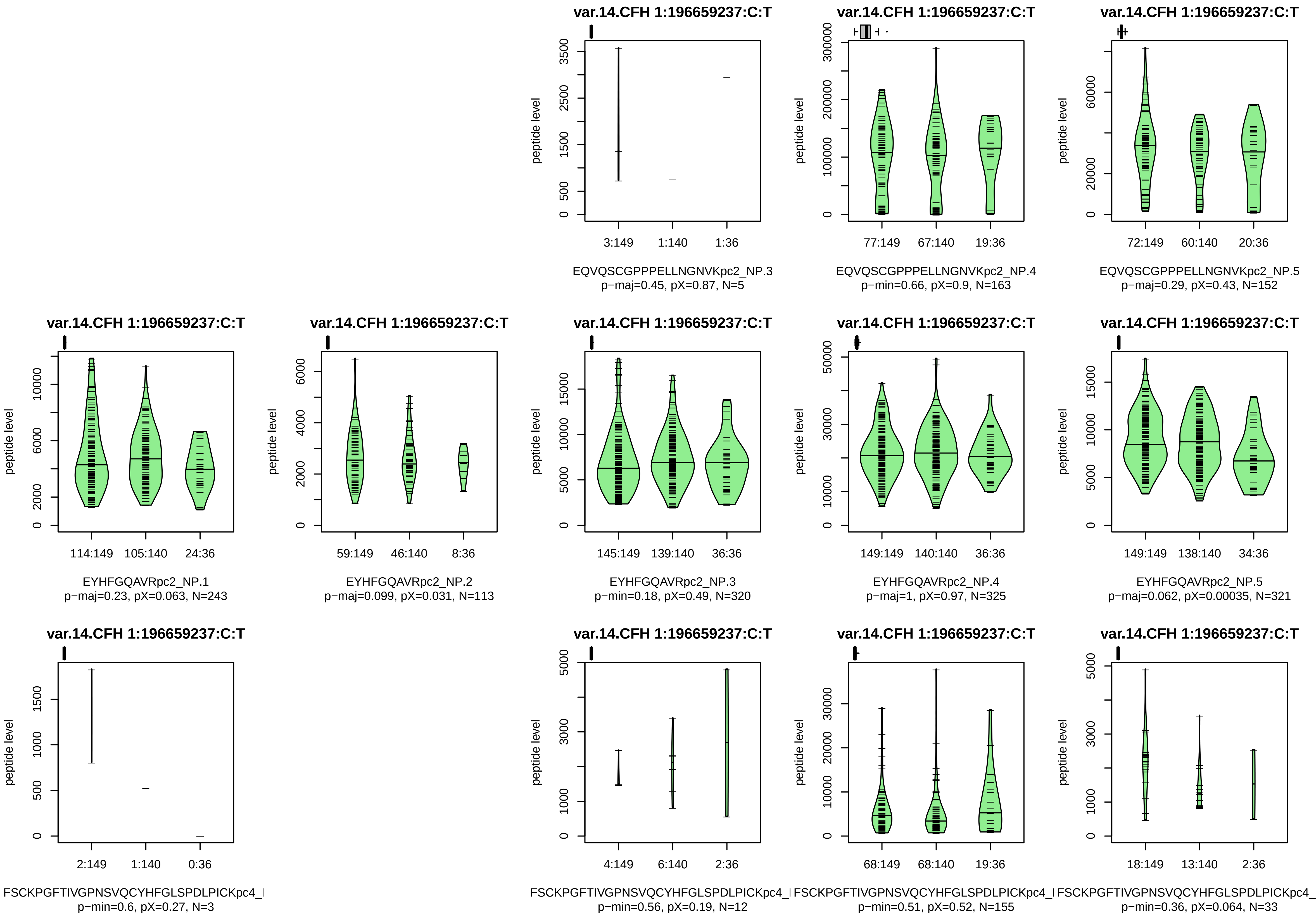
var.14.CFH 1:196659237:C:T
3:149
1:140
1:36
EQVQSCGPPPELLNGNVKpc2_NP.3
p−maj=0.45, pX=0.87, N=5
var.14.CFH 1:196659237:C:T
77:149
67:140
19:36
EQVQSCGPPPELLNGNVKpc2_NP.4
p−min=0.66, pX=0.9, N=163
var.14.CFH 1:196659237:C:T
72:149
60:140
20:36
EQVQSCGPPPELLNGNVKpc2_NP.5
p−maj=0.29, pX=0.43, N=152
var.14.CFH 1:196659237:C:T
114:149
105:140
24:36
EYHFGQAVRpc2_NP.1
p−maj=0.23, pX=0.063, N=243
var.14.CFH 1:196659237:C:T
59:149
46:140
8:36
EYHFGQAVRpc2_NP.2
p−maj=0.099, pX=0.031, N=113
var.14.CFH 1:196659237:C:T
145:149
139:140
36:36
EYHFGQAVRpc2_NP.3
p−min=0.18, pX=0.49, N=320
var.14.CFH 1:196659237:C:T
149:149
140:140
36:36
EYHFGQAVRpc2_NP.4
p−maj=1, pX=0.97, N=325
var.14.CFH 1:196659237:C:T
149:149
138:140
34:36
EYHFGQAVRpc2_NP.5
p−maj=0.062, pX=0.00035, N=321
var.14.CFH 1:196659237:C:T
2:149
1:140
0:36
FSCKPGFTIVGPNSVQCYHFGLSPDLPICKpc4_I
p−min=0.6, pX=0.27, N=3
var.14.CFH 1:196659237:C:T
4:149
6:140
2:36
FSCKPGFTIVGPNSVQCYHFGLSPDLPICKpc4_I
p−min=0.56, pX=0.19, N=12
var.14.CFH 1:196659237:C:T
68:149
68:140
19:36
FSCKPGFTIVGPNSVQCYHFGLSPDLPICKpc4_I
p−min=0.51, pX=0.52, N=155
var.14.CFH 1:196659237:C:T
18:149
13:140
2:36
FSCKPGFTIVGPNSVQCYHFGLSPDLPICKpc4_
p−min=0.36, pX=0.064, N=33
peptide level

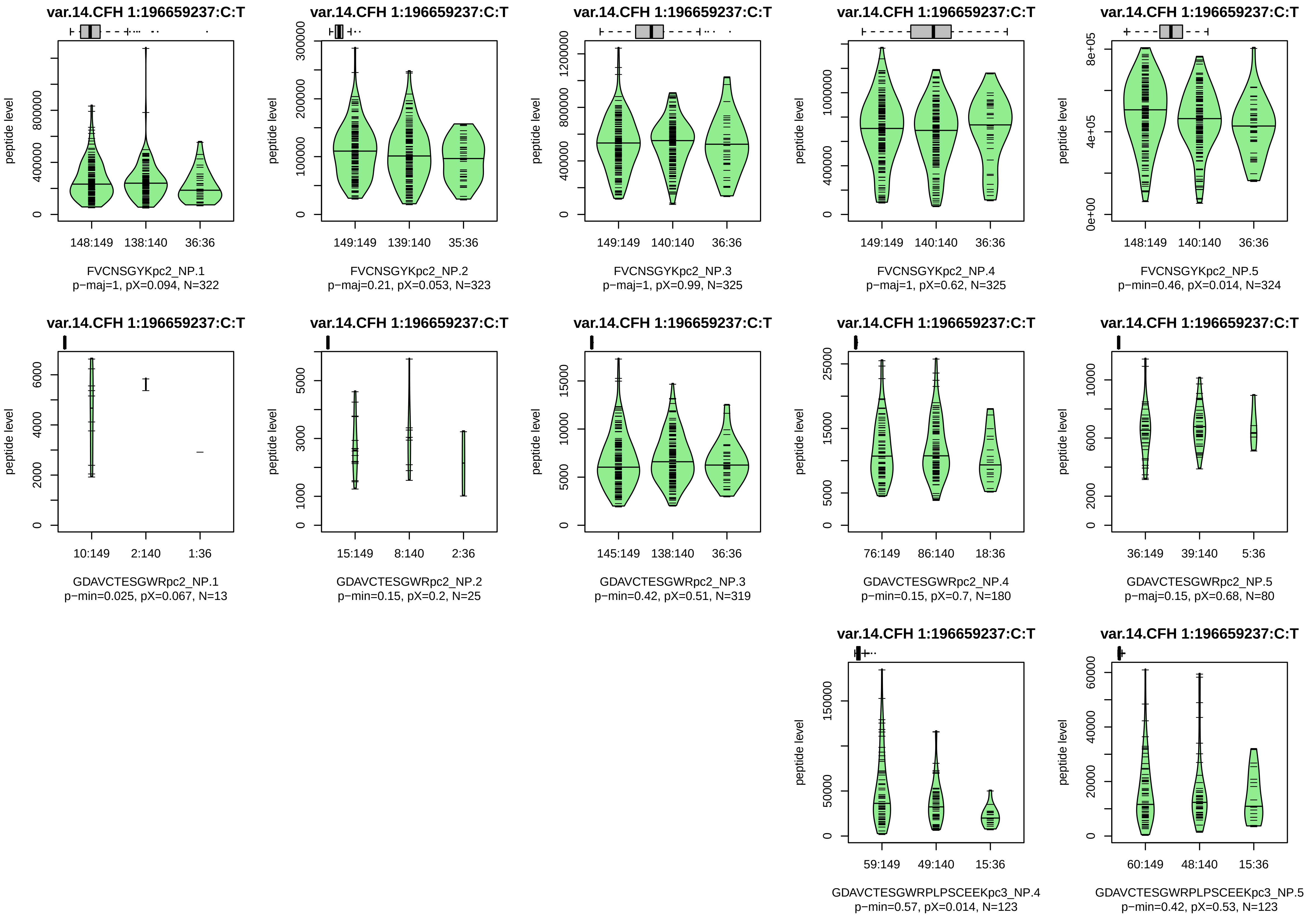
var.14.CFH 1:196659237:C:T
peptide level
148:149
138:140
36:36
FVCNSGYKpc2_NP.1
p−maj=1, pX=0.094, N=322
var.14.CFH 1:196659237:C:T
peptide level
149:149
139:140
35:36
FVCNSGYKpc2_NP.2
p−maj=0.21, pX=0.053, N=323
var.14.CFH 1:196659237:C:T
peptide level
149:149
140:140
36:36
FVCNSGYKpc2_NP.3
p−maj=1, pX=0.99, N=325
var.14.CFH 1:196659237:C:T
peptide level
149:149
140:140
36:36
FVCNSGYKpc2_NP.4
p−maj=1, pX=0.62, N=325
var.14.CFH 1:196659237:C:T
peptide level
148:149
140:140
36:36
FVCNSGYKpc2_NP.5
p−min=0.46, pX=0.014, N=324
var.14.CFH 1:196659237:C:T
peptide level
10:149
2:140
1:36
GDAVCTESGWRpc2_NP.1
p−min=0.025, pX=0.067, N=13
var.14.CFH 1:196659237:C:T
peptide level
15:149
8:140
2:36
GDAVCTESGWRpc2_NP.2
p−min=0.15, pX=0.2, N=25
var.14.CFH 1:196659237:C:T
peptide level
145:149
138:140
36:36
GDAVCTESGWRpc2_NP.3
p−min=0.42, pX=0.51, N=319
var.14.CFH 1:196659237:C:T
peptide level
76:149
86:140
18:36
GDAVCTESGWRpc2_NP.4
p−min=0.15, pX=0.7, N=180
var.14.CFH 1:196659237:C:T
peptide level
36:149
39:140
5:36
GDAVCTESGWRpc2_NP.5
p−maj=0.15, pX=0.68, N=80
var.14.CFH 1:196659237:C:T
peptide level
59:149
49:140
15:36
GDAVCTESGWRPLPSCEEKpc3_NP.4
p−min=0.57, pX=0.014, N=123
var.14.CFH 1:196659237:C:T
peptide level
60:149
48:140
15:36
GDAVCTESGWRPLPSCEEKpc3_NP.5
p−min=0.42, pX=0.53, N=123

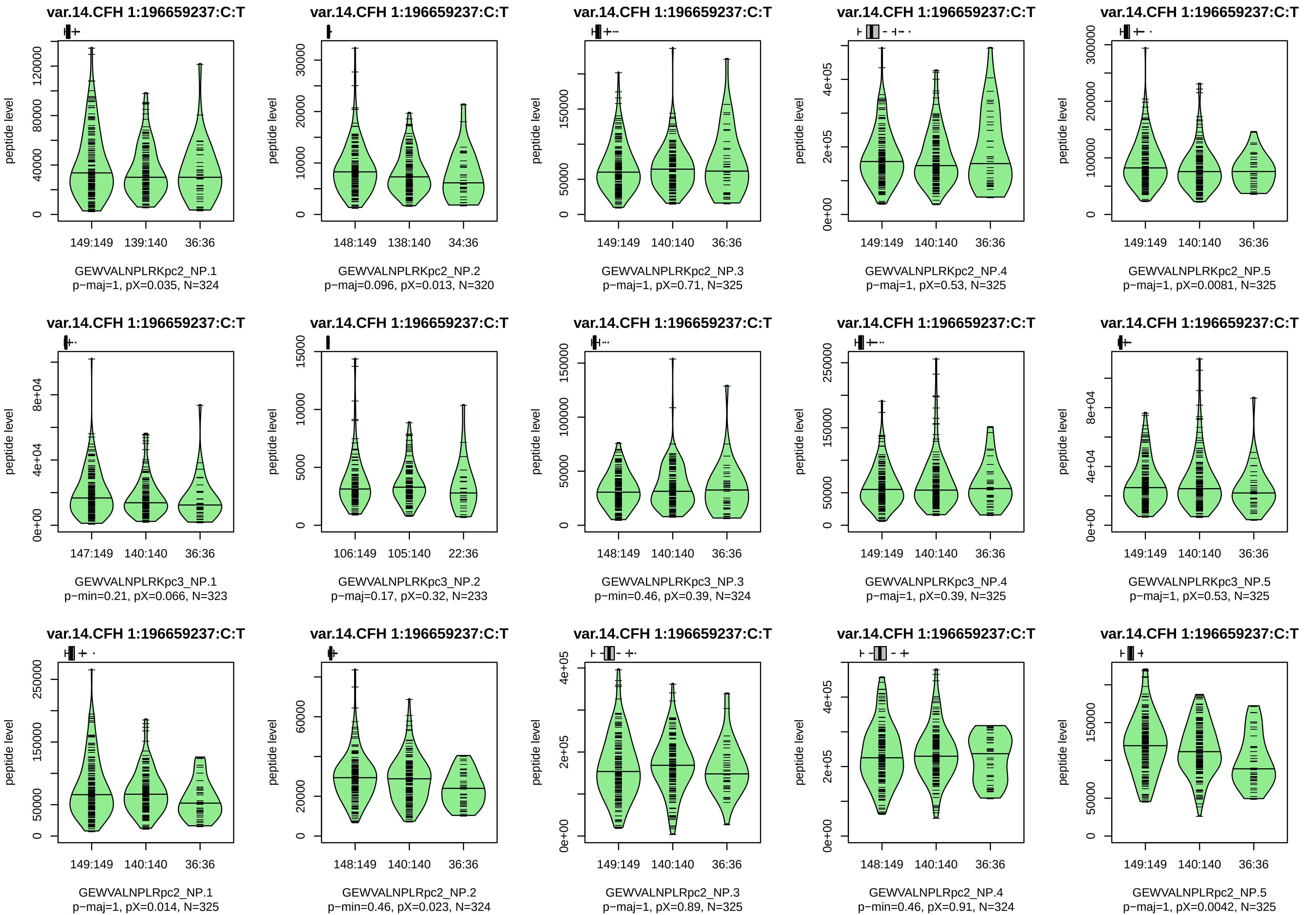

var.14.CFH 1:196659237:C:T
peptide level
149:149 139:140 36:36
GEWVALNPLRKpc2_NP.1
p−maj=1, pX=0.035, N=324
var.14.CFH 1:196659237:C:T
peptide level
148:149 138:140 34:36
GEWVALNPLRKpc2_NP.2
p−maj=0.096, pX=0.013, N=320
var.14.CFH 1:196659237:C:T
peptide level
149:149 140:140 36:36
GEWVALNPLRKpc2_NP.3
p−maj=1, pX=0.71, N=325
var.14.CFH 1:196659237:C:T
peptide level
149:149 140:140 36:36
GEWVALNPLRKpc2_NP.4
p−maj=1, pX=0.53, N=325
var.14.CFH 1:196659237:C:T
peptide level
149:149 140:140 36:36
GEWVALNPLRKpc2_NP.5
p−maj=1, pX=0.0081, N=325
var.14.CFH 1:196659237:C:T
peptide level
147:149 140:140 36:36
GEWVALNPLRKpc3_NP.1
p−min=0.21, pX=0.066, N=323
var.14.CFH 1:196659237:C:T
peptide level
106:149 105:140 22:36
GEWVALNPLRKpc3_NP.2
p−maj=0.17, pX=0.32, N=233
var.14.CFH 1:196659237:C:T
peptide level
148:149 140:140 36:36
GEWVALNPLRKpc3_NP.3
p−min=0.46, pX=0.39, N=324
var.14.CFH 1:196659237:C:T
peptide level
149:149 140:140 36:36
GEWVALNPLRKpc3_NP.4
p−maj=1, pX=0.39, N=325
var.14.CFH 1:196659237:C:T
peptide level
149:149 140:140 36:36
GEWVALNPLRKpc3_NP.5
p−maj=1, pX=0.53, N=325
var.14.CFH 1:196659237:C:T
peptide level
149:149 140:140 36:36
GEWVALNPLRpc2_NP.1
p−maj=1, pX=0.014, N=325
var.14.CFH 1:196659237:C:T
peptide level
148:149 140:140 36:36
GEWVALNPLRpc2_NP.2
p−min=0.46, pX=0.023, N=324
var.14.CFH 1:196659237:C:T
peptide level
149:149 140:140 36:36
GEWVALNPLRpc2_NP.3
p−maj=1, pX=0.89, N=325
var.14.CFH 1:196659237:C:T
peptide level
148:149 140:140 36:36
GEWVALNPLRpc2_NP.4
p−min=0.46, pX=0.91, N=324
var.14.CFH 1:196659237:C:T
peptide level
149:149 140:140 36:36
GEWVALNPLRpc2_NP.5
p−maj=1, pX=0.0042, N=325

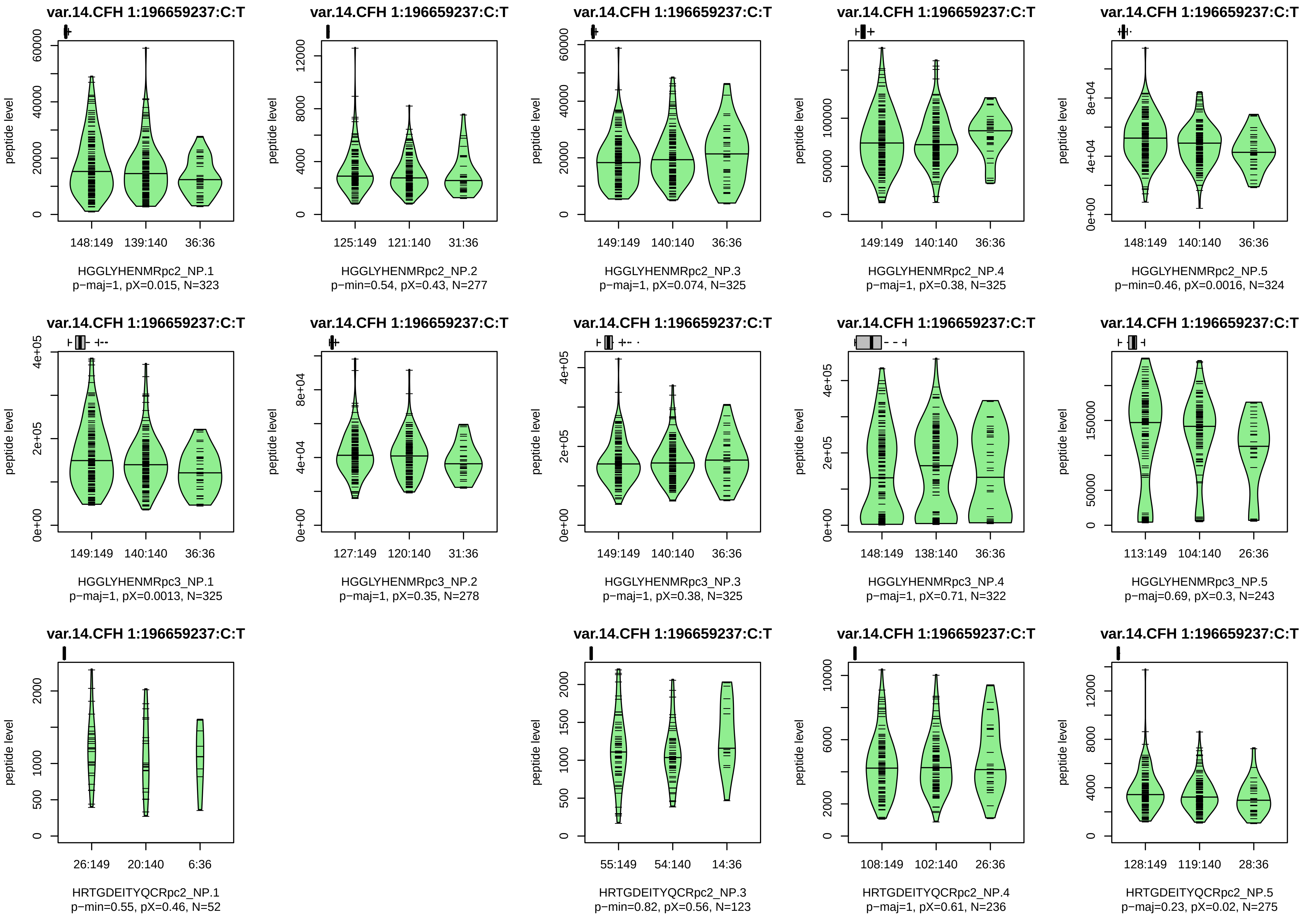
var.14.CFH 1:196659237:C:T
peptide level
148:149 139:140 36:36
HGGLYHENMRpc2_NP.1
p−maj=1, pX=0.015, N=323
var.14.CFH 1:196659237:C:T
125:149 121:140 31:36
HGGLYHENMRpc2_NP.2
p−min=0.54, pX=0.43, N=277
var.14.CFH 1:196659237:C:T
149:149 140:140 36:36
HGGLYHENMRpc2_NP.3
p−maj=1, pX=0.074, N=325
var.14.CFH 1:196659237:C:T
149:149 140:140 36:36
HGGLYHENMRpc2_NP.4
p−maj=1, pX=0.38, N=325
var.14.CFH 1:196659237:C:T
148:149 140:140 36:36
HGGLYHENMRpc2_NP.5
p−min=0.46, pX=0.0016, N=324
var.14.CFH 1:196659237:C:T
149:149 140:140 36:36
HGGLYHENMRpc3_NP.1
p−maj=1, pX=0.0013, N=325
var.14.CFH 1:196659237:C:T
127:149 120:140 31:36
HGGLYHENMRpc3_NP.2
p−maj=1, pX=0.35, N=278
var.14.CFH 1:196659237:C:T
149:149 140:140 36:36
HGGLYHENMRpc3_NP.3
p−maj=1, pX=0.38, N=325
var.14.CFH 1:196659237:C:T
148:149 138:140 36:36
HGGLYHENMRpc3_NP.4
p−maj=1, pX=0.71, N=322
var.14.CFH 1:196659237:C:T
113:149 104:140 26:36
HGGLYHENMRpc3_NP.5
p−maj=0.69, pX=0.3, N=243
var.14.CFH 1:196659237:C:T
26:149 20:140 6:36
HRTGDEITYQCRpc2_NP.1
p−min=0.55, pX=0.46, N=52
var.14.CFH 1:196659237:C:T
55:149 54:140 14:36
HRTGDEITYQCRpc2_NP.3
p−min=0.82, pX=0.56, N=123
var.14.CFH 1:196659237:C:T
108:149 102:140 26:36
HRTGDEITYQCRpc2_NP.4
p−maj=1, pX=0.61, N=236
var.14.CFH 1:196659237:C:T
128:149 119:140 28:36
HRTGDEITYQCRpc2_NP.5
p−maj=0.23, pX=0.02, N=275

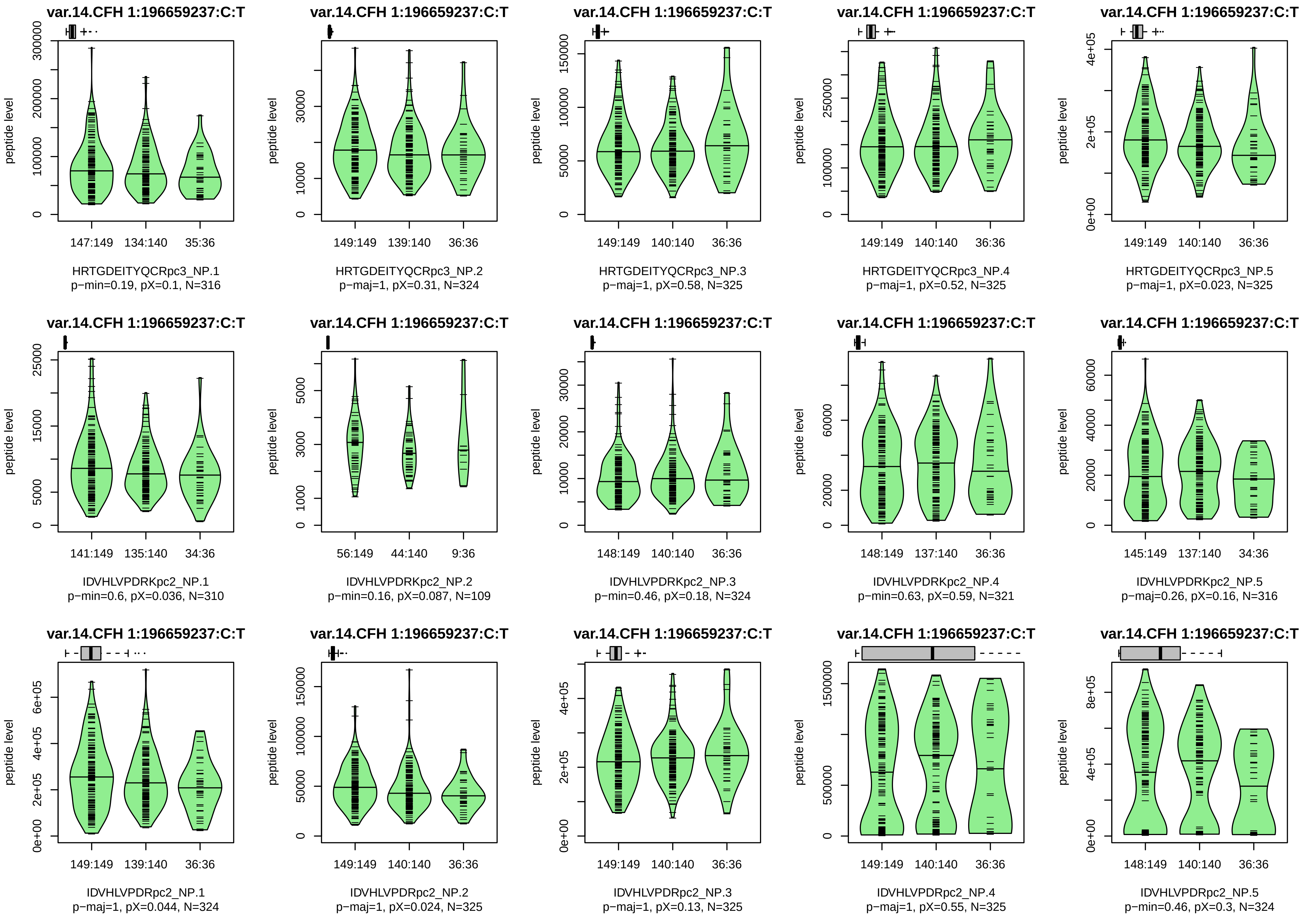
var.14.CFH 1:196659237:C:T
peptide level
0
100000
200000
300000
147:149
134:140
35:36
HRTGDEITYQCRpc3_NP.1
p−min=0.19, pX=0.1, N=316
var.14.CFH 1:196659237:C:T
peptide level
0
10000
30000
149:149
139:140
36:36
HRTGDEITYQCRpc3_NP.2
p−maj=1, pX=0.31, N=324
var.14.CFH 1:196659237:C:T
peptide level
0
50000
100000
150000
149:149
140:140
36:36
HRTGDEITYQCRpc3_NP.3
p−maj=1, pX=0.58, N=325
var.14.CFH 1:196659237:C:T
peptide level
0
100000
250000
149:149
140:140
36:36
HRTGDEITYQCRpc3_NP.4
p−maj=1, pX=0.52, N=325
var.14.CFH 1:196659237:C:T
peptide level
0e+00
2e+05
4e+05
149:149
140:140
36:36
HRTGDEITYQCRpc3_NP.5
p−maj=1, pX=0.023, N=325
var.14.CFH 1:196659237:C:T
peptide level
0
5000
15000
25000
141:149
135:140
34:36
IDVHLVPDRKpc2_NP.1
p−min=0.6, pX=0.036, N=310
var.14.CFH 1:196659237:C:T
peptide level
0
1000
3000
5000
56:149
44:140
9:36
IDVHLVPDRKpc2_NP.2
p−min=0.16, pX=0.087, N=109
var.14.CFH 1:196659237:C:T
peptide level
0
10000
20000
30000
148:149
140:140
36:36
IDVHLVPDRKpc2_NP.3
p−min=0.46, pX=0.18, N=324
var.14.CFH 1:196659237:C:T
peptide level
0
20000
60000
148:149
137:140
36:36
IDVHLVPDRKpc2_NP.4
p−min=0.63, pX=0.59, N=321
var.14.CFH 1:196659237:C:T
peptide level
0
20000
40000
60000
145:149
137:140
34:36
IDVHLVPDRKpc2_NP.5
p−maj=0.26, pX=0.16, N=316
var.14.CFH 1:196659237:C:T
peptide level
0e+00
2e+05
4e+05
6e+05
149:149
139:140
36:36
IDVHLVPDRpc2_NP.1
p−maj=1, pX=0.044, N=324
var.14.CFH 1:196659237:C:T
peptide level
0
50000
100000
150000
149:149
140:140
36:36
IDVHLVPDRpc2_NP.2
p−maj=1, pX=0.024, N=325
var.14.CFH 1:196659237:C:T
peptide level
0e+00
2e+05
4e+05
149:149
140:140
36:36
IDVHLVPDRpc2_NP.3
p−maj=1, pX=0.13, N=325
var.14.CFH 1:196659237:C:T
peptide level
0
500000
1500000
149:149
140:140
36:36
IDVHLVPDRpc2_NP.4
p−maj=1, pX=0.55, N=325
var.14.CFH 1:196659237:C:T
peptide level
0e+00
4e+05
8e+05
148:149
140:140
36:36
IDVHLVPDRpc2_NP.5
p−min=0.46, pX=0.3, N=324

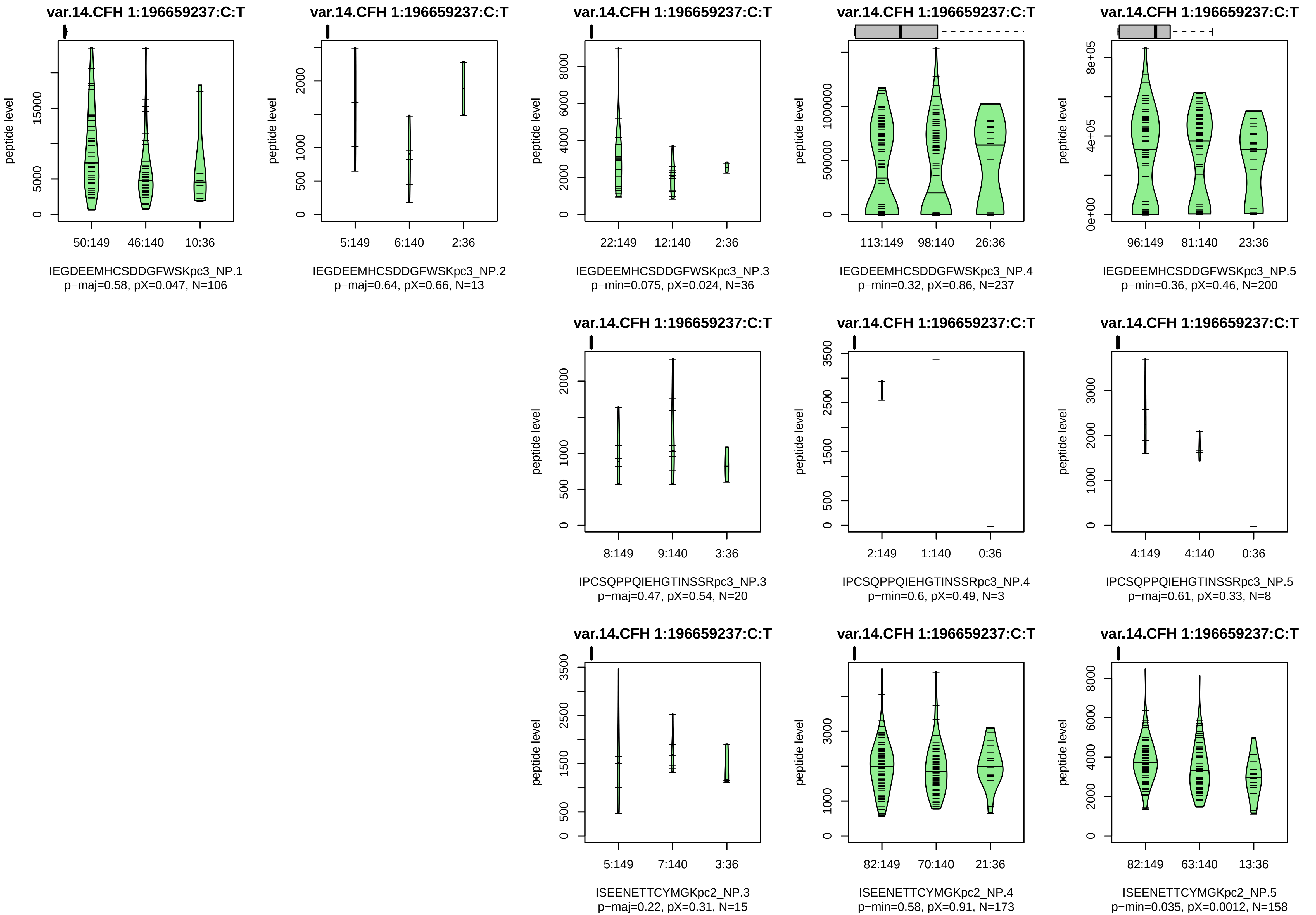
var.14.CFH 1:196659237:C:T
peptide level
50:149
46:140
10:36
IEGDEEMHCSDDGFWSKpc3_NP.1
p−maj=0.58, pX=0.047, N=106
var.14.CFH 1:196659237:C:T
peptide level
5:149
6:140
2:36
IEGDEEMHCSDDGFWSKpc3_NP.2
p−maj=0.64, pX=0.66, N=13
var.14.CFH 1:196659237:C:T
peptide level
22:149
12:140
2:36
IEGDEEMHCSDDGFWSKpc3_NP.3
p−min=0.075, pX=0.024, N=36
var.14.CFH 1:196659237:C:T
peptide level
113:149
98:140
26:36
IEGDEEMHCSDDGFWSKpc3_NP.4
p−min=0.32, pX=0.86, N=237
var.14.CFH 1:196659237:C:T
peptide level
96:149
81:140
23:36
IEGDEEMHCSDDGFWSKpc3_NP.5
p−min=0.36, pX=0.46, N=200
var.14.CFH 1:196659237:C:T
peptide level
8:149
9:140
3:36
IPCSQPPQIEHGTINSSRpc3_NP.3
p−maj=0.47, pX=0.54, N=20
var.14.CFH 1:196659237:C:T
peptide level
2:149
1:140
0:36
IPCSQPPQIEHGTINSSRpc3_NP.4
p−min=0.6, pX=0.49, N=3
var.14.CFH 1:196659237:C:T
peptide level
4:149
4:140
0:36
IPCSQPPQIEHGTINSSRpc3_NP.5
p−maj=0.61, pX=0.33, N=8
var.14.CFH 1:196659237:C:T
peptide level
5:149
7:140
3:36
ISEENETTCYMGKpc2_NP.3
p−maj=0.22, pX=0.31, N=15
var.14.CFH 1:196659237:C:T
peptide level
82:149
70:140
21:36
ISEENETTCYMGKpc2_NP.4
p−min=0.58, pX=0.91, N=173
var.14.CFH 1:196659237:C:T
peptide level
82:149
63:140
13:36
ISEENETTCYMGKpc2_NP.5
p−min=0.035, pX=0.0012, N=158

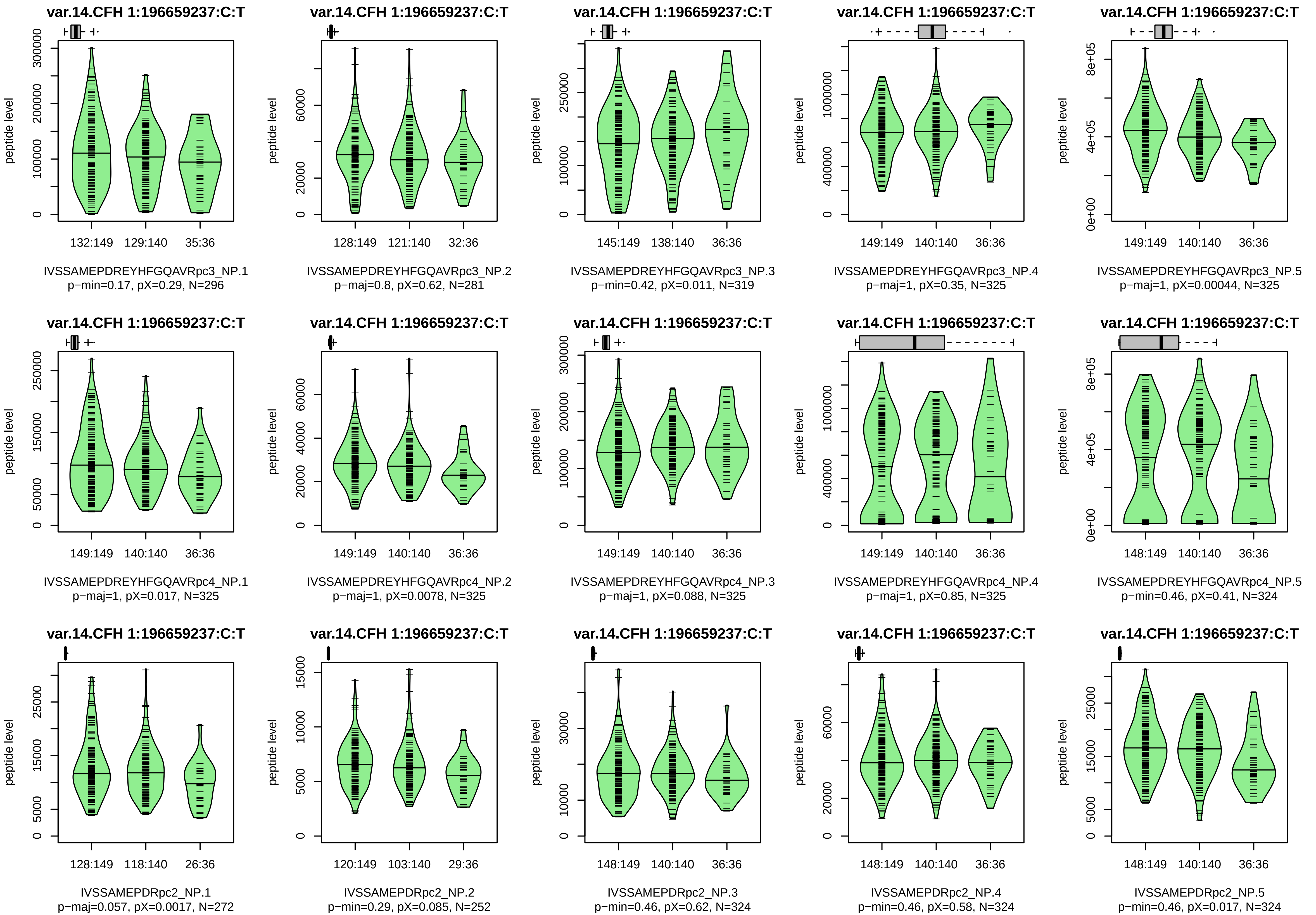
var.14.CFH 1:196659237:C:T
peptide level
132:149 129:140 35:36
IVSSAMEPDREYHFGQAVRpc3_NP.1
p−min=0.17, pX=0.29, N=296
var.14.CFH 1:196659237:C:T
peptide level
128:149 121:140 32:36
IVSSAMEPDREYHFGQAVRpc3_NP.2
p−maj=0.8, pX=0.62, N=281
var.14.CFH 1:196659237:C:T
peptide level
145:149 138:140 36:36
IVSSAMEPDREYHFGQAVRpc3_NP.3
p−min=0.42, pX=0.011, N=319
var.14.CFH 1:196659237:C:T
peptide level
149:149 140:140 36:36
IVSSAMEPDREYHFGQAVRpc3_NP.4
p−maj=1, pX=0.35, N=325
var.14.CFH 1:196659237:C:T
peptide level
149:149 140:140 36:36
IVSSAMEPDREYHFGQAVRpc3_NP.5
p−maj=1, pX=0.00044, N=325
var.14.CFH 1:196659237:C:T
peptide level
149:149 140:140 36:36
IVSSAMEPDREYHFGQAVRpc4_NP.1
p−maj=1, pX=0.017, N=325
var.14.CFH 1:196659237:C:T
peptide level
149:149 140:140 36:36
IVSSAMEPDREYHFGQAVRpc4_NP.2
p−maj=1, pX=0.0078, N=325
var.14.CFH 1:196659237:C:T
peptide level
149:149 140:140 36:36
IVSSAMEPDREYHFGQAVRpc4_NP.3
p−maj=1, pX=0.088, N=325
var.14.CFH 1:196659237:C:T
peptide level
149:149 140:140 36:36
IVSSAMEPDREYHFGQAVRpc4_NP.4
p−maj=1, pX=0.85, N=325
var.14.CFH 1:196659237:C:T
peptide level
148:149 140:140 36:36
IVSSAMEPDREYHFGQAVRpc4_NP.5
p−min=0.46, pX=0.41, N=324
var.14.CFH 1:196659237:C:T
peptide level
128:149 118:140 26:36
IVSSAMEPDRpc2_NP.1
p−maj=0.057, pX=0.0017, N=272
var.14.CFH 1:196659237:C:T
peptide level
120:149 103:140 29:36
IVSSAMEPDRpc2_NP.2
p−min=0.29, pX=0.085, N=252
var.14.CFH 1:196659237:C:T
peptide level
148:149 140:140 36:36
IVSSAMEPDRpc2_NP.3
p−min=0.46, pX=0.62, N=324
var.14.CFH 1:196659237:C:T
peptide level
148:149 140:140 36:36
IVSSAMEPDRpc2_NP.4
p−min=0.46, pX=0.58, N=324
var.14.CFH 1:196659237:C:T
peptide level
148:149 140:140 36:36
IVSSAMEPDRpc2_NP.5
p−min=0.46, pX=0.017, N=324

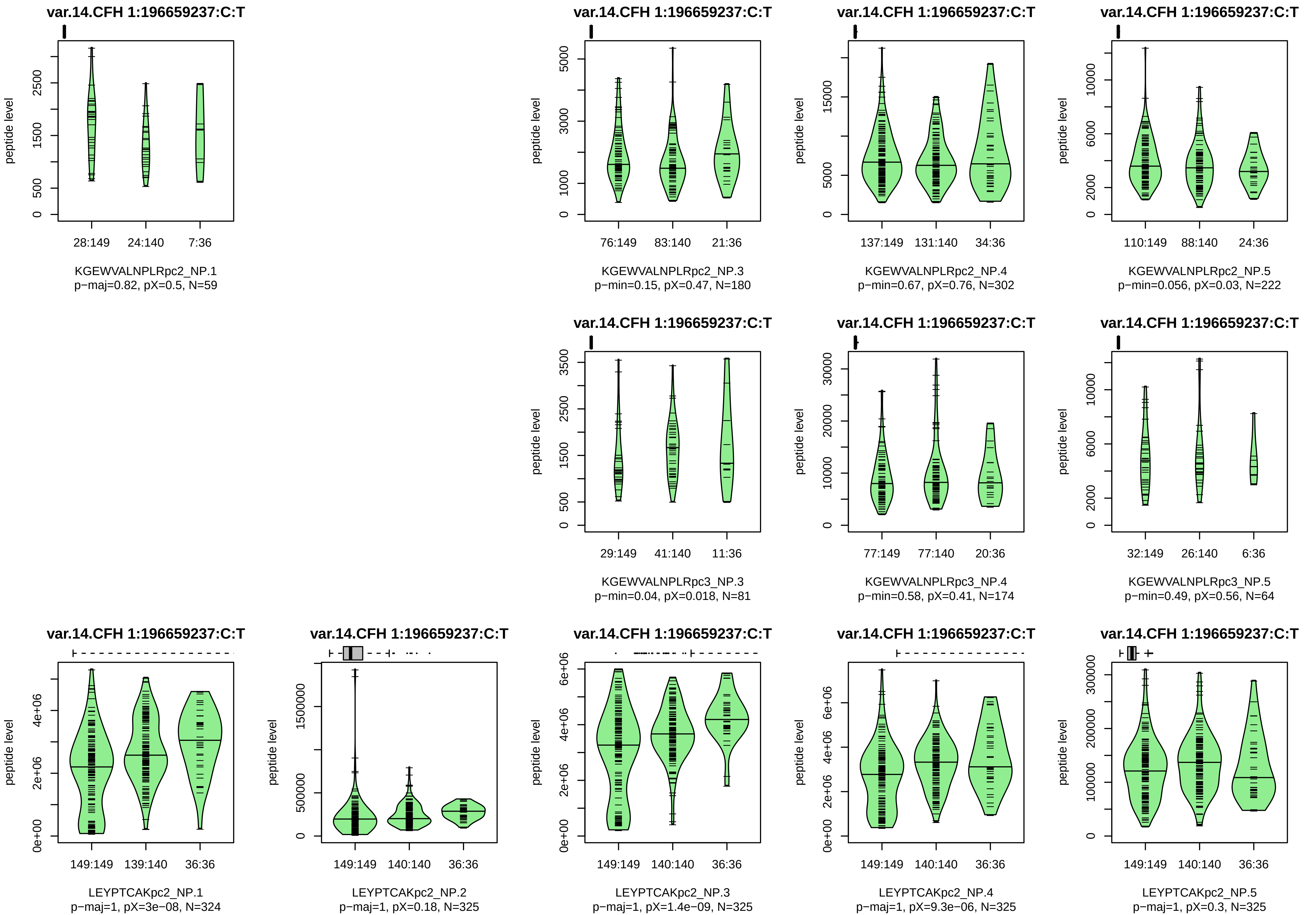
var.14.CFH 1:196659237:C:T
peptide level
0
500
1500
2500
28:149
24:140
7:36
KGEWVALNPLRpc2_NP.1
p−maj=0.82, pX=0.5, N=59
var.14.CFH 1:196659237:C:T
peptide level
0
1000
3000
5000
76:149
83:140
21:36
KGEWVALNPLRpc2_NP.3
p−min=0.15, pX=0.47, N=180
var.14.CFH 1:196659237:C:T
peptide level
0
5000
15000
137:149
131:140
34:36
KGEWVALNPLRpc2_NP.4
p−min=0.67, pX=0.76, N=302
var.14.CFH 1:196659237:C:T
peptide level
0
2000
6000
10000
110:149
88:140
24:36
KGEWVALNPLRpc2_NP.5
p−min=0.056, pX=0.03, N=222
var.14.CFH 1:196659237:C:T
peptide level
0
500
1500
2500
3500
29:149
41:140
11:36
KGEWVALNPLRpc3_NP.3
p−min=0.04, pX=0.018, N=81
var.14.CFH 1:196659237:C:T
peptide level
0
10000
20000
30000
77:149
77:140
20:36
KGEWVALNPLRpc3_NP.4
p−min=0.58, pX=0.41, N=174
var.14.CFH 1:196659237:C:T
peptide level
0
2000
6000
10000
32:149
26:140
6:36
KGEWVALNPLRpc3_NP.5
p−min=0.49, pX=0.56, N=64
var.14.CFH 1:196659237:C:T
peptide level
0e+00
2e+06
4e+06
149:149
139:140
36:36
LEYPTCAKpc2_NP.1
p−maj=1, pX=3e−08, N=324
var.14.CFH 1:196659237:C:T
peptide level
0
500000
1500000
149:149
140:140
36:36
LEYPTCAKpc2_NP.2
p−maj=1, pX=0.18, N=325
var.14.CFH 1:196659237:C:T
peptide level
0e+00
2e+06
4e+06
6e+06
149:149
140:140
36:36
LEYPTCAKpc2_NP.3
p−maj=1, pX=1.4e−09, N=325
var.14.CFH 1:196659237:C:T
peptide level
0e+00
2e+06
4e+06
6e+06
149:149
140:140
36:36
LEYPTCAKpc2_NP.4
p−maj=1, pX=9.3e−06, N=325
var.14.CFH 1:196659237:C:T
peptide level
0
100000
200000
300000
149:149
140:140
36:36
LEYPTCAKpc2_NP.5
p−maj=1, pX=0.3, N=325

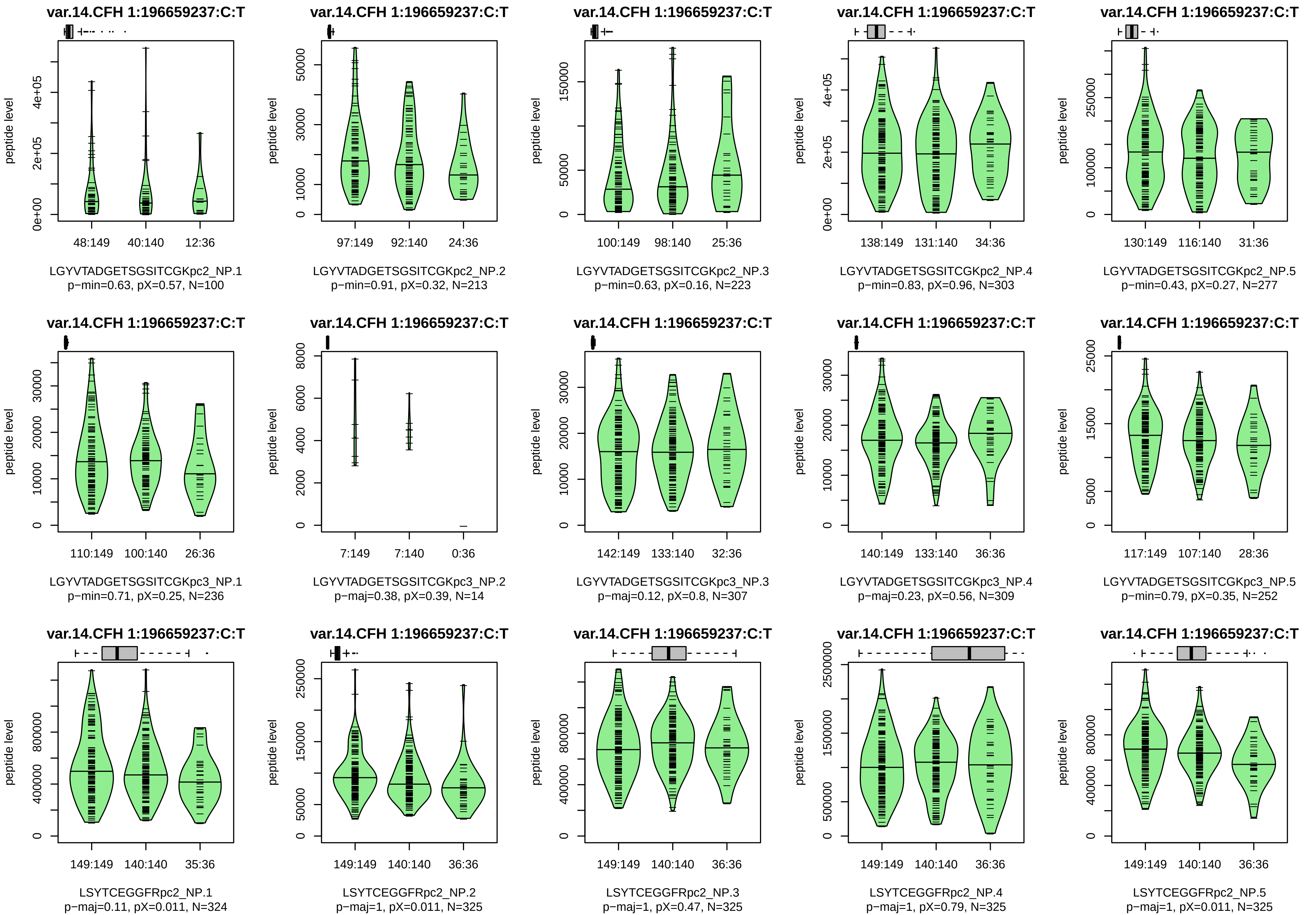
var.14.CFH 1:196659237:C:T
peptide level
48:149
40:140
12:36
LGYVTADGETSGSITCGKpc2_NP.1
p−min=0.63, pX=0.57, N=100
97:149
92:140
24:36
LGYVTADGETSGSITCGKpc2_NP.2
p−min=0.91, pX=0.32, N=213
100:149
98:140
25:36
LGYVTADGETSGSITCGKpc2_NP.3
p−min=0.63, pX=0.16, N=223
138:149
131:140
34:36
LGYVTADGETSGSITCGKpc2_NP.4
p−min=0.83, pX=0.96, N=303
130:149
116:140
31:36
LGYVTADGETSGSITCGKpc2_NP.5
p−min=0.43, pX=0.27, N=277
110:149
100:140
26:36
LGYVTADGETSGSITCGKpc3_NP.1
p−min=0.71, pX=0.25, N=236
7:149
7:140
0:36
LGYVTADGETSGSITCGKpc3_NP.2
p−maj=0.38, pX=0.39, N=14
142:149
133:140
32:36
LGYVTADGETSGSITCGKpc3_NP.3
p−maj=0.12, pX=0.8, N=307
140:149
133:140
36:36
LGYVTADGETSGSITCGKpc3_NP.4
p−maj=0.23, pX=0.56, N=309
117:149
107:140
28:36
LGYVTADGETSGSITCGKpc3_NP.5
p−min=0.79, pX=0.35, N=252
149:149
140:140
35:36
LSYTCEGGFRpc2_NP.1
p−maj=0.11, pX=0.011, N=324
149:149
140:140
36:36
LSYTCEGGFRpc2_NP.2
p−maj=1, pX=0.011, N=325
LSYTCEGGFRpc2_NP.3
p−maj=1, pX=0.47, N=325
LSYTCEGGFRpc2_NP.4
p−maj=1, pX=0.79, N=325
LSYTCEGGFRpc2_NP.5
p−maj=1, pX=0.011, N=325

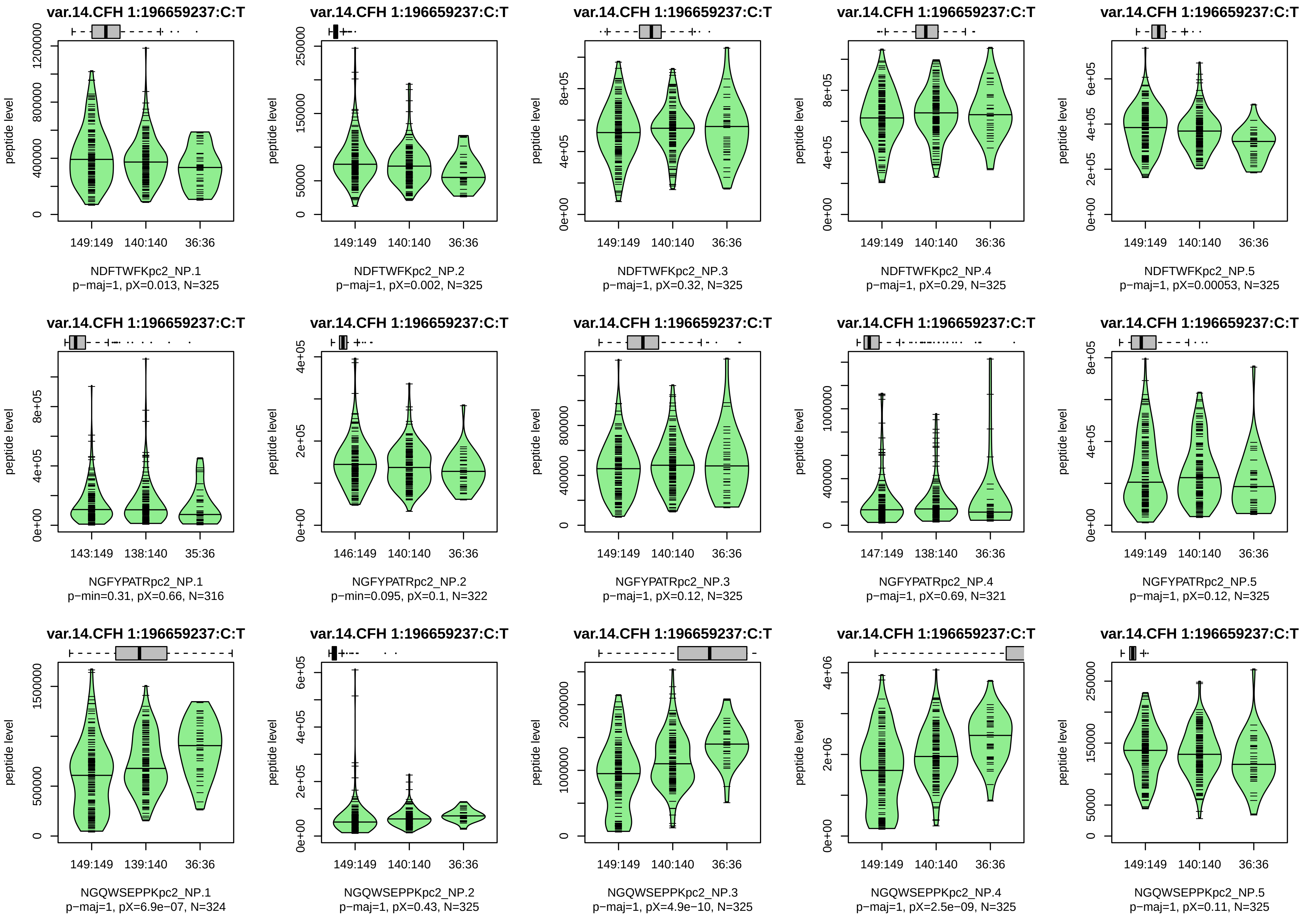

var.14.CFH 1:196659237:C:T
peptide level
149:149
140:140
36:36
NDFTWFKpc2_NP.1
p−maj=1, pX=0.013, N=325
var.14.CFH 1:196659237:C:T
peptide level
149:149
140:140
36:36
NDFTWFKpc2_NP.2
p−maj=1, pX=0.002, N=325
var.14.CFH 1:196659237:C:T
peptide level
149:149
140:140
36:36
NDFTWFKpc2_NP.3
p−maj=1, pX=0.32, N=325
var.14.CFH 1:196659237:C:T
peptide level
149:149
140:140
36:36
NDFTWFKpc2_NP.4
p−maj=1, pX=0.29, N=325
var.14.CFH 1:196659237:C:T
peptide level
149:149
140:140
36:36
NDFTWFKpc2_NP.5
p−maj=1, pX=0.00053, N=325
var.14.CFH 1:196659237:C:T
peptide level
143:149
138:140
35:36
NGFYPATRpc2_NP.1
p−min=0.31, pX=0.66, N=316
var.14.CFH 1:196659237:C:T
peptide level
146:149
140:140
36:36
NGFYPATRpc2_NP.2
p−min=0.095, pX=0.1, N=322
var.14.CFH 1:196659237:C:T
peptide level
149:149
140:140
36:36
NGFYPATRpc2_NP.3
p−maj=1, pX=0.12, N=325
var.14.CFH 1:196659237:C:T
peptide level
147:149
138:140
36:36
NGFYPATRpc2_NP.4
p−maj=1, pX=0.69, N=321
var.14.CFH 1:196659237:C:T
peptide level
149:149
140:140
36:36
NGFYPATRpc2_NP.5
p−maj=1, pX=0.12, N=325
var.14.CFH 1:196659237:C:T
peptide level
149:149
139:140
36:36
NGQWSEPPKpc2_NP.1
p−maj=1, pX=6.9e−07, N=324
var.14.CFH 1:196659237:C:T
peptide level
149:149
140:140
36:36
NGQWSEPPKpc2_NP.2
p−maj=1, pX=0.43, N=325
var.14.CFH 1:196659237:C:T
peptide level
149:149
140:140
36:36
NGQWSEPPKpc2_NP.3
p−maj=1, pX=4.9e−10, N=325
var.14.CFH 1:196659237:C:T
peptide level
149:149
140:140
36:36
NGQWSEPPKpc2_NP.4
p−maj=1, pX=2.5e−09, N=325
var.14.CFH 1:196659237:C:T
peptide level
149:149
140:140
36:36
NGQWSEPPKpc2_NP.5
p−maj=1, pX=0.11, N=325

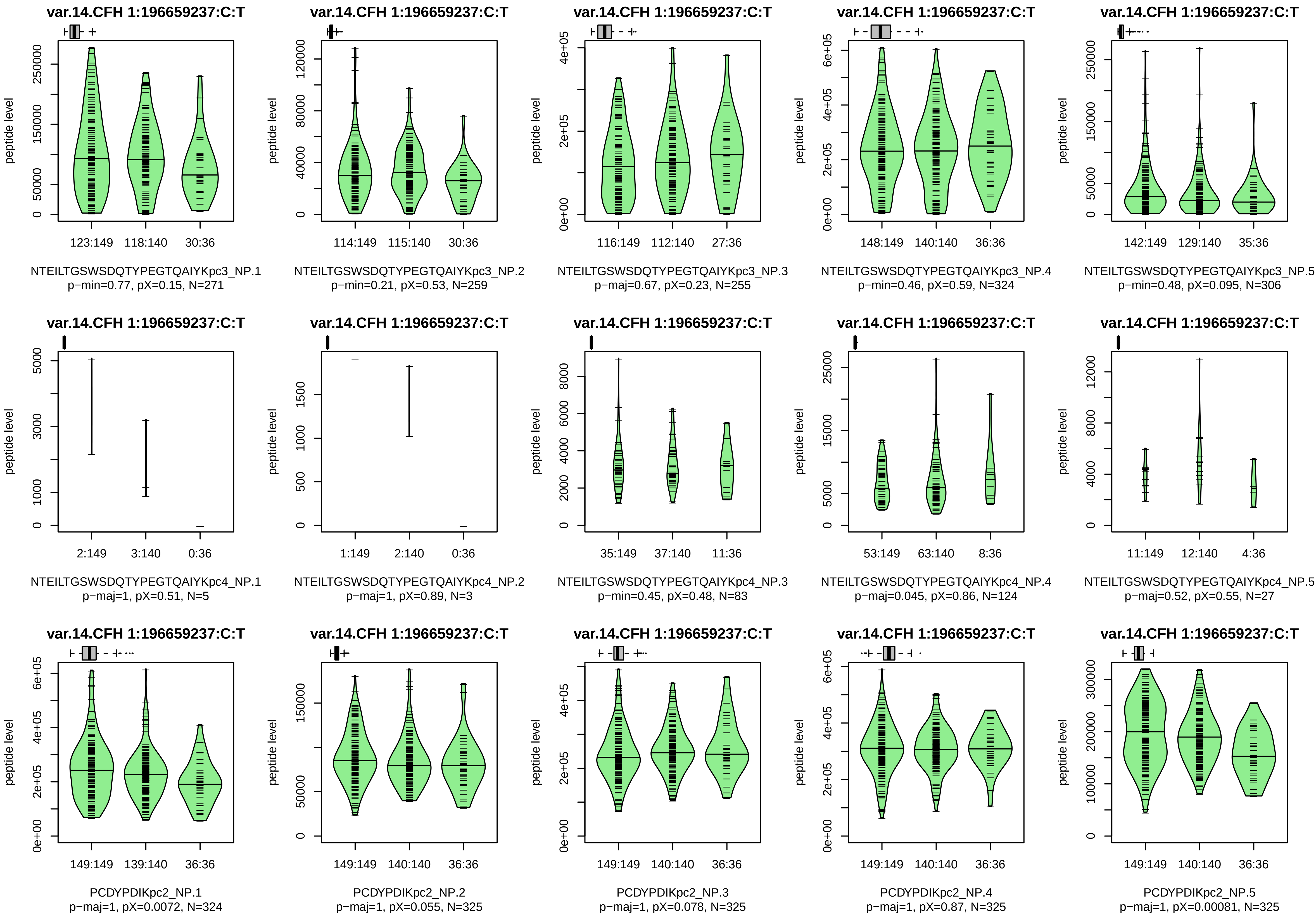

var.14.CFH 1:196659237:C:T
peptide level
0
50000
150000
250000
123:149
118:140
30:36
NTEILTGSWSDQTYPEGTQAIYKpc3_NP.1
p−min=0.77, pX=0.15, N=271
var.14.CFH 1:196659237:C:T
peptide level
0
40000
80000
120000
114:149
115:140
30:36
NTEILTGSWSDQTYPEGTQAIYKpc3_NP.2
p−min=0.21, pX=0.53, N=259
var.14.CFH 1:196659237:C:T
peptide level
0e+00
2e+05
4e+05
116:149
112:140
27:36
NTEILTGSWSDQTYPEGTQAIYKpc3_NP.3
p−maj=0.67, pX=0.23, N=255
var.14.CFH 1:196659237:C:T
peptide level
0e+00
2e+05
4e+05
6e+05
148:149
140:140
36:36
NTEILTGSWSDQTYPEGTQAIYKpc3_NP.4
p−min=0.46, pX=0.59, N=324
var.14.CFH 1:196659237:C:T
peptide level
0
50000
150000
250000
142:149
129:140
35:36
NTEILTGSWSDQTYPEGTQAIYKpc3_NP.5
p−min=0.48, pX=0.095, N=306
var.14.CFH 1:196659237:C:T
peptide level
0
1000
3000
5000
2:149
3:140
0:36
NTEILTGSWSDQTYPEGTQAIYKpc4_NP.1
p−maj=1, pX=0.51, N=5
var.14.CFH 1:196659237:C:T
peptide level
0
500
1000
1500
1:149
2:140
0:36
NTEILTGSWSDQTYPEGTQAIYKpc4_NP.2
p−maj=1, pX=0.89, N=3
var.14.CFH 1:196659237:C:T
peptide level
0
2000
4000
6000
8000
35:149
37:140
11:36
NTEILTGSWSDQTYPEGTQAIYKpc4_NP.3
p−min=0.45, pX=0.48, N=83
var.14.CFH 1:196659237:C:T
peptide level
0
5000
15000
25000
53:149
63:140
8:36
NTEILTGSWSDQTYPEGTQAIYKpc4_NP.4
p−maj=0.045, pX=0.86, N=124
var.14.CFH 1:196659237:C:T
peptide level
0
4000
8000
12000
11:149
12:140
4:36
NTEILTGSWSDQTYPEGTQAIYKpc4_NP.5
p−maj=0.52, pX=0.55, N=27
var.14.CFH 1:196659237:C:T
peptide level
0e+00
2e+05
4e+05
6e+05
149:149
139:140
36:36
PCDYPDIKpc2_NP.1
p−maj=1, pX=0.0072, N=324
var.14.CFH 1:196659237:C:T
peptide level
0
50000
150000
149:149
140:140
36:36
PCDYPDIKpc2_NP.2
p−maj=1, pX=0.055, N=325
var.14.CFH 1:196659237:C:T
peptide level
0e+00
2e+05
4e+05
149:149
140:140
36:36
PCDYPDIKpc2_NP.3
p−maj=1, pX=0.078, N=325
var.14.CFH 1:196659237:C:T
peptide level
0e+00
2e+05
4e+05
6e+05
149:149
140:140
36:36
PCDYPDIKpc2_NP.4
p−maj=1, pX=0.87, N=325
var.14.CFH 1:196659237:C:T
peptide level
0
100000
200000
300000
149:149
140:140
36:36
PCDYPDIKpc2_NP.5
p−maj=1, pX=0.00081, N=325

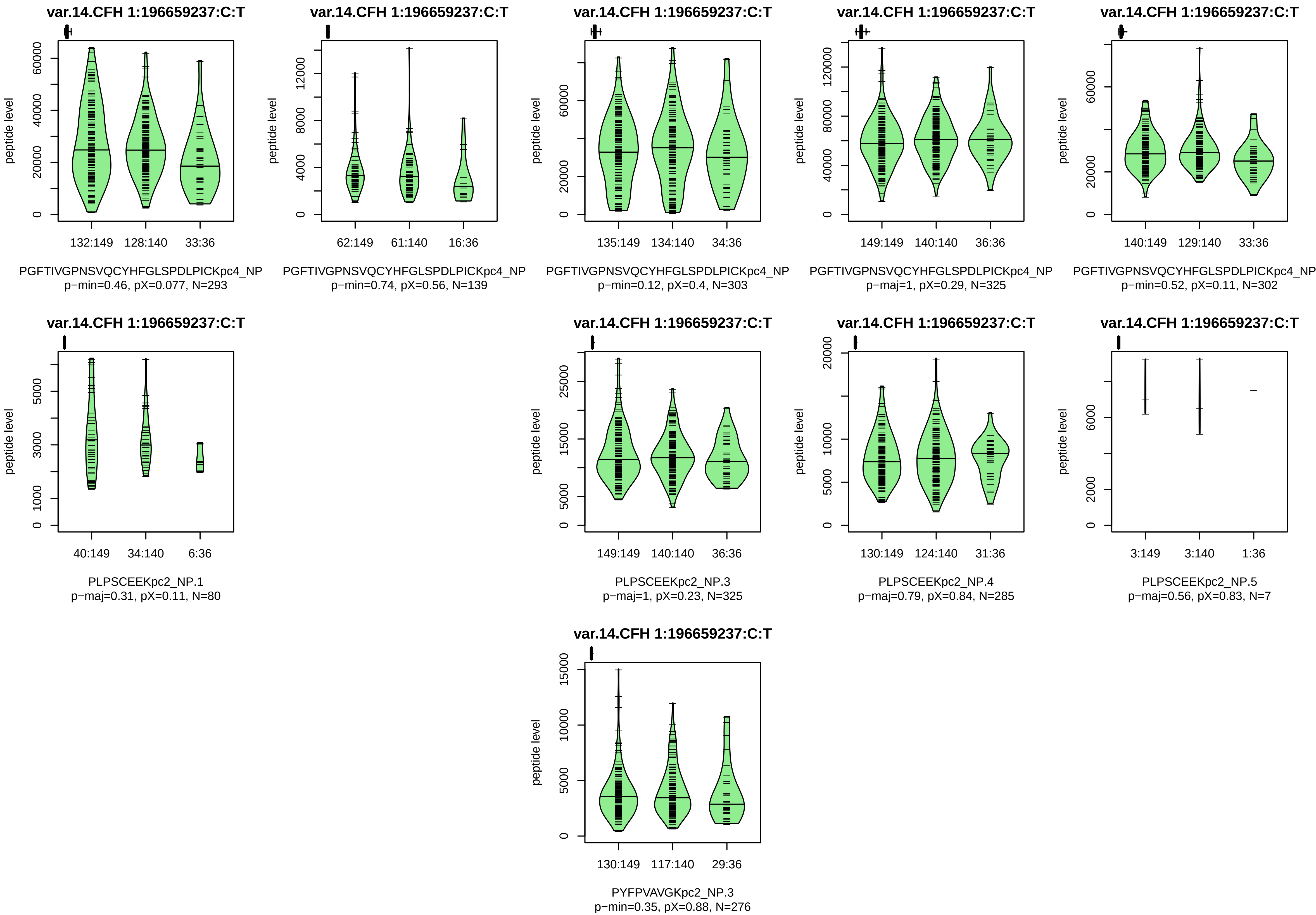
var.14.CFH 1:196659237:C:T
peptide level
132:149
128:140
33:36
PGFTIVGPNSVQCYHFGLSPDLPICKpc4_NP
p−min=0.46, pX=0.077, N=293
var.14.CFH 1:196659237:C:T
peptide level
62:149
61:140
16:36
PGFTIVGPNSVQCYHFGLSPDLPICKpc4_NP
p−min=0.74, pX=0.56, N=139
var.14.CFH 1:196659237:C:T
peptide level
135:149
134:140
34:36
PGFTIVGPNSVQCYHFGLSPDLPICKpc4_NP
p−min=0.12, pX=0.4, N=303
var.14.CFH 1:196659237:C:T
peptide level
149:149
140:140
36:36
PGFTIVGPNSVQCYHFGLSPDLPICKpc4_NP
p−maj=1, pX=0.29, N=325
var.14.CFH 1:196659237:C:T
peptide level
140:149
129:140
33:36
PGFTIVGPNSVQCYHFGLSPDLPICKpc4_NP
p−min=0.52, pX=0.11, N=302
var.14.CFH 1:196659237:C:T
peptide level
40:149
34:140
6:36
PLPSCEEKpc2_NP.1
p−maj=0.31, pX=0.11, N=80
var.14.CFH 1:196659237:C:T
peptide level
149:149
140:140
36:36
PLPSCEEKpc2_NP.3
p−maj=1, pX=0.23, N=325
var.14.CFH 1:196659237:C:T
peptide level
130:149
124:140
31:36
PLPSCEEKpc2_NP.4
p−maj=0.79, pX=0.84, N=285
var.14.CFH 1:196659237:C:T
peptide level
3:149
3:140
1:36
PLPSCEEKpc2_NP.5
p−maj=0.56, pX=0.83, N=7
var.14.CFH 1:196659237:C:T
peptide level
130:149
117:140
29:36
PYFPVAVGKpc2_NP.3
p−min=0.35, pX=0.88, N=276

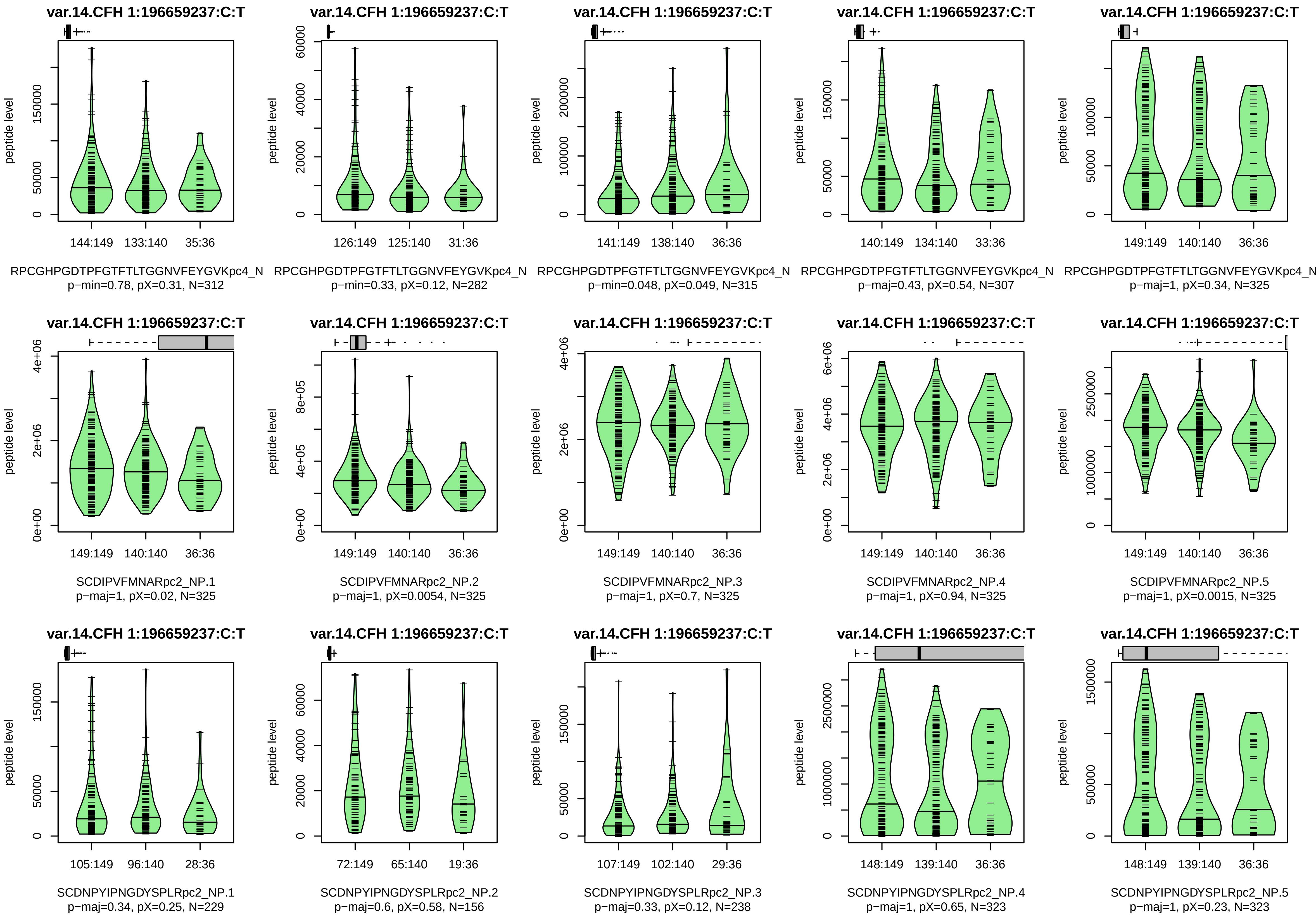
var.14.CFH 1:196659237:C:T
peptide level
144:149 133:140 35:36
RPCGHPGDTPFGTFTLTGGNVFEYGVKpc4_N
p−min=0.78, pX=0.31, N=312
var.14.CFH 1:196659237:C:T
peptide level
126:149 125:140 31:36
RPCGHPGDTPFGTFTLTGGNVFEYGVKpc4_N
p−min=0.33, pX=0.12, N=282
var.14.CFH 1:196659237:C:T
peptide level
141:149 138:140 36:36
RPCGHPGDTPFGTFTLTGGNVFEYGVKpc4_N
p−min=0.048, pX=0.049, N=315
var.14.CFH 1:196659237:C:T
peptide level
140:149 134:140 33:36
RPCGHPGDTPFGTFTLTGGNVFEYGVKpc4_N
p−maj=0.43, pX=0.54, N=307
var.14.CFH 1:196659237:C:T
peptide level
149:149 140:140 36:36
RPCGHPGDTPFGTFTLTGGNVFEYGVKpc4_N
p−maj=1, pX=0.34, N=325
var.14.CFH 1:196659237:C:T
peptide level
149:149 140:140 36:36
SCDIPVFMNARpc2_NP.1
p−maj=1, pX=0.02, N=325
var.14.CFH 1:196659237:C:T
peptide level
149:149 140:140 36:36
SCDIPVFMNARpc2_NP.2
p−maj=1, pX=0.0054, N=325
var.14.CFH 1:196659237:C:T
peptide level
149:149 140:140 36:36
SCDIPVFMNARpc2_NP.3
p−maj=1, pX=0.7, N=325
var.14.CFH 1:196659237:C:T
peptide level
149:149 140:140 36:36
SCDIPVFMNARpc2_NP.4
p−maj=1, pX=0.94, N=325
var.14.CFH 1:196659237:C:T
peptide level
149:149 140:140 36:36
SCDIPVFMNARpc2_NP.5
p−maj=1, pX=0.0015, N=325
var.14.CFH 1:196659237:C:T
peptide level
105:149 96:140 28:36
SCDNPYIPNGDYSPLRpc2_NP.1
p−maj=0.34, pX=0.25, N=229
var.14.CFH 1:196659237:C:T
peptide level
72:149 65:140 19:36
SCDNPYIPNGDYSPLRpc2_NP.2
p−maj=0.6, pX=0.58, N=156
var.14.CFH 1:196659237:C:T
peptide level
107:149 102:140 29:36
SCDNPYIPNGDYSPLRpc2_NP.3
p−maj=0.33, pX=0.12, N=238
var.14.CFH 1:196659237:C:T
peptide level
148:149 139:140 36:36
SCDNPYIPNGDYSPLRpc2_NP.4
p−maj=1, pX=0.65, N=323
var.14.CFH 1:196659237:C:T
peptide level
148:149 139:140 36:36
SCDNPYIPNGDYSPLRpc2_NP.5
p−maj=1, pX=0.23, N=323

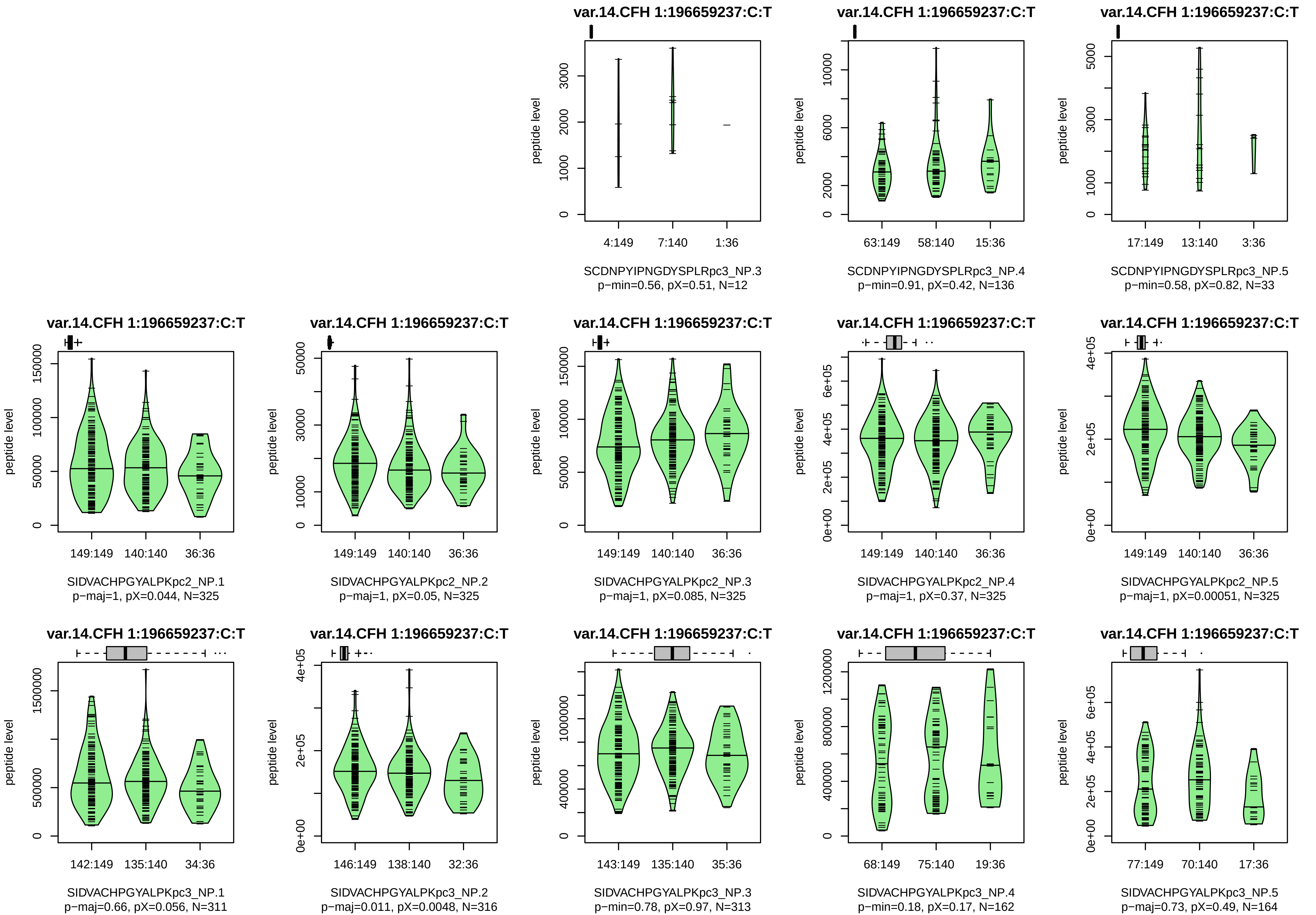
var.14.CFH 1:196659237:C:T
peptide level
4:149
7:140
1:36
SCDNPYIPNGDYSPLRpc3_NP.3
p−min=0.56, pX=0.51, N=12
var.14.CFH 1:196659237:C:T
peptide level
63:149
58:140
15:36
SCDNPYIPNGDYSPLRpc3_NP.4
p−min=0.91, pX=0.42, N=136
var.14.CFH 1:196659237:C:T
peptide level
17:149
13:140
3:36
SCDNPYIPNGDYSPLRpc3_NP.5
p−min=0.58, pX=0.82, N=33
var.14.CFH 1:196659237:C:T
peptide level
149:149
140:140
36:36
SIDVACHPGYALPKpc2_NP.1
p−maj=1, pX=0.044, N=325
var.14.CFH 1:196659237:C:T
peptide level
149:149
140:140
36:36
SIDVACHPGYALPKpc2_NP.2
p−maj=1, pX=0.05, N=325
var.14.CFH 1:196659237:C:T
peptide level
149:149
140:140
36:36
SIDVACHPGYALPKpc2_NP.3
p−maj=1, pX=0.085, N=325
var.14.CFH 1:196659237:C:T
peptide level
149:149
140:140
36:36
SIDVACHPGYALPKpc2_NP.4
p−maj=1, pX=0.37, N=325
var.14.CFH 1:196659237:C:T
peptide level
149:149
140:140
36:36
SIDVACHPGYALPKpc2_NP.5
p−maj=1, pX=0.00051, N=325
var.14.CFH 1:196659237:C:T
peptide level
142:149
135:140
34:36
SIDVACHPGYALPKpc3_NP.1
p−maj=0.66, pX=0.056, N=311
var.14.CFH 1:196659237:C:T
peptide level
146:149
138:140
32:36
SIDVACHPGYALPKpc3_NP.2
p−maj=0.011, pX=0.0048, N=316
var.14.CFH 1:196659237:C:T
peptide level
143:149
135:140
35:36
SIDVACHPGYALPKpc3_NP.3
p−min=0.78, pX=0.97, N=313
var.14.CFH 1:196659237:C:T
peptide level
68:149
75:140
19:36
SIDVACHPGYALPKpc3_NP.4
p−min=0.18, pX=0.17, N=162
var.14.CFH 1:196659237:C:T
peptide level
77:149
70:140
17:36
SIDVACHPGYALPKpc3_NP.5
p−maj=0.73, pX=0.49, N=164

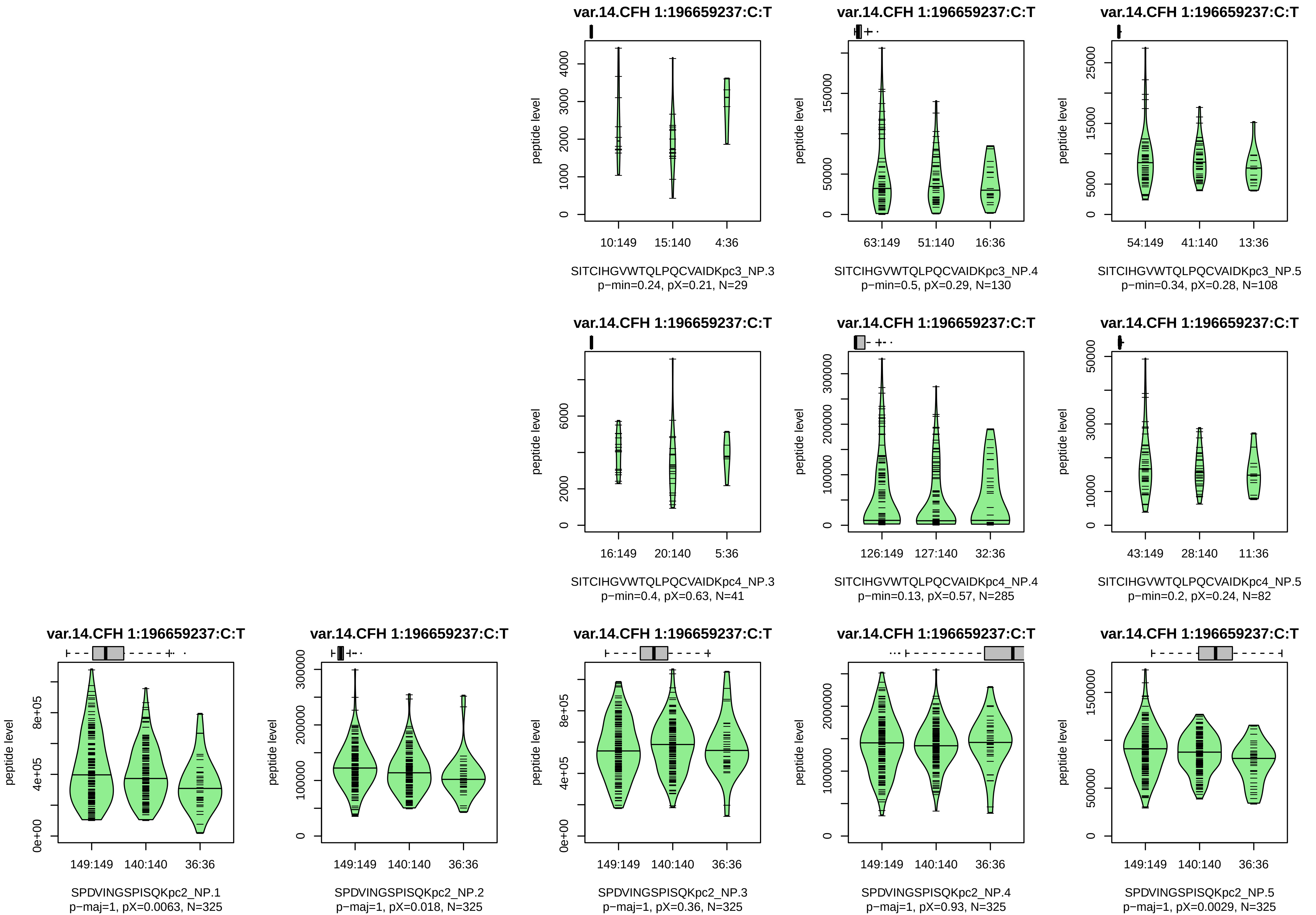
var.14.CFH 1:196659237:C:T
peptide level
10:149
15:140
4:36
SITCIHGVWTQLPQCVAIDKpc3_NP.3
p−min=0.24, pX=0.21, N=29
var.14.CFH 1:196659237:C:T
peptide level
63:149
51:140
16:36
SITCIHGVWTQLPQCVAIDKpc3_NP.4
p−min=0.5, pX=0.29, N=130
var.14.CFH 1:196659237:C:T
peptide level
54:149
41:140
13:36
SITCIHGVWTQLPQCVAIDKpc3_NP.5
p−min=0.34, pX=0.28, N=108
var.14.CFH 1:196659237:C:T
peptide level
16:149
20:140
5:36
SITCIHGVWTQLPQCVAIDKpc4_NP.3
p−min=0.4, pX=0.63, N=41
var.14.CFH 1:196659237:C:T
peptide level
126:149
127:140
32:36
SITCIHGVWTQLPQCVAIDKpc4_NP.4
p−min=0.13, pX=0.57, N=285
var.14.CFH 1:196659237:C:T
peptide level
43:149
28:140
11:36
SITCIHGVWTQLPQCVAIDKpc4_NP.5
p−min=0.2, pX=0.24, N=82
var.14.CFH 1:196659237:C:T
peptide level
149:149
140:140
36:36
SPDVINGSPISQKpc2_NP.1
p−maj=1, pX=0.0063, N=325
var.14.CFH 1:196659237:C:T
peptide level
149:149
140:140
36:36
SPDVINGSPISQKpc2_NP.2
p−maj=1, pX=0.018, N=325
var.14.CFH 1:196659237:C:T
peptide level
149:149
140:140
36:36
SPDVINGSPISQKpc2_NP.3
p−maj=1, pX=0.36, N=325
var.14.CFH 1:196659237:C:T
peptide level
149:149
140:140
36:36
SPDVINGSPISQKpc2_NP.4
p−maj=1, pX=0.93, N=325
var.14.CFH 1:196659237:C:T
peptide level
149:149
140:140
36:36
SPDVINGSPISQKpc2_NP.5
p−maj=1, pX=0.0029, N=325

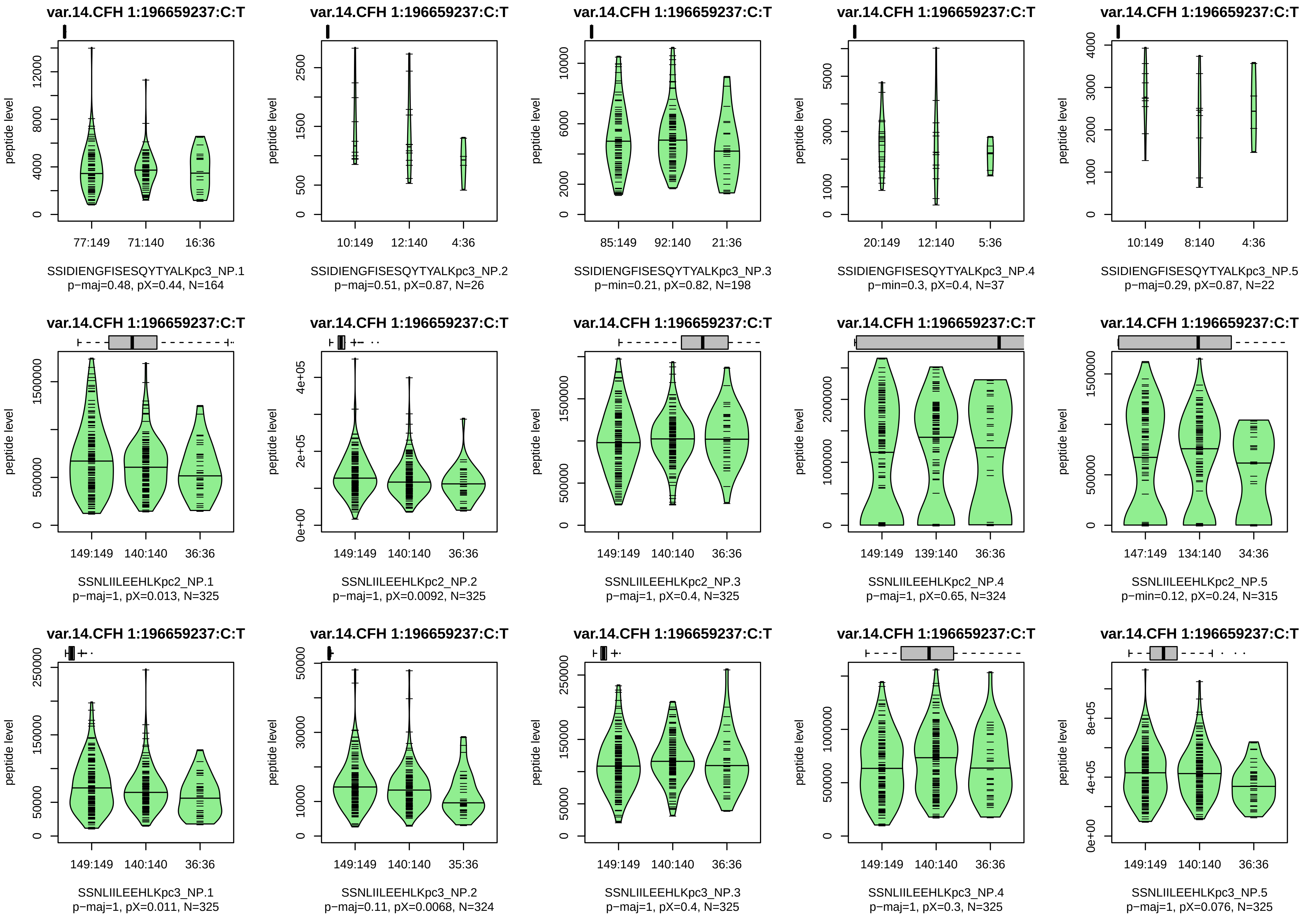
var.14.CFH 1:196659237:C:T
peptide level
77:149 71:140 16:36
SSIDIENGFISESQYTYALKpc3_NP.1
p−maj=0.48, pX=0.44, N=164
var.14.CFH 1:196659237:C:T
10:149 12:140 4:36
SSIDIENGFISESQYTYALKpc3_NP.2
p−maj=0.51, pX=0.87, N=26
var.14.CFH 1:196659237:C:T
85:149 92:140 21:36
SSIDIENGFISESQYTYALKpc3_NP.3
p−min=0.21, pX=0.82, N=198
var.14.CFH 1:196659237:C:T
20:149 12:140 5:36
SSIDIENGFISESQYTYALKpc3_NP.4
p−min=0.3, pX=0.4, N=37
var.14.CFH 1:196659237:C:T
10:149 8:140 4:36
SSIDIENGFISESQYTYALKpc3_NP.5
p−maj=0.29, pX=0.87, N=22
var.14.CFH 1:196659237:C:T
149:149 140:140 36:36
SSNLIILEEHLKpc2_NP.1
p−maj=1, pX=0.013, N=325
var.14.CFH 1:196659237:C:T
149:149 140:140 36:36
SSNLIILEEHLKpc2_NP.2
p−maj=1, pX=0.0092, N=325
var.14.CFH 1:196659237:C:T
149:149 140:140 36:36
SSNLIILEEHLKpc2_NP.3
p−maj=1, pX=0.4, N=325
var.14.CFH 1:196659237:C:T
149:149 139:140 36:36
SSNLIILEEHLKpc2_NP.4
p−maj=1, pX=0.65, N=324
var.14.CFH 1:196659237:C:T
147:149 134:140 34:36
SSNLIILEEHLKpc2_NP.5
p−min=0.12, pX=0.24, N=315
var.14.CFH 1:196659237:C:T
149:149 140:140 36:36
SSNLIILEEHLKpc3_NP.1
p−maj=1, pX=0.011, N=325
var.14.CFH 1:196659237:C:T
149:149 140:140 35:36
SSNLIILEEHLKpc3_NP.2
p−maj=0.11, pX=0.0068, N=324
var.14.CFH 1:196659237:C:T
149:149 140:140 36:36
SSNLIILEEHLKpc3_NP.3
p−maj=1, pX=0.4, N=325
var.14.CFH 1:196659237:C:T
149:149 140:140 36:36
SSNLIILEEHLKpc3_NP.4
p−maj=1, pX=0.3, N=325
var.14.CFH 1:196659237:C:T
149:149 140:140 36:36
SSNLIILEEHLKpc3_NP.5
p−maj=1, pX=0.076, N=325

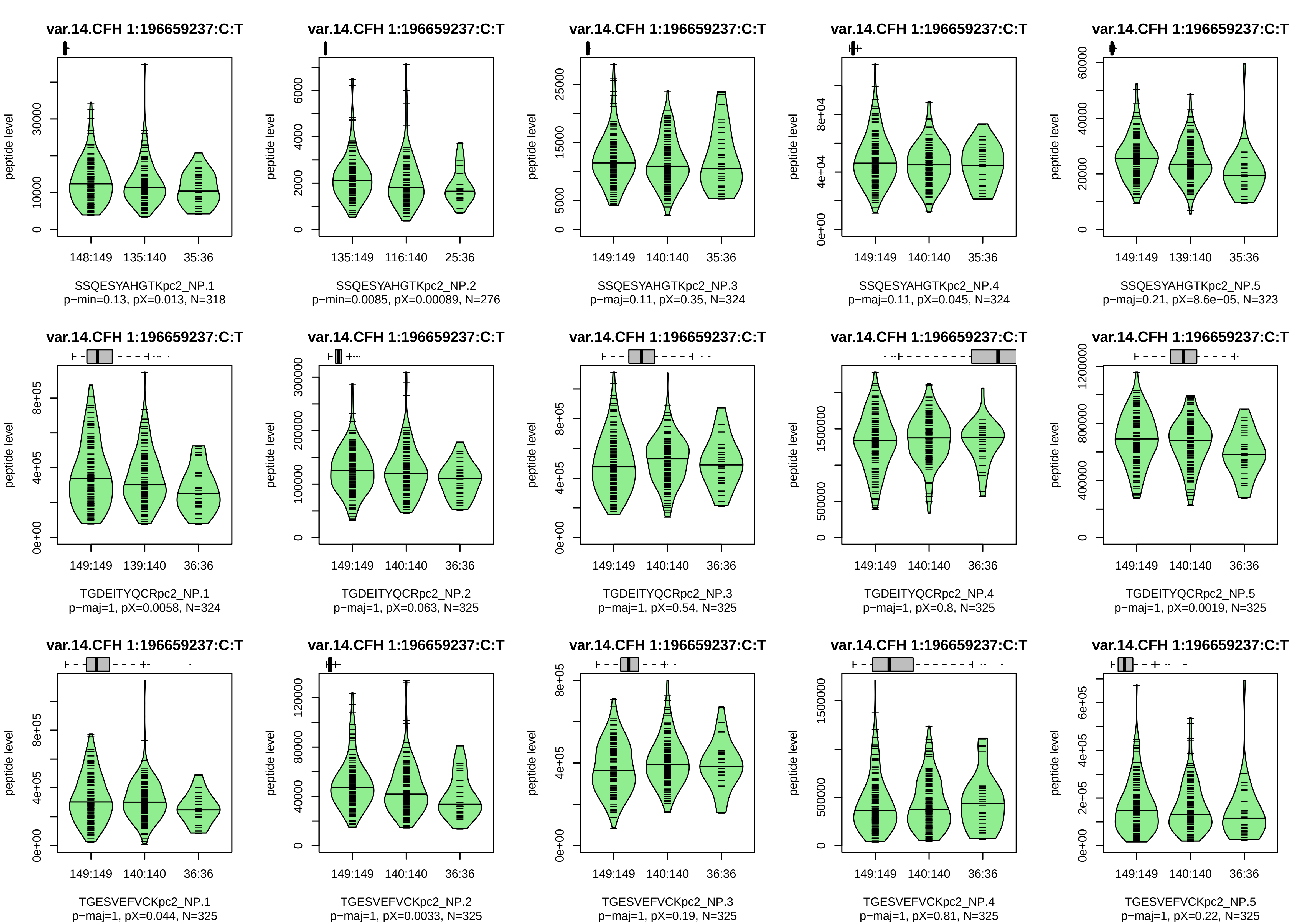

var.14.CFH 1:196659237:C:T
peptide level
148:149 135:140 35:36
SSQESYAHGTKpc2_NP.1
p−min=0.13, pX=0.013, N=318
var.14.CFH 1:196659237:C:T
peptide level
135:149 116:140 25:36
SSQESYAHGTKpc2_NP.2
p−min=0.0085, pX=0.00089, N=276
var.14.CFH 1:196659237:C:T
peptide level
149:149 140:140 35:36
SSQESYAHGTKpc2_NP.3
p−maj=0.11, pX=0.35, N=324
var.14.CFH 1:196659237:C:T
peptide level
149:149 140:140 35:36
SSQESYAHGTKpc2_NP.4
p−maj=0.11, pX=0.045, N=324
var.14.CFH 1:196659237:C:T
peptide level
149:149 139:140 35:36
SSQESYAHGTKpc2_NP.5
p−maj=0.21, pX=8.6e−05, N=323
var.14.CFH 1:196659237:C:T
peptide level
149:149 139:140 36:36
TGDEITYQCRpc2_NP.1
p−maj=1, pX=0.0058, N=324
var.14.CFH 1:196659237:C:T
peptide level
149:149 140:140 36:36
TGDEITYQCRpc2_NP.2
p−maj=1, pX=0.063, N=325
var.14.CFH 1:196659237:C:T
peptide level
149:149 140:140 36:36
TGDEITYQCRpc2_NP.3
p−maj=1, pX=0.54, N=325
var.14.CFH 1:196659237:C:T
peptide level
149:149 140:140 36:36
TGDEITYQCRpc2_NP.4
p−maj=1, pX=0.8, N=325
var.14.CFH 1:196659237:C:T
peptide level
149:149 140:140 36:36
TGDEITYQCRpc2_NP.5
p−maj=1, pX=0.0019, N=325
var.14.CFH 1:196659237:C:T
peptide level
149:149 140:140 36:36
TGESVEFVCKpc2_NP.1
p−maj=1, pX=0.044, N=325
var.14.CFH 1:196659237:C:T
peptide level
149:149 140:140 36:36
TGESVEFVCKpc2_NP.2
p−maj=1, pX=0.0033, N=325
var.14.CFH 1:196659237:C:T
peptide level
149:149 140:140 36:36
TGESVEFVCKpc2_NP.3
p−maj=1, pX=0.19, N=325
var.14.CFH 1:196659237:C:T
peptide level
149:149 140:140 36:36
TGESVEFVCKpc2_NP.4
p−maj=1, pX=0.81, N=325
var.14.CFH 1:196659237:C:T
peptide level
149:149 140:140 36:36
TGESVEFVCKpc2_NP.5
p−maj=1, pX=0.22, N=325

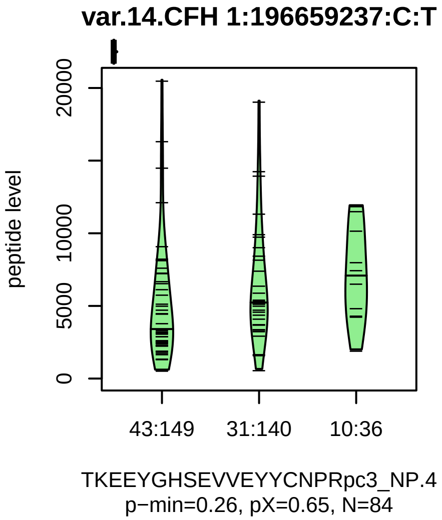
var.14.CFH 1:196659237:C:T
peptide level
0
5000
10000
20000
43:149
31:140
10:36
TKEEYGHSEVVEYYCNPRpc3_NP.4
p−min=0.26, pX=0.65, N=84

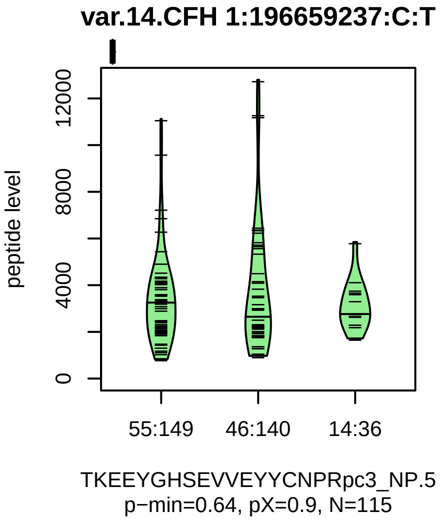
var.14.CFH 1:196659237:C:T
peptide level
0
4000
8000
12000
55:149
46:140
14:36
TKEEYGHSEVVEYYCNPRpc3_NP.5
p−min=0.64, pX=0.9, N=115

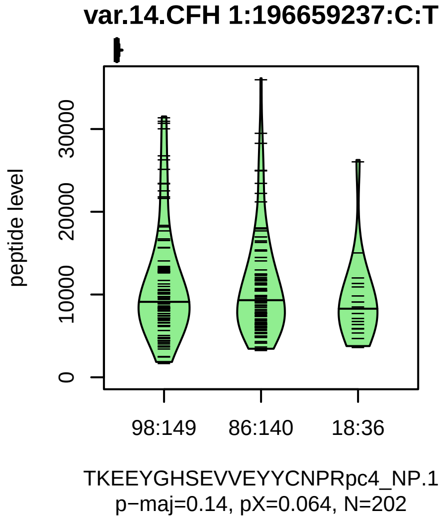
var.14.CFH 1:196659237:C:T
peptide level
0
10000
20000
30000
98:149
86:140
18:36
TKEEYGHSEVVEYYCNPRpc4_NP.1
p−maj=0.14, pX=0.064, N=202

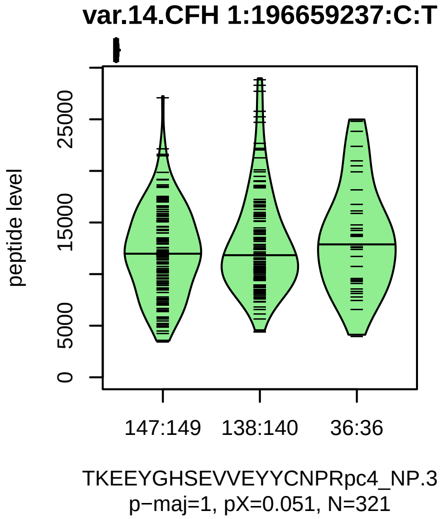
var.14.CFH 1:196659237:C:T
peptide level
0
5000
15000
25000
147:149
138:140
36:36
TKEEYGHSEVVEYYCNPRpc4_NP.3
p−maj=1, pX=0.051, N=321

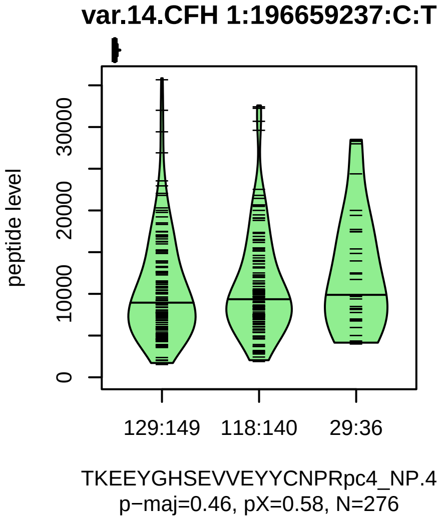
var.14.CFH 1:196659237:C:T
peptide level
0
10000
20000
30000
129:149
118:140
29:36
TKEEYGHSEVVEYYCNPRpc4_NP.4
p−maj=0.46, pX=0.58, N=276

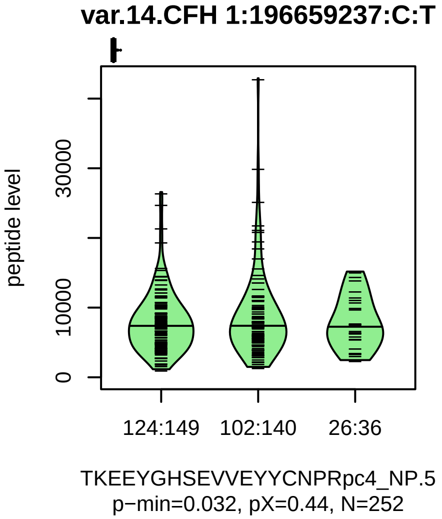
var.14.CFH 1:196659237:C:T
peptide level
0
10000
30000
124:149
102:140
26:36
TKEEYGHSEVVEYYCNPRpc4_NP.5
p−min=0.032, pX=0.44, N=252

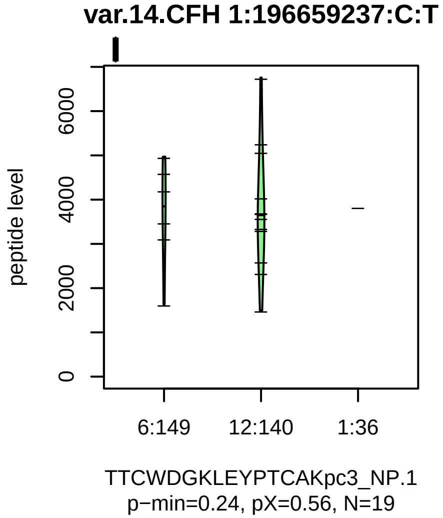
var.14.CFH 1:196659237:C:T
peptide level
0
2000
4000
6000
6:149
12:140
1:36
TTCWDGKLEYPTCAKpc3_NP.1
p−min=0.24, pX=0.56, N=19

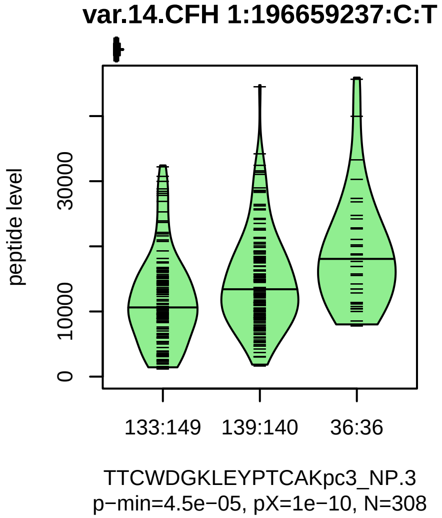
var.14.CFH 1:196659237:C:T
peptide level
0
10000
30000
133:149
139:140
36:36
TTCWDGKLEYPTCAKpc3_NP.3
p−min=4.5e−05, pX=1e−10, N=308

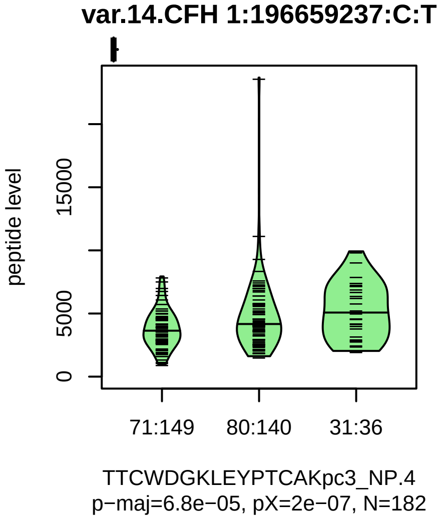
var.14.CFH 1:196659237:C:T
peptide level
0
5000
15000
71:149
80:140
31:36
TTCWDGKLEYPTCAKpc3_NP.4
p−maj=6.8e−05, pX=2e−07, N=182

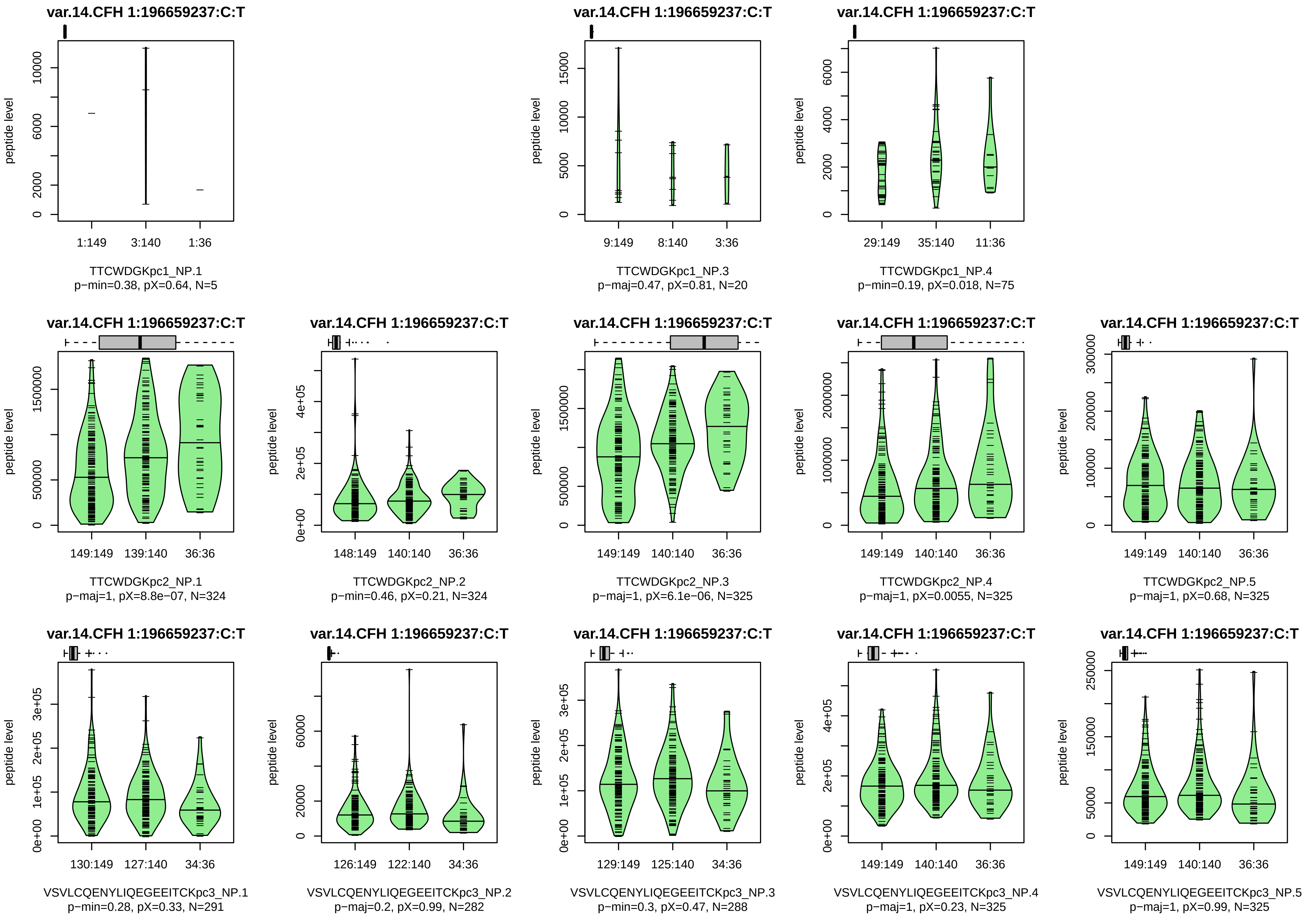
var.14.CFH 1:196659237:C:T
peptide level
1:149
3:140
1:36
TTCWDGKpc1_NP.1
p−min=0.38, pX=0.64, N=5
var.14.CFH 1:196659237:C:T
peptide level
9:149
8:140
3:36
TTCWDGKpc1_NP.3
p−maj=0.47, pX=0.81, N=20
var.14.CFH 1:196659237:C:T
peptide level
29:149
35:140
11:36
TTCWDGKpc1_NP.4
p−min=0.19, pX=0.018, N=75
var.14.CFH 1:196659237:C:T
peptide level
149:149
139:140
36:36
TTCWDGKpc2_NP.1
p−maj=1, pX=8.8e−07, N=324
var.14.CFH 1:196659237:C:T
peptide level
148:149
140:140
36:36
TTCWDGKpc2_NP.2
p−min=0.46, pX=0.21, N=324
var.14.CFH 1:196659237:C:T
peptide level
149:149
140:140
36:36
TTCWDGKpc2_NP.3
p−maj=1, pX=6.1e−06, N=325
var.14.CFH 1:196659237:C:T
peptide level
149:149
140:140
36:36
TTCWDGKpc2_NP.4
p−maj=1, pX=0.0055, N=325
var.14.CFH 1:196659237:C:T
peptide level
149:149
140:140
36:36
TTCWDGKpc2_NP.5
p−maj=1, pX=0.68, N=325
var.14.CFH 1:196659237:C:T
peptide level
130:149
127:140
34:36
VSVLCQENYLIQEGEEITCKpc3_NP.1
p−min=0.28, pX=0.33, N=291
var.14.CFH 1:196659237:C:T
peptide level
126:149
122:140
34:36
VSVLCQENYLIQEGEEITCKpc3_NP.2
p−maj=0.2, pX=0.99, N=282
var.14.CFH 1:196659237:C:T
peptide level
129:149
125:140
34:36
VSVLCQENYLIQEGEEITCKpc3_NP.3
p−min=0.3, pX=0.47, N=288
var.14.CFH 1:196659237:C:T
peptide level
149:149
140:140
36:36
VSVLCQENYLIQEGEEITCKpc3_NP.4
p−maj=1, pX=0.23, N=325
var.14.CFH 1:196659237:C:T
peptide level
149:149
140:140
36:36
VSVLCQENYLIQEGEEITCKpc3_NP.5
p−maj=1, pX=0.99, N=325

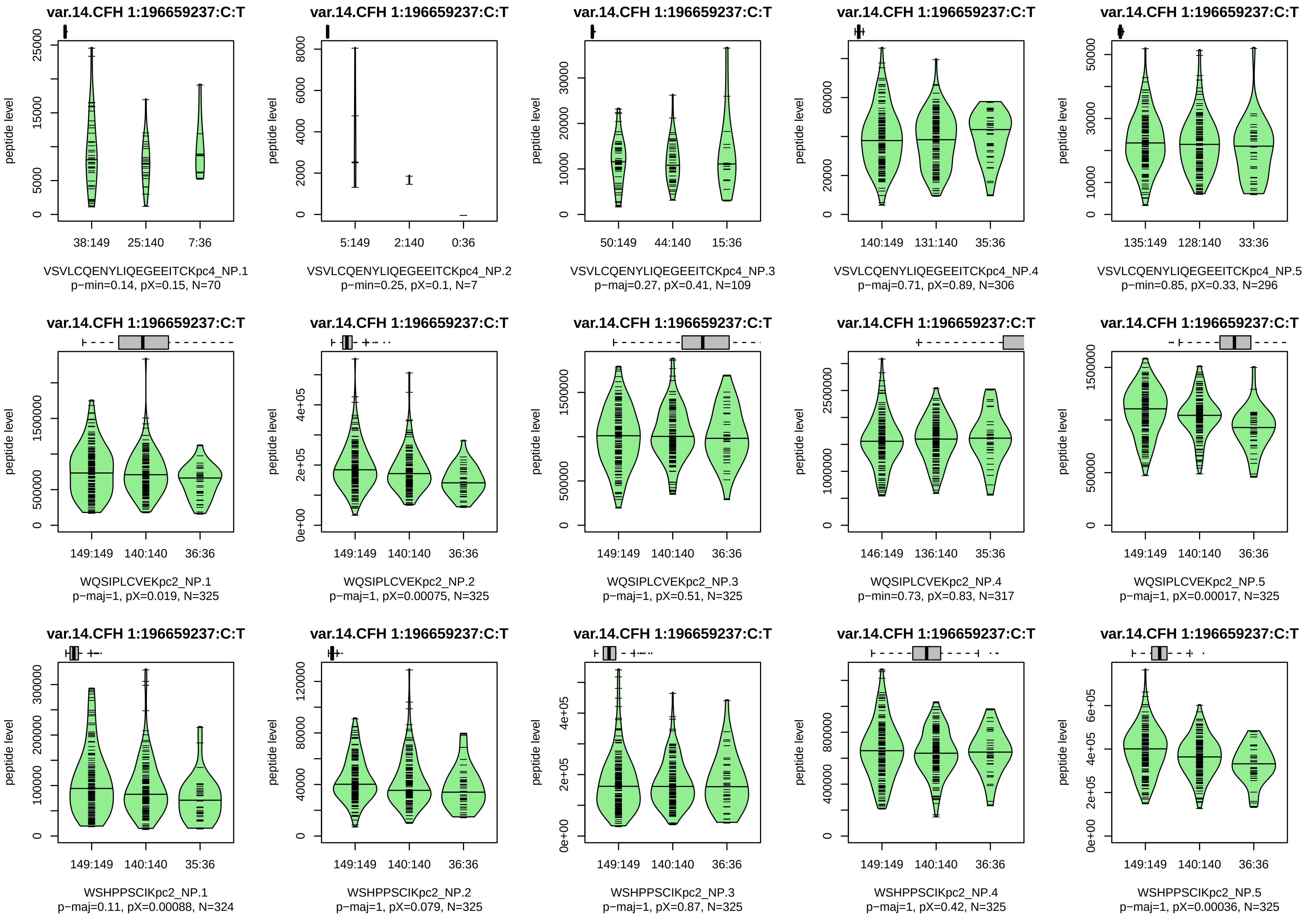
var.14.CFH 1:196659237:C:T
peptide level
38:149
25:140
7:36
VSVLCQENYLIQEGEEITCKpc4_NP.1
p−min=0.14, pX=0.15, N=70
var.14.CFH 1:196659237:C:T
peptide level
5:149
2:140
0:36
VSVLCQENYLIQEGEEITCKpc4_NP.2
p−min=0.25, pX=0.1, N=7
var.14.CFH 1:196659237:C:T
peptide level
50:149
44:140
15:36
VSVLCQENYLIQEGEEITCKpc4_NP.3
p−maj=0.27, pX=0.41, N=109
var.14.CFH 1:196659237:C:T
peptide level
140:149
131:140
35:36
VSVLCQENYLIQEGEEITCKpc4_NP.4
p−maj=0.71, pX=0.89, N=306
var.14.CFH 1:196659237:C:T
peptide level
135:149
128:140
33:36
VSVLCQENYLIQEGEEITCKpc4_NP.5
p−min=0.85, pX=0.33, N=296
var.14.CFH 1:196659237:C:T
peptide level
149:149
140:140
36:36
WQSIPLCVEKpc2_NP.1
p−maj=1, pX=0.019, N=325
var.14.CFH 1:196659237:C:T
peptide level
149:149
140:140
36:36
WQSIPLCVEKpc2_NP.2
p−maj=1, pX=0.00075, N=325
var.14.CFH 1:196659237:C:T
peptide level
149:149
140:140
36:36
WQSIPLCVEKpc2_NP.3
p−maj=1, pX=0.51, N=325
var.14.CFH 1:196659237:C:T
peptide level
146:149
136:140
35:36
WQSIPLCVEKpc2_NP.4
p−min=0.73, pX=0.83, N=317
var.14.CFH 1:196659237:C:T
peptide level
149:149
140:140
36:36
WQSIPLCVEKpc2_NP.5
p−maj=1, pX=0.00017, N=325
var.14.CFH 1:196659237:C:T
peptide level
149:149
140:140
35:36
WSHPPSCIKpc2_NP.1
p−maj=0.11, pX=0.00088, N=324
var.14.CFH 1:196659237:C:T
peptide level
149:149
140:140
36:36
WSHPPSCIKpc2_NP.2
p−maj=1, pX=0.079, N=325
var.14.CFH 1:196659237:C:T
peptide level
149:149
140:140
36:36
WSHPPSCIKpc2_NP.3
p−maj=1, pX=0.87, N=325
var.14.CFH 1:196659237:C:T
peptide level
149:149
140:140
36:36
WSHPPSCIKpc2_NP.4
p−maj=1, pX=0.42, N=325
var.14.CFH 1:196659237:C:T
peptide level
149:149
140:140
36:36
WSHPPSCIKpc2_NP.5
p−maj=1, pX=0.00036, N=325

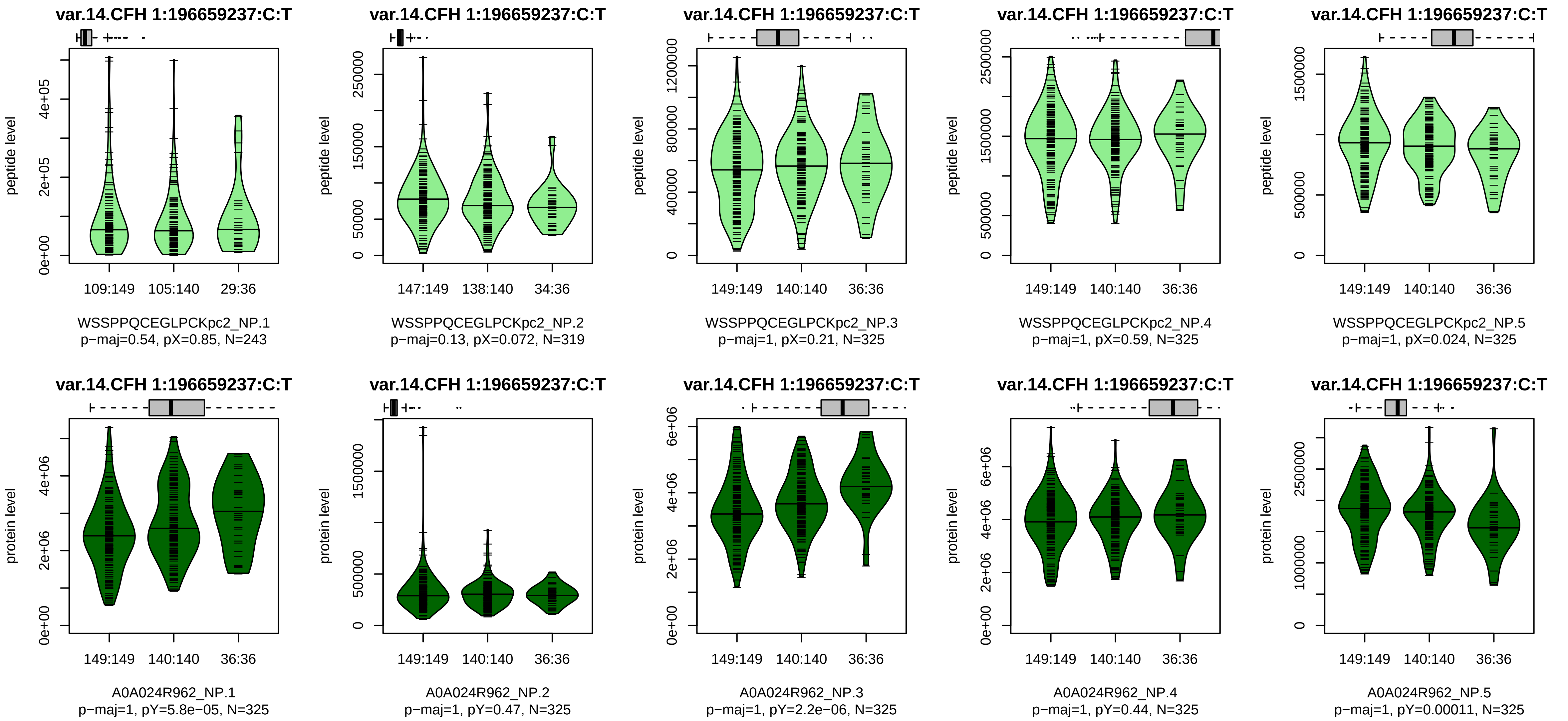

var.14.CFH 1:196659237:C:T
peptide level
0e+00
2e+05
4e+05
109:149
105:140
29:36
WSSPPQCEGLPCKpc2_NP.1
p−maj=0.54, pX=0.85, N=243
var.14.CFH 1:196659237:C:T
peptide level
0
50000
150000
250000
147:149
138:140
34:36
WSSPPQCEGLPCKpc2_NP.2
p−maj=0.13, pX=0.072, N=319
var.14.CFH 1:196659237:C:T
peptide level
0
400000
800000
1200000
149:149
140:140
36:36
WSSPPQCEGLPCKpc2_NP.3
p−maj=1, pX=0.21, N=325
var.14.CFH 1:196659237:C:T
peptide level
0
500000
1500000
2500000
149:149
140:140
36:36
WSSPPQCEGLPCKpc2_NP.4
p−maj=1, pX=0.59, N=325
var.14.CFH 1:196659237:C:T
peptide level
0
500000
1500000
149:149
140:140
36:36
WSSPPQCEGLPCKpc2_NP.5
p−maj=1, pX=0.024, N=325
var.14.CFH 1:196659237:C:T
protein level
0e+00
2e+06
4e+06
149:149
140:140
36:36
A0A024R962_NP.1
p−maj=1, pY=5.8e−05, N=325
var.14.CFH 1:196659237:C:T
protein level
0
500000
1500000
149:149
140:140
36:36
A0A024R962_NP.2
p−maj=1, pY=0.47, N=325
var.14.CFH 1:196659237:C:T
protein level
0e+00
2e+06
4e+06
6e+06
149:149
140:140
36:36
A0A024R962_NP.3
p−maj=1, pY=2.2e−06, N=325
var.14.CFH 1:196659237:C:T
protein level
0e+00
2e+06
4e+06
6e+06
149:149
140:140
36:36
A0A024R962_NP.4
p−maj=1, pY=0.44, N=325
var.14.CFH 1:196659237:C:T
protein level
0
1000000
2500000
149:149
140:140
36:36
A0A024R962_NP.5
p−maj=1, pY=0.00011, N=325

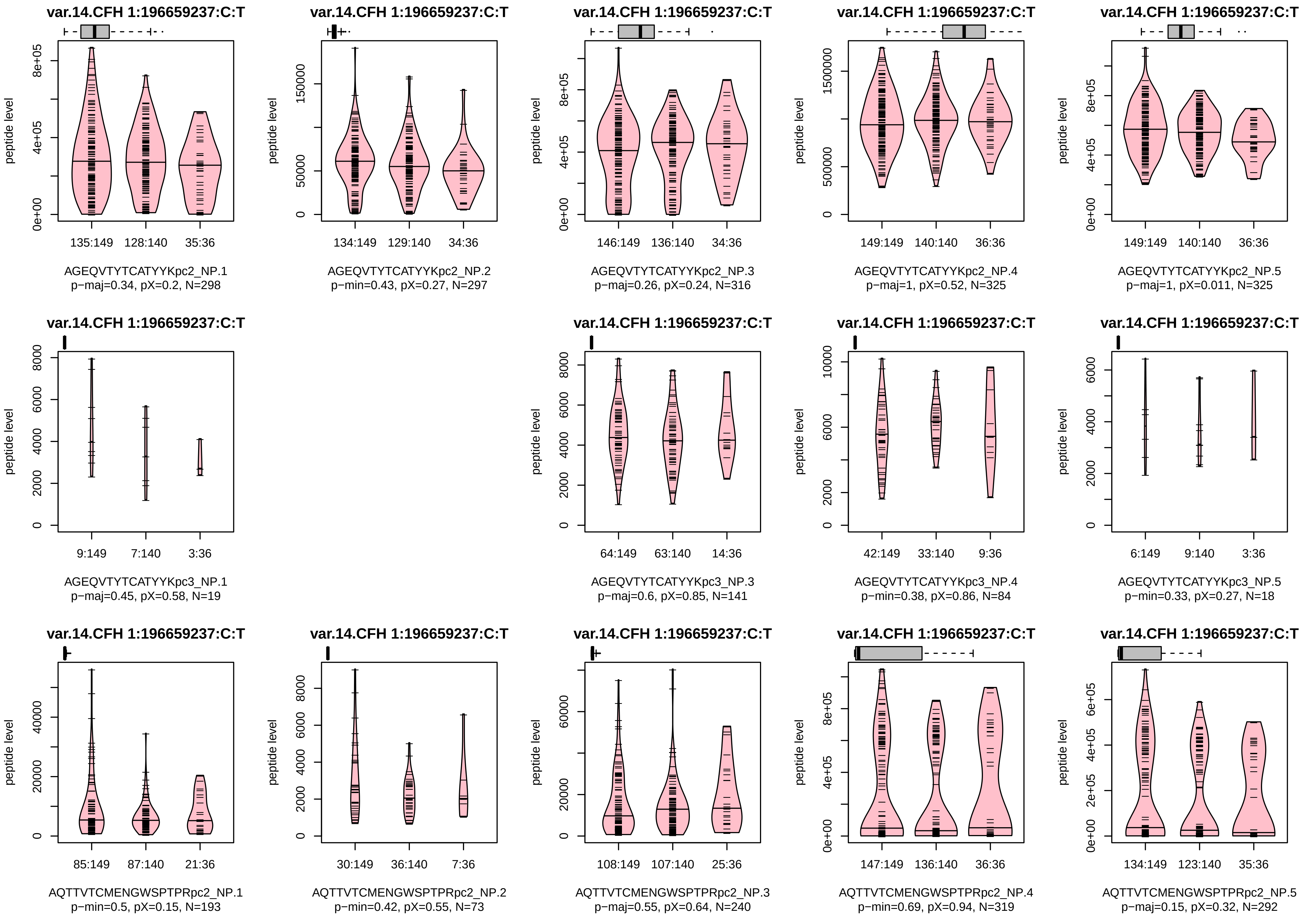
var.14.CFH 1:196659237:C:T
peptide level
135:149
128:140
35:36
AGEQVTYTCATYYKpc2_NP.1
p−maj=0.34, pX=0.2, N=298
var.14.CFH 1:196659237:C:T
peptide level
134:149
129:140
34:36
AGEQVTYTCATYYKpc2_NP.2
p−min=0.43, pX=0.27, N=297
var.14.CFH 1:196659237:C:T
peptide level
146:149
136:140
34:36
AGEQVTYTCATYYKpc2_NP.3
p−maj=0.26, pX=0.24, N=316
var.14.CFH 1:196659237:C:T
peptide level
149:149
140:140
36:36
AGEQVTYTCATYYKpc2_NP.4
p−maj=1, pX=0.52, N=325
var.14.CFH 1:196659237:C:T
peptide level
149:149
140:140
36:36
AGEQVTYTCATYYKpc2_NP.5
p−maj=1, pX=0.011, N=325
var.14.CFH 1:196659237:C:T
peptide level
9:149
7:140
3:36
AGEQVTYTCATYYKpc3_NP.1
p−maj=0.45, pX=0.58, N=19
var.14.CFH 1:196659237:C:T
peptide level
64:149
63:140
14:36
AGEQVTYTCATYYKpc3_NP.3
p−maj=0.6, pX=0.85, N=141
var.14.CFH 1:196659237:C:T
peptide level
42:149
33:140
9:36
AGEQVTYTCATYYKpc3_NP.4
p−min=0.38, pX=0.86, N=84
var.14.CFH 1:196659237:C:T
peptide level
6:149
9:140
3:36
AGEQVTYTCATYYKpc3_NP.5
p−min=0.33, pX=0.27, N=18
var.14.CFH 1:196659237:C:T
peptide level
85:149
87:140
21:36
AQTTVTCMENGWSPTPRpc2_NP.1
p−min=0.5, pX=0.15, N=193
var.14.CFH 1:196659237:C:T
peptide level
30:149
36:140
7:36
AQTTVTCMENGWSPTPRpc2_NP.2
p−min=0.42, pX=0.55, N=73
var.14.CFH 1:196659237:C:T
peptide level
108:149
107:140
25:36
AQTTVTCMENGWSPTPRpc2_NP.3
p−maj=0.55, pX=0.64, N=240
var.14.CFH 1:196659237:C:T
peptide level
147:149
136:140
36:36
AQTTVTCMENGWSPTPRpc2_NP.4
p−min=0.69, pX=0.94, N=319
var.14.CFH 1:196659237:C:T
peptide level
134:149
123:140
35:36
AQTTVTCMENGWSPTPRpc2_NP.5
p−maj=0.15, pX=0.32, N=292

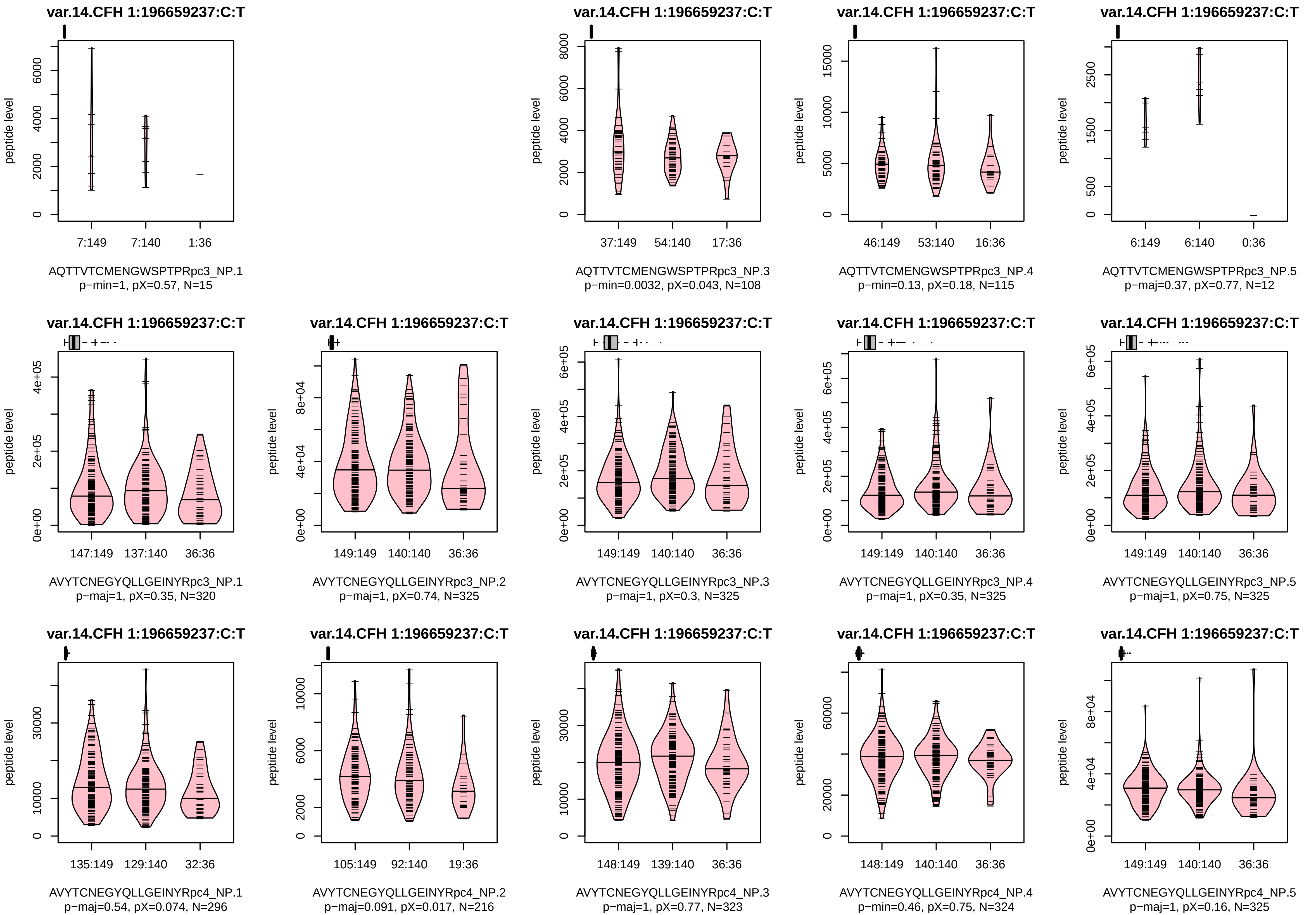

var.14.CFH 1:196659237:C:T
peptide level
7:149
7:140
1:36
AQTTVTCMENGWSPTPRpc3_NP.1
p−min=1, pX=0.57, N=15
var.14.CFH 1:196659237:C:T
peptide level
37:149
54:140
17:36
AQTTVTCMENGWSPTPRpc3_NP.3
p−min=0.0032, pX=0.043, N=108
var.14.CFH 1:196659237:C:T
peptide level
46:149
53:140
16:36
AQTTVTCMENGWSPTPRpc3_NP.4
p−min=0.13, pX=0.18, N=115
var.14.CFH 1:196659237:C:T
peptide level
6:149
6:140
0:36
AQTTVTCMENGWSPTPRpc3_NP.5
p−maj=0.37, pX=0.77, N=12
var.14.CFH 1:196659237:C:T
peptide level
147:149
137:140
36:36
AVYTCNEGYQLLGEINYRpc3_NP.1
p−maj=1, pX=0.35, N=320
var.14.CFH 1:196659237:C:T
peptide level
149:149
140:140
36:36
AVYTCNEGYQLLGEINYRpc3_NP.2
p−maj=1, pX=0.74, N=325
var.14.CFH 1:196659237:C:T
peptide level
149:149
140:140
36:36
AVYTCNEGYQLLGEINYRpc3_NP.3
p−maj=1, pX=0.3, N=325
var.14.CFH 1:196659237:C:T
peptide level
149:149
140:140
36:36
AVYTCNEGYQLLGEINYRpc3_NP.4
p−maj=1, pX=0.35, N=325
var.14.CFH 1:196659237:C:T
peptide level
149:149
140:140
36:36
AVYTCNEGYQLLGEINYRpc3_NP.5
p−maj=1, pX=0.75, N=325
var.14.CFH 1:196659237:C:T
peptide level
135:149
129:140
32:36
AVYTCNEGYQLLGEINYRpc4_NP.1
p−maj=0.54, pX=0.074, N=296
var.14.CFH 1:196659237:C:T
peptide level
105:149
92:140
19:36
AVYTCNEGYQLLGEINYRpc4_NP.2
p−maj=0.091, pX=0.017, N=216
var.14.CFH 1:196659237:C:T
peptide level
148:149
139:140
36:36
AVYTCNEGYQLLGEINYRpc4_NP.3
p−maj=1, pX=0.77, N=323
var.14.CFH 1:196659237:C:T
peptide level
148:149
140:140
36:36
AVYTCNEGYQLLGEINYRpc4_NP.4
p−min=0.46, pX=0.75, N=324
var.14.CFH 1:196659237:C:T
peptide level
149:149
140:140
36:36
AVYTCNEGYQLLGEINYRpc4_NP.5
p−maj=1, pX=0.16, N=325

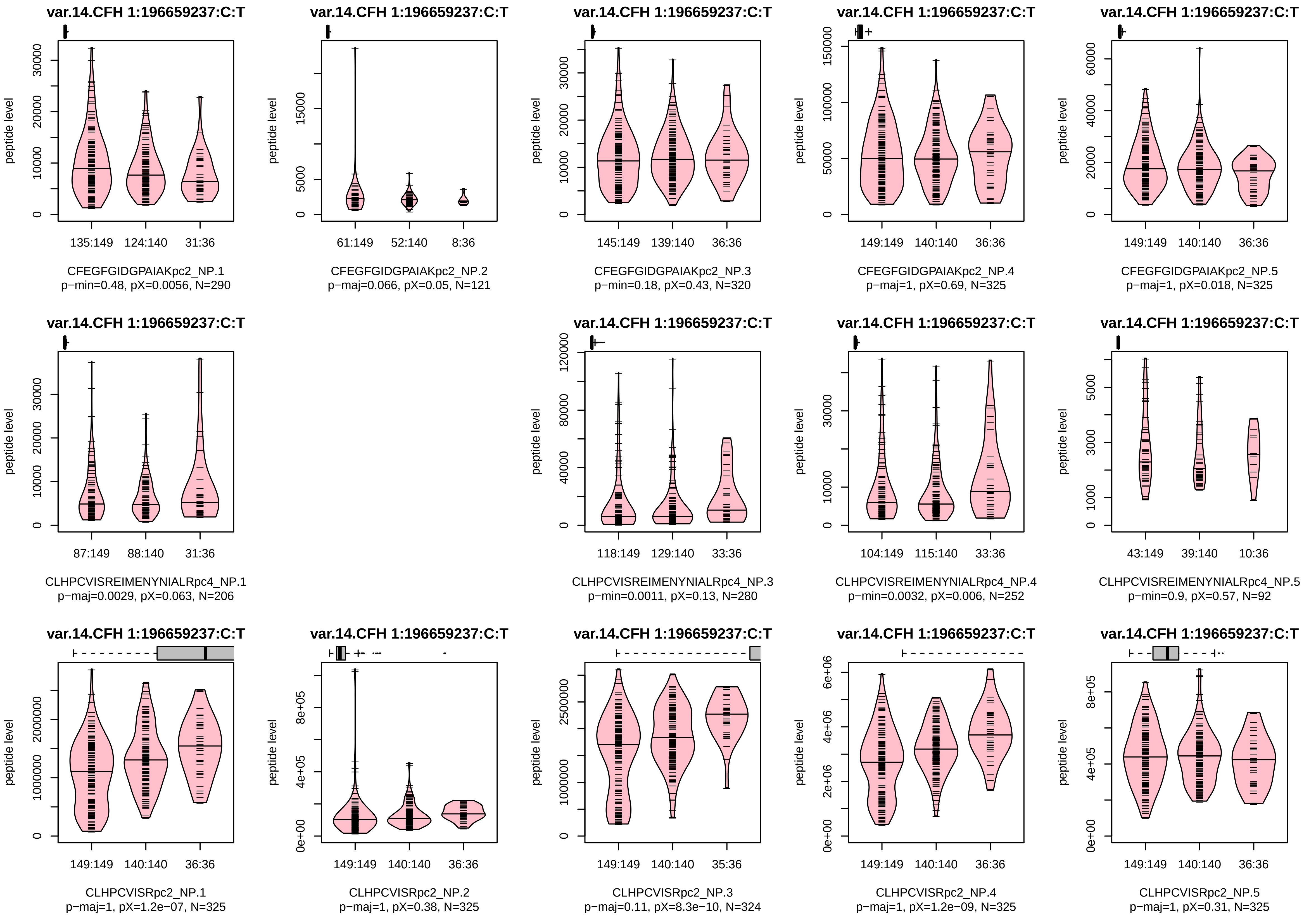
var.14.CFH 1:196659237:C:T
peptide level
135:149
124:140
31:36
CFEGFGIDGPAIAKpc2_NP.1
p−min=0.48, pX=0.0056, N=290
var.14.CFH 1:196659237:C:T
peptide level
61:149
52:140
8:36
CFEGFGIDGPAIAKpc2_NP.2
p−maj=0.066, pX=0.05, N=121
var.14.CFH 1:196659237:C:T
peptide level
145:149
139:140
36:36
CFEGFGIDGPAIAKpc2_NP.3
p−min=0.18, pX=0.43, N=320
var.14.CFH 1:196659237:C:T
peptide level
149:149
140:140
36:36
CFEGFGIDGPAIAKpc2_NP.4
p−maj=1, pX=0.69, N=325
var.14.CFH 1:196659237:C:T
peptide level
149:149
140:140
36:36
CFEGFGIDGPAIAKpc2_NP.5
p−maj=1, pX=0.018, N=325
var.14.CFH 1:196659237:C:T
peptide level
87:149
88:140
31:36
CLHPCVISREIMENYNIALRpc4_NP.1
p−maj=0.0029, pX=0.063, N=206
var.14.CFH 1:196659237:C:T
peptide level
118:149
129:140
33:36
CLHPCVISREIMENYNIALRpc4_NP.3
p−min=0.0011, pX=0.13, N=280
var.14.CFH 1:196659237:C:T
peptide level
104:149
115:140
33:36
CLHPCVISREIMENYNIALRpc4_NP.4
p−min=0.0032, pX=0.006, N=252
var.14.CFH 1:196659237:C:T
peptide level
43:149
39:140
10:36
CLHPCVISREIMENYNIALRpc4_NP.5
p−min=0.9, pX=0.57, N=92
var.14.CFH 1:196659237:C:T
peptide level
149:149
140:140
36:36
CLHPCVISRpc2_NP.1
p−maj=1, pX=1.2e−07, N=325
var.14.CFH 1:196659237:C:T
peptide level
149:149
140:140
36:36
CLHPCVISRpc2_NP.2
p−maj=1, pX=0.38, N=325
var.14.CFH 1:196659237:C:T
peptide level
149:149
140:140
35:36
CLHPCVISRpc2_NP.3
p−maj=0.11, pX=8.3e−10, N=324
var.14.CFH 1:196659237:C:T
peptide level
149:149
140:140
36:36
CLHPCVISRpc2_NP.4
p−maj=1, pX=1.2e−09, N=325
var.14.CFH 1:196659237:C:T
peptide level
149:149
140:140
36:36
CLHPCVISRpc2_NP.5
p−maj=1, pX=0.31, N=325

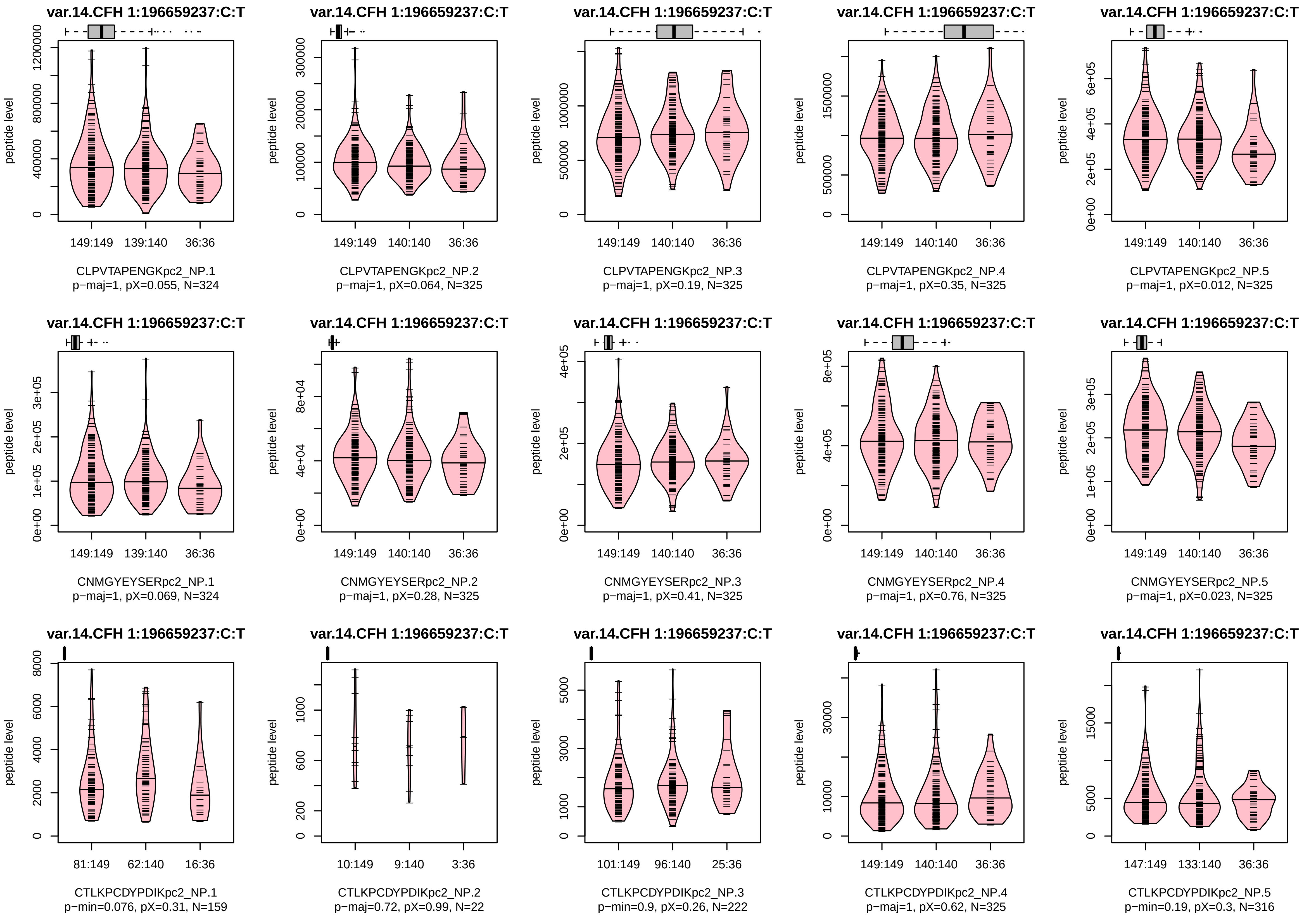
var.14.CFH 1:196659237:C:T
peptide level
0
400000
800000
1200000
149:149
139:140
36:36
CLPVTAPENGKpc2_NP.1
p−maj=1, pX=0.055, N=324
var.14.CFH 1:196659237:C:T
peptide level
0
100000
200000
300000
149:149
140:140
36:36
CLPVTAPENGKpc2_NP.2
p−maj=1, pX=0.064, N=325
var.14.CFH 1:196659237:C:T
peptide level
0
500000
1000000
149:149
140:140
36:36
CLPVTAPENGKpc2_NP.3
p−maj=1, pX=0.19, N=325
var.14.CFH 1:196659237:C:T
peptide level
0
500000
1500000
149:149
140:140
36:36
CLPVTAPENGKpc2_NP.4
p−maj=1, pX=0.35, N=325
var.14.CFH 1:196659237:C:T
peptide level
0e+00
2e+05
4e+05
6e+05
149:149
140:140
36:36
CLPVTAPENGKpc2_NP.5
p−maj=1, pX=0.012, N=325
var.14.CFH 1:196659237:C:T
peptide level
0e+00
1e+05
2e+05
3e+05
149:149
139:140
36:36
CNMGYEYSERpc2_NP.1
p−maj=1, pX=0.069, N=324
var.14.CFH 1:196659237:C:T
peptide level
0e+00
4e+04
8e+04
149:149
140:140
36:36
CNMGYEYSERpc2_NP.2
p−maj=1, pX=0.28, N=325
var.14.CFH 1:196659237:C:T
peptide level
0e+00
2e+05
4e+05
149:149
140:140
36:36
CNMGYEYSERpc2_NP.3
p−maj=1, pX=0.41, N=325
var.14.CFH 1:196659237:C:T
peptide level
0e+00
4e+05
8e+05
149:149
140:140
36:36
CNMGYEYSERpc2_NP.4
p−maj=1, pX=0.76, N=325
var.14.CFH 1:196659237:C:T
peptide level
0e+00
1e+05
2e+05
3e+05
149:149
140:140
36:36
CNMGYEYSERpc2_NP.5
p−maj=1, pX=0.023, N=325
var.14.CFH 1:196659237:C:T
peptide level
0
2000
4000
6000
8000
81:149
62:140
16:36
CTLKPCDYPDIKpc2_NP.1
p−min=0.076, pX=0.31, N=159
var.14.CFH 1:196659237:C:T
peptide level
0
200
600
1000
10:149
9:140
3:36
CTLKPCDYPDIKpc2_NP.2
p−maj=0.72, pX=0.99, N=22
var.14.CFH 1:196659237:C:T
peptide level
0
1000
3000
5000
101:149
96:140
25:36
CTLKPCDYPDIKpc2_NP.3
p−min=0.9, pX=0.26, N=222
var.14.CFH 1:196659237:C:T
peptide level
0
10000
30000
149:149
140:140
36:36
CTLKPCDYPDIKpc2_NP.4
p−maj=1, pX=0.62, N=325
var.14.CFH 1:196659237:C:T
peptide level
0
5000
15000
147:149
133:140
36:36
CTLKPCDYPDIKpc2_NP.5
p−min=0.19, pX=0.3, N=316

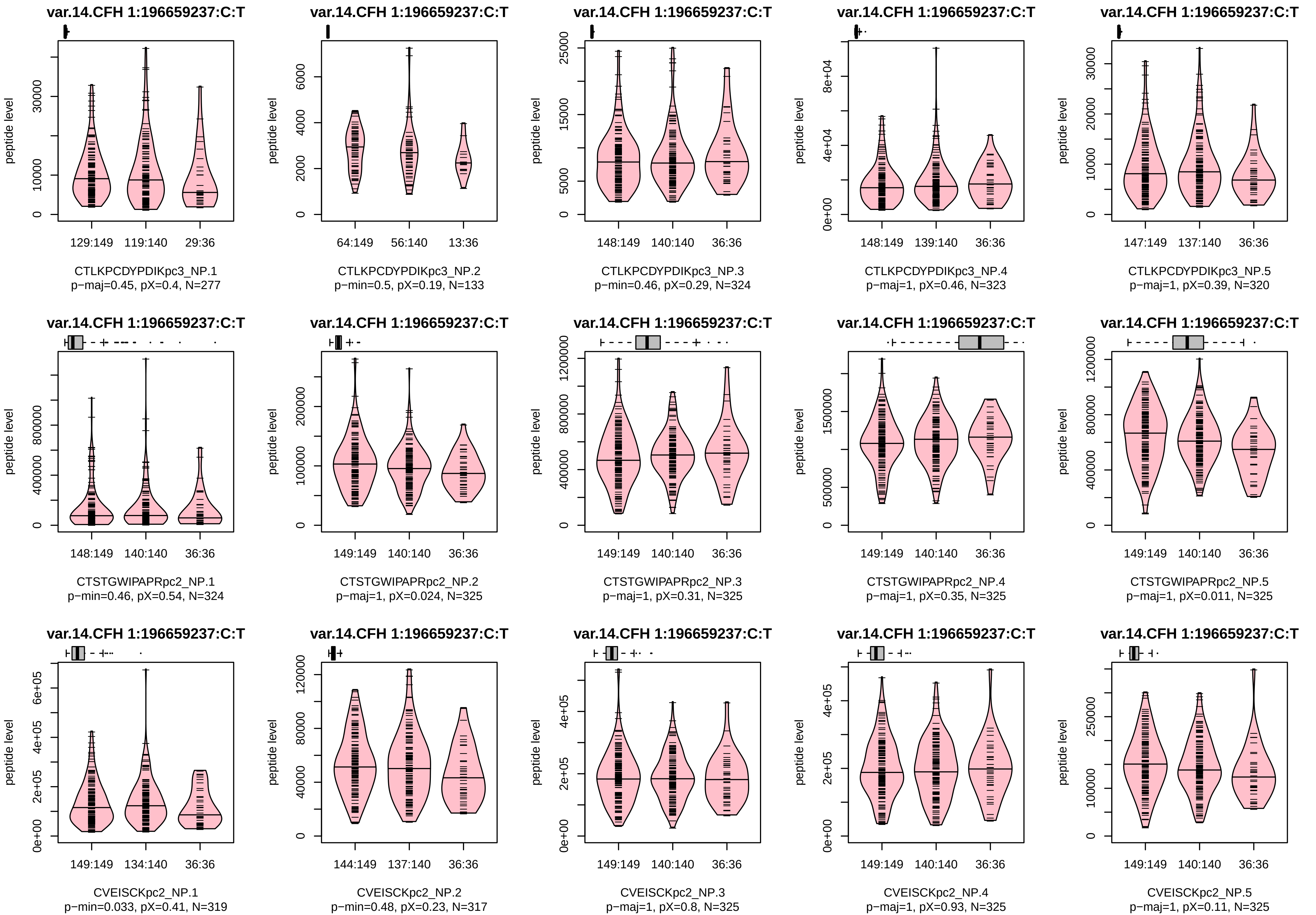

var.14.CFH 1:196659237:C:T
peptide level
129:149
119:140
29:36
CTLKPCDYPDIKpc3_NP.1
p−maj=0.45, pX=0.4, N=277
var.14.CFH 1:196659237:C:T
peptide level
64:149
56:140
13:36
CTLKPCDYPDIKpc3_NP.2
p−min=0.5, pX=0.19, N=133
var.14.CFH 1:196659237:C:T
peptide level
148:149
140:140
36:36
CTLKPCDYPDIKpc3_NP.3
p−min=0.46, pX=0.29, N=324
var.14.CFH 1:196659237:C:T
peptide level
148:149
139:140
36:36
CTLKPCDYPDIKpc3_NP.4
p−maj=1, pX=0.46, N=323
var.14.CFH 1:196659237:C:T
peptide level
147:149
137:140
36:36
CTLKPCDYPDIKpc3_NP.5
p−maj=1, pX=0.39, N=320
var.14.CFH 1:196659237:C:T
peptide level
148:149
140:140
36:36
CTSTGWIPAPRpc2_NP.1
p−min=0.46, pX=0.54, N=324
var.14.CFH 1:196659237:C:T
peptide level
149:149
140:140
36:36
CTSTGWIPAPRpc2_NP.2
p−maj=1, pX=0.024, N=325
var.14.CFH 1:196659237:C:T
peptide level
149:149
140:140
36:36
CTSTGWIPAPRpc2_NP.3
p−maj=1, pX=0.31, N=325
var.14.CFH 1:196659237:C:T
peptide level
149:149
140:140
36:36
CTSTGWIPAPRpc2_NP.4
p−maj=1, pX=0.35, N=325
var.14.CFH 1:196659237:C:T
peptide level
149:149
140:140
36:36
CTSTGWIPAPRpc2_NP.5
p−maj=1, pX=0.011, N=325
var.14.CFH 1:196659237:C:T
peptide level
149:149
134:140
36:36
CVEISCKpc2_NP.1
p−min=0.033, pX=0.41, N=319
var.14.CFH 1:196659237:C:T
peptide level
144:149
137:140
36:36
CVEISCKpc2_NP.2
p−min=0.48, pX=0.23, N=317
var.14.CFH 1:196659237:C:T
peptide level
149:149
140:140
36:36
CVEISCKpc2_NP.3
p−maj=1, pX=0.8, N=325
var.14.CFH 1:196659237:C:T
peptide level
149:149
140:140
36:36
CVEISCKpc2_NP.4
p−maj=1, pX=0.93, N=325
var.14.CFH 1:196659237:C:T
peptide level
149:149
140:140
36:36
CVEISCKpc2_NP.5
p−maj=1, pX=0.11, N=325

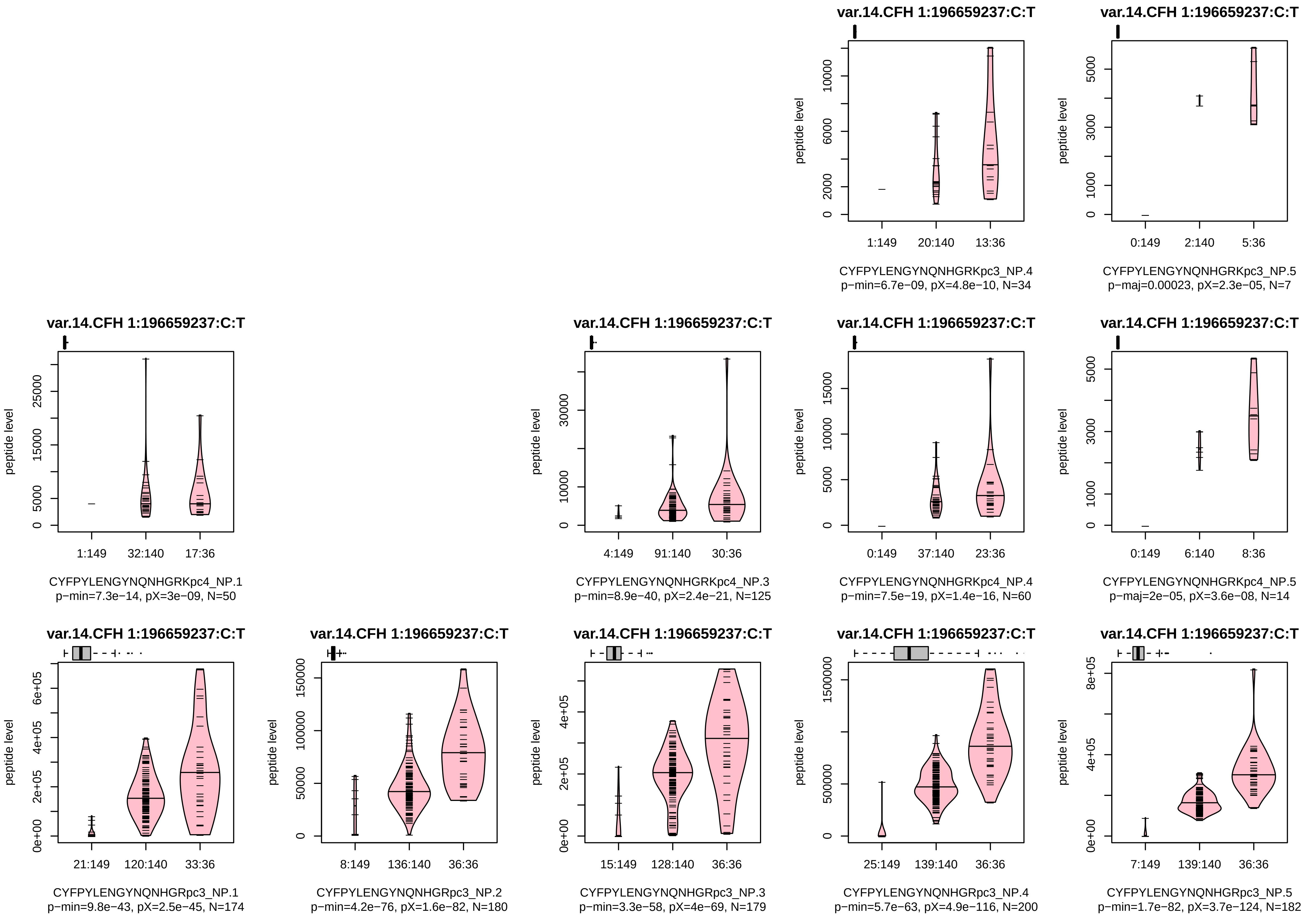
var.14.CFH 1:196659237:C:T
peptide level
1:149
20:140
13:36
CYFPYLENGYNQNHGRKpc3_NP.4
p−min=6.7e−09, pX=4.8e−10, N=34
var.14.CFH 1:196659237:C:T
peptide level
0:149
2:140
5:36
CYFPYLENGYNQNHGRKpc3_NP.5
p−maj=0.00023, pX=2.3e−05, N=7
var.14.CFH 1:196659237:C:T
peptide level
1:149
32:140
17:36
CYFPYLENGYNQNHGRKpc4_NP.1
p−min=7.3e−14, pX=3e−09, N=50
var.14.CFH 1:196659237:C:T
peptide level
4:149
91:140
30:36
CYFPYLENGYNQNHGRKpc4_NP.3
p−min=8.9e−40, pX=2.4e−21, N=125
var.14.CFH 1:196659237:C:T
peptide level
0:149
37:140
23:36
CYFPYLENGYNQNHGRKpc4_NP.4
p−min=7.5e−19, pX=1.4e−16, N=60
var.14.CFH 1:196659237:C:T
peptide level
0:149
6:140
8:36
CYFPYLENGYNQNHGRKpc4_NP.5
p−maj=2e−05, pX=3.6e−08, N=14
var.14.CFH 1:196659237:C:T
peptide level
21:149
120:140
33:36
CYFPYLENGYNQNHGRpc3_NP.1
p−min=9.8e−43, pX=2.5e−45, N=174
var.14.CFH 1:196659237:C:T
peptide level
8:149
136:140
36:36
CYFPYLENGYNQNHGRpc3_NP.2
p−min=4.2e−76, pX=1.6e−82, N=180
var.14.CFH 1:196659237:C:T
peptide level
15:149
128:140
36:36
CYFPYLENGYNQNHGRpc3_NP.3
p−min=3.3e−58, pX=4e−69, N=179
var.14.CFH 1:196659237:C:T
peptide level
25:149
139:140
36:36
CYFPYLENGYNQNHGRpc3_NP.4
p−min=5.7e−63, pX=4.9e−116, N=200
var.14.CFH 1:196659237:C:T
peptide level
7:149
139:140
36:36
CYFPYLENGYNQNHGRpc3_NP.5
p−min=1.7e−82, pX=3.7e−124, N=182

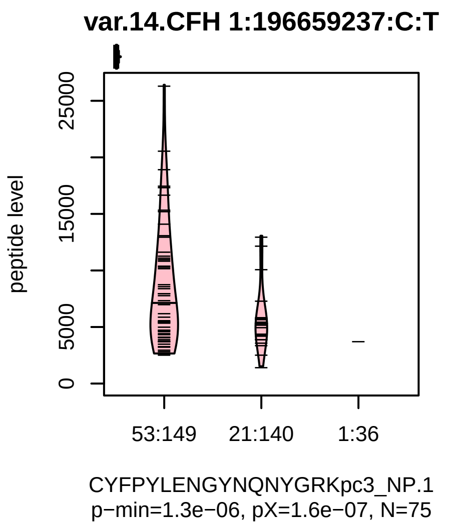
var.14.CFH 1:196659237:C:T
peptide level
0
5000
15000
25000
53:149
21:140
1:36
CYFPYLENGYNQNYGRKpc3_NP.1
p−min=1.3e−06, pX=1.6e−07, N=75

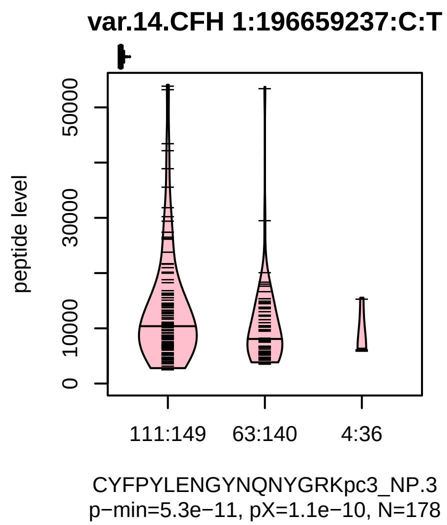
var.14.CFH 1:196659237:C:T
peptide level
0
10000
30000
50000
111:149
63:140
4:36
CYFPYLENGYNQNYGRKpc3_NP.3
p−min=5.3e−11, pX=1.1e−10, N=178

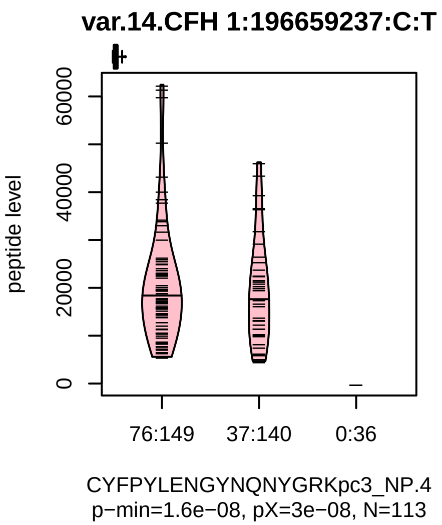
var.14.CFH 1:196659237:C:T
peptide level
0
20000
40000
60000
76:149
37:140
0:36
CYFPYLENGYNQNYGRKpc3_NP.4
p−min=1.6e−08, pX=3e−08, N=113

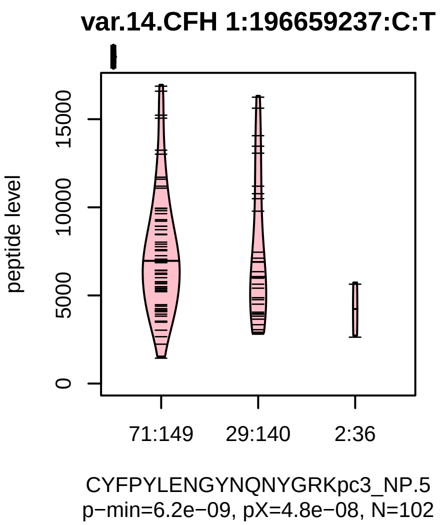
var.14.CFH 1:196659237:C:T
peptide level
0
5000
10000
15000
71:149
29:140
2:36
CYFPYLENGYNQNYGRKpc3_NP.5
p−min=6.2e−09, pX=4.8e−08, N=102

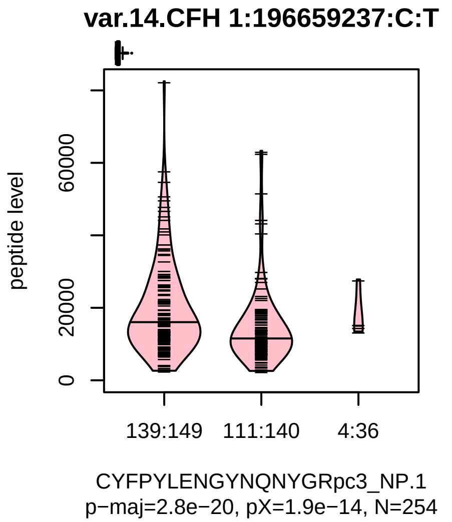
var.14.CFH 1:196659237:C:T
peptide level
0
20000
60000
139:149
111:140
4:36
CYFPYLENGYNQNYGRpc3_NP.1
p−maj=2.8e−20, pX=1.9e−14, N=254

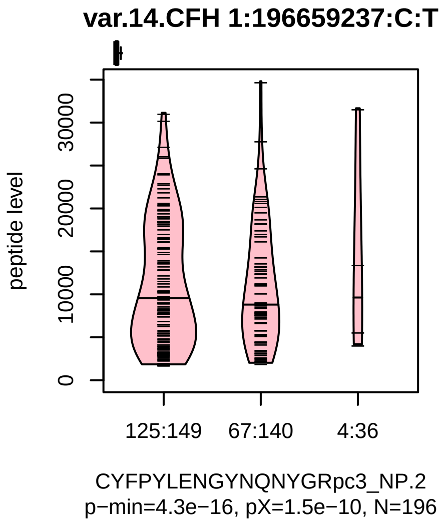
var.14.CFH 1:196659237:C:T
peptide level
0
10000
20000
30000
125:149
67:140
4:36
CYFPYLENGYNQNYGRpc3_NP.2
p−min=4.3e−16, pX=1.5e−10, N=196

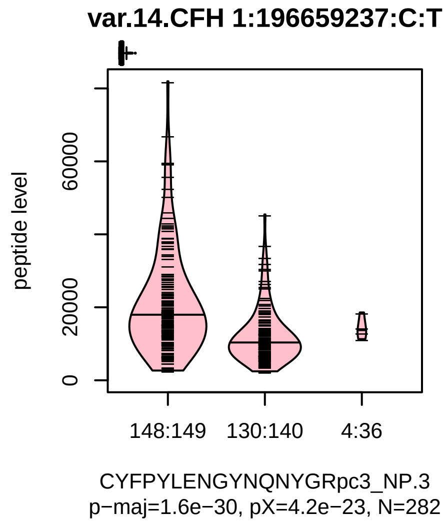
var.14.CFH 1:196659237:C:T
peptide level
0
20000
60000
148:149
130:140
4:36
CYFPYLENGYNQNYGRpc3_NP.3
p−maj=1.6e−30, pX=4.2e−23, N=282

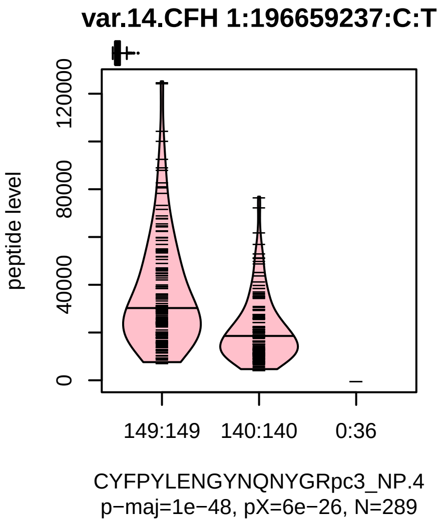
var.14.CFH 1:196659237:C:T
peptide level
0
40000
80000
120000
149:149
140:140
0:36
CYFPYLENGYNQNYGRpc3_NP.4
p−maj=1e−48, pX=6e−26, N=289

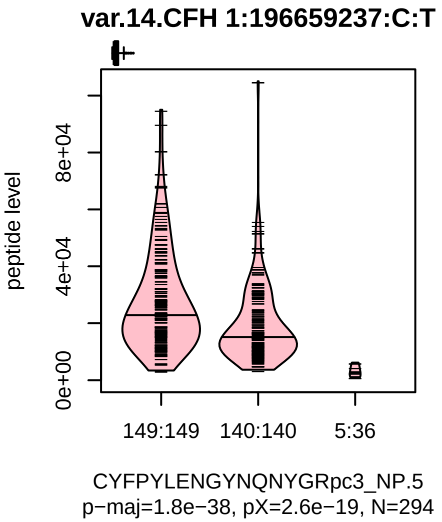
var.14.CFH 1:196659237:C:T
peptide level
0e+00
4e+04
8e+04
149:149
140:140
5:36
CYFPYLENGYNQNYGRpc3_NP.5
p−maj=1.8e−38, pX=2.6e−19, N=294

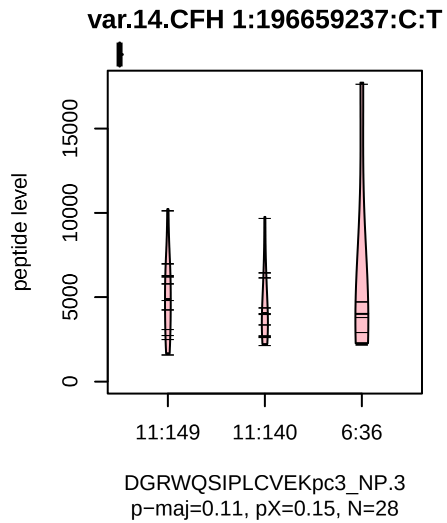
var.14.CFH 1:196659237:C:T
peptide level
0
5000
10000
15000
11:149
11:140
6:36
DGRWQSIPLCVEKpc3_NP.3
p−maj=0.11, pX=0.15, N=28

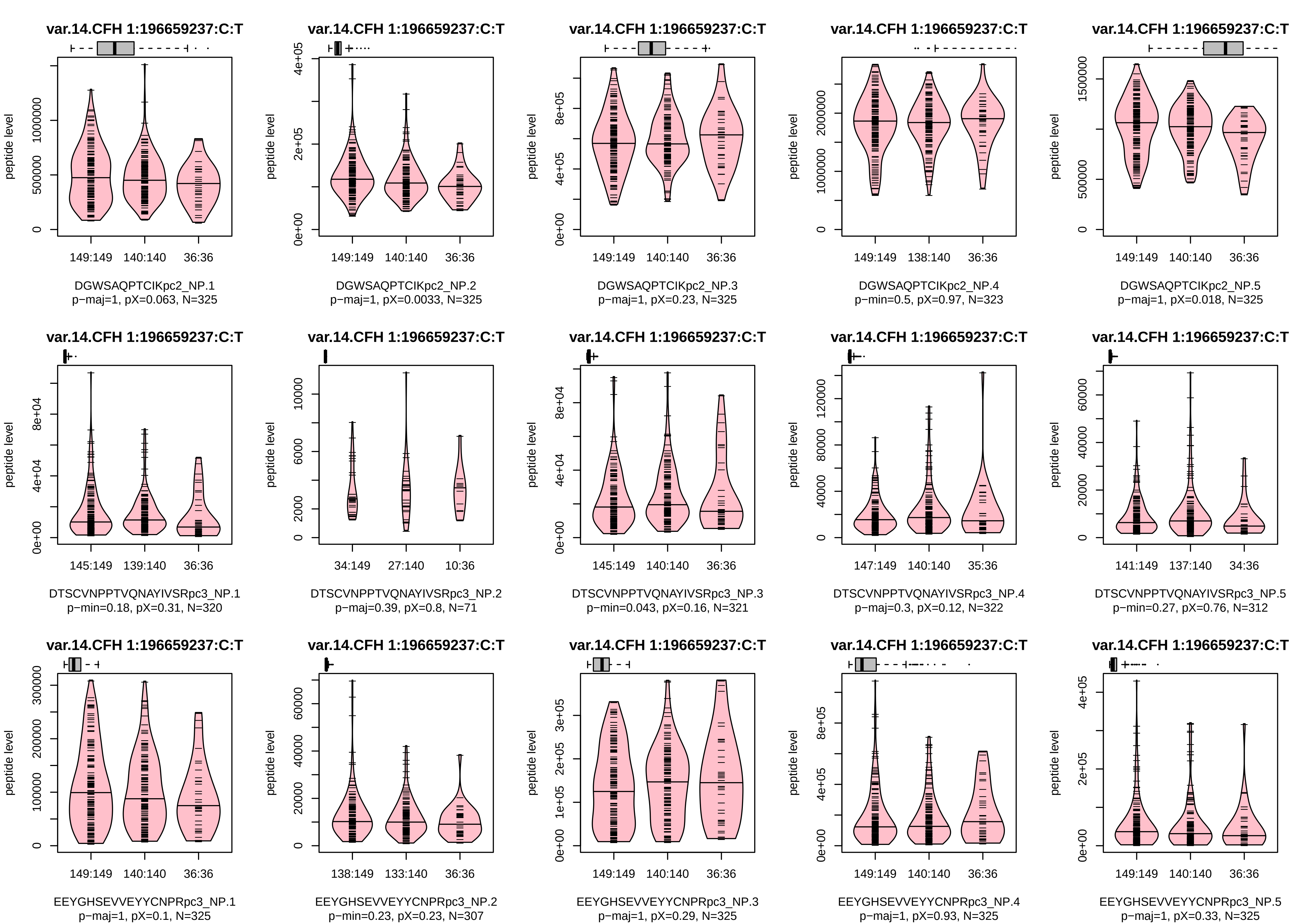
var.14.CFH 1:196659237:C:T
peptide level
149:149
140:140
36:36
DGWSAQPTCIKpc2_NP.1
p−maj=1, pX=0.063, N=325
var.14.CFH 1:196659237:C:T
peptide level
149:149
140:140
36:36
DGWSAQPTCIKpc2_NP.2
p−maj=1, pX=0.0033, N=325
var.14.CFH 1:196659237:C:T
peptide level
149:149
140:140
36:36
DGWSAQPTCIKpc2_NP.3
p−maj=1, pX=0.23, N=325
var.14.CFH 1:196659237:C:T
peptide level
149:149
138:140
36:36
DGWSAQPTCIKpc2_NP.4
p−min=0.5, pX=0.97, N=323
var.14.CFH 1:196659237:C:T
peptide level
149:149
140:140
36:36
DGWSAQPTCIKpc2_NP.5
p−maj=1, pX=0.018, N=325
var.14.CFH 1:196659237:C:T
peptide level
145:149
139:140
36:36
DTSCVNPPTVQNAYIVSRpc3_NP.1
p−min=0.18, pX=0.31, N=320
var.14.CFH 1:196659237:C:T
peptide level
34:149
27:140
10:36
DTSCVNPPTVQNAYIVSRpc3_NP.2
p−maj=0.39, pX=0.8, N=71
var.14.CFH 1:196659237:C:T
peptide level
145:149
140:140
36:36
DTSCVNPPTVQNAYIVSRpc3_NP.3
p−min=0.043, pX=0.16, N=321
var.14.CFH 1:196659237:C:T
peptide level
147:149
140:140
35:36
DTSCVNPPTVQNAYIVSRpc3_NP.4
p−maj=0.3, pX=0.12, N=322
var.14.CFH 1:196659237:C:T
peptide level
141:149
137:140
34:36
DTSCVNPPTVQNAYIVSRpc3_NP.5
p−min=0.27, pX=0.76, N=312
var.14.CFH 1:196659237:C:T
peptide level
149:149
140:140
36:36
EEYGHSEVVEYYCNPRpc3_NP.1
p−maj=1, pX=0.1, N=325
var.14.CFH 1:196659237:C:T
peptide level
138:149
133:140
36:36
EEYGHSEVVEYYCNPRpc3_NP.2
p−min=0.23, pX=0.23, N=307
var.14.CFH 1:196659237:C:T
peptide level
149:149
140:140
36:36
EEYGHSEVVEYYCNPRpc3_NP.3
p−maj=1, pX=0.29, N=325
var.14.CFH 1:196659237:C:T
peptide level
149:149
140:140
36:36
EEYGHSEVVEYYCNPRpc3_NP.4
p−maj=1, pX=0.93, N=325
var.14.CFH 1:196659237:C:T
peptide level
149:149
140:140
36:36
EEYGHSEVVEYYCNPRpc3_NP.5
p−maj=1, pX=0.33, N=325

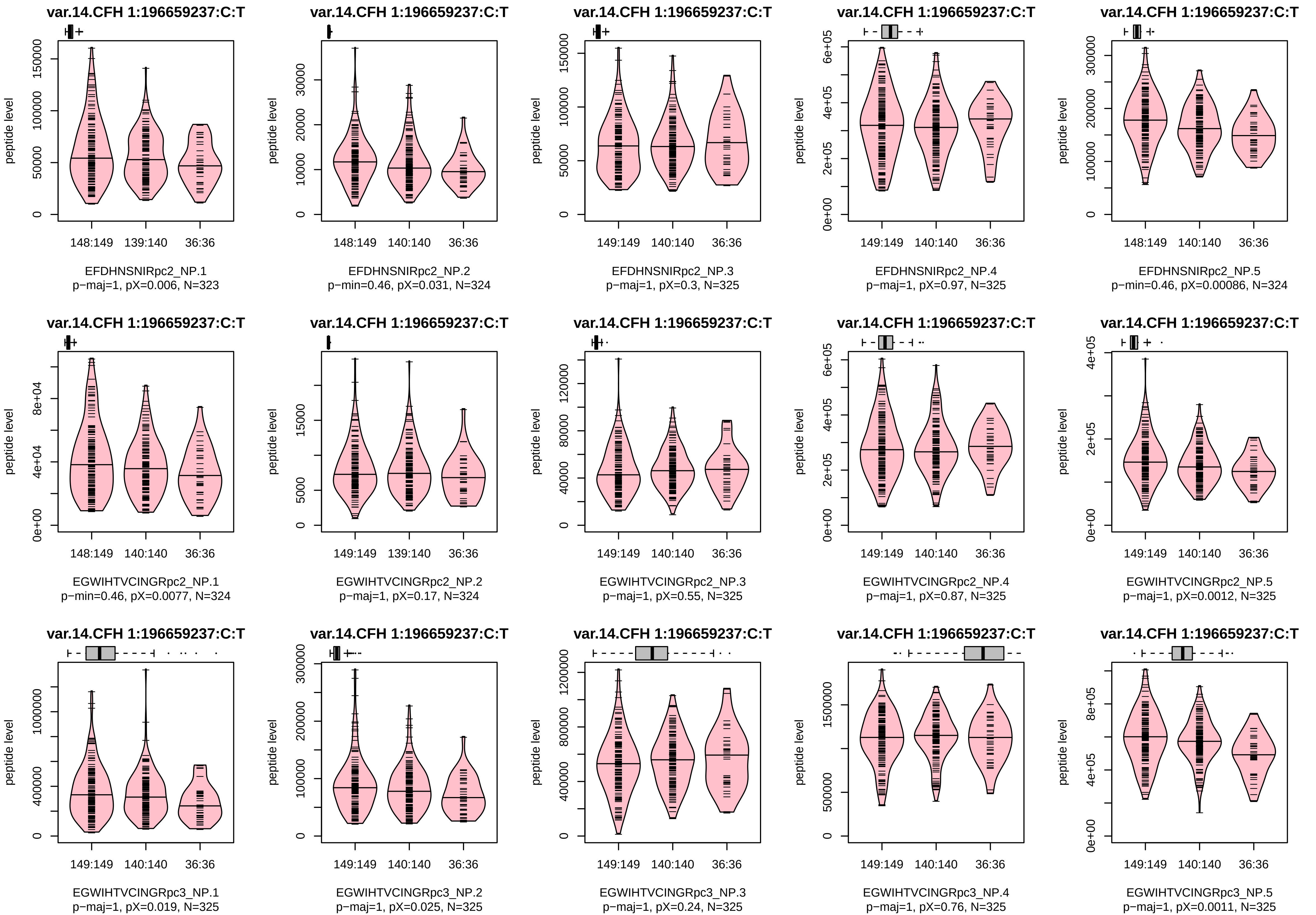
var.14.CFH 1:196659237:C:T
peptide level
148:149 139:140 36:36
EFDHNSNIRpc2_NP.1
p−maj=1, pX=0.006, N=323
var.14.CFH 1:196659237:C:T
peptide level
148:149 140:140 36:36
EFDHNSNIRpc2_NP.2
p−min=0.46, pX=0.031, N=324
var.14.CFH 1:196659237:C:T
peptide level
149:149 140:140 36:36
EFDHNSNIRpc2_NP.3
p−maj=1, pX=0.3, N=325
var.14.CFH 1:196659237:C:T
peptide level
149:149 140:140 36:36
EFDHNSNIRpc2_NP.4
p−maj=1, pX=0.97, N=325
var.14.CFH 1:196659237:C:T
peptide level
148:149 140:140 36:36
EFDHNSNIRpc2_NP.5
p−min=0.46, pX=0.00086, N=324
var.14.CFH 1:196659237:C:T
peptide level
148:149 140:140 36:36
EGWIHTVCINGRpc2_NP.1
p−min=0.46, pX=0.0077, N=324
var.14.CFH 1:196659237:C:T
peptide level
149:149 139:140 36:36
EGWIHTVCINGRpc2_NP.2
p−maj=1, pX=0.17, N=324
var.14.CFH 1:196659237:C:T
peptide level
149:149 140:140 36:36
EGWIHTVCINGRpc2_NP.3
p−maj=1, pX=0.55, N=325
var.14.CFH 1:196659237:C:T
peptide level
149:149 140:140 36:36
EGWIHTVCINGRpc2_NP.4
p−maj=1, pX=0.87, N=325
var.14.CFH 1:196659237:C:T
peptide level
149:149 140:140 36:36
EGWIHTVCINGRpc2_NP.5
p−maj=1, pX=0.0012, N=325
var.14.CFH 1:196659237:C:T
peptide level
149:149 140:140 36:36
EGWIHTVCINGRpc3_NP.1
p−maj=1, pX=0.019, N=325
var.14.CFH 1:196659237:C:T
peptide level
149:149 140:140 36:36
EGWIHTVCINGRpc3_NP.2
p−maj=1, pX=0.025, N=325
var.14.CFH 1:196659237:C:T
peptide level
149:149 140:140 36:36
EGWIHTVCINGRpc3_NP.3
p−maj=1, pX=0.24, N=325
var.14.CFH 1:196659237:C:T
peptide level
149:149 140:140 36:36
EGWIHTVCINGRpc3_NP.4
p−maj=1, pX=0.76, N=325
var.14.CFH 1:196659237:C:T
peptide level
149:149 140:140 36:36
EGWIHTVCINGRpc3_NP.5
p−maj=1, pX=0.0011, N=325

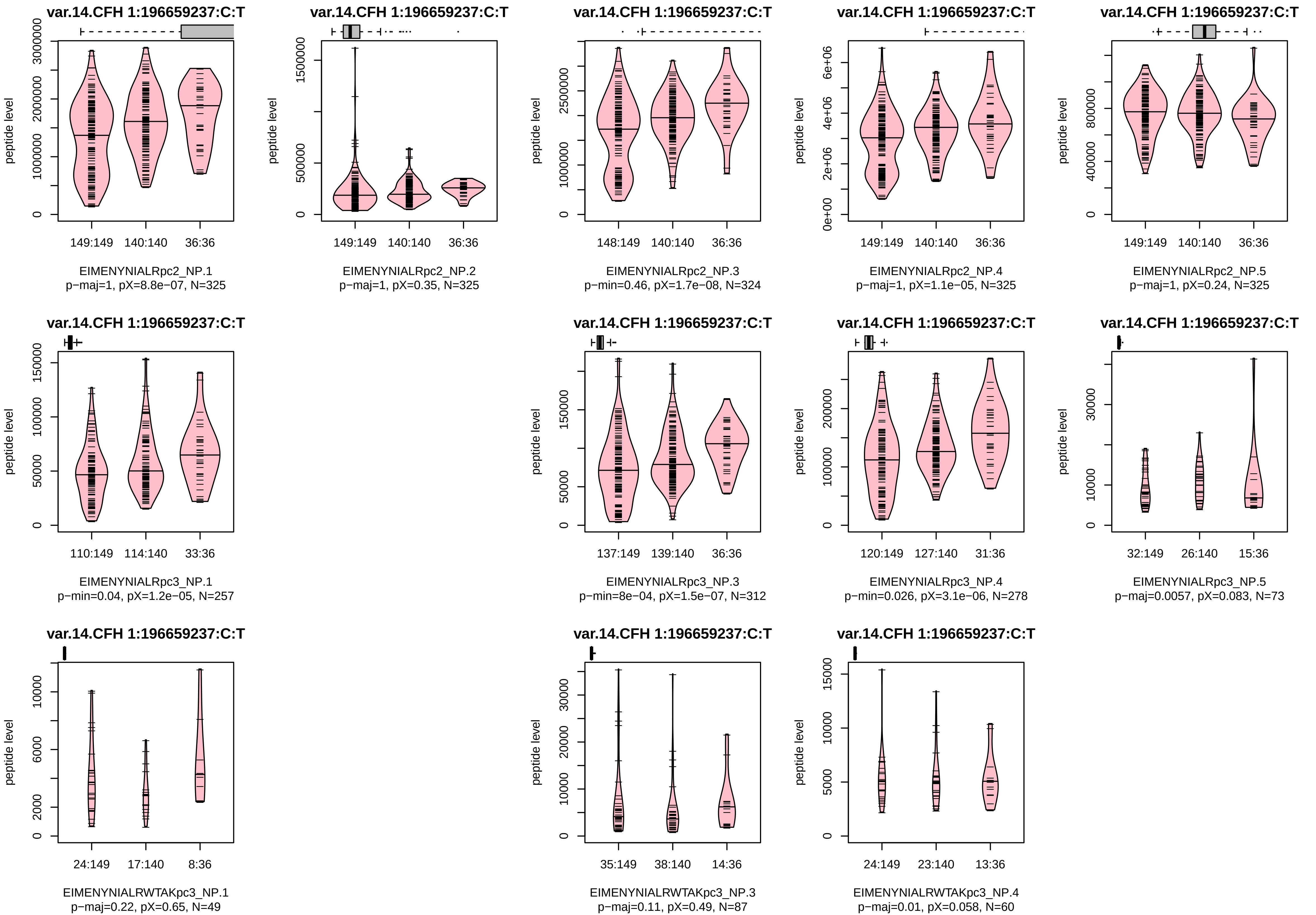

var.14.CFH 1:196659237:C:T
peptide level
149:149
140:140
36:36
EIMENYNIALRpc2_NP.1
p−maj=1, pX=8.8e−07, N=325
var.14.CFH 1:196659237:C:T
peptide level
149:149
140:140
36:36
EIMENYNIALRpc2_NP.2
p−maj=1, pX=0.35, N=325
var.14.CFH 1:196659237:C:T
peptide level
148:149
140:140
36:36
EIMENYNIALRpc2_NP.3
p−min=0.46, pX=1.7e−08, N=324
var.14.CFH 1:196659237:C:T
peptide level
149:149
140:140
36:36
EIMENYNIALRpc2_NP.4
p−maj=1, pX=1.1e−05, N=325
var.14.CFH 1:196659237:C:T
peptide level
149:149
140:140
36:36
EIMENYNIALRpc2_NP.5
p−maj=1, pX=0.24, N=325
var.14.CFH 1:196659237:C:T
peptide level
110:149
114:140
33:36
EIMENYNIALRpc3_NP.1
p−min=0.04, pX=1.2e−05, N=257
var.14.CFH 1:196659237:C:T
peptide level
137:149
139:140
36:36
EIMENYNIALRpc3_NP.3
p−min=8e−04, pX=1.5e−07, N=312
var.14.CFH 1:196659237:C:T
peptide level
120:149
127:140
31:36
EIMENYNIALRpc3_NP.4
p−min=0.026, pX=3.1e−06, N=278
var.14.CFH 1:196659237:C:T
peptide level
32:149
26:140
15:36
EIMENYNIALRpc3_NP.5
p−maj=0.0057, pX=0.083, N=73
var.14.CFH 1:196659237:C:T
peptide level
24:149
17:140
8:36
EIMENYNIALRWTAKpc3_NP.1
p−maj=0.22, pX=0.65, N=49
var.14.CFH 1:196659237:C:T
peptide level
35:149
38:140
14:36
EIMENYNIALRWTAKpc3_NP.3
p−maj=0.11, pX=0.49, N=87
var.14.CFH 1:196659237:C:T
peptide level
24:149
23:140
13:36
EIMENYNIALRWTAKpc3_NP.4
p−maj=0.01, pX=0.058, N=60

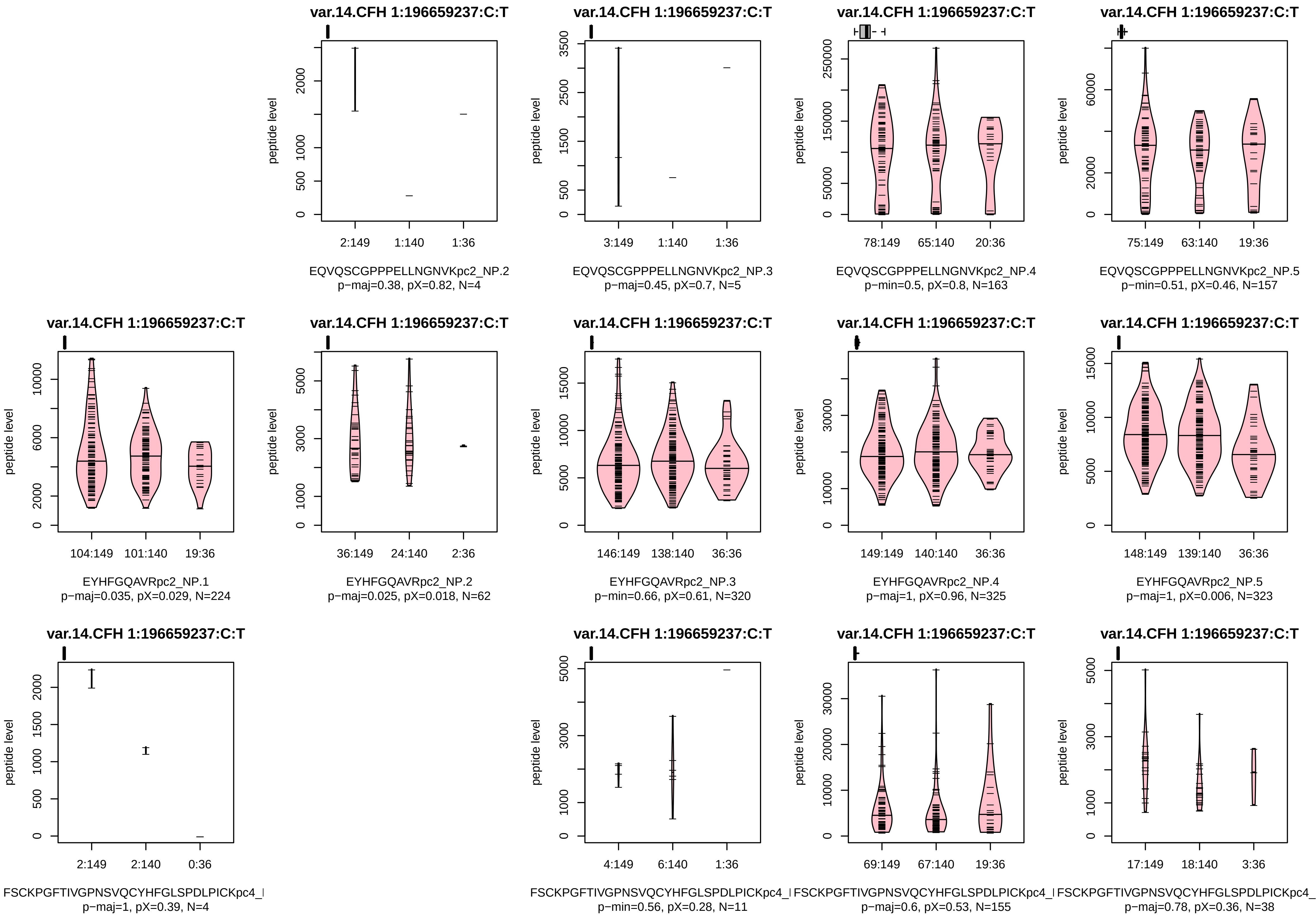
var.14.CFH 1:196659237:C:T
peptide level
2:149
1:140
1:36
EQVQSCGPPPELLNGNVKpc2_NP.2
p−maj=0.38, pX=0.82, N=4
var.14.CFH 1:196659237:C:T
peptide level
3:149
1:140
1:36
EQVQSCGPPPELLNGNVKpc2_NP.3
p−maj=0.45, pX=0.7, N=5
var.14.CFH 1:196659237:C:T
peptide level
78:149
65:140
20:36
EQVQSCGPPPELLNGNVKpc2_NP.4
p−min=0.5, pX=0.8, N=163
var.14.CFH 1:196659237:C:T
peptide level
75:149
63:140
19:36
EQVQSCGPPPELLNGNVKpc2_NP.5
p−min=0.51, pX=0.46, N=157
var.14.CFH 1:196659237:C:T
peptide level
104:149
101:140
19:36
EYHFGQAVRpc2_NP.1
p−maj=0.035, pX=0.029, N=224
var.14.CFH 1:196659237:C:T
peptide level
36:149
24:140
2:36
EYHFGQAVRpc2_NP.2
p−maj=0.025, pX=0.018, N=62
var.14.CFH 1:196659237:C:T
peptide level
146:149
138:140
36:36
EYHFGQAVRpc2_NP.3
p−min=0.66, pX=0.61, N=320
var.14.CFH 1:196659237:C:T
peptide level
149:149
140:140
36:36
EYHFGQAVRpc2_NP.4
p−maj=1, pX=0.96, N=325
var.14.CFH 1:196659237:C:T
peptide level
148:149
139:140
36:36
EYHFGQAVRpc2_NP.5
p−maj=1, pX=0.006, N=323
var.14.CFH 1:196659237:C:T
peptide level
2:149
2:140
0:36
FSCKPGFTIVGPNSVQCYHFGLSPDLPICKpc4_I
p−maj=1, pX=0.39, N=4
var.14.CFH 1:196659237:C:T
peptide level
4:149
6:140
1:36
FSCKPGFTIVGPNSVQCYHFGLSPDLPICKpc4_I
p−min=0.56, pX=0.28, N=11
var.14.CFH 1:196659237:C:T
peptide level
69:149
67:140
19:36
FSCKPGFTIVGPNSVQCYHFGLSPDLPICKpc4_I
p−maj=0.6, pX=0.53, N=155
var.14.CFH 1:196659237:C:T
peptide level
17:149
18:140
3:36
FSCKPGFTIVGPNSVQCYHFGLSPDLPICKpc4_
p−maj=0.78, pX=0.36, N=38

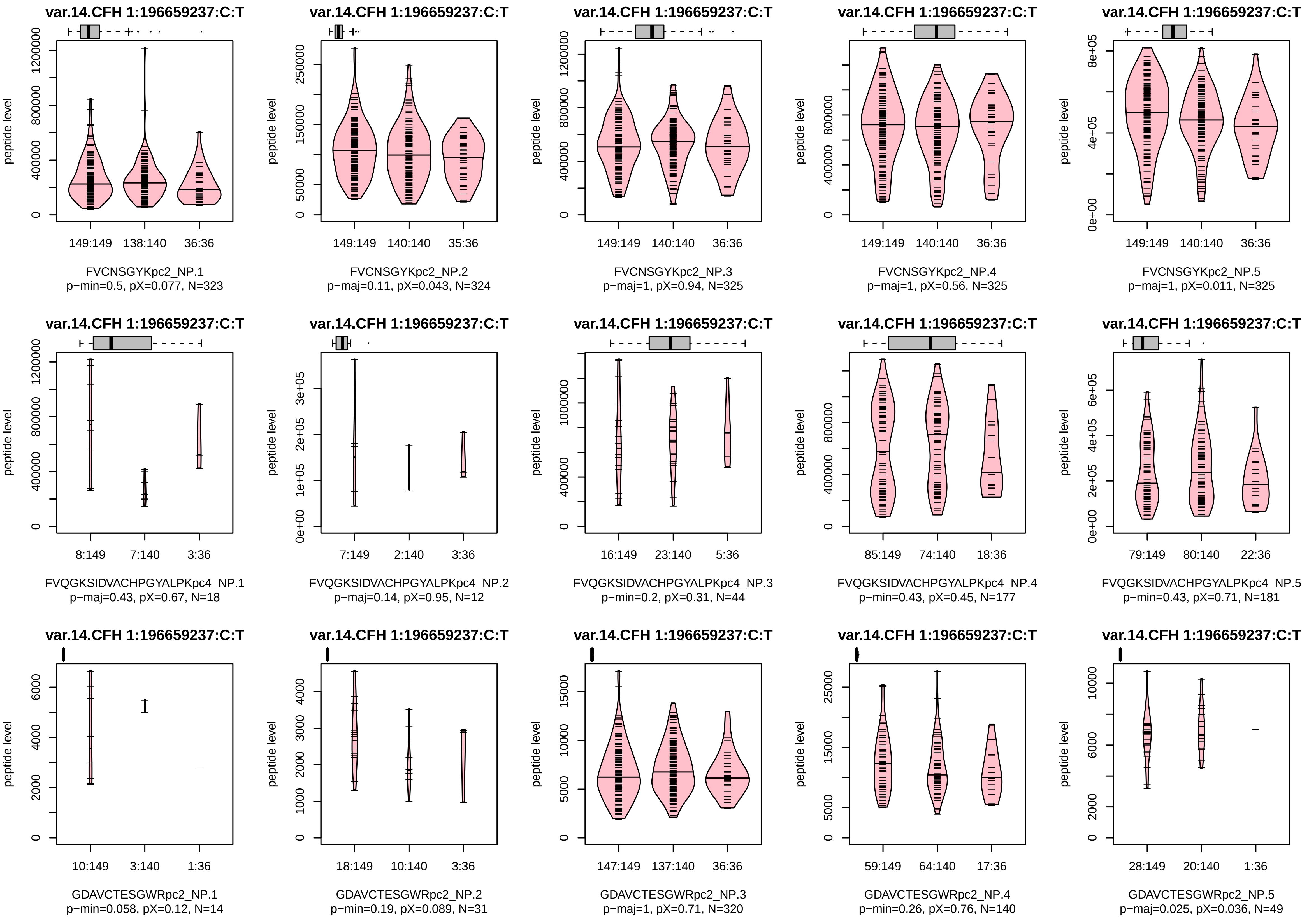
var.14.CFH 1:196659237:C:T
peptide level
149:149
138:140
36:36
FVCNSGYKpc2_NP.1
p−min=0.5, pX=0.077, N=323
var.14.CFH 1:196659237:C:T
peptide level
149:149
140:140
35:36
FVCNSGYKpc2_NP.2
p−maj=0.11, pX=0.043, N=324
var.14.CFH 1:196659237:C:T
peptide level
149:149
140:140
36:36
FVCNSGYKpc2_NP.3
p−maj=1, pX=0.94, N=325
var.14.CFH 1:196659237:C:T
peptide level
149:149
140:140
36:36
FVCNSGYKpc2_NP.4
p−maj=1, pX=0.56, N=325
var.14.CFH 1:196659237:C:T
peptide level
149:149
140:140
36:36
FVCNSGYKpc2_NP.5
p−maj=1, pX=0.011, N=325
var.14.CFH 1:196659237:C:T
peptide level
8:149
7:140
3:36
FVQGKSIDVACHPGYALPKpc4_NP.1
p−maj=0.43, pX=0.67, N=18
var.14.CFH 1:196659237:C:T
peptide level
7:149
2:140
3:36
FVQGKSIDVACHPGYALPKpc4_NP.2
p−maj=0.14, pX=0.95, N=12
var.14.CFH 1:196659237:C:T
peptide level
16:149
23:140
5:36
FVQGKSIDVACHPGYALPKpc4_NP.3
p−min=0.2, pX=0.31, N=44
var.14.CFH 1:196659237:C:T
peptide level
85:149
74:140
18:36
FVQGKSIDVACHPGYALPKpc4_NP.4
p−min=0.43, pX=0.45, N=177
var.14.CFH 1:196659237:C:T
peptide level
79:149
80:140
22:36
FVQGKSIDVACHPGYALPKpc4_NP.5
p−min=0.43, pX=0.71, N=181
var.14.CFH 1:196659237:C:T
peptide level
10:149
3:140
1:36
GDAVCTESGWRpc2_NP.1
p−min=0.058, pX=0.12, N=14
var.14.CFH 1:196659237:C:T
peptide level
18:149
10:140
3:36
GDAVCTESGWRpc2_NP.2
p−min=0.19, pX=0.089, N=31
var.14.CFH 1:196659237:C:T
peptide level
147:149
137:140
36:36
GDAVCTESGWRpc2_NP.3
p−maj=1, pX=0.71, N=320
var.14.CFH 1:196659237:C:T
peptide level
59:149
64:140
17:36
GDAVCTESGWRpc2_NP.4
p−min=0.26, pX=0.76, N=140
var.14.CFH 1:196659237:C:T
peptide level
28:149
20:140
1:36
GDAVCTESGWRpc2_NP.5
p−maj=0.025, pX=0.036, N=49

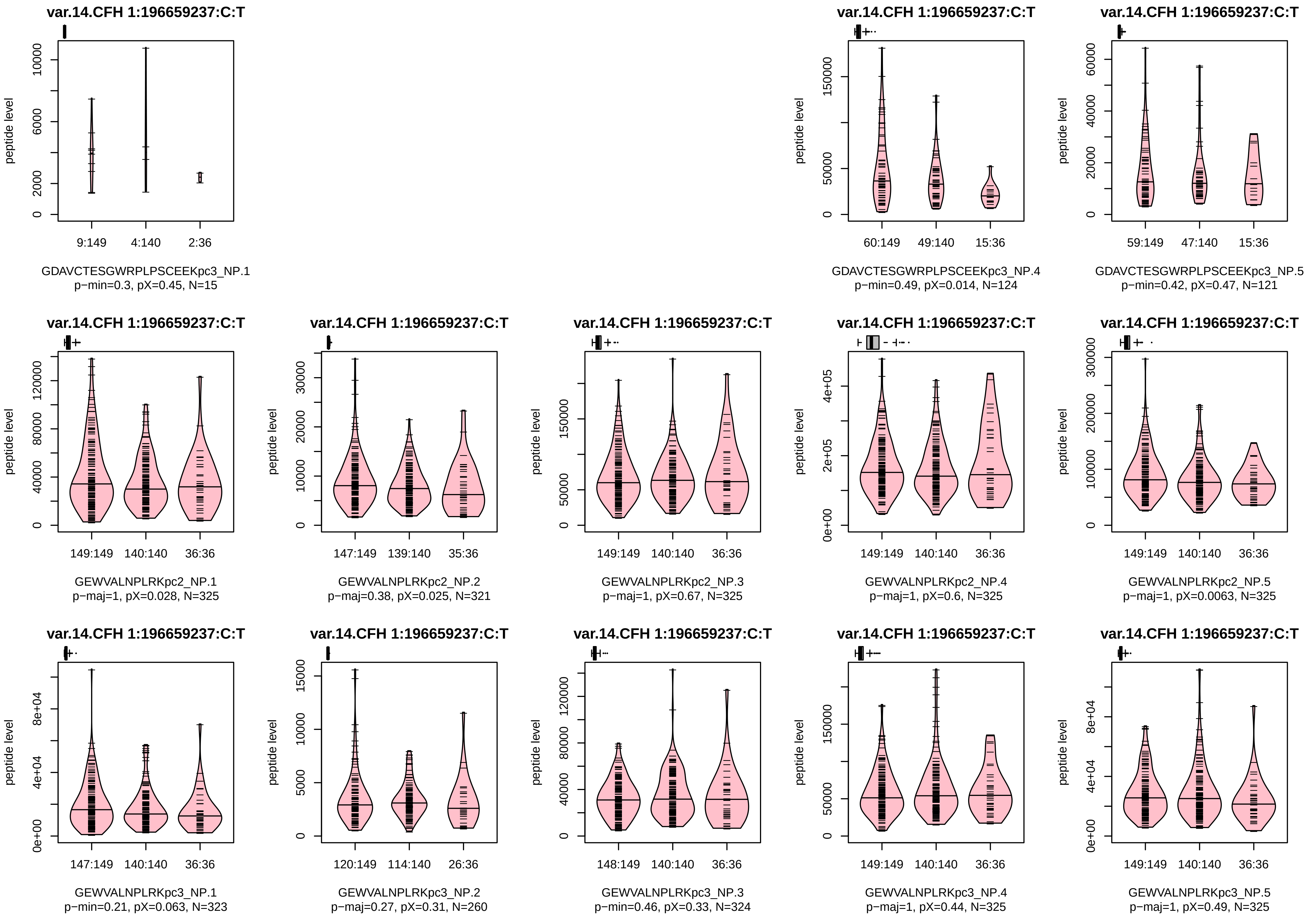
var.14.CFH 1:196659237:C:T
peptide level
9:149
4:140
2:36
GDAVCTESGWRPLPSCEEKpc3_NP.1
p−min=0.3, pX=0.45, N=15
var.14.CFH 1:196659237:C:T
peptide level
60:149
49:140
15:36
GDAVCTESGWRPLPSCEEKpc3_NP.4
p−min=0.49, pX=0.014, N=124
var.14.CFH 1:196659237:C:T
peptide level
59:149
47:140
15:36
GDAVCTESGWRPLPSCEEKpc3_NP.5
p−min=0.42, pX=0.47, N=121
var.14.CFH 1:196659237:C:T
peptide level
149:149
140:140
36:36
GEWVALNPLRKpc2_NP.1
p−maj=1, pX=0.028, N=325
var.14.CFH 1:196659237:C:T
peptide level
147:149
139:140
35:36
GEWVALNPLRKpc2_NP.2
p−maj=0.38, pX=0.025, N=321
var.14.CFH 1:196659237:C:T
peptide level
149:149
140:140
36:36
GEWVALNPLRKpc2_NP.3
p−maj=1, pX=0.67, N=325
var.14.CFH 1:196659237:C:T
peptide level
149:149
140:140
36:36
GEWVALNPLRKpc2_NP.4
p−maj=1, pX=0.6, N=325
var.14.CFH 1:196659237:C:T
peptide level
149:149
140:140
36:36
GEWVALNPLRKpc2_NP.5
p−maj=1, pX=0.0063, N=325
var.14.CFH 1:196659237:C:T
peptide level
147:149
140:140
36:36
GEWVALNPLRKpc3_NP.1
p−min=0.21, pX=0.063, N=323
var.14.CFH 1:196659237:C:T
peptide level
120:149
114:140
26:36
GEWVALNPLRKpc3_NP.2
p−maj=0.27, pX=0.31, N=260
var.14.CFH 1:196659237:C:T
peptide level
148:149
140:140
36:36
GEWVALNPLRKpc3_NP.3
p−min=0.46, pX=0.33, N=324
var.14.CFH 1:196659237:C:T
peptide level
149:149
140:140
36:36
GEWVALNPLRKpc3_NP.4
p−maj=1, pX=0.44, N=325
var.14.CFH 1:196659237:C:T
peptide level
149:149
140:140
36:36
GEWVALNPLRKpc3_NP.5
p−maj=1, pX=0.49, N=325

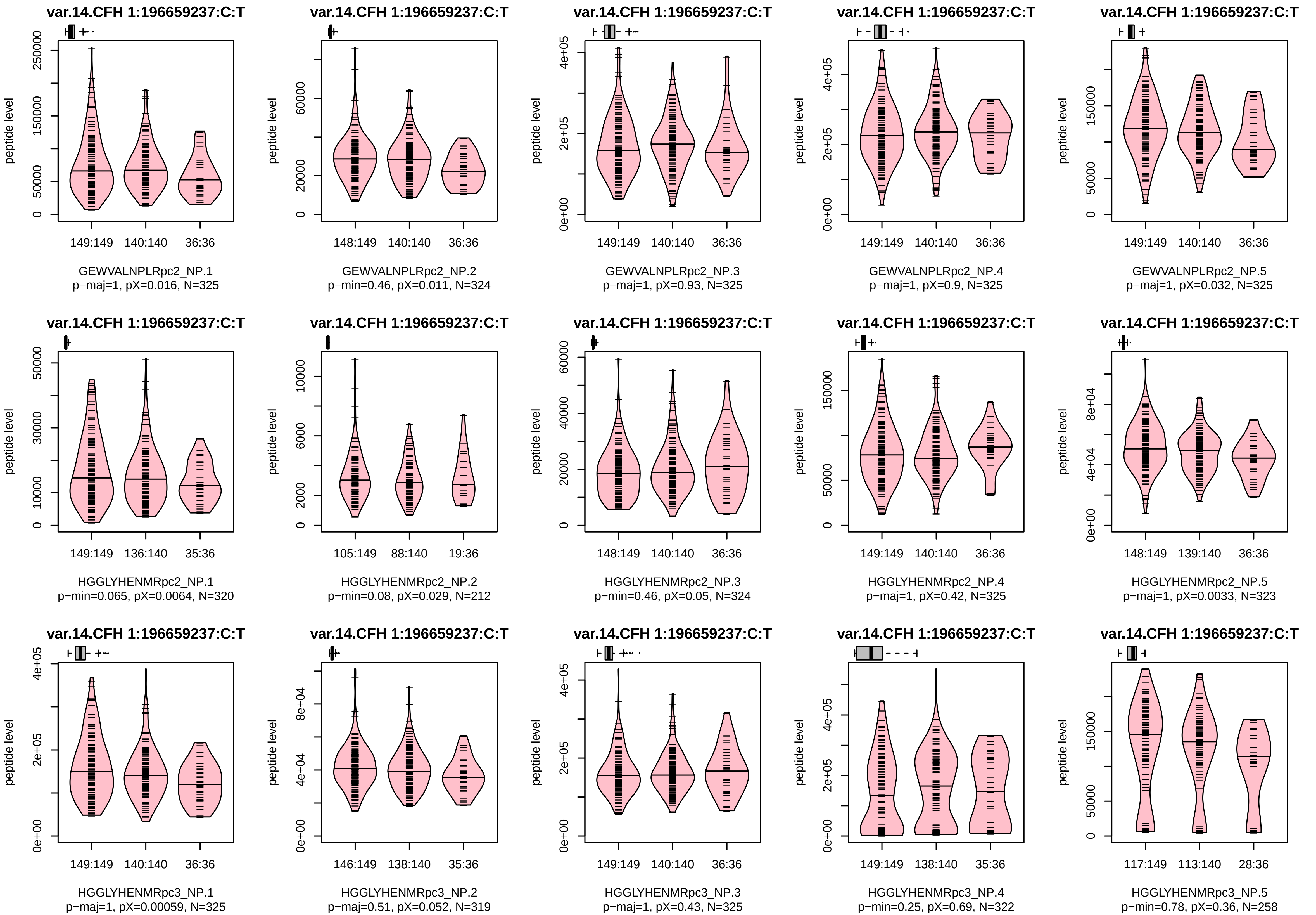
var.14.CFH 1:196659237:C:T
peptide level
149:149 140:140 36:36
GEWVALNPLRpc2_NP.1
p−maj=1, pX=0.016, N=325
var.14.CFH 1:196659237:C:T
peptide level
148:149 140:140 36:36
GEWVALNPLRpc2_NP.2
p−min=0.46, pX=0.011, N=324
var.14.CFH 1:196659237:C:T
peptide level
149:149 140:140 36:36
GEWVALNPLRpc2_NP.3
p−maj=1, pX=0.93, N=325
var.14.CFH 1:196659237:C:T
peptide level
149:149 140:140 36:36
GEWVALNPLRpc2_NP.4
p−maj=1, pX=0.9, N=325
var.14.CFH 1:196659237:C:T
peptide level
149:149 140:140 36:36
GEWVALNPLRpc2_NP.5
p−maj=1, pX=0.032, N=325
var.14.CFH 1:196659237:C:T
peptide level
149:149 136:140 35:36
HGGLYHENMRpc2_NP.1
p−min=0.065, pX=0.0064, N=320
var.14.CFH 1:196659237:C:T
peptide level
105:149 88:140 19:36
HGGLYHENMRpc2_NP.2
p−min=0.08, pX=0.029, N=212
var.14.CFH 1:196659237:C:T
peptide level
148:149 140:140 36:36
HGGLYHENMRpc2_NP.3
p−min=0.46, pX=0.05, N=324
var.14.CFH 1:196659237:C:T
peptide level
149:149 140:140 36:36
HGGLYHENMRpc2_NP.4
p−maj=1, pX=0.42, N=325
var.14.CFH 1:196659237:C:T
peptide level
148:149 139:140 36:36
HGGLYHENMRpc2_NP.5
p−maj=1, pX=0.0033, N=323
var.14.CFH 1:196659237:C:T
peptide level
149:149 140:140 36:36
HGGLYHENMRpc3_NP.1
p−maj=1, pX=0.00059, N=325
var.14.CFH 1:196659237:C:T
peptide level
146:149 138:140 35:36
HGGLYHENMRpc3_NP.2
p−maj=0.51, pX=0.052, N=319
var.14.CFH 1:196659237:C:T
peptide level
149:149 140:140 36:36
HGGLYHENMRpc3_NP.3
p−maj=1, pX=0.43, N=325
var.14.CFH 1:196659237:C:T
peptide level
149:149 138:140 35:36
HGGLYHENMRpc3_NP.4
p−min=0.25, pX=0.69, N=322
var.14.CFH 1:196659237:C:T
peptide level
117:149 113:140 28:36
HGGLYHENMRpc3_NP.5
p−min=0.78, pX=0.36, N=258

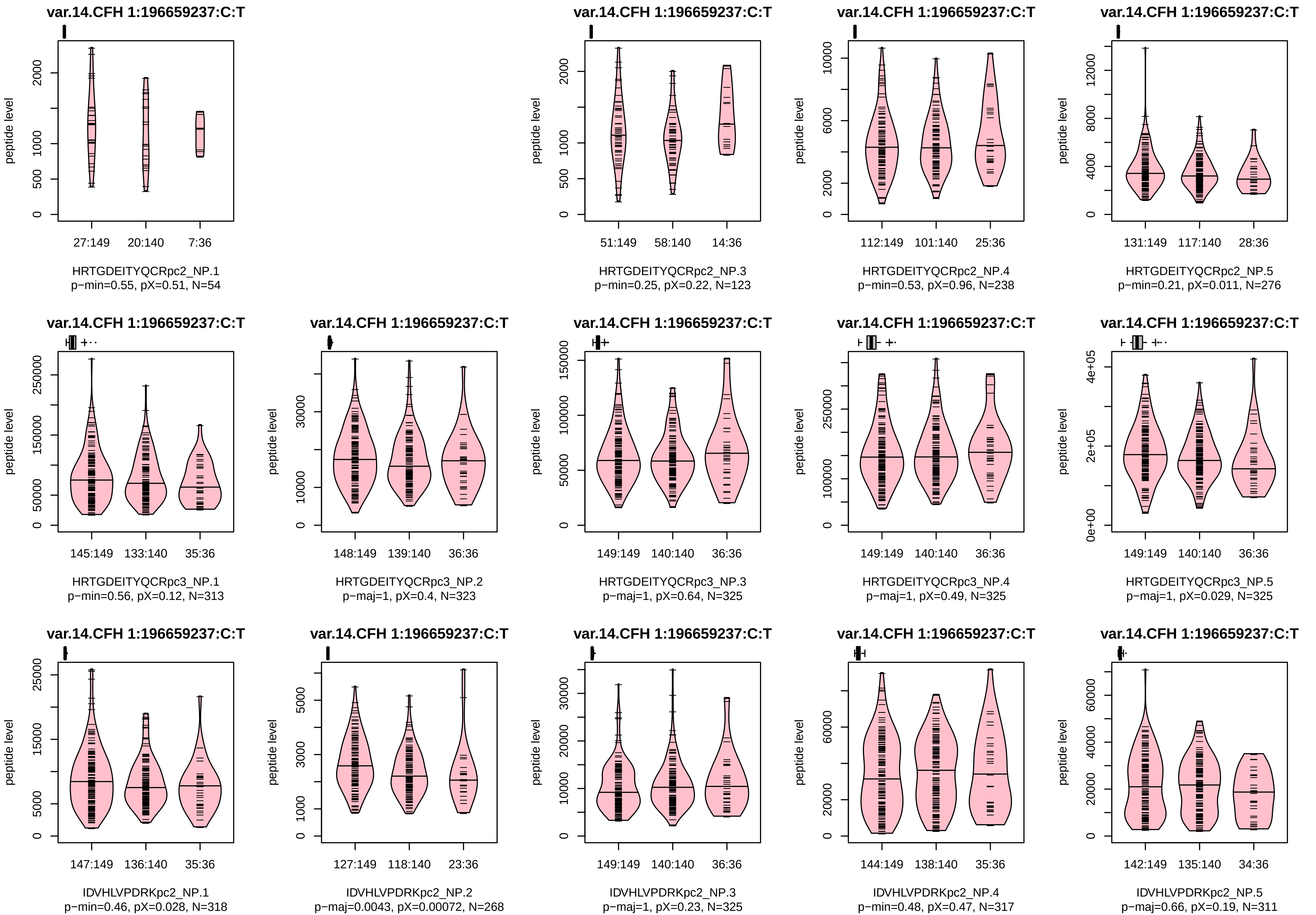
var.14.CFH 1:196659237:C:T
peptide level
27:149 20:140 7:36
HRTGDEITYQCRpc2_NP.1
p−min=0.55, pX=0.51, N=54
var.14.CFH 1:196659237:C:T
peptide level
51:149 58:140 14:36
HRTGDEITYQCRpc2_NP.3
p−min=0.25, pX=0.22, N=123
var.14.CFH 1:196659237:C:T
peptide level
112:149 101:140 25:36
HRTGDEITYQCRpc2_NP.4
p−min=0.53, pX=0.96, N=238
var.14.CFH 1:196659237:C:T
peptide level
131:149 117:140 28:36
HRTGDEITYQCRpc2_NP.5
p−min=0.21, pX=0.011, N=276
var.14.CFH 1:196659237:C:T
peptide level
145:149 133:140 35:36
HRTGDEITYQCRpc3_NP.1
p−min=0.56, pX=0.12, N=313
var.14.CFH 1:196659237:C:T
peptide level
148:149 139:140 36:36
HRTGDEITYQCRpc3_NP.2
p−maj=1, pX=0.4, N=323
var.14.CFH 1:196659237:C:T
peptide level
149:149 140:140 36:36
HRTGDEITYQCRpc3_NP.3
p−maj=1, pX=0.64, N=325
var.14.CFH 1:196659237:C:T
peptide level
149:149 140:140 36:36
HRTGDEITYQCRpc3_NP.4
p−maj=1, pX=0.49, N=325
var.14.CFH 1:196659237:C:T
peptide level
149:149 140:140 36:36
HRTGDEITYQCRpc3_NP.5
p−maj=1, pX=0.029, N=325
var.14.CFH 1:196659237:C:T
peptide level
147:149 136:140 35:36
IDVHLVPDRKpc2_NP.1
p−min=0.46, pX=0.028, N=318
var.14.CFH 1:196659237:C:T
peptide level
127:149 118:140 23:36
IDVHLVPDRKpc2_NP.2
p−maj=0.0043, pX=0.00072, N=268
var.14.CFH 1:196659237:C:T
peptide level
149:149 140:140 36:36
IDVHLVPDRKpc2_NP.3
p−maj=1, pX=0.23, N=325
var.14.CFH 1:196659237:C:T
peptide level
144:149 138:140 35:36
IDVHLVPDRKpc2_NP.4
p−min=0.48, pX=0.47, N=317
var.14.CFH 1:196659237:C:T
peptide level
142:149 135:140 34:36
IDVHLVPDRKpc2_NP.5
p−maj=0.66, pX=0.19, N=311

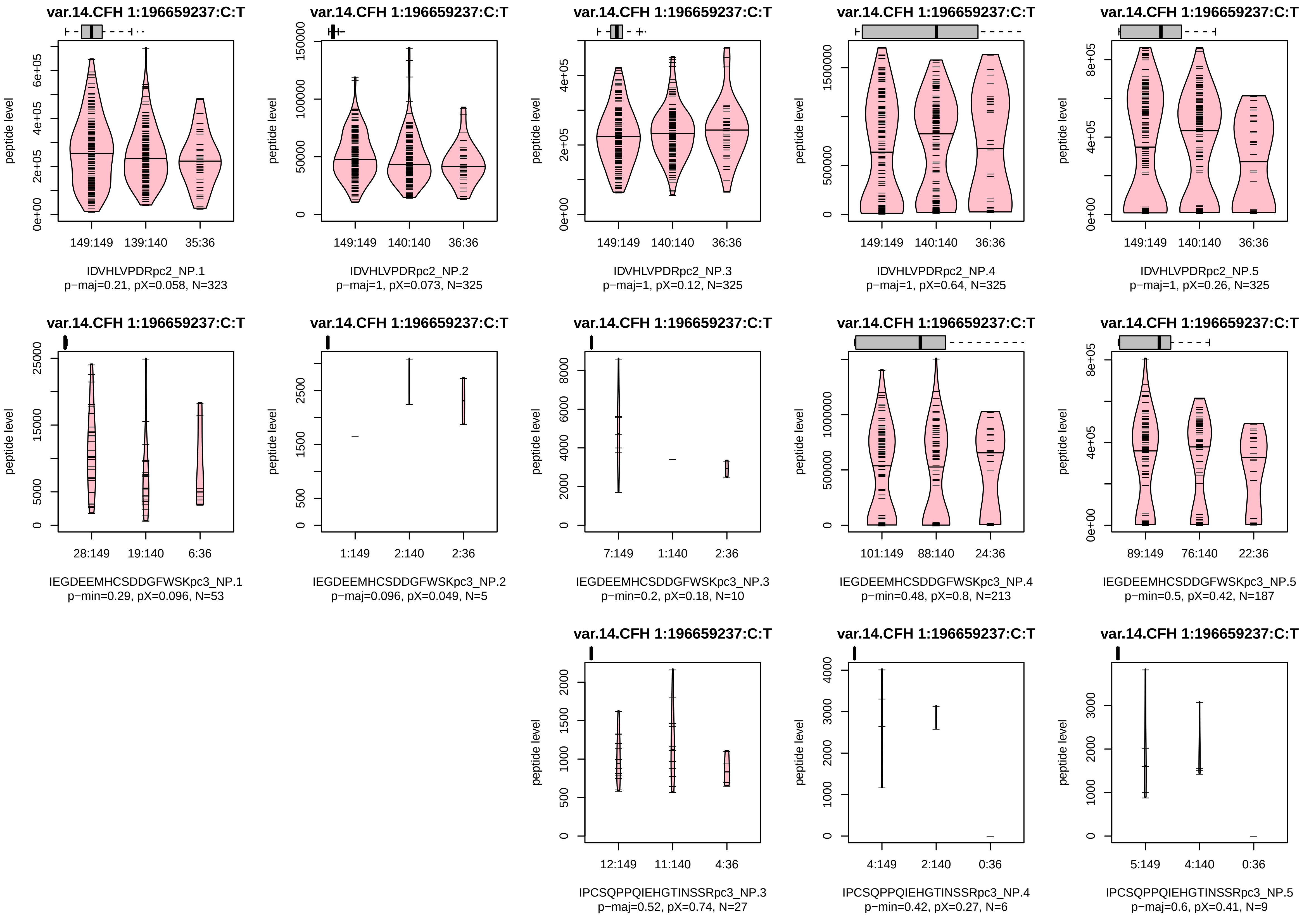
var.14.CFH 1:196659237:C:T
peptide level
149:149
139:140
35:36
IDVHLVPDRpc2_NP.1
p−maj=0.21, pX=0.058, N=323
var.14.CFH 1:196659237:C:T
peptide level
149:149
140:140
36:36
IDVHLVPDRpc2_NP.2
p−maj=1, pX=0.073, N=325
var.14.CFH 1:196659237:C:T
peptide level
149:149
140:140
36:36
IDVHLVPDRpc2_NP.3
p−maj=1, pX=0.12, N=325
var.14.CFH 1:196659237:C:T
peptide level
149:149
140:140
36:36
IDVHLVPDRpc2_NP.4
p−maj=1, pX=0.64, N=325
var.14.CFH 1:196659237:C:T
peptide level
149:149
140:140
36:36
IDVHLVPDRpc2_NP.5
p−maj=1, pX=0.26, N=325
var.14.CFH 1:196659237:C:T
peptide level
28:149
19:140
6:36
IEGDEEMHCSDDGFWSKpc3_NP.1
p−min=0.29, pX=0.096, N=53
var.14.CFH 1:196659237:C:T
peptide level
1:149
2:140
2:36
IEGDEEMHCSDDGFWSKpc3_NP.2
p−maj=0.096, pX=0.049, N=5
var.14.CFH 1:196659237:C:T
peptide level
7:149
1:140
2:36
IEGDEEMHCSDDGFWSKpc3_NP.3
p−min=0.2, pX=0.18, N=10
var.14.CFH 1:196659237:C:T
peptide level
101:149
88:140
24:36
IEGDEEMHCSDDGFWSKpc3_NP.4
p−min=0.48, pX=0.8, N=213
var.14.CFH 1:196659237:C:T
peptide level
89:149
76:140
22:36
IEGDEEMHCSDDGFWSKpc3_NP.5
p−min=0.5, pX=0.42, N=187
var.14.CFH 1:196659237:C:T
peptide level
12:149
11:140
4:36
IPCSQPPQIEHGTINSSRpc3_NP.3
p−maj=0.52, pX=0.74, N=27
var.14.CFH 1:196659237:C:T
peptide level
4:149
2:140
0:36
IPCSQPPQIEHGTINSSRpc3_NP.4
p−min=0.42, pX=0.27, N=6
var.14.CFH 1:196659237:C:T
peptide level
5:149
4:140
0:36
IPCSQPPQIEHGTINSSRpc3_NP.5
p−maj=0.6, pX=0.41, N=9

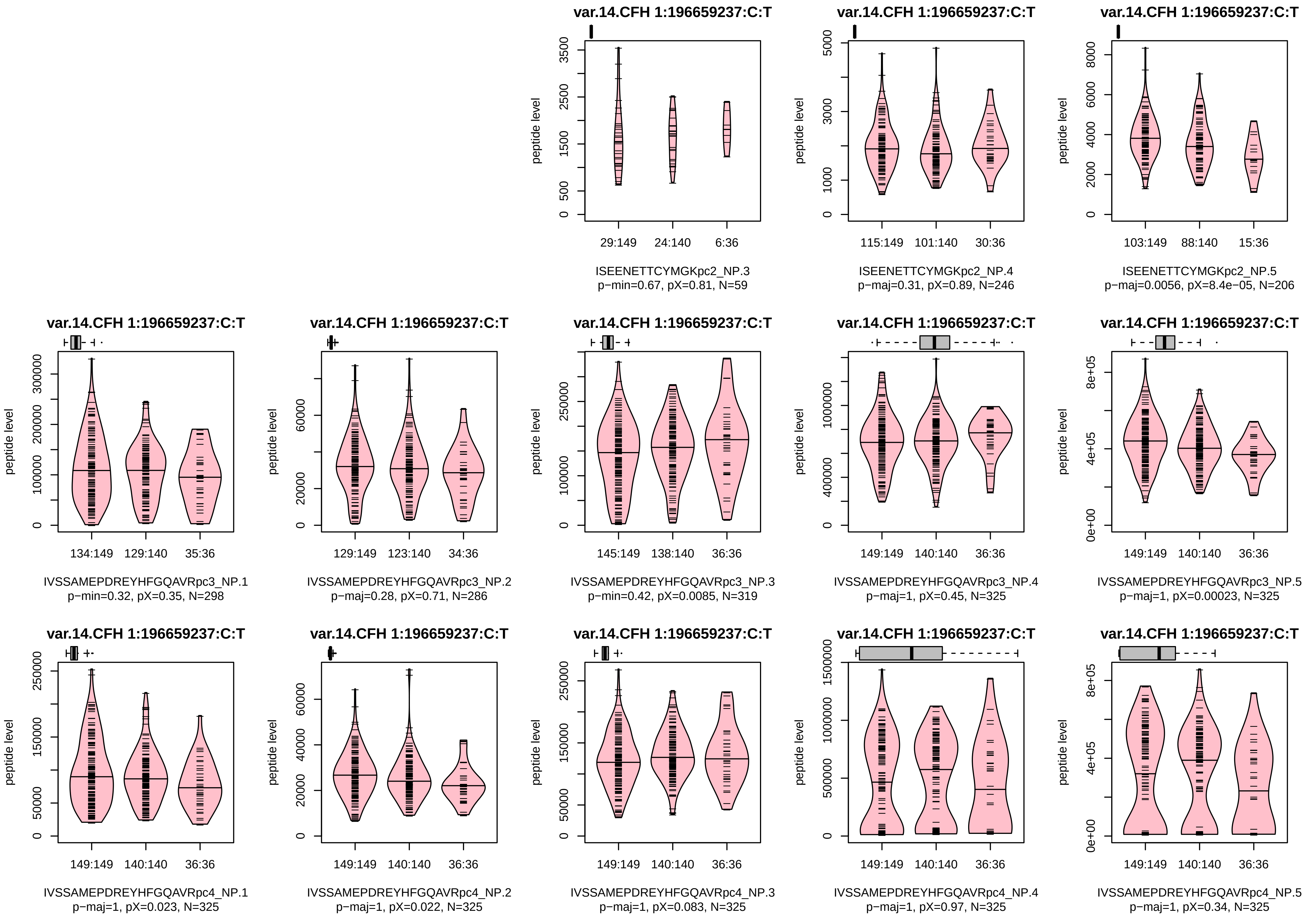
var.14.CFH 1:196659237:C:T
peptide level
29:149
24:140
6:36
ISEENETTCYMGKpc2_NP.3
p−min=0.67, pX=0.81, N=59
var.14.CFH 1:196659237:C:T
peptide level
115:149
101:140
30:36
ISEENETTCYMGKpc2_NP.4
p−maj=0.31, pX=0.89, N=246
var.14.CFH 1:196659237:C:T
peptide level
103:149
88:140
15:36
ISEENETTCYMGKpc2_NP.5
p−maj=0.0056, pX=8.4e−05, N=206
var.14.CFH 1:196659237:C:T
peptide level
134:149
129:140
35:36
IVSSAMEPDREYHFGQAVRpc3_NP.1
p−min=0.32, pX=0.35, N=298
var.14.CFH 1:196659237:C:T
peptide level
129:149
123:140
34:36
IVSSAMEPDREYHFGQAVRpc3_NP.2
p−maj=0.28, pX=0.71, N=286
var.14.CFH 1:196659237:C:T
peptide level
145:149
138:140
36:36
IVSSAMEPDREYHFGQAVRpc3_NP.3
p−min=0.42, pX=0.0085, N=319
var.14.CFH 1:196659237:C:T
peptide level
149:149
140:140
36:36
IVSSAMEPDREYHFGQAVRpc3_NP.4
p−maj=1, pX=0.45, N=325
var.14.CFH 1:196659237:C:T
peptide level
149:149
140:140
36:36
IVSSAMEPDREYHFGQAVRpc3_NP.5
p−maj=1, pX=0.00023, N=325
var.14.CFH 1:196659237:C:T
peptide level
149:149
140:140
36:36
IVSSAMEPDREYHFGQAVRpc4_NP.1
p−maj=1, pX=0.023, N=325
var.14.CFH 1:196659237:C:T
peptide level
149:149
140:140
36:36
IVSSAMEPDREYHFGQAVRpc4_NP.2
p−maj=1, pX=0.022, N=325
var.14.CFH 1:196659237:C:T
peptide level
149:149
140:140
36:36
IVSSAMEPDREYHFGQAVRpc4_NP.3
p−maj=1, pX=0.083, N=325
var.14.CFH 1:196659237:C:T
peptide level
149:149
140:140
36:36
IVSSAMEPDREYHFGQAVRpc4_NP.4
p−maj=1, pX=0.97, N=325
var.14.CFH 1:196659237:C:T
peptide level
149:149
140:140
36:36
IVSSAMEPDREYHFGQAVRpc4_NP.5
p−maj=1, pX=0.34, N=325

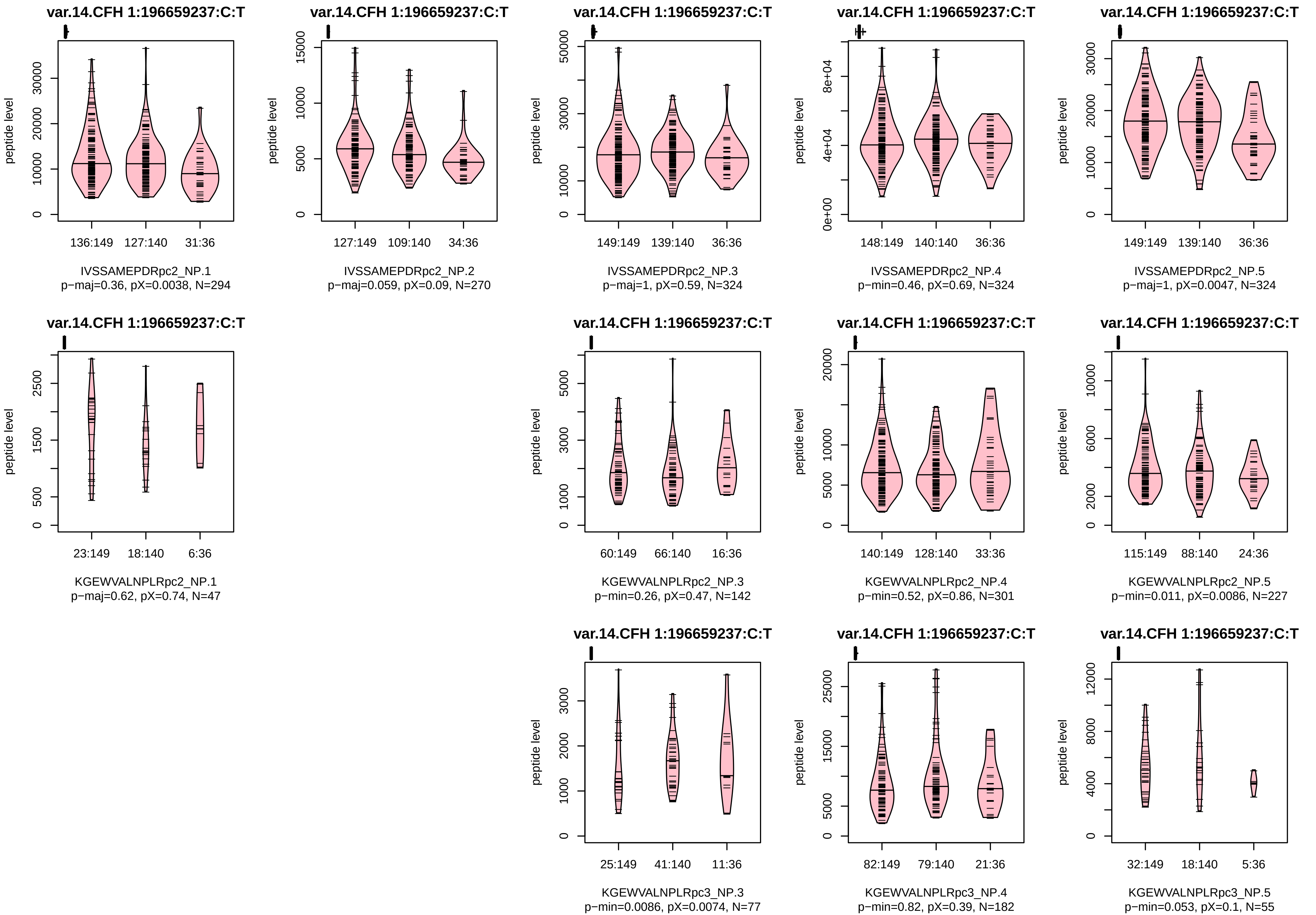

var.14.CFH 1:196659237:C:T
peptide level
136:149
127:140
31:36
IVSSAMEPDRpc2_NP.1
p−maj=0.36, pX=0.0038, N=294
var.14.CFH 1:196659237:C:T
127:149
109:140
34:36
IVSSAMEPDRpc2_NP.2
p−maj=0.059, pX=0.09, N=270
var.14.CFH 1:196659237:C:T
149:149
139:140
36:36
IVSSAMEPDRpc2_NP.3
p−maj=1, pX=0.59, N=324
var.14.CFH 1:196659237:C:T
148:149
140:140
36:36
IVSSAMEPDRpc2_NP.4
p−min=0.46, pX=0.69, N=324
var.14.CFH 1:196659237:C:T
149:149
139:140
36:36
IVSSAMEPDRpc2_NP.5
p−maj=1, pX=0.0047, N=324
var.14.CFH 1:196659237:C:T
23:149
18:140
6:36
KGEWVALNPLRpc2_NP.1
p−maj=0.62, pX=0.74, N=47
var.14.CFH 1:196659237:C:T
60:149
66:140
16:36
KGEWVALNPLRpc2_NP.3
p−min=0.26, pX=0.47, N=142
var.14.CFH 1:196659237:C:T
140:149
128:140
33:36
KGEWVALNPLRpc2_NP.4
p−min=0.52, pX=0.86, N=301
var.14.CFH 1:196659237:C:T
115:149
88:140
24:36
KGEWVALNPLRpc2_NP.5
p−min=0.011, pX=0.0086, N=227
var.14.CFH 1:196659237:C:T
25:149
41:140
11:36
KGEWVALNPLRpc3_NP.3
p−min=0.0086, pX=0.0074, N=77
var.14.CFH 1:196659237:C:T
82:149
79:140
21:36
KGEWVALNPLRpc3_NP.4
p−min=0.82, pX=0.39, N=182
var.14.CFH 1:196659237:C:T
32:149
18:140
5:36
KGEWVALNPLRpc3_NP.5
p−min=0.053, pX=0.1, N=55

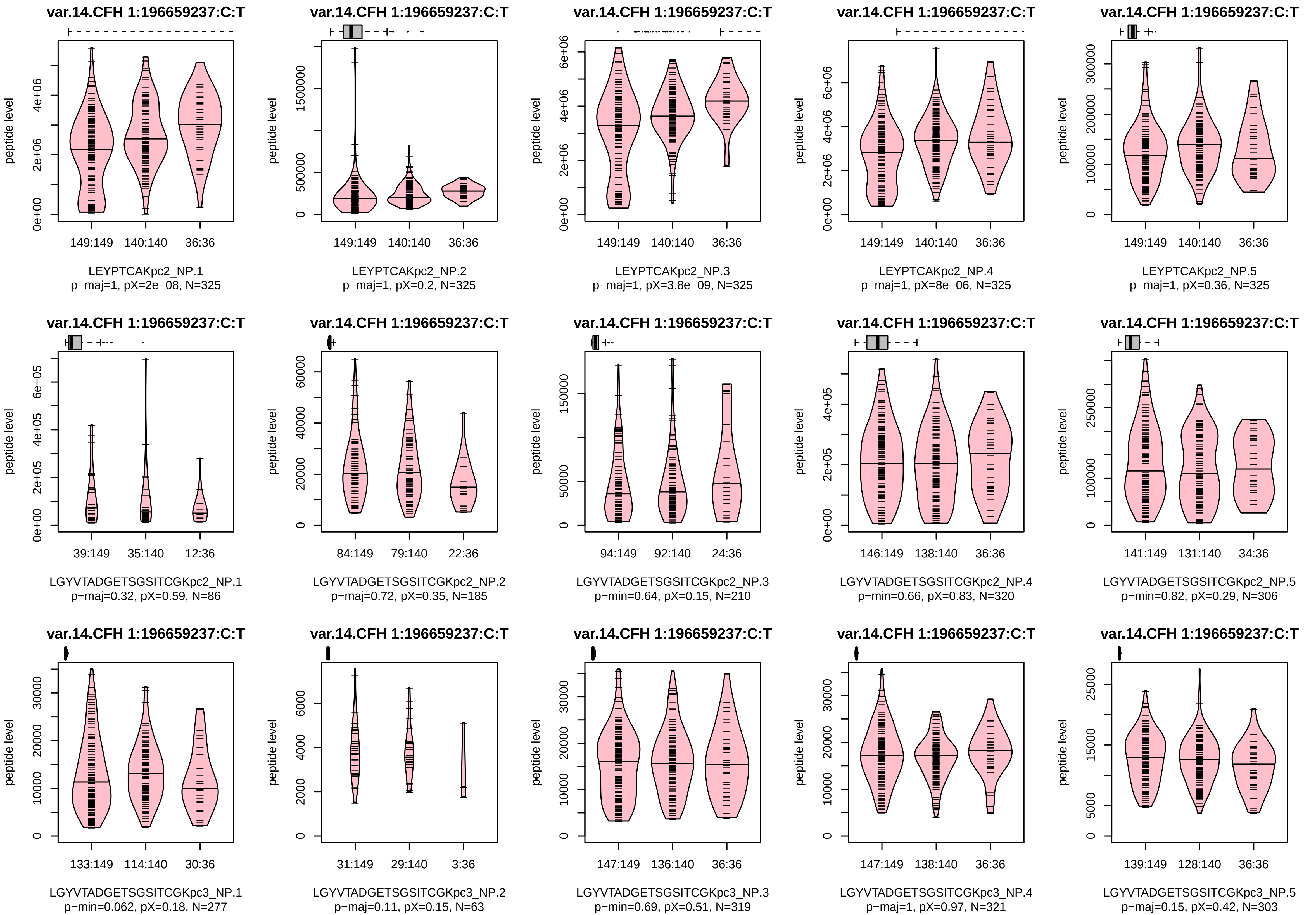
var.14.CFH 1:196659237:C:T
peptide level
149:149 140:140 36:36
LEYPTCAKpc2_NP.1
p−maj=1, pX=2e−08, N=325
var.14.CFH 1:196659237:C:T
peptide level
149:149 140:140 36:36
LEYPTCAKpc2_NP.2
p−maj=1, pX=0.2, N=325
var.14.CFH 1:196659237:C:T
peptide level
149:149 140:140 36:36
LEYPTCAKpc2_NP.3
p−maj=1, pX=3.8e−09, N=325
var.14.CFH 1:196659237:C:T
peptide level
149:149 140:140 36:36
LEYPTCAKpc2_NP.4
p−maj=1, pX=8e−06, N=325
var.14.CFH 1:196659237:C:T
peptide level
149:149 140:140 36:36
LEYPTCAKpc2_NP.5
p−maj=1, pX=0.36, N=325
var.14.CFH 1:196659237:C:T
peptide level
39:149 35:140 12:36
LGYVTADGETSGSITCGKpc2_NP.1
p−maj=0.32, pX=0.59, N=86
var.14.CFH 1:196659237:C:T
peptide level
84:149 79:140 22:36
LGYVTADGETSGSITCGKpc2_NP.2
p−maj=0.72, pX=0.35, N=185
var.14.CFH 1:196659237:C:T
peptide level
94:149 92:140 24:36
LGYVTADGETSGSITCGKpc2_NP.3
p−min=0.64, pX=0.15, N=210
var.14.CFH 1:196659237:C:T
peptide level
146:149 138:140 36:36
LGYVTADGETSGSITCGKpc2_NP.4
p−min=0.66, pX=0.83, N=320
var.14.CFH 1:196659237:C:T
peptide level
141:149 131:140 34:36
LGYVTADGETSGSITCGKpc2_NP.5
p−min=0.82, pX=0.29, N=306
var.14.CFH 1:196659237:C:T
peptide level
133:149 114:140 30:36
LGYVTADGETSGSITCGKpc3_NP.1
p−min=0.062, pX=0.18, N=277
var.14.CFH 1:196659237:C:T
peptide level
31:149 29:140 3:36
LGYVTADGETSGSITCGKpc3_NP.2
p−maj=0.11, pX=0.15, N=63
var.14.CFH 1:196659237:C:T
peptide level
147:149 136:140 36:36
LGYVTADGETSGSITCGKpc3_NP.3
p−min=0.69, pX=0.51, N=319
var.14.CFH 1:196659237:C:T
peptide level
147:149 138:140 36:36
LGYVTADGETSGSITCGKpc3_NP.4
p−maj=1, pX=0.97, N=321
var.14.CFH 1:196659237:C:T
peptide level
139:149 128:140 36:36
LGYVTADGETSGSITCGKpc3_NP.5
p−maj=0.15, pX=0.42, N=303

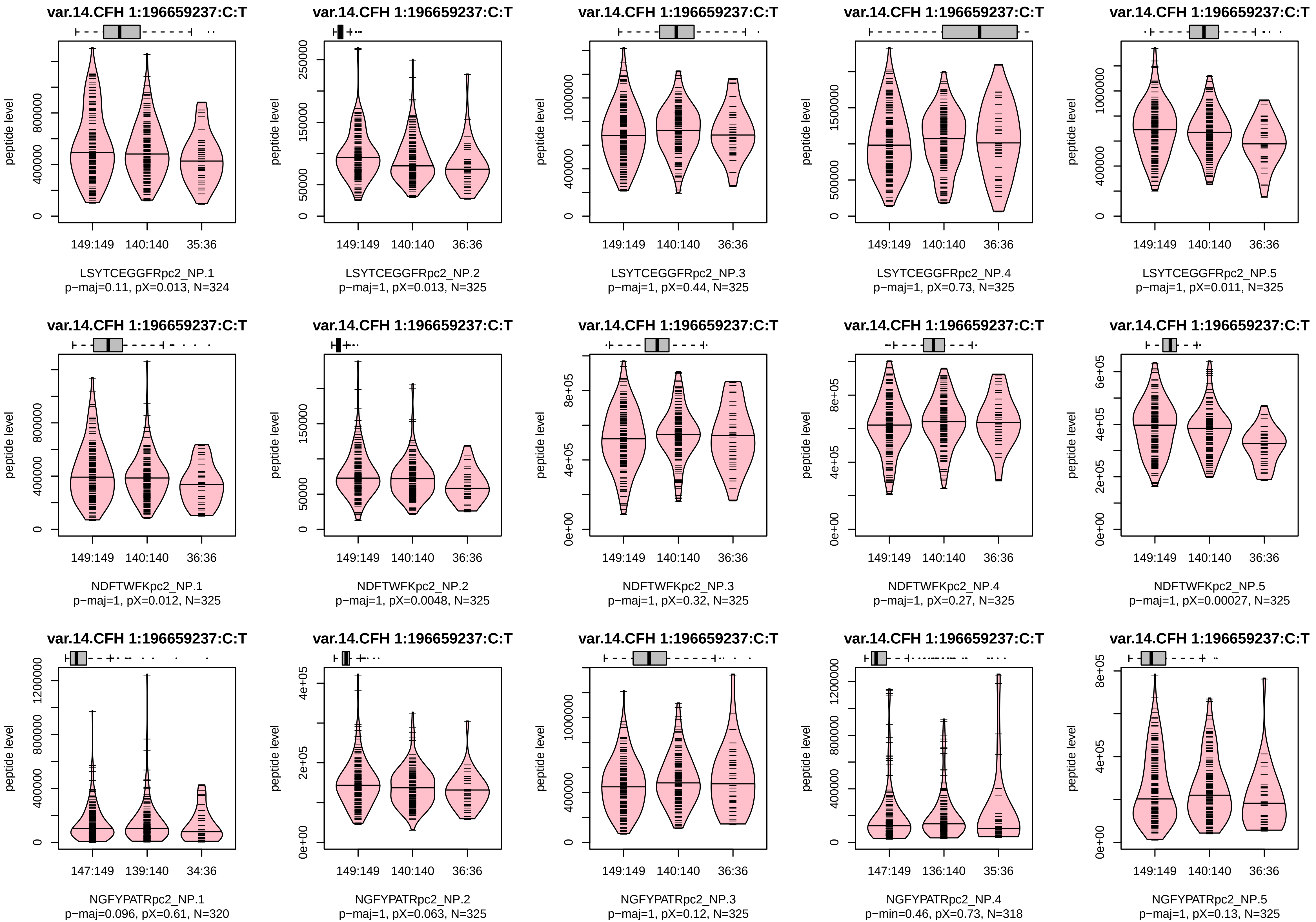
var.14.CFH 1:196659237:C:T
peptide level
149:149
140:140
35:36
LSYTCEGGFRpc2_NP.1
p−maj=0.11, pX=0.013, N=324
var.14.CFH 1:196659237:C:T
peptide level
149:149
140:140
36:36
LSYTCEGGFRpc2_NP.2
p−maj=1, pX=0.013, N=325
var.14.CFH 1:196659237:C:T
peptide level
149:149
140:140
36:36
LSYTCEGGFRpc2_NP.3
p−maj=1, pX=0.44, N=325
var.14.CFH 1:196659237:C:T
peptide level
149:149
140:140
36:36
LSYTCEGGFRpc2_NP.4
p−maj=1, pX=0.73, N=325
var.14.CFH 1:196659237:C:T
peptide level
149:149
140:140
36:36
LSYTCEGGFRpc2_NP.5
p−maj=1, pX=0.011, N=325
var.14.CFH 1:196659237:C:T
peptide level
149:149
140:140
36:36
NDFTWFKpc2_NP.1
p−maj=1, pX=0.012, N=325
var.14.CFH 1:196659237:C:T
peptide level
149:149
140:140
36:36
NDFTWFKpc2_NP.2
p−maj=1, pX=0.0048, N=325
var.14.CFH 1:196659237:C:T
peptide level
149:149
140:140
36:36
NDFTWFKpc2_NP.3
p−maj=1, pX=0.32, N=325
var.14.CFH 1:196659237:C:T
peptide level
149:149
140:140
36:36
NDFTWFKpc2_NP.4
p−maj=1, pX=0.27, N=325
var.14.CFH 1:196659237:C:T
peptide level
149:149
140:140
36:36
NDFTWFKpc2_NP.5
p−maj=1, pX=0.00027, N=325
var.14.CFH 1:196659237:C:T
peptide level
147:149
139:140
34:36
NGFYPATRpc2_NP.1
p−maj=0.096, pX=0.61, N=320
var.14.CFH 1:196659237:C:T
peptide level
149:149
140:140
36:36
NGFYPATRpc2_NP.2
p−maj=1, pX=0.063, N=325
var.14.CFH 1:196659237:C:T
peptide level
149:149
140:140
36:36
NGFYPATRpc2_NP.3
p−maj=1, pX=0.12, N=325
var.14.CFH 1:196659237:C:T
peptide level
147:149
136:140
35:36
NGFYPATRpc2_NP.4
p−min=0.46, pX=0.73, N=318
var.14.CFH 1:196659237:C:T
peptide level
149:149
140:140
36:36
NGFYPATRpc2_NP.5
p−maj=1, pX=0.13, N=325

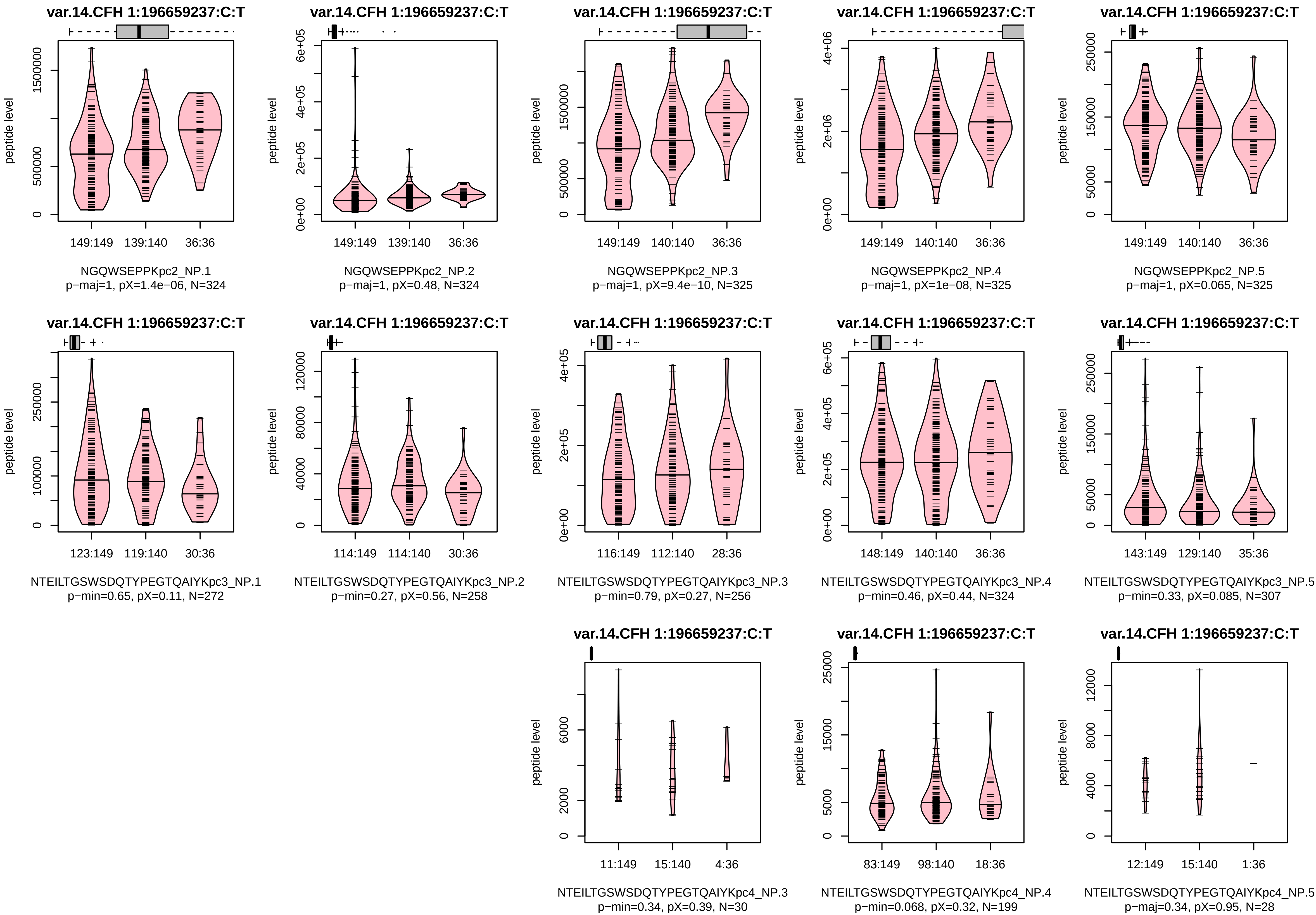
var.14.CFH 1:196659237:C:T
peptide level
149:149
139:140
36:36
NGQWSEPPKpc2_NP.1
p−maj=1, pX=1.4e−06, N=324
var.14.CFH 1:196659237:C:T
149:149
139:140
36:36
NGQWSEPPKpc2_NP.2
p−maj=1, pX=0.48, N=324
var.14.CFH 1:196659237:C:T
149:149
140:140
36:36
NGQWSEPPKpc2_NP.3
p−maj=1, pX=9.4e−10, N=325
var.14.CFH 1:196659237:C:T
149:149
140:140
36:36
NGQWSEPPKpc2_NP.4
p−maj=1, pX=1e−08, N=325
var.14.CFH 1:196659237:C:T
149:149
140:140
36:36
NGQWSEPPKpc2_NP.5
p−maj=1, pX=0.065, N=325
var.14.CFH 1:196659237:C:T
123:149
119:140
30:36
NTEILTGSWSDQTYPEGTQAIYKpc3_NP.1
p−min=0.65, pX=0.11, N=272
var.14.CFH 1:196659237:C:T
114:149
114:140
30:36
NTEILTGSWSDQTYPEGTQAIYKpc3_NP.2
p−min=0.27, pX=0.56, N=258
var.14.CFH 1:196659237:C:T
116:149
112:140
28:36
NTEILTGSWSDQTYPEGTQAIYKpc3_NP.3
p−min=0.79, pX=0.27, N=256
var.14.CFH 1:196659237:C:T
148:149
140:140
36:36
NTEILTGSWSDQTYPEGTQAIYKpc3_NP.4
p−min=0.46, pX=0.44, N=324
var.14.CFH 1:196659237:C:T
143:149
129:140
35:36
NTEILTGSWSDQTYPEGTQAIYKpc3_NP.5
p−min=0.33, pX=0.085, N=307
var.14.CFH 1:196659237:C:T
11:149
15:140
4:36
NTEILTGSWSDQTYPEGTQAIYKpc4_NP.3
p−min=0.34, pX=0.39, N=30
var.14.CFH 1:196659237:C:T
83:149
98:140
18:36
NTEILTGSWSDQTYPEGTQAIYKpc4_NP.4
p−min=0.068, pX=0.32, N=199
var.14.CFH 1:196659237:C:T
12:149
15:140
1:36
NTEILTGSWSDQTYPEGTQAIYKpc4_NP.5
p−maj=0.34, pX=0.95, N=28

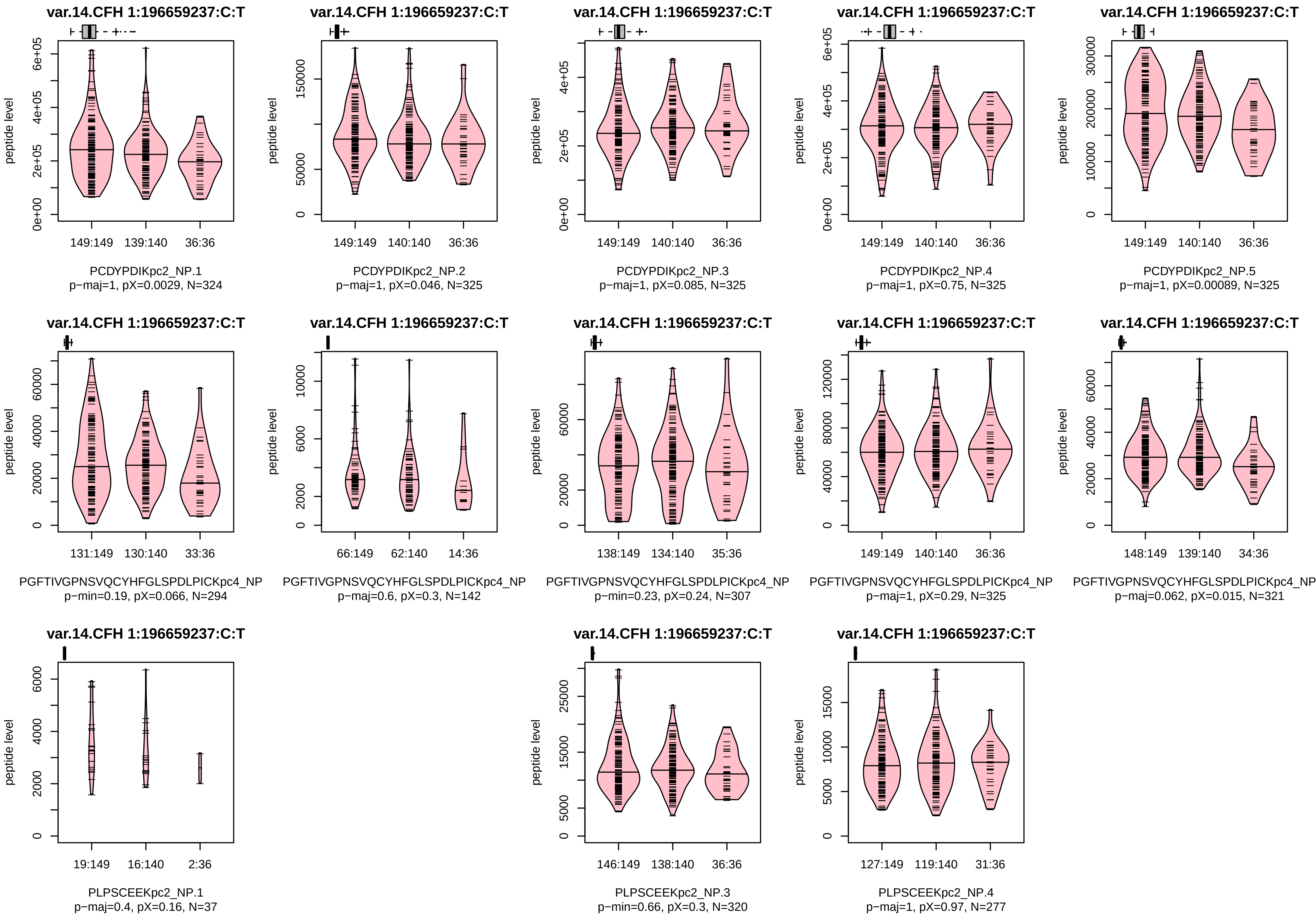
var.14.CFH 1:196659237:C:T
peptide level
0e+00
2e+05
4e+05
6e+05
149:149
139:140
36:36
PCDYPDIKpc2_NP.1
p−maj=1, pX=0.0029, N=324
var.14.CFH 1:196659237:C:T
peptide level
0
50000
150000
149:149
140:140
36:36
PCDYPDIKpc2_NP.2
p−maj=1, pX=0.046, N=325
var.14.CFH 1:196659237:C:T
peptide level
0e+00
2e+05
4e+05
149:149
140:140
36:36
PCDYPDIKpc2_NP.3
p−maj=1, pX=0.085, N=325
var.14.CFH 1:196659237:C:T
peptide level
0e+00
2e+05
4e+05
6e+05
149:149
140:140
36:36
PCDYPDIKpc2_NP.4
p−maj=1, pX=0.75, N=325
var.14.CFH 1:196659237:C:T
peptide level
0
100000
200000
300000
149:149
140:140
36:36
PCDYPDIKpc2_NP.5
p−maj=1, pX=0.00089, N=325
var.14.CFH 1:196659237:C:T
peptide level
0
20000
40000
60000
131:149
130:140
33:36
PGFTIVGPNSVQCYHFGLSPDLPICKpc4_NP
p−min=0.19, pX=0.066, N=294
var.14.CFH 1:196659237:C:T
peptide level
0
2000
6000
10000
66:149
62:140
14:36
PGFTIVGPNSVQCYHFGLSPDLPICKpc4_NP
p−maj=0.6, pX=0.3, N=142
var.14.CFH 1:196659237:C:T
peptide level
0
20000
60000
138:149
134:140
35:36
PGFTIVGPNSVQCYHFGLSPDLPICKpc4_NP
p−min=0.23, pX=0.24, N=307
var.14.CFH 1:196659237:C:T
peptide level
0
40000
80000
120000
149:149
140:140
36:36
PGFTIVGPNSVQCYHFGLSPDLPICKpc4_NP
p−maj=1, pX=0.29, N=325
var.14.CFH 1:196659237:C:T
peptide level
0
20000
40000
60000
148:149
139:140
34:36
PGFTIVGPNSVQCYHFGLSPDLPICKpc4_NP
p−maj=0.062, pX=0.015, N=321
var.14.CFH 1:196659237:C:T
peptide level
0
2000
4000
6000
19:149
16:140
2:36
PLPSCEEKpc2_NP.1
p−maj=0.4, pX=0.16, N=37
var.14.CFH 1:196659237:C:T
peptide level
0
5000
15000
25000
146:149
138:140
36:36
PLPSCEEKpc2_NP.3
p−min=0.66, pX=0.3, N=320
var.14.CFH 1:196659237:C:T
peptide level
0
5000
10000
15000
127:149
119:140
31:36
PLPSCEEKpc2_NP.4
p−maj=1, pX=0.97, N=277

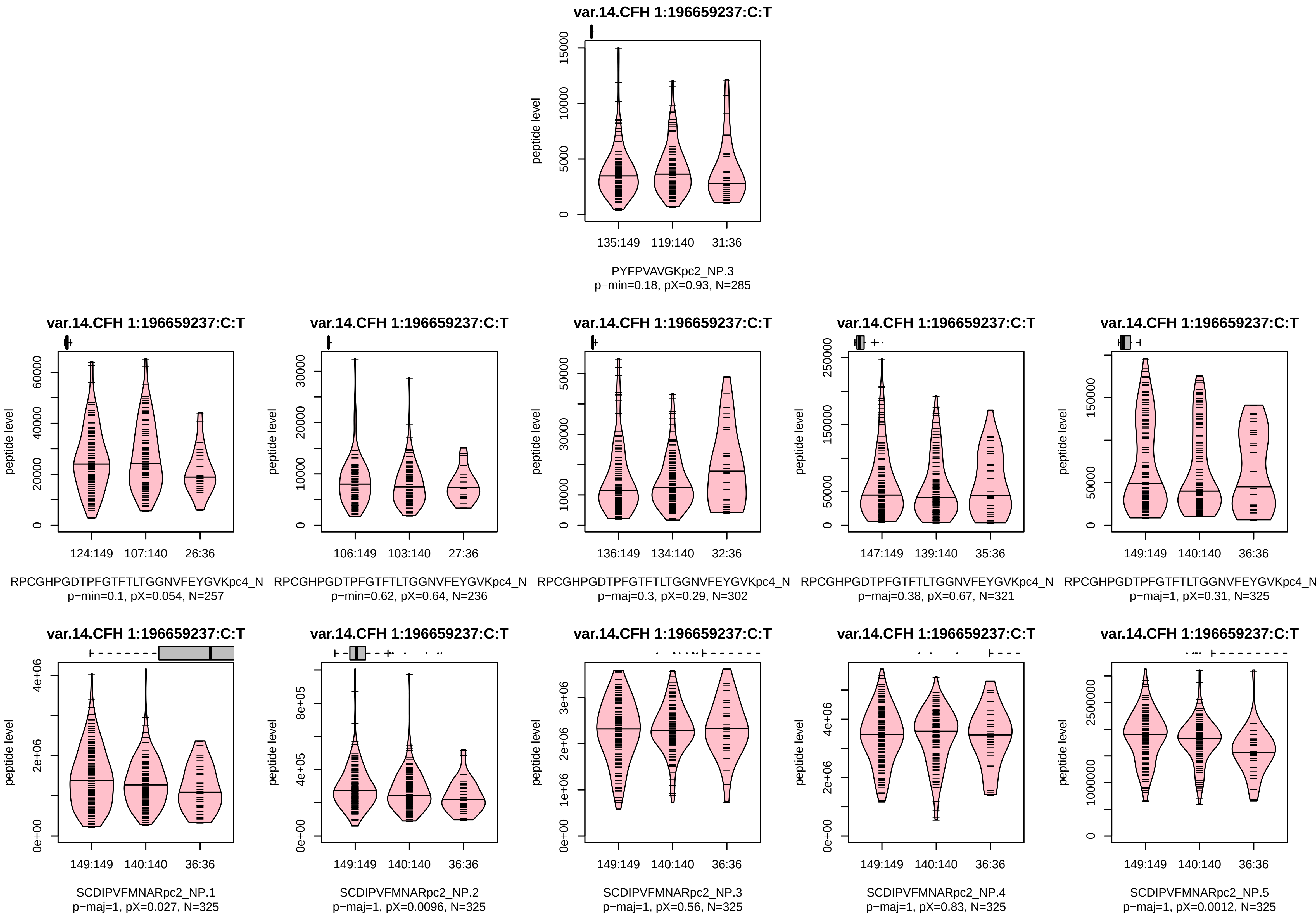

var.14.CFH 1:196659237:C:T
peptide level
135:149
119:140
31:36
PYFPVAVGKpc2_NP.3
p−min=0.18, pX=0.93, N=285
var.14.CFH 1:196659237:C:T
124:149
107:140
26:36
RPCGHPGDTPFGTFTLTGGNVFEYGVKpc4_N
p−min=0.1, pX=0.054, N=257
var.14.CFH 1:196659237:C:T
106:149
103:140
27:36
RPCGHPGDTPFGTFTLTGGNVFEYGVKpc4_N
p−min=0.62, pX=0.64, N=236
var.14.CFH 1:196659237:C:T
136:149
134:140
32:36
RPCGHPGDTPFGTFTLTGGNVFEYGVKpc4_N
p−maj=0.3, pX=0.29, N=302
var.14.CFH 1:196659237:C:T
147:149
139:140
35:36
RPCGHPGDTPFGTFTLTGGNVFEYGVKpc4_N
p−maj=0.38, pX=0.67, N=321
var.14.CFH 1:196659237:C:T
149:149
140:140
36:36
RPCGHPGDTPFGTFTLTGGNVFEYGVKpc4_N
p−maj=1, pX=0.31, N=325
var.14.CFH 1:196659237:C:T
149:149
140:140
36:36
SCDIPVFMNARpc2_NP.1
p−maj=1, pX=0.027, N=325
var.14.CFH 1:196659237:C:T
149:149
140:140
36:36
SCDIPVFMNARpc2_NP.2
p−maj=1, pX=0.0096, N=325
var.14.CFH 1:196659237:C:T
149:149
140:140
36:36
SCDIPVFMNARpc2_NP.3
p−maj=1, pX=0.56, N=325
var.14.CFH 1:196659237:C:T
149:149
140:140
36:36
SCDIPVFMNARpc2_NP.4
p−maj=1, pX=0.83, N=325
var.14.CFH 1:196659237:C:T
149:149
140:140
36:36
SCDIPVFMNARpc2_NP.5
p−maj=1, pX=0.0012, N=325

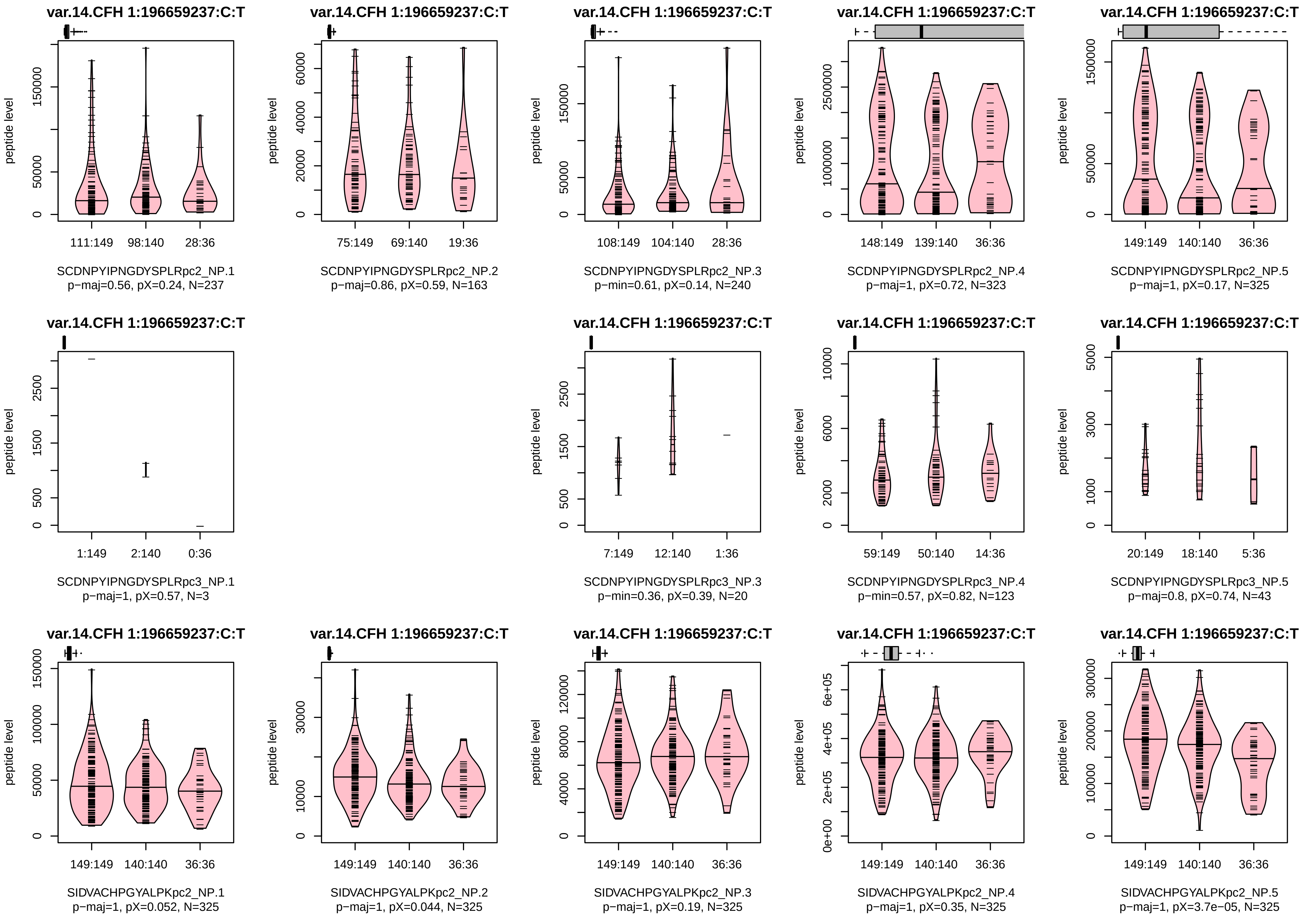
var.14.CFH 1:196659237:C:T
peptide level
111:149
98:140
28:36
SCDNPYIPNGDYSPLRpc2_NP.1
p−maj=0.56, pX=0.24, N=237
var.14.CFH 1:196659237:C:T
peptide level
75:149
69:140
19:36
SCDNPYIPNGDYSPLRpc2_NP.2
p−maj=0.86, pX=0.59, N=163
var.14.CFH 1:196659237:C:T
peptide level
108:149
104:140
28:36
SCDNPYIPNGDYSPLRpc2_NP.3
p−min=0.61, pX=0.14, N=240
var.14.CFH 1:196659237:C:T
peptide level
148:149
139:140
36:36
SCDNPYIPNGDYSPLRpc2_NP.4
p−maj=1, pX=0.72, N=323
var.14.CFH 1:196659237:C:T
peptide level
149:149
140:140
36:36
SCDNPYIPNGDYSPLRpc2_NP.5
p−maj=1, pX=0.17, N=325
var.14.CFH 1:196659237:C:T
peptide level
1:149
2:140
0:36
SCDNPYIPNGDYSPLRpc3_NP.1
p−maj=1, pX=0.57, N=3
var.14.CFH 1:196659237:C:T
peptide level
7:149
12:140
1:36
SCDNPYIPNGDYSPLRpc3_NP.3
p−min=0.36, pX=0.39, N=20
var.14.CFH 1:196659237:C:T
peptide level
59:149
50:140
14:36
SCDNPYIPNGDYSPLRpc3_NP.4
p−min=0.57, pX=0.82, N=123
var.14.CFH 1:196659237:C:T
peptide level
20:149
18:140
5:36
SCDNPYIPNGDYSPLRpc3_NP.5
p−maj=0.8, pX=0.74, N=43
var.14.CFH 1:196659237:C:T
peptide level
149:149
140:140
36:36
SIDVACHPGYALPKpc2_NP.1
p−maj=1, pX=0.052, N=325
var.14.CFH 1:196659237:C:T
peptide level
149:149
140:140
36:36
SIDVACHPGYALPKpc2_NP.2
p−maj=1, pX=0.044, N=325
var.14.CFH 1:196659237:C:T
peptide level
149:149
140:140
36:36
SIDVACHPGYALPKpc2_NP.3
p−maj=1, pX=0.19, N=325
var.14.CFH 1:196659237:C:T
peptide level
149:149
140:140
36:36
SIDVACHPGYALPKpc2_NP.4
p−maj=1, pX=0.35, N=325
var.14.CFH 1:196659237:C:T
peptide level
149:149
140:140
36:36
SIDVACHPGYALPKpc2_NP.5
p−maj=1, pX=3.7e−05, N=325

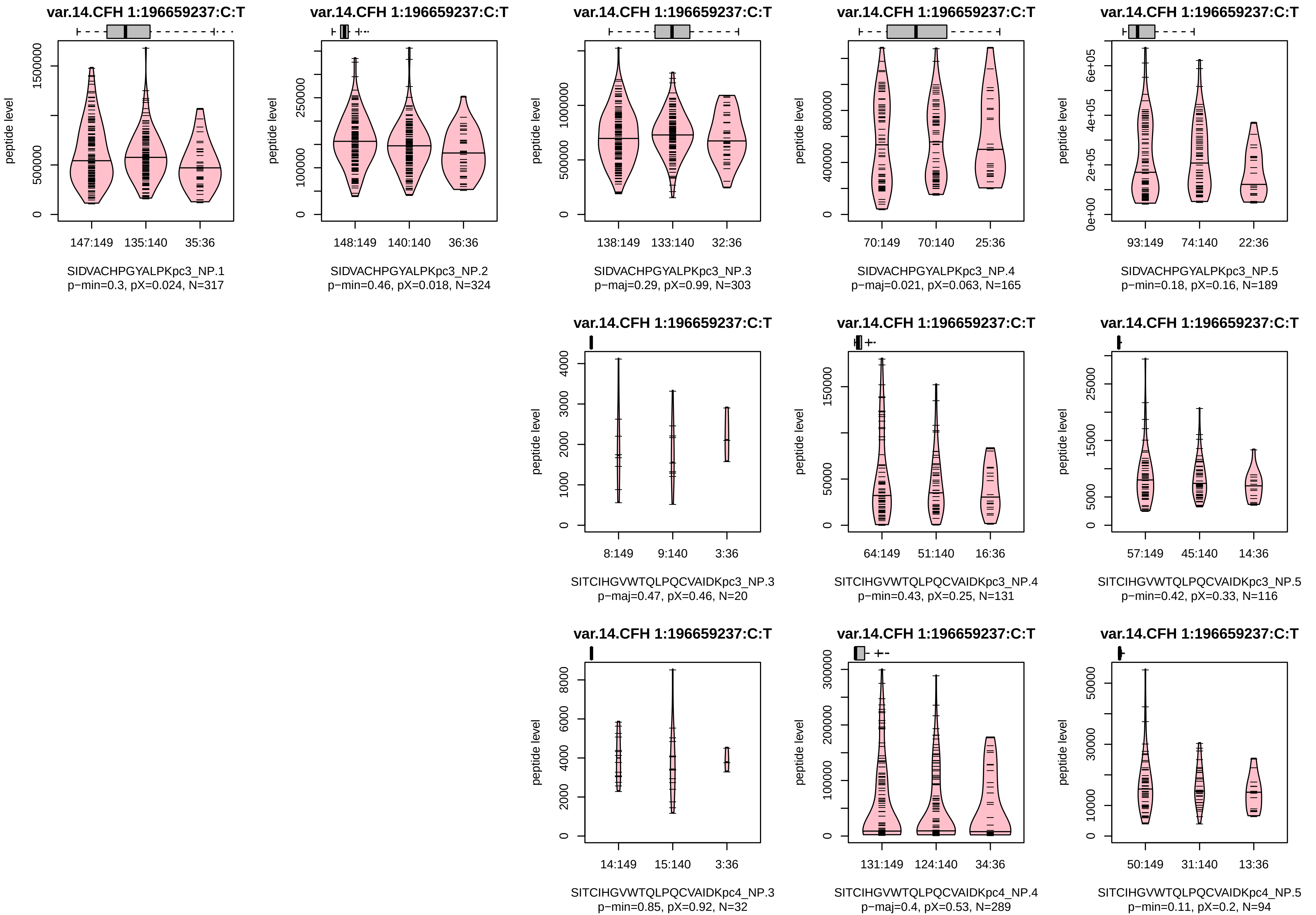
var.14.CFH 1:196659237:C:T
peptide level
147:149
135:140
35:36
SIDVACHPGYALPKpc3_NP.1
p−min=0.3, pX=0.024, N=317
var.14.CFH 1:196659237:C:T
peptide level
148:149
140:140
36:36
SIDVACHPGYALPKpc3_NP.2
p−min=0.46, pX=0.018, N=324
var.14.CFH 1:196659237:C:T
peptide level
138:149
133:140
32:36
SIDVACHPGYALPKpc3_NP.3
p−maj=0.29, pX=0.99, N=303
var.14.CFH 1:196659237:C:T
peptide level
70:149
70:140
25:36
SIDVACHPGYALPKpc3_NP.4
p−maj=0.021, pX=0.063, N=165
var.14.CFH 1:196659237:C:T
peptide level
93:149
74:140
22:36
SIDVACHPGYALPKpc3_NP.5
p−min=0.18, pX=0.16, N=189
var.14.CFH 1:196659237:C:T
peptide level
8:149
9:140
3:36
SITCIHGVWTQLPQCVAIDKpc3_NP.3
p−maj=0.47, pX=0.46, N=20
var.14.CFH 1:196659237:C:T
peptide level
64:149
51:140
16:36
SITCIHGVWTQLPQCVAIDKpc3_NP.4
p−min=0.43, pX=0.25, N=131
var.14.CFH 1:196659237:C:T
peptide level
57:149
45:140
14:36
SITCIHGVWTQLPQCVAIDKpc3_NP.5
p−min=0.42, pX=0.33, N=116
var.14.CFH 1:196659237:C:T
peptide level
14:149
15:140
3:36
SITCIHGVWTQLPQCVAIDKpc4_NP.3
p−min=0.85, pX=0.92, N=32
var.14.CFH 1:196659237:C:T
peptide level
131:149
124:140
34:36
SITCIHGVWTQLPQCVAIDKpc4_NP.4
p−maj=0.4, pX=0.53, N=289
var.14.CFH 1:196659237:C:T
peptide level
50:149
31:140
13:36
SITCIHGVWTQLPQCVAIDKpc4_NP.5
p−min=0.11, pX=0.2, N=94

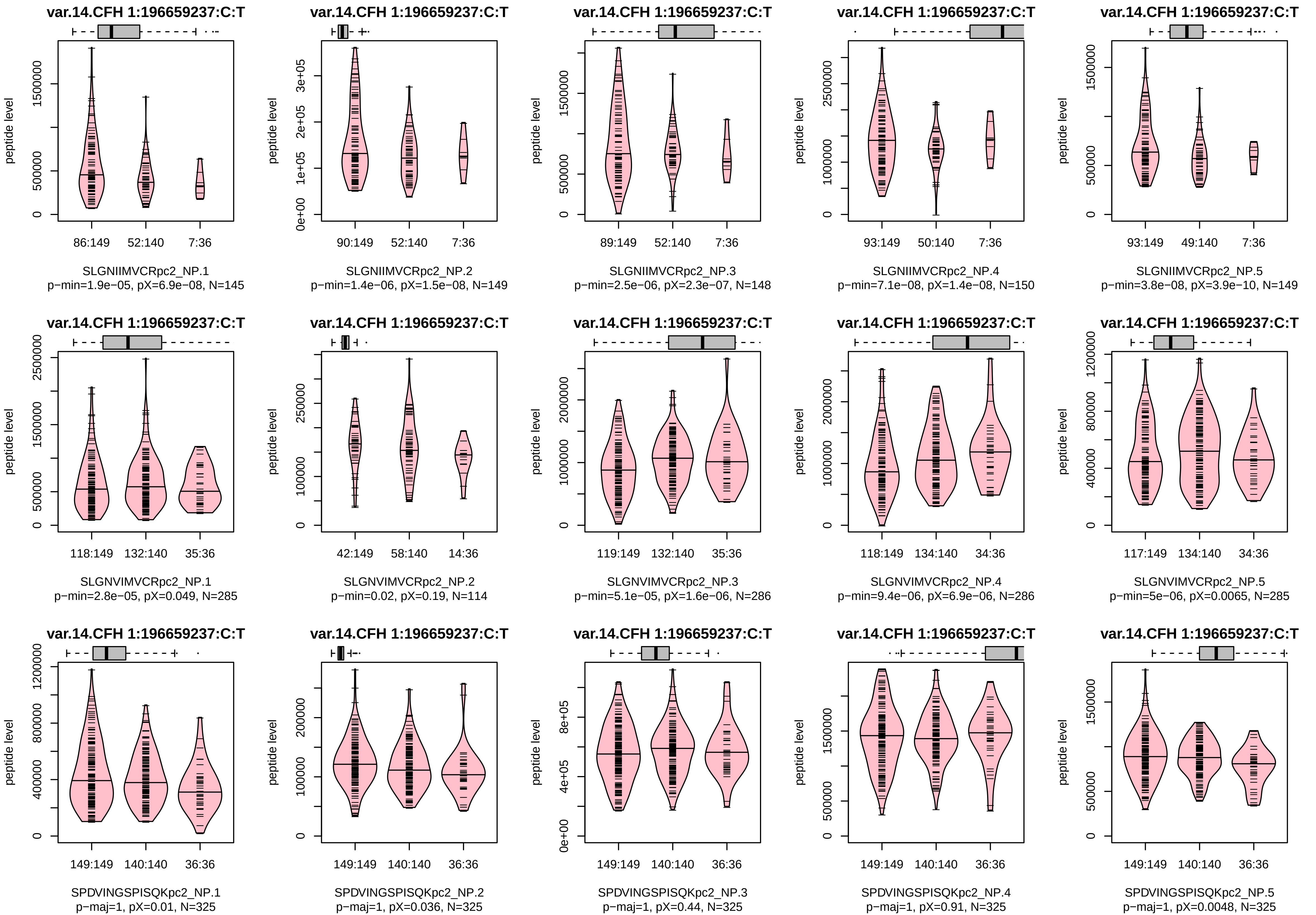

var.14.CFH 1:196659237:C:T
peptide level
86:149
52:140
7:36
SLGNIIMVCRpc2_NP.1
p−min=1.9e−05, pX=6.9e−08, N=145
var.14.CFH 1:196659237:C:T
peptide level
90:149
52:140
7:36
SLGNIIMVCRpc2_NP.2
p−min=1.4e−06, pX=1.5e−08, N=149
var.14.CFH 1:196659237:C:T
peptide level
89:149
52:140
7:36
SLGNIIMVCRpc2_NP.3
p−min=2.5e−06, pX=2.3e−07, N=148
var.14.CFH 1:196659237:C:T
peptide level
93:149
50:140
7:36
SLGNIIMVCRpc2_NP.4
p−min=7.1e−08, pX=1.4e−08, N=150
var.14.CFH 1:196659237:C:T
peptide level
93:149
49:140
7:36
SLGNIIMVCRpc2_NP.5
p−min=3.8e−08, pX=3.9e−10, N=149
var.14.CFH 1:196659237:C:T
peptide level
118:149
132:140
35:36
SLGNVIMVCRpc2_NP.1
p−min=2.8e−05, pX=0.049, N=285
var.14.CFH 1:196659237:C:T
peptide level
42:149
58:140
14:36
SLGNVIMVCRpc2_NP.2
p−min=0.02, pX=0.19, N=114
var.14.CFH 1:196659237:C:T
peptide level
119:149
132:140
35:36
SLGNVIMVCRpc2_NP.3
p−min=5.1e−05, pX=1.6e−06, N=286
var.14.CFH 1:196659237:C:T
peptide level
118:149
134:140
34:36
SLGNVIMVCRpc2_NP.4
p−min=9.4e−06, pX=6.9e−06, N=286
var.14.CFH 1:196659237:C:T
peptide level
117:149
134:140
34:36
SLGNVIMVCRpc2_NP.5
p−min=5e−06, pX=0.0065, N=285
var.14.CFH 1:196659237:C:T
peptide level
149:149
140:140
36:36
SPDVINGSPISQKpc2_NP.1
p−maj=1, pX=0.01, N=325
var.14.CFH 1:196659237:C:T
peptide level
149:149
140:140
36:36
SPDVINGSPISQKpc2_NP.2
p−maj=1, pX=0.036, N=325
var.14.CFH 1:196659237:C:T
peptide level
149:149
140:140
36:36
SPDVINGSPISQKpc2_NP.3
p−maj=1, pX=0.44, N=325
var.14.CFH 1:196659237:C:T
peptide level
149:149
140:140
36:36
SPDVINGSPISQKpc2_NP.4
p−maj=1, pX=0.91, N=325
var.14.CFH 1:196659237:C:T
peptide level
149:149
140:140
36:36
SPDVINGSPISQKpc2_NP.5
p−maj=1, pX=0.0048, N=325

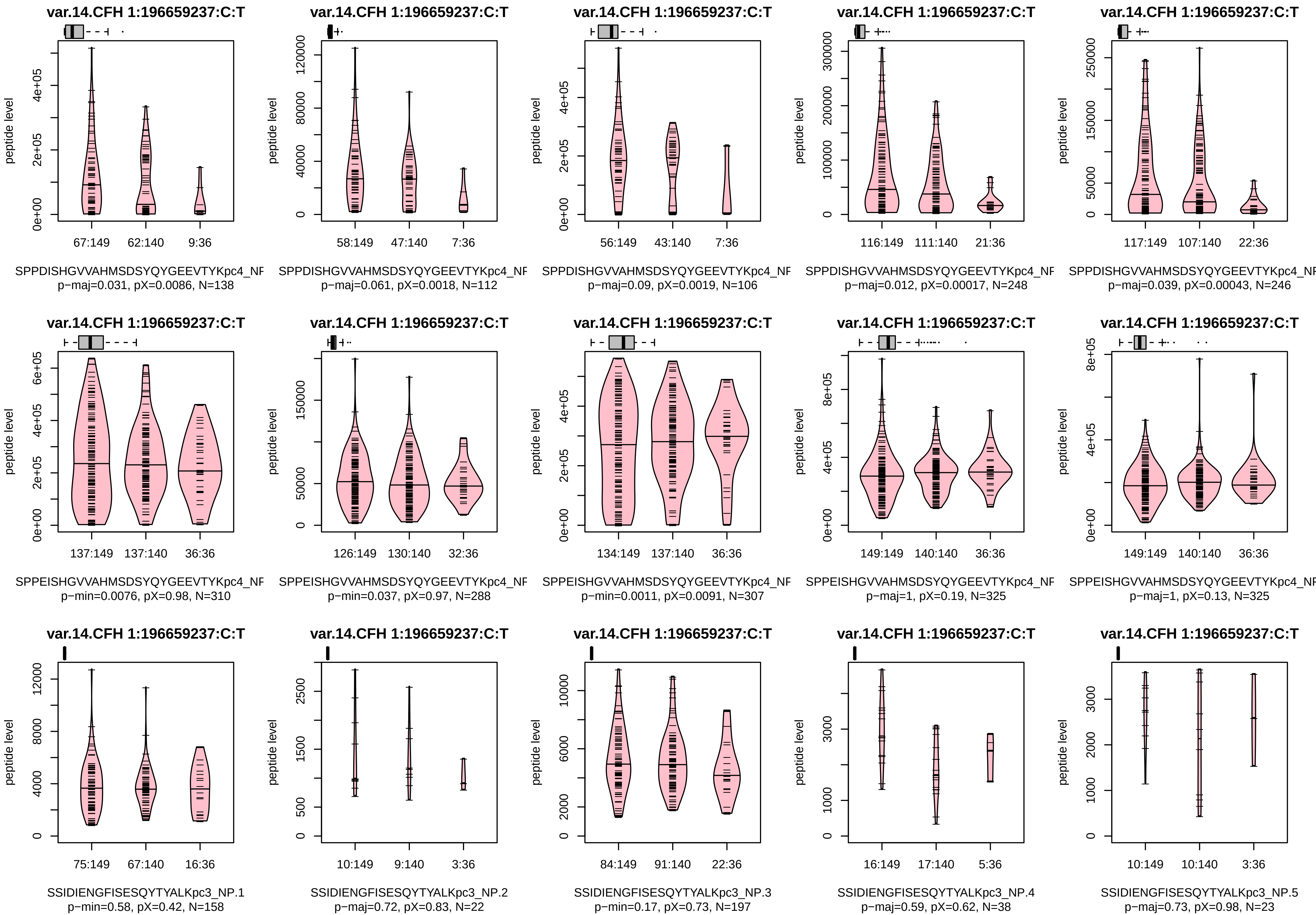
var.14.CFH 1:196659237:C:T
peptide level
67:149
62:140
9:36
SPPDISHGVVAHMSDSYQYGEEVTYKpc4_NF
p−maj=0.031, pX=0.0086, N=138
var.14.CFH 1:196659237:C:T
peptide level
58:149
47:140
7:36
SPPDISHGVVAHMSDSYQYGEEVTYKpc4_NF
p−maj=0.061, pX=0.0018, N=112
var.14.CFH 1:196659237:C:T
peptide level
56:149
43:140
7:36
SPPDISHGVVAHMSDSYQYGEEVTYKpc4_NF
p−maj=0.09, pX=0.0019, N=106
var.14.CFH 1:196659237:C:T
peptide level
116:149
111:140
21:36
SPPDISHGVVAHMSDSYQYGEEVTYKpc4_NF
p−maj=0.012, pX=0.00017, N=248
var.14.CFH 1:196659237:C:T
peptide level
117:149
107:140
22:36
SPPDISHGVVAHMSDSYQYGEEVTYKpc4_NF
p−maj=0.039, pX=0.00043, N=246
var.14.CFH 1:196659237:C:T
peptide level
137:149
137:140
36:36
SPPEISHGVVAHMSDSYQYGEEVTYKpc4_NF
p−min=0.0076, pX=0.98, N=310
var.14.CFH 1:196659237:C:T
peptide level
126:149
130:140
32:36
SPPEISHGVVAHMSDSYQYGEEVTYKpc4_NF
p−min=0.037, pX=0.97, N=288
var.14.CFH 1:196659237:C:T
peptide level
134:149
137:140
36:36
SPPEISHGVVAHMSDSYQYGEEVTYKpc4_NF
p−min=0.0011, pX=0.0091, N=307
var.14.CFH 1:196659237:C:T
peptide level
149:149
140:140
36:36
SPPEISHGVVAHMSDSYQYGEEVTYKpc4_NF
p−maj=1, pX=0.19, N=325
var.14.CFH 1:196659237:C:T
peptide level
149:149
140:140
36:36
SPPEISHGVVAHMSDSYQYGEEVTYKpc4_NF
p−maj=1, pX=0.13, N=325
var.14.CFH 1:196659237:C:T
peptide level
75:149
67:140
16:36
SSIDIENGFISESQYTYALKpc3_NP.1
p−min=0.58, pX=0.42, N=158
var.14.CFH 1:196659237:C:T
peptide level
10:149
9:140
3:36
SSIDIENGFISESQYTYALKpc3_NP.2
p−maj=0.72, pX=0.83, N=22
var.14.CFH 1:196659237:C:T
peptide level
84:149
91:140
22:36
SSIDIENGFISESQYTYALKpc3_NP.3
p−min=0.17, pX=0.73, N=197
var.14.CFH 1:196659237:C:T
peptide level
16:149
17:140
5:36
SSIDIENGFISESQYTYALKpc3_NP.4
p−maj=0.59, pX=0.62, N=38
var.14.CFH 1:196659237:C:T
peptide level
10:149
10:140
3:36
SSIDIENGFISESQYTYALKpc3_NP.5
p−maj=0.73, pX=0.98, N=23

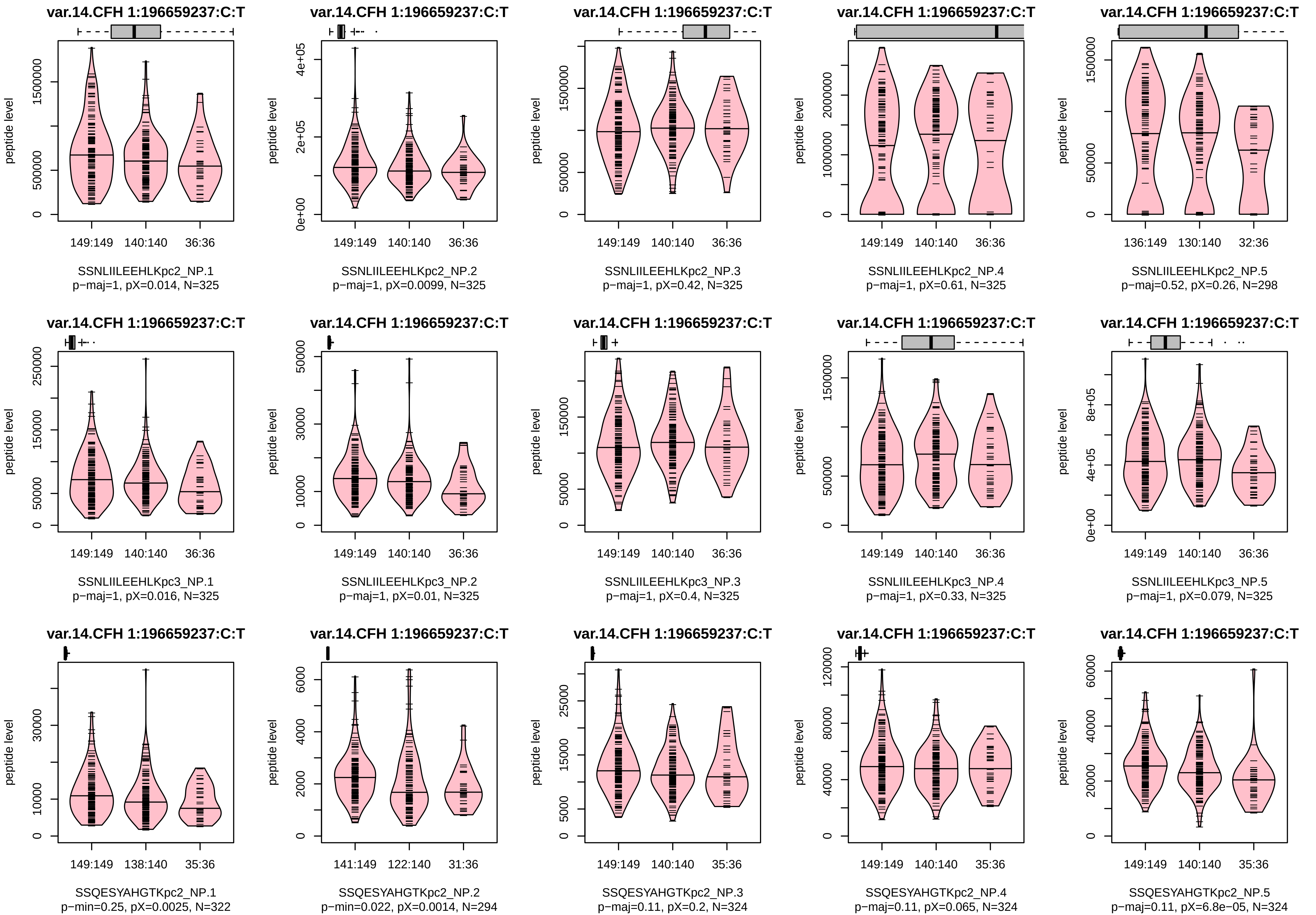
var.14.CFH 1:196659237:C:T
peptide level
149:149
140:140
36:36
SSNLIILEEHLKpc2_NP.1
p−maj=1, pX=0.014, N=325
SSNLIILEEHLKpc2_NP.2
p−maj=1, pX=0.0099, N=325
SSNLIILEEHLKpc2_NP.3
p−maj=1, pX=0.42, N=325
SSNLIILEEHLKpc2_NP.4
p−maj=1, pX=0.61, N=325
136:149
130:140
32:36
SSNLIILEEHLKpc2_NP.5
p−maj=0.52, pX=0.26, N=298
SSNLIILEEHLKpc3_NP.1
p−maj=1, pX=0.016, N=325
SSNLIILEEHLKpc3_NP.2
p−maj=1, pX=0.01, N=325
SSNLIILEEHLKpc3_NP.3
p−maj=1, pX=0.4, N=325
SSNLIILEEHLKpc3_NP.4
p−maj=1, pX=0.33, N=325
SSNLIILEEHLKpc3_NP.5
p−maj=1, pX=0.079, N=325
138:140
35:36
SSQESYAHGTKpc2_NP.1
p−min=0.25, pX=0.0025, N=322
141:149
122:140
31:36
SSQESYAHGTKpc2_NP.2
p−min=0.022, pX=0.0014, N=294
SSQESYAHGTKpc2_NP.3
p−maj=0.11, pX=0.2, N=324
SSQESYAHGTKpc2_NP.4
p−maj=0.11, pX=0.065, N=324
SSQESYAHGTKpc2_NP.5
p−maj=0.11, pX=6.8e−05, N=324

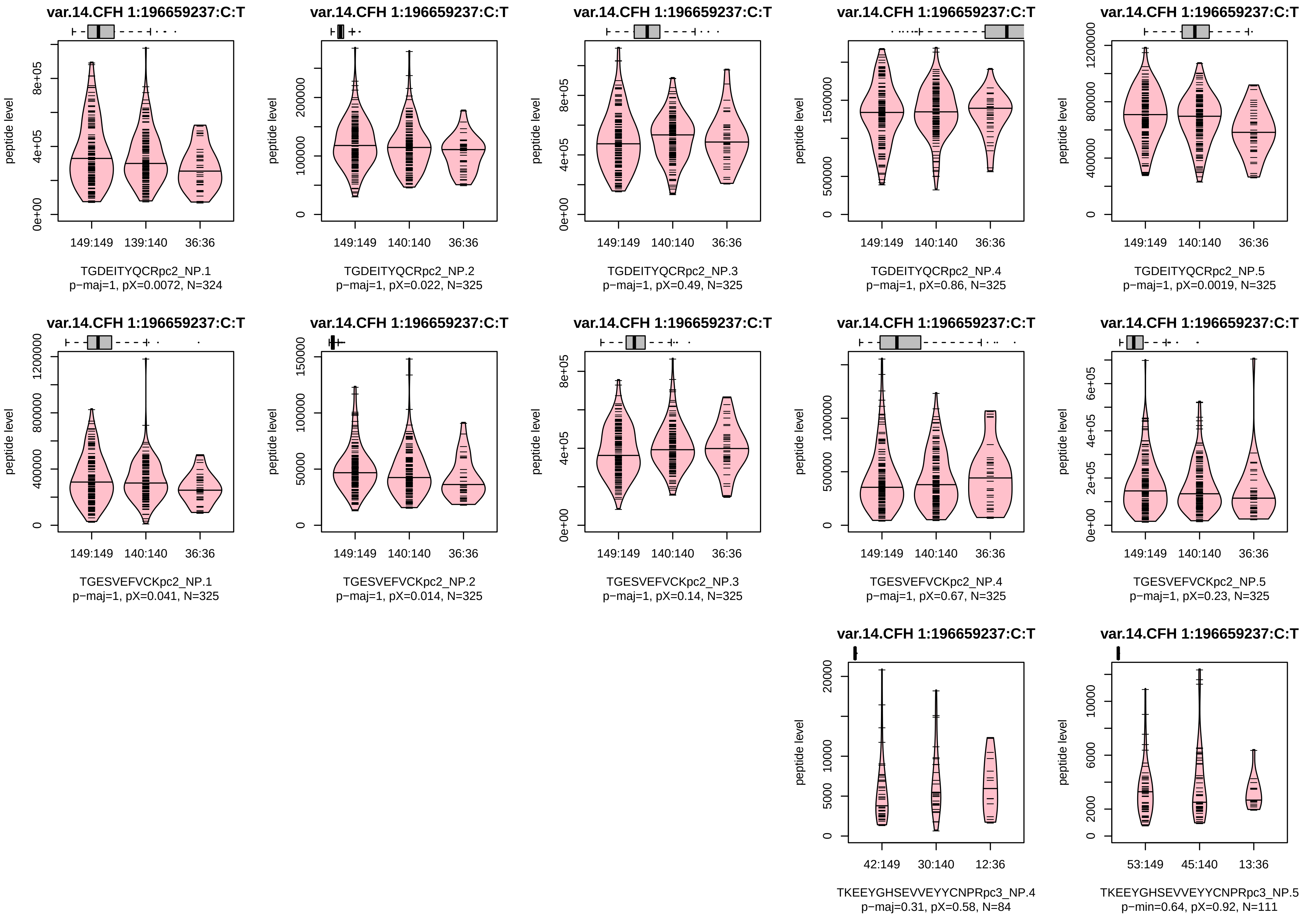
var.14.CFH 1:196659237:C:T
peptide level
149:149 139:140 36:36
TGDEITYQCRpc2_NP.1
p−maj=1, pX=0.0072, N=324
var.14.CFH 1:196659237:C:T
149:149 140:140 36:36
TGDEITYQCRpc2_NP.2
p−maj=1, pX=0.022, N=325
var.14.CFH 1:196659237:C:T
149:149 140:140 36:36
TGDEITYQCRpc2_NP.3
p−maj=1, pX=0.49, N=325
var.14.CFH 1:196659237:C:T
149:149 140:140 36:36
TGDEITYQCRpc2_NP.4
p−maj=1, pX=0.86, N=325
var.14.CFH 1:196659237:C:T
149:149 140:140 36:36
TGDEITYQCRpc2_NP.5
p−maj=1, pX=0.0019, N=325
var.14.CFH 1:196659237:C:T
149:149 140:140 36:36
TGESVEFVCKpc2_NP.1
p−maj=1, pX=0.041, N=325
var.14.CFH 1:196659237:C:T
149:149 140:140 36:36
TGESVEFVCKpc2_NP.2
p−maj=1, pX=0.014, N=325
var.14.CFH 1:196659237:C:T
149:149 140:140 36:36
TGESVEFVCKpc2_NP.3
p−maj=1, pX=0.14, N=325
var.14.CFH 1:196659237:C:T
149:149 140:140 36:36
TGESVEFVCKpc2_NP.4
p−maj=1, pX=0.67, N=325
var.14.CFH 1:196659237:C:T
149:149 140:140 36:36
TGESVEFVCKpc2_NP.5
p−maj=1, pX=0.23, N=325
var.14.CFH 1:196659237:C:T
42:149 30:140 12:36
TKEEYGHSEVVEYYCNPRpc3_NP.4
p−maj=0.31, pX=0.58, N=84
var.14.CFH 1:196659237:C:T
53:149 45:140 13:36
TKEEYGHSEVVEYYCNPRpc3_NP.5
p−min=0.64, pX=0.92, N=111

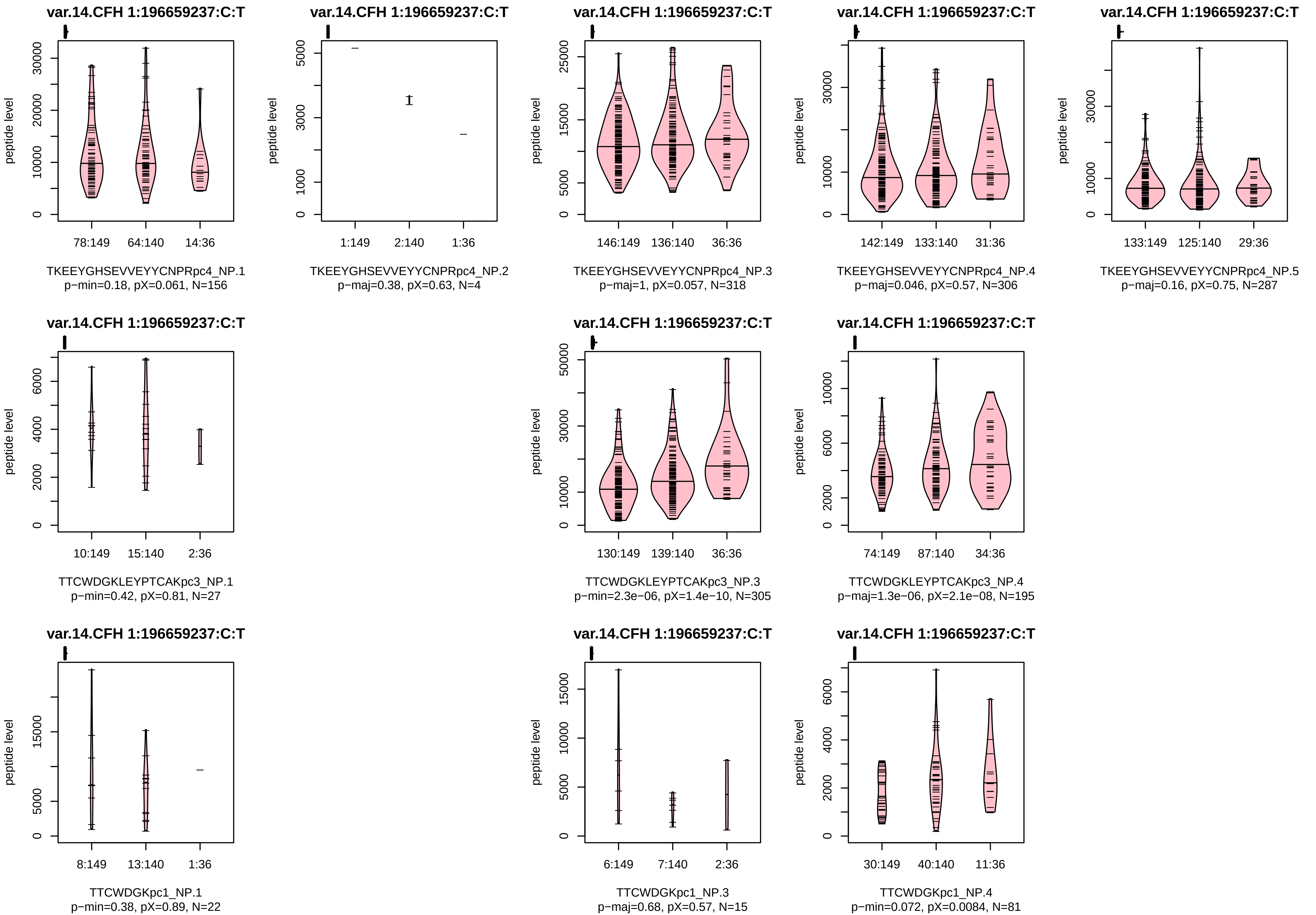

var.14.CFH 1:196659237:C:T
peptide level
78:149
64:140
14:36
TKEEYGHSEVVEYYCNPRpc4_NP.1
p−min=0.18, pX=0.061, N=156
var.14.CFH 1:196659237:C:T
peptide level
1:149
2:140
1:36
TKEEYGHSEVVEYYCNPRpc4_NP.2
p−maj=0.38, pX=0.63, N=4
var.14.CFH 1:196659237:C:T
peptide level
146:149
136:140
36:36
TKEEYGHSEVVEYYCNPRpc4_NP.3
p−maj=1, pX=0.057, N=318
var.14.CFH 1:196659237:C:T
peptide level
142:149
133:140
31:36
TKEEYGHSEVVEYYCNPRpc4_NP.4
p−maj=0.046, pX=0.57, N=306
var.14.CFH 1:196659237:C:T
peptide level
133:149
125:140
29:36
TKEEYGHSEVVEYYCNPRpc4_NP.5
p−maj=0.16, pX=0.75, N=287
var.14.CFH 1:196659237:C:T
peptide level
10:149
15:140
2:36
TTCWDGKLEYPTCAKpc3_NP.1
p−min=0.42, pX=0.81, N=27
var.14.CFH 1:196659237:C:T
peptide level
130:149
139:140
36:36
TTCWDGKLEYPTCAKpc3_NP.3
p−min=2.3e−06, pX=1.4e−10, N=305
var.14.CFH 1:196659237:C:T
peptide level
74:149
87:140
34:36
TTCWDGKLEYPTCAKpc3_NP.4
p−maj=1.3e−06, pX=2.1e−08, N=195
var.14.CFH 1:196659237:C:T
peptide level
8:149
13:140
1:36
TTCWDGKpc1_NP.1
p−min=0.38, pX=0.89, N=22
var.14.CFH 1:196659237:C:T
peptide level
6:149
7:140
2:36
TTCWDGKpc1_NP.3
p−maj=0.68, pX=0.57, N=15
var.14.CFH 1:196659237:C:T
peptide level
30:149
40:140
11:36
TTCWDGKpc1_NP.4
p−min=0.072, pX=0.0084, N=81

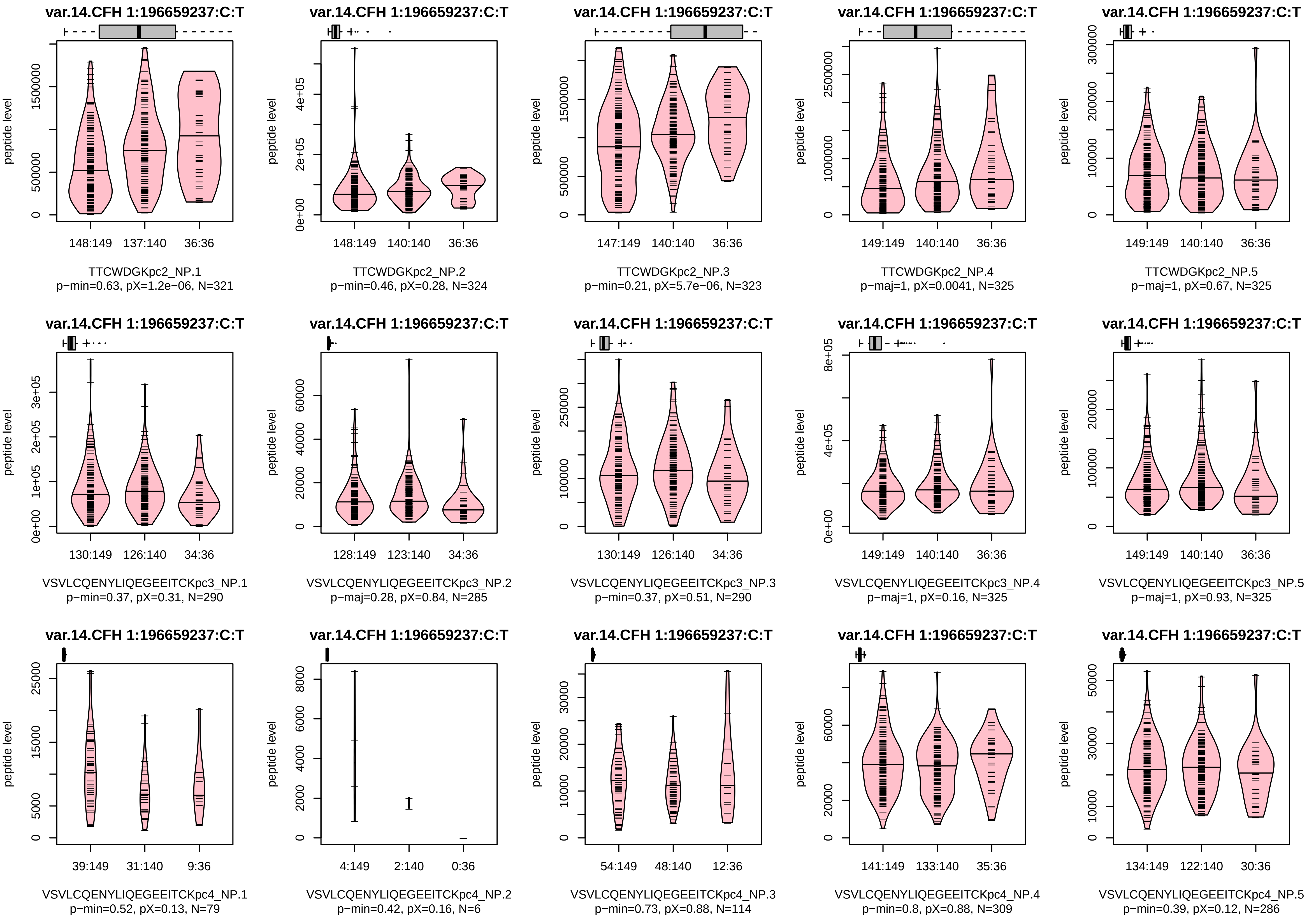
var.14.CFH 1:196659237:C:T
peptide level
148:149
137:140
36:36
TTCWDGKpc2_NP.1
p−min=0.63, pX=1.2e−06, N=321
148:149
140:140
36:36
TTCWDGKpc2_NP.2
p−min=0.46, pX=0.28, N=324
147:149
140:140
36:36
TTCWDGKpc2_NP.3
p−min=0.21, pX=5.7e−06, N=323
149:149
140:140
36:36
TTCWDGKpc2_NP.4
p−maj=1, pX=0.0041, N=325
149:149
140:140
36:36
TTCWDGKpc2_NP.5
p−maj=1, pX=0.67, N=325
130:149
126:140
34:36
VSVLCQENYLIQEGEEITCKpc3_NP.1
p−min=0.37, pX=0.31, N=290
128:149
123:140
34:36
VSVLCQENYLIQEGEEITCKpc3_NP.2
p−maj=0.28, pX=0.84, N=285
130:149
126:140
34:36
VSVLCQENYLIQEGEEITCKpc3_NP.3
p−min=0.37, pX=0.51, N=290
149:149
140:140
36:36
VSVLCQENYLIQEGEEITCKpc3_NP.4
p−maj=1, pX=0.16, N=325
149:149
140:140
36:36
VSVLCQENYLIQEGEEITCKpc3_NP.5
p−maj=1, pX=0.93, N=325
39:149
31:140
9:36
VSVLCQENYLIQEGEEITCKpc4_NP.1
p−min=0.52, pX=0.13, N=79
4:149
2:140
0:36
VSVLCQENYLIQEGEEITCKpc4_NP.2
p−min=0.42, pX=0.16, N=6
54:149
48:140
12:36
VSVLCQENYLIQEGEEITCKpc4_NP.3
p−min=0.73, pX=0.88, N=114
141:149
133:140
35:36
VSVLCQENYLIQEGEEITCKpc4_NP.4
p−min=0.8, pX=0.88, N=309
134:149
122:140
30:36
VSVLCQENYLIQEGEEITCKpc4_NP.5
p−min=0.39, pX=0.12, N=286

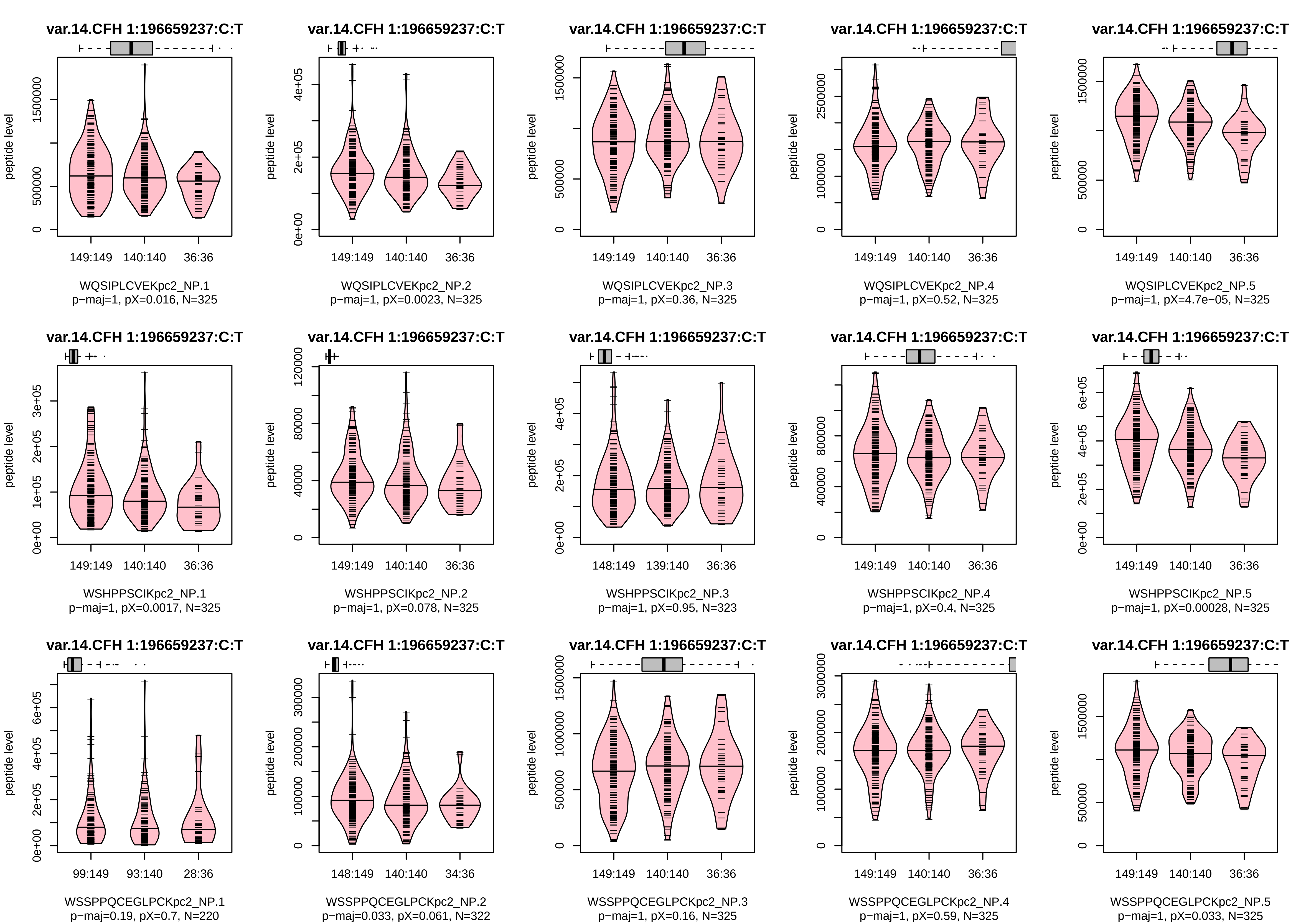

var.14.CFH 1:196659237:C:T
peptide level
149:149 140:140 36:36
WQSIPLCVEKpc2_NP.1
p−maj=1, pX=0.016, N=325
var.14.CFH 1:196659237:C:T
149:149 140:140 36:36
WQSIPLCVEKpc2_NP.2
p−maj=1, pX=0.0023, N=325
var.14.CFH 1:196659237:C:T
149:149 140:140 36:36
WQSIPLCVEKpc2_NP.3
p−maj=1, pX=0.36, N=325
var.14.CFH 1:196659237:C:T
149:149 140:140 36:36
WQSIPLCVEKpc2_NP.4
p−maj=1, pX=0.52, N=325
var.14.CFH 1:196659237:C:T
149:149 140:140 36:36
WQSIPLCVEKpc2_NP.5
p−maj=1, pX=4.7e−05, N=325
var.14.CFH 1:196659237:C:T
149:149 140:140 36:36
WSHPPSCIKpc2_NP.1
p−maj=1, pX=0.0017, N=325
var.14.CFH 1:196659237:C:T
149:149 140:140 36:36
WSHPPSCIKpc2_NP.2
p−maj=1, pX=0.078, N=325
var.14.CFH 1:196659237:C:T
148:149 139:140 36:36
WSHPPSCIKpc2_NP.3
p−maj=1, pX=0.95, N=323
var.14.CFH 1:196659237:C:T
149:149 140:140 36:36
WSHPPSCIKpc2_NP.4
p−maj=1, pX=0.4, N=325
var.14.CFH 1:196659237:C:T
149:149 140:140 36:36
WSHPPSCIKpc2_NP.5
p−maj=1, pX=0.00028, N=325
var.14.CFH 1:196659237:C:T
99:149 93:140 28:36
WSSPPQCEGLPCKpc2_NP.1
p−maj=0.19, pX=0.7, N=220
var.14.CFH 1:196659237:C:T
148:149 140:140 34:36
WSSPPQCEGLPCKpc2_NP.2
p−maj=0.033, pX=0.061, N=322
var.14.CFH 1:196659237:C:T
149:149 140:140 36:36
WSSPPQCEGLPCKpc2_NP.3
p−maj=1, pX=0.16, N=325
var.14.CFH 1:196659237:C:T
149:149 140:140 36:36
WSSPPQCEGLPCKpc2_NP.4
p−maj=1, pX=0.59, N=325
var.14.CFH 1:196659237:C:T
149:149 140:140 36:36
WSSPPQCEGLPCKpc2_NP.5
p−maj=1, pX=0.033, N=325

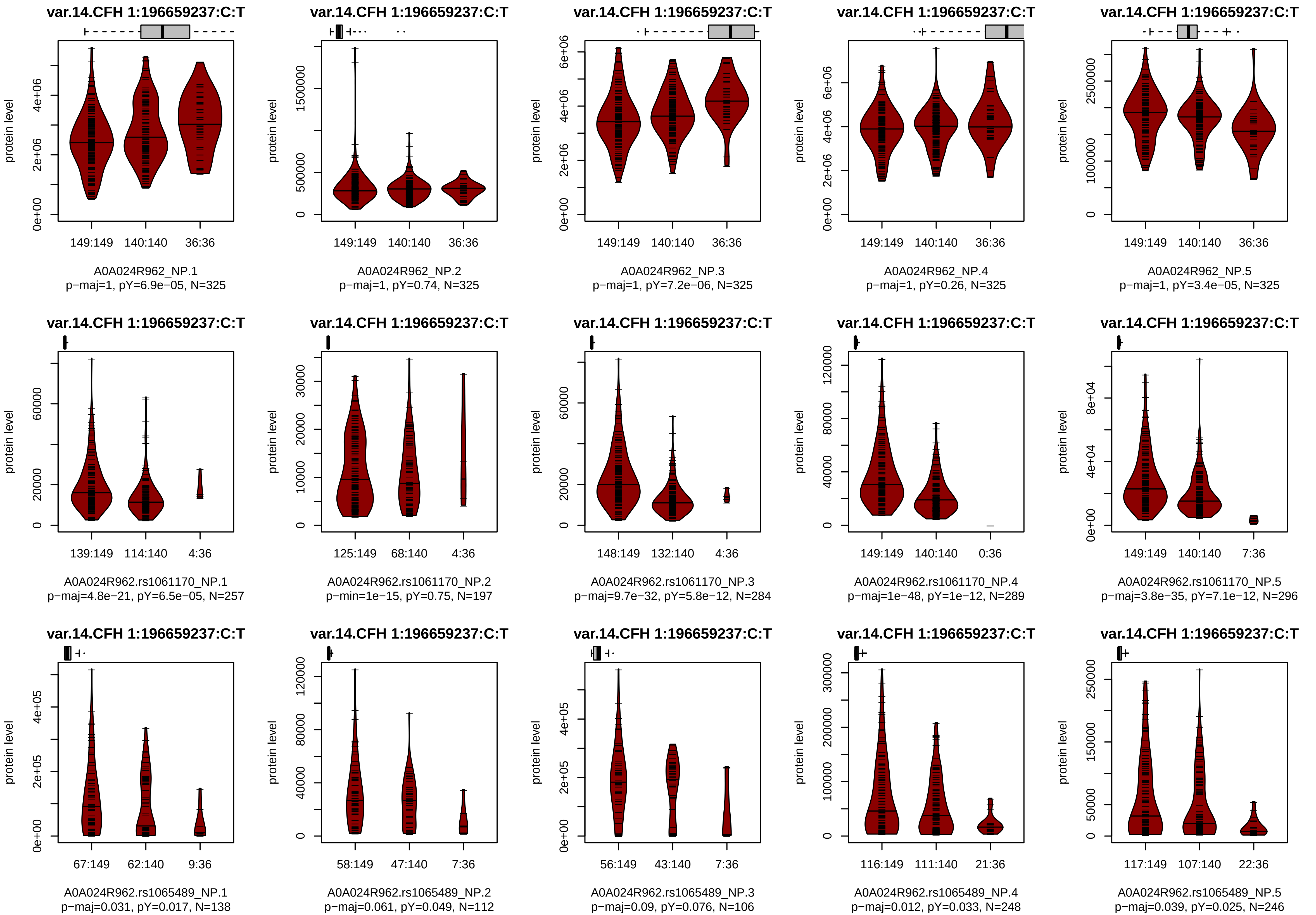
var.14.CFH 1:196659237:C:T
protein level
149:149
140:140
36:36
A0A024R962_NP.1
p−maj=1, pY=6.9e−05, N=325
A0A024R962_NP.2
p−maj=1, pY=0.74, N=325
A0A024R962_NP.3
p−maj=1, pY=7.2e−06, N=325
A0A024R962_NP.4
p−maj=1, pY=0.26, N=325
A0A024R962_NP.5
p−maj=1, pY=3.4e−05, N=325
139:149
114:140
4:36
A0A024R962.rs1061170_NP.1
p−maj=4.8e−21, pY=6.5e−05, N=257
125:149
68:140
4:36
A0A024R962.rs1061170_NP.2
p−min=1e−15, pY=0.75, N=197
148:149
132:140
4:36
A0A024R962.rs1061170_NP.3
p−maj=9.7e−32, pY=5.8e−12, N=284
149:149
140:140
0:36
A0A024R962.rs1061170_NP.4
p−maj=1e−48, pY=1e−12, N=289
149:149
140:140
7:36
A0A024R962.rs1061170_NP.5
p−maj=3.8e−35, pY=7.1e−12, N=296
67:149
62:140
9:36
A0A024R962.rs1065489_NP.1
p−maj=0.031, pY=0.017, N=138
58:149
47:140
7:36
A0A024R962.rs1065489_NP.2
p−maj=0.061, pY=0.049, N=112
56:149
43:140
7:36
A0A024R962.rs1065489_NP.3
p−maj=0.09, pY=0.076, N=106
116:149
111:140
21:36
A0A024R962.rs1065489_NP.4
p−maj=0.012, pY=0.033, N=248
117:149
107:140
22:36
A0A024R962.rs1065489_NP.5
p−maj=0.039, pY=0.025, N=246

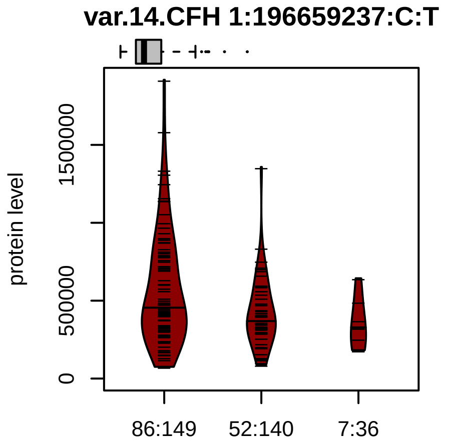

A0A024R962.rs800292_NP.1
p−min=1.9e−05, pY=0.00031, N=145

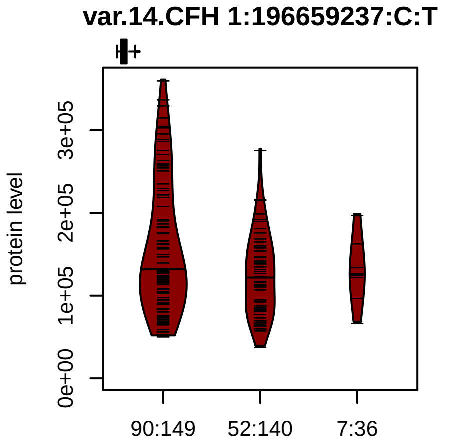

A0A024R962.rs800292_NP.2
p−min=1.4e−06, pY=0.044, N=149

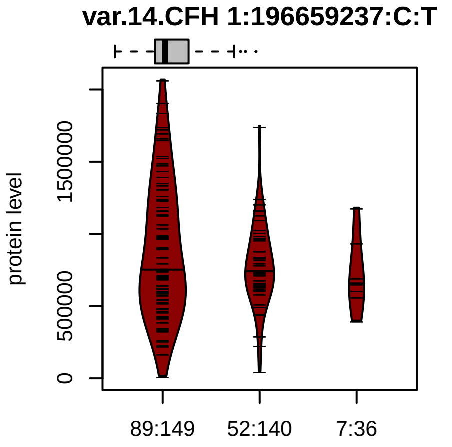

A0A024R962.rs800292_NP.3
p−min=2.5e−06, pY=0.045, N=148

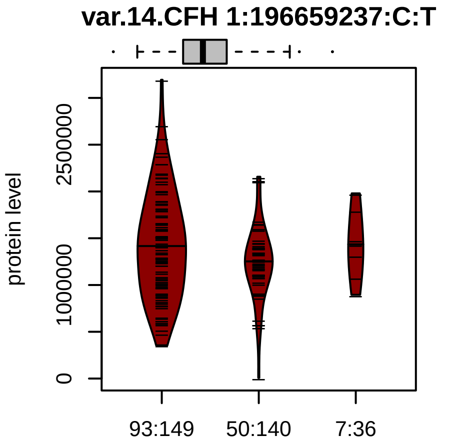

A0A024R962.rs800292_NP.4
p−min=7.1e−08, pY=0.048, N=150

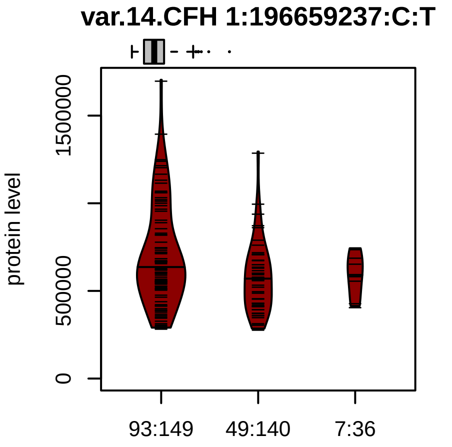

A0A024R962.rs800292_NP.5
p−min=3.8e−08, pY=0.018, N=149

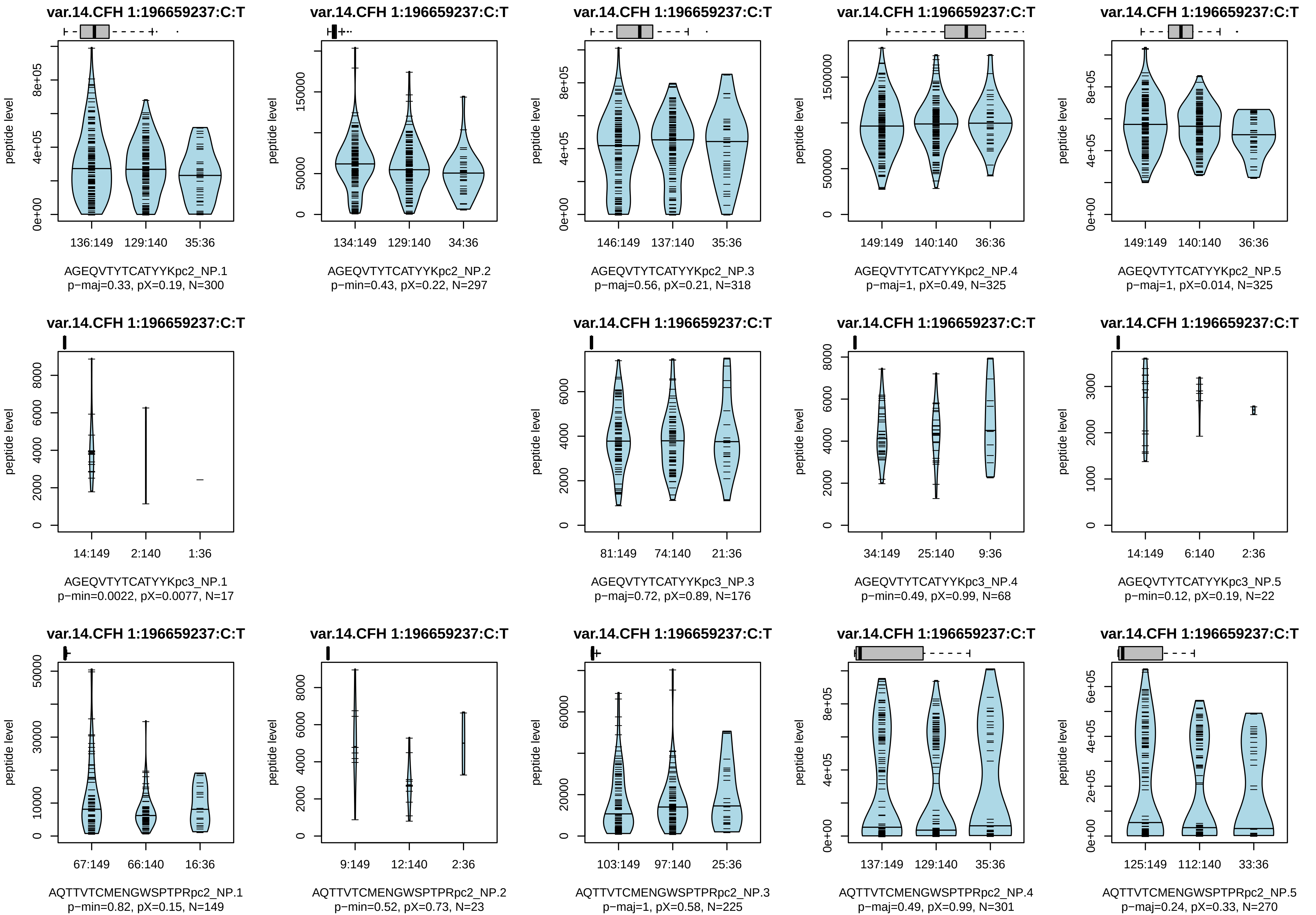
var.14.CFH 1:196659237:C:T
peptide level
136:149
129:140
35:36
AGEQVTYTCATYYKpc2_NP.1
p−maj=0.33, pX=0.19, N=300
var.14.CFH 1:196659237:C:T
peptide level
134:149
129:140
34:36
AGEQVTYTCATYYKpc2_NP.2
p−min=0.43, pX=0.22, N=297
var.14.CFH 1:196659237:C:T
peptide level
146:149
137:140
35:36
AGEQVTYTCATYYKpc2_NP.3
p−maj=0.56, pX=0.21, N=318
var.14.CFH 1:196659237:C:T
peptide level
149:149
140:140
36:36
AGEQVTYTCATYYKpc2_NP.4
p−maj=1, pX=0.49, N=325
var.14.CFH 1:196659237:C:T
peptide level
149:149
140:140
36:36
AGEQVTYTCATYYKpc2_NP.5
p−maj=1, pX=0.014, N=325
var.14.CFH 1:196659237:C:T
peptide level
14:149
2:140
1:36
AGEQVTYTCATYYKpc3_NP.1
p−min=0.0022, pX=0.0077, N=17
var.14.CFH 1:196659237:C:T
peptide level
81:149
74:140
21:36
AGEQVTYTCATYYKpc3_NP.3
p−maj=0.72, pX=0.89, N=176
var.14.CFH 1:196659237:C:T
peptide level
34:149
25:140
9:36
AGEQVTYTCATYYKpc3_NP.4
p−min=0.49, pX=0.99, N=68
var.14.CFH 1:196659237:C:T
peptide level
14:149
6:140
2:36
AGEQVTYTCATYYKpc3_NP.5
p−min=0.12, pX=0.19, N=22
var.14.CFH 1:196659237:C:T
peptide level
67:149
66:140
16:36
AQTTVTCMENGWSPTPRpc2_NP.1
p−min=0.82, pX=0.15, N=149
var.14.CFH 1:196659237:C:T
peptide level
9:149
12:140
2:36
AQTTVTCMENGWSPTPRpc2_NP.2
p−min=0.52, pX=0.73, N=23
var.14.CFH 1:196659237:C:T
peptide level
103:149
97:140
25:36
AQTTVTCMENGWSPTPRpc2_NP.3
p−maj=1, pX=0.58, N=225
var.14.CFH 1:196659237:C:T
peptide level
137:149
129:140
35:36
AQTTVTCMENGWSPTPRpc2_NP.4
p−maj=0.49, pX=0.99, N=301
var.14.CFH 1:196659237:C:T
peptide level
125:149
112:140
33:36
AQTTVTCMENGWSPTPRpc2_NP.5
p−maj=0.24, pX=0.33, N=270

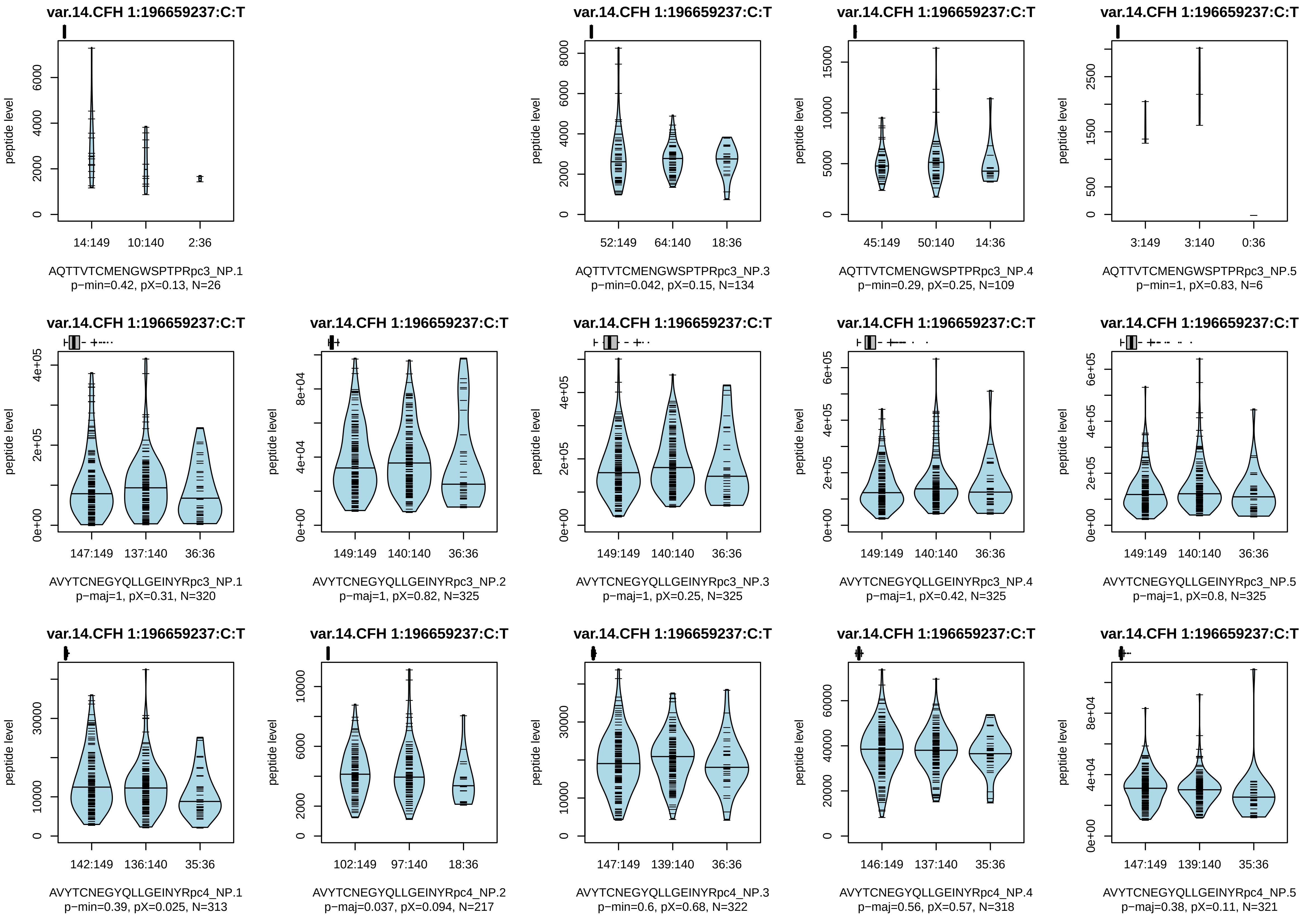
var.14.CFH 1:196659237:C:T
peptide level
14:149 10:140 2:36
AQTTVTCMENGWSPTPRpc3_NP.1
p−min=0.42, pX=0.13, N=26
var.14.CFH 1:196659237:C:T
peptide level
52:149 64:140 18:36
AQTTVTCMENGWSPTPRpc3_NP.3
p−min=0.042, pX=0.15, N=134
var.14.CFH 1:196659237:C:T
peptide level
45:149 50:140 14:36
AQTTVTCMENGWSPTPRpc3_NP.4
p−min=0.29, pX=0.25, N=109
var.14.CFH 1:196659237:C:T
peptide level
3:149 3:140 0:36
AQTTVTCMENGWSPTPRpc3_NP.5
p−min=1, pX=0.83, N=6
var.14.CFH 1:196659237:C:T
peptide level
147:149 137:140 36:36
AVYTCNEGYQLLGEINYRpc3_NP.1
p−maj=1, pX=0.31, N=320
var.14.CFH 1:196659237:C:T
peptide level
149:149 140:140 36:36
AVYTCNEGYQLLGEINYRpc3_NP.2
p−maj=1, pX=0.82, N=325
var.14.CFH 1:196659237:C:T
peptide level
149:149 140:140 36:36
AVYTCNEGYQLLGEINYRpc3_NP.3
p−maj=1, pX=0.25, N=325
var.14.CFH 1:196659237:C:T
peptide level
149:149 140:140 36:36
AVYTCNEGYQLLGEINYRpc3_NP.4
p−maj=1, pX=0.42, N=325
var.14.CFH 1:196659237:C:T
peptide level
149:149 140:140 36:36
AVYTCNEGYQLLGEINYRpc3_NP.5
p−maj=1, pX=0.8, N=325
var.14.CFH 1:196659237:C:T
peptide level
142:149 136:140 35:36
AVYTCNEGYQLLGEINYRpc4_NP.1
p−min=0.39, pX=0.025, N=313
var.14.CFH 1:196659237:C:T
peptide level
102:149 97:140 18:36
AVYTCNEGYQLLGEINYRpc4_NP.2
p−maj=0.037, pX=0.094, N=217
var.14.CFH 1:196659237:C:T
peptide level
147:149 139:140 36:36
AVYTCNEGYQLLGEINYRpc4_NP.3
p−min=0.6, pX=0.68, N=322
var.14.CFH 1:196659237:C:T
peptide level
146:149 137:140 35:36
AVYTCNEGYQLLGEINYRpc4_NP.4
p−maj=0.56, pX=0.57, N=318
var.14.CFH 1:196659237:C:T
peptide level
147:149 139:140 35:36
AVYTCNEGYQLLGEINYRpc4_NP.5
p−maj=0.38, pX=0.11, N=321

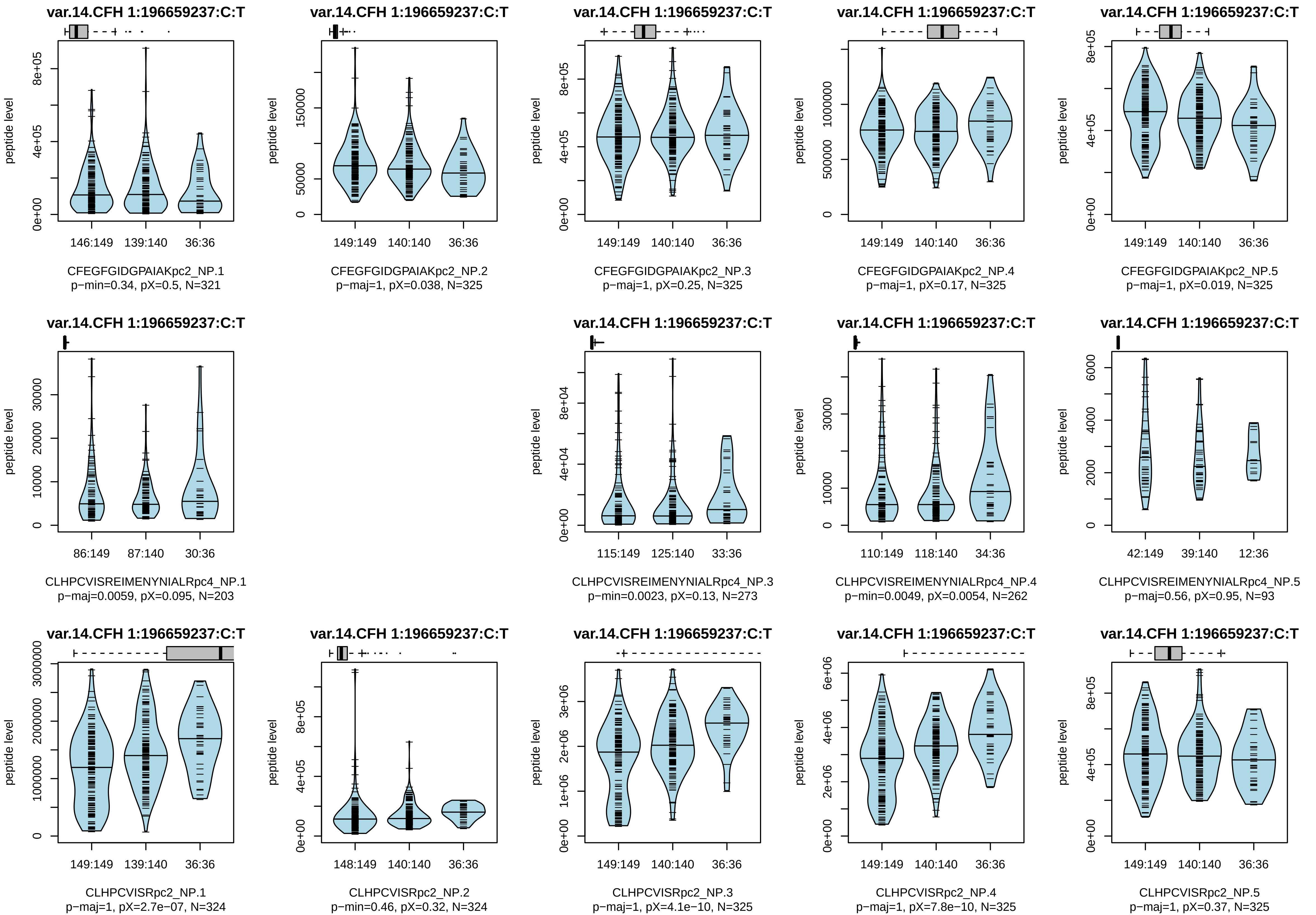
var.14.CFH 1:196659237:C:T
peptide level
146:149
139:140
36:36
CFEGFGIDGPAIAKpc2_NP.1
p−min=0.34, pX=0.5, N=321
var.14.CFH 1:196659237:C:T
149:149
140:140
36:36
CFEGFGIDGPAIAKpc2_NP.2
p−maj=1, pX=0.038, N=325
var.14.CFH 1:196659237:C:T
CFEGFGIDGPAIAKpc2_NP.3
p−maj=1, pX=0.25, N=325
var.14.CFH 1:196659237:C:T
CFEGFGIDGPAIAKpc2_NP.4
p−maj=1, pX=0.17, N=325
var.14.CFH 1:196659237:C:T
CFEGFGIDGPAIAKpc2_NP.5
p−maj=1, pX=0.019, N=325
var.14.CFH 1:196659237:C:T
86:149
87:140
30:36
CLHPCVISREIMENYNIALRpc4_NP.1
p−maj=0.0059, pX=0.095, N=203
var.14.CFH 1:196659237:C:T
115:149
125:140
33:36
CLHPCVISREIMENYNIALRpc4_NP.3
p−min=0.0023, pX=0.13, N=273
var.14.CFH 1:196659237:C:T
110:149
118:140
34:36
CLHPCVISREIMENYNIALRpc4_NP.4
p−min=0.0049, pX=0.0054, N=262
var.14.CFH 1:196659237:C:T
42:149
39:140
12:36
CLHPCVISREIMENYNIALRpc4_NP.5
p−maj=0.56, pX=0.95, N=93
var.14.CFH 1:196659237:C:T
149:149
139:140
36:36
CLHPCVISRpc2_NP.1
p−maj=1, pX=2.7e−07, N=324
var.14.CFH 1:196659237:C:T
148:149
140:140
36:36
CLHPCVISRpc2_NP.2
p−min=0.46, pX=0.32, N=324
var.14.CFH 1:196659237:C:T
CLHPCVISRpc2_NP.3
p−maj=1, pX=4.1e−10, N=325
var.14.CFH 1:196659237:C:T
CLHPCVISRpc2_NP.4
p−maj=1, pX=7.8e−10, N=325
var.14.CFH 1:196659237:C:T
CLHPCVISRpc2_NP.5
p−maj=1, pX=0.37, N=325

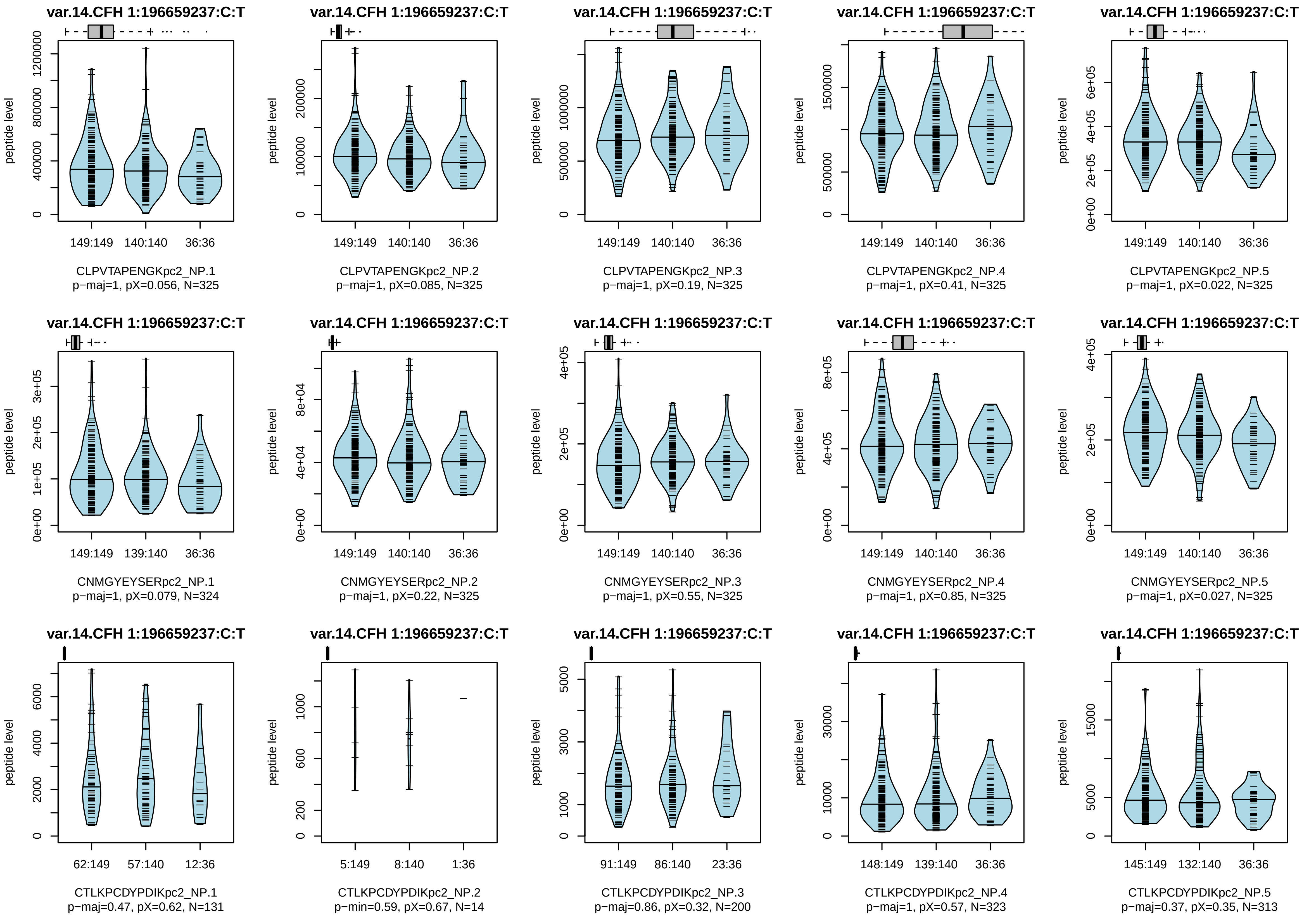
var.14.CFH 1:196659237:C:T
peptide level
149:149 140:140 36:36
CLPVTAPENGKpc2_NP.1
p−maj=1, pX=0.056, N=325
var.14.CFH 1:196659237:C:T
peptide level
149:149 140:140 36:36
CLPVTAPENGKpc2_NP.2
p−maj=1, pX=0.085, N=325
var.14.CFH 1:196659237:C:T
peptide level
149:149 140:140 36:36
CLPVTAPENGKpc2_NP.3
p−maj=1, pX=0.19, N=325
var.14.CFH 1:196659237:C:T
peptide level
149:149 140:140 36:36
CLPVTAPENGKpc2_NP.4
p−maj=1, pX=0.41, N=325
var.14.CFH 1:196659237:C:T
peptide level
149:149 140:140 36:36
CLPVTAPENGKpc2_NP.5
p−maj=1, pX=0.022, N=325
var.14.CFH 1:196659237:C:T
peptide level
149:149 139:140 36:36
CNMGYEYSERpc2_NP.1
p−maj=1, pX=0.079, N=324
var.14.CFH 1:196659237:C:T
peptide level
149:149 140:140 36:36
CNMGYEYSERpc2_NP.2
p−maj=1, pX=0.22, N=325
var.14.CFH 1:196659237:C:T
peptide level
149:149 140:140 36:36
CNMGYEYSERpc2_NP.3
p−maj=1, pX=0.55, N=325
var.14.CFH 1:196659237:C:T
peptide level
149:149 140:140 36:36
CNMGYEYSERpc2_NP.4
p−maj=1, pX=0.85, N=325
var.14.CFH 1:196659237:C:T
peptide level
149:149 140:140 36:36
CNMGYEYSERpc2_NP.5
p−maj=1, pX=0.027, N=325
var.14.CFH 1:196659237:C:T
peptide level
62:149 57:140 12:36
CTLKPCDYPDIKpc2_NP.1
p−maj=0.47, pX=0.62, N=131
var.14.CFH 1:196659237:C:T
peptide level
5:149 8:140 1:36
CTLKPCDYPDIKpc2_NP.2
p−min=0.59, pX=0.67, N=14
var.14.CFH 1:196659237:C:T
peptide level
91:149 86:140 23:36
CTLKPCDYPDIKpc2_NP.3
p−maj=0.86, pX=0.32, N=200
var.14.CFH 1:196659237:C:T
peptide level
148:149 139:140 36:36
CTLKPCDYPDIKpc2_NP.4
p−maj=1, pX=0.57, N=323
var.14.CFH 1:196659237:C:T
peptide level
145:149 132:140 36:36
CTLKPCDYPDIKpc2_NP.5
p−maj=0.37, pX=0.35, N=313

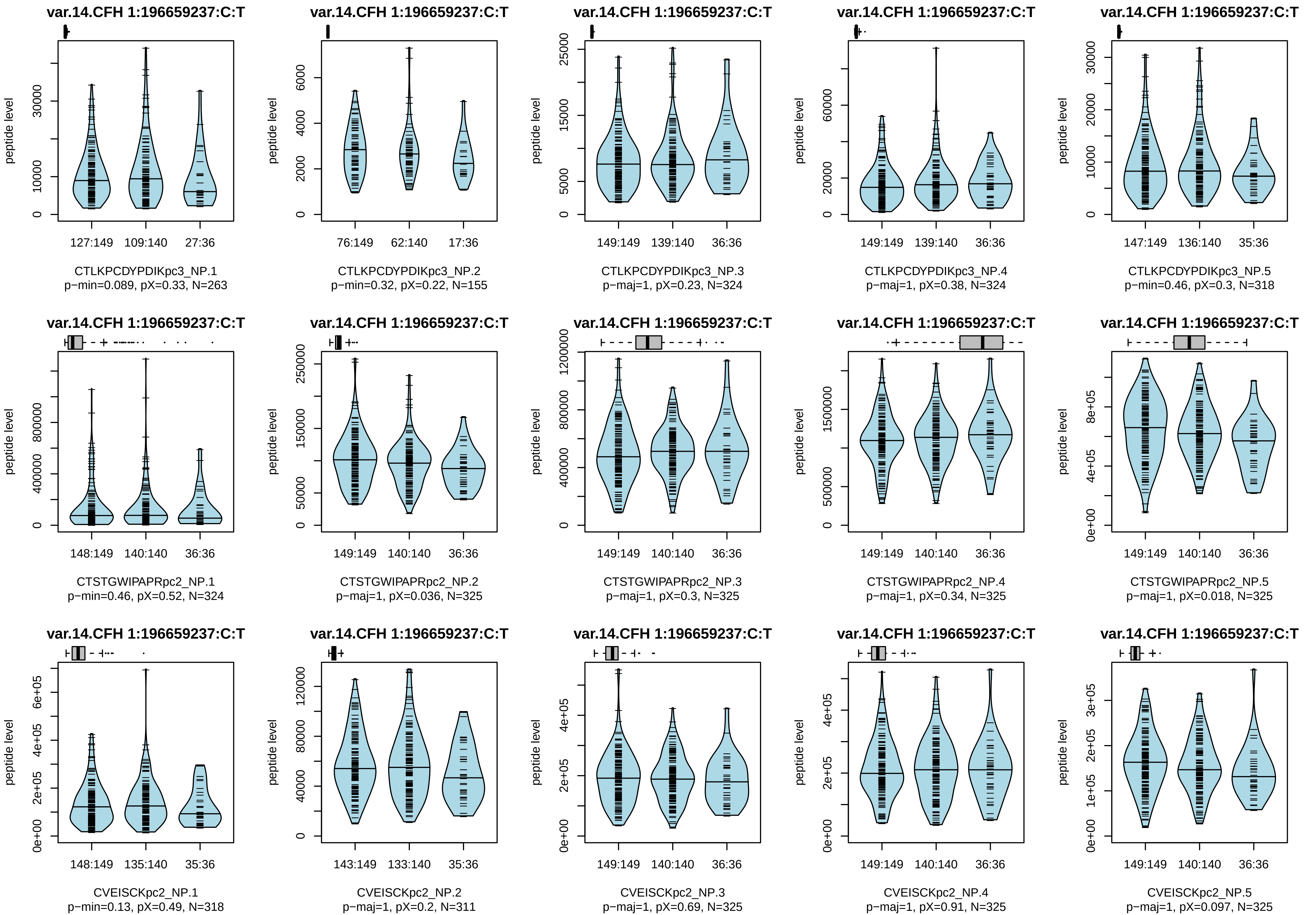
var.14.CFH 1:196659237:C:T
peptide level
127:149
109:140
27:36
CTLKPCDYPDIKpc3_NP.1
p−min=0.089, pX=0.33, N=263
var.14.CFH 1:196659237:C:T
peptide level
76:149
62:140
17:36
CTLKPCDYPDIKpc3_NP.2
p−min=0.32, pX=0.22, N=155
var.14.CFH 1:196659237:C:T
peptide level
149:149
139:140
36:36
CTLKPCDYPDIKpc3_NP.3
p−maj=1, pX=0.23, N=324
var.14.CFH 1:196659237:C:T
peptide level
149:149
139:140
36:36
CTLKPCDYPDIKpc3_NP.4
p−maj=1, pX=0.38, N=324
var.14.CFH 1:196659237:C:T
peptide level
147:149
136:140
35:36
CTLKPCDYPDIKpc3_NP.5
p−min=0.46, pX=0.3, N=318
var.14.CFH 1:196659237:C:T
peptide level
148:149
140:140
36:36
CTSTGWIPAPRpc2_NP.1
p−min=0.46, pX=0.52, N=324
var.14.CFH 1:196659237:C:T
peptide level
149:149
140:140
36:36
CTSTGWIPAPRpc2_NP.2
p−maj=1, pX=0.036, N=325
var.14.CFH 1:196659237:C:T
peptide level
149:149
140:140
36:36
CTSTGWIPAPRpc2_NP.3
p−maj=1, pX=0.3, N=325
var.14.CFH 1:196659237:C:T
peptide level
149:149
140:140
36:36
CTSTGWIPAPRpc2_NP.4
p−maj=1, pX=0.34, N=325
var.14.CFH 1:196659237:C:T
peptide level
149:149
140:140
36:36
CTSTGWIPAPRpc2_NP.5
p−maj=1, pX=0.018, N=325
var.14.CFH 1:196659237:C:T
peptide level
148:149
135:140
35:36
CVEISCKpc2_NP.1
p−min=0.13, pX=0.49, N=318
var.14.CFH 1:196659237:C:T
peptide level
143:149
133:140
35:36
CVEISCKpc2_NP.2
p−maj=1, pX=0.2, N=311
var.14.CFH 1:196659237:C:T
peptide level
149:149
140:140
36:36
CVEISCKpc2_NP.3
p−maj=1, pX=0.69, N=325
var.14.CFH 1:196659237:C:T
peptide level
149:149
140:140
36:36
CVEISCKpc2_NP.4
p−maj=1, pX=0.91, N=325
var.14.CFH 1:196659237:C:T
peptide level
149:149
140:140
36:36
CVEISCKpc2_NP.5
p−maj=1, pX=0.097, N=325

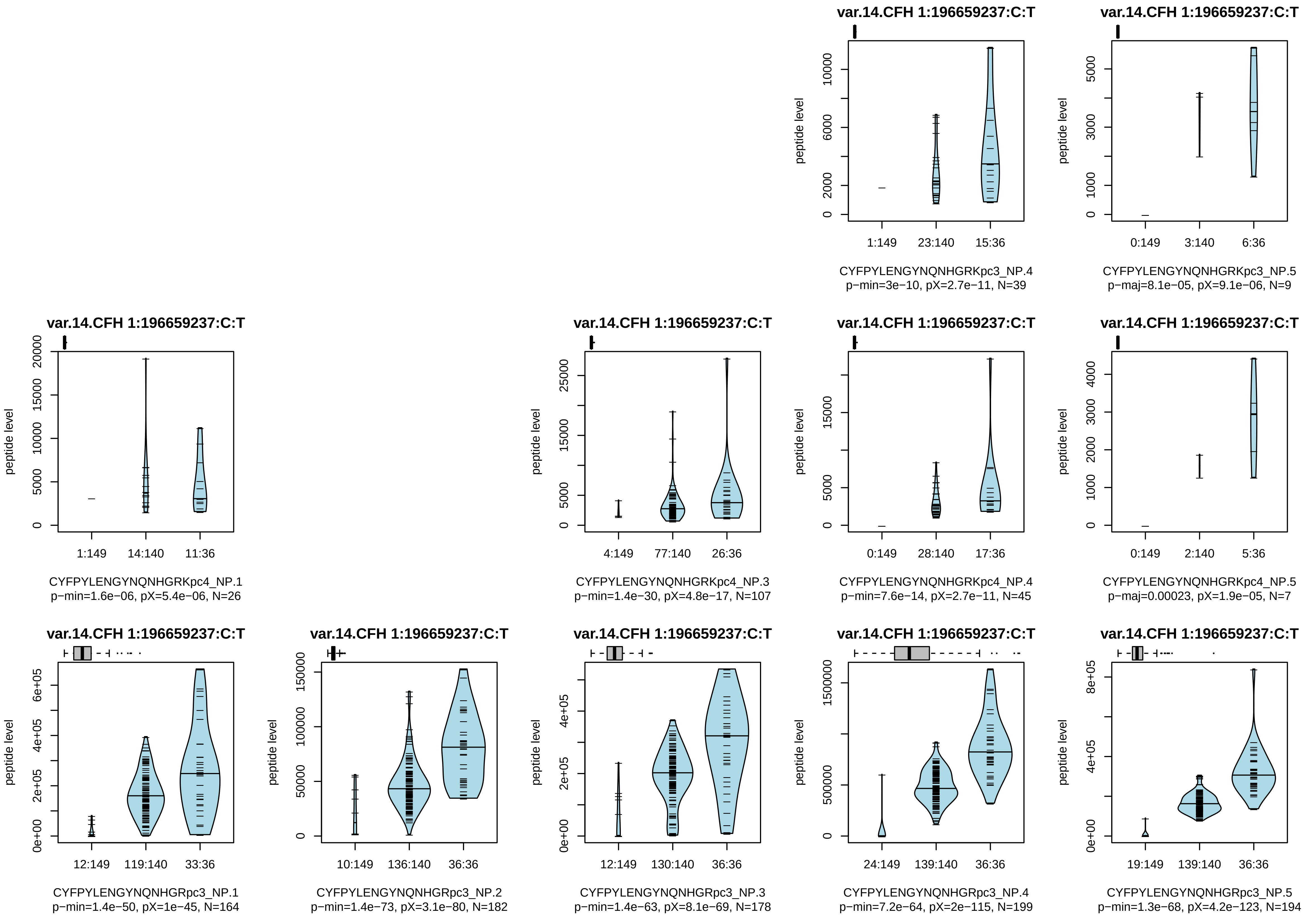
var.14.CFH 1:196659237:C:T
peptide level
1:149
23:140
15:36
CYFPYLENGYNQNHGRKpc3_NP.4
p−min=3e−10, pX=2.7e−11, N=39
var.14.CFH 1:196659237:C:T
peptide level
0:149
3:140
6:36
CYFPYLENGYNQNHGRKpc3_NP.5
p−maj=8.1e−05, pX=9.1e−06, N=9
var.14.CFH 1:196659237:C:T
peptide level
1:149
14:140
11:36
CYFPYLENGYNQNHGRKpc4_NP.1
p−min=1.6e−06, pX=5.4e−06, N=26
var.14.CFH 1:196659237:C:T
peptide level
4:149
77:140
26:36
CYFPYLENGYNQNHGRKpc4_NP.3
p−min=1.4e−30, pX=4.8e−17, N=107
var.14.CFH 1:196659237:C:T
peptide level
0:149
28:140
17:36
CYFPYLENGYNQNHGRKpc4_NP.4
p−min=7.6e−14, pX=2.7e−11, N=45
var.14.CFH 1:196659237:C:T
peptide level
0:149
2:140
5:36
CYFPYLENGYNQNHGRKpc4_NP.5
p−maj=0.00023, pX=1.9e−05, N=7
var.14.CFH 1:196659237:C:T
peptide level
12:149
119:140
33:36
CYFPYLENGYNQNHGRpc3_NP.1
p−min=1.4e−50, pX=1e−45, N=164
var.14.CFH 1:196659237:C:T
peptide level
10:149
136:140
36:36
CYFPYLENGYNQNHGRpc3_NP.2
p−min=1.4e−73, pX=3.1e−80, N=182
var.14.CFH 1:196659237:C:T
peptide level
12:149
130:140
36:36
CYFPYLENGYNQNHGRpc3_NP.3
p−min=1.4e−63, pX=8.1e−69, N=178
var.14.CFH 1:196659237:C:T
peptide level
24:149
139:140
36:36
CYFPYLENGYNQNHGRpc3_NP.4
p−min=7.2e−64, pX=2e−115, N=199
var.14.CFH 1:196659237:C:T
peptide level
19:149
139:140
36:36
CYFPYLENGYNQNHGRpc3_NP.5
p−min=1.3e−68, pX=4.2e−123, N=194

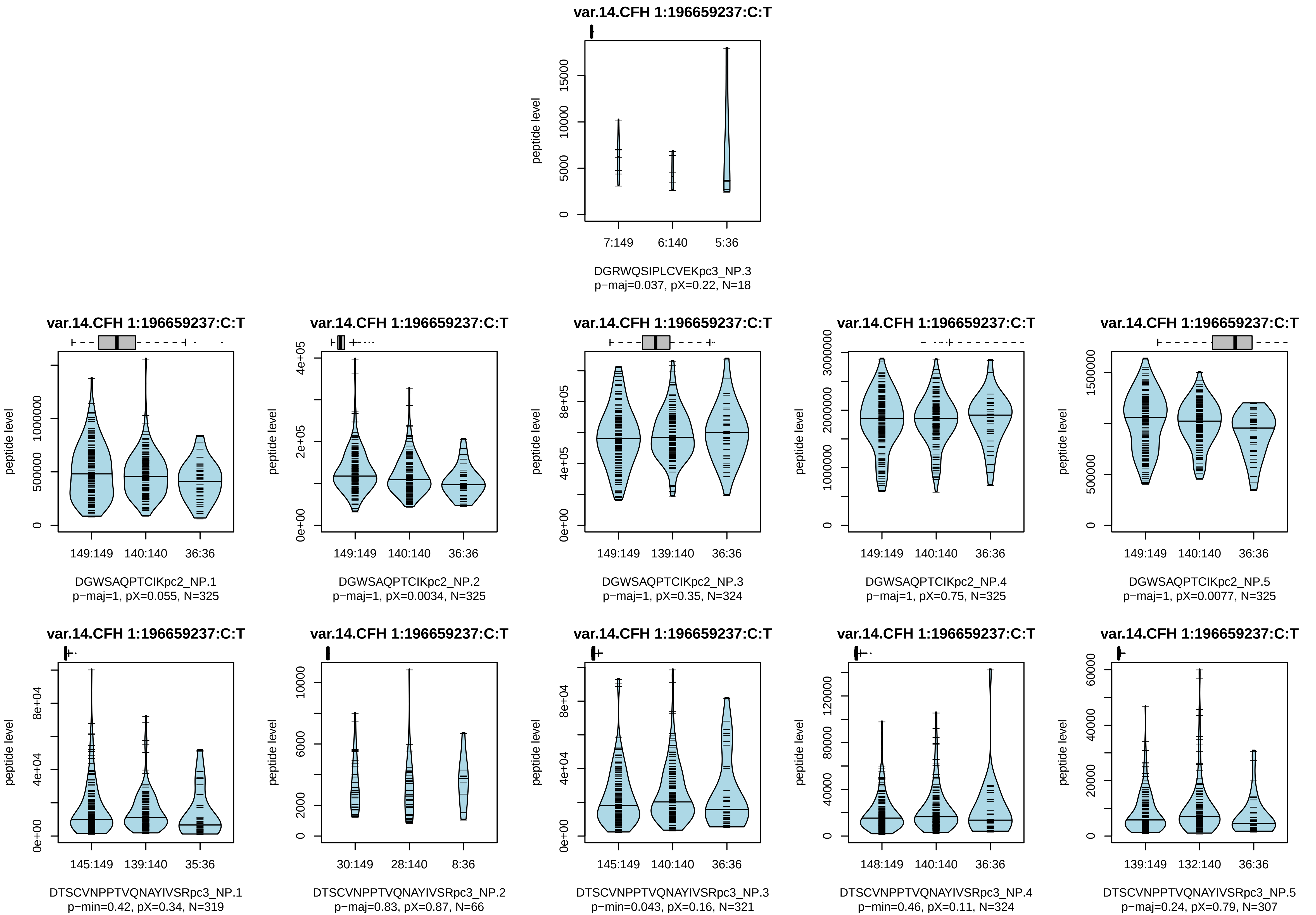
var.14.CFH 1:196659237:C:T
peptide level
0
5000
10000
15000
7:149
6:140
5:36
DGRWQSIPLCVEKpc3_NP.3
p−maj=0.037, pX=0.22, N=18
var.14.CFH 1:196659237:C:T
peptide level
0
500000
1000000
149:149
140:140
36:36
DGWSAQPTCIKpc2_NP.1
p−maj=1, pX=0.055, N=325
var.14.CFH 1:196659237:C:T
peptide level
0e+00
2e+05
4e+05
149:149
140:140
36:36
DGWSAQPTCIKpc2_NP.2
p−maj=1, pX=0.0034, N=325
var.14.CFH 1:196659237:C:T
peptide level
0e+00
4e+05
8e+05
149:149
139:140
36:36
DGWSAQPTCIKpc2_NP.3
p−maj=1, pX=0.35, N=324
var.14.CFH 1:196659237:C:T
peptide level
0
1000000
2000000
3000000
149:149
140:140
36:36
DGWSAQPTCIKpc2_NP.4
p−maj=1, pX=0.75, N=325
var.14.CFH 1:196659237:C:T
peptide level
0
500000
1500000
149:149
140:140
36:36
DGWSAQPTCIKpc2_NP.5
p−maj=1, pX=0.0077, N=325
var.14.CFH 1:196659237:C:T
peptide level
0e+00
4e+04
8e+04
145:149
139:140
35:36
DTSCVNPPTVQNAYIVSRpc3_NP.1
p−min=0.42, pX=0.34, N=319
var.14.CFH 1:196659237:C:T
peptide level
0
2000
6000
10000
30:149
28:140
8:36
DTSCVNPPTVQNAYIVSRpc3_NP.2
p−maj=0.83, pX=0.87, N=66
var.14.CFH 1:196659237:C:T
peptide level
0e+00
4e+04
8e+04
145:149
140:140
36:36
DTSCVNPPTVQNAYIVSRpc3_NP.3
p−min=0.043, pX=0.16, N=321
var.14.CFH 1:196659237:C:T
peptide level
0
40000
80000
120000
148:149
140:140
36:36
DTSCVNPPTVQNAYIVSRpc3_NP.4
p−min=0.46, pX=0.11, N=324
var.14.CFH 1:196659237:C:T
peptide level
0
20000
40000
60000
139:149
132:140
36:36
DTSCVNPPTVQNAYIVSRpc3_NP.5
p−maj=0.24, pX=0.79, N=307

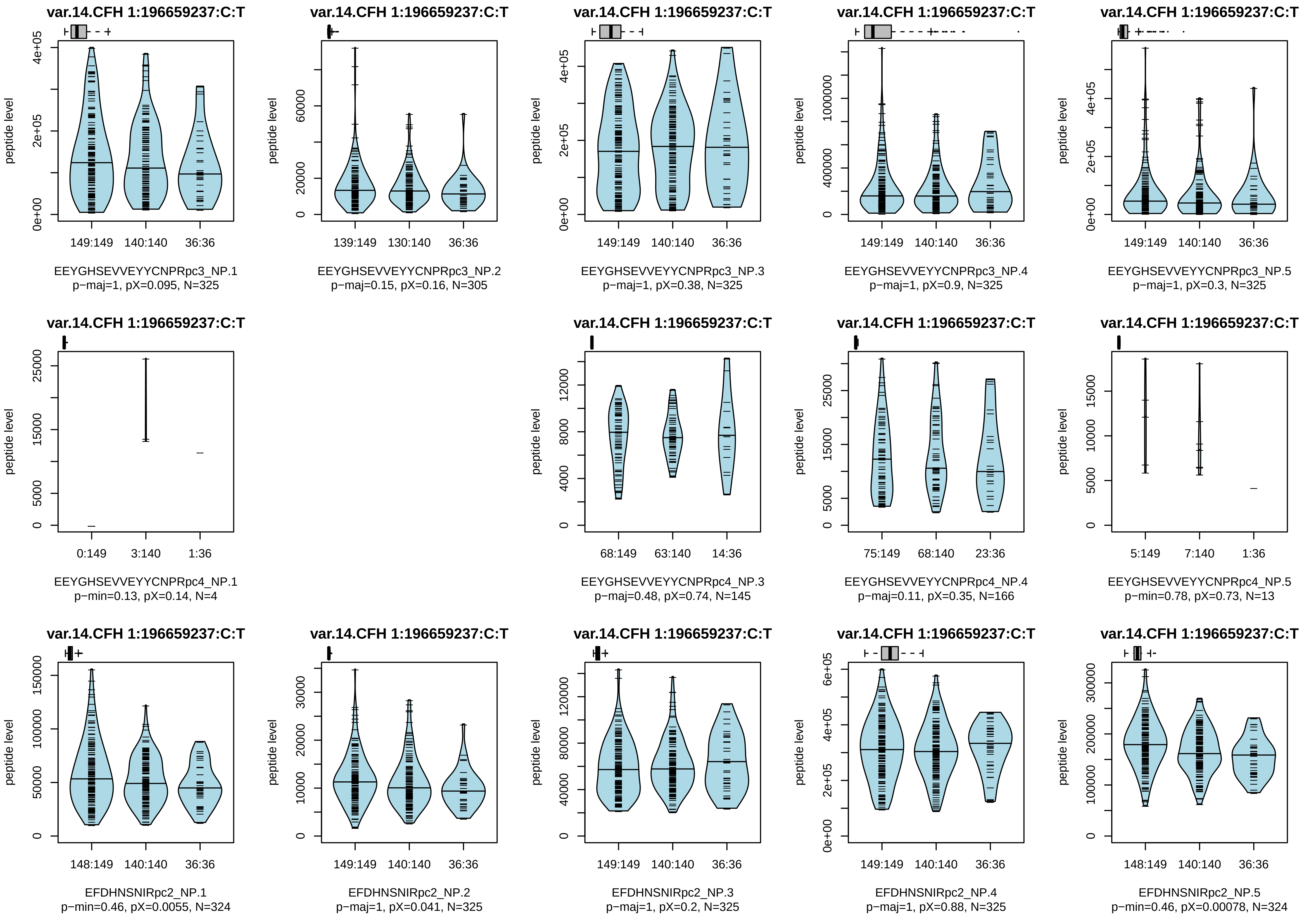
var.14.CFH 1:196659237:C:T
peptide level
149:149
140:140
36:36
EEYGHSEVVEYYCNPRpc3_NP.1
p−maj=1, pX=0.095, N=325
var.14.CFH 1:196659237:C:T
peptide level
139:149
130:140
36:36
EEYGHSEVVEYYCNPRpc3_NP.2
p−maj=0.15, pX=0.16, N=305
var.14.CFH 1:196659237:C:T
peptide level
149:149
140:140
36:36
EEYGHSEVVEYYCNPRpc3_NP.3
p−maj=1, pX=0.38, N=325
var.14.CFH 1:196659237:C:T
peptide level
149:149
140:140
36:36
EEYGHSEVVEYYCNPRpc3_NP.4
p−maj=1, pX=0.9, N=325
var.14.CFH 1:196659237:C:T
peptide level
149:149
140:140
36:36
EEYGHSEVVEYYCNPRpc3_NP.5
p−maj=1, pX=0.3, N=325
var.14.CFH 1:196659237:C:T
peptide level
0:149
3:140
1:36
EEYGHSEVVEYYCNPRpc4_NP.1
p−min=0.13, pX=0.14, N=4
var.14.CFH 1:196659237:C:T
peptide level
68:149
63:140
14:36
EEYGHSEVVEYYCNPRpc4_NP.3
p−maj=0.48, pX=0.74, N=145
var.14.CFH 1:196659237:C:T
peptide level
75:149
68:140
23:36
EEYGHSEVVEYYCNPRpc4_NP.4
p−maj=0.11, pX=0.35, N=166
var.14.CFH 1:196659237:C:T
peptide level
5:149
7:140
1:36
EEYGHSEVVEYYCNPRpc4_NP.5
p−min=0.78, pX=0.73, N=13
var.14.CFH 1:196659237:C:T
peptide level
148:149
140:140
36:36
EFDHNSNIRpc2_NP.1
p−min=0.46, pX=0.0055, N=324
var.14.CFH 1:196659237:C:T
peptide level
149:149
140:140
36:36
EFDHNSNIRpc2_NP.2
p−maj=1, pX=0.041, N=325
var.14.CFH 1:196659237:C:T
peptide level
149:149
140:140
36:36
EFDHNSNIRpc2_NP.3
p−maj=1, pX=0.2, N=325
var.14.CFH 1:196659237:C:T
peptide level
149:149
140:140
36:36
EFDHNSNIRpc2_NP.4
p−maj=1, pX=0.88, N=325
var.14.CFH 1:196659237:C:T
peptide level
148:149
140:140
36:36
EFDHNSNIRpc2_NP.5
p−min=0.46, pX=0.00078, N=324

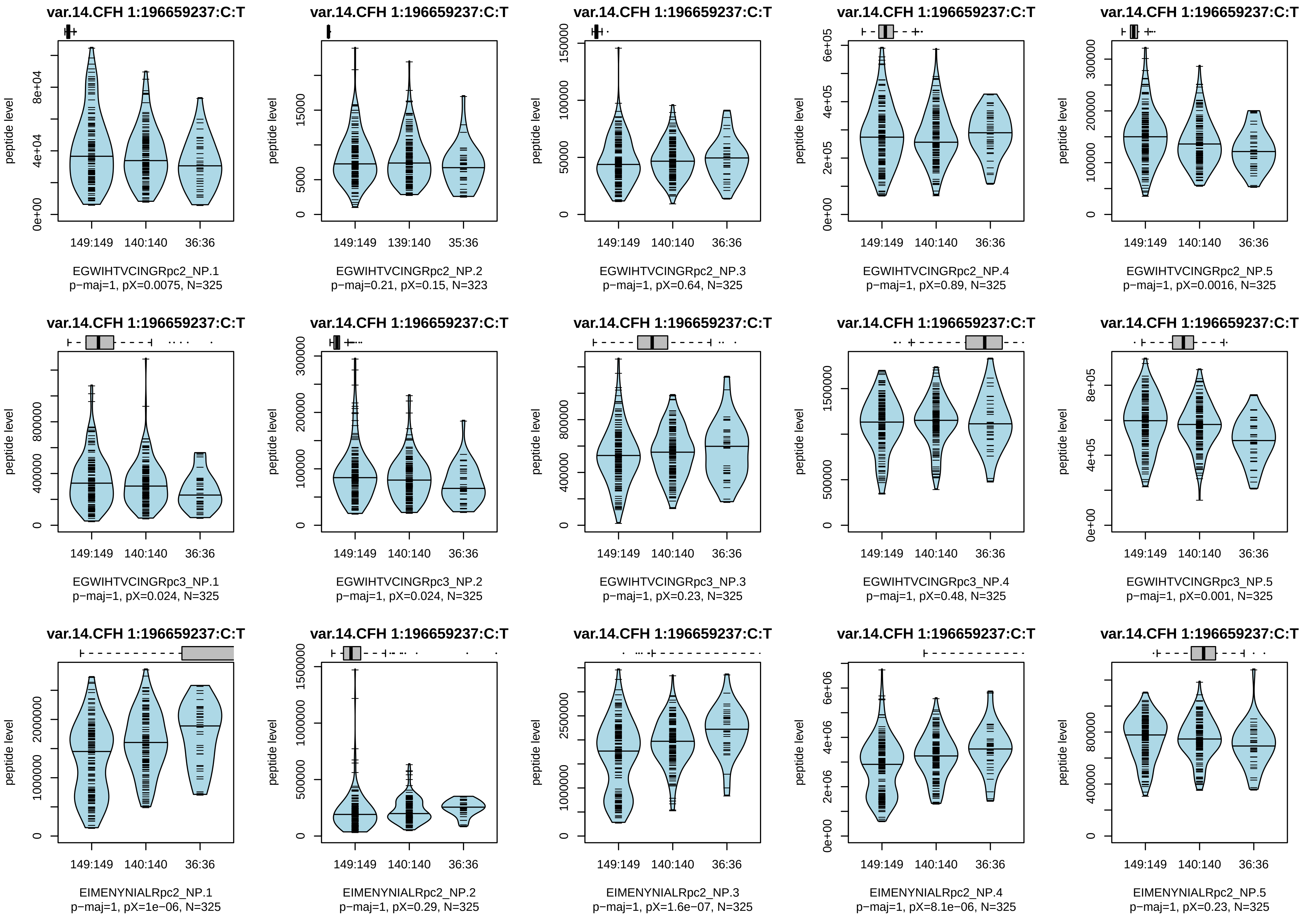
var.14.CFH 1:196659237:C:T
149:149
140:140
36:36
EGWIHTVCINGRpc2_NP.1
p−maj=1, pX=0.0075, N=325
var.14.CFH 1:196659237:C:T
149:149
139:140
35:36
EGWIHTVCINGRpc2_NP.2
p−maj=0.21, pX=0.15, N=323
var.14.CFH 1:196659237:C:T
149:149
140:140
36:36
EGWIHTVCINGRpc2_NP.3
p−maj=1, pX=0.64, N=325
var.14.CFH 1:196659237:C:T
149:149
140:140
36:36
EGWIHTVCINGRpc2_NP.4
p−maj=1, pX=0.89, N=325
var.14.CFH 1:196659237:C:T
149:149
140:140
36:36
EGWIHTVCINGRpc2_NP.5
p−maj=1, pX=0.0016, N=325
var.14.CFH 1:196659237:C:T
149:149
140:140
36:36
EGWIHTVCINGRpc3_NP.1
p−maj=1, pX=0.024, N=325
var.14.CFH 1:196659237:C:T
149:149
140:140
36:36
EGWIHTVCINGRpc3_NP.2
p−maj=1, pX=0.024, N=325
var.14.CFH 1:196659237:C:T
149:149
140:140
36:36
EGWIHTVCINGRpc3_NP.3
p−maj=1, pX=0.23, N=325
var.14.CFH 1:196659237:C:T
149:149
140:140
36:36
EGWIHTVCINGRpc3_NP.4
p−maj=1, pX=0.48, N=325
var.14.CFH 1:196659237:C:T
149:149
140:140
36:36
EGWIHTVCINGRpc3_NP.5
p−maj=1, pX=0.001, N=325
var.14.CFH 1:196659237:C:T
149:149
140:140
36:36
EIMENYNIALRpc2_NP.1
p−maj=1, pX=1e−06, N=325
var.14.CFH 1:196659237:C:T
149:149
140:140
36:36
EIMENYNIALRpc2_NP.2
p−maj=1, pX=0.29, N=325
var.14.CFH 1:196659237:C:T
149:149
140:140
36:36
EIMENYNIALRpc2_NP.3
p−maj=1, pX=1.6e−07, N=325
var.14.CFH 1:196659237:C:T
149:149
140:140
36:36
EIMENYNIALRpc2_NP.4
p−maj=1, pX=8.1e−06, N=325
var.14.CFH 1:196659237:C:T
149:149
140:140
36:36
EIMENYNIALRpc2_NP.5
p−maj=1, pX=0.23, N=325
peptide level

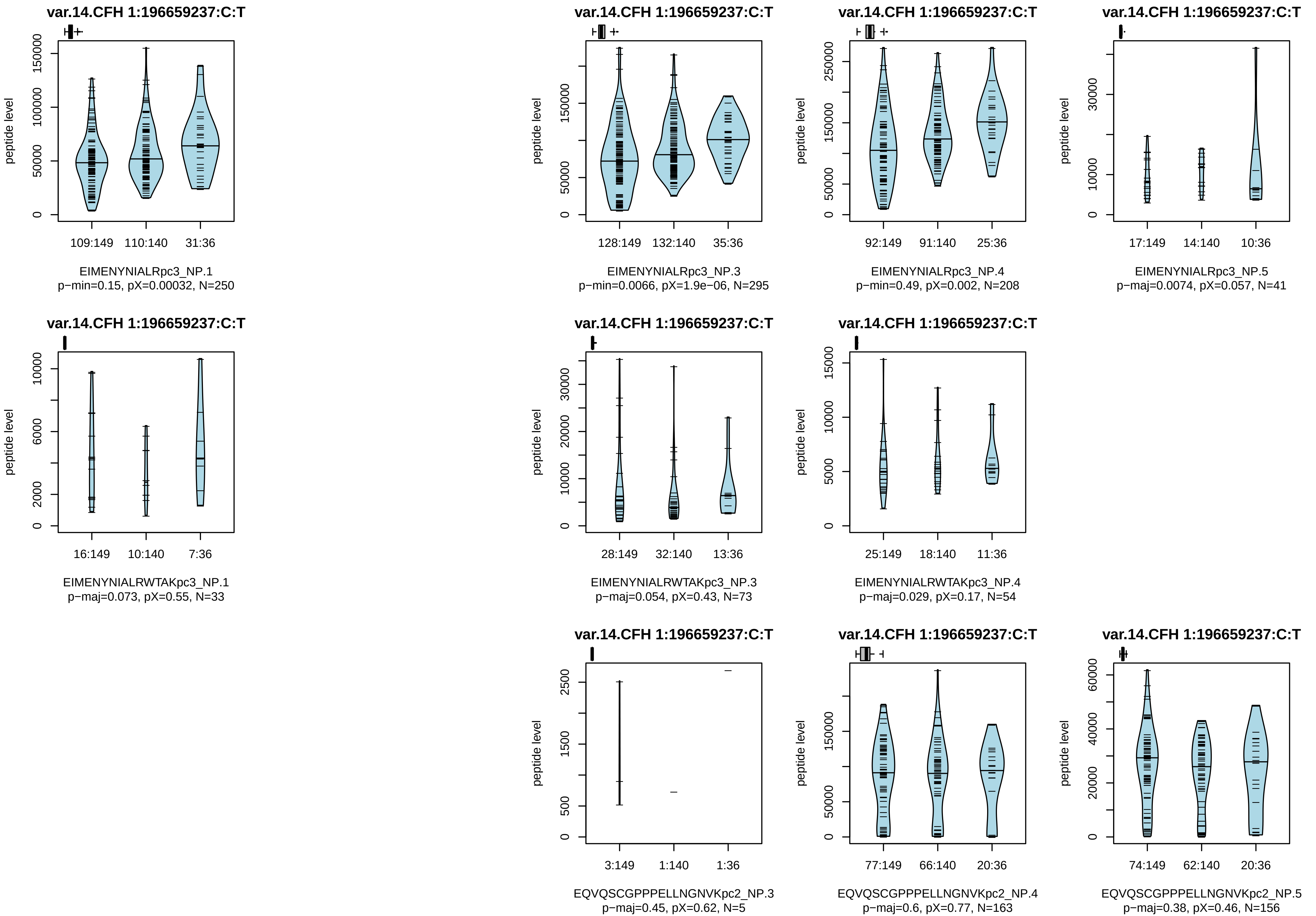
var.14.CFH 1:196659237:C:T
peptide level
109:149
110:140
31:36
EIMENYNIALRpc3_NP.1
p−min=0.15, pX=0.00032, N=250
var.14.CFH 1:196659237:C:T
128:149
132:140
35:36
EIMENYNIALRpc3_NP.3
p−min=0.0066, pX=1.9e−06, N=295
var.14.CFH 1:196659237:C:T
92:149
91:140
25:36
EIMENYNIALRpc3_NP.4
p−min=0.49, pX=0.002, N=208
var.14.CFH 1:196659237:C:T
17:149
14:140
10:36
EIMENYNIALRpc3_NP.5
p−maj=0.0074, pX=0.057, N=41
var.14.CFH 1:196659237:C:T
16:149
10:140
7:36
EIMENYNIALRWTAKpc3_NP.1
p−maj=0.073, pX=0.55, N=33
var.14.CFH 1:196659237:C:T
28:149
32:140
13:36
EIMENYNIALRWTAKpc3_NP.3
p−maj=0.054, pX=0.43, N=73
var.14.CFH 1:196659237:C:T
25:149
18:140
11:36
EIMENYNIALRWTAKpc3_NP.4
p−maj=0.029, pX=0.17, N=54
var.14.CFH 1:196659237:C:T
3:149
1:140
1:36
EQVQSCGPPPELLNGNVKpc2_NP.3
p−maj=0.45, pX=0.62, N=5
var.14.CFH 1:196659237:C:T
77:149
66:140
20:36
EQVQSCGPPPELLNGNVKpc2_NP.4
p−maj=0.6, pX=0.77, N=163
var.14.CFH 1:196659237:C:T
74:149
62:140
20:36
EQVQSCGPPPELLNGNVKpc2_NP.5
p−maj=0.38, pX=0.46, N=156

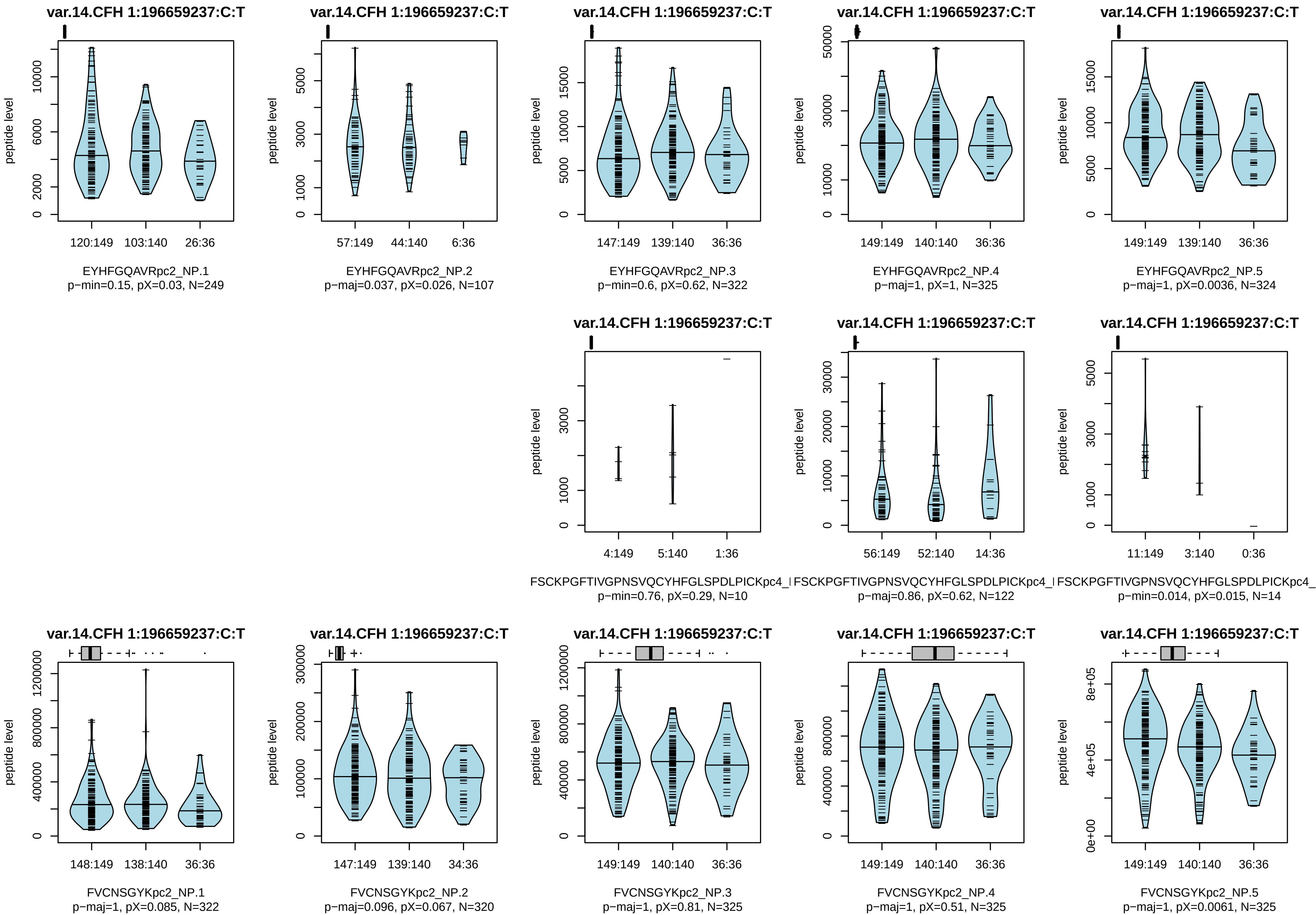

var.14.CFH 1:196659237:C:T
peptide level
0
2000
6000
10000
120:149
103:140
26:36
EYHFGQAVRpc2_NP.1
p−min=0.15, pX=0.03, N=249
var.14.CFH 1:196659237:C:T
peptide level
0
1000
3000
5000
57:149
44:140
6:36
EYHFGQAVRpc2_NP.2
p−maj=0.037, pX=0.026, N=107
var.14.CFH 1:196659237:C:T
peptide level
0
5000
10000
15000
147:149
139:140
36:36
EYHFGQAVRpc2_NP.3
p−min=0.6, pX=0.62, N=322
var.14.CFH 1:196659237:C:T
peptide level
0
10000
30000
50000
149:149
140:140
36:36
EYHFGQAVRpc2_NP.4
p−maj=1, pX=1, N=325
var.14.CFH 1:196659237:C:T
peptide level
0
5000
10000
15000
149:149
139:140
36:36
EYHFGQAVRpc2_NP.5
p−maj=1, pX=0.0036, N=324
var.14.CFH 1:196659237:C:T
peptide level
0
1000
3000
4:149
5:140
1:36
FSCKPGFTIVGPNSVQCYHFGLSPDLPICKpc4_I
p−min=0.76, pX=0.29, N=10
var.14.CFH 1:196659237:C:T
peptide level
0
10000
20000
30000
56:149
52:140
14:36
FSCKPGFTIVGPNSVQCYHFGLSPDLPICKpc4_I
p−maj=0.86, pX=0.62, N=122
var.14.CFH 1:196659237:C:T
peptide level
0
1000
3000
5000
11:149
3:140
0:36
FSCKPGFTIVGPNSVQCYHFGLSPDLPICKpc4_
p−min=0.014, pX=0.015, N=14
var.14.CFH 1:196659237:C:T
peptide level
0
400000
800000
1200000
148:149
138:140
36:36
FVCNSGYKpc2_NP.1
p−maj=1, pX=0.085, N=322
var.14.CFH 1:196659237:C:T
peptide level
0
100000
200000
300000
147:149
139:140
34:36
FVCNSGYKpc2_NP.2
p−maj=0.096, pX=0.067, N=320
var.14.CFH 1:196659237:C:T
peptide level
0
400000
800000
1200000
149:149
140:140
36:36
FVCNSGYKpc2_NP.3
p−maj=1, pX=0.81, N=325
var.14.CFH 1:196659237:C:T
peptide level
0
400000
800000
149:149
140:140
36:36
FVCNSGYKpc2_NP.4
p−maj=1, pX=0.51, N=325
var.14.CFH 1:196659237:C:T
peptide level
0e+00
4e+05
8e+05
149:149
140:140
36:36
FVCNSGYKpc2_NP.5
p−maj=1, pX=0.0061, N=325

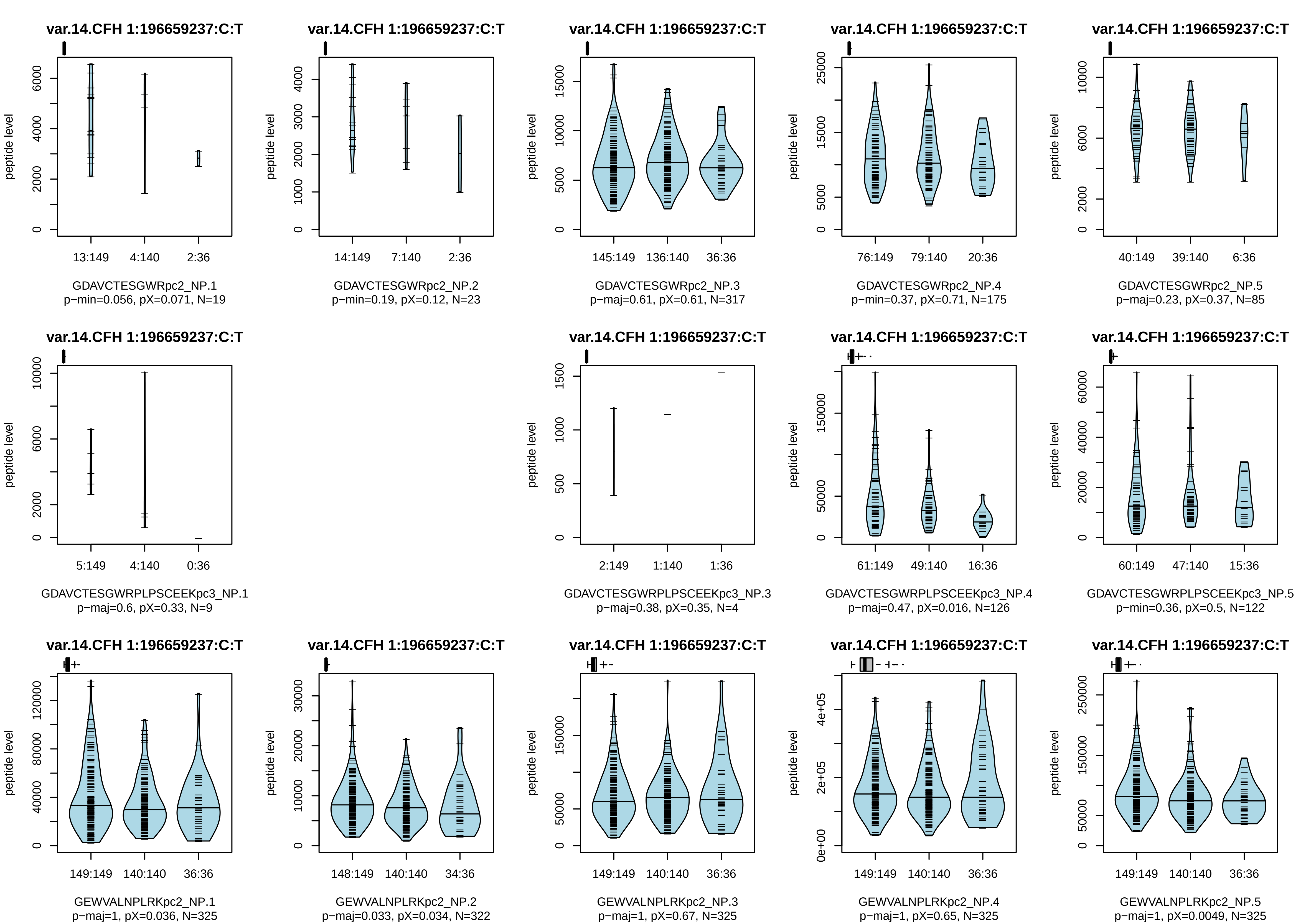
var.14.CFH 1:196659237:C:T
peptide level
13:149
4:140
2:36
GDAVCTESGWRpc2_NP.1
p−min=0.056, pX=0.071, N=19
var.14.CFH 1:196659237:C:T
peptide level
14:149
7:140
2:36
GDAVCTESGWRpc2_NP.2
p−min=0.19, pX=0.12, N=23
var.14.CFH 1:196659237:C:T
peptide level
145:149
136:140
36:36
GDAVCTESGWRpc2_NP.3
p−maj=0.61, pX=0.61, N=317
var.14.CFH 1:196659237:C:T
peptide level
76:149
79:140
20:36
GDAVCTESGWRpc2_NP.4
p−min=0.37, pX=0.71, N=175
var.14.CFH 1:196659237:C:T
peptide level
40:149
39:140
6:36
GDAVCTESGWRpc2_NP.5
p−maj=0.23, pX=0.37, N=85
var.14.CFH 1:196659237:C:T
peptide level
5:149
4:140
0:36
GDAVCTESGWRPLPSCEEKpc3_NP.1
p−maj=0.6, pX=0.33, N=9
var.14.CFH 1:196659237:C:T
peptide level
2:149
1:140
1:36
GDAVCTESGWRPLPSCEEKpc3_NP.3
p−maj=0.38, pX=0.35, N=4
var.14.CFH 1:196659237:C:T
peptide level
61:149
49:140
16:36
GDAVCTESGWRPLPSCEEKpc3_NP.4
p−maj=0.47, pX=0.016, N=126
var.14.CFH 1:196659237:C:T
peptide level
60:149
47:140
15:36
GDAVCTESGWRPLPSCEEKpc3_NP.5
p−min=0.36, pX=0.5, N=122
var.14.CFH 1:196659237:C:T
peptide level
149:149
140:140
36:36
GEWVALNPLRKpc2_NP.1
p−maj=1, pX=0.036, N=325
var.14.CFH 1:196659237:C:T
peptide level
148:149
140:140
34:36
GEWVALNPLRKpc2_NP.2
p−maj=0.033, pX=0.034, N=322
var.14.CFH 1:196659237:C:T
peptide level
149:149
140:140
36:36
GEWVALNPLRKpc2_NP.3
p−maj=1, pX=0.67, N=325
var.14.CFH 1:196659237:C:T
peptide level
149:149
140:140
36:36
GEWVALNPLRKpc2_NP.4
p−maj=1, pX=0.65, N=325
var.14.CFH 1:196659237:C:T
peptide level
149:149
140:140
36:36
GEWVALNPLRKpc2_NP.5
p−maj=1, pX=0.0049, N=325

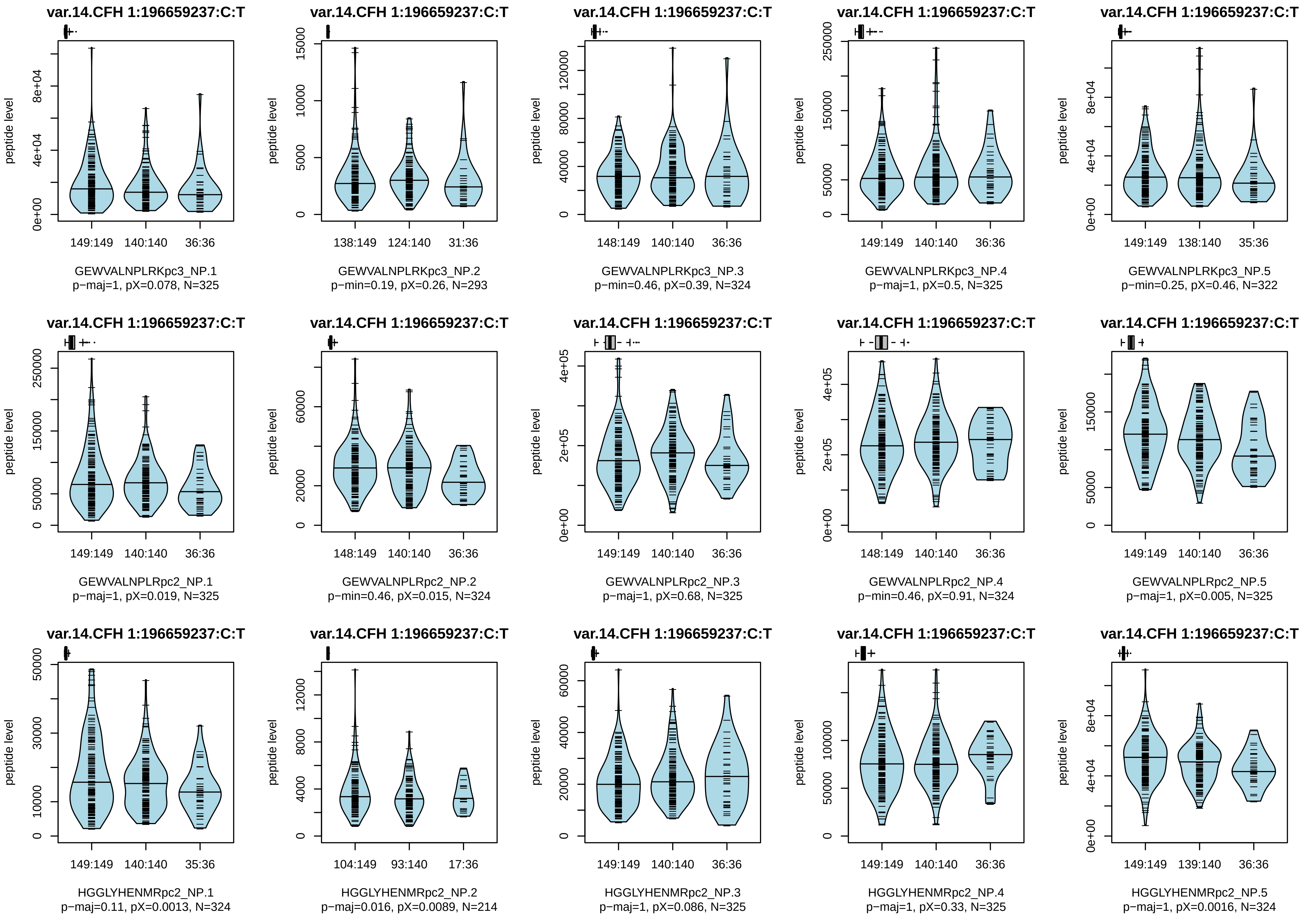
var.14.CFH 1:196659237:C:T
peptide level
149:149 140:140 36:36
GEWVALNPLRKpc3_NP.1
p−maj=1, pX=0.078, N=325
var.14.CFH 1:196659237:C:T
peptide level
138:149 124:140 31:36
GEWVALNPLRKpc3_NP.2
p−min=0.19, pX=0.26, N=293
var.14.CFH 1:196659237:C:T
peptide level
148:149 140:140 36:36
GEWVALNPLRKpc3_NP.3
p−min=0.46, pX=0.39, N=324
var.14.CFH 1:196659237:C:T
peptide level
149:149 140:140 36:36
GEWVALNPLRKpc3_NP.4
p−maj=1, pX=0.5, N=325
var.14.CFH 1:196659237:C:T
peptide level
149:149 138:140 35:36
GEWVALNPLRKpc3_NP.5
p−min=0.25, pX=0.46, N=322
var.14.CFH 1:196659237:C:T
peptide level
149:149 140:140 36:36
GEWVALNPLRpc2_NP.1
p−maj=1, pX=0.019, N=325
var.14.CFH 1:196659237:C:T
peptide level
148:149 140:140 36:36
GEWVALNPLRpc2_NP.2
p−min=0.46, pX=0.015, N=324
var.14.CFH 1:196659237:C:T
peptide level
149:149 140:140 36:36
GEWVALNPLRpc2_NP.3
p−maj=1, pX=0.68, N=325
var.14.CFH 1:196659237:C:T
peptide level
148:149 140:140 36:36
GEWVALNPLRpc2_NP.4
p−min=0.46, pX=0.91, N=324
var.14.CFH 1:196659237:C:T
peptide level
149:149 140:140 36:36
GEWVALNPLRpc2_NP.5
p−maj=1, pX=0.005, N=325
var.14.CFH 1:196659237:C:T
peptide level
149:149 140:140 35:36
HGGLYHENMRpc2_NP.1
p−maj=0.11, pX=0.0013, N=324
var.14.CFH 1:196659237:C:T
peptide level
104:149 93:140 17:36
HGGLYHENMRpc2_NP.2
p−maj=0.016, pX=0.0089, N=214
var.14.CFH 1:196659237:C:T
peptide level
149:149 140:140 36:36
HGGLYHENMRpc2_NP.3
p−maj=1, pX=0.086, N=325
var.14.CFH 1:196659237:C:T
peptide level
149:149 140:140 36:36
HGGLYHENMRpc2_NP.4
p−maj=1, pX=0.33, N=325
var.14.CFH 1:196659237:C:T
peptide level
149:149 139:140 36:36
HGGLYHENMRpc2_NP.5
p−maj=1, pX=0.0016, N=324

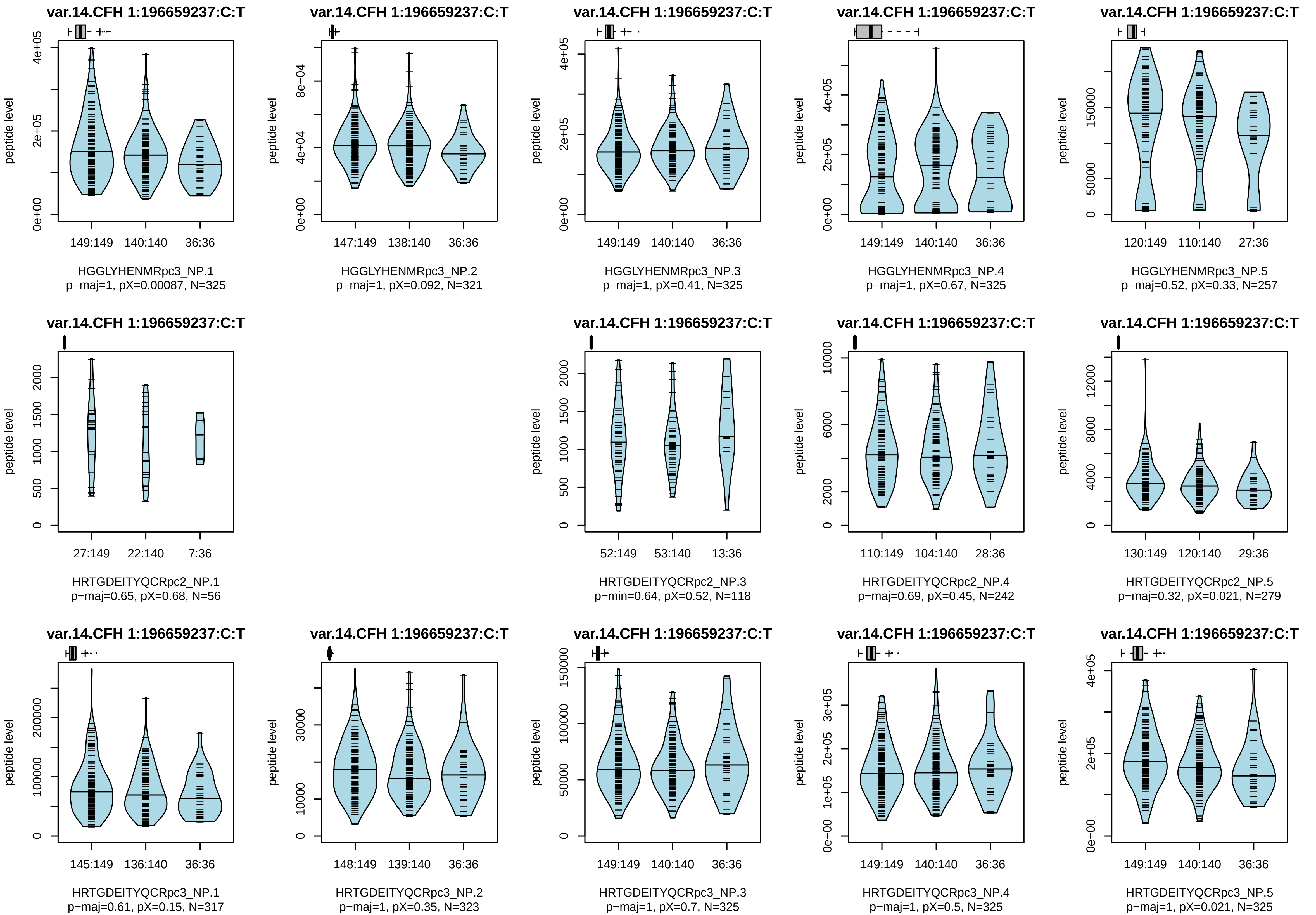
var.14.CFH 1:196659237:C:T
peptide level
149:149 140:140 36:36
HGGLYHENMRpc3_NP.1
p−maj=1, pX=0.00087, N=325
var.14.CFH 1:196659237:C:T
peptide level
147:149 138:140 36:36
HGGLYHENMRpc3_NP.2
p−maj=1, pX=0.092, N=321
var.14.CFH 1:196659237:C:T
peptide level
149:149 140:140 36:36
HGGLYHENMRpc3_NP.3
p−maj=1, pX=0.41, N=325
var.14.CFH 1:196659237:C:T
peptide level
149:149 140:140 36:36
HGGLYHENMRpc3_NP.4
p−maj=1, pX=0.67, N=325
var.14.CFH 1:196659237:C:T
peptide level
120:149 110:140 27:36
HGGLYHENMRpc3_NP.5
p−maj=0.52, pX=0.33, N=257
var.14.CFH 1:196659237:C:T
peptide level
27:149 22:140 7:36
HRTGDEITYQCRpc2_NP.1
p−maj=0.65, pX=0.68, N=56
var.14.CFH 1:196659237:C:T
peptide level
52:149 53:140 13:36
HRTGDEITYQCRpc2_NP.3
p−min=0.64, pX=0.52, N=118
var.14.CFH 1:196659237:C:T
peptide level
110:149 104:140 28:36
HRTGDEITYQCRpc2_NP.4
p−maj=0.69, pX=0.45, N=242
var.14.CFH 1:196659237:C:T
peptide level
130:149 120:140 29:36
HRTGDEITYQCRpc2_NP.5
p−maj=0.32, pX=0.021, N=279
var.14.CFH 1:196659237:C:T
peptide level
145:149 136:140 36:36
HRTGDEITYQCRpc3_NP.1
p−maj=0.61, pX=0.15, N=317
var.14.CFH 1:196659237:C:T
peptide level
148:149 139:140 36:36
HRTGDEITYQCRpc3_NP.2
p−maj=1, pX=0.35, N=323
var.14.CFH 1:196659237:C:T
peptide level
149:149 140:140 36:36
HRTGDEITYQCRpc3_NP.3
p−maj=1, pX=0.7, N=325
var.14.CFH 1:196659237:C:T
peptide level
149:149 140:140 36:36
HRTGDEITYQCRpc3_NP.4
p−maj=1, pX=0.5, N=325
var.14.CFH 1:196659237:C:T
peptide level
149:149 140:140 36:36
HRTGDEITYQCRpc3_NP.5
p−maj=1, pX=0.021, N=325

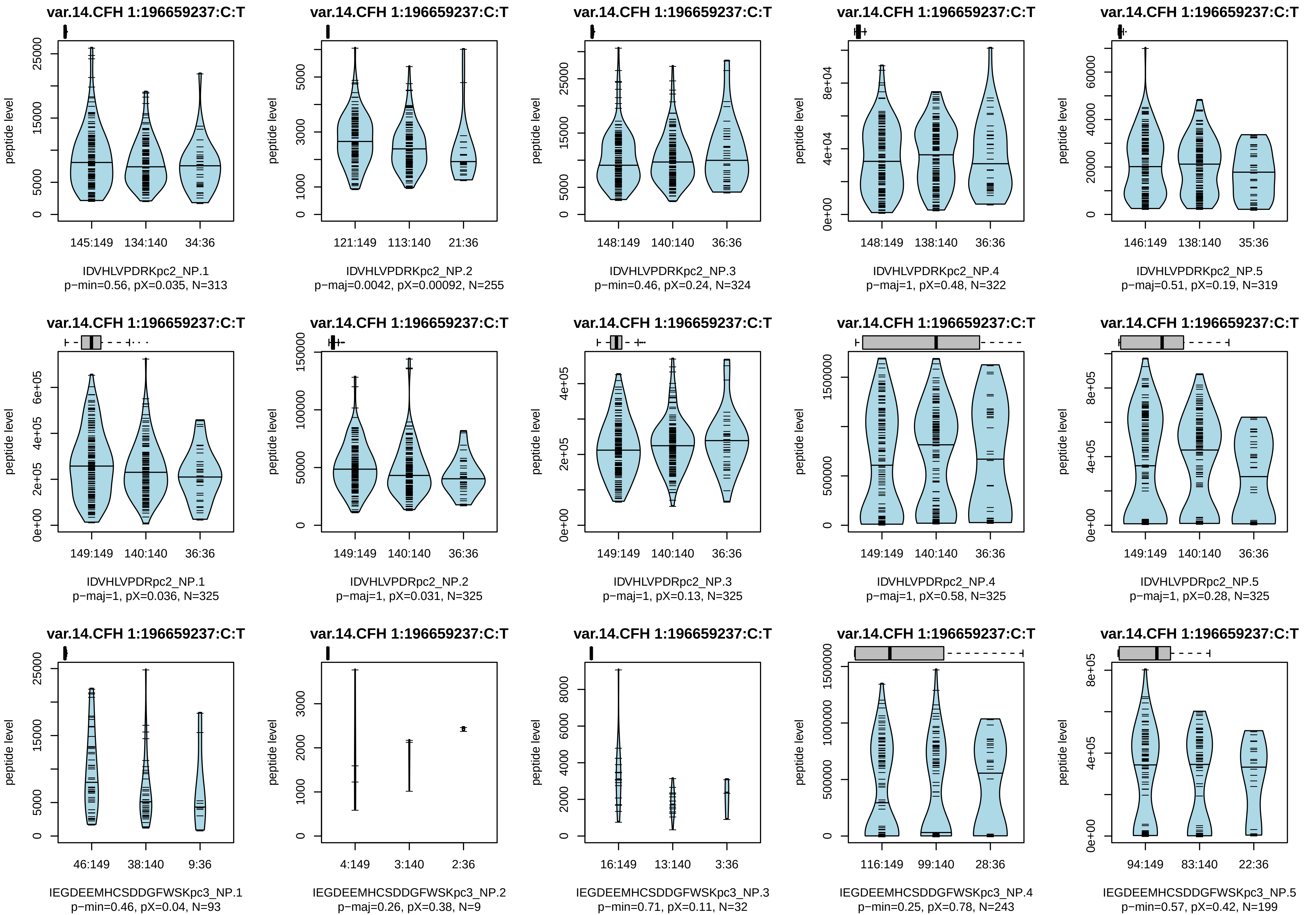

var.14.CFH 1:196659237:C:T
peptide level
145:149
134:140
34:36
IDVHLVPDRKpc2_NP.1
p−min=0.56, pX=0.035, N=313
var.14.CFH 1:196659237:C:T
peptide level
121:149
113:140
21:36
IDVHLVPDRKpc2_NP.2
p−maj=0.0042, pX=0.00092, N=255
var.14.CFH 1:196659237:C:T
peptide level
148:149
140:140
36:36
IDVHLVPDRKpc2_NP.3
p−min=0.46, pX=0.24, N=324
var.14.CFH 1:196659237:C:T
peptide level
148:149
138:140
36:36
IDVHLVPDRKpc2_NP.4
p−maj=1, pX=0.48, N=322
var.14.CFH 1:196659237:C:T
peptide level
146:149
138:140
35:36
IDVHLVPDRKpc2_NP.5
p−maj=0.51, pX=0.19, N=319
var.14.CFH 1:196659237:C:T
peptide level
149:149
140:140
36:36
IDVHLVPDRpc2_NP.1
p−maj=1, pX=0.036, N=325
var.14.CFH 1:196659237:C:T
peptide level
149:149
140:140
36:36
IDVHLVPDRpc2_NP.2
p−maj=1, pX=0.031, N=325
var.14.CFH 1:196659237:C:T
peptide level
149:149
140:140
36:36
IDVHLVPDRpc2_NP.3
p−maj=1, pX=0.13, N=325
var.14.CFH 1:196659237:C:T
peptide level
149:149
140:140
36:36
IDVHLVPDRpc2_NP.4
p−maj=1, pX=0.58, N=325
var.14.CFH 1:196659237:C:T
peptide level
149:149
140:140
36:36
IDVHLVPDRpc2_NP.5
p−maj=1, pX=0.28, N=325
var.14.CFH 1:196659237:C:T
peptide level
46:149
38:140
9:36
IEGDEEMHCSDDGFWSKpc3_NP.1
p−min=0.46, pX=0.04, N=93
var.14.CFH 1:196659237:C:T
peptide level
4:149
3:140
2:36
IEGDEEMHCSDDGFWSKpc3_NP.2
p−maj=0.26, pX=0.38, N=9
var.14.CFH 1:196659237:C:T
peptide level
16:149
13:140
3:36
IEGDEEMHCSDDGFWSKpc3_NP.3
p−min=0.71, pX=0.11, N=32
var.14.CFH 1:196659237:C:T
peptide level
116:149
99:140
28:36
IEGDEEMHCSDDGFWSKpc3_NP.4
p−min=0.25, pX=0.78, N=243
var.14.CFH 1:196659237:C:T
peptide level
94:149
83:140
22:36
IEGDEEMHCSDDGFWSKpc3_NP.5
p−min=0.57, pX=0.42, N=199

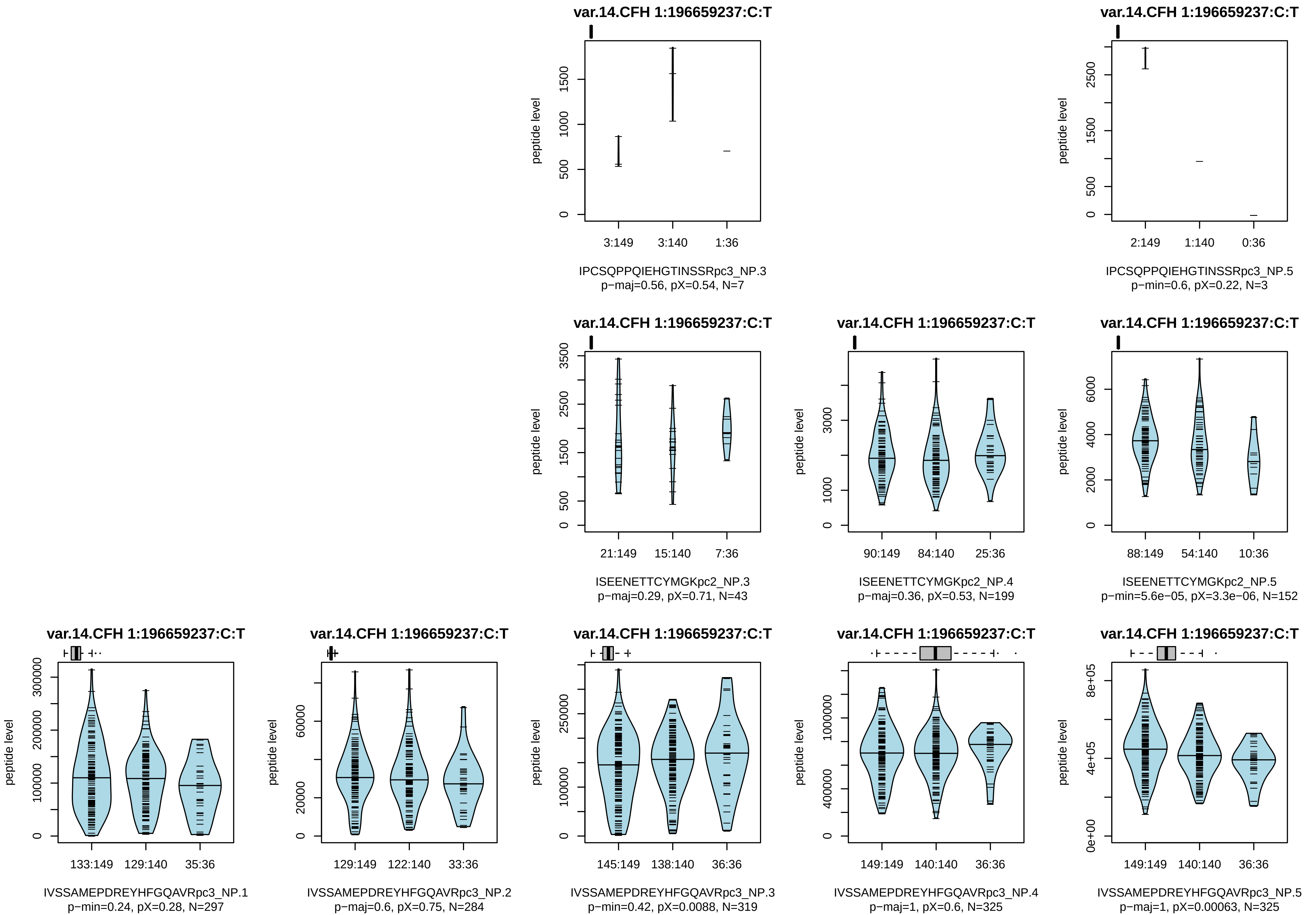
var.14.CFH 1:196659237:C:T
peptide level
3:149
3:140
1:36
IPCSQPPQIEHGTINSSRpc3_NP.3
p−maj=0.56, pX=0.54, N=7
var.14.CFH 1:196659237:C:T
peptide level
2:149
1:140
0:36
IPCSQPPQIEHGTINSSRpc3_NP.5
p−min=0.6, pX=0.22, N=3
var.14.CFH 1:196659237:C:T
peptide level
21:149
15:140
7:36
ISEENETTCYMGKpc2_NP.3
p−maj=0.29, pX=0.71, N=43
var.14.CFH 1:196659237:C:T
peptide level
90:149
84:140
25:36
ISEENETTCYMGKpc2_NP.4
p−maj=0.36, pX=0.53, N=199
var.14.CFH 1:196659237:C:T
peptide level
88:149
54:140
10:36
ISEENETTCYMGKpc2_NP.5
p−min=5.6e−05, pX=3.3e−06, N=152
var.14.CFH 1:196659237:C:T
peptide level
133:149
129:140
35:36
IVSSAMEPDREYHFGQAVRpc3_NP.1
p−min=0.24, pX=0.28, N=297
var.14.CFH 1:196659237:C:T
peptide level
129:149
122:140
33:36
IVSSAMEPDREYHFGQAVRpc3_NP.2
p−maj=0.6, pX=0.75, N=284
var.14.CFH 1:196659237:C:T
peptide level
145:149
138:140
36:36
IVSSAMEPDREYHFGQAVRpc3_NP.3
p−min=0.42, pX=0.0088, N=319
var.14.CFH 1:196659237:C:T
peptide level
149:149
140:140
36:36
IVSSAMEPDREYHFGQAVRpc3_NP.4
p−maj=1, pX=0.6, N=325
var.14.CFH 1:196659237:C:T
peptide level
149:149
140:140
36:36
IVSSAMEPDREYHFGQAVRpc3_NP.5
p−maj=1, pX=0.00063, N=325

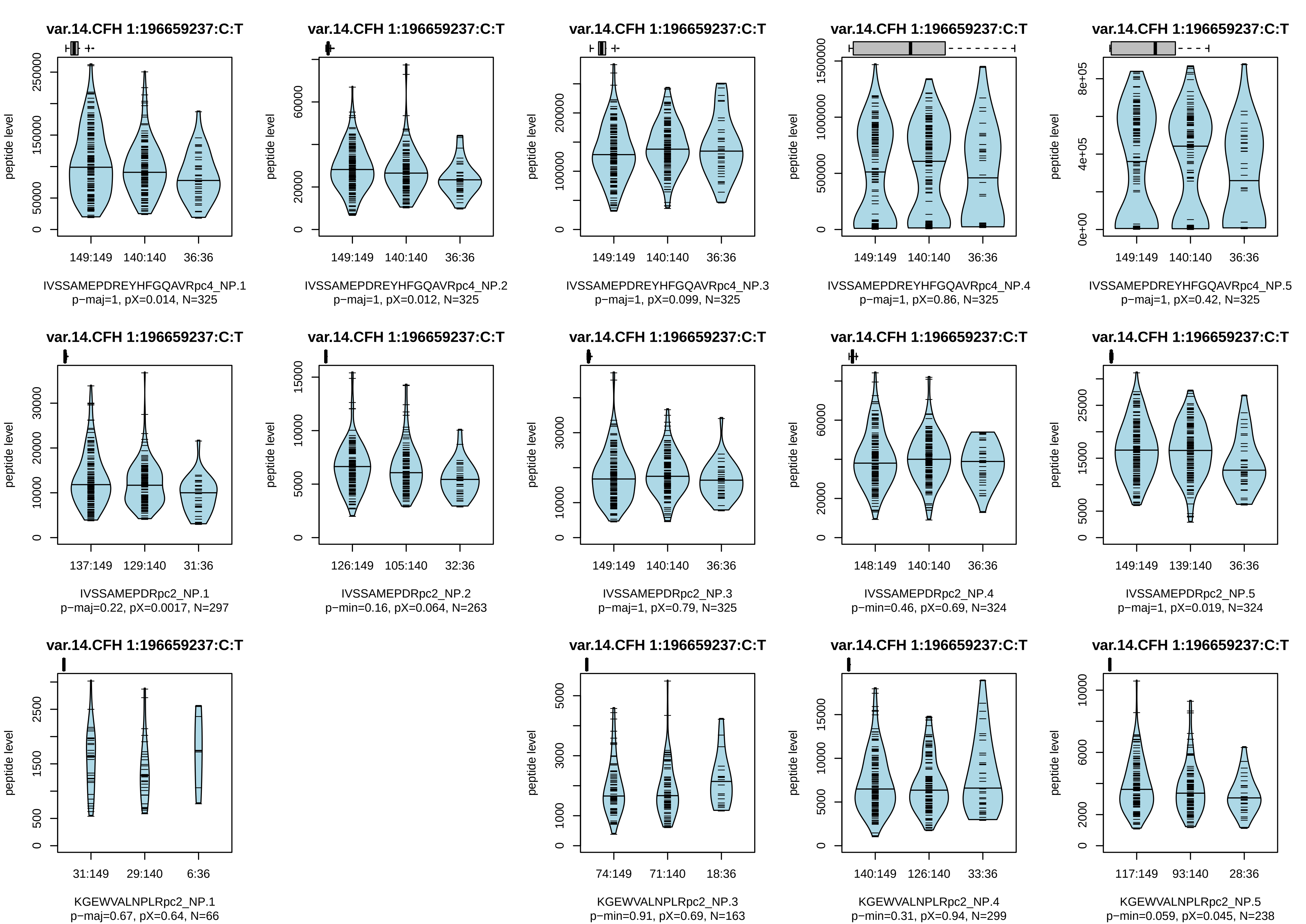
var.14.CFH 1:196659237:C:T
peptide level
149:149 140:140 36:36
IVSSAMEPDREYHFGQAVRpc4_NP.1
p−maj=1, pX=0.014, N=325
var.14.CFH 1:196659237:C:T
149:149 140:140 36:36
IVSSAMEPDREYHFGQAVRpc4_NP.2
p−maj=1, pX=0.012, N=325
var.14.CFH 1:196659237:C:T
149:149 140:140 36:36
IVSSAMEPDREYHFGQAVRpc4_NP.3
p−maj=1, pX=0.099, N=325
var.14.CFH 1:196659237:C:T
149:149 140:140 36:36
IVSSAMEPDREYHFGQAVRpc4_NP.4
p−maj=1, pX=0.86, N=325
var.14.CFH 1:196659237:C:T
149:149 140:140 36:36
IVSSAMEPDREYHFGQAVRpc4_NP.5
p−maj=1, pX=0.42, N=325
var.14.CFH 1:196659237:C:T
137:149 129:140 31:36
IVSSAMEPDRpc2_NP.1
p−maj=0.22, pX=0.0017, N=297
var.14.CFH 1:196659237:C:T
126:149 105:140 32:36
IVSSAMEPDRpc2_NP.2
p−min=0.16, pX=0.064, N=263
var.14.CFH 1:196659237:C:T
149:149 140:140 36:36
IVSSAMEPDRpc2_NP.3
p−maj=1, pX=0.79, N=325
var.14.CFH 1:196659237:C:T
148:149 140:140 36:36
IVSSAMEPDRpc2_NP.4
p−min=0.46, pX=0.69, N=324
var.14.CFH 1:196659237:C:T
149:149 139:140 36:36
IVSSAMEPDRpc2_NP.5
p−maj=1, pX=0.019, N=324
var.14.CFH 1:196659237:C:T
31:149 29:140 6:36
KGEWVALNPLRpc2_NP.1
p−maj=0.67, pX=0.64, N=66
var.14.CFH 1:196659237:C:T
74:149 71:140 18:36
KGEWVALNPLRpc2_NP.3
p−min=0.91, pX=0.69, N=163
var.14.CFH 1:196659237:C:T
140:149 126:140 33:36
KGEWVALNPLRpc2_NP.4
p−min=0.31, pX=0.94, N=299
var.14.CFH 1:196659237:C:T
117:149 93:140 28:36
KGEWVALNPLRpc2_NP.5
p−min=0.059, pX=0.045, N=238

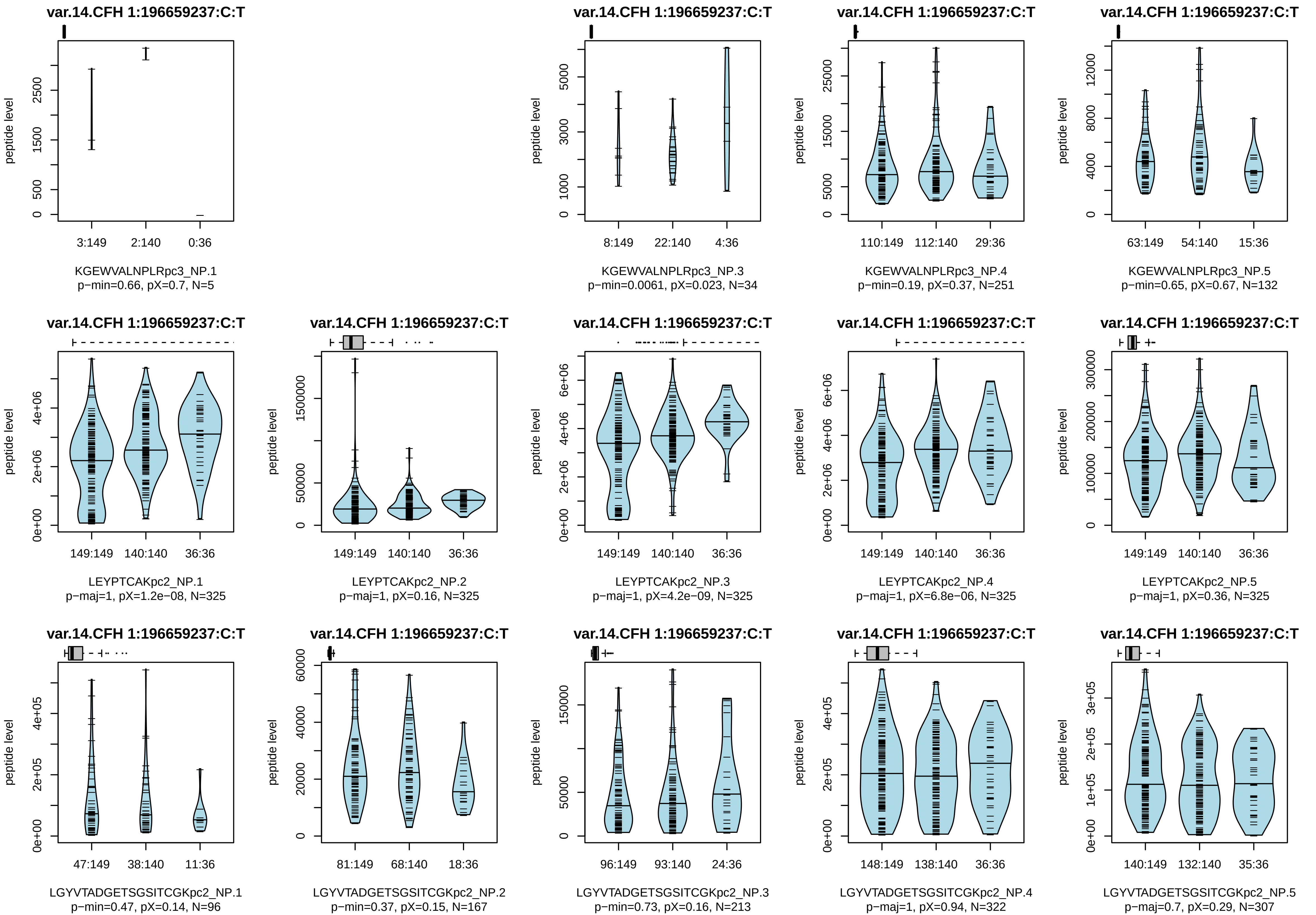
var.14.CFH 1:196659237:C:T
peptide level
3:149
2:140
0:36
KGEWVALNPLRpc3_NP.1
p−min=0.66, pX=0.7, N=5
var.14.CFH 1:196659237:C:T
8:149
22:140
4:36
KGEWVALNPLRpc3_NP.3
p−min=0.0061, pX=0.023, N=34
var.14.CFH 1:196659237:C:T
110:149
112:140
29:36
KGEWVALNPLRpc3_NP.4
p−min=0.19, pX=0.37, N=251
var.14.CFH 1:196659237:C:T
63:149
54:140
15:36
KGEWVALNPLRpc3_NP.5
p−min=0.65, pX=0.67, N=132
var.14.CFH 1:196659237:C:T
149:149
140:140
36:36
LEYPTCAKpc2_NP.1
p−maj=1, pX=1.2e−08, N=325
var.14.CFH 1:196659237:C:T
149:149
140:140
36:36
LEYPTCAKpc2_NP.2
p−maj=1, pX=0.16, N=325
var.14.CFH 1:196659237:C:T
149:149
140:140
36:36
LEYPTCAKpc2_NP.3
p−maj=1, pX=4.2e−09, N=325
var.14.CFH 1:196659237:C:T
149:149
140:140
36:36
LEYPTCAKpc2_NP.4
p−maj=1, pX=6.8e−06, N=325
var.14.CFH 1:196659237:C:T
149:149
140:140
36:36
LEYPTCAKpc2_NP.5
p−maj=1, pX=0.36, N=325
var.14.CFH 1:196659237:C:T
47:149
38:140
11:36
LGYVTADGETSGSITCGKpc2_NP.1
p−min=0.47, pX=0.14, N=96
var.14.CFH 1:196659237:C:T
81:149
68:140
18:36
LGYVTADGETSGSITCGKpc2_NP.2
p−min=0.37, pX=0.15, N=167
var.14.CFH 1:196659237:C:T
96:149
93:140
24:36
LGYVTADGETSGSITCGKpc2_NP.3
p−min=0.73, pX=0.16, N=213
var.14.CFH 1:196659237:C:T
148:149
138:140
36:36
LGYVTADGETSGSITCGKpc2_NP.4
p−maj=1, pX=0.94, N=322
var.14.CFH 1:196659237:C:T
140:149
132:140
35:36
LGYVTADGETSGSITCGKpc2_NP.5
p−maj=0.7, pX=0.29, N=307

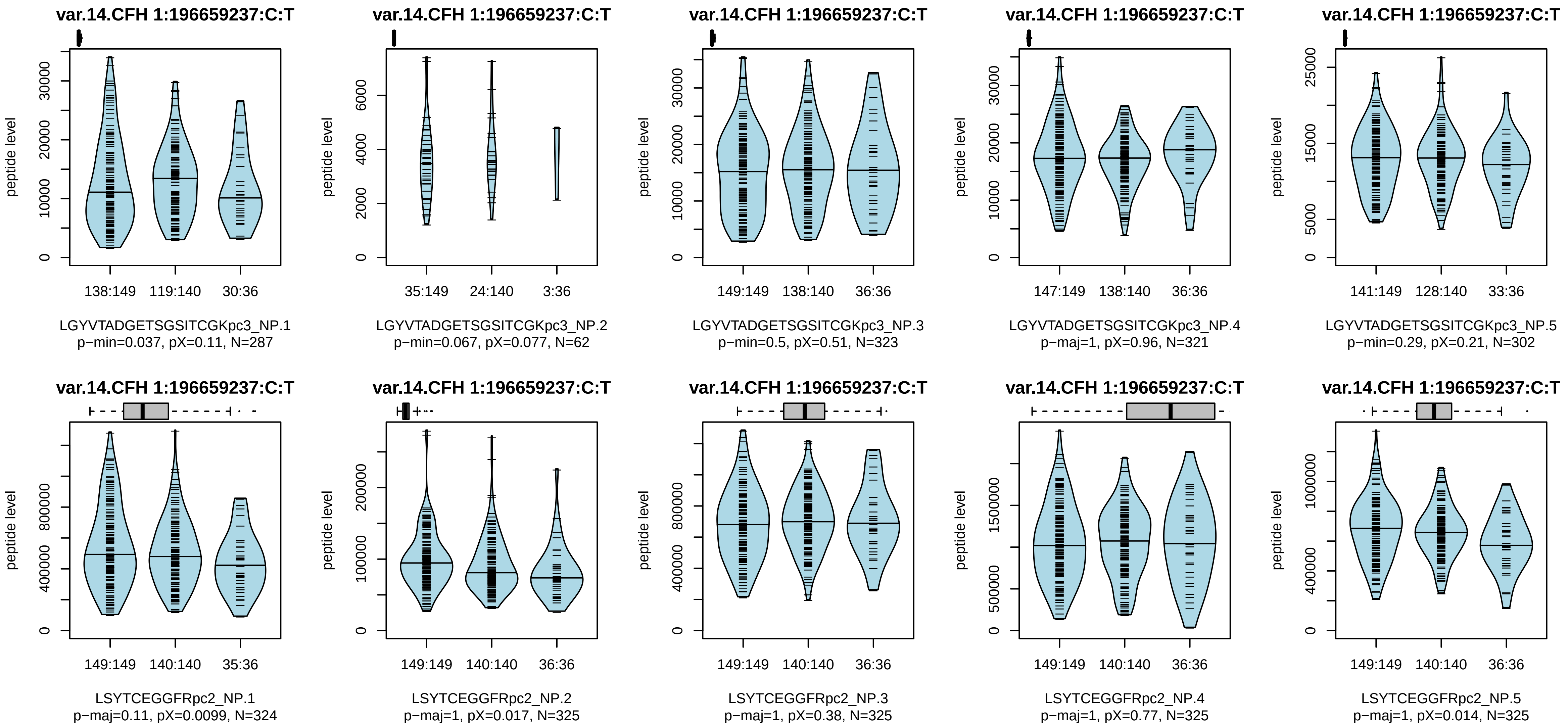

var.14.CFH 1:196659237:C:T
peptide level
138:149
119:140
30:36
LGYVTADGETSGSITCGKpc3_NP.1
p−min=0.037, pX=0.11, N=287
35:149
24:140
3:36
LGYVTADGETSGSITCGKpc3_NP.2
p−min=0.067, pX=0.077, N=62
149:149
138:140
36:36
LGYVTADGETSGSITCGKpc3_NP.3
p−min=0.5, pX=0.51, N=323
147:149
138:140
36:36
LGYVTADGETSGSITCGKpc3_NP.4
p−maj=1, pX=0.96, N=321
141:149
128:140
33:36
LGYVTADGETSGSITCGKpc3_NP.5
p−min=0.29, pX=0.21, N=302
149:149
140:140
35:36
LSYTCEGGFRpc2_NP.1
p−maj=0.11, pX=0.0099, N=324
149:149
140:140
36:36
LSYTCEGGFRpc2_NP.2
p−maj=1, pX=0.017, N=325
LSYTCEGGFRpc2_NP.3
p−maj=1, pX=0.38, N=325
LSYTCEGGFRpc2_NP.4
p−maj=1, pX=0.77, N=325
LSYTCEGGFRpc2_NP.5
p−maj=1, pX=0.014, N=325

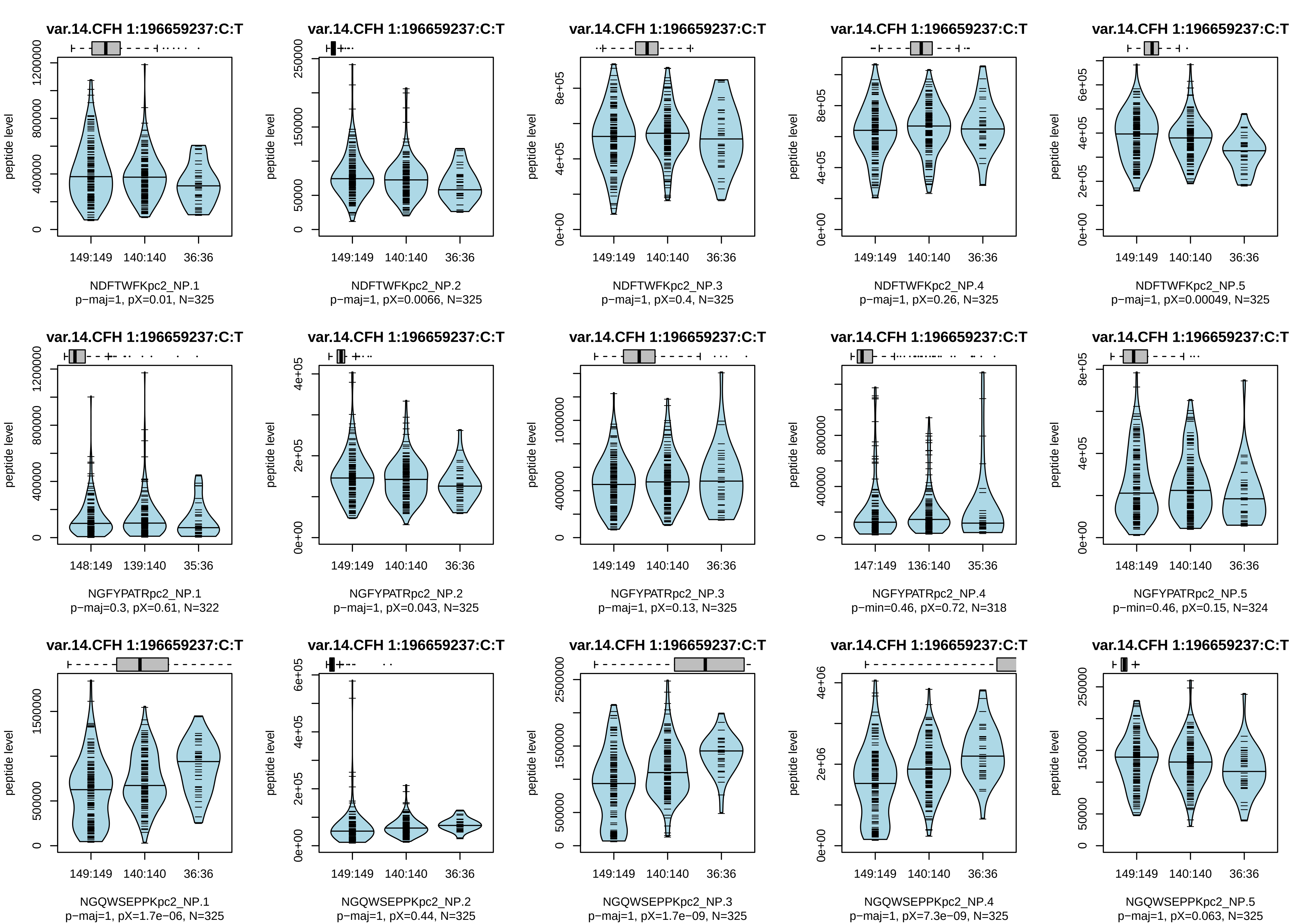
var.14.CFH 1:196659237:C:T
peptide level
149:149 140:140 36:36
NDFTWFKpc2_NP.1
p−maj=1, pX=0.01, N=325
var.14.CFH 1:196659237:C:T
149:149 140:140 36:36
NDFTWFKpc2_NP.2
p−maj=1, pX=0.0066, N=325
var.14.CFH 1:196659237:C:T
149:149 140:140 36:36
NDFTWFKpc2_NP.3
p−maj=1, pX=0.4, N=325
var.14.CFH 1:196659237:C:T
149:149 140:140 36:36
NDFTWFKpc2_NP.4
p−maj=1, pX=0.26, N=325
var.14.CFH 1:196659237:C:T
149:149 140:140 36:36
NDFTWFKpc2_NP.5
p−maj=1, pX=0.00049, N=325
var.14.CFH 1:196659237:C:T
148:149 139:140 35:36
NGFYPATRpc2_NP.1
p−maj=0.3, pX=0.61, N=322
var.14.CFH 1:196659237:C:T
149:149 140:140 36:36
NGFYPATRpc2_NP.2
p−maj=1, pX=0.043, N=325
var.14.CFH 1:196659237:C:T
149:149 140:140 36:36
NGFYPATRpc2_NP.3
p−maj=1, pX=0.13, N=325
var.14.CFH 1:196659237:C:T
147:149 136:140 35:36
NGFYPATRpc2_NP.4
p−min=0.46, pX=0.72, N=318
var.14.CFH 1:196659237:C:T
148:149 140:140 36:36
NGFYPATRpc2_NP.5
p−min=0.46, pX=0.15, N=324
var.14.CFH 1:196659237:C:T
149:149 140:140 36:36
NGQWSEPPKpc2_NP.1
p−maj=1, pX=1.7e−06, N=325
var.14.CFH 1:196659237:C:T
149:149 140:140 36:36
NGQWSEPPKpc2_NP.2
p−maj=1, pX=0.44, N=325
var.14.CFH 1:196659237:C:T
149:149 140:140 36:36
NGQWSEPPKpc2_NP.3
p−maj=1, pX=1.7e−09, N=325
var.14.CFH 1:196659237:C:T
149:149 140:140 36:36
NGQWSEPPKpc2_NP.4
p−maj=1, pX=7.3e−09, N=325
var.14.CFH 1:196659237:C:T
149:149 140:140 36:36
NGQWSEPPKpc2_NP.5
p−maj=1, pX=0.063, N=325

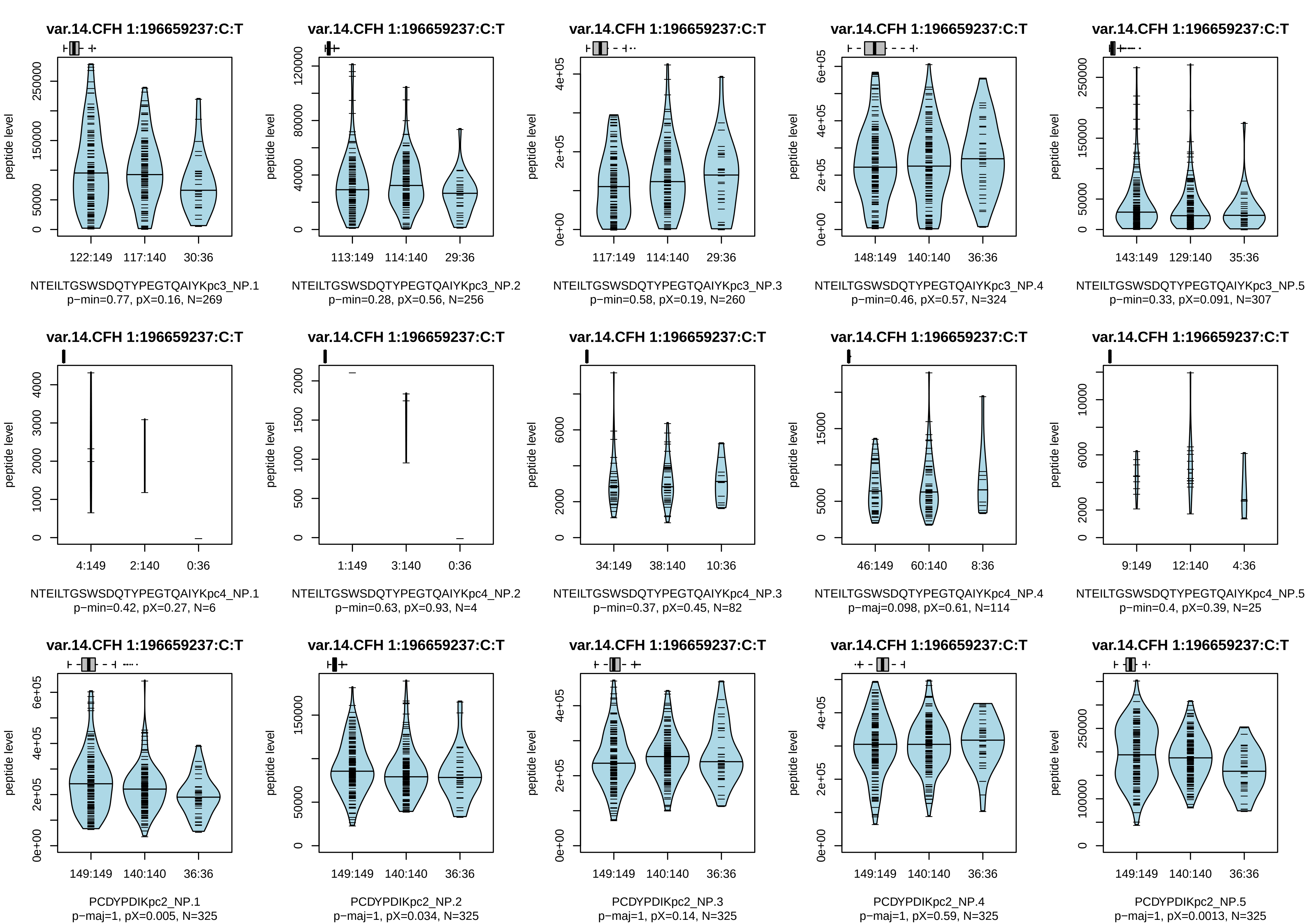

var.14.CFH 1:196659237:C:T
peptide level
122:149
117:140
30:36
NTEILTGSWSDQTYPEGTQAIYKpc3_NP.1
p−min=0.77, pX=0.16, N=269
var.14.CFH 1:196659237:C:T
peptide level
113:149
114:140
29:36
NTEILTGSWSDQTYPEGTQAIYKpc3_NP.2
p−min=0.28, pX=0.56, N=256
var.14.CFH 1:196659237:C:T
peptide level
117:149
114:140
29:36
NTEILTGSWSDQTYPEGTQAIYKpc3_NP.3
p−min=0.58, pX=0.19, N=260
var.14.CFH 1:196659237:C:T
peptide level
148:149
140:140
36:36
NTEILTGSWSDQTYPEGTQAIYKpc3_NP.4
p−min=0.46, pX=0.57, N=324
var.14.CFH 1:196659237:C:T
peptide level
143:149
129:140
35:36
NTEILTGSWSDQTYPEGTQAIYKpc3_NP.5
p−min=0.33, pX=0.091, N=307
var.14.CFH 1:196659237:C:T
peptide level
4:149
2:140
0:36
NTEILTGSWSDQTYPEGTQAIYKpc4_NP.1
p−min=0.42, pX=0.27, N=6
var.14.CFH 1:196659237:C:T
peptide level
1:149
3:140
0:36
NTEILTGSWSDQTYPEGTQAIYKpc4_NP.2
p−min=0.63, pX=0.93, N=4
var.14.CFH 1:196659237:C:T
peptide level
34:149
38:140
10:36
NTEILTGSWSDQTYPEGTQAIYKpc4_NP.3
p−min=0.37, pX=0.45, N=82
var.14.CFH 1:196659237:C:T
peptide level
46:149
60:140
8:36
NTEILTGSWSDQTYPEGTQAIYKpc4_NP.4
p−maj=0.098, pX=0.61, N=114
var.14.CFH 1:196659237:C:T
peptide level
9:149
12:140
4:36
NTEILTGSWSDQTYPEGTQAIYKpc4_NP.5
p−min=0.4, pX=0.39, N=25
var.14.CFH 1:196659237:C:T
peptide level
149:149
140:140
36:36
PCDYPDIKpc2_NP.1
p−maj=1, pX=0.005, N=325
var.14.CFH 1:196659237:C:T
peptide level
149:149
140:140
36:36
PCDYPDIKpc2_NP.2
p−maj=1, pX=0.034, N=325
var.14.CFH 1:196659237:C:T
peptide level
149:149
140:140
36:36
PCDYPDIKpc2_NP.3
p−maj=1, pX=0.14, N=325
var.14.CFH 1:196659237:C:T
peptide level
149:149
140:140
36:36
PCDYPDIKpc2_NP.4
p−maj=1, pX=0.59, N=325
var.14.CFH 1:196659237:C:T
peptide level
149:149
140:140
36:36
PCDYPDIKpc2_NP.5
p−maj=1, pX=0.0013, N=325

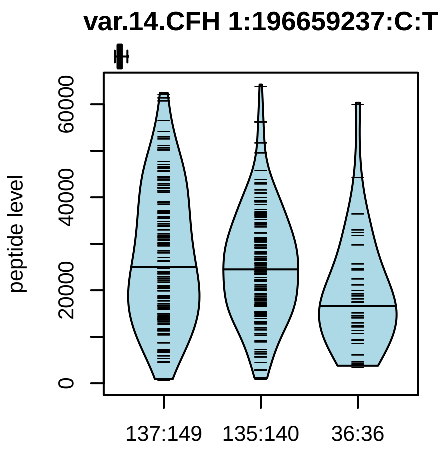

PGFTIVGPNSVQCYHFGLSPDLPICKpc4_NP
p−min=0.045, pX=0.055, N=308

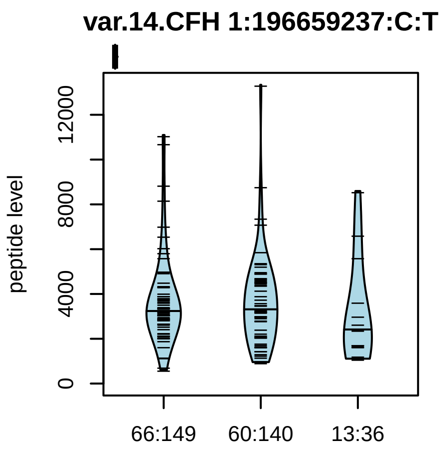

PGFTIVGPNSVQCYHFGLSPDLPICKpc4_NP
p−maj=0.48, pX=0.32, N=139

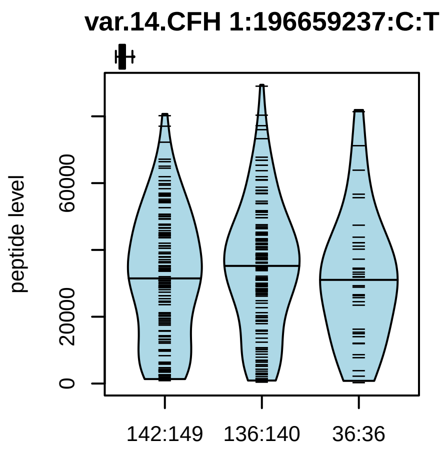

PGFTIVGPNSVQCYHFGLSPDLPICKpc4_NP
p−min=0.36, pX=0.39, N=314

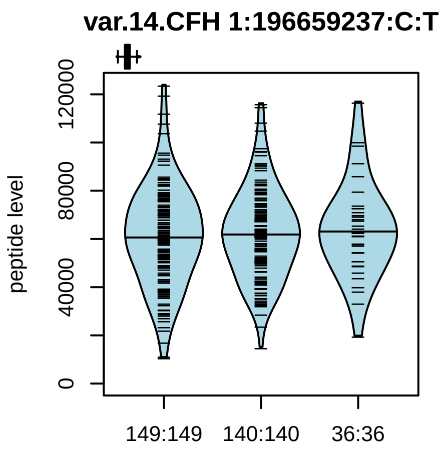

PGFTIVGPNSVQCYHFGLSPDLPICKpc4_NP
p−maj=1, pX=0.48, N=325

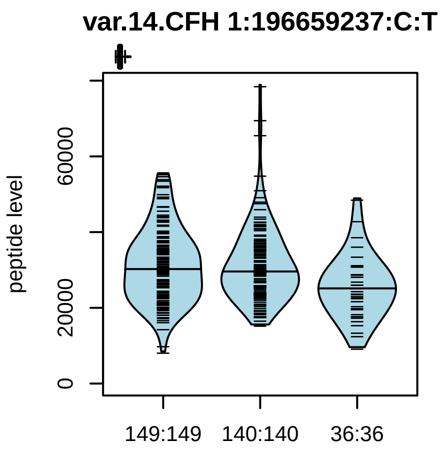

PGFTIVGPNSVQCYHFGLSPDLPICKpc4_NP
p−maj=1, pX=0.026, N=325

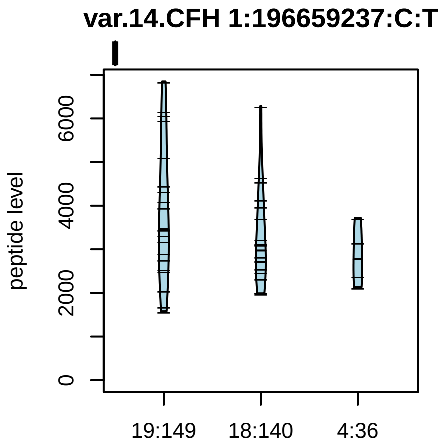

PLPSCEEKpc2_NP.1
p−maj=1, pX=0.43, N=41

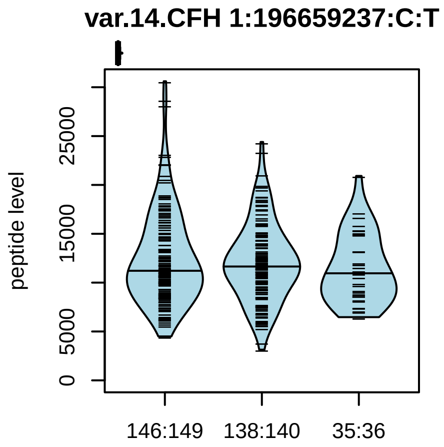

PLPSCEEKpc2_NP.3
p−maj=0.51, pX=0.19, N=319

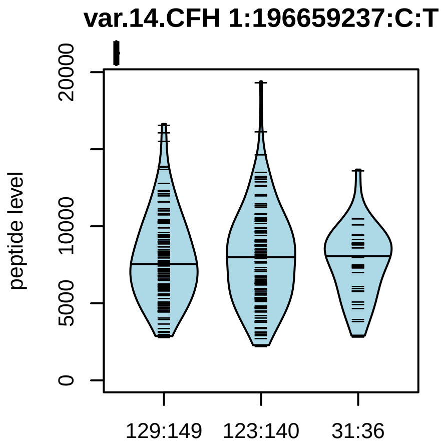

PLPSCEEKpc2_NP.4
p−maj=0.79, pX=0.89, N=283

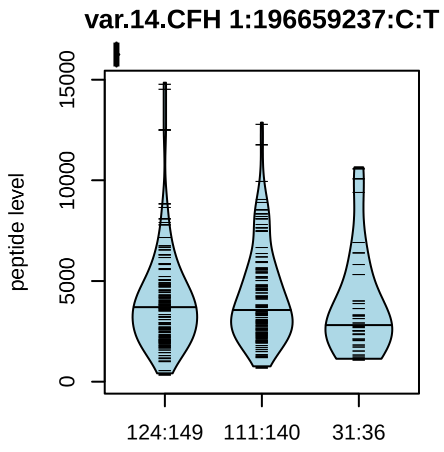

PYFPVAVGKpc2_NP.3
p−min=0.57, pX=0.98, N=266

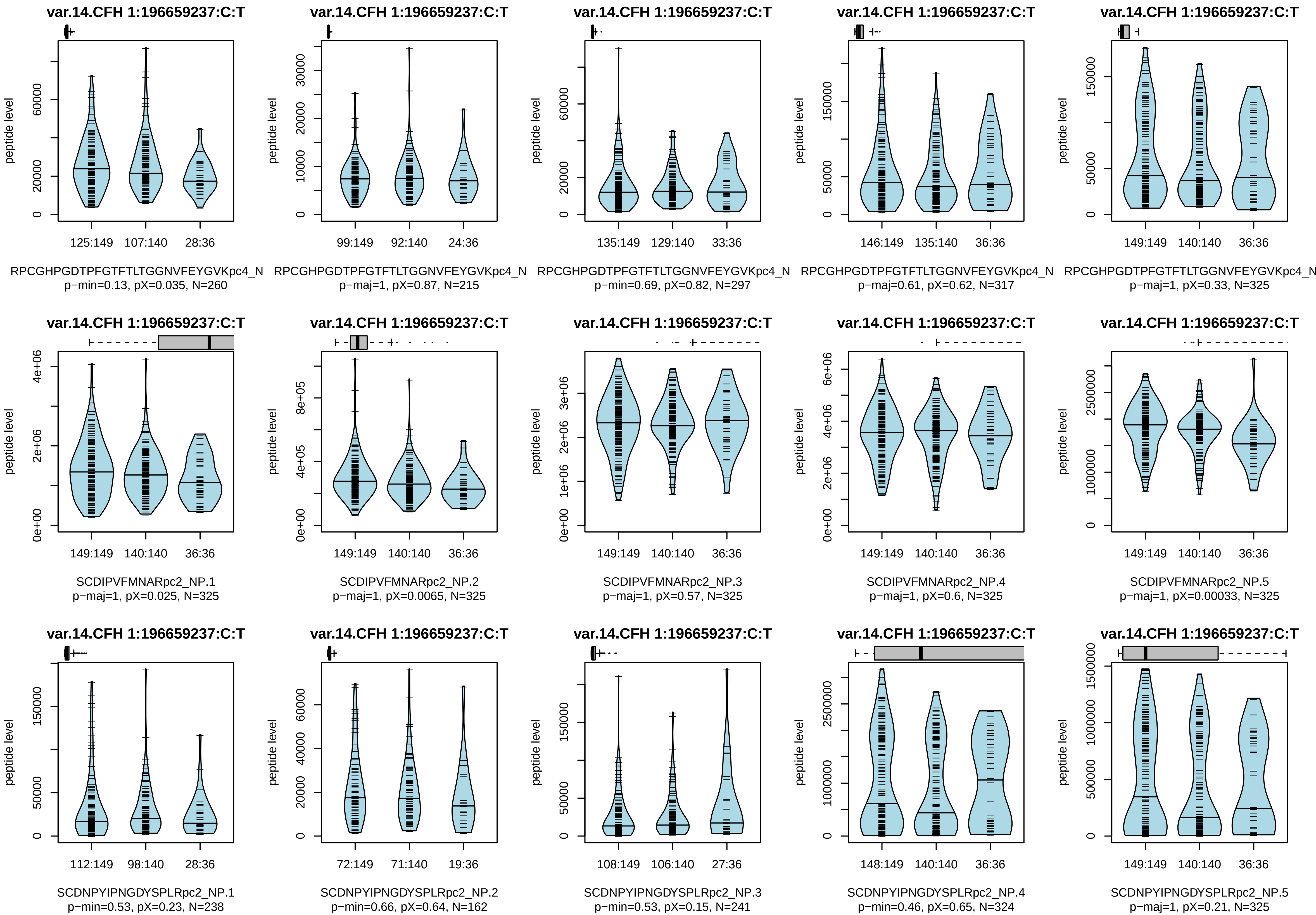
var.14.CFH 1:196659237:C:T
peptide level
125:149
107:140
28:36
RPCGHPGDTPFGTFTLTGGNVFEYGVKpc4_N
p−min=0.13, pX=0.035, N=260
var.14.CFH 1:196659237:C:T
peptide level
99:149
92:140
24:36
RPCGHPGDTPFGTFTLTGGNVFEYGVKpc4_N
p−maj=1, pX=0.87, N=215
var.14.CFH 1:196659237:C:T
peptide level
135:149
129:140
33:36
RPCGHPGDTPFGTFTLTGGNVFEYGVKpc4_N
p−min=0.69, pX=0.82, N=297
var.14.CFH 1:196659237:C:T
peptide level
146:149
135:140
36:36
RPCGHPGDTPFGTFTLTGGNVFEYGVKpc4_N
p−maj=0.61, pX=0.62, N=317
var.14.CFH 1:196659237:C:T
peptide level
149:149
140:140
36:36
RPCGHPGDTPFGTFTLTGGNVFEYGVKpc4_N
p−maj=1, pX=0.33, N=325
var.14.CFH 1:196659237:C:T
peptide level
149:149
140:140
36:36
SCDIPVFMNARpc2_NP.1
p−maj=1, pX=0.025, N=325
var.14.CFH 1:196659237:C:T
peptide level
149:149
140:140
36:36
SCDIPVFMNARpc2_NP.2
p−maj=1, pX=0.0065, N=325
var.14.CFH 1:196659237:C:T
peptide level
149:149
140:140
36:36
SCDIPVFMNARpc2_NP.3
p−maj=1, pX=0.57, N=325
var.14.CFH 1:196659237:C:T
peptide level
149:149
140:140
36:36
SCDIPVFMNARpc2_NP.4
p−maj=1, pX=0.6, N=325
var.14.CFH 1:196659237:C:T
peptide level
149:149
140:140
36:36
SCDIPVFMNARpc2_NP.5
p−maj=1, pX=0.00033, N=325
var.14.CFH 1:196659237:C:T
peptide level
112:149
98:140
28:36
SCDNPYIPNGDYSPLRpc2_NP.1
p−min=0.53, pX=0.23, N=238
var.14.CFH 1:196659237:C:T
peptide level
72:149
71:140
19:36
SCDNPYIPNGDYSPLRpc2_NP.2
p−min=0.66, pX=0.64, N=162
var.14.CFH 1:196659237:C:T
peptide level
108:149
106:140
27:36
SCDNPYIPNGDYSPLRpc2_NP.3
p−min=0.53, pX=0.15, N=241
var.14.CFH 1:196659237:C:T
peptide level
148:149
140:140
36:36
SCDNPYIPNGDYSPLRpc2_NP.4
p−min=0.46, pX=0.65, N=324
var.14.CFH 1:196659237:C:T
peptide level
149:149
140:140
36:36
SCDNPYIPNGDYSPLRpc2_NP.5
p−maj=1, pX=0.21, N=325

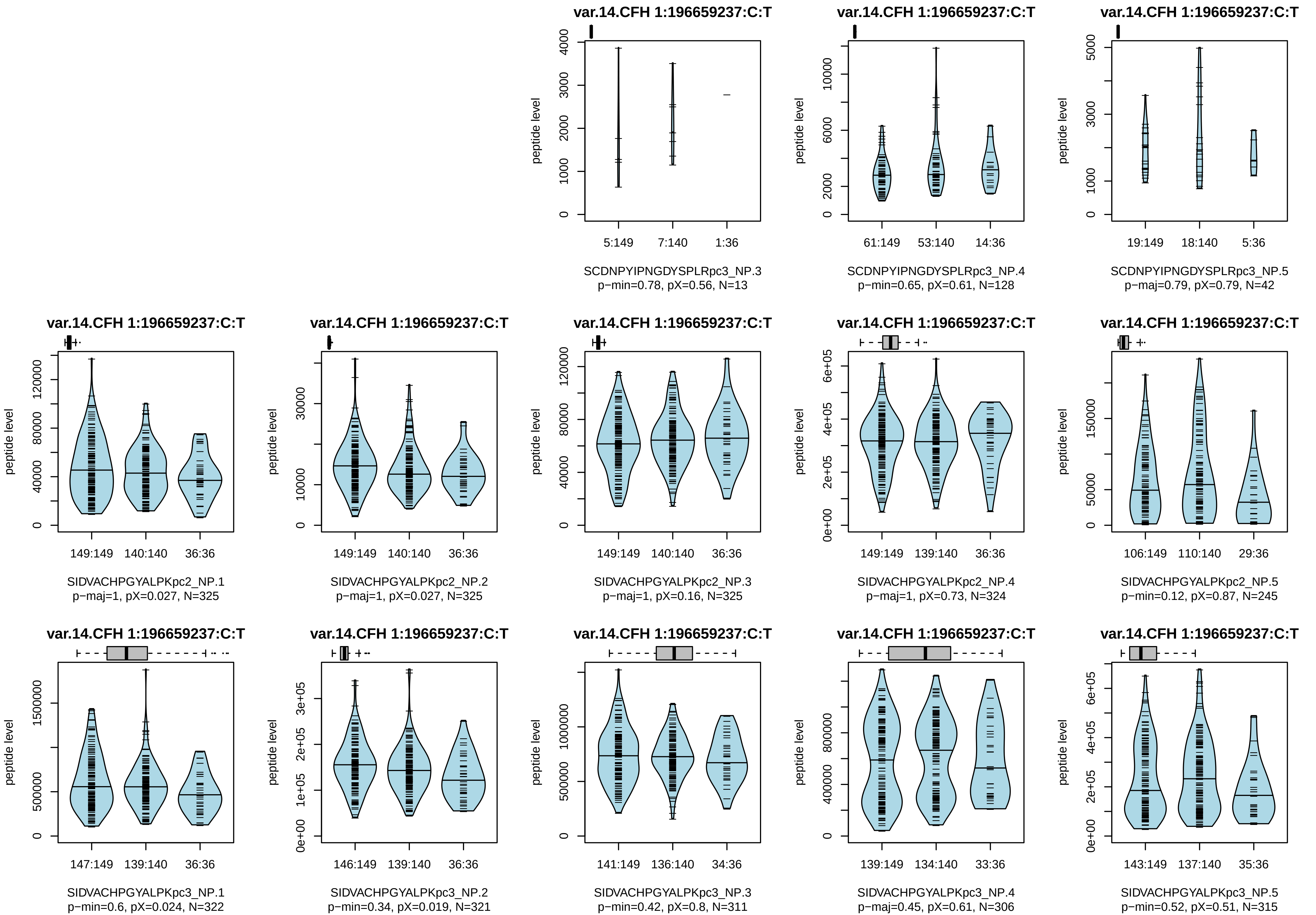
var.14.CFH 1:196659237:C:T
peptide level
5:149
7:140
1:36
SCDNPYIPNGDYSPLRpc3_NP.3
p−min=0.78, pX=0.56, N=13
var.14.CFH 1:196659237:C:T
peptide level
61:149
53:140
14:36
SCDNPYIPNGDYSPLRpc3_NP.4
p−min=0.65, pX=0.61, N=128
var.14.CFH 1:196659237:C:T
peptide level
19:149
18:140
5:36
SCDNPYIPNGDYSPLRpc3_NP.5
p−maj=0.79, pX=0.79, N=42
var.14.CFH 1:196659237:C:T
peptide level
149:149
140:140
36:36
SIDVACHPGYALPKpc2_NP.1
p−maj=1, pX=0.027, N=325
var.14.CFH 1:196659237:C:T
peptide level
149:149
140:140
36:36
SIDVACHPGYALPKpc2_NP.2
p−maj=1, pX=0.027, N=325
var.14.CFH 1:196659237:C:T
peptide level
149:149
140:140
36:36
SIDVACHPGYALPKpc2_NP.3
p−maj=1, pX=0.16, N=325
var.14.CFH 1:196659237:C:T
peptide level
149:149
139:140
36:36
SIDVACHPGYALPKpc2_NP.4
p−maj=1, pX=0.73, N=324
var.14.CFH 1:196659237:C:T
peptide level
106:149
110:140
29:36
SIDVACHPGYALPKpc2_NP.5
p−min=0.12, pX=0.87, N=245
var.14.CFH 1:196659237:C:T
peptide level
147:149
139:140
36:36
SIDVACHPGYALPKpc3_NP.1
p−min=0.6, pX=0.024, N=322
var.14.CFH 1:196659237:C:T
peptide level
146:149
139:140
36:36
SIDVACHPGYALPKpc3_NP.2
p−min=0.34, pX=0.019, N=321
var.14.CFH 1:196659237:C:T
peptide level
141:149
136:140
34:36
SIDVACHPGYALPKpc3_NP.3
p−min=0.42, pX=0.8, N=311
var.14.CFH 1:196659237:C:T
peptide level
139:149
134:140
33:36
SIDVACHPGYALPKpc3_NP.4
p−maj=0.45, pX=0.61, N=306
var.14.CFH 1:196659237:C:T
peptide level
143:149
137:140
35:36
SIDVACHPGYALPKpc3_NP.5
p−min=0.52, pX=0.51, N=315

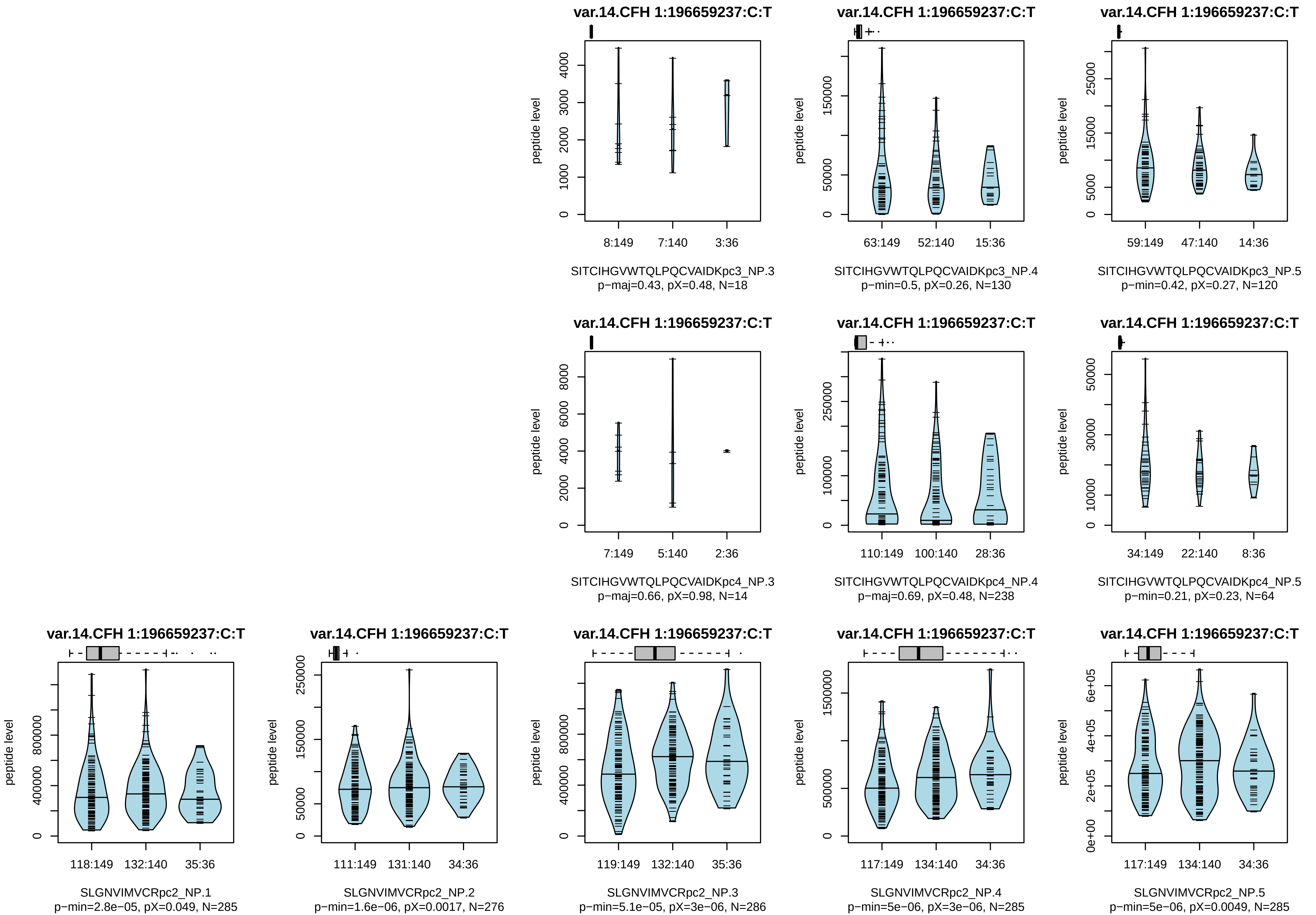

var.14.CFH 1:196659237:C:T
peptide level
8:149
7:140
3:36
SITCIHGVWTQLPQCVAIDKpc3_NP.3
p−maj=0.43, pX=0.48, N=18
var.14.CFH 1:196659237:C:T
peptide level
63:149
52:140
15:36
SITCIHGVWTQLPQCVAIDKpc3_NP.4
p−min=0.5, pX=0.26, N=130
var.14.CFH 1:196659237:C:T
peptide level
59:149
47:140
14:36
SITCIHGVWTQLPQCVAIDKpc3_NP.5
p−min=0.42, pX=0.27, N=120
var.14.CFH 1:196659237:C:T
peptide level
7:149
5:140
2:36
SITCIHGVWTQLPQCVAIDKpc4_NP.3
p−maj=0.66, pX=0.98, N=14
var.14.CFH 1:196659237:C:T
peptide level
110:149
100:140
28:36
SITCIHGVWTQLPQCVAIDKpc4_NP.4
p−maj=0.69, pX=0.48, N=238
var.14.CFH 1:196659237:C:T
peptide level
34:149
22:140
8:36
SITCIHGVWTQLPQCVAIDKpc4_NP.5
p−min=0.21, pX=0.23, N=64
var.14.CFH 1:196659237:C:T
peptide level
118:149
132:140
35:36
SLGNVIMVCRpc2_NP.1
p−min=2.8e−05, pX=0.049, N=285
var.14.CFH 1:196659237:C:T
peptide level
111:149
131:140
34:36
SLGNVIMVCRpc2_NP.2
p−min=1.6e−06, pX=0.0017, N=276
var.14.CFH 1:196659237:C:T
peptide level
119:149
132:140
35:36
SLGNVIMVCRpc2_NP.3
p−min=5.1e−05, pX=3e−06, N=286
var.14.CFH 1:196659237:C:T
peptide level
117:149
134:140
34:36
SLGNVIMVCRpc2_NP.4
p−min=5e−06, pX=3e−06, N=285
var.14.CFH 1:196659237:C:T
peptide level
117:149
134:140
34:36
SLGNVIMVCRpc2_NP.5
p−min=5e−06, pX=0.0049, N=285

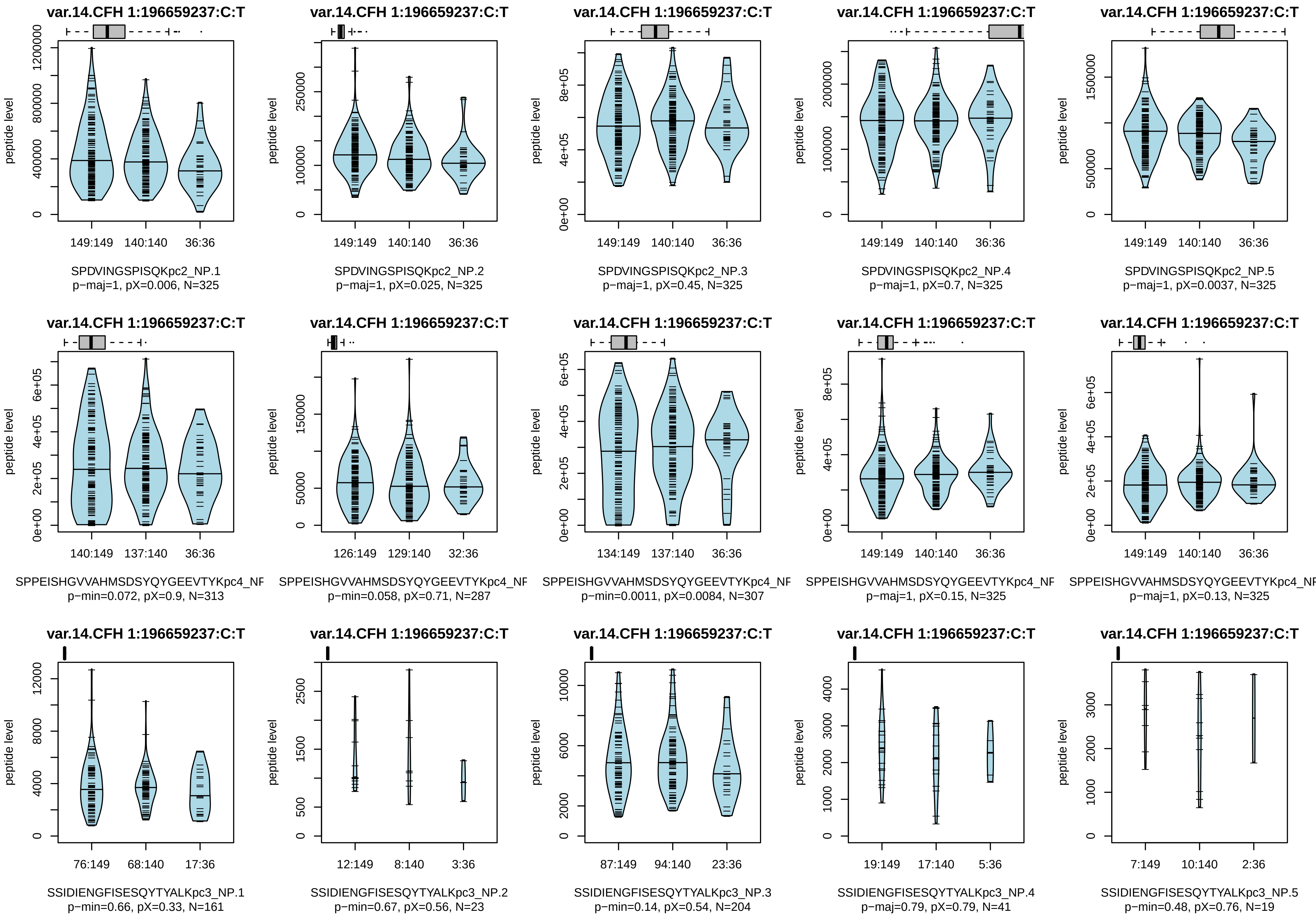
var.14.CFH 1:196659237:C:T
peptide level
149:149
140:140
36:36
SPDVINGSPISQKpc2_NP.1
p−maj=1, pX=0.006, N=325
var.14.CFH 1:196659237:C:T
peptide level
149:149
140:140
36:36
SPDVINGSPISQKpc2_NP.2
p−maj=1, pX=0.025, N=325
var.14.CFH 1:196659237:C:T
peptide level
149:149
140:140
36:36
SPDVINGSPISQKpc2_NP.3
p−maj=1, pX=0.45, N=325
var.14.CFH 1:196659237:C:T
peptide level
149:149
140:140
36:36
SPDVINGSPISQKpc2_NP.4
p−maj=1, pX=0.7, N=325
var.14.CFH 1:196659237:C:T
peptide level
149:149
140:140
36:36
SPDVINGSPISQKpc2_NP.5
p−maj=1, pX=0.0037, N=325
var.14.CFH 1:196659237:C:T
peptide level
140:149
137:140
36:36
SPPEISHGVVAHMSDSYQYGEEVTYKpc4_NF
p−min=0.072, pX=0.9, N=313
var.14.CFH 1:196659237:C:T
peptide level
126:149
129:140
32:36
SPPEISHGVVAHMSDSYQYGEEVTYKpc4_NF
p−min=0.058, pX=0.71, N=287
var.14.CFH 1:196659237:C:T
peptide level
134:149
137:140
36:36
SPPEISHGVVAHMSDSYQYGEEVTYKpc4_NF
p−min=0.0011, pX=0.0084, N=307
var.14.CFH 1:196659237:C:T
peptide level
149:149
140:140
36:36
SPPEISHGVVAHMSDSYQYGEEVTYKpc4_NF
p−maj=1, pX=0.15, N=325
var.14.CFH 1:196659237:C:T
peptide level
149:149
140:140
36:36
SPPEISHGVVAHMSDSYQYGEEVTYKpc4_NF
p−maj=1, pX=0.13, N=325
var.14.CFH 1:196659237:C:T
peptide level
76:149
68:140
17:36
SSIDIENGFISESQYTYALKpc3_NP.1
p−min=0.66, pX=0.33, N=161
var.14.CFH 1:196659237:C:T
peptide level
12:149
8:140
3:36
SSIDIENGFISESQYTYALKpc3_NP.2
p−min=0.67, pX=0.56, N=23
var.14.CFH 1:196659237:C:T
peptide level
87:149
94:140
23:36
SSIDIENGFISESQYTYALKpc3_NP.3
p−min=0.14, pX=0.54, N=204
var.14.CFH 1:196659237:C:T
peptide level
19:149
17:140
5:36
SSIDIENGFISESQYTYALKpc3_NP.4
p−maj=0.79, pX=0.79, N=41
var.14.CFH 1:196659237:C:T
peptide level
7:149
10:140
2:36
SSIDIENGFISESQYTYALKpc3_NP.5
p−min=0.48, pX=0.76, N=19

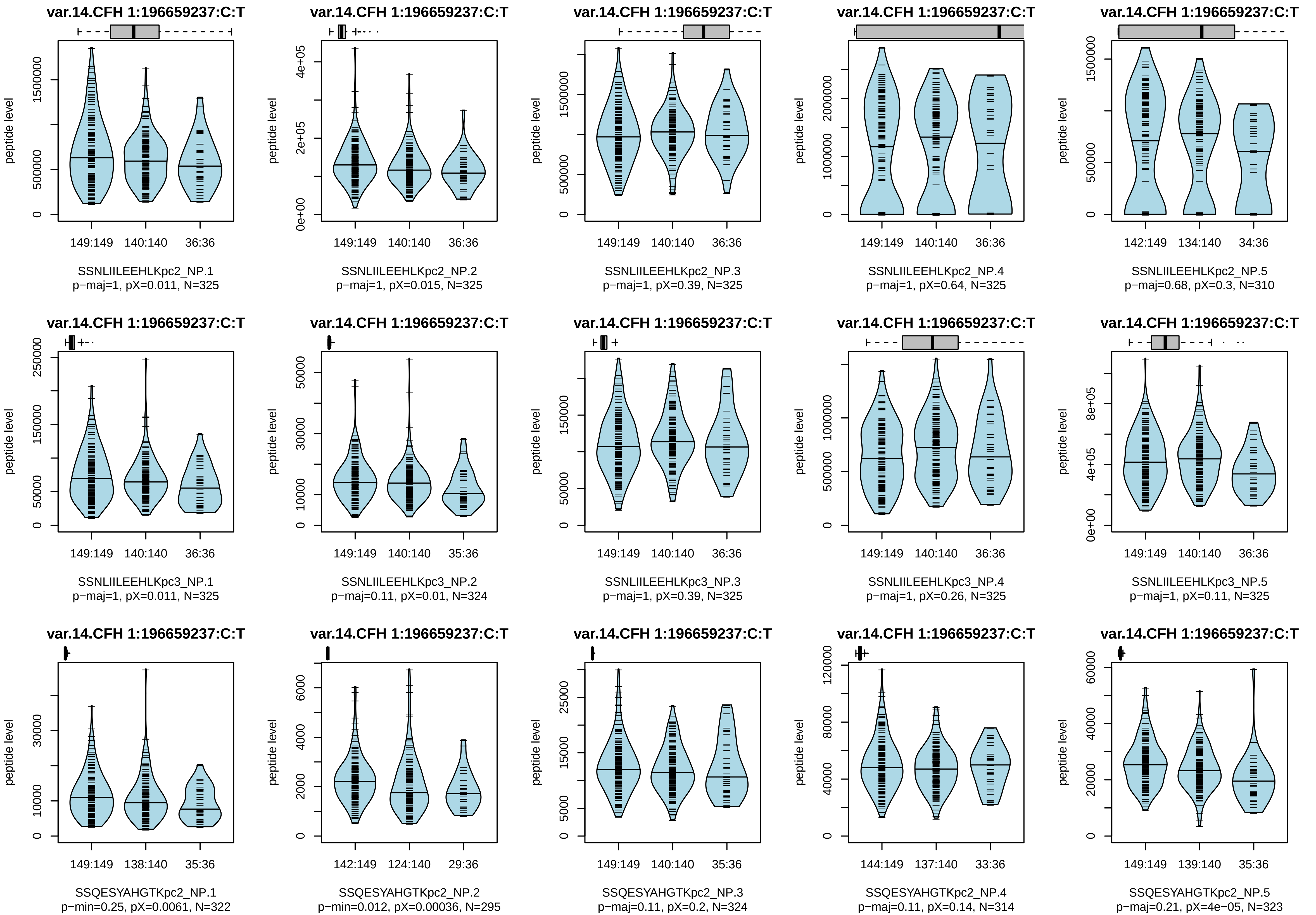
var.14.CFH 1:196659237:C:T
peptide level
149:149
140:140
36:36
SSNLIILEEHLKpc2_NP.1
p−maj=1, pX=0.011, N=325
var.14.CFH 1:196659237:C:T
peptide level
149:149
140:140
36:36
SSNLIILEEHLKpc2_NP.2
p−maj=1, pX=0.015, N=325
var.14.CFH 1:196659237:C:T
peptide level
149:149
140:140
36:36
SSNLIILEEHLKpc2_NP.3
p−maj=1, pX=0.39, N=325
var.14.CFH 1:196659237:C:T
peptide level
149:149
140:140
36:36
SSNLIILEEHLKpc2_NP.4
p−maj=1, pX=0.64, N=325
var.14.CFH 1:196659237:C:T
peptide level
142:149
134:140
34:36
SSNLIILEEHLKpc2_NP.5
p−maj=0.68, pX=0.3, N=310
var.14.CFH 1:196659237:C:T
peptide level
149:149
140:140
36:36
SSNLIILEEHLKpc3_NP.1
p−maj=1, pX=0.011, N=325
var.14.CFH 1:196659237:C:T
peptide level
149:149
140:140
35:36
SSNLIILEEHLKpc3_NP.2
p−maj=0.11, pX=0.01, N=324
var.14.CFH 1:196659237:C:T
peptide level
149:149
140:140
36:36
SSNLIILEEHLKpc3_NP.3
p−maj=1, pX=0.39, N=325
var.14.CFH 1:196659237:C:T
peptide level
149:149
140:140
36:36
SSNLIILEEHLKpc3_NP.4
p−maj=1, pX=0.26, N=325
var.14.CFH 1:196659237:C:T
peptide level
149:149
140:140
36:36
SSNLIILEEHLKpc3_NP.5
p−maj=1, pX=0.11, N=325
var.14.CFH 1:196659237:C:T
peptide level
149:149
138:140
35:36
SSQESYAHGTKpc2_NP.1
p−min=0.25, pX=0.0061, N=322
var.14.CFH 1:196659237:C:T
peptide level
142:149
124:140
29:36
SSQESYAHGTKpc2_NP.2
p−min=0.012, pX=0.00036, N=295
var.14.CFH 1:196659237:C:T
peptide level
149:149
140:140
35:36
SSQESYAHGTKpc2_NP.3
p−maj=0.11, pX=0.2, N=324
var.14.CFH 1:196659237:C:T
peptide level
144:149
137:140
33:36
SSQESYAHGTKpc2_NP.4
p−maj=0.11, pX=0.14, N=314
var.14.CFH 1:196659237:C:T
peptide level
149:149
139:140
35:36
SSQESYAHGTKpc2_NP.5
p−maj=0.21, pX=4e−05, N=323

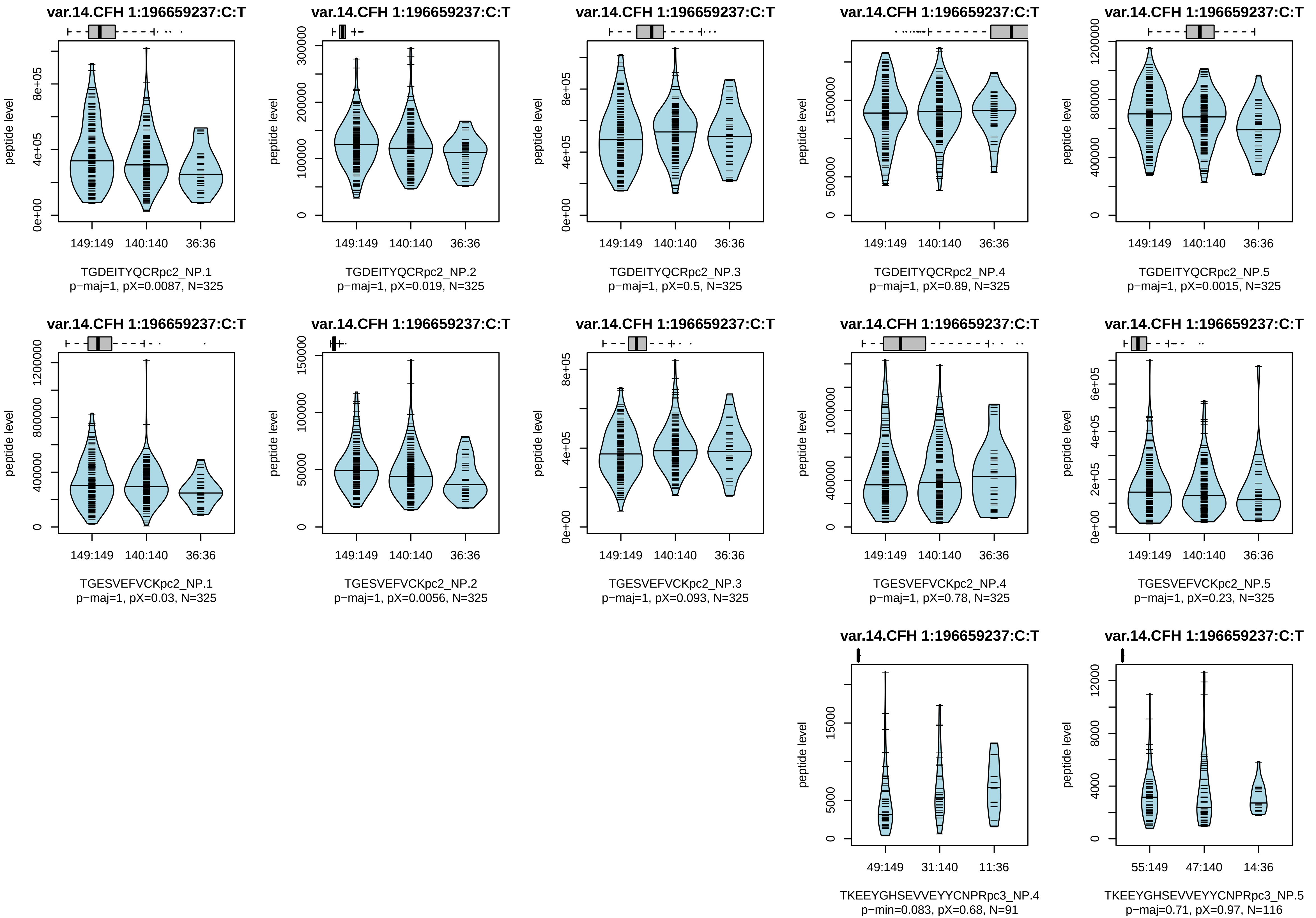

var.14.CFH 1:196659237:C:T
peptide level
149:149
140:140
36:36
TGDEITYQCRpc2_NP.1
p−maj=1, pX=0.0087, N=325
TGDEITYQCRpc2_NP.2
p−maj=1, pX=0.019, N=325
TGDEITYQCRpc2_NP.3
p−maj=1, pX=0.5, N=325
TGDEITYQCRpc2_NP.4
p−maj=1, pX=0.89, N=325
TGDEITYQCRpc2_NP.5
p−maj=1, pX=0.0015, N=325
TGESVEFVCKpc2_NP.1
p−maj=1, pX=0.03, N=325
TGESVEFVCKpc2_NP.2
p−maj=1, pX=0.0056, N=325
TGESVEFVCKpc2_NP.3
p−maj=1, pX=0.093, N=325
TGESVEFVCKpc2_NP.4
p−maj=1, pX=0.78, N=325
TGESVEFVCKpc2_NP.5
p−maj=1, pX=0.23, N=325
49:149
31:140
11:36
TKEEYGHSEVVEYYCNPRpc3_NP.4
p−min=0.083, pX=0.68, N=91
55:149
47:140
14:36
TKEEYGHSEVVEYYCNPRpc3_NP.5
p−maj=0.71, pX=0.97, N=116

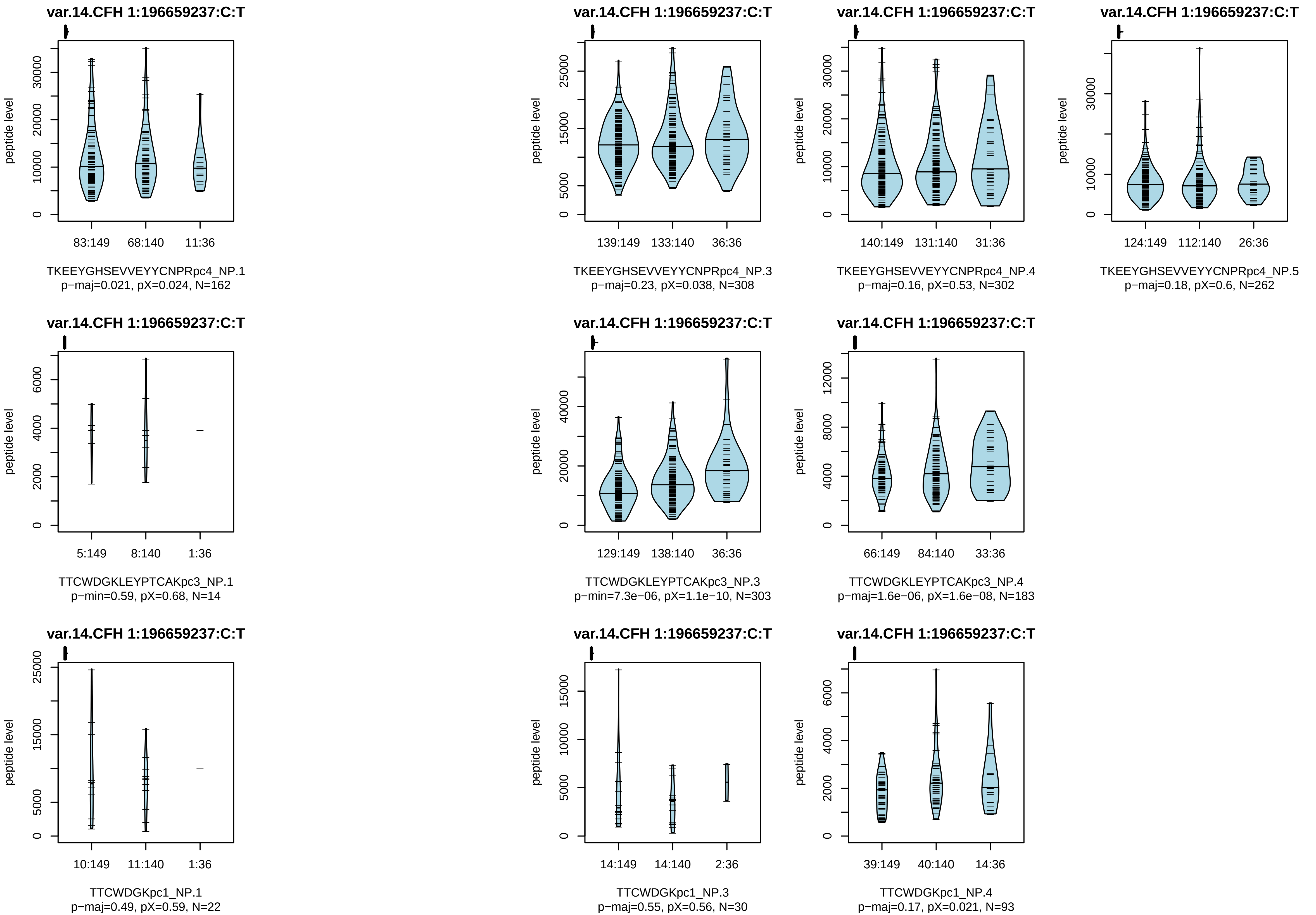
var.14.CFH 1:196659237:C:T
peptide level
83:149
68:140
11:36
TKEEYGHSEVVEYYCNPRpc4_NP.1
p−maj=0.021, pX=0.024, N=162
var.14.CFH 1:196659237:C:T
peptide level
139:149
133:140
36:36
TKEEYGHSEVVEYYCNPRpc4_NP.3
p−maj=0.23, pX=0.038, N=308
var.14.CFH 1:196659237:C:T
peptide level
140:149
131:140
31:36
TKEEYGHSEVVEYYCNPRpc4_NP.4
p−maj=0.16, pX=0.53, N=302
var.14.CFH 1:196659237:C:T
peptide level
124:149
112:140
26:36
TKEEYGHSEVVEYYCNPRpc4_NP.5
p−maj=0.18, pX=0.6, N=262
var.14.CFH 1:196659237:C:T
peptide level
5:149
8:140
1:36
TTCWDGKLEYPTCAKpc3_NP.1
p−min=0.59, pX=0.68, N=14
var.14.CFH 1:196659237:C:T
peptide level
129:149
138:140
36:36
TTCWDGKLEYPTCAKpc3_NP.3
p−min=7.3e−06, pX=1.1e−10, N=303
var.14.CFH 1:196659237:C:T
peptide level
66:149
84:140
33:36
TTCWDGKLEYPTCAKpc3_NP.4
p−maj=1.6e−06, pX=1.6e−08, N=183
var.14.CFH 1:196659237:C:T
peptide level
10:149
11:140
1:36
TTCWDGKpc1_NP.1
p−maj=0.49, pX=0.59, N=22
var.14.CFH 1:196659237:C:T
peptide level
14:149
14:140
2:36
TTCWDGKpc1_NP.3
p−maj=0.55, pX=0.56, N=30
var.14.CFH 1:196659237:C:T
peptide level
39:149
40:140
14:36
TTCWDGKpc1_NP.4
p−maj=0.17, pX=0.021, N=93

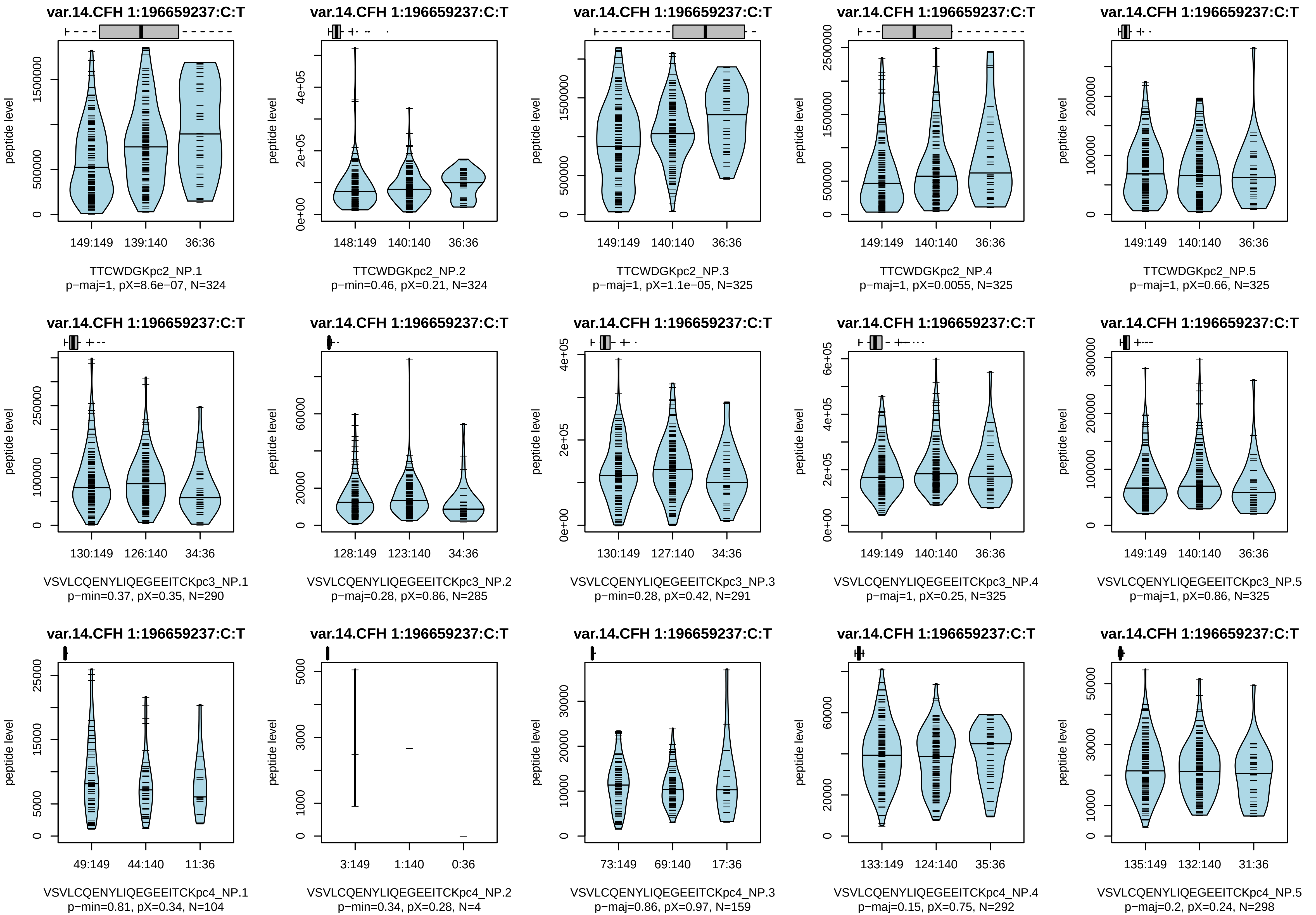
var.14.CFH 1:196659237:C:T
peptide level
149:149
139:140
36:36
TTCWDGKpc2_NP.1
p−maj=1, pX=8.6e−07, N=324
var.14.CFH 1:196659237:C:T
148:149
140:140
36:36
TTCWDGKpc2_NP.2
p−min=0.46, pX=0.21, N=324
var.14.CFH 1:196659237:C:T
149:149
140:140
36:36
TTCWDGKpc2_NP.3
p−maj=1, pX=1.1e−05, N=325
var.14.CFH 1:196659237:C:T
149:149
140:140
36:36
TTCWDGKpc2_NP.4
p−maj=1, pX=0.0055, N=325
var.14.CFH 1:196659237:C:T
149:149
140:140
36:36
TTCWDGKpc2_NP.5
p−maj=1, pX=0.66, N=325
var.14.CFH 1:196659237:C:T
130:149
126:140
34:36
VSVLCQENYLIQEGEEITCKpc3_NP.1
p−min=0.37, pX=0.35, N=290
var.14.CFH 1:196659237:C:T
128:149
123:140
34:36
VSVLCQENYLIQEGEEITCKpc3_NP.2
p−maj=0.28, pX=0.86, N=285
var.14.CFH 1:196659237:C:T
130:149
127:140
34:36
VSVLCQENYLIQEGEEITCKpc3_NP.3
p−min=0.28, pX=0.42, N=291
var.14.CFH 1:196659237:C:T
149:149
140:140
36:36
VSVLCQENYLIQEGEEITCKpc3_NP.4
p−maj=1, pX=0.25, N=325
var.14.CFH 1:196659237:C:T
149:149
140:140
36:36
VSVLCQENYLIQEGEEITCKpc3_NP.5
p−maj=1, pX=0.86, N=325
var.14.CFH 1:196659237:C:T
49:149
44:140
11:36
VSVLCQENYLIQEGEEITCKpc4_NP.1
p−min=0.81, pX=0.34, N=104
var.14.CFH 1:196659237:C:T
3:149
1:140
0:36
VSVLCQENYLIQEGEEITCKpc4_NP.2
p−min=0.34, pX=0.28, N=4
var.14.CFH 1:196659237:C:T
73:149
69:140
17:36
VSVLCQENYLIQEGEEITCKpc4_NP.3
p−maj=0.86, pX=0.97, N=159
var.14.CFH 1:196659237:C:T
133:149
124:140
35:36
VSVLCQENYLIQEGEEITCKpc4_NP.4
p−maj=0.15, pX=0.75, N=292
var.14.CFH 1:196659237:C:T
135:149
132:140
31:36
VSVLCQENYLIQEGEEITCKpc4_NP.5
p−maj=0.2, pX=0.24, N=298

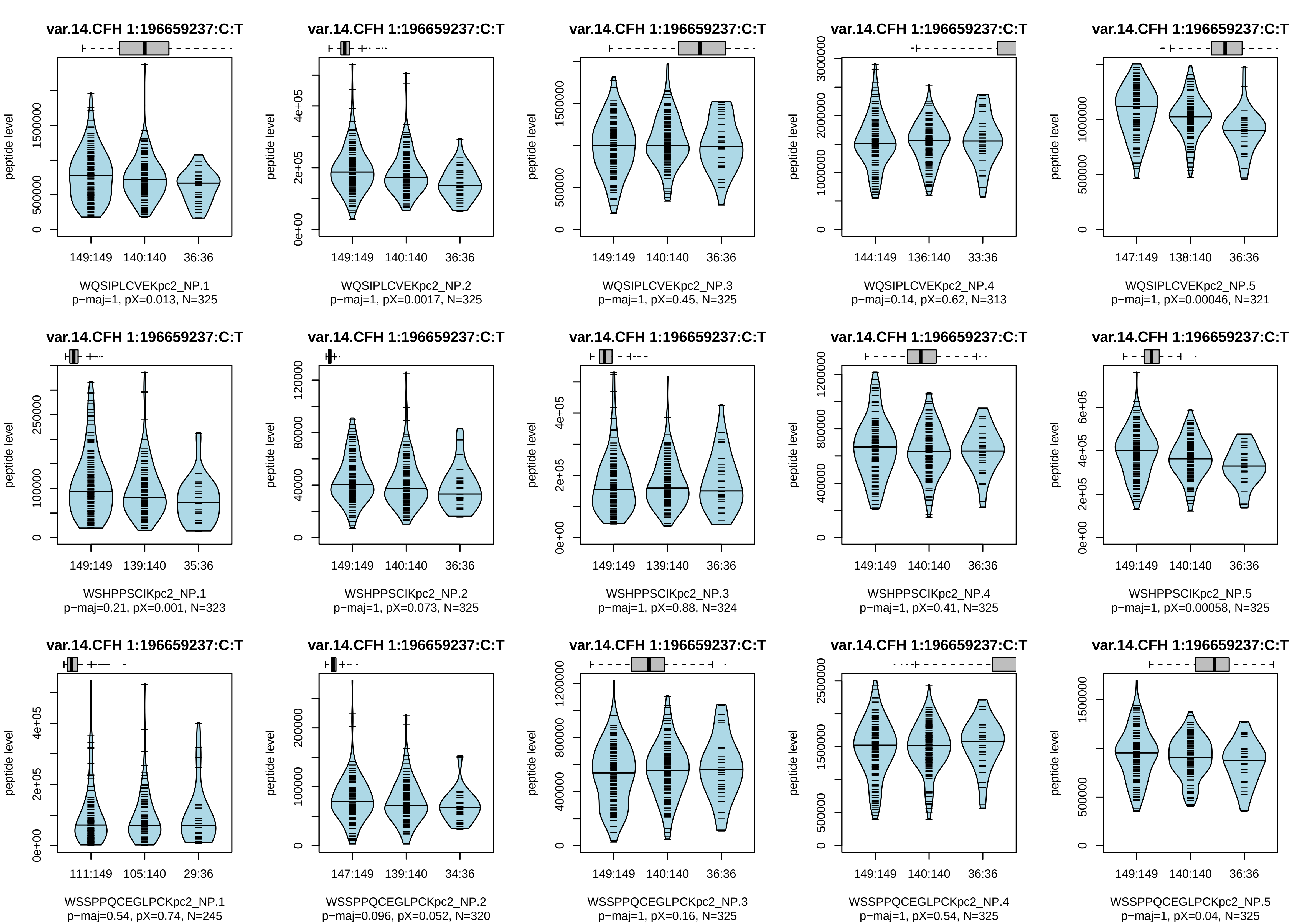
var.14.CFH 1:196659237:C:T
peptide level
149:149 140:140 36:36
WQSIPLCVEKpc2_NP.1
p−maj=1, pX=0.013, N=325
var.14.CFH 1:196659237:C:T
peptide level
149:149 140:140 36:36
WQSIPLCVEKpc2_NP.2
p−maj=1, pX=0.0017, N=325
var.14.CFH 1:196659237:C:T
peptide level
149:149 140:140 36:36
WQSIPLCVEKpc2_NP.3
p−maj=1, pX=0.45, N=325
var.14.CFH 1:196659237:C:T
peptide level
144:149 136:140 33:36
WQSIPLCVEKpc2_NP.4
p−maj=0.14, pX=0.62, N=313
var.14.CFH 1:196659237:C:T
peptide level
147:149 138:140 36:36
WQSIPLCVEKpc2_NP.5
p−maj=1, pX=0.00046, N=321
var.14.CFH 1:196659237:C:T
peptide level
149:149 139:140 35:36
WSHPPSCIKpc2_NP.1
p−maj=0.21, pX=0.001, N=323
var.14.CFH 1:196659237:C:T
peptide level
149:149 140:140 36:36
WSHPPSCIKpc2_NP.2
p−maj=1, pX=0.073, N=325
var.14.CFH 1:196659237:C:T
peptide level
149:149 139:140 36:36
WSHPPSCIKpc2_NP.3
p−maj=1, pX=0.88, N=324
var.14.CFH 1:196659237:C:T
peptide level
149:149 140:140 36:36
WSHPPSCIKpc2_NP.4
p−maj=1, pX=0.41, N=325
var.14.CFH 1:196659237:C:T
peptide level
149:149 140:140 36:36
WSHPPSCIKpc2_NP.5
p−maj=1, pX=0.00058, N=325
var.14.CFH 1:196659237:C:T
peptide level
111:149 105:140 29:36
WSSPPQCEGLPCKpc2_NP.1
p−maj=0.54, pX=0.74, N=245
var.14.CFH 1:196659237:C:T
peptide level
147:149 139:140 34:36
WSSPPQCEGLPCKpc2_NP.2
p−maj=0.096, pX=0.052, N=320
var.14.CFH 1:196659237:C:T
peptide level
149:149 140:140 36:36
WSSPPQCEGLPCKpc2_NP.3
p−maj=1, pX=0.16, N=325
var.14.CFH 1:196659237:C:T
peptide level
149:149 140:140 36:36
WSSPPQCEGLPCKpc2_NP.4
p−maj=1, pX=0.54, N=325
var.14.CFH 1:196659237:C:T
peptide level
149:149 140:140 36:36
WSSPPQCEGLPCKpc2_NP.5
p−maj=1, pX=0.04, N=325

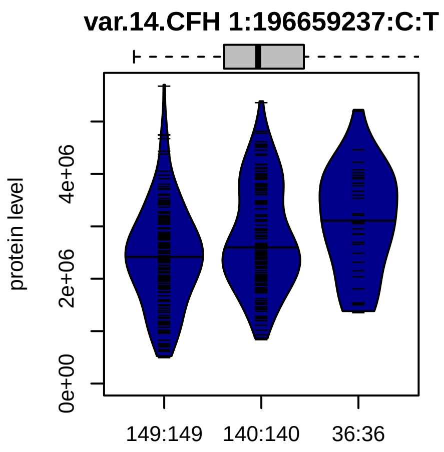

A0A024R962_NP.1
p−maj=1, pY=2.7e−05, N=325

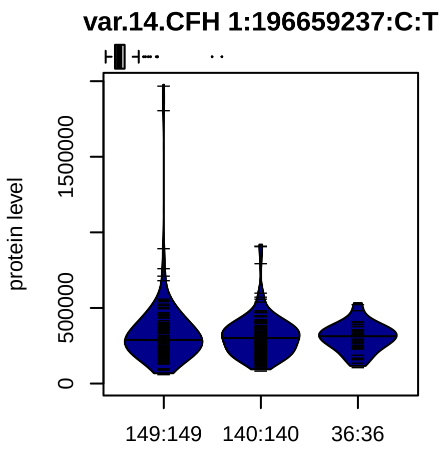

A0A024R962_NP.2
p−maj=1, pY=0.76, N=325

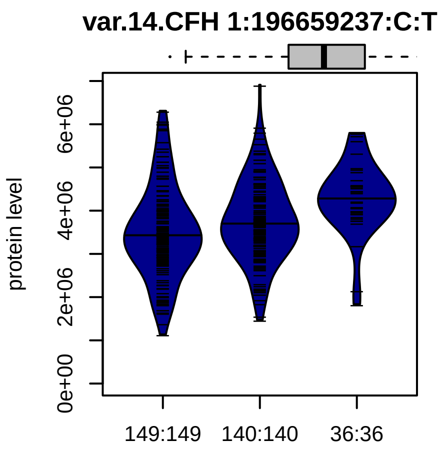

A0A024R962_NP.3
p−maj=1, pY=2.9e−06, N=325

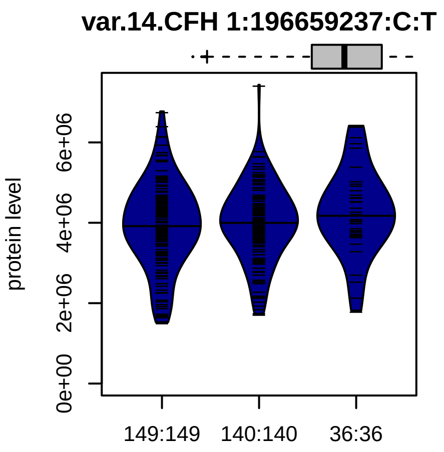

A0A024R962_NP.4
p−maj=1, pY=0.33, N=325

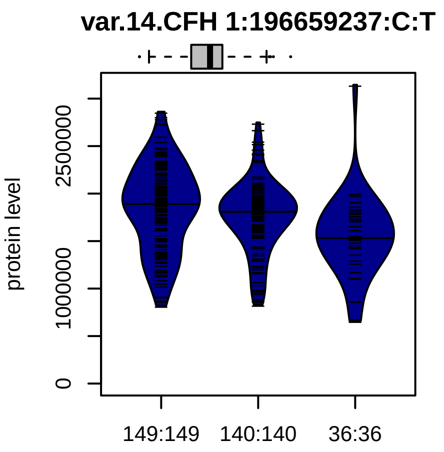

A0A024R962_NP.5
p−maj=1, pY=1e−05, N=325

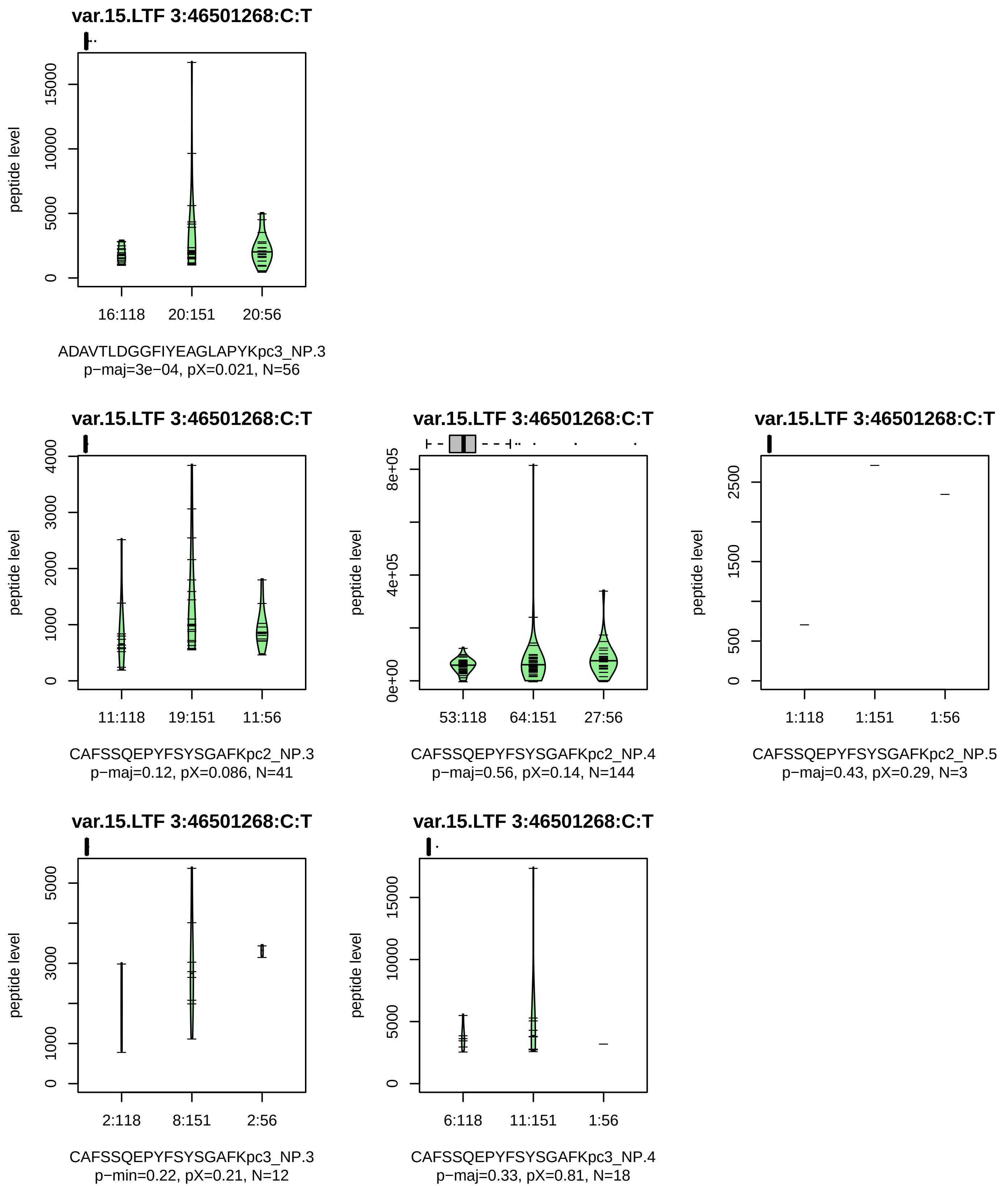
var.15.LTF 3:46501268:C:T
peptide level
16:118
20:151
20:56
ADAVTLDGGFIYEAGLAPYKpc3_NP.3
p−maj=3e−04, pX=0.021, N=56
var.15.LTF 3:46501268:C:T
peptide level
11:118
19:151
11:56
CAFSSQEPYFSYSGAFKpc2_NP.3
p−maj=0.12, pX=0.086, N=41
var.15.LTF 3:46501268:C:T
peptide level
53:118
64:151
27:56
CAFSSQEPYFSYSGAFKpc2_NP.4
p−maj=0.56, pX=0.14, N=144
var.15.LTF 3:46501268:C:T
peptide level
1:118
1:151
1:56
CAFSSQEPYFSYSGAFKpc2_NP.5
p−maj=0.43, pX=0.29, N=3
var.15.LTF 3:46501268:C:T
peptide level
2:118
8:151
2:56
CAFSSQEPYFSYSGAFKpc3_NP.3
p−min=0.22, pX=0.21, N=12
var.15.LTF 3:46501268:C:T
peptide level
6:118
11:151
1:56
CAFSSQEPYFSYSGAFKpc3_NP.4
p−maj=0.33, pX=0.81, N=18

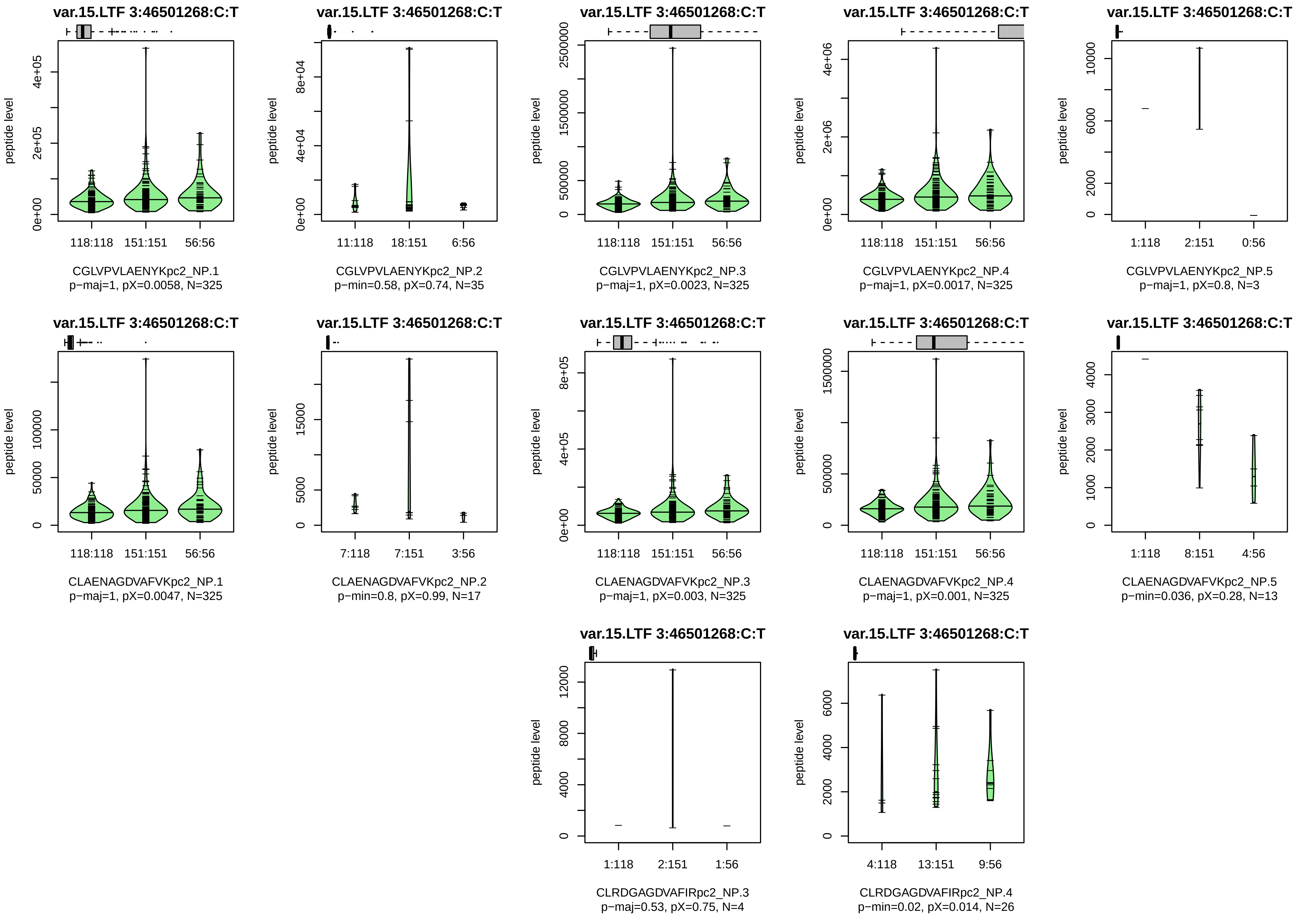
var.15.LTF 3:46501268:C:T
peptide level
118:118
151:151
56:56
CGLVPVLAENYKpc2_NP.1
p−maj=1, pX=0.0058, N=325
var.15.LTF 3:46501268:C:T
peptide level
11:118
18:151
6:56
CGLVPVLAENYKpc2_NP.2
p−min=0.58, pX=0.74, N=35
var.15.LTF 3:46501268:C:T
peptide level
118:118
151:151
56:56
CGLVPVLAENYKpc2_NP.3
p−maj=1, pX=0.0023, N=325
var.15.LTF 3:46501268:C:T
peptide level
118:118
151:151
56:56
CGLVPVLAENYKpc2_NP.4
p−maj=1, pX=0.0017, N=325
var.15.LTF 3:46501268:C:T
peptide level
1:118
2:151
0:56
CGLVPVLAENYKpc2_NP.5
p−maj=1, pX=0.8, N=3
var.15.LTF 3:46501268:C:T
peptide level
118:118
151:151
56:56
CLAENAGDVAFVKpc2_NP.1
p−maj=1, pX=0.0047, N=325
var.15.LTF 3:46501268:C:T
peptide level
7:118
7:151
3:56
CLAENAGDVAFVKpc2_NP.2
p−min=0.8, pX=0.99, N=17
var.15.LTF 3:46501268:C:T
peptide level
118:118
151:151
56:56
CLAENAGDVAFVKpc2_NP.3
p−maj=1, pX=0.003, N=325
var.15.LTF 3:46501268:C:T
peptide level
118:118
151:151
56:56
CLAENAGDVAFVKpc2_NP.4
p−maj=1, pX=0.001, N=325
var.15.LTF 3:46501268:C:T
peptide level
1:118
8:151
4:56
CLAENAGDVAFVKpc2_NP.5
p−min=0.036, pX=0.28, N=13
var.15.LTF 3:46501268:C:T
peptide level
1:118
2:151
1:56
CLRDGAGDVAFIRpc2_NP.3
p−maj=0.53, pX=0.75, N=4
var.15.LTF 3:46501268:C:T
peptide level
4:118
13:151
9:56
CLRDGAGDVAFIRpc2_NP.4
p−min=0.02, pX=0.014, N=26

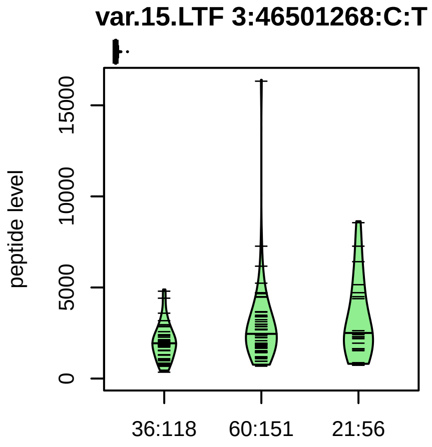

CLRDGAGDVAFIRpc3_NP.1
p−min=0.15, pX=0.012, N=117

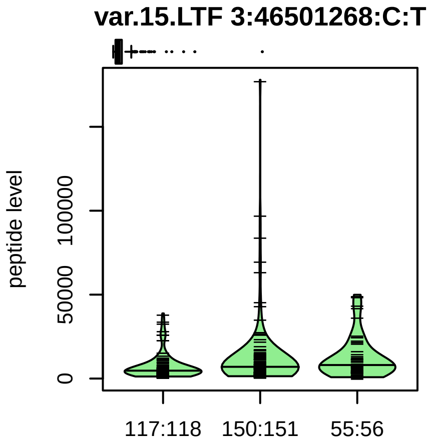

CLRDGAGDVAFIRpc3_NP.3
p−maj=0.43, pX=0.012, N=322

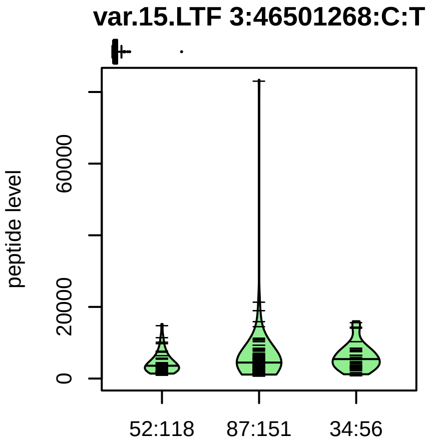

CLRDGAGDVAFIRpc3_NP.4
p−min=0.015, pX=0.026, N=173

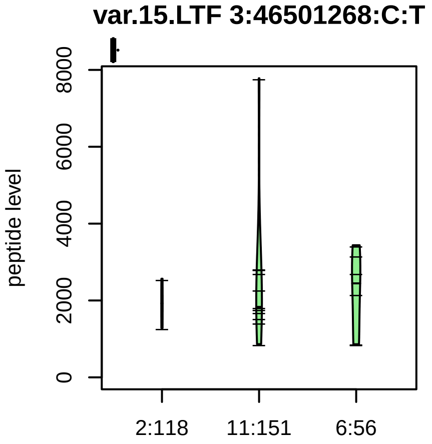

CSTSPLLEACEFLRKpc2_NP.4
p−min=0.014, pX=0.033, N=19

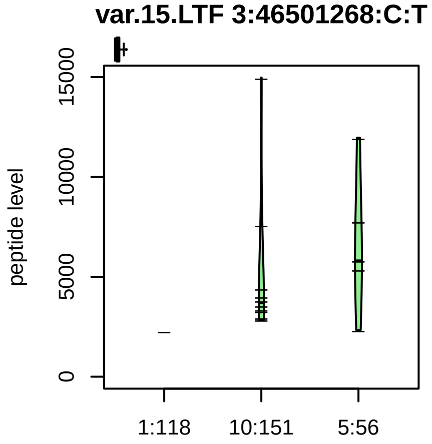

CSTSPLLEACEFLRKpc3_NP.1
p−min=0.014, pX=0.0077, N=16

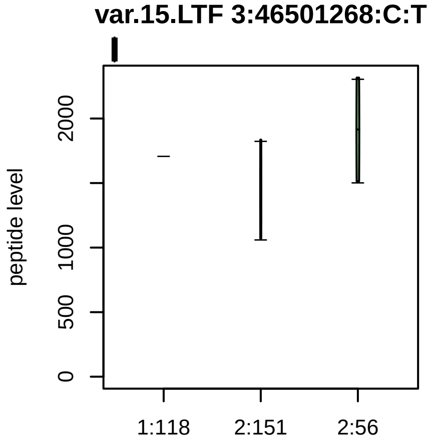

CSTSPLLEACEFLRKpc3_NP.2
p−maj=0.21, pX=0.17, N=5

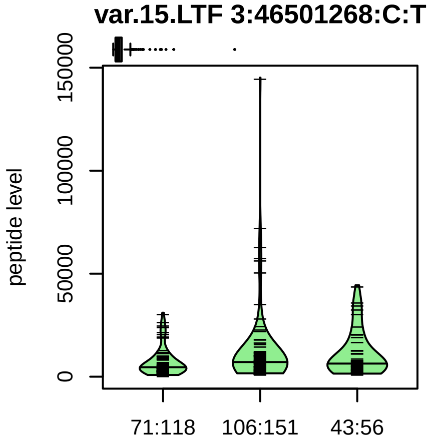

CSTSPLLEACEFLRKpc3_NP.3
p−min=0.036, pX=0.013, N=220

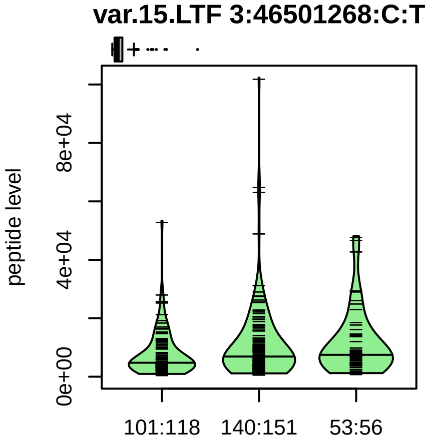

CSTSPLLEACEFLRKpc3_NP.4
p−min=0.03, pX=0.0043, N=294

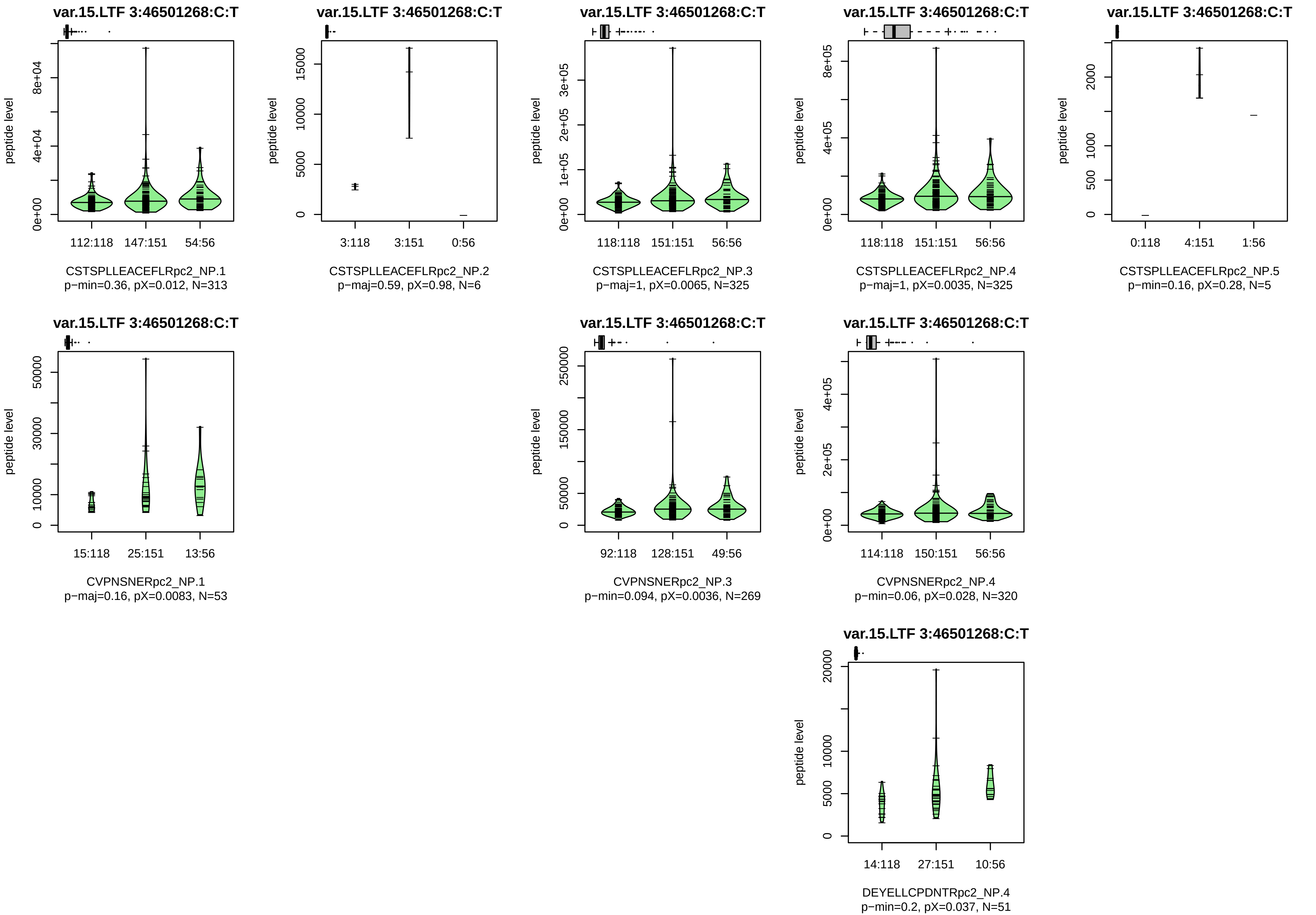

var.15.LTF 3:46501268:C:T
peptide level
112:118
147:151
54:56
CSTSPLLEACEFLRpc2_NP.1
p−min=0.36, pX=0.012, N=313
var.15.LTF 3:46501268:C:T
peptide level
3:118
3:151
0:56
CSTSPLLEACEFLRpc2_NP.2
p−maj=0.59, pX=0.98, N=6
var.15.LTF 3:46501268:C:T
peptide level
118:118
151:151
56:56
CSTSPLLEACEFLRpc2_NP.3
p−maj=1, pX=0.0065, N=325
var.15.LTF 3:46501268:C:T
peptide level
118:118
151:151
56:56
CSTSPLLEACEFLRpc2_NP.4
p−maj=1, pX=0.0035, N=325
var.15.LTF 3:46501268:C:T
peptide level
0:118
4:151
1:56
CSTSPLLEACEFLRpc2_NP.5
p−min=0.16, pX=0.28, N=5
var.15.LTF 3:46501268:C:T
peptide level
15:118
25:151
13:56
CVPNSNERpc2_NP.1
p−maj=0.16, pX=0.0083, N=53
var.15.LTF 3:46501268:C:T
peptide level
92:118
128:151
49:56
CVPNSNERpc2_NP.3
p−min=0.094, pX=0.0036, N=269
var.15.LTF 3:46501268:C:T
peptide level
114:118
150:151
56:56
CVPNSNERpc2_NP.4
p−min=0.06, pX=0.028, N=320
var.15.LTF 3:46501268:C:T
peptide level
14:118
27:151
10:56
DEYELLCPDNTRpc2_NP.4
p−min=0.2, pX=0.037, N=51

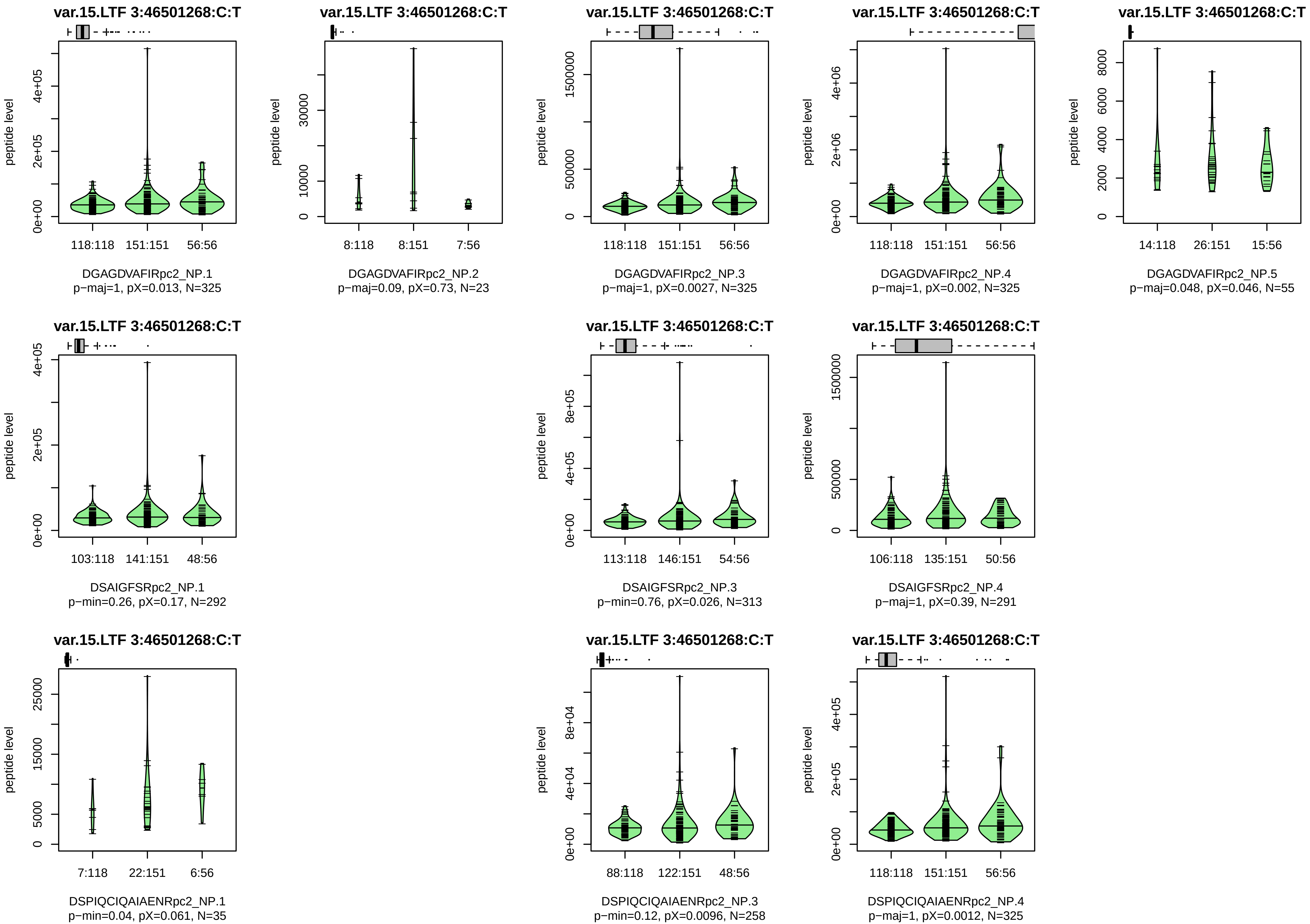

var.15.LTF 3:46501268:C:T
peptide level
118:118
151:151
56:56
DGAGDVAFIRpc2_NP.1
p−maj=1, pX=0.013, N=325
var.15.LTF 3:46501268:C:T
peptide level
8:118
8:151
7:56
DGAGDVAFIRpc2_NP.2
p−maj=0.09, pX=0.73, N=23
var.15.LTF 3:46501268:C:T
peptide level
118:118
151:151
56:56
DGAGDVAFIRpc2_NP.3
p−maj=1, pX=0.0027, N=325
var.15.LTF 3:46501268:C:T
peptide level
118:118
151:151
56:56
DGAGDVAFIRpc2_NP.4
p−maj=1, pX=0.002, N=325
var.15.LTF 3:46501268:C:T
peptide level
14:118
26:151
15:56
DGAGDVAFIRpc2_NP.5
p−maj=0.048, pX=0.046, N=55
var.15.LTF 3:46501268:C:T
peptide level
103:118
141:151
48:56
DSAIGFSRpc2_NP.1
p−min=0.26, pX=0.17, N=292
var.15.LTF 3:46501268:C:T
peptide level
113:118
146:151
54:56
DSAIGFSRpc2_NP.3
p−min=0.76, pX=0.026, N=313
var.15.LTF 3:46501268:C:T
peptide level
106:118
135:151
50:56
DSAIGFSRpc2_NP.4
p−maj=1, pX=0.39, N=291
var.15.LTF 3:46501268:C:T
peptide level
7:118
22:151
6:56
DSPIQCIQAIAENRpc2_NP.1
p−min=0.04, pX=0.061, N=35
var.15.LTF 3:46501268:C:T
peptide level
88:118
122:151
48:56
DSPIQCIQAIAENRpc2_NP.3
p−min=0.12, pX=0.0096, N=258
var.15.LTF 3:46501268:C:T
peptide level
118:118
151:151
56:56
DSPIQCIQAIAENRpc2_NP.4
p−maj=1, pX=0.0012, N=325

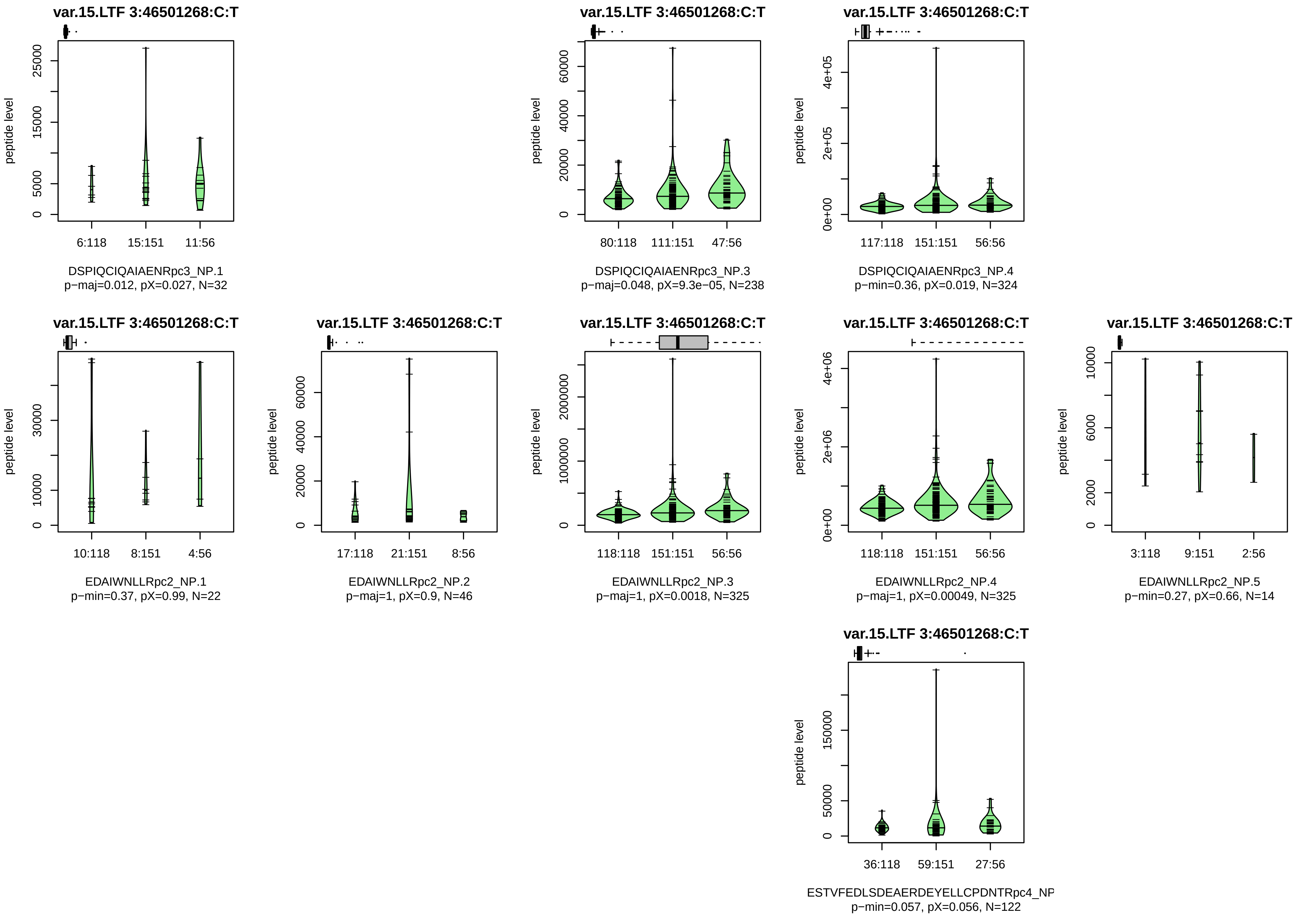
var.15.LTF 3:46501268:C:T
peptide level
6:118
15:151
11:56
DSPIQCIQAIAENRpc3_NP.1
p−maj=0.012, pX=0.027, N=32
var.15.LTF 3:46501268:C:T
peptide level
80:118
111:151
47:56
DSPIQCIQAIAENRpc3_NP.3
p−maj=0.048, pX=9.3e−05, N=238
var.15.LTF 3:46501268:C:T
peptide level
117:118
151:151
56:56
DSPIQCIQAIAENRpc3_NP.4
p−min=0.36, pX=0.019, N=324
var.15.LTF 3:46501268:C:T
peptide level
10:118
8:151
4:56
EDAIWNLLRpc2_NP.1
p−min=0.37, pX=0.99, N=22
var.15.LTF 3:46501268:C:T
peptide level
17:118
21:151
8:56
EDAIWNLLRpc2_NP.2
p−maj=1, pX=0.9, N=46
var.15.LTF 3:46501268:C:T
peptide level
118:118
151:151
56:56
EDAIWNLLRpc2_NP.3
p−maj=1, pX=0.0018, N=325
var.15.LTF 3:46501268:C:T
peptide level
118:118
151:151
56:56
EDAIWNLLRpc2_NP.4
p−maj=1, pX=0.00049, N=325
var.15.LTF 3:46501268:C:T
peptide level
3:118
9:151
2:56
EDAIWNLLRpc2_NP.5
p−min=0.27, pX=0.66, N=14
var.15.LTF 3:46501268:C:T
peptide level
36:118
59:151
27:56
ESTVFEDLSDEAERDEYELLCPDNTRpc4_NP
p−min=0.057, pX=0.056, N=122

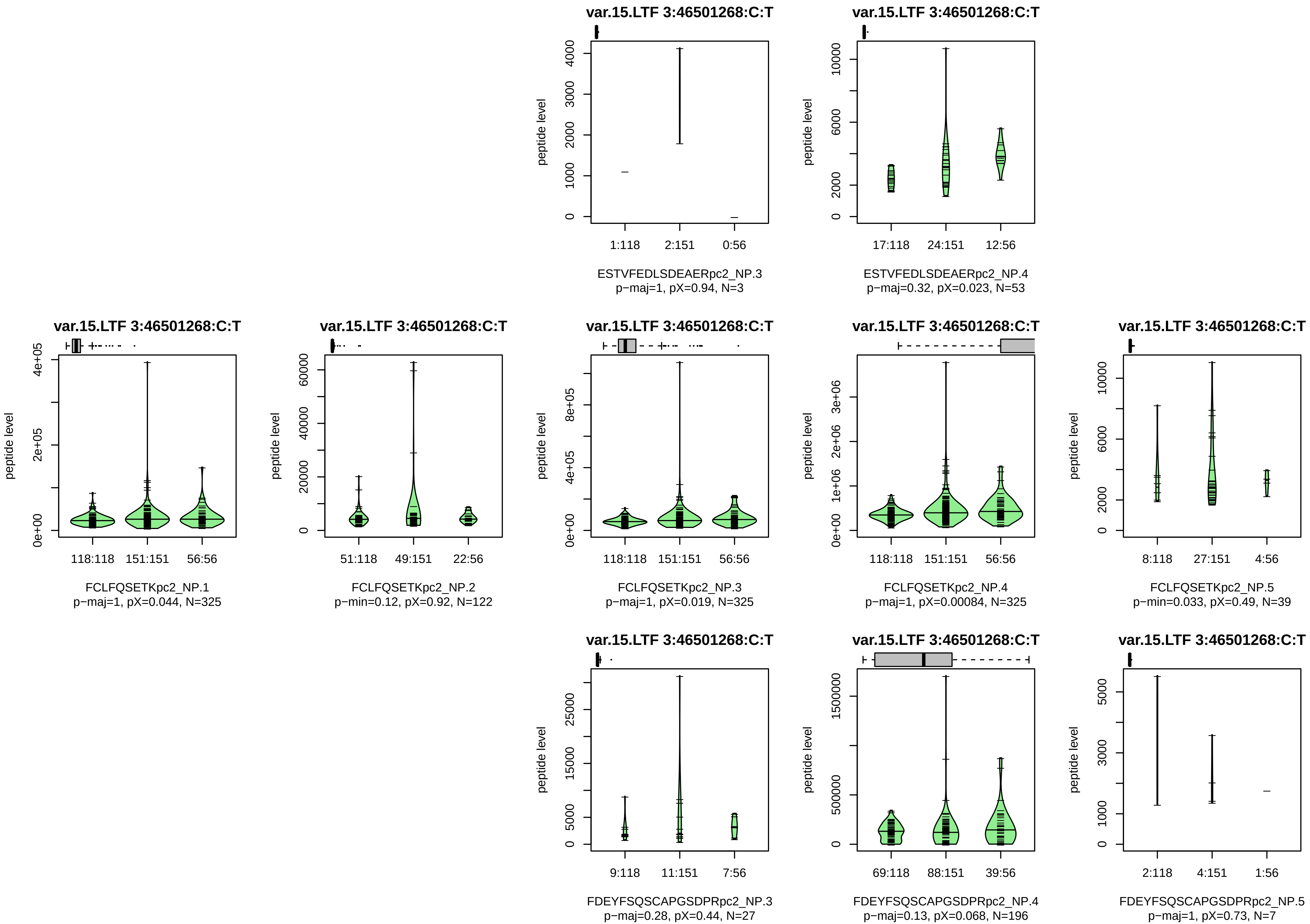
var.15.LTF 3:46501268:C:T
peptide level
1:118
2:151
0:56
ESTVFEDLSDEAERpc2_NP.3
p−maj=1, pX=0.94, N=3
var.15.LTF 3:46501268:C:T
peptide level
17:118
24:151
12:56
ESTVFEDLSDEAERpc2_NP.4
p−maj=0.32, pX=0.023, N=53
var.15.LTF 3:46501268:C:T
peptide level
118:118
151:151
56:56
FCLFQSETKpc2_NP.1
p−maj=1, pX=0.044, N=325
var.15.LTF 3:46501268:C:T
peptide level
51:118
49:151
22:56
FCLFQSETKpc2_NP.2
p−min=0.12, pX=0.92, N=122
var.15.LTF 3:46501268:C:T
peptide level
118:118
151:151
56:56
FCLFQSETKpc2_NP.3
p−maj=1, pX=0.019, N=325
var.15.LTF 3:46501268:C:T
peptide level
118:118
151:151
56:56
FCLFQSETKpc2_NP.4
p−maj=1, pX=0.00084, N=325
var.15.LTF 3:46501268:C:T
peptide level
8:118
27:151
4:56
FCLFQSETKpc2_NP.5
p−min=0.033, pX=0.49, N=39
var.15.LTF 3:46501268:C:T
peptide level
9:118
11:151
7:56
FDEYFSQSCAPGSDPRpc2_NP.3
p−maj=0.28, pX=0.44, N=27
var.15.LTF 3:46501268:C:T
peptide level
69:118
88:151
39:56
FDEYFSQSCAPGSDPRpc2_NP.4
p−maj=0.13, pX=0.068, N=196
var.15.LTF 3:46501268:C:T
peptide level
2:118
4:151
1:56
FDEYFSQSCAPGSDPRpc2_NP.5
p−maj=1, pX=0.73, N=7

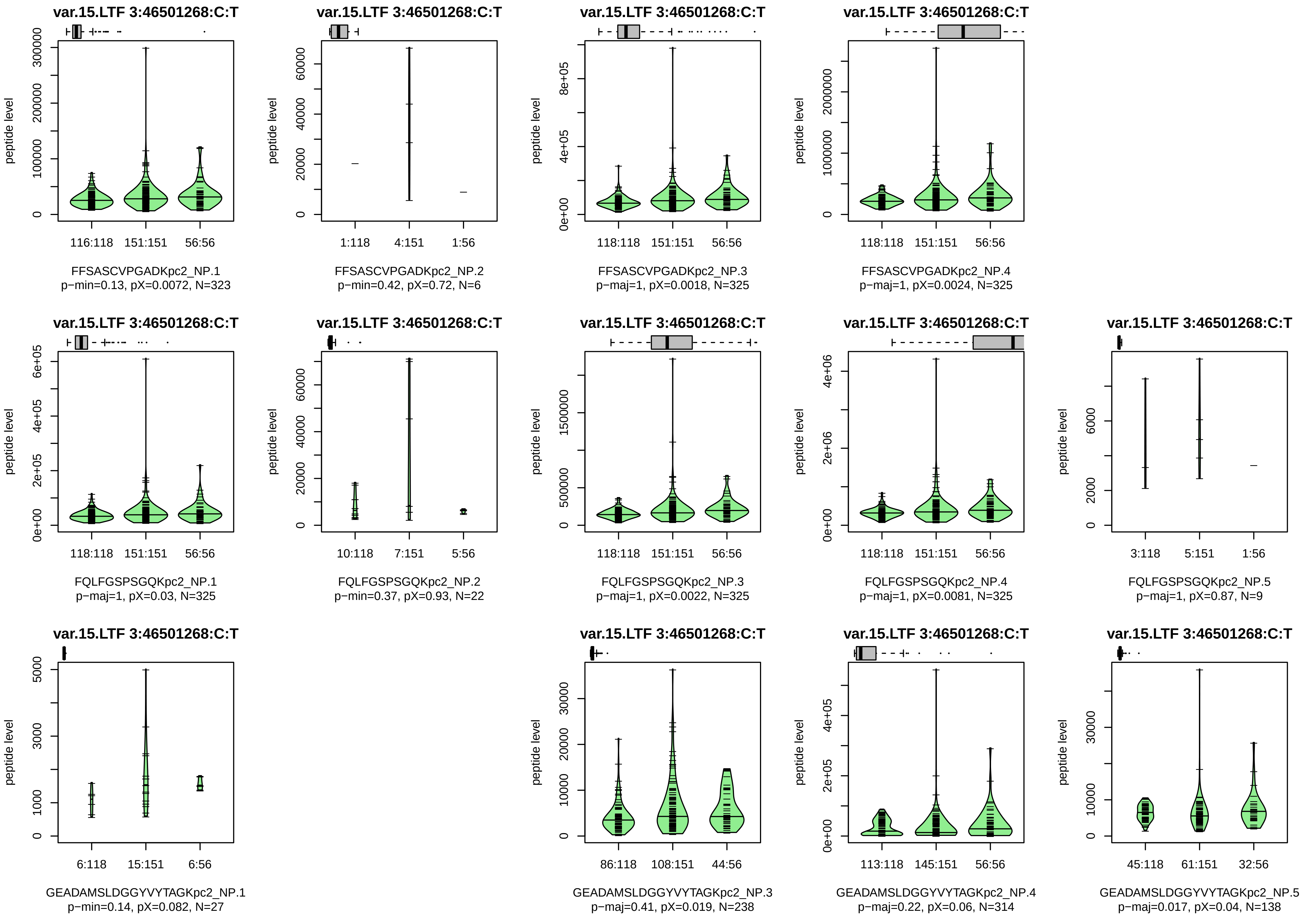
var.15.LTF 3:46501268:C:T
peptide level
116:118 151:151 56:56
FFSASCVPGADKpc2_NP.1
p−min=0.13, pX=0.0072, N=323
1:118 4:151 1:56
FFSASCVPGADKpc2_NP.2
p−min=0.42, pX=0.72, N=6
118:118 151:151 56:56
FFSASCVPGADKpc2_NP.3
p−maj=1, pX=0.0018, N=325
118:118 151:151 56:56
FFSASCVPGADKpc2_NP.4
p−maj=1, pX=0.0024, N=325
118:118 151:151 56:56
FQLFGSPSGQKpc2_NP.1
p−maj=1, pX=0.03, N=325
10:118 7:151 5:56
FQLFGSPSGQKpc2_NP.2
p−min=0.37, pX=0.93, N=22
118:118 151:151 56:56
FQLFGSPSGQKpc2_NP.3
p−maj=1, pX=0.0022, N=325
118:118 151:151 56:56
FQLFGSPSGQKpc2_NP.4
p−maj=1, pX=0.0081, N=325
3:118 5:151 1:56
FQLFGSPSGQKpc2_NP.5
p−maj=1, pX=0.87, N=9
6:118 15:151 6:56
GEADAMSLDGGYVYTAGKpc2_NP.1
p−min=0.14, pX=0.082, N=27
86:118 108:151 44:56
GEADAMSLDGGYVYTAGKpc2_NP.3
p−maj=0.41, pX=0.019, N=238
113:118 145:151 56:56
GEADAMSLDGGYVYTAGKpc2_NP.4
p−maj=0.22, pX=0.06, N=314
45:118 61:151 32:56
GEADAMSLDGGYVYTAGKpc2_NP.5
p−maj=0.017, pX=0.04, N=138

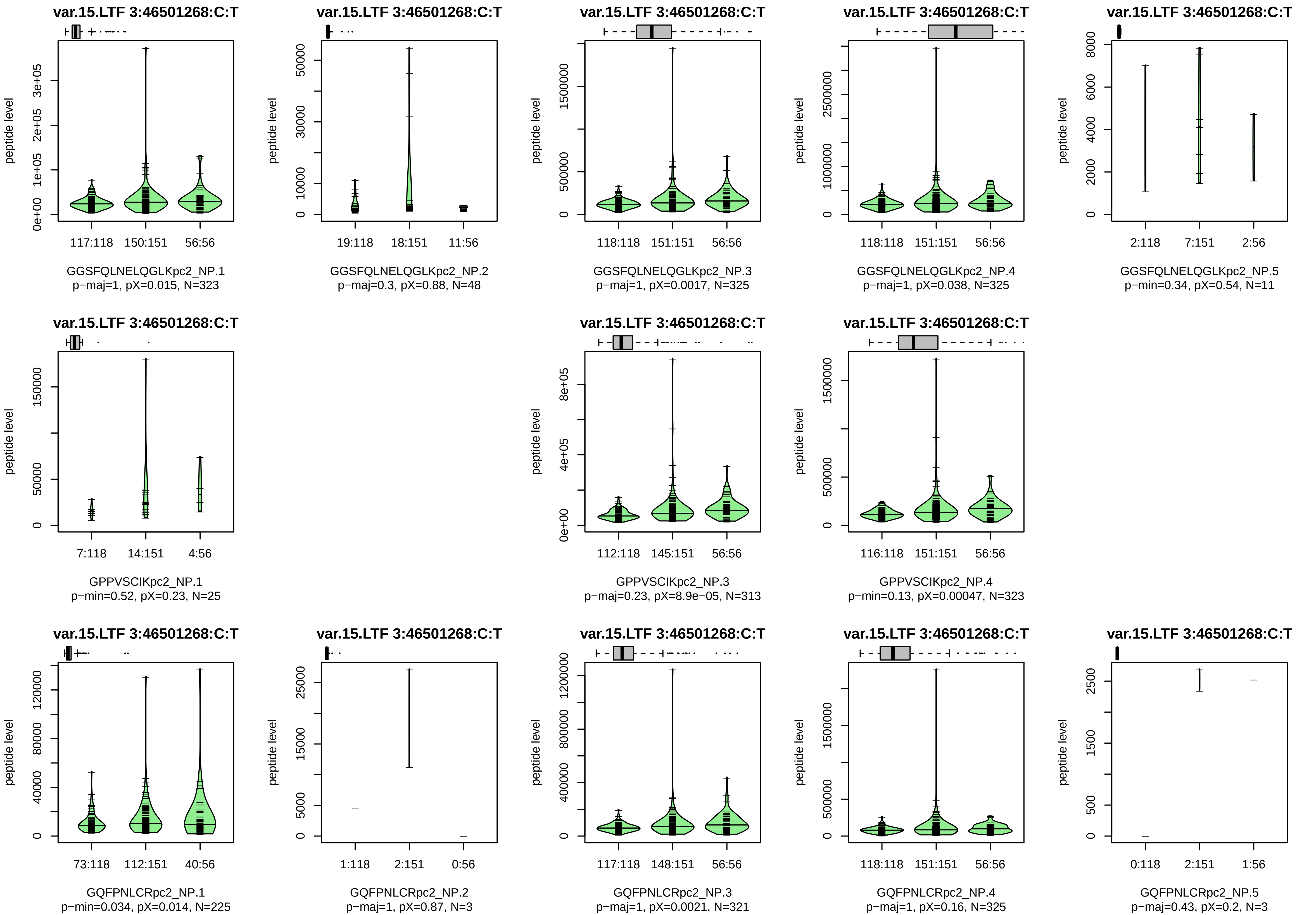
var.15.LTF 3:46501268:C:T
peptide level
117:118
150:151
56:56
GGSFQLNELQGLKpc2_NP.1
p−maj=1, pX=0.015, N=323
var.15.LTF 3:46501268:C:T
peptide level
19:118
18:151
11:56
GGSFQLNELQGLKpc2_NP.2
p−maj=0.3, pX=0.88, N=48
var.15.LTF 3:46501268:C:T
peptide level
118:118
151:151
56:56
GGSFQLNELQGLKpc2_NP.3
p−maj=1, pX=0.0017, N=325
var.15.LTF 3:46501268:C:T
peptide level
118:118
151:151
56:56
GGSFQLNELQGLKpc2_NP.4
p−maj=1, pX=0.038, N=325
var.15.LTF 3:46501268:C:T
peptide level
2:118
7:151
2:56
GGSFQLNELQGLKpc2_NP.5
p−min=0.34, pX=0.54, N=11
var.15.LTF 3:46501268:C:T
peptide level
7:118
14:151
4:56
GPPVSCIKpc2_NP.1
p−min=0.52, pX=0.23, N=25
var.15.LTF 3:46501268:C:T
peptide level
112:118
145:151
56:56
GPPVSCIKpc2_NP.3
p−maj=0.23, pX=8.9e−05, N=313
var.15.LTF 3:46501268:C:T
peptide level
116:118
151:151
56:56
GPPVSCIKpc2_NP.4
p−min=0.13, pX=0.00047, N=323
var.15.LTF 3:46501268:C:T
peptide level
73:118
112:151
40:56
GQFPNLCRpc2_NP.1
p−min=0.034, pX=0.014, N=225
var.15.LTF 3:46501268:C:T
peptide level
1:118
2:151
0:56
GQFPNLCRpc2_NP.2
p−maj=1, pX=0.87, N=3
var.15.LTF 3:46501268:C:T
peptide level
117:118
148:151
56:56
GQFPNLCRpc2_NP.3
p−maj=1, pX=0.0021, N=321
var.15.LTF 3:46501268:C:T
peptide level
118:118
151:151
56:56
GQFPNLCRpc2_NP.4
p−maj=1, pX=0.16, N=325
var.15.LTF 3:46501268:C:T
peptide level
0:118
2:151
1:56
GQFPNLCRpc2_NP.5
p−maj=0.43, pX=0.2, N=3

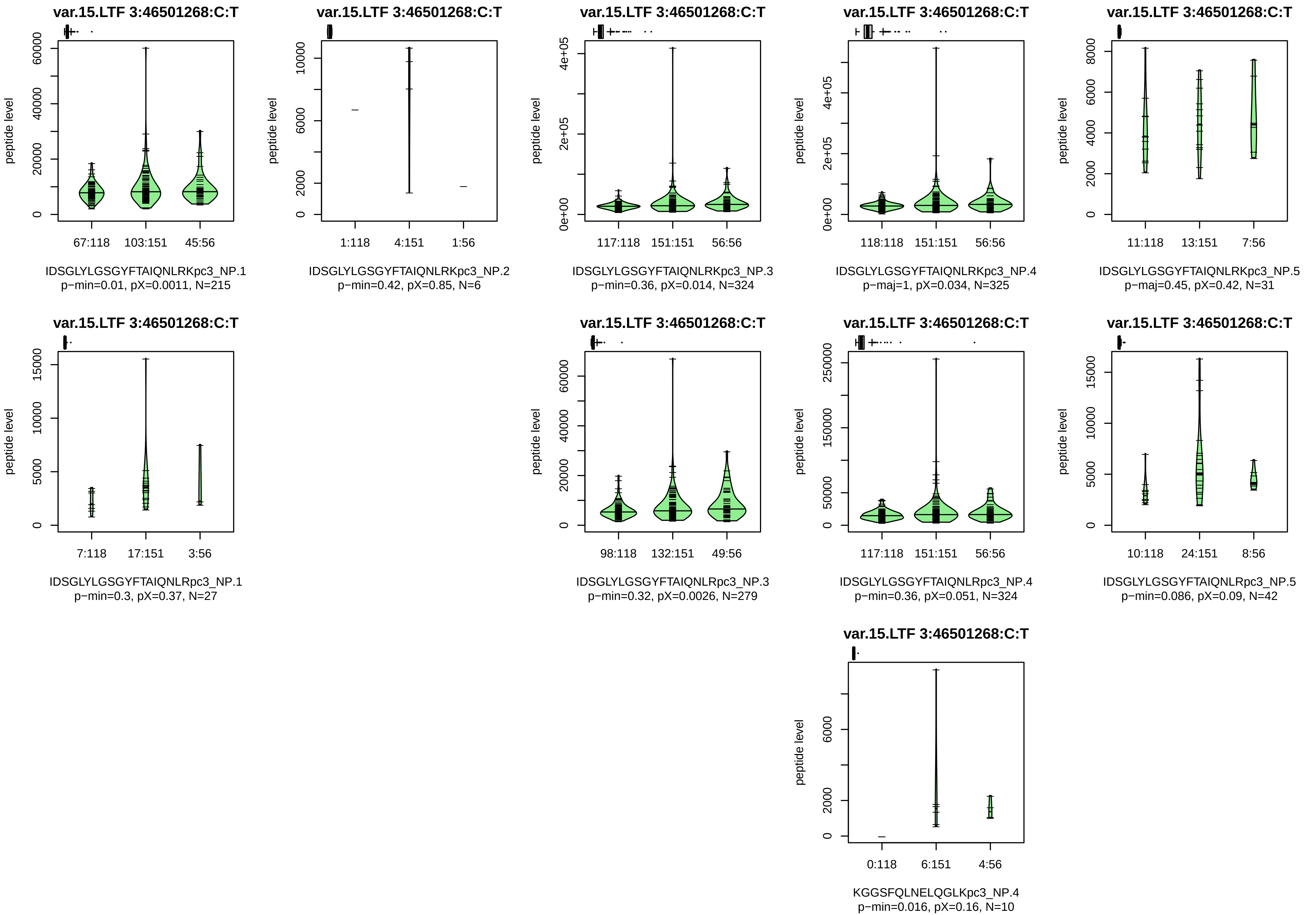
var.15.LTF 3:46501268:C:T
peptide level
67:118
103:151
45:56
IDSGLYLGSGYFTAIQNLRKpc3_NP.1
p−min=0.01, pX=0.0011, N=215
var.15.LTF 3:46501268:C:T
peptide level
1:118
4:151
1:56
IDSGLYLGSGYFTAIQNLRKpc3_NP.2
p−min=0.42, pX=0.85, N=6
var.15.LTF 3:46501268:C:T
peptide level
117:118
151:151
56:56
IDSGLYLGSGYFTAIQNLRKpc3_NP.3
p−min=0.36, pX=0.014, N=324
var.15.LTF 3:46501268:C:T
peptide level
118:118
151:151
56:56
IDSGLYLGSGYFTAIQNLRKpc3_NP.4
p−maj=1, pX=0.034, N=325
var.15.LTF 3:46501268:C:T
peptide level
11:118
13:151
7:56
IDSGLYLGSGYFTAIQNLRKpc3_NP.5
p−maj=0.45, pX=0.42, N=31
var.15.LTF 3:46501268:C:T
peptide level
7:118
17:151
3:56
IDSGLYLGSGYFTAIQNLRpc3_NP.1
p−min=0.3, pX=0.37, N=27
var.15.LTF 3:46501268:C:T
peptide level
98:118
132:151
49:56
IDSGLYLGSGYFTAIQNLRpc3_NP.3
p−min=0.32, pX=0.0026, N=279
var.15.LTF 3:46501268:C:T
peptide level
117:118
151:151
56:56
IDSGLYLGSGYFTAIQNLRpc3_NP.4
p−min=0.36, pX=0.051, N=324
var.15.LTF 3:46501268:C:T
peptide level
10:118
24:151
8:56
IDSGLYLGSGYFTAIQNLRpc3_NP.5
p−min=0.086, pX=0.09, N=42
var.15.LTF 3:46501268:C:T
peptide level
0:118
6:151
4:56
KGGSFQLNELQGLKpc3_NP.4
p−min=0.016, pX=0.16, N=10

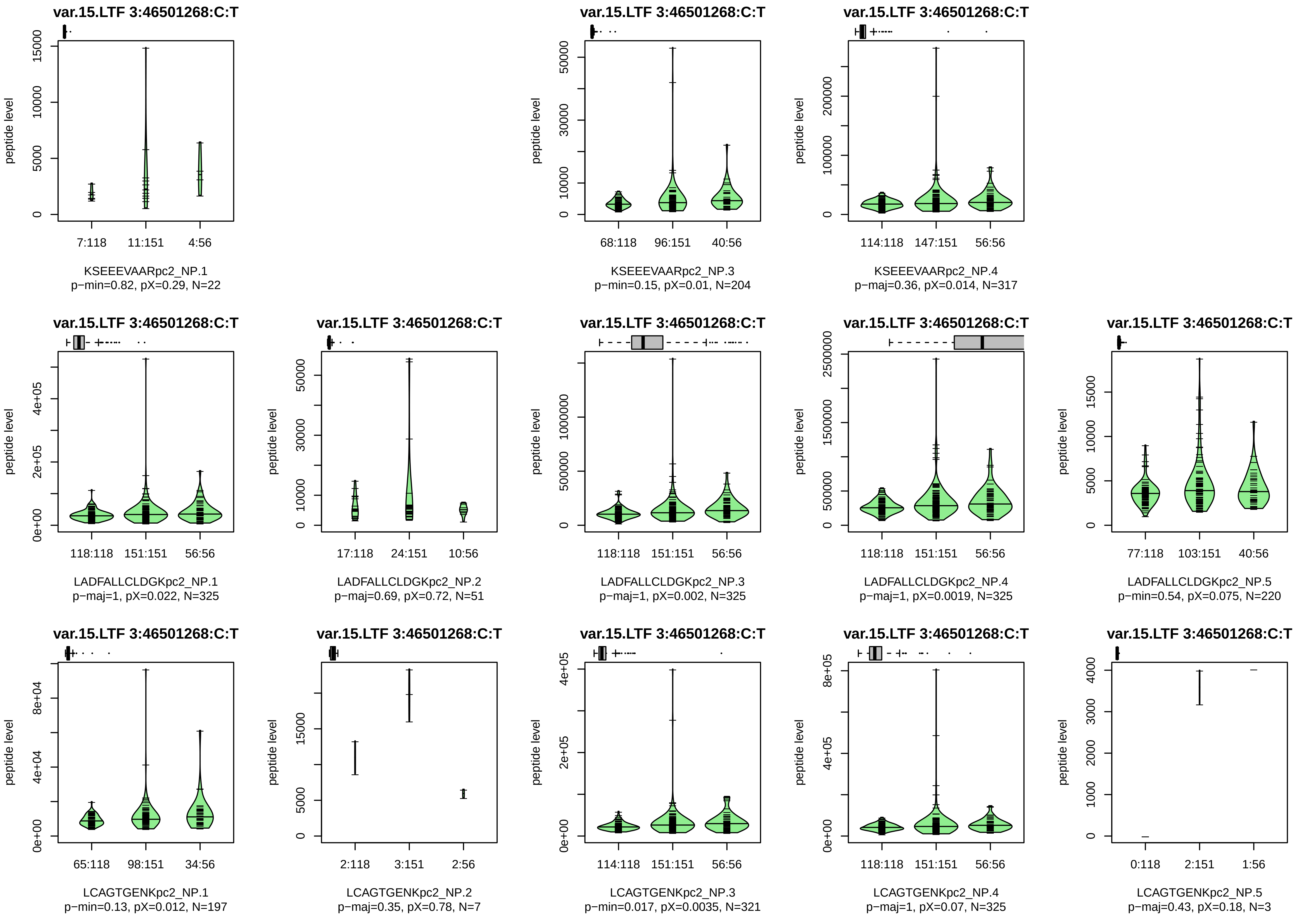

var.15.LTF 3:46501268:C:T
peptide level
7:118
11:151
4:56
KSEEEVAARpc2_NP.1
p−min=0.82, pX=0.29, N=22
var.15.LTF 3:46501268:C:T
peptide level
68:118
96:151
40:56
KSEEEVAARpc2_NP.3
p−min=0.15, pX=0.01, N=204
var.15.LTF 3:46501268:C:T
peptide level
114:118
147:151
56:56
KSEEEVAARpc2_NP.4
p−maj=0.36, pX=0.014, N=317
var.15.LTF 3:46501268:C:T
peptide level
118:118
151:151
56:56
LADFALLCLDGKpc2_NP.1
p−maj=1, pX=0.022, N=325
var.15.LTF 3:46501268:C:T
peptide level
17:118
24:151
10:56
LADFALLCLDGKpc2_NP.2
p−maj=0.69, pX=0.72, N=51
var.15.LTF 3:46501268:C:T
peptide level
118:118
151:151
56:56
LADFALLCLDGKpc2_NP.3
p−maj=1, pX=0.002, N=325
var.15.LTF 3:46501268:C:T
peptide level
118:118
151:151
56:56
LADFALLCLDGKpc2_NP.4
p−maj=1, pX=0.0019, N=325
var.15.LTF 3:46501268:C:T
peptide level
77:118
103:151
40:56
LADFALLCLDGKpc2_NP.5
p−min=0.54, pX=0.075, N=220
var.15.LTF 3:46501268:C:T
peptide level
65:118
98:151
34:56
LCAGTGENKpc2_NP.1
p−min=0.13, pX=0.012, N=197
var.15.LTF 3:46501268:C:T
peptide level
2:118
3:151
2:56
LCAGTGENKpc2_NP.2
p−maj=0.35, pX=0.78, N=7
var.15.LTF 3:46501268:C:T
peptide level
114:118
151:151
56:56
LCAGTGENKpc2_NP.3
p−min=0.017, pX=0.0035, N=321
var.15.LTF 3:46501268:C:T
peptide level
118:118
151:151
56:56
LCAGTGENKpc2_NP.4
p−maj=1, pX=0.07, N=325
var.15.LTF 3:46501268:C:T
peptide level
0:118
2:151
1:56
LCAGTGENKpc2_NP.5
p−maj=0.43, pX=0.18, N=3

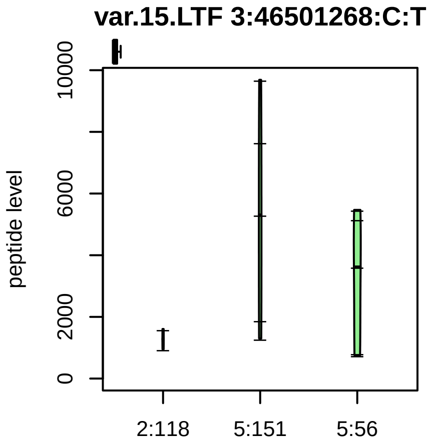

LRPVAAEVYGTERpc2_NP.3
p−maj=0.038, pX=0.054, N=12

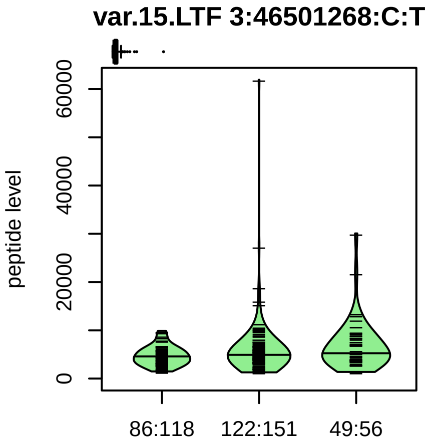

LRPVAAEVYGTERpc2_NP.4
p−min=0.047, pX=0.0011, N=257

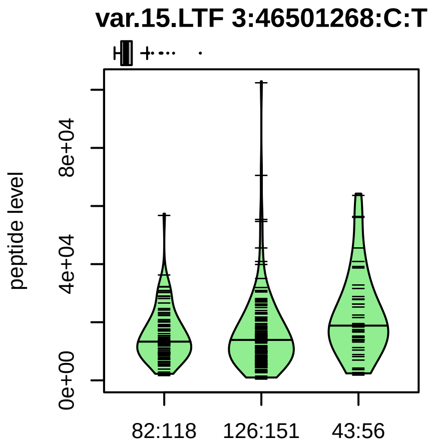

LRPVAAEVYGTERpc3_NP.1
p−min=0.014, pX=0.003, N=251

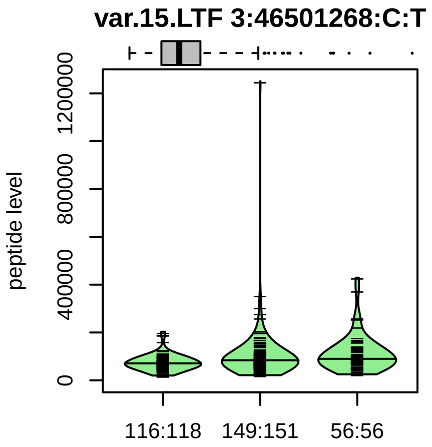

LRPVAAEVYGTERpc3_NP.3
p−min=0.62, pX=0.0015, N=321

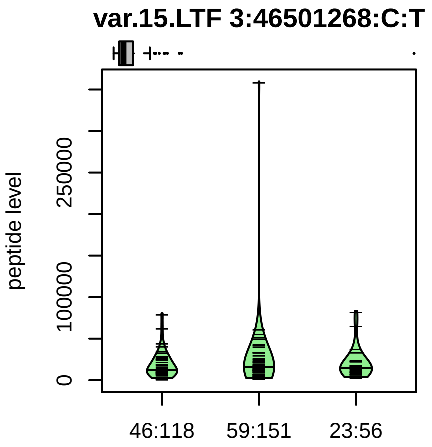

LRPVAAEVYGTERpc3_NP.4
p−maj=0.77, pX=0.58, N=128

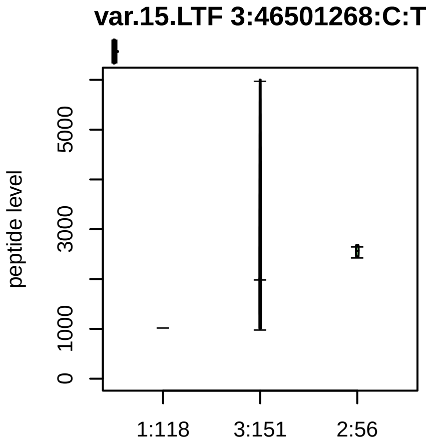

NGSDCPDKpc2_NP.3
p−maj=0.28, pX=0.18, N=6

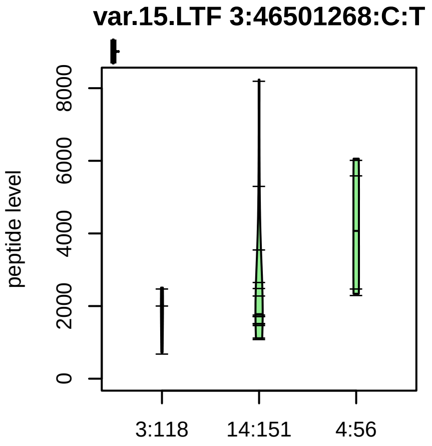

NGSDCPDKpc2_NP.4
p−min=0.034, pX=0.035, N=21

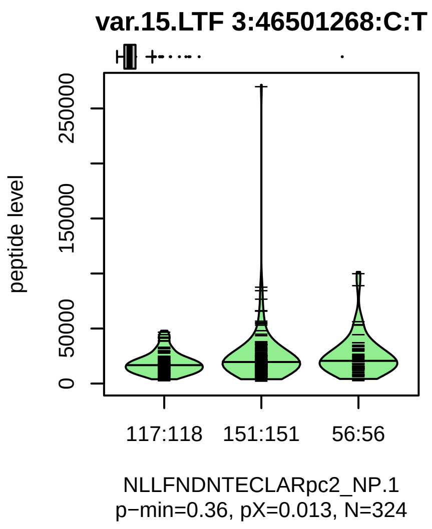
var.15.LTF 3:46501268:C:T
peptide level
0
50000
150000
250000
117:118
151:151
56:56
NLLFNDNTECLARpc2_NP.1
p−min=0.36, pX=0.013, N=324

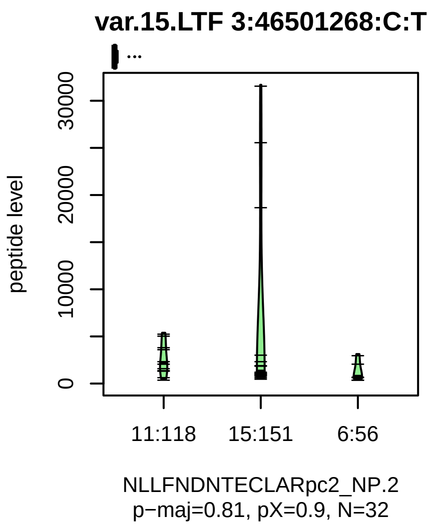
var.15.LTF 3:46501268:C:T
peptide level
0
10000
20000
30000
11:118
15:151
6:56
NLLFNDNTECLARpc2_NP.2
p−maj=0.81, pX=0.9, N=32

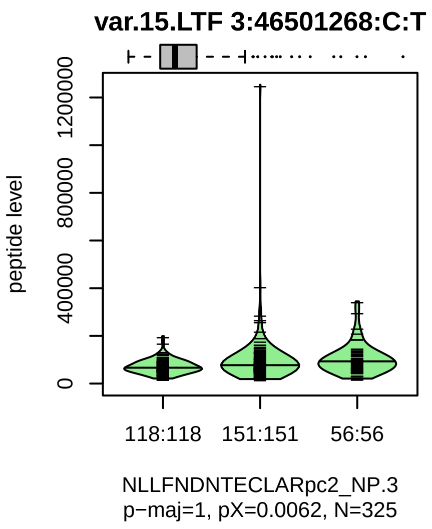
var.15.LTF 3:46501268:C:T
peptide level
0
400000
800000
1200000
118:118
151:151
56:56
NLLFNDNTECLARpc2_NP.3
p−maj=1, pX=0.0062, N=325

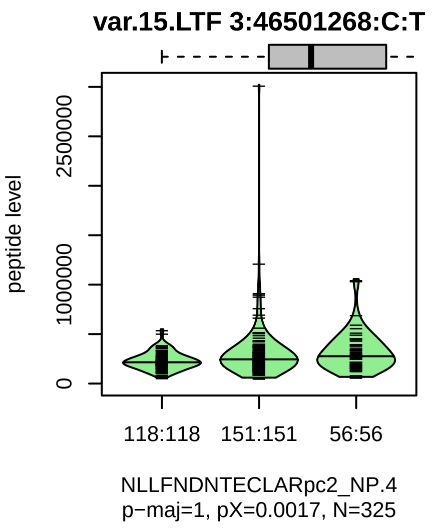
var.15.LTF 3:46501268:C:T
peptide level
0
1000000
2500000
118:118
151:151
56:56
NLLFNDNTECLARpc2_NP.4
p−maj=1, pX=0.0017, N=325

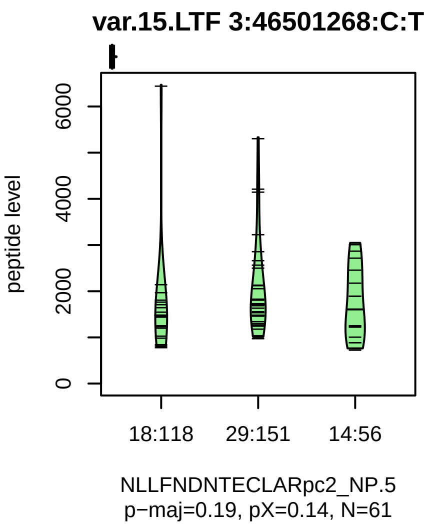
var.15.LTF 3:46501268:C:T
peptide level
0
2000
4000
6000
18:118
29:151
14:56
NLLFNDNTECLARpc2_NP.5
p−maj=0.19, pX=0.14, N=61

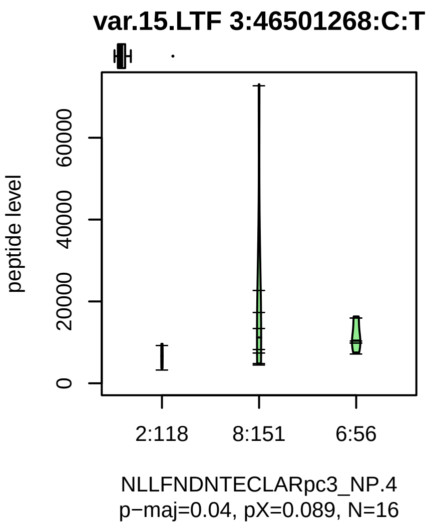
var.15.LTF 3:46501268:C:T
peptide level
0
20000
40000
60000
2:118
8:151
6:56
NLLFNDNTECLARpc3_NP.4
p−maj=0.04, pX=0.089, N=16

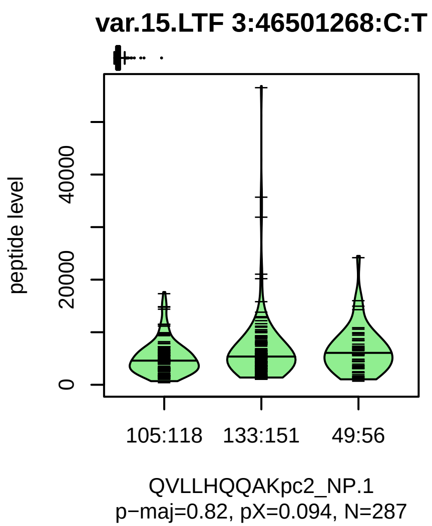
var.15.LTF 3:46501268:C:T
peptide level
0
20000
40000
105:118
133:151
49:56
QVLLHQQAKpc2_NP.1
p−maj=0.82, pX=0.094, N=287

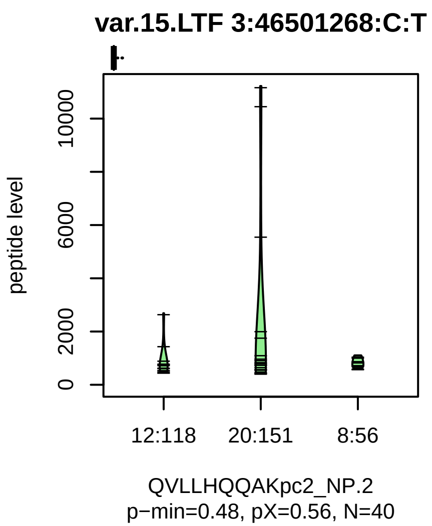
var.15.LTF 3:46501268:C:T
peptide level
0
2000
6000
10000
12:118
20:151
8:56
QVLLHQQAKpc2_NP.2
p−min=0.48, pX=0.56, N=40

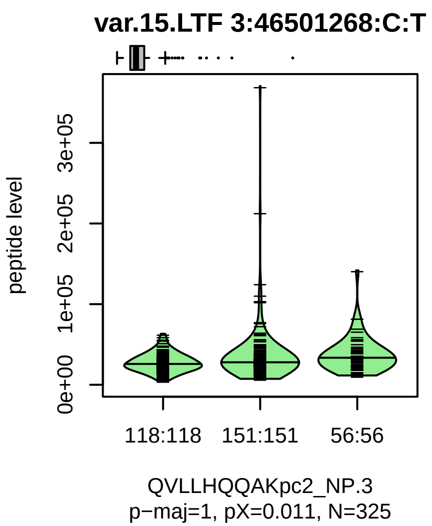
var.15.LTF 3:46501268:C:T
peptide level
0e+00
1e+05
2e+05
3e+05
118:118
151:151
56:56
QVLLHQQAKpc2_NP.3
p−maj=1, pX=0.011, N=325

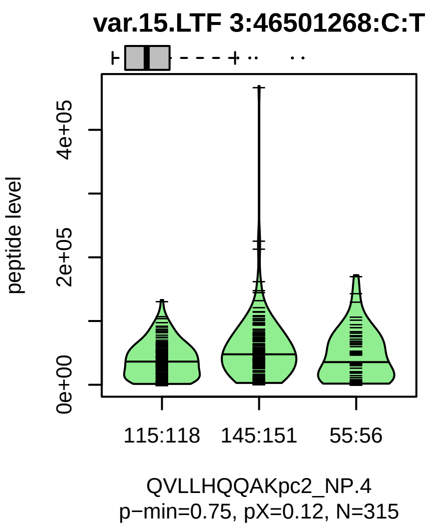
var.15.LTF 3:46501268:C:T
peptide level
0e+00
2e+05
4e+05
115:118
145:151
55:56
QVLLHQQAKpc2_NP.4
p−min=0.75, pX=0.12, N=315

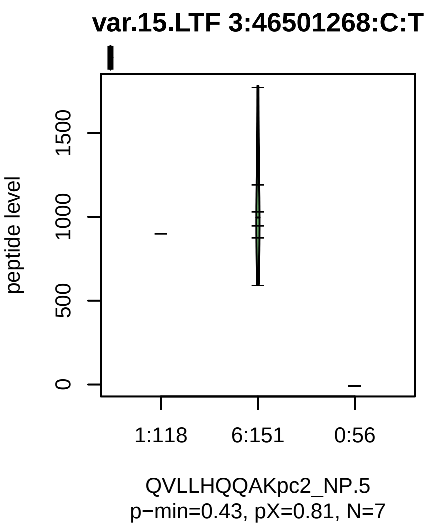
var.15.LTF 3:46501268:C:T
peptide level
0
500
1000
1500
1:118
6:151
0:56
QVLLHQQAKpc2_NP.5
p−min=0.43, pX=0.81, N=7

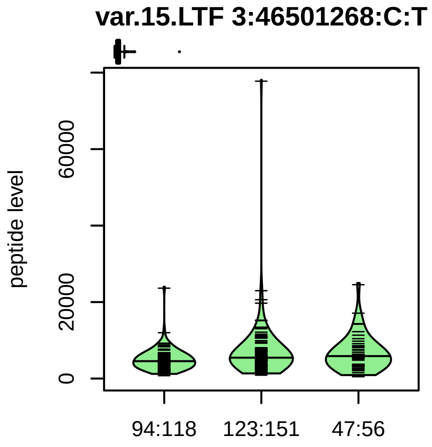

RDSPIQCIQAIAENRpc3_NP.1
p−min=0.66, pX=0.04, N=264

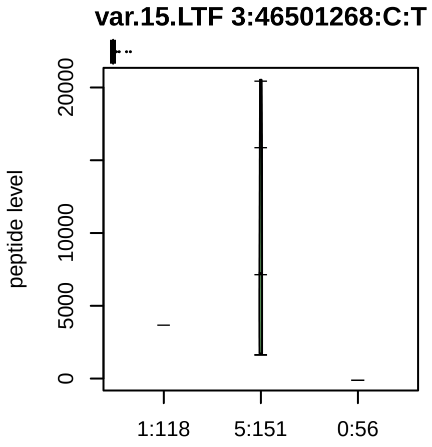

RDSPIQCIQAIAENRpc3_NP.2
p−min=0.42, pX=0.76, N=6

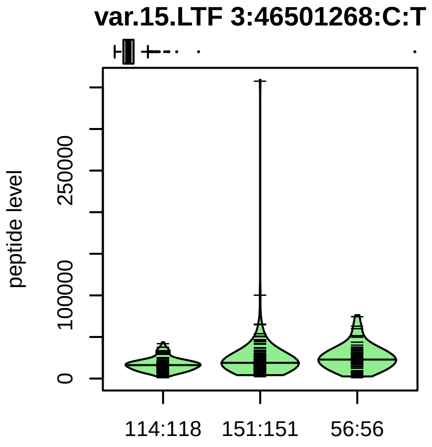

RDSPIQCIQAIAENRpc3_NP.3
p−min=0.017, pX=0.003, N=321

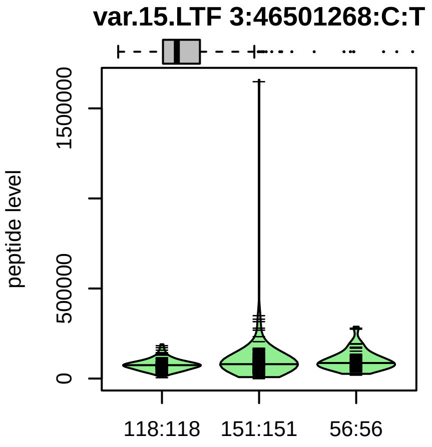

RDSPIQCIQAIAENRpc3_NP.4
p−maj=1, pX=0.056, N=325

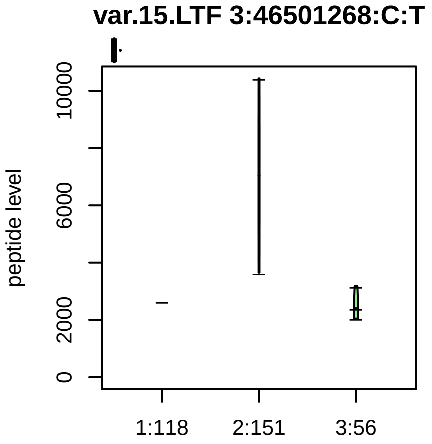

RSDTSLTWNSVKpc2_NP.4
p−maj=0.066, pX=0.26, N=6

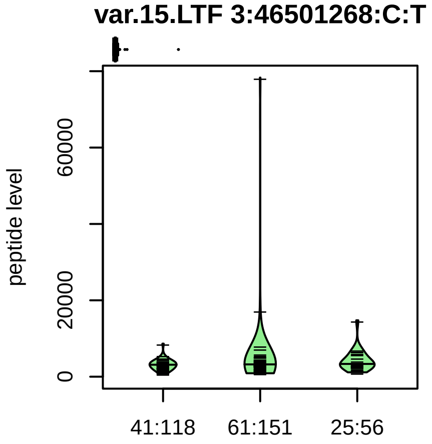

RSDTSLTWNSVKpc3_NP.3
p−min=0.24, pX=0.21, N=127

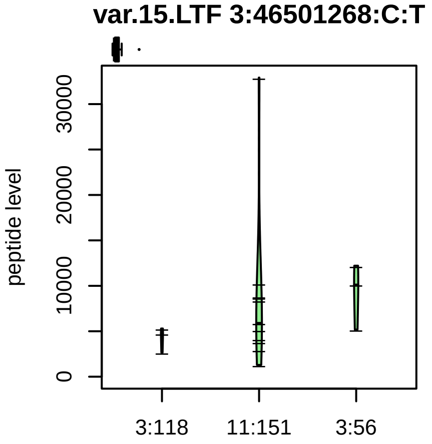

RSDTSLTWNSVKpc3_NP.4
p−min=0.12, pX=0.18, N=17

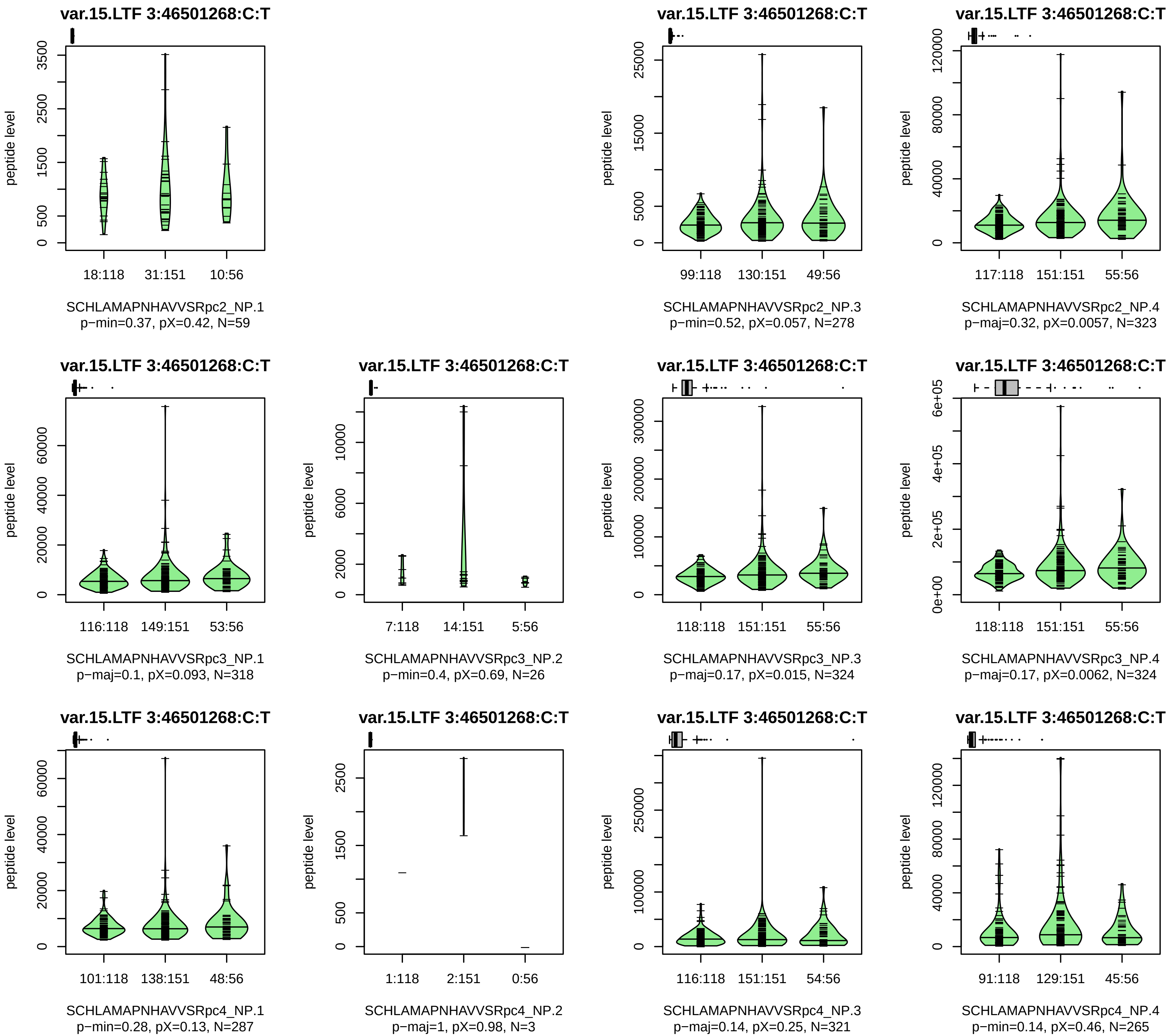
var.15.LTF 3:46501268:C:T
peptide level
18:118
31:151
10:56
SCHLAMAPNHAVVSRpc2_NP.1
p−min=0.37, pX=0.42, N=59
var.15.LTF 3:46501268:C:T
99:118
130:151
49:56
SCHLAMAPNHAVVSRpc2_NP.3
p−min=0.52, pX=0.057, N=278
var.15.LTF 3:46501268:C:T
117:118
151:151
55:56
SCHLAMAPNHAVVSRpc2_NP.4
p−maj=0.32, pX=0.0057, N=323
var.15.LTF 3:46501268:C:T
116:118
149:151
53:56
SCHLAMAPNHAVVSRpc3_NP.1
p−maj=0.1, pX=0.093, N=318
var.15.LTF 3:46501268:C:T
7:118
14:151
5:56
SCHLAMAPNHAVVSRpc3_NP.2
p−min=0.4, pX=0.69, N=26
var.15.LTF 3:46501268:C:T
118:118
151:151
55:56
SCHLAMAPNHAVVSRpc3_NP.3
p−maj=0.17, pX=0.015, N=324
var.15.LTF 3:46501268:C:T
118:118
151:151
55:56
SCHLAMAPNHAVVSRpc3_NP.4
p−maj=0.17, pX=0.0062, N=324
var.15.LTF 3:46501268:C:T
101:118
138:151
48:56
SCHLAMAPNHAVVSRpc4_NP.1
p−min=0.28, pX=0.13, N=287
var.15.LTF 3:46501268:C:T
1:118
2:151
0:56
SCHLAMAPNHAVVSRpc4_NP.2
p−maj=1, pX=0.98, N=3
var.15.LTF 3:46501268:C:T
116:118
151:151
54:56
SCHLAMAPNHAVVSRpc4_NP.3
p−maj=0.14, pX=0.25, N=321
var.15.LTF 3:46501268:C:T
91:118
129:151
45:56
SCHLAMAPNHAVVSRpc4_NP.4
p−min=0.14, pX=0.46, N=265

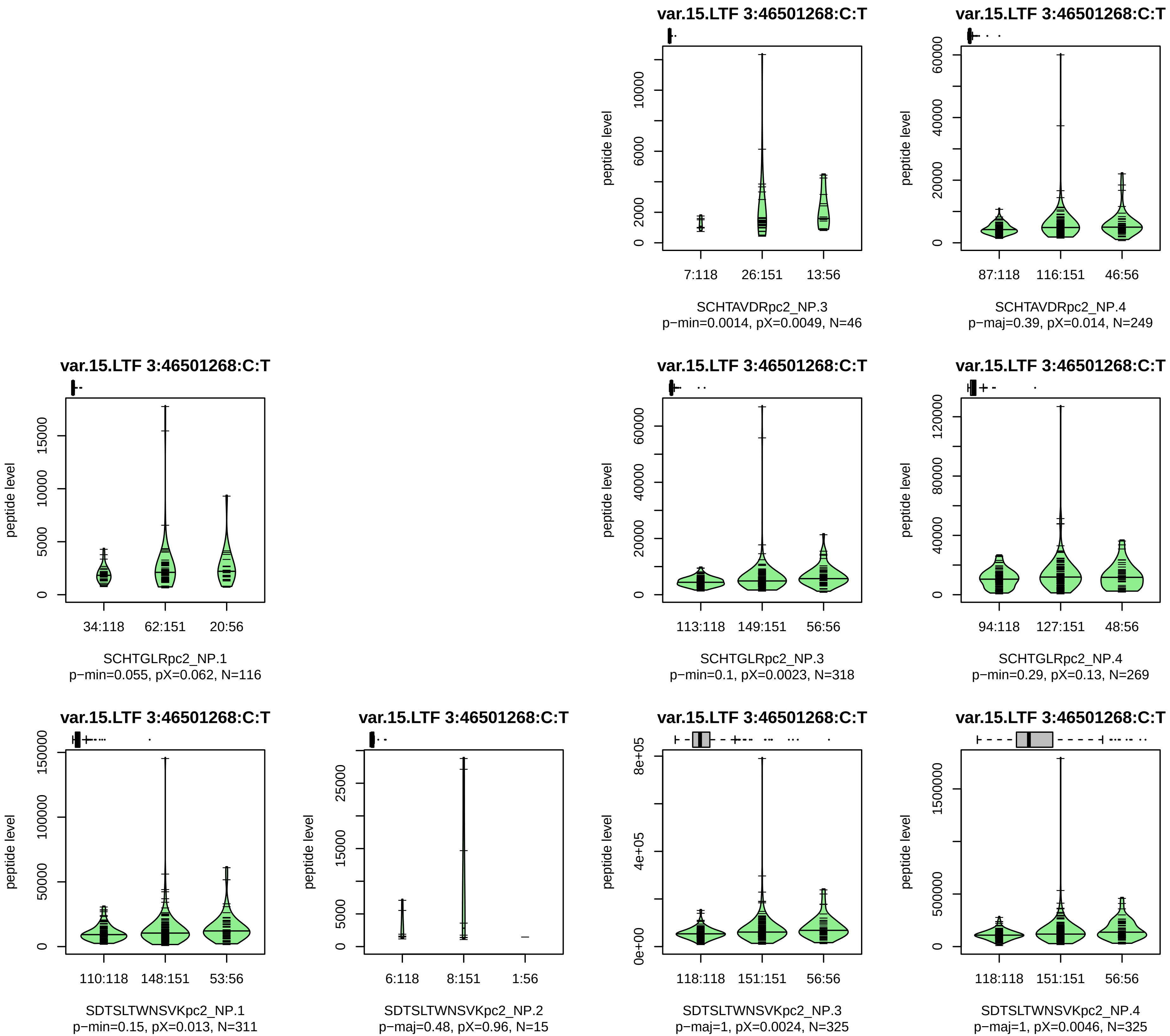
var.15.LTF 3:46501268:C:T
peptide level
7:118
26:151
13:56
SCHTAVDRpc2_NP.3
p−min=0.0014, pX=0.0049, N=46
var.15.LTF 3:46501268:C:T
peptide level
87:118
116:151
46:56
SCHTAVDRpc2_NP.4
p−maj=0.39, pX=0.014, N=249
var.15.LTF 3:46501268:C:T
peptide level
34:118
62:151
20:56
SCHTGLRpc2_NP.1
p−min=0.055, pX=0.062, N=116
var.15.LTF 3:46501268:C:T
peptide level
113:118
149:151
56:56
SCHTGLRpc2_NP.3
p−min=0.1, pX=0.0023, N=318
var.15.LTF 3:46501268:C:T
peptide level
94:118
127:151
48:56
SCHTGLRpc2_NP.4
p−min=0.29, pX=0.13, N=269
var.15.LTF 3:46501268:C:T
peptide level
110:118
148:151
53:56
SDTSLTWNSVKpc2_NP.1
p−min=0.15, pX=0.013, N=311
var.15.LTF 3:46501268:C:T
peptide level
6:118
8:151
1:56
SDTSLTWNSVKpc2_NP.2
p−maj=0.48, pX=0.96, N=15
var.15.LTF 3:46501268:C:T
peptide level
118:118
151:151
56:56
SDTSLTWNSVKpc2_NP.3
p−maj=1, pX=0.0024, N=325
var.15.LTF 3:46501268:C:T
peptide level
118:118
151:151
56:56
SDTSLTWNSVKpc2_NP.4
p−maj=1, pX=0.0046, N=325

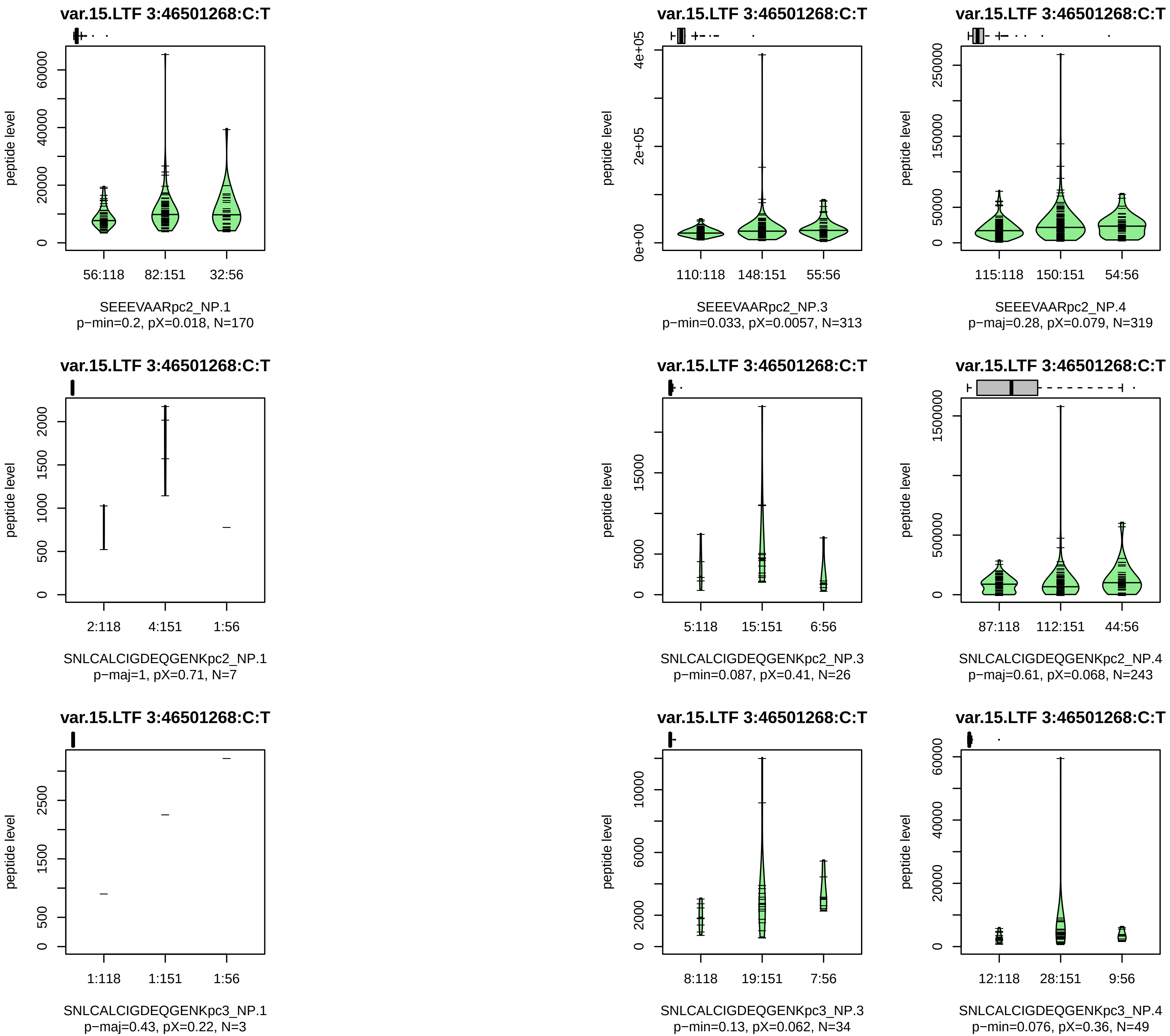

var.15.LTF 3:46501268:C:T
peptide level
56:118
82:151
32:56
SEEEVAARpc2_NP.1
p−min=0.2, pX=0.018, N=170
var.15.LTF 3:46501268:C:T
peptide level
110:118
148:151
55:56
SEEEVAARpc2_NP.3
p−min=0.033, pX=0.0057, N=313
var.15.LTF 3:46501268:C:T
peptide level
115:118
150:151
54:56
SEEEVAARpc2_NP.4
p−maj=0.28, pX=0.079, N=319
var.15.LTF 3:46501268:C:T
peptide level
2:118
4:151
1:56
SNLCALCIGDEQGENKpc2_NP.1
p−maj=1, pX=0.71, N=7
var.15.LTF 3:46501268:C:T
peptide level
5:118
15:151
6:56
SNLCALCIGDEQGENKpc2_NP.3
p−min=0.087, pX=0.41, N=26
var.15.LTF 3:46501268:C:T
peptide level
87:118
112:151
44:56
SNLCALCIGDEQGENKpc2_NP.4
p−maj=0.61, pX=0.068, N=243
var.15.LTF 3:46501268:C:T
peptide level
1:118
1:151
1:56
SNLCALCIGDEQGENKpc3_NP.1
p−maj=0.43, pX=0.22, N=3
var.15.LTF 3:46501268:C:T
peptide level
8:118
19:151
7:56
SNLCALCIGDEQGENKpc3_NP.3
p−min=0.13, pX=0.062, N=34
var.15.LTF 3:46501268:C:T
peptide level
12:118
28:151
9:56
SNLCALCIGDEQGENKpc3_NP.4
p−min=0.076, pX=0.36, N=49

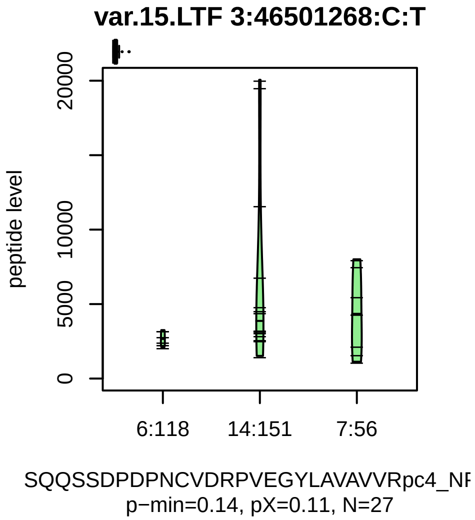
var.15.LTF 3:46501268:C:T
peptide level
0
5000
10000
20000
6:118
14:151
7:56
SQQSSDPDPNCVDRPVEGYLAVAVVRpc4_NF
p−min=0.14, pX=0.11, N=27

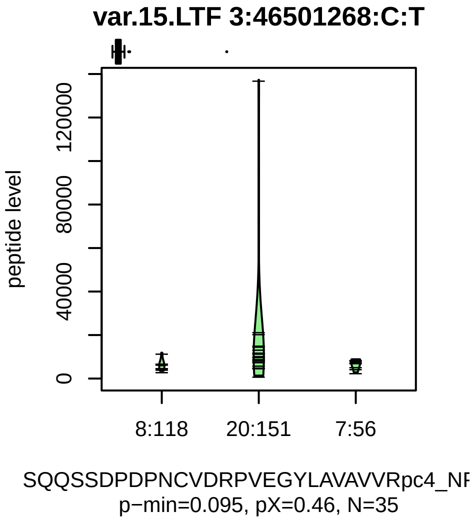
var.15.LTF 3:46501268:C:T
peptide level
0
40000
80000
120000
8:118
20:151
7:56
SQQSSDPDPNCVDRPVEGYLAVAVVRpc4_NF
p−min=0.095, pX=0.46, N=35

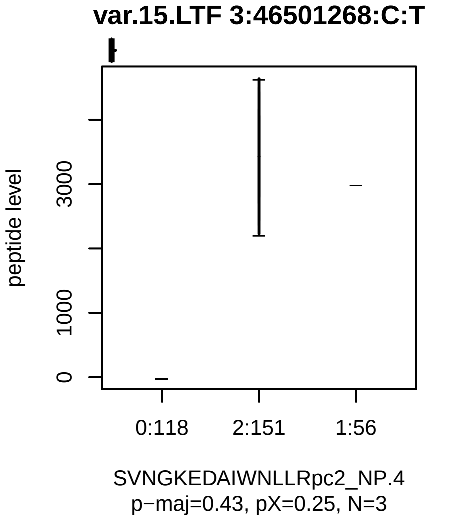
var.15.LTF 3:46501268:C:T
peptide level
0
1000
3000
0:118
2:151
1:56
SVNGKEDAIWNLLRpc2_NP.4
p−maj=0.43, pX=0.25, N=3

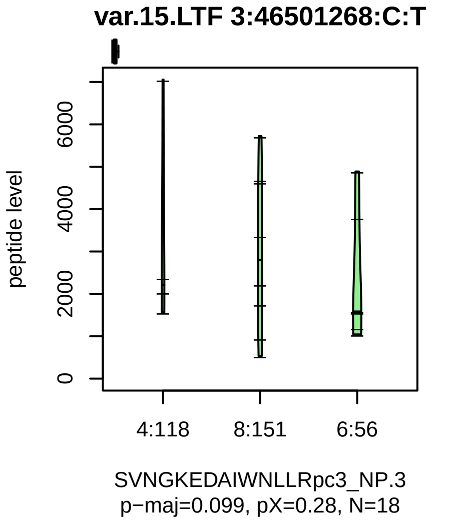
var.15.LTF 3:46501268:C:T
peptide level
0
2000
4000
6000
4:118
8:151
6:56
SVNGKEDAIWNLLRpc3_NP.3
p−maj=0.099, pX=0.28, N=18

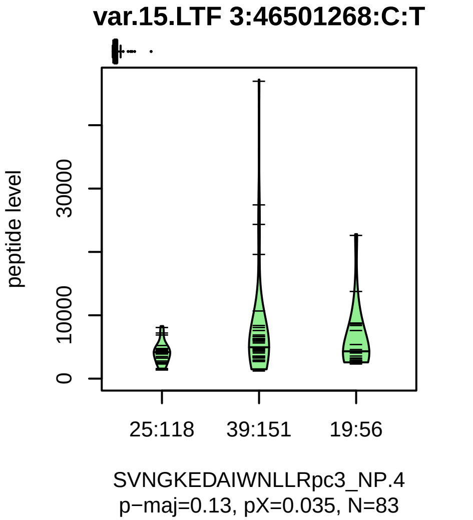
var.15.LTF 3:46501268:C:T
peptide level
0
10000
30000
25:118
39:151
19:56
SVNGKEDAIWNLLRpc3_NP.4
p−maj=0.13, pX=0.035, N=83

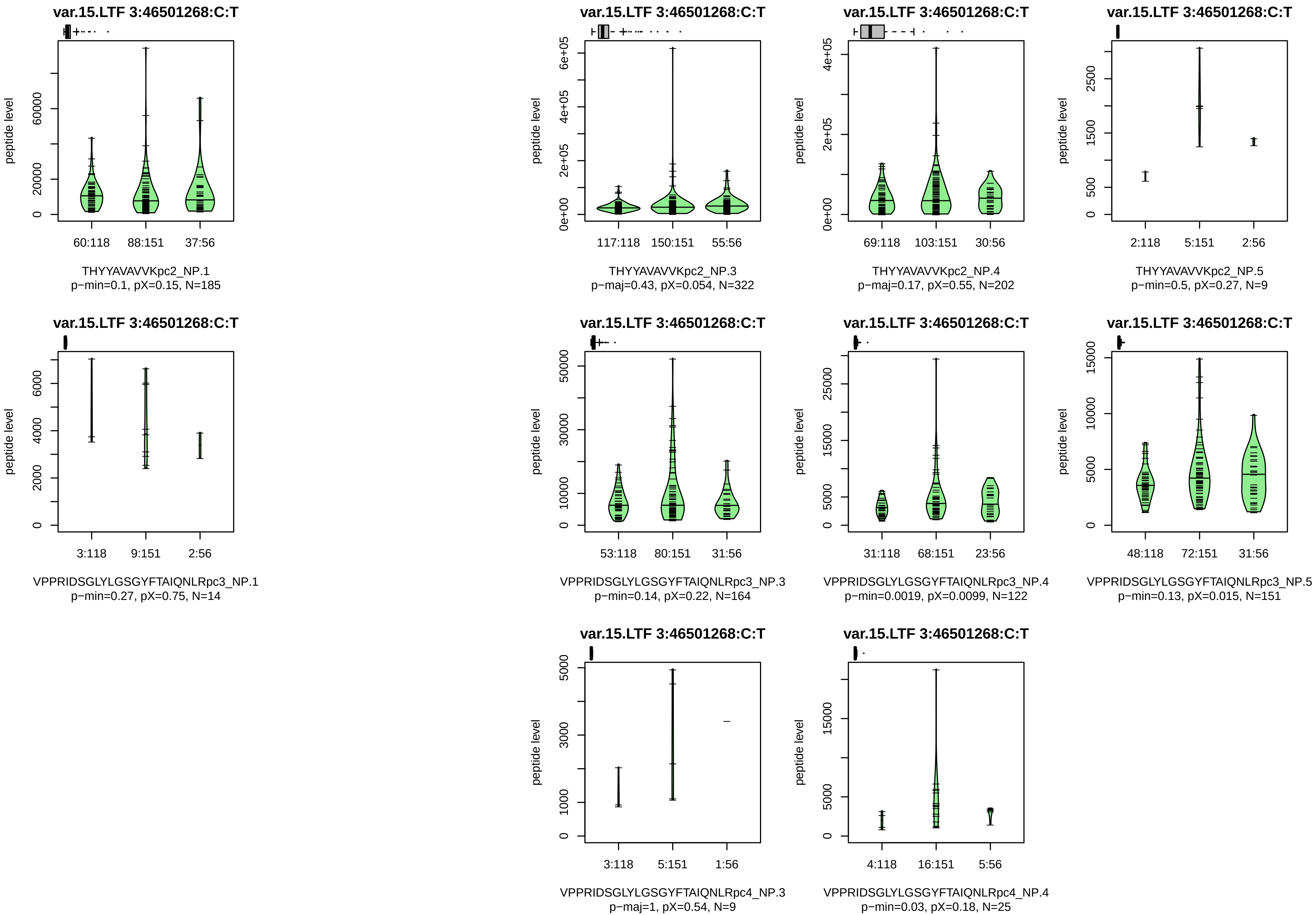
var.15.LTF 3:46501268:C:T
peptide level
60:118
88:151
37:56
THYYAVAVVKpc2_NP.1
p−min=0.1, pX=0.15, N=185
var.15.LTF 3:46501268:C:T
peptide level
117:118
150:151
55:56
THYYAVAVVKpc2_NP.3
p−maj=0.43, pX=0.054, N=322
var.15.LTF 3:46501268:C:T
peptide level
69:118
103:151
30:56
THYYAVAVVKpc2_NP.4
p−maj=0.17, pX=0.55, N=202
var.15.LTF 3:46501268:C:T
peptide level
2:118
5:151
2:56
THYYAVAVVKpc2_NP.5
p−min=0.5, pX=0.27, N=9
var.15.LTF 3:46501268:C:T
peptide level
3:118
9:151
2:56
VPPRIDSGLYLGSGYFTAIQNLRpc3_NP.1
p−min=0.27, pX=0.75, N=14
var.15.LTF 3:46501268:C:T
peptide level
53:118
80:151
31:56
VPPRIDSGLYLGSGYFTAIQNLRpc3_NP.3
p−min=0.14, pX=0.22, N=164
var.15.LTF 3:46501268:C:T
peptide level
31:118
68:151
23:56
VPPRIDSGLYLGSGYFTAIQNLRpc3_NP.4
p−min=0.0019, pX=0.0099, N=122
var.15.LTF 3:46501268:C:T
peptide level
48:118
72:151
31:56
VPPRIDSGLYLGSGYFTAIQNLRpc3_NP.5
p−min=0.13, pX=0.015, N=151
var.15.LTF 3:46501268:C:T
peptide level
3:118
5:151
1:56
VPPRIDSGLYLGSGYFTAIQNLRpc4_NP.3
p−maj=1, pX=0.54, N=9
var.15.LTF 3:46501268:C:T
peptide level
4:118
16:151
5:56
VPPRIDSGLYLGSGYFTAIQNLRpc4_NP.4
p−min=0.03, pX=0.18, N=25

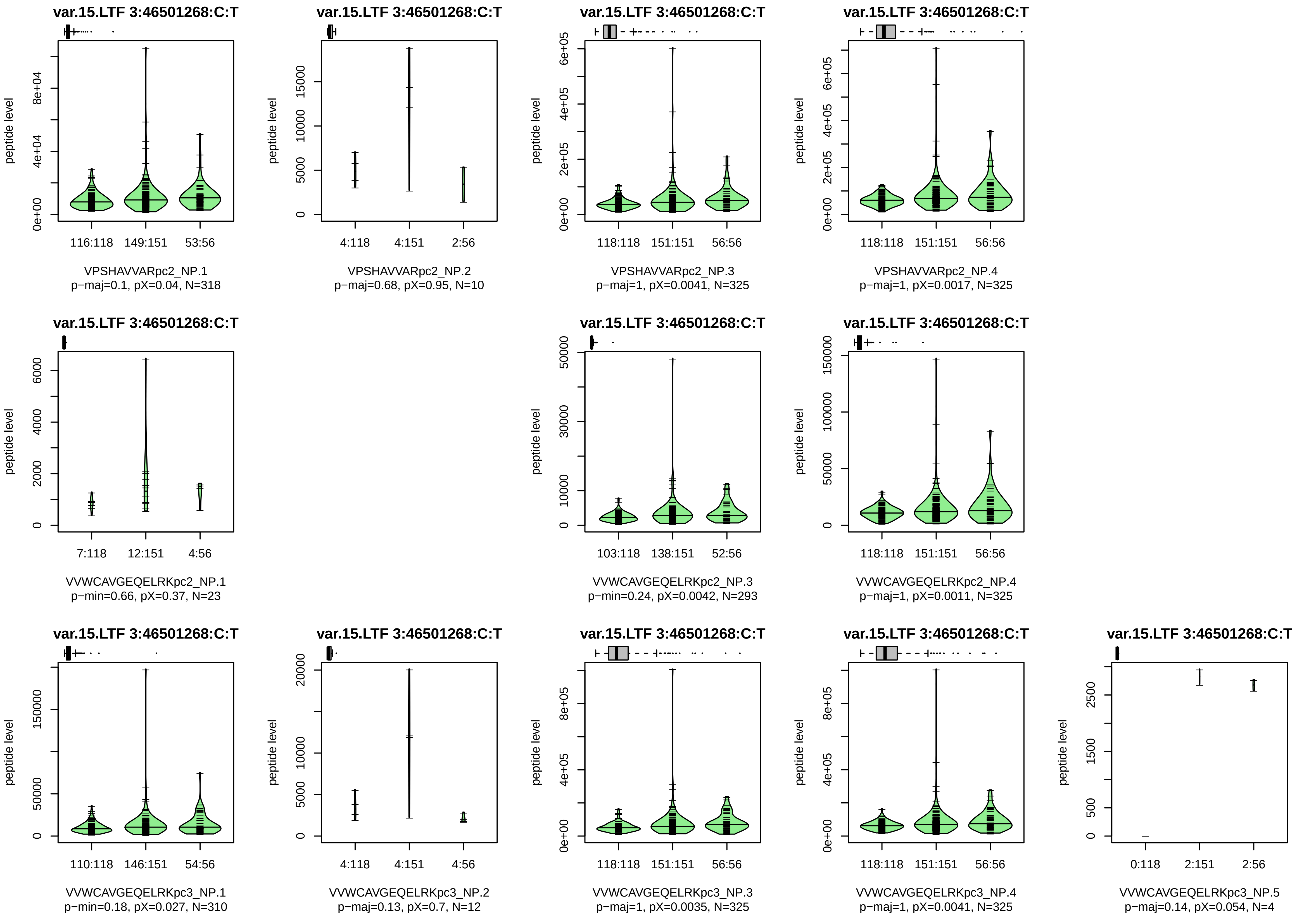
var.15.LTF 3:46501268:C:T
peptide level
116:118
149:151
53:56
VPSHAVVARpc2_NP.1
p−maj=0.1, pX=0.04, N=318
var.15.LTF 3:46501268:C:T
peptide level
4:118
4:151
2:56
VPSHAVVARpc2_NP.2
p−maj=0.68, pX=0.95, N=10
var.15.LTF 3:46501268:C:T
peptide level
118:118
151:151
56:56
VPSHAVVARpc2_NP.3
p−maj=1, pX=0.0041, N=325
var.15.LTF 3:46501268:C:T
peptide level
118:118
151:151
56:56
VPSHAVVARpc2_NP.4
p−maj=1, pX=0.0017, N=325
var.15.LTF 3:46501268:C:T
peptide level
7:118
12:151
4:56
VVWCAVGEQELRKpc2_NP.1
p−min=0.66, pX=0.37, N=23
var.15.LTF 3:46501268:C:T
peptide level
103:118
138:151
52:56
VVWCAVGEQELRKpc2_NP.3
p−min=0.24, pX=0.0042, N=293
var.15.LTF 3:46501268:C:T
peptide level
118:118
151:151
56:56
VVWCAVGEQELRKpc2_NP.4
p−maj=1, pX=0.0011, N=325
var.15.LTF 3:46501268:C:T
peptide level
110:118
146:151
54:56
VVWCAVGEQELRKpc3_NP.1
p−min=0.18, pX=0.027, N=310
var.15.LTF 3:46501268:C:T
peptide level
4:118
4:151
4:56
VVWCAVGEQELRKpc3_NP.2
p−maj=0.13, pX=0.7, N=12
var.15.LTF 3:46501268:C:T
peptide level
118:118
151:151
56:56
VVWCAVGEQELRKpc3_NP.3
p−maj=1, pX=0.0035, N=325
var.15.LTF 3:46501268:C:T
peptide level
118:118
151:151
56:56
VVWCAVGEQELRKpc3_NP.4
p−maj=1, pX=0.0041, N=325
var.15.LTF 3:46501268:C:T
peptide level
0:118
2:151
2:56
VVWCAVGEQELRKpc3_NP.5
p−maj=0.14, pX=0.054, N=4

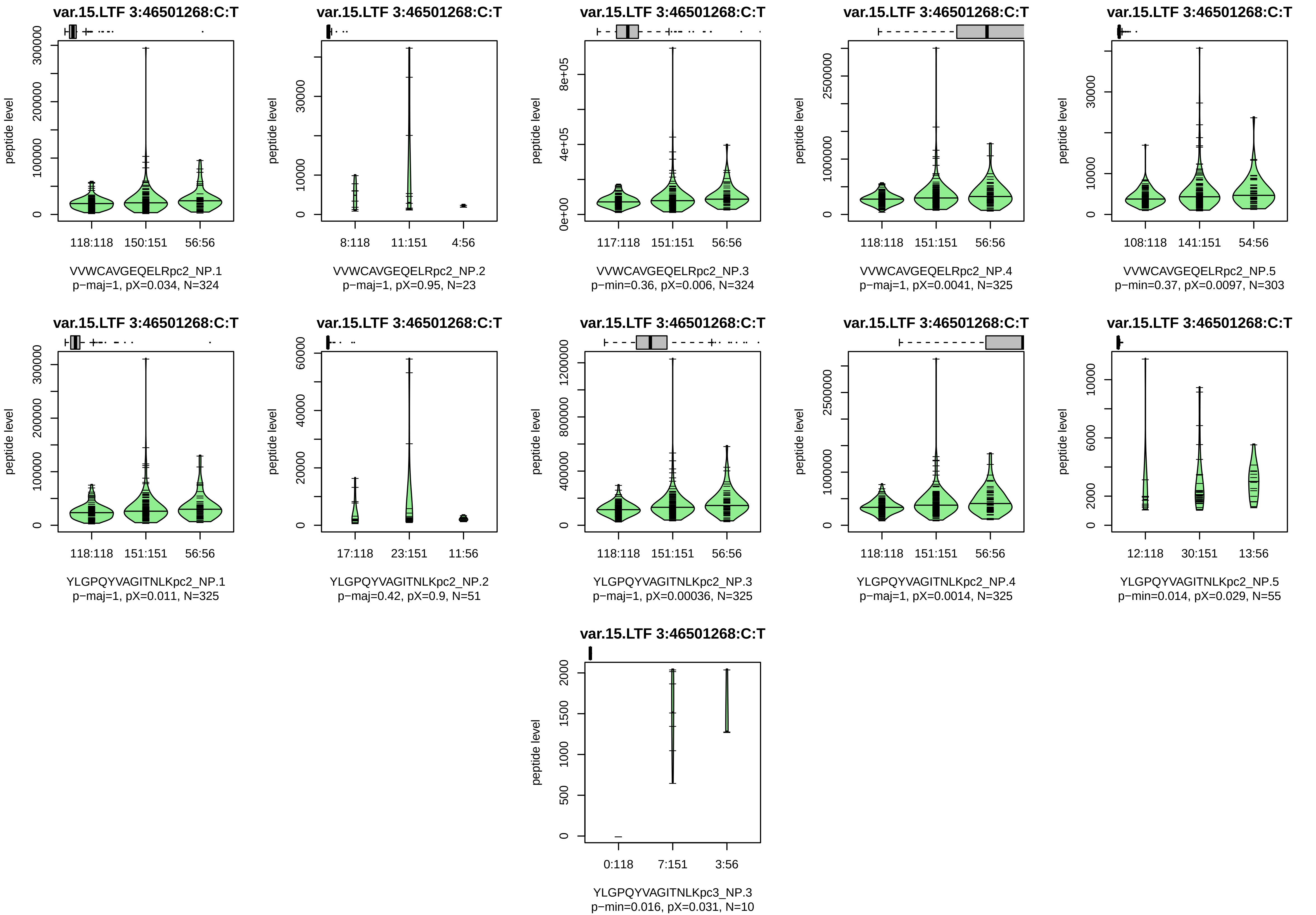

var.15.LTF 3:46501268:C:T
peptide level
0
100000
200000
300000
118:118
150:151
56:56
VVWCAVGEQELRpc2_NP.1
p−maj=1, pX=0.034, N=324
var.15.LTF 3:46501268:C:T
peptide level
0
10000
30000
8:118
11:151
4:56
VVWCAVGEQELRpc2_NP.2
p−maj=1, pX=0.95, N=23
var.15.LTF 3:46501268:C:T
peptide level
0e+00
4e+05
8e+05
117:118
151:151
56:56
VVWCAVGEQELRpc2_NP.3
p−min=0.36, pX=0.006, N=324
var.15.LTF 3:46501268:C:T
peptide level
0
1000000
2500000
118:118
151:151
56:56
VVWCAVGEQELRpc2_NP.4
p−maj=1, pX=0.0041, N=325
var.15.LTF 3:46501268:C:T
peptide level
0
10000
30000
108:118
141:151
54:56
VVWCAVGEQELRpc2_NP.5
p−min=0.37, pX=0.0097, N=303
var.15.LTF 3:46501268:C:T
peptide level
0
100000
200000
300000
118:118
151:151
56:56
YLGPQYVAGITNLKpc2_NP.1
p−maj=1, pX=0.011, N=325
var.15.LTF 3:46501268:C:T
peptide level
0
20000
40000
60000
17:118
23:151
11:56
YLGPQYVAGITNLKpc2_NP.2
p−maj=0.42, pX=0.9, N=51
var.15.LTF 3:46501268:C:T
peptide level
0
400000
800000
1200000
118:118
151:151
56:56
YLGPQYVAGITNLKpc2_NP.3
p−maj=1, pX=0.00036, N=325
var.15.LTF 3:46501268:C:T
peptide level
0
1000000
2500000
118:118
151:151
56:56
YLGPQYVAGITNLKpc2_NP.4
p−maj=1, pX=0.0014, N=325
var.15.LTF 3:46501268:C:T
peptide level
0
2000
6000
10000
12:118
30:151
13:56
YLGPQYVAGITNLKpc2_NP.5
p−min=0.014, pX=0.029, N=55
var.15.LTF 3:46501268:C:T
peptide level
0
500
1000
1500
2000
0:118
7:151
3:56
YLGPQYVAGITNLKpc3_NP.3
p−min=0.016, pX=0.031, N=10

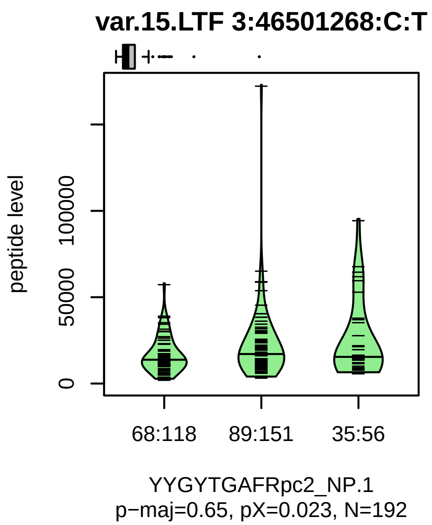

var.15.LTF 3:46501268:C:T
peptide level
0
50000
100000
68:118
89:151
35:56
YYGYTGAFRpc2_NP.1
p−maj=0.65, pX=0.023, N=192

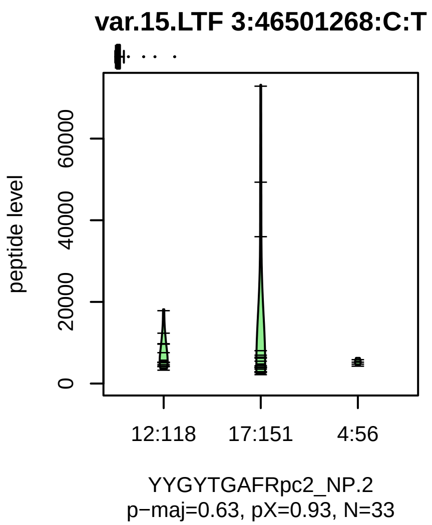

var.15.LTF 3:46501268:C:T
peptide level
0
20000
40000
60000
12:118
17:151
4:56
YYGYTGAFRpc2_NP.2
p−maj=0.63, pX=0.93, N=33

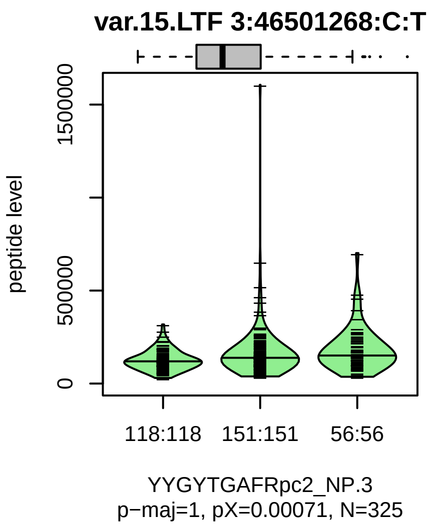

var.15.LTF 3:46501268:C:T
peptide level
0
500000
1500000
118:118
151:151
56:56
YYGYTGAFRpc2_NP.3
p−maj=1, pX=0.00071, N=325

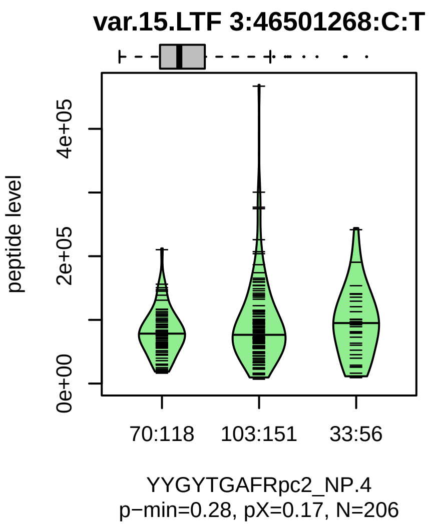

var.15.LTF 3:46501268:C:T
peptide level
0e+00
2e+05
4e+05
70:118
103:151
33:56
YYGYTGAFRpc2_NP.4
p−min=0.28, pX=0.17, N=206

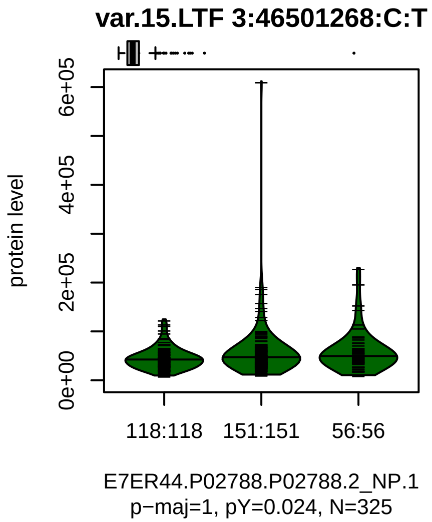

var.15.LTF 3:46501268:C:T
protein level
0e+00
2e+05
4e+05
6e+05
118:118
151:151
56:56
E7ER44.P02788.P02788.2_NP.1
p−maj=1, pY=0.024, N=325

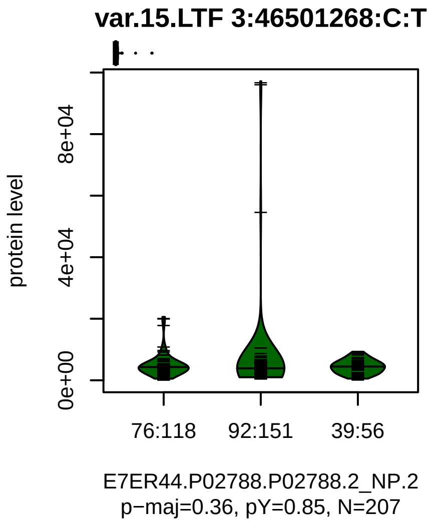

var.15.LTF 3:46501268:C:T
protein level
0e+00
4e+04
8e+04
76:118
92:151
39:56
E7ER44.P02788.P02788.2_NP.2
p−maj=0.36, pY=0.85, N=207

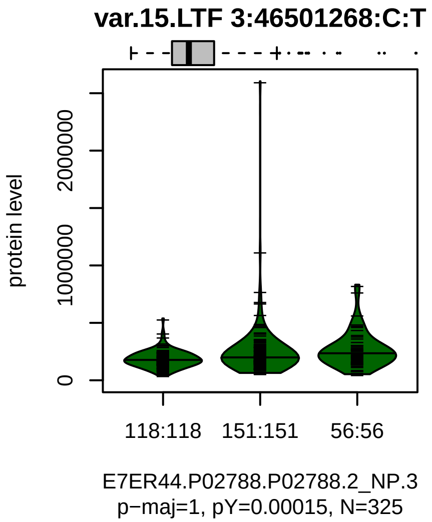

var.15.LTF 3:46501268:C:T
protein level
0
1000000
2000000
118:118
151:151
56:56
E7ER44.P02788.P02788.2_NP.3
p−maj=1, pY=0.00015, N=325

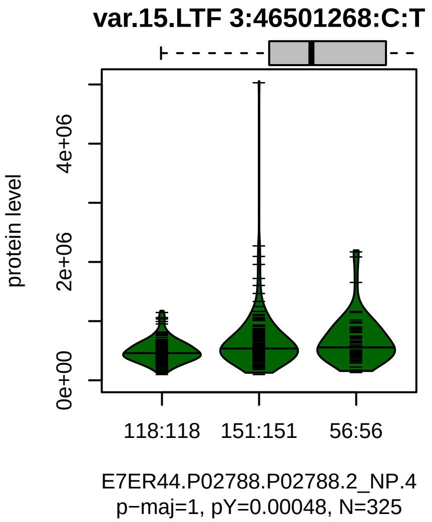

var.15.LTF 3:46501268:C:T
protein level
0e+00
2e+06
4e+06
118:118
151:151
56:56
E7ER44.P02788.P02788.2_NP.4
p−maj=1, pY=0.00048, N=325

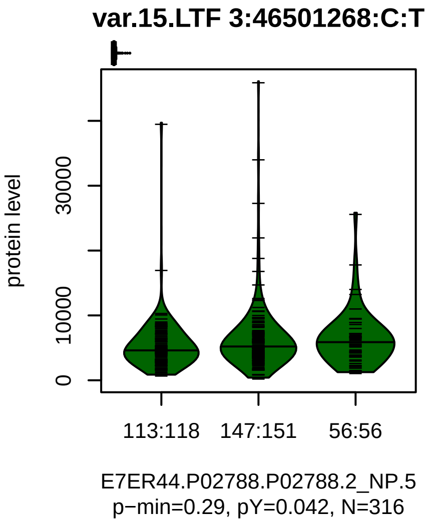

var.15.LTF 3:46501268:C:T
protein level
0
10000
30000
113:118
147:151
56:56
E7ER44.P02788.P02788.2_NP.5
p−min=0.29, pY=0.042, N=316

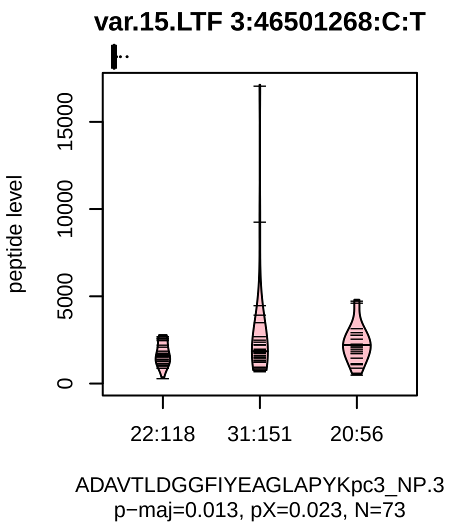
var.15.LTF 3:46501268:C:T
peptide level
0
5000
10000
15000
22:118
31:151
20:56
ADAVTLDGGFIYEAGLAPYKpc3_NP.3
p−maj=0.013, pX=0.023, N=73

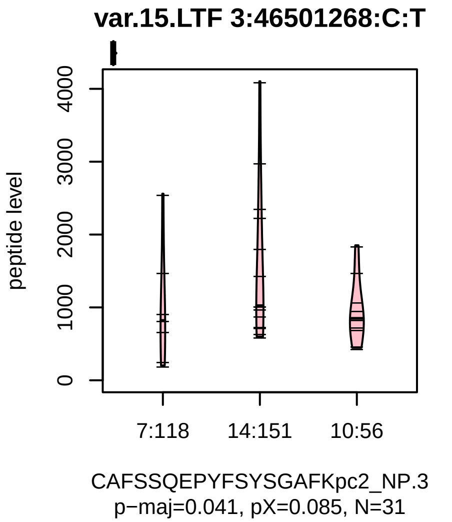
var.15.LTF 3:46501268:C:T
peptide level
0
1000
2000
3000
4000
7:118
14:151
10:56
CAFSSQEPYFSYSGAFKpc2_NP.3
p−maj=0.041, pX=0.085, N=31

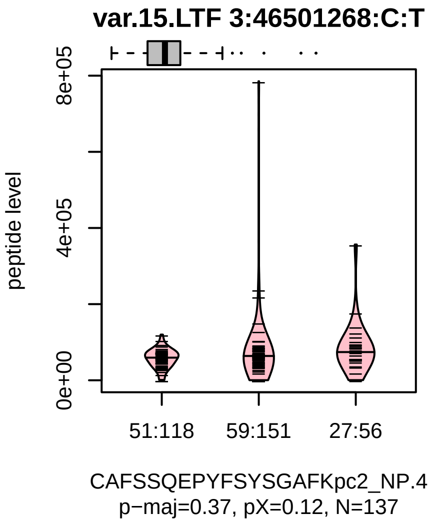
var.15.LTF 3:46501268:C:T
peptide level
0e+00
4e+05
8e+05
51:118
59:151
27:56
CAFSSQEPYFSYSGAFKpc2_NP.4
p−maj=0.37, pX=0.12, N=137

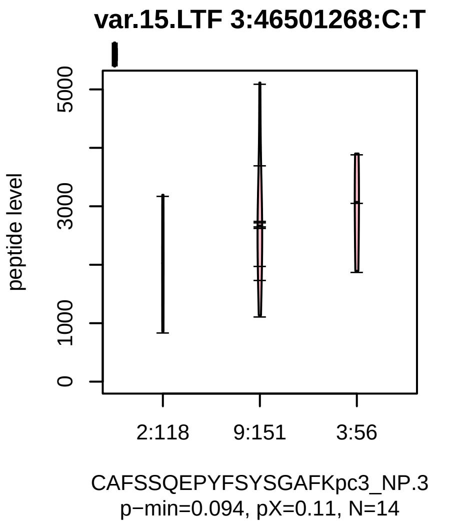
var.15.LTF 3:46501268:C:T
peptide level
0
1000
3000
5000
2:118
9:151
3:56
CAFSSQEPYFSYSGAFKpc3_NP.3
p−min=0.094, pX=0.11, N=14

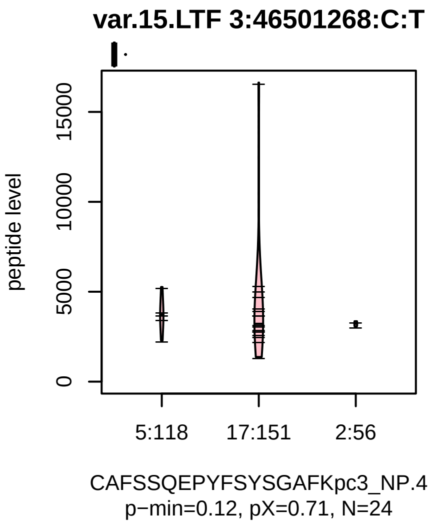
var.15.LTF 3:46501268:C:T
peptide level
0
5000
10000
15000
5:118
17:151
2:56
CAFSSQEPYFSYSGAFKpc3_NP.4
p−min=0.12, pX=0.71, N=24

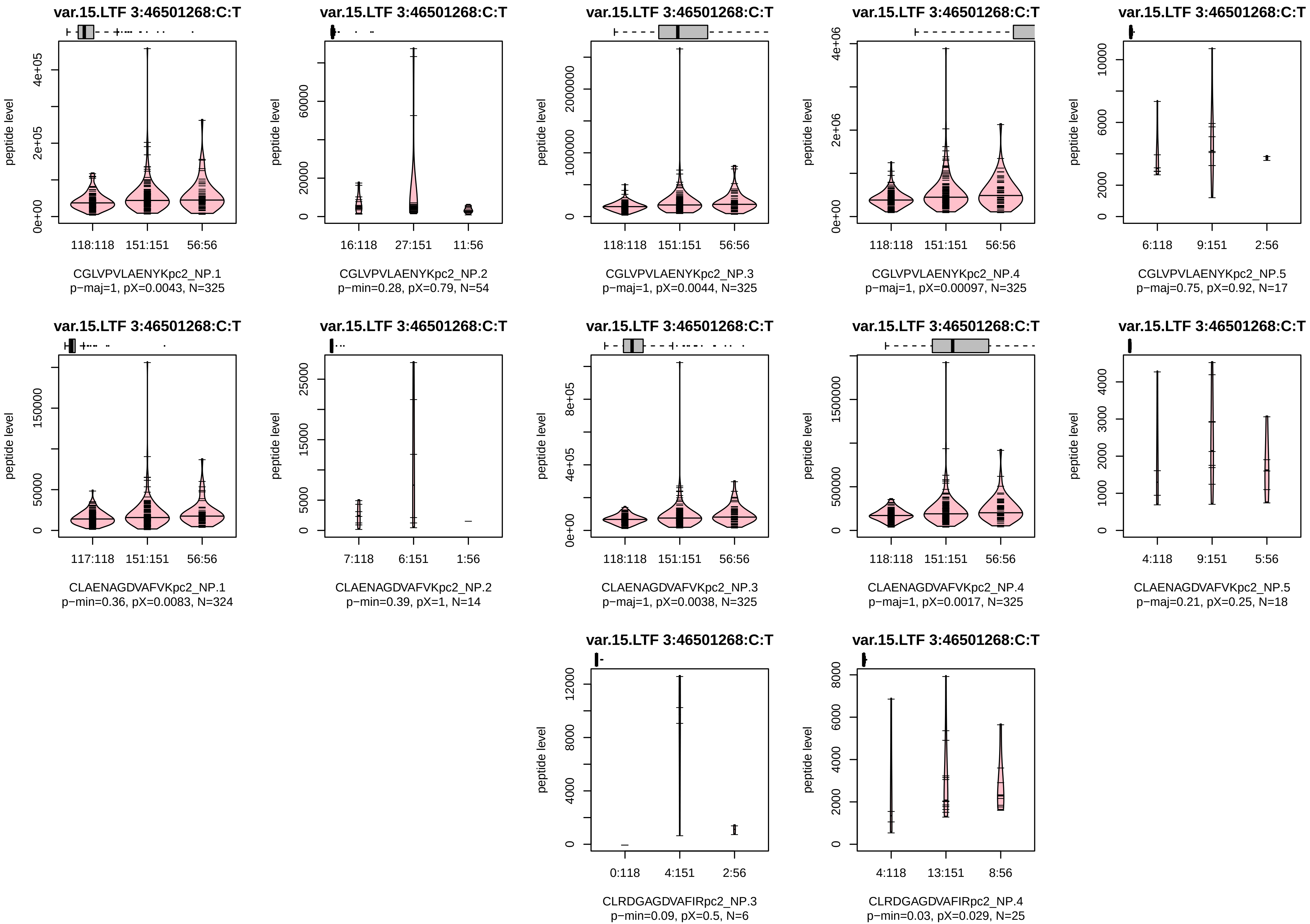
var.15.LTF 3:46501268:C:T
peptide level
118:118
151:151
56:56
CGLVPVLAENYKpc2_NP.1
p−maj=1, pX=0.0043, N=325
var.15.LTF 3:46501268:C:T
peptide level
16:118
27:151
11:56
CGLVPVLAENYKpc2_NP.2
p−min=0.28, pX=0.79, N=54
var.15.LTF 3:46501268:C:T
peptide level
118:118
151:151
56:56
CGLVPVLAENYKpc2_NP.3
p−maj=1, pX=0.0044, N=325
var.15.LTF 3:46501268:C:T
peptide level
118:118
151:151
56:56
CGLVPVLAENYKpc2_NP.4
p−maj=1, pX=0.00097, N=325
var.15.LTF 3:46501268:C:T
peptide level
6:118
9:151
2:56
CGLVPVLAENYKpc2_NP.5
p−maj=0.75, pX=0.92, N=17
var.15.LTF 3:46501268:C:T
peptide level
117:118
151:151
56:56
CLAENAGDVAFVKpc2_NP.1
p−min=0.36, pX=0.0083, N=324
var.15.LTF 3:46501268:C:T
peptide level
7:118
6:151
1:56
CLAENAGDVAFVKpc2_NP.2
p−min=0.39, pX=1, N=14
var.15.LTF 3:46501268:C:T
peptide level
118:118
151:151
56:56
CLAENAGDVAFVKpc2_NP.3
p−maj=1, pX=0.0038, N=325
var.15.LTF 3:46501268:C:T
peptide level
118:118
151:151
56:56
CLAENAGDVAFVKpc2_NP.4
p−maj=1, pX=0.0017, N=325
var.15.LTF 3:46501268:C:T
peptide level
4:118
9:151
5:56
CLAENAGDVAFVKpc2_NP.5
p−maj=0.21, pX=0.25, N=18
var.15.LTF 3:46501268:C:T
peptide level
0:118
4:151
2:56
CLRDGAGDVAFIRpc2_NP.3
p−min=0.09, pX=0.5, N=6
var.15.LTF 3:46501268:C:T
peptide level
4:118
13:151
8:56
CLRDGAGDVAFIRpc2_NP.4
p−min=0.03, pX=0.029, N=25

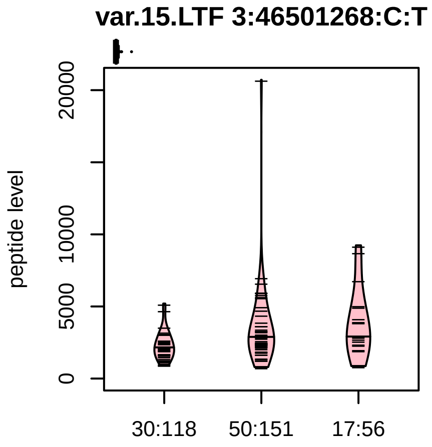

CLRDGAGDVAFIRpc3_NP.1
p−min=0.21, pX=0.027, N=97

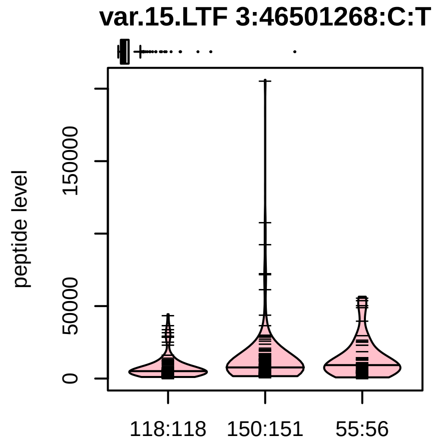

CLRDGAGDVAFIRpc3_NP.3
p−maj=0.32, pX=0.01, N=323

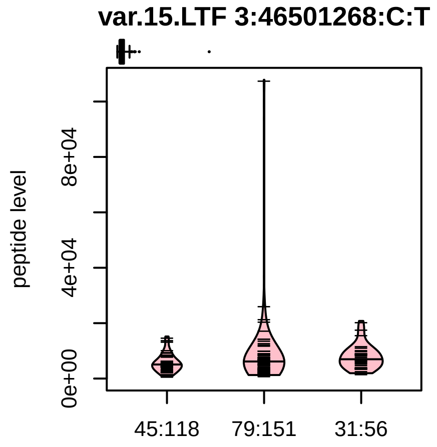

CLRDGAGDVAFIRpc3_NP.4
p−min=0.011, pX=0.026, N=155

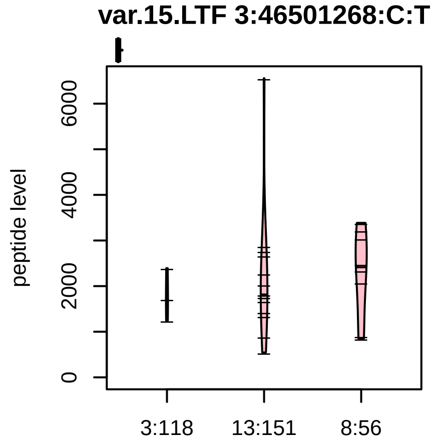

CSTSPLLEACEFLRKpc2_NP.4
p−min=0.014, pX=0.006, N=24

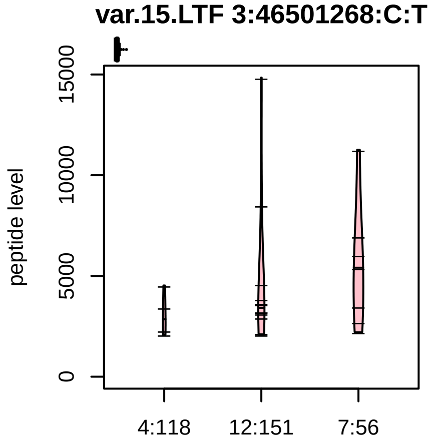

CSTSPLLEACEFLRKpc3_NP.1
p−min=0.07, pX=0.012, N=23

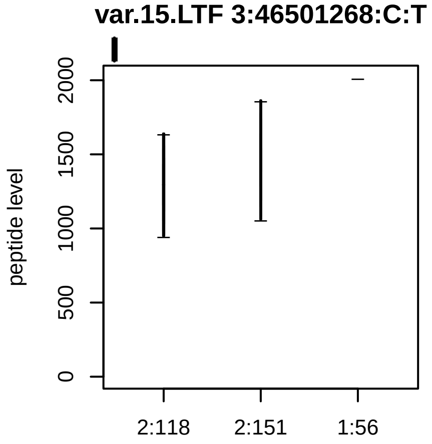

CSTSPLLEACEFLRKpc3_NP.2
p−min=1, pX=0.73, N=5

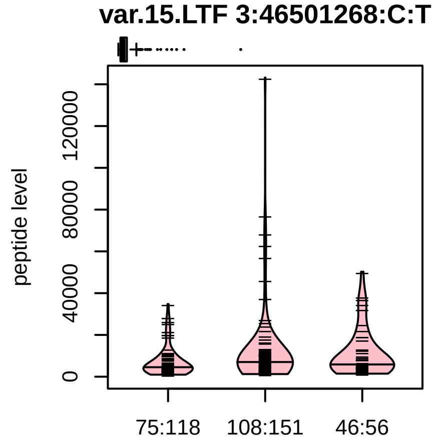

CSTSPLLEACEFLRKpc3_NP.3
p−maj=0.037, pX=0.009, N=229

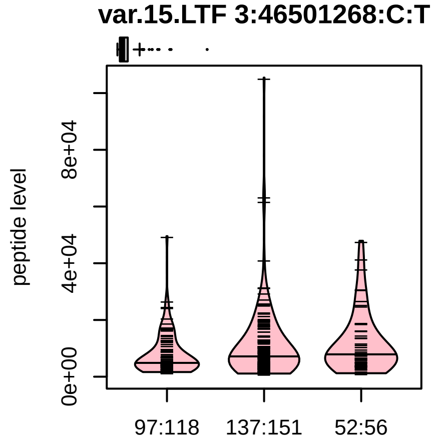

CSTSPLLEACEFLRKpc3_NP.4
p−min=0.02, pX=0.0027, N=286

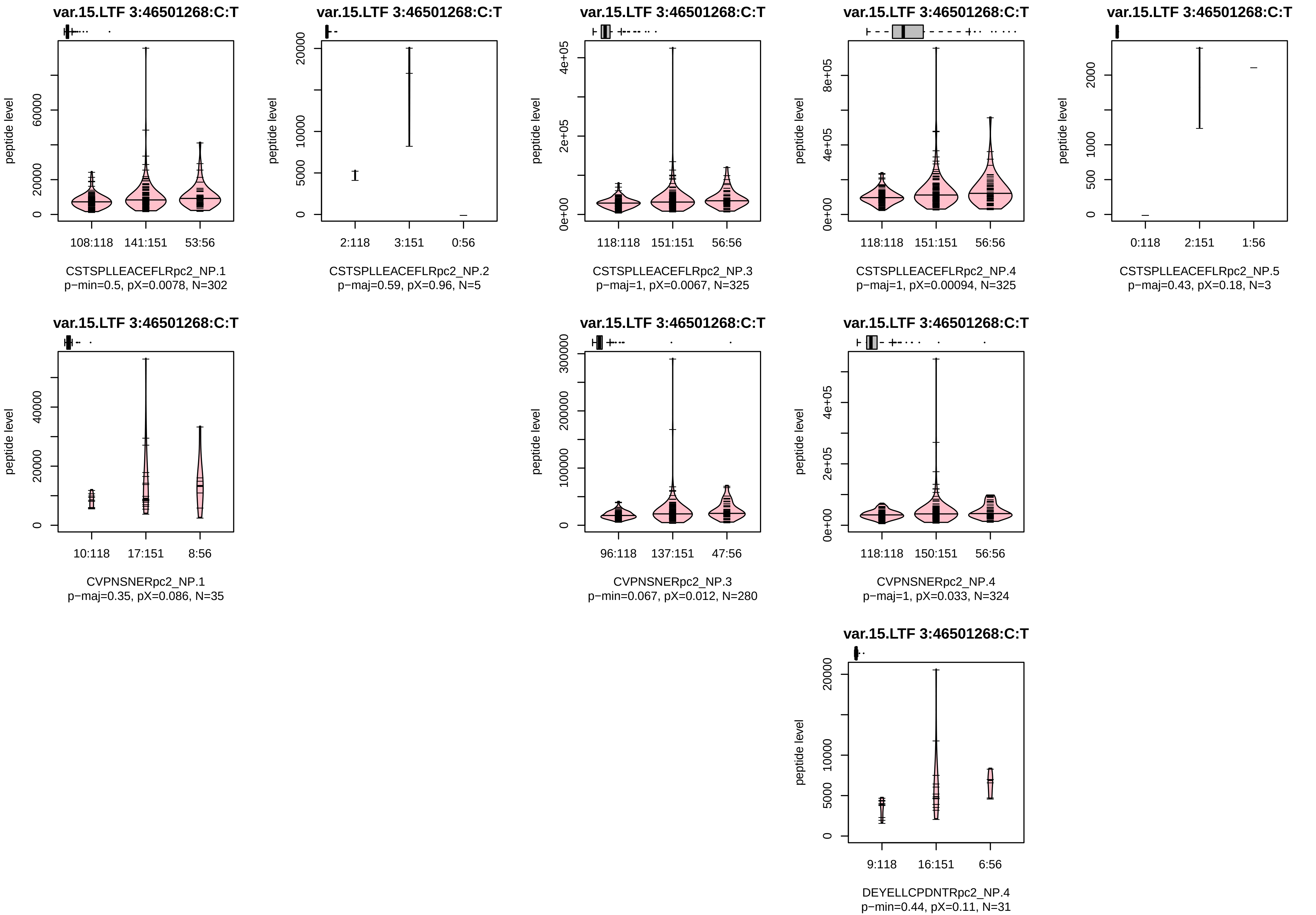
var.15.LTF 3:46501268:C:T
peptide level
108:118
141:151
53:56
CSTSPLLEACEFLRpc2_NP.1
p−min=0.5, pX=0.0078, N=302
var.15.LTF 3:46501268:C:T
peptide level
2:118
3:151
0:56
CSTSPLLEACEFLRpc2_NP.2
p−maj=0.59, pX=0.96, N=5
var.15.LTF 3:46501268:C:T
peptide level
118:118
151:151
56:56
CSTSPLLEACEFLRpc2_NP.3
p−maj=1, pX=0.0067, N=325
var.15.LTF 3:46501268:C:T
peptide level
118:118
151:151
56:56
CSTSPLLEACEFLRpc2_NP.4
p−maj=1, pX=0.00094, N=325
var.15.LTF 3:46501268:C:T
peptide level
0:118
2:151
1:56
CSTSPLLEACEFLRpc2_NP.5
p−maj=0.43, pX=0.18, N=3
var.15.LTF 3:46501268:C:T
peptide level
10:118
17:151
8:56
CVPNSNERpc2_NP.1
p−maj=0.35, pX=0.086, N=35
var.15.LTF 3:46501268:C:T
peptide level
96:118
137:151
47:56
CVPNSNERpc2_NP.3
p−min=0.067, pX=0.012, N=280
var.15.LTF 3:46501268:C:T
peptide level
118:118
150:151
56:56
CVPNSNERpc2_NP.4
p−maj=1, pX=0.033, N=324
var.15.LTF 3:46501268:C:T
peptide level
9:118
16:151
6:56
DEYELLCPDNTRpc2_NP.4
p−min=0.44, pX=0.11, N=31

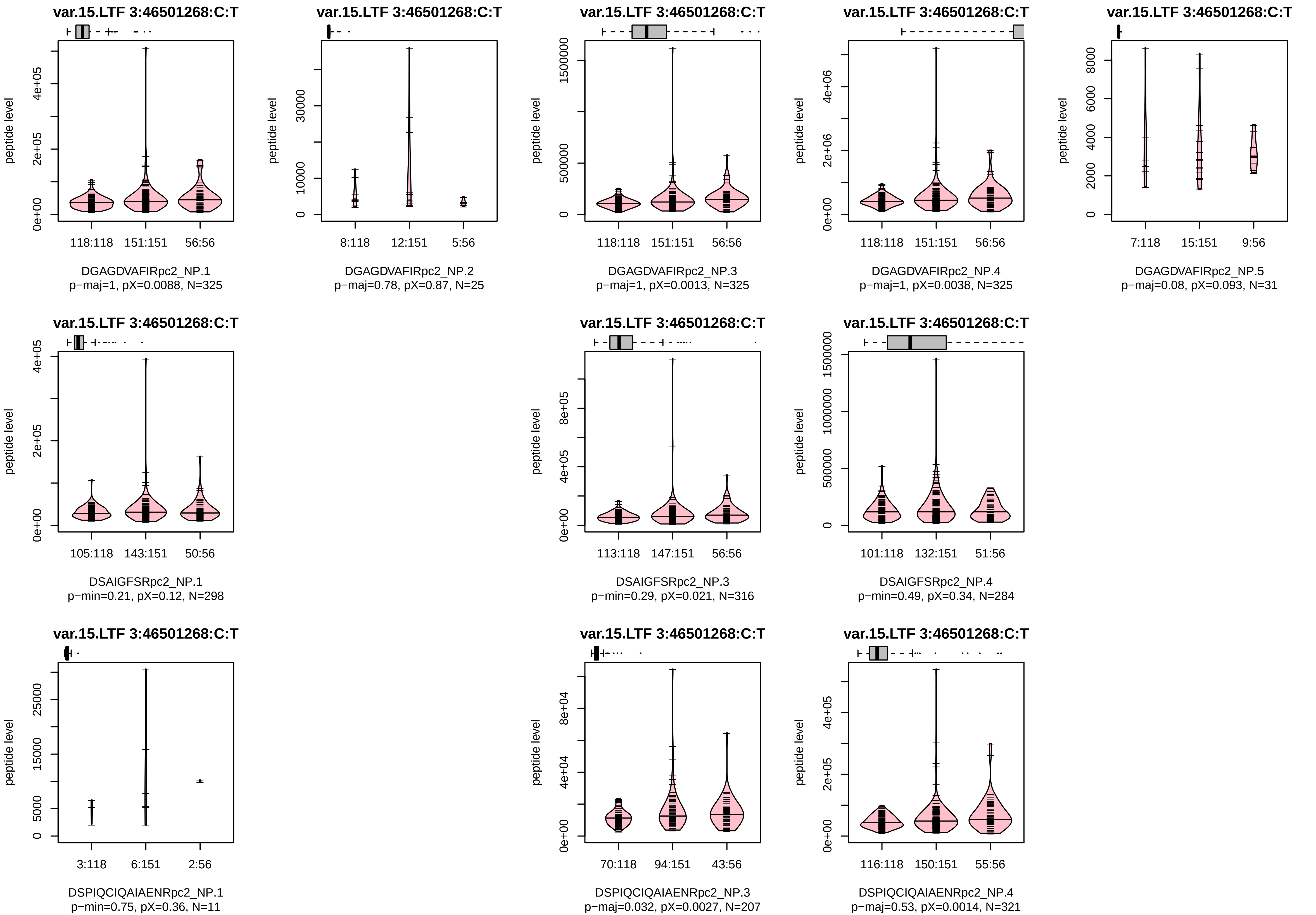

var.15.LTF 3:46501268:C:T
peptide level
118:118
151:151
56:56
DGAGDVAFIRpc2_NP.1
p−maj=1, pX=0.0088, N=325
var.15.LTF 3:46501268:C:T
peptide level
8:118
12:151
5:56
DGAGDVAFIRpc2_NP.2
p−maj=0.78, pX=0.87, N=25
var.15.LTF 3:46501268:C:T
peptide level
118:118
151:151
56:56
DGAGDVAFIRpc2_NP.3
p−maj=1, pX=0.0013, N=325
var.15.LTF 3:46501268:C:T
peptide level
118:118
151:151
56:56
DGAGDVAFIRpc2_NP.4
p−maj=1, pX=0.0038, N=325
var.15.LTF 3:46501268:C:T
peptide level
7:118
15:151
9:56
DGAGDVAFIRpc2_NP.5
p−maj=0.08, pX=0.093, N=31
var.15.LTF 3:46501268:C:T
peptide level
105:118
143:151
50:56
DSAIGFSRpc2_NP.1
p−min=0.21, pX=0.12, N=298
var.15.LTF 3:46501268:C:T
peptide level
113:118
147:151
56:56
DSAIGFSRpc2_NP.3
p−min=0.29, pX=0.021, N=316
var.15.LTF 3:46501268:C:T
peptide level
101:118
132:151
51:56
DSAIGFSRpc2_NP.4
p−min=0.49, pX=0.34, N=284
var.15.LTF 3:46501268:C:T
peptide level
3:118
6:151
2:56
DSPIQCIQAIAENRpc2_NP.1
p−min=0.75, pX=0.36, N=11
var.15.LTF 3:46501268:C:T
peptide level
70:118
94:151
43:56
DSPIQCIQAIAENRpc2_NP.3
p−maj=0.032, pX=0.0027, N=207
var.15.LTF 3:46501268:C:T
peptide level
116:118
150:151
55:56
DSPIQCIQAIAENRpc2_NP.4
p−maj=0.53, pX=0.0014, N=321

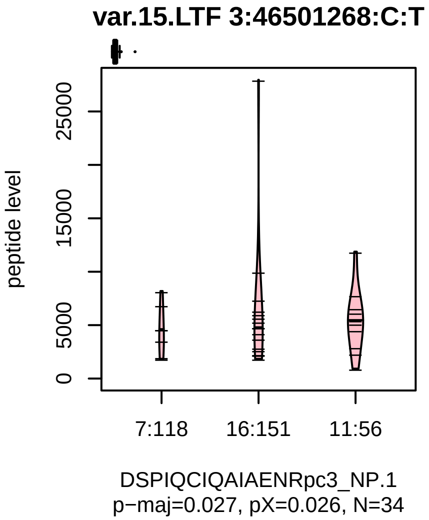
var.15.LTF 3:46501268:C:T
peptide level
0
5000
15000
25000
7:118
16:151
11:56
DSPIQCIQAIAENRpc3_NP.1
p−maj=0.027, pX=0.026, N=34

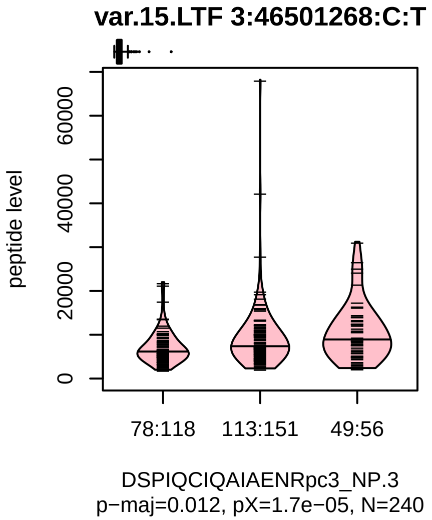
var.15.LTF 3:46501268:C:T
peptide level
0
20000
40000
60000
78:118
113:151
49:56
DSPIQCIQAIAENRpc3_NP.3
p−maj=0.012, pX=1.7e−05, N=240

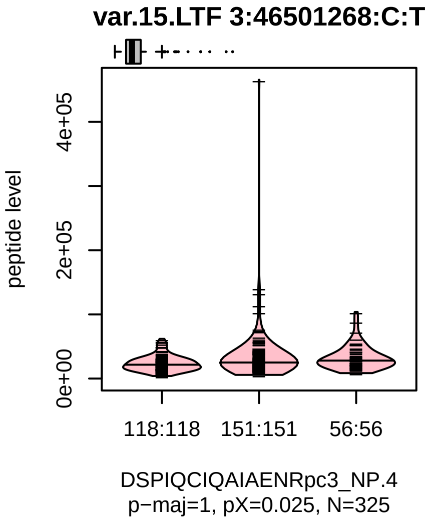
var.15.LTF 3:46501268:C:T
peptide level
0e+00
2e+05
4e+05
118:118
151:151
56:56
DSPIQCIQAIAENRpc3_NP.4
p−maj=1, pX=0.025, N=325

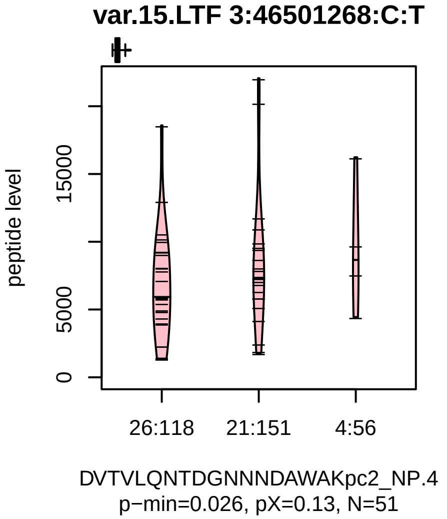
var.15.LTF 3:46501268:C:T
peptide level
0
5000
15000
26:118
21:151
4:56
DVTVLQNTDGNNNDAWAKpc2_NP.4
p−min=0.026, pX=0.13, N=51

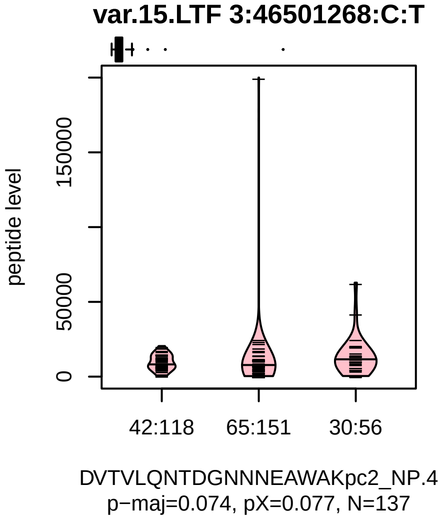
var.15.LTF 3:46501268:C:T
peptide level
0
50000
150000
42:118
65:151
30:56
DVTVLQNTDGNNNEAWAKpc2_NP.4
p−maj=0.074, pX=0.077, N=137

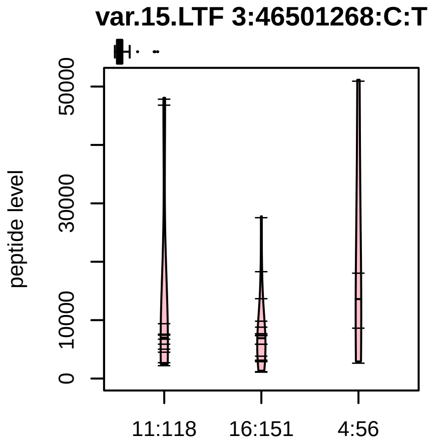

EDAIWNLLRpc2_NP.1
p−maj=0.62, pX=0.98, N=31

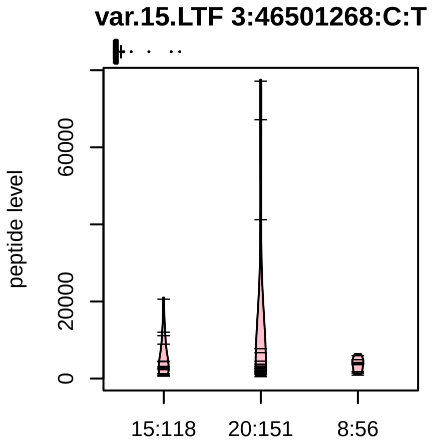

EDAIWNLLRpc2_NP.2
p−maj=0.83, pX=0.86, N=43

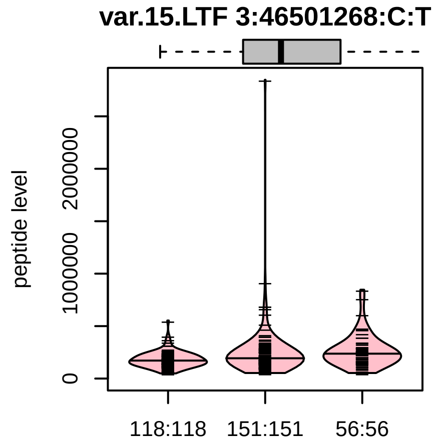

EDAIWNLLRpc2_NP.3
p−maj=1, pX=0.0023, N=325

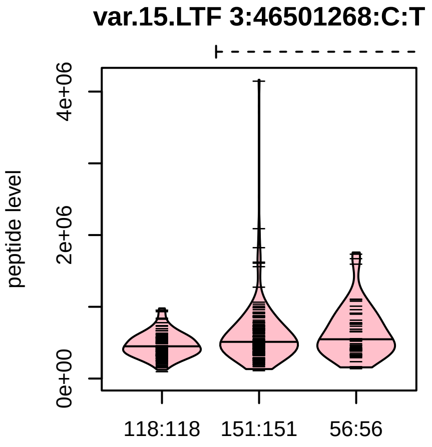

EDAIWNLLRpc2_NP.4
p−maj=1, pX=0.00033, N=325

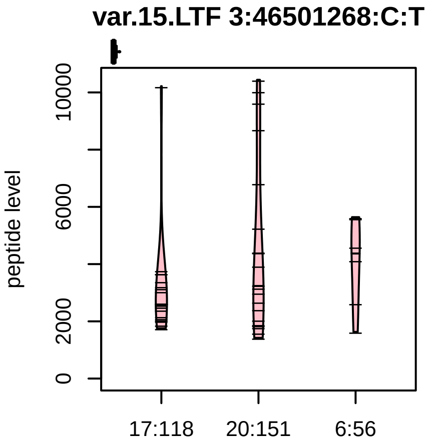

EDAIWNLLRpc2_NP.5
p−maj=0.67, pX=0.91, N=43

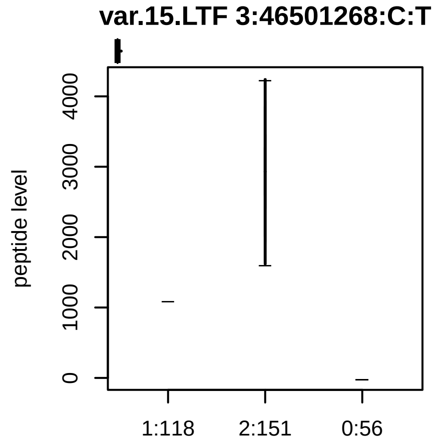

ESTVFEDLSDEAERpc2_NP.3
p−maj=1, pX=0.95, N=3

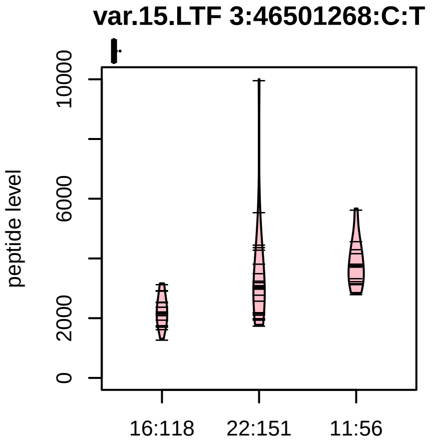

ESTVFEDLSDEAERpc2_NP.4
p−maj=0.31, pX=0.024, N=49

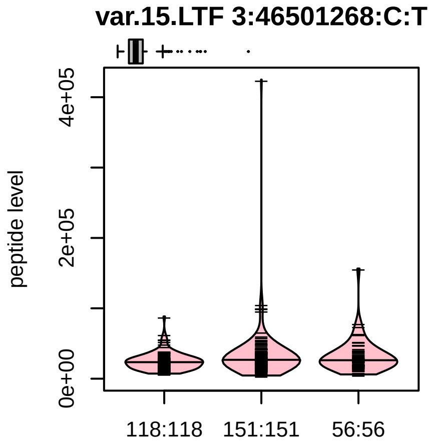

FCLFQSETKpc2_NP.1
p−maj=1, pX=0.048, N=325

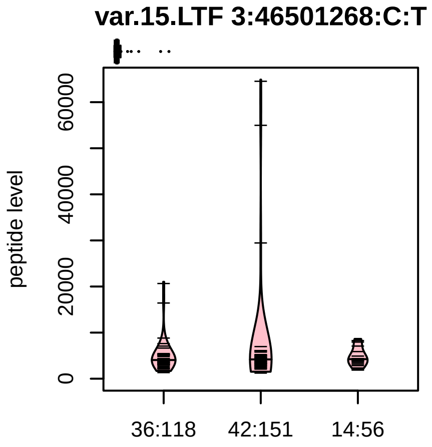

FCLFQSETKpc2_NP.2
p−min=0.52, pX=0.89, N=92

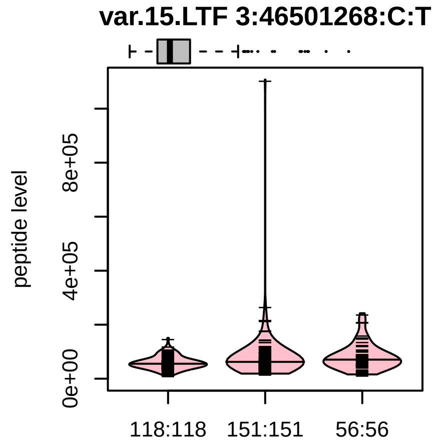

FCLFQSETKpc2_NP.3
p−maj=1, pX=0.019, N=325

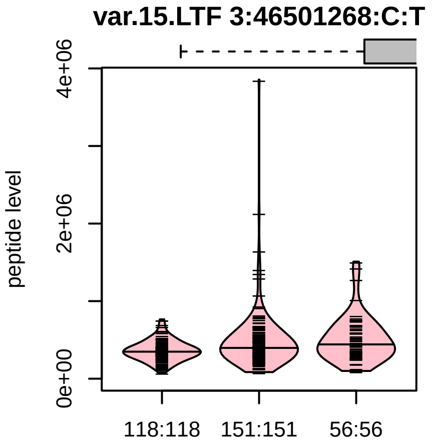

FCLFQSETKpc2_NP.4
p−maj=1, pX=0.00066, N=325

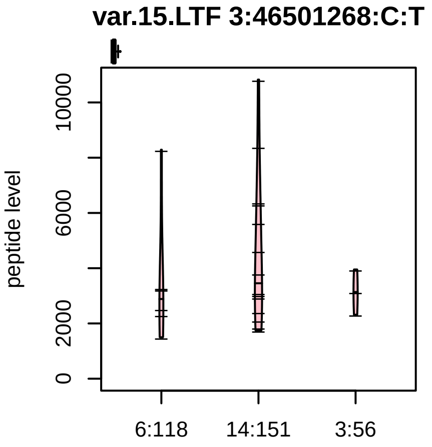

FCLFQSETKpc2_NP.5
p−min=0.37, pX=0.7, N=23

var.15.LTF 3:46501268:C:T

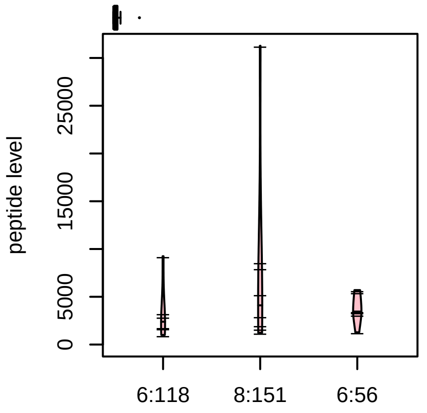

FDEYFSQSCAPGSDPRpc2_NP.3
p−maj=0.13, pX=0.39, N=20

var.15.LTF 3:46501268:C:T

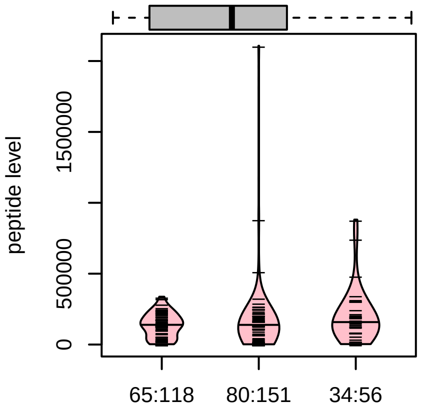

FDEYFSQSCAPGSDPRpc2_NP.4
p−maj=0.38, pX=0.1, N=179

var.15.LTF 3:46501268:C:T

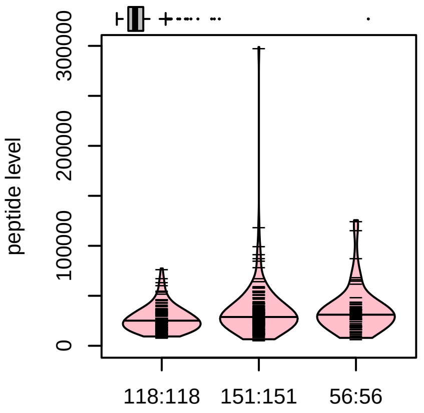

FFSASCVPGADKpc2_NP.1
p−maj=1, pX=0.009, N=325

var.15.LTF 3:46501268:C:T

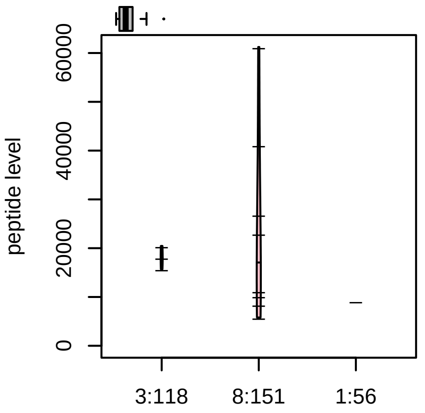

FFSASCVPGADKpc2_NP.2
p−min=0.55, pX=0.96, N=12

var.15.LTF 3:46501268:C:T

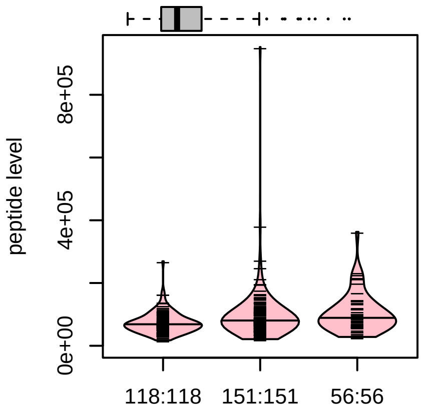

FFSASCVPGADKpc2_NP.3
p−maj=1, pX=0.0018, N=325

var.15.LTF 3:46501268:C:T

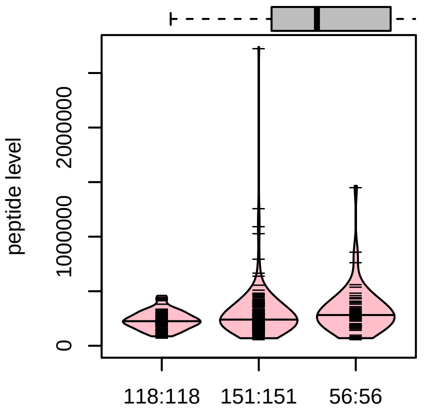

FFSASCVPGADKpc2_NP.4
p−maj=1, pX=0.0032, N=325

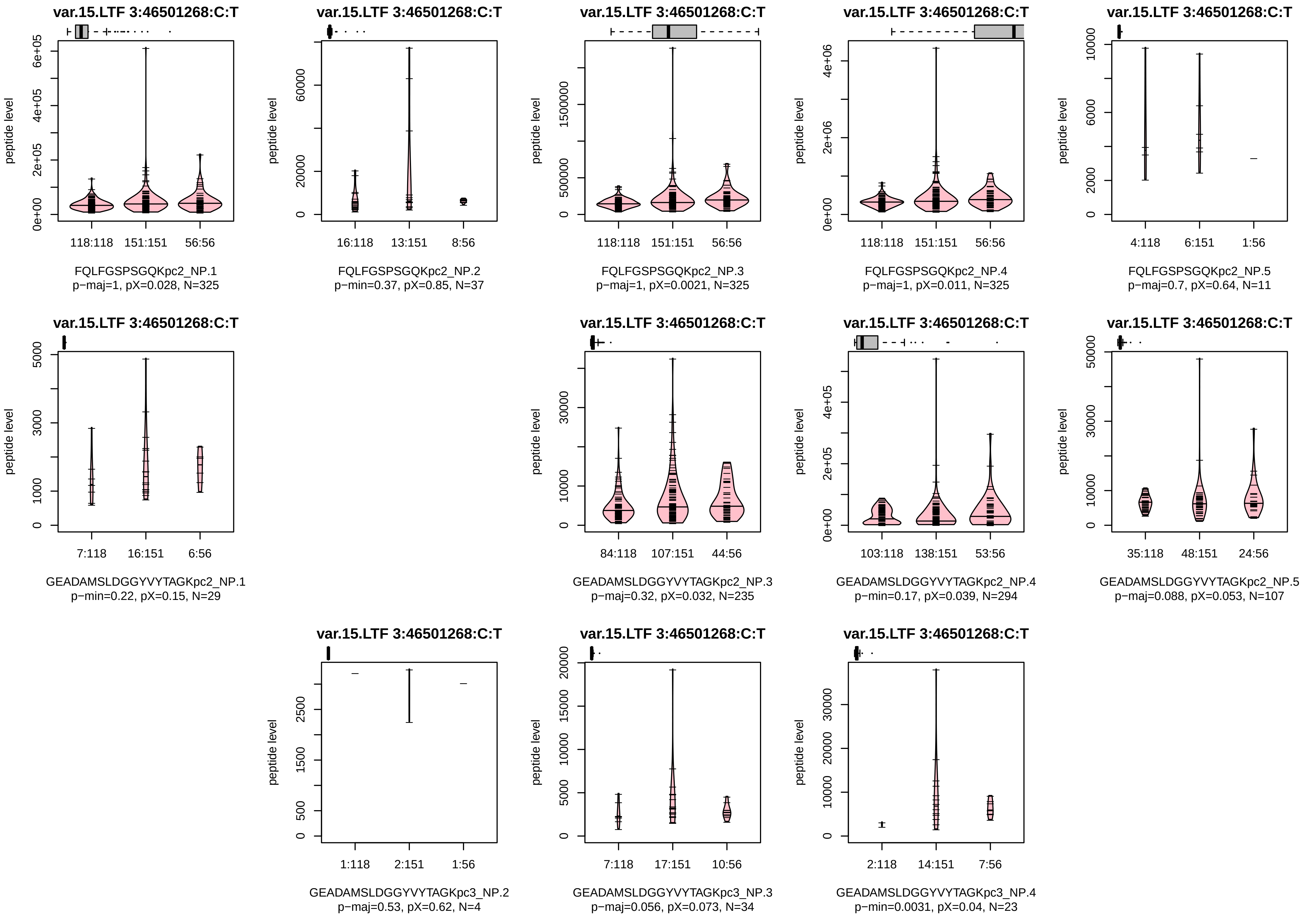

var.15.LTF 3:46501268:C:T
peptide level
118:118
151:151
56:56
FQLFGSPSGQKpc2_NP.1
p−maj=1, pX=0.028, N=325
var.15.LTF 3:46501268:C:T
16:118
13:151
8:56
FQLFGSPSGQKpc2_NP.2
p−min=0.37, pX=0.85, N=37
var.15.LTF 3:46501268:C:T
118:118
151:151
56:56
FQLFGSPSGQKpc2_NP.3
p−maj=1, pX=0.0021, N=325
var.15.LTF 3:46501268:C:T
118:118
151:151
56:56
FQLFGSPSGQKpc2_NP.4
p−maj=1, pX=0.011, N=325
var.15.LTF 3:46501268:C:T
4:118
6:151
1:56
FQLFGSPSGQKpc2_NP.5
p−maj=0.7, pX=0.64, N=11
var.15.LTF 3:46501268:C:T
7:118
16:151
6:56
GEADAMSLDGGYVYTAGKpc2_NP.1
p−min=0.22, pX=0.15, N=29
var.15.LTF 3:46501268:C:T
84:118
107:151
44:56
GEADAMSLDGGYVYTAGKpc2_NP.3
p−maj=0.32, pX=0.032, N=235
var.15.LTF 3:46501268:C:T
103:118
138:151
53:56
GEADAMSLDGGYVYTAGKpc2_NP.4
p−min=0.17, pX=0.039, N=294
var.15.LTF 3:46501268:C:T
35:118
48:151
24:56
GEADAMSLDGGYVYTAGKpc2_NP.5
p−maj=0.088, pX=0.053, N=107
var.15.LTF 3:46501268:C:T
1:118
2:151
1:56
GEADAMSLDGGYVYTAGKpc3_NP.2
p−maj=0.53, pX=0.62, N=4
var.15.LTF 3:46501268:C:T
7:118
17:151
10:56
GEADAMSLDGGYVYTAGKpc3_NP.3
p−maj=0.056, pX=0.073, N=34
var.15.LTF 3:46501268:C:T
2:118
14:151
7:56
GEADAMSLDGGYVYTAGKpc3_NP.4
p−min=0.0031, pX=0.04, N=23

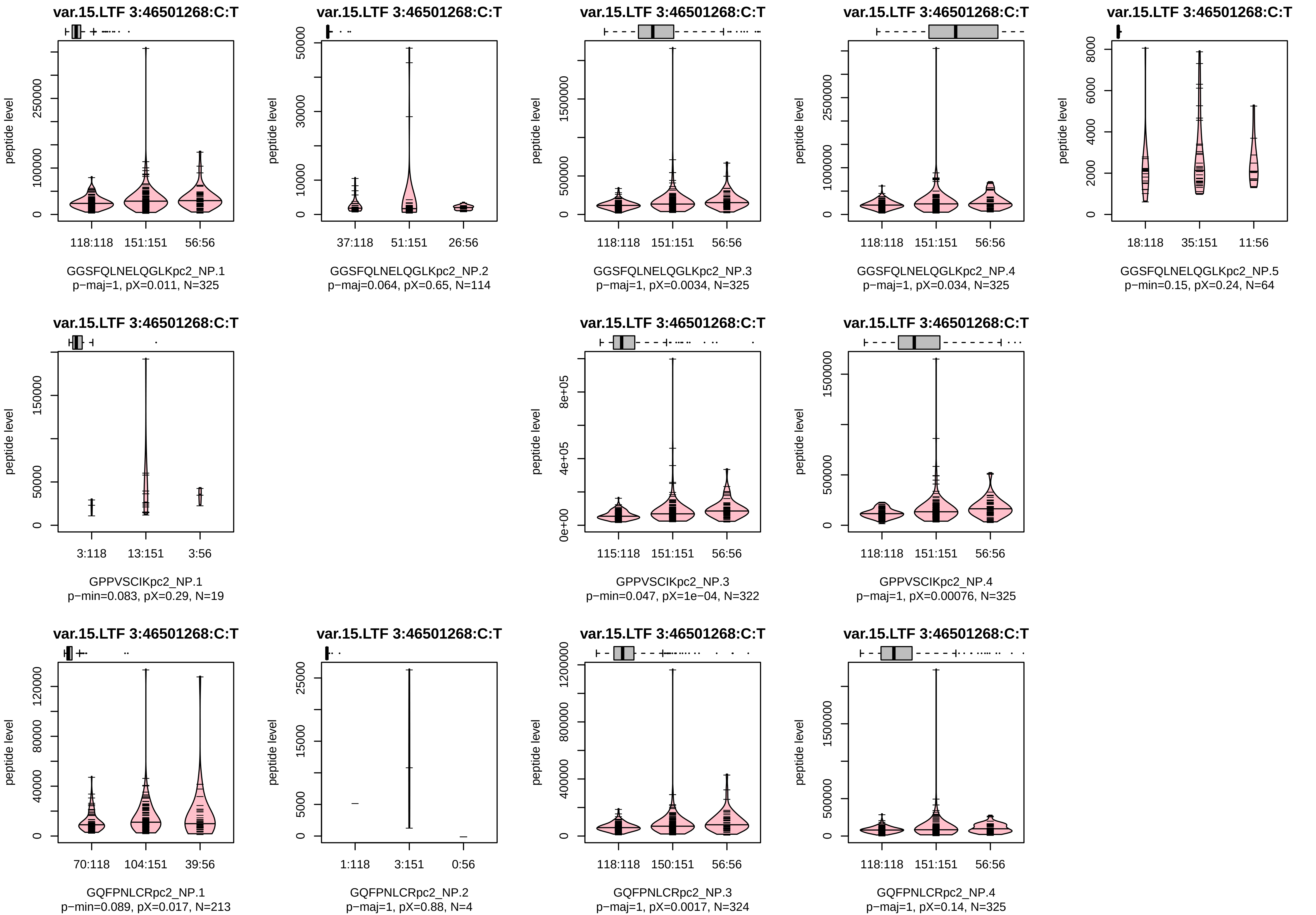

var.15.LTF 3:46501268:C:T
peptide level
118:118
151:151
56:56
GGSFQLNELQGLKpc2_NP.1
p−maj=1, pX=0.011, N=325
var.15.LTF 3:46501268:C:T
peptide level
37:118
51:151
26:56
GGSFQLNELQGLKpc2_NP.2
p−maj=0.064, pX=0.65, N=114
var.15.LTF 3:46501268:C:T
peptide level
118:118
151:151
56:56
GGSFQLNELQGLKpc2_NP.3
p−maj=1, pX=0.0034, N=325
var.15.LTF 3:46501268:C:T
peptide level
118:118
151:151
56:56
GGSFQLNELQGLKpc2_NP.4
p−maj=1, pX=0.034, N=325
var.15.LTF 3:46501268:C:T
peptide level
18:118
35:151
11:56
GGSFQLNELQGLKpc2_NP.5
p−min=0.15, pX=0.24, N=64
var.15.LTF 3:46501268:C:T
peptide level
3:118
13:151
3:56
GPPVSCIKpc2_NP.1
p−min=0.083, pX=0.29, N=19
var.15.LTF 3:46501268:C:T
peptide level
115:118
151:151
56:56
GPPVSCIKpc2_NP.3
p−min=0.047, pX=1e−04, N=322
var.15.LTF 3:46501268:C:T
peptide level
118:118
151:151
56:56
GPPVSCIKpc2_NP.4
p−maj=1, pX=0.00076, N=325
var.15.LTF 3:46501268:C:T
peptide level
70:118
104:151
39:56
GQFPNLCRpc2_NP.1
p−min=0.089, pX=0.017, N=213
var.15.LTF 3:46501268:C:T
peptide level
1:118
3:151
0:56
GQFPNLCRpc2_NP.2
p−maj=1, pX=0.88, N=4
var.15.LTF 3:46501268:C:T
peptide level
118:118
150:151
56:56
GQFPNLCRpc2_NP.3
p−maj=1, pX=0.0017, N=324
var.15.LTF 3:46501268:C:T
peptide level
118:118
151:151
56:56
GQFPNLCRpc2_NP.4
p−maj=1, pX=0.14, N=325

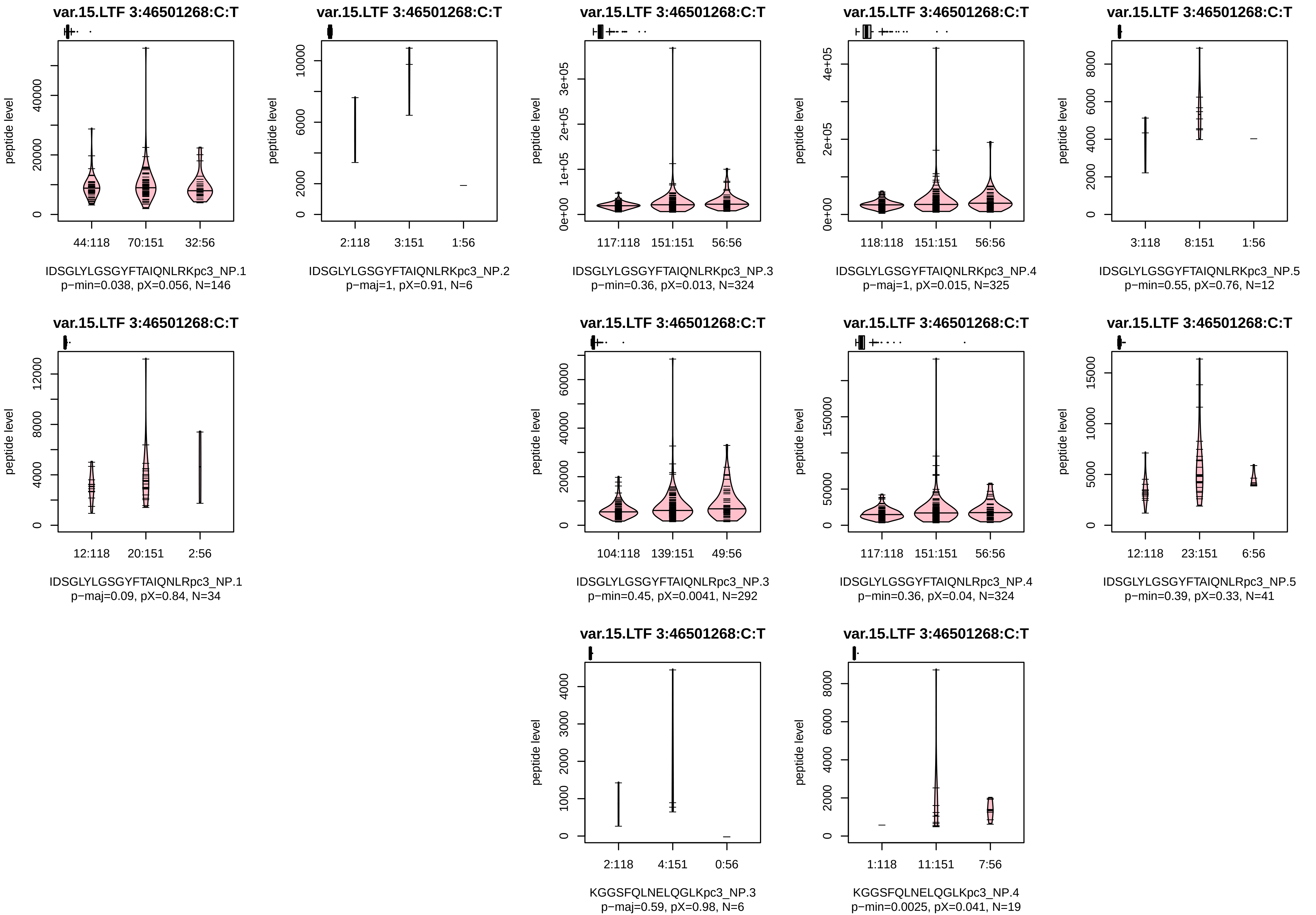

var.15.LTF 3:46501268:C:T
peptide level
44:118
70:151
32:56
IDSGLYLGSGYFTAIQNLRKpc3_NP.1
p−min=0.038, pX=0.056, N=146
var.15.LTF 3:46501268:C:T
peptide level
2:118
3:151
1:56
IDSGLYLGSGYFTAIQNLRKpc3_NP.2
p−maj=1, pX=0.91, N=6
var.15.LTF 3:46501268:C:T
peptide level
117:118
151:151
56:56
IDSGLYLGSGYFTAIQNLRKpc3_NP.3
p−min=0.36, pX=0.013, N=324
var.15.LTF 3:46501268:C:T
peptide level
118:118
151:151
56:56
IDSGLYLGSGYFTAIQNLRKpc3_NP.4
p−maj=1, pX=0.015, N=325
var.15.LTF 3:46501268:C:T
peptide level
3:118
8:151
1:56
IDSGLYLGSGYFTAIQNLRKpc3_NP.5
p−min=0.55, pX=0.76, N=12
var.15.LTF 3:46501268:C:T
peptide level
12:118
20:151
2:56
IDSGLYLGSGYFTAIQNLRpc3_NP.1
p−maj=0.09, pX=0.84, N=34
var.15.LTF 3:46501268:C:T
peptide level
104:118
139:151
49:56
IDSGLYLGSGYFTAIQNLRpc3_NP.3
p−min=0.45, pX=0.0041, N=292
var.15.LTF 3:46501268:C:T
peptide level
117:118
151:151
56:56
IDSGLYLGSGYFTAIQNLRpc3_NP.4
p−min=0.36, pX=0.04, N=324
var.15.LTF 3:46501268:C:T
peptide level
12:118
23:151
6:56
IDSGLYLGSGYFTAIQNLRpc3_NP.5
p−min=0.39, pX=0.33, N=41
var.15.LTF 3:46501268:C:T
peptide level
2:118
4:151
0:56
KGGSFQLNELQGLKpc3_NP.3
p−maj=0.59, pX=0.98, N=6
var.15.LTF 3:46501268:C:T
peptide level
1:118
11:151
7:56
KGGSFQLNELQGLKpc3_NP.4
p−min=0.0025, pX=0.041, N=19

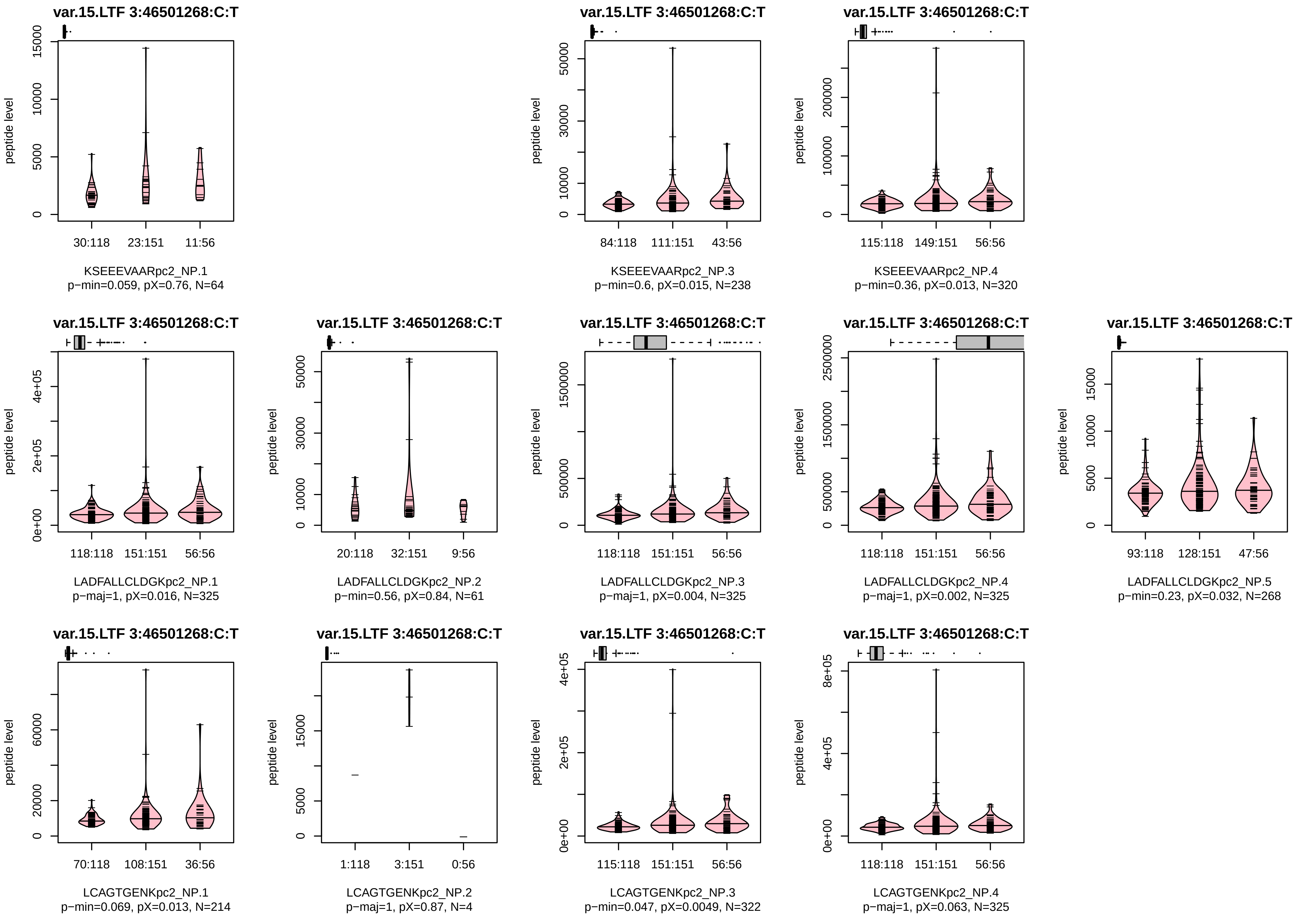
var.15.LTF 3:46501268:C:T
peptide level
30:118 23:151 11:56
KSEEEVAARpc2_NP.1
p−min=0.059, pX=0.76, N=64
var.15.LTF 3:46501268:C:T
84:118 111:151 43:56
KSEEEVAARpc2_NP.3
p−min=0.6, pX=0.015, N=238
var.15.LTF 3:46501268:C:T
115:118 149:151 56:56
KSEEEVAARpc2_NP.4
p−min=0.36, pX=0.013, N=320
var.15.LTF 3:46501268:C:T
118:118 151:151 56:56
LADFALLCLDGKpc2_NP.1
p−maj=1, pX=0.016, N=325
var.15.LTF 3:46501268:C:T
20:118 32:151 9:56
LADFALLCLDGKpc2_NP.2
p−min=0.56, pX=0.84, N=61
var.15.LTF 3:46501268:C:T
118:118 151:151 56:56
LADFALLCLDGKpc2_NP.3
p−maj=1, pX=0.004, N=325
var.15.LTF 3:46501268:C:T
118:118 151:151 56:56
LADFALLCLDGKpc2_NP.4
p−maj=1, pX=0.002, N=325
var.15.LTF 3:46501268:C:T
93:118 128:151 47:56
LADFALLCLDGKpc2_NP.5
p−min=0.23, pX=0.032, N=268
var.15.LTF 3:46501268:C:T
70:118 108:151 36:56
LCAGTGENKpc2_NP.1
p−min=0.069, pX=0.013, N=214
var.15.LTF 3:46501268:C:T
1:118 3:151 0:56
LCAGTGENKpc2_NP.2
p−maj=1, pX=0.87, N=4
var.15.LTF 3:46501268:C:T
115:118 151:151 56:56
LCAGTGENKpc2_NP.3
p−min=0.047, pX=0.0049, N=322
var.15.LTF 3:46501268:C:T
118:118 151:151 56:56
LCAGTGENKpc2_NP.4
p−maj=1, pX=0.063, N=325

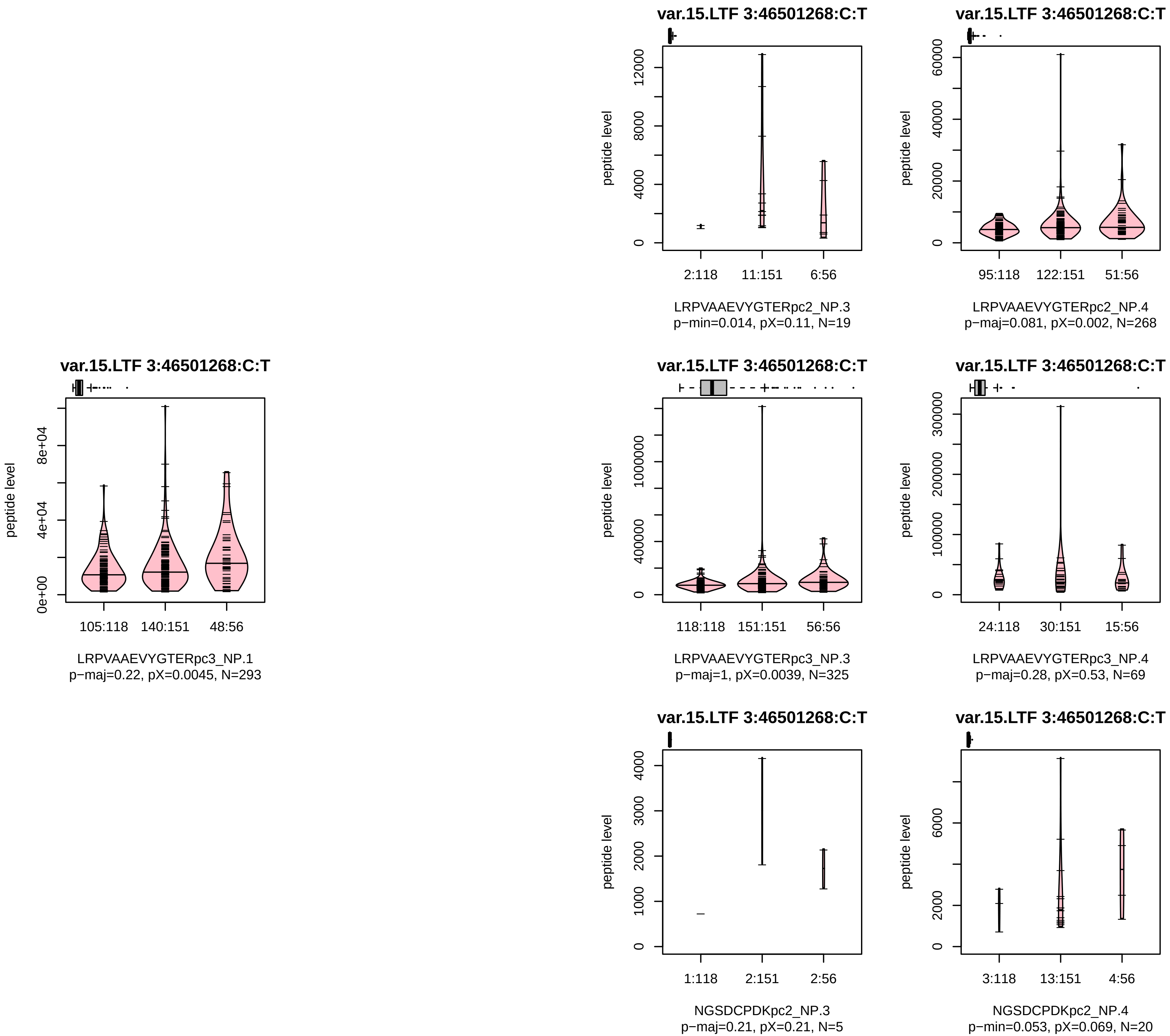
var.15.LTF 3:46501268:C:T
peptide level
2:118
11:151
6:56
LRPVAAEVYGTERpc2_NP.3
p−min=0.014, pX=0.11, N=19
var.15.LTF 3:46501268:C:T
peptide level
95:118
122:151
51:56
LRPVAAEVYGTERpc2_NP.4
p−maj=0.081, pX=0.002, N=268
var.15.LTF 3:46501268:C:T
peptide level
105:118
140:151
48:56
LRPVAAEVYGTERpc3_NP.1
p−maj=0.22, pX=0.0045, N=293
var.15.LTF 3:46501268:C:T
peptide level
118:118
151:151
56:56
LRPVAAEVYGTERpc3_NP.3
p−maj=1, pX=0.0039, N=325
var.15.LTF 3:46501268:C:T
peptide level
24:118
30:151
15:56
LRPVAAEVYGTERpc3_NP.4
p−maj=0.28, pX=0.53, N=69
var.15.LTF 3:46501268:C:T
peptide level
1:118
2:151
2:56
NGSDCPDKpc2_NP.3
p−maj=0.21, pX=0.21, N=5
var.15.LTF 3:46501268:C:T
peptide level
3:118
13:151
4:56
NGSDCPDKpc2_NP.4
p−min=0.053, pX=0.069, N=20

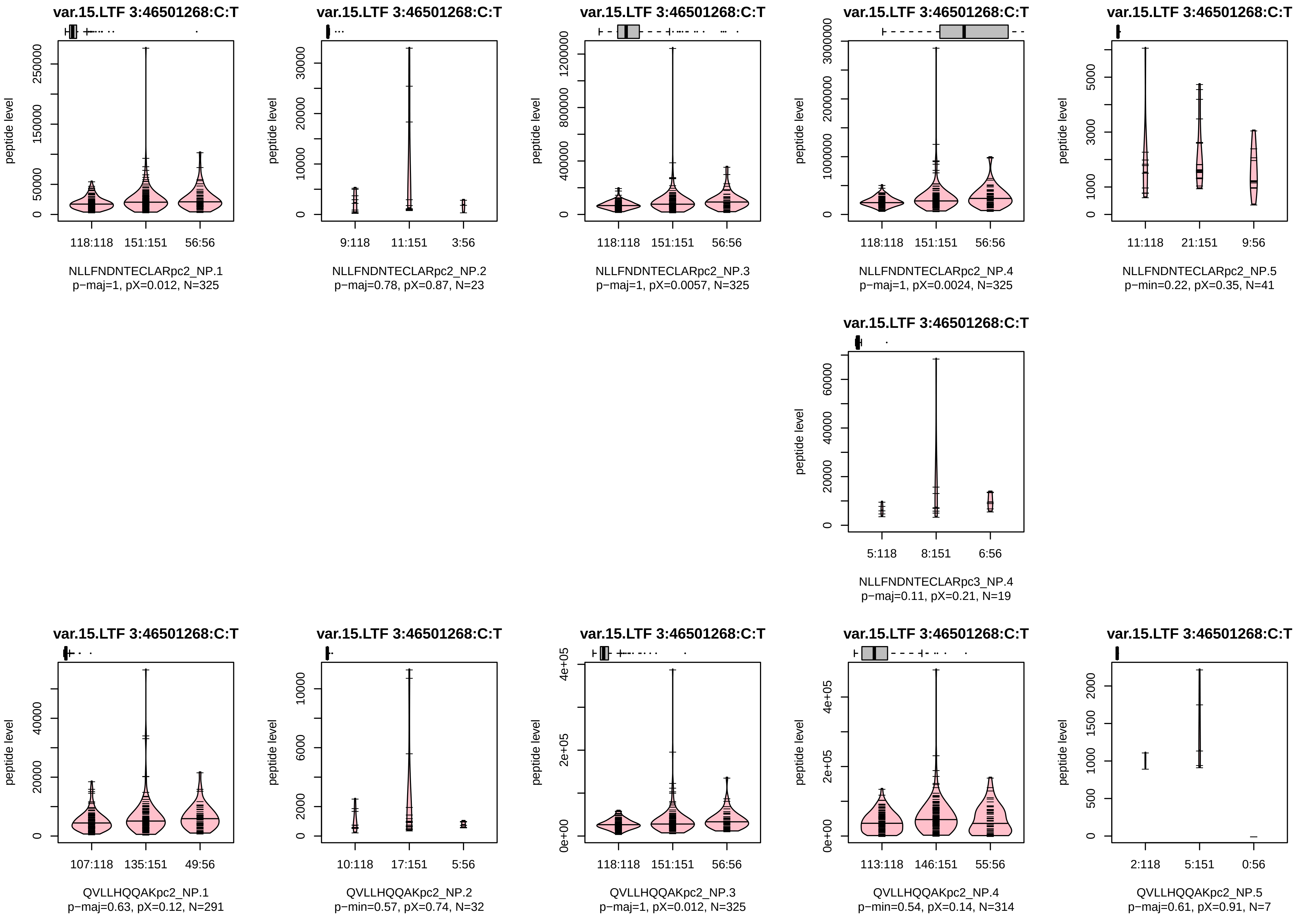

var.15.LTF 3:46501268:C:T
peptide level
118:118
151:151
56:56
NLLFNDNTECLARpc2_NP.1
p−maj=1, pX=0.012, N=325
var.15.LTF 3:46501268:C:T
peptide level
9:118
11:151
3:56
NLLFNDNTECLARpc2_NP.2
p−maj=0.78, pX=0.87, N=23
var.15.LTF 3:46501268:C:T
peptide level
118:118
151:151
56:56
NLLFNDNTECLARpc2_NP.3
p−maj=1, pX=0.0057, N=325
var.15.LTF 3:46501268:C:T
peptide level
118:118
151:151
56:56
NLLFNDNTECLARpc2_NP.4
p−maj=1, pX=0.0024, N=325
var.15.LTF 3:46501268:C:T
peptide level
11:118
21:151
9:56
NLLFNDNTECLARpc2_NP.5
p−min=0.22, pX=0.35, N=41
var.15.LTF 3:46501268:C:T
peptide level
5:118
8:151
6:56
NLLFNDNTECLARpc3_NP.4
p−maj=0.11, pX=0.21, N=19
var.15.LTF 3:46501268:C:T
peptide level
107:118
135:151
49:56
QVLLHQQAKpc2_NP.1
p−maj=0.63, pX=0.12, N=291
var.15.LTF 3:46501268:C:T
peptide level
10:118
17:151
5:56
QVLLHQQAKpc2_NP.2
p−min=0.57, pX=0.74, N=32
var.15.LTF 3:46501268:C:T
peptide level
118:118
151:151
56:56
QVLLHQQAKpc2_NP.3
p−maj=1, pX=0.012, N=325
var.15.LTF 3:46501268:C:T
peptide level
113:118
146:151
55:56
QVLLHQQAKpc2_NP.4
p−min=0.54, pX=0.14, N=314
var.15.LTF 3:46501268:C:T
peptide level
2:118
5:151
0:56
QVLLHQQAKpc2_NP.5
p−maj=0.61, pX=0.91, N=7

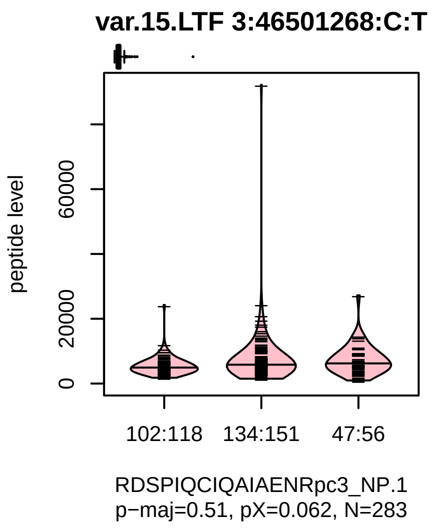
var.15.LTF 3:46501268:C:T
peptide level
0
20000
60000
102:118
134:151
47:56
RDSPIQCIQAIAENRpc3_NP.1
p−maj=0.51, pX=0.062, N=283

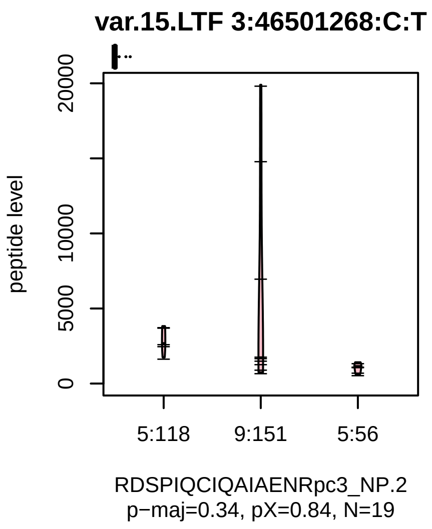
var.15.LTF 3:46501268:C:T
peptide level
0
5000
10000
20000
5:118
9:151
5:56
RDSPIQCIQAIAENRpc3_NP.2
p−maj=0.34, pX=0.84, N=19

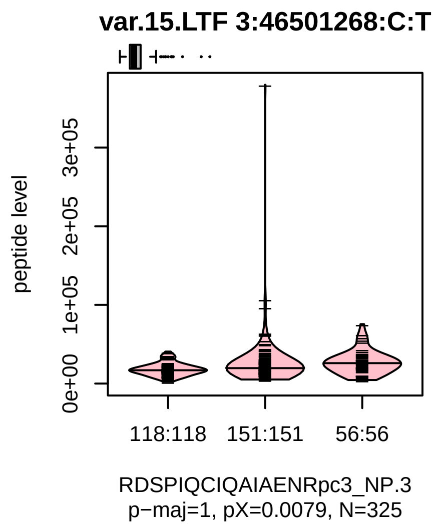
var.15.LTF 3:46501268:C:T
peptide level
0e+00
1e+05
2e+05
3e+05
118:118
151:151
56:56
RDSPIQCIQAIAENRpc3_NP.3
p−maj=1, pX=0.0079, N=325

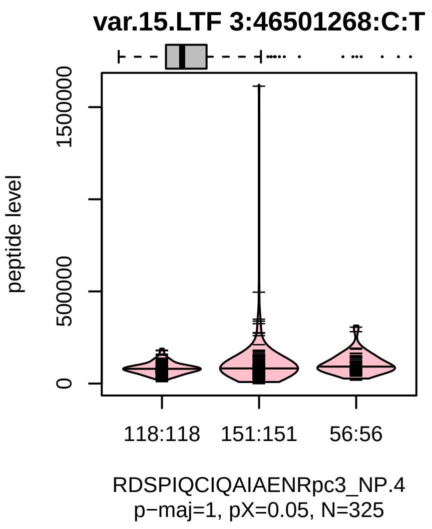
var.15.LTF 3:46501268:C:T
peptide level
0
500000
1500000
118:118
151:151
56:56
RDSPIQCIQAIAENRpc3_NP.4
p−maj=1, pX=0.05, N=325

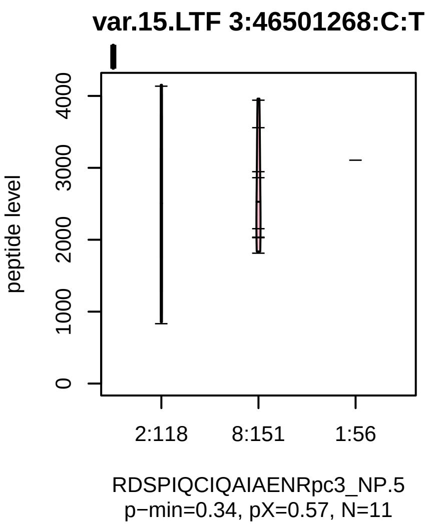
var.15.LTF 3:46501268:C:T
peptide level
0
1000
2000
3000
4000
2:118
8:151
1:56
RDSPIQCIQAIAENRpc3_NP.5
p−min=0.34, pX=0.57, N=11

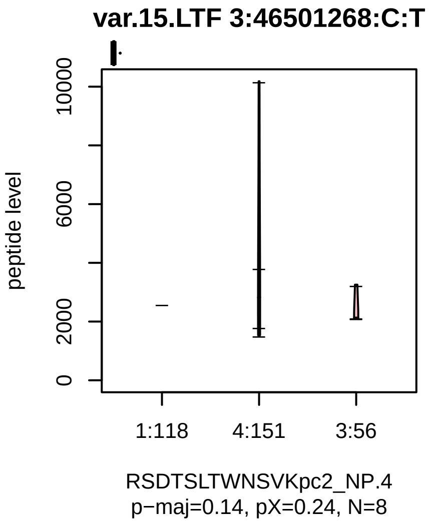
var.15.LTF 3:46501268:C:T
peptide level
0
2000
6000
10000
1:118
4:151
3:56
RSDTSLTWNSVKpc2_NP.4
p−maj=0.14, pX=0.24, N=8

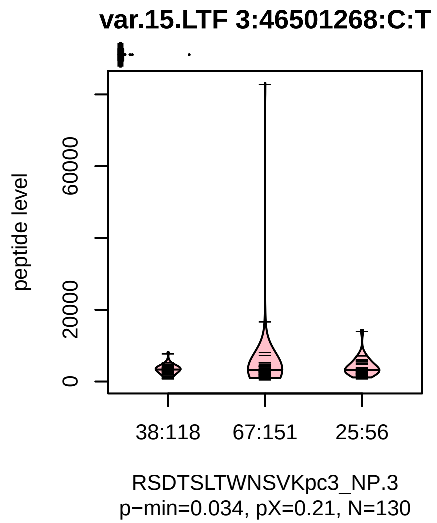
var.15.LTF 3:46501268:C:T
peptide level
0
20000
60000
38:118
67:151
25:56
RSDTSLTWNSVKpc3_NP.3
p−min=0.034, pX=0.21, N=130

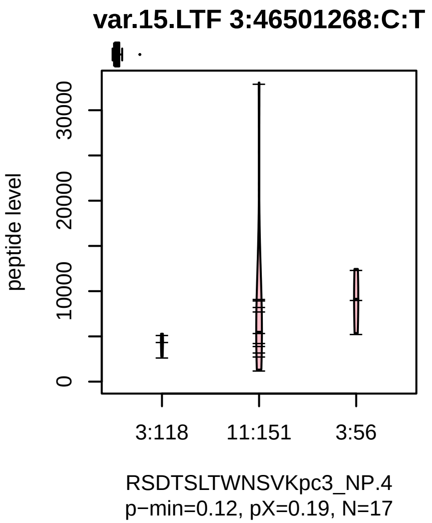
var.15.LTF 3:46501268:C:T
peptide level
0
10000
20000
30000
3:118
11:151
3:56
RSDTSLTWNSVKpc3_NP.4
p−min=0.12, pX=0.19, N=17

var.15.LTF 3:46501268:C:T

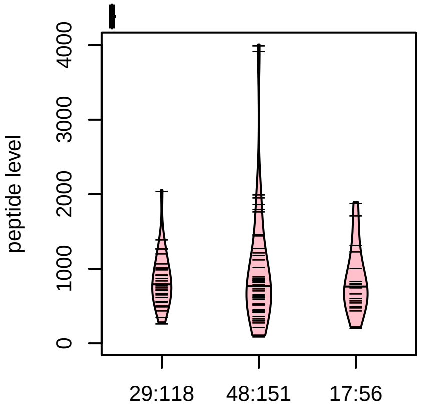

SCHLAMAPNHAVVSRpc2_NP.1
p−min=0.21, pX=0.36, N=94

var.15.LTF 3:46501268:C:T

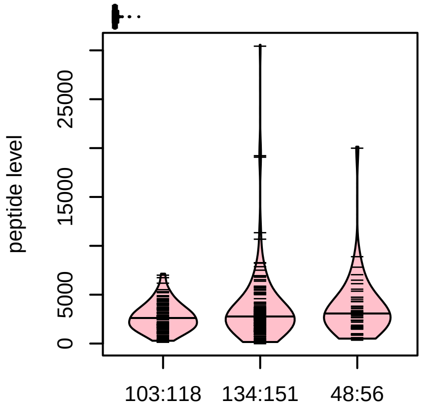

SCHLAMAPNHAVVSRpc2_NP.3
p−maj=0.65, pX=0.055, N=285

var.15.LTF 3:46501268:C:T

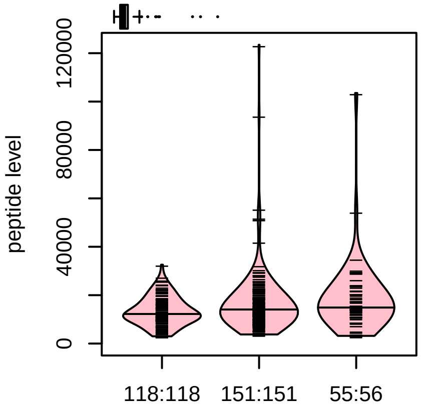

SCHLAMAPNHAVVSRpc2_NP.4
p−maj=0.17, pX=0.0045, N=324

var.15.LTF 3:46501268:C:T

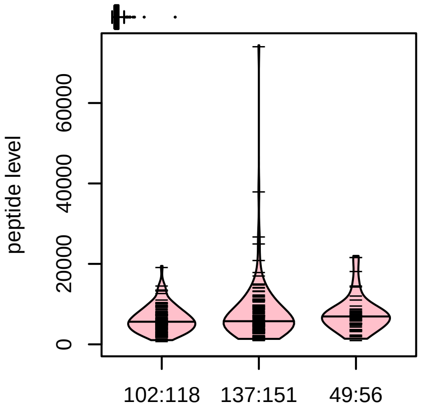

SCHLAMAPNHAVVSRpc3_NP.1
p−min=0.37, pX=0.11, N=288

var.15.LTF 3:46501268:C:T

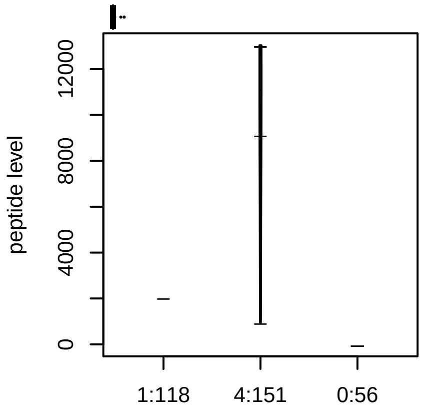

SCHLAMAPNHAVVSRpc3_NP.2
p−maj=0.59, pX=0.72, N=5

var.15.LTF 3:46501268:C:T

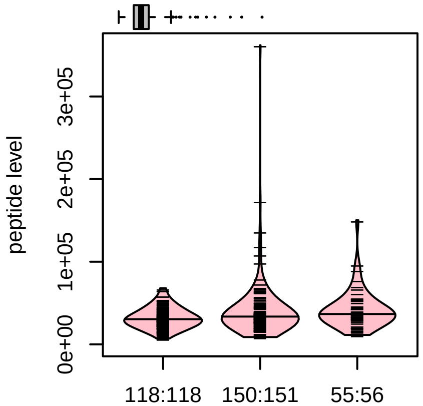

SCHLAMAPNHAVVSRpc3_NP.3
p−maj=0.32, pX=0.017, N=323

var.15.LTF 3:46501268:C:T

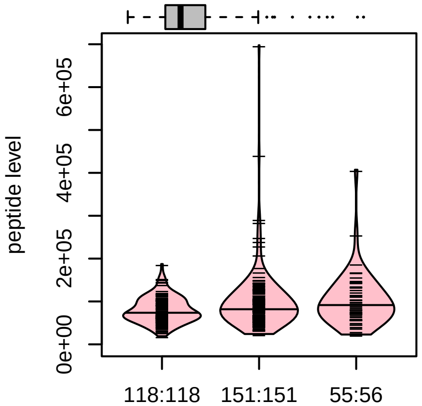

SCHLAMAPNHAVVSRpc3_NP.4
p−maj=0.17, pX=0.0093, N=324

var.15.LTF 3:46501268:C:T

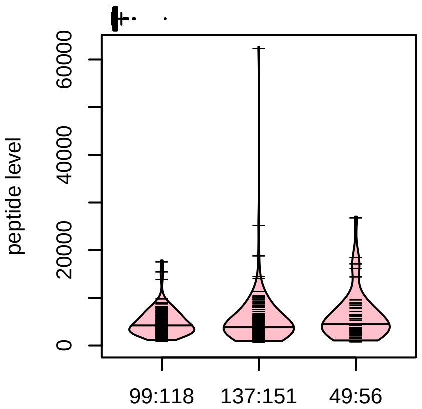

SCHLAMAPNHAVVSRpc4_NP.1
p−min=0.16, pX=0.11, N=285

var.15.LTF 3:46501268:C:T

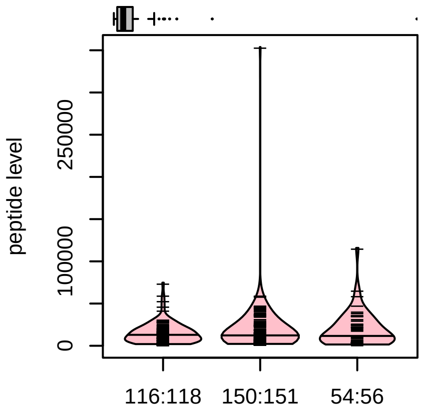

SCHLAMAPNHAVVSRpc4_NP.3
p−maj=0.21, pX=0.25, N=320

var.15.LTF 3:46501268:C:T

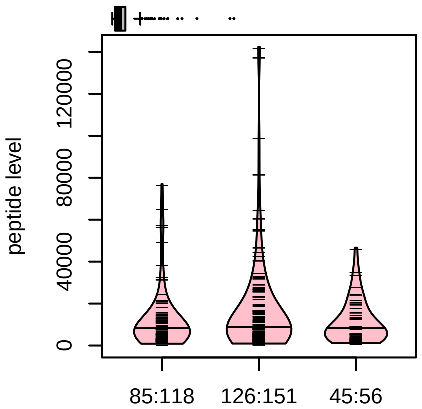

SCHLAMAPNHAVVSRpc4_NP.4
p−min=0.034, pX=0.46, N=256

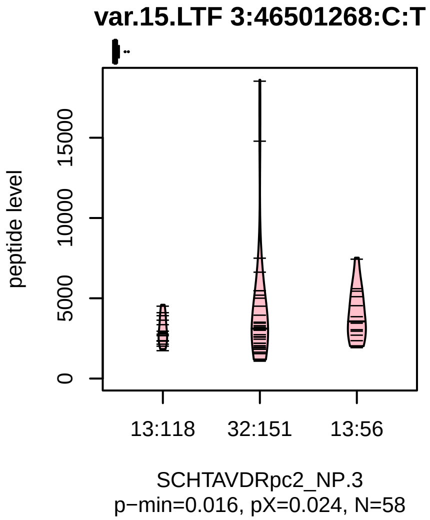
var.15.LTF 3:46501268:C:T
peptide level
0
5000
10000
15000
13:118
32:151
13:56
SCHTAVDRpc2_NP.3
p−min=0.016, pX=0.024, N=58

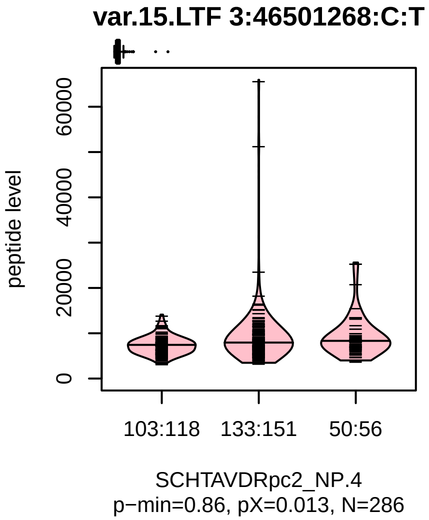
var.15.LTF 3:46501268:C:T
peptide level
0
20000
40000
60000
103:118
133:151
50:56
SCHTAVDRpc2_NP.4
p−min=0.86, pX=0.013, N=286

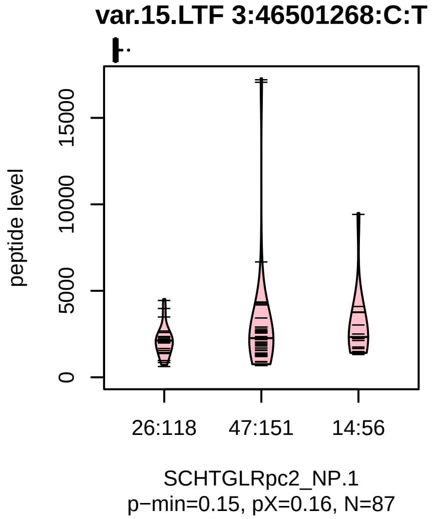
var.15.LTF 3:46501268:C:T
peptide level
0
5000
10000
15000
26:118
47:151
14:56
SCHTGLRpc2_NP.1
p−min=0.15, pX=0.16, N=87

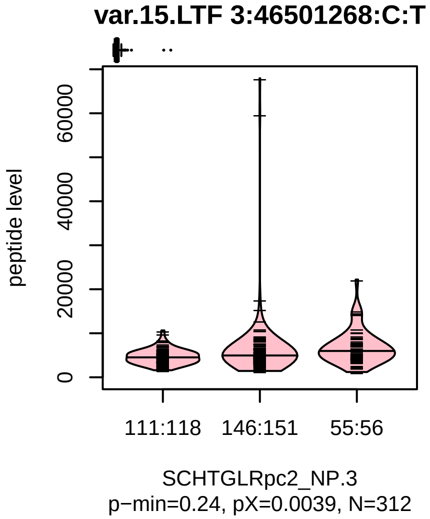
var.15.LTF 3:46501268:C:T
peptide level
0
20000
40000
60000
111:118
146:151
55:56
SCHTGLRpc2_NP.3
p−min=0.24, pX=0.0039, N=312

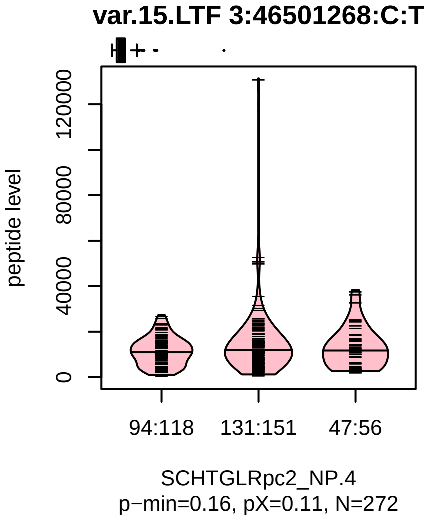
var.15.LTF 3:46501268:C:T
peptide level
0
40000
80000
120000
94:118
131:151
47:56
SCHTGLRpc2_NP.4
p−min=0.16, pX=0.11, N=272

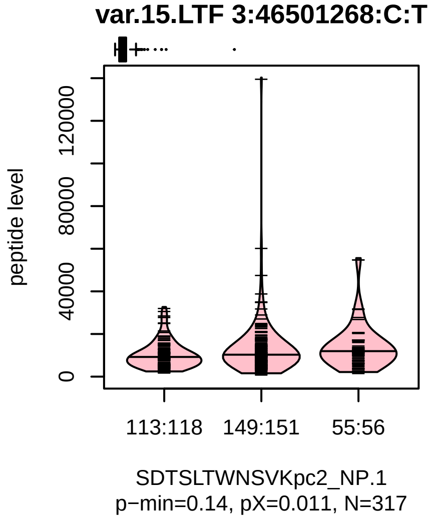
var.15.LTF 3:46501268:C:T
peptide level
0
40000
80000
120000
113:118
149:151
55:56
SDTSLTWNSVKpc2_NP.1
p−min=0.14, pX=0.011, N=317

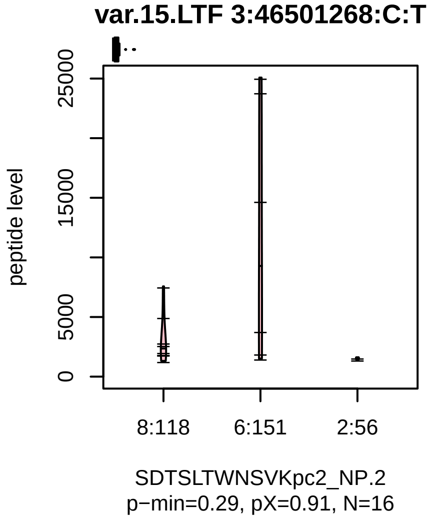
var.15.LTF 3:46501268:C:T
peptide level
0
5000
15000
25000
8:118
6:151
2:56
SDTSLTWNSVKpc2_NP.2
p−min=0.29, pX=0.91, N=16

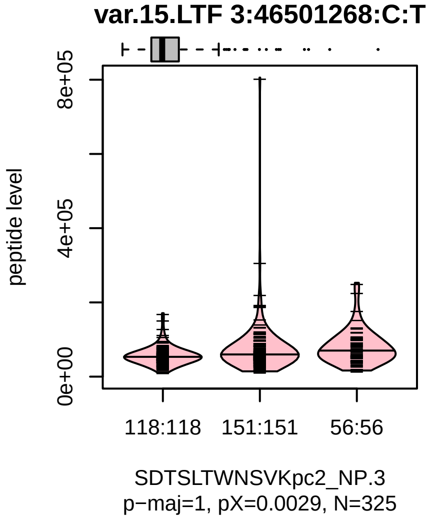
var.15.LTF 3:46501268:C:T
peptide level
0e+00
4e+05
8e+05
118:118
151:151
56:56
SDTSLTWNSVKpc2_NP.3
p−maj=1, pX=0.0029, N=325

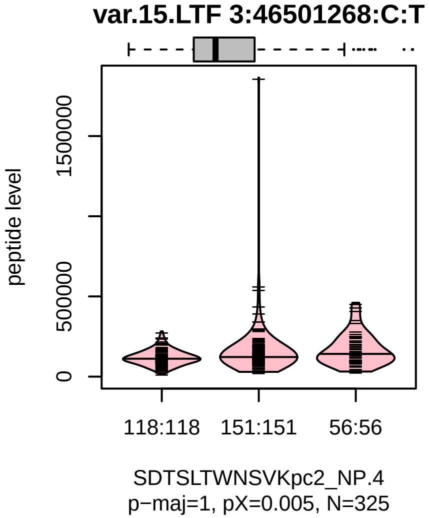
var.15.LTF 3:46501268:C:T
peptide level
0
500000
1500000
118:118
151:151
56:56
SDTSLTWNSVKpc2_NP.4
p−maj=1, pX=0.005, N=325

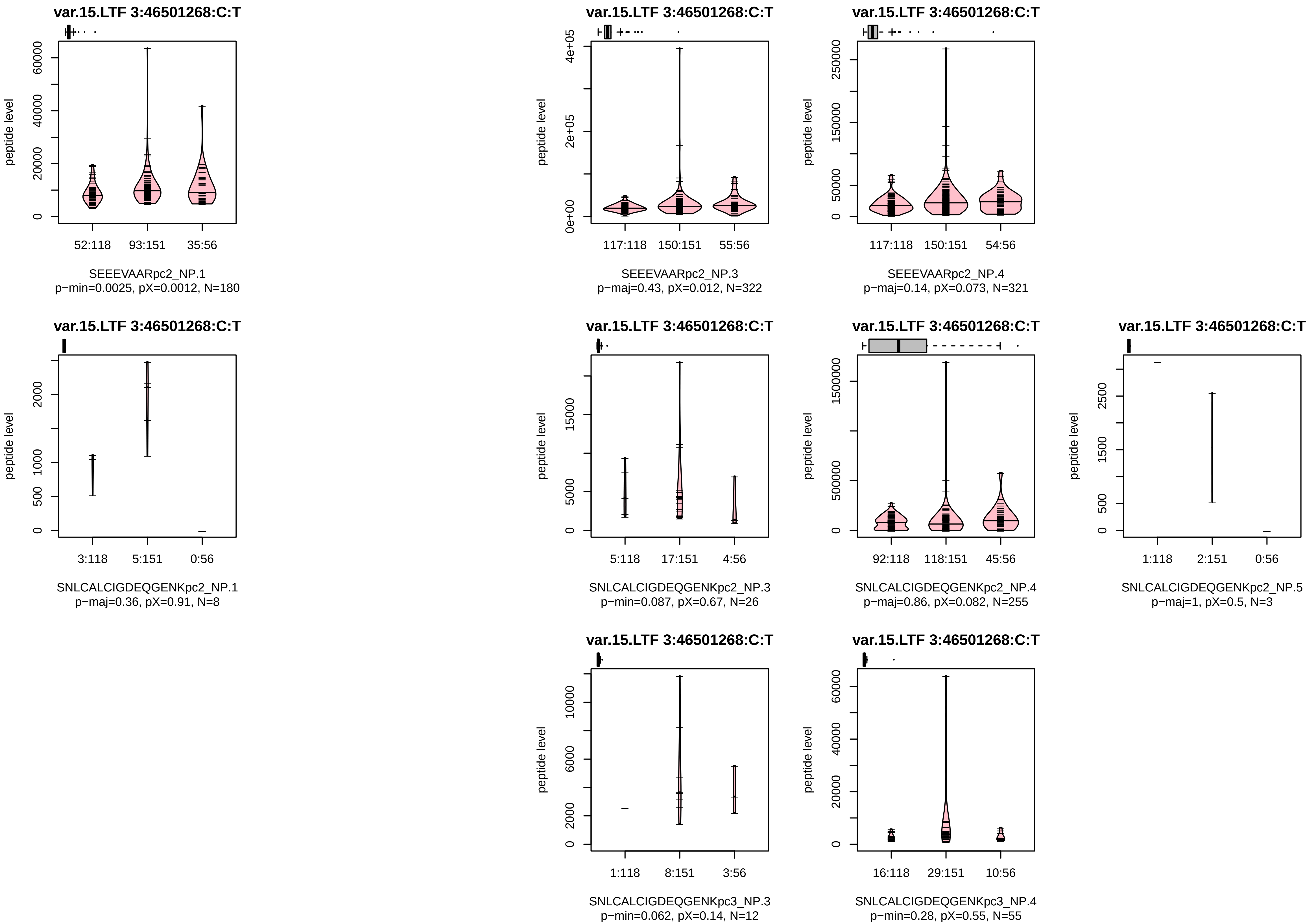
var.15.LTF 3:46501268:C:T
peptide level
52:118
93:151
35:56
SEEEVAARpc2_NP.1
p−min=0.0025, pX=0.0012, N=180
var.15.LTF 3:46501268:C:T
peptide level
117:118
150:151
55:56
SEEEVAARpc2_NP.3
p−maj=0.43, pX=0.012, N=322
var.15.LTF 3:46501268:C:T
peptide level
117:118
150:151
54:56
SEEEVAARpc2_NP.4
p−maj=0.14, pX=0.073, N=321
var.15.LTF 3:46501268:C:T
peptide level
3:118
5:151
0:56
SNLCALCIGDEQGENKpc2_NP.1
p−maj=0.36, pX=0.91, N=8
var.15.LTF 3:46501268:C:T
peptide level
5:118
17:151
4:56
SNLCALCIGDEQGENKpc2_NP.3
p−min=0.087, pX=0.67, N=26
var.15.LTF 3:46501268:C:T
peptide level
92:118
118:151
45:56
SNLCALCIGDEQGENKpc2_NP.4
p−maj=0.86, pX=0.082, N=255
var.15.LTF 3:46501268:C:T
peptide level
1:118
2:151
0:56
SNLCALCIGDEQGENKpc2_NP.5
p−maj=1, pX=0.5, N=3
var.15.LTF 3:46501268:C:T
peptide level
1:118
8:151
3:56
SNLCALCIGDEQGENKpc3_NP.3
p−min=0.062, pX=0.14, N=12
var.15.LTF 3:46501268:C:T
peptide level
16:118
29:151
10:56
SNLCALCIGDEQGENKpc3_NP.4
p−min=0.28, pX=0.55, N=55

var.15.LTF 3:46501268:C:T

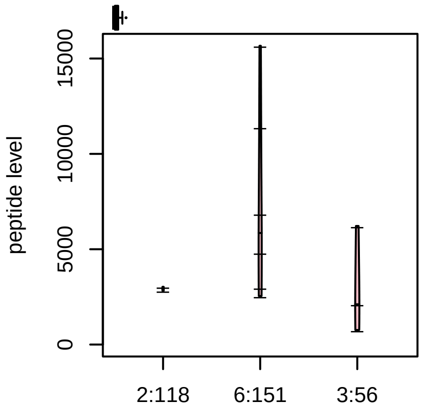

SQQSSDPDPNCVDRPVEGYLAVAVVRpc4_NP
p−min=0.34, pX=0.36, N=11

var.15.LTF 3:46501268:C:T

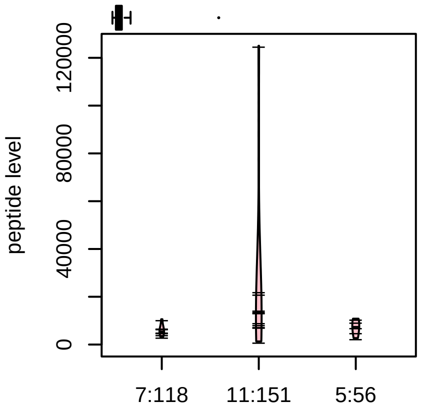

SQQSSDPDPNCVDRPVEGYLAVAVVRpc4_NP
p−maj=0.57, pX=0.56, N=23

var.15.LTF 3:46501268:C:T

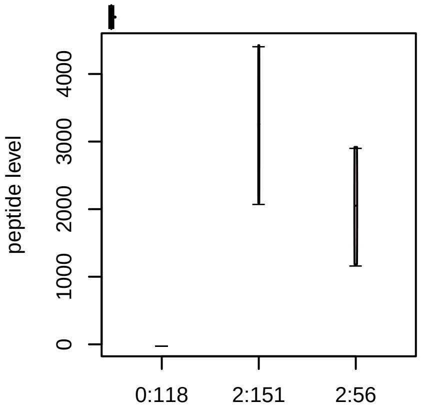

SVNGKEDAIWNLLRpc2_NP.4
p−maj=0.14, pX=0.13, N=4

var.15.LTF 3:46501268:C:T

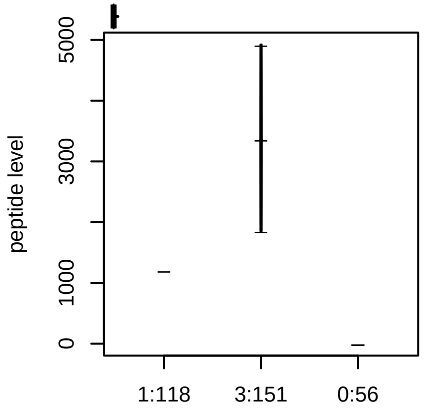

SVNGKEDAIWNLLRpc3_NP.1
p−maj=1, pX=0.83, N=4

var.15.LTF 3:46501268:C:T

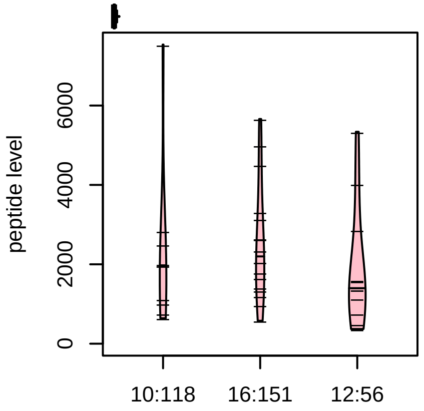

SVNGKEDAIWNLLRpc3_NP.3
p−maj=0.02, pX=0.16, N=38

var.15.LTF 3:46501268:C:T

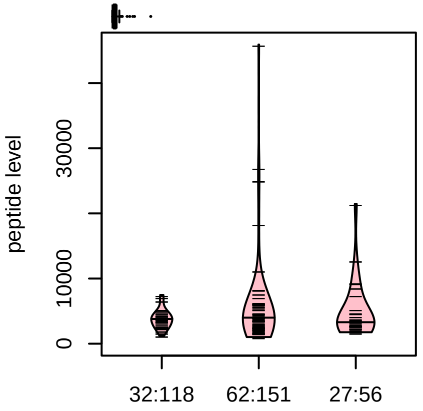

SVNGKEDAIWNLLRpc3_NP.4
p−min=0.0059, pX=0.013, N=121

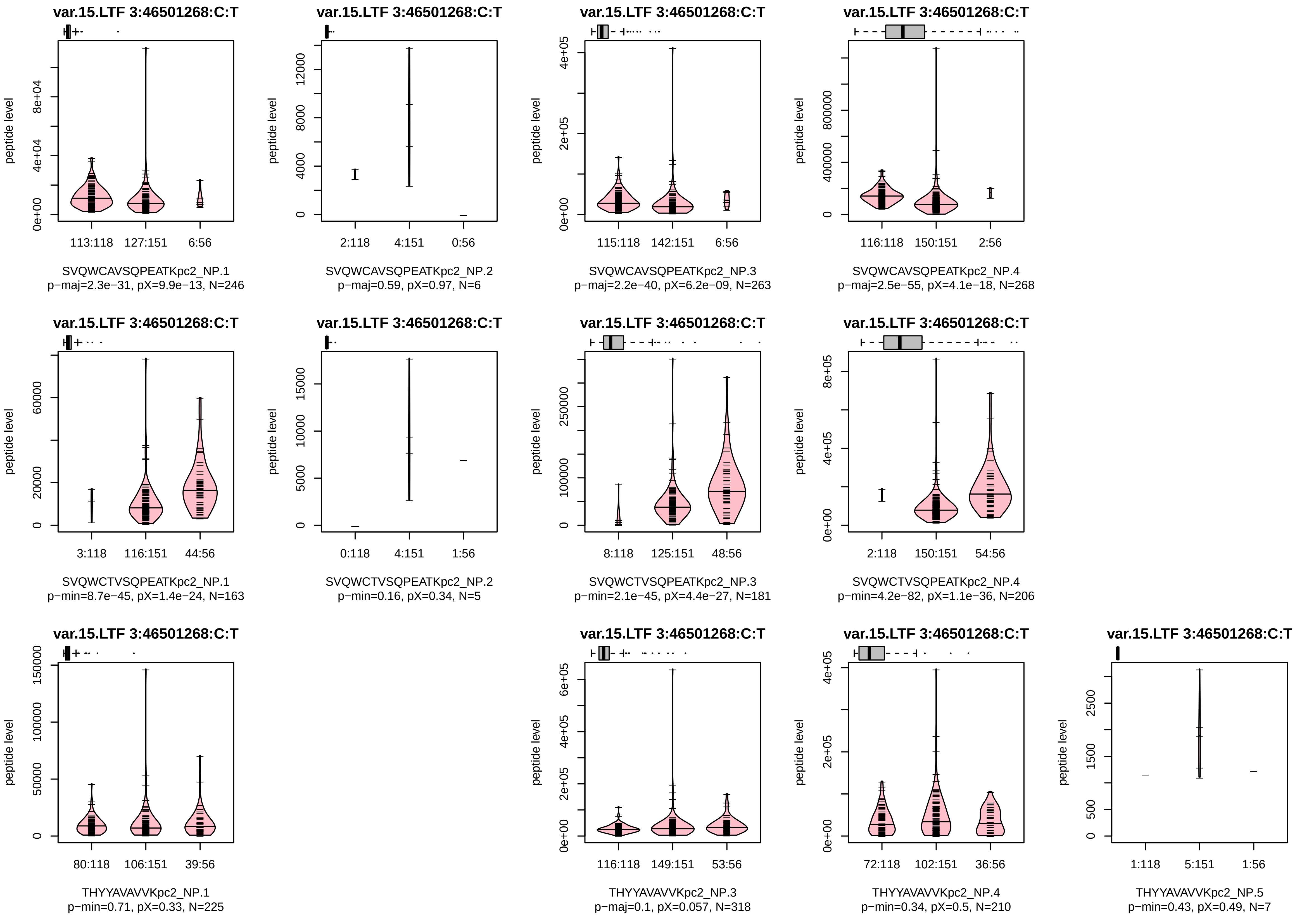
var.15.LTF 3:46501268:C:T
peptide level
113:118
127:151
6:56
SVQWCAVSQPEATKpc2_NP.1
p−maj=2.3e−31, pX=9.9e−13, N=246
var.15.LTF 3:46501268:C:T
peptide level
2:118
4:151
0:56
SVQWCAVSQPEATKpc2_NP.2
p−maj=0.59, pX=0.97, N=6
var.15.LTF 3:46501268:C:T
peptide level
115:118
142:151
6:56
SVQWCAVSQPEATKpc2_NP.3
p−maj=2.2e−40, pX=6.2e−09, N=263
var.15.LTF 3:46501268:C:T
peptide level
116:118
150:151
2:56
SVQWCAVSQPEATKpc2_NP.4
p−maj=2.5e−55, pX=4.1e−18, N=268
var.15.LTF 3:46501268:C:T
peptide level
3:118
116:151
44:56
SVQWCTVSQPEATKpc2_NP.1
p−min=8.7e−45, pX=1.4e−24, N=163
var.15.LTF 3:46501268:C:T
peptide level
0:118
4:151
1:56
SVQWCTVSQPEATKpc2_NP.2
p−min=0.16, pX=0.34, N=5
var.15.LTF 3:46501268:C:T
peptide level
8:118
125:151
48:56
SVQWCTVSQPEATKpc2_NP.3
p−min=2.1e−45, pX=4.4e−27, N=181
var.15.LTF 3:46501268:C:T
peptide level
2:118
150:151
54:56
SVQWCTVSQPEATKpc2_NP.4
p−min=4.2e−82, pX=1.1e−36, N=206
var.15.LTF 3:46501268:C:T
peptide level
80:118
106:151
39:56
THYYAVAVVKpc2_NP.1
p−min=0.71, pX=0.33, N=225
var.15.LTF 3:46501268:C:T
peptide level
116:118
149:151
53:56
THYYAVAVVKpc2_NP.3
p−maj=0.1, pX=0.057, N=318
var.15.LTF 3:46501268:C:T
peptide level
72:118
102:151
36:56
THYYAVAVVKpc2_NP.4
p−min=0.34, pX=0.5, N=210
var.15.LTF 3:46501268:C:T
peptide level
1:118
5:151
1:56
THYYAVAVVKpc2_NP.5
p−min=0.43, pX=0.49, N=7

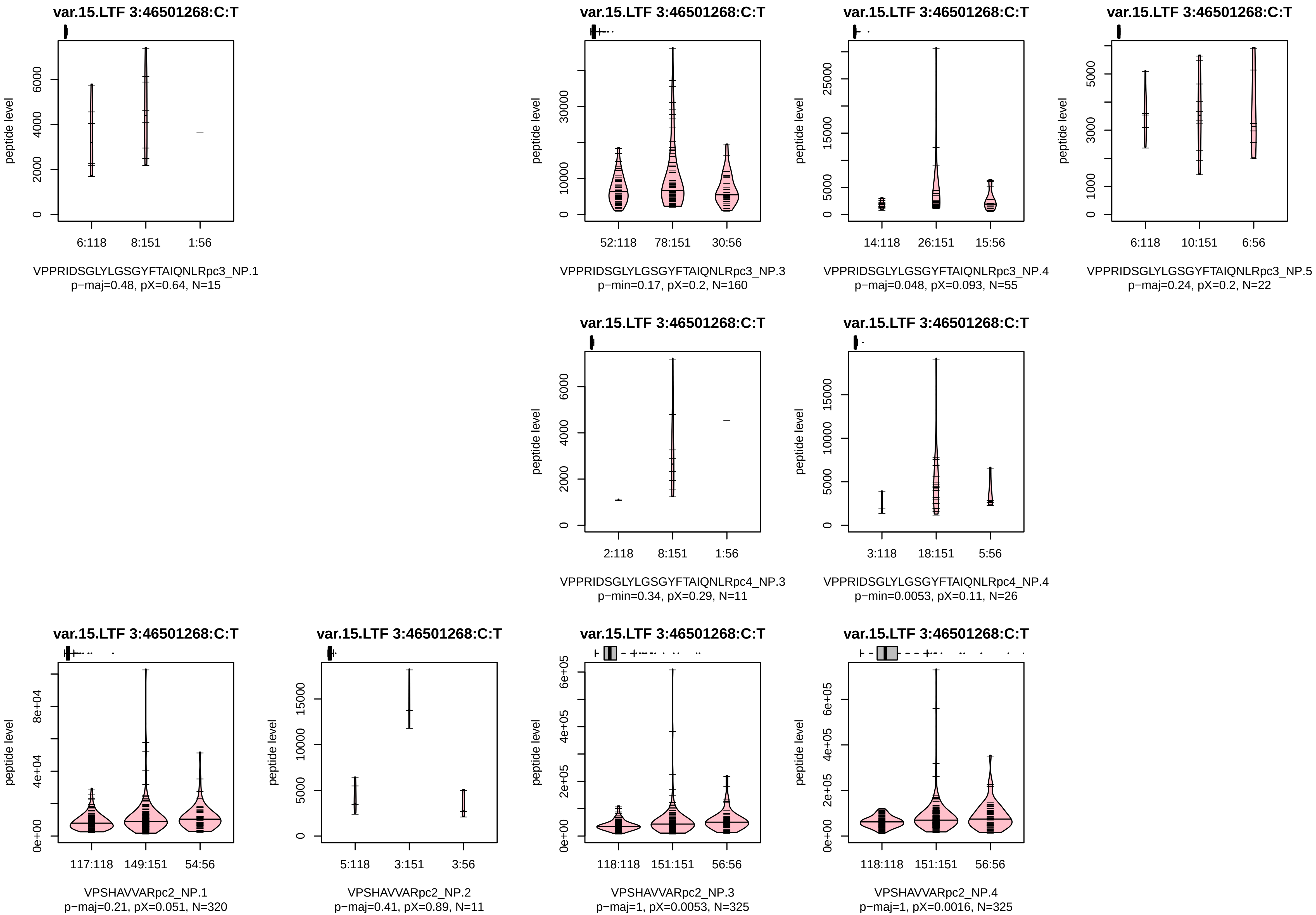
var.15.LTF 3:46501268:C:T
peptide level
6:118
8:151
1:56
VPPRIDSGLYLGSGYFTAIQNLRpc3_NP.1
p−maj=0.48, pX=0.64, N=15
var.15.LTF 3:46501268:C:T
peptide level
52:118
78:151
30:56
VPPRIDSGLYLGSGYFTAIQNLRpc3_NP.3
p−min=0.17, pX=0.2, N=160
var.15.LTF 3:46501268:C:T
peptide level
14:118
26:151
15:56
VPPRIDSGLYLGSGYFTAIQNLRpc3_NP.4
p−maj=0.048, pX=0.093, N=55
var.15.LTF 3:46501268:C:T
peptide level
6:118
10:151
6:56
VPPRIDSGLYLGSGYFTAIQNLRpc3_NP.5
p−maj=0.24, pX=0.2, N=22
var.15.LTF 3:46501268:C:T
peptide level
2:118
8:151
1:56
VPPRIDSGLYLGSGYFTAIQNLRpc4_NP.3
p−min=0.34, pX=0.29, N=11
var.15.LTF 3:46501268:C:T
peptide level
3:118
18:151
5:56
VPPRIDSGLYLGSGYFTAIQNLRpc4_NP.4
p−min=0.0053, pX=0.11, N=26
var.15.LTF 3:46501268:C:T
peptide level
117:118
149:151
54:56
VPSHAVVARpc2_NP.1
p−maj=0.21, pX=0.051, N=320
var.15.LTF 3:46501268:C:T
peptide level
5:118
3:151
3:56
VPSHAVVARpc2_NP.2
p−maj=0.41, pX=0.89, N=11
var.15.LTF 3:46501268:C:T
peptide level
118:118
151:151
56:56
VPSHAVVARpc2_NP.3
p−maj=1, pX=0.0053, N=325
var.15.LTF 3:46501268:C:T
peptide level
118:118
151:151
56:56
VPSHAVVARpc2_NP.4
p−maj=1, pX=0.0016, N=325

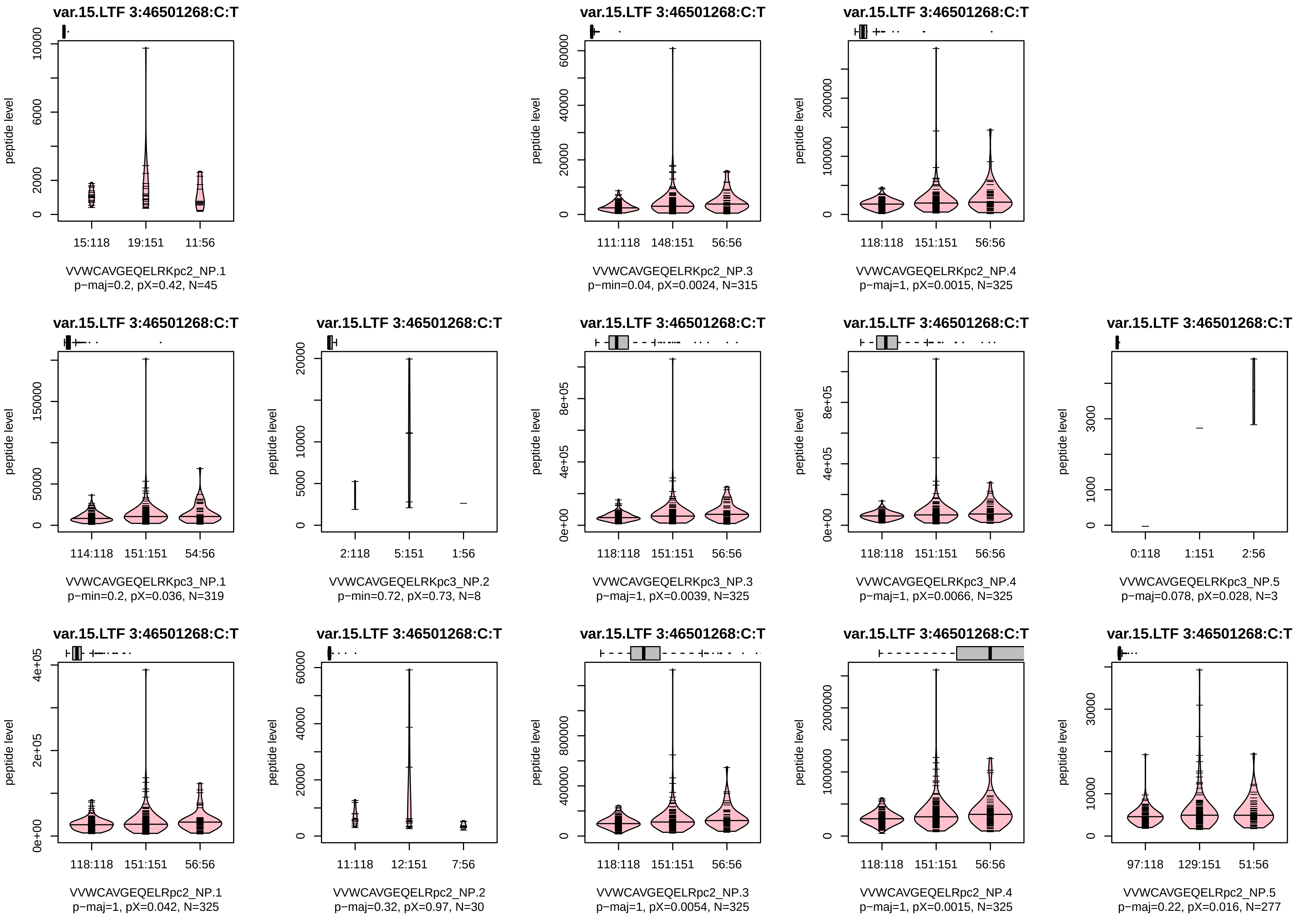
var.15.LTF 3:46501268:C:T
peptide level
15:118
19:151
11:56
VVWCAVGEQELRKpc2_NP.1
p−maj=0.2, pX=0.42, N=45
var.15.LTF 3:46501268:C:T
peptide level
111:118
148:151
56:56
VVWCAVGEQELRKpc2_NP.3
p−min=0.04, pX=0.0024, N=315
var.15.LTF 3:46501268:C:T
peptide level
118:118
151:151
56:56
VVWCAVGEQELRKpc2_NP.4
p−maj=1, pX=0.0015, N=325
var.15.LTF 3:46501268:C:T
peptide level
114:118
151:151
54:56
VVWCAVGEQELRKpc3_NP.1
p−min=0.2, pX=0.036, N=319
var.15.LTF 3:46501268:C:T
peptide level
2:118
5:151
1:56
VVWCAVGEQELRKpc3_NP.2
p−min=0.72, pX=0.73, N=8
var.15.LTF 3:46501268:C:T
peptide level
118:118
151:151
56:56
VVWCAVGEQELRKpc3_NP.3
p−maj=1, pX=0.0039, N=325
var.15.LTF 3:46501268:C:T
peptide level
118:118
151:151
56:56
VVWCAVGEQELRKpc3_NP.4
p−maj=1, pX=0.0066, N=325
var.15.LTF 3:46501268:C:T
peptide level
0:118
1:151
2:56
VVWCAVGEQELRKpc3_NP.5
p−maj=0.078, pX=0.028, N=3
var.15.LTF 3:46501268:C:T
peptide level
118:118
151:151
56:56
VVWCAVGEQELRpc2_NP.1
p−maj=1, pX=0.042, N=325
var.15.LTF 3:46501268:C:T
peptide level
11:118
12:151
7:56
VVWCAVGEQELRpc2_NP.2
p−maj=0.32, pX=0.97, N=30
var.15.LTF 3:46501268:C:T
peptide level
118:118
151:151
56:56
VVWCAVGEQELRpc2_NP.3
p−maj=1, pX=0.0054, N=325
var.15.LTF 3:46501268:C:T
peptide level
118:118
151:151
56:56
VVWCAVGEQELRpc2_NP.4
p−maj=1, pX=0.0015, N=325
var.15.LTF 3:46501268:C:T
peptide level
97:118
129:151
51:56
VVWCAVGEQELRpc2_NP.5
p−maj=0.22, pX=0.016, N=277

var.15.LTF 3:46501268:C:T

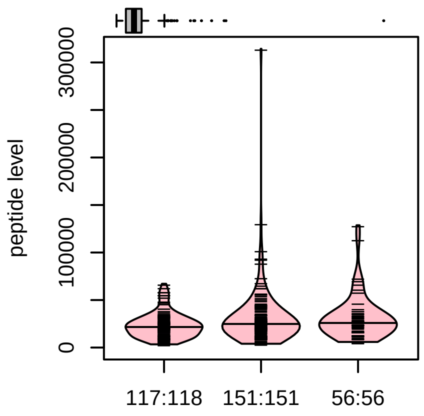

YLGPQYVAGITNLKpc2_NP.1
p−min=0.36, pX=0.016, N=324

var.15.LTF 3:46501268:C:T

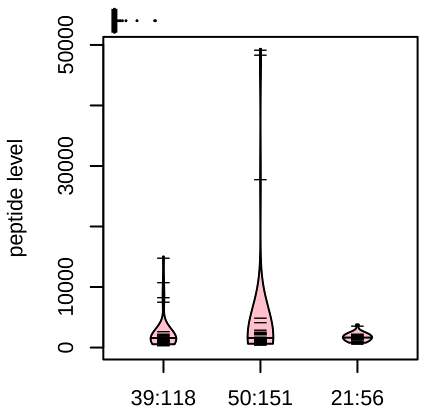

YLGPQYVAGITNLKpc2_NP.2
p−maj=0.54, pX=0.88, N=110

var.15.LTF 3:46501268:C:T

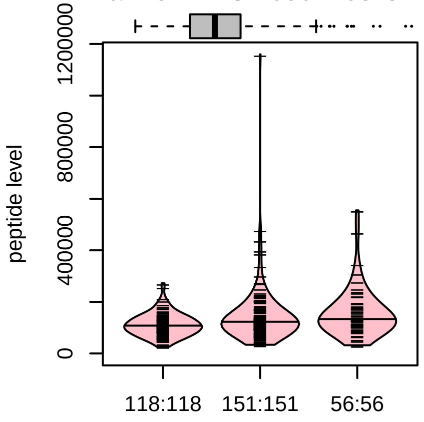

YLGPQYVAGITNLKpc2_NP.3
p−maj=1, pX=0.00048, N=325

var.15.LTF 3:46501268:C:T

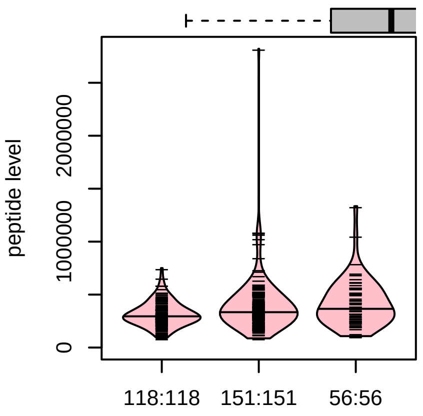

YLGPQYVAGITNLKpc2_NP.4
p−maj=1, pX=0.00092, N=325

var.15.LTF 3:46501268:C:T

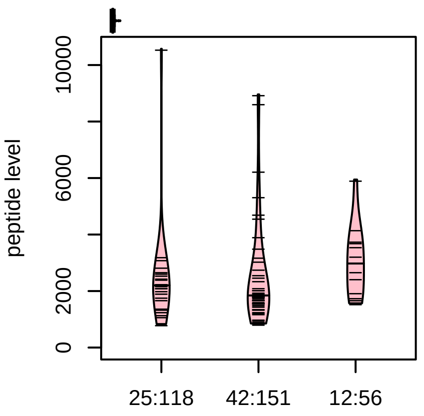

YLGPQYVAGITNLKpc2_NP.5
p−min=0.35, pX=0.4, N=79

var.15.LTF 3:46501268:C:T

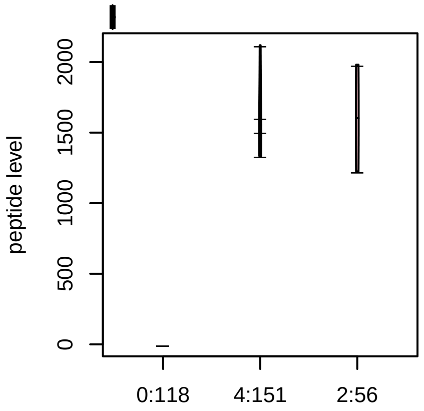

YLGPQYVAGITNLKpc3_NP.3
p−min=0.09, pX=0.075, N=6

var.15.LTF 3:46501268:C:T

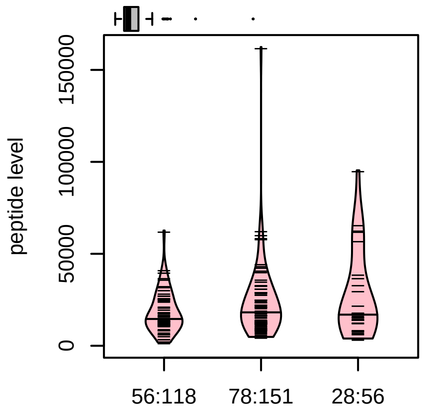

YYGYTGAFRpc2_NP.1
p−min=0.56, pX=0.041, N=162

var.15.LTF 3:46501268:C:T

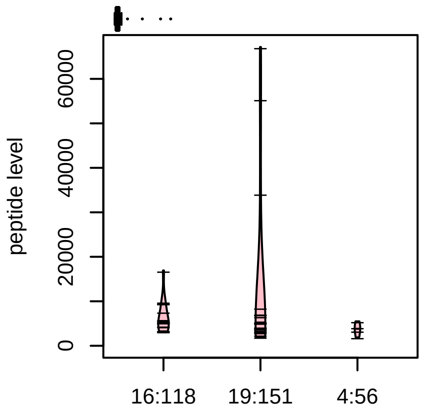

YYGYTGAFRpc2_NP.2
p−maj=0.26, pX=0.75, N=39

var.15.LTF 3:46501268:C:T

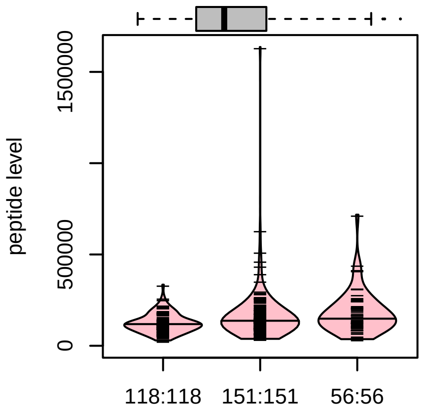

YYGYTGAFRpc2_NP.3
p−maj=1, pX=0.00092, N=325

var.15.LTF 3:46501268:C:T

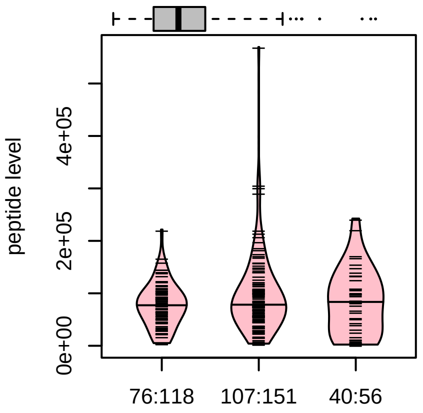

YYGYTGAFRpc2_NP.4
p−min=0.26, pX=0.17, N=223

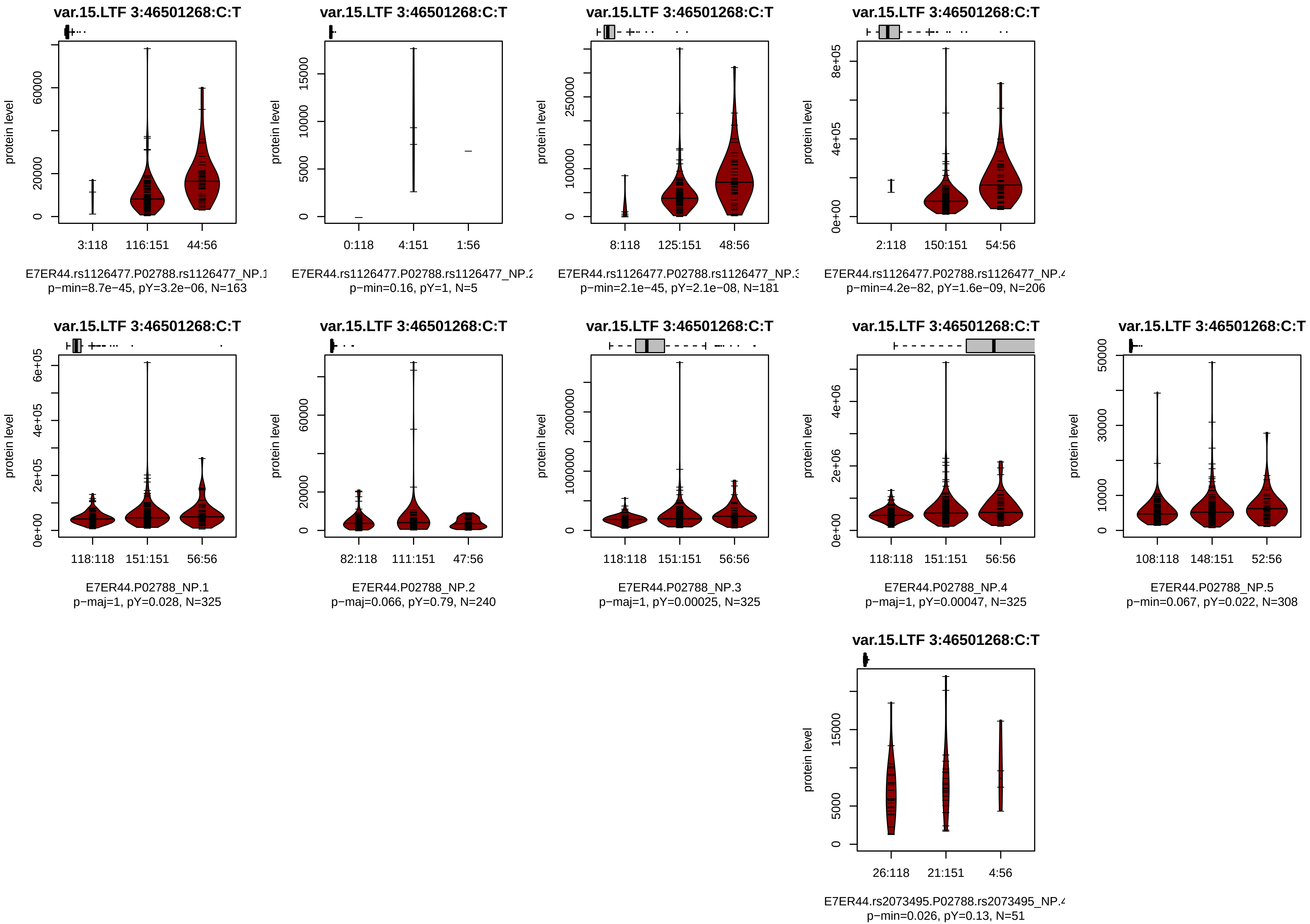
var.15.LTF 3:46501268:C:T
protein level
3:118
116:151
44:56
E7ER44.rs1126477.P02788.rs1126477_NP.1
p−min=8.7e−45, pY=3.2e−06, N=163
var.15.LTF 3:46501268:C:T
protein level
0:118
4:151
1:56
E7ER44.rs1126477.P02788.rs1126477_NP.2
p−min=0.16, pY=1, N=5
var.15.LTF 3:46501268:C:T
protein level
8:118
125:151
48:56
E7ER44.rs1126477.P02788.rs1126477_NP.3
p−min=2.1e−45, pY=2.1e−08, N=181
var.15.LTF 3:46501268:C:T
protein level
2:118
150:151
54:56
E7ER44.rs1126477.P02788.rs1126477_NP.4
p−min=4.2e−82, pY=1.6e−09, N=206
var.15.LTF 3:46501268:C:T
protein level
118:118
151:151
56:56
E7ER44.P02788_NP.1
p−maj=1, pY=0.028, N=325
var.15.LTF 3:46501268:C:T
protein level
82:118
111:151
47:56
E7ER44.P02788_NP.2
p−maj=0.066, pY=0.79, N=240
var.15.LTF 3:46501268:C:T
protein level
118:118
151:151
56:56
E7ER44.P02788_NP.3
p−maj=1, pY=0.00025, N=325
var.15.LTF 3:46501268:C:T
protein level
118:118
151:151
56:56
E7ER44.P02788_NP.4
p−maj=1, pY=0.00047, N=325
var.15.LTF 3:46501268:C:T
protein level
108:118
148:151
52:56
E7ER44.P02788_NP.5
p−min=0.067, pY=0.022, N=308
var.15.LTF 3:46501268:C:T
protein level
26:118
21:151
4:56
E7ER44.rs2073495.P02788.rs2073495_NP.4
p−min=0.026, pY=0.13, N=51

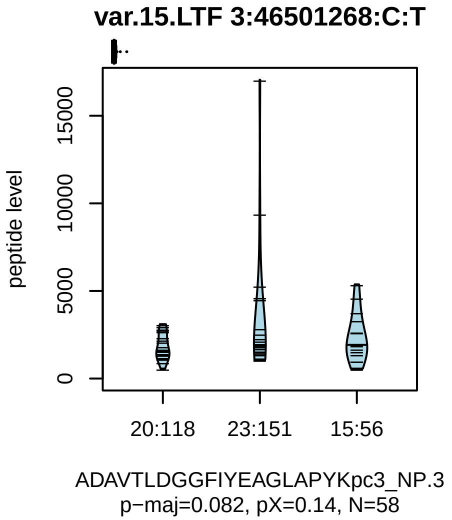
var.15.LTF 3:46501268:C:T
peptide level
0
5000
10000
15000
20:118
23:151
15:56
ADAVTLDGGFIYEAGLAPYKpc3_NP.3
p−maj=0.082, pX=0.14, N=58

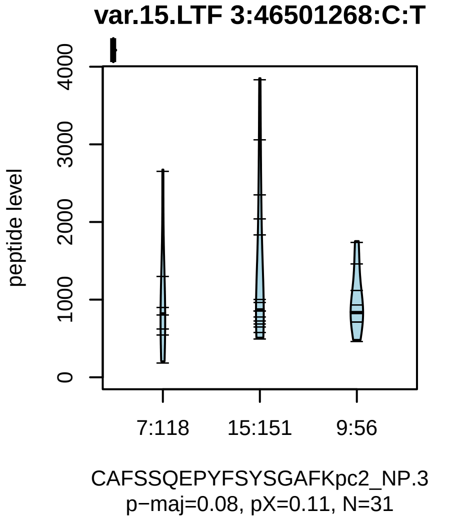
var.15.LTF 3:46501268:C:T
peptide level
0
1000
2000
3000
4000
7:118
15:151
9:56
CAFSSQEPYFSYSGAFKpc2_NP.3
p−maj=0.08, pX=0.11, N=31

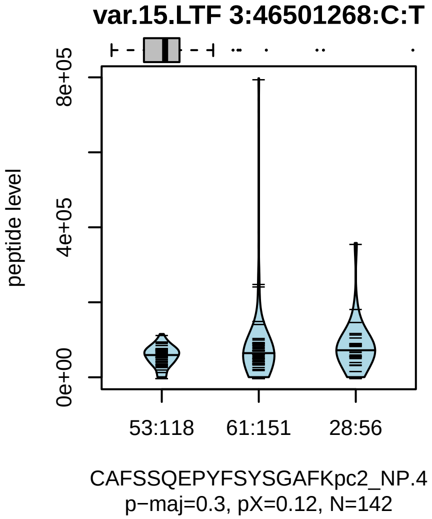
var.15.LTF 3:46501268:C:T
peptide level
0e+00
4e+05
8e+05
53:118
61:151
28:56
CAFSSQEPYFSYSGAFKpc2_NP.4
p−maj=0.3, pX=0.12, N=142

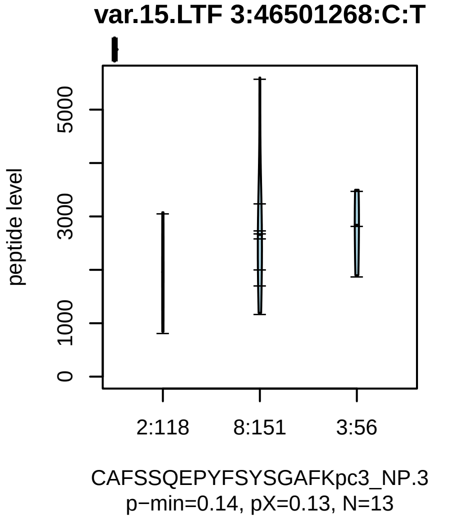
var.15.LTF 3:46501268:C:T
peptide level
0
1000
3000
5000
2:118
8:151
3:56
CAFSSQEPYFSYSGAFKpc3_NP.3
p−min=0.14, pX=0.13, N=13

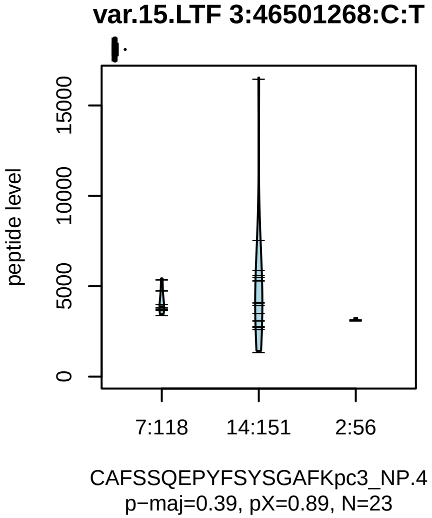
var.15.LTF 3:46501268:C:T
peptide level
0
5000
10000
15000
7:118
14:151
2:56
CAFSSQEPYFSYSGAFKpc3_NP.4
p−maj=0.39, pX=0.89, N=23

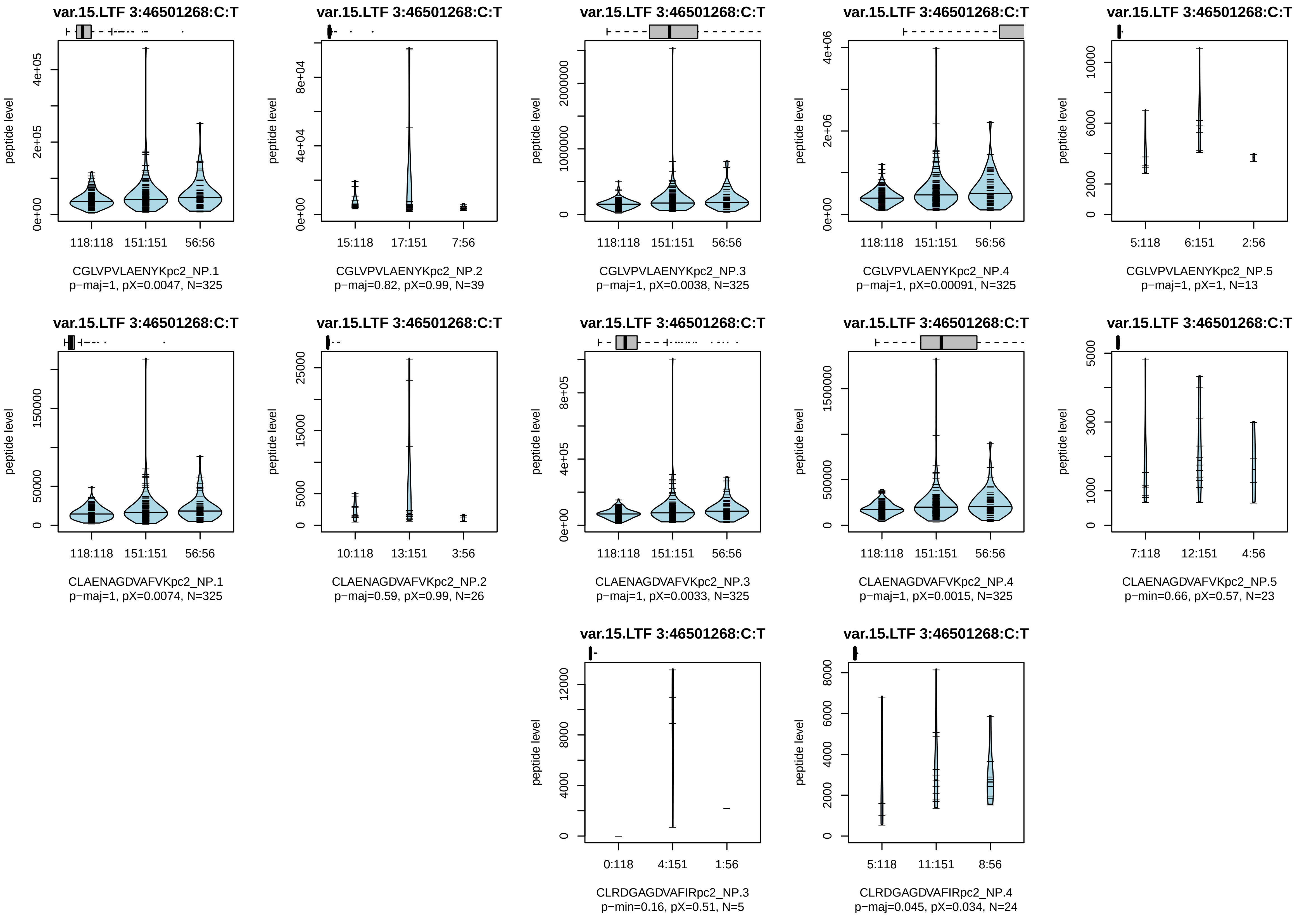
var.15.LTF 3:46501268:C:T
peptide level
118:118
151:151
56:56
CGLVPVLAENYKpc2_NP.1
p−maj=1, pX=0.0047, N=325
15:118
17:151
7:56
CGLVPVLAENYKpc2_NP.2
p−maj=0.82, pX=0.99, N=39
CGLVPVLAENYKpc2_NP.3
p−maj=1, pX=0.0038, N=325
CGLVPVLAENYKpc2_NP.4
p−maj=1, pX=0.00091, N=325
5:118
6:151
2:56
CGLVPVLAENYKpc2_NP.5
p−maj=1, pX=1, N=13
CLAENAGDVAFVKpc2_NP.1
p−maj=1, pX=0.0074, N=325
10:118
13:151
3:56
CLAENAGDVAFVKpc2_NP.2
p−maj=0.59, pX=0.99, N=26
CLAENAGDVAFVKpc2_NP.3
p−maj=1, pX=0.0033, N=325
CLAENAGDVAFVKpc2_NP.4
p−maj=1, pX=0.0015, N=325
7:118
12:151
4:56
CLAENAGDVAFVKpc2_NP.5
p−min=0.66, pX=0.57, N=23
0:118
4:151
1:56
CLRDGAGDVAFIRpc2_NP.3
p−min=0.16, pX=0.51, N=5
5:118
11:151
8:56
CLRDGAGDVAFIRpc2_NP.4
p−maj=0.045, pX=0.034, N=24

var.15.LTF 3:46501268:C:T

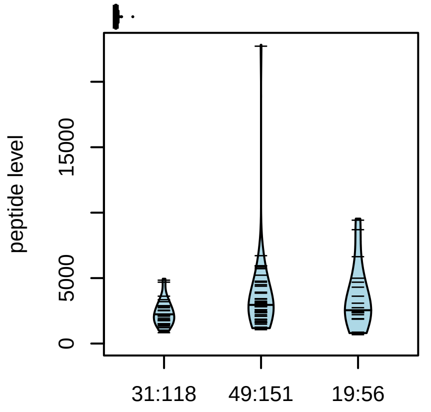

CLRDGAGDVAFIRpc3_NP.1
p−min=0.26, pX=0.032, N=99

var.15.LTF 3:46501268:C:T

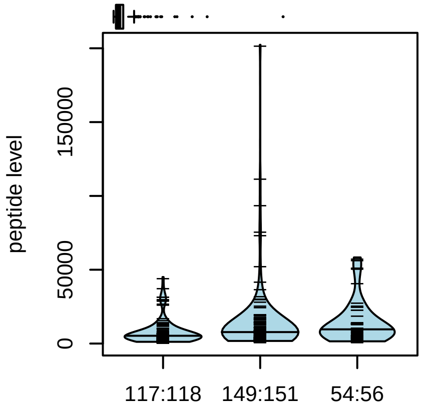

CLRDGAGDVAFIRpc3_NP.3
p−maj=0.21, pX=0.011, N=320

var.15.LTF 3:46501268:C:T

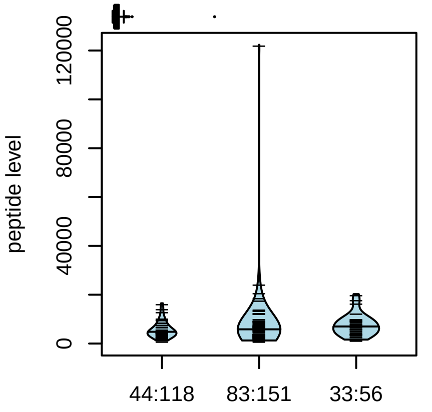

CLRDGAGDVAFIRpc3_NP.4
p−min=0.0013, pX=0.028, N=160

var.15.LTF 3:46501268:C:T

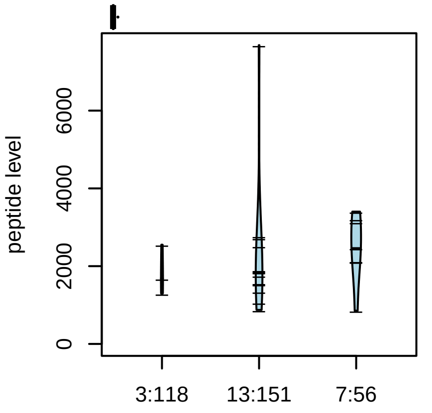

CSTSPLLEACEFLRKpc2_NP.4
p−min=0.022, pX=0.013, N=23

var.15.LTF 3:46501268:C:T

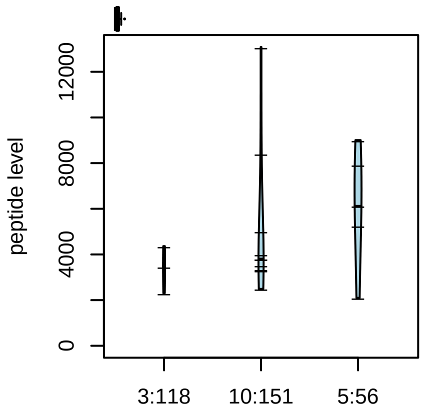

CSTSPLLEACEFLRKpc3_NP.1
p−min=0.083, pX=0.026, N=18

var.15.LTF 3:46501268:C:T

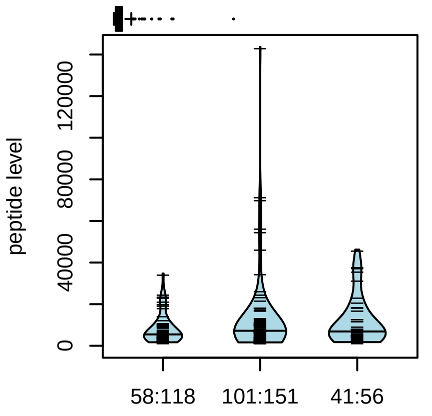

CSTSPLLEACEFLRKpc3_NP.3
p−min=6e−04, pX=0.0082, N=200

var.15.LTF 3:46501268:C:T

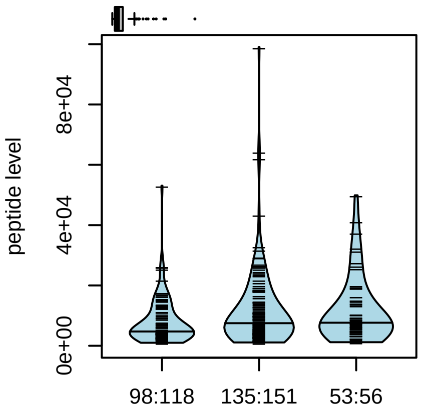

CSTSPLLEACEFLRKpc3_NP.4
p−min=0.05, pX=0.0022, N=286

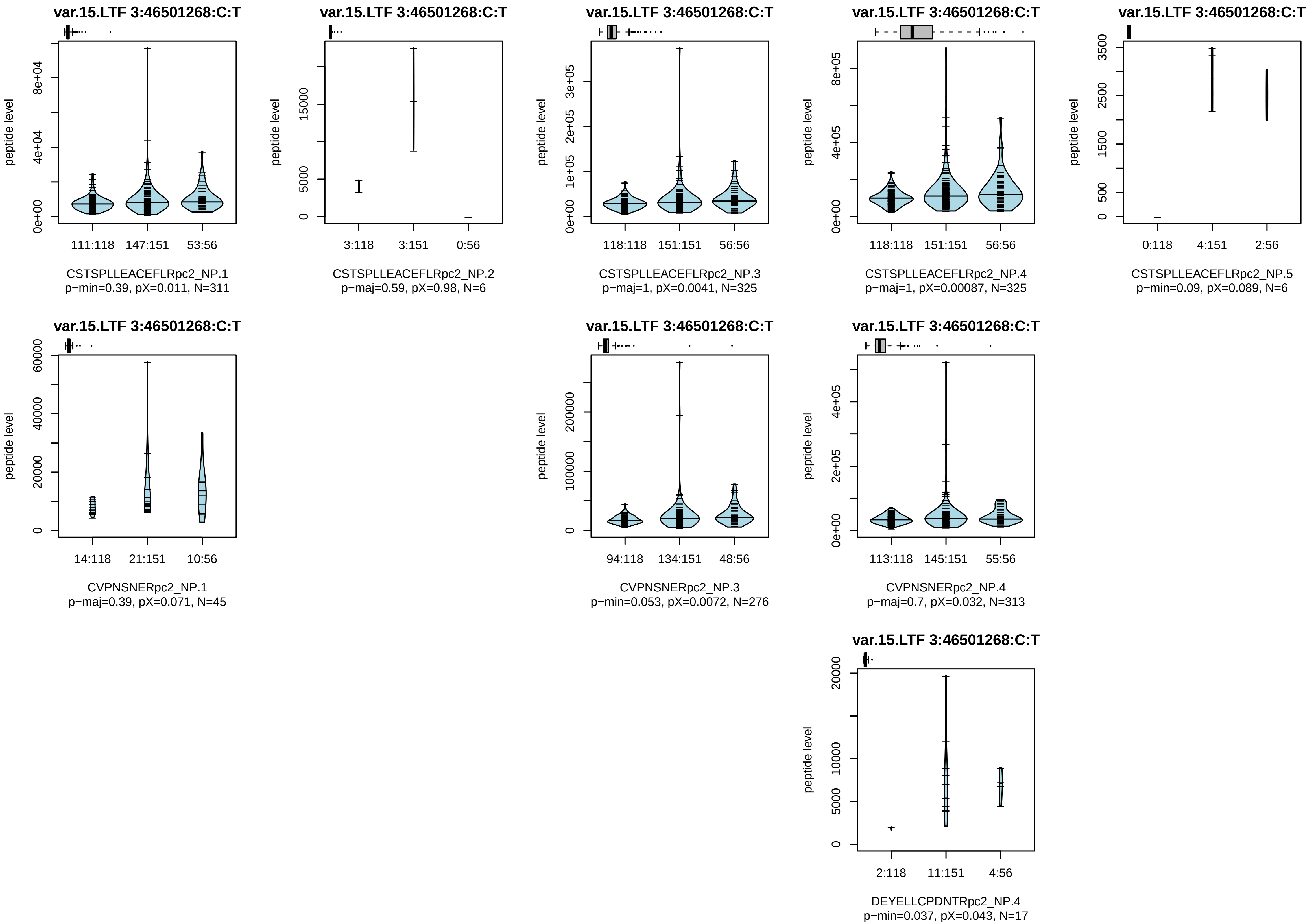

var.15.LTF 3:46501268:C:T
peptide level
0e+00
4e+04
8e+04
111:118
147:151
53:56
CSTSPLLEACEFLRpc2_NP.1
p−min=0.39, pX=0.011, N=311
var.15.LTF 3:46501268:C:T
peptide level
0
5000
15000
3:118
3:151
0:56
CSTSPLLEACEFLRpc2_NP.2
p−maj=0.59, pX=0.98, N=6
var.15.LTF 3:46501268:C:T
peptide level
0e+00
1e+05
2e+05
3e+05
118:118
151:151
56:56
CSTSPLLEACEFLRpc2_NP.3
p−maj=1, pX=0.0041, N=325
var.15.LTF 3:46501268:C:T
peptide level
0e+00
4e+05
8e+05
118:118
151:151
56:56
CSTSPLLEACEFLRpc2_NP.4
p−maj=1, pX=0.00087, N=325
var.15.LTF 3:46501268:C:T
peptide level
0
500
1500
2500
3500
0:118
4:151
2:56
CSTSPLLEACEFLRpc2_NP.5
p−min=0.09, pX=0.089, N=6
var.15.LTF 3:46501268:C:T
peptide level
0
20000
40000
60000
14:118
21:151
10:56
CVPNSNERpc2_NP.1
p−maj=0.39, pX=0.071, N=45
var.15.LTF 3:46501268:C:T
peptide level
0
100000
200000
94:118
134:151
48:56
CVPNSNERpc2_NP.3
p−min=0.053, pX=0.0072, N=276
var.15.LTF 3:46501268:C:T
peptide level
0e+00
2e+05
4e+05
113:118
145:151
55:56
CVPNSNERpc2_NP.4
p−maj=0.7, pX=0.032, N=313
var.15.LTF 3:46501268:C:T
peptide level
0
5000
10000
20000
2:118
11:151
4:56
DEYELLCPDNTRpc2_NP.4
p−min=0.037, pX=0.043, N=17

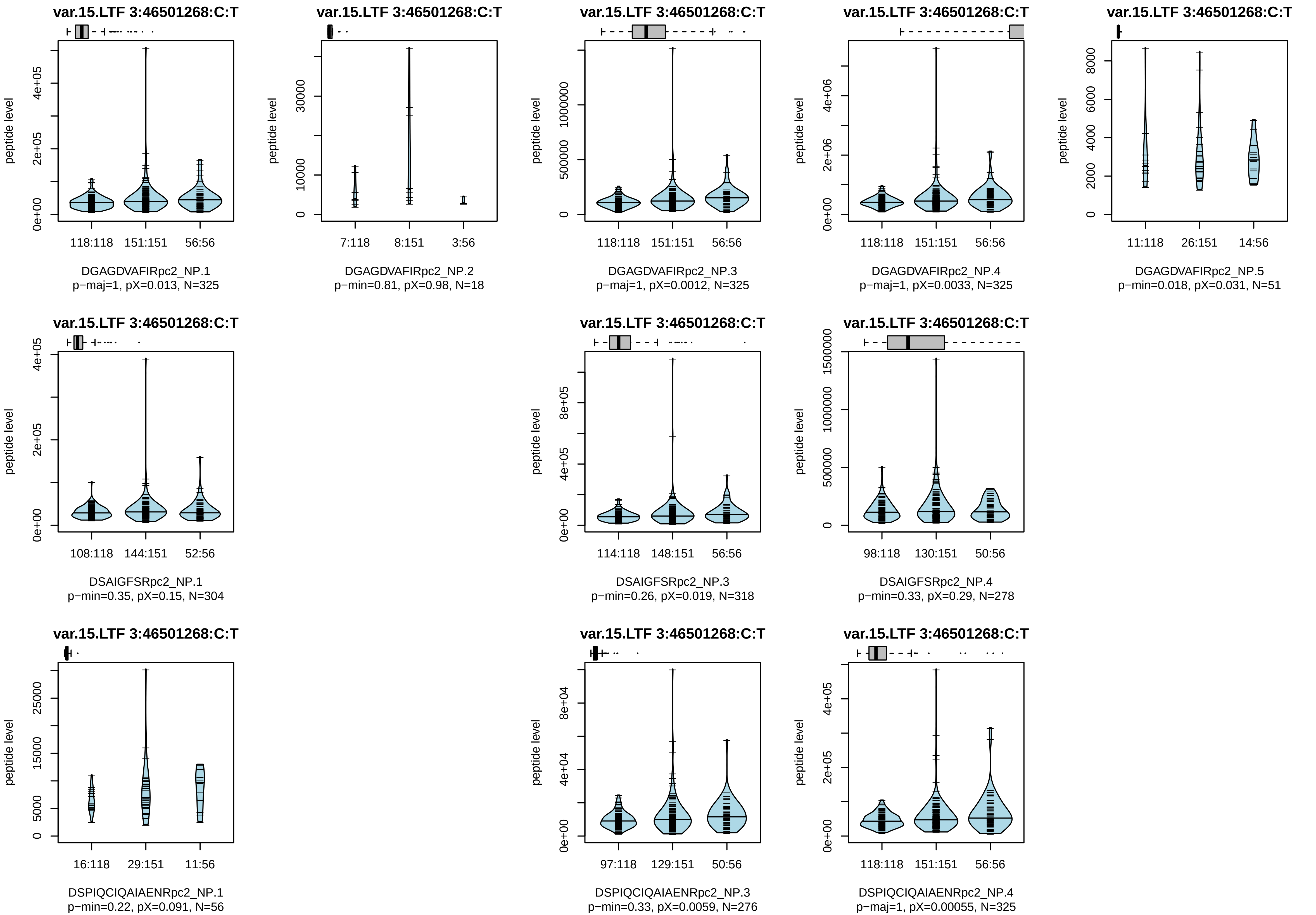
var.15.LTF 3:46501268:C:T
peptide level
118:118
151:151
56:56
DGAGDVAFIRpc2_NP.1
p−maj=1, pX=0.013, N=325
var.15.LTF 3:46501268:C:T
peptide level
7:118
8:151
3:56
DGAGDVAFIRpc2_NP.2
p−min=0.81, pX=0.98, N=18
var.15.LTF 3:46501268:C:T
peptide level
118:118
151:151
56:56
DGAGDVAFIRpc2_NP.3
p−maj=1, pX=0.0012, N=325
var.15.LTF 3:46501268:C:T
peptide level
118:118
151:151
56:56
DGAGDVAFIRpc2_NP.4
p−maj=1, pX=0.0033, N=325
var.15.LTF 3:46501268:C:T
peptide level
11:118
26:151
14:56
DGAGDVAFIRpc2_NP.5
p−min=0.018, pX=0.031, N=51
var.15.LTF 3:46501268:C:T
peptide level
108:118
144:151
52:56
DSAIGFSRpc2_NP.1
p−min=0.35, pX=0.15, N=304
var.15.LTF 3:46501268:C:T
peptide level
114:118
148:151
56:56
DSAIGFSRpc2_NP.3
p−min=0.26, pX=0.019, N=318
var.15.LTF 3:46501268:C:T
peptide level
98:118
130:151
50:56
DSAIGFSRpc2_NP.4
p−min=0.33, pX=0.29, N=278
var.15.LTF 3:46501268:C:T
peptide level
16:118
29:151
11:56
DSPIQCIQAIAENRpc2_NP.1
p−min=0.22, pX=0.091, N=56
var.15.LTF 3:46501268:C:T
peptide level
97:118
129:151
50:56
DSPIQCIQAIAENRpc2_NP.3
p−min=0.33, pX=0.0059, N=276
var.15.LTF 3:46501268:C:T
peptide level
118:118
151:151
56:56
DSPIQCIQAIAENRpc2_NP.4
p−maj=1, pX=0.00055, N=325

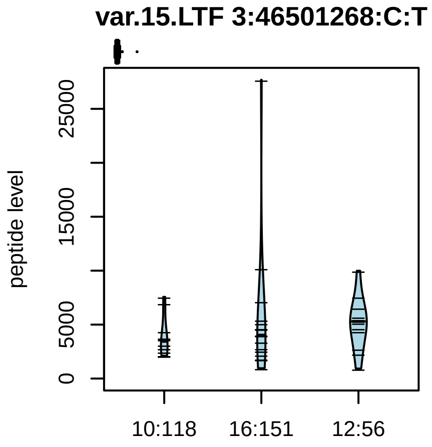

DSPIQCIQAIAENRpc3_NP.1
p−maj=0.02, pX=0.037, N=38

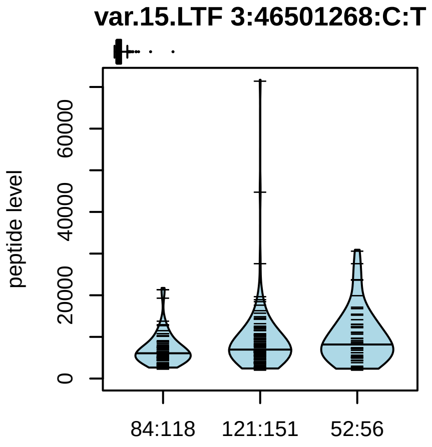

DSPIQCIQAIAENRpc3_NP.3
p−maj=0.0037, pX=3.2e−05, N=257

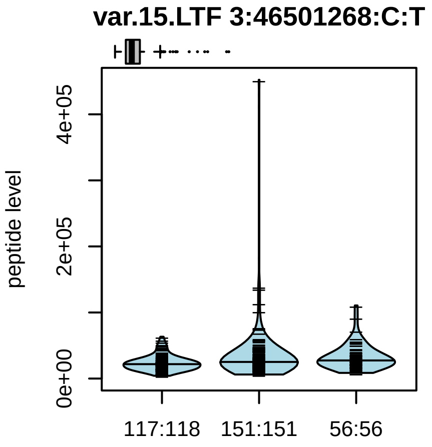

DSPIQCIQAIAENRpc3_NP.4
p−min=0.36, pX=0.015, N=324

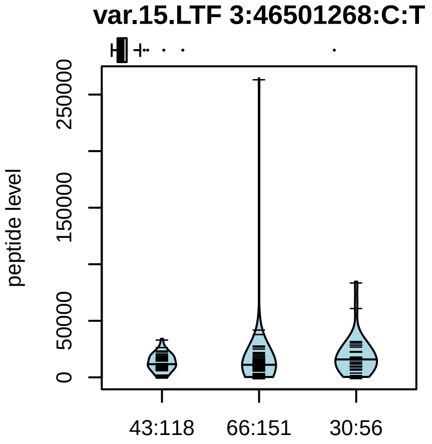

DVTVLQNTDGNNNEAWAKpc2_NP.4
p−maj=0.077, pX=0.062, N=139

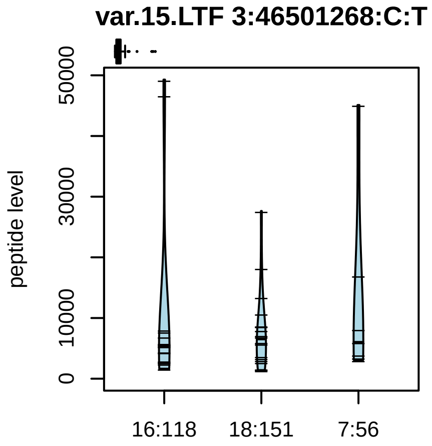

EDAIWNLLRpc2_NP.1
p−min=0.73, pX=0.98, N=41

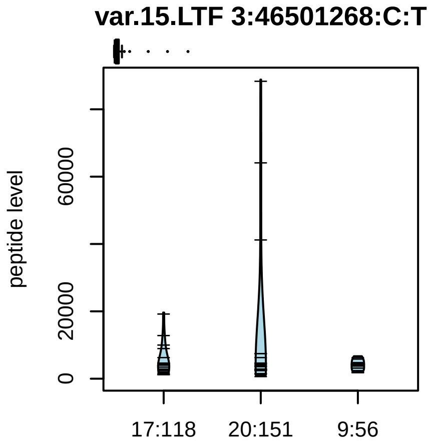

EDAIWNLLRpc2_NP.2
p−maj=0.67, pX=0.86, N=46

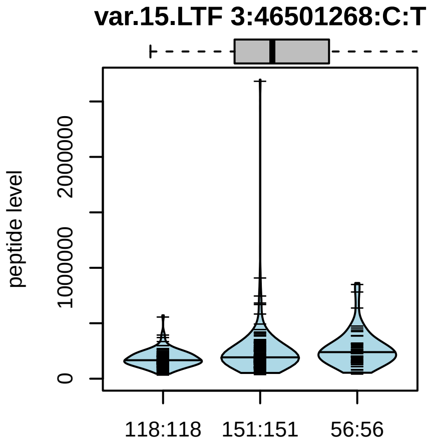

EDAIWNLLRpc2_NP.3
p−maj=1, pX=0.0015, N=325

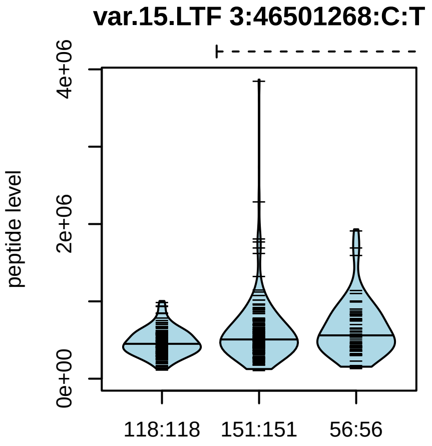

EDAIWNLLRpc2_NP.4
p−maj=1, pX=3e−04, N=325

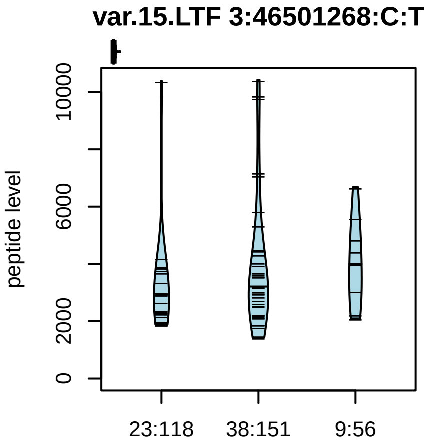

EDAIWNLLRpc2_NP.5
p−maj=0.37, pX=0.62, N=70

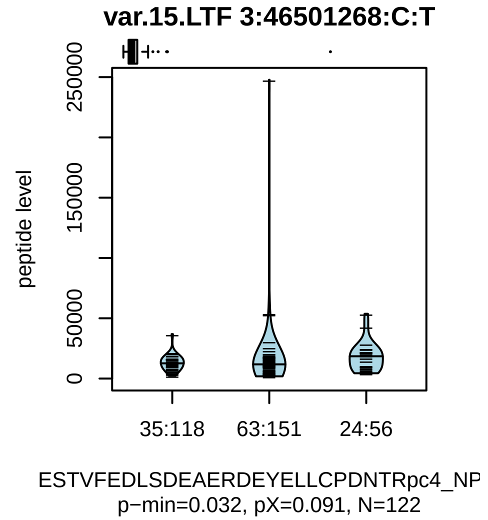
var.15.LTF 3:46501268:C:T
peptide level
0
50000
150000
250000
35:118
63:151
24:56
ESTVFEDLSDEAERDEYELLCPDNTRpc4_NP
p−min=0.032, pX=0.091, N=122

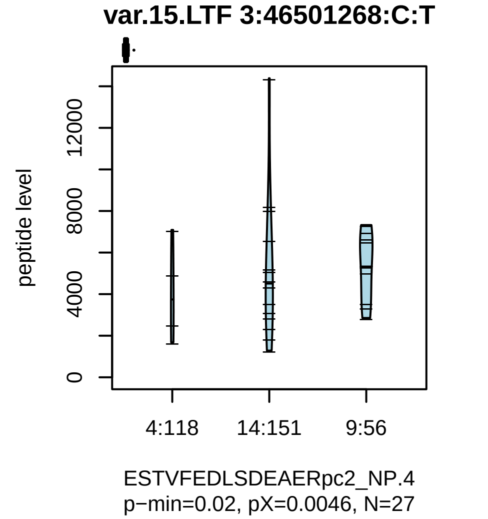
var.15.LTF 3:46501268:C:T
peptide level
0
4000
8000
12000
4:118
14:151
9:56
ESTVFEDLSDEAERpc2_NP.4
p−min=0.02, pX=0.0046, N=27

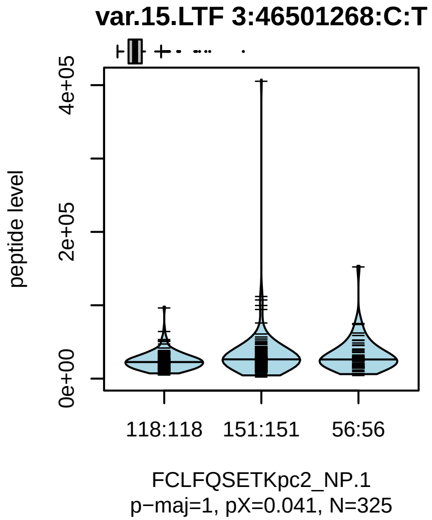
var.15.LTF 3:46501268:C:T
peptide level
0e+00
2e+05
4e+05
118:118
151:151
56:56
FCLFQSETKpc2_NP.1
p−maj=1, pX=0.041, N=325

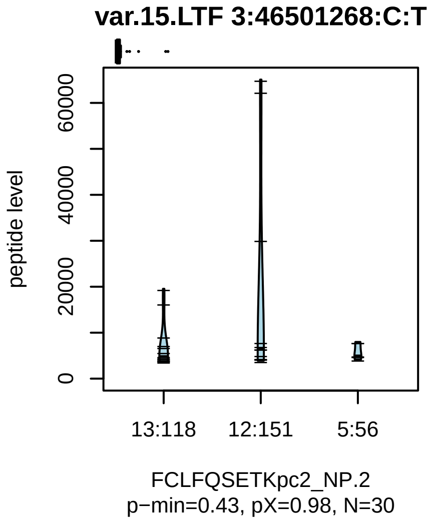
var.15.LTF 3:46501268:C:T
peptide level
0
20000
40000
60000
13:118
12:151
5:56
FCLFQSETKpc2_NP.2
p−min=0.43, pX=0.98, N=30

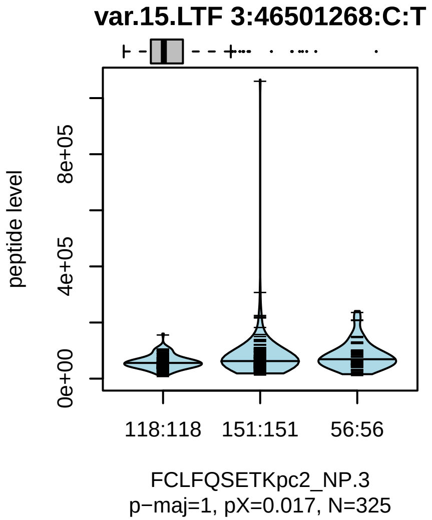
var.15.LTF 3:46501268:C:T
peptide level
0e+00
4e+05
8e+05
118:118
151:151
56:56
FCLFQSETKpc2_NP.3
p−maj=1, pX=0.017, N=325

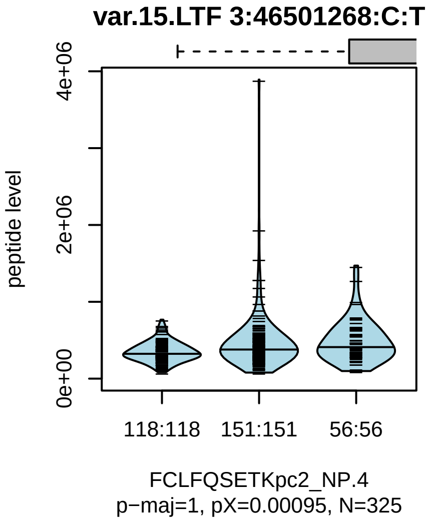
var.15.LTF 3:46501268:C:T
peptide level
0e+00
2e+06
4e+06
118:118
151:151
56:56
FCLFQSETKpc2_NP.4
p−maj=1, pX=0.00095, N=325

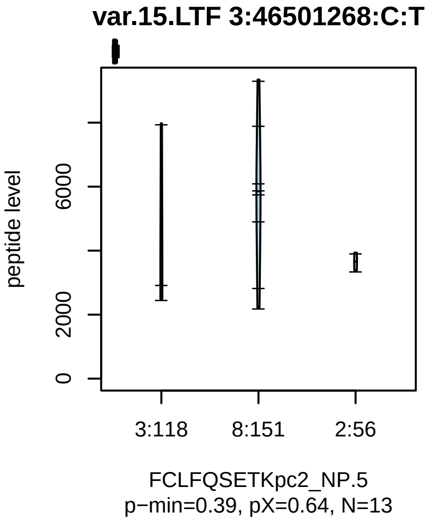
var.15.LTF 3:46501268:C:T
peptide level
0
2000
6000
3:118
8:151
2:56
FCLFQSETKpc2_NP.5
p−min=0.39, pX=0.64, N=13

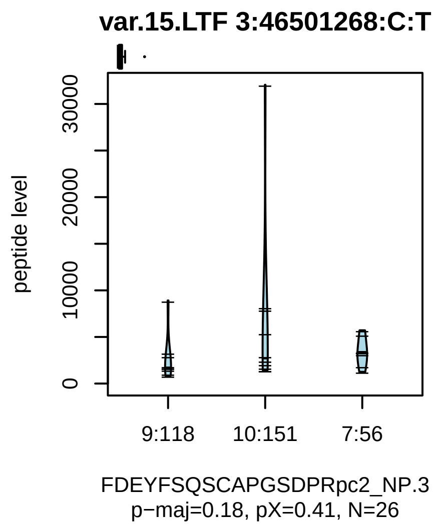
var.15.LTF 3:46501268:C:T
peptide level
0
10000
20000
30000
9:118
10:151
7:56
FDEYFSQSCAPGSDPRpc2_NP.3
p−maj=0.18, pX=0.41, N=26

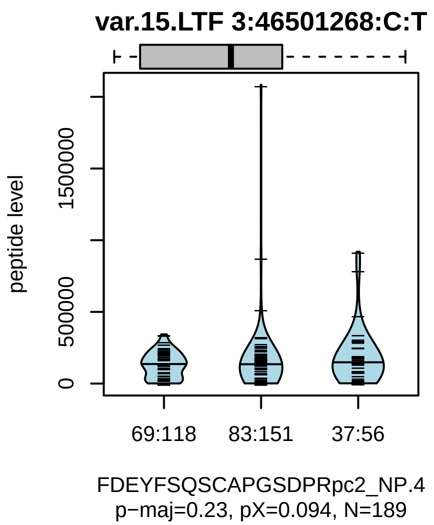
var.15.LTF 3:46501268:C:T
peptide level
0
500000
1500000
69:118
83:151
37:56
FDEYFSQSCAPGSDPRpc2_NP.4
p−maj=0.23, pX=0.094, N=189

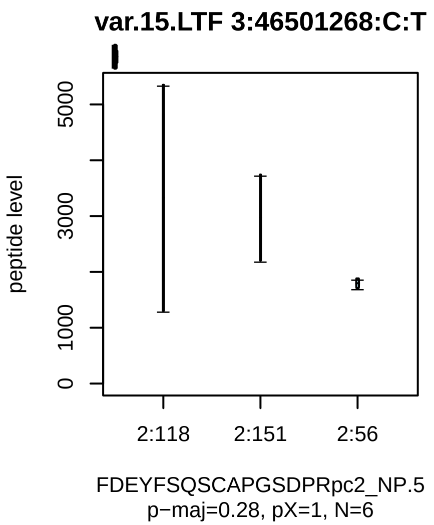
var.15.LTF 3:46501268:C:T
peptide level
0
1000
3000
5000
2:118
2:151
2:56
FDEYFSQSCAPGSDPRpc2_NP.5
p−maj=0.28, pX=1, N=6

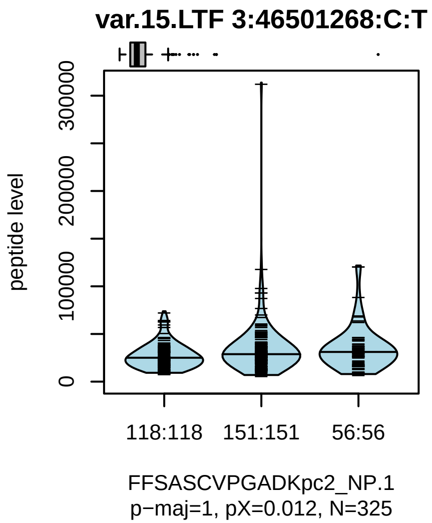
var.15.LTF 3:46501268:C:T
peptide level
0
100000
200000
300000
118:118
151:151
56:56
FFSASCVPGADKpc2_NP.1
p−maj=1, pX=0.012, N=325

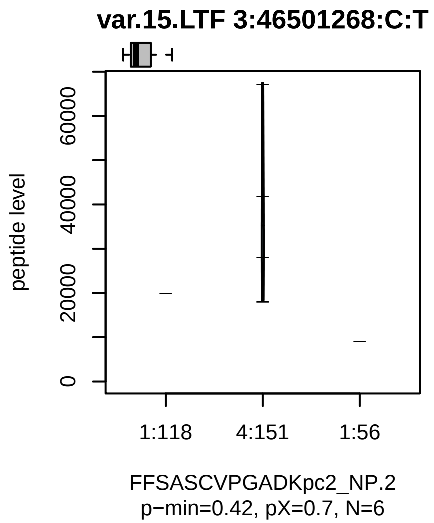
var.15.LTF 3:46501268:C:T
peptide level
0
20000
40000
60000
1:118
4:151
1:56
FFSASCVPGADKpc2_NP.2
p−min=0.42, pX=0.7, N=6

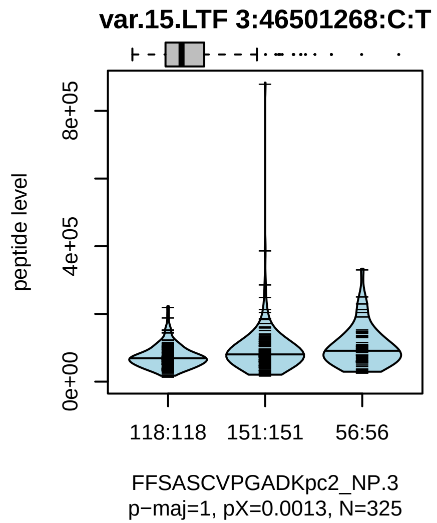
var.15.LTF 3:46501268:C:T
peptide level
0e+00
4e+05
8e+05
118:118
151:151
56:56
FFSASCVPGADKpc2_NP.3
p−maj=1, pX=0.0013, N=325

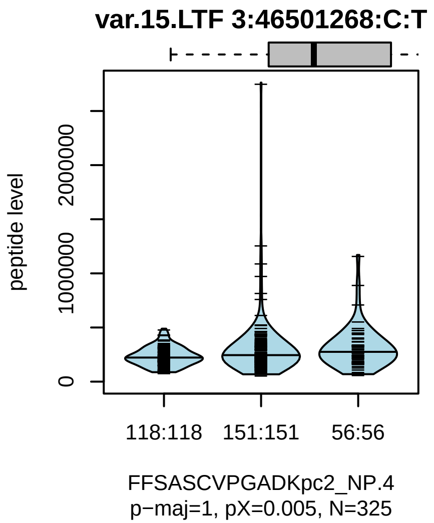
var.15.LTF 3:46501268:C:T
peptide level
0
1000000
2000000
118:118
151:151
56:56
FFSASCVPGADKpc2_NP.4
p−maj=1, pX=0.005, N=325

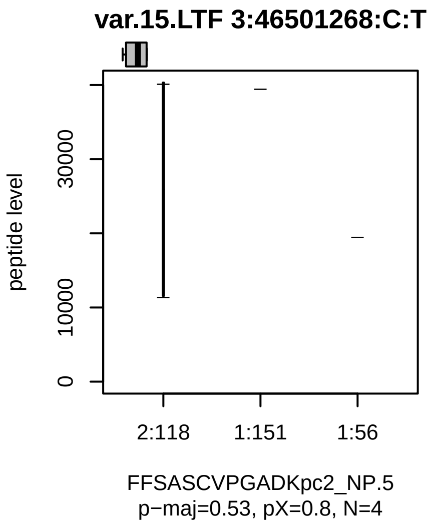
var.15.LTF 3:46501268:C:T
peptide level
0
10000
30000
2:118
1:151
1:56
FFSASCVPGADKpc2_NP.5
p−maj=0.53, pX=0.8, N=4

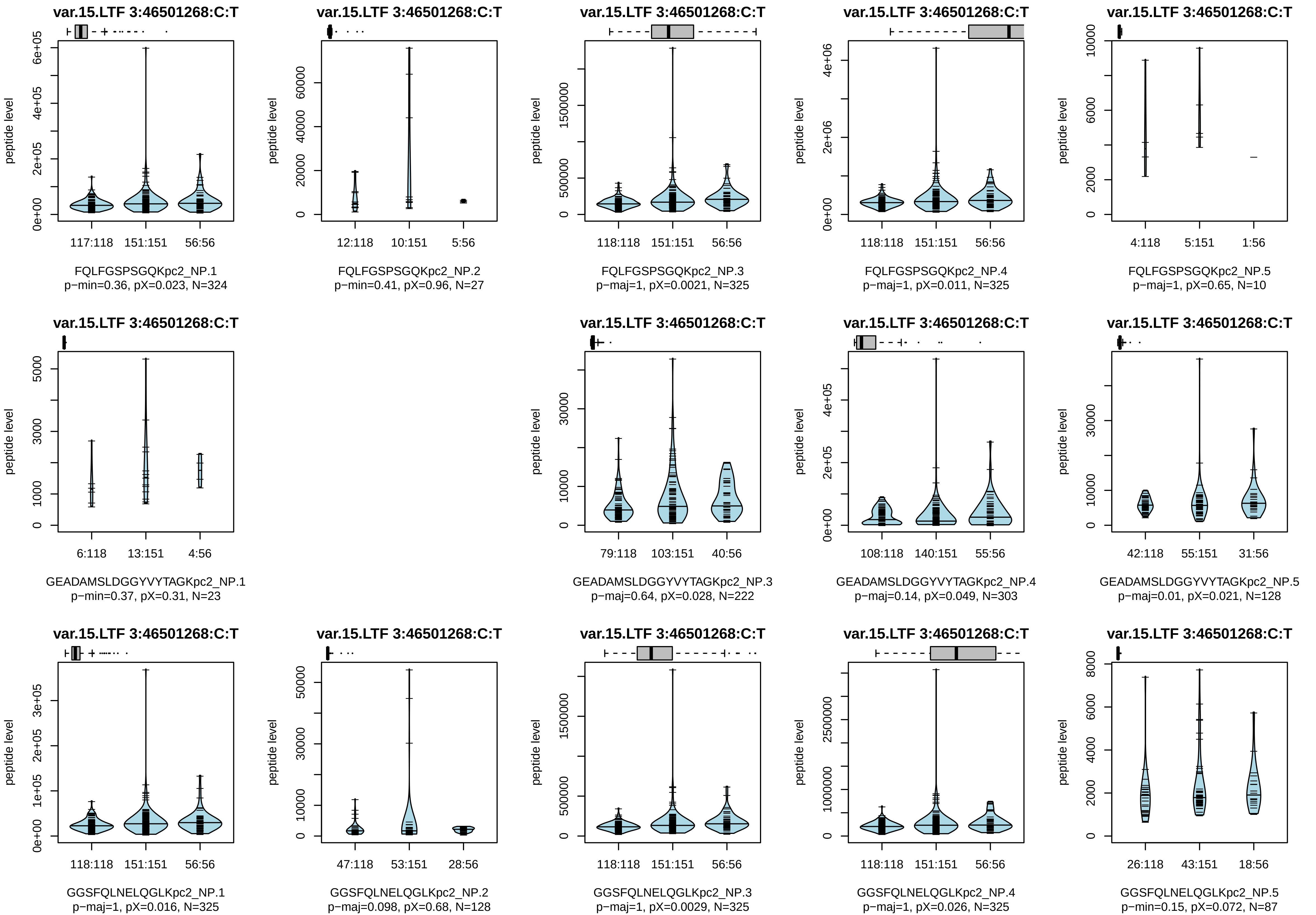
var.15.LTF 3:46501268:C:T
peptide level
117:118
151:151
56:56
FQLFGSPSGQKpc2_NP.1
p−min=0.36, pX=0.023, N=324
var.15.LTF 3:46501268:C:T
peptide level
12:118
10:151
5:56
FQLFGSPSGQKpc2_NP.2
p−min=0.41, pX=0.96, N=27
var.15.LTF 3:46501268:C:T
peptide level
118:118
151:151
56:56
FQLFGSPSGQKpc2_NP.3
p−maj=1, pX=0.0021, N=325
var.15.LTF 3:46501268:C:T
peptide level
118:118
151:151
56:56
FQLFGSPSGQKpc2_NP.4
p−maj=1, pX=0.011, N=325
var.15.LTF 3:46501268:C:T
peptide level
4:118
5:151
1:56
FQLFGSPSGQKpc2_NP.5
p−maj=1, pX=0.65, N=10
var.15.LTF 3:46501268:C:T
peptide level
6:118
13:151
4:56
GEADAMSLDGGYVYTAGKpc2_NP.1
p−min=0.37, pX=0.31, N=23
var.15.LTF 3:46501268:C:T
peptide level
79:118
103:151
40:56
GEADAMSLDGGYVYTAGKpc2_NP.3
p−maj=0.64, pX=0.028, N=222
var.15.LTF 3:46501268:C:T
peptide level
108:118
140:151
55:56
GEADAMSLDGGYVYTAGKpc2_NP.4
p−maj=0.14, pX=0.049, N=303
var.15.LTF 3:46501268:C:T
peptide level
42:118
55:151
31:56
GEADAMSLDGGYVYTAGKpc2_NP.5
p−maj=0.01, pX=0.021, N=128
var.15.LTF 3:46501268:C:T
peptide level
118:118
151:151
56:56
GGSFQLNELQGLKpc2_NP.1
p−maj=1, pX=0.016, N=325
var.15.LTF 3:46501268:C:T
peptide level
47:118
53:151
28:56
GGSFQLNELQGLKpc2_NP.2
p−maj=0.098, pX=0.68, N=128
var.15.LTF 3:46501268:C:T
peptide level
118:118
151:151
56:56
GGSFQLNELQGLKpc2_NP.3
p−maj=1, pX=0.0029, N=325
var.15.LTF 3:46501268:C:T
peptide level
118:118
151:151
56:56
GGSFQLNELQGLKpc2_NP.4
p−maj=1, pX=0.026, N=325
var.15.LTF 3:46501268:C:T
peptide level
26:118
43:151
18:56
GGSFQLNELQGLKpc2_NP.5
p−min=0.15, pX=0.072, N=87

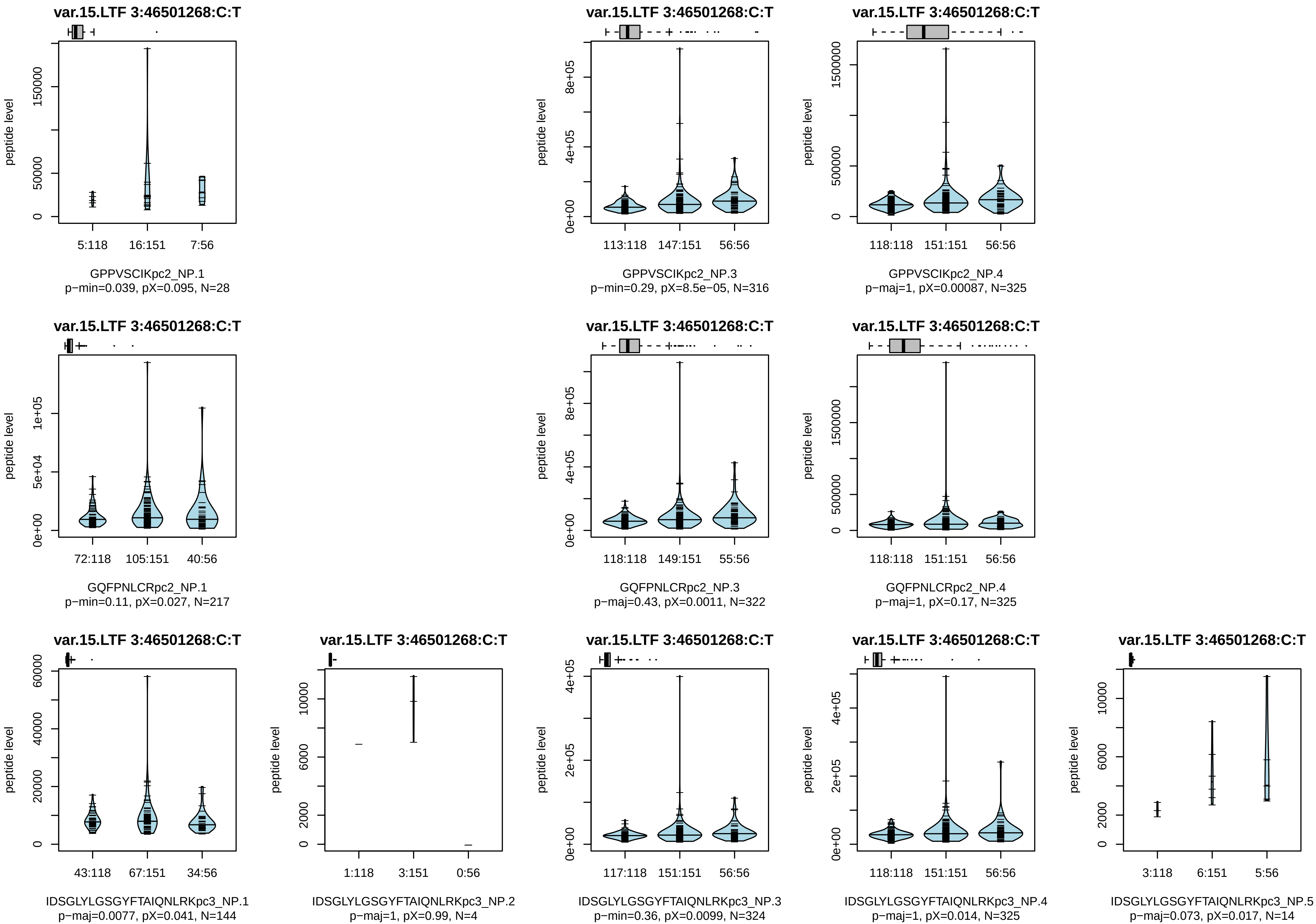
var.15.LTF 3:46501268:C:T
peptide level
150000
50000
0
5:118
16:151
7:56
GPPVSCIKpc2_NP.1
p−min=0.039, pX=0.095, N=28
var.15.LTF 3:46501268:C:T
peptide level
8e+05
4e+05
0e+00
113:118
147:151
56:56
GPPVSCIKpc2_NP.3
p−min=0.29, pX=8.5e−05, N=316
var.15.LTF 3:46501268:C:T
peptide level
1500000
500000
0
118:118
151:151
56:56
GPPVSCIKpc2_NP.4
p−maj=1, pX=0.00087, N=325
var.15.LTF 3:46501268:C:T
peptide level
1e+05
5e+04
0e+00
72:118
105:151
40:56
GQFPNLCRpc2_NP.1
p−min=0.11, pX=0.027, N=217
var.15.LTF 3:46501268:C:T
peptide level
8e+05
4e+05
0e+00
118:118
149:151
55:56
GQFPNLCRpc2_NP.3
p−maj=0.43, pX=0.0011, N=322
var.15.LTF 3:46501268:C:T
peptide level
1500000
500000
0
118:118
151:151
56:56
GQFPNLCRpc2_NP.4
p−maj=1, pX=0.17, N=325
var.15.LTF 3:46501268:C:T
peptide level
60000
40000
20000
0
43:118
67:151
34:56
IDSGLYLGSGYFTAIQNLRKpc3_NP.1
p−maj=0.0077, pX=0.041, N=144
var.15.LTF 3:46501268:C:T
peptide level
10000
6000
2000
0
1:118
3:151
0:56
IDSGLYLGSGYFTAIQNLRKpc3_NP.2
p−maj=1, pX=0.99, N=4
var.15.LTF 3:46501268:C:T
peptide level
4e+05
2e+05
0e+00
117:118
151:151
56:56
IDSGLYLGSGYFTAIQNLRKpc3_NP.3
p−min=0.36, pX=0.0099, N=324
var.15.LTF 3:46501268:C:T
peptide level
4e+05
2e+05
0e+00
118:118
151:151
56:56
IDSGLYLGSGYFTAIQNLRKpc3_NP.4
p−maj=1, pX=0.014, N=325
var.15.LTF 3:46501268:C:T
peptide level
10000
6000
2000
0
3:118
6:151
5:56
IDSGLYLGSGYFTAIQNLRKpc3_NP.5
p−maj=0.073, pX=0.017, N=14

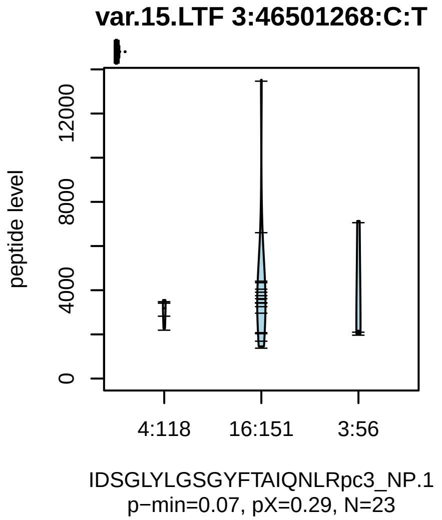
var.15.LTF 3:46501268:C:T
peptide level
0
4000
8000
12000
4:118
16:151
3:56
IDSGLYLGSGYFTAIQNLRpc3_NP.1
p−min=0.07, pX=0.29, N=23

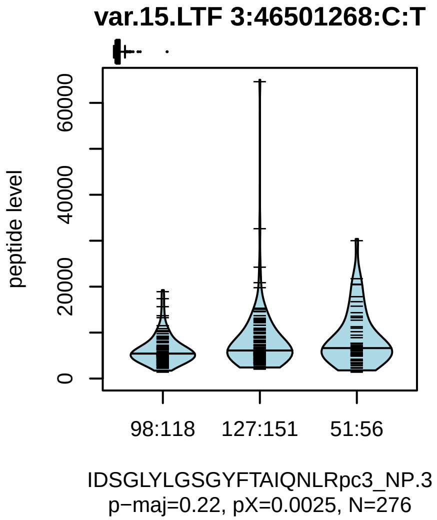
var.15.LTF 3:46501268:C:T
peptide level
0
20000
40000
60000
98:118
127:151
51:56
IDSGLYLGSGYFTAIQNLRpc3_NP.3
p−maj=0.22, pX=0.0025, N=276

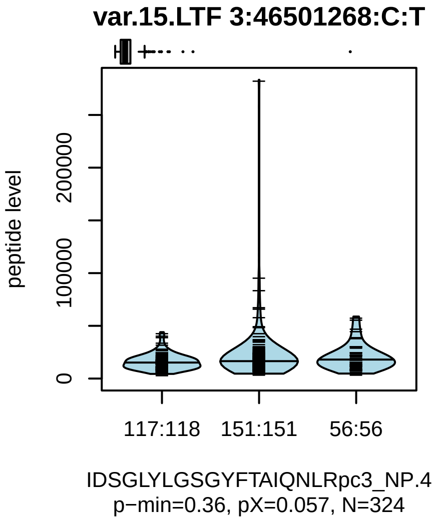
var.15.LTF 3:46501268:C:T
peptide level
0
100000
200000
117:118
151:151
56:56
IDSGLYLGSGYFTAIQNLRpc3_NP.4
p−min=0.36, pX=0.057, N=324

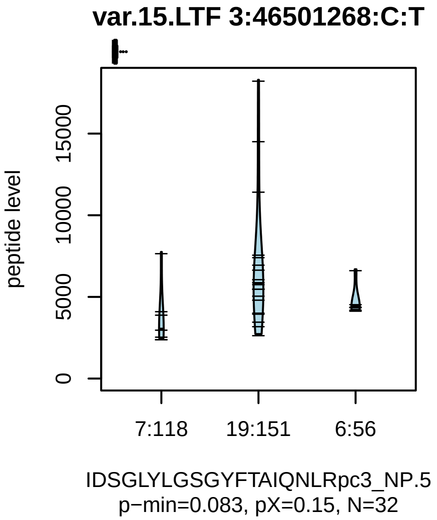
var.15.LTF 3:46501268:C:T
peptide level
0
5000
10000
15000
7:118
19:151
6:56
IDSGLYLGSGYFTAIQNLRpc3_NP.5
p−min=0.083, pX=0.15, N=32

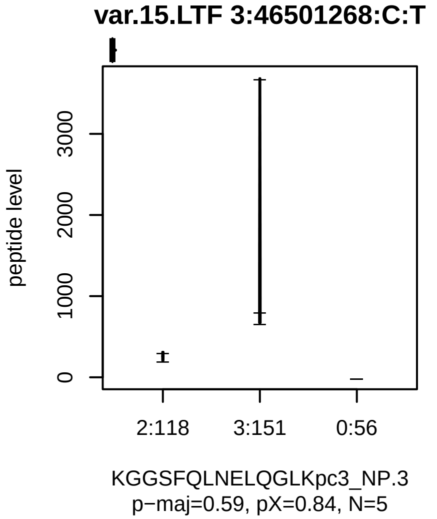
var.15.LTF 3:46501268:C:T
peptide level
0
1000
2000
3000
2:118
3:151
0:56
KGGSFQLNELQGLKpc3_NP.3
p−maj=0.59, pX=0.84, N=5

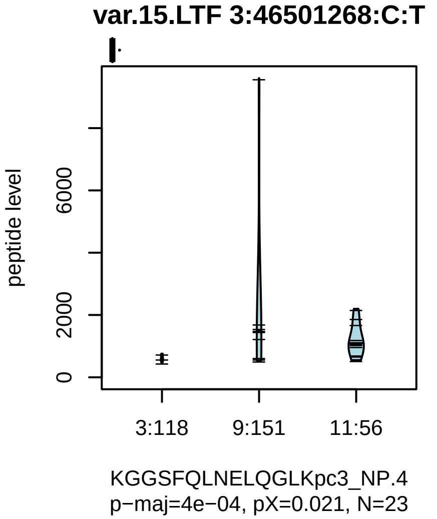
var.15.LTF 3:46501268:C:T
peptide level
0
2000
6000
3:118
9:151
11:56
KGGSFQLNELQGLKpc3_NP.4
p−maj=4e−04, pX=0.021, N=23

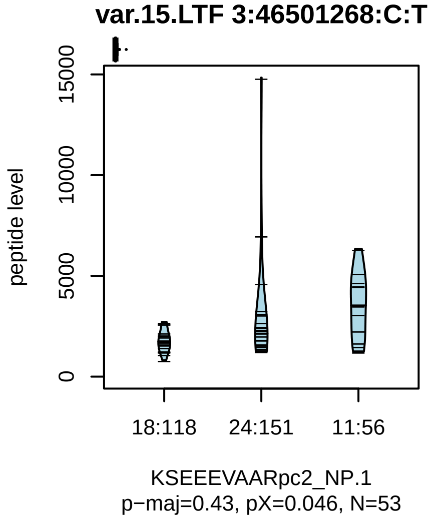
var.15.LTF 3:46501268:C:T
peptide level
0
5000
10000
15000
18:118
24:151
11:56
KSEEEVAARpc2_NP.1
p−maj=0.43, pX=0.046, N=53

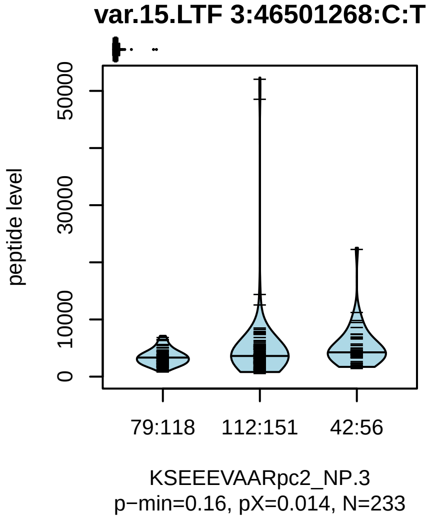
var.15.LTF 3:46501268:C:T
peptide level
0
10000
30000
50000
79:118
112:151
42:56
KSEEEVAARpc2_NP.3
p−min=0.16, pX=0.014, N=233

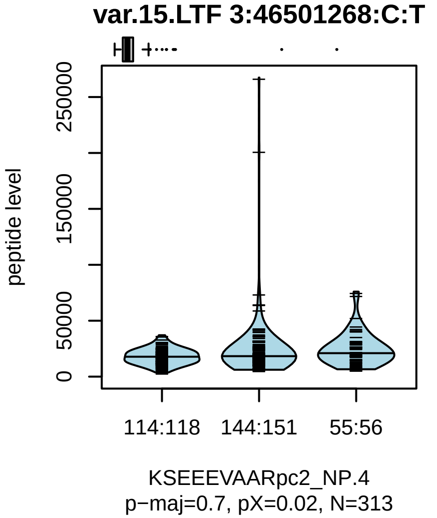
var.15.LTF 3:46501268:C:T
peptide level
0
50000
150000
250000
114:118
144:151
55:56
KSEEEVAARpc2_NP.4
p−maj=0.7, pX=0.02, N=313

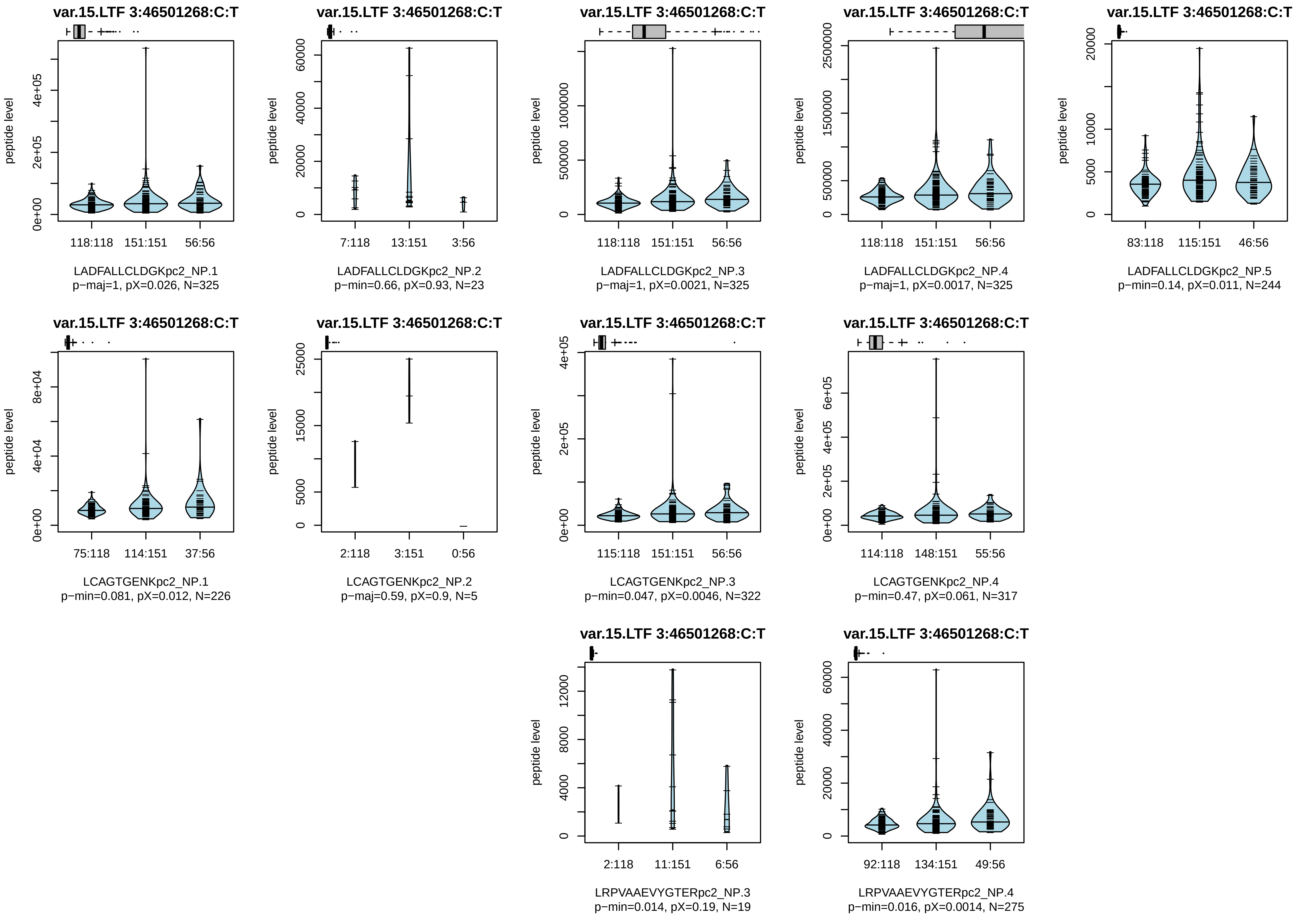
var.15.LTF 3:46501268:C:T
peptide level
118:118 151:151 56:56
LADFALLCLDGKpc2_NP.1
p−maj=1, pX=0.026, N=325
var.15.LTF 3:46501268:C:T
peptide level
7:118 13:151 3:56
LADFALLCLDGKpc2_NP.2
p−min=0.66, pX=0.93, N=23
var.15.LTF 3:46501268:C:T
peptide level
118:118 151:151 56:56
LADFALLCLDGKpc2_NP.3
p−maj=1, pX=0.0021, N=325
var.15.LTF 3:46501268:C:T
peptide level
118:118 151:151 56:56
LADFALLCLDGKpc2_NP.4
p−maj=1, pX=0.0017, N=325
var.15.LTF 3:46501268:C:T
peptide level
83:118 115:151 46:56
LADFALLCLDGKpc2_NP.5
p−min=0.14, pX=0.011, N=244
var.15.LTF 3:46501268:C:T
peptide level
75:118 114:151 37:56
LCAGTGENKpc2_NP.1
p−min=0.081, pX=0.012, N=226
var.15.LTF 3:46501268:C:T
peptide level
2:118 3:151 0:56
LCAGTGENKpc2_NP.2
p−maj=0.59, pX=0.9, N=5
var.15.LTF 3:46501268:C:T
peptide level
115:118 151:151 56:56
LCAGTGENKpc2_NP.3
p−min=0.047, pX=0.0046, N=322
var.15.LTF 3:46501268:C:T
peptide level
114:118 148:151 55:56
LCAGTGENKpc2_NP.4
p−min=0.47, pX=0.061, N=317
var.15.LTF 3:46501268:C:T
peptide level
2:118 11:151 6:56
LRPVAAEVYGTERpc2_NP.3
p−min=0.014, pX=0.19, N=19
var.15.LTF 3:46501268:C:T
peptide level
92:118 134:151 49:56
LRPVAAEVYGTERpc2_NP.4
p−min=0.016, pX=0.0014, N=275

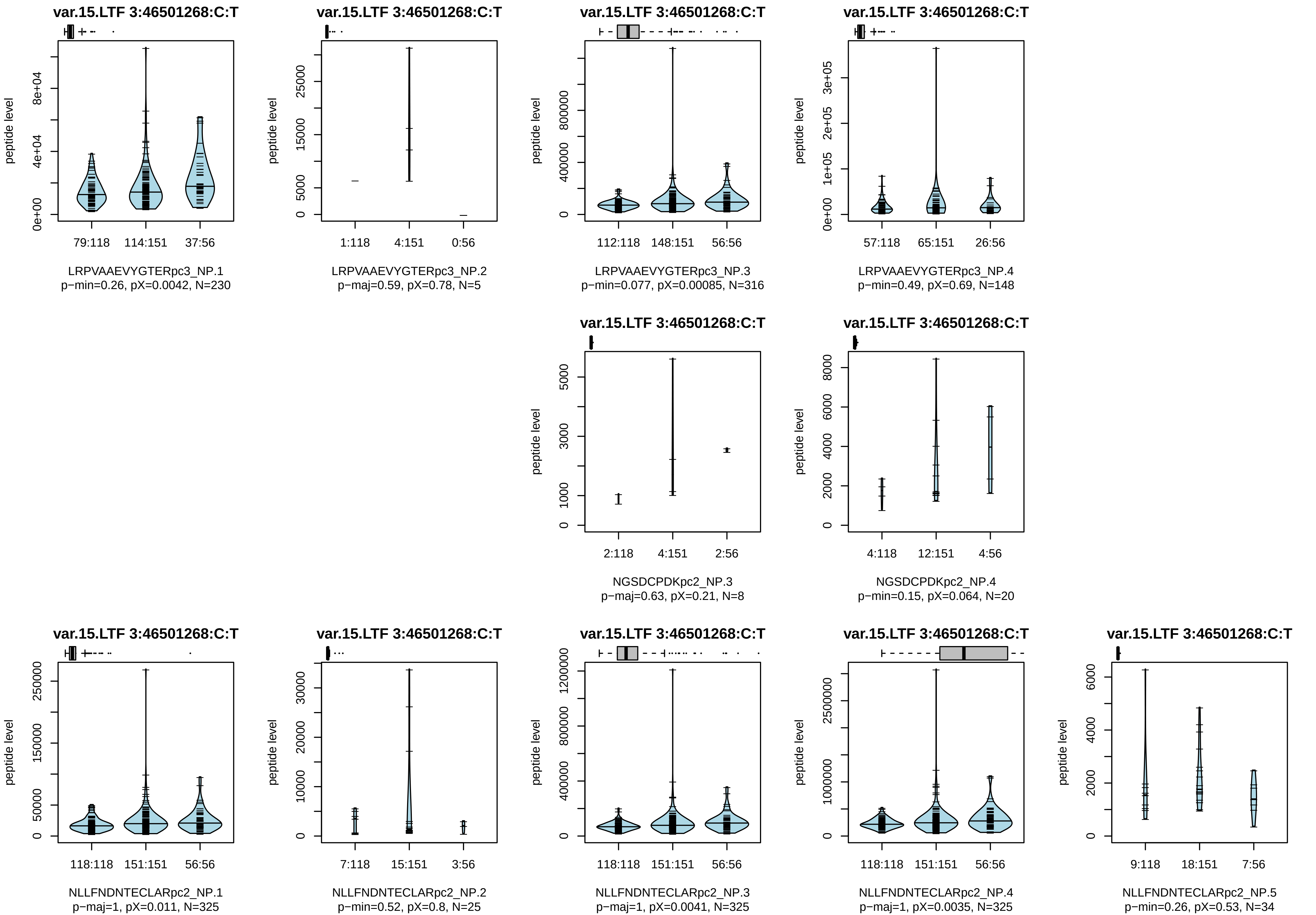

var.15.LTF 3:46501268:C:T
peptide level
79:118
114:151
37:56
LRPVAAEVYGTERpc3_NP.1
p−min=0.26, pX=0.0042, N=230
var.15.LTF 3:46501268:C:T
peptide level
1:118
4:151
0:56
LRPVAAEVYGTERpc3_NP.2
p−maj=0.59, pX=0.78, N=5
var.15.LTF 3:46501268:C:T
peptide level
112:118
148:151
56:56
LRPVAAEVYGTERpc3_NP.3
p−min=0.077, pX=0.00085, N=316
var.15.LTF 3:46501268:C:T
peptide level
57:118
65:151
26:56
LRPVAAEVYGTERpc3_NP.4
p−min=0.49, pX=0.69, N=148
var.15.LTF 3:46501268:C:T
peptide level
2:118
4:151
2:56
NGSDCPDKpc2_NP.3
p−maj=0.63, pX=0.21, N=8
var.15.LTF 3:46501268:C:T
peptide level
4:118
12:151
4:56
NGSDCPDKpc2_NP.4
p−min=0.15, pX=0.064, N=20
var.15.LTF 3:46501268:C:T
peptide level
118:118
151:151
56:56
NLLFNDNTECLARpc2_NP.1
p−maj=1, pX=0.011, N=325
var.15.LTF 3:46501268:C:T
peptide level
7:118
15:151
3:56
NLLFNDNTECLARpc2_NP.2
p−min=0.52, pX=0.8, N=25
var.15.LTF 3:46501268:C:T
peptide level
118:118
151:151
56:56
NLLFNDNTECLARpc2_NP.3
p−maj=1, pX=0.0041, N=325
var.15.LTF 3:46501268:C:T
peptide level
118:118
151:151
56:56
NLLFNDNTECLARpc2_NP.4
p−maj=1, pX=0.0035, N=325
var.15.LTF 3:46501268:C:T
peptide level
9:118
18:151
7:56
NLLFNDNTECLARpc2_NP.5
p−min=0.26, pX=0.53, N=34

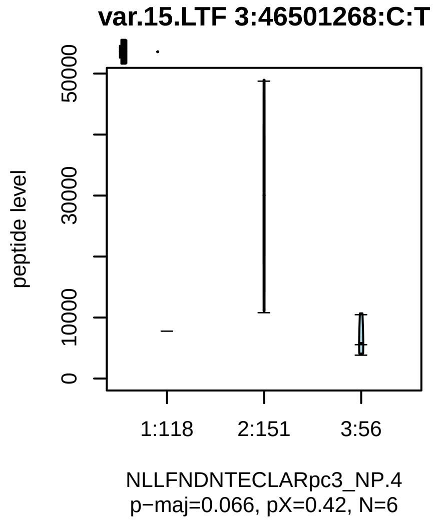
var.15.LTF 3:46501268:C:T
peptide level
0
10000
30000
50000
1:118
2:151
3:56
NLLFNDNTECLARpc3_NP.4
p−maj=0.066, pX=0.42, N=6

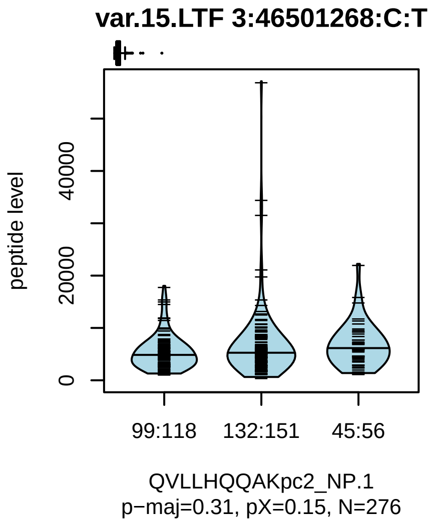
var.15.LTF 3:46501268:C:T
peptide level
0
20000
40000
99:118
132:151
45:56
QVLLHQQAKpc2_NP.1
p−maj=0.31, pX=0.15, N=276

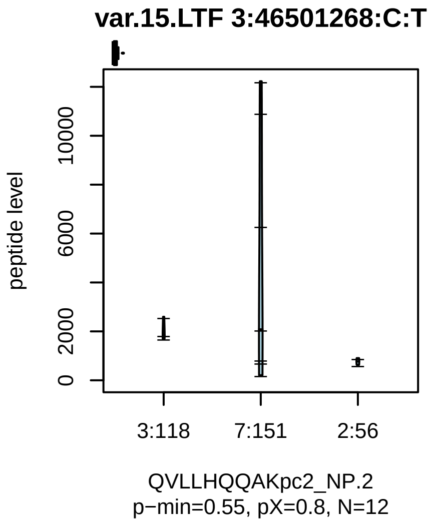
var.15.LTF 3:46501268:C:T
peptide level
0
2000
6000
10000
3:118
7:151
2:56
QVLLHQQAKpc2_NP.2
p−min=0.55, pX=0.8, N=12

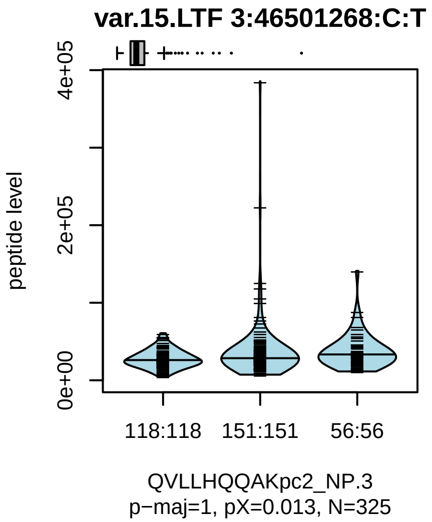
var.15.LTF 3:46501268:C:T
peptide level
0e+00
2e+05
4e+05
118:118
151:151
56:56
QVLLHQQAKpc2_NP.3
p−maj=1, pX=0.013, N=325

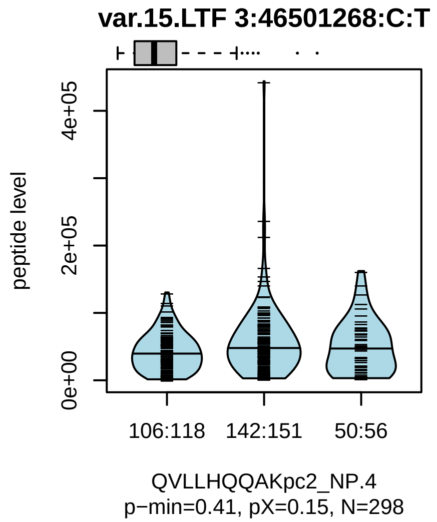
var.15.LTF 3:46501268:C:T
peptide level
0e+00
2e+05
4e+05
106:118
142:151
50:56
QVLLHQQAKpc2_NP.4
p−min=0.41, pX=0.15, N=298

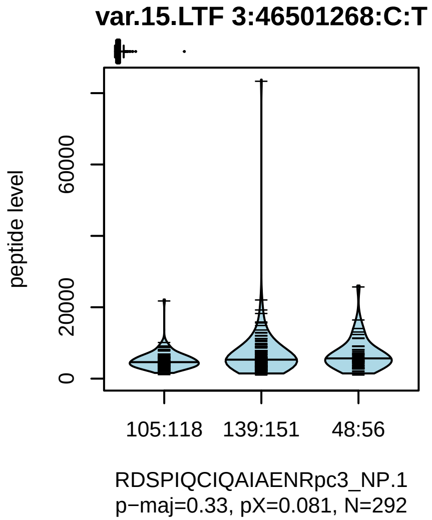
var.15.LTF 3:46501268:C:T
peptide level
0
20000
60000
105:118
139:151
48:56
RDSPIQCIQAIAENRpc3_NP.1
p−maj=0.33, pX=0.081, N=292

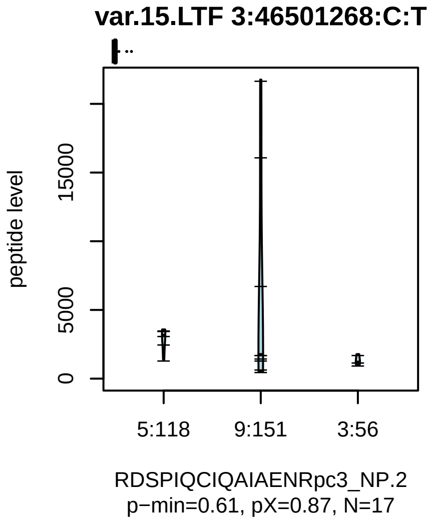
var.15.LTF 3:46501268:C:T
peptide level
0
5000
15000
5:118
9:151
3:56
RDSPIQCIQAIAENRpc3_NP.2
p−min=0.61, pX=0.87, N=17

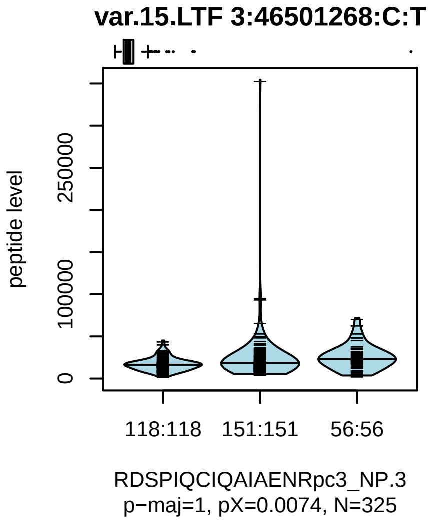
var.15.LTF 3:46501268:C:T
peptide level
0
100000
250000
118:118
151:151
56:56
RDSPIQCIQAIAENRpc3_NP.3
p−maj=1, pX=0.0074, N=325

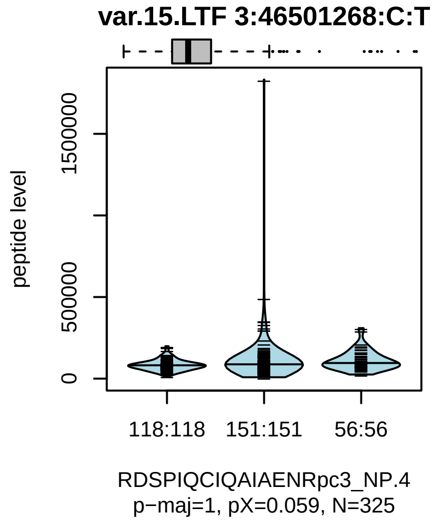
var.15.LTF 3:46501268:C:T
peptide level
0
500000
1500000
118:118
151:151
56:56
RDSPIQCIQAIAENRpc3_NP.4
p−maj=1, pX=0.059, N=325

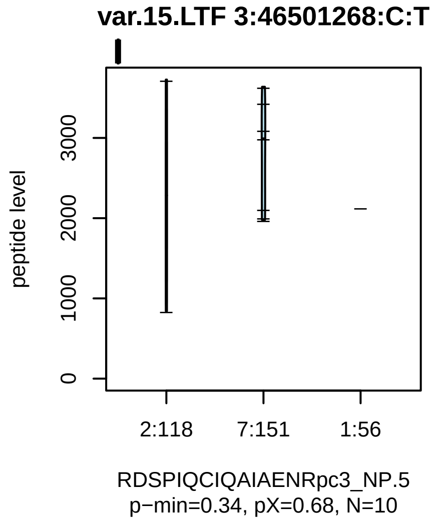
var.15.LTF 3:46501268:C:T
peptide level
0
1000
2000
3000
2:118
7:151
1:56
RDSPIQCIQAIAENRpc3_NP.5
p−min=0.34, pX=0.68, N=10

var.15.LTF 3:46501268:C:T

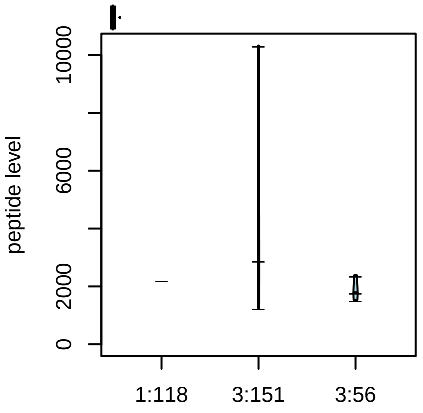

RSDTSLTWNSVKpc2_NP.4
p−maj=0.1, pX=0.34, N=7

var.15.LTF 3:46501268:C:T

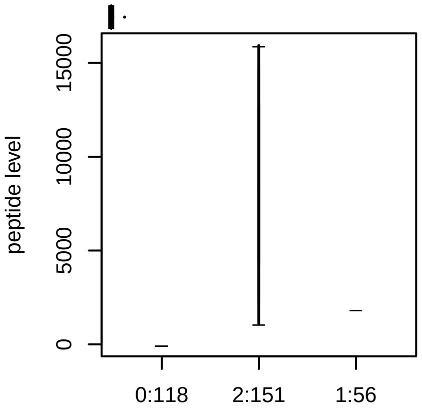

RSDTSLTWNSVKpc3_NP.1
p−maj=0.43, pX=0.63, N=3

var.15.LTF 3:46501268:C:T

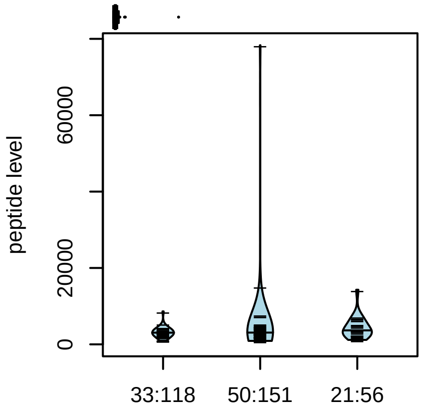

RSDTSLTWNSVKpc3_NP.3
p−min=0.27, pX=0.22, N=104

var.15.LTF 3:46501268:C:T

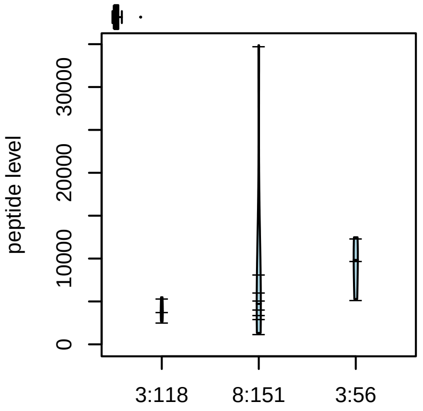

RSDTSLTWNSVKpc3_NP.4
p−min=0.27, pX=0.22, N=14

var.15.LTF 3:46501268:C:T

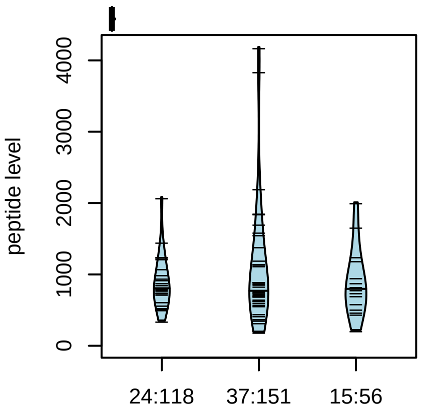

SCHLAMAPNHAVVSRpc2_NP.1
p−min=0.34, pX=0.35, N=76

var.15.LTF 3:46501268:C:T

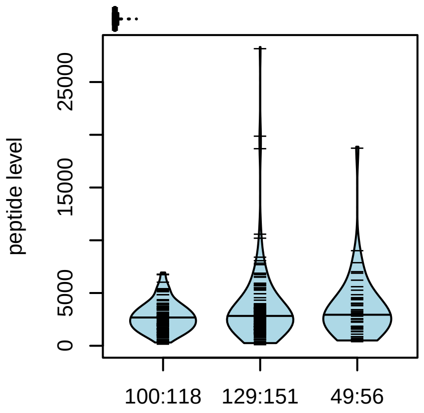

SCHLAMAPNHAVVSRpc2_NP.3
p−min=0.75, pX=0.054, N=278

var.15.LTF 3:46501268:C:T

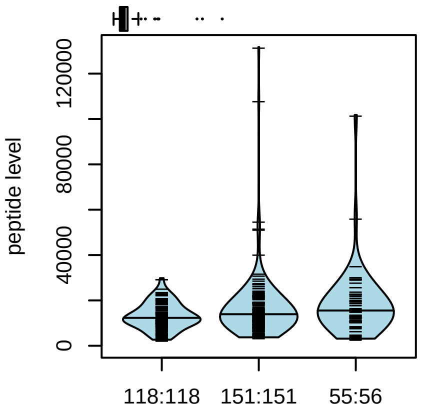

SCHLAMAPNHAVVSRpc2_NP.4
p−maj=0.17, pX=0.0057, N=324

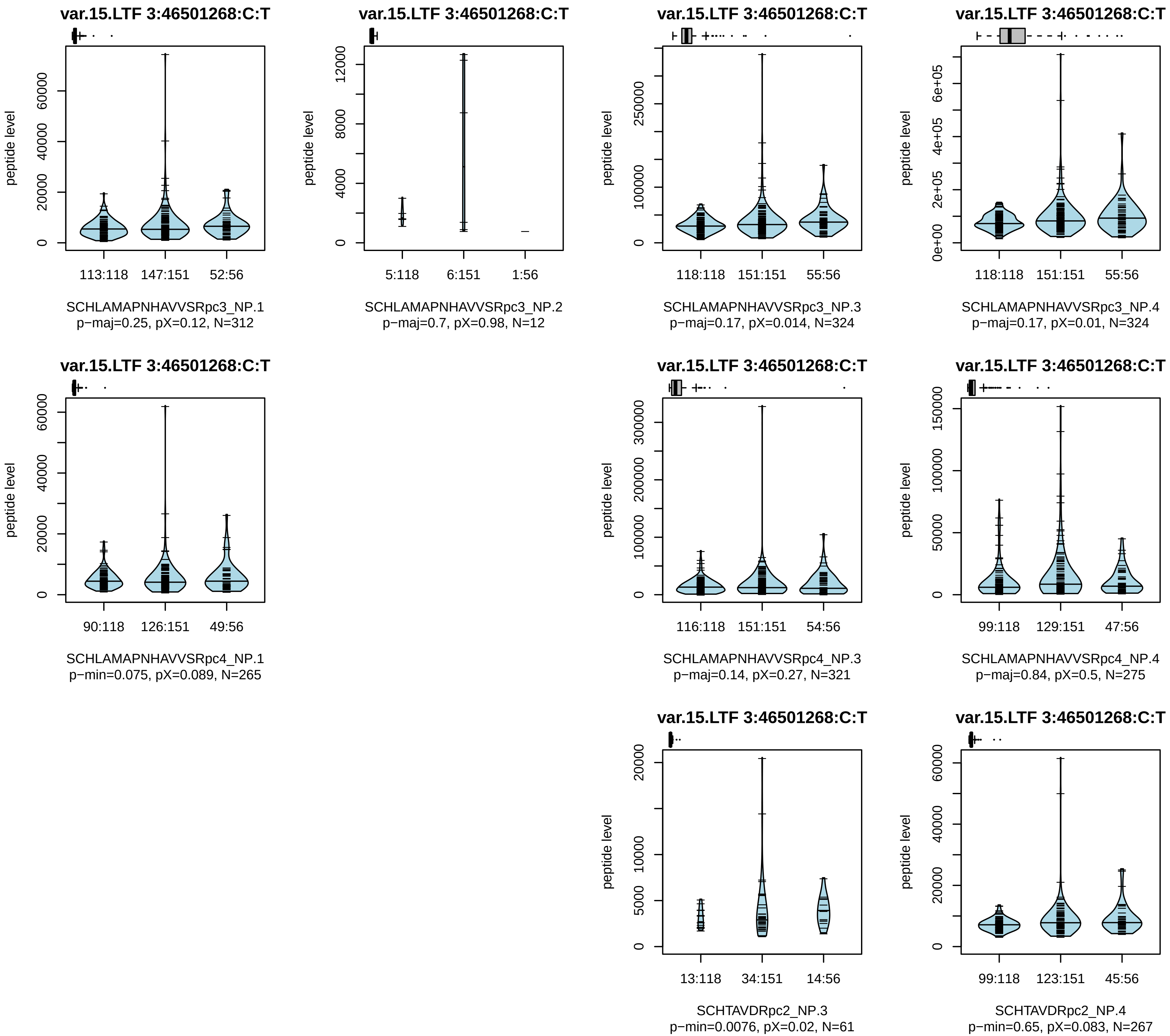
var.15.LTF 3:46501268:C:T
peptide level
0
20000
40000
60000
113:118
147:151
52:56
SCHLAMAPNHAVVSRpc3_NP.1
p−maj=0.25, pX=0.12, N=312
var.15.LTF 3:46501268:C:T
peptide level
0
4000
8000
12000
5:118
6:151
1:56
SCHLAMAPNHAVVSRpc3_NP.2
p−maj=0.7, pX=0.98, N=12
var.15.LTF 3:46501268:C:T
peptide level
0
100000
250000
118:118
151:151
55:56
SCHLAMAPNHAVVSRpc3_NP.3
p−maj=0.17, pX=0.014, N=324
var.15.LTF 3:46501268:C:T
peptide level
0e+00
2e+05
4e+05
6e+05
118:118
151:151
55:56
SCHLAMAPNHAVVSRpc3_NP.4
p−maj=0.17, pX=0.01, N=324
var.15.LTF 3:46501268:C:T
peptide level
0
20000
40000
60000
90:118
126:151
49:56
SCHLAMAPNHAVVSRpc4_NP.1
p−min=0.075, pX=0.089, N=265
var.15.LTF 3:46501268:C:T
peptide level
0
100000
200000
300000
116:118
151:151
54:56
SCHLAMAPNHAVVSRpc4_NP.3
p−maj=0.14, pX=0.27, N=321
var.15.LTF 3:46501268:C:T
peptide level
0
50000
100000
150000
99:118
129:151
47:56
SCHLAMAPNHAVVSRpc4_NP.4
p−maj=0.84, pX=0.5, N=275
var.15.LTF 3:46501268:C:T
peptide level
0
5000
10000
20000
13:118
34:151
14:56
SCHTAVDRpc2_NP.3
p−min=0.0076, pX=0.02, N=61
var.15.LTF 3:46501268:C:T
peptide level
0
20000
40000
60000
99:118
123:151
45:56
SCHTAVDRpc2_NP.4
p−min=0.65, pX=0.083, N=267

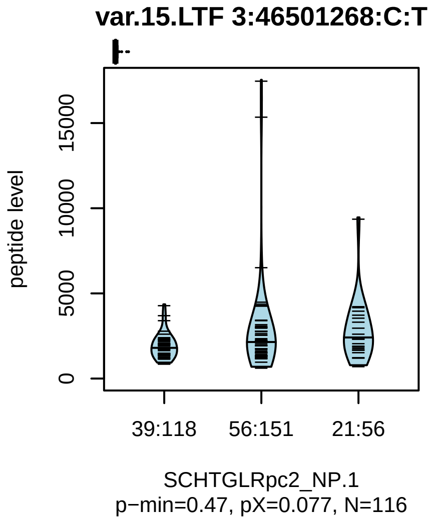
var.15.LTF 3:46501268:C:T
peptide level
0
5000
10000
15000
39:118
56:151
21:56
SCHTGLRpc2_NP.1
p−min=0.47, pX=0.077, N=116

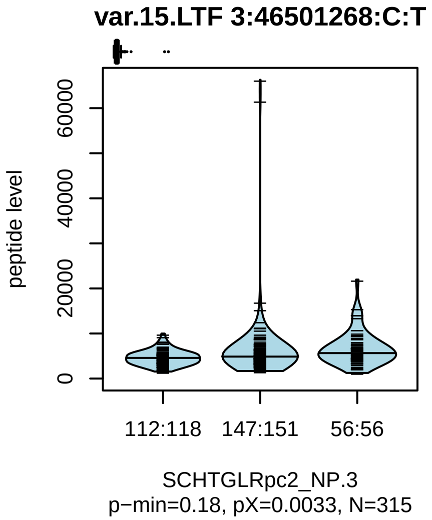
var.15.LTF 3:46501268:C:T
peptide level
0
20000
40000
60000
112:118
147:151
56:56
SCHTGLRpc2_NP.3
p−min=0.18, pX=0.0033, N=315

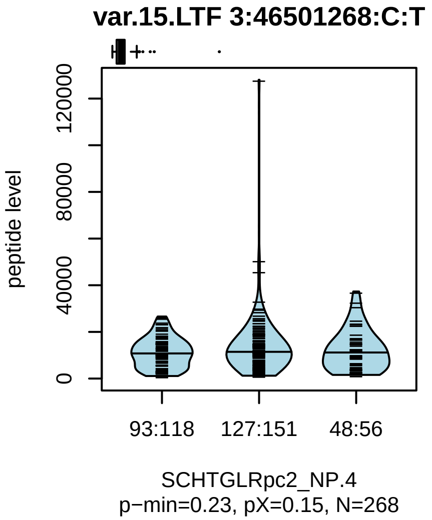
var.15.LTF 3:46501268:C:T
peptide level
0
40000
80000
120000
93:118
127:151
48:56
SCHTGLRpc2_NP.4
p−min=0.23, pX=0.15, N=268

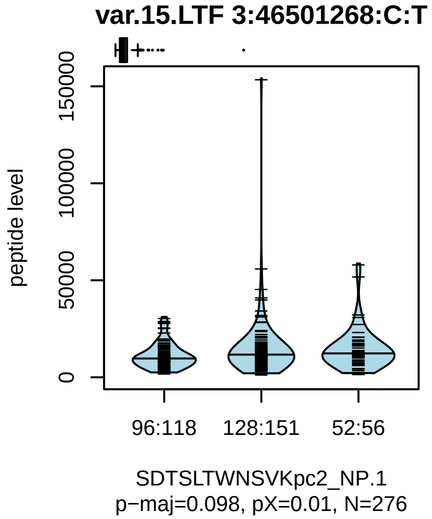
var.15.LTF 3:46501268:C:T
peptide level
0
50000
100000
150000
96:118
128:151
52:56
SDTSLTWNSVKpc2_NP.1
p−maj=0.098, pX=0.01, N=276

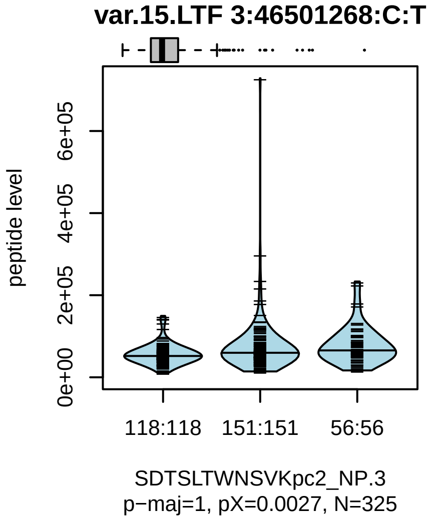
var.15.LTF 3:46501268:C:T
peptide level
0e+00
2e+05
4e+05
6e+05
118:118
151:151
56:56
SDTSLTWNSVKpc2_NP.3
p−maj=1, pX=0.0027, N=325

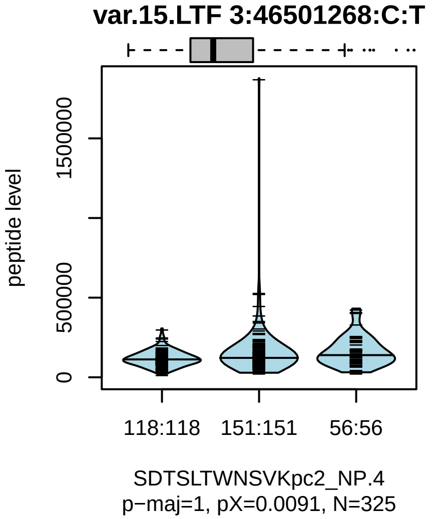
var.15.LTF 3:46501268:C:T
peptide level
0
500000
1500000
118:118
151:151
56:56
SDTSLTWNSVKpc2_NP.4
p−maj=1, pX=0.0091, N=325

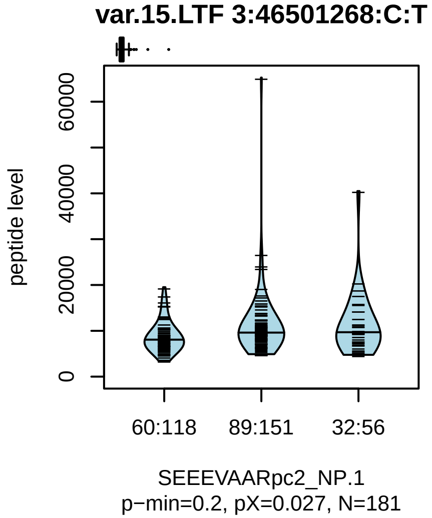
var.15.LTF 3:46501268:C:T
peptide level
0
20000
40000
60000
60:118
89:151
32:56
SEEEVAARpc2_NP.1
p−min=0.2, pX=0.027, N=181

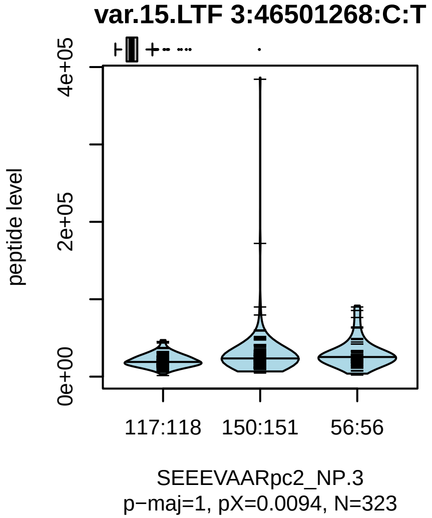
var.15.LTF 3:46501268:C:T
peptide level
0e+00
2e+05
4e+05
117:118
150:151
56:56
SEEEVAARpc2_NP.3
p−maj=1, pX=0.0094, N=323

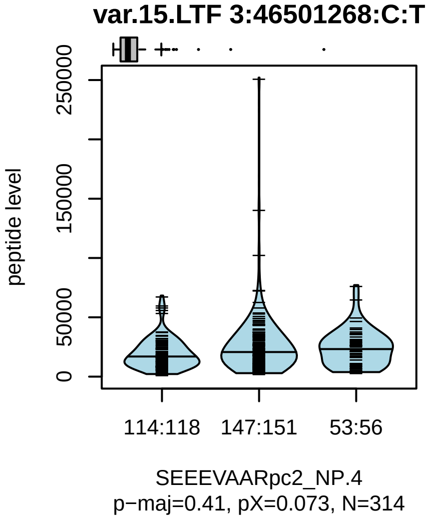
var.15.LTF 3:46501268:C:T
peptide level
0
50000
150000
250000
114:118
147:151
53:56
SEEEVAARpc2_NP.4
p−maj=0.41, pX=0.073, N=314

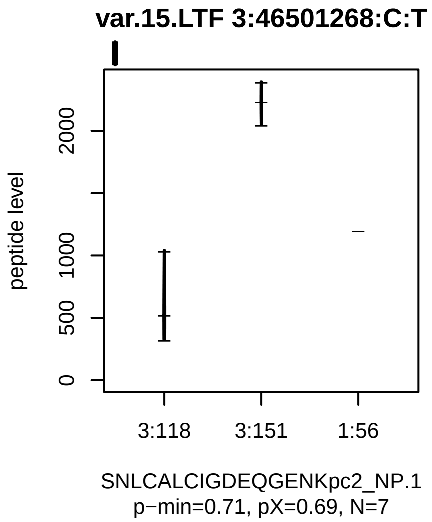
var.15.LTF 3:46501268:C:T
peptide level
0
500
1000
2000
3:118
3:151
1:56
SNLCALCIGDEQGENKpc2_NP.1
p−min=0.71, pX=0.69, N=7

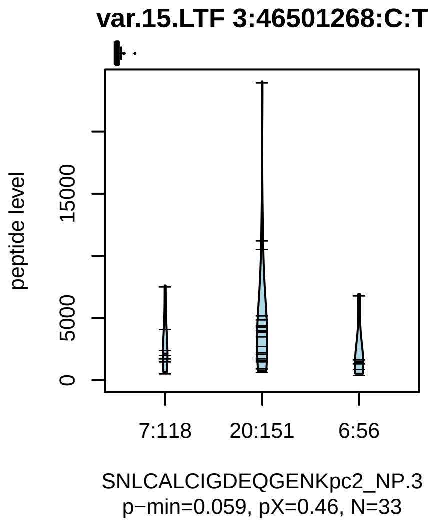
var.15.LTF 3:46501268:C:T
peptide level
0
5000
15000
7:118
20:151
6:56
SNLCALCIGDEQGENKpc2_NP.3
p−min=0.059, pX=0.46, N=33

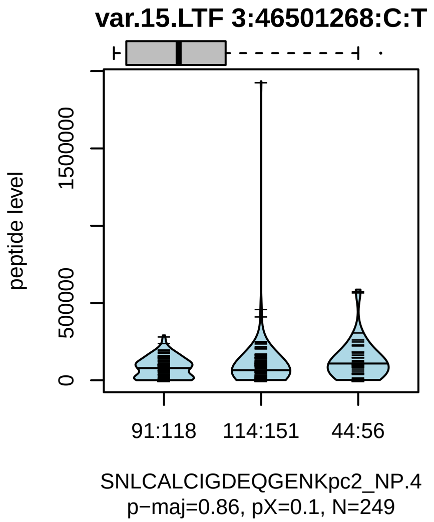
var.15.LTF 3:46501268:C:T
peptide level
0
500000
1500000
91:118
114:151
44:56
SNLCALCIGDEQGENKpc2_NP.4
p−maj=0.86, pX=0.1, N=249

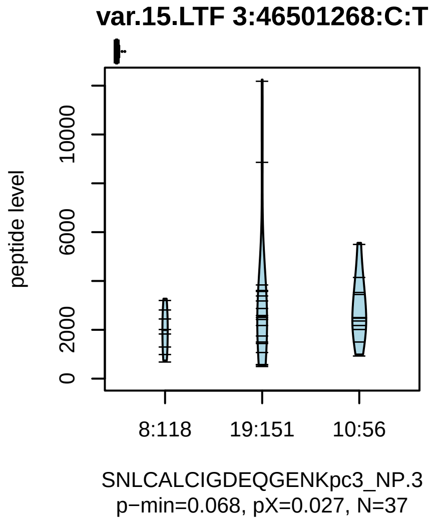
var.15.LTF 3:46501268:C:T
peptide level
0
2000
6000
10000
8:118
19:151
10:56
SNLCALCIGDEQGENKpc3_NP.3
p−min=0.068, pX=0.027, N=37

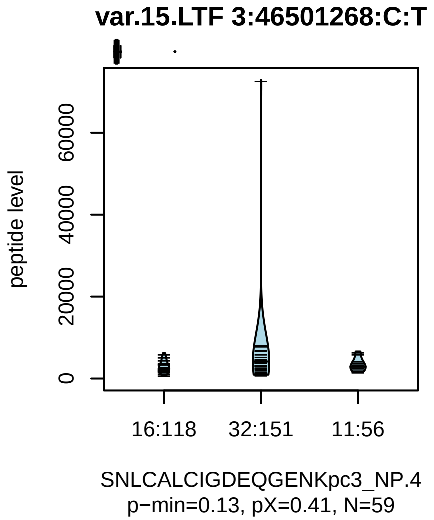
var.15.LTF 3:46501268:C:T
peptide level
0
20000
40000
60000
16:118
32:151
11:56
SNLCALCIGDEQGENKpc3_NP.4
p−min=0.13, pX=0.41, N=59

var.15.LTF 3:46501268:C:T

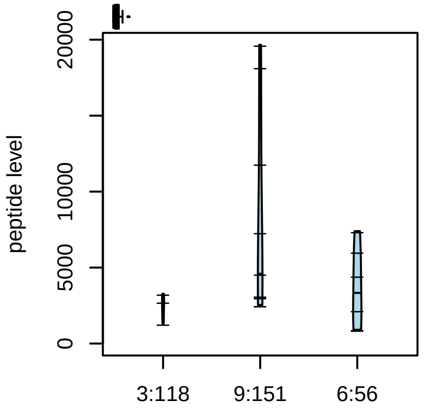

SQQSSDPDPNCVDRPVEGYLAVAVVRpc4_NF
p−min=0.083, pX=0.14, N=18

var.15.LTF 3:46501268:C:T

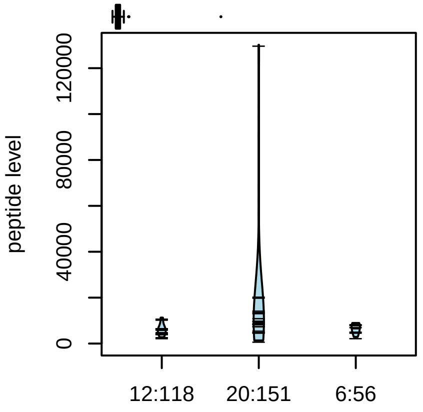

SQQSSDPDPNCVDRPVEGYLAVAVVRpc4_NF
p−min=0.59, pX=0.64, N=38

var.15.LTF 3:46501268:C:T

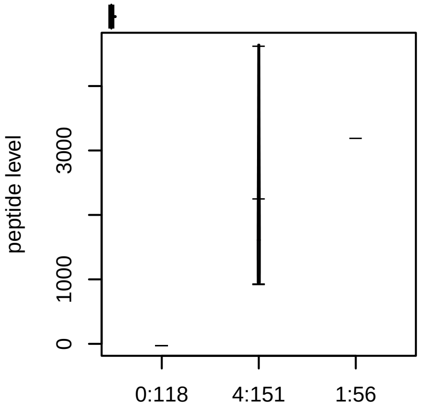

SVNGKEDAIWNLLRpc2_NP.4
p−min=0.16, pX=0.21, N=5

var.15.LTF 3:46501268:C:T

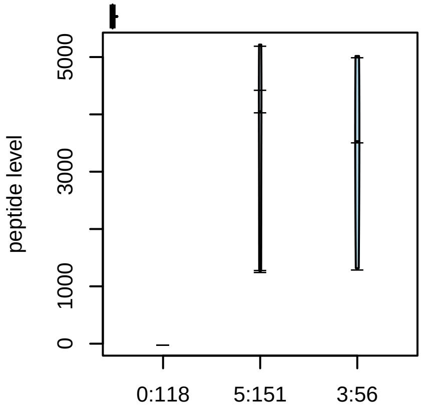

SVNGKEDAIWNLLRpc3_NP.3
p−min=0.055, pX=0.039, N=8

var.15.LTF 3:46501268:C:T

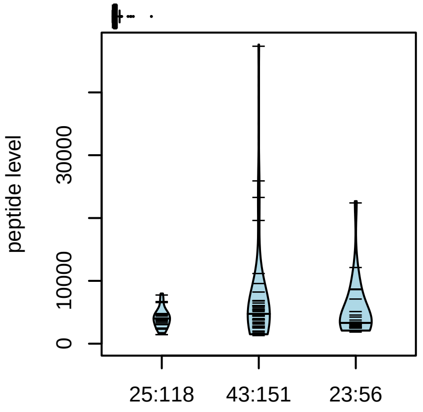

SVNGKEDAIWNLLRpc3_NP.4
p−maj=0.022, pX=0.019, N=91

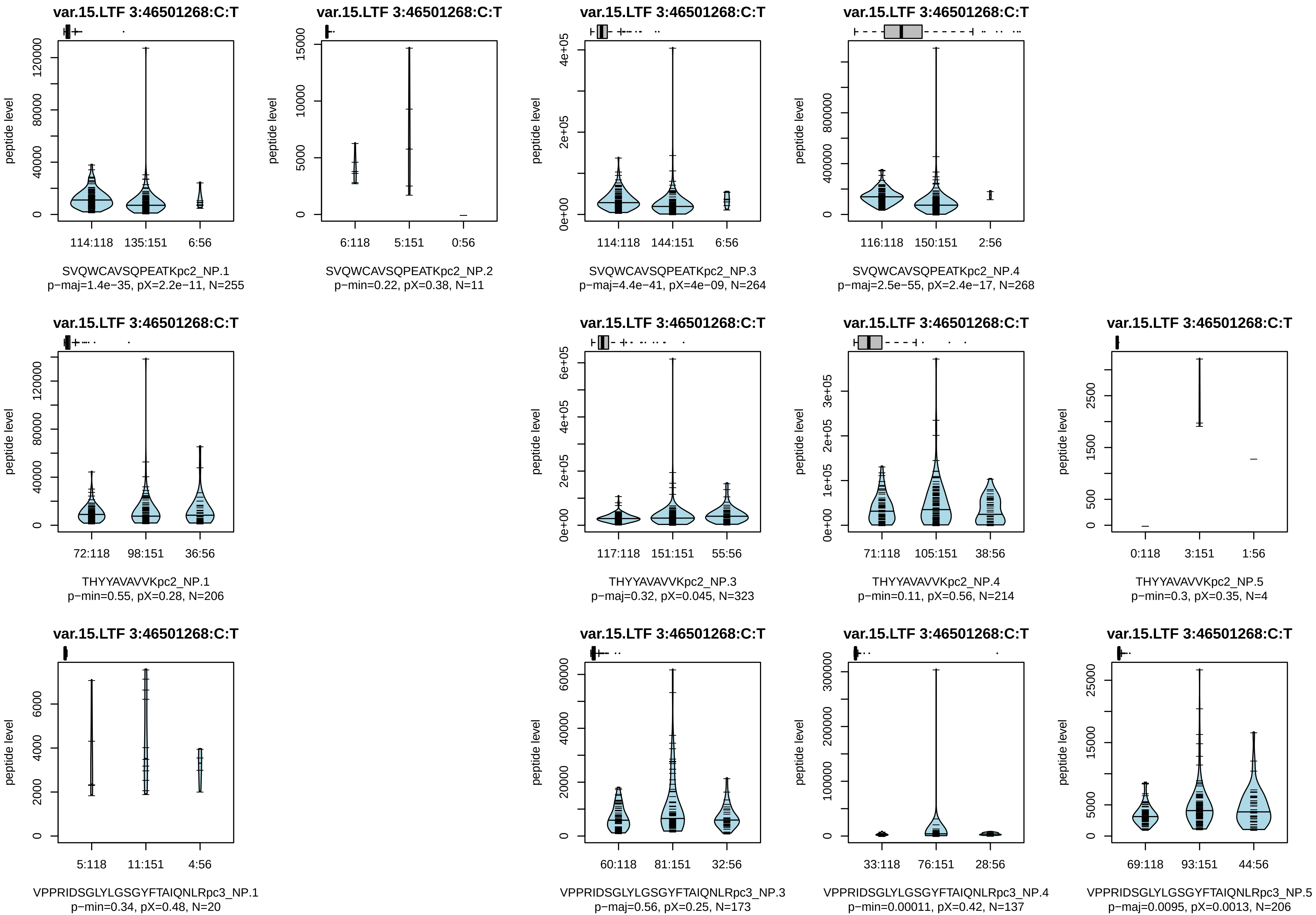
var.15.LTF 3:46501268:C:T
peptide level
114:118
135:151
6:56
SVQWCAVSQPEATKpc2_NP.1
p−maj=1.4e−35, pX=2.2e−11, N=255
var.15.LTF 3:46501268:C:T
peptide level
6:118
5:151
0:56
SVQWCAVSQPEATKpc2_NP.2
p−min=0.22, pX=0.38, N=11
var.15.LTF 3:46501268:C:T
peptide level
114:118
144:151
6:56
SVQWCAVSQPEATKpc2_NP.3
p−maj=4.4e−41, pX=4e−09, N=264
var.15.LTF 3:46501268:C:T
peptide level
116:118
150:151
2:56
SVQWCAVSQPEATKpc2_NP.4
p−maj=2.5e−55, pX=2.4e−17, N=268
var.15.LTF 3:46501268:C:T
peptide level
72:118
98:151
36:56
THYYAVAVVKpc2_NP.1
p−min=0.55, pX=0.28, N=206
var.15.LTF 3:46501268:C:T
peptide level
117:118
151:151
55:56
THYYAVAVVKpc2_NP.3
p−maj=0.32, pX=0.045, N=323
var.15.LTF 3:46501268:C:T
peptide level
71:118
105:151
38:56
THYYAVAVVKpc2_NP.4
p−min=0.11, pX=0.56, N=214
var.15.LTF 3:46501268:C:T
peptide level
0:118
3:151
1:56
THYYAVAVVKpc2_NP.5
p−min=0.3, pX=0.35, N=4
var.15.LTF 3:46501268:C:T
peptide level
5:118
11:151
4:56
VPPRIDSGLYLGSGYFTAIQNLRpc3_NP.1
p−min=0.34, pX=0.48, N=20
var.15.LTF 3:46501268:C:T
peptide level
60:118
81:151
32:56
VPPRIDSGLYLGSGYFTAIQNLRpc3_NP.3
p−maj=0.56, pX=0.25, N=173
var.15.LTF 3:46501268:C:T
peptide level
33:118
76:151
28:56
VPPRIDSGLYLGSGYFTAIQNLRpc3_NP.4
p−min=0.00011, pX=0.42, N=137
var.15.LTF 3:46501268:C:T
peptide level
69:118
93:151
44:56
VPPRIDSGLYLGSGYFTAIQNLRpc3_NP.5
p−maj=0.0095, pX=0.0013, N=206

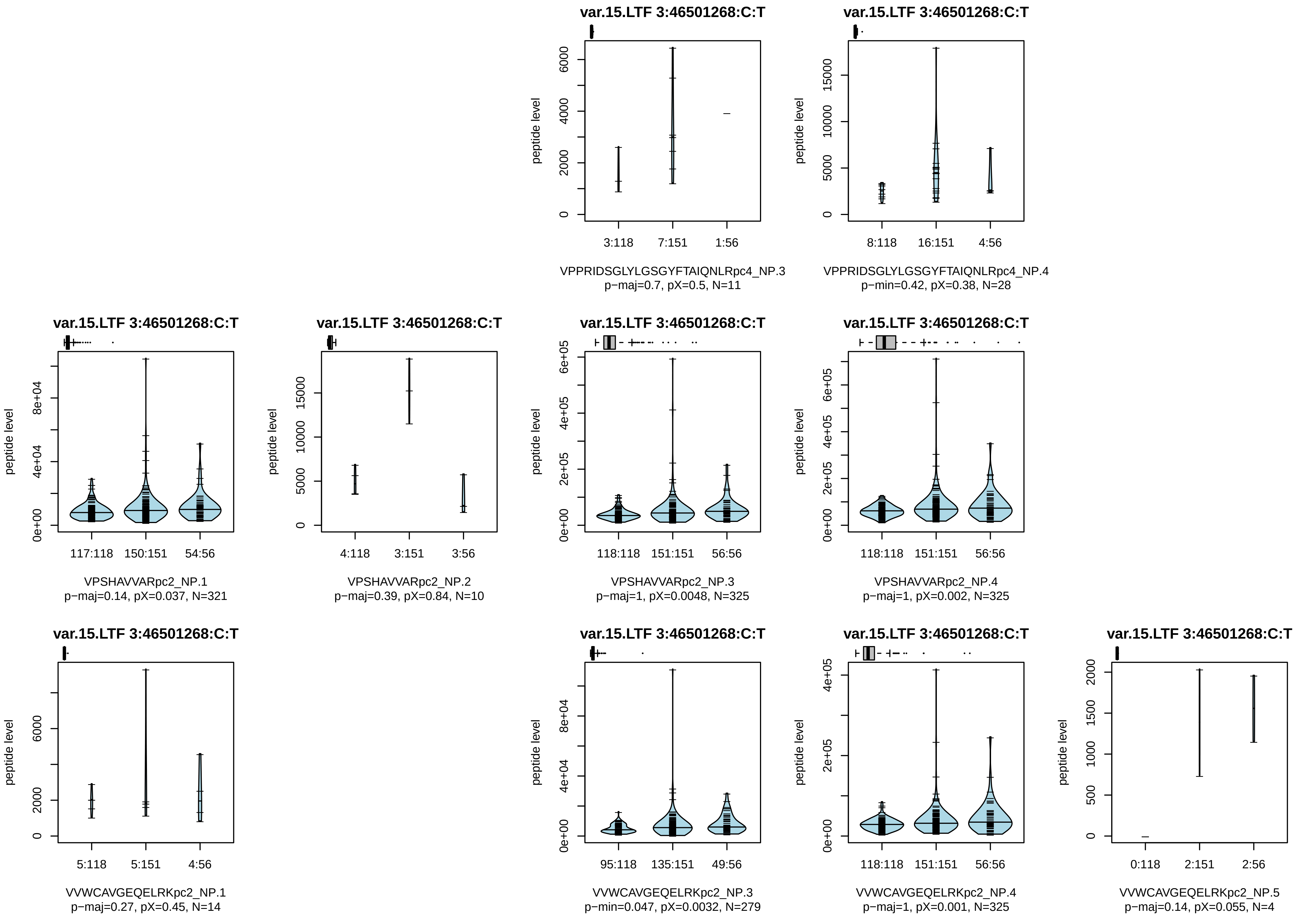
var.15.LTF 3:46501268:C:T
3:118
7:151
1:56
VPPRIDSGLYLGSGYFTAIQNLRpc4_NP.3
p−maj=0.7, pX=0.5, N=11
var.15.LTF 3:46501268:C:T
8:118
16:151
4:56
VPPRIDSGLYLGSGYFTAIQNLRpc4_NP.4
p−min=0.42, pX=0.38, N=28
var.15.LTF 3:46501268:C:T
117:118
150:151
54:56
VPSHAVVARpc2_NP.1
p−maj=0.14, pX=0.037, N=321
var.15.LTF 3:46501268:C:T
4:118
3:151
3:56
VPSHAVVARpc2_NP.2
p−maj=0.39, pX=0.84, N=10
var.15.LTF 3:46501268:C:T
118:118
151:151
56:56
VPSHAVVARpc2_NP.3
p−maj=1, pX=0.0048, N=325
var.15.LTF 3:46501268:C:T
118:118
151:151
56:56
VPSHAVVARpc2_NP.4
p−maj=1, pX=0.002, N=325
var.15.LTF 3:46501268:C:T
5:118
5:151
4:56
VVWCAVGEQELRKpc2_NP.1
p−maj=0.27, pX=0.45, N=14
var.15.LTF 3:46501268:C:T
95:118
135:151
49:56
VVWCAVGEQELRKpc2_NP.3
p−min=0.047, pX=0.0032, N=279
var.15.LTF 3:46501268:C:T
118:118
151:151
56:56
VVWCAVGEQELRKpc2_NP.4
p−maj=1, pX=0.001, N=325
var.15.LTF 3:46501268:C:T
0:118
2:151
2:56
VVWCAVGEQELRKpc2_NP.5
p−maj=0.14, pX=0.055, N=4
peptide level

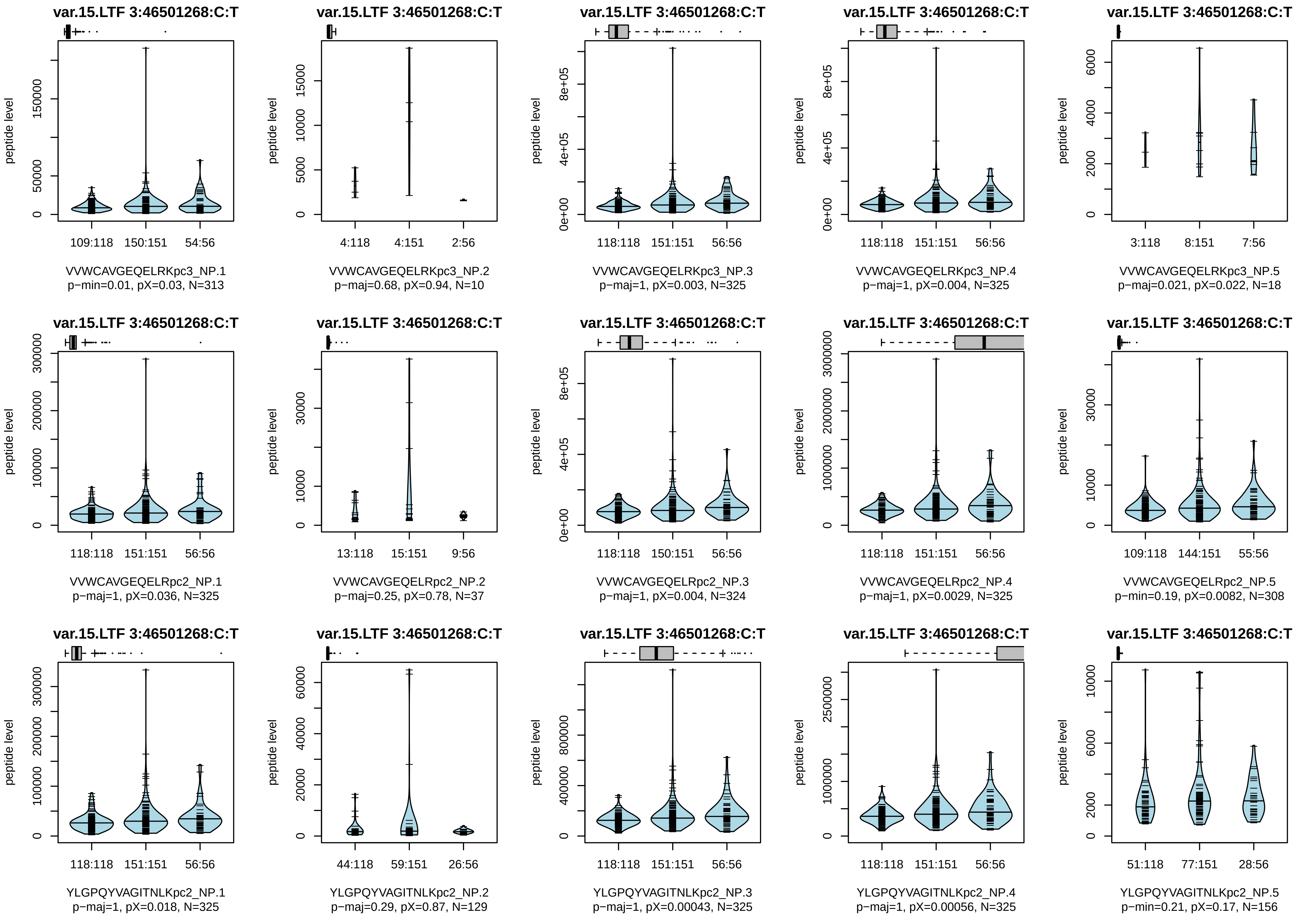

var.15.LTF 3:46501268:C:T
peptide level
109:118
150:151
54:56
VVWCAVGEQELRKpc3_NP.1
p−min=0.01, pX=0.03, N=313
var.15.LTF 3:46501268:C:T
peptide level
4:118
4:151
2:56
VVWCAVGEQELRKpc3_NP.2
p−maj=0.68, pX=0.94, N=10
var.15.LTF 3:46501268:C:T
peptide level
118:118
151:151
56:56
VVWCAVGEQELRKpc3_NP.3
p−maj=1, pX=0.003, N=325
var.15.LTF 3:46501268:C:T
peptide level
118:118
151:151
56:56
VVWCAVGEQELRKpc3_NP.4
p−maj=1, pX=0.004, N=325
var.15.LTF 3:46501268:C:T
peptide level
3:118
8:151
7:56
VVWCAVGEQELRKpc3_NP.5
p−maj=0.021, pX=0.022, N=18
var.15.LTF 3:46501268:C:T
peptide level
118:118
151:151
56:56
VVWCAVGEQELRpc2_NP.1
p−maj=1, pX=0.036, N=325
var.15.LTF 3:46501268:C:T
peptide level
13:118
15:151
9:56
VVWCAVGEQELRpc2_NP.2
p−maj=0.25, pX=0.78, N=37
var.15.LTF 3:46501268:C:T
peptide level
118:118
150:151
56:56
VVWCAVGEQELRpc2_NP.3
p−maj=1, pX=0.004, N=324
var.15.LTF 3:46501268:C:T
peptide level
118:118
151:151
56:56
VVWCAVGEQELRpc2_NP.4
p−maj=1, pX=0.0029, N=325
var.15.LTF 3:46501268:C:T
peptide level
109:118
144:151
55:56
VVWCAVGEQELRpc2_NP.5
p−min=0.19, pX=0.0082, N=308
var.15.LTF 3:46501268:C:T
peptide level
118:118
151:151
56:56
YLGPQYVAGITNLKpc2_NP.1
p−maj=1, pX=0.018, N=325
var.15.LTF 3:46501268:C:T
peptide level
44:118
59:151
26:56
YLGPQYVAGITNLKpc2_NP.2
p−maj=0.29, pX=0.87, N=129
var.15.LTF 3:46501268:C:T
peptide level
118:118
151:151
56:56
YLGPQYVAGITNLKpc2_NP.3
p−maj=1, pX=0.00043, N=325
var.15.LTF 3:46501268:C:T
peptide level
118:118
151:151
56:56
YLGPQYVAGITNLKpc2_NP.4
p−maj=1, pX=0.00056, N=325
var.15.LTF 3:46501268:C:T
peptide level
51:118
77:151
28:56
YLGPQYVAGITNLKpc2_NP.5
p−min=0.21, pX=0.17, N=156

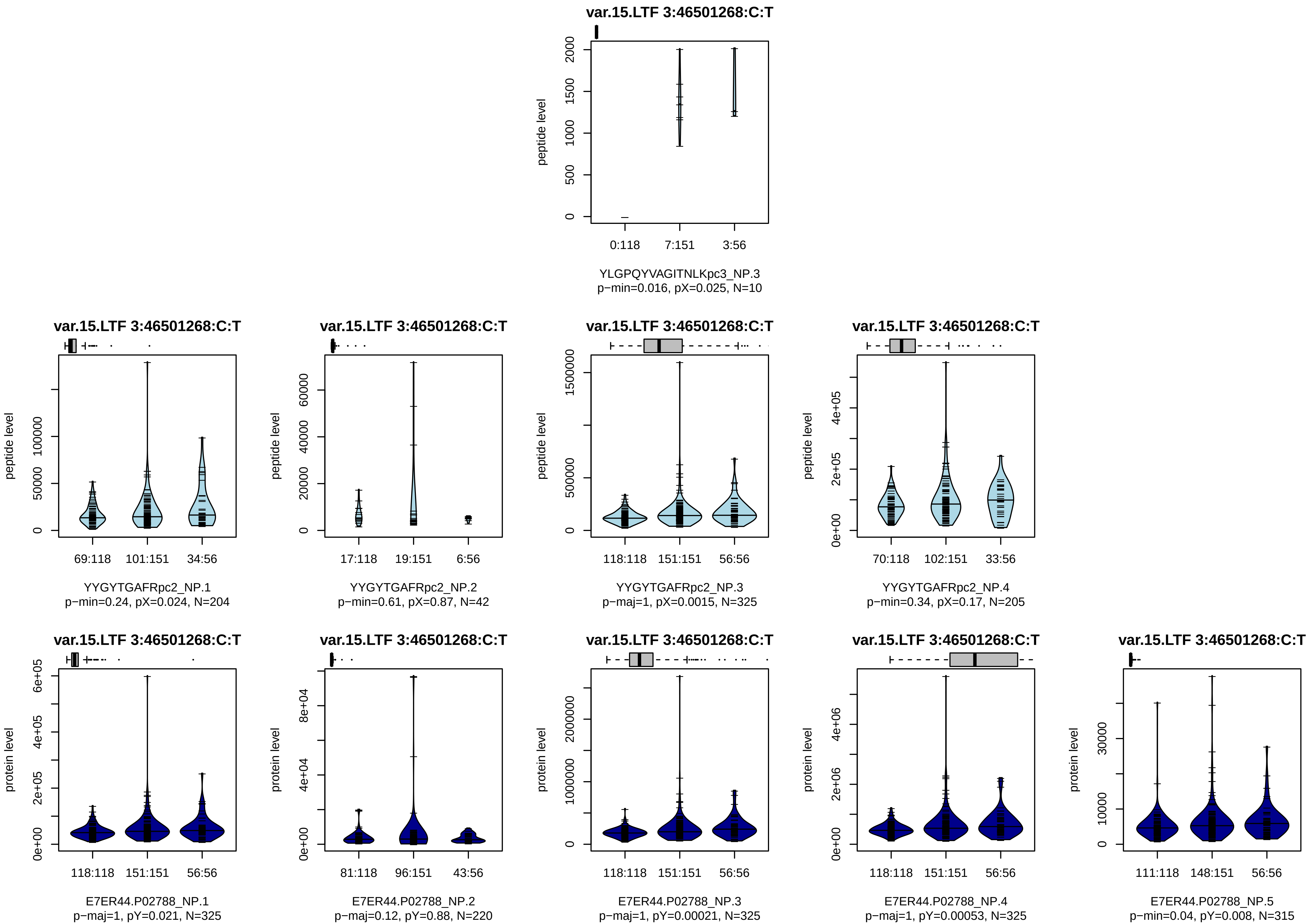
var.15.LTF 3:46501268:C:T
peptide level
0:118
7:151
3:56
YLGPQYVAGITNLKpc3_NP.3
p−min=0.016, pX=0.025, N=10
var.15.LTF 3:46501268:C:T
peptide level
69:118
101:151
34:56
YYGYTGAFRpc2_NP.1
p−min=0.24, pX=0.024, N=204
var.15.LTF 3:46501268:C:T
peptide level
17:118
19:151
6:56
YYGYTGAFRpc2_NP.2
p−min=0.61, pX=0.87, N=42
var.15.LTF 3:46501268:C:T
peptide level
118:118
151:151
56:56
YYGYTGAFRpc2_NP.3
p−maj=1, pX=0.0015, N=325
var.15.LTF 3:46501268:C:T
peptide level
70:118
102:151
33:56
YYGYTGAFRpc2_NP.4
p−min=0.34, pX=0.17, N=205
var.15.LTF 3:46501268:C:T
protein level
118:118
151:151
56:56
E7ER44.P02788_NP.1
p−maj=1, pY=0.021, N=325
var.15.LTF 3:46501268:C:T
protein level
81:118
96:151
43:56
E7ER44.P02788_NP.2
p−maj=0.12, pY=0.88, N=220
var.15.LTF 3:46501268:C:T
protein level
118:118
151:151
56:56
E7ER44.P02788_NP.3
p−maj=1, pY=0.00021, N=325
var.15.LTF 3:46501268:C:T
protein level
118:118
151:151
56:56
E7ER44.P02788_NP.4
p−maj=1, pY=0.00053, N=325
var.15.LTF 3:46501268:C:T
protein level
111:118
148:151
56:56
E7ER44.P02788_NP.5
p−min=0.04, pY=0.008, N=315

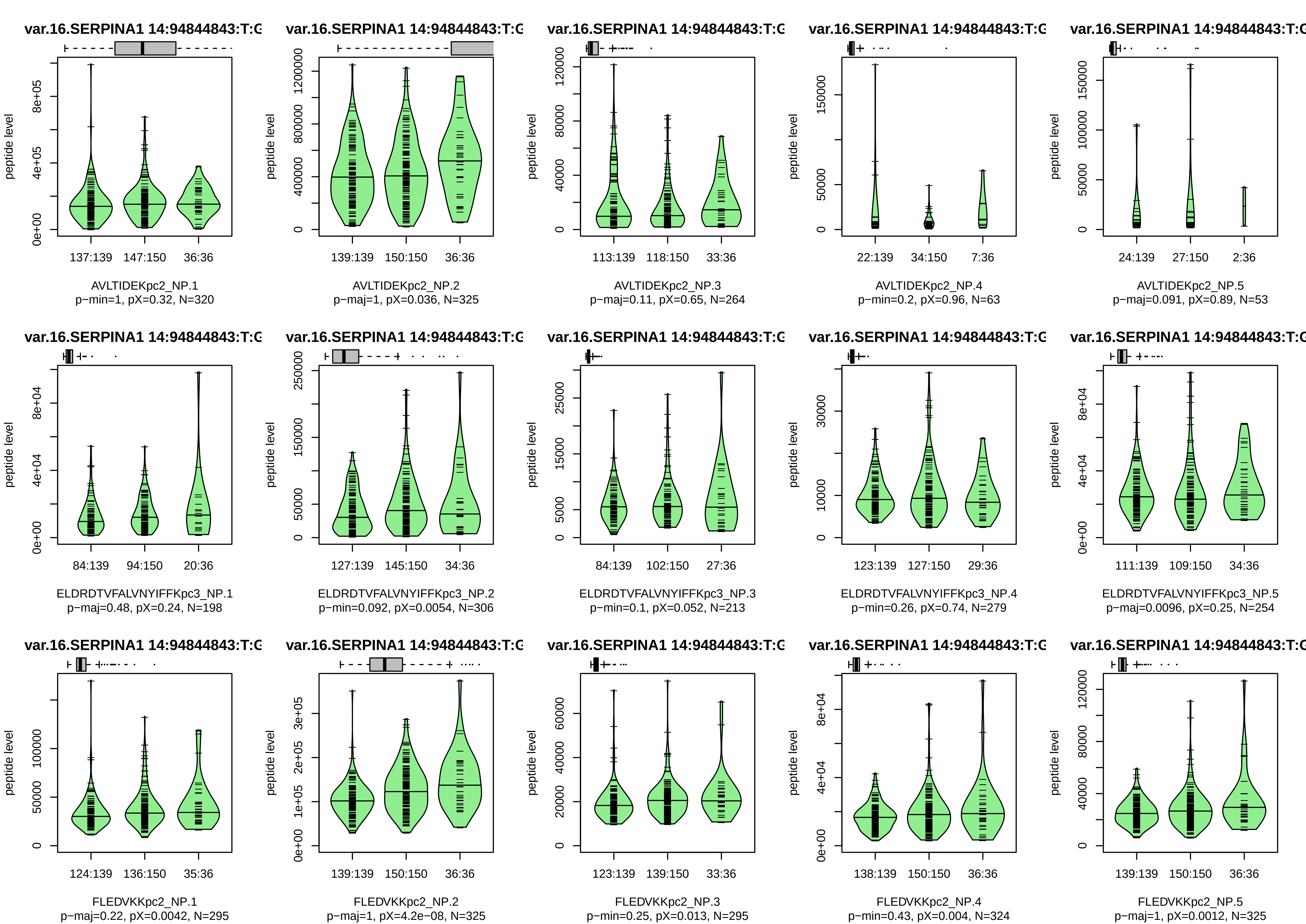
var.16.SERPINA1 14:94844843:T:G
peptide level
137:139 147:150 36:36
AVLTIDEKpc2_NP.1
p−min=1, pX=0.32, N=320
var.16.SERPINA1 14:94844843:T:G
peptide level
139:139 150:150 36:36
AVLTIDEKpc2_NP.2
p−maj=1, pX=0.036, N=325
var.16.SERPINA1 14:94844843:T:G
peptide level
113:139 118:150 33:36
AVLTIDEKpc2_NP.3
p−maj=0.11, pX=0.65, N=264
var.16.SERPINA1 14:94844843:T:G
peptide level
22:139 34:150 7:36
AVLTIDEKpc2_NP.4
p−min=0.2, pX=0.96, N=63
var.16.SERPINA1 14:94844843:T:G
peptide level
24:139 27:150 2:36
AVLTIDEKpc2_NP.5
p−maj=0.091, pX=0.89, N=53
var.16.SERPINA1 14:94844843:T:G
peptide level
84:139 94:150 20:36
ELDRDTVFALVNYIFFKpc3_NP.1
p−maj=0.48, pX=0.24, N=198
var.16.SERPINA1 14:94844843:T:G
peptide level
127:139 145:150 34:36
ELDRDTVFALVNYIFFKpc3_NP.2
p−min=0.092, pX=0.0054, N=306
var.16.SERPINA1 14:94844843:T:G
peptide level
84:139 102:150 27:36
ELDRDTVFALVNYIFFKpc3_NP.3
p−min=0.1, pX=0.052, N=213
var.16.SERPINA1 14:94844843:T:G
peptide level
123:139 127:150 29:36
ELDRDTVFALVNYIFFKpc3_NP.4
p−min=0.26, pX=0.74, N=279
var.16.SERPINA1 14:94844843:T:G
peptide level
111:139 109:150 34:36
ELDRDTVFALVNYIFFKpc3_NP.5
p−maj=0.0096, pX=0.25, N=254
var.16.SERPINA1 14:94844843:T:G
peptide level
124:139 136:150 35:36
FLEDVKKpc2_NP.1
p−maj=0.22, pX=0.0042, N=295
var.16.SERPINA1 14:94844843:T:G
peptide level
139:139 150:150 36:36
FLEDVKKpc2_NP.2
p−maj=1, pX=4.2e−08, N=325
var.16.SERPINA1 14:94844843:T:G
peptide level
123:139 139:150 33:36
FLEDVKKpc2_NP.3
p−min=0.25, pX=0.013, N=295
var.16.SERPINA1 14:94844843:T:G
peptide level
138:139 150:150 36:36
FLEDVKKpc2_NP.4
p−min=0.43, pX=0.004, N=324
var.16.SERPINA1 14:94844843:T:G
peptide level
139:139 150:150 36:36
FLEDVKKpc2_NP.5
p−maj=1, pX=0.0012, N=325

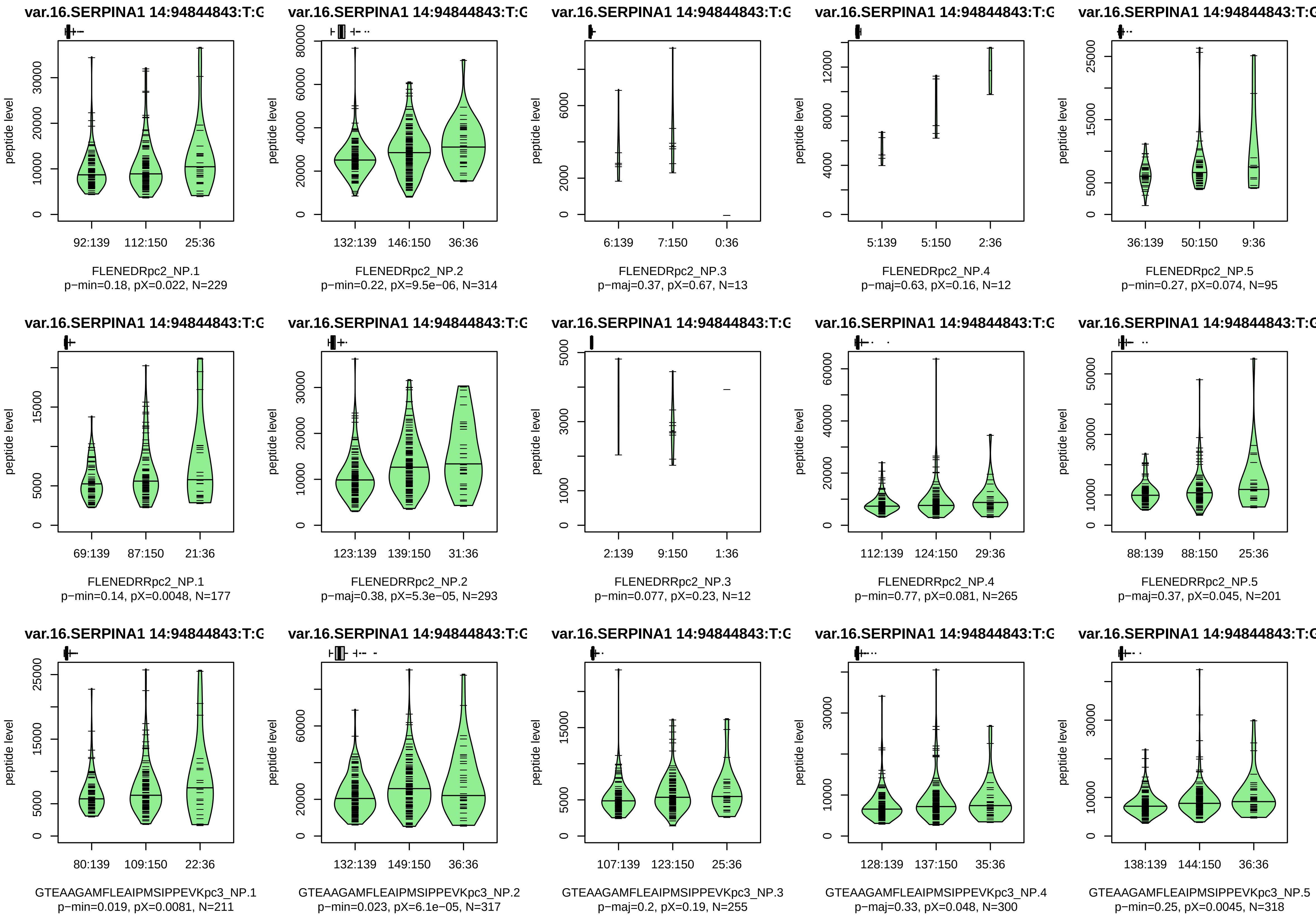
var.16.SERPINA1 14:94844843:T:G
peptide level
92:139
112:150
25:36
FLENEDRpc2_NP.1
p−min=0.18, pX=0.022, N=229
var.16.SERPINA1 14:94844843:T:G
peptide level
132:139
146:150
36:36
FLENEDRpc2_NP.2
p−min=0.22, pX=9.5e−06, N=314
var.16.SERPINA1 14:94844843:T:G
peptide level
6:139
7:150
0:36
FLENEDRpc2_NP.3
p−maj=0.37, pX=0.67, N=13
var.16.SERPINA1 14:94844843:T:G
peptide level
5:139
5:150
2:36
FLENEDRpc2_NP.4
p−maj=0.63, pX=0.16, N=12
var.16.SERPINA1 14:94844843:T:G
peptide level
36:139
50:150
9:36
FLENEDRpc2_NP.5
p−min=0.27, pX=0.074, N=95
var.16.SERPINA1 14:94844843:T:G
peptide level
69:139
87:150
21:36
FLENEDRRpc2_NP.1
p−min=0.14, pX=0.0048, N=177
var.16.SERPINA1 14:94844843:T:G
peptide level
123:139
139:150
31:36
FLENEDRRpc2_NP.2
p−maj=0.38, pX=5.3e−05, N=293
var.16.SERPINA1 14:94844843:T:G
peptide level
2:139
9:150
1:36
FLENEDRRpc2_NP.3
p−min=0.077, pX=0.23, N=12
var.16.SERPINA1 14:94844843:T:G
peptide level
112:139
124:150
29:36
FLENEDRRpc2_NP.4
p−min=0.77, pX=0.081, N=265
var.16.SERPINA1 14:94844843:T:G
peptide level
88:139
88:150
25:36
FLENEDRRpc2_NP.5
p−maj=0.37, pX=0.045, N=201
var.16.SERPINA1 14:94844843:T:G
peptide level
80:139
109:150
22:36
GTEAAGAMFLEAIPMSIPPEVKpc3_NP.1
p−min=0.019, pX=0.0081, N=211
var.16.SERPINA1 14:94844843:T:G
peptide level
132:139
149:150
36:36
GTEAAGAMFLEAIPMSIPPEVKpc3_NP.2
p−min=0.023, pX=6.1e−05, N=317
var.16.SERPINA1 14:94844843:T:G
peptide level
107:139
123:150
25:36
GTEAAGAMFLEAIPMSIPPEVKpc3_NP.3
p−maj=0.2, pX=0.19, N=255
var.16.SERPINA1 14:94844843:T:G
peptide level
128:139
137:150
35:36
GTEAAGAMFLEAIPMSIPPEVKpc3_NP.4
p−maj=0.33, pX=0.048, N=300
var.16.SERPINA1 14:94844843:T:G
peptide level
138:139
144:150
36:36
GTEAAGAMFLEAIPMSIPPEVKpc3_NP.5
p−min=0.25, pX=0.0045, N=318

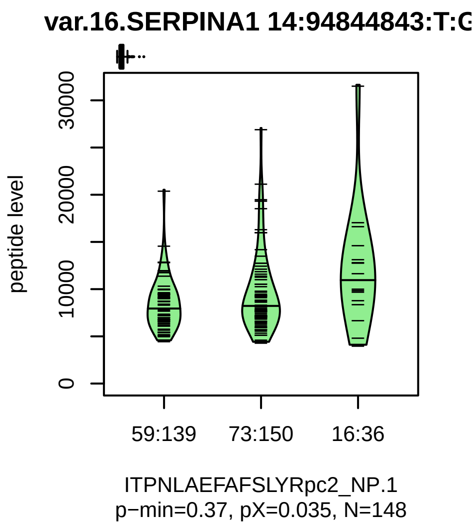
var.16.SERPINA1 14:94844843:T:G
peptide level
0
10000
20000
30000
59:139
73:150
16:36
ITPNLAEFAFSLYRpc2_NP.1
p−min=0.37, pX=0.035, N=148

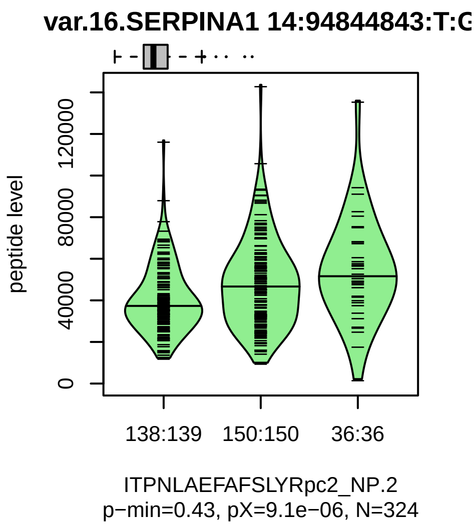
var.16.SERPINA1 14:94844843:T:G
peptide level
0
40000
80000
120000
138:139
150:150
36:36
ITPNLAEFAFSLYRpc2_NP.2
p−min=0.43, pX=9.1e−06, N=324

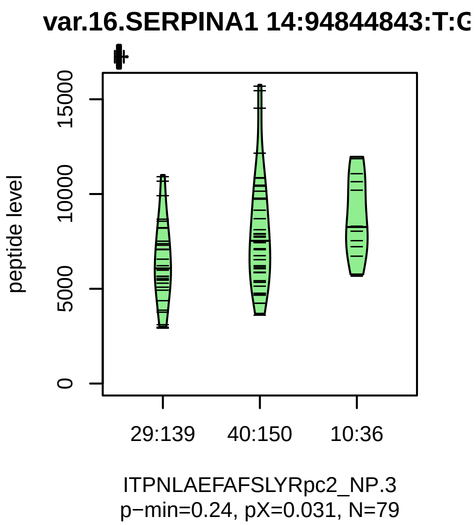
var.16.SERPINA1 14:94844843:T:G
peptide level
0
5000
10000
15000
29:139
40:150
10:36
ITPNLAEFAFSLYRpc2_NP.3
p−min=0.24, pX=0.031, N=79

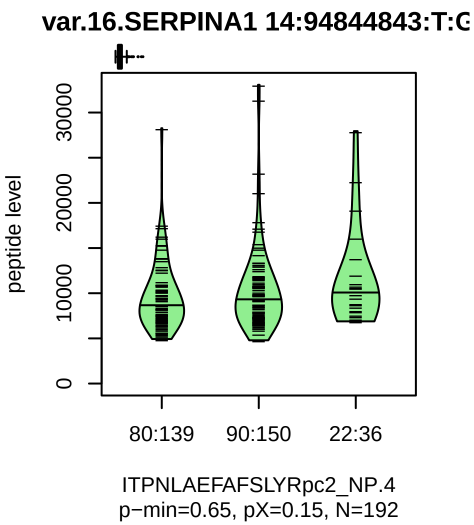
var.16.SERPINA1 14:94844843:T:G
peptide level
0
10000
20000
30000
80:139
90:150
22:36
ITPNLAEFAFSLYRpc2_NP.4
p−min=0.65, pX=0.15, N=192

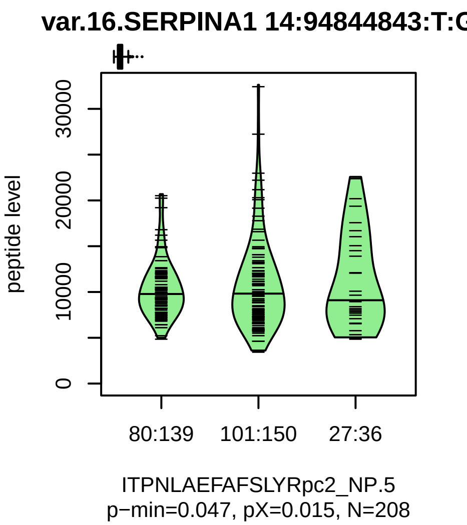
var.16.SERPINA1 14:94844843:T:G
peptide level
0
10000
20000
30000
80:139
101:150
27:36
ITPNLAEFAFSLYRpc2_NP.5
p−min=0.047, pX=0.015, N=208

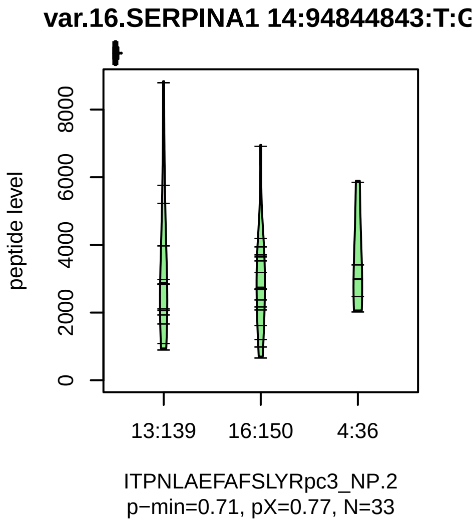
var.16.SERPINA1 14:94844843:T:G
peptide level
0
2000
4000
6000
8000
13:139
16:150
4:36
ITPNLAEFAFSLYRpc3_NP.2
p−min=0.71, pX=0.77, N=33

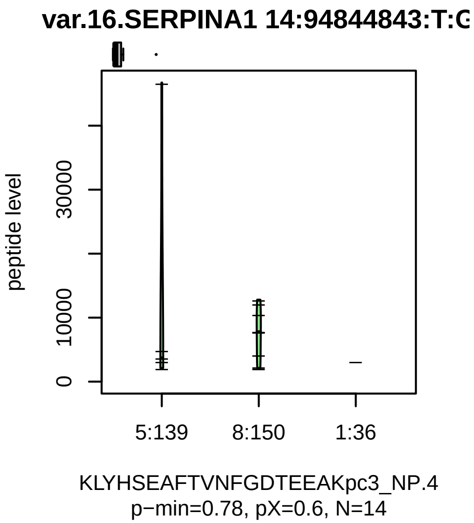
var.16.SERPINA1 14:94844843:T:G
peptide level
0
10000
30000
5:139
8:150
1:36
KLYHSEAFTVNFGDTEEAKpc3_NP.4
p−min=0.78, pX=0.6, N=14

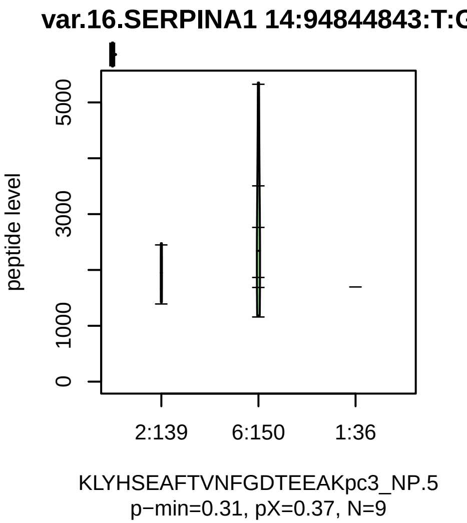
var.16.SERPINA1 14:94844843:T:G
peptide level
0
1000
3000
5000
2:139
6:150
1:36
KLYHSEAFTVNFGDTEEAKpc3_NP.5
p−min=0.31, pX=0.37, N=9

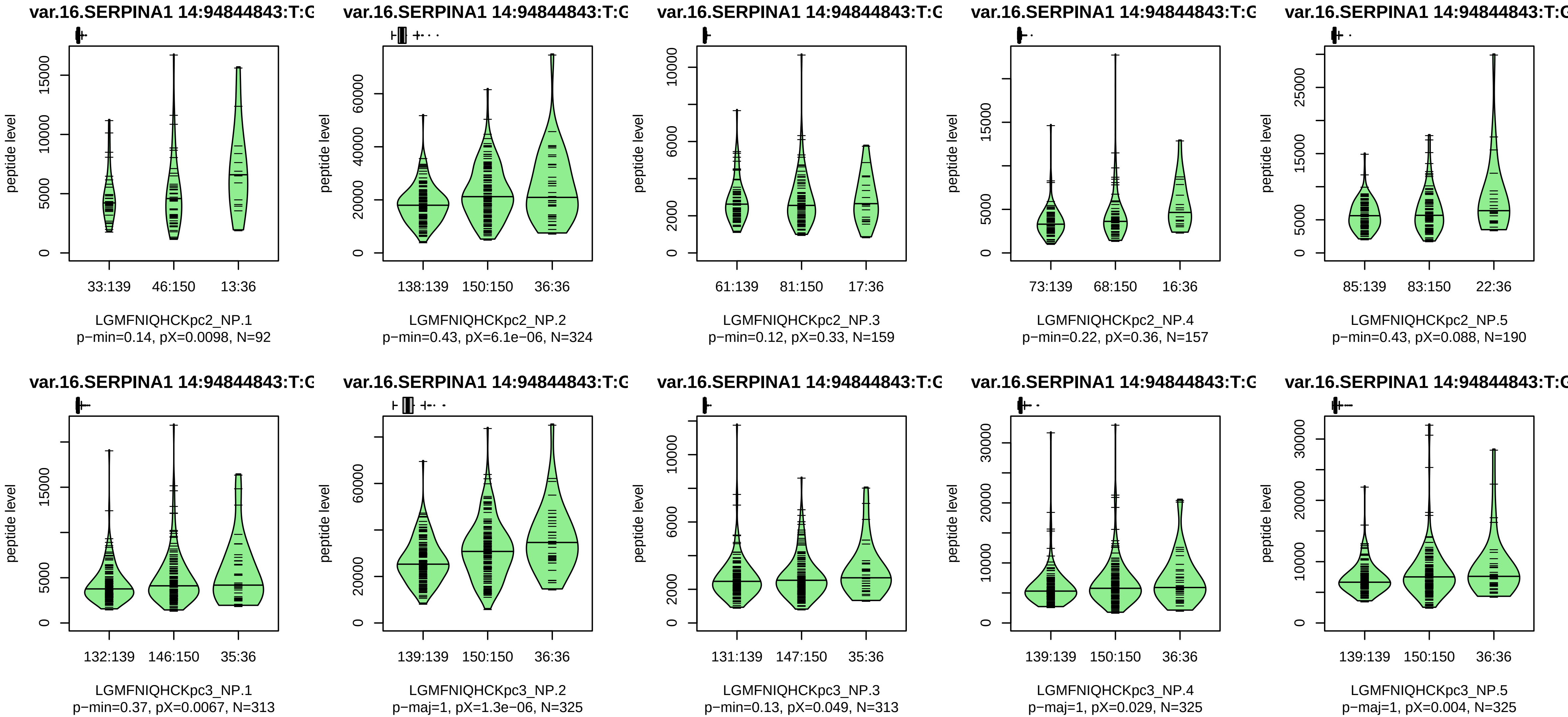
var.16.SERPINA1 14:94844843:T:G
peptide level
33:139
46:150
13:36
LGMFNIQHCKpc2_NP.1
p−min=0.14, pX=0.0098, N=92
var.16.SERPINA1 14:94844843:T:G
peptide level
138:139
150:150
36:36
LGMFNIQHCKpc2_NP.2
p−min=0.43, pX=6.1e−06, N=324
var.16.SERPINA1 14:94844843:T:G
peptide level
61:139
81:150
17:36
LGMFNIQHCKpc2_NP.3
p−min=0.12, pX=0.33, N=159
var.16.SERPINA1 14:94844843:T:G
peptide level
73:139
68:150
16:36
LGMFNIQHCKpc2_NP.4
p−min=0.22, pX=0.36, N=157
var.16.SERPINA1 14:94844843:T:G
peptide level
85:139
83:150
22:36
LGMFNIQHCKpc2_NP.5
p−min=0.43, pX=0.088, N=190
var.16.SERPINA1 14:94844843:T:G
peptide level
132:139
146:150
35:36
LGMFNIQHCKpc3_NP.1
p−min=0.37, pX=0.0067, N=313
var.16.SERPINA1 14:94844843:T:G
peptide level
139:139
150:150
36:36
LGMFNIQHCKpc3_NP.2
p−maj=1, pX=1.3e−06, N=325
var.16.SERPINA1 14:94844843:T:G
peptide level
131:139
147:150
35:36
LGMFNIQHCKpc3_NP.3
p−min=0.13, pX=0.049, N=313
var.16.SERPINA1 14:94844843:T:G
peptide level
139:139
150:150
36:36
LGMFNIQHCKpc3_NP.4
p−maj=1, pX=0.029, N=325
var.16.SERPINA1 14:94844843:T:G
peptide level
139:139
150:150
36:36
LGMFNIQHCKpc3_NP.5
p−maj=1, pX=0.004, N=325

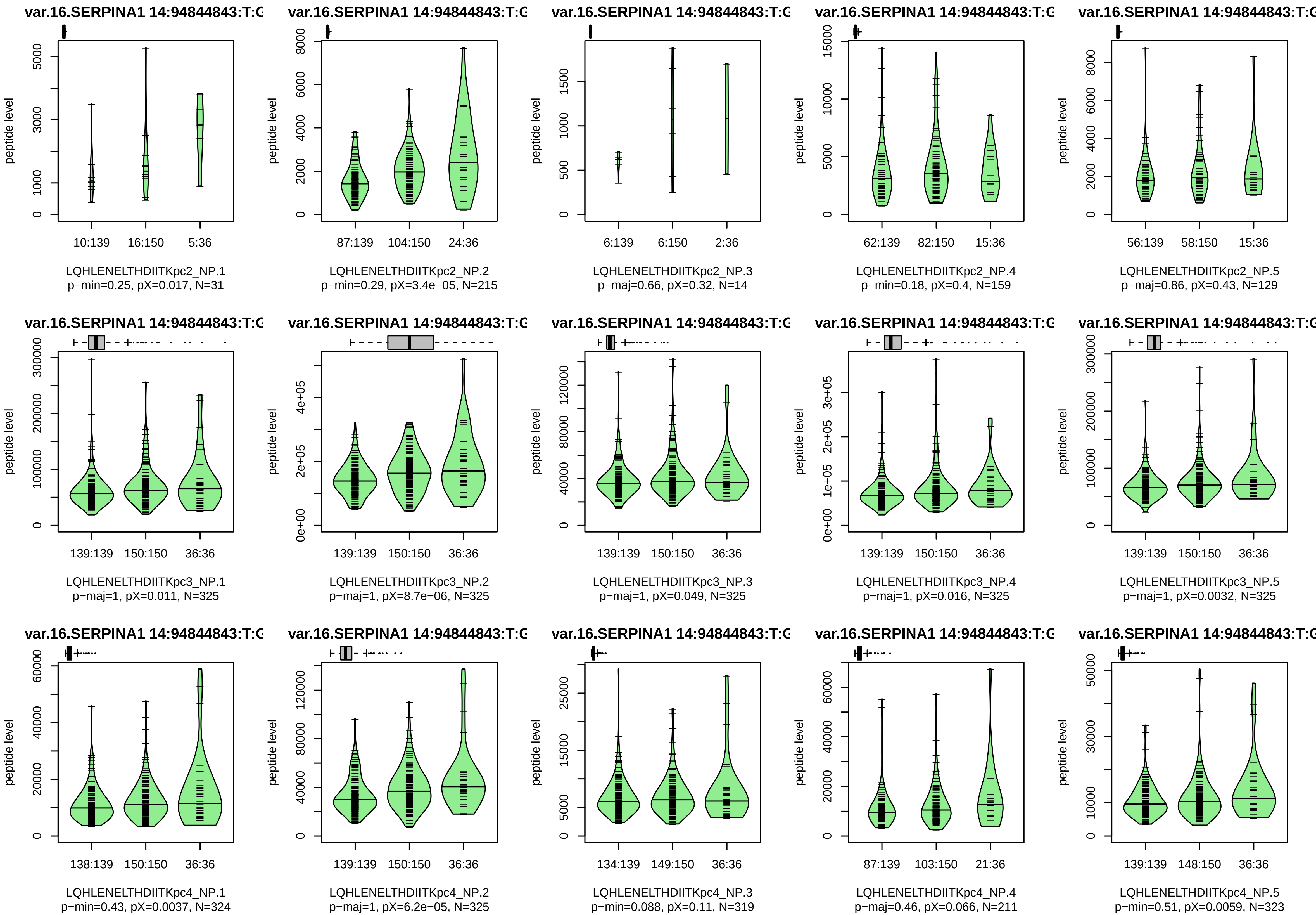

var.16.SERPINA1 14:94844843:T:G
peptide level
10:139
16:150
5:36
LQHLENELTHDIITKpc2_NP.1
p−min=0.25, pX=0.017, N=31
87:139
104:150
24:36
LQHLENELTHDIITKpc2_NP.2
p−min=0.29, pX=3.4e−05, N=215
6:139
6:150
2:36
LQHLENELTHDIITKpc2_NP.3
p−maj=0.66, pX=0.32, N=14
62:139
82:150
15:36
LQHLENELTHDIITKpc2_NP.4
p−min=0.18, pX=0.4, N=159
56:139
58:150
15:36
LQHLENELTHDIITKpc2_NP.5
p−maj=0.86, pX=0.43, N=129
139:139
150:150
36:36
LQHLENELTHDIITKpc3_NP.1
p−maj=1, pX=0.011, N=325
LQHLENELTHDIITKpc3_NP.2
p−maj=1, pX=8.7e−06, N=325
LQHLENELTHDIITKpc3_NP.3
p−maj=1, pX=0.049, N=325
LQHLENELTHDIITKpc3_NP.4
p−maj=1, pX=0.016, N=325
LQHLENELTHDIITKpc3_NP.5
p−maj=1, pX=0.0032, N=325
138:139
LQHLENELTHDIITKpc4_NP.1
p−min=0.43, pX=0.0037, N=324
LQHLENELTHDIITKpc4_NP.2
p−maj=1, pX=6.2e−05, N=325
134:139
149:150
LQHLENELTHDIITKpc4_NP.3
p−min=0.088, pX=0.11, N=319
87:139
103:150
21:36
LQHLENELTHDIITKpc4_NP.4
p−maj=0.46, pX=0.066, N=211
148:150
LQHLENELTHDIITKpc4_NP.5
p−min=0.51, pX=0.0059, N=323

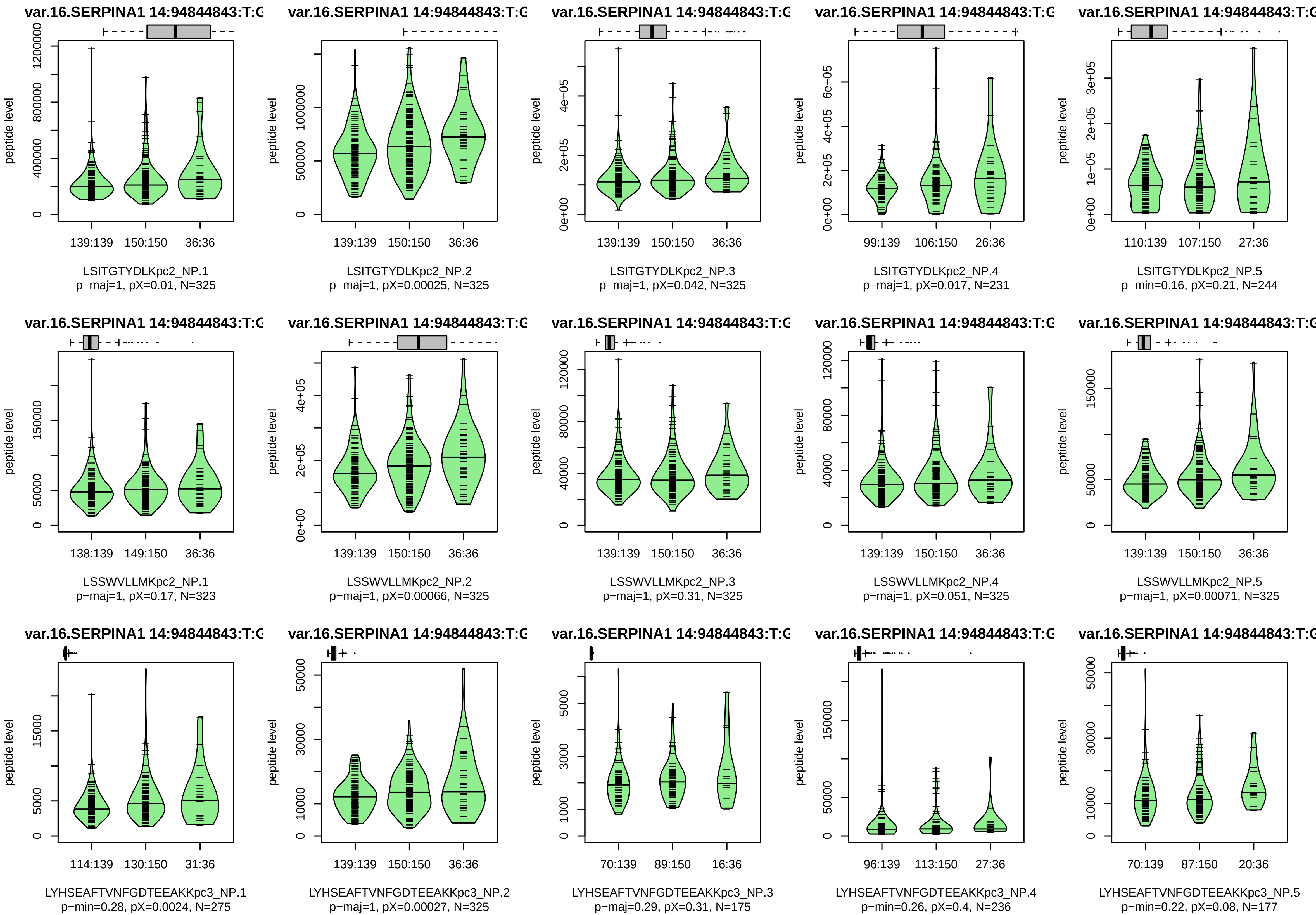
var.16.SERPINA1 14:94844843:T:G
peptide level
139:139 150:150 36:36
LSITGTYDLKpc2_NP.1
p−maj=1, pX=0.01, N=325
var.16.SERPINA1 14:94844843:T:G
139:139 150:150 36:36
LSITGTYDLKpc2_NP.2
p−maj=1, pX=0.00025, N=325
var.16.SERPINA1 14:94844843:T:G
139:139 150:150 36:36
LSITGTYDLKpc2_NP.3
p−maj=1, pX=0.042, N=325
var.16.SERPINA1 14:94844843:T:G
99:139 106:150 26:36
LSITGTYDLKpc2_NP.4
p−maj=1, pX=0.017, N=231
var.16.SERPINA1 14:94844843:T:G
110:139 107:150 27:36
LSITGTYDLKpc2_NP.5
p−min=0.16, pX=0.21, N=244
var.16.SERPINA1 14:94844843:T:G
138:139 149:150 36:36
LSSWVLLMKpc2_NP.1
p−maj=1, pX=0.17, N=323
var.16.SERPINA1 14:94844843:T:G
139:139 150:150 36:36
LSSWVLLMKpc2_NP.2
p−maj=1, pX=0.00066, N=325
var.16.SERPINA1 14:94844843:T:G
139:139 150:150 36:36
LSSWVLLMKpc2_NP.3
p−maj=1, pX=0.31, N=325
var.16.SERPINA1 14:94844843:T:G
139:139 150:150 36:36
LSSWVLLMKpc2_NP.4
p−maj=1, pX=0.051, N=325
var.16.SERPINA1 14:94844843:T:G
139:139 150:150 36:36
LSSWVLLMKpc2_NP.5
p−maj=1, pX=0.00071, N=325
var.16.SERPINA1 14:94844843:T:G
114:139 130:150 31:36
LYHSEAFTVNFGDTEEAKKpc3_NP.1
p−min=0.28, pX=0.0024, N=275
var.16.SERPINA1 14:94844843:T:G
139:139 150:150 36:36
LYHSEAFTVNFGDTEEAKKpc3_NP.2
p−maj=1, pX=0.00027, N=325
var.16.SERPINA1 14:94844843:T:G
70:139 89:150 16:36
LYHSEAFTVNFGDTEEAKKpc3_NP.3
p−maj=0.29, pX=0.31, N=175
var.16.SERPINA1 14:94844843:T:G
96:139 113:150 27:36
LYHSEAFTVNFGDTEEAKKpc3_NP.4
p−min=0.26, pX=0.4, N=236
var.16.SERPINA1 14:94844843:T:G
70:139 87:150 20:36
LYHSEAFTVNFGDTEEAKKpc3_NP.5
p−min=0.22, pX=0.08, N=177

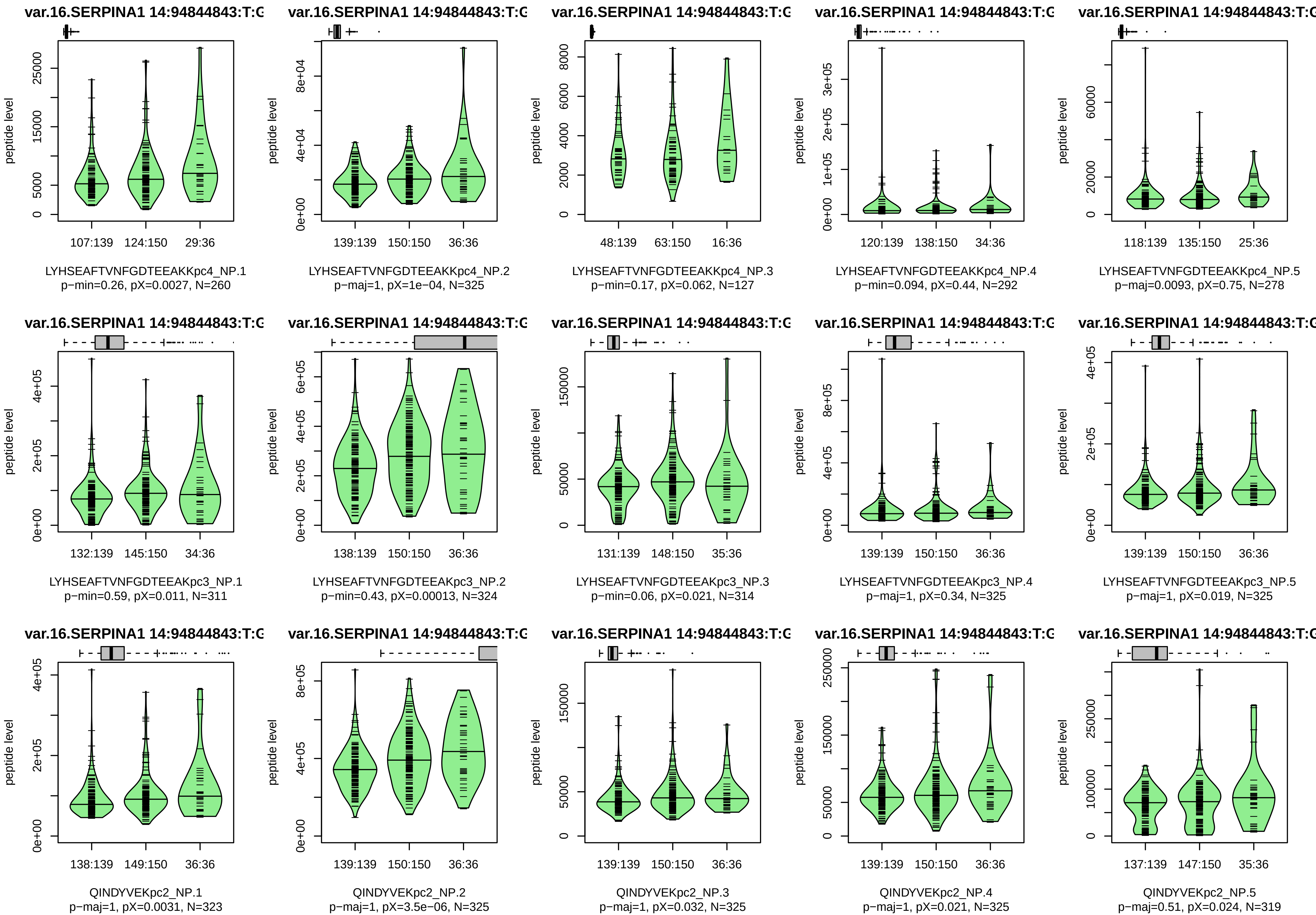
var.16.SERPINA1 14:94844843:T:G
peptide level
107:139
124:150
29:36
LYHSEAFTVNFGDTEEAKKpc4_NP.1
p−min=0.26, pX=0.0027, N=260
var.16.SERPINA1 14:94844843:T:G
peptide level
139:139
150:150
36:36
LYHSEAFTVNFGDTEEAKKpc4_NP.2
p−maj=1, pX=1e−04, N=325
var.16.SERPINA1 14:94844843:T:G
peptide level
48:139
63:150
16:36
LYHSEAFTVNFGDTEEAKKpc4_NP.3
p−min=0.17, pX=0.062, N=127
var.16.SERPINA1 14:94844843:T:G
peptide level
120:139
138:150
34:36
LYHSEAFTVNFGDTEEAKKpc4_NP.4
p−min=0.094, pX=0.44, N=292
var.16.SERPINA1 14:94844843:T:G
peptide level
118:139
135:150
25:36
LYHSEAFTVNFGDTEEAKKpc4_NP.5
p−maj=0.0093, pX=0.75, N=278
var.16.SERPINA1 14:94844843:T:G
peptide level
132:139
145:150
34:36
LYHSEAFTVNFGDTEEAKpc3_NP.1
p−min=0.59, pX=0.011, N=311
var.16.SERPINA1 14:94844843:T:G
peptide level
138:139
150:150
36:36
LYHSEAFTVNFGDTEEAKpc3_NP.2
p−min=0.43, pX=0.00013, N=324
var.16.SERPINA1 14:94844843:T:G
peptide level
131:139
148:150
35:36
LYHSEAFTVNFGDTEEAKpc3_NP.3
p−min=0.06, pX=0.021, N=314
var.16.SERPINA1 14:94844843:T:G
peptide level
139:139
150:150
36:36
LYHSEAFTVNFGDTEEAKpc3_NP.4
p−maj=1, pX=0.34, N=325
var.16.SERPINA1 14:94844843:T:G
peptide level
139:139
150:150
36:36
LYHSEAFTVNFGDTEEAKpc3_NP.5
p−maj=1, pX=0.019, N=325
var.16.SERPINA1 14:94844843:T:G
peptide level
138:139
149:150
36:36
QINDYVEKpc2_NP.1
p−maj=1, pX=0.0031, N=323
var.16.SERPINA1 14:94844843:T:G
peptide level
139:139
150:150
36:36
QINDYVEKpc2_NP.2
p−maj=1, pX=3.5e−06, N=325
var.16.SERPINA1 14:94844843:T:G
peptide level
139:139
150:150
36:36
QINDYVEKpc2_NP.3
p−maj=1, pX=0.032, N=325
var.16.SERPINA1 14:94844843:T:G
peptide level
139:139
150:150
36:36
QINDYVEKpc2_NP.4
p−maj=1, pX=0.021, N=325
var.16.SERPINA1 14:94844843:T:G
peptide level
137:139
147:150
35:36
QINDYVEKpc2_NP.5
p−maj=0.51, pX=0.024, N=319

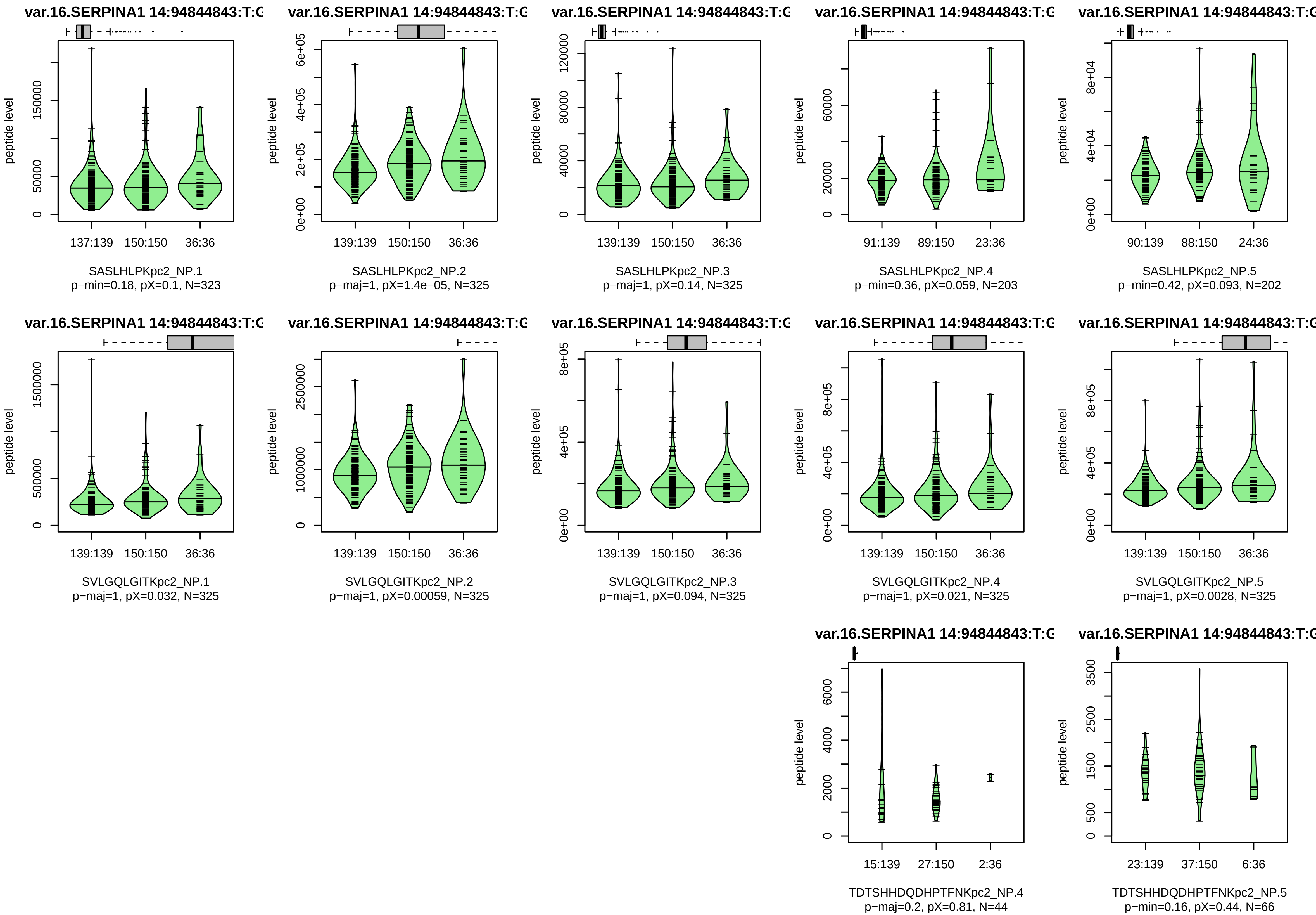
var.16.SERPINA1 14:94844843:T:G
peptide level
137:139 150:150 36:36
SASLHLPKpc2_NP.1
p−min=0.18, pX=0.1, N=323
var.16.SERPINA1 14:94844843:T:G
peptide level
139:139 150:150 36:36
SASLHLPKpc2_NP.2
p−maj=1, pX=1.4e−05, N=325
var.16.SERPINA1 14:94844843:T:G
peptide level
139:139 150:150 36:36
SASLHLPKpc2_NP.3
p−maj=1, pX=0.14, N=325
var.16.SERPINA1 14:94844843:T:G
peptide level
91:139 89:150 23:36
SASLHLPKpc2_NP.4
p−min=0.36, pX=0.059, N=203
var.16.SERPINA1 14:94844843:T:G
peptide level
90:139 88:150 24:36
SASLHLPKpc2_NP.5
p−min=0.42, pX=0.093, N=202
var.16.SERPINA1 14:94844843:T:G
peptide level
139:139 150:150 36:36
SVLGQLGITKpc2_NP.1
p−maj=1, pX=0.032, N=325
var.16.SERPINA1 14:94844843:T:G
peptide level
139:139 150:150 36:36
SVLGQLGITKpc2_NP.2
p−maj=1, pX=0.00059, N=325
var.16.SERPINA1 14:94844843:T:G
peptide level
139:139 150:150 36:36
SVLGQLGITKpc2_NP.3
p−maj=1, pX=0.094, N=325
var.16.SERPINA1 14:94844843:T:G
peptide level
139:139 150:150 36:36
SVLGQLGITKpc2_NP.4
p−maj=1, pX=0.021, N=325
var.16.SERPINA1 14:94844843:T:G
peptide level
139:139 150:150 36:36
SVLGQLGITKpc2_NP.5
p−maj=1, pX=0.0028, N=325
var.16.SERPINA1 14:94844843:T:G
peptide level
15:139 27:150 2:36
TDTSHHDQDHPTFNKpc2_NP.4
p−maj=0.2, pX=0.81, N=44
var.16.SERPINA1 14:94844843:T:G
peptide level
23:139 37:150 6:36
TDTSHHDQDHPTFNKpc2_NP.5
p−min=0.16, pX=0.44, N=66

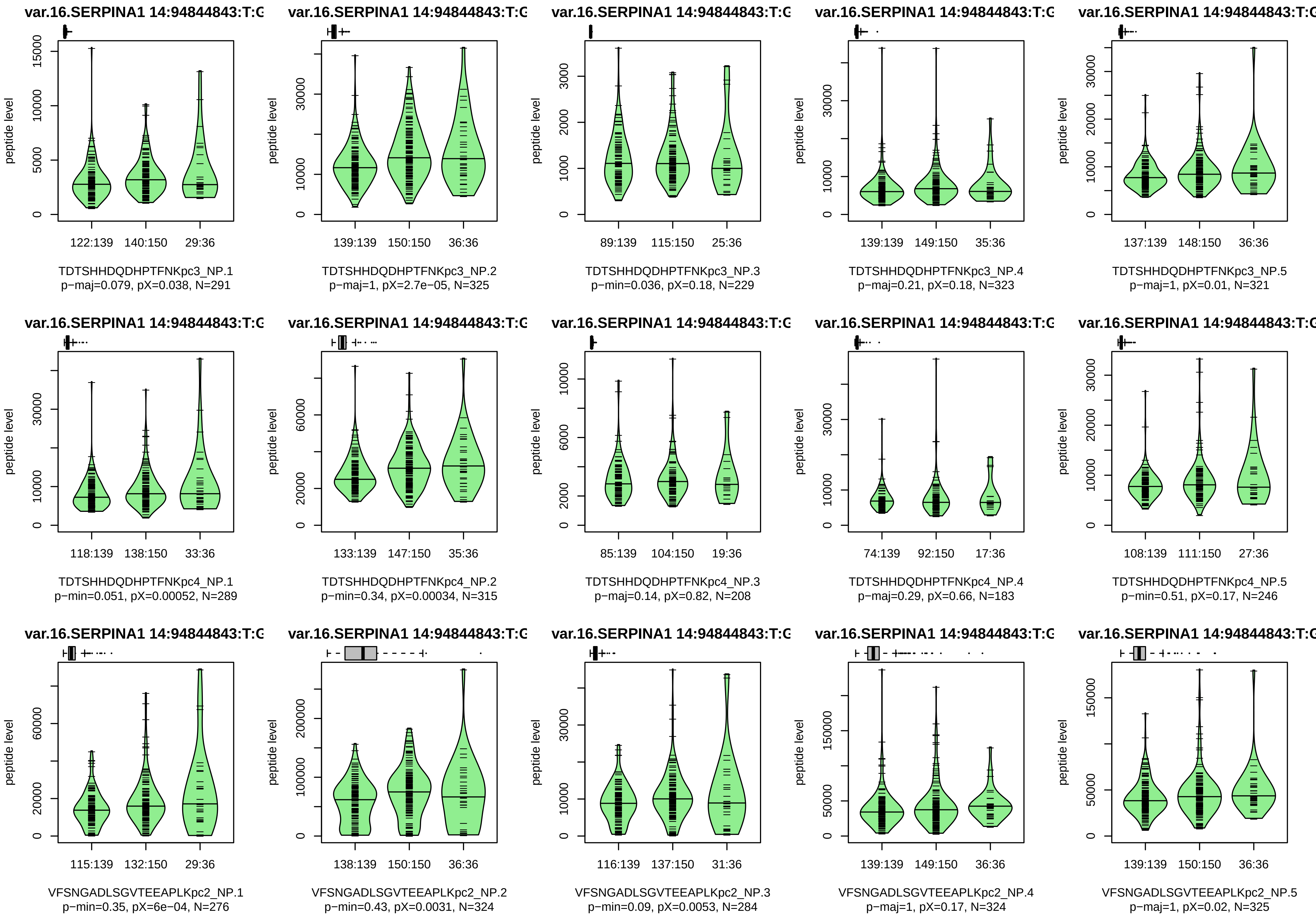

var.16.SERPINA1 14:94844843:T:
peptide level
122:139 140:150 29:36
TDTSHHDQDHPTFNKpc3_NP.1
p−maj=0.079, pX=0.038, N=291
var.16.SERPINA1 14:94844843:T:
peptide level
139:139 150:150 36:36
TDTSHHDQDHPTFNKpc3_NP.2
p−maj=1, pX=2.7e−05, N=325
var.16.SERPINA1 14:94844843:T:
peptide level
89:139 115:150 25:36
TDTSHHDQDHPTFNKpc3_NP.3
p−min=0.036, pX=0.18, N=229
var.16.SERPINA1 14:94844843:T:
peptide level
139:139 149:150 35:36
TDTSHHDQDHPTFNKpc3_NP.4
p−maj=0.21, pX=0.18, N=323
var.16.SERPINA1 14:94844843:T:
peptide level
137:139 148:150 36:36
TDTSHHDQDHPTFNKpc3_NP.5
p−maj=1, pX=0.01, N=321
var.16.SERPINA1 14:94844843:T:
peptide level
118:139 138:150 33:36
TDTSHHDQDHPTFNKpc4_NP.1
p−min=0.051, pX=0.00052, N=289
var.16.SERPINA1 14:94844843:T:
peptide level
133:139 147:150 35:36
TDTSHHDQDHPTFNKpc4_NP.2
p−min=0.34, pX=0.00034, N=315
var.16.SERPINA1 14:94844843:T:
peptide level
85:139 104:150 19:36
TDTSHHDQDHPTFNKpc4_NP.3
p−maj=0.14, pX=0.82, N=208
var.16.SERPINA1 14:94844843:T:
peptide level
74:139 92:150 17:36
TDTSHHDQDHPTFNKpc4_NP.4
p−maj=0.29, pX=0.66, N=183
var.16.SERPINA1 14:94844843:T:
peptide level
108:139 111:150 27:36
TDTSHHDQDHPTFNKpc4_NP.5
p−min=0.51, pX=0.17, N=246
var.16.SERPINA1 14:94844843:T:
peptide level
115:139 132:150 29:36
VFSNGADLSGVTEEAPLKpc2_NP.1
p−min=0.35, pX=6e−04, N=276
var.16.SERPINA1 14:94844843:T:
peptide level
138:139 150:150 36:36
VFSNGADLSGVTEEAPLKpc2_NP.2
p−min=0.43, pX=0.0031, N=324
var.16.SERPINA1 14:94844843:T:
peptide level
116:139 137:150 31:36
VFSNGADLSGVTEEAPLKpc2_NP.3
p−min=0.09, pX=0.0053, N=284
var.16.SERPINA1 14:94844843:T:
peptide level
139:139 149:150 36:36
VFSNGADLSGVTEEAPLKpc2_NP.4
p−maj=1, pX=0.17, N=324
var.16.SERPINA1 14:94844843:T:
peptide level
139:139 150:150 36:36
VFSNGADLSGVTEEAPLKpc2_NP.5
p−maj=1, pX=0.02, N=325

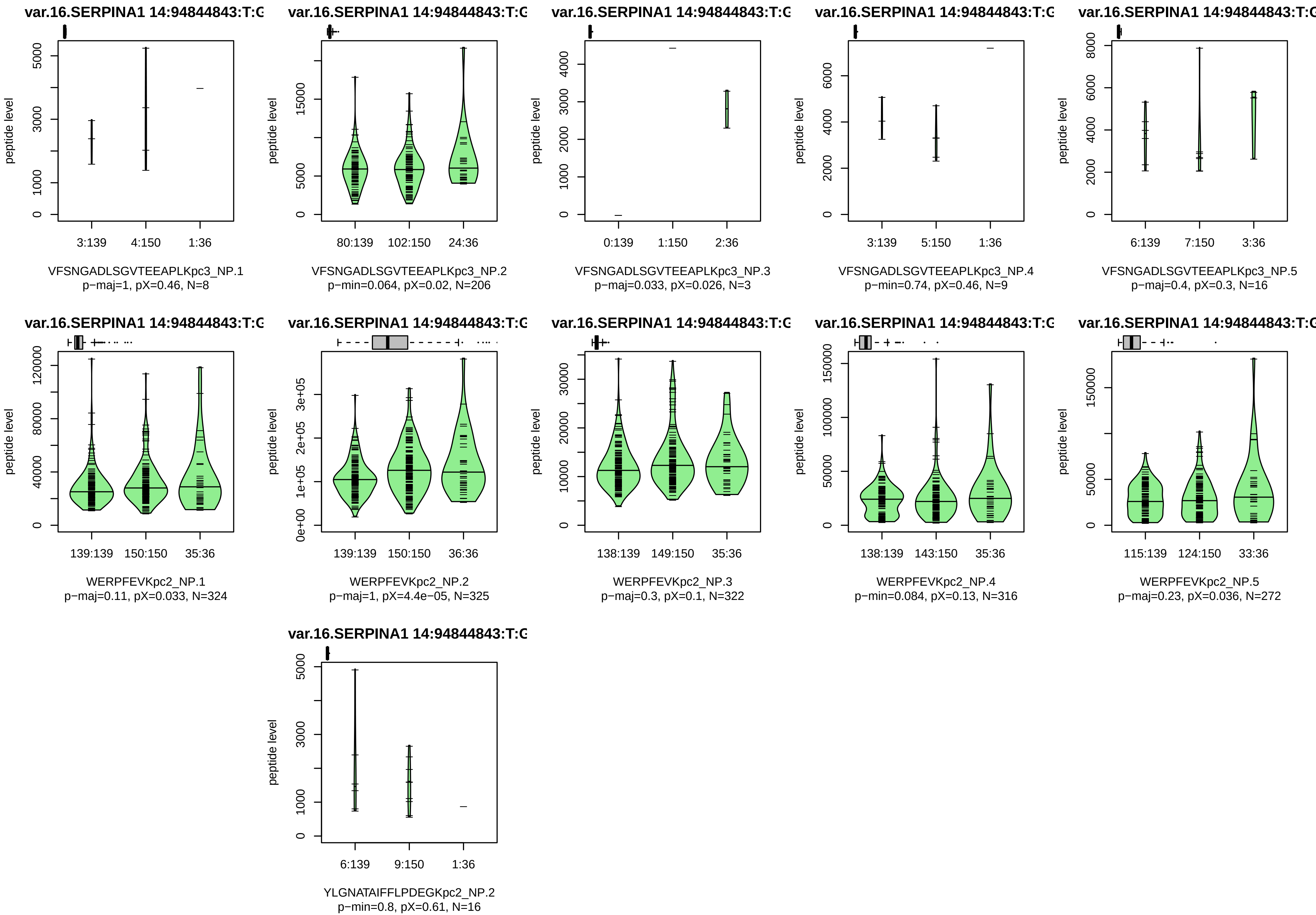
var.16.SERPINA1 14:94844843:T:G
peptide level
3:139
4:150
1:36
VFSNGADLSGVTEEAPLKpc3_NP.1
p−maj=1, pX=0.46, N=8
var.16.SERPINA1 14:94844843:T:G
80:139
102:150
24:36
VFSNGADLSGVTEEAPLKpc3_NP.2
p−min=0.064, pX=0.02, N=206
var.16.SERPINA1 14:94844843:T:G
0:139
1:150
2:36
VFSNGADLSGVTEEAPLKpc3_NP.3
p−maj=0.033, pX=0.026, N=3
var.16.SERPINA1 14:94844843:T:G
3:139
5:150
1:36
VFSNGADLSGVTEEAPLKpc3_NP.4
p−min=0.74, pX=0.46, N=9
var.16.SERPINA1 14:94844843:T:G
6:139
7:150
3:36
VFSNGADLSGVTEEAPLKpc3_NP.5
p−maj=0.4, pX=0.3, N=16
var.16.SERPINA1 14:94844843:T:G
139:139
150:150
35:36
WERPFEVKpc2_NP.1
p−maj=0.11, pX=0.033, N=324
var.16.SERPINA1 14:94844843:T:G
139:139
150:150
36:36
WERPFEVKpc2_NP.2
p−maj=1, pX=4.4e−05, N=325
var.16.SERPINA1 14:94844843:T:G
138:139
149:150
35:36
WERPFEVKpc2_NP.3
p−maj=0.3, pX=0.1, N=322
var.16.SERPINA1 14:94844843:T:G
138:139
143:150
35:36
WERPFEVKpc2_NP.4
p−min=0.084, pX=0.13, N=316
var.16.SERPINA1 14:94844843:T:G
115:139
124:150
33:36
WERPFEVKpc2_NP.5
p−maj=0.23, pX=0.036, N=272
var.16.SERPINA1 14:94844843:T:G
6:139
9:150
1:36
YLGNATAIFFLPDEGKpc2_NP.2
p−min=0.8, pX=0.61, N=16

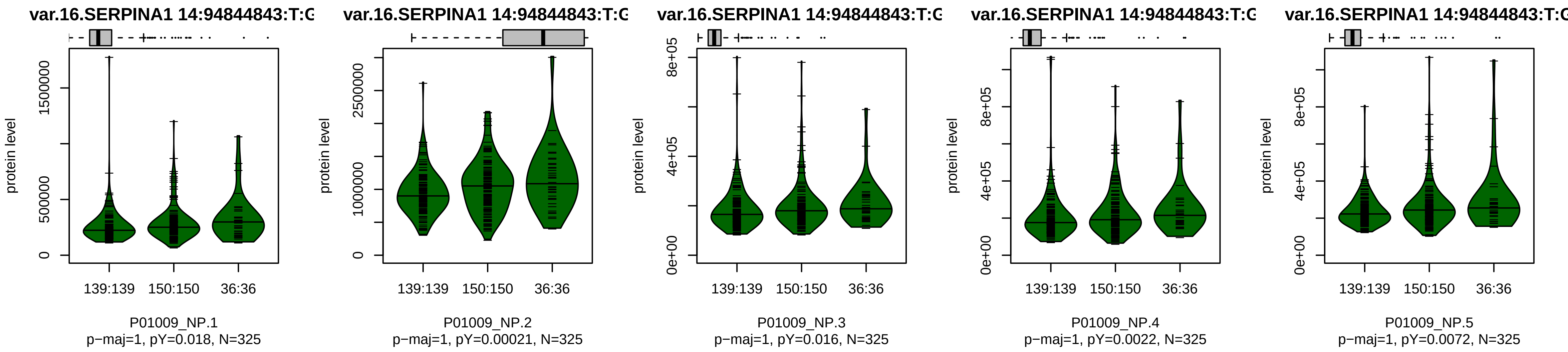

var.16.SERPINA1 14:94844843:T:G
protein level
0
500000
1500000
139:139
150:150
36:36
P01009_NP.1
p−maj=1, pY=0.018, N=325
var.16.SERPINA1 14:94844843:T:G
protein level
0
1000000
2500000
139:139
150:150
36:36
P01009_NP.2
p−maj=1, pY=0.00021, N=325
var.16.SERPINA1 14:94844843:T:G
protein level
0e+00
4e+05
8e+05
139:139
150:150
36:36
P01009_NP.3
p−maj=1, pY=0.016, N=325
var.16.SERPINA1 14:94844843:T:G
protein level
0e+00
4e+05
8e+05
139:139
150:150
36:36
P01009_NP.4
p−maj=1, pY=0.0022, N=325
var.16.SERPINA1 14:94844843:T:G
protein level
0e+00
4e+05
8e+05
139:139
150:150
36:36
P01009_NP.5
p−maj=1, pY=0.0072, N=325

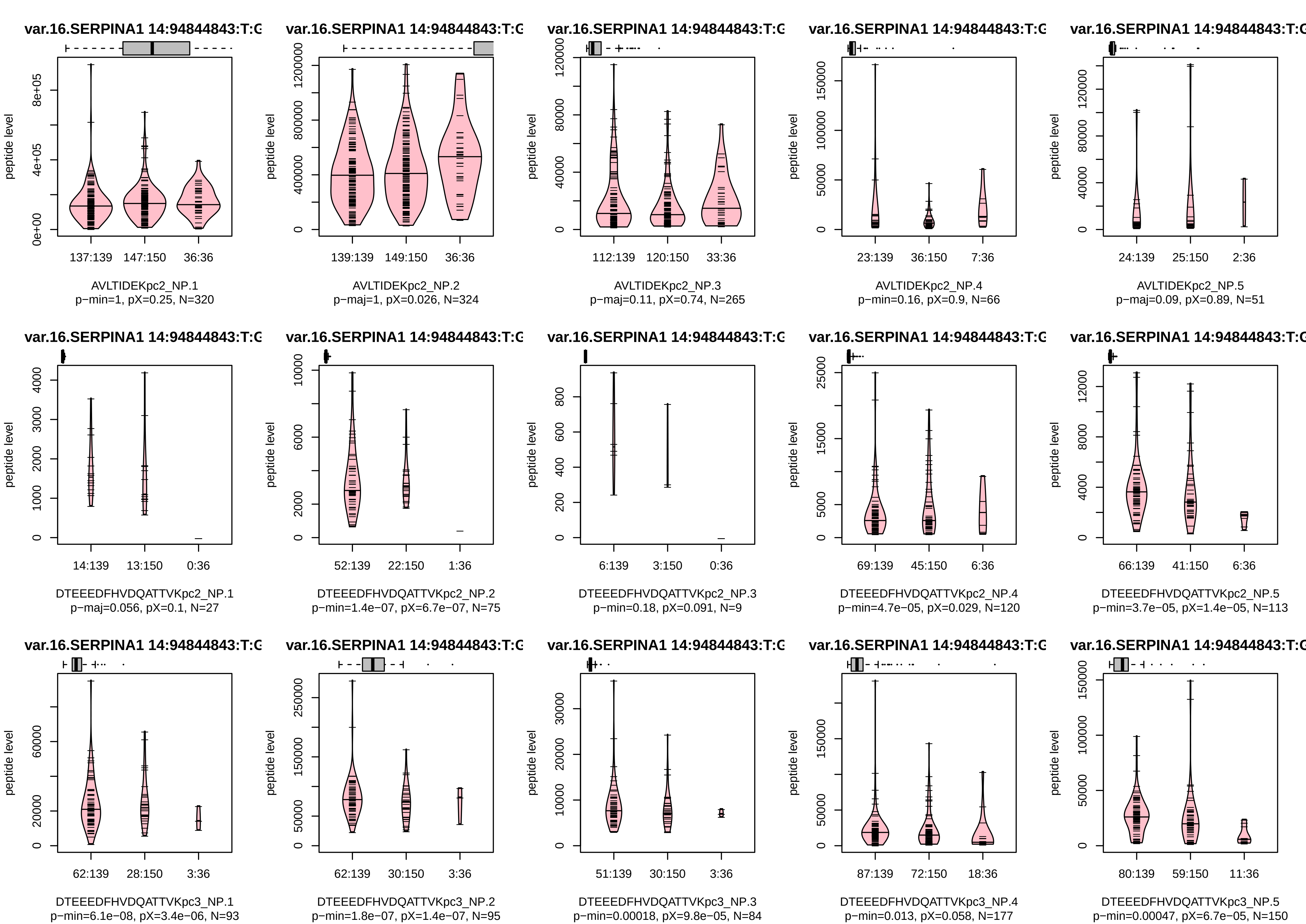
var.16.SERPINA1 14:94844843:T:G
peptide level
137:139
147:150
36:36
AVLTIDEKpc2_NP.1
p−min=1, pX=0.25, N=320
var.16.SERPINA1 14:94844843:T:G
peptide level
139:139
149:150
36:36
AVLTIDEKpc2_NP.2
p−maj=1, pX=0.026, N=324
var.16.SERPINA1 14:94844843:T:G
peptide level
112:139
120:150
33:36
AVLTIDEKpc2_NP.3
p−maj=0.11, pX=0.74, N=265
var.16.SERPINA1 14:94844843:T:G
peptide level
23:139
36:150
7:36
AVLTIDEKpc2_NP.4
p−min=0.16, pX=0.9, N=66
var.16.SERPINA1 14:94844843:T:G
peptide level
24:139
25:150
2:36
AVLTIDEKpc2_NP.5
p−maj=0.09, pX=0.89, N=51
var.16.SERPINA1 14:94844843:T:G
peptide level
14:139
13:150
0:36
DTEEEDFHVDQATTVKpc2_NP.1
p−maj=0.056, pX=0.1, N=27
var.16.SERPINA1 14:94844843:T:G
peptide level
52:139
22:150
1:36
DTEEEDFHVDQATTVKpc2_NP.2
p−min=1.4e−07, pX=6.7e−07, N=75
var.16.SERPINA1 14:94844843:T:G
peptide level
6:139
3:150
0:36
DTEEEDFHVDQATTVKpc2_NP.3
p−min=0.18, pX=0.091, N=9
var.16.SERPINA1 14:94844843:T:G
peptide level
69:139
45:150
6:36
DTEEEDFHVDQATTVKpc2_NP.4
p−min=4.7e−05, pX=0.029, N=120
var.16.SERPINA1 14:94844843:T:G
peptide level
66:139
41:150
6:36
DTEEEDFHVDQATTVKpc2_NP.5
p−min=3.7e−05, pX=1.4e−05, N=113
var.16.SERPINA1 14:94844843:T:G
peptide level
62:139
28:150
3:36
DTEEEDFHVDQATTVKpc3_NP.1
p−min=6.1e−08, pX=3.4e−06, N=93
var.16.SERPINA1 14:94844843:T:G
peptide level
62:139
30:150
3:36
DTEEEDFHVDQATTVKpc3_NP.2
p−min=1.8e−07, pX=1.4e−07, N=95
var.16.SERPINA1 14:94844843:T:G
peptide level
51:139
30:150
3:36
DTEEEDFHVDQATTVKpc3_NP.3
p−min=0.00018, pX=9.8e−05, N=84
var.16.SERPINA1 14:94844843:T:G
peptide level
87:139
72:150
18:36
DTEEEDFHVDQATTVKpc3_NP.4
p−min=0.013, pX=0.058, N=177
var.16.SERPINA1 14:94844843:T:G
peptide level
80:139
59:150
11:36
DTEEEDFHVDQATTVKpc3_NP.5
p−min=0.00047, pX=6.7e−05, N=150

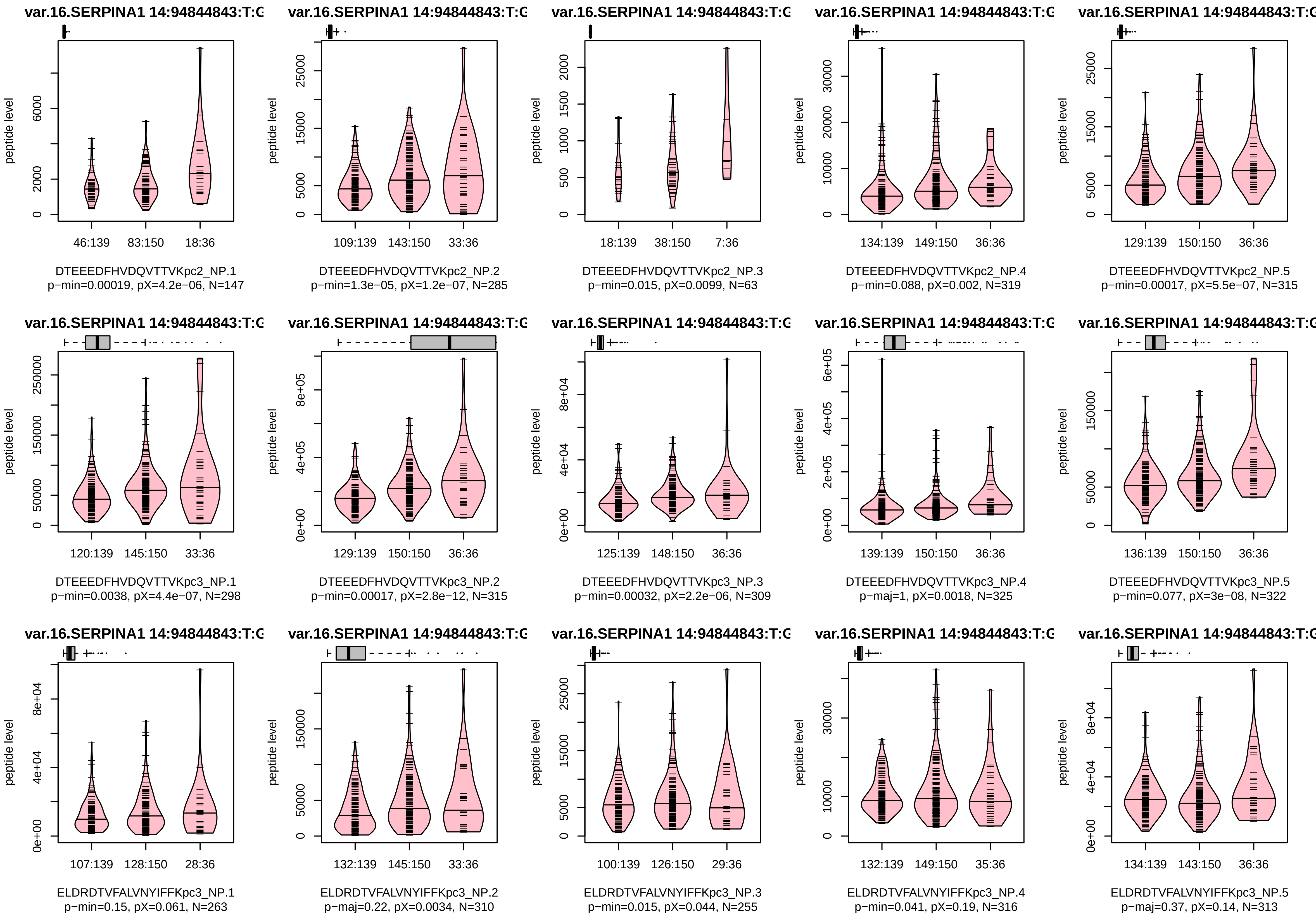
var.16.SERPINA1 14:94844843:T:G
peptide level
46:139 83:150 18:36
DTEEEDFHVDQVTTVKpc2_NP.1
p−min=0.00019, pX=4.2e−06, N=147
var.16.SERPINA1 14:94844843:T:G
peptide level
109:139 143:150 33:36
DTEEEDFHVDQVTTVKpc2_NP.2
p−min=1.3e−05, pX=1.2e−07, N=285
var.16.SERPINA1 14:94844843:T:G
peptide level
18:139 38:150 7:36
DTEEEDFHVDQVTTVKpc2_NP.3
p−min=0.015, pX=0.0099, N=63
var.16.SERPINA1 14:94844843:T:G
peptide level
134:139 149:150 36:36
DTEEEDFHVDQVTTVKpc2_NP.4
p−min=0.088, pX=0.002, N=319
var.16.SERPINA1 14:94844843:T:G
peptide level
129:139 150:150 36:36
DTEEEDFHVDQVTTVKpc2_NP.5
p−min=0.00017, pX=5.5e−07, N=315
var.16.SERPINA1 14:94844843:T:G
peptide level
120:139 145:150 33:36
DTEEEDFHVDQVTTVKpc3_NP.1
p−min=0.0038, pX=4.4e−07, N=298
var.16.SERPINA1 14:94844843:T:G
peptide level
129:139 150:150 36:36
DTEEEDFHVDQVTTVKpc3_NP.2
p−min=0.00017, pX=2.8e−12, N=315
var.16.SERPINA1 14:94844843:T:G
peptide level
125:139 148:150 36:36
DTEEEDFHVDQVTTVKpc3_NP.3
p−min=0.00032, pX=2.2e−06, N=309
var.16.SERPINA1 14:94844843:T:G
peptide level
139:139 150:150 36:36
DTEEEDFHVDQVTTVKpc3_NP.4
p−maj=1, pX=0.0018, N=325
var.16.SERPINA1 14:94844843:T:G
peptide level
136:139 150:150 36:36
DTEEEDFHVDQVTTVKpc3_NP.5
p−min=0.077, pX=3e−08, N=322
var.16.SERPINA1 14:94844843:T:G
peptide level
107:139 128:150 28:36
ELDRDTVFALVNYIFFKpc3_NP.1
p−min=0.15, pX=0.061, N=263
var.16.SERPINA1 14:94844843:T:G
peptide level
132:139 145:150 33:36
ELDRDTVFALVNYIFFKpc3_NP.2
p−maj=0.22, pX=0.0034, N=310
var.16.SERPINA1 14:94844843:T:G
peptide level
100:139 126:150 29:36
ELDRDTVFALVNYIFFKpc3_NP.3
p−min=0.015, pX=0.044, N=255
var.16.SERPINA1 14:94844843:T:G
peptide level
132:139 149:150 35:36
ELDRDTVFALVNYIFFKpc3_NP.4
p−min=0.041, pX=0.19, N=316
var.16.SERPINA1 14:94844843:T:G
peptide level
134:139 143:150 36:36
ELDRDTVFALVNYIFFKpc3_NP.5
p−maj=0.37, pX=0.14, N=313

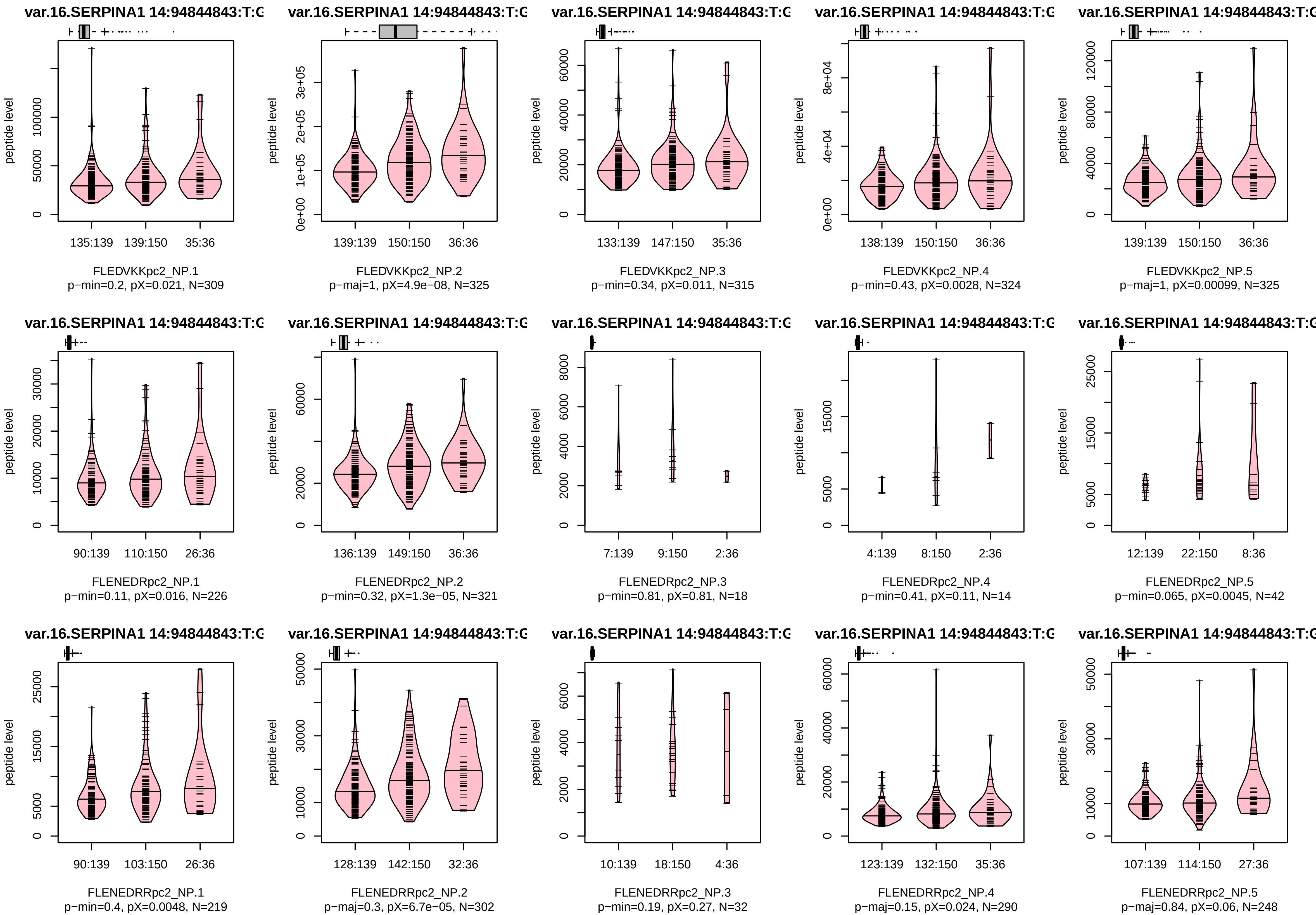
var.16.SERPINA1 14:94844843:T:G
peptide level
135:139
139:150
35:36
FLEDVKKpc2_NP.1
p−min=0.2, pX=0.021, N=309
139:139
150:150
36:36
FLEDVKKpc2_NP.2
p−maj=1, pX=4.9e−08, N=325
133:139
147:150
35:36
FLEDVKKpc2_NP.3
p−min=0.34, pX=0.011, N=315
138:139
150:150
36:36
FLEDVKKpc2_NP.4
p−min=0.43, pX=0.0028, N=324
139:139
150:150
36:36
FLEDVKKpc2_NP.5
p−maj=1, pX=0.00099, N=325
90:139
110:150
26:36
FLENEDRpc2_NP.1
p−min=0.11, pX=0.016, N=226
136:139
149:150
36:36
FLENEDRpc2_NP.2
p−min=0.32, pX=1.3e−05, N=321
7:139
9:150
2:36
FLENEDRpc2_NP.3
p−min=0.81, pX=0.81, N=18
4:139
8:150
2:36
FLENEDRpc2_NP.4
p−min=0.41, pX=0.11, N=14
12:139
22:150
8:36
FLENEDRpc2_NP.5
p−min=0.065, pX=0.0045, N=42
90:139
103:150
26:36
FLENEDRRpc2_NP.1
p−min=0.4, pX=0.0048, N=219
128:139
142:150
32:36
FLENEDRRpc2_NP.2
p−maj=0.3, pX=6.7e−05, N=302
10:139
18:150
4:36
FLENEDRRpc2_NP.3
p−min=0.19, pX=0.27, N=32
123:139
132:150
35:36
FLENEDRRpc2_NP.4
p−maj=0.15, pX=0.024, N=290
107:139
114:150
27:36
FLENEDRRpc2_NP.5
p−maj=0.84, pX=0.06, N=248

var.16.SERPINA1 14:94844843:T:G

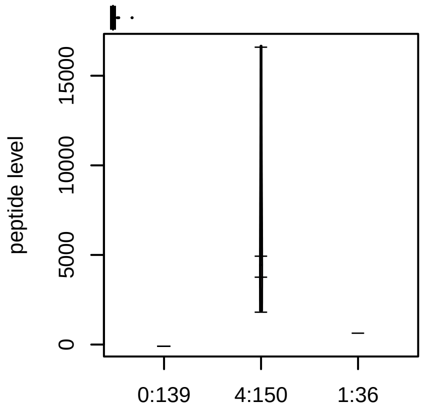

FNKPFVFLMIDQNTKpc3_NP.1
p−min=0.074, pX=0.42, N=5

var.16.SERPINA1 14:94844843:T:G

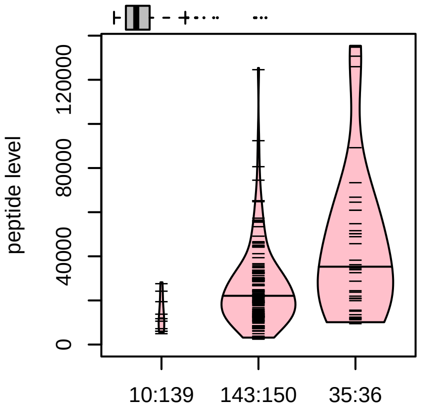

FNKPFVFLMIDQNTKpc3_NP.2
p−min=3e−67, pX=2.8e−40, N=188

var.16.SERPINA1 14:94844843:T:G

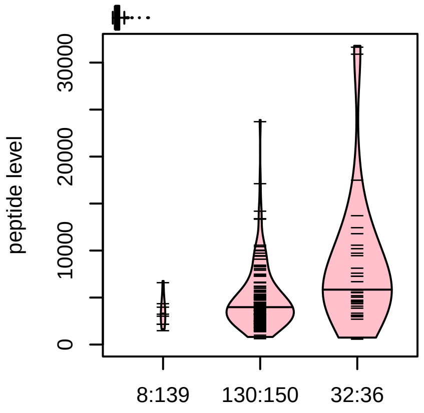

FNKPFVFLMIDQNTKpc3_NP.3
p−min=1.4e−54, pX=6.9e−32, N=170

var.16.SERPINA1 14:94844843:T:G

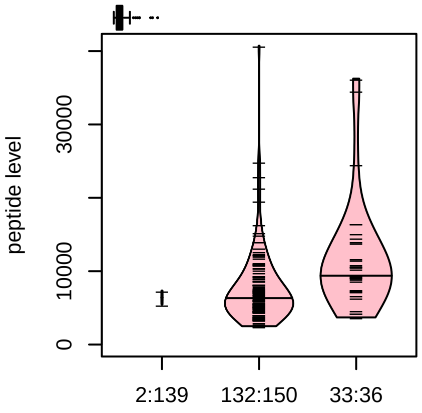

FNKPFVFLMIDQNTKpc3_NP.4
p−min=1e−65, pX=1.9e−40, N=167

var.16.SERPINA1 14:94844843:T:G

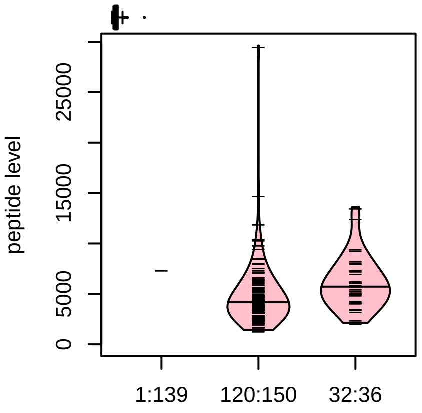

FNKPFVFLMIDQNTKpc3_NP.5
p−min=1.9e−57, pX=4.6e−34, N=153

var.16.SERPINA1 14:94844843:T:G

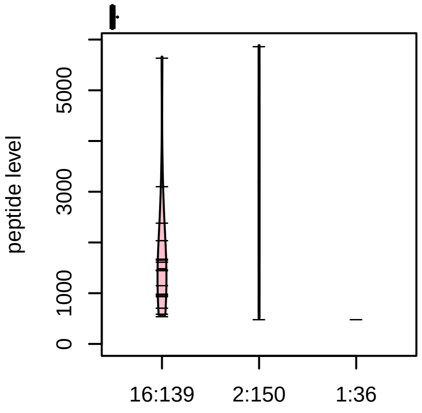

FNKPFVFLMIEQNTKpc2_NP.4
p−min=0.00019, pX=0.017, N=19

var.16.SERPINA1 14:94844843:T:G

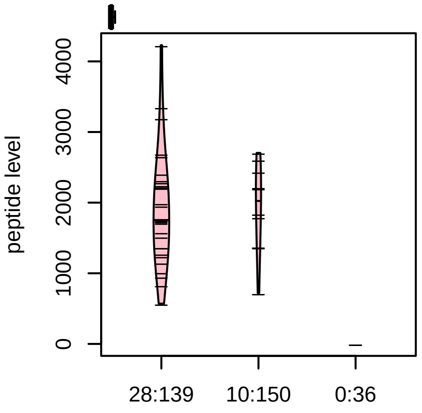

FNKPFVFLMIEQNTKpc2_NP.5
p−min=7.9e−05, pX=0.00012, N=38

var.16.SERPINA1 14:94844843:T:G

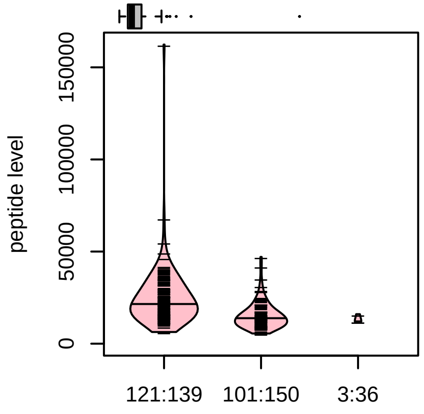

FNKPFVFLMIEQNTKpc3_NP.1
p−maj=5.9e−16, pX=8.6e−20, N=225

var.16.SERPINA1 14:94844843:T:G

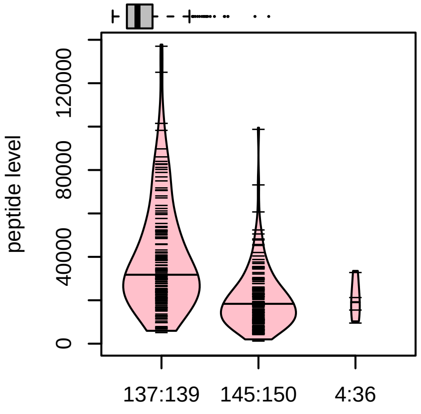

FNKPFVFLMIEQNTKpc3_NP.2
p−maj=4.4e−33, pX=7.2e−26, N=286

var.16.SERPINA1 14:94844843:T:G

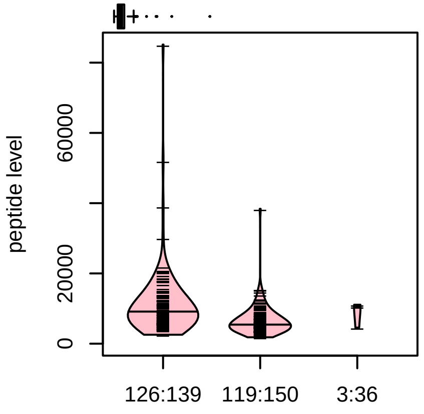

FNKPFVFLMIEQNTKpc3_NP.3
p−maj=1.7e−20, pX=1.8e−14, N=248

var.16.SERPINA1 14:94844843:T:G

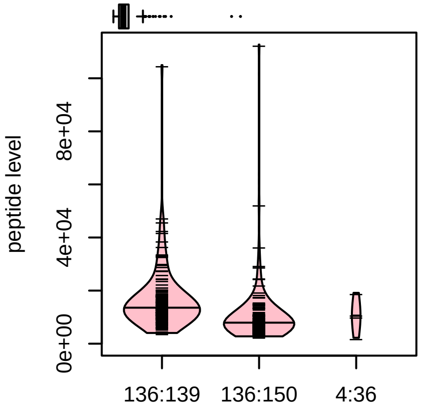

FNKPFVFLMIEQNTKpc3_NP.4
p−maj=1.6e−27, pX=1.7e−14, N=276

var.16.SERPINA1 14:94844843:T:G

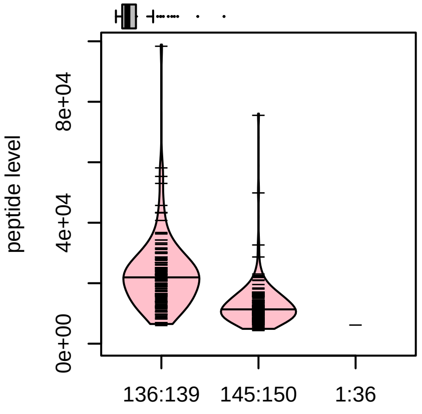

FNKPFVFLMIEQNTKpc3_NP.5
p−maj=4.3e−38, pX=6.3e−33, N=282

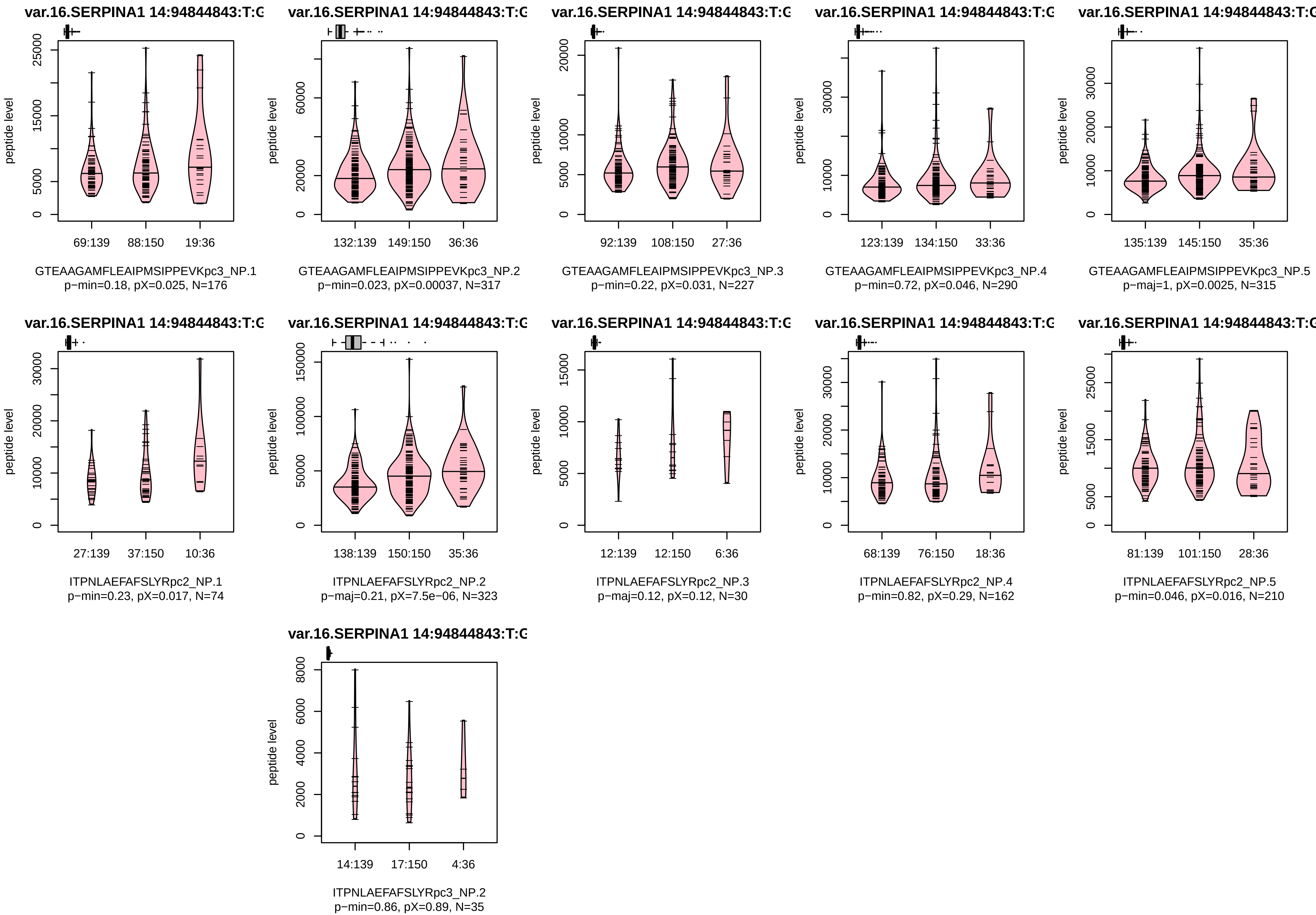
var.16.SERPINA1 14:94844843:T:G
peptide level
69:139 88:150 19:36
GTEAAGAMFLEAIPMSIPPEVKpc3_NP.1
p−min=0.18, pX=0.025, N=176
var.16.SERPINA1 14:94844843:T:G
peptide level
132:139 149:150 36:36
GTEAAGAMFLEAIPMSIPPEVKpc3_NP.2
p−min=0.023, pX=0.00037, N=317
var.16.SERPINA1 14:94844843:T:G
peptide level
92:139 108:150 27:36
GTEAAGAMFLEAIPMSIPPEVKpc3_NP.3
p−min=0.22, pX=0.031, N=227
var.16.SERPINA1 14:94844843:T:G
peptide level
123:139 134:150 33:36
GTEAAGAMFLEAIPMSIPPEVKpc3_NP.4
p−min=0.72, pX=0.046, N=290
var.16.SERPINA1 14:94844843:T:G
peptide level
135:139 145:150 35:36
GTEAAGAMFLEAIPMSIPPEVKpc3_NP.5
p−maj=1, pX=0.0025, N=315
var.16.SERPINA1 14:94844843:T:G
peptide level
27:139 37:150 10:36
ITPNLAEFAFSLYRpc2_NP.1
p−min=0.23, pX=0.017, N=74
var.16.SERPINA1 14:94844843:T:G
peptide level
138:139 150:150 35:36
ITPNLAEFAFSLYRpc2_NP.2
p−maj=0.21, pX=7.5e−06, N=323
var.16.SERPINA1 14:94844843:T:G
peptide level
12:139 12:150 6:36
ITPNLAEFAFSLYRpc2_NP.3
p−maj=0.12, pX=0.12, N=30
var.16.SERPINA1 14:94844843:T:G
peptide level
68:139 76:150 18:36
ITPNLAEFAFSLYRpc2_NP.4
p−min=0.82, pX=0.29, N=162
var.16.SERPINA1 14:94844843:T:G
peptide level
81:139 101:150 28:36
ITPNLAEFAFSLYRpc2_NP.5
p−min=0.046, pX=0.016, N=210
var.16.SERPINA1 14:94844843:T:G
peptide level
14:139 17:150 4:36
ITPNLAEFAFSLYRpc3_NP.2
p−min=0.86, pX=0.89, N=35

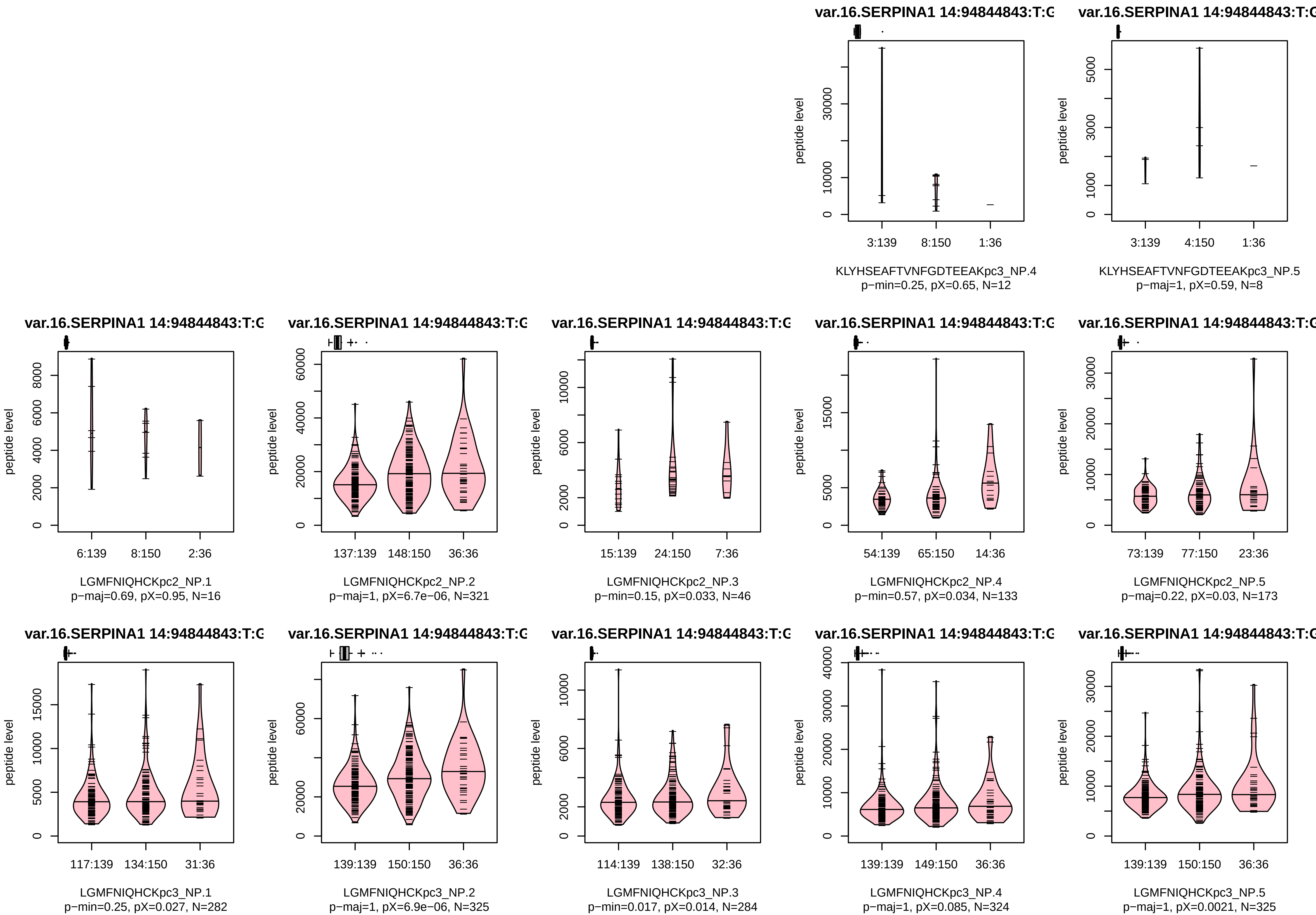
var.16.SERPINA1 14:94844843:T:G
peptide level
3:139
8:150
1:36
KLYHSEAFTVNFGDTEEAKpc3_NP.4
p−min=0.25, pX=0.65, N=12
var.16.SERPINA1 14:94844843:T:G
peptide level
3:139
4:150
1:36
KLYHSEAFTVNFGDTEEAKpc3_NP.5
p−maj=1, pX=0.59, N=8
var.16.SERPINA1 14:94844843:T:G
peptide level
6:139
8:150
2:36
LGMFNIQHCKpc2_NP.1
p−maj=0.69, pX=0.95, N=16
var.16.SERPINA1 14:94844843:T:G
peptide level
137:139
148:150
36:36
LGMFNIQHCKpc2_NP.2
p−maj=1, pX=6.7e−06, N=321
var.16.SERPINA1 14:94844843:T:G
peptide level
15:139
24:150
7:36
LGMFNIQHCKpc2_NP.3
p−min=0.15, pX=0.033, N=46
var.16.SERPINA1 14:94844843:T:G
peptide level
54:139
65:150
14:36
LGMFNIQHCKpc2_NP.4
p−min=0.57, pX=0.034, N=133
var.16.SERPINA1 14:94844843:T:G
peptide level
73:139
77:150
23:36
LGMFNIQHCKpc2_NP.5
p−maj=0.22, pX=0.03, N=173
var.16.SERPINA1 14:94844843:T:G
peptide level
117:139
134:150
31:36
LGMFNIQHCKpc3_NP.1
p−min=0.25, pX=0.027, N=282
var.16.SERPINA1 14:94844843:T:G
peptide level
139:139
150:150
36:36
LGMFNIQHCKpc3_NP.2
p−maj=1, pX=6.9e−06, N=325
var.16.SERPINA1 14:94844843:T:G
peptide level
114:139
138:150
32:36
LGMFNIQHCKpc3_NP.3
p−min=0.017, pX=0.014, N=284
var.16.SERPINA1 14:94844843:T:G
peptide level
139:139
149:150
36:36
LGMFNIQHCKpc3_NP.4
p−maj=1, pX=0.085, N=324
var.16.SERPINA1 14:94844843:T:G
peptide level
139:139
150:150
36:36
LGMFNIQHCKpc3_NP.5
p−maj=1, pX=0.0021, N=325

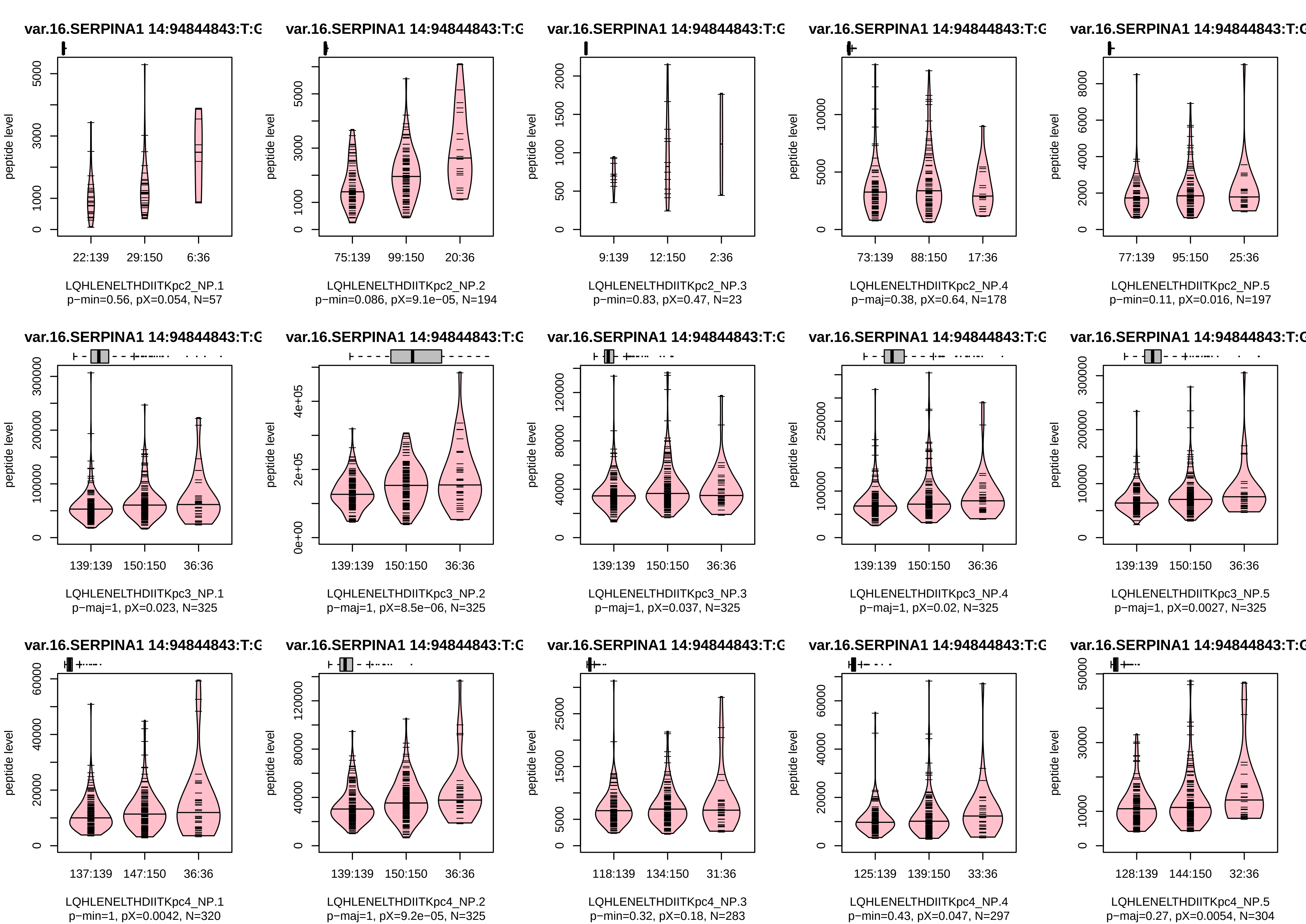

var.16.SERPINA1 14:94844843:T:G
peptide level
22:139
29:150
6:36
LQHLENELTHDIITKpc2_NP.1
p−min=0.56, pX=0.054, N=57
75:139
99:150
20:36
LQHLENELTHDIITKpc2_NP.2
p−min=0.086, pX=9.1e−05, N=194
9:139
12:150
2:36
LQHLENELTHDIITKpc2_NP.3
p−min=0.83, pX=0.47, N=23
73:139
88:150
17:36
LQHLENELTHDIITKpc2_NP.4
p−maj=0.38, pX=0.64, N=178
77:139
95:150
25:36
LQHLENELTHDIITKpc2_NP.5
p−min=0.11, pX=0.016, N=197
139:139
150:150
36:36
LQHLENELTHDIITKpc3_NP.1
p−maj=1, pX=0.023, N=325
LQHLENELTHDIITKpc3_NP.2
p−maj=1, pX=8.5e−06, N=325
LQHLENELTHDIITKpc3_NP.3
p−maj=1, pX=0.037, N=325
LQHLENELTHDIITKpc3_NP.4
p−maj=1, pX=0.02, N=325
LQHLENELTHDIITKpc3_NP.5
p−maj=1, pX=0.0027, N=325
137:139
147:150
36:36
LQHLENELTHDIITKpc4_NP.1
p−min=1, pX=0.0042, N=320
139:139
150:150
36:36
LQHLENELTHDIITKpc4_NP.2
p−maj=1, pX=9.2e−05, N=325
118:139
134:150
31:36
LQHLENELTHDIITKpc4_NP.3
p−min=0.32, pX=0.18, N=283
125:139
139:150
33:36
LQHLENELTHDIITKpc4_NP.4
p−min=0.43, pX=0.047, N=297
128:139
144:150
32:36
LQHLENELTHDIITKpc4_NP.5
p−maj=0.27, pX=0.0054, N=304

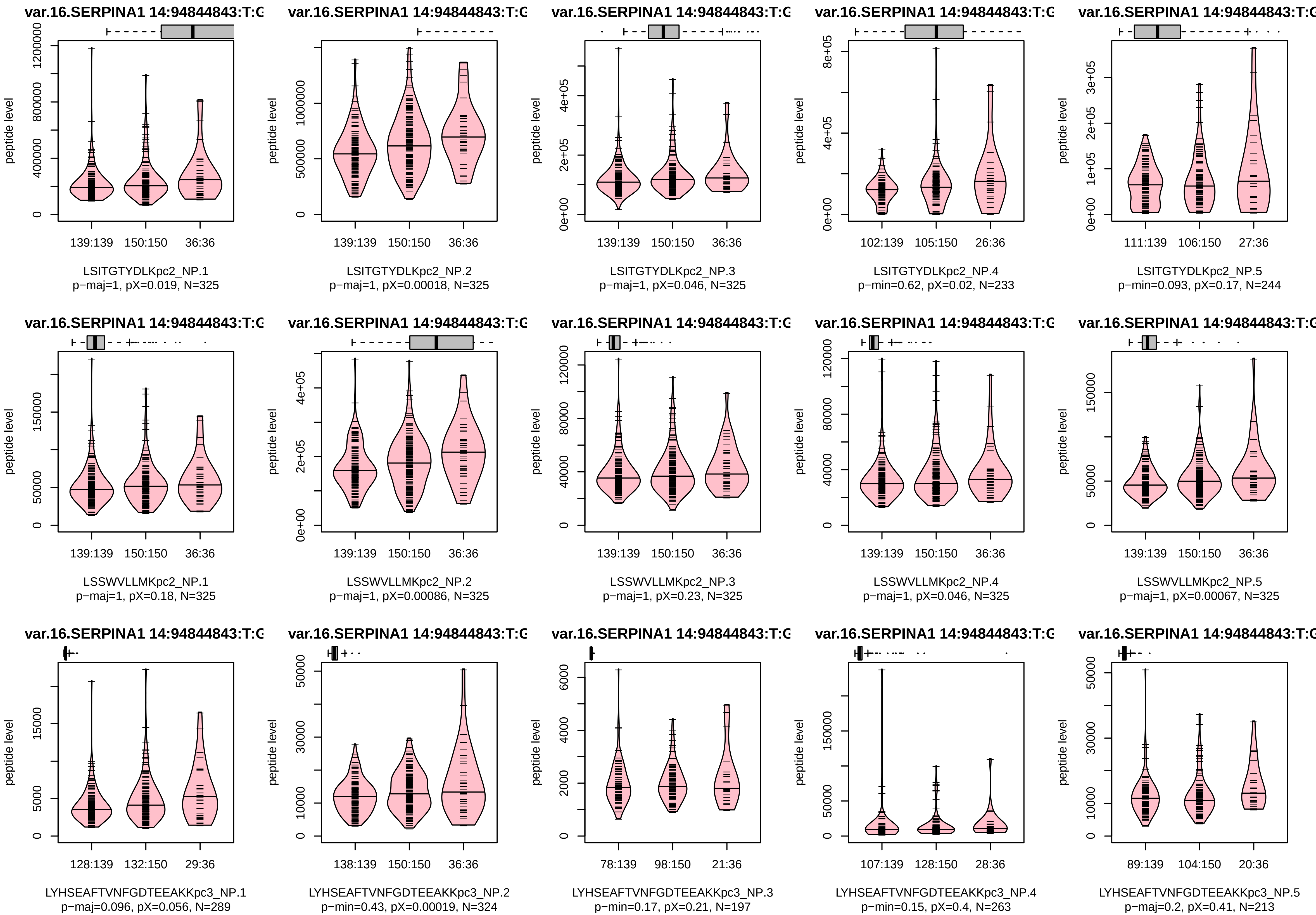
var.16.SERPINA1 14:94844843:T:G
peptide level
139:139
150:150
36:36
LSITGTYDLKpc2_NP.1
p−maj=1, pX=0.019, N=325
LSITGTYDLKpc2_NP.2
p−maj=1, pX=0.00018, N=325
LSITGTYDLKpc2_NP.3
p−maj=1, pX=0.046, N=325
102:139
105:150
26:36
LSITGTYDLKpc2_NP.4
p−min=0.62, pX=0.02, N=233
111:139
106:150
27:36
LSITGTYDLKpc2_NP.5
p−min=0.093, pX=0.17, N=244
LSSWVLLMKpc2_NP.1
p−maj=1, pX=0.18, N=325
LSSWVLLMKpc2_NP.2
p−maj=1, pX=0.00086, N=325
LSSWVLLMKpc2_NP.3
p−maj=1, pX=0.23, N=325
LSSWVLLMKpc2_NP.4
p−maj=1, pX=0.046, N=325
LSSWVLLMKpc2_NP.5
p−maj=1, pX=0.00067, N=325
128:139
132:150
29:36
LYHSEAFTVNFGDTEEAKKpc3_NP.1
p−maj=0.096, pX=0.056, N=289
138:139
150:150
36:36
LYHSEAFTVNFGDTEEAKKpc3_NP.2
p−min=0.43, pX=0.00019, N=324
78:139
98:150
21:36
LYHSEAFTVNFGDTEEAKKpc3_NP.3
p−min=0.17, pX=0.21, N=197
107:139
128:150
28:36
LYHSEAFTVNFGDTEEAKKpc3_NP.4
p−min=0.15, pX=0.4, N=263
89:139
104:150
20:36
LYHSEAFTVNFGDTEEAKKpc3_NP.5
p−maj=0.2, pX=0.41, N=213

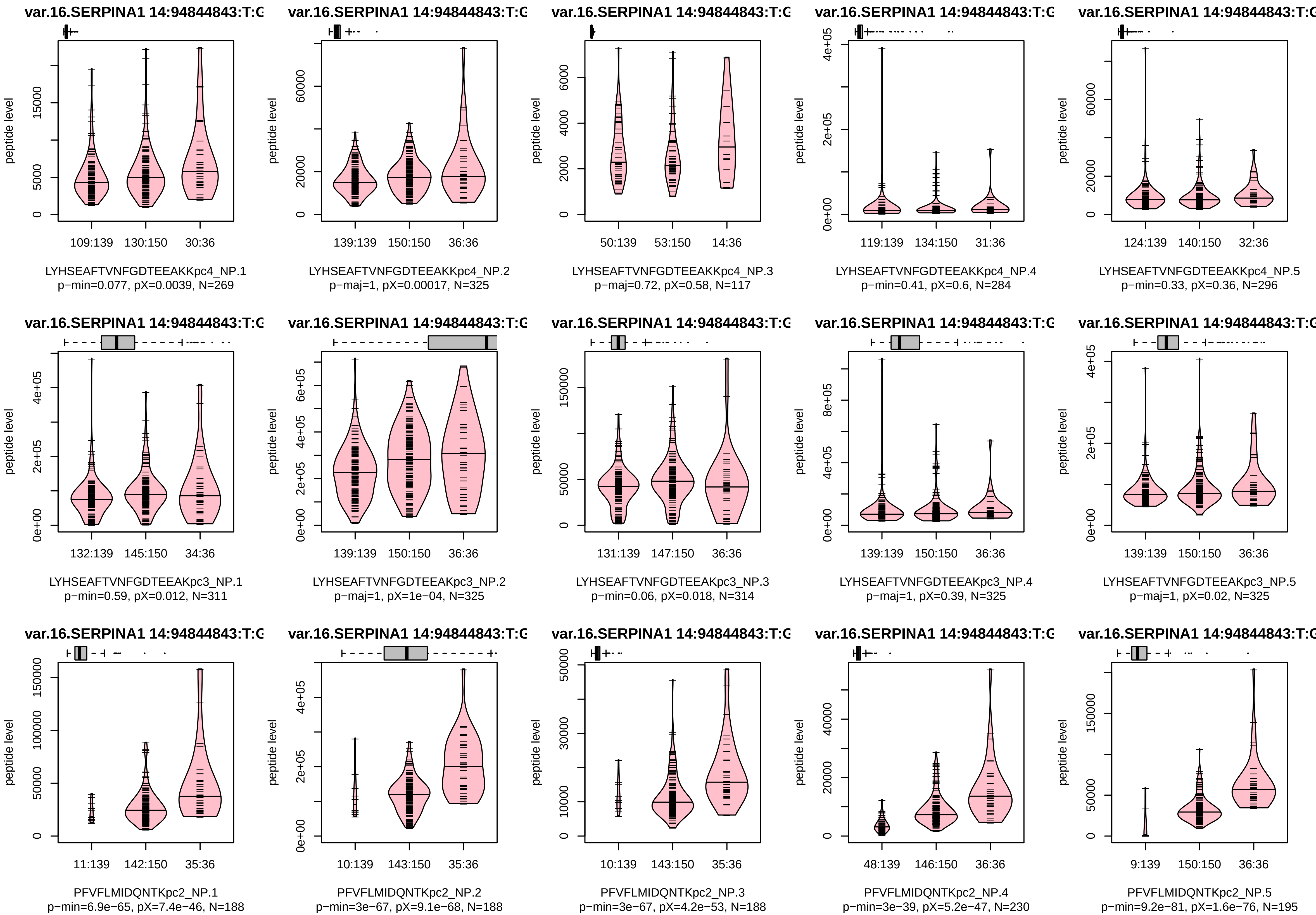
var.16.SERPINA1 14:94844843:T:G
peptide level
109:139 130:150 30:36
LYHSEAFTVNFGDTEEAKKpc4_NP.1
p−min=0.077, pX=0.0039, N=269
var.16.SERPINA1 14:94844843:T:G
peptide level
139:139 150:150 36:36
LYHSEAFTVNFGDTEEAKKpc4_NP.2
p−maj=1, pX=0.00017, N=325
var.16.SERPINA1 14:94844843:T:G
peptide level
50:139 53:150 14:36
LYHSEAFTVNFGDTEEAKKpc4_NP.3
p−maj=0.72, pX=0.58, N=117
var.16.SERPINA1 14:94844843:T:G
peptide level
119:139 134:150 31:36
LYHSEAFTVNFGDTEEAKKpc4_NP.4
p−min=0.41, pX=0.6, N=284
var.16.SERPINA1 14:94844843:T:G
peptide level
124:139 140:150 32:36
LYHSEAFTVNFGDTEEAKKpc4_NP.5
p−min=0.33, pX=0.36, N=296
var.16.SERPINA1 14:94844843:T:G
peptide level
132:139 145:150 34:36
LYHSEAFTVNFGDTEEAKpc3_NP.1
p−min=0.59, pX=0.012, N=311
var.16.SERPINA1 14:94844843:T:G
peptide level
139:139 150:150 36:36
LYHSEAFTVNFGDTEEAKpc3_NP.2
p−maj=1, pX=1e−04, N=325
var.16.SERPINA1 14:94844843:T:G
peptide level
131:139 147:150 36:36
LYHSEAFTVNFGDTEEAKpc3_NP.3
p−min=0.06, pX=0.018, N=314
var.16.SERPINA1 14:94844843:T:G
peptide level
139:139 150:150 36:36
LYHSEAFTVNFGDTEEAKpc3_NP.4
p−maj=1, pX=0.39, N=325
var.16.SERPINA1 14:94844843:T:G
peptide level
139:139 150:150 36:36
LYHSEAFTVNFGDTEEAKpc3_NP.5
p−maj=1, pX=0.02, N=325
var.16.SERPINA1 14:94844843:T:G
peptide level
11:139 142:150 35:36
PFVFLMIDQNTKpc2_NP.1
p−min=6.9e−65, pX=7.4e−46, N=188
var.16.SERPINA1 14:94844843:T:G
peptide level
10:139 143:150 35:36
PFVFLMIDQNTKpc2_NP.2
p−min=3e−67, pX=9.1e−68, N=188
var.16.SERPINA1 14:94844843:T:G
peptide level
10:139 143:150 35:36
PFVFLMIDQNTKpc2_NP.3
p−min=3e−67, pX=4.2e−53, N=188
var.16.SERPINA1 14:94844843:T:G
peptide level
48:139 146:150 36:36
PFVFLMIDQNTKpc2_NP.4
p−min=3e−39, pX=5.2e−47, N=230
var.16.SERPINA1 14:94844843:T:G
peptide level
9:139 150:150 36:36
PFVFLMIDQNTKpc2_NP.5
p−min=9.2e−81, pX=1.6e−76, N=195

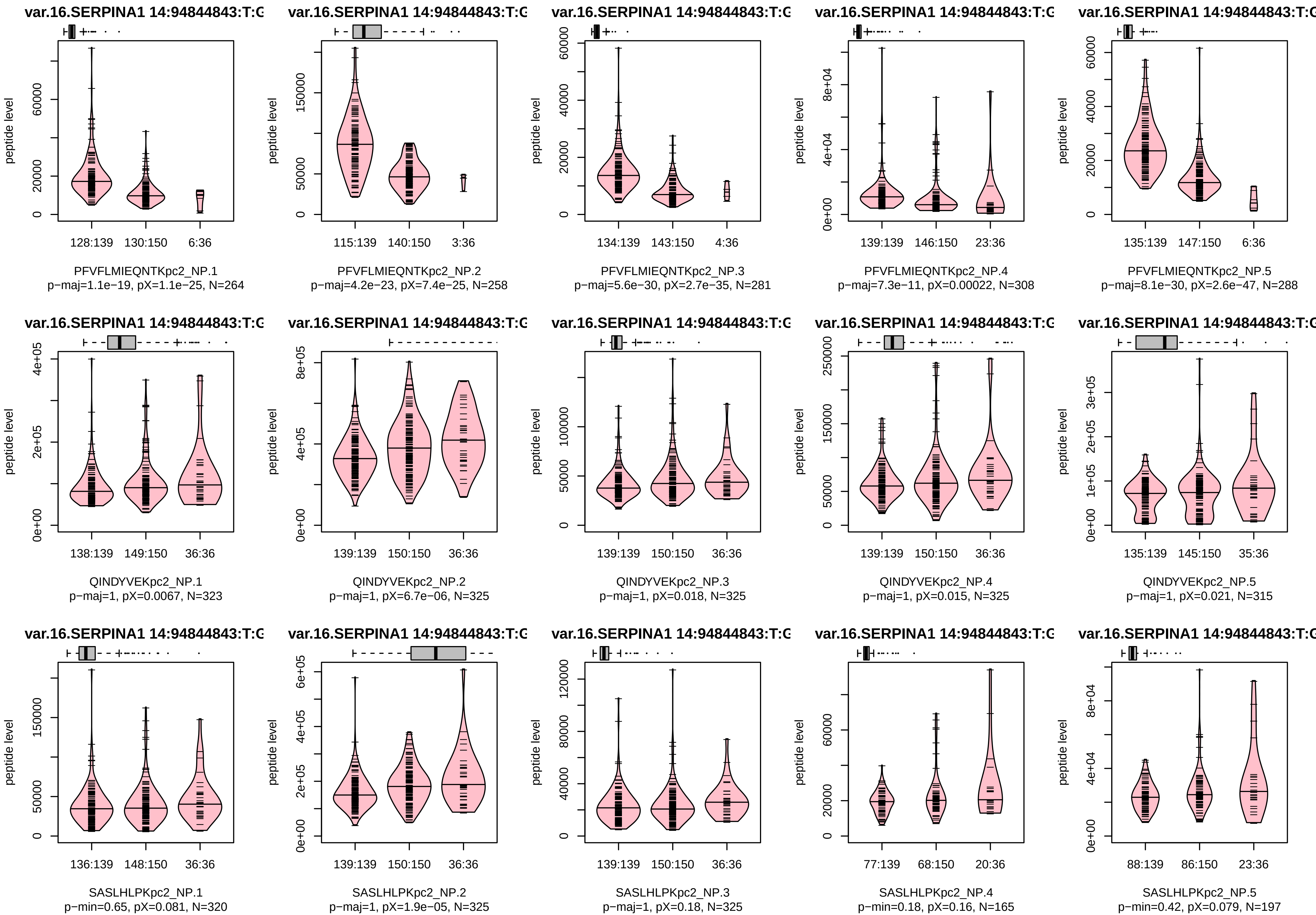
var.16.SERPINA1 14:94844843:T:G
peptide level
128:139 130:150 6:36
PFVFLMIEQNTKpc2_NP.1
p−maj=1.1e−19, pX=1.1e−25, N=264
var.16.SERPINA1 14:94844843:T:G
peptide level
115:139 140:150 3:36
PFVFLMIEQNTKpc2_NP.2
p−maj=4.2e−23, pX=7.4e−25, N=258
var.16.SERPINA1 14:94844843:T:G
peptide level
134:139 143:150 4:36
PFVFLMIEQNTKpc2_NP.3
p−maj=5.6e−30, pX=2.7e−35, N=281
var.16.SERPINA1 14:94844843:T:G
peptide level
139:139 146:150 23:36
PFVFLMIEQNTKpc2_NP.4
p−maj=7.3e−11, pX=0.00022, N=308
var.16.SERPINA1 14:94844843:T:G
peptide level
135:139 147:150 6:36
PFVFLMIEQNTKpc2_NP.5
p−maj=8.1e−30, pX=2.6e−47, N=288
var.16.SERPINA1 14:94844843:T:G
peptide level
138:139 149:150 36:36
QINDYVEKpc2_NP.1
p−maj=1, pX=0.0067, N=323
var.16.SERPINA1 14:94844843:T:G
peptide level
139:139 150:150 36:36
QINDYVEKpc2_NP.2
p−maj=1, pX=6.7e−06, N=325
var.16.SERPINA1 14:94844843:T:G
peptide level
139:139 150:150 36:36
QINDYVEKpc2_NP.3
p−maj=1, pX=0.018, N=325
var.16.SERPINA1 14:94844843:T:G
peptide level
139:139 150:150 36:36
QINDYVEKpc2_NP.4
p−maj=1, pX=0.015, N=325
var.16.SERPINA1 14:94844843:T:G
peptide level
135:139 145:150 35:36
QINDYVEKpc2_NP.5
p−maj=1, pX=0.021, N=315
var.16.SERPINA1 14:94844843:T:G
peptide level
136:139 148:150 36:36
SASLHLPKpc2_NP.1
p−min=0.65, pX=0.081, N=320
var.16.SERPINA1 14:94844843:T:G
peptide level
139:139 150:150 36:36
SASLHLPKpc2_NP.2
p−maj=1, pX=1.9e−05, N=325
var.16.SERPINA1 14:94844843:T:G
peptide level
139:139 150:150 36:36
SASLHLPKpc2_NP.3
p−maj=1, pX=0.18, N=325
var.16.SERPINA1 14:94844843:T:G
peptide level
77:139 68:150 20:36
SASLHLPKpc2_NP.4
p−min=0.18, pX=0.16, N=165
var.16.SERPINA1 14:94844843:T:G
peptide level
88:139 86:150 23:36
SASLHLPKpc2_NP.5
p−min=0.42, pX=0.079, N=197

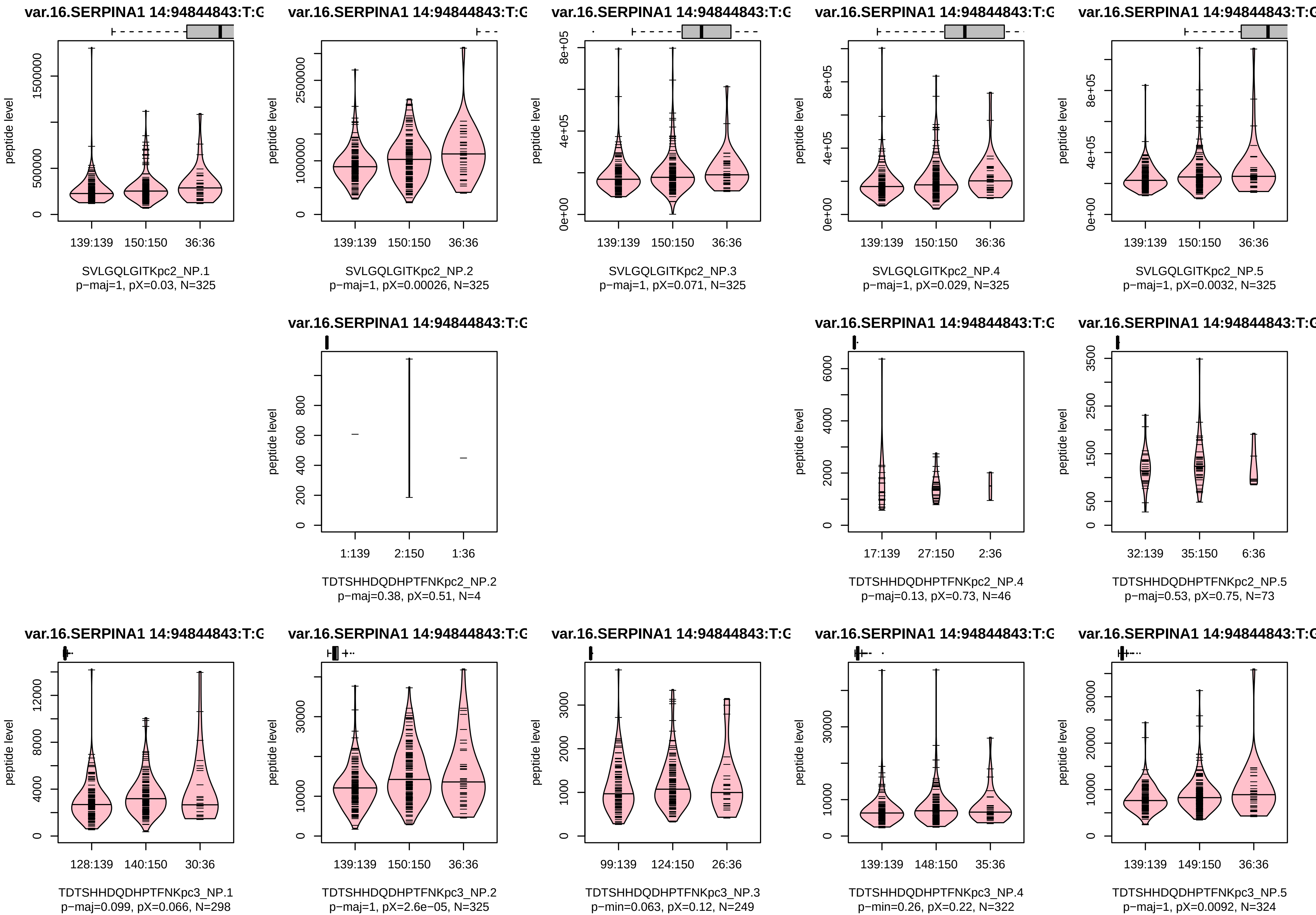
var.16.SERPINA1 14:94844843:T:G
peptide level
139:139
150:150
36:36
SVLGQLGITKpc2_NP.1
p−maj=1, pX=0.03, N=325
SVLGQLGITKpc2_NP.2
p−maj=1, pX=0.00026, N=325
SVLGQLGITKpc2_NP.3
p−maj=1, pX=0.071, N=325
SVLGQLGITKpc2_NP.4
p−maj=1, pX=0.029, N=325
SVLGQLGITKpc2_NP.5
p−maj=1, pX=0.0032, N=325
1:139
2:150
1:36
TDTSHHDQDHPTFNKpc2_NP.2
p−maj=0.38, pX=0.51, N=4
17:139
27:150
2:36
TDTSHHDQDHPTFNKpc2_NP.4
p−maj=0.13, pX=0.73, N=46
32:139
35:150
6:36
TDTSHHDQDHPTFNKpc2_NP.5
p−maj=0.53, pX=0.75, N=73
128:139
140:150
30:36
TDTSHHDQDHPTFNKpc3_NP.1
p−maj=0.099, pX=0.066, N=298
TDTSHHDQDHPTFNKpc3_NP.2
p−maj=1, pX=2.6e−05, N=325
99:139
124:150
26:36
TDTSHHDQDHPTFNKpc3_NP.3
p−min=0.063, pX=0.12, N=249
148:150
35:36
TDTSHHDQDHPTFNKpc3_NP.4
p−min=0.26, pX=0.22, N=322
149:150
TDTSHHDQDHPTFNKpc3_NP.5
p−maj=1, pX=0.0092, N=324

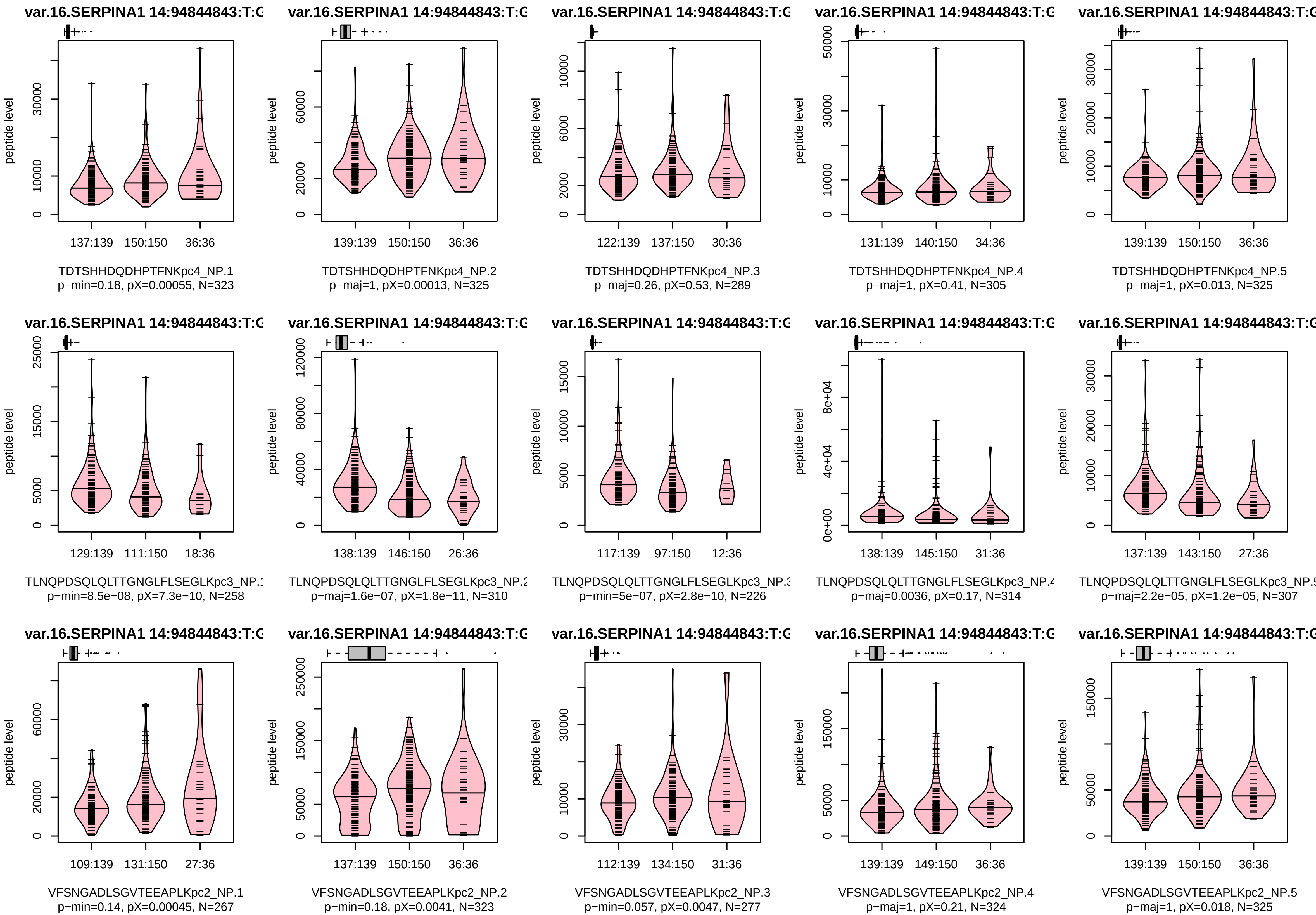

var.16.SERPINA1 14:94844843:T:G
peptide level
137:139
150:150
36:36
TDTSHHDQDHPTFNKpc4_NP.1
p−min=0.18, pX=0.00055, N=323
var.16.SERPINA1 14:94844843:T:G
peptide level
139:139
150:150
36:36
TDTSHHDQDHPTFNKpc4_NP.2
p−maj=1, pX=0.00013, N=325
var.16.SERPINA1 14:94844843:T:G
peptide level
122:139
137:150
30:36
TDTSHHDQDHPTFNKpc4_NP.3
p−maj=0.26, pX=0.53, N=289
var.16.SERPINA1 14:94844843:T:G
peptide level
131:139
140:150
34:36
TDTSHHDQDHPTFNKpc4_NP.4
p−maj=1, pX=0.41, N=305
var.16.SERPINA1 14:94844843:T:G
peptide level
139:139
150:150
36:36
TDTSHHDQDHPTFNKpc4_NP.5
p−maj=1, pX=0.013, N=325
var.16.SERPINA1 14:94844843:T:G
peptide level
129:139
111:150
18:36
TLNQPDSQLQLTTGNGLFLSEGLKpc3_NP.1
p−min=8.5e−08, pX=7.3e−10, N=258
var.16.SERPINA1 14:94844843:T:G
peptide level
138:139
146:150
26:36
TLNQPDSQLQLTTGNGLFLSEGLKpc3_NP.2
p−maj=1.6e−07, pX=1.8e−11, N=310
var.16.SERPINA1 14:94844843:T:G
peptide level
117:139
97:150
12:36
TLNQPDSQLQLTTGNGLFLSEGLKpc3_NP.3
p−min=5e−07, pX=2.8e−10, N=226
var.16.SERPINA1 14:94844843:T:G
peptide level
138:139
145:150
31:36
TLNQPDSQLQLTTGNGLFLSEGLKpc3_NP.4
p−maj=0.0036, pX=0.17, N=314
var.16.SERPINA1 14:94844843:T:G
peptide level
137:139
143:150
27:36
TLNQPDSQLQLTTGNGLFLSEGLKpc3_NP.5
p−maj=2.2e−05, pX=1.2e−05, N=307
var.16.SERPINA1 14:94844843:T:G
peptide level
109:139
131:150
27:36
VFSNGADLSGVTEEAPLKpc2_NP.1
p−min=0.14, pX=0.00045, N=267
var.16.SERPINA1 14:94844843:T:G
peptide level
137:139
150:150
36:36
VFSNGADLSGVTEEAPLKpc2_NP.2
p−min=0.18, pX=0.0041, N=323
var.16.SERPINA1 14:94844843:T:G
peptide level
112:139
134:150
31:36
VFSNGADLSGVTEEAPLKpc2_NP.3
p−min=0.057, pX=0.0047, N=277
var.16.SERPINA1 14:94844843:T:G
peptide level
139:139
149:150
36:36
VFSNGADLSGVTEEAPLKpc2_NP.4
p−maj=1, pX=0.21, N=324
var.16.SERPINA1 14:94844843:T:G
peptide level
139:139
150:150
36:36
VFSNGADLSGVTEEAPLKpc2_NP.5
p−maj=1, pX=0.018, N=325

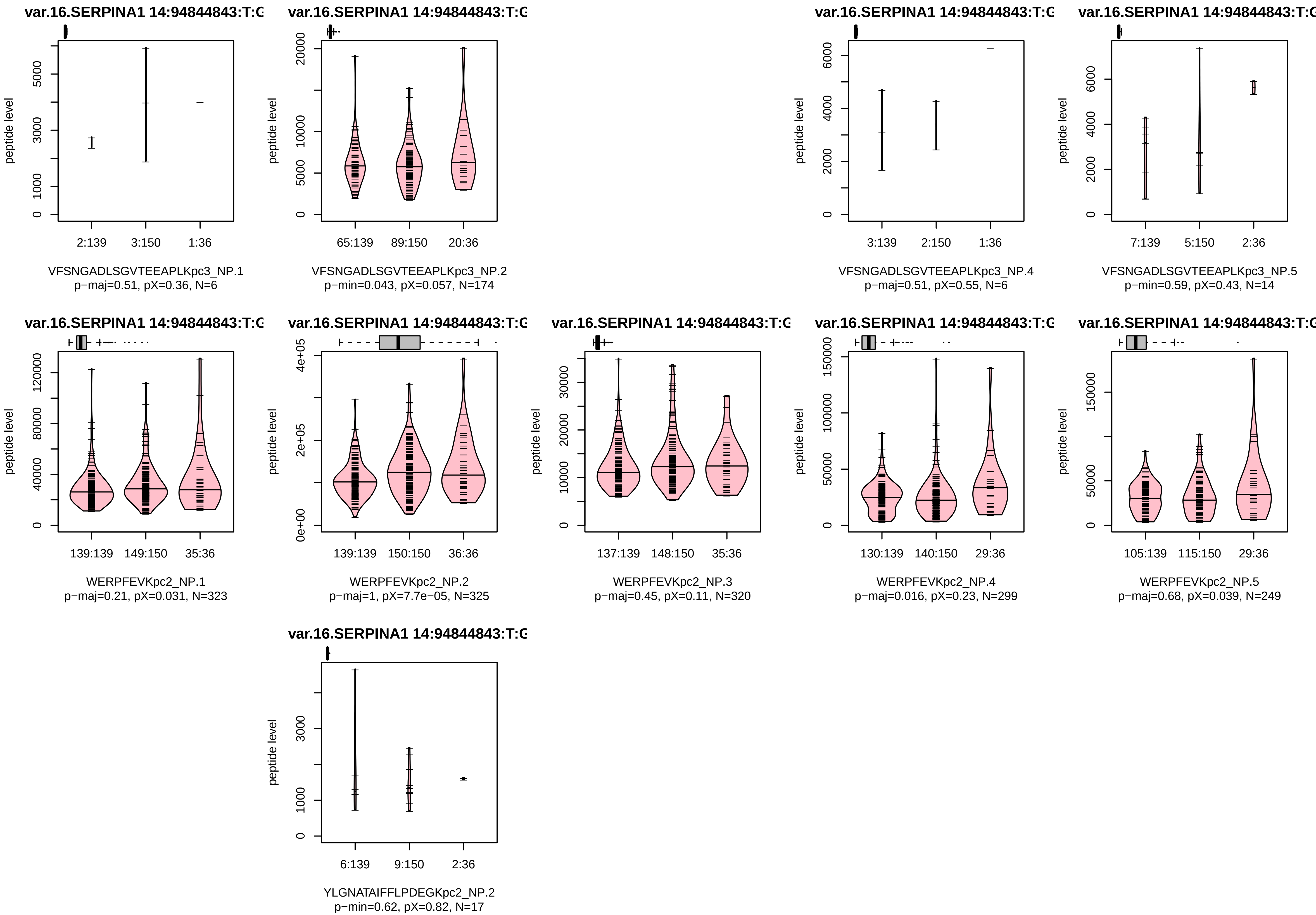
var.16.SERPINA1 14:94844843:T:G
peptide level
2:139
3:150
1:36
VFSNGADLSGVTEEAPLKpc3_NP.1
p−maj=0.51, pX=0.36, N=6
var.16.SERPINA1 14:94844843:T:G
peptide level
65:139
89:150
20:36
VFSNGADLSGVTEEAPLKpc3_NP.2
p−min=0.043, pX=0.057, N=174
var.16.SERPINA1 14:94844843:T:G
peptide level
3:139
2:150
1:36
VFSNGADLSGVTEEAPLKpc3_NP.4
p−maj=0.51, pX=0.55, N=6
var.16.SERPINA1 14:94844843:T:G
peptide level
7:139
5:150
2:36
VFSNGADLSGVTEEAPLKpc3_NP.5
p−min=0.59, pX=0.43, N=14
var.16.SERPINA1 14:94844843:T:G
peptide level
139:139
149:150
35:36
WERPFEVKpc2_NP.1
p−maj=0.21, pX=0.031, N=323
var.16.SERPINA1 14:94844843:T:G
peptide level
139:139
150:150
36:36
WERPFEVKpc2_NP.2
p−maj=1, pX=7.7e−05, N=325
var.16.SERPINA1 14:94844843:T:G
peptide level
137:139
148:150
35:36
WERPFEVKpc2_NP.3
p−maj=0.45, pX=0.11, N=320
var.16.SERPINA1 14:94844843:T:G
peptide level
130:139
140:150
29:36
WERPFEVKpc2_NP.4
p−maj=0.016, pX=0.23, N=299
var.16.SERPINA1 14:94844843:T:G
peptide level
105:139
115:150
29:36
WERPFEVKpc2_NP.5
p−maj=0.68, pX=0.039, N=249
var.16.SERPINA1 14:94844843:T:G
peptide level
6:139
9:150
2:36
YLGNATAIFFLPDEGKpc2_NP.2
p−min=0.62, pX=0.82, N=17

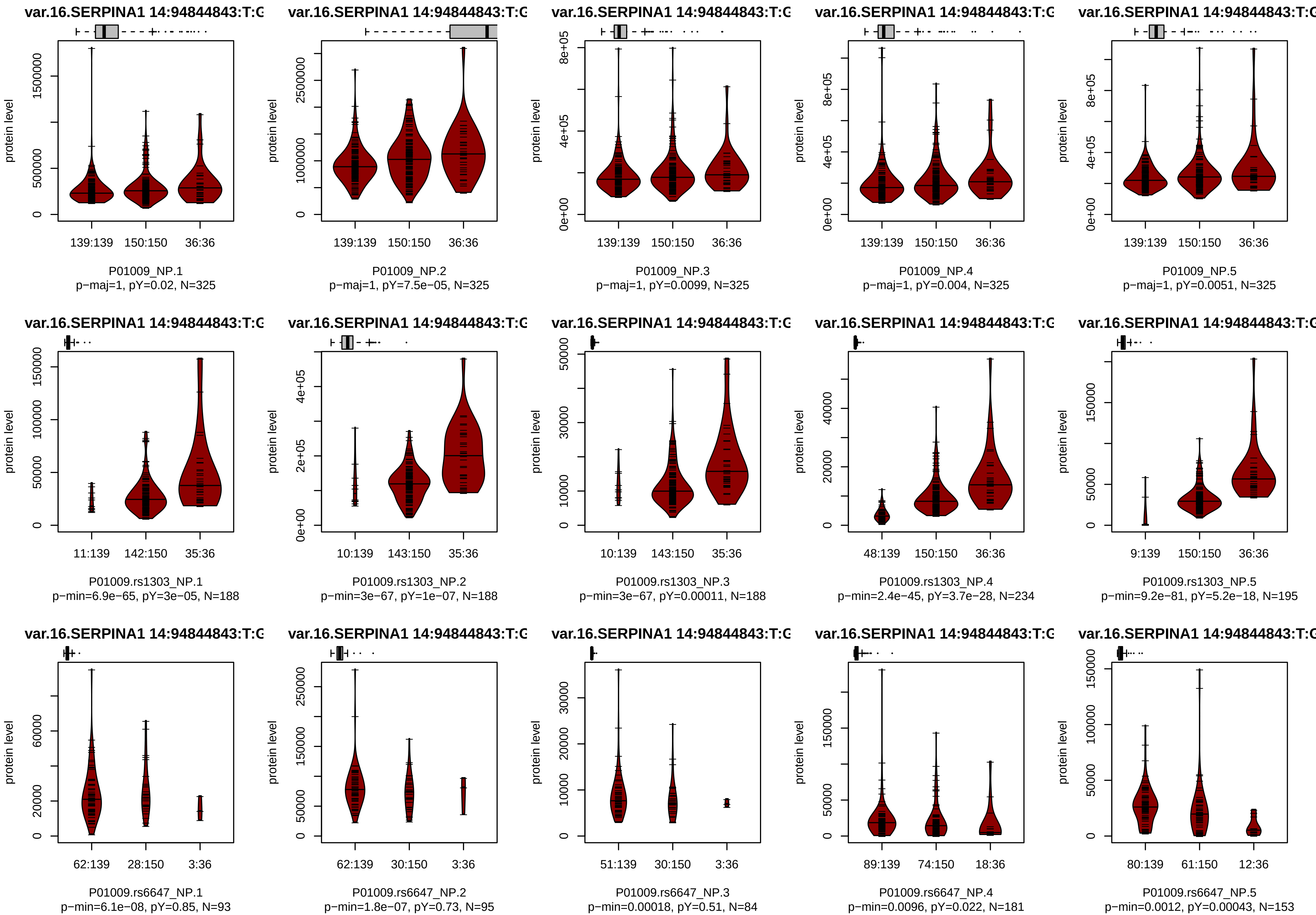
var.16.SERPINA1 14:94844843:T:C
protein level
0
500000
1500000
139:139
150:150
36:36
P01009_NP.1
p−maj=1, pY=0.02, N=325
var.16.SERPINA1 14:94844843:T:C
protein level
0
1000000
2500000
139:139
150:150
36:36
P01009_NP.2
p−maj=1, pY=7.5e−05, N=325
var.16.SERPINA1 14:94844843:T:C
protein level
0e+00
4e+05
8e+05
139:139
150:150
36:36
P01009_NP.3
p−maj=1, pY=0.0099, N=325
var.16.SERPINA1 14:94844843:T:C
protein level
0e+00
4e+05
8e+05
139:139
150:150
36:36
P01009_NP.4
p−maj=1, pY=0.004, N=325
var.16.SERPINA1 14:94844843:T:C
protein level
0e+00
4e+05
8e+05
139:139
150:150
36:36
P01009_NP.5
p−maj=1, pY=0.0051, N=325
var.16.SERPINA1 14:94844843:T:C
protein level
0
50000
100000
150000
11:139
142:150
35:36
P01009.rs1303_NP.1
p−min=6.9e−65, pY=3e−05, N=188
var.16.SERPINA1 14:94844843:T:C
protein level
0e+00
2e+05
4e+05
10:139
143:150
35:36
P01009.rs1303_NP.2
p−min=3e−67, pY=1e−07, N=188
var.16.SERPINA1 14:94844843:T:C
protein level
0
10000
30000
50000
10:139
143:150
35:36
P01009.rs1303_NP.3
p−min=3e−67, pY=0.00011, N=188
var.16.SERPINA1 14:94844843:T:C
protein level
0
20000
40000
48:139
150:150
36:36
P01009.rs1303_NP.4
p−min=2.4e−45, pY=3.7e−28, N=234
var.16.SERPINA1 14:94844843:T:C
protein level
0
50000
150000
9:139
150:150
36:36
P01009.rs1303_NP.5
p−min=9.2e−81, pY=5.2e−18, N=195
var.16.SERPINA1 14:94844843:T:C
protein level
0
20000
60000
62:139
28:150
3:36
P01009.rs6647_NP.1
p−min=6.1e−08, pY=0.85, N=93
var.16.SERPINA1 14:94844843:T:C
protein level
0
50000
150000
250000
62:139
30:150
3:36
P01009.rs6647_NP.2
p−min=1.8e−07, pY=0.73, N=95
var.16.SERPINA1 14:94844843:T:C
protein level
0
10000
20000
30000
51:139
30:150
3:36
P01009.rs6647_NP.3
p−min=0.00018, pY=0.51, N=84
var.16.SERPINA1 14:94844843:T:C
protein level
0
50000
150000
89:139
74:150
18:36
P01009.rs6647_NP.4
p−min=0.0096, pY=0.022, N=181
var.16.SERPINA1 14:94844843:T:C
protein level
0
50000
100000
150000
80:139
61:150
12:36
P01009.rs6647_NP.5
p−min=0.0012, pY=0.00043, N=153

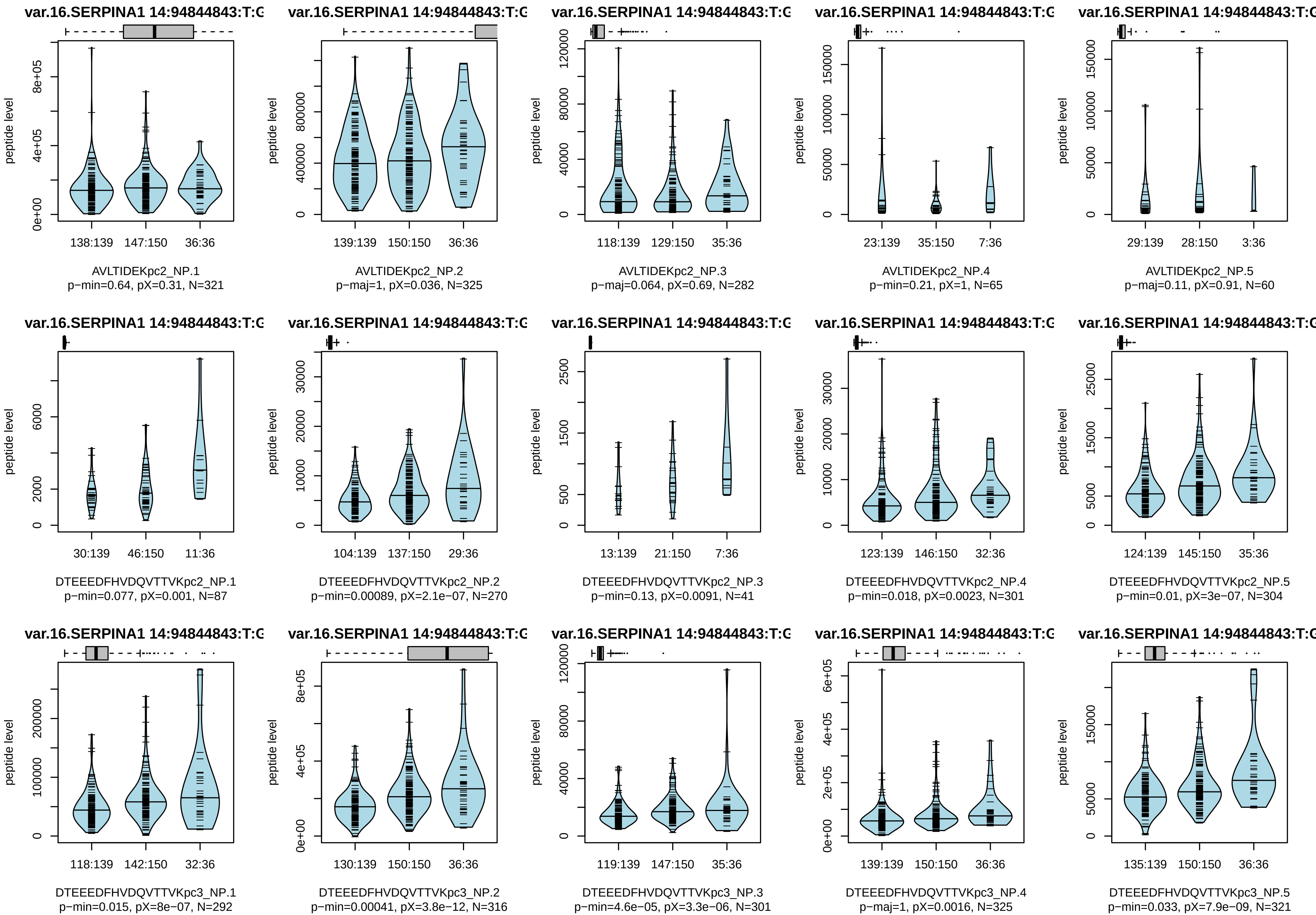
var.16.SERPINA1 14:94844843:T:G
peptide level
138:139 147:150 36:36
AVLTIDEKpc2_NP.1
p−min=0.64, pX=0.31, N=321
var.16.SERPINA1 14:94844843:T:G
139:139 150:150 36:36
AVLTIDEKpc2_NP.2
p−maj=1, pX=0.036, N=325
var.16.SERPINA1 14:94844843:T:G
118:139 129:150 35:36
AVLTIDEKpc2_NP.3
p−maj=0.064, pX=0.69, N=282
var.16.SERPINA1 14:94844843:T:G
23:139 35:150 7:36
AVLTIDEKpc2_NP.4
p−min=0.21, pX=1, N=65
var.16.SERPINA1 14:94844843:T:G
29:139 28:150 3:36
AVLTIDEKpc2_NP.5
p−maj=0.11, pX=0.91, N=60
var.16.SERPINA1 14:94844843:T:G
30:139 46:150 11:36
DTEEEDFHVDQVTTVKpc2_NP.1
p−min=0.077, pX=0.001, N=87
var.16.SERPINA1 14:94844843:T:G
104:139 137:150 29:36
DTEEEDFHVDQVTTVKpc2_NP.2
p−min=0.00089, pX=2.1e−07, N=270
var.16.SERPINA1 14:94844843:T:G
13:139 21:150 7:36
DTEEEDFHVDQVTTVKpc2_NP.3
p−min=0.13, pX=0.0091, N=41
var.16.SERPINA1 14:94844843:T:G
123:139 146:150 32:36
DTEEEDFHVDQVTTVKpc2_NP.4
p−min=0.018, pX=0.0023, N=301
var.16.SERPINA1 14:94844843:T:G
124:139 145:150 35:36
DTEEEDFHVDQVTTVKpc2_NP.5
p−min=0.01, pX=3e−07, N=304
var.16.SERPINA1 14:94844843:T:G
118:139 142:150 32:36
DTEEEDFHVDQVTTVKpc3_NP.1
p−min=0.015, pX=8e−07, N=292
var.16.SERPINA1 14:94844843:T:G
130:139 150:150 36:36
DTEEEDFHVDQVTTVKpc3_NP.2
p−min=0.00041, pX=3.8e−12, N=316
var.16.SERPINA1 14:94844843:T:G
119:139 147:150 35:36
DTEEEDFHVDQVTTVKpc3_NP.3
p−min=4.6e−05, pX=3.3e−06, N=301
var.16.SERPINA1 14:94844843:T:G
139:139 150:150 36:36
DTEEEDFHVDQVTTVKpc3_NP.4
p−maj=1, pX=0.0016, N=325
var.16.SERPINA1 14:94844843:T:G
135:139 150:150 36:36
DTEEEDFHVDQVTTVKpc3_NP.5
p−min=0.033, pX=7.9e−09, N=321

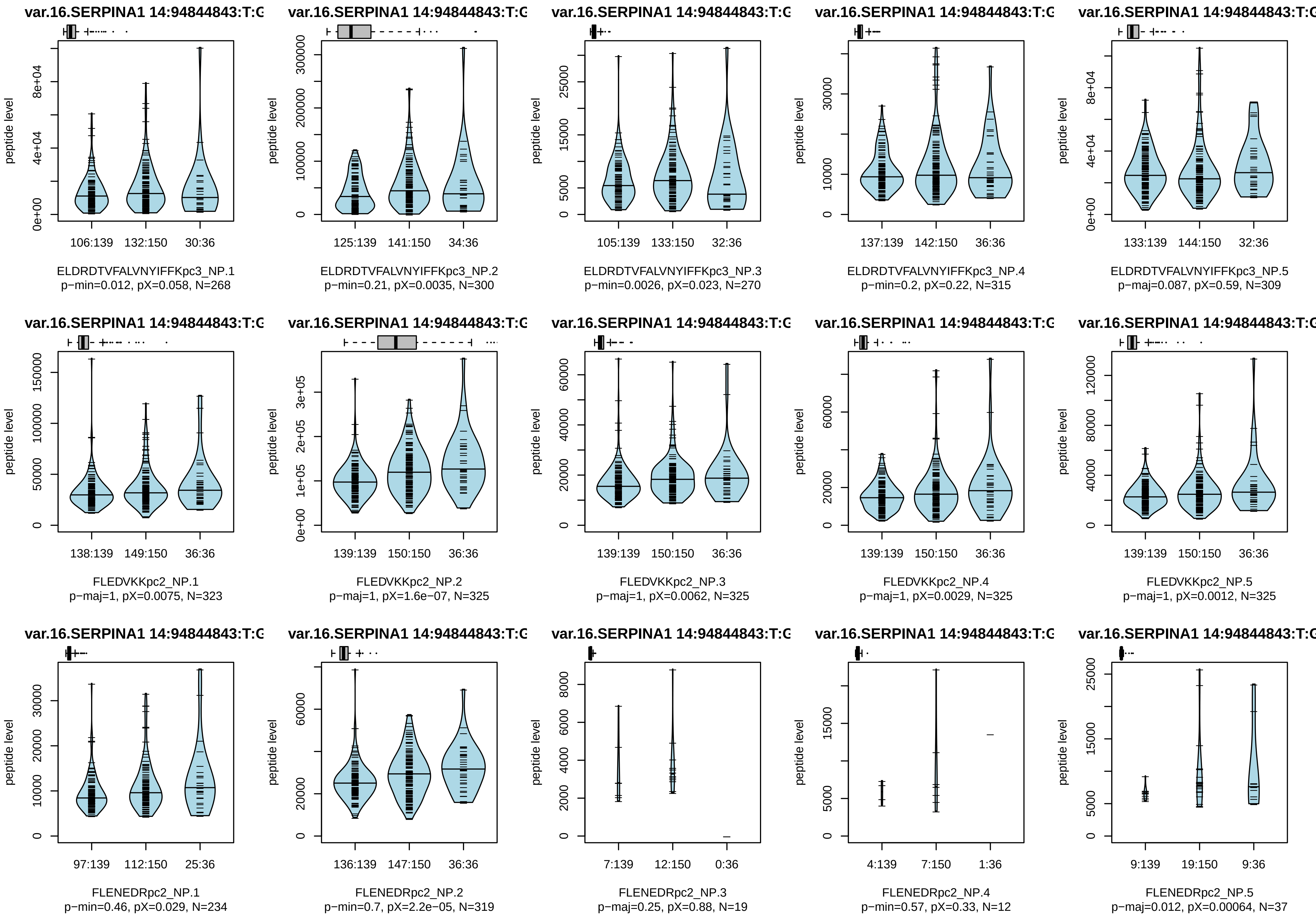

var.16.SERPINA1 14:94844843:T:G
peptide level
106:139
132:150
30:36
ELDRDTVFALVNYIFFKpc3_NP.1
p−min=0.012, pX=0.058, N=268
var.16.SERPINA1 14:94844843:T:G
peptide level
125:139
141:150
34:36
ELDRDTVFALVNYIFFKpc3_NP.2
p−min=0.21, pX=0.0035, N=300
var.16.SERPINA1 14:94844843:T:G
peptide level
105:139
133:150
32:36
ELDRDTVFALVNYIFFKpc3_NP.3
p−min=0.0026, pX=0.023, N=270
var.16.SERPINA1 14:94844843:T:G
peptide level
137:139
142:150
36:36
ELDRDTVFALVNYIFFKpc3_NP.4
p−min=0.2, pX=0.22, N=315
var.16.SERPINA1 14:94844843:T:G
peptide level
133:139
144:150
32:36
ELDRDTVFALVNYIFFKpc3_NP.5
p−maj=0.087, pX=0.59, N=309
var.16.SERPINA1 14:94844843:T:G
peptide level
138:139
149:150
36:36
FLEDVKKpc2_NP.1
p−maj=1, pX=0.0075, N=323
var.16.SERPINA1 14:94844843:T:G
peptide level
139:139
150:150
36:36
FLEDVKKpc2_NP.2
p−maj=1, pX=1.6e−07, N=325
var.16.SERPINA1 14:94844843:T:G
peptide level
139:139
150:150
36:36
FLEDVKKpc2_NP.3
p−maj=1, pX=0.0062, N=325
var.16.SERPINA1 14:94844843:T:G
peptide level
139:139
150:150
36:36
FLEDVKKpc2_NP.4
p−maj=1, pX=0.0029, N=325
var.16.SERPINA1 14:94844843:T:G
peptide level
139:139
150:150
36:36
FLEDVKKpc2_NP.5
p−maj=1, pX=0.0012, N=325
var.16.SERPINA1 14:94844843:T:G
peptide level
97:139
112:150
25:36
FLENEDRpc2_NP.1
p−min=0.46, pX=0.029, N=234
var.16.SERPINA1 14:94844843:T:G
peptide level
136:139
147:150
36:36
FLENEDRpc2_NP.2
p−min=0.7, pX=2.2e−05, N=319
var.16.SERPINA1 14:94844843:T:G
peptide level
7:139
12:150
0:36
FLENEDRpc2_NP.3
p−maj=0.25, pX=0.88, N=19
var.16.SERPINA1 14:94844843:T:G
peptide level
4:139
7:150
1:36
FLENEDRpc2_NP.4
p−min=0.57, pX=0.33, N=12
var.16.SERPINA1 14:94844843:T:G
peptide level
9:139
19:150
9:36
FLENEDRpc2_NP.5
p−maj=0.012, pX=0.00064, N=37

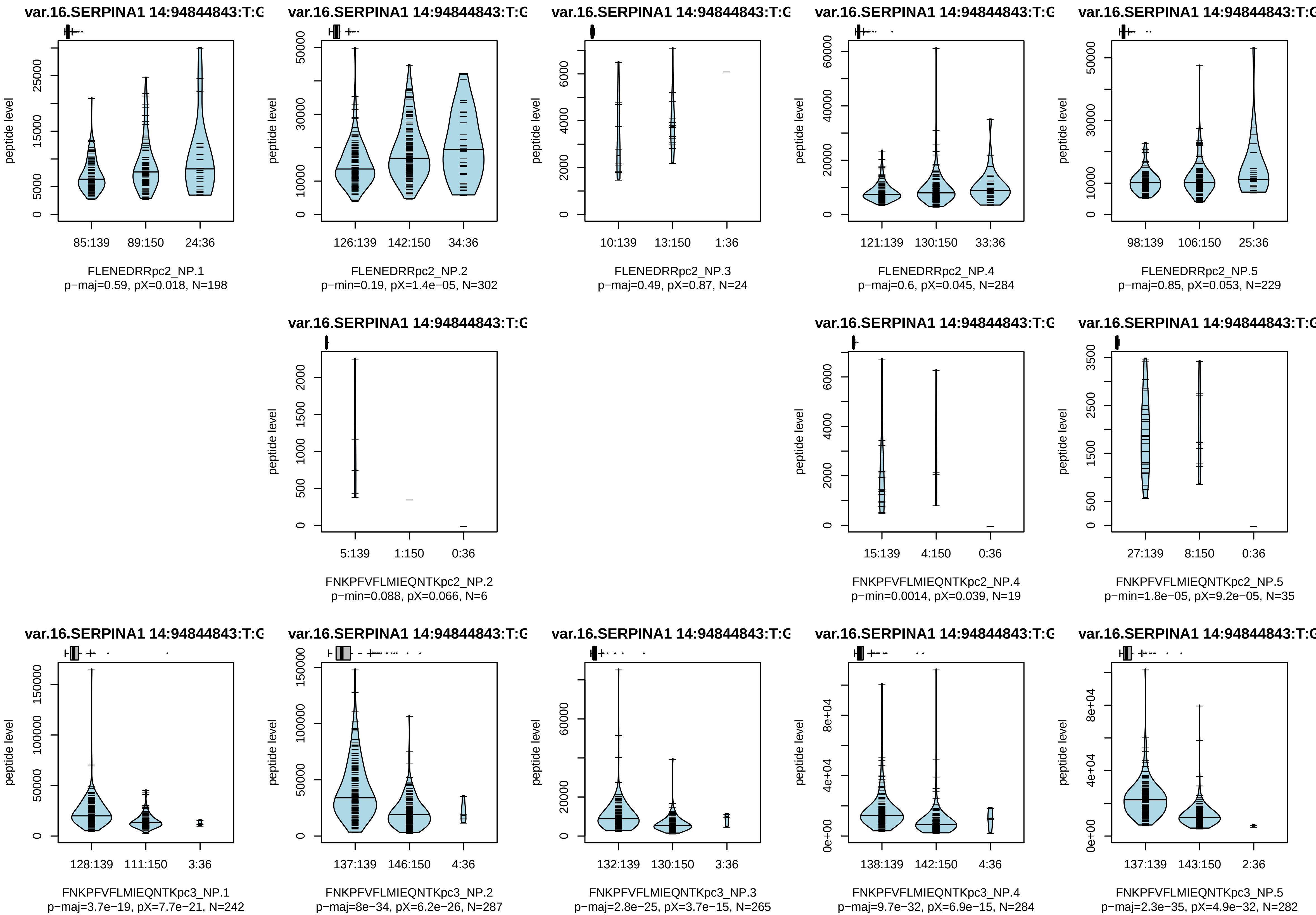
var.16.SERPINA1 14:94844843:T:G
85:139
89:150
24:36
FLENEDRRpc2_NP.1
p−maj=0.59, pX=0.018, N=198
126:139
142:150
34:36
FLENEDRRpc2_NP.2
p−min=0.19, pX=1.4e−05, N=302
10:139
13:150
1:36
FLENEDRRpc2_NP.3
p−maj=0.49, pX=0.87, N=24
121:139
130:150
33:36
FLENEDRRpc2_NP.4
p−maj=0.6, pX=0.045, N=284
98:139
106:150
25:36
FLENEDRRpc2_NP.5
p−maj=0.85, pX=0.053, N=229
5:139
1:150
0:36
FNKPFVFLMIEQNTKpc2_NP.2
p−min=0.088, pX=0.066, N=6
15:139
4:150
0:36
FNKPFVFLMIEQNTKpc2_NP.4
p−min=0.0014, pX=0.039, N=19
27:139
8:150
0:36
FNKPFVFLMIEQNTKpc2_NP.5
p−min=1.8e−05, pX=9.2e−05, N=35
128:139
111:150
3:36
FNKPFVFLMIEQNTKpc3_NP.1
p−maj=3.7e−19, pX=7.7e−21, N=242
137:139
146:150
4:36
FNKPFVFLMIEQNTKpc3_NP.2
p−maj=8e−34, pX=6.2e−26, N=287
132:139
130:150
3:36
FNKPFVFLMIEQNTKpc3_NP.3
p−maj=2.8e−25, pX=3.7e−15, N=265
138:139
142:150
4:36
FNKPFVFLMIEQNTKpc3_NP.4
p−maj=9.7e−32, pX=6.9e−15, N=284
137:139
143:150
2:36
FNKPFVFLMIEQNTKpc3_NP.5
p−maj=2.3e−35, pX=4.9e−32, N=282
peptide level

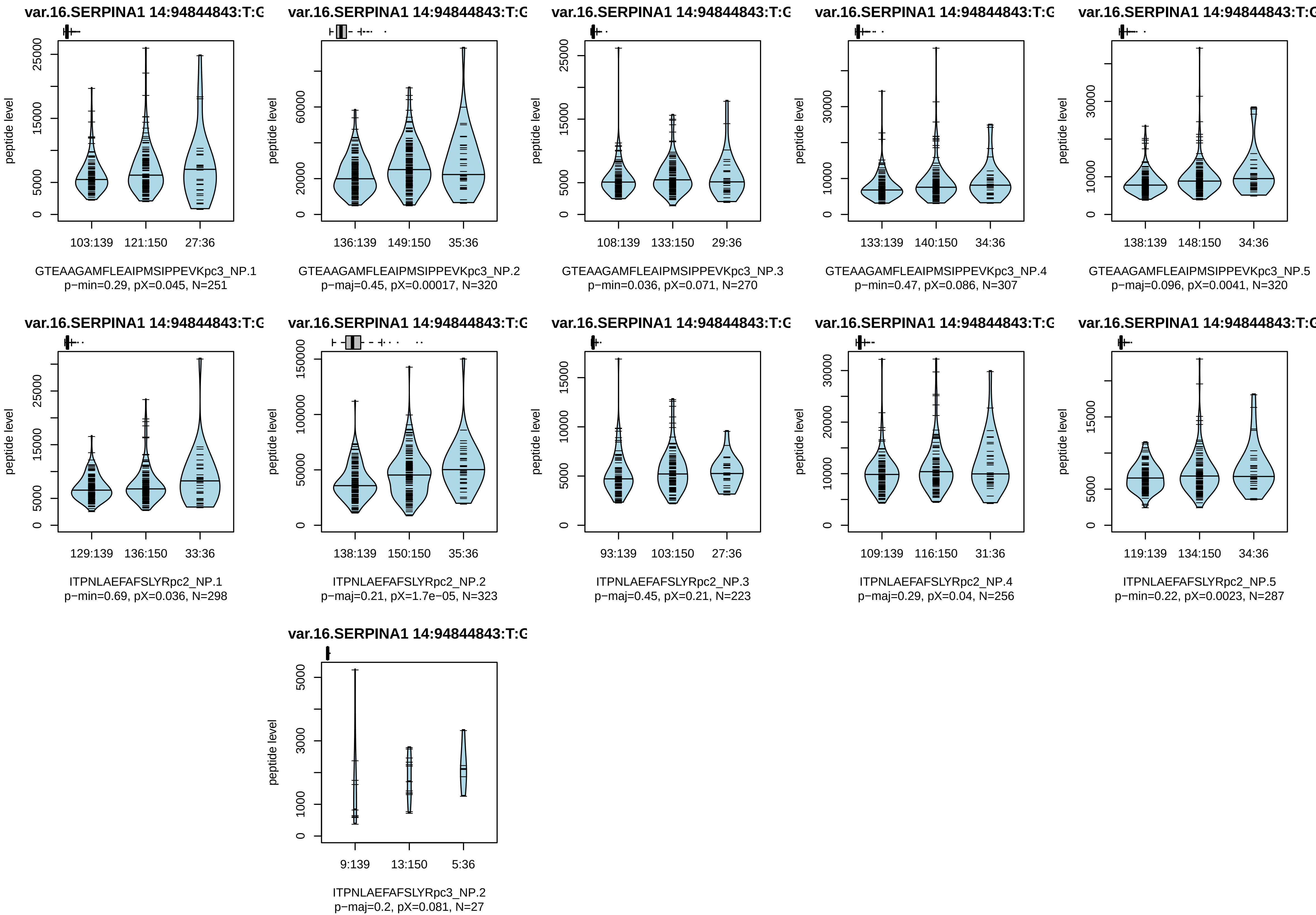
var.16.SERPINA1 14:94844843:T:G
peptide level
103:139
121:150
27:36
GTEAAGAMFLEAIPMSIPPEVKpc3_NP.1
p−min=0.29, pX=0.045, N=251
var.16.SERPINA1 14:94844843:T:G
peptide level
136:139
149:150
35:36
GTEAAGAMFLEAIPMSIPPEVKpc3_NP.2
p−maj=0.45, pX=0.00017, N=320
var.16.SERPINA1 14:94844843:T:G
peptide level
108:139
133:150
29:36
GTEAAGAMFLEAIPMSIPPEVKpc3_NP.3
p−min=0.036, pX=0.071, N=270
var.16.SERPINA1 14:94844843:T:G
peptide level
133:139
140:150
34:36
GTEAAGAMFLEAIPMSIPPEVKpc3_NP.4
p−min=0.47, pX=0.086, N=307
var.16.SERPINA1 14:94844843:T:G
peptide level
138:139
148:150
34:36
GTEAAGAMFLEAIPMSIPPEVKpc3_NP.5
p−maj=0.096, pX=0.0041, N=320
var.16.SERPINA1 14:94844843:T:G
peptide level
129:139
136:150
33:36
ITPNLAEFAFSLYRpc2_NP.1
p−min=0.69, pX=0.036, N=298
var.16.SERPINA1 14:94844843:T:G
peptide level
138:139
150:150
35:36
ITPNLAEFAFSLYRpc2_NP.2
p−maj=0.21, pX=1.7e−05, N=323
var.16.SERPINA1 14:94844843:T:G
peptide level
93:139
103:150
27:36
ITPNLAEFAFSLYRpc2_NP.3
p−maj=0.45, pX=0.21, N=223
var.16.SERPINA1 14:94844843:T:G
peptide level
109:139
116:150
31:36
ITPNLAEFAFSLYRpc2_NP.4
p−maj=0.29, pX=0.04, N=256
var.16.SERPINA1 14:94844843:T:G
peptide level
119:139
134:150
34:36
ITPNLAEFAFSLYRpc2_NP.5
p−min=0.22, pX=0.0023, N=287
var.16.SERPINA1 14:94844843:T:G
peptide level
9:139
13:150
5:36
ITPNLAEFAFSLYRpc3_NP.2
p−maj=0.2, pX=0.081, N=27

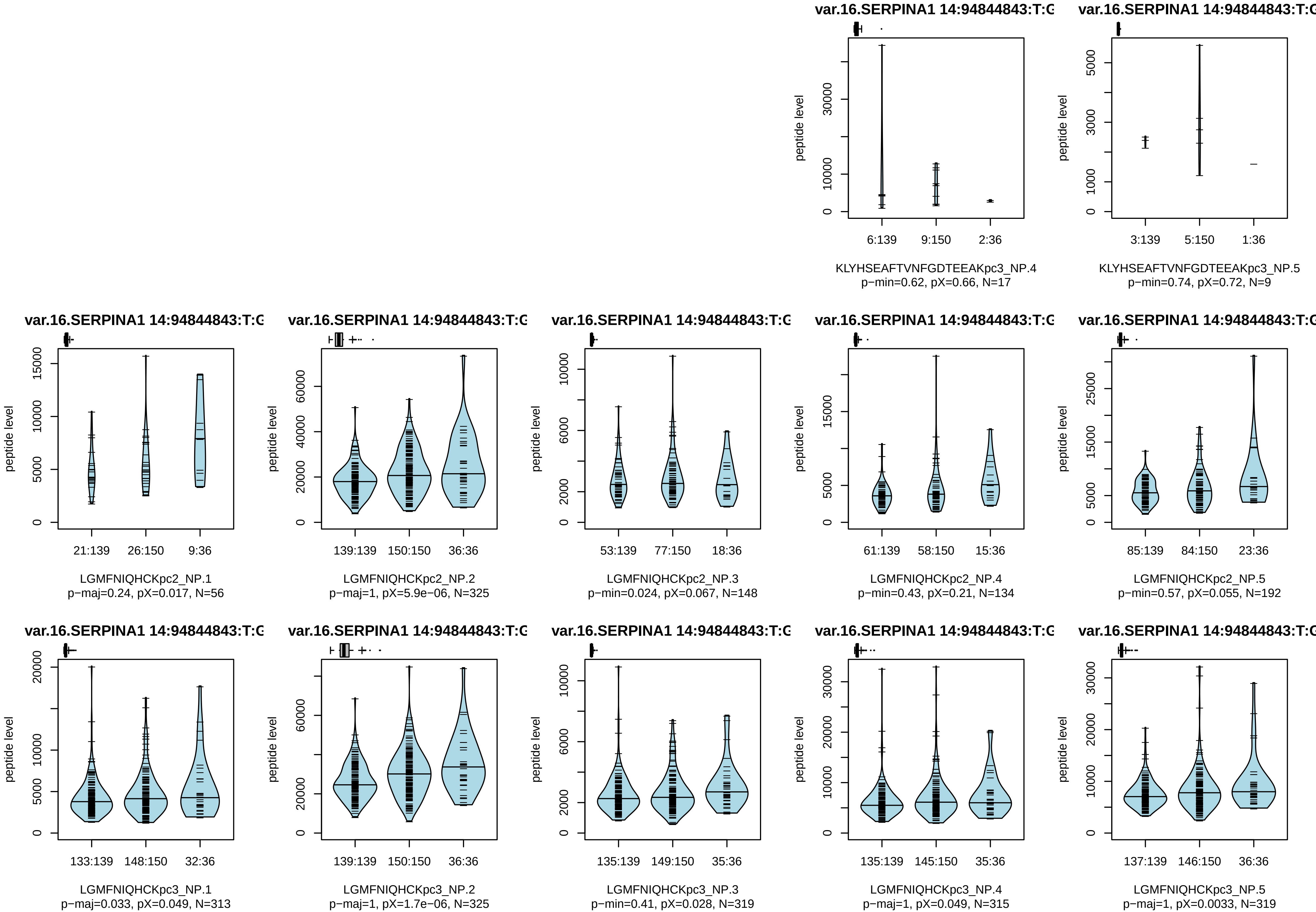
var.16.SERPINA1 14:94844843:T:G
peptide level
6:139
9:150
2:36
KLYHSEAFTVNFGDTEEAKpc3_NP.4
p−min=0.62, pX=0.66, N=17
var.16.SERPINA1 14:94844843:T:G
3:139
5:150
1:36
KLYHSEAFTVNFGDTEEAKpc3_NP.5
p−min=0.74, pX=0.72, N=9
var.16.SERPINA1 14:94844843:T:G
21:139
26:150
9:36
LGMFNIQHCKpc2_NP.1
p−maj=0.24, pX=0.017, N=56
var.16.SERPINA1 14:94844843:T:G
139:139
150:150
36:36
LGMFNIQHCKpc2_NP.2
p−maj=1, pX=5.9e−06, N=325
var.16.SERPINA1 14:94844843:T:G
53:139
77:150
18:36
LGMFNIQHCKpc2_NP.3
p−min=0.024, pX=0.067, N=148
var.16.SERPINA1 14:94844843:T:G
61:139
58:150
15:36
LGMFNIQHCKpc2_NP.4
p−min=0.43, pX=0.21, N=134
var.16.SERPINA1 14:94844843:T:G
85:139
84:150
23:36
LGMFNIQHCKpc2_NP.5
p−min=0.57, pX=0.055, N=192
var.16.SERPINA1 14:94844843:T:G
133:139
148:150
32:36
LGMFNIQHCKpc3_NP.1
p−maj=0.033, pX=0.049, N=313
var.16.SERPINA1 14:94844843:T:G
139:139
150:150
36:36
LGMFNIQHCKpc3_NP.2
p−maj=1, pX=1.7e−06, N=325
var.16.SERPINA1 14:94844843:T:G
135:139
149:150
35:36
LGMFNIQHCKpc3_NP.3
p−min=0.41, pX=0.028, N=319
var.16.SERPINA1 14:94844843:T:G
135:139
145:150
35:36
LGMFNIQHCKpc3_NP.4
p−maj=1, pX=0.049, N=315
var.16.SERPINA1 14:94844843:T:G
137:139
146:150
36:36
LGMFNIQHCKpc3_NP.5
p−maj=1, pX=0.0033, N=319

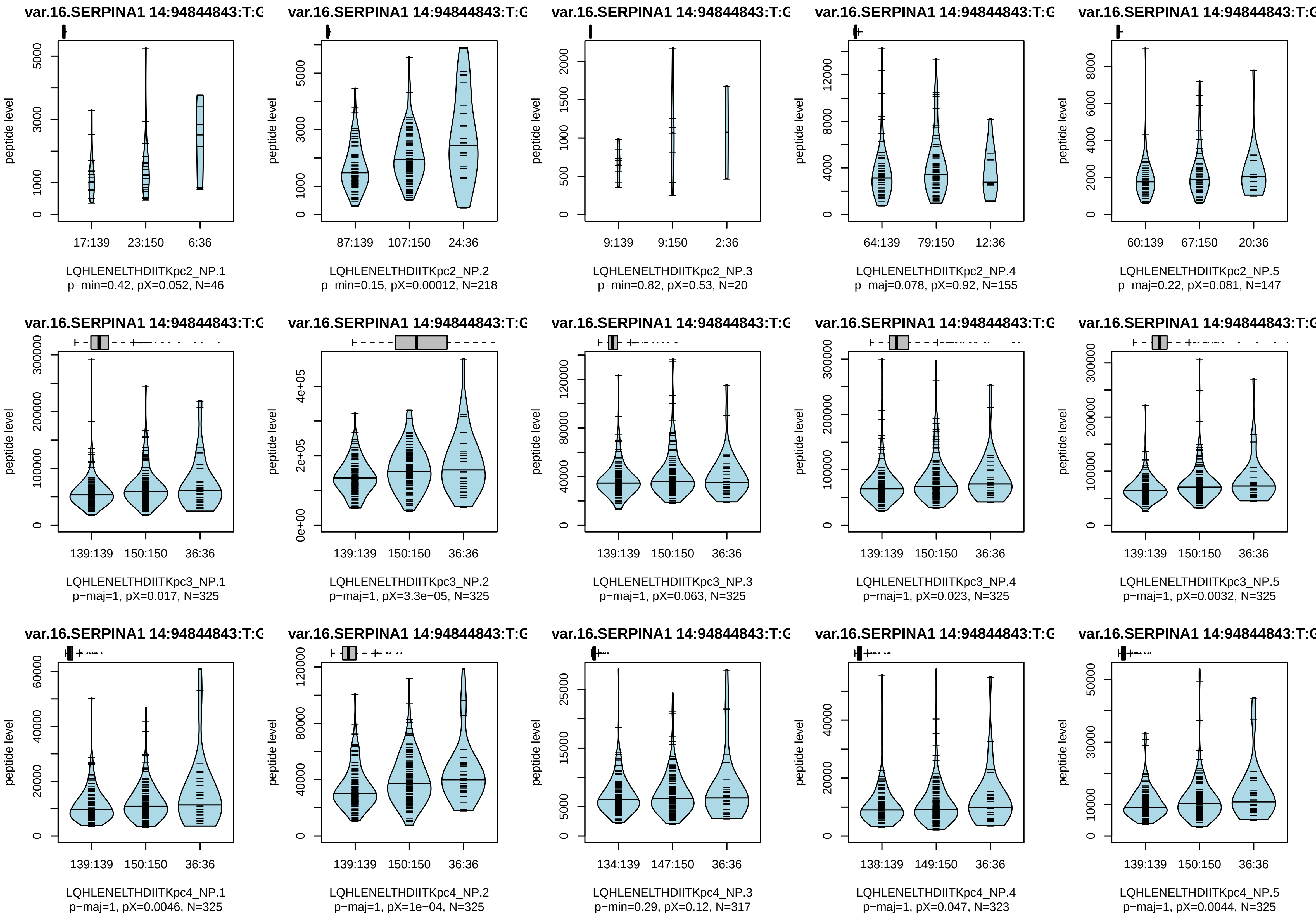
var.16.SERPINA1 14:94844843:T:G
peptide level
17:139
23:150
6:36
LQHLENELTHDIITKpc2_NP.1
p−min=0.42, pX=0.052, N=46
var.16.SERPINA1 14:94844843:T:G
peptide level
87:139
107:150
24:36
LQHLENELTHDIITKpc2_NP.2
p−min=0.15, pX=0.00012, N=218
var.16.SERPINA1 14:94844843:T:G
peptide level
9:139
9:150
2:36
LQHLENELTHDIITKpc2_NP.3
p−min=0.82, pX=0.53, N=20
var.16.SERPINA1 14:94844843:T:G
peptide level
64:139
79:150
12:36
LQHLENELTHDIITKpc2_NP.4
p−maj=0.078, pX=0.92, N=155
var.16.SERPINA1 14:94844843:T:G
peptide level
60:139
67:150
20:36
LQHLENELTHDIITKpc2_NP.5
p−maj=0.22, pX=0.081, N=147
var.16.SERPINA1 14:94844843:T:G
peptide level
139:139
150:150
36:36
LQHLENELTHDIITKpc3_NP.1
p−maj=1, pX=0.017, N=325
var.16.SERPINA1 14:94844843:T:G
peptide level
139:139
150:150
36:36
LQHLENELTHDIITKpc3_NP.2
p−maj=1, pX=3.3e−05, N=325
var.16.SERPINA1 14:94844843:T:G
peptide level
139:139
150:150
36:36
LQHLENELTHDIITKpc3_NP.3
p−maj=1, pX=0.063, N=325
var.16.SERPINA1 14:94844843:T:G
peptide level
139:139
150:150
36:36
LQHLENELTHDIITKpc3_NP.4
p−maj=1, pX=0.023, N=325
var.16.SERPINA1 14:94844843:T:G
peptide level
139:139
150:150
36:36
LQHLENELTHDIITKpc3_NP.5
p−maj=1, pX=0.0032, N=325
var.16.SERPINA1 14:94844843:T:G
peptide level
139:139
150:150
36:36
LQHLENELTHDIITKpc4_NP.1
p−maj=1, pX=0.0046, N=325
var.16.SERPINA1 14:94844843:T:G
peptide level
139:139
150:150
36:36
LQHLENELTHDIITKpc4_NP.2
p−maj=1, pX=1e−04, N=325
var.16.SERPINA1 14:94844843:T:G
peptide level
134:139
147:150
36:36
LQHLENELTHDIITKpc4_NP.3
p−min=0.29, pX=0.12, N=317
var.16.SERPINA1 14:94844843:T:G
peptide level
138:139
149:150
36:36
LQHLENELTHDIITKpc4_NP.4
p−maj=1, pX=0.047, N=323
var.16.SERPINA1 14:94844843:T:G
peptide level
139:139
150:150
36:36
LQHLENELTHDIITKpc4_NP.5
p−maj=1, pX=0.0044, N=325

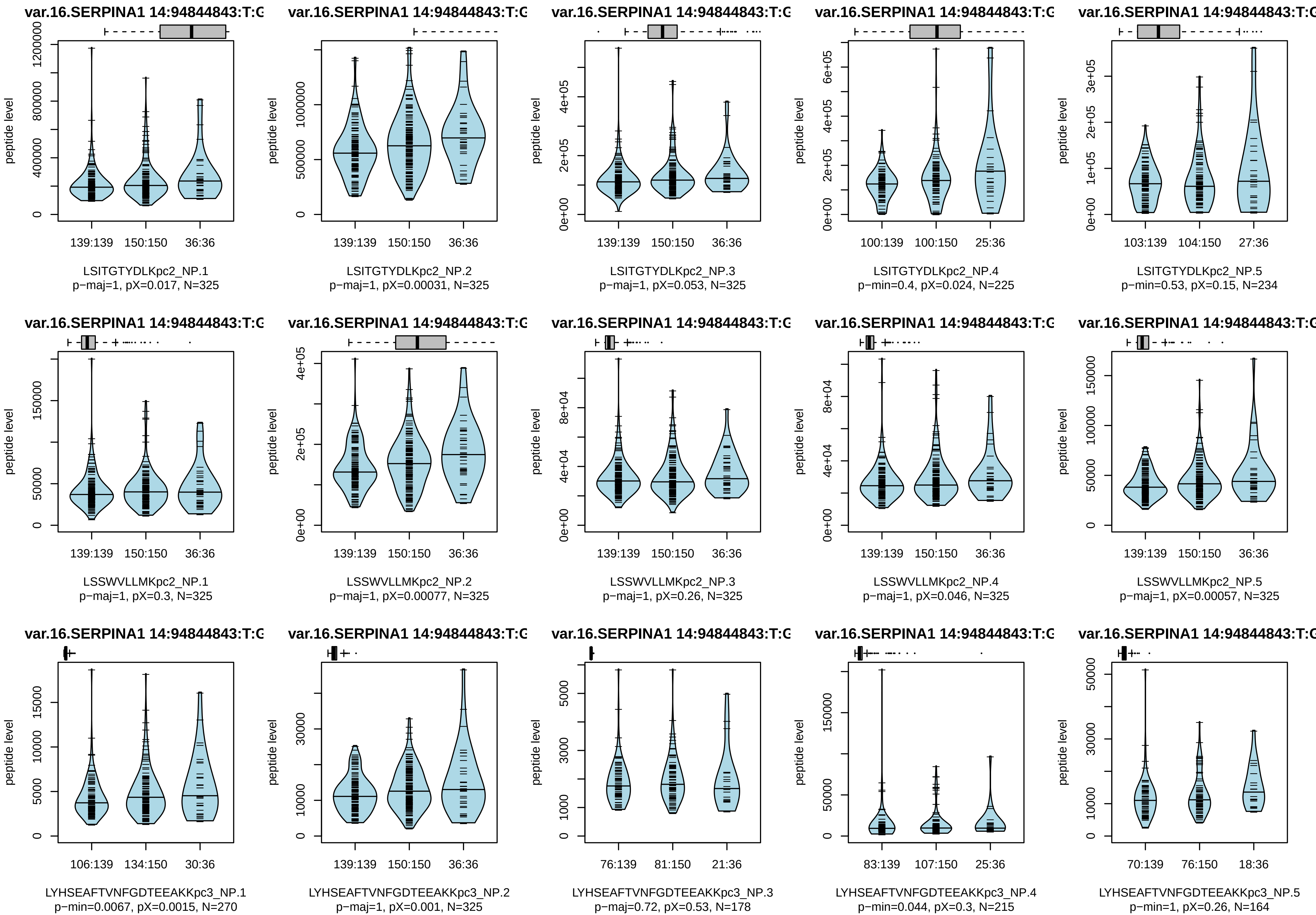
var.16.SERPINA1 14:94844843:T:G
peptide level
139:139 150:150 36:36
LSITGTYDLKpc2_NP.1
p−maj=1, pX=0.017, N=325
var.16.SERPINA1 14:94844843:T:G
peptide level
139:139 150:150 36:36
LSITGTYDLKpc2_NP.2
p−maj=1, pX=0.00031, N=325
var.16.SERPINA1 14:94844843:T:G
peptide level
139:139 150:150 36:36
LSITGTYDLKpc2_NP.3
p−maj=1, pX=0.053, N=325
var.16.SERPINA1 14:94844843:T:G
peptide level
100:139 100:150 25:36
LSITGTYDLKpc2_NP.4
p−min=0.4, pX=0.024, N=225
var.16.SERPINA1 14:94844843:T:G
peptide level
103:139 104:150 27:36
LSITGTYDLKpc2_NP.5
p−min=0.53, pX=0.15, N=234
var.16.SERPINA1 14:94844843:T:G
peptide level
139:139 150:150 36:36
LSSWVLLMKpc2_NP.1
p−maj=1, pX=0.3, N=325
var.16.SERPINA1 14:94844843:T:G
peptide level
139:139 150:150 36:36
LSSWVLLMKpc2_NP.2
p−maj=1, pX=0.00077, N=325
var.16.SERPINA1 14:94844843:T:G
peptide level
139:139 150:150 36:36
LSSWVLLMKpc2_NP.3
p−maj=1, pX=0.26, N=325
var.16.SERPINA1 14:94844843:T:G
peptide level
139:139 150:150 36:36
LSSWVLLMKpc2_NP.4
p−maj=1, pX=0.046, N=325
var.16.SERPINA1 14:94844843:T:G
peptide level
139:139 150:150 36:36
LSSWVLLMKpc2_NP.5
p−maj=1, pX=0.00057, N=325
var.16.SERPINA1 14:94844843:T:G
peptide level
106:139 134:150 30:36
LYHSEAFTVNFGDTEEAKKpc3_NP.1
p−min=0.0067, pX=0.0015, N=270
var.16.SERPINA1 14:94844843:T:G
peptide level
139:139 150:150 36:36
LYHSEAFTVNFGDTEEAKKpc3_NP.2
p−maj=1, pX=0.001, N=325
var.16.SERPINA1 14:94844843:T:G
peptide level
76:139 81:150 21:36
LYHSEAFTVNFGDTEEAKKpc3_NP.3
p−maj=0.72, pX=0.53, N=178
var.16.SERPINA1 14:94844843:T:G
peptide level
83:139 107:150 25:36
LYHSEAFTVNFGDTEEAKKpc3_NP.4
p−min=0.044, pX=0.3, N=215
var.16.SERPINA1 14:94844843:T:G
peptide level
70:139 76:150 18:36
LYHSEAFTVNFGDTEEAKKpc3_NP.5
p−min=1, pX=0.26, N=164

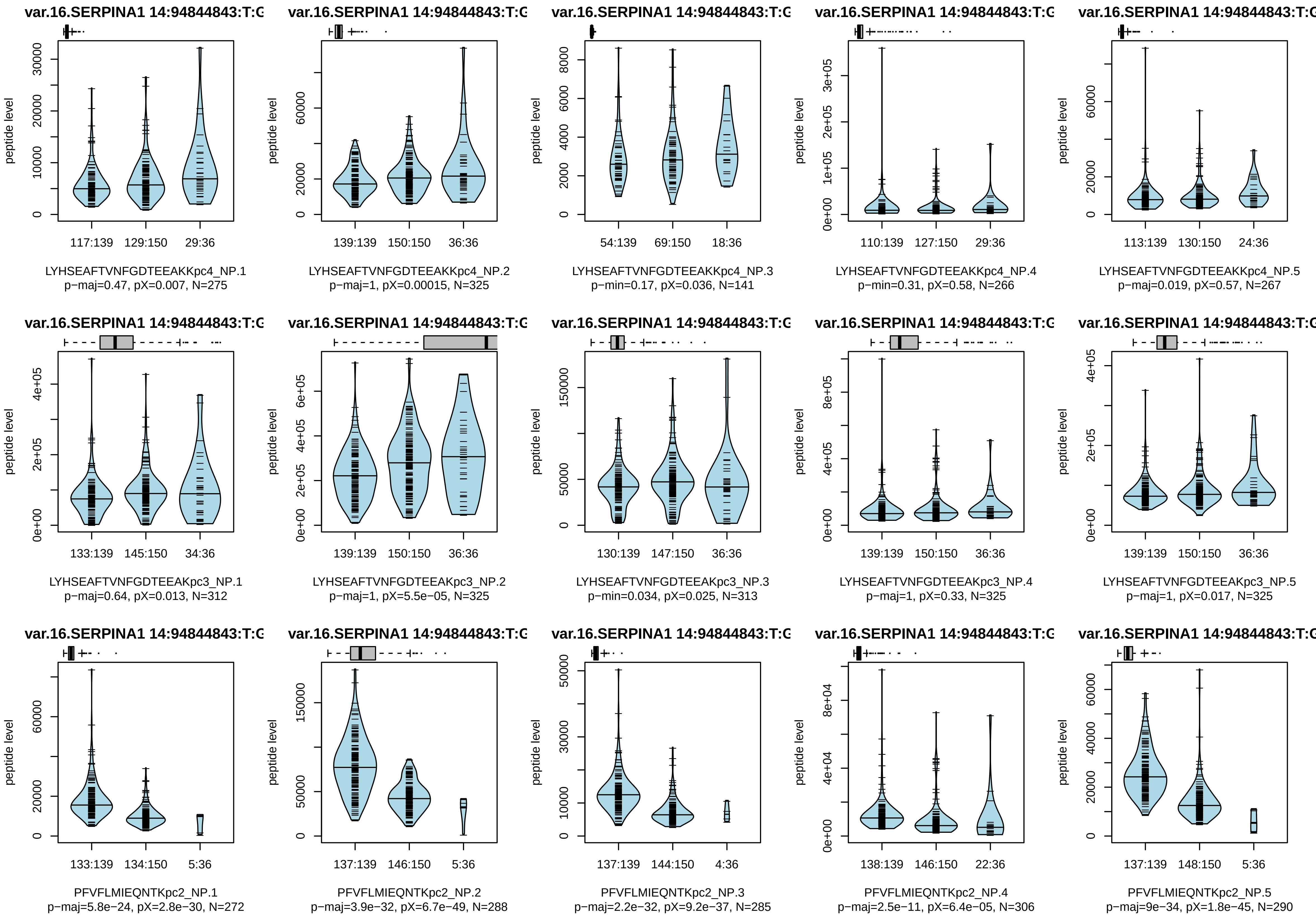
var.16.SERPINA1 14:94844843:T:G
peptide level
117:139 129:150 29:36
LYHSEAFTVNFGDTEEAKKpc4_NP.1
p−maj=0.47, pX=0.007, N=275
139:139 150:150 36:36
LYHSEAFTVNFGDTEEAKKpc4_NP.2
p−maj=1, pX=0.00015, N=325
54:139 69:150 18:36
LYHSEAFTVNFGDTEEAKKpc4_NP.3
p−min=0.17, pX=0.036, N=141
110:139 127:150 29:36
LYHSEAFTVNFGDTEEAKKpc4_NP.4
p−min=0.31, pX=0.58, N=266
113:139 130:150 24:36
LYHSEAFTVNFGDTEEAKKpc4_NP.5
p−maj=0.019, pX=0.57, N=267
133:139 145:150 34:36
LYHSEAFTVNFGDTEEAKpc3_NP.1
p−maj=0.64, pX=0.013, N=312
139:139 150:150 36:36
LYHSEAFTVNFGDTEEAKpc3_NP.2
p−maj=1, pX=5.5e−05, N=325
130:139 147:150 36:36
LYHSEAFTVNFGDTEEAKpc3_NP.3
p−min=0.034, pX=0.025, N=313
139:139 150:150 36:36
LYHSEAFTVNFGDTEEAKpc3_NP.4
p−maj=1, pX=0.33, N=325
139:139 150:150 36:36
LYHSEAFTVNFGDTEEAKpc3_NP.5
p−maj=1, pX=0.017, N=325
133:139 134:150 5:36
PFVFLMIEQNTKpc2_NP.1
p−maj=5.8e−24, pX=2.8e−30, N=272
137:139 146:150 5:36
PFVFLMIEQNTKpc2_NP.2
p−maj=3.9e−32, pX=6.7e−49, N=288
137:139 144:150 4:36
PFVFLMIEQNTKpc2_NP.3
p−maj=2.2e−32, pX=9.2e−37, N=285
138:139 146:150 22:36
PFVFLMIEQNTKpc2_NP.4
p−maj=2.5e−11, pX=6.4e−05, N=306
137:139 148:150 5:36
PFVFLMIEQNTKpc2_NP.5
p−maj=9e−34, pX=1.8e−45, N=290

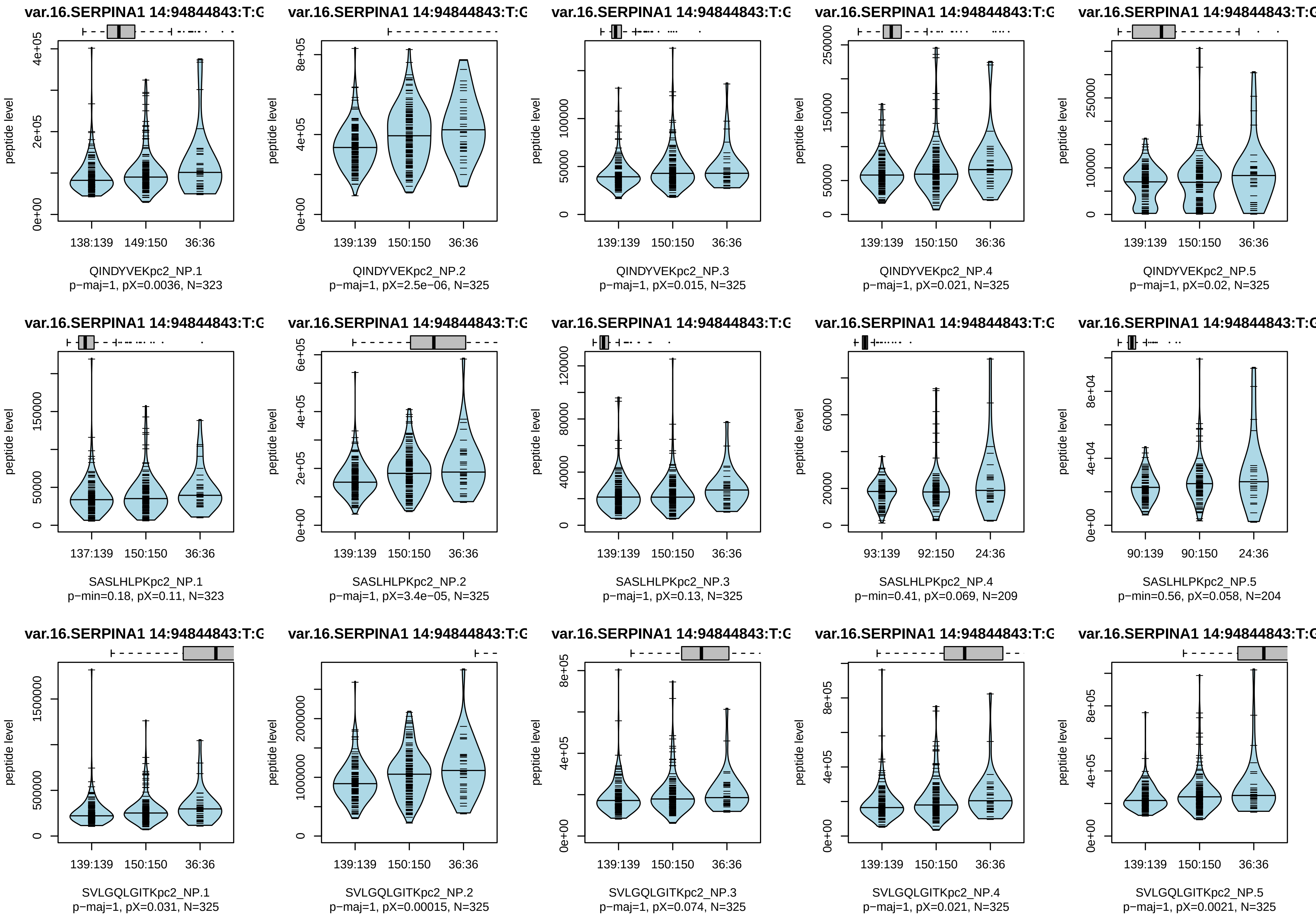
var.16.SERPINA1 14:94844843:T:G
peptide level
138:139 149:150 36:36
QINDYVEKpc2_NP.1
p−maj=1, pX=0.0036, N=323
var.16.SERPINA1 14:94844843:T:G
peptide level
139:139 150:150 36:36
QINDYVEKpc2_NP.2
p−maj=1, pX=2.5e−06, N=325
var.16.SERPINA1 14:94844843:T:G
peptide level
139:139 150:150 36:36
QINDYVEKpc2_NP.3
p−maj=1, pX=0.015, N=325
var.16.SERPINA1 14:94844843:T:G
peptide level
139:139 150:150 36:36
QINDYVEKpc2_NP.4
p−maj=1, pX=0.021, N=325
var.16.SERPINA1 14:94844843:T:G
peptide level
139:139 150:150 36:36
QINDYVEKpc2_NP.5
p−maj=1, pX=0.02, N=325
var.16.SERPINA1 14:94844843:T:G
peptide level
137:139 150:150 36:36
SASLHLPKpc2_NP.1
p−min=0.18, pX=0.11, N=323
var.16.SERPINA1 14:94844843:T:G
peptide level
139:139 150:150 36:36
SASLHLPKpc2_NP.2
p−maj=1, pX=3.4e−05, N=325
var.16.SERPINA1 14:94844843:T:G
peptide level
139:139 150:150 36:36
SASLHLPKpc2_NP.3
p−maj=1, pX=0.13, N=325
var.16.SERPINA1 14:94844843:T:G
peptide level
93:139 92:150 24:36
SASLHLPKpc2_NP.4
p−min=0.41, pX=0.069, N=209
var.16.SERPINA1 14:94844843:T:G
peptide level
90:139 90:150 24:36
SASLHLPKpc2_NP.5
p−min=0.56, pX=0.058, N=204
var.16.SERPINA1 14:94844843:T:G
peptide level
139:139 150:150 36:36
SVLGQLGITKpc2_NP.1
p−maj=1, pX=0.031, N=325
var.16.SERPINA1 14:94844843:T:G
peptide level
139:139 150:150 36:36
SVLGQLGITKpc2_NP.2
p−maj=1, pX=0.00015, N=325
var.16.SERPINA1 14:94844843:T:G
peptide level
139:139 150:150 36:36
SVLGQLGITKpc2_NP.3
p−maj=1, pX=0.074, N=325
var.16.SERPINA1 14:94844843:T:G
peptide level
139:139 150:150 36:36
SVLGQLGITKpc2_NP.4
p−maj=1, pX=0.021, N=325
var.16.SERPINA1 14:94844843:T:G
peptide level
139:139 150:150 36:36
SVLGQLGITKpc2_NP.5
p−maj=1, pX=0.0021, N=325

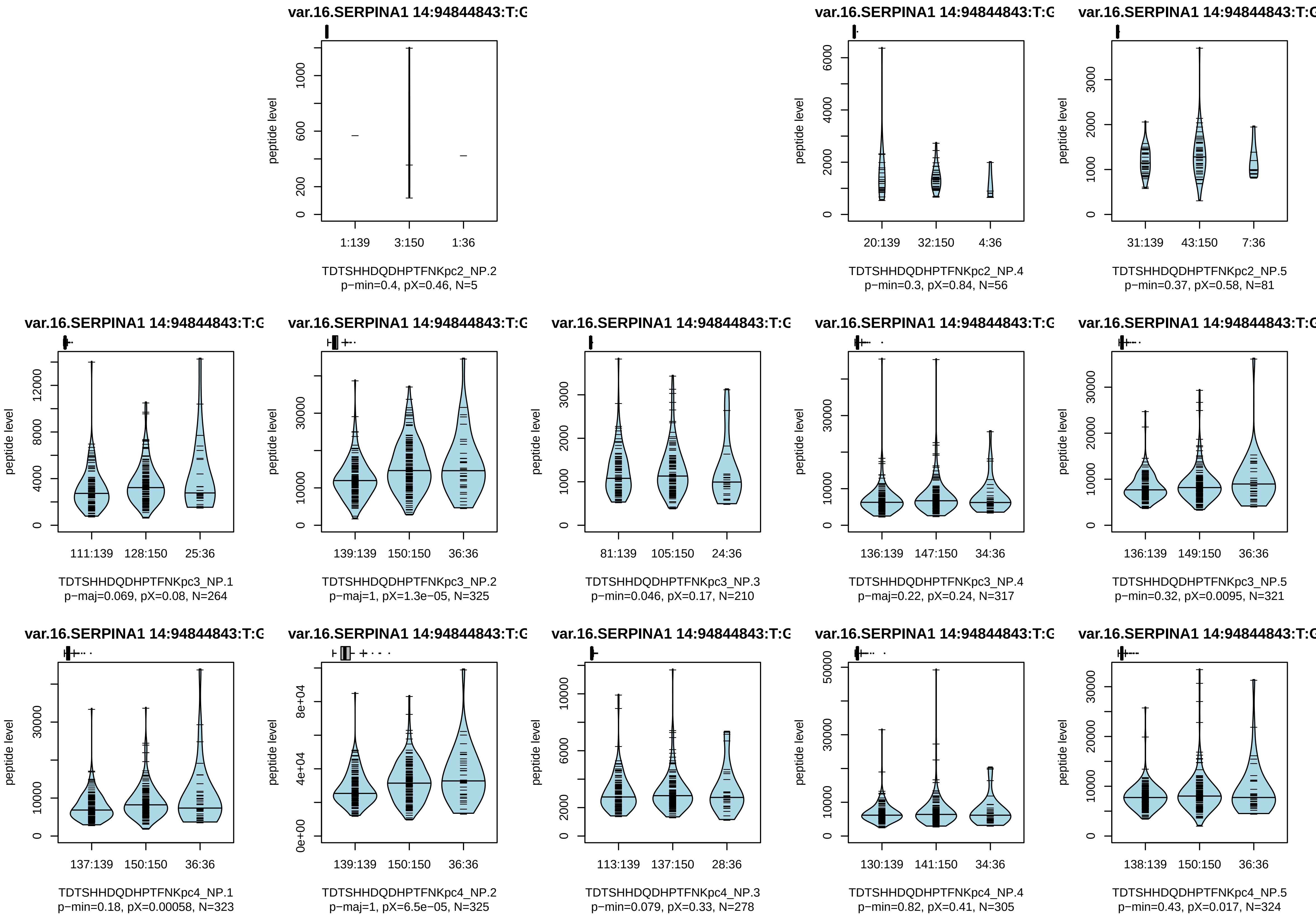
var.16.SERPINA1 14:94844843:T:G
TDTSHHDQDHPTFNKpc2_NP.2
p−min=0.4, pX=0.46, N=5
TDTSHHDQDHPTFNKpc2_NP.4
p−min=0.3, pX=0.84, N=56
TDTSHHDQDHPTFNKpc2_NP.5
p−min=0.37, pX=0.58, N=81
TDTSHHDQDHPTFNKpc3_NP.1
p−maj=0.069, pX=0.08, N=264
TDTSHHDQDHPTFNKpc3_NP.2
p−maj=1, pX=1.3e−05, N=325
TDTSHHDQDHPTFNKpc3_NP.3
p−min=0.046, pX=0.17, N=210
TDTSHHDQDHPTFNKpc3_NP.4
p−maj=0.22, pX=0.24, N=317
TDTSHHDQDHPTFNKpc3_NP.5
p−min=0.32, pX=0.0095, N=321
TDTSHHDQDHPTFNKpc4_NP.1
p−min=0.18, pX=0.00058, N=323
TDTSHHDQDHPTFNKpc4_NP.2
p−maj=1, pX=6.5e−05, N=325
TDTSHHDQDHPTFNKpc4_NP.3
p−min=0.079, pX=0.33, N=278
TDTSHHDQDHPTFNKpc4_NP.4
p−min=0.82, pX=0.41, N=305
TDTSHHDQDHPTFNKpc4_NP.5
p−min=0.43, pX=0.017, N=324
peptide level

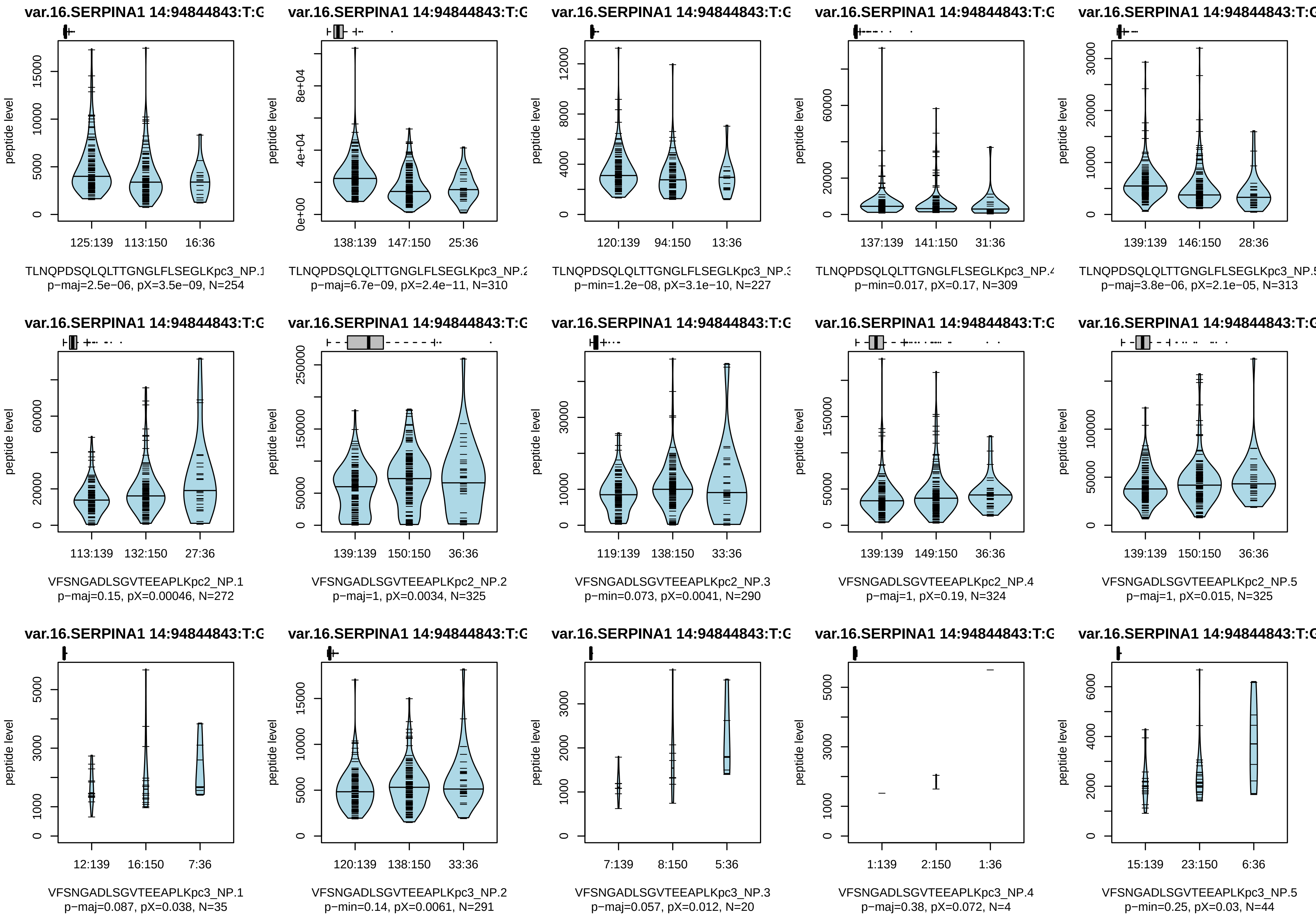
var.16.SERPINA1 14:94844843:T:G
peptide level
0 5000 10000 15000
125:139 113:150 16:36
TLNQPDSQLQLTTGNGLFLSEGLKpc3_NP.1
p−maj=2.5e−06, pX=3.5e−09, N=254
var.16.SERPINA1 14:94844843:T:G
peptide level
0e+00 4e+04 8e+04
138:139 147:150 25:36
TLNQPDSQLQLTTGNGLFLSEGLKpc3_NP.2
p−maj=6.7e−09, pX=2.4e−11, N=310
var.16.SERPINA1 14:94844843:T:G
peptide level
0 4000 8000 12000
120:139 94:150 13:36
TLNQPDSQLQLTTGNGLFLSEGLKpc3_NP.3
p−min=1.2e−08, pX=3.1e−10, N=227
var.16.SERPINA1 14:94844843:T:G
peptide level
0 20000 60000
137:139 141:150 31:36
TLNQPDSQLQLTTGNGLFLSEGLKpc3_NP.4
p−min=0.017, pX=0.17, N=309
var.16.SERPINA1 14:94844843:T:G
peptide level
0 10000 20000 30000
139:139 146:150 28:36
TLNQPDSQLQLTTGNGLFLSEGLKpc3_NP.5
p−maj=3.8e−06, pX=2.1e−05, N=313
var.16.SERPINA1 14:94844843:T:G
peptide level
0 20000 60000
113:139 132:150 27:36
VFSNGADLSGVTEEAPLKpc2_NP.1
p−maj=0.15, pX=0.00046, N=272
var.16.SERPINA1 14:94844843:T:G
peptide level
0 50000 150000 250000
139:139 150:150 36:36
VFSNGADLSGVTEEAPLKpc2_NP.2
p−maj=1, pX=0.0034, N=325
var.16.SERPINA1 14:94844843:T:G
peptide level
0 10000 20000 30000
119:139 138:150 33:36
VFSNGADLSGVTEEAPLKpc2_NP.3
p−min=0.073, pX=0.0041, N=290
var.16.SERPINA1 14:94844843:T:G
peptide level
0 50000 150000
139:139 149:150 36:36
VFSNGADLSGVTEEAPLKpc2_NP.4
p−maj=1, pX=0.19, N=324
var.16.SERPINA1 14:94844843:T:G
peptide level
0 50000 100000
139:139 150:150 36:36
VFSNGADLSGVTEEAPLKpc2_NP.5
p−maj=1, pX=0.015, N=325
var.16.SERPINA1 14:94844843:T:G
peptide level
0 1000 3000 5000
12:139 16:150 7:36
VFSNGADLSGVTEEAPLKpc3_NP.1
p−maj=0.087, pX=0.038, N=35
var.16.SERPINA1 14:94844843:T:G
peptide level
0 5000 10000 15000
120:139 138:150 33:36
VFSNGADLSGVTEEAPLKpc3_NP.2
p−min=0.14, pX=0.0061, N=291
var.16.SERPINA1 14:94844843:T:G
peptide level
0 1000 2000 3000
7:139 8:150 5:36
VFSNGADLSGVTEEAPLKpc3_NP.3
p−maj=0.057, pX=0.012, N=20
var.16.SERPINA1 14:94844843:T:G
peptide level
0 1000 3000 5000
1:139 2:150 1:36
VFSNGADLSGVTEEAPLKpc3_NP.4
p−maj=0.38, pX=0.072, N=4
var.16.SERPINA1 14:94844843:T:G
peptide level
0 2000 4000 6000
15:139 23:150 6:36
VFSNGADLSGVTEEAPLKpc3_NP.5
p−min=0.25, pX=0.03, N=44

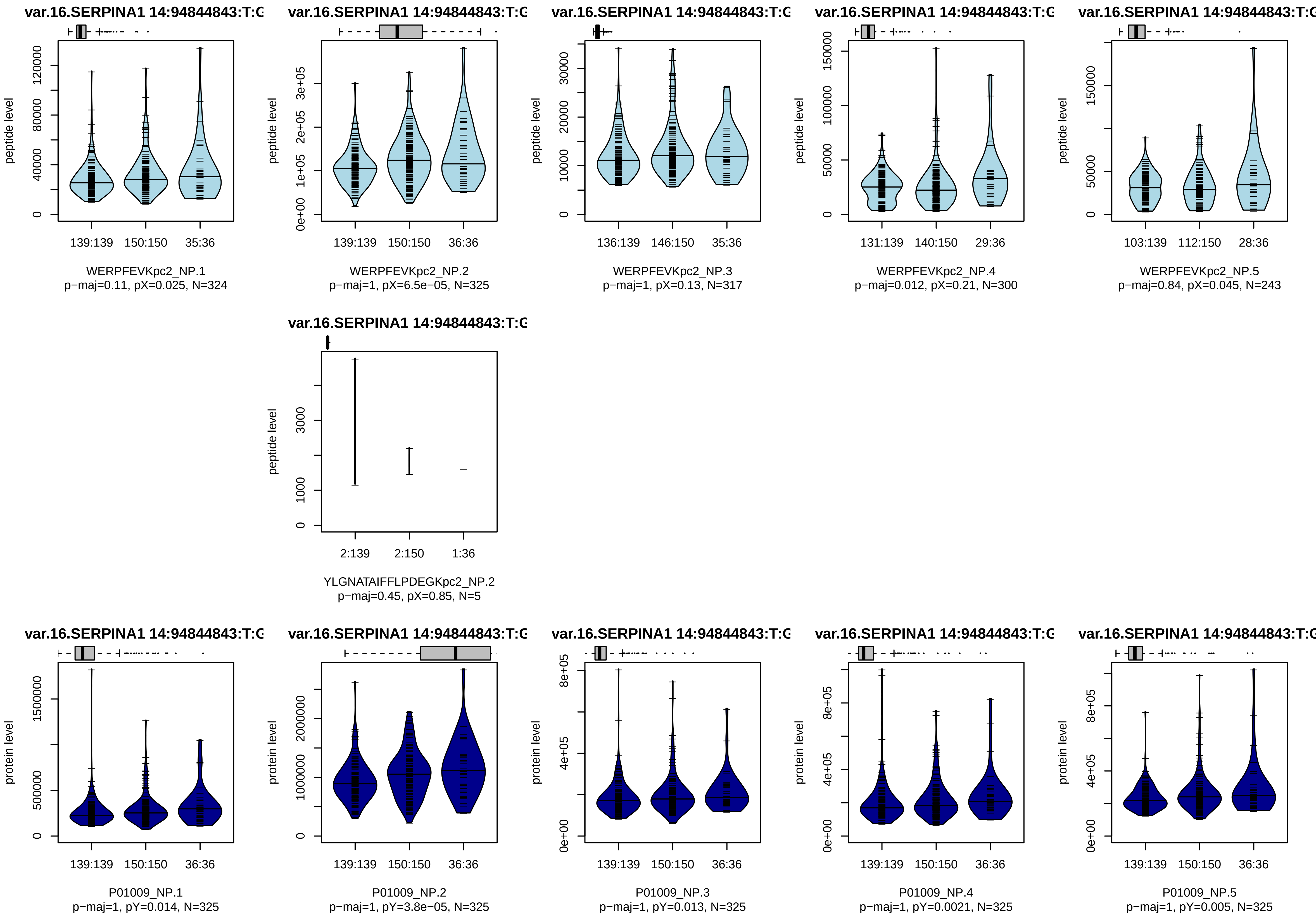

var.16.SERPINA1 14:94844843:T:G
peptide level
139:139
150:150
35:36
WERPFEVKpc2_NP.1
p−maj=0.11, pX=0.025, N=324
var.16.SERPINA1 14:94844843:T:G
peptide level
139:139
150:150
36:36
WERPFEVKpc2_NP.2
p−maj=1, pX=6.5e−05, N=325
var.16.SERPINA1 14:94844843:T:G
peptide level
136:139
146:150
35:36
WERPFEVKpc2_NP.3
p−maj=1, pX=0.13, N=317
var.16.SERPINA1 14:94844843:T:G
peptide level
131:139
140:150
29:36
WERPFEVKpc2_NP.4
p−maj=0.012, pX=0.21, N=300
var.16.SERPINA1 14:94844843:T:G
peptide level
103:139
112:150
28:36
WERPFEVKpc2_NP.5
p−maj=0.84, pX=0.045, N=243
var.16.SERPINA1 14:94844843:T:G
peptide level
2:139
2:150
1:36
YLGNATAIFFLPDEGKpc2_NP.2
p−maj=0.45, pX=0.85, N=5
var.16.SERPINA1 14:94844843:T:G
protein level
139:139
150:150
36:36
P01009_NP.1
p−maj=1, pY=0.014, N=325
var.16.SERPINA1 14:94844843:T:G
protein level
139:139
150:150
36:36
P01009_NP.2
p−maj=1, pY=3.8e−05, N=325
var.16.SERPINA1 14:94844843:T:G
protein level
139:139
150:150
36:36
P01009_NP.3
p−maj=1, pY=0.013, N=325
var.16.SERPINA1 14:94844843:T:G
protein level
139:139
150:150
36:36
P01009_NP.4
p−maj=1, pY=0.0021, N=325
var.16.SERPINA1 14:94844843:T:G
protein level
139:139
150:150
36:36
P01009_NP.5
p−maj=1, pY=0.005, N=325

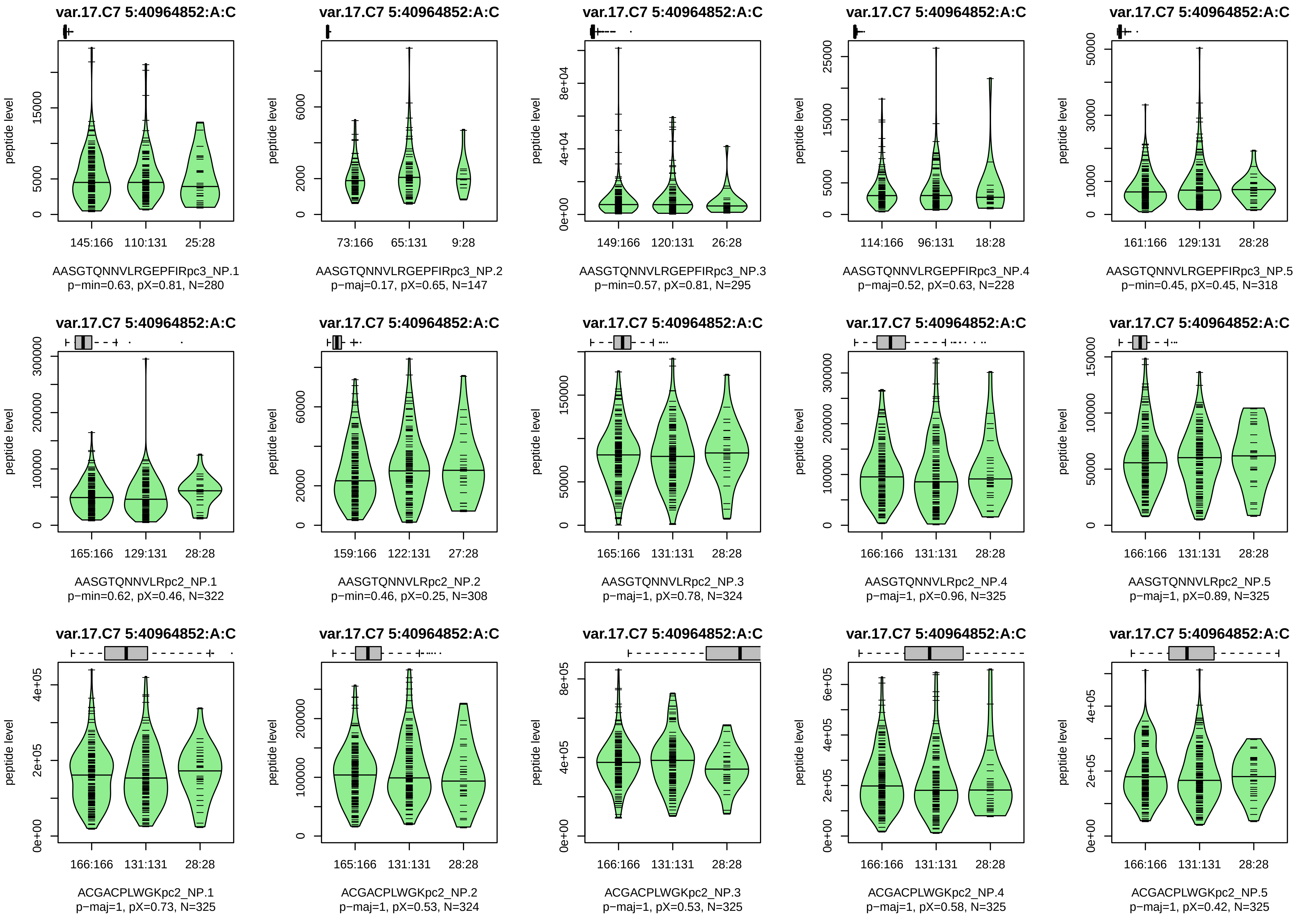

var.17.C7 5:40964852:A:C
peptide level
145:166 110:131 25:28
AASGTQNNVLRGEPFIRpc3_NP.1
p−min=0.63, pX=0.81, N=280
var.17.C7 5:40964852:A:C
peptide level
73:166 65:131 9:28
AASGTQNNVLRGEPFIRpc3_NP.2
p−maj=0.17, pX=0.65, N=147
var.17.C7 5:40964852:A:C
peptide level
149:166 120:131 26:28
AASGTQNNVLRGEPFIRpc3_NP.3
p−min=0.57, pX=0.81, N=295
var.17.C7 5:40964852:A:C
peptide level
114:166 96:131 18:28
AASGTQNNVLRGEPFIRpc3_NP.4
p−maj=0.52, pX=0.63, N=228
var.17.C7 5:40964852:A:C
peptide level
161:166 129:131 28:28
AASGTQNNVLRGEPFIRpc3_NP.5
p−min=0.45, pX=0.45, N=318
var.17.C7 5:40964852:A:C
peptide level
165:166 129:131 28:28
AASGTQNNVLRpc2_NP.1
p−min=0.62, pX=0.46, N=322
var.17.C7 5:40964852:A:C
peptide level
159:166 122:131 27:28
AASGTQNNVLRpc2_NP.2
p−min=0.46, pX=0.25, N=308
var.17.C7 5:40964852:A:C
peptide level
165:166 131:131 28:28
AASGTQNNVLRpc2_NP.3
p−maj=1, pX=0.78, N=324
var.17.C7 5:40964852:A:C
peptide level
166:166 131:131 28:28
AASGTQNNVLRpc2_NP.4
p−maj=1, pX=0.96, N=325
var.17.C7 5:40964852:A:C
peptide level
166:166 131:131 28:28
AASGTQNNVLRpc2_NP.5
p−maj=1, pX=0.89, N=325
var.17.C7 5:40964852:A:C
peptide level
166:166 131:131 28:28
ACGACPLWGKpc2_NP.1
p−maj=1, pX=0.73, N=325
var.17.C7 5:40964852:A:C
peptide level
165:166 131:131 28:28
ACGACPLWGKpc2_NP.2
p−maj=1, pX=0.53, N=324
var.17.C7 5:40964852:A:C
peptide level
166:166 131:131 28:28
ACGACPLWGKpc2_NP.3
p−maj=1, pX=0.53, N=325
var.17.C7 5:40964852:A:C
peptide level
166:166 131:131 28:28
ACGACPLWGKpc2_NP.4
p−maj=1, pX=0.58, N=325
var.17.C7 5:40964852:A:C
peptide level
166:166 131:131 28:28
ACGACPLWGKpc2_NP.5
p−maj=1, pX=0.42, N=325

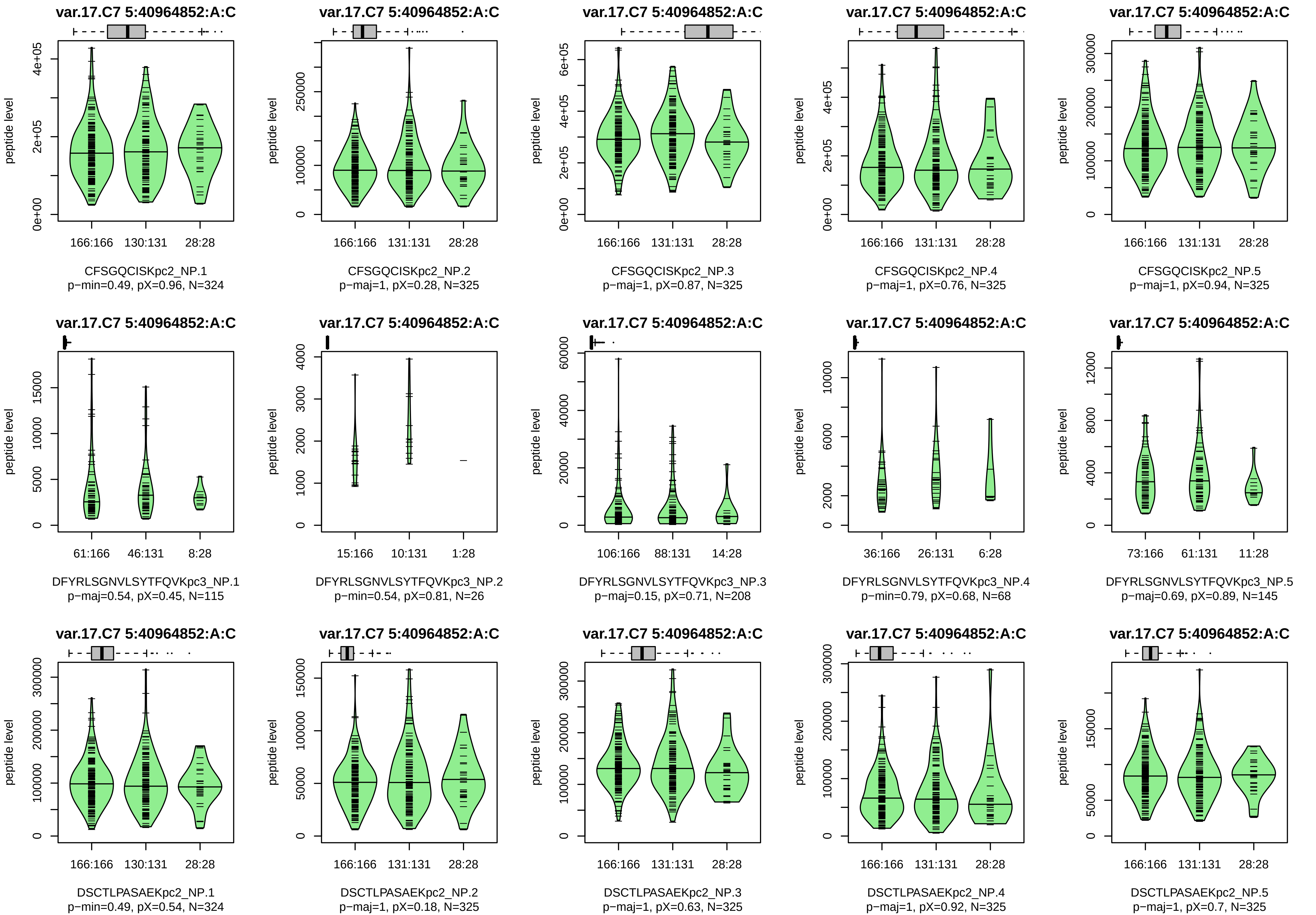
var.17.C7 5:40964852:A:C
peptide level
0e+00
2e+05
4e+05
166:166
130:131
28:28
CFSGQCISKpc2_NP.1
p−min=0.49, pX=0.96, N=324
var.17.C7 5:40964852:A:C
peptide level
0
100000
250000
166:166
131:131
28:28
CFSGQCISKpc2_NP.2
p−maj=1, pX=0.28, N=325
var.17.C7 5:40964852:A:C
peptide level
0e+00
2e+05
4e+05
6e+05
166:166
131:131
28:28
CFSGQCISKpc2_NP.3
p−maj=1, pX=0.87, N=325
var.17.C7 5:40964852:A:C
peptide level
0e+00
2e+05
4e+05
166:166
131:131
28:28
CFSGQCISKpc2_NP.4
p−maj=1, pX=0.76, N=325
var.17.C7 5:40964852:A:C
peptide level
0
100000
200000
300000
166:166
131:131
28:28
CFSGQCISKpc2_NP.5
p−maj=1, pX=0.94, N=325
var.17.C7 5:40964852:A:C
peptide level
0
5000
10000
15000
61:166
46:131
8:28
DFYRLSGNVLSYTFQVKpc3_NP.1
p−maj=0.54, pX=0.45, N=115
var.17.C7 5:40964852:A:C
peptide level
0
1000
2000
3000
4000
15:166
10:131
1:28
DFYRLSGNVLSYTFQVKpc3_NP.2
p−min=0.54, pX=0.81, N=26
var.17.C7 5:40964852:A:C
peptide level
0
20000
40000
60000
106:166
88:131
14:28
DFYRLSGNVLSYTFQVKpc3_NP.3
p−maj=0.15, pX=0.71, N=208
var.17.C7 5:40964852:A:C
peptide level
0
2000
6000
10000
36:166
26:131
6:28
DFYRLSGNVLSYTFQVKpc3_NP.4
p−min=0.79, pX=0.68, N=68
var.17.C7 5:40964852:A:C
peptide level
0
4000
8000
12000
73:166
61:131
11:28
DFYRLSGNVLSYTFQVKpc3_NP.5
p−maj=0.69, pX=0.89, N=145
var.17.C7 5:40964852:A:C
peptide level
0
100000
200000
300000
166:166
130:131
28:28
DSCTLPASAEKpc2_NP.1
p−min=0.49, pX=0.54, N=324
var.17.C7 5:40964852:A:C
peptide level
0
50000
100000
150000
166:166
131:131
28:28
DSCTLPASAEKpc2_NP.2
p−maj=1, pX=0.18, N=325
var.17.C7 5:40964852:A:C
peptide level
0
100000
200000
300000
166:166
131:131
28:28
DSCTLPASAEKpc2_NP.3
p−maj=1, pX=0.63, N=325
var.17.C7 5:40964852:A:C
peptide level
0
100000
200000
300000
166:166
131:131
28:28
DSCTLPASAEKpc2_NP.4
p−maj=1, pX=0.92, N=325
var.17.C7 5:40964852:A:C
peptide level
0
50000
150000
166:166
131:131
28:28
DSCTLPASAEKpc2_NP.5
p−maj=1, pX=0.7, N=325

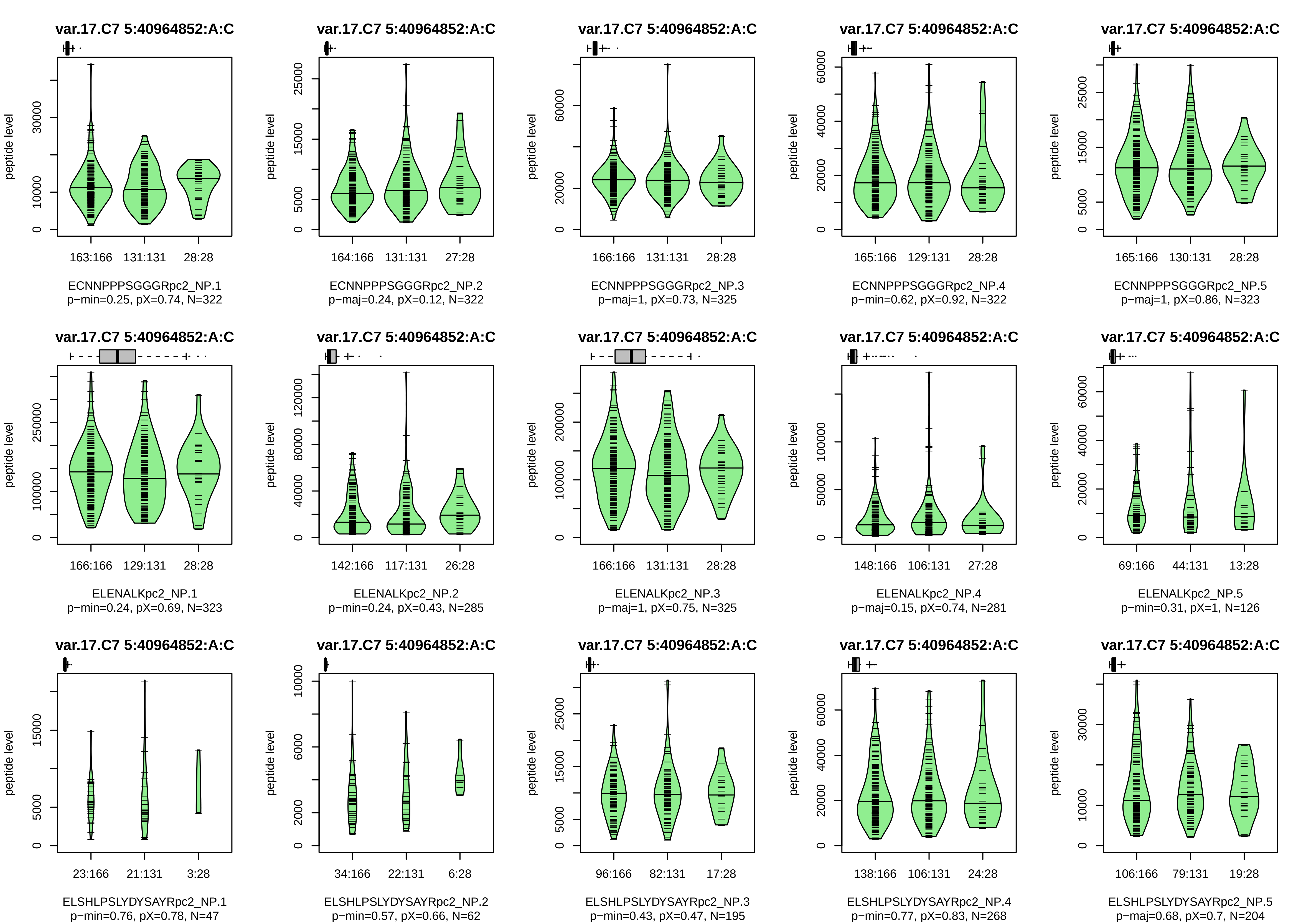
var.17.C7 5:40964852:A:C
peptide level
163:166
131:131
28:28
ECNNPPPSGGGRpc2_NP.1
p−min=0.25, pX=0.74, N=322
var.17.C7 5:40964852:A:C
peptide level
164:166
131:131
27:28
ECNNPPPSGGGRpc2_NP.2
p−maj=0.24, pX=0.12, N=322
var.17.C7 5:40964852:A:C
peptide level
166:166
131:131
28:28
ECNNPPPSGGGRpc2_NP.3
p−maj=1, pX=0.73, N=325
var.17.C7 5:40964852:A:C
peptide level
165:166
129:131
28:28
ECNNPPPSGGGRpc2_NP.4
p−min=0.62, pX=0.92, N=322
var.17.C7 5:40964852:A:C
peptide level
165:166
130:131
28:28
ECNNPPPSGGGRpc2_NP.5
p−maj=1, pX=0.86, N=323
var.17.C7 5:40964852:A:C
peptide level
166:166
129:131
28:28
ELENALKpc2_NP.1
p−min=0.24, pX=0.69, N=323
var.17.C7 5:40964852:A:C
peptide level
142:166
117:131
26:28
ELENALKpc2_NP.2
p−min=0.24, pX=0.43, N=285
var.17.C7 5:40964852:A:C
peptide level
166:166
131:131
28:28
ELENALKpc2_NP.3
p−maj=1, pX=0.75, N=325
var.17.C7 5:40964852:A:C
peptide level
148:166
106:131
27:28
ELENALKpc2_NP.4
p−maj=0.15, pX=0.74, N=281
var.17.C7 5:40964852:A:C
peptide level
69:166
44:131
13:28
ELENALKpc2_NP.5
p−min=0.31, pX=1, N=126
var.17.C7 5:40964852:A:C
peptide level
23:166
21:131
3:28
ELSHLPSLYDYSAYRpc2_NP.1
p−min=0.76, pX=0.78, N=47
var.17.C7 5:40964852:A:C
peptide level
34:166
22:131
6:28
ELSHLPSLYDYSAYRpc2_NP.2
p−min=0.57, pX=0.66, N=62
var.17.C7 5:40964852:A:C
peptide level
96:166
82:131
17:28
ELSHLPSLYDYSAYRpc2_NP.3
p−min=0.43, pX=0.47, N=195
var.17.C7 5:40964852:A:C
peptide level
138:166
106:131
24:28
ELSHLPSLYDYSAYRpc2_NP.4
p−min=0.77, pX=0.83, N=268
var.17.C7 5:40964852:A:C
peptide level
106:166
79:131
19:28
ELSHLPSLYDYSAYRpc2_NP.5
p−maj=0.68, pX=0.7, N=204

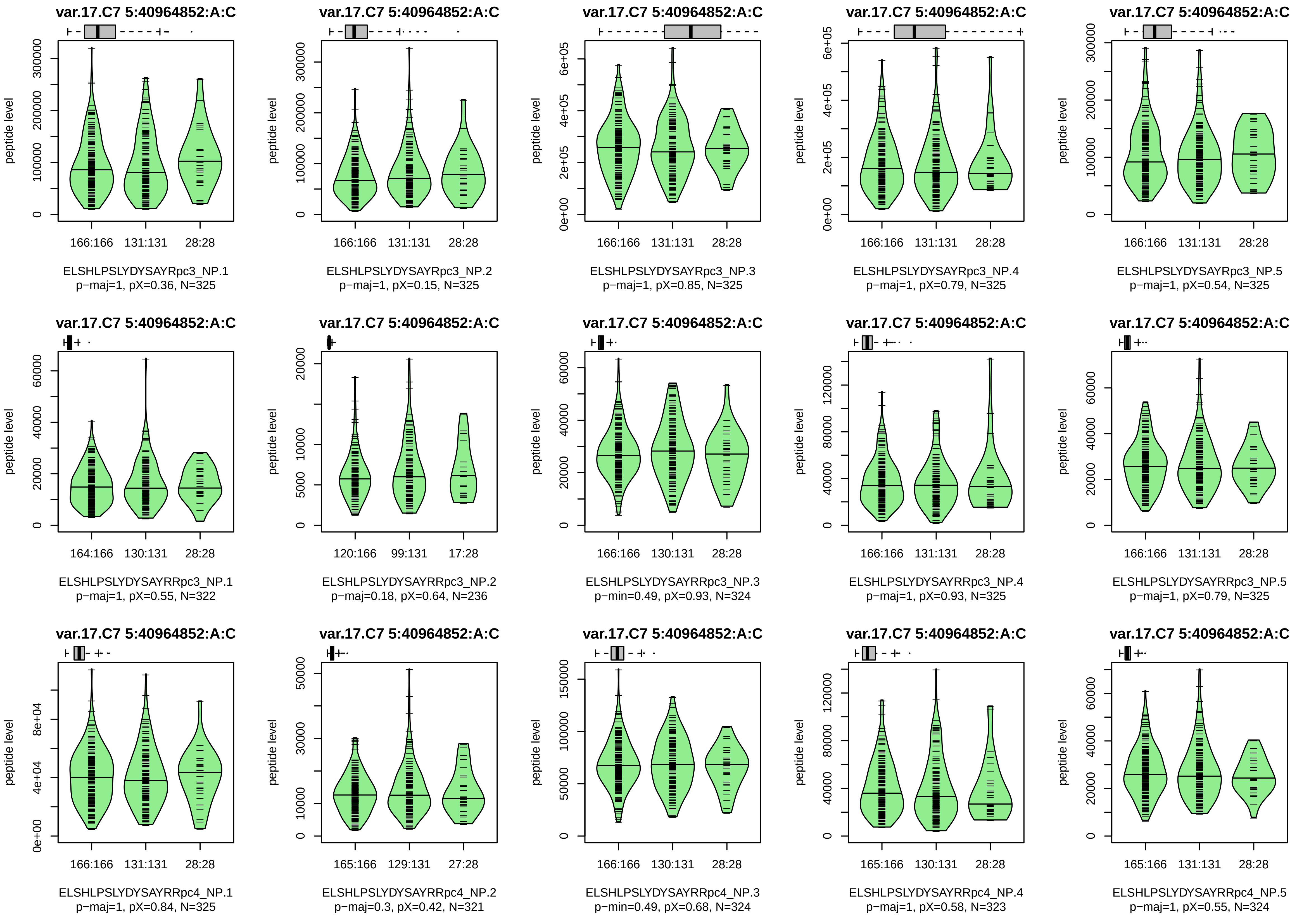
var.17.C7 5:40964852:A:C
peptide level
166:166
131:131
28:28
ELSHLPSLYDYSAYRpc3_NP.1
p−maj=1, pX=0.36, N=325
var.17.C7 5:40964852:A:C
166:166
131:131
28:28
ELSHLPSLYDYSAYRpc3_NP.2
p−maj=1, pX=0.15, N=325
var.17.C7 5:40964852:A:C
166:166
131:131
28:28
ELSHLPSLYDYSAYRpc3_NP.3
p−maj=1, pX=0.85, N=325
var.17.C7 5:40964852:A:C
166:166
131:131
28:28
ELSHLPSLYDYSAYRpc3_NP.4
p−maj=1, pX=0.79, N=325
var.17.C7 5:40964852:A:C
166:166
131:131
28:28
ELSHLPSLYDYSAYRpc3_NP.5
p−maj=1, pX=0.54, N=325
var.17.C7 5:40964852:A:C
164:166
130:131
28:28
ELSHLPSLYDYSAYRRpc3_NP.1
p−maj=1, pX=0.55, N=322
var.17.C7 5:40964852:A:C
120:166
99:131
17:28
ELSHLPSLYDYSAYRRpc3_NP.2
p−maj=0.18, pX=0.64, N=236
var.17.C7 5:40964852:A:C
166:166
130:131
28:28
ELSHLPSLYDYSAYRRpc3_NP.3
p−min=0.49, pX=0.93, N=324
var.17.C7 5:40964852:A:C
166:166
131:131
28:28
ELSHLPSLYDYSAYRRpc3_NP.4
p−maj=1, pX=0.93, N=325
var.17.C7 5:40964852:A:C
166:166
131:131
28:28
ELSHLPSLYDYSAYRRpc3_NP.5
p−maj=1, pX=0.79, N=325
var.17.C7 5:40964852:A:C
166:166
131:131
28:28
ELSHLPSLYDYSAYRRpc4_NP.1
p−maj=1, pX=0.84, N=325
var.17.C7 5:40964852:A:C
165:166
129:131
27:28
ELSHLPSLYDYSAYRRpc4_NP.2
p−maj=0.3, pX=0.42, N=321
var.17.C7 5:40964852:A:C
166:166
130:131
28:28
ELSHLPSLYDYSAYRRpc4_NP.3
p−min=0.49, pX=0.68, N=324
var.17.C7 5:40964852:A:C
165:166
130:131
28:28
ELSHLPSLYDYSAYRRpc4_NP.4
p−maj=1, pX=0.58, N=323
var.17.C7 5:40964852:A:C
165:166
131:131
28:28
ELSHLPSLYDYSAYRRpc4_NP.5
p−maj=1, pX=0.55, N=324

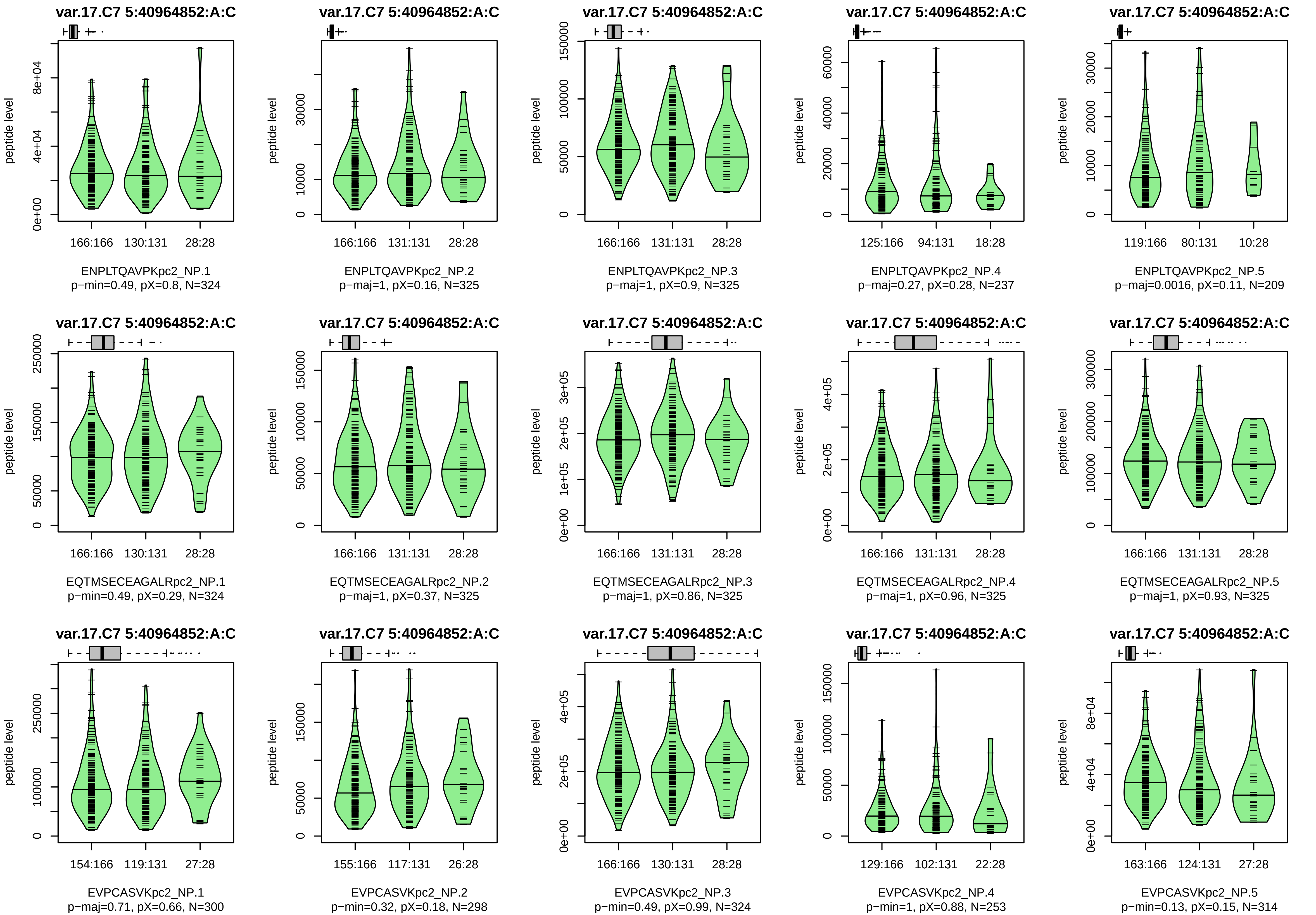

var.17.C7 5:40964852:A:C
peptide level
166:166 130:131 28:28
ENPLTQAVPKpc2_NP.1
p−min=0.49, pX=0.8, N=324
var.17.C7 5:40964852:A:C
peptide level
166:166 131:131 28:28
ENPLTQAVPKpc2_NP.2
p−maj=1, pX=0.16, N=325
var.17.C7 5:40964852:A:C
peptide level
166:166 131:131 28:28
ENPLTQAVPKpc2_NP.3
p−maj=1, pX=0.9, N=325
var.17.C7 5:40964852:A:C
peptide level
125:166 94:131 18:28
ENPLTQAVPKpc2_NP.4
p−maj=0.27, pX=0.28, N=237
var.17.C7 5:40964852:A:C
peptide level
119:166 80:131 10:28
ENPLTQAVPKpc2_NP.5
p−maj=0.0016, pX=0.11, N=209
var.17.C7 5:40964852:A:C
peptide level
166:166 130:131 28:28
EQTMSECEAGALRpc2_NP.1
p−min=0.49, pX=0.29, N=324
var.17.C7 5:40964852:A:C
peptide level
166:166 131:131 28:28
EQTMSECEAGALRpc2_NP.2
p−maj=1, pX=0.37, N=325
var.17.C7 5:40964852:A:C
peptide level
166:166 131:131 28:28
EQTMSECEAGALRpc2_NP.3
p−maj=1, pX=0.86, N=325
var.17.C7 5:40964852:A:C
peptide level
166:166 131:131 28:28
EQTMSECEAGALRpc2_NP.4
p−maj=1, pX=0.96, N=325
var.17.C7 5:40964852:A:C
peptide level
166:166 131:131 28:28
EQTMSECEAGALRpc2_NP.5
p−maj=1, pX=0.93, N=325
var.17.C7 5:40964852:A:C
peptide level
154:166 119:131 27:28
EVPCASVKpc2_NP.1
p−maj=0.71, pX=0.66, N=300
var.17.C7 5:40964852:A:C
peptide level
155:166 117:131 26:28
EVPCASVKpc2_NP.2
p−min=0.32, pX=0.18, N=298
var.17.C7 5:40964852:A:C
peptide level
166:166 130:131 28:28
EVPCASVKpc2_NP.3
p−min=0.49, pX=0.99, N=324
var.17.C7 5:40964852:A:C
peptide level
129:166 102:131 22:28
EVPCASVKpc2_NP.4
p−min=1, pX=0.88, N=253
var.17.C7 5:40964852:A:C
peptide level
163:166 124:131 27:28
EVPCASVKpc2_NP.5
p−min=0.13, pX=0.15, N=314

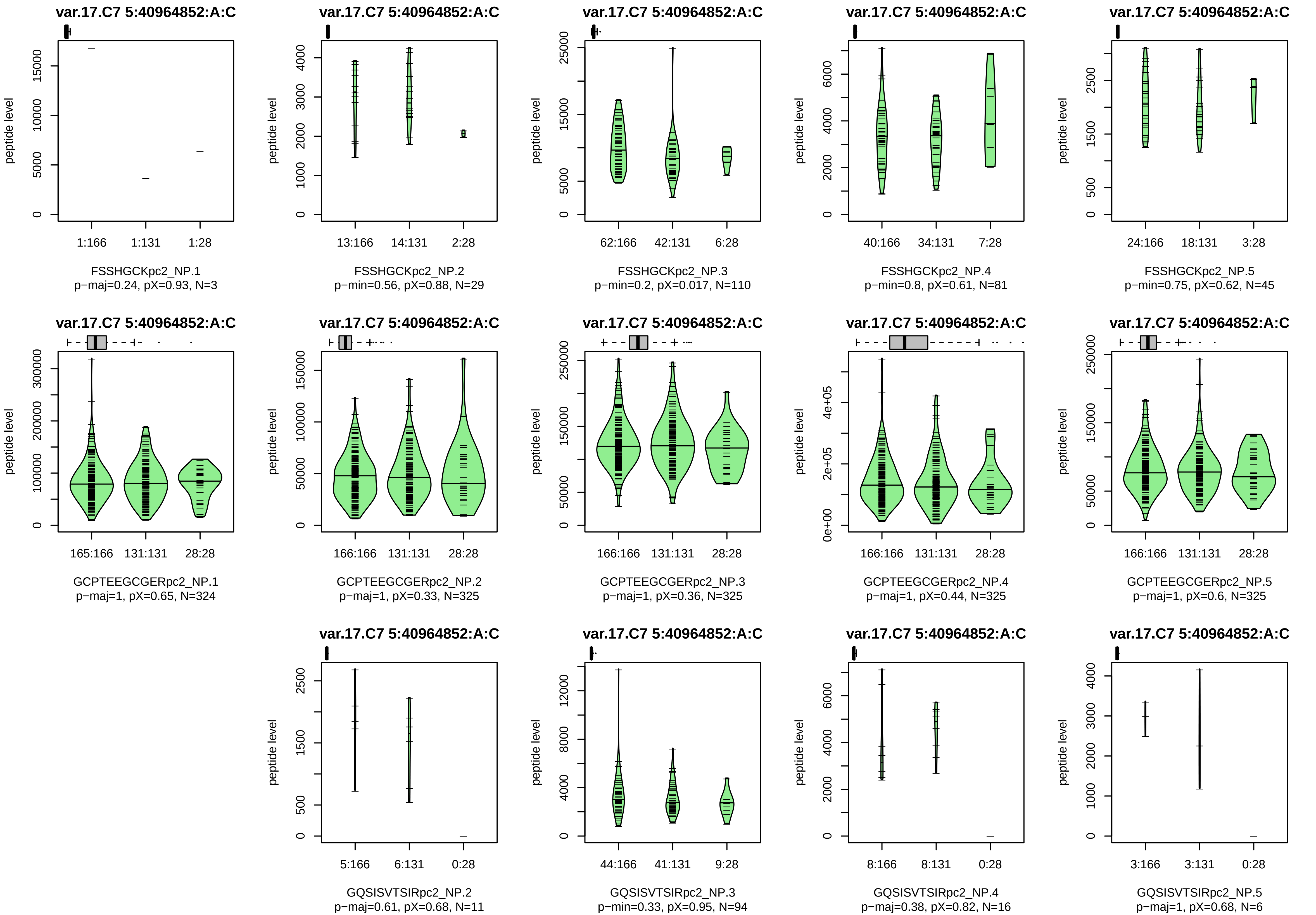

var.17.C7 5:40964852:A:C
peptide level
1:166
1:131
1:28
FSSHGCKpc2_NP.1
p−maj=0.24, pX=0.93, N=3
var.17.C7 5:40964852:A:C
peptide level
13:166
14:131
2:28
FSSHGCKpc2_NP.2
p−min=0.56, pX=0.88, N=29
var.17.C7 5:40964852:A:C
peptide level
62:166
42:131
6:28
FSSHGCKpc2_NP.3
p−min=0.2, pX=0.017, N=110
var.17.C7 5:40964852:A:C
peptide level
40:166
34:131
7:28
FSSHGCKpc2_NP.4
p−min=0.8, pX=0.61, N=81
var.17.C7 5:40964852:A:C
peptide level
24:166
18:131
3:28
FSSHGCKpc2_NP.5
p−min=0.75, pX=0.62, N=45
var.17.C7 5:40964852:A:C
peptide level
165:166
131:131
28:28
GCPTEEGCGERpc2_NP.1
p−maj=1, pX=0.65, N=324
var.17.C7 5:40964852:A:C
peptide level
166:166
131:131
28:28
GCPTEEGCGERpc2_NP.2
p−maj=1, pX=0.33, N=325
var.17.C7 5:40964852:A:C
peptide level
166:166
131:131
28:28
GCPTEEGCGERpc2_NP.3
p−maj=1, pX=0.36, N=325
var.17.C7 5:40964852:A:C
peptide level
166:166
131:131
28:28
GCPTEEGCGERpc2_NP.4
p−maj=1, pX=0.44, N=325
var.17.C7 5:40964852:A:C
peptide level
166:166
131:131
28:28
GCPTEEGCGERpc2_NP.5
p−maj=1, pX=0.6, N=325
var.17.C7 5:40964852:A:C
peptide level
5:166
6:131
0:28
GQSISVTSIRpc2_NP.2
p−maj=0.61, pX=0.68, N=11
var.17.C7 5:40964852:A:C
peptide level
44:166
41:131
9:28
GQSISVTSIRpc2_NP.3
p−min=0.33, pX=0.95, N=94
var.17.C7 5:40964852:A:C
peptide level
8:166
8:131
0:28
GQSISVTSIRpc2_NP.4
p−maj=0.38, pX=0.82, N=16
var.17.C7 5:40964852:A:C
peptide level
3:166
3:131
0:28
GQSISVTSIRpc2_NP.5
p−maj=1, pX=0.68, N=6

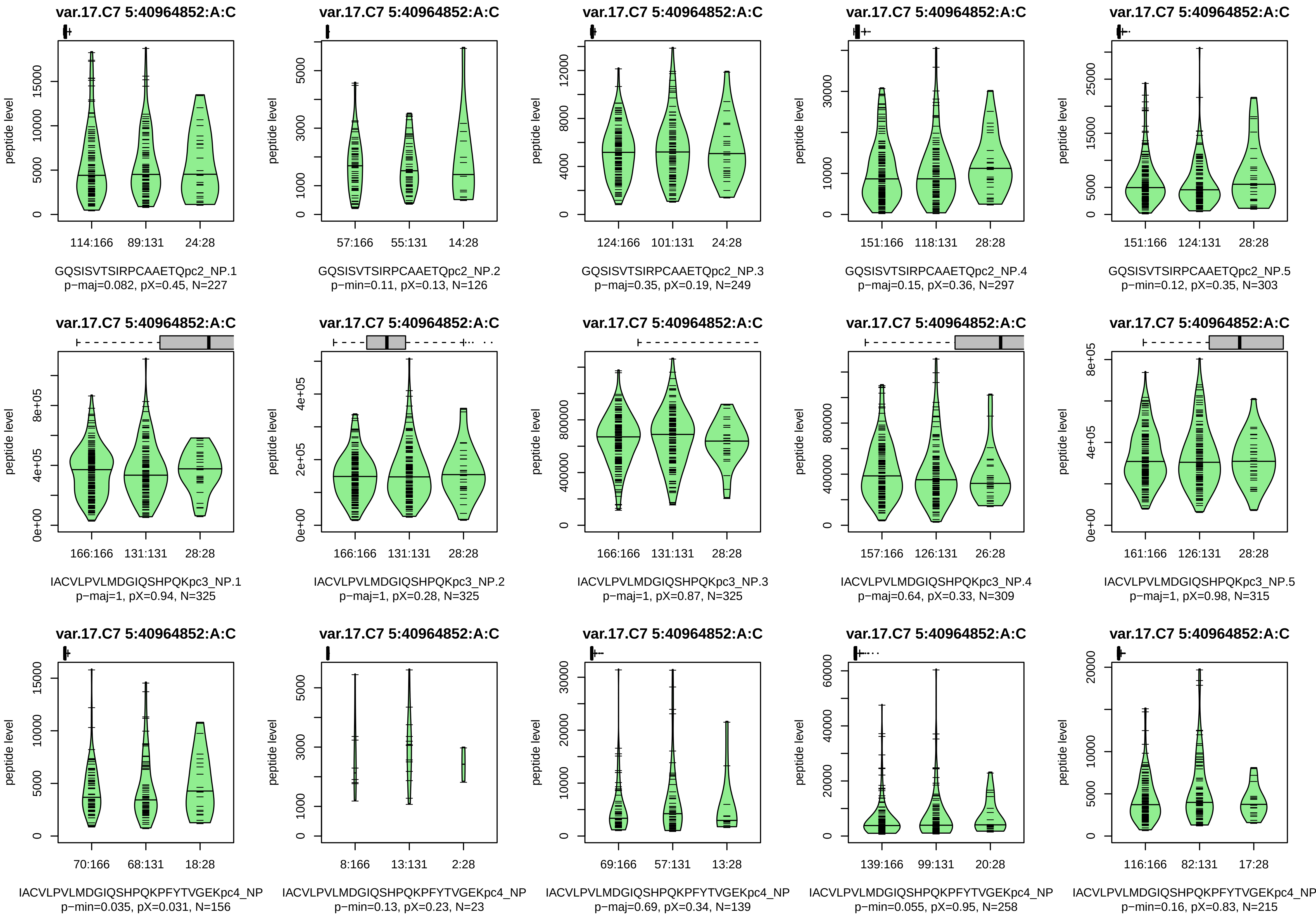
var.17.C7 5:40964852:A:C
peptide level
114:166
89:131
24:28
GQSISVTSIRPCAAETQpc2_NP.1
p−maj=0.082, pX=0.45, N=227
57:166
55:131
14:28
GQSISVTSIRPCAAETQpc2_NP.2
p−min=0.11, pX=0.13, N=126
124:166
101:131
24:28
GQSISVTSIRPCAAETQpc2_NP.3
p−maj=0.35, pX=0.19, N=249
151:166
118:131
28:28
GQSISVTSIRPCAAETQpc2_NP.4
p−maj=0.15, pX=0.36, N=297
151:166
124:131
28:28
GQSISVTSIRPCAAETQpc2_NP.5
p−min=0.12, pX=0.35, N=303
166:166
131:131
28:28
IACVLPVLMDGIQSHPQKpc3_NP.1
p−maj=1, pX=0.94, N=325
166:166
131:131
28:28
IACVLPVLMDGIQSHPQKpc3_NP.2
p−maj=1, pX=0.28, N=325
166:166
131:131
28:28
IACVLPVLMDGIQSHPQKpc3_NP.3
p−maj=1, pX=0.87, N=325
157:166
126:131
26:28
IACVLPVLMDGIQSHPQKpc3_NP.4
p−maj=0.64, pX=0.33, N=309
161:166
126:131
28:28
IACVLPVLMDGIQSHPQKpc3_NP.5
p−maj=1, pX=0.98, N=315
70:166
68:131
18:28
IACVLPVLMDGIQSHPQKPFYTVGEKpc4_NP
p−min=0.035, pX=0.031, N=156
8:166
13:131
2:28
IACVLPVLMDGIQSHPQKPFYTVGEKpc4_NP
p−min=0.13, pX=0.23, N=23
69:166
57:131
13:28
IACVLPVLMDGIQSHPQKPFYTVGEKpc4_NP
p−maj=0.69, pX=0.34, N=139
139:166
99:131
20:28
IACVLPVLMDGIQSHPQKPFYTVGEKpc4_NP
p−min=0.055, pX=0.95, N=258
116:166
82:131
17:28
IACVLPVLMDGIQSHPQKPFYTVGEKpc4_NP
p−min=0.16, pX=0.83, N=215

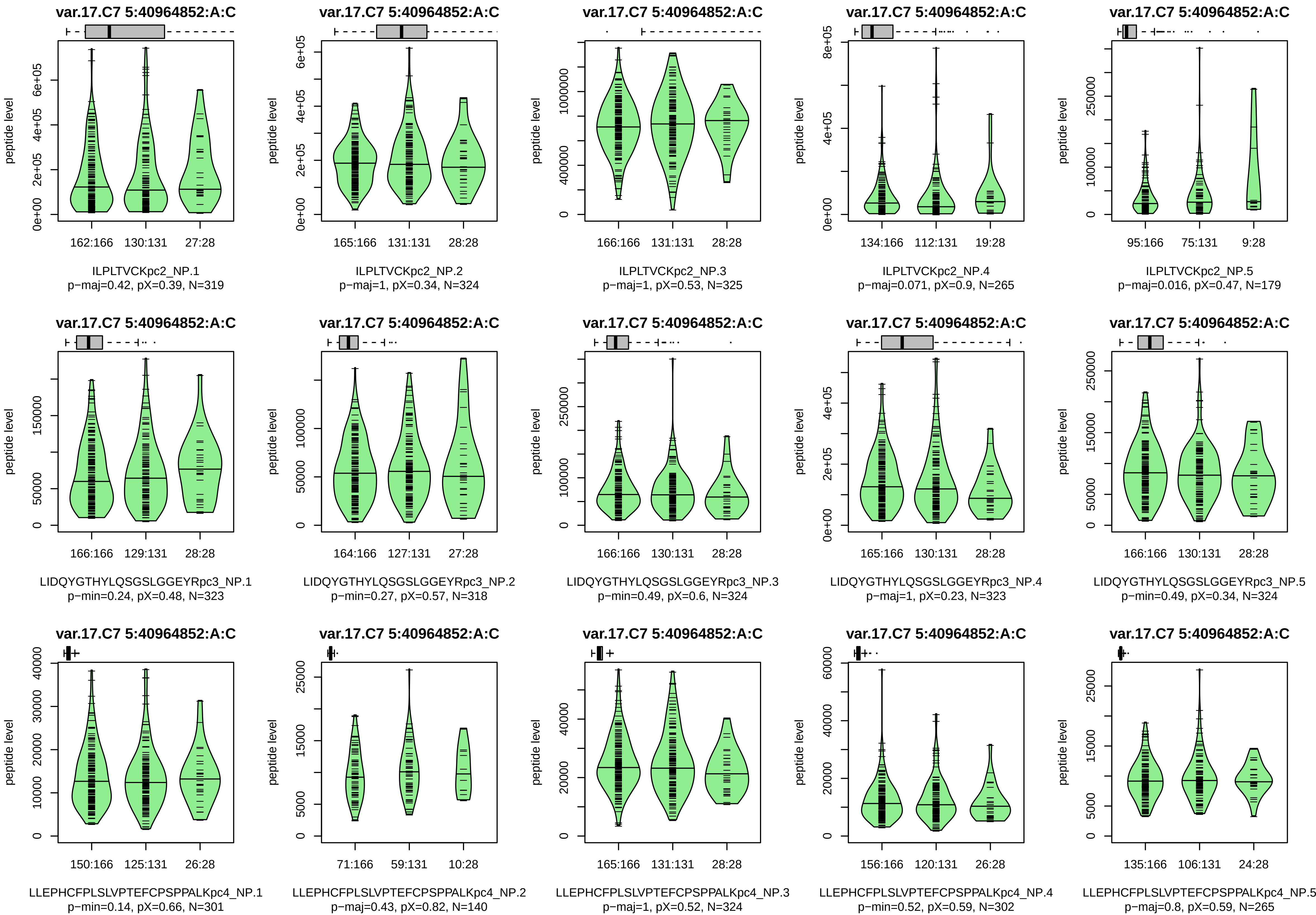
var.17.C7 5:40964852:A:C
peptide level
162:166
130:131
27:28
ILPLTVCKpc2_NP.1
p−maj=0.42, pX=0.39, N=319
var.17.C7 5:40964852:A:C
165:166
131:131
28:28
ILPLTVCKpc2_NP.2
p−maj=1, pX=0.34, N=324
var.17.C7 5:40964852:A:C
166:166
131:131
28:28
ILPLTVCKpc2_NP.3
p−maj=1, pX=0.53, N=325
var.17.C7 5:40964852:A:C
134:166
112:131
19:28
ILPLTVCKpc2_NP.4
p−maj=0.071, pX=0.9, N=265
var.17.C7 5:40964852:A:C
95:166
75:131
9:28
ILPLTVCKpc2_NP.5
p−maj=0.016, pX=0.47, N=179
var.17.C7 5:40964852:A:C
166:166
129:131
28:28
LIDQYGTHYLQSGSLGGEYRpc3_NP.1
p−min=0.24, pX=0.48, N=323
var.17.C7 5:40964852:A:C
164:166
127:131
27:28
LIDQYGTHYLQSGSLGGEYRpc3_NP.2
p−min=0.27, pX=0.57, N=318
var.17.C7 5:40964852:A:C
166:166
130:131
28:28
LIDQYGTHYLQSGSLGGEYRpc3_NP.3
p−min=0.49, pX=0.6, N=324
var.17.C7 5:40964852:A:C
165:166
130:131
28:28
LIDQYGTHYLQSGSLGGEYRpc3_NP.4
p−maj=1, pX=0.23, N=323
var.17.C7 5:40964852:A:C
166:166
130:131
28:28
LIDQYGTHYLQSGSLGGEYRpc3_NP.5
p−min=0.49, pX=0.34, N=324
var.17.C7 5:40964852:A:C
150:166
125:131
26:28
LLEPHCFPLSLVPTEFCPSPPALKpc4_NP.1
p−min=0.14, pX=0.66, N=301
var.17.C7 5:40964852:A:C
71:166
59:131
10:28
LLEPHCFPLSLVPTEFCPSPPALKpc4_NP.2
p−maj=0.43, pX=0.82, N=140
var.17.C7 5:40964852:A:C
165:166
131:131
28:28
LLEPHCFPLSLVPTEFCPSPPALKpc4_NP.3
p−maj=1, pX=0.52, N=324
var.17.C7 5:40964852:A:C
156:166
120:131
26:28
LLEPHCFPLSLVPTEFCPSPPALKpc4_NP.4
p−min=0.52, pX=0.59, N=302
var.17.C7 5:40964852:A:C
135:166
106:131
24:28
LLEPHCFPLSLVPTEFCPSPPALKpc4_NP.5
p−maj=0.8, pX=0.59, N=265

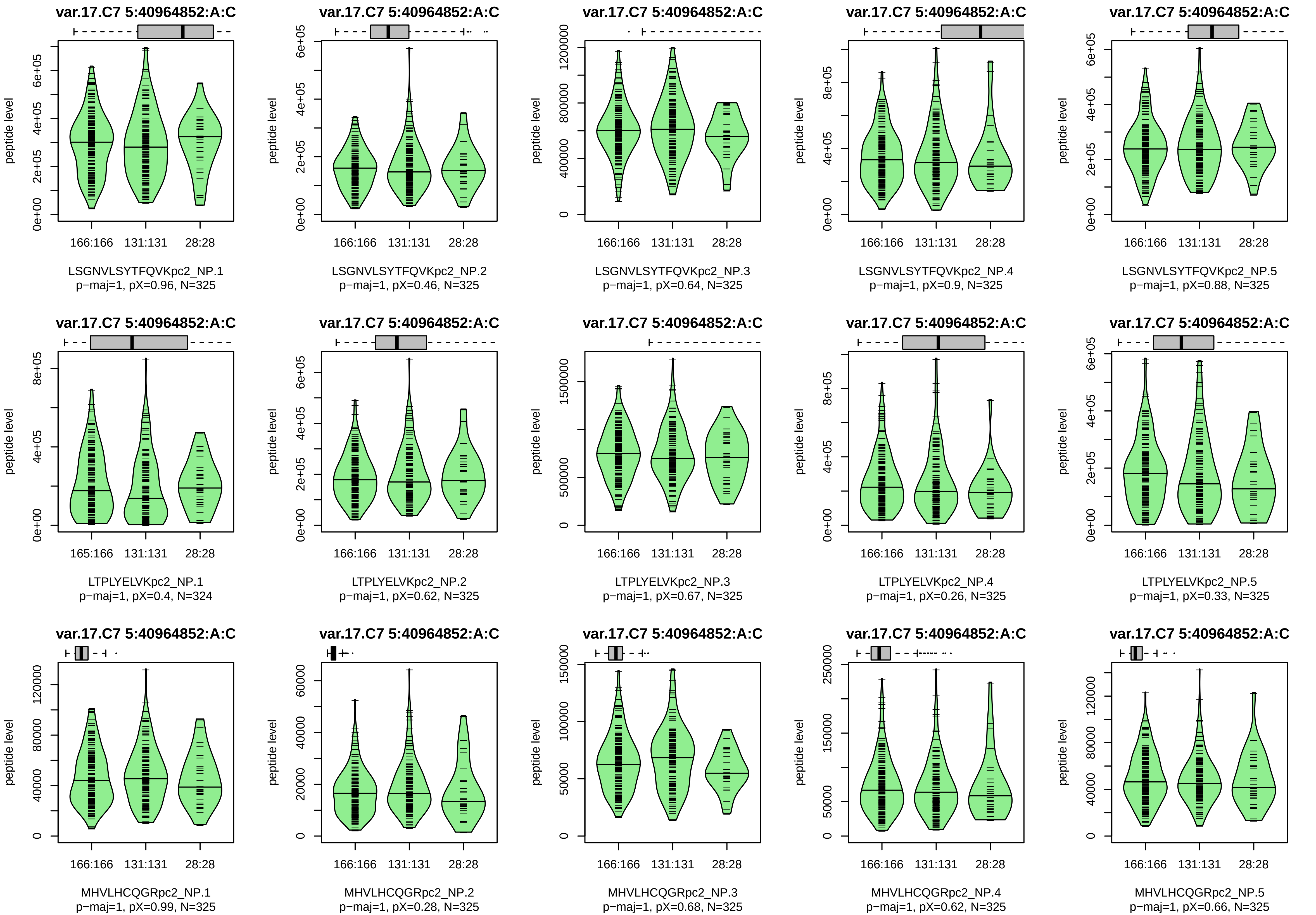
var.17.C7 5:40964852:A:C
peptide level
166:166
131:131
28:28
LSGNVLSYTFQVKpc2_NP.1
p−maj=1, pX=0.96, N=325
LSGNVLSYTFQVKpc2_NP.2
p−maj=1, pX=0.46, N=325
LSGNVLSYTFQVKpc2_NP.3
p−maj=1, pX=0.64, N=325
LSGNVLSYTFQVKpc2_NP.4
p−maj=1, pX=0.9, N=325
LSGNVLSYTFQVKpc2_NP.5
p−maj=1, pX=0.88, N=325
165:166
LTPLYELVKpc2_NP.1
p−maj=1, pX=0.4, N=324
LTPLYELVKpc2_NP.2
p−maj=1, pX=0.62, N=325
LTPLYELVKpc2_NP.3
p−maj=1, pX=0.67, N=325
LTPLYELVKpc2_NP.4
p−maj=1, pX=0.26, N=325
LTPLYELVKpc2_NP.5
p−maj=1, pX=0.33, N=325
MHVLHCQGRpc2_NP.1
p−maj=1, pX=0.99, N=325
MHVLHCQGRpc2_NP.2
p−maj=1, pX=0.28, N=325
MHVLHCQGRpc2_NP.3
p−maj=1, pX=0.68, N=325
MHVLHCQGRpc2_NP.4
p−maj=1, pX=0.62, N=325
MHVLHCQGRpc2_NP.5
p−maj=1, pX=0.66, N=325

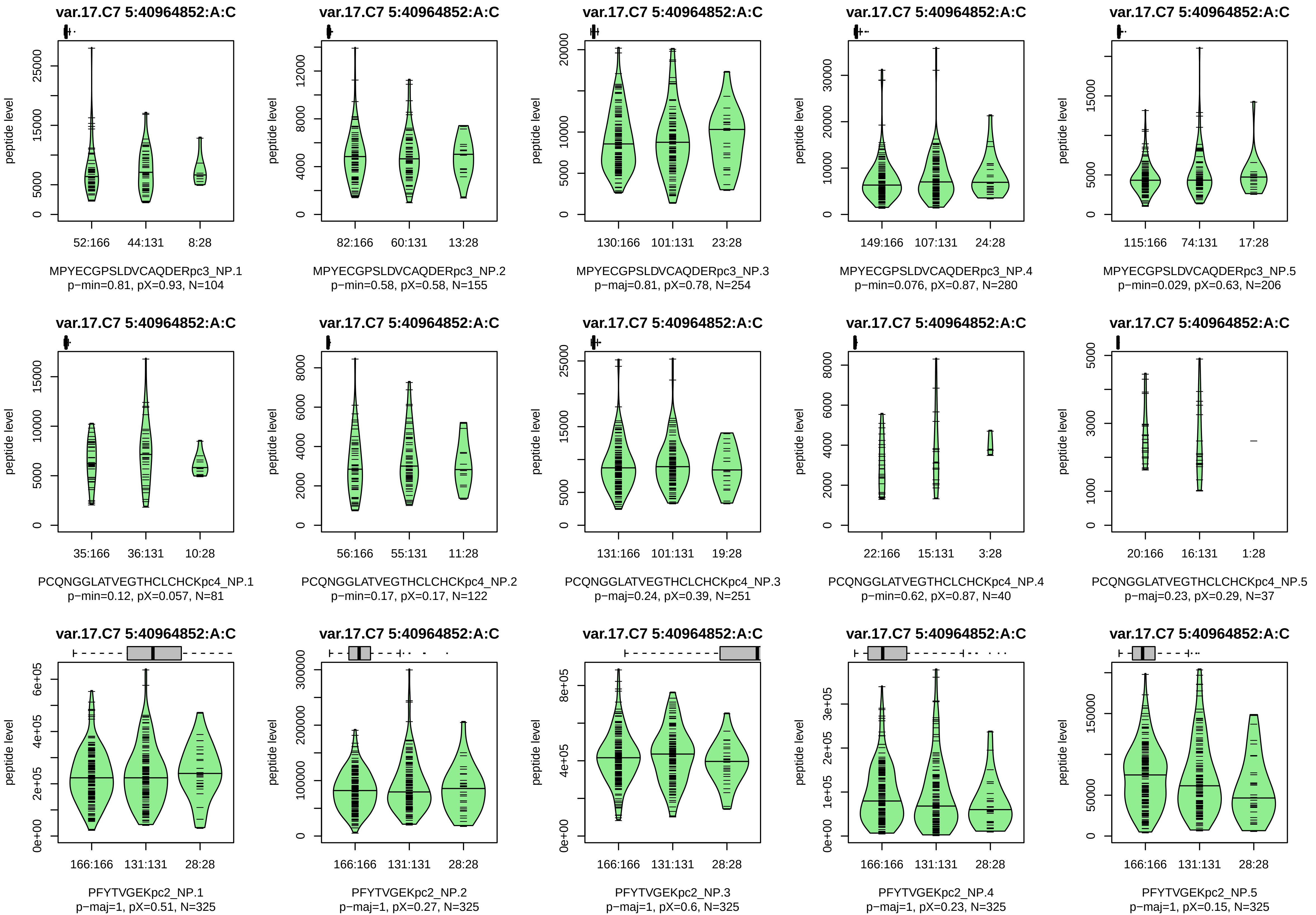
var.17.C7 5:40964852:A:C
peptide level
52:166 44:131 8:28
MPYECGPSLDVCAQDERpc3_NP.1
p−min=0.81, pX=0.93, N=104
var.17.C7 5:40964852:A:C
82:166 60:131 13:28
MPYECGPSLDVCAQDERpc3_NP.2
p−min=0.58, pX=0.58, N=155
var.17.C7 5:40964852:A:C
130:166 101:131 23:28
MPYECGPSLDVCAQDERpc3_NP.3
p−maj=0.81, pX=0.78, N=254
var.17.C7 5:40964852:A:C
149:166 107:131 24:28
MPYECGPSLDVCAQDERpc3_NP.4
p−min=0.076, pX=0.87, N=280
var.17.C7 5:40964852:A:C
115:166 74:131 17:28
MPYECGPSLDVCAQDERpc3_NP.5
p−min=0.029, pX=0.63, N=206
var.17.C7 5:40964852:A:C
35:166 36:131 10:28
PCQNGGLATVEGTHCLCHCKpc4_NP.1
p−min=0.12, pX=0.057, N=81
var.17.C7 5:40964852:A:C
56:166 55:131 11:28
PCQNGGLATVEGTHCLCHCKpc4_NP.2
p−min=0.17, pX=0.17, N=122
var.17.C7 5:40964852:A:C
131:166 101:131 19:28
PCQNGGLATVEGTHCLCHCKpc4_NP.3
p−maj=0.24, pX=0.39, N=251
var.17.C7 5:40964852:A:C
22:166 15:131 3:28
PCQNGGLATVEGTHCLCHCKpc4_NP.4
p−min=0.62, pX=0.87, N=40
var.17.C7 5:40964852:A:C
20:166 16:131 1:28
PCQNGGLATVEGTHCLCHCKpc4_NP.5
p−maj=0.23, pX=0.29, N=37
var.17.C7 5:40964852:A:C
166:166 131:131 28:28
PFYTVGEKpc2_NP.1
p−maj=1, pX=0.51, N=325
var.17.C7 5:40964852:A:C
166:166 131:131 28:28
PFYTVGEKpc2_NP.2
p−maj=1, pX=0.27, N=325
var.17.C7 5:40964852:A:C
166:166 131:131 28:28
PFYTVGEKpc2_NP.3
p−maj=1, pX=0.6, N=325
var.17.C7 5:40964852:A:C
166:166 131:131 28:28
PFYTVGEKpc2_NP.4
p−maj=1, pX=0.23, N=325
var.17.C7 5:40964852:A:C
166:166 131:131 28:28
PFYTVGEKpc2_NP.5
p−maj=1, pX=0.15, N=325

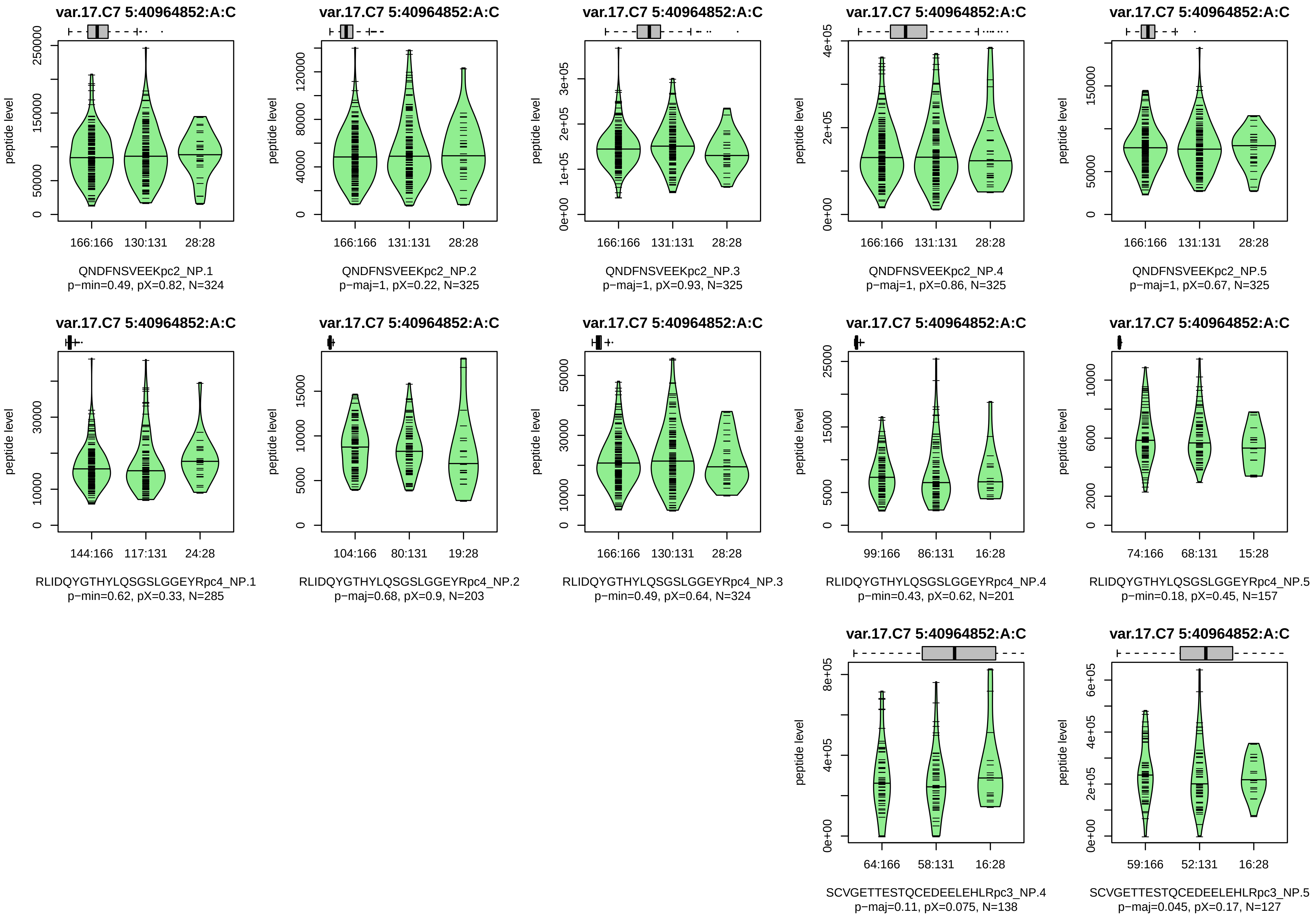
var.17.C7 5:40964852:A:C
peptide level
166:166
130:131
28:28
QNDFNSVEEKpc2_NP.1
p−min=0.49, pX=0.82, N=324
var.17.C7 5:40964852:A:C
peptide level
166:166
131:131
28:28
QNDFNSVEEKpc2_NP.2
p−maj=1, pX=0.22, N=325
var.17.C7 5:40964852:A:C
peptide level
166:166
131:131
28:28
QNDFNSVEEKpc2_NP.3
p−maj=1, pX=0.93, N=325
var.17.C7 5:40964852:A:C
peptide level
166:166
131:131
28:28
QNDFNSVEEKpc2_NP.4
p−maj=1, pX=0.86, N=325
var.17.C7 5:40964852:A:C
peptide level
166:166
131:131
28:28
QNDFNSVEEKpc2_NP.5
p−maj=1, pX=0.67, N=325
var.17.C7 5:40964852:A:C
peptide level
144:166
117:131
24:28
RLIDQYGTHYLQSGSLGGEYRpc4_NP.1
p−min=0.62, pX=0.33, N=285
var.17.C7 5:40964852:A:C
peptide level
104:166
80:131
19:28
RLIDQYGTHYLQSGSLGGEYRpc4_NP.2
p−maj=0.68, pX=0.9, N=203
var.17.C7 5:40964852:A:C
peptide level
166:166
130:131
28:28
RLIDQYGTHYLQSGSLGGEYRpc4_NP.3
p−min=0.49, pX=0.64, N=324
var.17.C7 5:40964852:A:C
peptide level
99:166
86:131
16:28
RLIDQYGTHYLQSGSLGGEYRpc4_NP.4
p−min=0.43, pX=0.62, N=201
var.17.C7 5:40964852:A:C
peptide level
74:166
68:131
15:28
RLIDQYGTHYLQSGSLGGEYRpc4_NP.5
p−min=0.18, pX=0.45, N=157
var.17.C7 5:40964852:A:C
peptide level
64:166
58:131
16:28
SCVGETTESTQCEDEELEHLRpc3_NP.4
p−maj=0.11, pX=0.075, N=138
var.17.C7 5:40964852:A:C
peptide level
59:166
52:131
16:28
SCVGETTESTQCEDEELEHLRpc3_NP.5
p−maj=0.045, pX=0.17, N=127

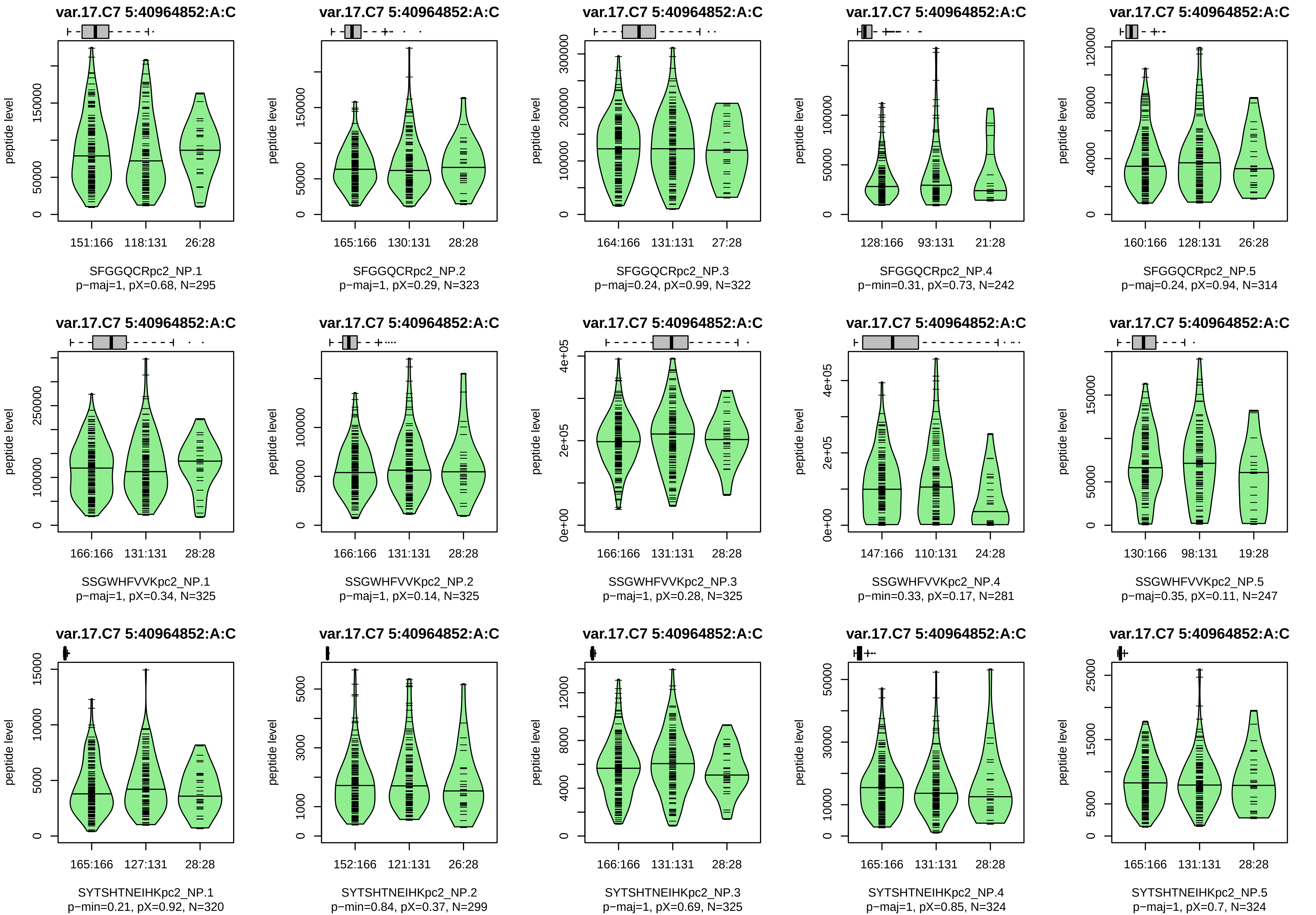
var.17.C7 5:40964852:A:C
peptide level
151:166
118:131
26:28
SFGGQCRpc2_NP.1
p−maj=1, pX=0.68, N=295
165:166
130:131
28:28
SFGGQCRpc2_NP.2
p−maj=1, pX=0.29, N=323
164:166
131:131
27:28
SFGGQCRpc2_NP.3
p−maj=0.24, pX=0.99, N=322
128:166
93:131
21:28
SFGGQCRpc2_NP.4
p−min=0.31, pX=0.73, N=242
160:166
128:131
26:28
SFGGQCRpc2_NP.5
p−maj=0.24, pX=0.94, N=314
166:166
131:131
28:28
SSGWHFVVKpc2_NP.1
p−maj=1, pX=0.34, N=325
SSGWHFVVKpc2_NP.2
p−maj=1, pX=0.14, N=325
SSGWHFVVKpc2_NP.3
p−maj=1, pX=0.28, N=325
147:166
110:131
24:28
SSGWHFVVKpc2_NP.4
p−min=0.33, pX=0.17, N=281
130:166
98:131
19:28
SSGWHFVVKpc2_NP.5
p−maj=0.35, pX=0.11, N=247
165:166
127:131
28:28
SYTSHTNEIHKpc2_NP.1
p−min=0.21, pX=0.92, N=320
152:166
121:131
26:28
SYTSHTNEIHKpc2_NP.2
p−min=0.84, pX=0.37, N=299
SYTSHTNEIHKpc2_NP.3
p−maj=1, pX=0.69, N=325
165:166
131:131
28:28
SYTSHTNEIHKpc2_NP.4
p−maj=1, pX=0.85, N=324
SYTSHTNEIHKpc2_NP.5
p−maj=1, pX=0.7, N=324

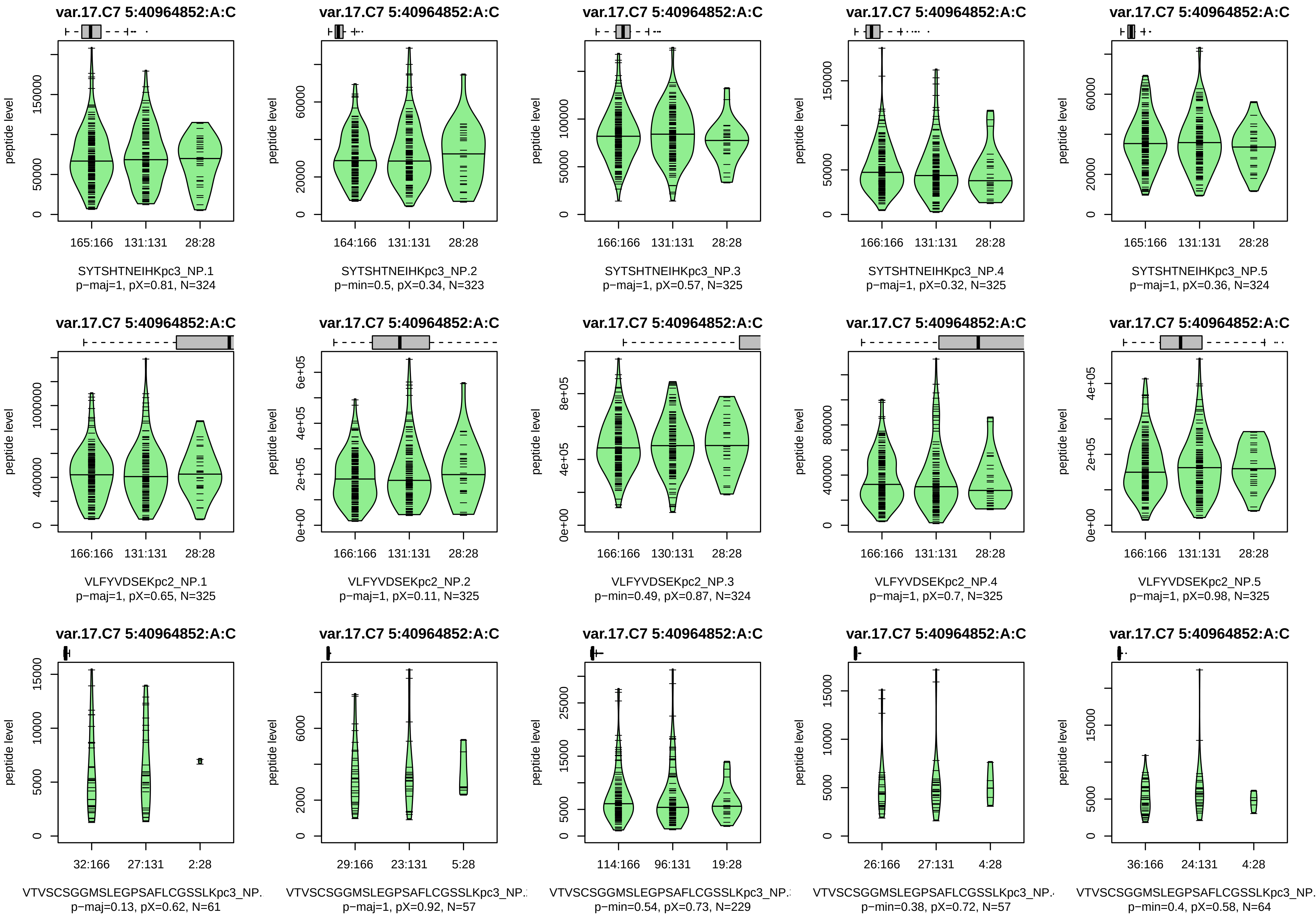

var.17.C7 5:40964852:A:C
peptide level
165:166
131:131
28:28
SYTSHTNEIHKpc3_NP.1
p−maj=1, pX=0.81, N=324
164:166
SYTSHTNEIHKpc3_NP.2
p−min=0.5, pX=0.34, N=323
166:166
SYTSHTNEIHKpc3_NP.3
p−maj=1, pX=0.57, N=325
SYTSHTNEIHKpc3_NP.4
p−maj=1, pX=0.32, N=325
SYTSHTNEIHKpc3_NP.5
p−maj=1, pX=0.36, N=324
VLFYVDSEKpc2_NP.1
p−maj=1, pX=0.65, N=325
VLFYVDSEKpc2_NP.2
p−maj=1, pX=0.11, N=325
130:131
VLFYVDSEKpc2_NP.3
p−min=0.49, pX=0.87, N=324
VLFYVDSEKpc2_NP.4
p−maj=1, pX=0.7, N=325
VLFYVDSEKpc2_NP.5
p−maj=1, pX=0.98, N=325
32:166
27:131
2:28
p−maj=0.13, pX=0.62, N=61
29:166
23:131
5:28
p−maj=1, pX=0.92, N=57
114:166
96:131
19:28
p−min=0.54, pX=0.73, N=229
26:166
4:28
p−min=0.38, pX=0.72, N=57
36:166
24:131
p−min=0.4, pX=0.58, N=64

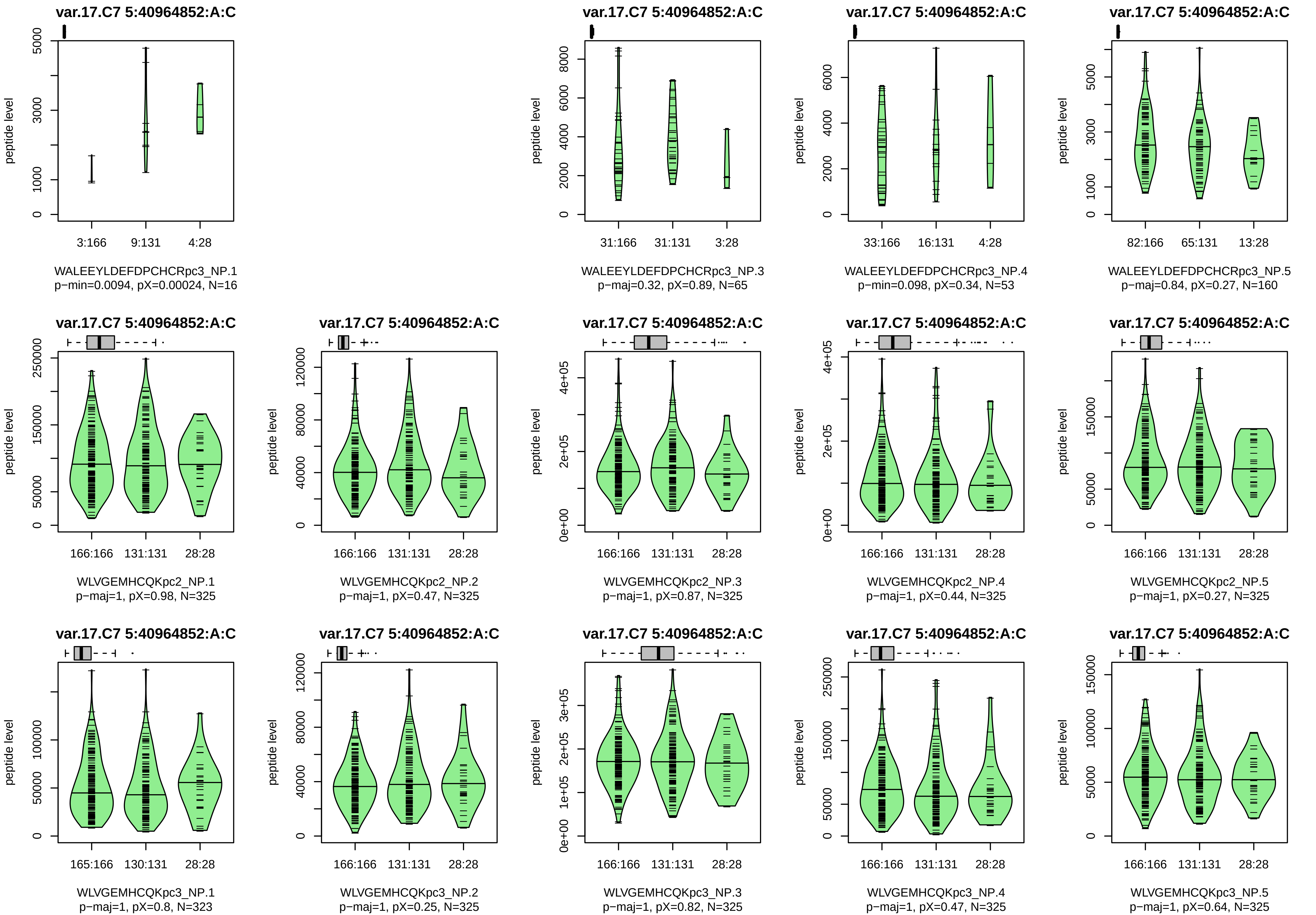
var.17.C7 5:40964852:A:C
peptide level
0
1000
3000
5000
3:166
9:131
4:28
WALEEYLDEFDPCHCRpc3_NP.1
p−min=0.0094, pX=0.00024, N=16
var.17.C7 5:40964852:A:C
peptide level
0
2000
4000
6000
8000
31:166
31:131
3:28
WALEEYLDEFDPCHCRpc3_NP.3
p−maj=0.32, pX=0.89, N=65
var.17.C7 5:40964852:A:C
peptide level
0
2000
4000
6000
33:166
16:131
4:28
WALEEYLDEFDPCHCRpc3_NP.4
p−min=0.098, pX=0.34, N=53
var.17.C7 5:40964852:A:C
peptide level
0
1000
3000
5000
82:166
65:131
13:28
WALEEYLDEFDPCHCRpc3_NP.5
p−maj=0.84, pX=0.27, N=160
var.17.C7 5:40964852:A:C
peptide level
0
50000
150000
250000
166:166
131:131
28:28
WLVGEMHCQKpc2_NP.1
p−maj=1, pX=0.98, N=325
var.17.C7 5:40964852:A:C
peptide level
0
40000
80000
120000
166:166
131:131
28:28
WLVGEMHCQKpc2_NP.2
p−maj=1, pX=0.47, N=325
var.17.C7 5:40964852:A:C
peptide level
0e+00
2e+05
4e+05
166:166
131:131
28:28
WLVGEMHCQKpc2_NP.3
p−maj=1, pX=0.87, N=325
var.17.C7 5:40964852:A:C
peptide level
0e+00
2e+05
4e+05
166:166
131:131
28:28
WLVGEMHCQKpc2_NP.4
p−maj=1, pX=0.44, N=325
var.17.C7 5:40964852:A:C
peptide level
0
50000
150000
166:166
131:131
28:28
WLVGEMHCQKpc2_NP.5
p−maj=1, pX=0.27, N=325
var.17.C7 5:40964852:A:C
peptide level
0
50000
100000
165:166
130:131
28:28
WLVGEMHCQKpc3_NP.1
p−maj=1, pX=0.8, N=323
var.17.C7 5:40964852:A:C
peptide level
0
40000
80000
120000
166:166
131:131
28:28
WLVGEMHCQKpc3_NP.2
p−maj=1, pX=0.25, N=325
var.17.C7 5:40964852:A:C
peptide level
0e+00
1e+05
2e+05
3e+05
166:166
131:131
28:28
WLVGEMHCQKpc3_NP.3
p−maj=1, pX=0.82, N=325
var.17.C7 5:40964852:A:C
peptide level
0
50000
150000
250000
166:166
131:131
28:28
WLVGEMHCQKpc3_NP.4
p−maj=1, pX=0.47, N=325
var.17.C7 5:40964852:A:C
peptide level
0
50000
100000
150000
166:166
131:131
28:28
WLVGEMHCQKpc3_NP.5
p−maj=1, pX=0.64, N=325

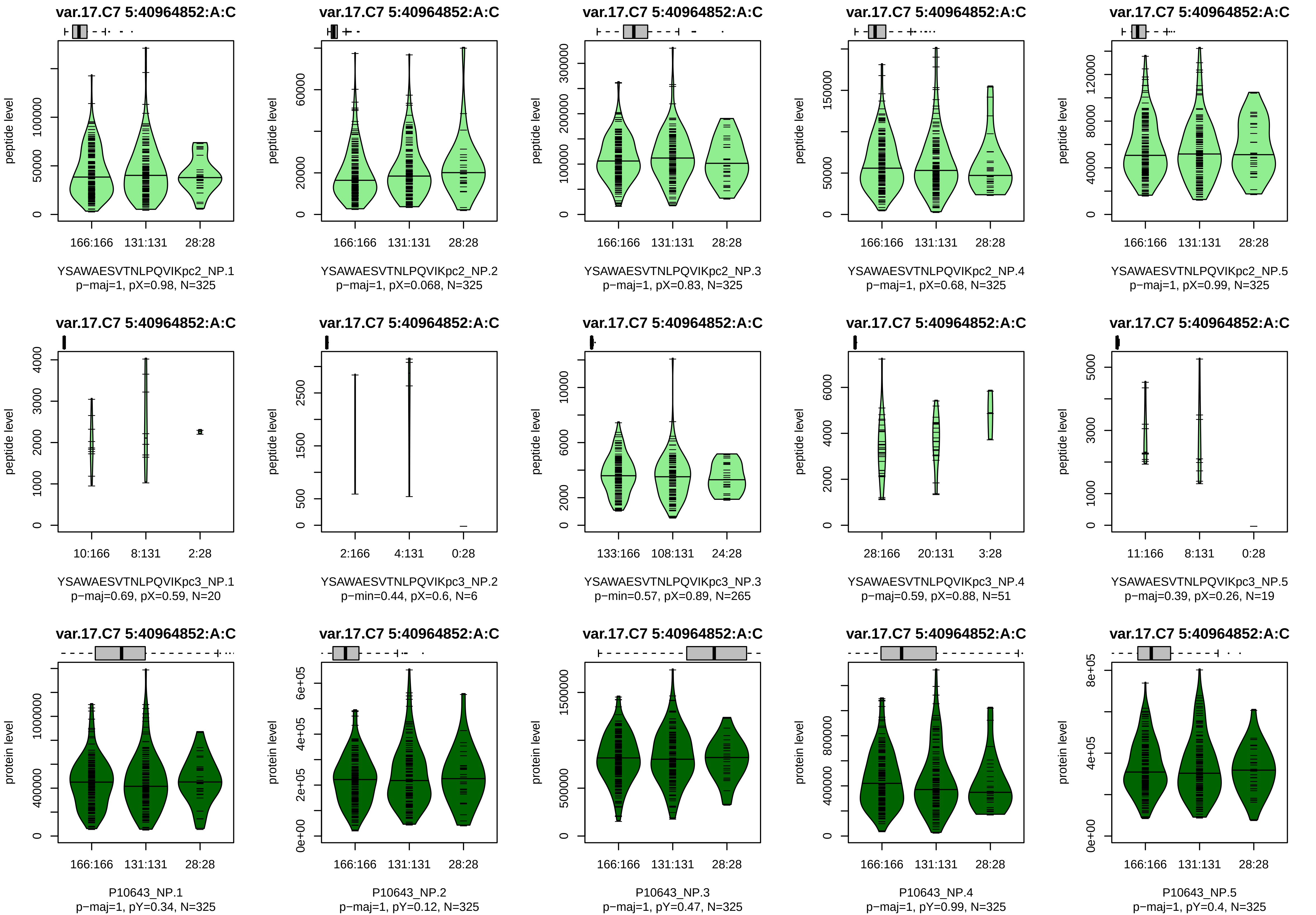
var.17.C7 5:40964852:A:C
peptide level
166:166
131:131
28:28
YSAWAESVTNLPQVIKpc2_NP.1
p−maj=1, pX=0.98, N=325
var.17.C7 5:40964852:A:C
peptide level
166:166
131:131
28:28
YSAWAESVTNLPQVIKpc2_NP.2
p−maj=1, pX=0.068, N=325
var.17.C7 5:40964852:A:C
peptide level
166:166
131:131
28:28
YSAWAESVTNLPQVIKpc2_NP.3
p−maj=1, pX=0.83, N=325
var.17.C7 5:40964852:A:C
peptide level
166:166
131:131
28:28
YSAWAESVTNLPQVIKpc2_NP.4
p−maj=1, pX=0.68, N=325
var.17.C7 5:40964852:A:C
peptide level
166:166
131:131
28:28
YSAWAESVTNLPQVIKpc2_NP.5
p−maj=1, pX=0.99, N=325
var.17.C7 5:40964852:A:C
peptide level
10:166
8:131
2:28
YSAWAESVTNLPQVIKpc3_NP.1
p−maj=0.69, pX=0.59, N=20
var.17.C7 5:40964852:A:C
peptide level
2:166
4:131
0:28
YSAWAESVTNLPQVIKpc3_NP.2
p−min=0.44, pX=0.6, N=6
var.17.C7 5:40964852:A:C
peptide level
133:166
108:131
24:28
YSAWAESVTNLPQVIKpc3_NP.3
p−min=0.57, pX=0.89, N=265
var.17.C7 5:40964852:A:C
peptide level
28:166
20:131
3:28
YSAWAESVTNLPQVIKpc3_NP.4
p−maj=0.59, pX=0.88, N=51
var.17.C7 5:40964852:A:C
peptide level
11:166
8:131
0:28
YSAWAESVTNLPQVIKpc3_NP.5
p−maj=0.39, pX=0.26, N=19
var.17.C7 5:40964852:A:C
protein level
166:166
131:131
28:28
P10643_NP.1
p−maj=1, pY=0.34, N=325
var.17.C7 5:40964852:A:C
protein level
166:166
131:131
28:28
P10643_NP.2
p−maj=1, pY=0.12, N=325
var.17.C7 5:40964852:A:C
protein level
166:166
131:131
28:28
P10643_NP.3
p−maj=1, pY=0.47, N=325
var.17.C7 5:40964852:A:C
protein level
166:166
131:131
28:28
P10643_NP.4
p−maj=1, pY=0.99, N=325
var.17.C7 5:40964852:A:C
protein level
166:166
131:131
28:28
P10643_NP.5
p−maj=1, pY=0.4, N=325

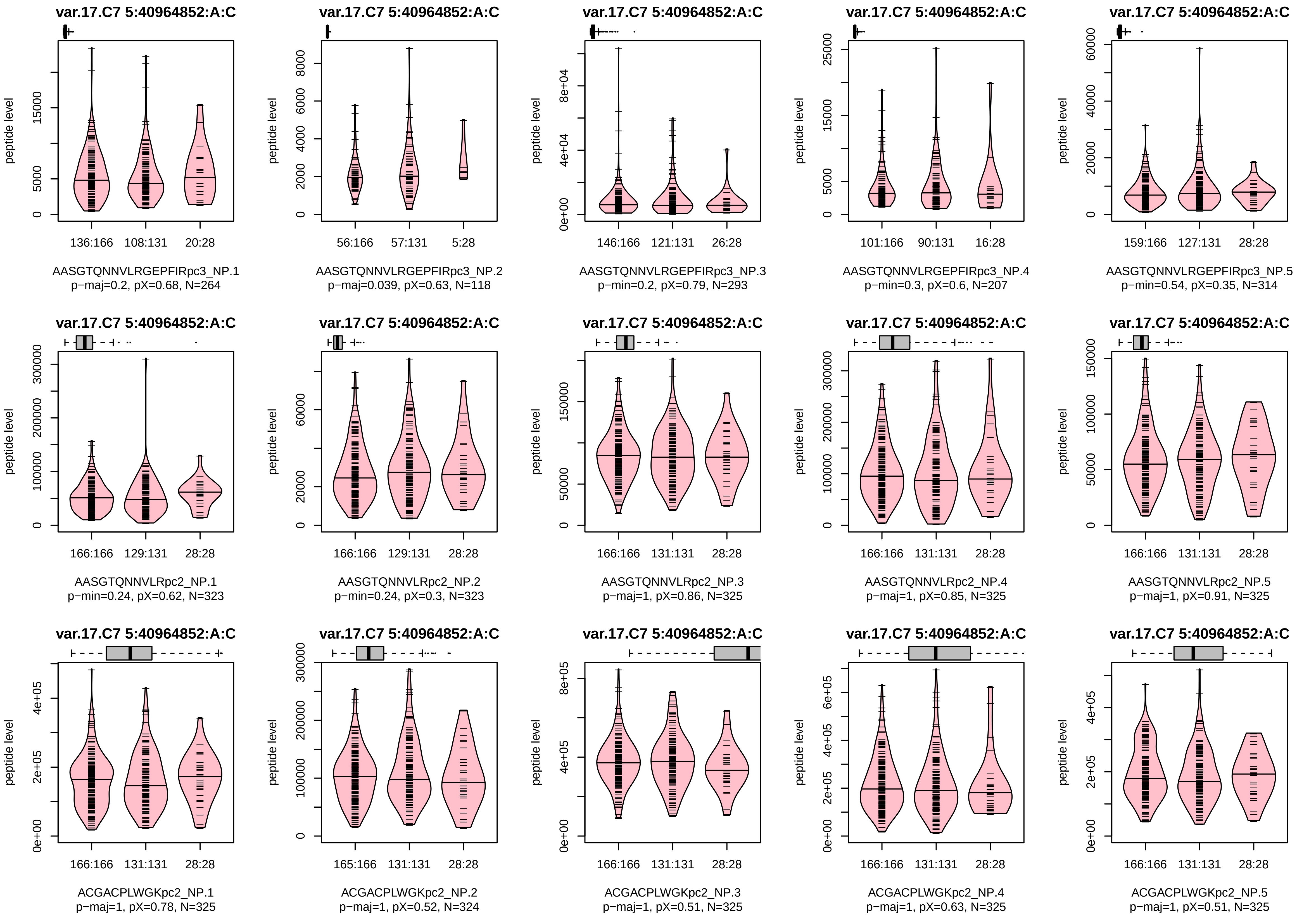
var.17.C7 5:40964852:A:C
peptide level
136:166
108:131
20:28
AASGTQNNVLRGEPFIRpc3_NP.1
p−maj=0.2, pX=0.68, N=264
var.17.C7 5:40964852:A:C
peptide level
56:166
57:131
5:28
AASGTQNNVLRGEPFIRpc3_NP.2
p−maj=0.039, pX=0.63, N=118
var.17.C7 5:40964852:A:C
peptide level
146:166
121:131
26:28
AASGTQNNVLRGEPFIRpc3_NP.3
p−min=0.2, pX=0.79, N=293
var.17.C7 5:40964852:A:C
peptide level
101:166
90:131
16:28
AASGTQNNVLRGEPFIRpc3_NP.4
p−min=0.3, pX=0.6, N=207
var.17.C7 5:40964852:A:C
peptide level
159:166
127:131
28:28
AASGTQNNVLRGEPFIRpc3_NP.5
p−min=0.54, pX=0.35, N=314
var.17.C7 5:40964852:A:C
peptide level
166:166
129:131
28:28
AASGTQNNVLRpc2_NP.1
p−min=0.24, pX=0.62, N=323
var.17.C7 5:40964852:A:C
peptide level
166:166
129:131
28:28
AASGTQNNVLRpc2_NP.2
p−min=0.24, pX=0.3, N=323
var.17.C7 5:40964852:A:C
peptide level
166:166
131:131
28:28
AASGTQNNVLRpc2_NP.3
p−maj=1, pX=0.86, N=325
var.17.C7 5:40964852:A:C
peptide level
166:166
131:131
28:28
AASGTQNNVLRpc2_NP.4
p−maj=1, pX=0.85, N=325
var.17.C7 5:40964852:A:C
peptide level
166:166
131:131
28:28
AASGTQNNVLRpc2_NP.5
p−maj=1, pX=0.91, N=325
var.17.C7 5:40964852:A:C
peptide level
166:166
131:131
28:28
ACGACPLWGKpc2_NP.1
p−maj=1, pX=0.78, N=325
var.17.C7 5:40964852:A:C
peptide level
165:166
131:131
28:28
ACGACPLWGKpc2_NP.2
p−maj=1, pX=0.52, N=324
var.17.C7 5:40964852:A:C
peptide level
166:166
131:131
28:28
ACGACPLWGKpc2_NP.3
p−maj=1, pX=0.51, N=325
var.17.C7 5:40964852:A:C
peptide level
166:166
131:131
28:28
ACGACPLWGKpc2_NP.4
p−maj=1, pX=0.63, N=325
var.17.C7 5:40964852:A:C
peptide level
166:166
131:131
28:28
ACGACPLWGKpc2_NP.5
p−maj=1, pX=0.51, N=325

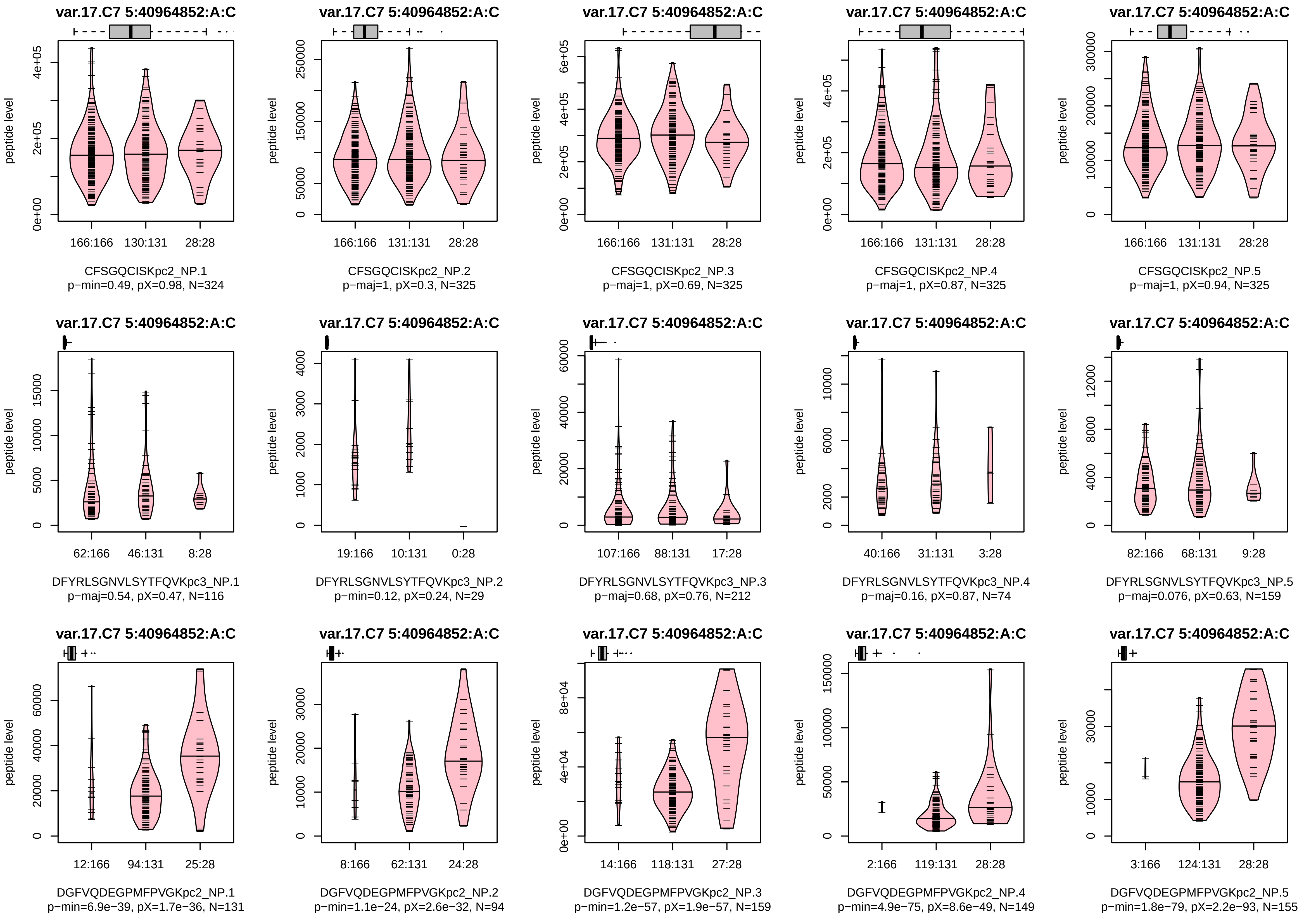

var.17.C7 5:40964852:A:C
peptide level
0e+00
2e+05
4e+05
166:166
130:131
28:28
CFSGQCISKpc2_NP.1
p−min=0.49, pX=0.98, N=324
var.17.C7 5:40964852:A:C
peptide level
0
50000
150000
250000
166:166
131:131
28:28
CFSGQCISKpc2_NP.2
p−maj=1, pX=0.3, N=325
var.17.C7 5:40964852:A:C
peptide level
0e+00
2e+05
4e+05
6e+05
166:166
131:131
28:28
CFSGQCISKpc2_NP.3
p−maj=1, pX=0.69, N=325
var.17.C7 5:40964852:A:C
peptide level
0e+00
2e+05
4e+05
166:166
131:131
28:28
CFSGQCISKpc2_NP.4
p−maj=1, pX=0.87, N=325
var.17.C7 5:40964852:A:C
peptide level
0
100000
200000
300000
166:166
131:131
28:28
CFSGQCISKpc2_NP.5
p−maj=1, pX=0.94, N=325
var.17.C7 5:40964852:A:C
peptide level
0
5000
10000
15000
62:166
46:131
8:28
DFYRLSGNVLSYTFQVKpc3_NP.1
p−maj=0.54, pX=0.47, N=116
var.17.C7 5:40964852:A:C
peptide level
0
1000
2000
3000
4000
19:166
10:131
0:28
DFYRLSGNVLSYTFQVKpc3_NP.2
p−min=0.12, pX=0.24, N=29
var.17.C7 5:40964852:A:C
peptide level
0
20000
40000
60000
107:166
88:131
17:28
DFYRLSGNVLSYTFQVKpc3_NP.3
p−maj=0.68, pX=0.76, N=212
var.17.C7 5:40964852:A:C
peptide level
0
2000
6000
10000
40:166
31:131
3:28
DFYRLSGNVLSYTFQVKpc3_NP.4
p−maj=0.16, pX=0.87, N=74
var.17.C7 5:40964852:A:C
peptide level
0
4000
8000
12000
82:166
68:131
9:28
DFYRLSGNVLSYTFQVKpc3_NP.5
p−maj=0.076, pX=0.63, N=159
var.17.C7 5:40964852:A:C
peptide level
0
20000
40000
60000
12:166
94:131
25:28
DGFVQDEGPMFPVGKpc2_NP.1
p−min=6.9e−39, pX=1.7e−36, N=131
var.17.C7 5:40964852:A:C
peptide level
0
10000
20000
30000
8:166
62:131
24:28
DGFVQDEGPMFPVGKpc2_NP.2
p−min=1.1e−24, pX=2.6e−32, N=94
var.17.C7 5:40964852:A:C
peptide level
0e+00
4e+04
8e+04
14:166
118:131
27:28
DGFVQDEGPMFPVGKpc2_NP.3
p−min=1.2e−57, pX=1.9e−57, N=159
var.17.C7 5:40964852:A:C
peptide level
0
50000
100000
150000
2:166
119:131
28:28
DGFVQDEGPMFPVGKpc2_NP.4
p−min=4.9e−75, pX=8.6e−49, N=149
var.17.C7 5:40964852:A:C
peptide level
0
10000
20000
30000
3:166
124:131
28:28
DGFVQDEGPMFPVGKpc2_NP.5
p−min=1.8e−79, pX=2.2e−93, N=155

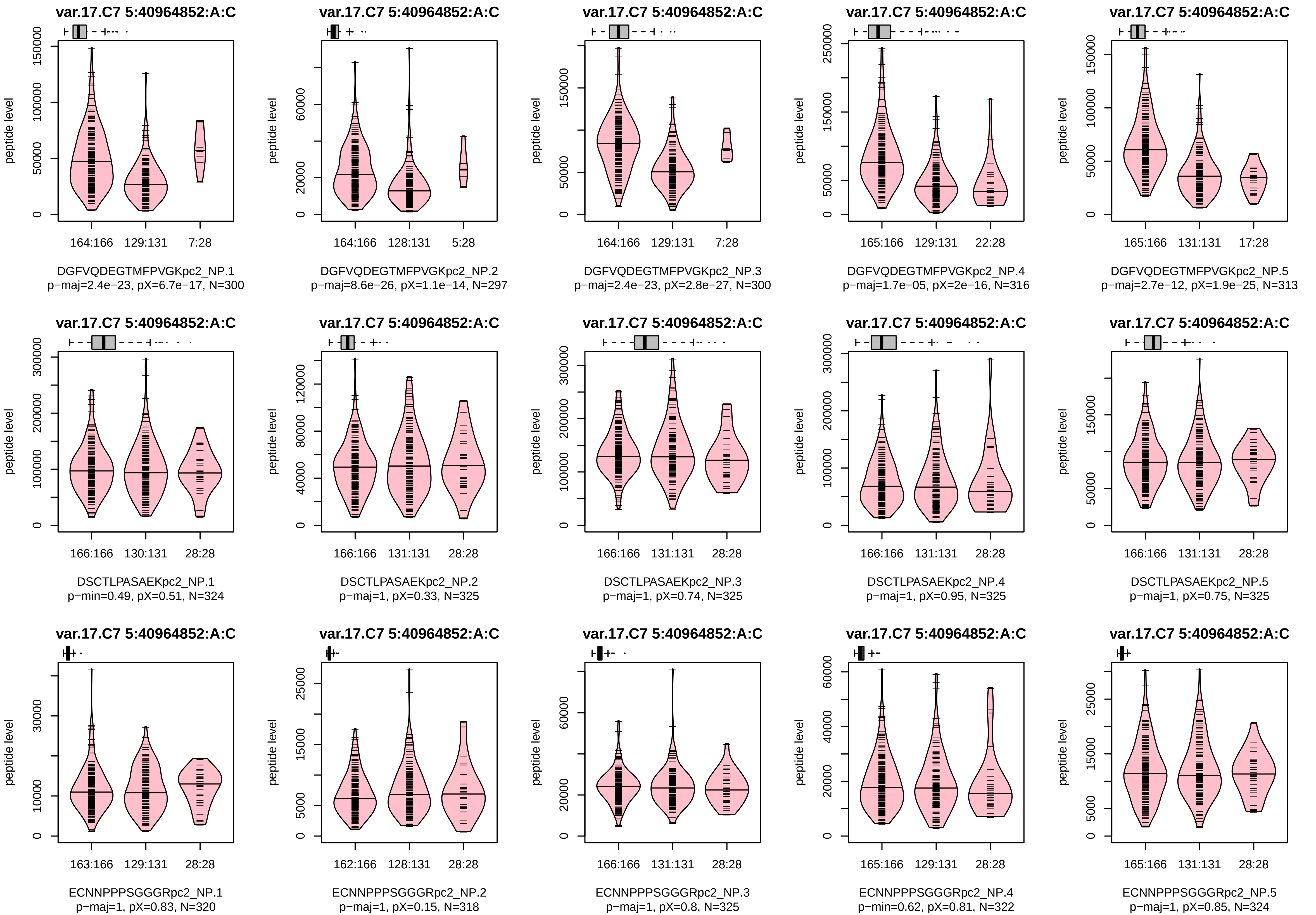
var.17.C7 5:40964852:A:C
peptide level
164:166
129:131
7:28
DGFVQDEGTMFPVGKpc2_NP.1
p−maj=2.4e−23, pX=6.7e−17, N=300
var.17.C7 5:40964852:A:C
peptide level
164:166
128:131
5:28
DGFVQDEGTMFPVGKpc2_NP.2
p−maj=8.6e−26, pX=1.1e−14, N=297
var.17.C7 5:40964852:A:C
peptide level
164:166
129:131
7:28
DGFVQDEGTMFPVGKpc2_NP.3
p−maj=2.4e−23, pX=2.8e−27, N=300
var.17.C7 5:40964852:A:C
peptide level
165:166
129:131
22:28
DGFVQDEGTMFPVGKpc2_NP.4
p−maj=1.7e−05, pX=2e−16, N=316
var.17.C7 5:40964852:A:C
peptide level
165:166
131:131
17:28
DGFVQDEGTMFPVGKpc2_NP.5
p−maj=2.7e−12, pX=1.9e−25, N=313
var.17.C7 5:40964852:A:C
peptide level
166:166
130:131
28:28
DSCTLPASAEKpc2_NP.1
p−min=0.49, pX=0.51, N=324
var.17.C7 5:40964852:A:C
peptide level
166:166
131:131
28:28
DSCTLPASAEKpc2_NP.2
p−maj=1, pX=0.33, N=325
var.17.C7 5:40964852:A:C
peptide level
166:166
131:131
28:28
DSCTLPASAEKpc2_NP.3
p−maj=1, pX=0.74, N=325
var.17.C7 5:40964852:A:C
peptide level
166:166
131:131
28:28
DSCTLPASAEKpc2_NP.4
p−maj=1, pX=0.95, N=325
var.17.C7 5:40964852:A:C
peptide level
166:166
131:131
28:28
DSCTLPASAEKpc2_NP.5
p−maj=1, pX=0.75, N=325
var.17.C7 5:40964852:A:C
peptide level
163:166
129:131
28:28
ECNNPPPSGGGRpc2_NP.1
p−maj=1, pX=0.83, N=320
var.17.C7 5:40964852:A:C
peptide level
162:166
128:131
28:28
ECNNPPPSGGGRpc2_NP.2
p−maj=1, pX=0.15, N=318
var.17.C7 5:40964852:A:C
peptide level
166:166
131:131
28:28
ECNNPPPSGGGRpc2_NP.3
p−maj=1, pX=0.8, N=325
var.17.C7 5:40964852:A:C
peptide level
165:166
129:131
28:28
ECNNPPPSGGGRpc2_NP.4
p−min=0.62, pX=0.81, N=322
var.17.C7 5:40964852:A:C
peptide level
165:166
131:131
28:28
ECNNPPPSGGGRpc2_NP.5
p−maj=1, pX=0.85, N=324

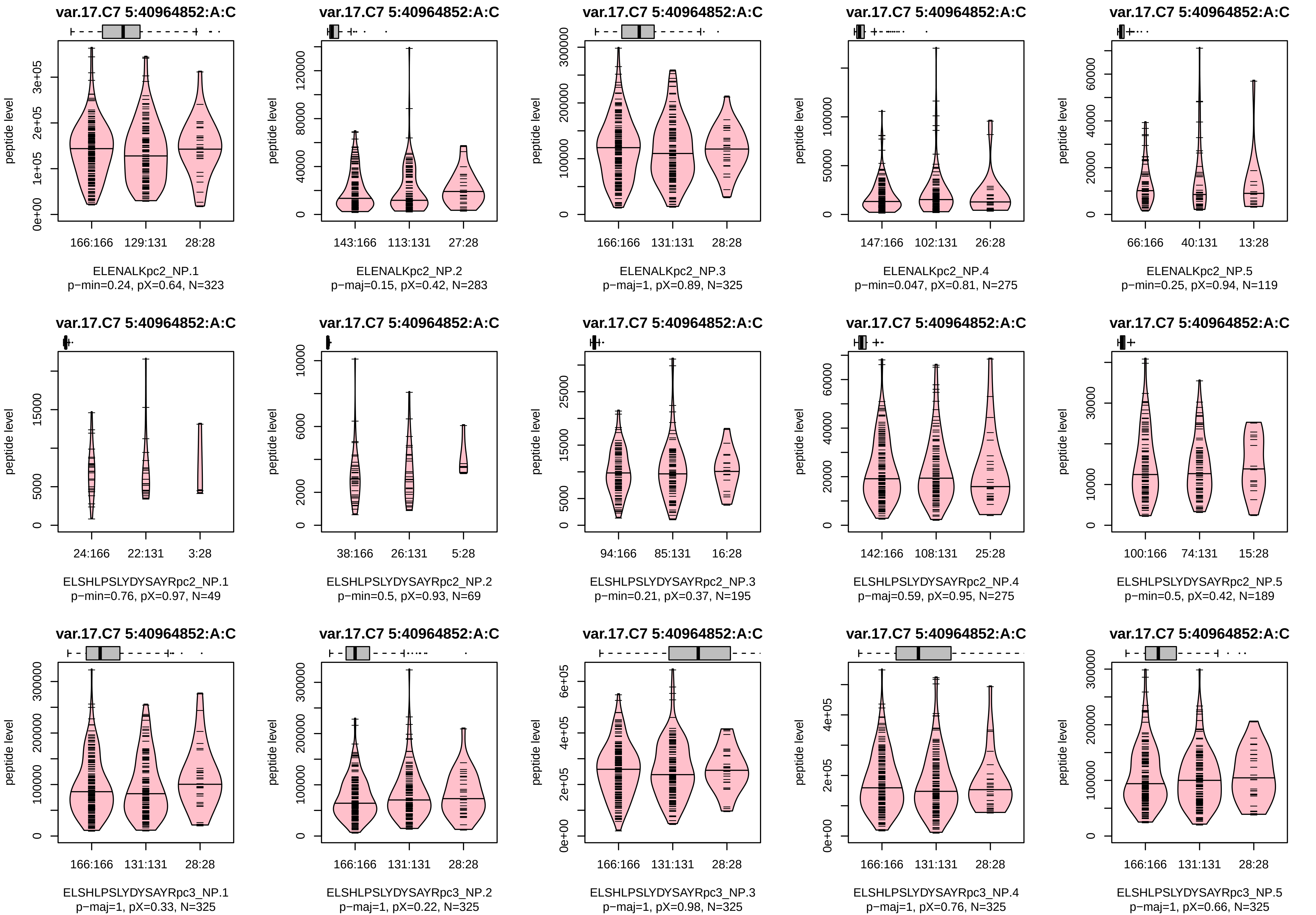

var.17.C7 5:40964852:A:C
peptide level
166:166
129:131
28:28
ELENALKpc2_NP.1
p−min=0.24, pX=0.64, N=323
var.17.C7 5:40964852:A:C
peptide level
143:166
113:131
27:28
ELENALKpc2_NP.2
p−maj=0.15, pX=0.42, N=283
var.17.C7 5:40964852:A:C
peptide level
166:166
131:131
28:28
ELENALKpc2_NP.3
p−maj=1, pX=0.89, N=325
var.17.C7 5:40964852:A:C
peptide level
147:166
102:131
26:28
ELENALKpc2_NP.4
p−min=0.047, pX=0.81, N=275
var.17.C7 5:40964852:A:C
peptide level
66:166
40:131
13:28
ELENALKpc2_NP.5
p−min=0.25, pX=0.94, N=119
var.17.C7 5:40964852:A:C
peptide level
24:166
22:131
3:28
ELSHLPSLYDYSAYRpc2_NP.1
p−min=0.76, pX=0.97, N=49
var.17.C7 5:40964852:A:C
peptide level
38:166
26:131
5:28
ELSHLPSLYDYSAYRpc2_NP.2
p−min=0.5, pX=0.93, N=69
var.17.C7 5:40964852:A:C
peptide level
94:166
85:131
16:28
ELSHLPSLYDYSAYRpc2_NP.3
p−min=0.21, pX=0.37, N=195
var.17.C7 5:40964852:A:C
peptide level
142:166
108:131
25:28
ELSHLPSLYDYSAYRpc2_NP.4
p−maj=0.59, pX=0.95, N=275
var.17.C7 5:40964852:A:C
peptide level
100:166
74:131
15:28
ELSHLPSLYDYSAYRpc2_NP.5
p−min=0.5, pX=0.42, N=189
var.17.C7 5:40964852:A:C
peptide level
166:166
131:131
28:28
ELSHLPSLYDYSAYRpc3_NP.1
p−maj=1, pX=0.33, N=325
var.17.C7 5:40964852:A:C
peptide level
166:166
131:131
28:28
ELSHLPSLYDYSAYRpc3_NP.2
p−maj=1, pX=0.22, N=325
var.17.C7 5:40964852:A:C
peptide level
166:166
131:131
28:28
ELSHLPSLYDYSAYRpc3_NP.3
p−maj=1, pX=0.98, N=325
var.17.C7 5:40964852:A:C
peptide level
166:166
131:131
28:28
ELSHLPSLYDYSAYRpc3_NP.4
p−maj=1, pX=0.76, N=325
var.17.C7 5:40964852:A:C
peptide level
166:166
131:131
28:28
ELSHLPSLYDYSAYRpc3_NP.5
p−maj=1, pX=0.66, N=325

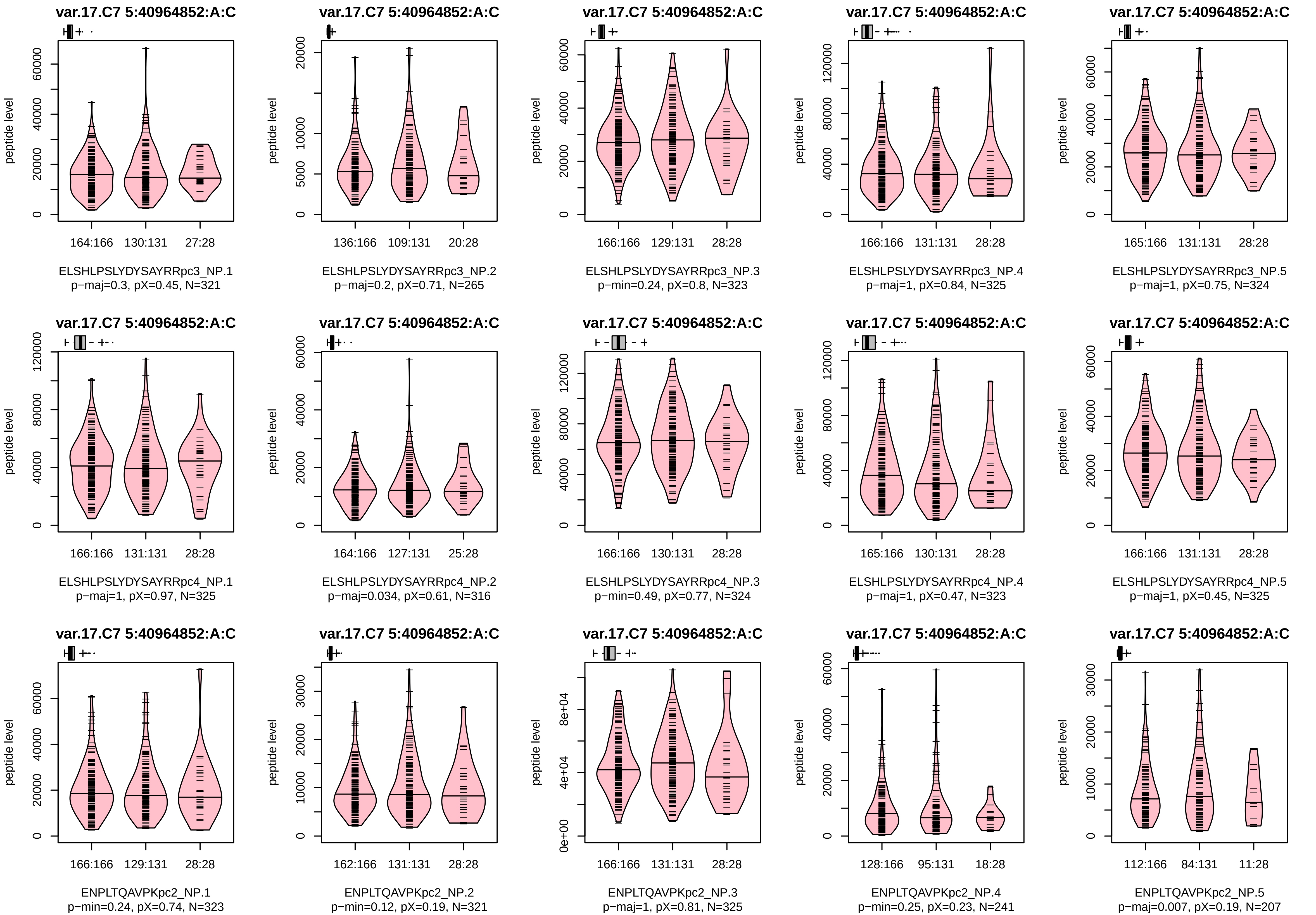
var.17.C7 5:40964852:A:C
peptide level
164:166 130:131 27:28
ELSHLPSLYDYSAYRRpc3_NP.1
p−maj=0.3, pX=0.45, N=321
var.17.C7 5:40964852:A:C
peptide level
136:166 109:131 20:28
ELSHLPSLYDYSAYRRpc3_NP.2
p−maj=0.2, pX=0.71, N=265
var.17.C7 5:40964852:A:C
peptide level
166:166 129:131 28:28
ELSHLPSLYDYSAYRRpc3_NP.3
p−min=0.24, pX=0.8, N=323
var.17.C7 5:40964852:A:C
peptide level
166:166 131:131 28:28
ELSHLPSLYDYSAYRRpc3_NP.4
p−maj=1, pX=0.84, N=325
var.17.C7 5:40964852:A:C
peptide level
165:166 131:131 28:28
ELSHLPSLYDYSAYRRpc3_NP.5
p−maj=1, pX=0.75, N=324
var.17.C7 5:40964852:A:C
peptide level
166:166 131:131 28:28
ELSHLPSLYDYSAYRRpc4_NP.1
p−maj=1, pX=0.97, N=325
var.17.C7 5:40964852:A:C
peptide level
164:166 127:131 25:28
ELSHLPSLYDYSAYRRpc4_NP.2
p−maj=0.034, pX=0.61, N=316
var.17.C7 5:40964852:A:C
peptide level
166:166 130:131 28:28
ELSHLPSLYDYSAYRRpc4_NP.3
p−min=0.49, pX=0.77, N=324
var.17.C7 5:40964852:A:C
peptide level
165:166 130:131 28:28
ELSHLPSLYDYSAYRRpc4_NP.4
p−maj=1, pX=0.47, N=323
var.17.C7 5:40964852:A:C
peptide level
166:166 131:131 28:28
ELSHLPSLYDYSAYRRpc4_NP.5
p−maj=1, pX=0.45, N=325
var.17.C7 5:40964852:A:C
peptide level
166:166 129:131 28:28
ENPLTQAVPKpc2_NP.1
p−min=0.24, pX=0.74, N=323
var.17.C7 5:40964852:A:C
peptide level
162:166 131:131 28:28
ENPLTQAVPKpc2_NP.2
p−min=0.12, pX=0.19, N=321
var.17.C7 5:40964852:A:C
peptide level
166:166 131:131 28:28
ENPLTQAVPKpc2_NP.3
p−maj=1, pX=0.81, N=325
var.17.C7 5:40964852:A:C
peptide level
128:166 95:131 18:28
ENPLTQAVPKpc2_NP.4
p−min=0.25, pX=0.23, N=241
var.17.C7 5:40964852:A:C
peptide level
112:166 84:131 11:28
ENPLTQAVPKpc2_NP.5
p−maj=0.007, pX=0.19, N=207

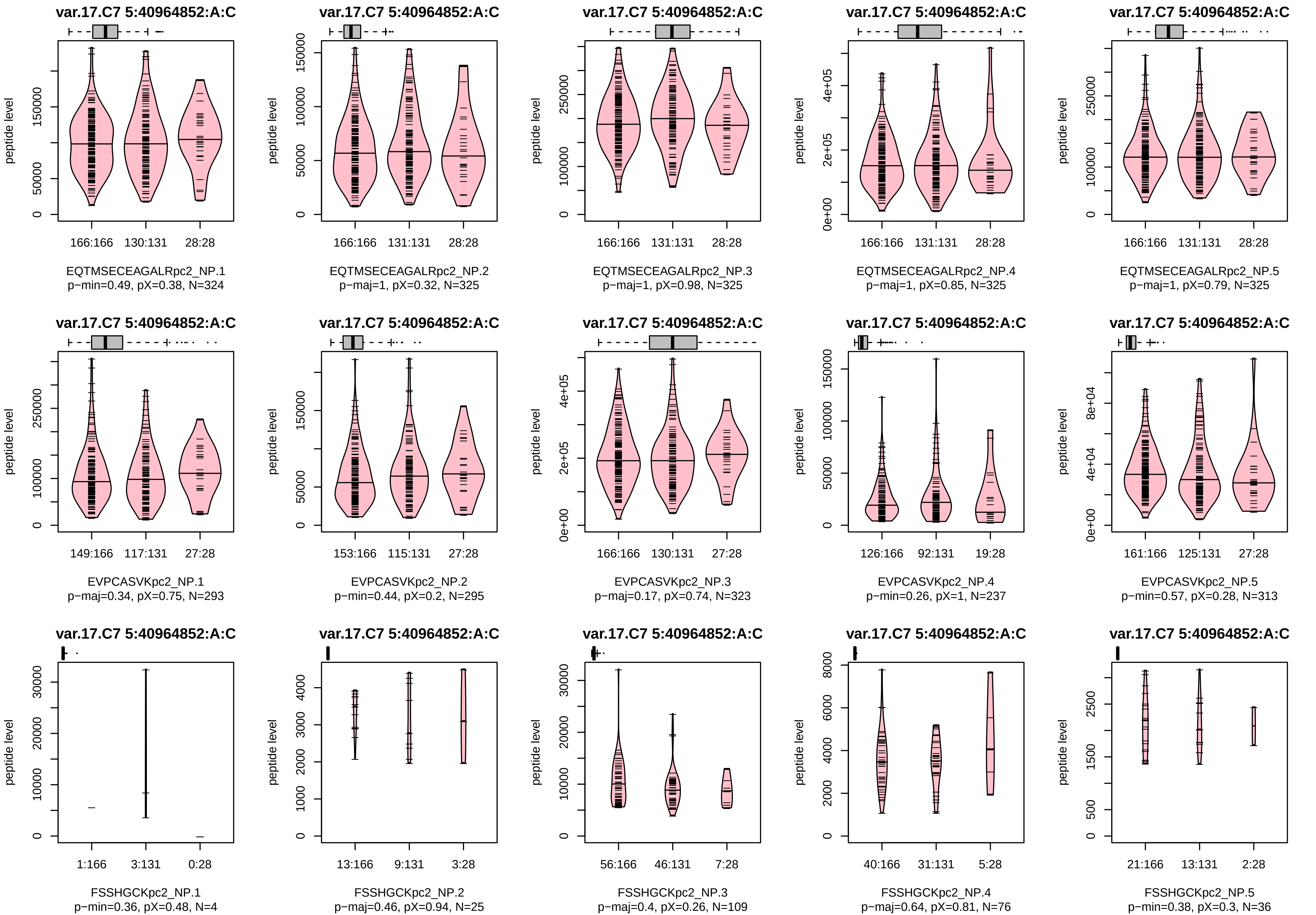
var.17.C7 5:40964852:A:C
peptide level
166:166
130:131
28:28
EQTMSECEAGALRpc2_NP.1
p−min=0.49, pX=0.38, N=324
var.17.C7 5:40964852:A:C
peptide level
166:166
131:131
28:28
EQTMSECEAGALRpc2_NP.2
p−maj=1, pX=0.32, N=325
var.17.C7 5:40964852:A:C
peptide level
166:166
131:131
28:28
EQTMSECEAGALRpc2_NP.3
p−maj=1, pX=0.98, N=325
var.17.C7 5:40964852:A:C
peptide level
166:166
131:131
28:28
EQTMSECEAGALRpc2_NP.4
p−maj=1, pX=0.85, N=325
var.17.C7 5:40964852:A:C
peptide level
166:166
131:131
28:28
EQTMSECEAGALRpc2_NP.5
p−maj=1, pX=0.79, N=325
var.17.C7 5:40964852:A:C
peptide level
149:166
117:131
27:28
EVPCASVKpc2_NP.1
p−maj=0.34, pX=0.75, N=293
var.17.C7 5:40964852:A:C
peptide level
153:166
115:131
27:28
EVPCASVKpc2_NP.2
p−min=0.44, pX=0.2, N=295
var.17.C7 5:40964852:A:C
peptide level
166:166
130:131
27:28
EVPCASVKpc2_NP.3
p−maj=0.17, pX=0.74, N=323
var.17.C7 5:40964852:A:C
peptide level
126:166
92:131
19:28
EVPCASVKpc2_NP.4
p−min=0.26, pX=1, N=237
var.17.C7 5:40964852:A:C
peptide level
161:166
125:131
27:28
EVPCASVKpc2_NP.5
p−min=0.57, pX=0.28, N=313
var.17.C7 5:40964852:A:C
peptide level
1:166
3:131
0:28
FSSHGCKpc2_NP.1
p−min=0.36, pX=0.48, N=4
var.17.C7 5:40964852:A:C
peptide level
13:166
9:131
3:28
FSSHGCKpc2_NP.2
p−maj=0.46, pX=0.94, N=25
var.17.C7 5:40964852:A:C
peptide level
56:166
46:131
7:28
FSSHGCKpc2_NP.3
p−maj=0.4, pX=0.26, N=109
var.17.C7 5:40964852:A:C
peptide level
40:166
31:131
5:28
FSSHGCKpc2_NP.4
p−maj=0.64, pX=0.81, N=76
var.17.C7 5:40964852:A:C
peptide level
21:166
13:131
2:28
FSSHGCKpc2_NP.5
p−min=0.38, pX=0.3, N=36

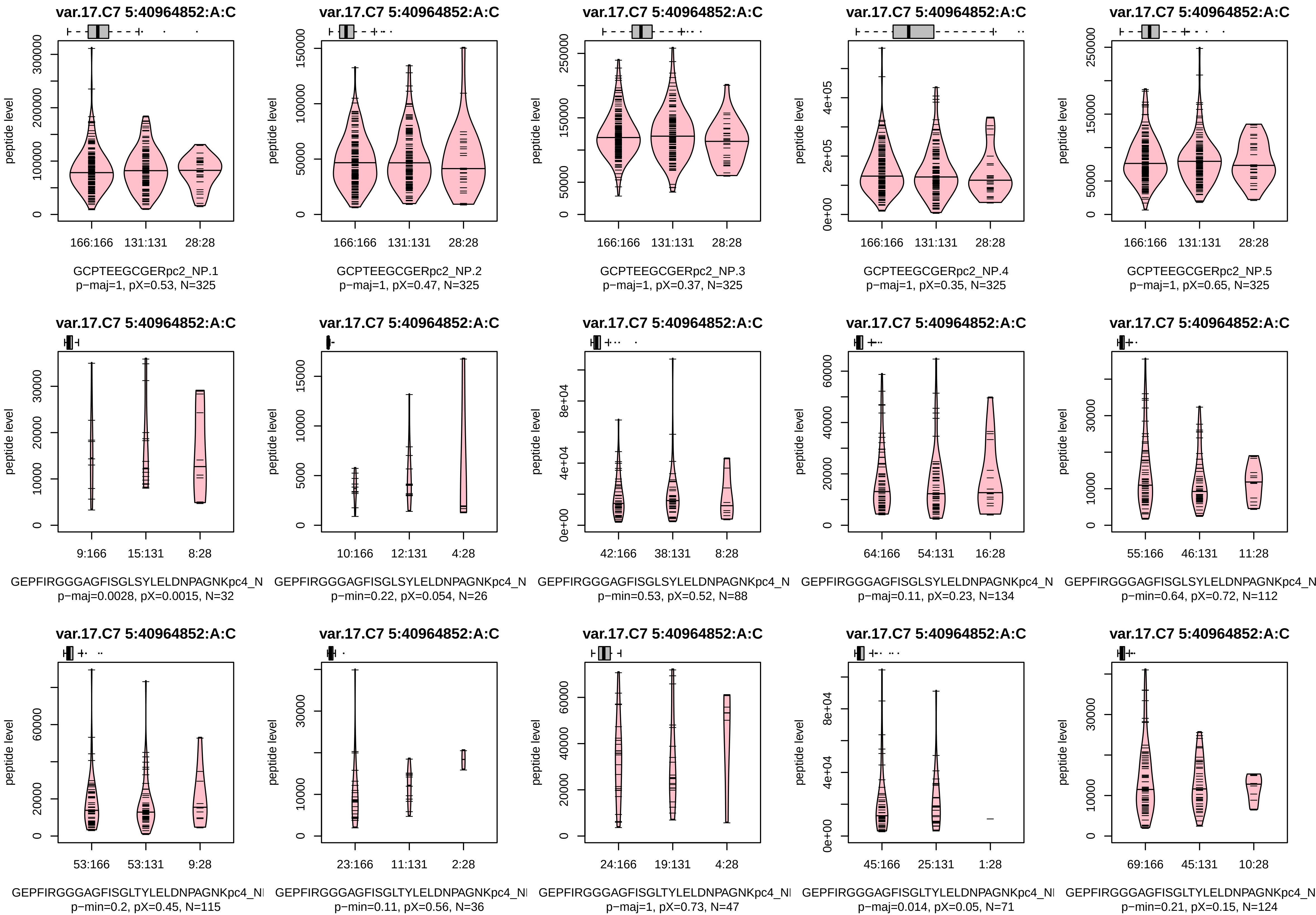

var.17.C7 5:40964852:A:C
peptide level
166:166
131:131
28:28
GCPTEEGCGERpc2_NP.1
p−maj=1, pX=0.53, N=325
var.17.C7 5:40964852:A:C
peptide level
166:166
131:131
28:28
GCPTEEGCGERpc2_NP.2
p−maj=1, pX=0.47, N=325
var.17.C7 5:40964852:A:C
peptide level
166:166
131:131
28:28
GCPTEEGCGERpc2_NP.3
p−maj=1, pX=0.37, N=325
var.17.C7 5:40964852:A:C
peptide level
166:166
131:131
28:28
GCPTEEGCGERpc2_NP.4
p−maj=1, pX=0.35, N=325
var.17.C7 5:40964852:A:C
peptide level
166:166
131:131
28:28
GCPTEEGCGERpc2_NP.5
p−maj=1, pX=0.65, N=325
var.17.C7 5:40964852:A:C
peptide level
9:166
15:131
8:28
GEPFIRGGGAGFISGLSYLELDNPAGNKpc4_N
p−maj=0.0028, pX=0.0015, N=32
var.17.C7 5:40964852:A:C
peptide level
10:166
12:131
4:28
GEPFIRGGGAGFISGLSYLELDNPAGNKpc4_N
p−min=0.22, pX=0.054, N=26
var.17.C7 5:40964852:A:C
peptide level
42:166
38:131
8:28
GEPFIRGGGAGFISGLSYLELDNPAGNKpc4_N
p−min=0.53, pX=0.52, N=88
var.17.C7 5:40964852:A:C
peptide level
64:166
54:131
16:28
GEPFIRGGGAGFISGLSYLELDNPAGNKpc4_N
p−maj=0.11, pX=0.23, N=134
var.17.C7 5:40964852:A:C
peptide level
55:166
46:131
11:28
GEPFIRGGGAGFISGLSYLELDNPAGNKpc4_N
p−min=0.64, pX=0.72, N=112
var.17.C7 5:40964852:A:C
peptide level
53:166
53:131
9:28
GEPFIRGGGAGFISGLTYLELDNPAGNKpc4_NI
p−min=0.2, pX=0.45, N=115
var.17.C7 5:40964852:A:C
peptide level
23:166
11:131
2:28
GEPFIRGGGAGFISGLTYLELDNPAGNKpc4_NI
p−min=0.11, pX=0.56, N=36
var.17.C7 5:40964852:A:C
peptide level
24:166
19:131
4:28
GEPFIRGGGAGFISGLTYLELDNPAGNKpc4_NI
p−maj=1, pX=0.73, N=47
var.17.C7 5:40964852:A:C
peptide level
45:166
25:131
1:28
GEPFIRGGGAGFISGLTYLELDNPAGNKpc4_NI
p−maj=0.014, pX=0.05, N=71
var.17.C7 5:40964852:A:C
peptide level
69:166
45:131
10:28
GEPFIRGGGAGFISGLTYLELDNPAGNKpc4_NI
p−min=0.21, pX=0.15, N=124

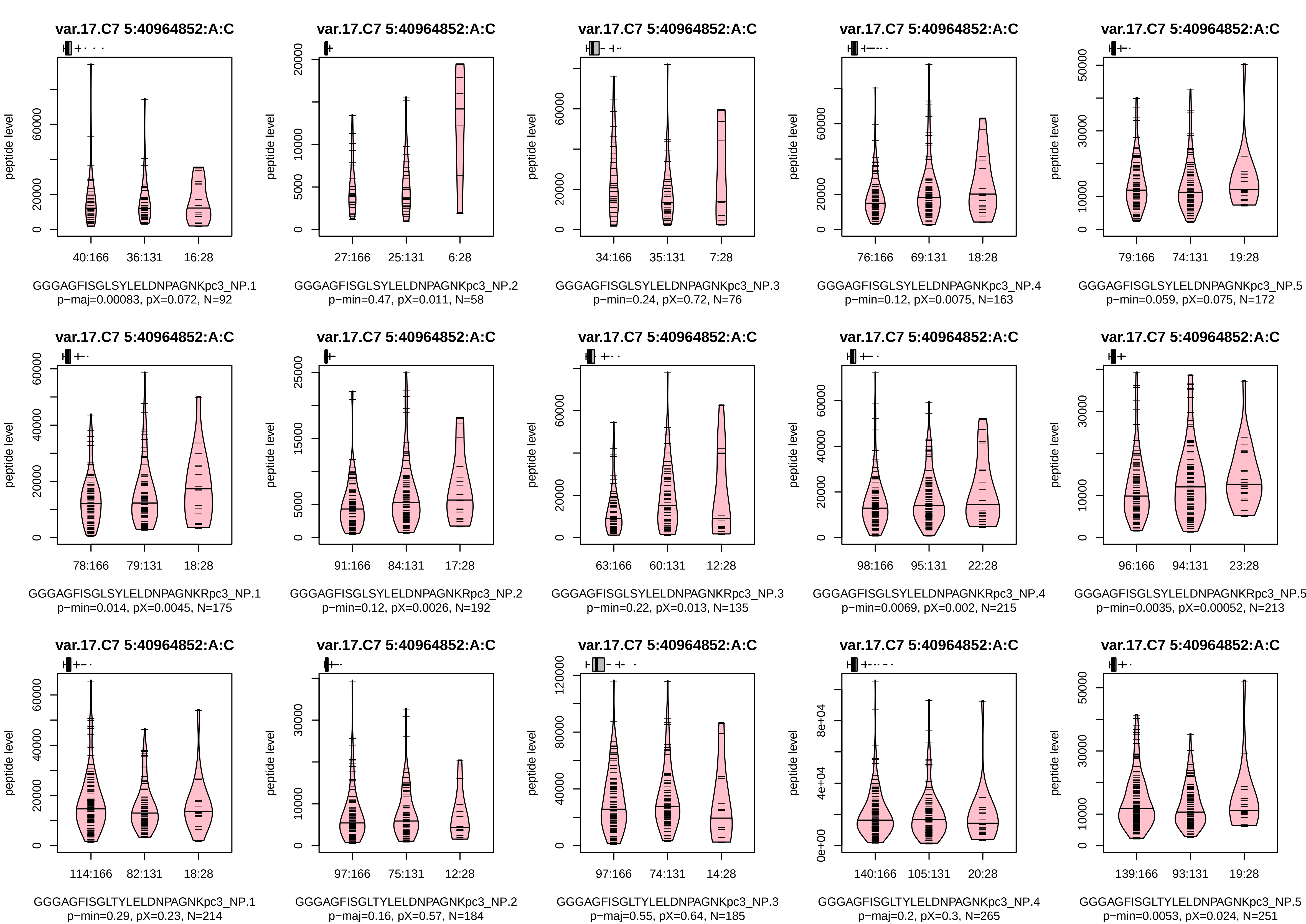
var.17.C7 5:40964852:A:C
peptide level
40:166
36:131
16:28
GGGAGFISGLSYLELDNPAGNKpc3_NP.1
p−maj=0.00083, pX=0.072, N=92
var.17.C7 5:40964852:A:C
peptide level
27:166
25:131
6:28
GGGAGFISGLSYLELDNPAGNKpc3_NP.2
p−min=0.47, pX=0.011, N=58
var.17.C7 5:40964852:A:C
peptide level
34:166
35:131
7:28
GGGAGFISGLSYLELDNPAGNKpc3_NP.3
p−min=0.24, pX=0.72, N=76
var.17.C7 5:40964852:A:C
peptide level
76:166
69:131
18:28
GGGAGFISGLSYLELDNPAGNKpc3_NP.4
p−min=0.12, pX=0.0075, N=163
var.17.C7 5:40964852:A:C
peptide level
79:166
74:131
19:28
GGGAGFISGLSYLELDNPAGNKpc3_NP.5
p−min=0.059, pX=0.075, N=172
var.17.C7 5:40964852:A:C
peptide level
78:166
79:131
18:28
GGGAGFISGLSYLELDNPAGNKRpc3_NP.1
p−min=0.014, pX=0.0045, N=175
var.17.C7 5:40964852:A:C
peptide level
91:166
84:131
17:28
GGGAGFISGLSYLELDNPAGNKRpc3_NP.2
p−min=0.12, pX=0.0026, N=192
var.17.C7 5:40964852:A:C
peptide level
63:166
60:131
12:28
GGGAGFISGLSYLELDNPAGNKRpc3_NP.3
p−min=0.22, pX=0.013, N=135
var.17.C7 5:40964852:A:C
peptide level
98:166
95:131
22:28
GGGAGFISGLSYLELDNPAGNKRpc3_NP.4
p−min=0.0069, pX=0.002, N=215
var.17.C7 5:40964852:A:C
peptide level
96:166
94:131
23:28
GGGAGFISGLSYLELDNPAGNKRpc3_NP.5
p−min=0.0035, pX=0.00052, N=213
var.17.C7 5:40964852:A:C
peptide level
114:166
82:131
18:28
GGGAGFISGLTYLELDNPAGNKpc3_NP.1
p−min=0.29, pX=0.23, N=214
var.17.C7 5:40964852:A:C
peptide level
97:166
75:131
12:28
GGGAGFISGLTYLELDNPAGNKpc3_NP.2
p−maj=0.16, pX=0.57, N=184
var.17.C7 5:40964852:A:C
peptide level
97:166
74:131
14:28
GGGAGFISGLTYLELDNPAGNKpc3_NP.3
p−maj=0.55, pX=0.64, N=185
var.17.C7 5:40964852:A:C
peptide level
140:166
105:131
20:28
GGGAGFISGLTYLELDNPAGNKpc3_NP.4
p−maj=0.2, pX=0.3, N=265
var.17.C7 5:40964852:A:C
peptide level
139:166
93:131
19:28
GGGAGFISGLTYLELDNPAGNKpc3_NP.5
p−min=0.0053, pX=0.024, N=251

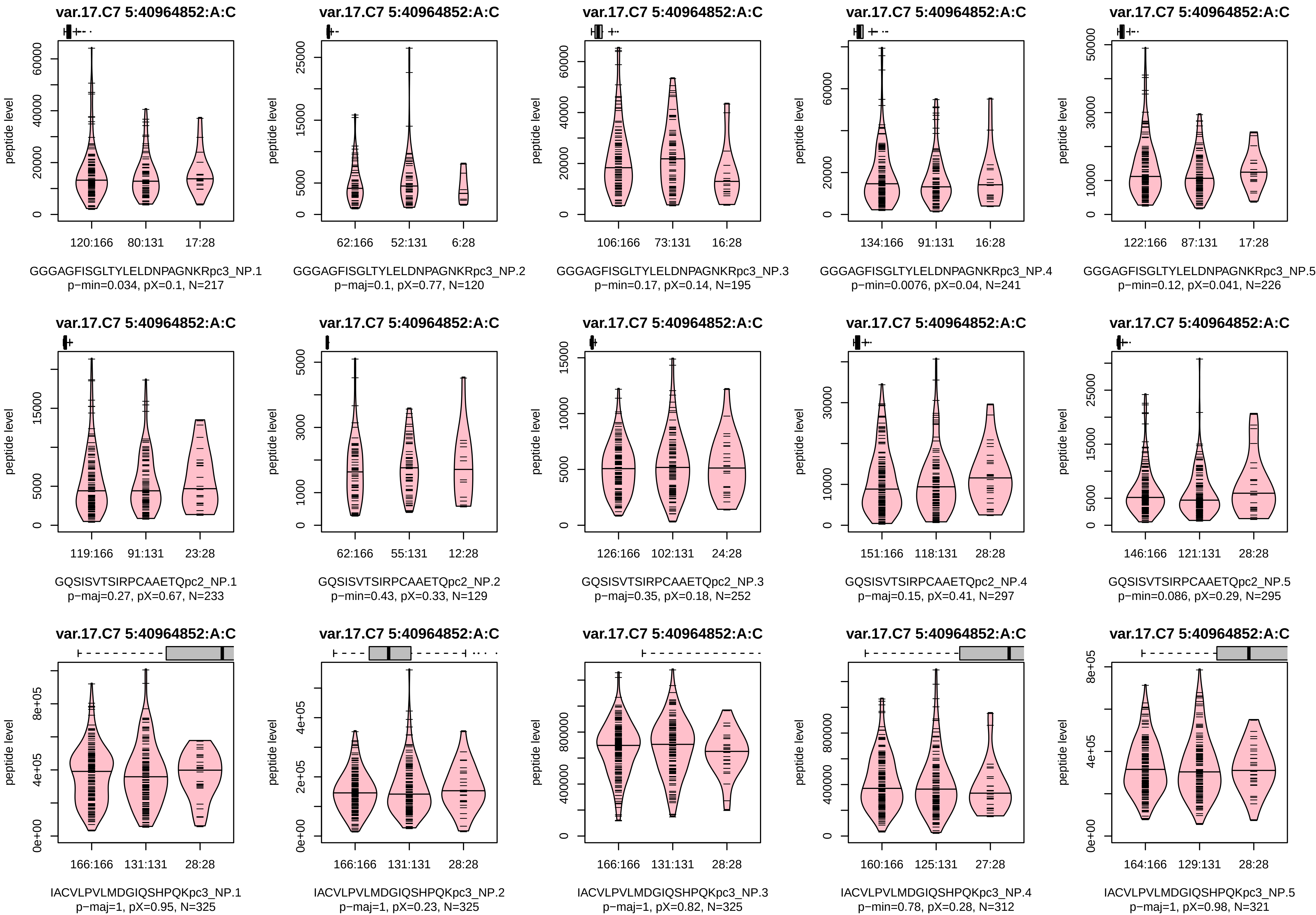
var.17.C7 5:40964852:A:C
peptide level
120:166
80:131
17:28
GGGAGFISGLTYLELDNPAGNKRpc3_NP.1
p−min=0.034, pX=0.1, N=217
var.17.C7 5:40964852:A:C
62:166
52:131
6:28
GGGAGFISGLTYLELDNPAGNKRpc3_NP.2
p−maj=0.1, pX=0.77, N=120
var.17.C7 5:40964852:A:C
106:166
73:131
16:28
GGGAGFISGLTYLELDNPAGNKRpc3_NP.3
p−min=0.17, pX=0.14, N=195
var.17.C7 5:40964852:A:C
134:166
91:131
16:28
GGGAGFISGLTYLELDNPAGNKRpc3_NP.4
p−min=0.0076, pX=0.04, N=241
var.17.C7 5:40964852:A:C
122:166
87:131
17:28
GGGAGFISGLTYLELDNPAGNKRpc3_NP.5
p−min=0.12, pX=0.041, N=226
var.17.C7 5:40964852:A:C
119:166
91:131
23:28
GQSISVTSIRPCAAETQpc2_NP.1
p−maj=0.27, pX=0.67, N=233
var.17.C7 5:40964852:A:C
62:166
55:131
12:28
GQSISVTSIRPCAAETQpc2_NP.2
p−min=0.43, pX=0.33, N=129
var.17.C7 5:40964852:A:C
126:166
102:131
24:28
GQSISVTSIRPCAAETQpc2_NP.3
p−maj=0.35, pX=0.18, N=252
var.17.C7 5:40964852:A:C
151:166
118:131
28:28
GQSISVTSIRPCAAETQpc2_NP.4
p−maj=0.15, pX=0.41, N=297
var.17.C7 5:40964852:A:C
146:166
121:131
28:28
GQSISVTSIRPCAAETQpc2_NP.5
p−min=0.086, pX=0.29, N=295
var.17.C7 5:40964852:A:C
166:166
131:131
28:28
IACVLPVLMDGIQSHPQKpc3_NP.1
p−maj=1, pX=0.95, N=325
var.17.C7 5:40964852:A:C
166:166
131:131
28:28
IACVLPVLMDGIQSHPQKpc3_NP.2
p−maj=1, pX=0.23, N=325
var.17.C7 5:40964852:A:C
166:166
131:131
28:28
IACVLPVLMDGIQSHPQKpc3_NP.3
p−maj=1, pX=0.82, N=325
var.17.C7 5:40964852:A:C
160:166
125:131
27:28
IACVLPVLMDGIQSHPQKpc3_NP.4
p−min=0.78, pX=0.28, N=312
var.17.C7 5:40964852:A:C
164:166
129:131
28:28
IACVLPVLMDGIQSHPQKpc3_NP.5
p−maj=1, pX=0.98, N=321

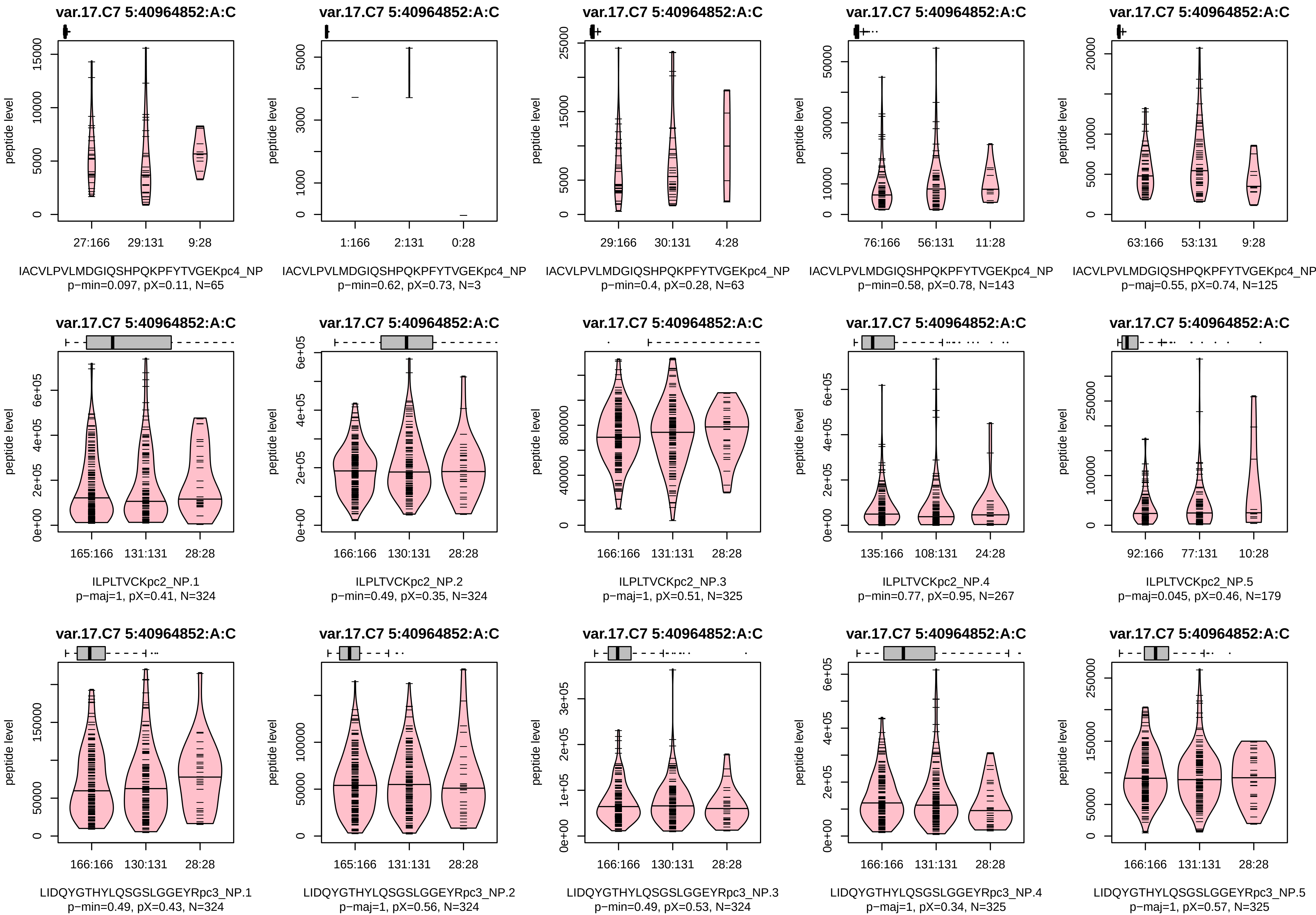
var.17.C7 5:40964852:A:C
peptide level
27:166
29:131
9:28
IACVLPVLMDGIQSHPQKPFYTVGEKpc4_NP
p−min=0.097, pX=0.11, N=65
var.17.C7 5:40964852:A:C
peptide level
1:166
2:131
0:28
IACVLPVLMDGIQSHPQKPFYTVGEKpc4_NP
p−min=0.62, pX=0.73, N=3
var.17.C7 5:40964852:A:C
peptide level
29:166
30:131
4:28
IACVLPVLMDGIQSHPQKPFYTVGEKpc4_NP
p−min=0.4, pX=0.28, N=63
var.17.C7 5:40964852:A:C
peptide level
76:166
56:131
11:28
IACVLPVLMDGIQSHPQKPFYTVGEKpc4_NP
p−min=0.58, pX=0.78, N=143
var.17.C7 5:40964852:A:C
peptide level
63:166
53:131
9:28
IACVLPVLMDGIQSHPQKPFYTVGEKpc4_NP
p−maj=0.55, pX=0.74, N=125
var.17.C7 5:40964852:A:C
peptide level
165:166
131:131
28:28
ILPLTVCKpc2_NP.1
p−maj=1, pX=0.41, N=324
var.17.C7 5:40964852:A:C
peptide level
166:166
130:131
28:28
ILPLTVCKpc2_NP.2
p−min=0.49, pX=0.35, N=324
var.17.C7 5:40964852:A:C
peptide level
166:166
131:131
28:28
ILPLTVCKpc2_NP.3
p−maj=1, pX=0.51, N=325
var.17.C7 5:40964852:A:C
peptide level
135:166
108:131
24:28
ILPLTVCKpc2_NP.4
p−min=0.77, pX=0.95, N=267
var.17.C7 5:40964852:A:C
peptide level
92:166
77:131
10:28
ILPLTVCKpc2_NP.5
p−maj=0.045, pX=0.46, N=179
var.17.C7 5:40964852:A:C
peptide level
166:166
130:131
28:28
LIDQYGTHYLQSGSLGGEYRpc3_NP.1
p−min=0.49, pX=0.43, N=324
var.17.C7 5:40964852:A:C
peptide level
165:166
131:131
28:28
LIDQYGTHYLQSGSLGGEYRpc3_NP.2
p−maj=1, pX=0.56, N=324
var.17.C7 5:40964852:A:C
peptide level
166:166
130:131
28:28
LIDQYGTHYLQSGSLGGEYRpc3_NP.3
p−min=0.49, pX=0.53, N=324
var.17.C7 5:40964852:A:C
peptide level
166:166
131:131
28:28
LIDQYGTHYLQSGSLGGEYRpc3_NP.4
p−maj=1, pX=0.34, N=325
var.17.C7 5:40964852:A:C
peptide level
166:166
131:131
28:28
LIDQYGTHYLQSGSLGGEYRpc3_NP.5
p−maj=1, pX=0.57, N=325

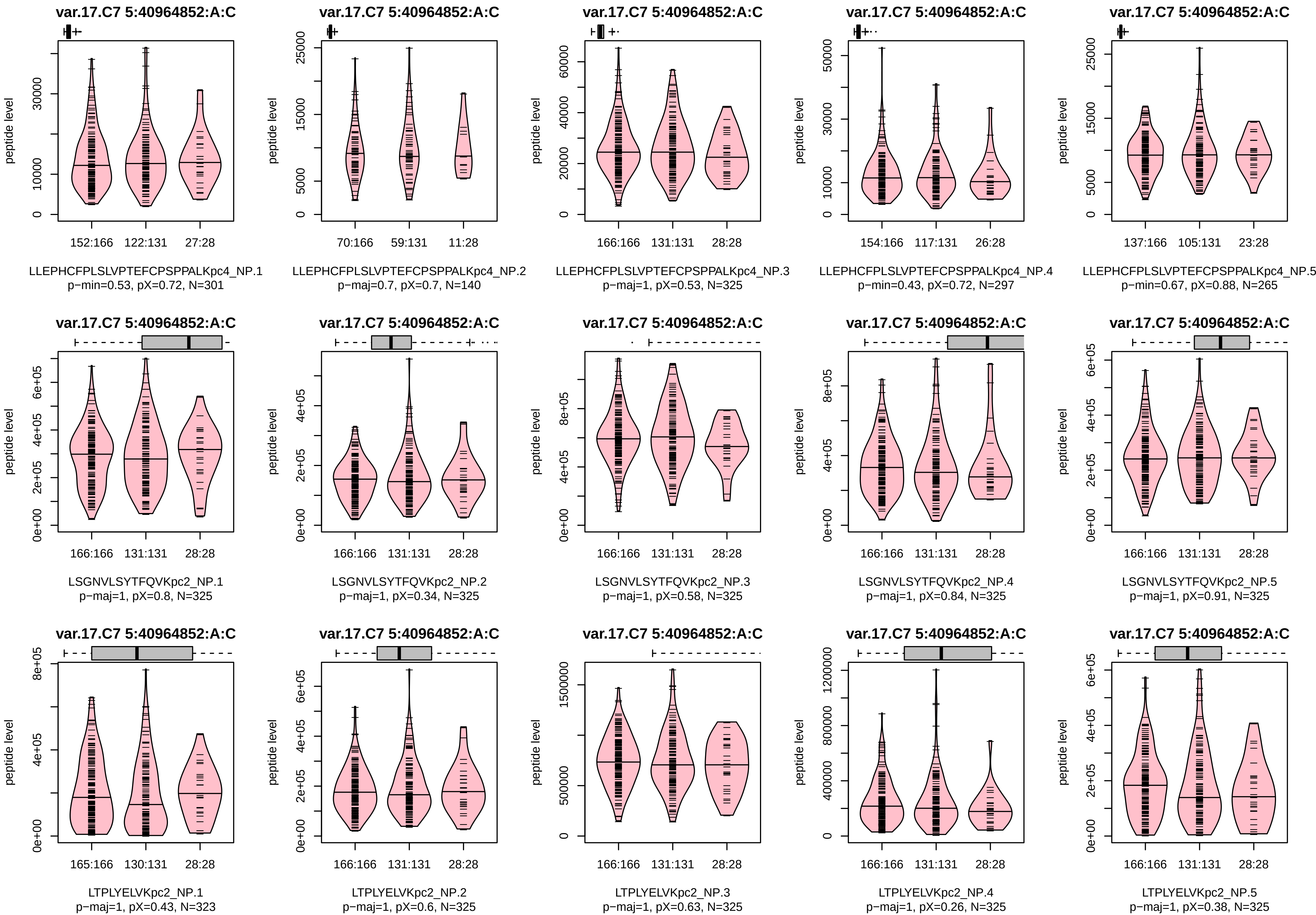
var.17.C7 5:40964852:A:C
peptide level
152:166
122:131
27:28
LLEPHCFPLSLVPTEFCPSPPALKpc4_NP.1
p−min=0.53, pX=0.72, N=301
var.17.C7 5:40964852:A:C
peptide level
70:166
59:131
11:28
LLEPHCFPLSLVPTEFCPSPPALKpc4_NP.2
p−maj=0.7, pX=0.7, N=140
var.17.C7 5:40964852:A:C
peptide level
166:166
131:131
28:28
LLEPHCFPLSLVPTEFCPSPPALKpc4_NP.3
p−maj=1, pX=0.53, N=325
var.17.C7 5:40964852:A:C
peptide level
154:166
117:131
26:28
LLEPHCFPLSLVPTEFCPSPPALKpc4_NP.4
p−min=0.43, pX=0.72, N=297
var.17.C7 5:40964852:A:C
peptide level
137:166
105:131
23:28
LLEPHCFPLSLVPTEFCPSPPALKpc4_NP.5
p−min=0.67, pX=0.88, N=265
var.17.C7 5:40964852:A:C
peptide level
166:166
131:131
28:28
LSGNVLSYTFQVKpc2_NP.1
p−maj=1, pX=0.8, N=325
var.17.C7 5:40964852:A:C
peptide level
166:166
131:131
28:28
LSGNVLSYTFQVKpc2_NP.2
p−maj=1, pX=0.34, N=325
var.17.C7 5:40964852:A:C
peptide level
166:166
131:131
28:28
LSGNVLSYTFQVKpc2_NP.3
p−maj=1, pX=0.58, N=325
var.17.C7 5:40964852:A:C
peptide level
166:166
131:131
28:28
LSGNVLSYTFQVKpc2_NP.4
p−maj=1, pX=0.84, N=325
var.17.C7 5:40964852:A:C
peptide level
166:166
131:131
28:28
LSGNVLSYTFQVKpc2_NP.5
p−maj=1, pX=0.91, N=325
var.17.C7 5:40964852:A:C
peptide level
165:166
130:131
28:28
LTPLYELVKpc2_NP.1
p−maj=1, pX=0.43, N=323
var.17.C7 5:40964852:A:C
peptide level
166:166
131:131
28:28
LTPLYELVKpc2_NP.2
p−maj=1, pX=0.6, N=325
var.17.C7 5:40964852:A:C
peptide level
166:166
131:131
28:28
LTPLYELVKpc2_NP.3
p−maj=1, pX=0.63, N=325
var.17.C7 5:40964852:A:C
peptide level
166:166
131:131
28:28
LTPLYELVKpc2_NP.4
p−maj=1, pX=0.26, N=325
var.17.C7 5:40964852:A:C
peptide level
166:166
131:131
28:28
LTPLYELVKpc2_NP.5
p−maj=1, pX=0.38, N=325

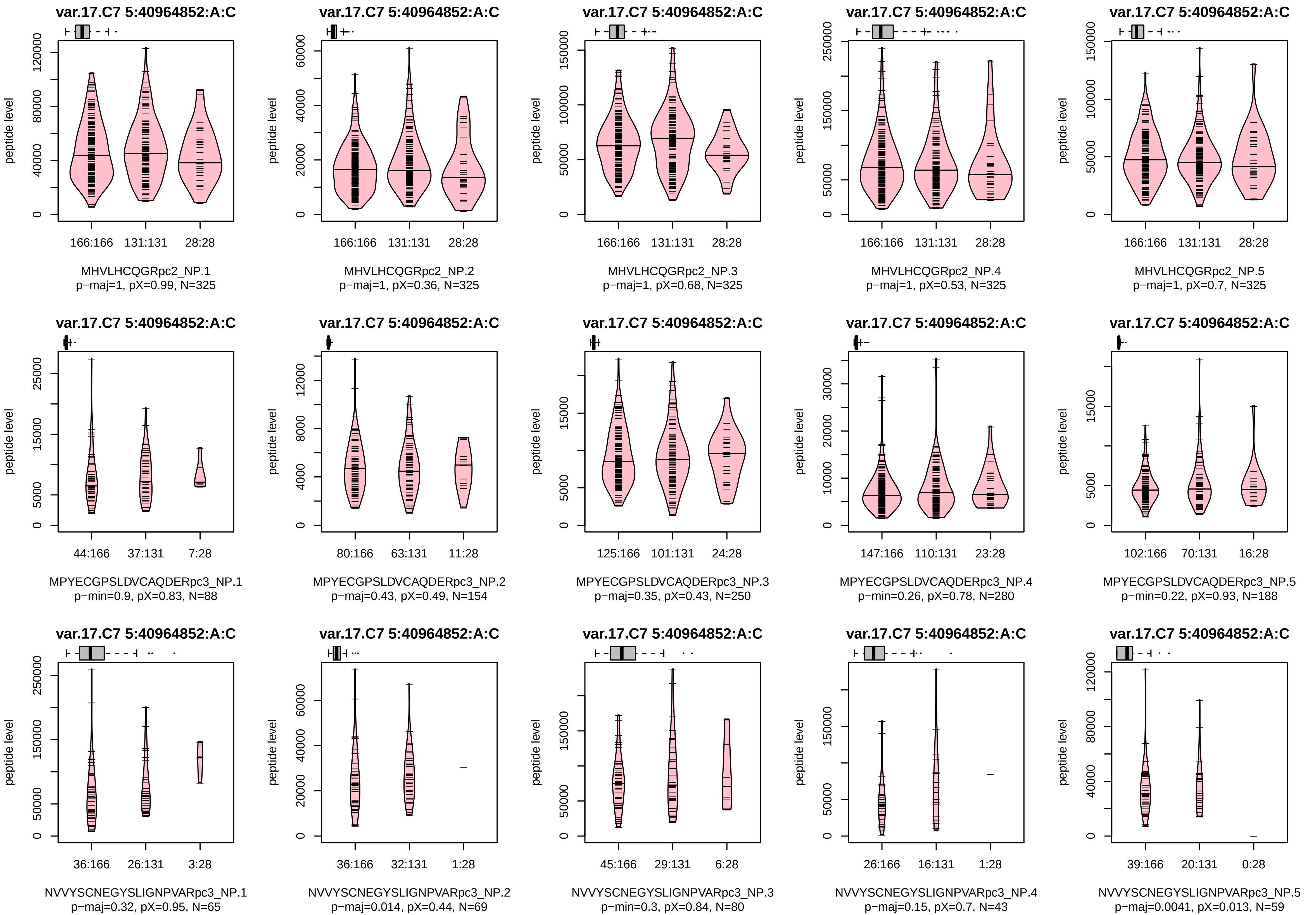
var.17.C7 5:40964852:A:C
peptide level
166:166
131:131
28:28
MHVLHCQGRpc2_NP.1
p−maj=1, pX=0.99, N=325
var.17.C7 5:40964852:A:C
peptide level
166:166
131:131
28:28
MHVLHCQGRpc2_NP.2
p−maj=1, pX=0.36, N=325
var.17.C7 5:40964852:A:C
peptide level
166:166
131:131
28:28
MHVLHCQGRpc2_NP.3
p−maj=1, pX=0.68, N=325
var.17.C7 5:40964852:A:C
peptide level
166:166
131:131
28:28
MHVLHCQGRpc2_NP.4
p−maj=1, pX=0.53, N=325
var.17.C7 5:40964852:A:C
peptide level
166:166
131:131
28:28
MHVLHCQGRpc2_NP.5
p−maj=1, pX=0.7, N=325
var.17.C7 5:40964852:A:C
peptide level
44:166
37:131
7:28
MPYECGPSLDVCAQDERpc3_NP.1
p−min=0.9, pX=0.83, N=88
var.17.C7 5:40964852:A:C
peptide level
80:166
63:131
11:28
MPYECGPSLDVCAQDERpc3_NP.2
p−maj=0.43, pX=0.49, N=154
var.17.C7 5:40964852:A:C
peptide level
125:166
101:131
24:28
MPYECGPSLDVCAQDERpc3_NP.3
p−maj=0.35, pX=0.43, N=250
var.17.C7 5:40964852:A:C
peptide level
147:166
110:131
23:28
MPYECGPSLDVCAQDERpc3_NP.4
p−min=0.26, pX=0.78, N=280
var.17.C7 5:40964852:A:C
peptide level
102:166
70:131
16:28
MPYECGPSLDVCAQDERpc3_NP.5
p−min=0.22, pX=0.93, N=188
var.17.C7 5:40964852:A:C
peptide level
36:166
26:131
3:28
NVVYSCNEGYSLIGNPVARpc3_NP.1
p−maj=0.32, pX=0.95, N=65
var.17.C7 5:40964852:A:C
peptide level
36:166
32:131
1:28
NVVYSCNEGYSLIGNPVARpc3_NP.2
p−maj=0.014, pX=0.44, N=69
var.17.C7 5:40964852:A:C
peptide level
45:166
29:131
6:28
NVVYSCNEGYSLIGNPVARpc3_NP.3
p−min=0.3, pX=0.84, N=80
var.17.C7 5:40964852:A:C
peptide level
26:166
16:131
1:28
NVVYSCNEGYSLIGNPVARpc3_NP.4
p−maj=0.15, pX=0.7, N=43
var.17.C7 5:40964852:A:C
peptide level
39:166
20:131
0:28
NVVYSCNEGYSLIGNPVARpc3_NP.5
p−maj=0.0041, pX=0.013, N=59

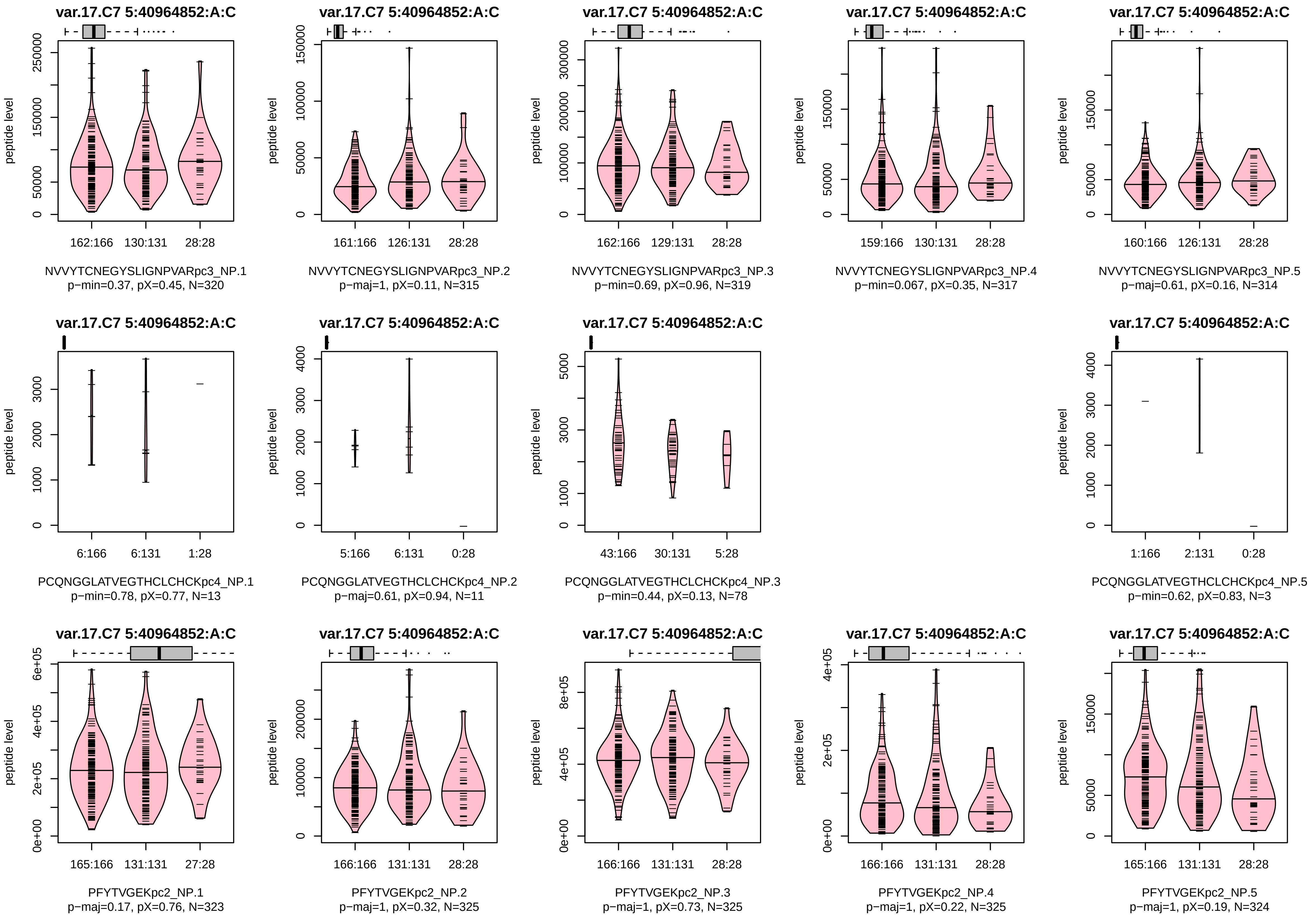
var.17.C7 5:40964852:A:C
peptide level
162:166
130:131
28:28
NVVYTCNEGYSLIGNPVARpc3_NP.1
p−min=0.37, pX=0.45, N=320
var.17.C7 5:40964852:A:C
peptide level
161:166
126:131
28:28
NVVYTCNEGYSLIGNPVARpc3_NP.2
p−maj=1, pX=0.11, N=315
var.17.C7 5:40964852:A:C
peptide level
162:166
129:131
28:28
NVVYTCNEGYSLIGNPVARpc3_NP.3
p−min=0.69, pX=0.96, N=319
var.17.C7 5:40964852:A:C
peptide level
159:166
130:131
28:28
NVVYTCNEGYSLIGNPVARpc3_NP.4
p−min=0.067, pX=0.35, N=317
var.17.C7 5:40964852:A:C
peptide level
160:166
126:131
28:28
NVVYTCNEGYSLIGNPVARpc3_NP.5
p−maj=0.61, pX=0.16, N=314
var.17.C7 5:40964852:A:C
peptide level
6:166
6:131
1:28
PCQNGGLATVEGTHCLCHCKpc4_NP.1
p−min=0.78, pX=0.77, N=13
var.17.C7 5:40964852:A:C
peptide level
5:166
6:131
0:28
PCQNGGLATVEGTHCLCHCKpc4_NP.2
p−maj=0.61, pX=0.94, N=11
var.17.C7 5:40964852:A:C
peptide level
43:166
30:131
5:28
PCQNGGLATVEGTHCLCHCKpc4_NP.3
p−min=0.44, pX=0.13, N=78
var.17.C7 5:40964852:A:C
peptide level
1:166
2:131
0:28
PCQNGGLATVEGTHCLCHCKpc4_NP.5
p−min=0.62, pX=0.83, N=3
var.17.C7 5:40964852:A:C
peptide level
165:166
131:131
27:28
PFYTVGEKpc2_NP.1
p−maj=0.17, pX=0.76, N=323
var.17.C7 5:40964852:A:C
peptide level
166:166
131:131
28:28
PFYTVGEKpc2_NP.2
p−maj=1, pX=0.32, N=325
var.17.C7 5:40964852:A:C
peptide level
166:166
131:131
28:28
PFYTVGEKpc2_NP.3
p−maj=1, pX=0.73, N=325
var.17.C7 5:40964852:A:C
peptide level
166:166
131:131
28:28
PFYTVGEKpc2_NP.4
p−maj=1, pX=0.22, N=325
var.17.C7 5:40964852:A:C
peptide level
165:166
131:131
28:28
PFYTVGEKpc2_NP.5
p−maj=1, pX=0.19, N=324

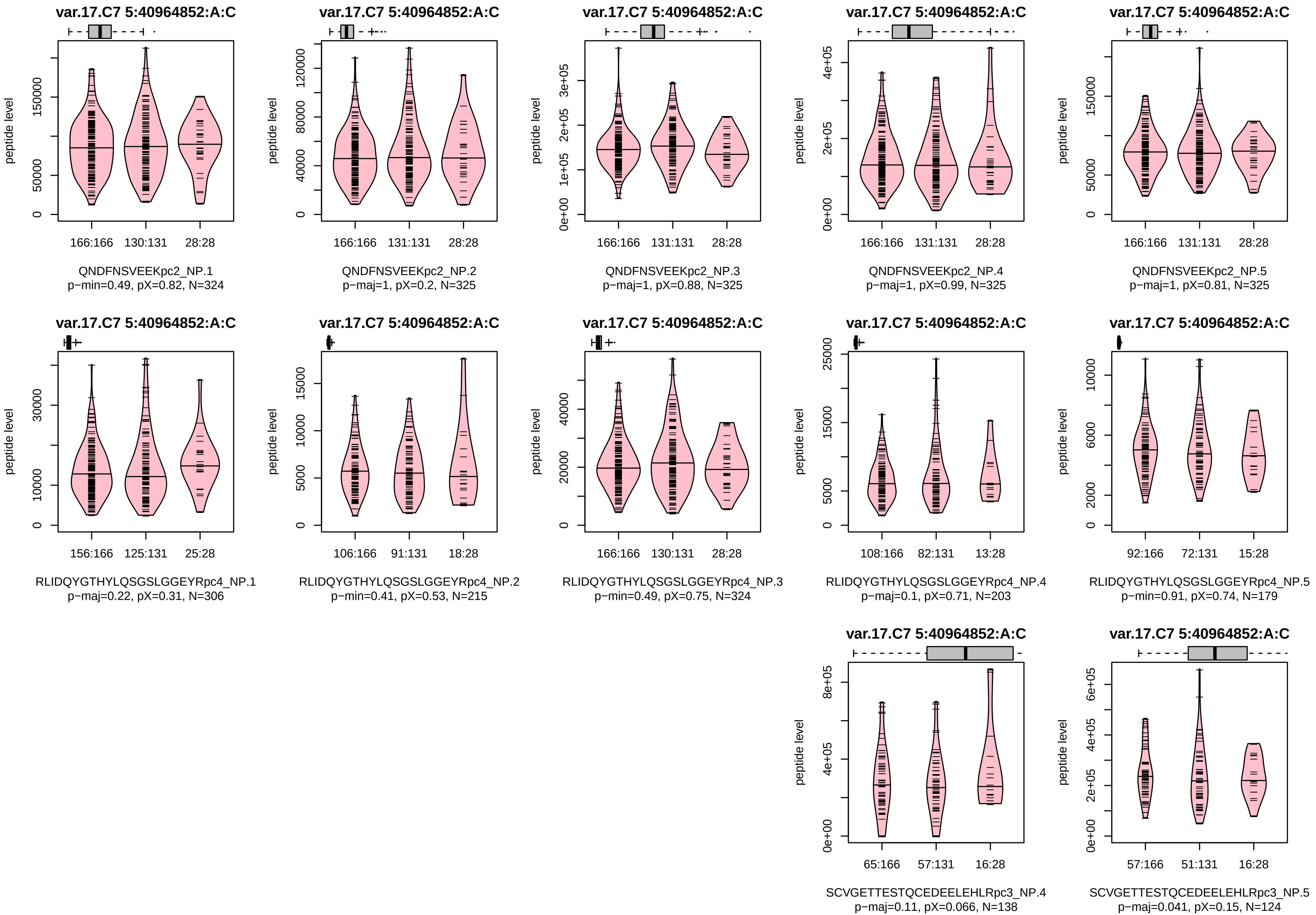
var.17.C7 5:40964852:A:C
peptide level
166:166
130:131
28:28
QNDFNSVEEKpc2_NP.1
p−min=0.49, pX=0.82, N=324
var.17.C7 5:40964852:A:C
166:166
131:131
28:28
QNDFNSVEEKpc2_NP.2
p−maj=1, pX=0.2, N=325
var.17.C7 5:40964852:A:C
166:166
131:131
28:28
QNDFNSVEEKpc2_NP.3
p−maj=1, pX=0.88, N=325
var.17.C7 5:40964852:A:C
166:166
131:131
28:28
QNDFNSVEEKpc2_NP.4
p−maj=1, pX=0.99, N=325
var.17.C7 5:40964852:A:C
166:166
131:131
28:28
QNDFNSVEEKpc2_NP.5
p−maj=1, pX=0.81, N=325
var.17.C7 5:40964852:A:C
156:166
125:131
25:28
RLIDQYGTHYLQSGSLGGEYRpc4_NP.1
p−maj=0.22, pX=0.31, N=306
var.17.C7 5:40964852:A:C
106:166
91:131
18:28
RLIDQYGTHYLQSGSLGGEYRpc4_NP.2
p−min=0.41, pX=0.53, N=215
var.17.C7 5:40964852:A:C
166:166
130:131
28:28
RLIDQYGTHYLQSGSLGGEYRpc4_NP.3
p−min=0.49, pX=0.75, N=324
var.17.C7 5:40964852:A:C
108:166
82:131
13:28
RLIDQYGTHYLQSGSLGGEYRpc4_NP.4
p−maj=0.1, pX=0.71, N=203
var.17.C7 5:40964852:A:C
92:166
72:131
15:28
RLIDQYGTHYLQSGSLGGEYRpc4_NP.5
p−min=0.91, pX=0.74, N=179
var.17.C7 5:40964852:A:C
65:166
57:131
16:28
SCVGETTESTQCEDEELEHLRpc3_NP.4
p−maj=0.11, pX=0.066, N=138
var.17.C7 5:40964852:A:C
57:166
51:131
16:28
SCVGETTESTQCEDEELEHLRpc3_NP.5
p−maj=0.041, pX=0.15, N=124

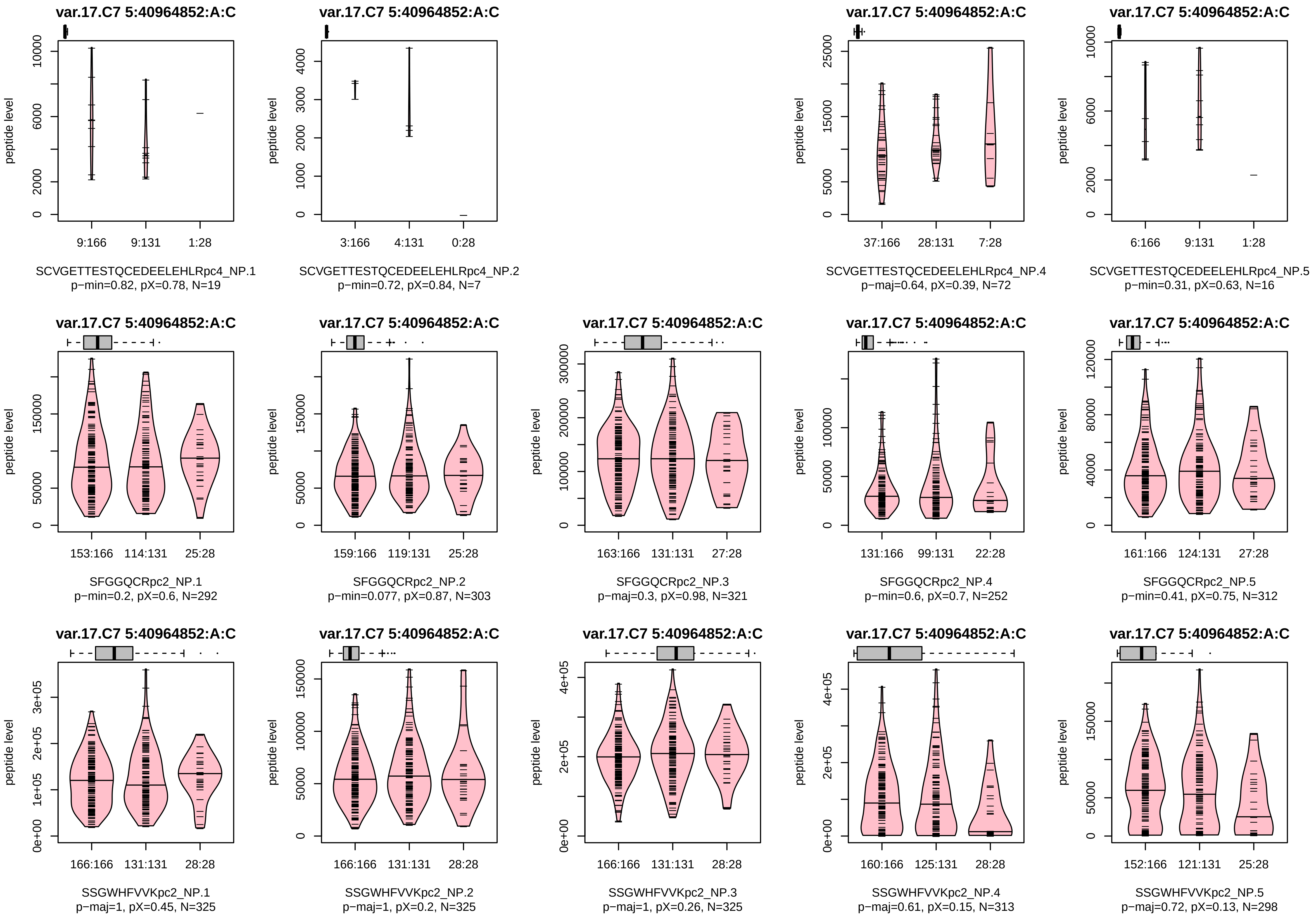
var.17.C7 5:40964852:A:C
peptide level
9:166
9:131
1:28
SCVGETTESTQCEDEELEHLRpc4_NP.1
p−min=0.82, pX=0.78, N=19
var.17.C7 5:40964852:A:C
peptide level
3:166
4:131
0:28
SCVGETTESTQCEDEELEHLRpc4_NP.2
p−min=0.72, pX=0.84, N=7
var.17.C7 5:40964852:A:C
peptide level
37:166
28:131
7:28
SCVGETTESTQCEDEELEHLRpc4_NP.4
p−maj=0.64, pX=0.39, N=72
var.17.C7 5:40964852:A:C
peptide level
6:166
9:131
1:28
SCVGETTESTQCEDEELEHLRpc4_NP.5
p−min=0.31, pX=0.63, N=16
var.17.C7 5:40964852:A:C
peptide level
153:166
114:131
25:28
SFGGQCRpc2_NP.1
p−min=0.2, pX=0.6, N=292
var.17.C7 5:40964852:A:C
peptide level
159:166
119:131
25:28
SFGGQCRpc2_NP.2
p−min=0.077, pX=0.87, N=303
var.17.C7 5:40964852:A:C
peptide level
163:166
131:131
27:28
SFGGQCRpc2_NP.3
p−maj=0.3, pX=0.98, N=321
var.17.C7 5:40964852:A:C
peptide level
131:166
99:131
22:28
SFGGQCRpc2_NP.4
p−min=0.6, pX=0.7, N=252
var.17.C7 5:40964852:A:C
peptide level
161:166
124:131
27:28
SFGGQCRpc2_NP.5
p−min=0.41, pX=0.75, N=312
var.17.C7 5:40964852:A:C
peptide level
166:166
131:131
28:28
SSGWHFVVKpc2_NP.1
p−maj=1, pX=0.45, N=325
var.17.C7 5:40964852:A:C
peptide level
166:166
131:131
28:28
SSGWHFVVKpc2_NP.2
p−maj=1, pX=0.2, N=325
var.17.C7 5:40964852:A:C
peptide level
166:166
131:131
28:28
SSGWHFVVKpc2_NP.3
p−maj=1, pX=0.26, N=325
var.17.C7 5:40964852:A:C
peptide level
160:166
125:131
28:28
SSGWHFVVKpc2_NP.4
p−maj=0.61, pX=0.15, N=313
var.17.C7 5:40964852:A:C
peptide level
152:166
121:131
25:28
SSGWHFVVKpc2_NP.5
p−maj=0.72, pX=0.13, N=298

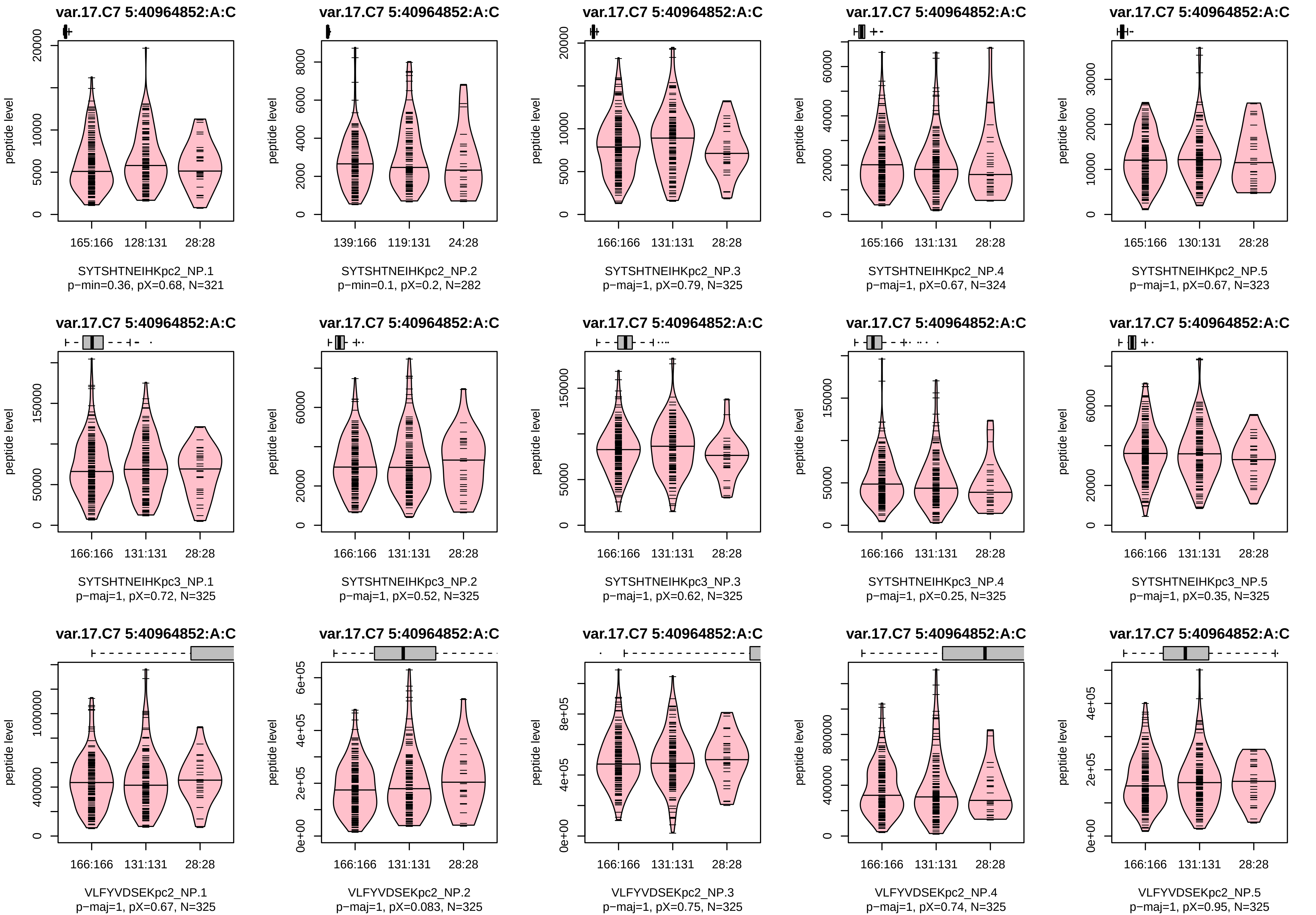
var.17.C7 5:40964852:A:C
peptide level
165:166
128:131
28:28
SYTSHTNEIHKpc2_NP.1
p−min=0.36, pX=0.68, N=321
var.17.C7 5:40964852:A:C
139:166
119:131
24:28
SYTSHTNEIHKpc2_NP.2
p−min=0.1, pX=0.2, N=282
var.17.C7 5:40964852:A:C
166:166
131:131
28:28
SYTSHTNEIHKpc2_NP.3
p−maj=1, pX=0.79, N=325
var.17.C7 5:40964852:A:C
165:166
131:131
28:28
SYTSHTNEIHKpc2_NP.4
p−maj=1, pX=0.67, N=324
var.17.C7 5:40964852:A:C
165:166
130:131
28:28
SYTSHTNEIHKpc2_NP.5
p−maj=1, pX=0.67, N=323
var.17.C7 5:40964852:A:C
SYTSHTNEIHKpc3_NP.1
p−maj=1, pX=0.72, N=325
SYTSHTNEIHKpc3_NP.2
p−maj=1, pX=0.52, N=325
SYTSHTNEIHKpc3_NP.3
p−maj=1, pX=0.62, N=325
SYTSHTNEIHKpc3_NP.4
p−maj=1, pX=0.25, N=325
SYTSHTNEIHKpc3_NP.5
p−maj=1, pX=0.35, N=325
VLFYVDSEKpc2_NP.1
p−maj=1, pX=0.67, N=325
VLFYVDSEKpc2_NP.2
p−maj=1, pX=0.083, N=325
VLFYVDSEKpc2_NP.3
p−maj=1, pX=0.75, N=325
VLFYVDSEKpc2_NP.4
p−maj=1, pX=0.74, N=325
VLFYVDSEKpc2_NP.5
p−maj=1, pX=0.95, N=325

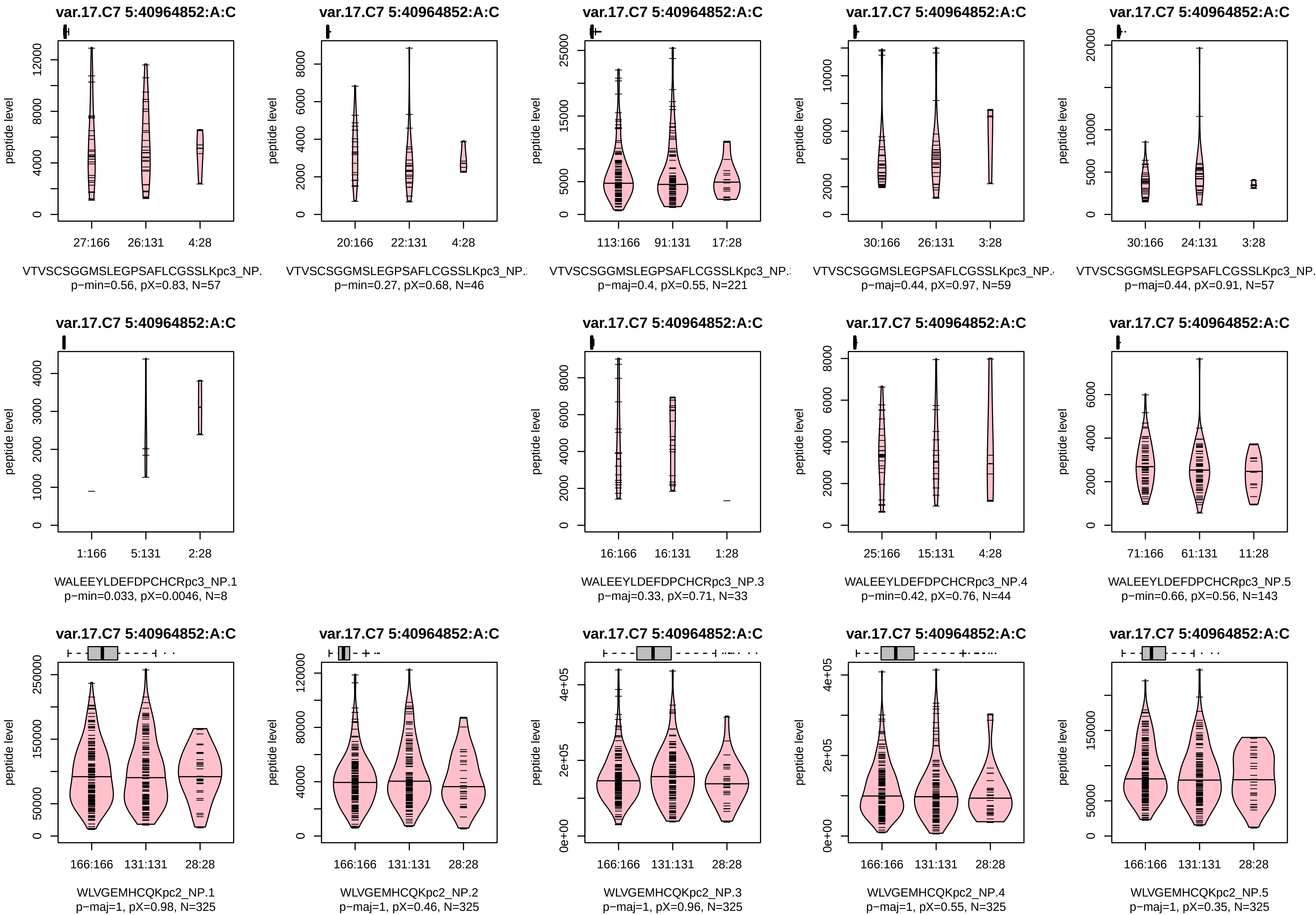
var.17.C7 5:40964852:A:C
peptide level
27:166
26:131
4:28
VTVSCSGGMSLEGPSAFLCGSSLKpc3_NP.
p−min=0.56, pX=0.83, N=57
20:166
22:131
4:28
VTVSCSGGMSLEGPSAFLCGSSLKpc3_NP.
p−min=0.27, pX=0.68, N=46
113:166
91:131
17:28
VTVSCSGGMSLEGPSAFLCGSSLKpc3_NP.
p−maj=0.4, pX=0.55, N=221
30:166
26:131
3:28
VTVSCSGGMSLEGPSAFLCGSSLKpc3_NP.
p−maj=0.44, pX=0.97, N=59
30:166
24:131
3:28
VTVSCSGGMSLEGPSAFLCGSSLKpc3_NP.
p−maj=0.44, pX=0.91, N=57
1:166
5:131
2:28
WALEEYLDEFDPCHCRpc3_NP.1
p−min=0.033, pX=0.0046, N=8
16:166
16:131
1:28
WALEEYLDEFDPCHCRpc3_NP.3
p−maj=0.33, pX=0.71, N=33
25:166
15:131
4:28
WALEEYLDEFDPCHCRpc3_NP.4
p−min=0.42, pX=0.76, N=44
71:166
61:131
11:28
WALEEYLDEFDPCHCRpc3_NP.5
p−min=0.66, pX=0.56, N=143
166:166
131:131
28:28
WLVGEMHCQKpc2_NP.1
p−maj=1, pX=0.98, N=325
WLVGEMHCQKpc2_NP.2
p−maj=1, pX=0.46, N=325
WLVGEMHCQKpc2_NP.3
p−maj=1, pX=0.96, N=325
WLVGEMHCQKpc2_NP.4
p−maj=1, pX=0.55, N=325
WLVGEMHCQKpc2_NP.5
p−maj=1, pX=0.35, N=325

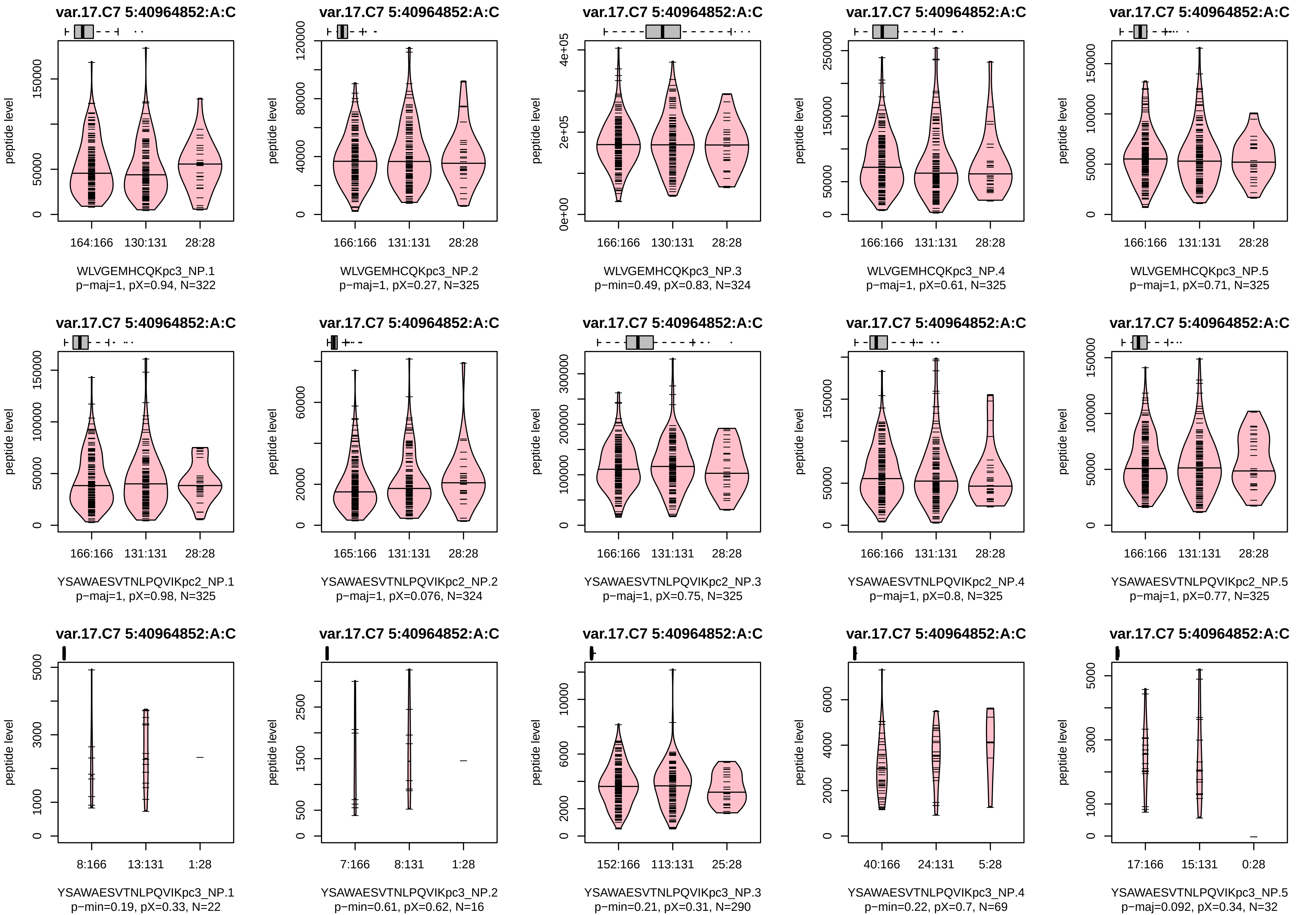
var.17.C7 5:40964852:A:C
peptide level
164:166
130:131
28:28
WLVGEMHCQKpc3_NP.1
p−maj=1, pX=0.94, N=322
var.17.C7 5:40964852:A:C
peptide level
166:166
131:131
28:28
WLVGEMHCQKpc3_NP.2
p−maj=1, pX=0.27, N=325
var.17.C7 5:40964852:A:C
peptide level
166:166
130:131
28:28
WLVGEMHCQKpc3_NP.3
p−min=0.49, pX=0.83, N=324
var.17.C7 5:40964852:A:C
peptide level
166:166
131:131
28:28
WLVGEMHCQKpc3_NP.4
p−maj=1, pX=0.61, N=325
var.17.C7 5:40964852:A:C
peptide level
166:166
131:131
28:28
WLVGEMHCQKpc3_NP.5
p−maj=1, pX=0.71, N=325
var.17.C7 5:40964852:A:C
peptide level
166:166
131:131
28:28
YSAWAESVTNLPQVIKpc2_NP.1
p−maj=1, pX=0.98, N=325
var.17.C7 5:40964852:A:C
peptide level
165:166
131:131
28:28
YSAWAESVTNLPQVIKpc2_NP.2
p−maj=1, pX=0.076, N=324
var.17.C7 5:40964852:A:C
peptide level
166:166
131:131
28:28
YSAWAESVTNLPQVIKpc2_NP.3
p−maj=1, pX=0.75, N=325
var.17.C7 5:40964852:A:C
peptide level
166:166
131:131
28:28
YSAWAESVTNLPQVIKpc2_NP.4
p−maj=1, pX=0.8, N=325
var.17.C7 5:40964852:A:C
peptide level
166:166
131:131
28:28
YSAWAESVTNLPQVIKpc2_NP.5
p−maj=1, pX=0.77, N=325
var.17.C7 5:40964852:A:C
peptide level
8:166
13:131
1:28
YSAWAESVTNLPQVIKpc3_NP.1
p−min=0.19, pX=0.33, N=22
var.17.C7 5:40964852:A:C
peptide level
7:166
8:131
1:28
YSAWAESVTNLPQVIKpc3_NP.2
p−min=0.61, pX=0.62, N=16
var.17.C7 5:40964852:A:C
peptide level
152:166
113:131
25:28
YSAWAESVTNLPQVIKpc3_NP.3
p−min=0.21, pX=0.31, N=290
var.17.C7 5:40964852:A:C
peptide level
40:166
24:131
5:28
YSAWAESVTNLPQVIKpc3_NP.4
p−min=0.22, pX=0.7, N=69
var.17.C7 5:40964852:A:C
peptide level
17:166
15:131
0:28
YSAWAESVTNLPQVIKpc3_NP.5
p−maj=0.092, pX=0.34, N=32

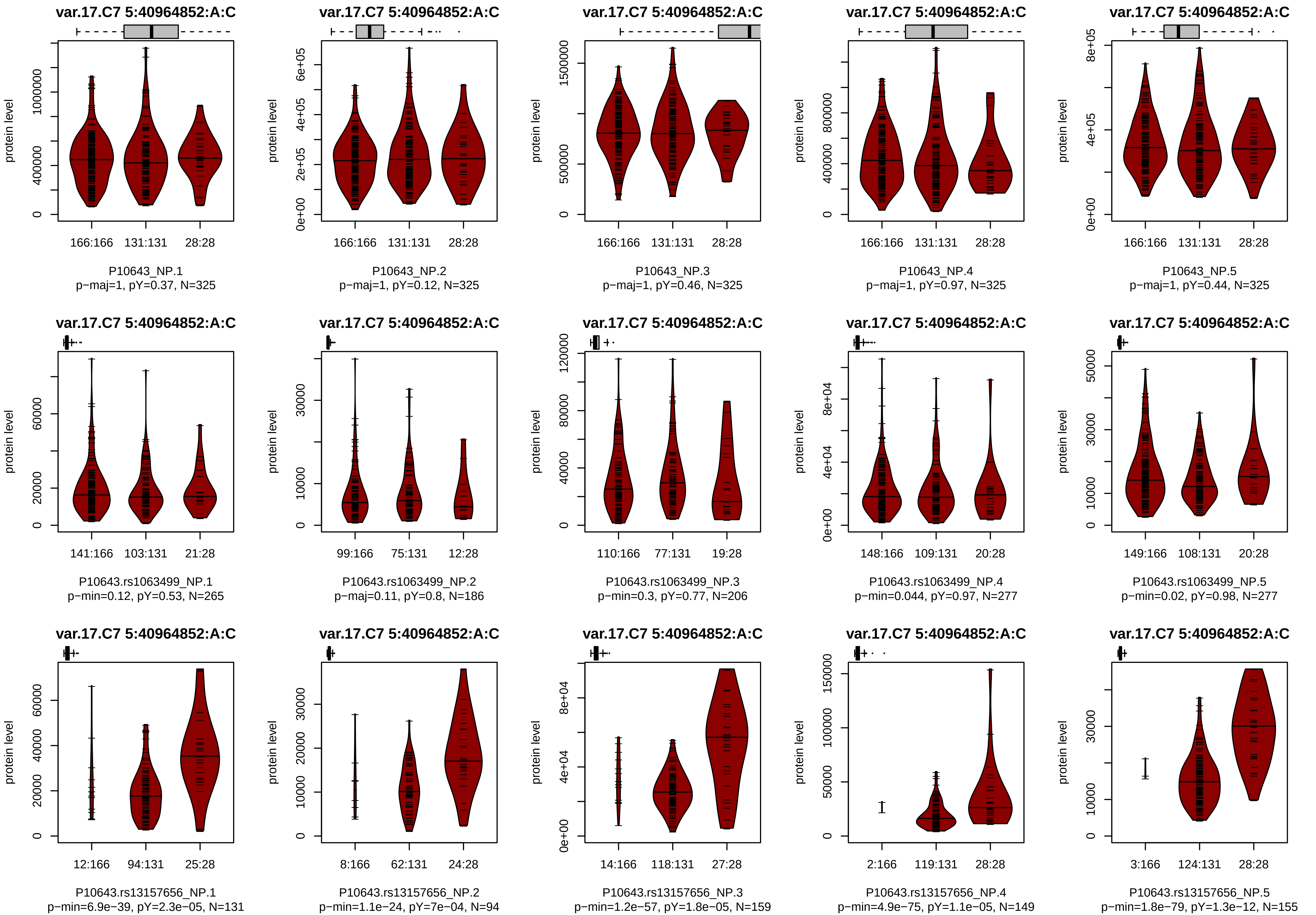
var.17.C7 5:40964852:A:C
protein level
166:166
131:131
28:28
P10643_NP.1
p−maj=1, pY=0.37, N=325
P10643_NP.2
p−maj=1, pY=0.12, N=325
P10643_NP.3
p−maj=1, pY=0.46, N=325
P10643_NP.4
p−maj=1, pY=0.97, N=325
P10643_NP.5
p−maj=1, pY=0.44, N=325
141:166
103:131
21:28
P10643.rs1063499_NP.1
p−min=0.12, pY=0.53, N=265
99:166
75:131
12:28
P10643.rs1063499_NP.2
p−maj=0.11, pY=0.8, N=186
110:166
77:131
19:28
P10643.rs1063499_NP.3
p−min=0.3, pY=0.77, N=206
148:166
109:131
20:28
P10643.rs1063499_NP.4
p−min=0.044, pY=0.97, N=277
149:166
108:131
20:28
P10643.rs1063499_NP.5
p−min=0.02, pY=0.98, N=277
12:166
94:131
25:28
P10643.rs13157656_NP.1
p−min=6.9e−39, pY=2.3e−05, N=131
8:166
62:131
24:28
P10643.rs13157656_NP.2
p−min=1.1e−24, pY=7e−04, N=94
14:166
118:131
27:28
P10643.rs13157656_NP.3
p−min=1.2e−57, pY=1.8e−05, N=159
2:166
119:131
28:28
P10643.rs13157656_NP.4
p−min=4.9e−75, pY=1.1e−05, N=149
3:166
124:131
28:28
P10643.rs13157656_NP.5
p−min=1.8e−79, pY=1.3e−12, N=155

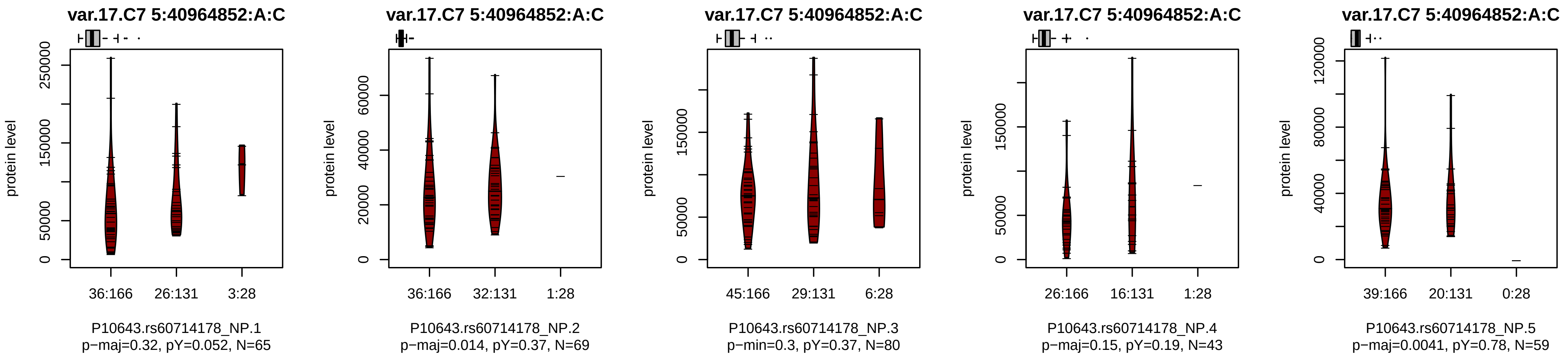

var.17.C7 5:40964852:A:C
protein level
0
50000
150000
250000
36:166
26:131
3:28
P10643.rs60714178_NP.1
p−maj=0.32, pY=0.052, N=65
var.17.C7 5:40964852:A:C
protein level
0
20000
40000
60000
36:166
32:131
1:28
P10643.rs60714178_NP.2
p−maj=0.014, pY=0.37, N=69
var.17.C7 5:40964852:A:C
protein level
0
50000
150000
45:166
29:131
6:28
P10643.rs60714178_NP.3
p−min=0.3, pY=0.37, N=80
var.17.C7 5:40964852:A:C
protein level
0
50000
150000
26:166
16:131
1:28
P10643.rs60714178_NP.4
p−maj=0.15, pY=0.19, N=43
var.17.C7 5:40964852:A:C
protein level
0
40000
80000
120000
39:166
20:131
0:28
P10643.rs60714178_NP.5
p−maj=0.0041, pY=0.78, N=59

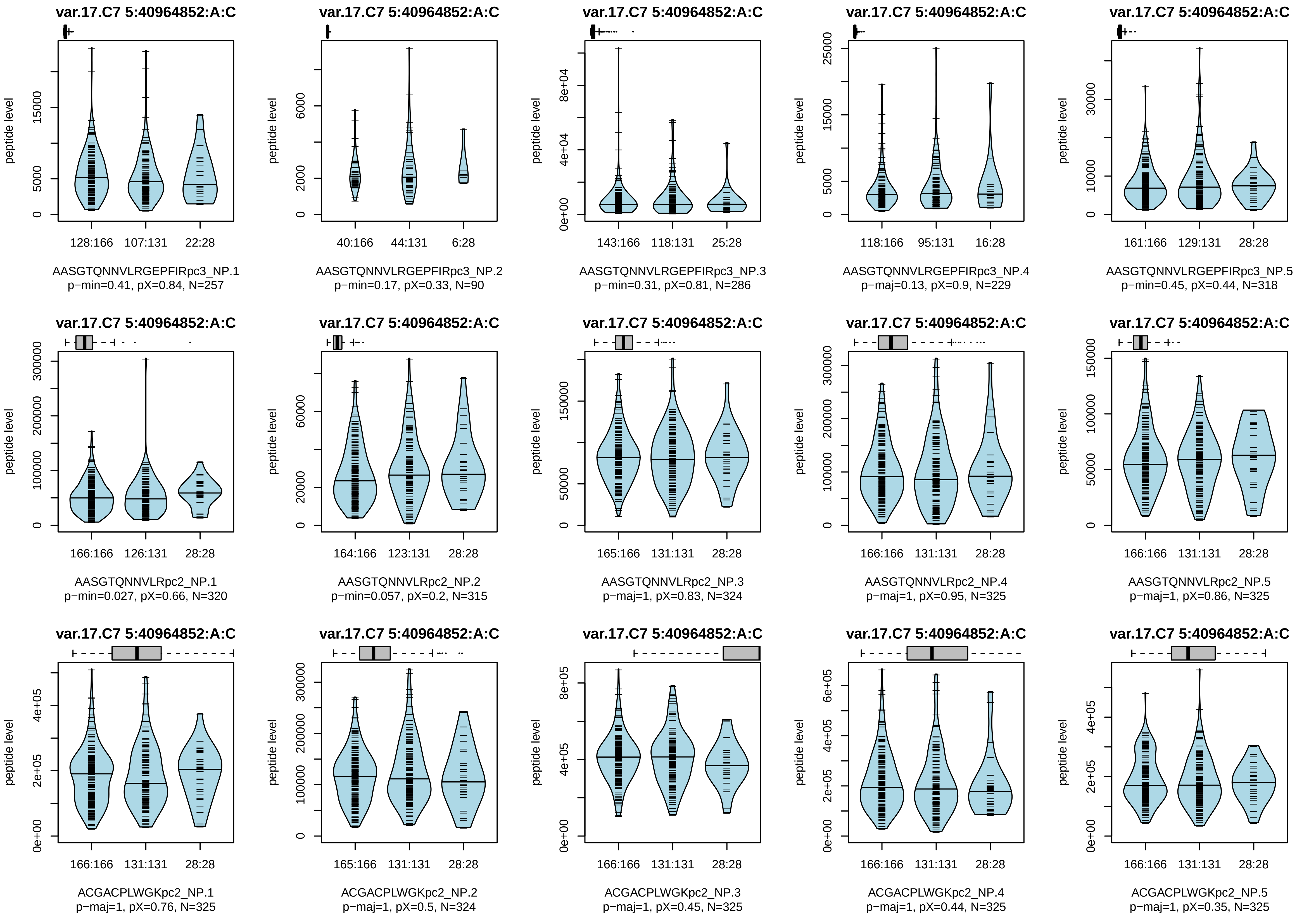

var.17.C7 5:40964852:A:C
peptide level
128:166 107:131 22:28
AASGTQNNVLRGEPFIRpc3_NP.1
p−min=0.41, pX=0.84, N=257
var.17.C7 5:40964852:A:C
peptide level
40:166 44:131 6:28
AASGTQNNVLRGEPFIRpc3_NP.2
p−min=0.17, pX=0.33, N=90
var.17.C7 5:40964852:A:C
peptide level
143:166 118:131 25:28
AASGTQNNVLRGEPFIRpc3_NP.3
p−min=0.31, pX=0.81, N=286
var.17.C7 5:40964852:A:C
peptide level
118:166 95:131 16:28
AASGTQNNVLRGEPFIRpc3_NP.4
p−maj=0.13, pX=0.9, N=229
var.17.C7 5:40964852:A:C
peptide level
161:166 129:131 28:28
AASGTQNNVLRGEPFIRpc3_NP.5
p−min=0.45, pX=0.44, N=318
var.17.C7 5:40964852:A:C
peptide level
166:166 126:131 28:28
AASGTQNNVLRpc2_NP.1
p−min=0.027, pX=0.66, N=320
var.17.C7 5:40964852:A:C
peptide level
164:166 123:131 28:28
AASGTQNNVLRpc2_NP.2
p−min=0.057, pX=0.2, N=315
var.17.C7 5:40964852:A:C
peptide level
165:166 131:131 28:28
AASGTQNNVLRpc2_NP.3
p−maj=1, pX=0.83, N=324
var.17.C7 5:40964852:A:C
peptide level
166:166 131:131 28:28
AASGTQNNVLRpc2_NP.4
p−maj=1, pX=0.95, N=325
var.17.C7 5:40964852:A:C
peptide level
166:166 131:131 28:28
AASGTQNNVLRpc2_NP.5
p−maj=1, pX=0.86, N=325
var.17.C7 5:40964852:A:C
peptide level
166:166 131:131 28:28
ACGACPLWGKpc2_NP.1
p−maj=1, pX=0.76, N=325
var.17.C7 5:40964852:A:C
peptide level
165:166 131:131 28:28
ACGACPLWGKpc2_NP.2
p−maj=1, pX=0.5, N=324
var.17.C7 5:40964852:A:C
peptide level
166:166 131:131 28:28
ACGACPLWGKpc2_NP.3
p−maj=1, pX=0.45, N=325
var.17.C7 5:40964852:A:C
peptide level
166:166 131:131 28:28
ACGACPLWGKpc2_NP.4
p−maj=1, pX=0.44, N=325
var.17.C7 5:40964852:A:C
peptide level
166:166 131:131 28:28
ACGACPLWGKpc2_NP.5
p−maj=1, pX=0.35, N=325

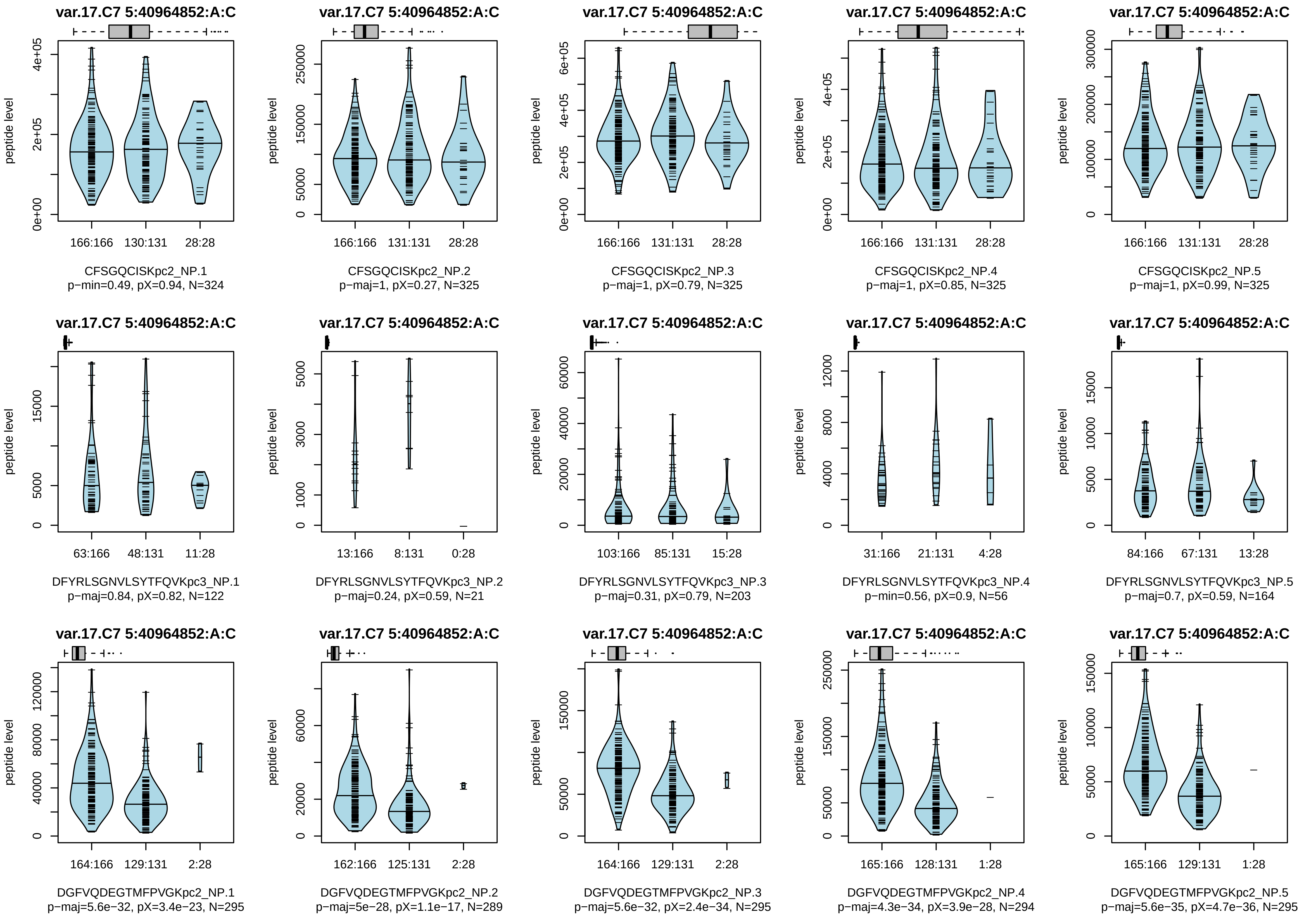
var.17.C7 5:40964852:A:C
peptide level
166:166
130:131
28:28
CFSGQCISKpc2_NP.1
p−min=0.49, pX=0.94, N=324
var.17.C7 5:40964852:A:C
peptide level
166:166
131:131
28:28
CFSGQCISKpc2_NP.2
p−maj=1, pX=0.27, N=325
var.17.C7 5:40964852:A:C
peptide level
166:166
131:131
28:28
CFSGQCISKpc2_NP.3
p−maj=1, pX=0.79, N=325
var.17.C7 5:40964852:A:C
peptide level
166:166
131:131
28:28
CFSGQCISKpc2_NP.4
p−maj=1, pX=0.85, N=325
var.17.C7 5:40964852:A:C
peptide level
166:166
131:131
28:28
CFSGQCISKpc2_NP.5
p−maj=1, pX=0.99, N=325
var.17.C7 5:40964852:A:C
peptide level
63:166
48:131
11:28
DFYRLSGNVLSYTFQVKpc3_NP.1
p−maj=0.84, pX=0.82, N=122
var.17.C7 5:40964852:A:C
peptide level
13:166
8:131
0:28
DFYRLSGNVLSYTFQVKpc3_NP.2
p−maj=0.24, pX=0.59, N=21
var.17.C7 5:40964852:A:C
peptide level
103:166
85:131
15:28
DFYRLSGNVLSYTFQVKpc3_NP.3
p−maj=0.31, pX=0.79, N=203
var.17.C7 5:40964852:A:C
peptide level
31:166
21:131
4:28
DFYRLSGNVLSYTFQVKpc3_NP.4
p−min=0.56, pX=0.9, N=56
var.17.C7 5:40964852:A:C
peptide level
84:166
67:131
13:28
DFYRLSGNVLSYTFQVKpc3_NP.5
p−maj=0.7, pX=0.59, N=164
var.17.C7 5:40964852:A:C
peptide level
164:166
129:131
2:28
DGFVQDEGTMFPVGKpc2_NP.1
p−maj=5.6e−32, pX=3.4e−23, N=295
var.17.C7 5:40964852:A:C
peptide level
162:166
125:131
2:28
DGFVQDEGTMFPVGKpc2_NP.2
p−maj=5e−28, pX=1.1e−17, N=289
var.17.C7 5:40964852:A:C
peptide level
164:166
129:131
2:28
DGFVQDEGTMFPVGKpc2_NP.3
p−maj=5.6e−32, pX=2.4e−34, N=295
var.17.C7 5:40964852:A:C
peptide level
165:166
128:131
1:28
DGFVQDEGTMFPVGKpc2_NP.4
p−maj=4.3e−34, pX=3.9e−28, N=294
var.17.C7 5:40964852:A:C
peptide level
165:166
129:131
1:28
DGFVQDEGTMFPVGKpc2_NP.5
p−maj=5.6e−35, pX=4.7e−36, N=295

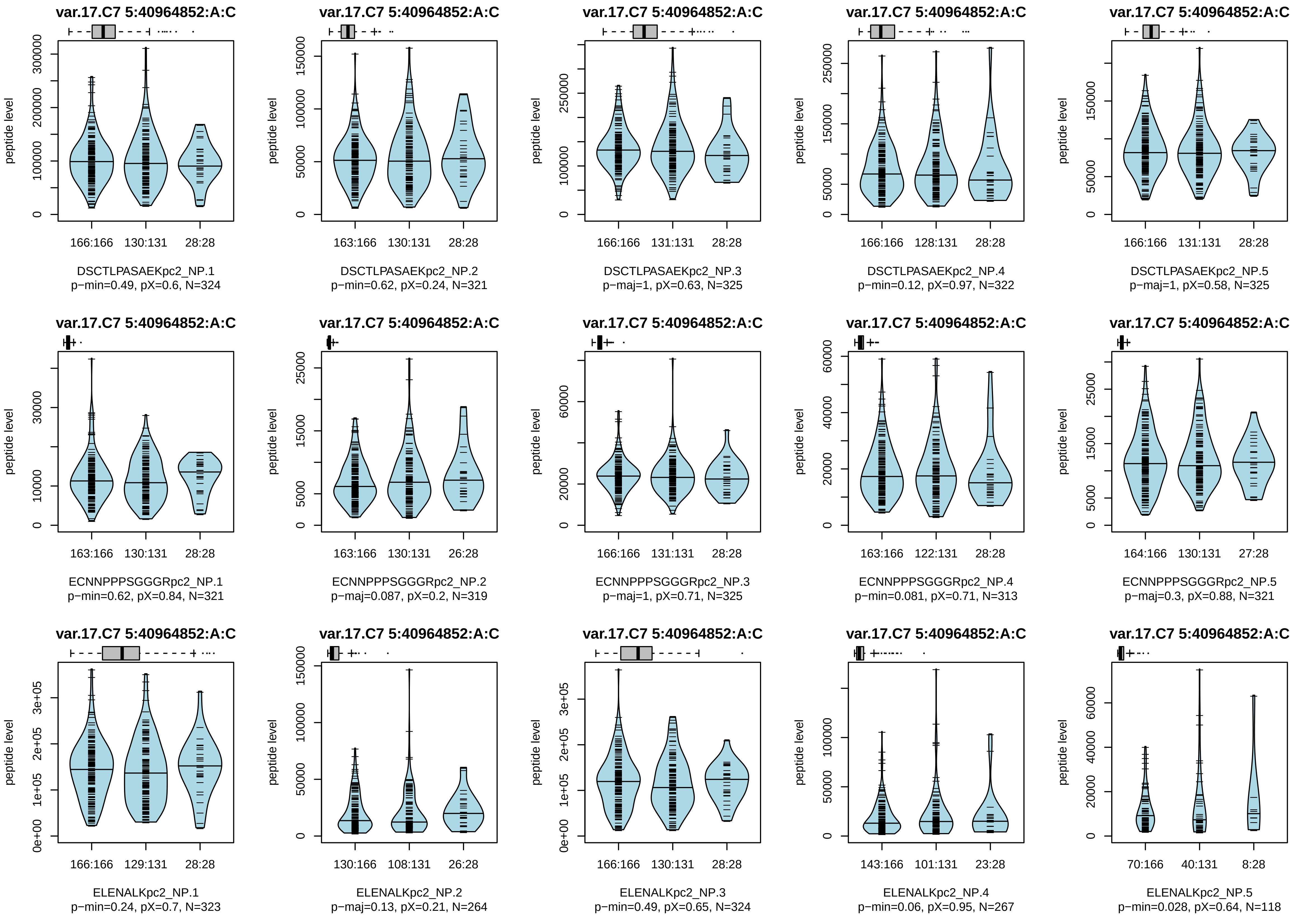
var.17.C7 5:40964852:A:C
peptide level
166:166 130:131 28:28
DSCTLPASAEKpc2_NP.1
p−min=0.49, pX=0.6, N=324
var.17.C7 5:40964852:A:C
163:166 130:131 28:28
DSCTLPASAEKpc2_NP.2
p−min=0.62, pX=0.24, N=321
var.17.C7 5:40964852:A:C
166:166 131:131 28:28
DSCTLPASAEKpc2_NP.3
p−maj=1, pX=0.63, N=325
var.17.C7 5:40964852:A:C
166:166 128:131 28:28
DSCTLPASAEKpc2_NP.4
p−min=0.12, pX=0.97, N=322
var.17.C7 5:40964852:A:C
166:166 131:131 28:28
DSCTLPASAEKpc2_NP.5
p−maj=1, pX=0.58, N=325
var.17.C7 5:40964852:A:C
163:166 130:131 28:28
ECNNPPPSGGGRpc2_NP.1
p−min=0.62, pX=0.84, N=321
var.17.C7 5:40964852:A:C
163:166 130:131 26:28
ECNNPPPSGGGRpc2_NP.2
p−maj=0.087, pX=0.2, N=319
var.17.C7 5:40964852:A:C
166:166 131:131 28:28
ECNNPPPSGGGRpc2_NP.3
p−maj=1, pX=0.71, N=325
var.17.C7 5:40964852:A:C
163:166 122:131 28:28
ECNNPPPSGGGRpc2_NP.4
p−min=0.081, pX=0.71, N=313
var.17.C7 5:40964852:A:C
164:166 130:131 27:28
ECNNPPPSGGGRpc2_NP.5
p−maj=0.3, pX=0.88, N=321
var.17.C7 5:40964852:A:C
166:166 129:131 28:28
ELENALKpc2_NP.1
p−min=0.24, pX=0.7, N=323
var.17.C7 5:40964852:A:C
130:166 108:131 26:28
ELENALKpc2_NP.2
p−maj=0.13, pX=0.21, N=264
var.17.C7 5:40964852:A:C
166:166 130:131 28:28
ELENALKpc2_NP.3
p−min=0.49, pX=0.65, N=324
var.17.C7 5:40964852:A:C
143:166 101:131 23:28
ELENALKpc2_NP.4
p−min=0.06, pX=0.95, N=267
var.17.C7 5:40964852:A:C
70:166 40:131 8:28
ELENALKpc2_NP.5
p−min=0.028, pX=0.64, N=118

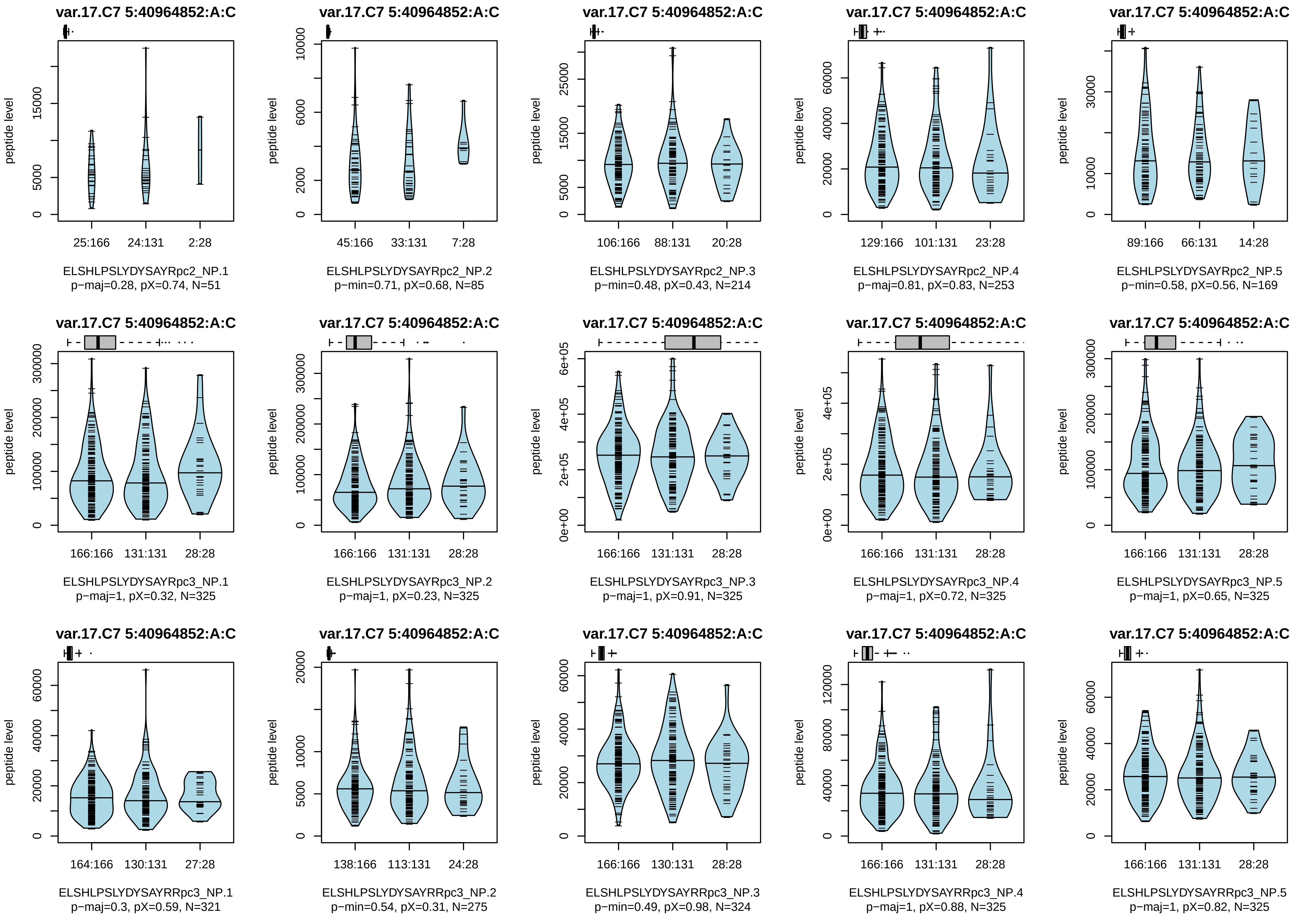
var.17.C7 5:40964852:A:C
peptide level
25:166
24:131
2:28
ELSHLPSLYDYSAYRpc2_NP.1
p−maj=0.28, pX=0.74, N=51
var.17.C7 5:40964852:A:C
45:166
33:131
7:28
ELSHLPSLYDYSAYRpc2_NP.2
p−min=0.71, pX=0.68, N=85
var.17.C7 5:40964852:A:C
106:166
88:131
20:28
ELSHLPSLYDYSAYRpc2_NP.3
p−min=0.48, pX=0.43, N=214
var.17.C7 5:40964852:A:C
129:166
101:131
23:28
ELSHLPSLYDYSAYRpc2_NP.4
p−maj=0.81, pX=0.83, N=253
var.17.C7 5:40964852:A:C
89:166
66:131
14:28
ELSHLPSLYDYSAYRpc2_NP.5
p−min=0.58, pX=0.56, N=169
var.17.C7 5:40964852:A:C
166:166
131:131
28:28
ELSHLPSLYDYSAYRpc3_NP.1
p−maj=1, pX=0.32, N=325
var.17.C7 5:40964852:A:C
166:166
131:131
28:28
ELSHLPSLYDYSAYRpc3_NP.2
p−maj=1, pX=0.23, N=325
var.17.C7 5:40964852:A:C
166:166
131:131
28:28
ELSHLPSLYDYSAYRpc3_NP.3
p−maj=1, pX=0.91, N=325
var.17.C7 5:40964852:A:C
166:166
131:131
28:28
ELSHLPSLYDYSAYRpc3_NP.4
p−maj=1, pX=0.72, N=325
var.17.C7 5:40964852:A:C
166:166
131:131
28:28
ELSHLPSLYDYSAYRpc3_NP.5
p−maj=1, pX=0.65, N=325
var.17.C7 5:40964852:A:C
164:166
130:131
27:28
ELSHLPSLYDYSAYRRpc3_NP.1
p−maj=0.3, pX=0.59, N=321
var.17.C7 5:40964852:A:C
138:166
113:131
24:28
ELSHLPSLYDYSAYRRpc3_NP.2
p−min=0.54, pX=0.31, N=275
var.17.C7 5:40964852:A:C
166:166
130:131
28:28
ELSHLPSLYDYSAYRRpc3_NP.3
p−min=0.49, pX=0.98, N=324
var.17.C7 5:40964852:A:C
166:166
131:131
28:28
ELSHLPSLYDYSAYRRpc3_NP.4
p−maj=1, pX=0.88, N=325
var.17.C7 5:40964852:A:C
166:166
131:131
28:28
ELSHLPSLYDYSAYRRpc3_NP.5
p−maj=1, pX=0.82, N=325

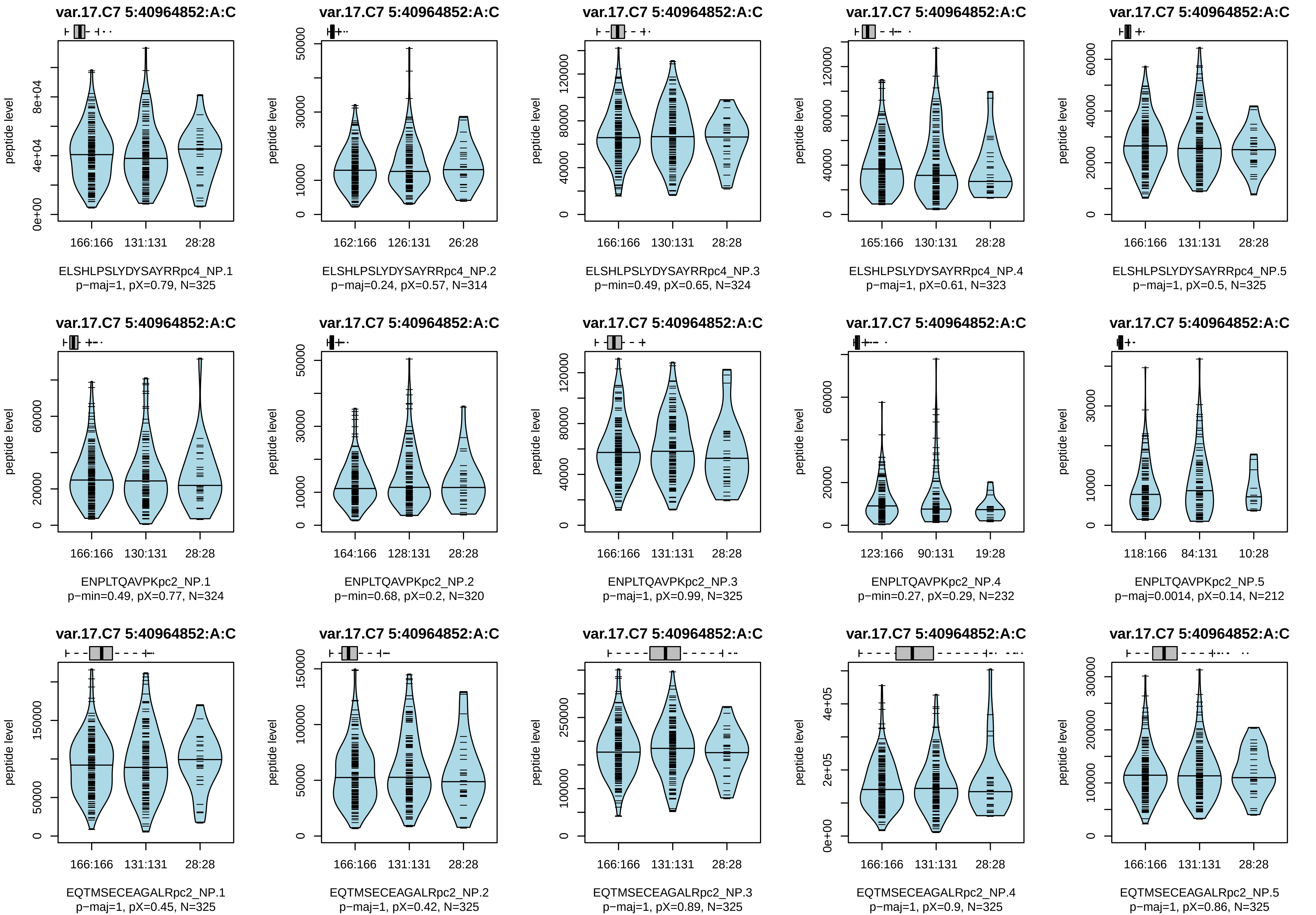
var.17.C7 5:40964852:A:C
peptide level
166:166
131:131
28:28
ELSHLPSLYDYSAYRRpc4_NP.1
p−maj=1, pX=0.79, N=325
var.17.C7 5:40964852:A:C
162:166
126:131
26:28
ELSHLPSLYDYSAYRRpc4_NP.2
p−maj=0.24, pX=0.57, N=314
var.17.C7 5:40964852:A:C
166:166
130:131
28:28
ELSHLPSLYDYSAYRRpc4_NP.3
p−min=0.49, pX=0.65, N=324
var.17.C7 5:40964852:A:C
165:166
130:131
28:28
ELSHLPSLYDYSAYRRpc4_NP.4
p−maj=1, pX=0.61, N=323
var.17.C7 5:40964852:A:C
166:166
131:131
28:28
ELSHLPSLYDYSAYRRpc4_NP.5
p−maj=1, pX=0.5, N=325
var.17.C7 5:40964852:A:C
166:166
130:131
28:28
ENPLTQAVPKpc2_NP.1
p−min=0.49, pX=0.77, N=324
var.17.C7 5:40964852:A:C
164:166
128:131
28:28
ENPLTQAVPKpc2_NP.2
p−min=0.68, pX=0.2, N=320
var.17.C7 5:40964852:A:C
166:166
131:131
28:28
ENPLTQAVPKpc2_NP.3
p−maj=1, pX=0.99, N=325
var.17.C7 5:40964852:A:C
123:166
90:131
19:28
ENPLTQAVPKpc2_NP.4
p−min=0.27, pX=0.29, N=232
var.17.C7 5:40964852:A:C
118:166
84:131
10:28
ENPLTQAVPKpc2_NP.5
p−maj=0.0014, pX=0.14, N=212
var.17.C7 5:40964852:A:C
166:166
131:131
28:28
EQTMSECEAGALRpc2_NP.1
p−maj=1, pX=0.45, N=325
var.17.C7 5:40964852:A:C
166:166
131:131
28:28
EQTMSECEAGALRpc2_NP.2
p−maj=1, pX=0.42, N=325
var.17.C7 5:40964852:A:C
166:166
131:131
28:28
EQTMSECEAGALRpc2_NP.3
p−maj=1, pX=0.89, N=325
var.17.C7 5:40964852:A:C
166:166
131:131
28:28
EQTMSECEAGALRpc2_NP.4
p−maj=1, pX=0.9, N=325
var.17.C7 5:40964852:A:C
166:166
131:131
28:28
EQTMSECEAGALRpc2_NP.5
p−maj=1, pX=0.86, N=325

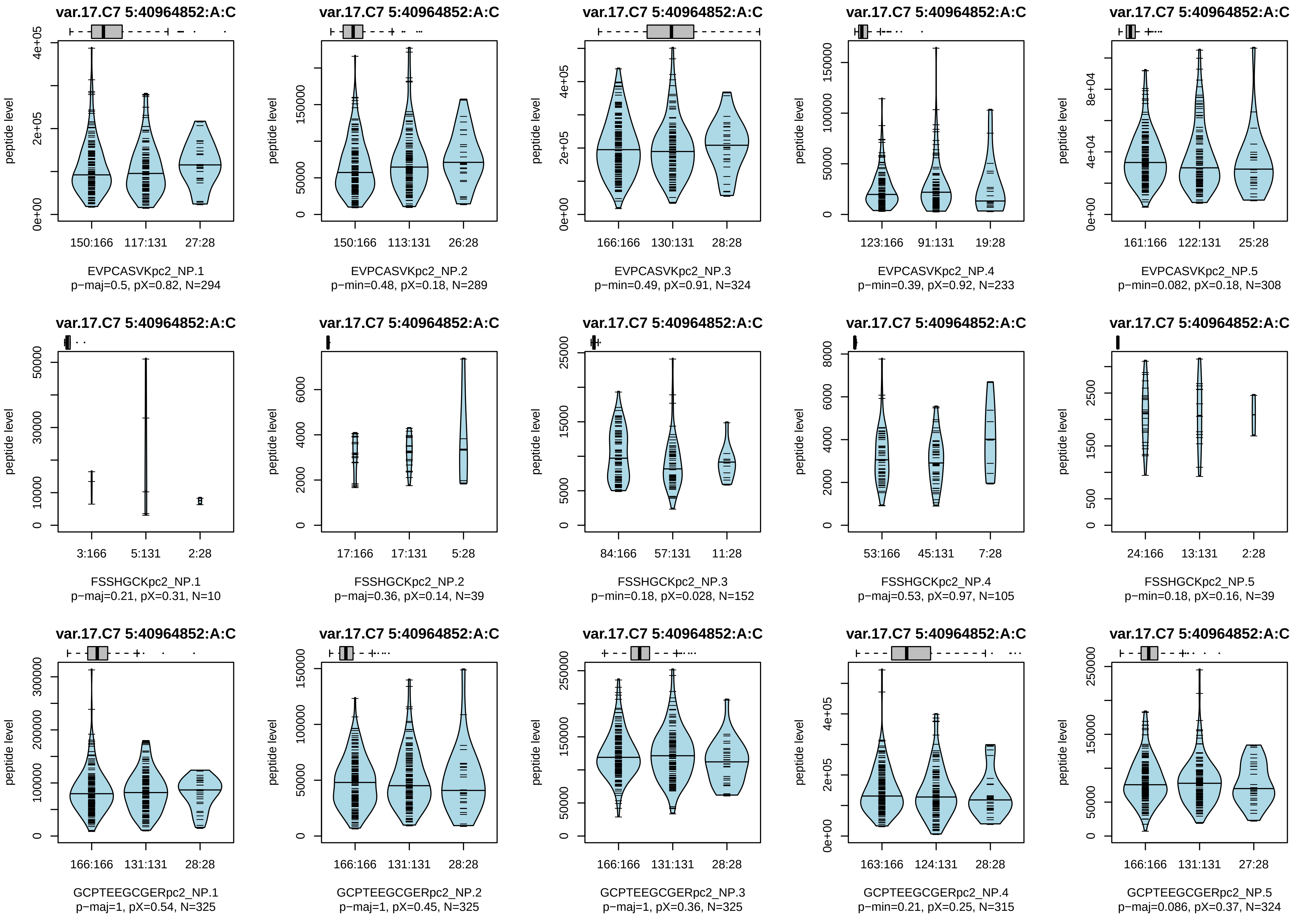

var.17.C7 5:40964852:A:C
peptide level
150:166
117:131
27:28
EVPCASVKpc2_NP.1
p−maj=0.5, pX=0.82, N=294
var.17.C7 5:40964852:A:C
150:166
113:131
26:28
EVPCASVKpc2_NP.2
p−min=0.48, pX=0.18, N=289
var.17.C7 5:40964852:A:C
166:166
130:131
28:28
EVPCASVKpc2_NP.3
p−min=0.49, pX=0.91, N=324
var.17.C7 5:40964852:A:C
123:166
91:131
19:28
EVPCASVKpc2_NP.4
p−min=0.39, pX=0.92, N=233
var.17.C7 5:40964852:A:C
161:166
122:131
25:28
EVPCASVKpc2_NP.5
p−min=0.082, pX=0.18, N=308
var.17.C7 5:40964852:A:C
3:166
5:131
2:28
FSSHGCKpc2_NP.1
p−maj=0.21, pX=0.31, N=10
var.17.C7 5:40964852:A:C
17:166
17:131
5:28
FSSHGCKpc2_NP.2
p−maj=0.36, pX=0.14, N=39
var.17.C7 5:40964852:A:C
84:166
57:131
11:28
FSSHGCKpc2_NP.3
p−min=0.18, pX=0.028, N=152
var.17.C7 5:40964852:A:C
53:166
45:131
7:28
FSSHGCKpc2_NP.4
p−maj=0.53, pX=0.97, N=105
var.17.C7 5:40964852:A:C
24:166
13:131
2:28
FSSHGCKpc2_NP.5
p−min=0.18, pX=0.16, N=39
var.17.C7 5:40964852:A:C
166:166
131:131
28:28
GCPTEEGCGERpc2_NP.1
p−maj=1, pX=0.54, N=325
var.17.C7 5:40964852:A:C
166:166
131:131
28:28
GCPTEEGCGERpc2_NP.2
p−maj=1, pX=0.45, N=325
var.17.C7 5:40964852:A:C
166:166
131:131
28:28
GCPTEEGCGERpc2_NP.3
p−maj=1, pX=0.36, N=325
var.17.C7 5:40964852:A:C
163:166
124:131
28:28
GCPTEEGCGERpc2_NP.4
p−min=0.21, pX=0.25, N=315
var.17.C7 5:40964852:A:C
166:166
131:131
27:28
GCPTEEGCGERpc2_NP.5
p−maj=0.086, pX=0.37, N=324

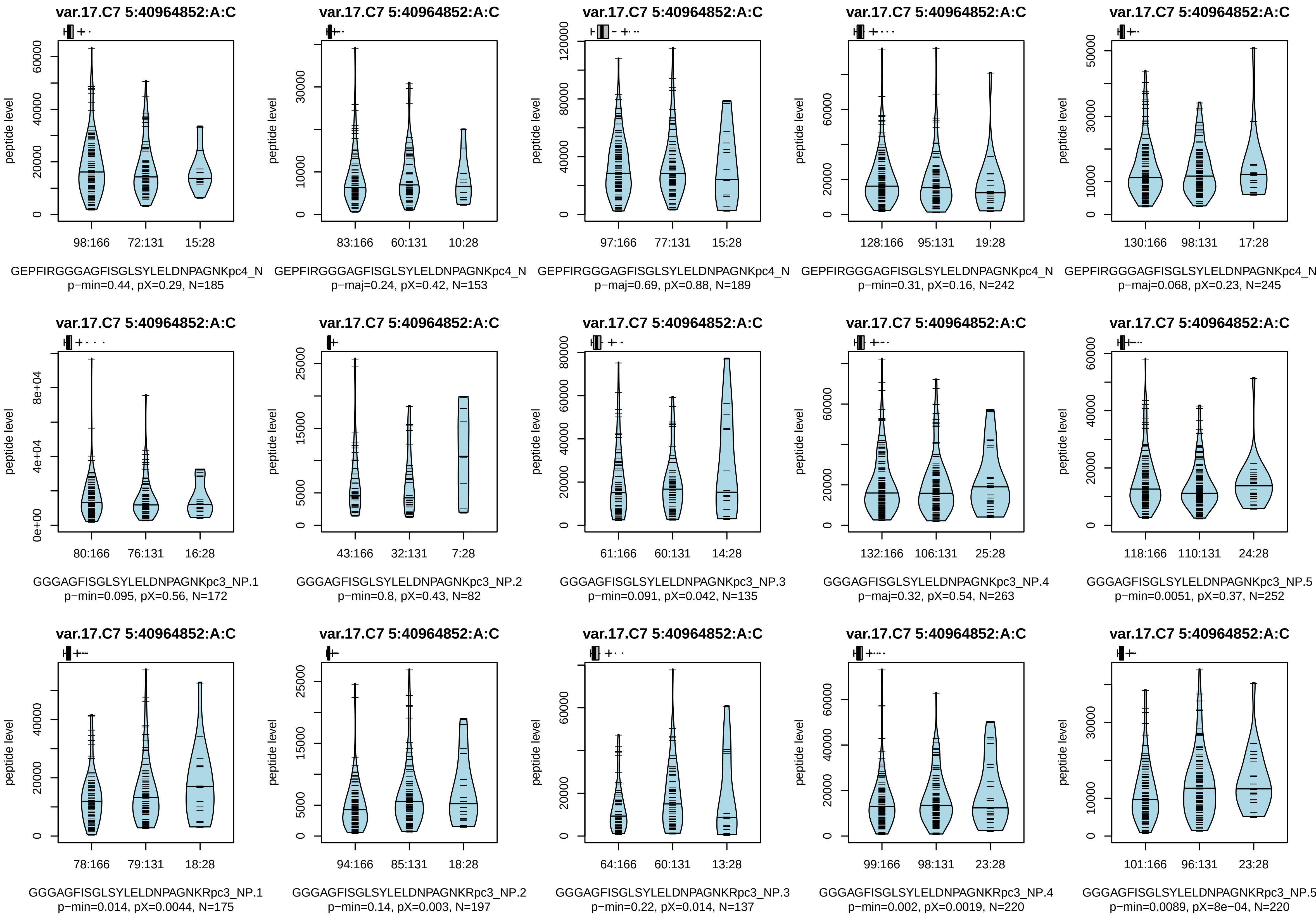
var.17.C7 5:40964852:A:C
peptide level
98:166
72:131
15:28
GEPFIRGGGAGFISGLSYLELDNPAGNKpc4_N
p−min=0.44, pX=0.29, N=185
var.17.C7 5:40964852:A:C
peptide level
83:166
60:131
10:28
GEPFIRGGGAGFISGLSYLELDNPAGNKpc4_N
p−maj=0.24, pX=0.42, N=153
var.17.C7 5:40964852:A:C
peptide level
97:166
77:131
15:28
GEPFIRGGGAGFISGLSYLELDNPAGNKpc4_N
p−maj=0.69, pX=0.88, N=189
var.17.C7 5:40964852:A:C
peptide level
128:166
95:131
19:28
GEPFIRGGGAGFISGLSYLELDNPAGNKpc4_N
p−min=0.31, pX=0.16, N=242
var.17.C7 5:40964852:A:C
peptide level
130:166
98:131
17:28
GEPFIRGGGAGFISGLSYLELDNPAGNKpc4_N
p−maj=0.068, pX=0.23, N=245
var.17.C7 5:40964852:A:C
peptide level
80:166
76:131
16:28
GGGAGFISGLSYLELDNPAGNKpc3_NP.1
p−min=0.095, pX=0.56, N=172
var.17.C7 5:40964852:A:C
peptide level
43:166
32:131
7:28
GGGAGFISGLSYLELDNPAGNKpc3_NP.2
p−min=0.8, pX=0.43, N=82
var.17.C7 5:40964852:A:C
peptide level
61:166
60:131
14:28
GGGAGFISGLSYLELDNPAGNKpc3_NP.3
p−min=0.091, pX=0.042, N=135
var.17.C7 5:40964852:A:C
peptide level
132:166
106:131
25:28
GGGAGFISGLSYLELDNPAGNKpc3_NP.4
p−maj=0.32, pX=0.54, N=263
var.17.C7 5:40964852:A:C
peptide level
118:166
110:131
24:28
GGGAGFISGLSYLELDNPAGNKpc3_NP.5
p−min=0.0051, pX=0.37, N=252
var.17.C7 5:40964852:A:C
peptide level
78:166
79:131
18:28
GGGAGFISGLSYLELDNPAGNKRpc3_NP.1
p−min=0.014, pX=0.0044, N=175
var.17.C7 5:40964852:A:C
peptide level
94:166
85:131
18:28
GGGAGFISGLSYLELDNPAGNKRpc3_NP.2
p−min=0.14, pX=0.003, N=197
var.17.C7 5:40964852:A:C
peptide level
64:166
60:131
13:28
GGGAGFISGLSYLELDNPAGNKRpc3_NP.3
p−min=0.22, pX=0.014, N=137
var.17.C7 5:40964852:A:C
peptide level
99:166
98:131
23:28
GGGAGFISGLSYLELDNPAGNKRpc3_NP.4
p−min=0.002, pX=0.0019, N=220
var.17.C7 5:40964852:A:C
peptide level
101:166
96:131
23:28
GGGAGFISGLSYLELDNPAGNKRpc3_NP.5
p−min=0.0089, pX=8e−04, N=220

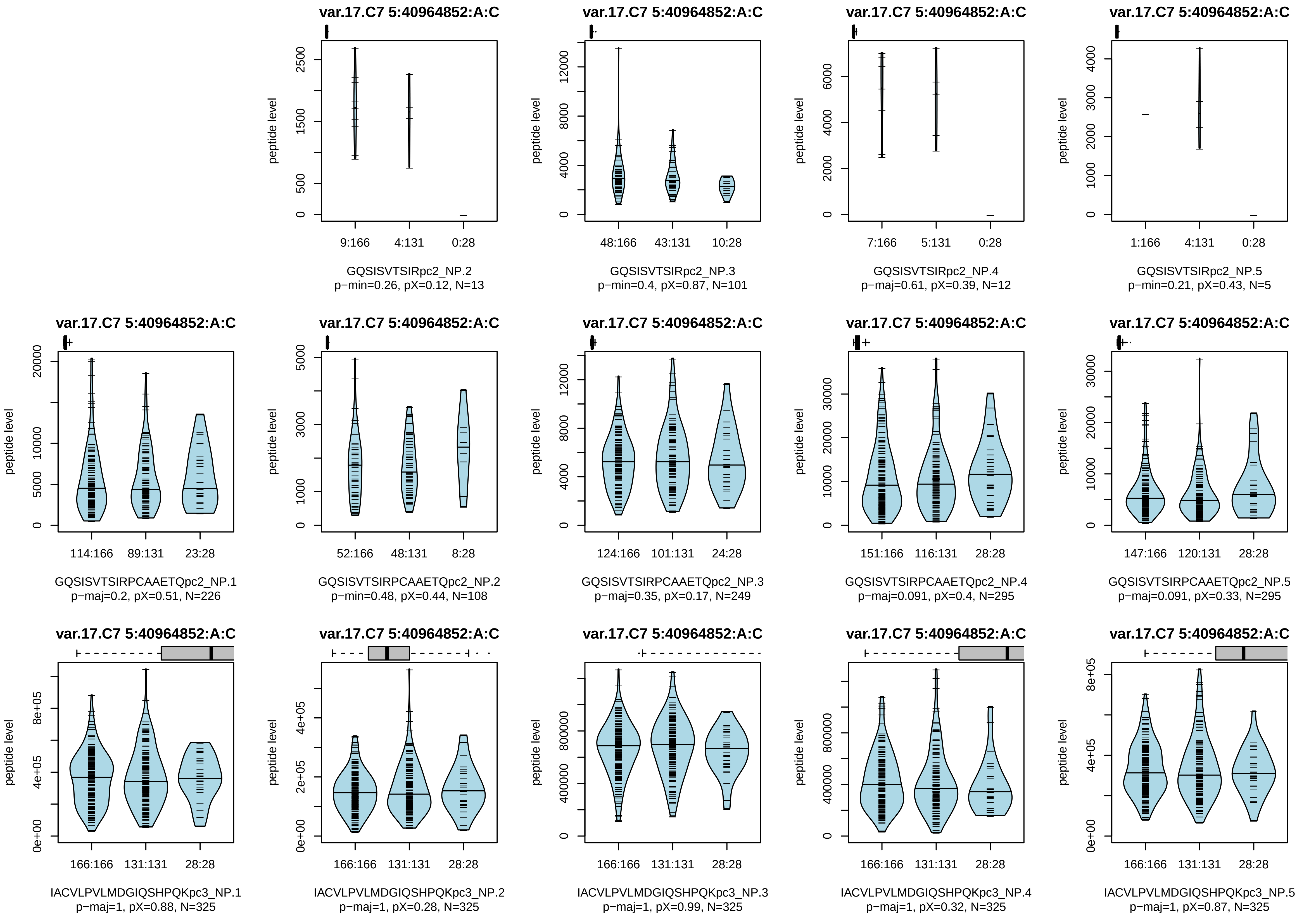
var.17.C7 5:40964852:A:C
peptide level
9:166 4:131 0:28
GQSISVTSIRpc2_NP.2
p−min=0.26, pX=0.12, N=13
var.17.C7 5:40964852:A:C
peptide level
48:166 43:131 10:28
GQSISVTSIRpc2_NP.3
p−min=0.4, pX=0.87, N=101
var.17.C7 5:40964852:A:C
peptide level
7:166 5:131 0:28
GQSISVTSIRpc2_NP.4
p−maj=0.61, pX=0.39, N=12
var.17.C7 5:40964852:A:C
peptide level
1:166 4:131 0:28
GQSISVTSIRpc2_NP.5
p−min=0.21, pX=0.43, N=5
var.17.C7 5:40964852:A:C
peptide level
114:166 89:131 23:28
GQSISVTSIRPCAAETQpc2_NP.1
p−maj=0.2, pX=0.51, N=226
var.17.C7 5:40964852:A:C
peptide level
52:166 48:131 8:28
GQSISVTSIRPCAAETQpc2_NP.2
p−min=0.48, pX=0.44, N=108
var.17.C7 5:40964852:A:C
peptide level
124:166 101:131 24:28
GQSISVTSIRPCAAETQpc2_NP.3
p−maj=0.35, pX=0.17, N=249
var.17.C7 5:40964852:A:C
peptide level
151:166 116:131 28:28
GQSISVTSIRPCAAETQpc2_NP.4
p−maj=0.091, pX=0.4, N=295
var.17.C7 5:40964852:A:C
peptide level
147:166 120:131 28:28
GQSISVTSIRPCAAETQpc2_NP.5
p−maj=0.091, pX=0.33, N=295
var.17.C7 5:40964852:A:C
peptide level
166:166 131:131 28:28
IACVLPVLMDGIQSHPQKpc3_NP.1
p−maj=1, pX=0.88, N=325
var.17.C7 5:40964852:A:C
peptide level
166:166 131:131 28:28
IACVLPVLMDGIQSHPQKpc3_NP.2
p−maj=1, pX=0.28, N=325
var.17.C7 5:40964852:A:C
peptide level
166:166 131:131 28:28
IACVLPVLMDGIQSHPQKpc3_NP.3
p−maj=1, pX=0.99, N=325
var.17.C7 5:40964852:A:C
peptide level
166:166 131:131 28:28
IACVLPVLMDGIQSHPQKpc3_NP.4
p−maj=1, pX=0.32, N=325
var.17.C7 5:40964852:A:C
peptide level
166:166 131:131 28:28
IACVLPVLMDGIQSHPQKpc3_NP.5
p−maj=1, pX=0.87, N=325

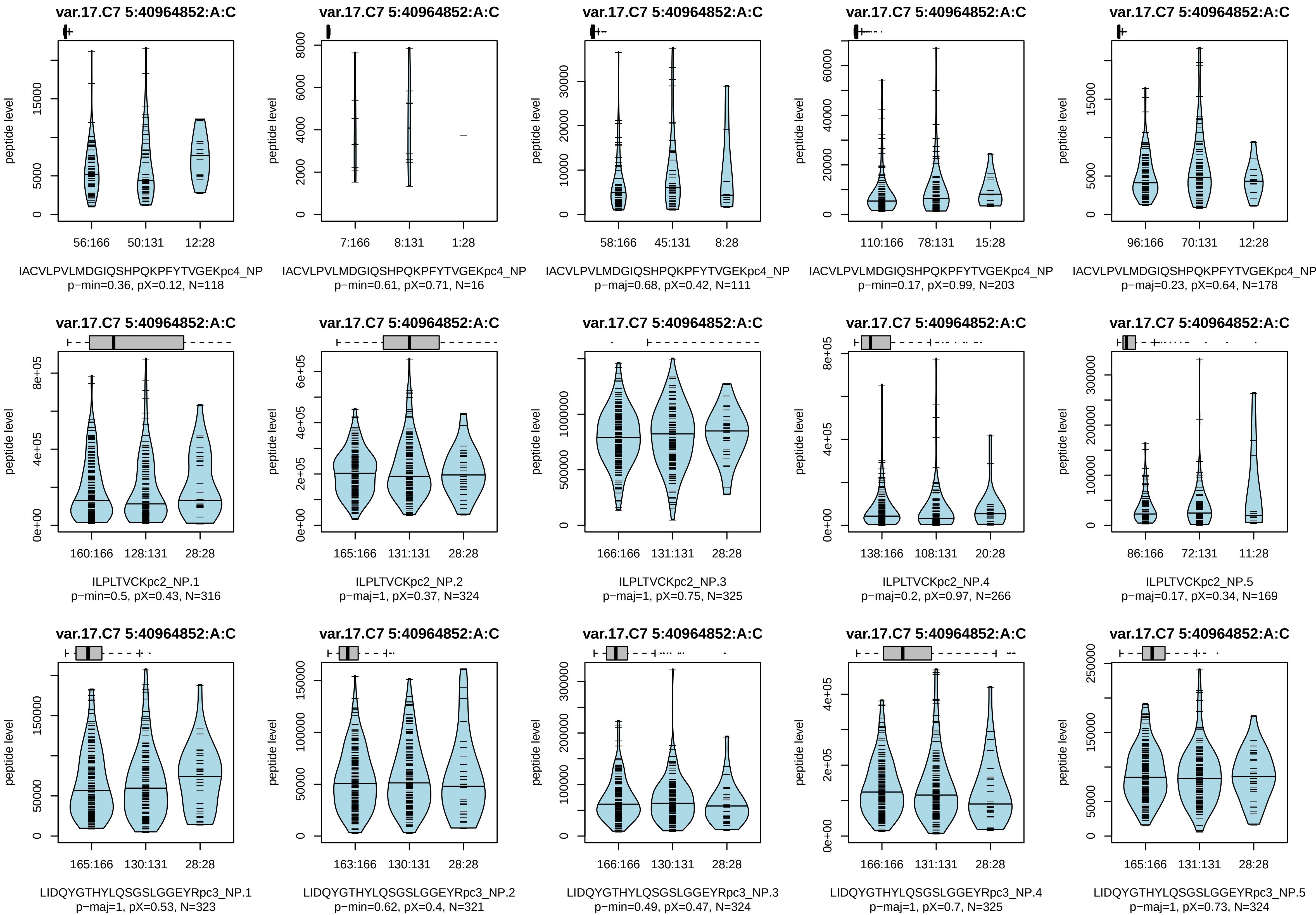

var.17.C7 5:40964852:A:C
peptide level
56:166
50:131
12:28
IACVLPVLMDGIQSHPQKPFYTVGEKpc4_NP
p−min=0.36, pX=0.12, N=118
var.17.C7 5:40964852:A:C
peptide level
7:166
8:131
1:28
IACVLPVLMDGIQSHPQKPFYTVGEKpc4_NP
p−min=0.61, pX=0.71, N=16
var.17.C7 5:40964852:A:C
peptide level
58:166
45:131
8:28
IACVLPVLMDGIQSHPQKPFYTVGEKpc4_NP
p−maj=0.68, pX=0.42, N=111
var.17.C7 5:40964852:A:C
peptide level
110:166
78:131
15:28
IACVLPVLMDGIQSHPQKPFYTVGEKpc4_NP
p−min=0.17, pX=0.99, N=203
var.17.C7 5:40964852:A:C
peptide level
96:166
70:131
12:28
IACVLPVLMDGIQSHPQKPFYTVGEKpc4_NP
p−maj=0.23, pX=0.64, N=178
var.17.C7 5:40964852:A:C
peptide level
160:166
128:131
28:28
ILPLTVCKpc2_NP.1
p−min=0.5, pX=0.43, N=316
var.17.C7 5:40964852:A:C
peptide level
165:166
131:131
28:28
ILPLTVCKpc2_NP.2
p−maj=1, pX=0.37, N=324
var.17.C7 5:40964852:A:C
peptide level
166:166
131:131
28:28
ILPLTVCKpc2_NP.3
p−maj=1, pX=0.75, N=325
var.17.C7 5:40964852:A:C
peptide level
138:166
108:131
20:28
ILPLTVCKpc2_NP.4
p−maj=0.2, pX=0.97, N=266
var.17.C7 5:40964852:A:C
peptide level
86:166
72:131
11:28
ILPLTVCKpc2_NP.5
p−maj=0.17, pX=0.34, N=169
var.17.C7 5:40964852:A:C
peptide level
165:166
130:131
28:28
LIDQYGTHYLQSGSLGGEYRpc3_NP.1
p−maj=1, pX=0.53, N=323
var.17.C7 5:40964852:A:C
peptide level
163:166
130:131
28:28
LIDQYGTHYLQSGSLGGEYRpc3_NP.2
p−min=0.62, pX=0.4, N=321
var.17.C7 5:40964852:A:C
peptide level
166:166
130:131
28:28
LIDQYGTHYLQSGSLGGEYRpc3_NP.3
p−min=0.49, pX=0.47, N=324
var.17.C7 5:40964852:A:C
peptide level
166:166
131:131
28:28
LIDQYGTHYLQSGSLGGEYRpc3_NP.4
p−maj=1, pX=0.7, N=325
var.17.C7 5:40964852:A:C
peptide level
165:166
131:131
28:28
LIDQYGTHYLQSGSLGGEYRpc3_NP.5
p−maj=1, pX=0.73, N=324

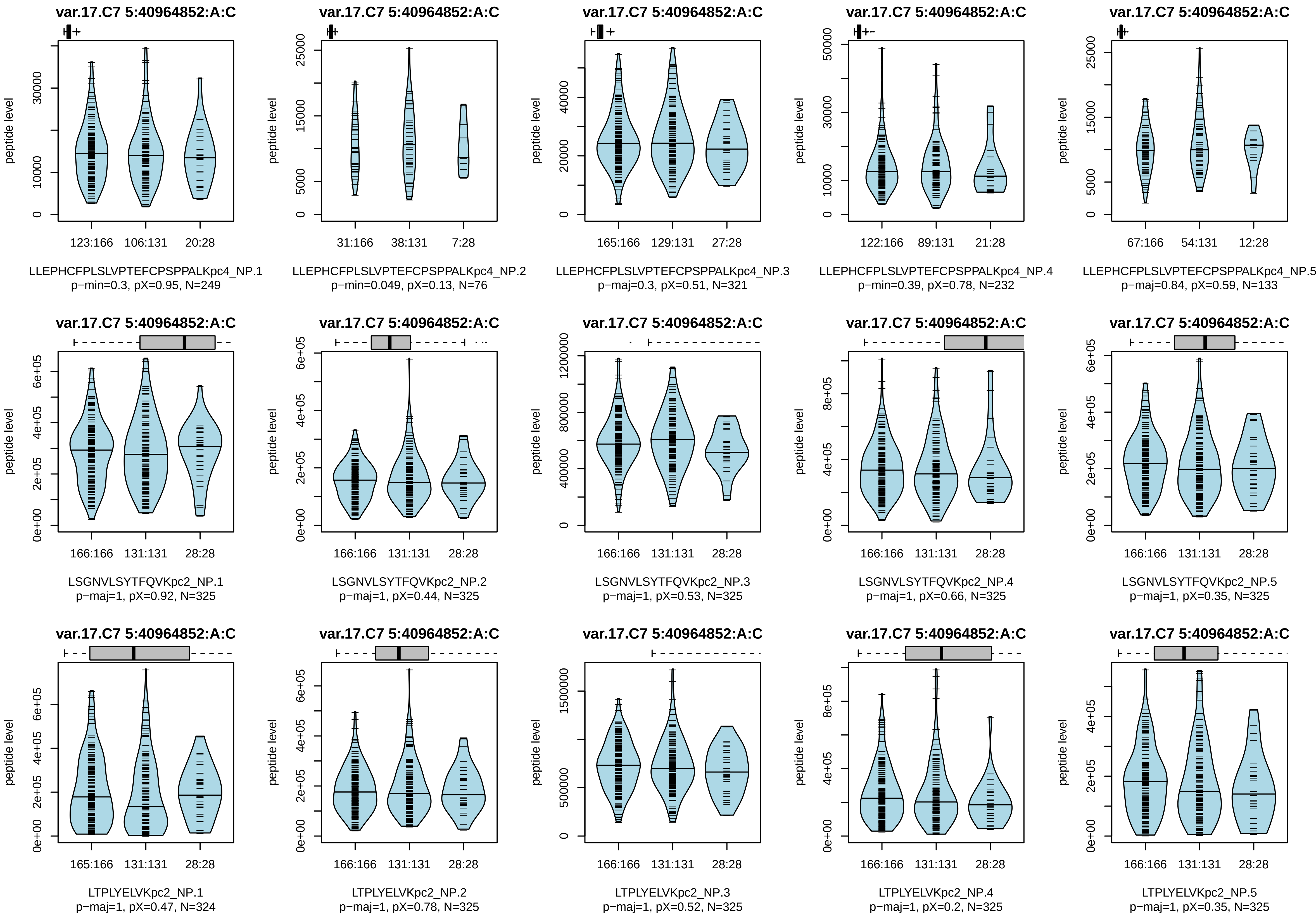
var.17.C7 5:40964852:A:C
peptide level
123:166
106:131
20:28
LLEPHCFPLSLVPTEFCPSPPALKpc4_NP.1
p−min=0.3, pX=0.95, N=249
var.17.C7 5:40964852:A:C
peptide level
31:166
38:131
7:28
LLEPHCFPLSLVPTEFCPSPPALKpc4_NP.2
p−min=0.049, pX=0.13, N=76
var.17.C7 5:40964852:A:C
peptide level
165:166
129:131
27:28
LLEPHCFPLSLVPTEFCPSPPALKpc4_NP.3
p−maj=0.3, pX=0.51, N=321
var.17.C7 5:40964852:A:C
peptide level
122:166
89:131
21:28
LLEPHCFPLSLVPTEFCPSPPALKpc4_NP.4
p−min=0.39, pX=0.78, N=232
var.17.C7 5:40964852:A:C
peptide level
67:166
54:131
12:28
LLEPHCFPLSLVPTEFCPSPPALKpc4_NP.5
p−maj=0.84, pX=0.59, N=133
var.17.C7 5:40964852:A:C
peptide level
166:166
131:131
28:28
LSGNVLSYTFQVKpc2_NP.1
p−maj=1, pX=0.92, N=325
var.17.C7 5:40964852:A:C
peptide level
166:166
131:131
28:28
LSGNVLSYTFQVKpc2_NP.2
p−maj=1, pX=0.44, N=325
var.17.C7 5:40964852:A:C
peptide level
166:166
131:131
28:28
LSGNVLSYTFQVKpc2_NP.3
p−maj=1, pX=0.53, N=325
var.17.C7 5:40964852:A:C
peptide level
166:166
131:131
28:28
LSGNVLSYTFQVKpc2_NP.4
p−maj=1, pX=0.66, N=325
var.17.C7 5:40964852:A:C
peptide level
166:166
131:131
28:28
LSGNVLSYTFQVKpc2_NP.5
p−maj=1, pX=0.35, N=325
var.17.C7 5:40964852:A:C
peptide level
165:166
131:131
28:28
LTPLYELVKpc2_NP.1
p−maj=1, pX=0.47, N=324
var.17.C7 5:40964852:A:C
peptide level
166:166
131:131
28:28
LTPLYELVKpc2_NP.2
p−maj=1, pX=0.78, N=325
var.17.C7 5:40964852:A:C
peptide level
166:166
131:131
28:28
LTPLYELVKpc2_NP.3
p−maj=1, pX=0.52, N=325
var.17.C7 5:40964852:A:C
peptide level
166:166
131:131
28:28
LTPLYELVKpc2_NP.4
p−maj=1, pX=0.2, N=325
var.17.C7 5:40964852:A:C
peptide level
166:166
131:131
28:28
LTPLYELVKpc2_NP.5
p−maj=1, pX=0.35, N=325

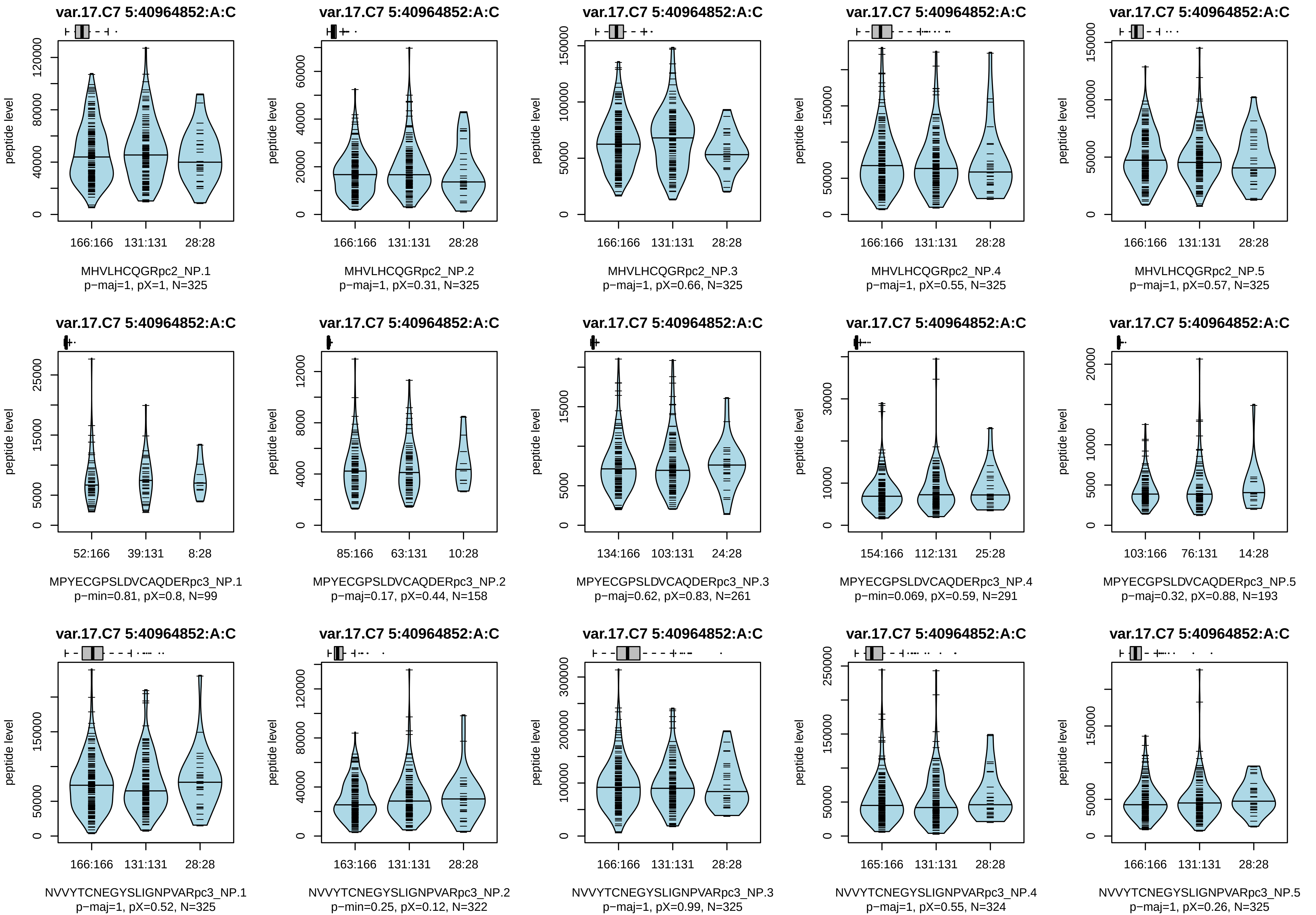
var.17.C7 5:40964852:A:C
peptide level
166:166
131:131
28:28
MHVLHCQGRpc2_NP.1
p−maj=1, pX=1, N=325
MHVLHCQGRpc2_NP.2
p−maj=1, pX=0.31, N=325
MHVLHCQGRpc2_NP.3
p−maj=1, pX=0.66, N=325
MHVLHCQGRpc2_NP.4
p−maj=1, pX=0.55, N=325
MHVLHCQGRpc2_NP.5
p−maj=1, pX=0.57, N=325
52:166
39:131
8:28
MPYECGPSLDVCAQDERpc3_NP.1
p−min=0.81, pX=0.8, N=99
85:166
63:131
10:28
MPYECGPSLDVCAQDERpc3_NP.2
p−maj=0.17, pX=0.44, N=158
134:166
103:131
24:28
MPYECGPSLDVCAQDERpc3_NP.3
p−maj=0.62, pX=0.83, N=261
154:166
112:131
25:28
MPYECGPSLDVCAQDERpc3_NP.4
p−min=0.069, pX=0.59, N=291
103:166
76:131
14:28
MPYECGPSLDVCAQDERpc3_NP.5
p−maj=0.32, pX=0.88, N=193
NVVYTCNEGYSLIGNPVARpc3_NP.1
p−maj=1, pX=0.52, N=325
163:166
NVVYTCNEGYSLIGNPVARpc3_NP.2
p−min=0.25, pX=0.12, N=322
NVVYTCNEGYSLIGNPVARpc3_NP.3
p−maj=1, pX=0.99, N=325
165:166
NVVYTCNEGYSLIGNPVARpc3_NP.4
p−maj=1, pX=0.55, N=324
NVVYTCNEGYSLIGNPVARpc3_NP.5
p−maj=1, pX=0.26, N=325

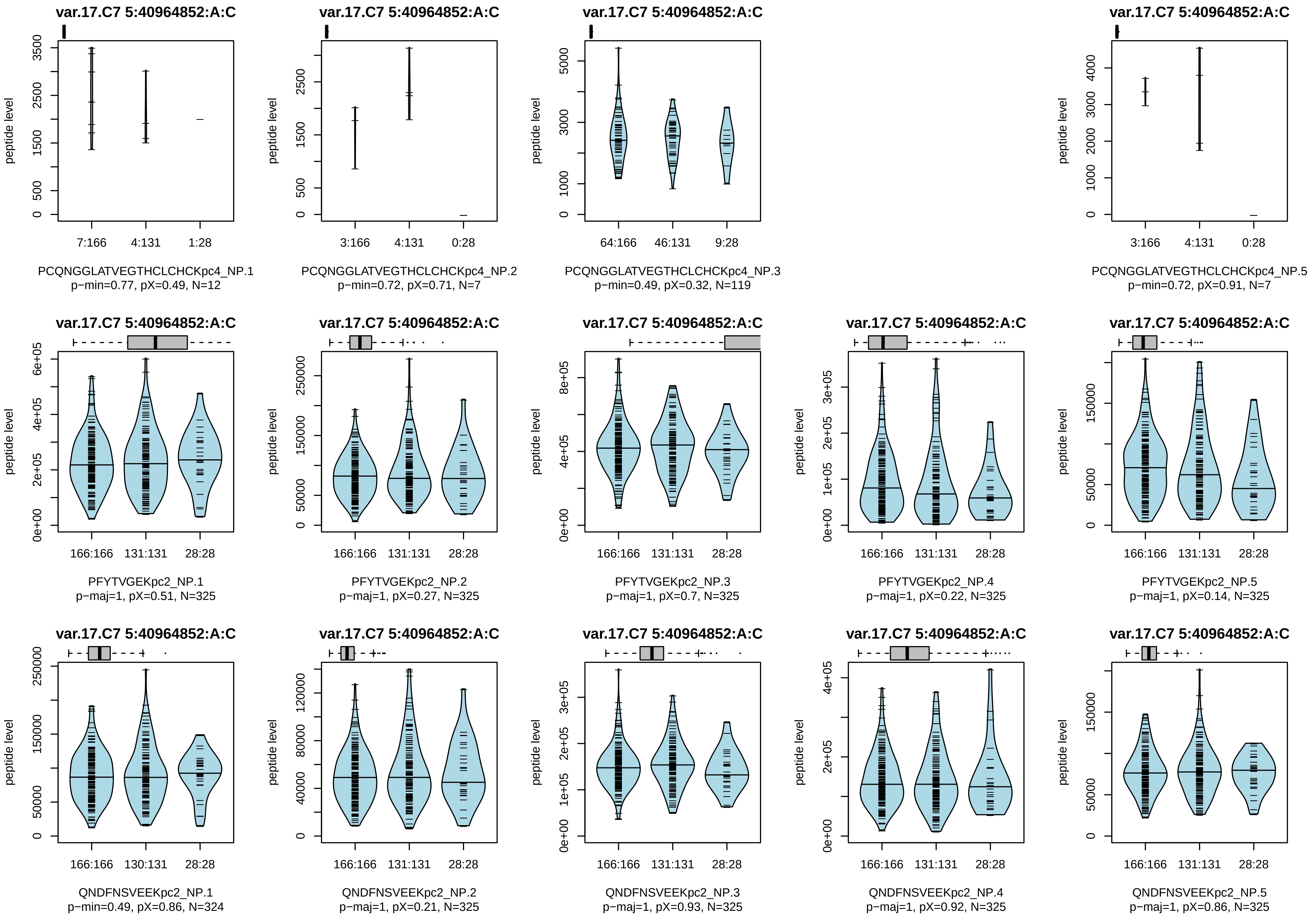

var.17.C7 5:40964852:A:C
peptide level
7:166
4:131
1:28
PCQNGGLATVEGTHCLCHCKpc4_NP.1
p−min=0.77, pX=0.49, N=12
var.17.C7 5:40964852:A:C
3:166
4:131
0:28
PCQNGGLATVEGTHCLCHCKpc4_NP.2
p−min=0.72, pX=0.71, N=7
var.17.C7 5:40964852:A:C
64:166
46:131
9:28
PCQNGGLATVEGTHCLCHCKpc4_NP.3
p−min=0.49, pX=0.32, N=119
var.17.C7 5:40964852:A:C
3:166
4:131
0:28
PCQNGGLATVEGTHCLCHCKpc4_NP.5
p−min=0.72, pX=0.91, N=7
var.17.C7 5:40964852:A:C
166:166
131:131
28:28
PFYTVGEKpc2_NP.1
p−maj=1, pX=0.51, N=325
PFYTVGEKpc2_NP.2
p−maj=1, pX=0.27, N=325
PFYTVGEKpc2_NP.3
p−maj=1, pX=0.7, N=325
PFYTVGEKpc2_NP.4
p−maj=1, pX=0.22, N=325
PFYTVGEKpc2_NP.5
p−maj=1, pX=0.14, N=325
130:131
QNDFNSVEEKpc2_NP.1
p−min=0.49, pX=0.86, N=324
QNDFNSVEEKpc2_NP.2
p−maj=1, pX=0.21, N=325
QNDFNSVEEKpc2_NP.3
p−maj=1, pX=0.93, N=325
QNDFNSVEEKpc2_NP.4
p−maj=1, pX=0.92, N=325
QNDFNSVEEKpc2_NP.5
p−maj=1, pX=0.86, N=325

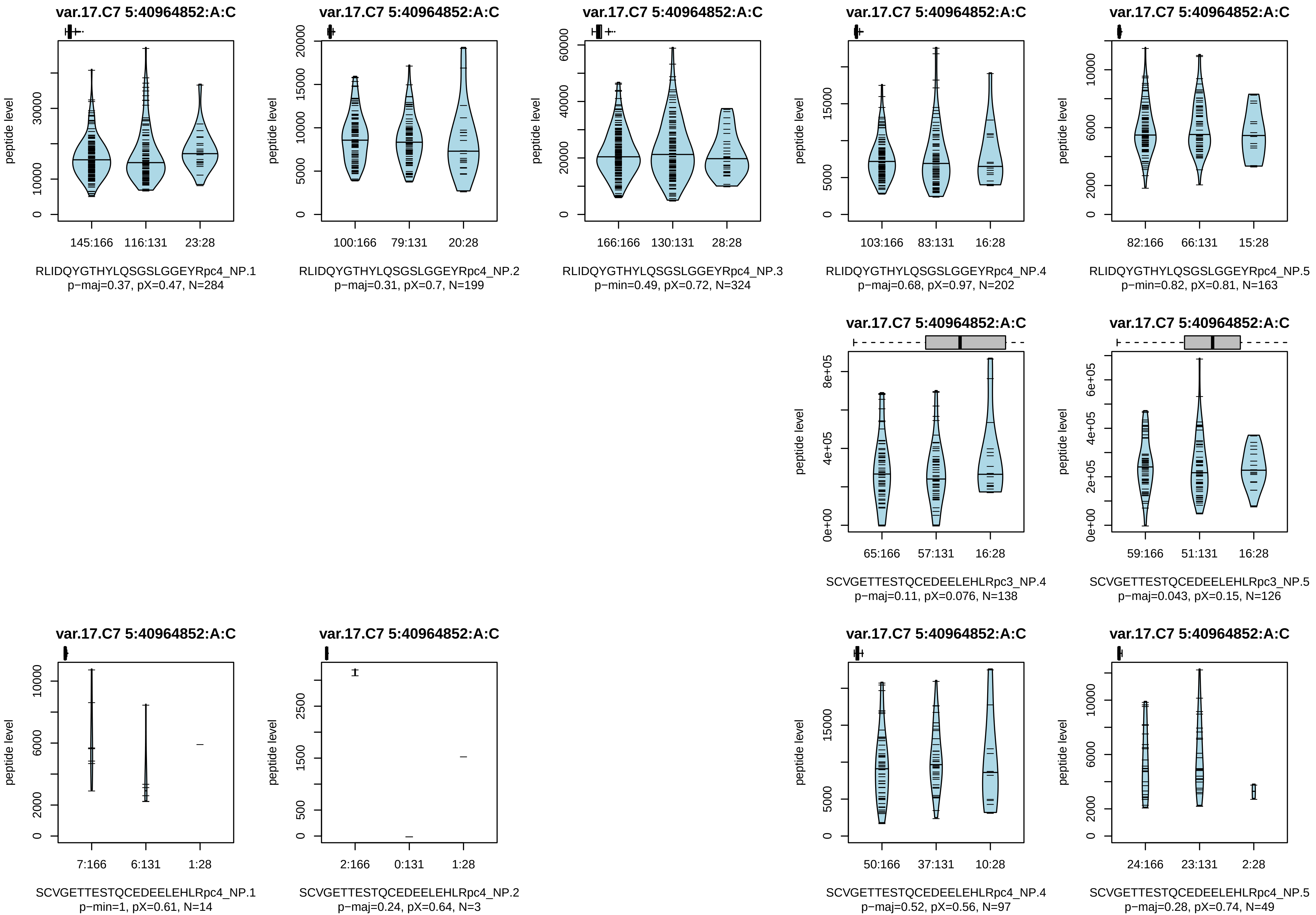
var.17.C7 5:40964852:A:C
peptide level
145:166
116:131
23:28
RLIDQYGTHYLQSGSLGGEYRpc4_NP.1
p−maj=0.37, pX=0.47, N=284
var.17.C7 5:40964852:A:C
peptide level
100:166
79:131
20:28
RLIDQYGTHYLQSGSLGGEYRpc4_NP.2
p−maj=0.31, pX=0.7, N=199
var.17.C7 5:40964852:A:C
peptide level
166:166
130:131
28:28
RLIDQYGTHYLQSGSLGGEYRpc4_NP.3
p−min=0.49, pX=0.72, N=324
var.17.C7 5:40964852:A:C
peptide level
103:166
83:131
16:28
RLIDQYGTHYLQSGSLGGEYRpc4_NP.4
p−maj=0.68, pX=0.97, N=202
var.17.C7 5:40964852:A:C
peptide level
82:166
66:131
15:28
RLIDQYGTHYLQSGSLGGEYRpc4_NP.5
p−min=0.82, pX=0.81, N=163
var.17.C7 5:40964852:A:C
peptide level
65:166
57:131
16:28
SCVGETTESTQCEDEELEHLRpc3_NP.4
p−maj=0.11, pX=0.076, N=138
var.17.C7 5:40964852:A:C
peptide level
59:166
51:131
16:28
SCVGETTESTQCEDEELEHLRpc3_NP.5
p−maj=0.043, pX=0.15, N=126
var.17.C7 5:40964852:A:C
peptide level
7:166
6:131
1:28
SCVGETTESTQCEDEELEHLRpc4_NP.1
p−min=1, pX=0.61, N=14
var.17.C7 5:40964852:A:C
peptide level
2:166
0:131
1:28
SCVGETTESTQCEDEELEHLRpc4_NP.2
p−maj=0.24, pX=0.64, N=3
var.17.C7 5:40964852:A:C
peptide level
50:166
37:131
10:28
SCVGETTESTQCEDEELEHLRpc4_NP.4
p−maj=0.52, pX=0.56, N=97
var.17.C7 5:40964852:A:C
peptide level
24:166
23:131
2:28
SCVGETTESTQCEDEELEHLRpc4_NP.5
p−maj=0.28, pX=0.74, N=49

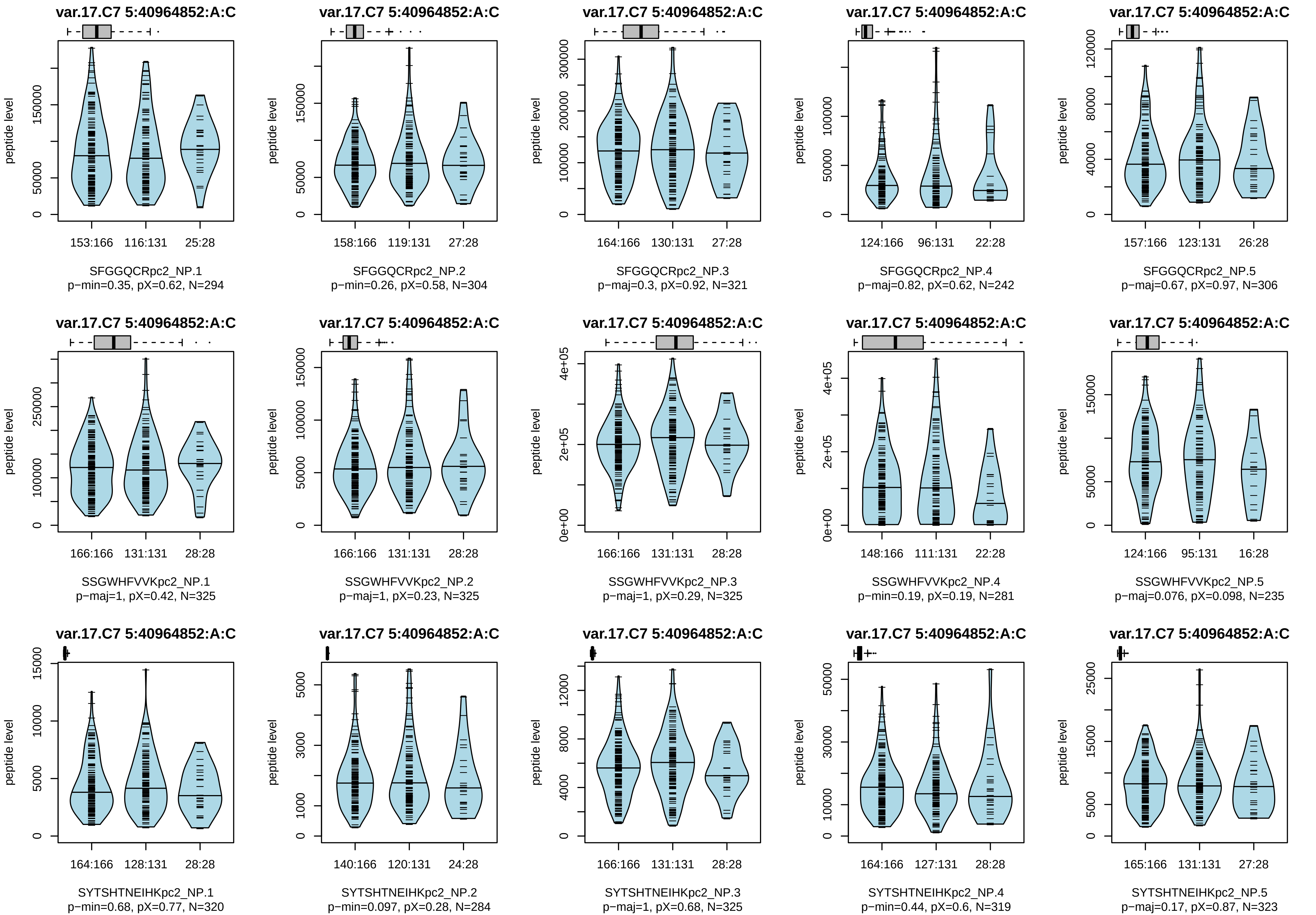
var.17.C7 5:40964852:A:C
peptide level
153:166 116:131 25:28
SFGGQCRpc2_NP.1
p−min=0.35, pX=0.62, N=294
var.17.C7 5:40964852:A:C
158:166 119:131 27:28
SFGGQCRpc2_NP.2
p−min=0.26, pX=0.58, N=304
var.17.C7 5:40964852:A:C
164:166 130:131 27:28
SFGGQCRpc2_NP.3
p−maj=0.3, pX=0.92, N=321
var.17.C7 5:40964852:A:C
124:166 96:131 22:28
SFGGQCRpc2_NP.4
p−maj=0.82, pX=0.62, N=242
var.17.C7 5:40964852:A:C
157:166 123:131 26:28
SFGGQCRpc2_NP.5
p−maj=0.67, pX=0.97, N=306
var.17.C7 5:40964852:A:C
166:166 131:131 28:28
SSGWHFVVKpc2_NP.1
p−maj=1, pX=0.42, N=325
var.17.C7 5:40964852:A:C
166:166 131:131 28:28
SSGWHFVVKpc2_NP.2
p−maj=1, pX=0.23, N=325
var.17.C7 5:40964852:A:C
166:166 131:131 28:28
SSGWHFVVKpc2_NP.3
p−maj=1, pX=0.29, N=325
var.17.C7 5:40964852:A:C
148:166 111:131 22:28
SSGWHFVVKpc2_NP.4
p−min=0.19, pX=0.19, N=281
var.17.C7 5:40964852:A:C
124:166 95:131 16:28
SSGWHFVVKpc2_NP.5
p−maj=0.076, pX=0.098, N=235
var.17.C7 5:40964852:A:C
164:166 128:131 28:28
SYTSHTNEIHKpc2_NP.1
p−min=0.68, pX=0.77, N=320
var.17.C7 5:40964852:A:C
140:166 120:131 24:28
SYTSHTNEIHKpc2_NP.2
p−min=0.097, pX=0.28, N=284
var.17.C7 5:40964852:A:C
166:166 131:131 28:28
SYTSHTNEIHKpc2_NP.3
p−maj=1, pX=0.68, N=325
var.17.C7 5:40964852:A:C
164:166 127:131 28:28
SYTSHTNEIHKpc2_NP.4
p−min=0.44, pX=0.6, N=319
var.17.C7 5:40964852:A:C
165:166 131:131 27:28
SYTSHTNEIHKpc2_NP.5
p−maj=0.17, pX=0.87, N=323

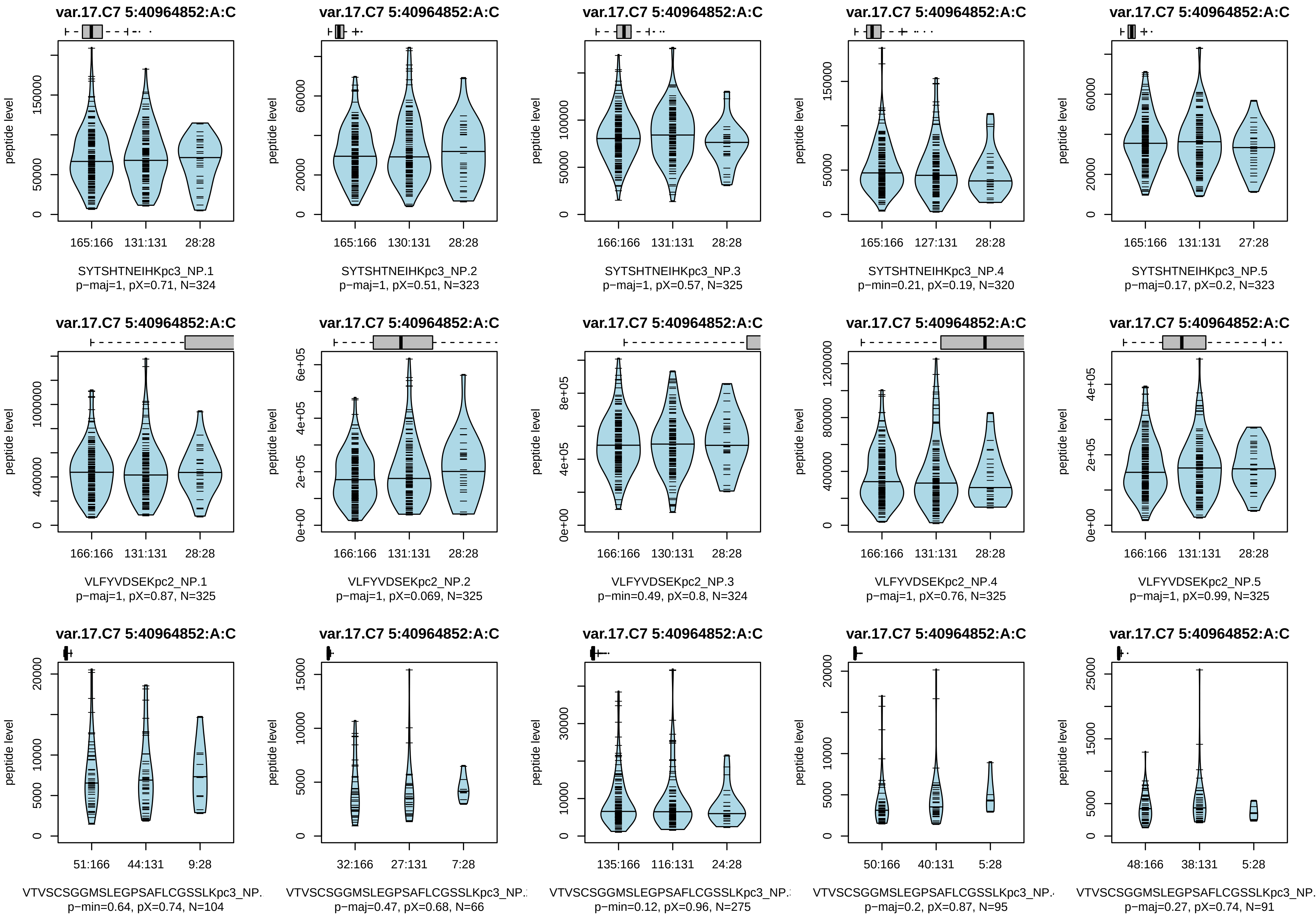
var.17.C7 5:40964852:A:C
peptide level
0
50000
150000
165:166
131:131
28:28
SYTSHTNEIHKpc3_NP.1
p−maj=1, pX=0.71, N=324
var.17.C7 5:40964852:A:C
peptide level
0
20000
60000
165:166
130:131
28:28
SYTSHTNEIHKpc3_NP.2
p−maj=1, pX=0.51, N=323
var.17.C7 5:40964852:A:C
peptide level
0
50000
100000
166:166
131:131
28:28
SYTSHTNEIHKpc3_NP.3
p−maj=1, pX=0.57, N=325
var.17.C7 5:40964852:A:C
peptide level
0
50000
150000
165:166
127:131
28:28
SYTSHTNEIHKpc3_NP.4
p−min=0.21, pX=0.19, N=320
var.17.C7 5:40964852:A:C
peptide level
0
20000
60000
165:166
131:131
27:28
SYTSHTNEIHKpc3_NP.5
p−maj=0.17, pX=0.2, N=323
var.17.C7 5:40964852:A:C
peptide level
0
400000
1000000
166:166
131:131
28:28
VLFYVDSEKpc2_NP.1
p−maj=1, pX=0.87, N=325
var.17.C7 5:40964852:A:C
peptide level
0e+00
2e+05
4e+05
6e+05
166:166
131:131
28:28
VLFYVDSEKpc2_NP.2
p−maj=1, pX=0.069, N=325
var.17.C7 5:40964852:A:C
peptide level
0e+00
4e+05
8e+05
166:166
130:131
28:28
VLFYVDSEKpc2_NP.3
p−min=0.49, pX=0.8, N=324
var.17.C7 5:40964852:A:C
peptide level
0
400000
800000
1200000
166:166
131:131
28:28
VLFYVDSEKpc2_NP.4
p−maj=1, pX=0.76, N=325
var.17.C7 5:40964852:A:C
peptide level
0e+00
2e+05
4e+05
166:166
131:131
28:28
VLFYVDSEKpc2_NP.5
p−maj=1, pX=0.99, N=325
var.17.C7 5:40964852:A:C
peptide level
0
5000
10000
20000
51:166
44:131
9:28
VTVSCSGGMSLEGPSAFLCGSSLKpc3_NP.
p−min=0.64, pX=0.74, N=104
var.17.C7 5:40964852:A:C
peptide level
0
5000
10000
15000
32:166
27:131
7:28
VTVSCSGGMSLEGPSAFLCGSSLKpc3_NP.
p−maj=0.47, pX=0.68, N=66
var.17.C7 5:40964852:A:C
peptide level
0
10000
30000
135:166
116:131
24:28
VTVSCSGGMSLEGPSAFLCGSSLKpc3_NP.
p−min=0.12, pX=0.96, N=275
var.17.C7 5:40964852:A:C
peptide level
0
5000
10000
20000
50:166
40:131
5:28
VTVSCSGGMSLEGPSAFLCGSSLKpc3_NP.
p−maj=0.2, pX=0.87, N=95
var.17.C7 5:40964852:A:C
peptide level
0
5000
15000
25000
48:166
38:131
5:28
VTVSCSGGMSLEGPSAFLCGSSLKpc3_NP.
p−maj=0.27, pX=0.74, N=91

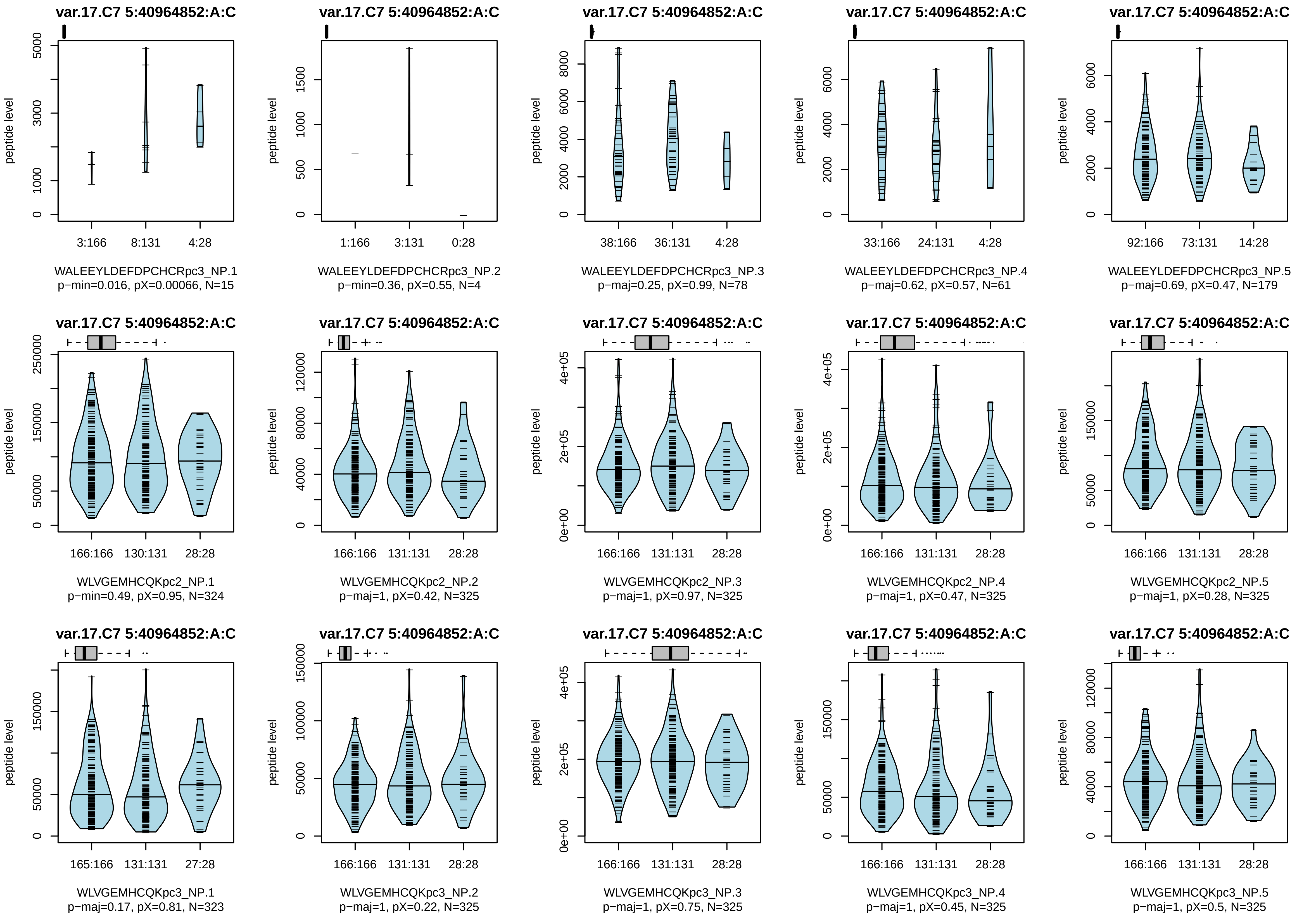
var.17.C7 5:40964852:A:C
peptide level
3:166
8:131
4:28
WALEEYLDEFDPCHCRpc3_NP.1
p−min=0.016, pX=0.00066, N=15
var.17.C7 5:40964852:A:C
1:166
3:131
0:28
WALEEYLDEFDPCHCRpc3_NP.2
p−min=0.36, pX=0.55, N=4
var.17.C7 5:40964852:A:C
38:166
36:131
4:28
WALEEYLDEFDPCHCRpc3_NP.3
p−maj=0.25, pX=0.99, N=78
var.17.C7 5:40964852:A:C
33:166
24:131
4:28
WALEEYLDEFDPCHCRpc3_NP.4
p−maj=0.62, pX=0.57, N=61
var.17.C7 5:40964852:A:C
92:166
73:131
14:28
WALEEYLDEFDPCHCRpc3_NP.5
p−maj=0.69, pX=0.47, N=179
var.17.C7 5:40964852:A:C
166:166
130:131
28:28
WLVGEMHCQKpc2_NP.1
p−min=0.49, pX=0.95, N=324
var.17.C7 5:40964852:A:C
166:166
131:131
28:28
WLVGEMHCQKpc2_NP.2
p−maj=1, pX=0.42, N=325
var.17.C7 5:40964852:A:C
166:166
131:131
28:28
WLVGEMHCQKpc2_NP.3
p−maj=1, pX=0.97, N=325
var.17.C7 5:40964852:A:C
166:166
131:131
28:28
WLVGEMHCQKpc2_NP.4
p−maj=1, pX=0.47, N=325
var.17.C7 5:40964852:A:C
166:166
131:131
28:28
WLVGEMHCQKpc2_NP.5
p−maj=1, pX=0.28, N=325
var.17.C7 5:40964852:A:C
165:166
131:131
27:28
WLVGEMHCQKpc3_NP.1
p−maj=0.17, pX=0.81, N=323
var.17.C7 5:40964852:A:C
166:166
131:131
28:28
WLVGEMHCQKpc3_NP.2
p−maj=1, pX=0.22, N=325
var.17.C7 5:40964852:A:C
166:166
131:131
28:28
WLVGEMHCQKpc3_NP.3
p−maj=1, pX=0.75, N=325
var.17.C7 5:40964852:A:C
166:166
131:131
28:28
WLVGEMHCQKpc3_NP.4
p−maj=1, pX=0.45, N=325
var.17.C7 5:40964852:A:C
166:166
131:131
28:28
WLVGEMHCQKpc3_NP.5
p−maj=1, pX=0.5, N=325

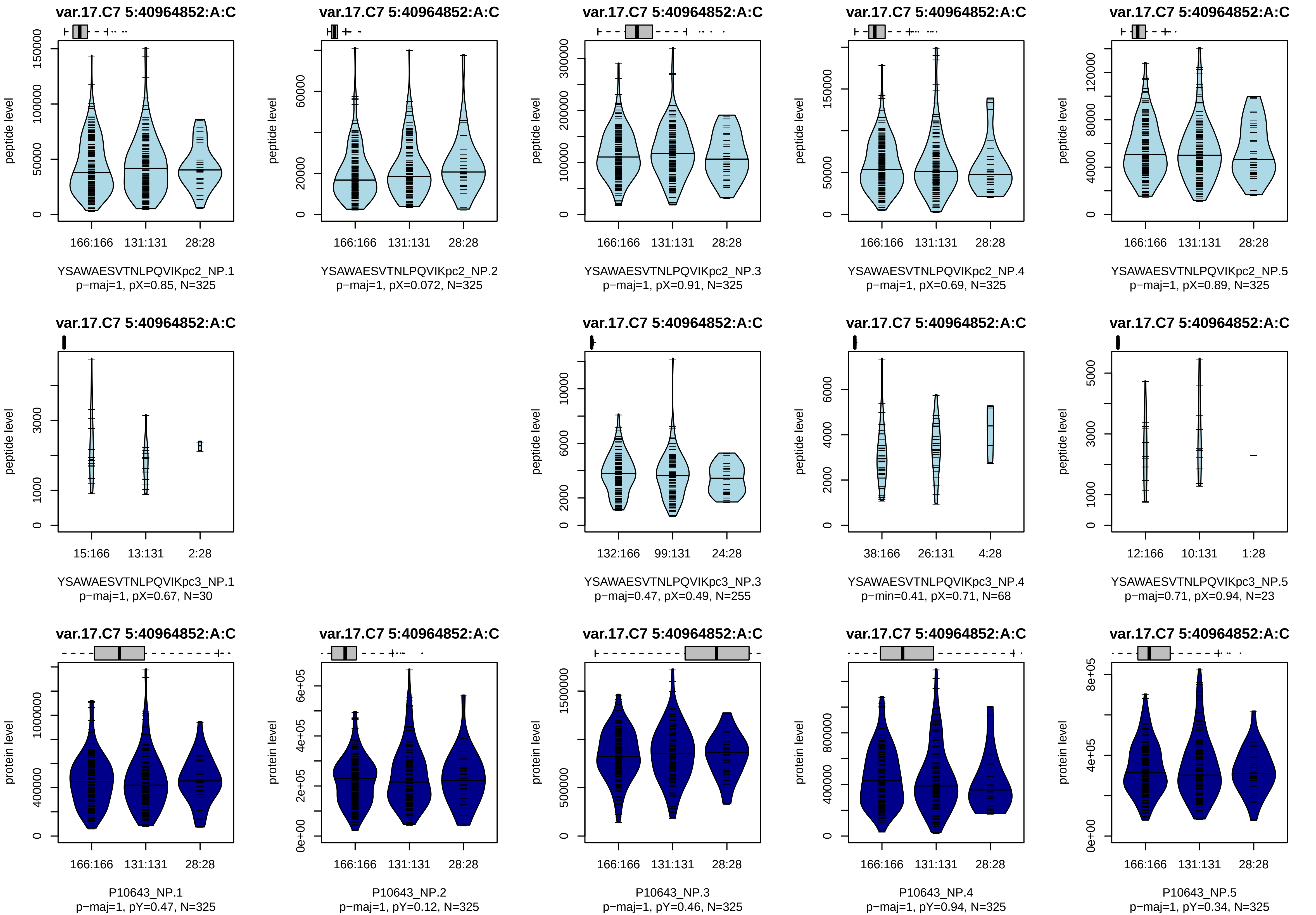
var.17.C7 5:40964852:A:C
peptide level
166:166
131:131
28:28
YSAWAESVTNLPQVIKpc2_NP.1
p−maj=1, pX=0.85, N=325
YSAWAESVTNLPQVIKpc2_NP.2
p−maj=1, pX=0.072, N=325
YSAWAESVTNLPQVIKpc2_NP.3
p−maj=1, pX=0.91, N=325
YSAWAESVTNLPQVIKpc2_NP.4
p−maj=1, pX=0.69, N=325
YSAWAESVTNLPQVIKpc2_NP.5
p−maj=1, pX=0.89, N=325
15:166
13:131
2:28
YSAWAESVTNLPQVIKpc3_NP.1
p−maj=1, pX=0.67, N=30
132:166
99:131
24:28
YSAWAESVTNLPQVIKpc3_NP.3
p−maj=0.47, pX=0.49, N=255
38:166
26:131
4:28
YSAWAESVTNLPQVIKpc3_NP.4
p−min=0.41, pX=0.71, N=68
12:166
10:131
1:28
YSAWAESVTNLPQVIKpc3_NP.5
p−maj=0.71, pX=0.94, N=23
protein level
P10643_NP.1
p−maj=1, pY=0.47, N=325
P10643_NP.2
p−maj=1, pY=0.12, N=325
P10643_NP.3
p−maj=1, pY=0.46, N=325
P10643_NP.4
p−maj=1, pY=0.94, N=325
P10643_NP.5
p−maj=1, pY=0.34, N=325

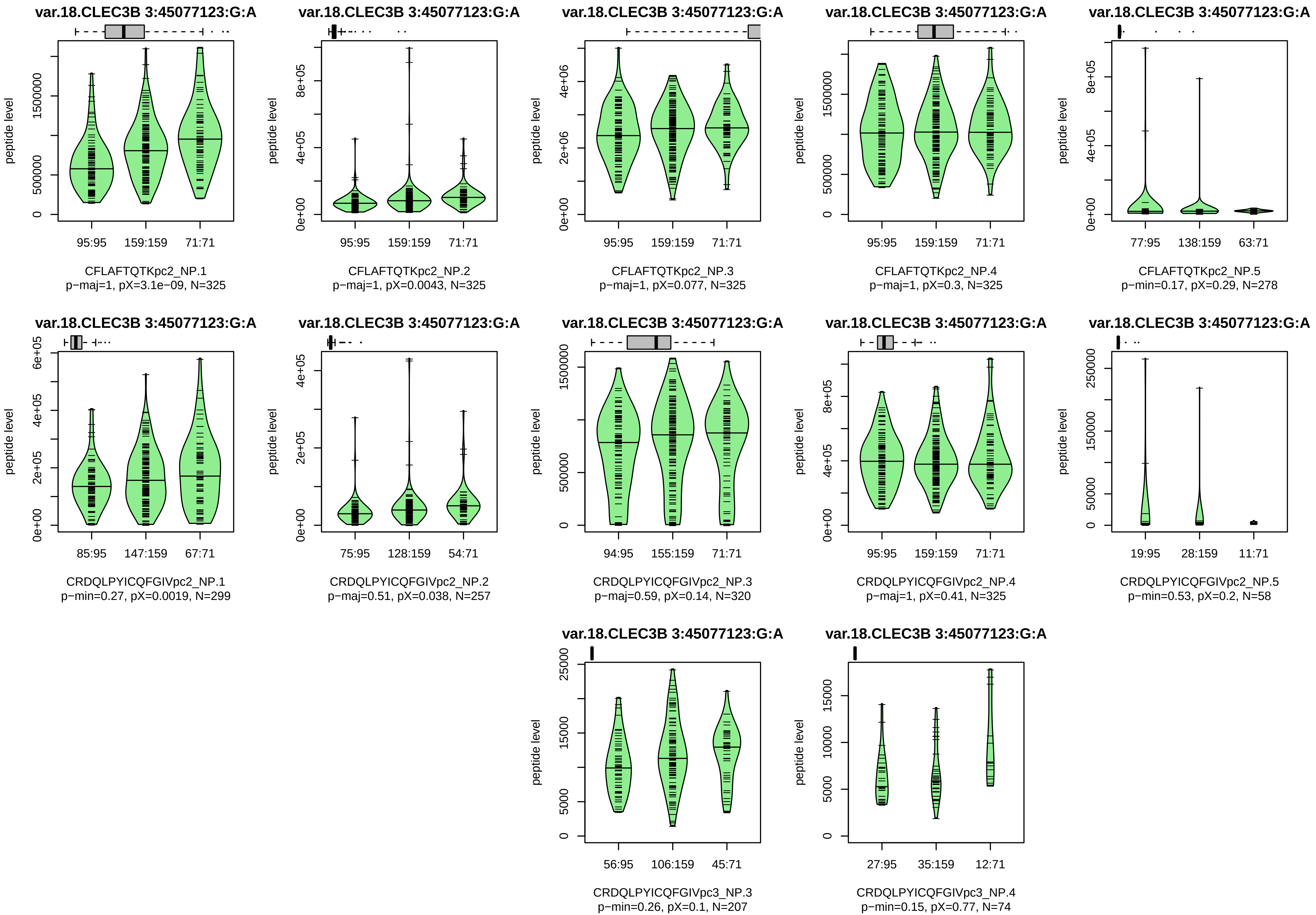
var.18.CLEC3B 3:45077123:G:A
peptide level
95:95
159:159
71:71
CFLAFTQTKpc2_NP.1
p−maj=1, pX=3.1e−09, N=325
var.18.CLEC3B 3:45077123:G:A
95:95
159:159
71:71
CFLAFTQTKpc2_NP.2
p−maj=1, pX=0.0043, N=325
var.18.CLEC3B 3:45077123:G:A
95:95
159:159
71:71
CFLAFTQTKpc2_NP.3
p−maj=1, pX=0.077, N=325
var.18.CLEC3B 3:45077123:G:A
95:95
159:159
71:71
CFLAFTQTKpc2_NP.4
p−maj=1, pX=0.3, N=325
var.18.CLEC3B 3:45077123:G:A
77:95
138:159
63:71
CFLAFTQTKpc2_NP.5
p−min=0.17, pX=0.29, N=278
var.18.CLEC3B 3:45077123:G:A
85:95
147:159
67:71
CRDQLPYICQFGIVpc2_NP.1
p−min=0.27, pX=0.0019, N=299
var.18.CLEC3B 3:45077123:G:A
75:95
128:159
54:71
CRDQLPYICQFGIVpc2_NP.2
p−maj=0.51, pX=0.038, N=257
var.18.CLEC3B 3:45077123:G:A
94:95
155:159
71:71
CRDQLPYICQFGIVpc2_NP.3
p−maj=0.59, pX=0.14, N=320
var.18.CLEC3B 3:45077123:G:A
95:95
159:159
71:71
CRDQLPYICQFGIVpc2_NP.4
p−maj=1, pX=0.41, N=325
var.18.CLEC3B 3:45077123:G:A
19:95
28:159
11:71
CRDQLPYICQFGIVpc2_NP.5
p−min=0.53, pX=0.2, N=58
var.18.CLEC3B 3:45077123:G:A
56:95
106:159
45:71
CRDQLPYICQFGIVpc3_NP.3
p−min=0.26, pX=0.1, N=207
var.18.CLEC3B 3:45077123:G:A
27:95
35:159
12:71
CRDQLPYICQFGIVpc3_NP.4
p−min=0.15, pX=0.77, N=74

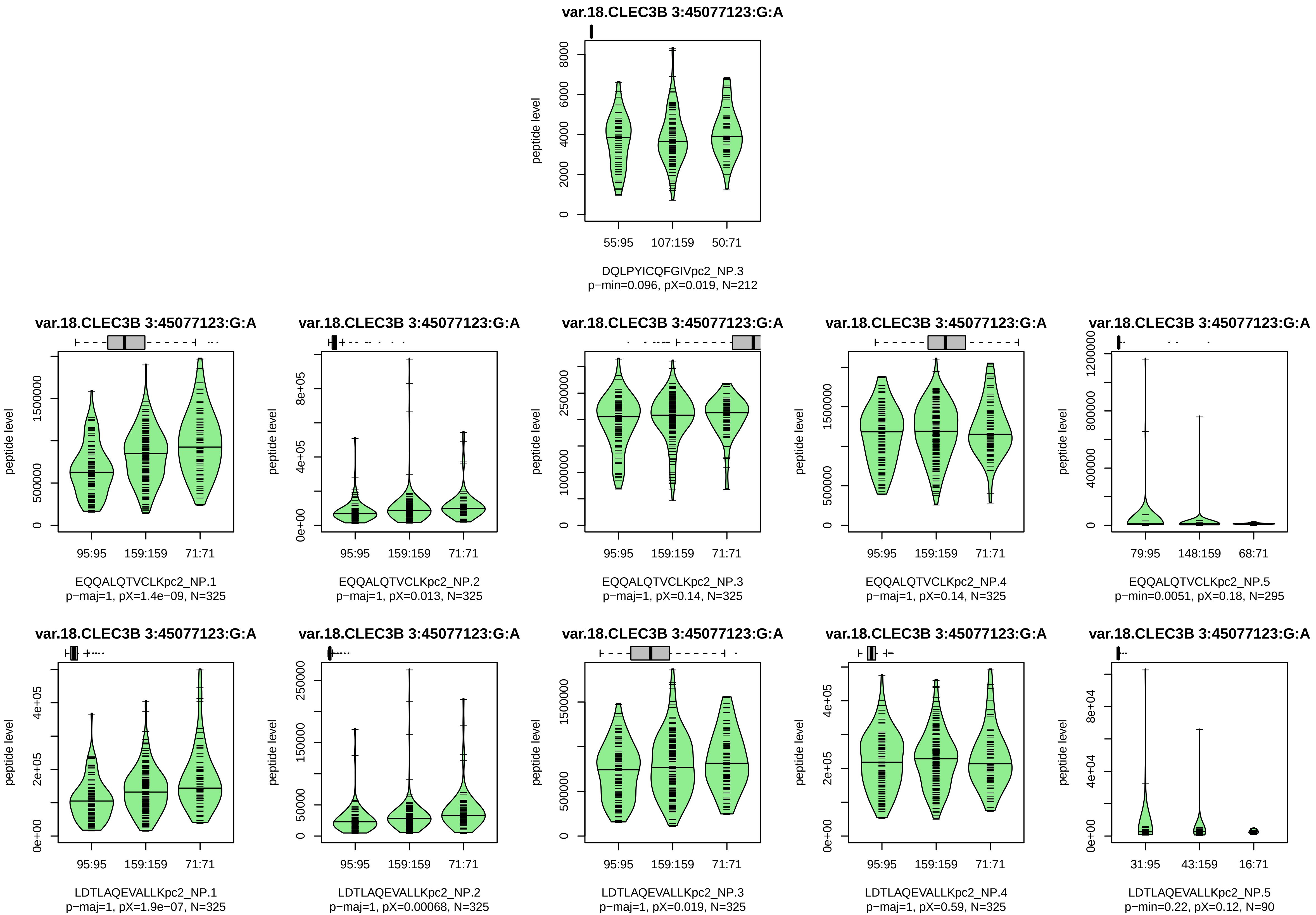

var.18.CLEC3B 3:45077123:G:A
peptide level
55:95
107:159
50:71
DQLPYICQFGIVpc2_NP.3
p−min=0.096, pX=0.019, N=212
var.18.CLEC3B 3:45077123:G:A
95:95
159:159
71:71
EQQALQTVCLKpc2_NP.1
p−maj=1, pX=1.4e−09, N=325
EQQALQTVCLKpc2_NP.2
p−maj=1, pX=0.013, N=325
EQQALQTVCLKpc2_NP.3
p−maj=1, pX=0.14, N=325
EQQALQTVCLKpc2_NP.4
p−maj=1, pX=0.14, N=325
79:95
148:159
68:71
EQQALQTVCLKpc2_NP.5
p−min=0.0051, pX=0.18, N=295
LDTLAQEVALLKpc2_NP.1
p−maj=1, pX=1.9e−07, N=325
LDTLAQEVALLKpc2_NP.2
p−maj=1, pX=0.00068, N=325
LDTLAQEVALLKpc2_NP.3
p−maj=1, pX=0.019, N=325
LDTLAQEVALLKpc2_NP.4
p−maj=1, pX=0.59, N=325
31:95
43:159
16:71
LDTLAQEVALLKpc2_NP.5
p−min=0.22, pX=0.12, N=90

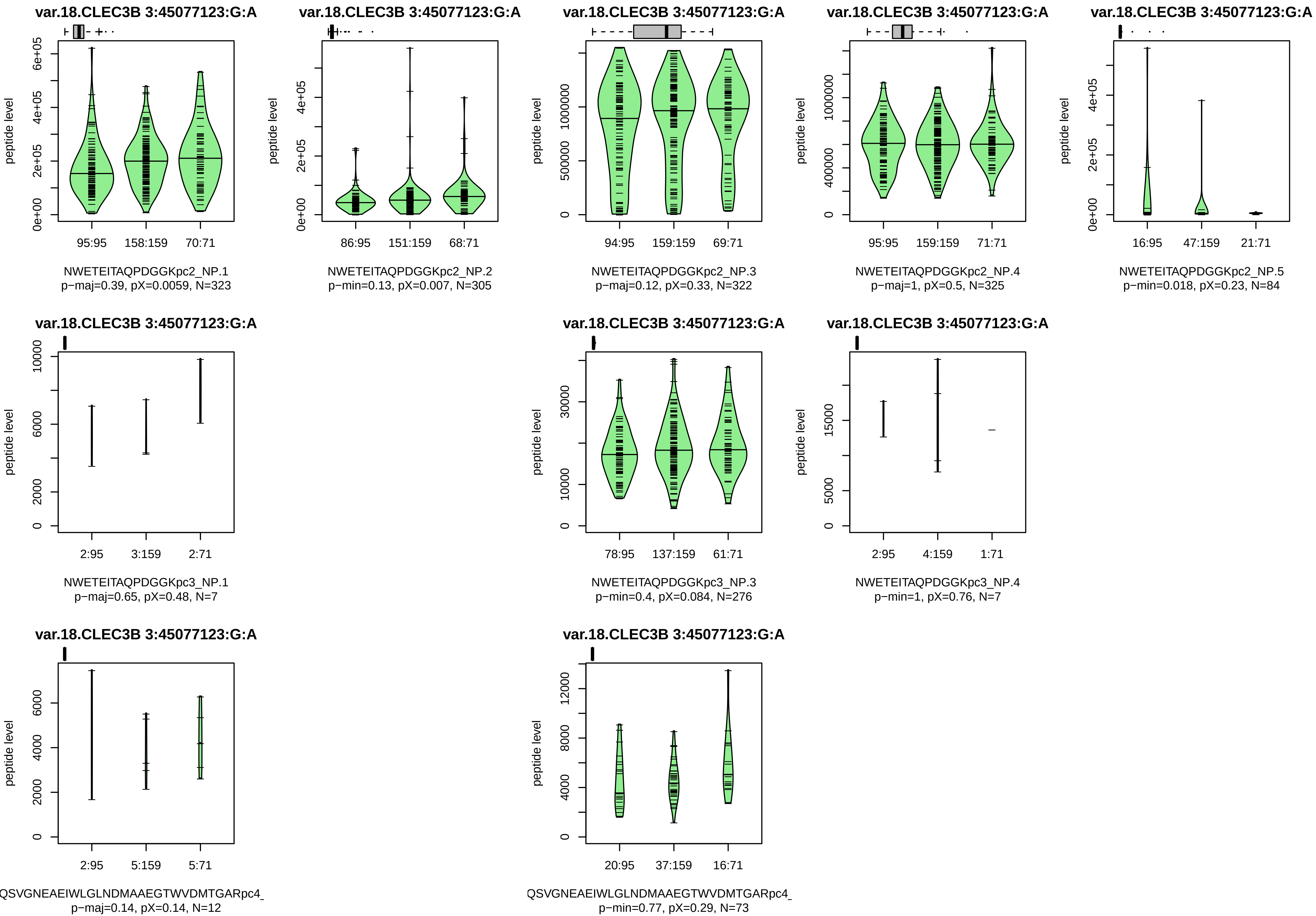
var.18.CLEC3B 3:45077123:G:A
peptide level
95:95
158:159
70:71
NWETEITAQPDGGKpc2_NP.1
p−maj=0.39, pX=0.0059, N=323
var.18.CLEC3B 3:45077123:G:A
peptide level
86:95
151:159
68:71
NWETEITAQPDGGKpc2_NP.2
p−min=0.13, pX=0.007, N=305
var.18.CLEC3B 3:45077123:G:A
peptide level
94:95
159:159
69:71
NWETEITAQPDGGKpc2_NP.3
p−maj=0.12, pX=0.33, N=322
var.18.CLEC3B 3:45077123:G:A
peptide level
95:95
159:159
71:71
NWETEITAQPDGGKpc2_NP.4
p−maj=1, pX=0.5, N=325
var.18.CLEC3B 3:45077123:G:A
peptide level
16:95
47:159
21:71
NWETEITAQPDGGKpc2_NP.5
p−min=0.018, pX=0.23, N=84
var.18.CLEC3B 3:45077123:G:A
peptide level
2:95
3:159
2:71
NWETEITAQPDGGKpc3_NP.1
p−maj=0.65, pX=0.48, N=7
var.18.CLEC3B 3:45077123:G:A
peptide level
78:95
137:159
61:71
NWETEITAQPDGGKpc3_NP.3
p−min=0.4, pX=0.084, N=276
var.18.CLEC3B 3:45077123:G:A
peptide level
2:95
4:159
1:71
NWETEITAQPDGGKpc3_NP.4
p−min=1, pX=0.76, N=7
var.18.CLEC3B 3:45077123:G:A
peptide level
2:95
5:159
5:71
QSVGNEAEIWLGLNDMAAEGTWVDMTGARpc4_
p−maj=0.14, pX=0.14, N=12
var.18.CLEC3B 3:45077123:G:A
peptide level
20:95
37:159
16:71
QSVGNEAEIWLGLNDMAAEGTWVDMTGARpc4_
p−min=0.77, pX=0.29, N=73

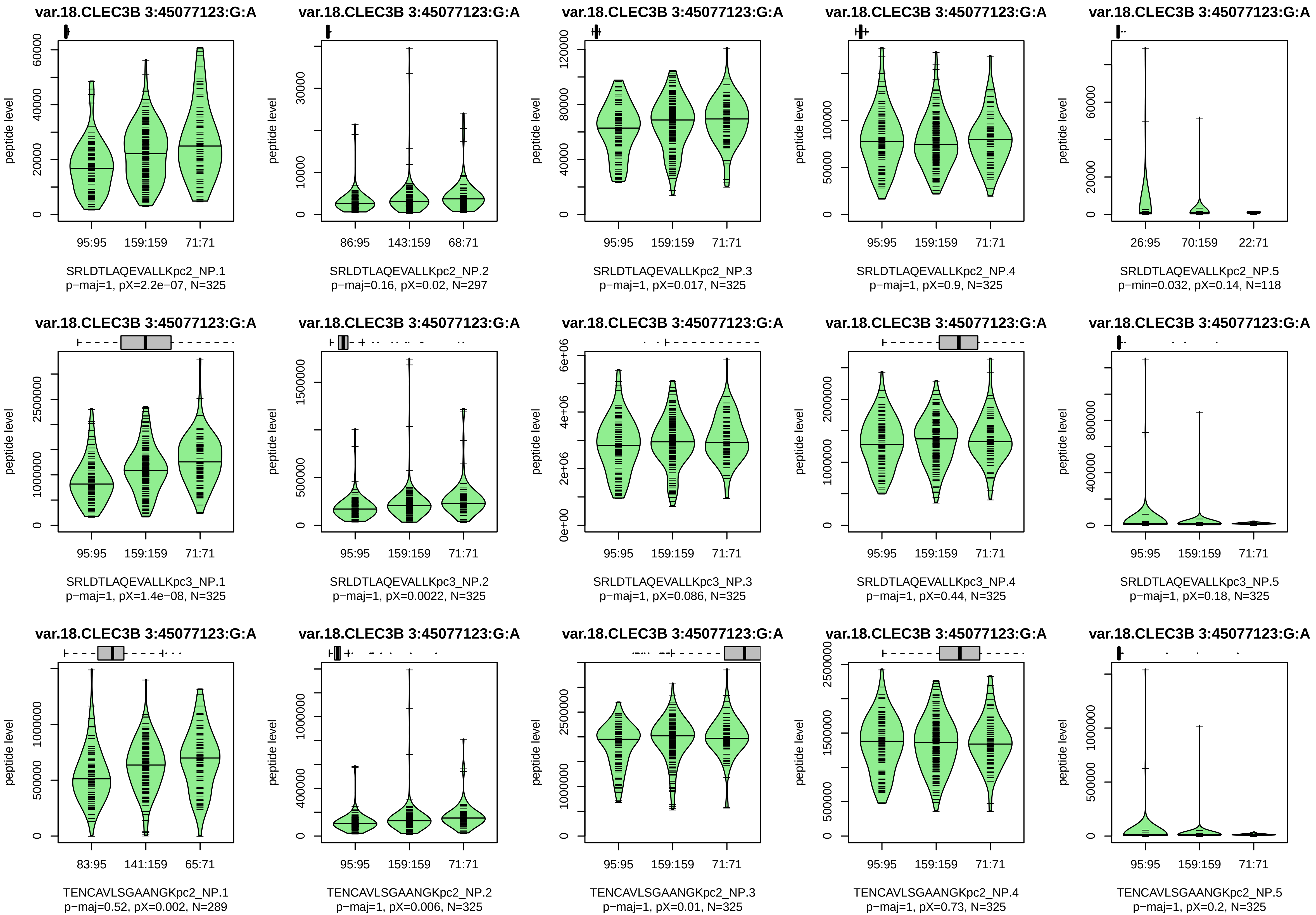
var.18.CLEC3B 3:45077123:G:A
peptide level
0
20000
40000
60000
95:95
159:159
71:71
SRLDTLAQEVALLKpc2_NP.1
p−maj=1, pX=2.2e−07, N=325
var.18.CLEC3B 3:45077123:G:A
peptide level
0
10000
30000
86:95
143:159
68:71
SRLDTLAQEVALLKpc2_NP.2
p−maj=0.16, pX=0.02, N=297
var.18.CLEC3B 3:45077123:G:A
peptide level
0
40000
80000
120000
95:95
159:159
71:71
SRLDTLAQEVALLKpc2_NP.3
p−maj=1, pX=0.017, N=325
var.18.CLEC3B 3:45077123:G:A
peptide level
0
50000
100000
95:95
159:159
71:71
SRLDTLAQEVALLKpc2_NP.4
p−maj=1, pX=0.9, N=325
var.18.CLEC3B 3:45077123:G:A
peptide level
0
20000
60000
26:95
70:159
22:71
SRLDTLAQEVALLKpc2_NP.5
p−min=0.032, pX=0.14, N=118
var.18.CLEC3B 3:45077123:G:A
peptide level
0
1000000
2500000
95:95
159:159
71:71
SRLDTLAQEVALLKpc3_NP.1
p−maj=1, pX=1.4e−08, N=325
var.18.CLEC3B 3:45077123:G:A
peptide level
0
500000
1500000
95:95
159:159
71:71
SRLDTLAQEVALLKpc3_NP.2
p−maj=1, pX=0.0022, N=325
var.18.CLEC3B 3:45077123:G:A
peptide level
0e+00
2e+06
4e+06
6e+06
95:95
159:159
71:71
SRLDTLAQEVALLKpc3_NP.3
p−maj=1, pX=0.086, N=325
var.18.CLEC3B 3:45077123:G:A
peptide level
0
1000000
2000000
95:95
159:159
71:71
SRLDTLAQEVALLKpc3_NP.4
p−maj=1, pX=0.44, N=325
var.18.CLEC3B 3:45077123:G:A
peptide level
0
400000
800000
95:95
159:159
71:71
SRLDTLAQEVALLKpc3_NP.5
p−maj=1, pX=0.18, N=325
var.18.CLEC3B 3:45077123:G:A
peptide level
0
500000
1000000
83:95
141:159
65:71
TENCAVLSGAANGKpc2_NP.1
p−maj=0.52, pX=0.002, N=289
var.18.CLEC3B 3:45077123:G:A
peptide level
0
400000
1000000
95:95
159:159
71:71
TENCAVLSGAANGKpc2_NP.2
p−maj=1, pX=0.006, N=325
var.18.CLEC3B 3:45077123:G:A
peptide level
0
1000000
2500000
95:95
159:159
71:71
TENCAVLSGAANGKpc2_NP.3
p−maj=1, pX=0.01, N=325
var.18.CLEC3B 3:45077123:G:A
peptide level
0
500000
1500000
2500000
95:95
159:159
71:71
TENCAVLSGAANGKpc2_NP.4
p−maj=1, pX=0.73, N=325
var.18.CLEC3B 3:45077123:G:A
peptide level
0
500000
1000000
95:95
159:159
71:71
TENCAVLSGAANGKpc2_NP.5
p−maj=1, pX=0.2, N=325

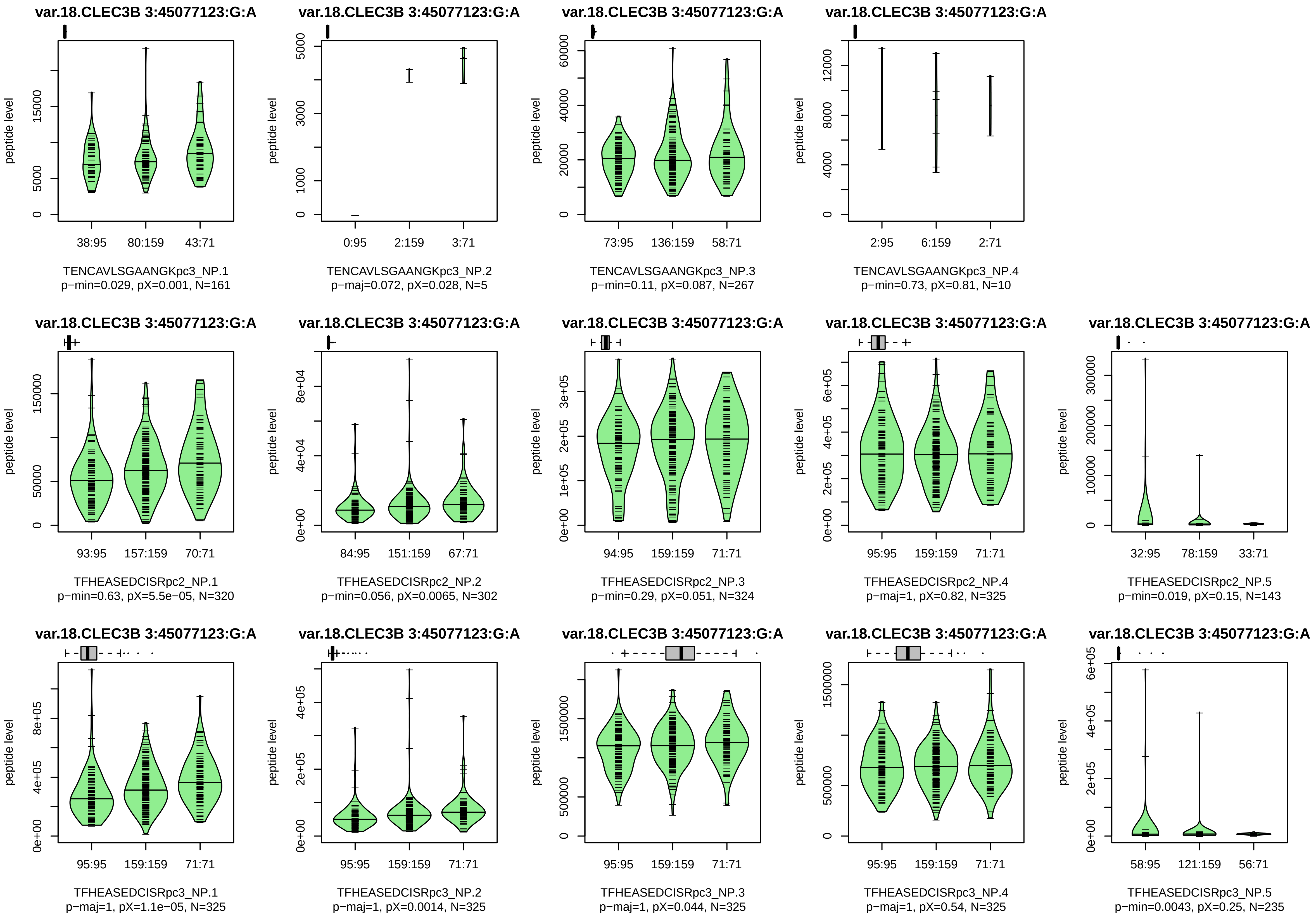

var.18.CLEC3B 3:45077123:G:A
peptide level
38:95
80:159
43:71
TENCAVLSGAANGKpc3_NP.1
p−min=0.029, pX=0.001, N=161
var.18.CLEC3B 3:45077123:G:A
peptide level
0:95
2:159
3:71
TENCAVLSGAANGKpc3_NP.2
p−maj=0.072, pX=0.028, N=5
var.18.CLEC3B 3:45077123:G:A
peptide level
73:95
136:159
58:71
TENCAVLSGAANGKpc3_NP.3
p−min=0.11, pX=0.087, N=267
var.18.CLEC3B 3:45077123:G:A
peptide level
2:95
6:159
2:71
TENCAVLSGAANGKpc3_NP.4
p−min=0.73, pX=0.81, N=10
var.18.CLEC3B 3:45077123:G:A
peptide level
93:95
157:159
70:71
TFHEASEDCISRpc2_NP.1
p−min=0.63, pX=5.5e−05, N=320
var.18.CLEC3B 3:45077123:G:A
peptide level
84:95
151:159
67:71
TFHEASEDCISRpc2_NP.2
p−min=0.056, pX=0.0065, N=302
var.18.CLEC3B 3:45077123:G:A
peptide level
94:95
159:159
71:71
TFHEASEDCISRpc2_NP.3
p−min=0.29, pX=0.051, N=324
var.18.CLEC3B 3:45077123:G:A
peptide level
95:95
159:159
71:71
TFHEASEDCISRpc2_NP.4
p−maj=1, pX=0.82, N=325
var.18.CLEC3B 3:45077123:G:A
peptide level
32:95
78:159
33:71
TFHEASEDCISRpc2_NP.5
p−min=0.019, pX=0.15, N=143
var.18.CLEC3B 3:45077123:G:A
peptide level
95:95
159:159
71:71
TFHEASEDCISRpc3_NP.1
p−maj=1, pX=1.1e−05, N=325
var.18.CLEC3B 3:45077123:G:A
peptide level
95:95
159:159
71:71
TFHEASEDCISRpc3_NP.2
p−maj=1, pX=0.0014, N=325
var.18.CLEC3B 3:45077123:G:A
peptide level
95:95
159:159
71:71
TFHEASEDCISRpc3_NP.3
p−maj=1, pX=0.044, N=325
var.18.CLEC3B 3:45077123:G:A
peptide level
95:95
159:159
71:71
TFHEASEDCISRpc3_NP.4
p−maj=1, pX=0.54, N=325
var.18.CLEC3B 3:45077123:G:A
peptide level
58:95
121:159
56:71
TFHEASEDCISRpc3_NP.5
p−min=0.0043, pX=0.25, N=235

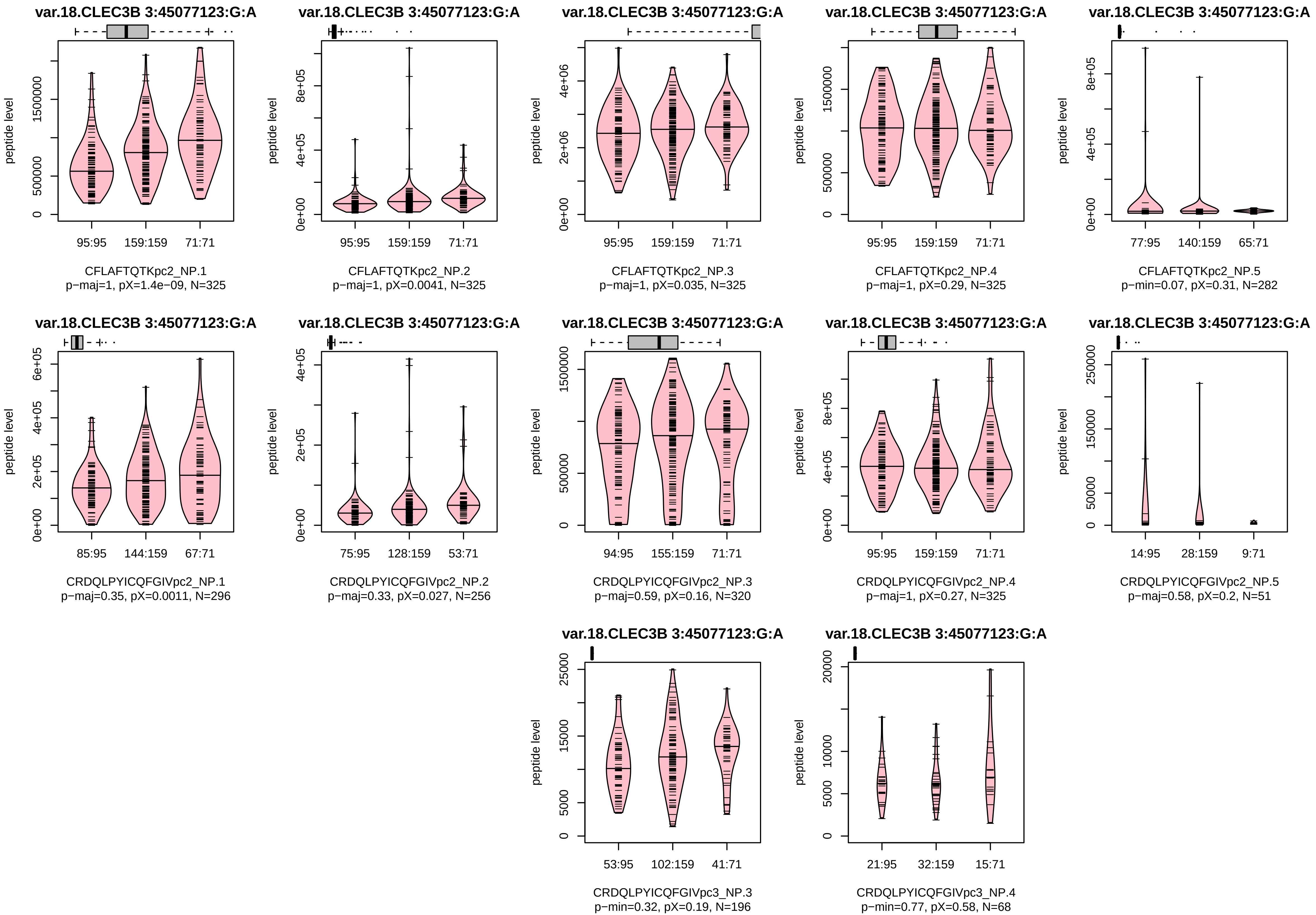
var.18.CLEC3B 3:45077123:G:A
peptide level
95:95
159:159
71:71
CFLAFTQTKpc2_NP.1
p−maj=1, pX=1.4e−09, N=325
var.18.CLEC3B 3:45077123:G:A
95:95
159:159
71:71
CFLAFTQTKpc2_NP.2
p−maj=1, pX=0.0041, N=325
var.18.CLEC3B 3:45077123:G:A
95:95
159:159
71:71
CFLAFTQTKpc2_NP.3
p−maj=1, pX=0.035, N=325
var.18.CLEC3B 3:45077123:G:A
95:95
159:159
71:71
CFLAFTQTKpc2_NP.4
p−maj=1, pX=0.29, N=325
var.18.CLEC3B 3:45077123:G:A
77:95
140:159
65:71
CFLAFTQTKpc2_NP.5
p−min=0.07, pX=0.31, N=282
var.18.CLEC3B 3:45077123:G:A
85:95
144:159
67:71
CRDQLPYICQFGIVpc2_NP.1
p−maj=0.35, pX=0.0011, N=296
var.18.CLEC3B 3:45077123:G:A
75:95
128:159
53:71
CRDQLPYICQFGIVpc2_NP.2
p−maj=0.33, pX=0.027, N=256
var.18.CLEC3B 3:45077123:G:A
94:95
155:159
71:71
CRDQLPYICQFGIVpc2_NP.3
p−maj=0.59, pX=0.16, N=320
var.18.CLEC3B 3:45077123:G:A
95:95
159:159
71:71
CRDQLPYICQFGIVpc2_NP.4
p−maj=1, pX=0.27, N=325
var.18.CLEC3B 3:45077123:G:A
14:95
28:159
9:71
CRDQLPYICQFGIVpc2_NP.5
p−maj=0.58, pX=0.2, N=51
var.18.CLEC3B 3:45077123:G:A
53:95
102:159
41:71
CRDQLPYICQFGIVpc3_NP.3
p−min=0.32, pX=0.19, N=196
var.18.CLEC3B 3:45077123:G:A
21:95
32:159
15:71
CRDQLPYICQFGIVpc3_NP.4
p−min=0.77, pX=0.58, N=68

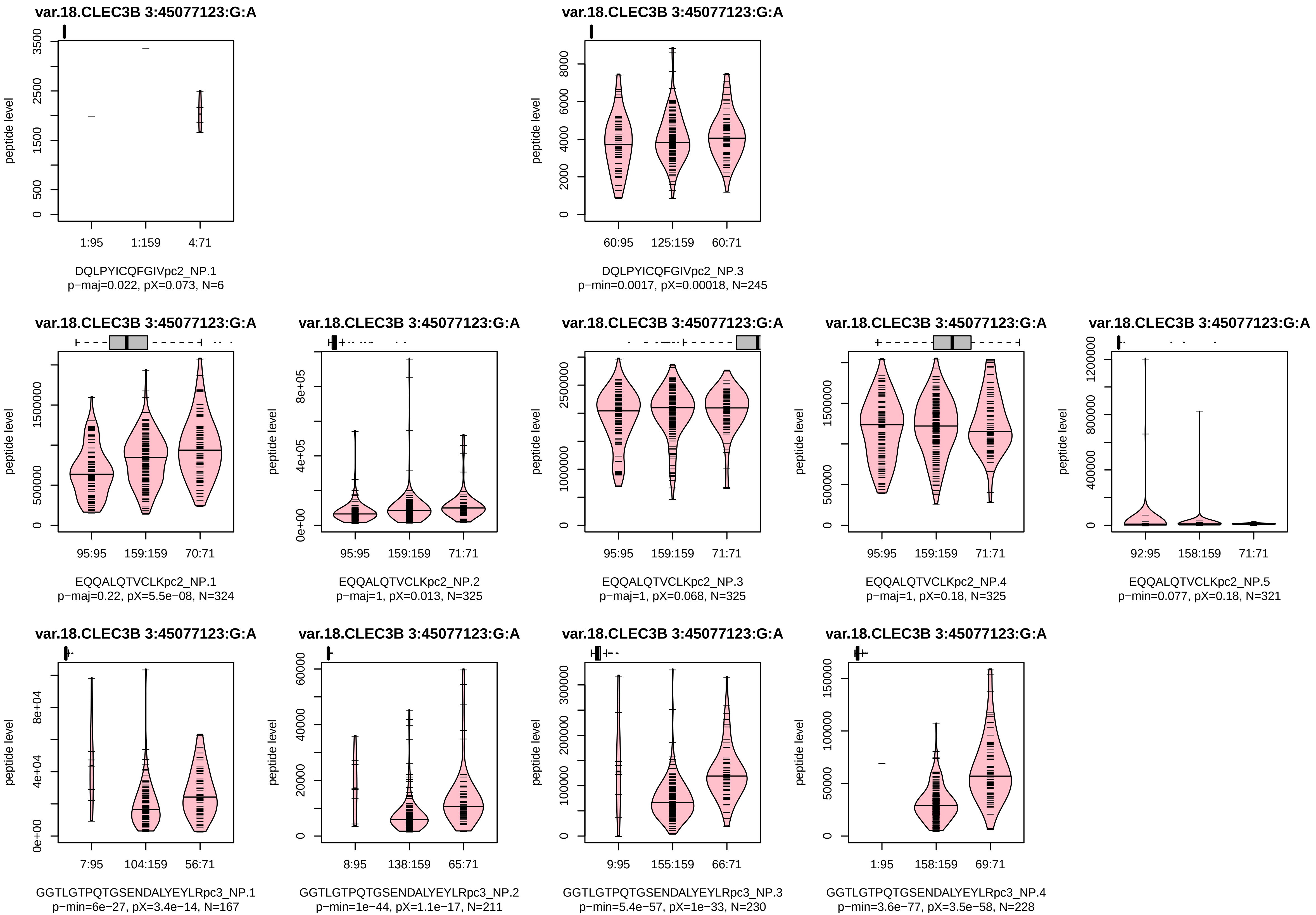

var.18.CLEC3B 3:45077123:G:A
peptide level
0 500 1500 2500 3500
1:95 1:159 4:71
DQLPYICQFGIVpc2_NP.1
p−maj=0.022, pX=0.073, N=6
var.18.CLEC3B 3:45077123:G:A
peptide level
0 2000 4000 6000 8000
60:95 125:159 60:71
DQLPYICQFGIVpc2_NP.3
p−min=0.0017, pX=0.00018, N=245
var.18.CLEC3B 3:45077123:G:A
peptide level
0 500000 1500000
95:95 159:159 70:71
EQQALQTVCLKpc2_NP.1
p−maj=0.22, pX=5.5e−08, N=324
var.18.CLEC3B 3:45077123:G:A
peptide level
0e+00 4e+05 8e+05
95:95 159:159 71:71
EQQALQTVCLKpc2_NP.2
p−maj=1, pX=0.013, N=325
var.18.CLEC3B 3:45077123:G:A
peptide level
0 1000000 2500000
95:95 159:159 71:71
EQQALQTVCLKpc2_NP.3
p−maj=1, pX=0.068, N=325
var.18.CLEC3B 3:45077123:G:A
peptide level
0 500000 1500000
95:95 159:159 71:71
EQQALQTVCLKpc2_NP.4
p−maj=1, pX=0.18, N=325
var.18.CLEC3B 3:45077123:G:A
peptide level
0 400000 800000 1200000
92:95 158:159 71:71
EQQALQTVCLKpc2_NP.5
p−min=0.077, pX=0.18, N=321
var.18.CLEC3B 3:45077123:G:A
peptide level
0e+00 4e+04 8e+04
7:95 104:159 56:71
GGTLGTPQTGSENDALYEYLRpc3_NP.1
p−min=6e−27, pX=3.4e−14, N=167
var.18.CLEC3B 3:45077123:G:A
peptide level
0 20000 40000 60000
8:95 138:159 65:71
GGTLGTPQTGSENDALYEYLRpc3_NP.2
p−min=1e−44, pX=1.1e−17, N=211
var.18.CLEC3B 3:45077123:G:A
peptide level
0 100000 200000 300000
9:95 155:159 66:71
GGTLGTPQTGSENDALYEYLRpc3_NP.3
p−min=5.4e−57, pX=1e−33, N=230
var.18.CLEC3B 3:45077123:G:A
peptide level
0 50000 100000 150000
1:95 158:159 69:71
GGTLGTPQTGSENDALYEYLRpc3_NP.4
p−min=3.6e−77, pX=3.5e−58, N=228

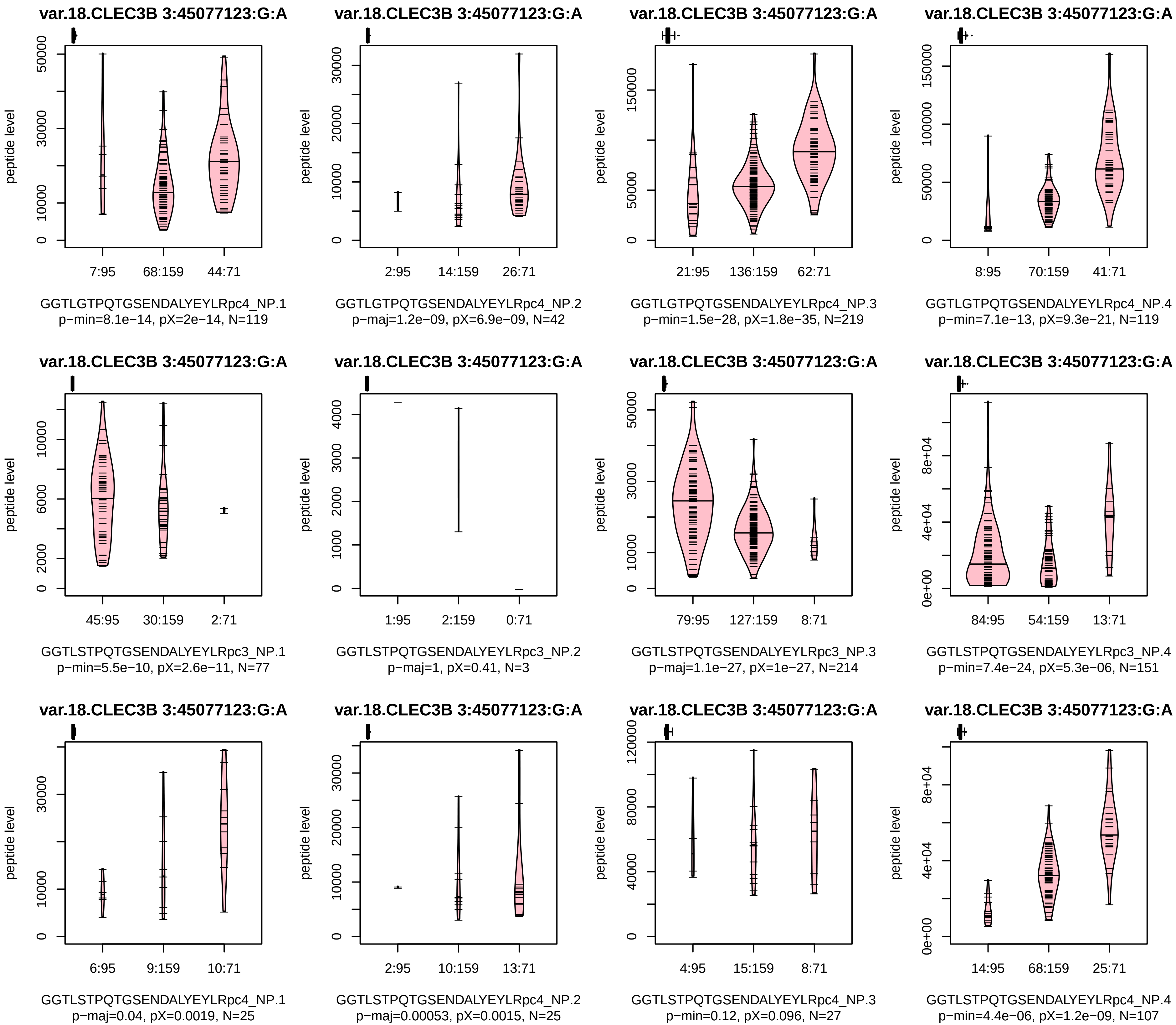
var.18.CLEC3B 3:45077123:G:A
peptide level
7:95
68:159
44:71
GGTLGTPQTGSENDALYEYLRpc4_NP.1
p−min=8.1e−14, pX=2e−14, N=119
var.18.CLEC3B 3:45077123:G:A
peptide level
2:95
14:159
26:71
GGTLGTPQTGSENDALYEYLRpc4_NP.2
p−maj=1.2e−09, pX=6.9e−09, N=42
var.18.CLEC3B 3:45077123:G:A
peptide level
21:95
136:159
62:71
GGTLGTPQTGSENDALYEYLRpc4_NP.3
p−min=1.5e−28, pX=1.8e−35, N=219
var.18.CLEC3B 3:45077123:G:A
peptide level
8:95
70:159
41:71
GGTLGTPQTGSENDALYEYLRpc4_NP.4
p−min=7.1e−13, pX=9.3e−21, N=119
var.18.CLEC3B 3:45077123:G:A
peptide level
45:95
30:159
2:71
GGTLSTPQTGSENDALYEYLRpc3_NP.1
p−min=5.5e−10, pX=2.6e−11, N=77
var.18.CLEC3B 3:45077123:G:A
peptide level
1:95
2:159
0:71
GGTLSTPQTGSENDALYEYLRpc3_NP.2
p−maj=1, pX=0.41, N=3
var.18.CLEC3B 3:45077123:G:A
peptide level
79:95
127:159
8:71
GGTLSTPQTGSENDALYEYLRpc3_NP.3
p−maj=1.1e−27, pX=1e−27, N=214
var.18.CLEC3B 3:45077123:G:A
peptide level
84:95
54:159
13:71
GGTLSTPQTGSENDALYEYLRpc3_NP.4
p−min=7.4e−24, pX=5.3e−06, N=151
var.18.CLEC3B 3:45077123:G:A
peptide level
6:95
9:159
10:71
GGTLSTPQTGSENDALYEYLRpc4_NP.1
p−maj=0.04, pX=0.0019, N=25
var.18.CLEC3B 3:45077123:G:A
peptide level
2:95
10:159
13:71
GGTLSTPQTGSENDALYEYLRpc4_NP.2
p−maj=0.00053, pX=0.0015, N=25
var.18.CLEC3B 3:45077123:G:A
peptide level
4:95
15:159
8:71
GGTLSTPQTGSENDALYEYLRpc4_NP.3
p−min=0.12, pX=0.096, N=27
var.18.CLEC3B 3:45077123:G:A
peptide level
14:95
68:159
25:71
GGTLSTPQTGSENDALYEYLRpc4_NP.4
p−min=4.4e−06, pX=1.2e−09, N=107

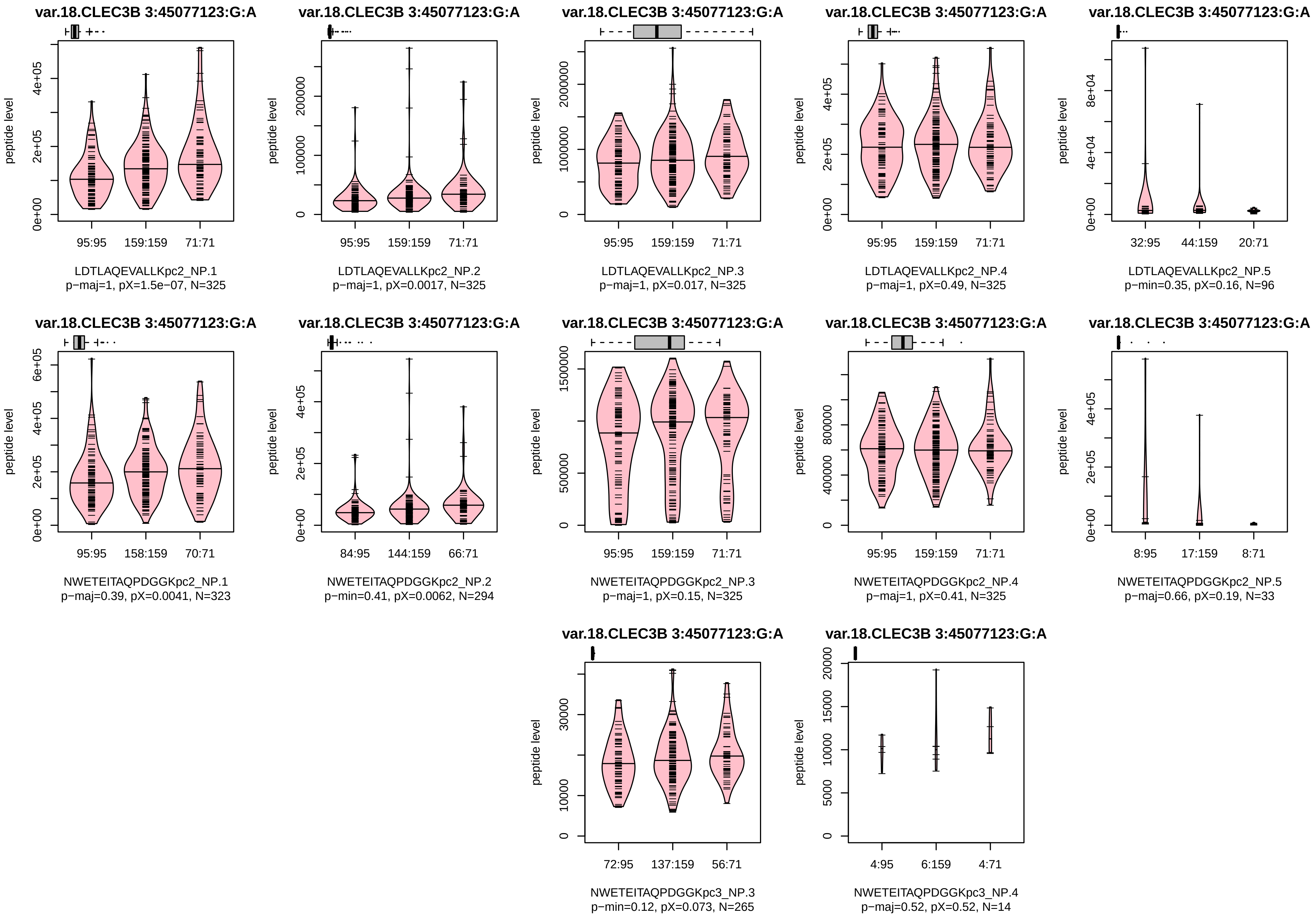
var.18.CLEC3B 3:45077123:G:A
peptide level
95:95
159:159
71:71
LDTLAQEVALLKpc2_NP.1
p−maj=1, pX=1.5e−07, N=325
var.18.CLEC3B 3:45077123:G:A
peptide level
95:95
159:159
71:71
LDTLAQEVALLKpc2_NP.2
p−maj=1, pX=0.0017, N=325
var.18.CLEC3B 3:45077123:G:A
peptide level
95:95
159:159
71:71
LDTLAQEVALLKpc2_NP.3
p−maj=1, pX=0.017, N=325
var.18.CLEC3B 3:45077123:G:A
peptide level
95:95
159:159
71:71
LDTLAQEVALLKpc2_NP.4
p−maj=1, pX=0.49, N=325
var.18.CLEC3B 3:45077123:G:A
peptide level
32:95
44:159
20:71
LDTLAQEVALLKpc2_NP.5
p−min=0.35, pX=0.16, N=96
var.18.CLEC3B 3:45077123:G:A
peptide level
95:95
158:159
70:71
NWETEITAQPDGGKpc2_NP.1
p−maj=0.39, pX=0.0041, N=323
var.18.CLEC3B 3:45077123:G:A
peptide level
84:95
144:159
66:71
NWETEITAQPDGGKpc2_NP.2
p−min=0.41, pX=0.0062, N=294
var.18.CLEC3B 3:45077123:G:A
peptide level
95:95
159:159
71:71
NWETEITAQPDGGKpc2_NP.3
p−maj=1, pX=0.15, N=325
var.18.CLEC3B 3:45077123:G:A
peptide level
95:95
159:159
71:71
NWETEITAQPDGGKpc2_NP.4
p−maj=1, pX=0.41, N=325
var.18.CLEC3B 3:45077123:G:A
peptide level
8:95
17:159
8:71
NWETEITAQPDGGKpc2_NP.5
p−maj=0.66, pX=0.19, N=33
var.18.CLEC3B 3:45077123:G:A
peptide level
72:95
137:159
56:71
NWETEITAQPDGGKpc3_NP.3
p−min=0.12, pX=0.073, N=265
var.18.CLEC3B 3:45077123:G:A
peptide level
4:95
6:159
4:71
NWETEITAQPDGGKpc3_NP.4
p−maj=0.52, pX=0.52, N=14

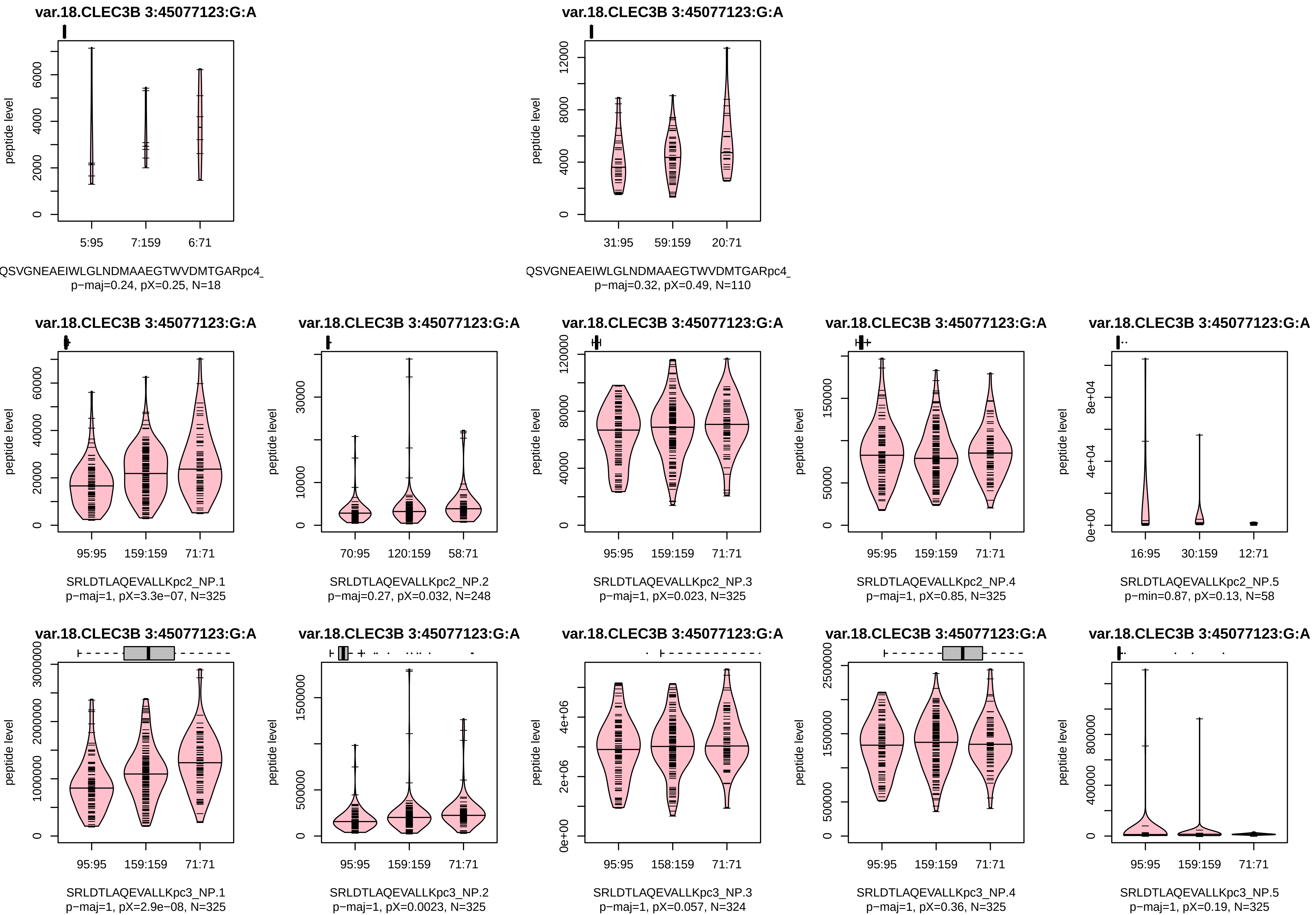
var.18.CLEC3B 3:45077123:G:A
peptide level
5:95
7:159
6:71
QSVGNEAEIWLGLNDMAAEGTWVDMTGARpc4_
p−maj=0.24, pX=0.25, N=18
var.18.CLEC3B 3:45077123:G:A
peptide level
31:95
59:159
20:71
QSVGNEAEIWLGLNDMAAEGTWVDMTGARpc4_
p−maj=0.32, pX=0.49, N=110
var.18.CLEC3B 3:45077123:G:A
95:95
159:159
71:71
SRLDTLAQEVALLKpc2_NP.1
p−maj=1, pX=3.3e−07, N=325
var.18.CLEC3B 3:45077123:G:A
70:95
120:159
58:71
SRLDTLAQEVALLKpc2_NP.2
p−maj=0.27, pX=0.032, N=248
var.18.CLEC3B 3:45077123:G:A
95:95
159:159
71:71
SRLDTLAQEVALLKpc2_NP.3
p−maj=1, pX=0.023, N=325
var.18.CLEC3B 3:45077123:G:A
95:95
159:159
71:71
SRLDTLAQEVALLKpc2_NP.4
p−maj=1, pX=0.85, N=325
var.18.CLEC3B 3:45077123:G:A
16:95
30:159
12:71
SRLDTLAQEVALLKpc2_NP.5
p−min=0.87, pX=0.13, N=58
var.18.CLEC3B 3:45077123:G:A
95:95
159:159
71:71
SRLDTLAQEVALLKpc3_NP.1
p−maj=1, pX=2.9e−08, N=325
var.18.CLEC3B 3:45077123:G:A
95:95
159:159
71:71
SRLDTLAQEVALLKpc3_NP.2
p−maj=1, pX=0.0023, N=325
var.18.CLEC3B 3:45077123:G:A
95:95
158:159
71:71
SRLDTLAQEVALLKpc3_NP.3
p−maj=1, pX=0.057, N=324
var.18.CLEC3B 3:45077123:G:A
95:95
159:159
71:71
SRLDTLAQEVALLKpc3_NP.4
p−maj=1, pX=0.36, N=325
var.18.CLEC3B 3:45077123:G:A
95:95
159:159
71:71
SRLDTLAQEVALLKpc3_NP.5
p−maj=1, pX=0.19, N=325

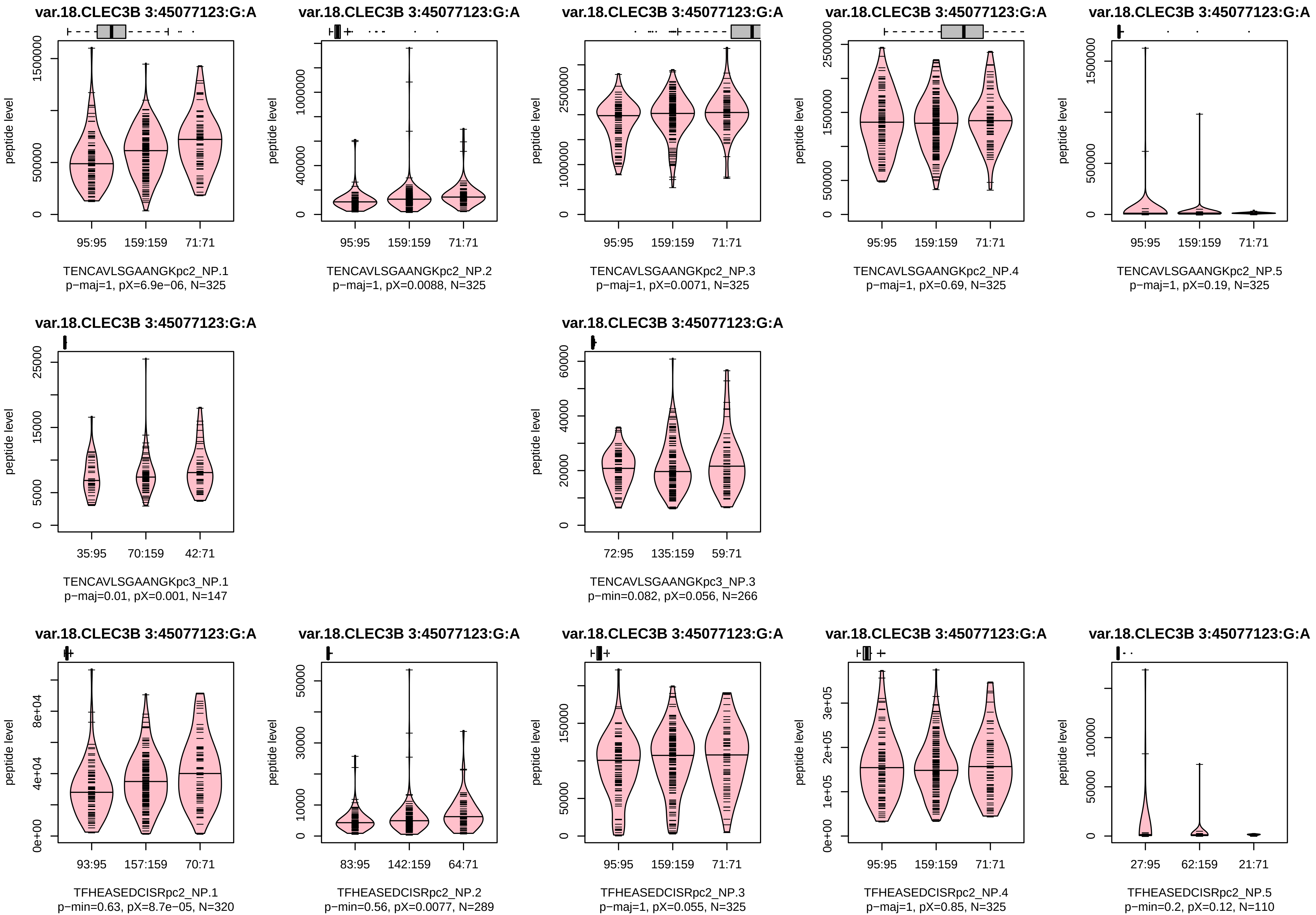

var.18.CLEC3B 3:45077123:G:A
peptide level
95:95
159:159
71:71
TENCAVLSGAANGKpc2_NP.1
p−maj=1, pX=6.9e−06, N=325
var.18.CLEC3B 3:45077123:G:A
peptide level
95:95
159:159
71:71
TENCAVLSGAANGKpc2_NP.2
p−maj=1, pX=0.0088, N=325
var.18.CLEC3B 3:45077123:G:A
peptide level
95:95
159:159
71:71
TENCAVLSGAANGKpc2_NP.3
p−maj=1, pX=0.0071, N=325
var.18.CLEC3B 3:45077123:G:A
peptide level
95:95
159:159
71:71
TENCAVLSGAANGKpc2_NP.4
p−maj=1, pX=0.69, N=325
var.18.CLEC3B 3:45077123:G:A
peptide level
95:95
159:159
71:71
TENCAVLSGAANGKpc2_NP.5
p−maj=1, pX=0.19, N=325
var.18.CLEC3B 3:45077123:G:A
peptide level
35:95
70:159
42:71
TENCAVLSGAANGKpc3_NP.1
p−maj=0.01, pX=0.001, N=147
var.18.CLEC3B 3:45077123:G:A
peptide level
72:95
135:159
59:71
TENCAVLSGAANGKpc3_NP.3
p−min=0.082, pX=0.056, N=266
var.18.CLEC3B 3:45077123:G:A
peptide level
93:95
157:159
70:71
TFHEASEDCISRpc2_NP.1
p−min=0.63, pX=8.7e−05, N=320
var.18.CLEC3B 3:45077123:G:A
peptide level
83:95
142:159
64:71
TFHEASEDCISRpc2_NP.2
p−min=0.56, pX=0.0077, N=289
var.18.CLEC3B 3:45077123:G:A
peptide level
95:95
159:159
71:71
TFHEASEDCISRpc2_NP.3
p−maj=1, pX=0.055, N=325
var.18.CLEC3B 3:45077123:G:A
peptide level
95:95
159:159
71:71
TFHEASEDCISRpc2_NP.4
p−maj=1, pX=0.85, N=325
var.18.CLEC3B 3:45077123:G:A
peptide level
27:95
62:159
21:71
TFHEASEDCISRpc2_NP.5
p−min=0.2, pX=0.12, N=110

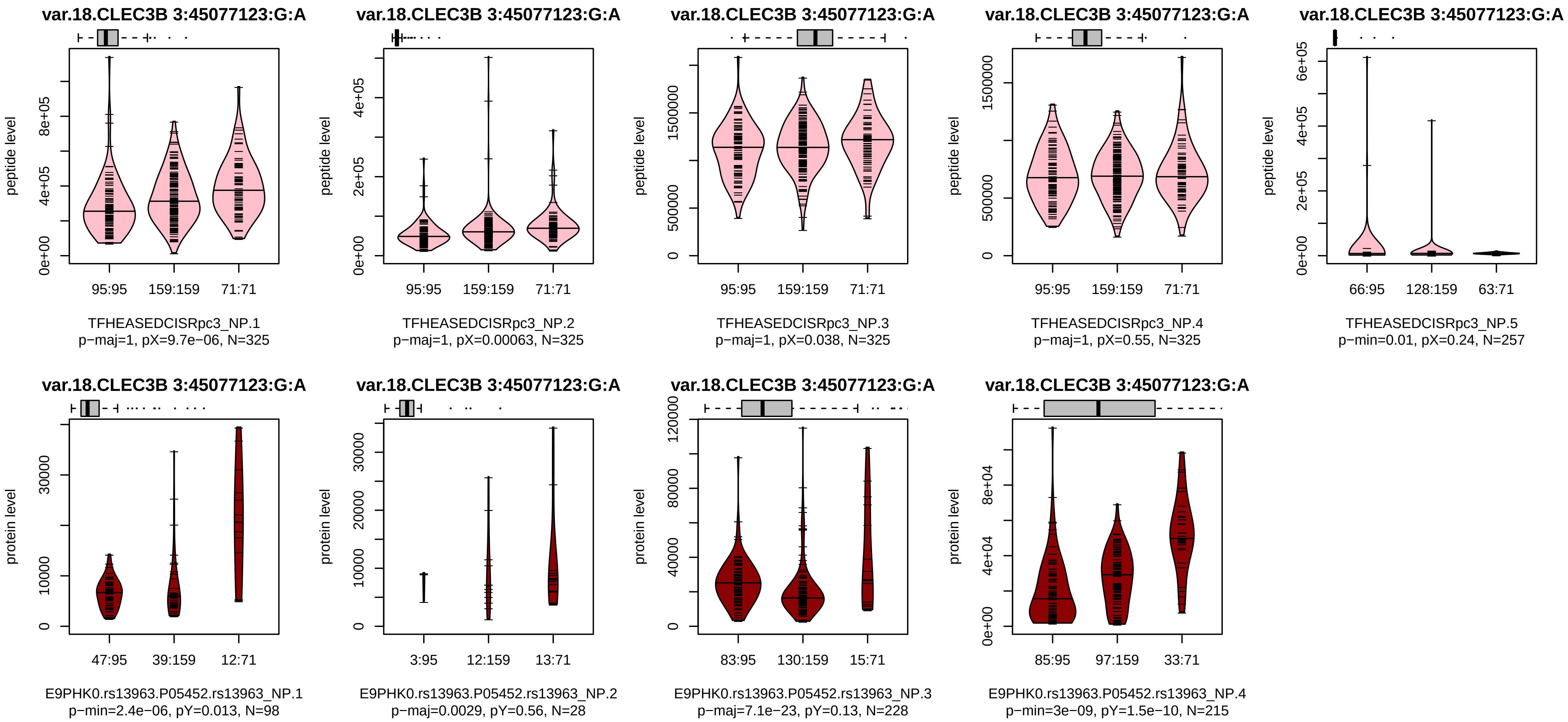
var.18.CLEC3B 3:45077123:G:A
peptide level
0e+00
4e+05
8e+05
95:95
159:159
71:71
TFHEASEDCISRpc3_NP.1
p−maj=1, pX=9.7e−06, N=325
var.18.CLEC3B 3:45077123:G:A
peptide level
0e+00
2e+05
4e+05
95:95
159:159
71:71
TFHEASEDCISRpc3_NP.2
p−maj=1, pX=0.00063, N=325
var.18.CLEC3B 3:45077123:G:A
peptide level
0
500000
1500000
95:95
159:159
71:71
TFHEASEDCISRpc3_NP.3
p−maj=1, pX=0.038, N=325
var.18.CLEC3B 3:45077123:G:A
peptide level
0
500000
1500000
95:95
159:159
71:71
TFHEASEDCISRpc3_NP.4
p−maj=1, pX=0.55, N=325
var.18.CLEC3B 3:45077123:G:A
peptide level
0e+00
2e+05
4e+05
6e+05
66:95
128:159
63:71
TFHEASEDCISRpc3_NP.5
p−min=0.01, pX=0.24, N=257
var.18.CLEC3B 3:45077123:G:A
protein level
0
10000
30000
47:95
39:159
12:71
E9PHK0.rs13963.P05452.rs13963_NP.1
p−min=2.4e−06, pY=0.013, N=98
var.18.CLEC3B 3:45077123:G:A
protein level
0
10000
20000
30000
3:95
12:159
13:71
E9PHK0.rs13963.P05452.rs13963_NP.2
p−maj=0.0029, pY=0.56, N=28
var.18.CLEC3B 3:45077123:G:A
protein level
0
40000
80000
120000
83:95
130:159
15:71
E9PHK0.rs13963.P05452.rs13963_NP.3
p−maj=7.1e−23, pY=0.13, N=228
var.18.CLEC3B 3:45077123:G:A
protein level
0e+00
4e+04
8e+04
85:95
97:159
33:71
E9PHK0.rs13963.P05452.rs13963_NP.4
p−min=3e−09, pY=1.5e−10, N=215

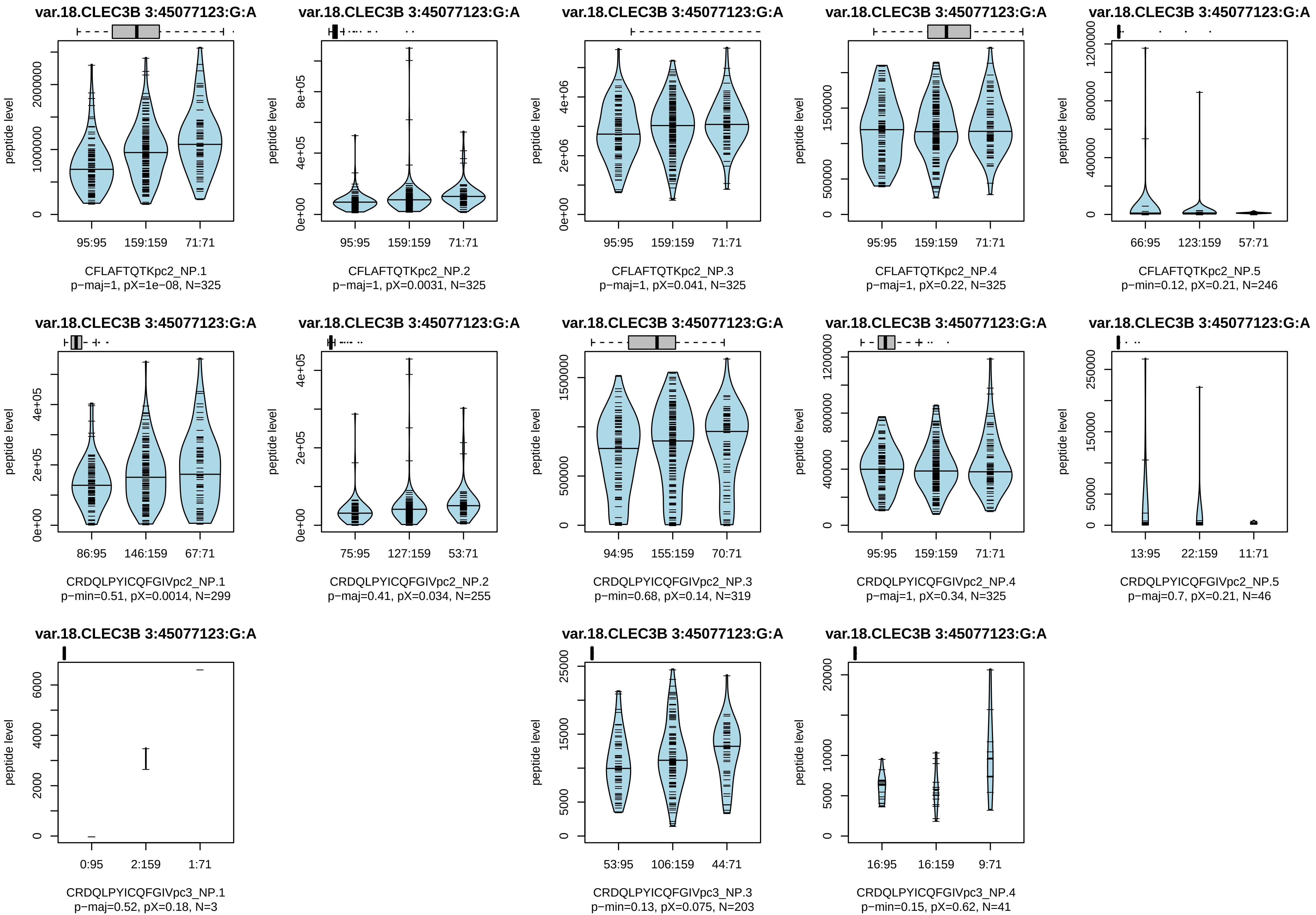
var.18.CLEC3B 3:45077123:G:A
peptide level
95:95 159:159 71:71
CFLAFTQTKpc2_NP.1
p−maj=1, pX=1e−08, N=325
var.18.CLEC3B 3:45077123:G:A
95:95 159:159 71:71
CFLAFTQTKpc2_NP.2
p−maj=1, pX=0.0031, N=325
var.18.CLEC3B 3:45077123:G:A
95:95 159:159 71:71
CFLAFTQTKpc2_NP.3
p−maj=1, pX=0.041, N=325
var.18.CLEC3B 3:45077123:G:A
95:95 159:159 71:71
CFLAFTQTKpc2_NP.4
p−maj=1, pX=0.22, N=325
var.18.CLEC3B 3:45077123:G:A
66:95 123:159 57:71
CFLAFTQTKpc2_NP.5
p−min=0.12, pX=0.21, N=246
var.18.CLEC3B 3:45077123:G:A
86:95 146:159 67:71
CRDQLPYICQFGIVpc2_NP.1
p−min=0.51, pX=0.0014, N=299
var.18.CLEC3B 3:45077123:G:A
75:95 127:159 53:71
CRDQLPYICQFGIVpc2_NP.2
p−maj=0.41, pX=0.034, N=255
var.18.CLEC3B 3:45077123:G:A
94:95 155:159 70:71
CRDQLPYICQFGIVpc2_NP.3
p−min=0.68, pX=0.14, N=319
var.18.CLEC3B 3:45077123:G:A
95:95 159:159 71:71
CRDQLPYICQFGIVpc2_NP.4
p−maj=1, pX=0.34, N=325
var.18.CLEC3B 3:45077123:G:A
13:95 22:159 11:71
CRDQLPYICQFGIVpc2_NP.5
p−maj=0.7, pX=0.21, N=46
var.18.CLEC3B 3:45077123:G:A
0:95 2:159 1:71
CRDQLPYICQFGIVpc3_NP.1
p−maj=0.52, pX=0.18, N=3
var.18.CLEC3B 3:45077123:G:A
53:95 106:159 44:71
CRDQLPYICQFGIVpc3_NP.3
p−min=0.13, pX=0.075, N=203
var.18.CLEC3B 3:45077123:G:A
16:95 16:159 9:71
CRDQLPYICQFGIVpc3_NP.4
p−min=0.15, pX=0.62, N=41

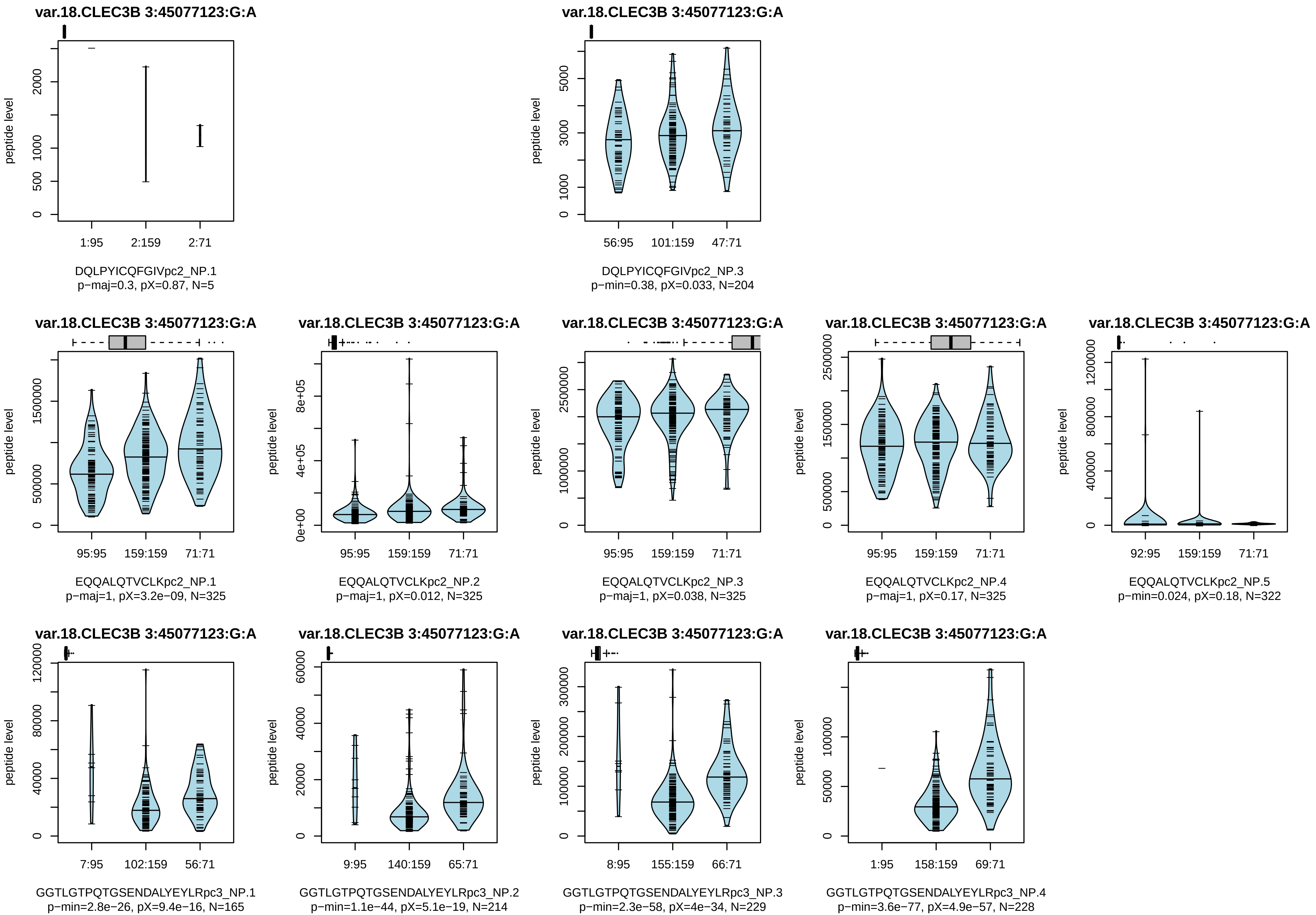
var.18.CLEC3B 3:45077123:G:A
peptide level
1:95
2:159
2:71
DQLPYICQFGIVpc2_NP.1
p−maj=0.3, pX=0.87, N=5
var.18.CLEC3B 3:45077123:G:A
peptide level
56:95
101:159
47:71
DQLPYICQFGIVpc2_NP.3
p−min=0.38, pX=0.033, N=204
var.18.CLEC3B 3:45077123:G:A
peptide level
95:95
159:159
71:71
EQQALQTVCLKpc2_NP.1
p−maj=1, pX=3.2e−09, N=325
var.18.CLEC3B 3:45077123:G:A
peptide level
95:95
159:159
71:71
EQQALQTVCLKpc2_NP.2
p−maj=1, pX=0.012, N=325
var.18.CLEC3B 3:45077123:G:A
peptide level
95:95
159:159
71:71
EQQALQTVCLKpc2_NP.3
p−maj=1, pX=0.038, N=325
var.18.CLEC3B 3:45077123:G:A
peptide level
95:95
159:159
71:71
EQQALQTVCLKpc2_NP.4
p−maj=1, pX=0.17, N=325
var.18.CLEC3B 3:45077123:G:A
peptide level
92:95
159:159
71:71
EQQALQTVCLKpc2_NP.5
p−min=0.024, pX=0.18, N=322
var.18.CLEC3B 3:45077123:G:A
peptide level
7:95
102:159
56:71
GGTLGTPQTGSENDALYEYLRpc3_NP.1
p−min=2.8e−26, pX=9.4e−16, N=165
var.18.CLEC3B 3:45077123:G:A
peptide level
9:95
140:159
65:71
GGTLGTPQTGSENDALYEYLRpc3_NP.2
p−min=1.1e−44, pX=5.1e−19, N=214
var.18.CLEC3B 3:45077123:G:A
peptide level
8:95
155:159
66:71
GGTLGTPQTGSENDALYEYLRpc3_NP.3
p−min=2.3e−58, pX=4e−34, N=229
var.18.CLEC3B 3:45077123:G:A
peptide level
1:95
158:159
69:71
GGTLGTPQTGSENDALYEYLRpc3_NP.4
p−min=3.6e−77, pX=4.9e−57, N=228

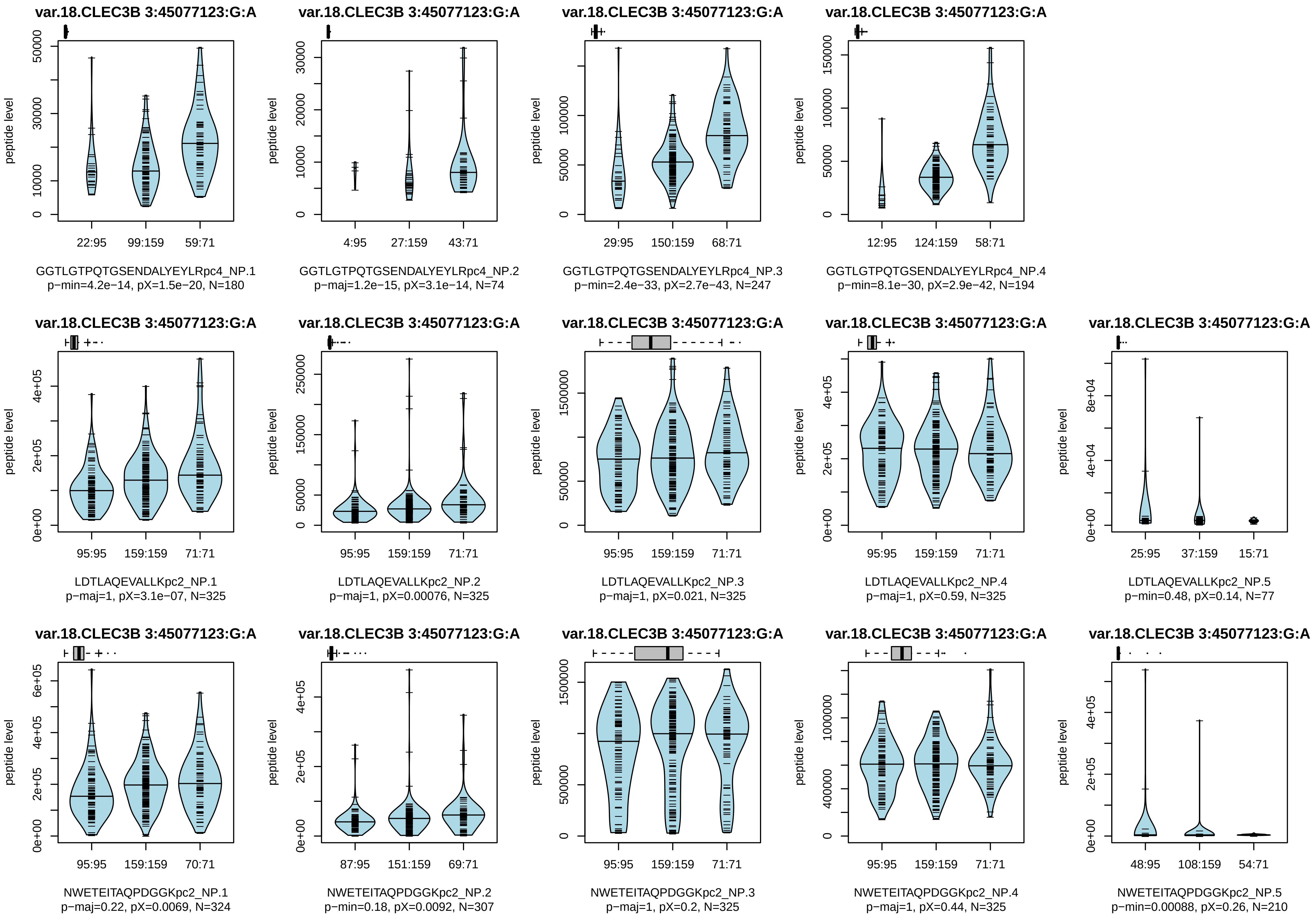

var.18.CLEC3B 3:45077123:G:A
peptide level
22:95
99:159
59:71
GGTLGTPQTGSENDALYEYLRpc4_NP.1
p−min=4.2e−14, pX=1.5e−20, N=180
var.18.CLEC3B 3:45077123:G:A
peptide level
4:95
27:159
43:71
GGTLGTPQTGSENDALYEYLRpc4_NP.2
p−maj=1.2e−15, pX=3.1e−14, N=74
var.18.CLEC3B 3:45077123:G:A
peptide level
29:95
150:159
68:71
GGTLGTPQTGSENDALYEYLRpc4_NP.3
p−min=2.4e−33, pX=2.7e−43, N=247
var.18.CLEC3B 3:45077123:G:A
peptide level
12:95
124:159
58:71
GGTLGTPQTGSENDALYEYLRpc4_NP.4
p−min=8.1e−30, pX=2.9e−42, N=194
var.18.CLEC3B 3:45077123:G:A
peptide level
95:95
159:159
71:71
LDTLAQEVALLKpc2_NP.1
p−maj=1, pX=3.1e−07, N=325
var.18.CLEC3B 3:45077123:G:A
peptide level
95:95
159:159
71:71
LDTLAQEVALLKpc2_NP.2
p−maj=1, pX=0.00076, N=325
var.18.CLEC3B 3:45077123:G:A
peptide level
95:95
159:159
71:71
LDTLAQEVALLKpc2_NP.3
p−maj=1, pX=0.021, N=325
var.18.CLEC3B 3:45077123:G:A
peptide level
95:95
159:159
71:71
LDTLAQEVALLKpc2_NP.4
p−maj=1, pX=0.59, N=325
var.18.CLEC3B 3:45077123:G:A
peptide level
25:95
37:159
15:71
LDTLAQEVALLKpc2_NP.5
p−min=0.48, pX=0.14, N=77
var.18.CLEC3B 3:45077123:G:A
peptide level
95:95
159:159
70:71
NWETEITAQPDGGKpc2_NP.1
p−maj=0.22, pX=0.0069, N=324
var.18.CLEC3B 3:45077123:G:A
peptide level
87:95
151:159
69:71
NWETEITAQPDGGKpc2_NP.2
p−min=0.18, pX=0.0092, N=307
var.18.CLEC3B 3:45077123:G:A
peptide level
95:95
159:159
71:71
NWETEITAQPDGGKpc2_NP.3
p−maj=1, pX=0.2, N=325
var.18.CLEC3B 3:45077123:G:A
peptide level
95:95
159:159
71:71
NWETEITAQPDGGKpc2_NP.4
p−maj=1, pX=0.44, N=325
var.18.CLEC3B 3:45077123:G:A
peptide level
48:95
108:159
54:71
NWETEITAQPDGGKpc2_NP.5
p−min=0.00088, pX=0.26, N=210

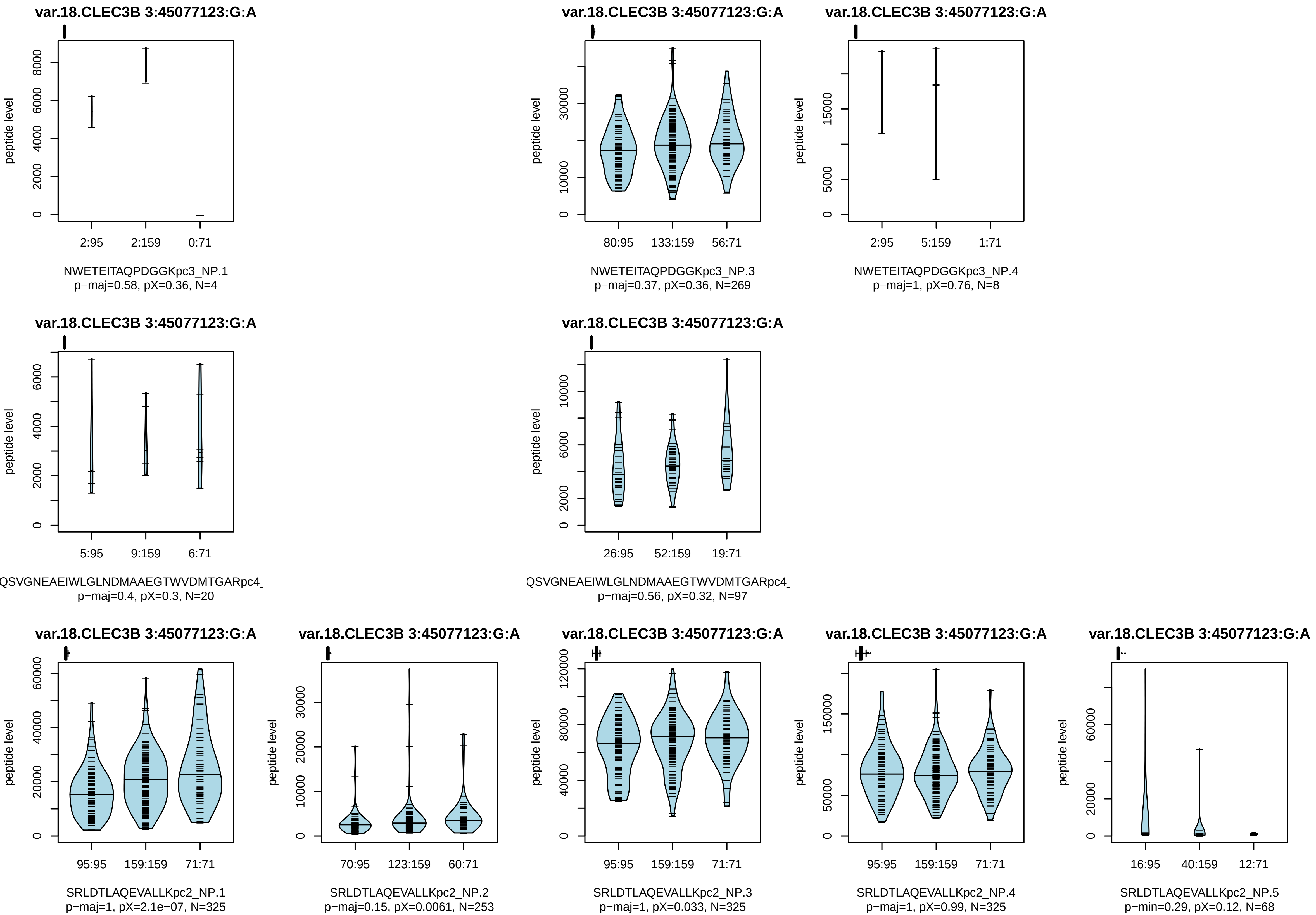
var.18.CLEC3B 3:45077123:G:A
peptide level
0
2000
4000
6000
8000
2:95
2:159
0:71
NWETEITAQPDGGKpc3_NP.1
p−maj=0.58, pX=0.36, N=4
var.18.CLEC3B 3:45077123:G:A
peptide level
0
10000
30000
80:95
133:159
56:71
NWETEITAQPDGGKpc3_NP.3
p−maj=0.37, pX=0.36, N=269
var.18.CLEC3B 3:45077123:G:A
peptide level
0
5000
15000
2:95
5:159
1:71
NWETEITAQPDGGKpc3_NP.4
p−maj=1, pX=0.76, N=8
var.18.CLEC3B 3:45077123:G:A
peptide level
0
2000
4000
6000
5:95
9:159
6:71
QSVGNEAEIWLGLNDMAAEGTWVDMTGARpc4_
p−maj=0.4, pX=0.3, N=20
var.18.CLEC3B 3:45077123:G:A
peptide level
0
2000
6000
10000
26:95
52:159
19:71
QSVGNEAEIWLGLNDMAAEGTWVDMTGARpc4_
p−maj=0.56, pX=0.32, N=97
var.18.CLEC3B 3:45077123:G:A
peptide level
0
20000
40000
60000
95:95
159:159
71:71
SRLDTLAQEVALLKpc2_NP.1
p−maj=1, pX=2.1e−07, N=325
var.18.CLEC3B 3:45077123:G:A
peptide level
0
10000
20000
30000
70:95
123:159
60:71
SRLDTLAQEVALLKpc2_NP.2
p−maj=0.15, pX=0.0061, N=253
var.18.CLEC3B 3:45077123:G:A
peptide level
0
40000
80000
120000
95:95
159:159
71:71
SRLDTLAQEVALLKpc2_NP.3
p−maj=1, pX=0.033, N=325
var.18.CLEC3B 3:45077123:G:A
peptide level
0
50000
150000
95:95
159:159
71:71
SRLDTLAQEVALLKpc2_NP.4
p−maj=1, pX=0.99, N=325
var.18.CLEC3B 3:45077123:G:A
peptide level
0
20000
60000
16:95
40:159
12:71
SRLDTLAQEVALLKpc2_NP.5
p−min=0.29, pX=0.12, N=68

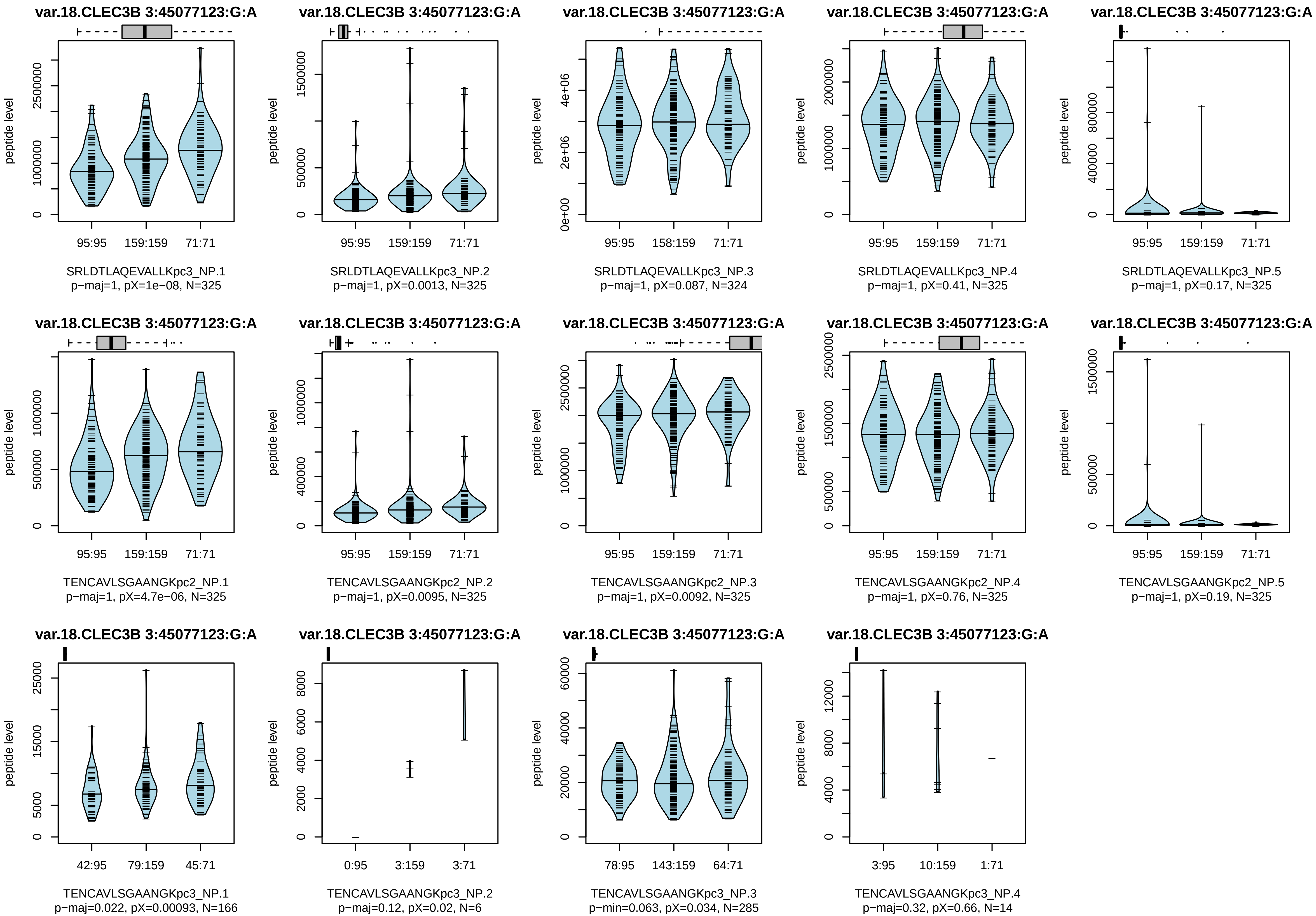
var.18.CLEC3B 3:45077123:G:A
peptide level
95:95
159:159
71:71
SRLDTLAQEVALLKpc3_NP.1
p−maj=1, pX=1e−08, N=325
var.18.CLEC3B 3:45077123:G:A
peptide level
95:95
159:159
71:71
SRLDTLAQEVALLKpc3_NP.2
p−maj=1, pX=0.0013, N=325
var.18.CLEC3B 3:45077123:G:A
peptide level
95:95
158:159
71:71
SRLDTLAQEVALLKpc3_NP.3
p−maj=1, pX=0.087, N=324
var.18.CLEC3B 3:45077123:G:A
peptide level
95:95
159:159
71:71
SRLDTLAQEVALLKpc3_NP.4
p−maj=1, pX=0.41, N=325
var.18.CLEC3B 3:45077123:G:A
peptide level
95:95
159:159
71:71
SRLDTLAQEVALLKpc3_NP.5
p−maj=1, pX=0.17, N=325
var.18.CLEC3B 3:45077123:G:A
peptide level
95:95
159:159
71:71
TENCAVLSGAANGKpc2_NP.1
p−maj=1, pX=4.7e−06, N=325
var.18.CLEC3B 3:45077123:G:A
peptide level
95:95
159:159
71:71
TENCAVLSGAANGKpc2_NP.2
p−maj=1, pX=0.0095, N=325
var.18.CLEC3B 3:45077123:G:A
peptide level
95:95
159:159
71:71
TENCAVLSGAANGKpc2_NP.3
p−maj=1, pX=0.0092, N=325
var.18.CLEC3B 3:45077123:G:A
peptide level
95:95
159:159
71:71
TENCAVLSGAANGKpc2_NP.4
p−maj=1, pX=0.76, N=325
var.18.CLEC3B 3:45077123:G:A
peptide level
95:95
159:159
71:71
TENCAVLSGAANGKpc2_NP.5
p−maj=1, pX=0.19, N=325
var.18.CLEC3B 3:45077123:G:A
peptide level
42:95
79:159
45:71
TENCAVLSGAANGKpc3_NP.1
p−maj=0.022, pX=0.00093, N=166
var.18.CLEC3B 3:45077123:G:A
peptide level
0:95
3:159
3:71
TENCAVLSGAANGKpc3_NP.2
p−maj=0.12, pX=0.02, N=6
var.18.CLEC3B 3:45077123:G:A
peptide level
78:95
143:159
64:71
TENCAVLSGAANGKpc3_NP.3
p−min=0.063, pX=0.034, N=285
var.18.CLEC3B 3:45077123:G:A
peptide level
3:95
10:159
1:71
TENCAVLSGAANGKpc3_NP.4
p−maj=0.32, pX=0.66, N=14

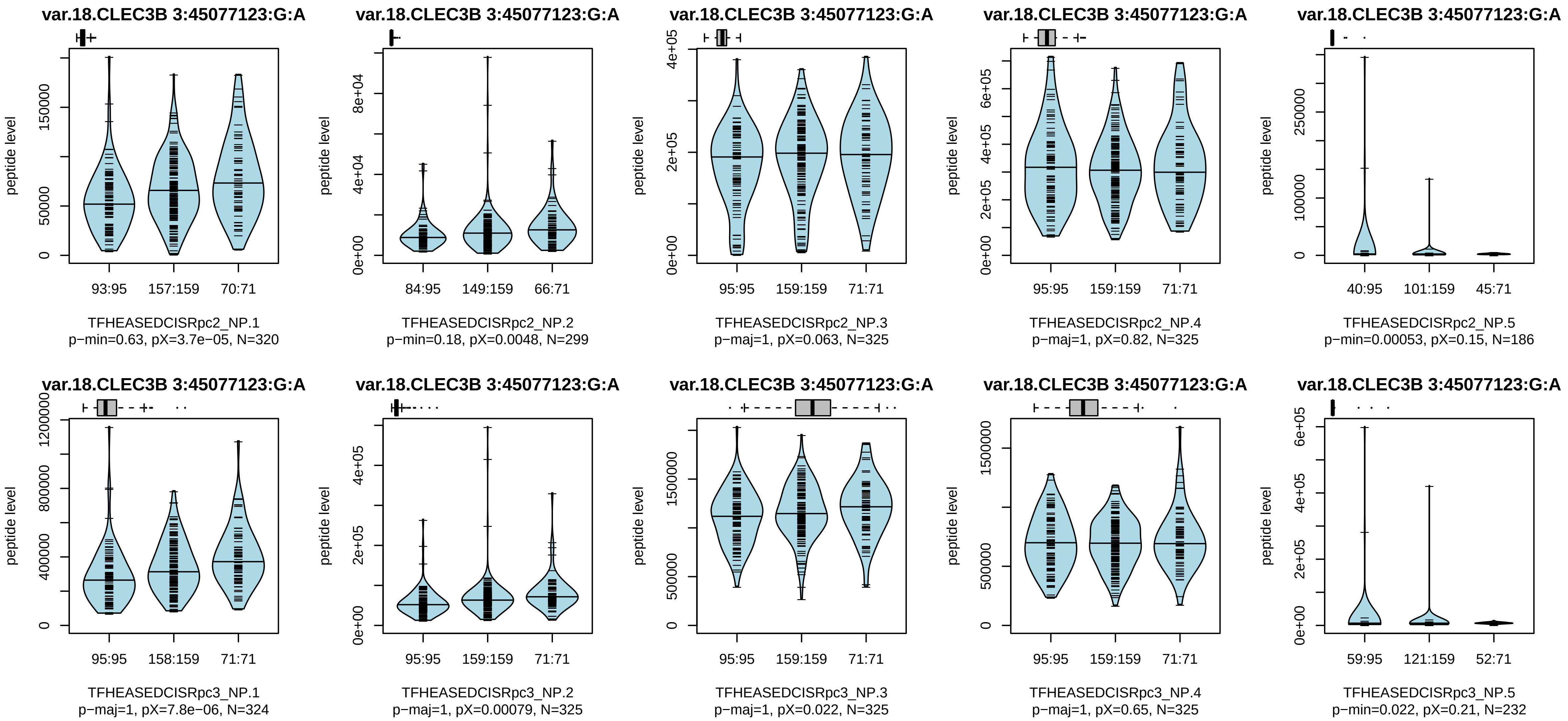

var.18.CLEC3B 3:45077123:G:A
peptide level
93:95
157:159
70:71
TFHEASEDCISRpc2_NP.1
p−min=0.63, pX=3.7e−05, N=320
var.18.CLEC3B 3:45077123:G:A
peptide level
84:95
149:159
66:71
TFHEASEDCISRpc2_NP.2
p−min=0.18, pX=0.0048, N=299
var.18.CLEC3B 3:45077123:G:A
peptide level
95:95
159:159
71:71
TFHEASEDCISRpc2_NP.3
p−maj=1, pX=0.063, N=325
var.18.CLEC3B 3:45077123:G:A
peptide level
95:95
159:159
71:71
TFHEASEDCISRpc2_NP.4
p−maj=1, pX=0.82, N=325
var.18.CLEC3B 3:45077123:G:A
peptide level
40:95
101:159
45:71
TFHEASEDCISRpc2_NP.5
p−min=0.00053, pX=0.15, N=186
var.18.CLEC3B 3:45077123:G:A
peptide level
95:95
158:159
71:71
TFHEASEDCISRpc3_NP.1
p−maj=1, pX=7.8e−06, N=324
var.18.CLEC3B 3:45077123:G:A
peptide level
95:95
159:159
71:71
TFHEASEDCISRpc3_NP.2
p−maj=1, pX=0.00079, N=325
var.18.CLEC3B 3:45077123:G:A
peptide level
95:95
159:159
71:71
TFHEASEDCISRpc3_NP.3
p−maj=1, pX=0.022, N=325
var.18.CLEC3B 3:45077123:G:A
peptide level
95:95
159:159
71:71
TFHEASEDCISRpc3_NP.4
p−maj=1, pX=0.65, N=325
var.18.CLEC3B 3:45077123:G:A
peptide level
59:95
121:159
52:71
TFHEASEDCISRpc3_NP.5
p−min=0.022, pX=0.21, N=232

var.19.FUCA2 6:143823112:G:A

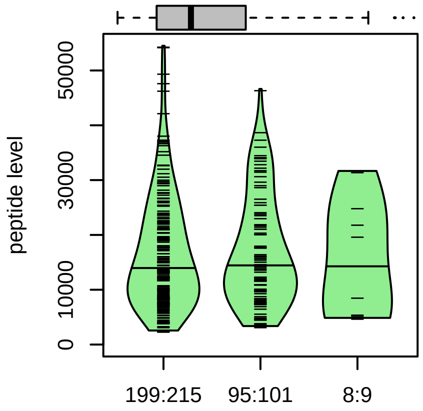

AILGATEVKpc2_NP.2
p−maj=0.49, pX=1, N=302

var.19.FUCA2 6:143823112:G:A

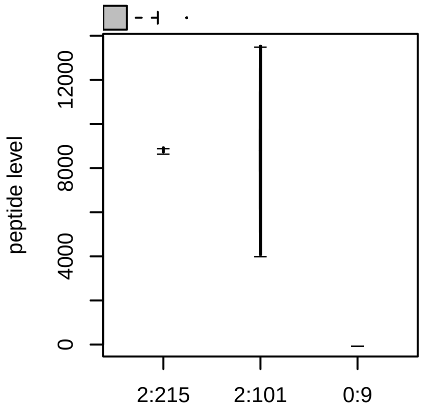

AILGATEVKpc2_NP.5
p−min=0.61, pX=0.64, N=4

var.19.FUCA2 6:143823112:G:A

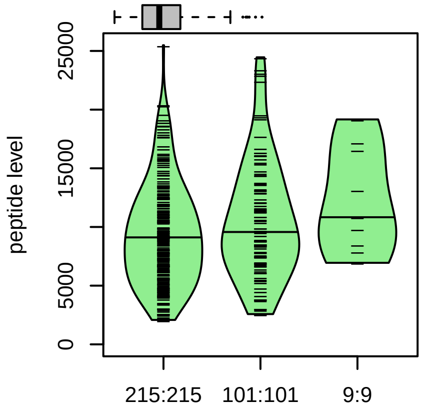

EAGISDYLTIEELVKpc2_NP.2
p−maj=1, pX=0.0086, N=325

var.19.FUCA2 6:143823112:G:A

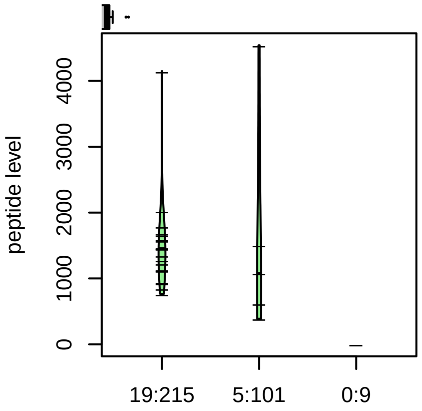

EAGISDYLTIEELVKpc2_NP.4
p−min=0.19, pX=0.25, N=24

var.19.FUCA2 6:143823112:G:A

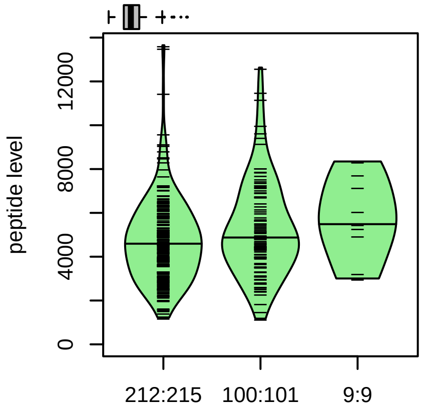

EAGISDYLTIEELVKpc2_NP.5
p−maj=1, pX=0.0095, N=321

var.19.FUCA2 6:143823112:G:A

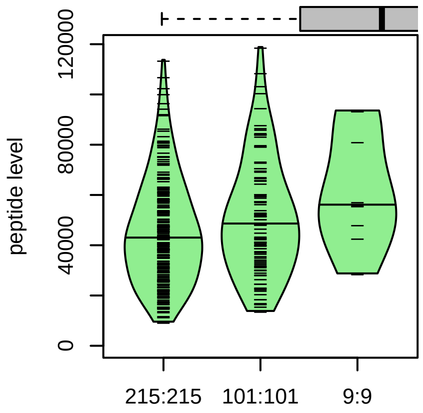

FDPTWESLDARpc2_NP.2
p−maj=1, pX=0.0072, N=325

var.19.FUCA2 6:143823112:G:A

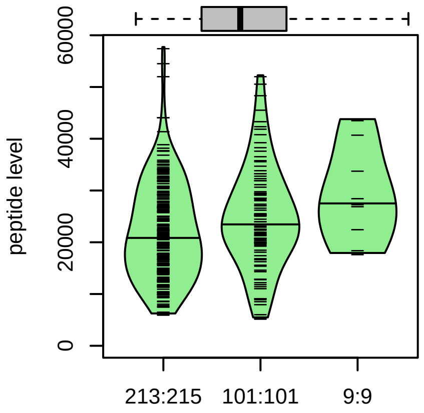

FDPTWESLDARpc2_NP.5
p−min=0.55, pX=0.0019, N=323

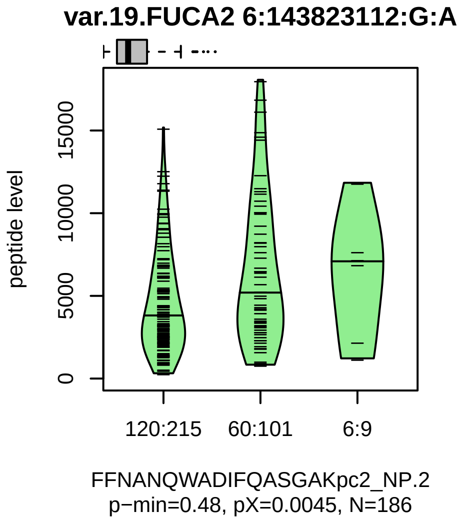
var.19.FUCA2 6:143823112:G:A
peptide level
0
5000
10000
15000
120:215
60:101
6:9
FFNANQWADIFQASGAKpc2_NP.2
p−min=0.48, pX=0.0045, N=186

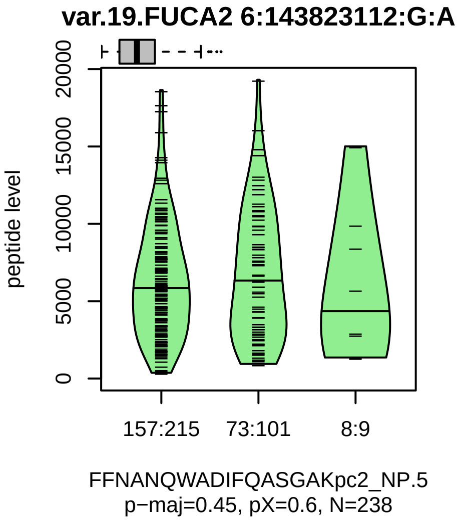
var.19.FUCA2 6:143823112:G:A
peptide level
0
5000
10000
15000
20000
157:215
73:101
8:9
FFNANQWADIFQASGAKpc2_NP.5
p−maj=0.45, pX=0.6, N=238

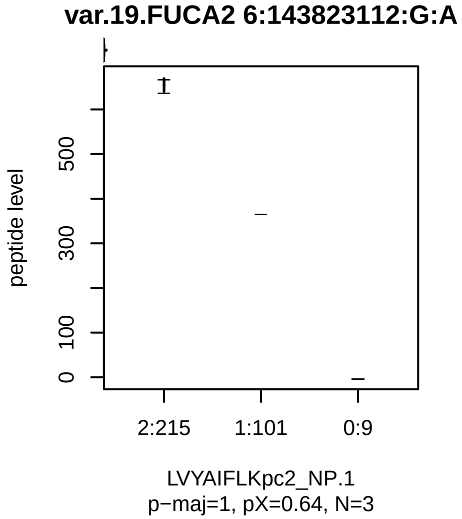
var.19.FUCA2 6:143823112:G:A
peptide level
0
100
300
500
2:215
1:101
0:9
LVYAIFLKpc2_NP.1
p−maj=1, pX=0.64, N=3

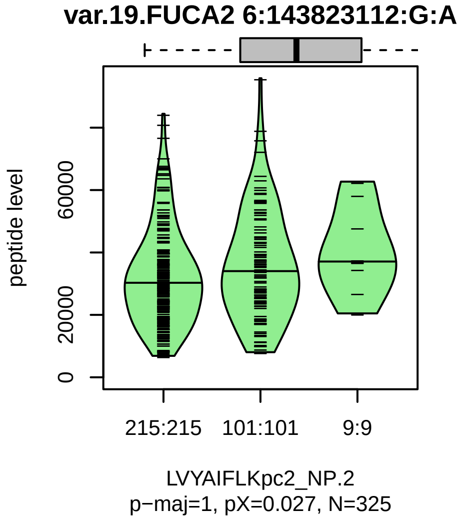
var.19.FUCA2 6:143823112:G:A
peptide level
0
20000
60000
215:215
101:101
9:9
LVYAIFLKpc2_NP.2
p−maj=1, pX=0.027, N=325

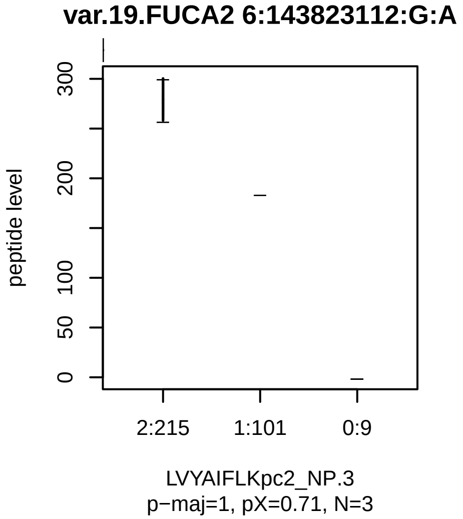
var.19.FUCA2 6:143823112:G:A
peptide level
0
50
100
200
300
2:215
1:101
0:9
LVYAIFLKpc2_NP.3
p−maj=1, pX=0.71, N=3

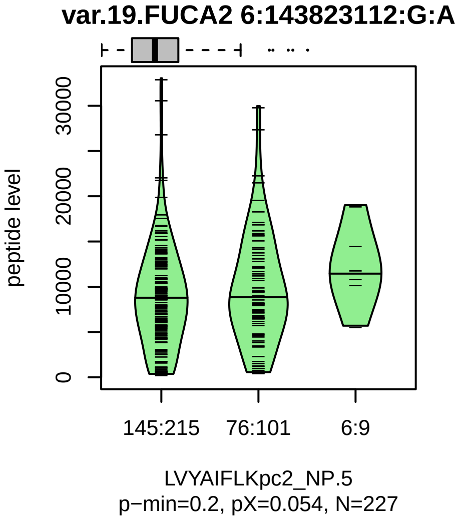
var.19.FUCA2 6:143823112:G:A
peptide level
0
10000
20000
30000
145:215
76:101
6:9
LVYAIFLKpc2_NP.5
p−min=0.2, pX=0.054, N=227

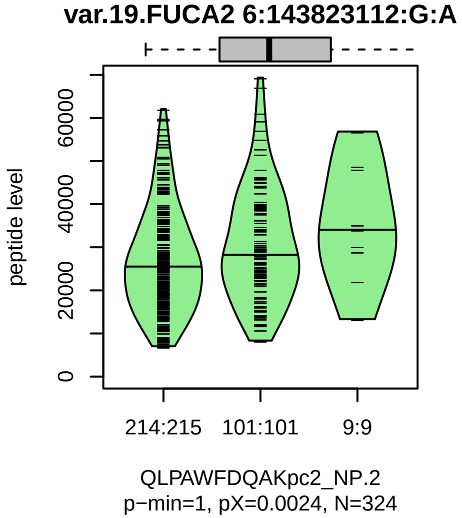
var.19.FUCA2 6:143823112:G:A
peptide level
0
20000
40000
60000
214:215
101:101
9:9
QLPAWFDQAKpc2_NP.2
p−min=1, pX=0.0024, N=324

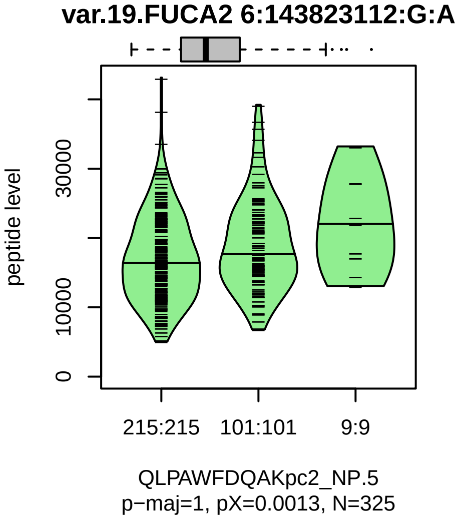
var.19.FUCA2 6:143823112:G:A
peptide level
0
10000
30000
215:215
101:101
9:9
QLPAWFDQAKpc2_NP.5
p−maj=1, pX=0.0013, N=325

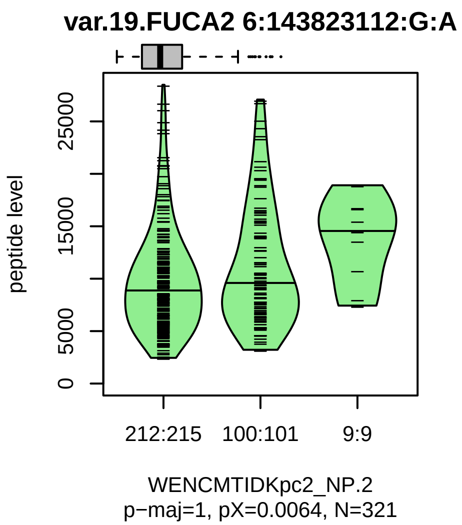
var.19.FUCA2 6:143823112:G:A
peptide level
0
5000
15000
25000
212:215
100:101
9:9
WENCMTIDKpc2_NP.2
p−maj=1, pX=0.0064, N=321

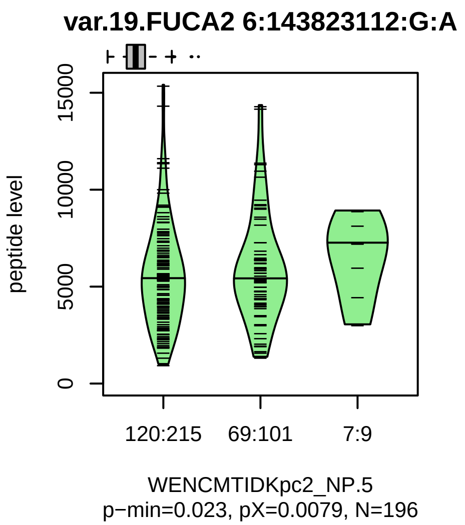
var.19.FUCA2 6:143823112:G:A
peptide level
0
5000
10000
15000
120:215
69:101
7:9
WENCMTIDKpc2_NP.5
p−min=0.023, pX=0.0079, N=196

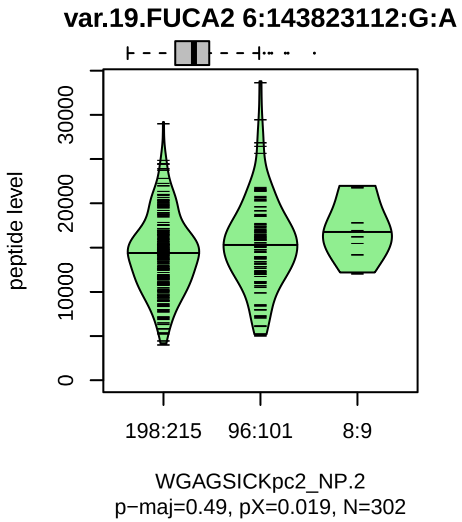
var.19.FUCA2 6:143823112:G:A
peptide level
0
10000
20000
30000
198:215
96:101
8:9
WGAGSICKpc2_NP.2
p−maj=0.49, pX=0.019, N=302

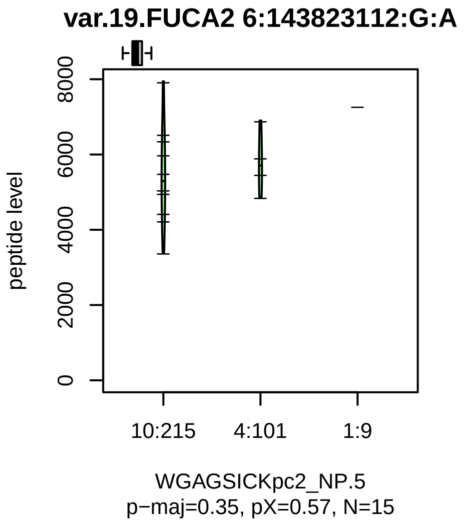
var.19.FUCA2 6:143823112:G:A
peptide level
0
2000
4000
6000
8000
10:215
4:101
1:9
WGAGSICKpc2_NP.5
p−maj=0.35, pX=0.57, N=15

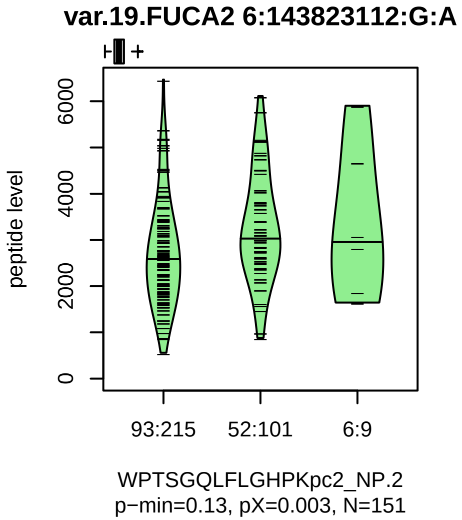
var.19.FUCA2 6:143823112:G:A
peptide level
0
2000
4000
6000
93:215
52:101
6:9
WPTSGQLFLGHPKpc2_NP.2
p−min=0.13, pX=0.003, N=151

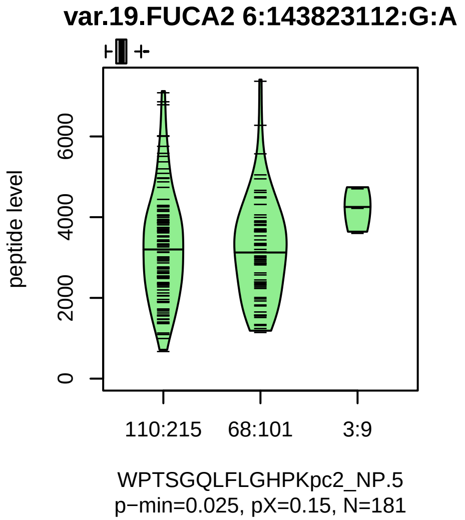
var.19.FUCA2 6:143823112:G:A
peptide level
0
2000
4000
6000
110:215
68:101
3:9
WPTSGQLFLGHPKpc2_NP.5
p−min=0.025, pX=0.15, N=181

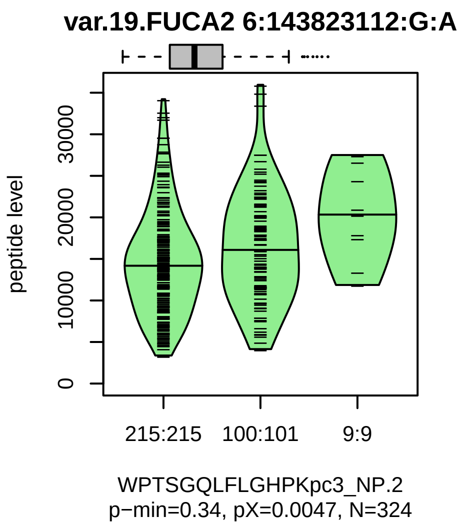
var.19.FUCA2 6:143823112:G:A
peptide level
0
10000
20000
30000
215:215
100:101
9:9
WPTSGQLFLGHPKpc3_NP.2
p−min=0.34, pX=0.0047, N=324

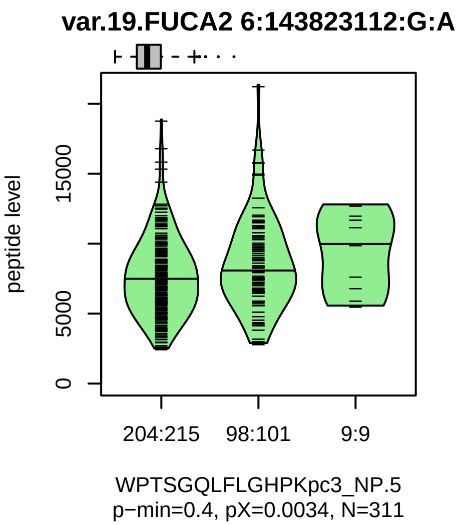
var.19.FUCA2 6:143823112:G:A
peptide level
0
5000
15000
204:215
98:101
9:9
WPTSGQLFLGHPKpc3_NP.5
p−min=0.4, pX=0.0034, N=311

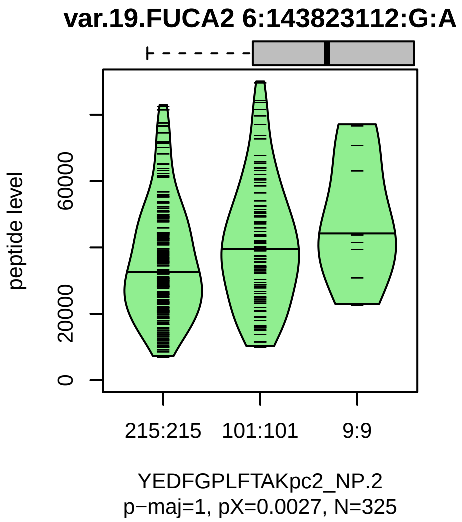
var.19.FUCA2 6:143823112:G:A
peptide level
0
20000
60000
215:215
101:101
9:9
YEDFGPLFTAKpc2_NP.2
p−maj=1, pX=0.0027, N=325

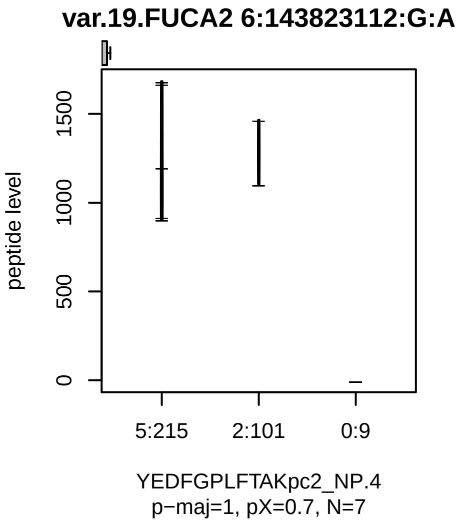
var.19.FUCA2 6:143823112:G:A
peptide level
0
500
1000
1500
5:215
2:101
0:9
YEDFGPLFTAKpc2_NP.4
p−maj=1, pX=0.7, N=7

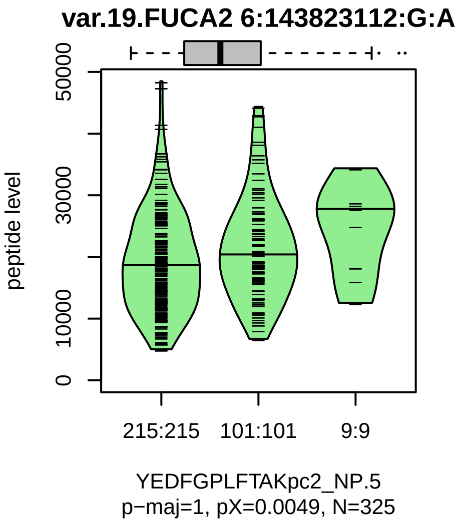
var.19.FUCA2 6:143823112:G:A
peptide level
0
10000
30000
50000
215:215
101:101
9:9
YEDFGPLFTAKpc2_NP.5
p−maj=1, pX=0.0049, N=325

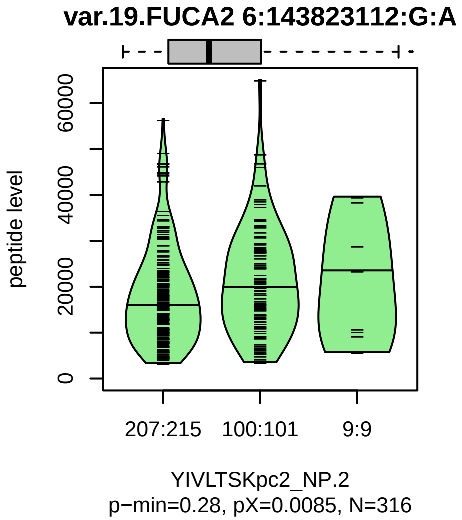
var.19.FUCA2 6:143823112:G:A
peptide level
0
20000
40000
60000
207:215
100:101
9:9
YIVLTSKpc2_NP.2
p−min=0.28, pX=0.0085, N=316

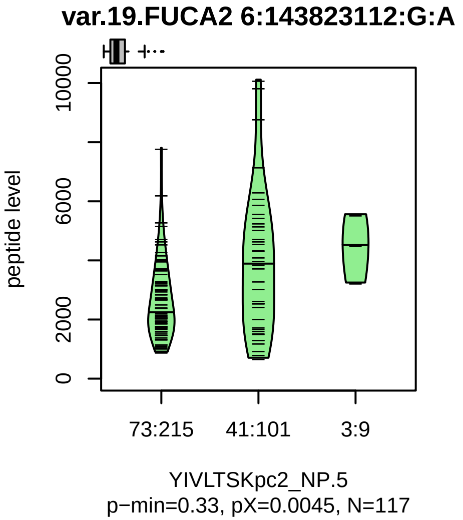
var.19.FUCA2 6:143823112:G:A
peptide level
0
2000
6000
10000
73:215
41:101
3:9
YIVLTSKpc2_NP.5
p−min=0.33, pX=0.0045, N=117

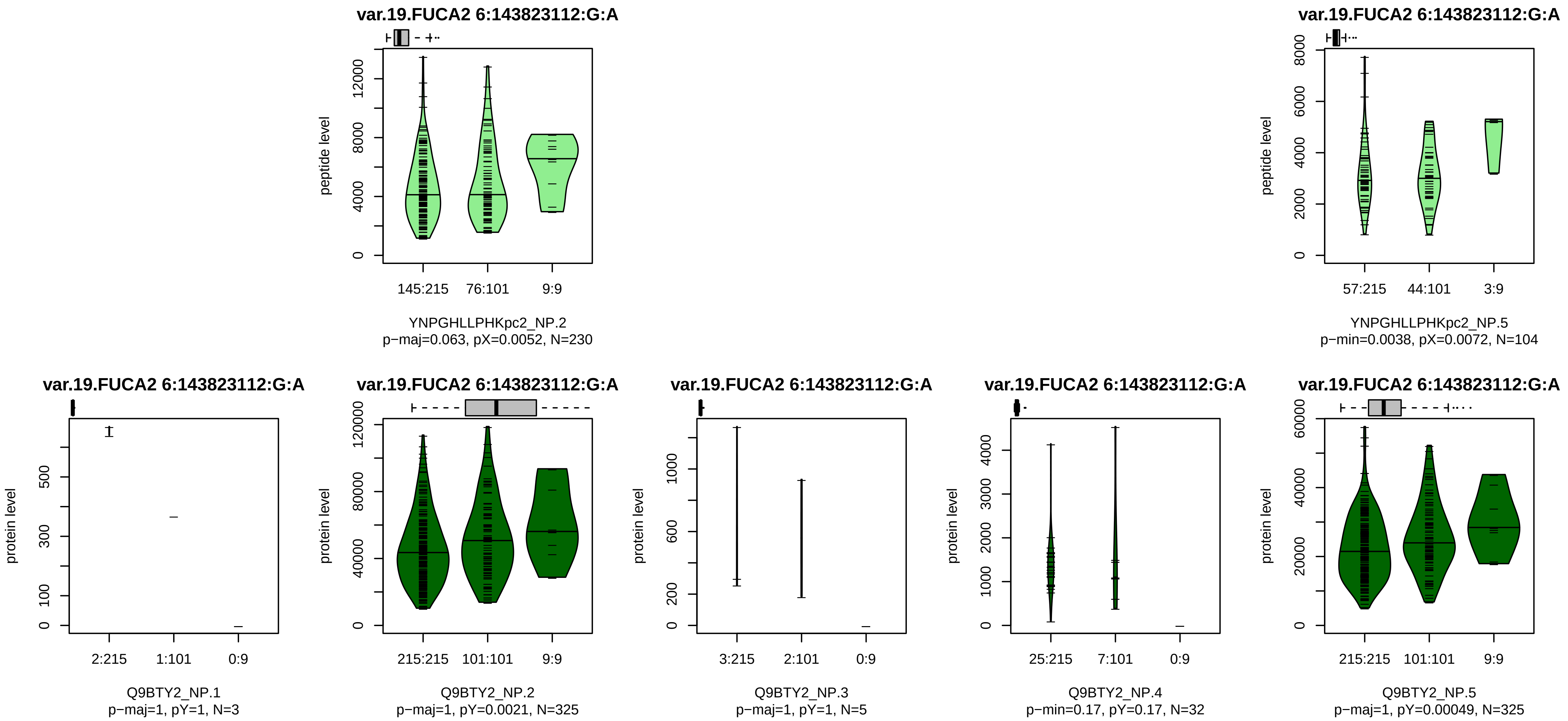
var.19.FUCA2 6:143823112:G:A
peptide level
0
4000
8000
12000
145:215
76:101
9:9
YNPGHLLPHKpc2_NP.2
p−maj=0.063, pX=0.0052, N=230
var.19.FUCA2 6:143823112:G:A
peptide level
0
2000
4000
6000
8000
57:215
44:101
3:9
YNPGHLLPHKpc2_NP.5
p−min=0.0038, pX=0.0072, N=104
var.19.FUCA2 6:143823112:G:A
protein level
0
100
300
500
2:215
1:101
0:9
Q9BTY2_NP.1
p−maj=1, pY=1, N=3
var.19.FUCA2 6:143823112:G:A
protein level
0
40000
80000
120000
215:215
101:101
9:9
Q9BTY2_NP.2
p−maj=1, pY=0.0021, N=325
var.19.FUCA2 6:143823112:G:A
protein level
0
200
600
1000
3:215
2:101
0:9
Q9BTY2_NP.3
p−maj=1, pY=1, N=5
var.19.FUCA2 6:143823112:G:A
protein level
0
1000
2000
3000
4000
25:215
7:101
0:9
Q9BTY2_NP.4
p−min=0.17, pY=0.17, N=32
var.19.FUCA2 6:143823112:G:A
protein level
0
20000
40000
60000
215:215
101:101
9:9
Q9BTY2_NP.5
p−maj=1, pY=0.00049, N=325

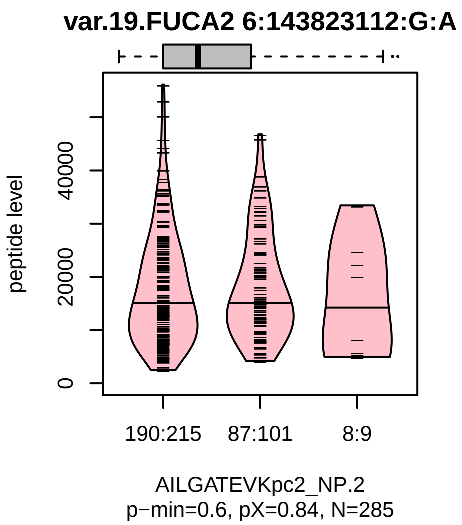
var.19.FUCA2 6:143823112:G:A
peptide level
0
20000
40000
190:215
87:101
8:9
AILGATEVKpc2_NP.2
p−min=0.6, pX=0.84, N=285

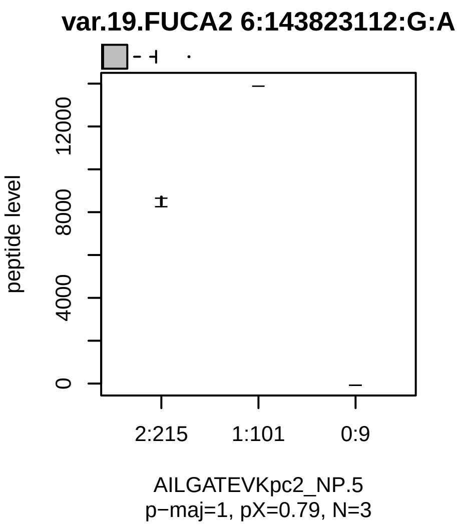
var.19.FUCA2 6:143823112:G:A
peptide level
0
4000
8000
12000
2:215
1:101
0:9
AILGATEVKpc2_NP.5
p−maj=1, pX=0.79, N=3

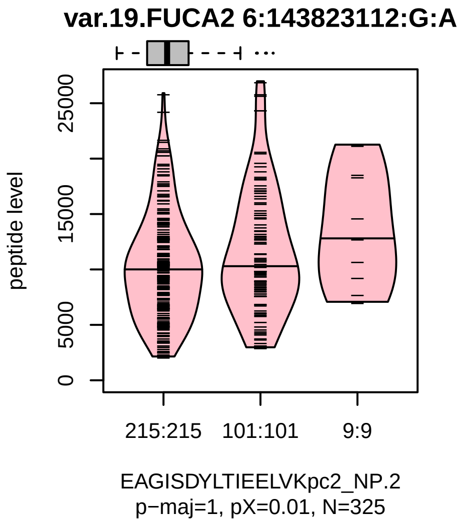
var.19.FUCA2 6:143823112:G:A
peptide level
0
5000
15000
25000
215:215
101:101
9:9
EAGISDYLTIEELVKpc2_NP.2
p−maj=1, pX=0.01, N=325

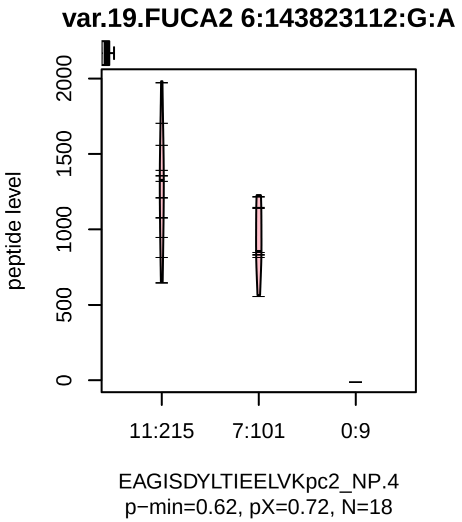
var.19.FUCA2 6:143823112:G:A
peptide level
0
500
1000
1500
2000
11:215
7:101
0:9
EAGISDYLTIEELVKpc2_NP.4
p−min=0.62, pX=0.72, N=18

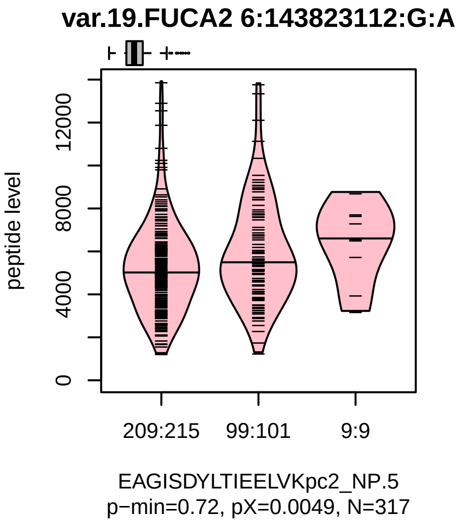
var.19.FUCA2 6:143823112:G:A
peptide level
0
4000
8000
12000
209:215
99:101
9:9
EAGISDYLTIEELVKpc2_NP.5
p−min=0.72, pX=0.0049, N=317

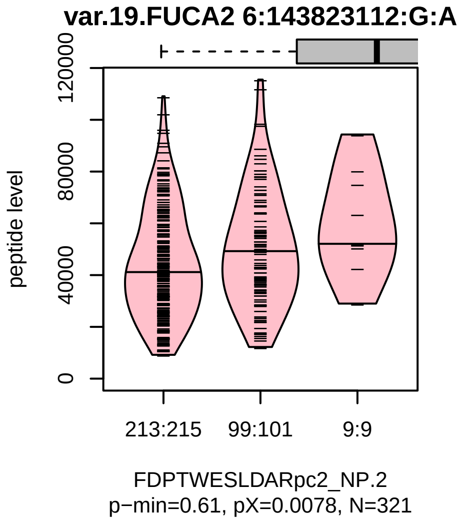
var.19.FUCA2 6:143823112:G:A
peptide level
0
40000
80000
120000
213:215
99:101
9:9
FDPTWESLDARpc2_NP.2
p−min=0.61, pX=0.0078, N=321

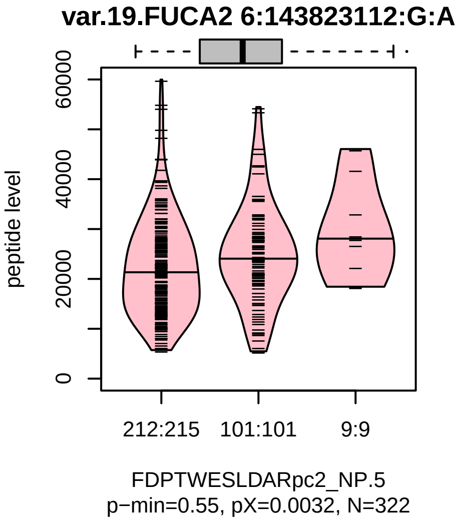
var.19.FUCA2 6:143823112:G:A
peptide level
0
20000
40000
60000
212:215
101:101
9:9
FDPTWESLDARpc2_NP.5
p−min=0.55, pX=0.0032, N=322

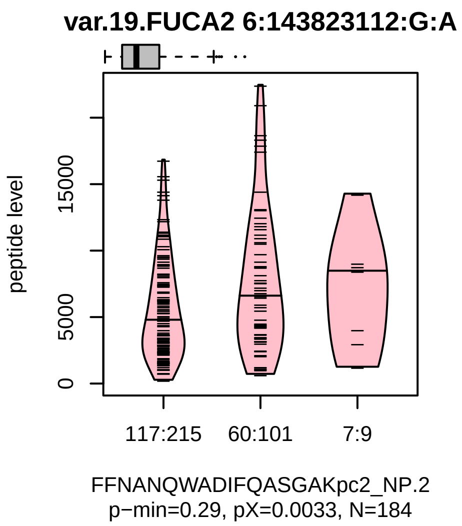
var.19.FUCA2 6:143823112:G:A
peptide level
0
5000
15000
117:215
60:101
7:9
FFNANQWADIFQASGAKpc2_NP.2
p−min=0.29, pX=0.0033, N=184

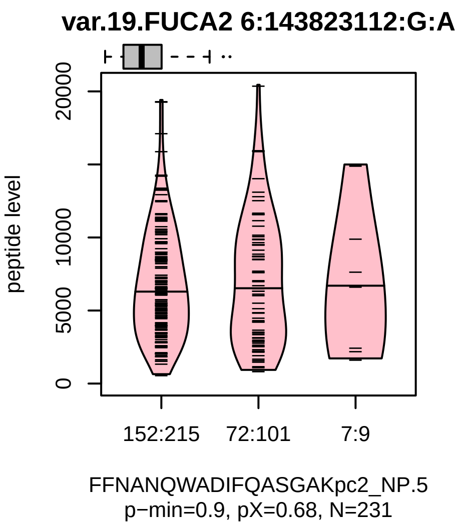
var.19.FUCA2 6:143823112:G:A
peptide level
0
5000
10000
20000
152:215
72:101
7:9
FFNANQWADIFQASGAKpc2_NP.5
p−min=0.9, pX=0.68, N=231

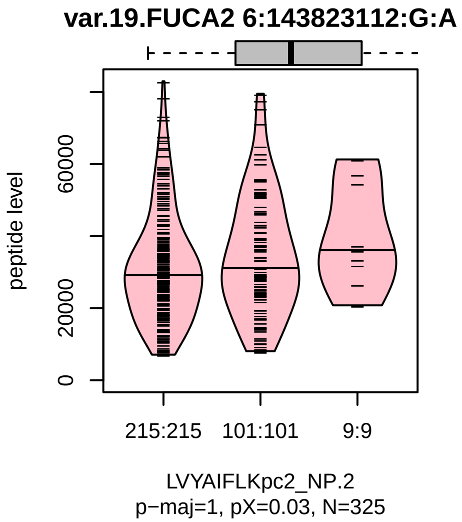
var.19.FUCA2 6:143823112:G:A
peptide level
0
20000
60000
215:215
101:101
9:9
LVYAIFLKpc2_NP.2
p−maj=1, pX=0.03, N=325

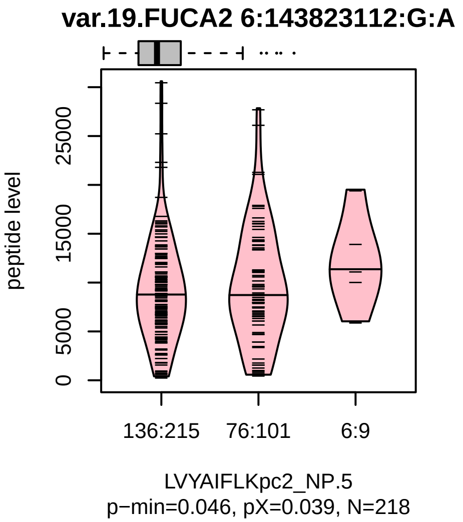
var.19.FUCA2 6:143823112:G:A
peptide level
0
5000
15000
25000
136:215
76:101
6:9
LVYAIFLKpc2_NP.5
p−min=0.046, pX=0.039, N=218

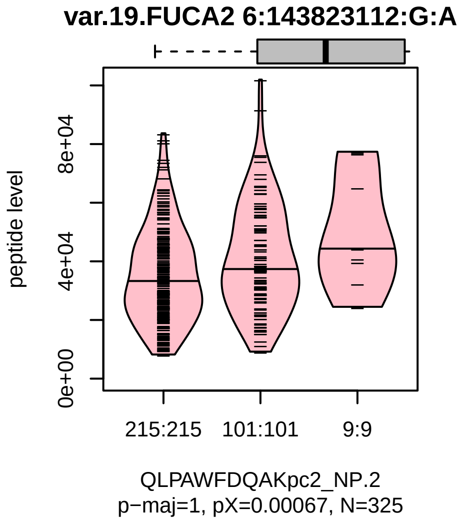
var.19.FUCA2 6:143823112:G:A
peptide level
0e+00
4e+04
8e+04
215:215
101:101
9:9
QLPAWFDQAKpc2_NP.2
p−maj=1, pX=0.00067, N=325

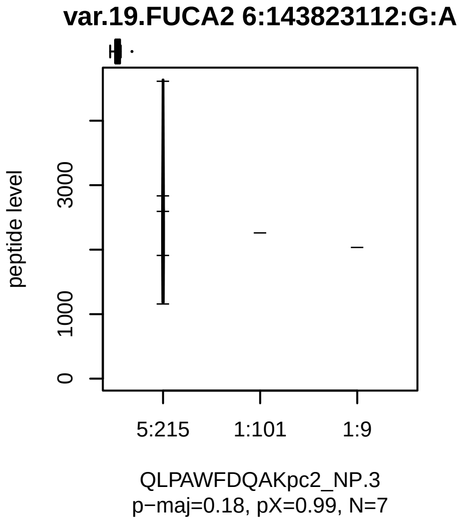
var.19.FUCA2 6:143823112:G:A
peptide level
0
1000
3000
5:215
1:101
1:9
QLPAWFDQAKpc2_NP.3
p−maj=0.18, pX=0.99, N=7

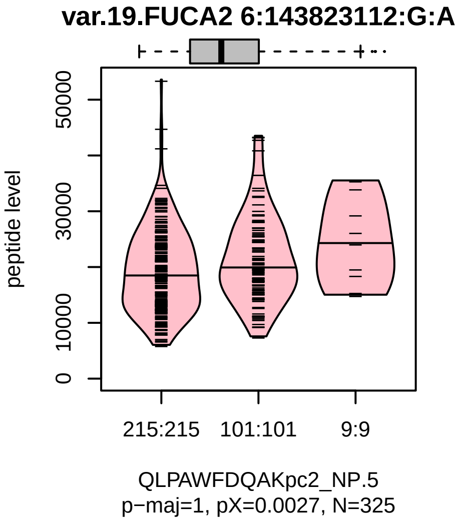
var.19.FUCA2 6:143823112:G:A
peptide level
0
10000
30000
50000
215:215
101:101
9:9
QLPAWFDQAKpc2_NP.5
p−maj=1, pX=0.0027, N=325

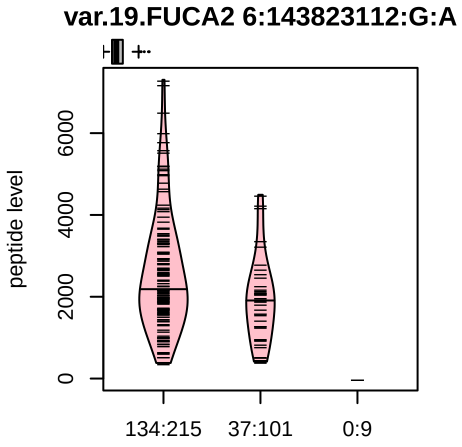

VNGEAIYETHTWRpc2_NP.2
p−min=1.2e−06, pX=4.7e−08, N=171

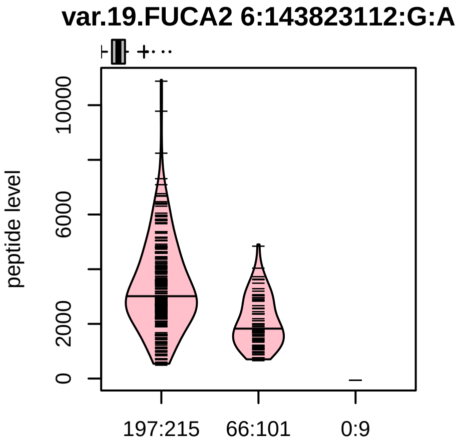

VNGEAIYETHTWRpc2_NP.5
p−min=3.6e−11, pX=8e−20, N=263

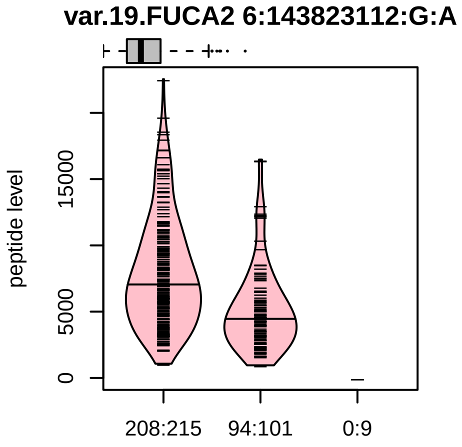

VNGEAIYETHTWRpc3_NP.2
p−maj=8.2e−12, pX=1.4e−13, N=302

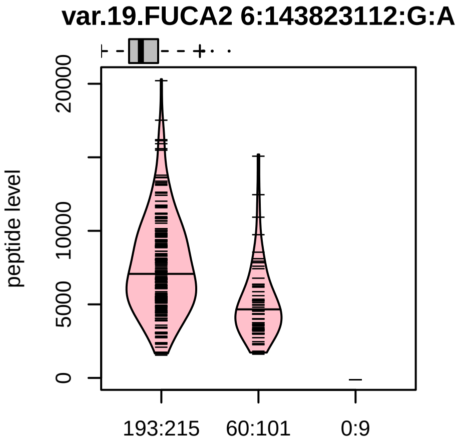

VNGEAIYETHTWRpc3_NP.5
p−min=3.1e−12, pX=5.5e−20, N=253

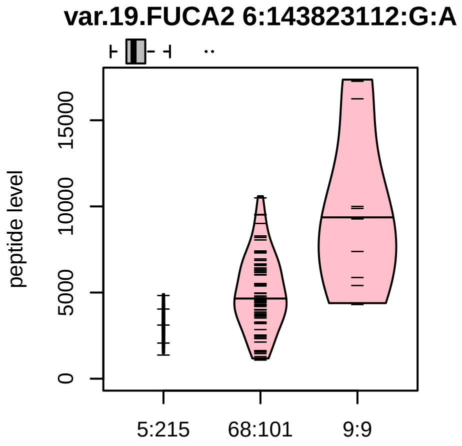

VNGEAIYETYTWRpc2_NP.2
p−min=1.7e−41, pX=1.6e−55, N=82

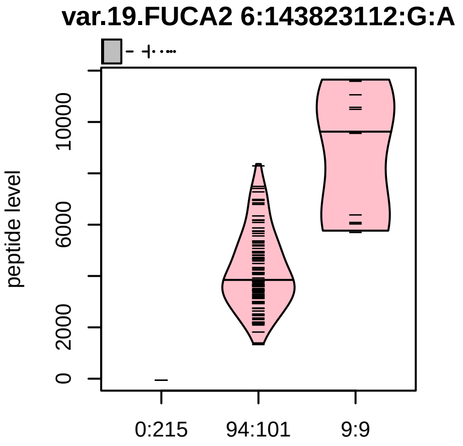

VNGEAIYETYTWRpc2_NP.5
p−min=4.7e−77, pX=4.8e−116, N=103

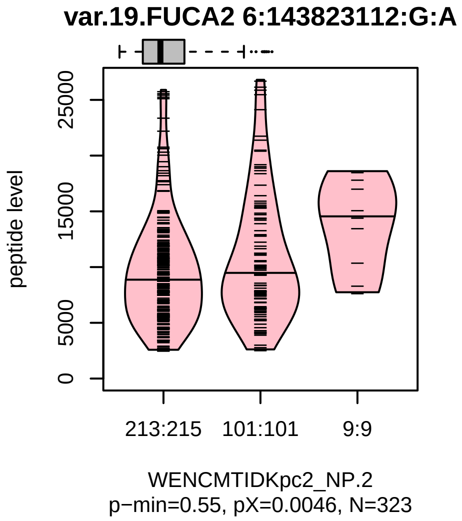
var.19.FUCA2 6:143823112:G:A
peptide level
0
5000
15000
25000
213:215
101:101
9:9
WENCMTIDKpc2_NP.2
p−min=0.55, pX=0.0046, N=323

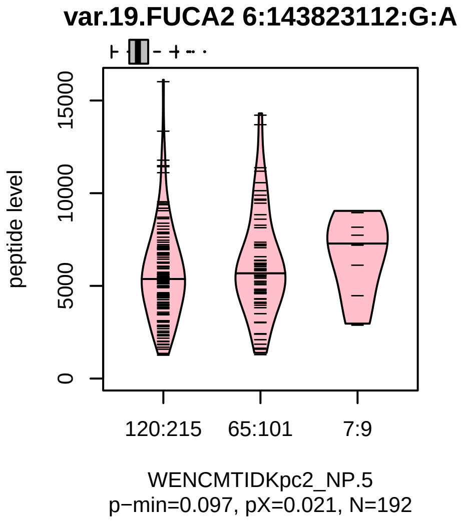
var.19.FUCA2 6:143823112:G:A
peptide level
0
5000
10000
15000
120:215
65:101
7:9
WENCMTIDKpc2_NP.5
p−min=0.097, pX=0.021, N=192

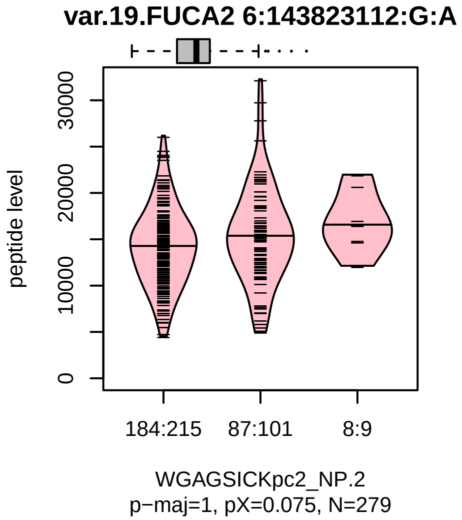
var.19.FUCA2 6:143823112:G:A
peptide level
0
10000
20000
30000
184:215
87:101
8:9
WGAGSICKpc2_NP.2
p−maj=1, pX=0.075, N=279

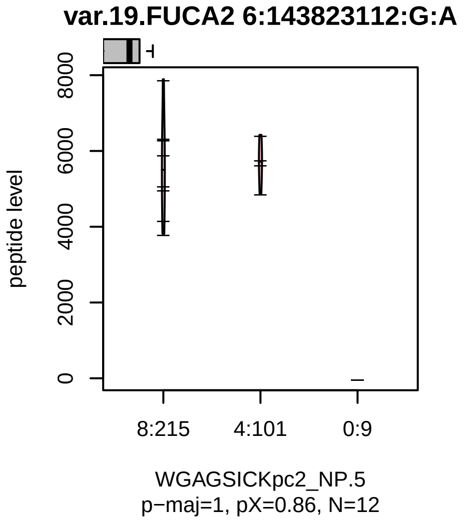
var.19.FUCA2 6:143823112:G:A
peptide level
0
2000
4000
6000
8000
8:215
4:101
0:9
WGAGSICKpc2_NP.5
p−maj=1, pX=0.86, N=12

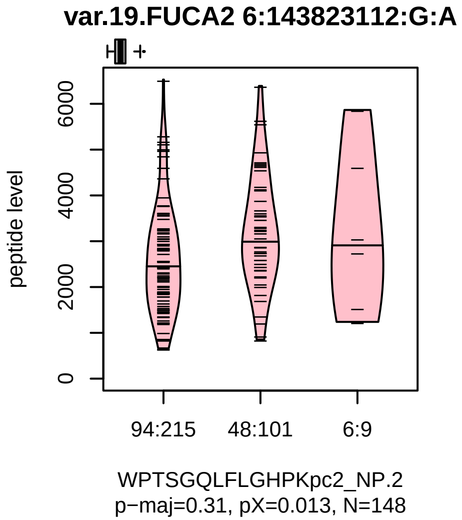
var.19.FUCA2 6:143823112:G:A
peptide level
0
2000
4000
6000
94:215
48:101
6:9
WPTSGQLFLGHPKpc2_NP.2
p−maj=0.31, pX=0.013, N=148

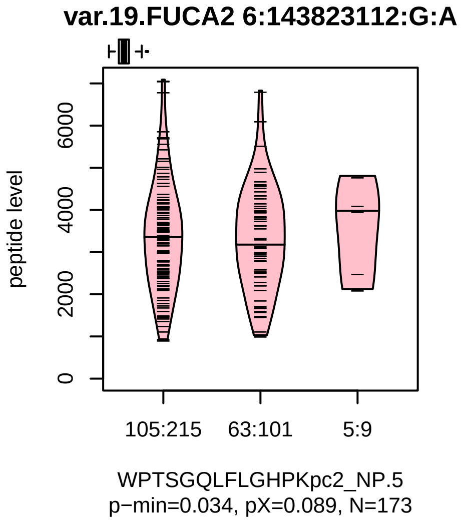
var.19.FUCA2 6:143823112:G:A
peptide level
0
2000
4000
6000
105:215
63:101
5:9
WPTSGQLFLGHPKpc2_NP.5
p−min=0.034, pX=0.089, N=173

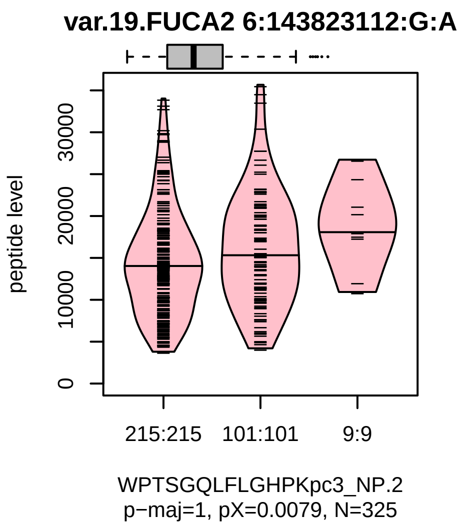
var.19.FUCA2 6:143823112:G:A
peptide level
0
10000
20000
30000
215:215
101:101
9:9
WPTSGQLFLGHPKpc3_NP.2
p−maj=1, pX=0.0079, N=325

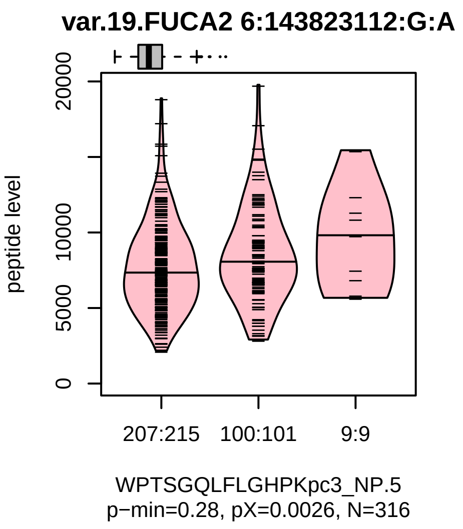
var.19.FUCA2 6:143823112:G:A
peptide level
0
5000
10000
20000
207:215
100:101
9:9
WPTSGQLFLGHPKpc3_NP.5
p−min=0.28, pX=0.0026, N=316

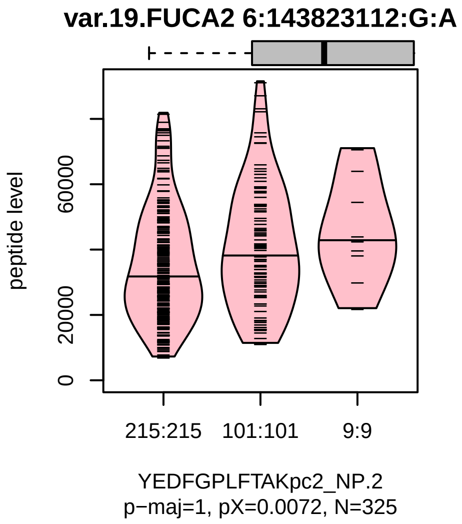
var.19.FUCA2 6:143823112:G:A
peptide level
0
20000
60000
215:215
101:101
9:9
YEDFGPLFTAKpc2_NP.2
p−maj=1, pX=0.0072, N=325

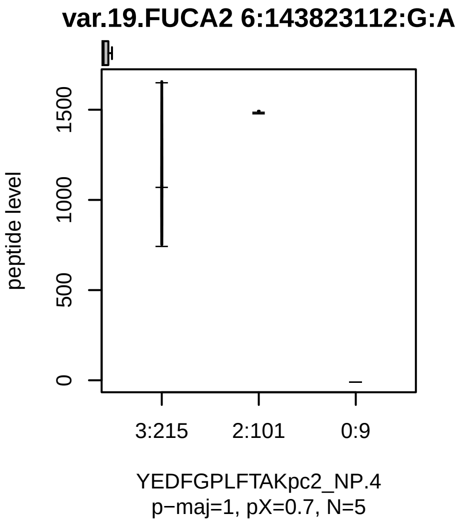
var.19.FUCA2 6:143823112:G:A
peptide level
0
500
1000
1500
3:215
2:101
0:9
YEDFGPLFTAKpc2_NP.4
p−maj=1, pX=0.7, N=5

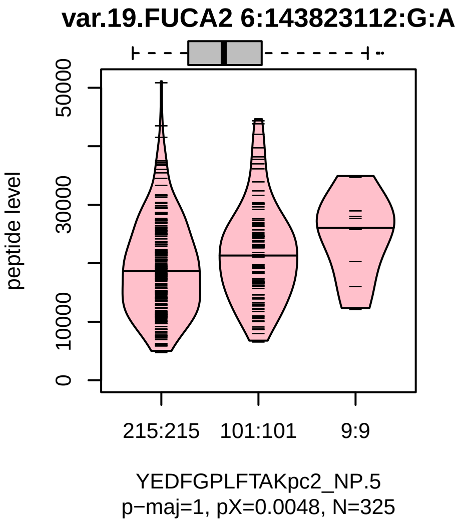
var.19.FUCA2 6:143823112:G:A
peptide level
0
10000
30000
50000
215:215
101:101
9:9
YEDFGPLFTAKpc2_NP.5
p−maj=1, pX=0.0048, N=325

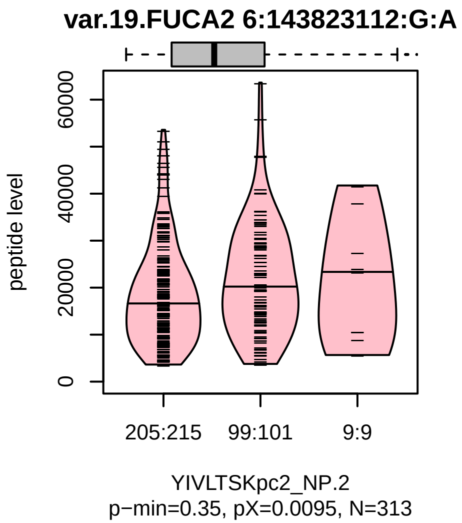
var.19.FUCA2 6:143823112:G:A
peptide level
0
20000
40000
60000
205:215
99:101
9:9
YIVLTSKpc2_NP.2
p−min=0.35, pX=0.0095, N=313

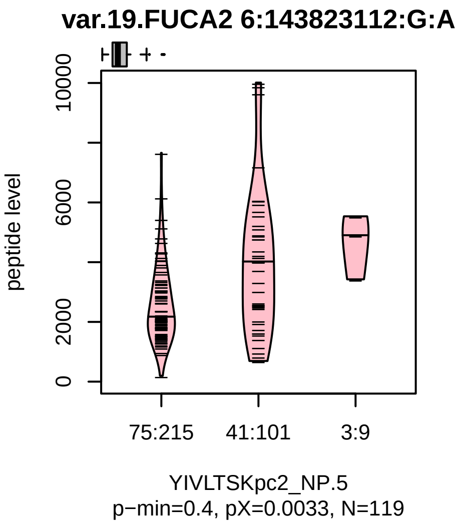
var.19.FUCA2 6:143823112:G:A
peptide level
0
2000
6000
10000
75:215
41:101
3:9
YIVLTSKpc2_NP.5
p−min=0.4, pX=0.0033, N=119

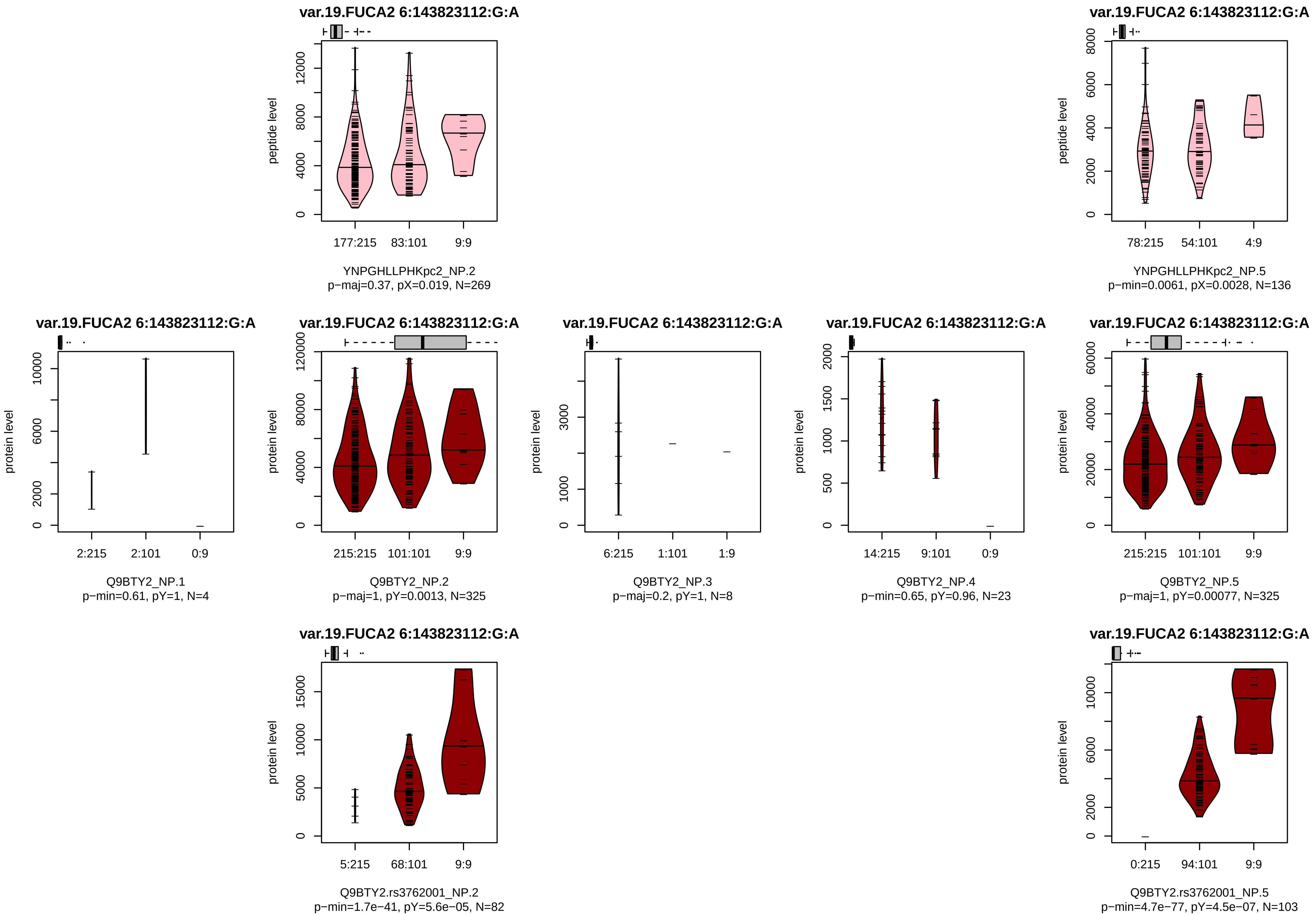
var.19.FUCA2 6:143823112:G:A
peptide level
177:215
83:101
9:9
YNPGHLLPHKpc2_NP.2
p−maj=0.37, pX=0.019, N=269
var.19.FUCA2 6:143823112:G:A
peptide level
78:215
54:101
4:9
YNPGHLLPHKpc2_NP.5
p−min=0.0061, pX=0.0028, N=136
var.19.FUCA2 6:143823112:G:A
protein level
2:215
2:101
0:9
Q9BTY2_NP.1
p−min=0.61, pY=1, N=4
var.19.FUCA2 6:143823112:G:A
protein level
215:215
101:101
9:9
Q9BTY2_NP.2
p−maj=1, pY=0.0013, N=325
var.19.FUCA2 6:143823112:G:A
protein level
6:215
1:101
1:9
Q9BTY2_NP.3
p−maj=0.2, pY=1, N=8
var.19.FUCA2 6:143823112:G:A
protein level
14:215
9:101
0:9
Q9BTY2_NP.4
p−min=0.65, pY=0.96, N=23
var.19.FUCA2 6:143823112:G:A
protein level
215:215
101:101
9:9
Q9BTY2_NP.5
p−maj=1, pY=0.00077, N=325
var.19.FUCA2 6:143823112:G:A
protein level
5:215
68:101
9:9
Q9BTY2.rs3762001_NP.2
p−min=1.7e−41, pY=5.6e−05, N=82
var.19.FUCA2 6:143823112:G:A
protein level
0:215
94:101
9:9
Q9BTY2.rs3762001_NP.5
p−min=4.7e−77, pY=4.5e−07, N=103

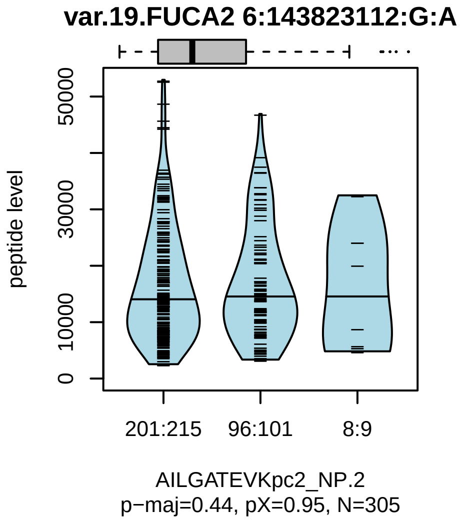
var.19.FUCA2 6:143823112:G:A
peptide level
0
10000
30000
50000
201:215
96:101
8:9
AILGATEVKpc2_NP.2
p−maj=0.44, pX=0.95, N=305

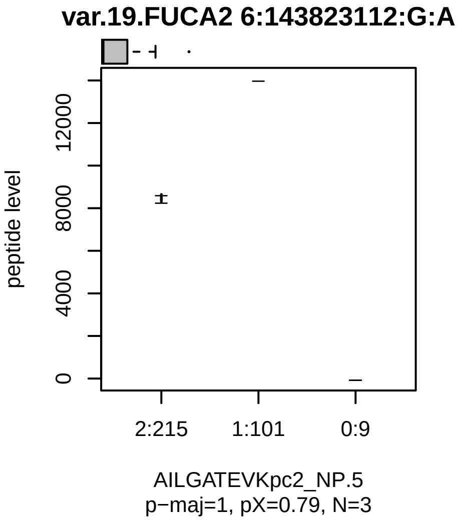
var.19.FUCA2 6:143823112:G:A
peptide level
0
4000
8000
12000
2:215
1:101
0:9
AILGATEVKpc2_NP.5
p−maj=1, pX=0.79, N=3

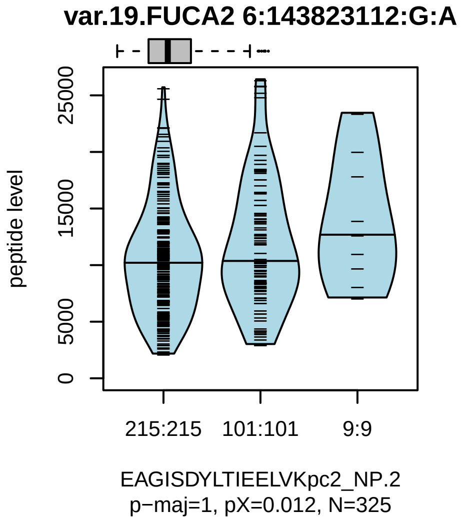
var.19.FUCA2 6:143823112:G:A
peptide level
0
5000
15000
25000
215:215
101:101
9:9
EAGISDYLTIEELVKpc2_NP.2
p−maj=1, pX=0.012, N=325

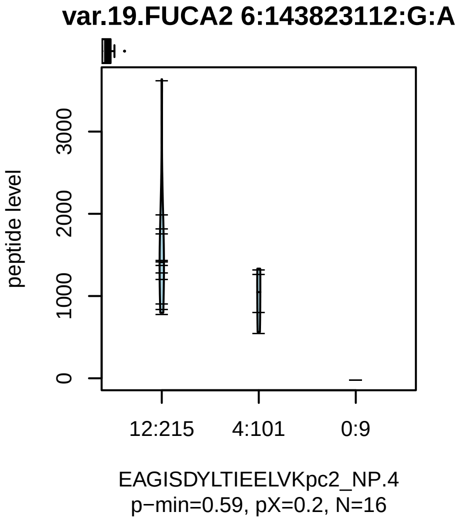
var.19.FUCA2 6:143823112:G:A
peptide level
0
1000
2000
3000
12:215
4:101
0:9
EAGISDYLTIEELVKpc2_NP.4
p−min=0.59, pX=0.2, N=16

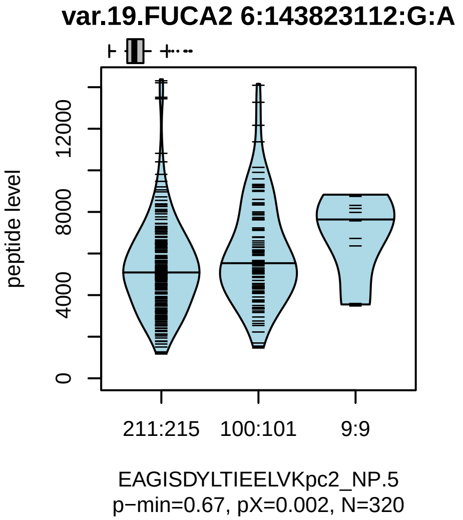
var.19.FUCA2 6:143823112:G:A
peptide level
0
4000
8000
12000
211:215
100:101
9:9
EAGISDYLTIEELVKpc2_NP.5
p−min=0.67, pX=0.002, N=320

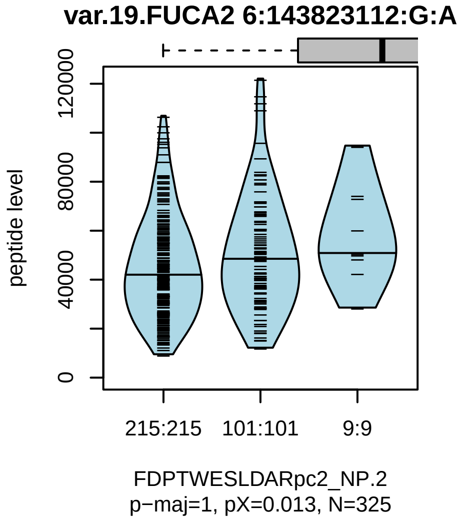
var.19.FUCA2 6:143823112:G:A
peptide level
0
40000
80000
120000
215:215
101:101
9:9
FDPTWESLDARpc2_NP.2
p−maj=1, pX=0.013, N=325

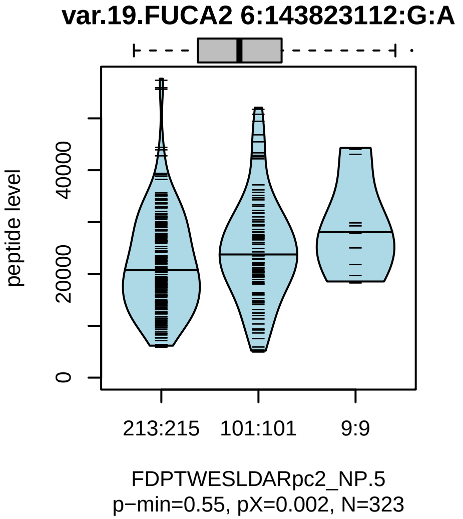
var.19.FUCA2 6:143823112:G:A
peptide level
0
20000
40000
213:215
101:101
9:9
FDPTWESLDARpc2_NP.5
p−min=0.55, pX=0.002, N=323

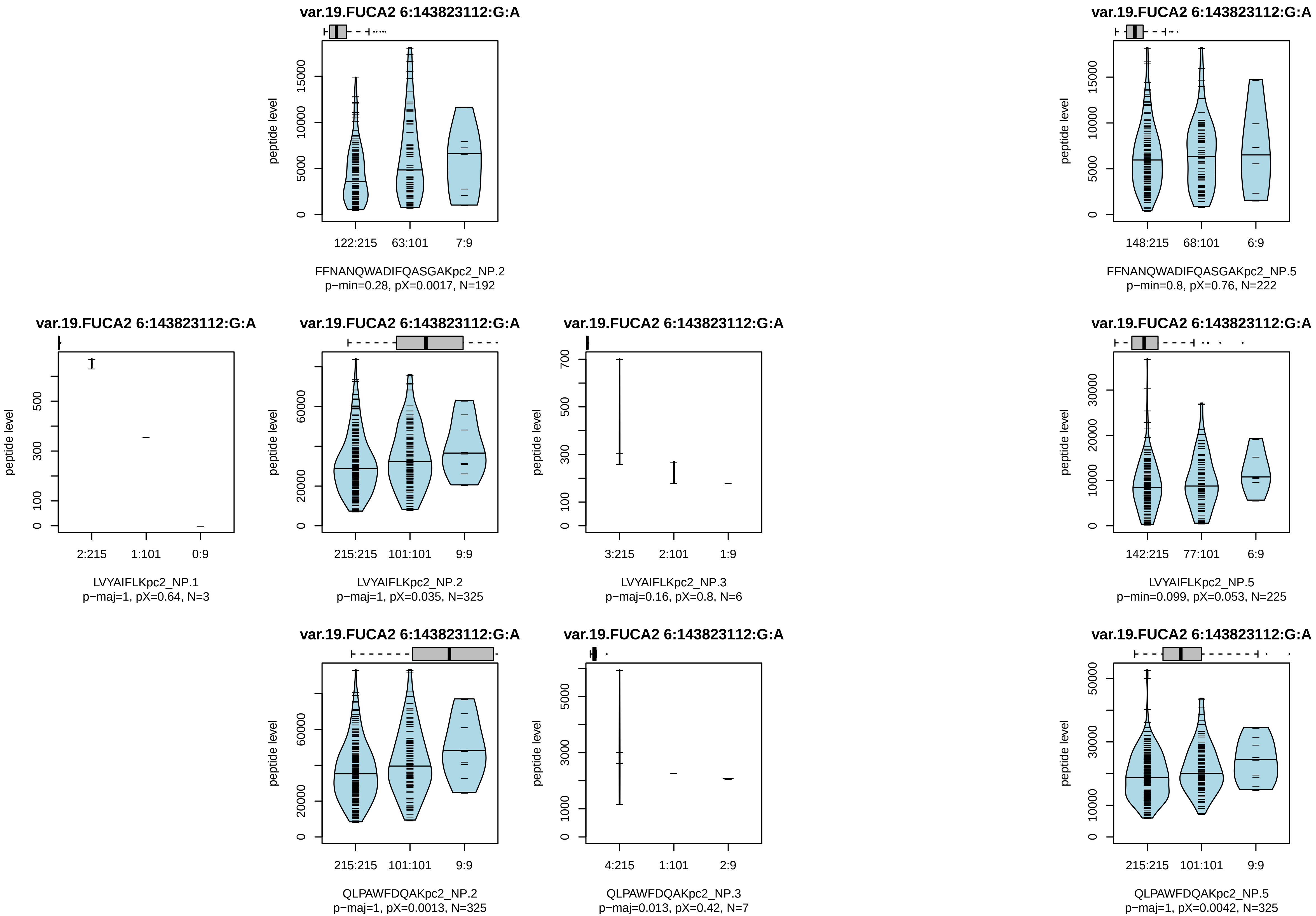
var.19.FUCA2 6:143823112:G:A
peptide level
122:215
63:101
7:9
FFNANQWADIFQASGAKpc2_NP.2
p−min=0.28, pX=0.0017, N=192
var.19.FUCA2 6:143823112:G:A
peptide level
148:215
68:101
6:9
FFNANQWADIFQASGAKpc2_NP.5
p−min=0.8, pX=0.76, N=222
var.19.FUCA2 6:143823112:G:A
peptide level
2:215
1:101
0:9
LVYAIFLKpc2_NP.1
p−maj=1, pX=0.64, N=3
var.19.FUCA2 6:143823112:G:A
peptide level
215:215
101:101
9:9
LVYAIFLKpc2_NP.2
p−maj=1, pX=0.035, N=325
var.19.FUCA2 6:143823112:G:A
peptide level
3:215
2:101
1:9
LVYAIFLKpc2_NP.3
p−maj=0.16, pX=0.8, N=6
var.19.FUCA2 6:143823112:G:A
peptide level
142:215
77:101
6:9
LVYAIFLKpc2_NP.5
p−min=0.099, pX=0.053, N=225
var.19.FUCA2 6:143823112:G:A
peptide level
215:215
101:101
9:9
QLPAWFDQAKpc2_NP.2
p−maj=1, pX=0.0013, N=325
var.19.FUCA2 6:143823112:G:A
peptide level
4:215
1:101
2:9
QLPAWFDQAKpc2_NP.3
p−maj=0.013, pX=0.42, N=7
var.19.FUCA2 6:143823112:G:A
peptide level
215:215
101:101
9:9
QLPAWFDQAKpc2_NP.5
p−maj=1, pX=0.0042, N=325

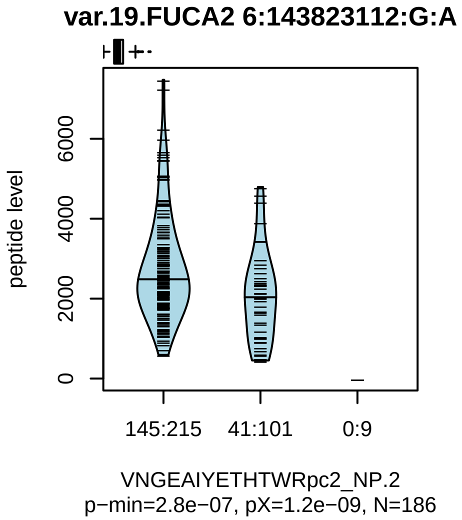
var.19.FUCA2 6:143823112:G:A
peptide level
0
2000
4000
6000
145:215
41:101
0:9
VNGEAIYETHTWRpc2_NP.2
p−min=2.8e−07, pX=1.2e−09, N=186

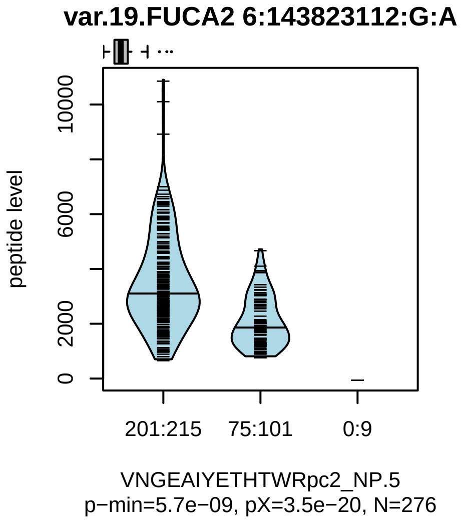
var.19.FUCA2 6:143823112:G:A
peptide level
0
2000
6000
10000
201:215
75:101
0:9
VNGEAIYETHTWRpc2_NP.5
p−min=5.7e−09, pX=3.5e−20, N=276

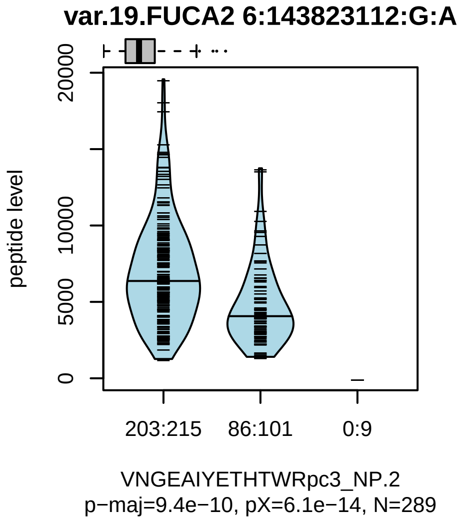
var.19.FUCA2 6:143823112:G:A
peptide level
0
5000
10000
20000
203:215
86:101
0:9
VNGEAIYETHTWRpc3_NP.2
p−maj=9.4e−10, pX=6.1e−14, N=289

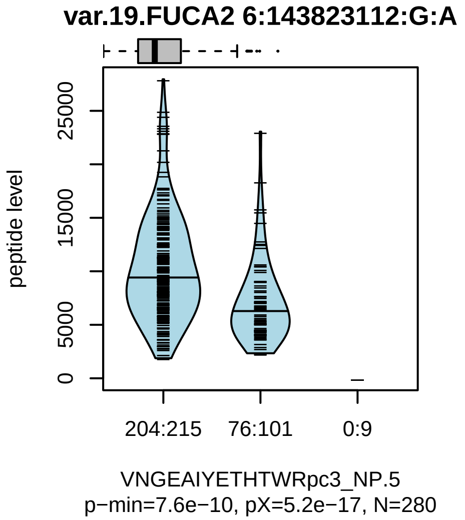
var.19.FUCA2 6:143823112:G:A
peptide level
0
5000
15000
25000
204:215
76:101
0:9
VNGEAIYETHTWRpc3_NP.5
p−min=7.6e−10, pX=5.2e−17, N=280

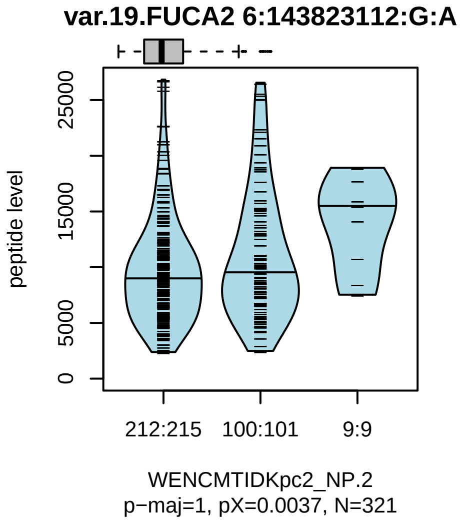
var.19.FUCA2 6:143823112:G:A
peptide level
0
5000
15000
25000
212:215
100:101
9:9
WENCMTIDKpc2_NP.2
p−maj=1, pX=0.0037, N=321

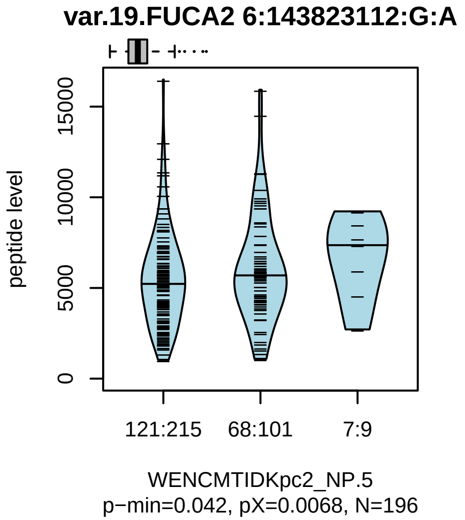
var.19.FUCA2 6:143823112:G:A
peptide level
0
5000
10000
15000
121:215
68:101
7:9
WENCMTIDKpc2_NP.5
p−min=0.042, pX=0.0068, N=196

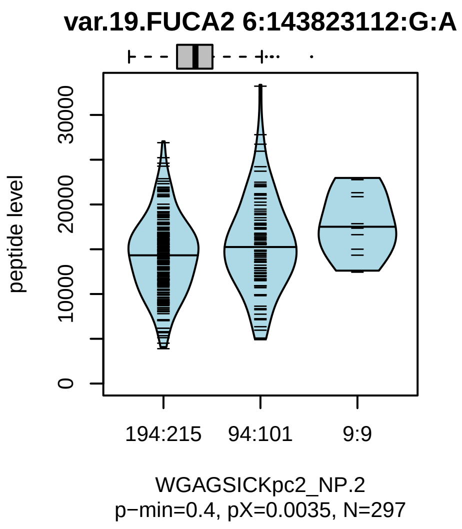
var.19.FUCA2 6:143823112:G:A
peptide level
0
10000
20000
30000
194:215
94:101
9:9
WGAGSICKpc2_NP.2
p−min=0.4, pX=0.0035, N=297

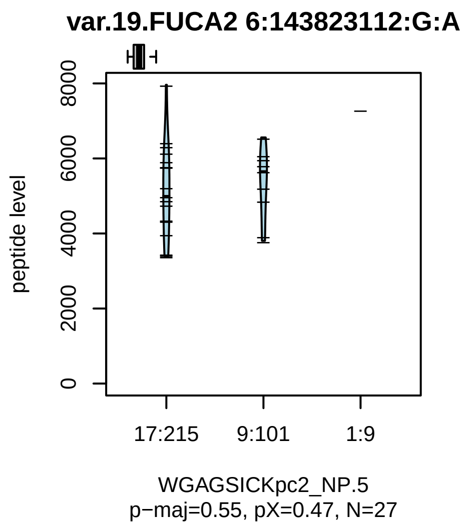
var.19.FUCA2 6:143823112:G:A
peptide level
0
2000
4000
6000
8000
17:215
9:101
1:9
WGAGSICKpc2_NP.5
p−maj=0.55, pX=0.47, N=27

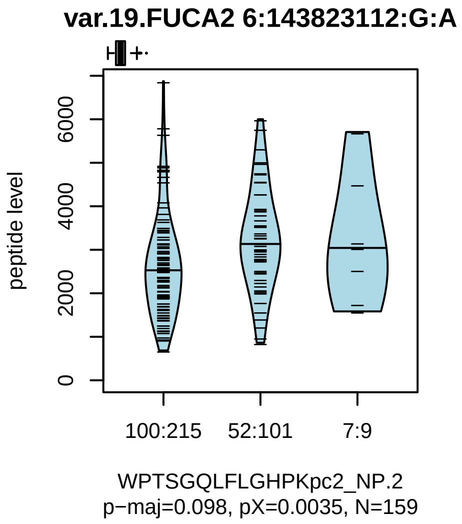
var.19.FUCA2 6:143823112:G:A
peptide level
0
2000
4000
6000
100:215
52:101
7:9
WPTSGQLFLGHPKpc2_NP.2
p−maj=0.098, pX=0.0035, N=159

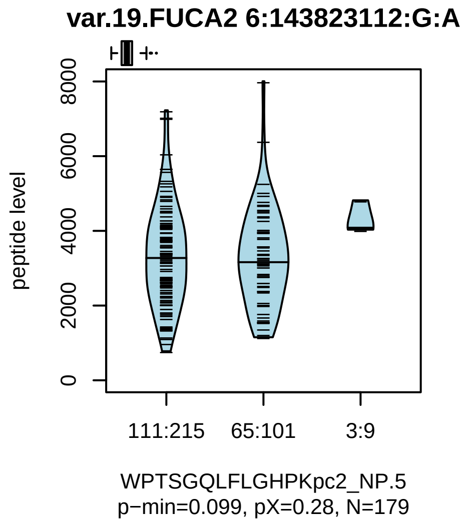
var.19.FUCA2 6:143823112:G:A
peptide level
0
2000
4000
6000
8000
111:215
65:101
3:9
WPTSGQLFLGHPKpc2_NP.5
p−min=0.099, pX=0.28, N=179

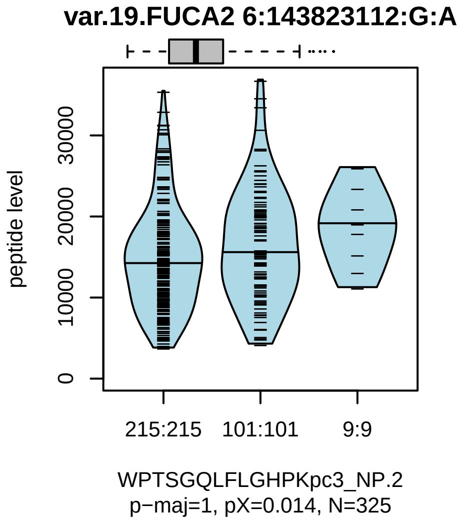
var.19.FUCA2 6:143823112:G:A
peptide level
0
10000
20000
30000
215:215
101:101
9:9
WPTSGQLFLGHPKpc3_NP.2
p−maj=1, pX=0.014, N=325

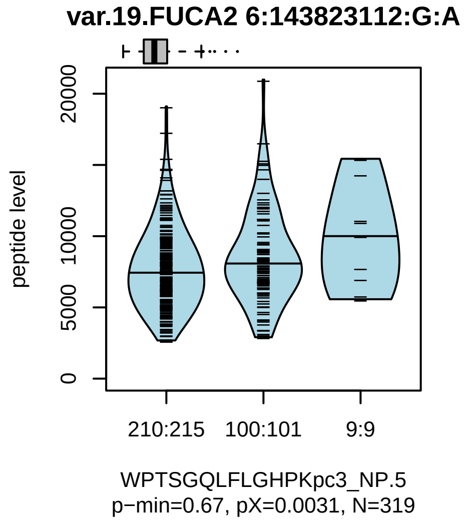
var.19.FUCA2 6:143823112:G:A
peptide level
0
5000
10000
20000
210:215
100:101
9:9
WPTSGQLFLGHPKpc3_NP.5
p−min=0.67, pX=0.0031, N=319

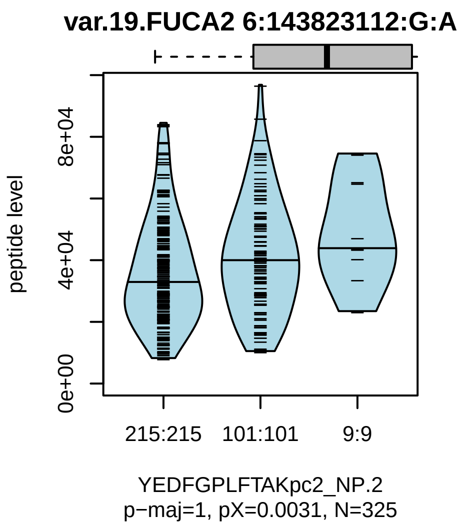
var.19.FUCA2 6:143823112:G:A
peptide level
0e+00
4e+04
8e+04
215:215
101:101
9:9
YEDFGPLFTAKpc2_NP.2
p−maj=1, pX=0.0031, N=325

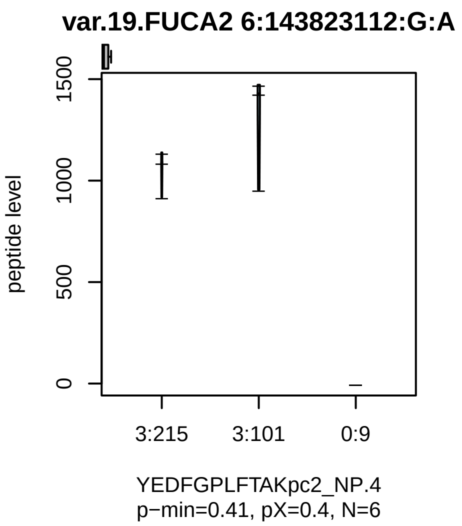
var.19.FUCA2 6:143823112:G:A
peptide level
0
500
1000
1500
3:215
3:101
0:9
YEDFGPLFTAKpc2_NP.4
p−min=0.41, pX=0.4, N=6

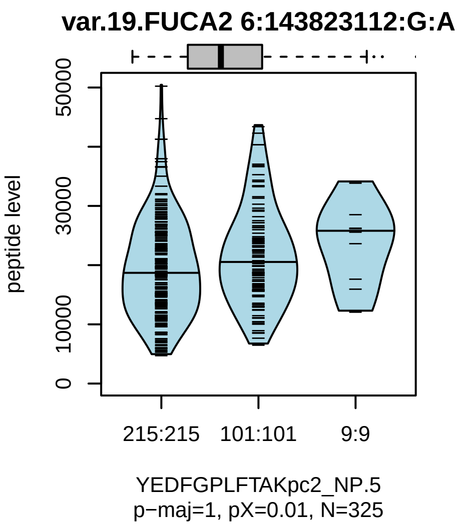
var.19.FUCA2 6:143823112:G:A
peptide level
0
10000
30000
50000
215:215
101:101
9:9
YEDFGPLFTAKpc2_NP.5
p−maj=1, pX=0.01, N=325

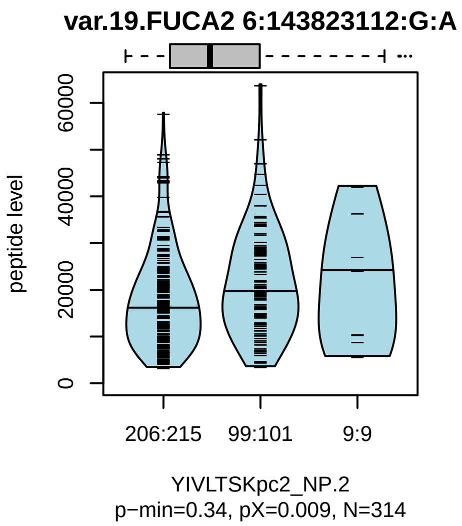
var.19.FUCA2 6:143823112:G:A
peptide level
0
20000
40000
60000
206:215
99:101
9:9
YIVLTSKpc2_NP.2
p−min=0.34, pX=0.009, N=314

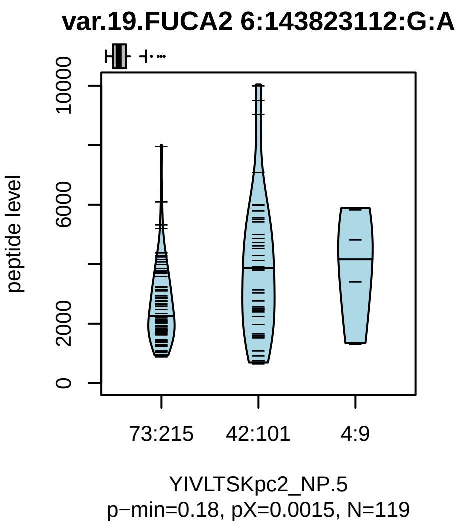
var.19.FUCA2 6:143823112:G:A
peptide level
0
2000
6000
10000
73:215
42:101
4:9
YIVLTSKpc2_NP.5
p−min=0.18, pX=0.0015, N=119

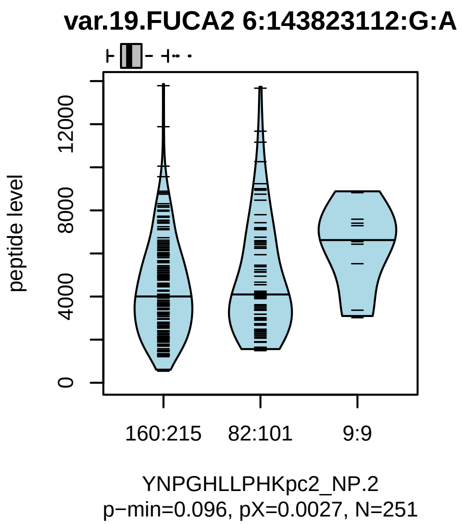
var.19.FUCA2 6:143823112:G:A
peptide level
0
4000
8000
12000
160:215
82:101
9:9
YNPGHLLPHKpc2_NP.2
p−min=0.096, pX=0.0027, N=251

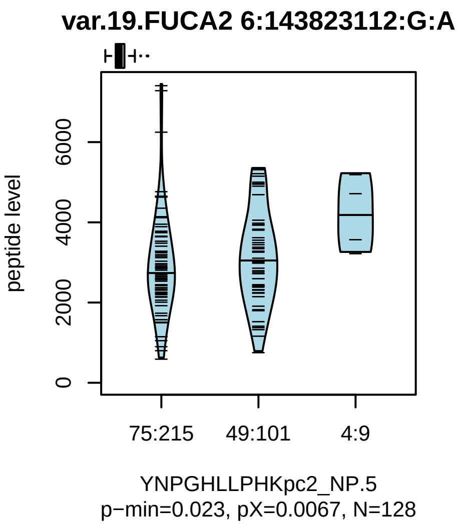
var.19.FUCA2 6:143823112:G:A
peptide level
0
2000
4000
6000
75:215
49:101
4:9
YNPGHLLPHKpc2_NP.5
p−min=0.023, pX=0.0067, N=128

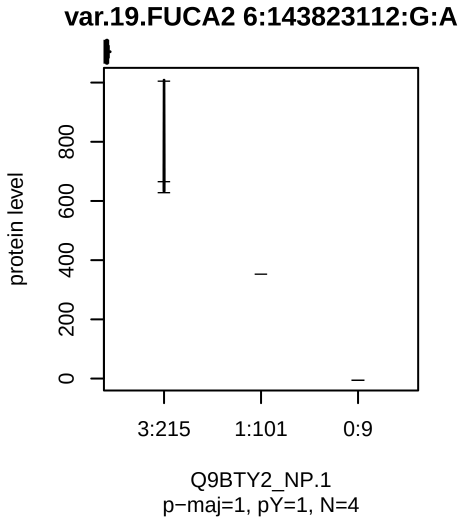
var.19.FUCA2 6:143823112:G:A
protein level
0
200
400
600
800
3:215
1:101
0:9
Q9BTY2_NP.1
p−maj=1, pY=1, N=4

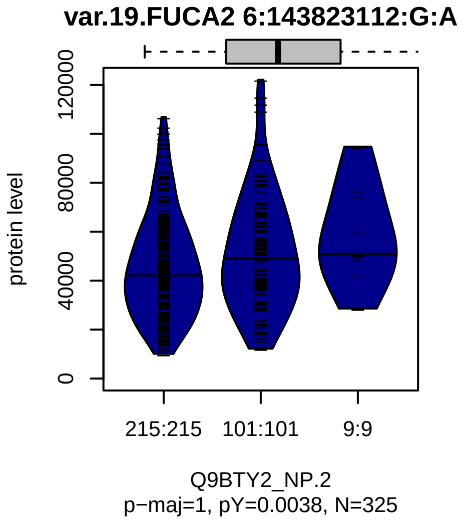
var.19.FUCA2 6:143823112:G:A
protein level
0
40000
80000
120000
215:215
101:101
9:9
Q9BTY2_NP.2
p−maj=1, pY=0.0038, N=325

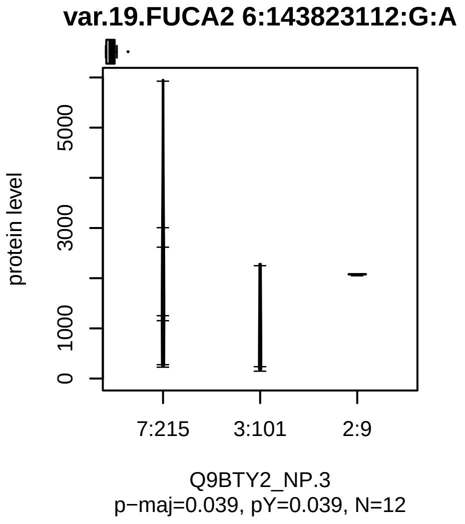
var.19.FUCA2 6:143823112:G:A
protein level
0
1000
3000
5000
7:215
3:101
2:9
Q9BTY2_NP.3
p−maj=0.039, pY=0.039, N=12

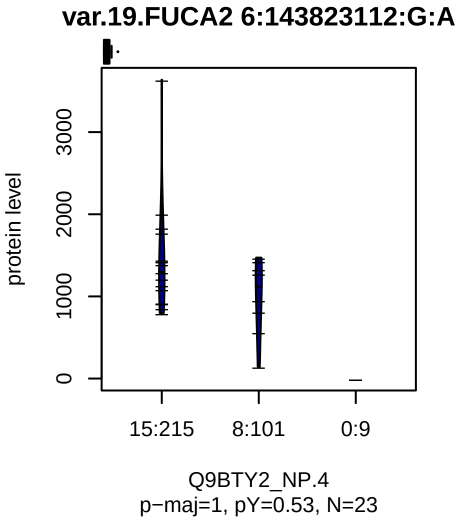
var.19.FUCA2 6:143823112:G:A
protein level
0
1000
2000
3000
15:215
8:101
0:9
Q9BTY2_NP.4
p−maj=1, pY=0.53, N=23

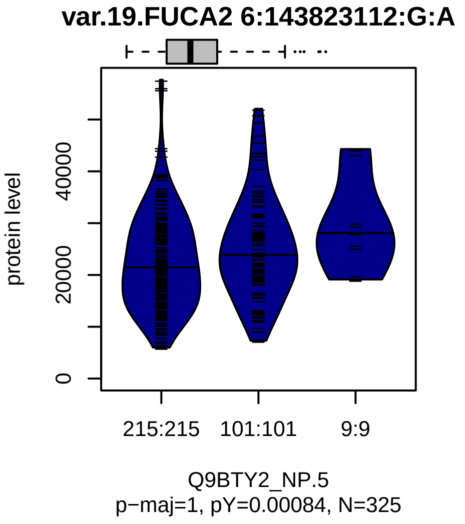
var.19.FUCA2 6:143823112:G:A
protein level
0
20000
40000
215:215
101:101
9:9
Q9BTY2_NP.5
p−maj=1, pY=0.00084, N=325

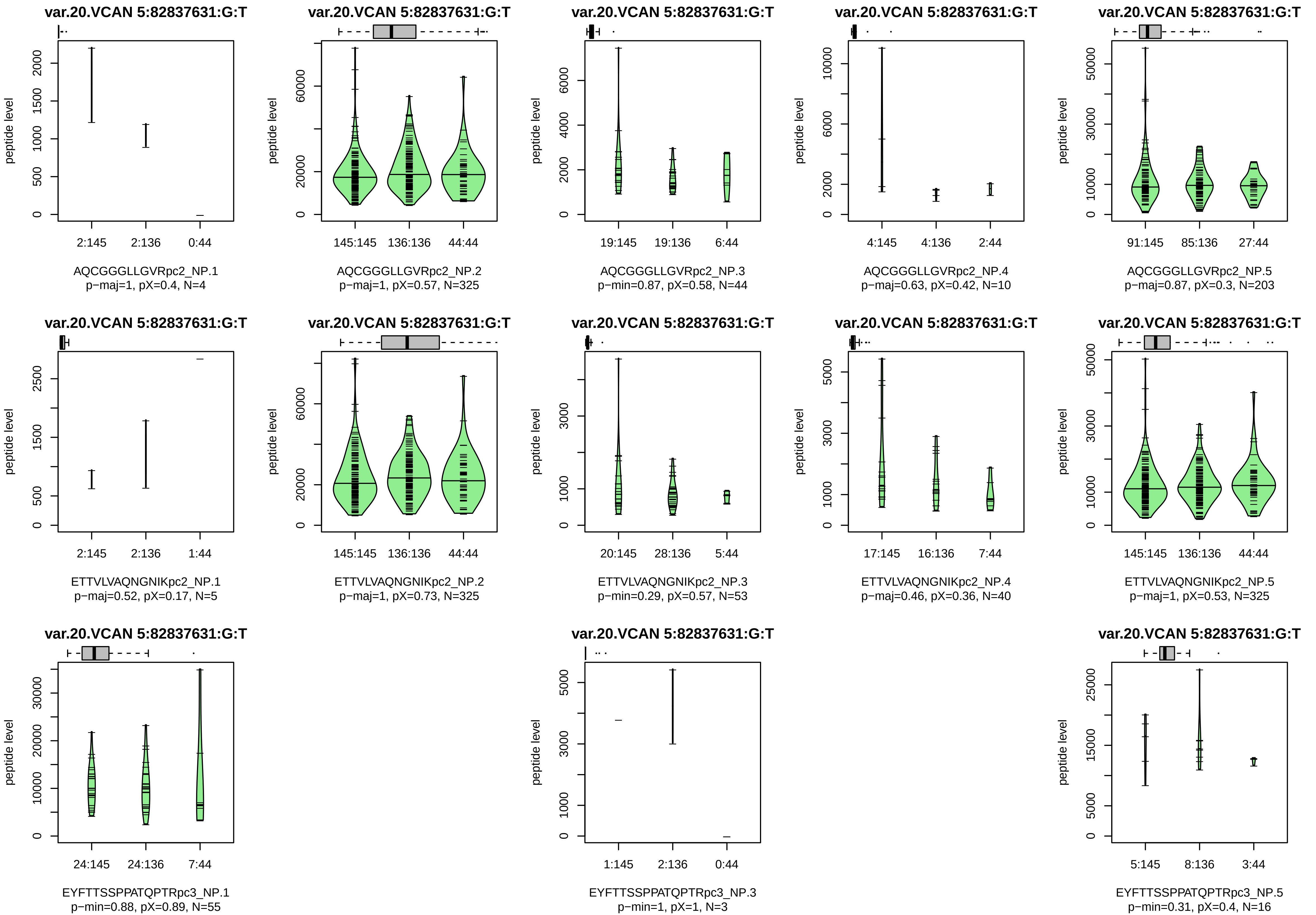
var.20.VCAN 5:82837631:G:T
peptide level
2:145
2:136
0:44
AQCGGGLLGVRpc2_NP.1
p−maj=1, pX=0.4, N=4
var.20.VCAN 5:82837631:G:T
peptide level
145:145
136:136
44:44
AQCGGGLLGVRpc2_NP.2
p−maj=1, pX=0.57, N=325
var.20.VCAN 5:82837631:G:T
peptide level
19:145
19:136
6:44
AQCGGGLLGVRpc2_NP.3
p−min=0.87, pX=0.58, N=44
var.20.VCAN 5:82837631:G:T
peptide level
4:145
4:136
2:44
AQCGGGLLGVRpc2_NP.4
p−maj=0.63, pX=0.42, N=10
var.20.VCAN 5:82837631:G:T
peptide level
91:145
85:136
27:44
AQCGGGLLGVRpc2_NP.5
p−maj=0.87, pX=0.3, N=203
var.20.VCAN 5:82837631:G:T
peptide level
2:145
2:136
1:44
ETTVLVAQNGNIKpc2_NP.1
p−maj=0.52, pX=0.17, N=5
var.20.VCAN 5:82837631:G:T
peptide level
145:145
136:136
44:44
ETTVLVAQNGNIKpc2_NP.2
p−maj=1, pX=0.73, N=325
var.20.VCAN 5:82837631:G:T
peptide level
20:145
28:136
5:44
ETTVLVAQNGNIKpc2_NP.3
p−min=0.29, pX=0.57, N=53
var.20.VCAN 5:82837631:G:T
peptide level
17:145
16:136
7:44
ETTVLVAQNGNIKpc2_NP.4
p−maj=0.46, pX=0.36, N=40
var.20.VCAN 5:82837631:G:T
peptide level
145:145
136:136
44:44
ETTVLVAQNGNIKpc2_NP.5
p−maj=1, pX=0.53, N=325
var.20.VCAN 5:82837631:G:T
peptide level
24:145
24:136
7:44
EYFTTSSPPATQPTRpc3_NP.1
p−min=0.88, pX=0.89, N=55
var.20.VCAN 5:82837631:G:T
peptide level
1:145
2:136
0:44
EYFTTSSPPATQPTRpc3_NP.3
p−min=1, pX=1, N=3
var.20.VCAN 5:82837631:G:T
peptide level
5:145
8:136
3:44
EYFTTSSPPATQPTRpc3_NP.5
p−min=0.31, pX=0.4, N=16

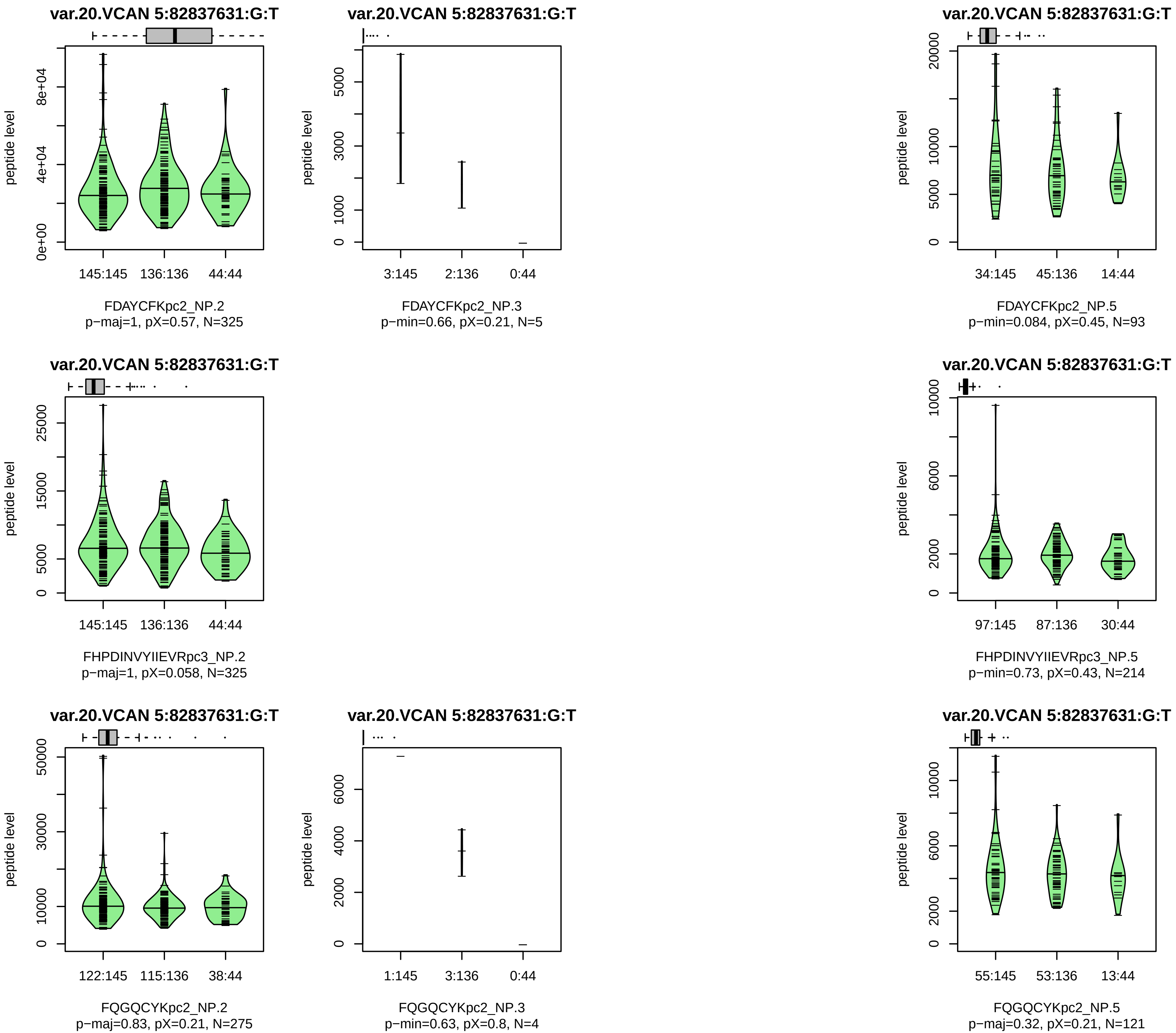
var.20.VCAN 5:82837631:G:T
peptide level
145:145
136:136
44:44
FDAYCFKpc2_NP.2
p−maj=1, pX=0.57, N=325
var.20.VCAN 5:82837631:G:T
peptide level
3:145
2:136
0:44
FDAYCFKpc2_NP.3
p−min=0.66, pX=0.21, N=5
var.20.VCAN 5:82837631:G:T
peptide level
34:145
45:136
14:44
FDAYCFKpc2_NP.5
p−min=0.084, pX=0.45, N=93
var.20.VCAN 5:82837631:G:T
peptide level
145:145
136:136
44:44
FHPDINVYIIEVRpc3_NP.2
p−maj=1, pX=0.058, N=325
var.20.VCAN 5:82837631:G:T
peptide level
97:145
87:136
30:44
FHPDINVYIIEVRpc3_NP.5
p−min=0.73, pX=0.43, N=214
var.20.VCAN 5:82837631:G:T
peptide level
122:145
115:136
38:44
FQGQCYKpc2_NP.2
p−maj=0.83, pX=0.21, N=275
var.20.VCAN 5:82837631:G:T
peptide level
1:145
3:136
0:44
FQGQCYKpc2_NP.3
p−min=0.63, pX=0.8, N=4
var.20.VCAN 5:82837631:G:T
peptide level
55:145
53:136
13:44
FQGQCYKpc2_NP.5
p−maj=0.32, pX=0.21, N=121

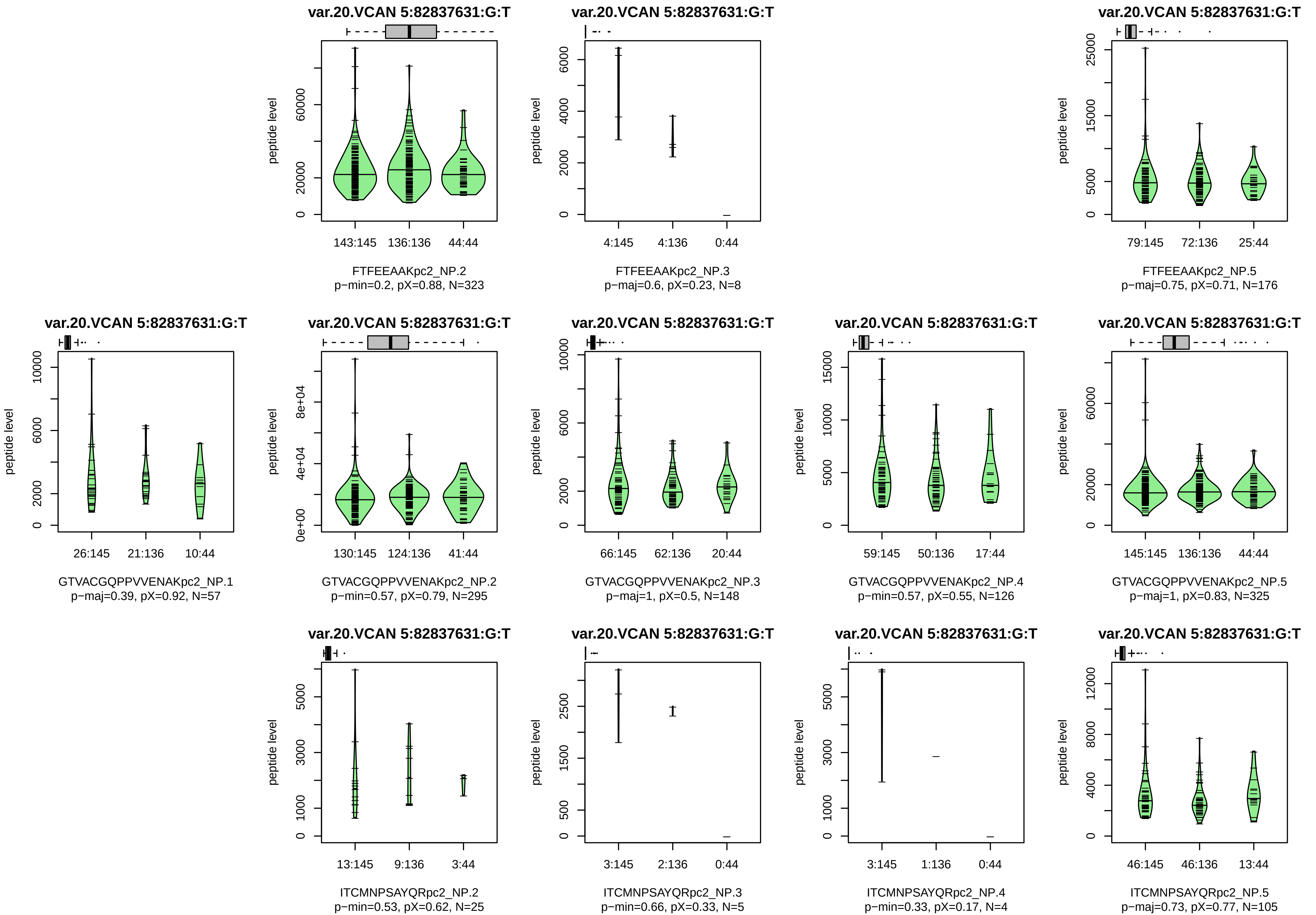
var.20.VCAN 5:82837631:G:T
peptide level
0
20000
60000
143:145
136:136
44:44
FTFEEAAKpc2_NP.2
p−min=0.2, pX=0.88, N=323
var.20.VCAN 5:82837631:G:T
peptide level
0
2000
4000
6000
4:145
4:136
0:44
FTFEEAAKpc2_NP.3
p−maj=0.6, pX=0.23, N=8
var.20.VCAN 5:82837631:G:T
peptide level
0
5000
15000
25000
79:145
72:136
25:44
FTFEEAAKpc2_NP.5
p−maj=0.75, pX=0.71, N=176
var.20.VCAN 5:82837631:G:T
peptide level
0
2000
6000
10000
26:145
21:136
10:44
GTVACGQPPVVENAKpc2_NP.1
p−maj=0.39, pX=0.92, N=57
var.20.VCAN 5:82837631:G:T
peptide level
0e+00
4e+04
8e+04
130:145
124:136
41:44
GTVACGQPPVVENAKpc2_NP.2
p−min=0.57, pX=0.79, N=295
var.20.VCAN 5:82837631:G:T
peptide level
0
2000
6000
10000
66:145
62:136
20:44
GTVACGQPPVVENAKpc2_NP.3
p−maj=1, pX=0.5, N=148
var.20.VCAN 5:82837631:G:T
peptide level
0
5000
10000
15000
59:145
50:136
17:44
GTVACGQPPVVENAKpc2_NP.4
p−min=0.57, pX=0.55, N=126
var.20.VCAN 5:82837631:G:T
peptide level
0
20000
60000
145:145
136:136
44:44
GTVACGQPPVVENAKpc2_NP.5
p−maj=1, pX=0.83, N=325
var.20.VCAN 5:82837631:G:T
peptide level
0
1000
3000
5000
13:145
9:136
3:44
ITCMNPSAYQRpc2_NP.2
p−min=0.53, pX=0.62, N=25
var.20.VCAN 5:82837631:G:T
peptide level
0
500
1500
2500
3:145
2:136
0:44
ITCMNPSAYQRpc2_NP.3
p−min=0.66, pX=0.33, N=5
var.20.VCAN 5:82837631:G:T
peptide level
0
1000
3000
5000
3:145
1:136
0:44
ITCMNPSAYQRpc2_NP.4
p−min=0.33, pX=0.17, N=4
var.20.VCAN 5:82837631:G:T
peptide level
0
4000
8000
12000
46:145
46:136
13:44
ITCMNPSAYQRpc2_NP.5
p−maj=0.73, pX=0.77, N=105

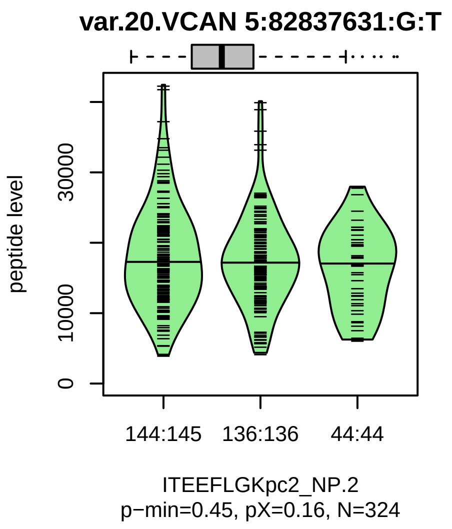
var.20.VCAN 5:82837631:G:T
peptide level
0
10000
30000
144:145
136:136
44:44
ITEEFLGKpc2_NP.2
p−min=0.45, pX=0.16, N=324

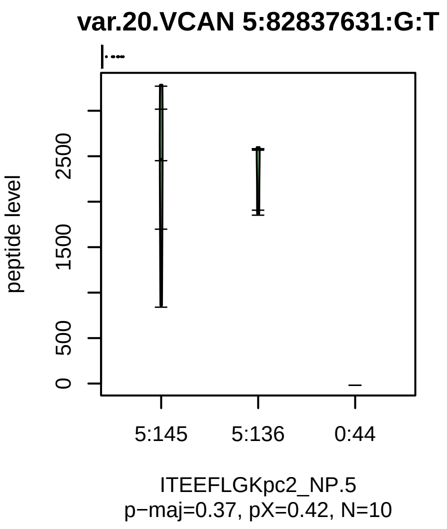
var.20.VCAN 5:82837631:G:T
peptide level
0
500
1500
2500
5:145
5:136
0:44
ITEEFLGKpc2_NP.5
p−maj=0.37, pX=0.42, N=10

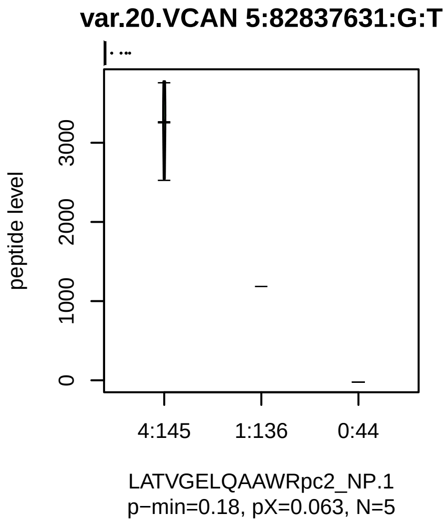
var.20.VCAN 5:82837631:G:T
peptide level
0
1000
2000
3000
4:145
1:136
0:44
LATVGELQAAWRpc2_NP.1
p−min=0.18, pX=0.063, N=5

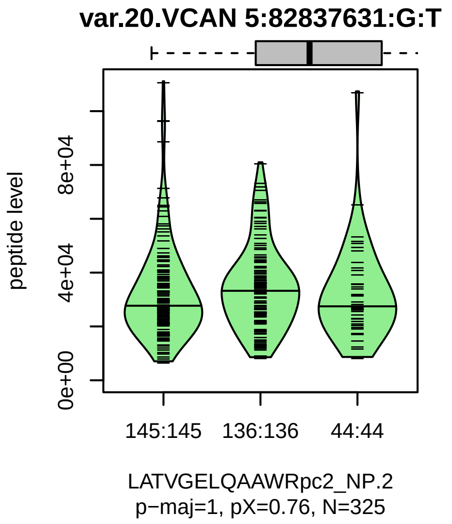
var.20.VCAN 5:82837631:G:T
peptide level
0e+00
4e+04
8e+04
145:145
136:136
44:44
LATVGELQAAWRpc2_NP.2
p−maj=1, pX=0.76, N=325

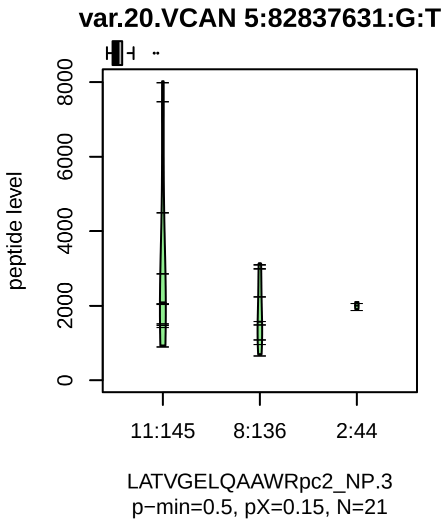
var.20.VCAN 5:82837631:G:T
peptide level
0
2000
4000
6000
8000
11:145
8:136
2:44
LATVGELQAAWRpc2_NP.3
p−min=0.5, pX=0.15, N=21

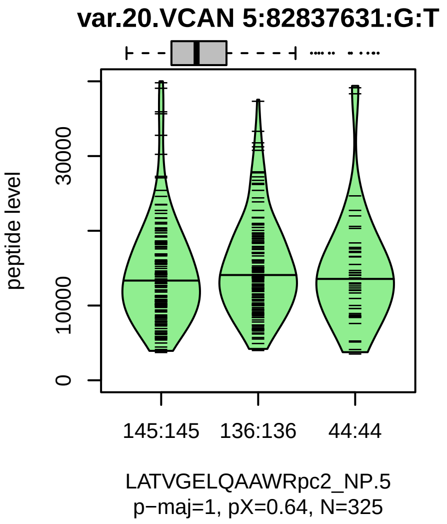
var.20.VCAN 5:82837631:G:T
peptide level
0
10000
30000
145:145
136:136
44:44
LATVGELQAAWRpc2_NP.5
p−maj=1, pX=0.64, N=325

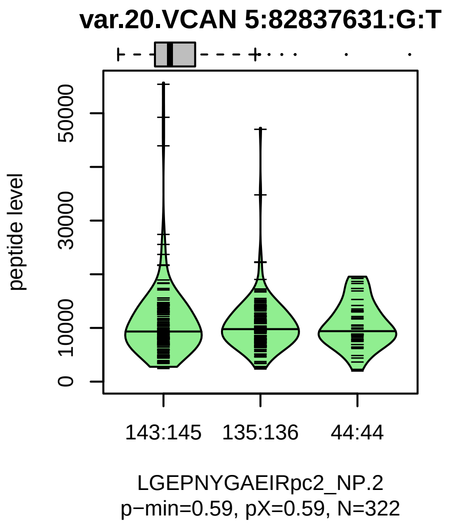
var.20.VCAN 5:82837631:G:T
peptide level
0
10000
30000
50000
143:145
135:136
44:44
LGEPNYGAEIRpc2_NP.2
p−min=0.59, pX=0.59, N=322

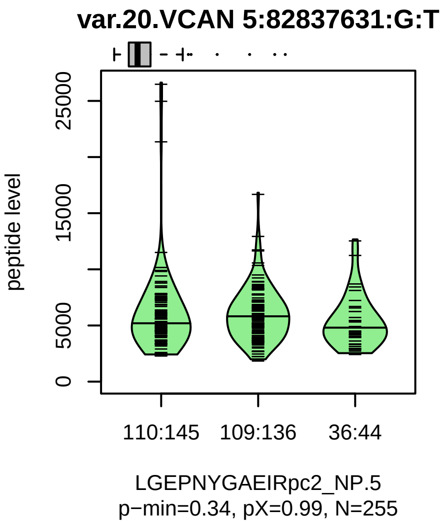
var.20.VCAN 5:82837631:G:T
peptide level
0
5000
15000
25000
110:145
109:136
36:44
LGEPNYGAEIRpc2_NP.5
p−min=0.34, pX=0.99, N=255

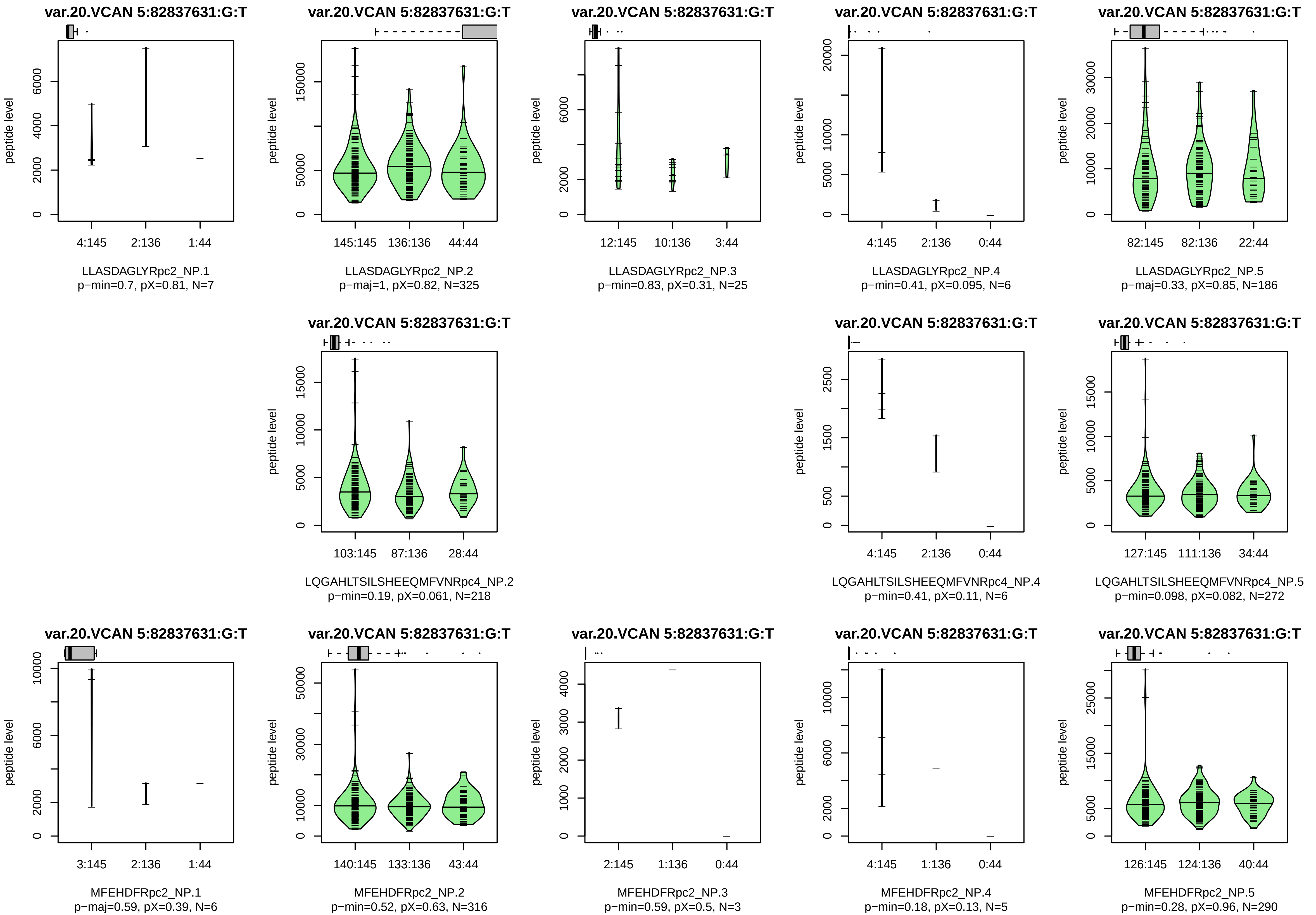
var.20.VCAN 5:82837631:G:T
peptide level
4:145
2:136
1:44
LLASDAGLYRpc2_NP.1
p−min=0.7, pX=0.81, N=7
var.20.VCAN 5:82837631:G:T
peptide level
145:145
136:136
44:44
LLASDAGLYRpc2_NP.2
p−maj=1, pX=0.82, N=325
var.20.VCAN 5:82837631:G:T
peptide level
12:145
10:136
3:44
LLASDAGLYRpc2_NP.3
p−min=0.83, pX=0.31, N=25
var.20.VCAN 5:82837631:G:T
peptide level
4:145
2:136
0:44
LLASDAGLYRpc2_NP.4
p−min=0.41, pX=0.095, N=6
var.20.VCAN 5:82837631:G:T
peptide level
82:145
82:136
22:44
LLASDAGLYRpc2_NP.5
p−maj=0.33, pX=0.85, N=186
var.20.VCAN 5:82837631:G:T
peptide level
103:145
87:136
28:44
LQGAHLTSILSHEEQMFVNRpc4_NP.2
p−min=0.19, pX=0.061, N=218
var.20.VCAN 5:82837631:G:T
peptide level
4:145
2:136
0:44
LQGAHLTSILSHEEQMFVNRpc4_NP.4
p−min=0.41, pX=0.11, N=6
var.20.VCAN 5:82837631:G:T
peptide level
127:145
111:136
34:44
LQGAHLTSILSHEEQMFVNRpc4_NP.5
p−min=0.098, pX=0.082, N=272
var.20.VCAN 5:82837631:G:T
peptide level
3:145
2:136
1:44
MFEHDFRpc2_NP.1
p−maj=0.59, pX=0.39, N=6
var.20.VCAN 5:82837631:G:T
peptide level
140:145
133:136
43:44
MFEHDFRpc2_NP.2
p−min=0.52, pX=0.63, N=316
var.20.VCAN 5:82837631:G:T
peptide level
2:145
1:136
0:44
MFEHDFRpc2_NP.3
p−min=0.59, pX=0.5, N=3
var.20.VCAN 5:82837631:G:T
peptide level
4:145
1:136
0:44
MFEHDFRpc2_NP.4
p−min=0.18, pX=0.13, N=5
var.20.VCAN 5:82837631:G:T
peptide level
126:145
124:136
40:44
MFEHDFRpc2_NP.5
p−min=0.28, pX=0.96, N=290

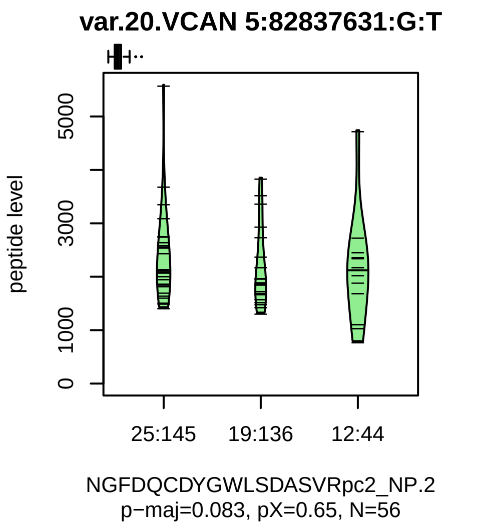
var.20.VCAN 5:82837631:G:T
peptide level
0
1000
3000
5000
25:145
19:136
12:44
NGFDQCDYGWLSDASVRpc2_NP.2
p−maj=0.083, pX=0.65, N=56

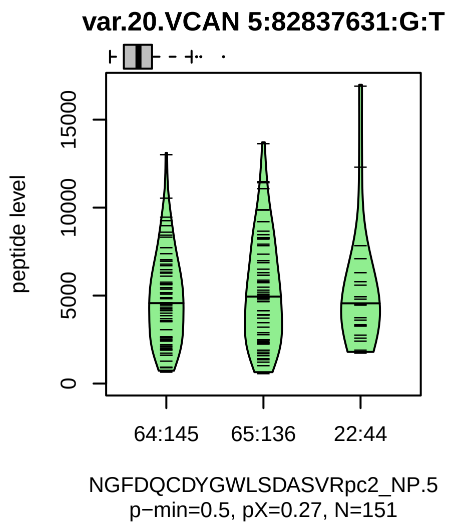
var.20.VCAN 5:82837631:G:T
peptide level
0
5000
10000
15000
64:145
65:136
22:44
NGFDQCDYGWLSDASVRpc2_NP.5
p−min=0.5, pX=0.27, N=151

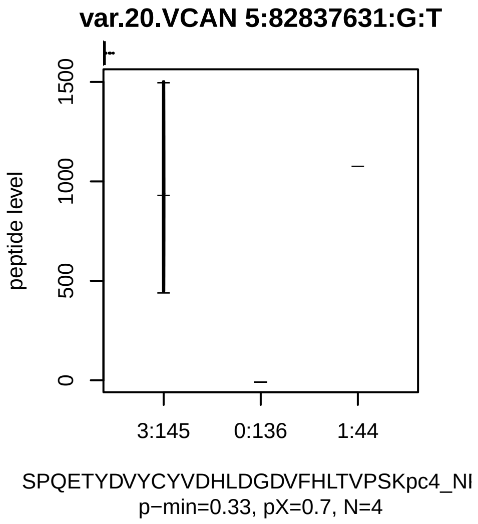
var.20.VCAN 5:82837631:G:T
peptide level
0
500
1000
1500
3:145
0:136
1:44
SPQETYDVYCYVDHLDGDVFHLTVPSKpc4_NI
p−min=0.33, pX=0.7, N=4

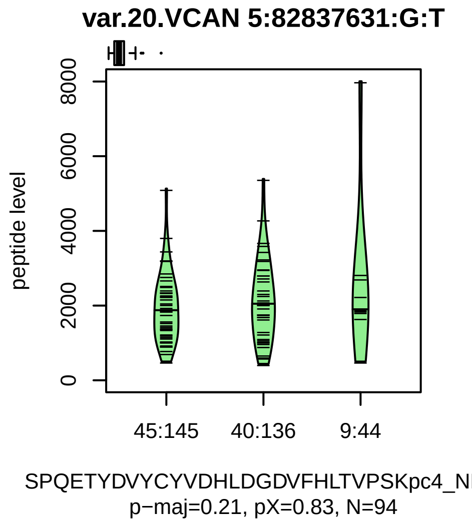
var.20.VCAN 5:82837631:G:T
peptide level
0
2000
4000
6000
8000
45:145
40:136
9:44
SPQETYDVYCYVDHLDGDVFHLTVPSKpc4_N
p−maj=0.21, pX=0.83, N=94

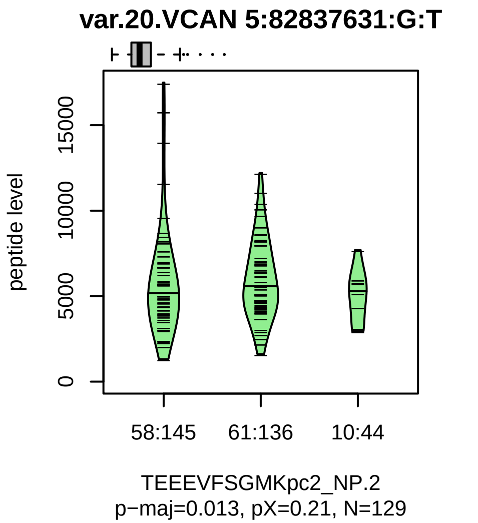
var.20.VCAN 5:82837631:G:T
peptide level
0
5000
10000
15000
58:145
61:136
10:44
TEEEVFSGMKpc2_NP.2
p−maj=0.013, pX=0.21, N=129

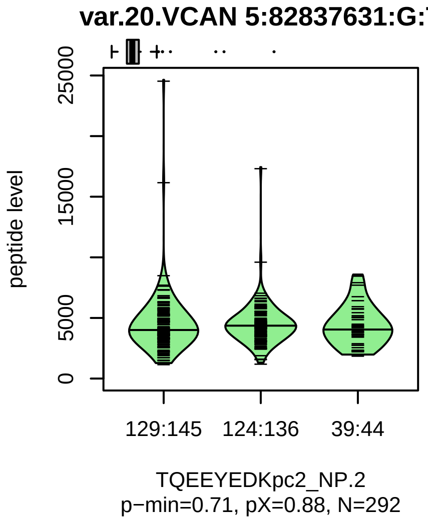
var.20.VCAN 5:82837631:G:T
peptide level
0
5000
15000
25000
129:145
124:136
39:44
TQEEYEDKpc2_NP.2
p−min=0.71, pX=0.88, N=292

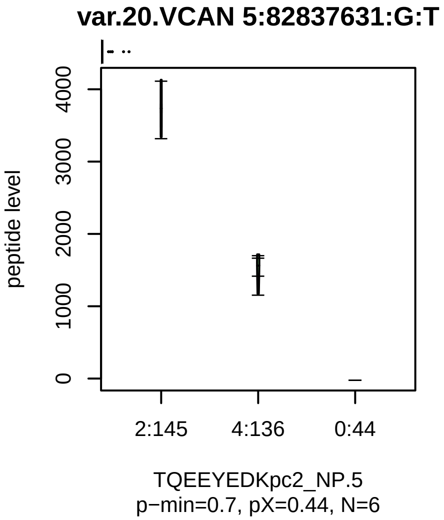
var.20.VCAN 5:82837631:G:T
peptide level
0
1000
2000
3000
4000
2:145
4:136
0:44
TQEEYEDKpc2_NP.5
p−min=0.7, pX=0.44, N=6

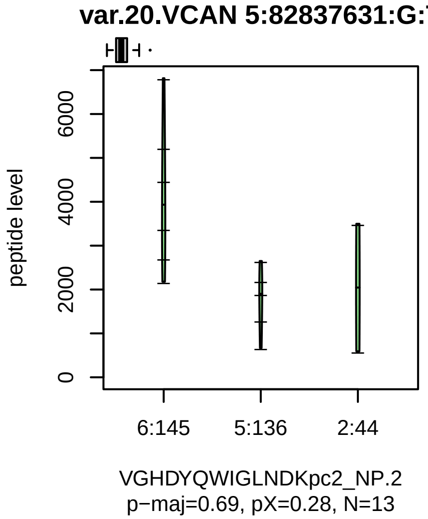
var.20.VCAN 5:82837631:G:T
peptide level
0
2000
4000
6000
6:145
5:136
2:44
VGHDYQWIGLNDKpc2_NP.2
p−maj=0.69, pX=0.28, N=13

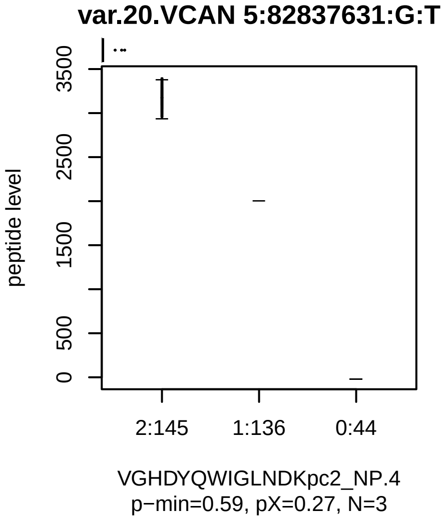
var.20.VCAN 5:82837631:G:T
peptide level
0
500
1500
2500
3500
2:145
1:136
0:44
VGHDYQWIGLNDKpc2_NP.4
p−min=0.59, pX=0.27, N=3

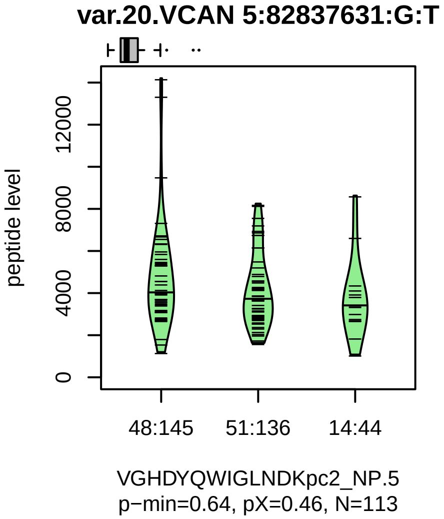
var.20.VCAN 5:82837631:G:T
peptide level
0
4000
8000
12000
48:145
51:136
14:44
VGHDYQWIGLNDKpc2_NP.5
p−min=0.64, pX=0.46, N=113

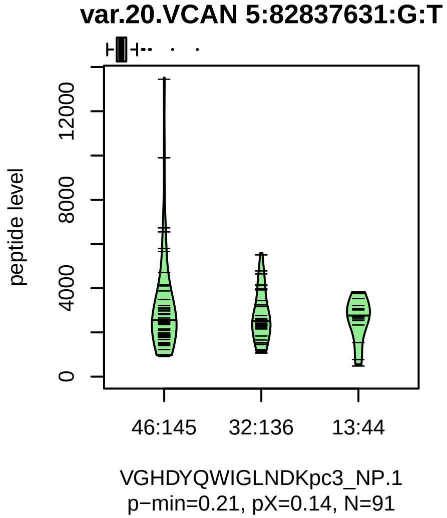
var.20.VCAN 5:82837631:G:T
peptide level
0
4000
8000
12000
46:145
32:136
13:44
VGHDYQWIGLNDKpc3_NP.1
p−min=0.21, pX=0.14, N=91

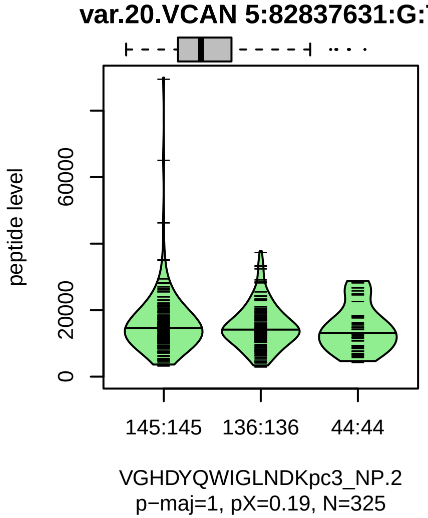
var.20.VCAN 5:82837631:G:T
peptide level
0
20000
60000
145:145
136:136
44:44
VGHDYQWIGLNDKpc3_NP.2
p−maj=1, pX=0.19, N=325

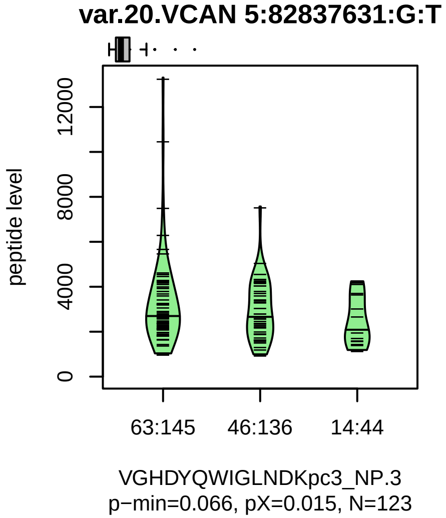
var.20.VCAN 5:82837631:G:T
peptide level
0
4000
8000
12000
63:145
46:136
14:44
VGHDYQWIGLNDKpc3_NP.3
p−min=0.066, pX=0.015, N=123

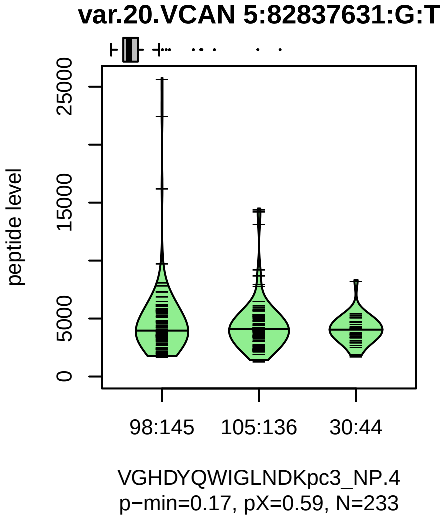
var.20.VCAN 5:82837631:G:T
peptide level
0
5000
15000
25000
98:145
105:136
30:44
VGHDYQWIGLNDKpc3_NP.4
p−min=0.17, pX=0.59, N=233

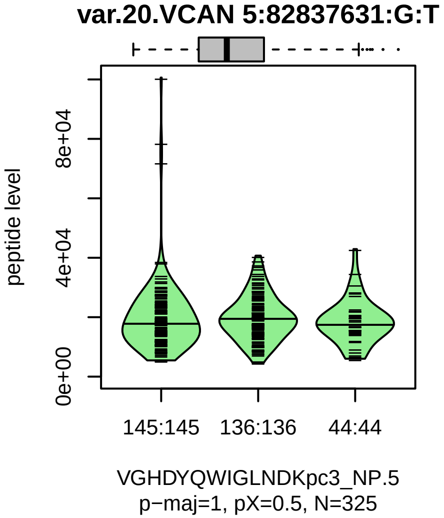
var.20.VCAN 5:82837631:G:T
peptide level
0e+00
4e+04
8e+04
145:145
136:136
44:44
VGHDYQWIGLNDKpc3_NP.5
p−maj=1, pX=0.5, N=325

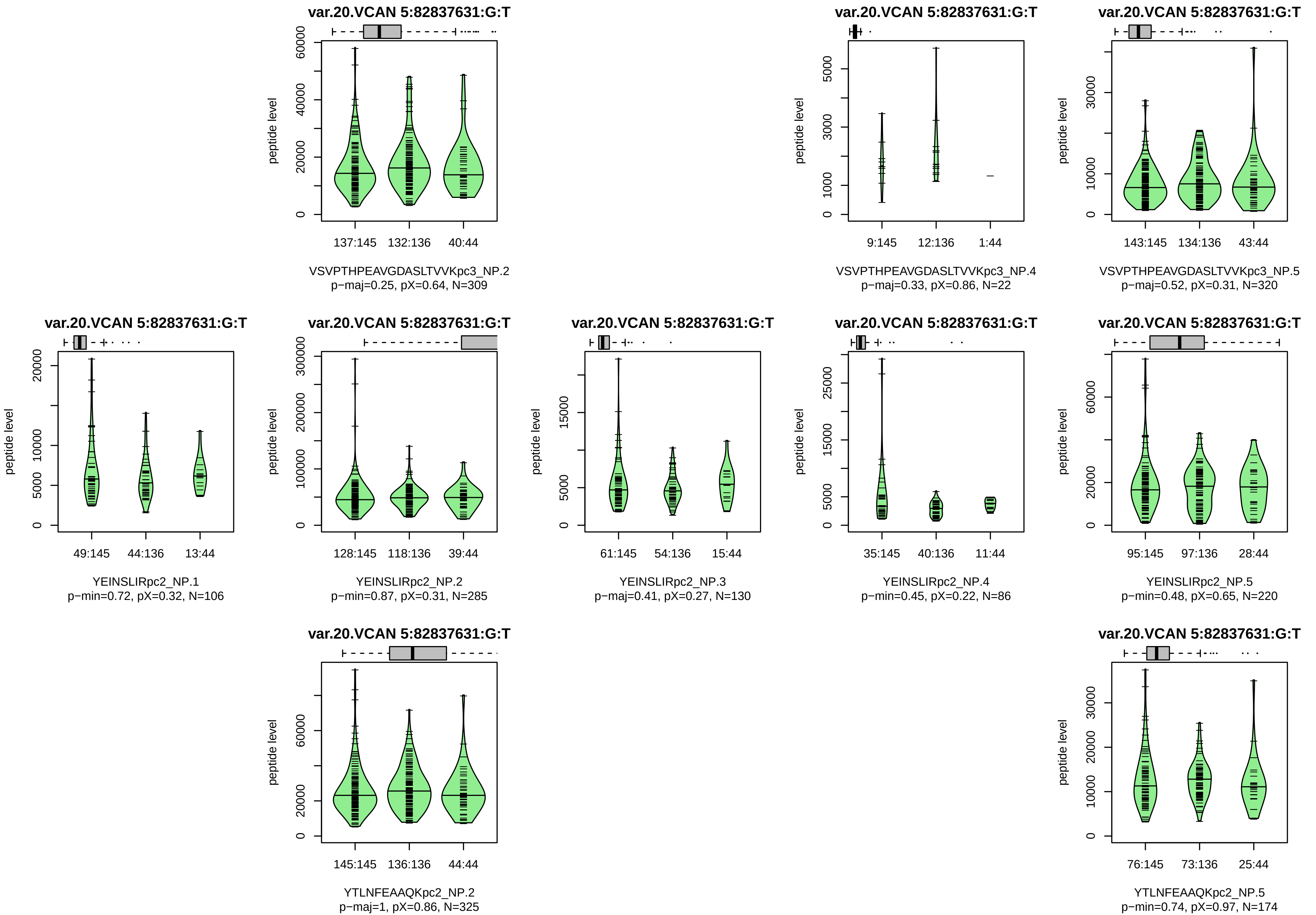
var.20.VCAN 5:82837631:G:T
peptide level
0
20000
40000
60000
137:145
132:136
40:44
VSVPTHPEAVGDASLTVVKpc3_NP.2
p−maj=0.25, pX=0.64, N=309
var.20.VCAN 5:82837631:G:T
peptide level
0
1000
3000
5000
9:145
12:136
1:44
VSVPTHPEAVGDASLTVVKpc3_NP.4
p−maj=0.33, pX=0.86, N=22
var.20.VCAN 5:82837631:G:T
peptide level
0
10000
20000
30000
143:145
134:136
43:44
VSVPTHPEAVGDASLTVVKpc3_NP.5
p−maj=0.52, pX=0.31, N=320
var.20.VCAN 5:82837631:G:T
peptide level
0
5000
10000
20000
49:145
44:136
13:44
YEINSLIRpc2_NP.1
p−min=0.72, pX=0.32, N=106
var.20.VCAN 5:82837631:G:T
peptide level
0
100000
200000
300000
128:145
118:136
39:44
YEINSLIRpc2_NP.2
p−min=0.87, pX=0.31, N=285
var.20.VCAN 5:82837631:G:T
peptide level
0
5000
15000
61:145
54:136
15:44
YEINSLIRpc2_NP.3
p−maj=0.41, pX=0.27, N=130
var.20.VCAN 5:82837631:G:T
peptide level
0
5000
15000
25000
35:145
40:136
11:44
YEINSLIRpc2_NP.4
p−min=0.45, pX=0.22, N=86
var.20.VCAN 5:82837631:G:T
peptide level
0
20000
60000
95:145
97:136
28:44
YEINSLIRpc2_NP.5
p−min=0.48, pX=0.65, N=220
var.20.VCAN 5:82837631:G:T
peptide level
0
20000
60000
145:145
136:136
44:44
YTLNFEAAQKpc2_NP.2
p−maj=1, pX=0.86, N=325
var.20.VCAN 5:82837631:G:T
peptide level
0
10000
20000
30000
76:145
73:136
25:44
YTLNFEAAQKpc2_NP.5
p−min=0.74, pX=0.97, N=174

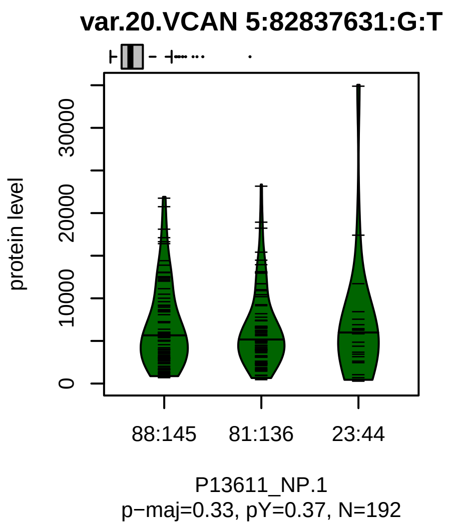
var.20.VCAN 5:82837631:G:T
protein level
0
10000
20000
30000
88:145
81:136
23:44
P13611_NP.1
p−maj=0.33, pY=0.37, N=192

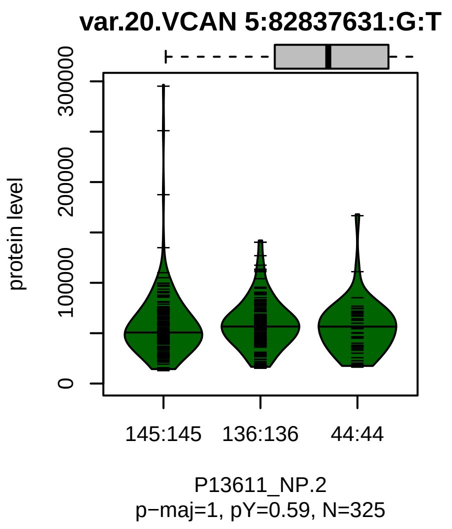
var.20.VCAN 5:82837631:G:T
protein level
0
100000
200000
300000
145:145
136:136
44:44
P13611_NP.2
p−maj=1, pY=0.59, N=325

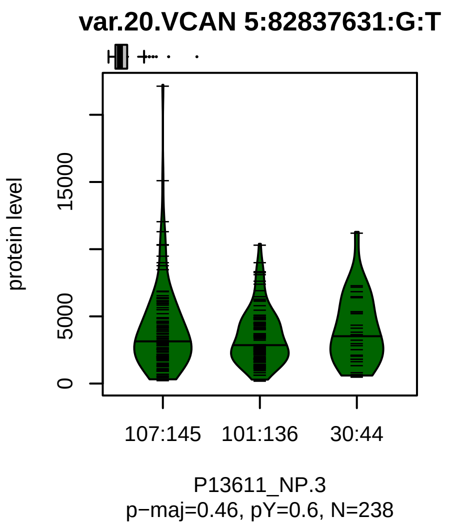
var.20.VCAN 5:82837631:G:T
protein level
0
5000
15000
107:145
101:136
30:44
P13611_NP.3
p−maj=0.46, pY=0.6, N=238

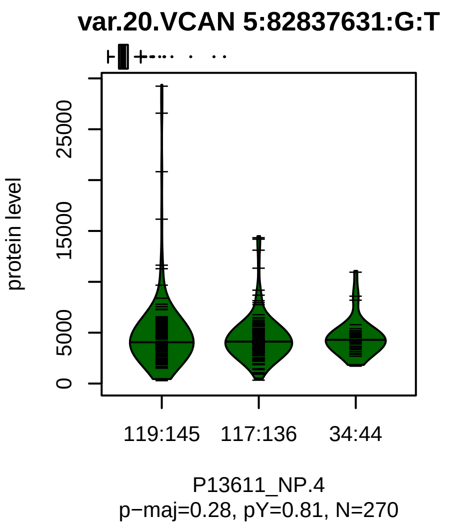
var.20.VCAN 5:82837631:G:T
protein level
0
5000
15000
25000
119:145
117:136
34:44
P13611_NP.4
p−maj=0.28, pY=0.81, N=270

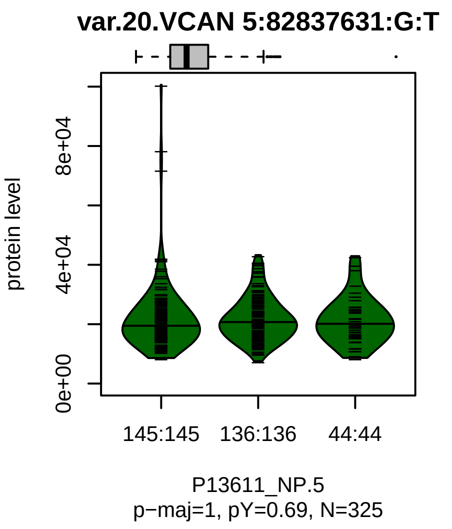
var.20.VCAN 5:82837631:G:T
protein level
0e+00
4e+04
8e+04
145:145
136:136
44:44
P13611_NP.5
p−maj=1, pY=0.69, N=325

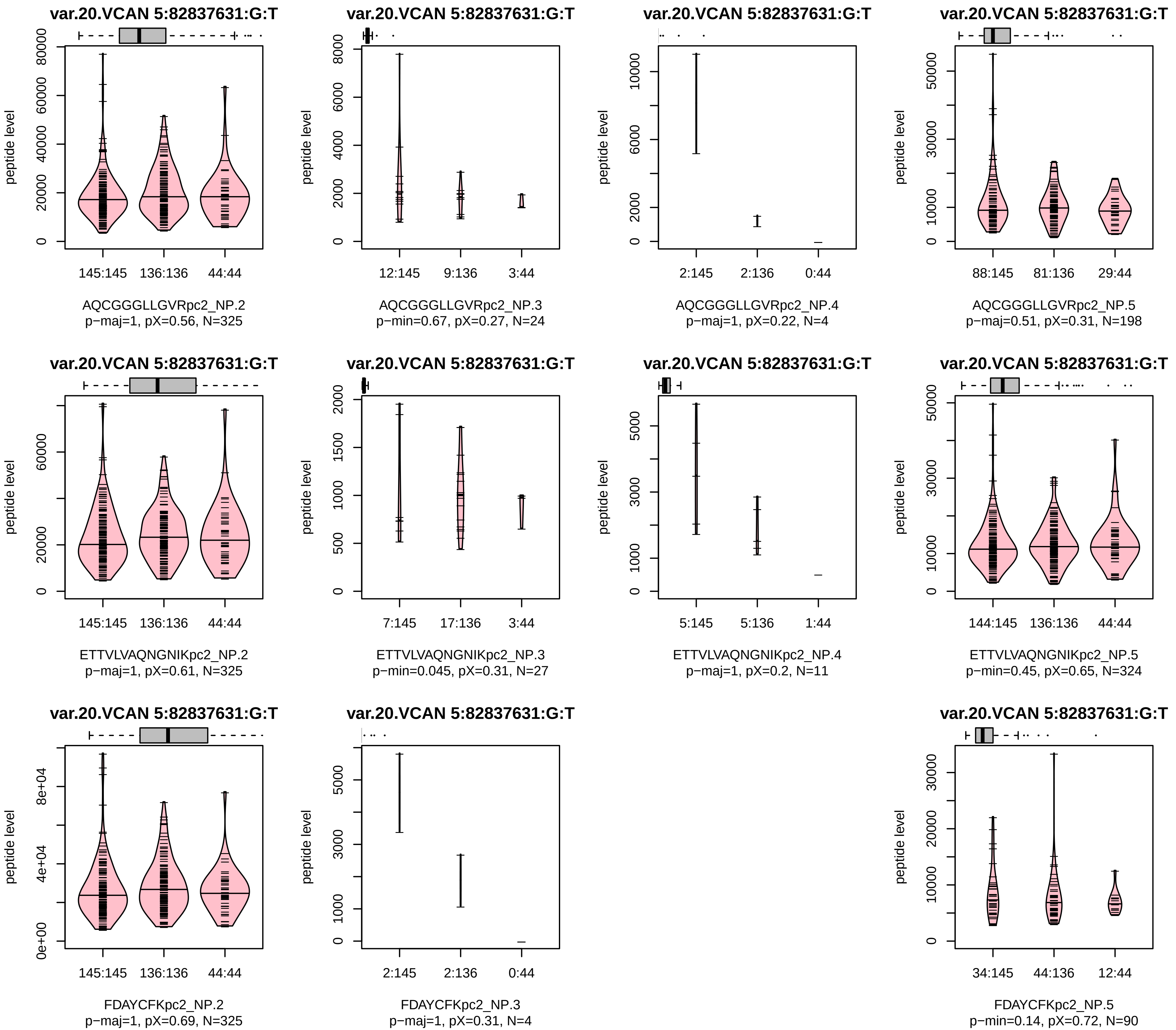
var.20.VCAN 5:82837631:G:T
peptide level
0
20000
40000
60000
80000
145:145
136:136
44:44
AQCGGGLLGVRpc2_NP.2
p−maj=1, pX=0.56, N=325
var.20.VCAN 5:82837631:G:T
peptide level
0
2000
4000
6000
8000
12:145
9:136
3:44
AQCGGGLLGVRpc2_NP.3
p−min=0.67, pX=0.27, N=24
var.20.VCAN 5:82837631:G:T
peptide level
0
2000
6000
10000
2:145
2:136
0:44
AQCGGGLLGVRpc2_NP.4
p−maj=1, pX=0.22, N=4
var.20.VCAN 5:82837631:G:T
peptide level
0
10000
30000
50000
88:145
81:136
29:44
AQCGGGLLGVRpc2_NP.5
p−maj=0.51, pX=0.31, N=198
var.20.VCAN 5:82837631:G:T
peptide level
0
20000
60000
145:145
136:136
44:44
ETTVLVAQNGNIKpc2_NP.2
p−maj=1, pX=0.61, N=325
var.20.VCAN 5:82837631:G:T
peptide level
0
500
1000
1500
2000
7:145
17:136
3:44
ETTVLVAQNGNIKpc2_NP.3
p−min=0.045, pX=0.31, N=27
var.20.VCAN 5:82837631:G:T
peptide level
0
1000
3000
5000
5:145
5:136
1:44
ETTVLVAQNGNIKpc2_NP.4
p−maj=1, pX=0.2, N=11
var.20.VCAN 5:82837631:G:T
peptide level
0
10000
30000
50000
144:145
136:136
44:44
ETTVLVAQNGNIKpc2_NP.5
p−min=0.45, pX=0.65, N=324
var.20.VCAN 5:82837631:G:T
peptide level
0e+00
4e+04
8e+04
145:145
136:136
44:44
FDAYCFKpc2_NP.2
p−maj=1, pX=0.69, N=325
var.20.VCAN 5:82837631:G:T
peptide level
0
1000
3000
5000
2:145
2:136
0:44
FDAYCFKpc2_NP.3
p−maj=1, pX=0.31, N=4
var.20.VCAN 5:82837631:G:T
peptide level
0
10000
20000
30000
34:145
44:136
12:44
FDAYCFKpc2_NP.5
p−min=0.14, pX=0.72, N=90

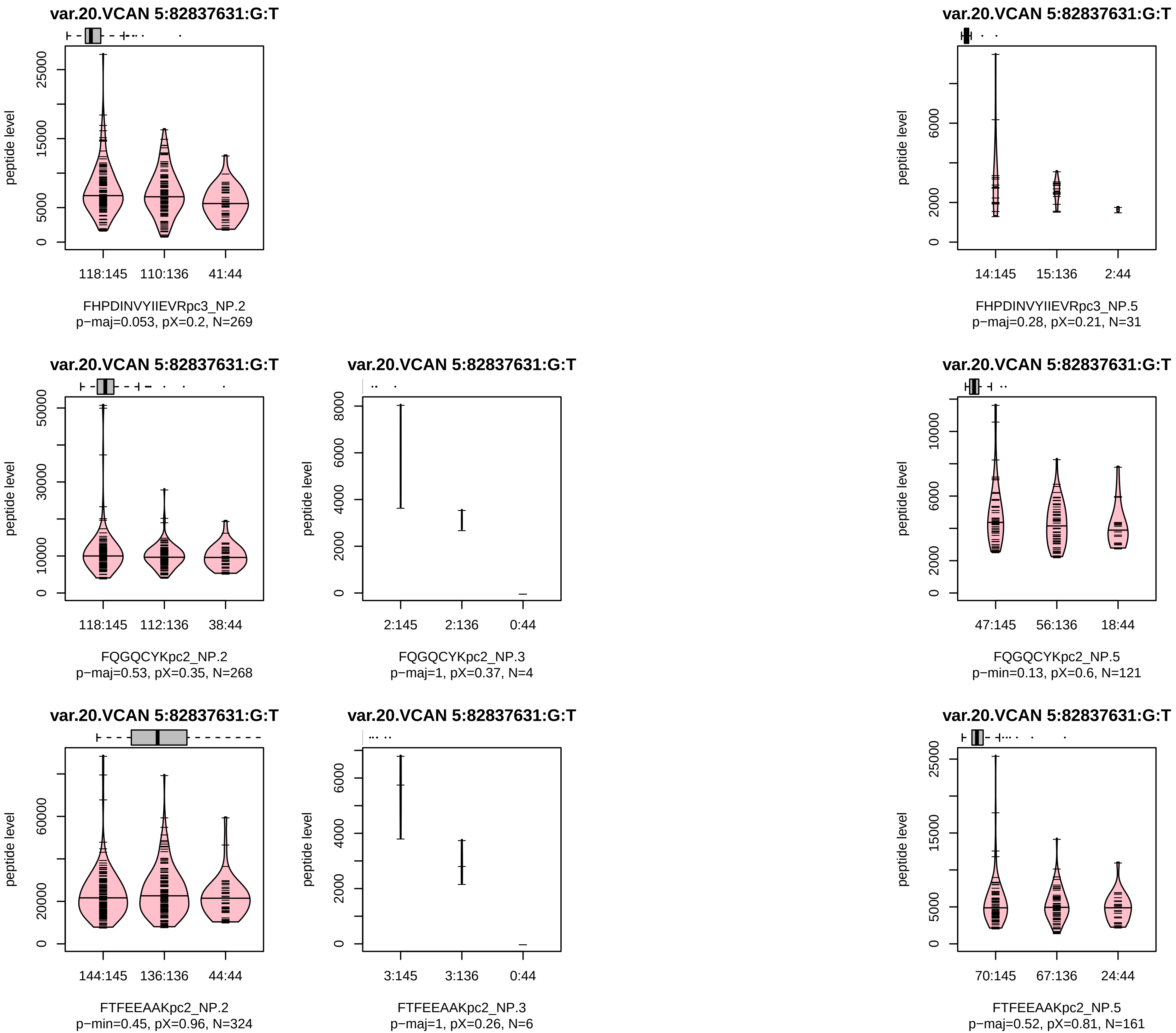
var.20.VCAN 5:82837631:G:T
peptide level
118:145
110:136
41:44
FHPDINVYIIEVRpc3_NP.2
p−maj=0.053, pX=0.2, N=269
var.20.VCAN 5:82837631:G:T
peptide level
14:145
15:136
2:44
FHPDINVYIIEVRpc3_NP.5
p−maj=0.28, pX=0.21, N=31
var.20.VCAN 5:82837631:G:T
peptide level
118:145
112:136
38:44
FQGQCYKpc2_NP.2
p−maj=0.53, pX=0.35, N=268
var.20.VCAN 5:82837631:G:T
peptide level
2:145
2:136
0:44
FQGQCYKpc2_NP.3
p−maj=1, pX=0.37, N=4
var.20.VCAN 5:82837631:G:T
peptide level
47:145
56:136
18:44
FQGQCYKpc2_NP.5
p−min=0.13, pX=0.6, N=121
var.20.VCAN 5:82837631:G:T
peptide level
144:145
136:136
44:44
FTFEEAAKpc2_NP.2
p−min=0.45, pX=0.96, N=324
var.20.VCAN 5:82837631:G:T
peptide level
3:145
3:136
0:44
FTFEEAAKpc2_NP.3
p−maj=1, pX=0.26, N=6
var.20.VCAN 5:82837631:G:T
peptide level
70:145
67:136
24:44
FTFEEAAKpc2_NP.5
p−maj=0.52, pX=0.81, N=161

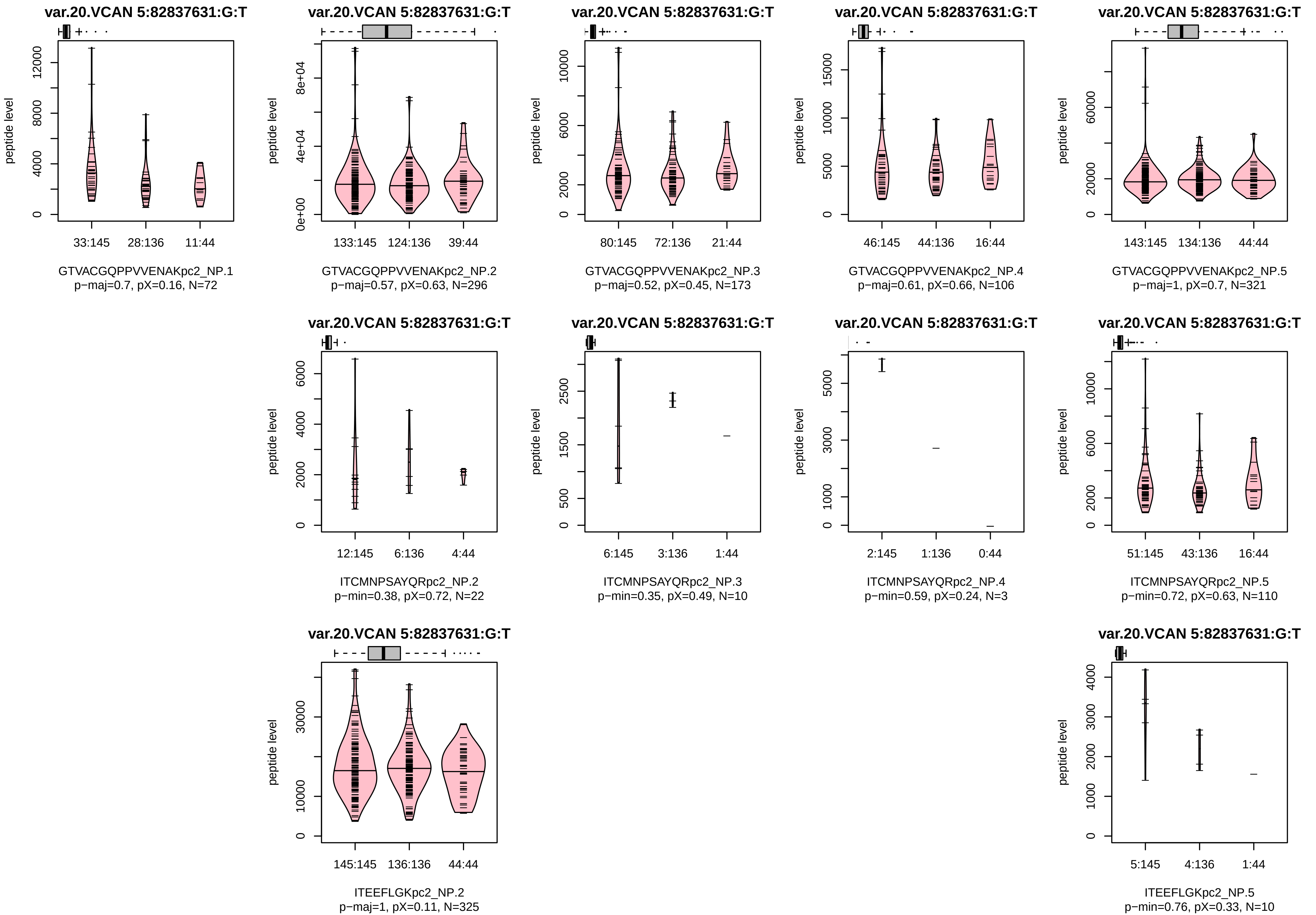

var.20.VCAN 5:82837631:G:T
peptide level
33:145
28:136
11:44
GTVACGQPPVVENAKpc2_NP.1
p−maj=0.7, pX=0.16, N=72
var.20.VCAN 5:82837631:G:T
133:145
124:136
39:44
GTVACGQPPVVENAKpc2_NP.2
p−maj=0.57, pX=0.63, N=296
var.20.VCAN 5:82837631:G:T
80:145
72:136
21:44
GTVACGQPPVVENAKpc2_NP.3
p−maj=0.52, pX=0.45, N=173
var.20.VCAN 5:82837631:G:T
46:145
44:136
16:44
GTVACGQPPVVENAKpc2_NP.4
p−maj=0.61, pX=0.66, N=106
var.20.VCAN 5:82837631:G:T
143:145
134:136
44:44
GTVACGQPPVVENAKpc2_NP.5
p−maj=1, pX=0.7, N=321
var.20.VCAN 5:82837631:G:T
12:145
6:136
4:44
ITCMNPSAYQRpc2_NP.2
p−min=0.38, pX=0.72, N=22
var.20.VCAN 5:82837631:G:T
6:145
3:136
1:44
ITCMNPSAYQRpc2_NP.3
p−min=0.35, pX=0.49, N=10
var.20.VCAN 5:82837631:G:T
2:145
1:136
0:44
ITCMNPSAYQRpc2_NP.4
p−min=0.59, pX=0.24, N=3
var.20.VCAN 5:82837631:G:T
51:145
43:136
16:44
ITCMNPSAYQRpc2_NP.5
p−min=0.72, pX=0.63, N=110
var.20.VCAN 5:82837631:G:T
145:145
136:136
44:44
ITEEFLGKpc2_NP.2
p−maj=1, pX=0.11, N=325
var.20.VCAN 5:82837631:G:T
5:145
4:136
1:44
ITEEFLGKpc2_NP.5
p−min=0.76, pX=0.33, N=10

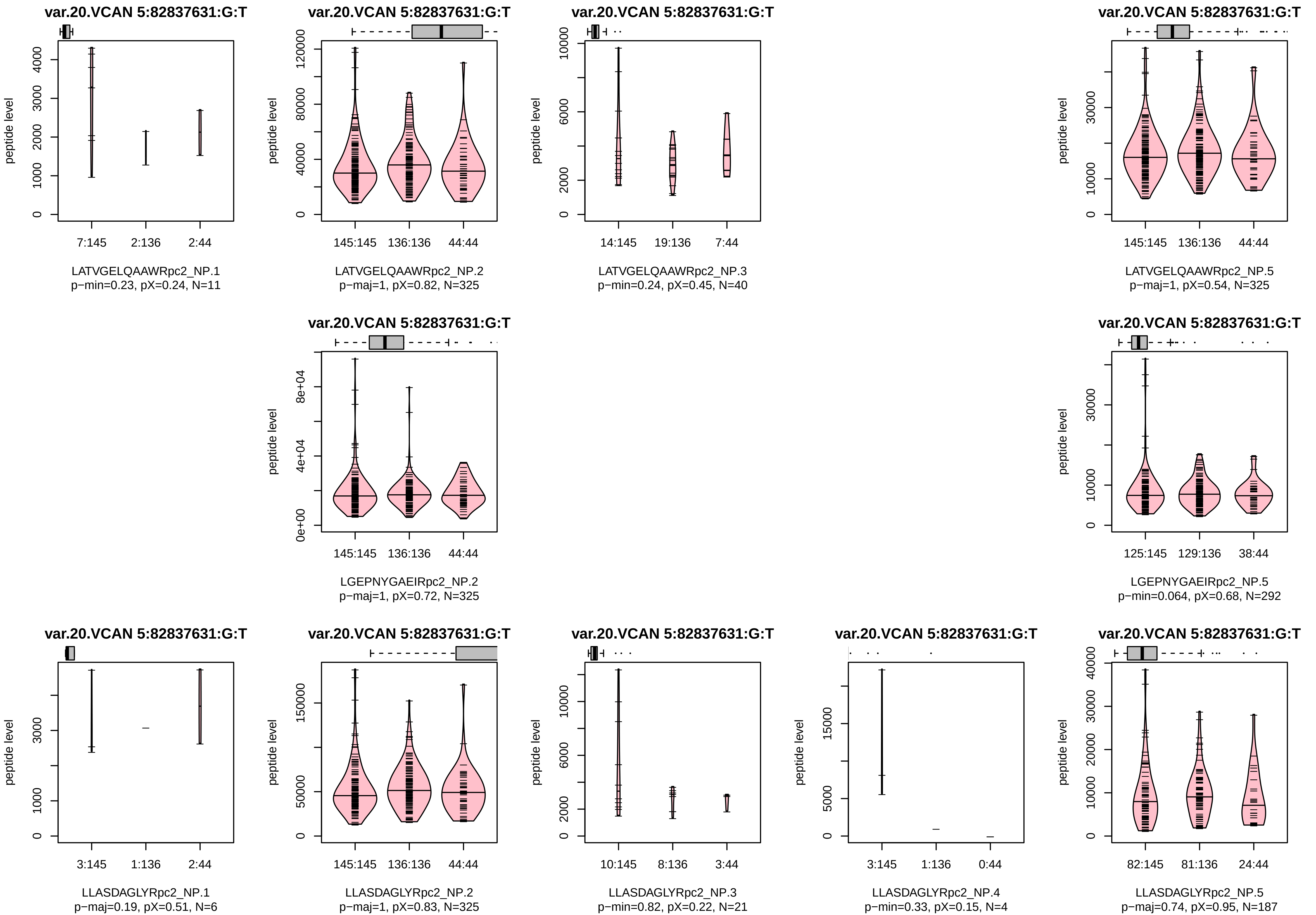
var.20.VCAN 5:82837631:G:T
peptide level
7:145
2:136
2:44
LATVGELQAAWRpc2_NP.1
p−min=0.23, pX=0.24, N=11
var.20.VCAN 5:82837631:G:T
peptide level
145:145
136:136
44:44
LATVGELQAAWRpc2_NP.2
p−maj=1, pX=0.82, N=325
var.20.VCAN 5:82837631:G:T
peptide level
14:145
19:136
7:44
LATVGELQAAWRpc2_NP.3
p−min=0.24, pX=0.45, N=40
var.20.VCAN 5:82837631:G:T
peptide level
145:145
136:136
44:44
LATVGELQAAWRpc2_NP.5
p−maj=1, pX=0.54, N=325
var.20.VCAN 5:82837631:G:T
peptide level
145:145
136:136
44:44
LGEPNYGAEIRpc2_NP.2
p−maj=1, pX=0.72, N=325
var.20.VCAN 5:82837631:G:T
peptide level
125:145
129:136
38:44
LGEPNYGAEIRpc2_NP.5
p−min=0.064, pX=0.68, N=292
var.20.VCAN 5:82837631:G:T
peptide level
3:145
1:136
2:44
LLASDAGLYRpc2_NP.1
p−maj=0.19, pX=0.51, N=6
var.20.VCAN 5:82837631:G:T
peptide level
145:145
136:136
44:44
LLASDAGLYRpc2_NP.2
p−maj=1, pX=0.83, N=325
var.20.VCAN 5:82837631:G:T
peptide level
10:145
8:136
3:44
LLASDAGLYRpc2_NP.3
p−min=0.82, pX=0.22, N=21
var.20.VCAN 5:82837631:G:T
peptide level
3:145
1:136
0:44
LLASDAGLYRpc2_NP.4
p−min=0.33, pX=0.15, N=4
var.20.VCAN 5:82837631:G:T
peptide level
82:145
81:136
24:44
LLASDAGLYRpc2_NP.5
p−maj=0.74, pX=0.95, N=187

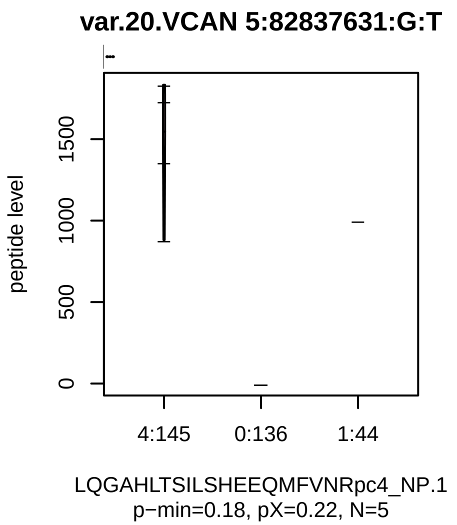
var.20.VCAN 5:82837631:G:T
peptide level
0
500
1000
1500
4:145
0:136
1:44
LQGAHLTSILSHEEQMFVNRpc4_NP.1
p−min=0.18, pX=0.22, N=5

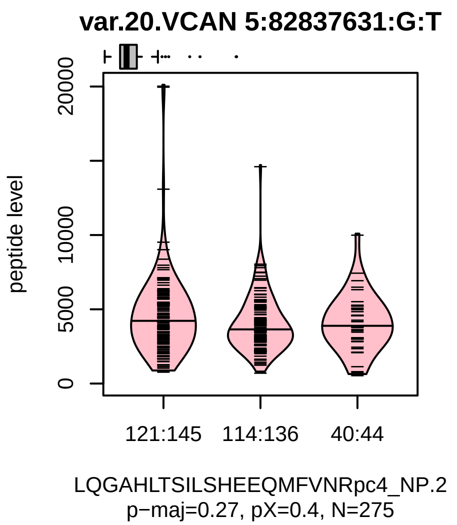
var.20.VCAN 5:82837631:G:T
peptide level
0
5000
10000
20000
121:145
114:136
40:44
LQGAHLTSILSHEEQMFVNRpc4_NP.2
p−maj=0.27, pX=0.4, N=275

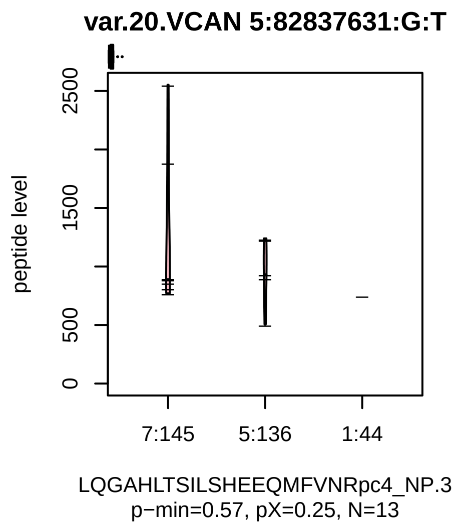
var.20.VCAN 5:82837631:G:T
peptide level
0
500
1500
2500
7:145
5:136
1:44
LQGAHLTSILSHEEQMFVNRpc4_NP.3
p−min=0.57, pX=0.25, N=13

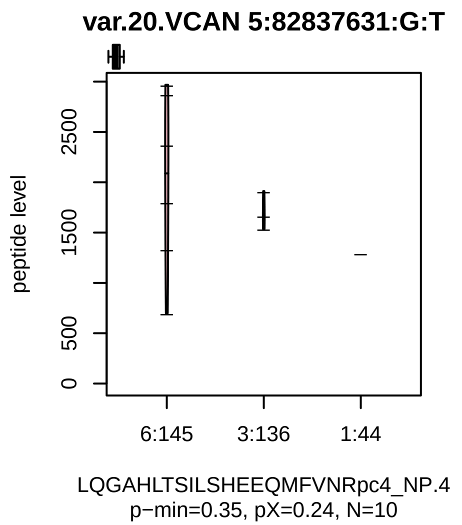
var.20.VCAN 5:82837631:G:T
peptide level
0
500
1500
2500
6:145
3:136
1:44
LQGAHLTSILSHEEQMFVNRpc4_NP.4
p−min=0.35, pX=0.24, N=10

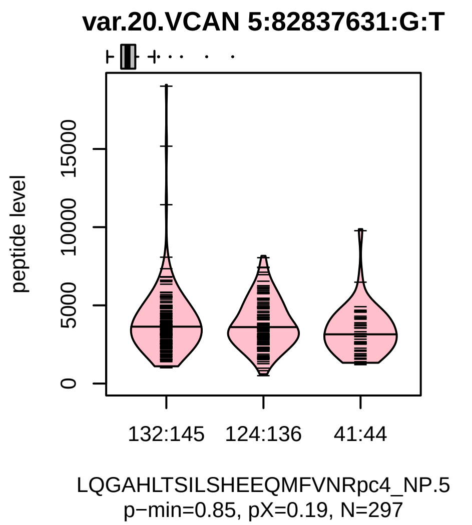
var.20.VCAN 5:82837631:G:T
peptide level
0
5000
10000
15000
132:145
124:136
41:44
LQGAHLTSILSHEEQMFVNRpc4_NP.5
p−min=0.85, pX=0.19, N=297

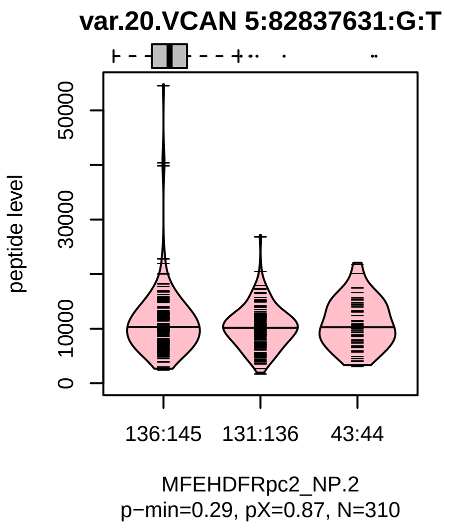
var.20.VCAN 5:82837631:G:T
peptide level
0
10000
30000
50000
136:145
131:136
43:44
MFEHDFRpc2_NP.2
p−min=0.29, pX=0.87, N=310

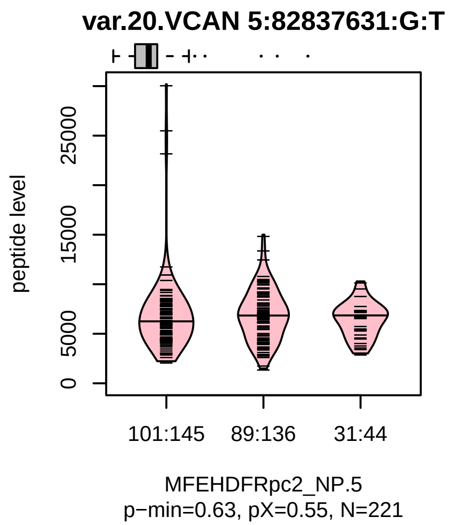
var.20.VCAN 5:82837631:G:T
peptide level
0
5000
15000
25000
101:145
89:136
31:44
MFEHDFRpc2_NP.5
p−min=0.63, pX=0.55, N=221

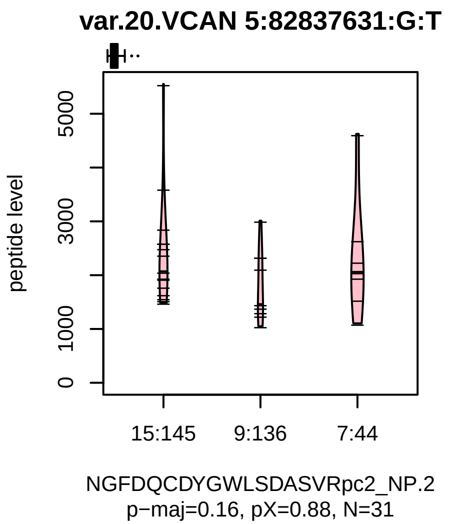
var.20.VCAN 5:82837631:G:T
peptide level
0
1000
3000
5000
15:145
9:136
7:44
NGFDQCDYGWLSDASVRpc2_NP.2
p−maj=0.16, pX=0.88, N=31

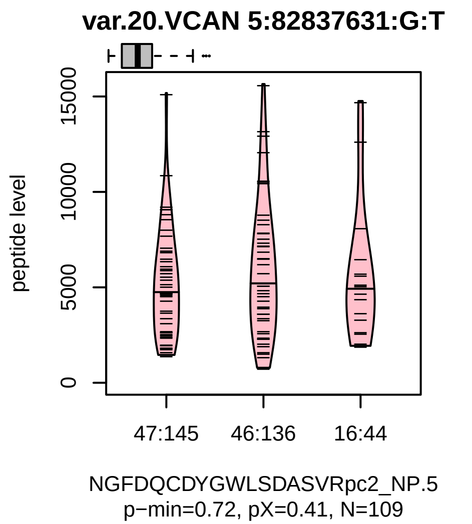
var.20.VCAN 5:82837631:G:T
peptide level
0
5000
10000
15000
47:145
46:136
16:44
NGFDQCDYGWLSDASVRpc2_NP.5
p−min=0.72, pX=0.41, N=109

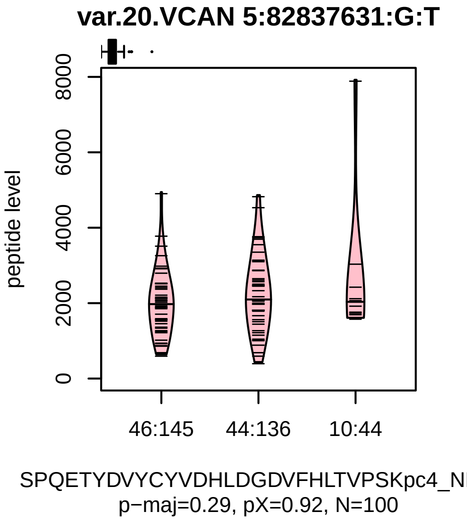
var.20.VCAN 5:82837631:G:T
peptide level
0
2000
4000
6000
8000
46:145
44:136
10:44
SPQETYDVYCYVDHLDGDVFHLTVPSKpc4_N
p−maj=0.29, pX=0.92, N=100

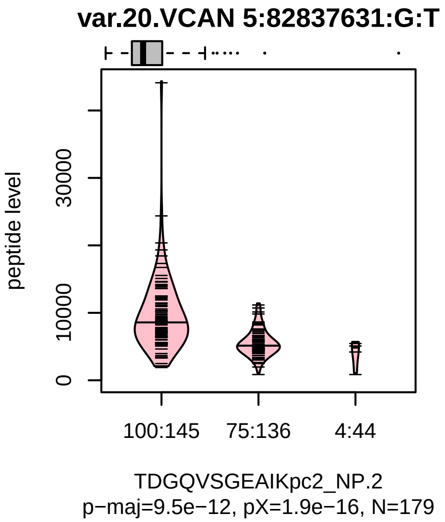
var.20.VCAN 5:82837631:G:T
peptide level
0
10000
30000
100:145
75:136
4:44
TDGQVSGEAIKpc2_NP.2
p−maj=9.5e−12, pX=1.9e−16, N=179

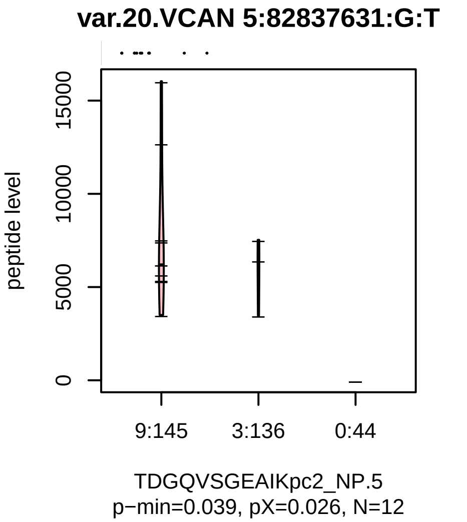
var.20.VCAN 5:82837631:G:T
peptide level
0
5000
10000
15000
9:145
3:136
0:44
TDGQVSGEAIKpc2_NP.5
p−min=0.039, pX=0.026, N=12

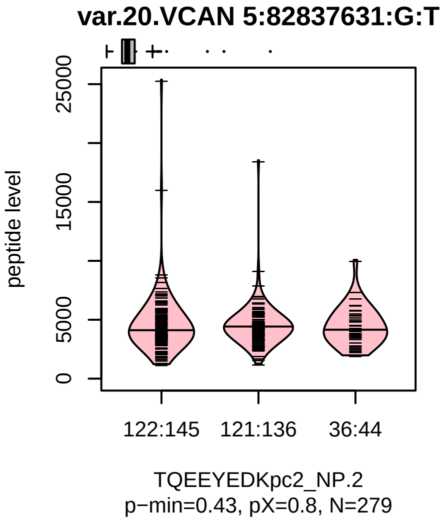
var.20.VCAN 5:82837631:G:T
peptide level
0
5000
15000
25000
122:145
121:136
36:44
TQEEYEDKpc2_NP.2
p−min=0.43, pX=0.8, N=279

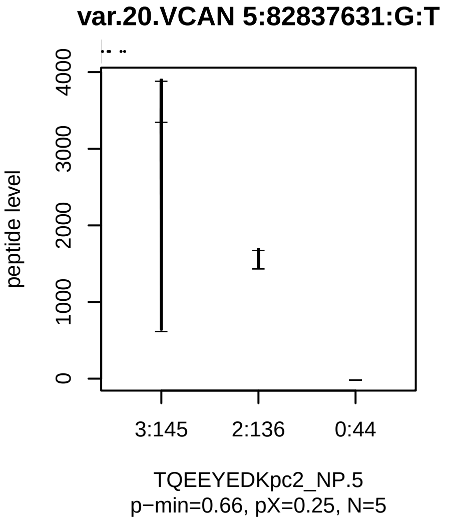
var.20.VCAN 5:82837631:G:T
peptide level
0
1000
2000
3000
4000
3:145
2:136
0:44
TQEEYEDKpc2_NP.5
p−min=0.66, pX=0.25, N=5

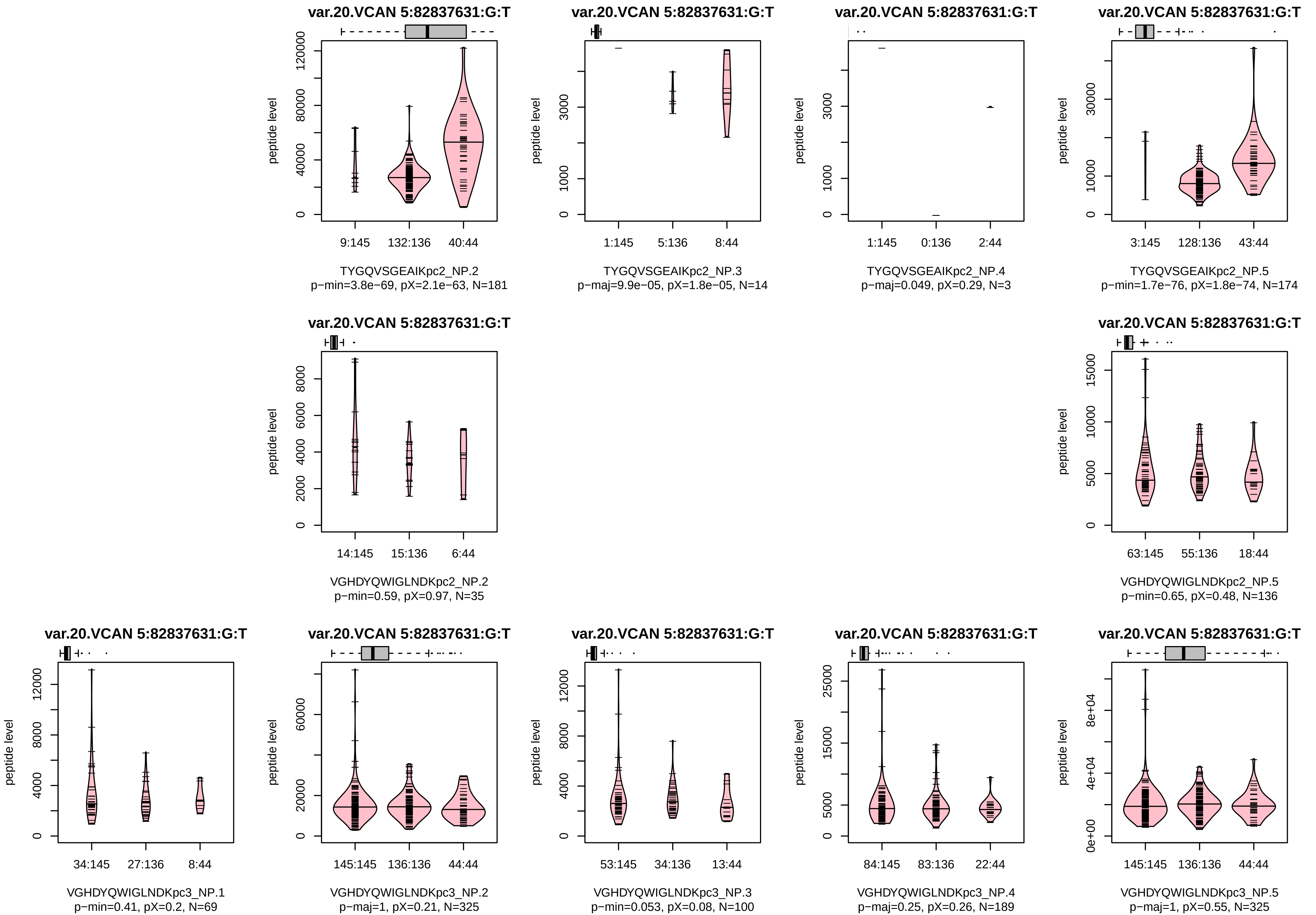
var.20.VCAN 5:82837631:G:T
peptide level
9:145
132:136
40:44
TYGQVSGEAIKpc2_NP.2
p−min=3.8e−69, pX=2.1e−63, N=181
1:145
5:136
8:44
TYGQVSGEAIKpc2_NP.3
p−maj=9.9e−05, pX=1.8e−05, N=14
1:145
0:136
2:44
TYGQVSGEAIKpc2_NP.4
p−maj=0.049, pX=0.29, N=3
3:145
128:136
43:44
TYGQVSGEAIKpc2_NP.5
p−min=1.7e−76, pX=1.8e−74, N=174
14:145
15:136
6:44
VGHDYQWIGLNDKpc2_NP.2
p−min=0.59, pX=0.97, N=35
63:145
55:136
18:44
VGHDYQWIGLNDKpc2_NP.5
p−min=0.65, pX=0.48, N=136
34:145
27:136
8:44
VGHDYQWIGLNDKpc3_NP.1
p−min=0.41, pX=0.2, N=69
145:145
136:136
44:44
VGHDYQWIGLNDKpc3_NP.2
p−maj=1, pX=0.21, N=325
53:145
34:136
13:44
VGHDYQWIGLNDKpc3_NP.3
p−min=0.053, pX=0.08, N=100
84:145
83:136
22:44
VGHDYQWIGLNDKpc3_NP.4
p−maj=0.25, pX=0.26, N=189
145:145
136:136
44:44
VGHDYQWIGLNDKpc3_NP.5
p−maj=1, pX=0.55, N=325

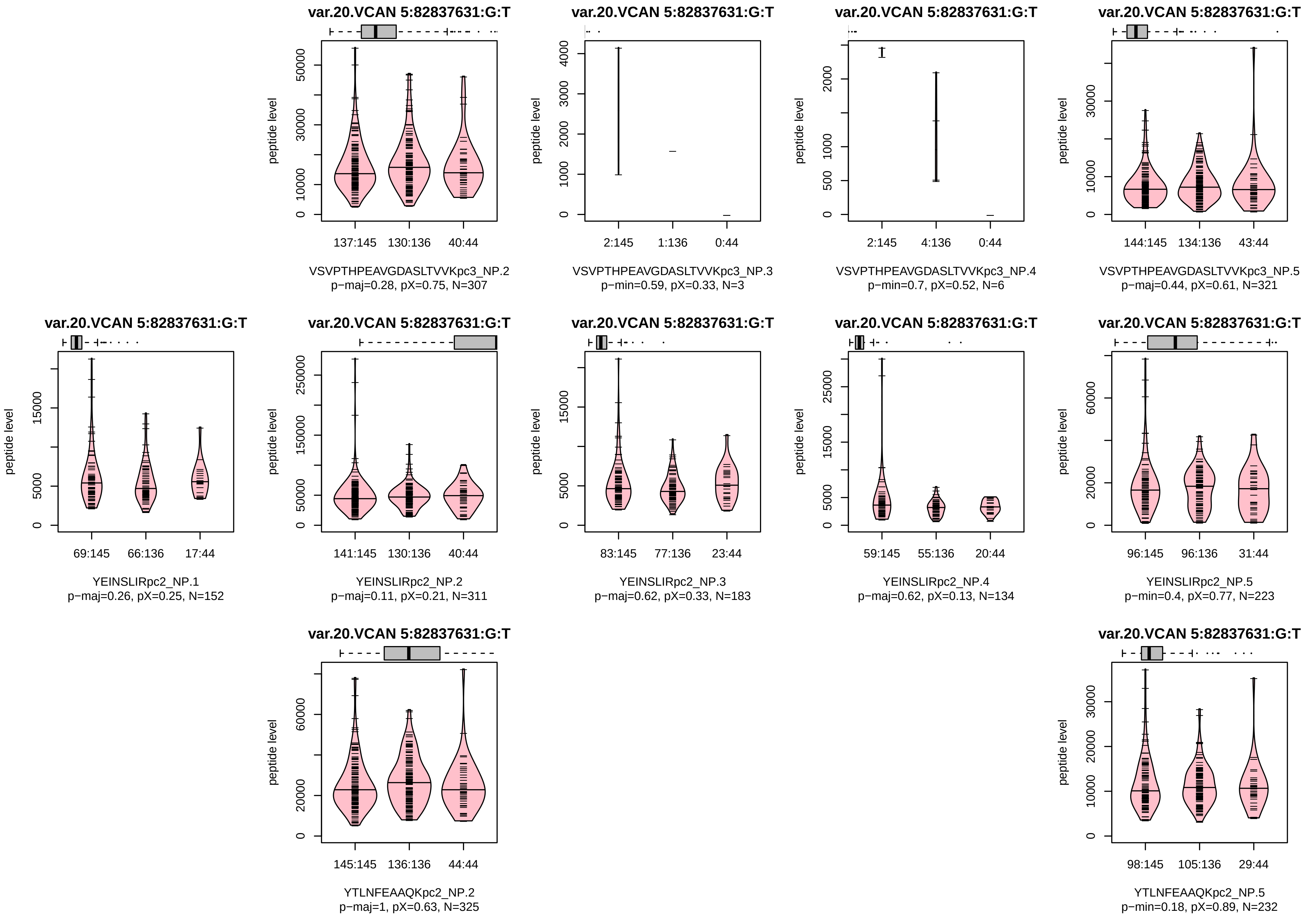
var.20.VCAN 5:82837631:G:T
peptide level
137:145
130:136
40:44
VSVPTHPEAVGDASLTVVKpc3_NP.2
p−maj=0.28, pX=0.75, N=307
var.20.VCAN 5:82837631:G:T
peptide level
2:145
1:136
0:44
VSVPTHPEAVGDASLTVVKpc3_NP.3
p−min=0.59, pX=0.33, N=3
var.20.VCAN 5:82837631:G:T
peptide level
2:145
4:136
0:44
VSVPTHPEAVGDASLTVVKpc3_NP.4
p−min=0.7, pX=0.52, N=6
var.20.VCAN 5:82837631:G:T
peptide level
144:145
134:136
43:44
VSVPTHPEAVGDASLTVVKpc3_NP.5
p−maj=0.44, pX=0.61, N=321
var.20.VCAN 5:82837631:G:T
peptide level
69:145
66:136
17:44
YEINSLIRpc2_NP.1
p−maj=0.26, pX=0.25, N=152
var.20.VCAN 5:82837631:G:T
peptide level
141:145
130:136
40:44
YEINSLIRpc2_NP.2
p−maj=0.11, pX=0.21, N=311
var.20.VCAN 5:82837631:G:T
peptide level
83:145
77:136
23:44
YEINSLIRpc2_NP.3
p−maj=0.62, pX=0.33, N=183
var.20.VCAN 5:82837631:G:T
peptide level
59:145
55:136
20:44
YEINSLIRpc2_NP.4
p−maj=0.62, pX=0.13, N=134
var.20.VCAN 5:82837631:G:T
peptide level
96:145
96:136
31:44
YEINSLIRpc2_NP.5
p−min=0.4, pX=0.77, N=223
var.20.VCAN 5:82837631:G:T
peptide level
145:145
136:136
44:44
YTLNFEAAQKpc2_NP.2
p−maj=1, pX=0.63, N=325
var.20.VCAN 5:82837631:G:T
peptide level
98:145
105:136
29:44
YTLNFEAAQKpc2_NP.5
p−min=0.18, pX=0.89, N=232

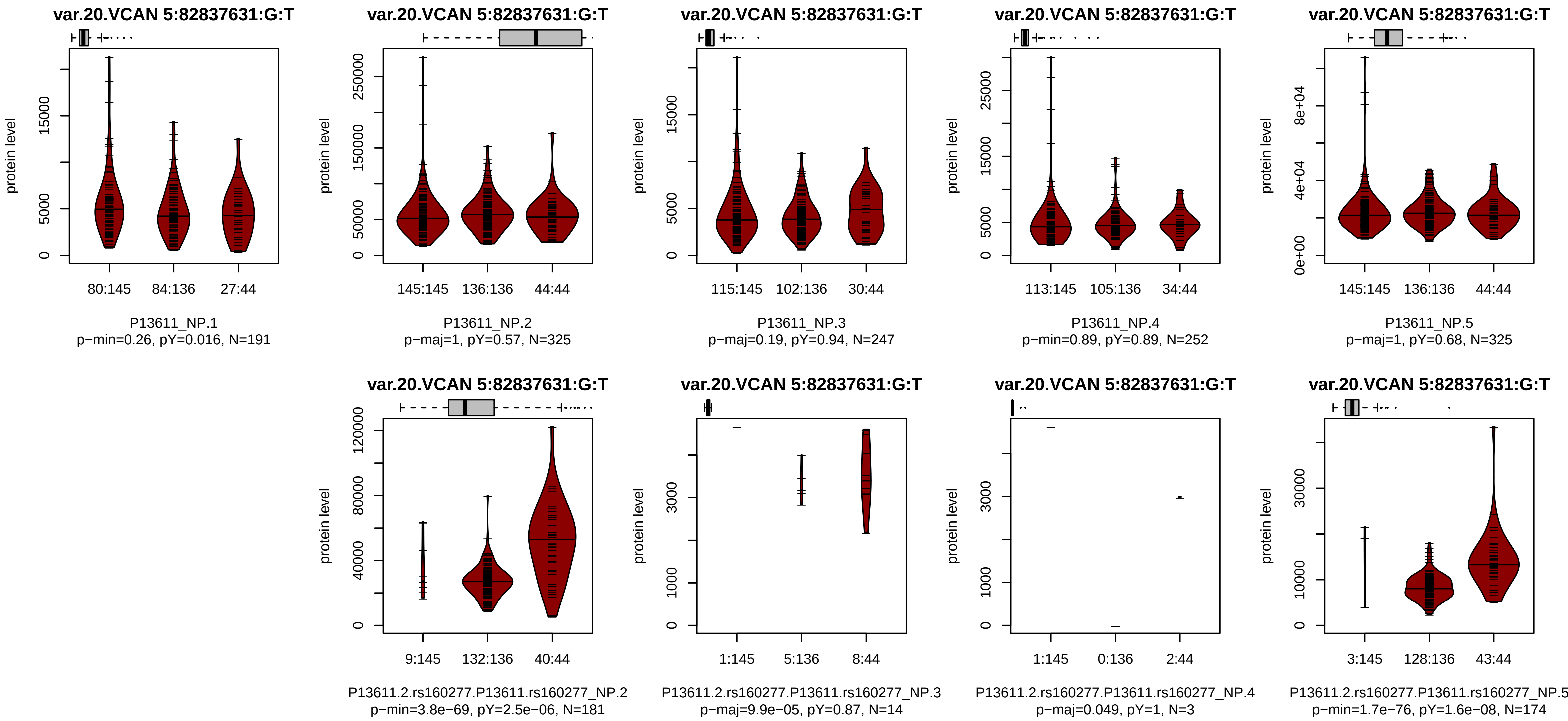
var.20.VCAN 5:82837631:G:T
protein level
80:145
84:136
27:44
P13611_NP.1
p−min=0.26, pY=0.016, N=191
var.20.VCAN 5:82837631:G:T
protein level
145:145
136:136
44:44
P13611_NP.2
p−maj=1, pY=0.57, N=325
var.20.VCAN 5:82837631:G:T
protein level
115:145
102:136
30:44
P13611_NP.3
p−maj=0.19, pY=0.94, N=247
var.20.VCAN 5:82837631:G:T
protein level
113:145
105:136
34:44
P13611_NP.4
p−min=0.89, pY=0.89, N=252
var.20.VCAN 5:82837631:G:T
protein level
145:145
136:136
44:44
P13611_NP.5
p−maj=1, pY=0.68, N=325
var.20.VCAN 5:82837631:G:T
protein level
9:145
132:136
40:44
P13611.2.rs160277.P13611.rs160277_NP.2
p−min=3.8e−69, pY=2.5e−06, N=181
var.20.VCAN 5:82837631:G:T
protein level
1:145
5:136
8:44
P13611.2.rs160277.P13611.rs160277_NP.3
p−maj=9.9e−05, pY=0.87, N=14
var.20.VCAN 5:82837631:G:T
protein level
1:145
0:136
2:44
P13611.2.rs160277.P13611.rs160277_NP.4
p−maj=0.049, pY=1, N=3
var.20.VCAN 5:82837631:G:T
protein level
3:145
128:136
43:44
P13611.2.rs160277.P13611.rs160277_NP.5
p−min=1.7e−76, pY=1.6e−08, N=174

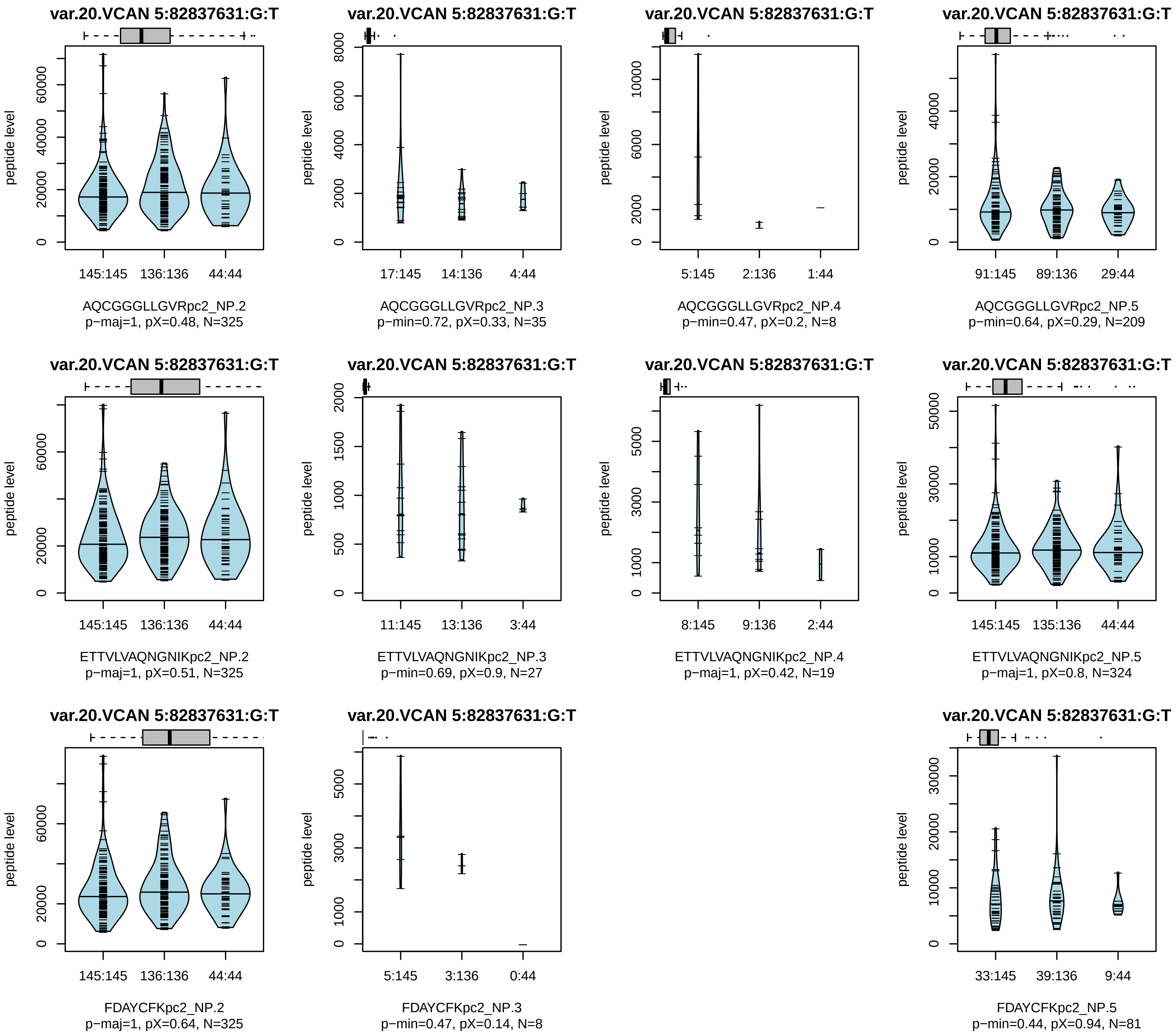
var.20.VCAN 5:82837631:G:T
peptide level
145:145
136:136
44:44
AQCGGGLLGVRpc2_NP.2
p−maj=1, pX=0.48, N=325
var.20.VCAN 5:82837631:G:T
peptide level
17:145
14:136
4:44
AQCGGGLLGVRpc2_NP.3
p−min=0.72, pX=0.33, N=35
var.20.VCAN 5:82837631:G:T
peptide level
5:145
2:136
1:44
AQCGGGLLGVRpc2_NP.4
p−min=0.47, pX=0.2, N=8
var.20.VCAN 5:82837631:G:T
peptide level
91:145
89:136
29:44
AQCGGGLLGVRpc2_NP.5
p−min=0.64, pX=0.29, N=209
var.20.VCAN 5:82837631:G:T
peptide level
145:145
136:136
44:44
ETTVLVAQNGNIKpc2_NP.2
p−maj=1, pX=0.51, N=325
var.20.VCAN 5:82837631:G:T
peptide level
11:145
13:136
3:44
ETTVLVAQNGNIKpc2_NP.3
p−min=0.69, pX=0.9, N=27
var.20.VCAN 5:82837631:G:T
peptide level
8:145
9:136
2:44
ETTVLVAQNGNIKpc2_NP.4
p−maj=1, pX=0.42, N=19
var.20.VCAN 5:82837631:G:T
peptide level
145:145
135:136
44:44
ETTVLVAQNGNIKpc2_NP.5
p−maj=1, pX=0.8, N=324
var.20.VCAN 5:82837631:G:T
peptide level
145:145
136:136
44:44
FDAYCFKpc2_NP.2
p−maj=1, pX=0.64, N=325
var.20.VCAN 5:82837631:G:T
peptide level
5:145
3:136
0:44
FDAYCFKpc2_NP.3
p−min=0.47, pX=0.14, N=8
var.20.VCAN 5:82837631:G:T
peptide level
33:145
39:136
9:44
FDAYCFKpc2_NP.5
p−min=0.44, pX=0.94, N=81

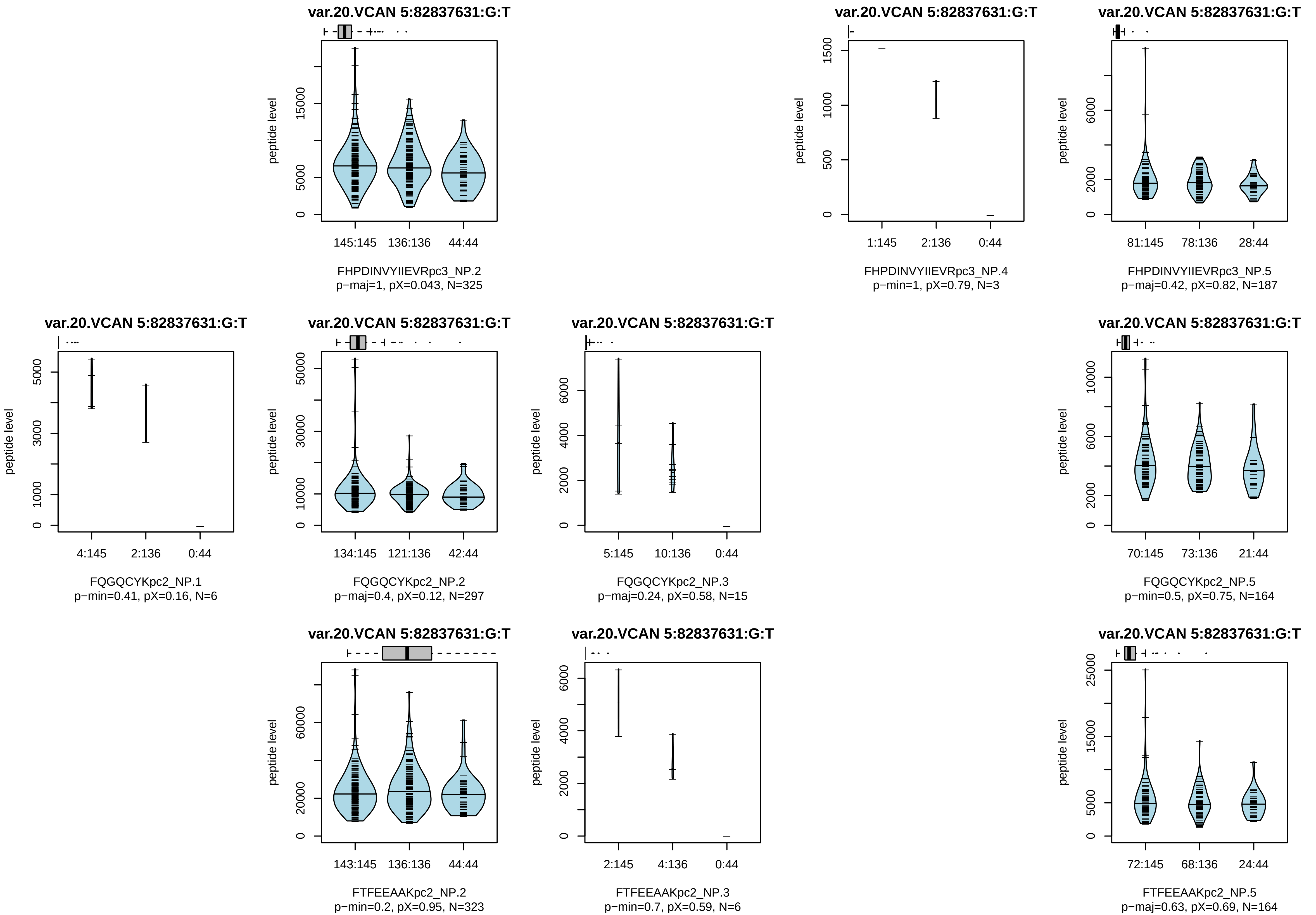

var.20.VCAN 5:82837631:G:T
peptide level
0
5000
15000
145:145
136:136
44:44
FHPDINVYIIEVRpc3_NP.2
p−maj=1, pX=0.043, N=325
var.20.VCAN 5:82837631:G:T
peptide level
0
500
1000
1500
1:145
2:136
0:44
FHPDINVYIIEVRpc3_NP.4
p−min=1, pX=0.79, N=3
var.20.VCAN 5:82837631:G:T
peptide level
0
2000
6000
81:145
78:136
28:44
FHPDINVYIIEVRpc3_NP.5
p−maj=0.42, pX=0.82, N=187
var.20.VCAN 5:82837631:G:T
peptide level
0
1000
3000
5000
4:145
2:136
0:44
FQGQCYKpc2_NP.1
p−min=0.41, pX=0.16, N=6
var.20.VCAN 5:82837631:G:T
peptide level
0
10000
30000
50000
134:145
121:136
42:44
FQGQCYKpc2_NP.2
p−maj=0.4, pX=0.12, N=297
var.20.VCAN 5:82837631:G:T
peptide level
0
2000
4000
6000
5:145
10:136
0:44
FQGQCYKpc2_NP.3
p−maj=0.24, pX=0.58, N=15
var.20.VCAN 5:82837631:G:T
peptide level
2000
6000
10000
70:145
73:136
21:44
FQGQCYKpc2_NP.5
p−min=0.5, pX=0.75, N=164
var.20.VCAN 5:82837631:G:T
peptide level
0
20000
60000
143:145
136:136
44:44
FTFEEAAKpc2_NP.2
p−min=0.2, pX=0.95, N=323
var.20.VCAN 5:82837631:G:T
peptide level
0
2000
4000
6000
2:145
4:136
0:44
FTFEEAAKpc2_NP.3
p−min=0.7, pX=0.59, N=6
var.20.VCAN 5:82837631:G:T
peptide level
0
5000
15000
25000
72:145
68:136
24:44
FTFEEAAKpc2_NP.5
p−maj=0.63, pX=0.69, N=164

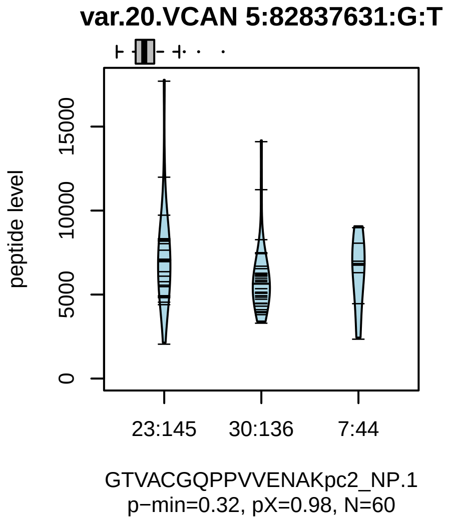
var.20.VCAN 5:82837631:G:T
peptide level
0
5000
10000
15000
23:145
30:136
7:44
GTVACGQPPVVENAKpc2_NP.1
p−min=0.32, pX=0.98, N=60

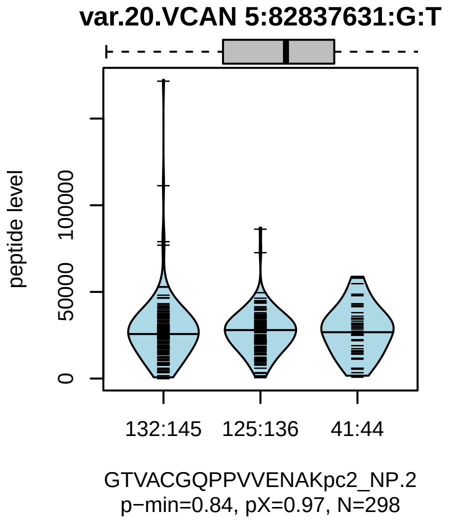
var.20.VCAN 5:82837631:G:T
peptide level
0
50000
100000
132:145
125:136
41:44
GTVACGQPPVVENAKpc2_NP.2
p−min=0.84, pX=0.97, N=298

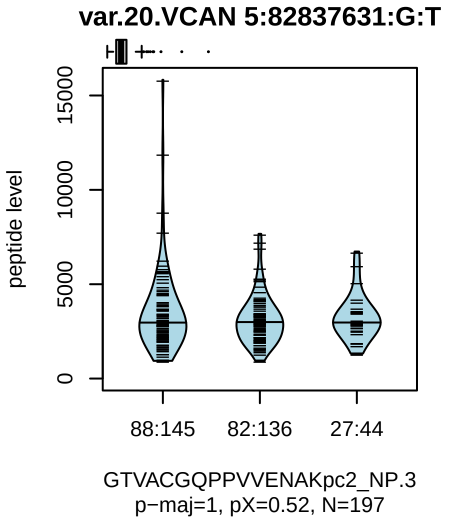
var.20.VCAN 5:82837631:G:T
peptide level
0
5000
10000
15000
88:145
82:136
27:44
GTVACGQPPVVENAKpc2_NP.3
p−maj=1, pX=0.52, N=197

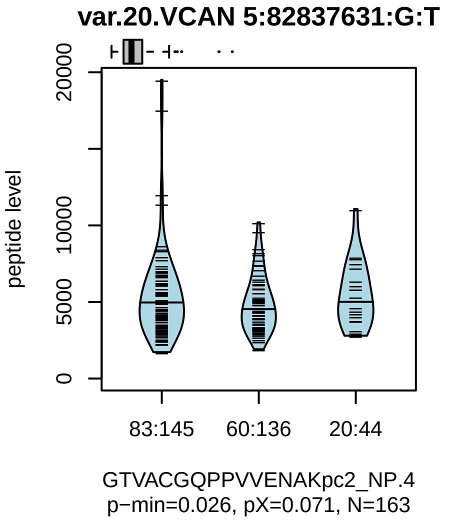
var.20.VCAN 5:82837631:G:T
peptide level
0
5000
10000
20000
83:145
60:136
20:44
GTVACGQPPVVENAKpc2_NP.4
p−min=0.026, pX=0.071, N=163

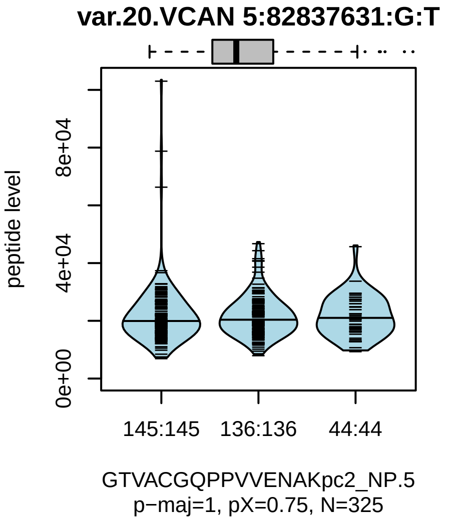
var.20.VCAN 5:82837631:G:T
peptide level
0e+00
4e+04
8e+04
145:145
136:136
44:44
GTVACGQPPVVENAKpc2_NP.5
p−maj=1, pX=0.75, N=325

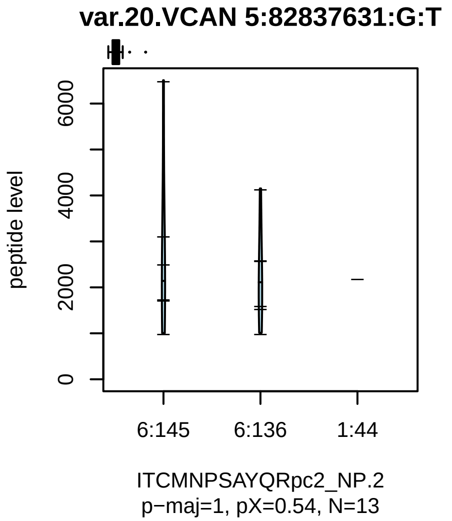
var.20.VCAN 5:82837631:G:T
peptide level
0
2000
4000
6000
6:145
6:136
1:44
ITCMNPSAYQRpc2_NP.2
p−maj=1, pX=0.54, N=13

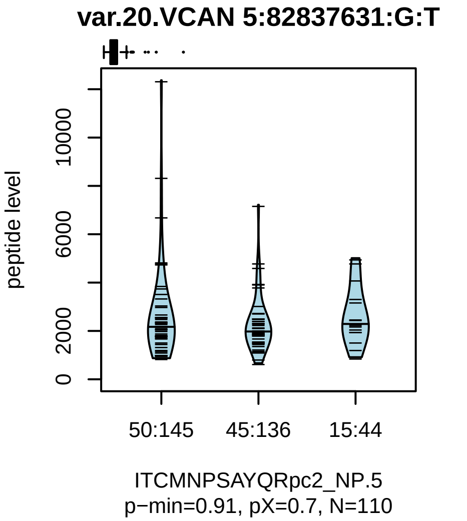
var.20.VCAN 5:82837631:G:T
peptide level
0
2000
6000
10000
50:145
45:136
15:44
ITCMNPSAYQRpc2_NP.5
p−min=0.91, pX=0.7, N=110

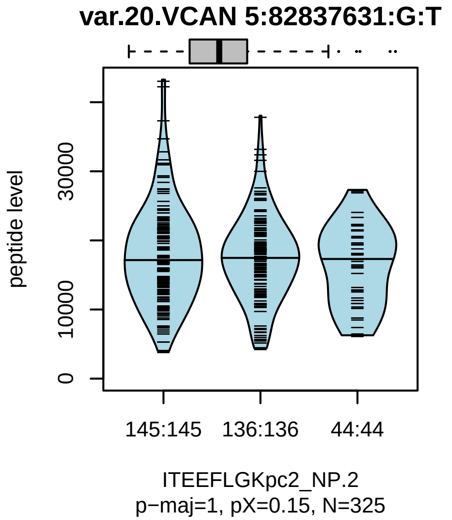
var.20.VCAN 5:82837631:G:T
peptide level
0
10000
30000
145:145
136:136
44:44
ITEEFLGKpc2_NP.2
p−maj=1, pX=0.15, N=325

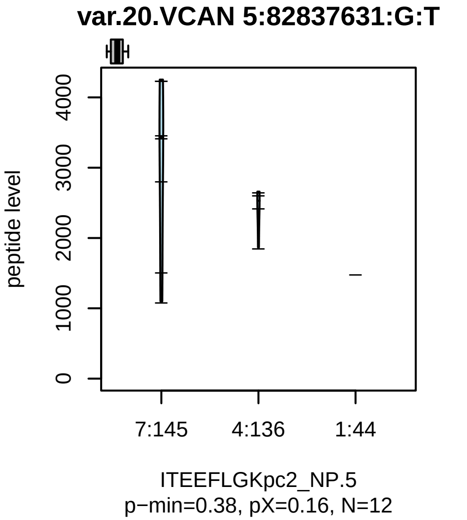
var.20.VCAN 5:82837631:G:T
peptide level
0
1000
2000
3000
4000
7:145
4:136
1:44
ITEEFLGKpc2_NP.5
p−min=0.38, pX=0.16, N=12

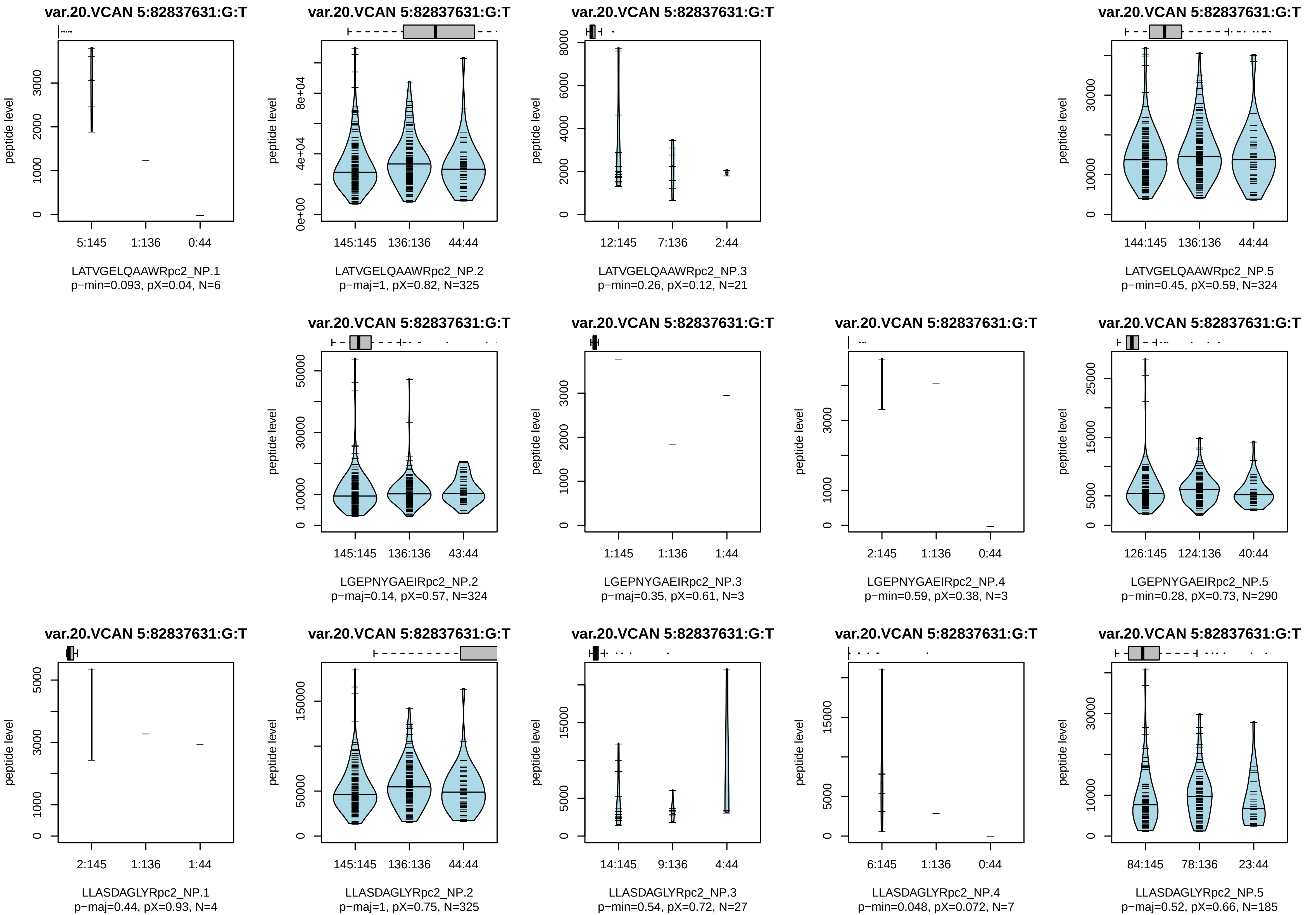

var.20.VCAN 5:82837631:G:T
peptide level
5:145
1:136
0:44
LATVGELQAAWRpc2_NP.1
p−min=0.093, pX=0.04, N=6
var.20.VCAN 5:82837631:G:T
peptide level
145:145
136:136
44:44
LATVGELQAAWRpc2_NP.2
p−maj=1, pX=0.82, N=325
var.20.VCAN 5:82837631:G:T
peptide level
12:145
7:136
2:44
LATVGELQAAWRpc2_NP.3
p−min=0.26, pX=0.12, N=21
var.20.VCAN 5:82837631:G:T
peptide level
144:145
136:136
44:44
LATVGELQAAWRpc2_NP.5
p−min=0.45, pX=0.59, N=324
var.20.VCAN 5:82837631:G:T
peptide level
145:145
136:136
43:44
LGEPNYGAEIRpc2_NP.2
p−maj=0.14, pX=0.57, N=324
var.20.VCAN 5:82837631:G:T
peptide level
1:145
1:136
1:44
LGEPNYGAEIRpc2_NP.3
p−maj=0.35, pX=0.61, N=3
var.20.VCAN 5:82837631:G:T
peptide level
2:145
1:136
0:44
LGEPNYGAEIRpc2_NP.4
p−min=0.59, pX=0.38, N=3
var.20.VCAN 5:82837631:G:T
peptide level
126:145
124:136
40:44
LGEPNYGAEIRpc2_NP.5
p−min=0.28, pX=0.73, N=290
var.20.VCAN 5:82837631:G:T
peptide level
2:145
1:136
1:44
LLASDAGLYRpc2_NP.1
p−maj=0.44, pX=0.93, N=4
var.20.VCAN 5:82837631:G:T
peptide level
145:145
136:136
44:44
LLASDAGLYRpc2_NP.2
p−maj=1, pX=0.75, N=325
var.20.VCAN 5:82837631:G:T
peptide level
14:145
9:136
4:44
LLASDAGLYRpc2_NP.3
p−min=0.54, pX=0.72, N=27
var.20.VCAN 5:82837631:G:T
peptide level
6:145
1:136
0:44
LLASDAGLYRpc2_NP.4
p−min=0.048, pX=0.072, N=7
var.20.VCAN 5:82837631:G:T
peptide level
84:145
78:136
23:44
LLASDAGLYRpc2_NP.5
p−maj=0.52, pX=0.66, N=185

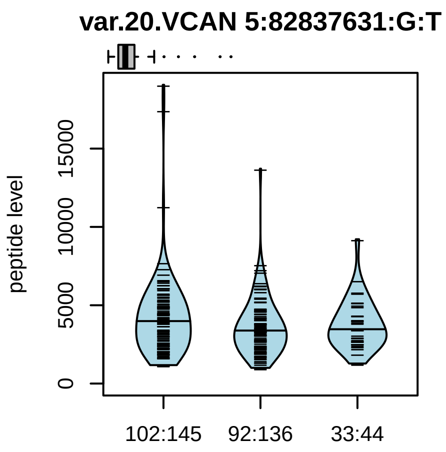

LQGAHLTSILSHEEQMFVNRpc4_NP.2
p−maj=0.48, pX=0.37, N=227

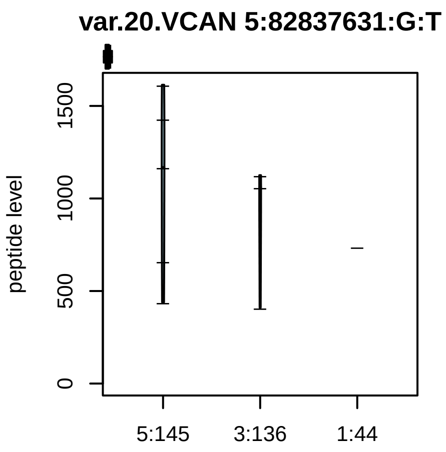

LQGAHLTSILSHEEQMFVNRpc4_NP.3
p−min=0.52, pX=0.38, N=9

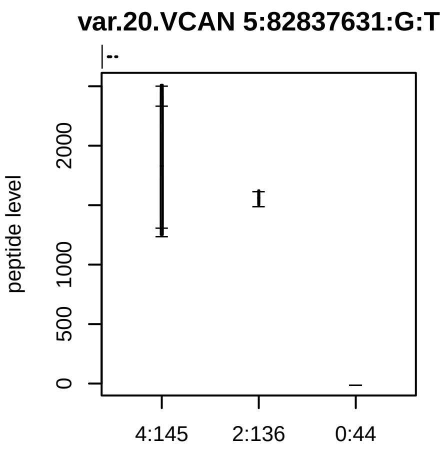

LQGAHLTSILSHEEQMFVNRpc4_NP.4
p−min=0.41, pX=0.18, N=6

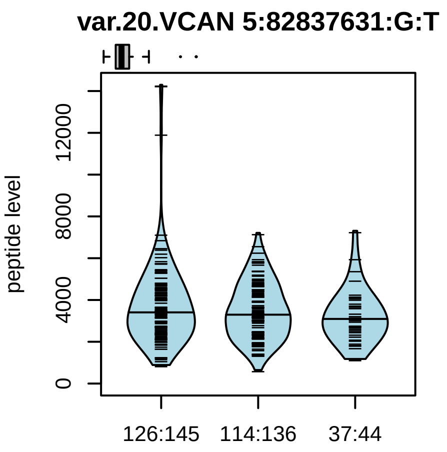

LQGAHLTSILSHEEQMFVNRpc4_NP.5
p−min=0.53, pX=0.065, N=277

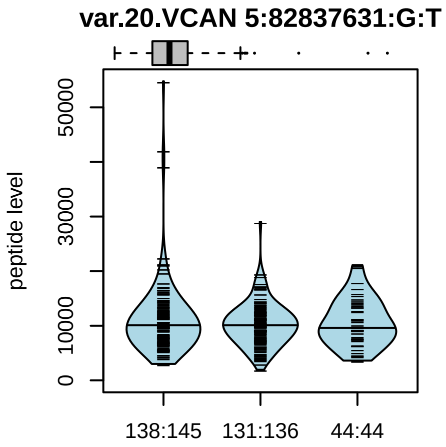

MFEHDFRpc2_NP.2
p−maj=0.38, pX=0.78, N=313

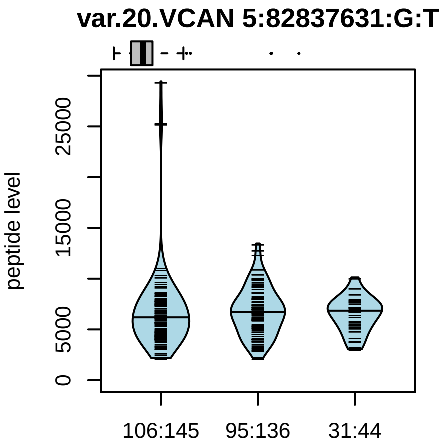

MFEHDFRpc2_NP.5
p−min=0.62, pX=0.55, N=232

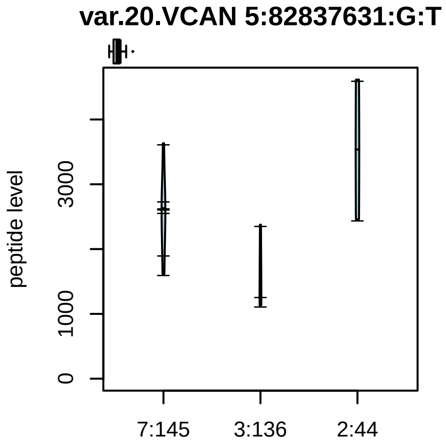

NGFDQCDYGWLSDASVRpc2_NP.2
p−min=0.38, pX=0.81, N=12

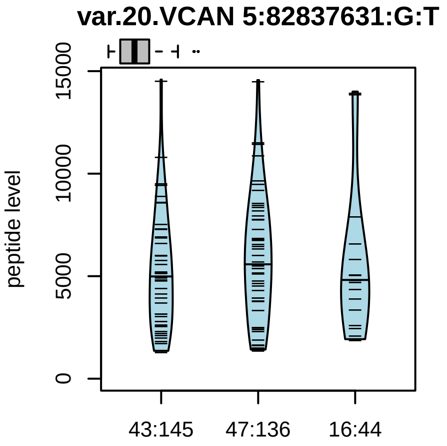

NGFDQCDYGWLSDASVRpc2_NP.5
p−min=0.34, pX=0.22, N=106

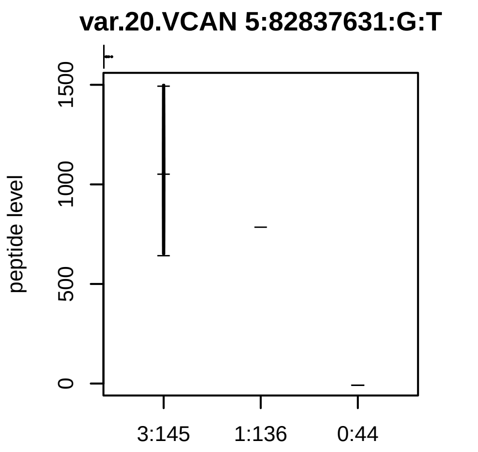

SPQETYDVYCYVDHLDGDVFHLTVPSKpc4_NI
p−min=0.33, pX=0.18, N=4

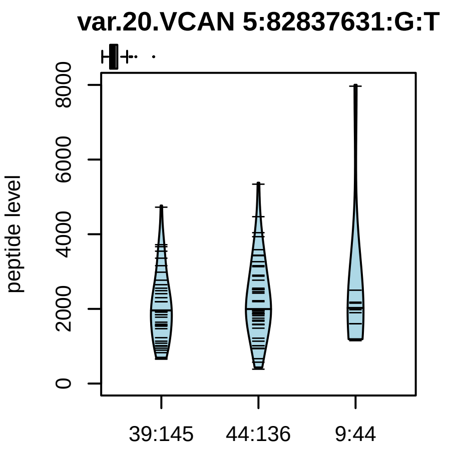

SPQETYDVYCYVDHLDGDVFHLTVPSKpc4_NI
p−maj=0.28, pX=0.67, N=92

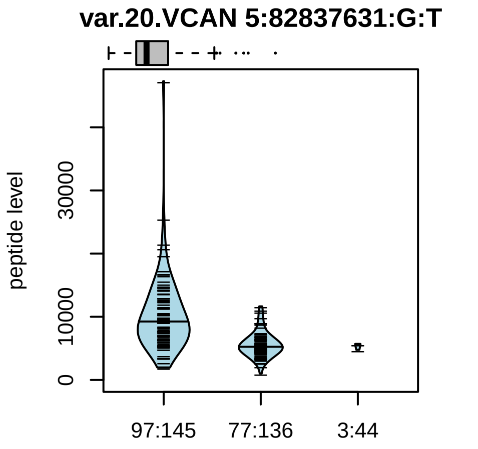

TDGQVSGEAIKpc2_NP.2
p−maj=1.1e−12, pX=1.2e−15, N=177

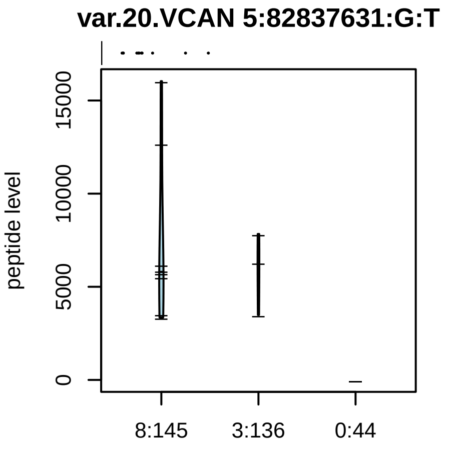

TDGQVSGEAIKpc2_NP.5
p−min=0.068, pX=0.052, N=11

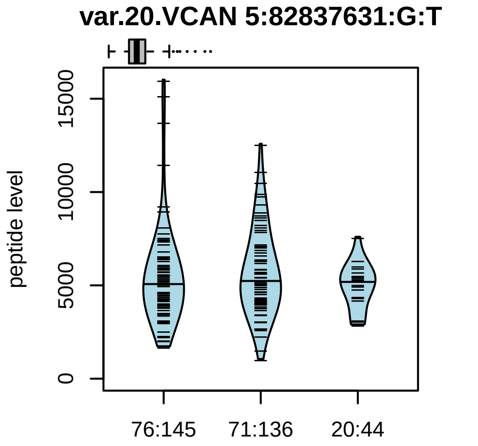

TEEEVFSGMKpc2_NP.2
p−maj=0.42, pX=0.44, N=167

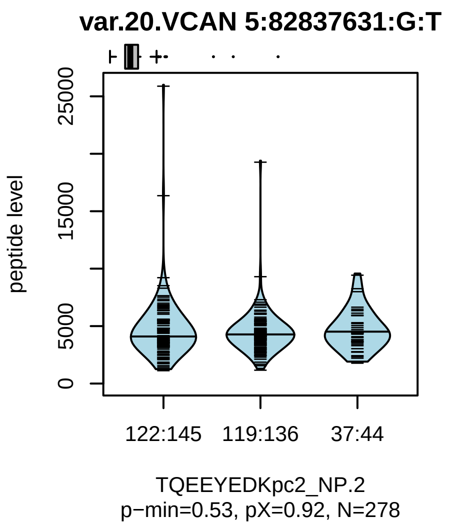
var.20.VCAN 5:82837631:G:T
peptide level
0
5000
15000
25000
122:145
119:136
37:44
TQEEYEDKpc2_NP.2
p−min=0.53, pX=0.92, N=278

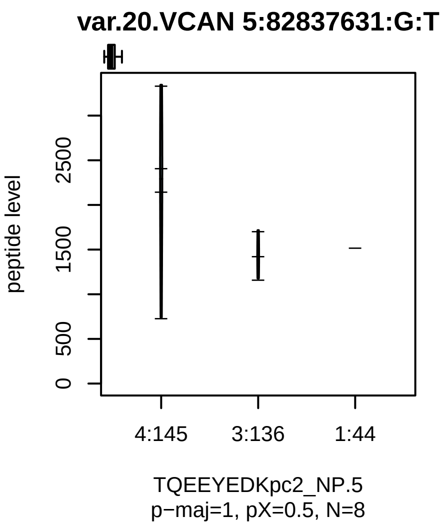
var.20.VCAN 5:82837631:G:T
peptide level
0
500
1500
2500
4:145
3:136
1:44
TQEEYEDKpc2_NP.5
p−maj=1, pX=0.5, N=8

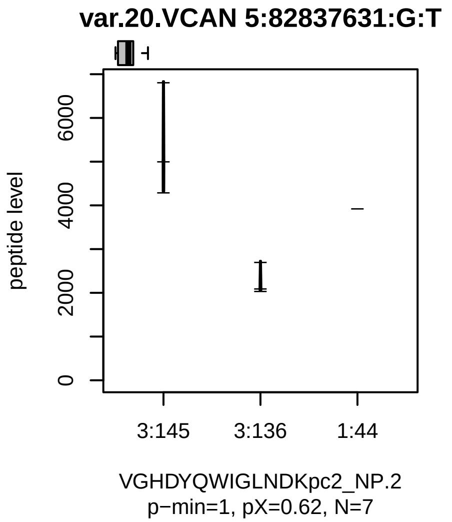
var.20.VCAN 5:82837631:G:T
peptide level
0
2000
4000
6000
3:145
3:136
1:44
VGHDYQWIGLNDKpc2_NP.2
p−min=1, pX=0.62, N=7

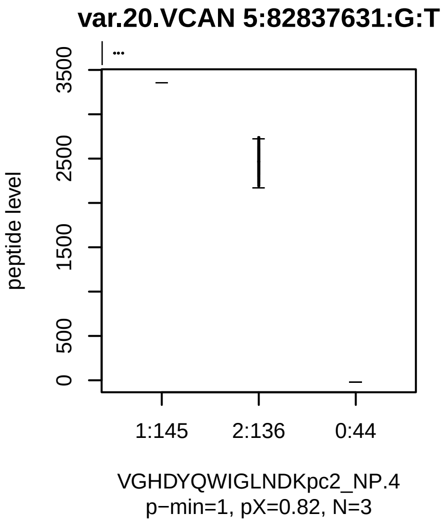
var.20.VCAN 5:82837631:G:T
peptide level
0
500
1500
2500
3500
1:145
2:136
0:44
VGHDYQWIGLNDKpc2_NP.4
p−min=1, pX=0.82, N=3

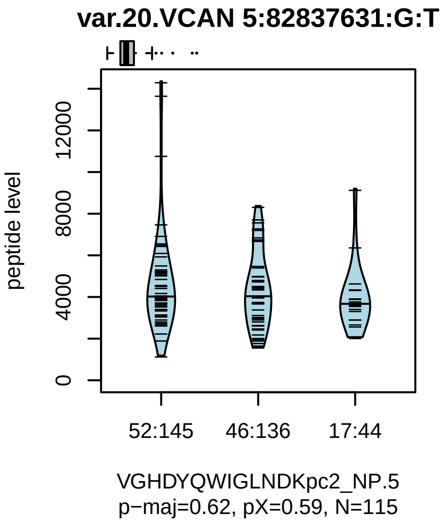
var.20.VCAN 5:82837631:G:T
peptide level
0
4000
8000
12000
52:145
46:136
17:44
VGHDYQWIGLNDKpc2_NP.5
p−maj=0.62, pX=0.59, N=115

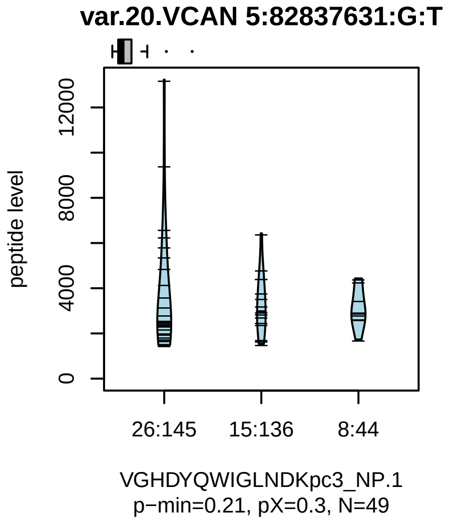
var.20.VCAN 5:82837631:G:T
peptide level
0
4000
8000
12000
26:145
15:136
8:44
VGHDYQWIGLNDKpc3_NP.1
p−min=0.21, pX=0.3, N=49

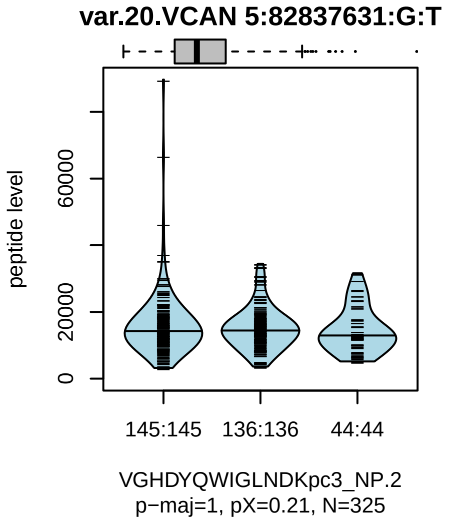
var.20.VCAN 5:82837631:G:T
peptide level
20000
60000
145:145
136:136
44:44
VGHDYQWIGLNDKpc3_NP.2
p−maj=1, pX=0.21, N=325

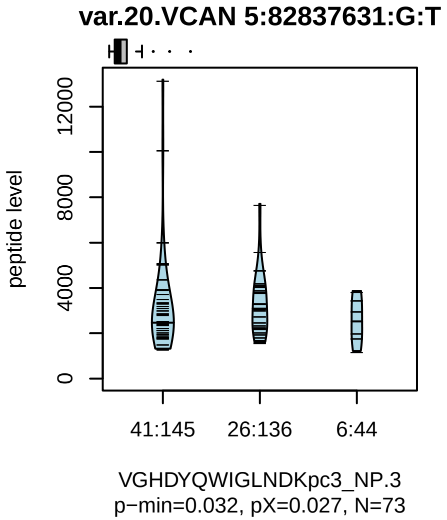
var.20.VCAN 5:82837631:G:T
peptide level
0
4000
8000
12000
41:145
26:136
6:44
VGHDYQWIGLNDKpc3_NP.3
p−min=0.032, pX=0.027, N=73

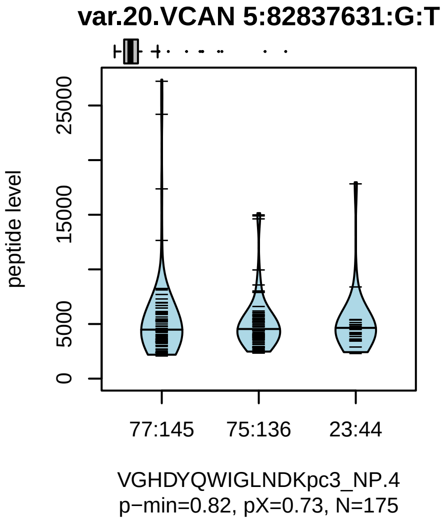
var.20.VCAN 5:82837631:G:T
peptide level
0
5000
15000
25000
77:145
75:136
23:44
VGHDYQWIGLNDKpc3_NP.4
p−min=0.82, pX=0.73, N=175

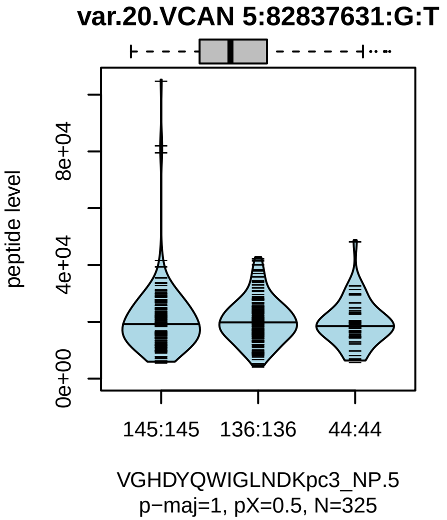
var.20.VCAN 5:82837631:G:T
peptide level
0e+00
4e+04
8e+04
145:145
136:136
44:44
VGHDYQWIGLNDKpc3_NP.5
p−maj=1, pX=0.5, N=325

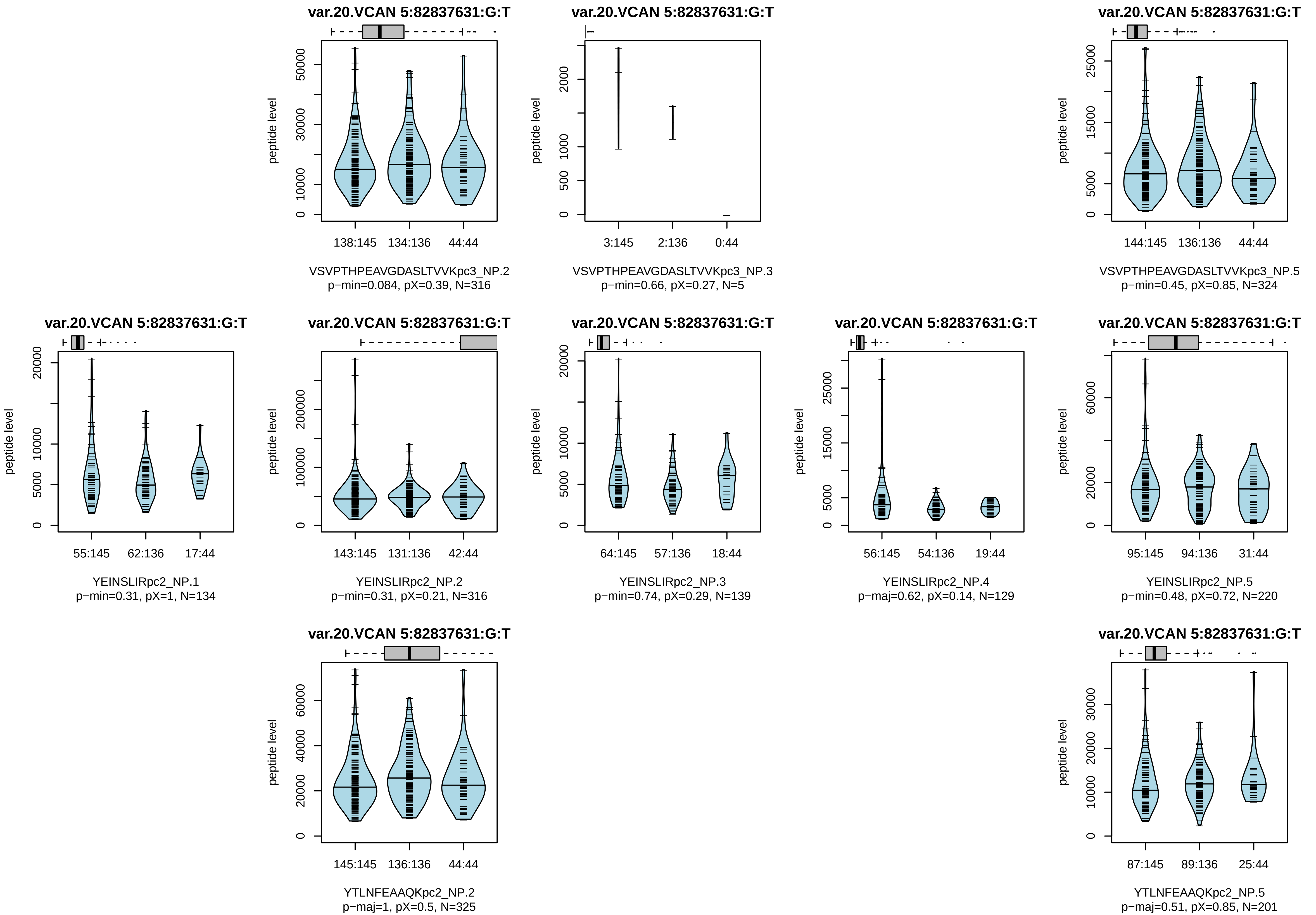

var.20.VCAN 5:82837631:G:T
peptide level
138:145
134:136
44:44
VSVPTHPEAVGDASLTVVKpc3_NP.2
p−min=0.084, pX=0.39, N=316
var.20.VCAN 5:82837631:G:T
peptide level
3:145
2:136
0:44
VSVPTHPEAVGDASLTVVKpc3_NP.3
p−min=0.66, pX=0.27, N=5
var.20.VCAN 5:82837631:G:T
peptide level
144:145
136:136
44:44
VSVPTHPEAVGDASLTVVKpc3_NP.5
p−min=0.45, pX=0.85, N=324
var.20.VCAN 5:82837631:G:T
peptide level
55:145
62:136
17:44
YEINSLIRpc2_NP.1
p−min=0.31, pX=1, N=134
var.20.VCAN 5:82837631:G:T
peptide level
143:145
131:136
42:44
YEINSLIRpc2_NP.2
p−min=0.31, pX=0.21, N=316
var.20.VCAN 5:82837631:G:T
peptide level
64:145
57:136
18:44
YEINSLIRpc2_NP.3
p−min=0.74, pX=0.29, N=139
var.20.VCAN 5:82837631:G:T
peptide level
56:145
54:136
19:44
YEINSLIRpc2_NP.4
p−maj=0.62, pX=0.14, N=129
var.20.VCAN 5:82837631:G:T
peptide level
95:145
94:136
31:44
YEINSLIRpc2_NP.5
p−min=0.48, pX=0.72, N=220
var.20.VCAN 5:82837631:G:T
peptide level
145:145
136:136
44:44
YTLNFEAAQKpc2_NP.2
p−maj=1, pX=0.5, N=325
var.20.VCAN 5:82837631:G:T
peptide level
87:145
89:136
25:44
YTLNFEAAQKpc2_NP.5
p−maj=0.51, pX=0.85, N=201

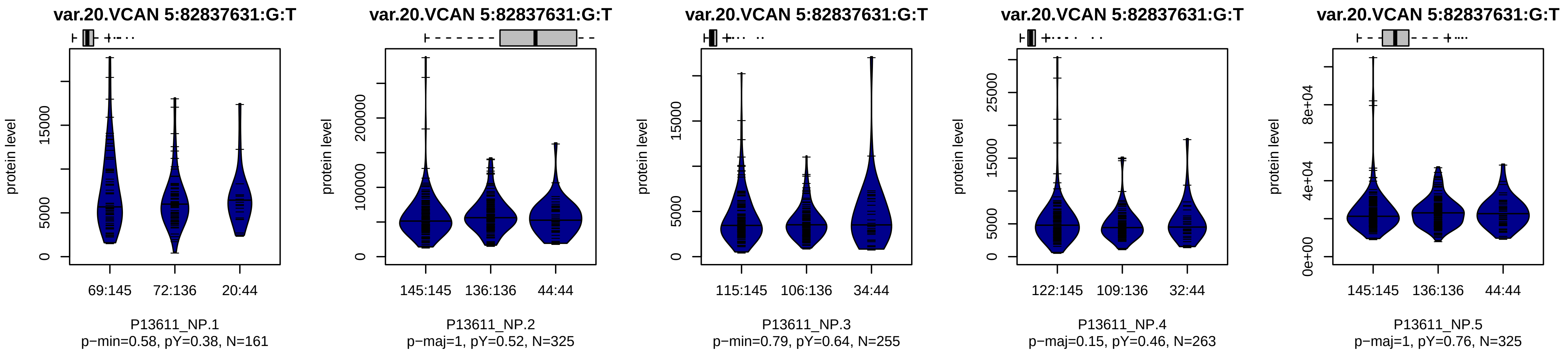
var.20.VCAN 5:82837631:G:T
protein level
69:145
72:136
20:44
P13611_NP.1
p−min=0.58, pY=0.38, N=161
var.20.VCAN 5:82837631:G:T
protein level
145:145
136:136
44:44
P13611_NP.2
p−maj=1, pY=0.52, N=325
var.20.VCAN 5:82837631:G:T
protein level
115:145
106:136
34:44
P13611_NP.3
p−min=0.79, pY=0.64, N=255
var.20.VCAN 5:82837631:G:T
protein level
122:145
109:136
32:44
P13611_NP.4
p−maj=0.15, pY=0.46, N=263
var.20.VCAN 5:82837631:G:T
protein level
145:145
136:136
44:44
P13611_NP.5
p−maj=1, pY=0.76, N=325

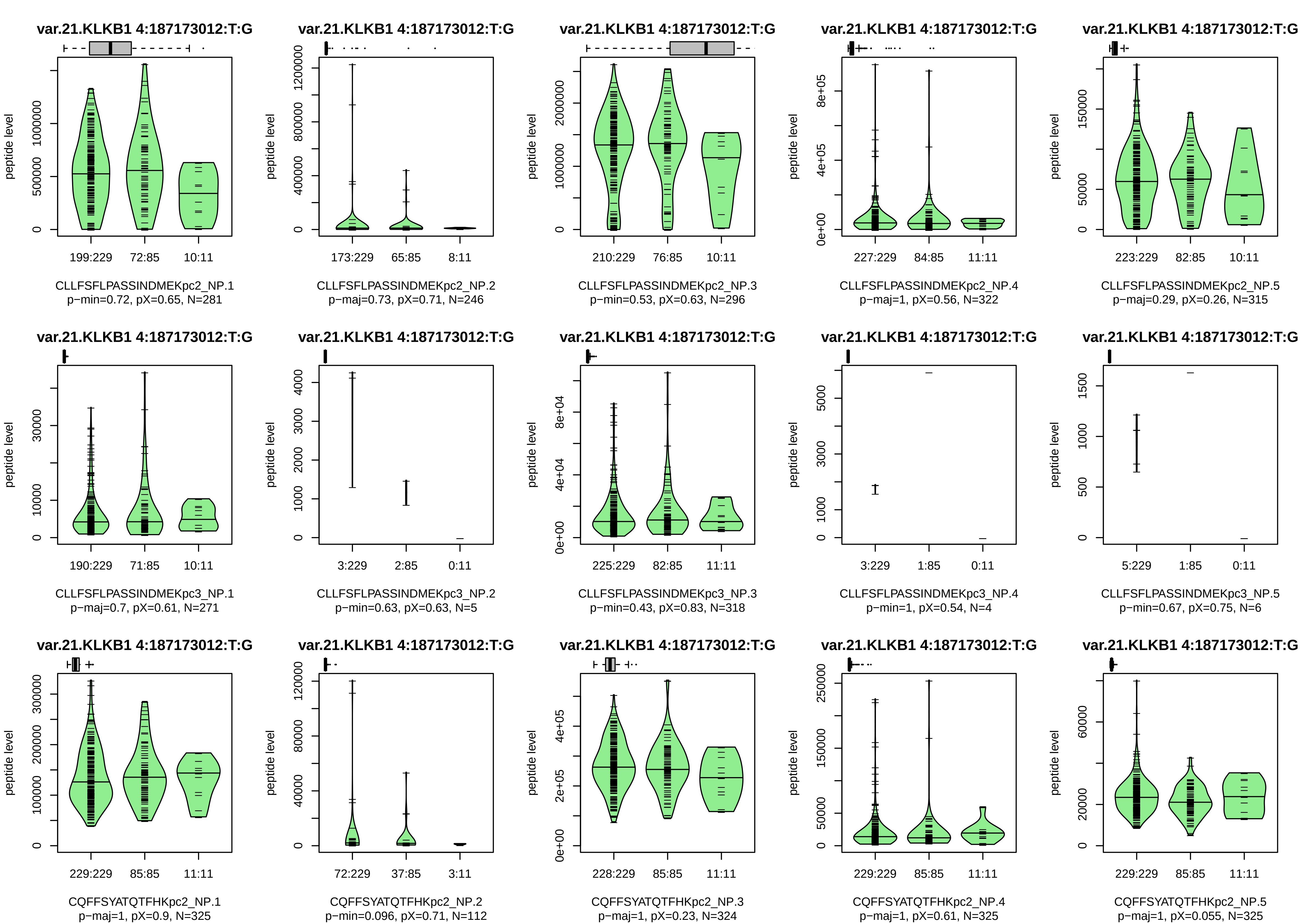

var.21.KLKB1 4:187173012:T:G
peptide level
199:229
72:85
10:11
CLLFSFLPASSINDMEKpc2_NP.1
p−min=0.72, pX=0.65, N=281
var.21.KLKB1 4:187173012:T:G
peptide level
173:229
65:85
8:11
CLLFSFLPASSINDMEKpc2_NP.2
p−maj=0.73, pX=0.71, N=246
var.21.KLKB1 4:187173012:T:G
peptide level
210:229
76:85
10:11
CLLFSFLPASSINDMEKpc2_NP.3
p−min=0.53, pX=0.63, N=296
var.21.KLKB1 4:187173012:T:G
peptide level
227:229
84:85
11:11
CLLFSFLPASSINDMEKpc2_NP.4
p−maj=1, pX=0.56, N=322
var.21.KLKB1 4:187173012:T:G
peptide level
223:229
82:85
10:11
CLLFSFLPASSINDMEKpc2_NP.5
p−maj=0.29, pX=0.26, N=315
var.21.KLKB1 4:187173012:T:G
peptide level
190:229
71:85
10:11
CLLFSFLPASSINDMEKpc3_NP.1
p−maj=0.7, pX=0.61, N=271
var.21.KLKB1 4:187173012:T:G
peptide level
3:229
2:85
0:11
CLLFSFLPASSINDMEKpc3_NP.2
p−min=0.63, pX=0.63, N=5
var.21.KLKB1 4:187173012:T:G
peptide level
225:229
82:85
11:11
CLLFSFLPASSINDMEKpc3_NP.3
p−min=0.43, pX=0.83, N=318
var.21.KLKB1 4:187173012:T:G
peptide level
3:229
1:85
0:11
CLLFSFLPASSINDMEKpc3_NP.4
p−min=1, pX=0.54, N=4
var.21.KLKB1 4:187173012:T:G
peptide level
5:229
1:85
0:11
CLLFSFLPASSINDMEKpc3_NP.5
p−min=0.67, pX=0.75, N=6
var.21.KLKB1 4:187173012:T:G
peptide level
229:229
85:85
11:11
CQFFSYATQTFHKpc2_NP.1
p−maj=1, pX=0.9, N=325
var.21.KLKB1 4:187173012:T:G
peptide level
72:229
37:85
3:11
CQFFSYATQTFHKpc2_NP.2
p−min=0.096, pX=0.71, N=112
var.21.KLKB1 4:187173012:T:G
peptide level
228:229
85:85
11:11
CQFFSYATQTFHKpc2_NP.3
p−maj=1, pX=0.23, N=324
var.21.KLKB1 4:187173012:T:G
peptide level
229:229
85:85
11:11
CQFFSYATQTFHKpc2_NP.4
p−maj=1, pX=0.61, N=325
var.21.KLKB1 4:187173012:T:G
peptide level
229:229
85:85
11:11
CQFFSYATQTFHKpc2_NP.5
p−maj=1, pX=0.055, N=325

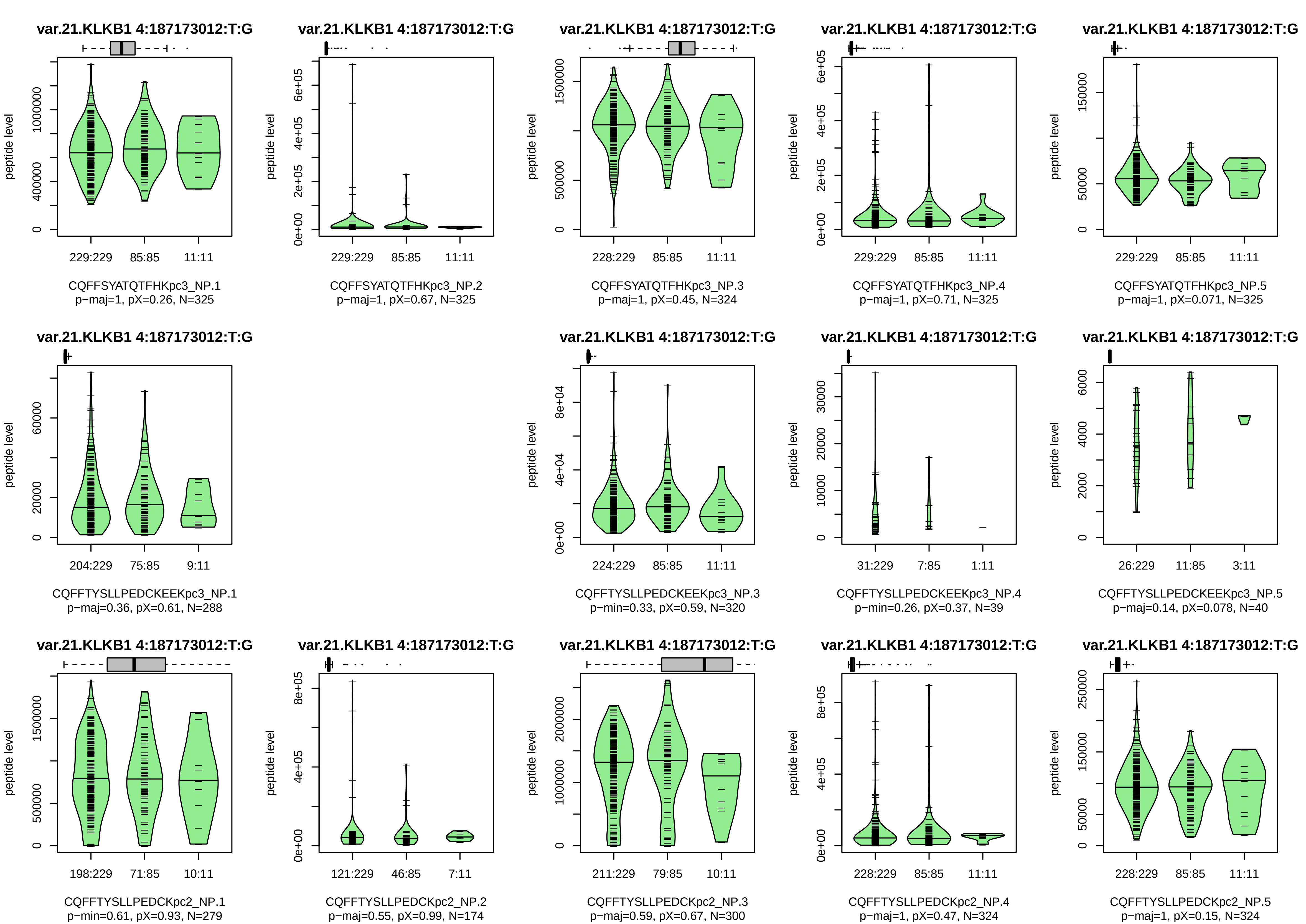

var.21.KLKB1 4:187173012:T:G
peptide level
229:229
85:85
11:11
CQFFSYATQTFHKpc3_NP.1
p−maj=1, pX=0.26, N=325
var.21.KLKB1 4:187173012:T:G
peptide level
229:229
85:85
11:11
CQFFSYATQTFHKpc3_NP.2
p−maj=1, pX=0.67, N=325
var.21.KLKB1 4:187173012:T:G
peptide level
228:229
85:85
11:11
CQFFSYATQTFHKpc3_NP.3
p−maj=1, pX=0.45, N=324
var.21.KLKB1 4:187173012:T:G
peptide level
229:229
85:85
11:11
CQFFSYATQTFHKpc3_NP.4
p−maj=1, pX=0.71, N=325
var.21.KLKB1 4:187173012:T:G
peptide level
229:229
85:85
11:11
CQFFSYATQTFHKpc3_NP.5
p−maj=1, pX=0.071, N=325
var.21.KLKB1 4:187173012:T:G
peptide level
204:229
75:85
9:11
CQFFTYSLLPEDCKEEKpc3_NP.1
p−maj=0.36, pX=0.61, N=288
var.21.KLKB1 4:187173012:T:G
peptide level
224:229
85:85
11:11
CQFFTYSLLPEDCKEEKpc3_NP.3
p−min=0.33, pX=0.59, N=320
var.21.KLKB1 4:187173012:T:G
peptide level
31:229
7:85
1:11
CQFFTYSLLPEDCKEEKpc3_NP.4
p−min=0.26, pX=0.37, N=39
var.21.KLKB1 4:187173012:T:G
peptide level
26:229
11:85
3:11
CQFFTYSLLPEDCKEEKpc3_NP.5
p−maj=0.14, pX=0.078, N=40
var.21.KLKB1 4:187173012:T:G
peptide level
198:229
71:85
10:11
CQFFTYSLLPEDCKpc2_NP.1
p−min=0.61, pX=0.93, N=279
var.21.KLKB1 4:187173012:T:G
peptide level
121:229
46:85
7:11
CQFFTYSLLPEDCKpc2_NP.2
p−maj=0.55, pX=0.99, N=174
var.21.KLKB1 4:187173012:T:G
peptide level
211:229
79:85
10:11
CQFFTYSLLPEDCKpc2_NP.3
p−maj=0.59, pX=0.67, N=300
var.21.KLKB1 4:187173012:T:G
peptide level
228:229
85:85
11:11
CQFFTYSLLPEDCKpc2_NP.4
p−maj=1, pX=0.47, N=324
var.21.KLKB1 4:187173012:T:G
peptide level
228:229
85:85
11:11
CQFFTYSLLPEDCKpc2_NP.5
p−maj=1, pX=0.15, N=324

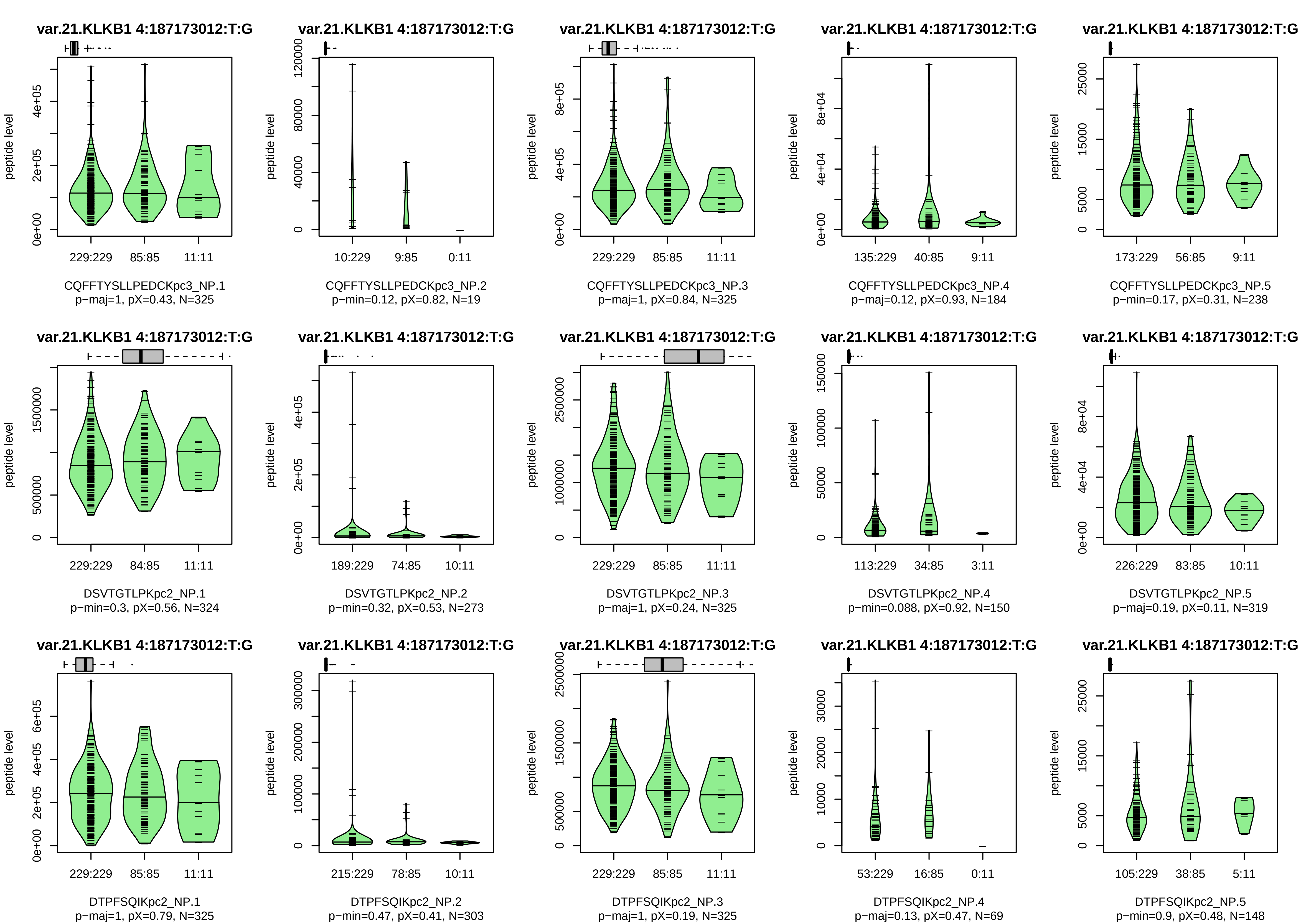
var.21.KLKB1 4:187173012:T:G
peptide level
229:229
85:85
11:11
CQFFTYSLLPEDCKpc3_NP.1
p−maj=1, pX=0.43, N=325
var.21.KLKB1 4:187173012:T:G
peptide level
10:229
9:85
0:11
CQFFTYSLLPEDCKpc3_NP.2
p−min=0.12, pX=0.82, N=19
var.21.KLKB1 4:187173012:T:G
peptide level
229:229
85:85
11:11
CQFFTYSLLPEDCKpc3_NP.3
p−maj=1, pX=0.84, N=325
var.21.KLKB1 4:187173012:T:G
peptide level
135:229
40:85
9:11
CQFFTYSLLPEDCKpc3_NP.4
p−maj=0.12, pX=0.93, N=184
var.21.KLKB1 4:187173012:T:G
peptide level
173:229
56:85
9:11
CQFFTYSLLPEDCKpc3_NP.5
p−min=0.17, pX=0.31, N=238
var.21.KLKB1 4:187173012:T:G
peptide level
229:229
84:85
11:11
DSVTGTLPKpc2_NP.1
p−min=0.3, pX=0.56, N=324
var.21.KLKB1 4:187173012:T:G
peptide level
189:229
74:85
10:11
DSVTGTLPKpc2_NP.2
p−min=0.32, pX=0.53, N=273
var.21.KLKB1 4:187173012:T:G
peptide level
229:229
85:85
11:11
DSVTGTLPKpc2_NP.3
p−maj=1, pX=0.24, N=325
var.21.KLKB1 4:187173012:T:G
peptide level
113:229
34:85
3:11
DSVTGTLPKpc2_NP.4
p−min=0.088, pX=0.92, N=150
var.21.KLKB1 4:187173012:T:G
peptide level
226:229
83:85
10:11
DSVTGTLPKpc2_NP.5
p−maj=0.19, pX=0.11, N=319
var.21.KLKB1 4:187173012:T:G
peptide level
229:229
85:85
11:11
DTPFSQIKpc2_NP.1
p−maj=1, pX=0.79, N=325
var.21.KLKB1 4:187173012:T:G
peptide level
215:229
78:85
10:11
DTPFSQIKpc2_NP.2
p−min=0.47, pX=0.41, N=303
var.21.KLKB1 4:187173012:T:G
peptide level
229:229
85:85
11:11
DTPFSQIKpc2_NP.3
p−maj=1, pX=0.19, N=325
var.21.KLKB1 4:187173012:T:G
peptide level
53:229
16:85
0:11
DTPFSQIKpc2_NP.4
p−maj=0.13, pX=0.47, N=69
var.21.KLKB1 4:187173012:T:G
peptide level
105:229
38:85
5:11
DTPFSQIKpc2_NP.5
p−min=0.9, pX=0.48, N=148

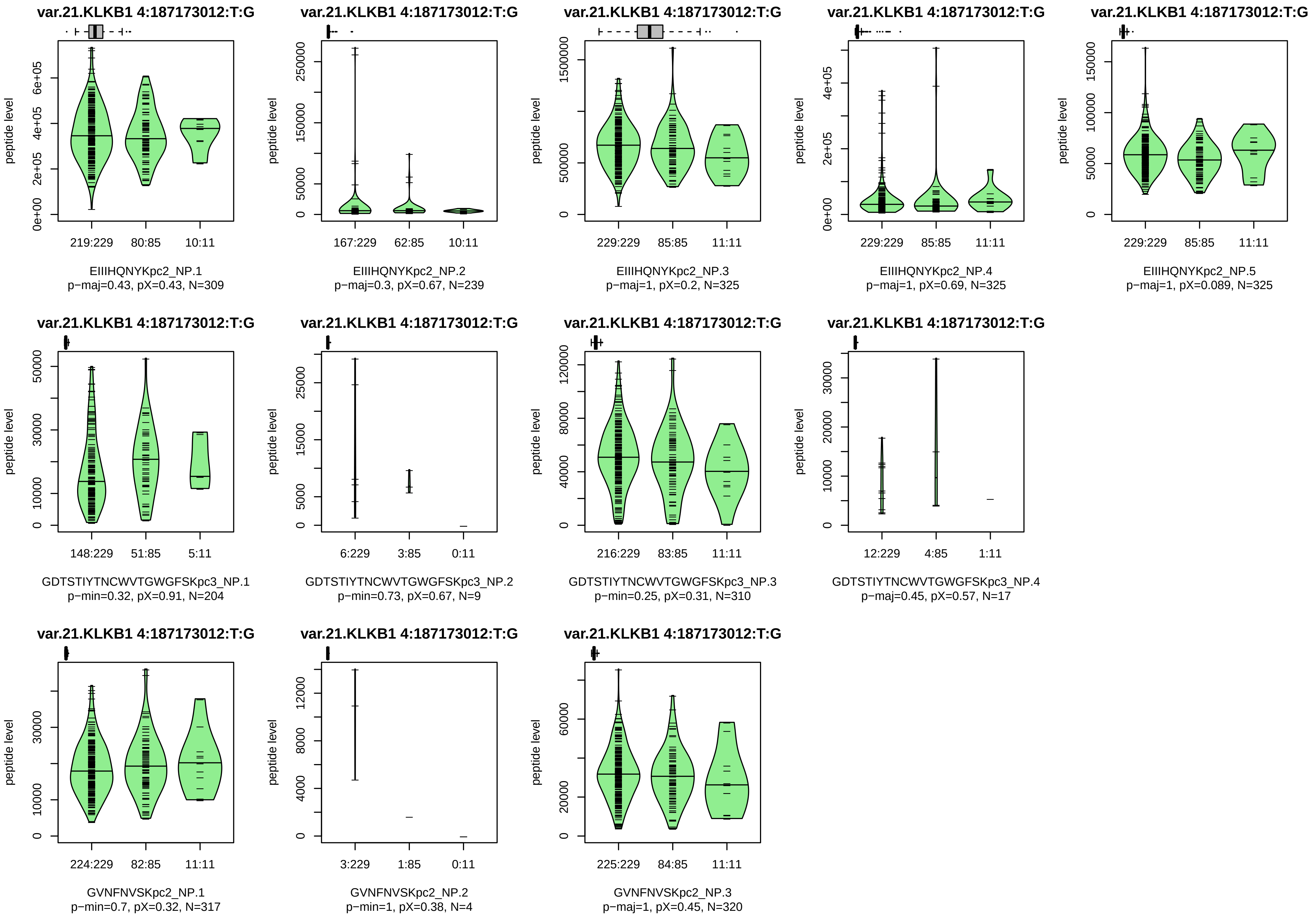
var.21.KLKB1 4:187173012:T:G
peptide level
0e+00
2e+05
4e+05
6e+05
219:229
80:85
10:11
EIIIHQNYKpc2_NP.1
p−maj=0.43, pX=0.43, N=309
var.21.KLKB1 4:187173012:T:G
peptide level
0
50000
150000
250000
167:229
62:85
10:11
EIIIHQNYKpc2_NP.2
p−maj=0.3, pX=0.67, N=239
var.21.KLKB1 4:187173012:T:G
peptide level
0
500000
1500000
229:229
85:85
11:11
EIIIHQNYKpc2_NP.3
p−maj=1, pX=0.2, N=325
var.21.KLKB1 4:187173012:T:G
peptide level
0e+00
2e+05
4e+05
229:229
85:85
11:11
EIIIHQNYKpc2_NP.4
p−maj=1, pX=0.69, N=325
var.21.KLKB1 4:187173012:T:G
peptide level
0
50000
100000
150000
229:229
85:85
11:11
EIIIHQNYKpc2_NP.5
p−maj=1, pX=0.089, N=325
var.21.KLKB1 4:187173012:T:G
peptide level
0
10000
30000
50000
148:229
51:85
5:11
GDTSTIYTNCWVTGWGFSKpc3_NP.1
p−min=0.32, pX=0.91, N=204
var.21.KLKB1 4:187173012:T:G
peptide level
0
5000
15000
25000
6:229
3:85
0:11
GDTSTIYTNCWVTGWGFSKpc3_NP.2
p−min=0.73, pX=0.67, N=9
var.21.KLKB1 4:187173012:T:G
peptide level
0
40000
80000
120000
216:229
83:85
11:11
GDTSTIYTNCWVTGWGFSKpc3_NP.3
p−min=0.25, pX=0.31, N=310
var.21.KLKB1 4:187173012:T:G
peptide level
0
10000
20000
30000
12:229
4:85
1:11
GDTSTIYTNCWVTGWGFSKpc3_NP.4
p−maj=0.45, pX=0.57, N=17
var.21.KLKB1 4:187173012:T:G
peptide level
0
10000
30000
224:229
82:85
11:11
GVNFNVSKpc2_NP.1
p−min=0.7, pX=0.32, N=317
var.21.KLKB1 4:187173012:T:G
peptide level
0
4000
8000
12000
3:229
1:85
0:11
GVNFNVSKpc2_NP.2
p−min=1, pX=0.38, N=4
var.21.KLKB1 4:187173012:T:G
peptide level
0
20000
60000
225:229
84:85
11:11
GVNFNVSKpc2_NP.3
p−maj=1, pX=0.45, N=320

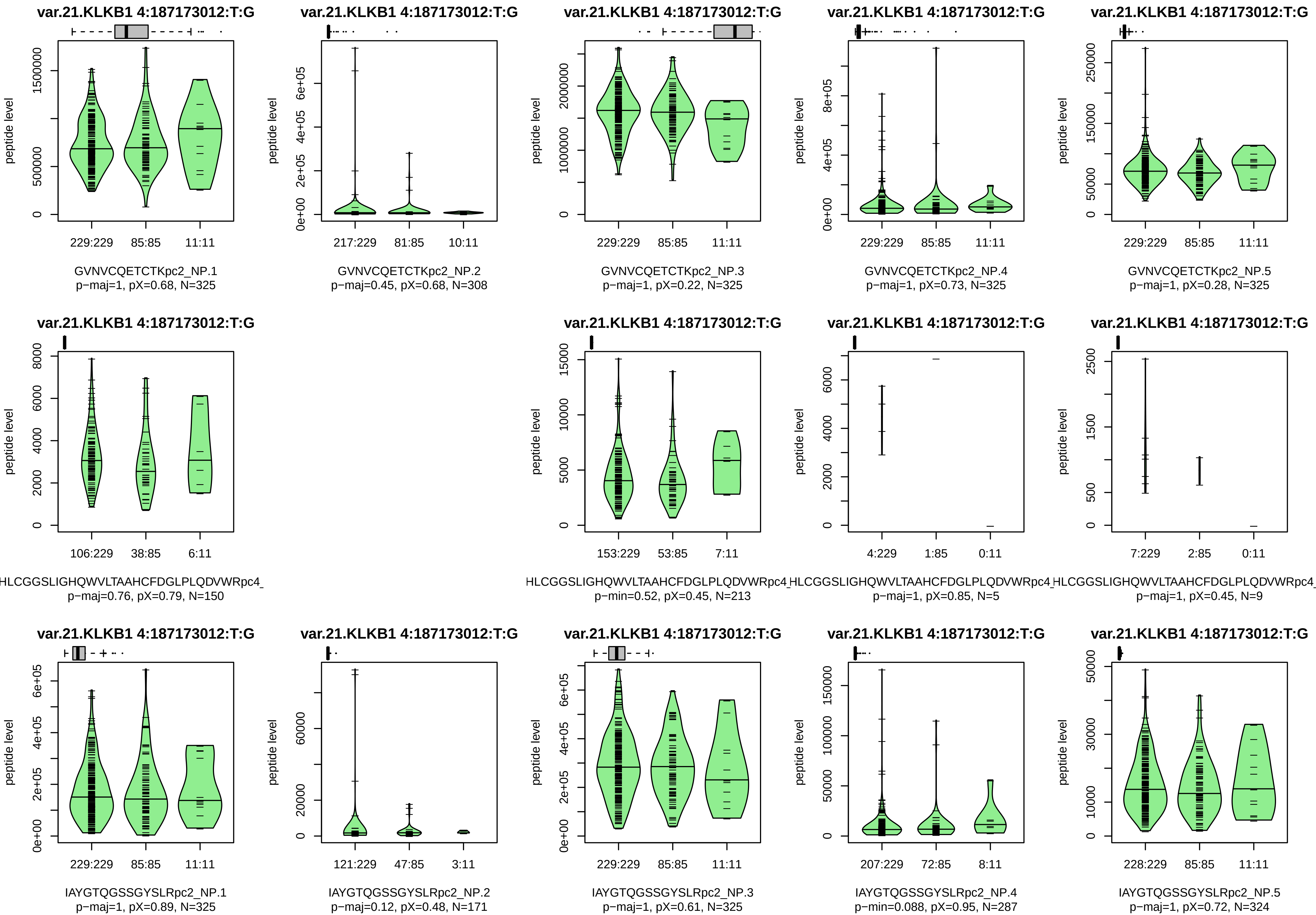

var.21.KLKB1 4:187173012:T:G
peptide level
229:229
85:85
11:11
GVNVCQETCTKpc2_NP.1
p−maj=1, pX=0.68, N=325
var.21.KLKB1 4:187173012:T:G
217:229
81:85
10:11
GVNVCQETCTKpc2_NP.2
p−maj=0.45, pX=0.68, N=308
var.21.KLKB1 4:187173012:T:G
229:229
85:85
11:11
GVNVCQETCTKpc2_NP.3
p−maj=1, pX=0.22, N=325
var.21.KLKB1 4:187173012:T:G
229:229
85:85
11:11
GVNVCQETCTKpc2_NP.4
p−maj=1, pX=0.73, N=325
var.21.KLKB1 4:187173012:T:G
229:229
85:85
11:11
GVNVCQETCTKpc2_NP.5
p−maj=1, pX=0.28, N=325
var.21.KLKB1 4:187173012:T:G
106:229
38:85
6:11
HLCGGSLIGHQWVLTAAHCFDGLPLQDVWRpc4_
p−maj=0.76, pX=0.79, N=150
var.21.KLKB1 4:187173012:T:G
153:229
53:85
7:11
HLCGGSLIGHQWVLTAAHCFDGLPLQDVWRpc4_
p−min=0.52, pX=0.45, N=213
var.21.KLKB1 4:187173012:T:G
4:229
1:85
0:11
HLCGGSLIGHQWVLTAAHCFDGLPLQDVWRpc4_
p−maj=1, pX=0.85, N=5
var.21.KLKB1 4:187173012:T:G
7:229
2:85
0:11
HLCGGSLIGHQWVLTAAHCFDGLPLQDVWRpc4_
p−maj=1, pX=0.45, N=9
var.21.KLKB1 4:187173012:T:G
229:229
85:85
11:11
IAYGTQGSSGYSLRpc2_NP.1
p−maj=1, pX=0.89, N=325
var.21.KLKB1 4:187173012:T:G
121:229
47:85
3:11
IAYGTQGSSGYSLRpc2_NP.2
p−maj=0.12, pX=0.48, N=171
var.21.KLKB1 4:187173012:T:G
229:229
85:85
11:11
IAYGTQGSSGYSLRpc2_NP.3
p−maj=1, pX=0.61, N=325
var.21.KLKB1 4:187173012:T:G
207:229
72:85
8:11
IAYGTQGSSGYSLRpc2_NP.4
p−min=0.088, pX=0.95, N=287
var.21.KLKB1 4:187173012:T:G
228:229
85:85
11:11
IAYGTQGSSGYSLRpc2_NP.5
p−maj=1, pX=0.72, N=324

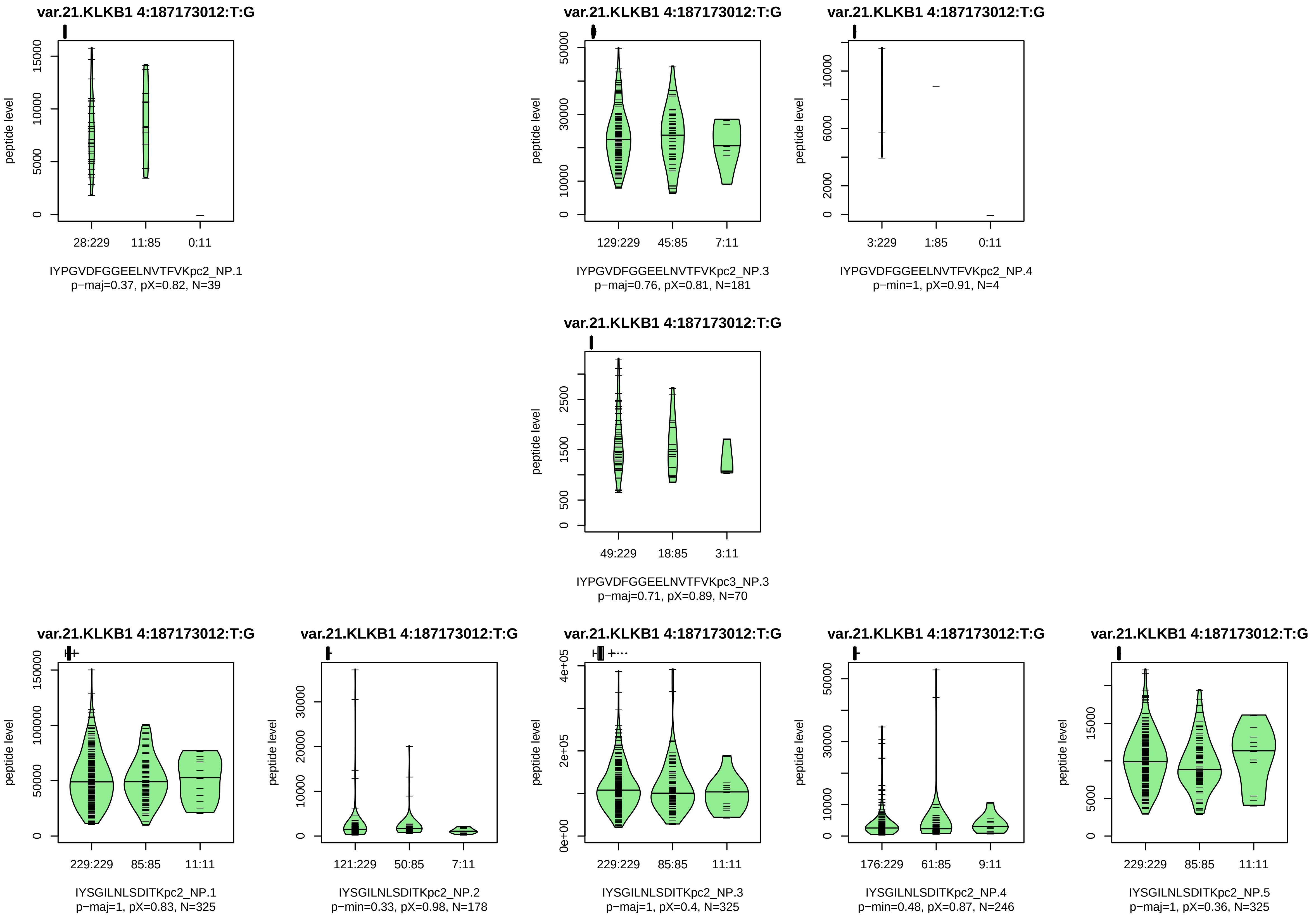
var.21.KLKB1 4:187173012:T:G
peptide level
0
5000
10000
15000
28:229
11:85
0:11
IYPGVDFGGEELNVTFVKpc2_NP.1
p−maj=0.37, pX=0.82, N=39
var.21.KLKB1 4:187173012:T:G
peptide level
0
10000
30000
50000
129:229
45:85
7:11
IYPGVDFGGEELNVTFVKpc2_NP.3
p−maj=0.76, pX=0.81, N=181
var.21.KLKB1 4:187173012:T:G
peptide level
0
2000
6000
10000
3:229
1:85
0:11
IYPGVDFGGEELNVTFVKpc2_NP.4
p−min=1, pX=0.91, N=4
var.21.KLKB1 4:187173012:T:G
peptide level
0
500
1500
2500
49:229
18:85
3:11
IYPGVDFGGEELNVTFVKpc3_NP.3
p−maj=0.71, pX=0.89, N=70
var.21.KLKB1 4:187173012:T:G
peptide level
0
50000
100000
150000
229:229
85:85
11:11
IYSGILNLSDITKpc2_NP.1
p−maj=1, pX=0.83, N=325
var.21.KLKB1 4:187173012:T:G
peptide level
0
10000
20000
30000
121:229
50:85
7:11
IYSGILNLSDITKpc2_NP.2
p−min=0.33, pX=0.98, N=178
var.21.KLKB1 4:187173012:T:G
peptide level
0e+00
2e+05
4e+05
229:229
85:85
11:11
IYSGILNLSDITKpc2_NP.3
p−maj=1, pX=0.4, N=325
var.21.KLKB1 4:187173012:T:G
peptide level
0
10000
30000
50000
176:229
61:85
9:11
IYSGILNLSDITKpc2_NP.4
p−min=0.48, pX=0.87, N=246
var.21.KLKB1 4:187173012:T:G
peptide level
0
5000
15000
229:229
85:85
11:11
IYSGILNLSDITKpc2_NP.5
p−maj=1, pX=0.36, N=325

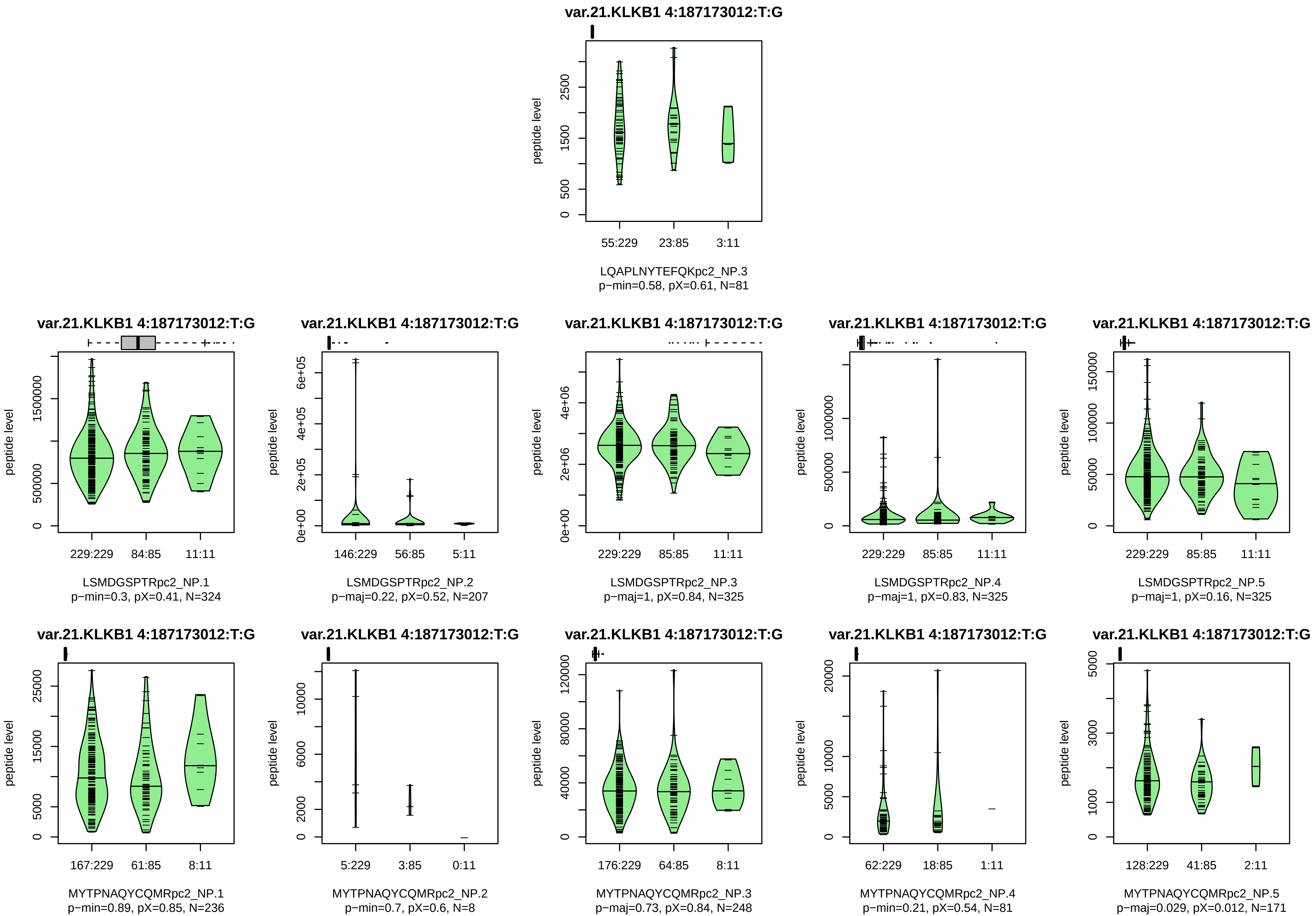
var.21.KLKB1 4:187173012:T:G
peptide level
0
500
1500
2500
55:229
23:85
3:11
LQAPLNYTEFQKpc2_NP.3
p−min=0.58, pX=0.61, N=81
var.21.KLKB1 4:187173012:T:G
peptide level
0
500000
1500000
229:229
84:85
11:11
LSMDGSPTRpc2_NP.1
p−min=0.3, pX=0.41, N=324
var.21.KLKB1 4:187173012:T:G
peptide level
0e+00
2e+05
4e+05
6e+05
146:229
56:85
5:11
LSMDGSPTRpc2_NP.2
p−maj=0.22, pX=0.52, N=207
var.21.KLKB1 4:187173012:T:G
peptide level
0e+00
2e+06
4e+06
229:229
85:85
11:11
LSMDGSPTRpc2_NP.3
p−maj=1, pX=0.84, N=325
var.21.KLKB1 4:187173012:T:G
peptide level
0
500000
1000000
229:229
85:85
11:11
LSMDGSPTRpc2_NP.4
p−maj=1, pX=0.83, N=325
var.21.KLKB1 4:187173012:T:G
peptide level
0
50000
100000
150000
229:229
85:85
11:11
LSMDGSPTRpc2_NP.5
p−maj=1, pX=0.16, N=325
var.21.KLKB1 4:187173012:T:G
peptide level
0
5000
15000
25000
167:229
61:85
8:11
MYTPNAQYCQMRpc2_NP.1
p−min=0.89, pX=0.85, N=236
var.21.KLKB1 4:187173012:T:G
peptide level
0
2000
6000
10000
5:229
3:85
0:11
MYTPNAQYCQMRpc2_NP.2
p−min=0.7, pX=0.6, N=8
var.21.KLKB1 4:187173012:T:G
peptide level
0
40000
80000
120000
176:229
64:85
8:11
MYTPNAQYCQMRpc2_NP.3
p−maj=0.73, pX=0.84, N=248
var.21.KLKB1 4:187173012:T:G
peptide level
0
5000
10000
20000
62:229
18:85
1:11
MYTPNAQYCQMRpc2_NP.4
p−min=0.21, pX=0.54, N=81
var.21.KLKB1 4:187173012:T:G
peptide level
0
1000
3000
5000
128:229
41:85
2:11
MYTPNAQYCQMRpc2_NP.5
p−maj=0.029, pX=0.012, N=171

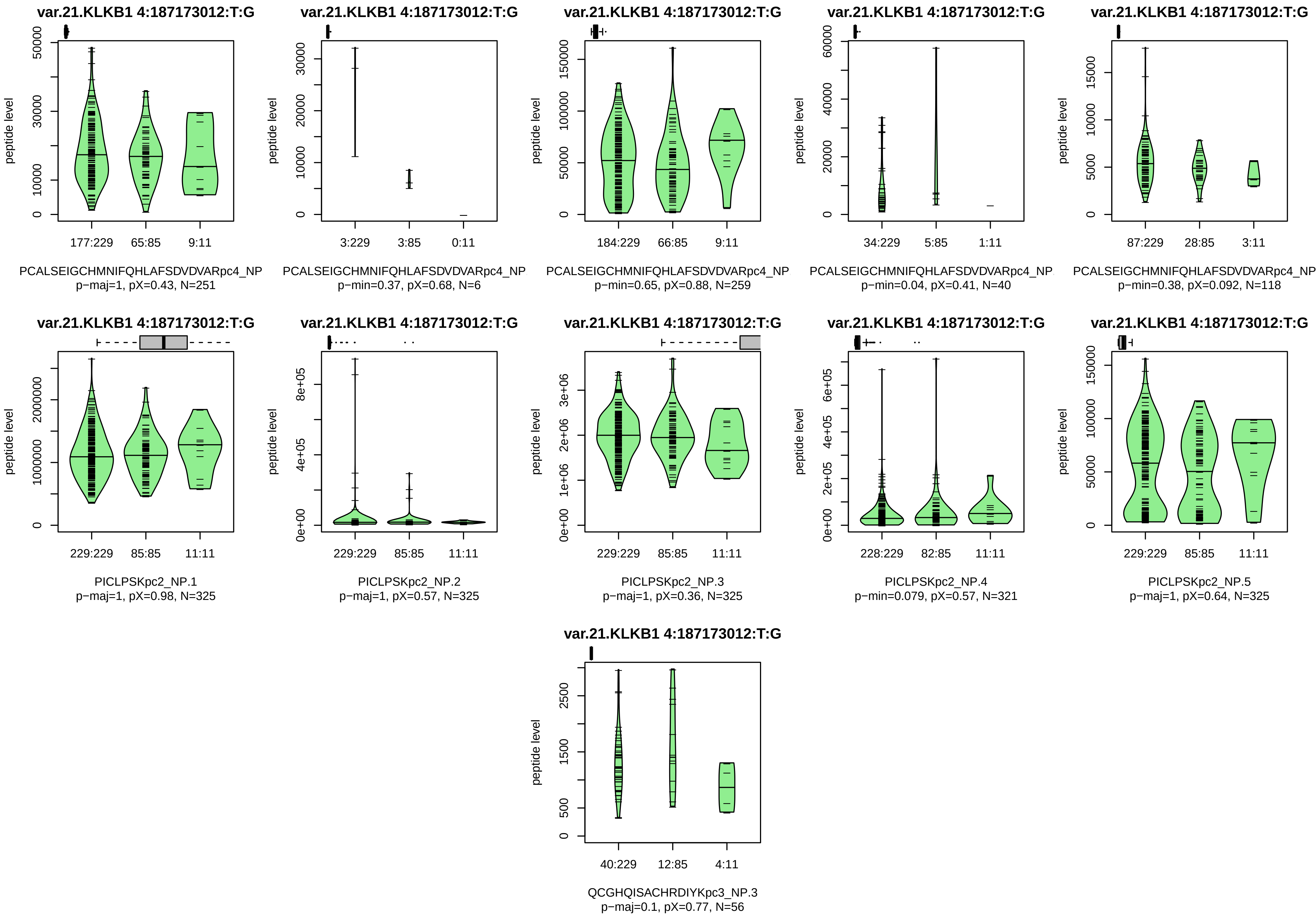
var.21.KLKB1 4:187173012:T:G
peptide level
177:229
65:85
9:11
PCALSEIGCHMNIFQHLAFSDVDVARpc4_NP
p−maj=1, pX=0.43, N=251
var.21.KLKB1 4:187173012:T:G
peptide level
3:229
3:85
0:11
PCALSEIGCHMNIFQHLAFSDVDVARpc4_NP
p−min=0.37, pX=0.68, N=6
var.21.KLKB1 4:187173012:T:G
peptide level
184:229
66:85
9:11
PCALSEIGCHMNIFQHLAFSDVDVARpc4_NP
p−min=0.65, pX=0.88, N=259
var.21.KLKB1 4:187173012:T:G
peptide level
34:229
5:85
1:11
PCALSEIGCHMNIFQHLAFSDVDVARpc4_NP
p−min=0.04, pX=0.41, N=40
var.21.KLKB1 4:187173012:T:G
peptide level
87:229
28:85
3:11
PCALSEIGCHMNIFQHLAFSDVDVARpc4_NP
p−min=0.38, pX=0.092, N=118
var.21.KLKB1 4:187173012:T:G
peptide level
229:229
85:85
11:11
PICLPSKpc2_NP.1
p−maj=1, pX=0.98, N=325
var.21.KLKB1 4:187173012:T:G
peptide level
229:229
85:85
11:11
PICLPSKpc2_NP.2
p−maj=1, pX=0.57, N=325
var.21.KLKB1 4:187173012:T:G
peptide level
229:229
85:85
11:11
PICLPSKpc2_NP.3
p−maj=1, pX=0.36, N=325
var.21.KLKB1 4:187173012:T:G
peptide level
228:229
82:85
11:11
PICLPSKpc2_NP.4
p−min=0.079, pX=0.57, N=321
var.21.KLKB1 4:187173012:T:G
peptide level
229:229
85:85
11:11
PICLPSKpc2_NP.5
p−maj=1, pX=0.64, N=325
var.21.KLKB1 4:187173012:T:G
peptide level
40:229
12:85
4:11
QCGHQISACHRDIYKpc3_NP.3
p−maj=0.1, pX=0.77, N=56

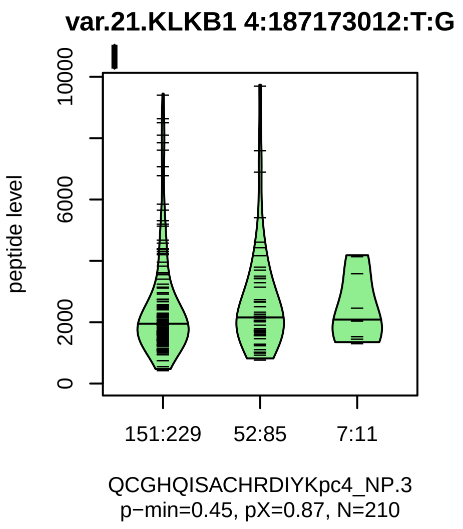
var.21.KLKB1 4:187173012:T:G
peptide level
0
2000
6000
10000
151:229
52:85
7:11
QCGHQISACHRDIYKpc4_NP.3
p−min=0.45, pX=0.87, N=210

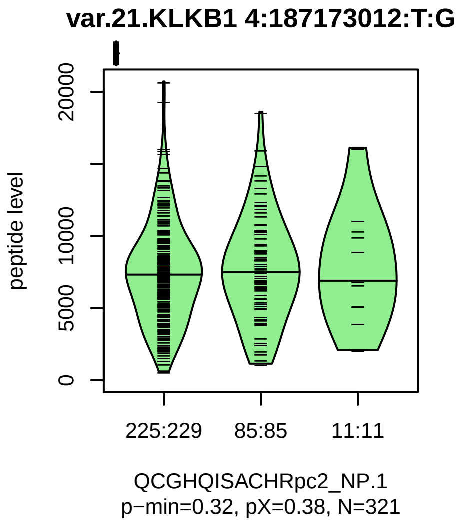
var.21.KLKB1 4:187173012:T:G
peptide level
0
5000
10000
20000
225:229
85:85
11:11
QCGHQISACHRpc2_NP.1
p−min=0.32, pX=0.38, N=321

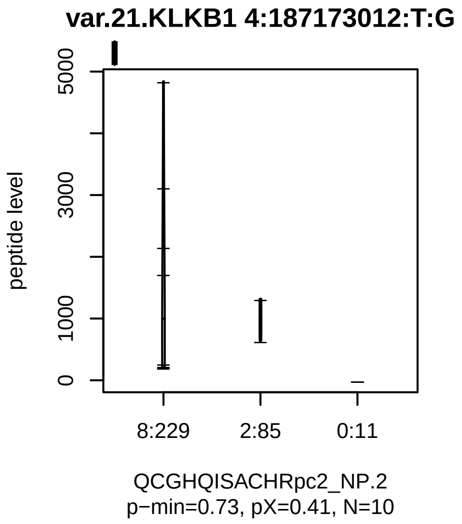
var.21.KLKB1 4:187173012:T:G
peptide level
0
1000
3000
5000
8:229
2:85
0:11
QCGHQISACHRpc2_NP.2
p−min=0.73, pX=0.41, N=10

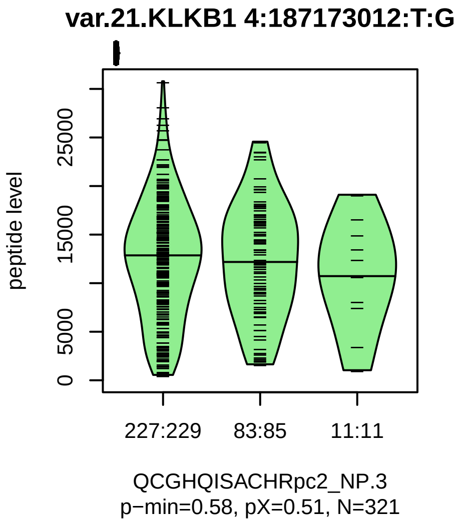
var.21.KLKB1 4:187173012:T:G
peptide level
0
5000
15000
25000
227:229
83:85
11:11
QCGHQISACHRpc2_NP.3
p−min=0.58, pX=0.51, N=321

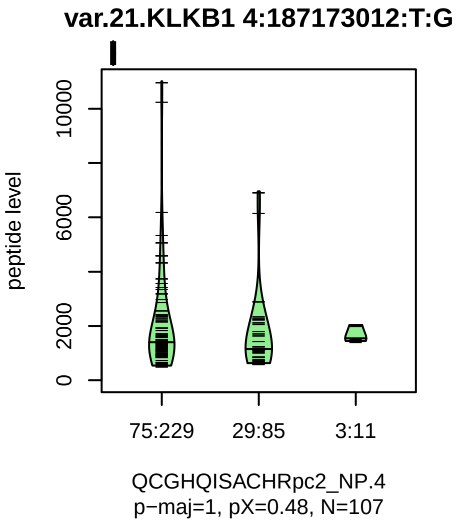
var.21.KLKB1 4:187173012:T:G
peptide level
0
2000
6000
10000
75:229
29:85
3:11
QCGHQISACHRpc2_NP.4
p−maj=1, pX=0.48, N=107

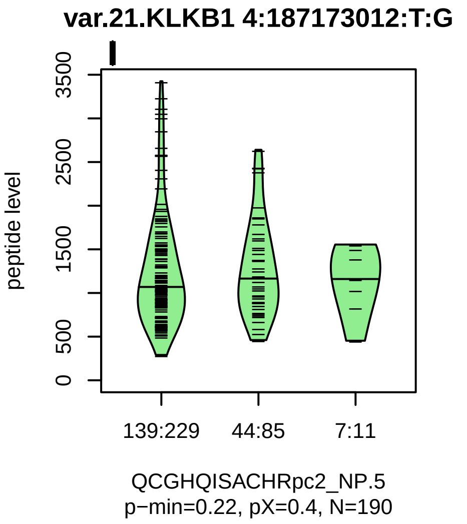
var.21.KLKB1 4:187173012:T:G
peptide level
0
500
1500
2500
3500
139:229
44:85
7:11
QCGHQISACHRpc2_NP.5
p−min=0.22, pX=0.4, N=190

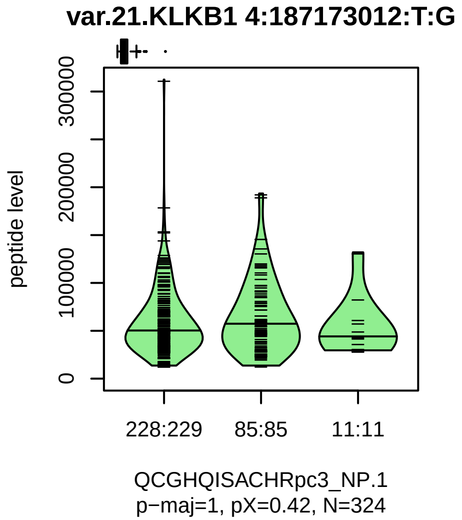
var.21.KLKB1 4:187173012:T:G
peptide level
0
100000
200000
300000
228:229
85:85
11:11
QCGHQISACHRpc3_NP.1
p−maj=1, pX=0.42, N=324

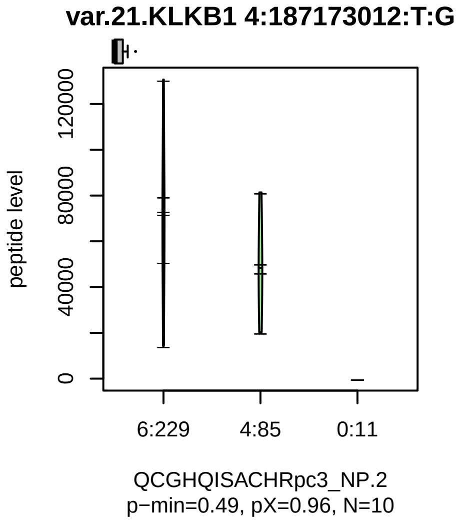
var.21.KLKB1 4:187173012:T:G
peptide level
0
40000
80000
120000
6:229
4:85
0:11
QCGHQISACHRpc3_NP.2
p−min=0.49, pX=0.96, N=10

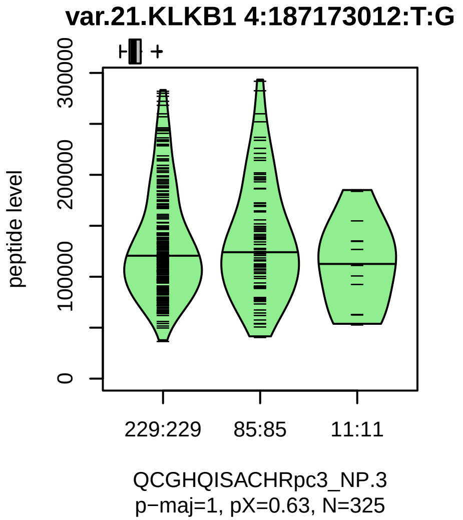
var.21.KLKB1 4:187173012:T:G
peptide level
0
100000
200000
300000
229:229
85:85
11:11
QCGHQISACHRpc3_NP.3
p−maj=1, pX=0.63, N=325

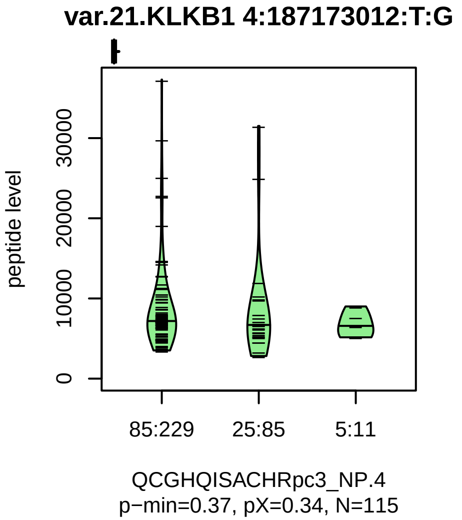
var.21.KLKB1 4:187173012:T:G
peptide level
0
10000
20000
30000
85:229
25:85
5:11
QCGHQISACHRpc3_NP.4
p−min=0.37, pX=0.34, N=115

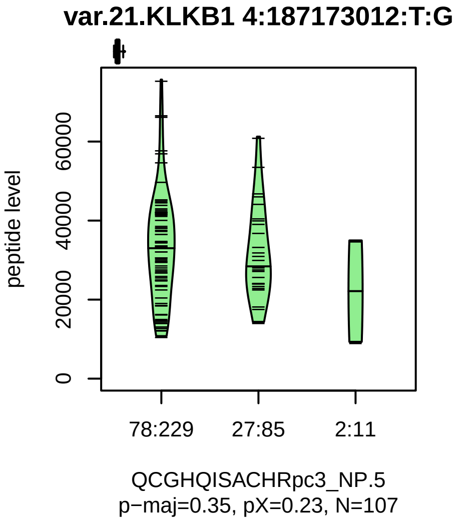
var.21.KLKB1 4:187173012:T:G
peptide level
0
20000
40000
60000
78:229
27:85
2:11
QCGHQISACHRpc3_NP.5
p−maj=0.35, pX=0.23, N=107

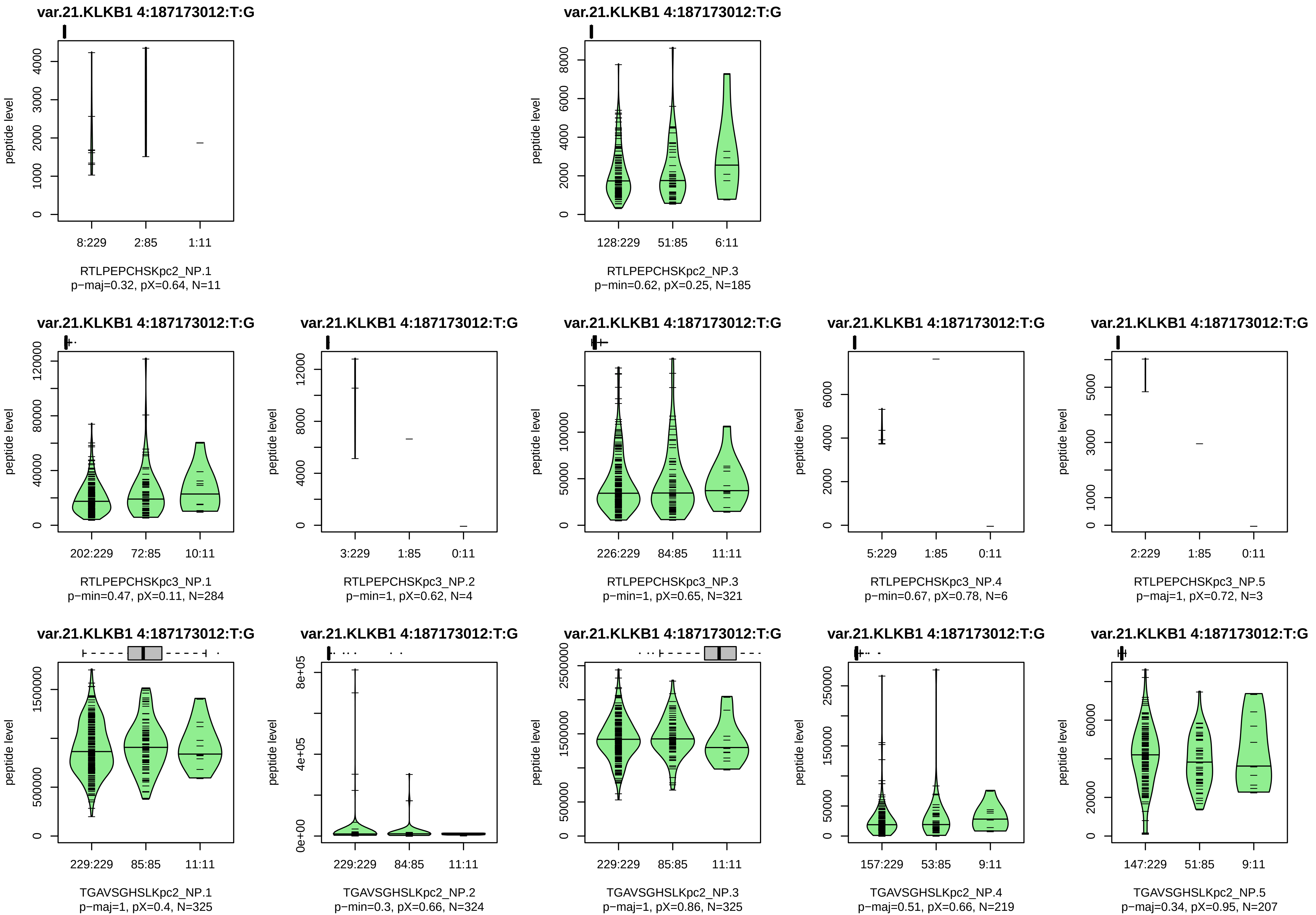

var.21.KLKB1 4:187173012:T:G
peptide level
0
1000
2000
3000
4000
8:229
2:85
1:11
RTLPEPCHSKpc2_NP.1
p−maj=0.32, pX=0.64, N=11
var.21.KLKB1 4:187173012:T:G
peptide level
0
2000
4000
6000
8000
128:229
51:85
6:11
RTLPEPCHSKpc2_NP.3
p−min=0.62, pX=0.25, N=185
var.21.KLKB1 4:187173012:T:G
peptide level
0
40000
80000
120000
202:229
72:85
10:11
RTLPEPCHSKpc3_NP.1
p−min=0.47, pX=0.11, N=284
var.21.KLKB1 4:187173012:T:G
peptide level
0
4000
8000
12000
3:229
1:85
0:11
RTLPEPCHSKpc3_NP.2
p−min=1, pX=0.62, N=4
var.21.KLKB1 4:187173012:T:G
peptide level
0
50000
100000
226:229
84:85
11:11
RTLPEPCHSKpc3_NP.3
p−min=1, pX=0.65, N=321
var.21.KLKB1 4:187173012:T:G
peptide level
0
2000
4000
6000
5:229
1:85
0:11
RTLPEPCHSKpc3_NP.4
p−min=0.67, pX=0.78, N=6
var.21.KLKB1 4:187173012:T:G
peptide level
0
1000
3000
5000
2:229
1:85
0:11
RTLPEPCHSKpc3_NP.5
p−maj=1, pX=0.72, N=3
var.21.KLKB1 4:187173012:T:G
peptide level
0
500000
1500000
229:229
85:85
11:11
TGAVSGHSLKpc2_NP.1
p−maj=1, pX=0.4, N=325
var.21.KLKB1 4:187173012:T:G
peptide level
0e+00
4e+05
8e+05
229:229
84:85
11:11
TGAVSGHSLKpc2_NP.2
p−min=0.3, pX=0.66, N=324
var.21.KLKB1 4:187173012:T:G
peptide level
0
500000
1500000
2500000
229:229
85:85
11:11
TGAVSGHSLKpc2_NP.3
p−maj=1, pX=0.86, N=325
var.21.KLKB1 4:187173012:T:G
peptide level
0
50000
150000
250000
157:229
53:85
9:11
TGAVSGHSLKpc2_NP.4
p−maj=0.51, pX=0.66, N=219
var.21.KLKB1 4:187173012:T:G
peptide level
0
20000
60000
147:229
51:85
9:11
TGAVSGHSLKpc2_NP.5
p−maj=0.34, pX=0.95, N=207

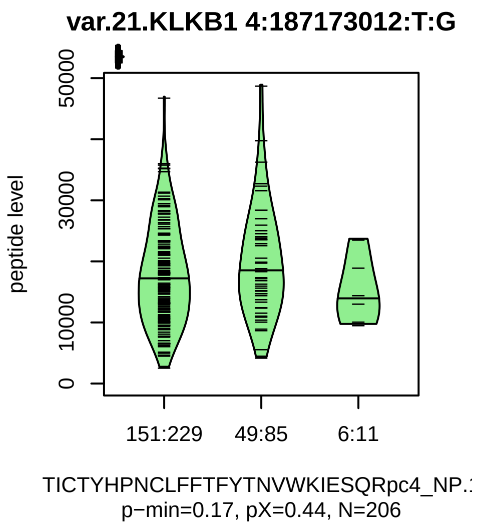
var.21.KLKB1 4:187173012:T:G
peptide level
0
10000
30000
50000
151:229
49:85
6:11
TICTYHPNCLFFTFYTNVWKIESQRpc4_NP.1
p−min=0.17, pX=0.44, N=206

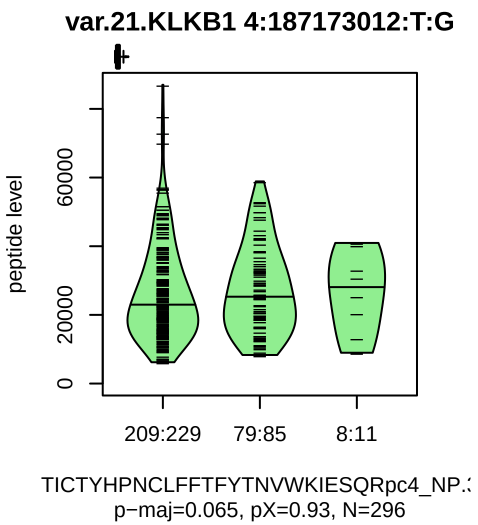
var.21.KLKB1 4:187173012:T:G
peptide level
0
20000
60000
209:229
79:85
8:11
TICTYHPNCLFFTFYTNVWKIESQRpc4_NP.3
p−maj=0.065, pX=0.93, N=296

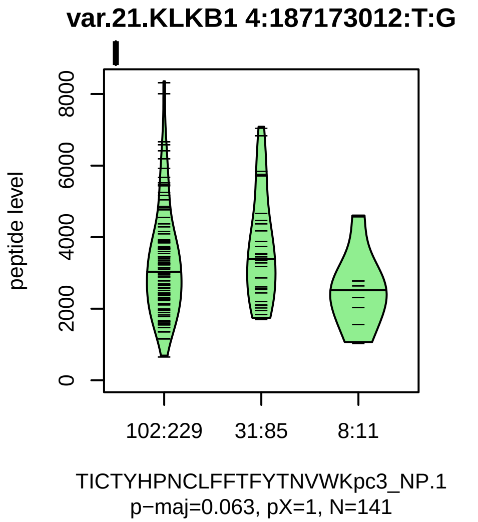
var.21.KLKB1 4:187173012:T:G
peptide level
0
2000
4000
6000
8000
102:229
31:85
8:11
TICTYHPNCLFFTFYTNVWKpc3_NP.1
p−maj=0.063, pX=1, N=141

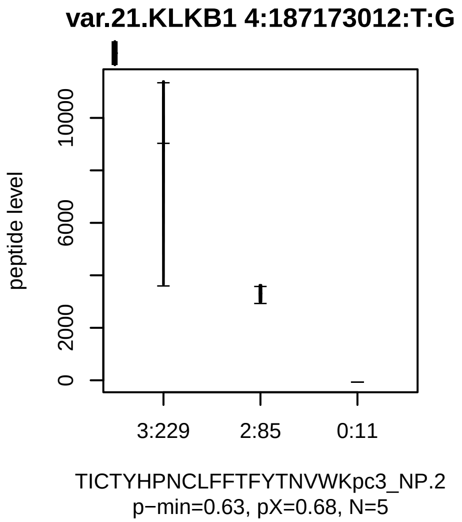
var.21.KLKB1 4:187173012:T:G
peptide level
0
2000
6000
10000
3:229
2:85
0:11
TICTYHPNCLFFTFYTNVWKpc3_NP.2
p−min=0.63, pX=0.68, N=5

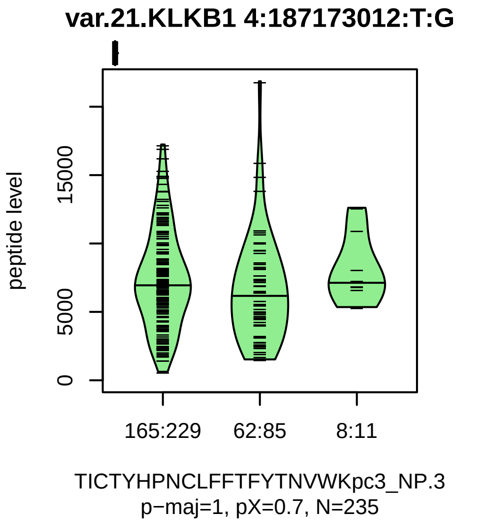
var.21.KLKB1 4:187173012:T:G
peptide level
0
5000
15000
165:229
62:85
8:11
TICTYHPNCLFFTFYTNVWKpc3_NP.3
p−maj=1, pX=0.7, N=235

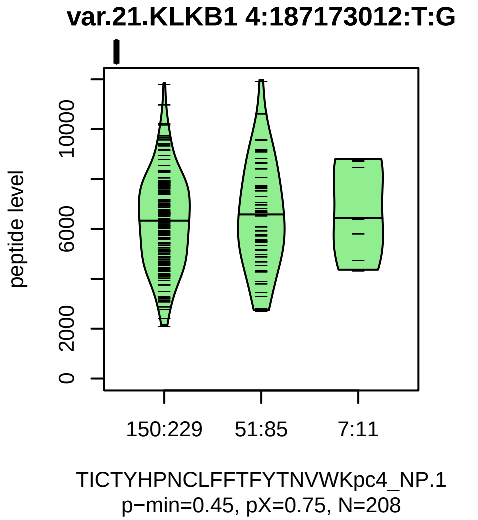
var.21.KLKB1 4:187173012:T:G
peptide level
0
2000
6000
10000
150:229
51:85
7:11
TICTYHPNCLFFTFYTNVWKpc4_NP.1
p−min=0.45, pX=0.75, N=208

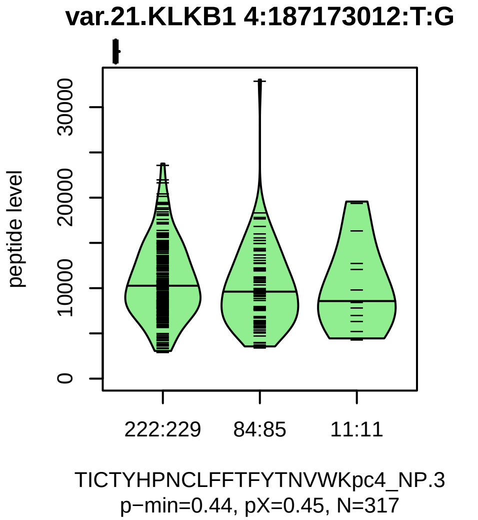
var.21.KLKB1 4:187173012:T:G
peptide level
0
10000
20000
30000
222:229
84:85
11:11
TICTYHPNCLFFTFYTNVWKpc4_NP.3
p−min=0.44, pX=0.45, N=317

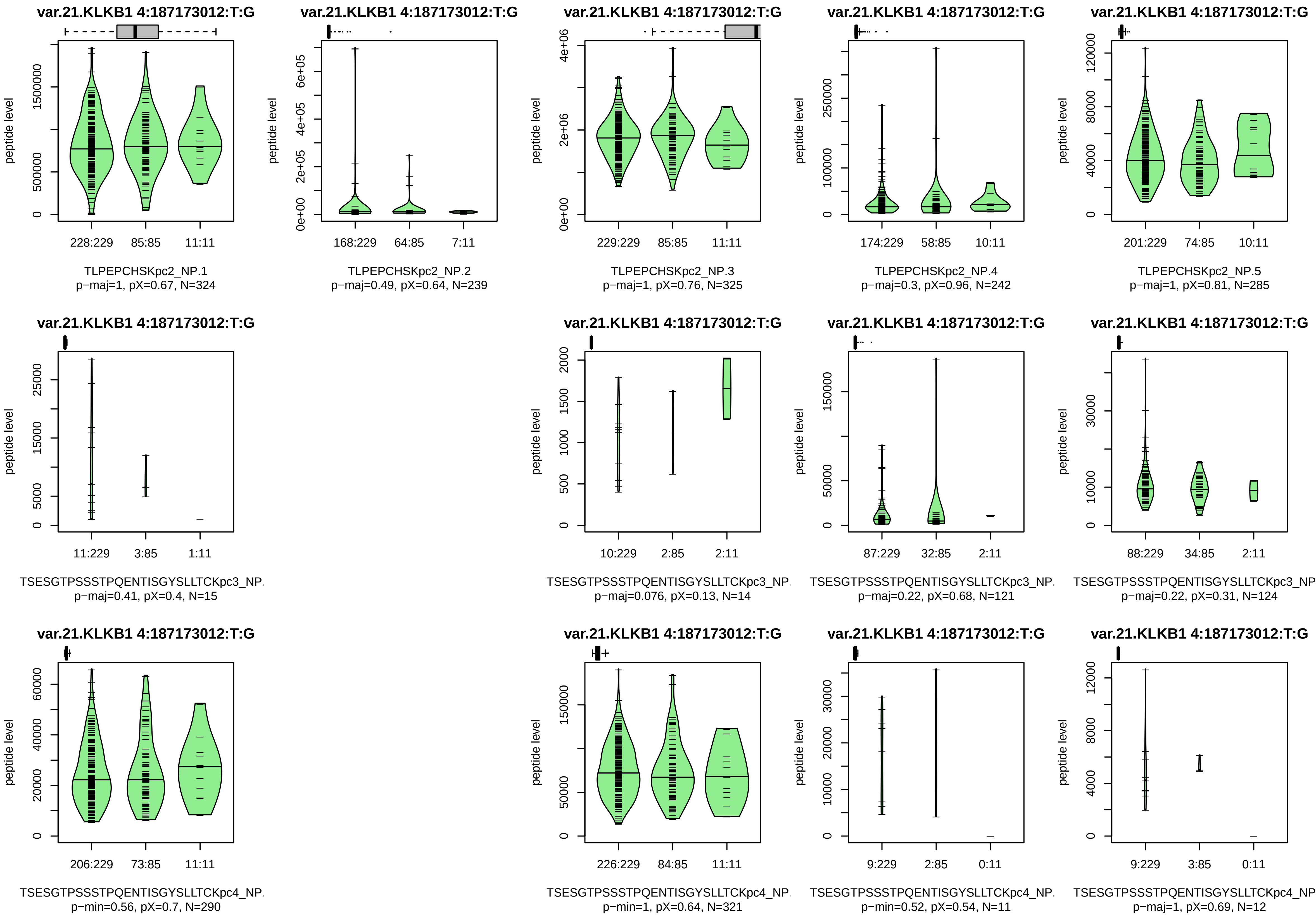
var.21.KLKB1 4:187173012:T:G
peptide level
228:229
85:85
11:11
TLPEPCHSKpc2_NP.1
p−maj=1, pX=0.67, N=324
var.21.KLKB1 4:187173012:T:G
peptide level
168:229
64:85
7:11
TLPEPCHSKpc2_NP.2
p−maj=0.49, pX=0.64, N=239
var.21.KLKB1 4:187173012:T:G
peptide level
229:229
85:85
11:11
TLPEPCHSKpc2_NP.3
p−maj=1, pX=0.76, N=325
var.21.KLKB1 4:187173012:T:G
peptide level
174:229
58:85
10:11
TLPEPCHSKpc2_NP.4
p−maj=0.3, pX=0.96, N=242
var.21.KLKB1 4:187173012:T:G
peptide level
201:229
74:85
10:11
TLPEPCHSKpc2_NP.5
p−maj=1, pX=0.81, N=285
var.21.KLKB1 4:187173012:T:G
peptide level
11:229
3:85
1:11
TSESGTPSSSTPQENTISGYSLLTCKpc3_NP.
p−maj=0.41, pX=0.4, N=15
var.21.KLKB1 4:187173012:T:G
peptide level
10:229
2:85
2:11
TSESGTPSSSTPQENTISGYSLLTCKpc3_NP.
p−maj=0.076, pX=0.13, N=14
var.21.KLKB1 4:187173012:T:G
peptide level
87:229
32:85
2:11
TSESGTPSSSTPQENTISGYSLLTCKpc3_NP.
p−maj=0.22, pX=0.68, N=121
var.21.KLKB1 4:187173012:T:G
peptide level
88:229
34:85
2:11
TSESGTPSSSTPQENTISGYSLLTCKpc3_NP
p−maj=0.22, pX=0.31, N=124
var.21.KLKB1 4:187173012:T:G
peptide level
206:229
73:85
11:11
TSESGTPSSSTPQENTISGYSLLTCKpc4_NP.
p−min=0.56, pX=0.7, N=290
var.21.KLKB1 4:187173012:T:G
peptide level
226:229
84:85
11:11
TSESGTPSSSTPQENTISGYSLLTCKpc4_NP.
p−min=1, pX=0.64, N=321
var.21.KLKB1 4:187173012:T:G
peptide level
9:229
2:85
0:11
TSESGTPSSSTPQENTISGYSLLTCKpc4_NP.
p−min=0.52, pX=0.54, N=11
var.21.KLKB1 4:187173012:T:G
peptide level
9:229
3:85
0:11
TSESGTPSSSTPQENTISGYSLLTCKpc4_NP
p−maj=1, pX=0.69, N=12

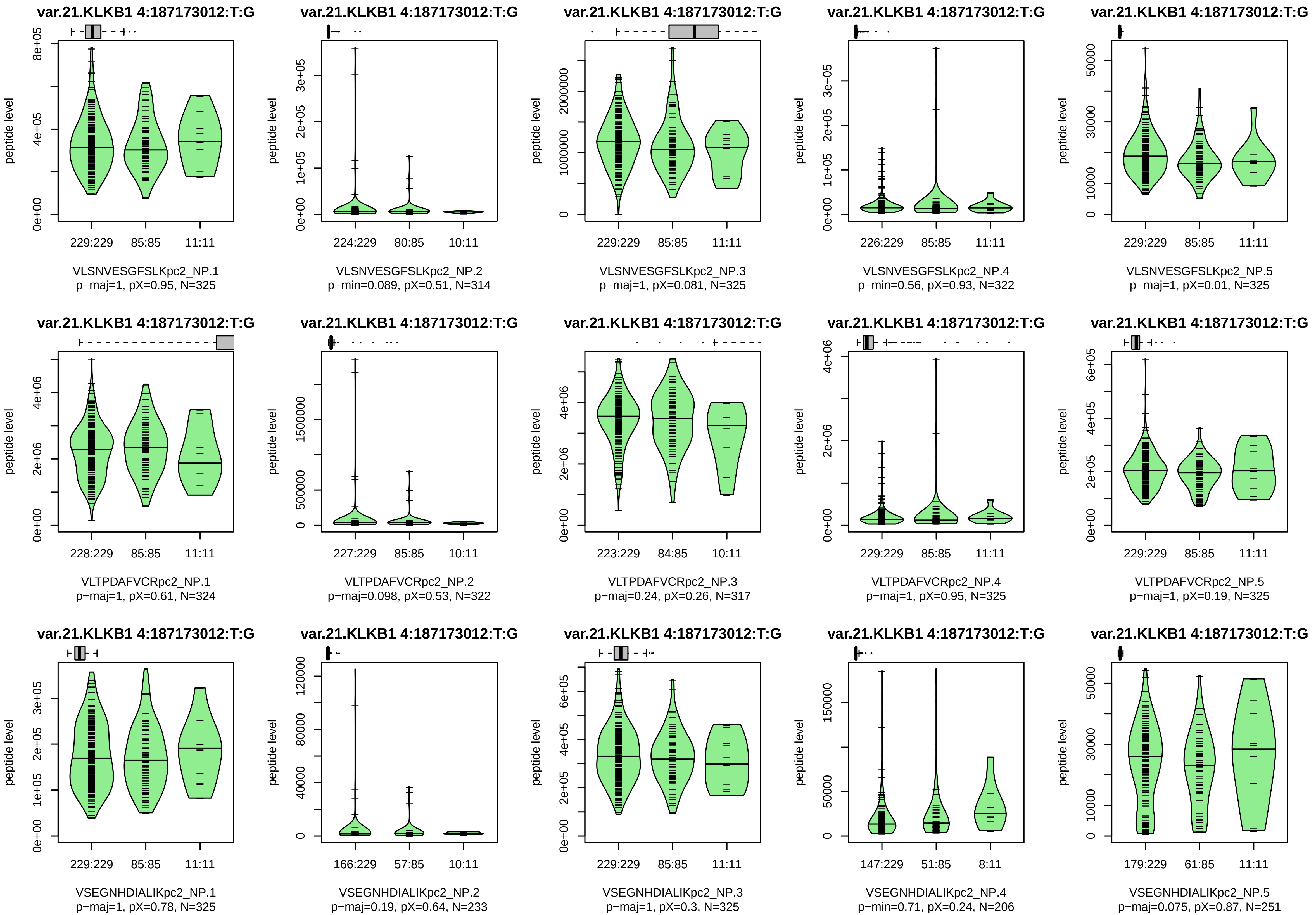
var.21.KLKB1 4:187173012:T:G
peptide level
229:229
85:85
11:11
VLSNVESGFSLKpc2_NP.1
p−maj=1, pX=0.95, N=325
224:229
80:85
10:11
VLSNVESGFSLKpc2_NP.2
p−min=0.089, pX=0.51, N=314
229:229
85:85
11:11
VLSNVESGFSLKpc2_NP.3
p−maj=1, pX=0.081, N=325
226:229
85:85
11:11
VLSNVESGFSLKpc2_NP.4
p−min=0.56, pX=0.93, N=322
229:229
85:85
11:11
VLSNVESGFSLKpc2_NP.5
p−maj=1, pX=0.01, N=325
228:229
85:85
11:11
VLTPDAFVCRpc2_NP.1
p−maj=1, pX=0.61, N=324
227:229
85:85
10:11
VLTPDAFVCRpc2_NP.2
p−maj=0.098, pX=0.53, N=322
223:229
84:85
10:11
VLTPDAFVCRpc2_NP.3
p−maj=0.24, pX=0.26, N=317
229:229
85:85
11:11
VLTPDAFVCRpc2_NP.4
p−maj=1, pX=0.95, N=325
229:229
85:85
11:11
VLTPDAFVCRpc2_NP.5
p−maj=1, pX=0.19, N=325
229:229
85:85
11:11
VSEGNHDIALIKpc2_NP.1
p−maj=1, pX=0.78, N=325
166:229
57:85
10:11
VSEGNHDIALIKpc2_NP.2
p−maj=0.19, pX=0.64, N=233
229:229
85:85
11:11
VSEGNHDIALIKpc2_NP.3
p−maj=1, pX=0.3, N=325
147:229
51:85
8:11
VSEGNHDIALIKpc2_NP.4
p−min=0.71, pX=0.24, N=206
179:229
61:85
11:11
VSEGNHDIALIKpc2_NP.5
p−maj=0.075, pX=0.87, N=251

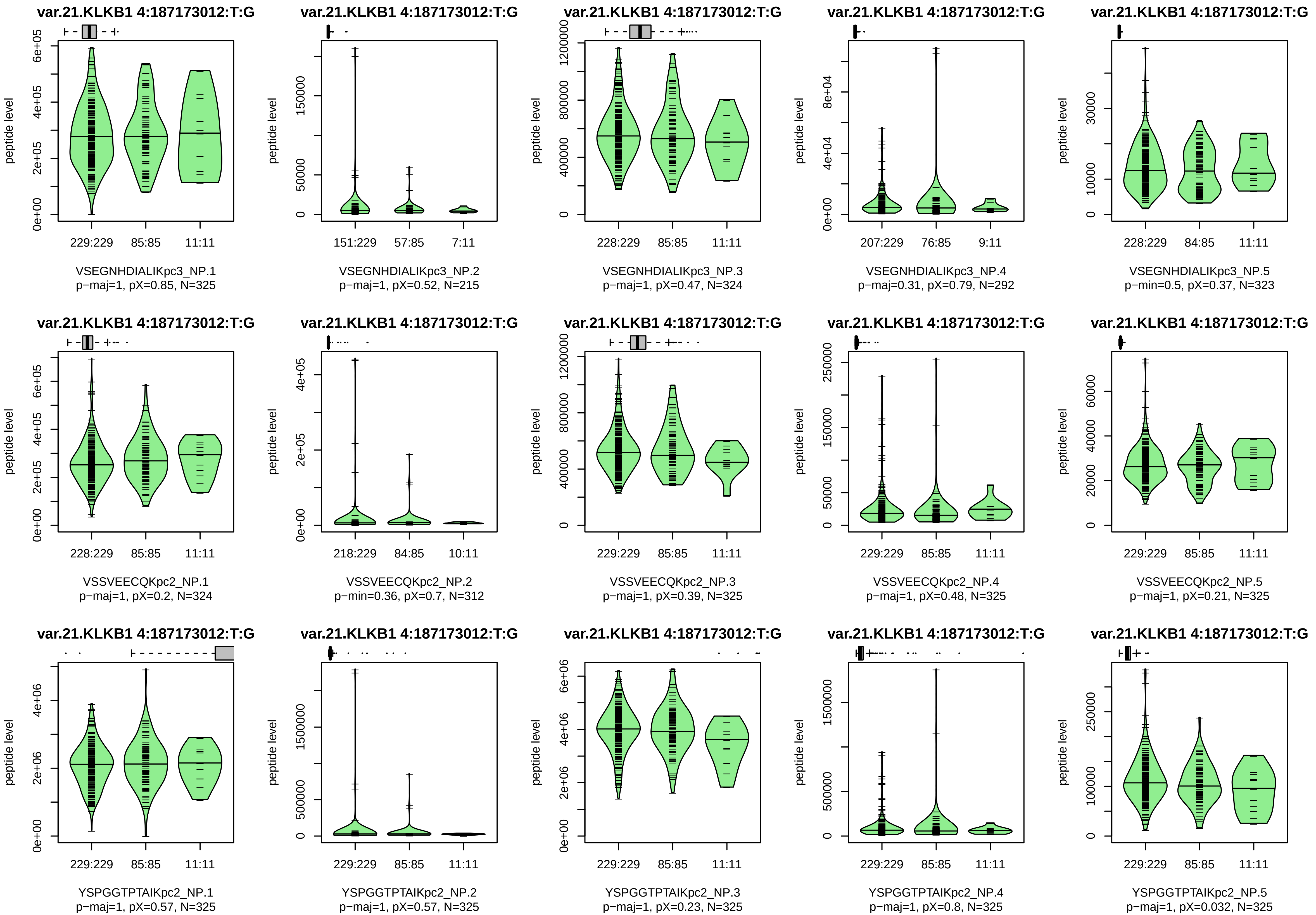

var.21.KLKB1 4:187173012:T:G
peptide level
229:229 85:85 11:11
VSEGNHDIALIKpc3_NP.1
p−maj=1, pX=0.85, N=325
var.21.KLKB1 4:187173012:T:G
peptide level
151:229 57:85 7:11
VSEGNHDIALIKpc3_NP.2
p−maj=1, pX=0.52, N=215
var.21.KLKB1 4:187173012:T:G
peptide level
228:229 85:85 11:11
VSEGNHDIALIKpc3_NP.3
p−maj=1, pX=0.47, N=324
var.21.KLKB1 4:187173012:T:G
peptide level
207:229 76:85 9:11
VSEGNHDIALIKpc3_NP.4
p−maj=0.31, pX=0.79, N=292
var.21.KLKB1 4:187173012:T:G
peptide level
228:229 84:85 11:11
VSEGNHDIALIKpc3_NP.5
p−min=0.5, pX=0.37, N=323
var.21.KLKB1 4:187173012:T:G
peptide level
228:229 85:85 11:11
VSSVEECQKpc2_NP.1
p−maj=1, pX=0.2, N=324
var.21.KLKB1 4:187173012:T:G
peptide level
218:229 84:85 10:11
VSSVEECQKpc2_NP.2
p−min=0.36, pX=0.7, N=312
var.21.KLKB1 4:187173012:T:G
peptide level
229:229 85:85 11:11
VSSVEECQKpc2_NP.3
p−maj=1, pX=0.39, N=325
var.21.KLKB1 4:187173012:T:G
peptide level
229:229 85:85 11:11
VSSVEECQKpc2_NP.4
p−maj=1, pX=0.48, N=325
var.21.KLKB1 4:187173012:T:G
peptide level
229:229 85:85 11:11
VSSVEECQKpc2_NP.5
p−maj=1, pX=0.21, N=325
var.21.KLKB1 4:187173012:T:G
peptide level
229:229 85:85 11:11
YSPGGTPTAIKpc2_NP.1
p−maj=1, pX=0.57, N=325
var.21.KLKB1 4:187173012:T:G
peptide level
229:229 85:85 11:11
YSPGGTPTAIKpc2_NP.2
p−maj=1, pX=0.57, N=325
var.21.KLKB1 4:187173012:T:G
peptide level
229:229 85:85 11:11
YSPGGTPTAIKpc2_NP.3
p−maj=1, pX=0.23, N=325
var.21.KLKB1 4:187173012:T:G
peptide level
229:229 85:85 11:11
YSPGGTPTAIKpc2_NP.4
p−maj=1, pX=0.8, N=325
var.21.KLKB1 4:187173012:T:G
peptide level
229:229 85:85 11:11
YSPGGTPTAIKpc2_NP.5
p−maj=1, pX=0.032, N=325

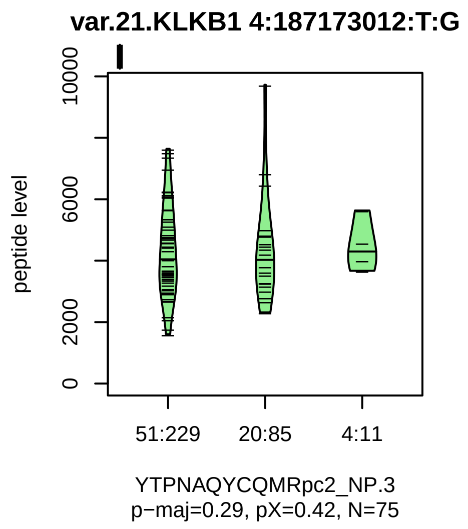

var.21.KLKB1 4:187173012:T:G
peptide level
0
2000
6000
10000
51:229
20:85
4:11
YTPNAQYCQMRpc2_NP.3
p−maj=0.29, pX=0.42, N=75

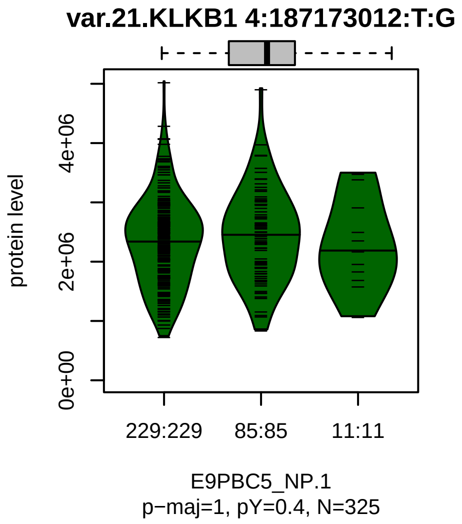

var.21.KLKB1 4:187173012:T:G
protein level
0e+00
2e+06
4e+06
229:229
85:85
11:11
E9PBC5_NP.1
p−maj=1, pY=0.4, N=325

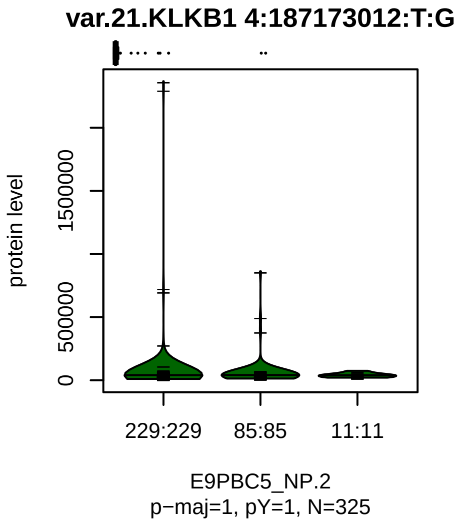

var.21.KLKB1 4:187173012:T:G
protein level
0
500000
1500000
229:229
85:85
11:11
E9PBC5_NP.2
p−maj=1, pY=1, N=325

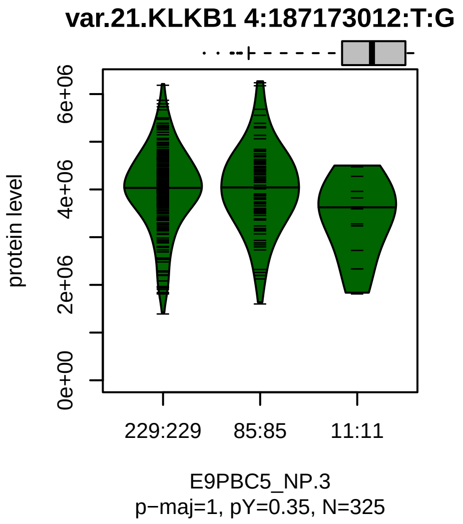

var.21.KLKB1 4:187173012:T:G
protein level
0e+00
2e+06
4e+06
6e+06
229:229
85:85
11:11
E9PBC5_NP.3
p−maj=1, pY=0.35, N=325

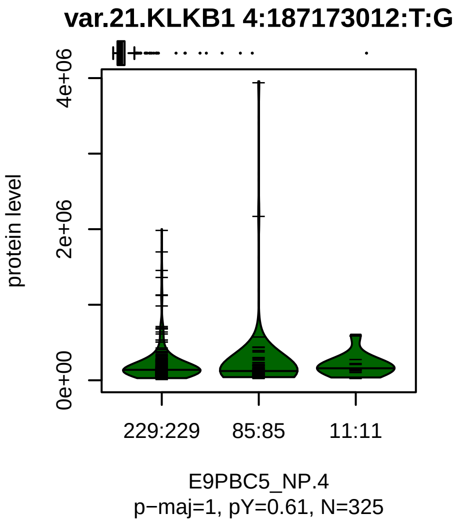

var.21.KLKB1 4:187173012:T:G
protein level
0e+00
2e+06
4e+06
229:229
85:85
11:11
E9PBC5_NP.4
p−maj=1, pY=0.61, N=325

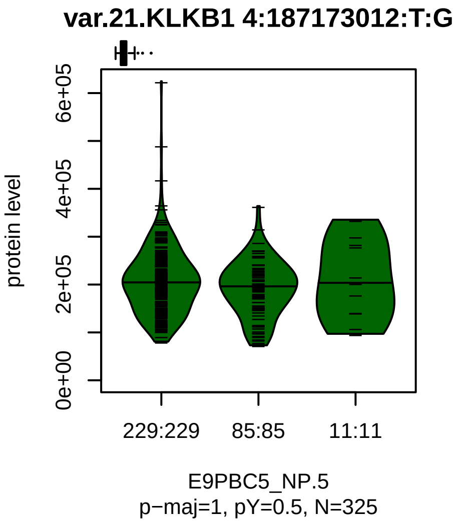

var.21.KLKB1 4:187173012:T:G
protein level
0e+00
2e+05
4e+05
6e+05
229:229
85:85
11:11
E9PBC5_NP.5
p−maj=1, pY=0.5, N=325

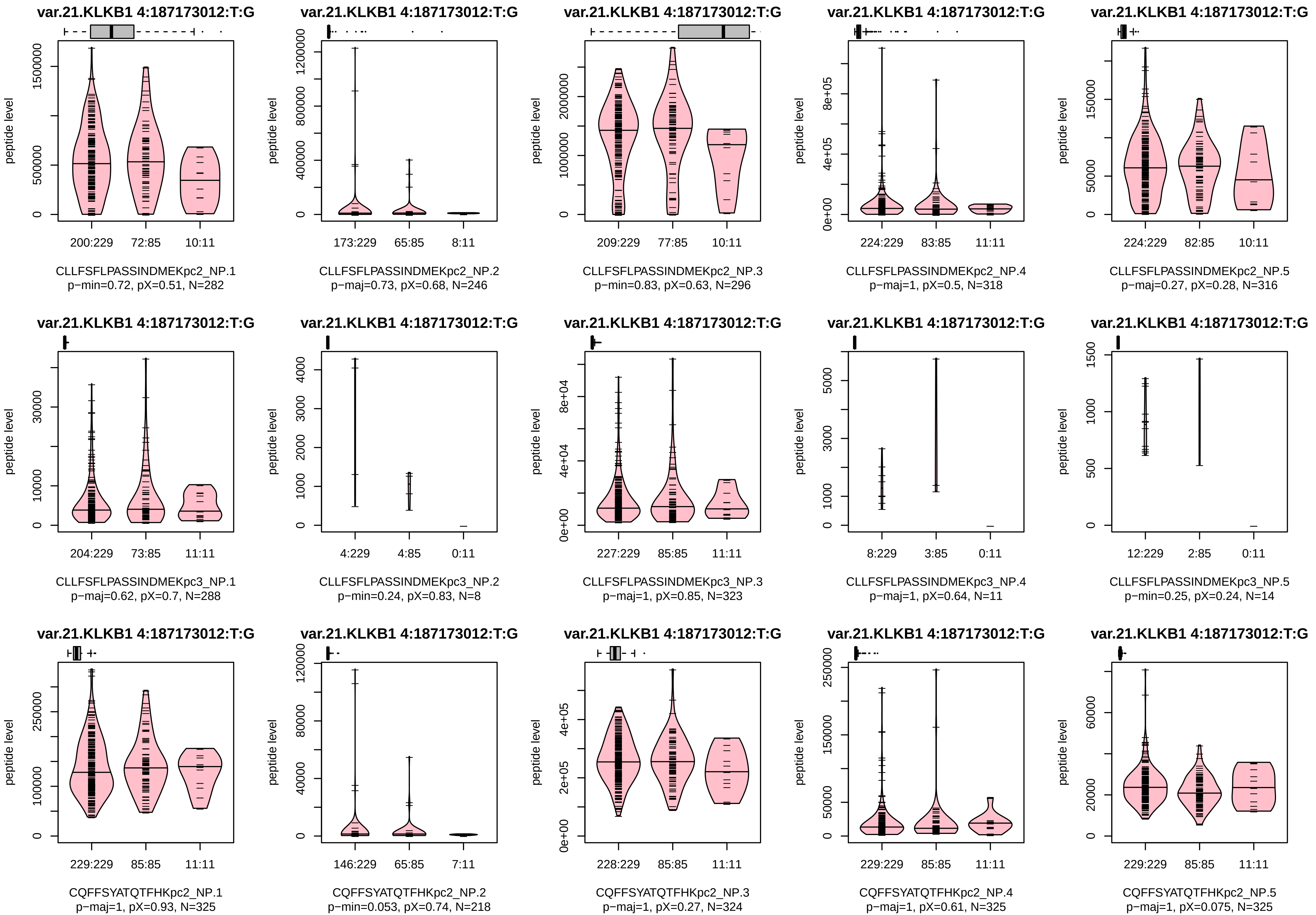
var.21.KLKB1 4:187173012:T:G
peptide level
200:229
72:85
10:11
CLLFSFLPASSINDMEKpc2_NP.1
p−min=0.72, pX=0.51, N=282
var.21.KLKB1 4:187173012:T:G
peptide level
173:229
65:85
8:11
CLLFSFLPASSINDMEKpc2_NP.2
p−maj=0.73, pX=0.68, N=246
var.21.KLKB1 4:187173012:T:G
peptide level
209:229
77:85
10:11
CLLFSFLPASSINDMEKpc2_NP.3
p−min=0.83, pX=0.63, N=296
var.21.KLKB1 4:187173012:T:G
peptide level
224:229
83:85
11:11
CLLFSFLPASSINDMEKpc2_NP.4
p−maj=1, pX=0.5, N=318
var.21.KLKB1 4:187173012:T:G
peptide level
224:229
82:85
10:11
CLLFSFLPASSINDMEKpc2_NP.5
p−maj=0.27, pX=0.28, N=316
var.21.KLKB1 4:187173012:T:G
peptide level
204:229
73:85
11:11
CLLFSFLPASSINDMEKpc3_NP.1
p−maj=0.62, pX=0.7, N=288
var.21.KLKB1 4:187173012:T:G
peptide level
4:229
4:85
0:11
CLLFSFLPASSINDMEKpc3_NP.2
p−min=0.24, pX=0.83, N=8
var.21.KLKB1 4:187173012:T:G
peptide level
227:229
85:85
11:11
CLLFSFLPASSINDMEKpc3_NP.3
p−maj=1, pX=0.85, N=323
var.21.KLKB1 4:187173012:T:G
peptide level
8:229
3:85
0:11
CLLFSFLPASSINDMEKpc3_NP.4
p−maj=1, pX=0.64, N=11
var.21.KLKB1 4:187173012:T:G
peptide level
12:229
2:85
0:11
CLLFSFLPASSINDMEKpc3_NP.5
p−min=0.25, pX=0.24, N=14
var.21.KLKB1 4:187173012:T:G
peptide level
229:229
85:85
11:11
CQFFSYATQTFHKpc2_NP.1
p−maj=1, pX=0.93, N=325
var.21.KLKB1 4:187173012:T:G
peptide level
146:229
65:85
7:11
CQFFSYATQTFHKpc2_NP.2
p−min=0.053, pX=0.74, N=218
var.21.KLKB1 4:187173012:T:G
peptide level
228:229
85:85
11:11
CQFFSYATQTFHKpc2_NP.3
p−maj=1, pX=0.27, N=324
var.21.KLKB1 4:187173012:T:G
peptide level
229:229
85:85
11:11
CQFFSYATQTFHKpc2_NP.4
p−maj=1, pX=0.61, N=325
var.21.KLKB1 4:187173012:T:G
peptide level
229:229
85:85
11:11
CQFFSYATQTFHKpc2_NP.5
p−maj=1, pX=0.075, N=325

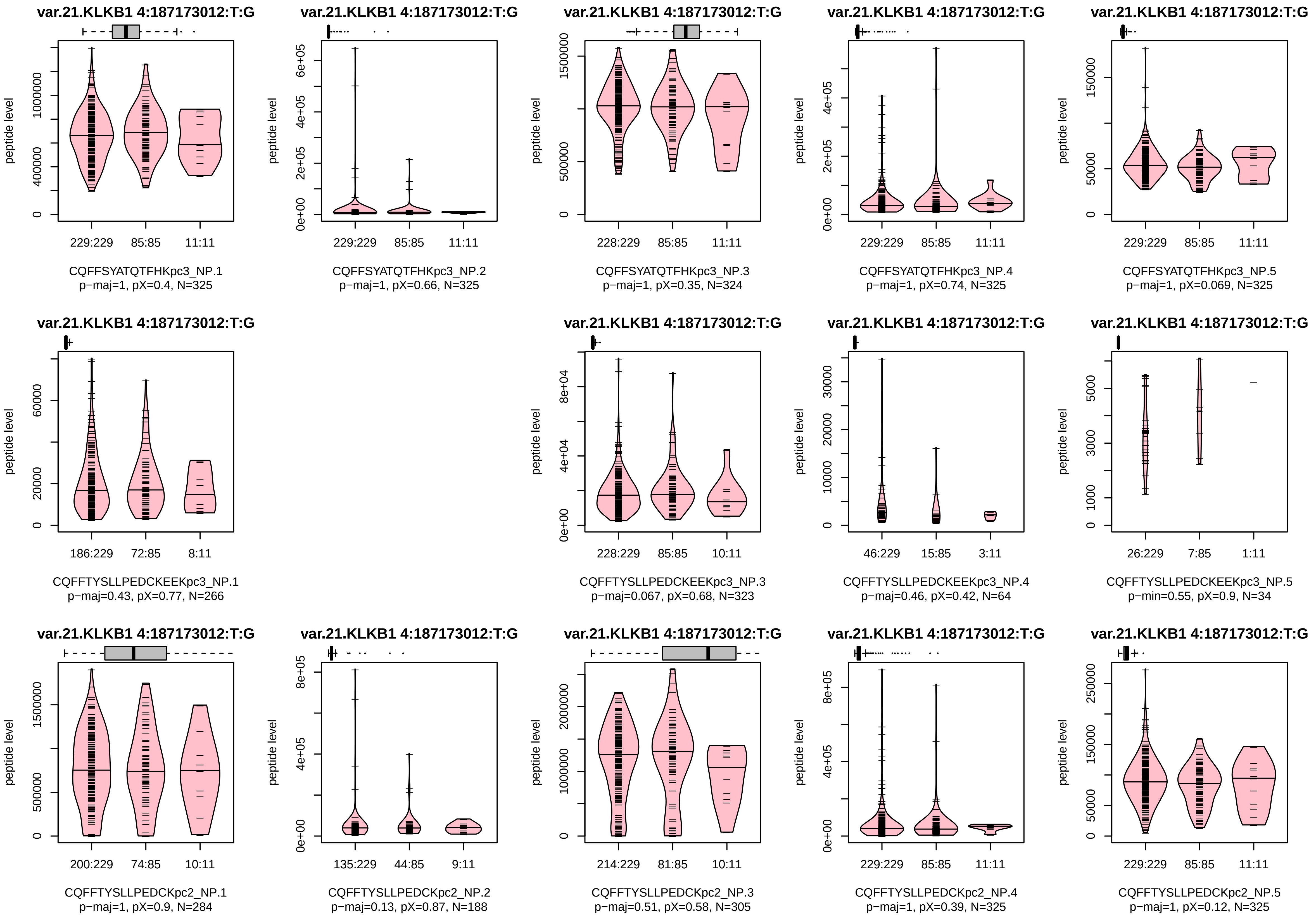
var.21.KLKB1 4:187173012:T:G
peptide level
229:229
85:85
11:11
CQFFSYATQTFHKpc3_NP.1
p−maj=1, pX=0.4, N=325
var.21.KLKB1 4:187173012:T:G
peptide level
229:229
85:85
11:11
CQFFSYATQTFHKpc3_NP.2
p−maj=1, pX=0.66, N=325
var.21.KLKB1 4:187173012:T:G
peptide level
228:229
85:85
11:11
CQFFSYATQTFHKpc3_NP.3
p−maj=1, pX=0.35, N=324
var.21.KLKB1 4:187173012:T:G
peptide level
229:229
85:85
11:11
CQFFSYATQTFHKpc3_NP.4
p−maj=1, pX=0.74, N=325
var.21.KLKB1 4:187173012:T:G
peptide level
229:229
85:85
11:11
CQFFSYATQTFHKpc3_NP.5
p−maj=1, pX=0.069, N=325
var.21.KLKB1 4:187173012:T:G
peptide level
186:229
72:85
8:11
CQFFTYSLLPEDCKEEKpc3_NP.1
p−maj=0.43, pX=0.77, N=266
var.21.KLKB1 4:187173012:T:G
peptide level
228:229
85:85
10:11
CQFFTYSLLPEDCKEEKpc3_NP.3
p−maj=0.067, pX=0.68, N=323
var.21.KLKB1 4:187173012:T:G
peptide level
46:229
15:85
3:11
CQFFTYSLLPEDCKEEKpc3_NP.4
p−maj=0.46, pX=0.42, N=64
var.21.KLKB1 4:187173012:T:G
peptide level
26:229
7:85
1:11
CQFFTYSLLPEDCKEEKpc3_NP.5
p−min=0.55, pX=0.9, N=34
var.21.KLKB1 4:187173012:T:G
peptide level
200:229
74:85
10:11
CQFFTYSLLPEDCKpc2_NP.1
p−maj=1, pX=0.9, N=284
var.21.KLKB1 4:187173012:T:G
peptide level
135:229
44:85
9:11
CQFFTYSLLPEDCKpc2_NP.2
p−maj=0.13, pX=0.87, N=188
var.21.KLKB1 4:187173012:T:G
peptide level
214:229
81:85
10:11
CQFFTYSLLPEDCKpc2_NP.3
p−maj=0.51, pX=0.58, N=305
var.21.KLKB1 4:187173012:T:G
peptide level
229:229
85:85
11:11
CQFFTYSLLPEDCKpc2_NP.4
p−maj=1, pX=0.39, N=325
var.21.KLKB1 4:187173012:T:G
peptide level
229:229
85:85
11:11
CQFFTYSLLPEDCKpc2_NP.5
p−maj=1, pX=0.12, N=325

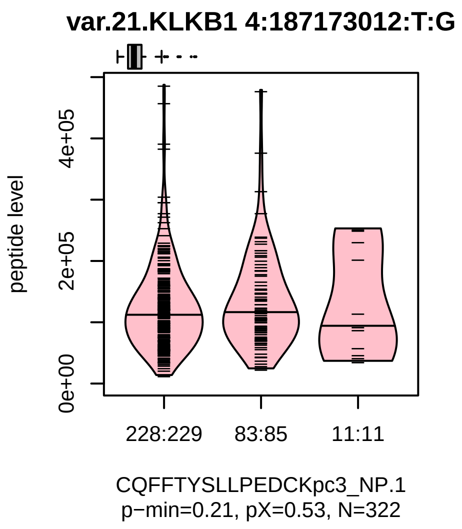
var.21.KLKB1 4:187173012:T:G
peptide level
228:229
83:85
11:11
CQFFTYSLLPEDCKpc3_NP.1
p−min=0.21, pX=0.53, N=322

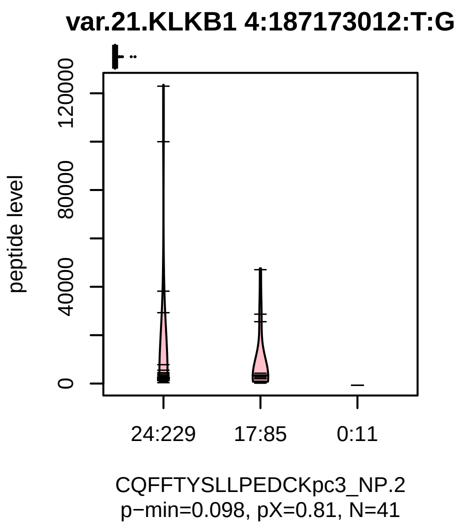
var.21.KLKB1 4:187173012:T:G
peptide level
24:229
17:85
0:11
CQFFTYSLLPEDCKpc3_NP.2
p−min=0.098, pX=0.81, N=41

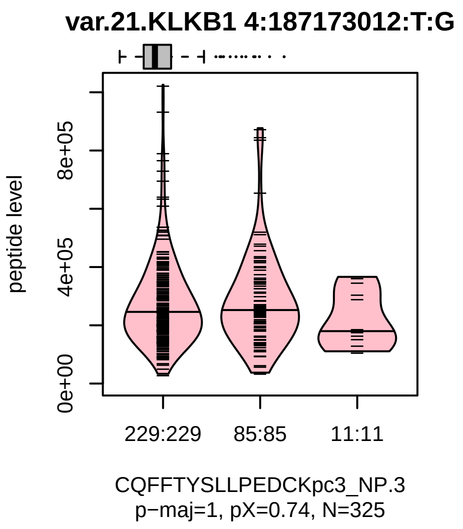
var.21.KLKB1 4:187173012:T:G
peptide level
229:229
85:85
11:11
CQFFTYSLLPEDCKpc3_NP.3
p−maj=1, pX=0.74, N=325

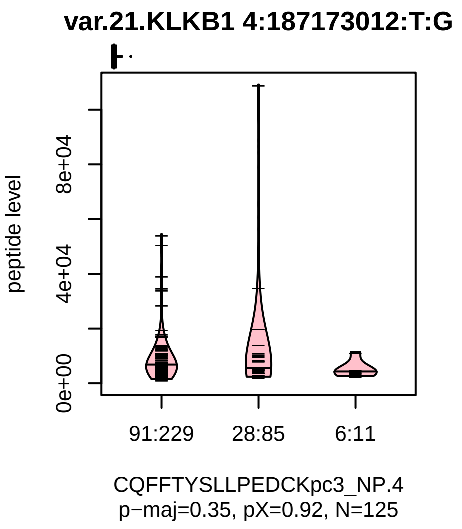
var.21.KLKB1 4:187173012:T:G
peptide level
91:229
28:85
6:11
CQFFTYSLLPEDCKpc3_NP.4
p−maj=0.35, pX=0.92, N=125

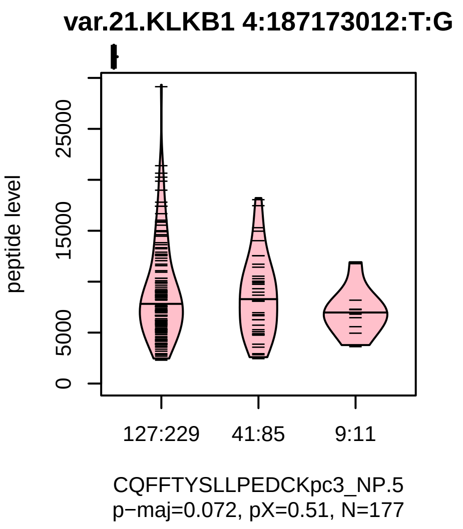
var.21.KLKB1 4:187173012:T:G
peptide level
127:229
41:85
9:11
CQFFTYSLLPEDCKpc3_NP.5
p−maj=0.072, pX=0.51, N=177

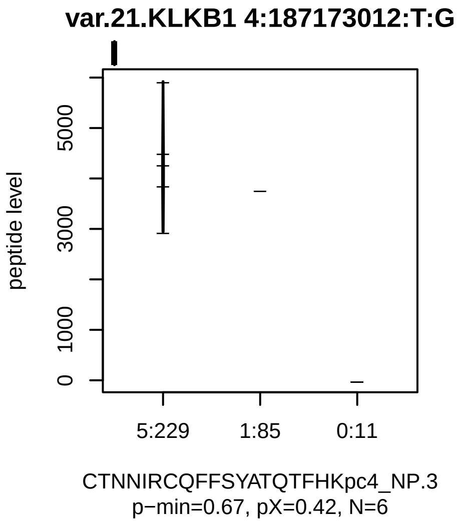
var.21.KLKB1 4:187173012:T:G
peptide level
5:229
1:85
0:11
CTNNIRCQFFSYATQTFHKpc4_NP.3
p−min=0.67, pX=0.42, N=6

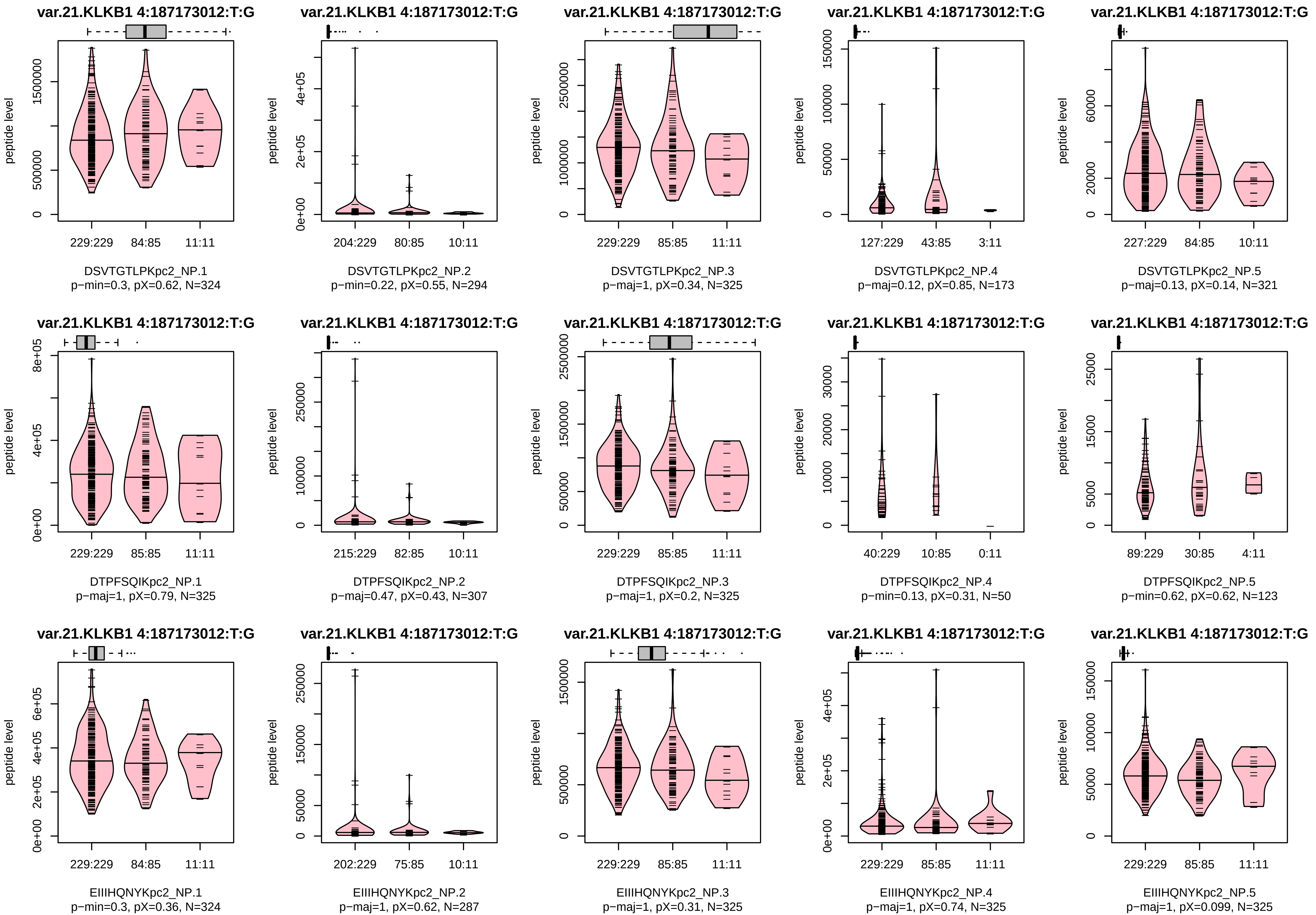

var.21.KLKB1 4:187173012:T:G
peptide level
229:229
84:85
11:11
DSVTGTLPKpc2_NP.1
p−min=0.3, pX=0.62, N=324
var.21.KLKB1 4:187173012:T:G
peptide level
204:229
80:85
10:11
DSVTGTLPKpc2_NP.2
p−min=0.22, pX=0.55, N=294
var.21.KLKB1 4:187173012:T:G
peptide level
229:229
85:85
11:11
DSVTGTLPKpc2_NP.3
p−maj=1, pX=0.34, N=325
var.21.KLKB1 4:187173012:T:G
peptide level
127:229
43:85
3:11
DSVTGTLPKpc2_NP.4
p−maj=0.12, pX=0.85, N=173
var.21.KLKB1 4:187173012:T:G
peptide level
227:229
84:85
10:11
DSVTGTLPKpc2_NP.5
p−maj=0.13, pX=0.14, N=321
var.21.KLKB1 4:187173012:T:G
peptide level
229:229
85:85
11:11
DTPFSQIKpc2_NP.1
p−maj=1, pX=0.79, N=325
var.21.KLKB1 4:187173012:T:G
peptide level
215:229
82:85
10:11
DTPFSQIKpc2_NP.2
p−maj=0.47, pX=0.43, N=307
var.21.KLKB1 4:187173012:T:G
peptide level
229:229
85:85
11:11
DTPFSQIKpc2_NP.3
p−maj=1, pX=0.2, N=325
var.21.KLKB1 4:187173012:T:G
peptide level
40:229
10:85
0:11
DTPFSQIKpc2_NP.4
p−min=0.13, pX=0.31, N=50
var.21.KLKB1 4:187173012:T:G
peptide level
89:229
30:85
4:11
DTPFSQIKpc2_NP.5
p−min=0.62, pX=0.62, N=123
var.21.KLKB1 4:187173012:T:G
peptide level
229:229
84:85
11:11
EIIIHQNYKpc2_NP.1
p−min=0.3, pX=0.36, N=324
var.21.KLKB1 4:187173012:T:G
peptide level
202:229
75:85
10:11
EIIIHQNYKpc2_NP.2
p−maj=1, pX=0.62, N=287
var.21.KLKB1 4:187173012:T:G
peptide level
229:229
85:85
11:11
EIIIHQNYKpc2_NP.3
p−maj=1, pX=0.31, N=325
var.21.KLKB1 4:187173012:T:G
peptide level
229:229
85:85
11:11
EIIIHQNYKpc2_NP.4
p−maj=1, pX=0.74, N=325
var.21.KLKB1 4:187173012:T:G
peptide level
229:229
85:85
11:11
EIIIHQNYKpc2_NP.5
p−maj=1, pX=0.099, N=325

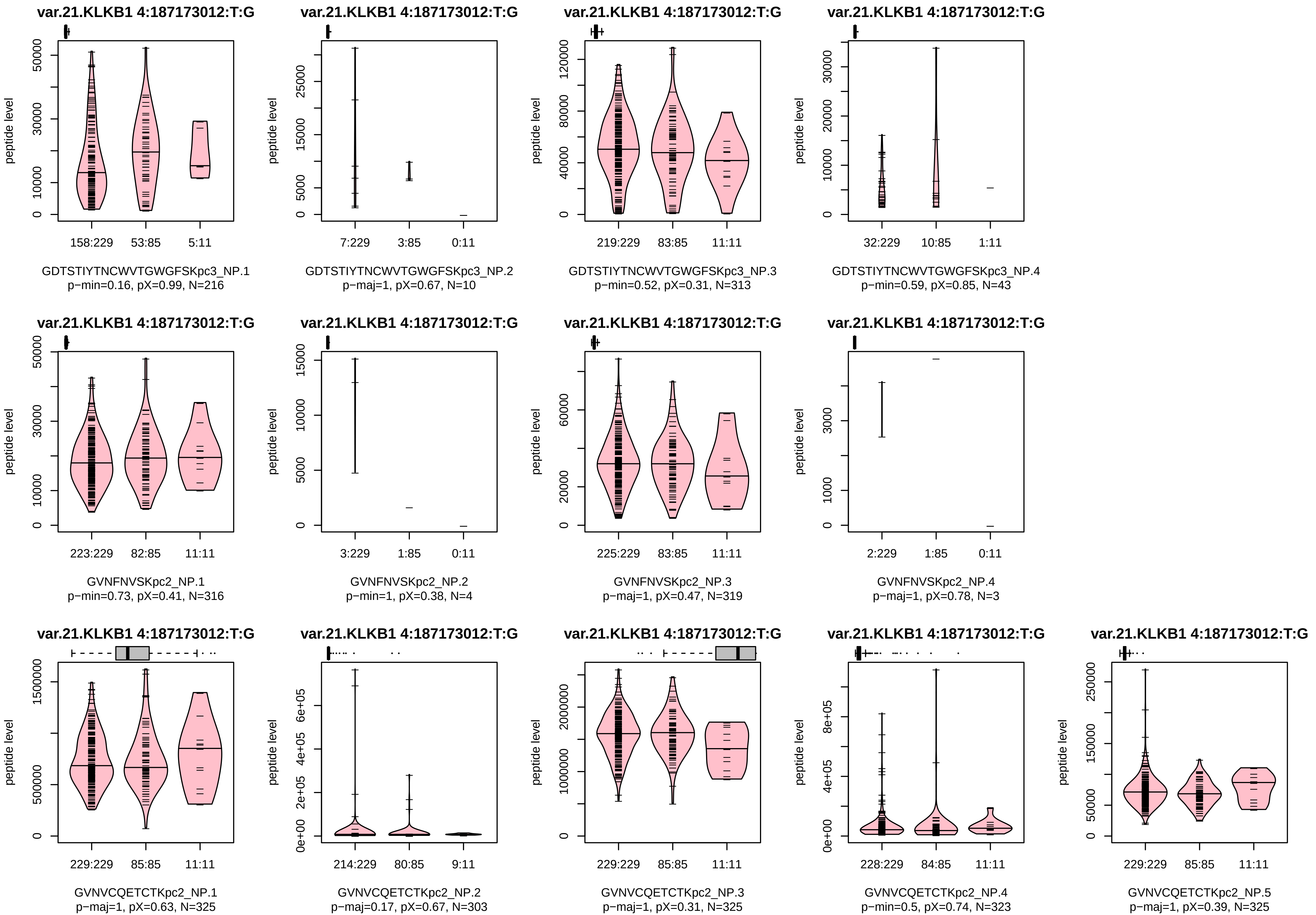
var.21.KLKB1 4:187173012:T:G
peptide level
158:229
53:85
5:11
GDTSTIYTNCWVTGWGFSKpc3_NP.1
p−min=0.16, pX=0.99, N=216
var.21.KLKB1 4:187173012:T:G
peptide level
7:229
3:85
0:11
GDTSTIYTNCWVTGWGFSKpc3_NP.2
p−maj=1, pX=0.67, N=10
var.21.KLKB1 4:187173012:T:G
peptide level
219:229
83:85
11:11
GDTSTIYTNCWVTGWGFSKpc3_NP.3
p−min=0.52, pX=0.31, N=313
var.21.KLKB1 4:187173012:T:G
peptide level
32:229
10:85
1:11
GDTSTIYTNCWVTGWGFSKpc3_NP.4
p−min=0.59, pX=0.85, N=43
var.21.KLKB1 4:187173012:T:G
peptide level
223:229
82:85
11:11
GVNFNVSKpc2_NP.1
p−min=0.73, pX=0.41, N=316
var.21.KLKB1 4:187173012:T:G
peptide level
3:229
1:85
0:11
GVNFNVSKpc2_NP.2
p−min=1, pX=0.38, N=4
var.21.KLKB1 4:187173012:T:G
peptide level
225:229
83:85
11:11
GVNFNVSKpc2_NP.3
p−maj=1, pX=0.47, N=319
var.21.KLKB1 4:187173012:T:G
peptide level
2:229
1:85
0:11
GVNFNVSKpc2_NP.4
p−maj=1, pX=0.78, N=3
var.21.KLKB1 4:187173012:T:G
peptide level
229:229
85:85
11:11
GVNVCQETCTKpc2_NP.1
p−maj=1, pX=0.63, N=325
var.21.KLKB1 4:187173012:T:G
peptide level
214:229
80:85
9:11
GVNVCQETCTKpc2_NP.2
p−maj=0.17, pX=0.67, N=303
var.21.KLKB1 4:187173012:T:G
peptide level
229:229
85:85
11:11
GVNVCQETCTKpc2_NP.3
p−maj=1, pX=0.31, N=325
var.21.KLKB1 4:187173012:T:G
peptide level
228:229
84:85
11:11
GVNVCQETCTKpc2_NP.4
p−min=0.5, pX=0.74, N=323
var.21.KLKB1 4:187173012:T:G
peptide level
229:229
85:85
11:11
GVNVCQETCTKpc2_NP.5
p−maj=1, pX=0.39, N=325

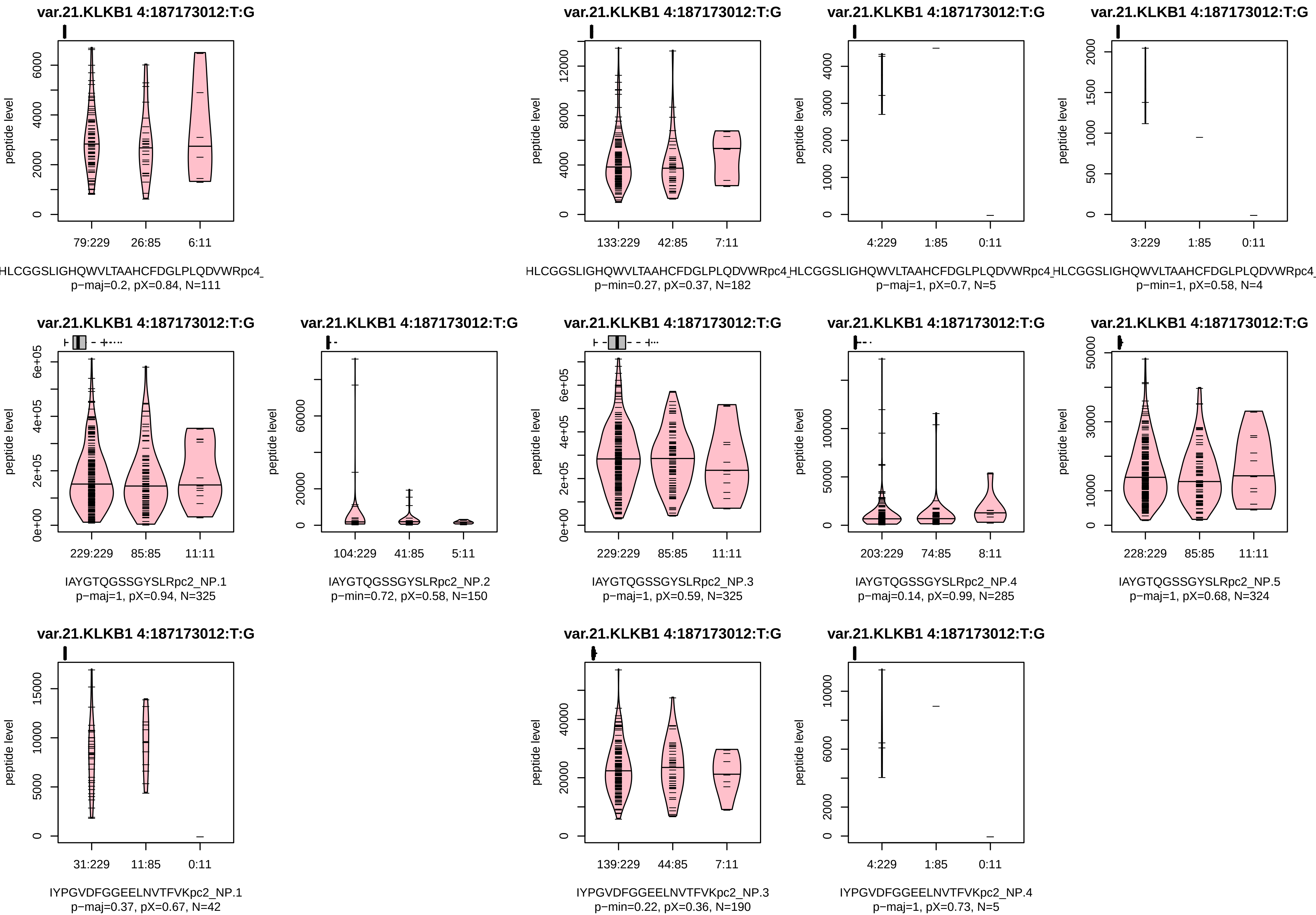
var.21.KLKB1 4:187173012:T:G
peptide level
79:229
26:85
6:11
HLCGGSLIGHQWVLTAAHCFDGLPLQDVWRpc4_
p−maj=0.2, pX=0.84, N=111
var.21.KLKB1 4:187173012:T:G
peptide level
133:229
42:85
7:11
HLCGGSLIGHQWVLTAAHCFDGLPLQDVWRpc4_
p−min=0.27, pX=0.37, N=182
var.21.KLKB1 4:187173012:T:G
peptide level
4:229
1:85
0:11
HLCGGSLIGHQWVLTAAHCFDGLPLQDVWRpc4_
p−maj=1, pX=0.7, N=5
var.21.KLKB1 4:187173012:T:G
peptide level
3:229
1:85
0:11
HLCGGSLIGHQWVLTAAHCFDGLPLQDVWRpc4_
p−min=1, pX=0.58, N=4
var.21.KLKB1 4:187173012:T:G
peptide level
229:229
85:85
11:11
IAYGTQGSSGYSLRpc2_NP.1
p−maj=1, pX=0.94, N=325
var.21.KLKB1 4:187173012:T:G
peptide level
104:229
41:85
5:11
IAYGTQGSSGYSLRpc2_NP.2
p−min=0.72, pX=0.58, N=150
var.21.KLKB1 4:187173012:T:G
peptide level
229:229
85:85
11:11
IAYGTQGSSGYSLRpc2_NP.3
p−maj=1, pX=0.59, N=325
var.21.KLKB1 4:187173012:T:G
peptide level
203:229
74:85
8:11
IAYGTQGSSGYSLRpc2_NP.4
p−maj=0.14, pX=0.99, N=285
var.21.KLKB1 4:187173012:T:G
peptide level
228:229
85:85
11:11
IAYGTQGSSGYSLRpc2_NP.5
p−maj=1, pX=0.68, N=324
var.21.KLKB1 4:187173012:T:G
peptide level
31:229
11:85
0:11
IYPGVDFGGEELNVTFVKpc2_NP.1
p−maj=0.37, pX=0.67, N=42
var.21.KLKB1 4:187173012:T:G
peptide level
139:229
44:85
7:11
IYPGVDFGGEELNVTFVKpc2_NP.3
p−min=0.22, pX=0.36, N=190
var.21.KLKB1 4:187173012:T:G
peptide level
4:229
1:85
0:11
IYPGVDFGGEELNVTFVKpc2_NP.4
p−maj=1, pX=0.73, N=5

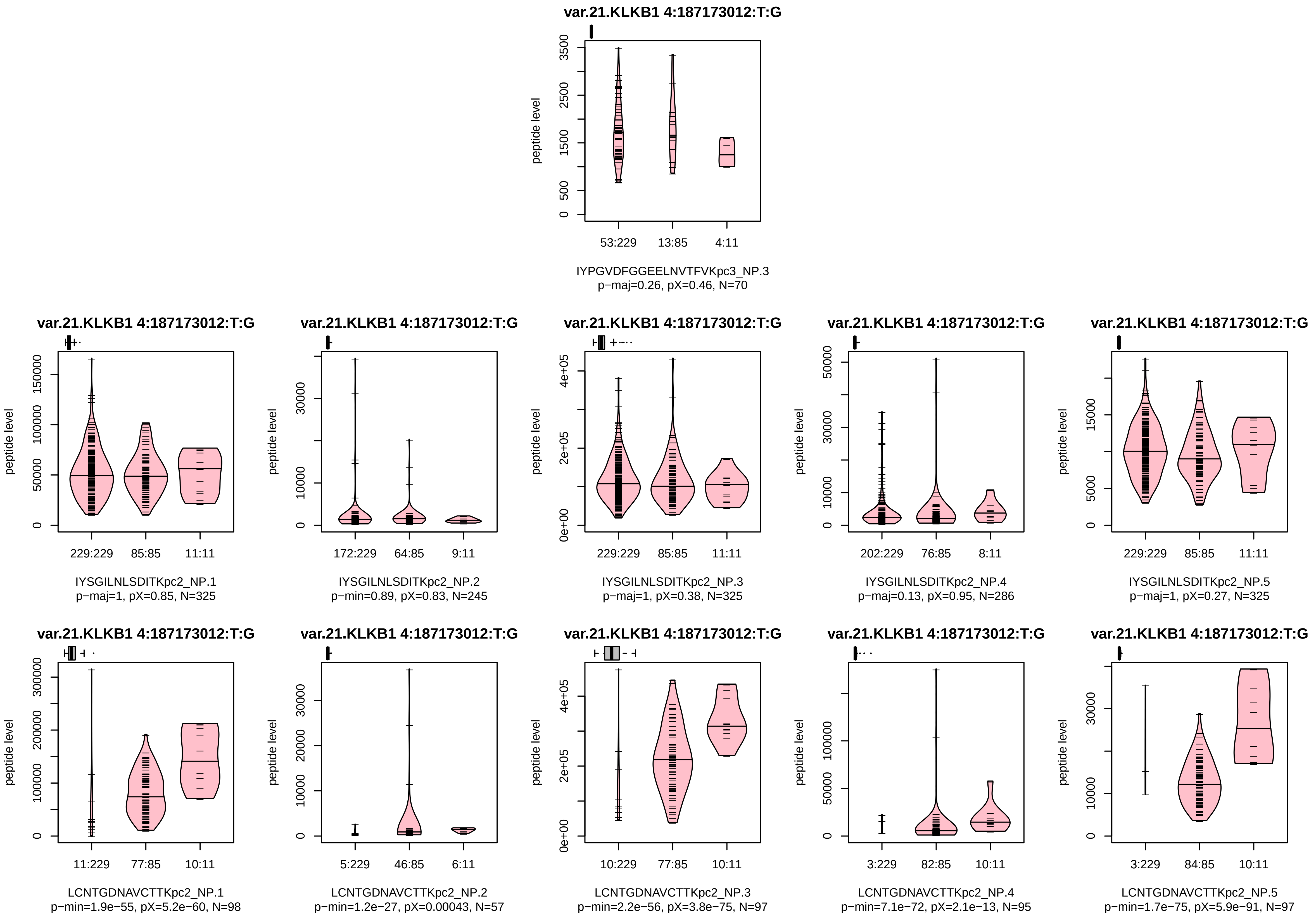

var.21.KLKB1 4:187173012:T:G
peptide level
53:229
13:85
4:11
IYPGVDFGGEELNVTFVKpc3_NP.3
p−maj=0.26, pX=0.46, N=70
var.21.KLKB1 4:187173012:T:G
229:229
85:85
11:11
IYSGILNLSDITKpc2_NP.1
p−maj=1, pX=0.85, N=325
var.21.KLKB1 4:187173012:T:G
172:229
64:85
9:11
IYSGILNLSDITKpc2_NP.2
p−min=0.89, pX=0.83, N=245
var.21.KLKB1 4:187173012:T:G
229:229
85:85
11:11
IYSGILNLSDITKpc2_NP.3
p−maj=1, pX=0.38, N=325
var.21.KLKB1 4:187173012:T:G
202:229
76:85
8:11
IYSGILNLSDITKpc2_NP.4
p−maj=0.13, pX=0.95, N=286
var.21.KLKB1 4:187173012:T:G
229:229
85:85
11:11
IYSGILNLSDITKpc2_NP.5
p−maj=1, pX=0.27, N=325
var.21.KLKB1 4:187173012:T:G
11:229
77:85
10:11
LCNTGDNAVCTTKpc2_NP.1
p−min=1.9e−55, pX=5.2e−60, N=98
var.21.KLKB1 4:187173012:T:G
5:229
46:85
6:11
LCNTGDNAVCTTKpc2_NP.2
p−min=1.2e−27, pX=0.00043, N=57
var.21.KLKB1 4:187173012:T:G
10:229
77:85
10:11
LCNTGDNAVCTTKpc2_NP.3
p−min=2.2e−56, pX=3.8e−75, N=97
var.21.KLKB1 4:187173012:T:G
3:229
82:85
10:11
LCNTGDNAVCTTKpc2_NP.4
p−min=7.1e−72, pX=2.1e−13, N=95
var.21.KLKB1 4:187173012:T:G
3:229
84:85
10:11
LCNTGDNAVCTTKpc2_NP.5
p−min=1.7e−75, pX=5.9e−91, N=97

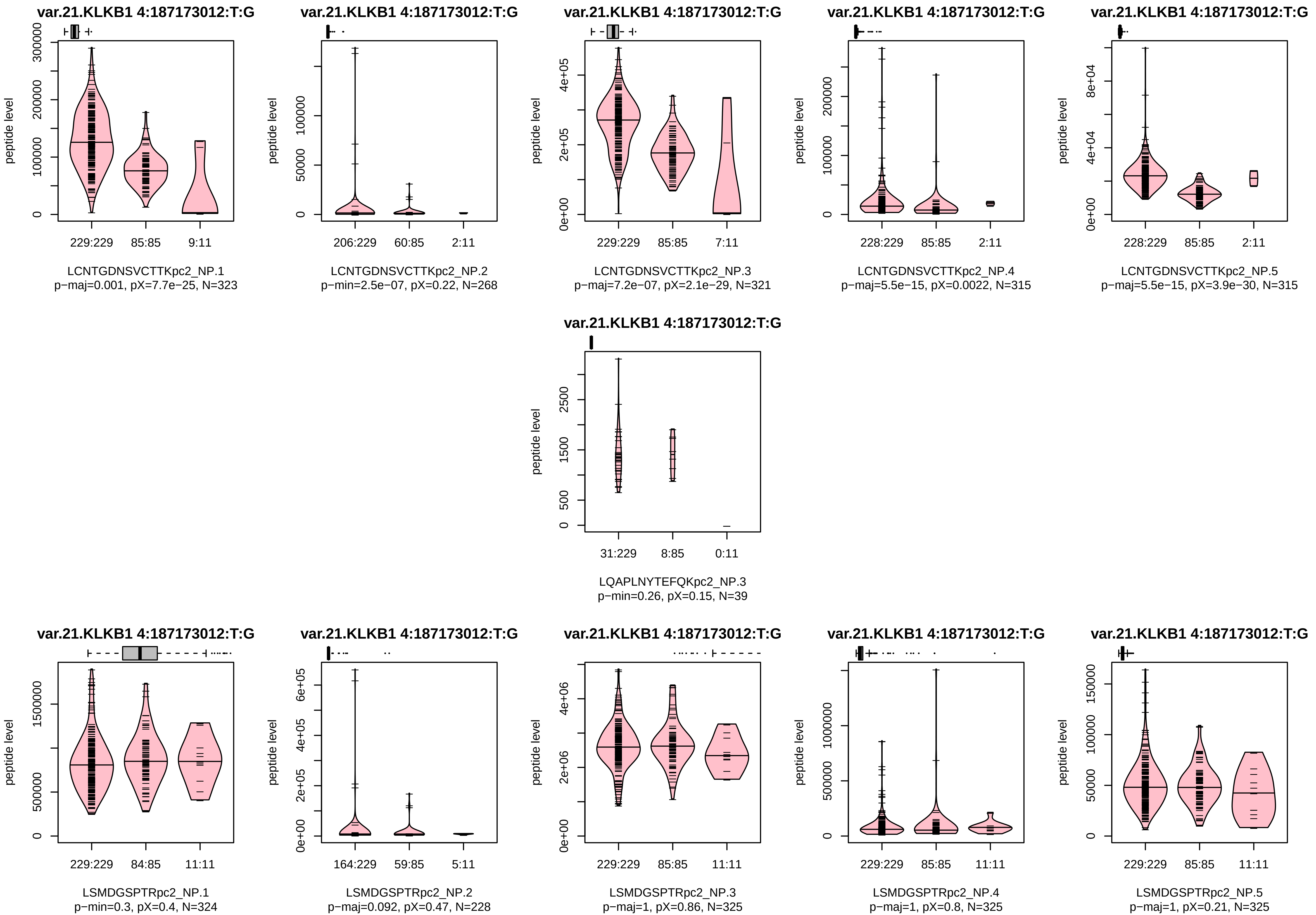
var.21.KLKB1 4:187173012:T:G
peptide level
229:229
85:85
9:11
LCNTGDNSVCTTKpc2_NP.1
p−maj=0.001, pX=7.7e−25, N=323
var.21.KLKB1 4:187173012:T:G
peptide level
206:229
60:85
2:11
LCNTGDNSVCTTKpc2_NP.2
p−min=2.5e−07, pX=0.22, N=268
var.21.KLKB1 4:187173012:T:G
peptide level
229:229
85:85
7:11
LCNTGDNSVCTTKpc2_NP.3
p−maj=7.2e−07, pX=2.1e−29, N=321
var.21.KLKB1 4:187173012:T:G
peptide level
228:229
85:85
2:11
LCNTGDNSVCTTKpc2_NP.4
p−maj=5.5e−15, pX=0.0022, N=315
var.21.KLKB1 4:187173012:T:G
peptide level
228:229
85:85
2:11
LCNTGDNSVCTTKpc2_NP.5
p−maj=5.5e−15, pX=3.9e−30, N=315
var.21.KLKB1 4:187173012:T:G
peptide level
31:229
8:85
0:11
LQAPLNYTEFQKpc2_NP.3
p−min=0.26, pX=0.15, N=39
var.21.KLKB1 4:187173012:T:G
peptide level
229:229
84:85
11:11
LSMDGSPTRpc2_NP.1
p−min=0.3, pX=0.4, N=324
var.21.KLKB1 4:187173012:T:G
peptide level
164:229
59:85
5:11
LSMDGSPTRpc2_NP.2
p−maj=0.092, pX=0.47, N=228
var.21.KLKB1 4:187173012:T:G
peptide level
229:229
85:85
11:11
LSMDGSPTRpc2_NP.3
p−maj=1, pX=0.86, N=325
var.21.KLKB1 4:187173012:T:G
peptide level
229:229
85:85
11:11
LSMDGSPTRpc2_NP.4
p−maj=1, pX=0.8, N=325
var.21.KLKB1 4:187173012:T:G
peptide level
229:229
85:85
11:11
LSMDGSPTRpc2_NP.5
p−maj=1, pX=0.21, N=325

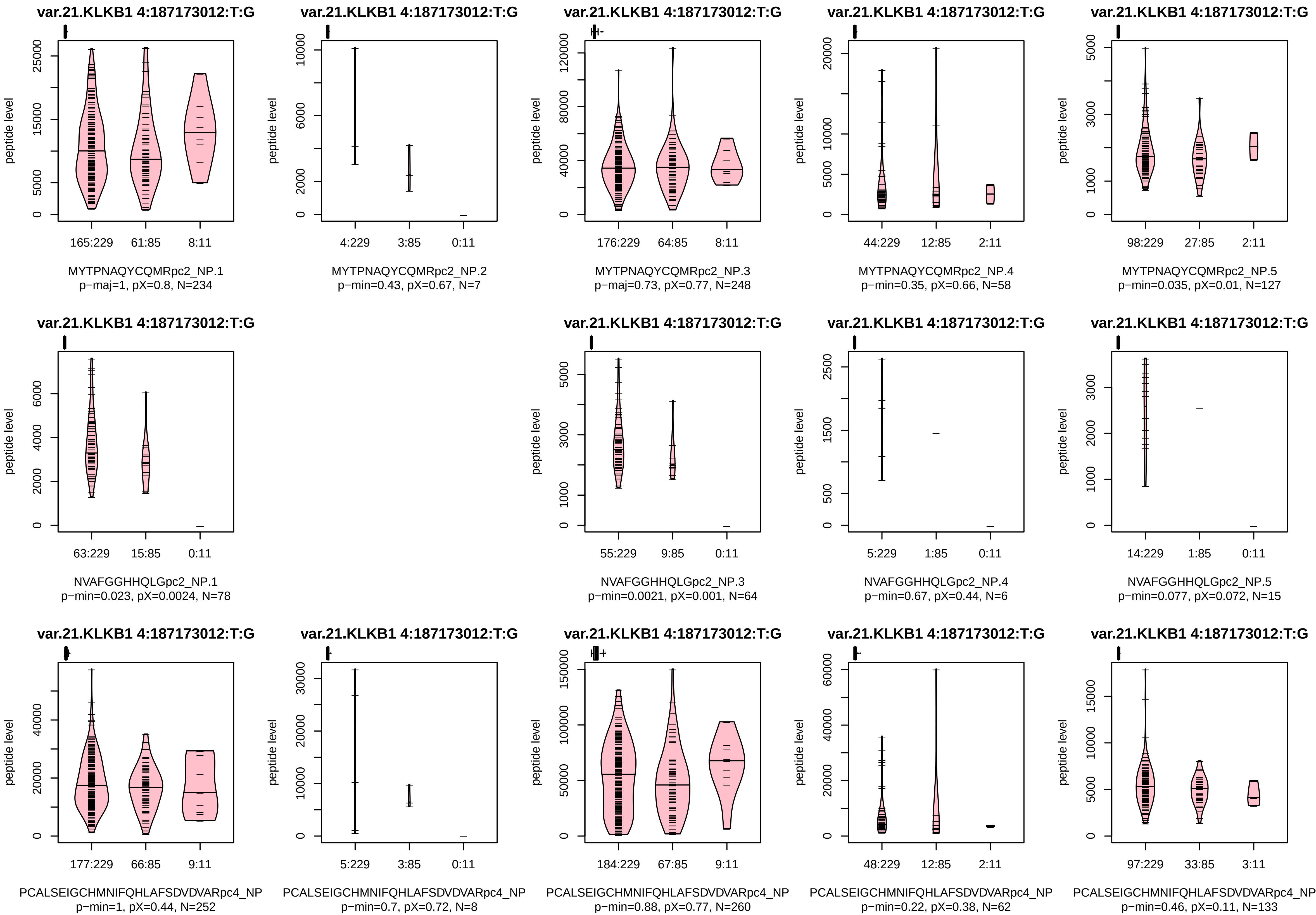
var.21.KLKB1 4:187173012:T:G
peptide level
165:229
61:85
8:11
MYTPNAQYCQMRpc2_NP.1
p−maj=1, pX=0.8, N=234
var.21.KLKB1 4:187173012:T:G
peptide level
4:229
3:85
0:11
MYTPNAQYCQMRpc2_NP.2
p−min=0.43, pX=0.67, N=7
var.21.KLKB1 4:187173012:T:G
peptide level
176:229
64:85
8:11
MYTPNAQYCQMRpc2_NP.3
p−maj=0.73, pX=0.77, N=248
var.21.KLKB1 4:187173012:T:G
peptide level
44:229
12:85
2:11
MYTPNAQYCQMRpc2_NP.4
p−min=0.35, pX=0.66, N=58
var.21.KLKB1 4:187173012:T:G
peptide level
98:229
27:85
2:11
MYTPNAQYCQMRpc2_NP.5
p−min=0.035, pX=0.01, N=127
var.21.KLKB1 4:187173012:T:G
peptide level
63:229
15:85
0:11
NVAFGGHHQLGpc2_NP.1
p−min=0.023, pX=0.0024, N=78
var.21.KLKB1 4:187173012:T:G
peptide level
55:229
9:85
0:11
NVAFGGHHQLGpc2_NP.3
p−min=0.0021, pX=0.001, N=64
var.21.KLKB1 4:187173012:T:G
peptide level
5:229
1:85
0:11
NVAFGGHHQLGpc2_NP.4
p−min=0.67, pX=0.44, N=6
var.21.KLKB1 4:187173012:T:G
peptide level
14:229
1:85
0:11
NVAFGGHHQLGpc2_NP.5
p−min=0.077, pX=0.072, N=15
var.21.KLKB1 4:187173012:T:G
peptide level
177:229
66:85
9:11
PCALSEIGCHMNIFQHLAFSDVDVARpc4_NP
p−min=1, pX=0.44, N=252
var.21.KLKB1 4:187173012:T:G
peptide level
5:229
3:85
0:11
PCALSEIGCHMNIFQHLAFSDVDVARpc4_NP
p−min=0.7, pX=0.72, N=8
var.21.KLKB1 4:187173012:T:G
peptide level
184:229
67:85
9:11
PCALSEIGCHMNIFQHLAFSDVDVARpc4_NP
p−min=0.88, pX=0.77, N=260
var.21.KLKB1 4:187173012:T:G
peptide level
48:229
12:85
2:11
PCALSEIGCHMNIFQHLAFSDVDVARpc4_NP
p−min=0.22, pX=0.38, N=62
var.21.KLKB1 4:187173012:T:G
peptide level
97:229
33:85
3:11
PCALSEIGCHMNIFQHLAFSDVDVARpc4_NP
p−min=0.46, pX=0.11, N=133

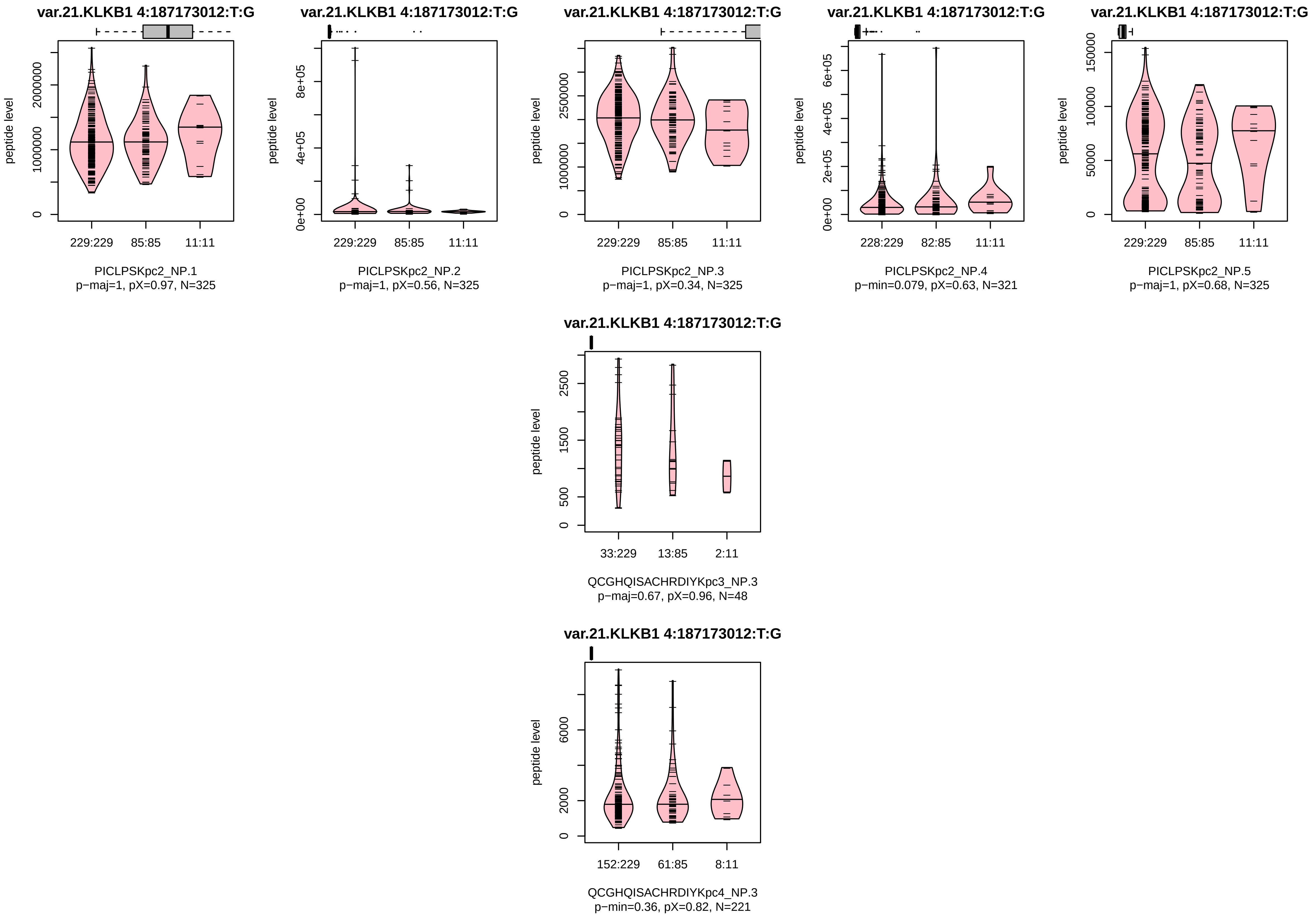
var.21.KLKB1 4:187173012:T:G
peptide level
229:229
85:85
11:11
PICLPSKpc2_NP.1
p−maj=1, pX=0.97, N=325
var.21.KLKB1 4:187173012:T:G
peptide level
229:229
85:85
11:11
PICLPSKpc2_NP.2
p−maj=1, pX=0.56, N=325
var.21.KLKB1 4:187173012:T:G
peptide level
229:229
85:85
11:11
PICLPSKpc2_NP.3
p−maj=1, pX=0.34, N=325
var.21.KLKB1 4:187173012:T:G
peptide level
228:229
82:85
11:11
PICLPSKpc2_NP.4
p−min=0.079, pX=0.63, N=321
var.21.KLKB1 4:187173012:T:G
peptide level
229:229
85:85
11:11
PICLPSKpc2_NP.5
p−maj=1, pX=0.68, N=325
var.21.KLKB1 4:187173012:T:G
peptide level
33:229
13:85
2:11
QCGHQISACHRDIYKpc3_NP.3
p−maj=0.67, pX=0.96, N=48
var.21.KLKB1 4:187173012:T:G
peptide level
152:229
61:85
8:11
QCGHQISACHRDIYKpc4_NP.3
p−min=0.36, pX=0.82, N=221

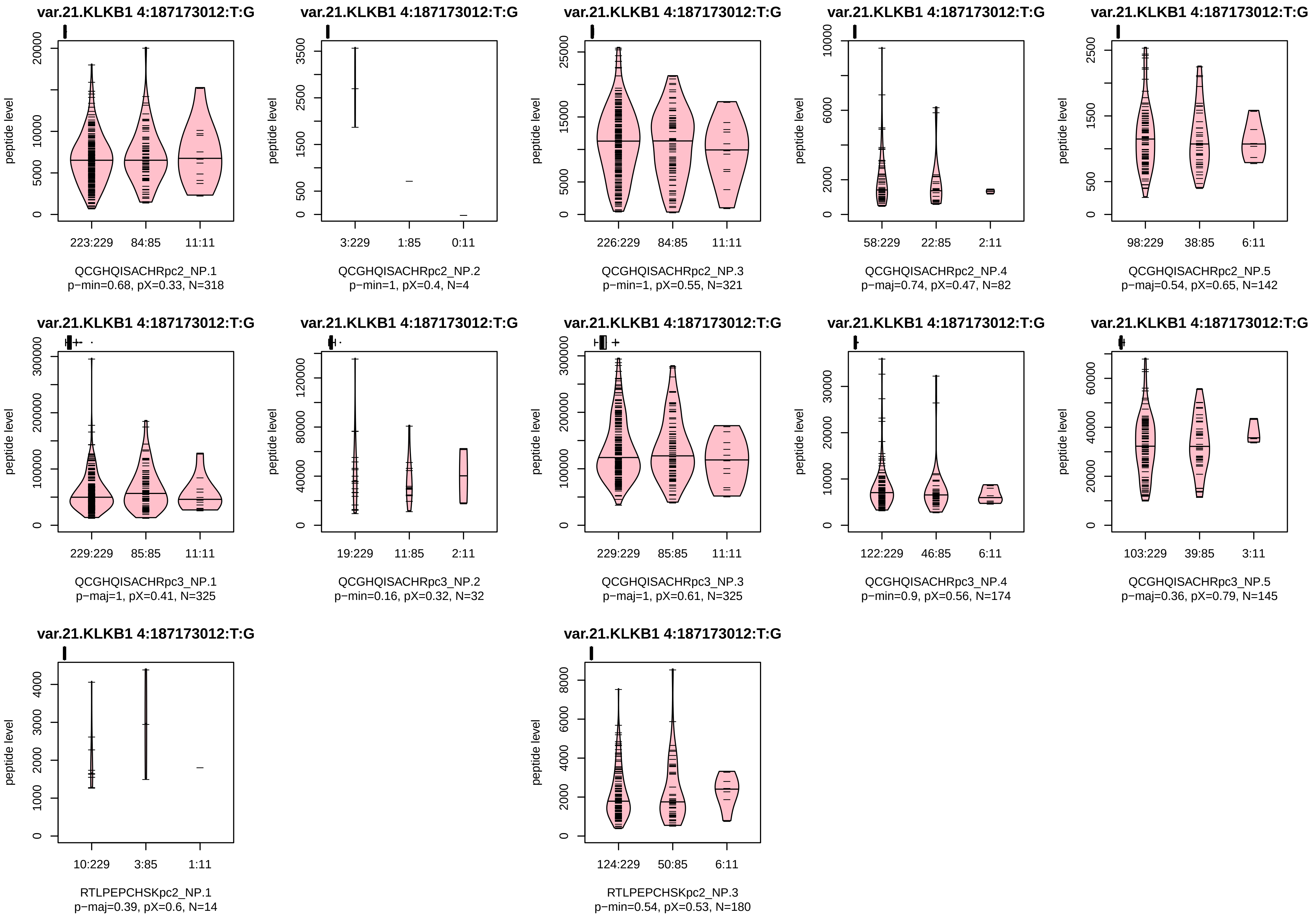
var.21.KLKB1 4:187173012:T:G
peptide level
223:229
84:85
11:11
QCGHQISACHRpc2_NP.1
p−min=0.68, pX=0.33, N=318
var.21.KLKB1 4:187173012:T:G
peptide level
3:229
1:85
0:11
QCGHQISACHRpc2_NP.2
p−min=1, pX=0.4, N=4
var.21.KLKB1 4:187173012:T:G
peptide level
226:229
84:85
11:11
QCGHQISACHRpc2_NP.3
p−min=1, pX=0.55, N=321
var.21.KLKB1 4:187173012:T:G
peptide level
58:229
22:85
2:11
QCGHQISACHRpc2_NP.4
p−maj=0.74, pX=0.47, N=82
var.21.KLKB1 4:187173012:T:G
peptide level
98:229
38:85
6:11
QCGHQISACHRpc2_NP.5
p−maj=0.54, pX=0.65, N=142
var.21.KLKB1 4:187173012:T:G
peptide level
229:229
85:85
11:11
QCGHQISACHRpc3_NP.1
p−maj=1, pX=0.41, N=325
var.21.KLKB1 4:187173012:T:G
peptide level
19:229
11:85
2:11
QCGHQISACHRpc3_NP.2
p−min=0.16, pX=0.32, N=32
var.21.KLKB1 4:187173012:T:G
peptide level
229:229
85:85
11:11
QCGHQISACHRpc3_NP.3
p−maj=1, pX=0.61, N=325
var.21.KLKB1 4:187173012:T:G
peptide level
122:229
46:85
6:11
QCGHQISACHRpc3_NP.4
p−min=0.9, pX=0.56, N=174
var.21.KLKB1 4:187173012:T:G
peptide level
103:229
39:85
3:11
QCGHQISACHRpc3_NP.5
p−maj=0.36, pX=0.79, N=145
var.21.KLKB1 4:187173012:T:G
peptide level
10:229
3:85
1:11
RTLPEPCHSKpc2_NP.1
p−maj=0.39, pX=0.6, N=14
var.21.KLKB1 4:187173012:T:G
peptide level
124:229
50:85
6:11
RTLPEPCHSKpc2_NP.3
p−min=0.54, pX=0.53, N=180

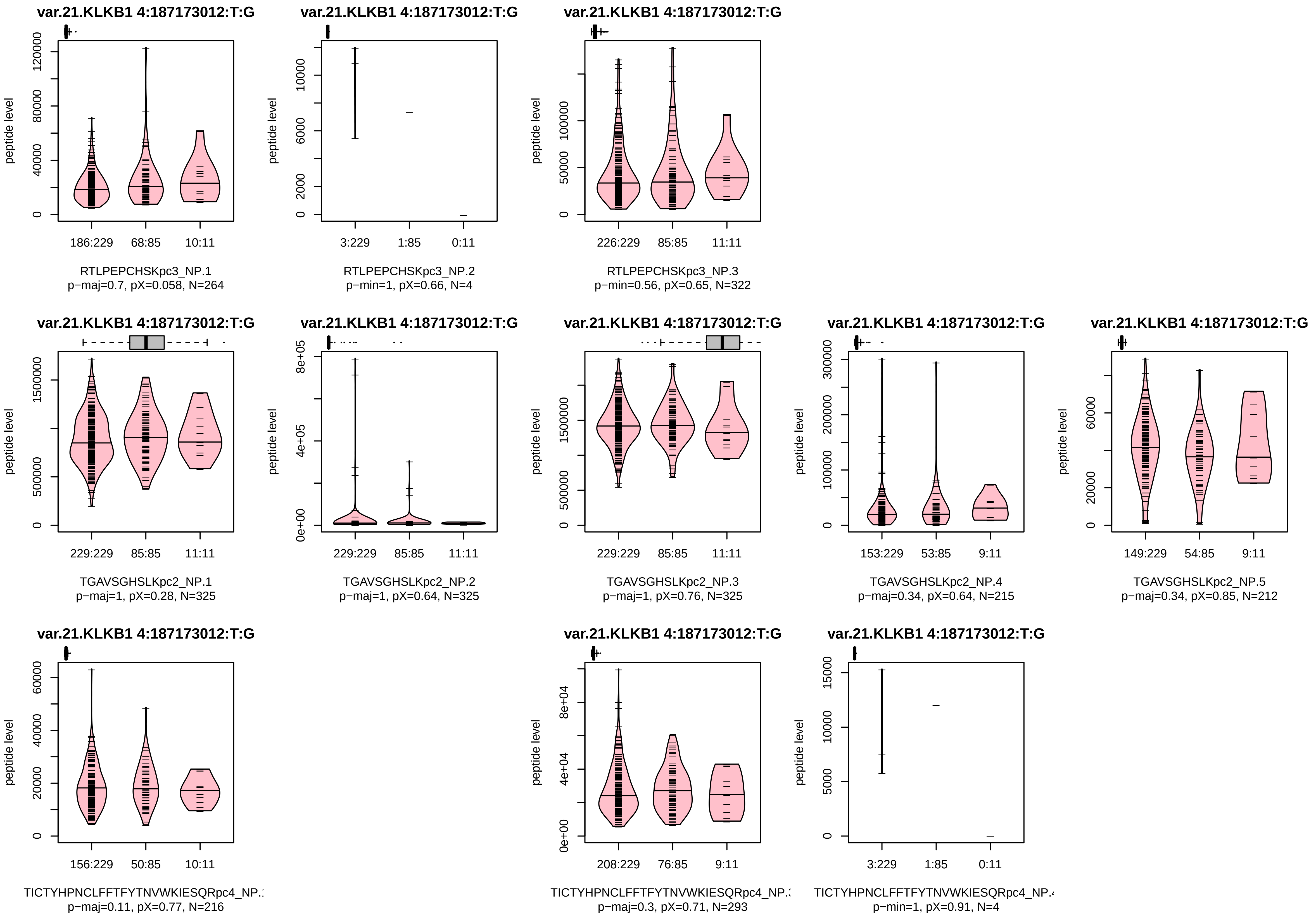
var.21.KLKB1 4:187173012:T:G
peptide level
186:229
68:85
10:11
RTLPEPCHSKpc3_NP.1
p−maj=0.7, pX=0.058, N=264
var.21.KLKB1 4:187173012:T:G
3:229
1:85
0:11
RTLPEPCHSKpc3_NP.2
p−min=1, pX=0.66, N=4
var.21.KLKB1 4:187173012:T:G
226:229
85:85
11:11
RTLPEPCHSKpc3_NP.3
p−min=0.56, pX=0.65, N=322
var.21.KLKB1 4:187173012:T:G
229:229
85:85
11:11
TGAVSGHSLKpc2_NP.1
p−maj=1, pX=0.28, N=325
var.21.KLKB1 4:187173012:T:G
229:229
85:85
11:11
TGAVSGHSLKpc2_NP.2
p−maj=1, pX=0.64, N=325
var.21.KLKB1 4:187173012:T:G
229:229
85:85
11:11
TGAVSGHSLKpc2_NP.3
p−maj=1, pX=0.76, N=325
var.21.KLKB1 4:187173012:T:G
153:229
53:85
9:11
TGAVSGHSLKpc2_NP.4
p−maj=0.34, pX=0.64, N=215
var.21.KLKB1 4:187173012:T:G
149:229
54:85
9:11
TGAVSGHSLKpc2_NP.5
p−maj=0.34, pX=0.85, N=212
var.21.KLKB1 4:187173012:T:G
156:229
50:85
10:11
TICTYHPNCLFFTFYTNVWKIESQRpc4_NP.
p−maj=0.11, pX=0.77, N=216
var.21.KLKB1 4:187173012:T:G
208:229
76:85
9:11
TICTYHPNCLFFTFYTNVWKIESQRpc4_NP.
p−maj=0.3, pX=0.71, N=293
var.21.KLKB1 4:187173012:T:G
3:229
1:85
0:11
TICTYHPNCLFFTFYTNVWKIESQRpc4_NP.
p−min=1, pX=0.91, N=4

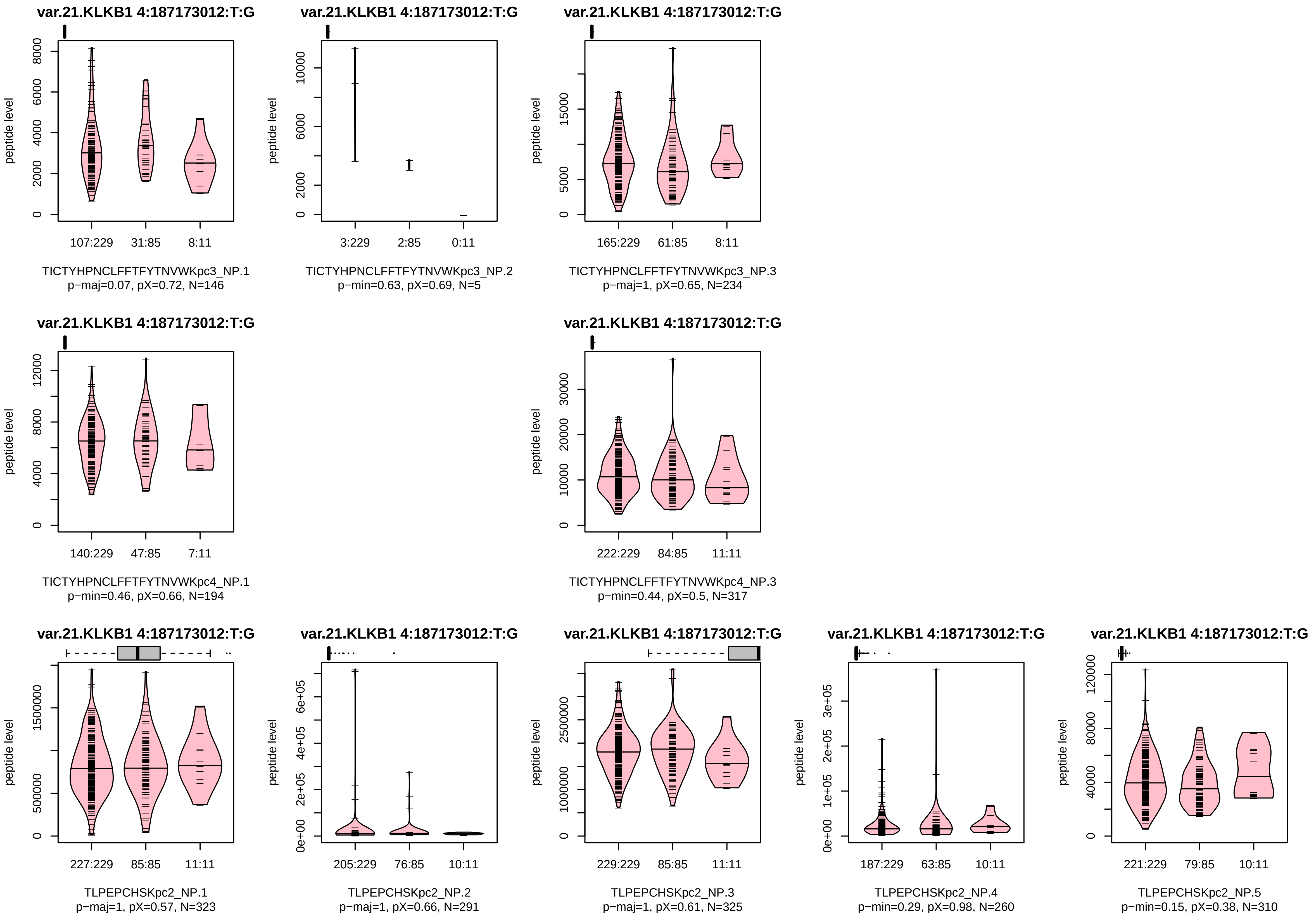
var.21.KLKB1 4:187173012:T:G
peptide level
107:229
31:85
8:11
TICTYHPNCLFFTFYTNVWKpc3_NP.1
p−maj=0.07, pX=0.72, N=146
var.21.KLKB1 4:187173012:T:G
peptide level
3:229
2:85
0:11
TICTYHPNCLFFTFYTNVWKpc3_NP.2
p−min=0.63, pX=0.69, N=5
var.21.KLKB1 4:187173012:T:G
peptide level
165:229
61:85
8:11
TICTYHPNCLFFTFYTNVWKpc3_NP.3
p−maj=1, pX=0.65, N=234
var.21.KLKB1 4:187173012:T:G
peptide level
140:229
47:85
7:11
TICTYHPNCLFFTFYTNVWKpc4_NP.1
p−min=0.46, pX=0.66, N=194
var.21.KLKB1 4:187173012:T:G
peptide level
222:229
84:85
11:11
TICTYHPNCLFFTFYTNVWKpc4_NP.3
p−min=0.44, pX=0.5, N=317
var.21.KLKB1 4:187173012:T:G
peptide level
227:229
85:85
11:11
TLPEPCHSKpc2_NP.1
p−maj=1, pX=0.57, N=323
var.21.KLKB1 4:187173012:T:G
peptide level
205:229
76:85
10:11
TLPEPCHSKpc2_NP.2
p−maj=1, pX=0.66, N=291
var.21.KLKB1 4:187173012:T:G
peptide level
229:229
85:85
11:11
TLPEPCHSKpc2_NP.3
p−maj=1, pX=0.61, N=325
var.21.KLKB1 4:187173012:T:G
peptide level
187:229
63:85
10:11
TLPEPCHSKpc2_NP.4
p−min=0.29, pX=0.98, N=260
var.21.KLKB1 4:187173012:T:G
peptide level
221:229
79:85
10:11
TLPEPCHSKpc2_NP.5
p−min=0.15, pX=0.38, N=310

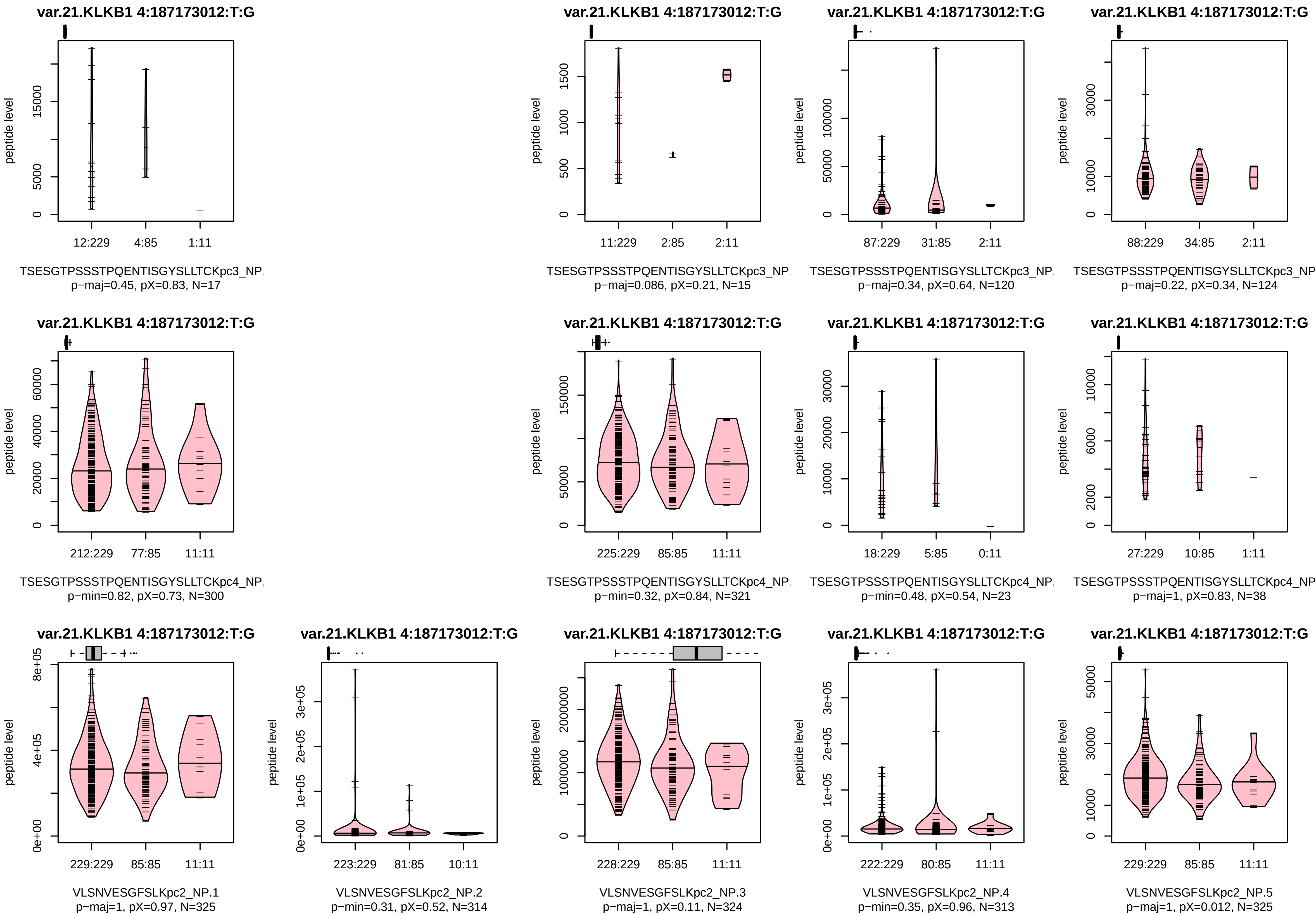

var.21.KLKB1 4:187173012:T:G
peptide level
12:229
4:85
1:11
TSESGTPSSSTPQENTISGYSLLTCKpc3_NP.
p−maj=0.45, pX=0.83, N=17
var.21.KLKB1 4:187173012:T:G
peptide level
11:229
2:85
2:11
TSESGTPSSSTPQENTISGYSLLTCKpc3_NP.
p−maj=0.086, pX=0.21, N=15
var.21.KLKB1 4:187173012:T:G
peptide level
87:229
31:85
2:11
TSESGTPSSSTPQENTISGYSLLTCKpc3_NP.
p−maj=0.34, pX=0.64, N=120
var.21.KLKB1 4:187173012:T:G
peptide level
88:229
34:85
2:11
TSESGTPSSSTPQENTISGYSLLTCKpc3_NP
p−maj=0.22, pX=0.34, N=124
var.21.KLKB1 4:187173012:T:G
peptide level
212:229
77:85
11:11
TSESGTPSSSTPQENTISGYSLLTCKpc4_NP.
p−min=0.82, pX=0.73, N=300
var.21.KLKB1 4:187173012:T:G
peptide level
225:229
85:85
11:11
TSESGTPSSSTPQENTISGYSLLTCKpc4_NP.
p−min=0.32, pX=0.84, N=321
var.21.KLKB1 4:187173012:T:G
peptide level
18:229
5:85
0:11
TSESGTPSSSTPQENTISGYSLLTCKpc4_NP.
p−min=0.48, pX=0.54, N=23
var.21.KLKB1 4:187173012:T:G
peptide level
27:229
10:85
1:11
TSESGTPSSSTPQENTISGYSLLTCKpc4_NP
p−maj=1, pX=0.83, N=38
var.21.KLKB1 4:187173012:T:G
peptide level
229:229
85:85
11:11
VLSNVESGFSLKpc2_NP.1
p−maj=1, pX=0.97, N=325
var.21.KLKB1 4:187173012:T:G
peptide level
223:229
81:85
10:11
VLSNVESGFSLKpc2_NP.2
p−min=0.31, pX=0.52, N=314
var.21.KLKB1 4:187173012:T:G
peptide level
228:229
85:85
11:11
VLSNVESGFSLKpc2_NP.3
p−maj=1, pX=0.11, N=324
var.21.KLKB1 4:187173012:T:G
peptide level
222:229
80:85
11:11
VLSNVESGFSLKpc2_NP.4
p−min=0.35, pX=0.96, N=313
var.21.KLKB1 4:187173012:T:G
peptide level
229:229
85:85
11:11
VLSNVESGFSLKpc2_NP.5
p−maj=1, pX=0.012, N=325

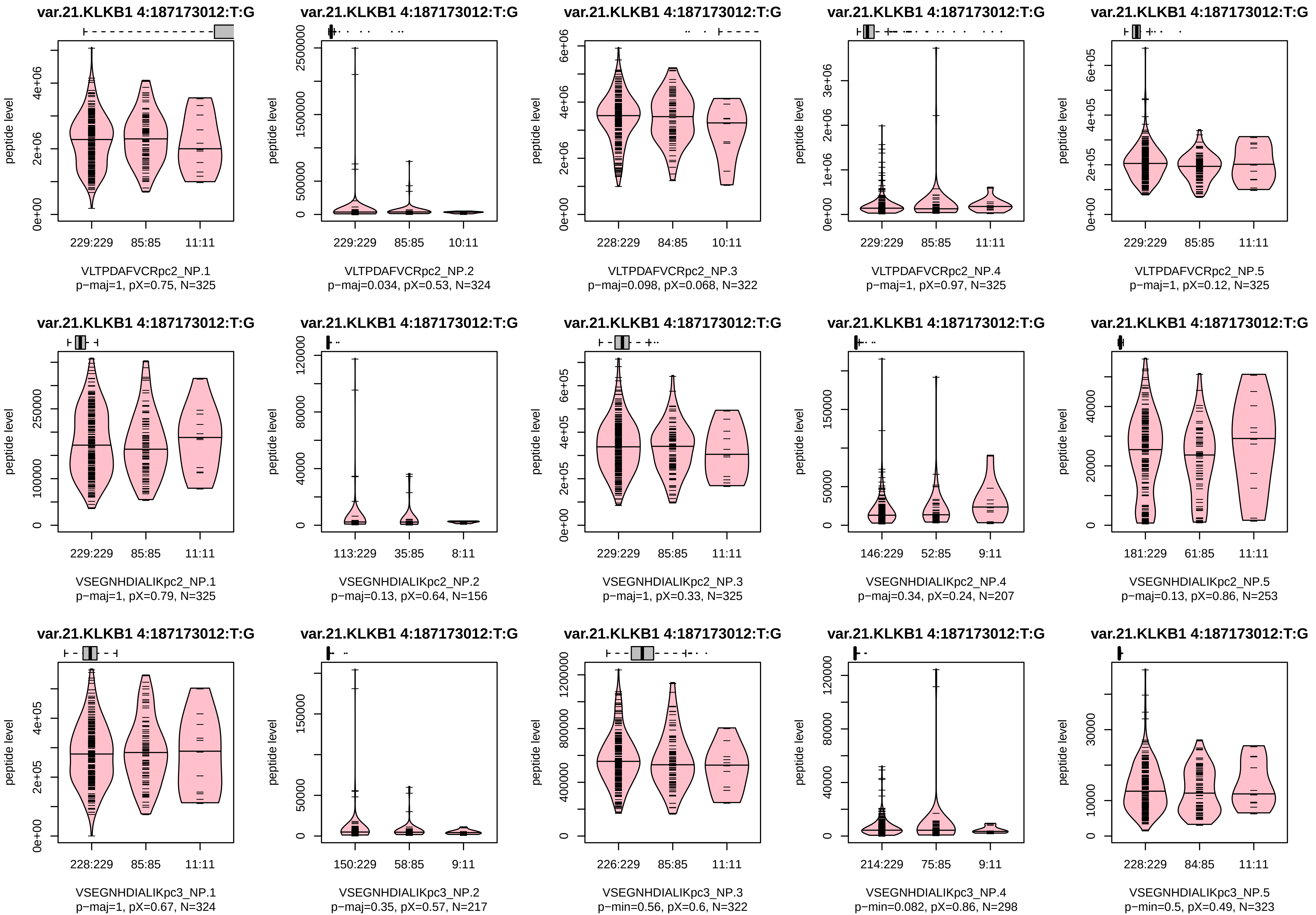

var.21.KLKB1 4:187173012:T:G
peptide level
0e+00
2e+06
4e+06
229:229
85:85
11:11
VLTPDAFVCRpc2_NP.1
p−maj=1, pX=0.75, N=325
var.21.KLKB1 4:187173012:T:G
peptide level
0
500000
1500000
2500000
229:229
85:85
10:11
VLTPDAFVCRpc2_NP.2
p−maj=0.034, pX=0.53, N=324
var.21.KLKB1 4:187173012:T:G
peptide level
0e+00
2e+06
4e+06
6e+06
228:229
84:85
10:11
VLTPDAFVCRpc2_NP.3
p−maj=0.098, pX=0.068, N=322
var.21.KLKB1 4:187173012:T:G
peptide level
0e+00
1e+06
2e+06
3e+06
229:229
85:85
11:11
VLTPDAFVCRpc2_NP.4
p−maj=1, pX=0.97, N=325
var.21.KLKB1 4:187173012:T:G
peptide level
0e+00
2e+05
4e+05
6e+05
229:229
85:85
11:11
VLTPDAFVCRpc2_NP.5
p−maj=1, pX=0.12, N=325
var.21.KLKB1 4:187173012:T:G
peptide level
0
100000
250000
229:229
85:85
11:11
VSEGNHDIALIKpc2_NP.1
p−maj=1, pX=0.79, N=325
var.21.KLKB1 4:187173012:T:G
peptide level
0
40000
80000
120000
113:229
35:85
8:11
VSEGNHDIALIKpc2_NP.2
p−maj=0.13, pX=0.64, N=156
var.21.KLKB1 4:187173012:T:G
peptide level
0e+00
2e+05
4e+05
6e+05
229:229
85:85
11:11
VSEGNHDIALIKpc2_NP.3
p−maj=1, pX=0.33, N=325
var.21.KLKB1 4:187173012:T:G
peptide level
0
50000
150000
146:229
52:85
9:11
VSEGNHDIALIKpc2_NP.4
p−maj=0.34, pX=0.24, N=207
var.21.KLKB1 4:187173012:T:G
peptide level
0
20000
40000
181:229
61:85
11:11
VSEGNHDIALIKpc2_NP.5
p−maj=0.13, pX=0.86, N=253
var.21.KLKB1 4:187173012:T:G
peptide level
0e+00
2e+05
4e+05
228:229
85:85
11:11
VSEGNHDIALIKpc3_NP.1
p−maj=1, pX=0.67, N=324
var.21.KLKB1 4:187173012:T:G
peptide level
0
50000
150000
150:229
58:85
9:11
VSEGNHDIALIKpc3_NP.2
p−maj=0.35, pX=0.57, N=217
var.21.KLKB1 4:187173012:T:G
peptide level
0
400000
800000
1200000
226:229
85:85
11:11
VSEGNHDIALIKpc3_NP.3
p−min=0.56, pX=0.6, N=322
var.21.KLKB1 4:187173012:T:G
peptide level
0
40000
80000
120000
214:229
75:85
9:11
VSEGNHDIALIKpc3_NP.4
p−min=0.082, pX=0.86, N=298
var.21.KLKB1 4:187173012:T:G
peptide level
0
10000
30000
228:229
84:85
11:11
VSEGNHDIALIKpc3_NP.5
p−min=0.5, pX=0.49, N=323

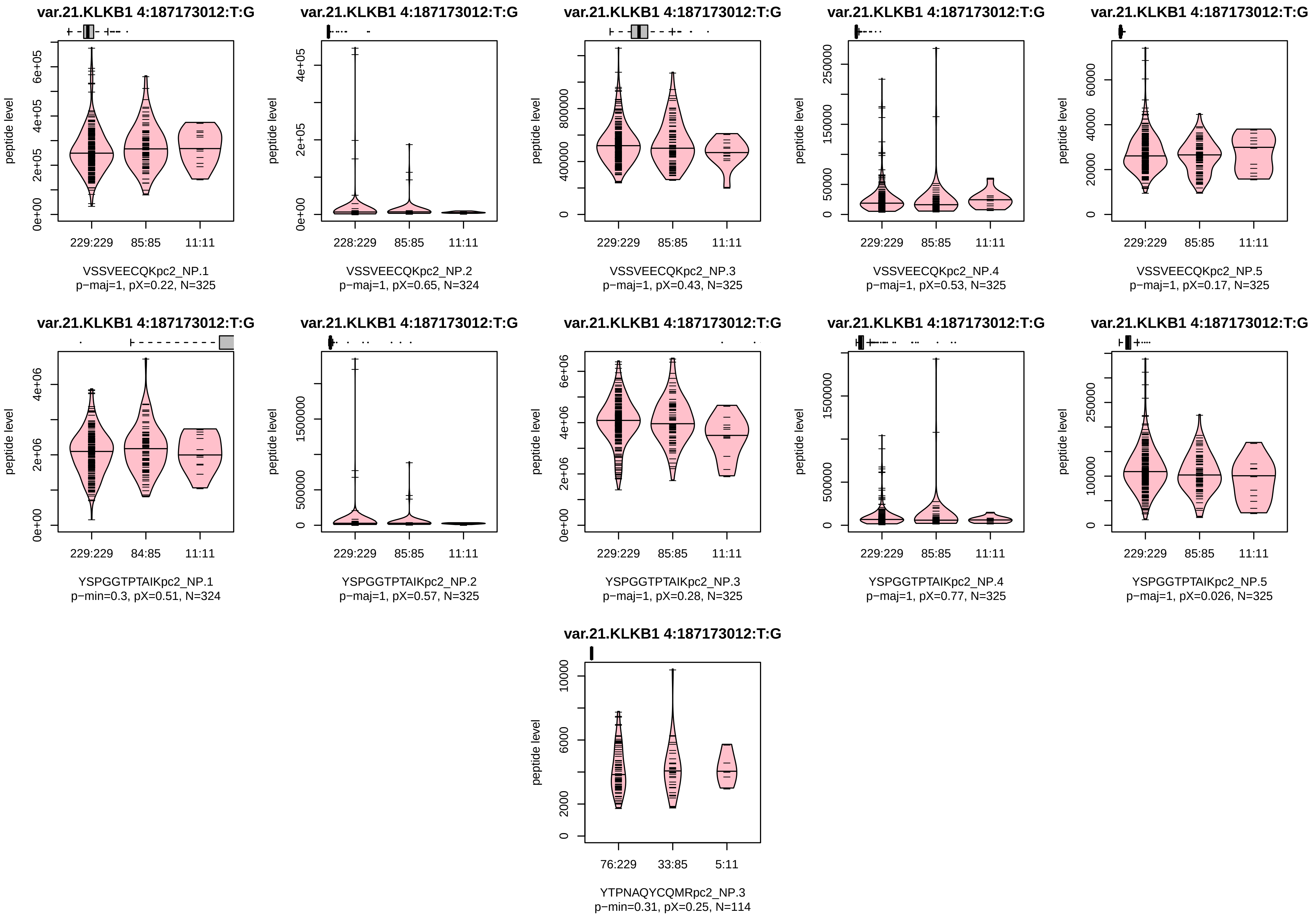
var.21.KLKB1 4:187173012:T:G
peptide level
0e+00
2e+05
4e+05
6e+05
229:229
85:85
11:11
VSSVEECQKpc2_NP.1
p−maj=1, pX=0.22, N=325
var.21.KLKB1 4:187173012:T:G
peptide level
0e+00
2e+05
4e+05
228:229
85:85
11:11
VSSVEECQKpc2_NP.2
p−maj=1, pX=0.65, N=324
var.21.KLKB1 4:187173012:T:G
peptide level
0
400000
800000
229:229
85:85
11:11
VSSVEECQKpc2_NP.3
p−maj=1, pX=0.43, N=325
var.21.KLKB1 4:187173012:T:G
peptide level
0
50000
150000
250000
229:229
85:85
11:11
VSSVEECQKpc2_NP.4
p−maj=1, pX=0.53, N=325
var.21.KLKB1 4:187173012:T:G
peptide level
0
20000
40000
60000
229:229
85:85
11:11
VSSVEECQKpc2_NP.5
p−maj=1, pX=0.17, N=325
var.21.KLKB1 4:187173012:T:G
peptide level
0e+00
2e+06
4e+06
229:229
84:85
11:11
YSPGGTPTAIKpc2_NP.1
p−min=0.3, pX=0.51, N=324
var.21.KLKB1 4:187173012:T:G
peptide level
0
500000
1500000
229:229
85:85
11:11
YSPGGTPTAIKpc2_NP.2
p−maj=1, pX=0.57, N=325
var.21.KLKB1 4:187173012:T:G
peptide level
0e+00
2e+06
4e+06
6e+06
229:229
85:85
11:11
YSPGGTPTAIKpc2_NP.3
p−maj=1, pX=0.28, N=325
var.21.KLKB1 4:187173012:T:G
peptide level
0
500000
1500000
229:229
85:85
11:11
YSPGGTPTAIKpc2_NP.4
p−maj=1, pX=0.77, N=325
var.21.KLKB1 4:187173012:T:G
peptide level
0
100000
250000
229:229
85:85
11:11
YSPGGTPTAIKpc2_NP.5
p−maj=1, pX=0.026, N=325
var.21.KLKB1 4:187173012:T:G
peptide level
0
2000
6000
10000
76:229
33:85
5:11
YTPNAQYCQMRpc2_NP.3
p−min=0.31, pX=0.25, N=114

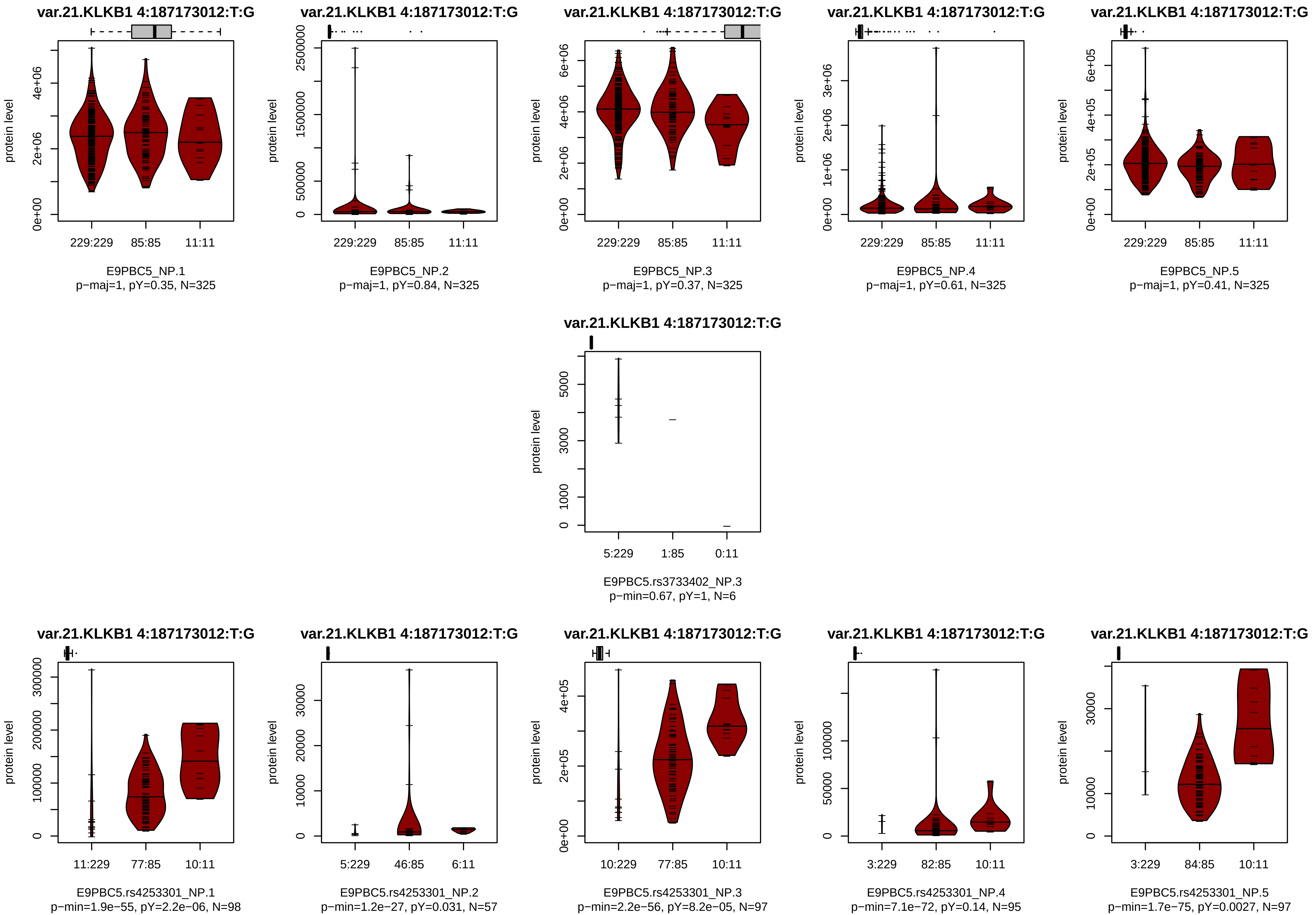
var.21.KLKB1 4:187173012:T:G
protein level
229:229
85:85
11:11
E9PBC5_NP.1
p−maj=1, pY=0.35, N=325
E9PBC5_NP.2
p−maj=1, pY=0.84, N=325
E9PBC5_NP.3
p−maj=1, pY=0.37, N=325
E9PBC5_NP.4
p−maj=1, pY=0.61, N=325
E9PBC5_NP.5
p−maj=1, pY=0.41, N=325
5:229
1:85
0:11
E9PBC5.rs3733402_NP.3
p−min=0.67, pY=1, N=6
11:229
77:85
10:11
E9PBC5.rs4253301_NP.1
p−min=1.9e−55, pY=2.2e−06, N=98
5:229
46:85
6:11
E9PBC5.rs4253301_NP.2
p−min=1.2e−27, pY=0.031, N=57
10:229
77:85
10:11
E9PBC5.rs4253301_NP.3
p−min=2.2e−56, pY=8.2e−05, N=97
3:229
82:85
10:11
E9PBC5.rs4253301_NP.4
p−min=7.1e−72, pY=0.14, N=95
3:229
84:85
10:11
E9PBC5.rs4253301_NP.5
p−min=1.7e−75, pY=0.0027, N=97

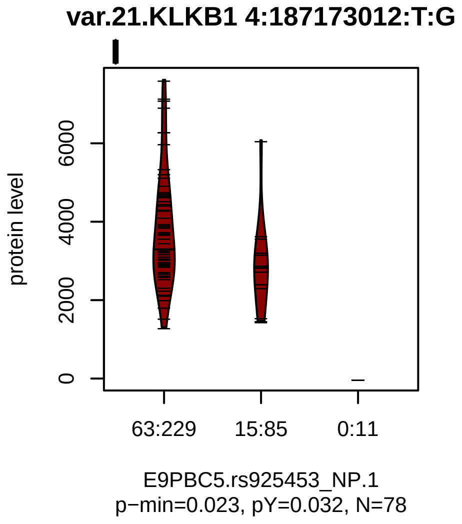
var.21.KLKB1 4:187173012:T:G
protein level
0
2000
4000
6000
63:229
15:85
0:11
E9PBC5.rs925453_NP.1
p−min=0.023, pY=0.032, N=78

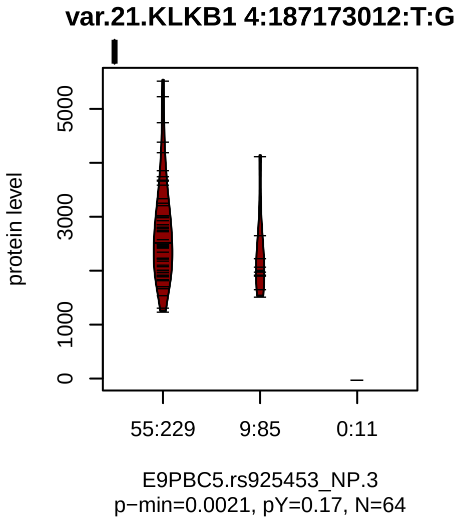
var.21.KLKB1 4:187173012:T:G
protein level
0
1000
3000
5000
55:229
9:85
0:11
E9PBC5.rs925453_NP.3
p−min=0.0021, pY=0.17, N=64

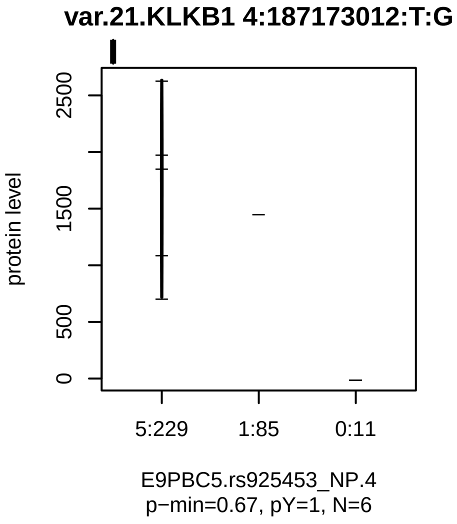
var.21.KLKB1 4:187173012:T:G
protein level
0
500
1500
2500
5:229
1:85
0:11
E9PBC5.rs925453_NP.4
p−min=0.67, pY=1, N=6

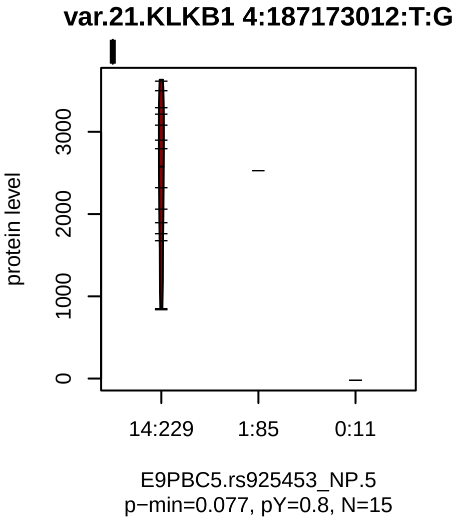
var.21.KLKB1 4:187173012:T:G
protein level
0
1000
2000
3000
14:229
1:85
0:11
E9PBC5.rs925453_NP.5
p−min=0.077, pY=0.8, N=15

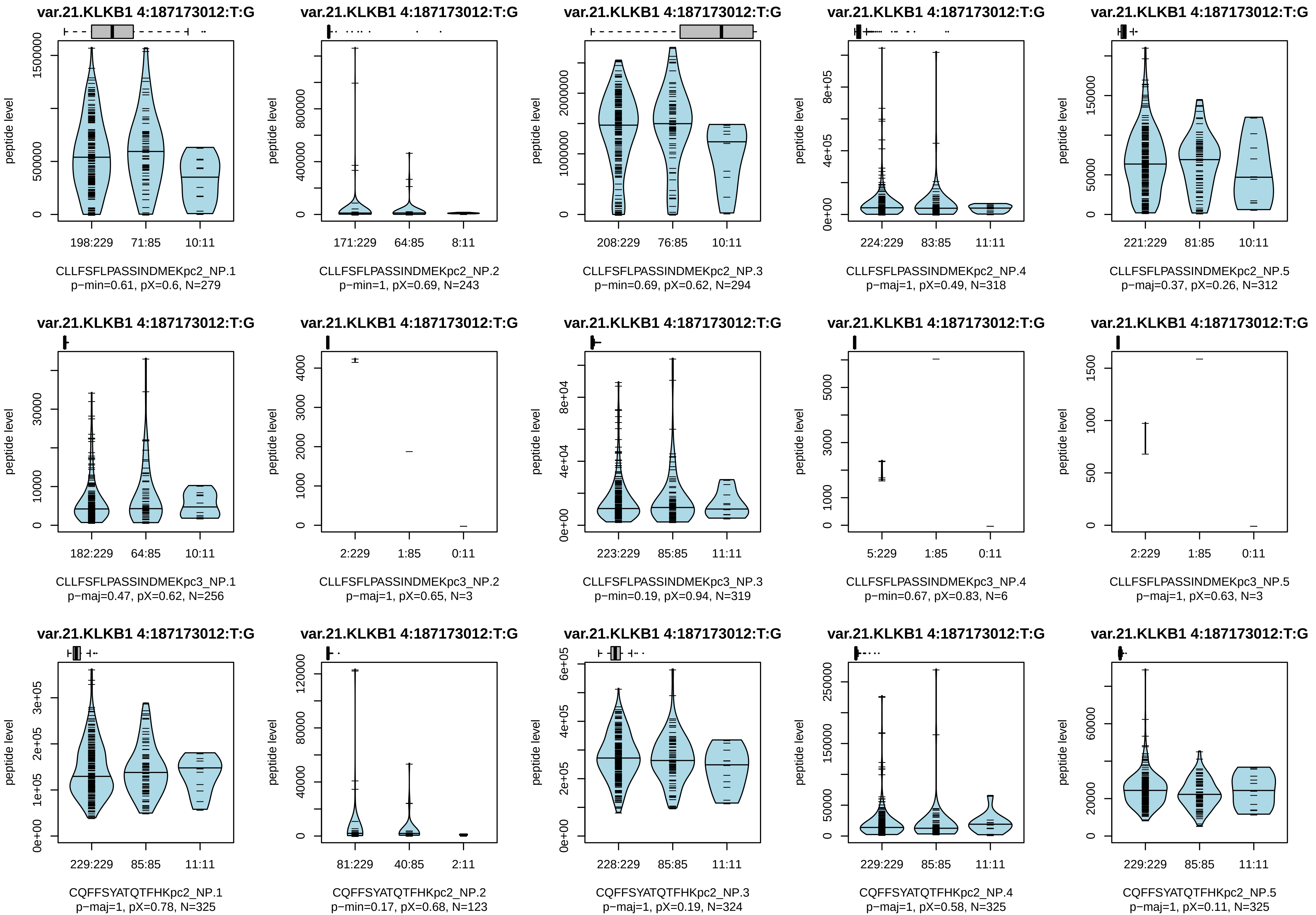
var.21.KLKB1 4:187173012:T:G
peptide level
198:229
71:85
10:11
CLLFSFLPASSINDMEKpc2_NP.1
p−min=0.61, pX=0.6, N=279
var.21.KLKB1 4:187173012:T:G
171:229
64:85
8:11
CLLFSFLPASSINDMEKpc2_NP.2
p−min=1, pX=0.69, N=243
var.21.KLKB1 4:187173012:T:G
208:229
76:85
10:11
CLLFSFLPASSINDMEKpc2_NP.3
p−min=0.69, pX=0.62, N=294
var.21.KLKB1 4:187173012:T:G
224:229
83:85
11:11
CLLFSFLPASSINDMEKpc2_NP.4
p−maj=1, pX=0.49, N=318
var.21.KLKB1 4:187173012:T:G
221:229
81:85
10:11
CLLFSFLPASSINDMEKpc2_NP.5
p−maj=0.37, pX=0.26, N=312
var.21.KLKB1 4:187173012:T:G
182:229
64:85
10:11
CLLFSFLPASSINDMEKpc3_NP.1
p−maj=0.47, pX=0.62, N=256
var.21.KLKB1 4:187173012:T:G
2:229
1:85
0:11
CLLFSFLPASSINDMEKpc3_NP.2
p−maj=1, pX=0.65, N=3
var.21.KLKB1 4:187173012:T:G
223:229
85:85
11:11
CLLFSFLPASSINDMEKpc3_NP.3
p−min=0.19, pX=0.94, N=319
var.21.KLKB1 4:187173012:T:G
5:229
1:85
0:11
CLLFSFLPASSINDMEKpc3_NP.4
p−min=0.67, pX=0.83, N=6
var.21.KLKB1 4:187173012:T:G
2:229
1:85
0:11
CLLFSFLPASSINDMEKpc3_NP.5
p−maj=1, pX=0.63, N=3
var.21.KLKB1 4:187173012:T:G
229:229
85:85
11:11
CQFFSYATQTFHKpc2_NP.1
p−maj=1, pX=0.78, N=325
var.21.KLKB1 4:187173012:T:G
81:229
40:85
2:11
CQFFSYATQTFHKpc2_NP.2
p−min=0.17, pX=0.68, N=123
var.21.KLKB1 4:187173012:T:G
228:229
85:85
11:11
CQFFSYATQTFHKpc2_NP.3
p−maj=1, pX=0.19, N=324
var.21.KLKB1 4:187173012:T:G
229:229
85:85
11:11
CQFFSYATQTFHKpc2_NP.4
p−maj=1, pX=0.58, N=325
var.21.KLKB1 4:187173012:T:G
229:229
85:85
11:11
CQFFSYATQTFHKpc2_NP.5
p−maj=1, pX=0.11, N=325

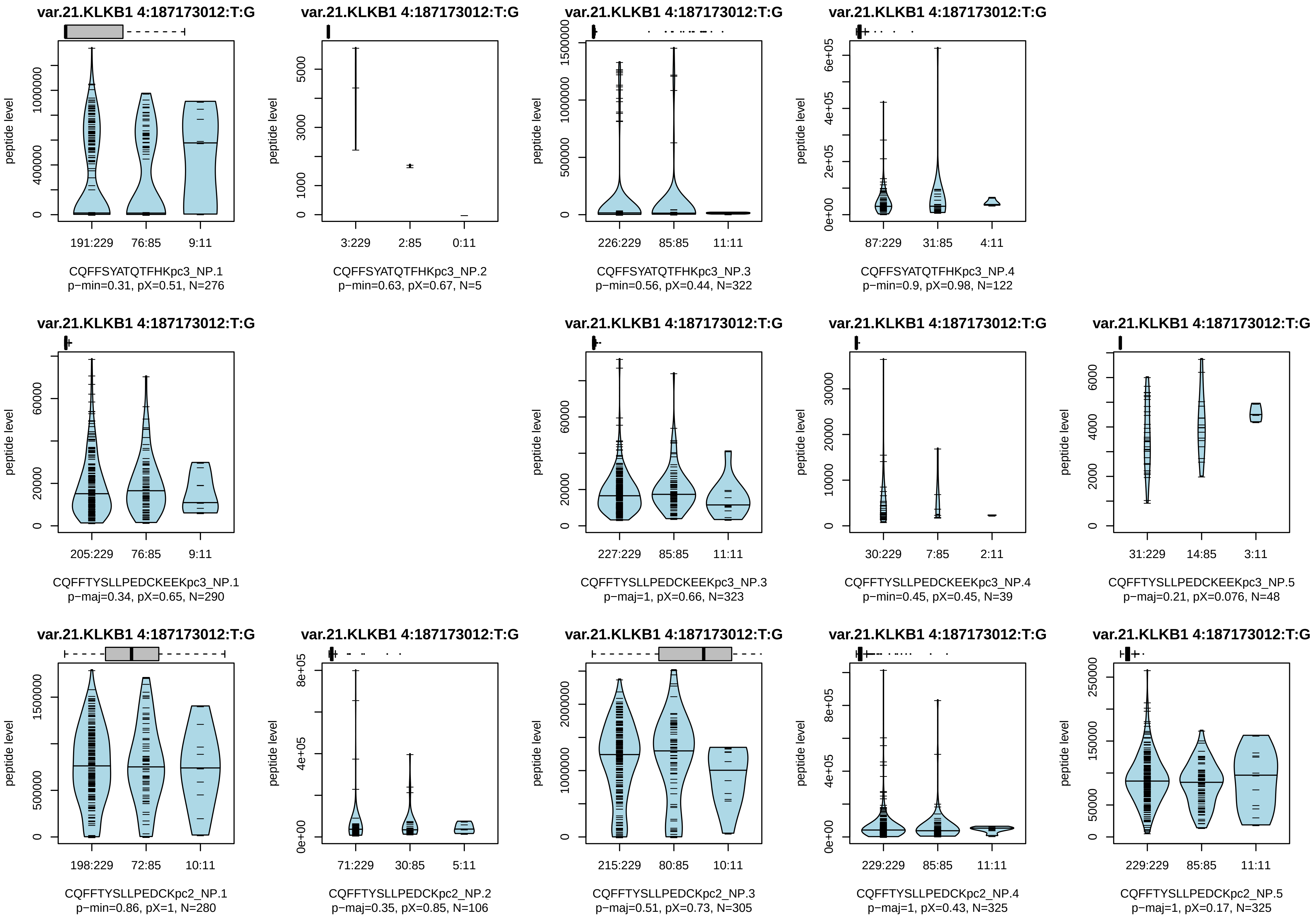
var.21.KLKB1 4:187173012:T:G
peptide level
191:229
76:85
9:11
CQFFSYATQTFHKpc3_NP.1
p−min=0.31, pX=0.51, N=276
var.21.KLKB1 4:187173012:T:G
peptide level
3:229
2:85
0:11
CQFFSYATQTFHKpc3_NP.2
p−min=0.63, pX=0.67, N=5
var.21.KLKB1 4:187173012:T:G
peptide level
226:229
85:85
11:11
CQFFSYATQTFHKpc3_NP.3
p−min=0.56, pX=0.44, N=322
var.21.KLKB1 4:187173012:T:G
peptide level
87:229
31:85
4:11
CQFFSYATQTFHKpc3_NP.4
p−min=0.9, pX=0.98, N=122
var.21.KLKB1 4:187173012:T:G
peptide level
205:229
76:85
9:11
CQFFTYSLLPEDCKEEKpc3_NP.1
p−maj=0.34, pX=0.65, N=290
var.21.KLKB1 4:187173012:T:G
peptide level
227:229
85:85
11:11
CQFFTYSLLPEDCKEEKpc3_NP.3
p−maj=1, pX=0.66, N=323
var.21.KLKB1 4:187173012:T:G
peptide level
30:229
7:85
2:11
CQFFTYSLLPEDCKEEKpc3_NP.4
p−min=0.45, pX=0.45, N=39
var.21.KLKB1 4:187173012:T:G
peptide level
31:229
14:85
3:11
CQFFTYSLLPEDCKEEKpc3_NP.5
p−maj=0.21, pX=0.076, N=48
var.21.KLKB1 4:187173012:T:G
peptide level
198:229
72:85
10:11
CQFFTYSLLPEDCKpc2_NP.1
p−min=0.86, pX=1, N=280
var.21.KLKB1 4:187173012:T:G
peptide level
71:229
30:85
5:11
CQFFTYSLLPEDCKpc2_NP.2
p−maj=0.35, pX=0.85, N=106
var.21.KLKB1 4:187173012:T:G
peptide level
215:229
80:85
10:11
CQFFTYSLLPEDCKpc2_NP.3
p−maj=0.51, pX=0.73, N=305
var.21.KLKB1 4:187173012:T:G
peptide level
229:229
85:85
11:11
CQFFTYSLLPEDCKpc2_NP.4
p−maj=1, pX=0.43, N=325
var.21.KLKB1 4:187173012:T:G
peptide level
229:229
85:85
11:11
CQFFTYSLLPEDCKpc2_NP.5
p−maj=1, pX=0.17, N=325

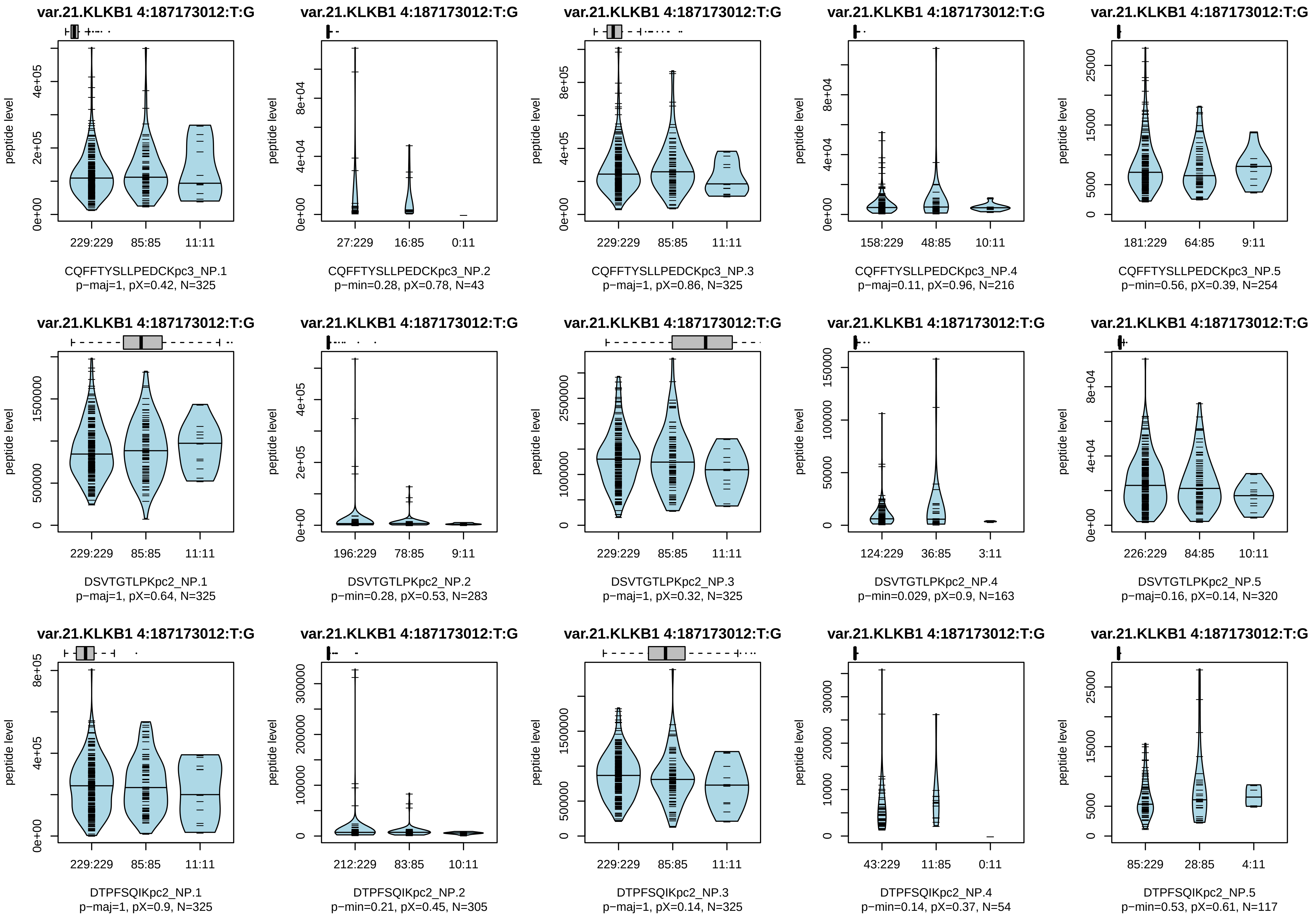
var.21.KLKB1 4:187173012:T:G
peptide level
229:229 85:85 11:11
CQFFTYSLLPEDCKpc3_NP.1
p−maj=1, pX=0.42, N=325
var.21.KLKB1 4:187173012:T:G
peptide level
27:229 16:85 0:11
CQFFTYSLLPEDCKpc3_NP.2
p−min=0.28, pX=0.78, N=43
var.21.KLKB1 4:187173012:T:G
peptide level
229:229 85:85 11:11
CQFFTYSLLPEDCKpc3_NP.3
p−maj=1, pX=0.86, N=325
var.21.KLKB1 4:187173012:T:G
peptide level
158:229 48:85 10:11
CQFFTYSLLPEDCKpc3_NP.4
p−maj=0.11, pX=0.96, N=216
var.21.KLKB1 4:187173012:T:G
peptide level
181:229 64:85 9:11
CQFFTYSLLPEDCKpc3_NP.5
p−min=0.56, pX=0.39, N=254
var.21.KLKB1 4:187173012:T:G
peptide level
229:229 85:85 11:11
DSVTGTLPKpc2_NP.1
p−maj=1, pX=0.64, N=325
var.21.KLKB1 4:187173012:T:G
peptide level
196:229 78:85 9:11
DSVTGTLPKpc2_NP.2
p−min=0.28, pX=0.53, N=283
var.21.KLKB1 4:187173012:T:G
peptide level
229:229 85:85 11:11
DSVTGTLPKpc2_NP.3
p−maj=1, pX=0.32, N=325
var.21.KLKB1 4:187173012:T:G
peptide level
124:229 36:85 3:11
DSVTGTLPKpc2_NP.4
p−min=0.029, pX=0.9, N=163
var.21.KLKB1 4:187173012:T:G
peptide level
226:229 84:85 10:11
DSVTGTLPKpc2_NP.5
p−maj=0.16, pX=0.14, N=320
var.21.KLKB1 4:187173012:T:G
peptide level
229:229 85:85 11:11
DTPFSQIKpc2_NP.1
p−maj=1, pX=0.9, N=325
var.21.KLKB1 4:187173012:T:G
peptide level
212:229 83:85 10:11
DTPFSQIKpc2_NP.2
p−min=0.21, pX=0.45, N=305
var.21.KLKB1 4:187173012:T:G
peptide level
229:229 85:85 11:11
DTPFSQIKpc2_NP.3
p−maj=1, pX=0.14, N=325
var.21.KLKB1 4:187173012:T:G
peptide level
43:229 11:85 0:11
DTPFSQIKpc2_NP.4
p−min=0.14, pX=0.37, N=54
var.21.KLKB1 4:187173012:T:G
peptide level
85:229 28:85 4:11
DTPFSQIKpc2_NP.5
p−min=0.53, pX=0.61, N=117

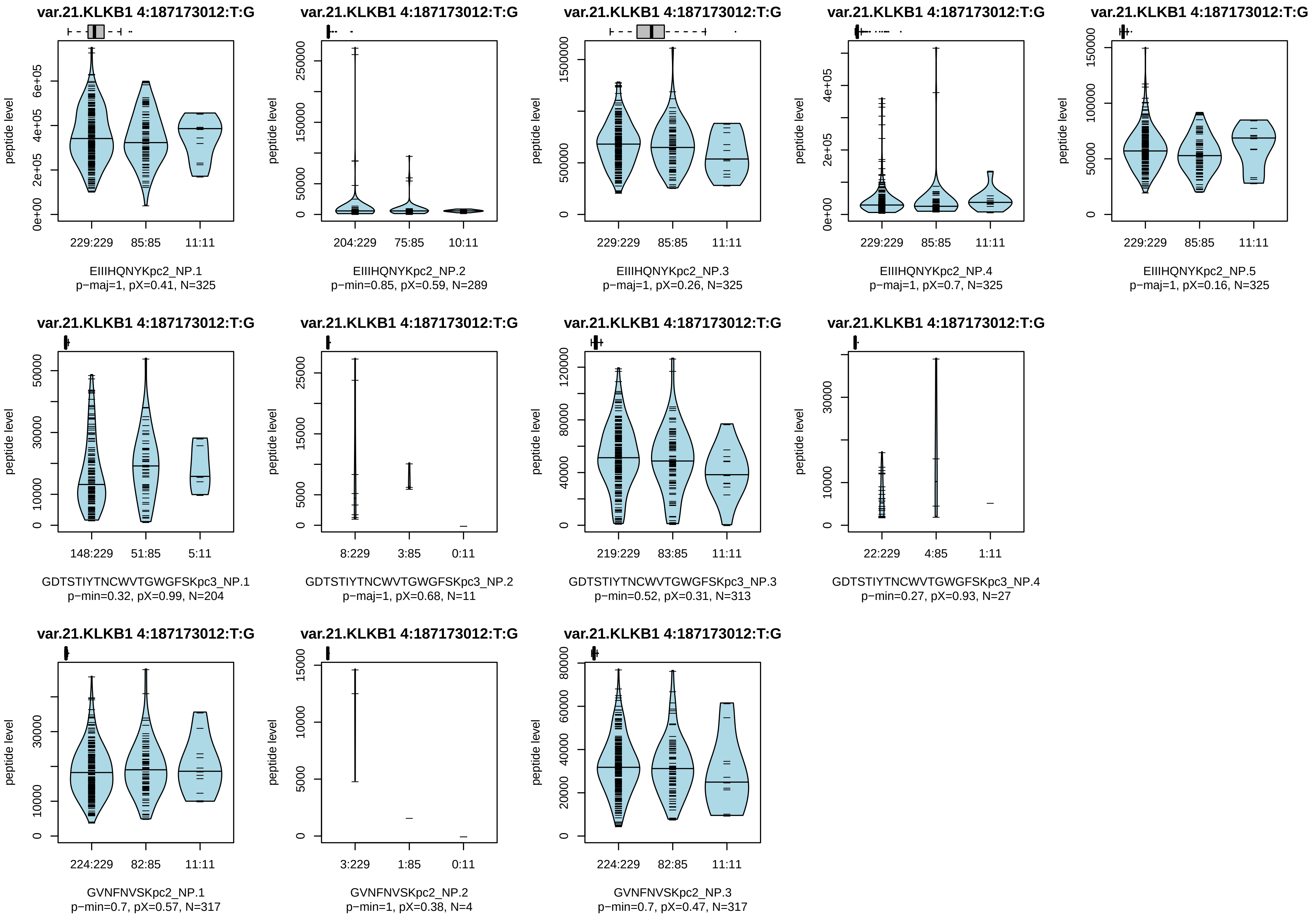
var.21.KLKB1 4:187173012:T:G
peptide level
229:229
85:85
11:11
EIIIHQNYKpc2_NP.1
p−maj=1, pX=0.41, N=325
var.21.KLKB1 4:187173012:T:G
peptide level
204:229
75:85
10:11
EIIIHQNYKpc2_NP.2
p−min=0.85, pX=0.59, N=289
var.21.KLKB1 4:187173012:T:G
peptide level
229:229
85:85
11:11
EIIIHQNYKpc2_NP.3
p−maj=1, pX=0.26, N=325
var.21.KLKB1 4:187173012:T:G
peptide level
229:229
85:85
11:11
EIIIHQNYKpc2_NP.4
p−maj=1, pX=0.7, N=325
var.21.KLKB1 4:187173012:T:G
peptide level
229:229
85:85
11:11
EIIIHQNYKpc2_NP.5
p−maj=1, pX=0.16, N=325
var.21.KLKB1 4:187173012:T:G
peptide level
148:229
51:85
5:11
GDTSTIYTNCWVTGWGFSKpc3_NP.1
p−min=0.32, pX=0.99, N=204
var.21.KLKB1 4:187173012:T:G
peptide level
8:229
3:85
0:11
GDTSTIYTNCWVTGWGFSKpc3_NP.2
p−maj=1, pX=0.68, N=11
var.21.KLKB1 4:187173012:T:G
peptide level
219:229
83:85
11:11
GDTSTIYTNCWVTGWGFSKpc3_NP.3
p−min=0.52, pX=0.31, N=313
var.21.KLKB1 4:187173012:T:G
peptide level
22:229
4:85
1:11
GDTSTIYTNCWVTGWGFSKpc3_NP.4
p−min=0.27, pX=0.93, N=27
var.21.KLKB1 4:187173012:T:G
peptide level
224:229
82:85
11:11
GVNFNVSKpc2_NP.1
p−min=0.7, pX=0.57, N=317
var.21.KLKB1 4:187173012:T:G
peptide level
3:229
1:85
0:11
GVNFNVSKpc2_NP.2
p−min=1, pX=0.38, N=4
var.21.KLKB1 4:187173012:T:G
peptide level
224:229
82:85
11:11
GVNFNVSKpc2_NP.3
p−min=0.7, pX=0.47, N=317

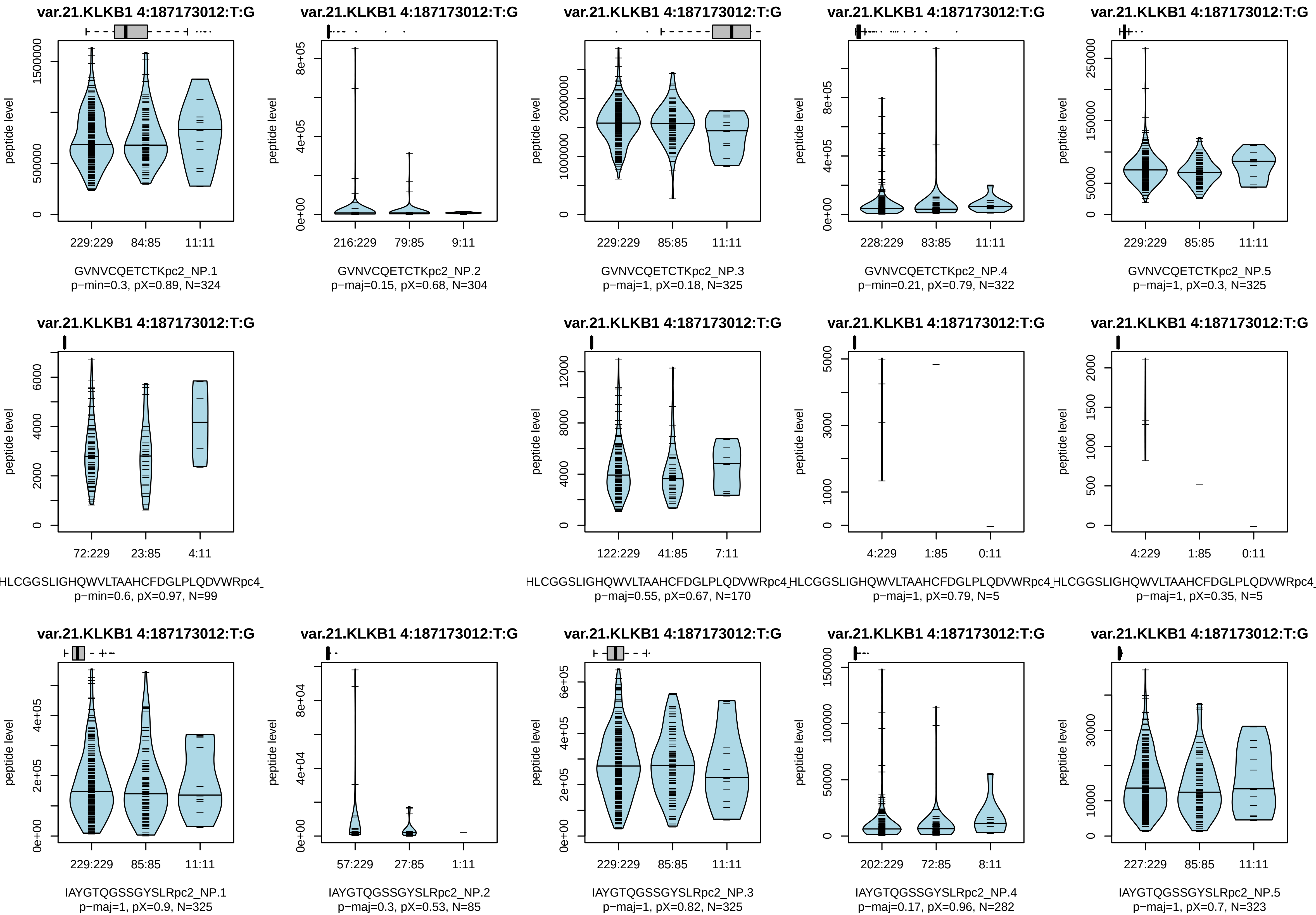
var.21.KLKB1 4:187173012:T:G
peptide level
229:229
84:85
11:11
GVNVCQETCTKpc2_NP.1
p−min=0.3, pX=0.89, N=324
var.21.KLKB1 4:187173012:T:G
216:229
79:85
9:11
GVNVCQETCTKpc2_NP.2
p−maj=0.15, pX=0.68, N=304
var.21.KLKB1 4:187173012:T:G
229:229
85:85
11:11
GVNVCQETCTKpc2_NP.3
p−maj=1, pX=0.18, N=325
var.21.KLKB1 4:187173012:T:G
228:229
83:85
11:11
GVNVCQETCTKpc2_NP.4
p−min=0.21, pX=0.79, N=322
var.21.KLKB1 4:187173012:T:G
229:229
85:85
11:11
GVNVCQETCTKpc2_NP.5
p−maj=1, pX=0.3, N=325
var.21.KLKB1 4:187173012:T:G
72:229
23:85
4:11
HLCGGSLIGHQWVLTAAHCFDGLPLQDVWRpc4_
p−min=0.6, pX=0.97, N=99
var.21.KLKB1 4:187173012:T:G
122:229
41:85
7:11
HLCGGSLIGHQWVLTAAHCFDGLPLQDVWRpc4_
p−maj=0.55, pX=0.67, N=170
var.21.KLKB1 4:187173012:T:G
4:229
1:85
0:11
HLCGGSLIGHQWVLTAAHCFDGLPLQDVWRpc4_
p−maj=1, pX=0.79, N=5
var.21.KLKB1 4:187173012:T:G
4:229
1:85
0:11
HLCGGSLIGHQWVLTAAHCFDGLPLQDVWRpc4_
p−maj=1, pX=0.35, N=5
var.21.KLKB1 4:187173012:T:G
229:229
85:85
11:11
IAYGTQGSSGYSLRpc2_NP.1
p−maj=1, pX=0.9, N=325
var.21.KLKB1 4:187173012:T:G
57:229
27:85
1:11
IAYGTQGSSGYSLRpc2_NP.2
p−maj=0.3, pX=0.53, N=85
var.21.KLKB1 4:187173012:T:G
229:229
85:85
11:11
IAYGTQGSSGYSLRpc2_NP.3
p−maj=1, pX=0.82, N=325
var.21.KLKB1 4:187173012:T:G
202:229
72:85
8:11
IAYGTQGSSGYSLRpc2_NP.4
p−maj=0.17, pX=0.96, N=282
var.21.KLKB1 4:187173012:T:G
227:229
85:85
11:11
IAYGTQGSSGYSLRpc2_NP.5
p−maj=1, pX=0.7, N=323

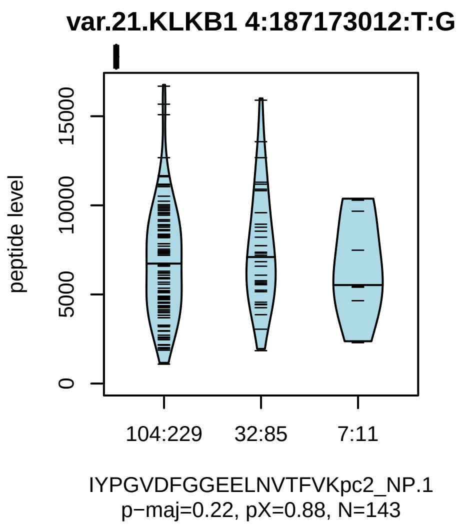
var.21.KLKB1 4:187173012:T:G
peptide level
0
5000
10000
15000
104:229
32:85
7:11
IYPGVDFGGEELNVTFVKpc2_NP.1
p−maj=0.22, pX=0.88, N=143

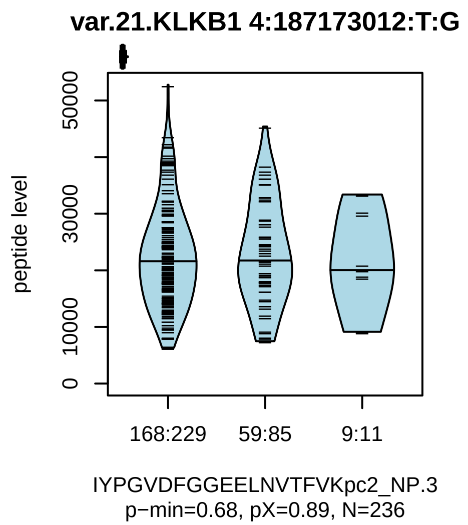
var.21.KLKB1 4:187173012:T:G
peptide level
0
10000
30000
50000
168:229
59:85
9:11
IYPGVDFGGEELNVTFVKpc2_NP.3
p−min=0.68, pX=0.89, N=236

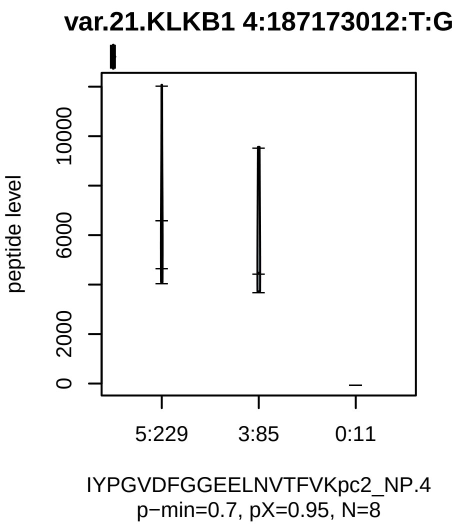
var.21.KLKB1 4:187173012:T:G
peptide level
0
2000
6000
10000
5:229
3:85
0:11
IYPGVDFGGEELNVTFVKpc2_NP.4
p−min=0.7, pX=0.95, N=8

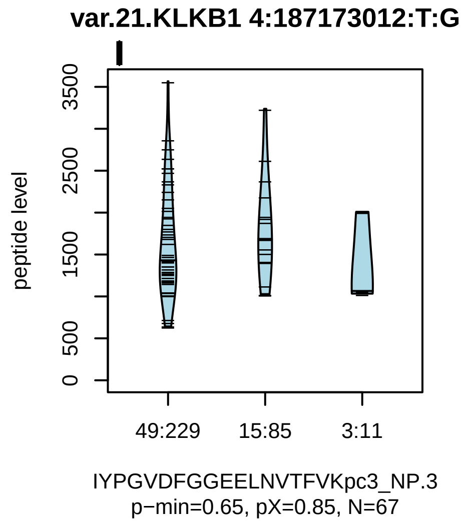
var.21.KLKB1 4:187173012:T:G
peptide level
0
500
1500
2500
3500
49:229
15:85
3:11
IYPGVDFGGEELNVTFVKpc3_NP.3
p−min=0.65, pX=0.85, N=67

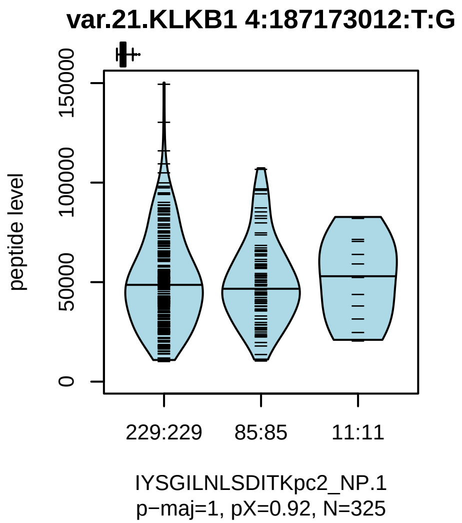
var.21.KLKB1 4:187173012:T:G
peptide level
0
50000
100000
150000
229:229
85:85
11:11
IYSGILNLSDITKpc2_NP.1
p−maj=1, pX=0.92, N=325

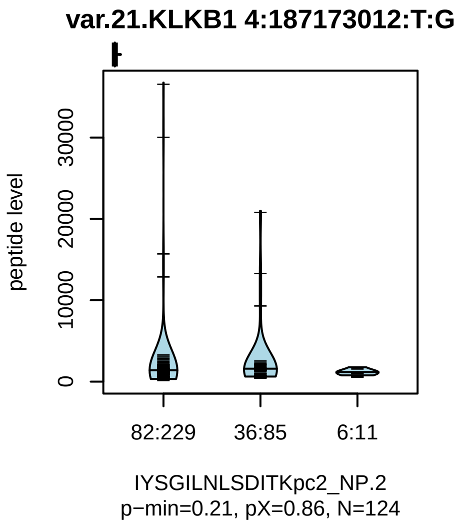
var.21.KLKB1 4:187173012:T:G
peptide level
0
10000
20000
30000
82:229
36:85
6:11
IYSGILNLSDITKpc2_NP.2
p−min=0.21, pX=0.86, N=124

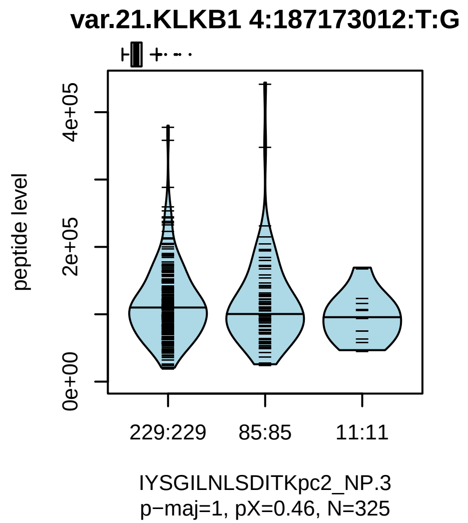
var.21.KLKB1 4:187173012:T:G
peptide level
0e+00
2e+05
4e+05
229:229
85:85
11:11
IYSGILNLSDITKpc2_NP.3
p−maj=1, pX=0.46, N=325

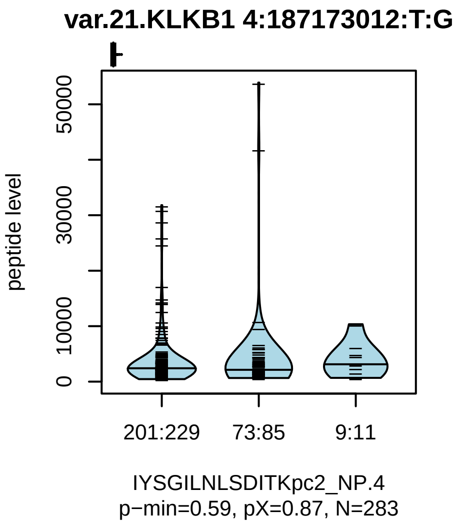
var.21.KLKB1 4:187173012:T:G
peptide level
0
10000
30000
50000
201:229
73:85
9:11
IYSGILNLSDITKpc2_NP.4
p−min=0.59, pX=0.87, N=283

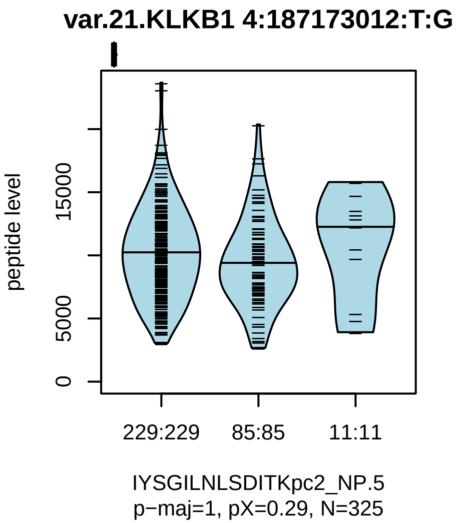
var.21.KLKB1 4:187173012:T:G
peptide level
0
5000
15000
229:229
85:85
11:11
IYSGILNLSDITKpc2_NP.5
p−maj=1, pX=0.29, N=325

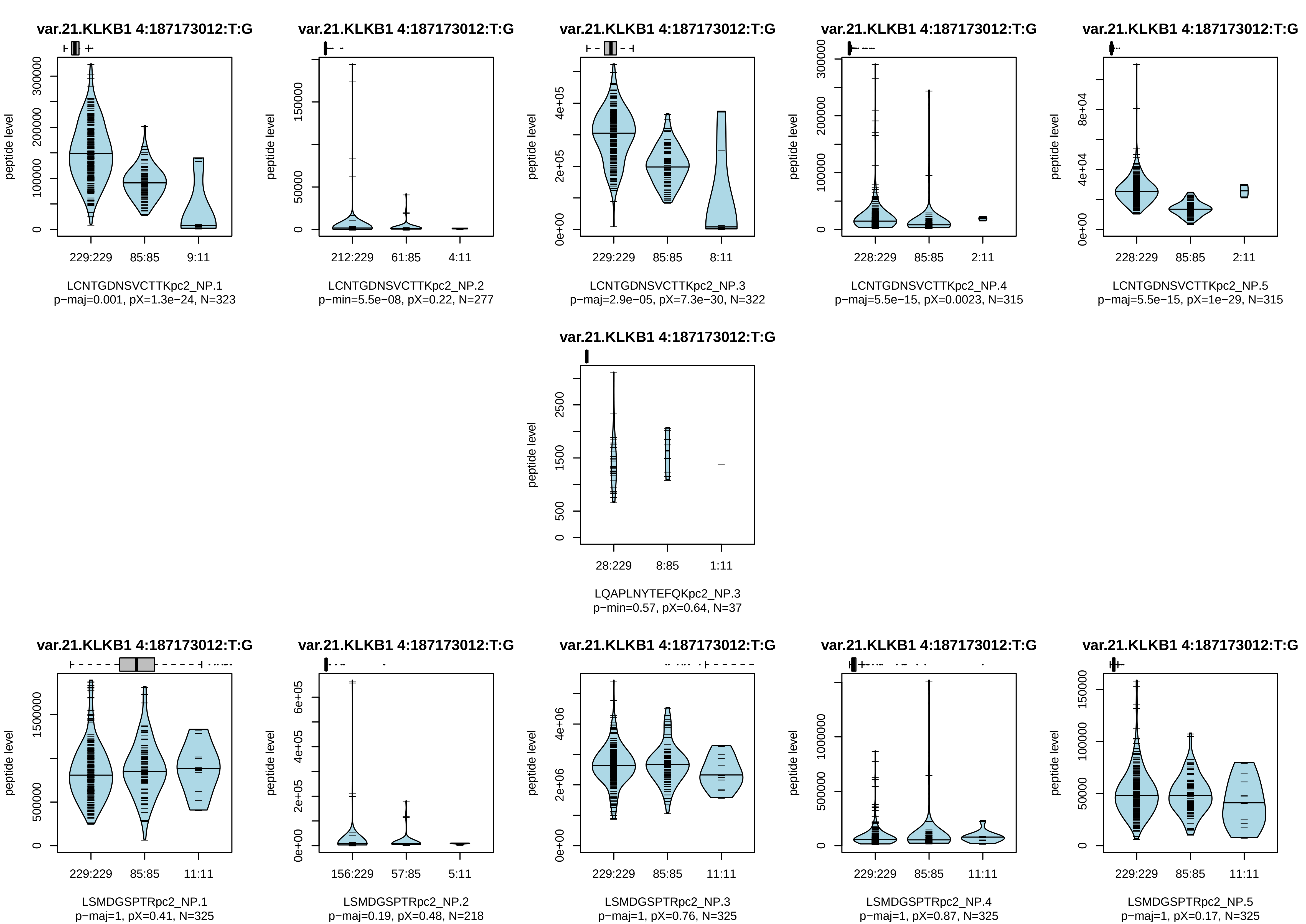
var.21.KLKB1 4:187173012:T:G
peptide level
229:229
85:85
9:11
LCNTGDNSVCTTKpc2_NP.1
p−maj=0.001, pX=1.3e−24, N=323
var.21.KLKB1 4:187173012:T:G
212:229
61:85
4:11
LCNTGDNSVCTTKpc2_NP.2
p−min=5.5e−08, pX=0.22, N=277
var.21.KLKB1 4:187173012:T:G
229:229
85:85
8:11
LCNTGDNSVCTTKpc2_NP.3
p−maj=2.9e−05, pX=7.3e−30, N=322
var.21.KLKB1 4:187173012:T:G
228:229
85:85
2:11
LCNTGDNSVCTTKpc2_NP.4
p−maj=5.5e−15, pX=0.0023, N=315
var.21.KLKB1 4:187173012:T:G
228:229
85:85
2:11
LCNTGDNSVCTTKpc2_NP.5
p−maj=5.5e−15, pX=1e−29, N=315
var.21.KLKB1 4:187173012:T:G
28:229
8:85
1:11
LQAPLNYTEFQKpc2_NP.3
p−min=0.57, pX=0.64, N=37
var.21.KLKB1 4:187173012:T:G
229:229
85:85
11:11
LSMDGSPTRpc2_NP.1
p−maj=1, pX=0.41, N=325
var.21.KLKB1 4:187173012:T:G
156:229
57:85
5:11
LSMDGSPTRpc2_NP.2
p−maj=0.19, pX=0.48, N=218
var.21.KLKB1 4:187173012:T:G
229:229
85:85
11:11
LSMDGSPTRpc2_NP.3
p−maj=1, pX=0.76, N=325
var.21.KLKB1 4:187173012:T:G
229:229
85:85
11:11
LSMDGSPTRpc2_NP.4
p−maj=1, pX=0.87, N=325
var.21.KLKB1 4:187173012:T:G
229:229
85:85
11:11
LSMDGSPTRpc2_NP.5
p−maj=1, pX=0.17, N=325

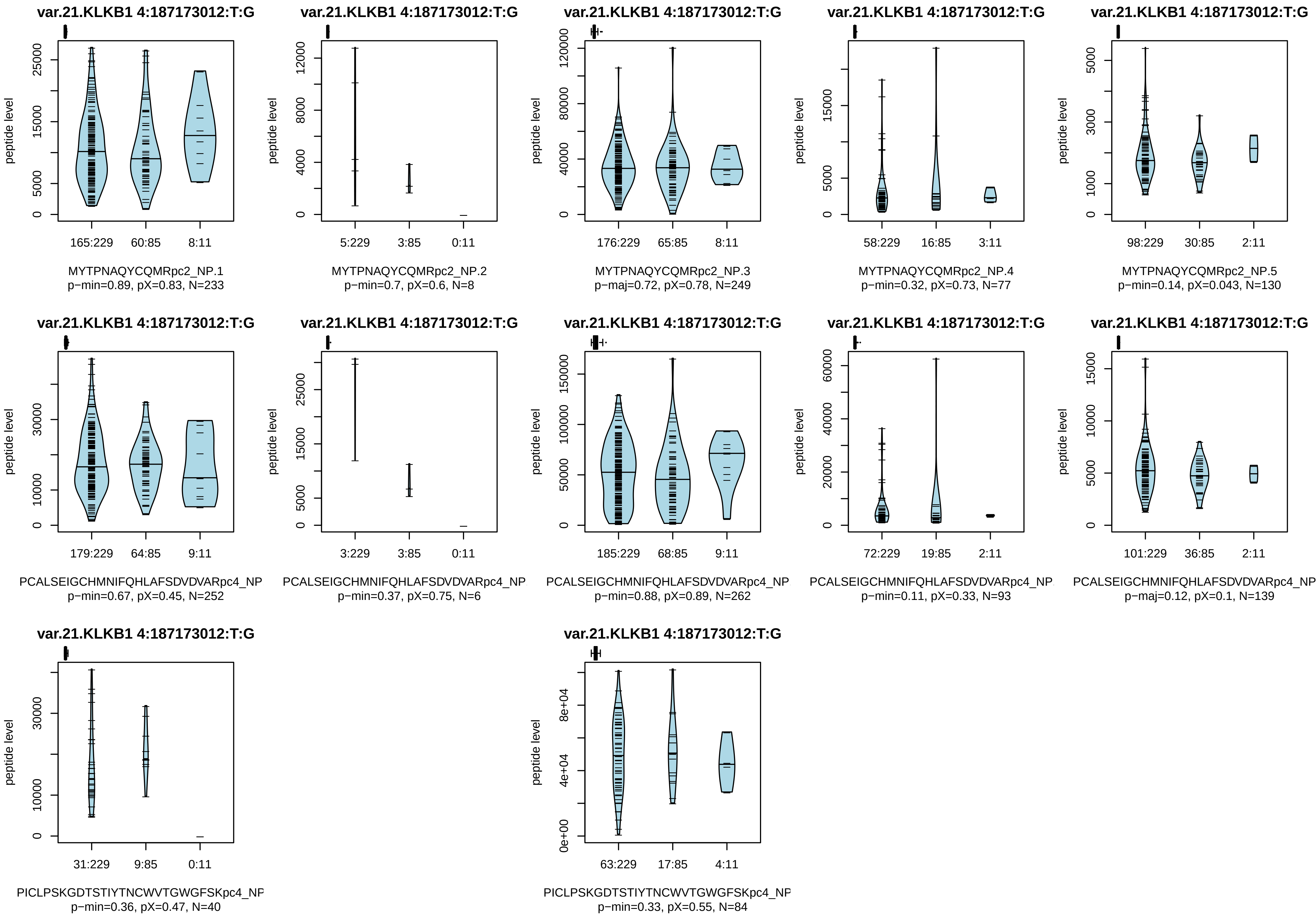
var.21.KLKB1 4:187173012:T:G
165:229
60:85
8:11
MYTPNAQYCQMRpc2_NP.1
p−min=0.89, pX=0.83, N=233
var.21.KLKB1 4:187173012:T:G
5:229
3:85
0:11
MYTPNAQYCQMRpc2_NP.2
p−min=0.7, pX=0.6, N=8
var.21.KLKB1 4:187173012:T:G
176:229
65:85
8:11
MYTPNAQYCQMRpc2_NP.3
p−maj=0.72, pX=0.78, N=249
var.21.KLKB1 4:187173012:T:G
58:229
16:85
3:11
MYTPNAQYCQMRpc2_NP.4
p−min=0.32, pX=0.73, N=77
var.21.KLKB1 4:187173012:T:G
98:229
30:85
2:11
MYTPNAQYCQMRpc2_NP.5
p−min=0.14, pX=0.043, N=130
var.21.KLKB1 4:187173012:T:G
179:229
64:85
9:11
PCALSEIGCHMNIFQHLAFSDVDVARpc4_NP
p−min=0.67, pX=0.45, N=252
var.21.KLKB1 4:187173012:T:G
3:229
3:85
0:11
PCALSEIGCHMNIFQHLAFSDVDVARpc4_NP
p−min=0.37, pX=0.75, N=6
var.21.KLKB1 4:187173012:T:G
185:229
68:85
9:11
PCALSEIGCHMNIFQHLAFSDVDVARpc4_NP
p−min=0.88, pX=0.89, N=262
var.21.KLKB1 4:187173012:T:G
72:229
19:85
2:11
PCALSEIGCHMNIFQHLAFSDVDVARpc4_NP
p−min=0.11, pX=0.33, N=93
var.21.KLKB1 4:187173012:T:G
101:229
36:85
2:11
PCALSEIGCHMNIFQHLAFSDVDVARpc4_NP
p−maj=0.12, pX=0.1, N=139
var.21.KLKB1 4:187173012:T:G
31:229
9:85
0:11
PICLPSKGDTSTIYTNCWVTGWGFSKpc4_NP
p−min=0.36, pX=0.47, N=40
var.21.KLKB1 4:187173012:T:G
63:229
17:85
4:11
PICLPSKGDTSTIYTNCWVTGWGFSKpc4_NP
p−min=0.33, pX=0.55, N=84
peptide level

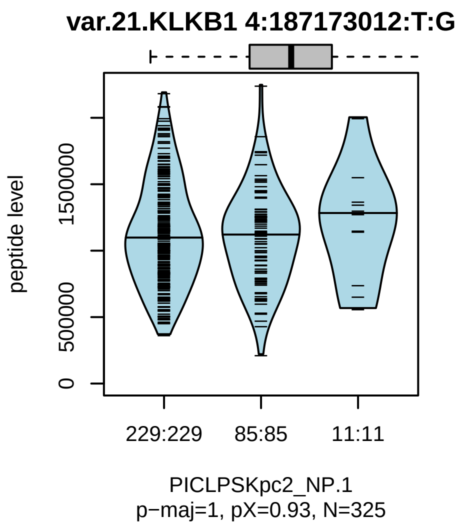
var.21.KLKB1 4:187173012:T:G
peptide level
0
500000
1500000
229:229
85:85
11:11
PICLPSKpc2_NP.1
p−maj=1, pX=0.93, N=325

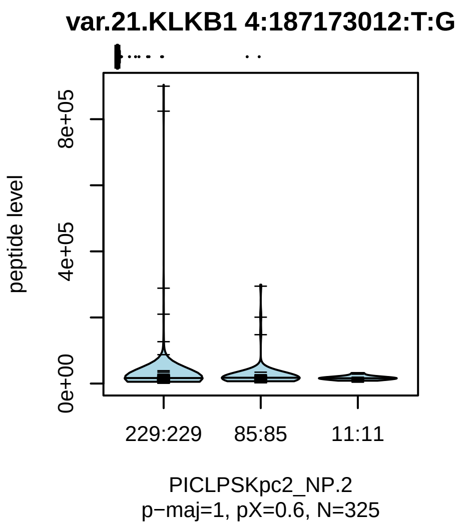
var.21.KLKB1 4:187173012:T:G
peptide level
0e+00
4e+05
8e+05
229:229
85:85
11:11
PICLPSKpc2_NP.2
p−maj=1, pX=0.6, N=325

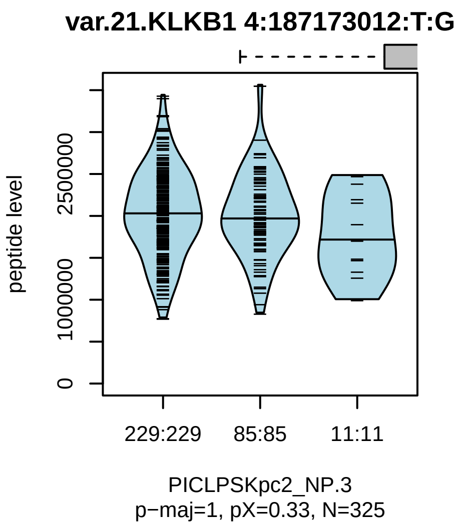
var.21.KLKB1 4:187173012:T:G
peptide level
0
1000000
2500000
229:229
85:85
11:11
PICLPSKpc2_NP.3
p−maj=1, pX=0.33, N=325

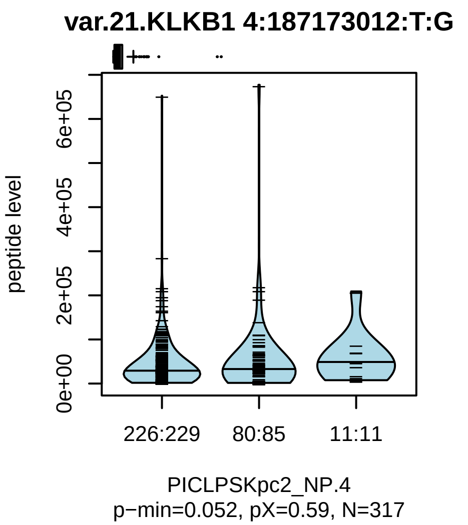
var.21.KLKB1 4:187173012:T:G
peptide level
0e+00
2e+05
4e+05
6e+05
226:229
80:85
11:11
PICLPSKpc2_NP.4
p−min=0.052, pX=0.59, N=317

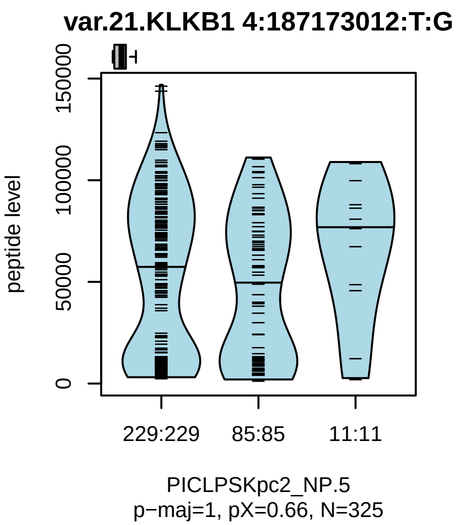
var.21.KLKB1 4:187173012:T:G
peptide level
0
50000
100000
150000
229:229
85:85
11:11
PICLPSKpc2_NP.5
p−maj=1, pX=0.66, N=325

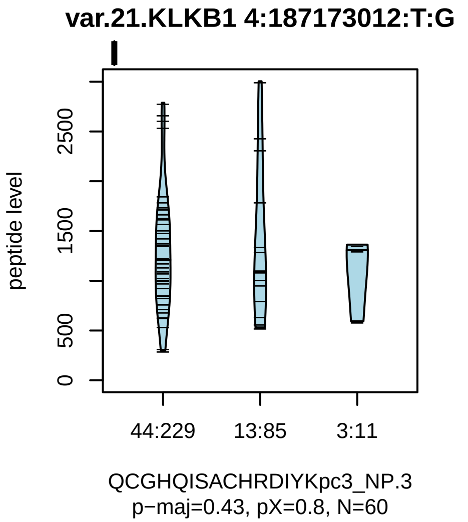
var.21.KLKB1 4:187173012:T:G
peptide level
0
500
1500
2500
44:229
13:85
3:11
QCGHQISACHRDIYKpc3_NP.3
p−maj=0.43, pX=0.8, N=60

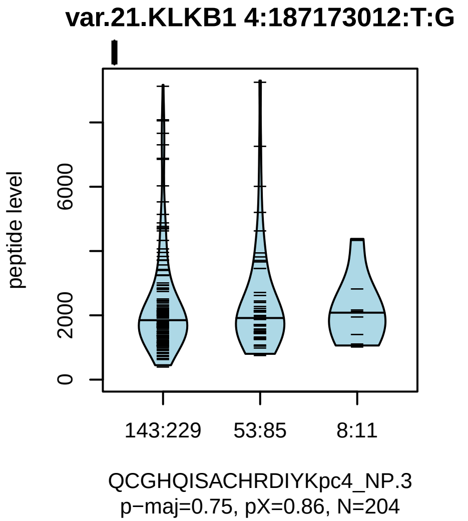
var.21.KLKB1 4:187173012:T:G
peptide level
0
2000
6000
143:229
53:85
8:11
QCGHQISACHRDIYKpc4_NP.3
p−maj=0.75, pX=0.86, N=204

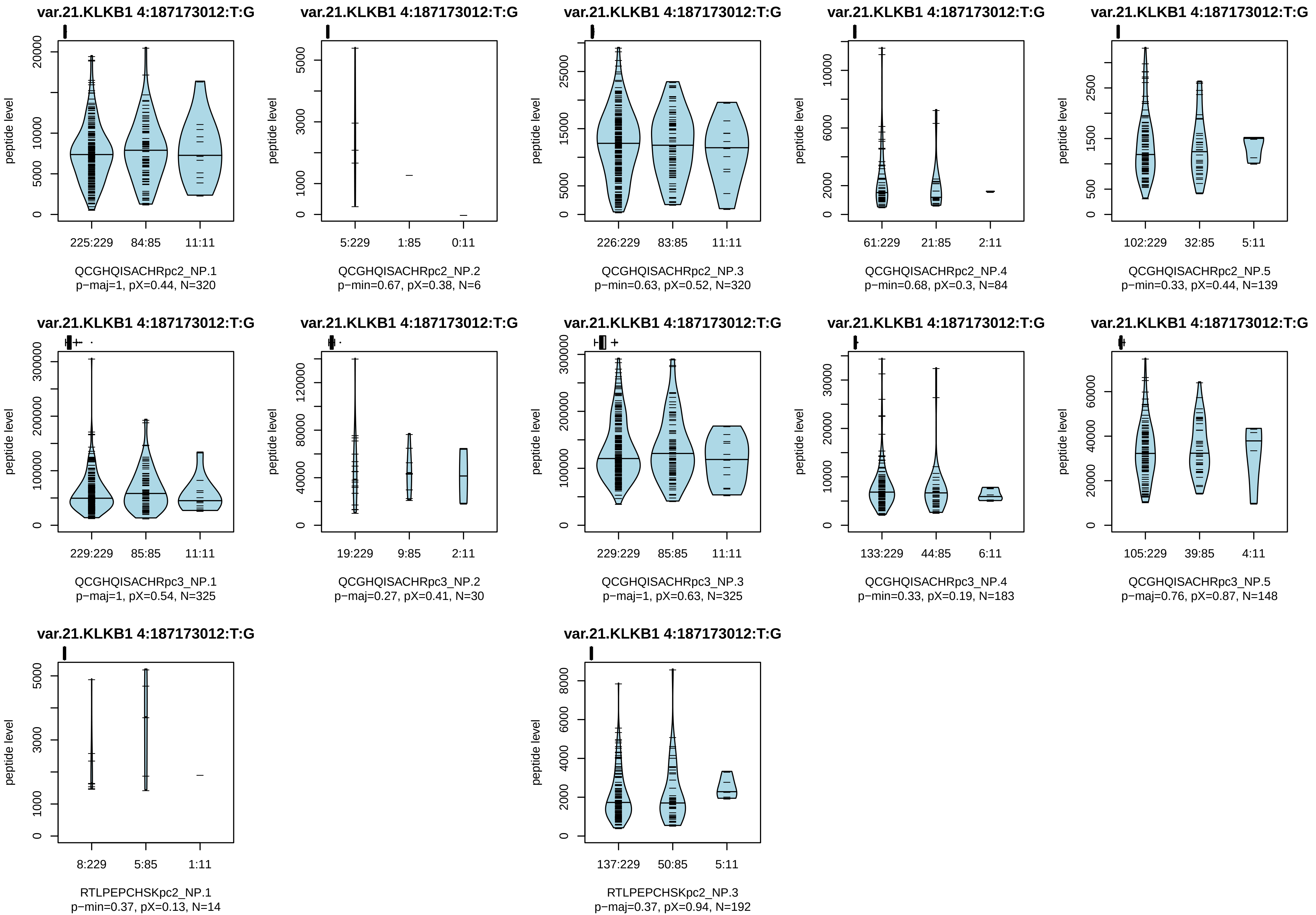
var.21.KLKB1 4:187173012:T:G
peptide level
225:229
84:85
11:11
QCGHQISACHRpc2_NP.1
p−maj=1, pX=0.44, N=320
var.21.KLKB1 4:187173012:T:G
peptide level
5:229
1:85
0:11
QCGHQISACHRpc2_NP.2
p−min=0.67, pX=0.38, N=6
var.21.KLKB1 4:187173012:T:G
peptide level
226:229
83:85
11:11
QCGHQISACHRpc2_NP.3
p−min=0.63, pX=0.52, N=320
var.21.KLKB1 4:187173012:T:G
peptide level
61:229
21:85
2:11
QCGHQISACHRpc2_NP.4
p−min=0.68, pX=0.3, N=84
var.21.KLKB1 4:187173012:T:G
peptide level
102:229
32:85
5:11
QCGHQISACHRpc2_NP.5
p−min=0.33, pX=0.44, N=139
var.21.KLKB1 4:187173012:T:G
peptide level
229:229
85:85
11:11
QCGHQISACHRpc3_NP.1
p−maj=1, pX=0.54, N=325
var.21.KLKB1 4:187173012:T:G
peptide level
19:229
9:85
2:11
QCGHQISACHRpc3_NP.2
p−maj=0.27, pX=0.41, N=30
var.21.KLKB1 4:187173012:T:G
peptide level
229:229
85:85
11:11
QCGHQISACHRpc3_NP.3
p−maj=1, pX=0.63, N=325
var.21.KLKB1 4:187173012:T:G
peptide level
133:229
44:85
6:11
QCGHQISACHRpc3_NP.4
p−min=0.33, pX=0.19, N=183
var.21.KLKB1 4:187173012:T:G
peptide level
105:229
39:85
4:11
QCGHQISACHRpc3_NP.5
p−maj=0.76, pX=0.87, N=148
var.21.KLKB1 4:187173012:T:G
peptide level
8:229
5:85
1:11
RTLPEPCHSKpc2_NP.1
p−min=0.37, pX=0.13, N=14
var.21.KLKB1 4:187173012:T:G
peptide level
137:229
50:85
5:11
RTLPEPCHSKpc2_NP.3
p−maj=0.37, pX=0.94, N=192

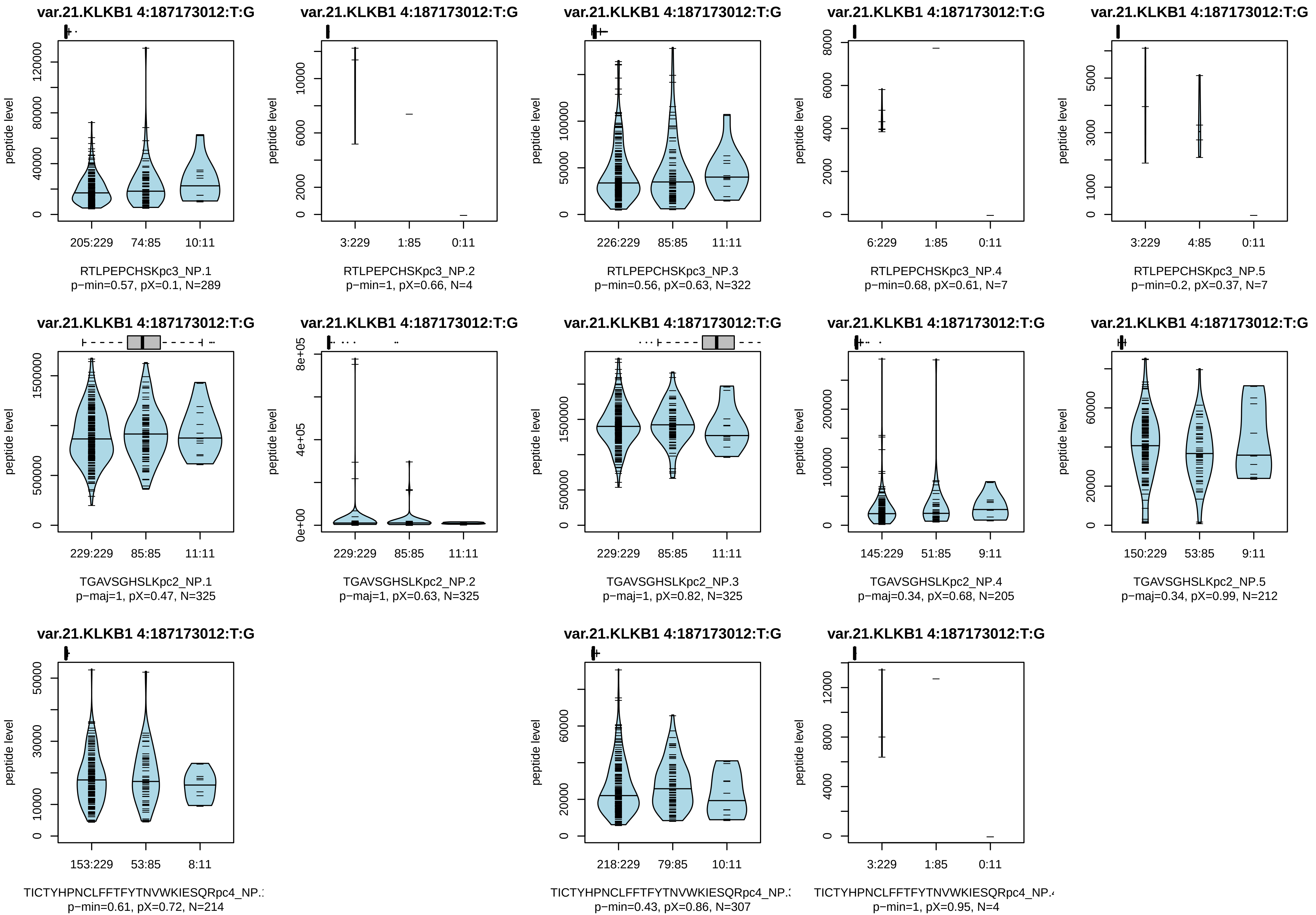
var.21.KLKB1 4:187173012:T:G
peptide level
205:229
74:85
10:11
RTLPEPCHSKpc3_NP.1
p−min=0.57, pX=0.1, N=289
3:229
1:85
0:11
RTLPEPCHSKpc3_NP.2
p−min=1, pX=0.66, N=4
226:229
85:85
11:11
RTLPEPCHSKpc3_NP.3
p−min=0.56, pX=0.63, N=322
6:229
1:85
0:11
RTLPEPCHSKpc3_NP.4
p−min=0.68, pX=0.61, N=7
3:229
4:85
0:11
RTLPEPCHSKpc3_NP.5
p−min=0.2, pX=0.37, N=7
229:229
85:85
11:11
TGAVSGHSLKpc2_NP.1
p−maj=1, pX=0.47, N=325
229:229
85:85
11:11
TGAVSGHSLKpc2_NP.2
p−maj=1, pX=0.63, N=325
229:229
85:85
11:11
TGAVSGHSLKpc2_NP.3
p−maj=1, pX=0.82, N=325
145:229
51:85
9:11
TGAVSGHSLKpc2_NP.4
p−maj=0.34, pX=0.68, N=205
150:229
53:85
9:11
TGAVSGHSLKpc2_NP.5
p−maj=0.34, pX=0.99, N=212
153:229
53:85
8:11
TICTYHPNCLFFTFYTNVWKIESQRpc4_NP.
p−min=0.61, pX=0.72, N=214
218:229
79:85
10:11
TICTYHPNCLFFTFYTNVWKIESQRpc4_NP.
p−min=0.43, pX=0.86, N=307
3:229
1:85
0:11
TICTYHPNCLFFTFYTNVWKIESQRpc4_NP.
p−min=1, pX=0.95, N=4

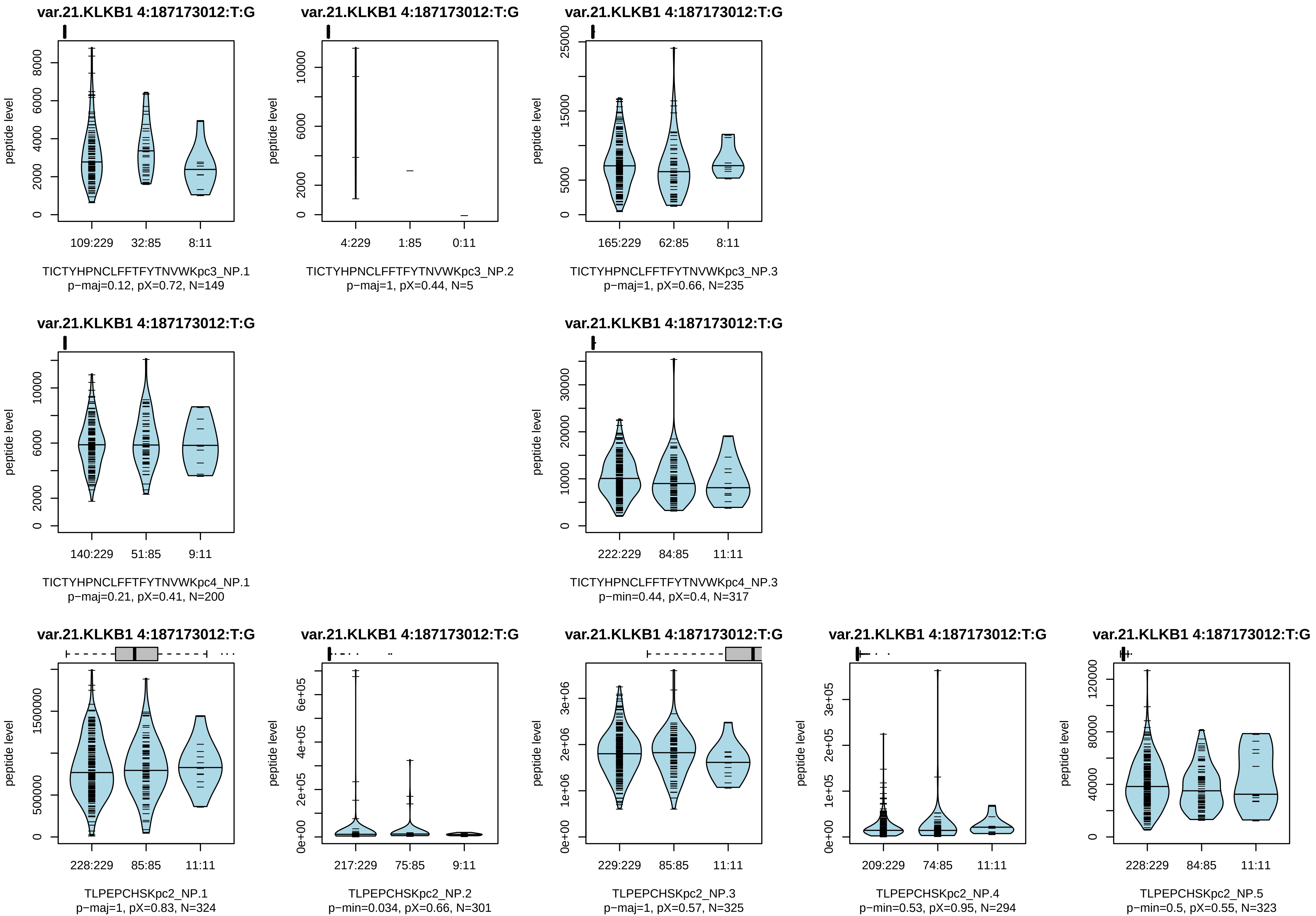
var.21.KLKB1 4:187173012:T:G
peptide level
109:229
32:85
8:11
TICTYHPNCLFFTFYTNVWKpc3_NP.1
p−maj=0.12, pX=0.72, N=149
var.21.KLKB1 4:187173012:T:G
peptide level
4:229
1:85
0:11
TICTYHPNCLFFTFYTNVWKpc3_NP.2
p−maj=1, pX=0.44, N=5
var.21.KLKB1 4:187173012:T:G
peptide level
165:229
62:85
8:11
TICTYHPNCLFFTFYTNVWKpc3_NP.3
p−maj=1, pX=0.66, N=235
var.21.KLKB1 4:187173012:T:G
peptide level
140:229
51:85
9:11
TICTYHPNCLFFTFYTNVWKpc4_NP.1
p−maj=0.21, pX=0.41, N=200
var.21.KLKB1 4:187173012:T:G
peptide level
222:229
84:85
11:11
TICTYHPNCLFFTFYTNVWKpc4_NP.3
p−min=0.44, pX=0.4, N=317
var.21.KLKB1 4:187173012:T:G
peptide level
228:229
85:85
11:11
TLPEPCHSKpc2_NP.1
p−maj=1, pX=0.83, N=324
var.21.KLKB1 4:187173012:T:G
peptide level
217:229
75:85
9:11
TLPEPCHSKpc2_NP.2
p−min=0.034, pX=0.66, N=301
var.21.KLKB1 4:187173012:T:G
peptide level
229:229
85:85
11:11
TLPEPCHSKpc2_NP.3
p−maj=1, pX=0.57, N=325
var.21.KLKB1 4:187173012:T:G
peptide level
209:229
74:85
11:11
TLPEPCHSKpc2_NP.4
p−min=0.53, pX=0.95, N=294
var.21.KLKB1 4:187173012:T:G
peptide level
228:229
84:85
11:11
TLPEPCHSKpc2_NP.5
p−min=0.5, pX=0.55, N=323

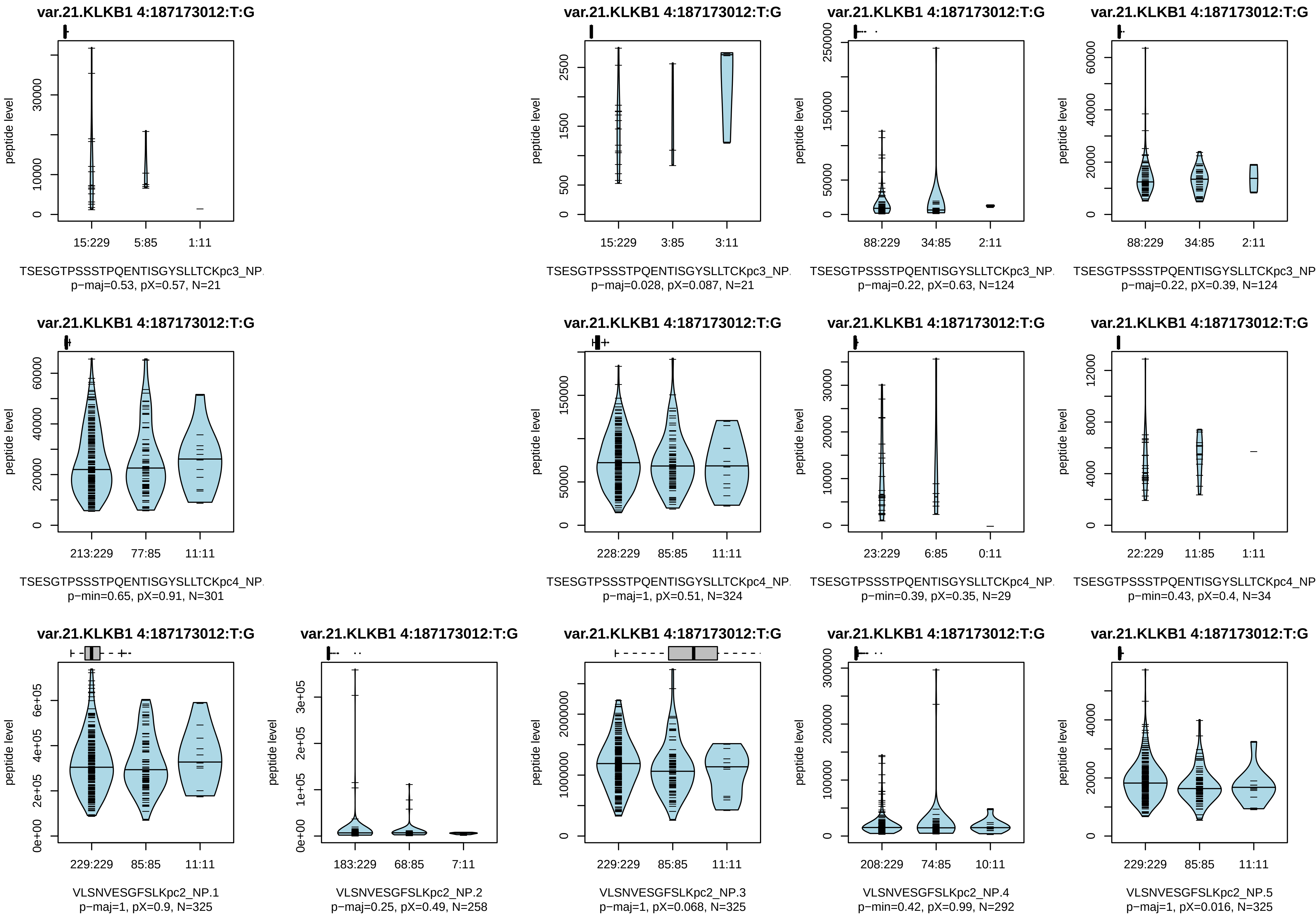
var.21.KLKB1 4:187173012:T:G
peptide level
15:229
5:85
1:11
TSESGTPSSSTPQENTISGYSLLTCKpc3_NP.
p−maj=0.53, pX=0.57, N=21
var.21.KLKB1 4:187173012:T:G
peptide level
15:229
3:85
3:11
TSESGTPSSSTPQENTISGYSLLTCKpc3_NP.
p−maj=0.028, pX=0.087, N=21
var.21.KLKB1 4:187173012:T:G
peptide level
88:229
34:85
2:11
TSESGTPSSSTPQENTISGYSLLTCKpc3_NP.
p−maj=0.22, pX=0.63, N=124
var.21.KLKB1 4:187173012:T:G
peptide level
88:229
34:85
2:11
TSESGTPSSSTPQENTISGYSLLTCKpc3_NP
p−maj=0.22, pX=0.39, N=124
var.21.KLKB1 4:187173012:T:G
peptide level
213:229
77:85
11:11
TSESGTPSSSTPQENTISGYSLLTCKpc4_NP.
p−min=0.65, pX=0.91, N=301
var.21.KLKB1 4:187173012:T:G
peptide level
228:229
85:85
11:11
TSESGTPSSSTPQENTISGYSLLTCKpc4_NP.
p−maj=1, pX=0.51, N=324
var.21.KLKB1 4:187173012:T:G
peptide level
23:229
6:85
0:11
TSESGTPSSSTPQENTISGYSLLTCKpc4_NP.
p−min=0.39, pX=0.35, N=29
var.21.KLKB1 4:187173012:T:G
peptide level
22:229
11:85
1:11
TSESGTPSSSTPQENTISGYSLLTCKpc4_NP
p−min=0.43, pX=0.4, N=34
var.21.KLKB1 4:187173012:T:G
peptide level
229:229
85:85
11:11
VLSNVESGFSLKpc2_NP.1
p−maj=1, pX=0.9, N=325
var.21.KLKB1 4:187173012:T:G
peptide level
183:229
68:85
7:11
VLSNVESGFSLKpc2_NP.2
p−maj=0.25, pX=0.49, N=258
var.21.KLKB1 4:187173012:T:G
peptide level
229:229
85:85
11:11
VLSNVESGFSLKpc2_NP.3
p−maj=1, pX=0.068, N=325
var.21.KLKB1 4:187173012:T:G
peptide level
208:229
74:85
10:11
VLSNVESGFSLKpc2_NP.4
p−min=0.42, pX=0.99, N=292
var.21.KLKB1 4:187173012:T:G
peptide level
229:229
85:85
11:11
VLSNVESGFSLKpc2_NP.5
p−maj=1, pX=0.016, N=325

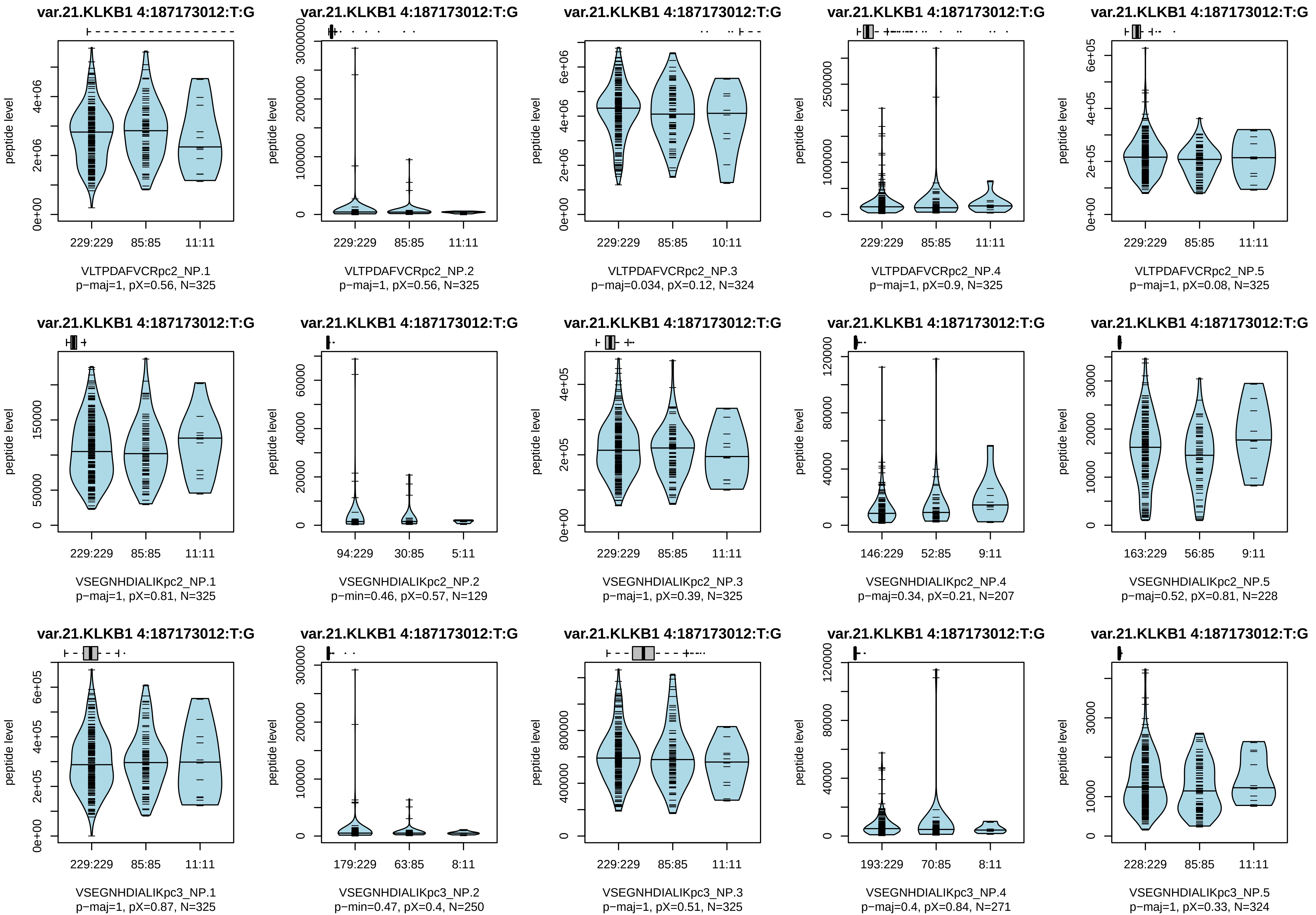
var.21.KLKB1 4:187173012:T:G
peptide level
0e+00
2e+06
4e+06
229:229
85:85
11:11
VLTPDAFVCRpc2_NP.1
p−maj=1, pX=0.56, N=325
var.21.KLKB1 4:187173012:T:G
peptide level
0
1000000
2000000
3000000
229:229
85:85
11:11
VLTPDAFVCRpc2_NP.2
p−maj=1, pX=0.56, N=325
var.21.KLKB1 4:187173012:T:G
peptide level
0e+00
2e+06
4e+06
6e+06
229:229
85:85
10:11
VLTPDAFVCRpc2_NP.3
p−maj=0.034, pX=0.12, N=324
var.21.KLKB1 4:187173012:T:G
peptide level
0
1000000
2500000
229:229
85:85
11:11
VLTPDAFVCRpc2_NP.4
p−maj=1, pX=0.9, N=325
var.21.KLKB1 4:187173012:T:G
peptide level
0e+00
2e+05
4e+05
6e+05
229:229
85:85
11:11
VLTPDAFVCRpc2_NP.5
p−maj=1, pX=0.08, N=325
var.21.KLKB1 4:187173012:T:G
peptide level
0
50000
150000
229:229
85:85
11:11
VSEGNHDIALIKpc2_NP.1
p−maj=1, pX=0.81, N=325
var.21.KLKB1 4:187173012:T:G
peptide level
0
20000
40000
60000
94:229
30:85
5:11
VSEGNHDIALIKpc2_NP.2
p−min=0.46, pX=0.57, N=129
var.21.KLKB1 4:187173012:T:G
peptide level
0e+00
2e+05
4e+05
229:229
85:85
11:11
VSEGNHDIALIKpc2_NP.3
p−maj=1, pX=0.39, N=325
var.21.KLKB1 4:187173012:T:G
peptide level
0
40000
80000
120000
146:229
52:85
9:11
VSEGNHDIALIKpc2_NP.4
p−maj=0.34, pX=0.21, N=207
var.21.KLKB1 4:187173012:T:G
peptide level
0
10000
20000
30000
163:229
56:85
9:11
VSEGNHDIALIKpc2_NP.5
p−maj=0.52, pX=0.81, N=228
var.21.KLKB1 4:187173012:T:G
peptide level
0e+00
2e+05
4e+05
6e+05
229:229
85:85
11:11
VSEGNHDIALIKpc3_NP.1
p−maj=1, pX=0.87, N=325
var.21.KLKB1 4:187173012:T:G
peptide level
0
100000
200000
300000
179:229
63:85
8:11
VSEGNHDIALIKpc3_NP.2
p−min=0.47, pX=0.4, N=250
var.21.KLKB1 4:187173012:T:G
peptide level
0
400000
800000
229:229
85:85
11:11
VSEGNHDIALIKpc3_NP.3
p−maj=1, pX=0.51, N=325
var.21.KLKB1 4:187173012:T:G
peptide level
0
40000
80000
120000
193:229
70:85
8:11
VSEGNHDIALIKpc3_NP.4
p−maj=0.4, pX=0.84, N=271
var.21.KLKB1 4:187173012:T:G
peptide level
0
10000
30000
228:229
85:85
11:11
VSEGNHDIALIKpc3_NP.5
p−maj=1, pX=0.33, N=324

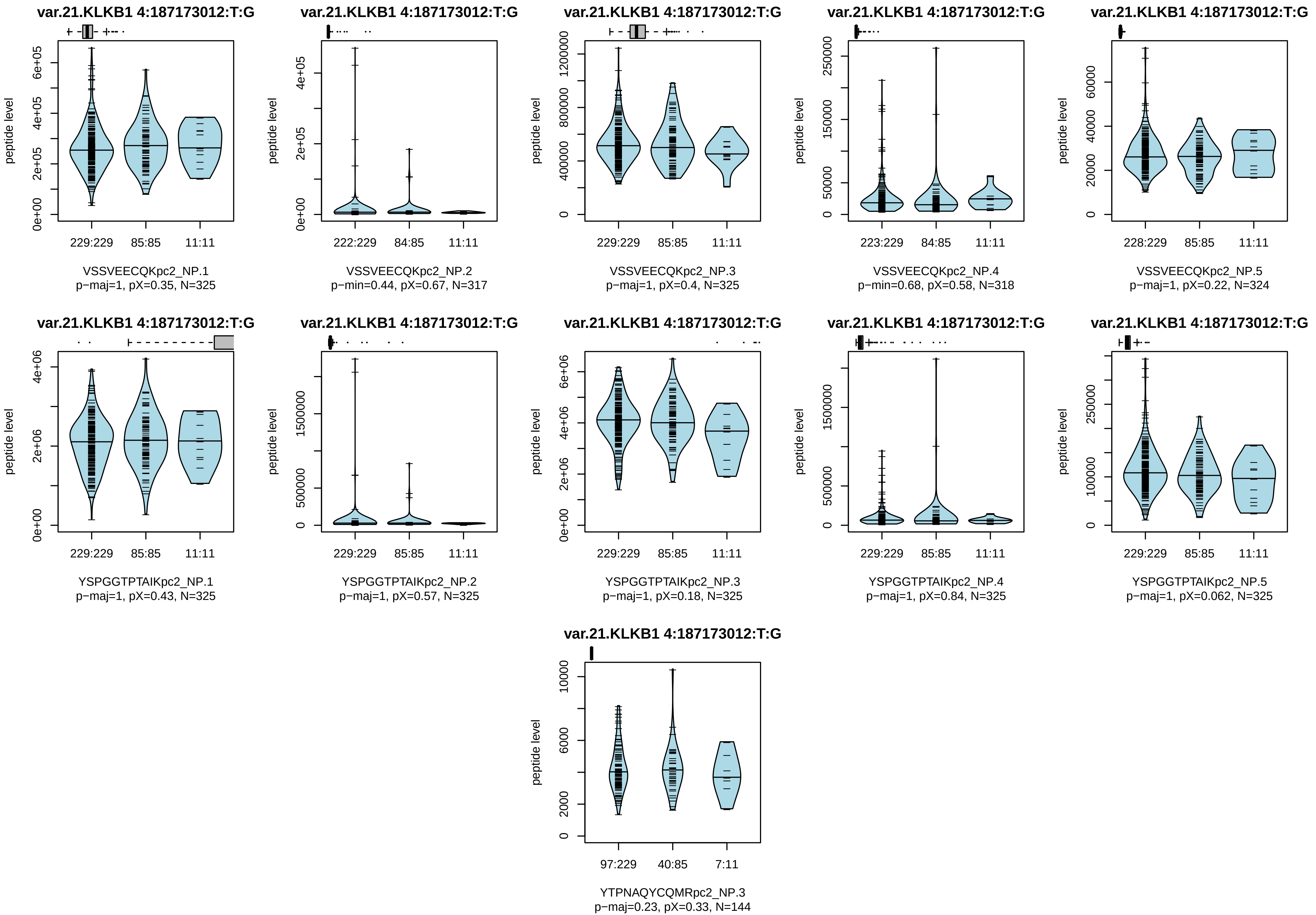
var.21.KLKB1 4:187173012:T:G
peptide level
229:229
85:85
11:11
VSSVEECQKpc2_NP.1
p−maj=1, pX=0.35, N=325
var.21.KLKB1 4:187173012:T:G
peptide level
222:229
84:85
11:11
VSSVEECQKpc2_NP.2
p−min=0.44, pX=0.67, N=317
var.21.KLKB1 4:187173012:T:G
peptide level
229:229
85:85
11:11
VSSVEECQKpc2_NP.3
p−maj=1, pX=0.4, N=325
var.21.KLKB1 4:187173012:T:G
peptide level
223:229
84:85
11:11
VSSVEECQKpc2_NP.4
p−min=0.68, pX=0.58, N=318
var.21.KLKB1 4:187173012:T:G
peptide level
228:229
85:85
11:11
VSSVEECQKpc2_NP.5
p−maj=1, pX=0.22, N=324
var.21.KLKB1 4:187173012:T:G
peptide level
229:229
85:85
11:11
YSPGGTPTAIKpc2_NP.1
p−maj=1, pX=0.43, N=325
var.21.KLKB1 4:187173012:T:G
peptide level
229:229
85:85
11:11
YSPGGTPTAIKpc2_NP.2
p−maj=1, pX=0.57, N=325
var.21.KLKB1 4:187173012:T:G
peptide level
229:229
85:85
11:11
YSPGGTPTAIKpc2_NP.3
p−maj=1, pX=0.18, N=325
var.21.KLKB1 4:187173012:T:G
peptide level
229:229
85:85
11:11
YSPGGTPTAIKpc2_NP.4
p−maj=1, pX=0.84, N=325
var.21.KLKB1 4:187173012:T:G
peptide level
229:229
85:85
11:11
YSPGGTPTAIKpc2_NP.5
p−maj=1, pX=0.062, N=325
var.21.KLKB1 4:187173012:T:G
peptide level
97:229
40:85
7:11
YTPNAQYCQMRpc2_NP.3
p−maj=0.23, pX=0.33, N=144

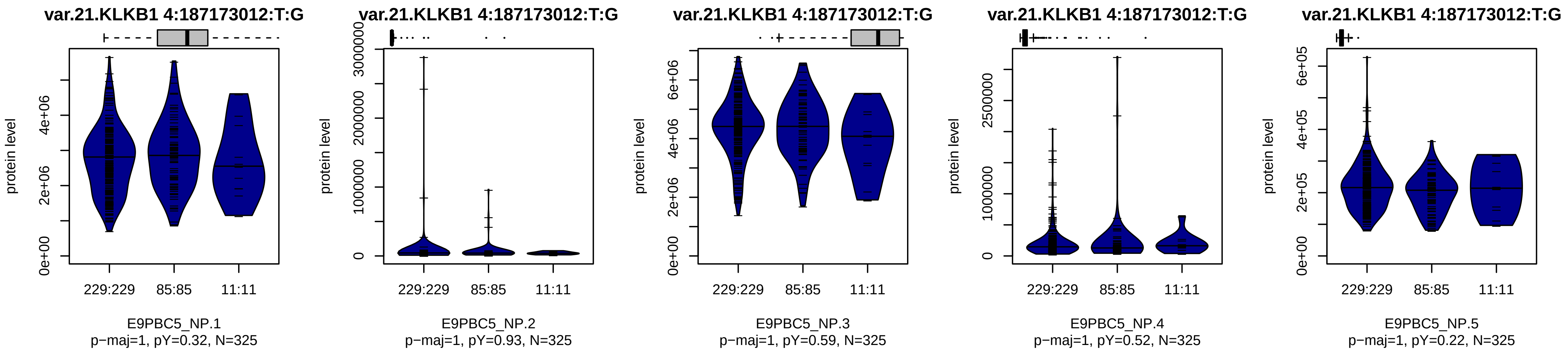
var.21.KLKB1 4:187173012:T:G
protein level
0e+00
2e+06
4e+06
229:229
85:85
11:11
E9PBC5_NP.1
p−maj=1, pY=0.32, N=325
var.21.KLKB1 4:187173012:T:G
protein level
0
1000000
2000000
3000000
229:229
85:85
11:11
E9PBC5_NP.2
p−maj=1, pY=0.93, N=325
var.21.KLKB1 4:187173012:T:G
protein level
0e+00
2e+06
4e+06
6e+06
229:229
85:85
11:11
E9PBC5_NP.3
p−maj=1, pY=0.59, N=325
var.21.KLKB1 4:187173012:T:G
protein level
0
1000000
2500000
229:229
85:85
11:11
E9PBC5_NP.4
p−maj=1, pY=0.52, N=325
var.21.KLKB1 4:187173012:T:G
protein level
0e+00
2e+05
4e+05
6e+05
229:229
85:85
11:11
E9PBC5_NP.5
p−maj=1, pY=0.22, N=325

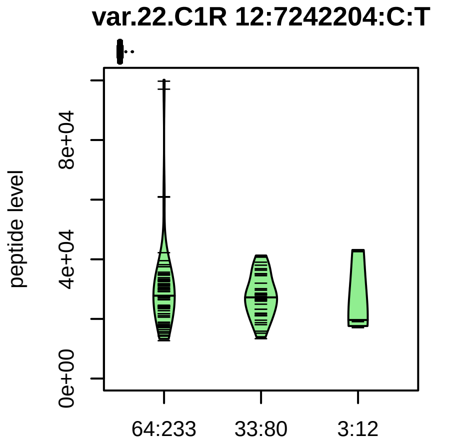

CLPVCGKpc2_NP.1
p−min=0.046, pX=0.39, N=100

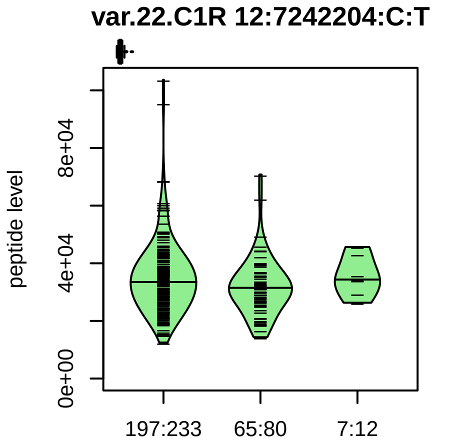

CLPVCGKpc2_NP.2
p−maj=0.038, pX=0.019, N=269

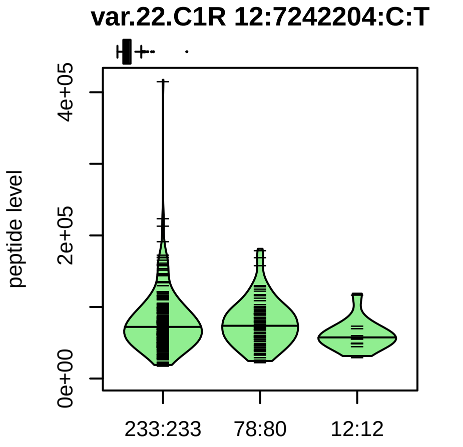

CLPVCGKpc2_NP.3
p−min=0.08, pX=0.13, N=323

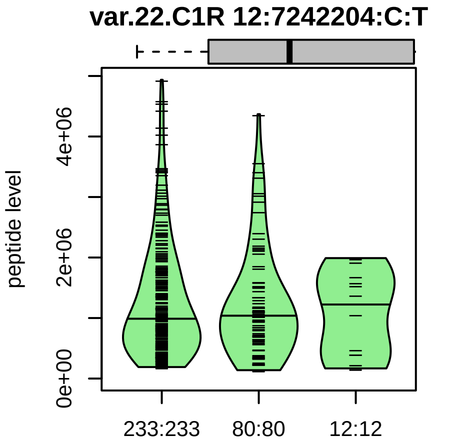

CLPVCGKpc2_NP.4
p−maj=1, pX=0.41, N=325

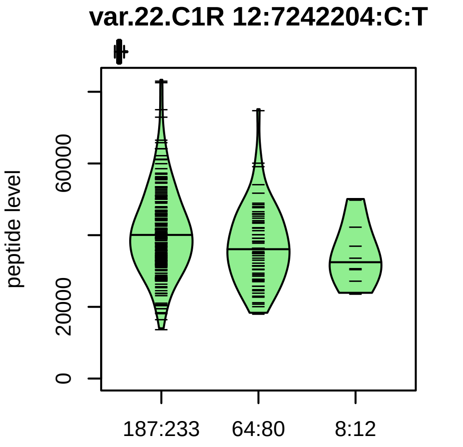

CLPVCGKpc2_NP.5
p−maj=0.27, pX=0.045, N=259

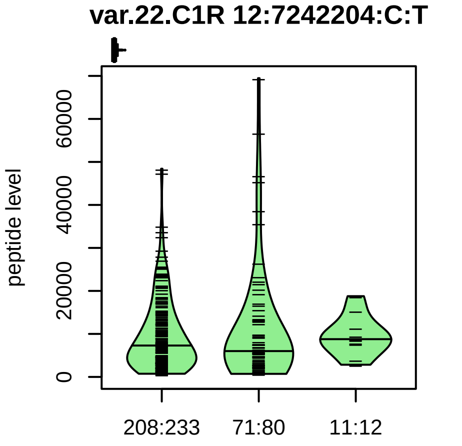

CLPVCGKPVNPVEQRpc2_NP.4
p−maj=1, pX=0.48, N=290

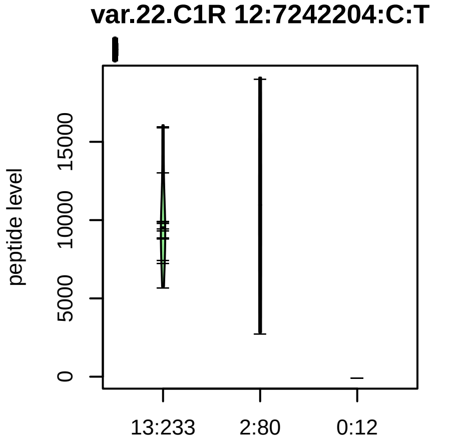

CLPVCGKPVNPVEQRpc3_NP.3
p−min=0.25, pX=0.23, N=15

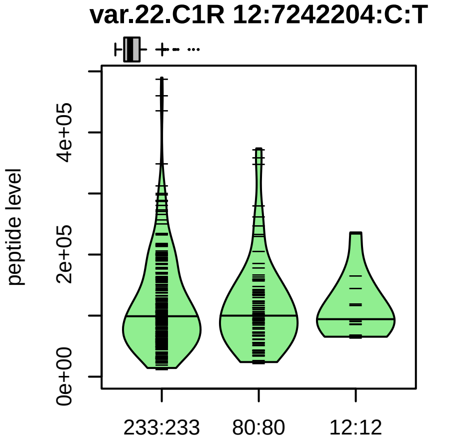

CLPVCGKPVNPVEQRpc3_NP.4
p−maj=1, pX=0.72, N=325

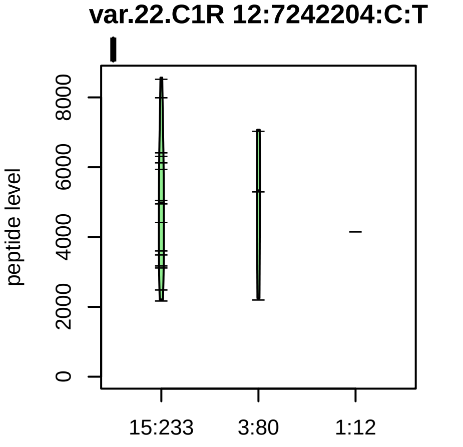

CLPVCGKPVNPVEQRpc3_NP.5
p−maj=0.52, pX=0.57, N=19

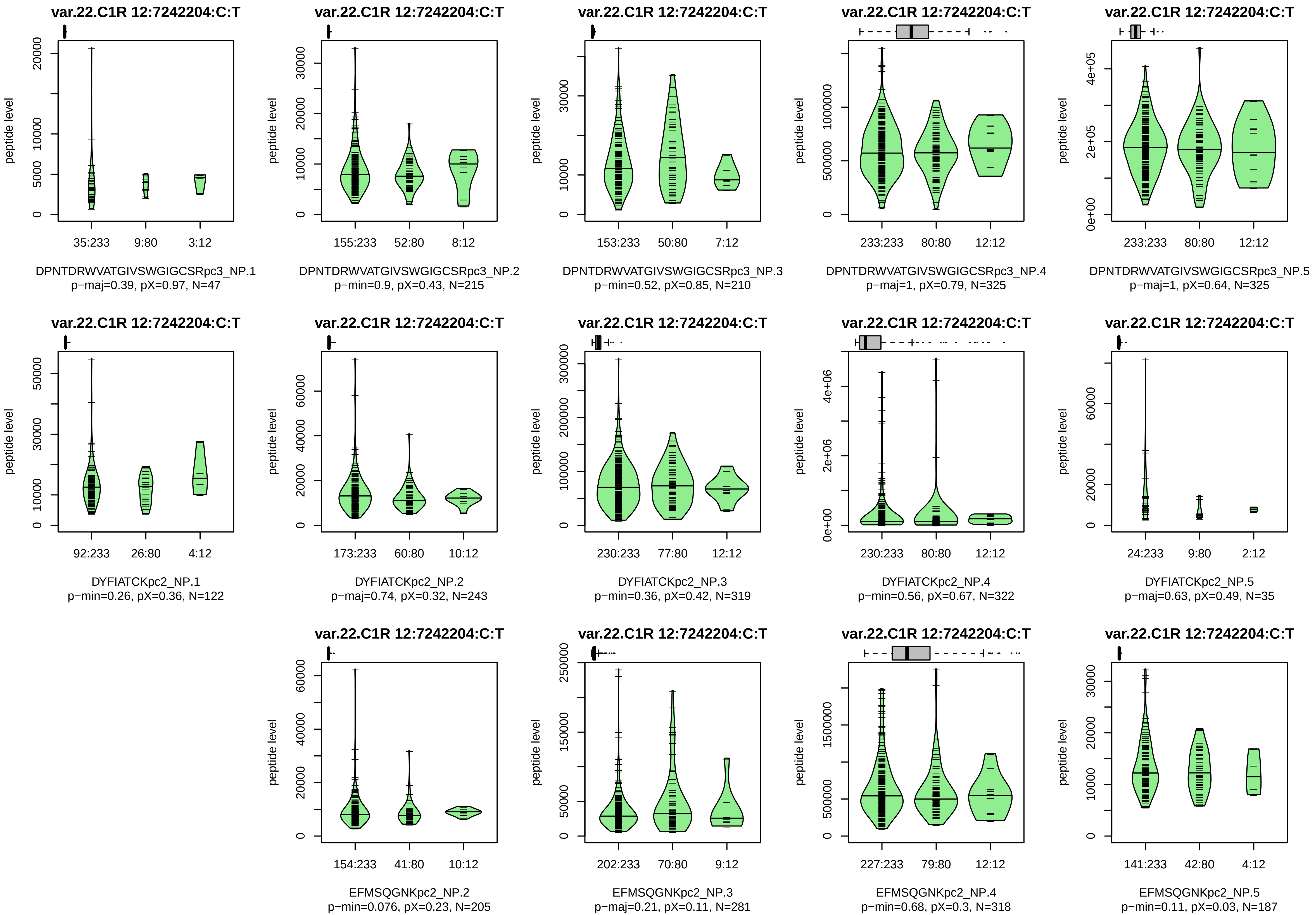
var.22.C1R 12:7242204:C:T
peptide level
35:233
9:80
3:12
DPNTDRWVATGIVSWGIGCSRpc3_NP.1
p−maj=0.39, pX=0.97, N=47
var.22.C1R 12:7242204:C:T
peptide level
155:233
52:80
8:12
DPNTDRWVATGIVSWGIGCSRpc3_NP.2
p−min=0.9, pX=0.43, N=215
var.22.C1R 12:7242204:C:T
peptide level
153:233
50:80
7:12
DPNTDRWVATGIVSWGIGCSRpc3_NP.3
p−min=0.52, pX=0.85, N=210
var.22.C1R 12:7242204:C:T
peptide level
233:233
80:80
12:12
DPNTDRWVATGIVSWGIGCSRpc3_NP.4
p−maj=1, pX=0.79, N=325
var.22.C1R 12:7242204:C:T
peptide level
233:233
80:80
12:12
DPNTDRWVATGIVSWGIGCSRpc3_NP.5
p−maj=1, pX=0.64, N=325
var.22.C1R 12:7242204:C:T
peptide level
92:233
26:80
4:12
DYFIATCKpc2_NP.1
p−min=0.26, pX=0.36, N=122
var.22.C1R 12:7242204:C:T
peptide level
173:233
60:80
10:12
DYFIATCKpc2_NP.2
p−maj=0.74, pX=0.32, N=243
var.22.C1R 12:7242204:C:T
peptide level
230:233
77:80
12:12
DYFIATCKpc2_NP.3
p−min=0.36, pX=0.42, N=319
var.22.C1R 12:7242204:C:T
peptide level
230:233
80:80
12:12
DYFIATCKpc2_NP.4
p−min=0.56, pX=0.67, N=322
var.22.C1R 12:7242204:C:T
peptide level
24:233
9:80
2:12
DYFIATCKpc2_NP.5
p−maj=0.63, pX=0.49, N=35
var.22.C1R 12:7242204:C:T
peptide level
154:233
41:80
10:12
EFMSQGNKpc2_NP.2
p−min=0.076, pX=0.23, N=205
var.22.C1R 12:7242204:C:T
peptide level
202:233
70:80
9:12
EFMSQGNKpc2_NP.3
p−maj=0.21, pX=0.11, N=281
var.22.C1R 12:7242204:C:T
peptide level
227:233
79:80
12:12
EFMSQGNKpc2_NP.4
p−min=0.68, pX=0.3, N=318
var.22.C1R 12:7242204:C:T
peptide level
141:233
42:80
4:12
EFMSQGNKpc2_NP.5
p−min=0.11, pX=0.03, N=187

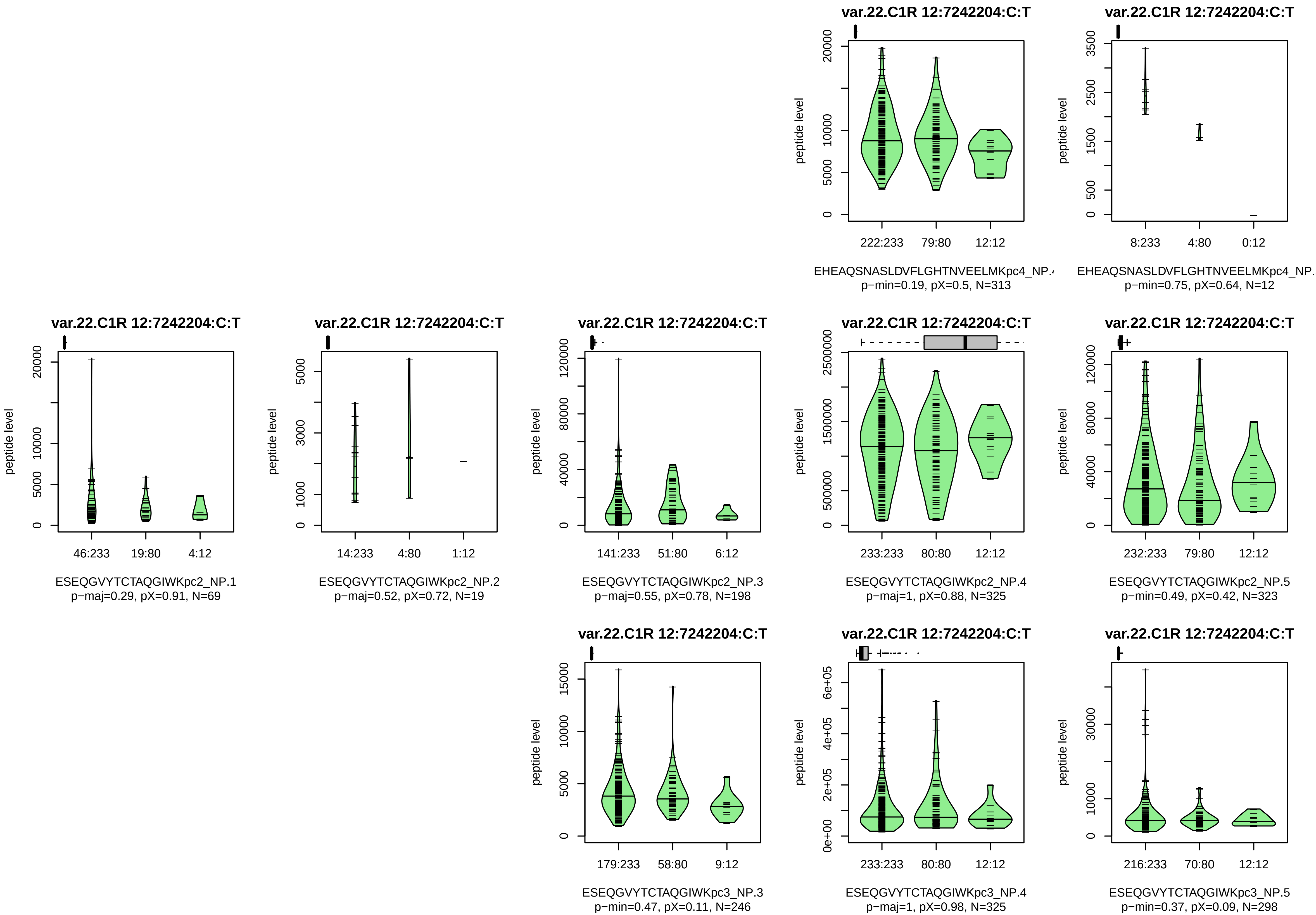
var.22.C1R 12:7242204:C:T
peptide level
222:233
79:80
12:12
EHEAQSNASLDVFLGHTNVEELMKpc4_NP.4
p−min=0.19, pX=0.5, N=313
var.22.C1R 12:7242204:C:T
peptide level
8:233
4:80
0:12
EHEAQSNASLDVFLGHTNVEELMKpc4_NP.
p−min=0.75, pX=0.64, N=12
var.22.C1R 12:7242204:C:T
peptide level
46:233
19:80
4:12
ESEQGVYTCTAQGIWKpc2_NP.1
p−maj=0.29, pX=0.91, N=69
var.22.C1R 12:7242204:C:T
peptide level
14:233
4:80
1:12
ESEQGVYTCTAQGIWKpc2_NP.2
p−maj=0.52, pX=0.72, N=19
var.22.C1R 12:7242204:C:T
peptide level
141:233
51:80
6:12
ESEQGVYTCTAQGIWKpc2_NP.3
p−maj=0.55, pX=0.78, N=198
var.22.C1R 12:7242204:C:T
peptide level
233:233
80:80
12:12
ESEQGVYTCTAQGIWKpc2_NP.4
p−maj=1, pX=0.88, N=325
var.22.C1R 12:7242204:C:T
peptide level
232:233
79:80
12:12
ESEQGVYTCTAQGIWKpc2_NP.5
p−min=0.49, pX=0.42, N=323
var.22.C1R 12:7242204:C:T
peptide level
179:233
58:80
9:12
ESEQGVYTCTAQGIWKpc3_NP.3
p−min=0.47, pX=0.11, N=246
var.22.C1R 12:7242204:C:T
peptide level
233:233
80:80
12:12
ESEQGVYTCTAQGIWKpc3_NP.4
p−maj=1, pX=0.98, N=325
var.22.C1R 12:7242204:C:T
peptide level
216:233
70:80
12:12
ESEQGVYTCTAQGIWKpc3_NP.5
p−min=0.37, pX=0.09, N=298

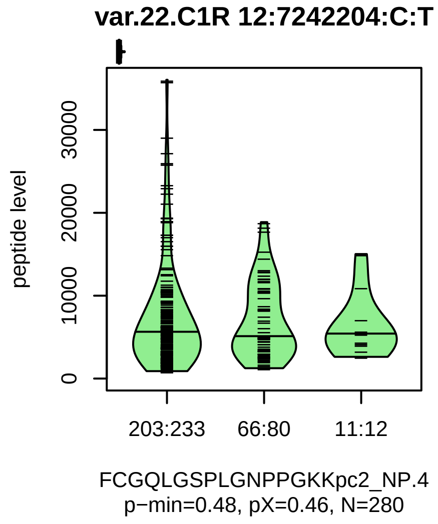
var.22.C1R 12:7242204:C:T
peptide level
0
10000
20000
30000
203:233
66:80
11:12
FCGQLGSPLGNPPGKKpc2_NP.4
p−min=0.48, pX=0.46, N=280

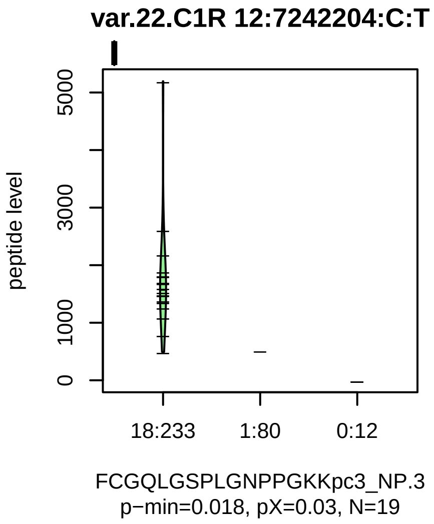
var.22.C1R 12:7242204:C:T
peptide level
0
1000
3000
5000
18:233
1:80
0:12
FCGQLGSPLGNPPGKKpc3_NP.3
p−min=0.018, pX=0.03, N=19

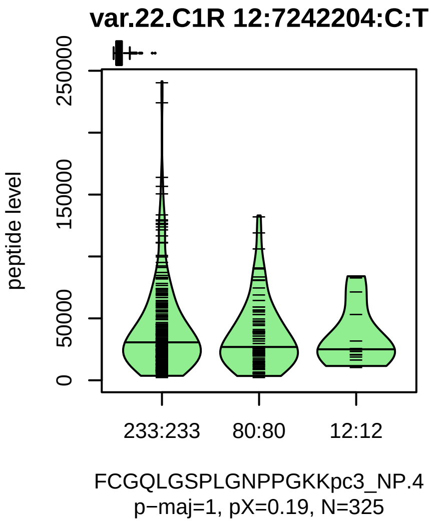
var.22.C1R 12:7242204:C:T
peptide level
0
50000
150000
250000
233:233
80:80
12:12
FCGQLGSPLGNPPGKKpc3_NP.4
p−maj=1, pX=0.19, N=325

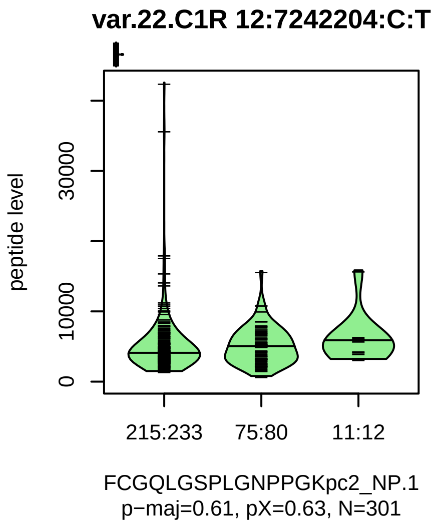
var.22.C1R 12:7242204:C:T
peptide level
0
10000
30000
215:233
75:80
11:12
FCGQLGSPLGNPPGKpc2_NP.1
p−maj=0.61, pX=0.63, N=301

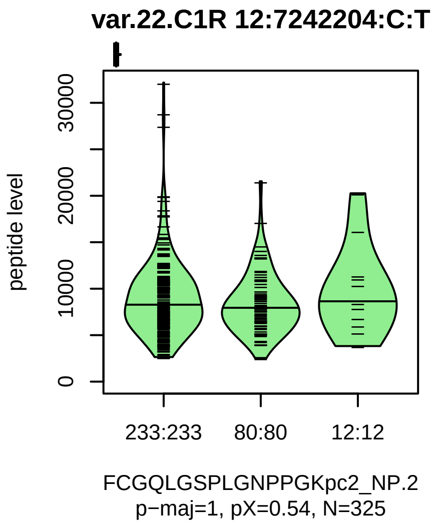
var.22.C1R 12:7242204:C:T
peptide level
0
10000
20000
30000
233:233
80:80
12:12
FCGQLGSPLGNPPGKpc2_NP.2
p−maj=1, pX=0.54, N=325

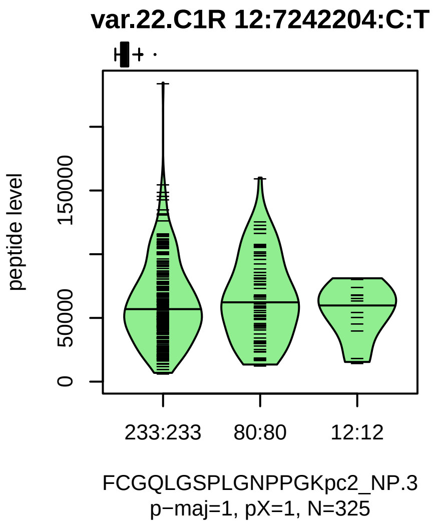
var.22.C1R 12:7242204:C:T
peptide level
0
50000
150000
233:233
80:80
12:12
FCGQLGSPLGNPPGKpc2_NP.3
p−maj=1, pX=1, N=325

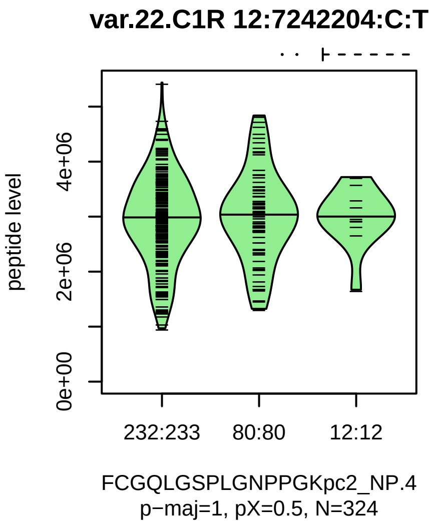
var.22.C1R 12:7242204:C:T
peptide level
0e+00
2e+06
4e+06
232:233
80:80
12:12
FCGQLGSPLGNPPGKpc2_NP.4
p−maj=1, pX=0.5, N=324

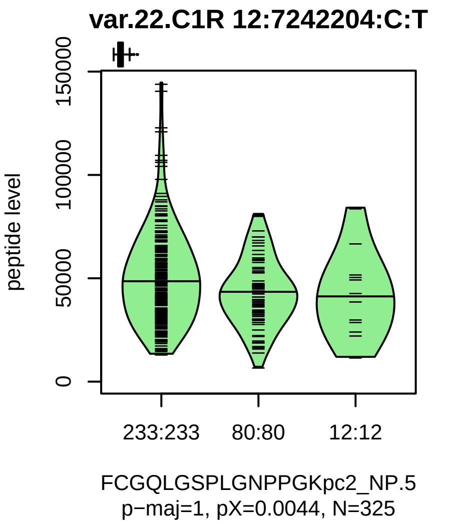
var.22.C1R 12:7242204:C:T
peptide level
0
50000
100000
150000
233:233
80:80
12:12
FCGQLGSPLGNPPGKpc2_NP.5
p−maj=1, pX=0.0044, N=325

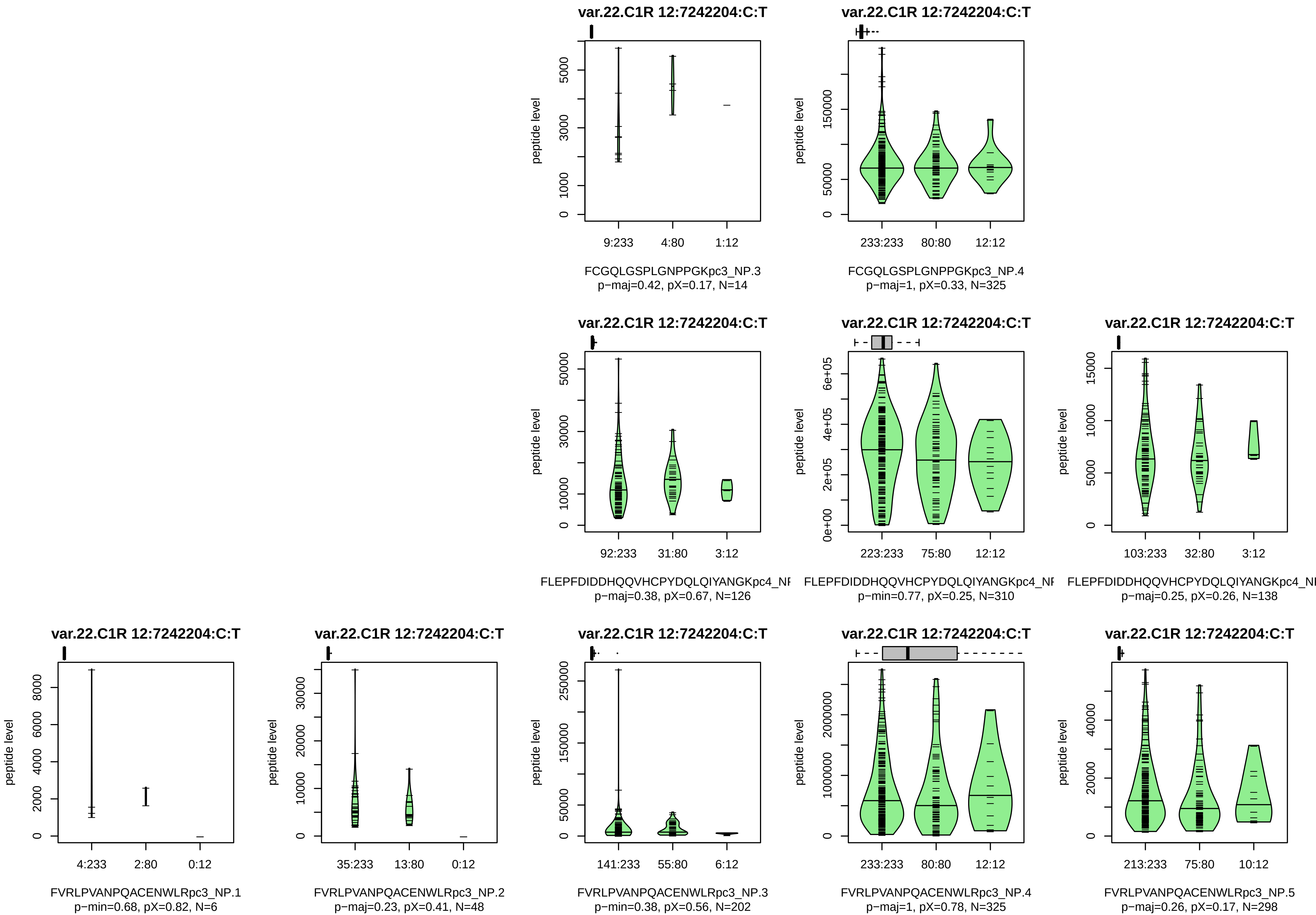
var.22.C1R 12:7242204:C:T
peptide level
9:233
4:80
1:12
FCGQLGSPLGNPPGKpc3_NP.3
p−maj=0.42, pX=0.17, N=14
var.22.C1R 12:7242204:C:T
peptide level
233:233
80:80
12:12
FCGQLGSPLGNPPGKpc3_NP.4
p−maj=1, pX=0.33, N=325
var.22.C1R 12:7242204:C:T
peptide level
92:233
31:80
3:12
FLEPFDIDDHQQVHCPYDQLQIYANGKpc4_NI
p−maj=0.38, pX=0.67, N=126
var.22.C1R 12:7242204:C:T
peptide level
223:233
75:80
12:12
FLEPFDIDDHQQVHCPYDQLQIYANGKpc4_NI
p−min=0.77, pX=0.25, N=310
var.22.C1R 12:7242204:C:T
peptide level
103:233
32:80
3:12
FLEPFDIDDHQQVHCPYDQLQIYANGKpc4_NI
p−maj=0.25, pX=0.26, N=138
var.22.C1R 12:7242204:C:T
peptide level
4:233
2:80
0:12
FVRLPVANPQACENWLRpc3_NP.1
p−min=0.68, pX=0.82, N=6
var.22.C1R 12:7242204:C:T
peptide level
35:233
13:80
0:12
FVRLPVANPQACENWLRpc3_NP.2
p−maj=0.23, pX=0.41, N=48
var.22.C1R 12:7242204:C:T
peptide level
141:233
55:80
6:12
FVRLPVANPQACENWLRpc3_NP.3
p−min=0.38, pX=0.56, N=202
var.22.C1R 12:7242204:C:T
peptide level
233:233
80:80
12:12
FVRLPVANPQACENWLRpc3_NP.4
p−maj=1, pX=0.78, N=325
var.22.C1R 12:7242204:C:T
peptide level
213:233
75:80
10:12
FVRLPVANPQACENWLRpc3_NP.5
p−maj=0.26, pX=0.17, N=298

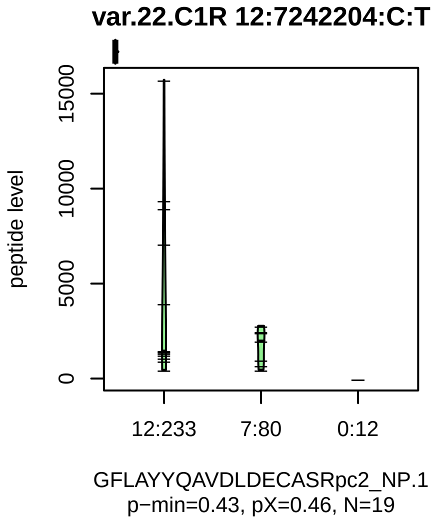
var.22.C1R 12:7242204:C:T
peptide level
0
5000
10000
15000
12:233
7:80
0:12
GFLAYYQAVDLDECASRpc2_NP.1
p−min=0.43, pX=0.46, N=19

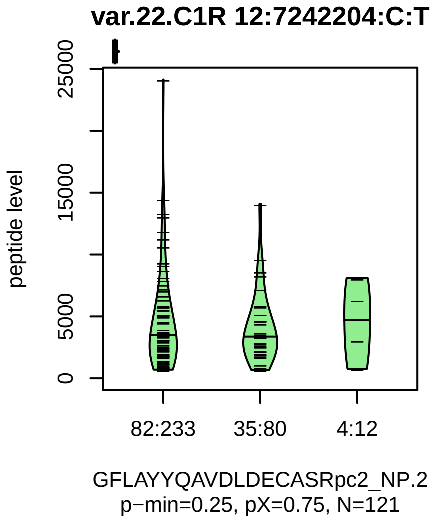
var.22.C1R 12:7242204:C:T
peptide level
0
5000
15000
25000
82:233
35:80
4:12
GFLAYYQAVDLDECASRpc2_NP.2
p−min=0.25, pX=0.75, N=121

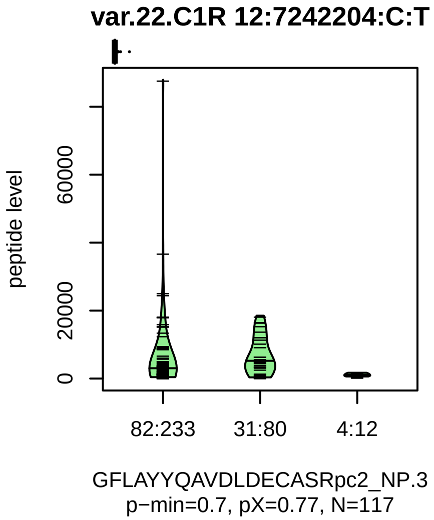
var.22.C1R 12:7242204:C:T
peptide level
0
20000
60000
82:233
31:80
4:12
GFLAYYQAVDLDECASRpc2_NP.3
p−min=0.7, pX=0.77, N=117

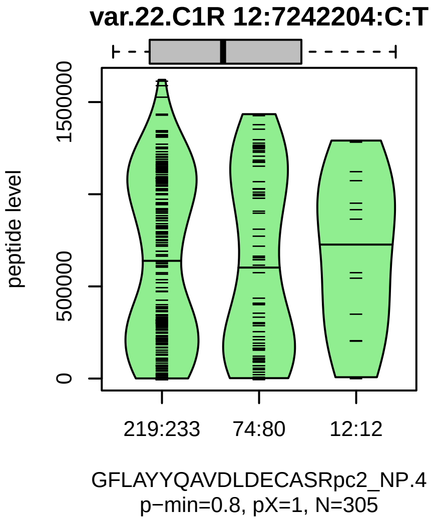
var.22.C1R 12:7242204:C:T
peptide level
0
500000
1500000
219:233
74:80
12:12
GFLAYYQAVDLDECASRpc2_NP.4
p−min=0.8, pX=1, N=305

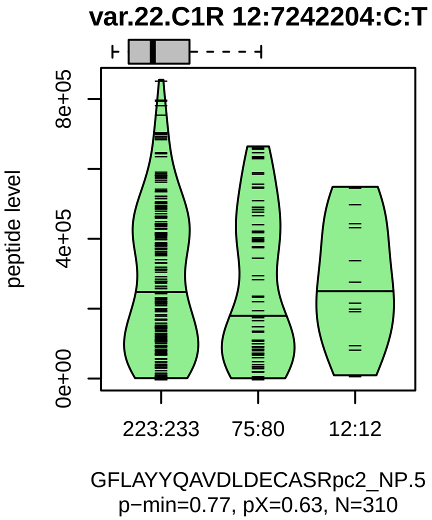
var.22.C1R 12:7242204:C:T
peptide level
0e+00
4e+05
8e+05
223:233
75:80
12:12
GFLAYYQAVDLDECASRpc2_NP.5
p−min=0.77, pX=0.63, N=310

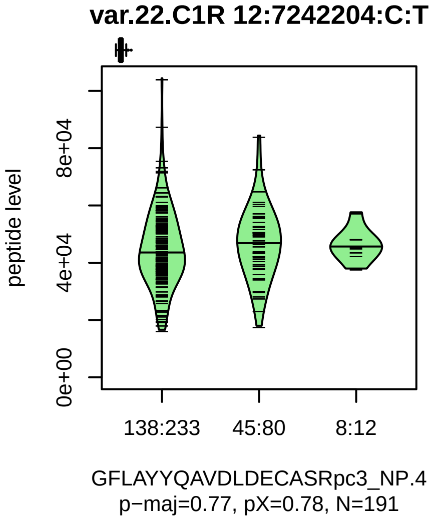
var.22.C1R 12:7242204:C:T
peptide level
0e+00
4e+04
8e+04
138:233
45:80
8:12
GFLAYYQAVDLDECASRpc3_NP.4
p−maj=0.77, pX=0.78, N=191

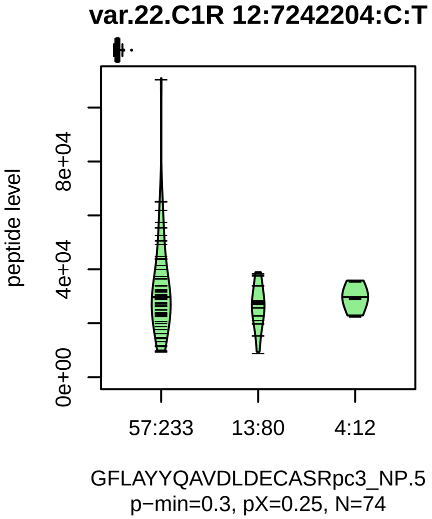
var.22.C1R 12:7242204:C:T
peptide level
0e+00
4e+04
8e+04
57:233
13:80
4:12
GFLAYYQAVDLDECASRpc3_NP.5
p−min=0.3, pX=0.25, N=74

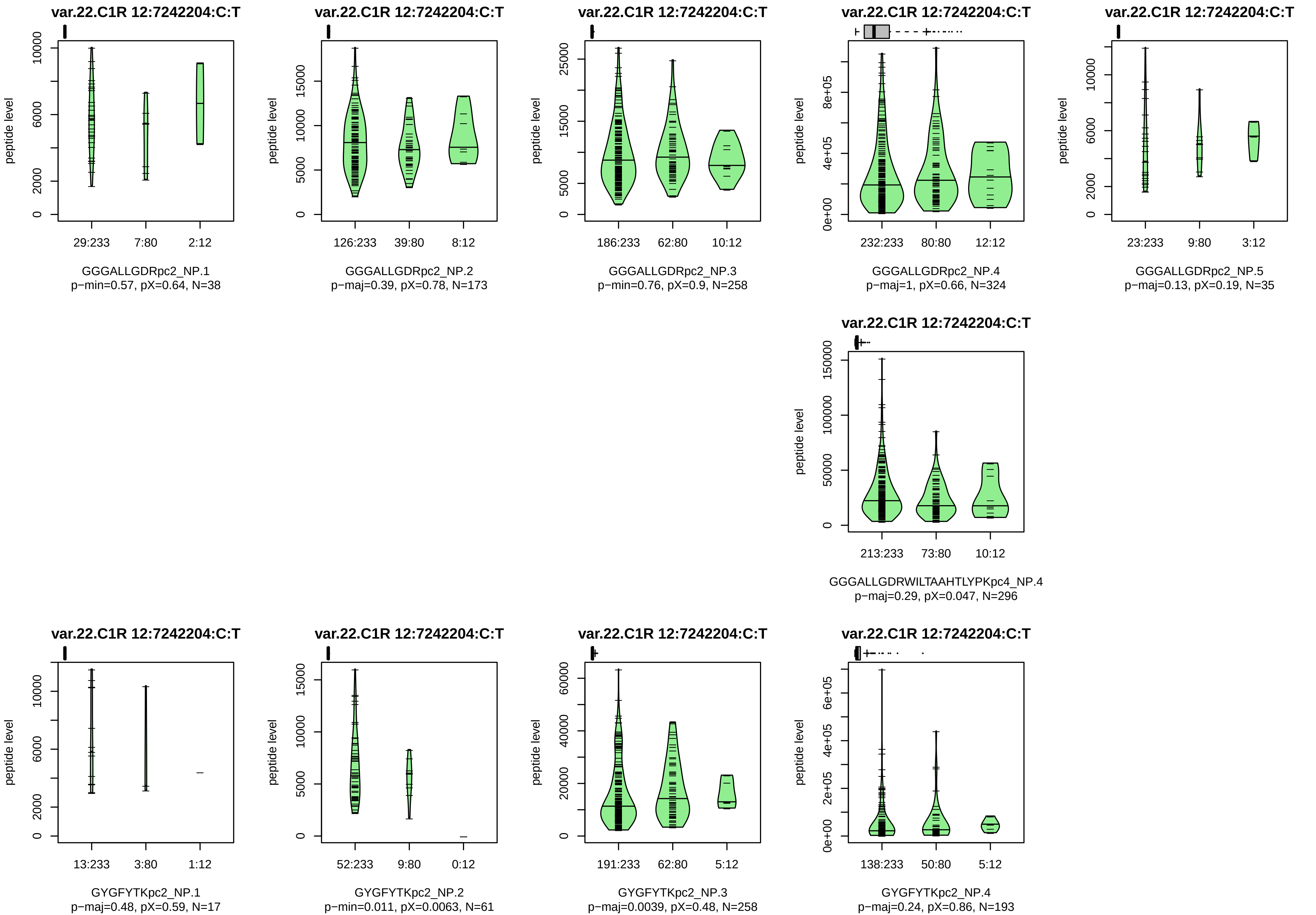

var.22.C1R 12:7242204:C:T
peptide level
29:233
7:80
2:12
GGGALLGDRpc2_NP.1
p−min=0.57, pX=0.64, N=38
var.22.C1R 12:7242204:C:T
126:233
39:80
8:12
GGGALLGDRpc2_NP.2
p−maj=0.39, pX=0.78, N=173
var.22.C1R 12:7242204:C:T
186:233
62:80
10:12
GGGALLGDRpc2_NP.3
p−min=0.76, pX=0.9, N=258
var.22.C1R 12:7242204:C:T
232:233
80:80
12:12
GGGALLGDRpc2_NP.4
p−maj=1, pX=0.66, N=324
var.22.C1R 12:7242204:C:T
23:233
9:80
3:12
GGGALLGDRpc2_NP.5
p−maj=0.13, pX=0.19, N=35
var.22.C1R 12:7242204:C:T
213:233
73:80
10:12
GGGALLGDRWILTAAHTLYPKpc4_NP.4
p−maj=0.29, pX=0.047, N=296
var.22.C1R 12:7242204:C:T
13:233
3:80
1:12
GYGFYTKpc2_NP.1
p−maj=0.48, pX=0.59, N=17
var.22.C1R 12:7242204:C:T
52:233
9:80
0:12
GYGFYTKpc2_NP.2
p−min=0.011, pX=0.0063, N=61
var.22.C1R 12:7242204:C:T
191:233
62:80
5:12
GYGFYTKpc2_NP.3
p−maj=0.0039, pX=0.48, N=258
var.22.C1R 12:7242204:C:T
138:233
50:80
5:12
GYGFYTKpc2_NP.4
p−maj=0.24, pX=0.86, N=193

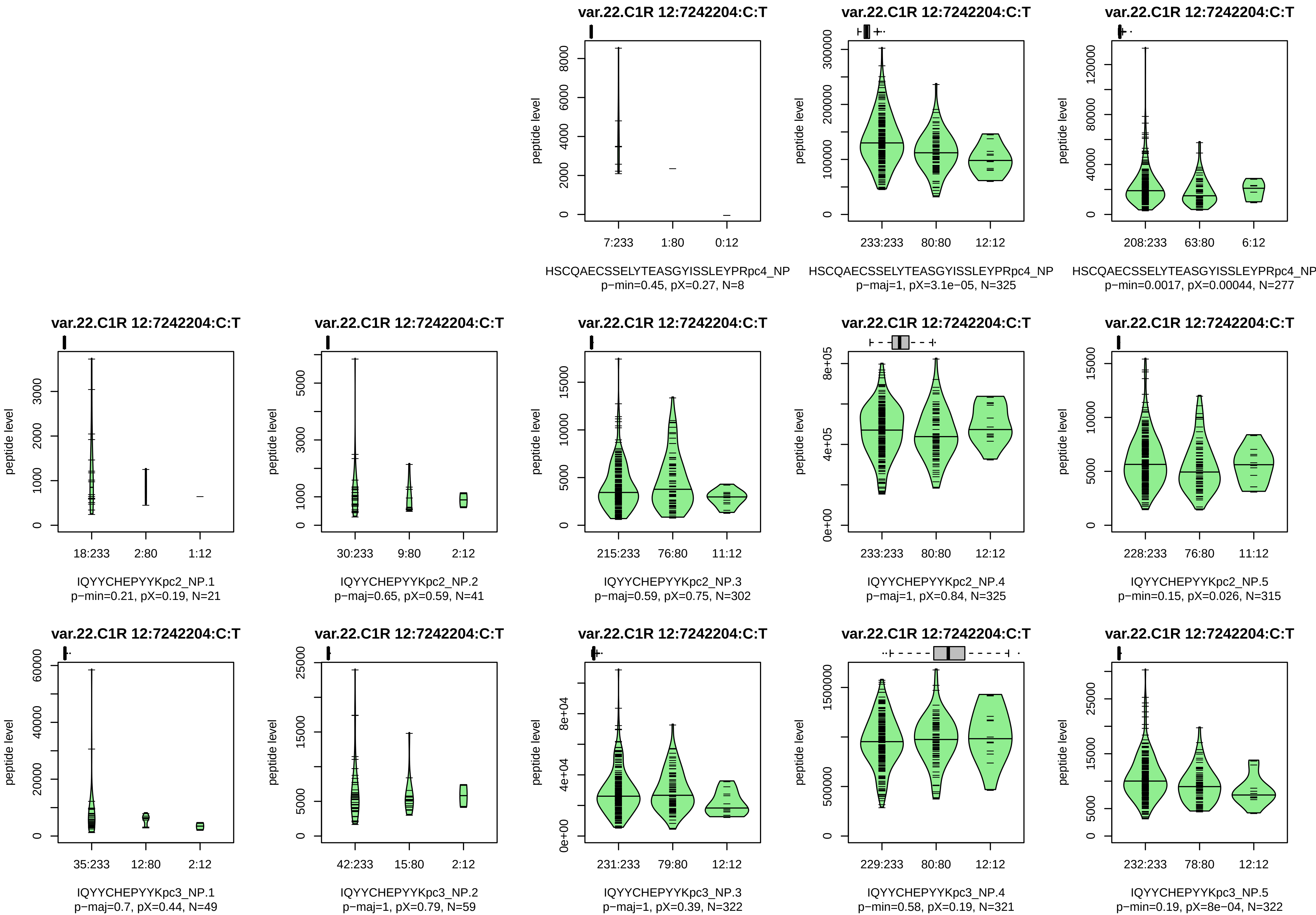
var.22.C1R 12:7242204:C:T
peptide level
7:233
1:80
0:12
HSCQAECSSELYTEASGYISSLEYPRpc4_NP
p−min=0.45, pX=0.27, N=8
var.22.C1R 12:7242204:C:T
peptide level
233:233
80:80
12:12
HSCQAECSSELYTEASGYISSLEYPRpc4_NP
p−maj=1, pX=3.1e−05, N=325
var.22.C1R 12:7242204:C:T
peptide level
208:233
63:80
6:12
HSCQAECSSELYTEASGYISSLEYPRpc4_NP
p−min=0.0017, pX=0.00044, N=277
var.22.C1R 12:7242204:C:T
peptide level
18:233
2:80
1:12
IQYYCHEPYYKpc2_NP.1
p−min=0.21, pX=0.19, N=21
var.22.C1R 12:7242204:C:T
peptide level
30:233
9:80
2:12
IQYYCHEPYYKpc2_NP.2
p−maj=0.65, pX=0.59, N=41
var.22.C1R 12:7242204:C:T
peptide level
215:233
76:80
11:12
IQYYCHEPYYKpc2_NP.3
p−maj=0.59, pX=0.75, N=302
var.22.C1R 12:7242204:C:T
peptide level
233:233
80:80
12:12
IQYYCHEPYYKpc2_NP.4
p−maj=1, pX=0.84, N=325
var.22.C1R 12:7242204:C:T
peptide level
228:233
76:80
11:12
IQYYCHEPYYKpc2_NP.5
p−min=0.15, pX=0.026, N=315
var.22.C1R 12:7242204:C:T
peptide level
35:233
12:80
2:12
IQYYCHEPYYKpc3_NP.1
p−maj=0.7, pX=0.44, N=49
var.22.C1R 12:7242204:C:T
peptide level
42:233
15:80
2:12
IQYYCHEPYYKpc3_NP.2
p−maj=1, pX=0.79, N=59
var.22.C1R 12:7242204:C:T
peptide level
231:233
79:80
12:12
IQYYCHEPYYKpc3_NP.3
p−maj=1, pX=0.39, N=322
var.22.C1R 12:7242204:C:T
peptide level
229:233
80:80
12:12
IQYYCHEPYYKpc3_NP.4
p−min=0.58, pX=0.19, N=321
var.22.C1R 12:7242204:C:T
peptide level
232:233
78:80
12:12
IQYYCHEPYYKpc3_NP.5
p−min=0.19, pX=8e−04, N=322

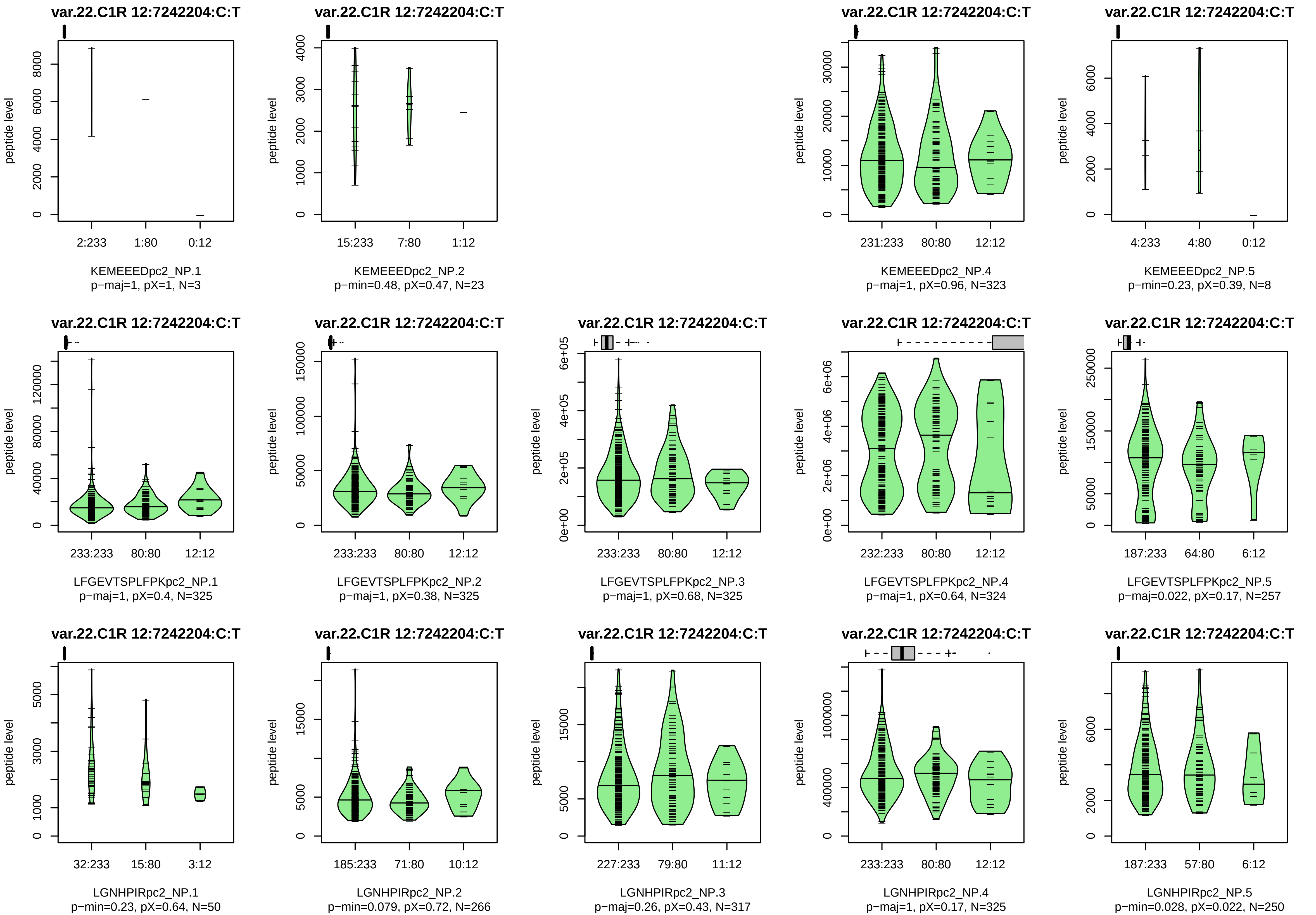
var.22.C1R 12:7242204:C:T
peptide level
2:233
1:80
0:12
KEMEEEDpc2_NP.1
p−maj=1, pX=1, N=3
var.22.C1R 12:7242204:C:T
peptide level
15:233
7:80
1:12
KEMEEEDpc2_NP.2
p−min=0.48, pX=0.47, N=23
var.22.C1R 12:7242204:C:T
peptide level
231:233
80:80
12:12
KEMEEEDpc2_NP.4
p−maj=1, pX=0.96, N=323
var.22.C1R 12:7242204:C:T
peptide level
4:233
4:80
0:12
KEMEEEDpc2_NP.5
p−min=0.23, pX=0.39, N=8
var.22.C1R 12:7242204:C:T
peptide level
233:233
80:80
12:12
LFGEVTSPLFPKpc2_NP.1
p−maj=1, pX=0.4, N=325
var.22.C1R 12:7242204:C:T
peptide level
233:233
80:80
12:12
LFGEVTSPLFPKpc2_NP.2
p−maj=1, pX=0.38, N=325
var.22.C1R 12:7242204:C:T
peptide level
233:233
80:80
12:12
LFGEVTSPLFPKpc2_NP.3
p−maj=1, pX=0.68, N=325
var.22.C1R 12:7242204:C:T
peptide level
232:233
80:80
12:12
LFGEVTSPLFPKpc2_NP.4
p−maj=1, pX=0.64, N=324
var.22.C1R 12:7242204:C:T
peptide level
187:233
64:80
6:12
LFGEVTSPLFPKpc2_NP.5
p−maj=0.022, pX=0.17, N=257
var.22.C1R 12:7242204:C:T
peptide level
32:233
15:80
3:12
LGNHPIRpc2_NP.1
p−min=0.23, pX=0.64, N=50
var.22.C1R 12:7242204:C:T
peptide level
185:233
71:80
10:12
LGNHPIRpc2_NP.2
p−min=0.079, pX=0.72, N=266
var.22.C1R 12:7242204:C:T
peptide level
227:233
79:80
11:12
LGNHPIRpc2_NP.3
p−maj=0.26, pX=0.43, N=317
var.22.C1R 12:7242204:C:T
peptide level
233:233
80:80
12:12
LGNHPIRpc2_NP.4
p−maj=1, pX=0.17, N=325
var.22.C1R 12:7242204:C:T
peptide level
187:233
57:80
6:12
LGNHPIRpc2_NP.5
p−min=0.028, pX=0.022, N=250

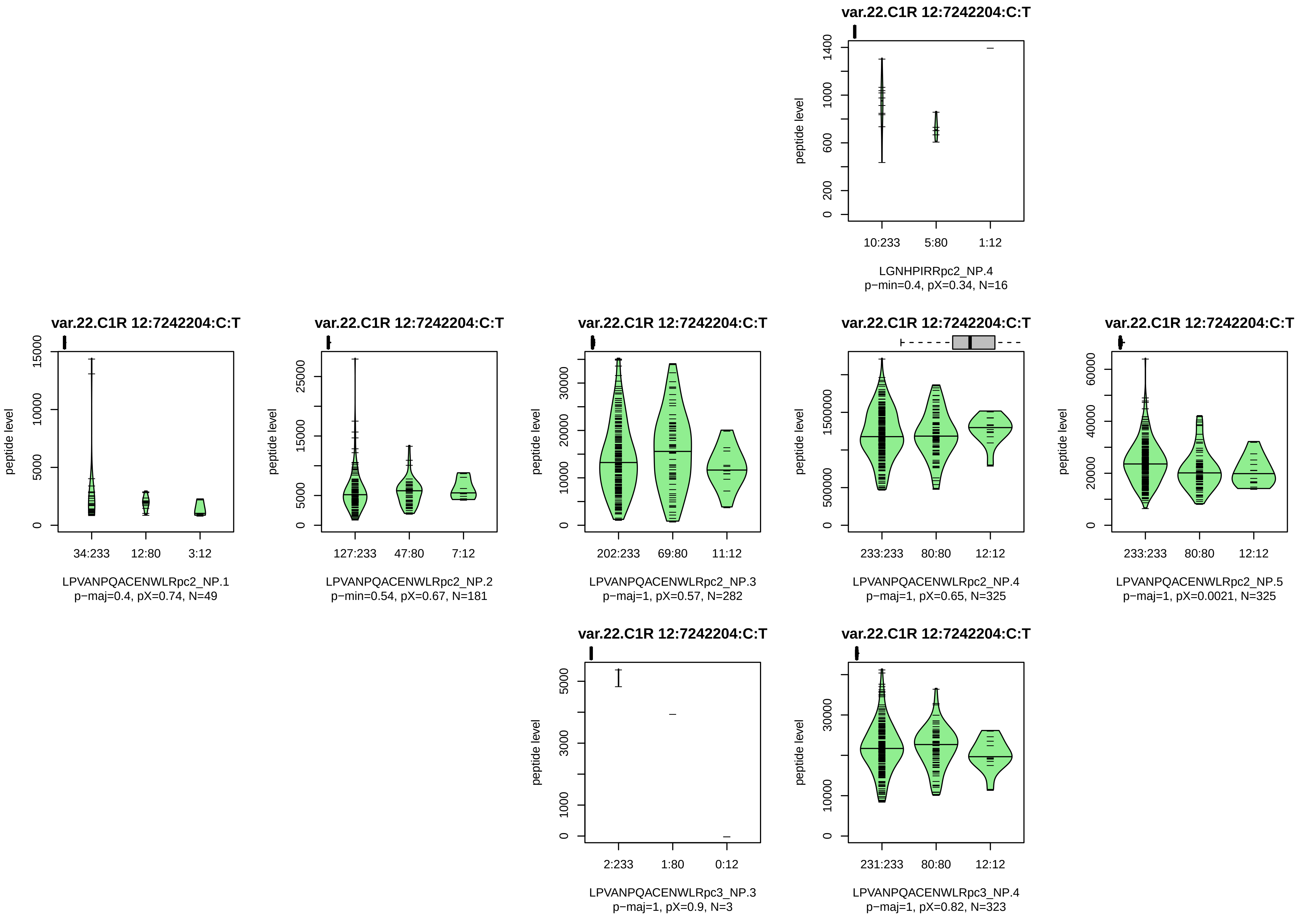
var.22.C1R 12:7242204:C:T
peptide level
10:233
5:80
1:12
LGNHPIRRpc2_NP.4
p−min=0.4, pX=0.34, N=16
var.22.C1R 12:7242204:C:T
34:233
12:80
3:12
LPVANPQACENWLRpc2_NP.1
p−maj=0.4, pX=0.74, N=49
var.22.C1R 12:7242204:C:T
127:233
47:80
7:12
LPVANPQACENWLRpc2_NP.2
p−min=0.54, pX=0.67, N=181
var.22.C1R 12:7242204:C:T
202:233
69:80
11:12
LPVANPQACENWLRpc2_NP.3
p−maj=1, pX=0.57, N=282
var.22.C1R 12:7242204:C:T
233:233
80:80
12:12
LPVANPQACENWLRpc2_NP.4
p−maj=1, pX=0.65, N=325
var.22.C1R 12:7242204:C:T
233:233
80:80
12:12
LPVANPQACENWLRpc2_NP.5
p−maj=1, pX=0.0021, N=325
var.22.C1R 12:7242204:C:T
2:233
1:80
0:12
LPVANPQACENWLRpc3_NP.3
p−maj=1, pX=0.9, N=3
var.22.C1R 12:7242204:C:T
231:233
80:80
12:12
LPVANPQACENWLRpc3_NP.4
p−maj=1, pX=0.82, N=323

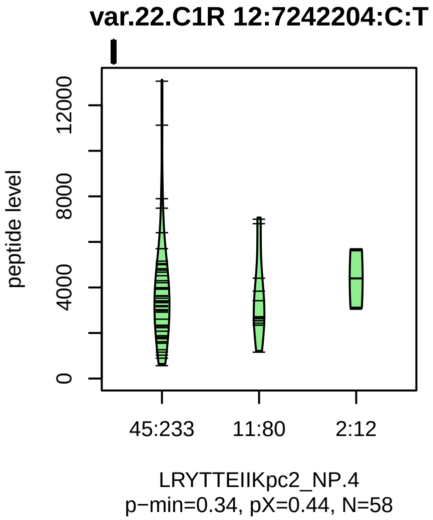
var.22.C1R 12:7242204:C:T
peptide level
0
4000
8000
12000
45:233
11:80
2:12
LRYTTEIIKpc2_NP.4
p−min=0.34, pX=0.44, N=58

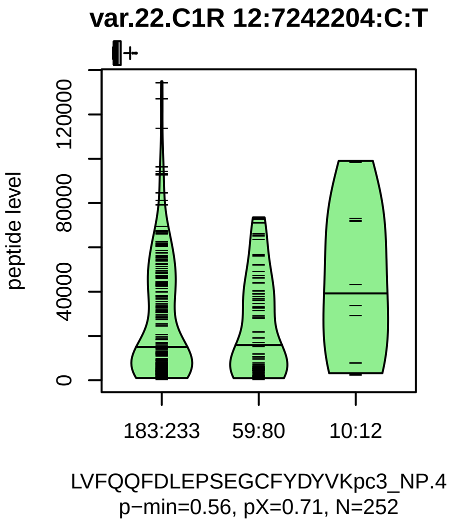
var.22.C1R 12:7242204:C:T
peptide level
0
40000
80000
120000
183:233
59:80
10:12
LVFQQFDLEPSEGCFYDYVKpc3_NP.4
p−min=0.56, pX=0.71, N=252

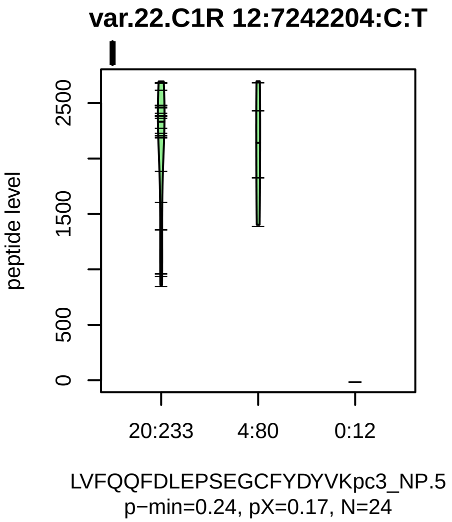
var.22.C1R 12:7242204:C:T
peptide level
0
500
1500
2500
20:233
4:80
0:12
LVFQQFDLEPSEGCFYDYVKpc3_NP.5
p−min=0.24, pX=0.17, N=24

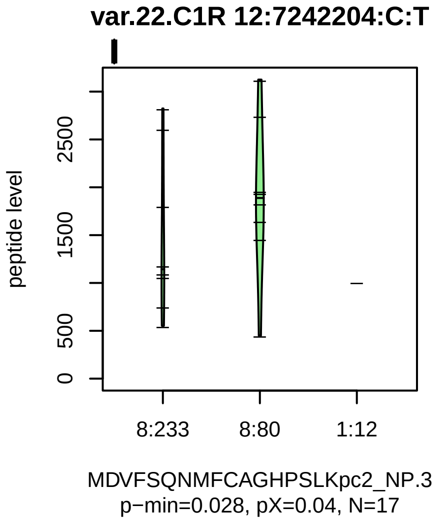
var.22.C1R 12:7242204:C:T
peptide level
0
500
1500
2500
8:233
8:80
1:12
MDVFSQNMFCAGHPSLKpc2_NP.3
p−min=0.028, pX=0.04, N=17

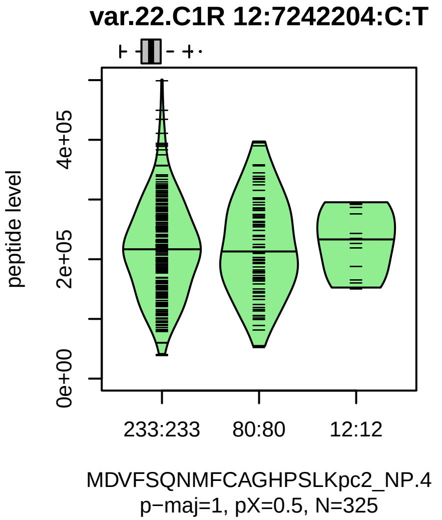
var.22.C1R 12:7242204:C:T
peptide level
0e+00
2e+05
4e+05
233:233
80:80
12:12
MDVFSQNMFCAGHPSLKpc2_NP.4
p−maj=1, pX=0.5, N=325

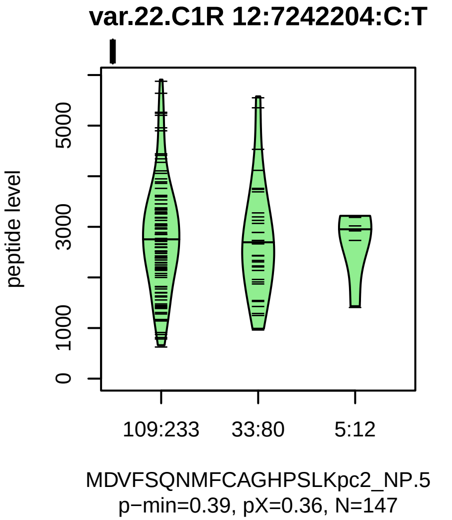
var.22.C1R 12:7242204:C:T
peptide level
0
1000
3000
5000
109:233
33:80
5:12
MDVFSQNMFCAGHPSLKpc2_NP.5
p−min=0.39, pX=0.36, N=147

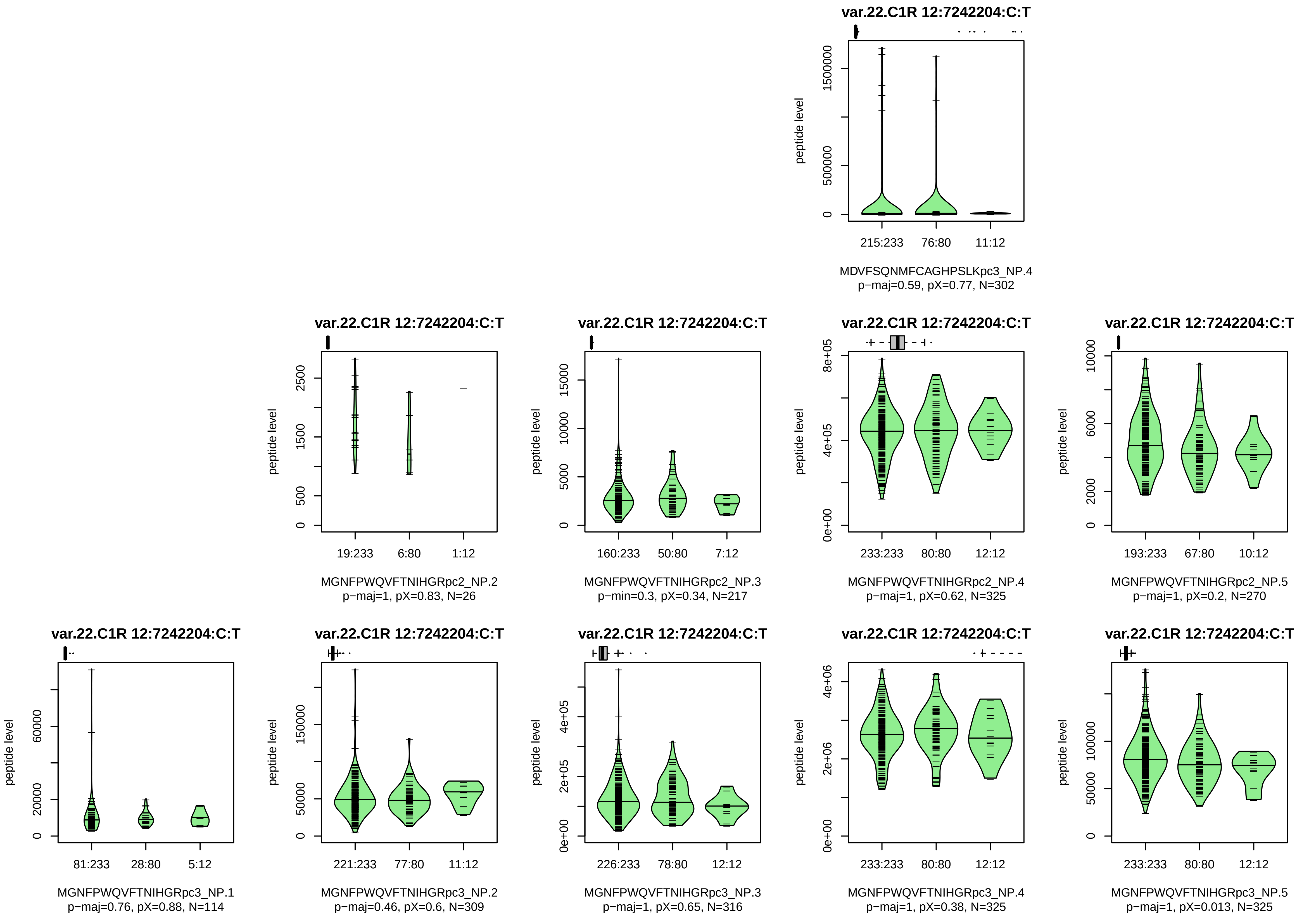

var.22.C1R 12:7242204:C:T
peptide level
0
500000
1500000
215:233
76:80
11:12
MDVFSQNMFCAGHPSLKpc3_NP.4
p−maj=0.59, pX=0.77, N=302
var.22.C1R 12:7242204:C:T
peptide level
0
500
1500
2500
19:233
6:80
1:12
MGNFPWQVFTNIHGRpc2_NP.2
p−maj=1, pX=0.83, N=26
var.22.C1R 12:7242204:C:T
peptide level
0
5000
10000
15000
160:233
50:80
7:12
MGNFPWQVFTNIHGRpc2_NP.3
p−min=0.3, pX=0.34, N=217
var.22.C1R 12:7242204:C:T
peptide level
0e+00
4e+05
8e+05
233:233
80:80
12:12
MGNFPWQVFTNIHGRpc2_NP.4
p−maj=1, pX=0.62, N=325
var.22.C1R 12:7242204:C:T
peptide level
0
2000
6000
10000
193:233
67:80
10:12
MGNFPWQVFTNIHGRpc2_NP.5
p−maj=1, pX=0.2, N=270
var.22.C1R 12:7242204:C:T
peptide level
0
20000
60000
81:233
28:80
5:12
MGNFPWQVFTNIHGRpc3_NP.1
p−maj=0.76, pX=0.88, N=114
var.22.C1R 12:7242204:C:T
peptide level
0
50000
150000
221:233
77:80
11:12
MGNFPWQVFTNIHGRpc3_NP.2
p−maj=0.46, pX=0.6, N=309
var.22.C1R 12:7242204:C:T
peptide level
0e+00
2e+05
4e+05
226:233
78:80
12:12
MGNFPWQVFTNIHGRpc3_NP.3
p−maj=1, pX=0.65, N=316
var.22.C1R 12:7242204:C:T
peptide level
0e+00
2e+06
4e+06
233:233
80:80
12:12
MGNFPWQVFTNIHGRpc3_NP.4
p−maj=1, pX=0.38, N=325
var.22.C1R 12:7242204:C:T
peptide level
0
50000
100000
233:233
80:80
12:12
MGNFPWQVFTNIHGRpc3_NP.5
p−maj=1, pX=0.013, N=325

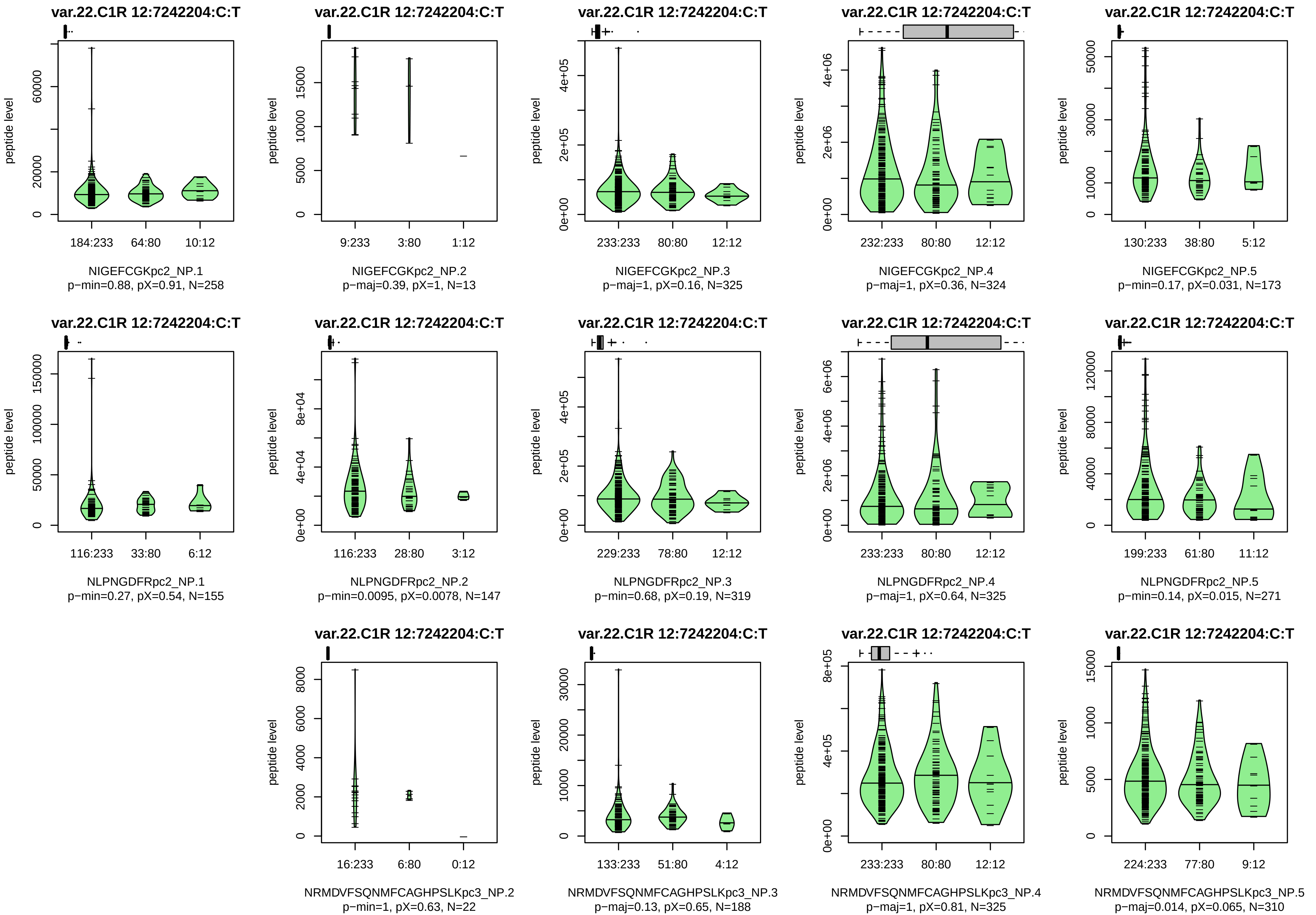

var.22.C1R 12:7242204:C:T
peptide level
184:233
64:80
10:12
NIGEFCGKpc2_NP.1
p−min=0.88, pX=0.91, N=258
var.22.C1R 12:7242204:C:T
peptide level
9:233
3:80
1:12
NIGEFCGKpc2_NP.2
p−maj=0.39, pX=1, N=13
var.22.C1R 12:7242204:C:T
peptide level
233:233
80:80
12:12
NIGEFCGKpc2_NP.3
p−maj=1, pX=0.16, N=325
var.22.C1R 12:7242204:C:T
peptide level
232:233
80:80
12:12
NIGEFCGKpc2_NP.4
p−maj=1, pX=0.36, N=324
var.22.C1R 12:7242204:C:T
peptide level
130:233
38:80
5:12
NIGEFCGKpc2_NP.5
p−min=0.17, pX=0.031, N=173
var.22.C1R 12:7242204:C:T
peptide level
116:233
33:80
6:12
NLPNGDFRpc2_NP.1
p−min=0.27, pX=0.54, N=155
var.22.C1R 12:7242204:C:T
peptide level
116:233
28:80
3:12
NLPNGDFRpc2_NP.2
p−min=0.0095, pX=0.0078, N=147
var.22.C1R 12:7242204:C:T
peptide level
229:233
78:80
12:12
NLPNGDFRpc2_NP.3
p−min=0.68, pX=0.19, N=319
var.22.C1R 12:7242204:C:T
peptide level
233:233
80:80
12:12
NLPNGDFRpc2_NP.4
p−maj=1, pX=0.64, N=325
var.22.C1R 12:7242204:C:T
peptide level
199:233
61:80
11:12
NLPNGDFRpc2_NP.5
p−min=0.14, pX=0.015, N=271
var.22.C1R 12:7242204:C:T
peptide level
16:233
6:80
0:12
NRMDVFSQNMFCAGHPSLKpc3_NP.2
p−min=1, pX=0.63, N=22
var.22.C1R 12:7242204:C:T
peptide level
133:233
51:80
4:12
NRMDVFSQNMFCAGHPSLKpc3_NP.3
p−maj=0.13, pX=0.65, N=188
var.22.C1R 12:7242204:C:T
peptide level
233:233
80:80
12:12
NRMDVFSQNMFCAGHPSLKpc3_NP.4
p−maj=1, pX=0.81, N=325
var.22.C1R 12:7242204:C:T
peptide level
224:233
77:80
9:12
NRMDVFSQNMFCAGHPSLKpc3_NP.5
p−maj=0.014, pX=0.065, N=310

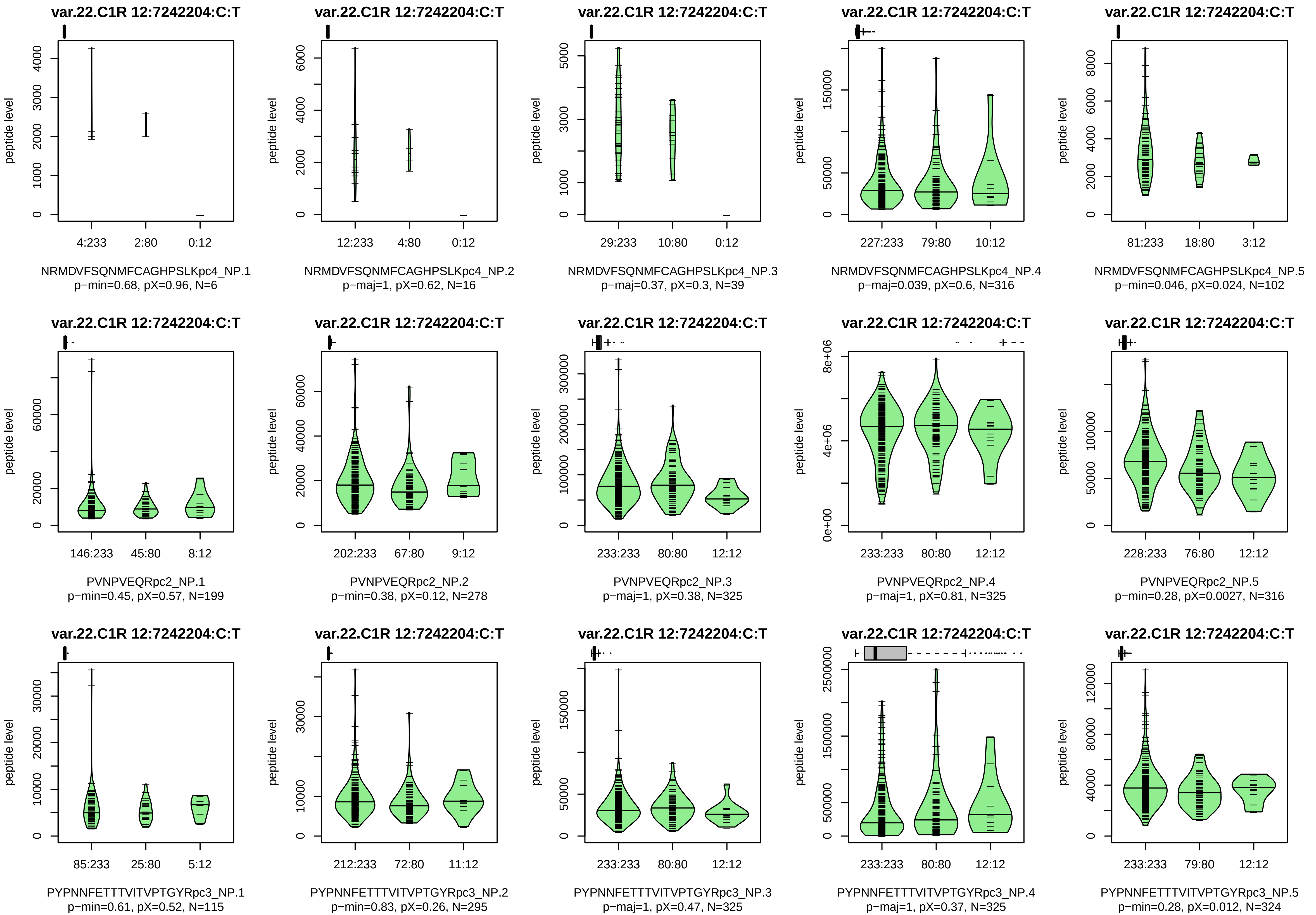

var.22.C1R 12:7242204:C:T
peptide level
4:233
2:80
0:12
NRMDVFSQNMFCAGHPSLKpc4_NP.1
p−min=0.68, pX=0.96, N=6
var.22.C1R 12:7242204:C:T
peptide level
12:233
4:80
0:12
NRMDVFSQNMFCAGHPSLKpc4_NP.2
p−maj=1, pX=0.62, N=16
var.22.C1R 12:7242204:C:T
peptide level
29:233
10:80
0:12
NRMDVFSQNMFCAGHPSLKpc4_NP.3
p−maj=0.37, pX=0.3, N=39
var.22.C1R 12:7242204:C:T
peptide level
227:233
79:80
10:12
NRMDVFSQNMFCAGHPSLKpc4_NP.4
p−maj=0.039, pX=0.6, N=316
var.22.C1R 12:7242204:C:T
peptide level
81:233
18:80
3:12
NRMDVFSQNMFCAGHPSLKpc4_NP.5
p−min=0.046, pX=0.024, N=102
var.22.C1R 12:7242204:C:T
peptide level
146:233
45:80
8:12
PVNPVEQRpc2_NP.1
p−min=0.45, pX=0.57, N=199
var.22.C1R 12:7242204:C:T
peptide level
202:233
67:80
9:12
PVNPVEQRpc2_NP.2
p−min=0.38, pX=0.12, N=278
var.22.C1R 12:7242204:C:T
peptide level
233:233
80:80
12:12
PVNPVEQRpc2_NP.3
p−maj=1, pX=0.38, N=325
var.22.C1R 12:7242204:C:T
peptide level
233:233
80:80
12:12
PVNPVEQRpc2_NP.4
p−maj=1, pX=0.81, N=325
var.22.C1R 12:7242204:C:T
peptide level
228:233
76:80
12:12
PVNPVEQRpc2_NP.5
p−min=0.28, pX=0.0027, N=316
var.22.C1R 12:7242204:C:T
peptide level
85:233
25:80
5:12
PYPNNFETTTVITVPTGYRpc3_NP.1
p−min=0.61, pX=0.52, N=115
var.22.C1R 12:7242204:C:T
peptide level
212:233
72:80
11:12
PYPNNFETTTVITVPTGYRpc3_NP.2
p−min=0.83, pX=0.26, N=295
var.22.C1R 12:7242204:C:T
peptide level
233:233
80:80
12:12
PYPNNFETTTVITVPTGYRpc3_NP.3
p−maj=1, pX=0.47, N=325
var.22.C1R 12:7242204:C:T
peptide level
233:233
80:80
12:12
PYPNNFETTTVITVPTGYRpc3_NP.4
p−maj=1, pX=0.37, N=325
var.22.C1R 12:7242204:C:T
peptide level
233:233
79:80
12:12
PYPNNFETTTVITVPTGYRpc3_NP.5
p−min=0.28, pX=0.012, N=324

var.22.C1R 12:7242204:C:T

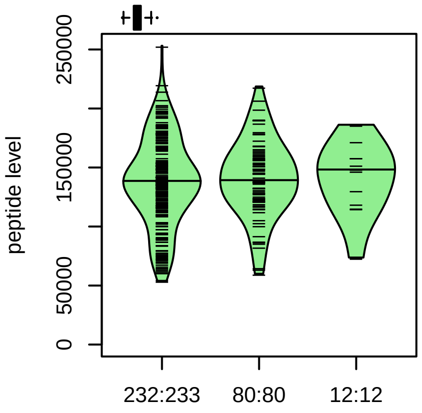

PYPNNFETTTVITVPTGYRpc4_NP.4
p−maj=1, pX=0.48, N=324

var.22.C1R 12:7242204:C:T

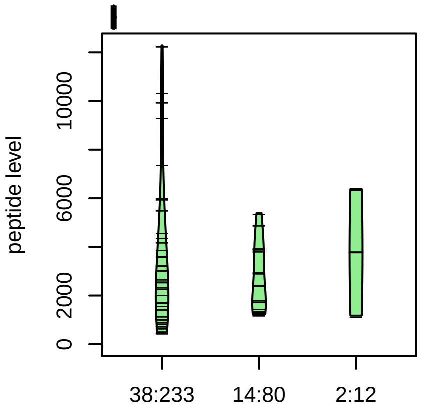

PYPNNFETTTVITVPTGYRVKpc3_NP.4
p−min=0.87, pX=0.84, N=54

var.22.C1R 12:7242204:C:T

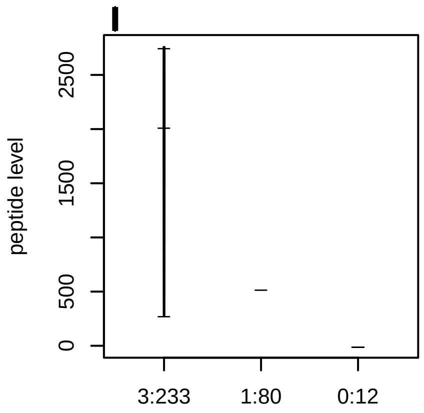

QDACQGDSGGVFAVRDPNTDRpc3_NP.1
p−maj=1, pX=0.5, N=4

var.22.C1R 12:7242204:C:T

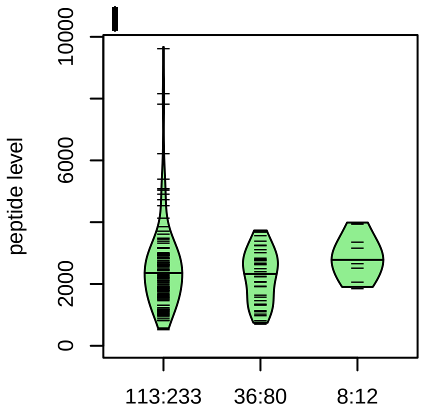

QDACQGDSGGVFAVRDPNTDRpc3_NP.2
p−maj=0.24, pX=0.99, N=157

var.22.C1R 12:7242204:C:T

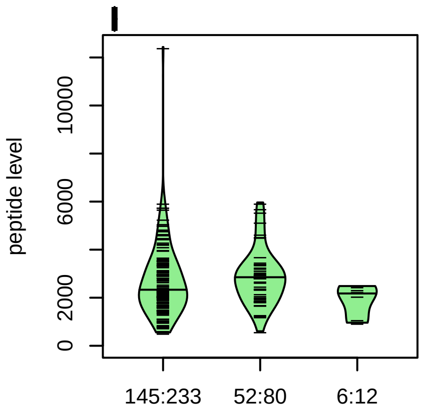

QDACQGDSGGVFAVRDPNTDRpc3_NP.3
p−maj=0.38, pX=0.82, N=203

var.22.C1R 12:7242204:C:T

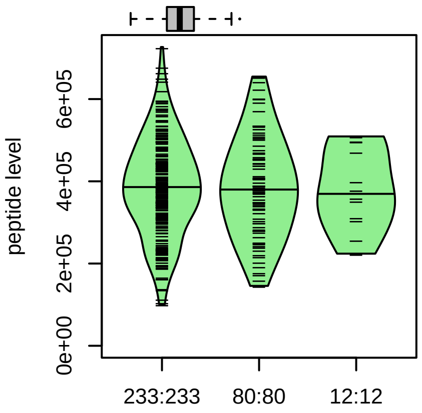

QDACQGDSGGVFAVRDPNTDRpc3_NP.4
p−maj=1, pX=0.86, N=325

var.22.C1R 12:7242204:C:T

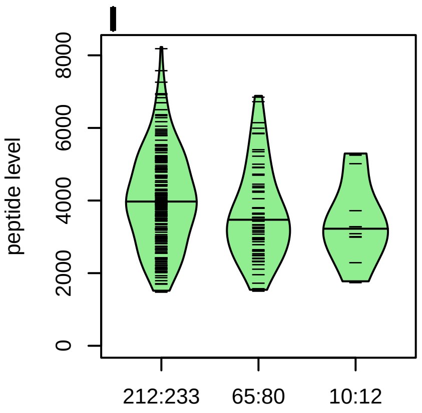

QDACQGDSGGVFAVRDPNTDRpc3_NP.5
p−min=0.021, pX=0.0013, N=287

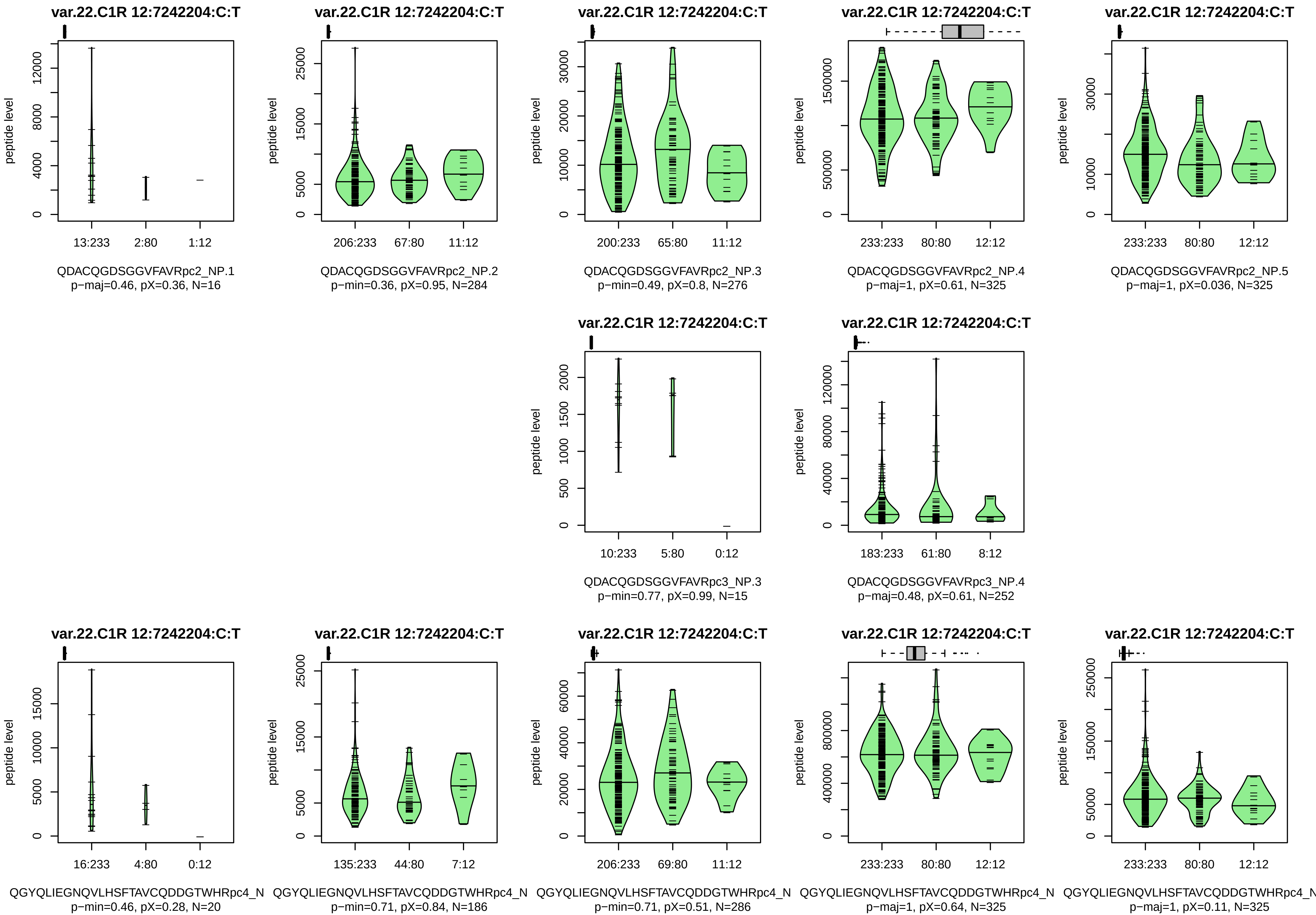
var.22.C1R 12:7242204:C:T
peptide level
13:233
2:80
1:12
QDACQGDSGGVFAVRpc2_NP.1
p−maj=0.46, pX=0.36, N=16
var.22.C1R 12:7242204:C:T
peptide level
206:233
67:80
11:12
QDACQGDSGGVFAVRpc2_NP.2
p−min=0.36, pX=0.95, N=284
var.22.C1R 12:7242204:C:T
peptide level
200:233
65:80
11:12
QDACQGDSGGVFAVRpc2_NP.3
p−min=0.49, pX=0.8, N=276
var.22.C1R 12:7242204:C:T
peptide level
233:233
80:80
12:12
QDACQGDSGGVFAVRpc2_NP.4
p−maj=1, pX=0.61, N=325
var.22.C1R 12:7242204:C:T
peptide level
233:233
80:80
12:12
QDACQGDSGGVFAVRpc2_NP.5
p−maj=1, pX=0.036, N=325
var.22.C1R 12:7242204:C:T
peptide level
10:233
5:80
0:12
QDACQGDSGGVFAVRpc3_NP.3
p−min=0.77, pX=0.99, N=15
var.22.C1R 12:7242204:C:T
peptide level
183:233
61:80
8:12
QDACQGDSGGVFAVRpc3_NP.4
p−maj=0.48, pX=0.61, N=252
var.22.C1R 12:7242204:C:T
peptide level
16:233
4:80
0:12
QGYQLIEGNQVLHSFTAVCQDDGTWHRpc4_N
p−min=0.46, pX=0.28, N=20
var.22.C1R 12:7242204:C:T
peptide level
135:233
44:80
7:12
QGYQLIEGNQVLHSFTAVCQDDGTWHRpc4_N
p−min=0.71, pX=0.84, N=186
var.22.C1R 12:7242204:C:T
peptide level
206:233
69:80
11:12
QGYQLIEGNQVLHSFTAVCQDDGTWHRpc4_N
p−min=0.71, pX=0.51, N=286
var.22.C1R 12:7242204:C:T
peptide level
233:233
80:80
12:12
QGYQLIEGNQVLHSFTAVCQDDGTWHRpc4_N
p−maj=1, pX=0.64, N=325
var.22.C1R 12:7242204:C:T
peptide level
233:233
80:80
12:12
QGYQLIEGNQVLHSFTAVCQDDGTWHRpc4_N
p−maj=1, pX=0.11, N=325

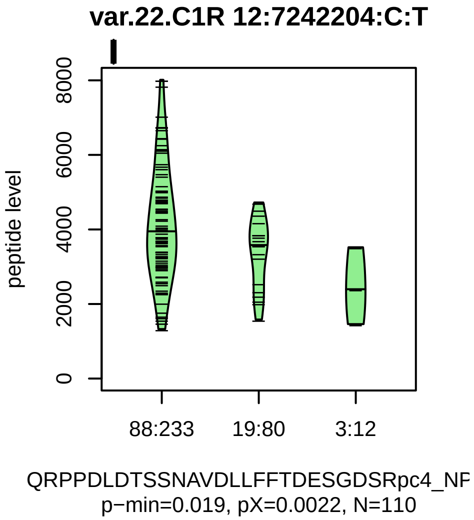
var.22.C1R 12:7242204:C:T
peptide level
0
2000
4000
6000
8000
88:233
19:80
3:12
QRPPDLDTSSNAVDLLFFTDESGDSRpc4_NP
p−min=0.019, pX=0.0022, N=110

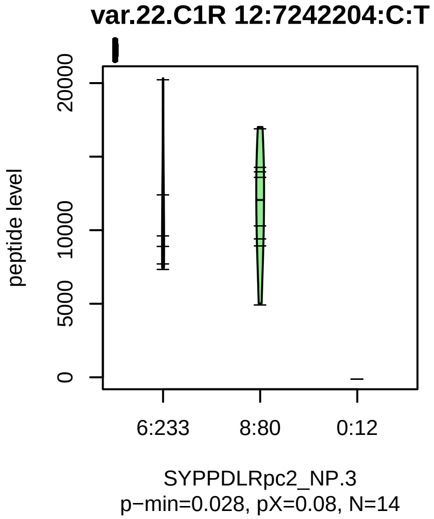
var.22.C1R 12:7242204:C:T
peptide level
0
5000
10000
20000
6:233
8:80
0:12
SYPPDLRpc2_NP.3
p−min=0.028, pX=0.08, N=14

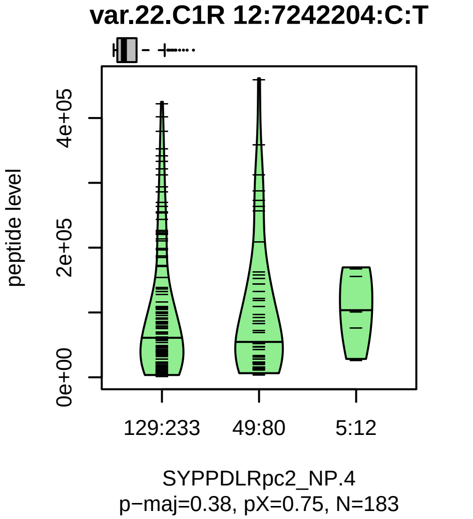
var.22.C1R 12:7242204:C:T
peptide level
0e+00
2e+05
4e+05
129:233
49:80
5:12
SYPPDLRpc2_NP.4
p−maj=0.38, pX=0.75, N=183

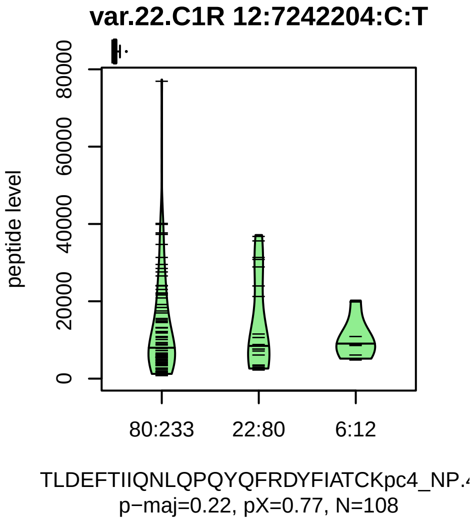
var.22.C1R 12:7242204:C:T
peptide level
0
20000
40000
60000
80000
80:233
22:80
6:12
TLDEFTIIQNLQPQYQFRDYFIATCKpc4_NP.
p−maj=0.22, pX=0.77, N=108

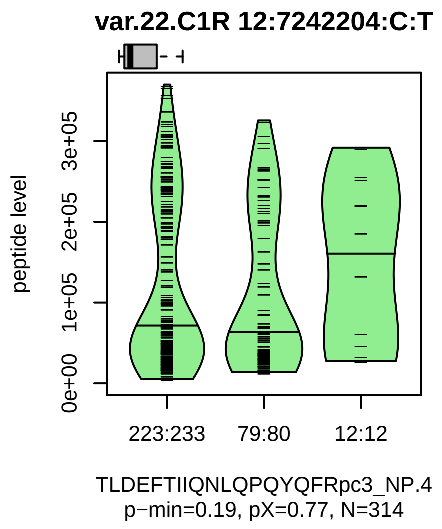
var.22.C1R 12:7242204:C:T
peptide level
0e+00
1e+05
2e+05
3e+05
223:233
79:80
12:12
TLDEFTIIQNLQPQYQFRpc3_NP.4
p−min=0.19, pX=0.77, N=314

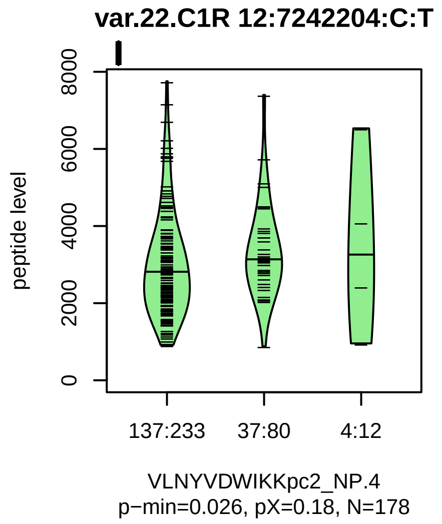
var.22.C1R 12:7242204:C:T
peptide level
0
2000
4000
6000
8000
137:233
37:80
4:12
VLNYVDWIKKpc2_NP.4
p−min=0.026, pX=0.18, N=178

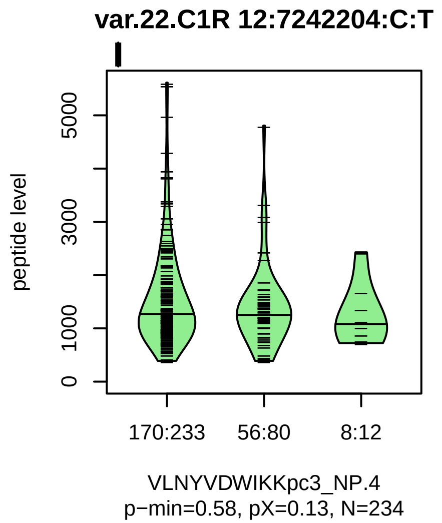
var.22.C1R 12:7242204:C:T
peptide level
0
1000
3000
5000
170:233
56:80
8:12
VLNYVDWIKKpc3_NP.4
p−min=0.58, pX=0.13, N=234

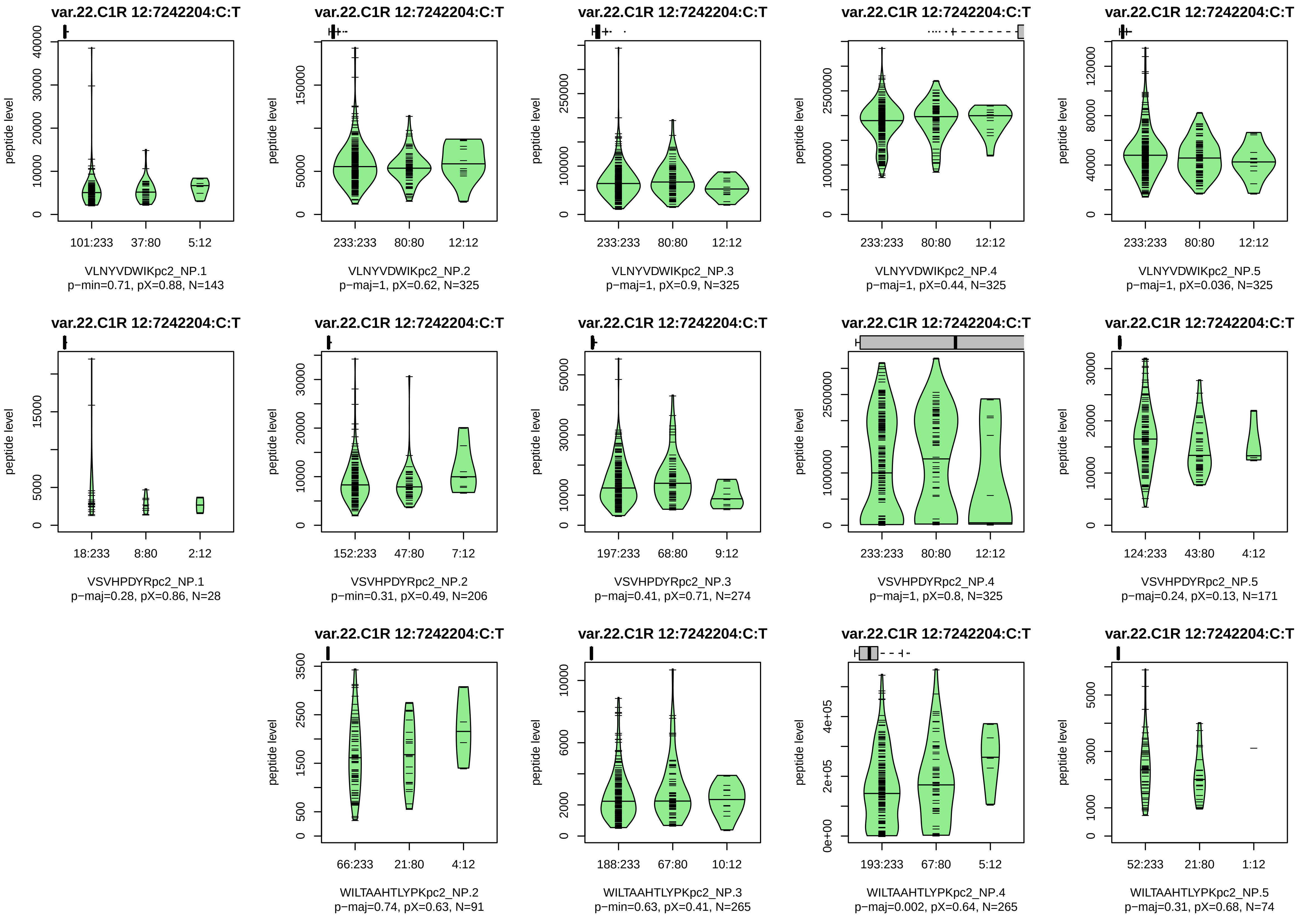
var.22.C1R 12:7242204:C:T
peptide level
101:233
37:80
5:12
VLNYVDWIKpc2_NP.1
p−min=0.71, pX=0.88, N=143
233:233
80:80
12:12
VLNYVDWIKpc2_NP.2
p−maj=1, pX=0.62, N=325
VLNYVDWIKpc2_NP.3
p−maj=1, pX=0.9, N=325
VLNYVDWIKpc2_NP.4
p−maj=1, pX=0.44, N=325
VLNYVDWIKpc2_NP.5
p−maj=1, pX=0.036, N=325
18:233
8:80
2:12
VSVHPDYRpc2_NP.1
p−maj=0.28, pX=0.86, N=28
152:233
47:80
7:12
VSVHPDYRpc2_NP.2
p−min=0.31, pX=0.49, N=206
197:233
68:80
9:12
VSVHPDYRpc2_NP.3
p−maj=0.41, pX=0.71, N=274
VSVHPDYRpc2_NP.4
p−maj=1, pX=0.8, N=325
124:233
43:80
4:12
VSVHPDYRpc2_NP.5
p−maj=0.24, pX=0.13, N=171
66:233
21:80
4:12
WILTAAHTLYPKpc2_NP.2
p−maj=0.74, pX=0.63, N=91
188:233
67:80
10:12
WILTAAHTLYPKpc2_NP.3
p−min=0.63, pX=0.41, N=265
193:233
67:80
5:12
WILTAAHTLYPKpc2_NP.4
p−maj=0.002, pX=0.64, N=265
52:233
21:80
1:12
WILTAAHTLYPKpc2_NP.5
p−maj=0.31, pX=0.68, N=74

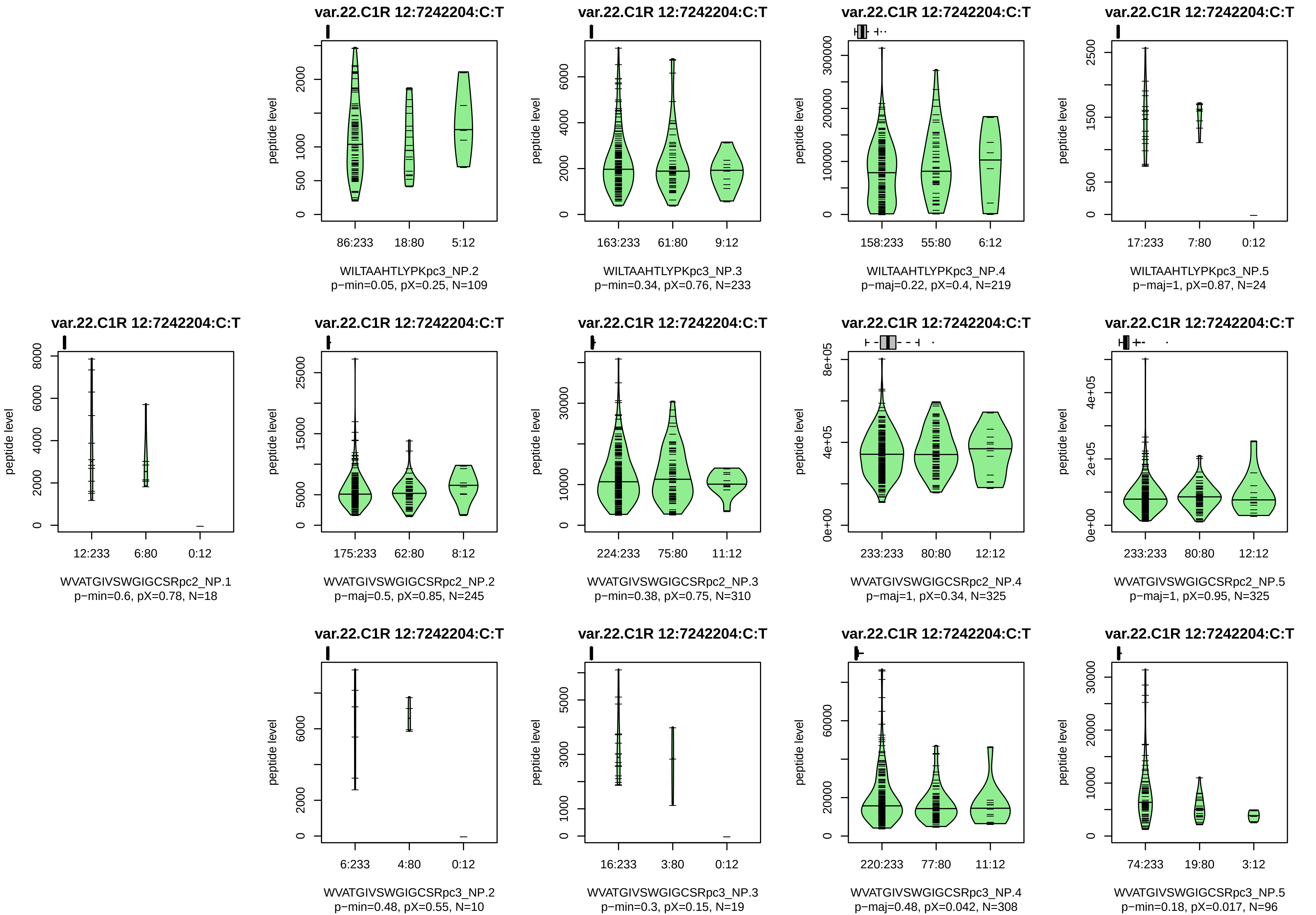
var.22.C1R 12:7242204:C:T
peptide level
86:233
18:80
5:12
WILTAAHTLYPKpc3_NP.2
p−min=0.05, pX=0.25, N=109
var.22.C1R 12:7242204:C:T
peptide level
163:233
61:80
9:12
WILTAAHTLYPKpc3_NP.3
p−min=0.34, pX=0.76, N=233
var.22.C1R 12:7242204:C:T
peptide level
158:233
55:80
6:12
WILTAAHTLYPKpc3_NP.4
p−maj=0.22, pX=0.4, N=219
var.22.C1R 12:7242204:C:T
peptide level
17:233
7:80
0:12
WILTAAHTLYPKpc3_NP.5
p−maj=1, pX=0.87, N=24
var.22.C1R 12:7242204:C:T
peptide level
12:233
6:80
0:12
WVATGIVSWGIGCSRpc2_NP.1
p−min=0.6, pX=0.78, N=18
var.22.C1R 12:7242204:C:T
peptide level
175:233
62:80
8:12
WVATGIVSWGIGCSRpc2_NP.2
p−maj=0.5, pX=0.85, N=245
var.22.C1R 12:7242204:C:T
peptide level
224:233
75:80
11:12
WVATGIVSWGIGCSRpc2_NP.3
p−min=0.38, pX=0.75, N=310
var.22.C1R 12:7242204:C:T
peptide level
233:233
80:80
12:12
WVATGIVSWGIGCSRpc2_NP.4
p−maj=1, pX=0.34, N=325
var.22.C1R 12:7242204:C:T
peptide level
233:233
80:80
12:12
WVATGIVSWGIGCSRpc2_NP.5
p−maj=1, pX=0.95, N=325
var.22.C1R 12:7242204:C:T
peptide level
6:233
4:80
0:12
WVATGIVSWGIGCSRpc3_NP.2
p−min=0.48, pX=0.55, N=10
var.22.C1R 12:7242204:C:T
peptide level
16:233
3:80
0:12
WVATGIVSWGIGCSRpc3_NP.3
p−min=0.3, pX=0.15, N=19
var.22.C1R 12:7242204:C:T
peptide level
220:233
77:80
11:12
WVATGIVSWGIGCSRpc3_NP.4
p−maj=0.48, pX=0.042, N=308
var.22.C1R 12:7242204:C:T
peptide level
74:233
19:80
3:12
WVATGIVSWGIGCSRpc3_NP.5
p−min=0.18, pX=0.017, N=96

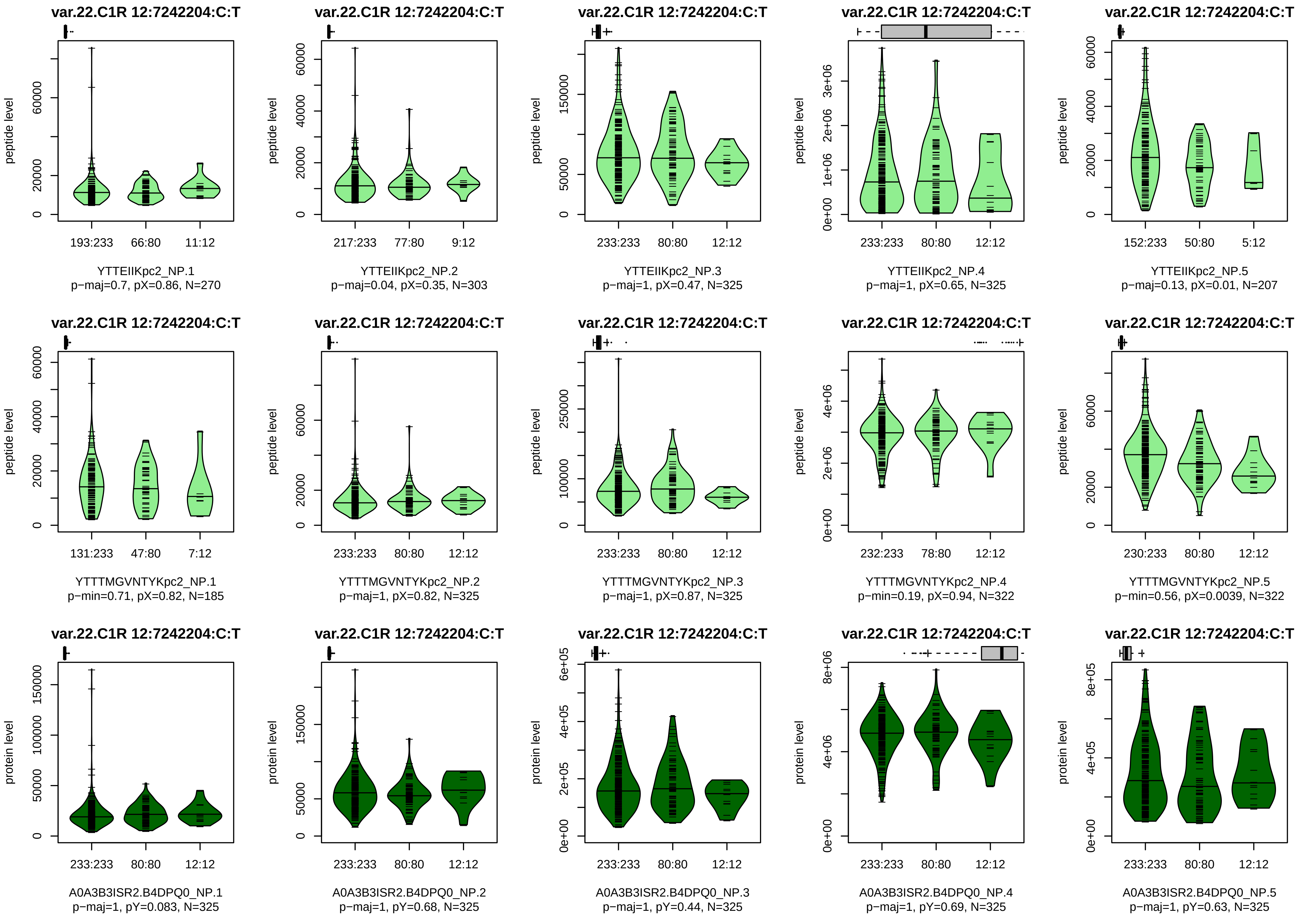

var.22.C1R 12:7242204:C:T
peptide level
193:233
66:80
11:12
YTTEIIKpc2_NP.1
p−maj=0.7, pX=0.86, N=270
var.22.C1R 12:7242204:C:T
peptide level
217:233
77:80
9:12
YTTEIIKpc2_NP.2
p−maj=0.04, pX=0.35, N=303
var.22.C1R 12:7242204:C:T
peptide level
233:233
80:80
12:12
YTTEIIKpc2_NP.3
p−maj=1, pX=0.47, N=325
var.22.C1R 12:7242204:C:T
peptide level
233:233
80:80
12:12
YTTEIIKpc2_NP.4
p−maj=1, pX=0.65, N=325
var.22.C1R 12:7242204:C:T
peptide level
152:233
50:80
5:12
YTTEIIKpc2_NP.5
p−maj=0.13, pX=0.01, N=207
var.22.C1R 12:7242204:C:T
peptide level
131:233
47:80
7:12
YTTTMGVNTYKpc2_NP.1
p−min=0.71, pX=0.82, N=185
var.22.C1R 12:7242204:C:T
peptide level
233:233
80:80
12:12
YTTTMGVNTYKpc2_NP.2
p−maj=1, pX=0.82, N=325
var.22.C1R 12:7242204:C:T
peptide level
233:233
80:80
12:12
YTTTMGVNTYKpc2_NP.3
p−maj=1, pX=0.87, N=325
var.22.C1R 12:7242204:C:T
peptide level
232:233
78:80
12:12
YTTTMGVNTYKpc2_NP.4
p−min=0.19, pX=0.94, N=322
var.22.C1R 12:7242204:C:T
peptide level
230:233
80:80
12:12
YTTTMGVNTYKpc2_NP.5
p−min=0.56, pX=0.0039, N=322
var.22.C1R 12:7242204:C:T
protein level
233:233
80:80
12:12
A0A3B3ISR2.B4DPQ0_NP.1
p−maj=1, pY=0.083, N=325
var.22.C1R 12:7242204:C:T
protein level
233:233
80:80
12:12
A0A3B3ISR2.B4DPQ0_NP.2
p−maj=1, pY=0.68, N=325
var.22.C1R 12:7242204:C:T
protein level
233:233
80:80
12:12
A0A3B3ISR2.B4DPQ0_NP.3
p−maj=1, pY=0.44, N=325
var.22.C1R 12:7242204:C:T
protein level
233:233
80:80
12:12
A0A3B3ISR2.B4DPQ0_NP.4
p−maj=1, pY=0.69, N=325
var.22.C1R 12:7242204:C:T
protein level
233:233
80:80
12:12
A0A3B3ISR2.B4DPQ0_NP.5
p−maj=1, pY=0.63, N=325

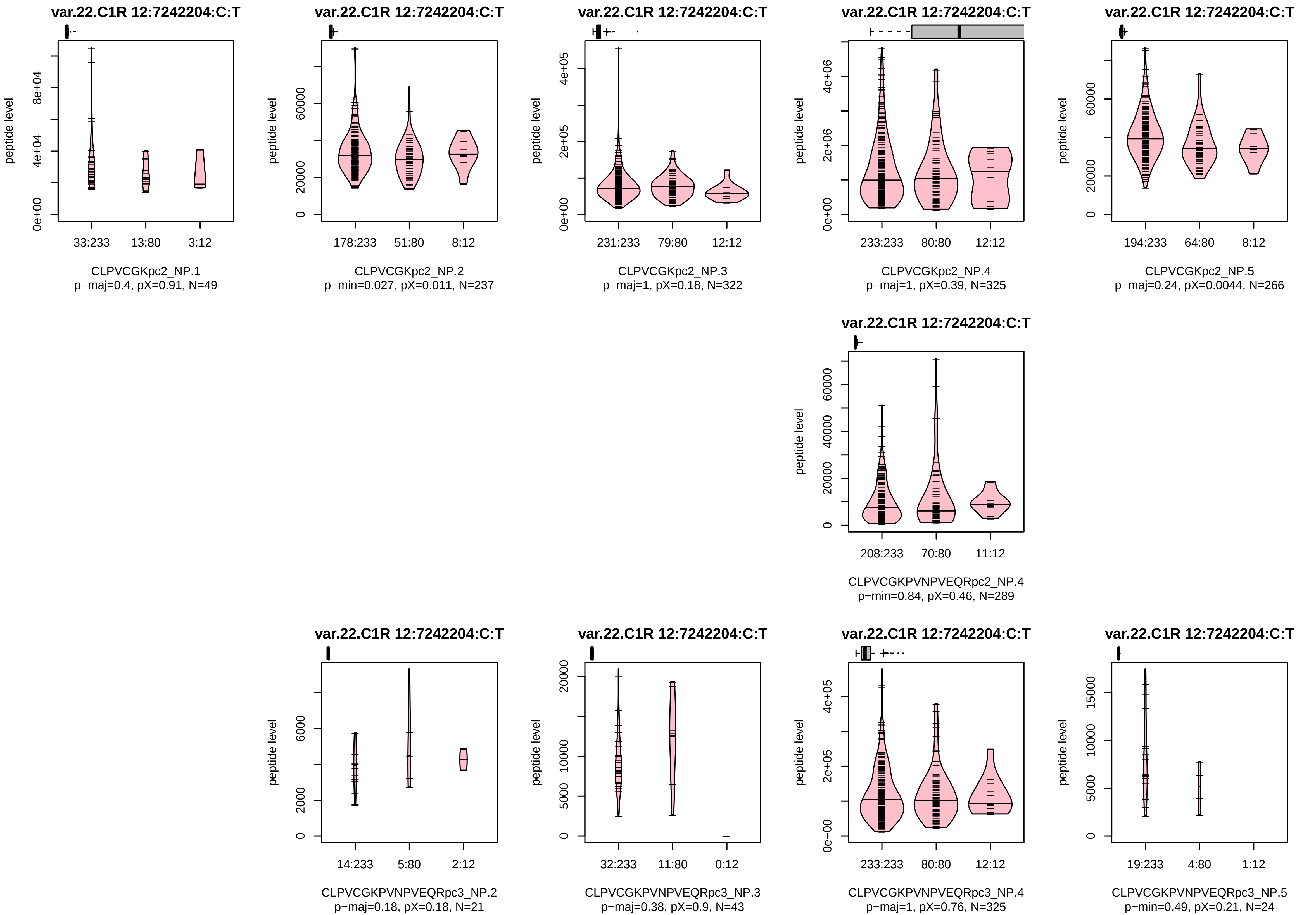
var.22.C1R 12:7242204:C:T
peptide level
33:233
13:80
3:12
CLPVCGKpc2_NP.1
p−maj=0.4, pX=0.91, N=49
var.22.C1R 12:7242204:C:T
peptide level
178:233
51:80
8:12
CLPVCGKpc2_NP.2
p−min=0.027, pX=0.011, N=237
var.22.C1R 12:7242204:C:T
peptide level
231:233
79:80
12:12
CLPVCGKpc2_NP.3
p−maj=1, pX=0.18, N=322
var.22.C1R 12:7242204:C:T
peptide level
233:233
80:80
12:12
CLPVCGKpc2_NP.4
p−maj=1, pX=0.39, N=325
var.22.C1R 12:7242204:C:T
peptide level
194:233
64:80
8:12
CLPVCGKpc2_NP.5
p−maj=0.24, pX=0.0044, N=266
var.22.C1R 12:7242204:C:T
peptide level
208:233
70:80
11:12
CLPVCGKPVNPVEQRpc2_NP.4
p−min=0.84, pX=0.46, N=289
var.22.C1R 12:7242204:C:T
peptide level
14:233
5:80
2:12
CLPVCGKPVNPVEQRpc3_NP.2
p−maj=0.18, pX=0.18, N=21
var.22.C1R 12:7242204:C:T
peptide level
32:233
11:80
0:12
CLPVCGKPVNPVEQRpc3_NP.3
p−maj=0.38, pX=0.9, N=43
var.22.C1R 12:7242204:C:T
peptide level
233:233
80:80
12:12
CLPVCGKPVNPVEQRpc3_NP.4
p−maj=1, pX=0.76, N=325
var.22.C1R 12:7242204:C:T
peptide level
19:233
4:80
1:12
CLPVCGKPVNPVEQRpc3_NP.5
p−min=0.49, pX=0.21, N=24

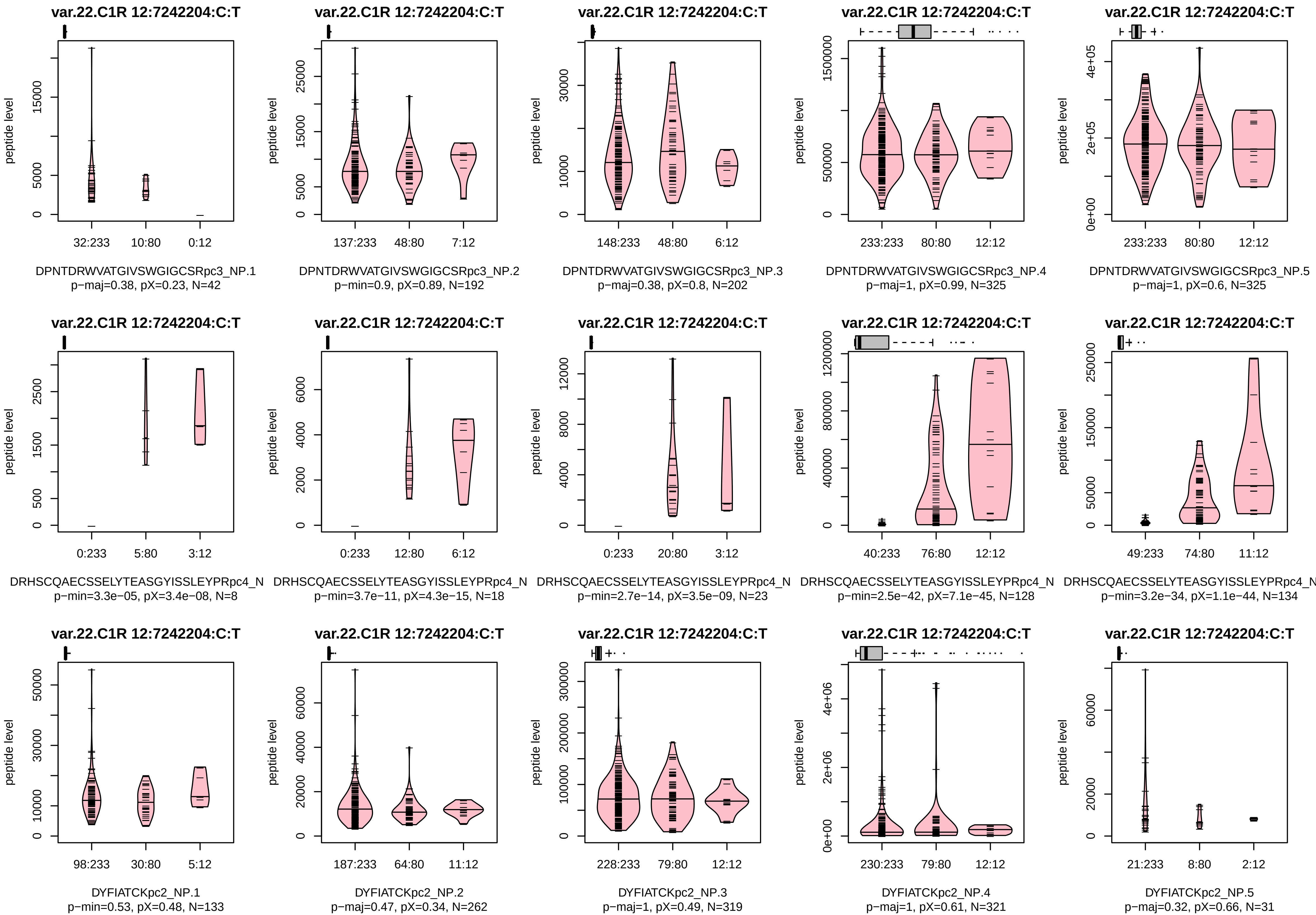
var.22.C1R 12:7242204:C:T
peptide level
32:233
10:80
0:12
DPNTDRWVATGIVSWGIGCSRpc3_NP.1
p−maj=0.38, pX=0.23, N=42
var.22.C1R 12:7242204:C:T
137:233
48:80
7:12
DPNTDRWVATGIVSWGIGCSRpc3_NP.2
p−min=0.9, pX=0.89, N=192
var.22.C1R 12:7242204:C:T
148:233
48:80
6:12
DPNTDRWVATGIVSWGIGCSRpc3_NP.3
p−maj=0.38, pX=0.8, N=202
var.22.C1R 12:7242204:C:T
233:233
80:80
12:12
DPNTDRWVATGIVSWGIGCSRpc3_NP.4
p−maj=1, pX=0.99, N=325
var.22.C1R 12:7242204:C:T
233:233
80:80
12:12
DPNTDRWVATGIVSWGIGCSRpc3_NP.5
p−maj=1, pX=0.6, N=325
var.22.C1R 12:7242204:C:T
0:233
5:80
3:12
DRHSCQAECSSELYTEASGYISSLEYPRpc4_N
p−min=3.3e−05, pX=3.4e−08, N=8
var.22.C1R 12:7242204:C:T
0:233
12:80
6:12
DRHSCQAECSSELYTEASGYISSLEYPRpc4_N
p−min=3.7e−11, pX=4.3e−15, N=18
var.22.C1R 12:7242204:C:T
0:233
20:80
3:12
DRHSCQAECSSELYTEASGYISSLEYPRpc4_N
p−min=2.7e−14, pX=3.5e−09, N=23
var.22.C1R 12:7242204:C:T
40:233
76:80
12:12
DRHSCQAECSSELYTEASGYISSLEYPRpc4_N
p−min=2.5e−42, pX=7.1e−45, N=128
var.22.C1R 12:7242204:C:T
49:233
74:80
11:12
DRHSCQAECSSELYTEASGYISSLEYPRpc4_N
p−min=3.2e−34, pX=1.1e−44, N=134
var.22.C1R 12:7242204:C:T
98:233
30:80
5:12
DYFIATCKpc2_NP.1
p−min=0.53, pX=0.48, N=133
var.22.C1R 12:7242204:C:T
187:233
64:80
11:12
DYFIATCKpc2_NP.2
p−maj=0.47, pX=0.34, N=262
var.22.C1R 12:7242204:C:T
228:233
79:80
12:12
DYFIATCKpc2_NP.3
p−maj=1, pX=0.49, N=319
var.22.C1R 12:7242204:C:T
230:233
79:80
12:12
DYFIATCKpc2_NP.4
p−maj=1, pX=0.61, N=321
var.22.C1R 12:7242204:C:T
21:233
8:80
2:12
DYFIATCKpc2_NP.5
p−maj=0.32, pX=0.66, N=31

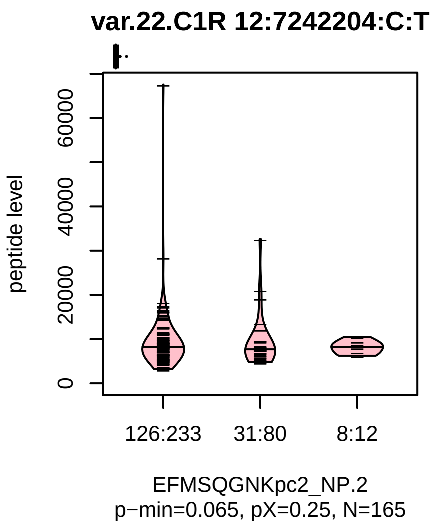
var.22.C1R 12:7242204:C:T
peptide level
0
20000
40000
60000
126:233
31:80
8:12
EFMSQGNKpc2_NP.2
p−min=0.065, pX=0.25, N=165

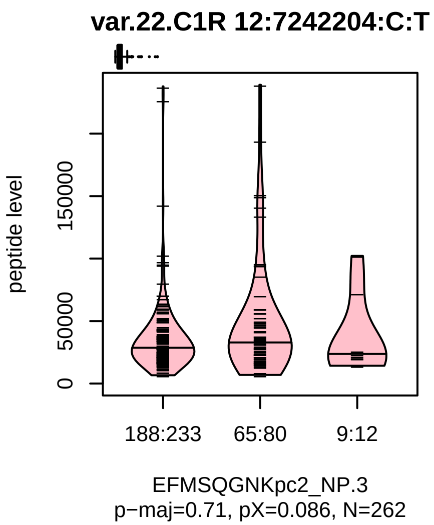
var.22.C1R 12:7242204:C:T
peptide level
0
50000
150000
188:233
65:80
9:12
EFMSQGNKpc2_NP.3
p−maj=0.71, pX=0.086, N=262

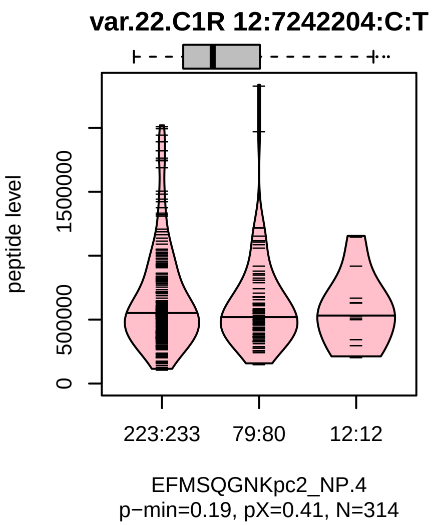
var.22.C1R 12:7242204:C:T
peptide level
0
500000
1500000
223:233
79:80
12:12
EFMSQGNKpc2_NP.4
p−min=0.19, pX=0.41, N=314

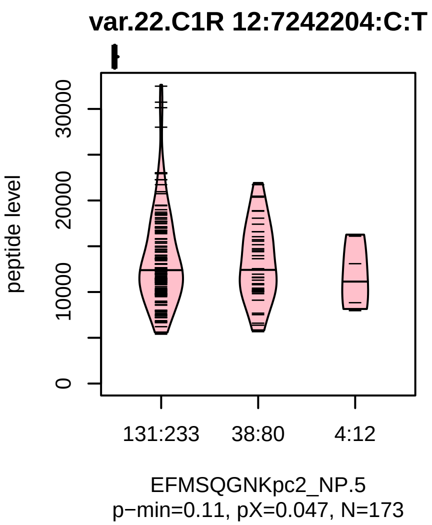
var.22.C1R 12:7242204:C:T
peptide level
0
10000
20000
30000
131:233
38:80
4:12
EFMSQGNKpc2_NP.5
p−min=0.11, pX=0.047, N=173

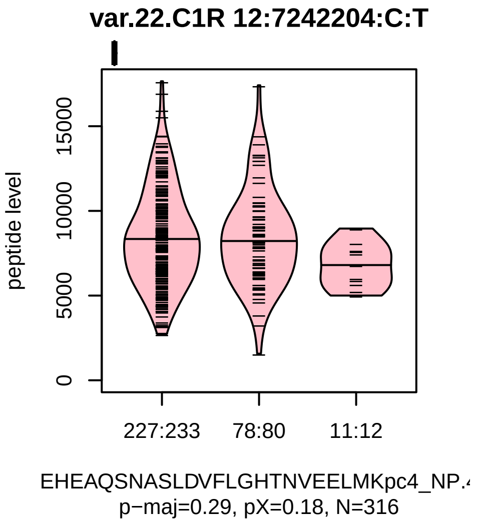
var.22.C1R 12:7242204:C:T
peptide level
0
5000
10000
15000
227:233
78:80
11:12
EHEAQSNASLDVFLGHTNVEELMKpc4_NP.4
p−maj=0.29, pX=0.18, N=316

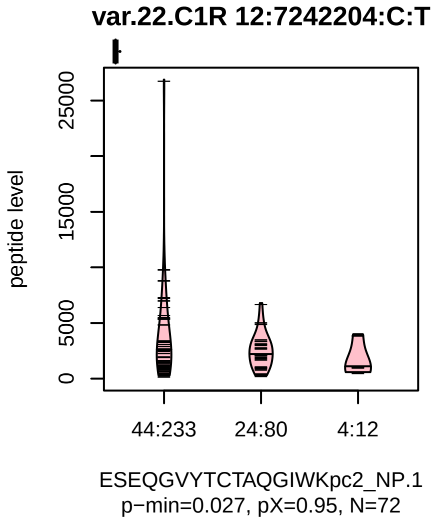
var.22.C1R 12:7242204:C:T
peptide level
0
5000
15000
25000
44:233
24:80
4:12
ESEQGVYTCTAQGIWKpc2_NP.1
p−min=0.027, pX=0.95, N=72

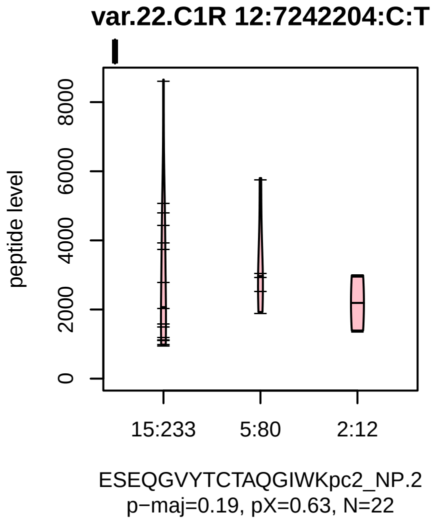
var.22.C1R 12:7242204:C:T
peptide level
0
2000
4000
6000
8000
15:233
5:80
2:12
ESEQGVYTCTAQGIWKpc2_NP.2
p−maj=0.19, pX=0.63, N=22

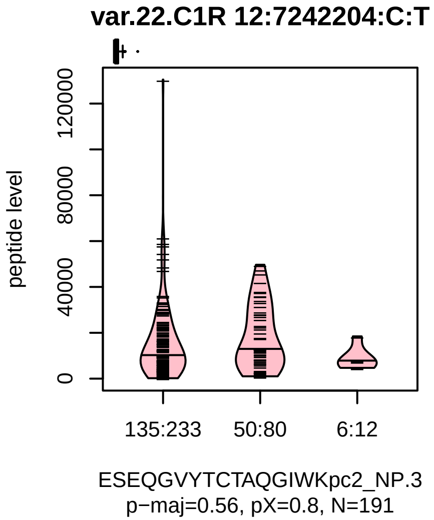
var.22.C1R 12:7242204:C:T
peptide level
0
40000
80000
120000
135:233
50:80
6:12
ESEQGVYTCTAQGIWKpc2_NP.3
p−maj=0.56, pX=0.8, N=191

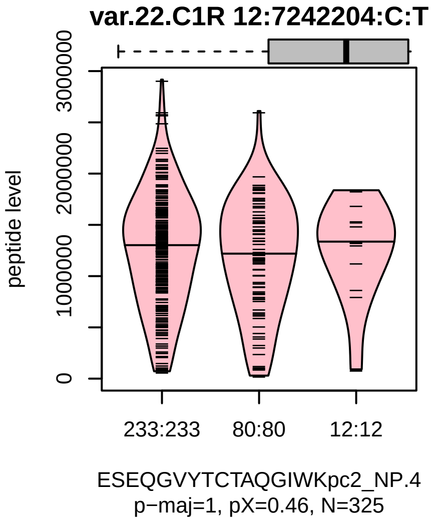
var.22.C1R 12:7242204:C:T
peptide level
0
1000000
2000000
3000000
233:233
80:80
12:12
ESEQGVYTCTAQGIWKpc2_NP.4
p−maj=1, pX=0.46, N=325

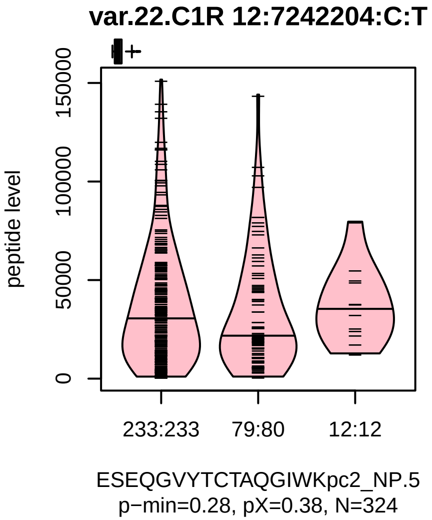
var.22.C1R 12:7242204:C:T
peptide level
0
50000
100000
150000
233:233
79:80
12:12
ESEQGVYTCTAQGIWKpc2_NP.5
p−min=0.28, pX=0.38, N=324

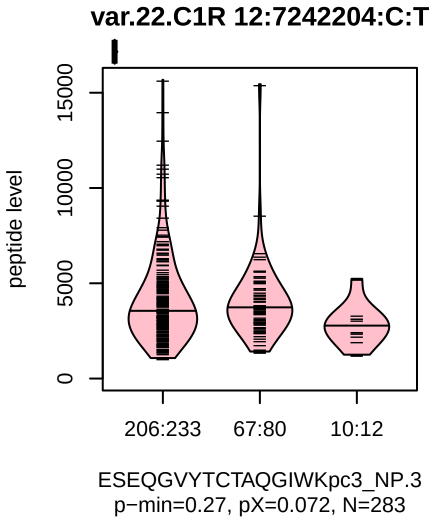
var.22.C1R 12:7242204:C:T
peptide level
0
5000
10000
15000
206:233
67:80
10:12
ESEQGVYTCTAQGIWKpc3_NP.3
p−min=0.27, pX=0.072, N=283

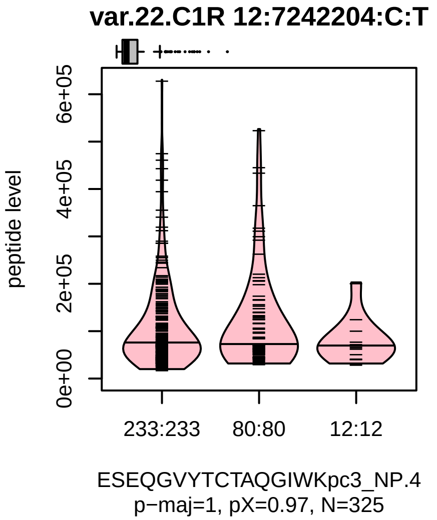
var.22.C1R 12:7242204:C:T
peptide level
0e+00
2e+05
4e+05
6e+05
233:233
80:80
12:12
ESEQGVYTCTAQGIWKpc3_NP.4
p−maj=1, pX=0.97, N=325

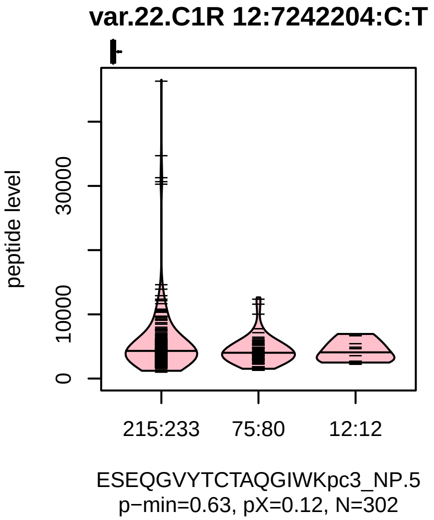
var.22.C1R 12:7242204:C:T
peptide level
0
10000
30000
215:233
75:80
12:12
ESEQGVYTCTAQGIWKpc3_NP.5
p−min=0.63, pX=0.12, N=302

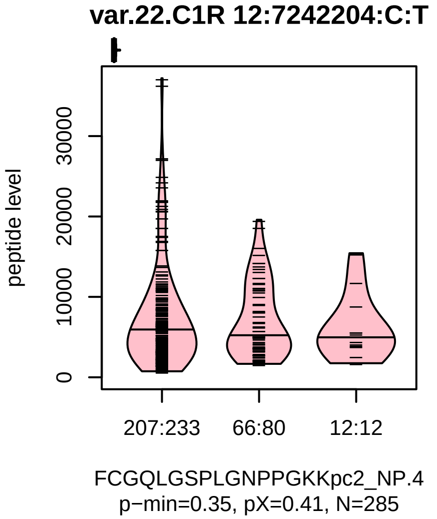
var.22.C1R 12:7242204:C:T
peptide level
0
10000
20000
30000
207:233
66:80
12:12
FCGQLGSPLGNPPGKKpc2_NP.4
p−min=0.35, pX=0.41, N=285

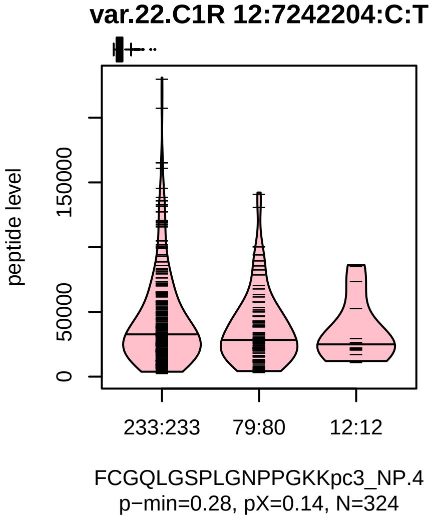
var.22.C1R 12:7242204:C:T
peptide level
0
50000
150000
233:233
79:80
12:12
FCGQLGSPLGNPPGKKpc3_NP.4
p−min=0.28, pX=0.14, N=324

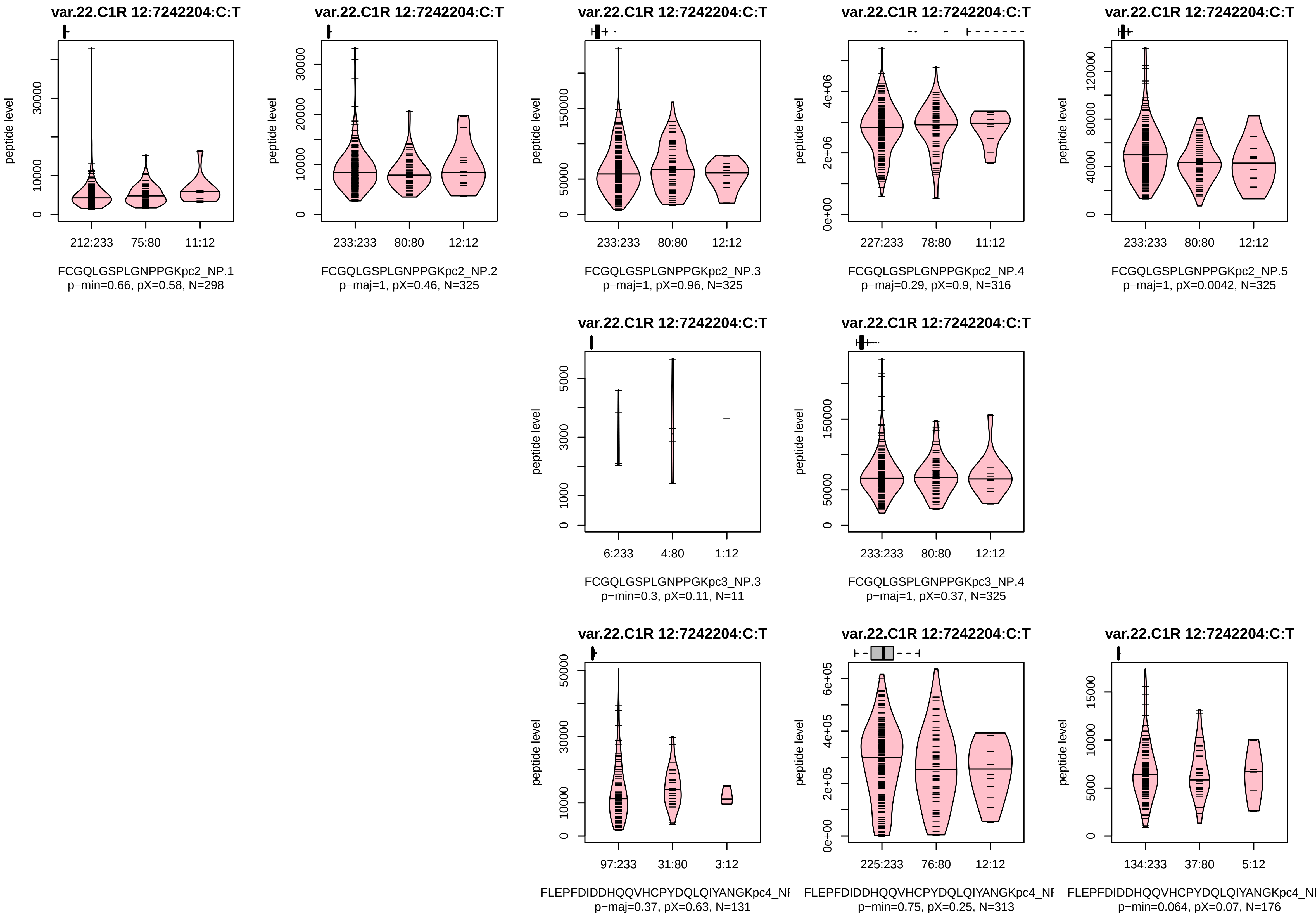
var.22.C1R 12:7242204:C:T
peptide level
212:233
75:80
11:12
FCGQLGSPLGNPPGKpc2_NP.1
p−min=0.66, pX=0.58, N=298
var.22.C1R 12:7242204:C:T
peptide level
233:233
80:80
12:12
FCGQLGSPLGNPPGKpc2_NP.2
p−maj=1, pX=0.46, N=325
var.22.C1R 12:7242204:C:T
peptide level
233:233
80:80
12:12
FCGQLGSPLGNPPGKpc2_NP.3
p−maj=1, pX=0.96, N=325
var.22.C1R 12:7242204:C:T
peptide level
227:233
78:80
11:12
FCGQLGSPLGNPPGKpc2_NP.4
p−maj=0.29, pX=0.9, N=316
var.22.C1R 12:7242204:C:T
peptide level
233:233
80:80
12:12
FCGQLGSPLGNPPGKpc2_NP.5
p−maj=1, pX=0.0042, N=325
var.22.C1R 12:7242204:C:T
peptide level
6:233
4:80
1:12
FCGQLGSPLGNPPGKpc3_NP.3
p−min=0.3, pX=0.11, N=11
var.22.C1R 12:7242204:C:T
peptide level
233:233
80:80
12:12
FCGQLGSPLGNPPGKpc3_NP.4
p−maj=1, pX=0.37, N=325
var.22.C1R 12:7242204:C:T
peptide level
97:233
31:80
3:12
FLEPFDIDDHQQVHCPYDQLQIYANGKpc4_NI
p−maj=0.37, pX=0.63, N=131
var.22.C1R 12:7242204:C:T
peptide level
225:233
76:80
12:12
FLEPFDIDDHQQVHCPYDQLQIYANGKpc4_NI
p−min=0.75, pX=0.25, N=313
var.22.C1R 12:7242204:C:T
peptide level
134:233
37:80
5:12
FLEPFDIDDHQQVHCPYDQLQIYANGKpc4_NI
p−min=0.064, pX=0.07, N=176

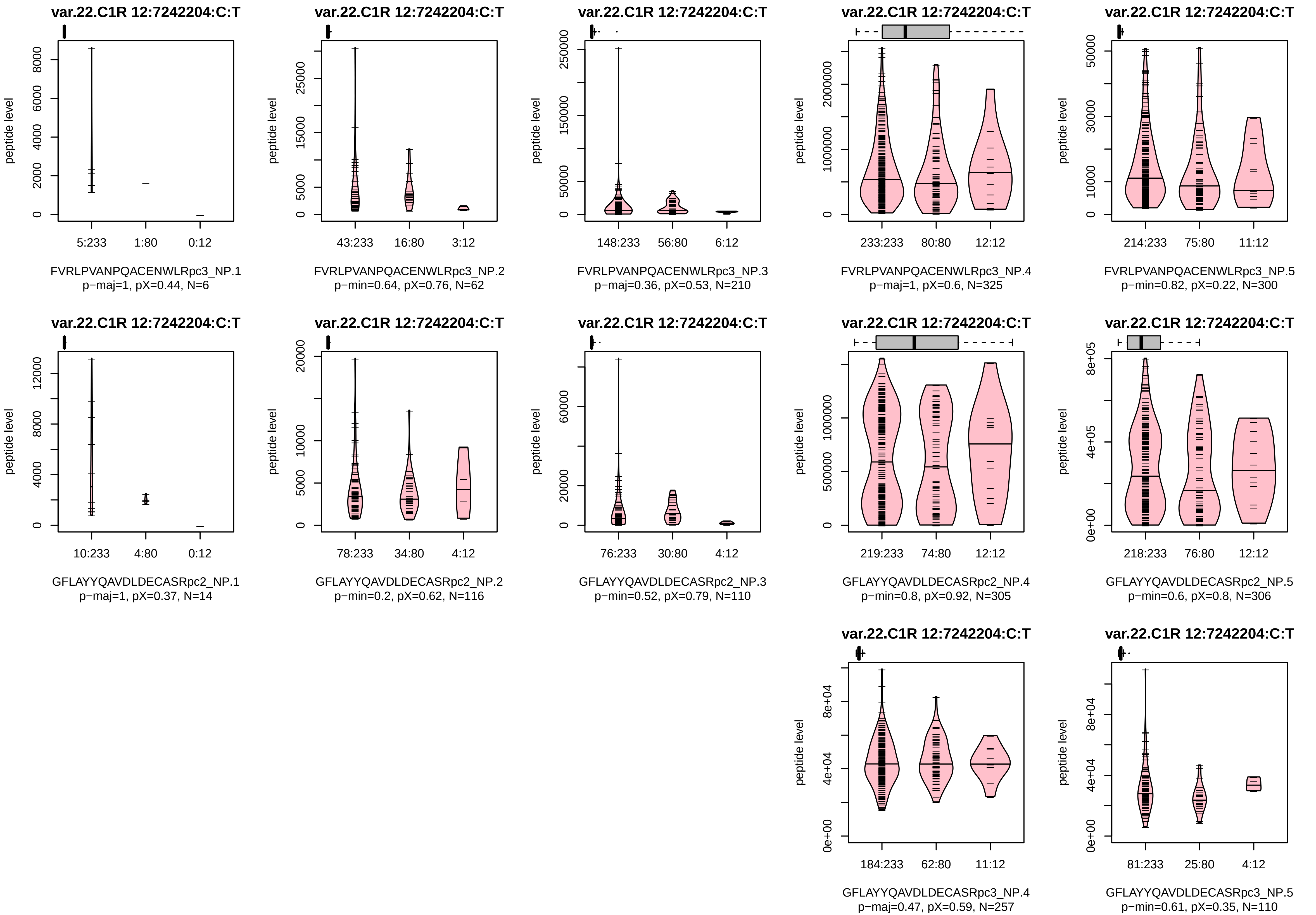

var.22.C1R 12:7242204:C:T
peptide level
5:233
1:80
0:12
FVRLPVANPQACENWLRpc3_NP.1
p−maj=1, pX=0.44, N=6
var.22.C1R 12:7242204:C:T
peptide level
43:233
16:80
3:12
FVRLPVANPQACENWLRpc3_NP.2
p−min=0.64, pX=0.76, N=62
var.22.C1R 12:7242204:C:T
peptide level
148:233
56:80
6:12
FVRLPVANPQACENWLRpc3_NP.3
p−maj=0.36, pX=0.53, N=210
var.22.C1R 12:7242204:C:T
peptide level
233:233
80:80
12:12
FVRLPVANPQACENWLRpc3_NP.4
p−maj=1, pX=0.6, N=325
var.22.C1R 12:7242204:C:T
peptide level
214:233
75:80
11:12
FVRLPVANPQACENWLRpc3_NP.5
p−min=0.82, pX=0.22, N=300
var.22.C1R 12:7242204:C:T
peptide level
10:233
4:80
0:12
GFLAYYQAVDLDECASRpc2_NP.1
p−maj=1, pX=0.37, N=14
var.22.C1R 12:7242204:C:T
peptide level
78:233
34:80
4:12
GFLAYYQAVDLDECASRpc2_NP.2
p−min=0.2, pX=0.62, N=116
var.22.C1R 12:7242204:C:T
peptide level
76:233
30:80
4:12
GFLAYYQAVDLDECASRpc2_NP.3
p−min=0.52, pX=0.79, N=110
var.22.C1R 12:7242204:C:T
peptide level
219:233
74:80
12:12
GFLAYYQAVDLDECASRpc2_NP.4
p−min=0.8, pX=0.92, N=305
var.22.C1R 12:7242204:C:T
peptide level
218:233
76:80
12:12
GFLAYYQAVDLDECASRpc2_NP.5
p−min=0.6, pX=0.8, N=306
var.22.C1R 12:7242204:C:T
peptide level
184:233
62:80
11:12
GFLAYYQAVDLDECASRpc3_NP.4
p−maj=0.47, pX=0.59, N=257
var.22.C1R 12:7242204:C:T
peptide level
81:233
25:80
4:12
GFLAYYQAVDLDECASRpc3_NP.5
p−min=0.61, pX=0.35, N=110

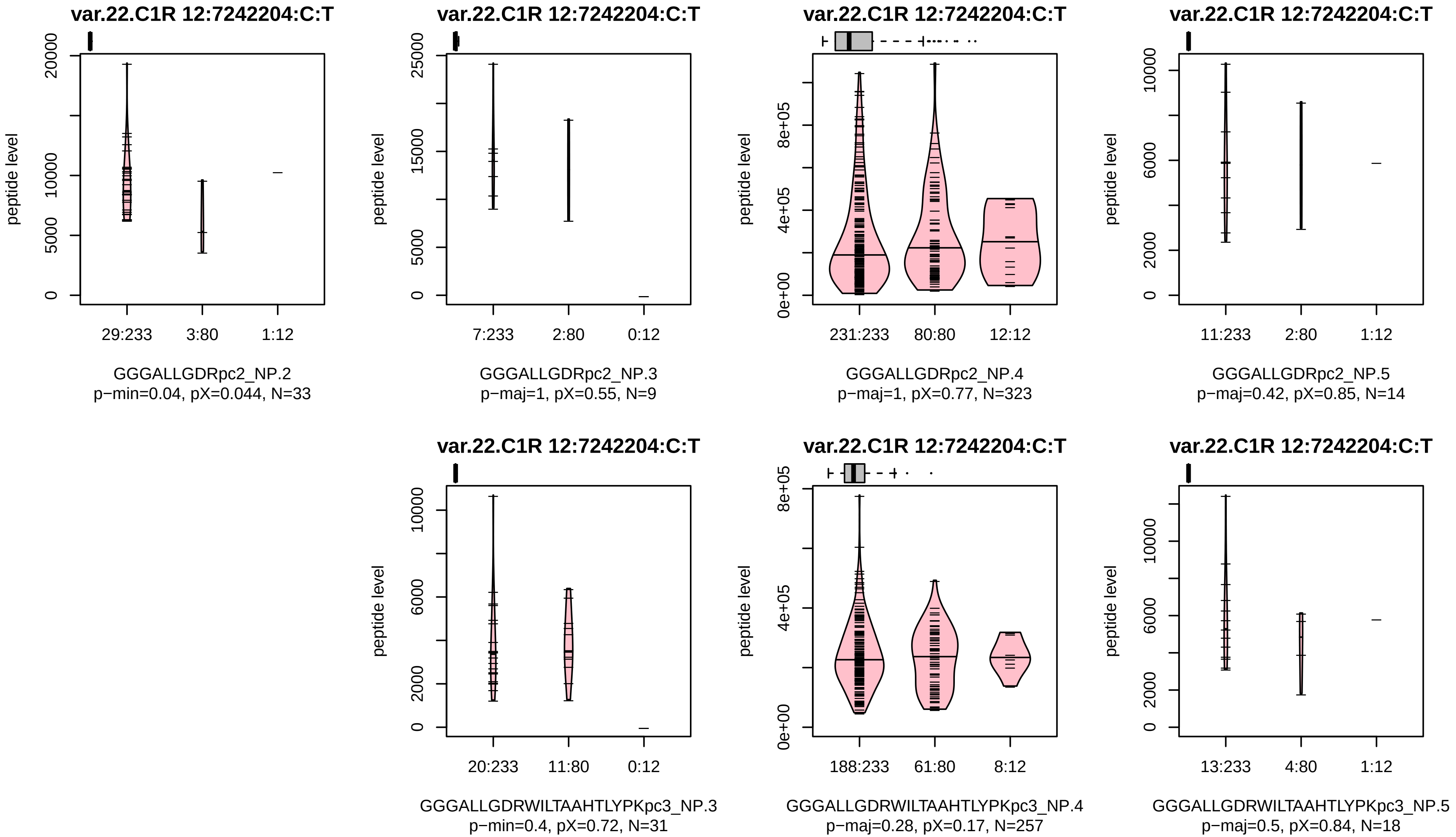
var.22.C1R 12:7242204:C:T
peptide level
29:233
3:80
1:12
GGGALLGDRpc2_NP.2
p−min=0.04, pX=0.044, N=33
var.22.C1R 12:7242204:C:T
peptide level
7:233
2:80
0:12
GGGALLGDRpc2_NP.3
p−maj=1, pX=0.55, N=9
var.22.C1R 12:7242204:C:T
peptide level
231:233
80:80
12:12
GGGALLGDRpc2_NP.4
p−maj=1, pX=0.77, N=323
var.22.C1R 12:7242204:C:T
peptide level
11:233
2:80
1:12
GGGALLGDRpc2_NP.5
p−maj=0.42, pX=0.85, N=14
var.22.C1R 12:7242204:C:T
peptide level
20:233
11:80
0:12
GGGALLGDRWILTAAHTLYPKpc3_NP.3
p−min=0.4, pX=0.72, N=31
var.22.C1R 12:7242204:C:T
peptide level
188:233
61:80
8:12
GGGALLGDRWILTAAHTLYPKpc3_NP.4
p−maj=0.28, pX=0.17, N=257
var.22.C1R 12:7242204:C:T
peptide level
13:233
4:80
1:12
GGGALLGDRWILTAAHTLYPKpc3_NP.5
p−maj=0.5, pX=0.84, N=18

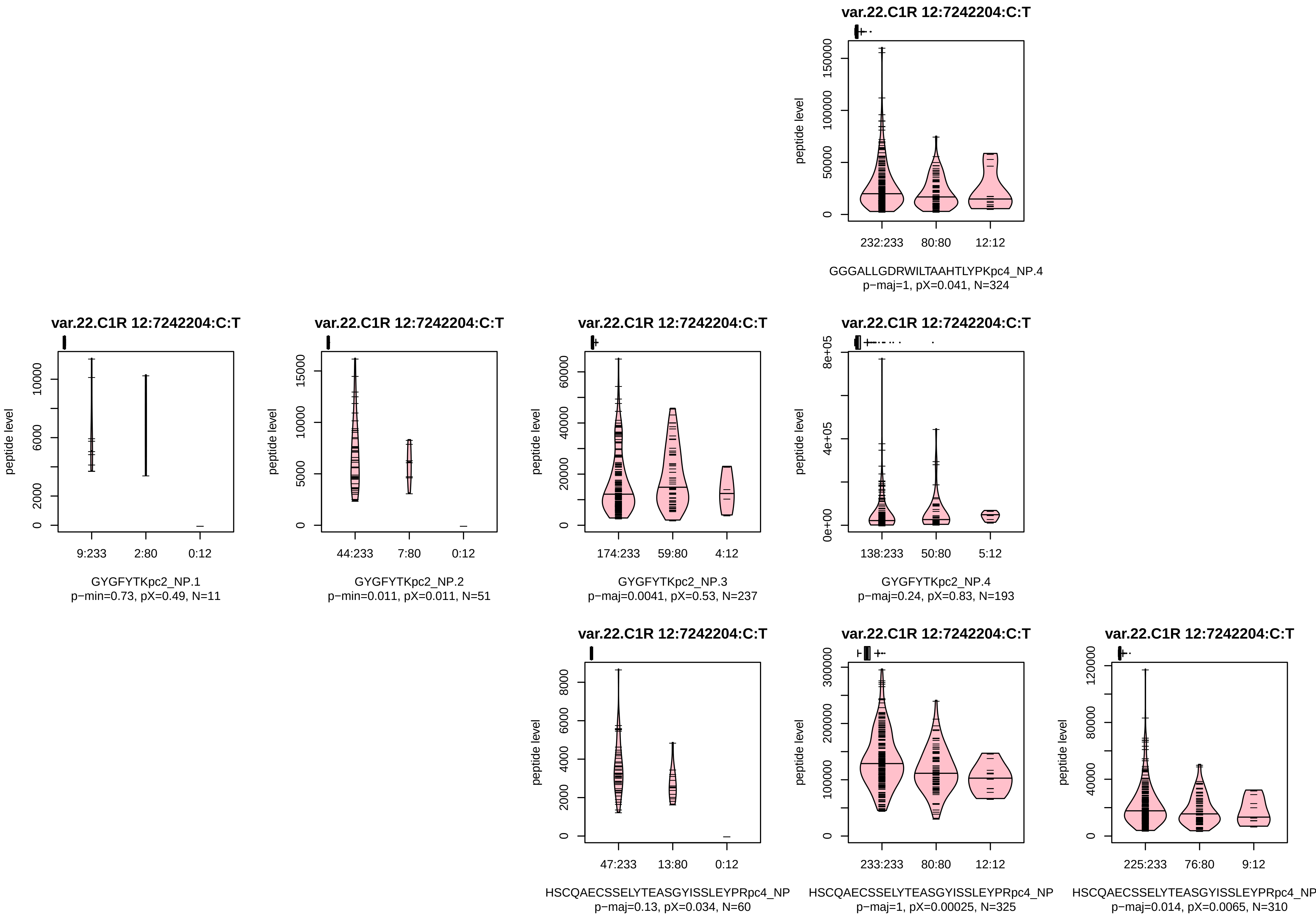
var.22.C1R 12:7242204:C:T
peptide level
232:233
80:80
12:12
GGGALLGDRWILTAAHTLYPKpc4_NP.4
p−maj=1, pX=0.041, N=324
var.22.C1R 12:7242204:C:T
9:233
2:80
0:12
GYGFYTKpc2_NP.1
p−min=0.73, pX=0.49, N=11
var.22.C1R 12:7242204:C:T
44:233
7:80
0:12
GYGFYTKpc2_NP.2
p−min=0.011, pX=0.011, N=51
var.22.C1R 12:7242204:C:T
174:233
59:80
4:12
GYGFYTKpc2_NP.3
p−maj=0.0041, pX=0.53, N=237
var.22.C1R 12:7242204:C:T
138:233
50:80
5:12
GYGFYTKpc2_NP.4
p−maj=0.24, pX=0.83, N=193
var.22.C1R 12:7242204:C:T
47:233
13:80
0:12
HSCQAECSSELYTEASGYISSLEYPRpc4_NP
p−maj=0.13, pX=0.034, N=60
var.22.C1R 12:7242204:C:T
233:233
80:80
12:12
HSCQAECSSELYTEASGYISSLEYPRpc4_NP
p−maj=1, pX=0.00025, N=325
var.22.C1R 12:7242204:C:T
225:233
76:80
9:12
HSCQAECSSELYTEASGYISSLEYPRpc4_NP
p−maj=0.014, pX=0.0065, N=310

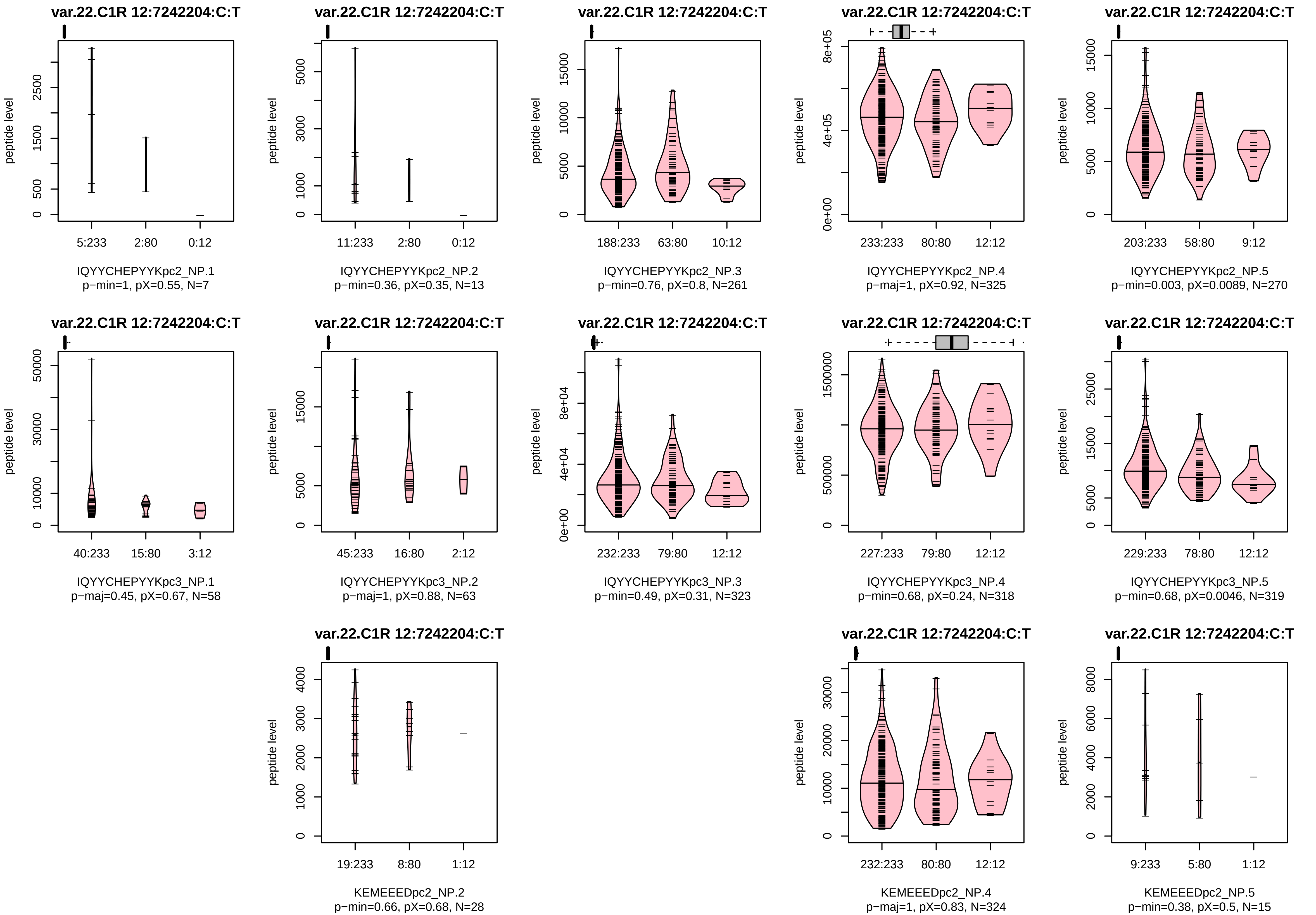
var.22.C1R 12:7242204:C:T
peptide level
5:233
2:80
0:12
IQYYCHEPYYKpc2_NP.1
p−min=1, pX=0.55, N=7
var.22.C1R 12:7242204:C:T
peptide level
11:233
2:80
0:12
IQYYCHEPYYKpc2_NP.2
p−min=0.36, pX=0.35, N=13
var.22.C1R 12:7242204:C:T
peptide level
188:233
63:80
10:12
IQYYCHEPYYKpc2_NP.3
p−min=0.76, pX=0.8, N=261
var.22.C1R 12:7242204:C:T
peptide level
233:233
80:80
12:12
IQYYCHEPYYKpc2_NP.4
p−maj=1, pX=0.92, N=325
var.22.C1R 12:7242204:C:T
peptide level
203:233
58:80
9:12
IQYYCHEPYYKpc2_NP.5
p−min=0.003, pX=0.0089, N=270
var.22.C1R 12:7242204:C:T
peptide level
40:233
15:80
3:12
IQYYCHEPYYKpc3_NP.1
p−maj=0.45, pX=0.67, N=58
var.22.C1R 12:7242204:C:T
peptide level
45:233
16:80
2:12
IQYYCHEPYYKpc3_NP.2
p−maj=1, pX=0.88, N=63
var.22.C1R 12:7242204:C:T
peptide level
232:233
79:80
12:12
IQYYCHEPYYKpc3_NP.3
p−min=0.49, pX=0.31, N=323
var.22.C1R 12:7242204:C:T
peptide level
227:233
79:80
12:12
IQYYCHEPYYKpc3_NP.4
p−min=0.68, pX=0.24, N=318
var.22.C1R 12:7242204:C:T
peptide level
229:233
78:80
12:12
IQYYCHEPYYKpc3_NP.5
p−min=0.68, pX=0.0046, N=319
var.22.C1R 12:7242204:C:T
peptide level
19:233
8:80
1:12
KEMEEEDpc2_NP.2
p−min=0.66, pX=0.68, N=28
var.22.C1R 12:7242204:C:T
peptide level
232:233
80:80
12:12
KEMEEEDpc2_NP.4
p−maj=1, pX=0.83, N=324
var.22.C1R 12:7242204:C:T
peptide level
9:233
5:80
1:12
KEMEEEDpc2_NP.5
p−min=0.38, pX=0.5, N=15

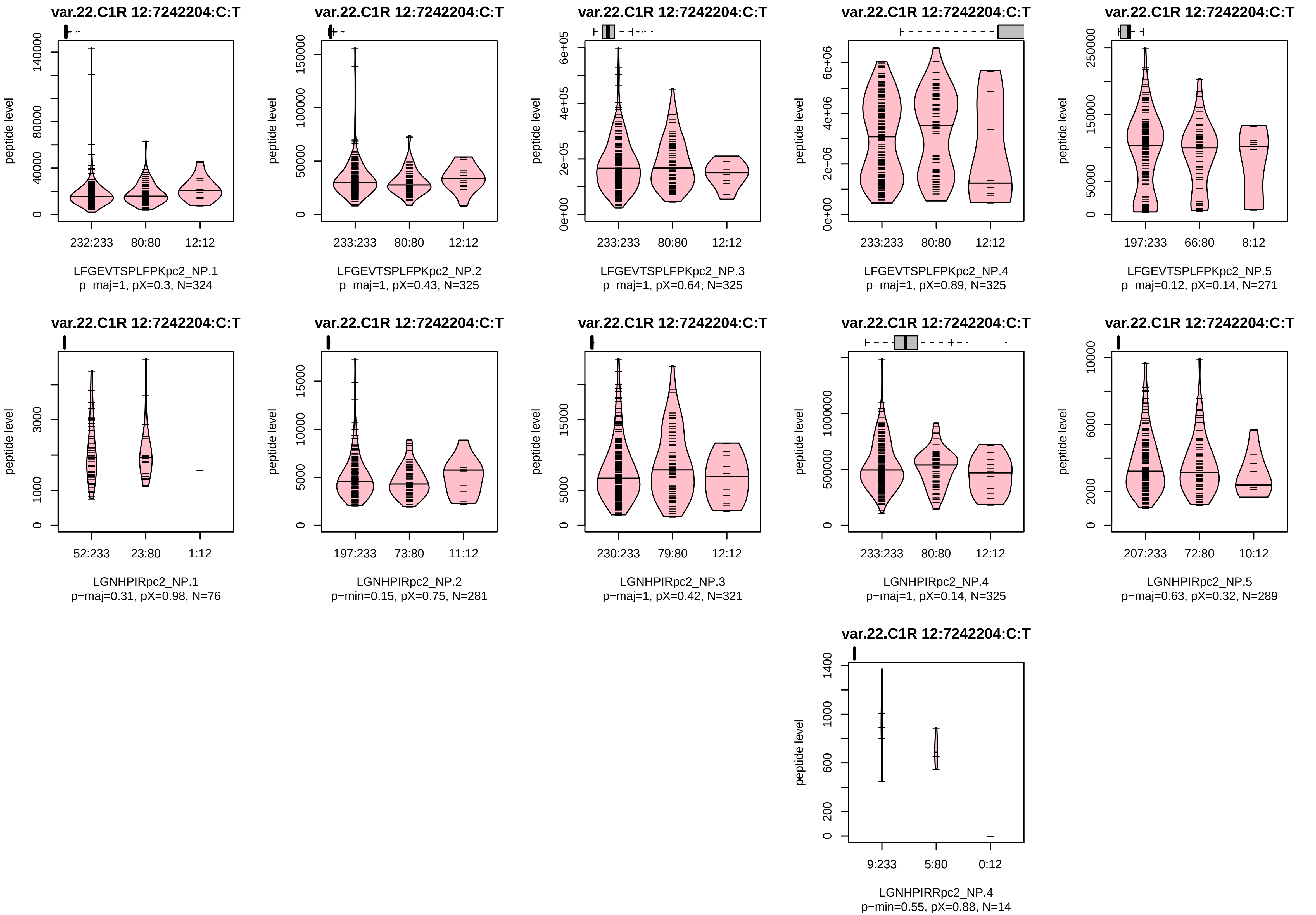
var.22.C1R 12:7242204:C:T
peptide level
232:233
80:80
12:12
LFGEVTSPLFPKpc2_NP.1
p−maj=1, pX=0.3, N=324
var.22.C1R 12:7242204:C:T
233:233
80:80
12:12
LFGEVTSPLFPKpc2_NP.2
p−maj=1, pX=0.43, N=325
var.22.C1R 12:7242204:C:T
233:233
80:80
12:12
LFGEVTSPLFPKpc2_NP.3
p−maj=1, pX=0.64, N=325
var.22.C1R 12:7242204:C:T
233:233
80:80
12:12
LFGEVTSPLFPKpc2_NP.4
p−maj=1, pX=0.89, N=325
var.22.C1R 12:7242204:C:T
197:233
66:80
8:12
LFGEVTSPLFPKpc2_NP.5
p−maj=0.12, pX=0.14, N=271
var.22.C1R 12:7242204:C:T
52:233
23:80
1:12
LGNHPIRpc2_NP.1
p−maj=0.31, pX=0.98, N=76
var.22.C1R 12:7242204:C:T
197:233
73:80
11:12
LGNHPIRpc2_NP.2
p−min=0.15, pX=0.75, N=281
var.22.C1R 12:7242204:C:T
230:233
79:80
12:12
LGNHPIRpc2_NP.3
p−maj=1, pX=0.42, N=321
var.22.C1R 12:7242204:C:T
233:233
80:80
12:12
LGNHPIRpc2_NP.4
p−maj=1, pX=0.14, N=325
var.22.C1R 12:7242204:C:T
207:233
72:80
10:12
LGNHPIRpc2_NP.5
p−maj=0.63, pX=0.32, N=289
var.22.C1R 12:7242204:C:T
9:233
5:80
0:12
LGNHPIRRpc2_NP.4
p−min=0.55, pX=0.88, N=14

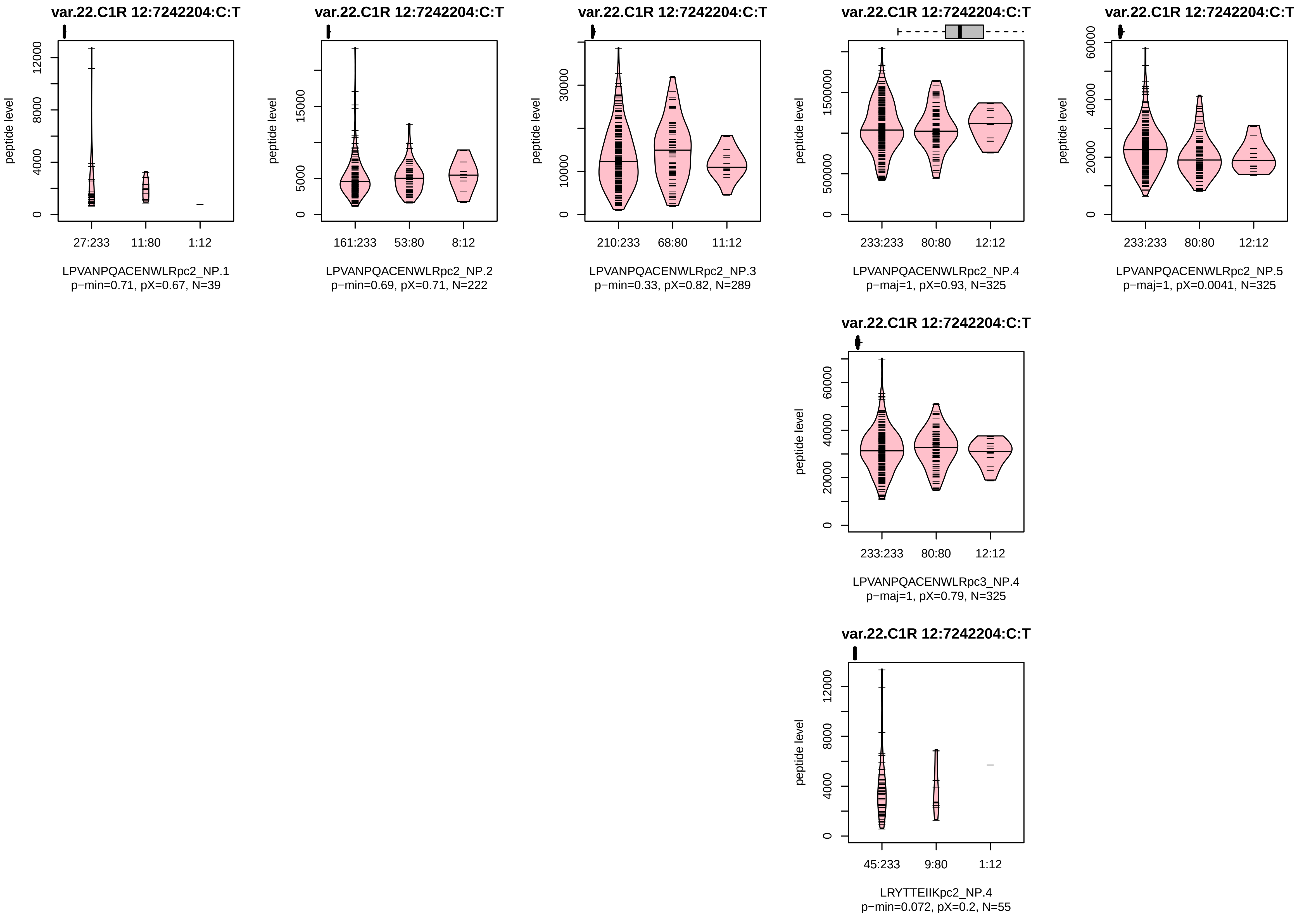

var.22.C1R 12:7242204:C:T
peptide level
0
4000
8000
12000
27:233
11:80
1:12
LPVANPQACENWLRpc2_NP.1
p−min=0.71, pX=0.67, N=39
var.22.C1R 12:7242204:C:T
peptide level
0
5000
15000
161:233
53:80
8:12
LPVANPQACENWLRpc2_NP.2
p−min=0.69, pX=0.71, N=222
var.22.C1R 12:7242204:C:T
peptide level
0
10000
30000
210:233
68:80
11:12
LPVANPQACENWLRpc2_NP.3
p−min=0.33, pX=0.82, N=289
var.22.C1R 12:7242204:C:T
peptide level
0
500000
1500000
233:233
80:80
12:12
LPVANPQACENWLRpc2_NP.4
p−maj=1, pX=0.93, N=325
var.22.C1R 12:7242204:C:T
peptide level
0
20000
40000
60000
233:233
80:80
12:12
LPVANPQACENWLRpc2_NP.5
p−maj=1, pX=0.0041, N=325
var.22.C1R 12:7242204:C:T
peptide level
0
20000
40000
60000
233:233
80:80
12:12
LPVANPQACENWLRpc3_NP.4
p−maj=1, pX=0.79, N=325
var.22.C1R 12:7242204:C:T
peptide level
0
4000
8000
12000
45:233
9:80
1:12
LRYTTEIIKpc2_NP.4
p−min=0.072, pX=0.2, N=55

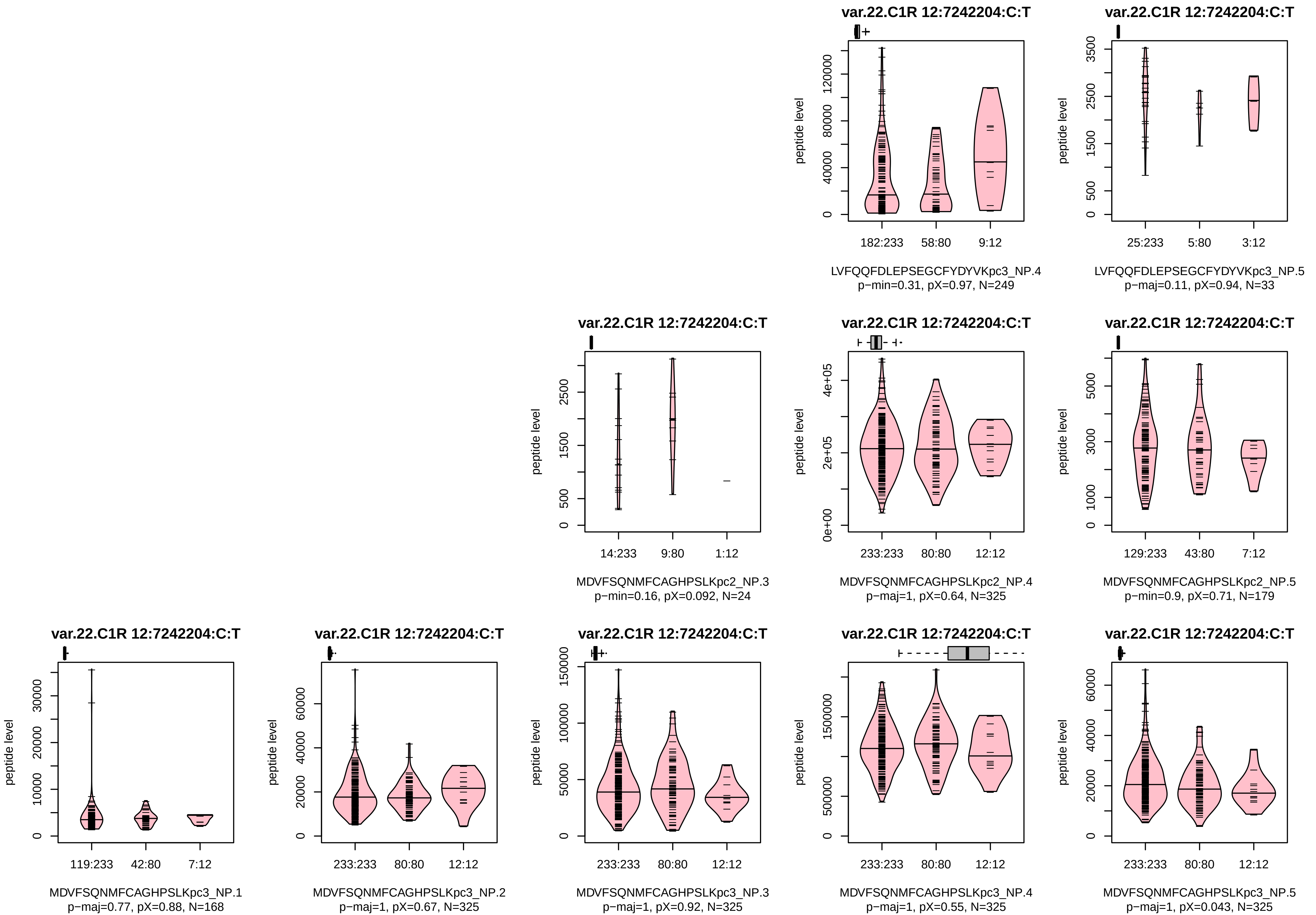
var.22.C1R 12:7242204:C:T
LVFQQFDLEPSEGCFYDYVKpc3_NP.4
p−min=0.31, pX=0.97, N=249
182:233
58:80
9:12
var.22.C1R 12:7242204:C:T
LVFQQFDLEPSEGCFYDYVKpc3_NP.5
p−maj=0.11, pX=0.94, N=33
25:233
5:80
3:12
var.22.C1R 12:7242204:C:T
MDVFSQNMFCAGHPSLKpc2_NP.3
p−min=0.16, pX=0.092, N=24
14:233
9:80
1:12
var.22.C1R 12:7242204:C:T
MDVFSQNMFCAGHPSLKpc2_NP.4
p−maj=1, pX=0.64, N=325
233:233
80:80
12:12
var.22.C1R 12:7242204:C:T
MDVFSQNMFCAGHPSLKpc2_NP.5
p−min=0.9, pX=0.71, N=179
129:233
43:80
7:12
var.22.C1R 12:7242204:C:T
MDVFSQNMFCAGHPSLKpc3_NP.1
p−maj=0.77, pX=0.88, N=168
119:233
42:80
7:12
var.22.C1R 12:7242204:C:T
MDVFSQNMFCAGHPSLKpc3_NP.2
p−maj=1, pX=0.67, N=325
233:233
80:80
12:12
var.22.C1R 12:7242204:C:T
MDVFSQNMFCAGHPSLKpc3_NP.3
p−maj=1, pX=0.92, N=325
233:233
80:80
12:12
var.22.C1R 12:7242204:C:T
MDVFSQNMFCAGHPSLKpc3_NP.4
p−maj=1, pX=0.55, N=325
233:233
80:80
12:12
var.22.C1R 12:7242204:C:T
MDVFSQNMFCAGHPSLKpc3_NP.5
p−maj=1, pX=0.043, N=325
233:233
80:80
12:12
peptide level

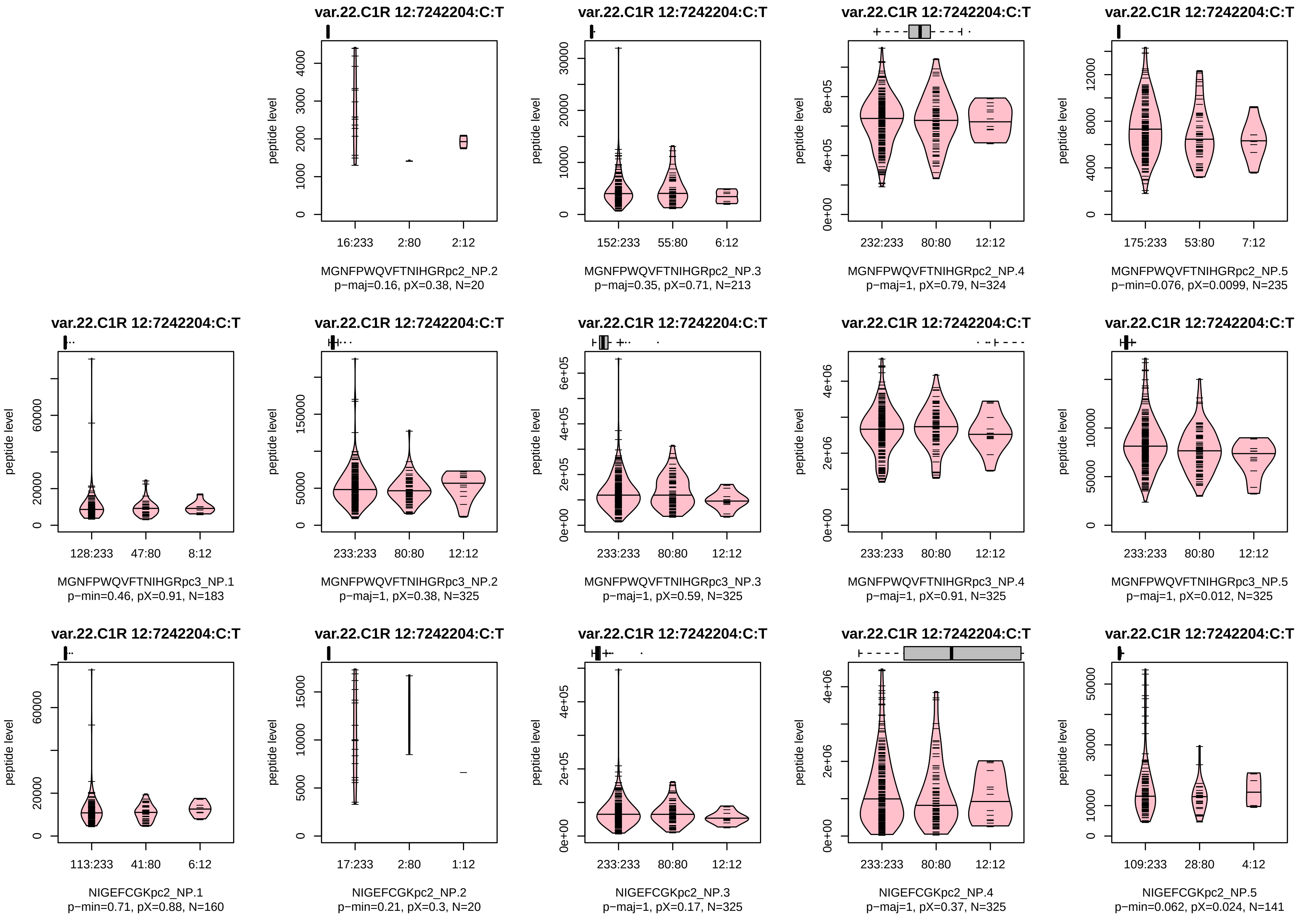
var.22.C1R 12:7242204:C:T
peptide level
16:233
2:80
2:12
MGNFPWQVFTNIHGRpc2_NP.2
p−maj=0.16, pX=0.38, N=20
var.22.C1R 12:7242204:C:T
152:233
55:80
6:12
MGNFPWQVFTNIHGRpc2_NP.3
p−maj=0.35, pX=0.71, N=213
var.22.C1R 12:7242204:C:T
232:233
80:80
12:12
MGNFPWQVFTNIHGRpc2_NP.4
p−maj=1, pX=0.79, N=324
var.22.C1R 12:7242204:C:T
175:233
53:80
7:12
MGNFPWQVFTNIHGRpc2_NP.5
p−min=0.076, pX=0.0099, N=235
var.22.C1R 12:7242204:C:T
128:233
47:80
8:12
MGNFPWQVFTNIHGRpc3_NP.1
p−min=0.46, pX=0.91, N=183
var.22.C1R 12:7242204:C:T
233:233
80:80
12:12
MGNFPWQVFTNIHGRpc3_NP.2
p−maj=1, pX=0.38, N=325
var.22.C1R 12:7242204:C:T
233:233
80:80
12:12
MGNFPWQVFTNIHGRpc3_NP.3
p−maj=1, pX=0.59, N=325
var.22.C1R 12:7242204:C:T
233:233
80:80
12:12
MGNFPWQVFTNIHGRpc3_NP.4
p−maj=1, pX=0.91, N=325
var.22.C1R 12:7242204:C:T
233:233
80:80
12:12
MGNFPWQVFTNIHGRpc3_NP.5
p−maj=1, pX=0.012, N=325
var.22.C1R 12:7242204:C:T
113:233
41:80
6:12
NIGEFCGKpc2_NP.1
p−min=0.71, pX=0.88, N=160
var.22.C1R 12:7242204:C:T
17:233
2:80
1:12
NIGEFCGKpc2_NP.2
p−min=0.21, pX=0.3, N=20
var.22.C1R 12:7242204:C:T
233:233
80:80
12:12
NIGEFCGKpc2_NP.3
p−maj=1, pX=0.17, N=325
var.22.C1R 12:7242204:C:T
233:233
80:80
12:12
NIGEFCGKpc2_NP.4
p−maj=1, pX=0.37, N=325
var.22.C1R 12:7242204:C:T
109:233
28:80
4:12
NIGEFCGKpc2_NP.5
p−min=0.062, pX=0.024, N=141

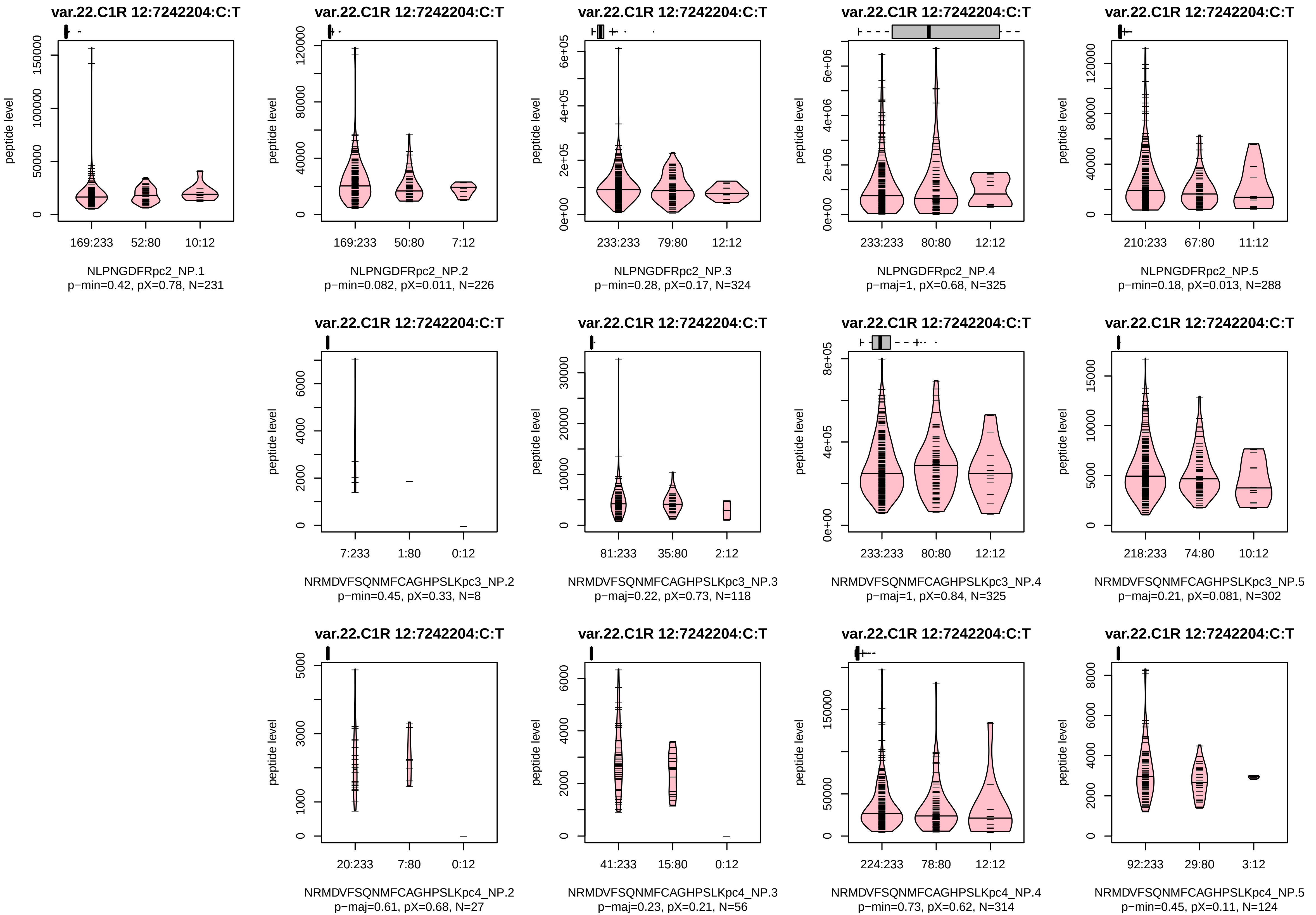

var.22.C1R 12:7242204:C:T
peptide level
169:233
52:80
10:12
NLPNGDFRpc2_NP.1
p−min=0.42, pX=0.78, N=231
var.22.C1R 12:7242204:C:T
peptide level
169:233
50:80
7:12
NLPNGDFRpc2_NP.2
p−min=0.082, pX=0.011, N=226
var.22.C1R 12:7242204:C:T
peptide level
233:233
79:80
12:12
NLPNGDFRpc2_NP.3
p−min=0.28, pX=0.17, N=324
var.22.C1R 12:7242204:C:T
peptide level
233:233
80:80
12:12
NLPNGDFRpc2_NP.4
p−maj=1, pX=0.68, N=325
var.22.C1R 12:7242204:C:T
peptide level
210:233
67:80
11:12
NLPNGDFRpc2_NP.5
p−min=0.18, pX=0.013, N=288
var.22.C1R 12:7242204:C:T
peptide level
7:233
1:80
0:12
NRMDVFSQNMFCAGHPSLKpc3_NP.2
p−min=0.45, pX=0.33, N=8
var.22.C1R 12:7242204:C:T
peptide level
81:233
35:80
2:12
NRMDVFSQNMFCAGHPSLKpc3_NP.3
p−maj=0.22, pX=0.73, N=118
var.22.C1R 12:7242204:C:T
peptide level
233:233
80:80
12:12
NRMDVFSQNMFCAGHPSLKpc3_NP.4
p−maj=1, pX=0.84, N=325
var.22.C1R 12:7242204:C:T
peptide level
218:233
74:80
10:12
NRMDVFSQNMFCAGHPSLKpc3_NP.5
p−maj=0.21, pX=0.081, N=302
var.22.C1R 12:7242204:C:T
peptide level
20:233
7:80
0:12
NRMDVFSQNMFCAGHPSLKpc4_NP.2
p−maj=0.61, pX=0.68, N=27
var.22.C1R 12:7242204:C:T
peptide level
41:233
15:80
0:12
NRMDVFSQNMFCAGHPSLKpc4_NP.3
p−maj=0.23, pX=0.21, N=56
var.22.C1R 12:7242204:C:T
peptide level
224:233
78:80
12:12
NRMDVFSQNMFCAGHPSLKpc4_NP.4
p−min=0.73, pX=0.62, N=314
var.22.C1R 12:7242204:C:T
peptide level
92:233
29:80
3:12
NRMDVFSQNMFCAGHPSLKpc4_NP.5
p−min=0.45, pX=0.11, N=124

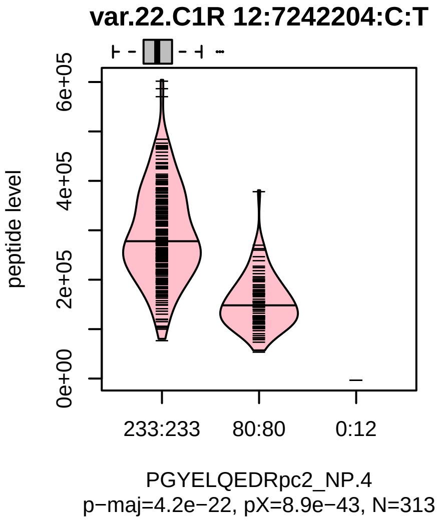
var.22.C1R 12:7242204:C:T
peptide level
0e+00
2e+05
4e+05
6e+05
233:233
80:80
0:12
PGYELQEDRpc2_NP.4
p−maj=4.2e−22, pX=8.9e−43, N=313

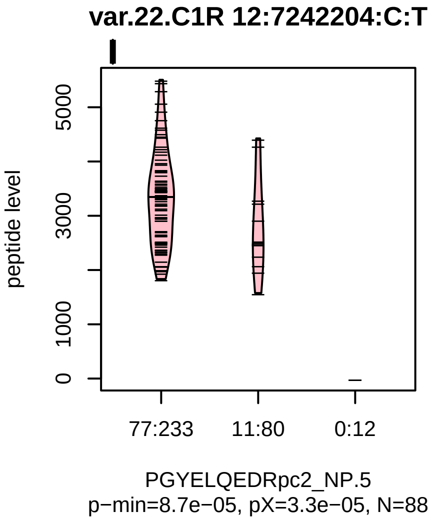
var.22.C1R 12:7242204:C:T
peptide level
0
1000
3000
5000
77:233
11:80
0:12
PGYELQEDRpc2_NP.5
p−min=8.7e−05, pX=3.3e−05, N=88

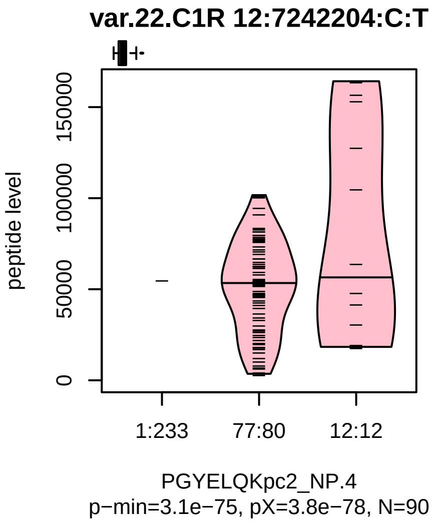
var.22.C1R 12:7242204:C:T
peptide level
0
50000
100000
150000
1:233
77:80
12:12
PGYELQKpc2_NP.4
p−min=3.1e−75, pX=3.8e−78, N=90

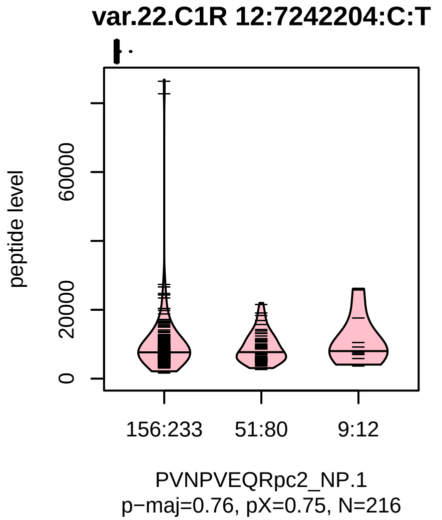
var.22.C1R 12:7242204:C:T
peptide level
0
20000
60000
156:233
51:80
9:12
PVNPVEQRpc2_NP.1
p−maj=0.76, pX=0.75, N=216

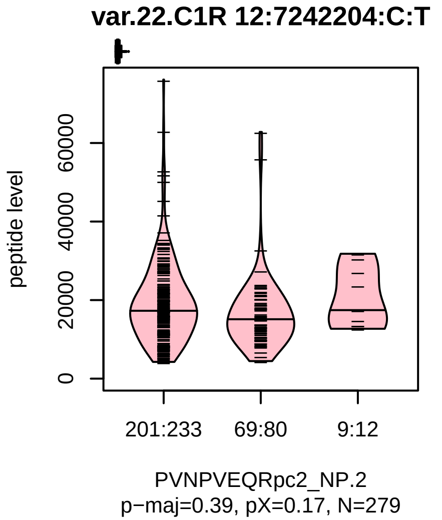
var.22.C1R 12:7242204:C:T
peptide level
0
20000
40000
60000
201:233
69:80
9:12
PVNPVEQRpc2_NP.2
p−maj=0.39, pX=0.17, N=279

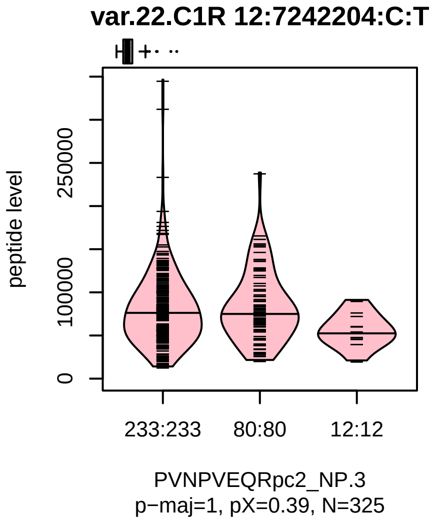
var.22.C1R 12:7242204:C:T
peptide level
0
100000
250000
233:233
80:80
12:12
PVNPVEQRpc2_NP.3
p−maj=1, pX=0.39, N=325

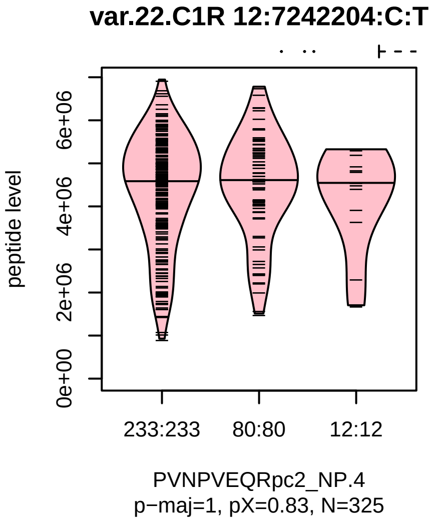
var.22.C1R 12:7242204:C:T
peptide level
0e+00
2e+06
4e+06
6e+06
233:233
80:80
12:12
PVNPVEQRpc2_NP.4
p−maj=1, pX=0.83, N=325

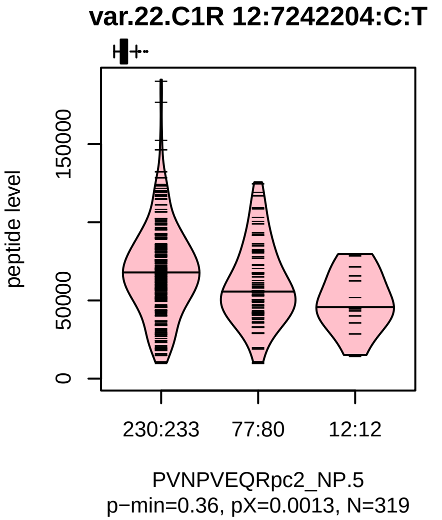
var.22.C1R 12:7242204:C:T
peptide level
0
50000
150000
230:233
77:80
12:12
PVNPVEQRpc2_NP.5
p−min=0.36, pX=0.0013, N=319

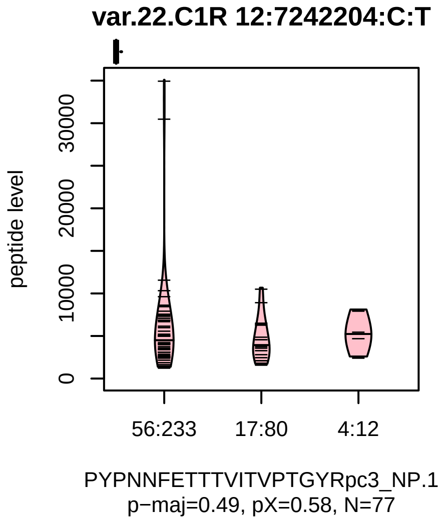
var.22.C1R 12:7242204:C:T
peptide level
0
10000
20000
30000
56:233
17:80
4:12
PYPNNFETTTVITVPTGYRpc3_NP.1
p−maj=0.49, pX=0.58, N=77

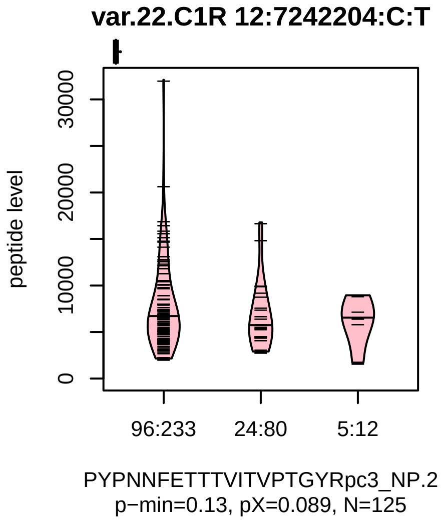
var.22.C1R 12:7242204:C:T
peptide level
0
10000
20000
30000
96:233
24:80
5:12
PYPNNFETTTVITVPTGYRpc3_NP.2
p−min=0.13, pX=0.089, N=125

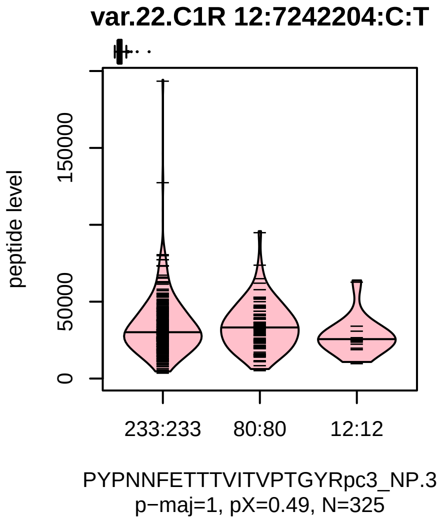
var.22.C1R 12:7242204:C:T
peptide level
0
50000
150000
233:233
80:80
12:12
PYPNNFETTTVITVPTGYRpc3_NP.3
p−maj=1, pX=0.49, N=325

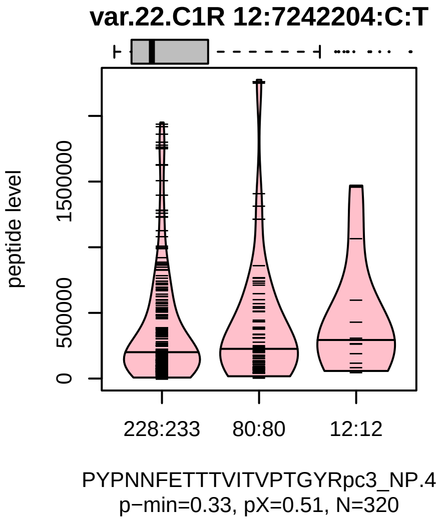
var.22.C1R 12:7242204:C:T
peptide level
0
500000
1500000
228:233
80:80
12:12
PYPNNFETTTVITVPTGYRpc3_NP.4
p−min=0.33, pX=0.51, N=320

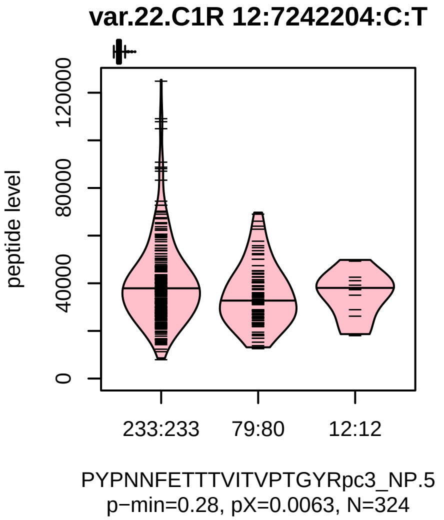
var.22.C1R 12:7242204:C:T
peptide level
0
40000
80000
120000
233:233
79:80
12:12
PYPNNFETTTVITVPTGYRpc3_NP.5
p−min=0.28, pX=0.0063, N=324

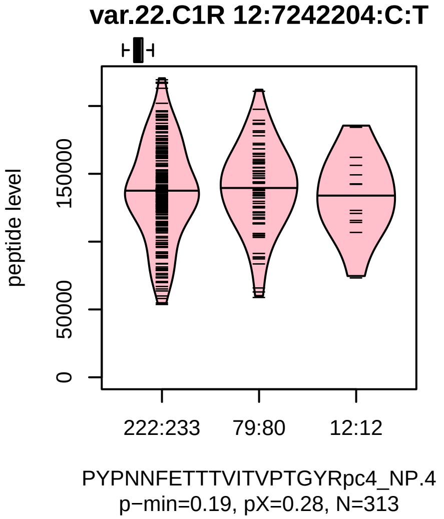
var.22.C1R 12:7242204:C:T
peptide level
0
50000
150000
222:233
79:80
12:12
PYPNNFETTTVITVPTGYRpc4_NP.4
p−min=0.19, pX=0.28, N=313

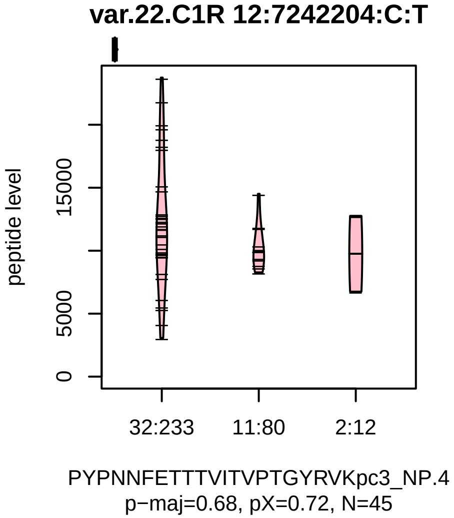
var.22.C1R 12:7242204:C:T
peptide level
0
5000
15000
32:233
11:80
2:12
PYPNNFETTTVITVPTGYRVKpc3_NP.4
p−maj=0.68, pX=0.72, N=45

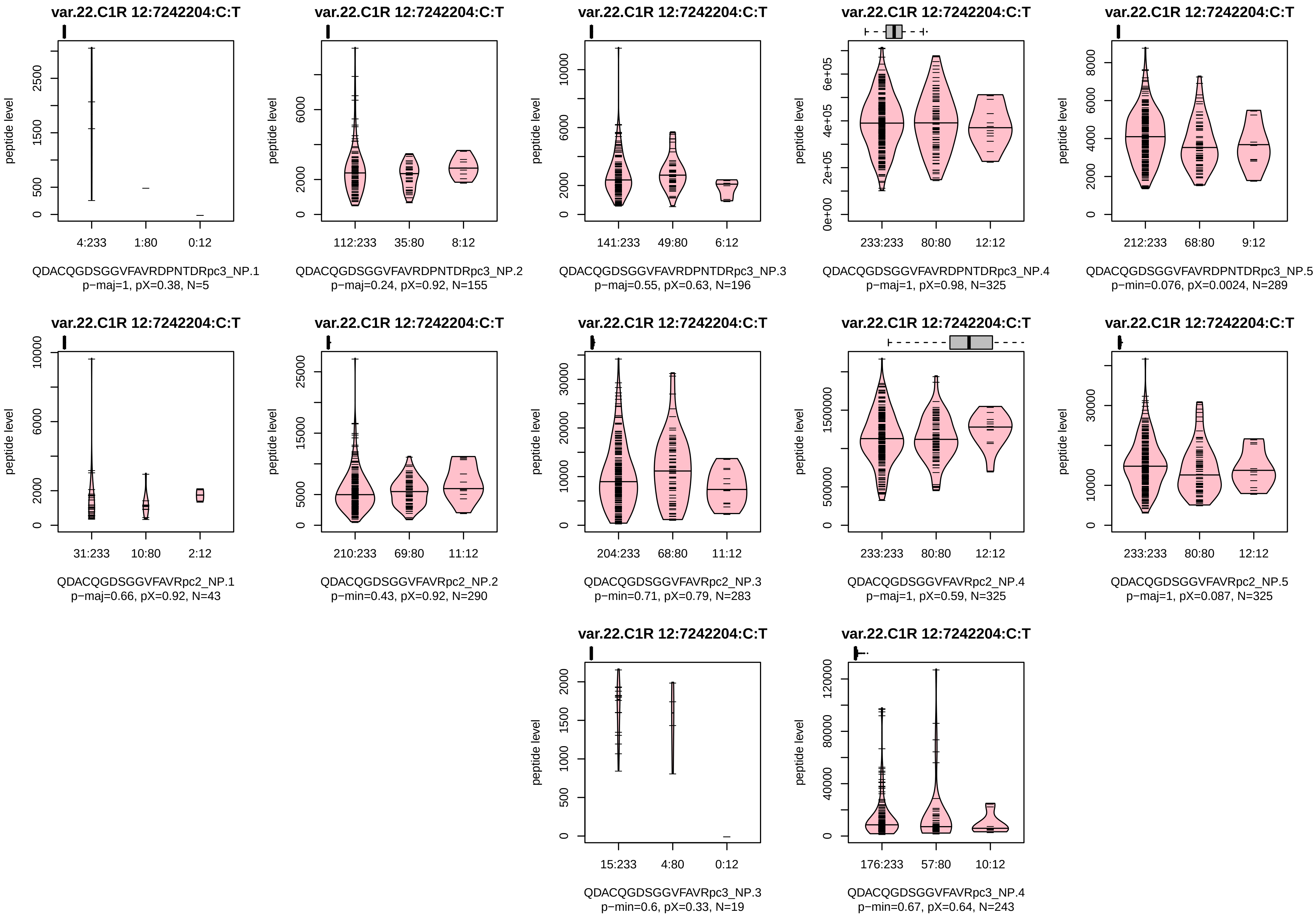
var.22.C1R 12:7242204:C:T
peptide level
4:233
1:80
0:12
QDACQGDSGGVFAVRDPNTDRpc3_NP.1
p−maj=1, pX=0.38, N=5
112:233
35:80
8:12
QDACQGDSGGVFAVRDPNTDRpc3_NP.2
p−maj=0.24, pX=0.92, N=155
141:233
49:80
6:12
QDACQGDSGGVFAVRDPNTDRpc3_NP.3
p−maj=0.55, pX=0.63, N=196
233:233
80:80
12:12
QDACQGDSGGVFAVRDPNTDRpc3_NP.4
p−maj=1, pX=0.98, N=325
212:233
68:80
9:12
QDACQGDSGGVFAVRDPNTDRpc3_NP.5
p−min=0.076, pX=0.0024, N=289
31:233
10:80
2:12
QDACQGDSGGVFAVRpc2_NP.1
p−maj=0.66, pX=0.92, N=43
210:233
69:80
11:12
QDACQGDSGGVFAVRpc2_NP.2
p−min=0.43, pX=0.92, N=290
204:233
68:80
11:12
QDACQGDSGGVFAVRpc2_NP.3
p−min=0.71, pX=0.79, N=283
233:233
80:80
12:12
QDACQGDSGGVFAVRpc2_NP.4
p−maj=1, pX=0.59, N=325
233:233
80:80
12:12
QDACQGDSGGVFAVRpc2_NP.5
p−maj=1, pX=0.087, N=325
15:233
4:80
0:12
QDACQGDSGGVFAVRpc3_NP.3
p−min=0.6, pX=0.33, N=19
176:233
57:80
10:12
QDACQGDSGGVFAVRpc3_NP.4
p−min=0.67, pX=0.64, N=243

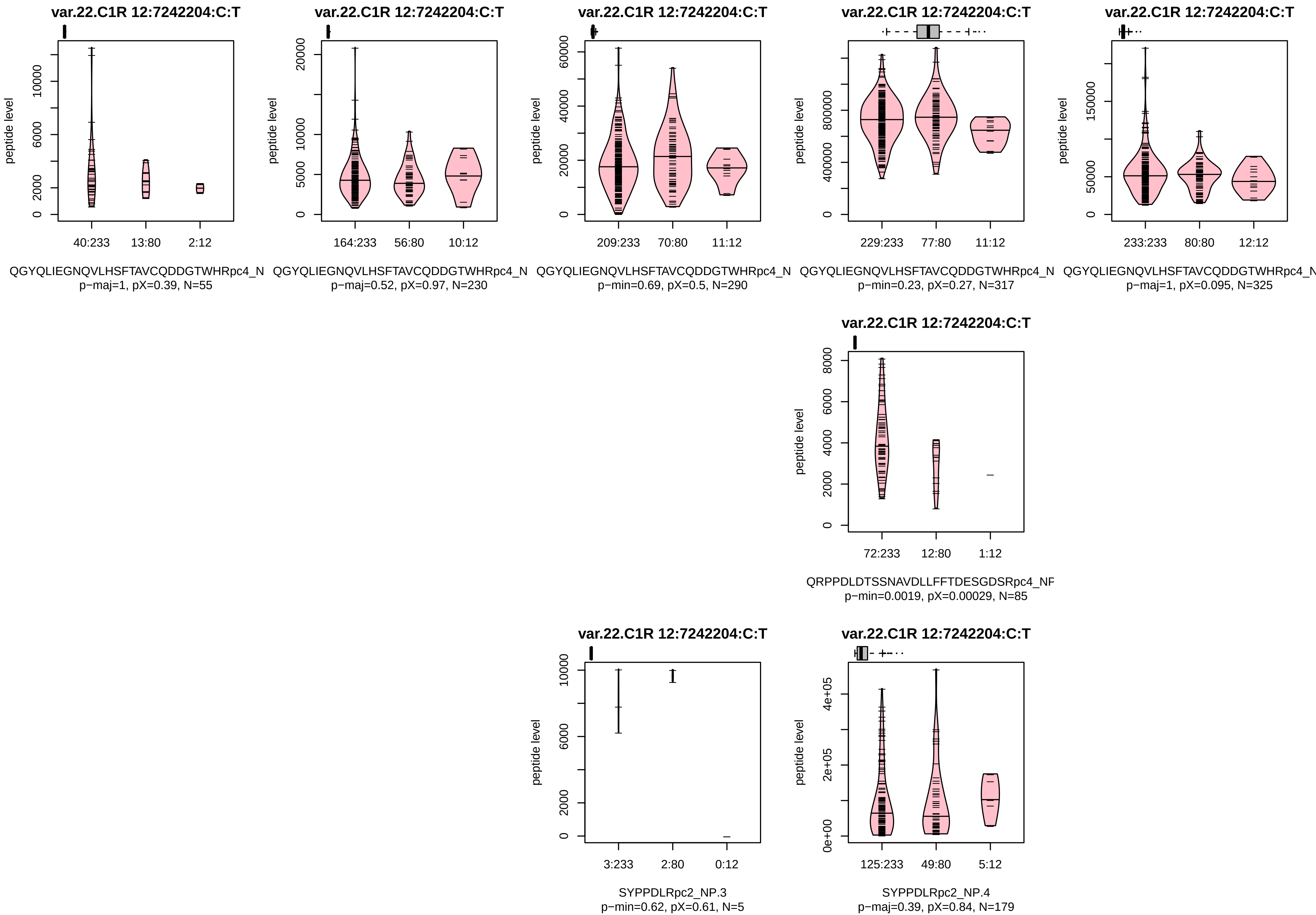
var.22.C1R 12:7242204:C:T
peptide level
40:233
13:80
2:12
QGYQLIEGNQVLHSFTAVCQDDGTWHRpc4_N
p−maj=1, pX=0.39, N=55
var.22.C1R 12:7242204:C:T
peptide level
164:233
56:80
10:12
QGYQLIEGNQVLHSFTAVCQDDGTWHRpc4_N
p−maj=0.52, pX=0.97, N=230
var.22.C1R 12:7242204:C:T
peptide level
209:233
70:80
11:12
QGYQLIEGNQVLHSFTAVCQDDGTWHRpc4_N
p−min=0.69, pX=0.5, N=290
var.22.C1R 12:7242204:C:T
peptide level
229:233
77:80
11:12
QGYQLIEGNQVLHSFTAVCQDDGTWHRpc4_N
p−min=0.23, pX=0.27, N=317
var.22.C1R 12:7242204:C:T
peptide level
233:233
80:80
12:12
QGYQLIEGNQVLHSFTAVCQDDGTWHRpc4_N
p−maj=1, pX=0.095, N=325
var.22.C1R 12:7242204:C:T
peptide level
72:233
12:80
1:12
QRPPDLDTSSNAVDLLFFTDESGDSRpc4_NF
p−min=0.0019, pX=0.00029, N=85
var.22.C1R 12:7242204:C:T
peptide level
3:233
2:80
0:12
SYPPDLRpc2_NP.3
p−min=0.62, pX=0.61, N=5
var.22.C1R 12:7242204:C:T
peptide level
125:233
49:80
5:12
SYPPDLRpc2_NP.4
p−maj=0.39, pX=0.84, N=179

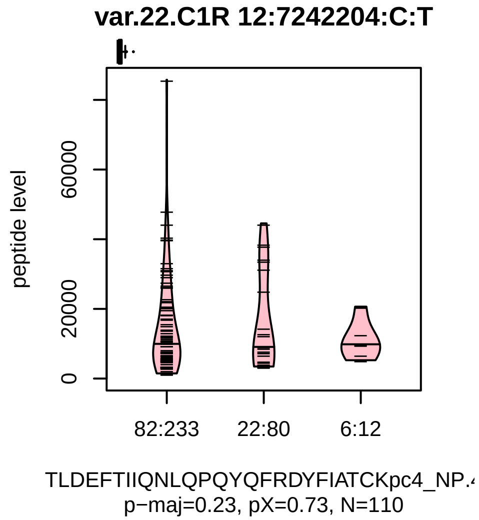
var.22.C1R 12:7242204:C:T
peptide level
0
20000
60000
82:233
22:80
6:12
TLDEFTIIQNLQPQYQFRDYFIATCKpc4_NP.4
p−maj=0.23, pX=0.73, N=110

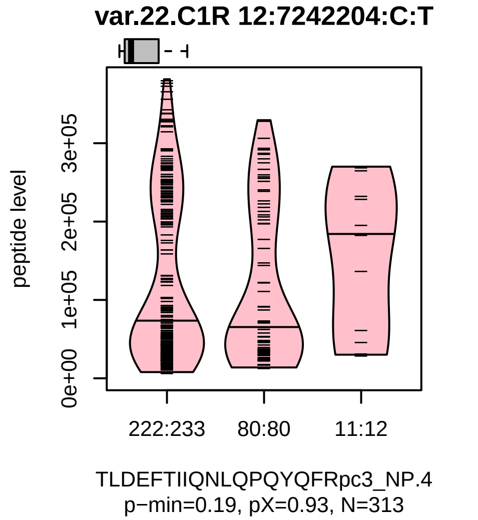
var.22.C1R 12:7242204:C:T
peptide level
0e+00
1e+05
2e+05
3e+05
222:233
80:80
11:12
TLDEFTIIQNLQPQYQFRpc3_NP.4
p−min=0.19, pX=0.93, N=313

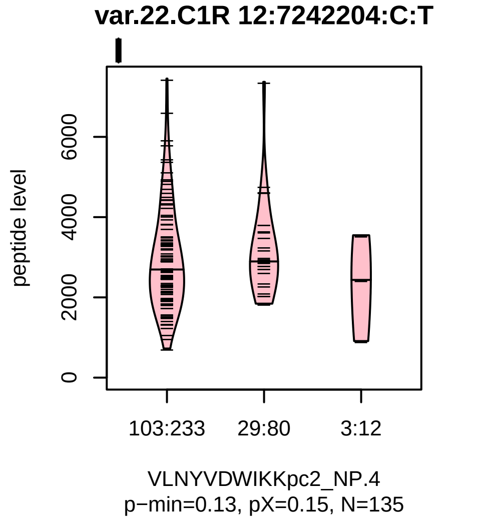
var.22.C1R 12:7242204:C:T
peptide level
0
2000
4000
6000
103:233
29:80
3:12
VLNYVDWIKKpc2_NP.4
p−min=0.13, pX=0.15, N=135

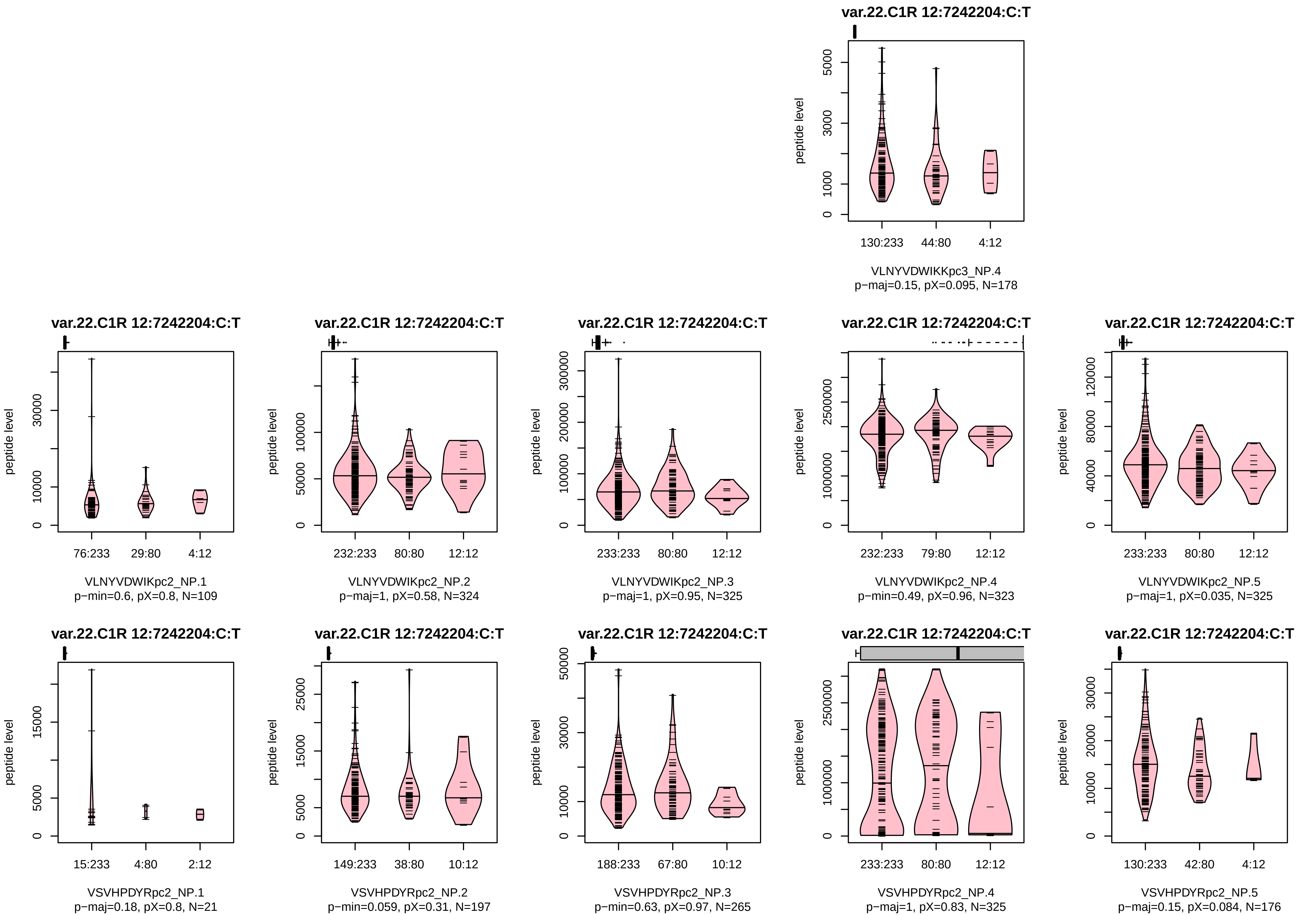
var.22.C1R 12:7242204:C:T
peptide level
130:233
44:80
4:12
VLNYVDWIKKpc3_NP.4
p−maj=0.15, pX=0.095, N=178
var.22.C1R 12:7242204:C:T
76:233
29:80
4:12
VLNYVDWIKpc2_NP.1
p−min=0.6, pX=0.8, N=109
var.22.C1R 12:7242204:C:T
232:233
80:80
12:12
VLNYVDWIKpc2_NP.2
p−maj=1, pX=0.58, N=324
var.22.C1R 12:7242204:C:T
233:233
80:80
12:12
VLNYVDWIKpc2_NP.3
p−maj=1, pX=0.95, N=325
var.22.C1R 12:7242204:C:T
232:233
79:80
12:12
VLNYVDWIKpc2_NP.4
p−min=0.49, pX=0.96, N=323
var.22.C1R 12:7242204:C:T
233:233
80:80
12:12
VLNYVDWIKpc2_NP.5
p−maj=1, pX=0.035, N=325
var.22.C1R 12:7242204:C:T
15:233
4:80
2:12
VSVHPDYRpc2_NP.1
p−maj=0.18, pX=0.8, N=21
var.22.C1R 12:7242204:C:T
149:233
38:80
10:12
VSVHPDYRpc2_NP.2
p−min=0.059, pX=0.31, N=197
var.22.C1R 12:7242204:C:T
188:233
67:80
10:12
VSVHPDYRpc2_NP.3
p−min=0.63, pX=0.97, N=265
var.22.C1R 12:7242204:C:T
233:233
80:80
12:12
VSVHPDYRpc2_NP.4
p−maj=1, pX=0.83, N=325
var.22.C1R 12:7242204:C:T
130:233
42:80
4:12
VSVHPDYRpc2_NP.5
p−maj=0.15, pX=0.084, N=176

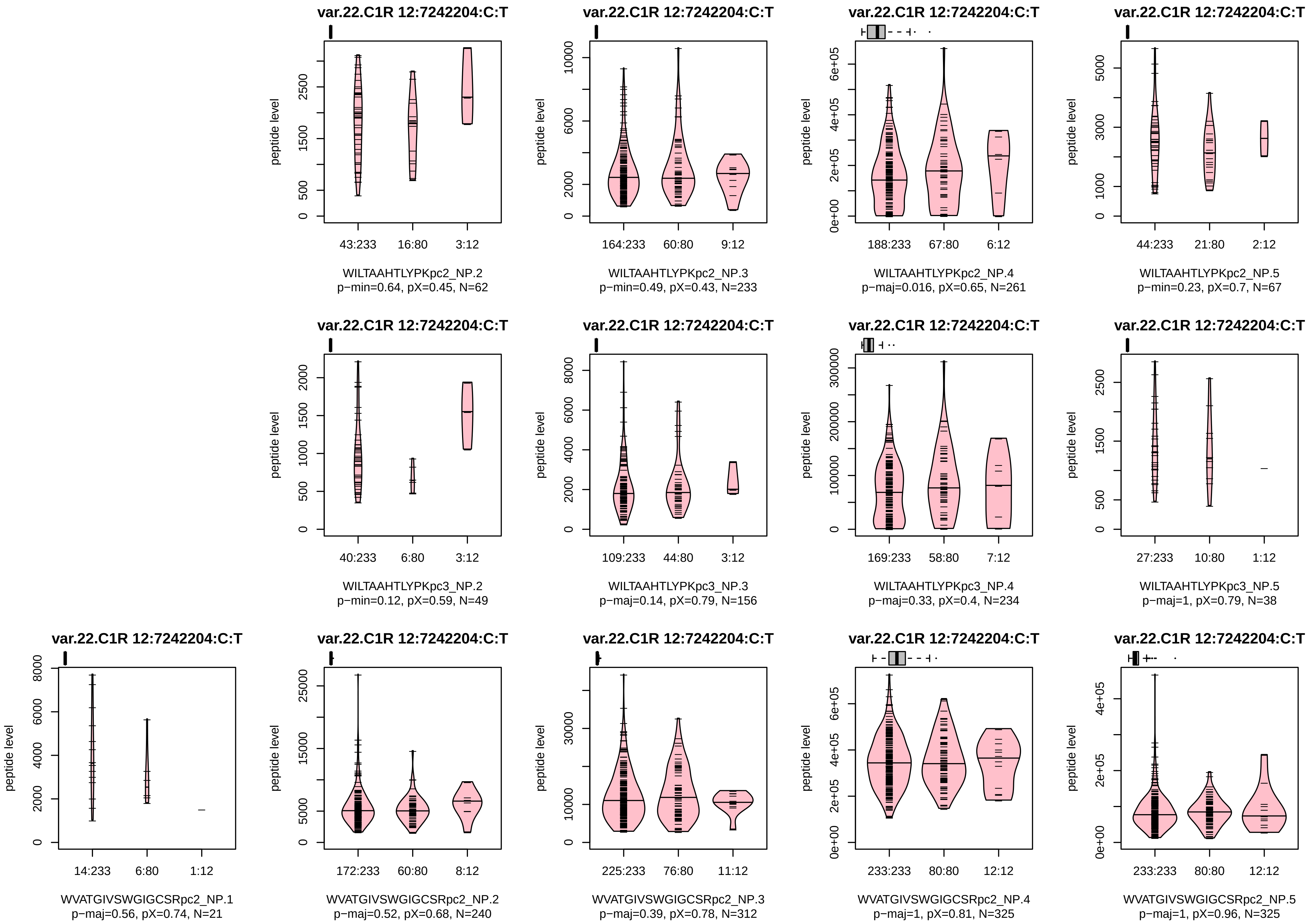
var.22.C1R 12:7242204:C:T
43:233
16:80
3:12
WILTAAHTLYPKpc2_NP.2
p−min=0.64, pX=0.45, N=62
var.22.C1R 12:7242204:C:T
164:233
60:80
9:12
WILTAAHTLYPKpc2_NP.3
p−min=0.49, pX=0.43, N=233
var.22.C1R 12:7242204:C:T
188:233
67:80
6:12
WILTAAHTLYPKpc2_NP.4
p−maj=0.016, pX=0.65, N=261
var.22.C1R 12:7242204:C:T
44:233
21:80
2:12
WILTAAHTLYPKpc2_NP.5
p−min=0.23, pX=0.7, N=67
var.22.C1R 12:7242204:C:T
40:233
6:80
3:12
WILTAAHTLYPKpc3_NP.2
p−min=0.12, pX=0.59, N=49
var.22.C1R 12:7242204:C:T
109:233
44:80
3:12
WILTAAHTLYPKpc3_NP.3
p−maj=0.14, pX=0.79, N=156
var.22.C1R 12:7242204:C:T
169:233
58:80
7:12
WILTAAHTLYPKpc3_NP.4
p−maj=0.33, pX=0.4, N=234
var.22.C1R 12:7242204:C:T
27:233
10:80
1:12
WILTAAHTLYPKpc3_NP.5
p−maj=1, pX=0.79, N=38
var.22.C1R 12:7242204:C:T
14:233
6:80
1:12
WVATGIVSWGIGCSRpc2_NP.1
p−maj=0.56, pX=0.74, N=21
var.22.C1R 12:7242204:C:T
172:233
60:80
8:12
WVATGIVSWGIGCSRpc2_NP.2
p−maj=0.52, pX=0.68, N=240
var.22.C1R 12:7242204:C:T
225:233
76:80
11:12
WVATGIVSWGIGCSRpc2_NP.3
p−maj=0.39, pX=0.78, N=312
var.22.C1R 12:7242204:C:T
233:233
80:80
12:12
WVATGIVSWGIGCSRpc2_NP.4
p−maj=1, pX=0.81, N=325
var.22.C1R 12:7242204:C:T
233:233
80:80
12:12
WVATGIVSWGIGCSRpc2_NP.5
p−maj=1, pX=0.96, N=325
peptide level

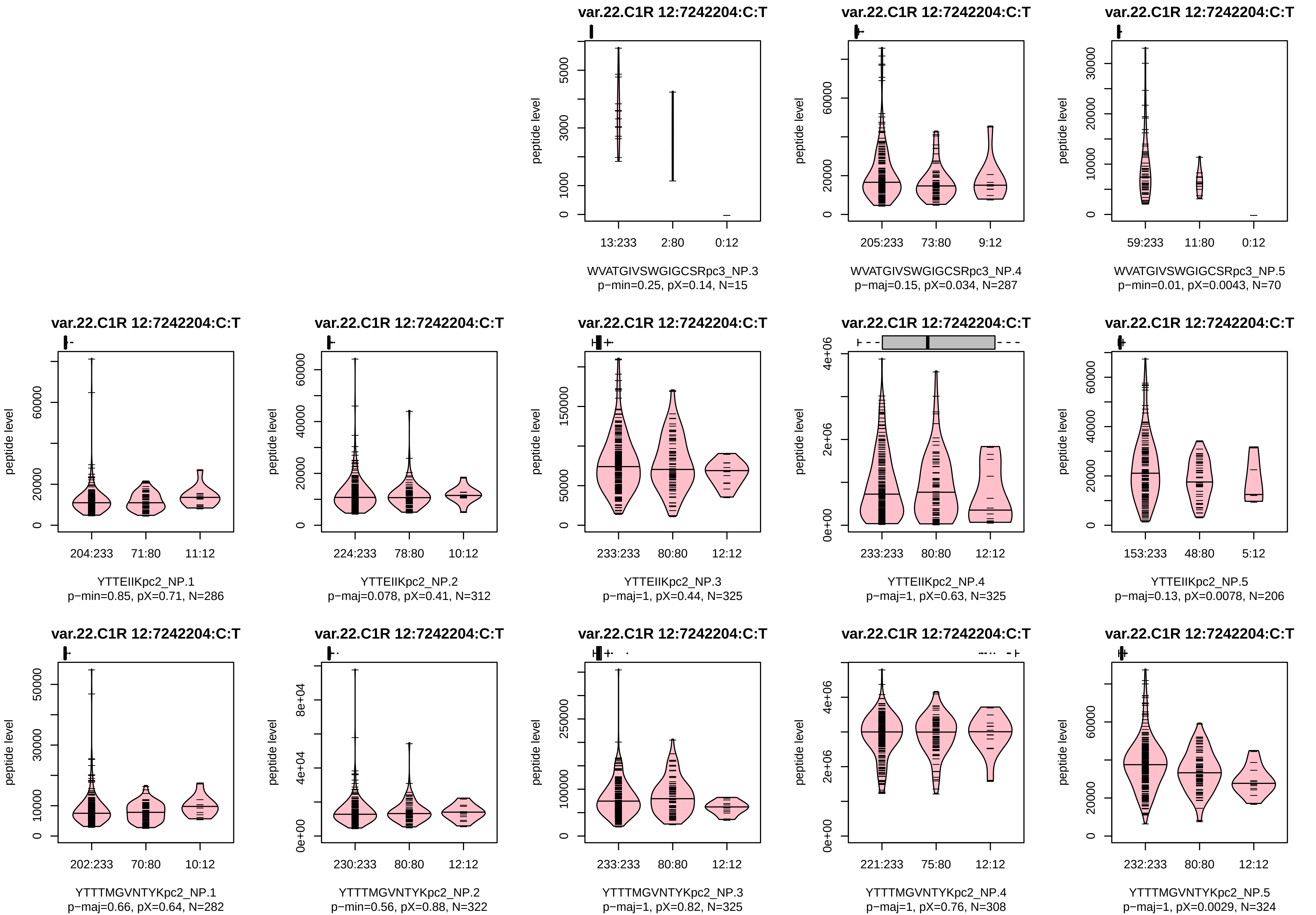
var.22.C1R 12:7242204:C:T
peptide level
13:233
2:80
0:12
WVATGIVSWGIGCSRpc3_NP.3
p−min=0.25, pX=0.14, N=15
var.22.C1R 12:7242204:C:T
205:233
73:80
9:12
WVATGIVSWGIGCSRpc3_NP.4
p−maj=0.15, pX=0.034, N=287
var.22.C1R 12:7242204:C:T
59:233
11:80
0:12
WVATGIVSWGIGCSRpc3_NP.5
p−min=0.01, pX=0.0043, N=70
var.22.C1R 12:7242204:C:T
204:233
71:80
11:12
YTTEIIKpc2_NP.1
p−min=0.85, pX=0.71, N=286
var.22.C1R 12:7242204:C:T
224:233
78:80
10:12
YTTEIIKpc2_NP.2
p−maj=0.078, pX=0.41, N=312
var.22.C1R 12:7242204:C:T
233:233
80:80
12:12
YTTEIIKpc2_NP.3
p−maj=1, pX=0.44, N=325
var.22.C1R 12:7242204:C:T
233:233
80:80
12:12
YTTEIIKpc2_NP.4
p−maj=1, pX=0.63, N=325
var.22.C1R 12:7242204:C:T
153:233
48:80
5:12
YTTEIIKpc2_NP.5
p−maj=0.13, pX=0.0078, N=206
var.22.C1R 12:7242204:C:T
202:233
70:80
10:12
YTTTMGVNTYKpc2_NP.1
p−maj=0.66, pX=0.64, N=282
var.22.C1R 12:7242204:C:T
230:233
80:80
12:12
YTTTMGVNTYKpc2_NP.2
p−min=0.56, pX=0.88, N=322
var.22.C1R 12:7242204:C:T
233:233
80:80
12:12
YTTTMGVNTYKpc2_NP.3
p−maj=1, pX=0.82, N=325
var.22.C1R 12:7242204:C:T
221:233
75:80
12:12
YTTTMGVNTYKpc2_NP.4
p−maj=1, pX=0.76, N=308
var.22.C1R 12:7242204:C:T
232:233
80:80
12:12
YTTTMGVNTYKpc2_NP.5
p−maj=1, pX=0.0029, N=324

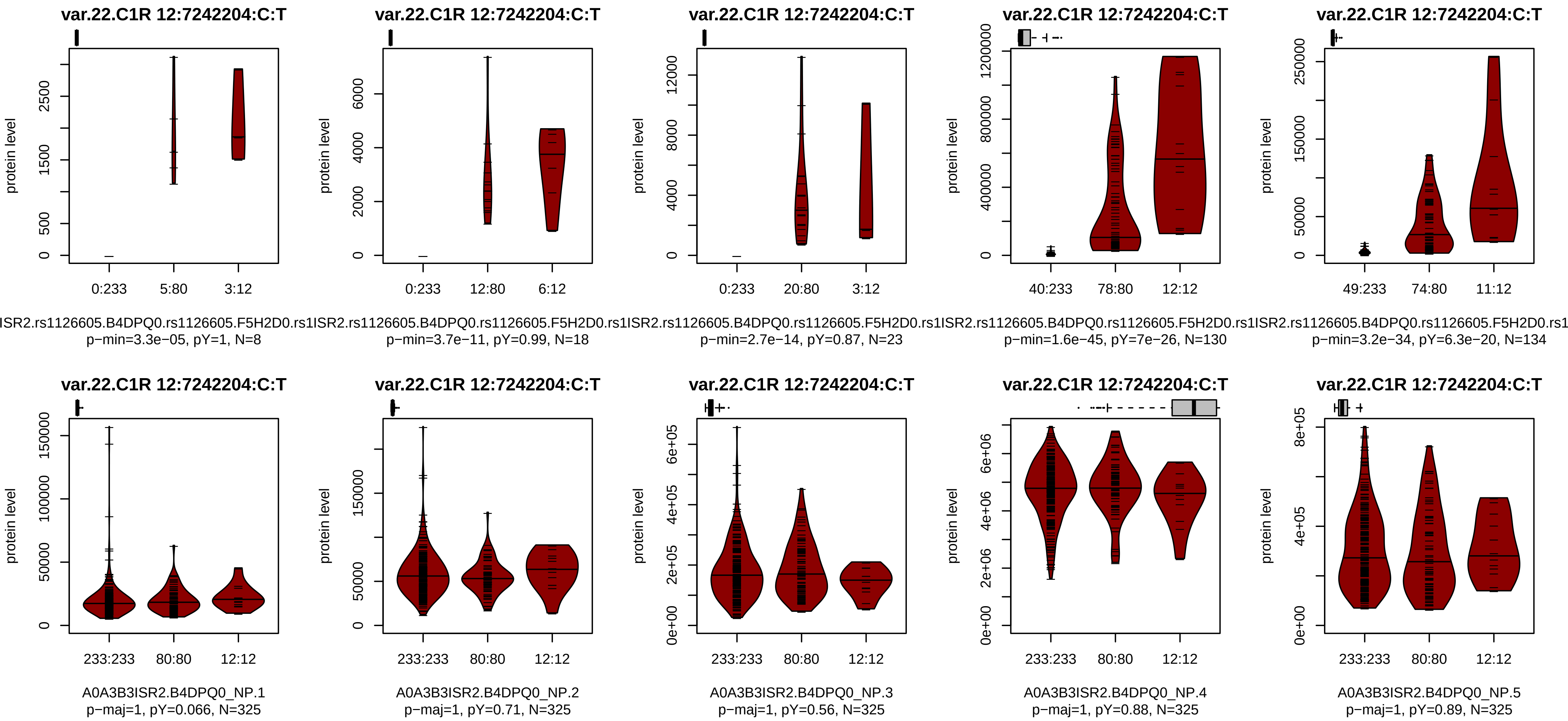
var.22.C1R 12:7242204:C:T
protein level
0:233
5:80
3:12
ISR2.rs1126605.B4DPQ0.rs1126605.F5H2D0.rs1
p−min=3.3e−05, pY=1, N=8
var.22.C1R 12:7242204:C:T
protein level
0:233
12:80
6:12
ISR2.rs1126605.B4DPQ0.rs1126605.F5H2D0.rs1
p−min=3.7e−11, pY=0.99, N=18
var.22.C1R 12:7242204:C:T
protein level
0:233
20:80
3:12
ISR2.rs1126605.B4DPQ0.rs1126605.F5H2D0.rs1
p−min=2.7e−14, pY=0.87, N=23
var.22.C1R 12:7242204:C:T
protein level
40:233
78:80
12:12
ISR2.rs1126605.B4DPQ0.rs1126605.F5H2D0.rs1
p−min=1.6e−45, pY=7e−26, N=130
var.22.C1R 12:7242204:C:T
protein level
49:233
74:80
11:12
ISR2.rs1126605.B4DPQ0.rs1126605.F5H2D0.rs1
p−min=3.2e−34, pY=6.3e−20, N=134
var.22.C1R 12:7242204:C:T
protein level
233:233
80:80
12:12
A0A3B3ISR2.B4DPQ0_NP.1
p−maj=1, pY=0.066, N=325
var.22.C1R 12:7242204:C:T
protein level
233:233
80:80
12:12
A0A3B3ISR2.B4DPQ0_NP.2
p−maj=1, pY=0.71, N=325
var.22.C1R 12:7242204:C:T
protein level
233:233
80:80
12:12
A0A3B3ISR2.B4DPQ0_NP.3
p−maj=1, pY=0.56, N=325
var.22.C1R 12:7242204:C:T
protein level
233:233
80:80
12:12
A0A3B3ISR2.B4DPQ0_NP.4
p−maj=1, pY=0.88, N=325
var.22.C1R 12:7242204:C:T
protein level
233:233
80:80
12:12
A0A3B3ISR2.B4DPQ0_NP.5
p−maj=1, pY=0.89, N=325

var.22.C1R 12:7242204:C:T

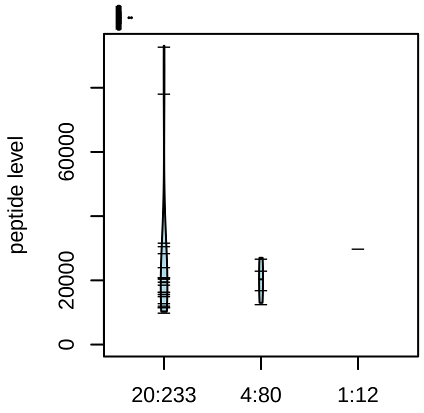

CLPVCGKpc2_NP.1
p−min=0.49, pX=0.48, N=25

var.22.C1R 12:7242204:C:T

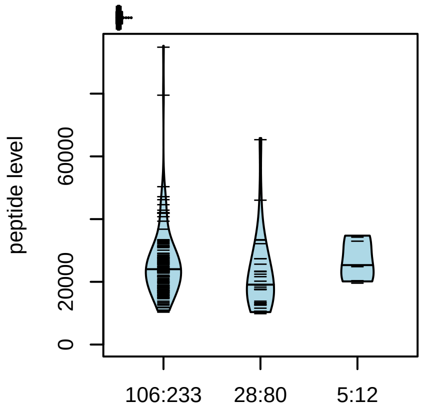

CLPVCGKpc2_NP.2
p−min=0.14, pX=0.1, N=139

var.22.C1R 12:7242204:C:T

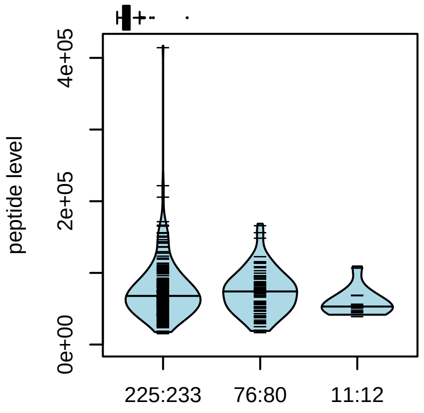

CLPVCGKpc2_NP.3
p−maj=0.39, pX=0.15, N=312

var.22.C1R 12:7242204:C:T

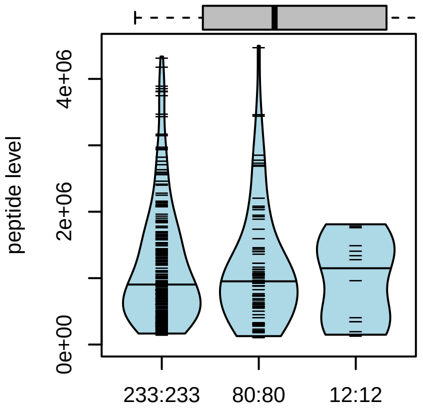

CLPVCGKpc2_NP.4
p−maj=1, pX=0.46, N=325

var.22.C1R 12:7242204:C:T

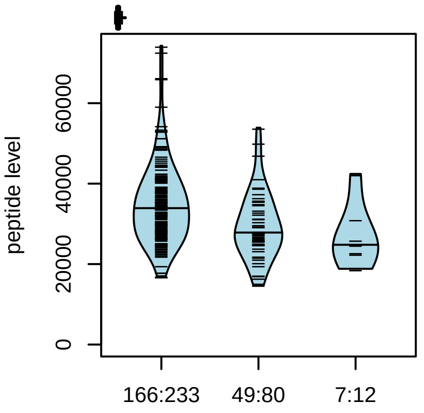

CLPVCGKpc2_NP.5
p−min=0.085, pX=0.0013, N=222

var.22.C1R 12:7242204:C:T

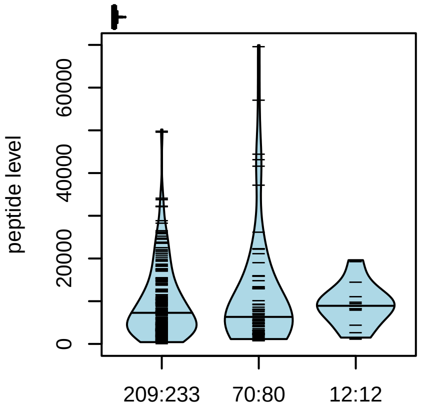

CLPVCGKPVNPVEQRpc2_NP.4
p−maj=0.62, pX=0.48, N=291

var.22.C1R 12:7242204:C:T

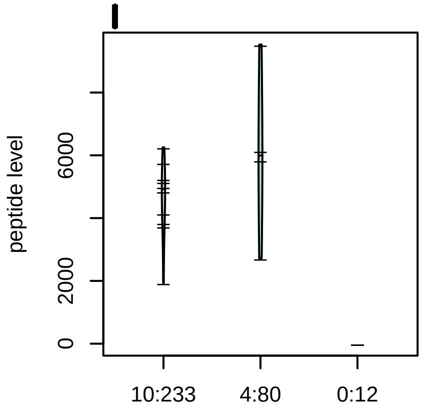

CLPVCGKPVNPVEQRpc3_NP.2
p−maj=1, pX=0.87, N=14

var.22.C1R 12:7242204:C:T

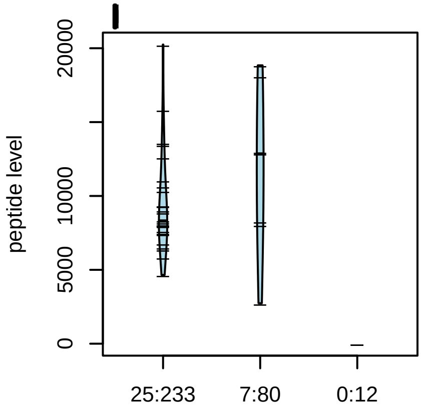

CLPVCGKPVNPVEQRpc3_NP.3
p−min=0.54, pX=0.52, N=32

var.22.C1R 12:7242204:C:T

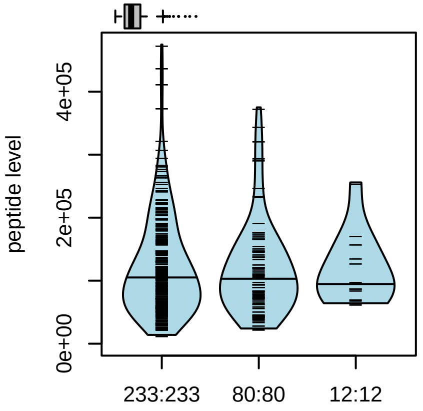

CLPVCGKPVNPVEQRpc3_NP.4
p−maj=1, pX=0.81, N=325

var.22.C1R 12:7242204:C:T

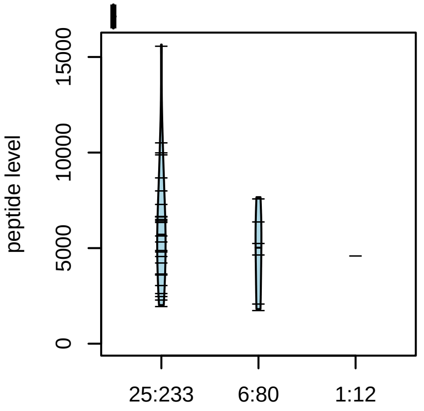

CLPVCGKPVNPVEQRpc3_NP.5
p−min=0.54, pX=0.25, N=32

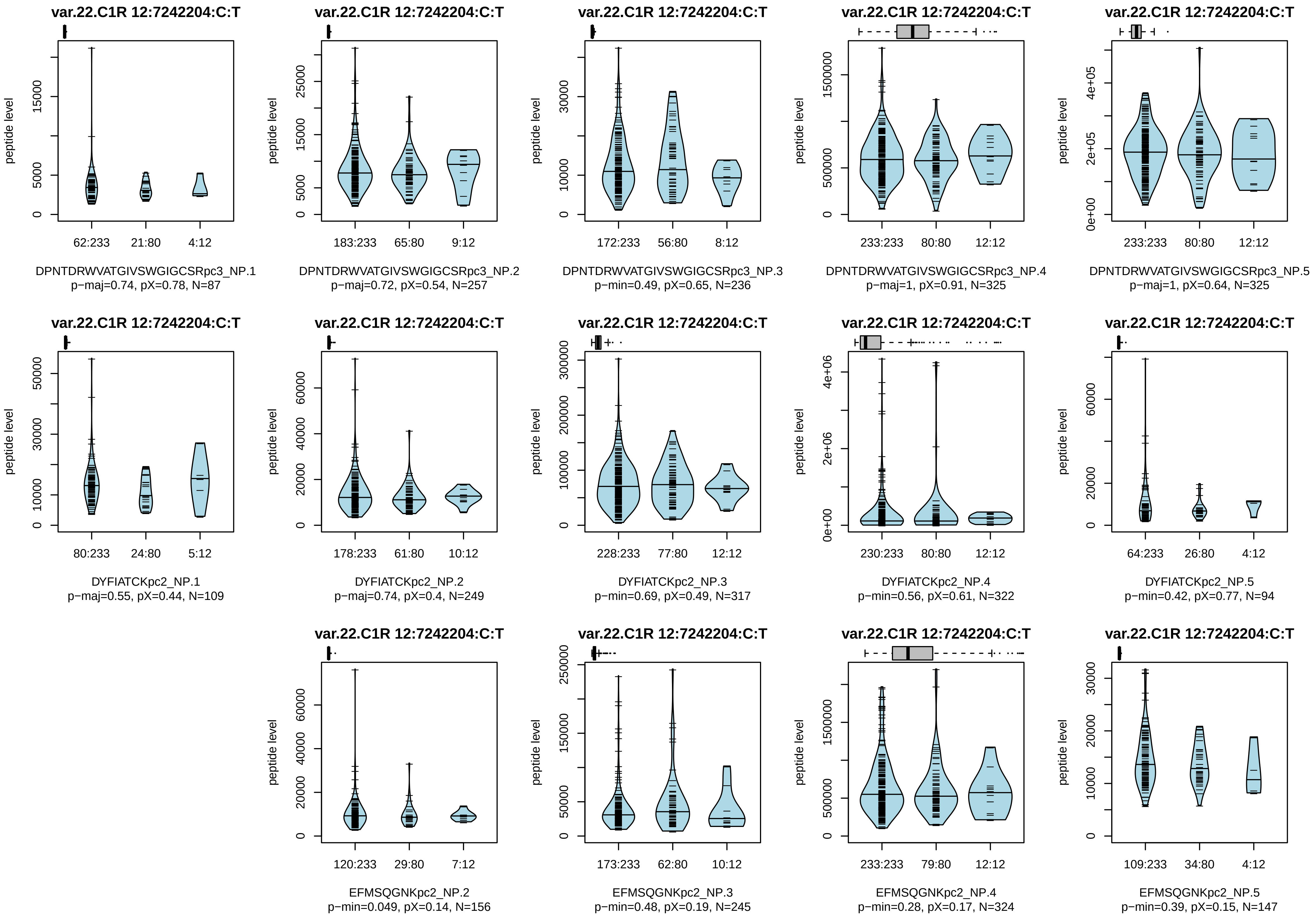
var.22.C1R 12:7242204:C:T
peptide level
62:233
21:80
4:12
DPNTDRWVATGIVSWGIGCSRpc3_NP.1
p−maj=0.74, pX=0.78, N=87
var.22.C1R 12:7242204:C:T
peptide level
183:233
65:80
9:12
DPNTDRWVATGIVSWGIGCSRpc3_NP.2
p−maj=0.72, pX=0.54, N=257
var.22.C1R 12:7242204:C:T
peptide level
172:233
56:80
8:12
DPNTDRWVATGIVSWGIGCSRpc3_NP.3
p−min=0.49, pX=0.65, N=236
var.22.C1R 12:7242204:C:T
peptide level
233:233
80:80
12:12
DPNTDRWVATGIVSWGIGCSRpc3_NP.4
p−maj=1, pX=0.91, N=325
var.22.C1R 12:7242204:C:T
peptide level
233:233
80:80
12:12
DPNTDRWVATGIVSWGIGCSRpc3_NP.5
p−maj=1, pX=0.64, N=325
var.22.C1R 12:7242204:C:T
peptide level
80:233
24:80
5:12
DYFIATCKpc2_NP.1
p−maj=0.55, pX=0.44, N=109
var.22.C1R 12:7242204:C:T
peptide level
178:233
61:80
10:12
DYFIATCKpc2_NP.2
p−maj=0.74, pX=0.4, N=249
var.22.C1R 12:7242204:C:T
peptide level
228:233
77:80
12:12
DYFIATCKpc2_NP.3
p−min=0.69, pX=0.49, N=317
var.22.C1R 12:7242204:C:T
peptide level
230:233
80:80
12:12
DYFIATCKpc2_NP.4
p−min=0.56, pX=0.61, N=322
var.22.C1R 12:7242204:C:T
peptide level
64:233
26:80
4:12
DYFIATCKpc2_NP.5
p−min=0.42, pX=0.77, N=94
var.22.C1R 12:7242204:C:T
peptide level
120:233
29:80
7:12
EFMSQGNKpc2_NP.2
p−min=0.049, pX=0.14, N=156
var.22.C1R 12:7242204:C:T
peptide level
173:233
62:80
10:12
EFMSQGNKpc2_NP.3
p−min=0.48, pX=0.19, N=245
var.22.C1R 12:7242204:C:T
peptide level
233:233
79:80
12:12
EFMSQGNKpc2_NP.4
p−min=0.28, pX=0.17, N=324
var.22.C1R 12:7242204:C:T
peptide level
109:233
34:80
4:12
EFMSQGNKpc2_NP.5
p−min=0.39, pX=0.15, N=147

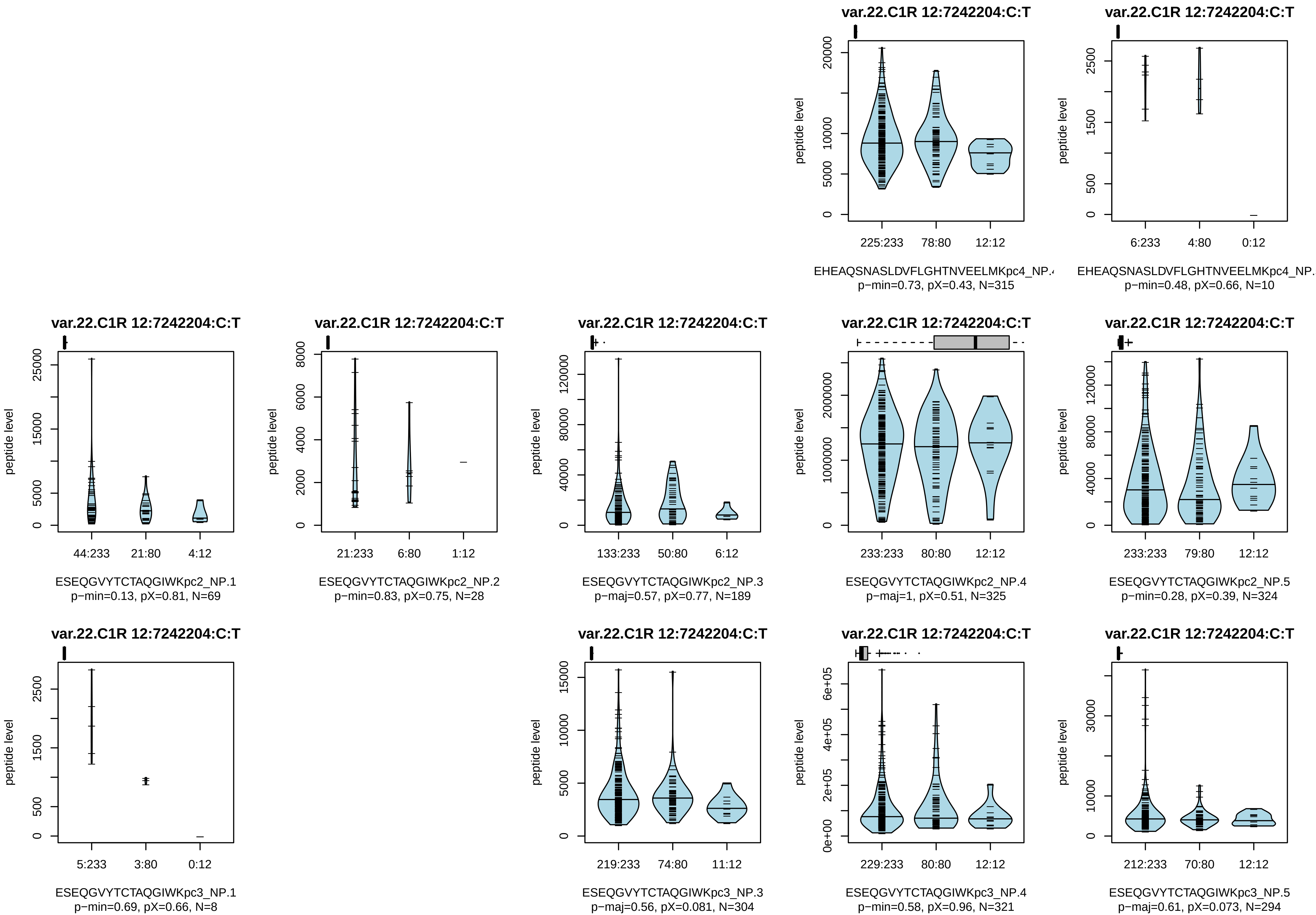
var.22.C1R 12:7242204:C:T
peptide level
225:233
78:80
12:12
EHEAQSNASLDVFLGHTNVEELMKpc4_NP.4
p−min=0.73, pX=0.43, N=315
var.22.C1R 12:7242204:C:T
peptide level
6:233
4:80
0:12
EHEAQSNASLDVFLGHTNVEELMKpc4_NP.
p−min=0.48, pX=0.66, N=10
var.22.C1R 12:7242204:C:T
peptide level
44:233
21:80
4:12
ESEQGVYTCTAQGIWKpc2_NP.1
p−min=0.13, pX=0.81, N=69
var.22.C1R 12:7242204:C:T
peptide level
21:233
6:80
1:12
ESEQGVYTCTAQGIWKpc2_NP.2
p−min=0.83, pX=0.75, N=28
var.22.C1R 12:7242204:C:T
peptide level
133:233
50:80
6:12
ESEQGVYTCTAQGIWKpc2_NP.3
p−maj=0.57, pX=0.77, N=189
var.22.C1R 12:7242204:C:T
peptide level
233:233
80:80
12:12
ESEQGVYTCTAQGIWKpc2_NP.4
p−maj=1, pX=0.51, N=325
var.22.C1R 12:7242204:C:T
peptide level
233:233
79:80
12:12
ESEQGVYTCTAQGIWKpc2_NP.5
p−min=0.28, pX=0.39, N=324
var.22.C1R 12:7242204:C:T
peptide level
5:233
3:80
0:12
ESEQGVYTCTAQGIWKpc3_NP.1
p−min=0.69, pX=0.66, N=8
var.22.C1R 12:7242204:C:T
peptide level
219:233
74:80
11:12
ESEQGVYTCTAQGIWKpc3_NP.3
p−maj=0.56, pX=0.081, N=304
var.22.C1R 12:7242204:C:T
peptide level
229:233
80:80
12:12
ESEQGVYTCTAQGIWKpc3_NP.4
p−min=0.58, pX=0.96, N=321
var.22.C1R 12:7242204:C:T
peptide level
212:233
70:80
12:12
ESEQGVYTCTAQGIWKpc3_NP.5
p−maj=0.61, pX=0.073, N=294

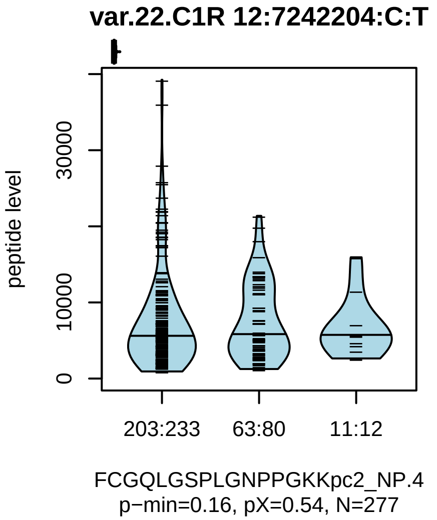
var.22.C1R 12:7242204:C:T
peptide level
0
10000
30000
203:233
63:80
11:12
FCGQLGSPLGNPPGKKpc2_NP.4
p−min=0.16, pX=0.54, N=277

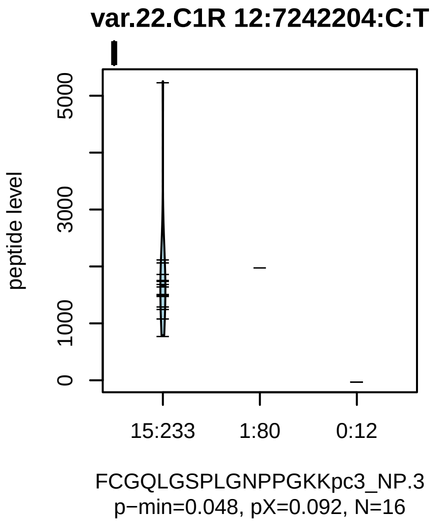
var.22.C1R 12:7242204:C:T
peptide level
0
1000
3000
5000
15:233
1:80
0:12
FCGQLGSPLGNPPGKKpc3_NP.3
p−min=0.048, pX=0.092, N=16

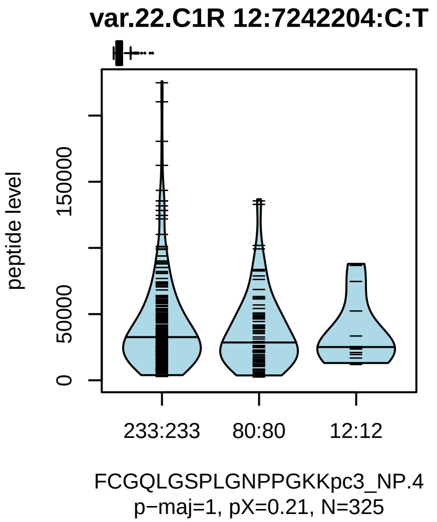
var.22.C1R 12:7242204:C:T
peptide level
0
50000
150000
233:233
80:80
12:12
FCGQLGSPLGNPPGKKpc3_NP.4
p−maj=1, pX=0.21, N=325

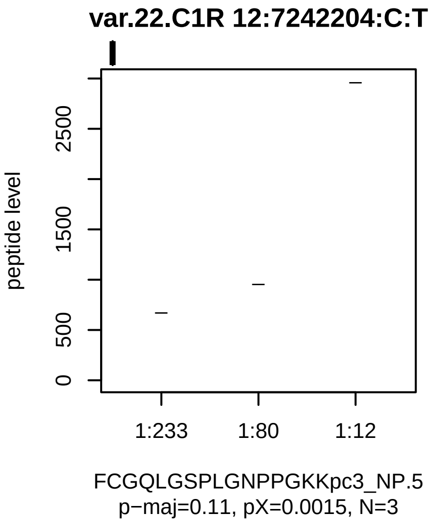
var.22.C1R 12:7242204:C:T
peptide level
0
500
1500
2500
1:233
1:80
1:12
FCGQLGSPLGNPPGKKpc3_NP.5
p−maj=0.11, pX=0.0015, N=3

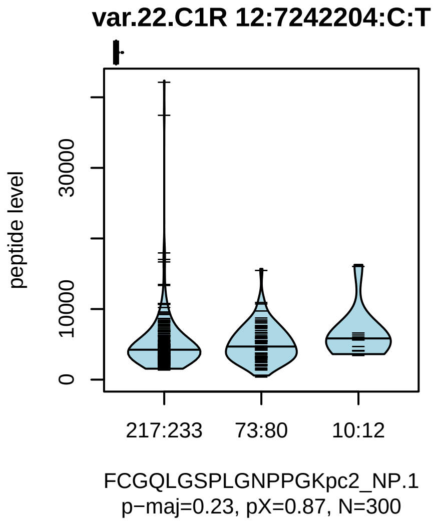
var.22.C1R 12:7242204:C:T
peptide level
0
10000
30000
217:233
73:80
10:12
FCGQLGSPLGNPPGKpc2_NP.1
p−maj=0.23, pX=0.87, N=300

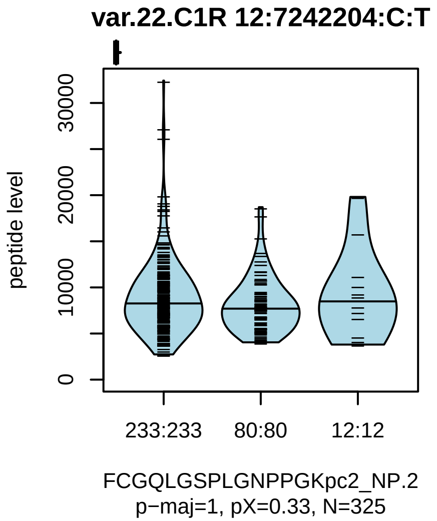
var.22.C1R 12:7242204:C:T
peptide level
0
10000
20000
30000
233:233
80:80
12:12
FCGQLGSPLGNPPGKpc2_NP.2
p−maj=1, pX=0.33, N=325

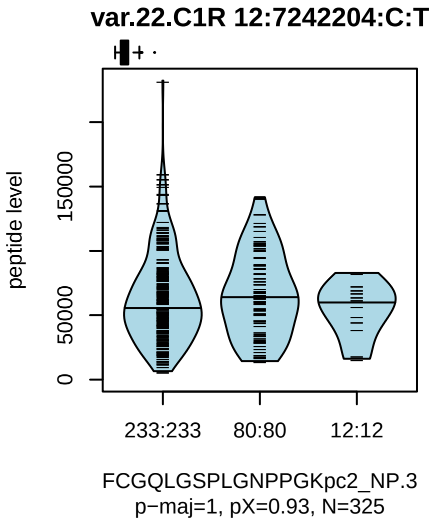
var.22.C1R 12:7242204:C:T
peptide level
0
50000
150000
233:233
80:80
12:12
FCGQLGSPLGNPPGKpc2_NP.3
p−maj=1, pX=0.93, N=325

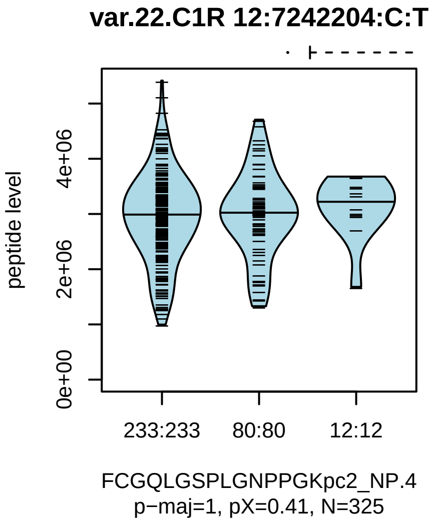
var.22.C1R 12:7242204:C:T
peptide level
0e+00
2e+06
4e+06
233:233
80:80
12:12
FCGQLGSPLGNPPGKpc2_NP.4
p−maj=1, pX=0.41, N=325

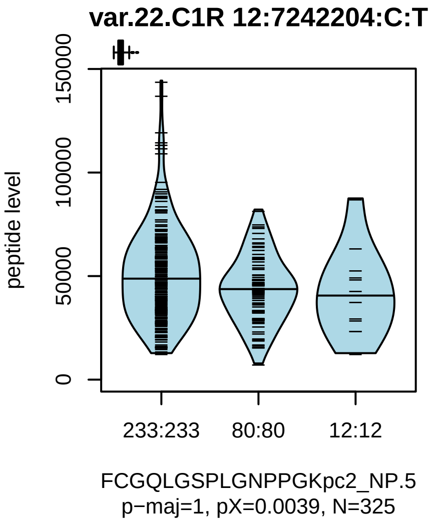
var.22.C1R 12:7242204:C:T
peptide level
0
50000
100000
150000
233:233
80:80
12:12
FCGQLGSPLGNPPGKpc2_NP.5
p−maj=1, pX=0.0039, N=325

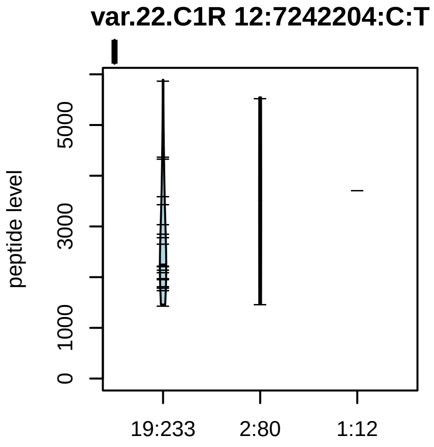

FCGQLGSPLGNPPGKpc3_NP.3
p−min=0.14, pX=0.45, N=22

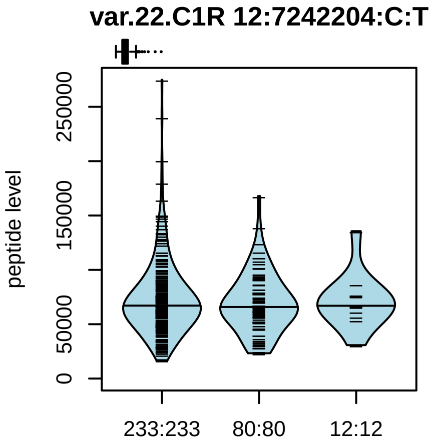

FCGQLGSPLGNPPGKpc3_NP.4
p−maj=1, pX=0.4, N=325

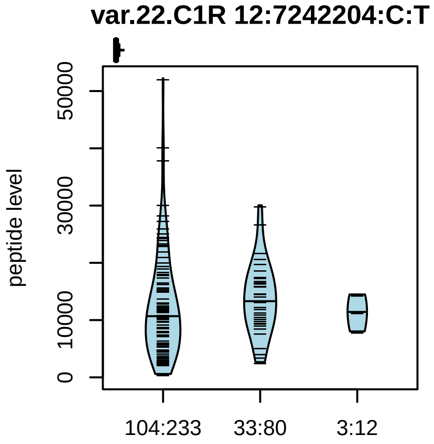

FLEPFDIDDHQQVHCPYDQLQIYANGKpc4_N[illegible]
p−maj=0.24, pX=0.59, N=140

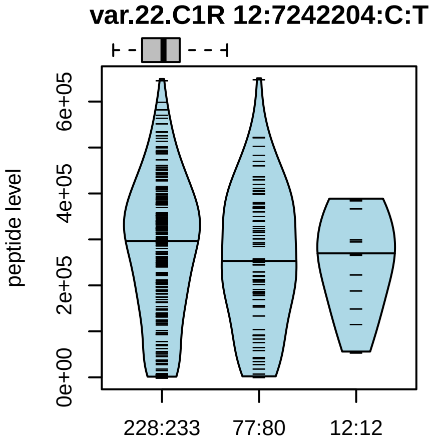

FLEPFDIDDHQQVHCPYDQLQIYANGKpc4_N[illegible]
p−min=0.69, pX=0.28, N=317

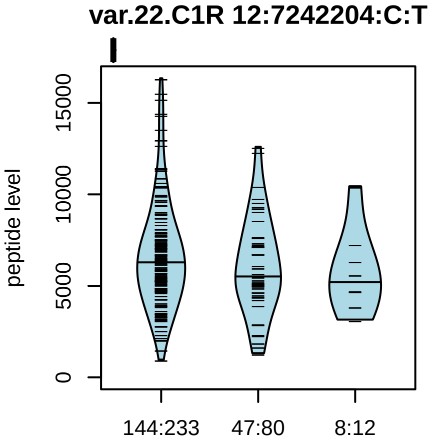

FLEPFDIDDHQQVHCPYDQLQIYANGKpc4_N[illegible]
p−maj=0.77, pX=0.38, N=199

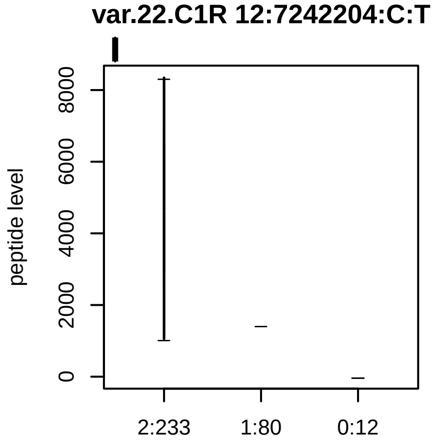

FVRLPVANPQACENWLRpc3_NP.1
p−maj=1, pX=0.66, N=3

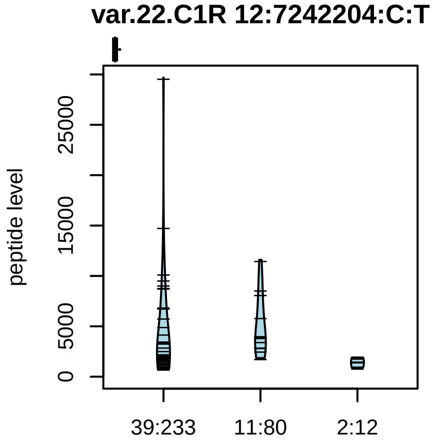

FVRLPVANPQACENWLRpc3_NP.2
p−min=0.62, pX=0.6, N=52

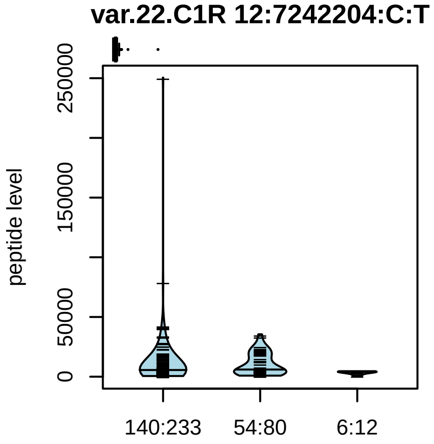

FVRLPVANPQACENWLRpc3_NP.3
p−min=0.45, pX=0.55, N=200

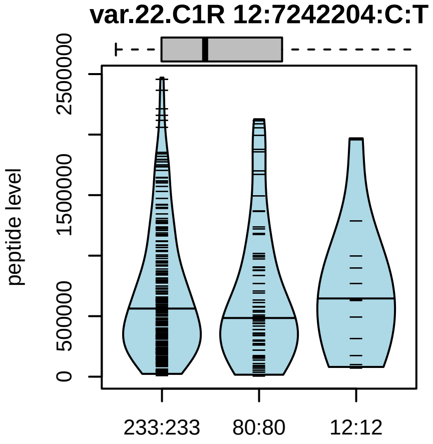

FVRLPVANPQACENWLRpc3_NP.4
p−maj=1, pX=0.61, N=325

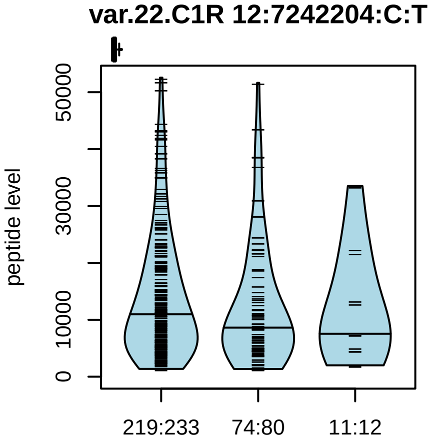

FVRLPVANPQACENWLRpc3_NP.5
p−maj=0.56, pX=0.2, N=304

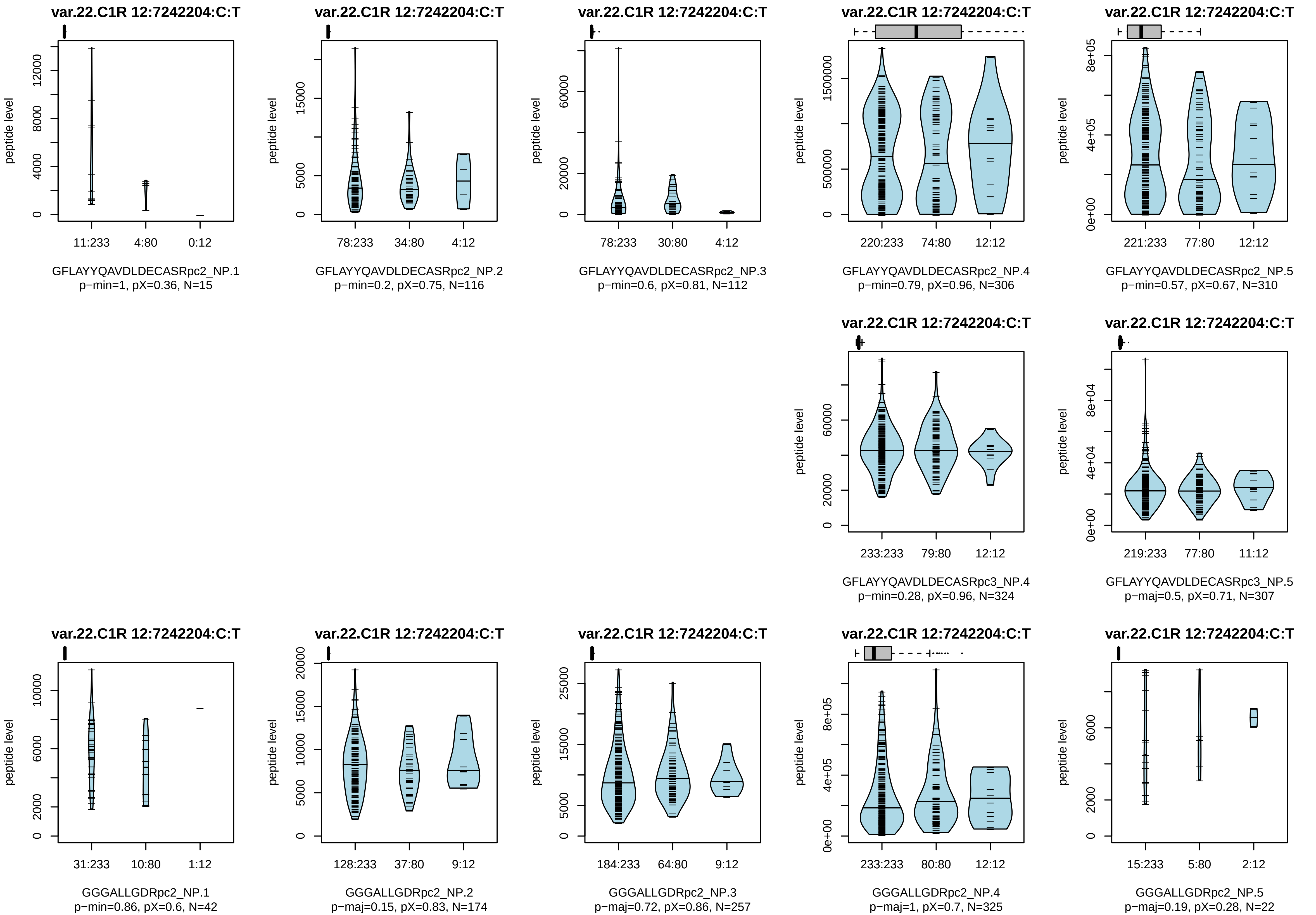

var.22.C1R 12:7242204:C:T
peptide level
11:233
4:80
0:12
GFLAYYQAVDLDECASRpc2_NP.1
p−min=1, pX=0.36, N=15
var.22.C1R 12:7242204:C:T
peptide level
78:233
34:80
4:12
GFLAYYQAVDLDECASRpc2_NP.2
p−min=0.2, pX=0.75, N=116
var.22.C1R 12:7242204:C:T
peptide level
78:233
30:80
4:12
GFLAYYQAVDLDECASRpc2_NP.3
p−min=0.6, pX=0.81, N=112
var.22.C1R 12:7242204:C:T
peptide level
220:233
74:80
12:12
GFLAYYQAVDLDECASRpc2_NP.4
p−min=0.79, pX=0.96, N=306
var.22.C1R 12:7242204:C:T
peptide level
221:233
77:80
12:12
GFLAYYQAVDLDECASRpc2_NP.5
p−min=0.57, pX=0.67, N=310
var.22.C1R 12:7242204:C:T
peptide level
233:233
79:80
12:12
GFLAYYQAVDLDECASRpc3_NP.4
p−min=0.28, pX=0.96, N=324
var.22.C1R 12:7242204:C:T
peptide level
219:233
77:80
11:12
GFLAYYQAVDLDECASRpc3_NP.5
p−maj=0.5, pX=0.71, N=307
var.22.C1R 12:7242204:C:T
peptide level
31:233
10:80
1:12
GGGALLGDRpc2_NP.1
p−min=0.86, pX=0.6, N=42
var.22.C1R 12:7242204:C:T
peptide level
128:233
37:80
9:12
GGGALLGDRpc2_NP.2
p−maj=0.15, pX=0.83, N=174
var.22.C1R 12:7242204:C:T
peptide level
184:233
64:80
9:12
GGGALLGDRpc2_NP.3
p−maj=0.72, pX=0.86, N=257
var.22.C1R 12:7242204:C:T
peptide level
233:233
80:80
12:12
GGGALLGDRpc2_NP.4
p−maj=1, pX=0.7, N=325
var.22.C1R 12:7242204:C:T
peptide level
15:233
5:80
2:12
GGGALLGDRpc2_NP.5
p−maj=0.19, pX=0.28, N=22

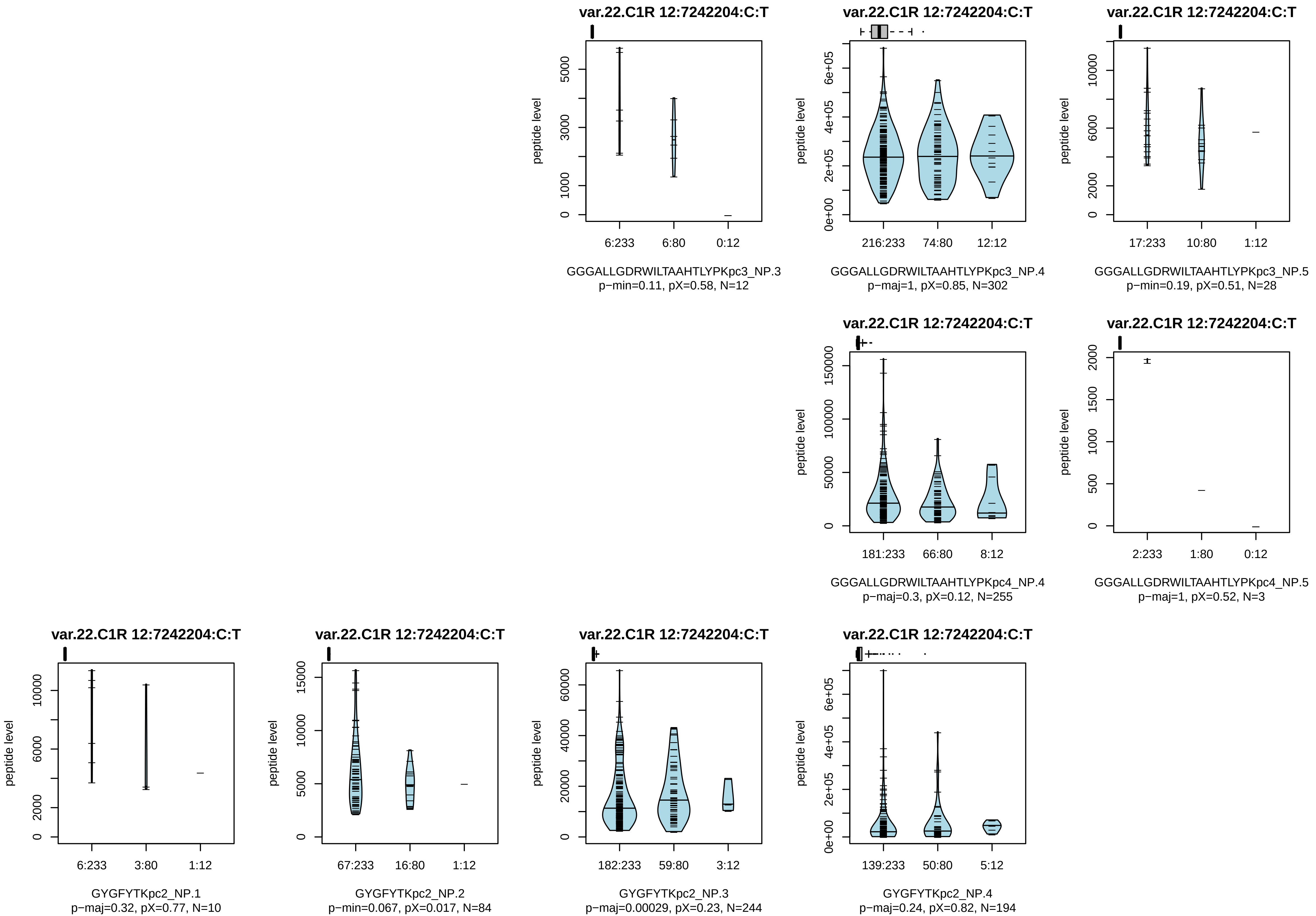

var.22.C1R 12:7242204:C:T
peptide level
6:233
6:80
0:12
GGGALLGDRWILTAAHTLYPKpc3_NP.3
p−min=0.11, pX=0.58, N=12
var.22.C1R 12:7242204:C:T
peptide level
216:233
74:80
12:12
GGGALLGDRWILTAAHTLYPKpc3_NP.4
p−maj=1, pX=0.85, N=302
var.22.C1R 12:7242204:C:T
peptide level
17:233
10:80
1:12
GGGALLGDRWILTAAHTLYPKpc3_NP.5
p−min=0.19, pX=0.51, N=28
var.22.C1R 12:7242204:C:T
peptide level
181:233
66:80
8:12
GGGALLGDRWILTAAHTLYPKpc4_NP.4
p−maj=0.3, pX=0.12, N=255
var.22.C1R 12:7242204:C:T
peptide level
2:233
1:80
0:12
GGGALLGDRWILTAAHTLYPKpc4_NP.5
p−maj=1, pX=0.52, N=3
var.22.C1R 12:7242204:C:T
peptide level
6:233
3:80
1:12
GYGFYTKpc2_NP.1
p−maj=0.32, pX=0.77, N=10
var.22.C1R 12:7242204:C:T
peptide level
67:233
16:80
1:12
GYGFYTKpc2_NP.2
p−min=0.067, pX=0.017, N=84
var.22.C1R 12:7242204:C:T
peptide level
182:233
59:80
3:12
GYGFYTKpc2_NP.3
p−maj=0.00029, pX=0.23, N=244
var.22.C1R 12:7242204:C:T
peptide level
139:233
50:80
5:12
GYGFYTKpc2_NP.4
p−maj=0.24, pX=0.82, N=194

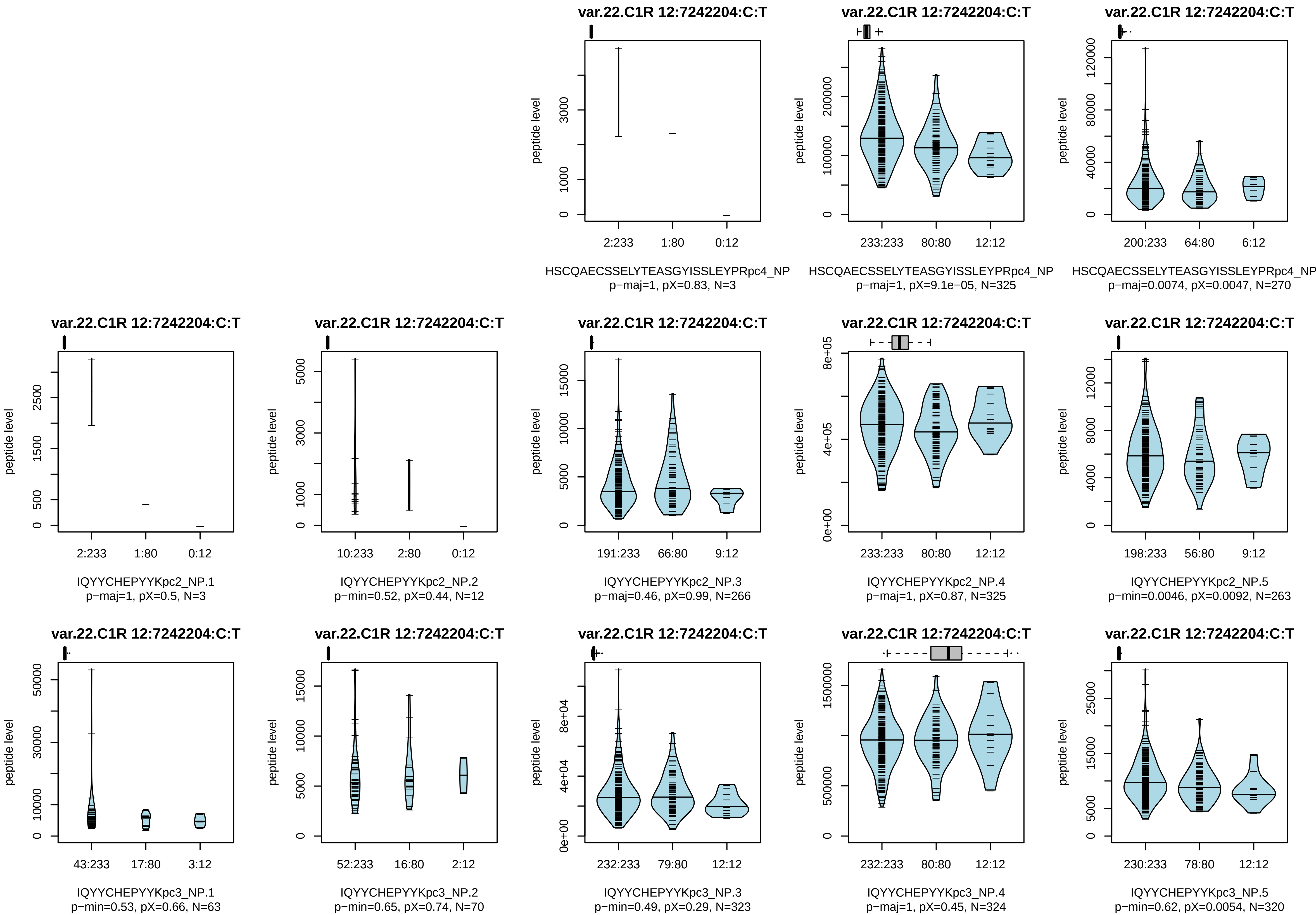
var.22.C1R 12:7242204:C:T
peptide level
2:233
1:80
0:12
HSCQAECSSELYTEASGYISSLEYPRpc4_NP
p−maj=1, pX=0.83, N=3
var.22.C1R 12:7242204:C:T
peptide level
233:233
80:80
12:12
HSCQAECSSELYTEASGYISSLEYPRpc4_NP
p−maj=1, pX=9.1e−05, N=325
var.22.C1R 12:7242204:C:T
peptide level
200:233
64:80
6:12
HSCQAECSSELYTEASGYISSLEYPRpc4_NP
p−maj=0.0074, pX=0.0047, N=270
var.22.C1R 12:7242204:C:T
peptide level
2:233
1:80
0:12
IQYYCHEPYYKpc2_NP.1
p−maj=1, pX=0.5, N=3
var.22.C1R 12:7242204:C:T
peptide level
10:233
2:80
0:12
IQYYCHEPYYKpc2_NP.2
p−min=0.52, pX=0.44, N=12
var.22.C1R 12:7242204:C:T
peptide level
191:233
66:80
9:12
IQYYCHEPYYKpc2_NP.3
p−maj=0.46, pX=0.99, N=266
var.22.C1R 12:7242204:C:T
peptide level
233:233
80:80
12:12
IQYYCHEPYYKpc2_NP.4
p−maj=1, pX=0.87, N=325
var.22.C1R 12:7242204:C:T
peptide level
198:233
56:80
9:12
IQYYCHEPYYKpc2_NP.5
p−min=0.0046, pX=0.0092, N=263
var.22.C1R 12:7242204:C:T
peptide level
43:233
17:80
3:12
IQYYCHEPYYKpc3_NP.1
p−min=0.53, pX=0.66, N=63
var.22.C1R 12:7242204:C:T
peptide level
52:233
16:80
2:12
IQYYCHEPYYKpc3_NP.2
p−min=0.65, pX=0.74, N=70
var.22.C1R 12:7242204:C:T
peptide level
232:233
79:80
12:12
IQYYCHEPYYKpc3_NP.3
p−min=0.49, pX=0.29, N=323
var.22.C1R 12:7242204:C:T
peptide level
232:233
80:80
12:12
IQYYCHEPYYKpc3_NP.4
p−maj=1, pX=0.45, N=324
var.22.C1R 12:7242204:C:T
peptide level
230:233
78:80
12:12
IQYYCHEPYYKpc3_NP.5
p−min=0.62, pX=0.0054, N=320

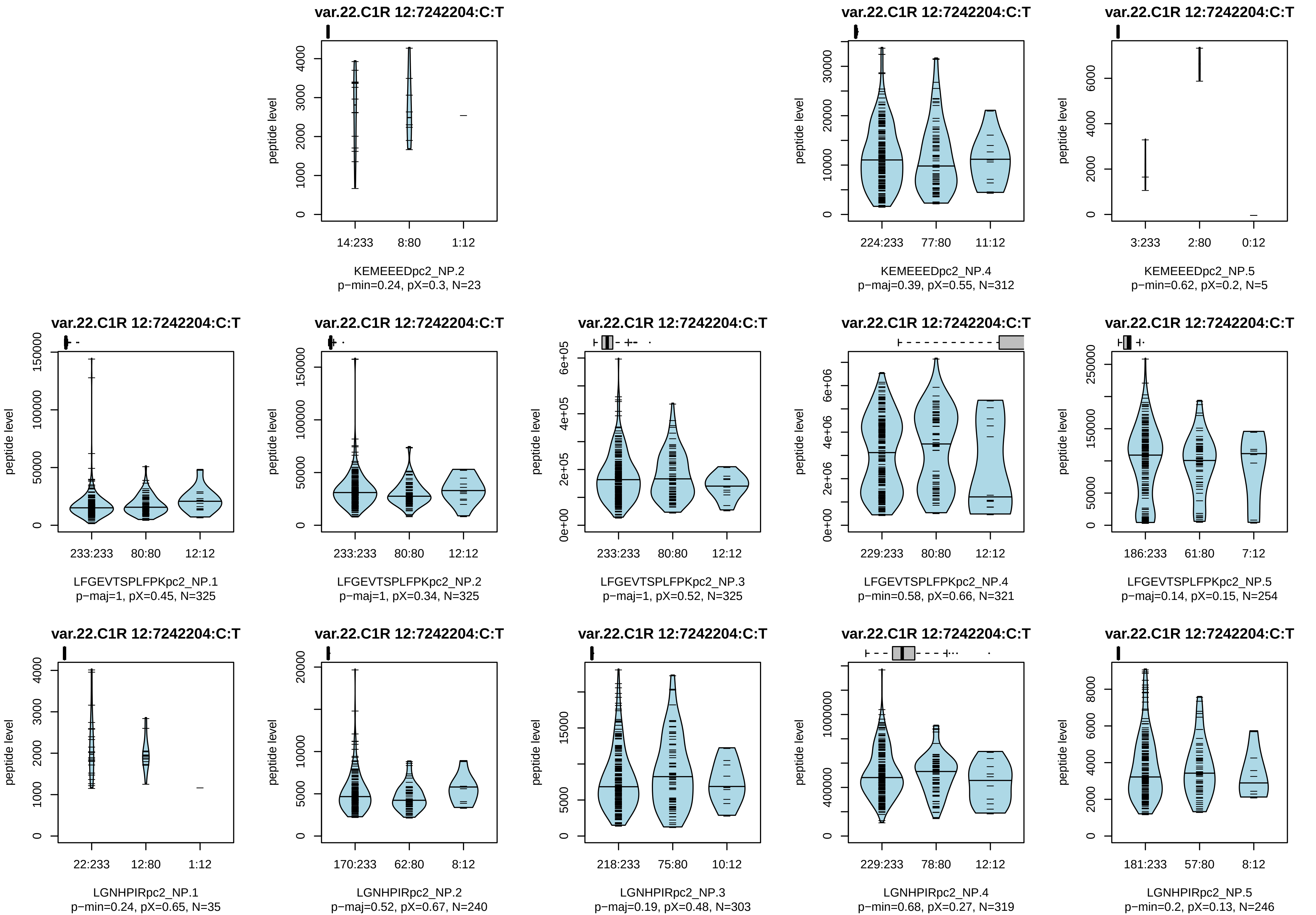
var.22.C1R 12:7242204:C:T
peptide level
14:233
8:80
1:12
KEMEEEDpc2_NP.2
p−min=0.24, pX=0.3, N=23
var.22.C1R 12:7242204:C:T
224:233
77:80
11:12
KEMEEEDpc2_NP.4
p−maj=0.39, pX=0.55, N=312
var.22.C1R 12:7242204:C:T
3:233
2:80
0:12
KEMEEEDpc2_NP.5
p−min=0.62, pX=0.2, N=5
var.22.C1R 12:7242204:C:T
233:233
80:80
12:12
LFGEVTSPLFPKpc2_NP.1
p−maj=1, pX=0.45, N=325
var.22.C1R 12:7242204:C:T
233:233
80:80
12:12
LFGEVTSPLFPKpc2_NP.2
p−maj=1, pX=0.34, N=325
var.22.C1R 12:7242204:C:T
233:233
80:80
12:12
LFGEVTSPLFPKpc2_NP.3
p−maj=1, pX=0.52, N=325
var.22.C1R 12:7242204:C:T
229:233
80:80
12:12
LFGEVTSPLFPKpc2_NP.4
p−min=0.58, pX=0.66, N=321
var.22.C1R 12:7242204:C:T
186:233
61:80
7:12
LFGEVTSPLFPKpc2_NP.5
p−maj=0.14, pX=0.15, N=254
var.22.C1R 12:7242204:C:T
22:233
12:80
1:12
LGNHPIRpc2_NP.1
p−min=0.24, pX=0.65, N=35
var.22.C1R 12:7242204:C:T
170:233
62:80
8:12
LGNHPIRpc2_NP.2
p−maj=0.52, pX=0.67, N=240
var.22.C1R 12:7242204:C:T
218:233
75:80
10:12
LGNHPIRpc2_NP.3
p−maj=0.19, pX=0.48, N=303
var.22.C1R 12:7242204:C:T
229:233
78:80
12:12
LGNHPIRpc2_NP.4
p−min=0.68, pX=0.27, N=319
var.22.C1R 12:7242204:C:T
181:233
57:80
8:12
LGNHPIRpc2_NP.5
p−min=0.2, pX=0.13, N=246

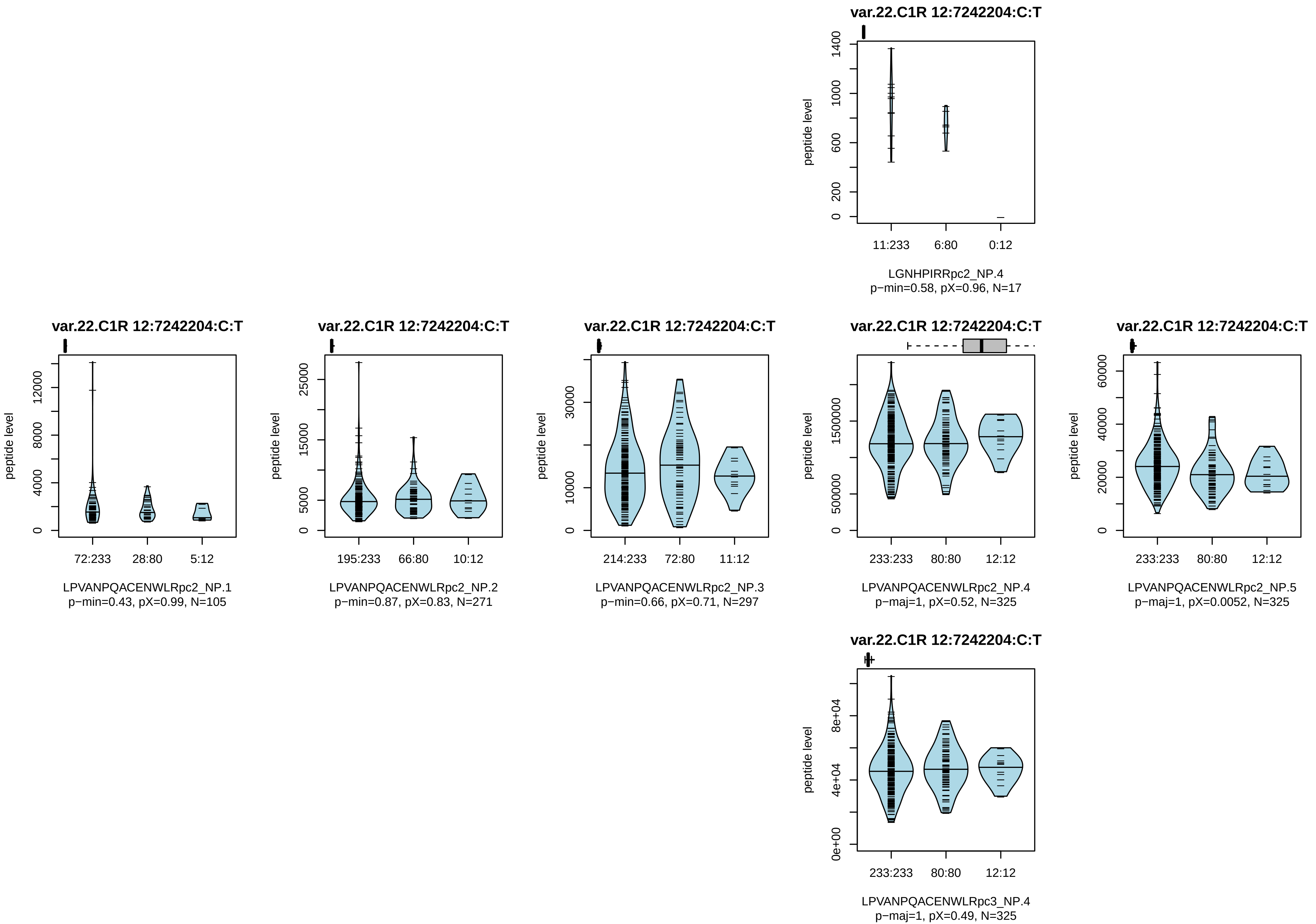
var.22.C1R 12:7242204:C:T
peptide level
0
200
600
1000
1400
11:233
6:80
0:12
LGNHPIRRpc2_NP.4
p−min=0.58, pX=0.96, N=17
var.22.C1R 12:7242204:C:T
peptide level
0
4000
8000
12000
72:233
28:80
5:12
LPVANPQACENWLRpc2_NP.1
p−min=0.43, pX=0.99, N=105
var.22.C1R 12:7242204:C:T
peptide level
0
5000
15000
25000
195:233
66:80
10:12
LPVANPQACENWLRpc2_NP.2
p−min=0.87, pX=0.83, N=271
var.22.C1R 12:7242204:C:T
peptide level
0
10000
30000
214:233
72:80
11:12
LPVANPQACENWLRpc2_NP.3
p−min=0.66, pX=0.71, N=297
var.22.C1R 12:7242204:C:T
peptide level
0
500000
1500000
233:233
80:80
12:12
LPVANPQACENWLRpc2_NP.4
p−maj=1, pX=0.52, N=325
var.22.C1R 12:7242204:C:T
peptide level
0
20000
40000
60000
233:233
80:80
12:12
LPVANPQACENWLRpc2_NP.5
p−maj=1, pX=0.0052, N=325
var.22.C1R 12:7242204:C:T
peptide level
0e+00
4e+04
8e+04
233:233
80:80
12:12
LPVANPQACENWLRpc3_NP.4
p−maj=1, pX=0.49, N=325

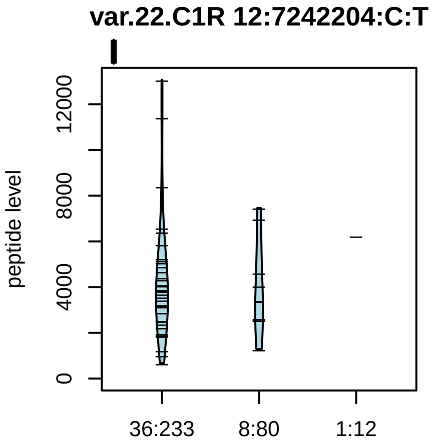

LRYTTEIIKpc2_NP.4
p−min=0.21, pX=0.34, N=45

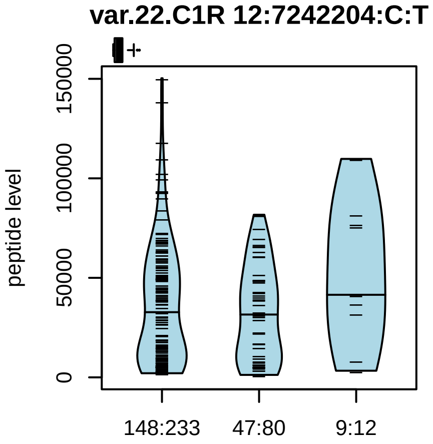

LVFQQFDLEPSEGCFYDYVKpc3_NP.4
p−maj=0.55, pX=0.79, N=204

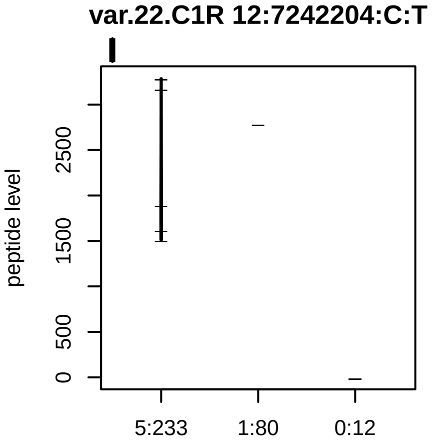

LVFQQFDLEPSEGCFYDYVKpc3_NP.5
p−maj=1, pX=0.59, N=6

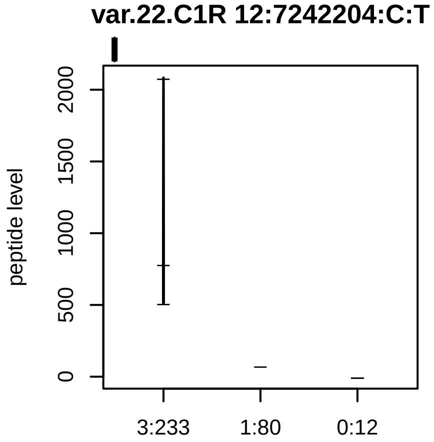

MDVFSQNMFCAGHPSLKpc2_NP.2
p−maj=1, pX=0.4, N=4

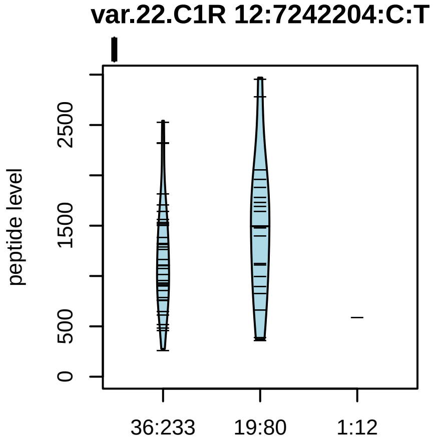

MDVFSQNMFCAGHPSLKpc2_NP.3
p−min=0.19, pX=0.23, N=56

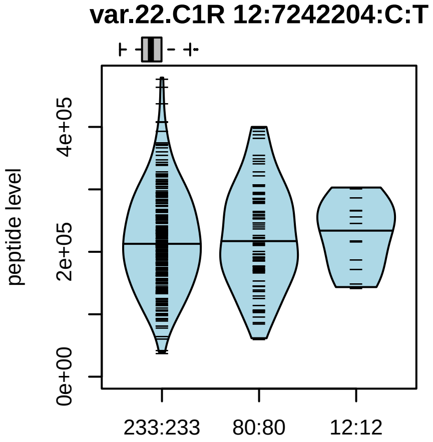

MDVFSQNMFCAGHPSLKpc2_NP.4
p−maj=1, pX=0.59, N=325

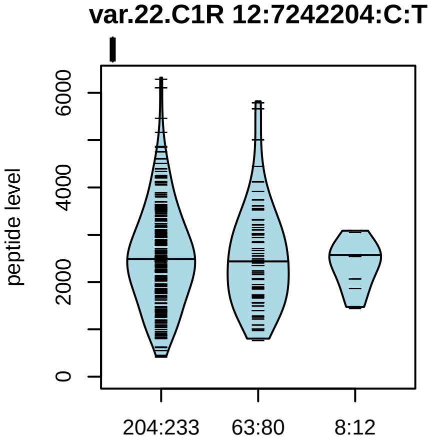

MDVFSQNMFCAGHPSLKpc2_NP.5
p−min=0.026, pX=0.042, N=275

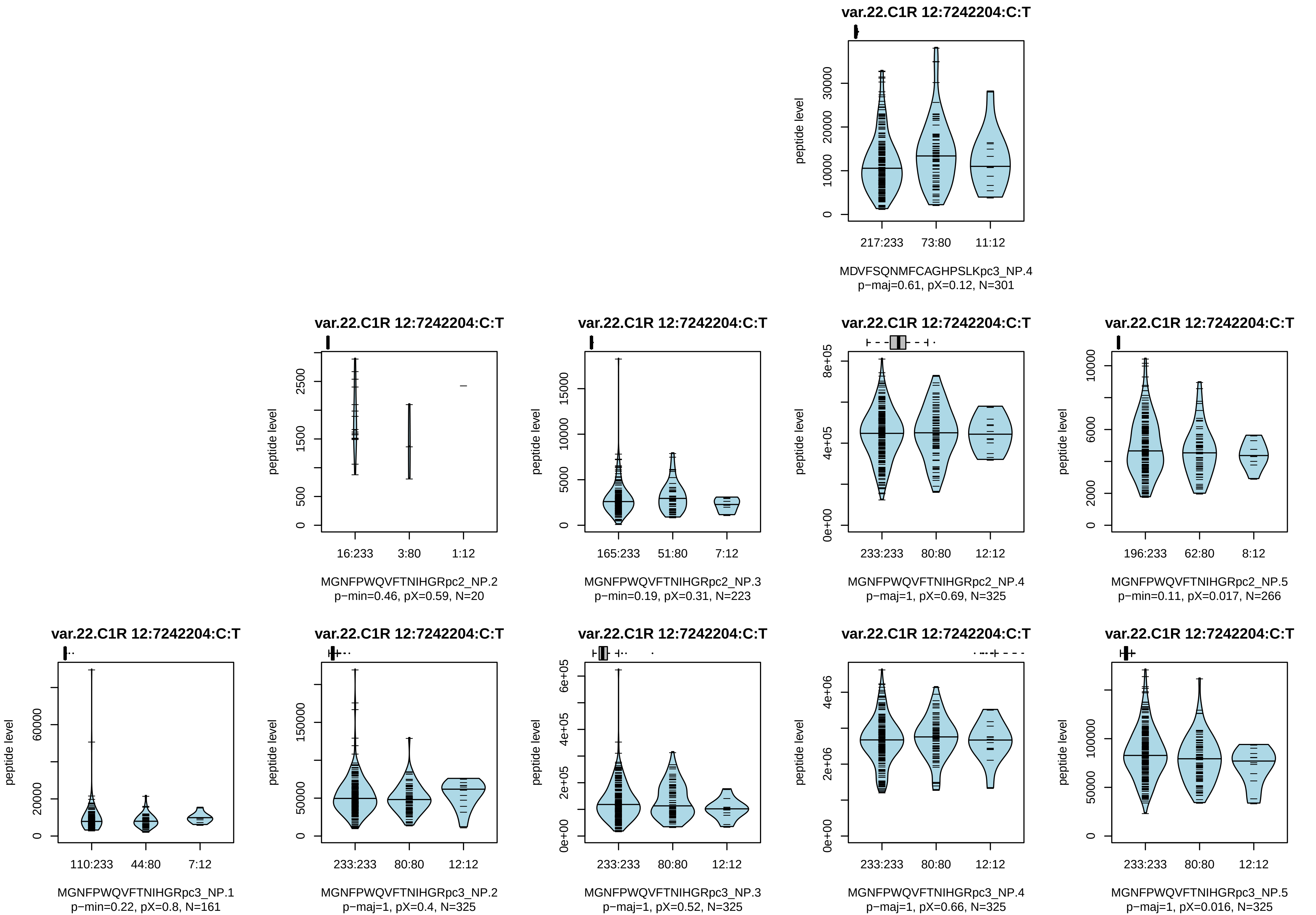

var.22.C1R 12:7242204:C:T
peptide level
217:233
73:80
11:12
MDVFSQNMFCAGHPSLKpc3_NP.4
p−maj=0.61, pX=0.12, N=301
var.22.C1R 12:7242204:C:T
16:233
3:80
1:12
MGNFPWQVFTNIHGRpc2_NP.2
p−min=0.46, pX=0.59, N=20
var.22.C1R 12:7242204:C:T
165:233
51:80
7:12
MGNFPWQVFTNIHGRpc2_NP.3
p−min=0.19, pX=0.31, N=223
var.22.C1R 12:7242204:C:T
233:233
80:80
12:12
MGNFPWQVFTNIHGRpc2_NP.4
p−maj=1, pX=0.69, N=325
var.22.C1R 12:7242204:C:T
196:233
62:80
8:12
MGNFPWQVFTNIHGRpc2_NP.5
p−min=0.11, pX=0.017, N=266
var.22.C1R 12:7242204:C:T
110:233
44:80
7:12
MGNFPWQVFTNIHGRpc3_NP.1
p−min=0.22, pX=0.8, N=161
var.22.C1R 12:7242204:C:T
233:233
80:80
12:12
MGNFPWQVFTNIHGRpc3_NP.2
p−maj=1, pX=0.4, N=325
var.22.C1R 12:7242204:C:T
233:233
80:80
12:12
MGNFPWQVFTNIHGRpc3_NP.3
p−maj=1, pX=0.52, N=325
var.22.C1R 12:7242204:C:T
233:233
80:80
12:12
MGNFPWQVFTNIHGRpc3_NP.4
p−maj=1, pX=0.66, N=325
var.22.C1R 12:7242204:C:T
233:233
80:80
12:12
MGNFPWQVFTNIHGRpc3_NP.5
p−maj=1, pX=0.016, N=325

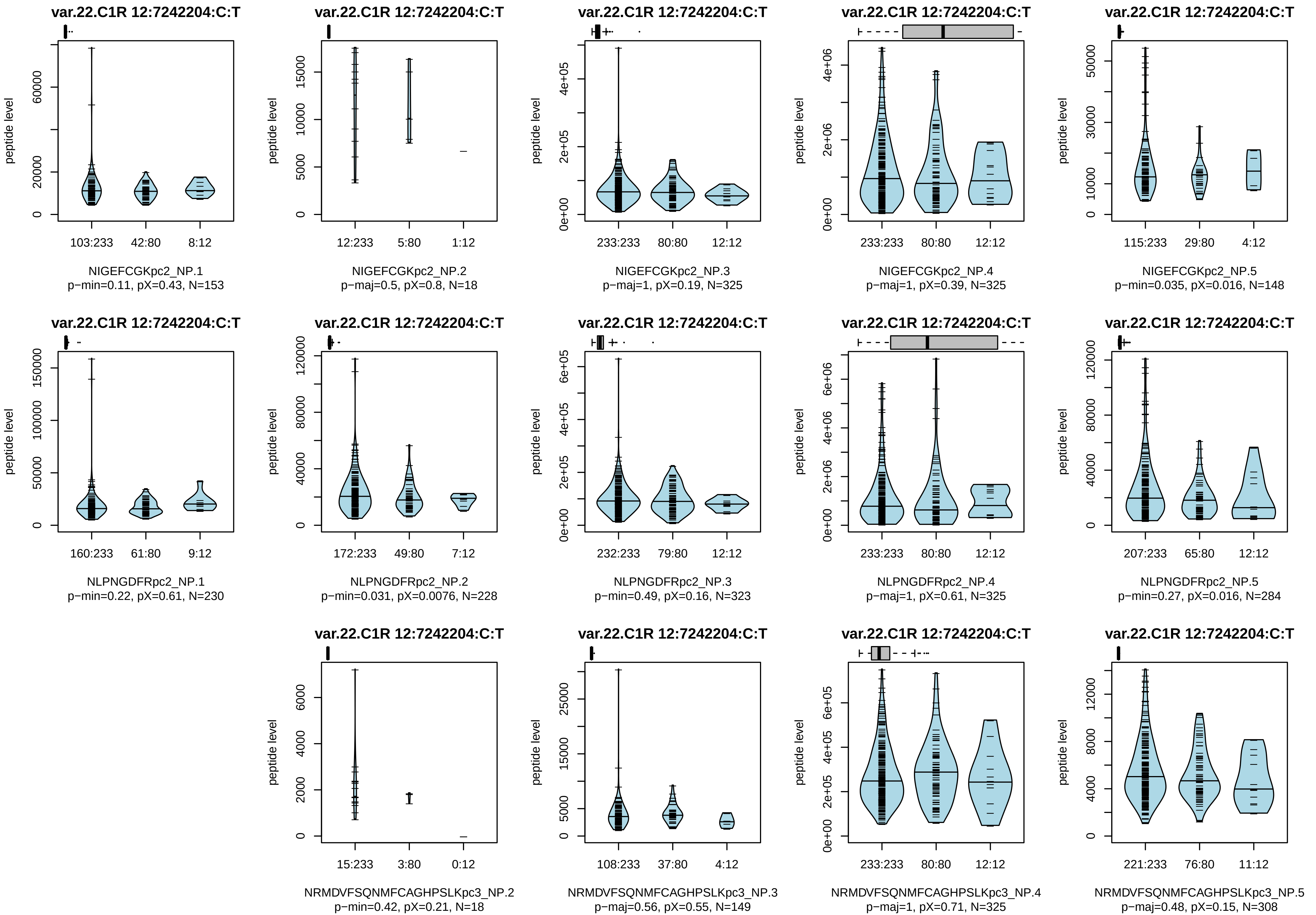

var.22.C1R 12:7242204:C:T
peptide level
103:233
42:80
8:12
NIGEFCGKpc2_NP.1
p−min=0.11, pX=0.43, N=153
12:233
5:80
1:12
NIGEFCGKpc2_NP.2
p−maj=0.5, pX=0.8, N=18
233:233
80:80
12:12
NIGEFCGKpc2_NP.3
p−maj=1, pX=0.19, N=325
NIGEFCGKpc2_NP.4
p−maj=1, pX=0.39, N=325
115:233
29:80
4:12
NIGEFCGKpc2_NP.5
p−min=0.035, pX=0.016, N=148
160:233
61:80
9:12
NLPNGDFRpc2_NP.1
p−min=0.22, pX=0.61, N=230
172:233
49:80
7:12
NLPNGDFRpc2_NP.2
p−min=0.031, pX=0.0076, N=228
232:233
79:80
NLPNGDFRpc2_NP.3
p−min=0.49, pX=0.16, N=323
NLPNGDFRpc2_NP.4
p−maj=1, pX=0.61, N=325
207:233
65:80
NLPNGDFRpc2_NP.5
p−min=0.27, pX=0.016, N=284
15:233
3:80
0:12
NRMDVFSQNMFCAGHPSLKpc3_NP.2
p−min=0.42, pX=0.21, N=18
108:233
37:80
4:12
NRMDVFSQNMFCAGHPSLKpc3_NP.3
p−maj=0.56, pX=0.55, N=149
NRMDVFSQNMFCAGHPSLKpc3_NP.4
p−maj=1, pX=0.71, N=325
221:233
76:80
11:12
NRMDVFSQNMFCAGHPSLKpc3_NP.5
p−maj=0.48, pX=0.15, N=308

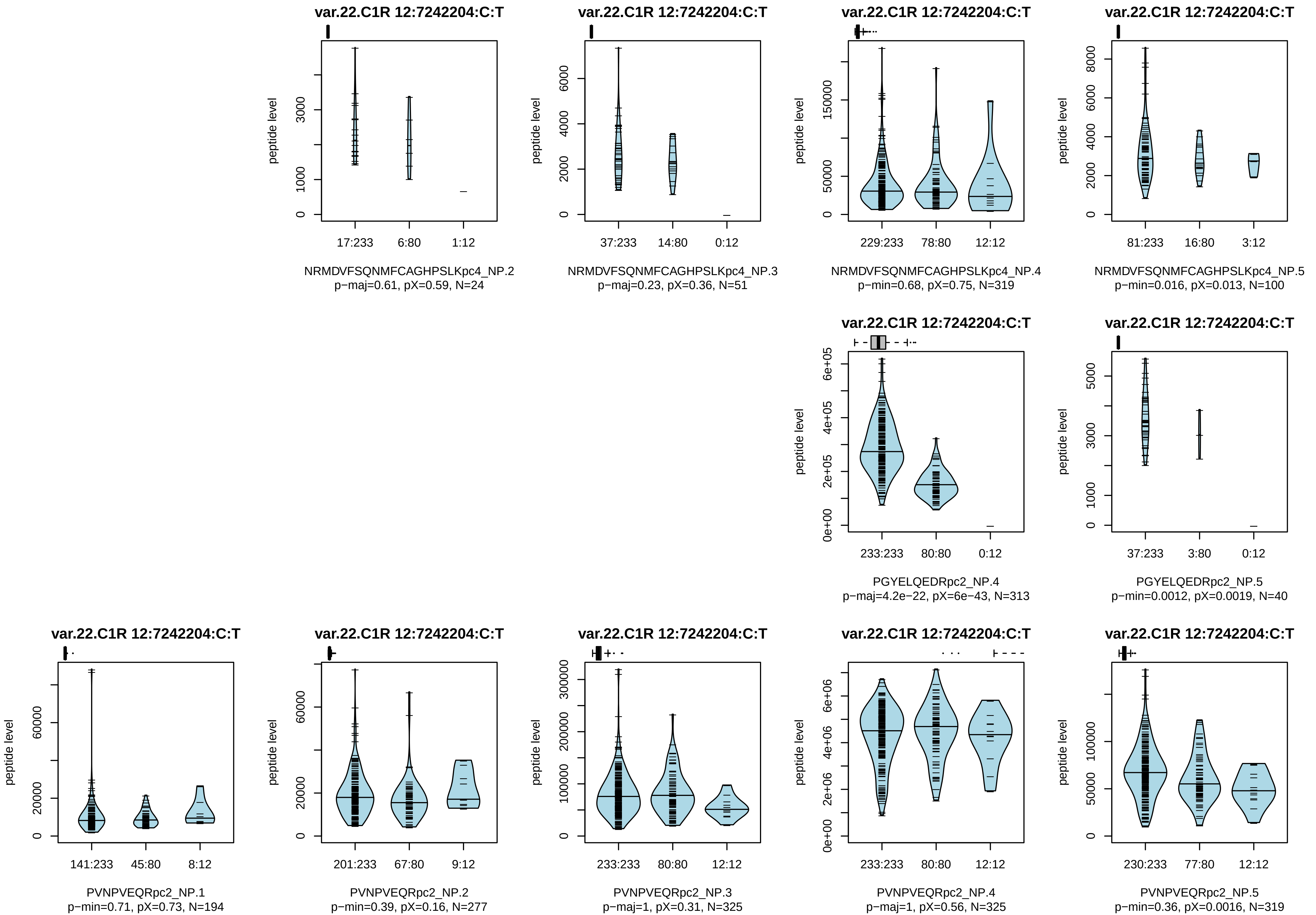
var.22.C1R 12:7242204:C:T
peptide level
17:233
6:80
1:12
NRMDVFSQNMFCAGHPSLKpc4_NP.2
p−maj=0.61, pX=0.59, N=24
var.22.C1R 12:7242204:C:T
peptide level
37:233
14:80
0:12
NRMDVFSQNMFCAGHPSLKpc4_NP.3
p−maj=0.23, pX=0.36, N=51
var.22.C1R 12:7242204:C:T
peptide level
229:233
78:80
12:12
NRMDVFSQNMFCAGHPSLKpc4_NP.4
p−min=0.68, pX=0.75, N=319
var.22.C1R 12:7242204:C:T
peptide level
81:233
16:80
3:12
NRMDVFSQNMFCAGHPSLKpc4_NP.5
p−min=0.016, pX=0.013, N=100
var.22.C1R 12:7242204:C:T
peptide level
233:233
80:80
0:12
PGYELQEDRpc2_NP.4
p−maj=4.2e−22, pX=6e−43, N=313
var.22.C1R 12:7242204:C:T
peptide level
37:233
3:80
0:12
PGYELQEDRpc2_NP.5
p−min=0.0012, pX=0.0019, N=40
var.22.C1R 12:7242204:C:T
peptide level
141:233
45:80
8:12
PVNPVEQRpc2_NP.1
p−min=0.71, pX=0.73, N=194
var.22.C1R 12:7242204:C:T
peptide level
201:233
67:80
9:12
PVNPVEQRpc2_NP.2
p−min=0.39, pX=0.16, N=277
var.22.C1R 12:7242204:C:T
peptide level
233:233
80:80
12:12
PVNPVEQRpc2_NP.3
p−maj=1, pX=0.31, N=325
var.22.C1R 12:7242204:C:T
peptide level
233:233
80:80
12:12
PVNPVEQRpc2_NP.4
p−maj=1, pX=0.56, N=325
var.22.C1R 12:7242204:C:T
peptide level
230:233
77:80
12:12
PVNPVEQRpc2_NP.5
p−min=0.36, pX=0.0016, N=319

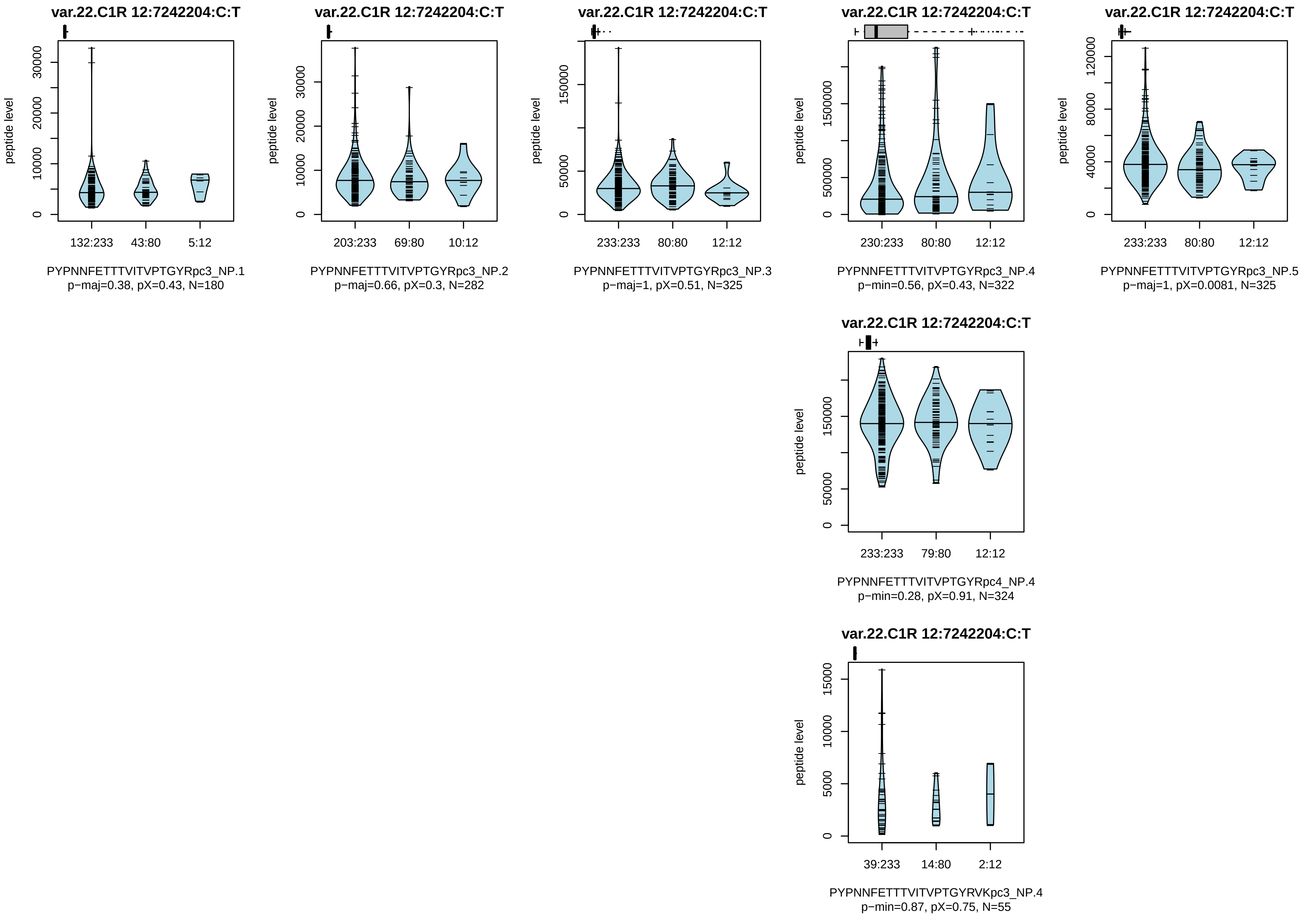
var.22.C1R 12:7242204:C:T
peptide level
0
10000
20000
30000
132:233
43:80
5:12
PYPNNFETTTVITVPTGYRpc3_NP.1
p−maj=0.38, pX=0.43, N=180
var.22.C1R 12:7242204:C:T
peptide level
0
10000
20000
30000
203:233
69:80
10:12
PYPNNFETTTVITVPTGYRpc3_NP.2
p−maj=0.66, pX=0.3, N=282
var.22.C1R 12:7242204:C:T
peptide level
0
50000
150000
233:233
80:80
12:12
PYPNNFETTTVITVPTGYRpc3_NP.3
p−maj=1, pX=0.51, N=325
var.22.C1R 12:7242204:C:T
peptide level
0
500000
1500000
230:233
80:80
12:12
PYPNNFETTTVITVPTGYRpc3_NP.4
p−min=0.56, pX=0.43, N=322
var.22.C1R 12:7242204:C:T
peptide level
0
40000
80000
120000
233:233
80:80
12:12
PYPNNFETTTVITVPTGYRpc3_NP.5
p−maj=1, pX=0.0081, N=325
var.22.C1R 12:7242204:C:T
peptide level
0
50000
150000
233:233
79:80
12:12
PYPNNFETTTVITVPTGYRpc4_NP.4
p−min=0.28, pX=0.91, N=324
var.22.C1R 12:7242204:C:T
peptide level
0
5000
10000
15000
39:233
14:80
2:12
PYPNNFETTTVITVPTGYRVKpc3_NP.4
p−min=0.87, pX=0.75, N=55

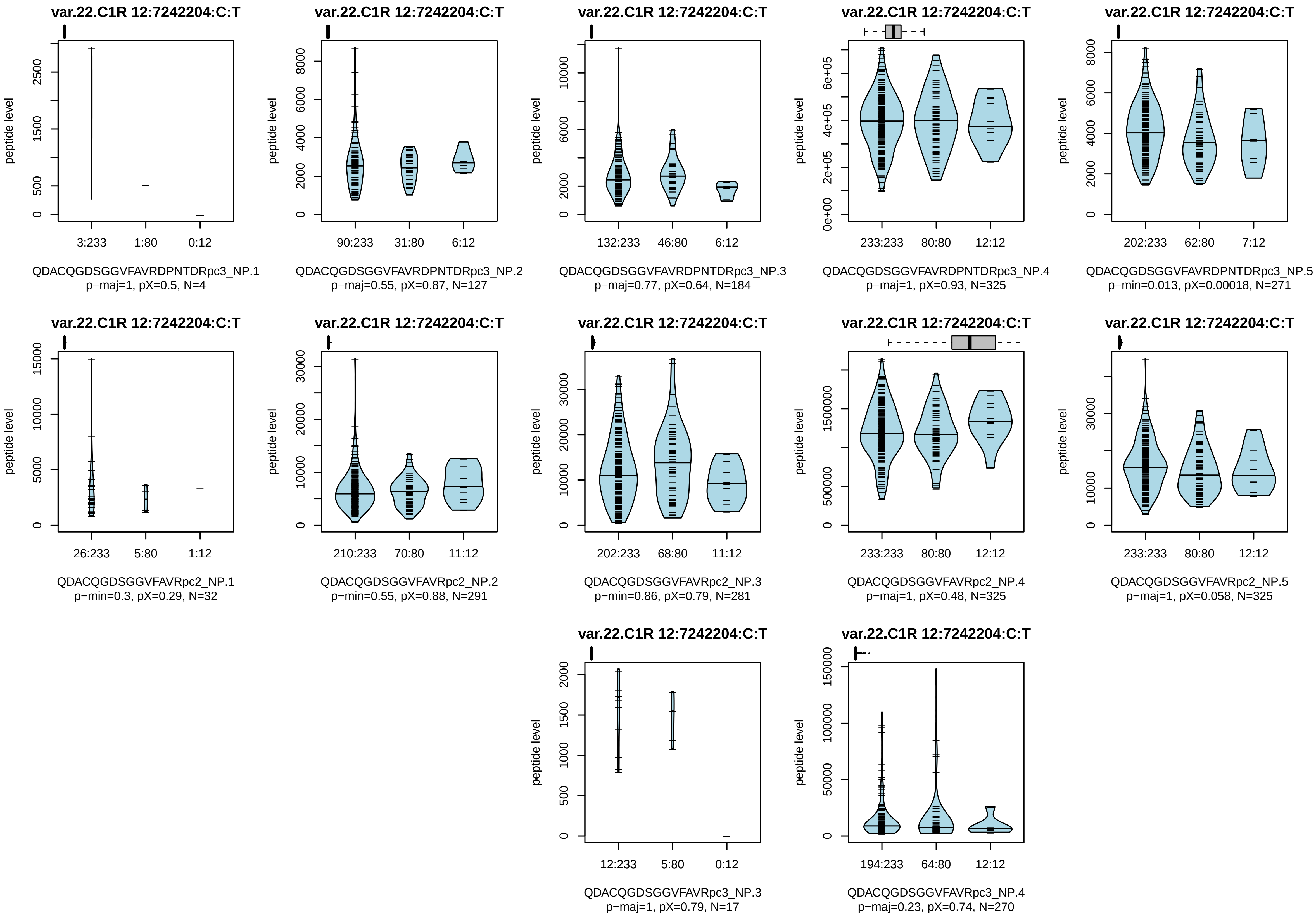
var.22.C1R 12:7242204:C:T
peptide level
3:233
1:80
0:12
QDACQGDSGGVFAVRDPNTDRpc3_NP.1
p−maj=1, pX=0.5, N=4
90:233
31:80
6:12
QDACQGDSGGVFAVRDPNTDRpc3_NP.2
p−maj=0.55, pX=0.87, N=127
132:233
46:80
6:12
QDACQGDSGGVFAVRDPNTDRpc3_NP.3
p−maj=0.77, pX=0.64, N=184
233:233
80:80
12:12
QDACQGDSGGVFAVRDPNTDRpc3_NP.4
p−maj=1, pX=0.93, N=325
202:233
62:80
7:12
QDACQGDSGGVFAVRDPNTDRpc3_NP.5
p−min=0.013, pX=0.00018, N=271
26:233
5:80
1:12
QDACQGDSGGVFAVRpc2_NP.1
p−min=0.3, pX=0.29, N=32
210:233
70:80
11:12
QDACQGDSGGVFAVRpc2_NP.2
p−min=0.55, pX=0.88, N=291
202:233
68:80
11:12
QDACQGDSGGVFAVRpc2_NP.3
p−min=0.86, pX=0.79, N=281
233:233
80:80
12:12
QDACQGDSGGVFAVRpc2_NP.4
p−maj=1, pX=0.48, N=325
233:233
80:80
12:12
QDACQGDSGGVFAVRpc2_NP.5
p−maj=1, pX=0.058, N=325
12:233
5:80
0:12
QDACQGDSGGVFAVRpc3_NP.3
p−maj=1, pX=0.79, N=17
194:233
64:80
12:12
QDACQGDSGGVFAVRpc3_NP.4
p−maj=0.23, pX=0.74, N=270

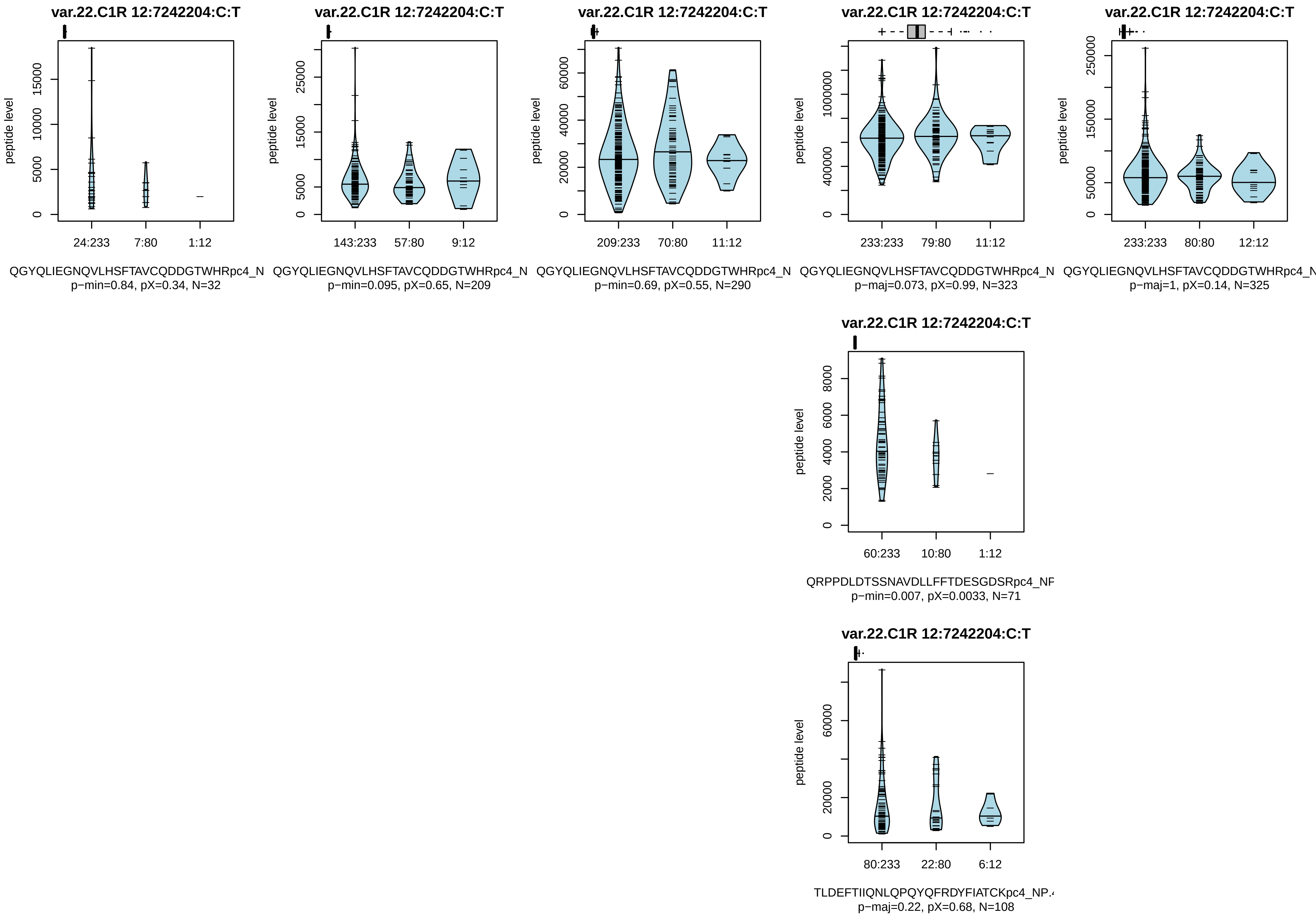

var.22.C1R 12:7242204:C:T
peptide level
24:233
7:80
1:12
QGYQLIEGNQVLHSFTAVCQDDGTWHRpc4_N
p−min=0.84, pX=0.34, N=32
var.22.C1R 12:7242204:C:T
peptide level
143:233
57:80
9:12
QGYQLIEGNQVLHSFTAVCQDDGTWHRpc4_N
p−min=0.095, pX=0.65, N=209
var.22.C1R 12:7242204:C:T
peptide level
209:233
70:80
11:12
QGYQLIEGNQVLHSFTAVCQDDGTWHRpc4_N
p−min=0.69, pX=0.55, N=290
var.22.C1R 12:7242204:C:T
peptide level
233:233
79:80
11:12
QGYQLIEGNQVLHSFTAVCQDDGTWHRpc4_N
p−maj=0.073, pX=0.99, N=323
var.22.C1R 12:7242204:C:T
peptide level
233:233
80:80
12:12
QGYQLIEGNQVLHSFTAVCQDDGTWHRpc4_N
p−maj=1, pX=0.14, N=325
var.22.C1R 12:7242204:C:T
peptide level
60:233
10:80
1:12
QRPPDLDTSSNAVDLLFFTDESGDSRpc4_NF
p−min=0.007, pX=0.0033, N=71
var.22.C1R 12:7242204:C:T
peptide level
80:233
22:80
6:12
TLDEFTIIQNLQPQYQFRDYFIATCKpc4_NP.
p−maj=0.22, pX=0.68, N=108

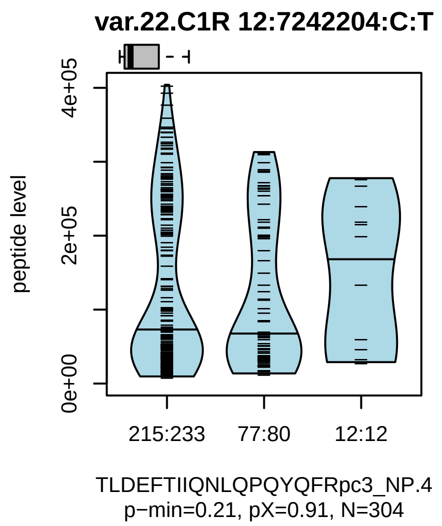
var.22.C1R 12:7242204:C:T
peptide level
4e+05
2e+05
0e+00
215:233
77:80
12:12
TLDEFTIIQNLQPQYQFRpc3_NP.4
p−min=0.21, pX=0.91, N=304

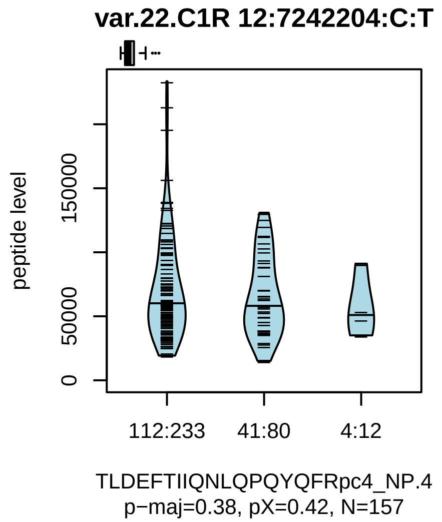
var.22.C1R 12:7242204:C:T
peptide level
150000
50000
0
112:233
41:80
4:12
TLDEFTIIQNLQPQYQFRpc4_NP.4
p−maj=0.38, pX=0.42, N=157

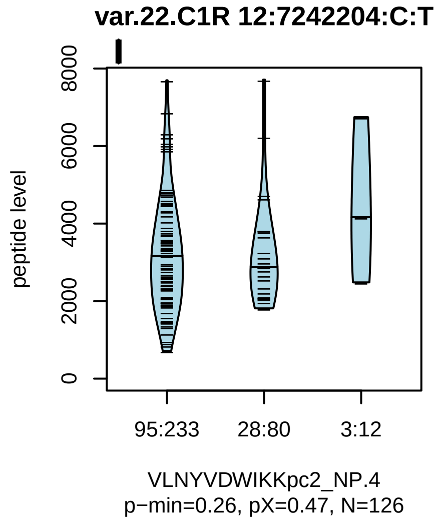
var.22.C1R 12:7242204:C:T
peptide level
8000
6000
4000
2000
0
95:233
28:80
3:12
VLNYVDWIKKpc2_NP.4
p−min=0.26, pX=0.47, N=126

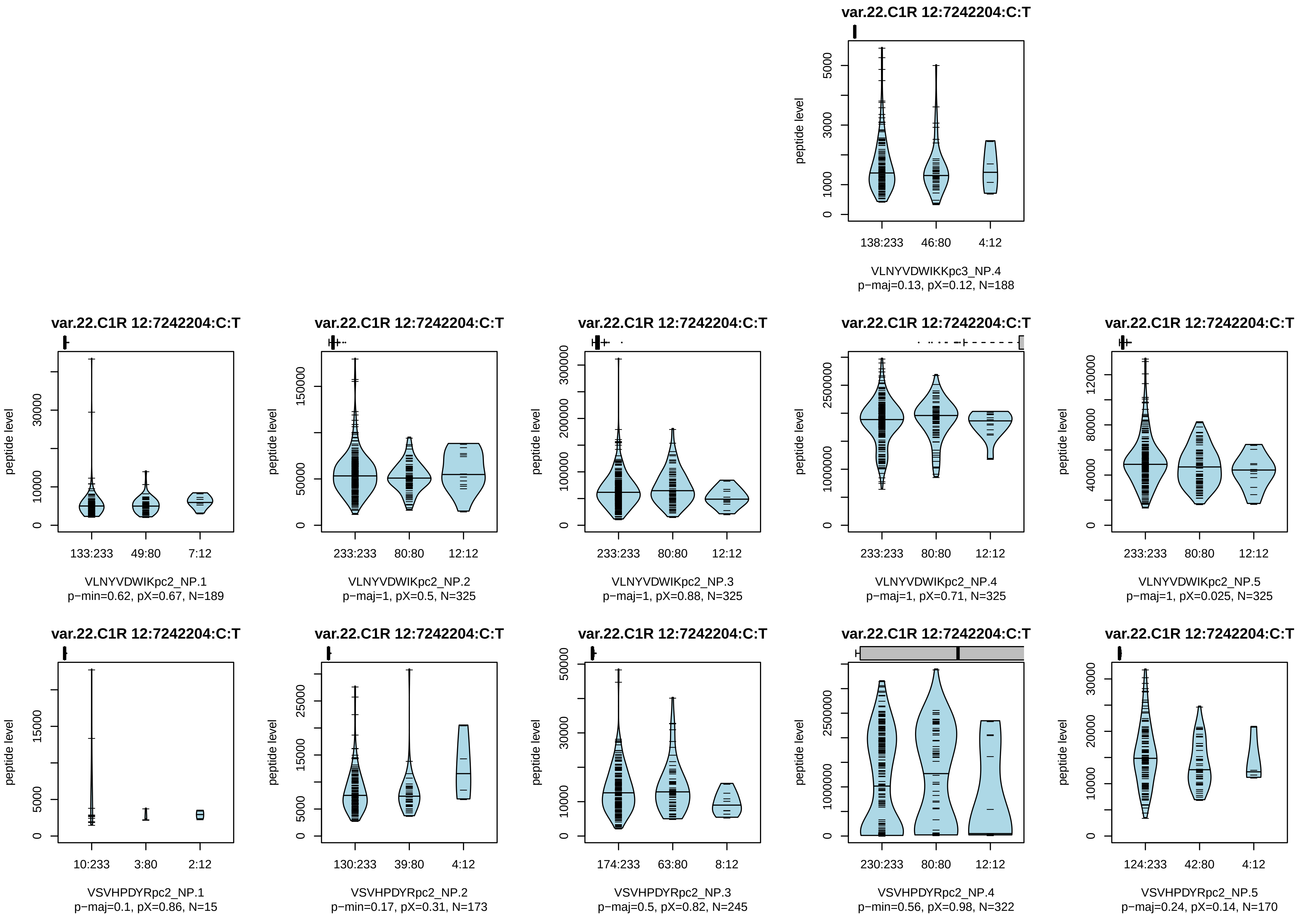
var.22.C1R 12:7242204:C:T
peptide level
138:233
46:80
4:12
VLNYVDWIKKpc3_NP.4
p−maj=0.13, pX=0.12, N=188
var.22.C1R 12:7242204:C:T
133:233
49:80
7:12
VLNYVDWIKpc2_NP.1
p−min=0.62, pX=0.67, N=189
var.22.C1R 12:7242204:C:T
233:233
80:80
12:12
VLNYVDWIKpc2_NP.2
p−maj=1, pX=0.5, N=325
var.22.C1R 12:7242204:C:T
233:233
80:80
12:12
VLNYVDWIKpc2_NP.3
p−maj=1, pX=0.88, N=325
var.22.C1R 12:7242204:C:T
233:233
80:80
12:12
VLNYVDWIKpc2_NP.4
p−maj=1, pX=0.71, N=325
var.22.C1R 12:7242204:C:T
233:233
80:80
12:12
VLNYVDWIKpc2_NP.5
p−maj=1, pX=0.025, N=325
var.22.C1R 12:7242204:C:T
10:233
3:80
2:12
VSVHPDYRpc2_NP.1
p−maj=0.1, pX=0.86, N=15
var.22.C1R 12:7242204:C:T
130:233
39:80
4:12
VSVHPDYRpc2_NP.2
p−min=0.17, pX=0.31, N=173
var.22.C1R 12:7242204:C:T
174:233
63:80
8:12
VSVHPDYRpc2_NP.3
p−maj=0.5, pX=0.82, N=245
var.22.C1R 12:7242204:C:T
230:233
80:80
12:12
VSVHPDYRpc2_NP.4
p−min=0.56, pX=0.98, N=322
var.22.C1R 12:7242204:C:T
124:233
42:80
4:12
VSVHPDYRpc2_NP.5
p−maj=0.24, pX=0.14, N=170

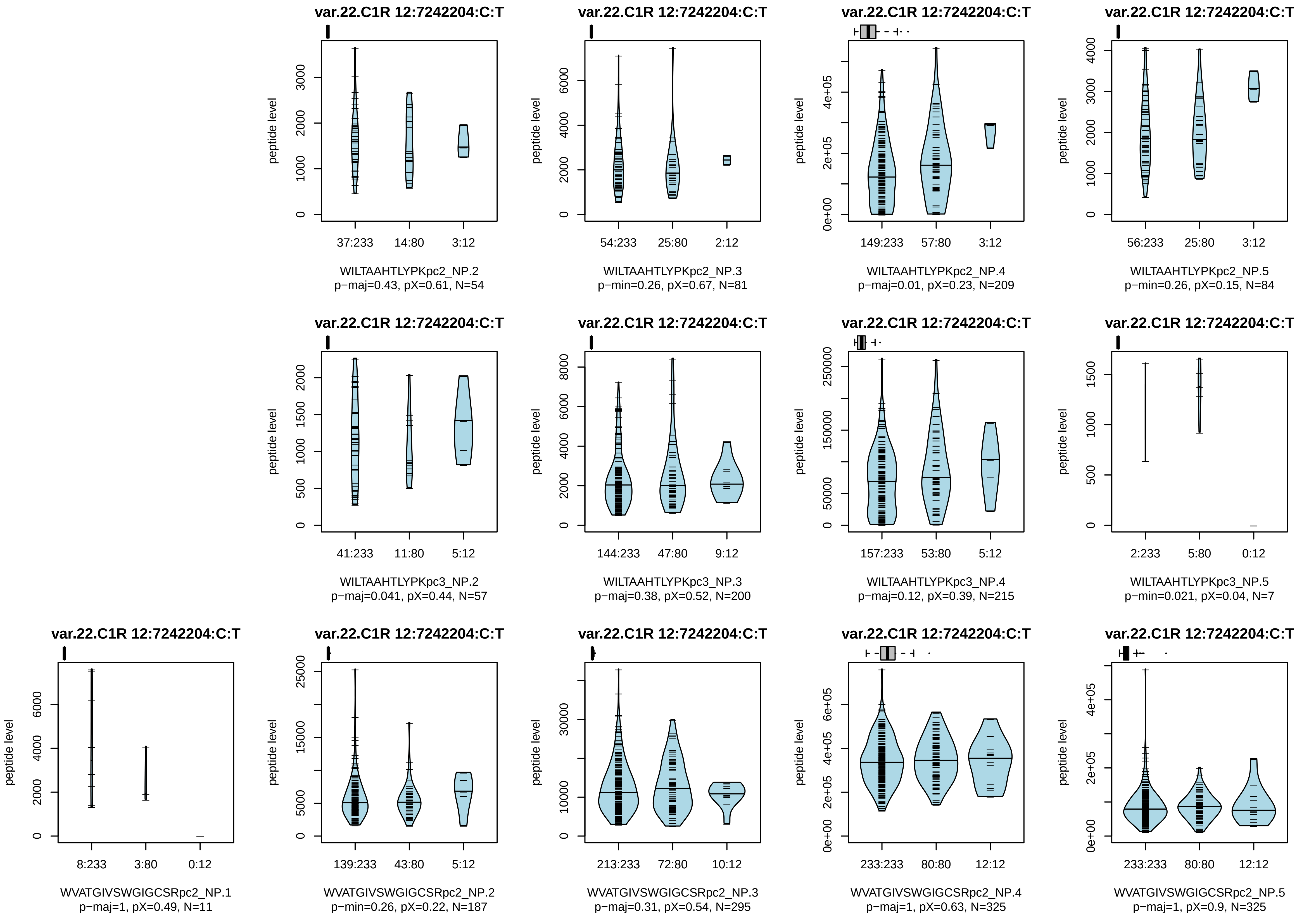
var.22.C1R 12:7242204:C:T
peptide level
37:233
14:80
3:12
WILTAAHTLYPKpc2_NP.2
p−maj=0.43, pX=0.61, N=54
var.22.C1R 12:7242204:C:T
peptide level
54:233
25:80
2:12
WILTAAHTLYPKpc2_NP.3
p−min=0.26, pX=0.67, N=81
var.22.C1R 12:7242204:C:T
peptide level
149:233
57:80
3:12
WILTAAHTLYPKpc2_NP.4
p−maj=0.01, pX=0.23, N=209
var.22.C1R 12:7242204:C:T
peptide level
56:233
25:80
3:12
WILTAAHTLYPKpc2_NP.5
p−min=0.26, pX=0.15, N=84
var.22.C1R 12:7242204:C:T
peptide level
41:233
11:80
5:12
WILTAAHTLYPKpc3_NP.2
p−maj=0.041, pX=0.44, N=57
var.22.C1R 12:7242204:C:T
peptide level
144:233
47:80
9:12
WILTAAHTLYPKpc3_NP.3
p−maj=0.38, pX=0.52, N=200
var.22.C1R 12:7242204:C:T
peptide level
157:233
53:80
5:12
WILTAAHTLYPKpc3_NP.4
p−maj=0.12, pX=0.39, N=215
var.22.C1R 12:7242204:C:T
peptide level
2:233
5:80
0:12
WILTAAHTLYPKpc3_NP.5
p−min=0.021, pX=0.04, N=7
var.22.C1R 12:7242204:C:T
peptide level
8:233
3:80
0:12
WVATGIVSWGIGCSRpc2_NP.1
p−maj=1, pX=0.49, N=11
var.22.C1R 12:7242204:C:T
peptide level
139:233
43:80
5:12
WVATGIVSWGIGCSRpc2_NP.2
p−min=0.26, pX=0.22, N=187
var.22.C1R 12:7242204:C:T
peptide level
213:233
72:80
10:12
WVATGIVSWGIGCSRpc2_NP.3
p−maj=0.31, pX=0.54, N=295
var.22.C1R 12:7242204:C:T
peptide level
233:233
80:80
12:12
WVATGIVSWGIGCSRpc2_NP.4
p−maj=1, pX=0.63, N=325
var.22.C1R 12:7242204:C:T
peptide level
233:233
80:80
12:12
WVATGIVSWGIGCSRpc2_NP.5
p−maj=1, pX=0.9, N=325

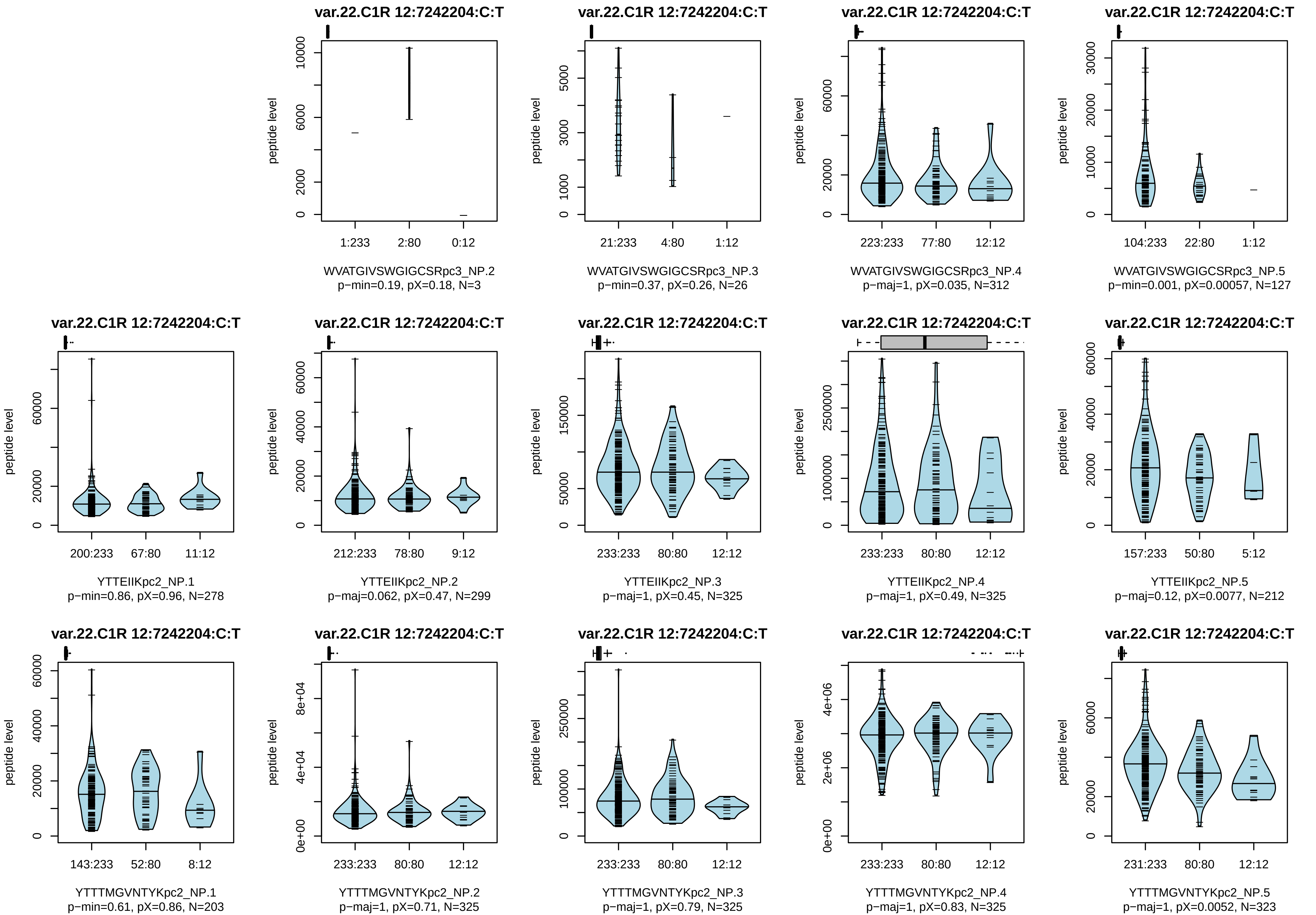
var.22.C1R 12:7242204:C:T
peptide level
1:233
2:80
0:12
WVATGIVSWGIGCSRpc3_NP.2
p−min=0.19, pX=0.18, N=3
var.22.C1R 12:7242204:C:T
21:233
4:80
1:12
WVATGIVSWGIGCSRpc3_NP.3
p−min=0.37, pX=0.26, N=26
var.22.C1R 12:7242204:C:T
223:233
77:80
12:12
WVATGIVSWGIGCSRpc3_NP.4
p−maj=1, pX=0.035, N=312
var.22.C1R 12:7242204:C:T
104:233
22:80
1:12
WVATGIVSWGIGCSRpc3_NP.5
p−min=0.001, pX=0.00057, N=127
var.22.C1R 12:7242204:C:T
200:233
67:80
11:12
YTTEIIKpc2_NP.1
p−min=0.86, pX=0.96, N=278
var.22.C1R 12:7242204:C:T
212:233
78:80
9:12
YTTEIIKpc2_NP.2
p−maj=0.062, pX=0.47, N=299
var.22.C1R 12:7242204:C:T
233:233
80:80
12:12
YTTEIIKpc2_NP.3
p−maj=1, pX=0.45, N=325
var.22.C1R 12:7242204:C:T
233:233
80:80
12:12
YTTEIIKpc2_NP.4
p−maj=1, pX=0.49, N=325
var.22.C1R 12:7242204:C:T
157:233
50:80
5:12
YTTEIIKpc2_NP.5
p−maj=0.12, pX=0.0077, N=212
var.22.C1R 12:7242204:C:T
143:233
52:80
8:12
YTTTMGVNTYKpc2_NP.1
p−min=0.61, pX=0.86, N=203
var.22.C1R 12:7242204:C:T
233:233
80:80
12:12
YTTTMGVNTYKpc2_NP.2
p−maj=1, pX=0.71, N=325
var.22.C1R 12:7242204:C:T
233:233
80:80
12:12
YTTTMGVNTYKpc2_NP.3
p−maj=1, pX=0.79, N=325
var.22.C1R 12:7242204:C:T
233:233
80:80
12:12
YTTTMGVNTYKpc2_NP.4
p−maj=1, pX=0.83, N=325
var.22.C1R 12:7242204:C:T
231:233
80:80
12:12
YTTTMGVNTYKpc2_NP.5
p−maj=1, pX=0.0052, N=323

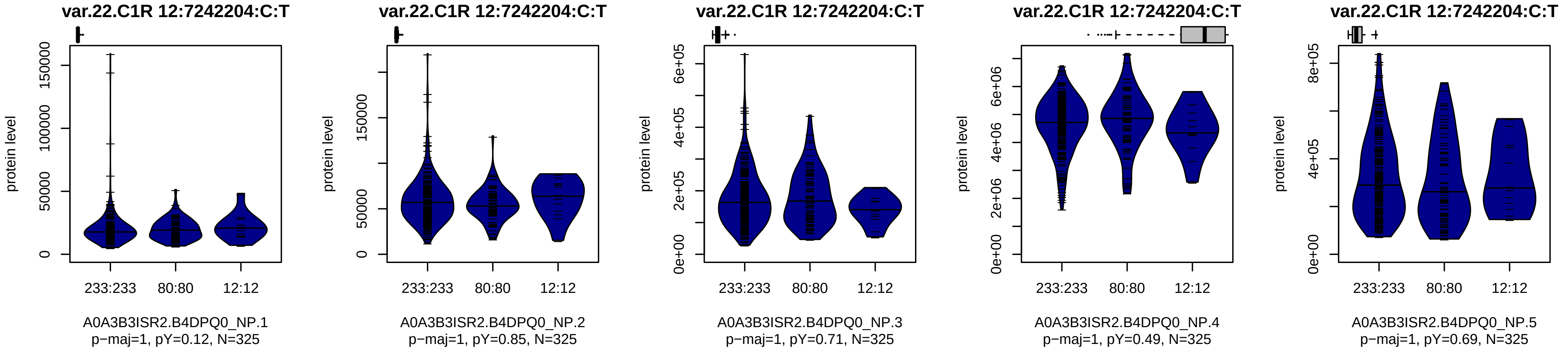

var.22.C1R 12:7242204:C:T
protein level
233:233
80:80
12:12
A0A3B3ISR2.B4DPQ0_NP.1
p−maj=1, pY=0.12, N=325
var.22.C1R 12:7242204:C:T
A0A3B3ISR2.B4DPQ0_NP.2
p−maj=1, pY=0.85, N=325
var.22.C1R 12:7242204:C:T
A0A3B3ISR2.B4DPQ0_NP.3
p−maj=1, pY=0.71, N=325
var.22.C1R 12:7242204:C:T
A0A3B3ISR2.B4DPQ0_NP.4
p−maj=1, pY=0.49, N=325
var.22.C1R 12:7242204:C:T
A0A3B3ISR2.B4DPQ0_NP.5
p−maj=1, pY=0.69, N=325

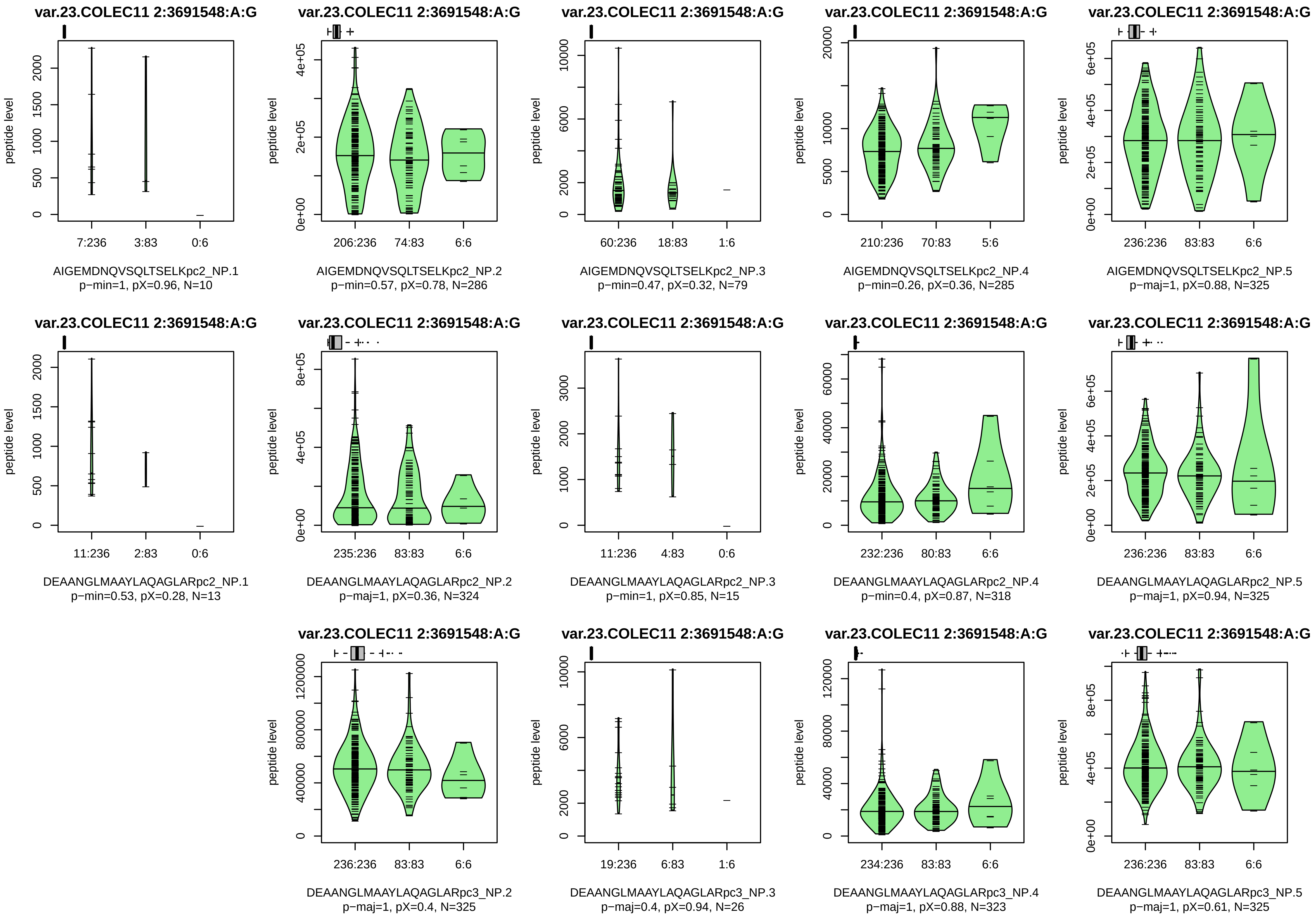
var.23.COLEC11 2:3691548:A:G
peptide level
7:236
3:83
0:6
AIGEMDNQVSQLTSELKpc2_NP.1
p−min=1, pX=0.96, N=10
var.23.COLEC11 2:3691548:A:G
peptide level
206:236
74:83
6:6
AIGEMDNQVSQLTSELKpc2_NP.2
p−min=0.57, pX=0.78, N=286
var.23.COLEC11 2:3691548:A:G
peptide level
60:236
18:83
1:6
AIGEMDNQVSQLTSELKpc2_NP.3
p−min=0.47, pX=0.32, N=79
var.23.COLEC11 2:3691548:A:G
peptide level
210:236
70:83
5:6
AIGEMDNQVSQLTSELKpc2_NP.4
p−min=0.26, pX=0.36, N=285
var.23.COLEC11 2:3691548:A:G
peptide level
236:236
83:83
6:6
AIGEMDNQVSQLTSELKpc2_NP.5
p−maj=1, pX=0.88, N=325
var.23.COLEC11 2:3691548:A:G
peptide level
11:236
2:83
0:6
DEAANGLMAAYLAQAGLARpc2_NP.1
p−min=0.53, pX=0.28, N=13
var.23.COLEC11 2:3691548:A:G
peptide level
235:236
83:83
6:6
DEAANGLMAAYLAQAGLARpc2_NP.2
p−maj=1, pX=0.36, N=324
var.23.COLEC11 2:3691548:A:G
peptide level
11:236
4:83
0:6
DEAANGLMAAYLAQAGLARpc2_NP.3
p−min=1, pX=0.85, N=15
var.23.COLEC11 2:3691548:A:G
peptide level
232:236
80:83
6:6
DEAANGLMAAYLAQAGLARpc2_NP.4
p−min=0.4, pX=0.87, N=318
var.23.COLEC11 2:3691548:A:G
peptide level
236:236
83:83
6:6
DEAANGLMAAYLAQAGLARpc2_NP.5
p−maj=1, pX=0.94, N=325
var.23.COLEC11 2:3691548:A:G
peptide level
236:236
83:83
6:6
DEAANGLMAAYLAQAGLARpc3_NP.2
p−maj=1, pX=0.4, N=325
var.23.COLEC11 2:3691548:A:G
peptide level
19:236
6:83
1:6
DEAANGLMAAYLAQAGLARpc3_NP.3
p−maj=0.4, pX=0.94, N=26
var.23.COLEC11 2:3691548:A:G
peptide level
234:236
83:83
6:6
DEAANGLMAAYLAQAGLARpc3_NP.4
p−maj=1, pX=0.88, N=323
var.23.COLEC11 2:3691548:A:G
peptide level
236:236
83:83
6:6
DEAANGLMAAYLAQAGLARpc3_NP.5
p−maj=1, pX=0.61, N=325

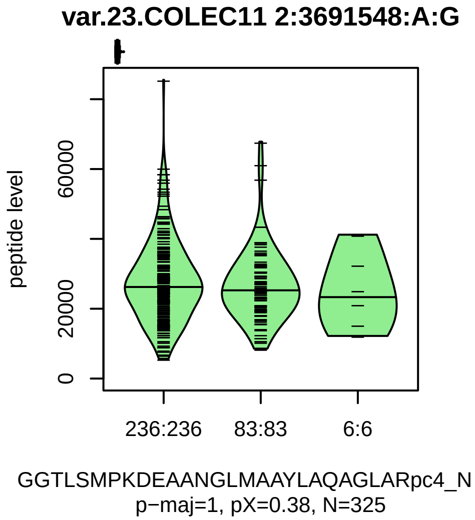
var.23.COLEC11 2:3691548:A:G
peptide level
0
20000
60000
236:236
83:83
6:6
GGTLSMPKDEAANGLMAAYLAQAGLARpc4_N
p−maj=1, pX=0.38, N=325

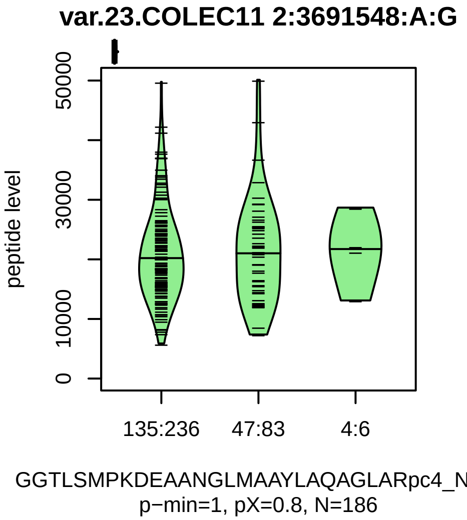
var.23.COLEC11 2:3691548:A:G
peptide level
0
10000
30000
50000
135:236
47:83
4:6
GGTLSMPKDEAANGLMAAYLAQAGLARpc4_N
p−min=1, pX=0.8, N=186

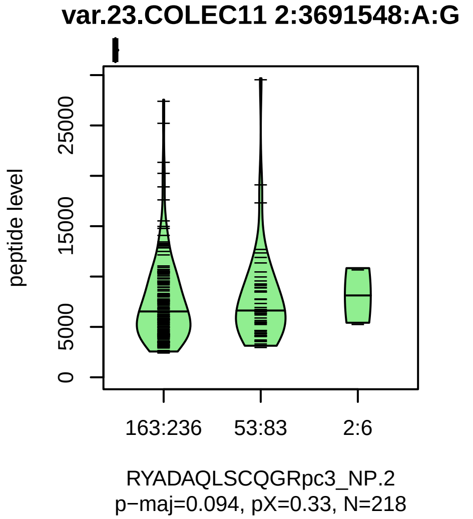
var.23.COLEC11 2:3691548:A:G
peptide level
0
5000
15000
25000
163:236
53:83
2:6
RYADAQLSCQGRpc3_NP.2
p−maj=0.094, pX=0.33, N=218

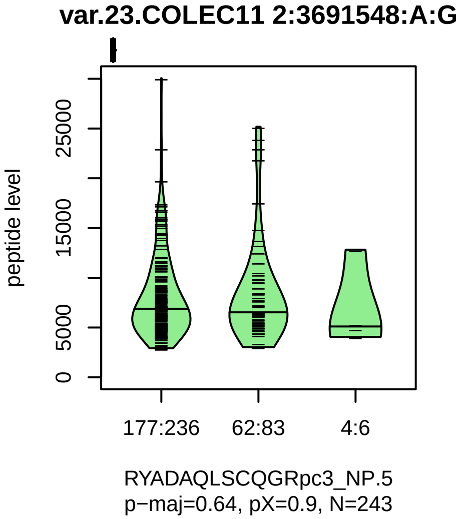
var.23.COLEC11 2:3691548:A:G
peptide level
0
5000
15000
25000
177:236
62:83
4:6
RYADAQLSCQGRpc3_NP.5
p−maj=0.64, pX=0.9, N=243

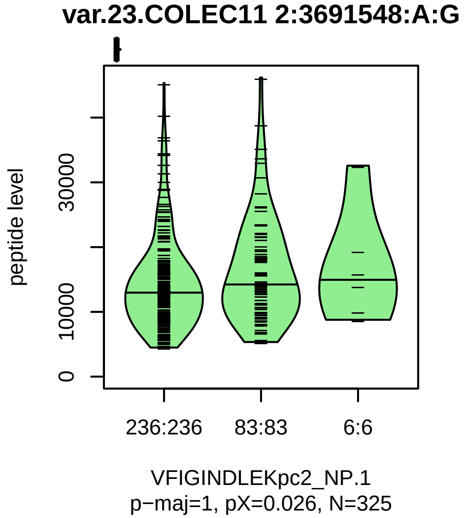
var.23.COLEC11 2:3691548:A:G
peptide level
0
10000
30000
236:236
83:83
6:6
VFIGINDLEKpc2_NP.1
p−maj=1, pX=0.026, N=325

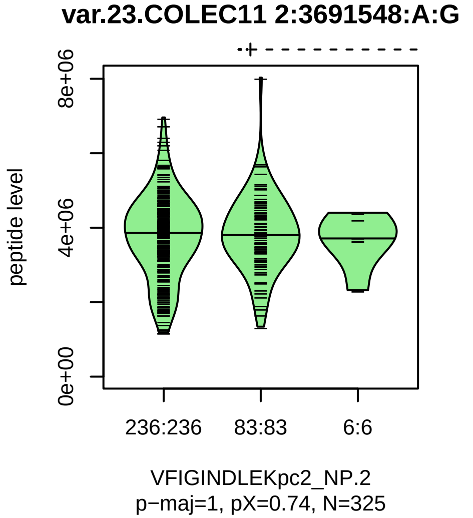
var.23.COLEC11 2:3691548:A:G
peptide level
0e+00
4e+06
8e+06
236:236
83:83
6:6
VFIGINDLEKpc2_NP.2
p−maj=1, pX=0.74, N=325

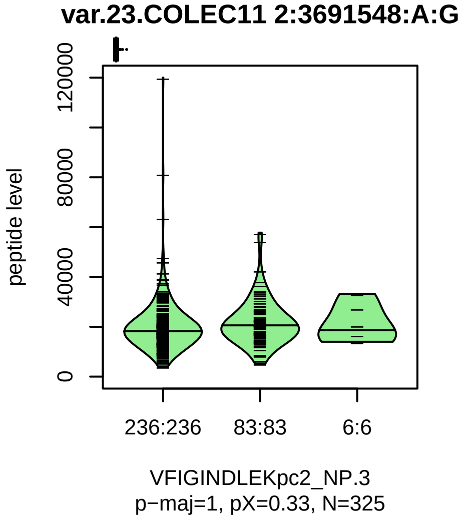
var.23.COLEC11 2:3691548:A:G
peptide level
0
40000
80000
120000
236:236
83:83
6:6
VFIGINDLEKpc2_NP.3
p−maj=1, pX=0.33, N=325

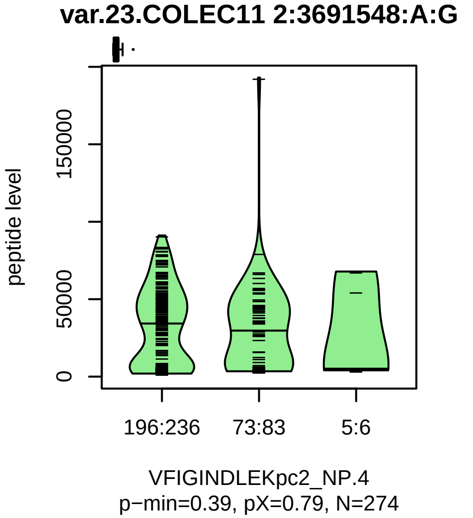
var.23.COLEC11 2:3691548:A:G
peptide level
0
50000
150000
196:236
73:83
5:6
VFIGINDLEKpc2_NP.4
p−min=0.39, pX=0.79, N=274

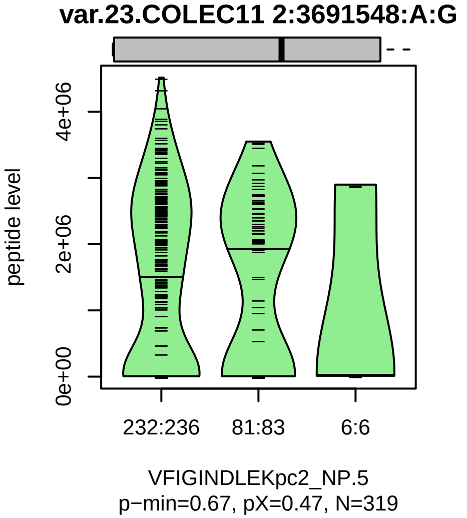
var.23.COLEC11 2:3691548:A:G
peptide level
0e+00
2e+06
4e+06
232:236
81:83
6:6
VFIGINDLEKpc2_NP.5
p−min=0.67, pX=0.47, N=319

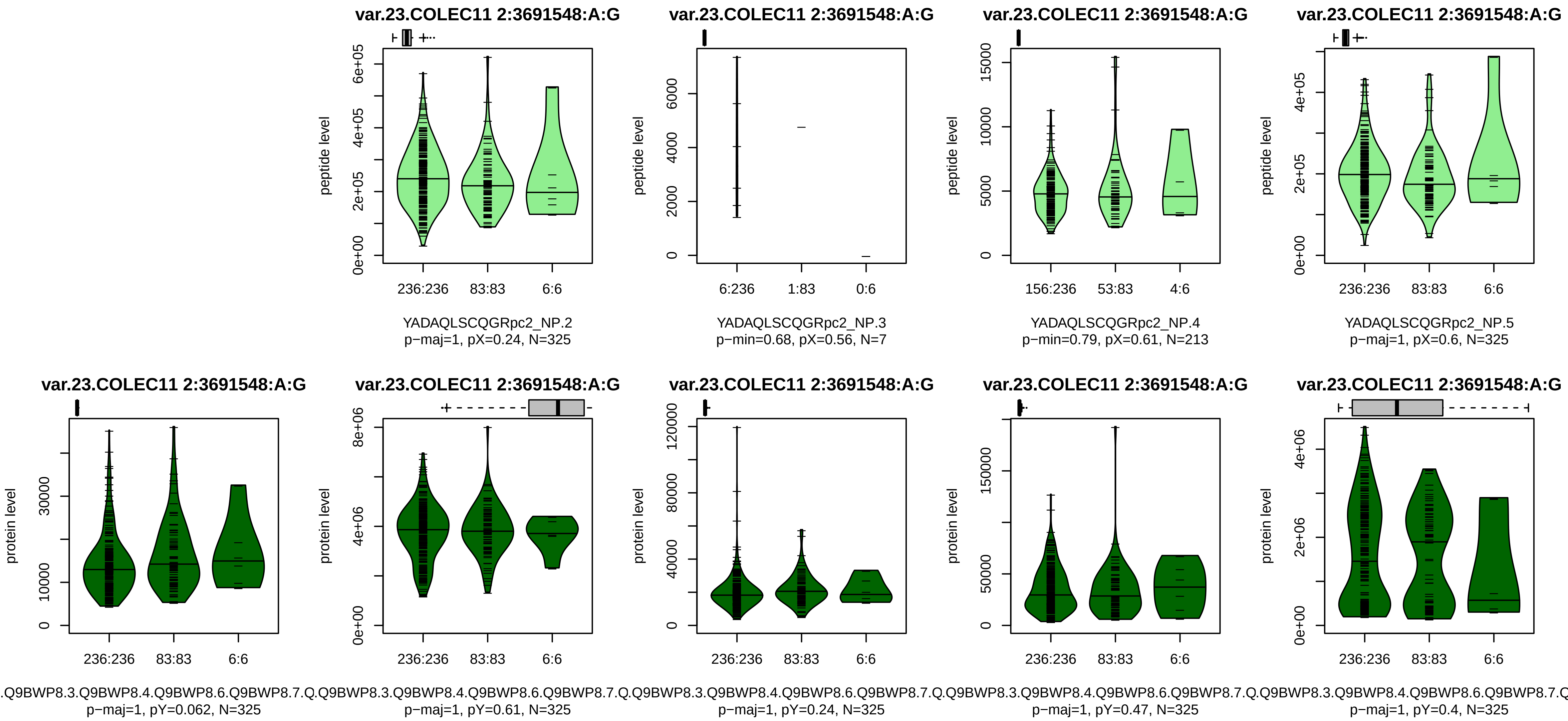
var.23.COLEC11 2:3691548:A:G
peptide level
236:236
83:83
6:6
YADAQLSCQGRpc2_NP.2
p−maj=1, pX=0.24, N=325
var.23.COLEC11 2:3691548:A:G
peptide level
6:236
1:83
0:6
YADAQLSCQGRpc2_NP.3
p−min=0.68, pX=0.56, N=7
var.23.COLEC11 2:3691548:A:G
peptide level
156:236
53:83
4:6
YADAQLSCQGRpc2_NP.4
p−min=0.79, pX=0.61, N=213
var.23.COLEC11 2:3691548:A:G
peptide level
236:236
83:83
6:6
YADAQLSCQGRpc2_NP.5
p−maj=1, pX=0.6, N=325
var.23.COLEC11 2:3691548:A:G
protein level
236:236
83:83
6:6
.Q9BWP8.3.Q9BWP8.4.Q9BWP8.6.Q9BWP8.7.Q
p−maj=1, pY=0.062, N=325
var.23.COLEC11 2:3691548:A:G
protein level
236:236
83:83
6:6
.Q9BWP8.3.Q9BWP8.4.Q9BWP8.6.Q9BWP8.7.Q
p−maj=1, pY=0.61, N=325
var.23.COLEC11 2:3691548:A:G
protein level
236:236
83:83
6:6
.Q9BWP8.3.Q9BWP8.4.Q9BWP8.6.Q9BWP8.7.Q
p−maj=1, pY=0.24, N=325
var.23.COLEC11 2:3691548:A:G
protein level
236:236
83:83
6:6
.Q9BWP8.3.Q9BWP8.4.Q9BWP8.6.Q9BWP8.7.Q
p−maj=1, pY=0.47, N=325
var.23.COLEC11 2:3691548:A:G
protein level
236:236
83:83
6:6
.Q9BWP8.3.Q9BWP8.4.Q9BWP8.6.Q9BWP8.7.Q
p−maj=1, pY=0.4, N=325

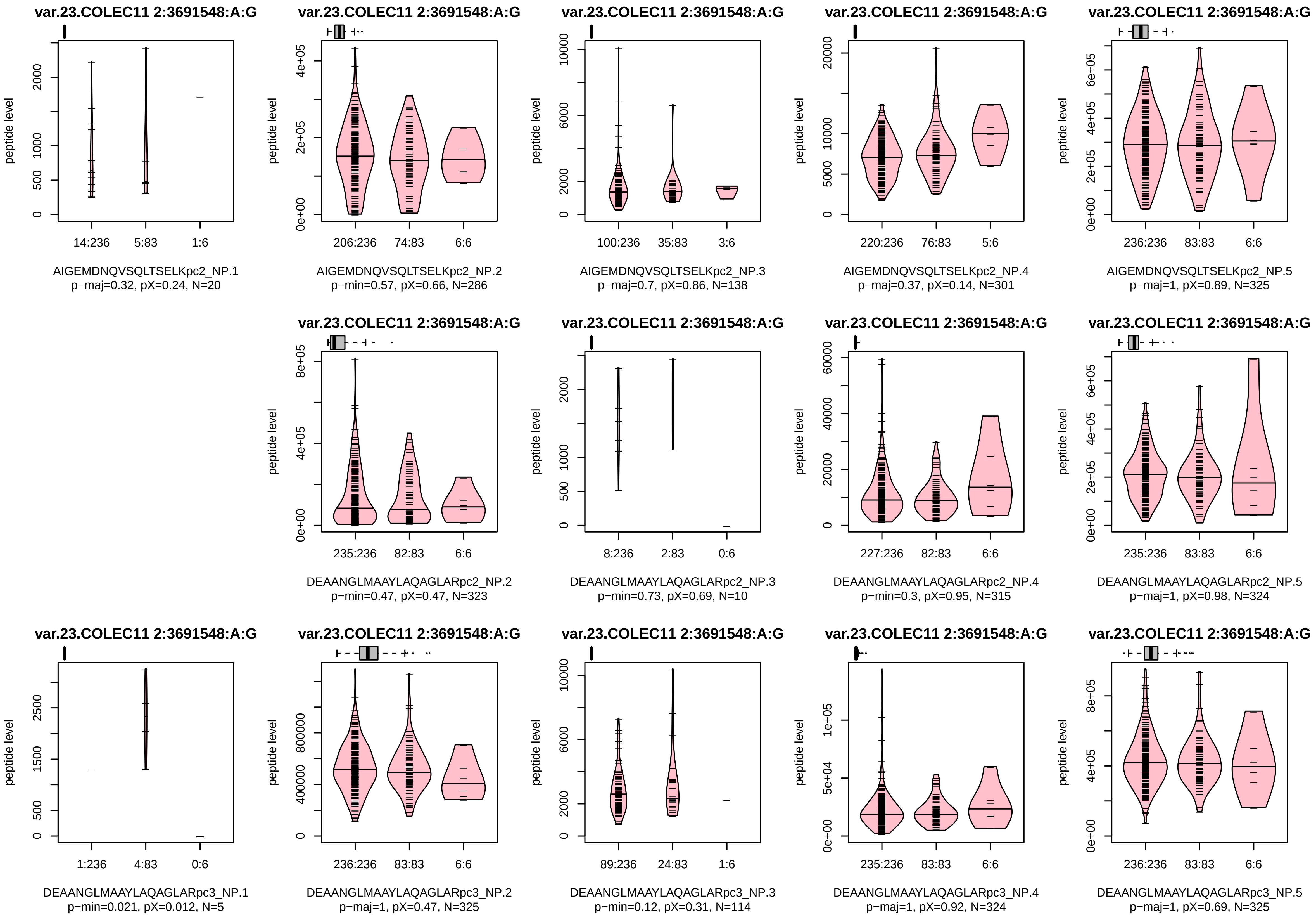
var.23.COLEC11 2:3691548:A:G
peptide level
14:236
5:83
1:6
AIGEMDNQVSQLTSELKpc2_NP.1
p−maj=0.32, pX=0.24, N=20
var.23.COLEC11 2:3691548:A:G
peptide level
206:236
74:83
6:6
AIGEMDNQVSQLTSELKpc2_NP.2
p−min=0.57, pX=0.66, N=286
var.23.COLEC11 2:3691548:A:G
peptide level
100:236
35:83
3:6
AIGEMDNQVSQLTSELKpc2_NP.3
p−maj=0.7, pX=0.86, N=138
var.23.COLEC11 2:3691548:A:G
peptide level
220:236
76:83
5:6
AIGEMDNQVSQLTSELKpc2_NP.4
p−maj=0.37, pX=0.14, N=301
var.23.COLEC11 2:3691548:A:G
peptide level
236:236
83:83
6:6
AIGEMDNQVSQLTSELKpc2_NP.5
p−maj=1, pX=0.89, N=325
var.23.COLEC11 2:3691548:A:G
peptide level
235:236
82:83
6:6
DEAANGLMAAYLAQAGLARpc2_NP.2
p−min=0.47, pX=0.47, N=323
var.23.COLEC11 2:3691548:A:G
peptide level
8:236
2:83
0:6
DEAANGLMAAYLAQAGLARpc2_NP.3
p−min=0.73, pX=0.69, N=10
var.23.COLEC11 2:3691548:A:G
peptide level
227:236
82:83
6:6
DEAANGLMAAYLAQAGLARpc2_NP.4
p−min=0.3, pX=0.95, N=315
var.23.COLEC11 2:3691548:A:G
peptide level
235:236
83:83
6:6
DEAANGLMAAYLAQAGLARpc2_NP.5
p−maj=1, pX=0.98, N=324
var.23.COLEC11 2:3691548:A:G
peptide level
1:236
4:83
0:6
DEAANGLMAAYLAQAGLARpc3_NP.1
p−min=0.021, pX=0.012, N=5
var.23.COLEC11 2:3691548:A:G
peptide level
236:236
83:83
6:6
DEAANGLMAAYLAQAGLARpc3_NP.2
p−maj=1, pX=0.47, N=325
var.23.COLEC11 2:3691548:A:G
peptide level
89:236
24:83
1:6
DEAANGLMAAYLAQAGLARpc3_NP.3
p−min=0.12, pX=0.31, N=114
var.23.COLEC11 2:3691548:A:G
peptide level
235:236
83:83
6:6
DEAANGLMAAYLAQAGLARpc3_NP.4
p−maj=1, pX=0.92, N=324
var.23.COLEC11 2:3691548:A:G
peptide level
236:236
83:83
6:6
DEAANGLMAAYLAQAGLARpc3_NP.5
p−maj=1, pX=0.69, N=325

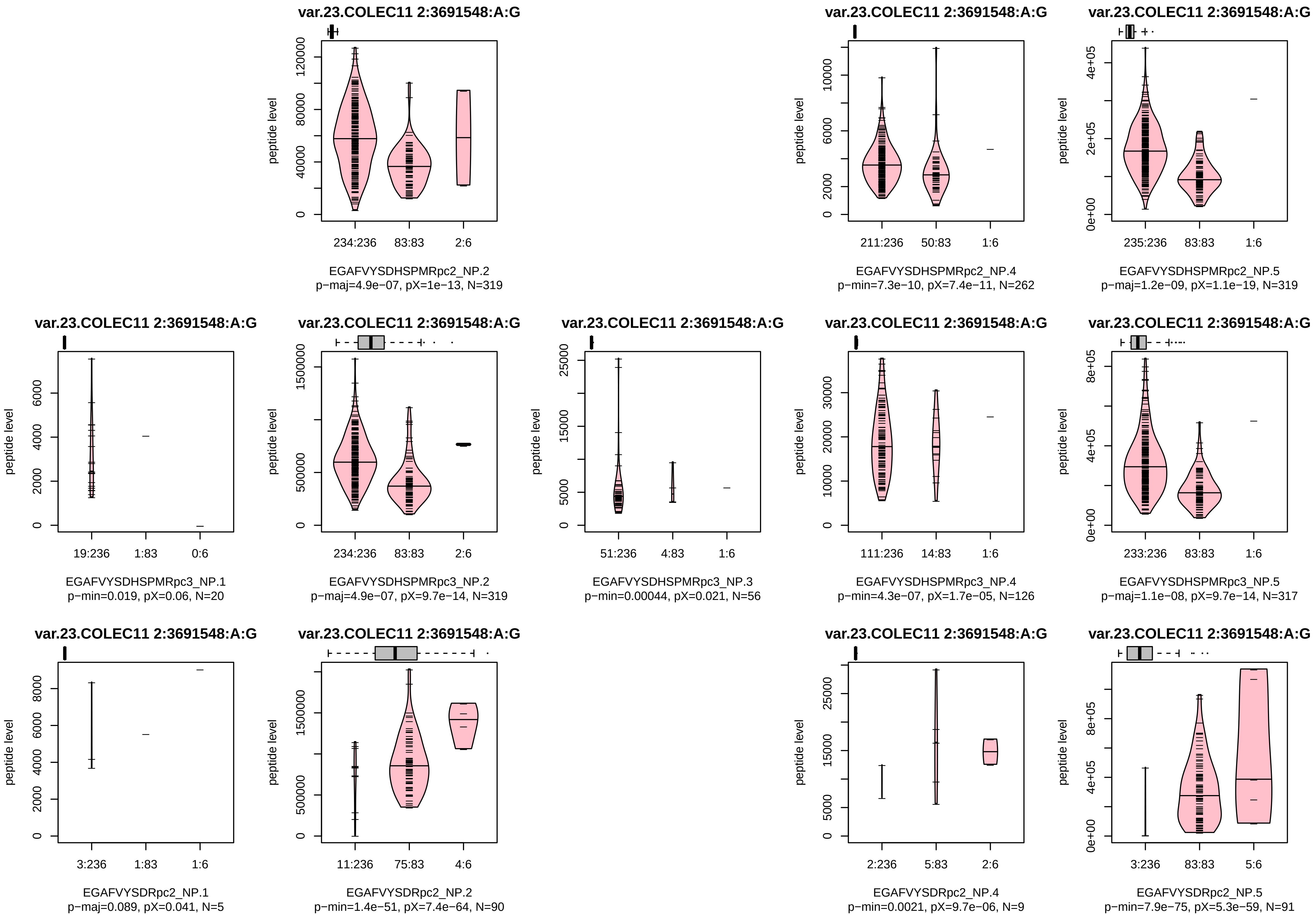
var.23.COLEC11 2:3691548:A:G
peptide level
0
40000
80000
120000
234:236
83:83
2:6
EGAFVYSDHSPMRpc2_NP.2
p−maj=4.9e−07, pX=1e−13, N=319
var.23.COLEC11 2:3691548:A:G
peptide level
0
2000
6000
10000
211:236
50:83
1:6
EGAFVYSDHSPMRpc2_NP.4
p−min=7.3e−10, pX=7.4e−11, N=262
var.23.COLEC11 2:3691548:A:G
peptide level
0e+00
2e+05
4e+05
235:236
83:83
1:6
EGAFVYSDHSPMRpc2_NP.5
p−maj=1.2e−09, pX=1.1e−19, N=319
var.23.COLEC11 2:3691548:A:G
peptide level
0
2000
4000
6000
19:236
1:83
0:6
EGAFVYSDHSPMRpc3_NP.1
p−min=0.019, pX=0.06, N=20
var.23.COLEC11 2:3691548:A:G
peptide level
0
500000
1500000
234:236
83:83
2:6
EGAFVYSDHSPMRpc3_NP.2
p−maj=4.9e−07, pX=9.7e−14, N=319
var.23.COLEC11 2:3691548:A:G
peptide level
0
5000
15000
25000
51:236
4:83
1:6
EGAFVYSDHSPMRpc3_NP.3
p−min=0.00044, pX=0.021, N=56
var.23.COLEC11 2:3691548:A:G
peptide level
0
10000
20000
30000
111:236
14:83
1:6
EGAFVYSDHSPMRpc3_NP.4
p−min=4.3e−07, pX=1.7e−05, N=126
var.23.COLEC11 2:3691548:A:G
peptide level
0e+00
4e+05
8e+05
233:236
83:83
1:6
EGAFVYSDHSPMRpc3_NP.5
p−maj=1.1e−08, pX=9.7e−14, N=317
var.23.COLEC11 2:3691548:A:G
peptide level
0
2000
4000
6000
8000
3:236
1:83
1:6
EGAFVYSDRpc2_NP.1
p−maj=0.089, pX=0.041, N=5
var.23.COLEC11 2:3691548:A:G
peptide level
0
500000
1500000
11:236
75:83
4:6
EGAFVYSDRpc2_NP.2
p−min=1.4e−51, pX=7.4e−64, N=90
var.23.COLEC11 2:3691548:A:G
peptide level
0
5000
15000
25000
2:236
5:83
2:6
EGAFVYSDRpc2_NP.4
p−min=0.0021, pX=9.7e−06, N=9
var.23.COLEC11 2:3691548:A:G
peptide level
0e+00
4e+05
8e+05
3:236
83:83
5:6
EGAFVYSDRpc2_NP.5
p−min=7.9e−75, pX=5.3e−59, N=91

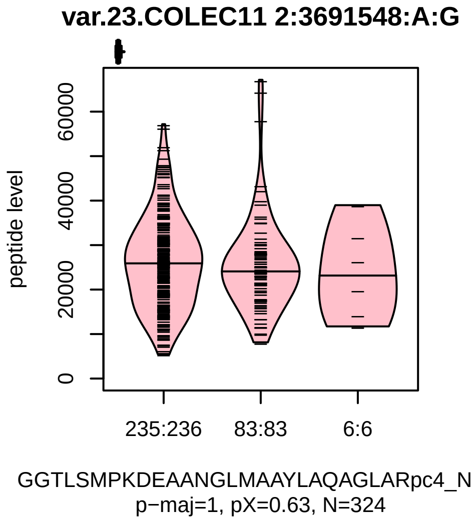
var.23.COLEC11 2:3691548:A:G
peptide level
0
20000
40000
60000
235:236
83:83
6:6
GGTLSMPKDEAANGLMAAYLAQAGLARpc4_N
p−maj=1, pX=0.63, N=324

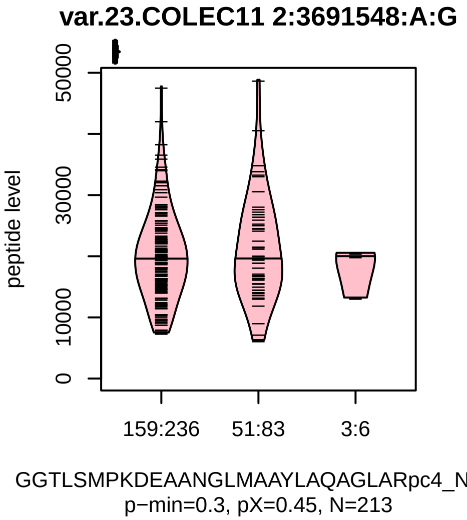
var.23.COLEC11 2:3691548:A:G
peptide level
0
10000
30000
50000
159:236
51:83
3:6
GGTLSMPKDEAANGLMAAYLAQAGLARpc4_N
p−min=0.3, pX=0.45, N=213

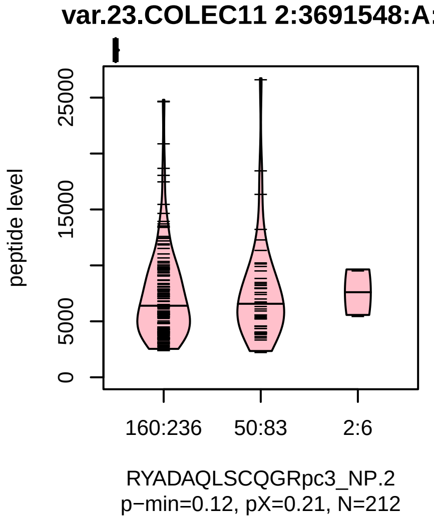
var.23.COLEC11 2:3691548:A:G
peptide level
0
5000
15000
25000
160:236
50:83
2:6
RYADAQLSCQGRpc3_NP.2
p−min=0.12, pX=0.21, N=212

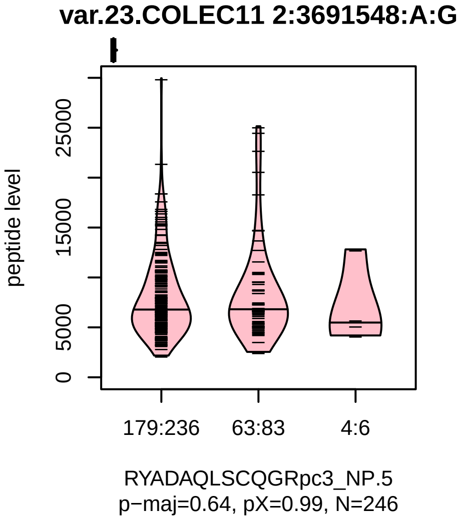
var.23.COLEC11 2:3691548:A:G
peptide level
0
5000
15000
25000
179:236
63:83
4:6
RYADAQLSCQGRpc3_NP.5
p−maj=0.64, pX=0.99, N=246

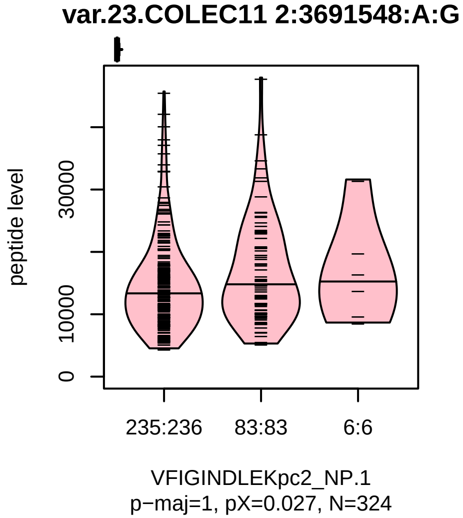
var.23.COLEC11 2:3691548:A:G
peptide level
0
10000
30000
235:236
83:83
6:6
VFIGINDLEKpc2_NP.1
p−maj=1, pX=0.027, N=324

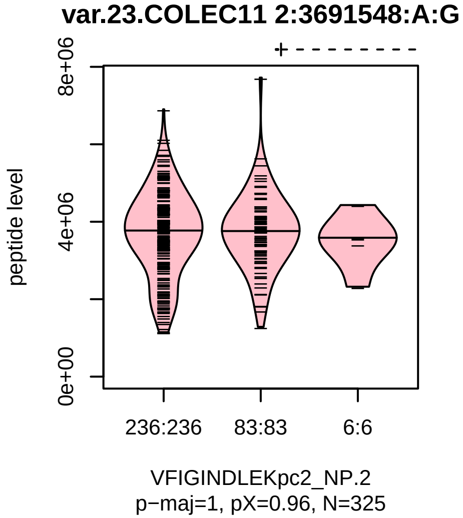
var.23.COLEC11 2:3691548:A:G
peptide level
0e+00
4e+06
8e+06
236:236
83:83
6:6
VFIGINDLEKpc2_NP.2
p−maj=1, pX=0.96, N=325

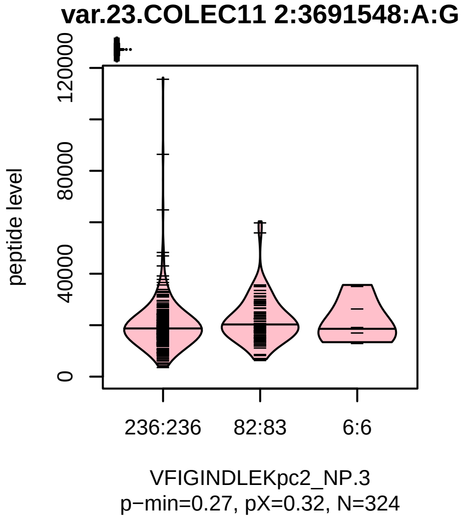
var.23.COLEC11 2:3691548:A:G
peptide level
0
40000
80000
120000
236:236
82:83
6:6
VFIGINDLEKpc2_NP.3
p−min=0.27, pX=0.32, N=324

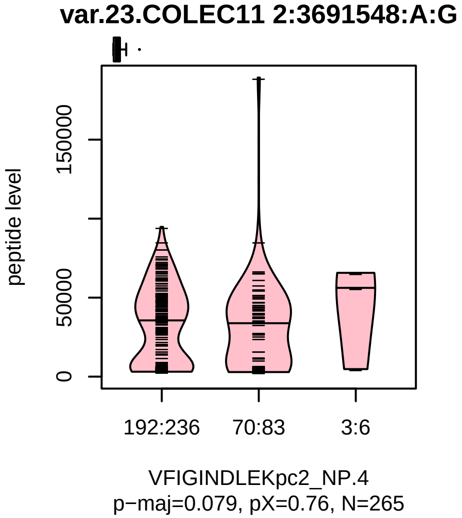
var.23.COLEC11 2:3691548:A:G
peptide level
0
50000
150000
192:236
70:83
3:6
VFIGINDLEKpc2_NP.4
p−maj=0.079, pX=0.76, N=265

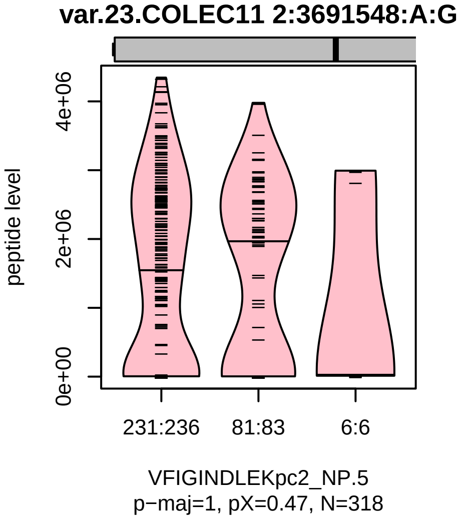
var.23.COLEC11 2:3691548:A:G
peptide level
0e+00
2e+06
4e+06
231:236
81:83
6:6
VFIGINDLEKpc2_NP.5
p−maj=1, pX=0.47, N=318

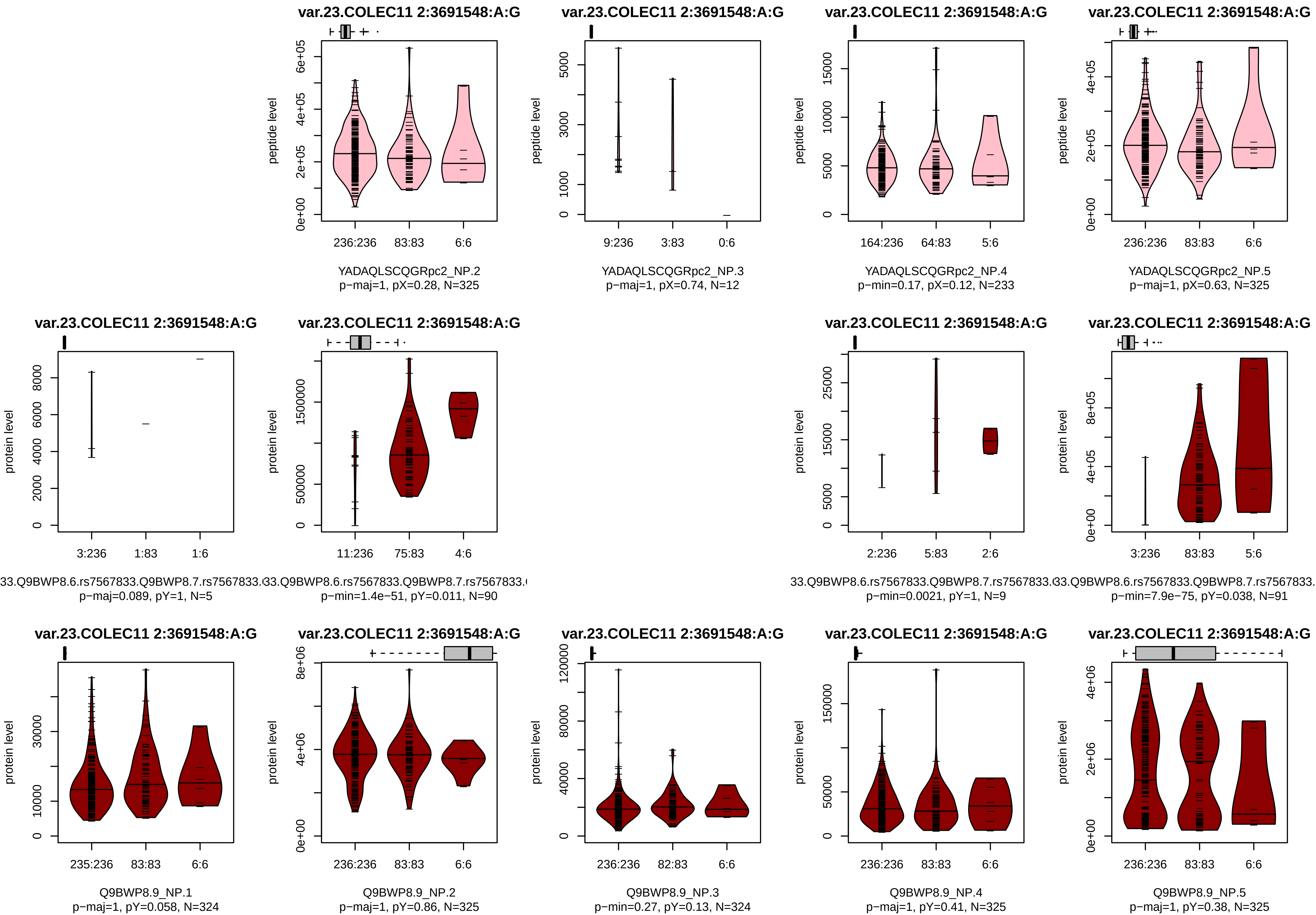

var.23.COLEC11 2:3691548:A:G
peptide level
236:236
83:83
6:6
YADAQLSCQGRpc2_NP.2
p−maj=1, pX=0.28, N=325
var.23.COLEC11 2:3691548:A:G
peptide level
9:236
3:83
0:6
YADAQLSCQGRpc2_NP.3
p−maj=1, pX=0.74, N=12
var.23.COLEC11 2:3691548:A:G
peptide level
164:236
64:83
5:6
YADAQLSCQGRpc2_NP.4
p−min=0.17, pX=0.12, N=233
var.23.COLEC11 2:3691548:A:G
peptide level
236:236
83:83
6:6
YADAQLSCQGRpc2_NP.5
p−maj=1, pX=0.63, N=325
var.23.COLEC11 2:3691548:A:G
protein level
3:236
1:83
1:6
33.Q9BWP8.6.rs7567833.Q9BWP8.7.rs7567833.
p−maj=0.089, pY=1, N=5
var.23.COLEC11 2:3691548:A:G
protein level
11:236
75:83
4:6
33.Q9BWP8.6.rs7567833.Q9BWP8.7.rs7567833.
p−min=1.4e−51, pY=0.011, N=90
var.23.COLEC11 2:3691548:A:G
protein level
2:236
5:83
2:6
33.Q9BWP8.6.rs7567833.Q9BWP8.7.rs7567833.
p−min=0.0021, pY=1, N=9
var.23.COLEC11 2:3691548:A:G
protein level
3:236
83:83
5:6
33.Q9BWP8.6.rs7567833.Q9BWP8.7.rs7567833.
p−min=7.9e−75, pY=0.038, N=91
var.23.COLEC11 2:3691548:A:G
protein level
235:236
83:83
6:6
Q9BWP8.9_NP.1
p−maj=1, pY=0.058, N=324
var.23.COLEC11 2:3691548:A:G
protein level
236:236
83:83
6:6
Q9BWP8.9_NP.2
p−maj=1, pY=0.86, N=325
var.23.COLEC11 2:3691548:A:G
protein level
236:236
82:83
6:6
Q9BWP8.9_NP.3
p−min=0.27, pY=0.13, N=324
var.23.COLEC11 2:3691548:A:G
protein level
236:236
83:83
6:6
Q9BWP8.9_NP.4
p−maj=1, pY=0.41, N=325
var.23.COLEC11 2:3691548:A:G
protein level
236:236
83:83
6:6
Q9BWP8.9_NP.5
p−maj=1, pY=0.38, N=325

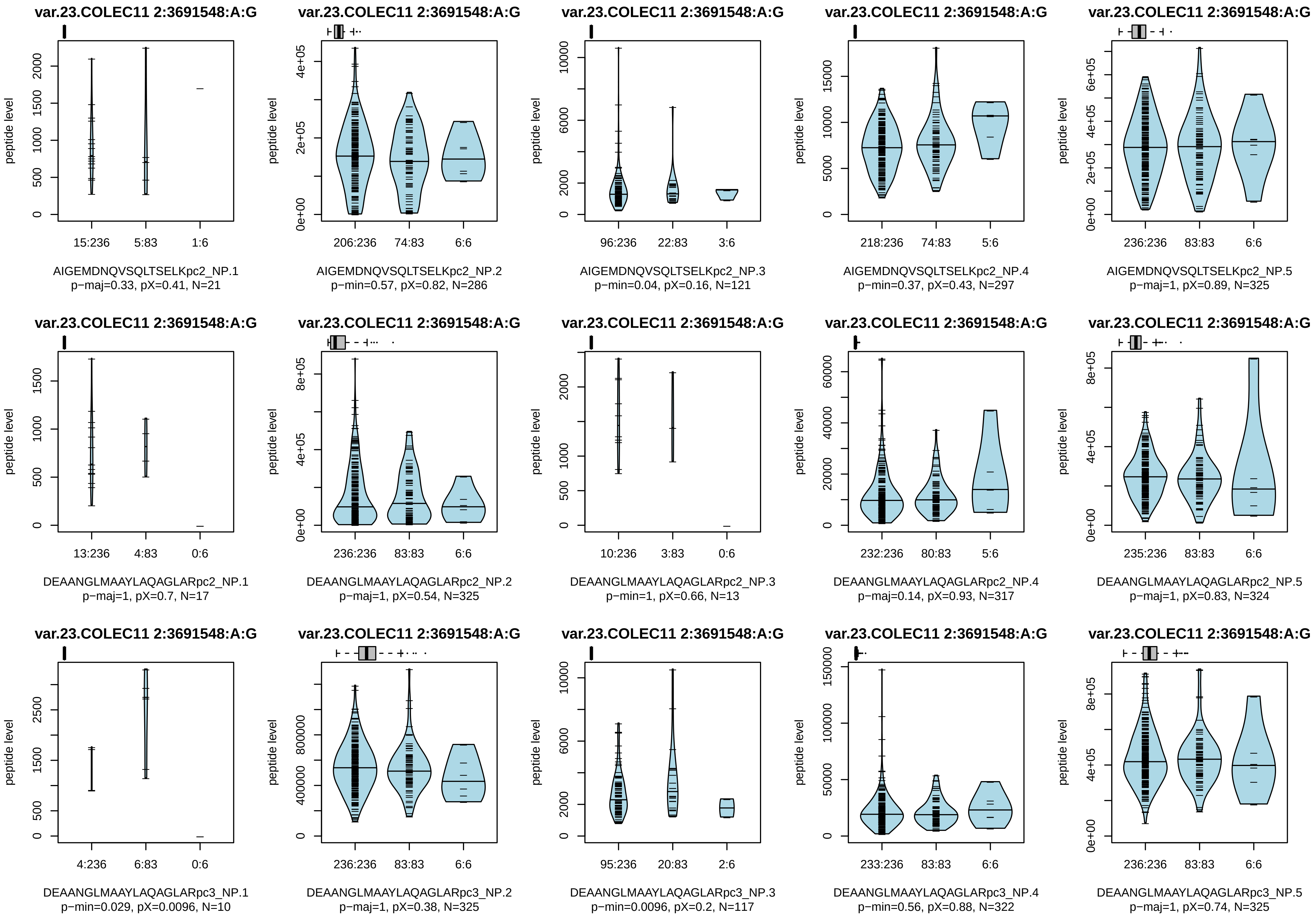
var.23.COLEC11 2:3691548:A:G
peptide level
15:236
5:83
1:6
AIGEMDNQVSQLTSELKpc2_NP.1
p−maj=0.33, pX=0.41, N=21
var.23.COLEC11 2:3691548:A:G
peptide level
206:236
74:83
6:6
AIGEMDNQVSQLTSELKpc2_NP.2
p−min=0.57, pX=0.82, N=286
var.23.COLEC11 2:3691548:A:G
peptide level
96:236
22:83
3:6
AIGEMDNQVSQLTSELKpc2_NP.3
p−min=0.04, pX=0.16, N=121
var.23.COLEC11 2:3691548:A:G
peptide level
218:236
74:83
5:6
AIGEMDNQVSQLTSELKpc2_NP.4
p−min=0.37, pX=0.43, N=297
var.23.COLEC11 2:3691548:A:G
peptide level
236:236
83:83
6:6
AIGEMDNQVSQLTSELKpc2_NP.5
p−maj=1, pX=0.89, N=325
var.23.COLEC11 2:3691548:A:G
peptide level
13:236
4:83
0:6
DEAANGLMAAYLAQAGLARpc2_NP.1
p−maj=1, pX=0.7, N=17
var.23.COLEC11 2:3691548:A:G
peptide level
236:236
83:83
6:6
DEAANGLMAAYLAQAGLARpc2_NP.2
p−maj=1, pX=0.54, N=325
var.23.COLEC11 2:3691548:A:G
peptide level
10:236
3:83
0:6
DEAANGLMAAYLAQAGLARpc2_NP.3
p−min=1, pX=0.66, N=13
var.23.COLEC11 2:3691548:A:G
peptide level
232:236
80:83
5:6
DEAANGLMAAYLAQAGLARpc2_NP.4
p−maj=0.14, pX=0.93, N=317
var.23.COLEC11 2:3691548:A:G
peptide level
235:236
83:83
6:6
DEAANGLMAAYLAQAGLARpc2_NP.5
p−maj=1, pX=0.83, N=324
var.23.COLEC11 2:3691548:A:G
peptide level
4:236
6:83
0:6
DEAANGLMAAYLAQAGLARpc3_NP.1
p−min=0.029, pX=0.0096, N=10
var.23.COLEC11 2:3691548:A:G
peptide level
236:236
83:83
6:6
DEAANGLMAAYLAQAGLARpc3_NP.2
p−maj=1, pX=0.38, N=325
var.23.COLEC11 2:3691548:A:G
peptide level
95:236
20:83
2:6
DEAANGLMAAYLAQAGLARpc3_NP.3
p−min=0.0096, pX=0.2, N=117
var.23.COLEC11 2:3691548:A:G
peptide level
233:236
83:83
6:6
DEAANGLMAAYLAQAGLARpc3_NP.4
p−min=0.56, pX=0.88, N=322
var.23.COLEC11 2:3691548:A:G
peptide level
236:236
83:83
6:6
DEAANGLMAAYLAQAGLARpc3_NP.5
p−maj=1, pX=0.74, N=325

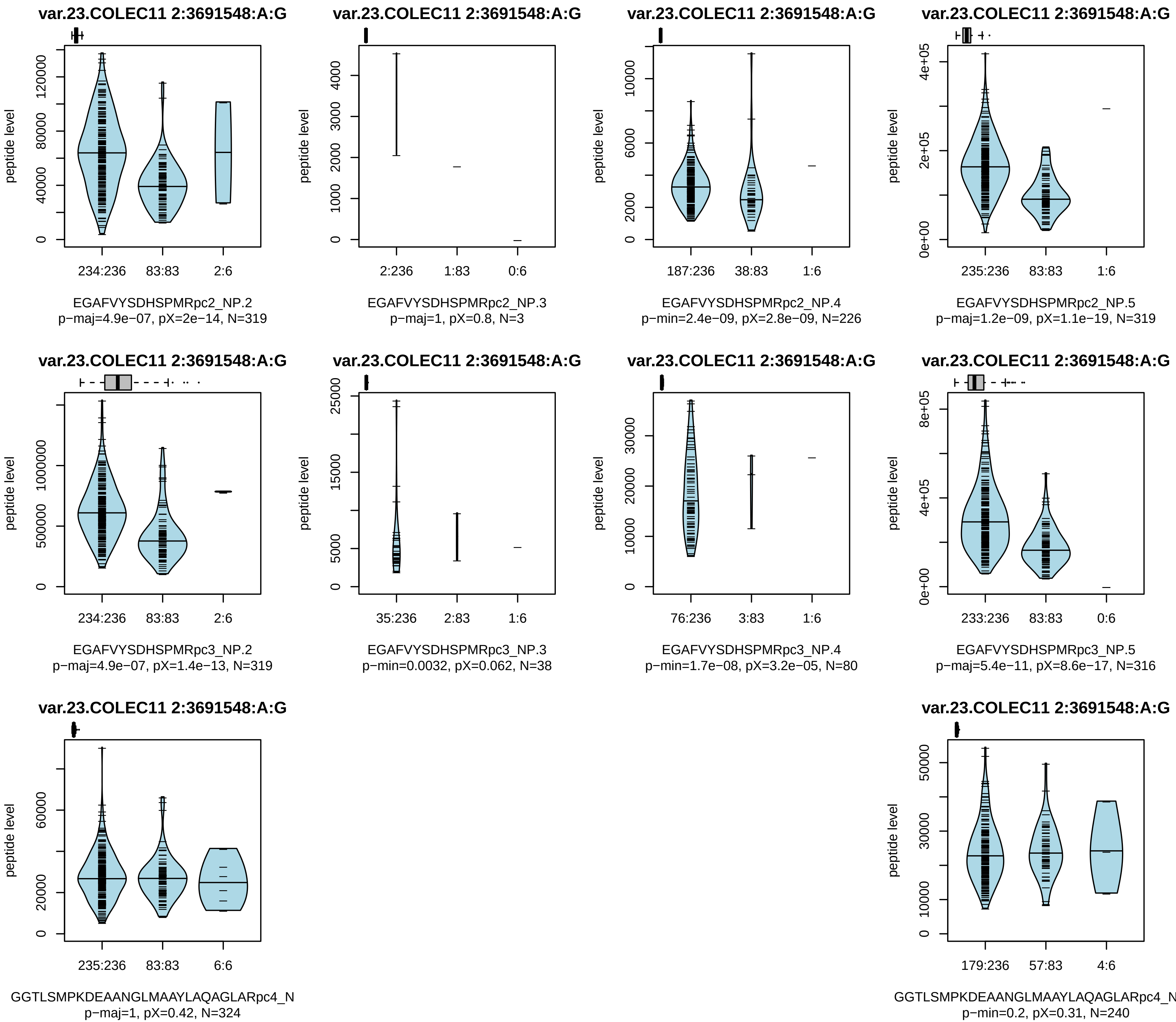

var.23.COLEC11 2:3691548:A:G
peptide level
234:236
83:83
2:6
EGAFVYSDHSPMRpc2_NP.2
p−maj=4.9e−07, pX=2e−14, N=319
var.23.COLEC11 2:3691548:A:G
peptide level
2:236
1:83
0:6
EGAFVYSDHSPMRpc2_NP.3
p−maj=1, pX=0.8, N=3
var.23.COLEC11 2:3691548:A:G
peptide level
187:236
38:83
1:6
EGAFVYSDHSPMRpc2_NP.4
p−min=2.4e−09, pX=2.8e−09, N=226
var.23.COLEC11 2:3691548:A:G
peptide level
235:236
83:83
1:6
EGAFVYSDHSPMRpc2_NP.5
p−maj=1.2e−09, pX=1.1e−19, N=319
var.23.COLEC11 2:3691548:A:G
peptide level
234:236
83:83
2:6
EGAFVYSDHSPMRpc3_NP.2
p−maj=4.9e−07, pX=1.4e−13, N=319
var.23.COLEC11 2:3691548:A:G
peptide level
35:236
2:83
1:6
EGAFVYSDHSPMRpc3_NP.3
p−min=0.0032, pX=0.062, N=38
var.23.COLEC11 2:3691548:A:G
peptide level
76:236
3:83
1:6
EGAFVYSDHSPMRpc3_NP.4
p−min=1.7e−08, pX=3.2e−05, N=80
var.23.COLEC11 2:3691548:A:G
peptide level
233:236
83:83
0:6
EGAFVYSDHSPMRpc3_NP.5
p−maj=5.4e−11, pX=8.6e−17, N=316
var.23.COLEC11 2:3691548:A:G
peptide level
235:236
83:83
6:6
GGTLSMPKDEAANGLMAAYLAQAGLARpc4_N
p−maj=1, pX=0.42, N=324
var.23.COLEC11 2:3691548:A:G
peptide level
179:236
57:83
4:6
GGTLSMPKDEAANGLMAAYLAQAGLARpc4_N
p−min=0.2, pX=0.31, N=240

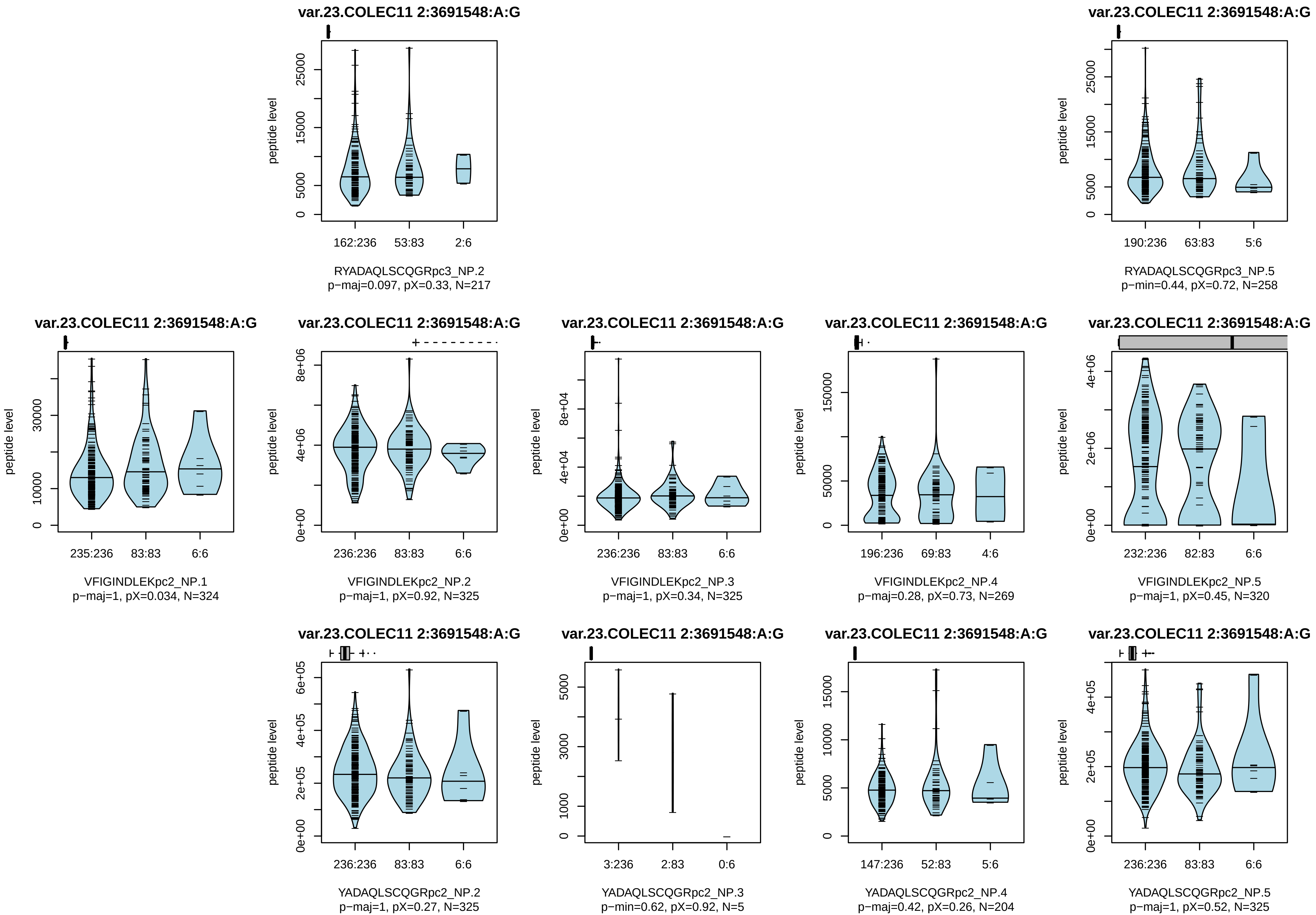
var.23.COLEC11 2:3691548:A:G
peptide level
162:236
53:83
2:6
RYADAQLSCQGRpc3_NP.2
p−maj=0.097, pX=0.33, N=217
var.23.COLEC11 2:3691548:A:G
peptide level
190:236
63:83
5:6
RYADAQLSCQGRpc3_NP.5
p−min=0.44, pX=0.72, N=258
var.23.COLEC11 2:3691548:A:G
peptide level
235:236
83:83
6:6
VFIGINDLEKpc2_NP.1
p−maj=1, pX=0.034, N=324
var.23.COLEC11 2:3691548:A:G
peptide level
236:236
83:83
6:6
VFIGINDLEKpc2_NP.2
p−maj=1, pX=0.92, N=325
var.23.COLEC11 2:3691548:A:G
peptide level
236:236
83:83
6:6
VFIGINDLEKpc2_NP.3
p−maj=1, pX=0.34, N=325
var.23.COLEC11 2:3691548:A:G
peptide level
196:236
69:83
4:6
VFIGINDLEKpc2_NP.4
p−maj=0.28, pX=0.73, N=269
var.23.COLEC11 2:3691548:A:G
peptide level
232:236
82:83
6:6
VFIGINDLEKpc2_NP.5
p−maj=1, pX=0.45, N=320
var.23.COLEC11 2:3691548:A:G
peptide level
236:236
83:83
6:6
YADAQLSCQGRpc2_NP.2
p−maj=1, pX=0.27, N=325
var.23.COLEC11 2:3691548:A:G
peptide level
3:236
2:83
0:6
YADAQLSCQGRpc2_NP.3
p−min=0.62, pX=0.92, N=5
var.23.COLEC11 2:3691548:A:G
peptide level
147:236
52:83
5:6
YADAQLSCQGRpc2_NP.4
p−maj=0.42, pX=0.26, N=204
var.23.COLEC11 2:3691548:A:G
peptide level
236:236
83:83
6:6
YADAQLSCQGRpc2_NP.5
p−maj=1, pX=0.52, N=325

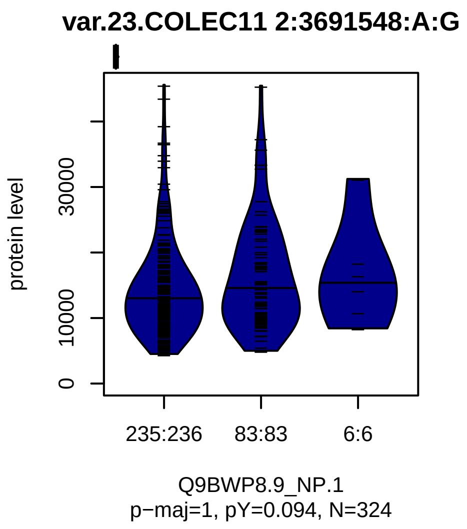
var.23.COLEC11 2:3691548:A:G
protein level
0
10000
30000
235:236
83:83
6:6
Q9BWP8.9_NP.1
p−maj=1, pY=0.094, N=324

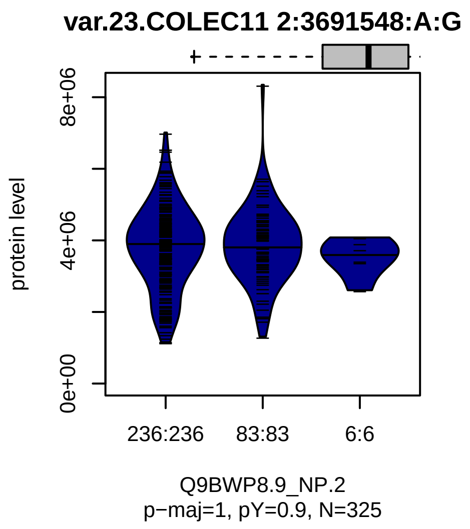
var.23.COLEC11 2:3691548:A:G
protein level
0e+00
4e+06
8e+06
236:236
83:83
6:6
Q9BWP8.9_NP.2
p−maj=1, pY=0.9, N=325

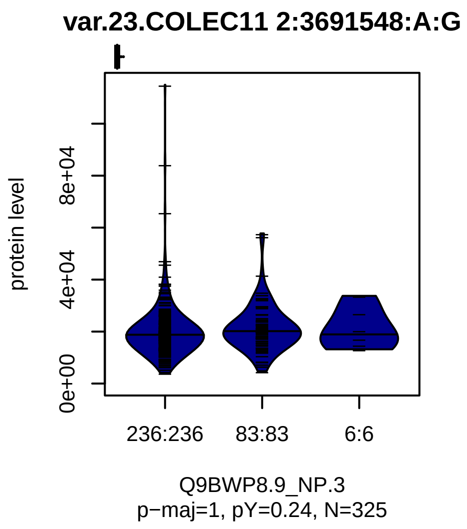
var.23.COLEC11 2:3691548:A:G
protein level
0e+00
4e+04
8e+04
236:236
83:83
6:6
Q9BWP8.9_NP.3
p−maj=1, pY=0.24, N=325

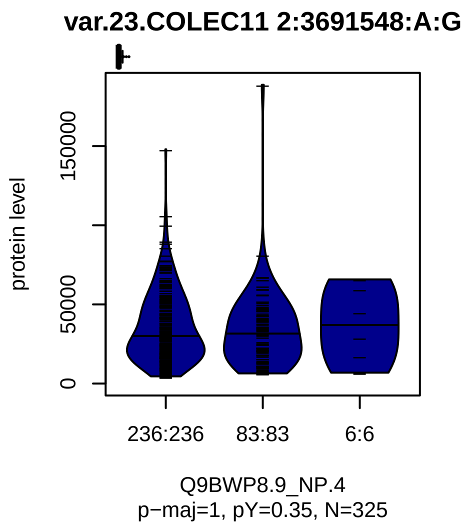
var.23.COLEC11 2:3691548:A:G
protein level
0
50000
150000
236:236
83:83
6:6
Q9BWP8.9_NP.4
p−maj=1, pY=0.35, N=325

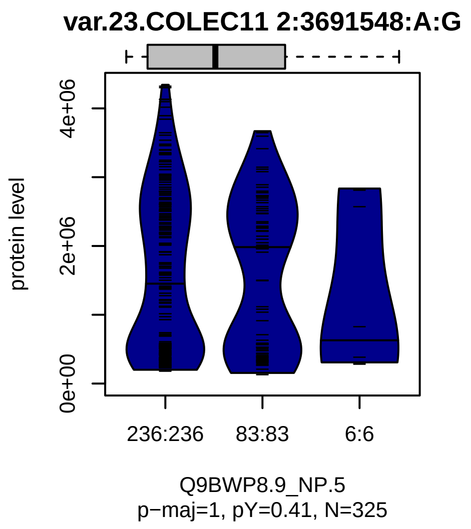
var.23.COLEC11 2:3691548:A:G
protein level
0e+00
2e+06
4e+06
236:236
83:83
6:6
Q9BWP8.9_NP.5
p−maj=1, pY=0.41, N=325

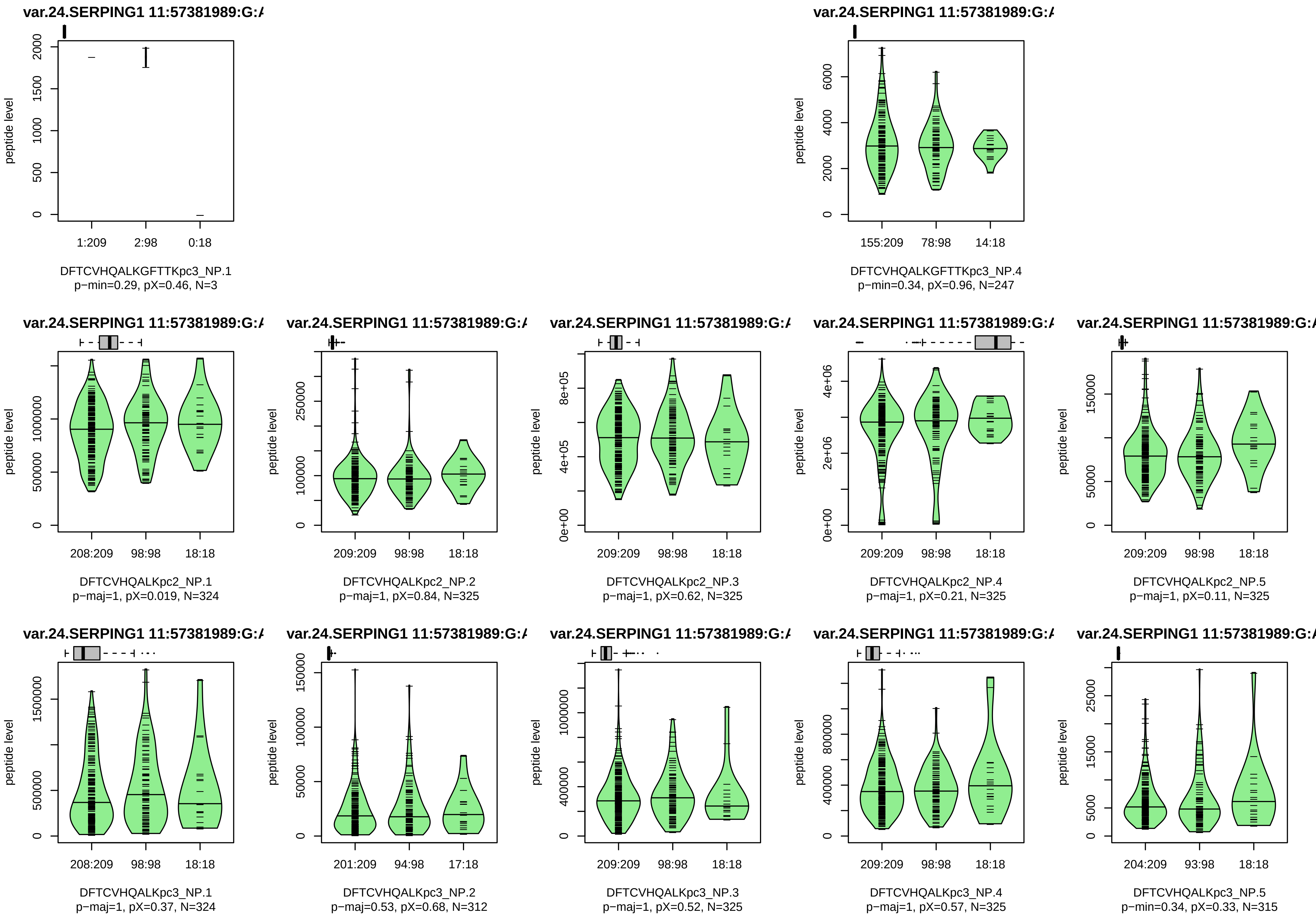
var.24.SERPING1 11:57381989:G:A
peptide level
1:209
2:98
0:18
DFTCVHQALKGFTTKpc3_NP.1
p−min=0.29, pX=0.46, N=3
var.24.SERPING1 11:57381989:G:A
peptide level
155:209
78:98
14:18
DFTCVHQALKGFTTKpc3_NP.4
p−min=0.34, pX=0.96, N=247
var.24.SERPING1 11:57381989:G:A
peptide level
208:209
98:98
18:18
DFTCVHQALKpc2_NP.1
p−maj=1, pX=0.019, N=324
var.24.SERPING1 11:57381989:G:A
peptide level
209:209
98:98
18:18
DFTCVHQALKpc2_NP.2
p−maj=1, pX=0.84, N=325
var.24.SERPING1 11:57381989:G:A
peptide level
209:209
98:98
18:18
DFTCVHQALKpc2_NP.3
p−maj=1, pX=0.62, N=325
var.24.SERPING1 11:57381989:G:A
peptide level
209:209
98:98
18:18
DFTCVHQALKpc2_NP.4
p−maj=1, pX=0.21, N=325
var.24.SERPING1 11:57381989:G:A
peptide level
209:209
98:98
18:18
DFTCVHQALKpc2_NP.5
p−maj=1, pX=0.11, N=325
var.24.SERPING1 11:57381989:G:A
peptide level
208:209
98:98
18:18
DFTCVHQALKpc3_NP.1
p−maj=1, pX=0.37, N=324
var.24.SERPING1 11:57381989:G:A
peptide level
201:209
94:98
17:18
DFTCVHQALKpc3_NP.2
p−maj=0.53, pX=0.68, N=312
var.24.SERPING1 11:57381989:G:A
peptide level
209:209
98:98
18:18
DFTCVHQALKpc3_NP.3
p−maj=1, pX=0.52, N=325
var.24.SERPING1 11:57381989:G:A
peptide level
209:209
98:98
18:18
DFTCVHQALKpc3_NP.4
p−maj=1, pX=0.57, N=325
var.24.SERPING1 11:57381989:G:A
peptide level
204:209
93:98
18:18
DFTCVHQALKpc3_NP.5
p−min=0.34, pX=0.33, N=315

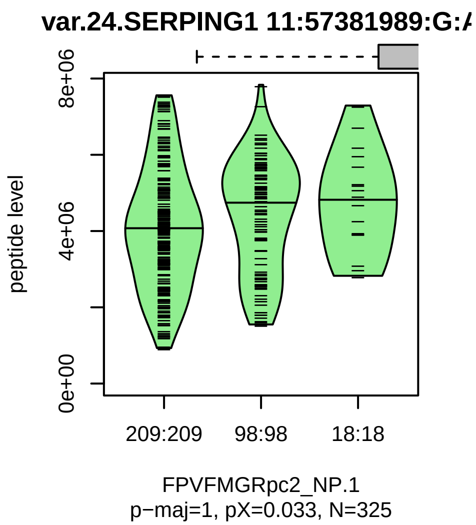
var.24.SERPING1 11:57381989:G:A
peptide level
0e+00
4e+06
8e+06
209:209
98:98
18:18
FPVFMGRpc2_NP.1
p−maj=1, pX=0.033, N=325

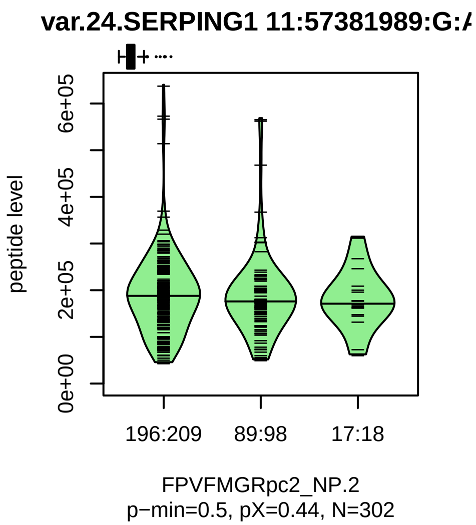
var.24.SERPING1 11:57381989:G:A
peptide level
0e+00
2e+05
4e+05
6e+05
196:209
89:98
17:18
FPVFMGRpc2_NP.2
p−min=0.5, pX=0.44, N=302

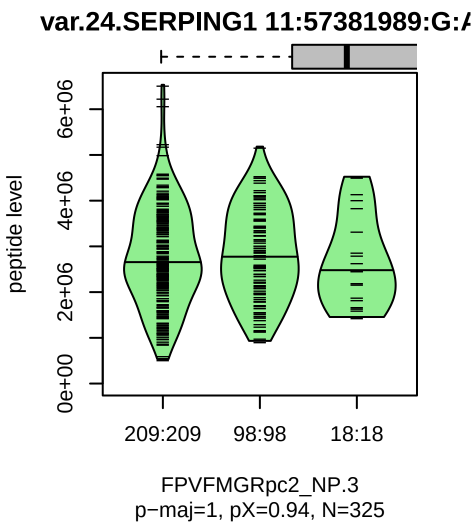
var.24.SERPING1 11:57381989:G:A
peptide level
0e+00
2e+06
4e+06
6e+06
209:209
98:98
18:18
FPVFMGRpc2_NP.3
p−maj=1, pX=0.94, N=325

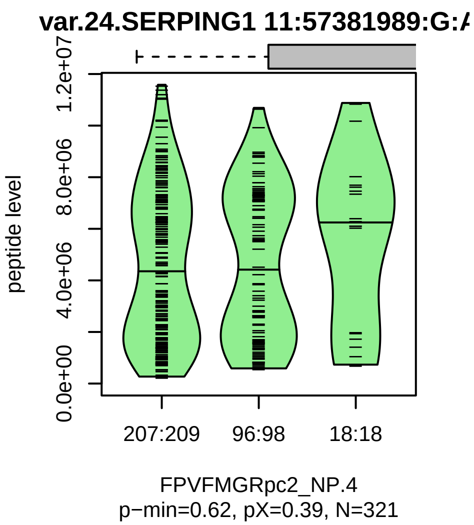
var.24.SERPING1 11:57381989:G:A
peptide level
0.0e+00
4.0e+06
8.0e+06
1.2e+07
207:209
96:98
18:18
FPVFMGRpc2_NP.4
p−min=0.62, pX=0.39, N=321

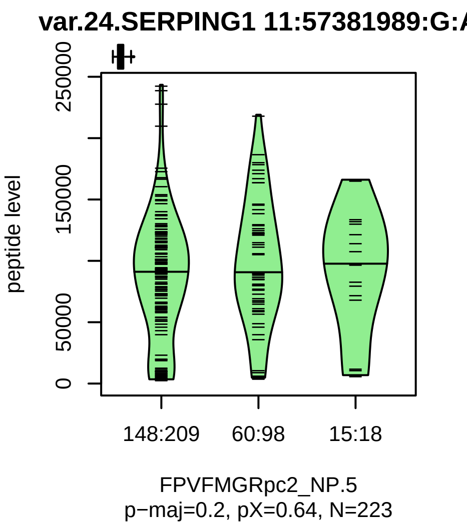
var.24.SERPING1 11:57381989:G:A
peptide level
0
50000
150000
250000
148:209
60:98
15:18
FPVFMGRpc2_NP.5
p−maj=0.2, pX=0.64, N=223

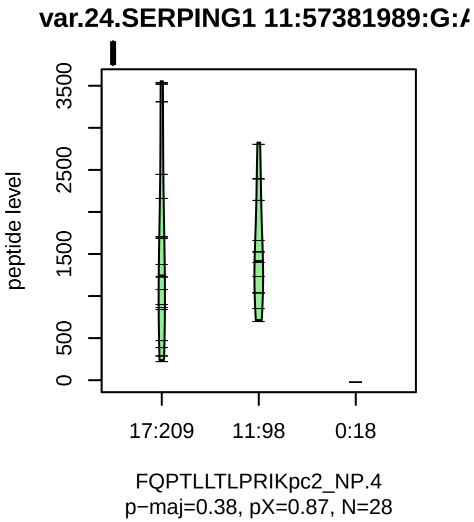
var.24.SERPING1 11:57381989:G:A
peptide level
0
500
1500
2500
3500
17:209
11:98
0:18
FQPTLLTLPRIKpc2_NP.4
p−maj=0.38, pX=0.87, N=28

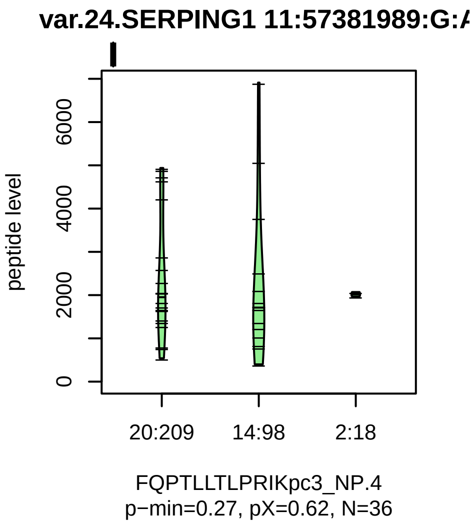
var.24.SERPING1 11:57381989:G:A
peptide level
0
2000
4000
6000
20:209
14:98
2:18
FQPTLLTLPRIKpc3_NP.4
p−min=0.27, pX=0.62, N=36

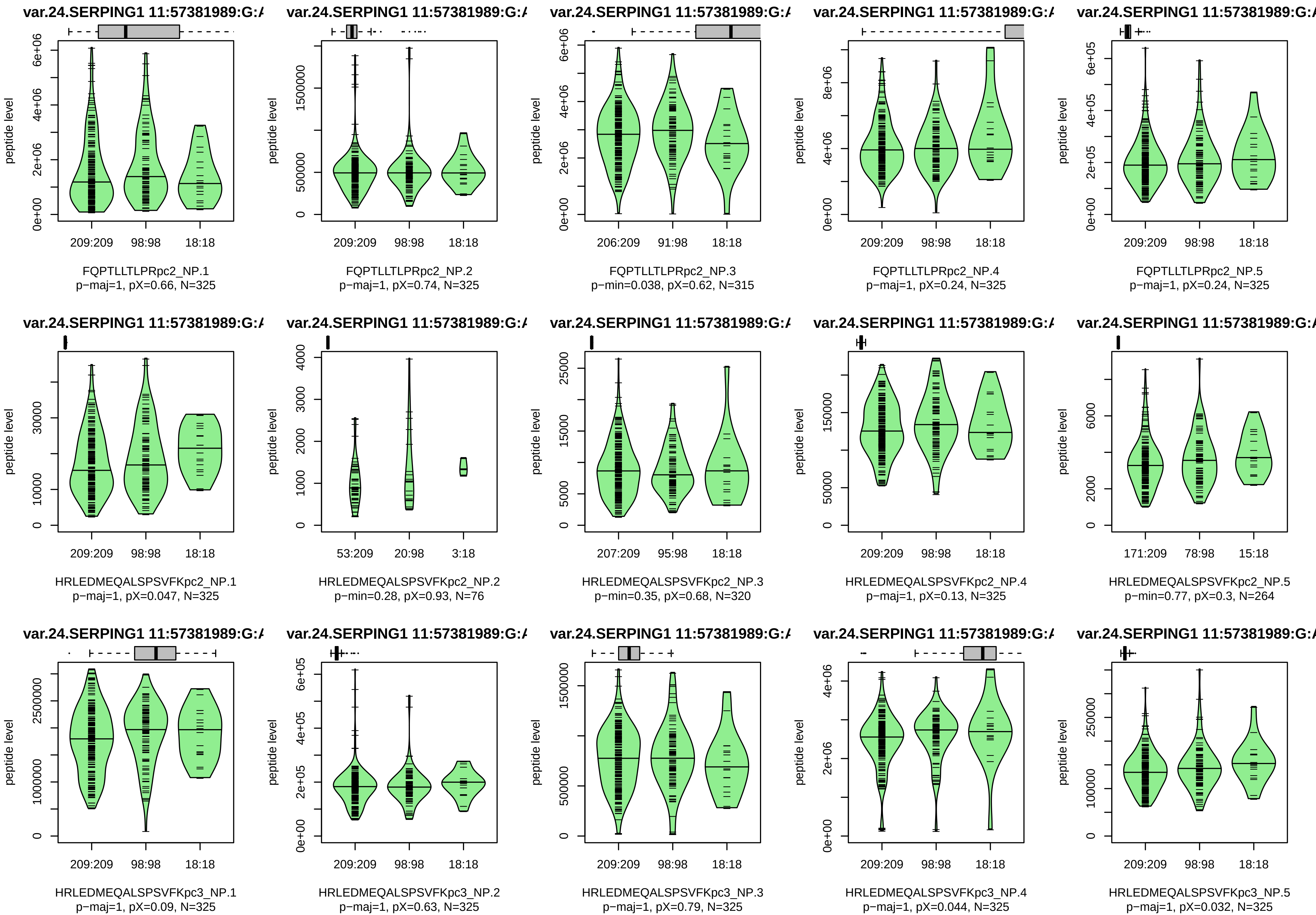
var.24.SERPING1 11:57381989:G:/
peptide level
209:209
98:98
18:18
FQPTLLTLPRpc2_NP.1
p−maj=1, pX=0.66, N=325
var.24.SERPING1 11:57381989:G:/
peptide level
209:209
98:98
18:18
FQPTLLTLPRpc2_NP.2
p−maj=1, pX=0.74, N=325
var.24.SERPING1 11:57381989:G:/
peptide level
206:209
91:98
18:18
FQPTLLTLPRpc2_NP.3
p−min=0.038, pX=0.62, N=315
var.24.SERPING1 11:57381989:G:/
peptide level
209:209
98:98
18:18
FQPTLLTLPRpc2_NP.4
p−maj=1, pX=0.24, N=325
var.24.SERPING1 11:57381989:G:/
peptide level
209:209
98:98
18:18
FQPTLLTLPRpc2_NP.5
p−maj=1, pX=0.24, N=325
var.24.SERPING1 11:57381989:G:/
peptide level
209:209
98:98
18:18
HRLEDMEQALSPSVFKpc2_NP.1
p−maj=1, pX=0.047, N=325
var.24.SERPING1 11:57381989:G:/
peptide level
53:209
20:98
3:18
HRLEDMEQALSPSVFKpc2_NP.2
p−min=0.28, pX=0.93, N=76
var.24.SERPING1 11:57381989:G:/
peptide level
207:209
95:98
18:18
HRLEDMEQALSPSVFKpc2_NP.3
p−min=0.35, pX=0.68, N=320
var.24.SERPING1 11:57381989:G:/
peptide level
209:209
98:98
18:18
HRLEDMEQALSPSVFKpc2_NP.4
p−maj=1, pX=0.13, N=325
var.24.SERPING1 11:57381989:G:/
peptide level
171:209
78:98
15:18
HRLEDMEQALSPSVFKpc2_NP.5
p−min=0.77, pX=0.3, N=264
var.24.SERPING1 11:57381989:G:/
peptide level
209:209
98:98
18:18
HRLEDMEQALSPSVFKpc3_NP.1
p−maj=1, pX=0.09, N=325
var.24.SERPING1 11:57381989:G:/
peptide level
209:209
98:98
18:18
HRLEDMEQALSPSVFKpc3_NP.2
p−maj=1, pX=0.63, N=325
var.24.SERPING1 11:57381989:G:/
peptide level
209:209
98:98
18:18
HRLEDMEQALSPSVFKpc3_NP.3
p−maj=1, pX=0.79, N=325
var.24.SERPING1 11:57381989:G:/
peptide level
209:209
98:98
18:18
HRLEDMEQALSPSVFKpc3_NP.4
p−maj=1, pX=0.044, N=325
var.24.SERPING1 11:57381989:G:/
peptide level
209:209
98:98
18:18
HRLEDMEQALSPSVFKpc3_NP.5
p−maj=1, pX=0.032, N=325

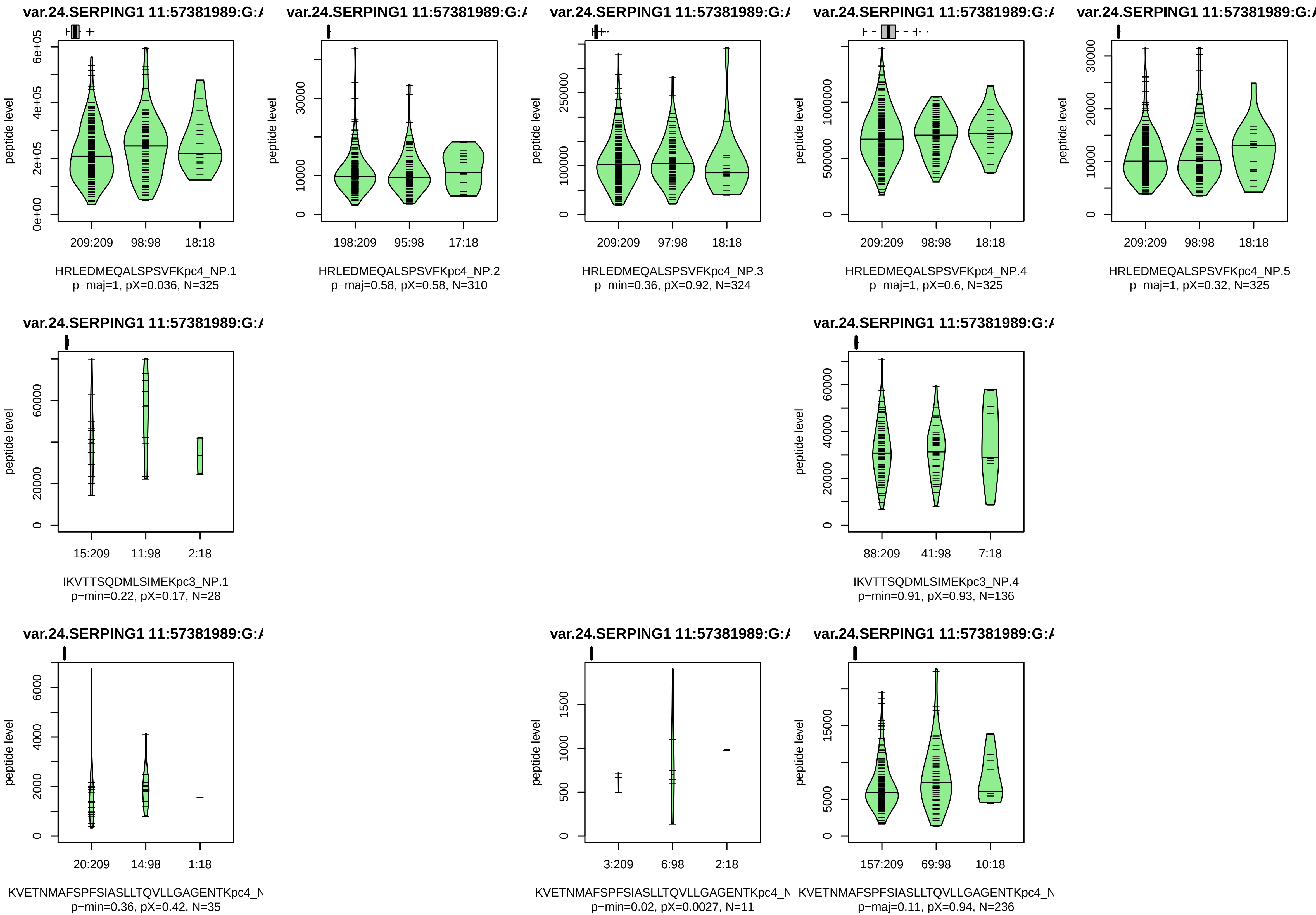
var.24.SERPING1 11:57381989:G:A
peptide level
0e+00
2e+05
4e+05
6e+05
209:209
98:98
18:18
HRLEDMEQALSPSVFKpc4_NP.1
p−maj=1, pX=0.036, N=325
var.24.SERPING1 11:57381989:G:A
peptide level
0
10000
30000
198:209
95:98
17:18
HRLEDMEQALSPSVFKpc4_NP.2
p−maj=0.58, pX=0.58, N=310
var.24.SERPING1 11:57381989:G:A
peptide level
0
100000
250000
209:209
97:98
18:18
HRLEDMEQALSPSVFKpc4_NP.3
p−min=0.36, pX=0.92, N=324
var.24.SERPING1 11:57381989:G:A
peptide level
0
500000
1000000
209:209
98:98
18:18
HRLEDMEQALSPSVFKpc4_NP.4
p−maj=1, pX=0.6, N=325
var.24.SERPING1 11:57381989:G:A
peptide level
0
10000
20000
30000
209:209
98:98
18:18
HRLEDMEQALSPSVFKpc4_NP.5
p−maj=1, pX=0.32, N=325
var.24.SERPING1 11:57381989:G:A
peptide level
0
20000
60000
15:209
11:98
2:18
IKVTTSQDMLSIMEKpc3_NP.1
p−min=0.22, pX=0.17, N=28
var.24.SERPING1 11:57381989:G:A
peptide level
0
20000
40000
60000
88:209
41:98
7:18
IKVTTSQDMLSIMEKpc3_NP.4
p−min=0.91, pX=0.93, N=136
var.24.SERPING1 11:57381989:G:A
peptide level
0
2000
4000
6000
20:209
14:98
1:18
KVETNMAFSPFSIASLLTQVLLGAGENTKpc4_N
p−min=0.36, pX=0.42, N=35
var.24.SERPING1 11:57381989:G:A
peptide level
0
500
1000
1500
3:209
6:98
2:18
KVETNMAFSPFSIASLLTQVLLGAGENTKpc4_N
p−min=0.02, pX=0.0027, N=11
var.24.SERPING1 11:57381989:G:A
peptide level
0
5000
15000
157:209
69:98
10:18
KVETNMAFSPFSIASLLTQVLLGAGENTKpc4_N
p−maj=0.11, pX=0.94, N=236

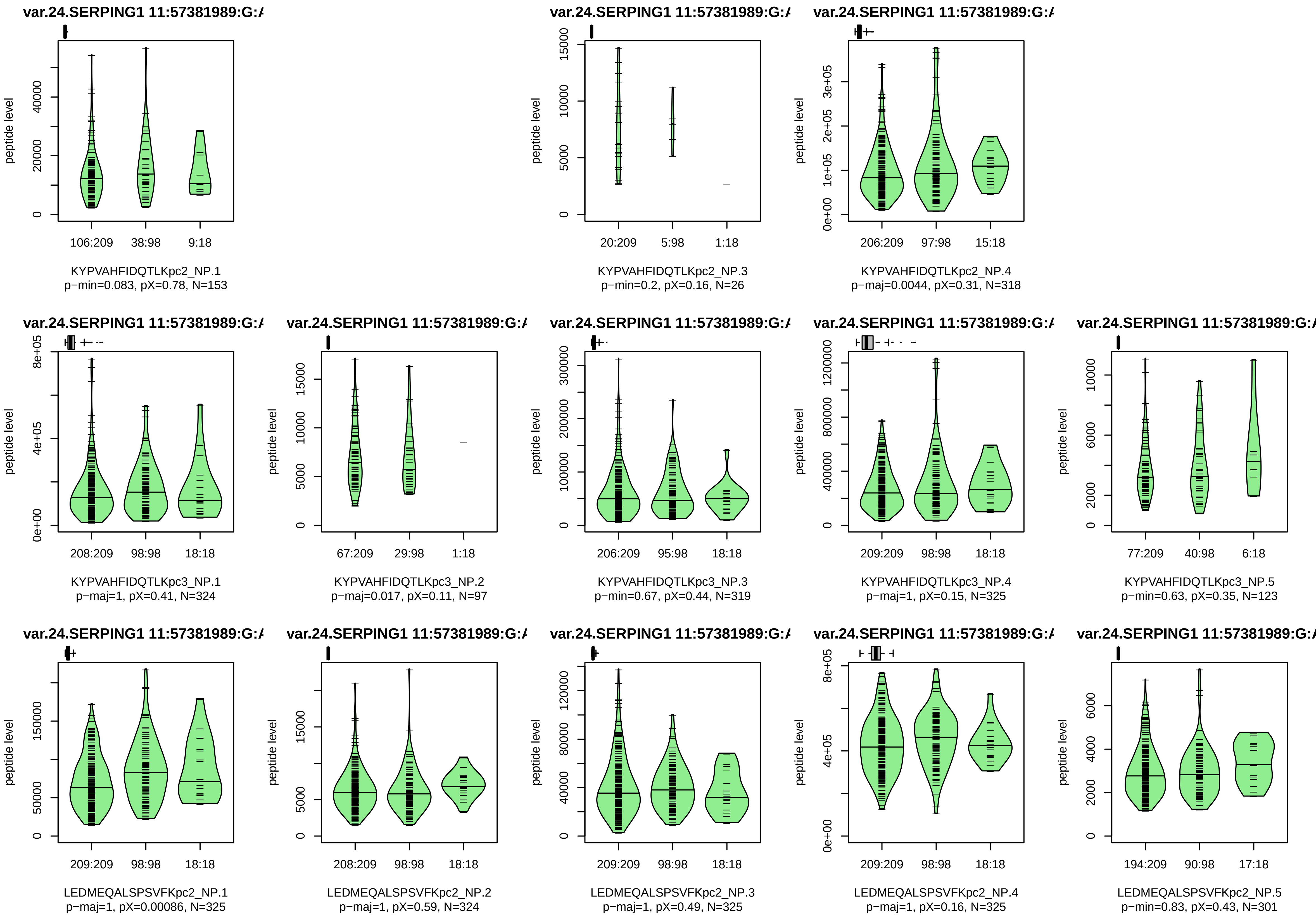
var.24.SERPING1 11:57381989:G:/
peptide level
106:209
38:98
9:18
KYPVAHFIDQTLKpc2_NP.1
p−min=0.083, pX=0.78, N=153
var.24.SERPING1 11:57381989:G:/
peptide level
20:209
5:98
1:18
KYPVAHFIDQTLKpc2_NP.3
p−min=0.2, pX=0.16, N=26
var.24.SERPING1 11:57381989:G:/
peptide level
206:209
97:98
15:18
KYPVAHFIDQTLKpc2_NP.4
p−maj=0.0044, pX=0.31, N=318
var.24.SERPING1 11:57381989:G:/
peptide level
208:209
98:98
18:18
KYPVAHFIDQTLKpc3_NP.1
p−maj=1, pX=0.41, N=324
var.24.SERPING1 11:57381989:G:/
peptide level
67:209
29:98
1:18
KYPVAHFIDQTLKpc3_NP.2
p−maj=0.017, pX=0.11, N=97
var.24.SERPING1 11:57381989:G:/
peptide level
206:209
95:98
18:18
KYPVAHFIDQTLKpc3_NP.3
p−min=0.67, pX=0.44, N=319
var.24.SERPING1 11:57381989:G:/
peptide level
209:209
98:98
18:18
KYPVAHFIDQTLKpc3_NP.4
p−maj=1, pX=0.15, N=325
var.24.SERPING1 11:57381989:G:/
peptide level
77:209
40:98
6:18
KYPVAHFIDQTLKpc3_NP.5
p−min=0.63, pX=0.35, N=123
var.24.SERPING1 11:57381989:G:/
peptide level
209:209
98:98
18:18
LEDMEQALSPSVFKpc2_NP.1
p−maj=1, pX=0.00086, N=325
var.24.SERPING1 11:57381989:G:/
peptide level
208:209
98:98
18:18
LEDMEQALSPSVFKpc2_NP.2
p−maj=1, pX=0.59, N=324
var.24.SERPING1 11:57381989:G:/
peptide level
209:209
98:98
18:18
LEDMEQALSPSVFKpc2_NP.3
p−maj=1, pX=0.49, N=325
var.24.SERPING1 11:57381989:G:/
peptide level
209:209
98:98
18:18
LEDMEQALSPSVFKpc2_NP.4
p−maj=1, pX=0.16, N=325
var.24.SERPING1 11:57381989:G:/
peptide level
194:209
90:98
17:18
LEDMEQALSPSVFKpc2_NP.5
p−min=0.83, pX=0.43, N=301

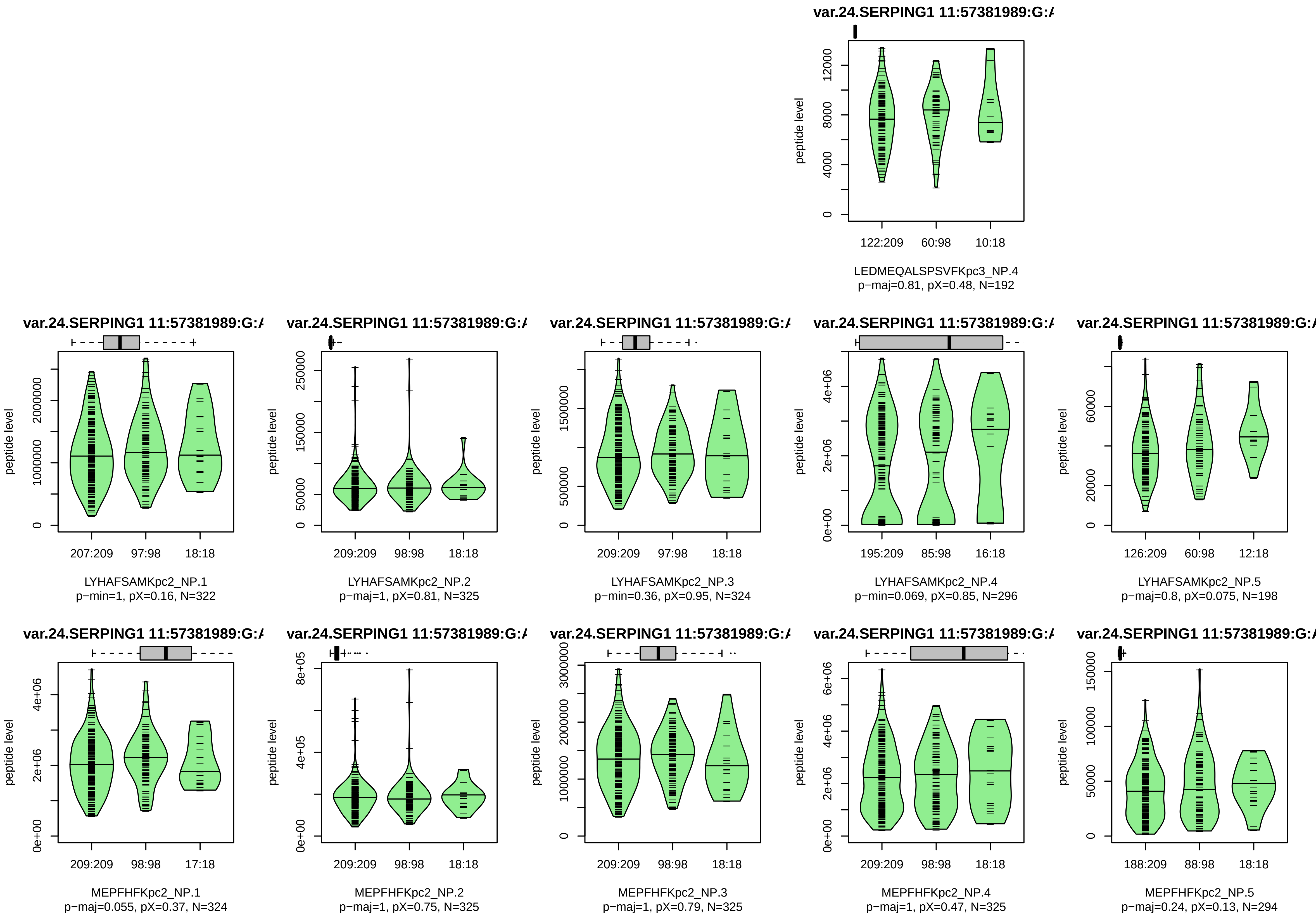
var.24.SERPING1 11:57381989:G:A
peptide level
122:209
60:98
10:18
LEDMEQALSPSVFKpc3_NP.4
p−maj=0.81, pX=0.48, N=192
var.24.SERPING1 11:57381989:G:A
peptide level
207:209
97:98
18:18
LYHAFSAMKpc2_NP.1
p−min=1, pX=0.16, N=322
var.24.SERPING1 11:57381989:G:A
peptide level
209:209
98:98
18:18
LYHAFSAMKpc2_NP.2
p−maj=1, pX=0.81, N=325
var.24.SERPING1 11:57381989:G:A
peptide level
209:209
97:98
18:18
LYHAFSAMKpc2_NP.3
p−min=0.36, pX=0.95, N=324
var.24.SERPING1 11:57381989:G:A
peptide level
195:209
85:98
16:18
LYHAFSAMKpc2_NP.4
p−min=0.069, pX=0.85, N=296
var.24.SERPING1 11:57381989:G:A
peptide level
126:209
60:98
12:18
LYHAFSAMKpc2_NP.5
p−maj=0.8, pX=0.075, N=198
var.24.SERPING1 11:57381989:G:A
peptide level
209:209
98:98
17:18
MEPFHFKpc2_NP.1
p−maj=0.055, pX=0.37, N=324
var.24.SERPING1 11:57381989:G:A
peptide level
209:209
98:98
18:18
MEPFHFKpc2_NP.2
p−maj=1, pX=0.75, N=325
var.24.SERPING1 11:57381989:G:A
peptide level
209:209
97:98
18:18
MEPFHFKpc2_NP.3
p−maj=1, pX=0.79, N=325
var.24.SERPING1 11:57381989:G:A
peptide level
209:209
98:98
18:18
MEPFHFKpc2_NP.4
p−maj=1, pX=0.47, N=325
var.24.SERPING1 11:57381989:G:A
peptide level
188:209
88:98
18:18
MEPFHFKpc2_NP.5
p−maj=0.24, pX=0.13, N=294

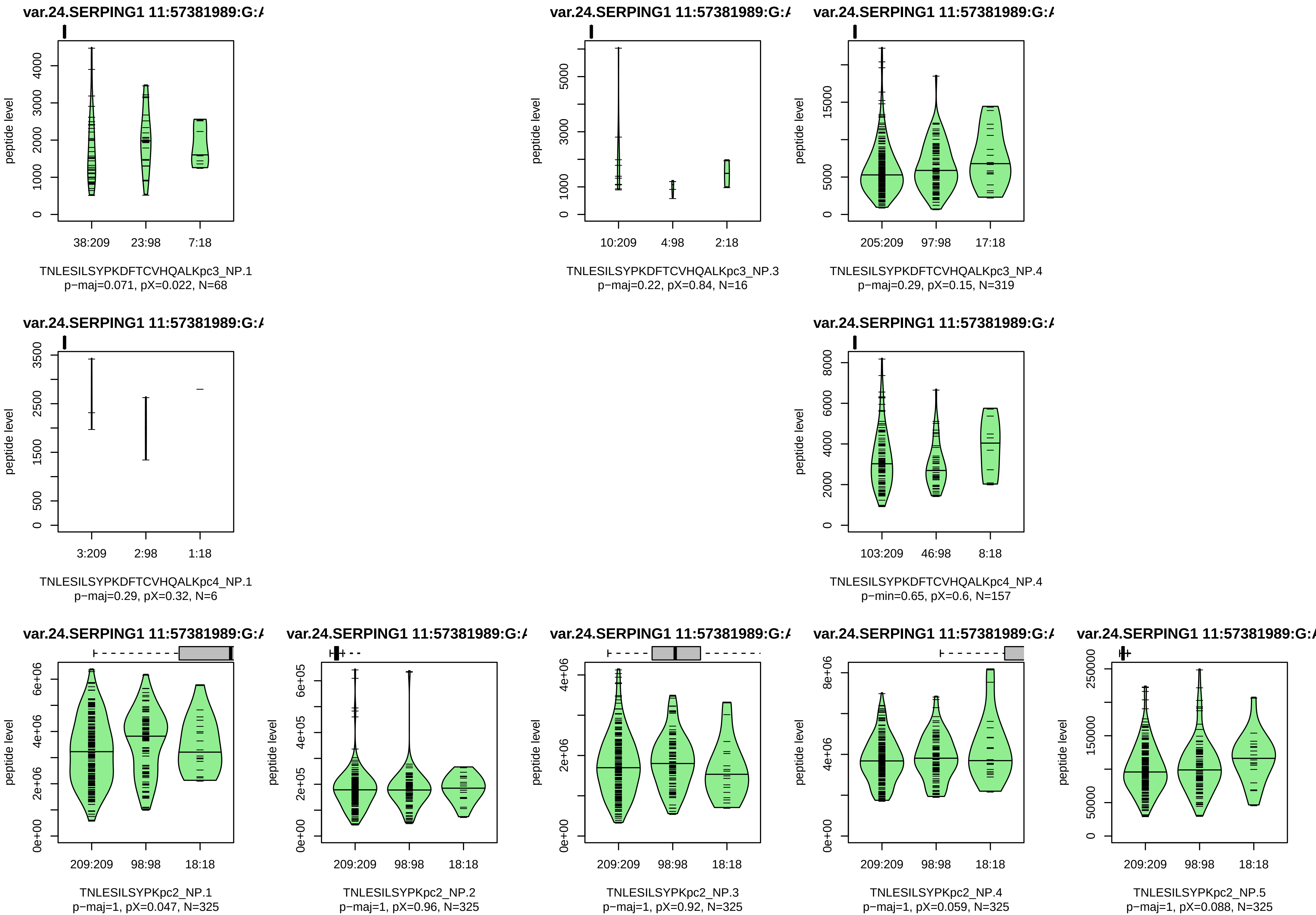
var.24.SERPING1 11:57381989:G:A
peptide level
38:209
23:98
7:18
TNLESILSYPKDFTCVHQALKpc3_NP.1
p−maj=0.071, pX=0.022, N=68
var.24.SERPING1 11:57381989:G:A
peptide level
10:209
4:98
2:18
TNLESILSYPKDFTCVHQALKpc3_NP.3
p−maj=0.22, pX=0.84, N=16
var.24.SERPING1 11:57381989:G:A
peptide level
205:209
97:98
17:18
TNLESILSYPKDFTCVHQALKpc3_NP.4
p−maj=0.29, pX=0.15, N=319
var.24.SERPING1 11:57381989:G:A
peptide level
3:209
2:98
1:18
TNLESILSYPKDFTCVHQALKpc4_NP.1
p−maj=0.29, pX=0.32, N=6
var.24.SERPING1 11:57381989:G:A
peptide level
103:209
46:98
8:18
TNLESILSYPKDFTCVHQALKpc4_NP.4
p−min=0.65, pX=0.6, N=157
var.24.SERPING1 11:57381989:G:A
peptide level
209:209
98:98
18:18
TNLESILSYPKpc2_NP.1
p−maj=1, pX=0.047, N=325
var.24.SERPING1 11:57381989:G:A
peptide level
209:209
98:98
18:18
TNLESILSYPKpc2_NP.2
p−maj=1, pX=0.96, N=325
var.24.SERPING1 11:57381989:G:A
peptide level
209:209
98:98
18:18
TNLESILSYPKpc2_NP.3
p−maj=1, pX=0.92, N=325
var.24.SERPING1 11:57381989:G:A
peptide level
209:209
98:98
18:18
TNLESILSYPKpc2_NP.4
p−maj=1, pX=0.059, N=325
var.24.SERPING1 11:57381989:G:A
peptide level
209:209
98:98
18:18
TNLESILSYPKpc2_NP.5
p−maj=1, pX=0.088, N=325

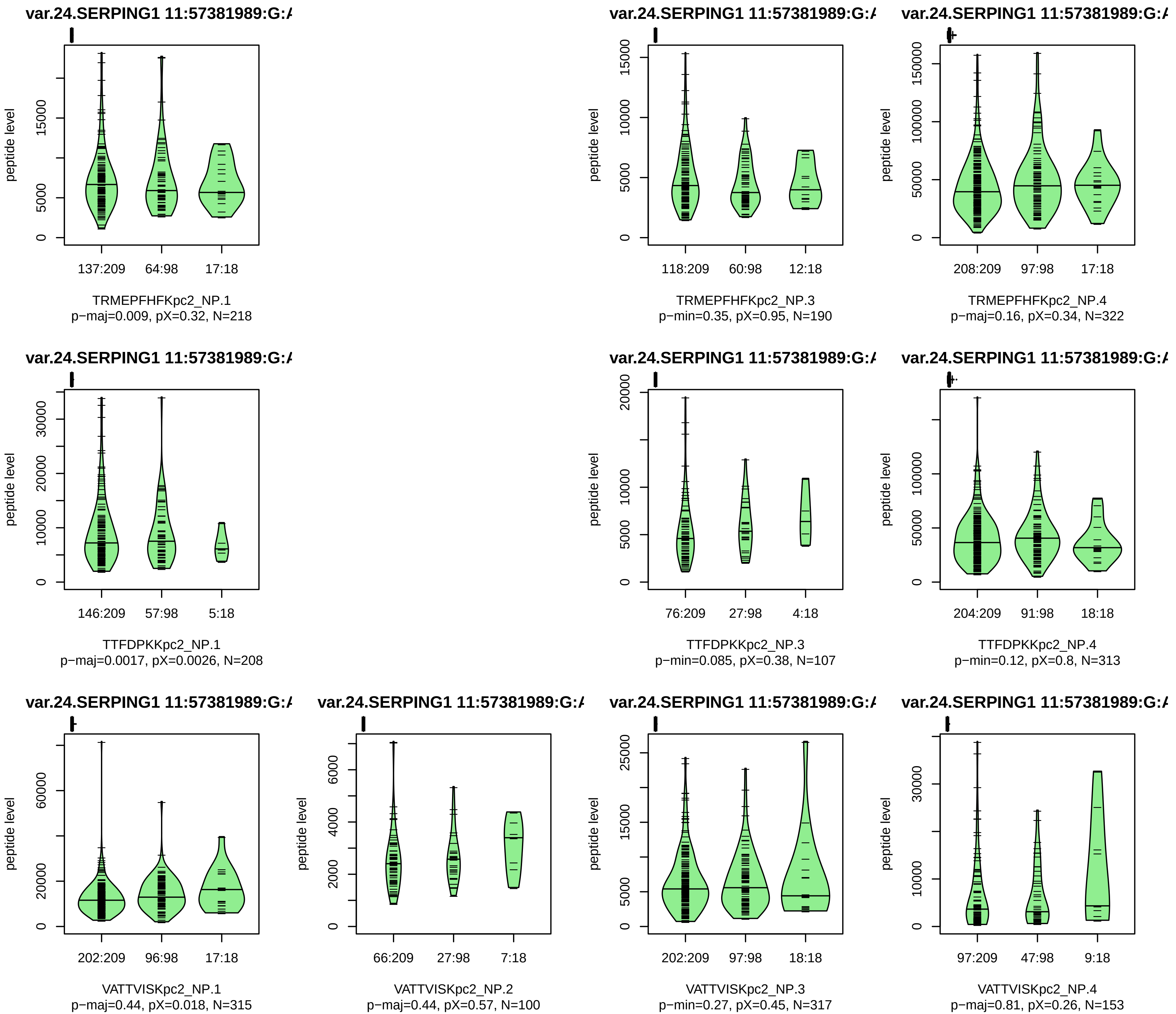
var.24.SERPING1 11:57381989:G:A
peptide level
0
5000
15000
137:209
64:98
17:18
TRMEPFHFKpc2_NP.1
p−maj=0.009, pX=0.32, N=218
var.24.SERPING1 11:57381989:G:A
peptide level
0
5000
10000
15000
118:209
60:98
12:18
TRMEPFHFKpc2_NP.3
p−min=0.35, pX=0.95, N=190
var.24.SERPING1 11:57381989:G:A
peptide level
0
50000
100000
150000
208:209
97:98
17:18
TRMEPFHFKpc2_NP.4
p−maj=0.16, pX=0.34, N=322
var.24.SERPING1 11:57381989:G:A
peptide level
0
10000
20000
30000
146:209
57:98
5:18
TTFDPKKpc2_NP.1
p−maj=0.0017, pX=0.0026, N=208
var.24.SERPING1 11:57381989:G:A
peptide level
0
5000
10000
20000
76:209
27:98
4:18
TTFDPKKpc2_NP.3
p−min=0.085, pX=0.38, N=107
var.24.SERPING1 11:57381989:G:A
peptide level
0
50000
100000
204:209
91:98
18:18
TTFDPKKpc2_NP.4
p−min=0.12, pX=0.8, N=313
var.24.SERPING1 11:57381989:G:A
peptide level
0
20000
60000
202:209
96:98
17:18
VATTVISKpc2_NP.1
p−maj=0.44, pX=0.018, N=315
var.24.SERPING1 11:57381989:G:A
peptide level
0
2000
4000
6000
66:209
27:98
7:18
VATTVISKpc2_NP.2
p−maj=0.44, pX=0.57, N=100
var.24.SERPING1 11:57381989:G:A
peptide level
0
5000
15000
25000
202:209
97:98
18:18
VATTVISKpc2_NP.3
p−min=0.27, pX=0.45, N=317
var.24.SERPING1 11:57381989:G:A
peptide level
0
10000
30000
97:209
47:98
9:18
VATTVISKpc2_NP.4
p−maj=0.81, pX=0.26, N=153

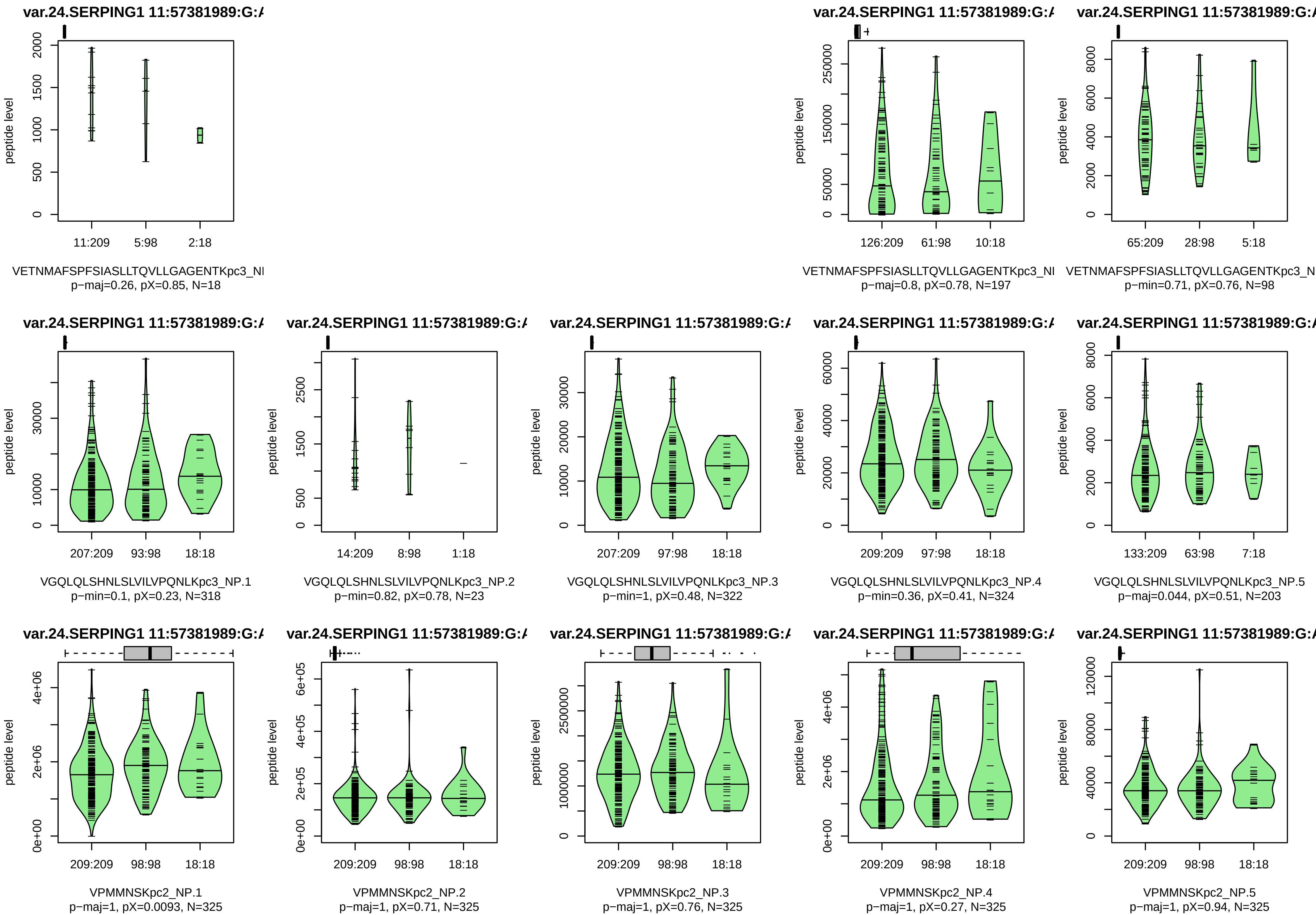
var.24.SERPING1 11:57381989:G:/
peptide level
11:209
5:98
2:18
VETNMAFSPFSIASLLTQVLLGAGENTKpc3_NI
p−maj=0.26, pX=0.85, N=18
var.24.SERPING1 11:57381989:G:/
peptide level
126:209
61:98
10:18
VETNMAFSPFSIASLLTQVLLGAGENTKpc3_NI
p−maj=0.8, pX=0.78, N=197
var.24.SERPING1 11:57381989:G:/
peptide level
65:209
28:98
5:18
VETNMAFSPFSIASLLTQVLLGAGENTKpc3_N
p−min=0.71, pX=0.76, N=98
var.24.SERPING1 11:57381989:G:/
peptide level
207:209
93:98
18:18
VGQLQLSHNLSLVILVPQNLKpc3_NP.1
p−min=0.1, pX=0.23, N=318
var.24.SERPING1 11:57381989:G:/
peptide level
14:209
8:98
1:18
VGQLQLSHNLSLVILVPQNLKpc3_NP.2
p−min=0.82, pX=0.78, N=23
var.24.SERPING1 11:57381989:G:/
peptide level
207:209
97:98
18:18
VGQLQLSHNLSLVILVPQNLKpc3_NP.3
p−min=1, pX=0.48, N=322
var.24.SERPING1 11:57381989:G:/
peptide level
209:209
97:98
18:18
VGQLQLSHNLSLVILVPQNLKpc3_NP.4
p−min=0.36, pX=0.41, N=324
var.24.SERPING1 11:57381989:G:/
peptide level
133:209
63:98
7:18
VGQLQLSHNLSLVILVPQNLKpc3_NP.5
p−maj=0.044, pX=0.51, N=203
var.24.SERPING1 11:57381989:G:/
peptide level
209:209
98:98
18:18
VPMMNSKpc2_NP.1
p−maj=1, pX=0.0093, N=325
var.24.SERPING1 11:57381989:G:/
peptide level
209:209
98:98
18:18
VPMMNSKpc2_NP.2
p−maj=1, pX=0.71, N=325
var.24.SERPING1 11:57381989:G:/
peptide level
209:209
98:98
18:18
VPMMNSKpc2_NP.3
p−maj=1, pX=0.76, N=325
var.24.SERPING1 11:57381989:G:/
peptide level
209:209
98:98
18:18
VPMMNSKpc2_NP.4
p−maj=1, pX=0.27, N=325
var.24.SERPING1 11:57381989:G:/
peptide level
209:209
98:98
18:18
VPMMNSKpc2_NP.5
p−maj=1, pX=0.94, N=325

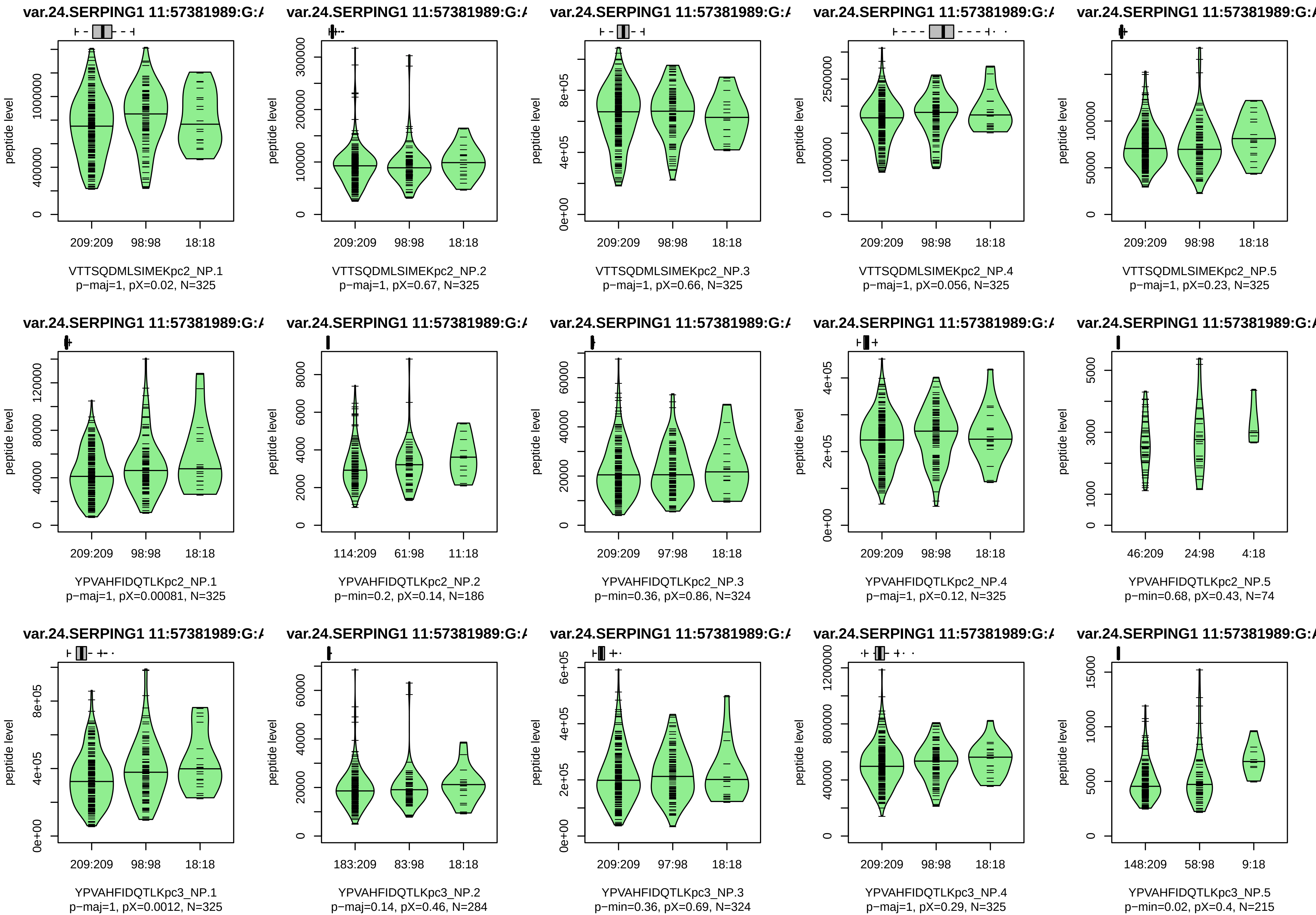
var.24.SERPING1 11:57381989:G:A
peptide level
209:209
98:98
18:18
VTTSQDMLSIMEKpc2_NP.1
p−maj=1, pX=0.02, N=325
VTTSQDMLSIMEKpc2_NP.2
p−maj=1, pX=0.67, N=325
VTTSQDMLSIMEKpc2_NP.3
p−maj=1, pX=0.66, N=325
VTTSQDMLSIMEKpc2_NP.4
p−maj=1, pX=0.056, N=325
VTTSQDMLSIMEKpc2_NP.5
p−maj=1, pX=0.23, N=325
YPVAHFIDQTLKpc2_NP.1
p−maj=1, pX=0.00081, N=325
114:209
61:98
11:18
YPVAHFIDQTLKpc2_NP.2
p−min=0.2, pX=0.14, N=186
97:98
YPVAHFIDQTLKpc2_NP.3
p−min=0.36, pX=0.86, N=324
YPVAHFIDQTLKpc2_NP.4
p−maj=1, pX=0.12, N=325
46:209
24:98
4:18
YPVAHFIDQTLKpc2_NP.5
p−min=0.68, pX=0.43, N=74
YPVAHFIDQTLKpc3_NP.1
p−maj=1, pX=0.0012, N=325
183:209
83:98
YPVAHFIDQTLKpc3_NP.2
p−maj=0.14, pX=0.46, N=284
YPVAHFIDQTLKpc3_NP.3
p−min=0.36, pX=0.69, N=324
YPVAHFIDQTLKpc3_NP.4
p−maj=1, pX=0.29, N=325
148:209
58:98
9:18
YPVAHFIDQTLKpc3_NP.5
p−min=0.02, pX=0.4, N=215

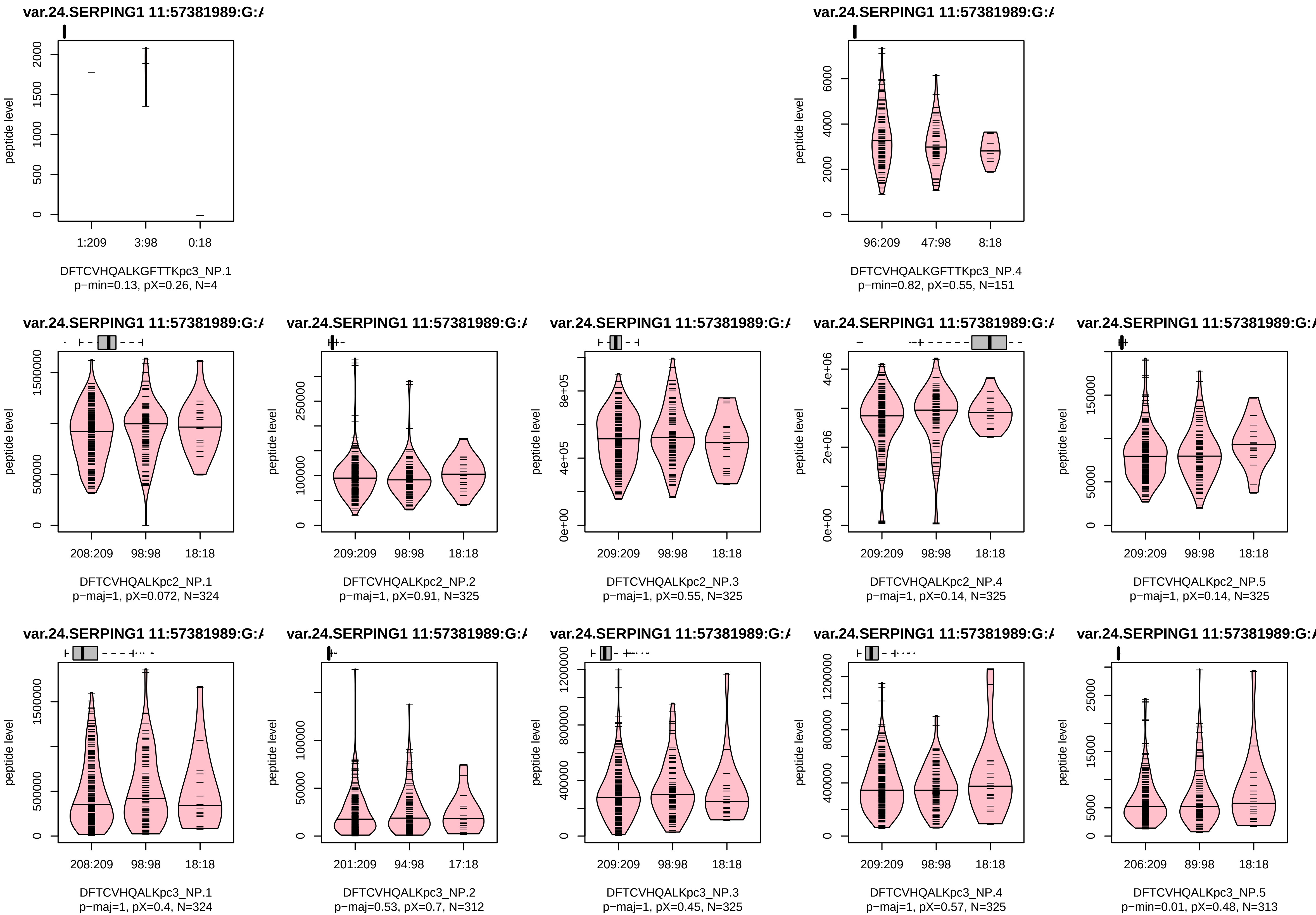
var.24.SERPING1 11:57381989:G:A
peptide level
1:209
3:98
0:18
DFTCVHQALKGFTTKpc3_NP.1
p−min=0.13, pX=0.26, N=4
var.24.SERPING1 11:57381989:G:A
peptide level
96:209
47:98
8:18
DFTCVHQALKGFTTKpc3_NP.4
p−min=0.82, pX=0.55, N=151
var.24.SERPING1 11:57381989:G:A
peptide level
208:209
98:98
18:18
DFTCVHQALKpc2_NP.1
p−maj=1, pX=0.072, N=324
var.24.SERPING1 11:57381989:G:A
peptide level
209:209
98:98
18:18
DFTCVHQALKpc2_NP.2
p−maj=1, pX=0.91, N=325
var.24.SERPING1 11:57381989:G:A
peptide level
209:209
98:98
18:18
DFTCVHQALKpc2_NP.3
p−maj=1, pX=0.55, N=325
var.24.SERPING1 11:57381989:G:A
peptide level
209:209
98:98
18:18
DFTCVHQALKpc2_NP.4
p−maj=1, pX=0.14, N=325
var.24.SERPING1 11:57381989:G:A
peptide level
209:209
98:98
18:18
DFTCVHQALKpc2_NP.5
p−maj=1, pX=0.14, N=325
var.24.SERPING1 11:57381989:G:A
peptide level
208:209
98:98
18:18
DFTCVHQALKpc3_NP.1
p−maj=1, pX=0.4, N=324
var.24.SERPING1 11:57381989:G:A
peptide level
201:209
94:98
17:18
DFTCVHQALKpc3_NP.2
p−maj=0.53, pX=0.7, N=312
var.24.SERPING1 11:57381989:G:A
peptide level
209:209
98:98
18:18
DFTCVHQALKpc3_NP.3
p−maj=1, pX=0.45, N=325
var.24.SERPING1 11:57381989:G:A
peptide level
209:209
98:98
18:18
DFTCVHQALKpc3_NP.4
p−maj=1, pX=0.57, N=325
var.24.SERPING1 11:57381989:G:A
peptide level
206:209
89:98
18:18
DFTCVHQALKpc3_NP.5
p−min=0.01, pX=0.48, N=313

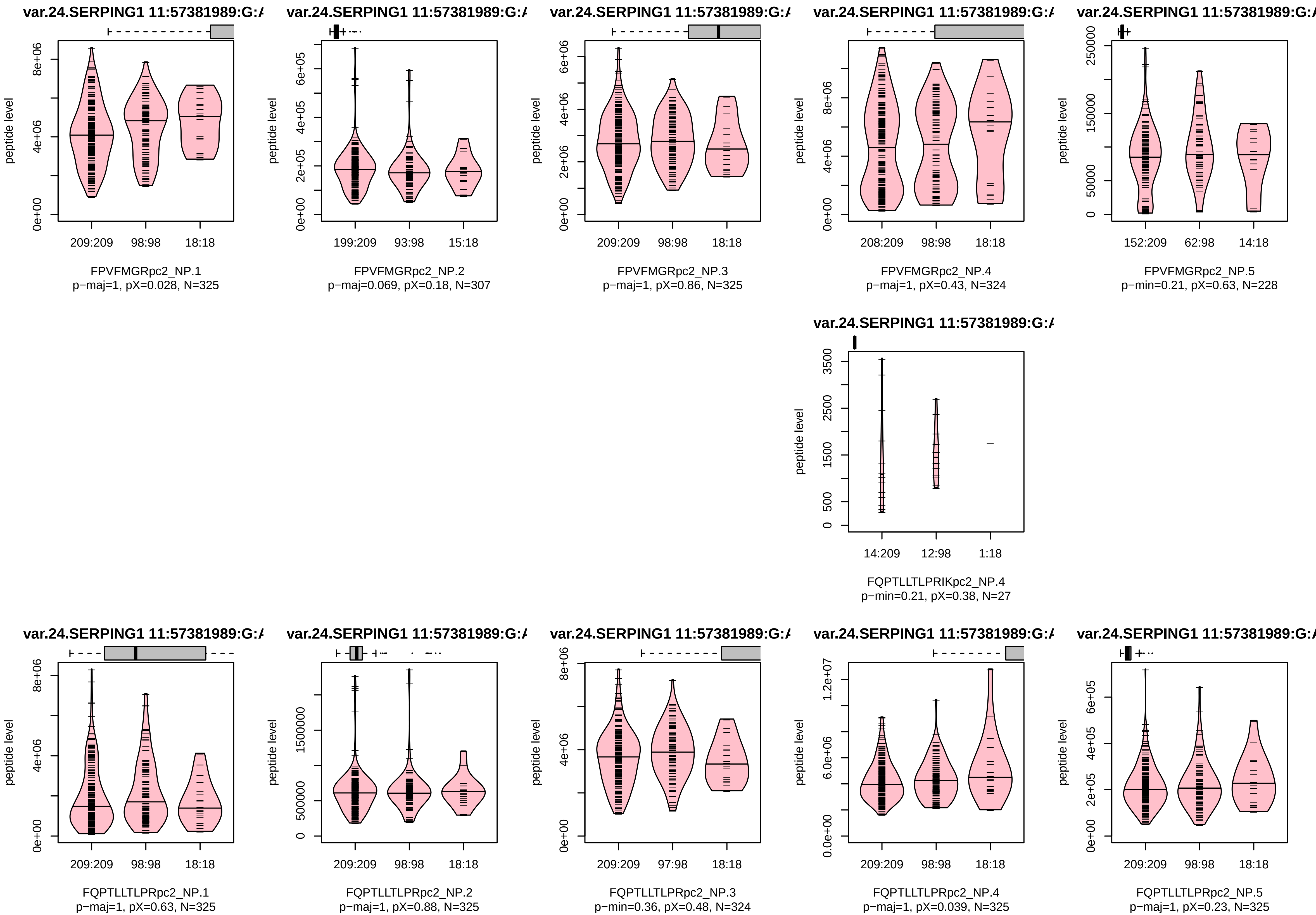

var.24.SERPING1 11:57381989:G:/
peptide level
0e+00
4e+06
8e+06
209:209
98:98
18:18
FPVFMGRpc2_NP.1
p−maj=1, pX=0.028, N=325
var.24.SERPING1 11:57381989:G:/
peptide level
0e+00
2e+05
4e+05
6e+05
199:209
93:98
15:18
FPVFMGRpc2_NP.2
p−maj=0.069, pX=0.18, N=307
var.24.SERPING1 11:57381989:G:/
peptide level
0e+00
2e+06
4e+06
6e+06
209:209
98:98
18:18
FPVFMGRpc2_NP.3
p−maj=1, pX=0.86, N=325
var.24.SERPING1 11:57381989:G:/
peptide level
0e+00
4e+06
8e+06
208:209
98:98
18:18
FPVFMGRpc2_NP.4
p−maj=1, pX=0.43, N=324
var.24.SERPING1 11:57381989:G:/
peptide level
0
50000
150000
250000
152:209
62:98
14:18
FPVFMGRpc2_NP.5
p−min=0.21, pX=0.63, N=228
var.24.SERPING1 11:57381989:G:/
peptide level
0
500
1500
2500
3500
14:209
12:98
1:18
FQPTLLTLPRIKpc2_NP.4
p−min=0.21, pX=0.38, N=27
var.24.SERPING1 11:57381989:G:/
peptide level
0e+00
4e+06
8e+06
209:209
98:98
18:18
FQPTLLTLPRpc2_NP.1
p−maj=1, pX=0.63, N=325
var.24.SERPING1 11:57381989:G:/
peptide level
0
500000
1500000
209:209
98:98
18:18
FQPTLLTLPRpc2_NP.2
p−maj=1, pX=0.88, N=325
var.24.SERPING1 11:57381989:G:/
peptide level
0e+00
4e+06
8e+06
209:209
97:98
18:18
FQPTLLTLPRpc2_NP.3
p−min=0.36, pX=0.48, N=324
var.24.SERPING1 11:57381989:G:/
peptide level
0.0e+00
6.0e+06
1.2e+07
209:209
98:98
18:18
FQPTLLTLPRpc2_NP.4
p−maj=1, pX=0.039, N=325
var.24.SERPING1 11:57381989:G:/
peptide level
0e+00
2e+05
4e+05
6e+05
209:209
98:98
18:18
FQPTLLTLPRpc2_NP.5
p−maj=1, pX=0.23, N=325

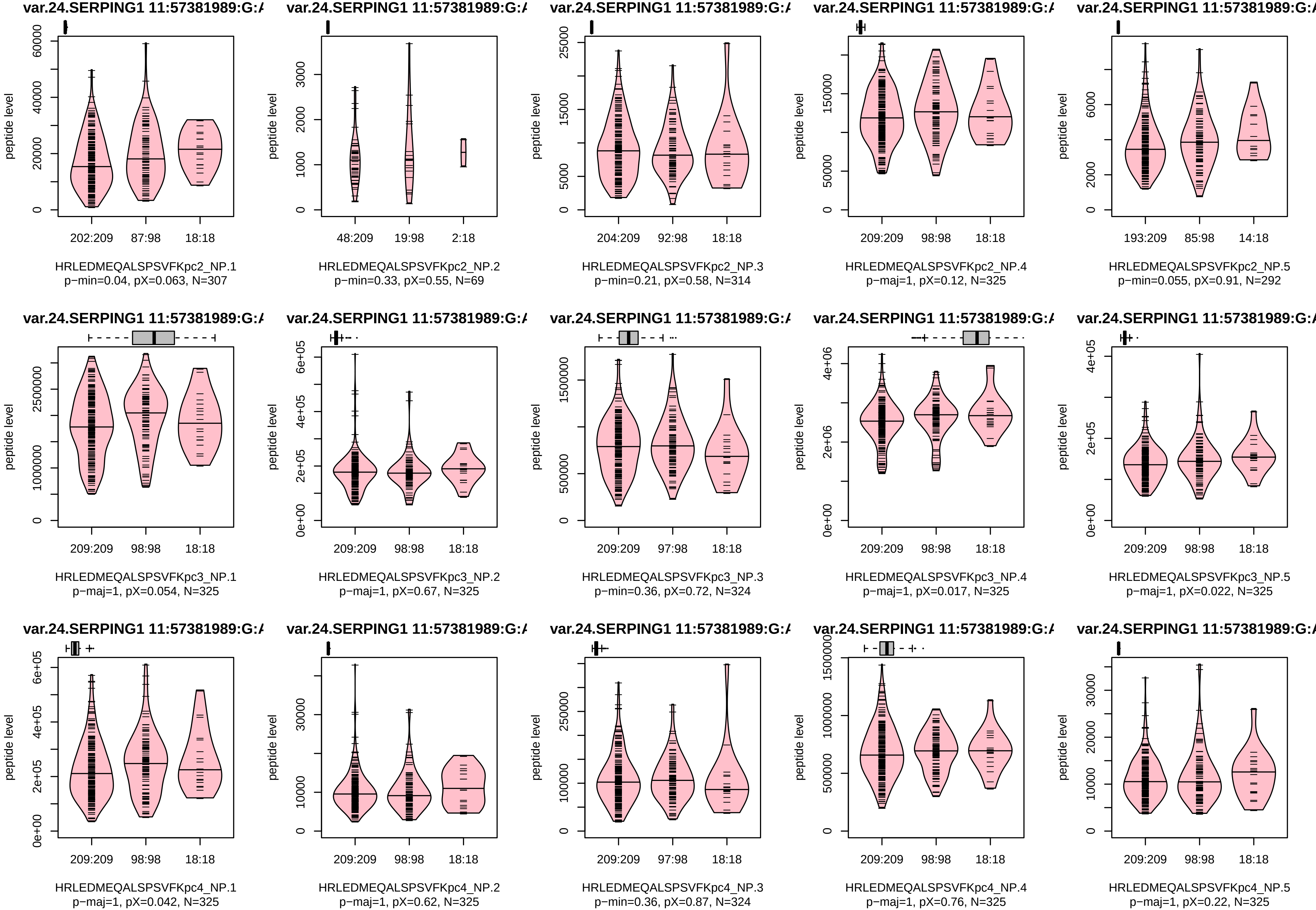
var.24.SERPING1 11:57381989:G:A
peptide level
202:209 87:98 18:18
HRLEDMEQALSPSVFKpc2_NP.1
p−min=0.04, pX=0.063, N=307
var.24.SERPING1 11:57381989:G:A
peptide level
48:209 19:98 2:18
HRLEDMEQALSPSVFKpc2_NP.2
p−min=0.33, pX=0.55, N=69
var.24.SERPING1 11:57381989:G:A
peptide level
204:209 92:98 18:18
HRLEDMEQALSPSVFKpc2_NP.3
p−min=0.21, pX=0.58, N=314
var.24.SERPING1 11:57381989:G:A
peptide level
209:209 98:98 18:18
HRLEDMEQALSPSVFKpc2_NP.4
p−maj=1, pX=0.12, N=325
var.24.SERPING1 11:57381989:G:A
peptide level
193:209 85:98 14:18
HRLEDMEQALSPSVFKpc2_NP.5
p−min=0.055, pX=0.91, N=292
var.24.SERPING1 11:57381989:G:A
peptide level
209:209 98:98 18:18
HRLEDMEQALSPSVFKpc3_NP.1
p−maj=1, pX=0.054, N=325
var.24.SERPING1 11:57381989:G:A
peptide level
209:209 98:98 18:18
HRLEDMEQALSPSVFKpc3_NP.2
p−maj=1, pX=0.67, N=325
var.24.SERPING1 11:57381989:G:A
peptide level
209:209 97:98 18:18
HRLEDMEQALSPSVFKpc3_NP.3
p−min=0.36, pX=0.72, N=324
var.24.SERPING1 11:57381989:G:A
peptide level
209:209 98:98 18:18
HRLEDMEQALSPSVFKpc3_NP.4
p−maj=1, pX=0.017, N=325
var.24.SERPING1 11:57381989:G:A
peptide level
209:209 98:98 18:18
HRLEDMEQALSPSVFKpc3_NP.5
p−maj=1, pX=0.022, N=325
var.24.SERPING1 11:57381989:G:A
peptide level
209:209 98:98 18:18
HRLEDMEQALSPSVFKpc4_NP.1
p−maj=1, pX=0.042, N=325
var.24.SERPING1 11:57381989:G:A
peptide level
209:209 98:98 18:18
HRLEDMEQALSPSVFKpc4_NP.2
p−maj=1, pX=0.62, N=325
var.24.SERPING1 11:57381989:G:A
peptide level
209:209 97:98 18:18
HRLEDMEQALSPSVFKpc4_NP.3
p−min=0.36, pX=0.87, N=324
var.24.SERPING1 11:57381989:G:A
peptide level
209:209 98:98 18:18
HRLEDMEQALSPSVFKpc4_NP.4
p−maj=1, pX=0.76, N=325
var.24.SERPING1 11:57381989:G:A
peptide level
209:209 98:98 18:18
HRLEDMEQALSPSVFKpc4_NP.5
p−maj=1, pX=0.22, N=325

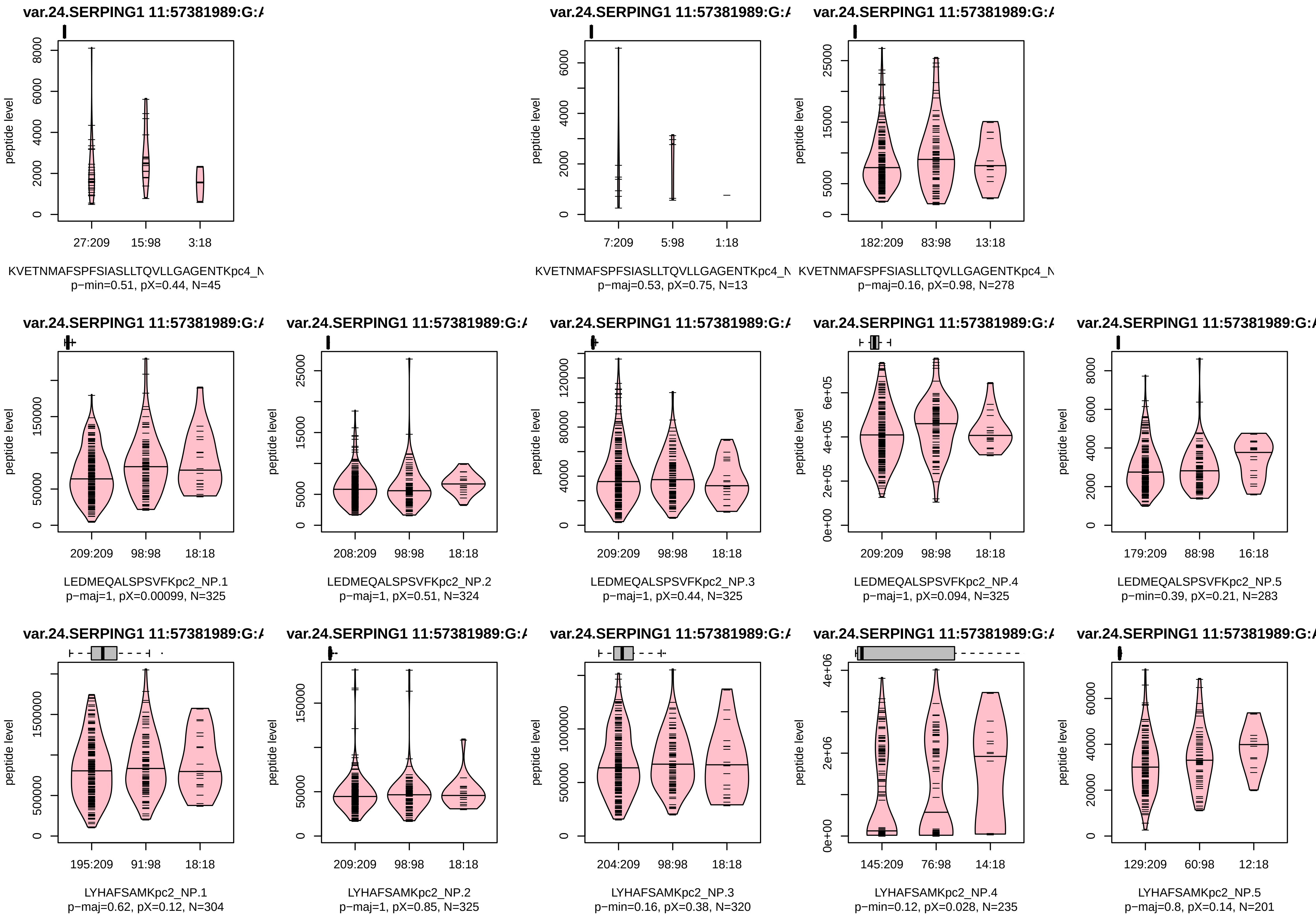
var.24.SERPING1 11:57381989:G:A
peptide level
27:209
15:98
3:18
KVETNMAFSPFSIASLLTQVLLGAGENTKpc4_N
p−min=0.51, pX=0.44, N=45
var.24.SERPING1 11:57381989:G:A
peptide level
7:209
5:98
1:18
KVETNMAFSPFSIASLLTQVLLGAGENTKpc4_N
p−maj=0.53, pX=0.75, N=13
var.24.SERPING1 11:57381989:G:A
peptide level
182:209
83:98
13:18
KVETNMAFSPFSIASLLTQVLLGAGENTKpc4_N
p−maj=0.16, pX=0.98, N=278
var.24.SERPING1 11:57381989:G:A
peptide level
209:209
98:98
18:18
LEDMEQALSPSVFKpc2_NP.1
p−maj=1, pX=0.00099, N=325
var.24.SERPING1 11:57381989:G:A
peptide level
208:209
98:98
18:18
LEDMEQALSPSVFKpc2_NP.2
p−maj=1, pX=0.51, N=324
var.24.SERPING1 11:57381989:G:A
peptide level
209:209
98:98
18:18
LEDMEQALSPSVFKpc2_NP.3
p−maj=1, pX=0.44, N=325
var.24.SERPING1 11:57381989:G:A
peptide level
209:209
98:98
18:18
LEDMEQALSPSVFKpc2_NP.4
p−maj=1, pX=0.094, N=325
var.24.SERPING1 11:57381989:G:A
peptide level
179:209
88:98
16:18
LEDMEQALSPSVFKpc2_NP.5
p−min=0.39, pX=0.21, N=283
var.24.SERPING1 11:57381989:G:A
peptide level
195:209
91:98
18:18
LYHAFSAMKpc2_NP.1
p−maj=0.62, pX=0.12, N=304
var.24.SERPING1 11:57381989:G:A
peptide level
209:209
98:98
18:18
LYHAFSAMKpc2_NP.2
p−maj=1, pX=0.85, N=325
var.24.SERPING1 11:57381989:G:A
peptide level
204:209
98:98
18:18
LYHAFSAMKpc2_NP.3
p−min=0.16, pX=0.38, N=320
var.24.SERPING1 11:57381989:G:A
peptide level
145:209
76:98
14:18
LYHAFSAMKpc2_NP.4
p−min=0.12, pX=0.028, N=235
var.24.SERPING1 11:57381989:G:A
peptide level
129:209
60:98
12:18
LYHAFSAMKpc2_NP.5
p−maj=0.8, pX=0.14, N=201

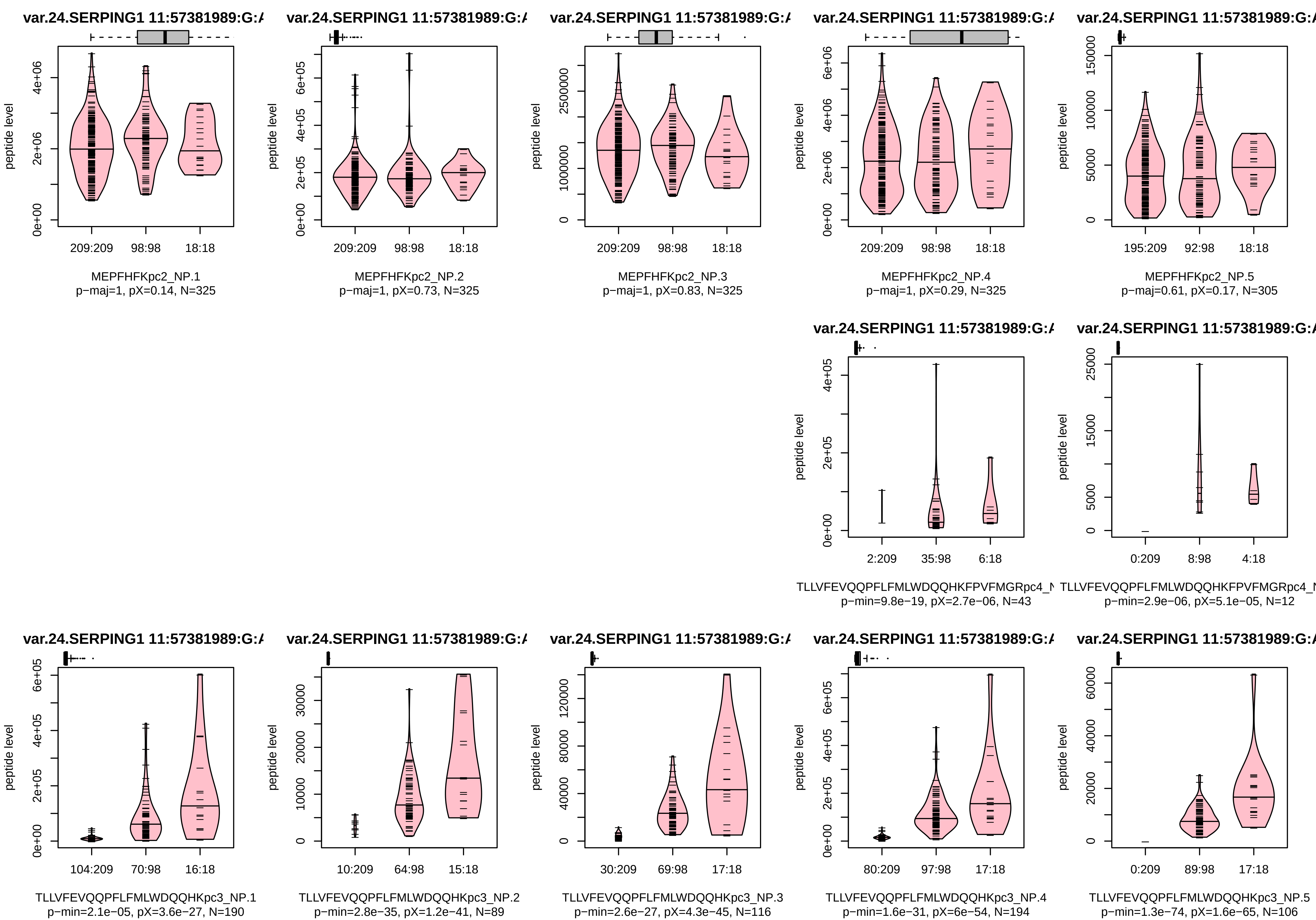
var.24.SERPING1 11:57381989:G:A
peptide level
209:209
98:98
18:18
MEPFHFKpc2_NP.1
p−maj=1, pX=0.14, N=325
var.24.SERPING1 11:57381989:G:A
peptide level
209:209
98:98
18:18
MEPFHFKpc2_NP.2
p−maj=1, pX=0.73, N=325
var.24.SERPING1 11:57381989:G:A
peptide level
209:209
98:98
18:18
MEPFHFKpc2_NP.3
p−maj=1, pX=0.83, N=325
var.24.SERPING1 11:57381989:G:A
peptide level
209:209
98:98
18:18
MEPFHFKpc2_NP.4
p−maj=1, pX=0.29, N=325
var.24.SERPING1 11:57381989:G:A
peptide level
195:209
92:98
18:18
MEPFHFKpc2_NP.5
p−maj=0.61, pX=0.17, N=305
var.24.SERPING1 11:57381989:G:A
peptide level
2:209
35:98
6:18
TLLVFEVQQPFLFMLWDQQHKFPVFMGRpc4_N
p−min=9.8e−19, pX=2.7e−06, N=43
var.24.SERPING1 11:57381989:G:A
peptide level
0:209
8:98
4:18
TLLVFEVQQPFLFMLWDQQHKFPVFMGRpc4_N
p−min=2.9e−06, pX=5.1e−05, N=12
var.24.SERPING1 11:57381989:G:A
peptide level
104:209
70:98
16:18
TLLVFEVQQPFLFMLWDQQHKpc3_NP.1
p−min=2.1e−05, pX=3.6e−27, N=190
var.24.SERPING1 11:57381989:G:A
peptide level
10:209
64:98
15:18
TLLVFEVQQPFLFMLWDQQHKpc3_NP.2
p−min=2.8e−35, pX=1.2e−41, N=89
var.24.SERPING1 11:57381989:G:A
peptide level
30:209
69:98
17:18
TLLVFEVQQPFLFMLWDQQHKpc3_NP.3
p−min=2.6e−27, pX=4.3e−45, N=116
var.24.SERPING1 11:57381989:G:A
peptide level
80:209
97:98
17:18
TLLVFEVQQPFLFMLWDQQHKpc3_NP.4
p−min=1.6e−31, pX=6e−54, N=194
var.24.SERPING1 11:57381989:G:A
peptide level
0:209
89:98
17:18
TLLVFEVQQPFLFMLWDQQHKpc3_NP.5
p−min=1.3e−74, pX=1.6e−65, N=106

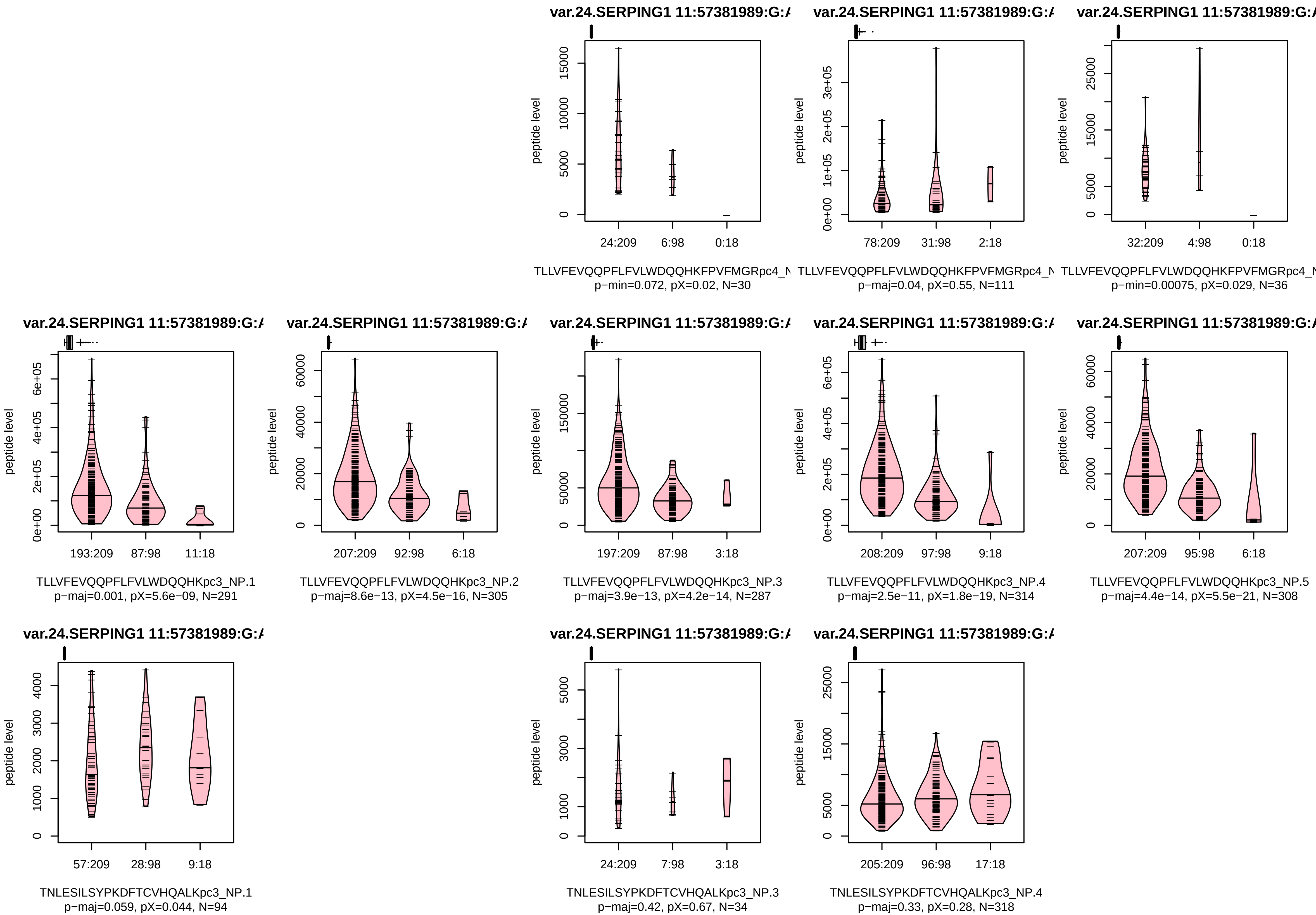

var.24.SERPING1 11:57381989:G:/
peptide level
24:209
6:98
0:18
TLLVFEVQQPFLFVLWDQQHKFPVFMGRpc4_N
p−min=0.072, pX=0.02, N=30
var.24.SERPING1 11:57381989:G:/
peptide level
78:209
31:98
2:18
TLLVFEVQQPFLFVLWDQQHKFPVFMGRpc4_N
p−maj=0.04, pX=0.55, N=111
var.24.SERPING1 11:57381989:G:/
peptide level
32:209
4:98
0:18
TLLVFEVQQPFLFVLWDQQHKFPVFMGRpc4_N
p−min=0.00075, pX=0.029, N=36
var.24.SERPING1 11:57381989:G:/
peptide level
193:209
87:98
11:18
TLLVFEVQQPFLFVLWDQQHKpc3_NP.1
p−maj=0.001, pX=5.6e−09, N=291
var.24.SERPING1 11:57381989:G:/
peptide level
207:209
92:98
6:18
TLLVFEVQQPFLFVLWDQQHKpc3_NP.2
p−maj=8.6e−13, pX=4.5e−16, N=305
var.24.SERPING1 11:57381989:G:/
peptide level
197:209
87:98
3:18
TLLVFEVQQPFLFVLWDQQHKpc3_NP.3
p−maj=3.9e−13, pX=4.2e−14, N=287
var.24.SERPING1 11:57381989:G:/
peptide level
208:209
97:98
9:18
TLLVFEVQQPFLFVLWDQQHKpc3_NP.4
p−maj=2.5e−11, pX=1.8e−19, N=314
var.24.SERPING1 11:57381989:G:/
peptide level
207:209
95:98
6:18
TLLVFEVQQPFLFVLWDQQHKpc3_NP.5
p−maj=4.4e−14, pX=5.5e−21, N=308
var.24.SERPING1 11:57381989:G:/
peptide level
57:209
28:98
9:18
TNLESILSYPKDFTCVHQALKpc3_NP.1
p−maj=0.059, pX=0.044, N=94
var.24.SERPING1 11:57381989:G:/
peptide level
24:209
7:98
3:18
TNLESILSYPKDFTCVHQALKpc3_NP.3
p−maj=0.42, pX=0.67, N=34
var.24.SERPING1 11:57381989:G:/
peptide level
205:209
96:98
17:18
TNLESILSYPKDFTCVHQALKpc3_NP.4
p−maj=0.33, pX=0.28, N=318

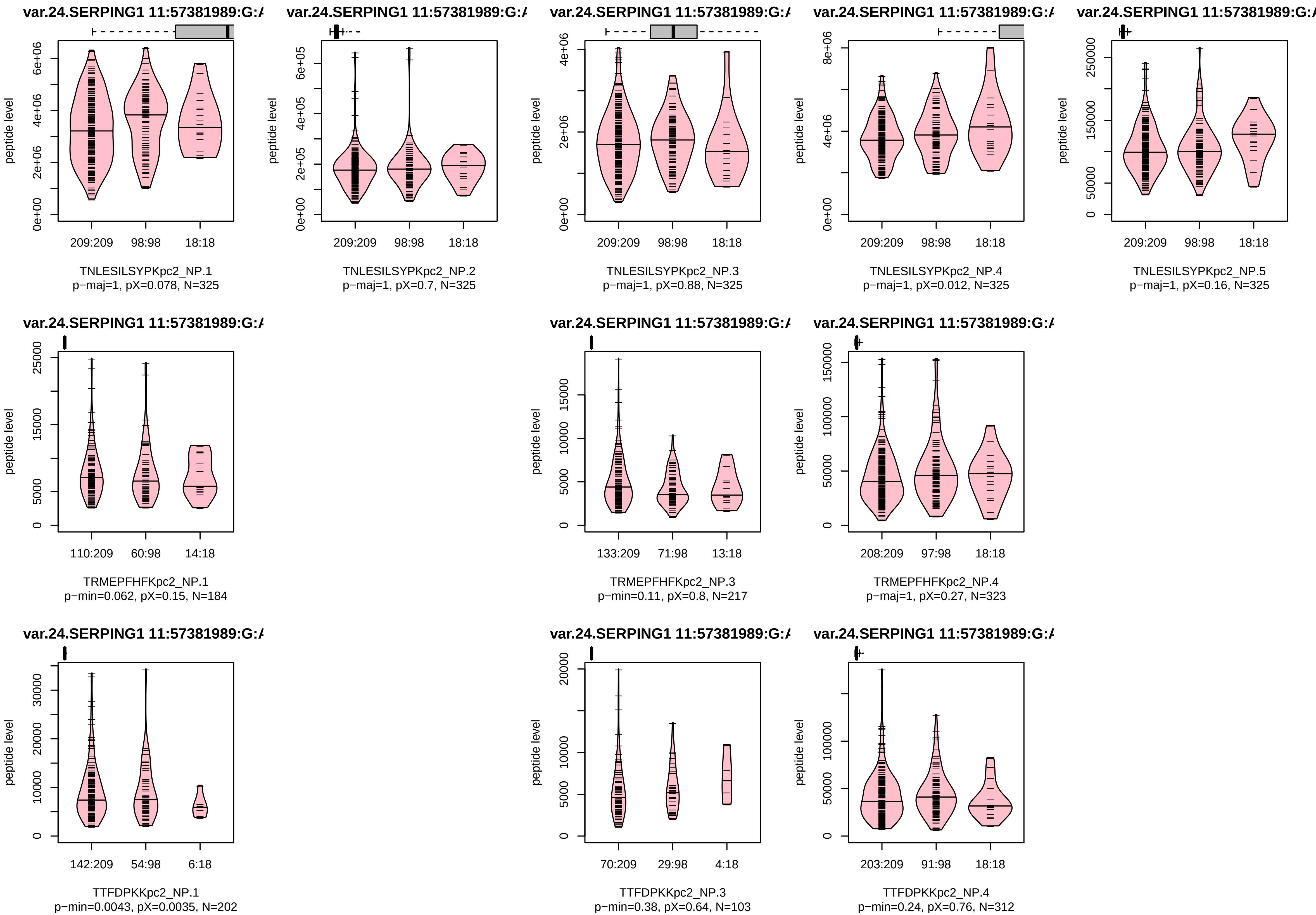
var.24.SERPING1 11:57381989:G:A
peptide level
209:209
98:98
18:18
TNLESILSYPKpc2_NP.1
p−maj=1, pX=0.078, N=325
var.24.SERPING1 11:57381989:G:A
peptide level
209:209
98:98
18:18
TNLESILSYPKpc2_NP.2
p−maj=1, pX=0.7, N=325
var.24.SERPING1 11:57381989:G:A
peptide level
209:209
98:98
18:18
TNLESILSYPKpc2_NP.3
p−maj=1, pX=0.88, N=325
var.24.SERPING1 11:57381989:G:A
peptide level
209:209
98:98
18:18
TNLESILSYPKpc2_NP.4
p−maj=1, pX=0.012, N=325
var.24.SERPING1 11:57381989:G:A
peptide level
209:209
98:98
18:18
TNLESILSYPKpc2_NP.5
p−maj=1, pX=0.16, N=325
var.24.SERPING1 11:57381989:G:A
peptide level
110:209
60:98
14:18
TRMEPFHFKpc2_NP.1
p−min=0.062, pX=0.15, N=184
var.24.SERPING1 11:57381989:G:A
peptide level
133:209
71:98
13:18
TRMEPFHFKpc2_NP.3
p−min=0.11, pX=0.8, N=217
var.24.SERPING1 11:57381989:G:A
peptide level
208:209
97:98
18:18
TRMEPFHFKpc2_NP.4
p−maj=1, pX=0.27, N=323
var.24.SERPING1 11:57381989:G:A
peptide level
142:209
54:98
6:18
TTFDPKKpc2_NP.1
p−min=0.0043, pX=0.0035, N=202
var.24.SERPING1 11:57381989:G:A
peptide level
70:209
29:98
4:18
TTFDPKKpc2_NP.3
p−min=0.38, pX=0.64, N=103
var.24.SERPING1 11:57381989:G:A
peptide level
203:209
91:98
18:18
TTFDPKKpc2_NP.4
p−min=0.24, pX=0.76, N=312

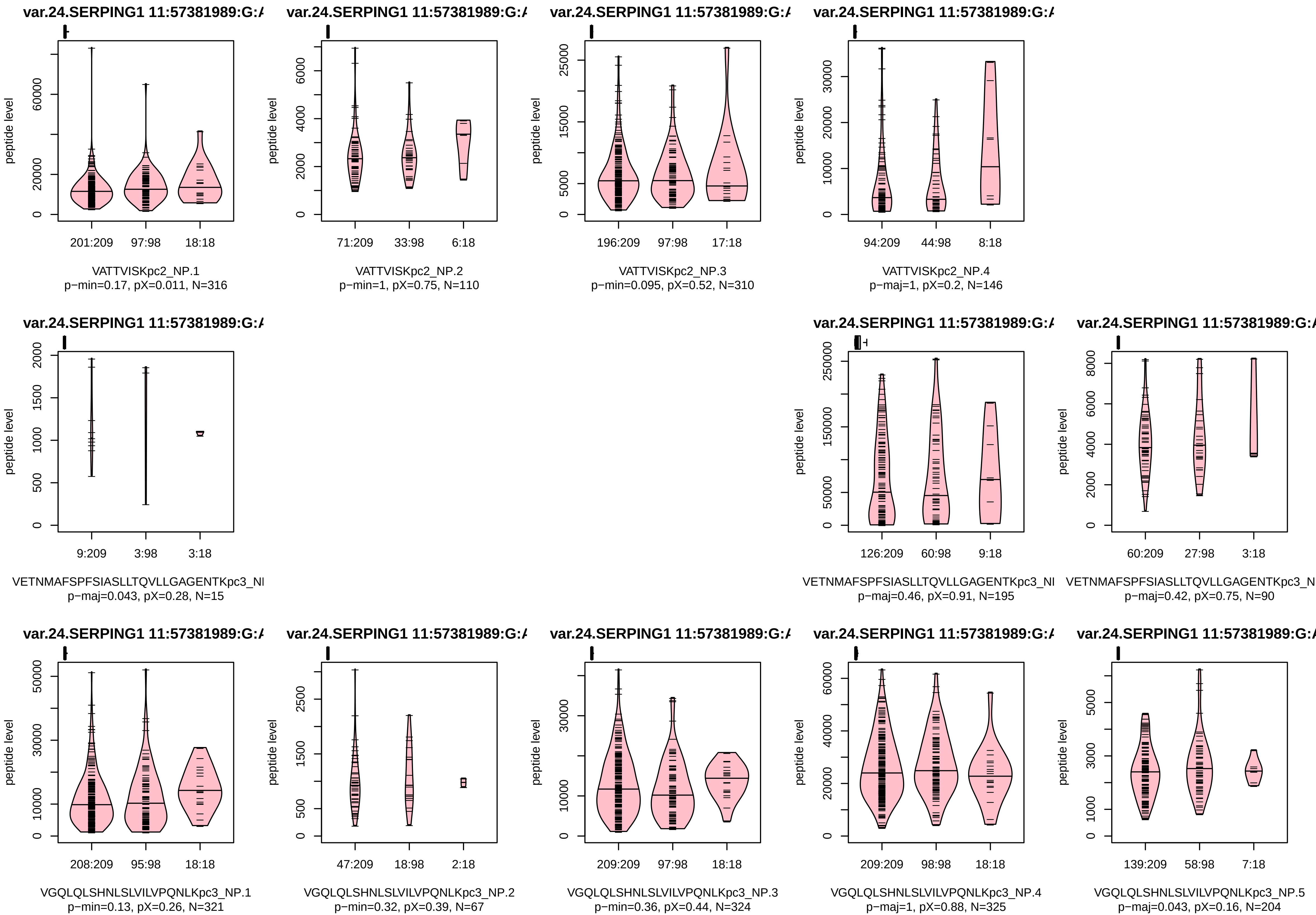
var.24.SERPING1 11:57381989:G:A
peptide level
201:209
97:98
18:18
VATTVISKpc2_NP.1
p−min=0.17, pX=0.011, N=316
var.24.SERPING1 11:57381989:G:A
peptide level
71:209
33:98
6:18
VATTVISKpc2_NP.2
p−min=1, pX=0.75, N=110
var.24.SERPING1 11:57381989:G:A
peptide level
196:209
97:98
17:18
VATTVISKpc2_NP.3
p−min=0.095, pX=0.52, N=310
var.24.SERPING1 11:57381989:G:A
peptide level
94:209
44:98
8:18
VATTVISKpc2_NP.4
p−maj=1, pX=0.2, N=146
var.24.SERPING1 11:57381989:G:A
peptide level
9:209
3:98
3:18
VETNMAFSPFSIASLLTQVLLGAGENTKpc3_NI
p−maj=0.043, pX=0.28, N=15
var.24.SERPING1 11:57381989:G:A
peptide level
126:209
60:98
9:18
VETNMAFSPFSIASLLTQVLLGAGENTKpc3_NI
p−maj=0.46, pX=0.91, N=195
var.24.SERPING1 11:57381989:G:A
peptide level
60:209
27:98
3:18
VETNMAFSPFSIASLLTQVLLGAGENTKpc3_N
p−maj=0.42, pX=0.75, N=90
var.24.SERPING1 11:57381989:G:A
peptide level
208:209
95:98
18:18
VGQLQLSHNLSLVILVPQNLKpc3_NP.1
p−min=0.13, pX=0.26, N=321
var.24.SERPING1 11:57381989:G:A
peptide level
47:209
18:98
2:18
VGQLQLSHNLSLVILVPQNLKpc3_NP.2
p−min=0.32, pX=0.39, N=67
var.24.SERPING1 11:57381989:G:A
peptide level
209:209
97:98
18:18
VGQLQLSHNLSLVILVPQNLKpc3_NP.3
p−min=0.36, pX=0.44, N=324
var.24.SERPING1 11:57381989:G:A
peptide level
209:209
98:98
18:18
VGQLQLSHNLSLVILVPQNLKpc3_NP.4
p−maj=1, pX=0.88, N=325
var.24.SERPING1 11:57381989:G:A
peptide level
139:209
58:98
7:18
VGQLQLSHNLSLVILVPQNLKpc3_NP.5
p−maj=0.043, pX=0.16, N=204

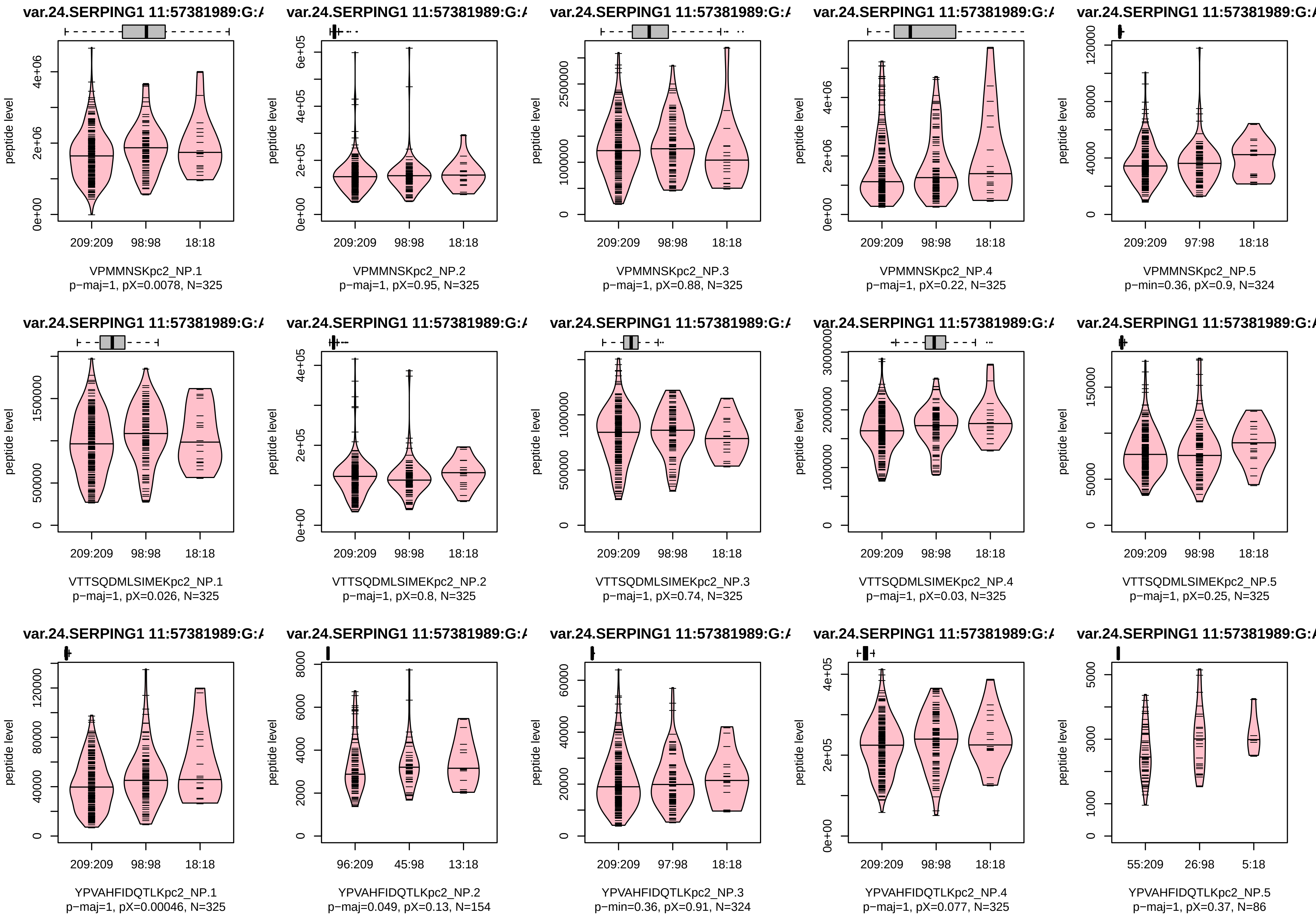
var.24.SERPING1 11:57381989:G:A
peptide level
209:209 98:98 18:18
VPMMNSKpc2_NP.1
p−maj=1, pX=0.0078, N=325
var.24.SERPING1 11:57381989:G:A
209:209 98:98 18:18
VPMMNSKpc2_NP.2
p−maj=1, pX=0.95, N=325
var.24.SERPING1 11:57381989:G:A
209:209 98:98 18:18
VPMMNSKpc2_NP.3
p−maj=1, pX=0.88, N=325
var.24.SERPING1 11:57381989:G:A
209:209 98:98 18:18
VPMMNSKpc2_NP.4
p−maj=1, pX=0.22, N=325
var.24.SERPING1 11:57381989:G:A
209:209 97:98 18:18
VPMMNSKpc2_NP.5
p−min=0.36, pX=0.9, N=324
var.24.SERPING1 11:57381989:G:A
209:209 98:98 18:18
VTTSQDMLSIMEKpc2_NP.1
p−maj=1, pX=0.026, N=325
var.24.SERPING1 11:57381989:G:A
209:209 98:98 18:18
VTTSQDMLSIMEKpc2_NP.2
p−maj=1, pX=0.8, N=325
var.24.SERPING1 11:57381989:G:A
209:209 98:98 18:18
VTTSQDMLSIMEKpc2_NP.3
p−maj=1, pX=0.74, N=325
var.24.SERPING1 11:57381989:G:A
209:209 98:98 18:18
VTTSQDMLSIMEKpc2_NP.4
p−maj=1, pX=0.03, N=325
var.24.SERPING1 11:57381989:G:A
209:209 98:98 18:18
VTTSQDMLSIMEKpc2_NP.5
p−maj=1, pX=0.25, N=325
var.24.SERPING1 11:57381989:G:A
209:209 98:98 18:18
YPVAHFIDQTLKpc2_NP.1
p−maj=1, pX=0.00046, N=325
var.24.SERPING1 11:57381989:G:A
96:209 45:98 13:18
YPVAHFIDQTLKpc2_NP.2
p−maj=0.049, pX=0.13, N=154
var.24.SERPING1 11:57381989:G:A
209:209 97:98 18:18
YPVAHFIDQTLKpc2_NP.3
p−min=0.36, pX=0.91, N=324
var.24.SERPING1 11:57381989:G:A
209:209 98:98 18:18
YPVAHFIDQTLKpc2_NP.4
p−maj=1, pX=0.077, N=325
var.24.SERPING1 11:57381989:G:A
55:209 26:98 5:18
YPVAHFIDQTLKpc2_NP.5
p−maj=1, pX=0.37, N=86

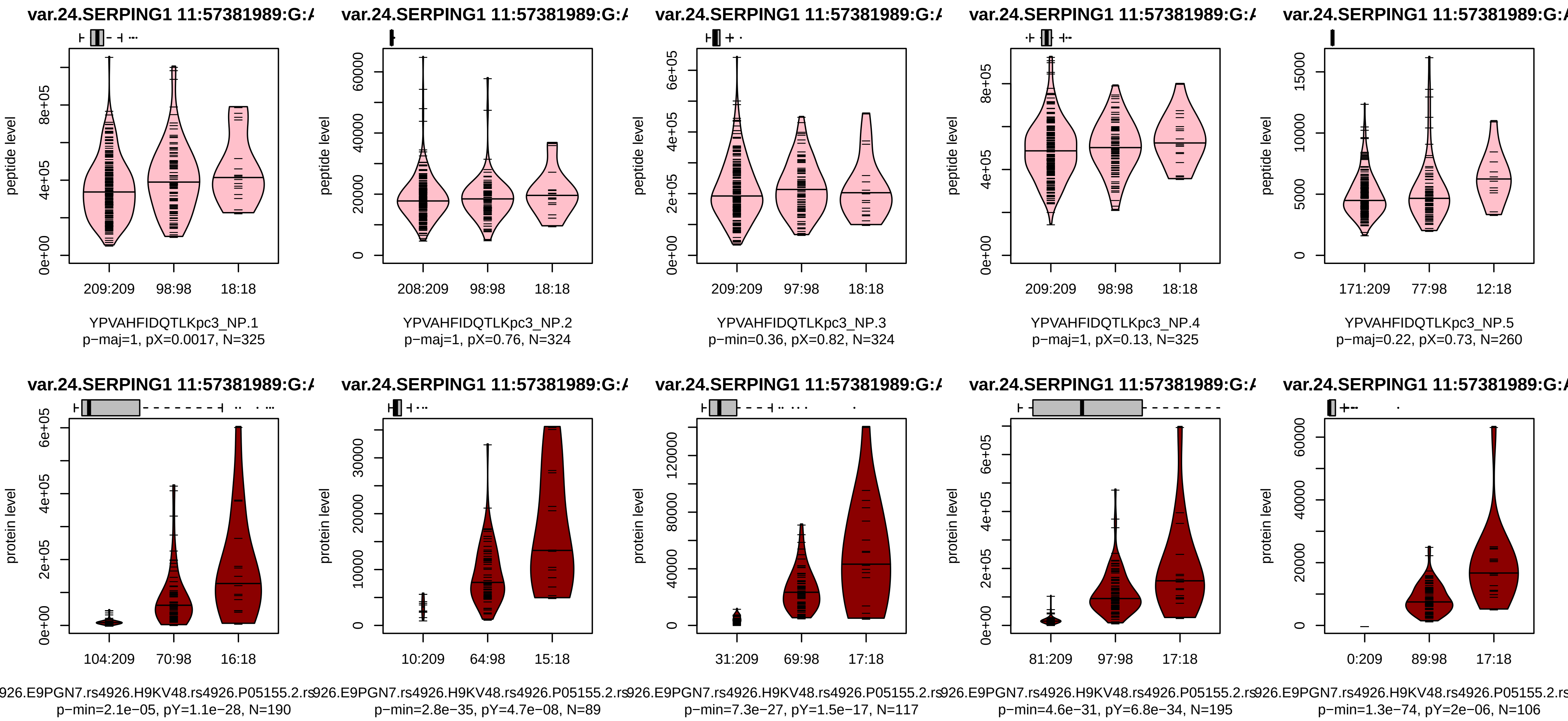
var.24.SERPING1 11:57381989:G:A
peptide level
209:209
98:98
18:18
YPVAHFIDQTLKpc3_NP.1
p−maj=1, pX=0.0017, N=325
208:209
98:98
18:18
YPVAHFIDQTLKpc3_NP.2
p−maj=1, pX=0.76, N=324
209:209
97:98
18:18
YPVAHFIDQTLKpc3_NP.3
p−min=0.36, pX=0.82, N=324
209:209
98:98
18:18
YPVAHFIDQTLKpc3_NP.4
p−maj=1, pX=0.13, N=325
171:209
77:98
12:18
YPVAHFIDQTLKpc3_NP.5
p−maj=0.22, pX=0.73, N=260
protein level
104:209
70:98
16:18
p−min=2.1e−05, pY=1.1e−28, N=190
10:209
64:98
15:18
p−min=2.8e−35, pY=4.7e−08, N=89
31:209
69:98
17:18
p−min=7.3e−27, pY=1.5e−17, N=117
81:209
97:98
17:18
p−min=4.6e−31, pY=6.8e−34, N=195
0:209
89:98
17:18
p−min=1.3e−74, pY=2e−06, N=106
926.E9PGN7.rs4926.H9KV48.rs4926.P05155.2.rs

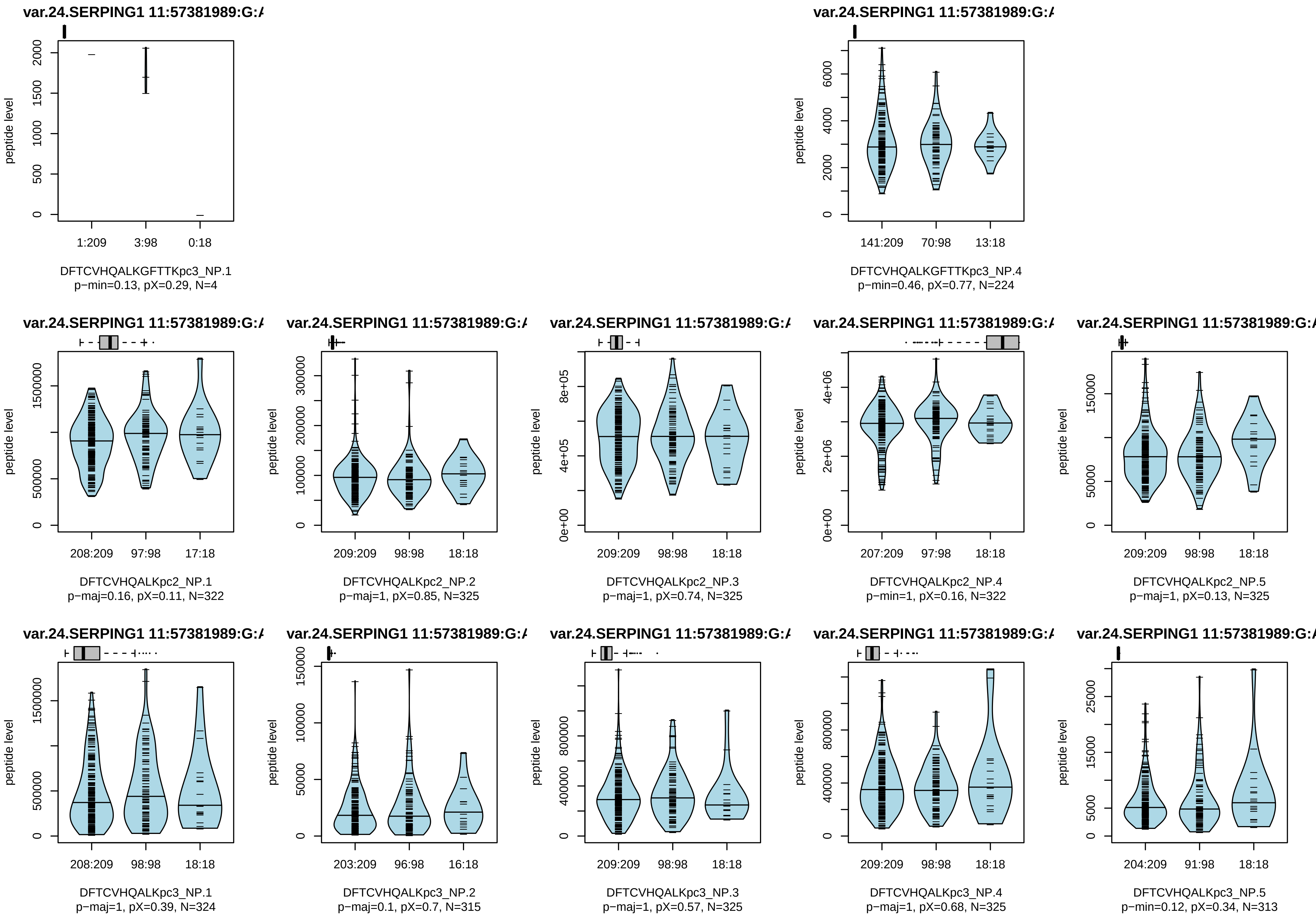
var.24.SERPING1 11:57381989:G:/
peptide level
1:209
3:98
0:18
DFTCVHQALKGFTTKpc3_NP.1
p−min=0.13, pX=0.29, N=4
var.24.SERPING1 11:57381989:G:/
peptide level
141:209
70:98
13:18
DFTCVHQALKGFTTKpc3_NP.4
p−min=0.46, pX=0.77, N=224
var.24.SERPING1 11:57381989:G:/
peptide level
208:209
97:98
17:18
DFTCVHQALKpc2_NP.1
p−maj=0.16, pX=0.11, N=322
var.24.SERPING1 11:57381989:G:/
peptide level
209:209
98:98
18:18
DFTCVHQALKpc2_NP.2
p−maj=1, pX=0.85, N=325
var.24.SERPING1 11:57381989:G:/
peptide level
209:209
98:98
18:18
DFTCVHQALKpc2_NP.3
p−maj=1, pX=0.74, N=325
var.24.SERPING1 11:57381989:G:/
peptide level
207:209
97:98
18:18
DFTCVHQALKpc2_NP.4
p−min=1, pX=0.16, N=322
var.24.SERPING1 11:57381989:G:/
peptide level
209:209
98:98
18:18
DFTCVHQALKpc2_NP.5
p−maj=1, pX=0.13, N=325
var.24.SERPING1 11:57381989:G:/
peptide level
208:209
98:98
18:18
DFTCVHQALKpc3_NP.1
p−maj=1, pX=0.39, N=324
var.24.SERPING1 11:57381989:G:/
peptide level
203:209
96:98
16:18
DFTCVHQALKpc3_NP.2
p−maj=0.1, pX=0.7, N=315
var.24.SERPING1 11:57381989:G:/
peptide level
209:209
98:98
18:18
DFTCVHQALKpc3_NP.3
p−maj=1, pX=0.57, N=325
var.24.SERPING1 11:57381989:G:/
peptide level
209:209
98:98
18:18
DFTCVHQALKpc3_NP.4
p−maj=1, pX=0.68, N=325
var.24.SERPING1 11:57381989:G:/
peptide level
204:209
91:98
18:18
DFTCVHQALKpc3_NP.5
p−min=0.12, pX=0.34, N=313

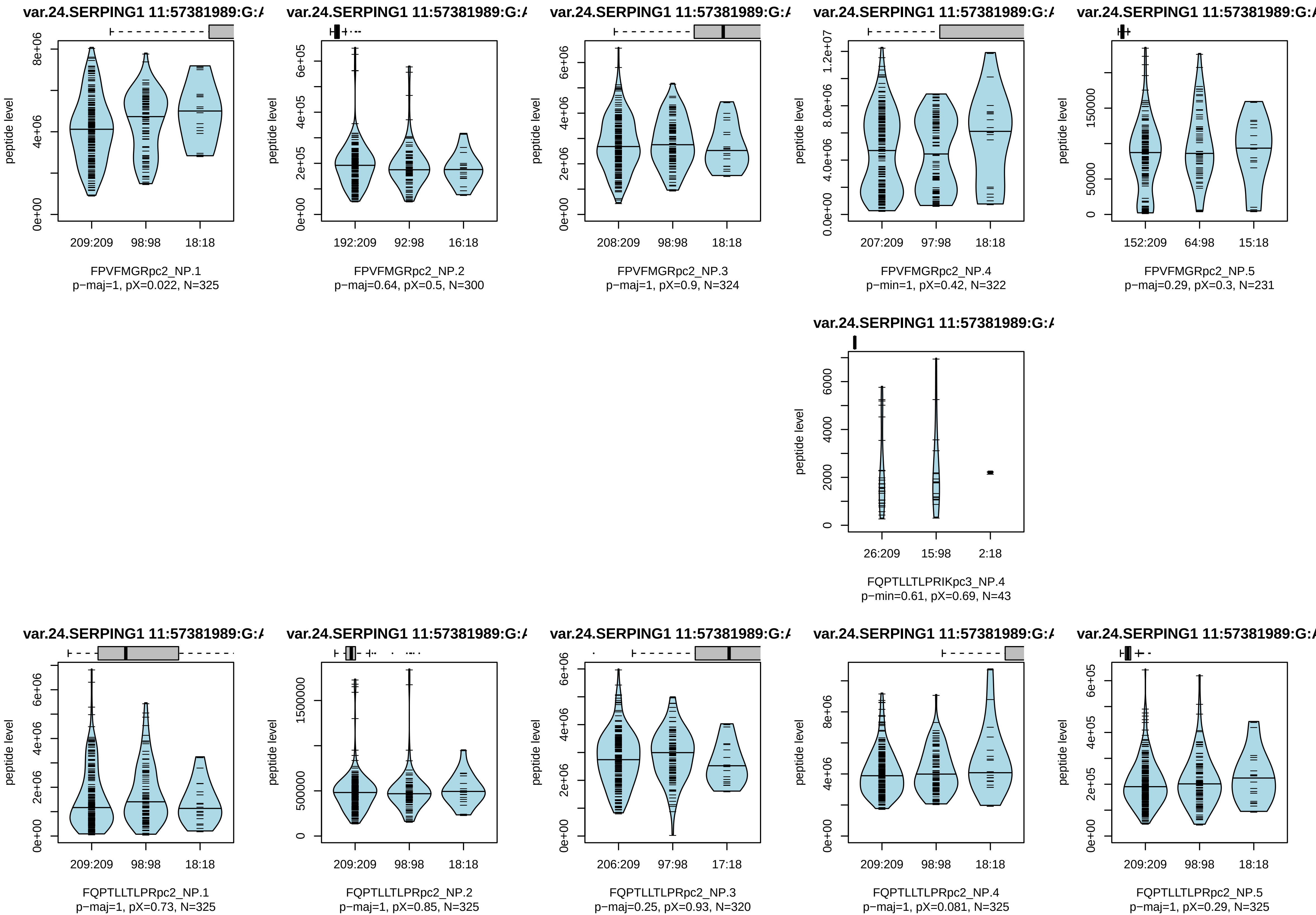

var.24.SERPING1 11:57381989:G:A
peptide level
209:209
98:98
18:18
FPVFMGRpc2_NP.1
p−maj=1, pX=0.022, N=325
var.24.SERPING1 11:57381989:G:A
peptide level
192:209
92:98
16:18
FPVFMGRpc2_NP.2
p−maj=0.64, pX=0.5, N=300
var.24.SERPING1 11:57381989:G:A
peptide level
208:209
98:98
18:18
FPVFMGRpc2_NP.3
p−maj=1, pX=0.9, N=324
var.24.SERPING1 11:57381989:G:A
peptide level
207:209
97:98
18:18
FPVFMGRpc2_NP.4
p−min=1, pX=0.42, N=322
var.24.SERPING1 11:57381989:G:A
peptide level
152:209
64:98
15:18
FPVFMGRpc2_NP.5
p−maj=0.29, pX=0.3, N=231
var.24.SERPING1 11:57381989:G:A
peptide level
26:209
15:98
2:18
FQPTLLTLPRIKpc3_NP.4
p−min=0.61, pX=0.69, N=43
var.24.SERPING1 11:57381989:G:A
peptide level
209:209
98:98
18:18
FQPTLLTLPRpc2_NP.1
p−maj=1, pX=0.73, N=325
var.24.SERPING1 11:57381989:G:A
peptide level
209:209
98:98
18:18
FQPTLLTLPRpc2_NP.2
p−maj=1, pX=0.85, N=325
var.24.SERPING1 11:57381989:G:A
peptide level
206:209
97:98
17:18
FQPTLLTLPRpc2_NP.3
p−maj=0.25, pX=0.93, N=320
var.24.SERPING1 11:57381989:G:A
peptide level
209:209
98:98
18:18
FQPTLLTLPRpc2_NP.4
p−maj=1, pX=0.081, N=325
var.24.SERPING1 11:57381989:G:A
peptide level
209:209
98:98
18:18
FQPTLLTLPRpc2_NP.5
p−maj=1, pX=0.29, N=325

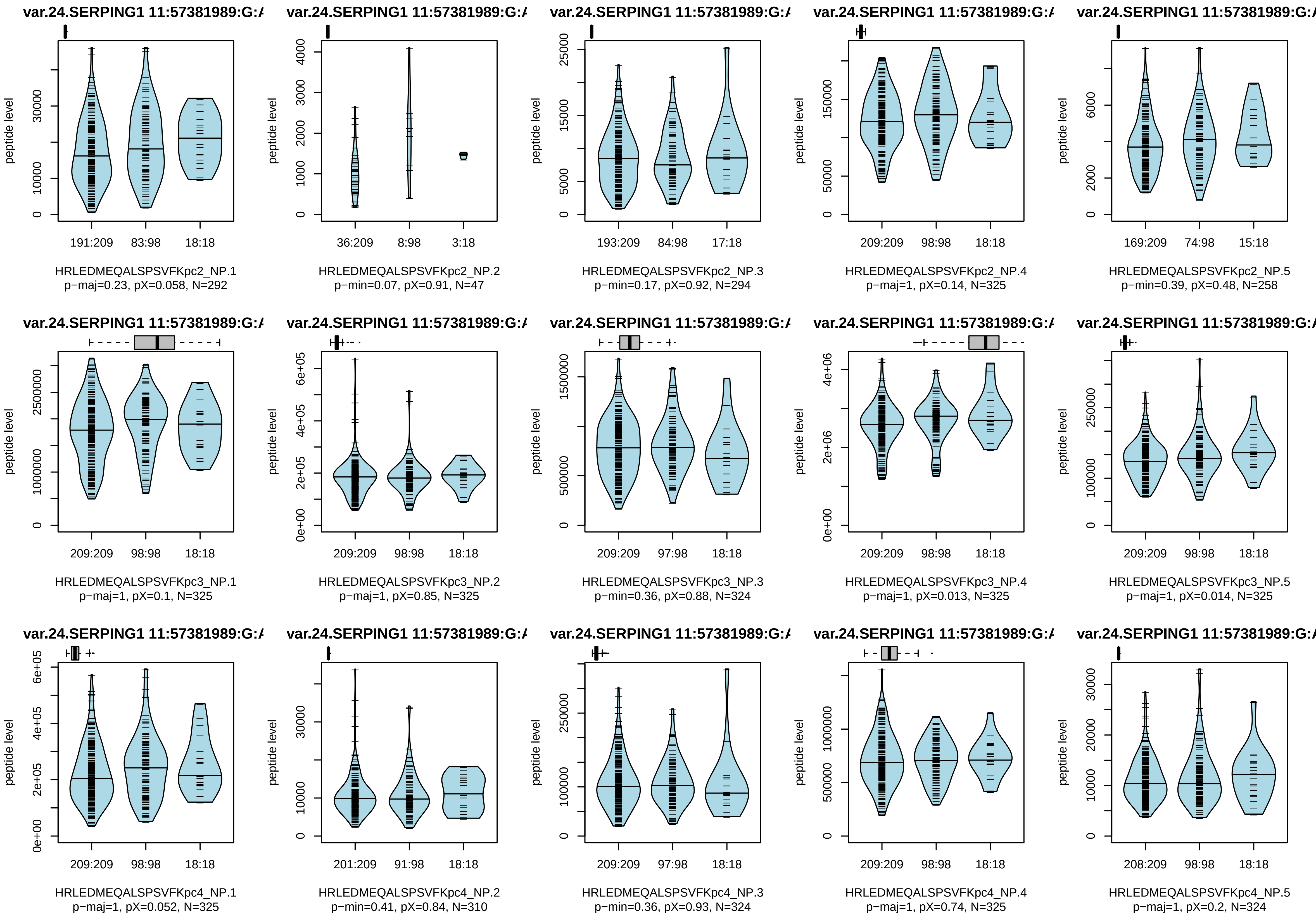

var.24.SERPING1 11:57381989:G:
peptide level
191:209 83:98 18:18
HRLEDMEQALSPSVFKpc2_NP.1
p−maj=0.23, pX=0.058, N=292
36:209 8:98 3:18
HRLEDMEQALSPSVFKpc2_NP.2
p−min=0.07, pX=0.91, N=47
193:209 84:98 17:18
HRLEDMEQALSPSVFKpc2_NP.3
p−min=0.17, pX=0.92, N=294
209:209 98:98 18:18
HRLEDMEQALSPSVFKpc2_NP.4
p−maj=1, pX=0.14, N=325
169:209 74:98 15:18
HRLEDMEQALSPSVFKpc2_NP.5
p−min=0.39, pX=0.48, N=258
209:209 98:98 18:18
HRLEDMEQALSPSVFKpc3_NP.1
p−maj=1, pX=0.1, N=325
209:209 98:98 18:18
HRLEDMEQALSPSVFKpc3_NP.2
p−maj=1, pX=0.85, N=325
209:209 97:98 18:18
HRLEDMEQALSPSVFKpc3_NP.3
p−min=0.36, pX=0.88, N=324
209:209 98:98 18:18
HRLEDMEQALSPSVFKpc3_NP.4
p−maj=1, pX=0.013, N=325
209:209 98:98 18:18
HRLEDMEQALSPSVFKpc3_NP.5
p−maj=1, pX=0.014, N=325
209:209 98:98 18:18
HRLEDMEQALSPSVFKpc4_NP.1
p−maj=1, pX=0.052, N=325
201:209 91:98 18:18
HRLEDMEQALSPSVFKpc4_NP.2
p−min=0.41, pX=0.84, N=310
209:209 97:98 18:18
HRLEDMEQALSPSVFKpc4_NP.3
p−min=0.36, pX=0.93, N=324
209:209 98:98 18:18
HRLEDMEQALSPSVFKpc4_NP.4
p−maj=1, pX=0.74, N=325
208:209 98:98 18:18
HRLEDMEQALSPSVFKpc4_NP.5
p−maj=1, pX=0.2, N=324

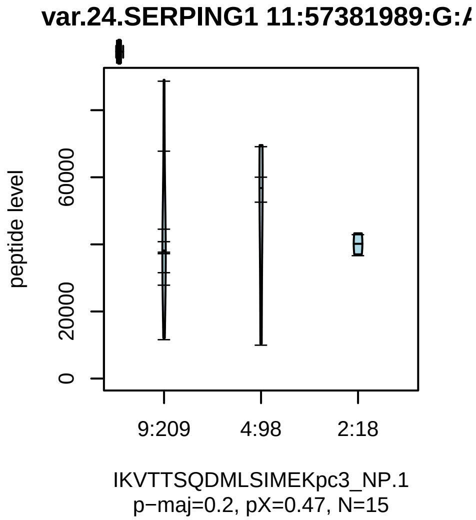
var.24.SERPING1 11:57381989:G:/
peptide level
0
20000
60000
9:209
4:98
2:18
IKVTTSQDMLSIMEKpc3_NP.1
p−maj=0.2, pX=0.47, N=15

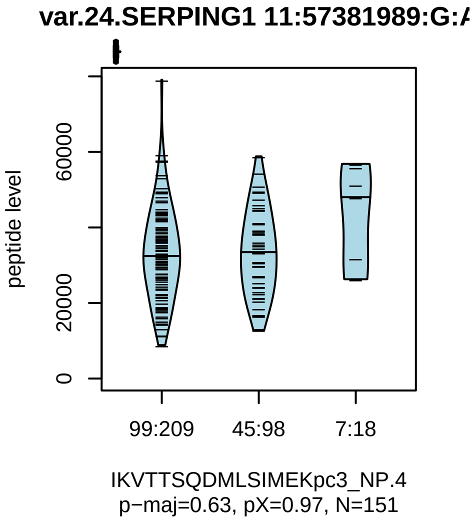
var.24.SERPING1 11:57381989:G:/
peptide level
0
20000
60000
99:209
45:98
7:18
IKVTTSQDMLSIMEKpc3_NP.4
p−maj=0.63, pX=0.97, N=151

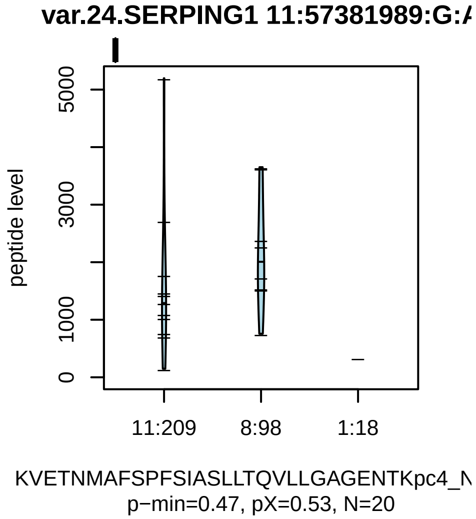
var.24.SERPING1 11:57381989:G:/
peptide level
0
1000
3000
5000
11:209
8:98
1:18
KVETNMAFSPFSIASLLTQVLLGAGENTKpc4_N
p−min=0.47, pX=0.53, N=20

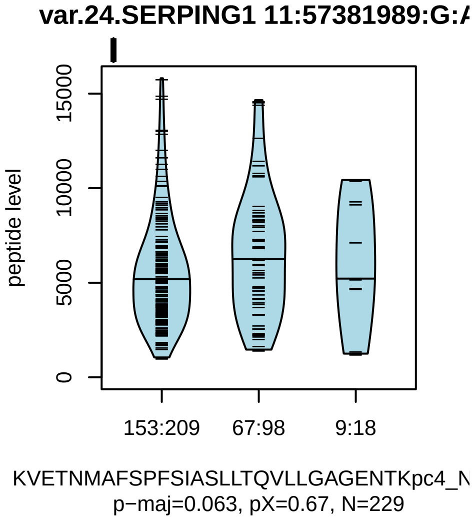
var.24.SERPING1 11:57381989:G:/
peptide level
0
5000
10000
15000
153:209
67:98
9:18
KVETNMAFSPFSIASLLTQVLLGAGENTKpc4_N
p−maj=0.063, pX=0.67, N=229

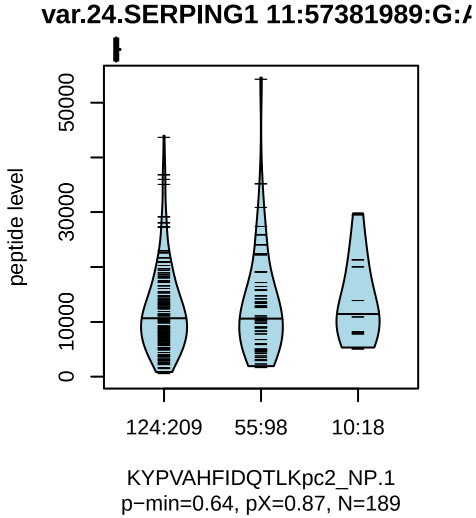
var.24.SERPING1 11:57381989:G:/
peptide level
0
10000
30000
50000
124:209
55:98
10:18
KYPVAHFIDQTLKpc2_NP.1
p−min=0.64, pX=0.87, N=189

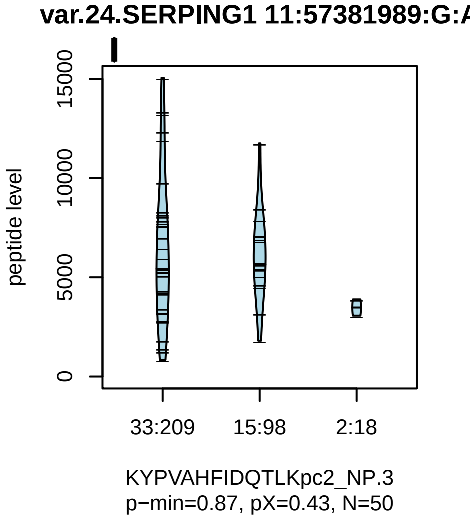
var.24.SERPING1 11:57381989:G:/
peptide level
0
5000
10000
15000
33:209
15:98
2:18
KYPVAHFIDQTLKpc2_NP.3
p−min=0.87, pX=0.43, N=50

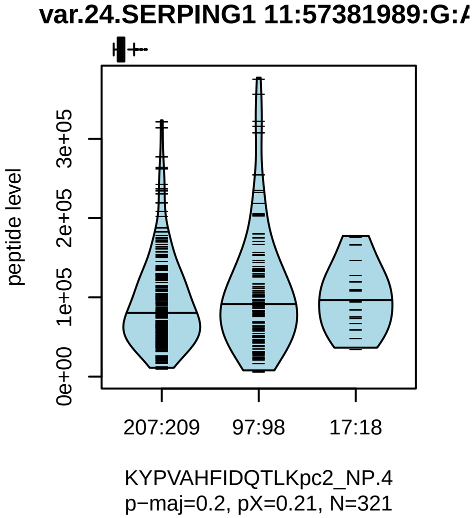
var.24.SERPING1 11:57381989:G:/
peptide level
0e+00
1e+05
2e+05
3e+05
207:209
97:98
17:18
KYPVAHFIDQTLKpc2_NP.4
p−maj=0.2, pX=0.21, N=321

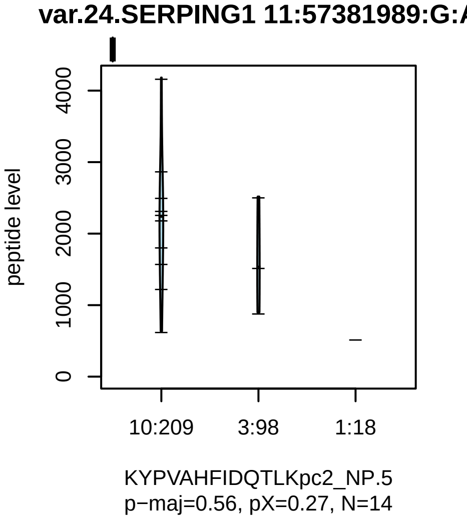
var.24.SERPING1 11:57381989:G:/
peptide level
0
1000
2000
3000
4000
10:209
3:98
1:18
KYPVAHFIDQTLKpc2_NP.5
p−maj=0.56, pX=0.27, N=14

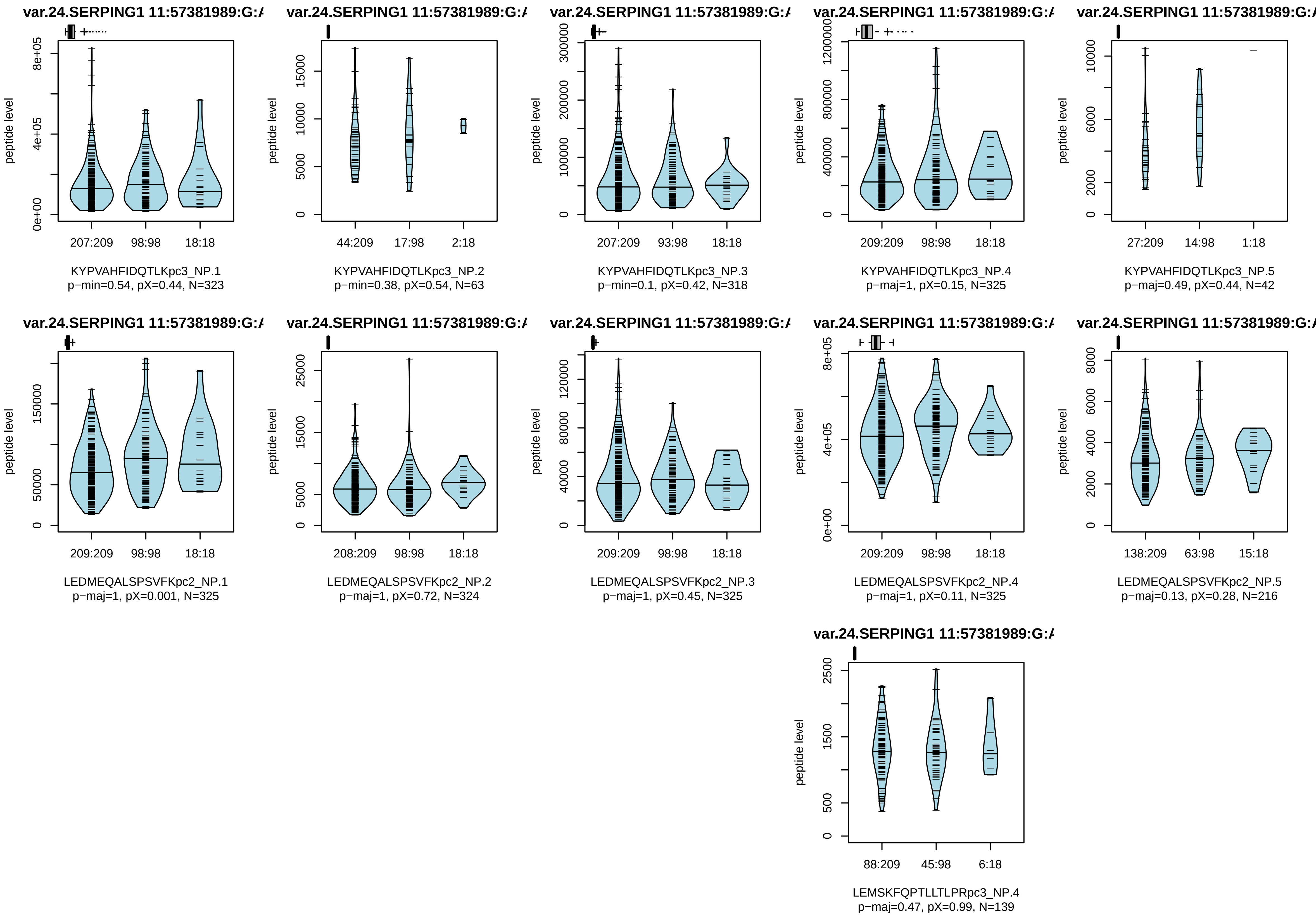

var.24.SERPING1 11:57381989:G:/
peptide level
0e+00
4e+05
8e+05
207:209
98:98
18:18
KYPVAHFIDQTLKpc3_NP.1
p−min=0.54, pX=0.44, N=323
var.24.SERPING1 11:57381989:G:/
peptide level
0
5000
10000
15000
44:209
17:98
2:18
KYPVAHFIDQTLKpc3_NP.2
p−min=0.38, pX=0.54, N=63
var.24.SERPING1 11:57381989:G:/
peptide level
0
100000
200000
300000
207:209
93:98
18:18
KYPVAHFIDQTLKpc3_NP.3
p−min=0.1, pX=0.42, N=318
var.24.SERPING1 11:57381989:G:/
peptide level
0
400000
800000
1200000
209:209
98:98
18:18
KYPVAHFIDQTLKpc3_NP.4
p−maj=1, pX=0.15, N=325
var.24.SERPING1 11:57381989:G:/
peptide level
0
2000
6000
10000
27:209
14:98
1:18
KYPVAHFIDQTLKpc3_NP.5
p−maj=0.49, pX=0.44, N=42
var.24.SERPING1 11:57381989:G:/
peptide level
0
50000
150000
209:209
98:98
18:18
LEDMEQALSPSVFKpc2_NP.1
p−maj=1, pX=0.001, N=325
var.24.SERPING1 11:57381989:G:/
peptide level
0
5000
15000
25000
208:209
98:98
18:18
LEDMEQALSPSVFKpc2_NP.2
p−maj=1, pX=0.72, N=324
var.24.SERPING1 11:57381989:G:/
peptide level
0
40000
80000
120000
209:209
98:98
18:18
LEDMEQALSPSVFKpc2_NP.3
p−maj=1, pX=0.45, N=325
var.24.SERPING1 11:57381989:G:/
peptide level
0e+00
4e+05
8e+05
209:209
98:98
18:18
LEDMEQALSPSVFKpc2_NP.4
p−maj=1, pX=0.11, N=325
var.24.SERPING1 11:57381989:G:/
peptide level
0
2000
4000
6000
8000
138:209
63:98
15:18
LEDMEQALSPSVFKpc2_NP.5
p−maj=0.13, pX=0.28, N=216
var.24.SERPING1 11:57381989:G:/
peptide level
0
500
1500
2500
88:209
45:98
6:18
LEMSKFQPTLLTLPRpc3_NP.4
p−maj=0.47, pX=0.99, N=139

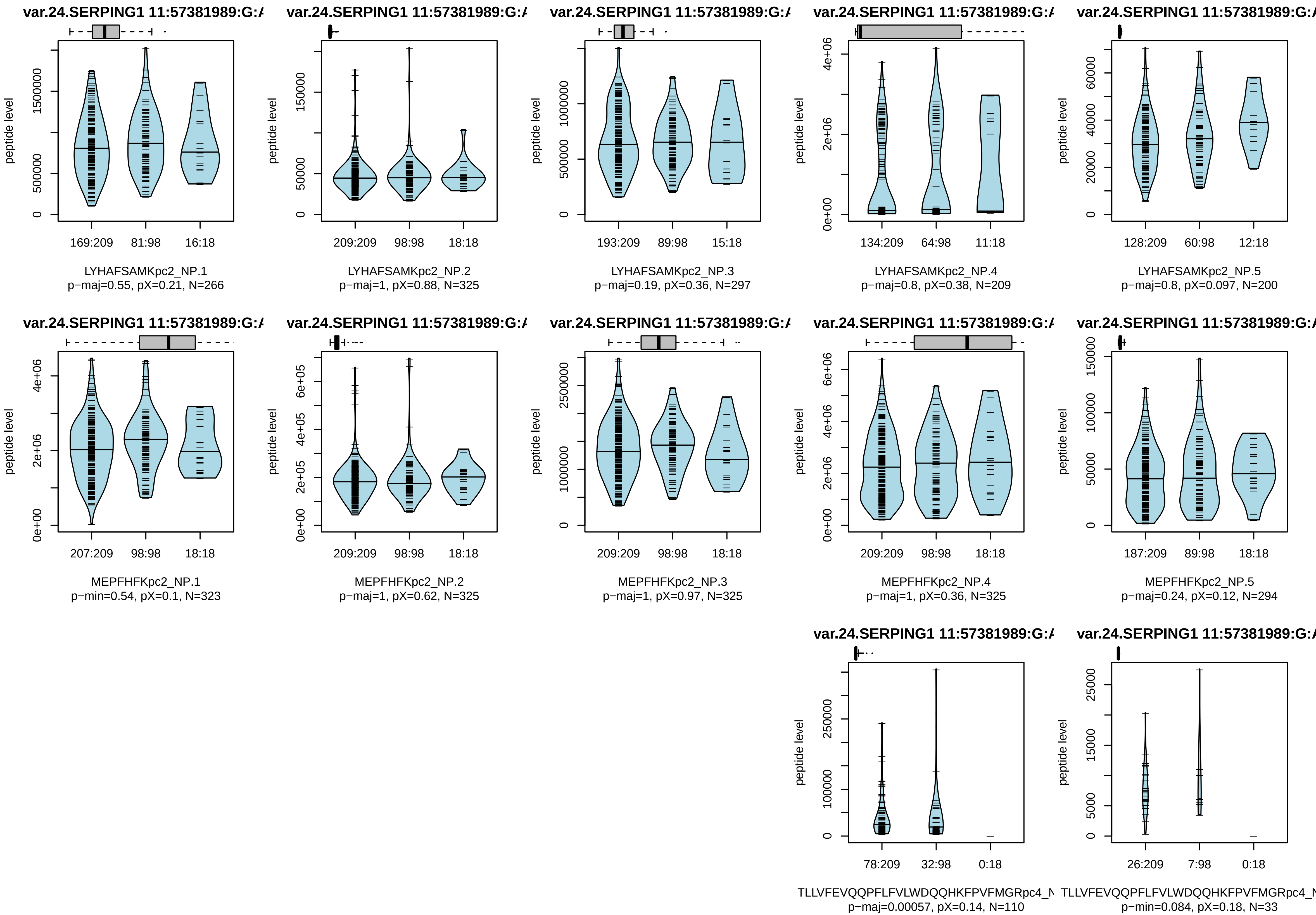
var.24.SERPING1 11:57381989:G:A
peptide level
169:209
81:98
16:18
LYHAFSAMKpc2_NP.1
p−maj=0.55, pX=0.21, N=266
var.24.SERPING1 11:57381989:G:A
peptide level
209:209
98:98
18:18
LYHAFSAMKpc2_NP.2
p−maj=1, pX=0.88, N=325
var.24.SERPING1 11:57381989:G:A
peptide level
193:209
89:98
15:18
LYHAFSAMKpc2_NP.3
p−maj=0.19, pX=0.36, N=297
var.24.SERPING1 11:57381989:G:A
peptide level
134:209
64:98
11:18
LYHAFSAMKpc2_NP.4
p−maj=0.8, pX=0.38, N=209
var.24.SERPING1 11:57381989:G:A
peptide level
128:209
60:98
12:18
LYHAFSAMKpc2_NP.5
p−maj=0.8, pX=0.097, N=200
var.24.SERPING1 11:57381989:G:A
peptide level
207:209
98:98
18:18
MEPFHFKpc2_NP.1
p−min=0.54, pX=0.1, N=323
var.24.SERPING1 11:57381989:G:A
peptide level
209:209
98:98
18:18
MEPFHFKpc2_NP.2
p−maj=1, pX=0.62, N=325
var.24.SERPING1 11:57381989:G:A
peptide level
209:209
98:98
18:18
MEPFHFKpc2_NP.3
p−maj=1, pX=0.97, N=325
var.24.SERPING1 11:57381989:G:A
peptide level
209:209
98:98
18:18
MEPFHFKpc2_NP.4
p−maj=1, pX=0.36, N=325
var.24.SERPING1 11:57381989:G:A
peptide level
187:209
89:98
18:18
MEPFHFKpc2_NP.5
p−maj=0.24, pX=0.12, N=294
var.24.SERPING1 11:57381989:G:A
peptide level
78:209
32:98
0:18
TLLVFEVQQPFLFVLWDQQHKFPVFMGRpc4_N
p−maj=0.00057, pX=0.14, N=110
var.24.SERPING1 11:57381989:G:A
peptide level
26:209
7:98
0:18
TLLVFEVQQPFLFVLWDQQHKFPVFMGRpc4_N
p−min=0.084, pX=0.18, N=33

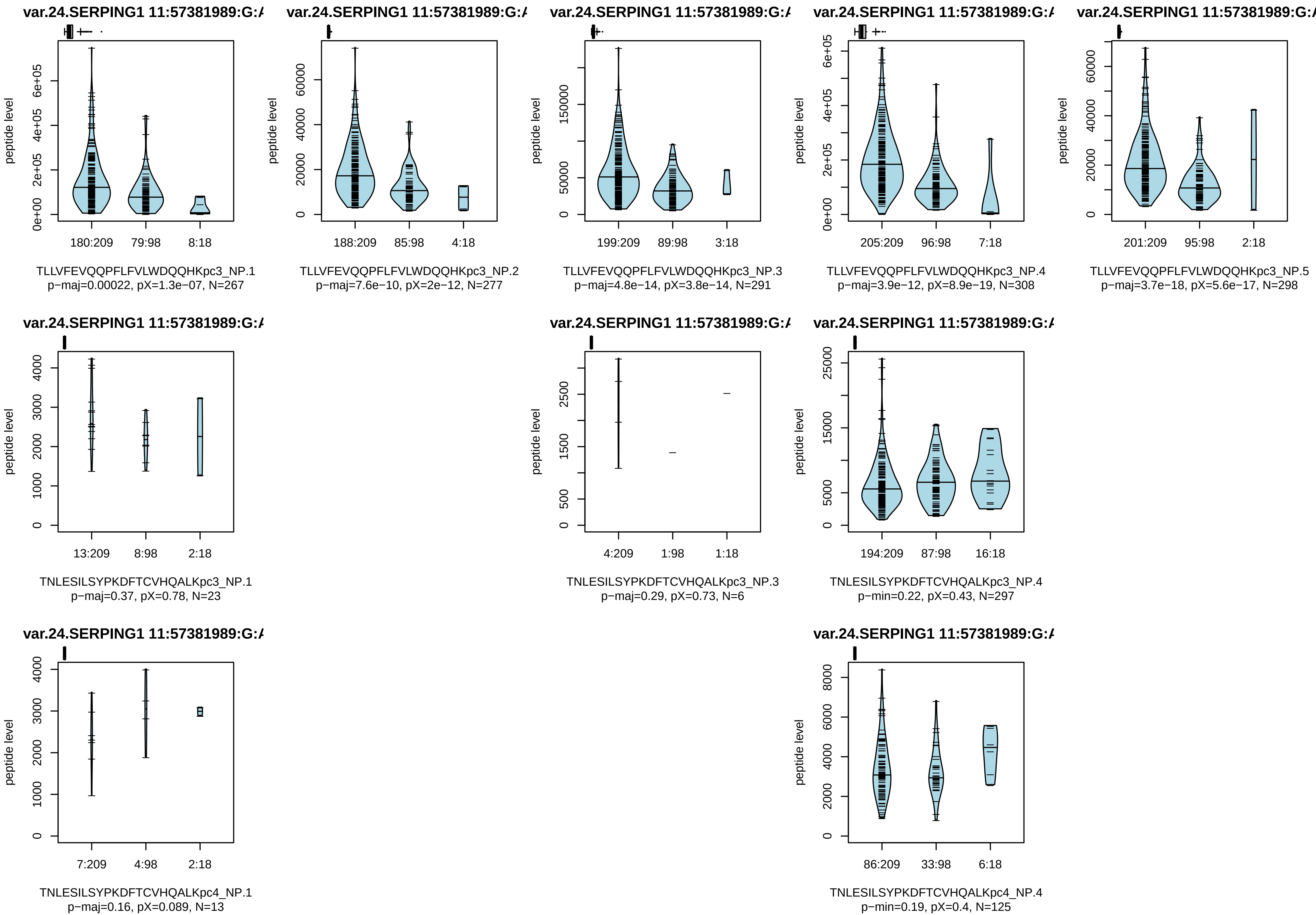
var.24.SERPING1 11:57381989:G:A
180:209
79:98
8:18
TLLVFEVQQPFLFVLWDQQHKpc3_NP.1
p−maj=0.00022, pX=1.3e−07, N=267
var.24.SERPING1 11:57381989:G:A
188:209
85:98
4:18
TLLVFEVQQPFLFVLWDQQHKpc3_NP.2
p−maj=7.6e−10, pX=2e−12, N=277
var.24.SERPING1 11:57381989:G:A
199:209
89:98
3:18
TLLVFEVQQPFLFVLWDQQHKpc3_NP.3
p−maj=4.8e−14, pX=3.8e−14, N=291
var.24.SERPING1 11:57381989:G:A
205:209
96:98
7:18
TLLVFEVQQPFLFVLWDQQHKpc3_NP.4
p−maj=3.9e−12, pX=8.9e−19, N=308
var.24.SERPING1 11:57381989:G:A
201:209
95:98
2:18
TLLVFEVQQPFLFVLWDQQHKpc3_NP.5
p−maj=3.7e−18, pX=5.6e−17, N=298
var.24.SERPING1 11:57381989:G:A
13:209
8:98
2:18
TNLESILSYPKDFTCVHQALKpc3_NP.1
p−maj=0.37, pX=0.78, N=23
var.24.SERPING1 11:57381989:G:A
4:209
1:98
1:18
TNLESILSYPKDFTCVHQALKpc3_NP.3
p−maj=0.29, pX=0.73, N=6
var.24.SERPING1 11:57381989:G:A
194:209
87:98
16:18
TNLESILSYPKDFTCVHQALKpc3_NP.4
p−min=0.22, pX=0.43, N=297
var.24.SERPING1 11:57381989:G:A
7:209
4:98
2:18
TNLESILSYPKDFTCVHQALKpc4_NP.1
p−maj=0.16, pX=0.089, N=13
var.24.SERPING1 11:57381989:G:A
86:209
33:98
6:18
TNLESILSYPKDFTCVHQALKpc4_NP.4
p−min=0.19, pX=0.4, N=125
peptide level

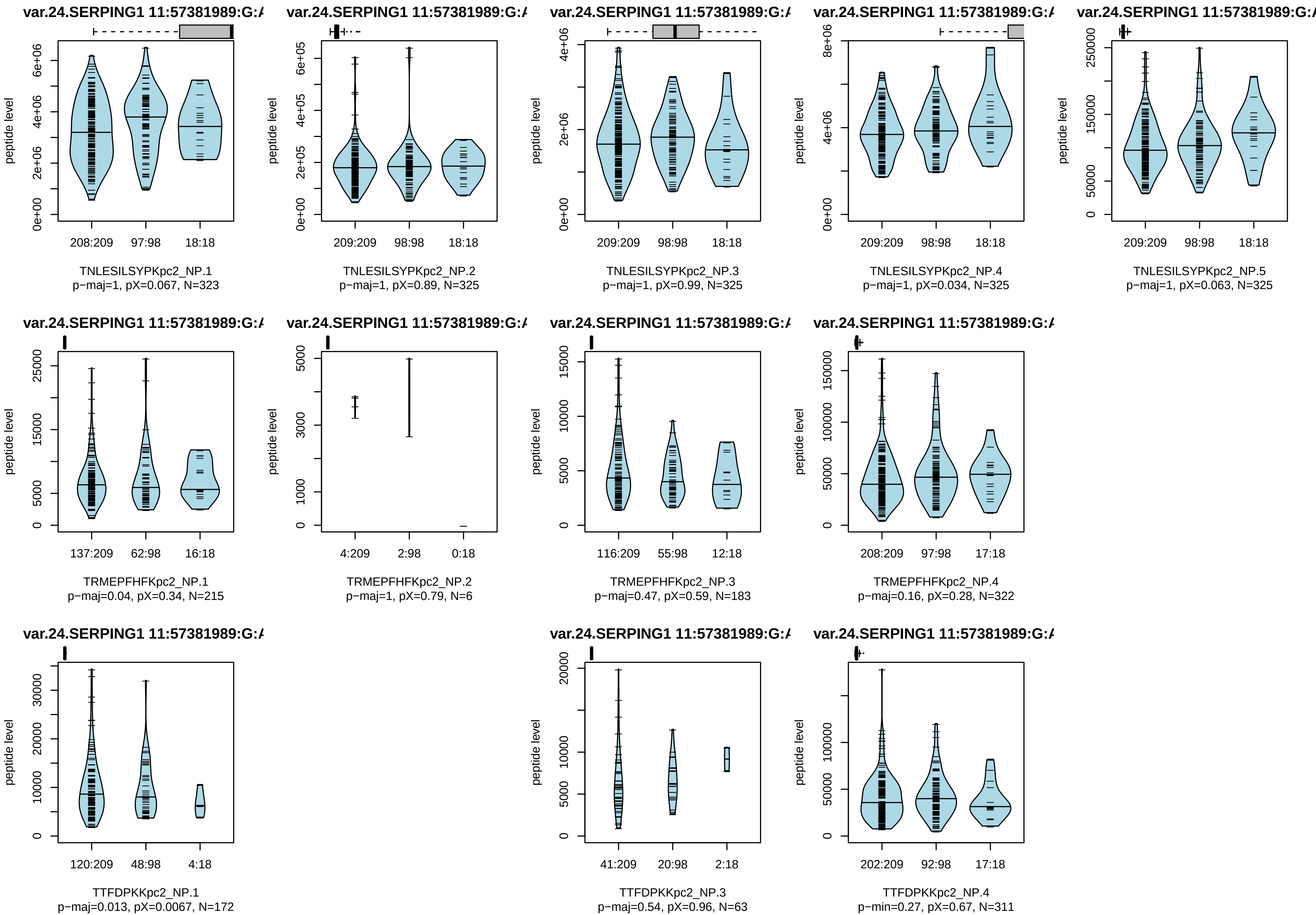
var.24.SERPING1 11:57381989:G:A
peptide level
208:209 97:98 18:18
TNLESILSYPKpc2_NP.1
p−maj=1, pX=0.067, N=323
var.24.SERPING1 11:57381989:G:A
peptide level
209:209 98:98 18:18
TNLESILSYPKpc2_NP.2
p−maj=1, pX=0.89, N=325
var.24.SERPING1 11:57381989:G:A
peptide level
209:209 98:98 18:18
TNLESILSYPKpc2_NP.3
p−maj=1, pX=0.99, N=325
var.24.SERPING1 11:57381989:G:A
peptide level
209:209 98:98 18:18
TNLESILSYPKpc2_NP.4
p−maj=1, pX=0.034, N=325
var.24.SERPING1 11:57381989:G:A
peptide level
209:209 98:98 18:18
TNLESILSYPKpc2_NP.5
p−maj=1, pX=0.063, N=325
var.24.SERPING1 11:57381989:G:A
peptide level
137:209 62:98 16:18
TRMEPFHFKpc2_NP.1
p−maj=0.04, pX=0.34, N=215
var.24.SERPING1 11:57381989:G:A
peptide level
4:209 2:98 0:18
TRMEPFHFKpc2_NP.2
p−maj=1, pX=0.79, N=6
var.24.SERPING1 11:57381989:G:A
peptide level
116:209 55:98 12:18
TRMEPFHFKpc2_NP.3
p−maj=0.47, pX=0.59, N=183
var.24.SERPING1 11:57381989:G:A
peptide level
208:209 97:98 17:18
TRMEPFHFKpc2_NP.4
p−maj=0.16, pX=0.28, N=322
var.24.SERPING1 11:57381989:G:A
peptide level
120:209 48:98 4:18
TTFDPKKpc2_NP.1
p−maj=0.013, pX=0.0067, N=172
var.24.SERPING1 11:57381989:G:A
peptide level
41:209 20:98 2:18
TTFDPKKpc2_NP.3
p−maj=0.54, pX=0.96, N=63
var.24.SERPING1 11:57381989:G:A
peptide level
202:209 92:98 17:18
TTFDPKKpc2_NP.4
p−min=0.27, pX=0.67, N=311

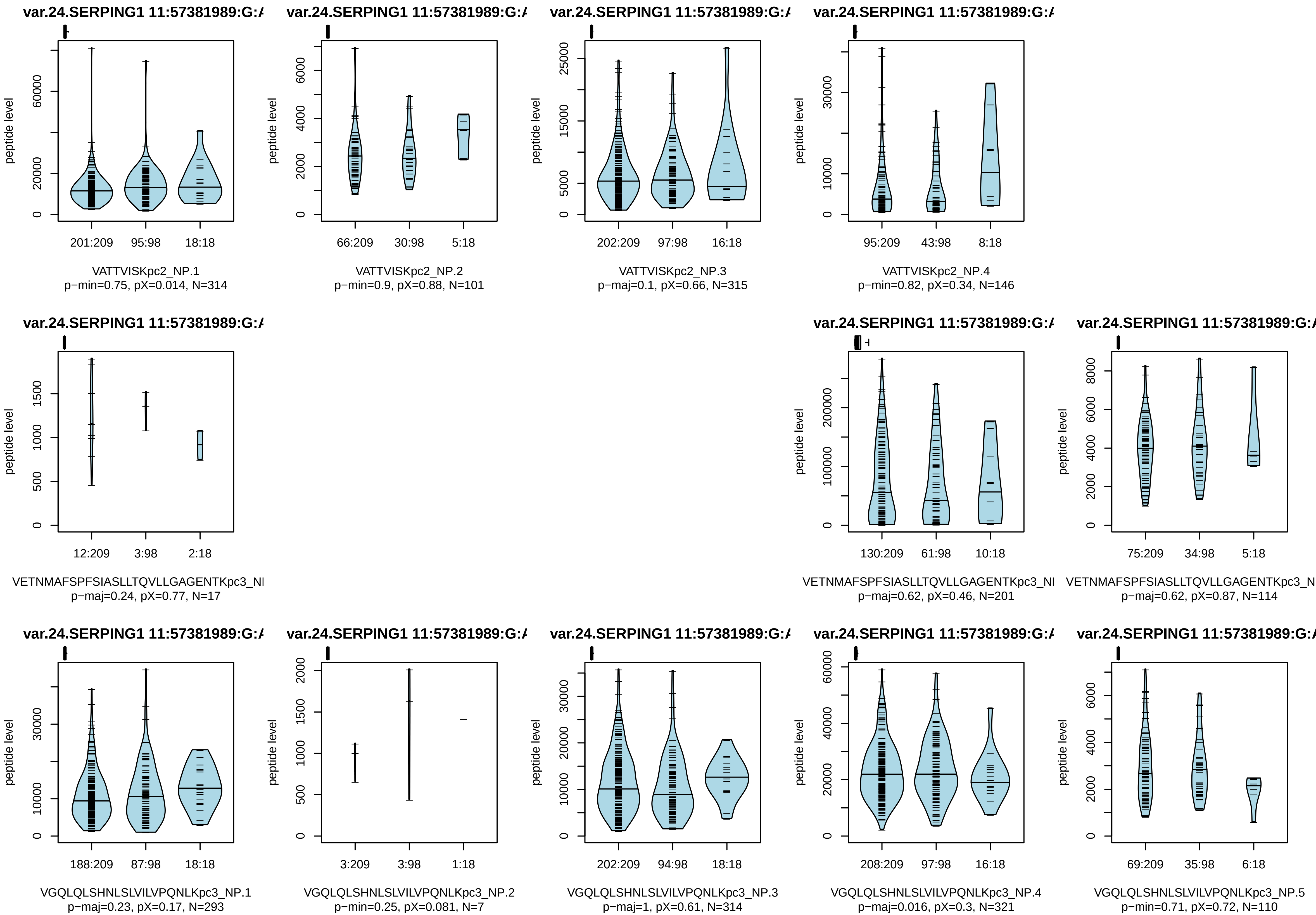

var.24.SERPING1 11:57381989:G:A
peptide level
201:209
95:98
18:18
VATTVISKpc2_NP.1
p−min=0.75, pX=0.014, N=314
var.24.SERPING1 11:57381989:G:A
peptide level
66:209
30:98
5:18
VATTVISKpc2_NP.2
p−min=0.9, pX=0.88, N=101
var.24.SERPING1 11:57381989:G:A
peptide level
202:209
97:98
16:18
VATTVISKpc2_NP.3
p−maj=0.1, pX=0.66, N=315
var.24.SERPING1 11:57381989:G:A
peptide level
95:209
43:98
8:18
VATTVISKpc2_NP.4
p−min=0.82, pX=0.34, N=146
var.24.SERPING1 11:57381989:G:A
peptide level
12:209
3:98
2:18
VETNMAFSPFSIASLLTQVLLGAGENTKpc3_NI
p−maj=0.24, pX=0.77, N=17
var.24.SERPING1 11:57381989:G:A
peptide level
130:209
61:98
10:18
VETNMAFSPFSIASLLTQVLLGAGENTKpc3_NI
p−maj=0.62, pX=0.46, N=201
var.24.SERPING1 11:57381989:G:A
peptide level
75:209
34:98
5:18
VETNMAFSPFSIASLLTQVLLGAGENTKpc3_N
p−maj=0.62, pX=0.87, N=114
var.24.SERPING1 11:57381989:G:A
peptide level
188:209
87:98
18:18
VGQLQLSHNLSLVILVPQNLKpc3_NP.1
p−maj=0.23, pX=0.17, N=293
var.24.SERPING1 11:57381989:G:A
peptide level
3:209
3:98
1:18
VGQLQLSHNLSLVILVPQNLKpc3_NP.2
p−min=0.25, pX=0.081, N=7
var.24.SERPING1 11:57381989:G:A
peptide level
202:209
94:98
18:18
VGQLQLSHNLSLVILVPQNLKpc3_NP.3
p−maj=1, pX=0.61, N=314
var.24.SERPING1 11:57381989:G:A
peptide level
208:209
97:98
16:18
VGQLQLSHNLSLVILVPQNLKpc3_NP.4
p−maj=0.016, pX=0.3, N=321
var.24.SERPING1 11:57381989:G:A
peptide level
69:209
35:98
6:18
VGQLQLSHNLSLVILVPQNLKpc3_NP.5
p−min=0.71, pX=0.72, N=110

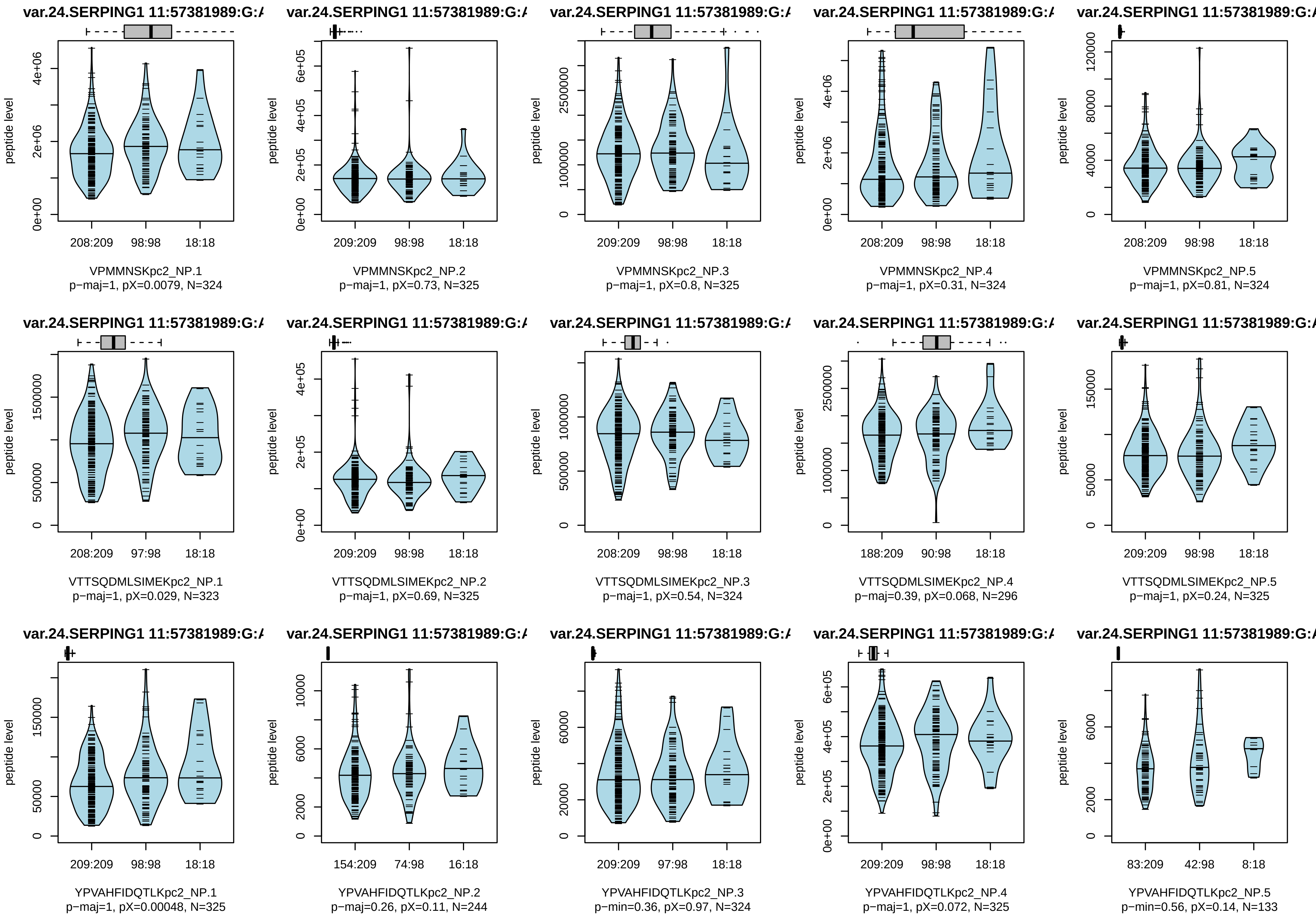
var.24.SERPING1 11:57381989:G:A
peptide level
208:209
98:98
18:18
VPMMNSKpc2_NP.1
p−maj=1, pX=0.0079, N=324
var.24.SERPING1 11:57381989:G:A
peptide level
209:209
98:98
18:18
VPMMNSKpc2_NP.2
p−maj=1, pX=0.73, N=325
var.24.SERPING1 11:57381989:G:A
peptide level
209:209
98:98
18:18
VPMMNSKpc2_NP.3
p−maj=1, pX=0.8, N=325
var.24.SERPING1 11:57381989:G:A
peptide level
208:209
98:98
18:18
VPMMNSKpc2_NP.4
p−maj=1, pX=0.31, N=324
var.24.SERPING1 11:57381989:G:A
peptide level
208:209
98:98
18:18
VPMMNSKpc2_NP.5
p−maj=1, pX=0.81, N=324
var.24.SERPING1 11:57381989:G:A
peptide level
208:209
97:98
18:18
VTTSQDMLSIMEKpc2_NP.1
p−maj=1, pX=0.029, N=323
var.24.SERPING1 11:57381989:G:A
peptide level
209:209
98:98
18:18
VTTSQDMLSIMEKpc2_NP.2
p−maj=1, pX=0.69, N=325
var.24.SERPING1 11:57381989:G:A
peptide level
208:209
98:98
18:18
VTTSQDMLSIMEKpc2_NP.3
p−maj=1, pX=0.54, N=324
var.24.SERPING1 11:57381989:G:A
peptide level
188:209
90:98
18:18
VTTSQDMLSIMEKpc2_NP.4
p−maj=0.39, pX=0.068, N=296
var.24.SERPING1 11:57381989:G:A
peptide level
209:209
98:98
18:18
VTTSQDMLSIMEKpc2_NP.5
p−maj=1, pX=0.24, N=325
var.24.SERPING1 11:57381989:G:A
peptide level
209:209
98:98
18:18
YPVAHFIDQTLKpc2_NP.1
p−maj=1, pX=0.00048, N=325
var.24.SERPING1 11:57381989:G:A
peptide level
154:209
74:98
16:18
YPVAHFIDQTLKpc2_NP.2
p−maj=0.26, pX=0.11, N=244
var.24.SERPING1 11:57381989:G:A
peptide level
209:209
97:98
18:18
YPVAHFIDQTLKpc2_NP.3
p−min=0.36, pX=0.97, N=324
var.24.SERPING1 11:57381989:G:A
peptide level
209:209
98:98
18:18
YPVAHFIDQTLKpc2_NP.4
p−maj=1, pX=0.072, N=325
var.24.SERPING1 11:57381989:G:A
peptide level
83:209
42:98
8:18
YPVAHFIDQTLKpc2_NP.5
p−min=0.56, pX=0.14, N=133

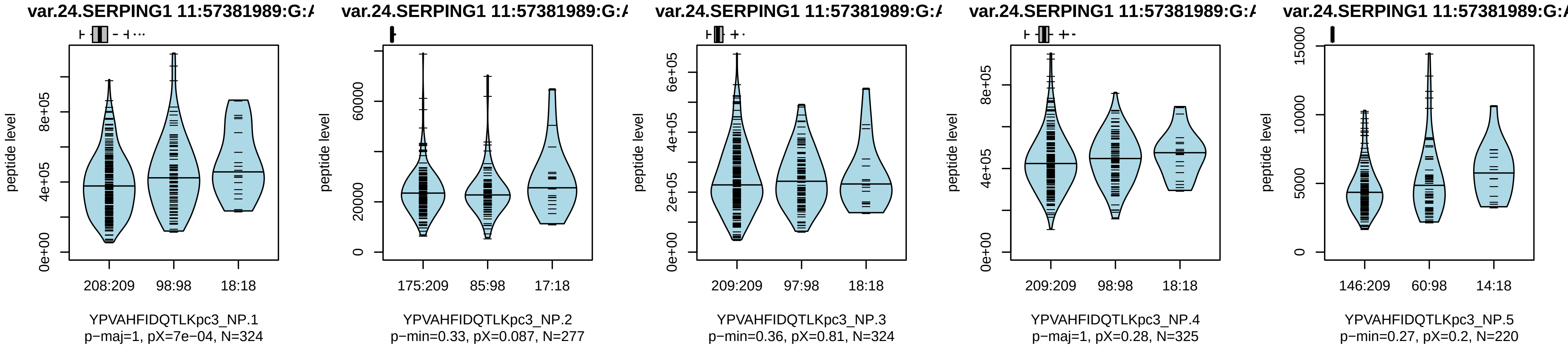
var.24.SERPING1 11:57381989:G:A
peptide level
208:209
98:98
18:18
YPVAHFIDQTLKpc3_NP.1
p−maj=1, pX=7e−04, N=324
var.24.SERPING1 11:57381989:G:A
peptide level
175:209
85:98
17:18
YPVAHFIDQTLKpc3_NP.2
p−min=0.33, pX=0.087, N=277
var.24.SERPING1 11:57381989:G:A
peptide level
209:209
97:98
18:18
YPVAHFIDQTLKpc3_NP.3
p−min=0.36, pX=0.81, N=324
var.24.SERPING1 11:57381989:G:A
peptide level
209:209
98:98
18:18
YPVAHFIDQTLKpc3_NP.4
p−maj=1, pX=0.28, N=325
var.24.SERPING1 11:57381989:G:A
peptide level
146:209
60:98
14:18
YPVAHFIDQTLKpc3_NP.5
p−min=0.27, pX=0.2, N=220

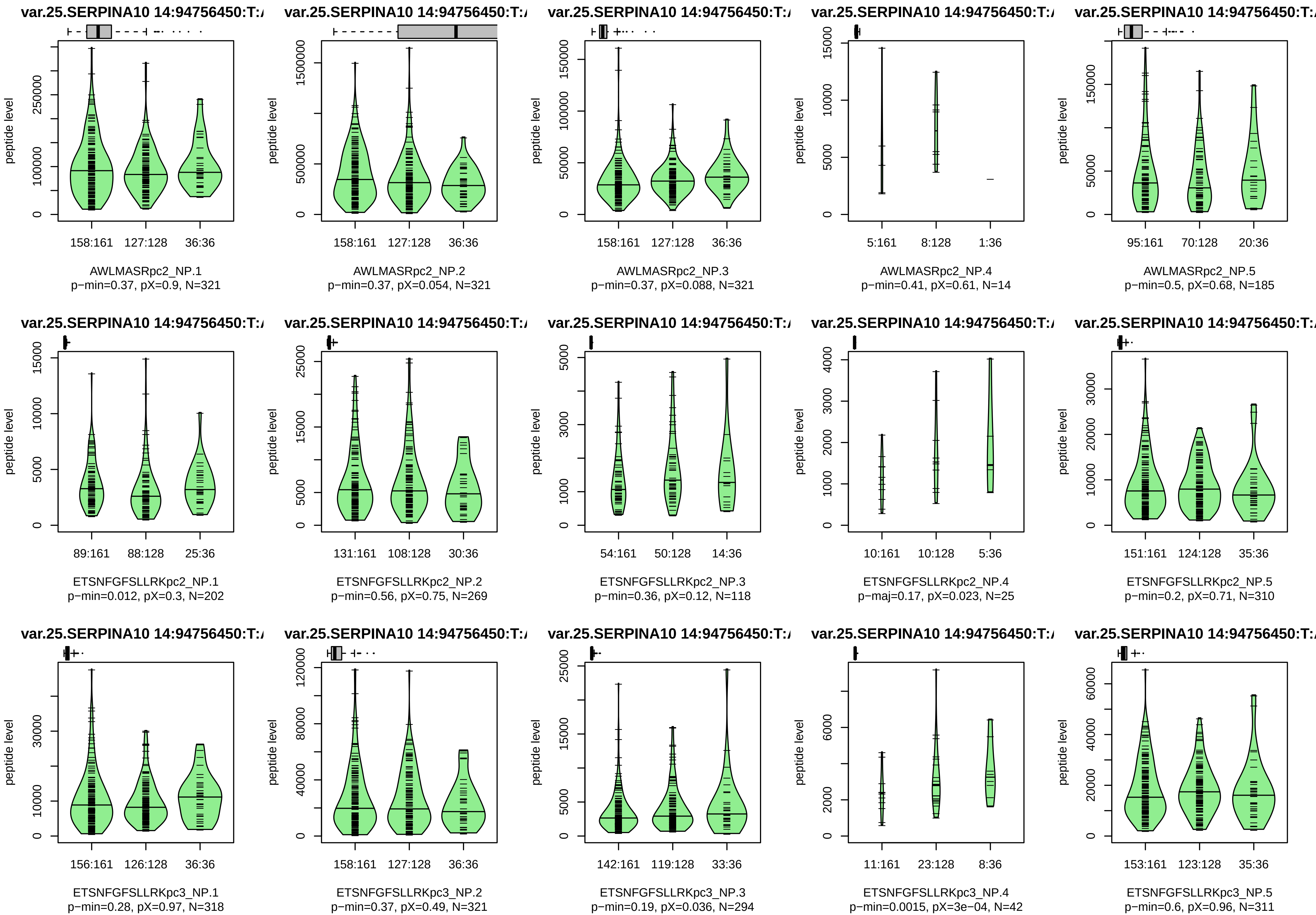
var.25.SERPINA10 14:94756450:T:
peptide level
158:161
127:128
36:36
AWLMASRpc2_NP.1
p−min=0.37, pX=0.9, N=321
158:161
127:128
36:36
AWLMASRpc2_NP.2
p−min=0.37, pX=0.054, N=321
158:161
127:128
36:36
AWLMASRpc2_NP.3
p−min=0.37, pX=0.088, N=321
5:161
8:128
1:36
AWLMASRpc2_NP.4
p−min=0.41, pX=0.61, N=14
95:161
70:128
20:36
AWLMASRpc2_NP.5
p−min=0.5, pX=0.68, N=185
89:161
88:128
25:36
ETSNFGFSLLRKpc2_NP.1
p−min=0.012, pX=0.3, N=202
131:161
108:128
30:36
ETSNFGFSLLRKpc2_NP.2
p−min=0.56, pX=0.75, N=269
54:161
50:128
14:36
ETSNFGFSLLRKpc2_NP.3
p−min=0.36, pX=0.12, N=118
10:161
10:128
5:36
ETSNFGFSLLRKpc2_NP.4
p−maj=0.17, pX=0.023, N=25
151:161
124:128
35:36
ETSNFGFSLLRKpc2_NP.5
p−min=0.2, pX=0.71, N=310
156:161
126:128
36:36
ETSNFGFSLLRKpc3_NP.1
p−min=0.28, pX=0.97, N=318
158:161
127:128
36:36
ETSNFGFSLLRKpc3_NP.2
p−min=0.37, pX=0.49, N=321
142:161
119:128
33:36
ETSNFGFSLLRKpc3_NP.3
p−min=0.19, pX=0.036, N=294
11:161
23:128
8:36
ETSNFGFSLLRKpc3_NP.4
p−min=0.0015, pX=3e−04, N=42
153:161
123:128
35:36
ETSNFGFSLLRKpc3_NP.5
p−min=0.6, pX=0.96, N=311

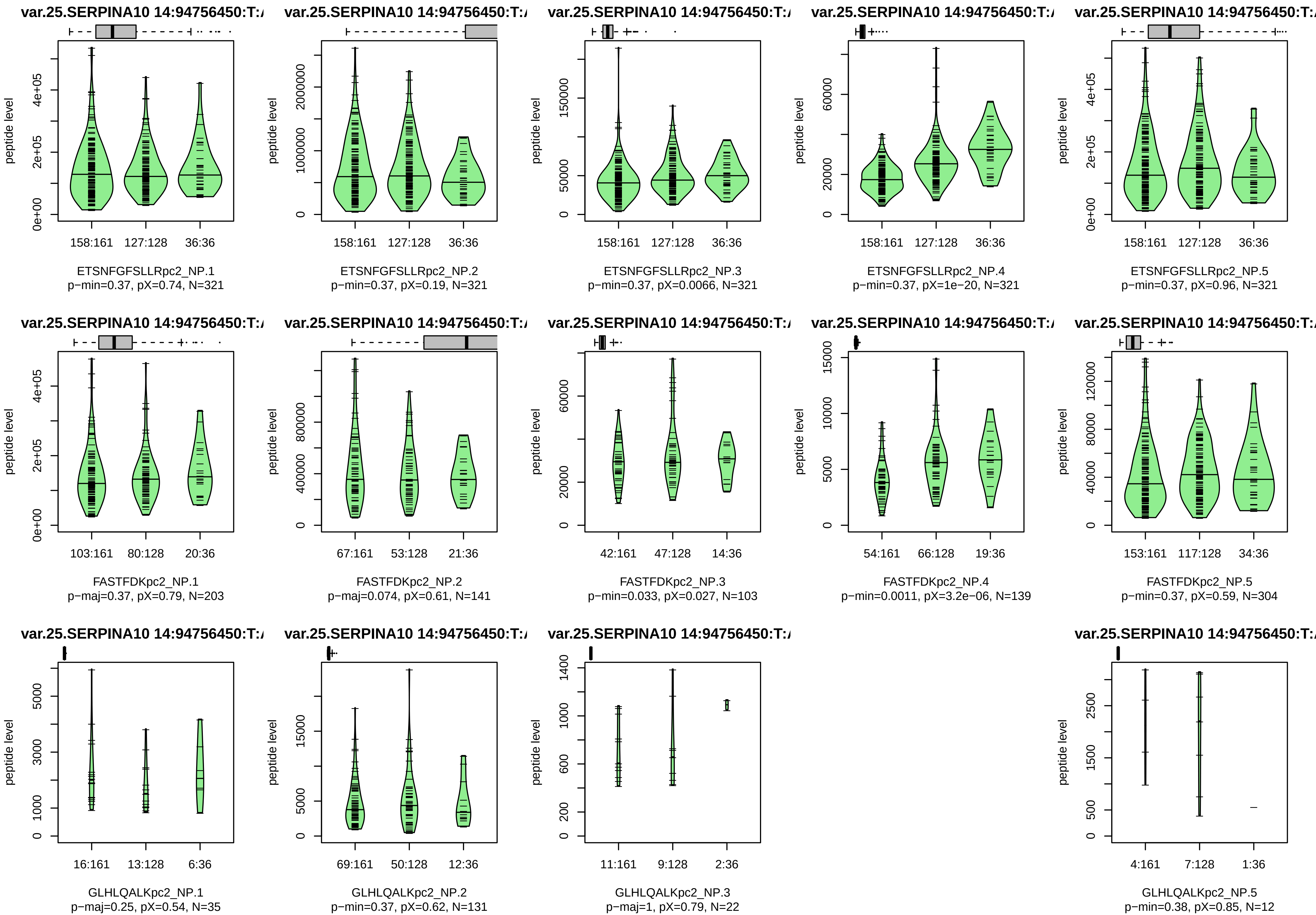

var.25.SERPINA10 14:94756450:T:/
peptide level
158:161
127:128
36:36
ETSNFGFSLLRpc2_NP.1
p−min=0.37, pX=0.74, N=321
ETSNFGFSLLRpc2_NP.2
p−min=0.37, pX=0.19, N=321
ETSNFGFSLLRpc2_NP.3
p−min=0.37, pX=0.0066, N=321
ETSNFGFSLLRpc2_NP.4
p−min=0.37, pX=1e−20, N=321
ETSNFGFSLLRpc2_NP.5
p−min=0.37, pX=0.96, N=321
103:161
80:128
20:36
FASTFDKpc2_NP.1
p−maj=0.37, pX=0.79, N=203
67:161
53:128
21:36
FASTFDKpc2_NP.2
p−maj=0.074, pX=0.61, N=141
42:161
47:128
14:36
FASTFDKpc2_NP.3
p−min=0.033, pX=0.027, N=103
54:161
66:128
19:36
FASTFDKpc2_NP.4
p−min=0.0011, pX=3.2e−06, N=139
153:161
117:128
34:36
FASTFDKpc2_NP.5
p−min=0.37, pX=0.59, N=304
16:161
13:128
6:36
GLHLQALKpc2_NP.1
p−maj=0.25, pX=0.54, N=35
69:161
50:128
12:36
GLHLQALKpc2_NP.2
p−min=0.37, pX=0.62, N=131
11:161
9:128
2:36
GLHLQALKpc2_NP.3
p−maj=1, pX=0.79, N=22
4:161
7:128
1:36
GLHLQALKpc2_NP.5
p−min=0.38, pX=0.85, N=12

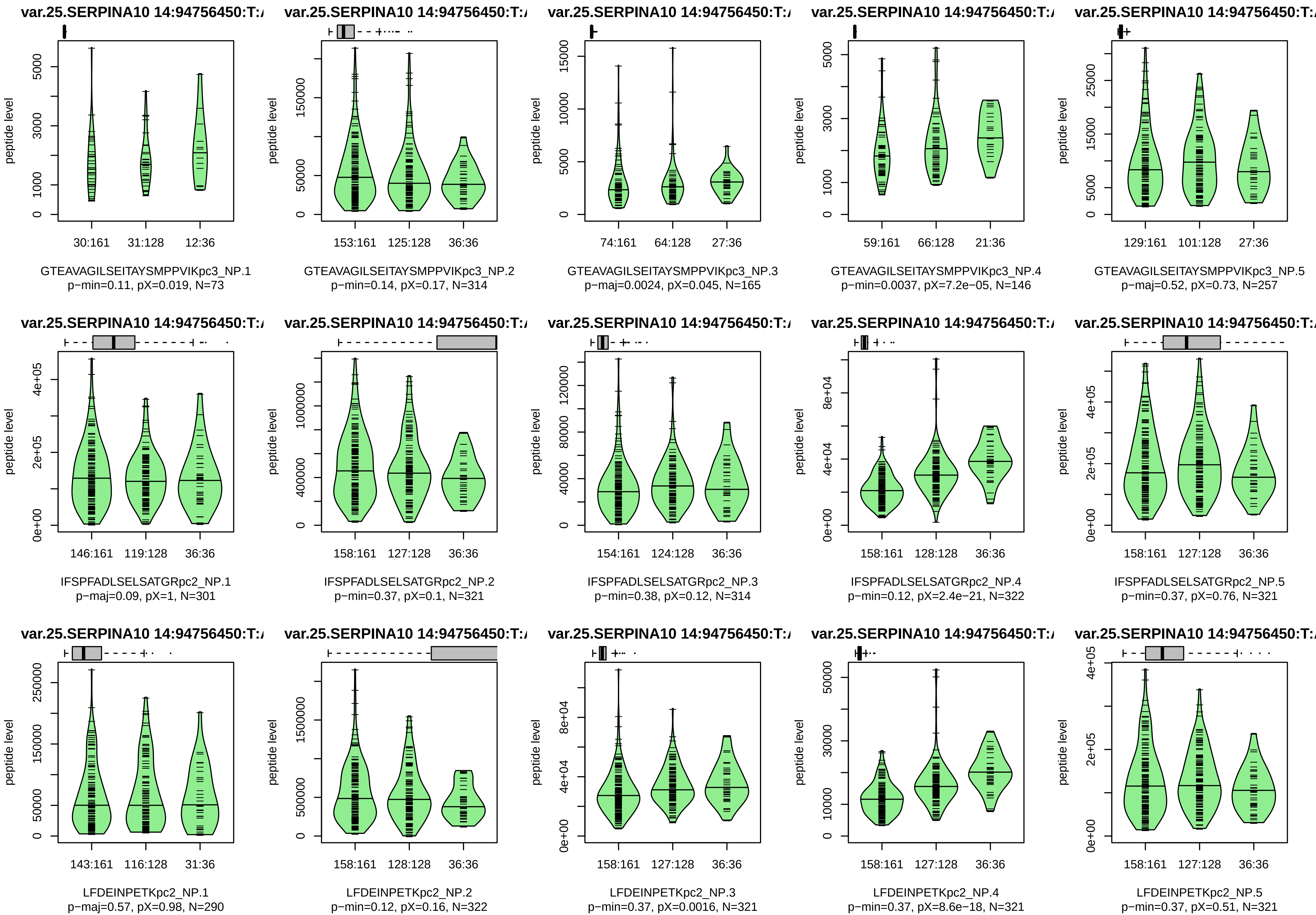
var.25.SERPINA10 14:94756450:T:/
peptide level
30:161
31:128
12:36
GTEAVAGILSEITAYSMPPVIKpc3_NP.1
p−min=0.11, pX=0.019, N=73
var.25.SERPINA10 14:94756450:T:/
peptide level
153:161
125:128
36:36
GTEAVAGILSEITAYSMPPVIKpc3_NP.2
p−min=0.14, pX=0.17, N=314
var.25.SERPINA10 14:94756450:T:/
peptide level
74:161
64:128
27:36
GTEAVAGILSEITAYSMPPVIKpc3_NP.3
p−maj=0.0024, pX=0.045, N=165
var.25.SERPINA10 14:94756450:T:/
peptide level
59:161
66:128
21:36
GTEAVAGILSEITAYSMPPVIKpc3_NP.4
p−min=0.0037, pX=7.2e−05, N=146
var.25.SERPINA10 14:94756450:T:/
peptide level
129:161
101:128
27:36
GTEAVAGILSEITAYSMPPVIKpc3_NP.5
p−maj=0.52, pX=0.73, N=257
var.25.SERPINA10 14:94756450:T:/
peptide level
146:161
119:128
36:36
IFSPFADLSELSATGRpc2_NP.1
p−maj=0.09, pX=1, N=301
var.25.SERPINA10 14:94756450:T:/
peptide level
158:161
127:128
36:36
IFSPFADLSELSATGRpc2_NP.2
p−min=0.37, pX=0.1, N=321
var.25.SERPINA10 14:94756450:T:/
peptide level
154:161
124:128
36:36
IFSPFADLSELSATGRpc2_NP.3
p−min=0.38, pX=0.12, N=314
var.25.SERPINA10 14:94756450:T:/
peptide level
158:161
128:128
36:36
IFSPFADLSELSATGRpc2_NP.4
p−min=0.12, pX=2.4e−21, N=322
var.25.SERPINA10 14:94756450:T:/
peptide level
158:161
127:128
36:36
IFSPFADLSELSATGRpc2_NP.5
p−min=0.37, pX=0.76, N=321
var.25.SERPINA10 14:94756450:T:/
peptide level
143:161
116:128
31:36
LFDEINPETKpc2_NP.1
p−maj=0.57, pX=0.98, N=290
var.25.SERPINA10 14:94756450:T:/
peptide level
158:161
128:128
36:36
LFDEINPETKpc2_NP.2
p−min=0.12, pX=0.16, N=322
var.25.SERPINA10 14:94756450:T:/
peptide level
158:161
127:128
36:36
LFDEINPETKpc2_NP.3
p−min=0.37, pX=0.0016, N=321
var.25.SERPINA10 14:94756450:T:/
peptide level
158:161
127:128
36:36
LFDEINPETKpc2_NP.4
p−min=0.37, pX=8.6e−18, N=321
var.25.SERPINA10 14:94756450:T:/
peptide level
158:161
127:128
36:36
LFDEINPETKpc2_NP.5
p−min=0.37, pX=0.51, N=321

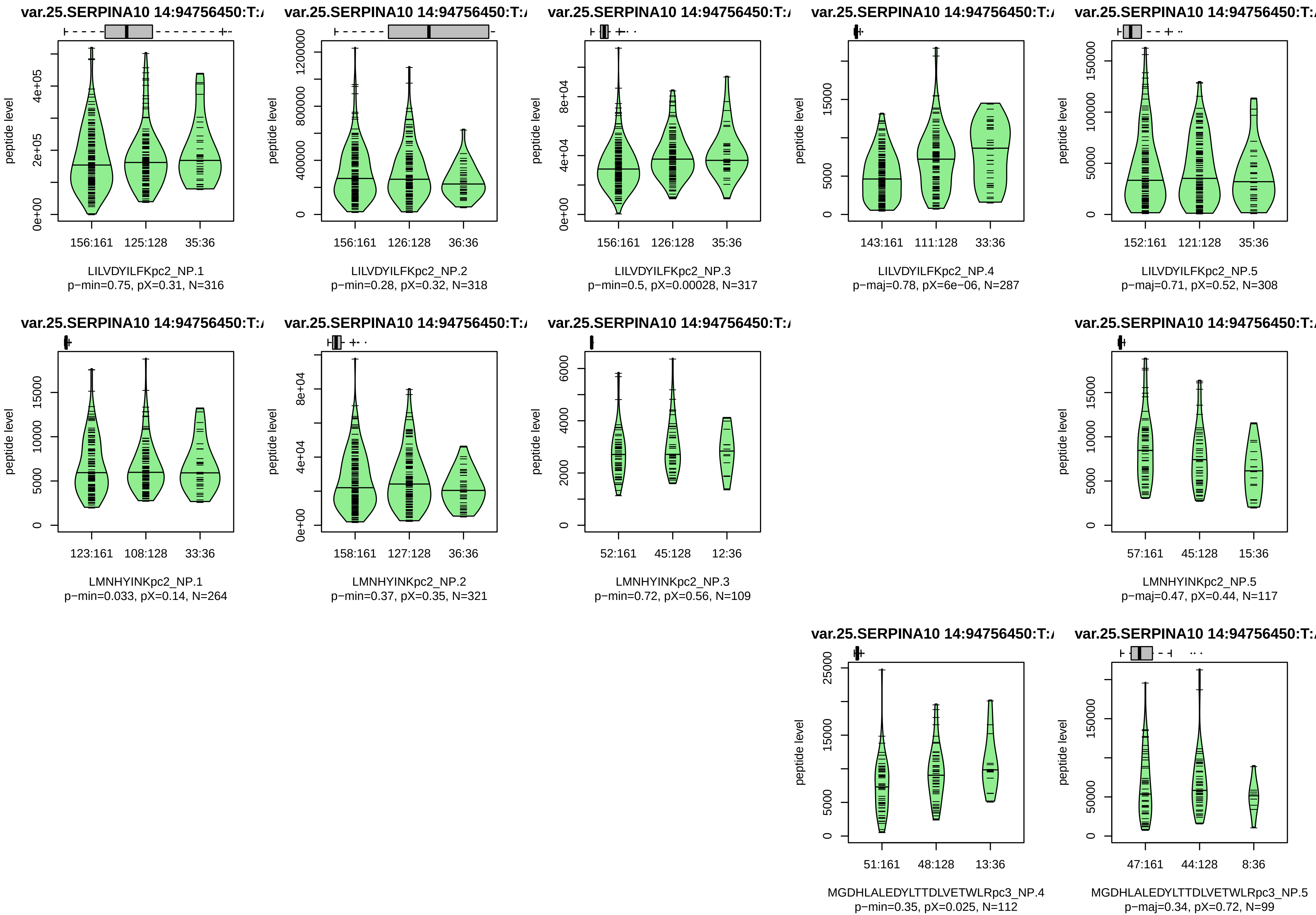

var.25.SERPINA10 14:94756450:T:/
peptide level
156:161 125:128 35:36
LILVDYILFKpc2_NP.1
p−min=0.75, pX=0.31, N=316
var.25.SERPINA10 14:94756450:T:/
peptide level
156:161 126:128 36:36
LILVDYILFKpc2_NP.2
p−min=0.28, pX=0.32, N=318
var.25.SERPINA10 14:94756450:T:/
peptide level
156:161 126:128 35:36
LILVDYILFKpc2_NP.3
p−min=0.5, pX=0.00028, N=317
var.25.SERPINA10 14:94756450:T:/
peptide level
143:161 111:128 33:36
LILVDYILFKpc2_NP.4
p−maj=0.78, pX=6e−06, N=287
var.25.SERPINA10 14:94756450:T:/
peptide level
152:161 121:128 35:36
LILVDYILFKpc2_NP.5
p−maj=0.71, pX=0.52, N=308
var.25.SERPINA10 14:94756450:T:/
peptide level
123:161 108:128 33:36
LMNHYINKpc2_NP.1
p−min=0.033, pX=0.14, N=264
var.25.SERPINA10 14:94756450:T:/
peptide level
158:161 127:128 36:36
LMNHYINKpc2_NP.2
p−min=0.37, pX=0.35, N=321
var.25.SERPINA10 14:94756450:T:/
peptide level
52:161 45:128 12:36
LMNHYINKpc2_NP.3
p−min=0.72, pX=0.56, N=109
var.25.SERPINA10 14:94756450:T:/
peptide level
57:161 45:128 15:36
LMNHYINKpc2_NP.5
p−maj=0.47, pX=0.44, N=117
var.25.SERPINA10 14:94756450:T:/
peptide level
51:161 48:128 13:36
MGDHLALEDYLTTDLVETWLRpc3_NP.4
p−min=0.35, pX=0.025, N=112
var.25.SERPINA10 14:94756450:T:/
peptide level
47:161 44:128 8:36
MGDHLALEDYLTTDLVETWLRpc3_NP.5
p−maj=0.34, pX=0.72, N=99

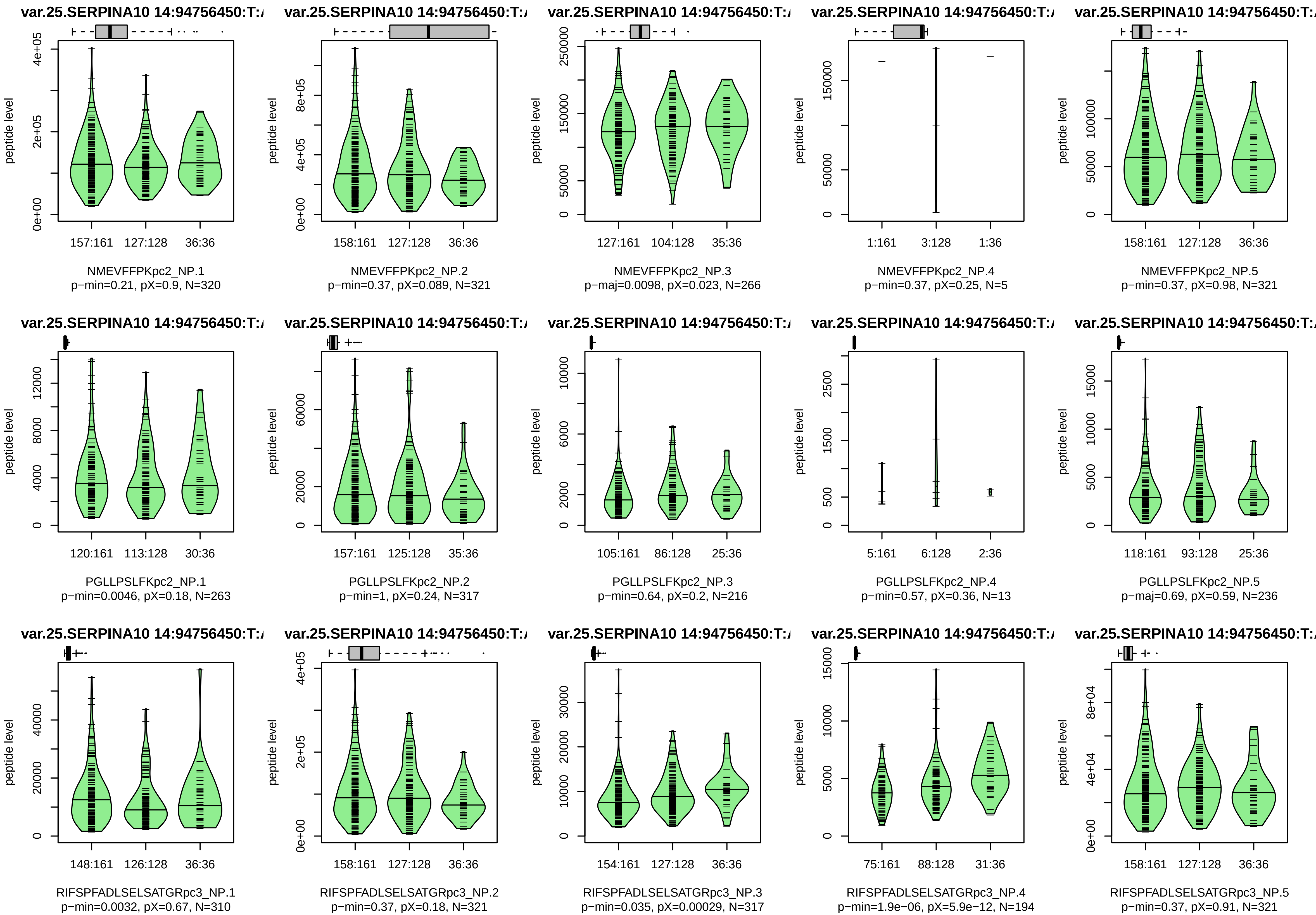

var.25.SERPINA10 14:94756450:T:
peptide level
157:161
127:128
36:36
NMEVFFPKpc2_NP.1
p−min=0.21, pX=0.9, N=320
var.25.SERPINA10 14:94756450:T:
peptide level
158:161
127:128
36:36
NMEVFFPKpc2_NP.2
p−min=0.37, pX=0.089, N=321
var.25.SERPINA10 14:94756450:T:
peptide level
127:161
104:128
35:36
NMEVFFPKpc2_NP.3
p−maj=0.0098, pX=0.023, N=266
var.25.SERPINA10 14:94756450:T:
peptide level
1:161
3:128
1:36
NMEVFFPKpc2_NP.4
p−min=0.37, pX=0.25, N=5
var.25.SERPINA10 14:94756450:T:
peptide level
158:161
127:128
36:36
NMEVFFPKpc2_NP.5
p−min=0.37, pX=0.98, N=321
var.25.SERPINA10 14:94756450:T:
peptide level
120:161
113:128
30:36
PGLLPSLFKpc2_NP.1
p−min=0.0046, pX=0.18, N=263
var.25.SERPINA10 14:94756450:T:
peptide level
157:161
125:128
35:36
PGLLPSLFKpc2_NP.2
p−min=1, pX=0.24, N=317
var.25.SERPINA10 14:94756450:T:
peptide level
105:161
86:128
25:36
PGLLPSLFKpc2_NP.3
p−min=0.64, pX=0.2, N=216
var.25.SERPINA10 14:94756450:T:
peptide level
5:161
6:128
2:36
PGLLPSLFKpc2_NP.4
p−min=0.57, pX=0.36, N=13
var.25.SERPINA10 14:94756450:T:
peptide level
118:161
93:128
25:36
PGLLPSLFKpc2_NP.5
p−maj=0.69, pX=0.59, N=236
var.25.SERPINA10 14:94756450:T:
peptide level
148:161
126:128
36:36
RIFSPFADLSELSATGRpc3_NP.1
p−min=0.0032, pX=0.67, N=310
var.25.SERPINA10 14:94756450:T:
peptide level
158:161
127:128
36:36
RIFSPFADLSELSATGRpc3_NP.2
p−min=0.37, pX=0.18, N=321
var.25.SERPINA10 14:94756450:T:
peptide level
154:161
127:128
36:36
RIFSPFADLSELSATGRpc3_NP.3
p−min=0.035, pX=0.00029, N=317
var.25.SERPINA10 14:94756450:T:
peptide level
75:161
88:128
31:36
RIFSPFADLSELSATGRpc3_NP.4
p−min=1.9e−06, pX=5.9e−12, N=194
var.25.SERPINA10 14:94756450:T:
peptide level
158:161
127:128
36:36
RIFSPFADLSELSATGRpc3_NP.5
p−min=0.37, pX=0.91, N=321

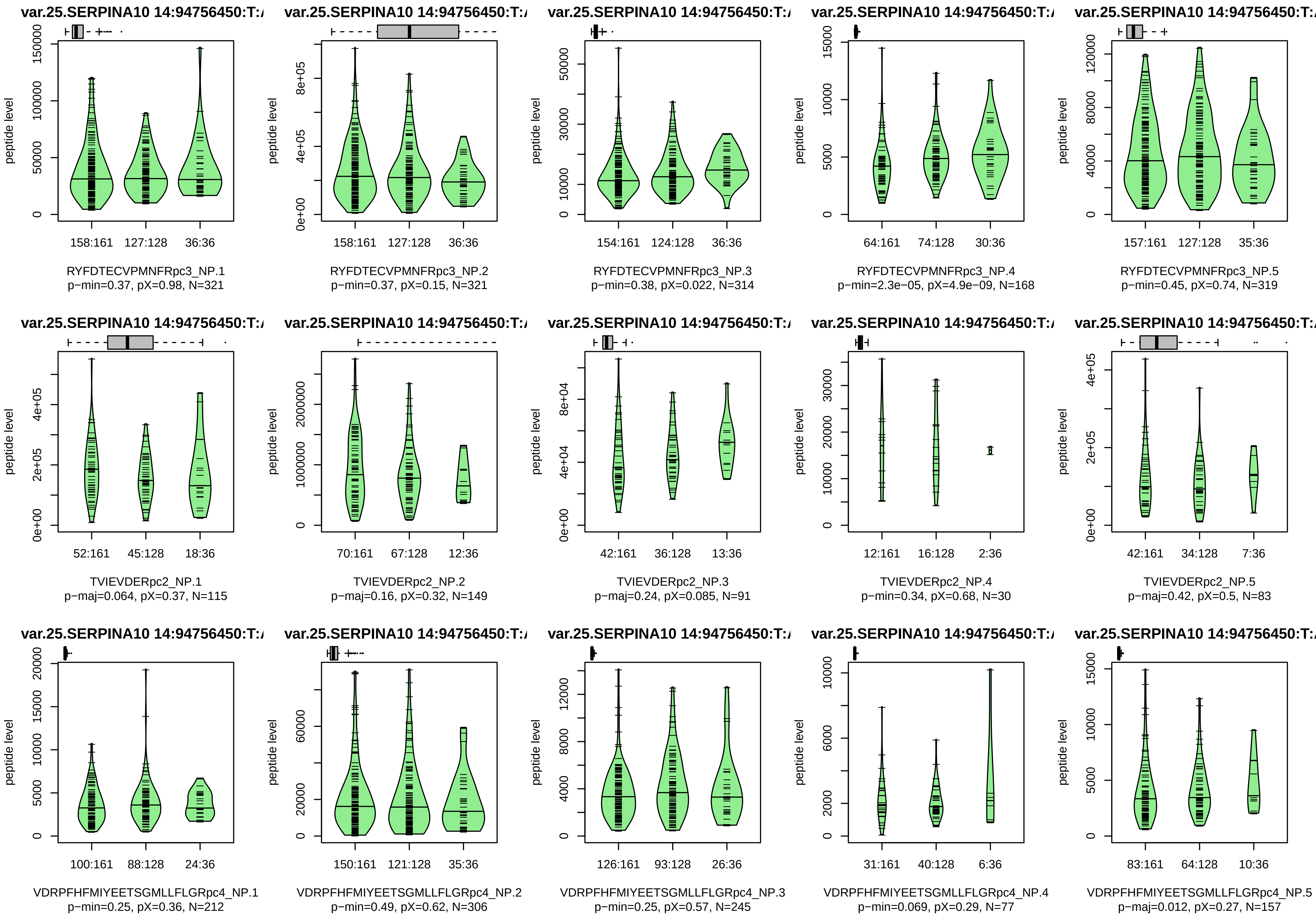

var.25.SERPINA10 14:94756450:T:/
peptide level
158:161
127:128
36:36
RYFDTECVPMNFRpc3_NP.1
p−min=0.37, pX=0.98, N=321
var.25.SERPINA10 14:94756450:T:/
158:161
127:128
36:36
RYFDTECVPMNFRpc3_NP.2
p−min=0.37, pX=0.15, N=321
var.25.SERPINA10 14:94756450:T:/
154:161
124:128
36:36
RYFDTECVPMNFRpc3_NP.3
p−min=0.38, pX=0.022, N=314
var.25.SERPINA10 14:94756450:T:/
64:161
74:128
30:36
RYFDTECVPMNFRpc3_NP.4
p−min=2.3e−05, pX=4.9e−09, N=168
var.25.SERPINA10 14:94756450:T:/
157:161
127:128
35:36
RYFDTECVPMNFRpc3_NP.5
p−min=0.45, pX=0.74, N=319
var.25.SERPINA10 14:94756450:T:/
52:161
45:128
18:36
TVIEVDERpc2_NP.1
p−maj=0.064, pX=0.37, N=115
var.25.SERPINA10 14:94756450:T:/
70:161
67:128
12:36
TVIEVDERpc2_NP.2
p−maj=0.16, pX=0.32, N=149
var.25.SERPINA10 14:94756450:T:/
42:161
36:128
13:36
TVIEVDERpc2_NP.3
p−maj=0.24, pX=0.085, N=91
var.25.SERPINA10 14:94756450:T:/
12:161
16:128
2:36
TVIEVDERpc2_NP.4
p−min=0.34, pX=0.68, N=30
var.25.SERPINA10 14:94756450:T:/
42:161
34:128
7:36
TVIEVDERpc2_NP.5
p−maj=0.42, pX=0.5, N=83
var.25.SERPINA10 14:94756450:T:/
100:161
88:128
24:36
VDRPFHFMIYEETSGMLLFLGRpc4_NP.1
p−min=0.25, pX=0.36, N=212
var.25.SERPINA10 14:94756450:T:/
150:161
121:128
35:36
VDRPFHFMIYEETSGMLLFLGRpc4_NP.2
p−min=0.49, pX=0.62, N=306
var.25.SERPINA10 14:94756450:T:/
126:161
93:128
26:36
VDRPFHFMIYEETSGMLLFLGRpc4_NP.3
p−min=0.25, pX=0.57, N=245
var.25.SERPINA10 14:94756450:T:/
31:161
40:128
6:36
VDRPFHFMIYEETSGMLLFLGRpc4_NP.4
p−min=0.069, pX=0.29, N=77
var.25.SERPINA10 14:94756450:T:/
83:161
64:128
10:36
VDRPFHFMIYEETSGMLLFLGRpc4_NP.5
p−maj=0.012, pX=0.27, N=157

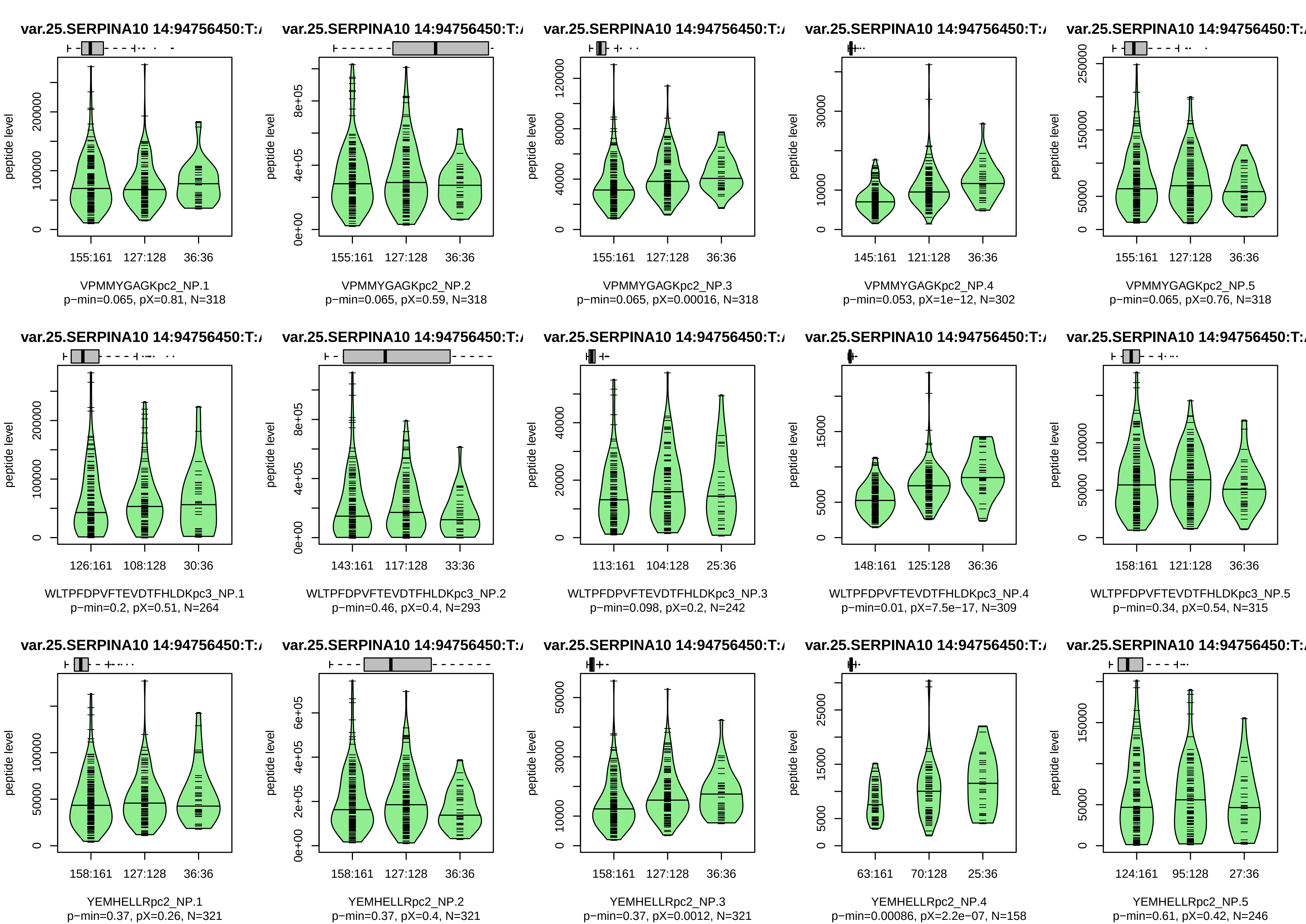
var.25.SERPINA10 14:94756450:T:/
155:161
127:128
36:36
VPMMYGAGKpc2_NP.1
p−min=0.065, pX=0.81, N=318
var.25.SERPINA10 14:94756450:T:/
155:161
127:128
36:36
VPMMYGAGKpc2_NP.2
p−min=0.065, pX=0.59, N=318
var.25.SERPINA10 14:94756450:T:/
155:161
127:128
36:36
VPMMYGAGKpc2_NP.3
p−min=0.065, pX=0.00016, N=318
var.25.SERPINA10 14:94756450:T:/
145:161
121:128
36:36
VPMMYGAGKpc2_NP.4
p−min=0.053, pX=1e−12, N=302
var.25.SERPINA10 14:94756450:T:/
155:161
127:128
36:36
VPMMYGAGKpc2_NP.5
p−min=0.065, pX=0.76, N=318
var.25.SERPINA10 14:94756450:T:/
126:161
108:128
30:36
WLTPFDPVFTEVDTFHLDKpc3_NP.1
p−min=0.2, pX=0.51, N=264
var.25.SERPINA10 14:94756450:T:/
143:161
117:128
33:36
WLTPFDPVFTEVDTFHLDKpc3_NP.2
p−min=0.46, pX=0.4, N=293
var.25.SERPINA10 14:94756450:T:/
113:161
104:128
25:36
WLTPFDPVFTEVDTFHLDKpc3_NP.3
p−min=0.098, pX=0.2, N=242
var.25.SERPINA10 14:94756450:T:/
148:161
125:128
36:36
WLTPFDPVFTEVDTFHLDKpc3_NP.4
p−min=0.01, pX=7.5e−17, N=309
var.25.SERPINA10 14:94756450:T:/
158:161
121:128
36:36
WLTPFDPVFTEVDTFHLDKpc3_NP.5
p−min=0.34, pX=0.54, N=315
var.25.SERPINA10 14:94756450:T:/
158:161
127:128
36:36
YEMHELLRpc2_NP.1
p−min=0.37, pX=0.26, N=321
var.25.SERPINA10 14:94756450:T:/
158:161
127:128
36:36
YEMHELLRpc2_NP.2
p−min=0.37, pX=0.4, N=321
var.25.SERPINA10 14:94756450:T:/
158:161
127:128
36:36
YEMHELLRpc2_NP.3
p−min=0.37, pX=0.0012, N=321
var.25.SERPINA10 14:94756450:T:/
63:161
70:128
25:36
YEMHELLRpc2_NP.4
p−min=0.00086, pX=2.2e−07, N=158
var.25.SERPINA10 14:94756450:T:/
124:161
95:128
27:36
YEMHELLRpc2_NP.5
p−min=0.61, pX=0.42, N=246
peptide level

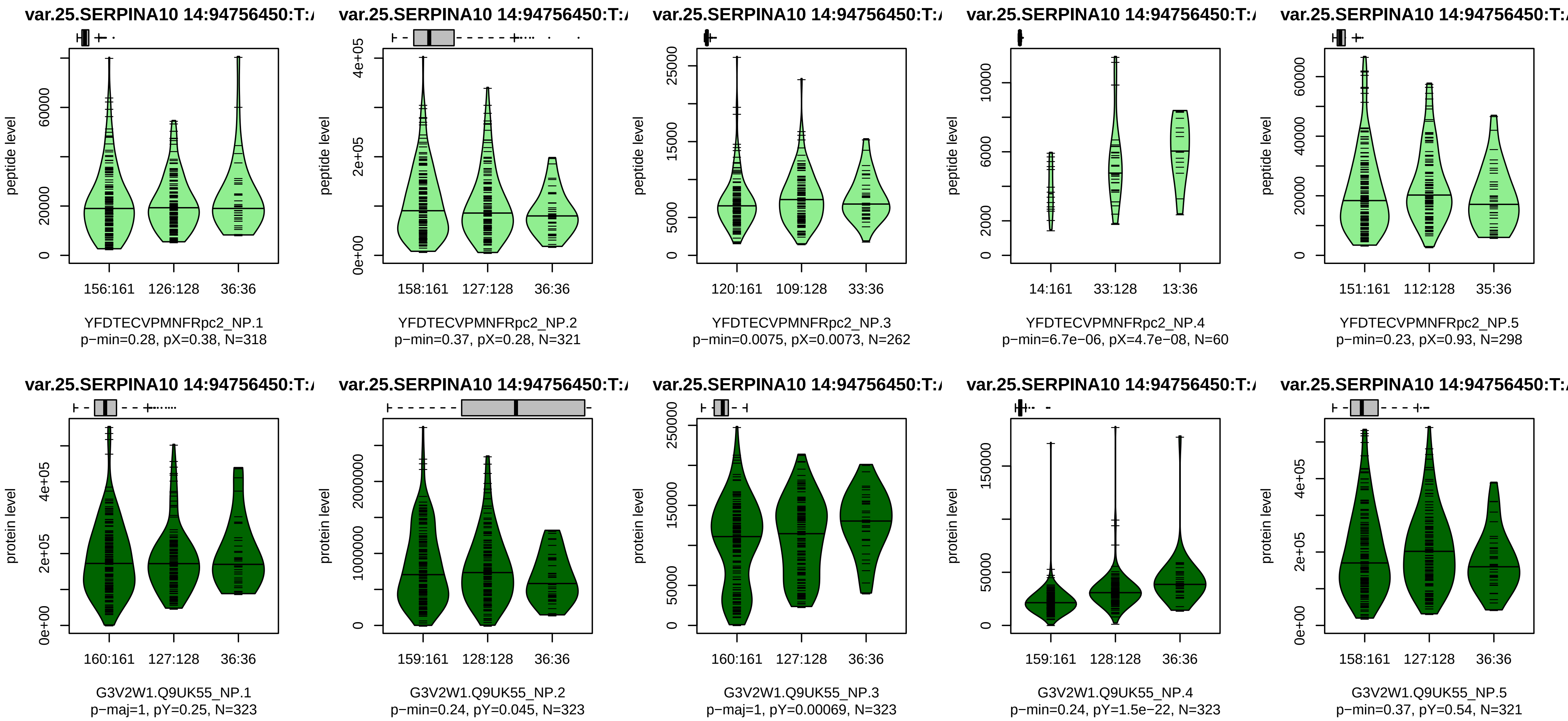

var.25.SERPINA10 14:94756450:T:A
peptide level
156:161
126:128
36:36
YFDTECVPMNFRpc2_NP.1
p−min=0.28, pX=0.38, N=318
var.25.SERPINA10 14:94756450:T:A
peptide level
158:161
127:128
36:36
YFDTECVPMNFRpc2_NP.2
p−min=0.37, pX=0.28, N=321
var.25.SERPINA10 14:94756450:T:A
peptide level
120:161
109:128
33:36
YFDTECVPMNFRpc2_NP.3
p−min=0.0075, pX=0.0073, N=262
var.25.SERPINA10 14:94756450:T:A
peptide level
14:161
33:128
13:36
YFDTECVPMNFRpc2_NP.4
p−min=6.7e−06, pX=4.7e−08, N=60
var.25.SERPINA10 14:94756450:T:A
peptide level
151:161
112:128
35:36
YFDTECVPMNFRpc2_NP.5
p−min=0.23, pX=0.93, N=298
var.25.SERPINA10 14:94756450:T:A
protein level
160:161
127:128
36:36
G3V2W1.Q9UK55_NP.1
p−maj=1, pY=0.25, N=323
var.25.SERPINA10 14:94756450:T:A
protein level
159:161
128:128
36:36
G3V2W1.Q9UK55_NP.2
p−min=0.24, pY=0.045, N=323
var.25.SERPINA10 14:94756450:T:A
protein level
160:161
127:128
36:36
G3V2W1.Q9UK55_NP.3
p−maj=1, pY=0.00069, N=323
var.25.SERPINA10 14:94756450:T:A
protein level
159:161
128:128
36:36
G3V2W1.Q9UK55_NP.4
p−min=0.24, pY=1.5e−22, N=323
var.25.SERPINA10 14:94756450:T:A
protein level
158:161
127:128
36:36
G3V2W1.Q9UK55_NP.5
p−min=0.37, pY=0.54, N=321

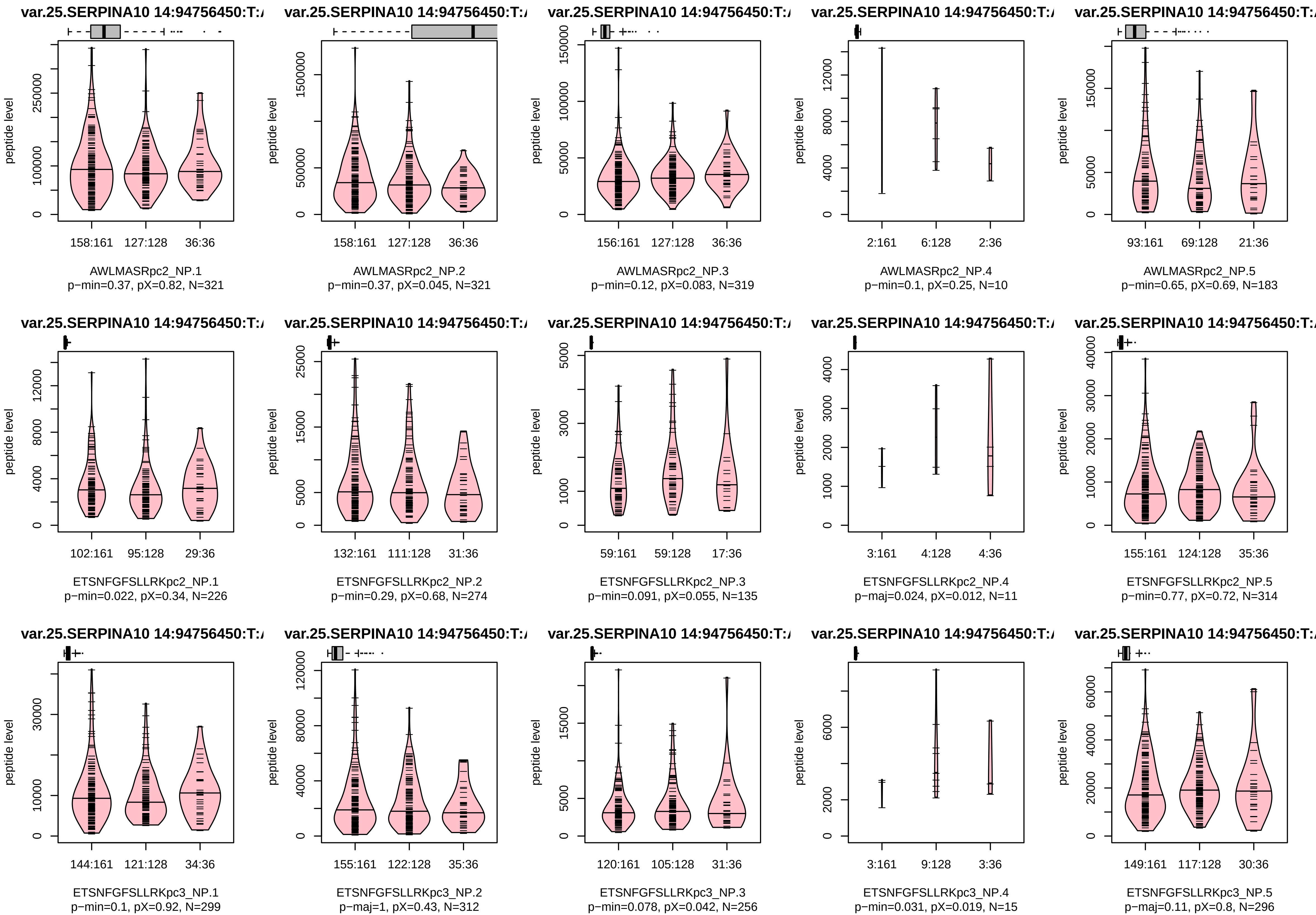
var.25.SERPINA10 14:94756450:T:
peptide level
158:161
127:128
36:36
AWLMASRpc2_NP.1
p−min=0.37, pX=0.82, N=321
var.25.SERPINA10 14:94756450:T:
peptide level
158:161
127:128
36:36
AWLMASRpc2_NP.2
p−min=0.37, pX=0.045, N=321
var.25.SERPINA10 14:94756450:T:
peptide level
156:161
127:128
36:36
AWLMASRpc2_NP.3
p−min=0.12, pX=0.083, N=319
var.25.SERPINA10 14:94756450:T:
peptide level
2:161
6:128
2:36
AWLMASRpc2_NP.4
p−min=0.1, pX=0.25, N=10
var.25.SERPINA10 14:94756450:T:
peptide level
93:161
69:128
21:36
AWLMASRpc2_NP.5
p−min=0.65, pX=0.69, N=183
var.25.SERPINA10 14:94756450:T:
peptide level
102:161
95:128
29:36
ETSNFGFSLLRKpc2_NP.1
p−min=0.022, pX=0.34, N=226
var.25.SERPINA10 14:94756450:T:
peptide level
132:161
111:128
31:36
ETSNFGFSLLRKpc2_NP.2
p−min=0.29, pX=0.68, N=274
var.25.SERPINA10 14:94756450:T:
peptide level
59:161
59:128
17:36
ETSNFGFSLLRKpc2_NP.3
p−min=0.091, pX=0.055, N=135
var.25.SERPINA10 14:94756450:T:
peptide level
3:161
4:128
4:36
ETSNFGFSLLRKpc2_NP.4
p−maj=0.024, pX=0.012, N=11
var.25.SERPINA10 14:94756450:T:
peptide level
155:161
124:128
35:36
ETSNFGFSLLRKpc2_NP.5
p−min=0.77, pX=0.72, N=314
var.25.SERPINA10 14:94756450:T:
peptide level
144:161
121:128
34:36
ETSNFGFSLLRKpc3_NP.1
p−min=0.1, pX=0.92, N=299
var.25.SERPINA10 14:94756450:T:
peptide level
155:161
122:128
35:36
ETSNFGFSLLRKpc3_NP.2
p−maj=1, pX=0.43, N=312
var.25.SERPINA10 14:94756450:T:
peptide level
120:161
105:128
31:36
ETSNFGFSLLRKpc3_NP.3
p−min=0.078, pX=0.042, N=256
var.25.SERPINA10 14:94756450:T:
peptide level
3:161
9:128
3:36
ETSNFGFSLLRKpc3_NP.4
p−min=0.031, pX=0.019, N=15
var.25.SERPINA10 14:94756450:T:
peptide level
149:161
117:128
30:36
ETSNFGFSLLRKpc3_NP.5
p−maj=0.11, pX=0.8, N=296

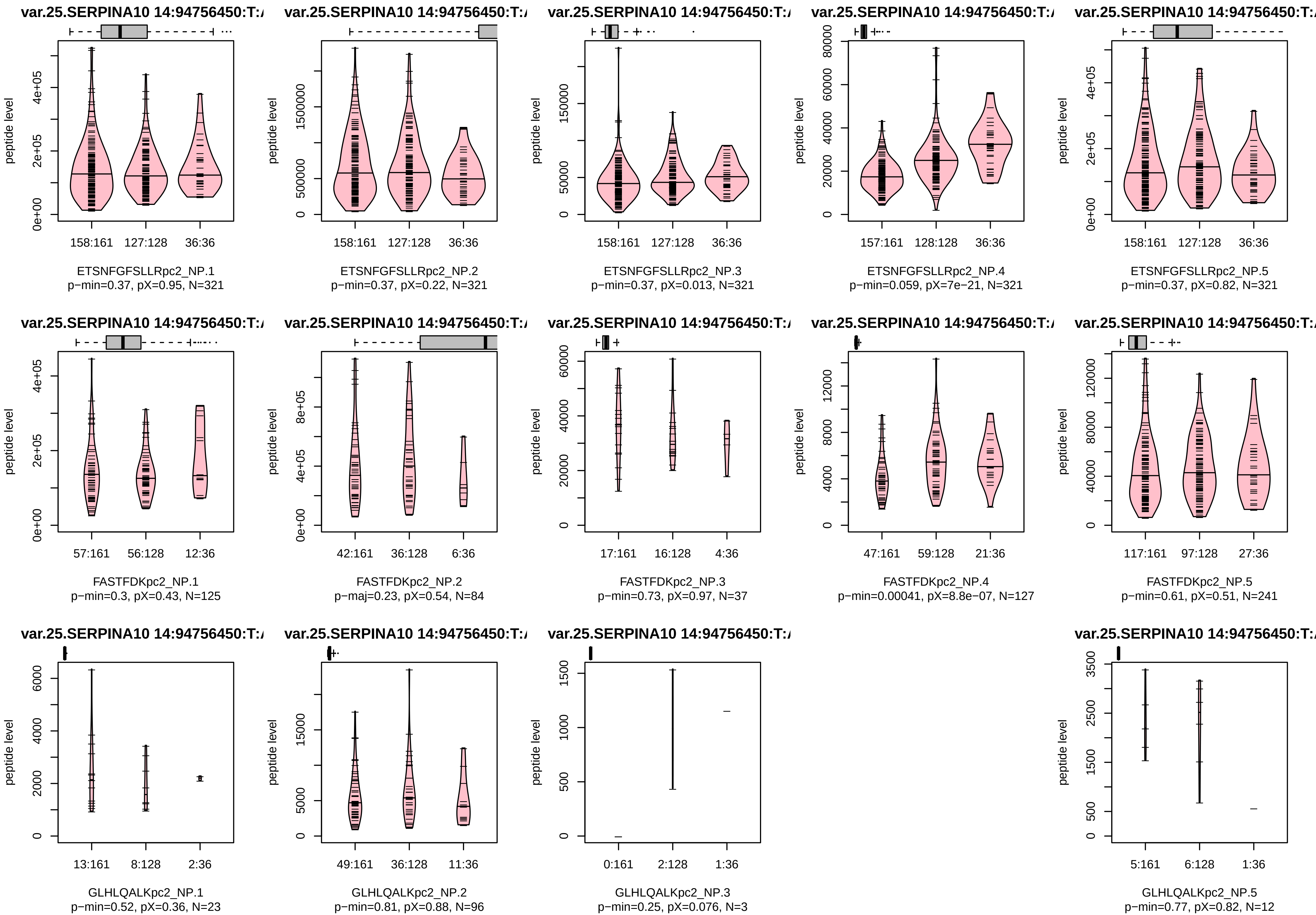

var.25.SERPINA10 14:94756450:T:/
peptide level
158:161
127:128
36:36
ETSNFGFSLLRpc2_NP.1
p−min=0.37, pX=0.95, N=321
var.25.SERPINA10 14:94756450:T:/
peptide level
158:161
127:128
36:36
ETSNFGFSLLRpc2_NP.2
p−min=0.37, pX=0.22, N=321
var.25.SERPINA10 14:94756450:T:/
peptide level
158:161
127:128
36:36
ETSNFGFSLLRpc2_NP.3
p−min=0.37, pX=0.013, N=321
var.25.SERPINA10 14:94756450:T:/
peptide level
157:161
128:128
36:36
ETSNFGFSLLRpc2_NP.4
p−min=0.059, pX=7e−21, N=321
var.25.SERPINA10 14:94756450:T:/
peptide level
158:161
127:128
36:36
ETSNFGFSLLRpc2_NP.5
p−min=0.37, pX=0.82, N=321
var.25.SERPINA10 14:94756450:T:/
peptide level
57:161
56:128
12:36
FASTFDKpc2_NP.1
p−min=0.3, pX=0.43, N=125
var.25.SERPINA10 14:94756450:T:/
peptide level
42:161
36:128
6:36
FASTFDKpc2_NP.2
p−maj=0.23, pX=0.54, N=84
var.25.SERPINA10 14:94756450:T:/
peptide level
17:161
16:128
4:36
FASTFDKpc2_NP.3
p−min=0.73, pX=0.97, N=37
var.25.SERPINA10 14:94756450:T:/
peptide level
47:161
59:128
21:36
FASTFDKpc2_NP.4
p−min=0.00041, pX=8.8e−07, N=127
var.25.SERPINA10 14:94756450:T:/
peptide level
117:161
97:128
27:36
FASTFDKpc2_NP.5
p−min=0.61, pX=0.51, N=241
var.25.SERPINA10 14:94756450:T:/
peptide level
13:161
8:128
2:36
GLHLQALKpc2_NP.1
p−min=0.52, pX=0.36, N=23
var.25.SERPINA10 14:94756450:T:/
peptide level
49:161
36:128
11:36
GLHLQALKpc2_NP.2
p−min=0.81, pX=0.88, N=96
var.25.SERPINA10 14:94756450:T:/
peptide level
0:161
2:128
1:36
GLHLQALKpc2_NP.3
p−min=0.25, pX=0.076, N=3
var.25.SERPINA10 14:94756450:T:/
peptide level
5:161
6:128
1:36
GLHLQALKpc2_NP.5
p−min=0.77, pX=0.82, N=12

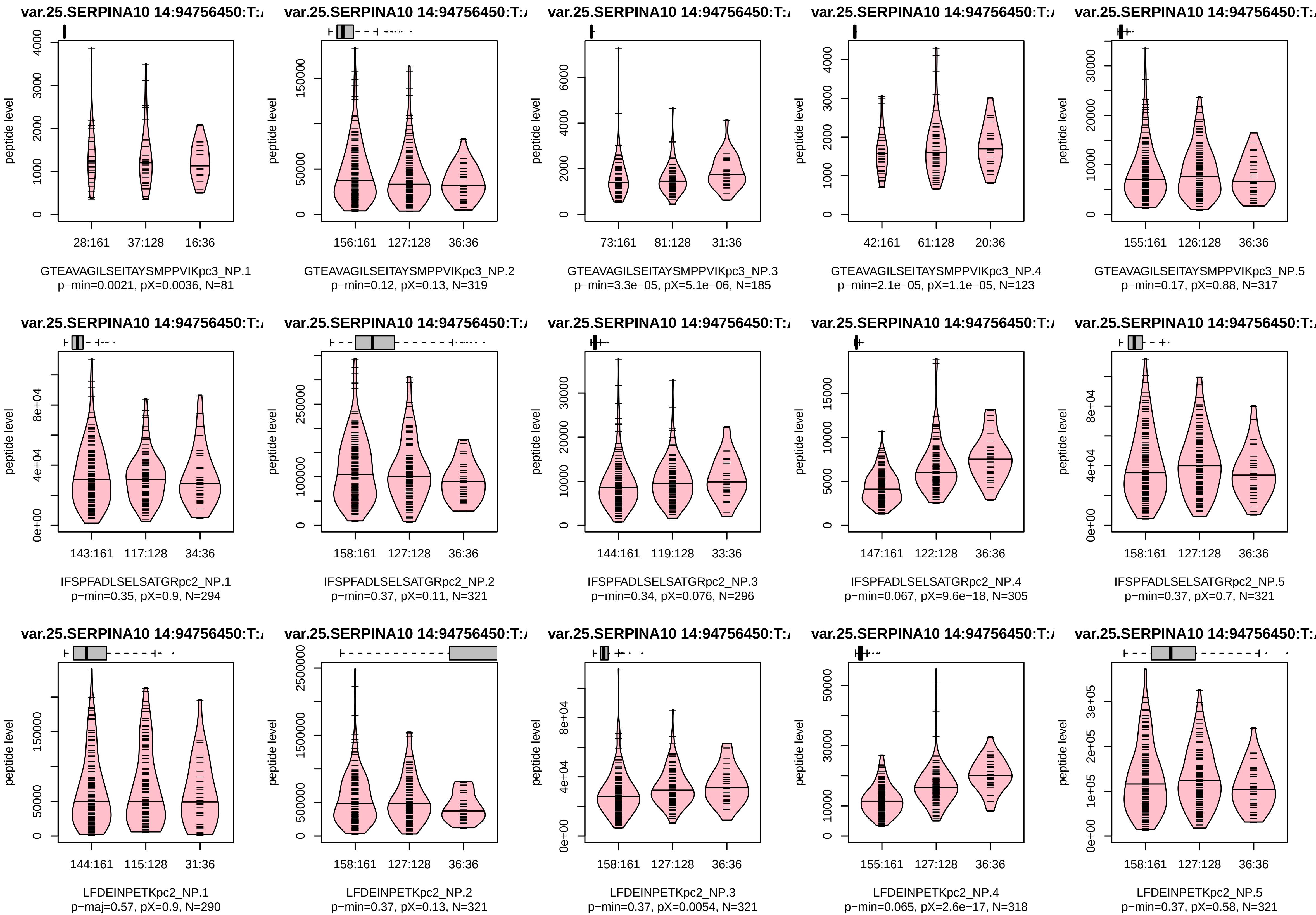
var.25.SERPINA10 14:94756450:T:/
peptide level
28:161
37:128
16:36
GTEAVAGILSEITAYSMPPVIKpc3_NP.1
p−min=0.0021, pX=0.0036, N=81
156:161
127:128
36:36
GTEAVAGILSEITAYSMPPVIKpc3_NP.2
p−min=0.12, pX=0.13, N=319
73:161
81:128
31:36
GTEAVAGILSEITAYSMPPVIKpc3_NP.3
p−min=3.3e−05, pX=5.1e−06, N=185
42:161
61:128
20:36
GTEAVAGILSEITAYSMPPVIKpc3_NP.4
p−min=2.1e−05, pX=1.1e−05, N=123
155:161
126:128
36:36
GTEAVAGILSEITAYSMPPVIKpc3_NP.5
p−min=0.17, pX=0.88, N=317
143:161
117:128
34:36
IFSPFADLSELSATGRpc2_NP.1
p−min=0.35, pX=0.9, N=294
158:161
127:128
36:36
IFSPFADLSELSATGRpc2_NP.2
p−min=0.37, pX=0.11, N=321
144:161
119:128
33:36
IFSPFADLSELSATGRpc2_NP.3
p−min=0.34, pX=0.076, N=296
147:161
122:128
36:36
IFSPFADLSELSATGRpc2_NP.4
p−min=0.067, pX=9.6e−18, N=305
158:161
127:128
36:36
IFSPFADLSELSATGRpc2_NP.5
p−min=0.37, pX=0.7, N=321
144:161
115:128
31:36
LFDEINPETKpc2_NP.1
p−maj=0.57, pX=0.9, N=290
158:161
127:128
36:36
LFDEINPETKpc2_NP.2
p−min=0.37, pX=0.13, N=321
158:161
127:128
36:36
LFDEINPETKpc2_NP.3
p−min=0.37, pX=0.0054, N=321
155:161
127:128
36:36
LFDEINPETKpc2_NP.4
p−min=0.065, pX=2.6e−17, N=318
158:161
127:128
36:36
LFDEINPETKpc2_NP.5
p−min=0.37, pX=0.58, N=321

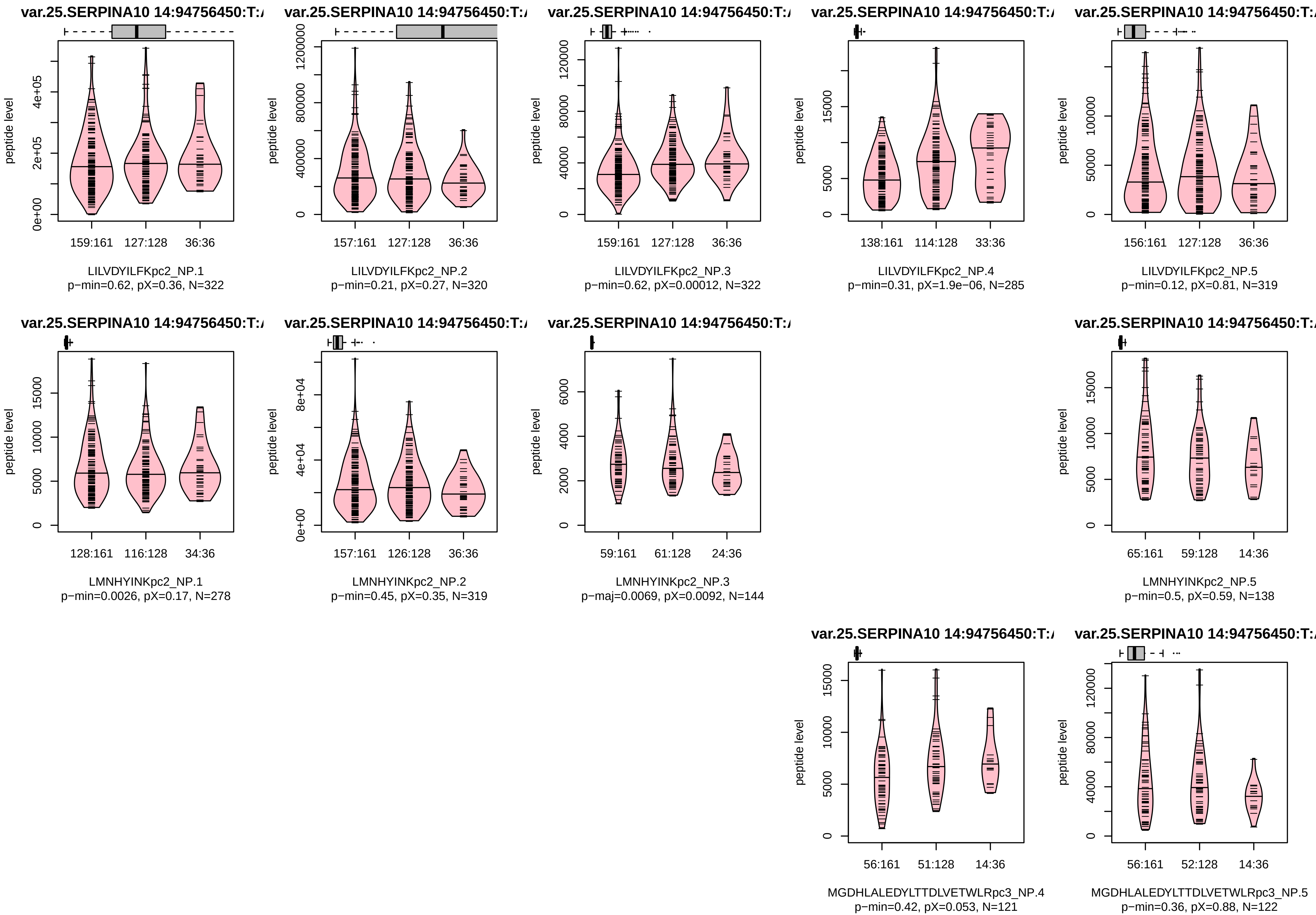
var.25.SERPINA10 14:94756450:T:/
peptide level
159:161
127:128
36:36
LILVDYILFKpc2_NP.1
p−min=0.62, pX=0.36, N=322
var.25.SERPINA10 14:94756450:T:/
peptide level
157:161
127:128
36:36
LILVDYILFKpc2_NP.2
p−min=0.21, pX=0.27, N=320
var.25.SERPINA10 14:94756450:T:/
peptide level
159:161
127:128
36:36
LILVDYILFKpc2_NP.3
p−min=0.62, pX=0.00012, N=322
var.25.SERPINA10 14:94756450:T:/
peptide level
138:161
114:128
33:36
LILVDYILFKpc2_NP.4
p−min=0.31, pX=1.9e−06, N=285
var.25.SERPINA10 14:94756450:T:/
peptide level
156:161
127:128
36:36
LILVDYILFKpc2_NP.5
p−min=0.12, pX=0.81, N=319
var.25.SERPINA10 14:94756450:T:/
peptide level
128:161
116:128
34:36
LMNHYINKpc2_NP.1
p−min=0.0026, pX=0.17, N=278
var.25.SERPINA10 14:94756450:T:/
peptide level
157:161
126:128
36:36
LMNHYINKpc2_NP.2
p−min=0.45, pX=0.35, N=319
var.25.SERPINA10 14:94756450:T:/
peptide level
59:161
61:128
24:36
LMNHYINKpc2_NP.3
p−maj=0.0069, pX=0.0092, N=144
var.25.SERPINA10 14:94756450:T:/
peptide level
65:161
59:128
14:36
LMNHYINKpc2_NP.5
p−min=0.5, pX=0.59, N=138
var.25.SERPINA10 14:94756450:T:/
peptide level
56:161
51:128
14:36
MGDHLALEDYLTTDLVETWLRpc3_NP.4
p−min=0.42, pX=0.053, N=121
var.25.SERPINA10 14:94756450:T:/
peptide level
56:161
52:128
14:36
MGDHLALEDYLTTDLVETWLRpc3_NP.5
p−min=0.36, pX=0.88, N=122

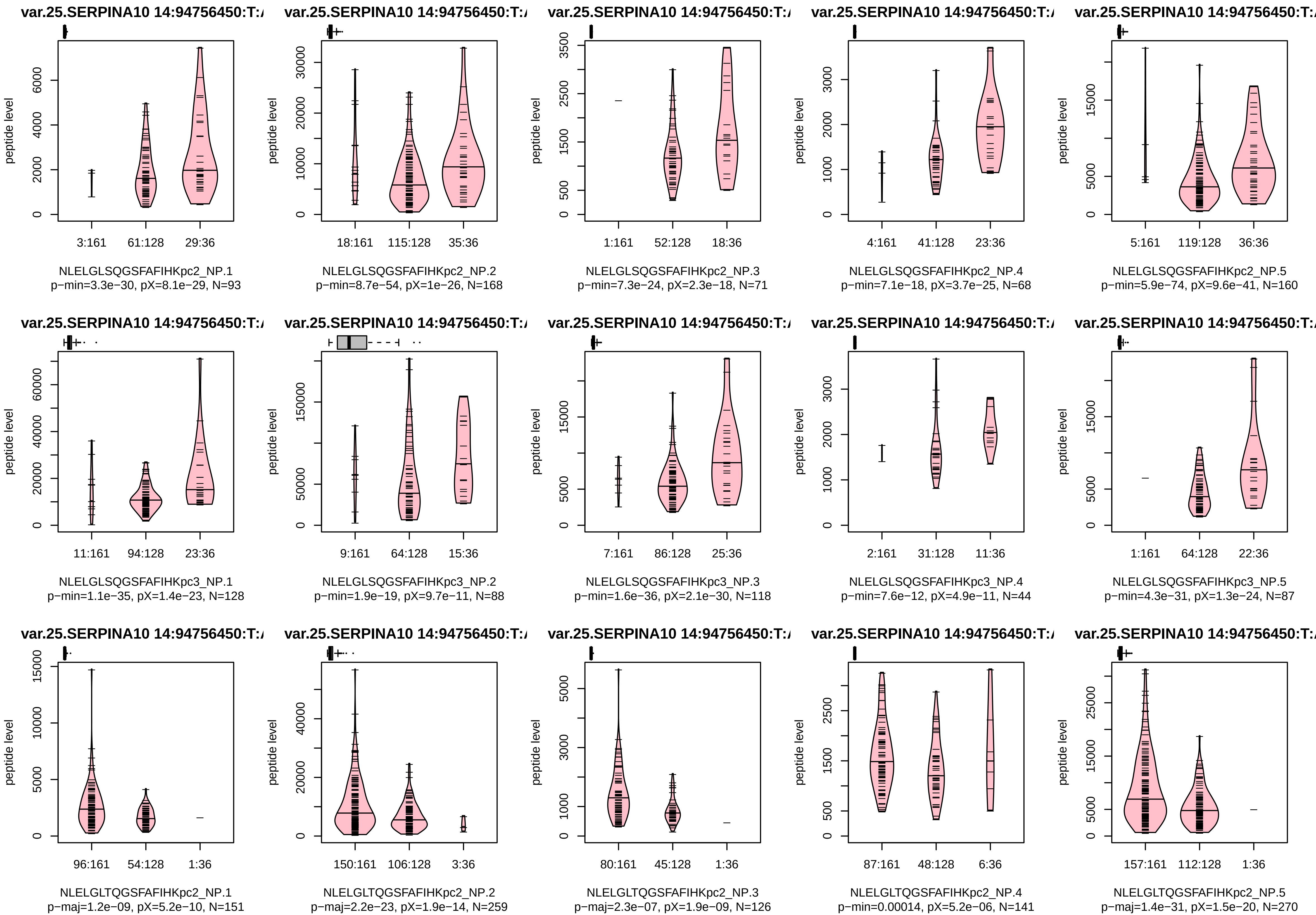

var.25.SERPINA10 14:94756450:T:
peptide level
3:161
61:128
29:36
NLELGLSQGSFAFIHKpc2_NP.1
p−min=3.3e−30, pX=8.1e−29, N=93
var.25.SERPINA10 14:94756450:T:
peptide level
18:161
115:128
35:36
NLELGLSQGSFAFIHKpc2_NP.2
p−min=8.7e−54, pX=1e−26, N=168
var.25.SERPINA10 14:94756450:T:
peptide level
1:161
52:128
18:36
NLELGLSQGSFAFIHKpc2_NP.3
p−min=7.3e−24, pX=2.3e−18, N=71
var.25.SERPINA10 14:94756450:T:
peptide level
4:161
41:128
23:36
NLELGLSQGSFAFIHKpc2_NP.4
p−min=7.1e−18, pX=3.7e−25, N=68
var.25.SERPINA10 14:94756450:T:
peptide level
5:161
119:128
36:36
NLELGLSQGSFAFIHKpc2_NP.5
p−min=5.9e−74, pX=9.6e−41, N=160
var.25.SERPINA10 14:94756450:T:
peptide level
11:161
94:128
23:36
NLELGLSQGSFAFIHKpc3_NP.1
p−min=1.1e−35, pX=1.4e−23, N=128
var.25.SERPINA10 14:94756450:T:
peptide level
9:161
64:128
15:36
NLELGLSQGSFAFIHKpc3_NP.2
p−min=1.9e−19, pX=9.7e−11, N=88
var.25.SERPINA10 14:94756450:T:
peptide level
7:161
86:128
25:36
NLELGLSQGSFAFIHKpc3_NP.3
p−min=1.6e−36, pX=2.1e−30, N=118
var.25.SERPINA10 14:94756450:T:
peptide level
2:161
31:128
11:36
NLELGLSQGSFAFIHKpc3_NP.4
p−min=7.6e−12, pX=4.9e−11, N=44
var.25.SERPINA10 14:94756450:T:
peptide level
1:161
64:128
22:36
NLELGLSQGSFAFIHKpc3_NP.5
p−min=4.3e−31, pX=1.3e−24, N=87
var.25.SERPINA10 14:94756450:T:
peptide level
96:161
54:128
1:36
NLELGLTQGSFAFIHKpc2_NP.1
p−maj=1.2e−09, pX=5.2e−10, N=151
var.25.SERPINA10 14:94756450:T:
peptide level
150:161
106:128
3:36
NLELGLTQGSFAFIHKpc2_NP.2
p−maj=2.2e−23, pX=1.9e−14, N=259
var.25.SERPINA10 14:94756450:T:
peptide level
80:161
45:128
1:36
NLELGLTQGSFAFIHKpc2_NP.3
p−maj=2.3e−07, pX=1.9e−09, N=126
var.25.SERPINA10 14:94756450:T:
peptide level
87:161
48:128
6:36
NLELGLTQGSFAFIHKpc2_NP.4
p−min=0.00014, pX=5.2e−06, N=141
var.25.SERPINA10 14:94756450:T:
peptide level
157:161
112:128
1:36
NLELGLTQGSFAFIHKpc2_NP.5
p−maj=1.4e−31, pX=1.5e−20, N=270

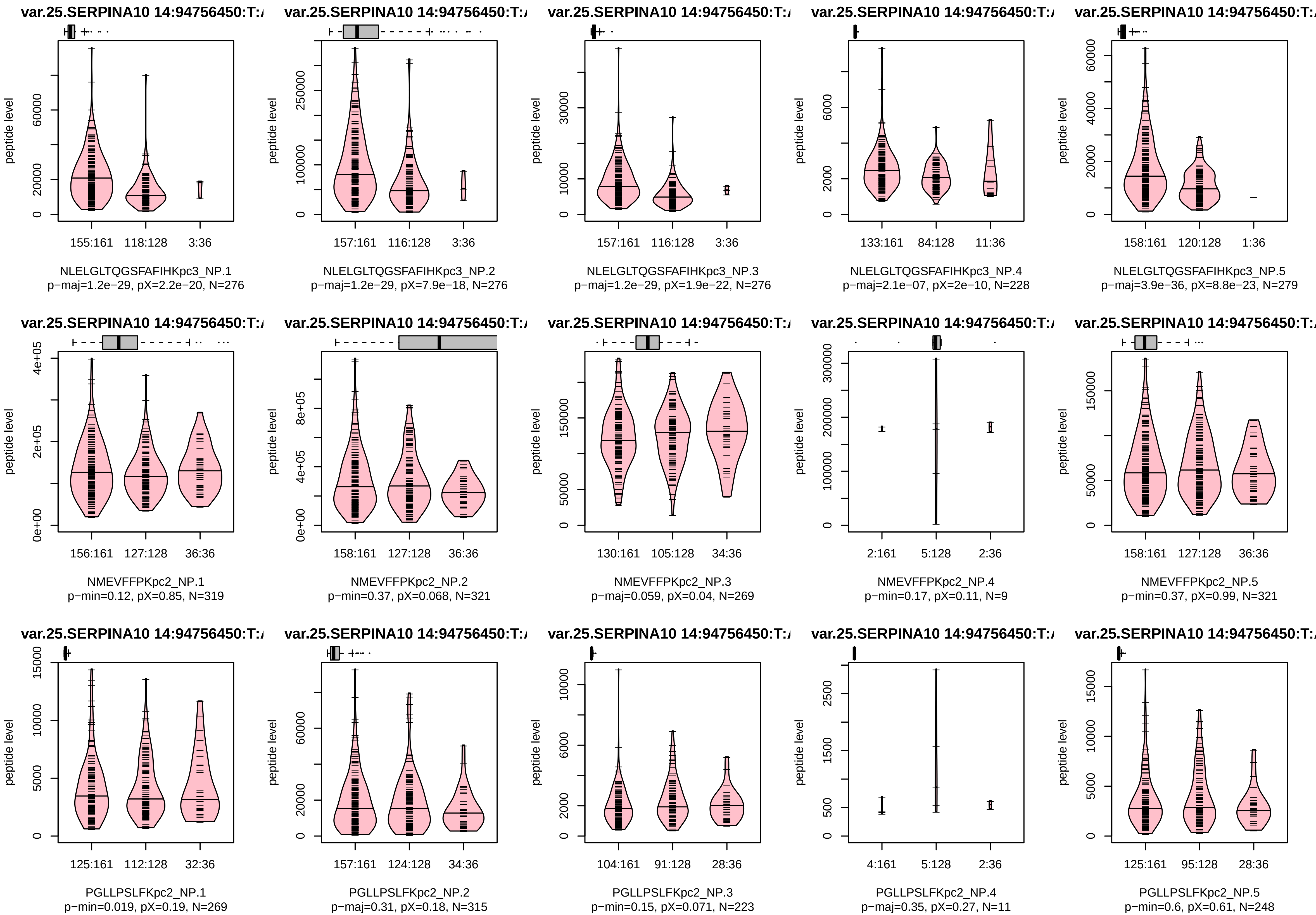

var.25.SERPINA10 14:94756450:T:/
peptide level
155:161 118:128 3:36
NLELGLTQGSFAFIHKpc3_NP.1
p−maj=1.2e−29, pX=2.2e−20, N=276
var.25.SERPINA10 14:94756450:T:/
peptide level
157:161 116:128 3:36
NLELGLTQGSFAFIHKpc3_NP.2
p−maj=1.2e−29, pX=7.9e−18, N=276
var.25.SERPINA10 14:94756450:T:/
peptide level
157:161 116:128 3:36
NLELGLTQGSFAFIHKpc3_NP.3
p−maj=1.2e−29, pX=1.9e−22, N=276
var.25.SERPINA10 14:94756450:T:/
peptide level
133:161 84:128 11:36
NLELGLTQGSFAFIHKpc3_NP.4
p−maj=2.1e−07, pX=2e−10, N=228
var.25.SERPINA10 14:94756450:T:/
peptide level
158:161 120:128 1:36
NLELGLTQGSFAFIHKpc3_NP.5
p−maj=3.9e−36, pX=8.8e−23, N=279
var.25.SERPINA10 14:94756450:T:/
peptide level
156:161 127:128 36:36
NMEVFFPKpc2_NP.1
p−min=0.12, pX=0.85, N=319
var.25.SERPINA10 14:94756450:T:/
peptide level
158:161 127:128 36:36
NMEVFFPKpc2_NP.2
p−min=0.37, pX=0.068, N=321
var.25.SERPINA10 14:94756450:T:/
peptide level
130:161 105:128 34:36
NMEVFFPKpc2_NP.3
p−maj=0.059, pX=0.04, N=269
var.25.SERPINA10 14:94756450:T:/
peptide level
2:161 5:128 2:36
NMEVFFPKpc2_NP.4
p−min=0.17, pX=0.11, N=9
var.25.SERPINA10 14:94756450:T:/
peptide level
158:161 127:128 36:36
NMEVFFPKpc2_NP.5
p−min=0.37, pX=0.99, N=321
var.25.SERPINA10 14:94756450:T:/
peptide level
125:161 112:128 32:36
PGLLPSLFKpc2_NP.1
p−min=0.019, pX=0.19, N=269
var.25.SERPINA10 14:94756450:T:/
peptide level
157:161 124:128 34:36
PGLLPSLFKpc2_NP.2
p−maj=0.31, pX=0.18, N=315
var.25.SERPINA10 14:94756450:T:/
peptide level
104:161 91:128 28:36
PGLLPSLFKpc2_NP.3
p−min=0.15, pX=0.071, N=223
var.25.SERPINA10 14:94756450:T:/
peptide level
4:161 5:128 2:36
PGLLPSLFKpc2_NP.4
p−maj=0.35, pX=0.27, N=11
var.25.SERPINA10 14:94756450:T:/
peptide level
125:161 95:128 28:36
PGLLPSLFKpc2_NP.5
p−min=0.6, pX=0.61, N=248

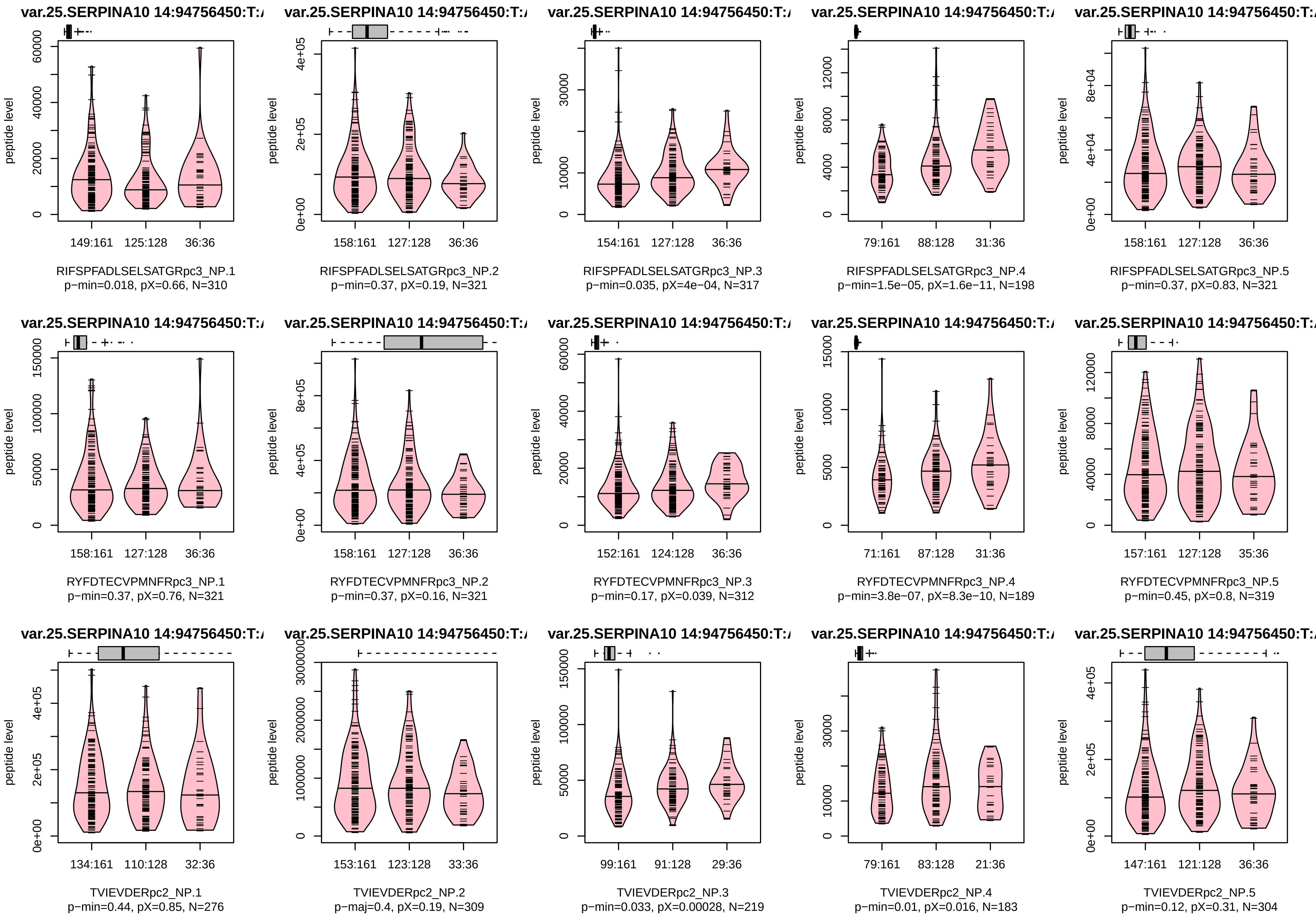
var.25.SERPINA10 14:94756450:T:/
peptide level
149:161
125:128
36:36
RIFSPFADLSELSATGRpc3_NP.1
p−min=0.018, pX=0.66, N=310
var.25.SERPINA10 14:94756450:T:/
158:161
127:128
36:36
RIFSPFADLSELSATGRpc3_NP.2
p−min=0.37, pX=0.19, N=321
var.25.SERPINA10 14:94756450:T:/
154:161
127:128
36:36
RIFSPFADLSELSATGRpc3_NP.3
p−min=0.035, pX=4e−04, N=317
var.25.SERPINA10 14:94756450:T:/
79:161
88:128
31:36
RIFSPFADLSELSATGRpc3_NP.4
p−min=1.5e−05, pX=1.6e−11, N=198
var.25.SERPINA10 14:94756450:T:/
158:161
127:128
36:36
RIFSPFADLSELSATGRpc3_NP.5
p−min=0.37, pX=0.83, N=321
var.25.SERPINA10 14:94756450:T:/
158:161
127:128
36:36
RYFDTECVPMNFRpc3_NP.1
p−min=0.37, pX=0.76, N=321
var.25.SERPINA10 14:94756450:T:/
158:161
127:128
36:36
RYFDTECVPMNFRpc3_NP.2
p−min=0.37, pX=0.16, N=321
var.25.SERPINA10 14:94756450:T:/
152:161
124:128
36:36
RYFDTECVPMNFRpc3_NP.3
p−min=0.17, pX=0.039, N=312
var.25.SERPINA10 14:94756450:T:/
71:161
87:128
31:36
RYFDTECVPMNFRpc3_NP.4
p−min=3.8e−07, pX=8.3e−10, N=189
var.25.SERPINA10 14:94756450:T:/
157:161
127:128
35:36
RYFDTECVPMNFRpc3_NP.5
p−min=0.45, pX=0.8, N=319
var.25.SERPINA10 14:94756450:T:/
134:161
110:128
32:36
TVIEVDERpc2_NP.1
p−min=0.44, pX=0.85, N=276
var.25.SERPINA10 14:94756450:T:/
153:161
123:128
33:36
TVIEVDERpc2_NP.2
p−maj=0.4, pX=0.19, N=309
var.25.SERPINA10 14:94756450:T:/
99:161
91:128
29:36
TVIEVDERpc2_NP.3
p−min=0.033, pX=0.00028, N=219
var.25.SERPINA10 14:94756450:T:/
79:161
83:128
21:36
TVIEVDERpc2_NP.4
p−min=0.01, pX=0.016, N=183
var.25.SERPINA10 14:94756450:T:/
147:161
121:128
36:36
TVIEVDERpc2_NP.5
p−min=0.12, pX=0.31, N=304

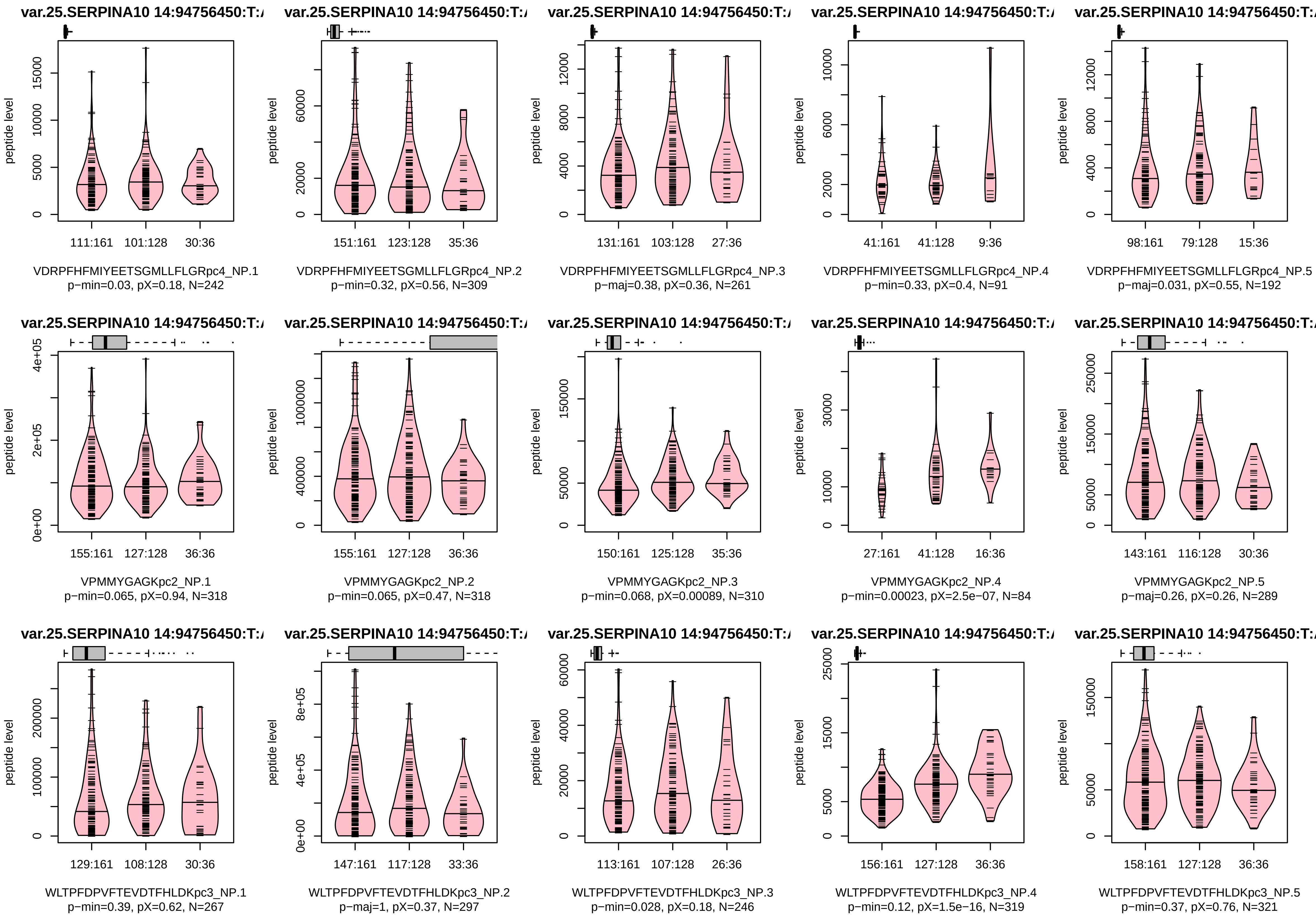
var.25.SERPINA10 14:94756450:T:/
peptide level
111:161
101:128
30:36
VDRPFHFMIYEETSGMLLFLGRpc4_NP.1
p−min=0.03, pX=0.18, N=242
var.25.SERPINA10 14:94756450:T:/
peptide level
151:161
123:128
35:36
VDRPFHFMIYEETSGMLLFLGRpc4_NP.2
p−min=0.32, pX=0.56, N=309
var.25.SERPINA10 14:94756450:T:/
peptide level
131:161
103:128
27:36
VDRPFHFMIYEETSGMLLFLGRpc4_NP.3
p−maj=0.38, pX=0.36, N=261
var.25.SERPINA10 14:94756450:T:/
peptide level
41:161
41:128
9:36
VDRPFHFMIYEETSGMLLFLGRpc4_NP.4
p−min=0.33, pX=0.4, N=91
var.25.SERPINA10 14:94756450:T:/
peptide level
98:161
79:128
15:36
VDRPFHFMIYEETSGMLLFLGRpc4_NP.5
p−maj=0.031, pX=0.55, N=192
var.25.SERPINA10 14:94756450:T:/
peptide level
155:161
127:128
36:36
VPMMYGAGKpc2_NP.1
p−min=0.065, pX=0.94, N=318
var.25.SERPINA10 14:94756450:T:/
peptide level
155:161
127:128
36:36
VPMMYGAGKpc2_NP.2
p−min=0.065, pX=0.47, N=318
var.25.SERPINA10 14:94756450:T:/
peptide level
150:161
125:128
35:36
VPMMYGAGKpc2_NP.3
p−min=0.068, pX=0.00089, N=310
var.25.SERPINA10 14:94756450:T:/
peptide level
27:161
41:128
16:36
VPMMYGAGKpc2_NP.4
p−min=0.00023, pX=2.5e−07, N=84
var.25.SERPINA10 14:94756450:T:/
peptide level
143:161
116:128
30:36
VPMMYGAGKpc2_NP.5
p−maj=0.26, pX=0.26, N=289
var.25.SERPINA10 14:94756450:T:/
peptide level
129:161
108:128
30:36
WLTPFDPVFTEVDTFHLDKpc3_NP.1
p−min=0.39, pX=0.62, N=267
var.25.SERPINA10 14:94756450:T:/
peptide level
147:161
117:128
33:36
WLTPFDPVFTEVDTFHLDKpc3_NP.2
p−maj=1, pX=0.37, N=297
var.25.SERPINA10 14:94756450:T:/
peptide level
113:161
107:128
26:36
WLTPFDPVFTEVDTFHLDKpc3_NP.3
p−min=0.028, pX=0.18, N=246
var.25.SERPINA10 14:94756450:T:/
peptide level
156:161
127:128
36:36
WLTPFDPVFTEVDTFHLDKpc3_NP.4
p−min=0.12, pX=1.5e−16, N=319
var.25.SERPINA10 14:94756450:T:/
peptide level
158:161
127:128
36:36
WLTPFDPVFTEVDTFHLDKpc3_NP.5
p−min=0.37, pX=0.76, N=321

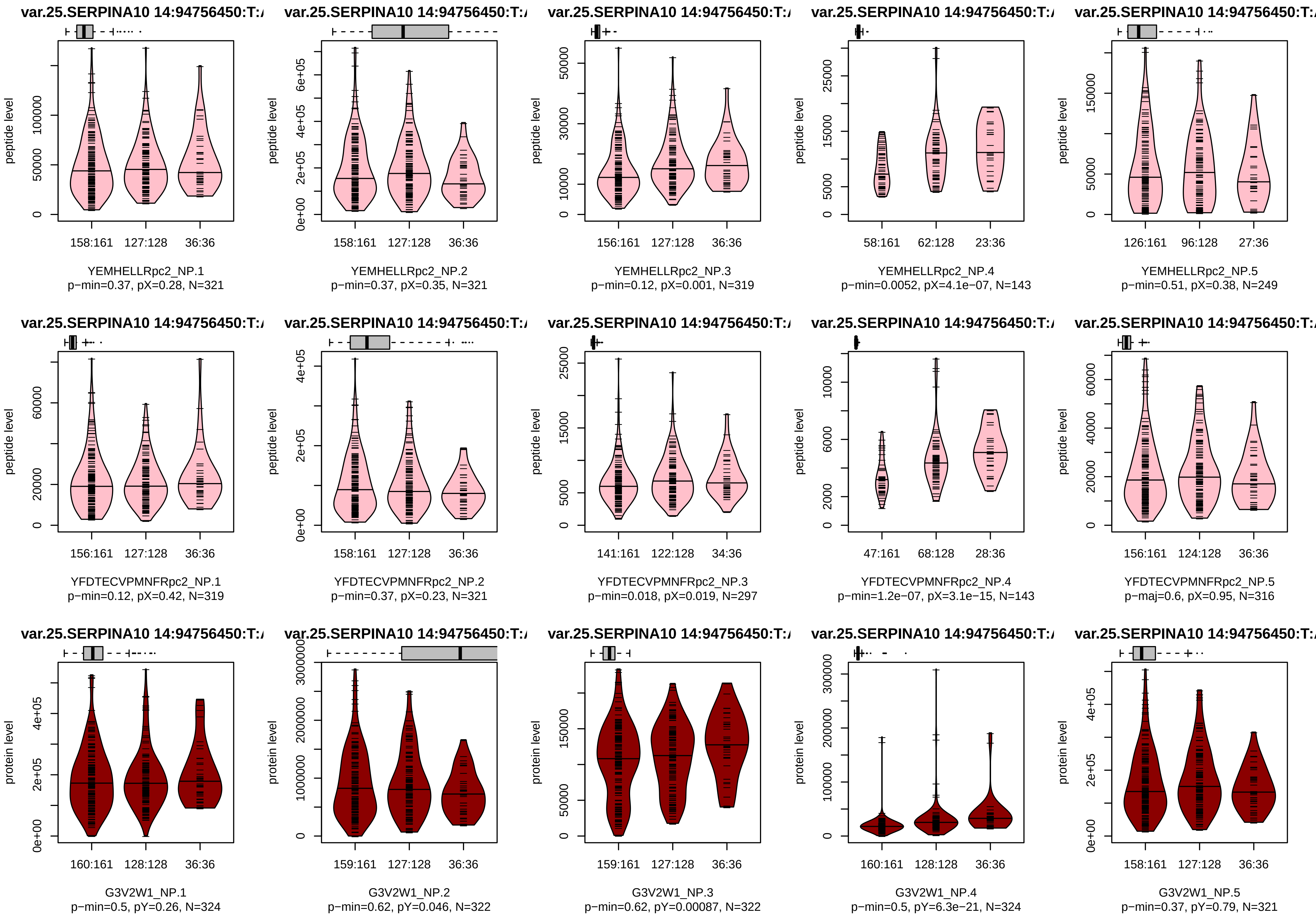

var.25.SERPINA10 14:94756450:T:/
158:161 127:128 36:36
YEMHELLRpc2_NP.1
p−min=0.37, pX=0.28, N=321
158:161 127:128 36:36
YEMHELLRpc2_NP.2
p−min=0.37, pX=0.35, N=321
156:161 127:128 36:36
YEMHELLRpc2_NP.3
p−min=0.12, pX=0.001, N=319
58:161 62:128 23:36
YEMHELLRpc2_NP.4
p−min=0.0052, pX=4.1e−07, N=143
126:161 96:128 27:36
YEMHELLRpc2_NP.5
p−min=0.51, pX=0.38, N=249
156:161 127:128 36:36
YFDTECVPMNFRpc2_NP.1
p−min=0.12, pX=0.42, N=319
158:161 127:128 36:36
YFDTECVPMNFRpc2_NP.2
p−min=0.37, pX=0.23, N=321
141:161 122:128 34:36
YFDTECVPMNFRpc2_NP.3
p−min=0.018, pX=0.019, N=297
47:161 68:128 28:36
YFDTECVPMNFRpc2_NP.4
p−min=1.2e−07, pX=3.1e−15, N=143
156:161 124:128 36:36
YFDTECVPMNFRpc2_NP.5
p−maj=0.6, pX=0.95, N=316
160:161 128:128 36:36
G3V2W1_NP.1
p−min=0.5, pY=0.26, N=324
159:161 127:128 36:36
G3V2W1_NP.2
p−min=0.62, pY=0.046, N=322
159:161 127:128 36:36
G3V2W1_NP.3
p−min=0.62, pY=0.00087, N=322
160:161 128:128 36:36
G3V2W1_NP.4
p−min=0.5, pY=6.3e−21, N=324
158:161 127:128 36:36
G3V2W1_NP.5
p−min=0.37, pY=0.79, N=321
peptide level
protein level

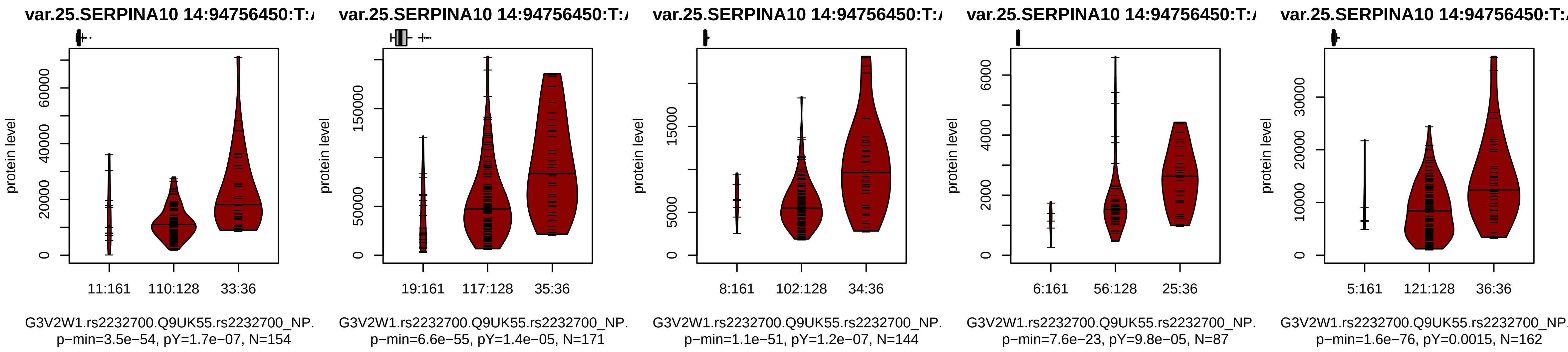
var.25.SERPINA10 14:94756450:T:A
protein level
0
20000
40000
60000
11:161
110:128
33:36
G3V2W1.rs2232700.Q9UK55.rs2232700_NP.
p−min=3.5e−54, pY=1.7e−07, N=154
var.25.SERPINA10 14:94756450:T:A
protein level
0
50000
150000
19:161
117:128
35:36
G3V2W1.rs2232700.Q9UK55.rs2232700_NP.
p−min=6.6e−55, pY=1.4e−05, N=171
var.25.SERPINA10 14:94756450:T:A
protein level
0
5000
15000
8:161
102:128
34:36
G3V2W1.rs2232700.Q9UK55.rs2232700_NP.
p−min=1.1e−51, pY=1.2e−07, N=144
var.25.SERPINA10 14:94756450:T:A
protein level
0
2000
4000
6000
6:161
56:128
25:36
G3V2W1.rs2232700.Q9UK55.rs2232700_NP.
p−min=7.6e−23, pY=9.8e−05, N=87
var.25.SERPINA10 14:94756450:T:A
protein level
0
10000
20000
30000
5:161
121:128
36:36
G3V2W1.rs2232700.Q9UK55.rs2232700_NP.
p−min=1.6e−76, pY=0.0015, N=162

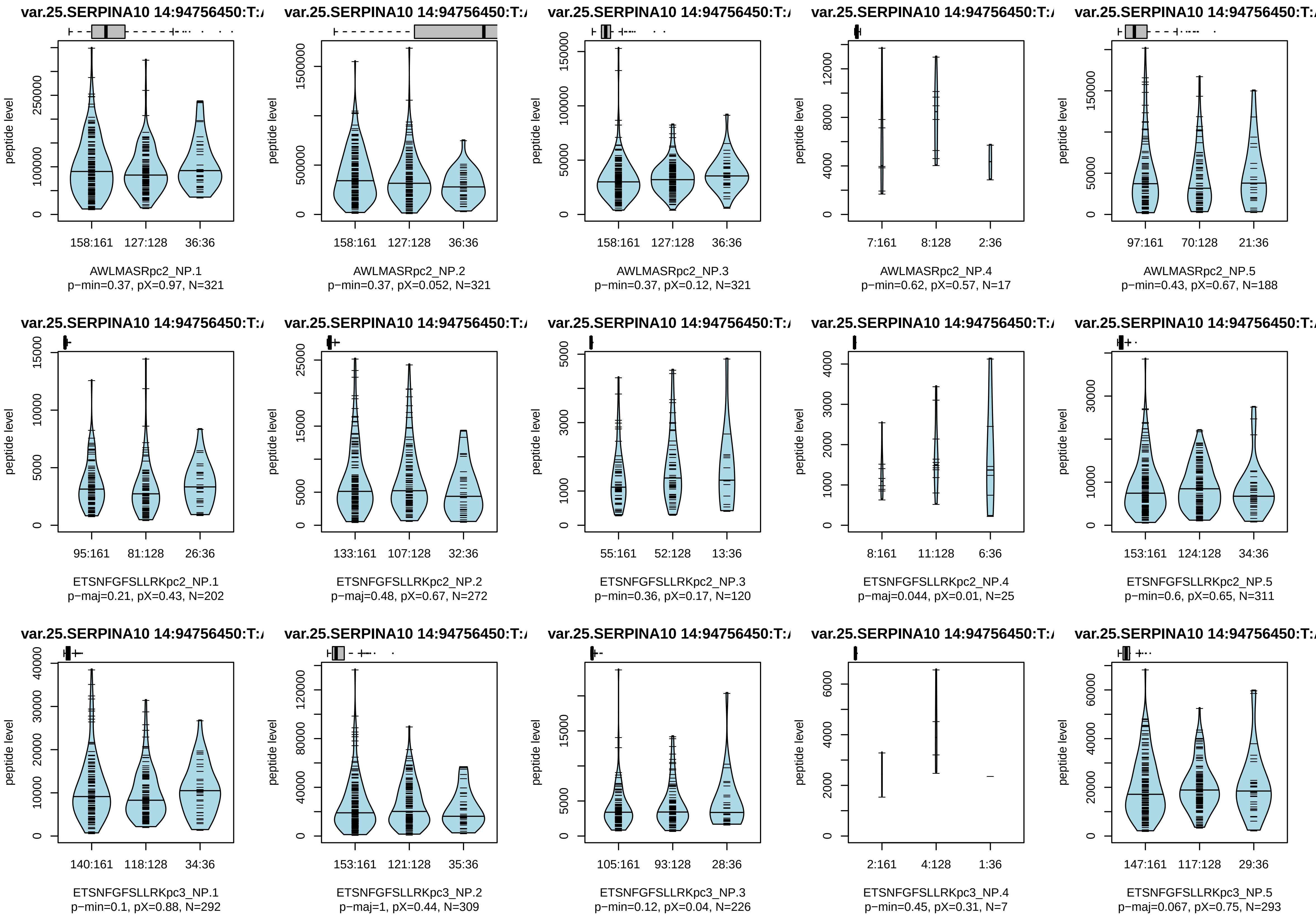
var.25.SERPINA10 14:94756450:T:/
peptide level
158:161
127:128
36:36
AWLMASRpc2_NP.1
p−min=0.37, pX=0.97, N=321
var.25.SERPINA10 14:94756450:T:/
peptide level
158:161
127:128
36:36
AWLMASRpc2_NP.2
p−min=0.37, pX=0.052, N=321
var.25.SERPINA10 14:94756450:T:/
peptide level
158:161
127:128
36:36
AWLMASRpc2_NP.3
p−min=0.37, pX=0.12, N=321
var.25.SERPINA10 14:94756450:T:/
peptide level
7:161
8:128
2:36
AWLMASRpc2_NP.4
p−min=0.62, pX=0.57, N=17
var.25.SERPINA10 14:94756450:T:/
peptide level
97:161
70:128
21:36
AWLMASRpc2_NP.5
p−min=0.43, pX=0.67, N=188
var.25.SERPINA10 14:94756450:T:/
peptide level
95:161
81:128
26:36
ETSNFGFSLLRKpc2_NP.1
p−maj=0.21, pX=0.43, N=202
var.25.SERPINA10 14:94756450:T:/
peptide level
133:161
107:128
32:36
ETSNFGFSLLRKpc2_NP.2
p−maj=0.48, pX=0.67, N=272
var.25.SERPINA10 14:94756450:T:/
peptide level
55:161
52:128
13:36
ETSNFGFSLLRKpc2_NP.3
p−min=0.36, pX=0.17, N=120
var.25.SERPINA10 14:94756450:T:/
peptide level
8:161
11:128
6:36
ETSNFGFSLLRKpc2_NP.4
p−maj=0.044, pX=0.01, N=25
var.25.SERPINA10 14:94756450:T:/
peptide level
153:161
124:128
34:36
ETSNFGFSLLRKpc2_NP.5
p−min=0.6, pX=0.65, N=311
var.25.SERPINA10 14:94756450:T:/
peptide level
140:161
118:128
34:36
ETSNFGFSLLRKpc3_NP.1
p−min=0.1, pX=0.88, N=292
var.25.SERPINA10 14:94756450:T:/
peptide level
153:161
121:128
35:36
ETSNFGFSLLRKpc3_NP.2
p−maj=1, pX=0.44, N=309
var.25.SERPINA10 14:94756450:T:/
peptide level
105:161
93:128
28:36
ETSNFGFSLLRKpc3_NP.3
p−min=0.12, pX=0.04, N=226
var.25.SERPINA10 14:94756450:T:/
peptide level
2:161
4:128
1:36
ETSNFGFSLLRKpc3_NP.4
p−min=0.45, pX=0.31, N=7
var.25.SERPINA10 14:94756450:T:/
peptide level
147:161
117:128
29:36
ETSNFGFSLLRKpc3_NP.5
p−maj=0.067, pX=0.75, N=293

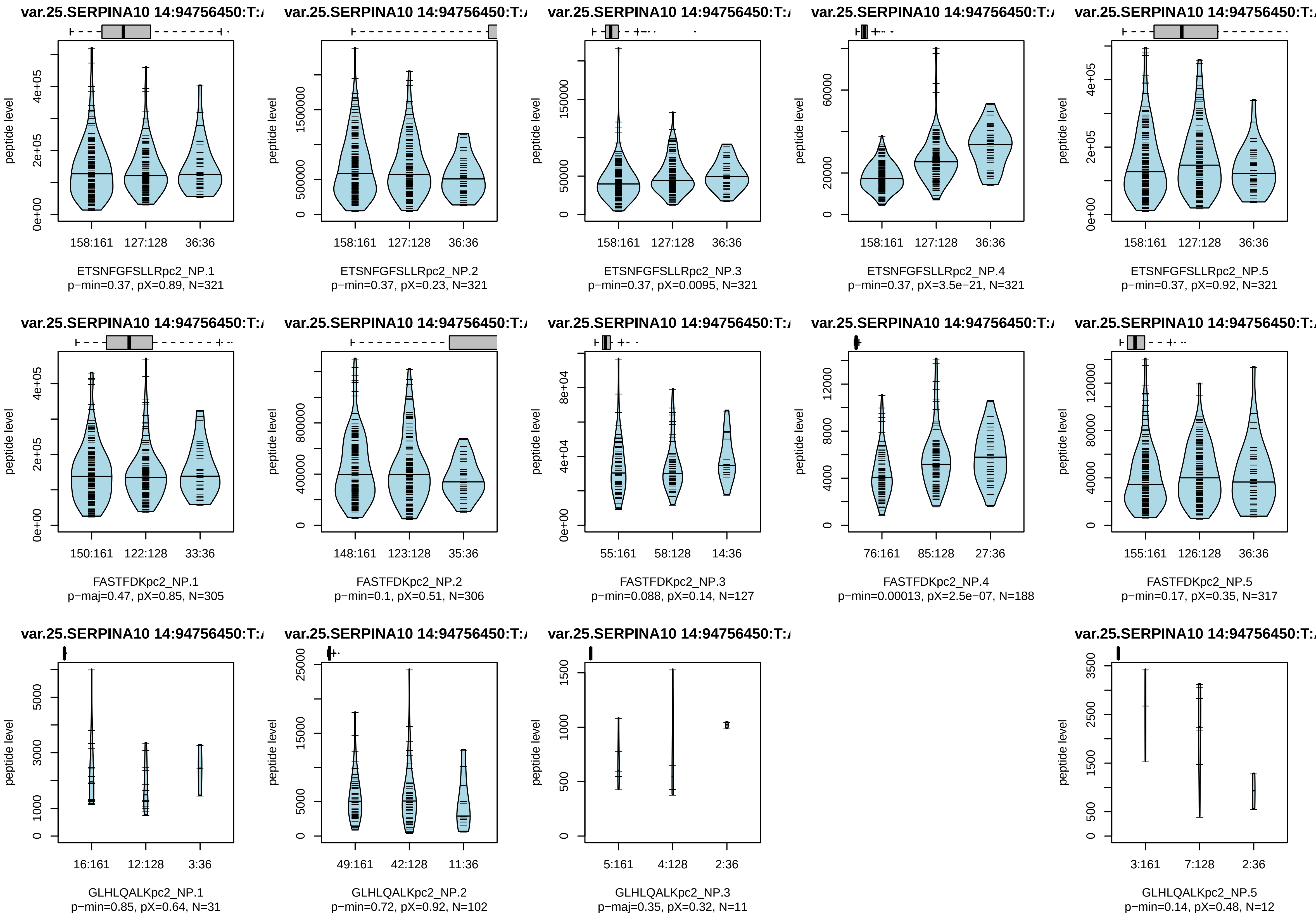

var.25.SERPINA10 14:94756450:T:/
ETSNFGFSLLRpc2_NP.1
p−min=0.37, pX=0.89, N=321
ETSNFGFSLLRpc2_NP.2
p−min=0.37, pX=0.23, N=321
ETSNFGFSLLRpc2_NP.3
p−min=0.37, pX=0.0095, N=321
ETSNFGFSLLRpc2_NP.4
p−min=0.37, pX=3.5e−21, N=321
ETSNFGFSLLRpc2_NP.5
p−min=0.37, pX=0.92, N=321
FASTFDKpc2_NP.1
p−maj=0.47, pX=0.85, N=305
FASTFDKpc2_NP.2
p−min=0.1, pX=0.51, N=306
FASTFDKpc2_NP.3
p−min=0.088, pX=0.14, N=127
FASTFDKpc2_NP.4
p−min=0.00013, pX=2.5e−07, N=188
FASTFDKpc2_NP.5
p−min=0.17, pX=0.35, N=317
GLHLQALKpc2_NP.1
p−min=0.85, pX=0.64, N=31
GLHLQALKpc2_NP.2
p−min=0.72, pX=0.92, N=102
GLHLQALKpc2_NP.3
p−maj=0.35, pX=0.32, N=11
GLHLQALKpc2_NP.5
p−min=0.14, pX=0.48, N=12
peptide level
158:161
127:128
36:36
150:161
122:128
33:36
148:161
123:128
35:36
55:161
58:128
14:36
76:161
85:128
27:36
155:161
126:128
36:36
16:161
12:128
3:36
49:161
42:128
11:36
5:161
4:128
2:36
3:161
7:128
2:36

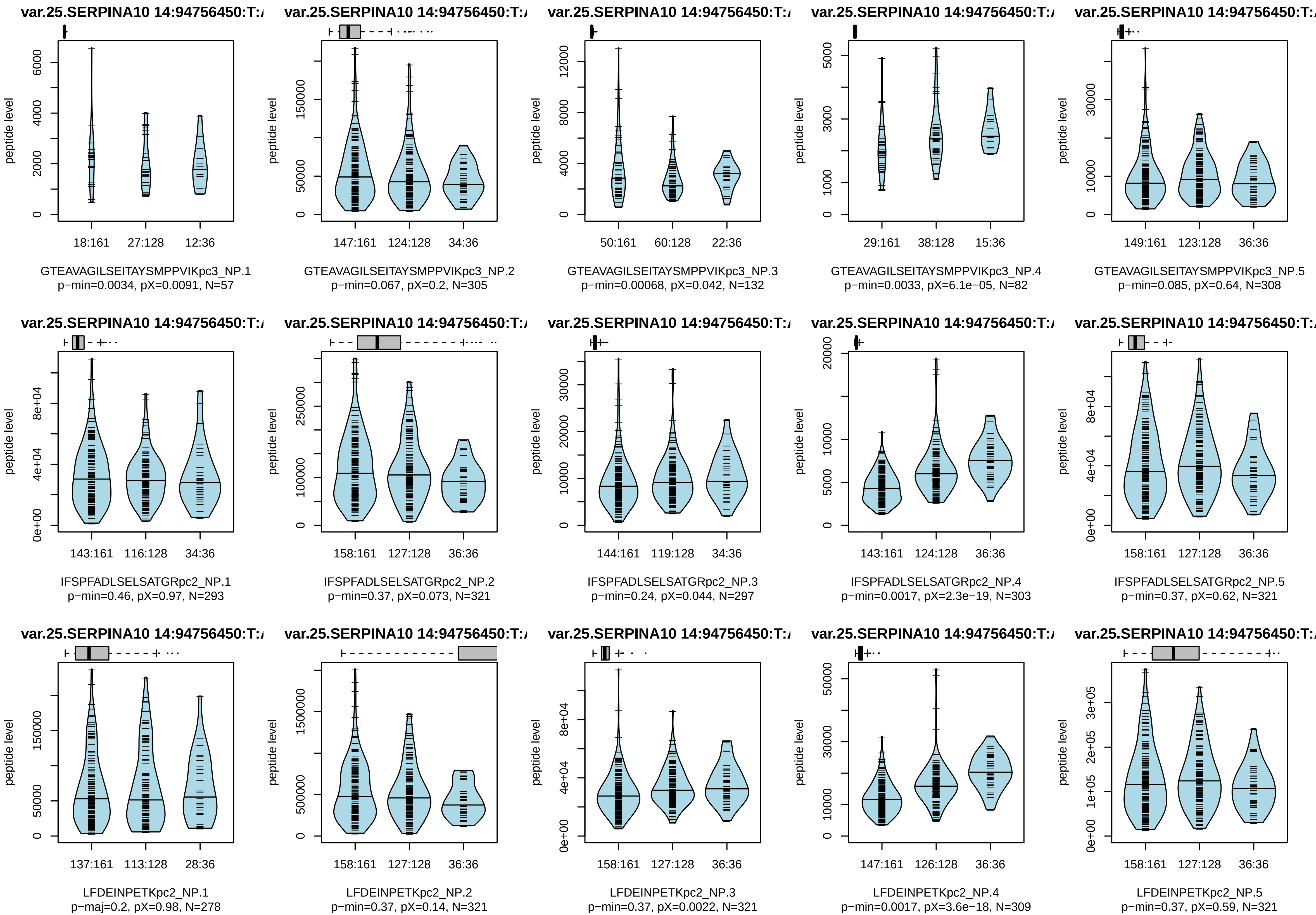
var.25.SERPINA10 14:94756450:T:/
18:161 27:128 12:36
GTEAVAGILSEITAYSMPPVIKpc3_NP.1
p−min=0.0034, pX=0.0091, N=57
var.25.SERPINA10 14:94756450:T:/
147:161 124:128 34:36
GTEAVAGILSEITAYSMPPVIKpc3_NP.2
p−min=0.067, pX=0.2, N=305
var.25.SERPINA10 14:94756450:T:/
50:161 60:128 22:36
GTEAVAGILSEITAYSMPPVIKpc3_NP.3
p−min=0.00068, pX=0.042, N=132
var.25.SERPINA10 14:94756450:T:/
29:161 38:128 15:36
GTEAVAGILSEITAYSMPPVIKpc3_NP.4
p−min=0.0033, pX=6.1e−05, N=82
var.25.SERPINA10 14:94756450:T:/
149:161 123:128 36:36
GTEAVAGILSEITAYSMPPVIKpc3_NP.5
p−min=0.085, pX=0.64, N=308
var.25.SERPINA10 14:94756450:T:/
143:161 116:128 34:36
IFSPFADLSELSATGRpc2_NP.1
p−min=0.46, pX=0.97, N=293
var.25.SERPINA10 14:94756450:T:/
158:161 127:128 36:36
IFSPFADLSELSATGRpc2_NP.2
p−min=0.37, pX=0.073, N=321
var.25.SERPINA10 14:94756450:T:/
144:161 119:128 34:36
IFSPFADLSELSATGRpc2_NP.3
p−min=0.24, pX=0.044, N=297
var.25.SERPINA10 14:94756450:T:/
143:161 124:128 36:36
IFSPFADLSELSATGRpc2_NP.4
p−min=0.0017, pX=2.3e−19, N=303
var.25.SERPINA10 14:94756450:T:/
158:161 127:128 36:36
IFSPFADLSELSATGRpc2_NP.5
p−min=0.37, pX=0.62, N=321
var.25.SERPINA10 14:94756450:T:/
137:161 113:128 28:36
LFDEINPETKpc2_NP.1
p−maj=0.2, pX=0.98, N=278
var.25.SERPINA10 14:94756450:T:/
158:161 127:128 36:36
LFDEINPETKpc2_NP.2
p−min=0.37, pX=0.14, N=321
var.25.SERPINA10 14:94756450:T:/
158:161 127:128 36:36
LFDEINPETKpc2_NP.3
p−min=0.37, pX=0.0022, N=321
var.25.SERPINA10 14:94756450:T:/
147:161 126:128 36:36
LFDEINPETKpc2_NP.4
p−min=0.0017, pX=3.6e−18, N=309
var.25.SERPINA10 14:94756450:T:/
158:161 127:128 36:36
LFDEINPETKpc2_NP.5
p−min=0.37, pX=0.59, N=321
peptide level

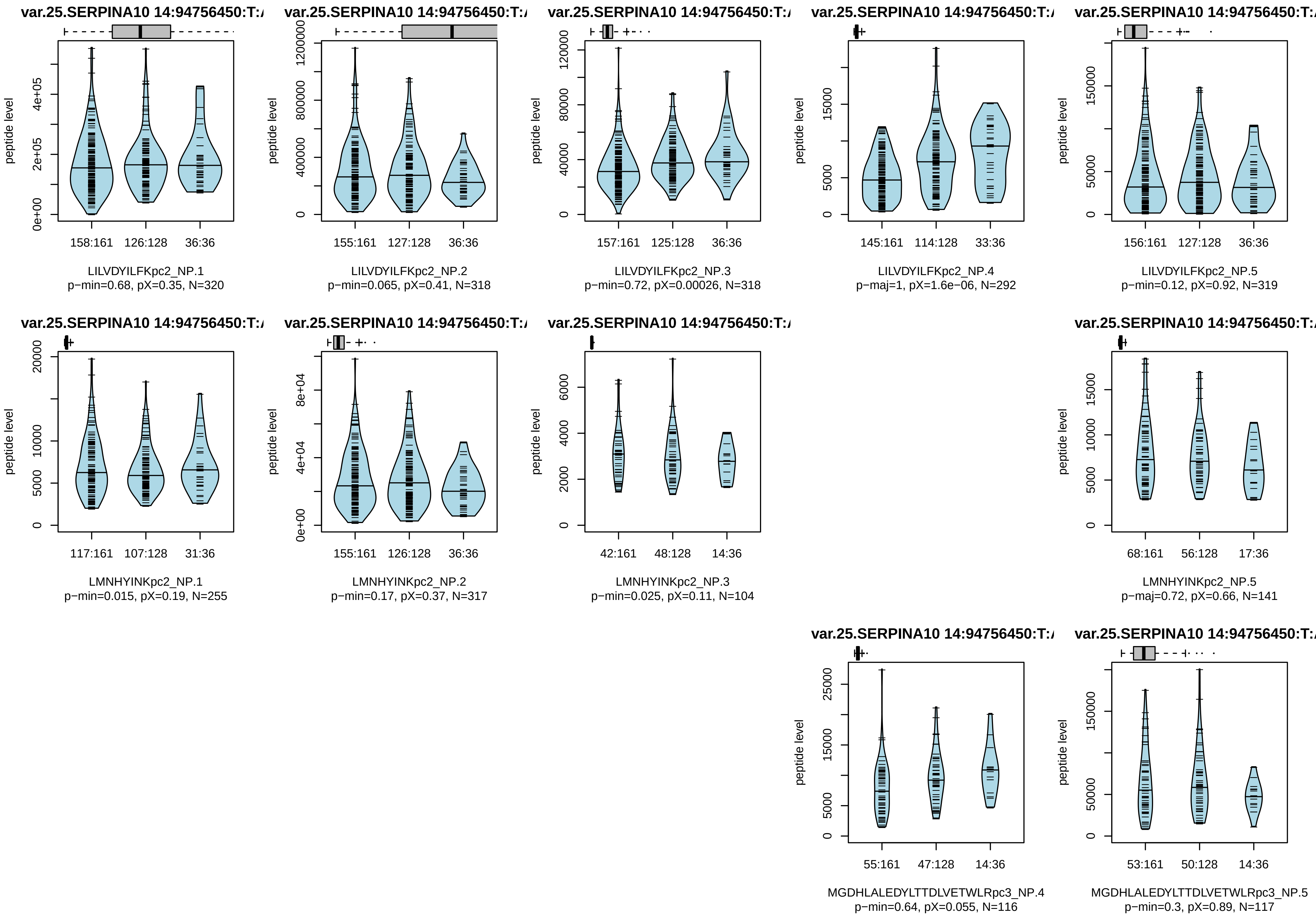
var.25.SERPINA10 14:94756450:T:/
LILVDYILFKpc2_NP.1
p−min=0.68, pX=0.35, N=320
LILVDYILFKpc2_NP.2
p−min=0.065, pX=0.41, N=318
LILVDYILFKpc2_NP.3
p−min=0.72, pX=0.00026, N=318
LILVDYILFKpc2_NP.4
p−maj=1, pX=1.6e−06, N=292
LILVDYILFKpc2_NP.5
p−min=0.12, pX=0.92, N=319
LMNHYINKpc2_NP.1
p−min=0.015, pX=0.19, N=255
LMNHYINKpc2_NP.2
p−min=0.17, pX=0.37, N=317
LMNHYINKpc2_NP.3
p−min=0.025, pX=0.11, N=104
LMNHYINKpc2_NP.5
p−maj=0.72, pX=0.66, N=141
MGDHLALEDYLTTDLVETWLRpc3_NP.4
p−min=0.64, pX=0.055, N=116
MGDHLALEDYLTTDLVETWLRpc3_NP.5
p−min=0.3, pX=0.89, N=117
peptide level

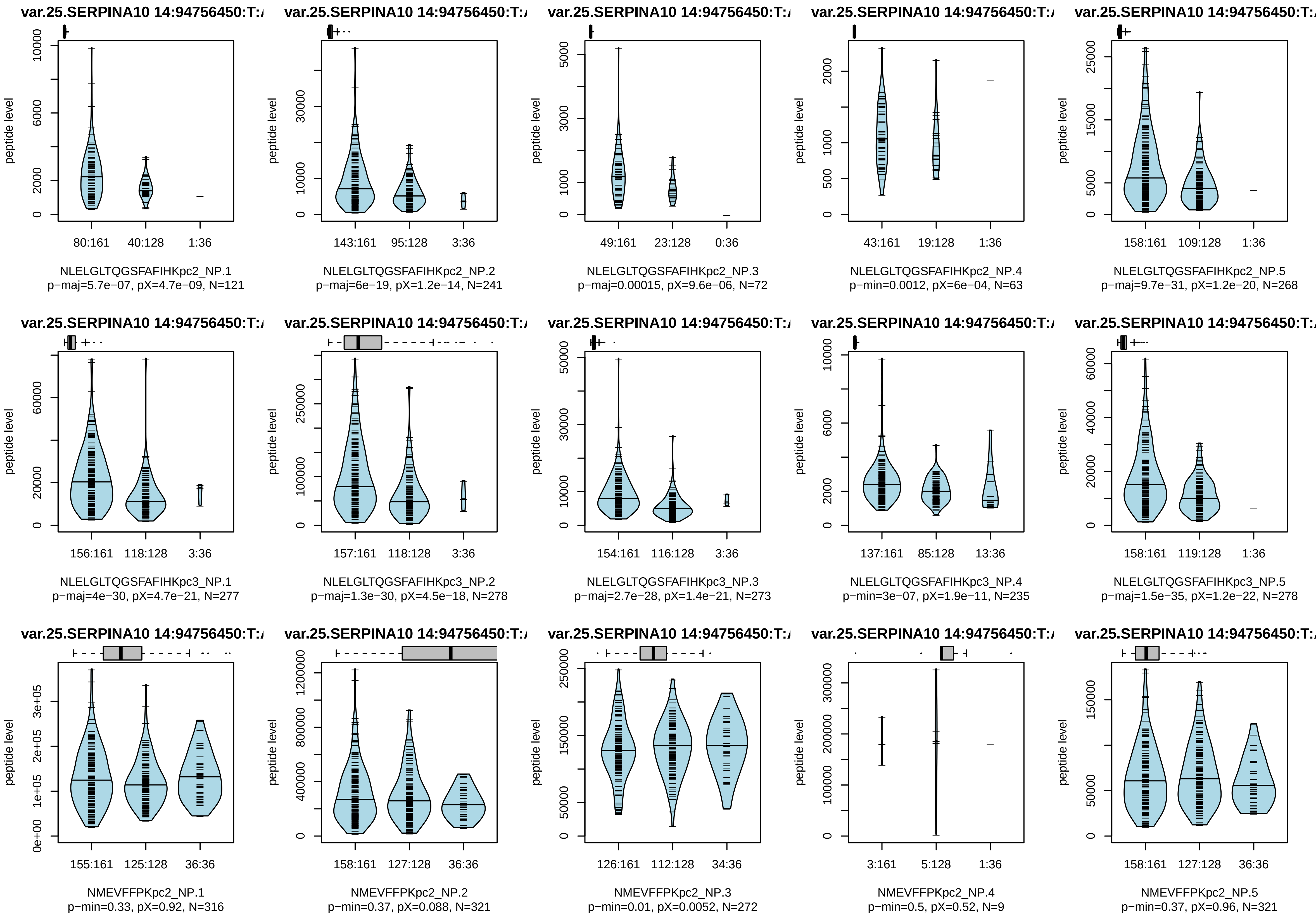

var.25.SERPINA10 14:94756450:T:/
peptide level
80:161
40:128
1:36
NLELGLTQGSFAFIHKpc2_NP.1
p−maj=5.7e−07, pX=4.7e−09, N=121
var.25.SERPINA10 14:94756450:T:/
peptide level
143:161
95:128
3:36
NLELGLTQGSFAFIHKpc2_NP.2
p−maj=6e−19, pX=1.2e−14, N=241
var.25.SERPINA10 14:94756450:T:/
peptide level
49:161
23:128
0:36
NLELGLTQGSFAFIHKpc2_NP.3
p−maj=0.00015, pX=9.6e−06, N=72
var.25.SERPINA10 14:94756450:T:/
peptide level
43:161
19:128
1:36
NLELGLTQGSFAFIHKpc2_NP.4
p−min=0.0012, pX=6e−04, N=63
var.25.SERPINA10 14:94756450:T:/
peptide level
158:161
109:128
1:36
NLELGLTQGSFAFIHKpc2_NP.5
p−maj=9.7e−31, pX=1.2e−20, N=268
var.25.SERPINA10 14:94756450:T:/
peptide level
156:161
118:128
3:36
NLELGLTQGSFAFIHKpc3_NP.1
p−maj=4e−30, pX=4.7e−21, N=277
var.25.SERPINA10 14:94756450:T:/
peptide level
157:161
118:128
3:36
NLELGLTQGSFAFIHKpc3_NP.2
p−maj=1.3e−30, pX=4.5e−18, N=278
var.25.SERPINA10 14:94756450:T:/
peptide level
154:161
116:128
3:36
NLELGLTQGSFAFIHKpc3_NP.3
p−maj=2.7e−28, pX=1.4e−21, N=273
var.25.SERPINA10 14:94756450:T:/
peptide level
137:161
85:128
13:36
NLELGLTQGSFAFIHKpc3_NP.4
p−min=3e−07, pX=1.9e−11, N=235
var.25.SERPINA10 14:94756450:T:/
peptide level
158:161
119:128
1:36
NLELGLTQGSFAFIHKpc3_NP.5
p−maj=1.5e−35, pX=1.2e−22, N=278
var.25.SERPINA10 14:94756450:T:/
peptide level
155:161
125:128
36:36
NMEVFFPKpc2_NP.1
p−min=0.33, pX=0.92, N=316
var.25.SERPINA10 14:94756450:T:/
peptide level
158:161
127:128
36:36
NMEVFFPKpc2_NP.2
p−min=0.37, pX=0.088, N=321
var.25.SERPINA10 14:94756450:T:/
peptide level
126:161
112:128
34:36
NMEVFFPKpc2_NP.3
p−min=0.01, pX=0.0052, N=272
var.25.SERPINA10 14:94756450:T:/
peptide level
3:161
5:128
1:36
NMEVFFPKpc2_NP.4
p−min=0.5, pX=0.52, N=9
var.25.SERPINA10 14:94756450:T:/
peptide level
158:161
127:128
36:36
NMEVFFPKpc2_NP.5
p−min=0.37, pX=0.96, N=321

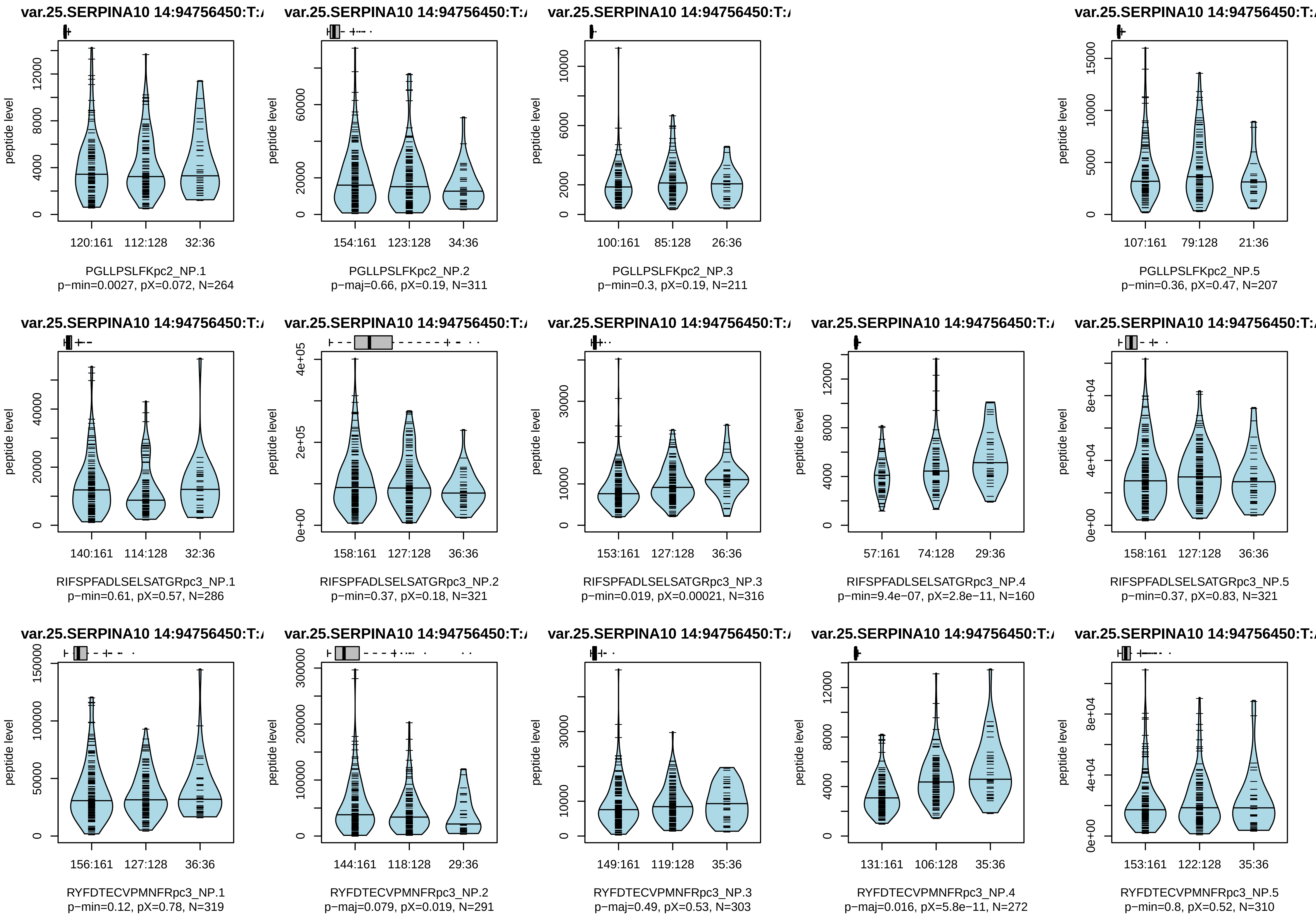
var.25.SERPINA10 14:94756450:T:/
peptide level
120:161
112:128
32:36
PGLLPSLFKpc2_NP.1
p−min=0.0027, pX=0.072, N=264
var.25.SERPINA10 14:94756450:T:/
peptide level
154:161
123:128
34:36
PGLLPSLFKpc2_NP.2
p−maj=0.66, pX=0.19, N=311
var.25.SERPINA10 14:94756450:T:/
peptide level
100:161
85:128
26:36
PGLLPSLFKpc2_NP.3
p−min=0.3, pX=0.19, N=211
var.25.SERPINA10 14:94756450:T:/
peptide level
107:161
79:128
21:36
PGLLPSLFKpc2_NP.5
p−min=0.36, pX=0.47, N=207
var.25.SERPINA10 14:94756450:T:/
peptide level
140:161
114:128
32:36
RIFSPFADLSELSATGRpc3_NP.1
p−min=0.61, pX=0.57, N=286
var.25.SERPINA10 14:94756450:T:/
peptide level
158:161
127:128
36:36
RIFSPFADLSELSATGRpc3_NP.2
p−min=0.37, pX=0.18, N=321
var.25.SERPINA10 14:94756450:T:/
peptide level
153:161
127:128
36:36
RIFSPFADLSELSATGRpc3_NP.3
p−min=0.019, pX=0.00021, N=316
var.25.SERPINA10 14:94756450:T:/
peptide level
57:161
74:128
29:36
RIFSPFADLSELSATGRpc3_NP.4
p−min=9.4e−07, pX=2.8e−11, N=160
var.25.SERPINA10 14:94756450:T:/
peptide level
158:161
127:128
36:36
RIFSPFADLSELSATGRpc3_NP.5
p−min=0.37, pX=0.83, N=321
var.25.SERPINA10 14:94756450:T:/
peptide level
156:161
127:128
36:36
RYFDTECVPMNFRpc3_NP.1
p−min=0.12, pX=0.78, N=319
var.25.SERPINA10 14:94756450:T:/
peptide level
144:161
118:128
29:36
RYFDTECVPMNFRpc3_NP.2
p−maj=0.079, pX=0.019, N=291
var.25.SERPINA10 14:94756450:T:/
peptide level
149:161
119:128
35:36
RYFDTECVPMNFRpc3_NP.3
p−maj=0.49, pX=0.53, N=303
var.25.SERPINA10 14:94756450:T:/
peptide level
131:161
106:128
35:36
RYFDTECVPMNFRpc3_NP.4
p−maj=0.016, pX=5.8e−11, N=272
var.25.SERPINA10 14:94756450:T:/
peptide level
153:161
122:128
35:36
RYFDTECVPMNFRpc3_NP.5
p−min=0.8, pX=0.52, N=310

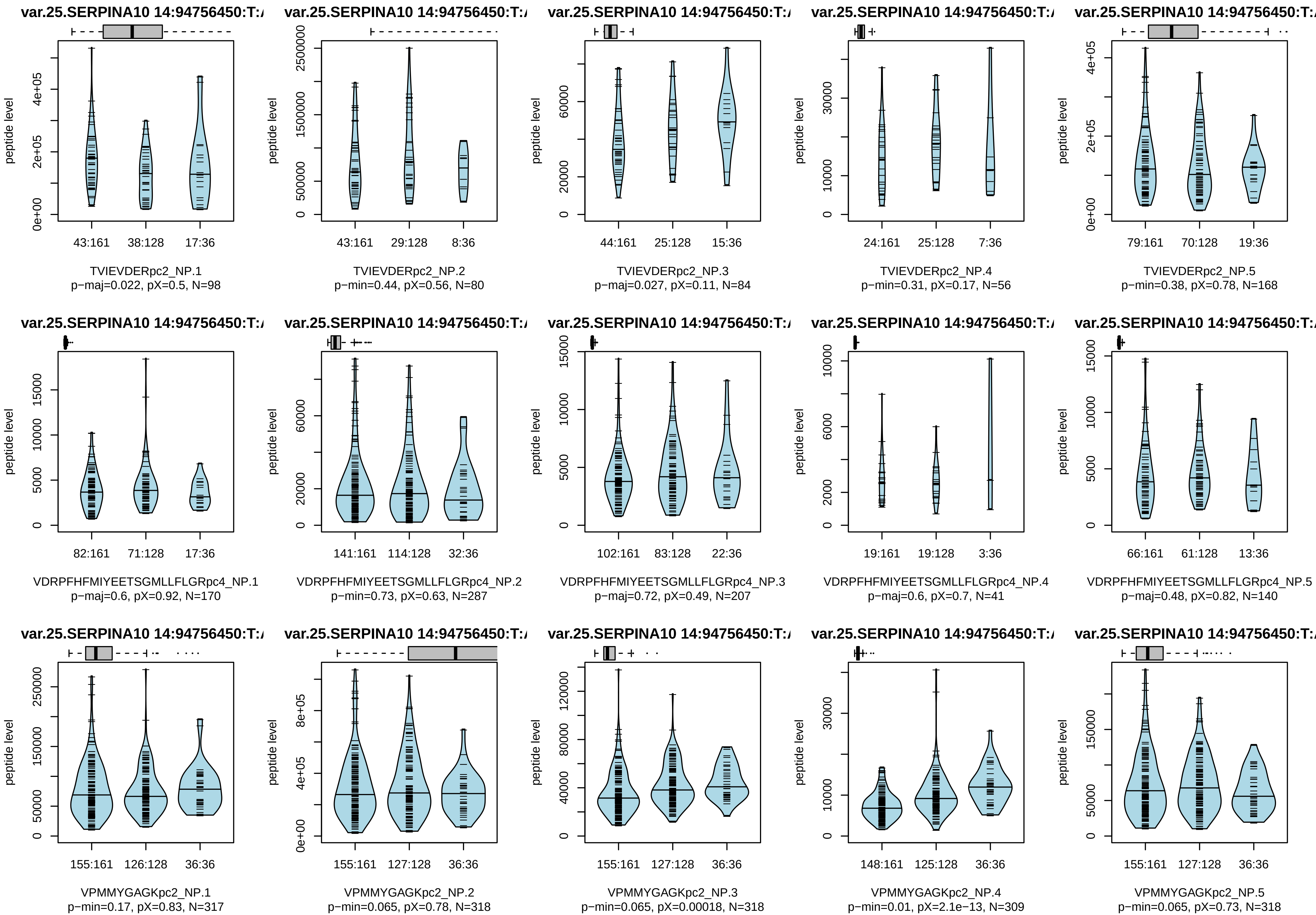
var.25.SERPINA10 14:94756450:T:/
peptide level
43:161 38:128 17:36
TVIEVDERpc2_NP.1
p−maj=0.022, pX=0.5, N=98
var.25.SERPINA10 14:94756450:T:/
peptide level
43:161 29:128 8:36
TVIEVDERpc2_NP.2
p−min=0.44, pX=0.56, N=80
var.25.SERPINA10 14:94756450:T:/
peptide level
44:161 25:128 15:36
TVIEVDERpc2_NP.3
p−maj=0.027, pX=0.11, N=84
var.25.SERPINA10 14:94756450:T:/
peptide level
24:161 25:128 7:36
TVIEVDERpc2_NP.4
p−min=0.31, pX=0.17, N=56
var.25.SERPINA10 14:94756450:T:/
peptide level
79:161 70:128 19:36
TVIEVDERpc2_NP.5
p−min=0.38, pX=0.78, N=168
var.25.SERPINA10 14:94756450:T:/
peptide level
82:161 71:128 17:36
VDRPFHFMIYEETSGMLLFLGRpc4_NP.1
p−maj=0.6, pX=0.92, N=170
var.25.SERPINA10 14:94756450:T:/
peptide level
141:161 114:128 32:36
VDRPFHFMIYEETSGMLLFLGRpc4_NP.2
p−min=0.73, pX=0.63, N=287
var.25.SERPINA10 14:94756450:T:/
peptide level
102:161 83:128 22:36
VDRPFHFMIYEETSGMLLFLGRpc4_NP.3
p−maj=0.72, pX=0.49, N=207
var.25.SERPINA10 14:94756450:T:/
peptide level
19:161 19:128 3:36
VDRPFHFMIYEETSGMLLFLGRpc4_NP.4
p−maj=0.6, pX=0.7, N=41
var.25.SERPINA10 14:94756450:T:/
peptide level
66:161 61:128 13:36
VDRPFHFMIYEETSGMLLFLGRpc4_NP.5
p−maj=0.48, pX=0.82, N=140
var.25.SERPINA10 14:94756450:T:/
peptide level
155:161 126:128 36:36
VPMMYGAGKpc2_NP.1
p−min=0.17, pX=0.83, N=317
var.25.SERPINA10 14:94756450:T:/
peptide level
155:161 127:128 36:36
VPMMYGAGKpc2_NP.2
p−min=0.065, pX=0.78, N=318
var.25.SERPINA10 14:94756450:T:/
peptide level
155:161 127:128 36:36
VPMMYGAGKpc2_NP.3
p−min=0.065, pX=0.00018, N=318
var.25.SERPINA10 14:94756450:T:/
peptide level
148:161 125:128 36:36
VPMMYGAGKpc2_NP.4
p−min=0.01, pX=2.1e−13, N=309
var.25.SERPINA10 14:94756450:T:/
peptide level
155:161 127:128 36:36
VPMMYGAGKpc2_NP.5
p−min=0.065, pX=0.73, N=318

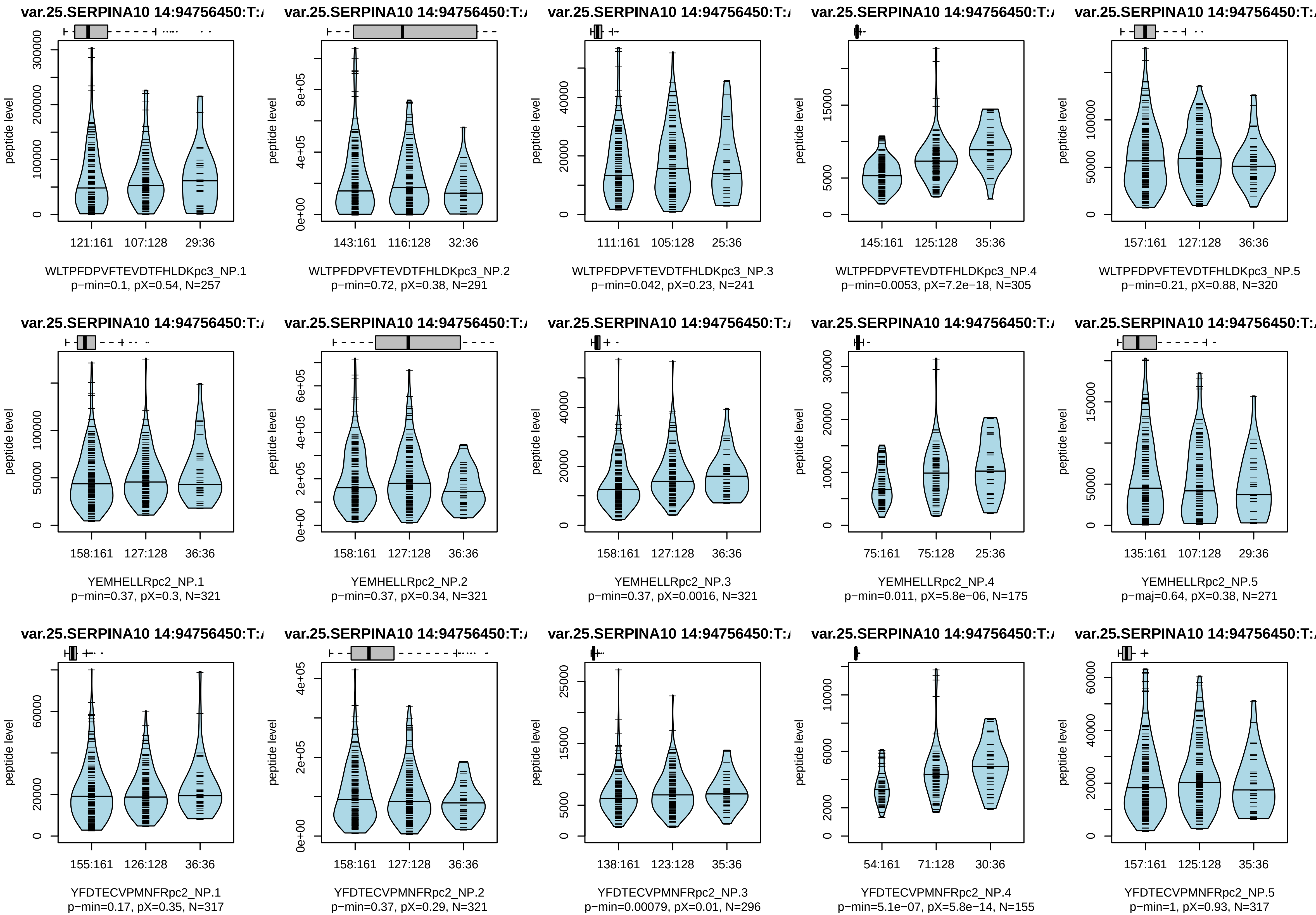
var.25.SERPINA10 14:94756450:T:
peptide level
121:161 107:128 29:36
WLTPFDPVFTEVDTFHLDKpc3_NP.1
p−min=0.1, pX=0.54, N=257
var.25.SERPINA10 14:94756450:T:
peptide level
143:161 116:128 32:36
WLTPFDPVFTEVDTFHLDKpc3_NP.2
p−min=0.72, pX=0.38, N=291
var.25.SERPINA10 14:94756450:T:
peptide level
111:161 105:128 25:36
WLTPFDPVFTEVDTFHLDKpc3_NP.3
p−min=0.042, pX=0.23, N=241
var.25.SERPINA10 14:94756450:T:
peptide level
145:161 125:128 35:36
WLTPFDPVFTEVDTFHLDKpc3_NP.4
p−min=0.0053, pX=7.2e−18, N=305
var.25.SERPINA10 14:94756450:T:
peptide level
157:161 127:128 36:36
WLTPFDPVFTEVDTFHLDKpc3_NP.5
p−min=0.21, pX=0.88, N=320
var.25.SERPINA10 14:94756450:T:
peptide level
158:161 127:128 36:36
YEMHELLRpc2_NP.1
p−min=0.37, pX=0.3, N=321
var.25.SERPINA10 14:94756450:T:
peptide level
158:161 127:128 36:36
YEMHELLRpc2_NP.2
p−min=0.37, pX=0.34, N=321
var.25.SERPINA10 14:94756450:T:
peptide level
158:161 127:128 36:36
YEMHELLRpc2_NP.3
p−min=0.37, pX=0.0016, N=321
var.25.SERPINA10 14:94756450:T:
peptide level
75:161 75:128 25:36
YEMHELLRpc2_NP.4
p−min=0.011, pX=5.8e−06, N=175
var.25.SERPINA10 14:94756450:T:
peptide level
135:161 107:128 29:36
YEMHELLRpc2_NP.5
p−maj=0.64, pX=0.38, N=271
var.25.SERPINA10 14:94756450:T:
peptide level
155:161 126:128 36:36
YFDTECVPMNFRpc2_NP.1
p−min=0.17, pX=0.35, N=317
var.25.SERPINA10 14:94756450:T:
peptide level
158:161 127:128 36:36
YFDTECVPMNFRpc2_NP.2
p−min=0.37, pX=0.29, N=321
var.25.SERPINA10 14:94756450:T:
peptide level
138:161 123:128 35:36
YFDTECVPMNFRpc2_NP.3
p−min=0.00079, pX=0.01, N=296
var.25.SERPINA10 14:94756450:T:
peptide level
54:161 71:128 30:36
YFDTECVPMNFRpc2_NP.4
p−min=5.1e−07, pX=5.8e−14, N=155
var.25.SERPINA10 14:94756450:T:
peptide level
157:161 125:128 35:36
YFDTECVPMNFRpc2_NP.5
p−min=1, pX=0.93, N=317

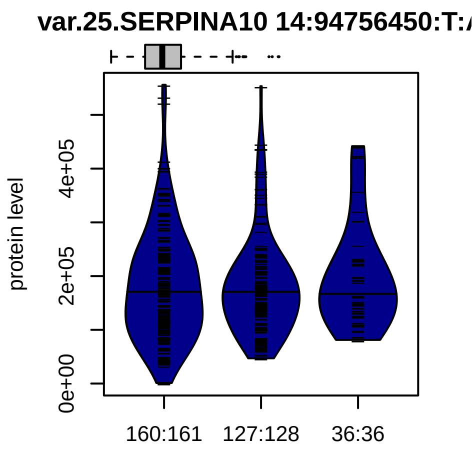

G3V2W1.Q9UK55_NP.1
p−maj=1, pY=0.26, N=323

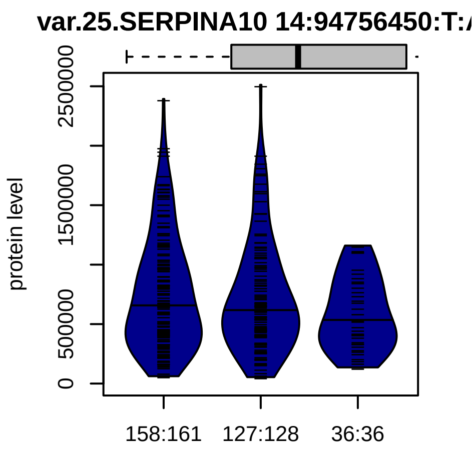

G3V2W1.Q9UK55_NP.2
p−min=0.37, pY=0.02, N=321

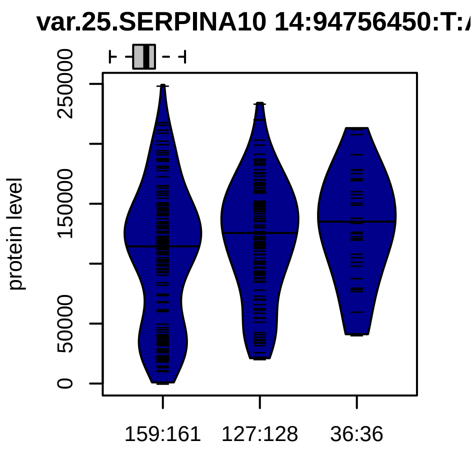

G3V2W1.Q9UK55_NP.3
p−min=0.62, pY=0.00012, N=322

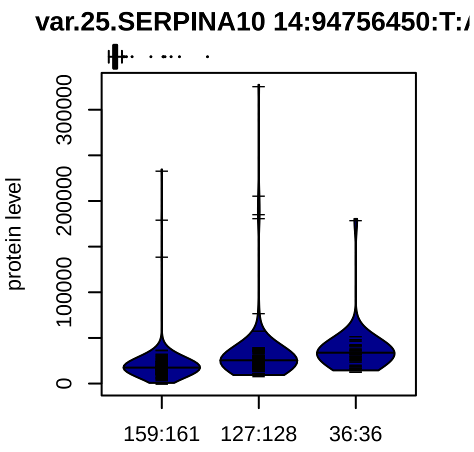

G3V2W1.Q9UK55_NP.4
p−min=0.62, pY=3.2e−20, N=322

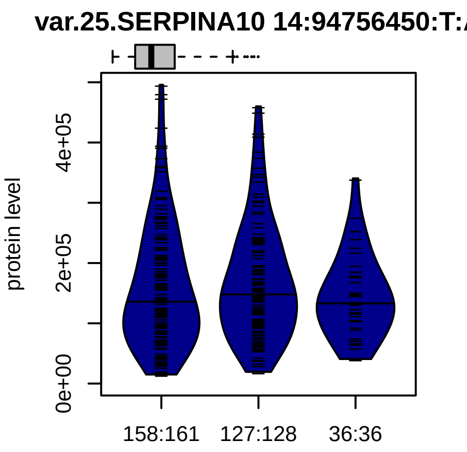

G3V2W1.Q9UK55_NP.5
p−min=0.37, pY=0.75, N=321

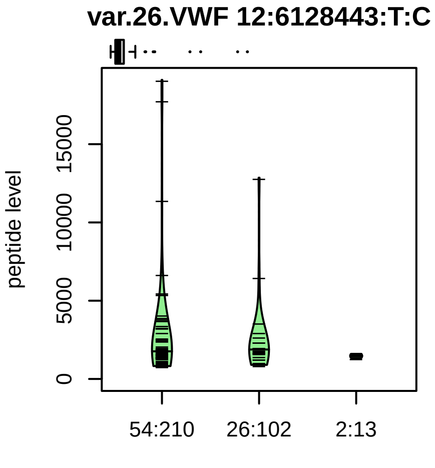

AFVLSSVDELEQQRpc2_NP.4
p−maj=0.53, pX=0.35, N=82

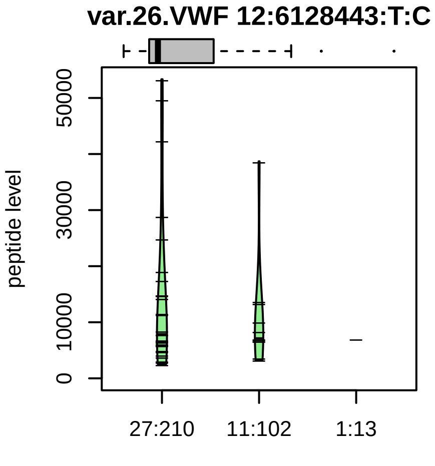

AFVVDMMERpc2_NP.4
p−min=0.59, pX=0.28, N=39

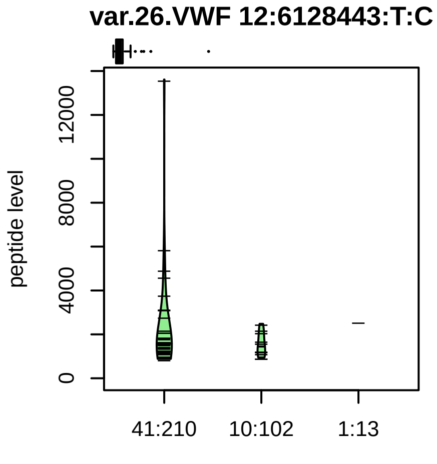

AHLLSLVDVMQRpc2_NP.1
p−min=0.026, pX=0.048, N=52

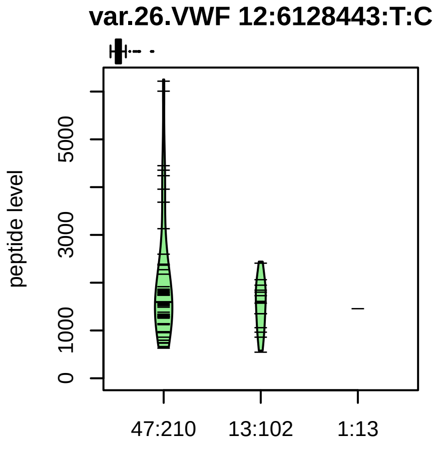

AHLLSLVDVMQRpc2_NP.2
p−min=0.026, pX=0.012, N=61

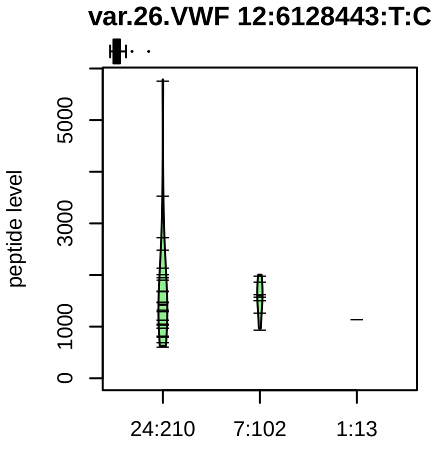

AHLLSLVDVMQRpc2_NP.3
p−min=0.24, pX=0.19, N=32

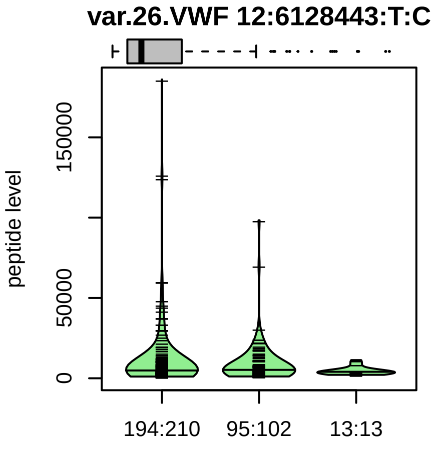

AHLLSLVDVMQRpc2_NP.4
p−maj=0.61, pX=0.22, N=302

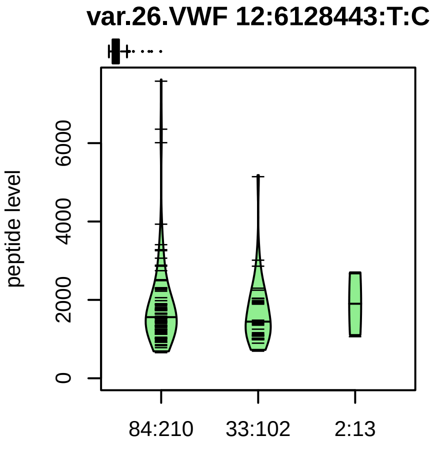

AHLLSLVDVMQRpc2_NP.5
p−min=0.093, pX=0.049, N=119

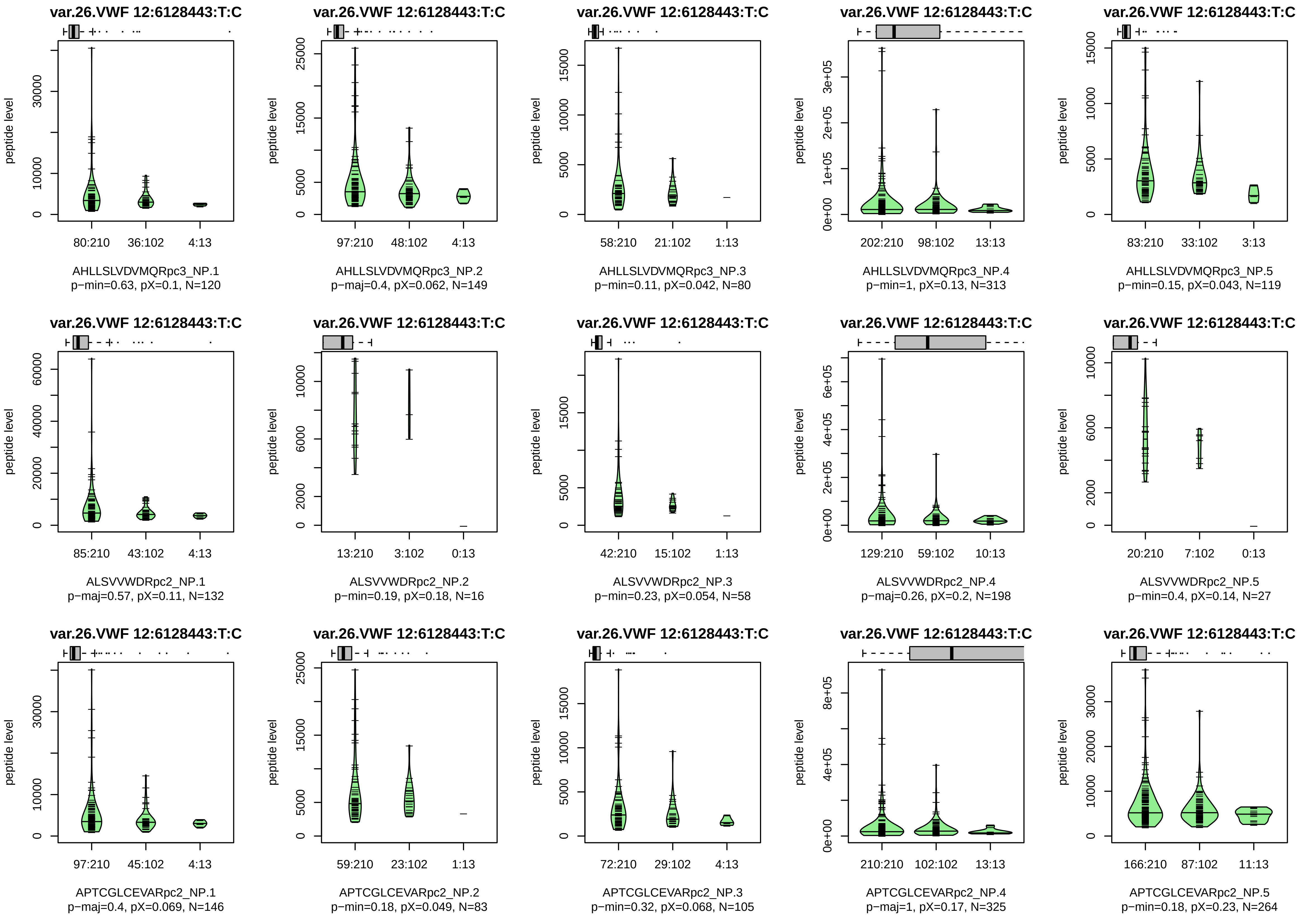
var.26.VWF 12:6128443:T:C
peptide level
80:210
36:102
4:13
AHLLSLVDVMQRpc3_NP.1
p−min=0.63, pX=0.1, N=120
var.26.VWF 12:6128443:T:C
peptide level
97:210
48:102
4:13
AHLLSLVDVMQRpc3_NP.2
p−maj=0.4, pX=0.062, N=149
var.26.VWF 12:6128443:T:C
peptide level
58:210
21:102
1:13
AHLLSLVDVMQRpc3_NP.3
p−min=0.11, pX=0.042, N=80
var.26.VWF 12:6128443:T:C
peptide level
202:210
98:102
13:13
AHLLSLVDVMQRpc3_NP.4
p−min=1, pX=0.13, N=313
var.26.VWF 12:6128443:T:C
peptide level
83:210
33:102
3:13
AHLLSLVDVMQRpc3_NP.5
p−min=0.15, pX=0.043, N=119
var.26.VWF 12:6128443:T:C
peptide level
85:210
43:102
4:13
ALSVVWDRpc2_NP.1
p−maj=0.57, pX=0.11, N=132
var.26.VWF 12:6128443:T:C
peptide level
13:210
3:102
0:13
ALSVVWDRpc2_NP.2
p−min=0.19, pX=0.18, N=16
var.26.VWF 12:6128443:T:C
peptide level
42:210
15:102
1:13
ALSVVWDRpc2_NP.3
p−min=0.23, pX=0.054, N=58
var.26.VWF 12:6128443:T:C
peptide level
129:210
59:102
10:13
ALSVVWDRpc2_NP.4
p−maj=0.26, pX=0.2, N=198
var.26.VWF 12:6128443:T:C
peptide level
20:210
7:102
0:13
ALSVVWDRpc2_NP.5
p−min=0.4, pX=0.14, N=27
var.26.VWF 12:6128443:T:C
peptide level
97:210
45:102
4:13
APTCGLCEVARpc2_NP.1
p−maj=0.4, pX=0.069, N=146
var.26.VWF 12:6128443:T:C
peptide level
59:210
23:102
1:13
APTCGLCEVARpc2_NP.2
p−min=0.18, pX=0.049, N=83
var.26.VWF 12:6128443:T:C
peptide level
72:210
29:102
4:13
APTCGLCEVARpc2_NP.3
p−min=0.32, pX=0.068, N=105
var.26.VWF 12:6128443:T:C
peptide level
210:210
102:102
13:13
APTCGLCEVARpc2_NP.4
p−maj=1, pX=0.17, N=325
var.26.VWF 12:6128443:T:C
peptide level
166:210
87:102
11:13
APTCGLCEVARpc2_NP.5
p−min=0.18, pX=0.23, N=264

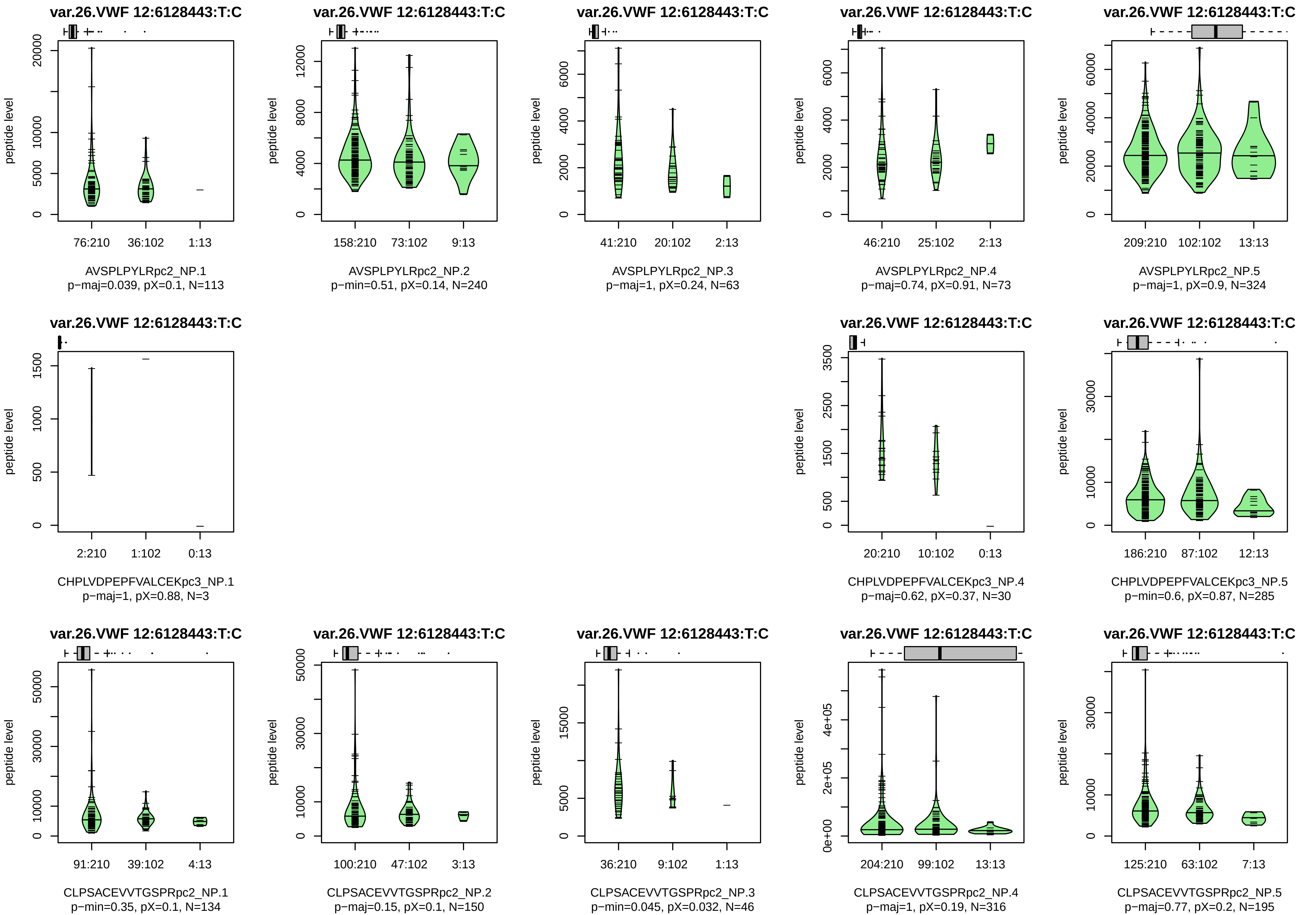
var.26.VWF 12:6128443:T:C
peptide level
76:210
36:102
1:13
AVSPLPYLRpc2_NP.1
p−maj=0.039, pX=0.1, N=113
var.26.VWF 12:6128443:T:C
peptide level
158:210
73:102
9:13
AVSPLPYLRpc2_NP.2
p−min=0.51, pX=0.14, N=240
var.26.VWF 12:6128443:T:C
peptide level
41:210
20:102
2:13
AVSPLPYLRpc2_NP.3
p−maj=1, pX=0.24, N=63
var.26.VWF 12:6128443:T:C
peptide level
46:210
25:102
2:13
AVSPLPYLRpc2_NP.4
p−maj=0.74, pX=0.91, N=73
var.26.VWF 12:6128443:T:C
peptide level
209:210
102:102
13:13
AVSPLPYLRpc2_NP.5
p−maj=1, pX=0.9, N=324
var.26.VWF 12:6128443:T:C
peptide level
2:210
1:102
0:13
CHPLVDPEPFVALCEKpc3_NP.1
p−maj=1, pX=0.88, N=3
var.26.VWF 12:6128443:T:C
peptide level
20:210
10:102
0:13
CHPLVDPEPFVALCEKpc3_NP.4
p−maj=0.62, pX=0.37, N=30
var.26.VWF 12:6128443:T:C
peptide level
186:210
87:102
12:13
CHPLVDPEPFVALCEKpc3_NP.5
p−min=0.6, pX=0.87, N=285
var.26.VWF 12:6128443:T:C
peptide level
91:210
39:102
4:13
CLPSACEVVTGSPRpc2_NP.1
p−min=0.35, pX=0.1, N=134
var.26.VWF 12:6128443:T:C
peptide level
100:210
47:102
3:13
CLPSACEVVTGSPRpc2_NP.2
p−maj=0.15, pX=0.1, N=150
var.26.VWF 12:6128443:T:C
peptide level
36:210
9:102
1:13
CLPSACEVVTGSPRpc2_NP.3
p−min=0.045, pX=0.032, N=46
var.26.VWF 12:6128443:T:C
peptide level
204:210
99:102
13:13
CLPSACEVVTGSPRpc2_NP.4
p−maj=1, pX=0.19, N=316
var.26.VWF 12:6128443:T:C
peptide level
125:210
63:102
7:13
CLPSACEVVTGSPRpc2_NP.5
p−maj=0.77, pX=0.2, N=195

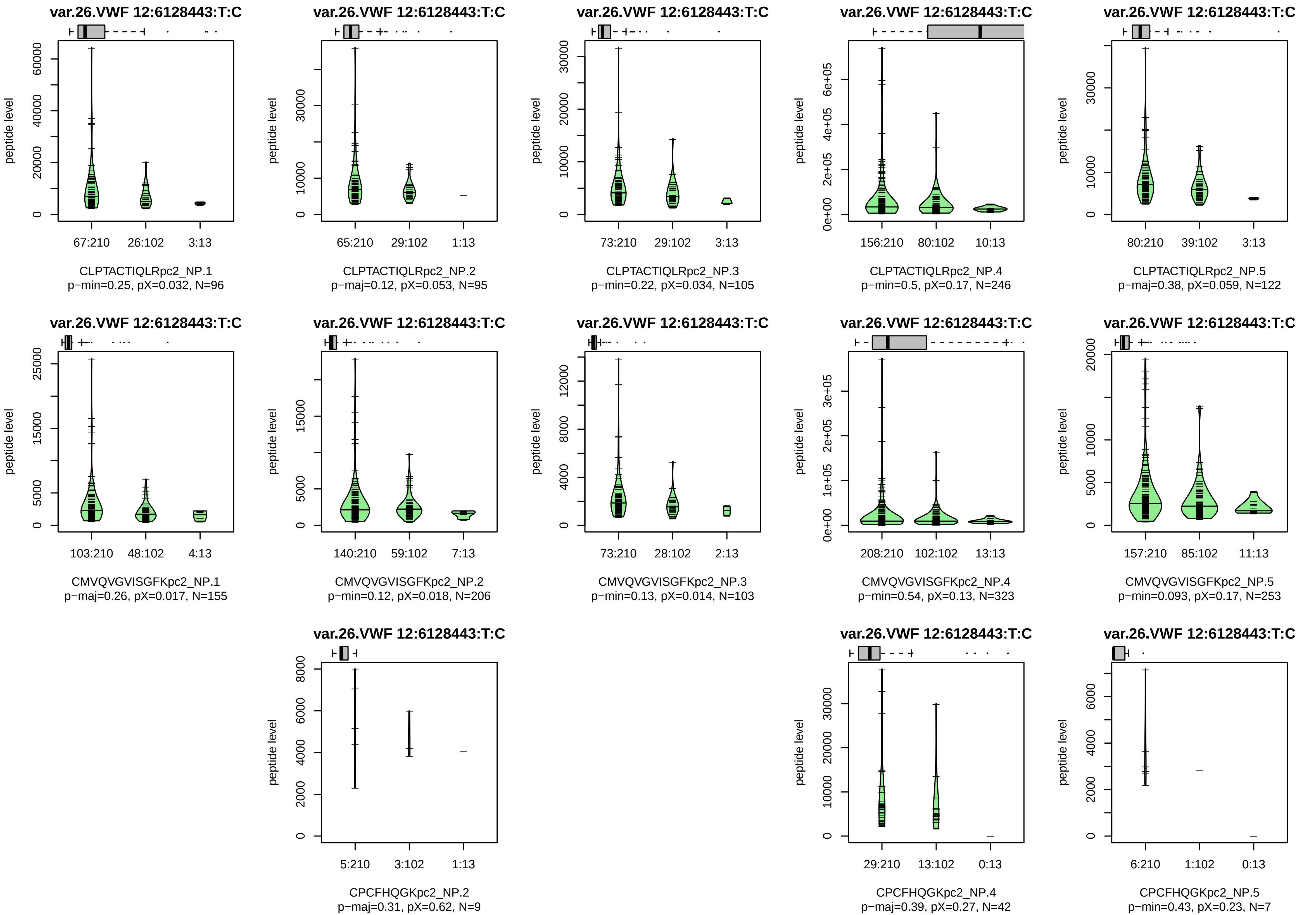
var.26.VWF 12:6128443:T:C
peptide level
67:210
26:102
3:13
CLPTACTIQLRpc2_NP.1
p−min=0.25, pX=0.032, N=96
var.26.VWF 12:6128443:T:C
65:210
29:102
1:13
CLPTACTIQLRpc2_NP.2
p−maj=0.12, pX=0.053, N=95
var.26.VWF 12:6128443:T:C
73:210
29:102
3:13
CLPTACTIQLRpc2_NP.3
p−min=0.22, pX=0.034, N=105
var.26.VWF 12:6128443:T:C
156:210
80:102
10:13
CLPTACTIQLRpc2_NP.4
p−min=0.5, pX=0.17, N=246
var.26.VWF 12:6128443:T:C
80:210
39:102
3:13
CLPTACTIQLRpc2_NP.5
p−maj=0.38, pX=0.059, N=122
var.26.VWF 12:6128443:T:C
103:210
48:102
4:13
CMVQVGVISGFKpc2_NP.1
p−maj=0.26, pX=0.017, N=155
var.26.VWF 12:6128443:T:C
140:210
59:102
7:13
CMVQVGVISGFKpc2_NP.2
p−min=0.12, pX=0.018, N=206
var.26.VWF 12:6128443:T:C
73:210
28:102
2:13
CMVQVGVISGFKpc2_NP.3
p−min=0.13, pX=0.014, N=103
var.26.VWF 12:6128443:T:C
208:210
102:102
13:13
CMVQVGVISGFKpc2_NP.4
p−min=0.54, pX=0.13, N=323
var.26.VWF 12:6128443:T:C
157:210
85:102
11:13
CMVQVGVISGFKpc2_NP.5
p−min=0.093, pX=0.17, N=253
var.26.VWF 12:6128443:T:C
5:210
3:102
1:13
CPCFHQGKpc2_NP.2
p−maj=0.31, pX=0.62, N=9
var.26.VWF 12:6128443:T:C
29:210
13:102
0:13
CPCFHQGKpc2_NP.4
p−maj=0.39, pX=0.27, N=42
var.26.VWF 12:6128443:T:C
6:210
1:102
0:13
CPCFHQGKpc2_NP.5
p−min=0.43, pX=0.23, N=7

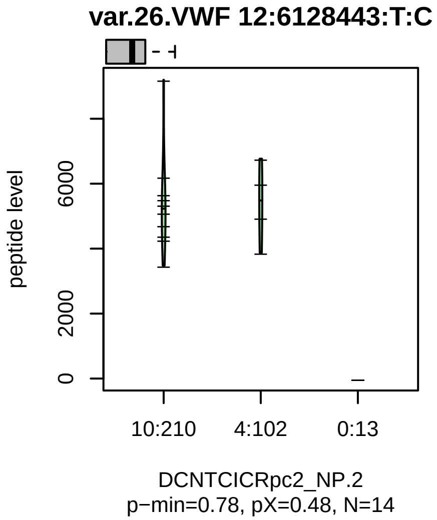
var.26.VWF 12:6128443:T:C
peptide level
0
2000
6000
10:210
4:102
0:13
DCNTCICRpc2_NP.2
p−min=0.78, pX=0.48, N=14

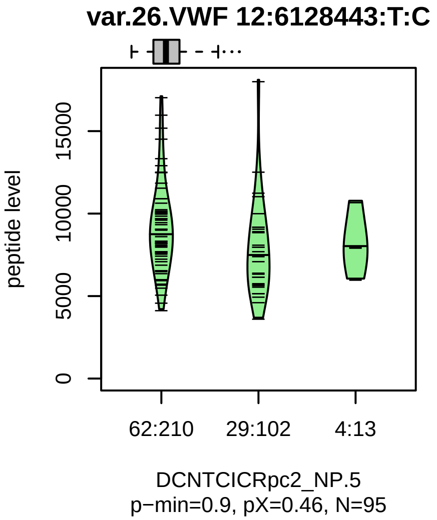
var.26.VWF 12:6128443:T:C
peptide level
0
5000
10000
15000
62:210
29:102
4:13
DCNTCICRpc2_NP.5
p−min=0.9, pX=0.46, N=95

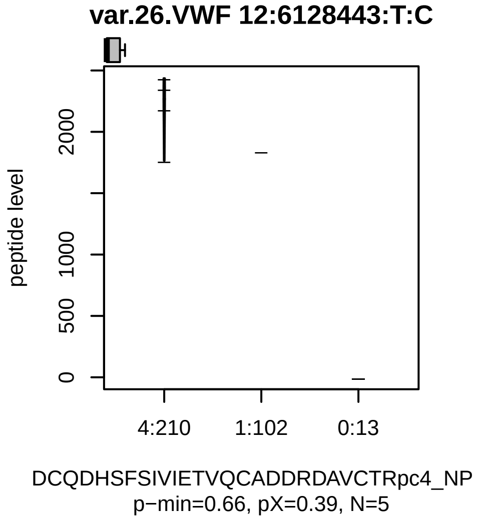
var.26.VWF 12:6128443:T:C
peptide level
0
500
1000
2000
4:210
1:102
0:13
DCQDHSFSIVIETVQCADDRDAVCTRpc4_NP
p−min=0.66, pX=0.39, N=5

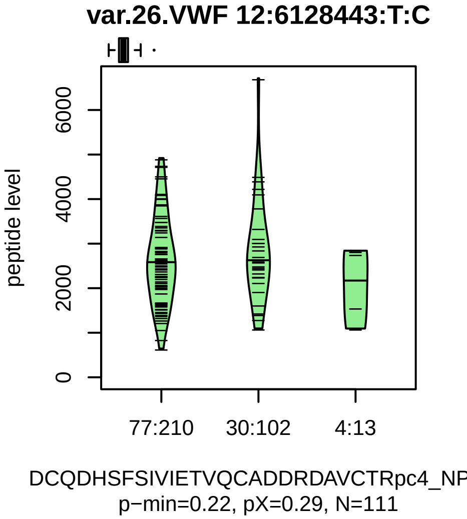
var.26.VWF 12:6128443:T:C
peptide level
0
2000
4000
6000
77:210
30:102
4:13
DCQDHSFSIVIETVQCADDRDAVCTRpc4_NP
p−min=0.22, pX=0.29, N=111

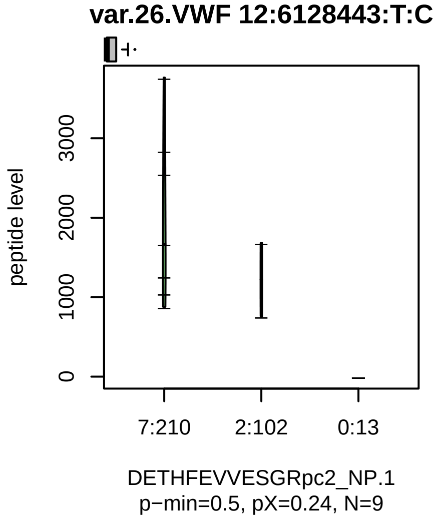
var.26.VWF 12:6128443:T:C
peptide level
0
1000
2000
3000
7:210
2:102
0:13
DETHFEVVESGRpc2_NP.1
p−min=0.5, pX=0.24, N=9

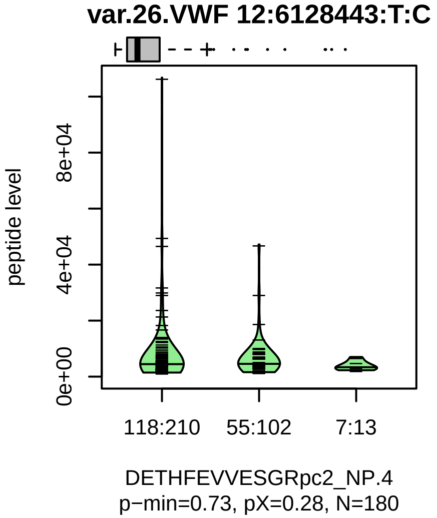
var.26.VWF 12:6128443:T:C
peptide level
0e+00
4e+04
8e+04
118:210
55:102
7:13
DETHFEVVESGRpc2_NP.4
p−min=0.73, pX=0.28, N=180

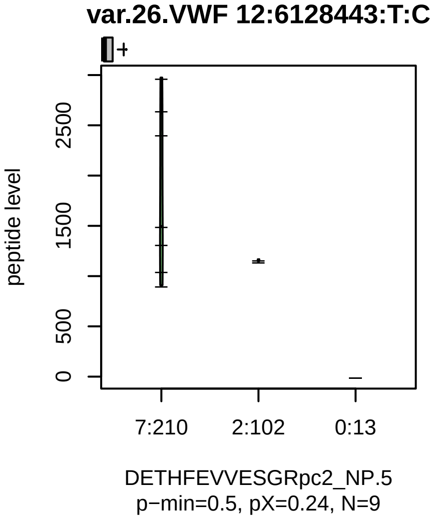
var.26.VWF 12:6128443:T:C
peptide level
0
500
1500
2500
7:210
2:102
0:13
DETHFEVVESGRpc2_NP.5
p−min=0.5, pX=0.24, N=9

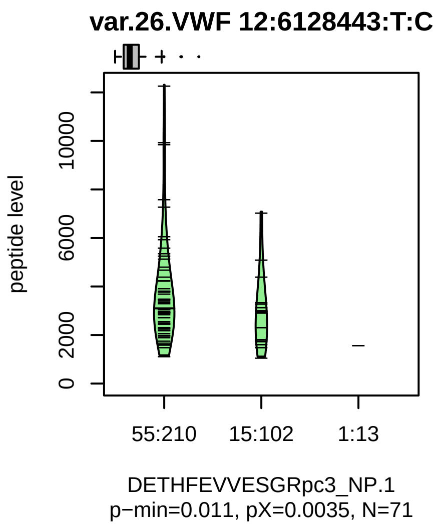
var.26.VWF 12:6128443:T:C
peptide level
0
2000
6000
10000
55:210
15:102
1:13
DETHFEVVESGRpc3_NP.1
p−min=0.011, pX=0.0035, N=71

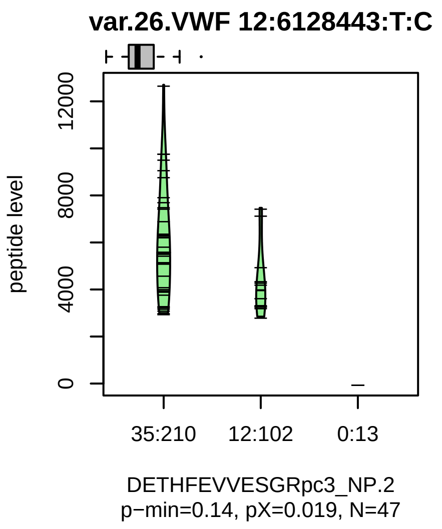
var.26.VWF 12:6128443:T:C
peptide level
0
4000
8000
12000
35:210
12:102
0:13
DETHFEVVESGRpc3_NP.2
p−min=0.14, pX=0.019, N=47

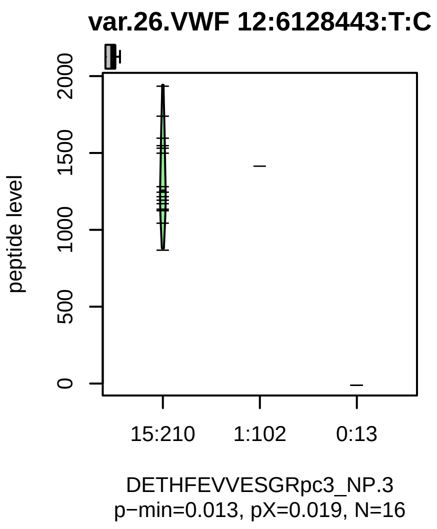
var.26.VWF 12:6128443:T:C
peptide level
0
500
1000
1500
2000
15:210
1:102
0:13
DETHFEVVESGRpc3_NP.3
p−min=0.013, pX=0.019, N=16

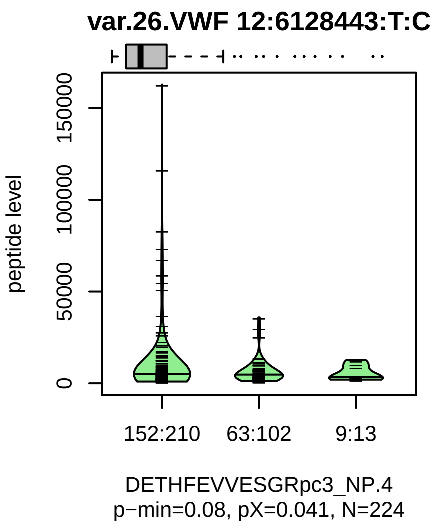
var.26.VWF 12:6128443:T:C
peptide level
0
50000
100000
150000
152:210
63:102
9:13
DETHFEVVESGRpc3_NP.4
p−min=0.08, pX=0.041, N=224

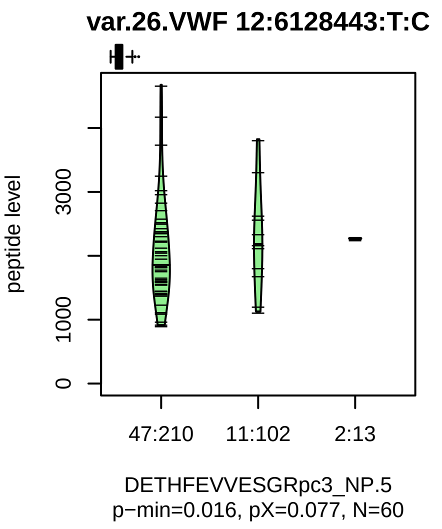
var.26.VWF 12:6128443:T:C
peptide level
0
1000
3000
47:210
11:102
2:13
DETHFEVVESGRpc3_NP.5
p−min=0.016, pX=0.077, N=60

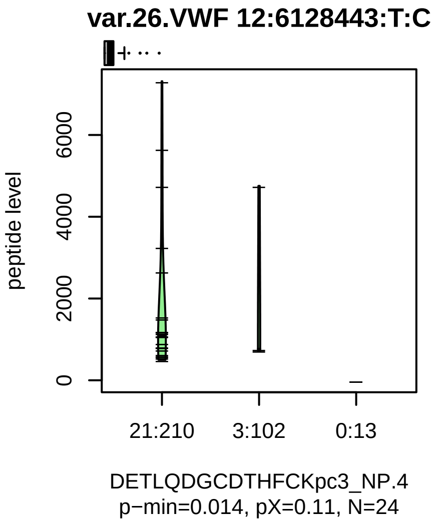
var.26.VWF 12:6128443:T:C
peptide level
0
2000
4000
6000
21:210
3:102
0:13
DETLQDGCDTHFCKpc3_NP.4
p−min=0.014, pX=0.11, N=24

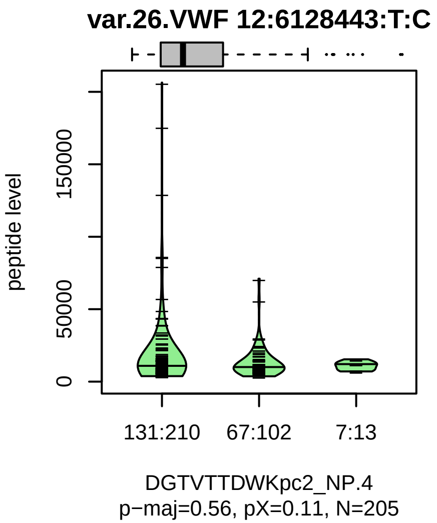
var.26.VWF 12:6128443:T:C
peptide level
0
50000
150000
131:210
67:102
7:13
DGTVTTDWKpc2_NP.4
p−maj=0.56, pX=0.11, N=205

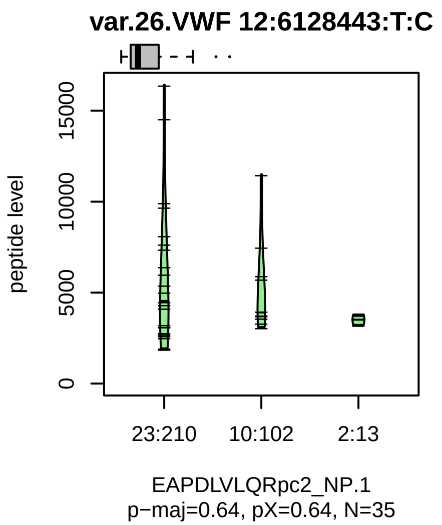
var.26.VWF 12:6128443:T:C
peptide level
23:210
10:102
2:13
EAPDLVLQRpc2_NP.1
p−maj=0.64, pX=0.64, N=35

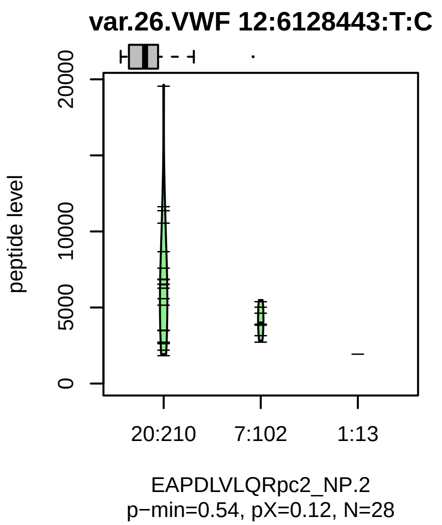
var.26.VWF 12:6128443:T:C
peptide level
20:210
7:102
1:13
EAPDLVLQRpc2_NP.2
p−min=0.54, pX=0.12, N=28

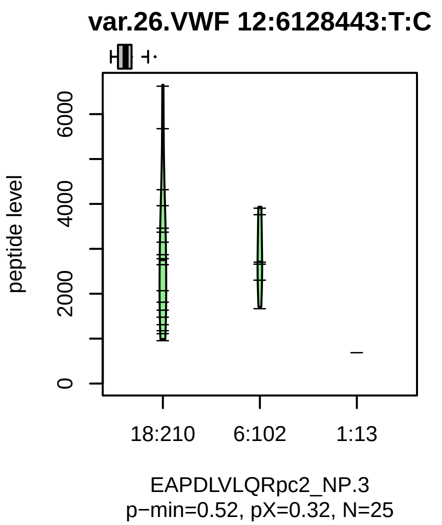
var.26.VWF 12:6128443:T:C
peptide level
18:210
6:102
1:13
EAPDLVLQRpc2_NP.3
p−min=0.52, pX=0.32, N=25

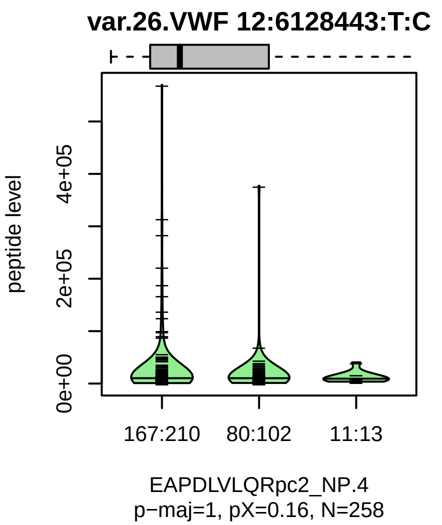
var.26.VWF 12:6128443:T:C
peptide level
167:210
80:102
11:13
EAPDLVLQRpc2_NP.4
p−maj=1, pX=0.16, N=258

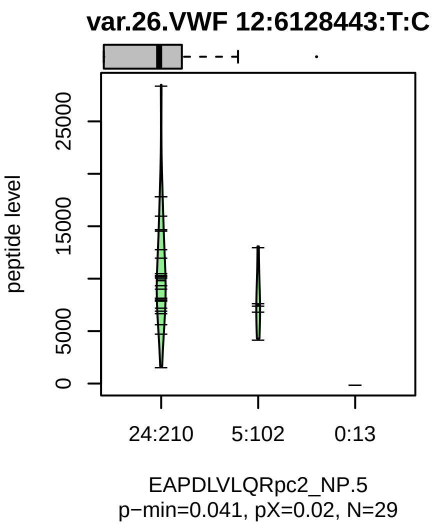
var.26.VWF 12:6128443:T:C
peptide level
24:210
5:102
0:13
EAPDLVLQRpc2_NP.5
p−min=0.041, pX=0.02, N=29

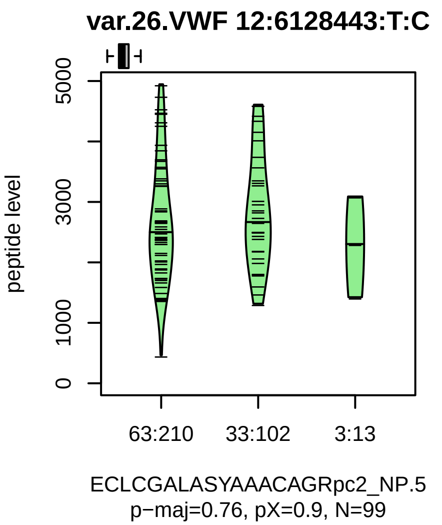
var.26.VWF 12:6128443:T:C
peptide level
63:210
33:102
3:13
ECLCGALASYAAACAGRpc2_NP.5
p−maj=0.76, pX=0.9, N=99

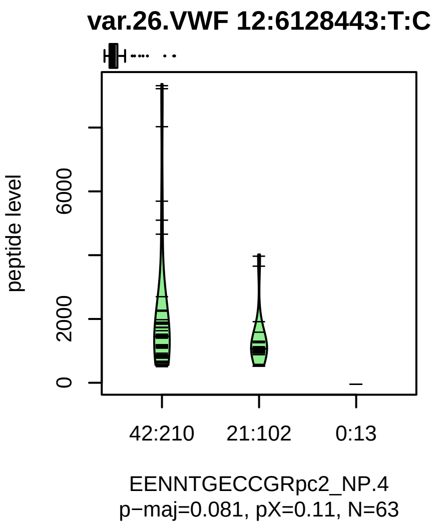
var.26.VWF 12:6128443:T:C
peptide level
42:210
21:102
0:13
EENNTGECCGRpc2_NP.4
p−maj=0.081, pX=0.11, N=63

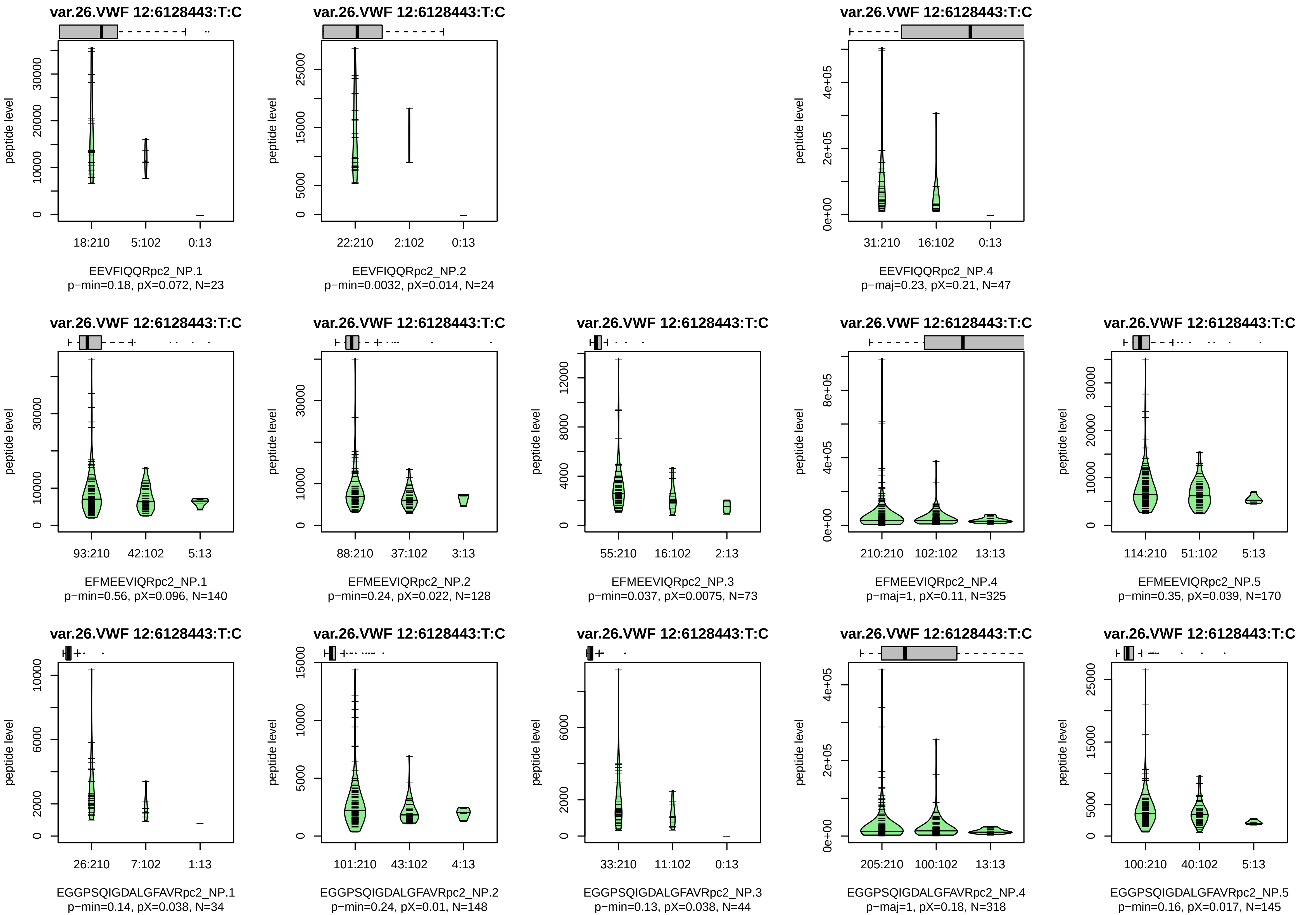
var.26.VWF 12:6128443:T:C
peptide level
18:210
5:102
0:13
EEVFIQQRpc2_NP.1
p−min=0.18, pX=0.072, N=23
var.26.VWF 12:6128443:T:C
peptide level
22:210
2:102
0:13
EEVFIQQRpc2_NP.2
p−min=0.0032, pX=0.014, N=24
var.26.VWF 12:6128443:T:C
peptide level
31:210
16:102
0:13
EEVFIQQRpc2_NP.4
p−maj=0.23, pX=0.21, N=47
var.26.VWF 12:6128443:T:C
peptide level
93:210
42:102
5:13
EFMEEVIQRpc2_NP.1
p−min=0.56, pX=0.096, N=140
var.26.VWF 12:6128443:T:C
peptide level
88:210
37:102
3:13
EFMEEVIQRpc2_NP.2
p−min=0.24, pX=0.022, N=128
var.26.VWF 12:6128443:T:C
peptide level
55:210
16:102
2:13
EFMEEVIQRpc2_NP.3
p−min=0.037, pX=0.0075, N=73
var.26.VWF 12:6128443:T:C
peptide level
210:210
102:102
13:13
EFMEEVIQRpc2_NP.4
p−maj=1, pX=0.11, N=325
var.26.VWF 12:6128443:T:C
peptide level
114:210
51:102
5:13
EFMEEVIQRpc2_NP.5
p−min=0.35, pX=0.039, N=170
var.26.VWF 12:6128443:T:C
peptide level
26:210
7:102
1:13
EGGPSQIGDALGFAVRpc2_NP.1
p−min=0.14, pX=0.038, N=34
var.26.VWF 12:6128443:T:C
peptide level
101:210
43:102
4:13
EGGPSQIGDALGFAVRpc2_NP.2
p−min=0.24, pX=0.01, N=148
var.26.VWF 12:6128443:T:C
peptide level
33:210
11:102
0:13
EGGPSQIGDALGFAVRpc2_NP.3
p−min=0.13, pX=0.038, N=44
var.26.VWF 12:6128443:T:C
peptide level
205:210
100:102
13:13
EGGPSQIGDALGFAVRpc2_NP.4
p−maj=1, pX=0.18, N=318
var.26.VWF 12:6128443:T:C
peptide level
100:210
40:102
5:13
EGGPSQIGDALGFAVRpc2_NP.5
p−min=0.16, pX=0.017, N=145

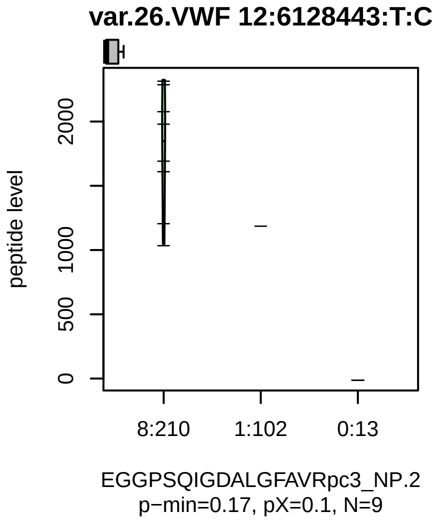
var.26.VWF 12:6128443:T:C
peptide level
8:210
1:102
0:13
EGGPSQIGDALGFAVRpc3_NP.2
p−min=0.17, pX=0.1, N=9

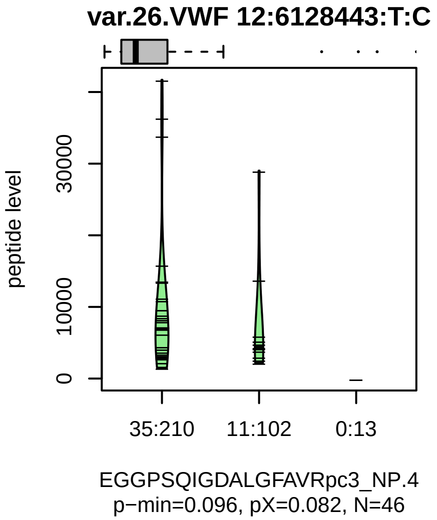
var.26.VWF 12:6128443:T:C
peptide level
35:210
11:102
0:13
EGGPSQIGDALGFAVRpc3_NP.4
p−min=0.096, pX=0.082, N=46

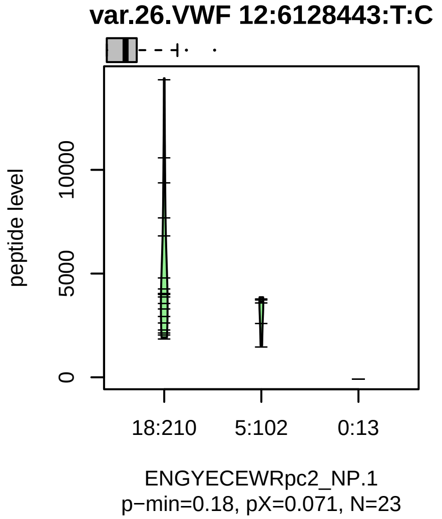
var.26.VWF 12:6128443:T:C
peptide level
18:210
5:102
0:13
ENGYECEWRpc2_NP.1
p−min=0.18, pX=0.071, N=23

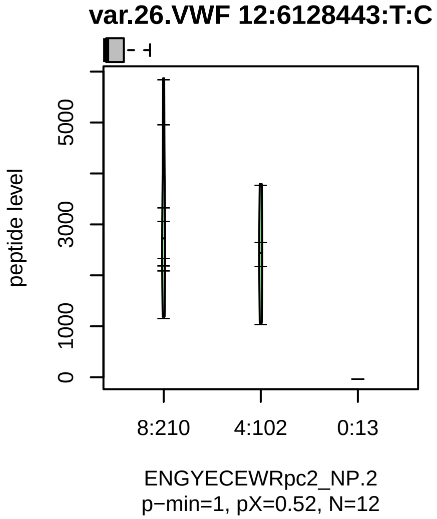
var.26.VWF 12:6128443:T:C
peptide level
8:210
4:102
0:13
ENGYECEWRpc2_NP.2
p−min=1, pX=0.52, N=12

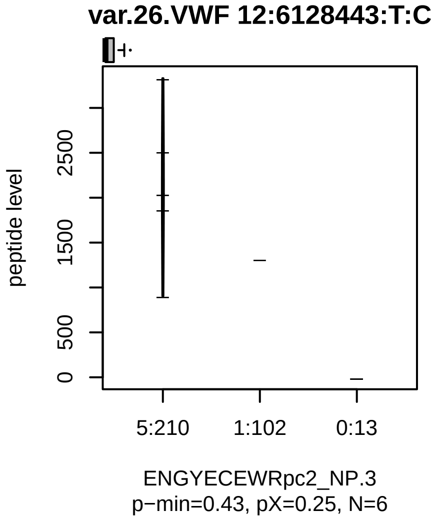
var.26.VWF 12:6128443:T:C
peptide level
5:210
1:102
0:13
ENGYECEWRpc2_NP.3
p−min=0.43, pX=0.25, N=6

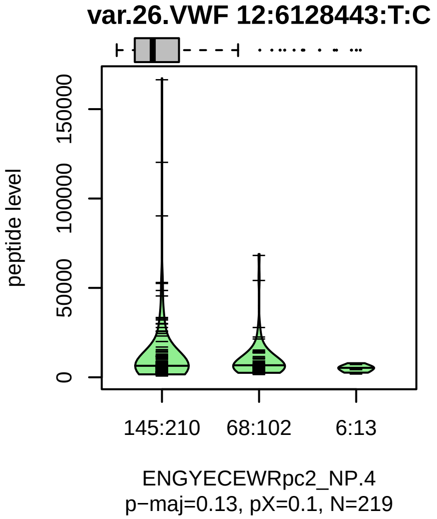
var.26.VWF 12:6128443:T:C
peptide level
145:210
68:102
6:13
ENGYECEWRpc2_NP.4
p−maj=0.13, pX=0.1, N=219

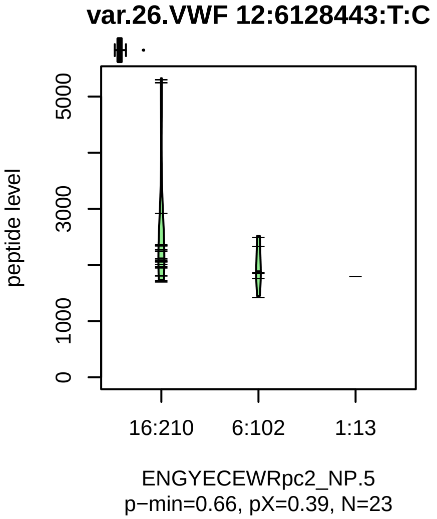
var.26.VWF 12:6128443:T:C
peptide level
16:210
6:102
1:13
ENGYECEWRpc2_NP.5
p−min=0.66, pX=0.39, N=23

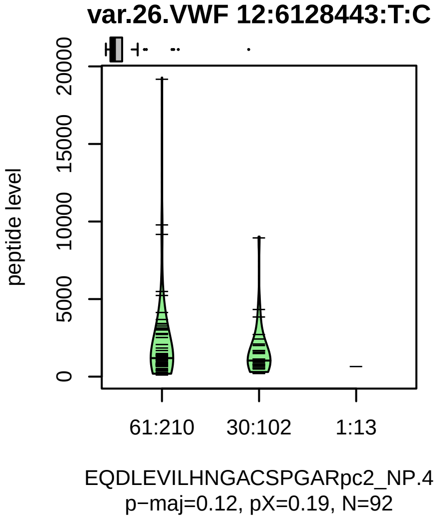
var.26.VWF 12:6128443:T:C
peptide level
61:210
30:102
1:13
EQDLEVILHNGACSPGARpc2_NP.4
p−maj=0.12, pX=0.19, N=92

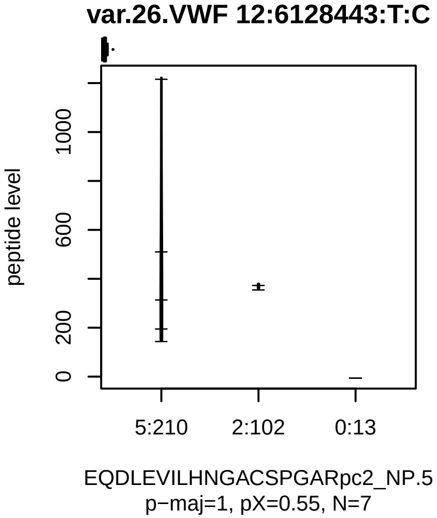
var.26.VWF 12:6128443:T:C
peptide level
5:210
2:102
0:13
EQDLEVILHNGACSPGARpc2_NP.5
p−maj=1, pX=0.55, N=7

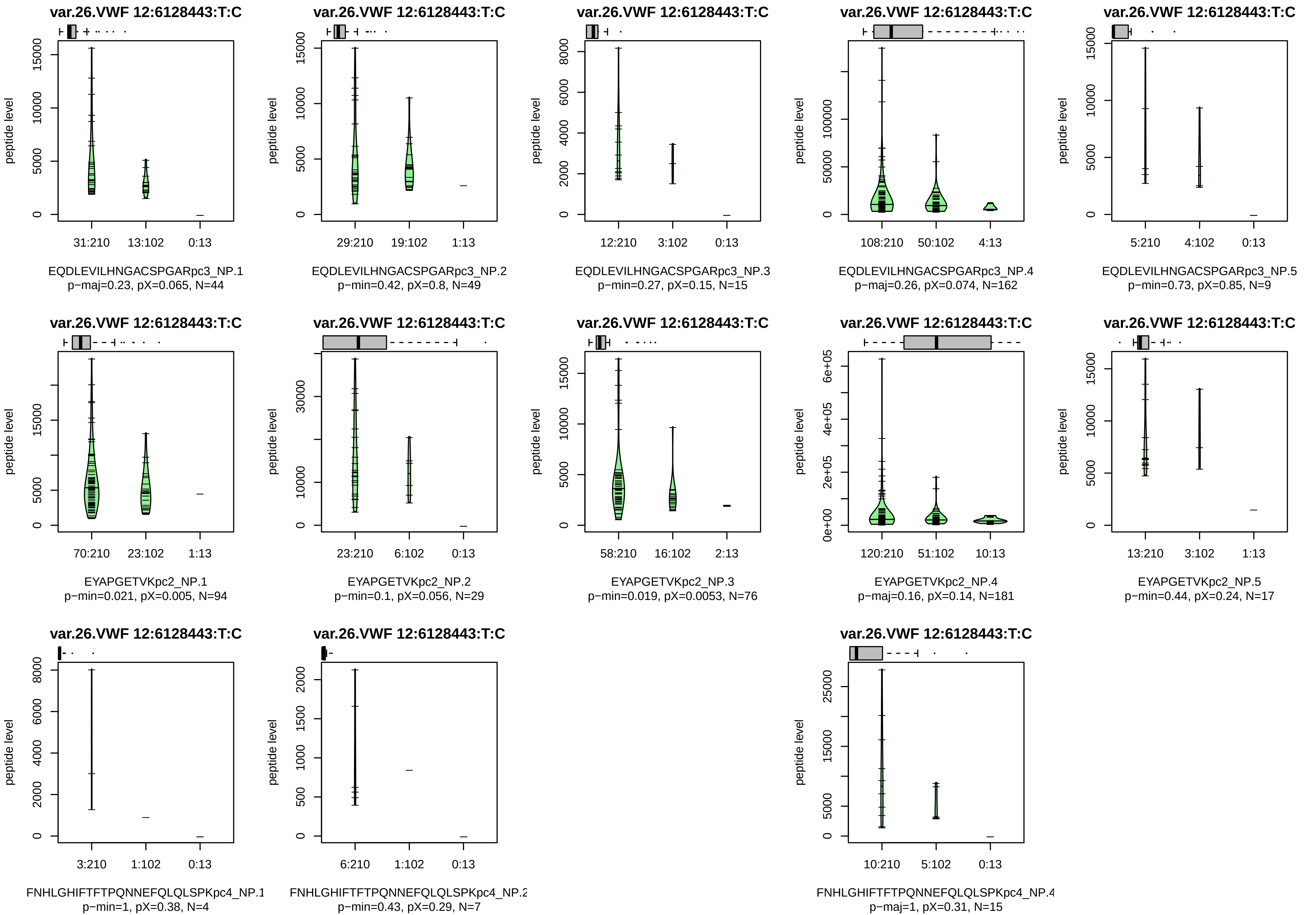
var.26.VWF 12:6128443:T:C
peptide level
31:210
13:102
0:13
EQDLEVILHNGACSPGARpc3_NP.1
p−maj=0.23, pX=0.065, N=44
var.26.VWF 12:6128443:T:C
peptide level
29:210
19:102
1:13
EQDLEVILHNGACSPGARpc3_NP.2
p−min=0.42, pX=0.8, N=49
var.26.VWF 12:6128443:T:C
peptide level
12:210
3:102
0:13
EQDLEVILHNGACSPGARpc3_NP.3
p−min=0.27, pX=0.15, N=15
var.26.VWF 12:6128443:T:C
peptide level
108:210
50:102
4:13
EQDLEVILHNGACSPGARpc3_NP.4
p−maj=0.26, pX=0.074, N=162
var.26.VWF 12:6128443:T:C
peptide level
5:210
4:102
0:13
EQDLEVILHNGACSPGARpc3_NP.5
p−min=0.73, pX=0.85, N=9
var.26.VWF 12:6128443:T:C
peptide level
70:210
23:102
1:13
EYAPGETVKpc2_NP.1
p−min=0.021, pX=0.005, N=94
var.26.VWF 12:6128443:T:C
peptide level
23:210
6:102
0:13
EYAPGETVKpc2_NP.2
p−min=0.1, pX=0.056, N=29
var.26.VWF 12:6128443:T:C
peptide level
58:210
16:102
2:13
EYAPGETVKpc2_NP.3
p−min=0.019, pX=0.0053, N=76
var.26.VWF 12:6128443:T:C
peptide level
120:210
51:102
10:13
EYAPGETVKpc2_NP.4
p−maj=0.16, pX=0.14, N=181
var.26.VWF 12:6128443:T:C
peptide level
13:210
3:102
1:13
EYAPGETVKpc2_NP.5
p−min=0.44, pX=0.24, N=17
var.26.VWF 12:6128443:T:C
peptide level
3:210
1:102
0:13
FNHLGHIFTFTPQNNEFQLQLSPKpc4_NP.1
p−min=1, pX=0.38, N=4
var.26.VWF 12:6128443:T:C
peptide level
6:210
1:102
0:13
FNHLGHIFTFTPQNNEFQLQLSPKpc4_NP.2
p−min=0.43, pX=0.29, N=7
var.26.VWF 12:6128443:T:C
peptide level
10:210
5:102
0:13
FNHLGHIFTFTPQNNEFQLQLSPKpc4_NP.4
p−maj=1, pX=0.31, N=15

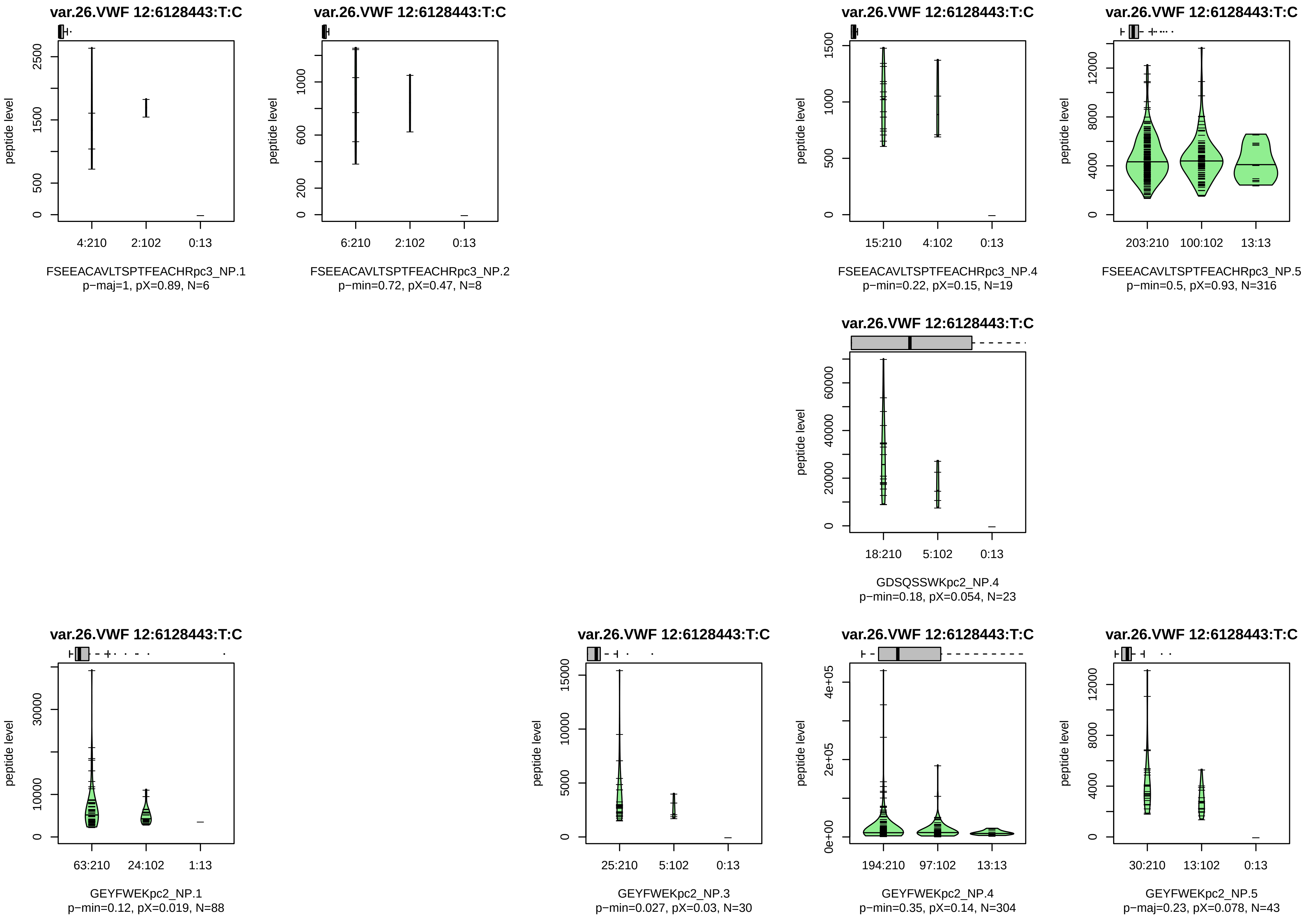

var.26.VWF 12:6128443:T:C
peptide level
4:210
2:102
0:13
FSEEACAVLTSPTFEACHRpc3_NP.1
p−maj=1, pX=0.89, N=6
var.26.VWF 12:6128443:T:C
peptide level
6:210
2:102
0:13
FSEEACAVLTSPTFEACHRpc3_NP.2
p−min=0.72, pX=0.47, N=8
var.26.VWF 12:6128443:T:C
peptide level
15:210
4:102
0:13
FSEEACAVLTSPTFEACHRpc3_NP.4
p−min=0.22, pX=0.15, N=19
var.26.VWF 12:6128443:T:C
peptide level
203:210
100:102
13:13
FSEEACAVLTSPTFEACHRpc3_NP.5
p−min=0.5, pX=0.93, N=316
var.26.VWF 12:6128443:T:C
peptide level
18:210
5:102
0:13
GDSQSSWKpc2_NP.4
p−min=0.18, pX=0.054, N=23
var.26.VWF 12:6128443:T:C
peptide level
63:210
24:102
1:13
GEYFWEKpc2_NP.1
p−min=0.12, pX=0.019, N=88
var.26.VWF 12:6128443:T:C
peptide level
25:210
5:102
0:13
GEYFWEKpc2_NP.3
p−min=0.027, pX=0.03, N=30
var.26.VWF 12:6128443:T:C
peptide level
194:210
97:102
13:13
GEYFWEKpc2_NP.4
p−min=0.35, pX=0.14, N=304
var.26.VWF 12:6128443:T:C
peptide level
30:210
13:102
0:13
GEYFWEKpc2_NP.5
p−maj=0.23, pX=0.078, N=43

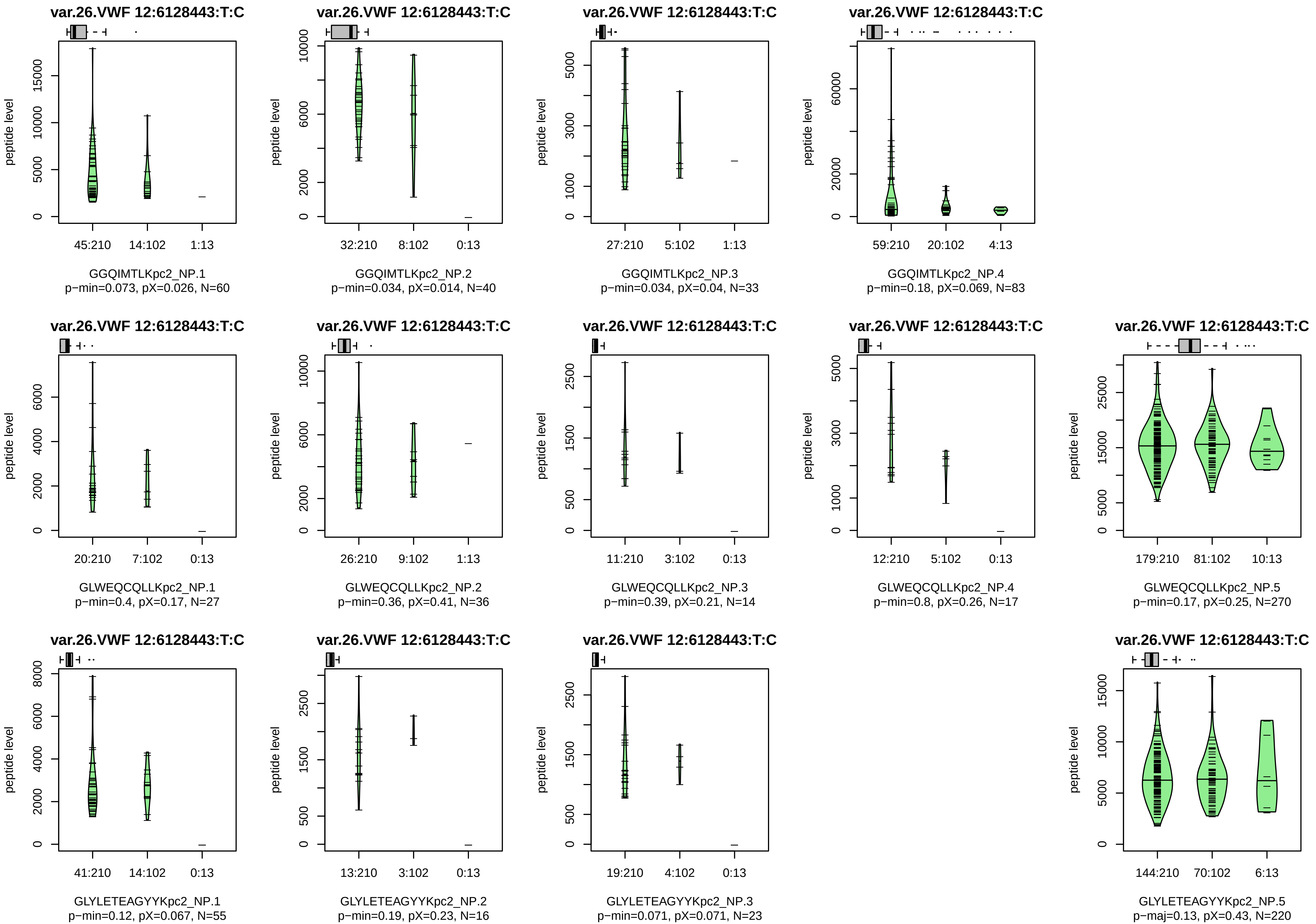
var.26.VWF 12:6128443:T:C
peptide level
45:210
14:102
1:13
GGQIMTLKpc2_NP.1
p−min=0.073, pX=0.026, N=60
var.26.VWF 12:6128443:T:C
peptide level
32:210
8:102
0:13
GGQIMTLKpc2_NP.2
p−min=0.034, pX=0.014, N=40
var.26.VWF 12:6128443:T:C
peptide level
27:210
5:102
1:13
GGQIMTLKpc2_NP.3
p−min=0.034, pX=0.04, N=33
var.26.VWF 12:6128443:T:C
peptide level
59:210
20:102
4:13
GGQIMTLKpc2_NP.4
p−min=0.18, pX=0.069, N=83
var.26.VWF 12:6128443:T:C
peptide level
20:210
7:102
0:13
GLWEQCQLLKpc2_NP.1
p−min=0.4, pX=0.17, N=27
var.26.VWF 12:6128443:T:C
peptide level
26:210
9:102
1:13
GLWEQCQLLKpc2_NP.2
p−min=0.36, pX=0.41, N=36
var.26.VWF 12:6128443:T:C
peptide level
11:210
3:102
0:13
GLWEQCQLLKpc2_NP.3
p−min=0.39, pX=0.21, N=14
var.26.VWF 12:6128443:T:C
peptide level
12:210
5:102
0:13
GLWEQCQLLKpc2_NP.4
p−min=0.8, pX=0.26, N=17
var.26.VWF 12:6128443:T:C
peptide level
179:210
81:102
10:13
GLWEQCQLLKpc2_NP.5
p−min=0.17, pX=0.25, N=270
var.26.VWF 12:6128443:T:C
peptide level
41:210
14:102
0:13
GLYLETEAGYYKpc2_NP.1
p−min=0.12, pX=0.067, N=55
var.26.VWF 12:6128443:T:C
peptide level
13:210
3:102
0:13
GLYLETEAGYYKpc2_NP.2
p−min=0.19, pX=0.23, N=16
var.26.VWF 12:6128443:T:C
peptide level
19:210
4:102
0:13
GLYLETEAGYYKpc2_NP.3
p−min=0.071, pX=0.071, N=23
var.26.VWF 12:6128443:T:C
peptide level
144:210
70:102
6:13
GLYLETEAGYYKpc2_NP.5
p−maj=0.13, pX=0.43, N=220

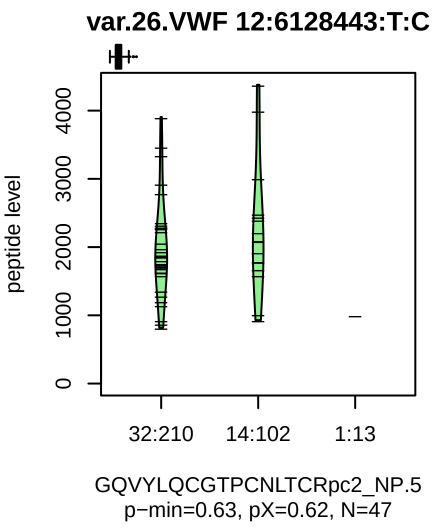
var.26.VWF 12:6128443:T:C
peptide level
0
1000
2000
3000
4000
32:210
14:102
1:13
GQVYLQCGTPCNLTCRpc2_NP.5
p−min=0.63, pX=0.62, N=47

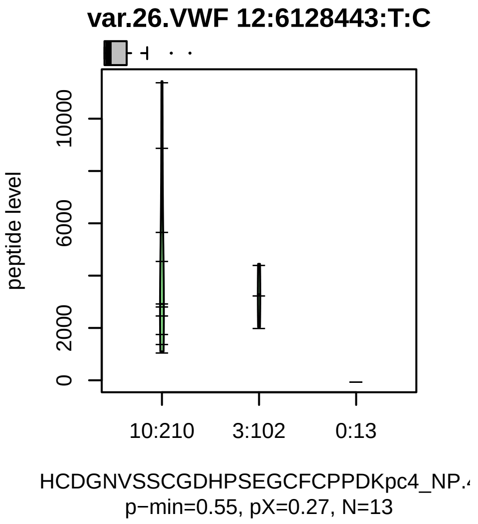
var.26.VWF 12:6128443:T:C
peptide level
0
2000
6000
10000
10:210
3:102
0:13
HCDGNVSSCGDHPSEGCFCPPDKpc4_NP.
p−min=0.55, pX=0.27, N=13

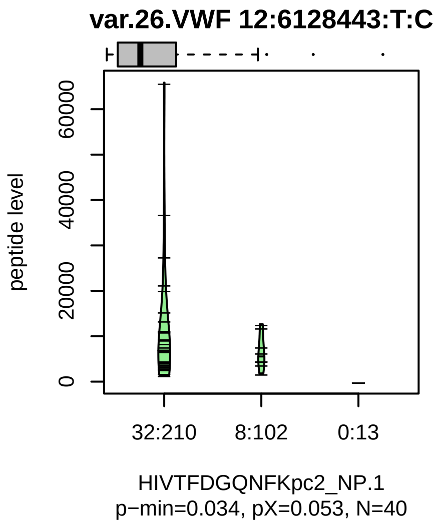
var.26.VWF 12:6128443:T:C
peptide level
0
20000
40000
60000
32:210
8:102
0:13
HIVTFDGQNFKpc2_NP.1
p−min=0.034, pX=0.053, N=40

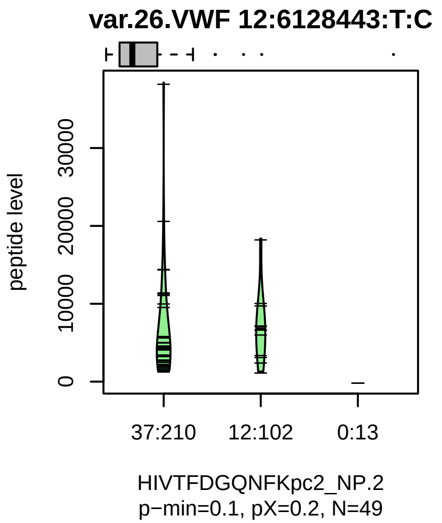
var.26.VWF 12:6128443:T:C
peptide level
0
10000
20000
30000
37:210
12:102
0:13
HIVTFDGQNFKpc2_NP.2
p−min=0.1, pX=0.2, N=49

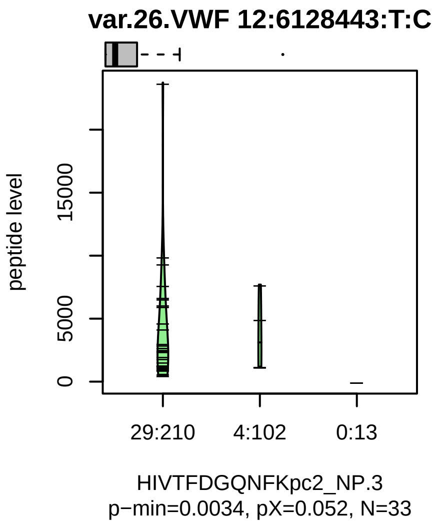
var.26.VWF 12:6128443:T:C
peptide level
0
5000
15000
29:210
4:102
0:13
HIVTFDGQNFKpc2_NP.3
p−min=0.0034, pX=0.052, N=33

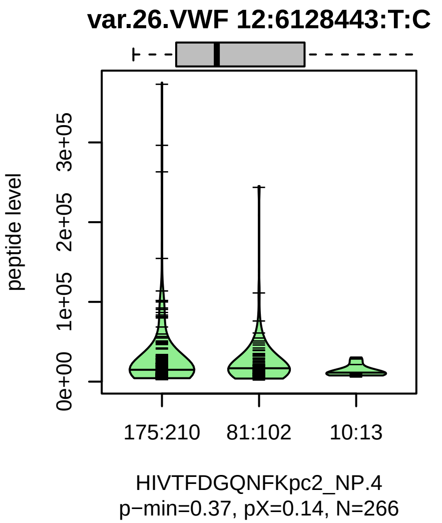
var.26.VWF 12:6128443:T:C
peptide level
0e+00
1e+05
2e+05
3e+05
175:210
81:102
10:13
HIVTFDGQNFKpc2_NP.4
p−min=0.37, pX=0.14, N=266

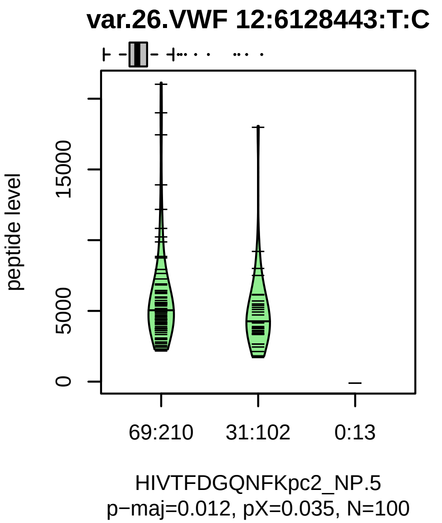
var.26.VWF 12:6128443:T:C
peptide level
0
5000
15000
69:210
31:102
0:13
HIVTFDGQNFKpc2_NP.5
p−maj=0.012, pX=0.035, N=100

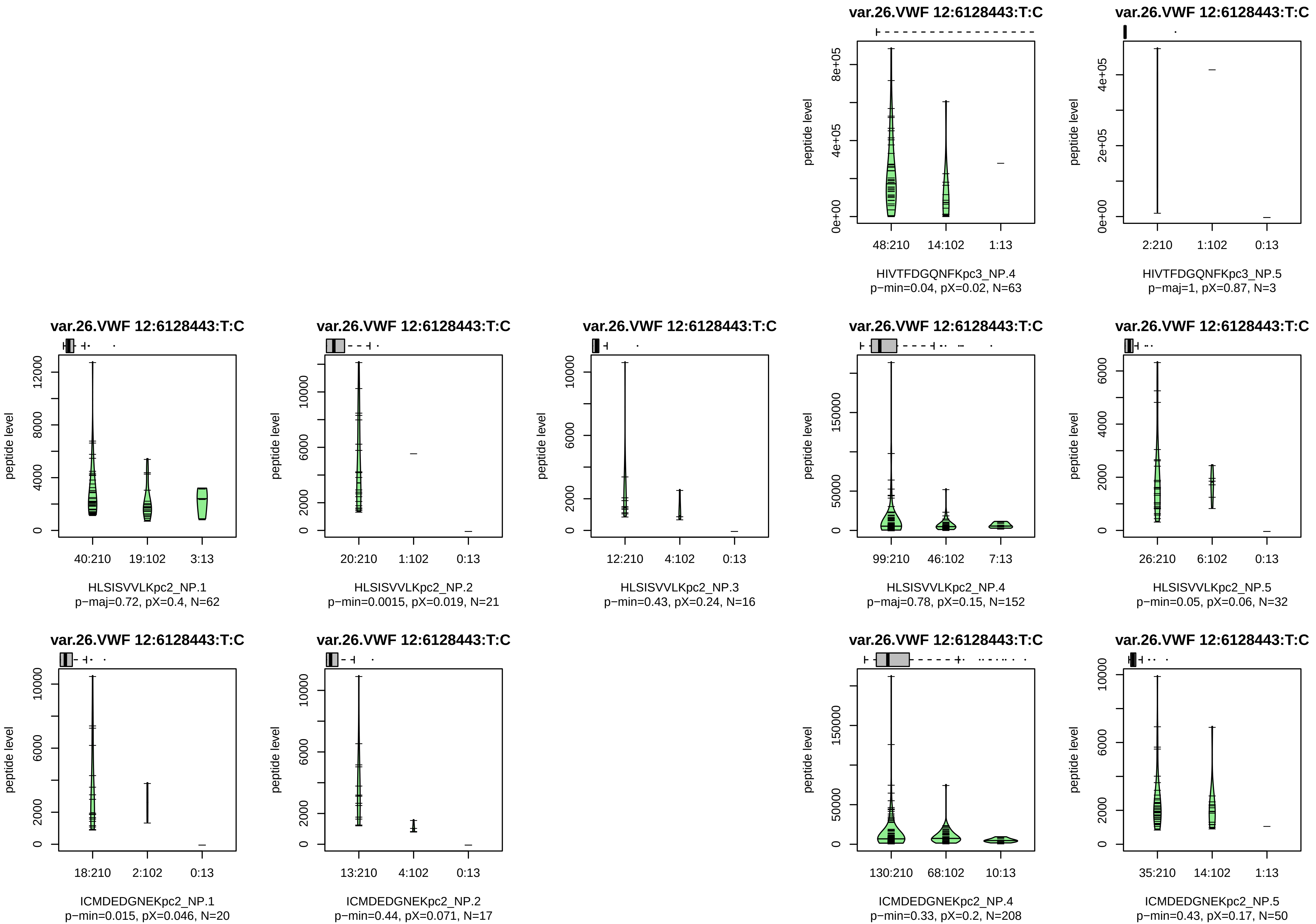

var.26.VWF 12:6128443:T:C
peptide level
0e+00
4e+05
8e+05
48:210
14:102
1:13
HIVTFDGQNFKpc3_NP.4
p−min=0.04, pX=0.02, N=63
var.26.VWF 12:6128443:T:C
peptide level
0e+00
2e+05
4e+05
2:210
1:102
0:13
HIVTFDGQNFKpc3_NP.5
p−maj=1, pX=0.87, N=3
var.26.VWF 12:6128443:T:C
peptide level
0
4000
8000
12000
40:210
19:102
3:13
HLSISVVLKpc2_NP.1
p−maj=0.72, pX=0.4, N=62
var.26.VWF 12:6128443:T:C
peptide level
0
2000
6000
10000
20:210
1:102
0:13
HLSISVVLKpc2_NP.2
p−min=0.0015, pX=0.019, N=21
var.26.VWF 12:6128443:T:C
peptide level
0
2000
6000
10000
12:210
4:102
0:13
HLSISVVLKpc2_NP.3
p−min=0.43, pX=0.24, N=16
var.26.VWF 12:6128443:T:C
peptide level
0
50000
150000
99:210
46:102
7:13
HLSISVVLKpc2_NP.4
p−maj=0.78, pX=0.15, N=152
var.26.VWF 12:6128443:T:C
peptide level
0
2000
4000
6000
26:210
6:102
0:13
HLSISVVLKpc2_NP.5
p−min=0.05, pX=0.06, N=32
var.26.VWF 12:6128443:T:C
peptide level
0
2000
6000
10000
18:210
2:102
0:13
ICMDEDGNEKpc2_NP.1
p−min=0.015, pX=0.046, N=20
var.26.VWF 12:6128443:T:C
peptide level
0
2000
6000
10000
13:210
4:102
0:13
ICMDEDGNEKpc2_NP.2
p−min=0.44, pX=0.071, N=17
var.26.VWF 12:6128443:T:C
peptide level
0
50000
150000
130:210
68:102
10:13
ICMDEDGNEKpc2_NP.4
p−min=0.33, pX=0.2, N=208
var.26.VWF 12:6128443:T:C
peptide level
0
2000
6000
10000
35:210
14:102
1:13
ICMDEDGNEKpc2_NP.5
p−min=0.43, pX=0.17, N=50

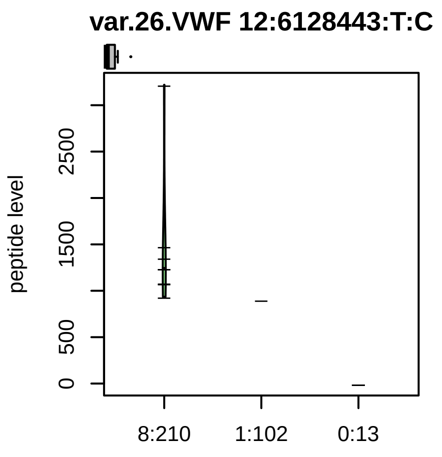

IDGSGNFQVLLSDRpc2_NP.1
p−min=0.17, pX=0.12, N=9

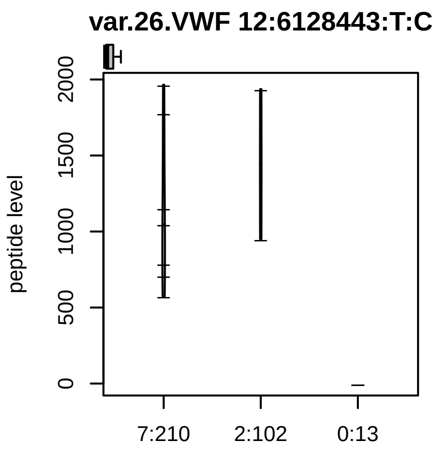

IDGSGNFQVLLSDRpc2_NP.2
p−min=0.5, pX=0.52, N=9

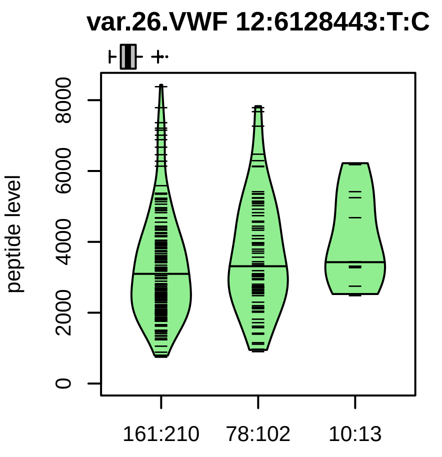

IDGSGNFQVLLSDRpc2_NP.5
p−maj=1, pX=0.17, N=249

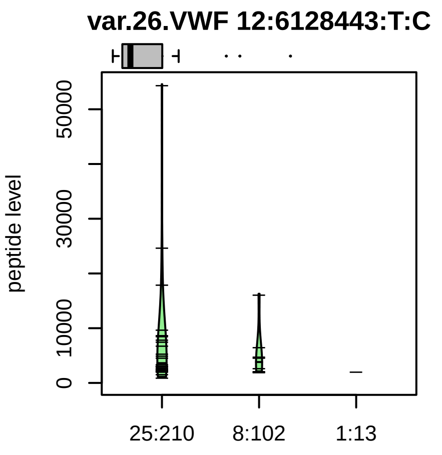

IDRPEASRpc2_NP.4
p−min=0.34, pX=0.21, N=34

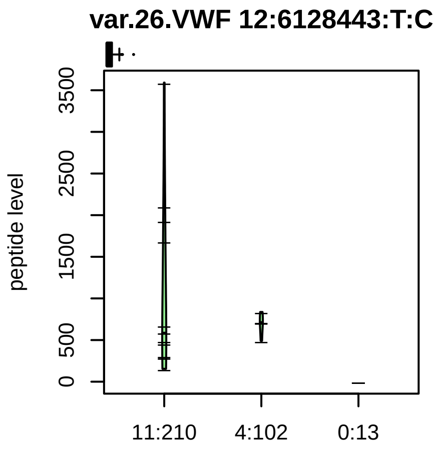

IEDLPTMVTLGNSFLHKpc2_NP.1
p−min=0.59, pX=0.28, N=15

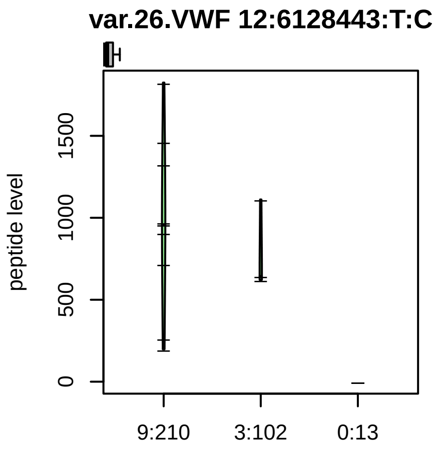

IEDLPTMVTLGNSFLHKpc2_NP.2
p−min=0.55, pX=0.32, N=12

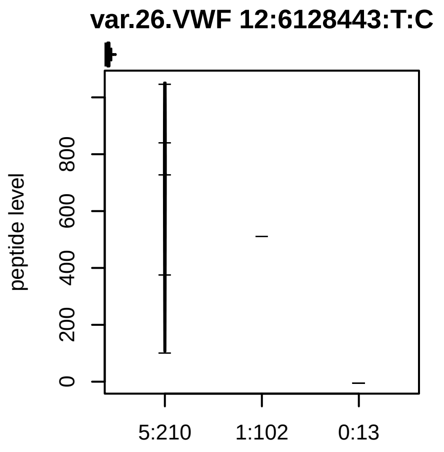

IEDLPTMVTLGNSFLHKpc2_NP.3
p−min=0.43, pX=0.33, N=6

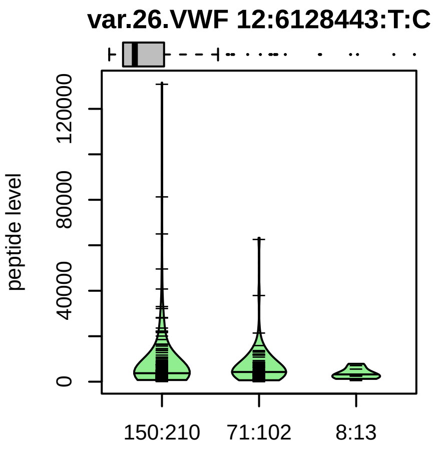

IEDLPTMVTLGNSFLHKpc2_NP.4
p−maj=0.54, pX=0.19, N=229

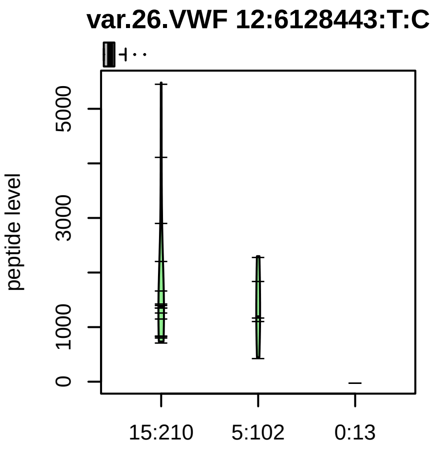

IEDLPTMVTLGNSFLHKpc2_NP.5
p−min=0.47, pX=0.2, N=20

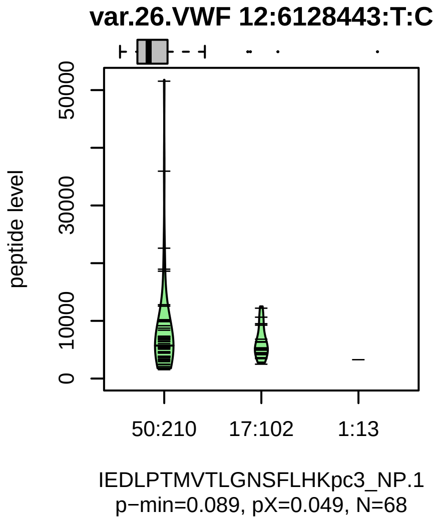
var.26.VWF 12:6128443:T:C
peptide level
0
10000
30000
50000
50:210
17:102
1:13
IEDLPTMVTLGNSFLHKpc3_NP.1
p−min=0.089, pX=0.049, N=68

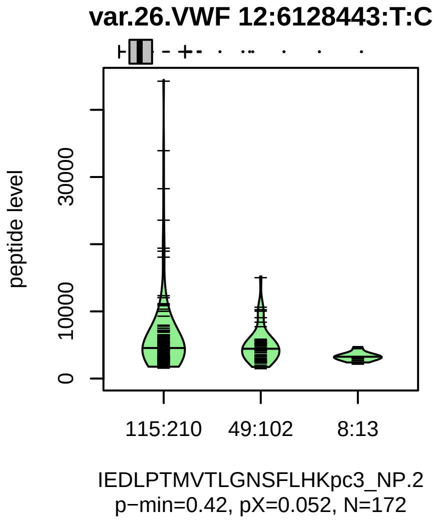
var.26.VWF 12:6128443:T:C
peptide level
0
10000
30000
115:210
49:102
8:13
IEDLPTMVTLGNSFLHKpc3_NP.2
p−min=0.42, pX=0.052, N=172

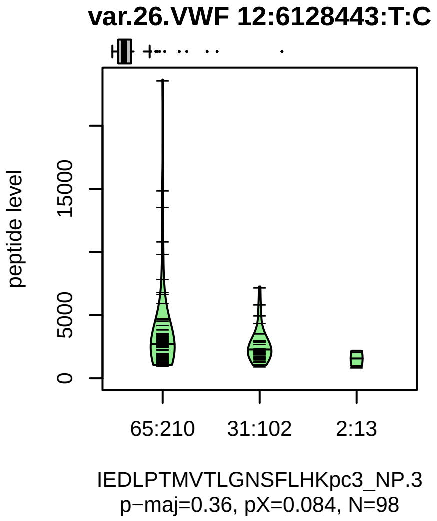
var.26.VWF 12:6128443:T:C
peptide level
0
5000
15000
65:210
31:102
2:13
IEDLPTMVTLGNSFLHKpc3_NP.3
p−maj=0.36, pX=0.084, N=98

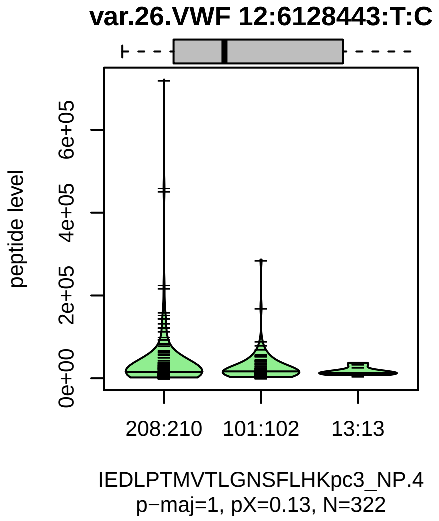
var.26.VWF 12:6128443:T:C
peptide level
0e+00
2e+05
4e+05
6e+05
208:210
101:102
13:13
IEDLPTMVTLGNSFLHKpc3_NP.4
p−maj=1, pX=0.13, N=322

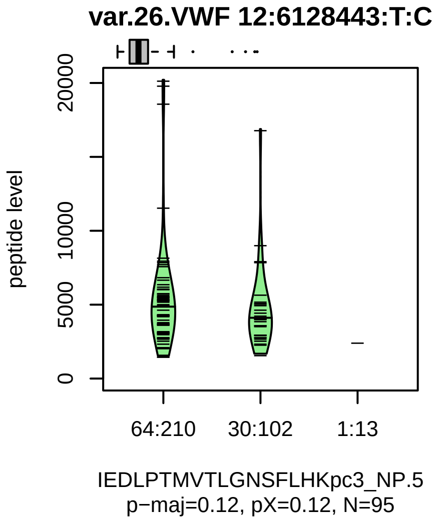
var.26.VWF 12:6128443:T:C
peptide level
0
5000
10000
20000
64:210
30:102
1:13
IEDLPTMVTLGNSFLHKpc3_NP.5
p−maj=0.12, pX=0.12, N=95

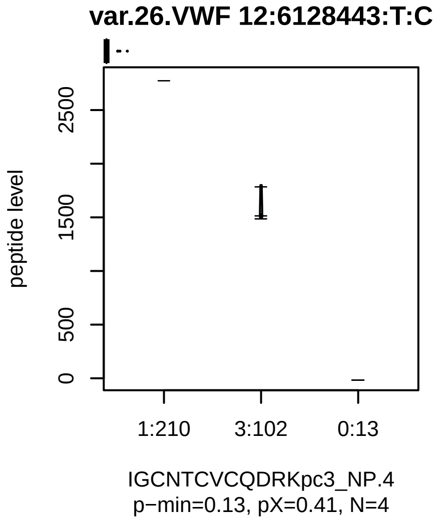
var.26.VWF 12:6128443:T:C
peptide level
0
500
1500
2500
1:210
3:102
0:13
IGCNTCVCQDRKpc3_NP.4
p−min=0.13, pX=0.41, N=4

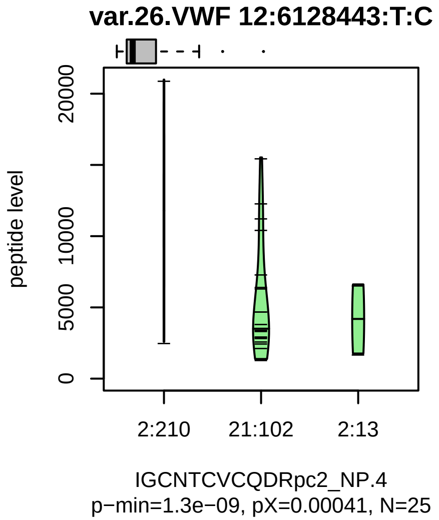
var.26.VWF 12:6128443:T:C
peptide level
0
5000
10000
20000
2:210
21:102
2:13
IGCNTCVCQDRpc2_NP.4
p−min=1.3e−09, pX=0.00041, N=25

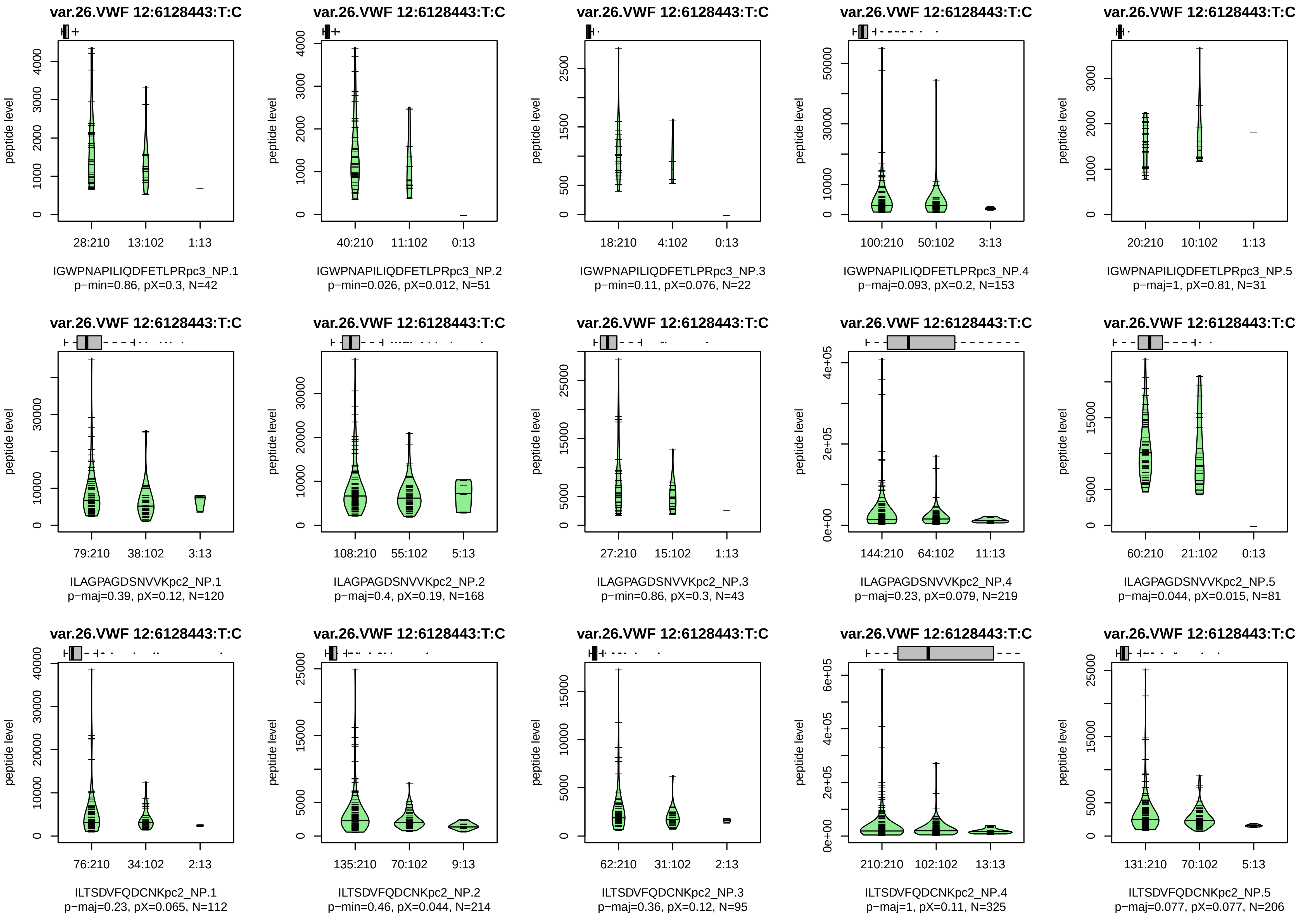
var.26.VWF 12:6128443:T:C
peptide level
28:210
13:102
1:13
IGWPNAPILIQDFETLPRpc3_NP.1
p−min=0.86, pX=0.3, N=42
var.26.VWF 12:6128443:T:C
peptide level
40:210
11:102
0:13
IGWPNAPILIQDFETLPRpc3_NP.2
p−min=0.026, pX=0.012, N=51
var.26.VWF 12:6128443:T:C
peptide level
18:210
4:102
0:13
IGWPNAPILIQDFETLPRpc3_NP.3
p−min=0.11, pX=0.076, N=22
var.26.VWF 12:6128443:T:C
peptide level
100:210
50:102
3:13
IGWPNAPILIQDFETLPRpc3_NP.4
p−maj=0.093, pX=0.2, N=153
var.26.VWF 12:6128443:T:C
peptide level
20:210
10:102
1:13
IGWPNAPILIQDFETLPRpc3_NP.5
p−maj=1, pX=0.81, N=31
var.26.VWF 12:6128443:T:C
peptide level
79:210
38:102
3:13
ILAGPAGDSNVVKpc2_NP.1
p−maj=0.39, pX=0.12, N=120
var.26.VWF 12:6128443:T:C
peptide level
108:210
55:102
5:13
ILAGPAGDSNVVKpc2_NP.2
p−maj=0.4, pX=0.19, N=168
var.26.VWF 12:6128443:T:C
peptide level
27:210
15:102
1:13
ILAGPAGDSNVVKpc2_NP.3
p−min=0.86, pX=0.3, N=43
var.26.VWF 12:6128443:T:C
peptide level
144:210
64:102
11:13
ILAGPAGDSNVVKpc2_NP.4
p−maj=0.23, pX=0.079, N=219
var.26.VWF 12:6128443:T:C
peptide level
60:210
21:102
0:13
ILAGPAGDSNVVKpc2_NP.5
p−maj=0.044, pX=0.015, N=81
var.26.VWF 12:6128443:T:C
peptide level
76:210
34:102
2:13
ILTSDVFQDCNKpc2_NP.1
p−maj=0.23, pX=0.065, N=112
var.26.VWF 12:6128443:T:C
peptide level
135:210
70:102
9:13
ILTSDVFQDCNKpc2_NP.2
p−min=0.46, pX=0.044, N=214
var.26.VWF 12:6128443:T:C
peptide level
62:210
31:102
2:13
ILTSDVFQDCNKpc2_NP.3
p−maj=0.36, pX=0.12, N=95
var.26.VWF 12:6128443:T:C
peptide level
210:210
102:102
13:13
ILTSDVFQDCNKpc2_NP.4
p−maj=1, pX=0.11, N=325
var.26.VWF 12:6128443:T:C
peptide level
131:210
70:102
5:13
ILTSDVFQDCNKpc2_NP.5
p−maj=0.077, pX=0.077, N=206

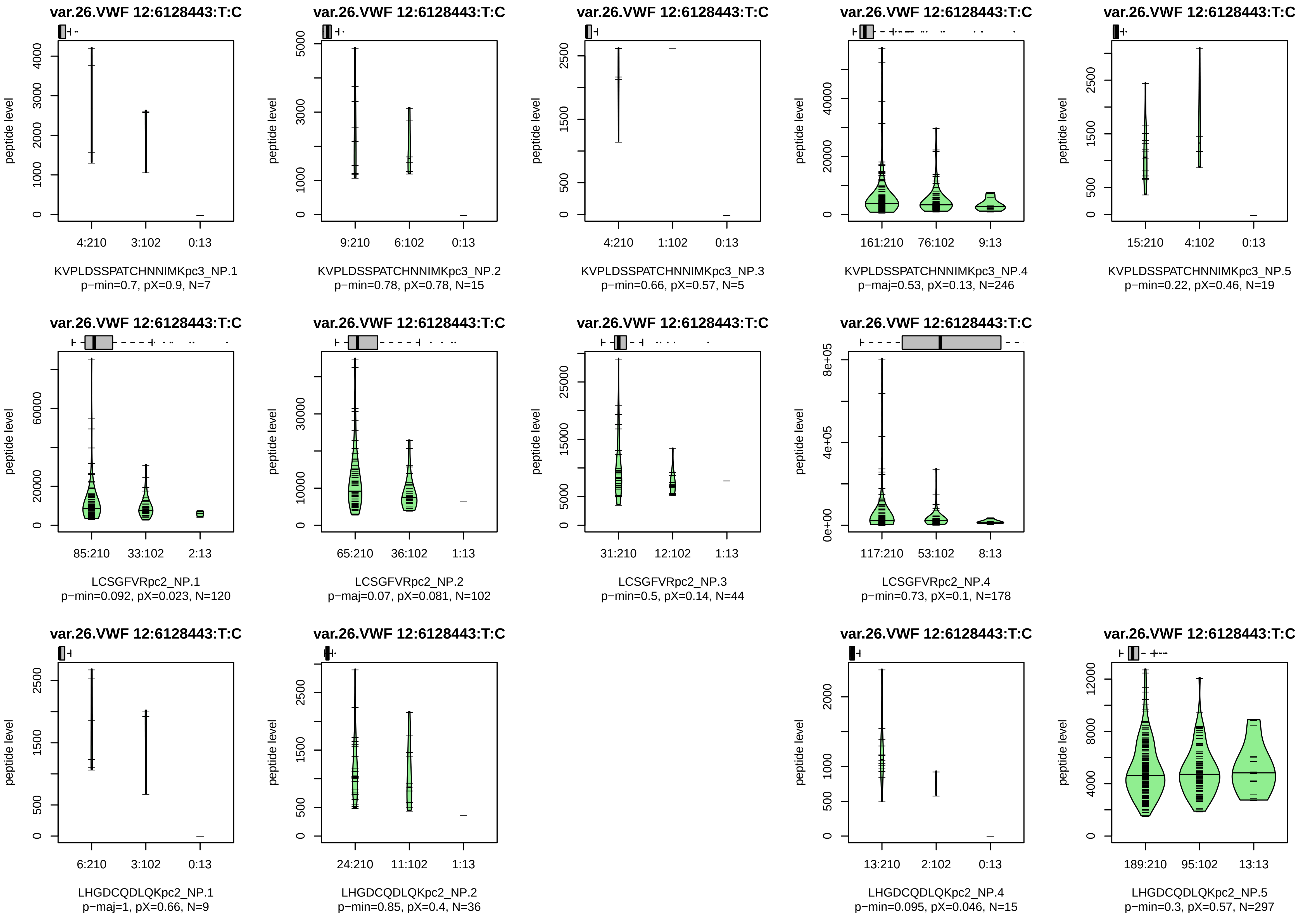
var.26.VWF 12:6128443:T:C
peptide level
4:210
3:102
0:13
KVPLDSSPATCHNNIMKpc3_NP.1
p−min=0.7, pX=0.9, N=7
var.26.VWF 12:6128443:T:C
peptide level
9:210
6:102
0:13
KVPLDSSPATCHNNIMKpc3_NP.2
p−min=0.78, pX=0.78, N=15
var.26.VWF 12:6128443:T:C
peptide level
4:210
1:102
0:13
KVPLDSSPATCHNNIMKpc3_NP.3
p−min=0.66, pX=0.57, N=5
var.26.VWF 12:6128443:T:C
peptide level
161:210
76:102
9:13
KVPLDSSPATCHNNIMKpc3_NP.4
p−maj=0.53, pX=0.13, N=246
var.26.VWF 12:6128443:T:C
peptide level
15:210
4:102
0:13
KVPLDSSPATCHNNIMKpc3_NP.5
p−min=0.22, pX=0.46, N=19
var.26.VWF 12:6128443:T:C
peptide level
85:210
33:102
2:13
LCSGFVRpc2_NP.1
p−min=0.092, pX=0.023, N=120
var.26.VWF 12:6128443:T:C
peptide level
65:210
36:102
1:13
LCSGFVRpc2_NP.2
p−maj=0.07, pX=0.081, N=102
var.26.VWF 12:6128443:T:C
peptide level
31:210
12:102
1:13
LCSGFVRpc2_NP.3
p−min=0.5, pX=0.14, N=44
var.26.VWF 12:6128443:T:C
peptide level
117:210
53:102
8:13
LCSGFVRpc2_NP.4
p−min=0.73, pX=0.1, N=178
var.26.VWF 12:6128443:T:C
peptide level
6:210
3:102
0:13
LHGDCQDLQKpc2_NP.1
p−maj=1, pX=0.66, N=9
var.26.VWF 12:6128443:T:C
peptide level
24:210
11:102
1:13
LHGDCQDLQKpc2_NP.2
p−min=0.85, pX=0.4, N=36
var.26.VWF 12:6128443:T:C
peptide level
13:210
2:102
0:13
LHGDCQDLQKpc2_NP.4
p−min=0.095, pX=0.046, N=15
var.26.VWF 12:6128443:T:C
peptide level
189:210
95:102
13:13
LHGDCQDLQKpc2_NP.5
p−min=0.3, pX=0.57, N=297

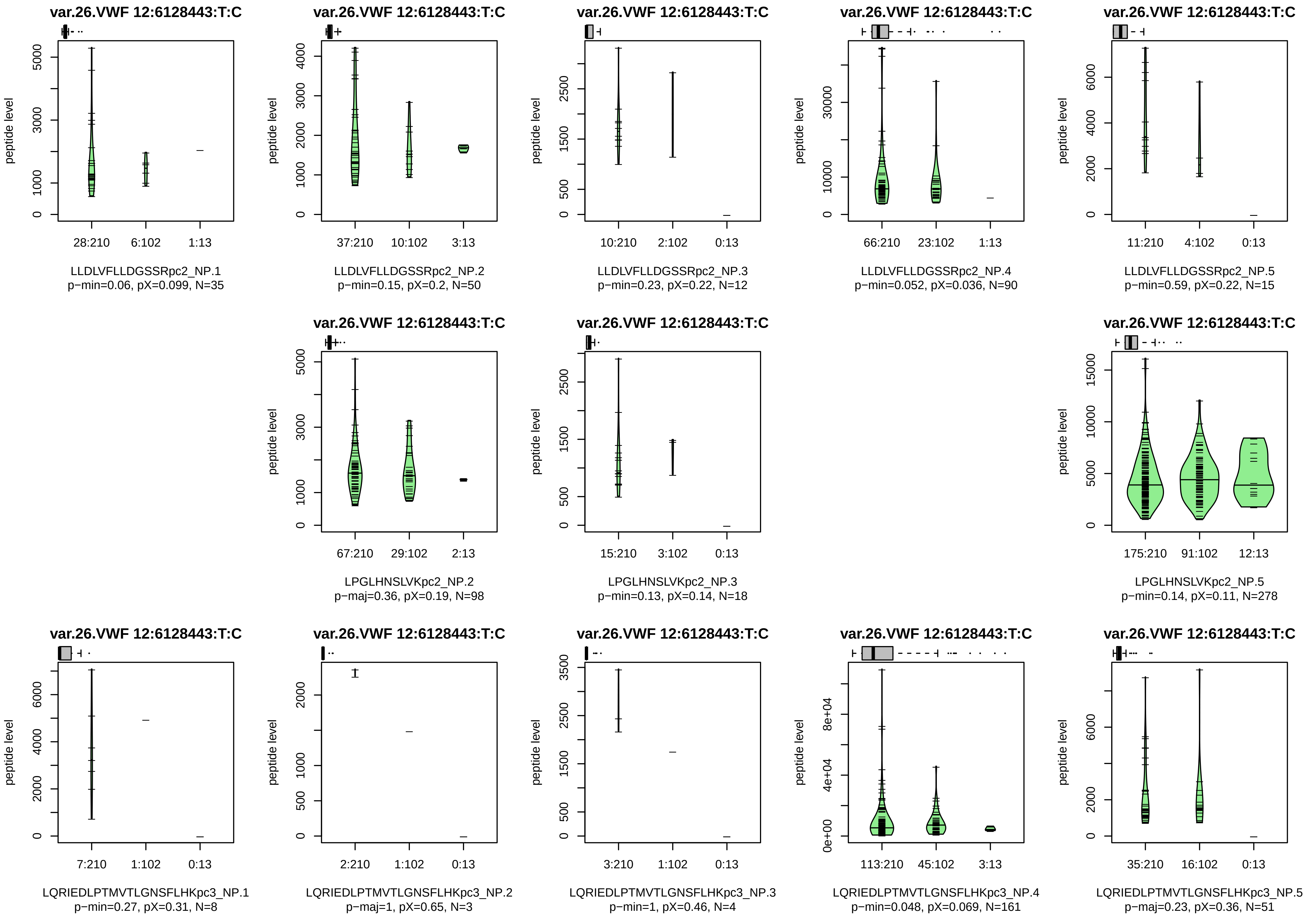
var.26.VWF 12:6128443:T:C
peptide level
28:210
6:102
1:13
LLDLVFLLDGSSRpc2_NP.1
p−min=0.06, pX=0.099, N=35
var.26.VWF 12:6128443:T:C
peptide level
37:210
10:102
3:13
LLDLVFLLDGSSRpc2_NP.2
p−min=0.15, pX=0.2, N=50
var.26.VWF 12:6128443:T:C
peptide level
10:210
2:102
0:13
LLDLVFLLDGSSRpc2_NP.3
p−min=0.23, pX=0.22, N=12
var.26.VWF 12:6128443:T:C
peptide level
66:210
23:102
1:13
LLDLVFLLDGSSRpc2_NP.4
p−min=0.052, pX=0.036, N=90
var.26.VWF 12:6128443:T:C
peptide level
11:210
4:102
0:13
LLDLVFLLDGSSRpc2_NP.5
p−min=0.59, pX=0.22, N=15
var.26.VWF 12:6128443:T:C
peptide level
67:210
29:102
2:13
LPGLHNSLVKpc2_NP.2
p−maj=0.36, pX=0.19, N=98
var.26.VWF 12:6128443:T:C
peptide level
15:210
3:102
0:13
LPGLHNSLVKpc2_NP.3
p−min=0.13, pX=0.14, N=18
var.26.VWF 12:6128443:T:C
peptide level
175:210
91:102
12:13
LPGLHNSLVKpc2_NP.5
p−min=0.14, pX=0.11, N=278
var.26.VWF 12:6128443:T:C
peptide level
7:210
1:102
0:13
LQRIEDLPTMVTLGNSFLHKpc3_NP.1
p−min=0.27, pX=0.31, N=8
var.26.VWF 12:6128443:T:C
peptide level
2:210
1:102
0:13
LQRIEDLPTMVTLGNSFLHKpc3_NP.2
p−maj=1, pX=0.65, N=3
var.26.VWF 12:6128443:T:C
peptide level
3:210
1:102
0:13
LQRIEDLPTMVTLGNSFLHKpc3_NP.3
p−min=1, pX=0.46, N=4
var.26.VWF 12:6128443:T:C
peptide level
113:210
45:102
3:13
LQRIEDLPTMVTLGNSFLHKpc3_NP.4
p−min=0.048, pX=0.069, N=161
var.26.VWF 12:6128443:T:C
peptide level
35:210
16:102
0:13
LQRIEDLPTMVTLGNSFLHKpc3_NP.5
p−maj=0.23, pX=0.36, N=51

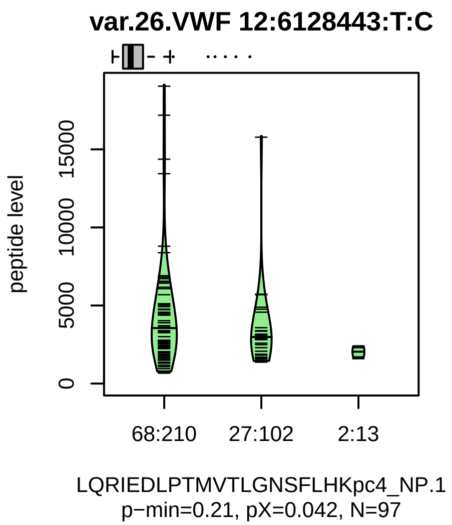
var.26.VWF 12:6128443:T:C
peptide level
68:210
27:102
2:13
LQRIEDLPTMVTLGNSFLHKpc4_NP.1
p−min=0.21, pX=0.042, N=97

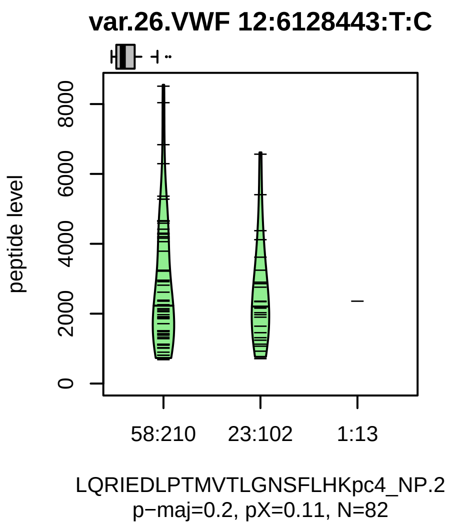
var.26.VWF 12:6128443:T:C
peptide level
58:210
23:102
1:13
LQRIEDLPTMVTLGNSFLHKpc4_NP.2
p−maj=0.2, pX=0.11, N=82

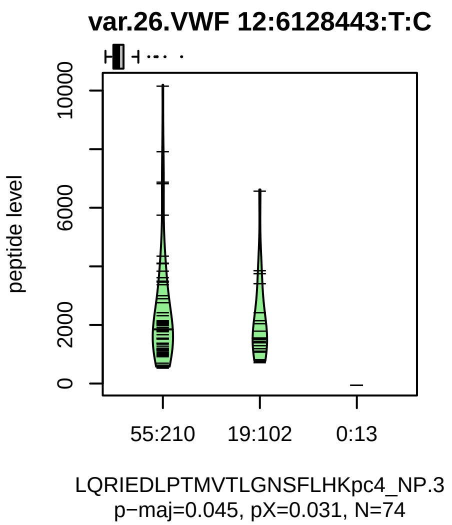
var.26.VWF 12:6128443:T:C
peptide level
55:210
19:102
0:13
LQRIEDLPTMVTLGNSFLHKpc4_NP.3
p−maj=0.045, pX=0.031, N=74

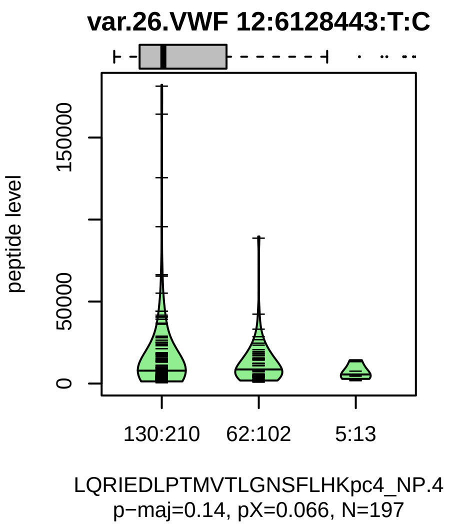
var.26.VWF 12:6128443:T:C
peptide level
130:210
62:102
5:13
LQRIEDLPTMVTLGNSFLHKpc4_NP.4
p−maj=0.14, pX=0.066, N=197

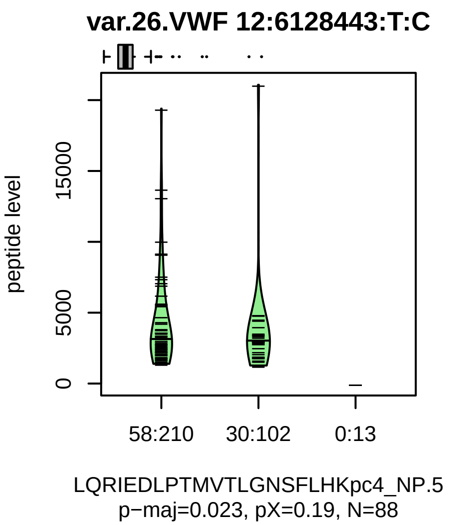
var.26.VWF 12:6128443:T:C
peptide level
58:210
30:102
0:13
LQRIEDLPTMVTLGNSFLHKpc4_NP.5
p−maj=0.023, pX=0.19, N=88

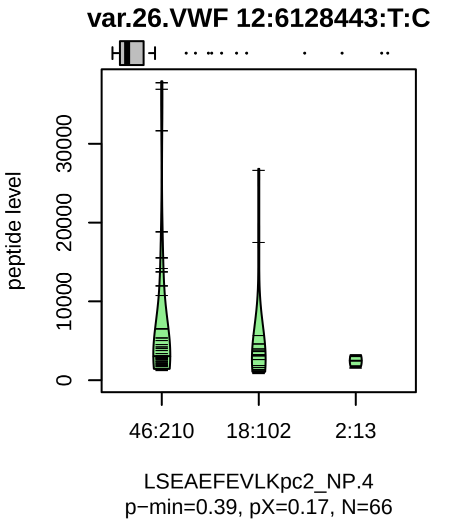
var.26.VWF 12:6128443:T:C
peptide level
46:210
18:102
2:13
LSEAEFEVLKpc2_NP.4
p−min=0.39, pX=0.17, N=66

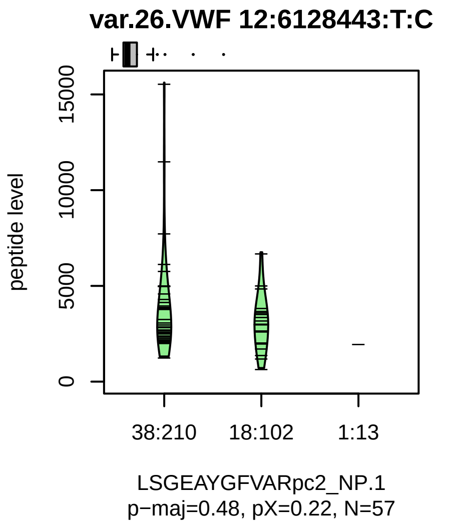
var.26.VWF 12:6128443:T:C
peptide level
38:210
18:102
1:13
LSGEAYGFVARpc2_NP.1
p−maj=0.48, pX=0.22, N=57

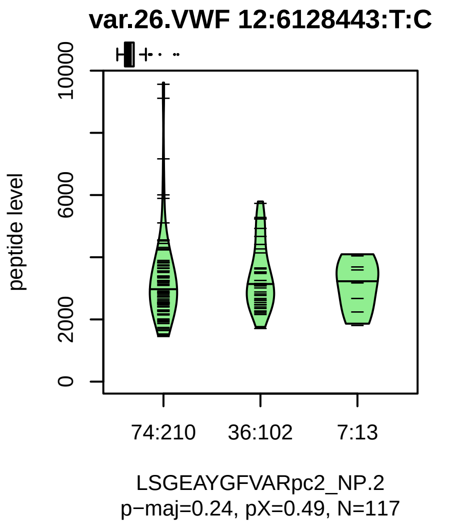
var.26.VWF 12:6128443:T:C
peptide level
74:210
36:102
7:13
LSGEAYGFVARpc2_NP.2
p−maj=0.24, pX=0.49, N=117

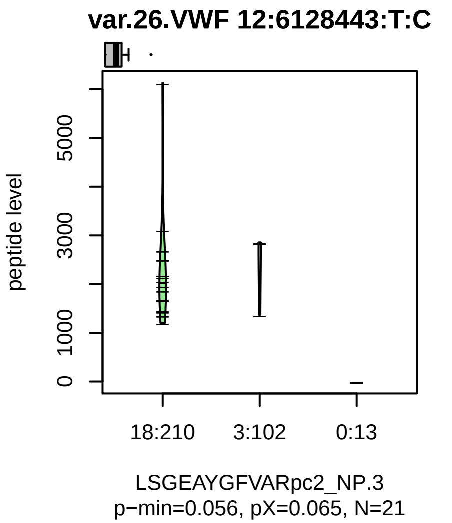
var.26.VWF 12:6128443:T:C
peptide level
18:210
3:102
0:13
LSGEAYGFVARpc2_NP.3
p−min=0.056, pX=0.065, N=21

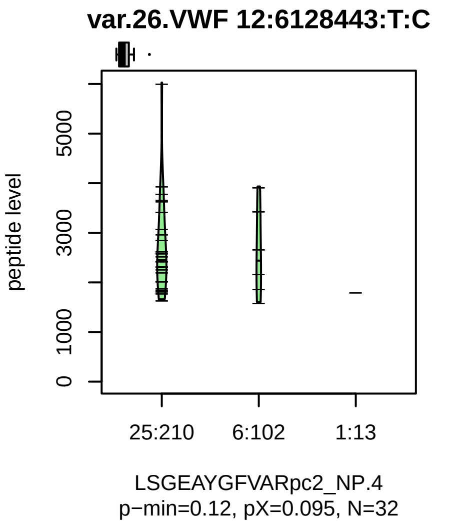
var.26.VWF 12:6128443:T:C
peptide level
25:210
6:102
1:13
LSGEAYGFVARpc2_NP.4
p−min=0.12, pX=0.095, N=32

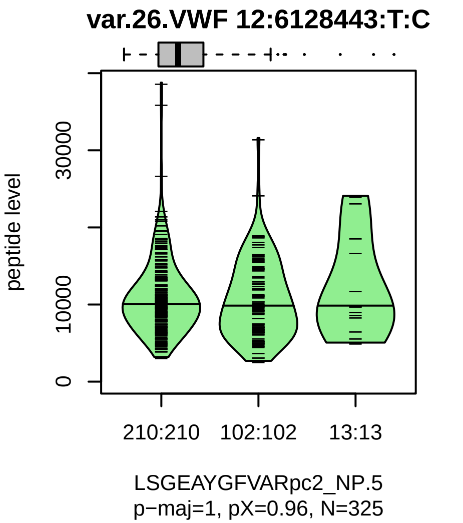
var.26.VWF 12:6128443:T:C
peptide level
210:210
102:102
13:13
LSGEAYGFVARpc2_NP.5
p−maj=1, pX=0.96, N=325

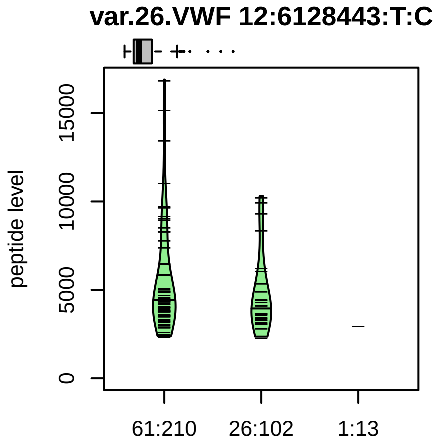

LSPVYAGKpc2_NP.1
p−maj=0.2, pX=0.066, N=88

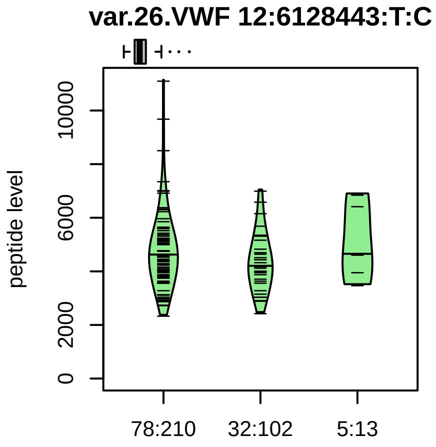

LSPVYAGKpc2_NP.2
p−min=0.4, pX=0.37, N=115

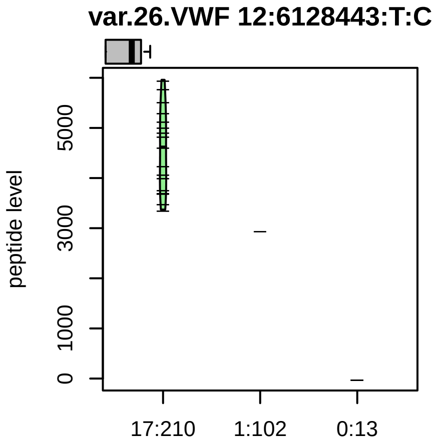

LSPVYAGKpc2_NP.3
p−min=0.0047, pX=0.0067, N=18

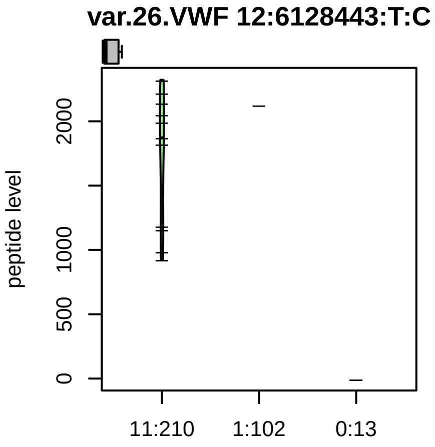

LSPVYAGKpc2_NP.4
p−min=0.062, pX=0.08, N=12

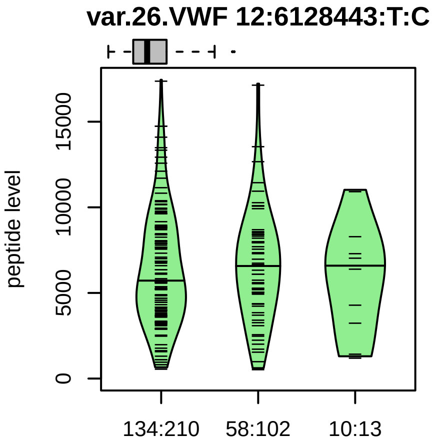

LSPVYAGKpc2_NP.5
p−maj=0.38, pX=0.78, N=202

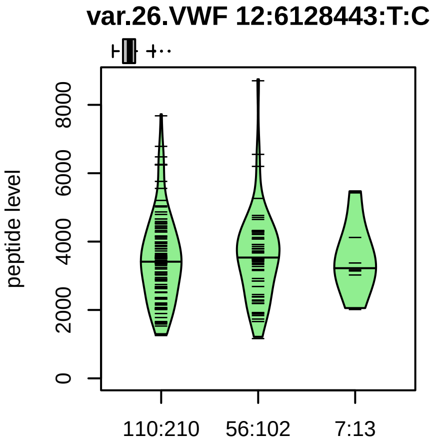

LSYGEDLQMDWDGRpc2_NP.5
p−min=0.73, pX=0.59, N=173

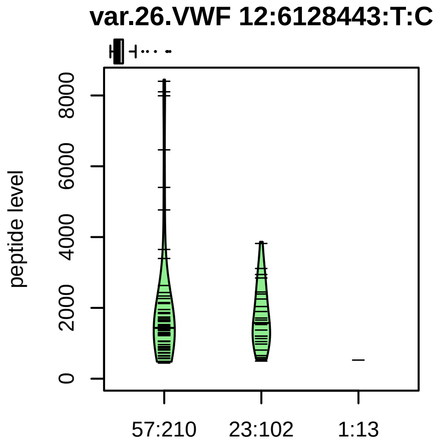

LTGSCSYVLFQNKpc2_NP.1
p−maj=0.2, pX=0.086, N=81

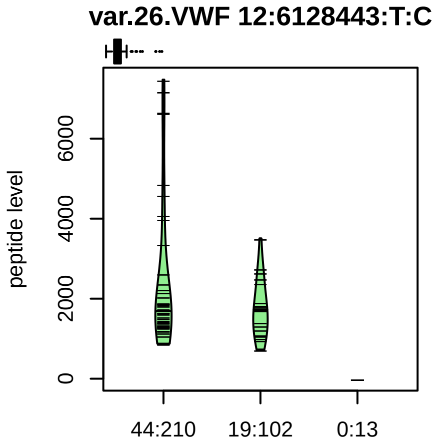

LTGSCSYVLFQNKpc2_NP.2
p−maj=0.081, pX=0.061, N=63

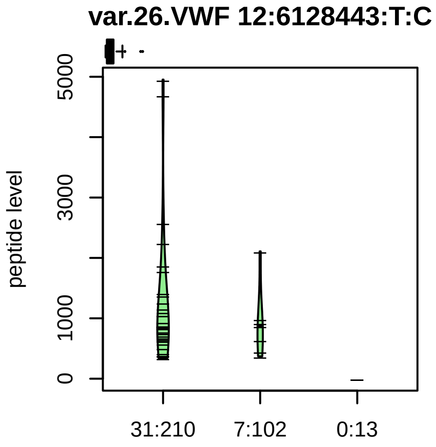

LTGSCSYVLFQNKpc2_NP.3
p−min=0.019, pX=0.031, N=38

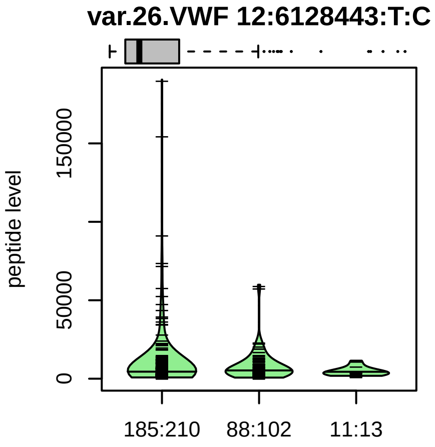

LTGSCSYVLFQNKpc2_NP.4
p−min=0.6, pX=0.095, N=284

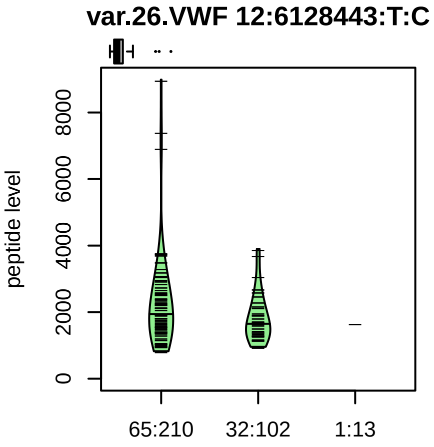

LTGSCSYVLFQNKpc2_NP.5
p−maj=0.12, pX=0.099, N=98

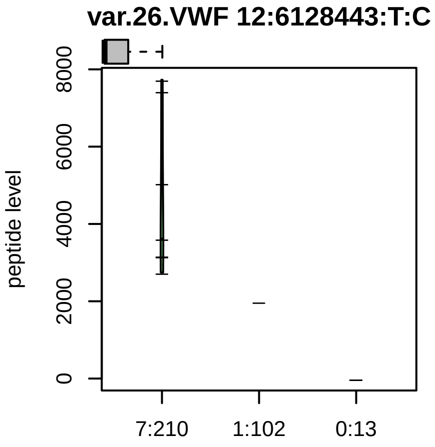

LTGSCSYVLFQNKpc3_NP.4
p−min=0.27, pX=0.12, N=8

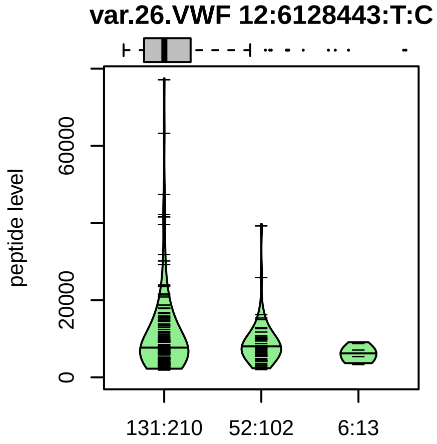

LVCPADNLRpc2_NP.1
p−min=0.045, pX=0.0097, N=189

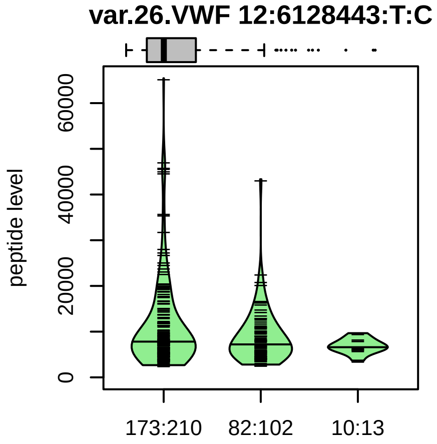

LVCPADNLRpc2_NP.2
p−min=0.65, pX=0.017, N=265

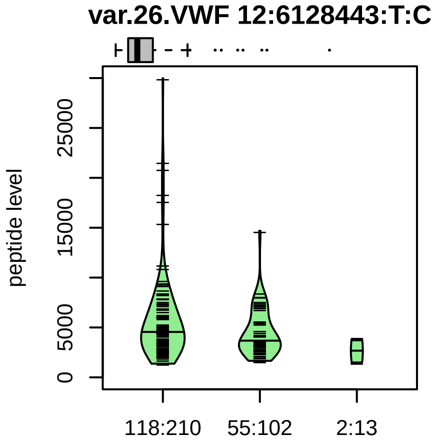

LVCPADNLRpc2_NP.3
p−maj=0.0079, pX=0.0068, N=175

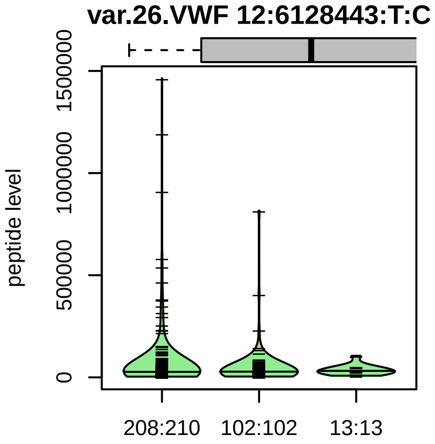

LVCPADNLRpc2_NP.4
p−min=0.54, pX=0.13, N=323

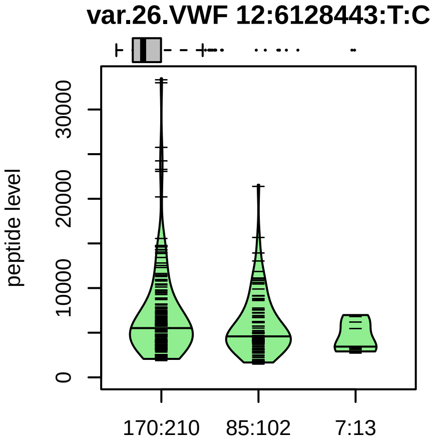

LVCPADNLRpc2_NP.5
p−maj=0.023, pX=0.017, N=262

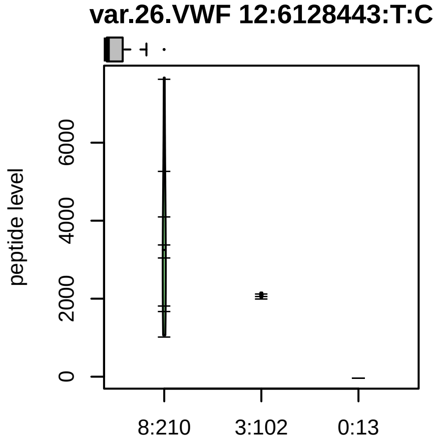

LVSVPYVGGNMEVNVYGAIMHEVRpc3_NP.
p−min=0.75, pX=0.28, N=11

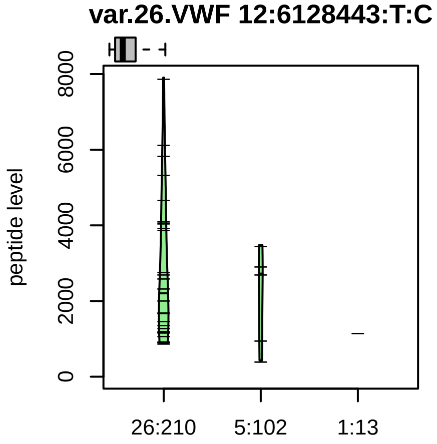

LVSVPYVGGNMEVNVYGAIMHEVRpc3_NP.
p−min=0.05, pX=0.037, N=32

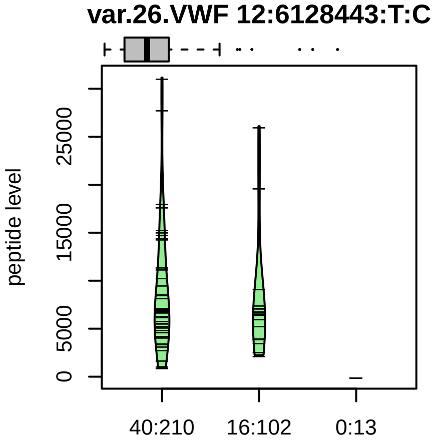

LVSVPYVGGNMEVNVYGAIMHEVRpc3_NP.
p−maj=0.14, pX=0.14, N=56

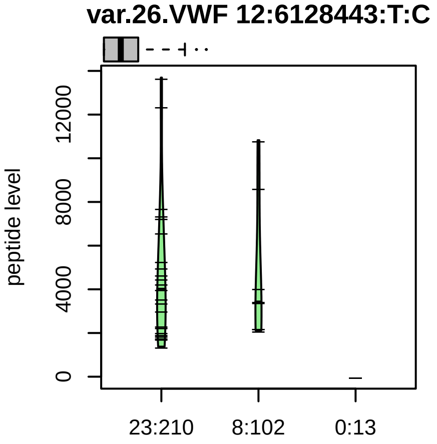

LVSVPYVGGNMEVNVYGAIMHEVRpc3_NP.
p−min=0.32, pX=0.24, N=31

var.26.VWF 12:6128443:T:C

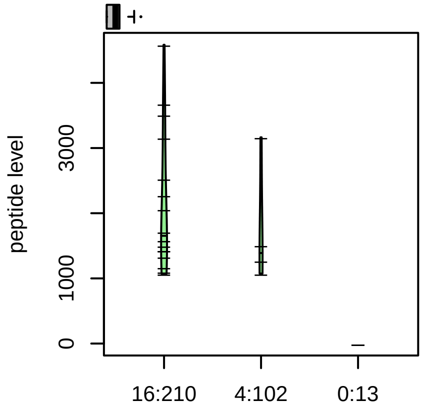

NLRENGYECEWRpc3_NP.1
p−min=0.16, pX=0.1, N=20

var.26.VWF 12:6128443:T:C

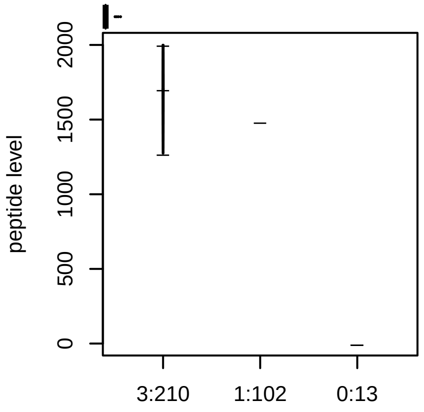

NLRENGYECEWRpc3_NP.3
p−min=1, pX=0.57, N=4

var.26.VWF 12:6128443:T:C

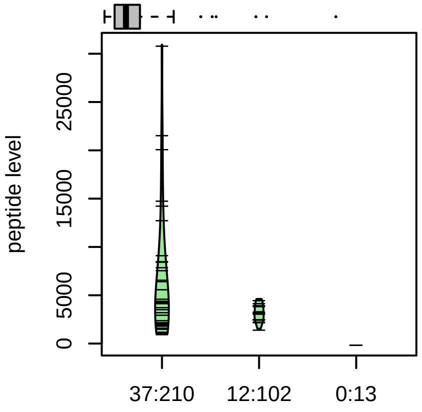

NLRENGYECEWRpc3_NP.4
p−min=0.1, pX=0.021, N=49

var.26.VWF 12:6128443:T:C

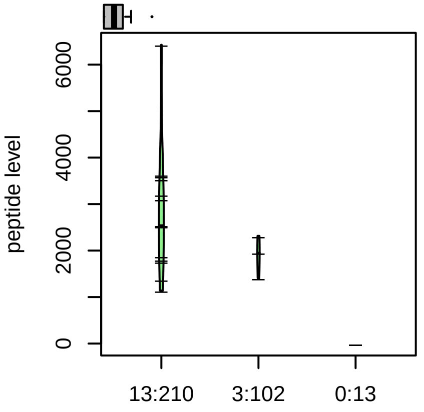

NLRENGYECEWRpc3_NP.5
p−min=0.19, pX=0.091, N=16

var.26.VWF 12:6128443:T:C

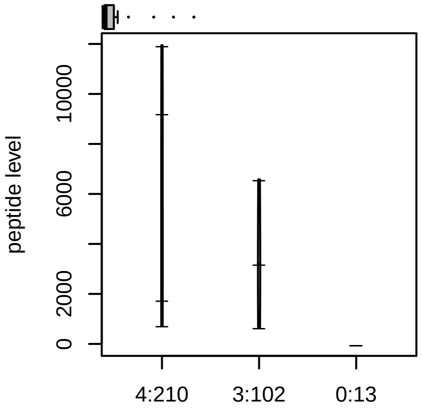

NSMVLDVAFVLEGSDKpc2_NP.4
p−min=0.7, pX=0.75, N=7

var.26.VWF 12:6128443:T:C

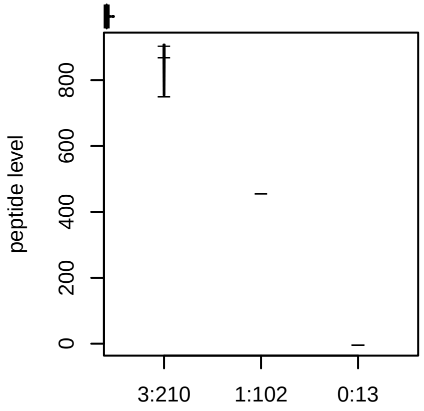

QHSDPCALNPRpc2_NP.1
p−min=1, pX=0.41, N=4

var.26.VWF 12:6128443:T:C

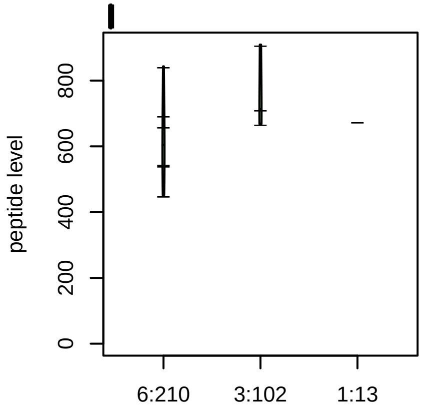

QHSDPCALNPRpc2_NP.2
p−maj=0.34, pX=0.41, N=10

var.26.VWF 12:6128443:T:C

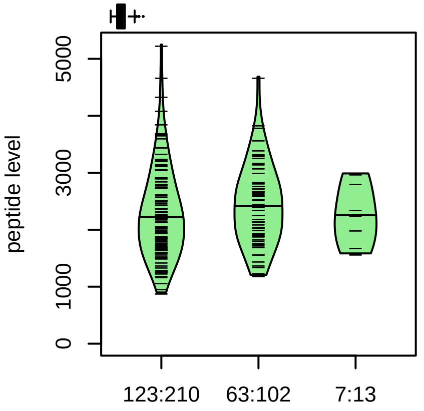

QHSDPCALNPRpc2_NP.5
p−min=0.72, pX=0.58, N=193

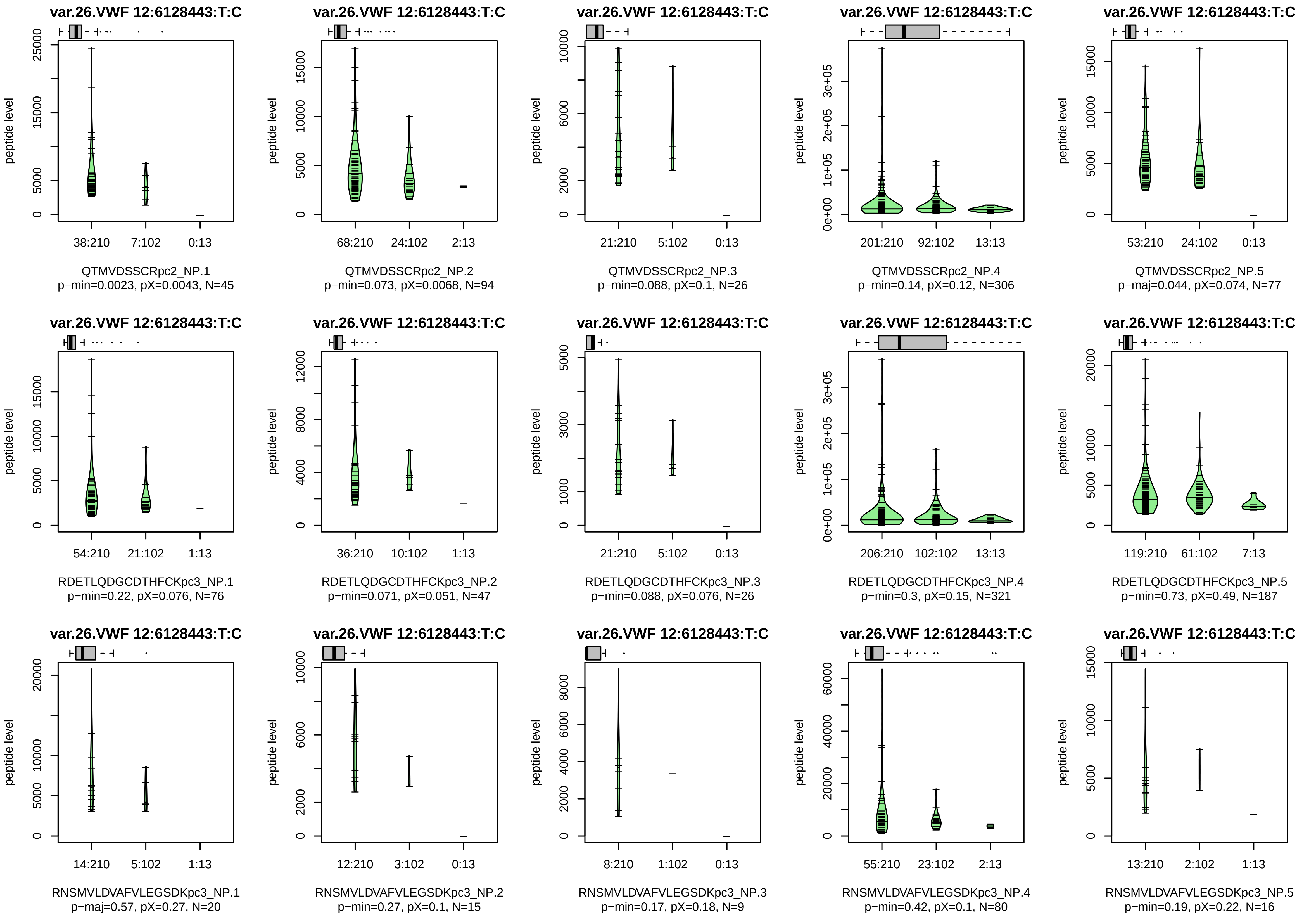
var.26.VWF 12:6128443:T:C
peptide level
38:210
7:102
0:13
QTMVDSSCRpc2_NP.1
p−min=0.0023, pX=0.0043, N=45
var.26.VWF 12:6128443:T:C
peptide level
68:210
24:102
2:13
QTMVDSSCRpc2_NP.2
p−min=0.073, pX=0.0068, N=94
var.26.VWF 12:6128443:T:C
peptide level
21:210
5:102
0:13
QTMVDSSCRpc2_NP.3
p−min=0.088, pX=0.1, N=26
var.26.VWF 12:6128443:T:C
peptide level
201:210
92:102
13:13
QTMVDSSCRpc2_NP.4
p−min=0.14, pX=0.12, N=306
var.26.VWF 12:6128443:T:C
peptide level
53:210
24:102
0:13
QTMVDSSCRpc2_NP.5
p−maj=0.044, pX=0.074, N=77
var.26.VWF 12:6128443:T:C
peptide level
54:210
21:102
1:13
RDETLQDGCDTHFCKpc3_NP.1
p−min=0.22, pX=0.076, N=76
var.26.VWF 12:6128443:T:C
peptide level
36:210
10:102
1:13
RDETLQDGCDTHFCKpc3_NP.2
p−min=0.071, pX=0.051, N=47
var.26.VWF 12:6128443:T:C
peptide level
21:210
5:102
0:13
RDETLQDGCDTHFCKpc3_NP.3
p−min=0.088, pX=0.076, N=26
var.26.VWF 12:6128443:T:C
peptide level
206:210
102:102
13:13
RDETLQDGCDTHFCKpc3_NP.4
p−min=0.3, pX=0.15, N=321
var.26.VWF 12:6128443:T:C
peptide level
119:210
61:102
7:13
RDETLQDGCDTHFCKpc3_NP.5
p−min=0.73, pX=0.49, N=187
var.26.VWF 12:6128443:T:C
peptide level
14:210
5:102
1:13
RNSMVLDVAFVLEGSDKpc3_NP.1
p−maj=0.57, pX=0.27, N=20
var.26.VWF 12:6128443:T:C
peptide level
12:210
3:102
0:13
RNSMVLDVAFVLEGSDKpc3_NP.2
p−min=0.27, pX=0.1, N=15
var.26.VWF 12:6128443:T:C
peptide level
8:210
1:102
0:13
RNSMVLDVAFVLEGSDKpc3_NP.3
p−min=0.17, pX=0.18, N=9
var.26.VWF 12:6128443:T:C
peptide level
55:210
23:102
2:13
RNSMVLDVAFVLEGSDKpc3_NP.4
p−min=0.42, pX=0.1, N=80
var.26.VWF 12:6128443:T:C
peptide level
13:210
2:102
1:13
RNSMVLDVAFVLEGSDKpc3_NP.5
p−min=0.19, pX=0.22, N=16

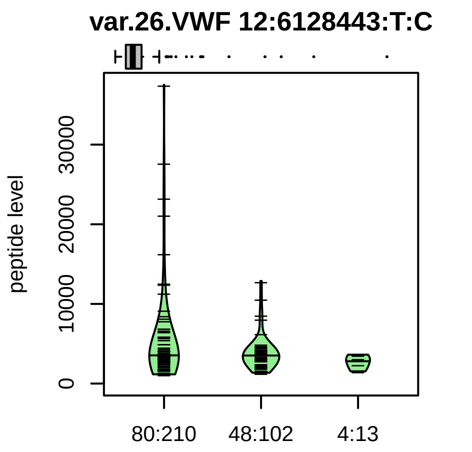

RPGDVWTLPDQCHTVTCQPDGQTLLKpc4_NI
p−min=0.24, pX=0.28, N=132

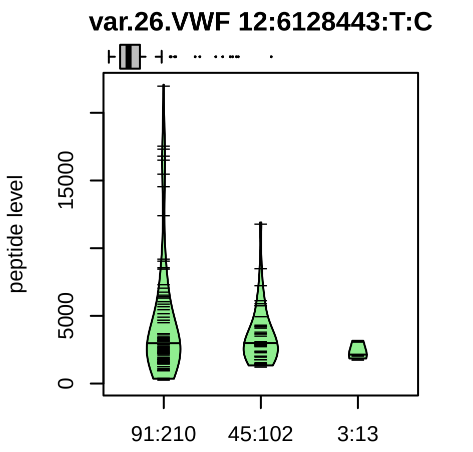

RPGDVWTLPDQCHTVTCQPDGQTLLKpc4_NI
p−maj=0.16, pX=0.094, N=139

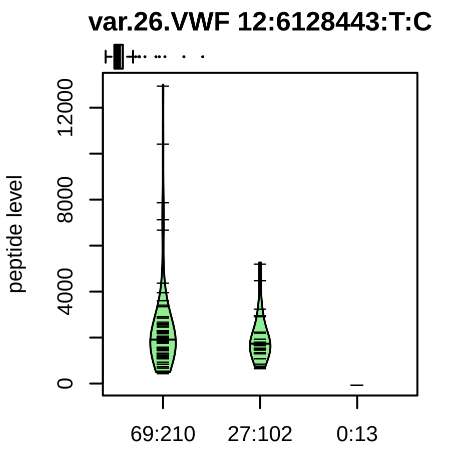

RPGDVWTLPDQCHTVTCQPDGQTLLKpc4_NI
p−maj=0.012, pX=0.026, N=96

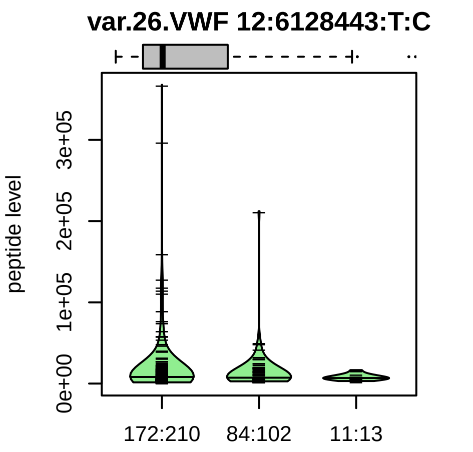

RPGDVWTLPDQCHTVTCQPDGQTLLKpc4_NI
p−maj=1, pX=0.13, N=267

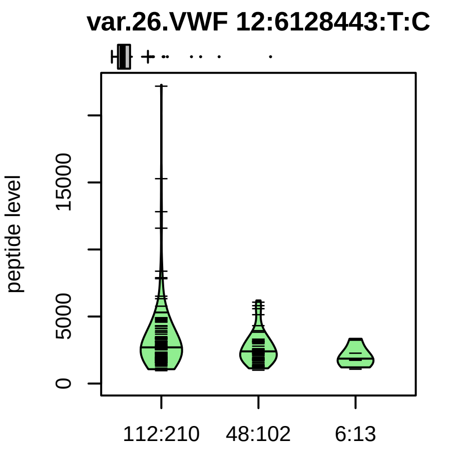

RPGDVWTLPDQCHTVTCQPDGQTLLKpc4_NI
p−min=0.3, pX=0.022, N=166

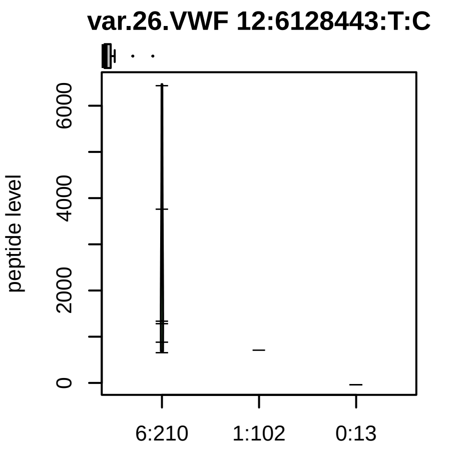

RVTGCPPFDEHKpc2_NP.4
p−min=0.43, pX=0.23, N=7

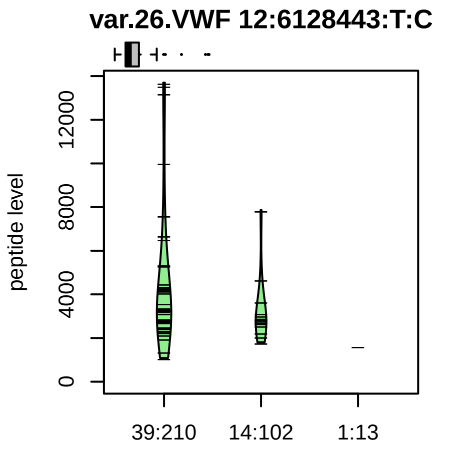

RVTGCPPFDEHKpc3_NP.1
p−min=0.22, pX=0.05, N=54

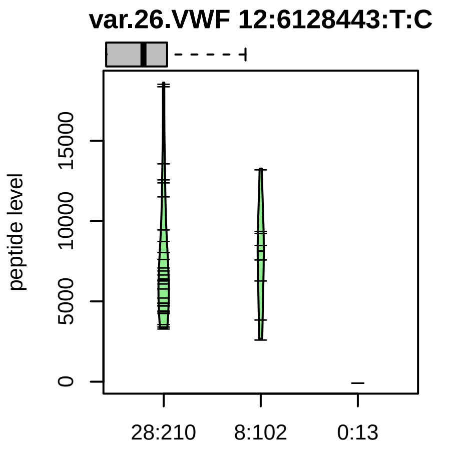

RVTGCPPFDEHKpc3_NP.2
p−min=0.097, pX=0.087, N=36

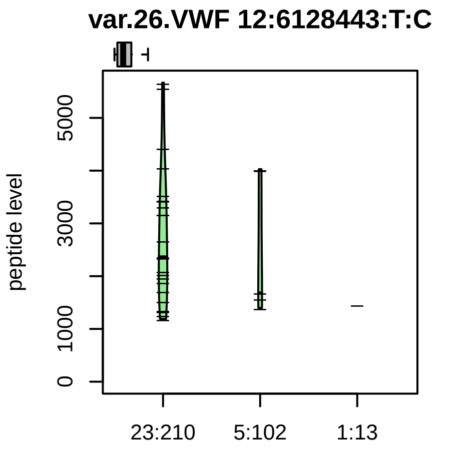

RVTGCPPFDEHKpc3_NP.3
p−min=0.1, pX=0.084, N=29

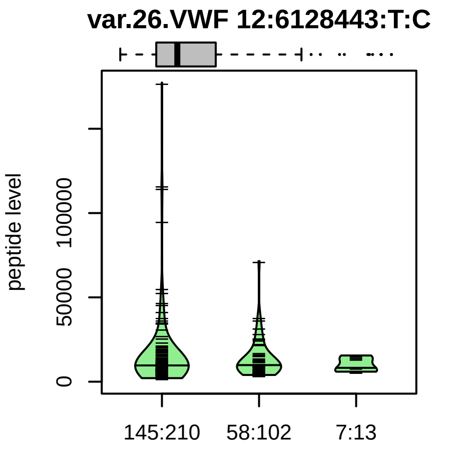

RVTGCPPFDEHKpc3_NP.4
p−min=0.029, pX=0.068, N=210

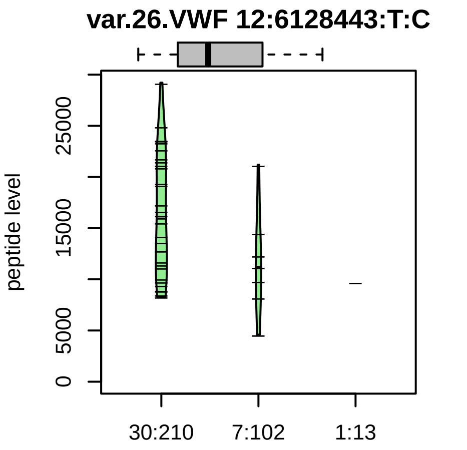

RVTGCPPFDEHKpc3_NP.5
p−min=0.07, pX=0.019, N=38

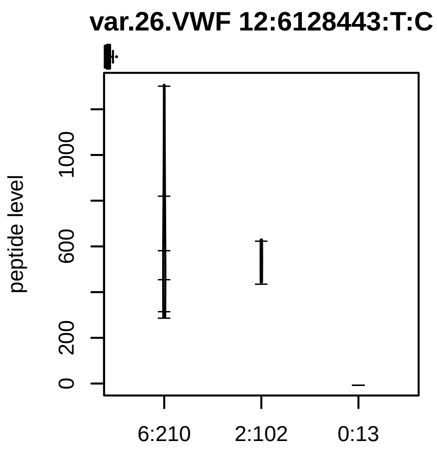

SEVEVDIHYCQGKpc2_NP.1
p−min=0.72, pX=0.44, N=8

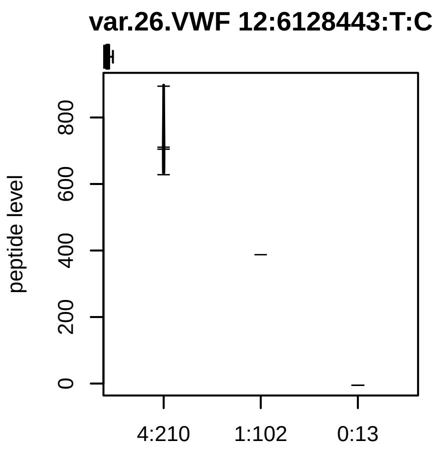

SEVEVDIHYCQGKpc2_NP.2
p−min=0.66, pX=0.29, N=5

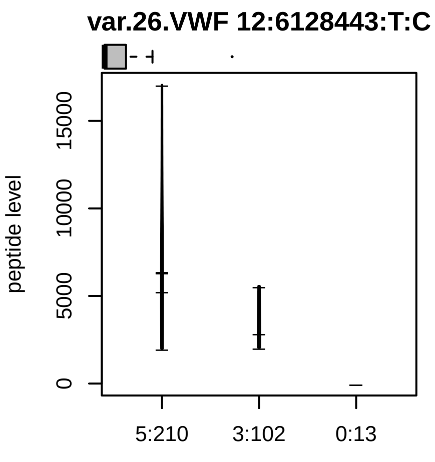

SEVEVDIHYCQGKpc2_NP.4
p−min=1, pX=0.49, N=8

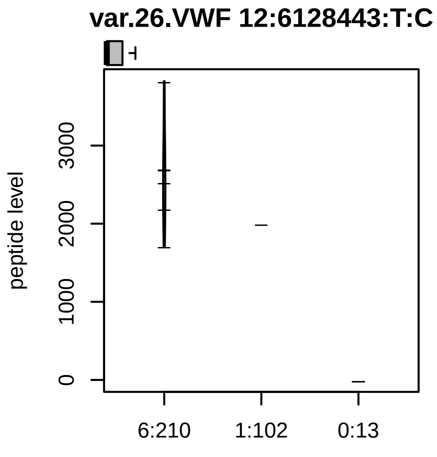

SEVEVDIHYCQGKpc3_NP.1
p−min=0.43, pX=0.2, N=7

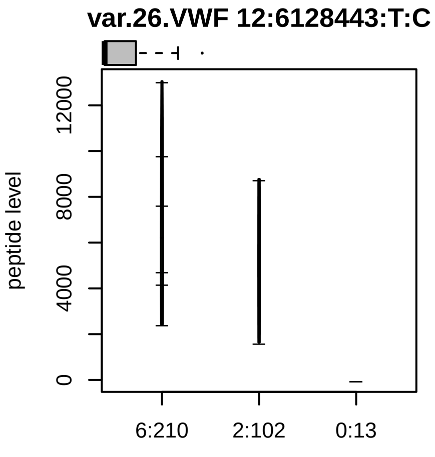

SEVEVDIHYCQGKpc3_NP.4
p−min=0.72, pX=0.39, N=8

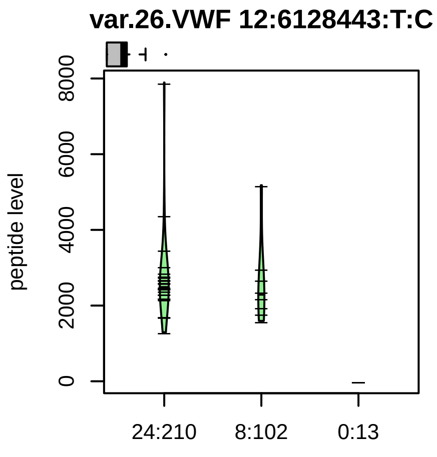

SFSIIGDFQNGKpc2_NP.1
p−min=0.24, pX=0.14, N=32

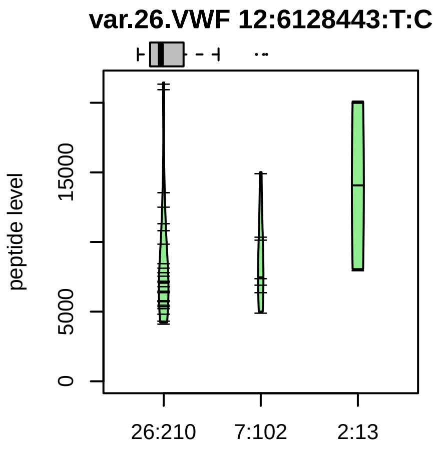

SFSIIGDFQNGKpc2_NP.2
p−min=0.26, pX=0.88, N=35

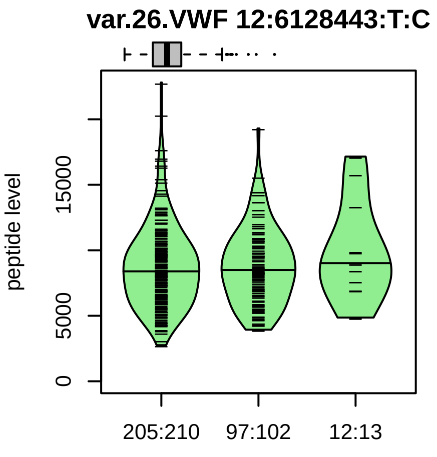

SFSIIGDFQNGKpc2_NP.5
p−min=0.21, pX=1, N=314

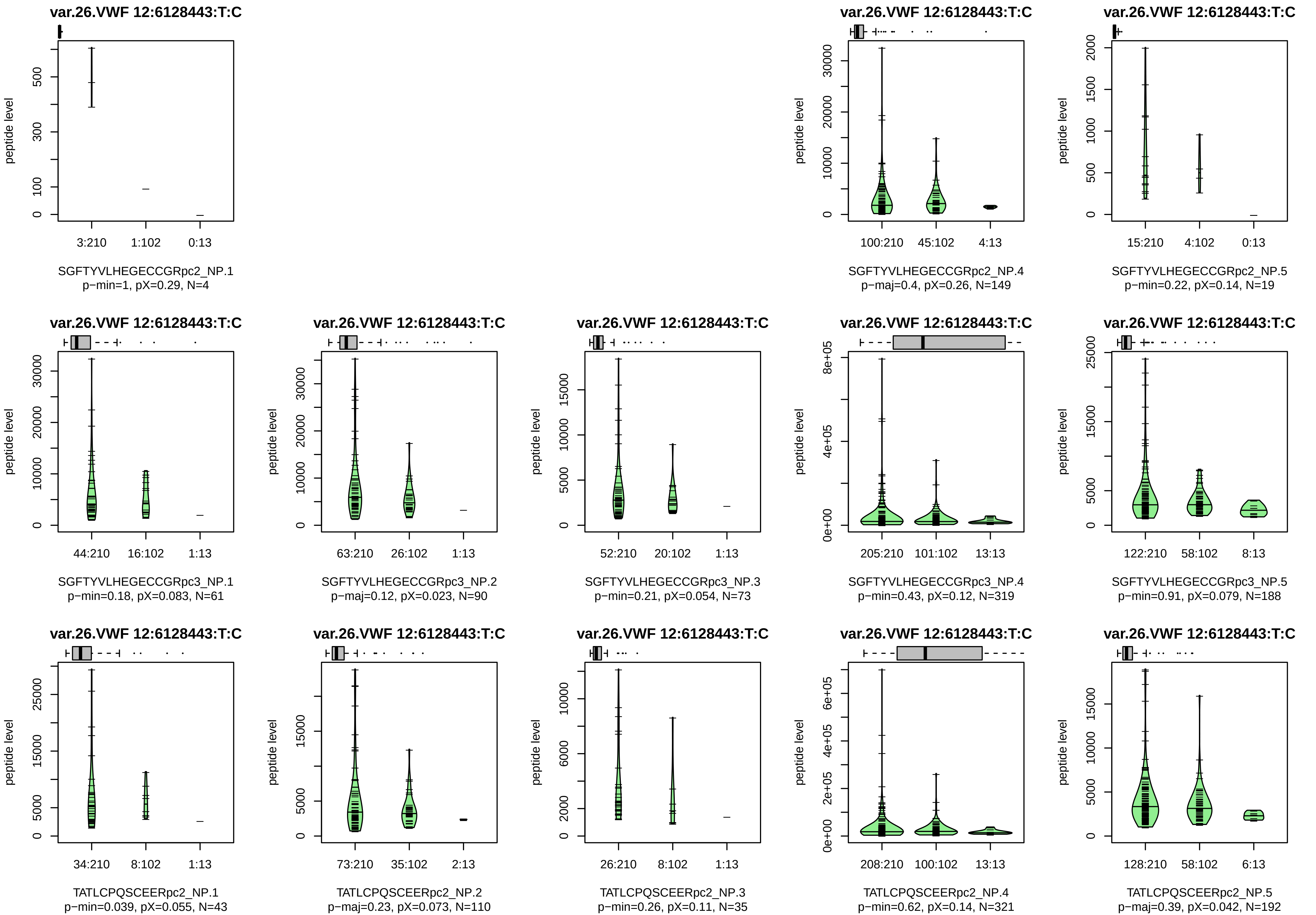
var.26.VWF 12:6128443:T:C
peptide level
3:210
1:102
0:13
SGFTYVLHEGECCGRpc2_NP.1
p−min=1, pX=0.29, N=4
var.26.VWF 12:6128443:T:C
peptide level
100:210
45:102
4:13
SGFTYVLHEGECCGRpc2_NP.4
p−maj=0.4, pX=0.26, N=149
var.26.VWF 12:6128443:T:C
peptide level
15:210
4:102
0:13
SGFTYVLHEGECCGRpc2_NP.5
p−min=0.22, pX=0.14, N=19
var.26.VWF 12:6128443:T:C
peptide level
44:210
16:102
1:13
SGFTYVLHEGECCGRpc3_NP.1
p−min=0.18, pX=0.083, N=61
var.26.VWF 12:6128443:T:C
peptide level
63:210
26:102
1:13
SGFTYVLHEGECCGRpc3_NP.2
p−maj=0.12, pX=0.023, N=90
var.26.VWF 12:6128443:T:C
peptide level
52:210
20:102
1:13
SGFTYVLHEGECCGRpc3_NP.3
p−min=0.21, pX=0.054, N=73
var.26.VWF 12:6128443:T:C
peptide level
205:210
101:102
13:13
SGFTYVLHEGECCGRpc3_NP.4
p−min=0.43, pX=0.12, N=319
var.26.VWF 12:6128443:T:C
peptide level
122:210
58:102
8:13
SGFTYVLHEGECCGRpc3_NP.5
p−min=0.91, pX=0.079, N=188
var.26.VWF 12:6128443:T:C
peptide level
34:210
8:102
1:13
TATLCPQSCEERpc2_NP.1
p−min=0.039, pX=0.055, N=43
var.26.VWF 12:6128443:T:C
peptide level
73:210
35:102
2:13
TATLCPQSCEERpc2_NP.2
p−maj=0.23, pX=0.073, N=110
var.26.VWF 12:6128443:T:C
peptide level
26:210
8:102
1:13
TATLCPQSCEERpc2_NP.3
p−min=0.26, pX=0.11, N=35
var.26.VWF 12:6128443:T:C
peptide level
208:210
100:102
13:13
TATLCPQSCEERpc2_NP.4
p−min=0.62, pX=0.14, N=321
var.26.VWF 12:6128443:T:C
peptide level
128:210
58:102
6:13
TATLCPQSCEERpc2_NP.5
p−maj=0.39, pX=0.042, N=192

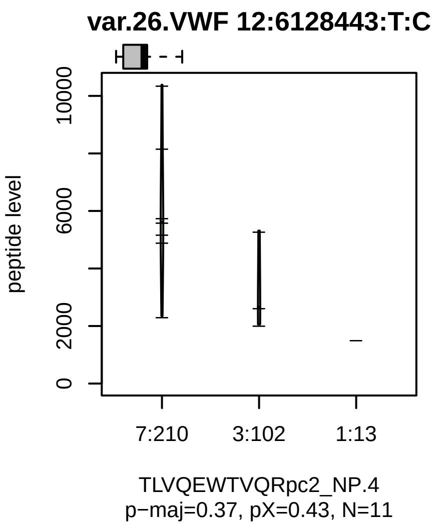
var.26.VWF 12:6128443:T:C
peptide level
0
2000
6000
10000
7:210
3:102
1:13
TLVQEWTVQRpc2_NP.4
p−maj=0.37, pX=0.43, N=11

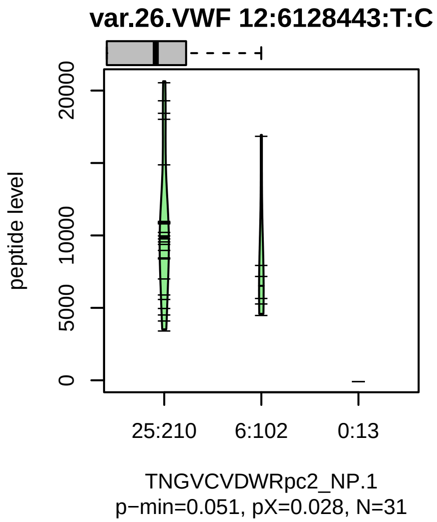
var.26.VWF 12:6128443:T:C
peptide level
0
5000
10000
20000
25:210
6:102
0:13
TNGVCVDWRpc2_NP.1
p−min=0.051, pX=0.028, N=31

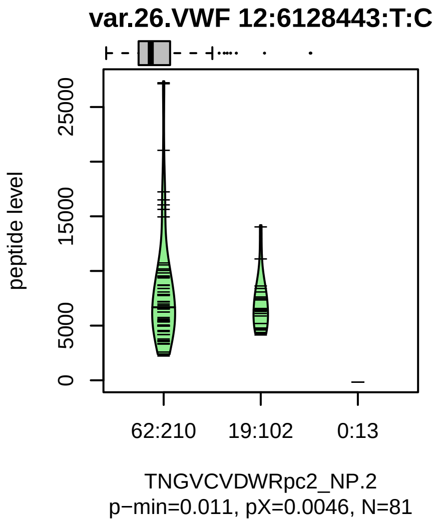
var.26.VWF 12:6128443:T:C
peptide level
0
5000
15000
25000
62:210
19:102
0:13
TNGVCVDWRpc2_NP.2
p−min=0.011, pX=0.0046, N=81

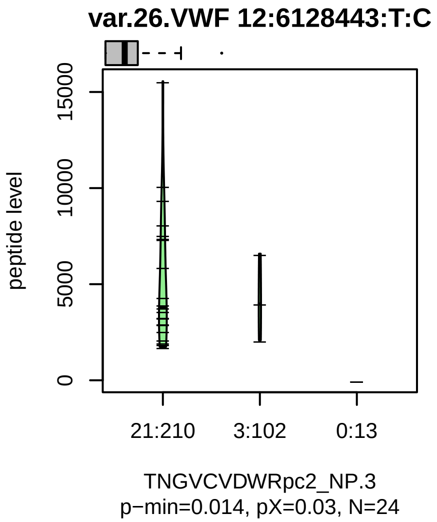
var.26.VWF 12:6128443:T:C
peptide level
0
5000
10000
15000
21:210
3:102
0:13
TNGVCVDWRpc2_NP.3
p−min=0.014, pX=0.03, N=24

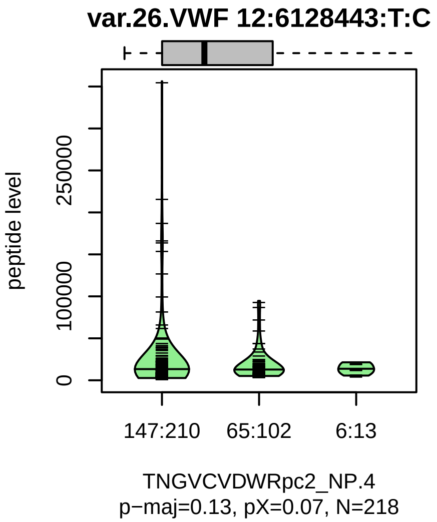
var.26.VWF 12:6128443:T:C
peptide level
0
100000
250000
147:210
65:102
6:13
TNGVCVDWRpc2_NP.4
p−maj=0.13, pX=0.07, N=218

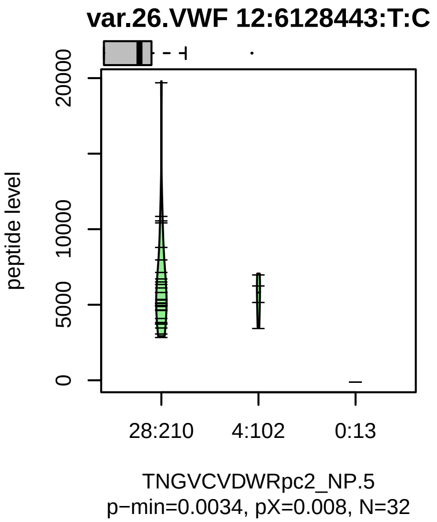
var.26.VWF 12:6128443:T:C
peptide level
0
5000
10000
20000
28:210
4:102
0:13
TNGVCVDWRpc2_NP.5
p−min=0.0034, pX=0.008, N=32

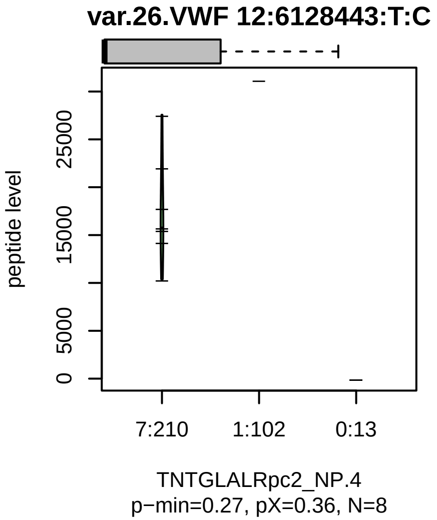
var.26.VWF 12:6128443:T:C
peptide level
0
5000
15000
25000
7:210
1:102
0:13
TNTGLALRpc2_NP.4
p−min=0.27, pX=0.36, N=8

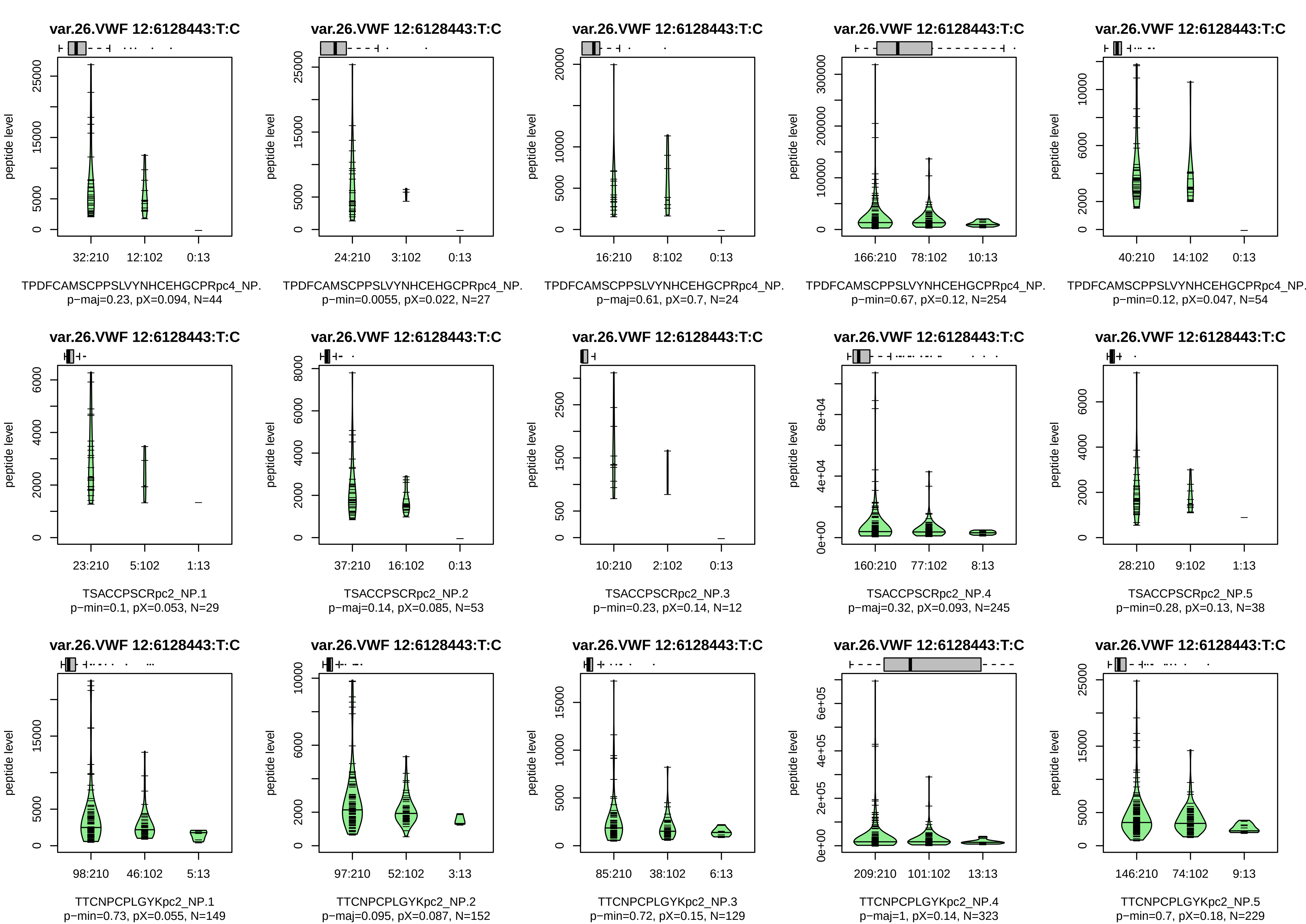
var.26.VWF 12:6128443:T:C
peptide level
32:210 12:102 0:13
TPDFCAMSCPPSLVYNHCEHGCPRpc4_NP.
p−maj=0.23, pX=0.094, N=44
var.26.VWF 12:6128443:T:C
peptide level
24:210 3:102 0:13
TPDFCAMSCPPSLVYNHCEHGCPRpc4_NP.
p−min=0.0055, pX=0.022, N=27
var.26.VWF 12:6128443:T:C
peptide level
16:210 8:102 0:13
TPDFCAMSCPPSLVYNHCEHGCPRpc4_NP.
p−maj=0.61, pX=0.7, N=24
var.26.VWF 12:6128443:T:C
peptide level
166:210 78:102 10:13
TPDFCAMSCPPSLVYNHCEHGCPRpc4_NP.
p−min=0.67, pX=0.12, N=254
var.26.VWF 12:6128443:T:C
peptide level
40:210 14:102 0:13
TPDFCAMSCPPSLVYNHCEHGCPRpc4_NP.
p−min=0.12, pX=0.047, N=54
var.26.VWF 12:6128443:T:C
peptide level
23:210 5:102 1:13
TSACCPSCRpc2_NP.1
p−min=0.1, pX=0.053, N=29
var.26.VWF 12:6128443:T:C
peptide level
37:210 16:102 0:13
TSACCPSCRpc2_NP.2
p−maj=0.14, pX=0.085, N=53
var.26.VWF 12:6128443:T:C
peptide level
10:210 2:102 0:13
TSACCPSCRpc2_NP.3
p−min=0.23, pX=0.14, N=12
var.26.VWF 12:6128443:T:C
peptide level
160:210 77:102 8:13
TSACCPSCRpc2_NP.4
p−maj=0.32, pX=0.093, N=245
var.26.VWF 12:6128443:T:C
peptide level
28:210 9:102 1:13
TSACCPSCRpc2_NP.5
p−min=0.28, pX=0.13, N=38
var.26.VWF 12:6128443:T:C
peptide level
98:210 46:102 5:13
TTCNPCPLGYKpc2_NP.1
p−min=0.73, pX=0.055, N=149
var.26.VWF 12:6128443:T:C
peptide level
97:210 52:102 3:13
TTCNPCPLGYKpc2_NP.2
p−maj=0.095, pX=0.087, N=152
var.26.VWF 12:6128443:T:C
peptide level
85:210 38:102 6:13
TTCNPCPLGYKpc2_NP.3
p−min=0.72, pX=0.15, N=129
var.26.VWF 12:6128443:T:C
peptide level
209:210 101:102 13:13
TTCNPCPLGYKpc2_NP.4
p−maj=1, pX=0.14, N=323
var.26.VWF 12:6128443:T:C
peptide level
146:210 74:102 9:13
TTCNPCPLGYKpc2_NP.5
p−min=0.7, pX=0.18, N=229

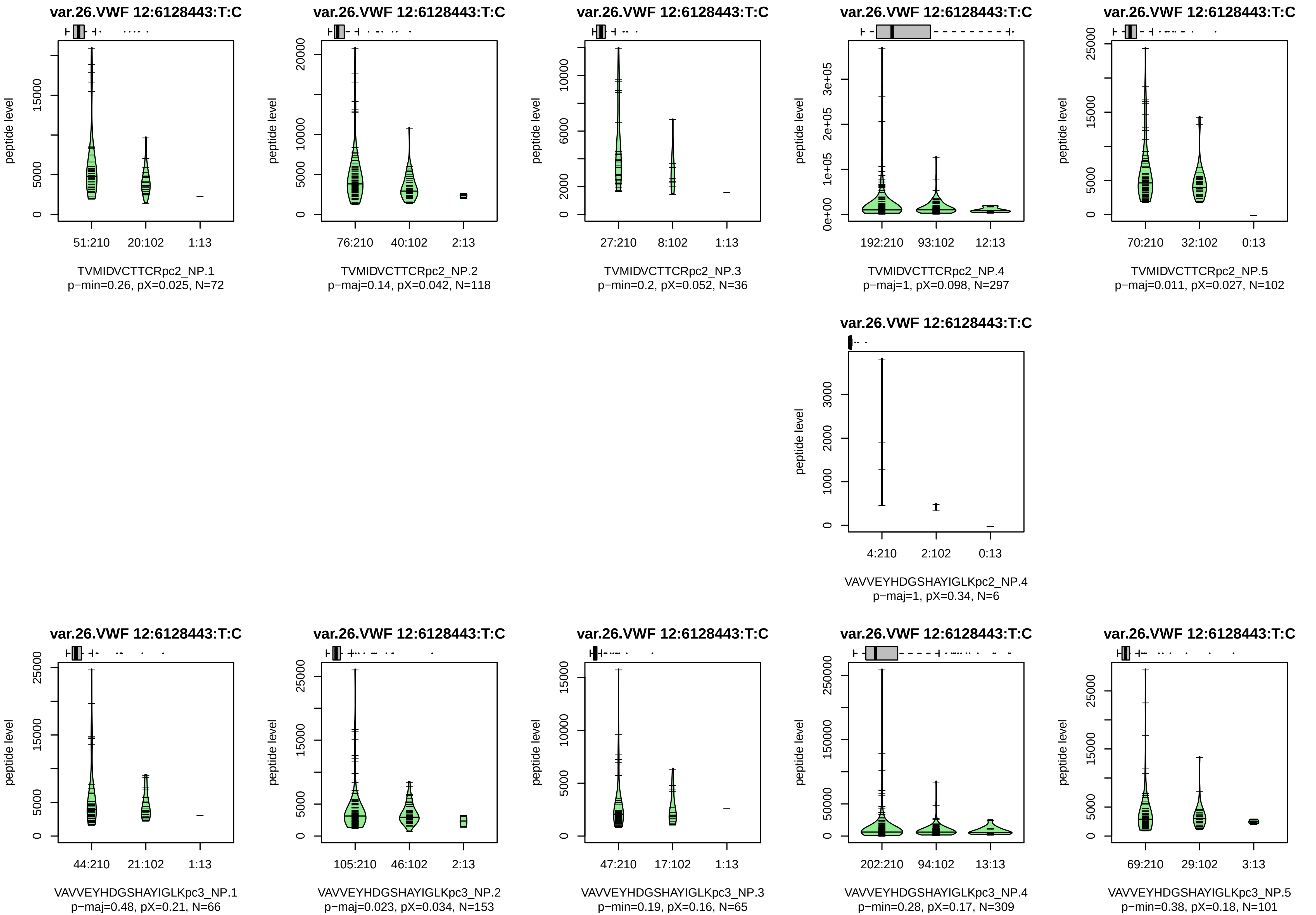

var.26.VWF 12:6128443:T:C
peptide level
0
5000
15000
51:210
20:102
1:13
TVMIDVCTTCRpc2_NP.1
p−min=0.26, pX=0.025, N=72
var.26.VWF 12:6128443:T:C
peptide level
0
5000
10000
20000
76:210
40:102
2:13
TVMIDVCTTCRpc2_NP.2
p−maj=0.14, pX=0.042, N=118
var.26.VWF 12:6128443:T:C
peptide level
0
2000
6000
10000
27:210
8:102
1:13
TVMIDVCTTCRpc2_NP.3
p−min=0.2, pX=0.052, N=36
var.26.VWF 12:6128443:T:C
peptide level
0e+00
1e+05
2e+05
3e+05
192:210
93:102
12:13
TVMIDVCTTCRpc2_NP.4
p−maj=1, pX=0.098, N=297
var.26.VWF 12:6128443:T:C
peptide level
0
5000
15000
25000
70:210
32:102
0:13
TVMIDVCTTCRpc2_NP.5
p−maj=0.011, pX=0.027, N=102
var.26.VWF 12:6128443:T:C
peptide level
0
1000
2000
3000
4:210
2:102
0:13
VAVVEYHDGSHAYIGLKpc2_NP.4
p−maj=1, pX=0.34, N=6
var.26.VWF 12:6128443:T:C
peptide level
0
5000
15000
25000
44:210
21:102
1:13
VAVVEYHDGSHAYIGLKpc3_NP.1
p−maj=0.48, pX=0.21, N=66
var.26.VWF 12:6128443:T:C
peptide level
0
5000
15000
25000
105:210
46:102
2:13
VAVVEYHDGSHAYIGLKpc3_NP.2
p−maj=0.023, pX=0.034, N=153
var.26.VWF 12:6128443:T:C
peptide level
0
5000
10000
15000
47:210
17:102
1:13
VAVVEYHDGSHAYIGLKpc3_NP.3
p−min=0.19, pX=0.16, N=65
var.26.VWF 12:6128443:T:C
peptide level
0
50000
150000
250000
202:210
94:102
13:13
VAVVEYHDGSHAYIGLKpc3_NP.4
p−min=0.28, pX=0.17, N=309
var.26.VWF 12:6128443:T:C
peptide level
0
5000
15000
25000
69:210
29:102
3:13
VAVVEYHDGSHAYIGLKpc3_NP.5
p−min=0.38, pX=0.18, N=101

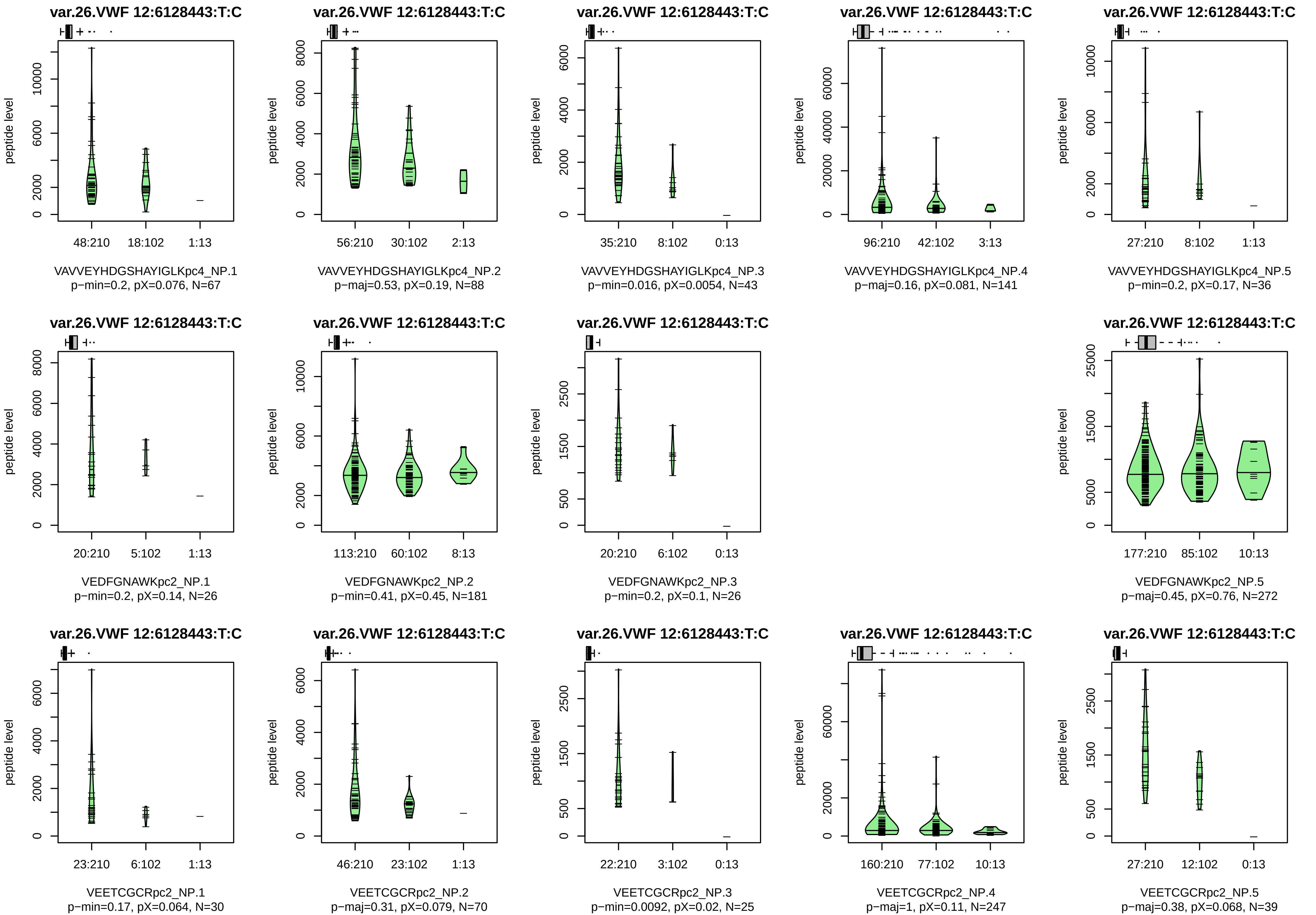

var.26.VWF 12:6128443:T:C
peptide level
48:210
18:102
1:13
VAVVEYHDGSHAYIGLKpc4_NP.1
p−min=0.2, pX=0.076, N=67
var.26.VWF 12:6128443:T:C
peptide level
56:210
30:102
2:13
VAVVEYHDGSHAYIGLKpc4_NP.2
p−maj=0.53, pX=0.19, N=88
var.26.VWF 12:6128443:T:C
peptide level
35:210
8:102
0:13
VAVVEYHDGSHAYIGLKpc4_NP.3
p−min=0.016, pX=0.0054, N=43
var.26.VWF 12:6128443:T:C
peptide level
96:210
42:102
3:13
VAVVEYHDGSHAYIGLKpc4_NP.4
p−maj=0.16, pX=0.081, N=141
var.26.VWF 12:6128443:T:C
peptide level
27:210
8:102
1:13
VAVVEYHDGSHAYIGLKpc4_NP.5
p−min=0.2, pX=0.17, N=36
var.26.VWF 12:6128443:T:C
peptide level
20:210
5:102
1:13
VEDFGNAWKpc2_NP.1
p−min=0.2, pX=0.14, N=26
var.26.VWF 12:6128443:T:C
peptide level
113:210
60:102
8:13
VEDFGNAWKpc2_NP.2
p−min=0.41, pX=0.45, N=181
var.26.VWF 12:6128443:T:C
peptide level
20:210
6:102
0:13
VEDFGNAWKpc2_NP.3
p−min=0.2, pX=0.1, N=26
var.26.VWF 12:6128443:T:C
peptide level
177:210
85:102
10:13
VEDFGNAWKpc2_NP.5
p−maj=0.45, pX=0.76, N=272
var.26.VWF 12:6128443:T:C
peptide level
23:210
6:102
1:13
VEETCGCRpc2_NP.1
p−min=0.17, pX=0.064, N=30
var.26.VWF 12:6128443:T:C
peptide level
46:210
23:102
1:13
VEETCGCRpc2_NP.2
p−maj=0.31, pX=0.079, N=70
var.26.VWF 12:6128443:T:C
peptide level
22:210
3:102
0:13
VEETCGCRpc2_NP.3
p−min=0.0092, pX=0.02, N=25
var.26.VWF 12:6128443:T:C
peptide level
160:210
77:102
10:13
VEETCGCRpc2_NP.4
p−maj=1, pX=0.11, N=247
var.26.VWF 12:6128443:T:C
peptide level
27:210
12:102
0:13
VEETCGCRpc2_NP.5
p−maj=0.38, pX=0.068, N=39

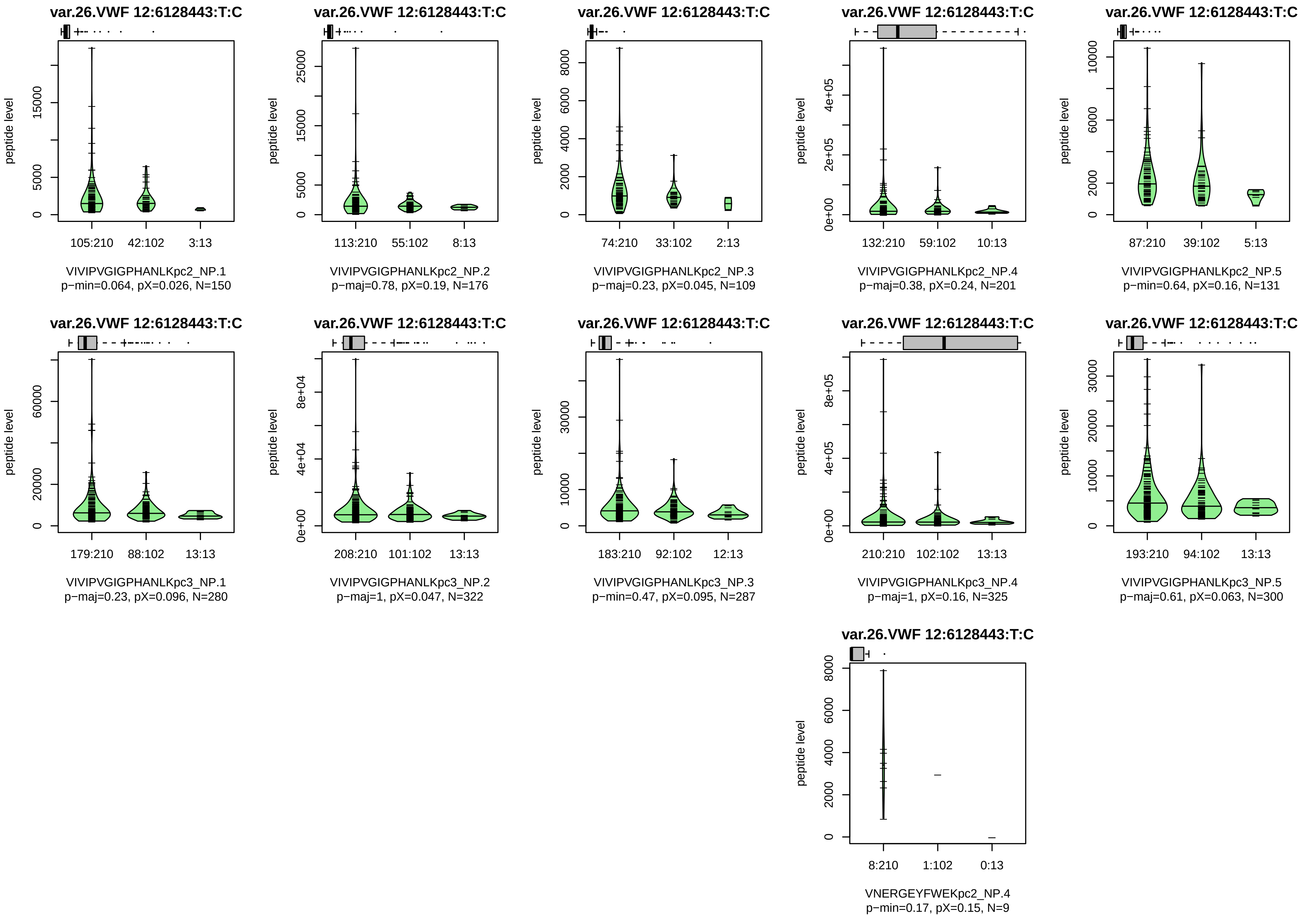

var.26.VWF 12:6128443:T:C
peptide level
105:210
42:102
3:13
VIVIPVGIGPHANLKpc2_NP.1
p−min=0.064, pX=0.026, N=150
var.26.VWF 12:6128443:T:C
peptide level
113:210
55:102
8:13
VIVIPVGIGPHANLKpc2_NP.2
p−maj=0.78, pX=0.19, N=176
var.26.VWF 12:6128443:T:C
peptide level
74:210
33:102
2:13
VIVIPVGIGPHANLKpc2_NP.3
p−maj=0.23, pX=0.045, N=109
var.26.VWF 12:6128443:T:C
peptide level
132:210
59:102
10:13
VIVIPVGIGPHANLKpc2_NP.4
p−maj=0.38, pX=0.24, N=201
var.26.VWF 12:6128443:T:C
peptide level
87:210
39:102
5:13
VIVIPVGIGPHANLKpc2_NP.5
p−min=0.64, pX=0.16, N=131
var.26.VWF 12:6128443:T:C
peptide level
179:210
88:102
13:13
VIVIPVGIGPHANLKpc3_NP.1
p−maj=0.23, pX=0.096, N=280
var.26.VWF 12:6128443:T:C
peptide level
208:210
101:102
13:13
VIVIPVGIGPHANLKpc3_NP.2
p−maj=1, pX=0.047, N=322
var.26.VWF 12:6128443:T:C
peptide level
183:210
92:102
12:13
VIVIPVGIGPHANLKpc3_NP.3
p−min=0.47, pX=0.095, N=287
var.26.VWF 12:6128443:T:C
peptide level
210:210
102:102
13:13
VIVIPVGIGPHANLKpc3_NP.4
p−maj=1, pX=0.16, N=325
var.26.VWF 12:6128443:T:C
peptide level
193:210
94:102
13:13
VIVIPVGIGPHANLKpc3_NP.5
p−maj=0.61, pX=0.063, N=300
var.26.VWF 12:6128443:T:C
peptide level
8:210
1:102
0:13
VNERGEYFWEKpc2_NP.4
p−min=0.17, pX=0.15, N=9

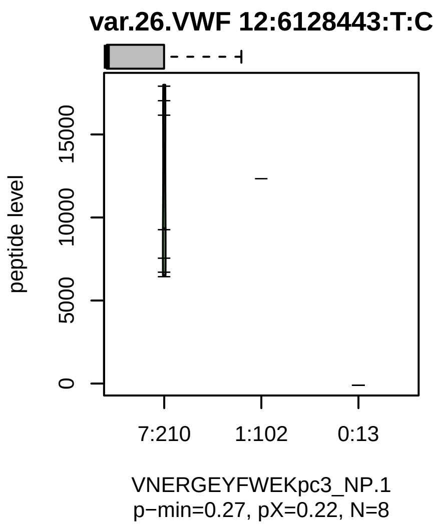
var.26.VWF 12:6128443:T:C
peptide level
7:210
1:102
0:13
VNERGEYFWEKpc3_NP.1
p−min=0.27, pX=0.22, N=8

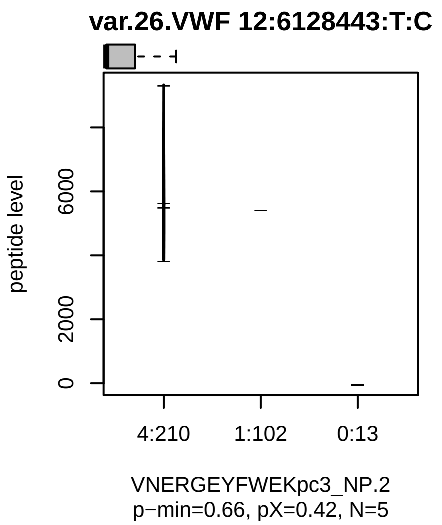
var.26.VWF 12:6128443:T:C
peptide level
4:210
1:102
0:13
VNERGEYFWEKpc3_NP.2
p−min=0.66, pX=0.42, N=5

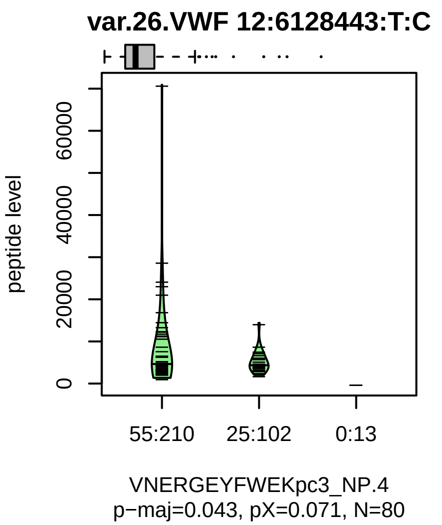
var.26.VWF 12:6128443:T:C
peptide level
55:210
25:102
0:13
VNERGEYFWEKpc3_NP.4
p−maj=0.043, pX=0.071, N=80

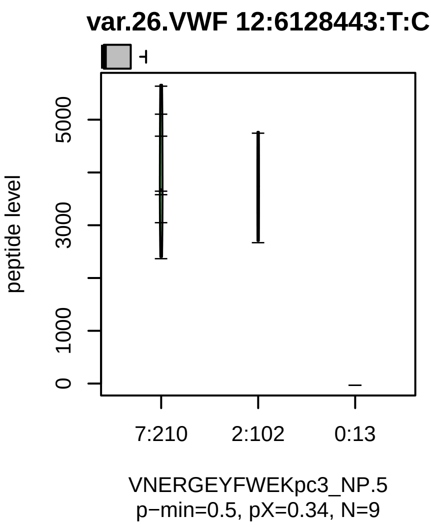
var.26.VWF 12:6128443:T:C
peptide level
7:210
2:102
0:13
VNERGEYFWEKpc3_NP.5
p−min=0.5, pX=0.34, N=9

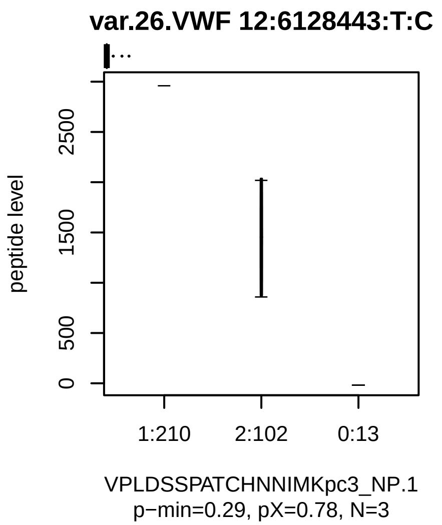
var.26.VWF 12:6128443:T:C
peptide level
1:210
2:102
0:13
VPLDSSPATCHNNIMKpc3_NP.1
p−min=0.29, pX=0.78, N=3

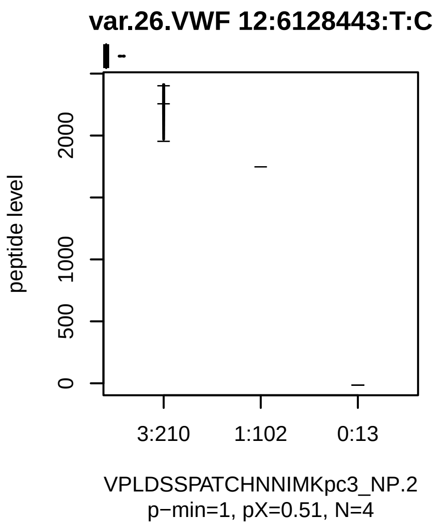
var.26.VWF 12:6128443:T:C
peptide level
3:210
1:102
0:13
VPLDSSPATCHNNIMKpc3_NP.2
p−min=1, pX=0.51, N=4

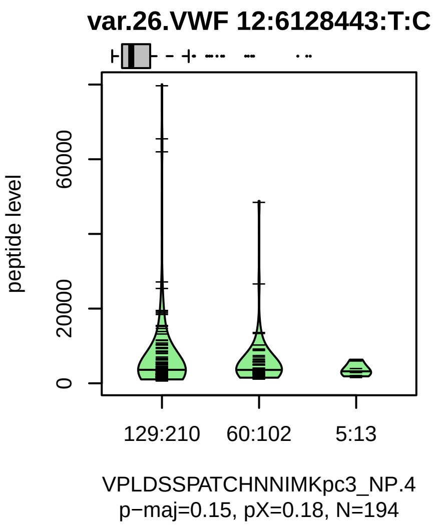
var.26.VWF 12:6128443:T:C
peptide level
129:210
60:102
5:13
VPLDSSPATCHNNIMKpc3_NP.4
p−maj=0.15, pX=0.18, N=194

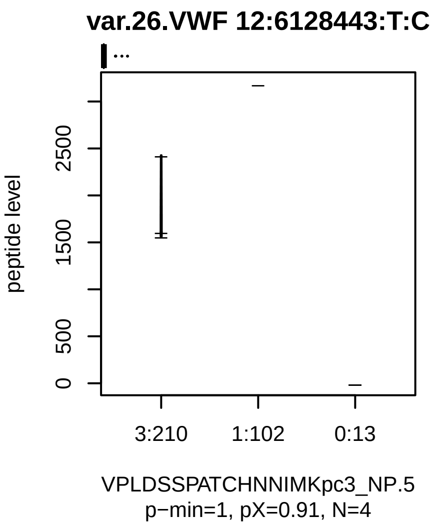
var.26.VWF 12:6128443:T:C
peptide level
3:210
1:102
0:13
VPLDSSPATCHNNIMKpc3_NP.5
p−min=1, pX=0.91, N=4

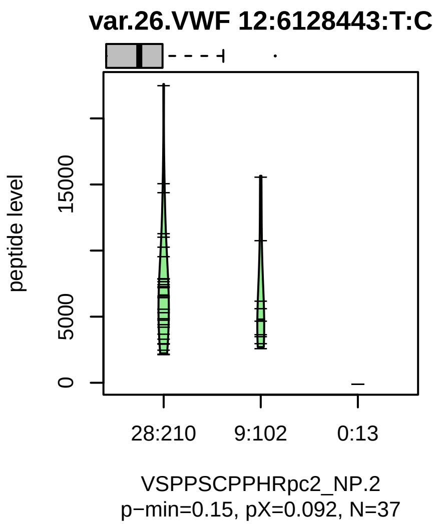
var.26.VWF 12:6128443:T:C
peptide level
28:210
9:102
0:13
VSPPSCPPHRpc2_NP.2
p−min=0.15, pX=0.092, N=37

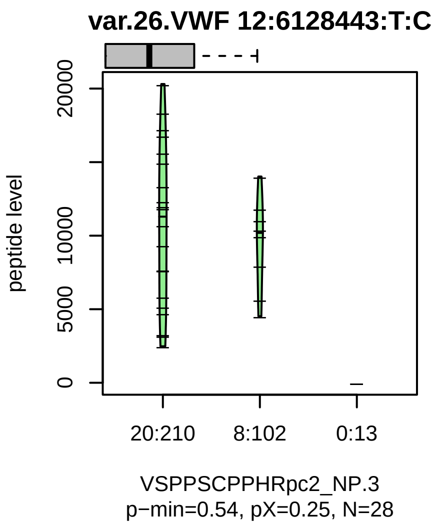
var.26.VWF 12:6128443:T:C
peptide level
20:210
8:102
0:13
VSPPSCPPHRpc2_NP.3
p−min=0.54, pX=0.25, N=28

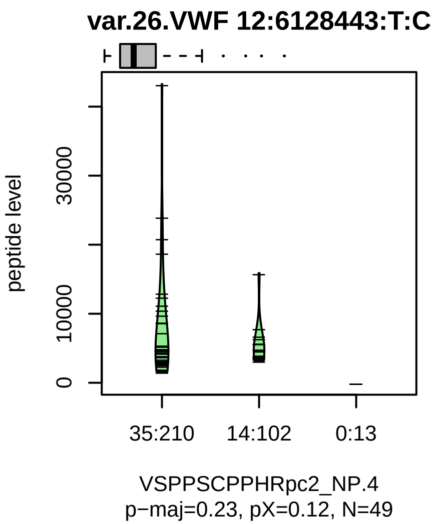
var.26.VWF 12:6128443:T:C
peptide level
35:210
14:102
0:13
VSPPSCPPHRpc2_NP.4
p−maj=0.23, pX=0.12, N=49

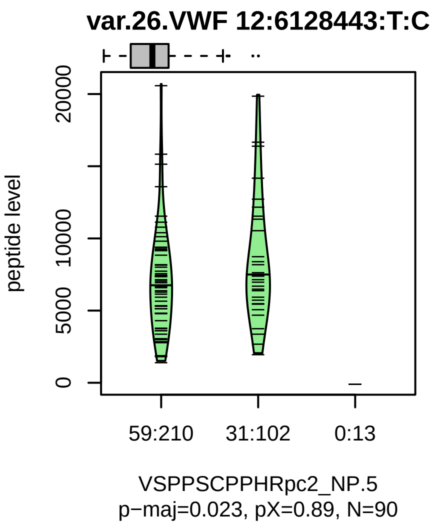
var.26.VWF 12:6128443:T:C
peptide level
59:210
31:102
0:13
VSPPSCPPHRpc2_NP.5
p−maj=0.023, pX=0.89, N=90

var.26.VWF 12:6128443:T:C

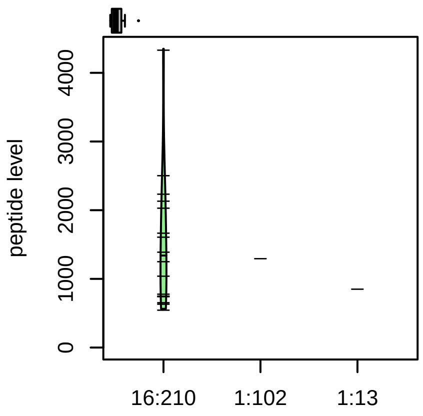

VSSQCADTRpc2_NP.2
p−min=0.039, pX=0.068, N=18

var.26.VWF 12:6128443:T:C

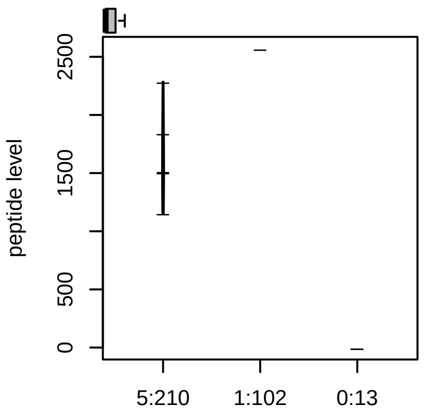

VSSQCADTRpc2_NP.3
p−min=0.43, pX=0.51, N=6

var.26.VWF 12:6128443:T:C

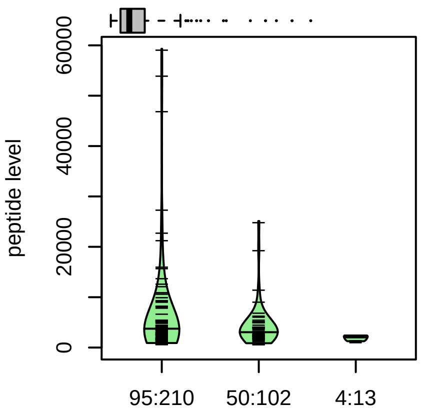

VSSQCADTRpc2_NP.4
p−maj=0.4, pX=0.1, N=149

var.26.VWF 12:6128443:T:C

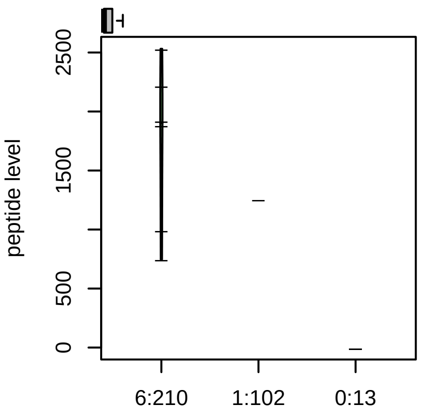

VSSQCADTRpc2_NP.5
p−min=0.43, pX=0.21, N=7

var.26.VWF 12:6128443:T:C

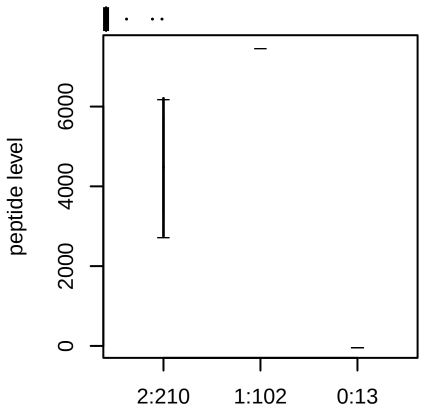

VTGCPPFDEHKpc2_NP.2
p−maj=1, pX=0.86, N=3

var.26.VWF 12:6128443:T:C

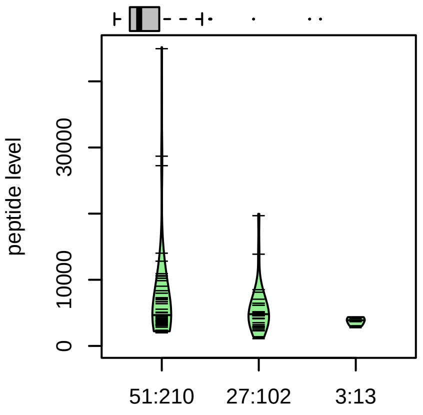

VTGCPPFDEHKpc2_NP.4
p−min=0.79, pX=0.39, N=81

var.26.VWF 12:6128443:T:C

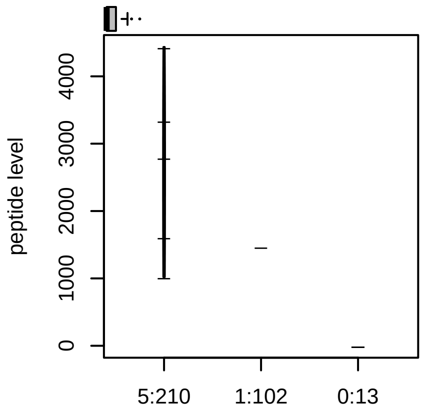

VTGCPPFDEHKpc3_NP.1
p−min=0.43, pX=0.25, N=6

var.26.VWF 12:6128443:T:C

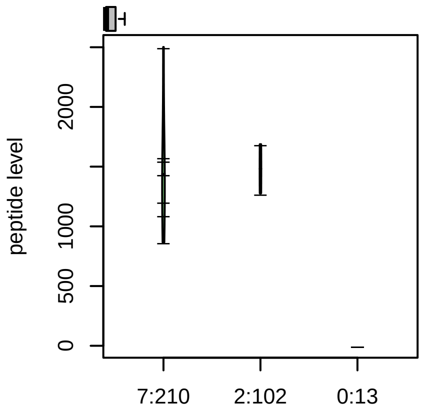

VTGCPPFDEHKpc3_NP.2
p−min=0.5, pX=0.38, N=9

var.26.VWF 12:6128443:T:C

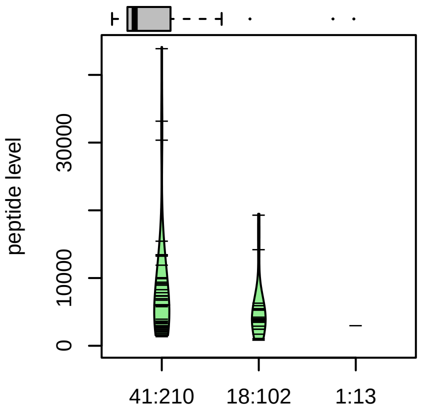

VTGCPPFDEHKpc3_NP.4
p−maj=0.48, pX=0.11, N=60

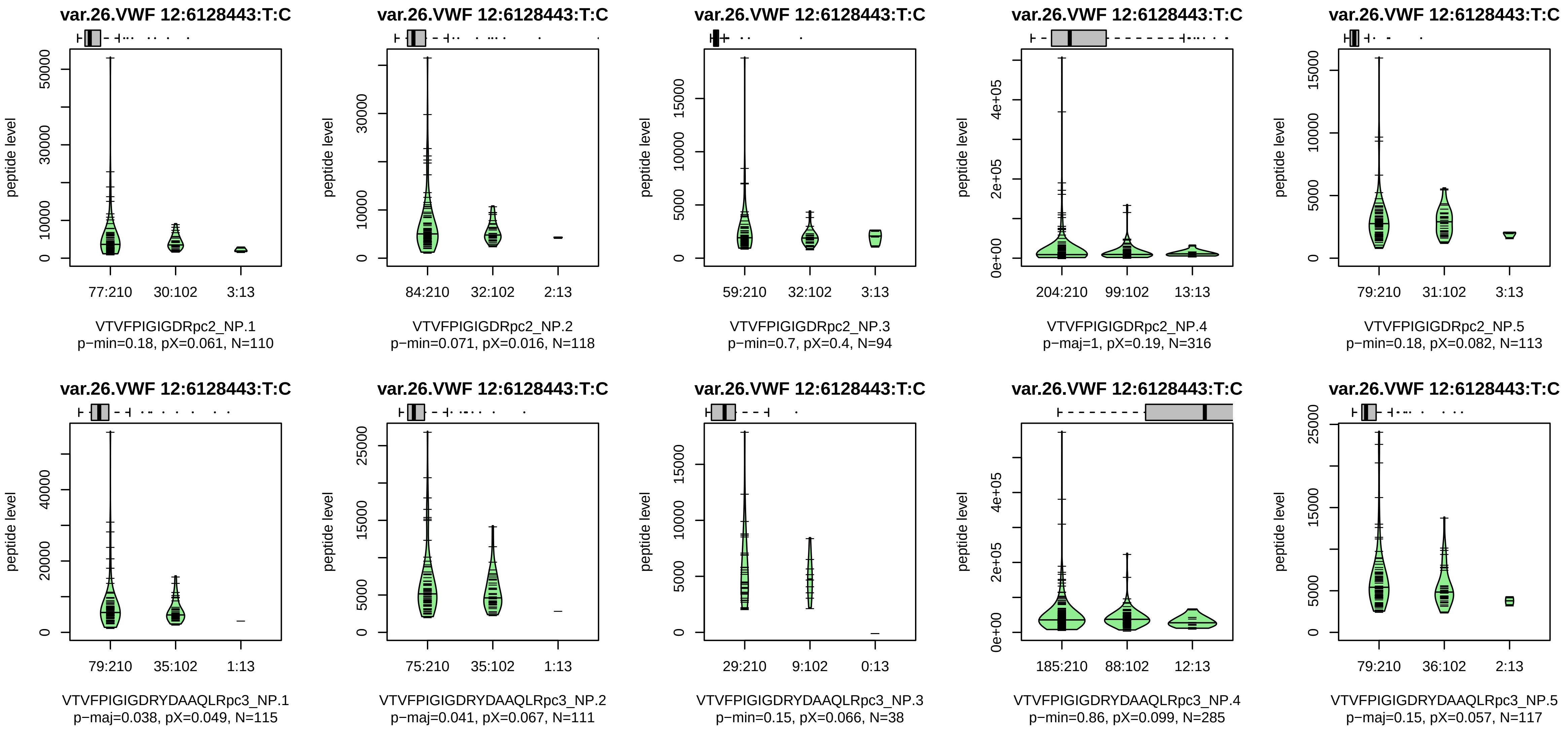
var.26.VWF 12:6128443:T:C
peptide level
0
10000
30000
50000
77:210
30:102
3:13
VTVFPIGIGDRpc2_NP.1
p−min=0.18, pX=0.061, N=110
var.26.VWF 12:6128443:T:C
peptide level
0
10000
30000
84:210
32:102
2:13
VTVFPIGIGDRpc2_NP.2
p−min=0.071, pX=0.016, N=118
var.26.VWF 12:6128443:T:C
peptide level
0
5000
10000
15000
59:210
32:102
3:13
VTVFPIGIGDRpc2_NP.3
p−min=0.7, pX=0.4, N=94
var.26.VWF 12:6128443:T:C
peptide level
0e+00
2e+05
4e+05
204:210
99:102
13:13
VTVFPIGIGDRpc2_NP.4
p−maj=1, pX=0.19, N=316
var.26.VWF 12:6128443:T:C
peptide level
0
5000
10000
15000
79:210
31:102
3:13
VTVFPIGIGDRpc2_NP.5
p−min=0.18, pX=0.082, N=113
var.26.VWF 12:6128443:T:C
peptide level
0
20000
40000
79:210
35:102
1:13
VTVFPIGIGDRYDAAQLRpc3_NP.1
p−maj=0.038, pX=0.049, N=115
var.26.VWF 12:6128443:T:C
peptide level
0
5000
15000
25000
75:210
35:102
1:13
VTVFPIGIGDRYDAAQLRpc3_NP.2
p−maj=0.041, pX=0.067, N=111
var.26.VWF 12:6128443:T:C
peptide level
0
5000
10000
15000
29:210
9:102
0:13
VTVFPIGIGDRYDAAQLRpc3_NP.3
p−min=0.15, pX=0.066, N=38
var.26.VWF 12:6128443:T:C
peptide level
0e+00
2e+05
4e+05
185:210
88:102
12:13
VTVFPIGIGDRYDAAQLRpc3_NP.4
p−min=0.86, pX=0.099, N=285
var.26.VWF 12:6128443:T:C
peptide level
0
5000
15000
25000
79:210
36:102
2:13
VTVFPIGIGDRYDAAQLRpc3_NP.5
p−maj=0.15, pX=0.057, N=117

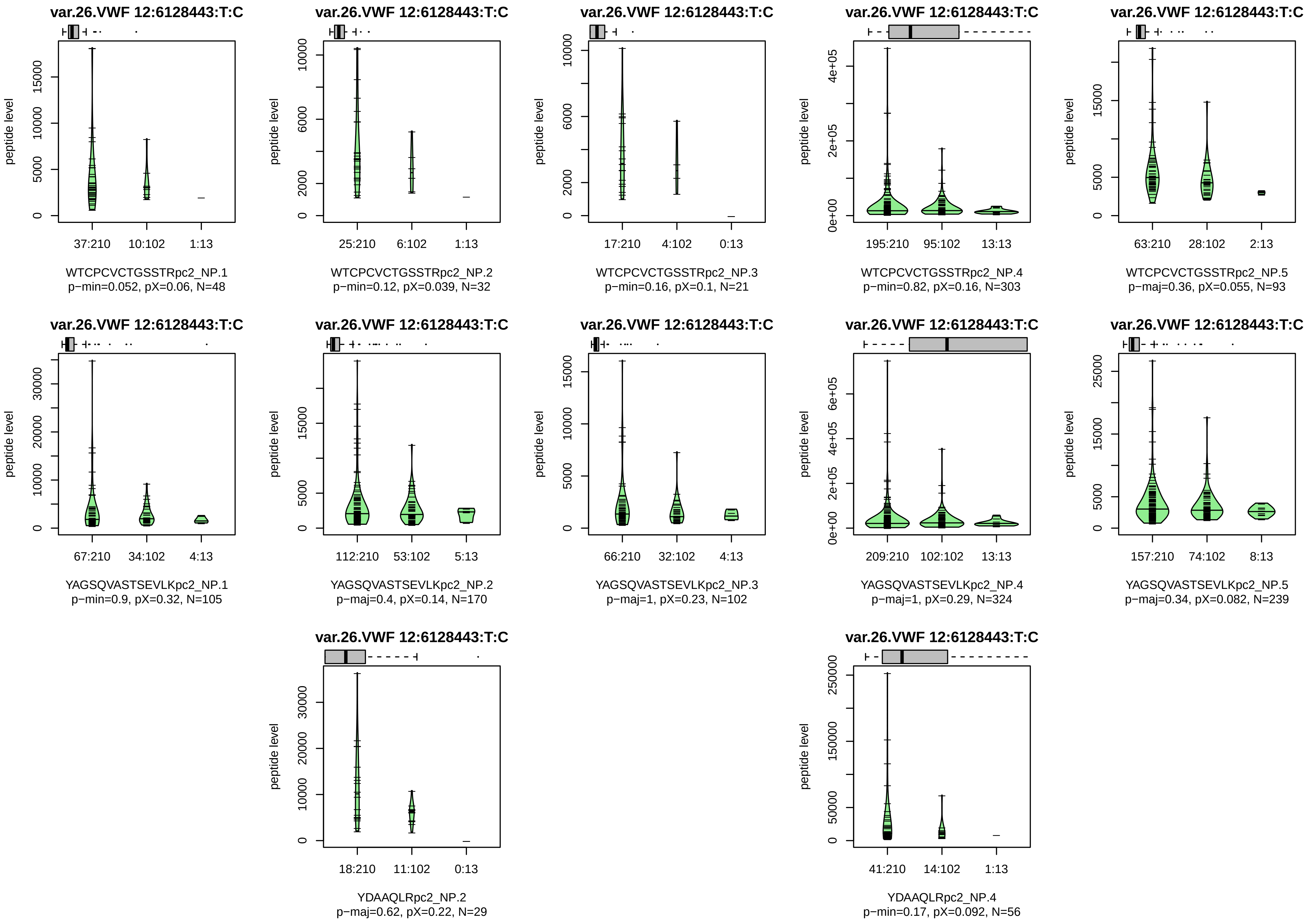

var.26.VWF 12:6128443:T:C
peptide level
37:210
10:102
1:13
WTCPCVCTGSSTRpc2_NP.1
p−min=0.052, pX=0.06, N=48
var.26.VWF 12:6128443:T:C
peptide level
25:210
6:102
1:13
WTCPCVCTGSSTRpc2_NP.2
p−min=0.12, pX=0.039, N=32
var.26.VWF 12:6128443:T:C
peptide level
17:210
4:102
0:13
WTCPCVCTGSSTRpc2_NP.3
p−min=0.16, pX=0.1, N=21
var.26.VWF 12:6128443:T:C
peptide level
195:210
95:102
13:13
WTCPCVCTGSSTRpc2_NP.4
p−min=0.82, pX=0.16, N=303
var.26.VWF 12:6128443:T:C
peptide level
63:210
28:102
2:13
WTCPCVCTGSSTRpc2_NP.5
p−maj=0.36, pX=0.055, N=93
var.26.VWF 12:6128443:T:C
peptide level
67:210
34:102
4:13
YAGSQVASTSEVLKpc2_NP.1
p−min=0.9, pX=0.32, N=105
var.26.VWF 12:6128443:T:C
peptide level
112:210
53:102
5:13
YAGSQVASTSEVLKpc2_NP.2
p−maj=0.4, pX=0.14, N=170
var.26.VWF 12:6128443:T:C
peptide level
66:210
32:102
4:13
YAGSQVASTSEVLKpc2_NP.3
p−maj=1, pX=0.23, N=102
var.26.VWF 12:6128443:T:C
peptide level
209:210
102:102
13:13
YAGSQVASTSEVLKpc2_NP.4
p−maj=1, pX=0.29, N=324
var.26.VWF 12:6128443:T:C
peptide level
157:210
74:102
8:13
YAGSQVASTSEVLKpc2_NP.5
p−maj=0.34, pX=0.082, N=239
var.26.VWF 12:6128443:T:C
peptide level
18:210
11:102
0:13
YDAAQLRpc2_NP.2
p−maj=0.62, pX=0.22, N=29
var.26.VWF 12:6128443:T:C
peptide level
41:210
14:102
1:13
YDAAQLRpc2_NP.4
p−min=0.17, pX=0.092, N=56

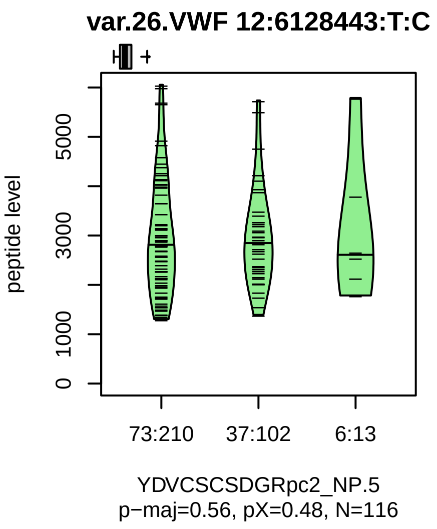
var.26.VWF 12:6128443:T:C
peptide level
0
1000
3000
5000
73:210
37:102
6:13
YDVCSCSDGRpc2_NP.5
p−maj=0.56, pX=0.48, N=116

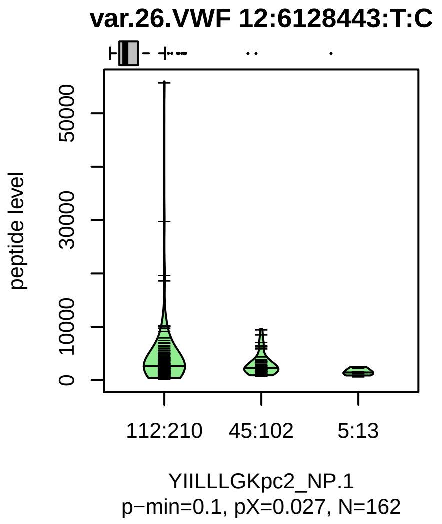
var.26.VWF 12:6128443:T:C
peptide level
0
10000
30000
50000
112:210
45:102
5:13
YIILLLGKpc2_NP.1
p−min=0.1, pX=0.027, N=162

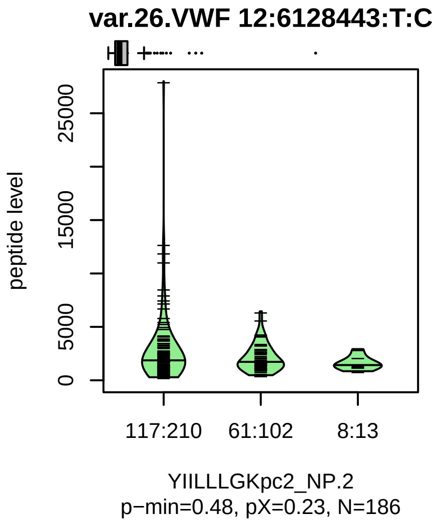
var.26.VWF 12:6128443:T:C
peptide level
0
5000
15000
25000
117:210
61:102
8:13
YIILLLGKpc2_NP.2
p−min=0.48, pX=0.23, N=186

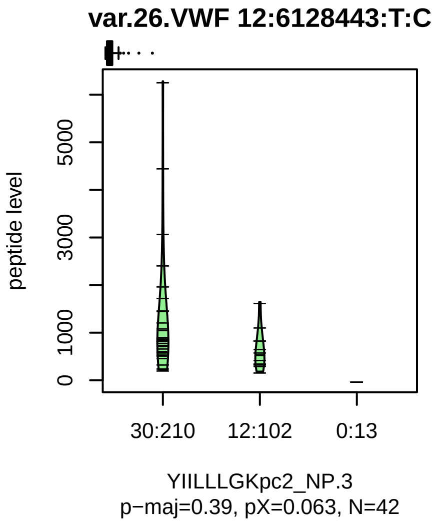
var.26.VWF 12:6128443:T:C
peptide level
0
1000
3000
5000
30:210
12:102
0:13
YIILLLGKpc2_NP.3
p−maj=0.39, pX=0.063, N=42

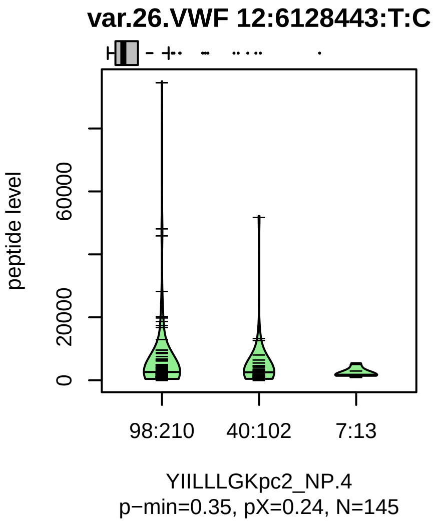
var.26.VWF 12:6128443:T:C
peptide level
0
20000
60000
98:210
40:102
7:13
YIILLLGKpc2_NP.4
p−min=0.35, pX=0.24, N=145

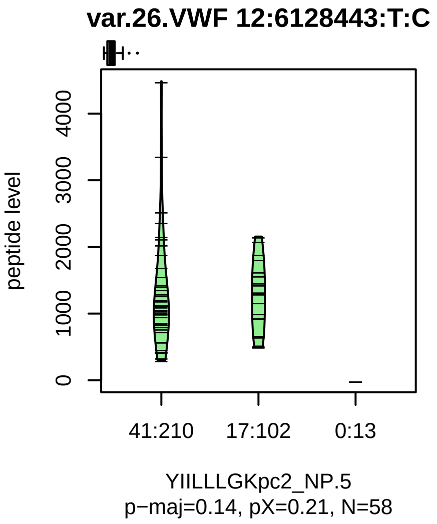
var.26.VWF 12:6128443:T:C
peptide level
0
1000
2000
3000
4000
41:210
17:102
0:13
YIILLLGKpc2_NP.5
p−maj=0.14, pX=0.21, N=58

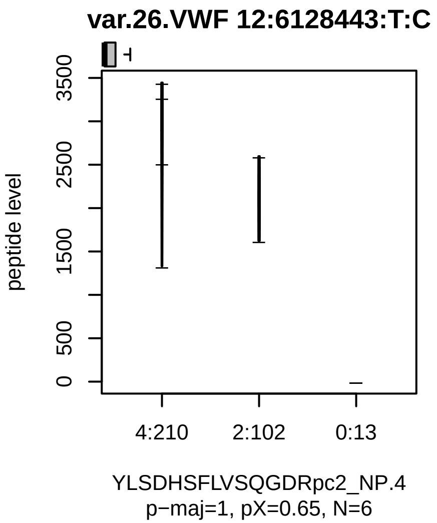
var.26.VWF 12:6128443:T:C
peptide level
0
500
1500
2500
3500
4:210
2:102
0:13
YLSDHSFLVSQGDRpc2_NP.4
p−maj=1, pX=0.65, N=6

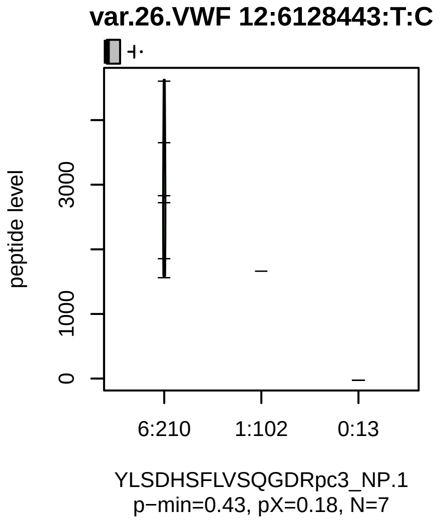
var.26.VWF 12:6128443:T:C
peptide level
0
1000
3000
6:210
1:102
0:13
YLSDHSFLVSQGDRpc3_NP.1
p−min=0.43, pX=0.18, N=7

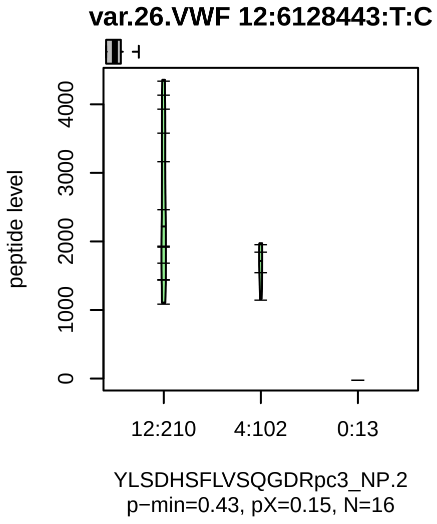
var.26.VWF 12:6128443:T:C
peptide level
0
1000
2000
3000
4000
12:210
4:102
0:13
YLSDHSFLVSQGDRpc3_NP.2
p−min=0.43, pX=0.15, N=16

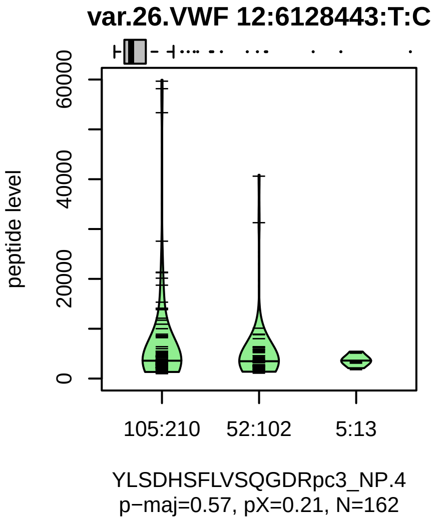
var.26.VWF 12:6128443:T:C
peptide level
0
20000
40000
60000
105:210
52:102
5:13
YLSDHSFLVSQGDRpc3_NP.4
p−maj=0.57, pX=0.21, N=162

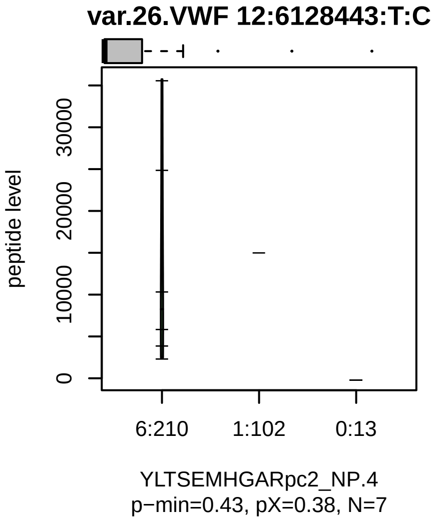
var.26.VWF 12:6128443:T:C
peptide level
0
10000
20000
30000
6:210
1:102
0:13
YLTSEMHGARpc2_NP.4
p−min=0.43, pX=0.38, N=7

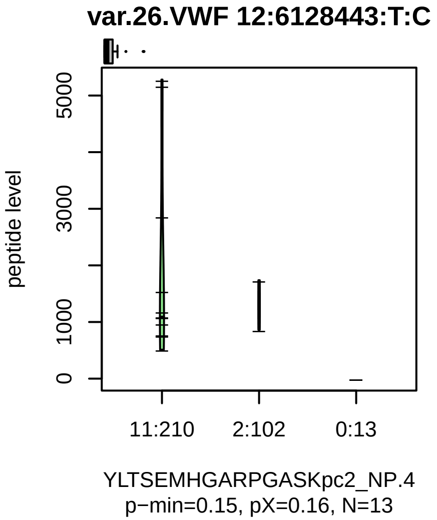
var.26.VWF 12:6128443:T:C
peptide level
0
1000
3000
5000
11:210
2:102
0:13
YLTSEMHGARPGASKpc2_NP.4
p−min=0.15, pX=0.16, N=13

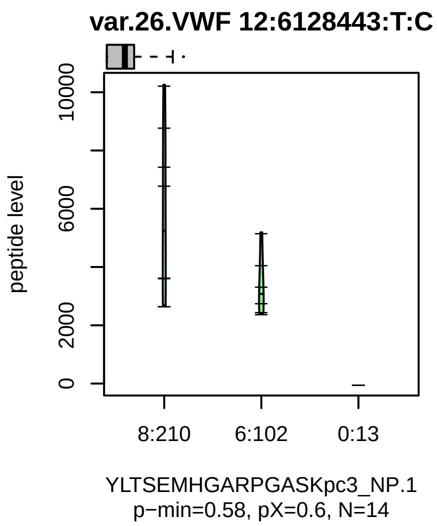
var.26.VWF 12:6128443:T:C
peptide level
8:210
6:102
0:13
YLTSEMHGARPGASKpc3_NP.1
p−min=0.58, pX=0.6, N=14

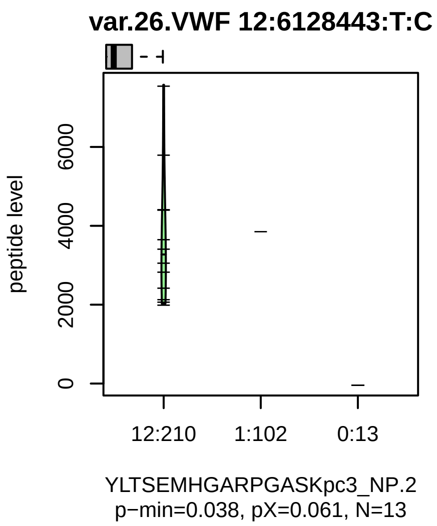
var.26.VWF 12:6128443:T:C
peptide level
12:210
1:102
0:13
YLTSEMHGARPGASKpc3_NP.2
p−min=0.038, pX=0.061, N=13

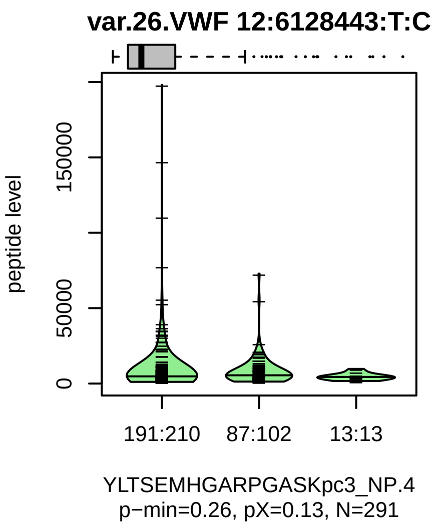
var.26.VWF 12:6128443:T:C
peptide level
191:210
87:102
13:13
YLTSEMHGARPGASKpc3_NP.4
p−min=0.26, pX=0.13, N=291

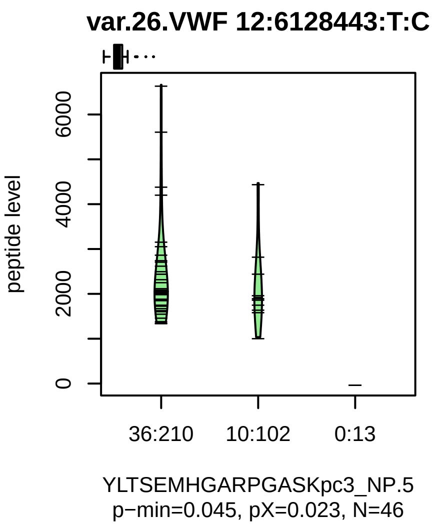
var.26.VWF 12:6128443:T:C
peptide level
36:210
10:102
0:13
YLTSEMHGARPGASKpc3_NP.5
p−min=0.045, pX=0.023, N=46

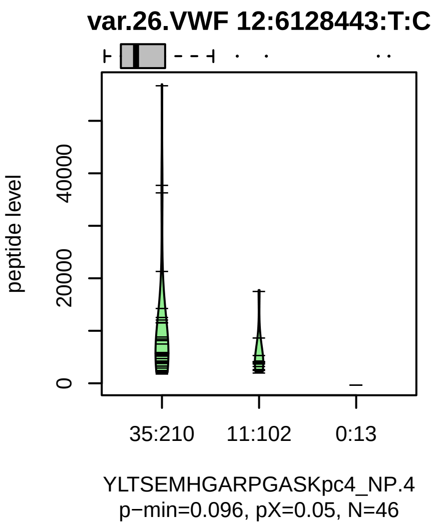
var.26.VWF 12:6128443:T:C
peptide level
35:210
11:102
0:13
YLTSEMHGARPGASKpc4_NP.4
p−min=0.096, pX=0.05, N=46

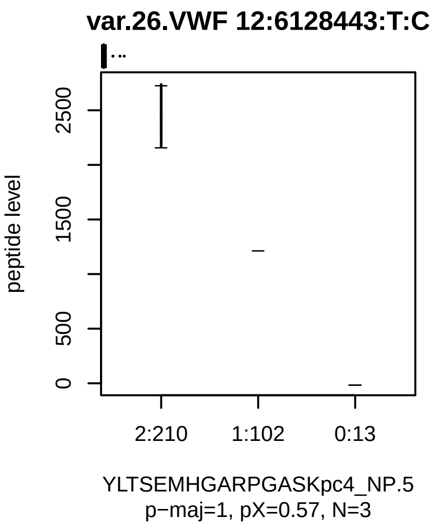
var.26.VWF 12:6128443:T:C
peptide level
2:210
1:102
0:13
YLTSEMHGARPGASKpc4_NP.5
p−maj=1, pX=0.57, N=3

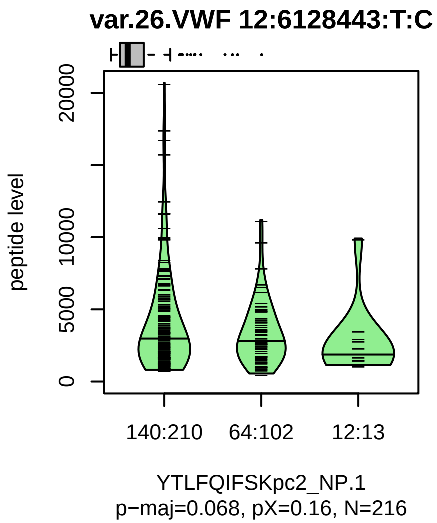
var.26.VWF 12:6128443:T:C
peptide level
140:210
64:102
12:13
YTLFQIFSKpc2_NP.1
p−maj=0.068, pX=0.16, N=216

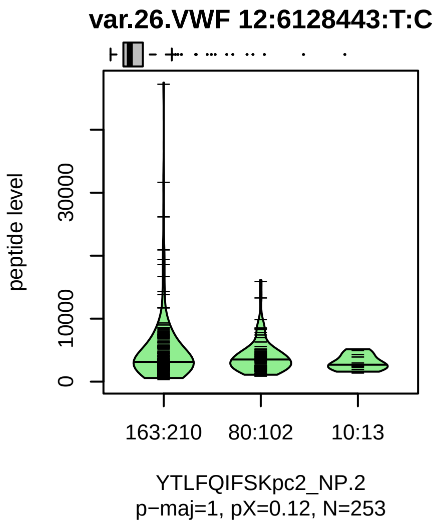
var.26.VWF 12:6128443:T:C
peptide level
163:210
80:102
10:13
YTLFQIFSKpc2_NP.2
p−maj=1, pX=0.12, N=253

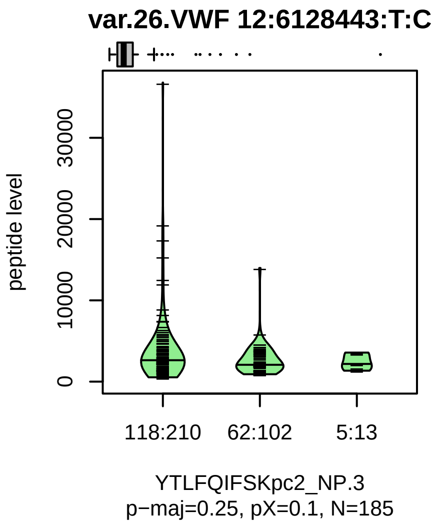
var.26.VWF 12:6128443:T:C
peptide level
118:210
62:102
5:13
YTLFQIFSKpc2_NP.3
p−maj=0.25, pX=0.1, N=185

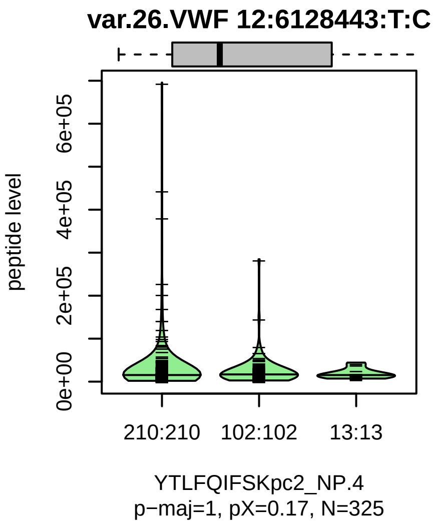
var.26.VWF 12:6128443:T:C
peptide level
210:210
102:102
13:13
YTLFQIFSKpc2_NP.4
p−maj=1, pX=0.17, N=325

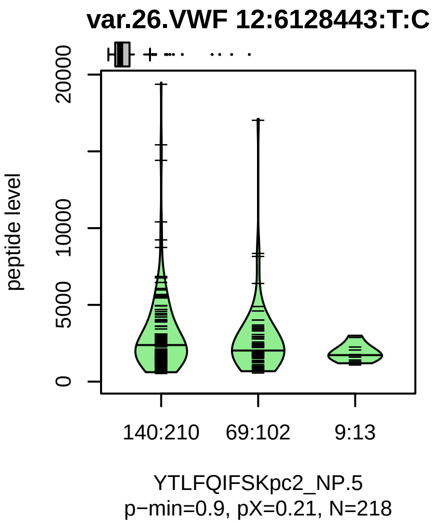
var.26.VWF 12:6128443:T:C
peptide level
140:210
69:102
9:13
YTLFQIFSKpc2_NP.5
p−min=0.9, pX=0.21, N=218

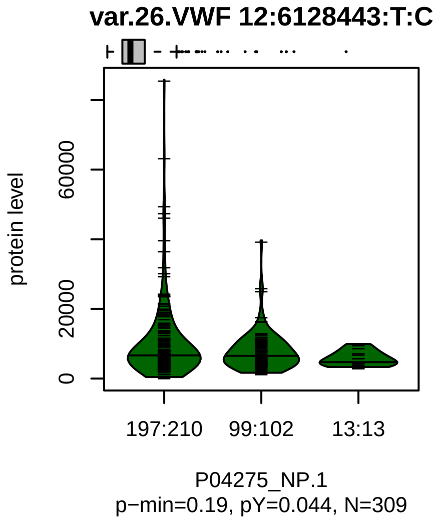
var.26.VWF 12:6128443:T:C
protein level
0
20000
60000
197:210
99:102
13:13
P04275_NP.1
p−min=0.19, pY=0.044, N=309

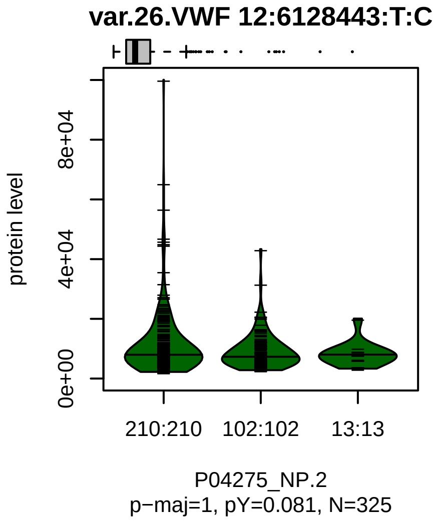
var.26.VWF 12:6128443:T:C
protein level
0e+00
4e+04
8e+04
210:210
102:102
13:13
P04275_NP.2
p−maj=1, pY=0.081, N=325

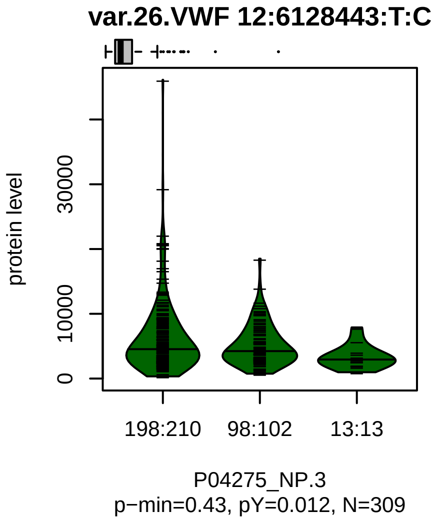
var.26.VWF 12:6128443:T:C
protein level
0
10000
30000
198:210
98:102
13:13
P04275_NP.3
p−min=0.43, pY=0.012, N=309

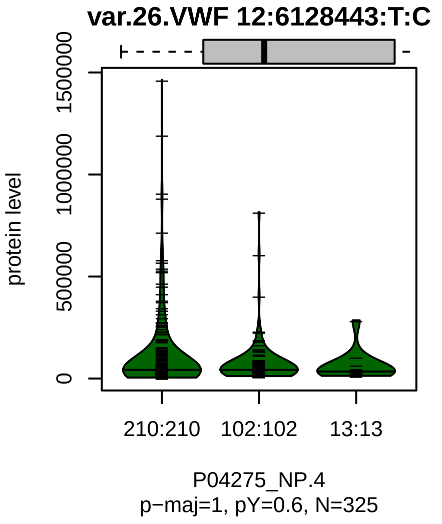
var.26.VWF 12:6128443:T:C
protein level
0
500000
1000000
1500000
210:210
102:102
13:13
P04275_NP.4
p−maj=1, pY=0.6, N=325

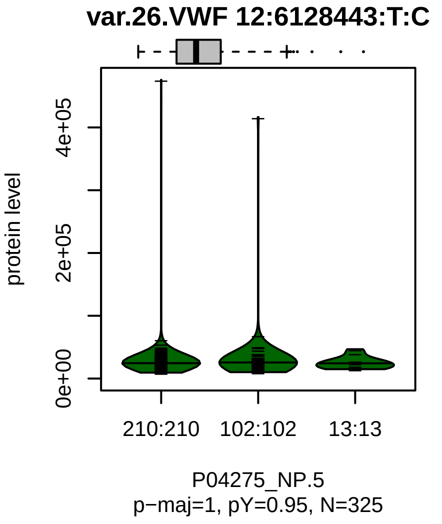
var.26.VWF 12:6128443:T:C
protein level
0e+00
2e+05
4e+05
210:210
102:102
13:13
P04275_NP.5
p−maj=1, pY=0.95, N=325

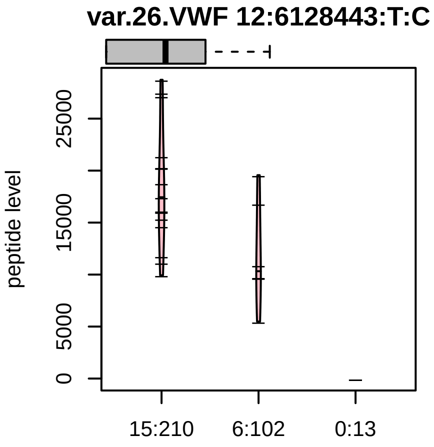

AEGLECTKpc2_NP.1
p−min=0.64, pX=0.14, N=21

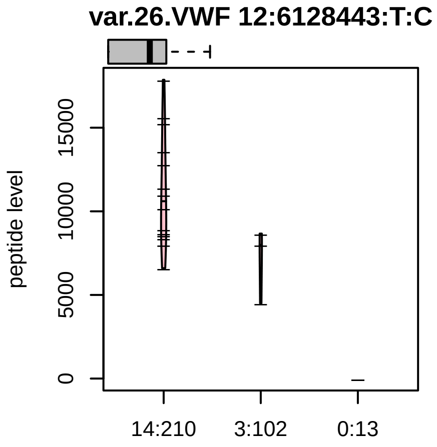

AEGLECTKpc2_NP.2
p−min=0.19, pX=0.051, N=17

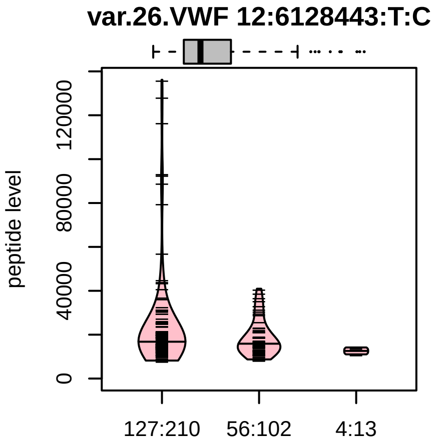

AEGLECTKpc2_NP.4
p−maj=0.082, pX=0.0074, N=187

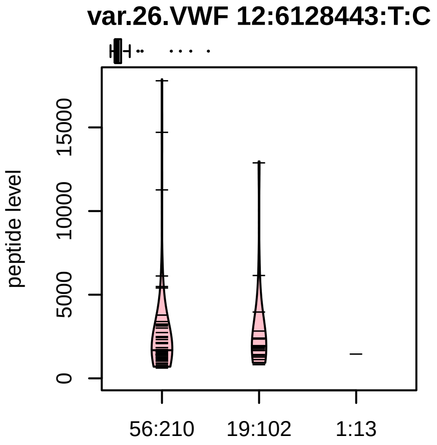

AFVLSSVDELEQQRpc2_NP.4
p−min=0.074, pX=0.16, N=76

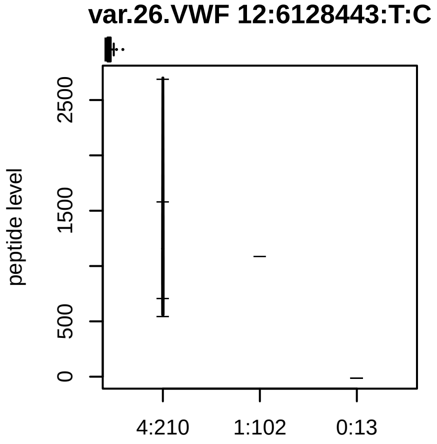

AFVVDMMERpc2_NP.3
p−min=0.66, pX=0.43, N=5

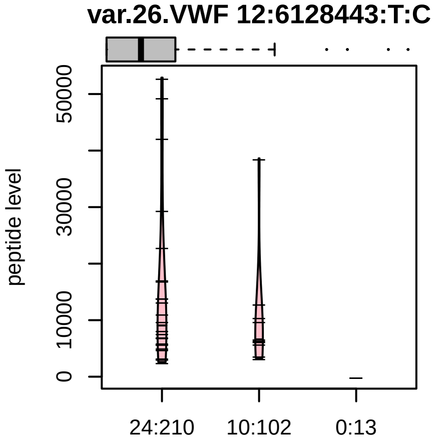

AFVVDMMERpc2_NP.4
p−maj=0.38, pX=0.2, N=34

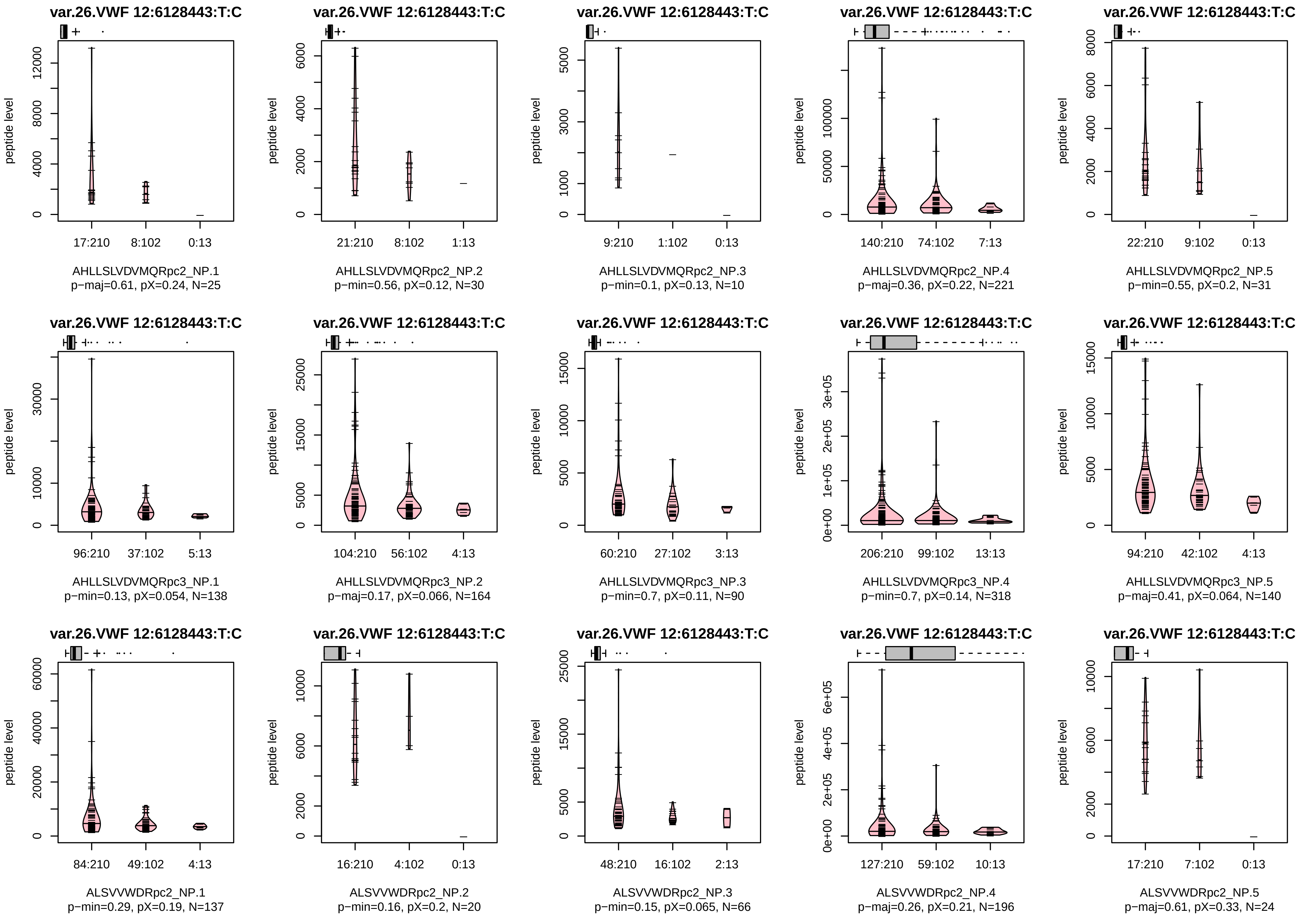

var.26.VWF 12:6128443:T:C
peptide level
17:210 8:102 0:13
AHLLSLVDVMQRpc2_NP.1
p−maj=0.61, pX=0.24, N=25
var.26.VWF 12:6128443:T:C
peptide level
21:210 8:102 1:13
AHLLSLVDVMQRpc2_NP.2
p−min=0.56, pX=0.12, N=30
var.26.VWF 12:6128443:T:C
peptide level
9:210 1:102 0:13
AHLLSLVDVMQRpc2_NP.3
p−min=0.1, pX=0.13, N=10
var.26.VWF 12:6128443:T:C
peptide level
140:210 74:102 7:13
AHLLSLVDVMQRpc2_NP.4
p−maj=0.36, pX=0.22, N=221
var.26.VWF 12:6128443:T:C
peptide level
22:210 9:102 0:13
AHLLSLVDVMQRpc2_NP.5
p−min=0.55, pX=0.2, N=31
var.26.VWF 12:6128443:T:C
peptide level
96:210 37:102 5:13
AHLLSLVDVMQRpc3_NP.1
p−min=0.13, pX=0.054, N=138
var.26.VWF 12:6128443:T:C
peptide level
104:210 56:102 4:13
AHLLSLVDVMQRpc3_NP.2
p−maj=0.17, pX=0.066, N=164
var.26.VWF 12:6128443:T:C
peptide level
60:210 27:102 3:13
AHLLSLVDVMQRpc3_NP.3
p−min=0.7, pX=0.11, N=90
var.26.VWF 12:6128443:T:C
peptide level
206:210 99:102 13:13
AHLLSLVDVMQRpc3_NP.4
p−min=0.7, pX=0.14, N=318
var.26.VWF 12:6128443:T:C
peptide level
94:210 42:102 4:13
AHLLSLVDVMQRpc3_NP.5
p−maj=0.41, pX=0.064, N=140
var.26.VWF 12:6128443:T:C
peptide level
84:210 49:102 4:13
ALSVVWDRpc2_NP.1
p−min=0.29, pX=0.19, N=137
var.26.VWF 12:6128443:T:C
peptide level
16:210 4:102 0:13
ALSVVWDRpc2_NP.2
p−min=0.16, pX=0.2, N=20
var.26.VWF 12:6128443:T:C
peptide level
48:210 16:102 2:13
ALSVVWDRpc2_NP.3
p−min=0.15, pX=0.065, N=66
var.26.VWF 12:6128443:T:C
peptide level
127:210 59:102 10:13
ALSVVWDRpc2_NP.4
p−maj=0.26, pX=0.21, N=196
var.26.VWF 12:6128443:T:C
peptide level
17:210 7:102 0:13
ALSVVWDRpc2_NP.5
p−maj=0.61, pX=0.33, N=24

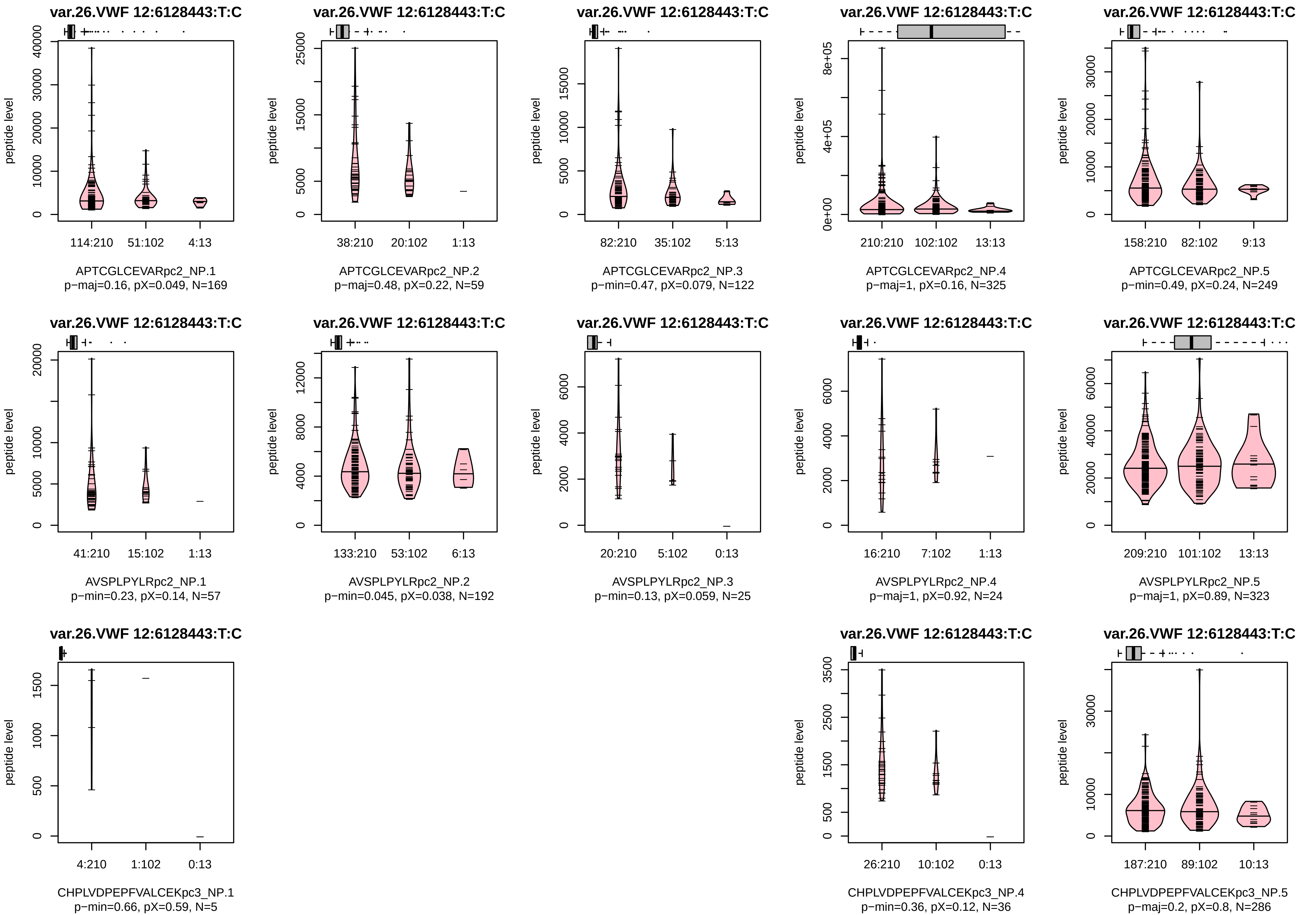

var.26.VWF 12:6128443:T:C
peptide level
114:210 51:102 4:13
APTCGLCEVARpc2_NP.1
p−maj=0.16, pX=0.049, N=169
var.26.VWF 12:6128443:T:C
peptide level
38:210 20:102 1:13
APTCGLCEVARpc2_NP.2
p−maj=0.48, pX=0.22, N=59
var.26.VWF 12:6128443:T:C
peptide level
82:210 35:102 5:13
APTCGLCEVARpc2_NP.3
p−min=0.47, pX=0.079, N=122
var.26.VWF 12:6128443:T:C
peptide level
210:210 102:102 13:13
APTCGLCEVARpc2_NP.4
p−maj=1, pX=0.16, N=325
var.26.VWF 12:6128443:T:C
peptide level
158:210 82:102 9:13
APTCGLCEVARpc2_NP.5
p−min=0.49, pX=0.24, N=249
var.26.VWF 12:6128443:T:C
peptide level
41:210 15:102 1:13
AVSPLPYLRpc2_NP.1
p−min=0.23, pX=0.14, N=57
var.26.VWF 12:6128443:T:C
peptide level
133:210 53:102 6:13
AVSPLPYLRpc2_NP.2
p−min=0.045, pX=0.038, N=192
var.26.VWF 12:6128443:T:C
peptide level
20:210 5:102 0:13
AVSPLPYLRpc2_NP.3
p−min=0.13, pX=0.059, N=25
var.26.VWF 12:6128443:T:C
peptide level
16:210 7:102 1:13
AVSPLPYLRpc2_NP.4
p−maj=1, pX=0.92, N=24
var.26.VWF 12:6128443:T:C
peptide level
209:210 101:102 13:13
AVSPLPYLRpc2_NP.5
p−maj=1, pX=0.89, N=323
var.26.VWF 12:6128443:T:C
peptide level
4:210 1:102 0:13
CHPLVDPEPFVALCEKpc3_NP.1
p−min=0.66, pX=0.59, N=5
var.26.VWF 12:6128443:T:C
peptide level
26:210 10:102 0:13
CHPLVDPEPFVALCEKpc3_NP.4
p−min=0.36, pX=0.12, N=36
var.26.VWF 12:6128443:T:C
peptide level
187:210 89:102 10:13
CHPLVDPEPFVALCEKpc3_NP.5
p−maj=0.2, pX=0.8, N=286

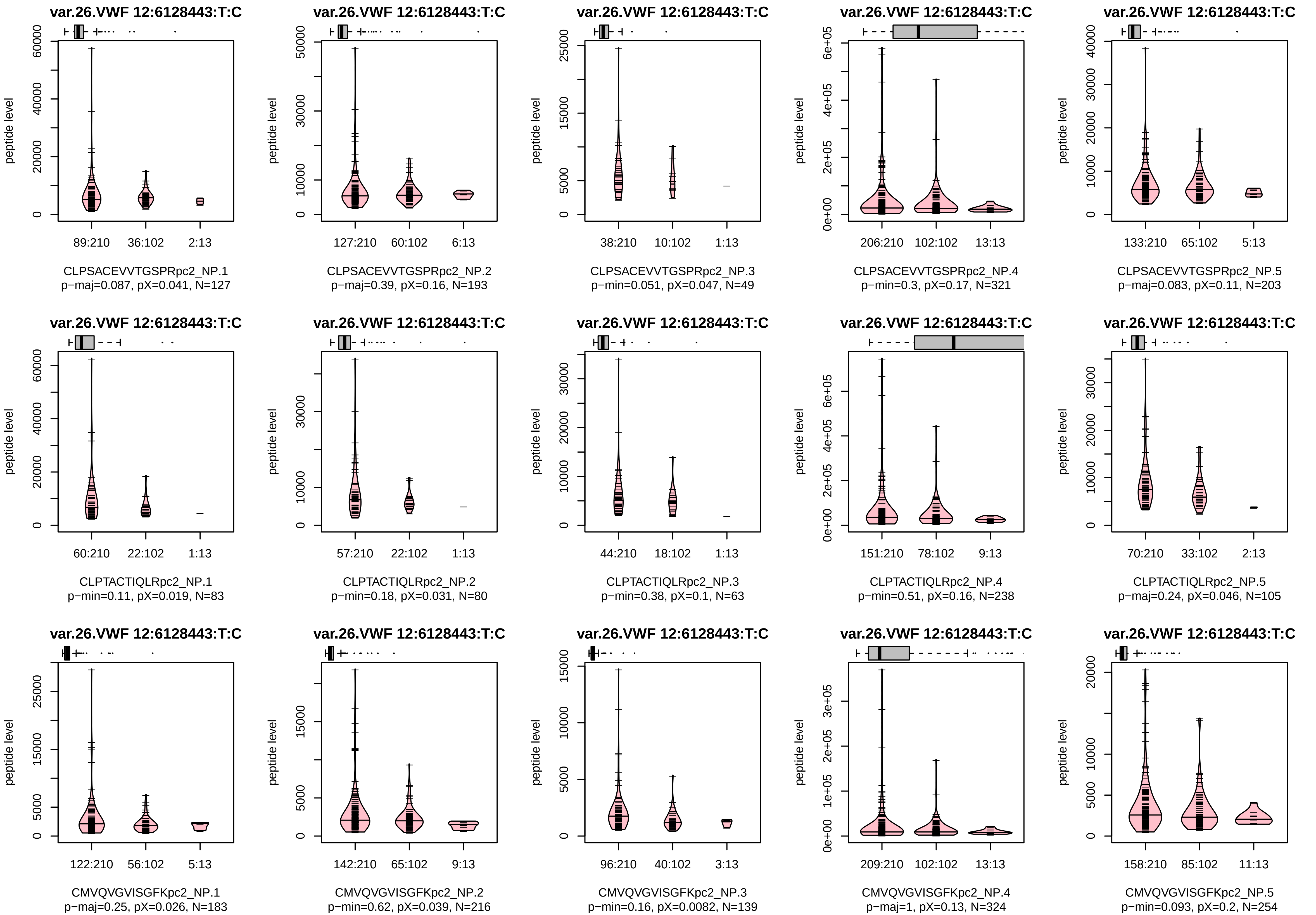
var.26.VWF 12:6128443:T:C
peptide level
89:210
36:102
2:13
CLPSACEVVTGSPRpc2_NP.1
p−maj=0.087, pX=0.041, N=127
var.26.VWF 12:6128443:T:C
peptide level
127:210
60:102
6:13
CLPSACEVVTGSPRpc2_NP.2
p−maj=0.39, pX=0.16, N=193
var.26.VWF 12:6128443:T:C
peptide level
38:210
10:102
1:13
CLPSACEVVTGSPRpc2_NP.3
p−min=0.051, pX=0.047, N=49
var.26.VWF 12:6128443:T:C
peptide level
206:210
102:102
13:13
CLPSACEVVTGSPRpc2_NP.4
p−min=0.3, pX=0.17, N=321
var.26.VWF 12:6128443:T:C
peptide level
133:210
65:102
5:13
CLPSACEVVTGSPRpc2_NP.5
p−maj=0.083, pX=0.11, N=203
var.26.VWF 12:6128443:T:C
peptide level
60:210
22:102
1:13
CLPTACTIQLRpc2_NP.1
p−min=0.11, pX=0.019, N=83
var.26.VWF 12:6128443:T:C
peptide level
57:210
22:102
1:13
CLPTACTIQLRpc2_NP.2
p−min=0.18, pX=0.031, N=80
var.26.VWF 12:6128443:T:C
peptide level
44:210
18:102
1:13
CLPTACTIQLRpc2_NP.3
p−min=0.38, pX=0.1, N=63
var.26.VWF 12:6128443:T:C
peptide level
151:210
78:102
9:13
CLPTACTIQLRpc2_NP.4
p−min=0.51, pX=0.16, N=238
var.26.VWF 12:6128443:T:C
peptide level
70:210
33:102
2:13
CLPTACTIQLRpc2_NP.5
p−maj=0.24, pX=0.046, N=105
var.26.VWF 12:6128443:T:C
peptide level
122:210
56:102
5:13
CMVQVGVISGFKpc2_NP.1
p−maj=0.25, pX=0.026, N=183
var.26.VWF 12:6128443:T:C
peptide level
142:210
65:102
9:13
CMVQVGVISGFKpc2_NP.2
p−min=0.62, pX=0.039, N=216
var.26.VWF 12:6128443:T:C
peptide level
96:210
40:102
3:13
CMVQVGVISGFKpc2_NP.3
p−min=0.16, pX=0.0082, N=139
var.26.VWF 12:6128443:T:C
peptide level
209:210
102:102
13:13
CMVQVGVISGFKpc2_NP.4
p−maj=1, pX=0.13, N=324
var.26.VWF 12:6128443:T:C
peptide level
158:210
85:102
11:13
CMVQVGVISGFKpc2_NP.5
p−min=0.093, pX=0.2, N=254

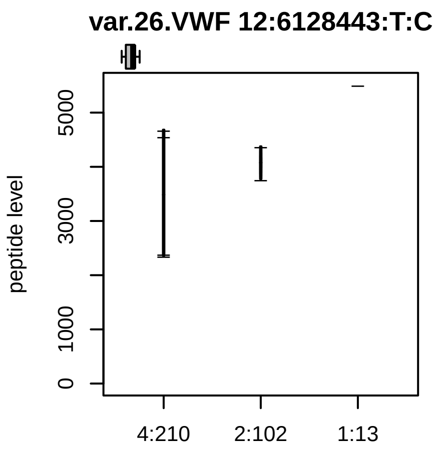

CPCFHQGKpc2_NP.2
p−maj=0.25, pX=0.17, N=7

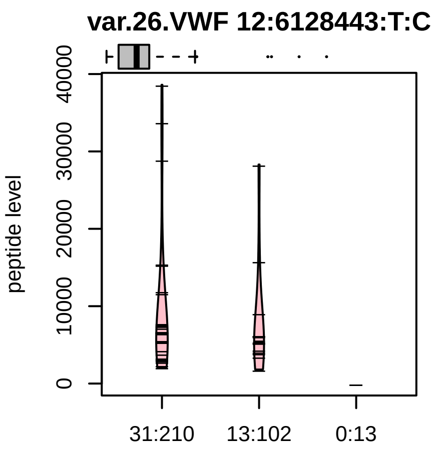

CPCFHQGKpc2_NP.4
p−maj=0.23, pX=0.25, N=44

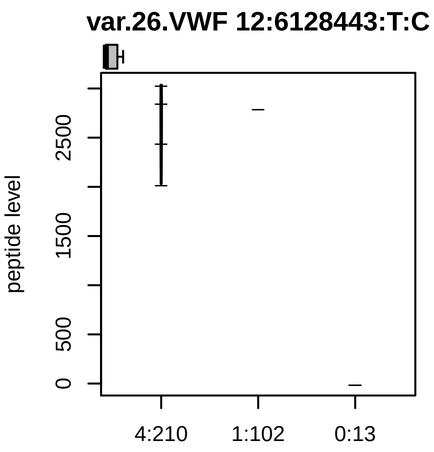

CPCFHQGKpc2_NP.5
p−min=0.66, pX=0.48, N=5

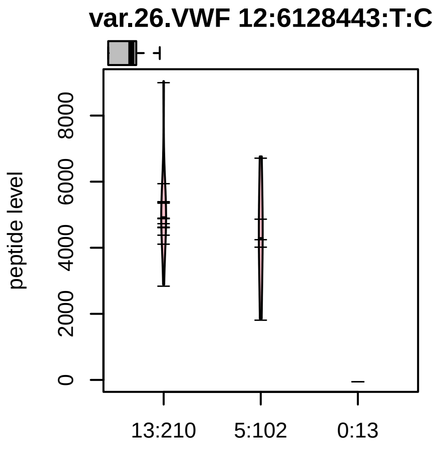

DCNTCICRpc2_NP.2
p−min=0.62, pX=0.28, N=18

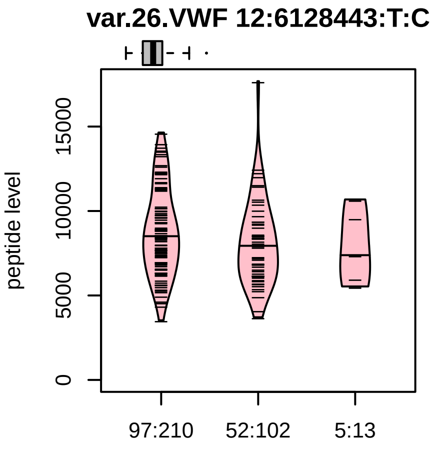

DCNTCICRpc2_NP.5
p−min=0.56, pX=0.72, N=154

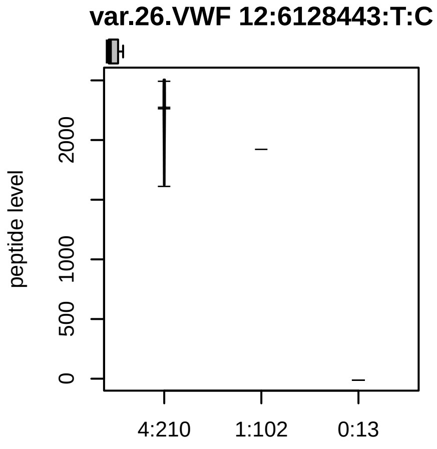

DCQDHSFSIVIETVQCADDRDAVCTRpc4_NP
p−min=0.66, pX=0.4, N=5

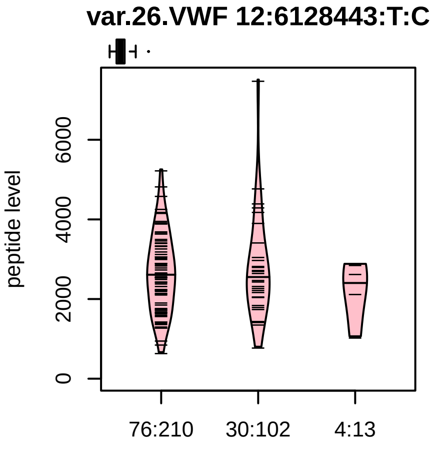

DCQDHSFSIVIETVQCADDRDAVCTRpc4_NP
p−min=0.27, pX=0.31, N=110

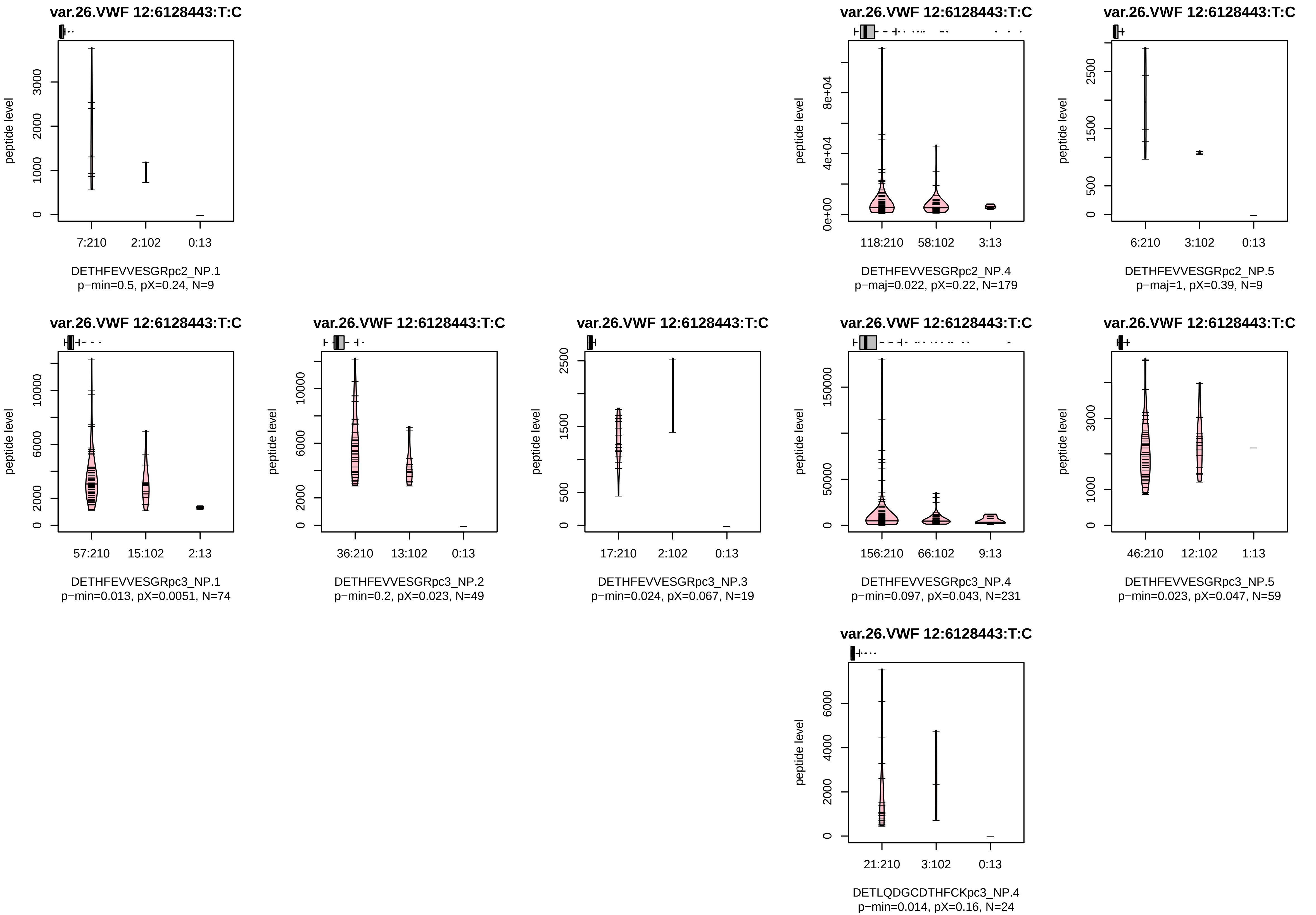
var.26.VWF 12:6128443:T:C
peptide level
0
1000
2000
3000
7:210
2:102
0:13
DETHFEVVESGRpc2_NP.1
p−min=0.5, pX=0.24, N=9
var.26.VWF 12:6128443:T:C
peptide level
0e+00
4e+04
8e+04
118:210
58:102
3:13
DETHFEVVESGRpc2_NP.4
p−maj=0.022, pX=0.22, N=179
var.26.VWF 12:6128443:T:C
peptide level
0
500
1500
2500
6:210
3:102
0:13
DETHFEVVESGRpc2_NP.5
p−maj=1, pX=0.39, N=9
var.26.VWF 12:6128443:T:C
peptide level
0
2000
6000
10000
57:210
15:102
2:13
DETHFEVVESGRpc3_NP.1
p−min=0.013, pX=0.0051, N=74
var.26.VWF 12:6128443:T:C
peptide level
0
2000
6000
10000
36:210
13:102
0:13
DETHFEVVESGRpc3_NP.2
p−min=0.2, pX=0.023, N=49
var.26.VWF 12:6128443:T:C
peptide level
0
500
1500
2500
17:210
2:102
0:13
DETHFEVVESGRpc3_NP.3
p−min=0.024, pX=0.067, N=19
var.26.VWF 12:6128443:T:C
peptide level
0
50000
150000
156:210
66:102
9:13
DETHFEVVESGRpc3_NP.4
p−min=0.097, pX=0.043, N=231
var.26.VWF 12:6128443:T:C
peptide level
0
1000
3000
46:210
12:102
1:13
DETHFEVVESGRpc3_NP.5
p−min=0.023, pX=0.047, N=59
var.26.VWF 12:6128443:T:C
peptide level
0
2000
4000
6000
21:210
3:102
0:13
DETLQDGCDTHFCKpc3_NP.4
p−min=0.014, pX=0.16, N=24

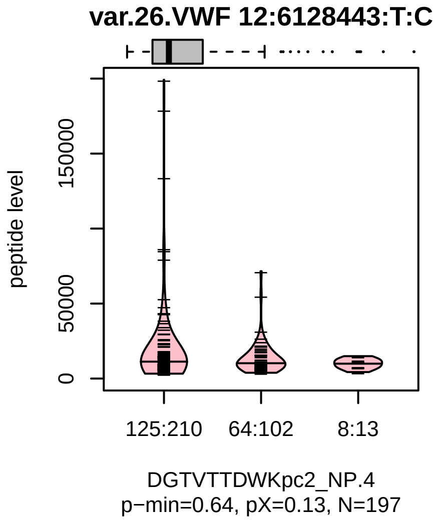
var.26.VWF 12:6128443:T:C
peptide level
0
50000
150000
125:210
64:102
8:13
DGTVTTDWKpc2_NP.4
p−min=0.64, pX=0.13, N=197

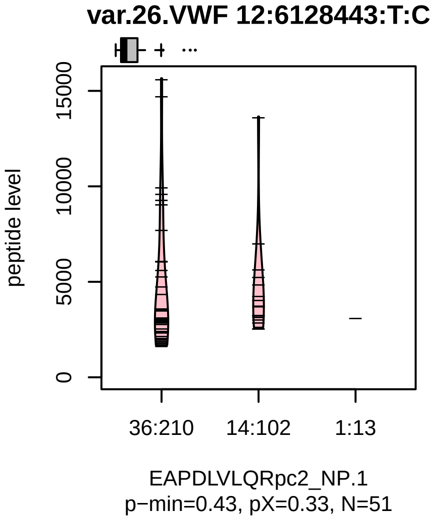
var.26.VWF 12:6128443:T:C
peptide level
0
5000
10000
15000
36:210
14:102
1:13
EAPDLVLQRpc2_NP.1
p−min=0.43, pX=0.33, N=51

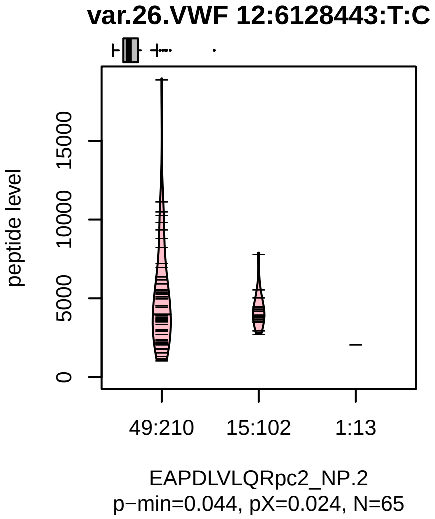
var.26.VWF 12:6128443:T:C
peptide level
0
5000
10000
15000
49:210
15:102
1:13
EAPDLVLQRpc2_NP.2
p−min=0.044, pX=0.024, N=65

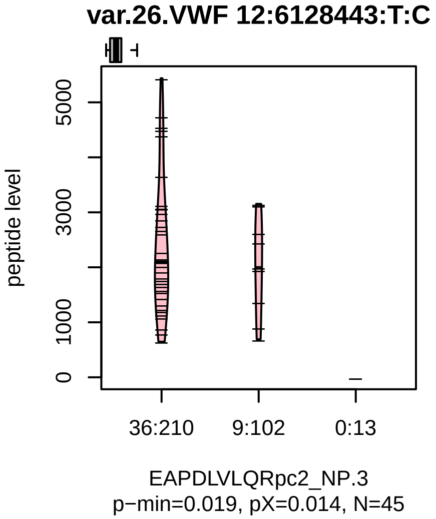
var.26.VWF 12:6128443:T:C
peptide level
0
1000
3000
5000
36:210
9:102
0:13
EAPDLVLQRpc2_NP.3
p−min=0.019, pX=0.014, N=45

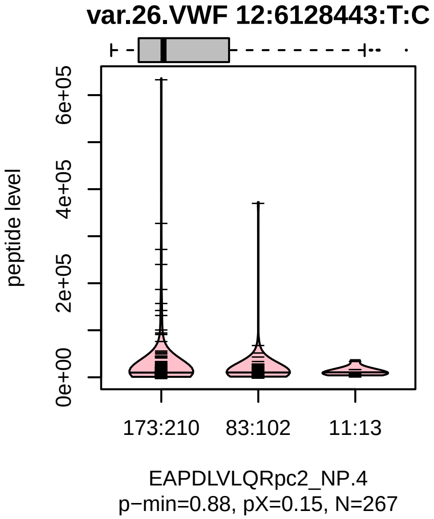
var.26.VWF 12:6128443:T:C
peptide level
0e+00
2e+05
4e+05
6e+05
173:210
83:102
11:13
EAPDLVLQRpc2_NP.4
p−min=0.88, pX=0.15, N=267

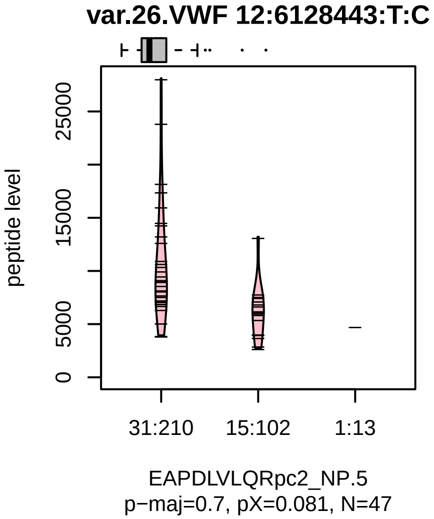
var.26.VWF 12:6128443:T:C
peptide level
0
5000
15000
25000
31:210
15:102
1:13
EAPDLVLQRpc2_NP.5
p−maj=0.7, pX=0.081, N=47

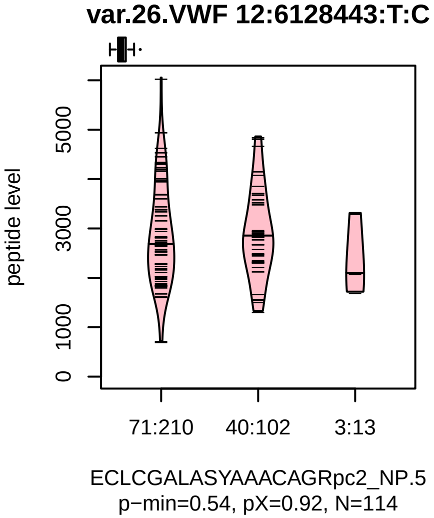
var.26.VWF 12:6128443:T:C
peptide level
0
1000
3000
5000
71:210
40:102
3:13
ECLCGALASYAAACAGRpc2_NP.5
p−min=0.54, pX=0.92, N=114

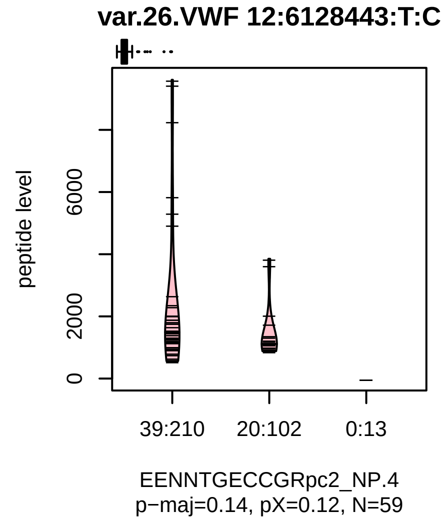
var.26.VWF 12:6128443:T:C
peptide level
0
2000
6000
39:210
20:102
0:13
EENNTGECCGRpc2_NP.4
p−maj=0.14, pX=0.12, N=59

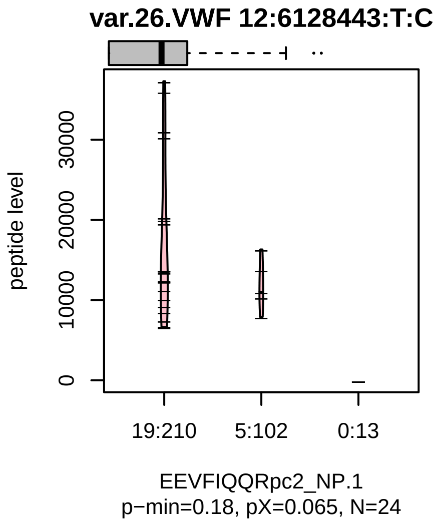
var.26.VWF 12:6128443:T:C
peptide level
0
10000
20000
30000
19:210
5:102
0:13
EEVFIQQRpc2_NP.1
p−min=0.18, pX=0.065, N=24

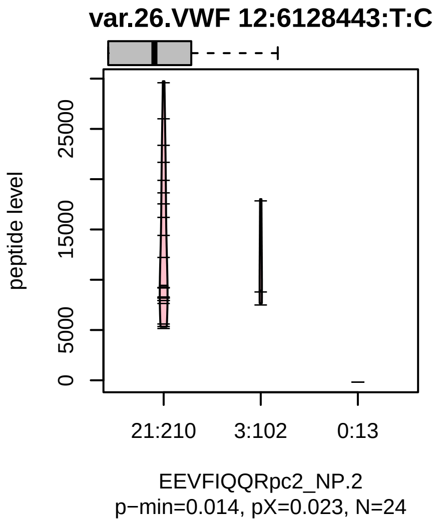
var.26.VWF 12:6128443:T:C
peptide level
0
5000
15000
25000
21:210
3:102
0:13
EEVFIQQRpc2_NP.2
p−min=0.014, pX=0.023, N=24

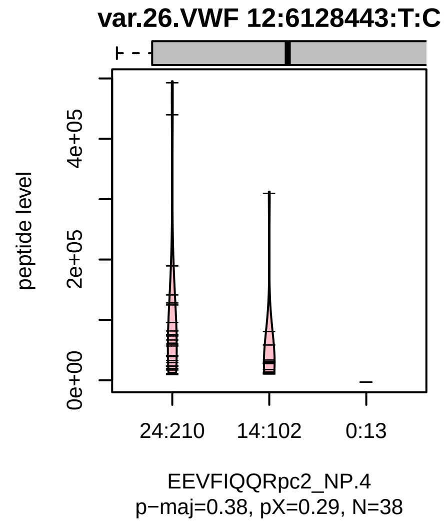
var.26.VWF 12:6128443:T:C
peptide level
0e+00
2e+05
4e+05
24:210
14:102
0:13
EEVFIQQRpc2_NP.4
p−maj=0.38, pX=0.29, N=38

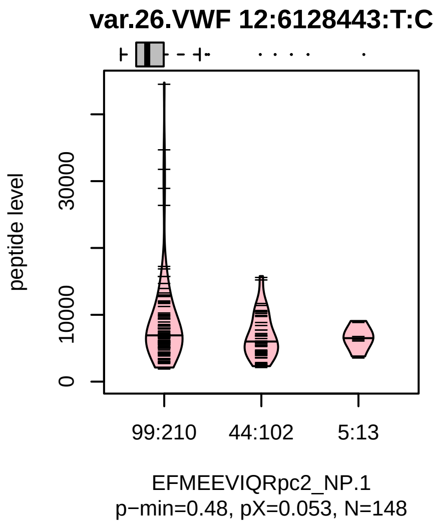
var.26.VWF 12:6128443:T:C
peptide level
0
10000
30000
99:210
44:102
5:13
EFMEEVIQRpc2_NP.1
p−min=0.48, pX=0.053, N=148

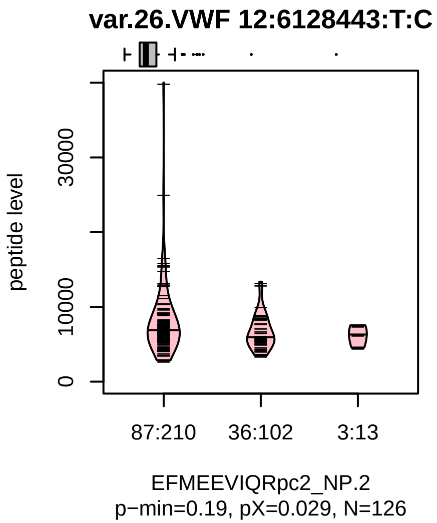
var.26.VWF 12:6128443:T:C
peptide level
0
10000
30000
87:210
36:102
3:13
EFMEEVIQRpc2_NP.2
p−min=0.19, pX=0.029, N=126

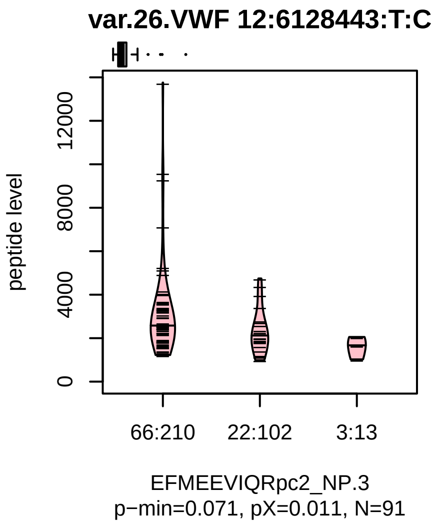
var.26.VWF 12:6128443:T:C
peptide level
0
4000
8000
12000
66:210
22:102
3:13
EFMEEVIQRpc2_NP.3
p−min=0.071, pX=0.011, N=91

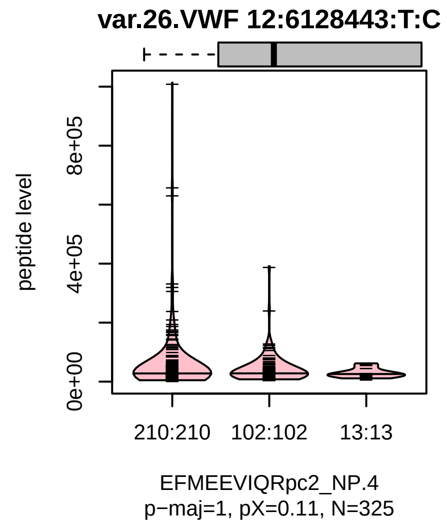
var.26.VWF 12:6128443:T:C
peptide level
0e+00
4e+05
8e+05
210:210
102:102
13:13
EFMEEVIQRpc2_NP.4
p−maj=1, pX=0.11, N=325

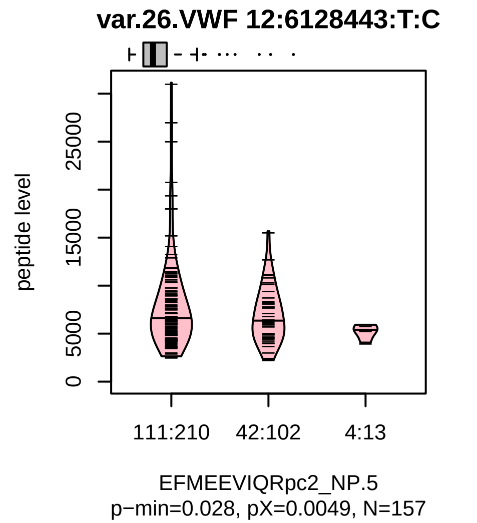
var.26.VWF 12:6128443:T:C
peptide level
0
5000
15000
25000
111:210
42:102
4:13
EFMEEVIQRpc2_NP.5
p−min=0.028, pX=0.0049, N=157

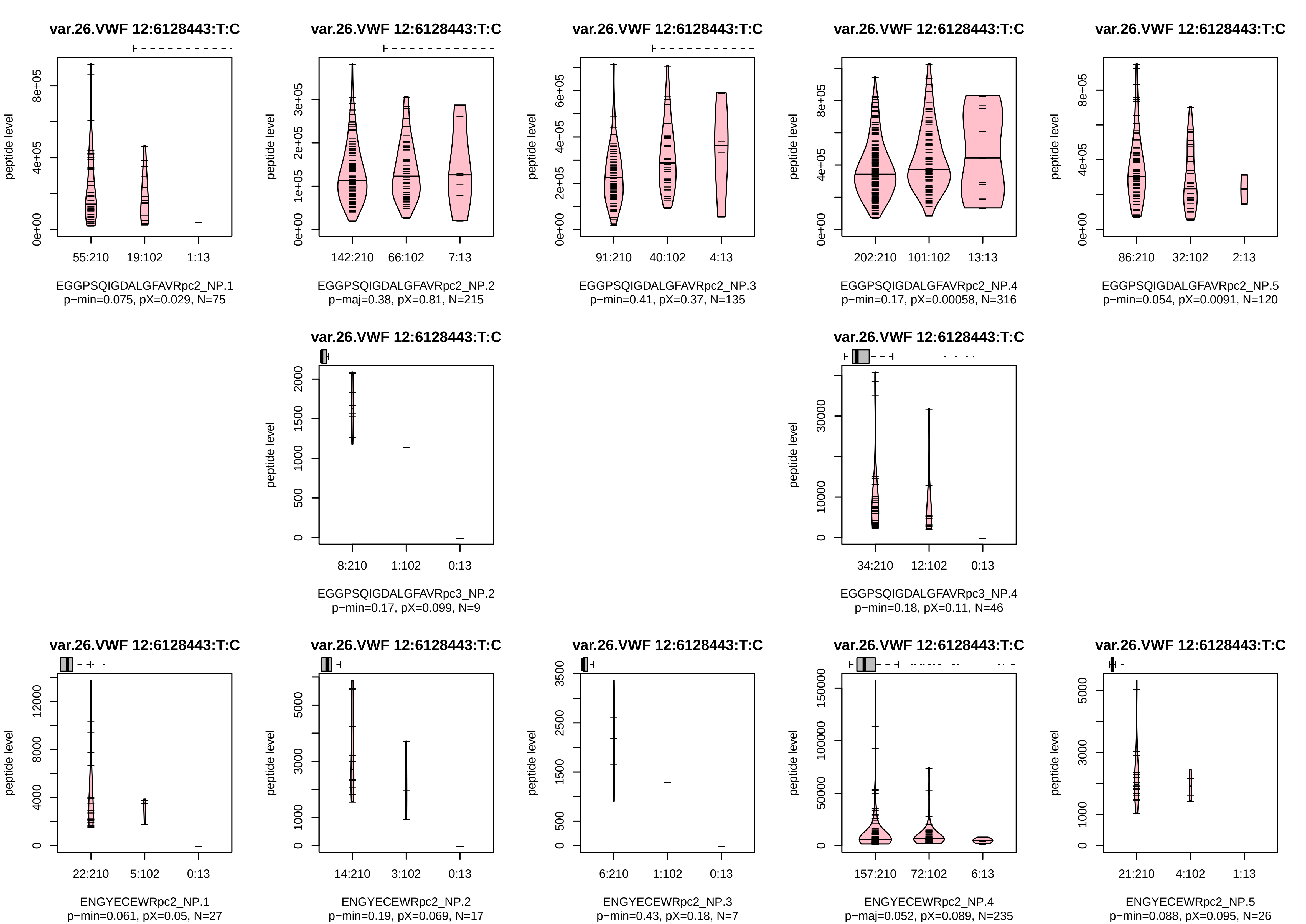
var.26.VWF 12:6128443:T:C
peptide level
55:210
19:102
1:13
EGGPSQIGDALGFAVRpc2_NP.1
p−min=0.075, pX=0.029, N=75
var.26.VWF 12:6128443:T:C
142:210
66:102
7:13
EGGPSQIGDALGFAVRpc2_NP.2
p−maj=0.38, pX=0.81, N=215
var.26.VWF 12:6128443:T:C
91:210
40:102
4:13
EGGPSQIGDALGFAVRpc2_NP.3
p−min=0.41, pX=0.37, N=135
var.26.VWF 12:6128443:T:C
202:210
101:102
13:13
EGGPSQIGDALGFAVRpc2_NP.4
p−min=0.17, pX=0.00058, N=316
var.26.VWF 12:6128443:T:C
86:210
32:102
2:13
EGGPSQIGDALGFAVRpc2_NP.5
p−min=0.054, pX=0.0091, N=120
var.26.VWF 12:6128443:T:C
8:210
1:102
0:13
EGGPSQIGDALGFAVRpc3_NP.2
p−min=0.17, pX=0.099, N=9
var.26.VWF 12:6128443:T:C
34:210
12:102
0:13
EGGPSQIGDALGFAVRpc3_NP.4
p−min=0.18, pX=0.11, N=46
var.26.VWF 12:6128443:T:C
22:210
5:102
0:13
ENGYECEWRpc2_NP.1
p−min=0.061, pX=0.05, N=27
var.26.VWF 12:6128443:T:C
14:210
3:102
0:13
ENGYECEWRpc2_NP.2
p−min=0.19, pX=0.069, N=17
var.26.VWF 12:6128443:T:C
6:210
1:102
0:13
ENGYECEWRpc2_NP.3
p−min=0.43, pX=0.18, N=7
var.26.VWF 12:6128443:T:C
157:210
72:102
6:13
ENGYECEWRpc2_NP.4
p−maj=0.052, pX=0.089, N=235
var.26.VWF 12:6128443:T:C
21:210
4:102
1:13
ENGYECEWRpc2_NP.5
p−min=0.088, pX=0.095, N=26

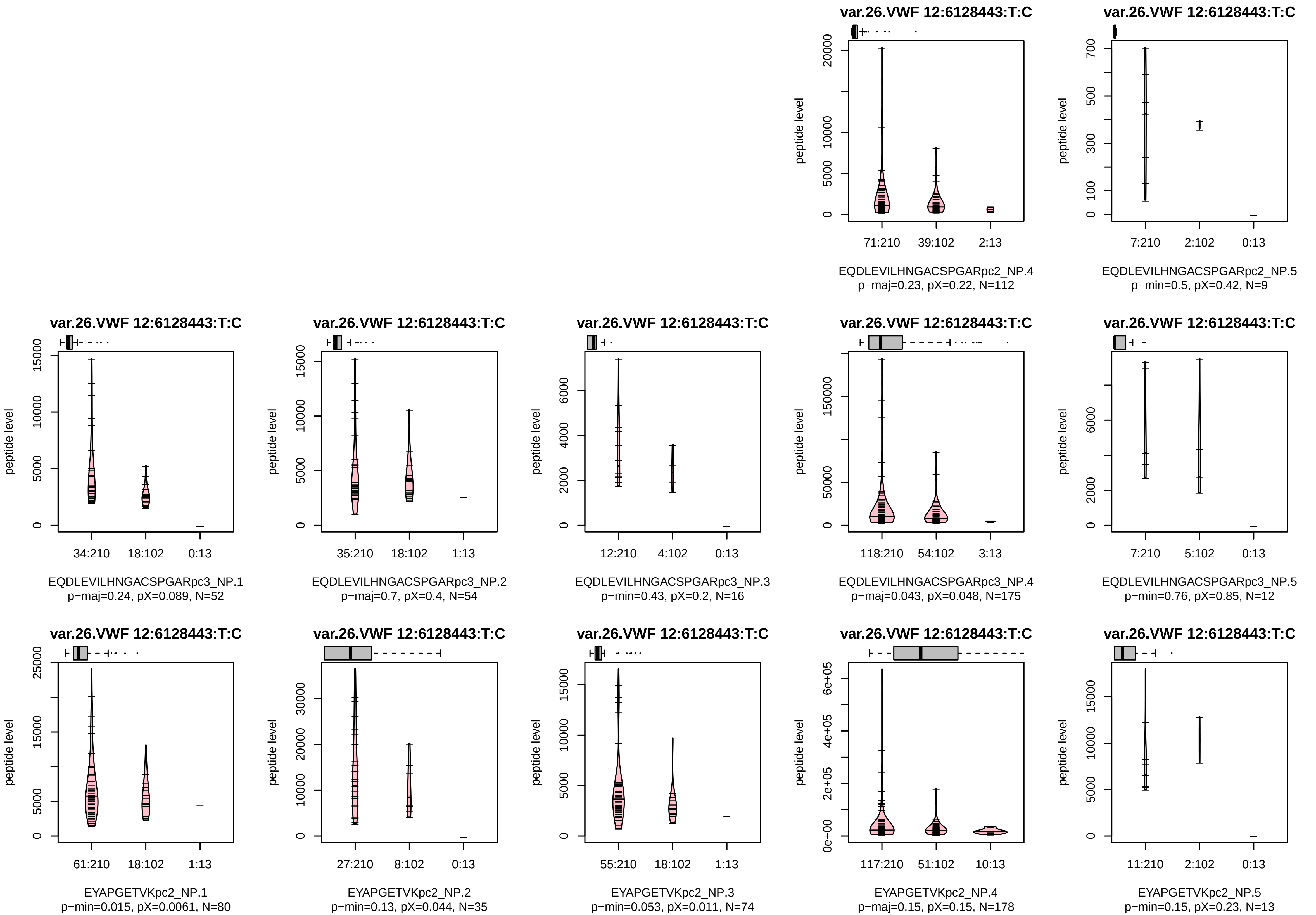

var.26.VWF 12:6128443:T:C
peptide level
71:210
39:102
2:13
EQDLEVILHNGACSPGARpc2_NP.4
p−maj=0.23, pX=0.22, N=112
var.26.VWF 12:6128443:T:C
peptide level
7:210
2:102
0:13
EQDLEVILHNGACSPGARpc2_NP.5
p−min=0.5, pX=0.42, N=9
var.26.VWF 12:6128443:T:C
peptide level
34:210
18:102
0:13
EQDLEVILHNGACSPGARpc3_NP.1
p−maj=0.24, pX=0.089, N=52
var.26.VWF 12:6128443:T:C
peptide level
35:210
18:102
1:13
EQDLEVILHNGACSPGARpc3_NP.2
p−maj=0.7, pX=0.4, N=54
var.26.VWF 12:6128443:T:C
peptide level
12:210
4:102
0:13
EQDLEVILHNGACSPGARpc3_NP.3
p−min=0.43, pX=0.2, N=16
var.26.VWF 12:6128443:T:C
peptide level
118:210
54:102
3:13
EQDLEVILHNGACSPGARpc3_NP.4
p−maj=0.043, pX=0.048, N=175
var.26.VWF 12:6128443:T:C
peptide level
7:210
5:102
0:13
EQDLEVILHNGACSPGARpc3_NP.5
p−min=0.76, pX=0.85, N=12
var.26.VWF 12:6128443:T:C
peptide level
61:210
18:102
1:13
EYAPGETVKpc2_NP.1
p−min=0.015, pX=0.0061, N=80
var.26.VWF 12:6128443:T:C
peptide level
27:210
8:102
0:13
EYAPGETVKpc2_NP.2
p−min=0.13, pX=0.044, N=35
var.26.VWF 12:6128443:T:C
peptide level
55:210
18:102
1:13
EYAPGETVKpc2_NP.3
p−min=0.053, pX=0.011, N=74
var.26.VWF 12:6128443:T:C
peptide level
117:210
51:102
10:13
EYAPGETVKpc2_NP.4
p−maj=0.15, pX=0.15, N=178
var.26.VWF 12:6128443:T:C
peptide level
11:210
2:102
0:13
EYAPGETVKpc2_NP.5
p−min=0.15, pX=0.23, N=13

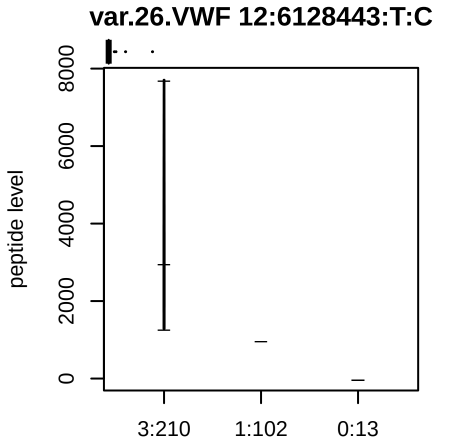

FNHLGHIFTFTPQNNEFQLQLSPKpc4_NP.1
p−min=1, pX=0.39, N=4

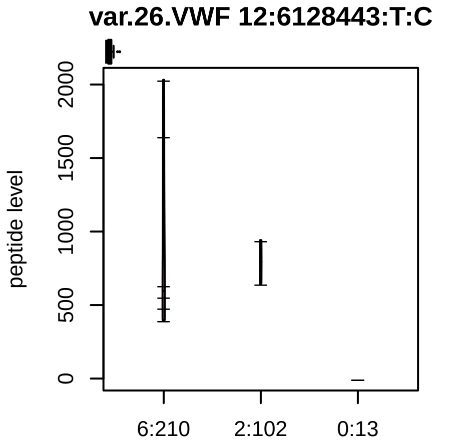

FNHLGHIFTFTPQNNEFQLQLSPKpc4_NP.2
p−min=0.72, pX=0.45, N=8

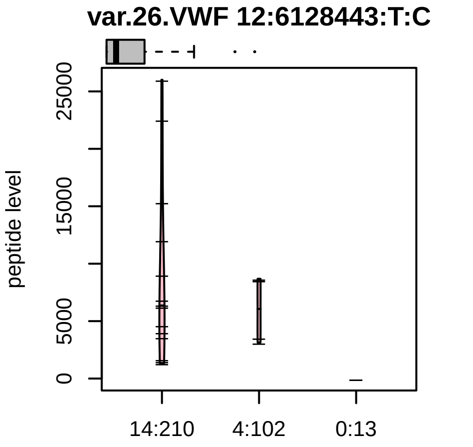

FNHLGHIFTFTPQNNEFQLQLSPKpc4_NP.4
p−min=0.31, pX=0.18, N=18

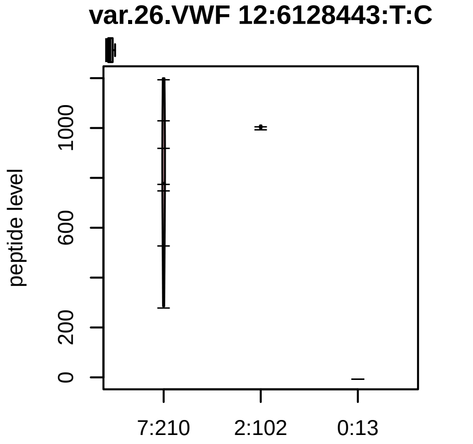

FSEEACAVLTSPTFEACHRpc3_NP.2
p−min=0.5, pX=0.52, N=9

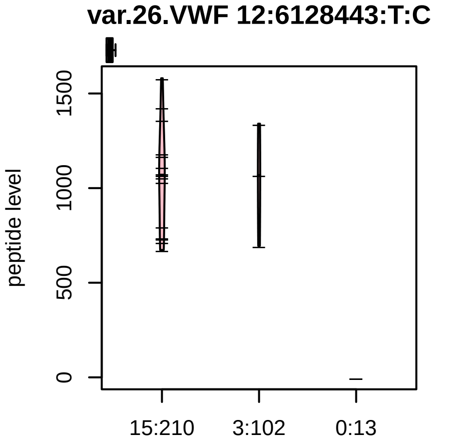

FSEEACAVLTSPTFEACHRpc3_NP.4
p−min=0.13, pX=0.087, N=18

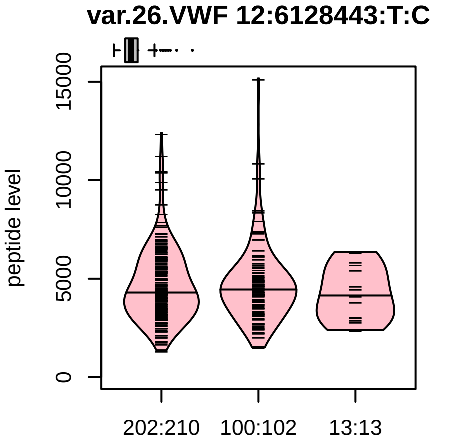

FSEEACAVLTSPTFEACHRpc3_NP.5
p−min=0.5, pX=0.88, N=315

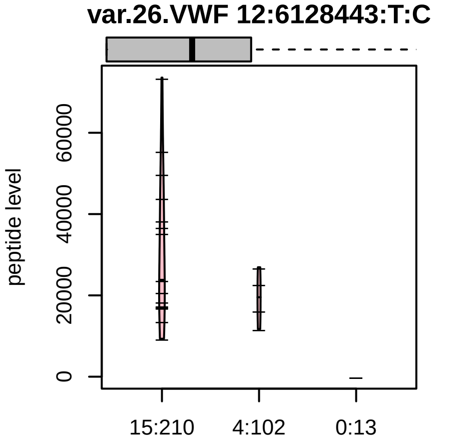

GDSQSSWKpc2_NP.4
p−min=0.22, pX=0.085, N=19

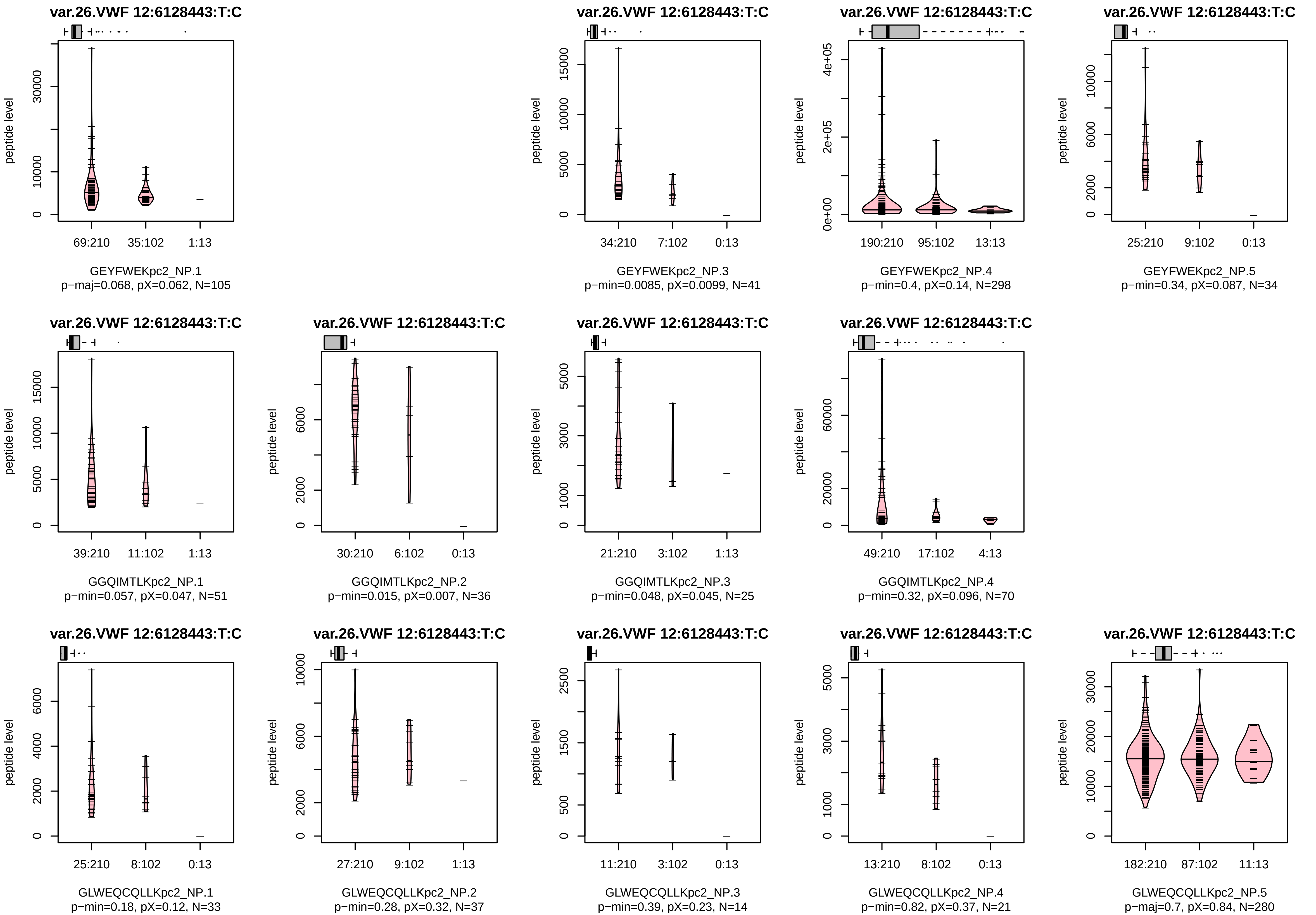
var.26.VWF 12:6128443:T:C
peptide level
69:210
35:102
1:13
GEYFWEKpc2_NP.1
p−maj=0.068, pX=0.062, N=105
var.26.VWF 12:6128443:T:C
peptide level
34:210
7:102
0:13
GEYFWEKpc2_NP.3
p−min=0.0085, pX=0.0099, N=41
var.26.VWF 12:6128443:T:C
peptide level
190:210
95:102
13:13
GEYFWEKpc2_NP.4
p−min=0.4, pX=0.14, N=298
var.26.VWF 12:6128443:T:C
peptide level
25:210
9:102
0:13
GEYFWEKpc2_NP.5
p−min=0.34, pX=0.087, N=34
var.26.VWF 12:6128443:T:C
peptide level
39:210
11:102
1:13
GGQIMTLKpc2_NP.1
p−min=0.057, pX=0.047, N=51
var.26.VWF 12:6128443:T:C
peptide level
30:210
6:102
0:13
GGQIMTLKpc2_NP.2
p−min=0.015, pX=0.007, N=36
var.26.VWF 12:6128443:T:C
peptide level
21:210
3:102
1:13
GGQIMTLKpc2_NP.3
p−min=0.048, pX=0.045, N=25
var.26.VWF 12:6128443:T:C
peptide level
49:210
17:102
4:13
GGQIMTLKpc2_NP.4
p−min=0.32, pX=0.096, N=70
var.26.VWF 12:6128443:T:C
peptide level
25:210
8:102
0:13
GLWEQCQLLKpc2_NP.1
p−min=0.18, pX=0.12, N=33
var.26.VWF 12:6128443:T:C
peptide level
27:210
9:102
1:13
GLWEQCQLLKpc2_NP.2
p−min=0.28, pX=0.32, N=37
var.26.VWF 12:6128443:T:C
peptide level
11:210
3:102
0:13
GLWEQCQLLKpc2_NP.3
p−min=0.39, pX=0.23, N=14
var.26.VWF 12:6128443:T:C
peptide level
13:210
8:102
0:13
GLWEQCQLLKpc2_NP.4
p−min=0.82, pX=0.37, N=21
var.26.VWF 12:6128443:T:C
peptide level
182:210
87:102
11:13
GLWEQCQLLKpc2_NP.5
p−maj=0.7, pX=0.84, N=280

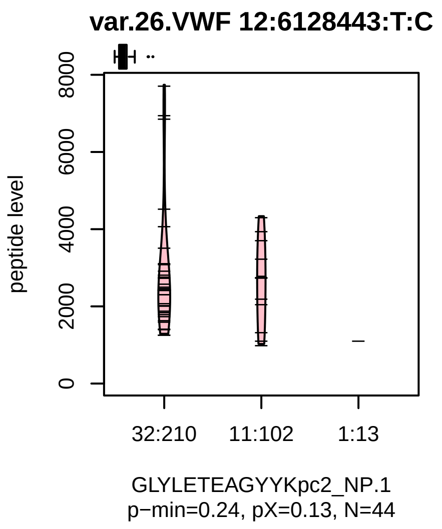
var.26.VWF 12:6128443:T:C
peptide level
0
2000
4000
6000
8000
32:210
11:102
1:13
GLYLETEAGYYKpc2_NP.1
p−min=0.24, pX=0.13, N=44

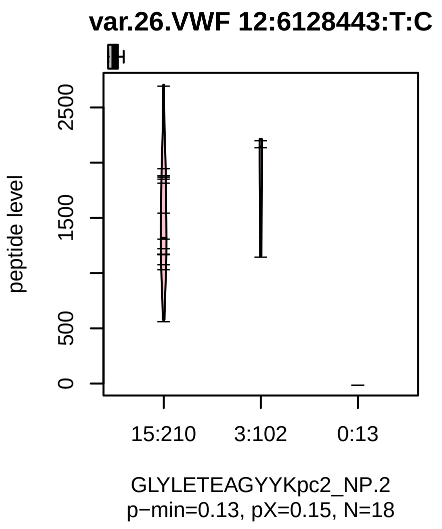
var.26.VWF 12:6128443:T:C
peptide level
0
500
1500
2500
15:210
3:102
0:13
GLYLETEAGYYKpc2_NP.2
p−min=0.13, pX=0.15, N=18

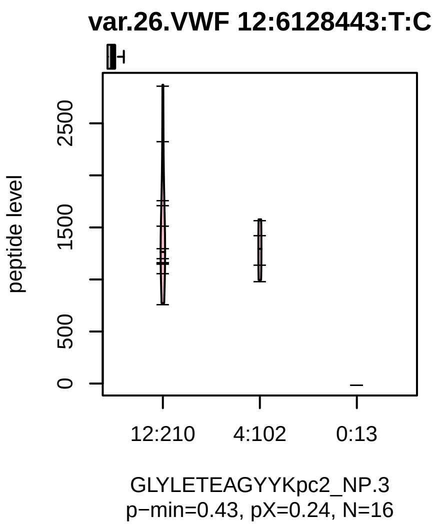
var.26.VWF 12:6128443:T:C
peptide level
0
500
1500
2500
12:210
4:102
0:13
GLYLETEAGYYKpc2_NP.3
p−min=0.43, pX=0.24, N=16

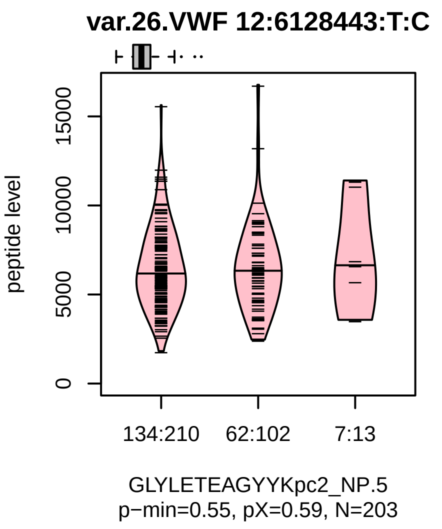
var.26.VWF 12:6128443:T:C
peptide level
0
5000
10000
15000
134:210
62:102
7:13
GLYLETEAGYYKpc2_NP.5
p−min=0.55, pX=0.59, N=203

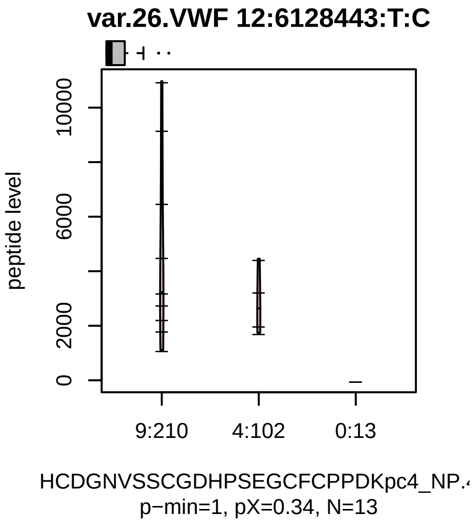
var.26.VWF 12:6128443:T:C
peptide level
0
2000
6000
10000
9:210
4:102
0:13
HCDGNVSSCGDHPSEGCFCPPDKpc4_NP.4
p−min=1, pX=0.34, N=13

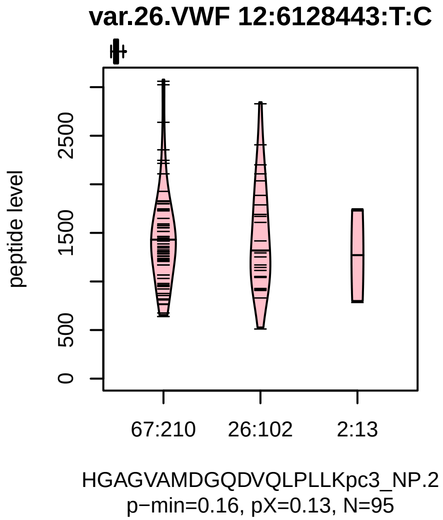
var.26.VWF 12:6128443:T:C
peptide level
0
500
1500
2500
67:210
26:102
2:13
HGAGVAMDGQDVQLPLLKpc3_NP.2
p−min=0.16, pX=0.13, N=95

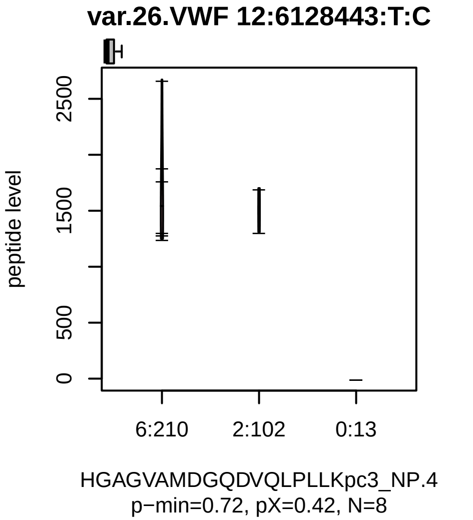
var.26.VWF 12:6128443:T:C
peptide level
0
500
1500
2500
6:210
2:102
0:13
HGAGVAMDGQDVQLPLLKpc3_NP.4
p−min=0.72, pX=0.42, N=8

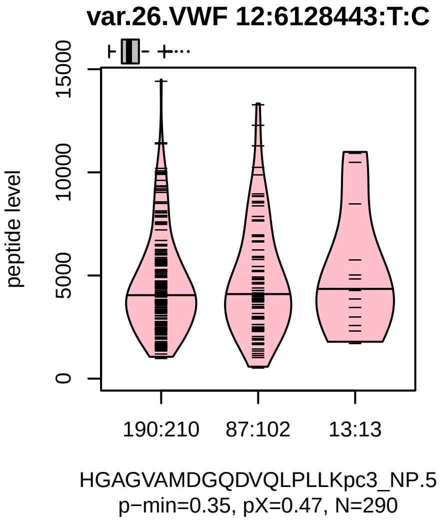
var.26.VWF 12:6128443:T:C
peptide level
0
5000
10000
15000
190:210
87:102
13:13
HGAGVAMDGQDVQLPLLKpc3_NP.5
p−min=0.35, pX=0.47, N=290

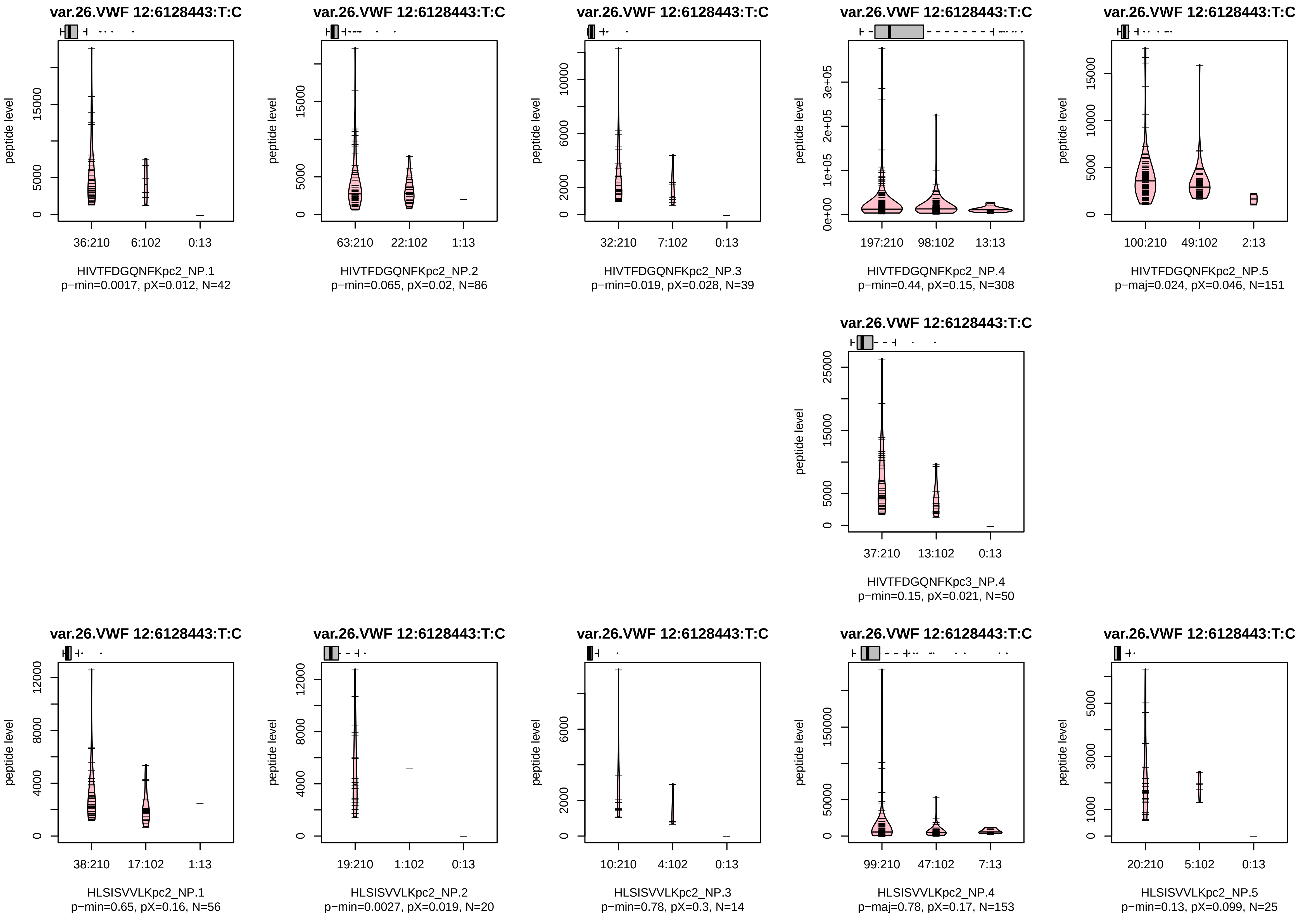
var.26.VWF 12:6128443:T:C
peptide level
36:210
6:102
0:13
HIVTFDGQNFKpc2_NP.1
p−min=0.0017, pX=0.012, N=42
var.26.VWF 12:6128443:T:C
peptide level
63:210
22:102
1:13
HIVTFDGQNFKpc2_NP.2
p−min=0.065, pX=0.02, N=86
var.26.VWF 12:6128443:T:C
peptide level
32:210
7:102
0:13
HIVTFDGQNFKpc2_NP.3
p−min=0.019, pX=0.028, N=39
var.26.VWF 12:6128443:T:C
peptide level
197:210
98:102
13:13
HIVTFDGQNFKpc2_NP.4
p−min=0.44, pX=0.15, N=308
var.26.VWF 12:6128443:T:C
peptide level
100:210
49:102
2:13
HIVTFDGQNFKpc2_NP.5
p−maj=0.024, pX=0.046, N=151
var.26.VWF 12:6128443:T:C
peptide level
37:210
13:102
0:13
HIVTFDGQNFKpc3_NP.4
p−min=0.15, pX=0.021, N=50
var.26.VWF 12:6128443:T:C
peptide level
38:210
17:102
1:13
HLSISVVLKpc2_NP.1
p−min=0.65, pX=0.16, N=56
var.26.VWF 12:6128443:T:C
peptide level
19:210
1:102
0:13
HLSISVVLKpc2_NP.2
p−min=0.0027, pX=0.019, N=20
var.26.VWF 12:6128443:T:C
peptide level
10:210
4:102
0:13
HLSISVVLKpc2_NP.3
p−min=0.78, pX=0.3, N=14
var.26.VWF 12:6128443:T:C
peptide level
99:210
47:102
7:13
HLSISVVLKpc2_NP.4
p−maj=0.78, pX=0.17, N=153
var.26.VWF 12:6128443:T:C
peptide level
20:210
5:102
0:13
HLSISVVLKpc2_NP.5
p−min=0.13, pX=0.099, N=25

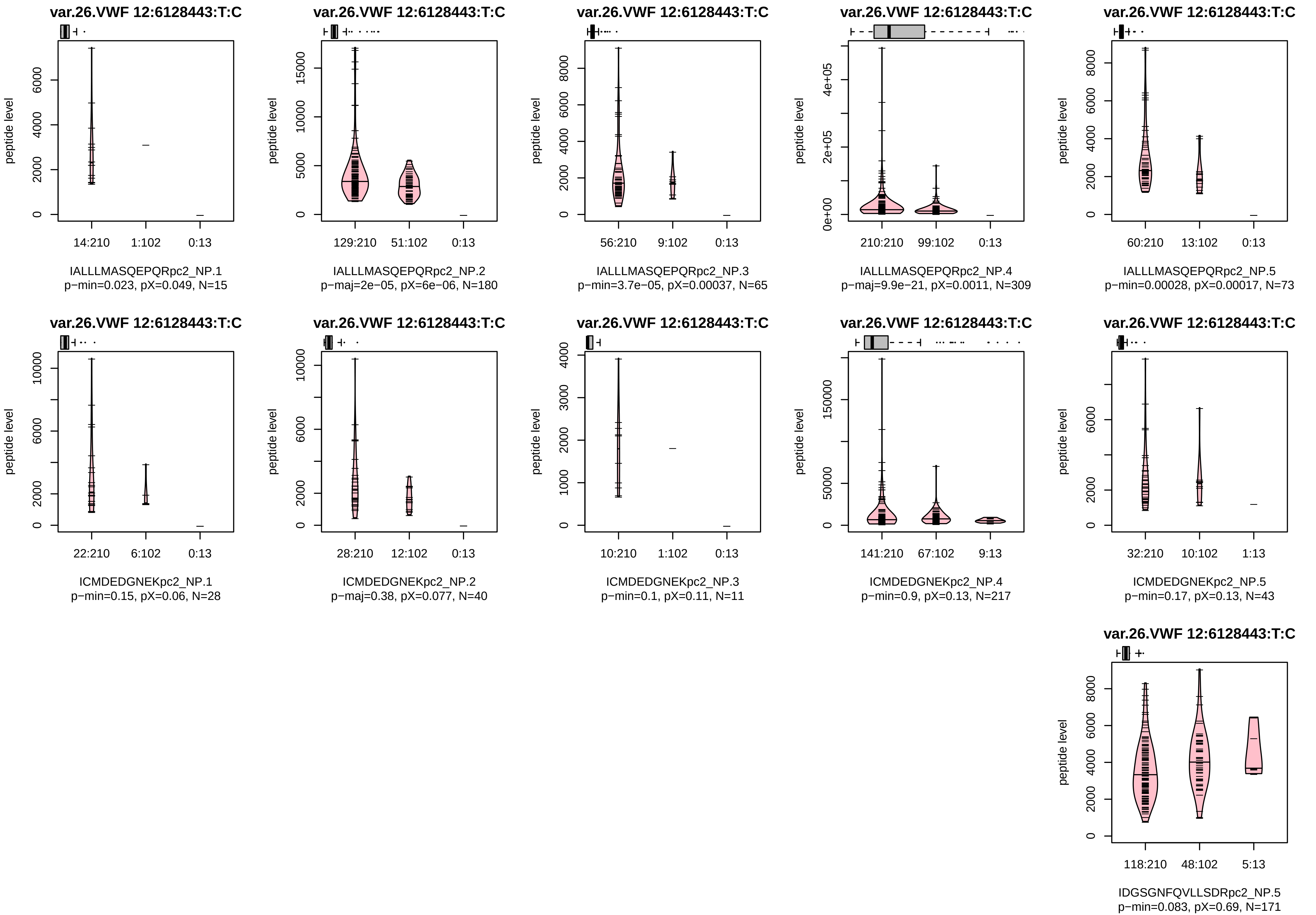
var.26.VWF 12:6128443:T:C
peptide level
14:210
1:102
0:13
IALLLMASQEPQRpc2_NP.1
p−min=0.023, pX=0.049, N=15
var.26.VWF 12:6128443:T:C
peptide level
129:210
51:102
0:13
IALLLMASQEPQRpc2_NP.2
p−maj=2e−05, pX=6e−06, N=180
var.26.VWF 12:6128443:T:C
peptide level
56:210
9:102
0:13
IALLLMASQEPQRpc2_NP.3
p−min=3.7e−05, pX=0.00037, N=65
var.26.VWF 12:6128443:T:C
peptide level
210:210
99:102
0:13
IALLLMASQEPQRpc2_NP.4
p−maj=9.9e−21, pX=0.0011, N=309
var.26.VWF 12:6128443:T:C
peptide level
60:210
13:102
0:13
IALLLMASQEPQRpc2_NP.5
p−min=0.00028, pX=0.00017, N=73
var.26.VWF 12:6128443:T:C
peptide level
22:210
6:102
0:13
ICMDEDGNEKpc2_NP.1
p−min=0.15, pX=0.06, N=28
var.26.VWF 12:6128443:T:C
peptide level
28:210
12:102
0:13
ICMDEDGNEKpc2_NP.2
p−maj=0.38, pX=0.077, N=40
var.26.VWF 12:6128443:T:C
peptide level
10:210
1:102
0:13
ICMDEDGNEKpc2_NP.3
p−min=0.1, pX=0.11, N=11
var.26.VWF 12:6128443:T:C
peptide level
141:210
67:102
9:13
ICMDEDGNEKpc2_NP.4
p−min=0.9, pX=0.13, N=217
var.26.VWF 12:6128443:T:C
peptide level
32:210
10:102
1:13
ICMDEDGNEKpc2_NP.5
p−min=0.17, pX=0.13, N=43
var.26.VWF 12:6128443:T:C
peptide level
118:210
48:102
5:13
IDGSGNFQVLLSDRpc2_NP.5
p−min=0.083, pX=0.69, N=171

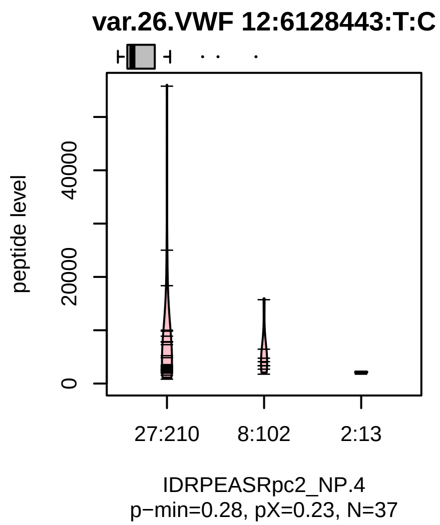
var.26.VWF 12:6128443:T:C
peptide level
27:210
8:102
2:13
IDRPEASRpc2_NP.4
p−min=0.28, pX=0.23, N=37

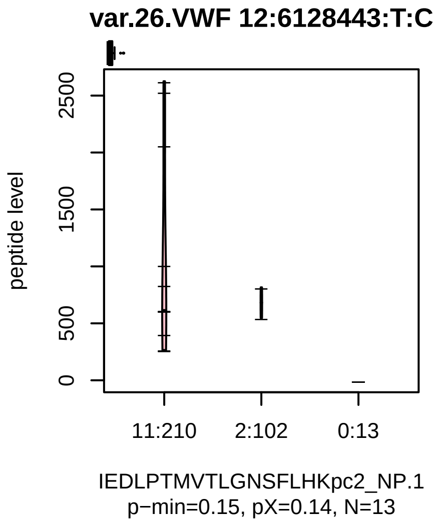
var.26.VWF 12:6128443:T:C
peptide level
11:210
2:102
0:13
IEDLPTMVTLGNSFLHKpc2_NP.1
p−min=0.15, pX=0.14, N=13

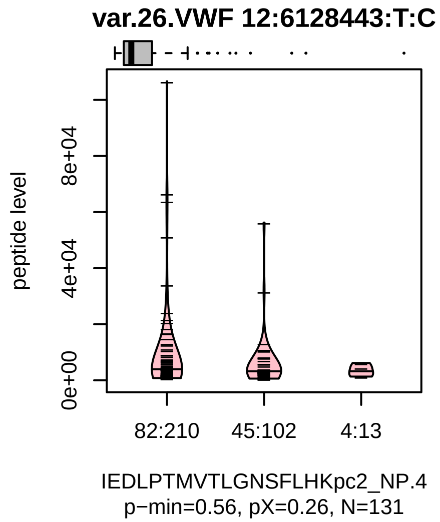
var.26.VWF 12:6128443:T:C
peptide level
82:210
45:102
4:13
IEDLPTMVTLGNSFLHKpc2_NP.4
p−min=0.56, pX=0.26, N=131

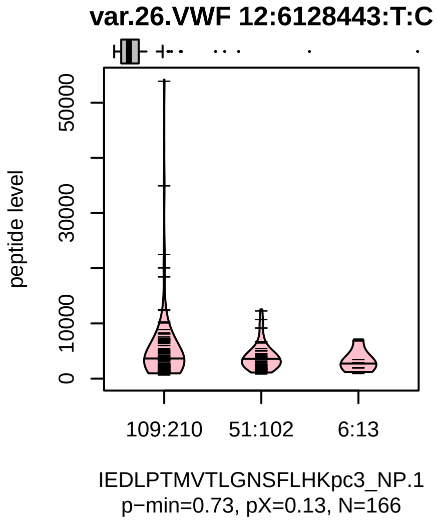
var.26.VWF 12:6128443:T:C
peptide level
109:210
51:102
6:13
IEDLPTMVTLGNSFLHKpc3_NP.1
p−min=0.73, pX=0.13, N=166

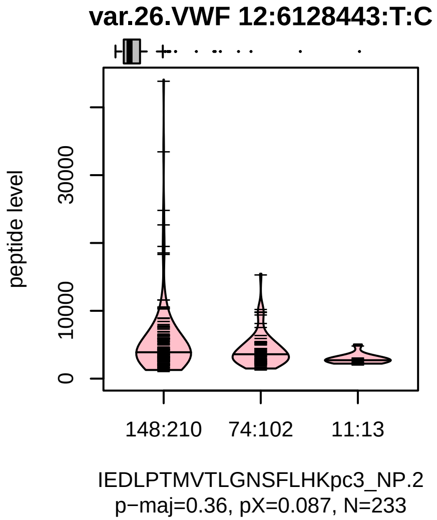
var.26.VWF 12:6128443:T:C
peptide level
148:210
74:102
11:13
IEDLPTMVTLGNSFLHKpc3_NP.2
p−maj=0.36, pX=0.087, N=233

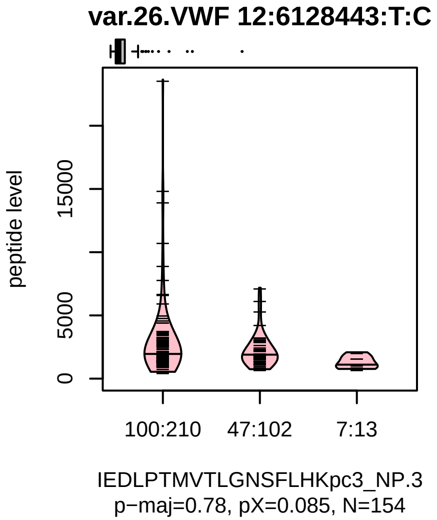
var.26.VWF 12:6128443:T:C
peptide level
100:210
47:102
7:13
IEDLPTMVTLGNSFLHKpc3_NP.3
p−maj=0.78, pX=0.085, N=154

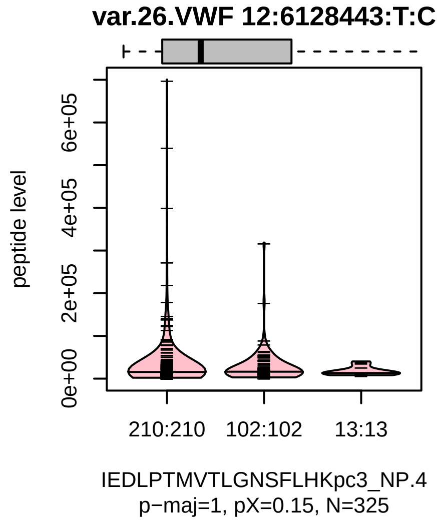
var.26.VWF 12:6128443:T:C
peptide level
210:210
102:102
13:13
IEDLPTMVTLGNSFLHKpc3_NP.4
p−maj=1, pX=0.15, N=325

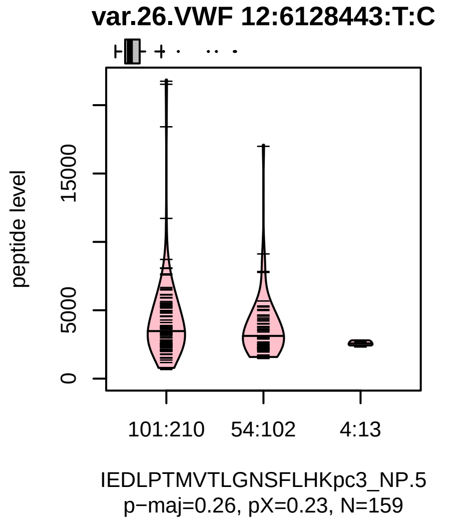
var.26.VWF 12:6128443:T:C
peptide level
101:210
54:102
4:13
IEDLPTMVTLGNSFLHKpc3_NP.5
p−maj=0.26, pX=0.23, N=159

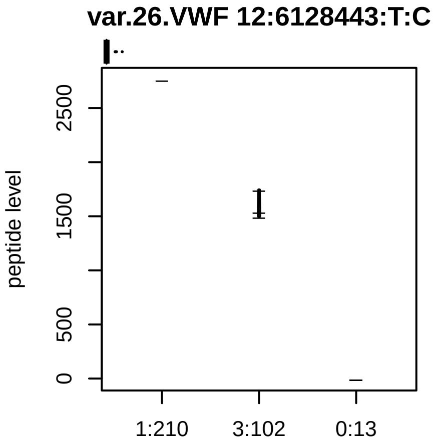

IGCNTCVCQDRKpc3_NP.4
p−min=0.13, pX=0.41, N=4

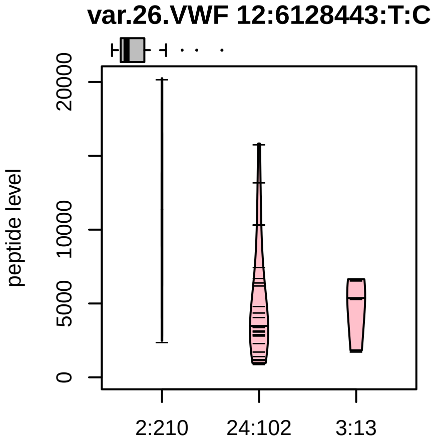

IGCNTCVCQDRpc2_NP.4
p−min=1.5e−11, pX=2.9e−05, N=29

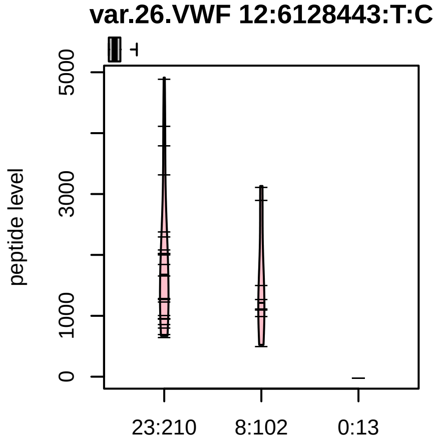

IGWPNAPILIQDFETLPRpc3_NP.1
p−min=0.32, pX=0.14, N=31

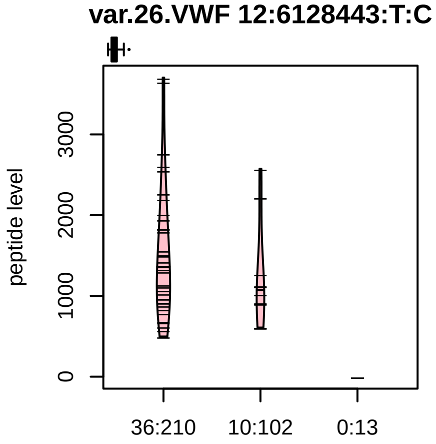

IGWPNAPILIQDFETLPRpc3_NP.2
p−min=0.045, pX=0.019, N=46

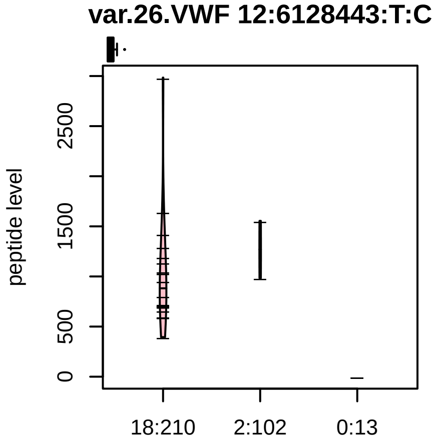

IGWPNAPILIQDFETLPRpc3_NP.3
p−min=0.015, pX=0.049, N=20

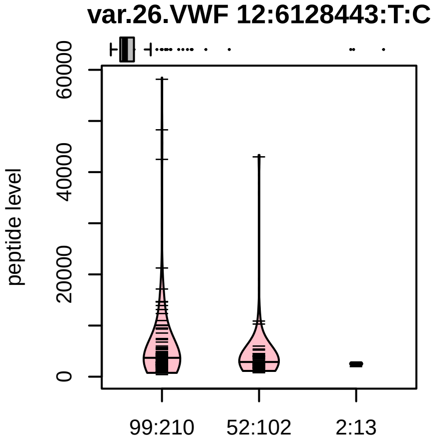

IGWPNAPILIQDFETLPRpc3_NP.4
p−maj=0.023, pX=0.099, N=153

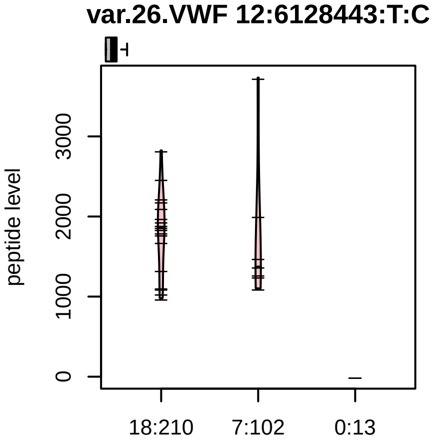

IGWPNAPILIQDFETLPRpc3_NP.5
p−min=0.52, pX=0.31, N=25

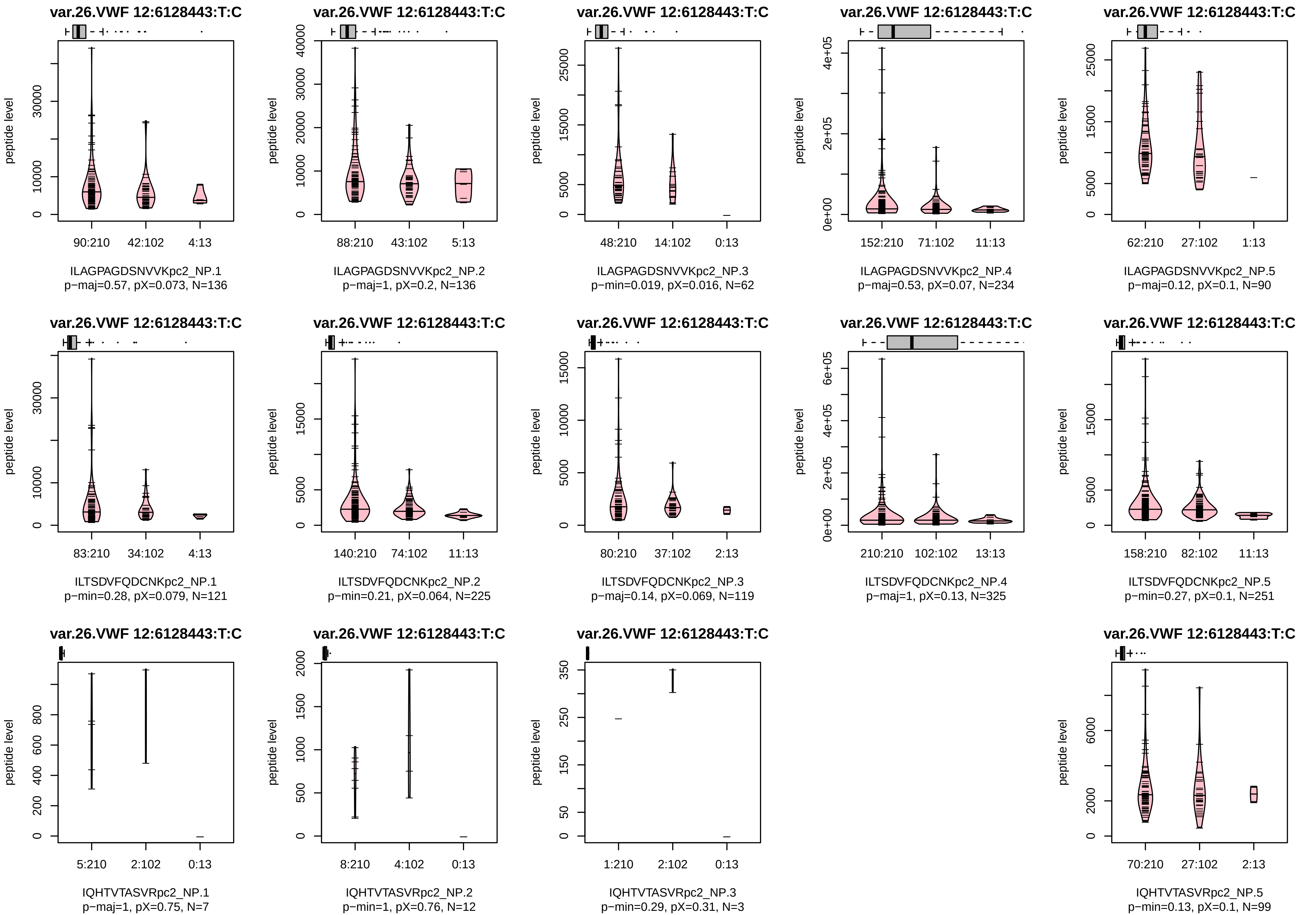
var.26.VWF 12:6128443:T:C
peptide level
90:210
42:102
4:13
ILAGPAGDSNVVKpc2_NP.1
p−maj=0.57, pX=0.073, N=136
var.26.VWF 12:6128443:T:C
peptide level
88:210
43:102
5:13
ILAGPAGDSNVVKpc2_NP.2
p−maj=1, pX=0.2, N=136
var.26.VWF 12:6128443:T:C
peptide level
48:210
14:102
0:13
ILAGPAGDSNVVKpc2_NP.3
p−min=0.019, pX=0.016, N=62
var.26.VWF 12:6128443:T:C
peptide level
152:210
71:102
11:13
ILAGPAGDSNVVKpc2_NP.4
p−maj=0.53, pX=0.07, N=234
var.26.VWF 12:6128443:T:C
peptide level
62:210
27:102
1:13
ILAGPAGDSNVVKpc2_NP.5
p−maj=0.12, pX=0.1, N=90
var.26.VWF 12:6128443:T:C
peptide level
83:210
34:102
4:13
ILTSDVFQDCNKpc2_NP.1
p−min=0.28, pX=0.079, N=121
var.26.VWF 12:6128443:T:C
peptide level
140:210
74:102
11:13
ILTSDVFQDCNKpc2_NP.2
p−min=0.21, pX=0.064, N=225
var.26.VWF 12:6128443:T:C
peptide level
80:210
37:102
2:13
ILTSDVFQDCNKpc2_NP.3
p−maj=0.14, pX=0.069, N=119
var.26.VWF 12:6128443:T:C
peptide level
210:210
102:102
13:13
ILTSDVFQDCNKpc2_NP.4
p−maj=1, pX=0.13, N=325
var.26.VWF 12:6128443:T:C
peptide level
158:210
82:102
11:13
ILTSDVFQDCNKpc2_NP.5
p−min=0.27, pX=0.1, N=251
var.26.VWF 12:6128443:T:C
peptide level
5:210
2:102
0:13
IQHTVTASVRpc2_NP.1
p−maj=1, pX=0.75, N=7
var.26.VWF 12:6128443:T:C
peptide level
8:210
4:102
0:13
IQHTVTASVRpc2_NP.2
p−min=1, pX=0.76, N=12
var.26.VWF 12:6128443:T:C
peptide level
1:210
2:102
0:13
IQHTVTASVRpc2_NP.3
p−min=0.29, pX=0.31, N=3
var.26.VWF 12:6128443:T:C
peptide level
70:210
27:102
2:13
IQHTVTASVRpc2_NP.5
p−min=0.13, pX=0.1, N=99

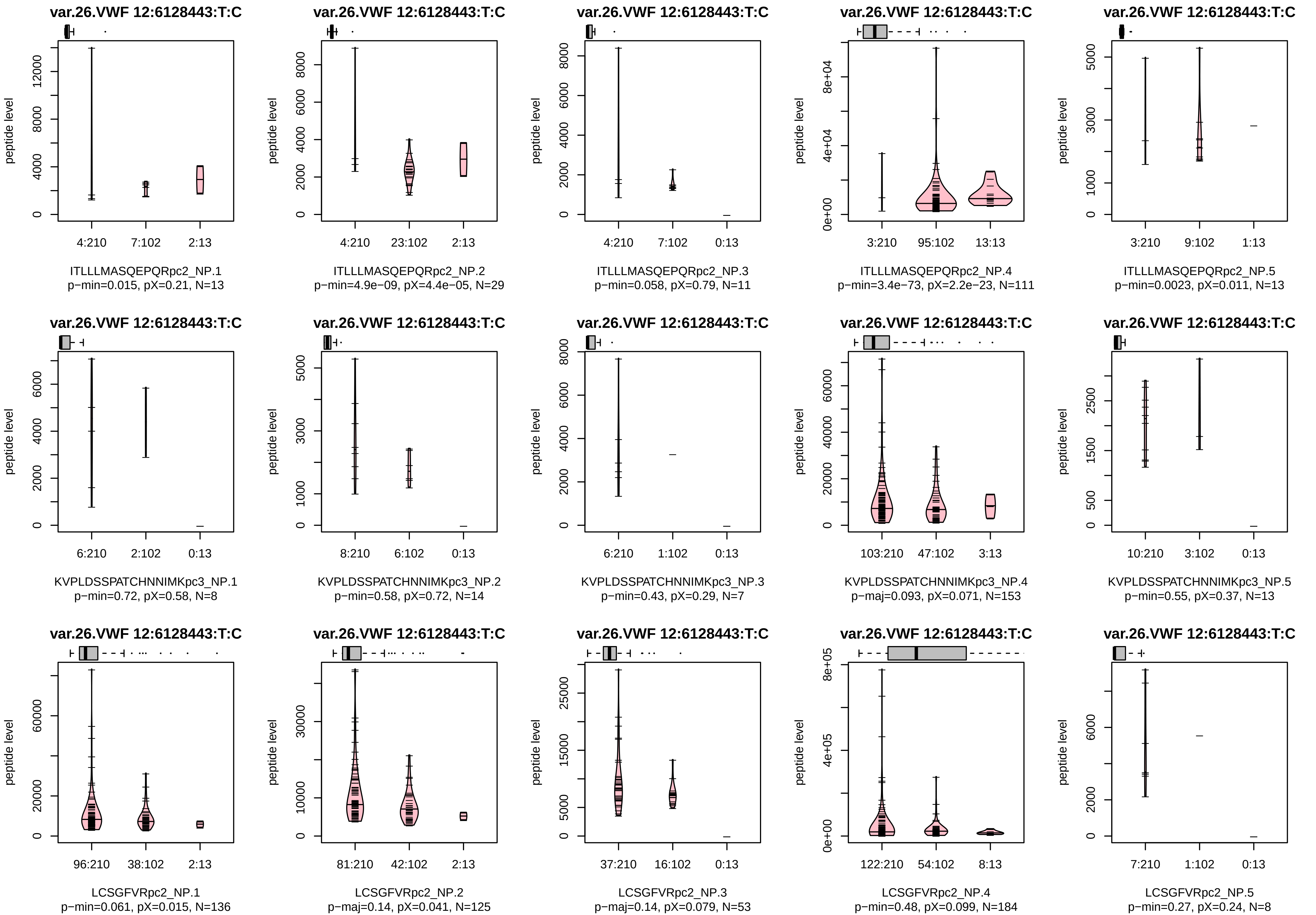
var.26.VWF 12:6128443:T:C
peptide level
4:210
7:102
2:13
ITLLLMASQEPQRpc2_NP.1
p−min=0.015, pX=0.21, N=13
var.26.VWF 12:6128443:T:C
peptide level
4:210
23:102
2:13
ITLLLMASQEPQRpc2_NP.2
p−min=4.9e−09, pX=4.4e−05, N=29
var.26.VWF 12:6128443:T:C
peptide level
4:210
7:102
0:13
ITLLLMASQEPQRpc2_NP.3
p−min=0.058, pX=0.79, N=11
var.26.VWF 12:6128443:T:C
peptide level
3:210
95:102
13:13
ITLLLMASQEPQRpc2_NP.4
p−min=3.4e−73, pX=2.2e−23, N=111
var.26.VWF 12:6128443:T:C
peptide level
3:210
9:102
1:13
ITLLLMASQEPQRpc2_NP.5
p−min=0.0023, pX=0.011, N=13
var.26.VWF 12:6128443:T:C
peptide level
6:210
2:102
0:13
KVPLDSSPATCHNNIMKpc3_NP.1
p−min=0.72, pX=0.58, N=8
var.26.VWF 12:6128443:T:C
peptide level
8:210
6:102
0:13
KVPLDSSPATCHNNIMKpc3_NP.2
p−min=0.58, pX=0.72, N=14
var.26.VWF 12:6128443:T:C
peptide level
6:210
1:102
0:13
KVPLDSSPATCHNNIMKpc3_NP.3
p−min=0.43, pX=0.29, N=7
var.26.VWF 12:6128443:T:C
peptide level
103:210
47:102
3:13
KVPLDSSPATCHNNIMKpc3_NP.4
p−maj=0.093, pX=0.071, N=153
var.26.VWF 12:6128443:T:C
peptide level
10:210
3:102
0:13
KVPLDSSPATCHNNIMKpc3_NP.5
p−min=0.55, pX=0.37, N=13
var.26.VWF 12:6128443:T:C
peptide level
96:210
38:102
2:13
LCSGFVRpc2_NP.1
p−min=0.061, pX=0.015, N=136
var.26.VWF 12:6128443:T:C
peptide level
81:210
42:102
2:13
LCSGFVRpc2_NP.2
p−maj=0.14, pX=0.041, N=125
var.26.VWF 12:6128443:T:C
peptide level
37:210
16:102
0:13
LCSGFVRpc2_NP.3
p−maj=0.14, pX=0.079, N=53
var.26.VWF 12:6128443:T:C
peptide level
122:210
54:102
8:13
LCSGFVRpc2_NP.4
p−min=0.48, pX=0.099, N=184
var.26.VWF 12:6128443:T:C
peptide level
7:210
1:102
0:13
LCSGFVRpc2_NP.5
p−min=0.27, pX=0.24, N=8

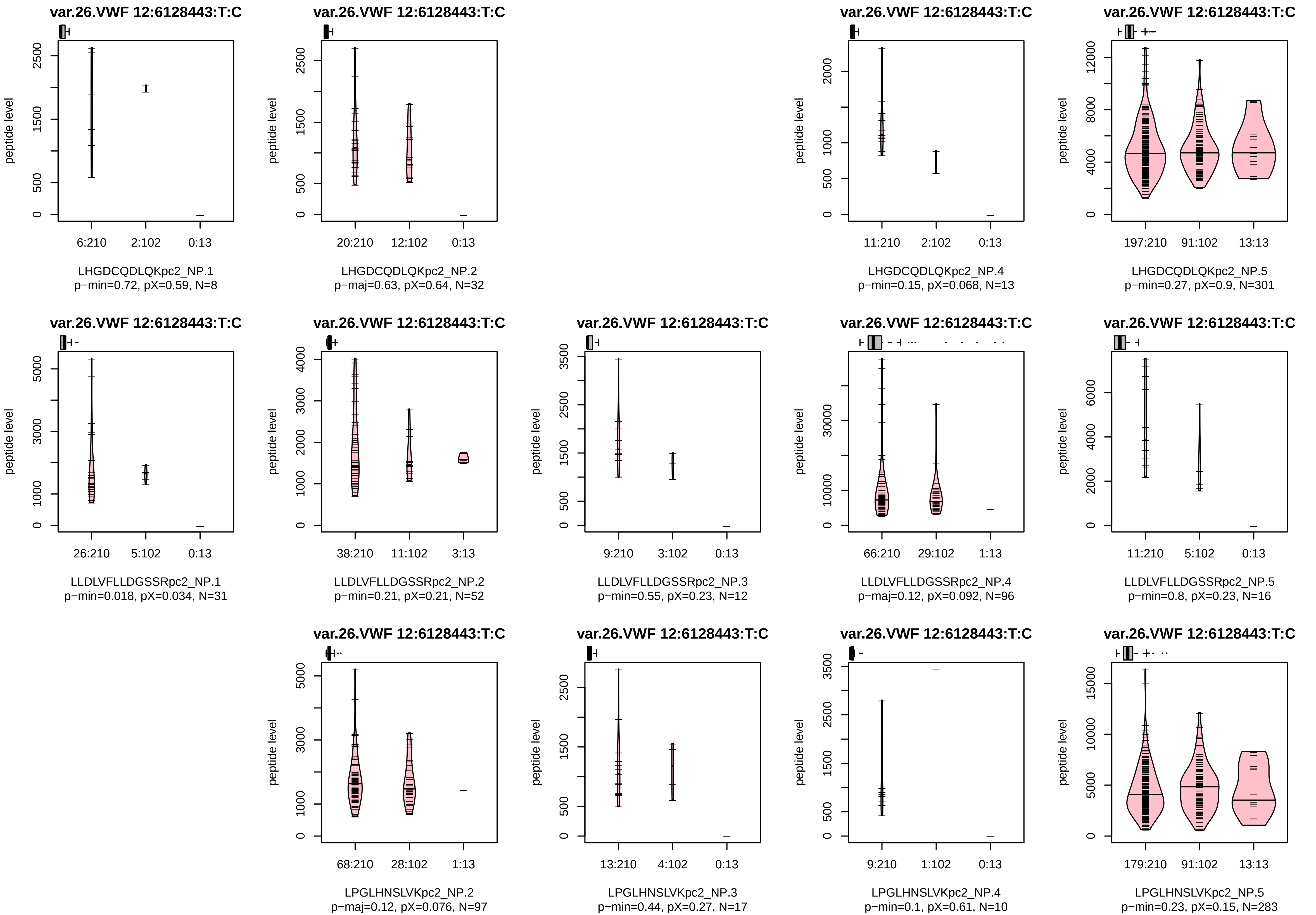
var.26.VWF 12:6128443:T:C
peptide level
6:210
2:102
0:13
LHGDCQDLQKpc2_NP.1
p−min=0.72, pX=0.59, N=8
var.26.VWF 12:6128443:T:C
peptide level
20:210
12:102
0:13
LHGDCQDLQKpc2_NP.2
p−maj=0.63, pX=0.64, N=32
var.26.VWF 12:6128443:T:C
peptide level
11:210
2:102
0:13
LHGDCQDLQKpc2_NP.4
p−min=0.15, pX=0.068, N=13
var.26.VWF 12:6128443:T:C
peptide level
197:210
91:102
13:13
LHGDCQDLQKpc2_NP.5
p−min=0.27, pX=0.9, N=301
var.26.VWF 12:6128443:T:C
peptide level
26:210
5:102
0:13
LLDLVFLLDGSSRpc2_NP.1
p−min=0.018, pX=0.034, N=31
var.26.VWF 12:6128443:T:C
peptide level
38:210
11:102
3:13
LLDLVFLLDGSSRpc2_NP.2
p−min=0.21, pX=0.21, N=52
var.26.VWF 12:6128443:T:C
peptide level
9:210
3:102
0:13
LLDLVFLLDGSSRpc2_NP.3
p−min=0.55, pX=0.23, N=12
var.26.VWF 12:6128443:T:C
peptide level
66:210
29:102
1:13
LLDLVFLLDGSSRpc2_NP.4
p−maj=0.12, pX=0.092, N=96
var.26.VWF 12:6128443:T:C
peptide level
11:210
5:102
0:13
LLDLVFLLDGSSRpc2_NP.5
p−min=0.8, pX=0.23, N=16
var.26.VWF 12:6128443:T:C
peptide level
68:210
28:102
1:13
LPGLHNSLVKpc2_NP.2
p−maj=0.12, pX=0.076, N=97
var.26.VWF 12:6128443:T:C
peptide level
13:210
4:102
0:13
LPGLHNSLVKpc2_NP.3
p−min=0.44, pX=0.27, N=17
var.26.VWF 12:6128443:T:C
peptide level
9:210
1:102
0:13
LPGLHNSLVKpc2_NP.4
p−min=0.1, pX=0.61, N=10
var.26.VWF 12:6128443:T:C
peptide level
179:210
91:102
13:13
LPGLHNSLVKpc2_NP.5
p−min=0.23, pX=0.15, N=283

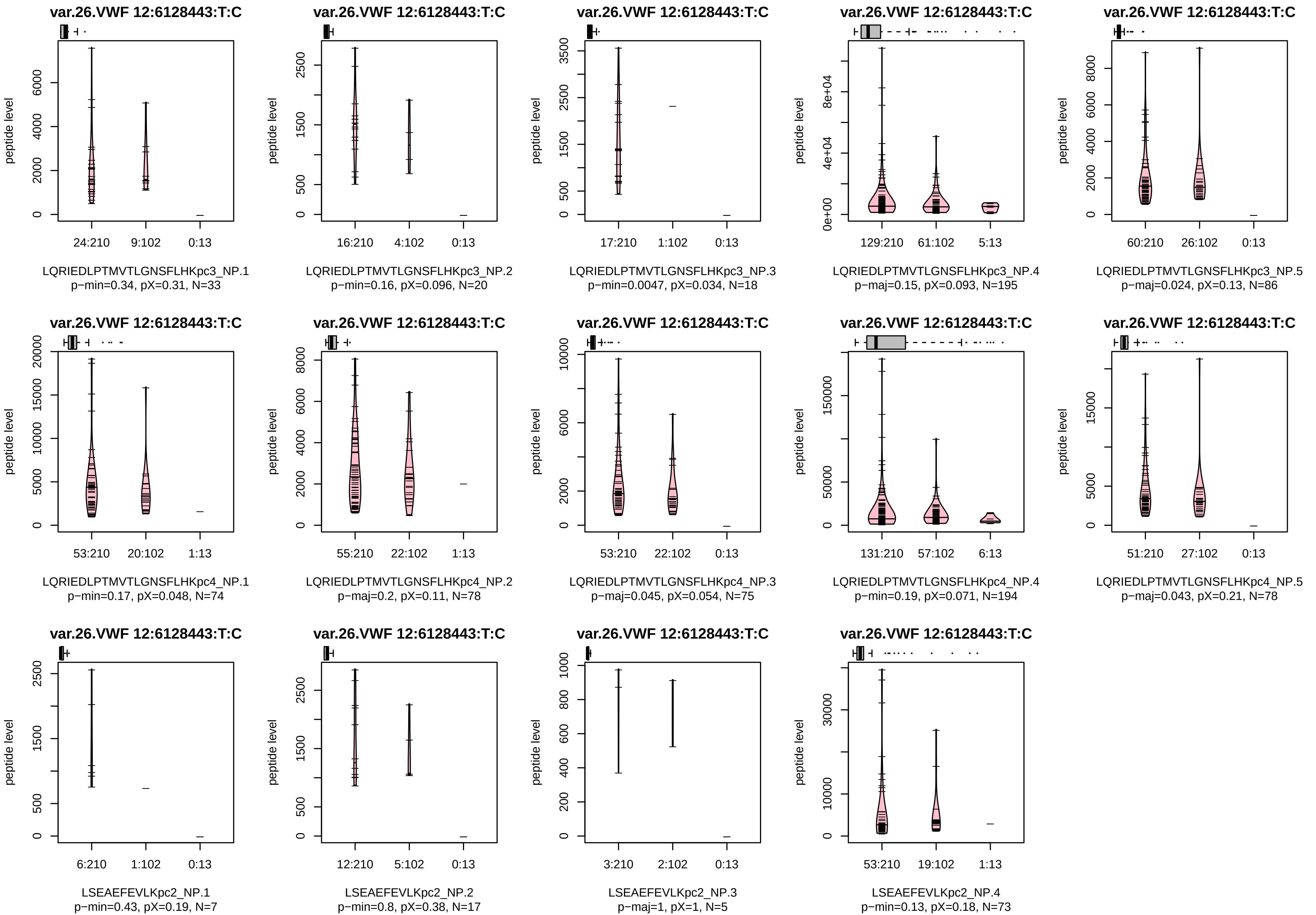

var.26.VWF 12:6128443:T:C
peptide level
24:210
9:102
0:13
LQRIEDLPTMVTLGNSFLHKpc3_NP.1
p−min=0.34, pX=0.31, N=33
var.26.VWF 12:6128443:T:C
peptide level
16:210
4:102
0:13
LQRIEDLPTMVTLGNSFLHKpc3_NP.2
p−min=0.16, pX=0.096, N=20
var.26.VWF 12:6128443:T:C
peptide level
17:210
1:102
0:13
LQRIEDLPTMVTLGNSFLHKpc3_NP.3
p−min=0.0047, pX=0.034, N=18
var.26.VWF 12:6128443:T:C
peptide level
129:210
61:102
5:13
LQRIEDLPTMVTLGNSFLHKpc3_NP.4
p−maj=0.15, pX=0.093, N=195
var.26.VWF 12:6128443:T:C
peptide level
60:210
26:102
0:13
LQRIEDLPTMVTLGNSFLHKpc3_NP.5
p−maj=0.024, pX=0.13, N=86
var.26.VWF 12:6128443:T:C
peptide level
53:210
20:102
1:13
LQRIEDLPTMVTLGNSFLHKpc4_NP.1
p−min=0.17, pX=0.048, N=74
var.26.VWF 12:6128443:T:C
peptide level
55:210
22:102
1:13
LQRIEDLPTMVTLGNSFLHKpc4_NP.2
p−maj=0.2, pX=0.11, N=78
var.26.VWF 12:6128443:T:C
peptide level
53:210
22:102
0:13
LQRIEDLPTMVTLGNSFLHKpc4_NP.3
p−maj=0.045, pX=0.054, N=75
var.26.VWF 12:6128443:T:C
peptide level
131:210
57:102
6:13
LQRIEDLPTMVTLGNSFLHKpc4_NP.4
p−min=0.19, pX=0.071, N=194
var.26.VWF 12:6128443:T:C
peptide level
51:210
27:102
0:13
LQRIEDLPTMVTLGNSFLHKpc4_NP.5
p−maj=0.043, pX=0.21, N=78
var.26.VWF 12:6128443:T:C
peptide level
6:210
1:102
0:13
LSEAEFEVLKpc2_NP.1
p−min=0.43, pX=0.19, N=7
var.26.VWF 12:6128443:T:C
peptide level
12:210
5:102
0:13
LSEAEFEVLKpc2_NP.2
p−min=0.8, pX=0.38, N=17
var.26.VWF 12:6128443:T:C
peptide level
3:210
2:102
0:13
LSEAEFEVLKpc2_NP.3
p−maj=1, pX=1, N=5
var.26.VWF 12:6128443:T:C
peptide level
53:210
19:102
1:13
LSEAEFEVLKpc2_NP.4
p−min=0.13, pX=0.18, N=73

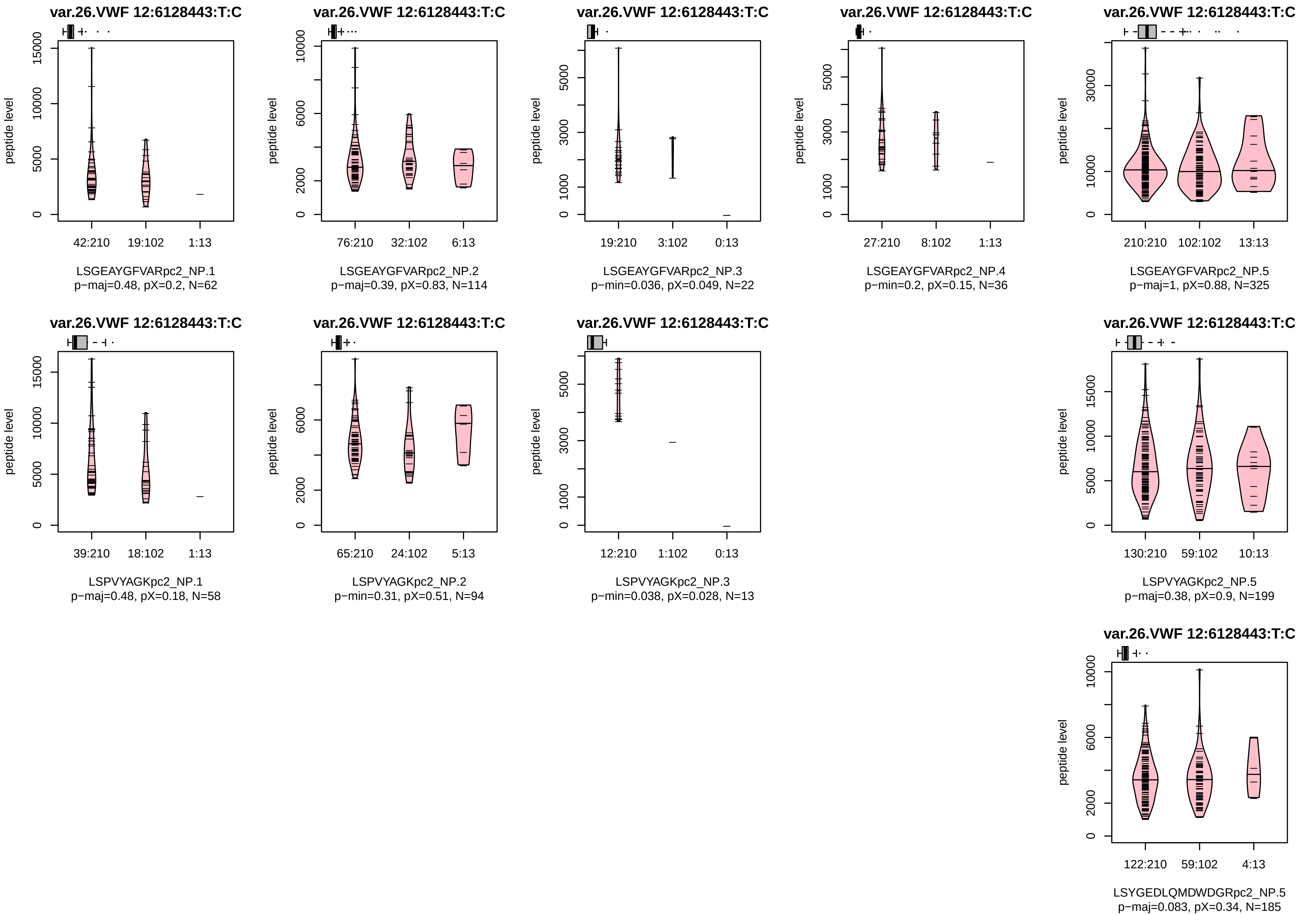
var.26.VWF 12:6128443:T:C
peptide level
42:210
19:102
1:13
LSGEAYGFVARpc2_NP.1
p−maj=0.48, pX=0.2, N=62
var.26.VWF 12:6128443:T:C
peptide level
76:210
32:102
6:13
LSGEAYGFVARpc2_NP.2
p−maj=0.39, pX=0.83, N=114
var.26.VWF 12:6128443:T:C
peptide level
19:210
3:102
0:13
LSGEAYGFVARpc2_NP.3
p−min=0.036, pX=0.049, N=22
var.26.VWF 12:6128443:T:C
peptide level
27:210
8:102
1:13
LSGEAYGFVARpc2_NP.4
p−min=0.2, pX=0.15, N=36
var.26.VWF 12:6128443:T:C
peptide level
210:210
102:102
13:13
LSGEAYGFVARpc2_NP.5
p−maj=1, pX=0.88, N=325
var.26.VWF 12:6128443:T:C
peptide level
39:210
18:102
1:13
LSPVYAGKpc2_NP.1
p−maj=0.48, pX=0.18, N=58
var.26.VWF 12:6128443:T:C
peptide level
65:210
24:102
5:13
LSPVYAGKpc2_NP.2
p−min=0.31, pX=0.51, N=94
var.26.VWF 12:6128443:T:C
peptide level
12:210
1:102
0:13
LSPVYAGKpc2_NP.3
p−min=0.038, pX=0.028, N=13
var.26.VWF 12:6128443:T:C
peptide level
130:210
59:102
10:13
LSPVYAGKpc2_NP.5
p−maj=0.38, pX=0.9, N=199
var.26.VWF 12:6128443:T:C
peptide level
122:210
59:102
4:13
LSYGEDLQMDWDGRpc2_NP.5
p−maj=0.083, pX=0.34, N=185

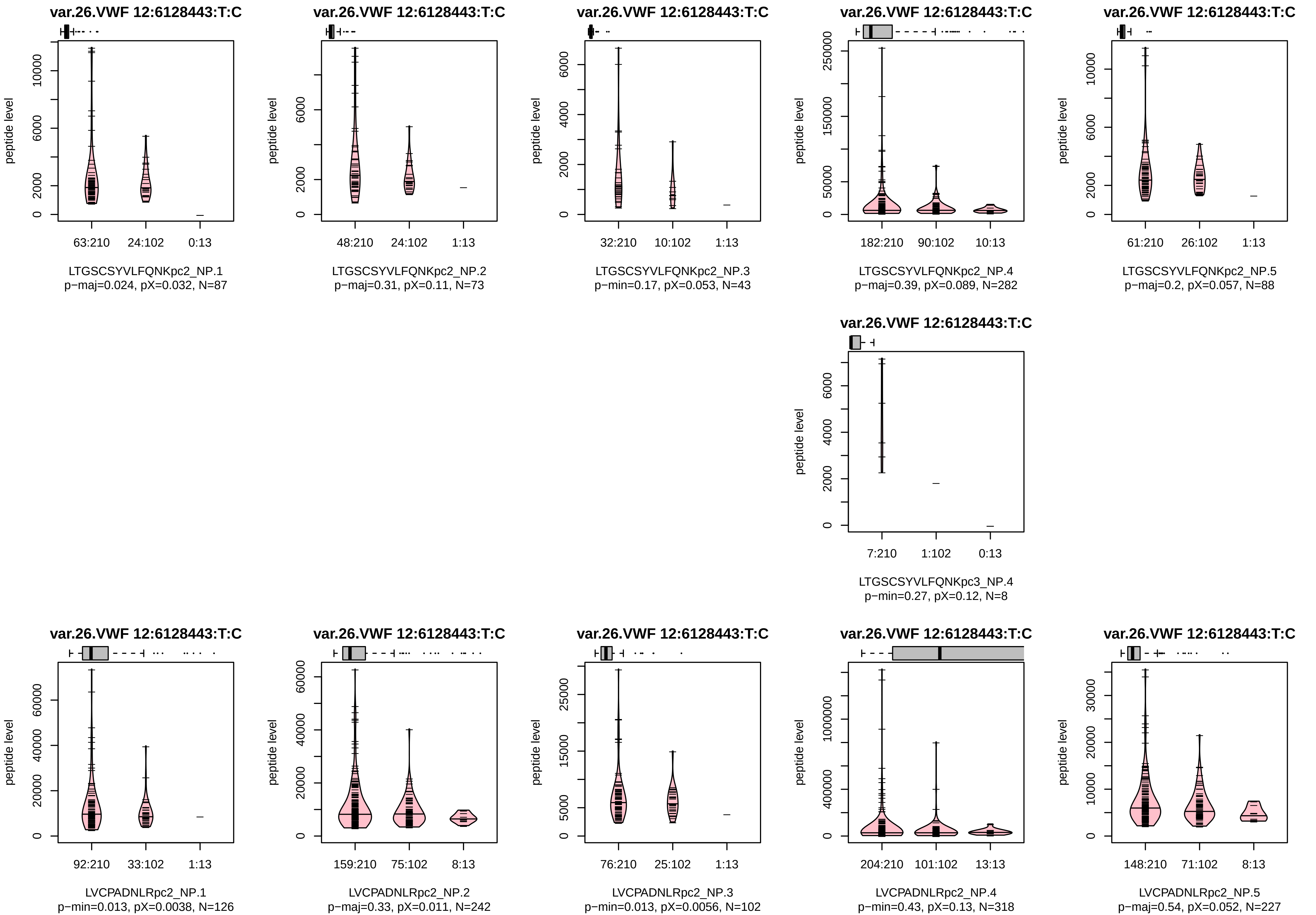
var.26.VWF 12:6128443:T:C
peptide level
63:210
24:102
0:13
LTGSCSYVLFQNKpc2_NP.1
p−maj=0.024, pX=0.032, N=87
var.26.VWF 12:6128443:T:C
peptide level
48:210
24:102
1:13
LTGSCSYVLFQNKpc2_NP.2
p−maj=0.31, pX=0.11, N=73
var.26.VWF 12:6128443:T:C
peptide level
32:210
10:102
1:13
LTGSCSYVLFQNKpc2_NP.3
p−min=0.17, pX=0.053, N=43
var.26.VWF 12:6128443:T:C
peptide level
182:210
90:102
10:13
LTGSCSYVLFQNKpc2_NP.4
p−maj=0.39, pX=0.089, N=282
var.26.VWF 12:6128443:T:C
peptide level
61:210
26:102
1:13
LTGSCSYVLFQNKpc2_NP.5
p−maj=0.2, pX=0.057, N=88
var.26.VWF 12:6128443:T:C
peptide level
7:210
1:102
0:13
LTGSCSYVLFQNKpc3_NP.4
p−min=0.27, pX=0.12, N=8
var.26.VWF 12:6128443:T:C
peptide level
92:210
33:102
1:13
LVCPADNLRpc2_NP.1
p−min=0.013, pX=0.0038, N=126
var.26.VWF 12:6128443:T:C
peptide level
159:210
75:102
8:13
LVCPADNLRpc2_NP.2
p−maj=0.33, pX=0.011, N=242
var.26.VWF 12:6128443:T:C
peptide level
76:210
25:102
1:13
LVCPADNLRpc2_NP.3
p−min=0.013, pX=0.0056, N=102
var.26.VWF 12:6128443:T:C
peptide level
204:210
101:102
13:13
LVCPADNLRpc2_NP.4
p−min=0.43, pX=0.13, N=318
var.26.VWF 12:6128443:T:C
peptide level
148:210
71:102
8:13
LVCPADNLRpc2_NP.5
p−maj=0.54, pX=0.052, N=227

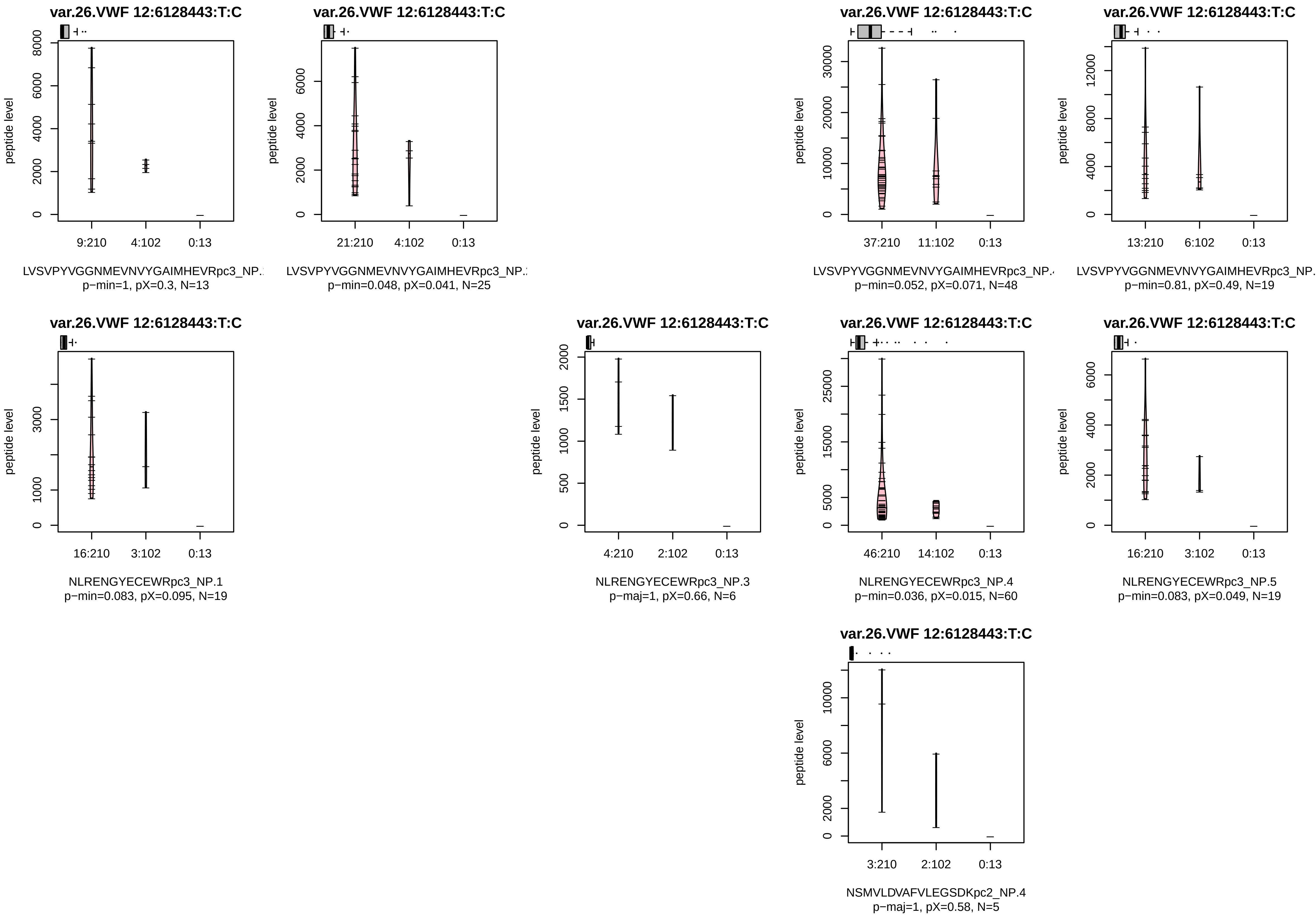
var.26.VWF 12:6128443:T:C
peptide level
9:210
4:102
0:13
p−min=1, pX=0.3, N=13
var.26.VWF 12:6128443:T:C
peptide level
21:210
4:102
0:13
LVSVPYVGGNMEVNVYGAIMHEVRpc3_NP.
p−min=0.048, pX=0.041, N=25
var.26.VWF 12:6128443:T:C
peptide level
37:210
11:102
0:13
LVSVPYVGGNMEVNVYGAIMHEVRpc3_NP.
p−min=0.052, pX=0.071, N=48
var.26.VWF 12:6128443:T:C
peptide level
13:210
6:102
0:13
LVSVPYVGGNMEVNVYGAIMHEVRpc3_NP.
p−min=0.81, pX=0.49, N=19
var.26.VWF 12:6128443:T:C
peptide level
16:210
3:102
0:13
NLRENGYECEWRpc3_NP.1
p−min=0.083, pX=0.095, N=19
var.26.VWF 12:6128443:T:C
peptide level
4:210
2:102
0:13
NLRENGYECEWRpc3_NP.3
p−maj=1, pX=0.66, N=6
var.26.VWF 12:6128443:T:C
peptide level
46:210
14:102
0:13
NLRENGYECEWRpc3_NP.4
p−min=0.036, pX=0.015, N=60
var.26.VWF 12:6128443:T:C
peptide level
16:210
3:102
0:13
NLRENGYECEWRpc3_NP.5
p−min=0.083, pX=0.049, N=19
var.26.VWF 12:6128443:T:C
peptide level
3:210
2:102
0:13
NSMVLDVAFVLEGSDKpc2_NP.4
p−maj=1, pX=0.58, N=5

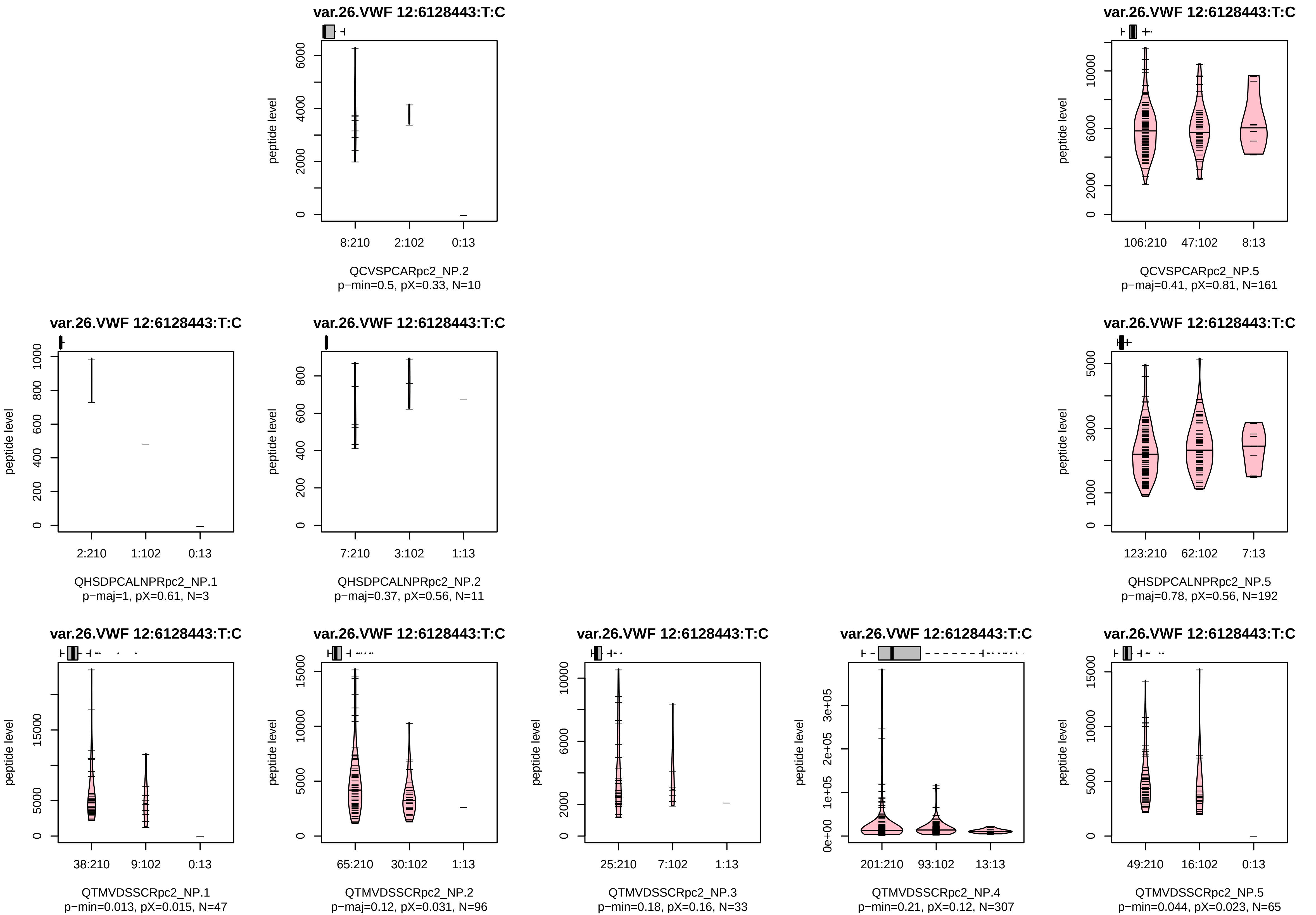

var.26.VWF 12:6128443:T:C
peptide level
8:210
2:102
0:13
QCVSPCARpc2_NP.2
p−min=0.5, pX=0.33, N=10
var.26.VWF 12:6128443:T:C
peptide level
106:210
47:102
8:13
QCVSPCARpc2_NP.5
p−maj=0.41, pX=0.81, N=161
var.26.VWF 12:6128443:T:C
peptide level
2:210
1:102
0:13
QHSDPCALNPRpc2_NP.1
p−maj=1, pX=0.61, N=3
var.26.VWF 12:6128443:T:C
peptide level
7:210
3:102
1:13
QHSDPCALNPRpc2_NP.2
p−maj=0.37, pX=0.56, N=11
var.26.VWF 12:6128443:T:C
peptide level
123:210
62:102
7:13
QHSDPCALNPRpc2_NP.5
p−maj=0.78, pX=0.56, N=192
var.26.VWF 12:6128443:T:C
peptide level
38:210
9:102
0:13
QTMVDSSCRpc2_NP.1
p−min=0.013, pX=0.015, N=47
var.26.VWF 12:6128443:T:C
peptide level
65:210
30:102
1:13
QTMVDSSCRpc2_NP.2
p−maj=0.12, pX=0.031, N=96
var.26.VWF 12:6128443:T:C
peptide level
25:210
7:102
1:13
QTMVDSSCRpc2_NP.3
p−min=0.18, pX=0.16, N=33
var.26.VWF 12:6128443:T:C
peptide level
201:210
93:102
13:13
QTMVDSSCRpc2_NP.4
p−min=0.21, pX=0.12, N=307
var.26.VWF 12:6128443:T:C
peptide level
49:210
16:102
0:13
QTMVDSSCRpc2_NP.5
p−min=0.044, pX=0.023, N=65

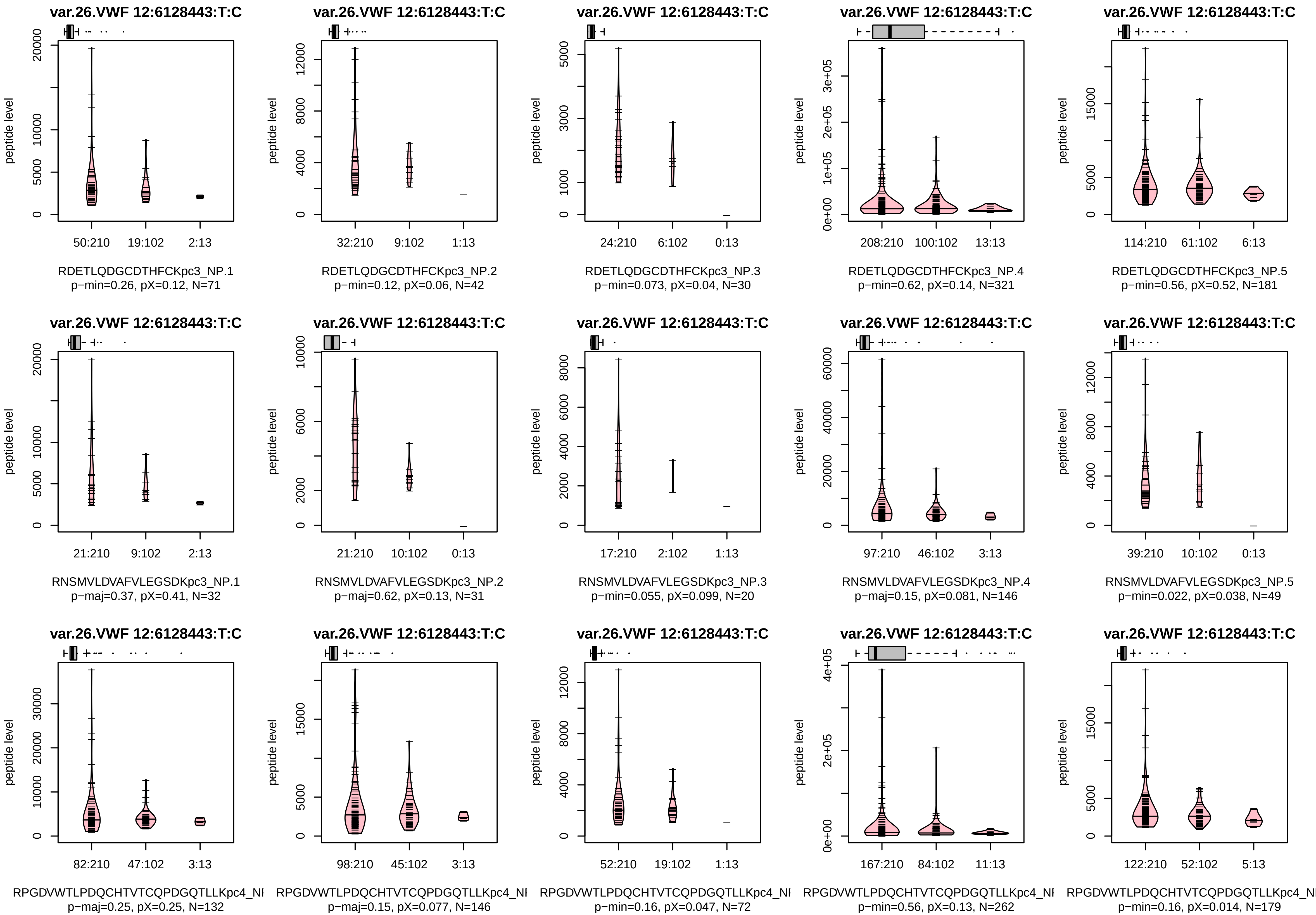

var.26.VWF 12:6128443:T:C
peptide level
50:210
19:102
2:13
RDETLQDGCDTHFCKpc3_NP.1
p−min=0.26, pX=0.12, N=71
var.26.VWF 12:6128443:T:C
peptide level
32:210
9:102
1:13
RDETLQDGCDTHFCKpc3_NP.2
p−min=0.12, pX=0.06, N=42
var.26.VWF 12:6128443:T:C
peptide level
24:210
6:102
0:13
RDETLQDGCDTHFCKpc3_NP.3
p−min=0.073, pX=0.04, N=30
var.26.VWF 12:6128443:T:C
peptide level
208:210
100:102
13:13
RDETLQDGCDTHFCKpc3_NP.4
p−min=0.62, pX=0.14, N=321
var.26.VWF 12:6128443:T:C
peptide level
114:210
61:102
6:13
RDETLQDGCDTHFCKpc3_NP.5
p−min=0.56, pX=0.52, N=181
var.26.VWF 12:6128443:T:C
peptide level
21:210
9:102
2:13
RNSMVLDVAFVLEGSDKpc3_NP.1
p−maj=0.37, pX=0.41, N=32
var.26.VWF 12:6128443:T:C
peptide level
21:210
10:102
0:13
RNSMVLDVAFVLEGSDKpc3_NP.2
p−maj=0.62, pX=0.13, N=31
var.26.VWF 12:6128443:T:C
peptide level
17:210
2:102
1:13
RNSMVLDVAFVLEGSDKpc3_NP.3
p−min=0.055, pX=0.099, N=20
var.26.VWF 12:6128443:T:C
peptide level
97:210
46:102
3:13
RNSMVLDVAFVLEGSDKpc3_NP.4
p−maj=0.15, pX=0.081, N=146
var.26.VWF 12:6128443:T:C
peptide level
39:210
10:102
0:13
RNSMVLDVAFVLEGSDKpc3_NP.5
p−min=0.022, pX=0.038, N=49
var.26.VWF 12:6128443:T:C
peptide level
82:210
47:102
3:13
RPGDVWTLPDQCHTVTCQPDGQTLLKpc4_NI
p−maj=0.25, pX=0.25, N=132
var.26.VWF 12:6128443:T:C
peptide level
98:210
45:102
3:13
RPGDVWTLPDQCHTVTCQPDGQTLLKpc4_NI
p−maj=0.15, pX=0.077, N=146
var.26.VWF 12:6128443:T:C
peptide level
52:210
19:102
1:13
RPGDVWTLPDQCHTVTCQPDGQTLLKpc4_NI
p−min=0.16, pX=0.047, N=72
var.26.VWF 12:6128443:T:C
peptide level
167:210
84:102
11:13
RPGDVWTLPDQCHTVTCQPDGQTLLKpc4_NI
p−min=0.56, pX=0.13, N=262
var.26.VWF 12:6128443:T:C
peptide level
122:210
52:102
5:13
RPGDVWTLPDQCHTVTCQPDGQTLLKpc4_NI
p−min=0.16, pX=0.014, N=179

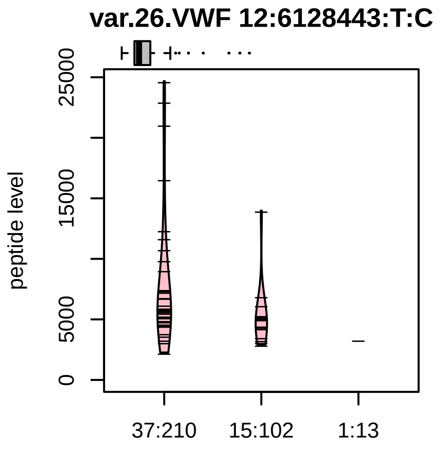

RVTGCPPFDEHKpc3_NP.1
p−min=0.43, pX=0.069, N=53

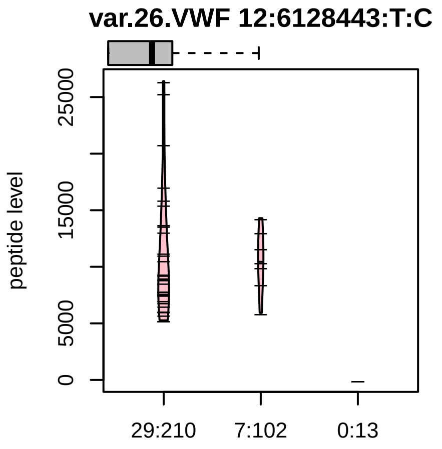

RVTGCPPFDEHKpc3_NP.2
p−min=0.041, pX=0.038, N=36

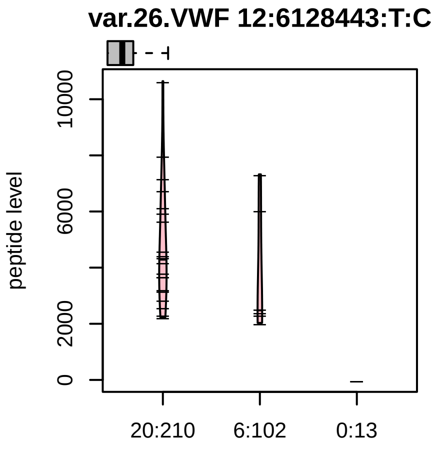

RVTGCPPFDEHKpc3_NP.3
p−min=0.2, pX=0.088, N=26

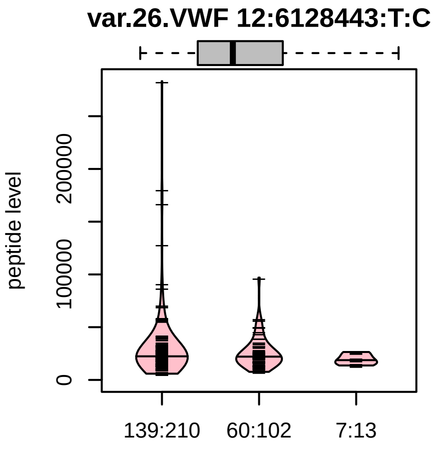

RVTGCPPFDEHKpc3_NP.4
p−min=0.19, pX=0.055, N=206

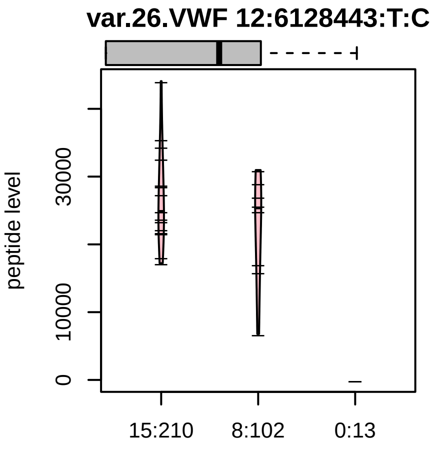

RVTGCPPFDEHKpc3_NP.5
p−maj=0.61, pX=0.46, N=23

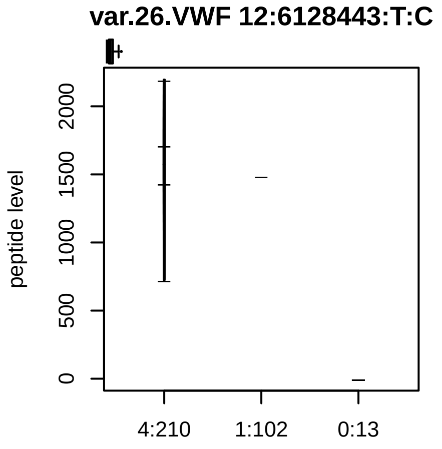

SEVEVDIHYCQGKpc2_NP.1
p−min=0.66, pX=0.46, N=5

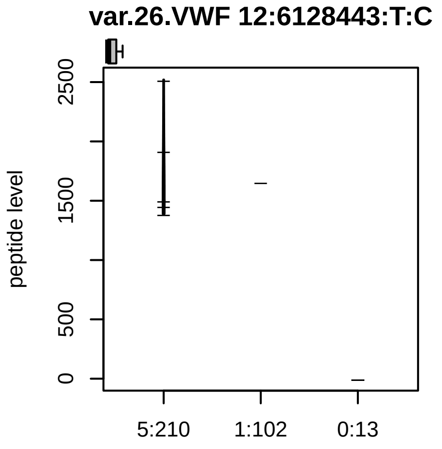

SEVEVDIHYCQGKpc2_NP.2
p−min=0.43, pX=0.32, N=6

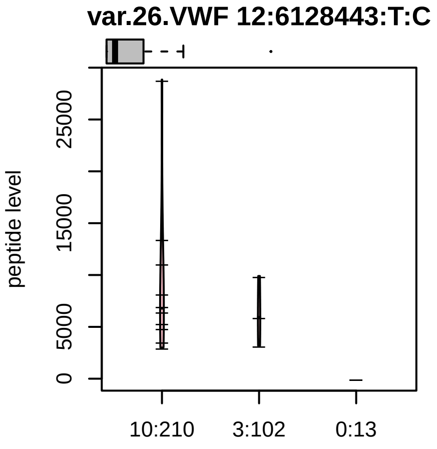

SEVEVDIHYCQGKpc2_NP.4
p−min=0.55, pX=0.26, N=13

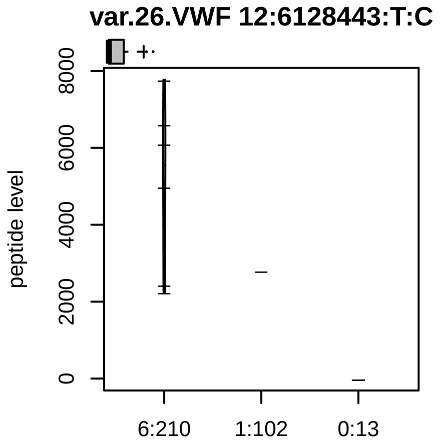

SEVEVDIHYCQGKpc3_NP.1
p−min=0.43, pX=0.18, N=7

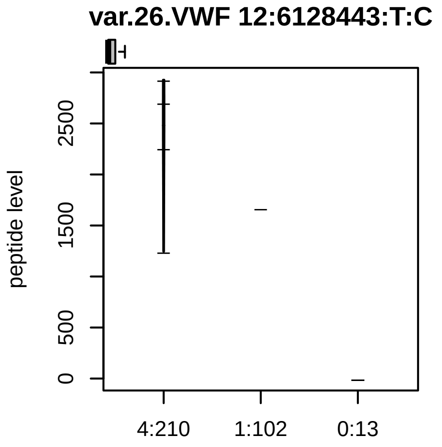

SEVEVDIHYCQGKpc3_NP.2
p−min=0.66, pX=0.36, N=5

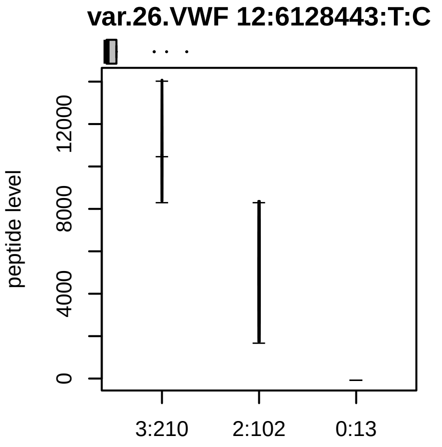

SEVEVDIHYCQGKpc3_NP.4
p−maj=1, pX=0.57, N=5

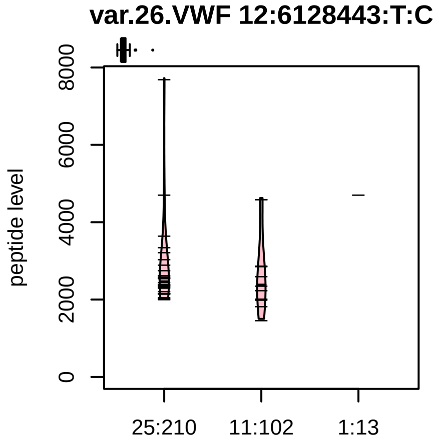

SFSIIGDFQNGKpc2_NP.1
p−min=0.86, pX=0.81, N=37

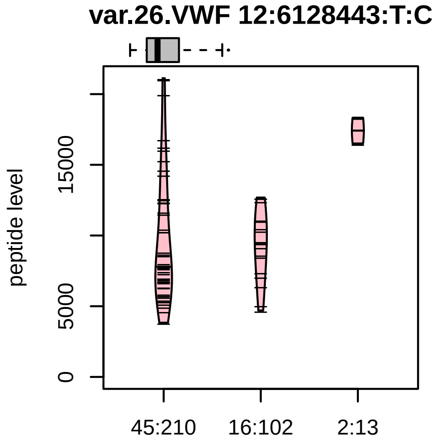

SFSIIGDFQNGKpc2_NP.2
p−min=0.24, pX=0.53, N=63

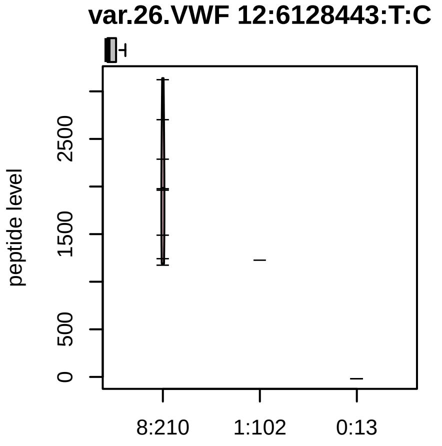

SFSIIGDFQNGKpc2_NP.3
p−min=0.17, pX=0.1, N=9

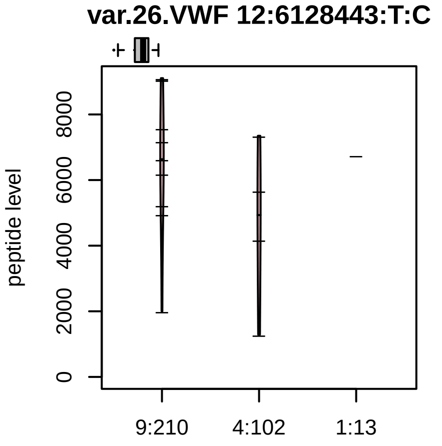

SFSIIGDFQNGKpc2_NP.4
p−maj=0.44, pX=0.95, N=14

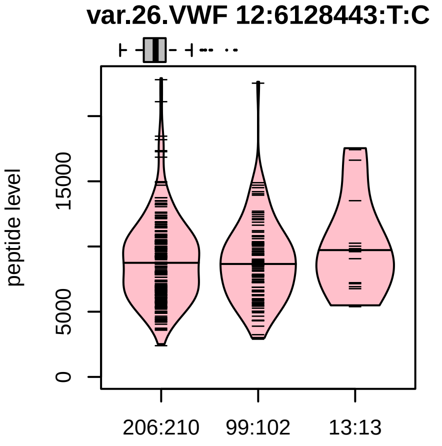

SFSIIGDFQNGKpc2_NP.5
p−min=0.7, pX=0.71, N=318

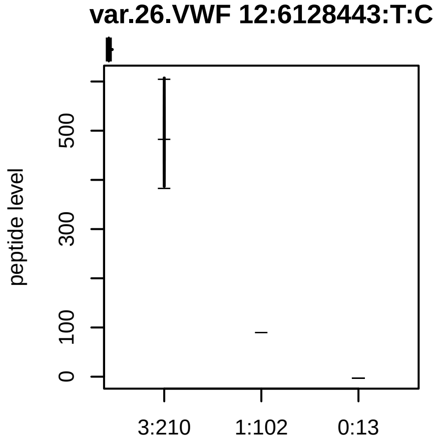

SGFTYVLHEGECCGRpc2_NP.1
p−min=1, pX=0.29, N=4

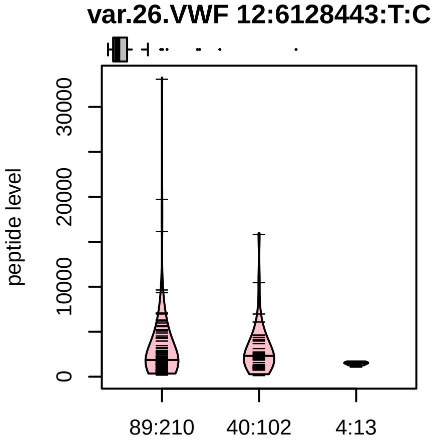

SGFTYVLHEGECCGRpc2_NP.4
p−min=0.48, pX=0.31, N=133

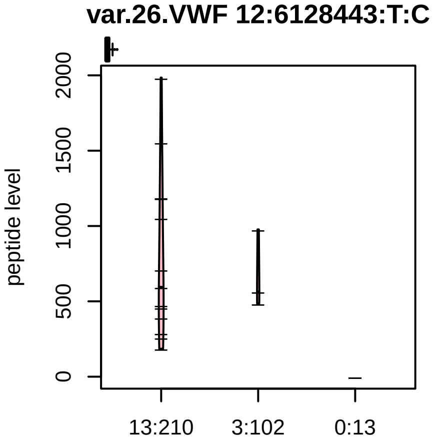

SGFTYVLHEGECCGRpc2_NP.5
p−min=0.19, pX=0.16, N=16

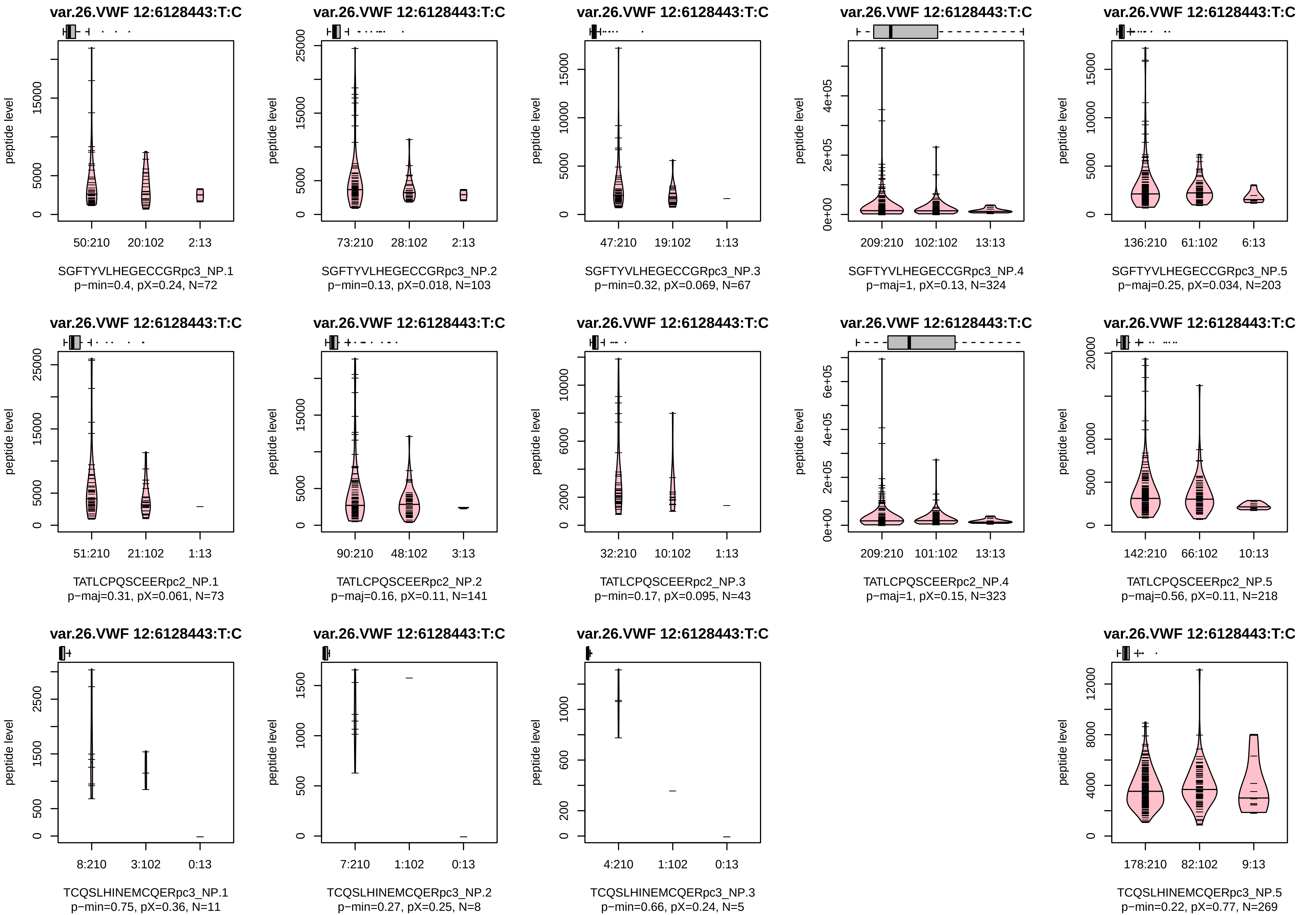

var.26.VWF 12:6128443:T:C
peptide level
50:210
20:102
2:13
SGFTYVLHEGECCGRpc3_NP.1
p−min=0.4, pX=0.24, N=72
var.26.VWF 12:6128443:T:C
peptide level
73:210
28:102
2:13
SGFTYVLHEGECCGRpc3_NP.2
p−min=0.13, pX=0.018, N=103
var.26.VWF 12:6128443:T:C
peptide level
47:210
19:102
1:13
SGFTYVLHEGECCGRpc3_NP.3
p−min=0.32, pX=0.069, N=67
var.26.VWF 12:6128443:T:C
peptide level
209:210
102:102
13:13
SGFTYVLHEGECCGRpc3_NP.4
p−maj=1, pX=0.13, N=324
var.26.VWF 12:6128443:T:C
peptide level
136:210
61:102
6:13
SGFTYVLHEGECCGRpc3_NP.5
p−maj=0.25, pX=0.034, N=203
var.26.VWF 12:6128443:T:C
peptide level
51:210
21:102
1:13
TATLCPQSCEERpc2_NP.1
p−maj=0.31, pX=0.061, N=73
var.26.VWF 12:6128443:T:C
peptide level
90:210
48:102
3:13
TATLCPQSCEERpc2_NP.2
p−maj=0.16, pX=0.11, N=141
var.26.VWF 12:6128443:T:C
peptide level
32:210
10:102
1:13
TATLCPQSCEERpc2_NP.3
p−min=0.17, pX=0.095, N=43
var.26.VWF 12:6128443:T:C
peptide level
209:210
101:102
13:13
TATLCPQSCEERpc2_NP.4
p−maj=1, pX=0.15, N=323
var.26.VWF 12:6128443:T:C
peptide level
142:210
66:102
10:13
TATLCPQSCEERpc2_NP.5
p−maj=0.56, pX=0.11, N=218
var.26.VWF 12:6128443:T:C
peptide level
8:210
3:102
0:13
TCQSLHINEMCQERpc3_NP.1
p−min=0.75, pX=0.36, N=11
var.26.VWF 12:6128443:T:C
peptide level
7:210
1:102
0:13
TCQSLHINEMCQERpc3_NP.2
p−min=0.27, pX=0.25, N=8
var.26.VWF 12:6128443:T:C
peptide level
4:210
1:102
0:13
TCQSLHINEMCQERpc3_NP.3
p−min=0.66, pX=0.24, N=5
var.26.VWF 12:6128443:T:C
peptide level
178:210
82:102
9:13
TCQSLHINEMCQERpc3_NP.5
p−min=0.22, pX=0.77, N=269

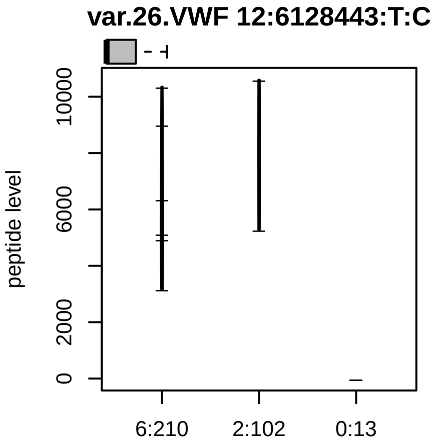

TLVQEWTVQRpc2_NP.4
p−min=0.72, pX=0.62, N=8

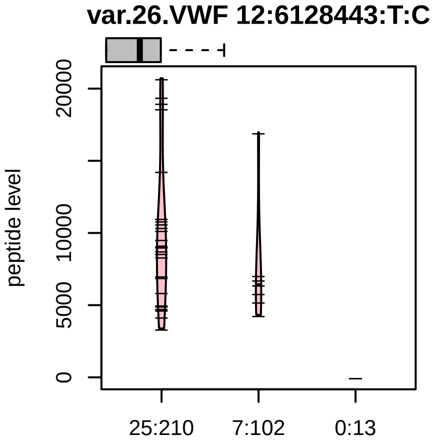

TNGVCVDWRpc2_NP.1
p−min=0.12, pX=0.044, N=32

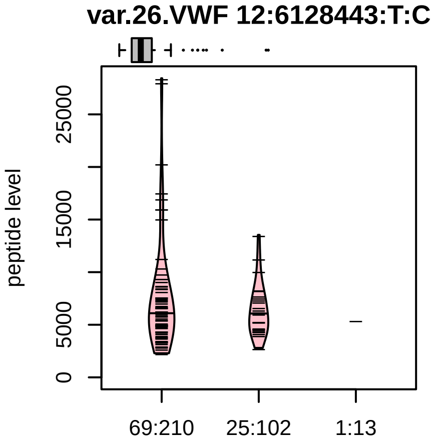

TNGVCVDWRpc2_NP.2
p−min=0.056, pX=0.019, N=95

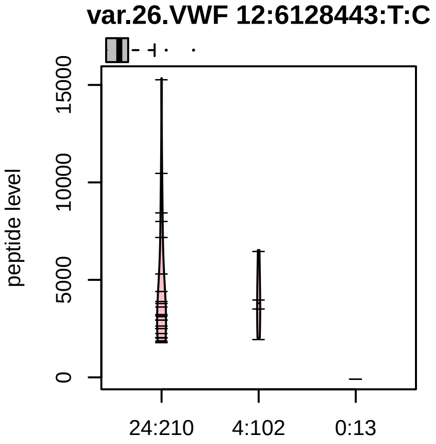

TNGVCVDWRpc2_NP.3
p−min=0.013, pX=0.039, N=28

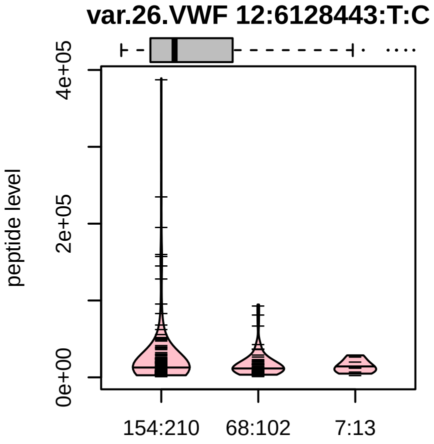

TNGVCVDWRpc2_NP.4
p−min=0.13, pX=0.045, N=229

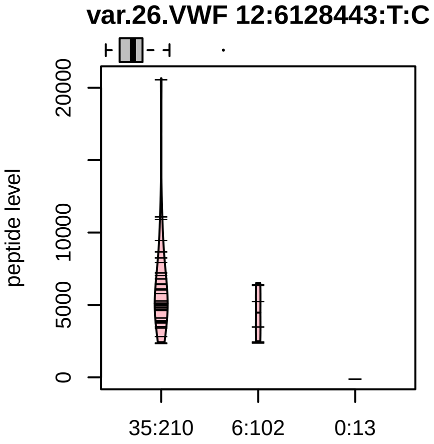

TNGVCVDWRpc2_NP.5
p−min=0.0027, pX=0.0028, N=41

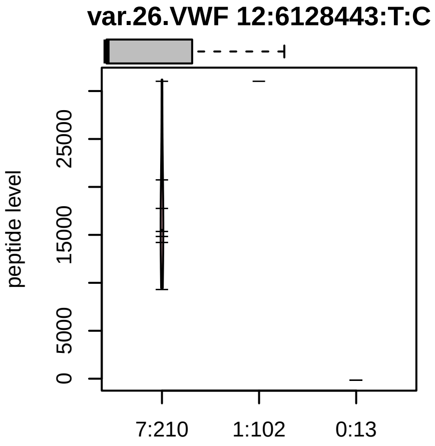

TNTGLALRpc2_NP.4
p−min=0.27, pX=0.36, N=8

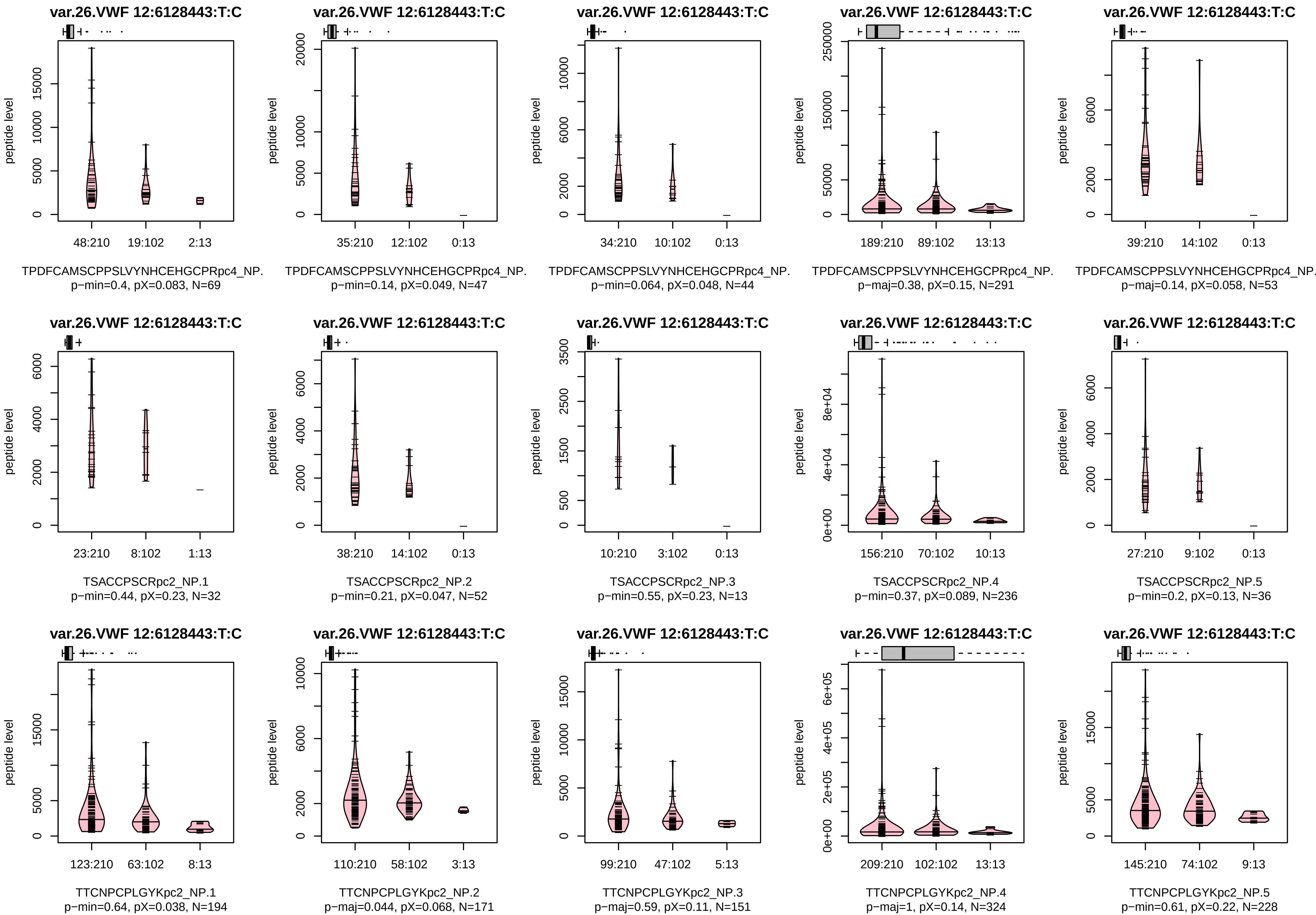
var.26.VWF 12:6128443:T:C
peptide level
48:210
19:102
2:13
TPDFCAMSCPPSLVYNHCEHGCPRpc4_NP.
p−min=0.4, pX=0.083, N=69
var.26.VWF 12:6128443:T:C
peptide level
35:210
12:102
0:13
TPDFCAMSCPPSLVYNHCEHGCPRpc4_NP.
p−min=0.14, pX=0.049, N=47
var.26.VWF 12:6128443:T:C
peptide level
34:210
10:102
0:13
TPDFCAMSCPPSLVYNHCEHGCPRpc4_NP.
p−min=0.064, pX=0.048, N=44
var.26.VWF 12:6128443:T:C
peptide level
189:210
89:102
13:13
TPDFCAMSCPPSLVYNHCEHGCPRpc4_NP.
p−maj=0.38, pX=0.15, N=291
var.26.VWF 12:6128443:T:C
peptide level
39:210
14:102
0:13
TPDFCAMSCPPSLVYNHCEHGCPRpc4_NP.
p−maj=0.14, pX=0.058, N=53
var.26.VWF 12:6128443:T:C
peptide level
23:210
8:102
1:13
TSACCPSCRpc2_NP.1
p−min=0.44, pX=0.23, N=32
var.26.VWF 12:6128443:T:C
peptide level
38:210
14:102
0:13
TSACCPSCRpc2_NP.2
p−min=0.21, pX=0.047, N=52
var.26.VWF 12:6128443:T:C
peptide level
10:210
3:102
0:13
TSACCPSCRpc2_NP.3
p−min=0.55, pX=0.23, N=13
var.26.VWF 12:6128443:T:C
peptide level
156:210
70:102
10:13
TSACCPSCRpc2_NP.4
p−min=0.37, pX=0.089, N=236
var.26.VWF 12:6128443:T:C
peptide level
27:210
9:102
0:13
TSACCPSCRpc2_NP.5
p−min=0.2, pX=0.13, N=36
var.26.VWF 12:6128443:T:C
peptide level
123:210
63:102
8:13
TTCNPCPLGYKpc2_NP.1
p−min=0.64, pX=0.038, N=194
var.26.VWF 12:6128443:T:C
peptide level
110:210
58:102
3:13
TTCNPCPLGYKpc2_NP.2
p−maj=0.044, pX=0.068, N=171
var.26.VWF 12:6128443:T:C
peptide level
99:210
47:102
5:13
TTCNPCPLGYKpc2_NP.3
p−maj=0.59, pX=0.11, N=151
var.26.VWF 12:6128443:T:C
peptide level
209:210
102:102
13:13
TTCNPCPLGYKpc2_NP.4
p−maj=1, pX=0.14, N=324
var.26.VWF 12:6128443:T:C
peptide level
145:210
74:102
9:13
TTCNPCPLGYKpc2_NP.5
p−min=0.61, pX=0.22, N=228

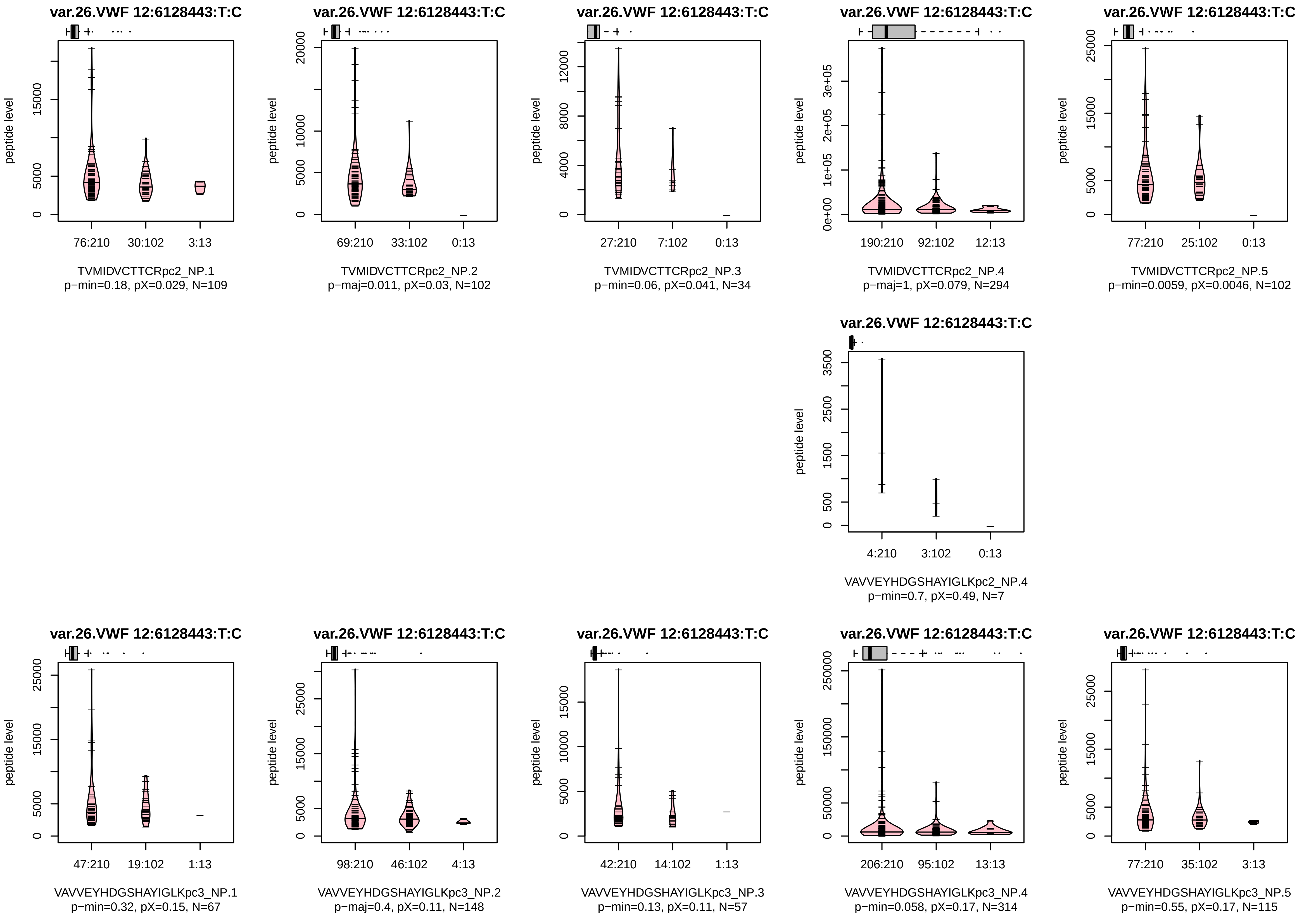
var.26.VWF 12:6128443:T:C
peptide level
76:210
30:102
3:13
TVMIDVCTTCRpc2_NP.1
p−min=0.18, pX=0.029, N=109
var.26.VWF 12:6128443:T:C
peptide level
69:210
33:102
0:13
TVMIDVCTTCRpc2_NP.2
p−maj=0.011, pX=0.03, N=102
var.26.VWF 12:6128443:T:C
peptide level
27:210
7:102
0:13
TVMIDVCTTCRpc2_NP.3
p−min=0.06, pX=0.041, N=34
var.26.VWF 12:6128443:T:C
peptide level
190:210
92:102
12:13
TVMIDVCTTCRpc2_NP.4
p−maj=1, pX=0.079, N=294
var.26.VWF 12:6128443:T:C
peptide level
77:210
25:102
0:13
TVMIDVCTTCRpc2_NP.5
p−min=0.0059, pX=0.0046, N=102
var.26.VWF 12:6128443:T:C
peptide level
4:210
3:102
0:13
VAVVEYHDGSHAYIGLKpc2_NP.4
p−min=0.7, pX=0.49, N=7
var.26.VWF 12:6128443:T:C
peptide level
47:210
19:102
1:13
VAVVEYHDGSHAYIGLKpc3_NP.1
p−min=0.32, pX=0.15, N=67
var.26.VWF 12:6128443:T:C
peptide level
98:210
46:102
4:13
VAVVEYHDGSHAYIGLKpc3_NP.2
p−maj=0.4, pX=0.11, N=148
var.26.VWF 12:6128443:T:C
peptide level
42:210
14:102
1:13
VAVVEYHDGSHAYIGLKpc3_NP.3
p−min=0.13, pX=0.11, N=57
var.26.VWF 12:6128443:T:C
peptide level
206:210
95:102
13:13
VAVVEYHDGSHAYIGLKpc3_NP.4
p−min=0.058, pX=0.17, N=314
var.26.VWF 12:6128443:T:C
peptide level
77:210
35:102
3:13
VAVVEYHDGSHAYIGLKpc3_NP.5
p−min=0.55, pX=0.17, N=115

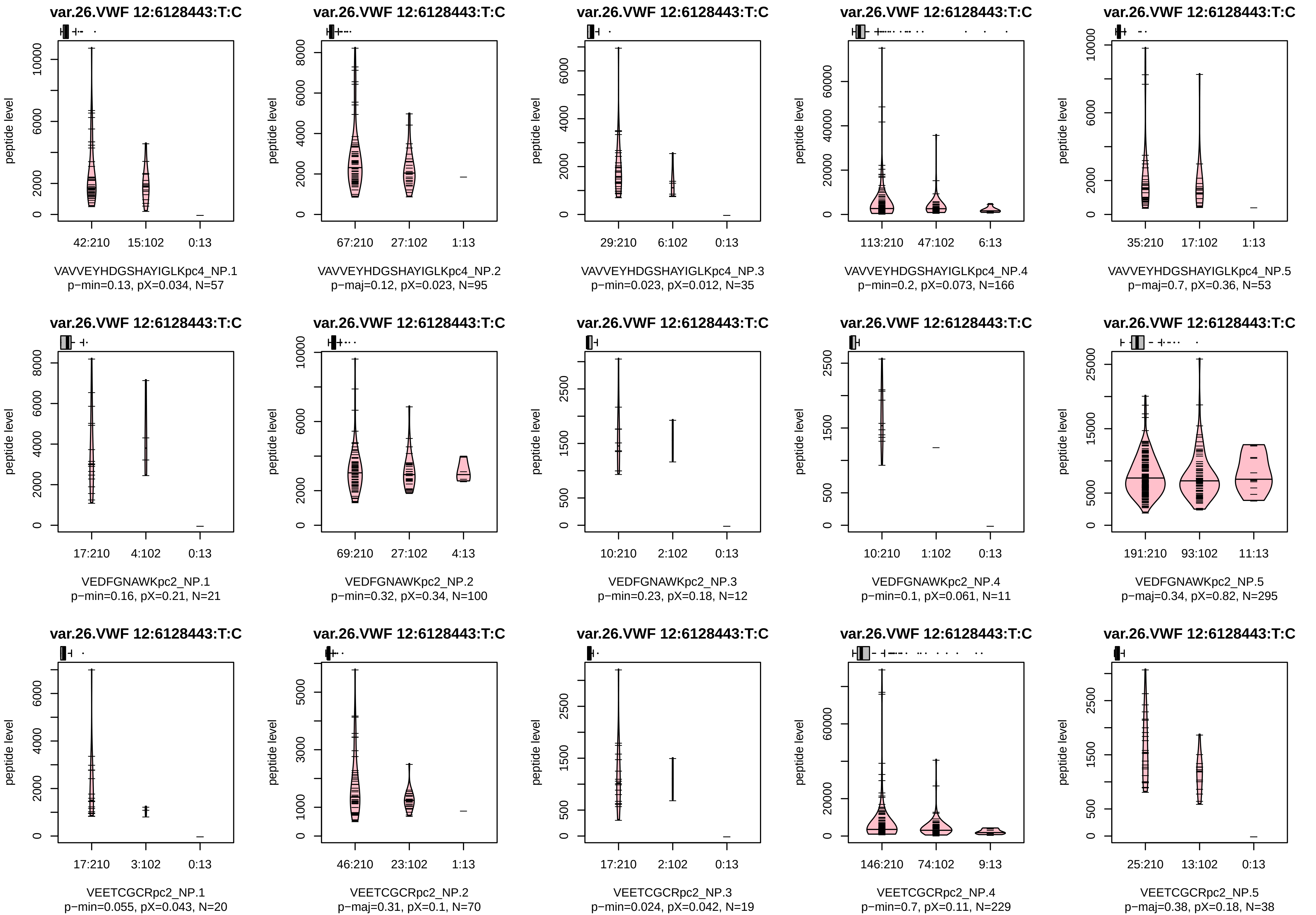

var.26.VWF 12:6128443:T:C
peptide level
42:210
15:102
0:13
VAVVEYHDGSHAYIGLKpc4_NP.1
p−min=0.13, pX=0.034, N=57
var.26.VWF 12:6128443:T:C
peptide level
67:210
27:102
1:13
VAVVEYHDGSHAYIGLKpc4_NP.2
p−maj=0.12, pX=0.023, N=95
var.26.VWF 12:6128443:T:C
peptide level
29:210
6:102
0:13
VAVVEYHDGSHAYIGLKpc4_NP.3
p−min=0.023, pX=0.012, N=35
var.26.VWF 12:6128443:T:C
peptide level
113:210
47:102
6:13
VAVVEYHDGSHAYIGLKpc4_NP.4
p−min=0.2, pX=0.073, N=166
var.26.VWF 12:6128443:T:C
peptide level
35:210
17:102
1:13
VAVVEYHDGSHAYIGLKpc4_NP.5
p−maj=0.7, pX=0.36, N=53
var.26.VWF 12:6128443:T:C
peptide level
17:210
4:102
0:13
VEDFGNAWKpc2_NP.1
p−min=0.16, pX=0.21, N=21
var.26.VWF 12:6128443:T:C
peptide level
69:210
27:102
4:13
VEDFGNAWKpc2_NP.2
p−min=0.32, pX=0.34, N=100
var.26.VWF 12:6128443:T:C
peptide level
10:210
2:102
0:13
VEDFGNAWKpc2_NP.3
p−min=0.23, pX=0.18, N=12
var.26.VWF 12:6128443:T:C
peptide level
10:210
1:102
0:13
VEDFGNAWKpc2_NP.4
p−min=0.1, pX=0.061, N=11
var.26.VWF 12:6128443:T:C
peptide level
191:210
93:102
11:13
VEDFGNAWKpc2_NP.5
p−maj=0.34, pX=0.82, N=295
var.26.VWF 12:6128443:T:C
peptide level
17:210
3:102
0:13
VEETCGCRpc2_NP.1
p−min=0.055, pX=0.043, N=20
var.26.VWF 12:6128443:T:C
peptide level
46:210
23:102
1:13
VEETCGCRpc2_NP.2
p−maj=0.31, pX=0.1, N=70
var.26.VWF 12:6128443:T:C
peptide level
17:210
2:102
0:13
VEETCGCRpc2_NP.3
p−min=0.024, pX=0.042, N=19
var.26.VWF 12:6128443:T:C
peptide level
146:210
74:102
9:13
VEETCGCRpc2_NP.4
p−min=0.7, pX=0.11, N=229
var.26.VWF 12:6128443:T:C
peptide level
25:210
13:102
0:13
VEETCGCRpc2_NP.5
p−maj=0.38, pX=0.18, N=38

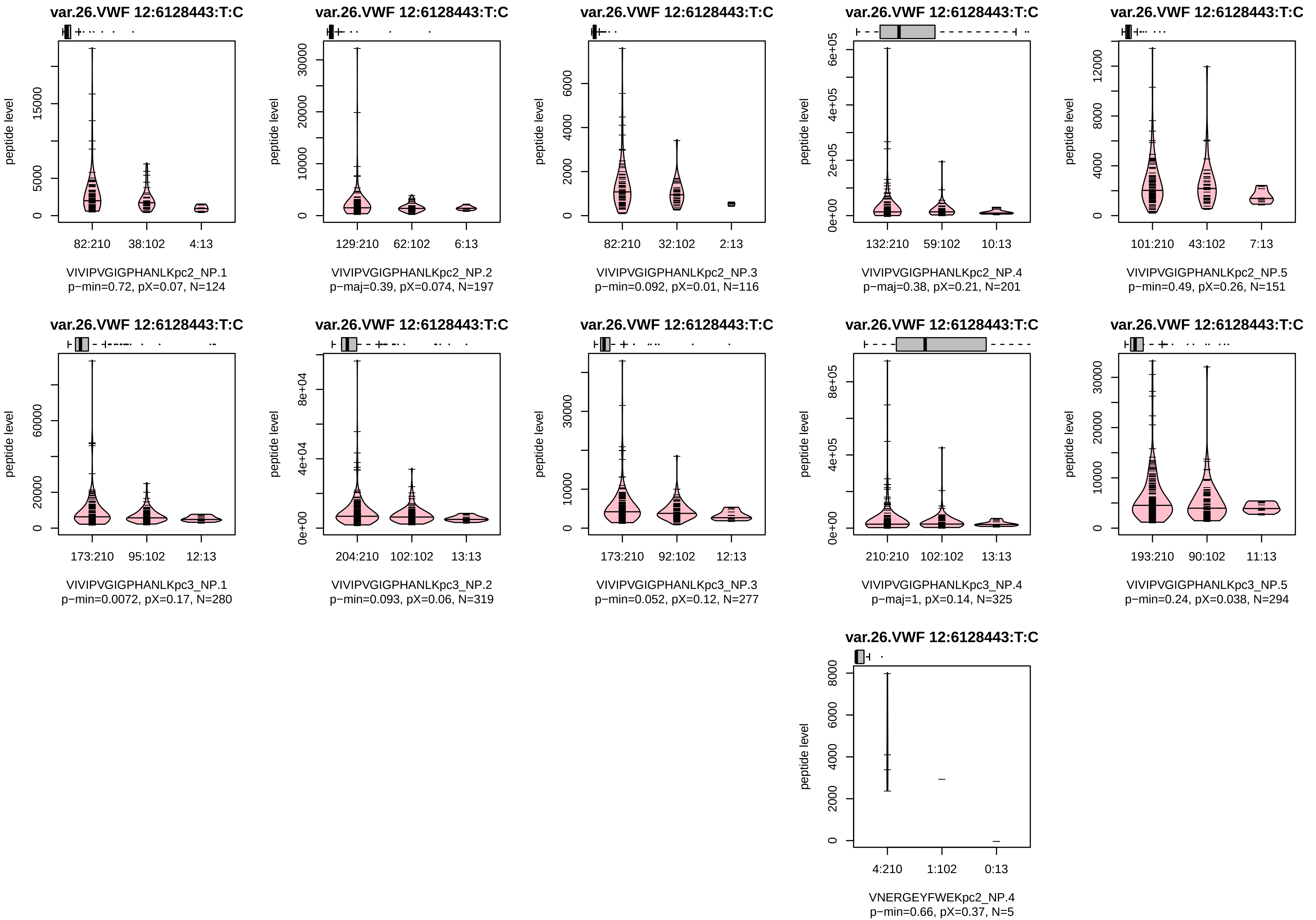
var.26.VWF 12:6128443:T:C
peptide level
82:210
38:102
4:13
VIVIPVGIGPHANLKpc2_NP.1
p−min=0.72, pX=0.07, N=124
var.26.VWF 12:6128443:T:C
peptide level
129:210
62:102
6:13
VIVIPVGIGPHANLKpc2_NP.2
p−maj=0.39, pX=0.074, N=197
var.26.VWF 12:6128443:T:C
peptide level
82:210
32:102
2:13
VIVIPVGIGPHANLKpc2_NP.3
p−min=0.092, pX=0.01, N=116
var.26.VWF 12:6128443:T:C
peptide level
132:210
59:102
10:13
VIVIPVGIGPHANLKpc2_NP.4
p−maj=0.38, pX=0.21, N=201
var.26.VWF 12:6128443:T:C
peptide level
101:210
43:102
7:13
VIVIPVGIGPHANLKpc2_NP.5
p−min=0.49, pX=0.26, N=151
var.26.VWF 12:6128443:T:C
peptide level
173:210
95:102
12:13
VIVIPVGIGPHANLKpc3_NP.1
p−min=0.0072, pX=0.17, N=280
var.26.VWF 12:6128443:T:C
peptide level
204:210
102:102
13:13
VIVIPVGIGPHANLKpc3_NP.2
p−min=0.093, pX=0.06, N=319
var.26.VWF 12:6128443:T:C
peptide level
173:210
92:102
12:13
VIVIPVGIGPHANLKpc3_NP.3
p−min=0.052, pX=0.12, N=277
var.26.VWF 12:6128443:T:C
peptide level
210:210
102:102
13:13
VIVIPVGIGPHANLKpc3_NP.4
p−maj=1, pX=0.14, N=325
var.26.VWF 12:6128443:T:C
peptide level
193:210
90:102
11:13
VIVIPVGIGPHANLKpc3_NP.5
p−min=0.24, pX=0.038, N=294
var.26.VWF 12:6128443:T:C
peptide level
4:210
1:102
0:13
VNERGEYFWEKpc2_NP.4
p−min=0.66, pX=0.37, N=5

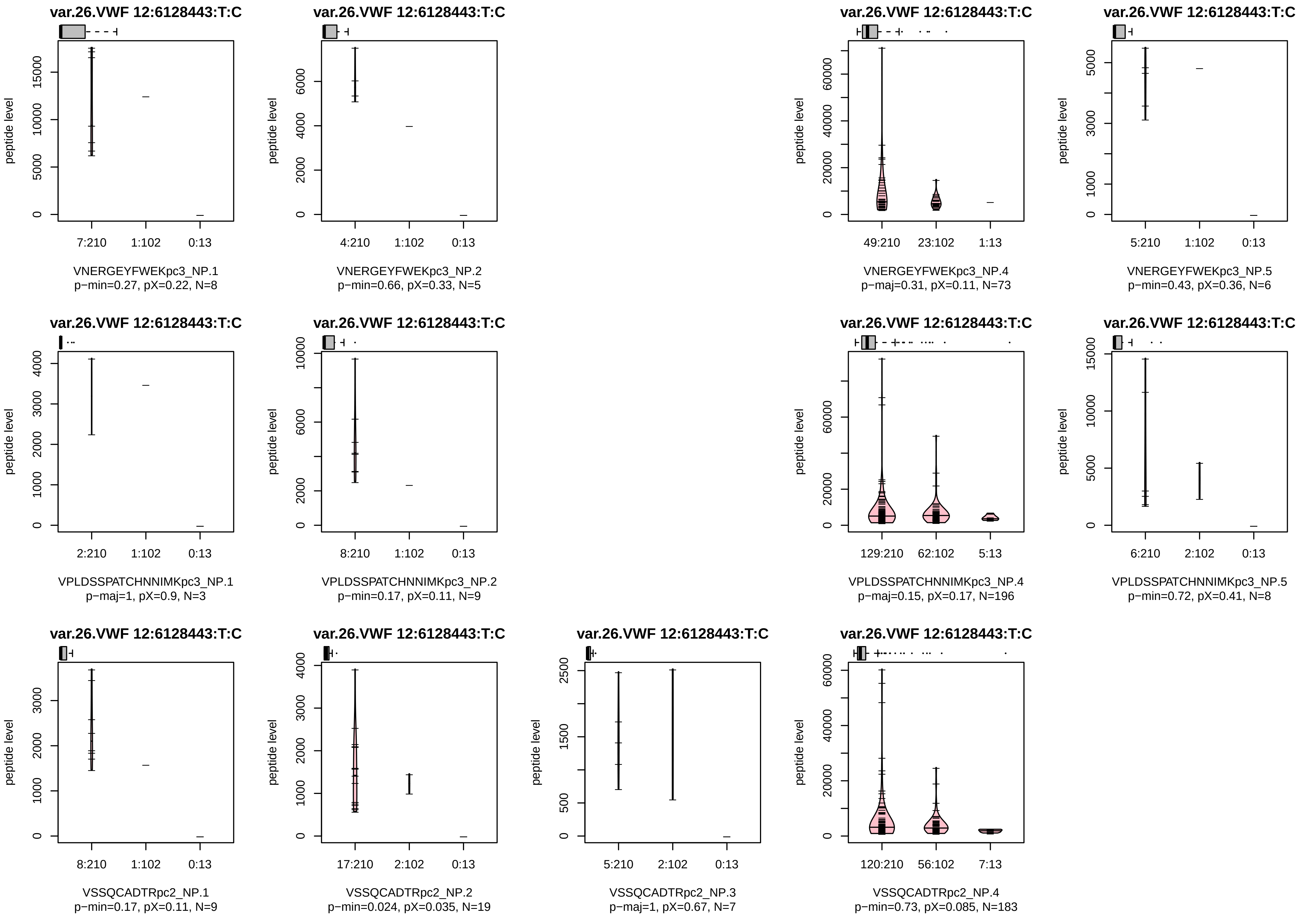
var.26.VWF 12:6128443:T:C
peptide level
7:210
1:102
0:13
VNERGEYFWEKpc3_NP.1
p−min=0.27, pX=0.22, N=8
4:210
1:102
0:13
VNERGEYFWEKpc3_NP.2
p−min=0.66, pX=0.33, N=5
49:210
23:102
1:13
VNERGEYFWEKpc3_NP.4
p−maj=0.31, pX=0.11, N=73
5:210
1:102
0:13
VNERGEYFWEKpc3_NP.5
p−min=0.43, pX=0.36, N=6
2:210
1:102
0:13
VPLDSSPATCHNNIMKpc3_NP.1
p−maj=1, pX=0.9, N=3
8:210
1:102
0:13
VPLDSSPATCHNNIMKpc3_NP.2
p−min=0.17, pX=0.11, N=9
129:210
62:102
5:13
VPLDSSPATCHNNIMKpc3_NP.4
p−maj=0.15, pX=0.17, N=196
6:210
2:102
0:13
VPLDSSPATCHNNIMKpc3_NP.5
p−min=0.72, pX=0.41, N=8
8:210
1:102
0:13
VSSQCADTRpc2_NP.1
p−min=0.17, pX=0.11, N=9
17:210
2:102
0:13
VSSQCADTRpc2_NP.2
p−min=0.024, pX=0.035, N=19
5:210
2:102
0:13
VSSQCADTRpc2_NP.3
p−maj=1, pX=0.67, N=7
120:210
56:102
7:13
VSSQCADTRpc2_NP.4
p−min=0.73, pX=0.085, N=183

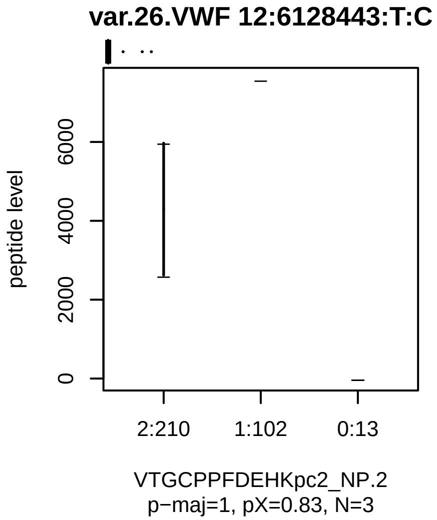
var.26.VWF 12:6128443:T:C
peptide level
0
2000
4000
6000
2:210
1:102
0:13
VTGCPPFDEHKpc2_NP.2
p−maj=1, pX=0.83, N=3

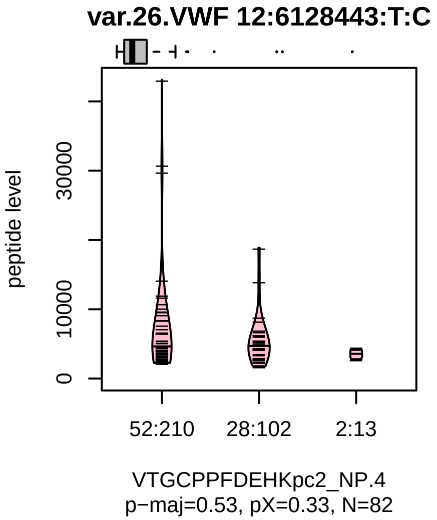
var.26.VWF 12:6128443:T:C
peptide level
0
10000
30000
52:210
28:102
2:13
VTGCPPFDEHKpc2_NP.4
p−maj=0.53, pX=0.33, N=82

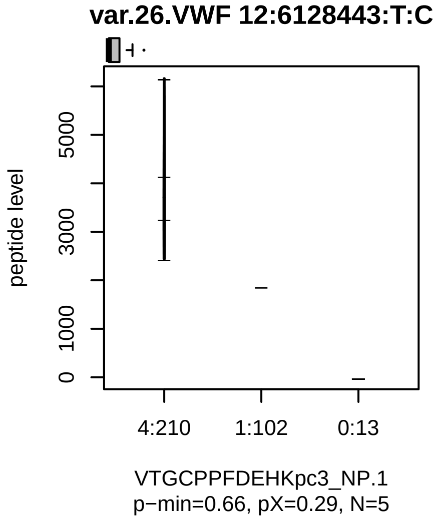
var.26.VWF 12:6128443:T:C
peptide level
0
1000
3000
5000
4:210
1:102
0:13
VTGCPPFDEHKpc3_NP.1
p−min=0.66, pX=0.29, N=5

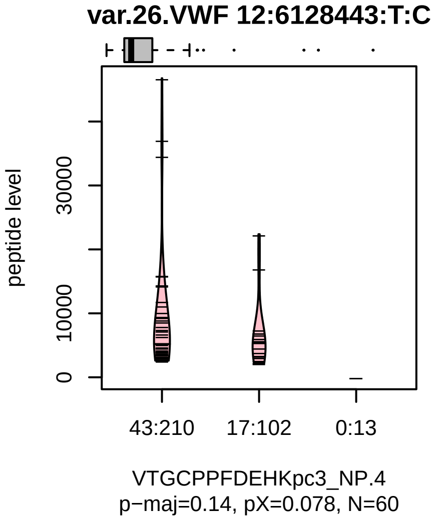
var.26.VWF 12:6128443:T:C
peptide level
0
10000
30000
43:210
17:102
0:13
VTGCPPFDEHKpc3_NP.4
p−maj=0.14, pX=0.078, N=60

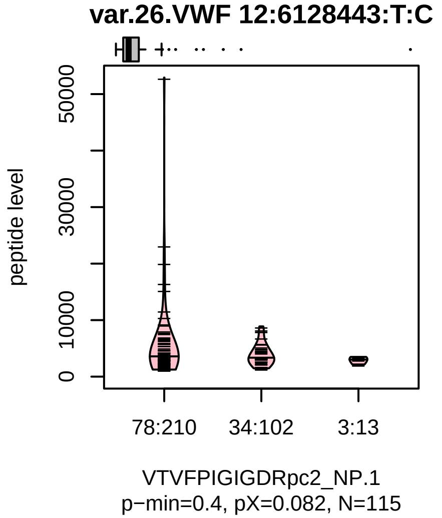
var.26.VWF 12:6128443:T:C
peptide level
0
10000
30000
50000
78:210
34:102
3:13
VTVFPIGIGDRpc2_NP.1
p−min=0.4, pX=0.082, N=115

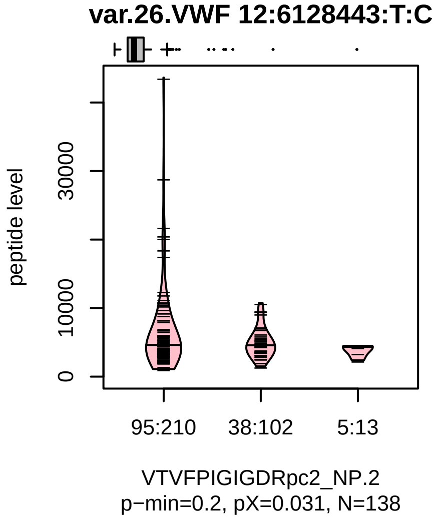
var.26.VWF 12:6128443:T:C
peptide level
0
10000
30000
95:210
38:102
5:13
VTVFPIGIGDRpc2_NP.2
p−min=0.2, pX=0.031, N=138

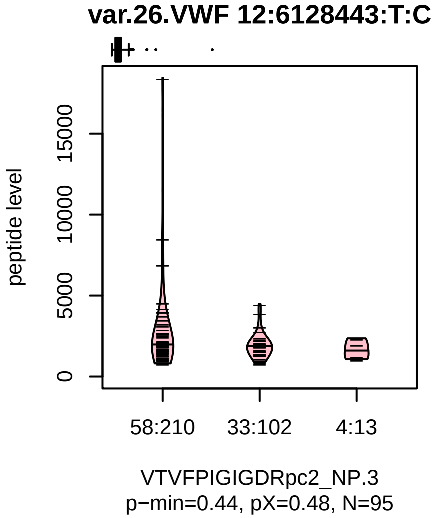
var.26.VWF 12:6128443:T:C
peptide level
0
5000
10000
15000
58:210
33:102
4:13
VTVFPIGIGDRpc2_NP.3
p−min=0.44, pX=0.48, N=95

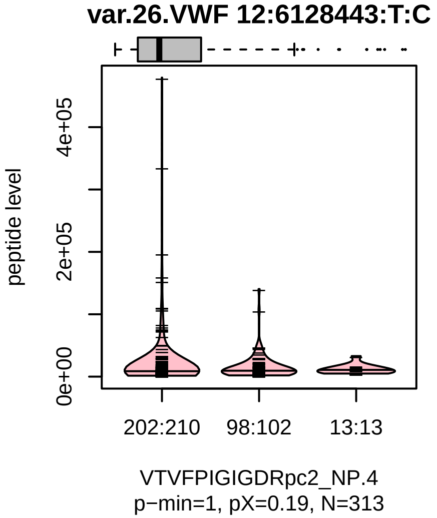
var.26.VWF 12:6128443:T:C
peptide level
0e+00
2e+05
4e+05
202:210
98:102
13:13
VTVFPIGIGDRpc2_NP.4
p−min=1, pX=0.19, N=313

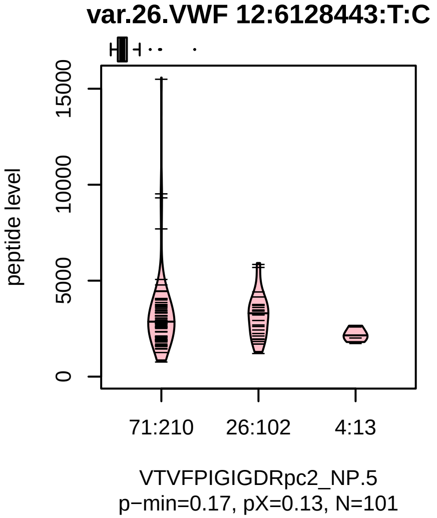
var.26.VWF 12:6128443:T:C
peptide level
0
5000
10000
15000
71:210
26:102
4:13
VTVFPIGIGDRpc2_NP.5
p−min=0.17, pX=0.13, N=101

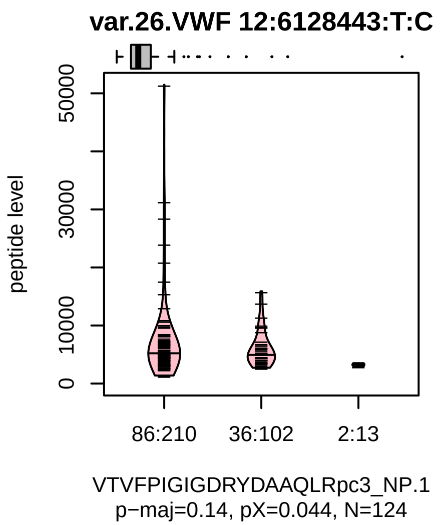
var.26.VWF 12:6128443:T:C
peptide level
0
10000
30000
50000
86:210
36:102
2:13
VTVFPIGIGDRYDAAQLRpc3_NP.1
p−maj=0.14, pX=0.044, N=124

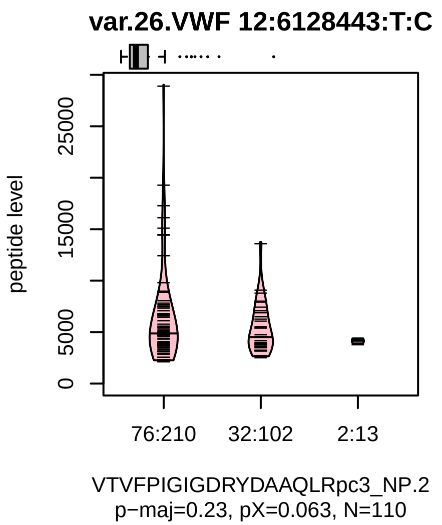
var.26.VWF 12:6128443:T:C
peptide level
0
5000
15000
25000
76:210
32:102
2:13
VTVFPIGIGDRYDAAQLRpc3_NP.2
p−maj=0.23, pX=0.063, N=110

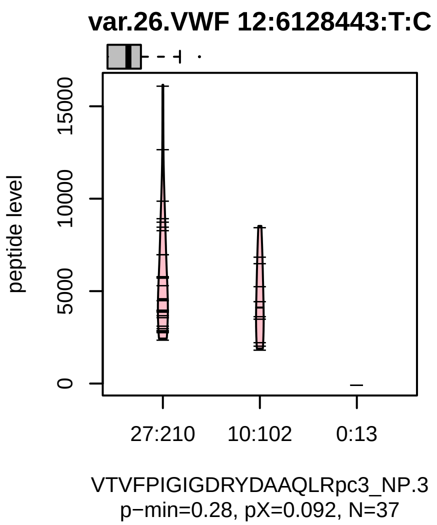
var.26.VWF 12:6128443:T:C
peptide level
0
5000
10000
15000
27:210
10:102
0:13
VTVFPIGIGDRYDAAQLRpc3_NP.3
p−min=0.28, pX=0.092, N=37

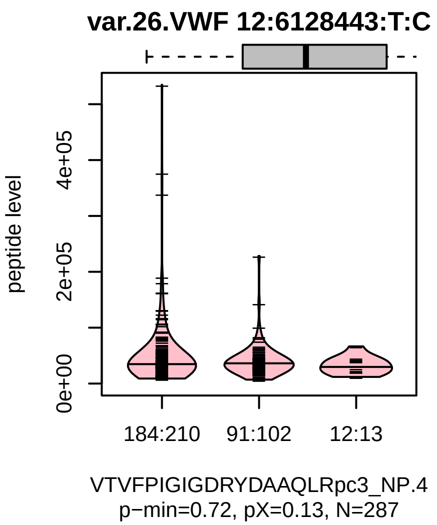
var.26.VWF 12:6128443:T:C
peptide level
0e+00
2e+05
4e+05
184:210
91:102
12:13
VTVFPIGIGDRYDAAQLRpc3_NP.4
p−min=0.72, pX=0.13, N=287

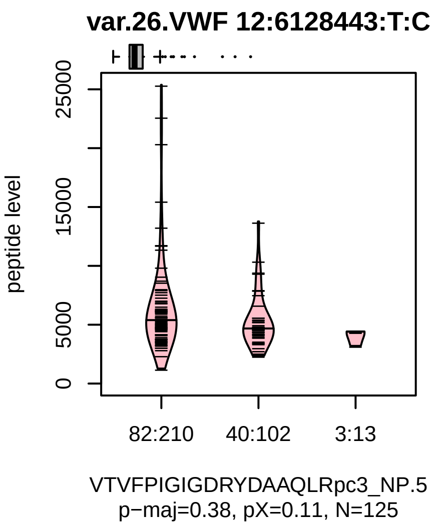
var.26.VWF 12:6128443:T:C
peptide level
0
5000
15000
25000
82:210
40:102
3:13
VTVFPIGIGDRYDAAQLRpc3_NP.5
p−maj=0.38, pX=0.11, N=125

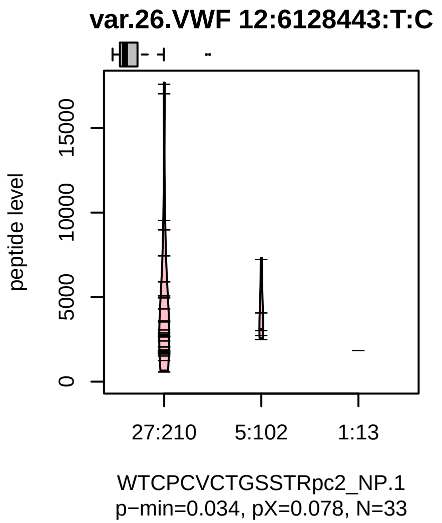
var.26.VWF 12:6128443:T:C
peptide level
0
5000
10000
15000
27:210
5:102
1:13
WTCPCVCTGSSTRpc2_NP.1
p−min=0.034, pX=0.078, N=33

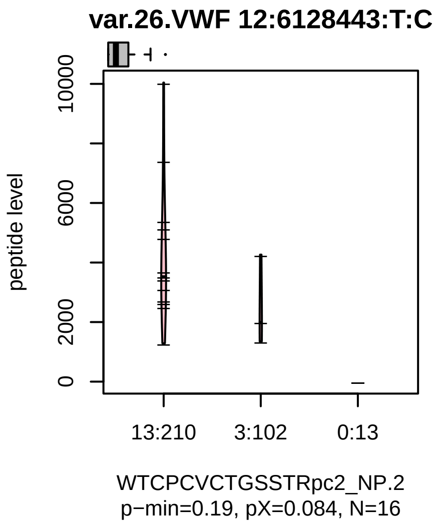
var.26.VWF 12:6128443:T:C
peptide level
0
2000
6000
10000
13:210
3:102
0:13
WTCPCVCTGSSTRpc2_NP.2
p−min=0.19, pX=0.084, N=16

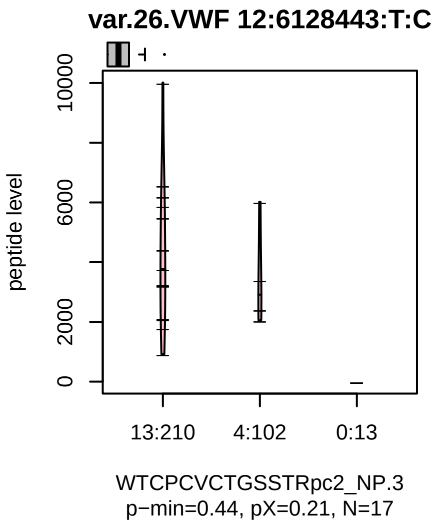
var.26.VWF 12:6128443:T:C
peptide level
0
2000
6000
10000
13:210
4:102
0:13
WTCPCVCTGSSTRpc2_NP.3
p−min=0.44, pX=0.21, N=17

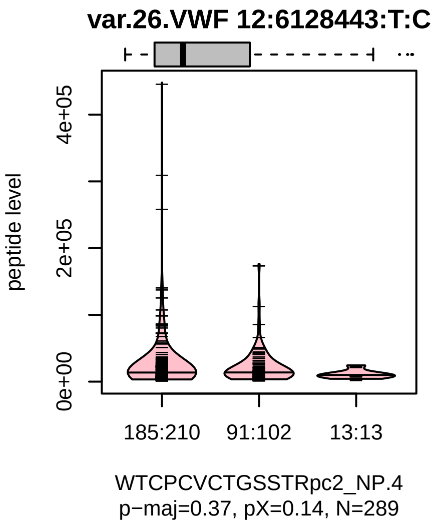
var.26.VWF 12:6128443:T:C
peptide level
0e+00
2e+05
4e+05
185:210
91:102
13:13
WTCPCVCTGSSTRpc2_NP.4
p−maj=0.37, pX=0.14, N=289

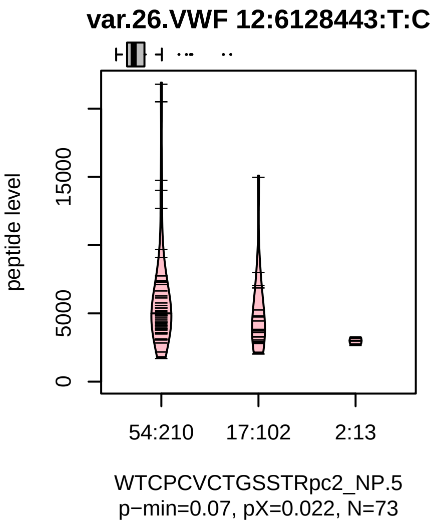
var.26.VWF 12:6128443:T:C
peptide level
0
5000
15000
54:210
17:102
2:13
WTCPCVCTGSSTRpc2_NP.5
p−min=0.07, pX=0.022, N=73

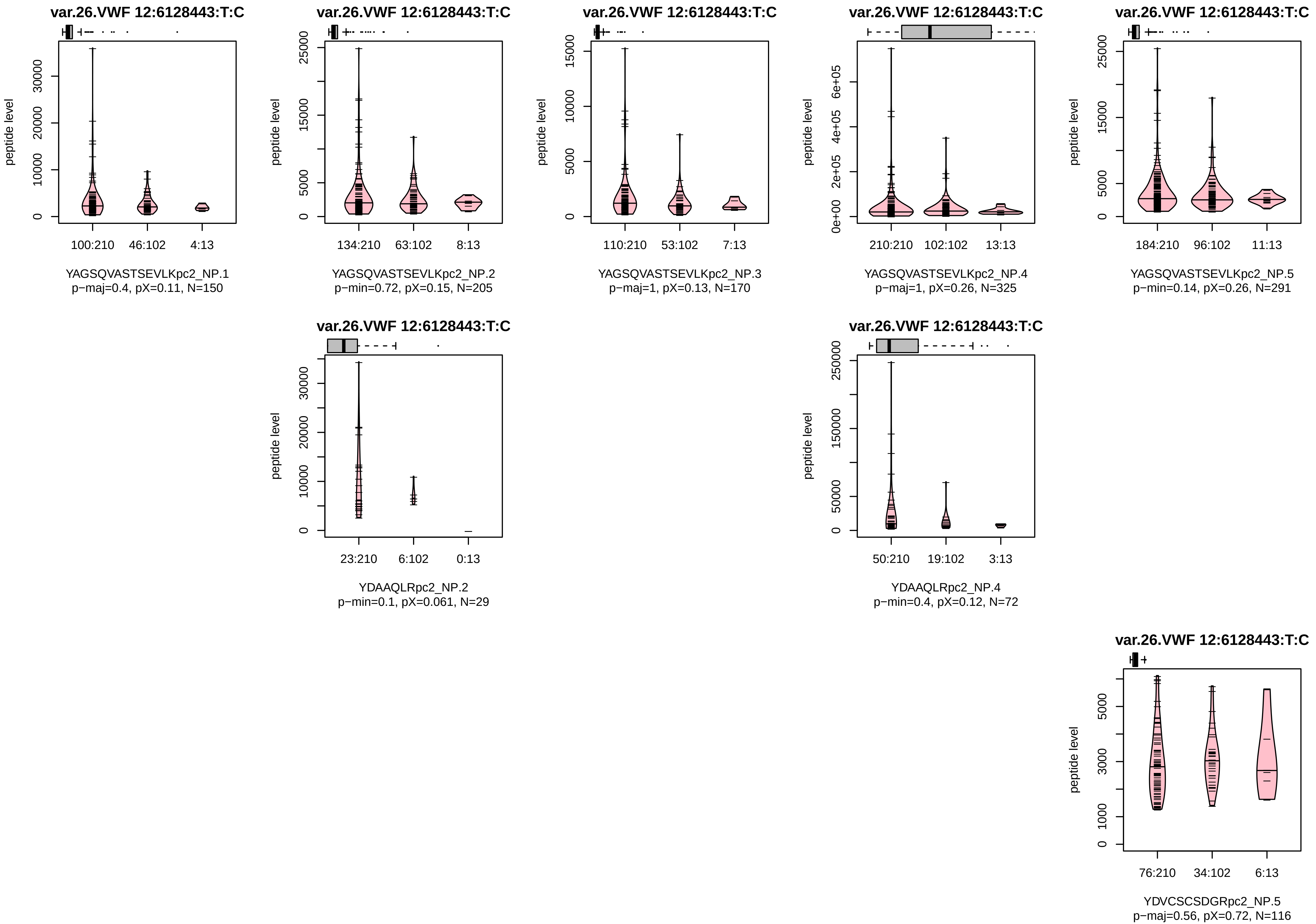

var.26.VWF 12:6128443:T:C
peptide level
100:210
46:102
4:13
YAGSQVASTSEVLKpc2_NP.1
p−maj=0.4, pX=0.11, N=150
var.26.VWF 12:6128443:T:C
peptide level
134:210
63:102
8:13
YAGSQVASTSEVLKpc2_NP.2
p−min=0.72, pX=0.15, N=205
var.26.VWF 12:6128443:T:C
peptide level
110:210
53:102
7:13
YAGSQVASTSEVLKpc2_NP.3
p−maj=1, pX=0.13, N=170
var.26.VWF 12:6128443:T:C
peptide level
210:210
102:102
13:13
YAGSQVASTSEVLKpc2_NP.4
p−maj=1, pX=0.26, N=325
var.26.VWF 12:6128443:T:C
peptide level
184:210
96:102
11:13
YAGSQVASTSEVLKpc2_NP.5
p−min=0.14, pX=0.26, N=291
var.26.VWF 12:6128443:T:C
peptide level
23:210
6:102
0:13
YDAAQLRpc2_NP.2
p−min=0.1, pX=0.061, N=29
var.26.VWF 12:6128443:T:C
peptide level
50:210
19:102
3:13
YDAAQLRpc2_NP.4
p−min=0.4, pX=0.12, N=72
var.26.VWF 12:6128443:T:C
peptide level
76:210
34:102
6:13
YDVCSCSDGRpc2_NP.5
p−maj=0.56, pX=0.72, N=116

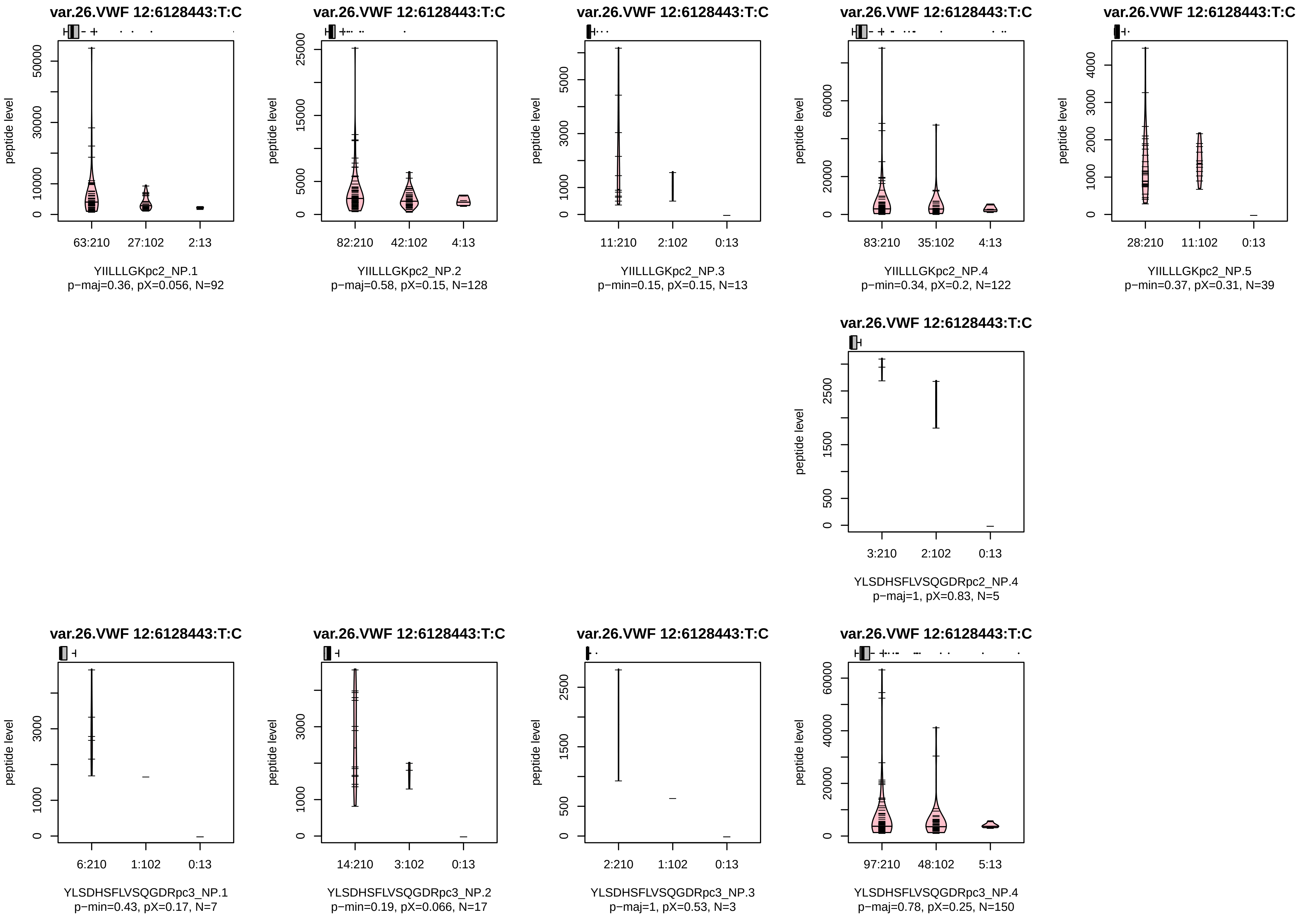

var.26.VWF 12:6128443:T:C
63:210
27:102
2:13
YIILLLGKpc2_NP.1
p−maj=0.36, pX=0.056, N=92
var.26.VWF 12:6128443:T:C
82:210
42:102
4:13
YIILLLGKpc2_NP.2
p−maj=0.58, pX=0.15, N=128
var.26.VWF 12:6128443:T:C
11:210
2:102
0:13
YIILLLGKpc2_NP.3
p−min=0.15, pX=0.15, N=13
var.26.VWF 12:6128443:T:C
83:210
35:102
4:13
YIILLLGKpc2_NP.4
p−min=0.34, pX=0.2, N=122
var.26.VWF 12:6128443:T:C
28:210
11:102
0:13
YIILLLGKpc2_NP.5
p−min=0.37, pX=0.31, N=39
var.26.VWF 12:6128443:T:C
3:210
2:102
0:13
YLSDHSFLVSQGDRpc2_NP.4
p−maj=1, pX=0.83, N=5
var.26.VWF 12:6128443:T:C
6:210
1:102
0:13
YLSDHSFLVSQGDRpc3_NP.1
p−min=0.43, pX=0.17, N=7
var.26.VWF 12:6128443:T:C
14:210
3:102
0:13
YLSDHSFLVSQGDRpc3_NP.2
p−min=0.19, pX=0.066, N=17
var.26.VWF 12:6128443:T:C
2:210
1:102
0:13
YLSDHSFLVSQGDRpc3_NP.3
p−maj=1, pX=0.53, N=3
var.26.VWF 12:6128443:T:C
97:210
48:102
5:13
YLSDHSFLVSQGDRpc3_NP.4
p−maj=0.78, pX=0.25, N=150

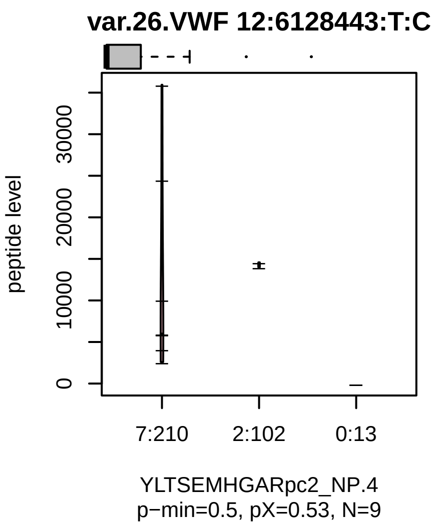
var.26.VWF 12:6128443:T:C
peptide level
0
10000
20000
30000
7:210
2:102
0:13
YLTSEMHGARpc2_NP.4
p−min=0.5, pX=0.53, N=9

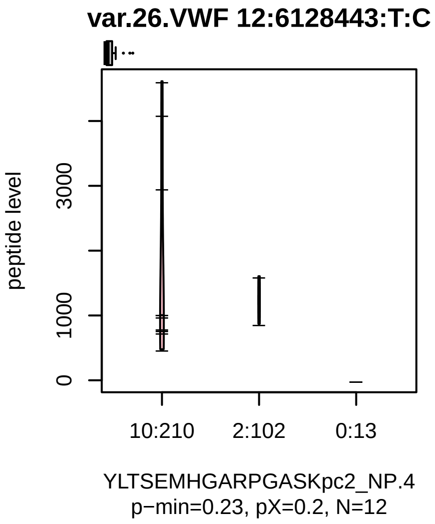
var.26.VWF 12:6128443:T:C
peptide level
0
1000
3000
10:210
2:102
0:13
YLTSEMHGARPGASKpc2_NP.4
p−min=0.23, pX=0.2, N=12

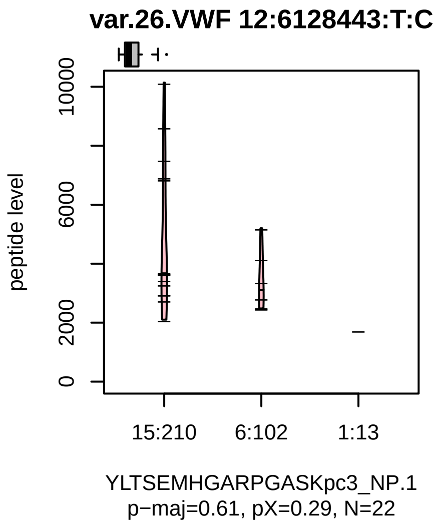
var.26.VWF 12:6128443:T:C
peptide level
0
2000
6000
10000
15:210
6:102
1:13
YLTSEMHGARPGASKpc3_NP.1
p−maj=0.61, pX=0.29, N=22

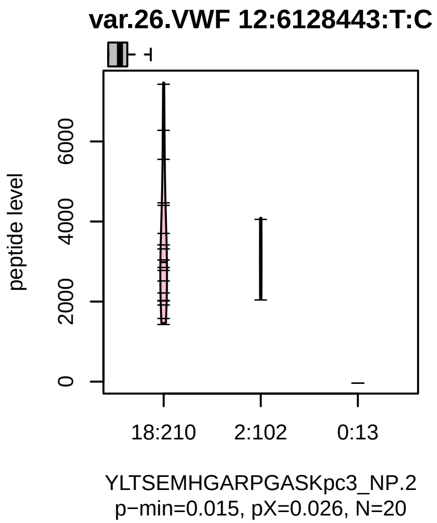
var.26.VWF 12:6128443:T:C
peptide level
0
2000
4000
6000
18:210
2:102
0:13
YLTSEMHGARPGASKpc3_NP.2
p−min=0.015, pX=0.026, N=20

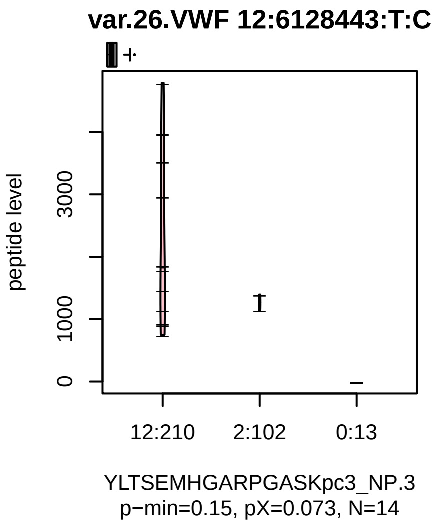
var.26.VWF 12:6128443:T:C
peptide level
0
1000
3000
12:210
2:102
0:13
YLTSEMHGARPGASKpc3_NP.3
p−min=0.15, pX=0.073, N=14

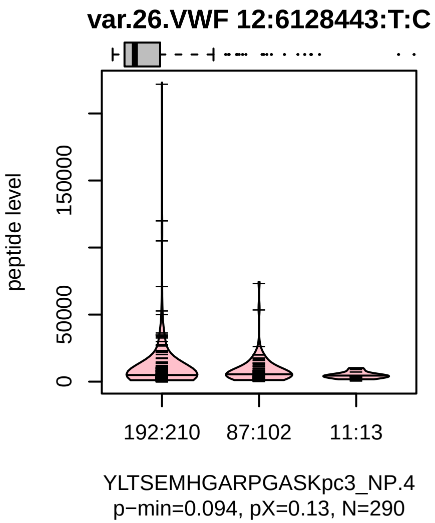
var.26.VWF 12:6128443:T:C
peptide level
0
50000
150000
192:210
87:102
11:13
YLTSEMHGARPGASKpc3_NP.4
p−min=0.094, pX=0.13, N=290

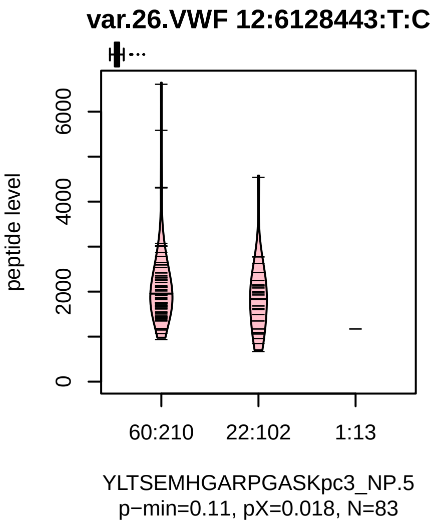
var.26.VWF 12:6128443:T:C
peptide level
0
2000
4000
6000
60:210
22:102
1:13
YLTSEMHGARPGASKpc3_NP.5
p−min=0.11, pX=0.018, N=83

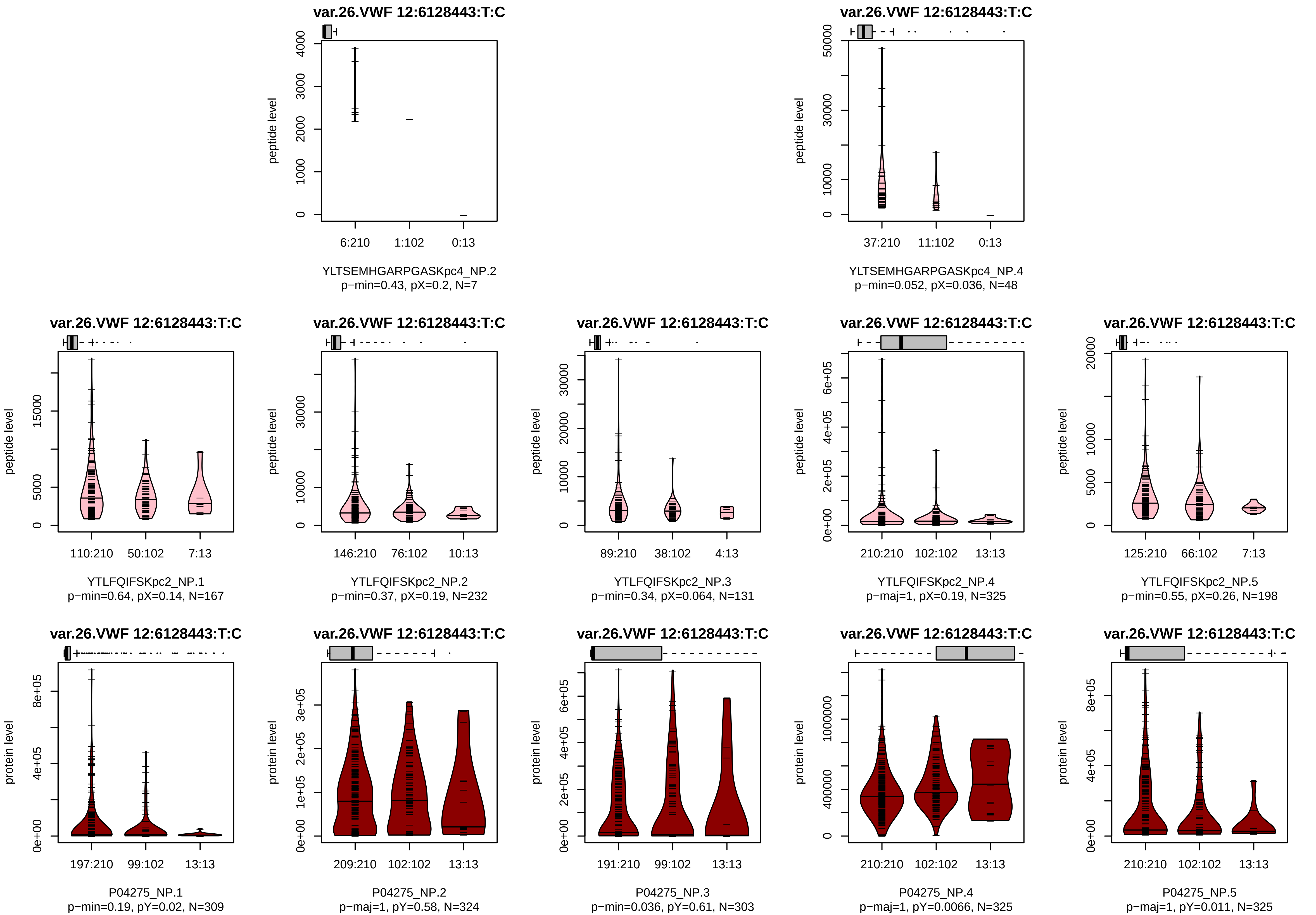
var.26.VWF 12:6128443:T:C
peptide level
6:210
1:102
0:13
YLTSEMHGARPGASKpc4_NP.2
p−min=0.43, pX=0.2, N=7
var.26.VWF 12:6128443:T:C
peptide level
37:210
11:102
0:13
YLTSEMHGARPGASKpc4_NP.4
p−min=0.052, pX=0.036, N=48
var.26.VWF 12:6128443:T:C
peptide level
110:210
50:102
7:13
YTLFQIFSKpc2_NP.1
p−min=0.64, pX=0.14, N=167
var.26.VWF 12:6128443:T:C
peptide level
146:210
76:102
10:13
YTLFQIFSKpc2_NP.2
p−min=0.37, pX=0.19, N=232
var.26.VWF 12:6128443:T:C
peptide level
89:210
38:102
4:13
YTLFQIFSKpc2_NP.3
p−min=0.34, pX=0.064, N=131
var.26.VWF 12:6128443:T:C
peptide level
210:210
102:102
13:13
YTLFQIFSKpc2_NP.4
p−maj=1, pX=0.19, N=325
var.26.VWF 12:6128443:T:C
peptide level
125:210
66:102
7:13
YTLFQIFSKpc2_NP.5
p−min=0.55, pX=0.26, N=198
var.26.VWF 12:6128443:T:C
protein level
197:210
99:102
13:13
P04275_NP.1
p−min=0.19, pY=0.02, N=309
var.26.VWF 12:6128443:T:C
protein level
209:210
102:102
13:13
P04275_NP.2
p−maj=1, pY=0.58, N=324
var.26.VWF 12:6128443:T:C
protein level
191:210
99:102
13:13
P04275_NP.3
p−min=0.036, pY=0.61, N=303
var.26.VWF 12:6128443:T:C
protein level
210:210
102:102
13:13
P04275_NP.4
p−maj=1, pY=0.0066, N=325
var.26.VWF 12:6128443:T:C
protein level
210:210
102:102
13:13
P04275_NP.5
p−maj=1, pY=0.011, N=325

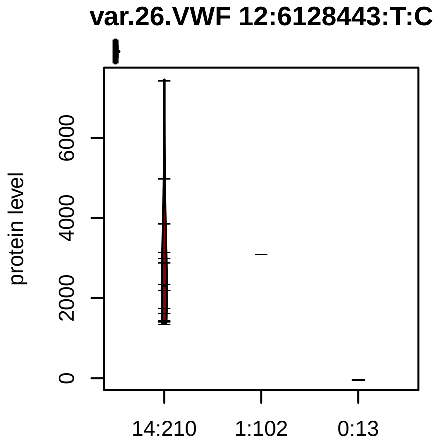

P04275.rs216311_NP.1
p−min=0.023, pY=0.9, N=15

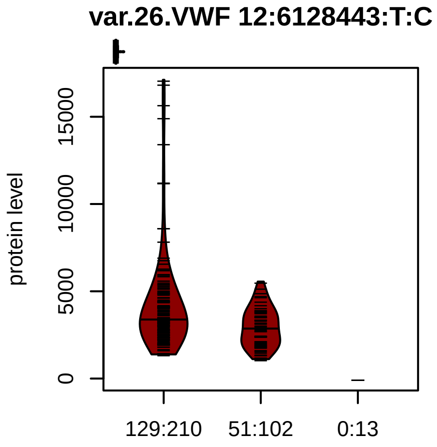

P04275.rs216311_NP.2
p−maj=2e−05, pY=0.00024, N=180

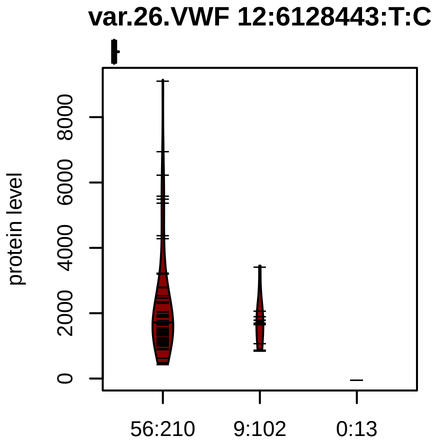

P04275.rs216311_NP.3
p−min=3.7e−05, pY=0.43, N=65

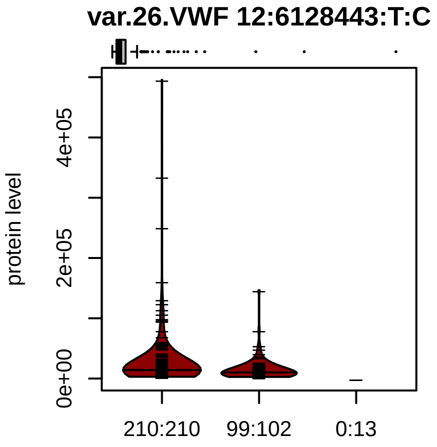

P04275.rs216311_NP.4
p−maj=9.9e−21, pY=1.6e−06, N=309

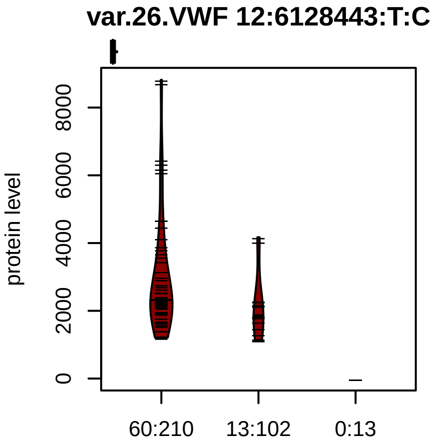

P04275.rs216311_NP.5
p−min=0.00028, pY=0.02, N=73

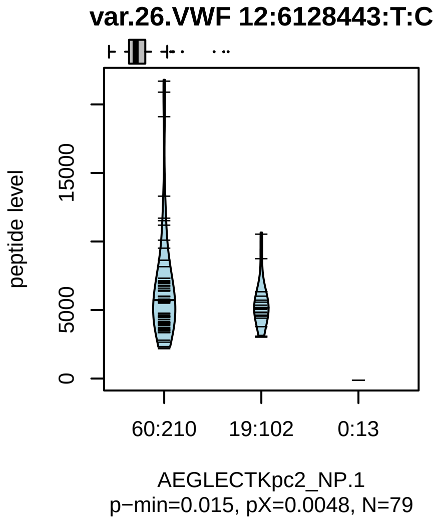
var.26.VWF 12:6128443:T:C
peptide level
0
5000
15000
60:210
19:102
0:13
AEGLECTKpc2_NP.1
p−min=0.015, pX=0.0048, N=79

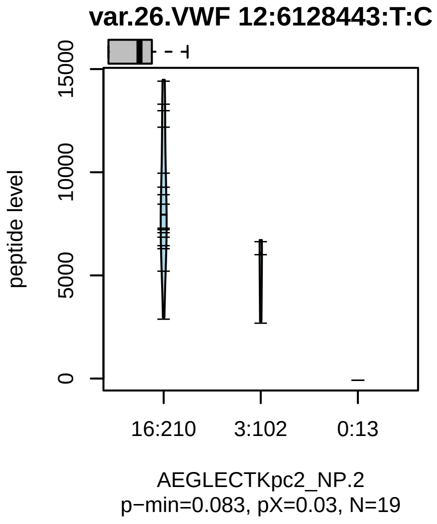
var.26.VWF 12:6128443:T:C
peptide level
0
5000
10000
15000
16:210
3:102
0:13
AEGLECTKpc2_NP.2
p−min=0.083, pX=0.03, N=19

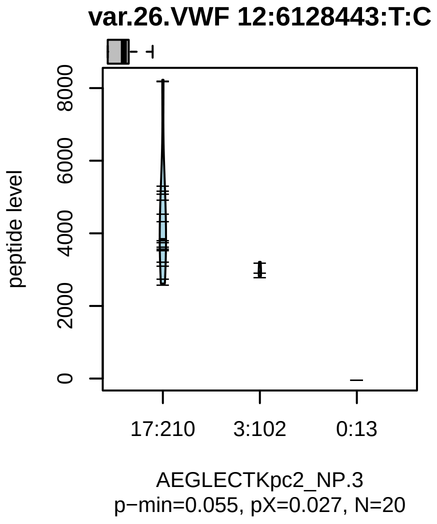
var.26.VWF 12:6128443:T:C
peptide level
0
2000
4000
6000
8000
17:210
3:102
0:13
AEGLECTKpc2_NP.3
p−min=0.055, pX=0.027, N=20

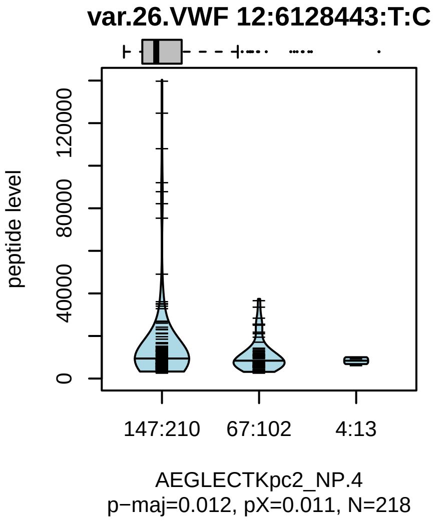
var.26.VWF 12:6128443:T:C
peptide level
0
40000
80000
120000
147:210
67:102
4:13
AEGLECTKpc2_NP.4
p−maj=0.012, pX=0.011, N=218

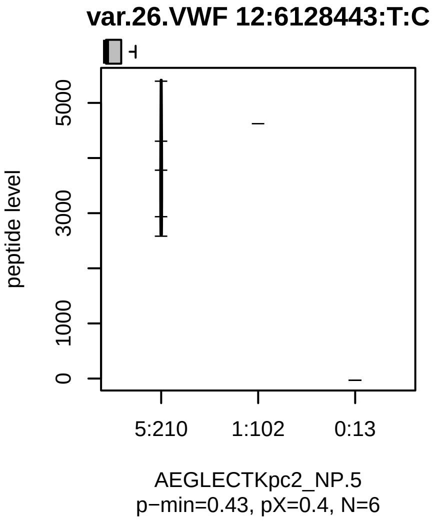
var.26.VWF 12:6128443:T:C
peptide level
0
1000
3000
5000
5:210
1:102
0:13
AEGLECTKpc2_NP.5
p−min=0.43, pX=0.4, N=6

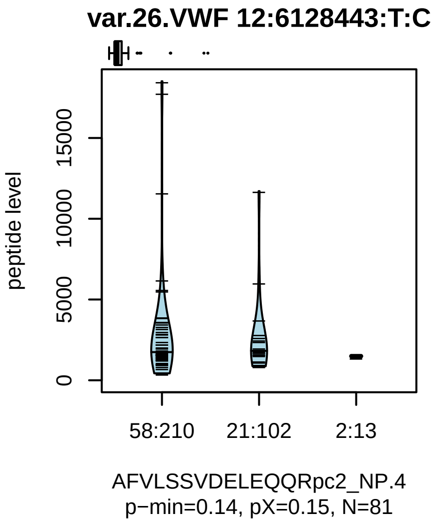
var.26.VWF 12:6128443:T:C
peptide level
0
5000
10000
15000
58:210
21:102
2:13
AFVLSSVDELEQQRpc2_NP.4
p−min=0.14, pX=0.15, N=81

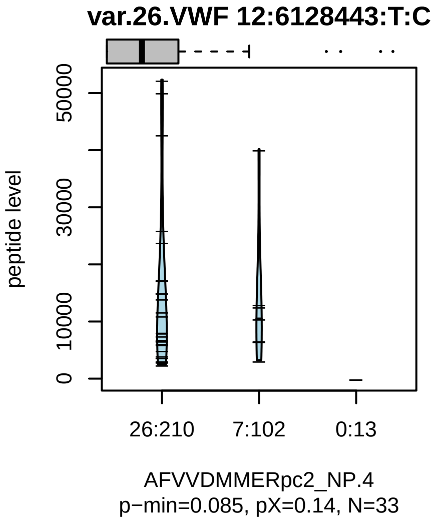
var.26.VWF 12:6128443:T:C
peptide level
0
10000
30000
50000
26:210
7:102
0:13
AFVVDMMERpc2_NP.4
p−min=0.085, pX=0.14, N=33

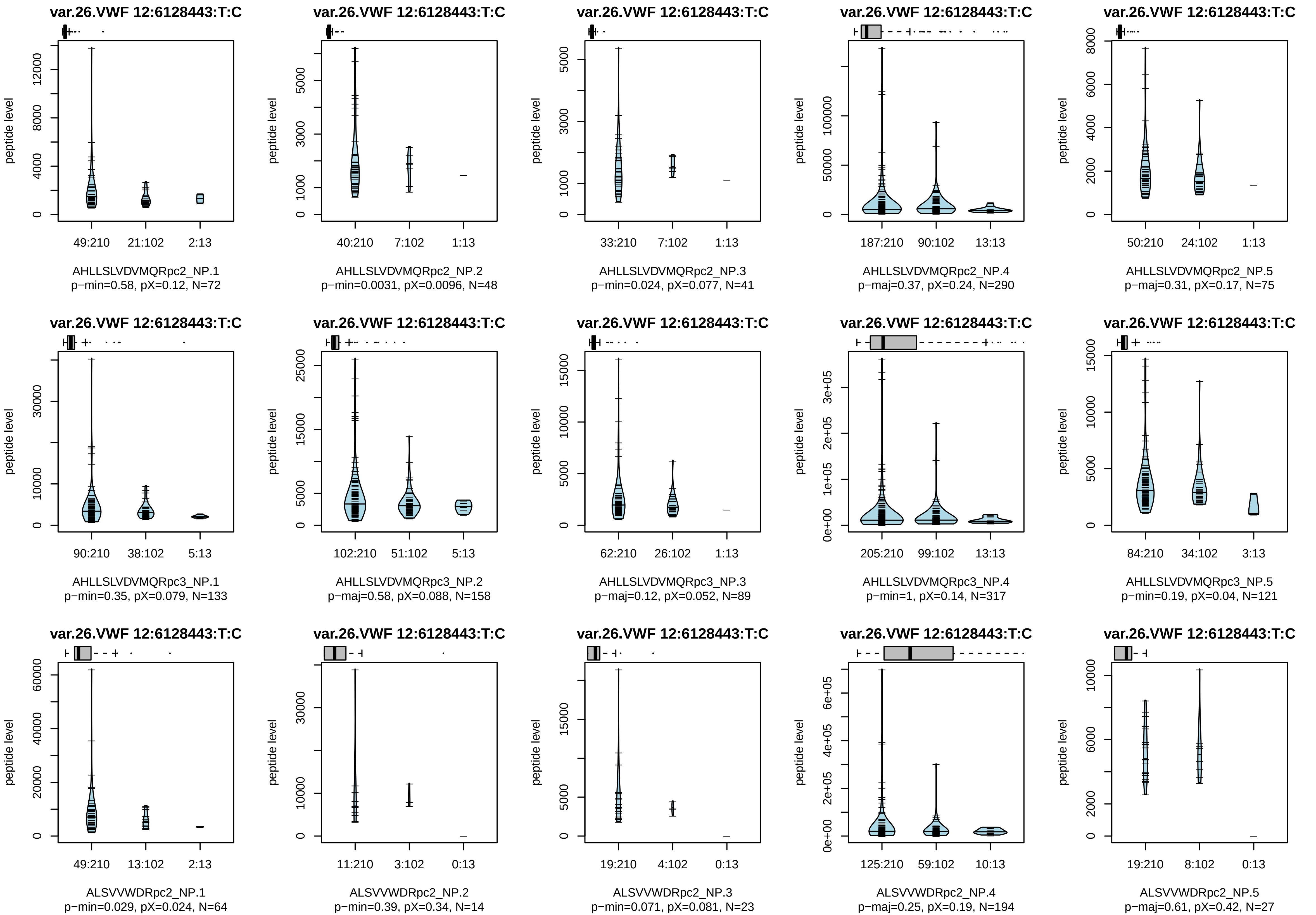
var.26.VWF 12:6128443:T:C
peptide level
49:210
21:102
2:13
AHLLSLVDVMQRpc2_NP.1
p−min=0.58, pX=0.12, N=72
var.26.VWF 12:6128443:T:C
peptide level
40:210
7:102
1:13
AHLLSLVDVMQRpc2_NP.2
p−min=0.0031, pX=0.0096, N=48
var.26.VWF 12:6128443:T:C
peptide level
33:210
7:102
1:13
AHLLSLVDVMQRpc2_NP.3
p−min=0.024, pX=0.077, N=41
var.26.VWF 12:6128443:T:C
peptide level
187:210
90:102
13:13
AHLLSLVDVMQRpc2_NP.4
p−maj=0.37, pX=0.24, N=290
var.26.VWF 12:6128443:T:C
peptide level
50:210
24:102
1:13
AHLLSLVDVMQRpc2_NP.5
p−maj=0.31, pX=0.17, N=75
var.26.VWF 12:6128443:T:C
peptide level
90:210
38:102
5:13
AHLLSLVDVMQRpc3_NP.1
p−min=0.35, pX=0.079, N=133
var.26.VWF 12:6128443:T:C
peptide level
102:210
51:102
5:13
AHLLSLVDVMQRpc3_NP.2
p−maj=0.58, pX=0.088, N=158
var.26.VWF 12:6128443:T:C
peptide level
62:210
26:102
1:13
AHLLSLVDVMQRpc3_NP.3
p−maj=0.12, pX=0.052, N=89
var.26.VWF 12:6128443:T:C
peptide level
205:210
99:102
13:13
AHLLSLVDVMQRpc3_NP.4
p−min=1, pX=0.14, N=317
var.26.VWF 12:6128443:T:C
peptide level
84:210
34:102
3:13
AHLLSLVDVMQRpc3_NP.5
p−min=0.19, pX=0.04, N=121
var.26.VWF 12:6128443:T:C
peptide level
49:210
13:102
2:13
ALSVVWDRpc2_NP.1
p−min=0.029, pX=0.024, N=64
var.26.VWF 12:6128443:T:C
peptide level
11:210
3:102
0:13
ALSVVWDRpc2_NP.2
p−min=0.39, pX=0.34, N=14
var.26.VWF 12:6128443:T:C
peptide level
19:210
4:102
0:13
ALSVVWDRpc2_NP.3
p−min=0.071, pX=0.081, N=23
var.26.VWF 12:6128443:T:C
peptide level
125:210
59:102
10:13
ALSVVWDRpc2_NP.4
p−maj=0.25, pX=0.19, N=194
var.26.VWF 12:6128443:T:C
peptide level
19:210
8:102
0:13
ALSVVWDRpc2_NP.5
p−maj=0.61, pX=0.42, N=27

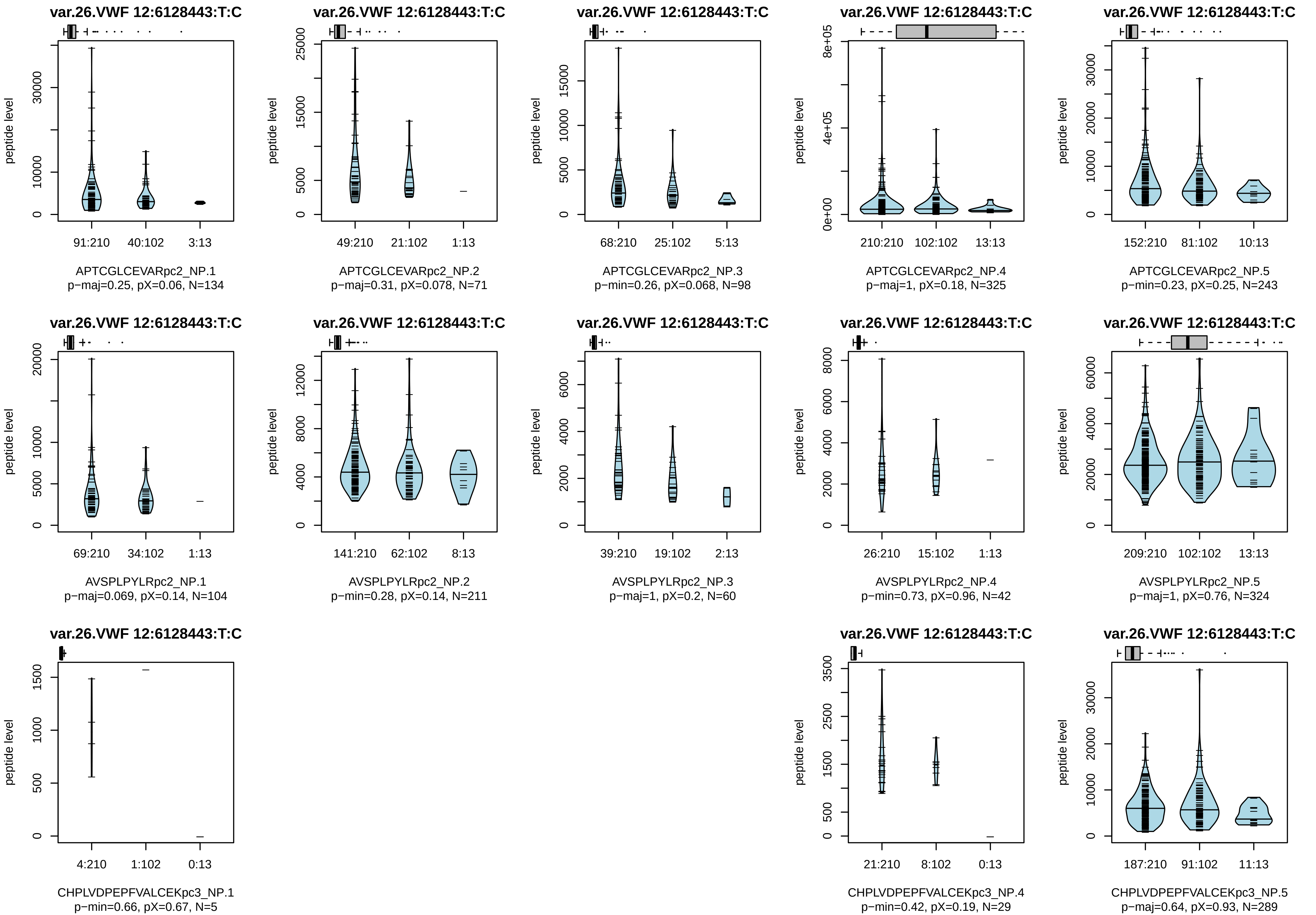
var.26.VWF 12:6128443:T:C
peptide level
91:210
40:102
3:13
APTCGLCEVARpc2_NP.1
p−maj=0.25, pX=0.06, N=134
var.26.VWF 12:6128443:T:C
peptide level
49:210
21:102
1:13
APTCGLCEVARpc2_NP.2
p−maj=0.31, pX=0.078, N=71
var.26.VWF 12:6128443:T:C
peptide level
68:210
25:102
5:13
APTCGLCEVARpc2_NP.3
p−min=0.26, pX=0.068, N=98
var.26.VWF 12:6128443:T:C
peptide level
210:210
102:102
13:13
APTCGLCEVARpc2_NP.4
p−maj=1, pX=0.18, N=325
var.26.VWF 12:6128443:T:C
peptide level
152:210
81:102
10:13
APTCGLCEVARpc2_NP.5
p−min=0.23, pX=0.25, N=243
var.26.VWF 12:6128443:T:C
peptide level
69:210
34:102
1:13
AVSPLPYLRpc2_NP.1
p−maj=0.069, pX=0.14, N=104
var.26.VWF 12:6128443:T:C
peptide level
141:210
62:102
8:13
AVSPLPYLRpc2_NP.2
p−min=0.28, pX=0.14, N=211
var.26.VWF 12:6128443:T:C
peptide level
39:210
19:102
2:13
AVSPLPYLRpc2_NP.3
p−maj=1, pX=0.2, N=60
var.26.VWF 12:6128443:T:C
peptide level
26:210
15:102
1:13
AVSPLPYLRpc2_NP.4
p−min=0.73, pX=0.96, N=42
var.26.VWF 12:6128443:T:C
peptide level
209:210
102:102
13:13
AVSPLPYLRpc2_NP.5
p−maj=1, pX=0.76, N=324
var.26.VWF 12:6128443:T:C
peptide level
4:210
1:102
0:13
CHPLVDPEPFVALCEKpc3_NP.1
p−min=0.66, pX=0.67, N=5
var.26.VWF 12:6128443:T:C
peptide level
21:210
8:102
0:13
CHPLVDPEPFVALCEKpc3_NP.4
p−min=0.42, pX=0.19, N=29
var.26.VWF 12:6128443:T:C
peptide level
187:210
91:102
11:13
CHPLVDPEPFVALCEKpc3_NP.5
p−maj=0.64, pX=0.93, N=289

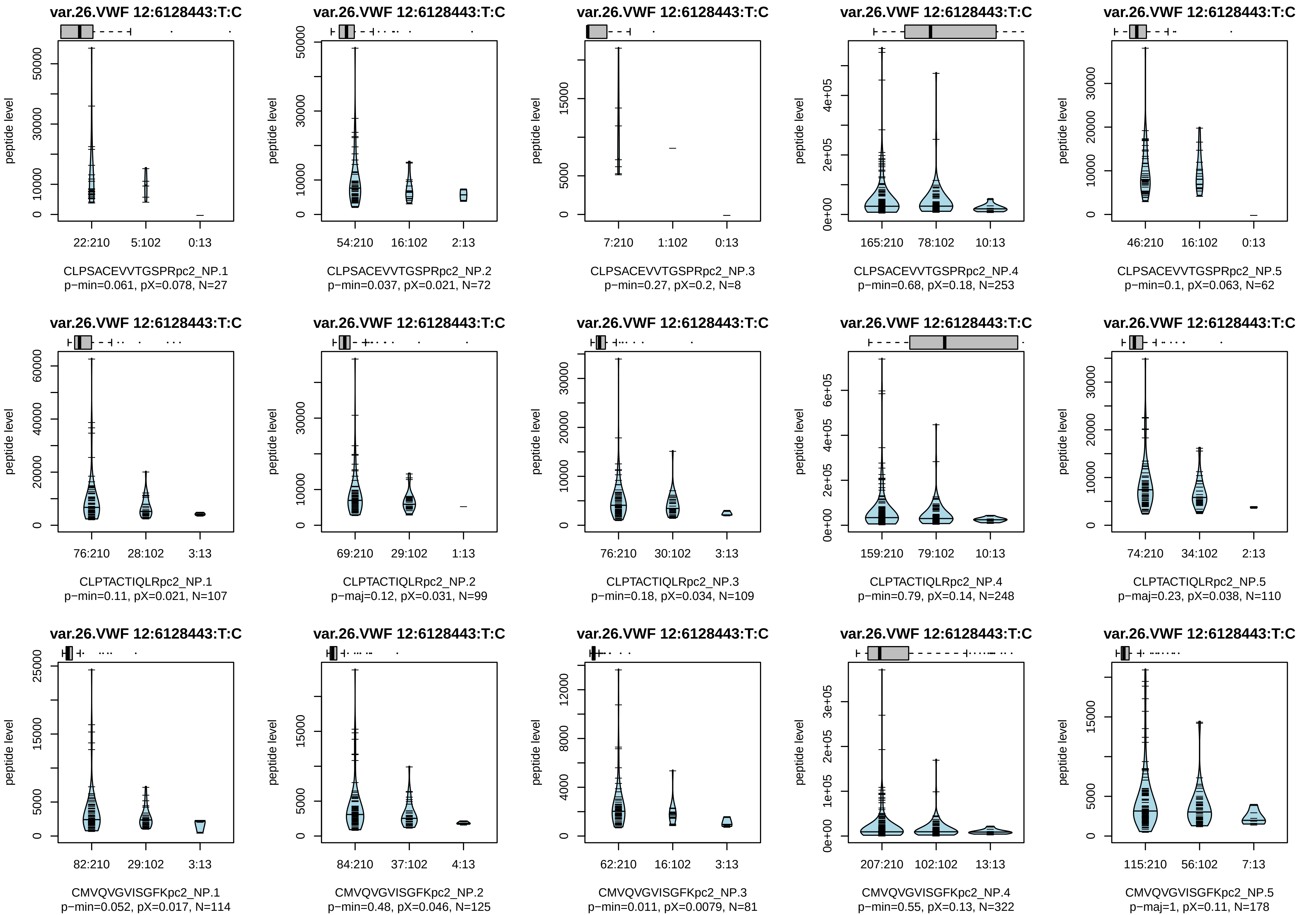
var.26.VWF 12:6128443:T:C
peptide level
22:210
5:102
0:13
CLPSACEVVTGSPRpc2_NP.1
p−min=0.061, pX=0.078, N=27
54:210
16:102
2:13
CLPSACEVVTGSPRpc2_NP.2
p−min=0.037, pX=0.021, N=72
7:210
1:102
0:13
CLPSACEVVTGSPRpc2_NP.3
p−min=0.27, pX=0.2, N=8
165:210
78:102
10:13
CLPSACEVVTGSPRpc2_NP.4
p−min=0.68, pX=0.18, N=253
46:210
16:102
0:13
CLPSACEVVTGSPRpc2_NP.5
p−min=0.1, pX=0.063, N=62
76:210
28:102
3:13
CLPTACTIQLRpc2_NP.1
p−min=0.11, pX=0.021, N=107
69:210
29:102
1:13
CLPTACTIQLRpc2_NP.2
p−maj=0.12, pX=0.031, N=99
76:210
30:102
3:13
CLPTACTIQLRpc2_NP.3
p−min=0.18, pX=0.034, N=109
159:210
79:102
10:13
CLPTACTIQLRpc2_NP.4
p−min=0.79, pX=0.14, N=248
74:210
34:102
2:13
CLPTACTIQLRpc2_NP.5
p−maj=0.23, pX=0.038, N=110
82:210
29:102
3:13
CMVQVGVISGFKpc2_NP.1
p−min=0.052, pX=0.017, N=114
84:210
37:102
4:13
CMVQVGVISGFKpc2_NP.2
p−min=0.48, pX=0.046, N=125
62:210
16:102
3:13
CMVQVGVISGFKpc2_NP.3
p−min=0.011, pX=0.0079, N=81
207:210
102:102
13:13
CMVQVGVISGFKpc2_NP.4
p−min=0.55, pX=0.13, N=322
115:210
56:102
7:13
CMVQVGVISGFKpc2_NP.5
p−maj=1, pX=0.11, N=178

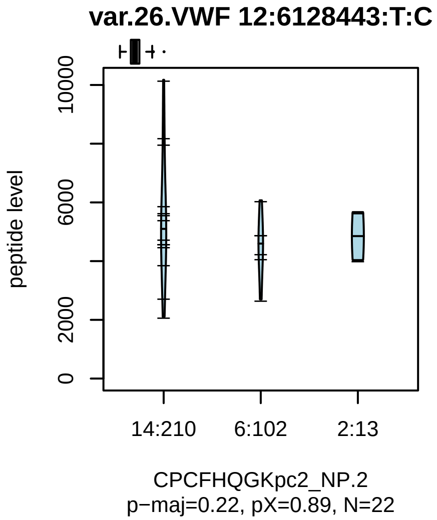
var.26.VWF 12:6128443:T:C
peptide level
0
2000
6000
10000
14:210
6:102
2:13
CPCFHQGKpc2_NP.2
p−maj=0.22, pX=0.89, N=22

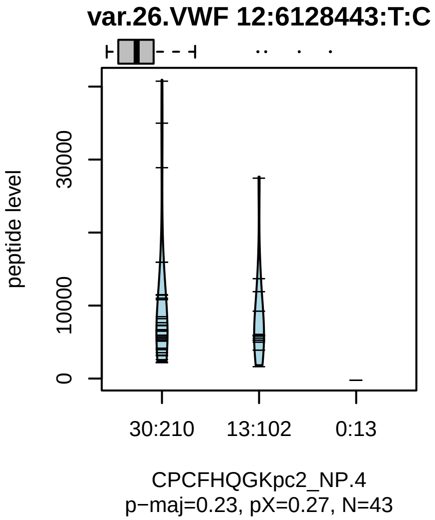
var.26.VWF 12:6128443:T:C
peptide level
0
10000
30000
30:210
13:102
0:13
CPCFHQGKpc2_NP.4
p−maj=0.23, pX=0.27, N=43

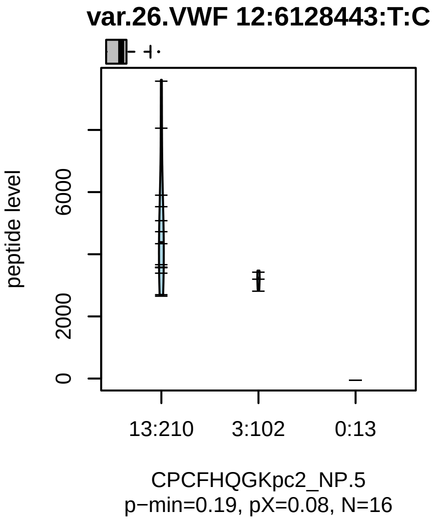
var.26.VWF 12:6128443:T:C
peptide level
0
2000
6000
13:210
3:102
0:13
CPCFHQGKpc2_NP.5
p−min=0.19, pX=0.08, N=16

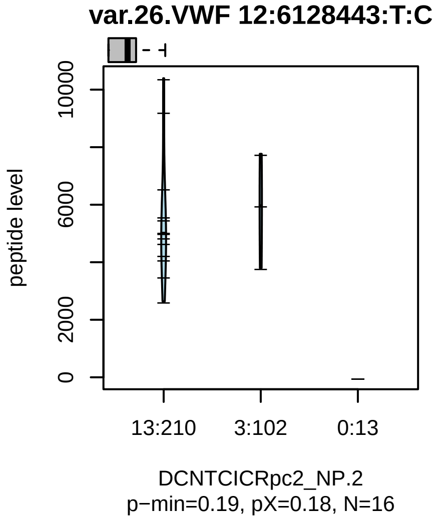
var.26.VWF 12:6128443:T:C
peptide level
0
2000
6000
10000
13:210
3:102
0:13
DCNTCICRpc2_NP.2
p−min=0.19, pX=0.18, N=16

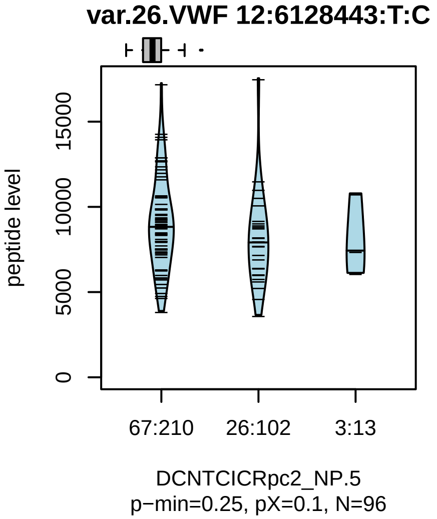
var.26.VWF 12:6128443:T:C
peptide level
0
5000
10000
15000
67:210
26:102
3:13
DCNTCICRpc2_NP.5
p−min=0.25, pX=0.1, N=96

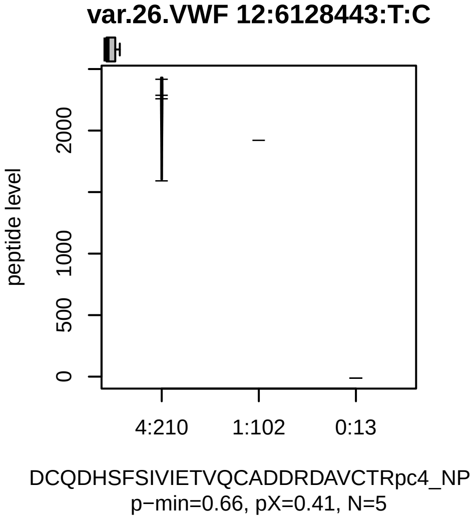
var.26.VWF 12:6128443:T:C
peptide level
0
500
1000
2000
4:210
1:102
0:13
DCQDHSFSIVIETVQCADDRDAVCTRpc4_NP
p−min=0.66, pX=0.41, N=5

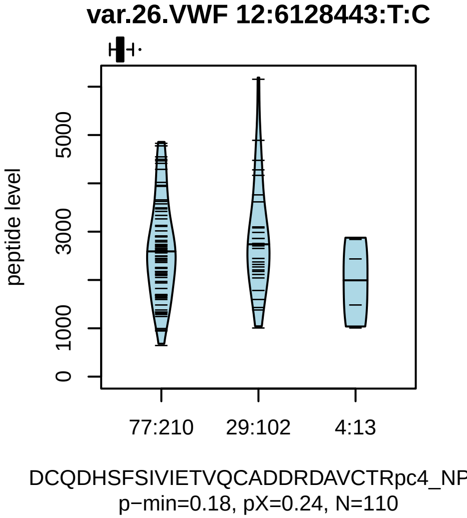
var.26.VWF 12:6128443:T:C
peptide level
0
1000
3000
5000
77:210
29:102
4:13
DCQDHSFSIVIETVQCADDRDAVCTRpc4_NP
p−min=0.18, pX=0.24, N=110

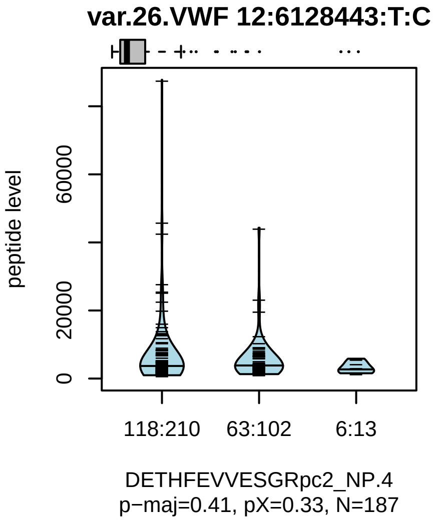
var.26.VWF 12:6128443:T:C
peptide level
0
20000
60000
118:210
63:102
6:13
DETHFEVVESGRpc2_NP.4
p−maj=0.41, pX=0.33, N=187

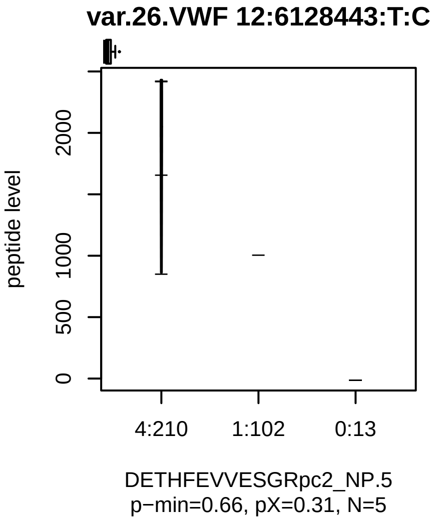
var.26.VWF 12:6128443:T:C
peptide level
0
500
1000
2000
4:210
1:102
0:13
DETHFEVVESGRpc2_NP.5
p−min=0.66, pX=0.31, N=5

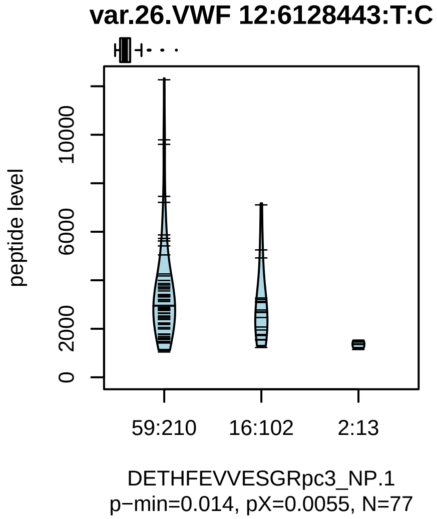
var.26.VWF 12:6128443:T:C
peptide level
0
2000
6000
10000
59:210
16:102
2:13
DETHFEVVESGRpc3_NP.1
p−min=0.014, pX=0.0055, N=77

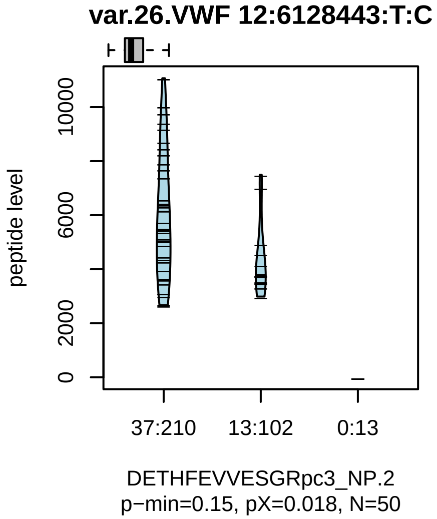
var.26.VWF 12:6128443:T:C
peptide level
0
2000
6000
10000
37:210
13:102
0:13
DETHFEVVESGRpc3_NP.2
p−min=0.15, pX=0.018, N=50

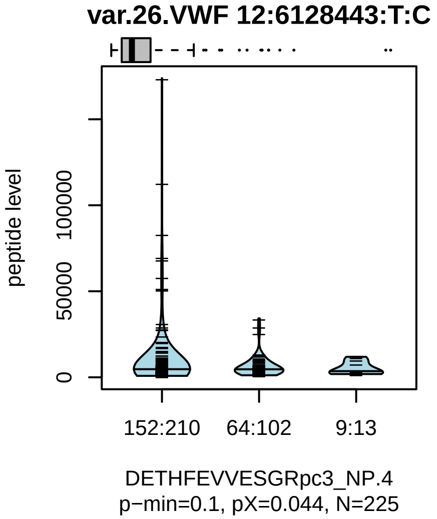
var.26.VWF 12:6128443:T:C
peptide level
0
50000
100000
152:210
64:102
9:13
DETHFEVVESGRpc3_NP.4
p−min=0.1, pX=0.044, N=225

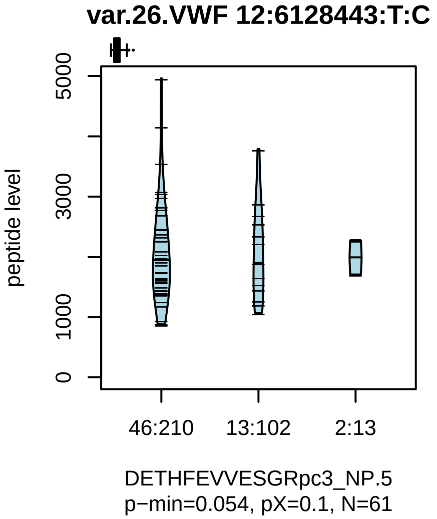
var.26.VWF 12:6128443:T:C
peptide level
0
1000
3000
5000
46:210
13:102
2:13
DETHFEVVESGRpc3_NP.5
p−min=0.054, pX=0.1, N=61

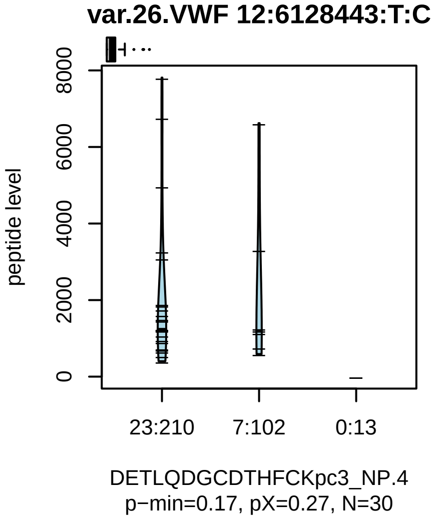
var.26.VWF 12:6128443:T:C
peptide level
0
2000
4000
6000
8000
23:210
7:102
0:13
DETLQDGCDTHFCKpc3_NP.4
p−min=0.17, pX=0.27, N=30

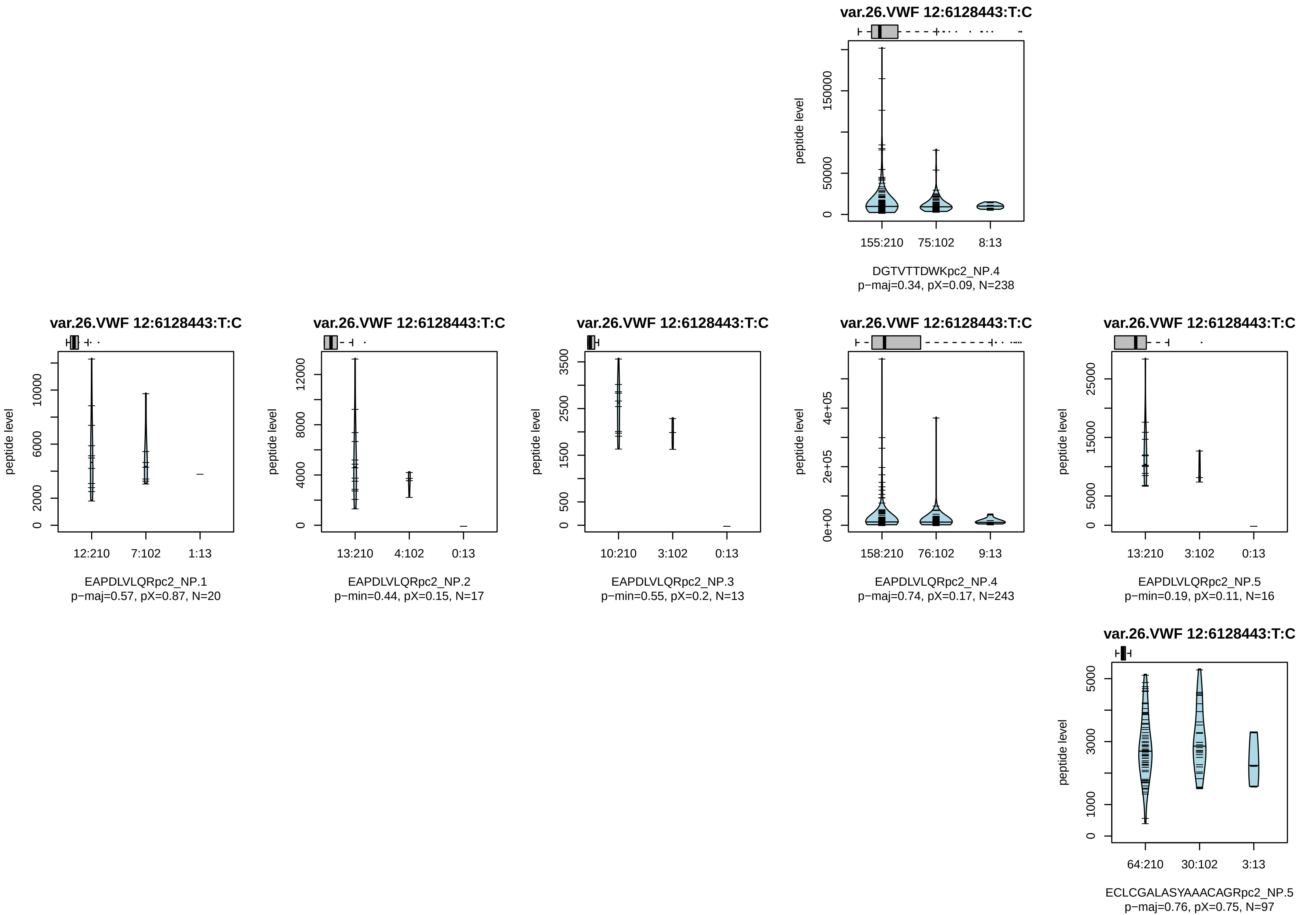
var.26.VWF 12:6128443:T:C
peptide level
155:210
75:102
8:13
DGTVTTDWKpc2_NP.4
p−maj=0.34, pX=0.09, N=238
var.26.VWF 12:6128443:T:C
12:210
7:102
1:13
EAPDLVLQRpc2_NP.1
p−maj=0.57, pX=0.87, N=20
var.26.VWF 12:6128443:T:C
13:210
4:102
0:13
EAPDLVLQRpc2_NP.2
p−min=0.44, pX=0.15, N=17
var.26.VWF 12:6128443:T:C
10:210
3:102
0:13
EAPDLVLQRpc2_NP.3
p−min=0.55, pX=0.2, N=13
var.26.VWF 12:6128443:T:C
158:210
76:102
9:13
EAPDLVLQRpc2_NP.4
p−maj=0.74, pX=0.17, N=243
var.26.VWF 12:6128443:T:C
13:210
3:102
0:13
EAPDLVLQRpc2_NP.5
p−min=0.19, pX=0.11, N=16
var.26.VWF 12:6128443:T:C
64:210
30:102
3:13
ECLCGALASYAAACAGRpc2_NP.5
p−maj=0.76, pX=0.75, N=97

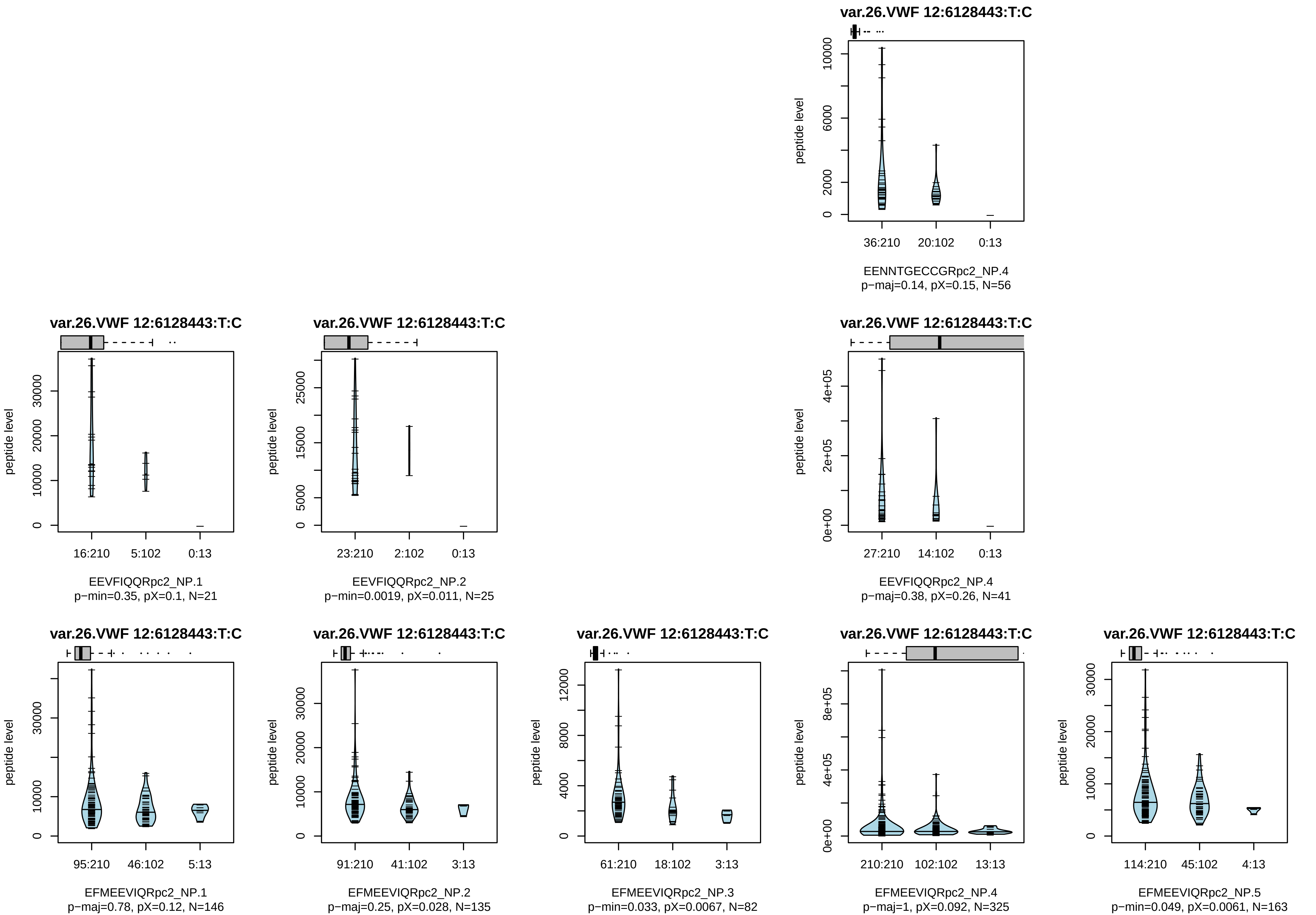

var.26.VWF 12:6128443:T:C
peptide level
36:210
20:102
0:13
EENNTGECCGRpc2_NP.4
p−maj=0.14, pX=0.15, N=56
var.26.VWF 12:6128443:T:C
peptide level
16:210
5:102
0:13
EEVFIQQRpc2_NP.1
p−min=0.35, pX=0.1, N=21
var.26.VWF 12:6128443:T:C
peptide level
23:210
2:102
0:13
EEVFIQQRpc2_NP.2
p−min=0.0019, pX=0.011, N=25
var.26.VWF 12:6128443:T:C
peptide level
27:210
14:102
0:13
EEVFIQQRpc2_NP.4
p−maj=0.38, pX=0.26, N=41
var.26.VWF 12:6128443:T:C
peptide level
95:210
46:102
5:13
EFMEEVIQRpc2_NP.1
p−maj=0.78, pX=0.12, N=146
var.26.VWF 12:6128443:T:C
peptide level
91:210
41:102
3:13
EFMEEVIQRpc2_NP.2
p−maj=0.25, pX=0.028, N=135
var.26.VWF 12:6128443:T:C
peptide level
61:210
18:102
3:13
EFMEEVIQRpc2_NP.3
p−min=0.033, pX=0.0067, N=82
var.26.VWF 12:6128443:T:C
peptide level
210:210
102:102
13:13
EFMEEVIQRpc2_NP.4
p−maj=1, pX=0.092, N=325
var.26.VWF 12:6128443:T:C
peptide level
114:210
45:102
4:13
EFMEEVIQRpc2_NP.5
p−min=0.049, pX=0.0061, N=163

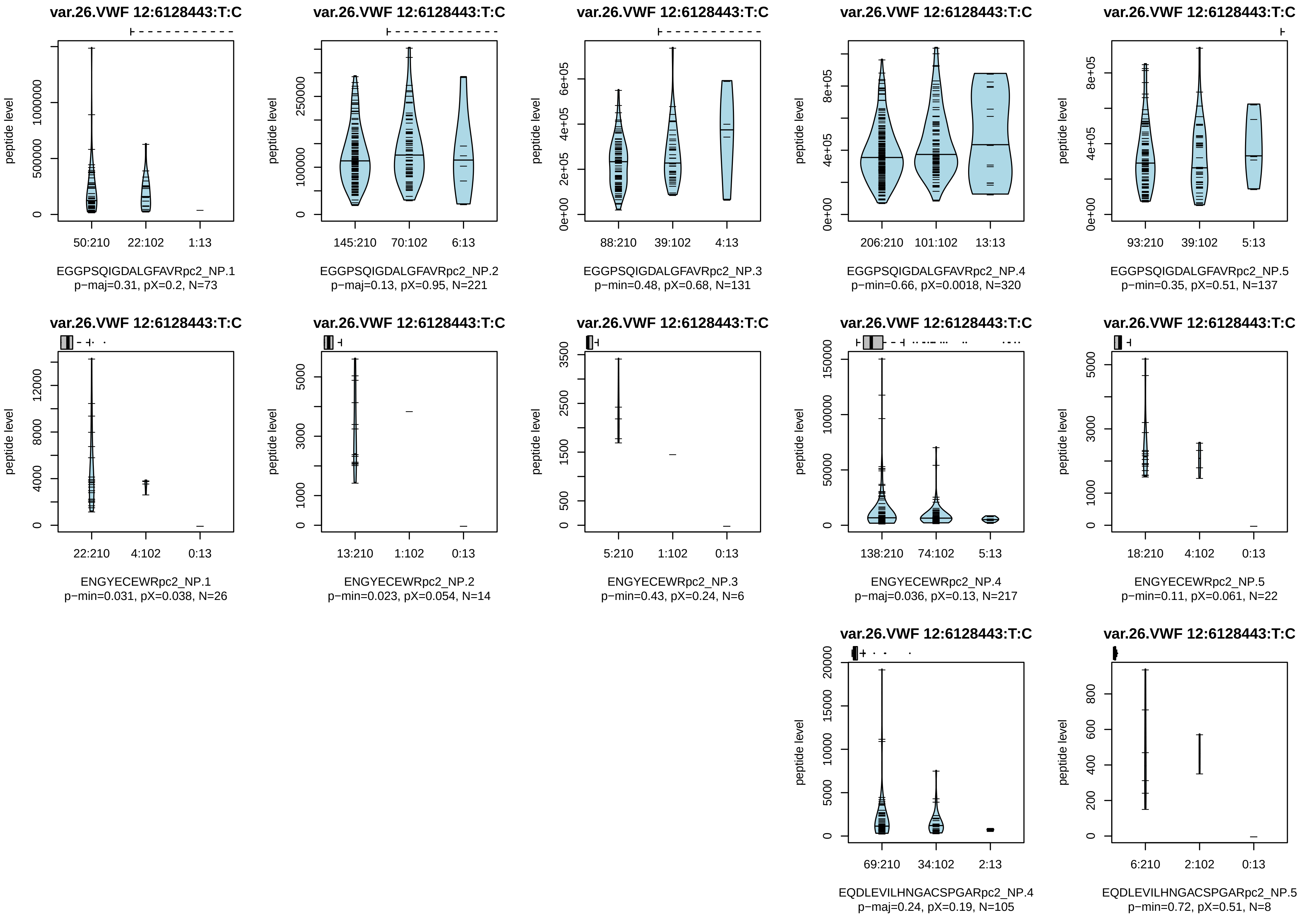

var.26.VWF 12:6128443:T:C
peptide level
50:210
22:102
1:13
EGGPSQIGDALGFAVRpc2_NP.1
p−maj=0.31, pX=0.2, N=73
var.26.VWF 12:6128443:T:C
peptide level
145:210
70:102
6:13
EGGPSQIGDALGFAVRpc2_NP.2
p−maj=0.13, pX=0.95, N=221
var.26.VWF 12:6128443:T:C
peptide level
88:210
39:102
4:13
EGGPSQIGDALGFAVRpc2_NP.3
p−min=0.48, pX=0.68, N=131
var.26.VWF 12:6128443:T:C
peptide level
206:210
101:102
13:13
EGGPSQIGDALGFAVRpc2_NP.4
p−min=0.66, pX=0.0018, N=320
var.26.VWF 12:6128443:T:C
peptide level
93:210
39:102
5:13
EGGPSQIGDALGFAVRpc2_NP.5
p−min=0.35, pX=0.51, N=137
var.26.VWF 12:6128443:T:C
peptide level
22:210
4:102
0:13
ENGYECEWRpc2_NP.1
p−min=0.031, pX=0.038, N=26
var.26.VWF 12:6128443:T:C
peptide level
13:210
1:102
0:13
ENGYECEWRpc2_NP.2
p−min=0.023, pX=0.054, N=14
var.26.VWF 12:6128443:T:C
peptide level
5:210
1:102
0:13
ENGYECEWRpc2_NP.3
p−min=0.43, pX=0.24, N=6
var.26.VWF 12:6128443:T:C
peptide level
138:210
74:102
5:13
ENGYECEWRpc2_NP.4
p−maj=0.036, pX=0.13, N=217
var.26.VWF 12:6128443:T:C
peptide level
18:210
4:102
0:13
ENGYECEWRpc2_NP.5
p−min=0.11, pX=0.061, N=22
var.26.VWF 12:6128443:T:C
peptide level
69:210
34:102
2:13
EQDLEVILHNGACSPGARpc2_NP.4
p−maj=0.24, pX=0.19, N=105
var.26.VWF 12:6128443:T:C
peptide level
6:210
2:102
0:13
EQDLEVILHNGACSPGARpc2_NP.5
p−min=0.72, pX=0.51, N=8

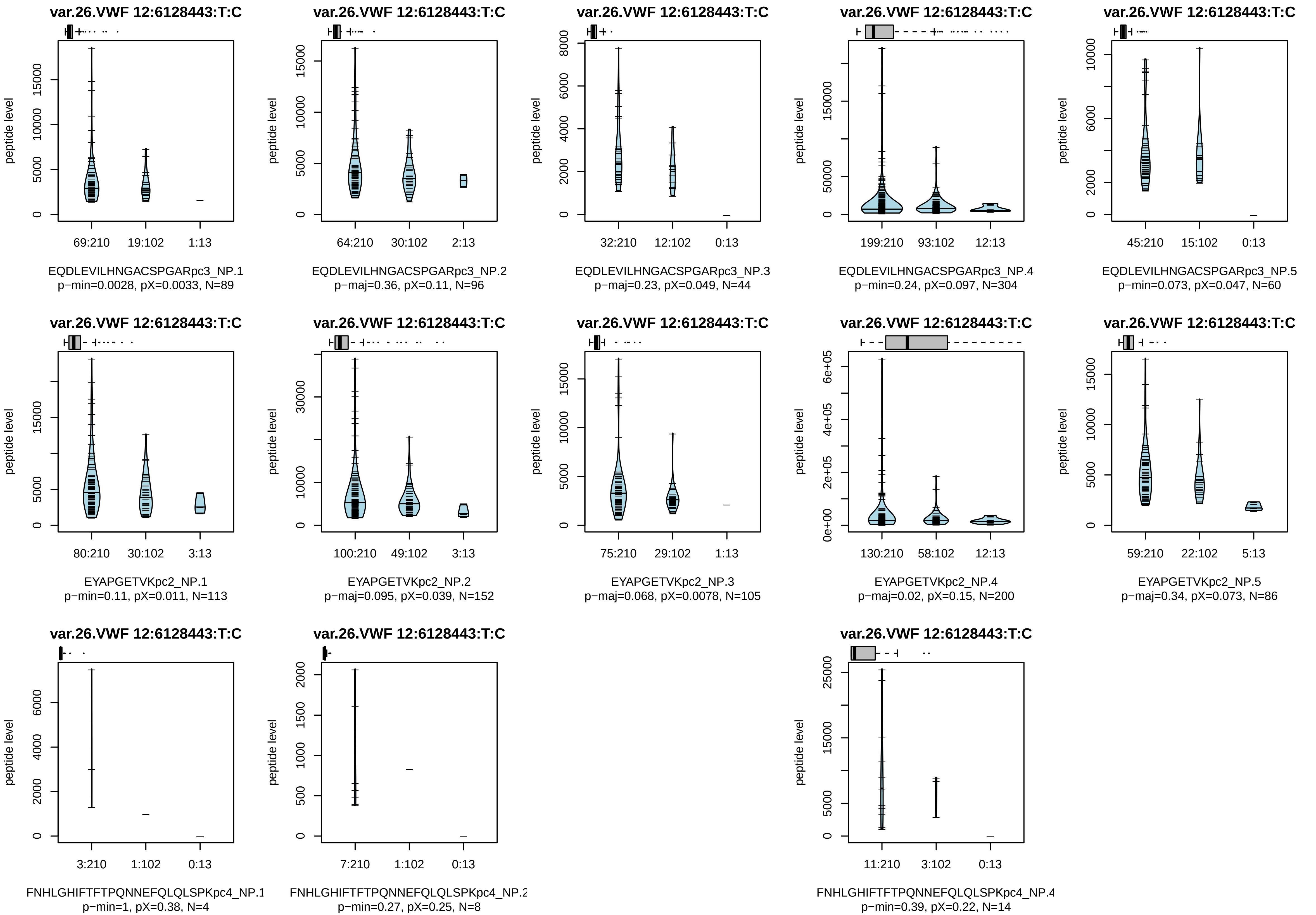

var.26.VWF 12:6128443:T:C
peptide level
69:210
19:102
1:13
EQDLEVILHNGACSPGARpc3_NP.1
p−min=0.0028, pX=0.0033, N=89
var.26.VWF 12:6128443:T:C
peptide level
64:210
30:102
2:13
EQDLEVILHNGACSPGARpc3_NP.2
p−maj=0.36, pX=0.11, N=96
var.26.VWF 12:6128443:T:C
peptide level
32:210
12:102
0:13
EQDLEVILHNGACSPGARpc3_NP.3
p−maj=0.23, pX=0.049, N=44
var.26.VWF 12:6128443:T:C
peptide level
199:210
93:102
12:13
EQDLEVILHNGACSPGARpc3_NP.4
p−min=0.24, pX=0.097, N=304
var.26.VWF 12:6128443:T:C
peptide level
45:210
15:102
0:13
EQDLEVILHNGACSPGARpc3_NP.5
p−min=0.073, pX=0.047, N=60
var.26.VWF 12:6128443:T:C
peptide level
80:210
30:102
3:13
EYAPGETVKpc2_NP.1
p−min=0.11, pX=0.011, N=113
var.26.VWF 12:6128443:T:C
peptide level
100:210
49:102
3:13
EYAPGETVKpc2_NP.2
p−maj=0.095, pX=0.039, N=152
var.26.VWF 12:6128443:T:C
peptide level
75:210
29:102
1:13
EYAPGETVKpc2_NP.3
p−maj=0.068, pX=0.0078, N=105
var.26.VWF 12:6128443:T:C
peptide level
130:210
58:102
12:13
EYAPGETVKpc2_NP.4
p−maj=0.02, pX=0.15, N=200
var.26.VWF 12:6128443:T:C
peptide level
59:210
22:102
5:13
EYAPGETVKpc2_NP.5
p−maj=0.34, pX=0.073, N=86
var.26.VWF 12:6128443:T:C
peptide level
3:210
1:102
0:13
FNHLGHIFTFTPQNNEFQLQLSPKpc4_NP.1
p−min=1, pX=0.38, N=4
var.26.VWF 12:6128443:T:C
peptide level
7:210
1:102
0:13
FNHLGHIFTFTPQNNEFQLQLSPKpc4_NP.2
p−min=0.27, pX=0.25, N=8
var.26.VWF 12:6128443:T:C
peptide level
11:210
3:102
0:13
FNHLGHIFTFTPQNNEFQLQLSPKpc4_NP.4
p−min=0.39, pX=0.22, N=14

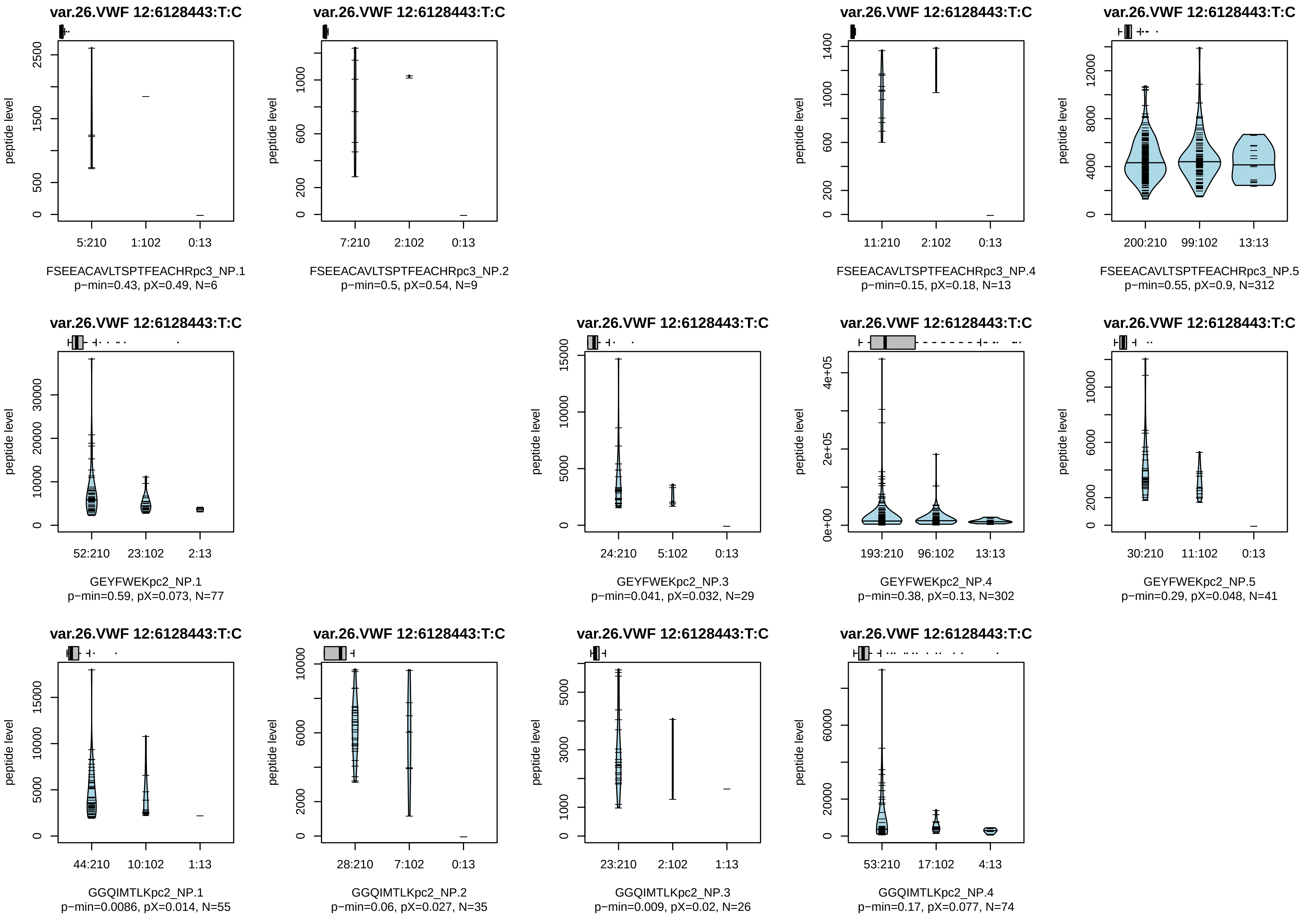

var.26.VWF 12:6128443:T:C
peptide level
5:210
1:102
0:13
FSEEACAVLTSPTFEACHRpc3_NP.1
p−min=0.43, pX=0.49, N=6
var.26.VWF 12:6128443:T:C
peptide level
7:210
2:102
0:13
FSEEACAVLTSPTFEACHRpc3_NP.2
p−min=0.5, pX=0.54, N=9
var.26.VWF 12:6128443:T:C
peptide level
11:210
2:102
0:13
FSEEACAVLTSPTFEACHRpc3_NP.4
p−min=0.15, pX=0.18, N=13
var.26.VWF 12:6128443:T:C
peptide level
200:210
99:102
13:13
FSEEACAVLTSPTFEACHRpc3_NP.5
p−min=0.55, pX=0.9, N=312
var.26.VWF 12:6128443:T:C
peptide level
52:210
23:102
2:13
GEYFWEKpc2_NP.1
p−min=0.59, pX=0.073, N=77
var.26.VWF 12:6128443:T:C
peptide level
24:210
5:102
0:13
GEYFWEKpc2_NP.3
p−min=0.041, pX=0.032, N=29
var.26.VWF 12:6128443:T:C
peptide level
193:210
96:102
13:13
GEYFWEKpc2_NP.4
p−min=0.38, pX=0.13, N=302
var.26.VWF 12:6128443:T:C
peptide level
30:210
11:102
0:13
GEYFWEKpc2_NP.5
p−min=0.29, pX=0.048, N=41
var.26.VWF 12:6128443:T:C
peptide level
44:210
10:102
1:13
GGQIMTLKpc2_NP.1
p−min=0.0086, pX=0.014, N=55
var.26.VWF 12:6128443:T:C
peptide level
28:210
7:102
0:13
GGQIMTLKpc2_NP.2
p−min=0.06, pX=0.027, N=35
var.26.VWF 12:6128443:T:C
peptide level
23:210
2:102
1:13
GGQIMTLKpc2_NP.3
p−min=0.009, pX=0.02, N=26
var.26.VWF 12:6128443:T:C
peptide level
53:210
17:102
4:13
GGQIMTLKpc2_NP.4
p−min=0.17, pX=0.077, N=74

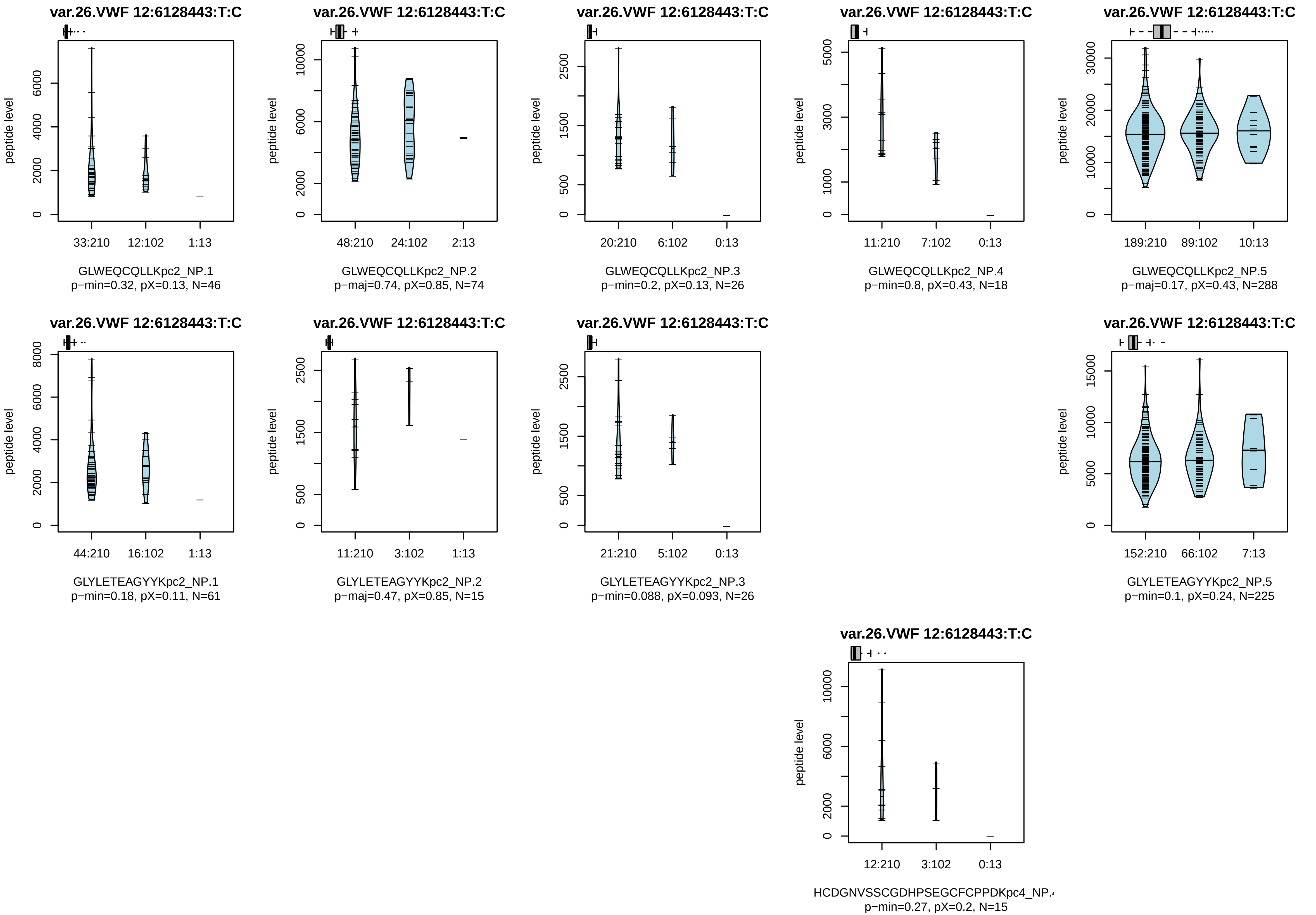
var.26.VWF 12:6128443:T:C
peptide level
33:210
12:102
1:13
GLWEQCQLLKpc2_NP.1
p−min=0.32, pX=0.13, N=46
var.26.VWF 12:6128443:T:C
peptide level
48:210
24:102
2:13
GLWEQCQLLKpc2_NP.2
p−maj=0.74, pX=0.85, N=74
var.26.VWF 12:6128443:T:C
peptide level
20:210
6:102
0:13
GLWEQCQLLKpc2_NP.3
p−min=0.2, pX=0.13, N=26
var.26.VWF 12:6128443:T:C
peptide level
11:210
7:102
0:13
GLWEQCQLLKpc2_NP.4
p−min=0.8, pX=0.43, N=18
var.26.VWF 12:6128443:T:C
peptide level
189:210
89:102
10:13
GLWEQCQLLKpc2_NP.5
p−maj=0.17, pX=0.43, N=288
var.26.VWF 12:6128443:T:C
peptide level
44:210
16:102
1:13
GLYLETEAGYYKpc2_NP.1
p−min=0.18, pX=0.11, N=61
var.26.VWF 12:6128443:T:C
peptide level
11:210
3:102
1:13
GLYLETEAGYYKpc2_NP.2
p−maj=0.47, pX=0.85, N=15
var.26.VWF 12:6128443:T:C
peptide level
21:210
5:102
0:13
GLYLETEAGYYKpc2_NP.3
p−min=0.088, pX=0.093, N=26
var.26.VWF 12:6128443:T:C
peptide level
152:210
66:102
7:13
GLYLETEAGYYKpc2_NP.5
p−min=0.1, pX=0.24, N=225
var.26.VWF 12:6128443:T:C
peptide level
12:210
3:102
0:13
HCDGNVSSCGDHPSEGCFCPPDKpc4_NP.4
p−min=0.27, pX=0.2, N=15

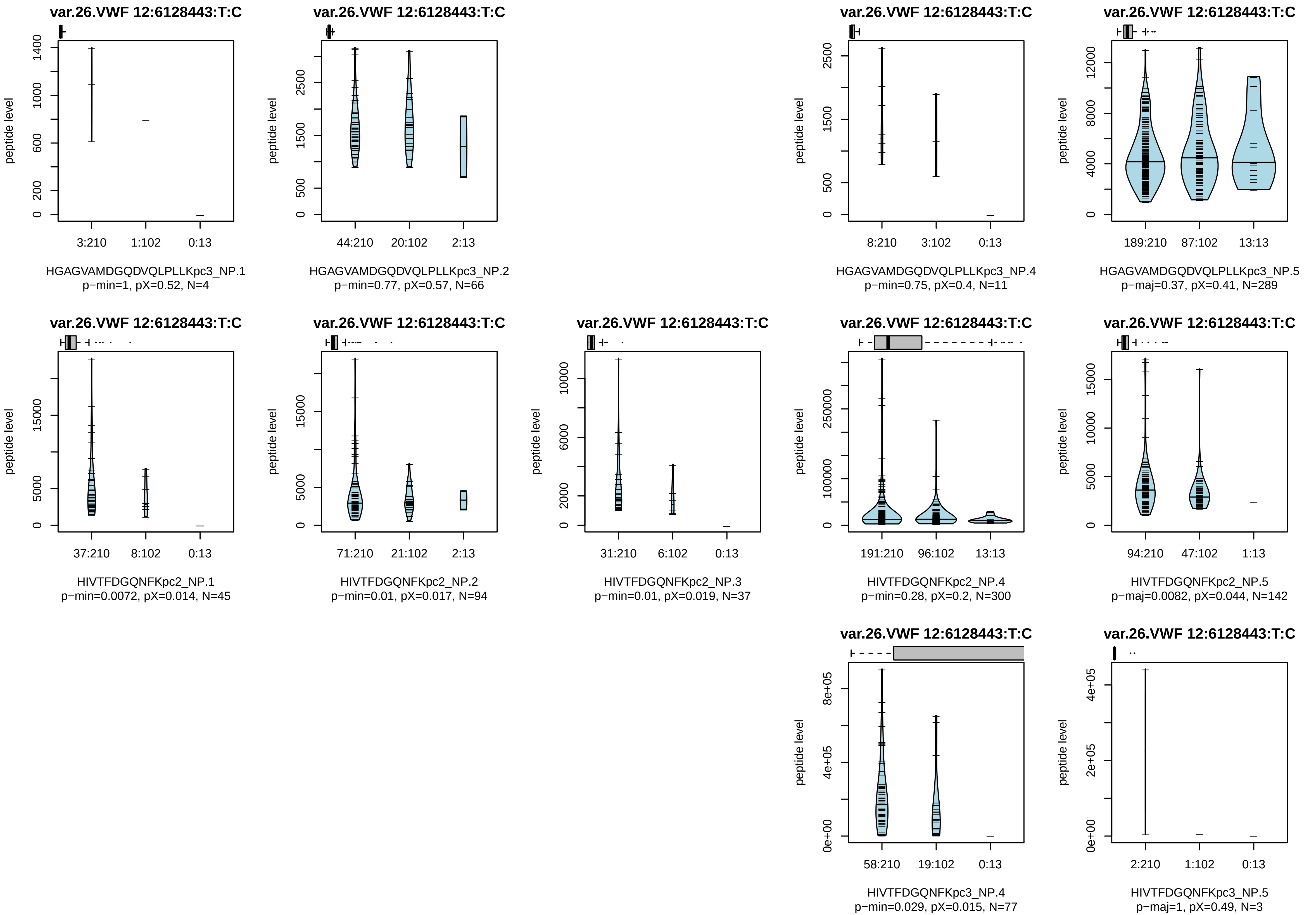
var.26.VWF 12:6128443:T:C
peptide level
0
200
600
1000
1400
3:210
1:102
0:13
HGAGVAMDGQDVQLPLLKpc3_NP.1
p−min=1, pX=0.52, N=4
var.26.VWF 12:6128443:T:C
peptide level
0
500
1500
2500
44:210
20:102
2:13
HGAGVAMDGQDVQLPLLKpc3_NP.2
p−min=0.77, pX=0.57, N=66
var.26.VWF 12:6128443:T:C
peptide level
0
500
1500
2500
8:210
3:102
0:13
HGAGVAMDGQDVQLPLLKpc3_NP.4
p−min=0.75, pX=0.4, N=11
var.26.VWF 12:6128443:T:C
peptide level
0
4000
8000
12000
189:210
87:102
13:13
HGAGVAMDGQDVQLPLLKpc3_NP.5
p−maj=0.37, pX=0.41, N=289
var.26.VWF 12:6128443:T:C
peptide level
0
5000
15000
37:210
8:102
0:13
HIVTFDGQNFKpc2_NP.1
p−min=0.0072, pX=0.014, N=45
var.26.VWF 12:6128443:T:C
peptide level
0
5000
15000
71:210
21:102
2:13
HIVTFDGQNFKpc2_NP.2
p−min=0.01, pX=0.017, N=94
var.26.VWF 12:6128443:T:C
peptide level
0
2000
6000
10000
31:210
6:102
0:13
HIVTFDGQNFKpc2_NP.3
p−min=0.01, pX=0.019, N=37
var.26.VWF 12:6128443:T:C
peptide level
0
100000
250000
191:210
96:102
13:13
HIVTFDGQNFKpc2_NP.4
p−min=0.28, pX=0.2, N=300
var.26.VWF 12:6128443:T:C
peptide level
0
5000
10000
15000
94:210
47:102
1:13
HIVTFDGQNFKpc2_NP.5
p−maj=0.0082, pX=0.044, N=142
var.26.VWF 12:6128443:T:C
peptide level
0e+00
4e+05
8e+05
58:210
19:102
0:13
HIVTFDGQNFKpc3_NP.4
p−min=0.029, pX=0.015, N=77
var.26.VWF 12:6128443:T:C
peptide level
0e+00
2e+05
4e+05
2:210
1:102
0:13
HIVTFDGQNFKpc3_NP.5
p−maj=1, pX=0.49, N=3

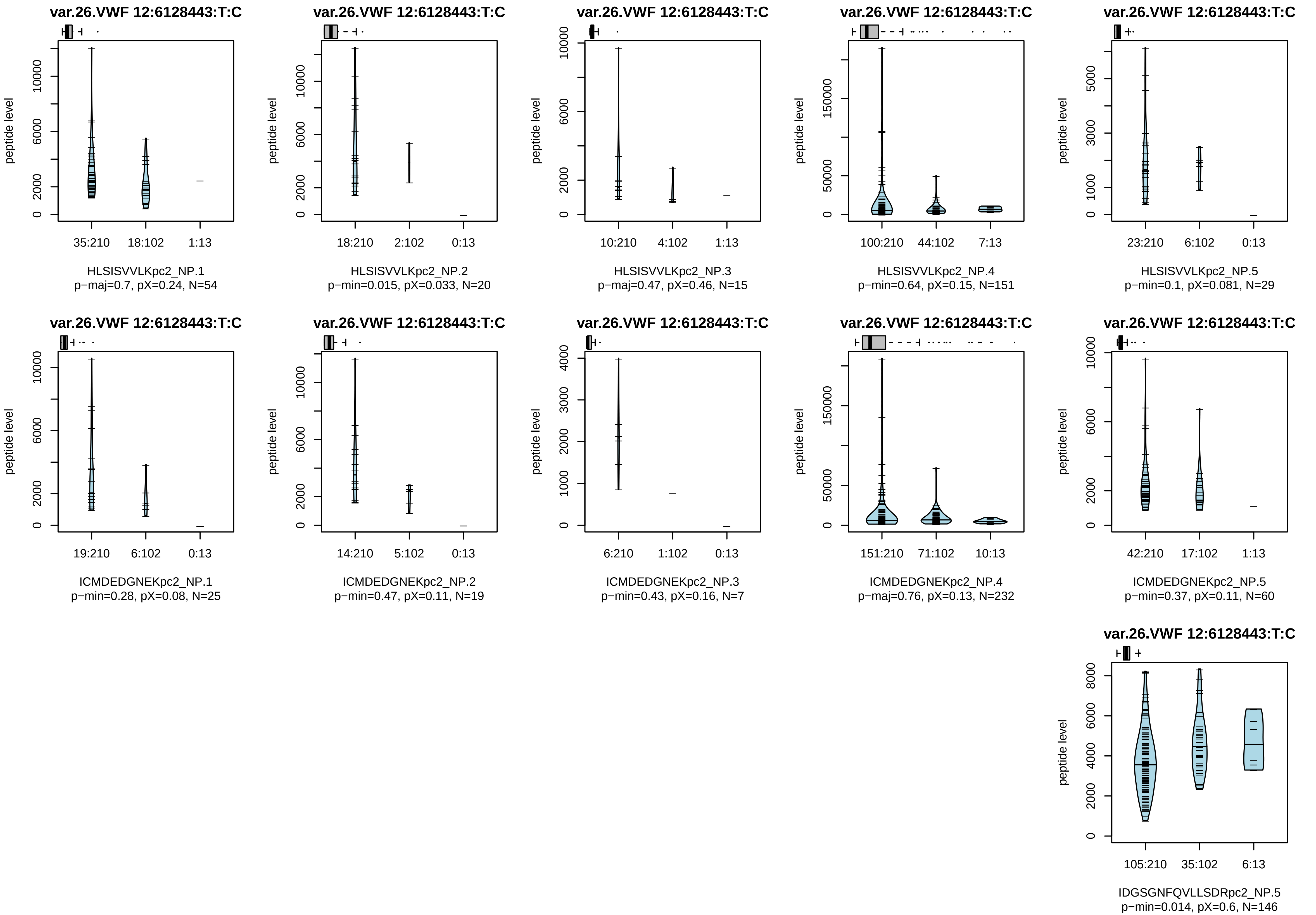
var.26.VWF 12:6128443:T:C
peptide level
35:210
18:102
1:13
HLSISVVLKpc2_NP.1
p−maj=0.7, pX=0.24, N=54
var.26.VWF 12:6128443:T:C
peptide level
18:210
2:102
0:13
HLSISVVLKpc2_NP.2
p−min=0.015, pX=0.033, N=20
var.26.VWF 12:6128443:T:C
peptide level
10:210
4:102
1:13
HLSISVVLKpc2_NP.3
p−maj=0.47, pX=0.46, N=15
var.26.VWF 12:6128443:T:C
peptide level
100:210
44:102
7:13
HLSISVVLKpc2_NP.4
p−min=0.64, pX=0.15, N=151
var.26.VWF 12:6128443:T:C
peptide level
23:210
6:102
0:13
HLSISVVLKpc2_NP.5
p−min=0.1, pX=0.081, N=29
var.26.VWF 12:6128443:T:C
peptide level
19:210
6:102
0:13
ICMDEDGNEKpc2_NP.1
p−min=0.28, pX=0.08, N=25
var.26.VWF 12:6128443:T:C
peptide level
14:210
5:102
0:13
ICMDEDGNEKpc2_NP.2
p−min=0.47, pX=0.11, N=19
var.26.VWF 12:6128443:T:C
peptide level
6:210
1:102
0:13
ICMDEDGNEKpc2_NP.3
p−min=0.43, pX=0.16, N=7
var.26.VWF 12:6128443:T:C
peptide level
151:210
71:102
10:13
ICMDEDGNEKpc2_NP.4
p−maj=0.76, pX=0.13, N=232
var.26.VWF 12:6128443:T:C
peptide level
42:210
17:102
1:13
ICMDEDGNEKpc2_NP.5
p−min=0.37, pX=0.11, N=60
var.26.VWF 12:6128443:T:C
peptide level
105:210
35:102
6:13
IDGSGNFQVLLSDRpc2_NP.5
p−min=0.014, pX=0.6, N=146

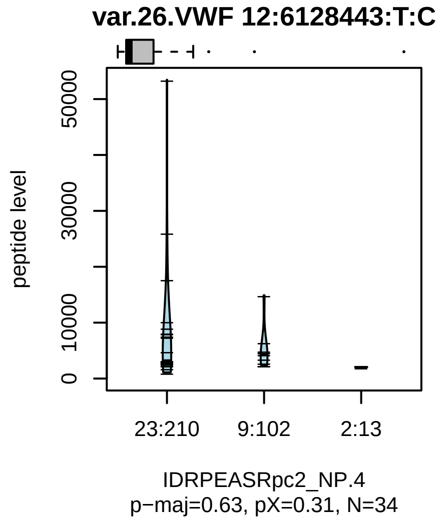
var.26.VWF 12:6128443:T:C
peptide level
0
10000
30000
50000
23:210
9:102
2:13
IDRPEASRpc2_NP.4
p−maj=0.63, pX=0.31, N=34

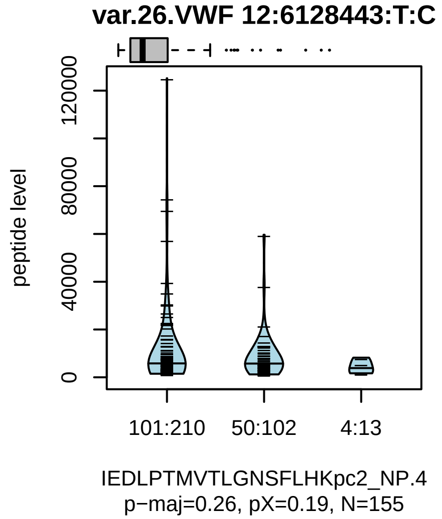
var.26.VWF 12:6128443:T:C
peptide level
0
40000
80000
120000
101:210
50:102
4:13
IEDLPTMVTLGNSFLHKpc2_NP.4
p−maj=0.26, pX=0.19, N=155

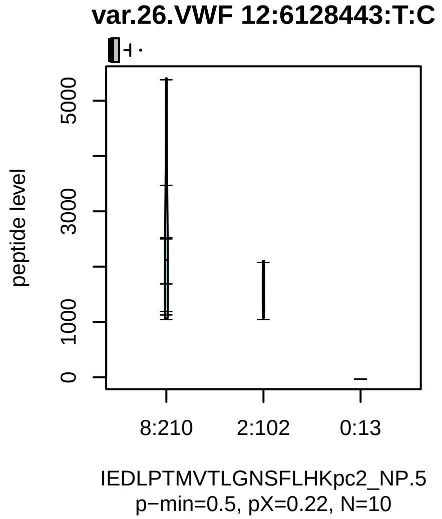
var.26.VWF 12:6128443:T:C
peptide level
0
1000
3000
5000
8:210
2:102
0:13
IEDLPTMVTLGNSFLHKpc2_NP.5
p−min=0.5, pX=0.22, N=10

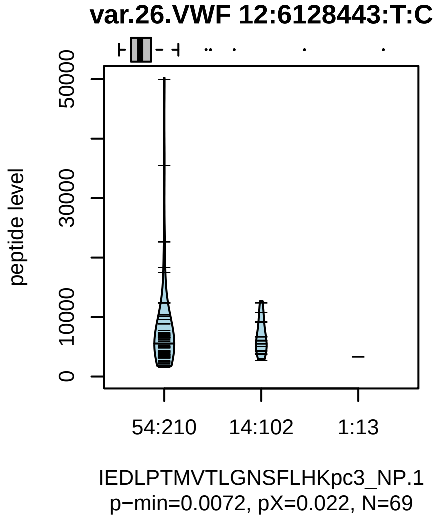
var.26.VWF 12:6128443:T:C
peptide level
0
10000
30000
50000
54:210
14:102
1:13
IEDLPTMVTLGNSFLHKpc3_NP.1
p−min=0.0072, pX=0.022, N=69

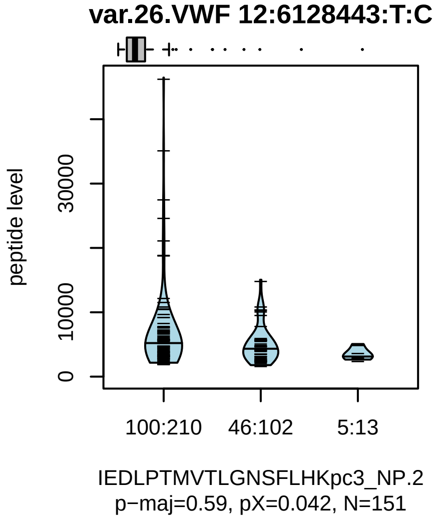
var.26.VWF 12:6128443:T:C
peptide level
0
10000
30000
100:210
46:102
5:13
IEDLPTMVTLGNSFLHKpc3_NP.2
p−maj=0.59, pX=0.042, N=151

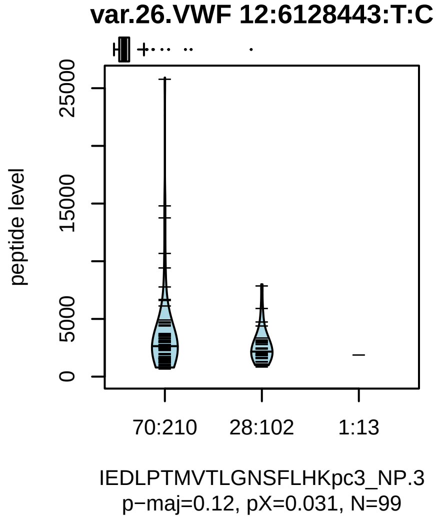
var.26.VWF 12:6128443:T:C
peptide level
0
5000
15000
25000
70:210
28:102
1:13
IEDLPTMVTLGNSFLHKpc3_NP.3
p−maj=0.12, pX=0.031, N=99

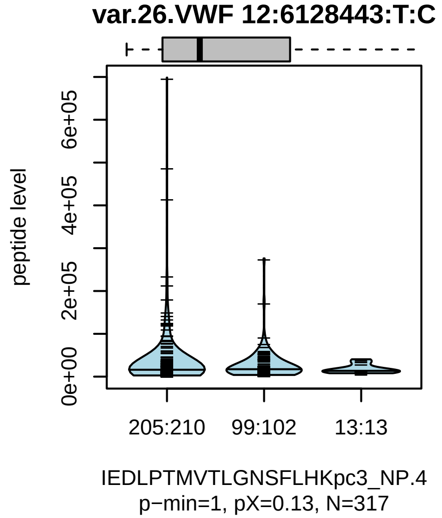
var.26.VWF 12:6128443:T:C
peptide level
0e+00
2e+05
4e+05
6e+05
205:210
99:102
13:13
IEDLPTMVTLGNSFLHKpc3_NP.4
p−min=1, pX=0.13, N=317

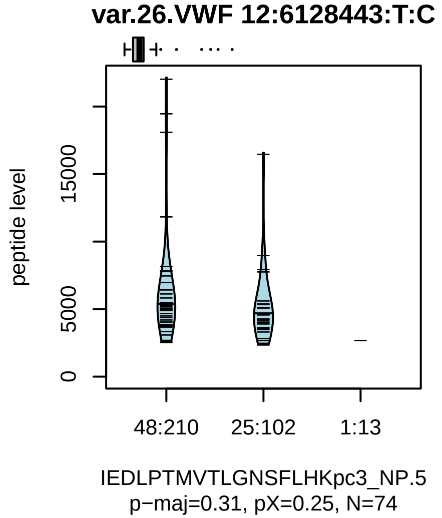
var.26.VWF 12:6128443:T:C
peptide level
0
5000
15000
48:210
25:102
1:13
IEDLPTMVTLGNSFLHKpc3_NP.5
p−maj=0.31, pX=0.25, N=74

var.26.VWF 12:6128443:T:C

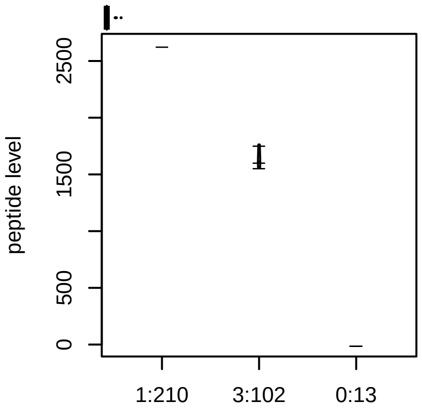

IGCNTCVCQDRKpc3_NP.4
p−min=0.13, pX=0.37, N=4

var.26.VWF 12:6128443:T:C

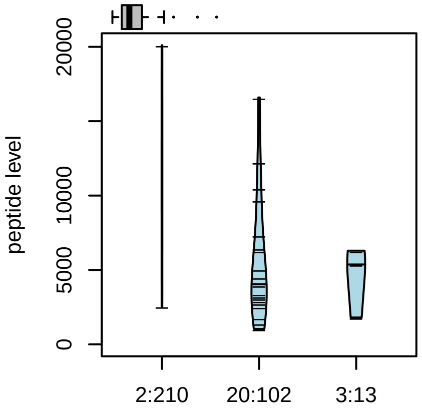

IGCNTCVCQDRpc2_NP.4
p−min=1.3e−09, pX=0.00011, N=25

var.26.VWF 12:6128443:T:C

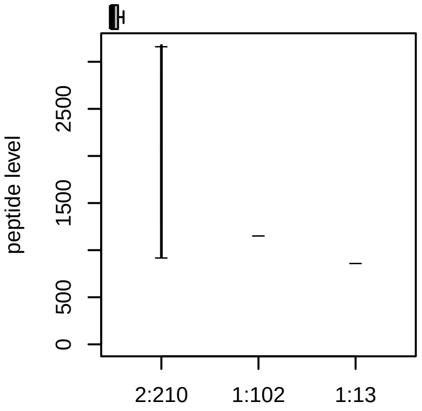

IGCNTCVCQDRpc2_NP.5
p−maj=0.15, pX=0.81, N=4

var.26.VWF 12:6128443:T:C

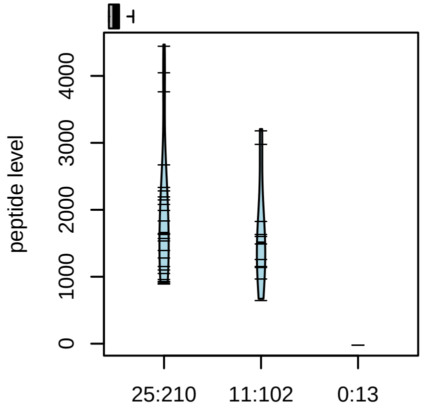

IGWPNAPILIQDFETLPRpc3_NP.1
p−maj=0.37, pX=0.25, N=36

var.26.VWF 12:6128443:T:C

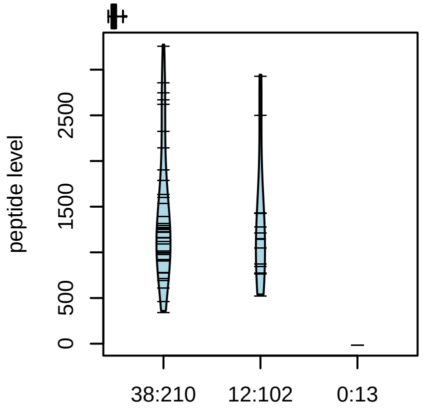

IGWPNAPILIQDFETLPRpc3_NP.2
p−min=0.077, pX=0.048, N=50

var.26.VWF 12:6128443:T:C

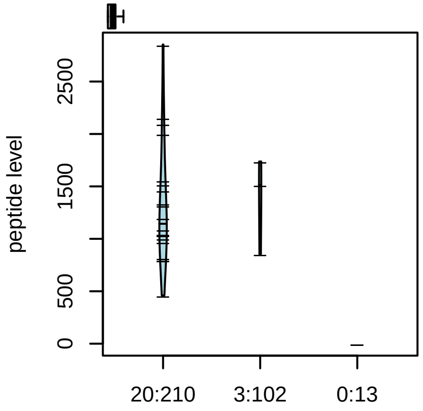

IGWPNAPILIQDFETLPRpc3_NP.3
p−min=0.023, pX=0.035, N=23

var.26.VWF 12:6128443:T:C

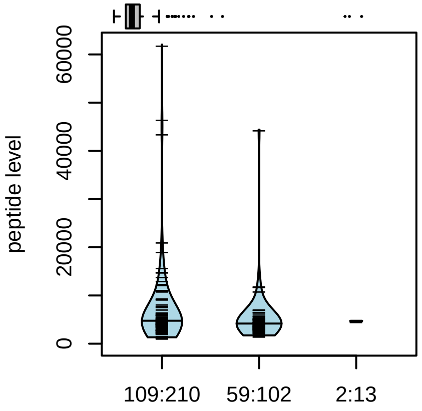

IGWPNAPILIQDFETLPRpc3_NP.4
p−maj=0.0087, pX=0.13, N=170

var.26.VWF 12:6128443:T:C

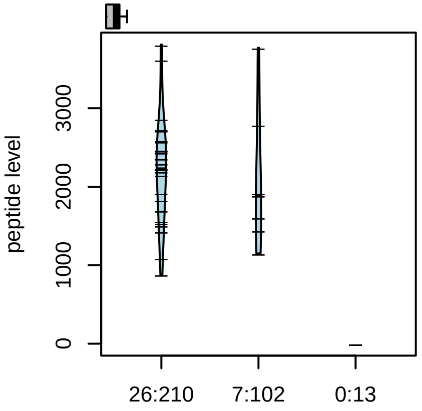

IGWPNAPILIQDFETLPRpc3_NP.5
p−min=0.085, pX=0.052, N=33

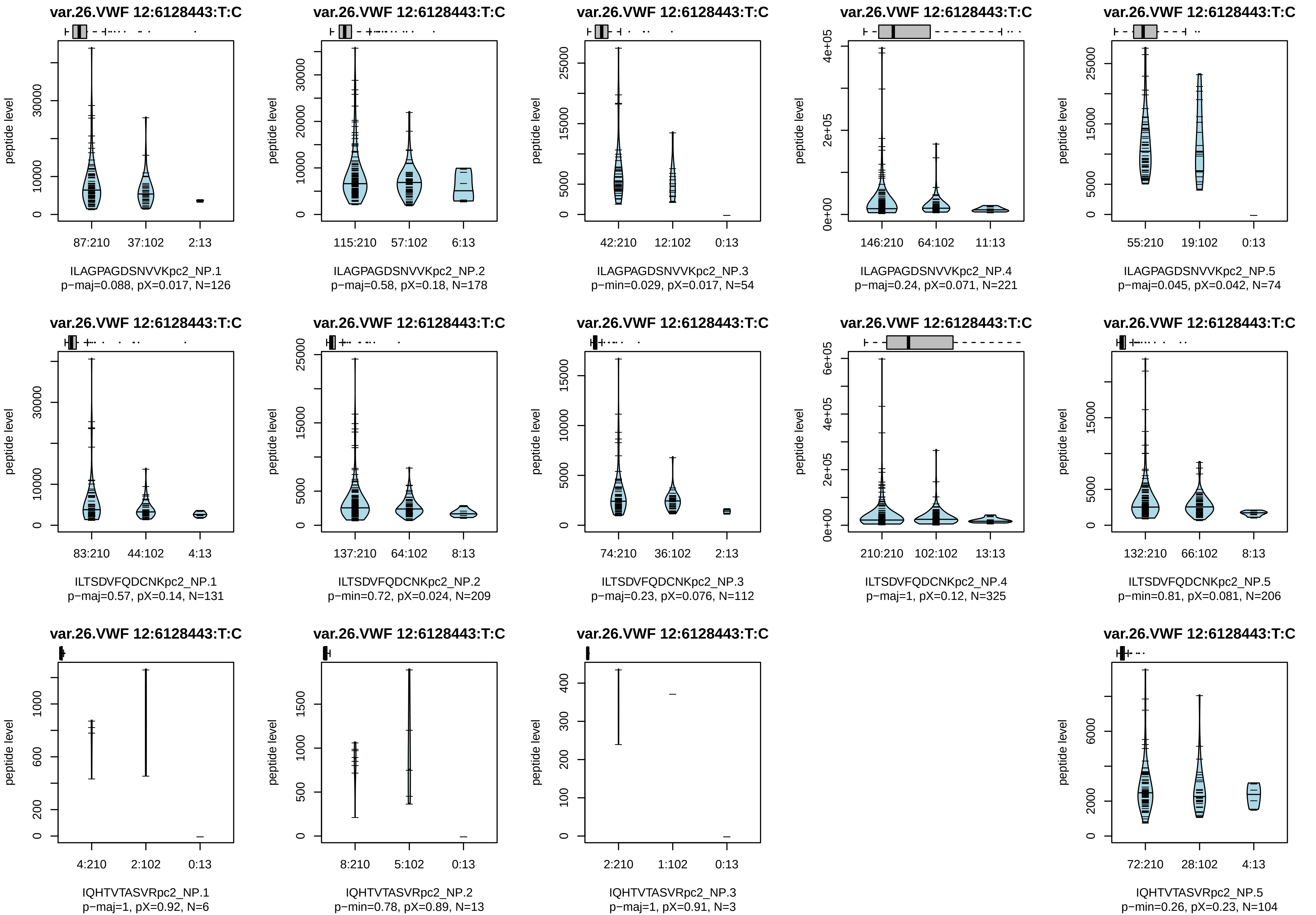
var.26.VWF 12:6128443:T:C
peptide level
87:210
37:102
2:13
ILAGPAGDSNVVKpc2_NP.1
p−maj=0.088, pX=0.017, N=126
var.26.VWF 12:6128443:T:C
peptide level
115:210
57:102
6:13
ILAGPAGDSNVVKpc2_NP.2
p−maj=0.58, pX=0.18, N=178
var.26.VWF 12:6128443:T:C
peptide level
42:210
12:102
0:13
ILAGPAGDSNVVKpc2_NP.3
p−min=0.029, pX=0.017, N=54
var.26.VWF 12:6128443:T:C
peptide level
146:210
64:102
11:13
ILAGPAGDSNVVKpc2_NP.4
p−maj=0.24, pX=0.071, N=221
var.26.VWF 12:6128443:T:C
peptide level
55:210
19:102
0:13
ILAGPAGDSNVVKpc2_NP.5
p−maj=0.045, pX=0.042, N=74
var.26.VWF 12:6128443:T:C
peptide level
83:210
44:102
4:13
ILTSDVFQDCNKpc2_NP.1
p−maj=0.57, pX=0.14, N=131
var.26.VWF 12:6128443:T:C
peptide level
137:210
64:102
8:13
ILTSDVFQDCNKpc2_NP.2
p−min=0.72, pX=0.024, N=209
var.26.VWF 12:6128443:T:C
peptide level
74:210
36:102
2:13
ILTSDVFQDCNKpc2_NP.3
p−maj=0.23, pX=0.076, N=112
var.26.VWF 12:6128443:T:C
peptide level
210:210
102:102
13:13
ILTSDVFQDCNKpc2_NP.4
p−maj=1, pX=0.12, N=325
var.26.VWF 12:6128443:T:C
peptide level
132:210
66:102
8:13
ILTSDVFQDCNKpc2_NP.5
p−min=0.81, pX=0.081, N=206
var.26.VWF 12:6128443:T:C
peptide level
4:210
2:102
0:13
IQHTVTASVRpc2_NP.1
p−maj=1, pX=0.92, N=6
var.26.VWF 12:6128443:T:C
peptide level
8:210
5:102
0:13
IQHTVTASVRpc2_NP.2
p−min=0.78, pX=0.89, N=13
var.26.VWF 12:6128443:T:C
peptide level
2:210
1:102
0:13
IQHTVTASVRpc2_NP.3
p−maj=1, pX=0.91, N=3
var.26.VWF 12:6128443:T:C
peptide level
72:210
28:102
4:13
IQHTVTASVRpc2_NP.5
p−min=0.26, pX=0.23, N=104

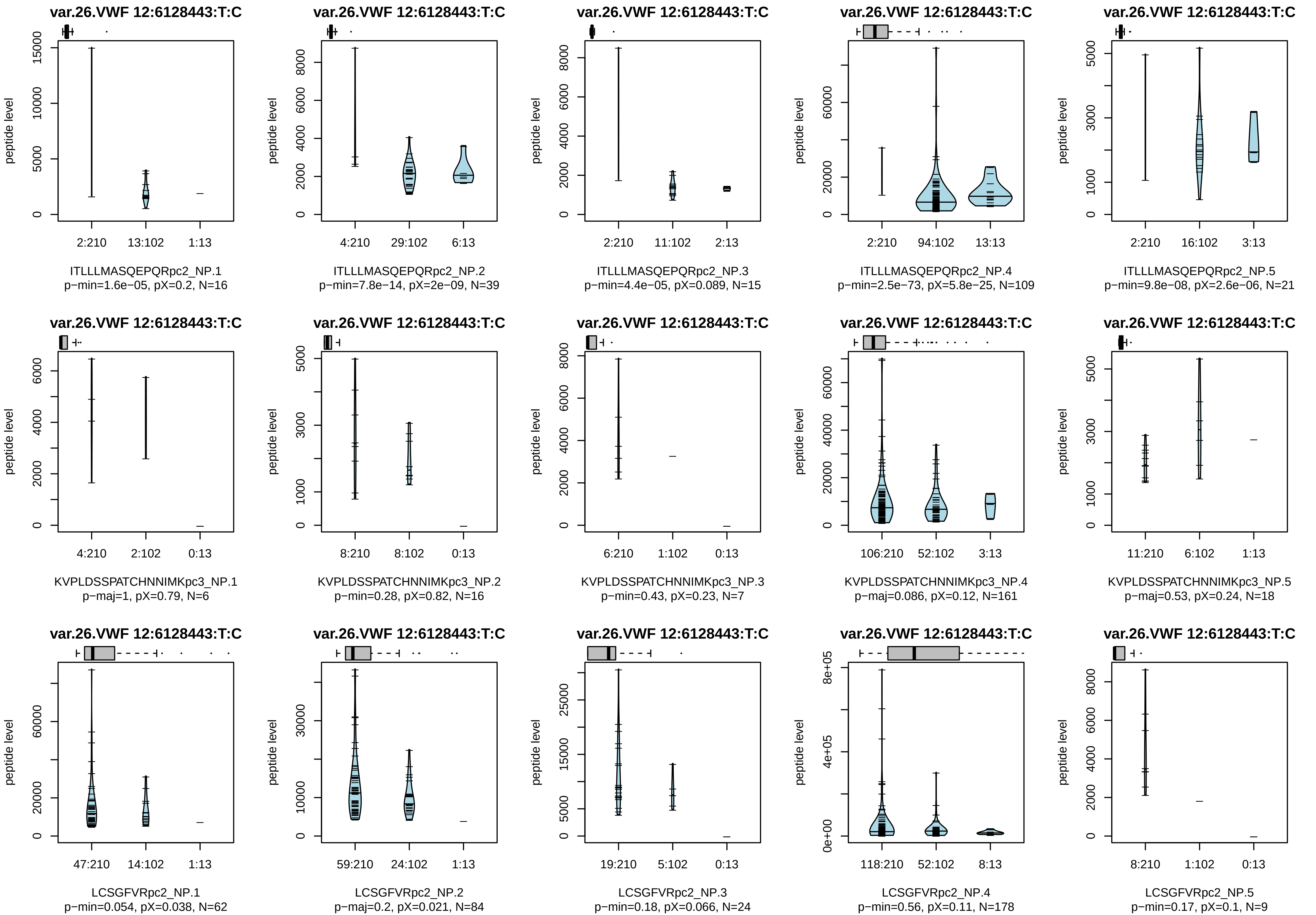
var.26.VWF 12:6128443:T:C
peptide level
2:210
13:102
1:13
ITLLLMASQEPQRpc2_NP.1
p−min=1.6e−05, pX=0.2, N=16
var.26.VWF 12:6128443:T:C
peptide level
4:210
29:102
6:13
ITLLLMASQEPQRpc2_NP.2
p−min=7.8e−14, pX=2e−09, N=39
var.26.VWF 12:6128443:T:C
peptide level
2:210
11:102
2:13
ITLLLMASQEPQRpc2_NP.3
p−min=4.4e−05, pX=0.089, N=15
var.26.VWF 12:6128443:T:C
peptide level
2:210
94:102
13:13
ITLLLMASQEPQRpc2_NP.4
p−min=2.5e−73, pX=5.8e−25, N=109
var.26.VWF 12:6128443:T:C
peptide level
2:210
16:102
3:13
ITLLLMASQEPQRpc2_NP.5
p−min=9.8e−08, pX=2.6e−06, N=21
var.26.VWF 12:6128443:T:C
peptide level
4:210
2:102
0:13
KVPLDSSPATCHNNIMKpc3_NP.1
p−maj=1, pX=0.79, N=6
var.26.VWF 12:6128443:T:C
peptide level
8:210
8:102
0:13
KVPLDSSPATCHNNIMKpc3_NP.2
p−min=0.28, pX=0.82, N=16
var.26.VWF 12:6128443:T:C
peptide level
6:210
1:102
0:13
KVPLDSSPATCHNNIMKpc3_NP.3
p−min=0.43, pX=0.23, N=7
var.26.VWF 12:6128443:T:C
peptide level
106:210
52:102
3:13
KVPLDSSPATCHNNIMKpc3_NP.4
p−maj=0.086, pX=0.12, N=161
var.26.VWF 12:6128443:T:C
peptide level
11:210
6:102
1:13
KVPLDSSPATCHNNIMKpc3_NP.5
p−maj=0.53, pX=0.24, N=18
var.26.VWF 12:6128443:T:C
peptide level
47:210
14:102
1:13
LCSGFVRpc2_NP.1
p−min=0.054, pX=0.038, N=62
var.26.VWF 12:6128443:T:C
peptide level
59:210
24:102
1:13
LCSGFVRpc2_NP.2
p−maj=0.2, pX=0.021, N=84
var.26.VWF 12:6128443:T:C
peptide level
19:210
5:102
0:13
LCSGFVRpc2_NP.3
p−min=0.18, pX=0.066, N=24
var.26.VWF 12:6128443:T:C
peptide level
118:210
52:102
8:13
LCSGFVRpc2_NP.4
p−min=0.56, pX=0.11, N=178
var.26.VWF 12:6128443:T:C
peptide level
8:210
1:102
0:13
LCSGFVRpc2_NP.5
p−min=0.17, pX=0.1, N=9

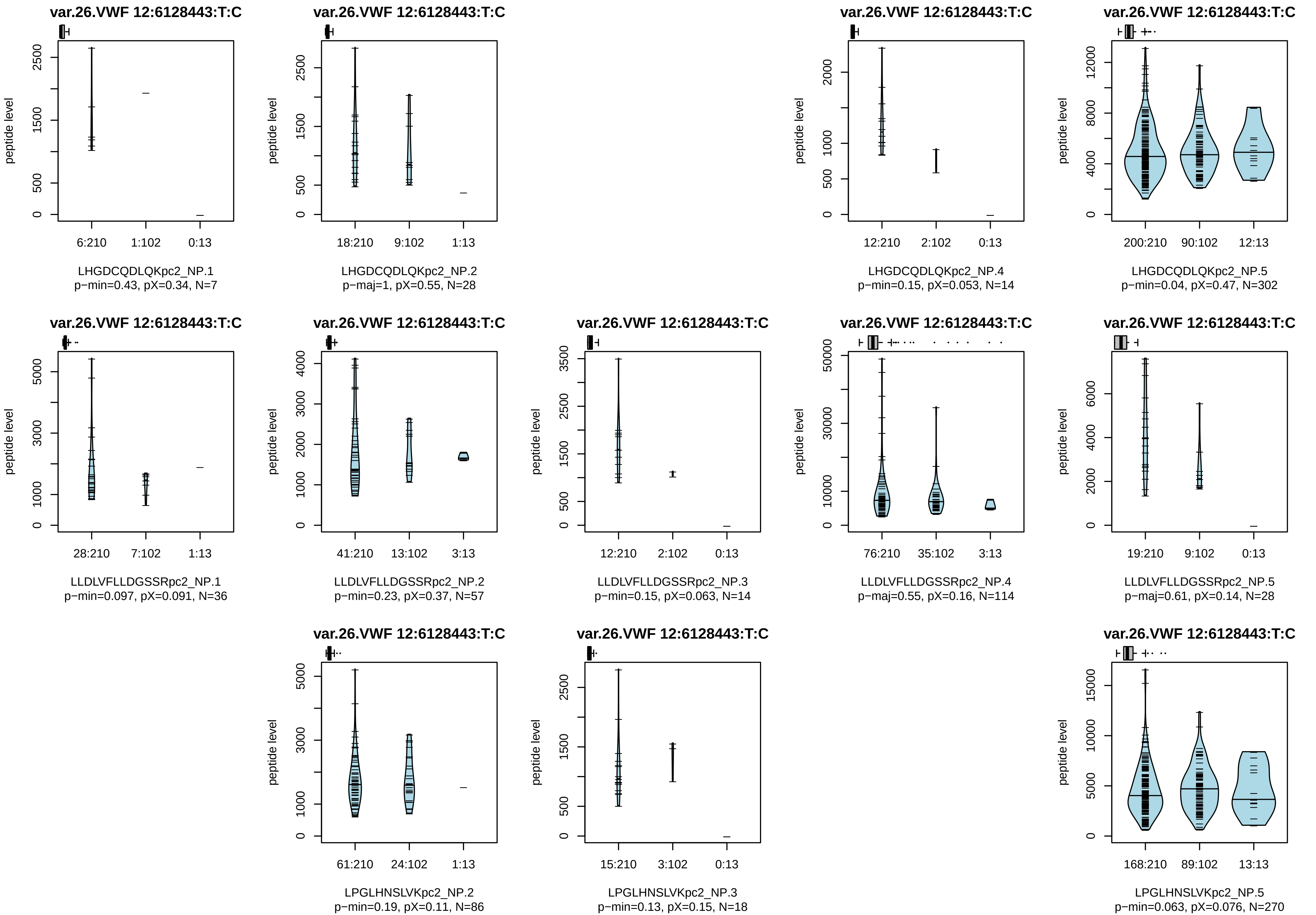
var.26.VWF 12:6128443:T:C
peptide level
6:210
1:102
0:13
LHGDCQDLQKpc2_NP.1
p−min=0.43, pX=0.34, N=7
var.26.VWF 12:6128443:T:C
peptide level
18:210
9:102
1:13
LHGDCQDLQKpc2_NP.2
p−maj=1, pX=0.55, N=28
var.26.VWF 12:6128443:T:C
peptide level
12:210
2:102
0:13
LHGDCQDLQKpc2_NP.4
p−min=0.15, pX=0.053, N=14
var.26.VWF 12:6128443:T:C
peptide level
200:210
90:102
12:13
LHGDCQDLQKpc2_NP.5
p−min=0.04, pX=0.47, N=302
var.26.VWF 12:6128443:T:C
peptide level
28:210
7:102
1:13
LLDLVFLLDGSSRpc2_NP.1
p−min=0.097, pX=0.091, N=36
var.26.VWF 12:6128443:T:C
peptide level
41:210
13:102
3:13
LLDLVFLLDGSSRpc2_NP.2
p−min=0.23, pX=0.37, N=57
var.26.VWF 12:6128443:T:C
peptide level
12:210
2:102
0:13
LLDLVFLLDGSSRpc2_NP.3
p−min=0.15, pX=0.063, N=14
var.26.VWF 12:6128443:T:C
peptide level
76:210
35:102
3:13
LLDLVFLLDGSSRpc2_NP.4
p−maj=0.55, pX=0.16, N=114
var.26.VWF 12:6128443:T:C
peptide level
19:210
9:102
0:13
LLDLVFLLDGSSRpc2_NP.5
p−maj=0.61, pX=0.14, N=28
var.26.VWF 12:6128443:T:C
peptide level
61:210
24:102
1:13
LPGLHNSLVKpc2_NP.2
p−min=0.19, pX=0.11, N=86
var.26.VWF 12:6128443:T:C
peptide level
15:210
3:102
0:13
LPGLHNSLVKpc2_NP.3
p−min=0.13, pX=0.15, N=18
var.26.VWF 12:6128443:T:C
peptide level
168:210
89:102
13:13
LPGLHNSLVKpc2_NP.5
p−min=0.063, pX=0.076, N=270

var.26.VWF 12:6128443:T:C
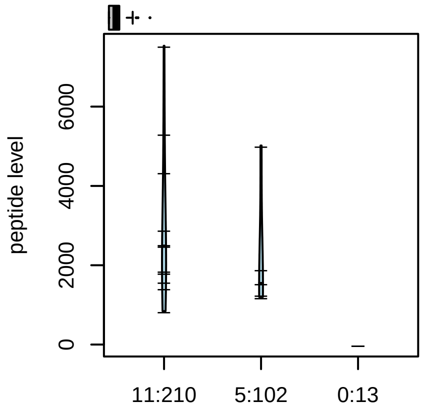

LQRIEDLPTMVTLGNSFLHKpc3_NP.1
p−min=0.8, pX=0.39, N=16

var.26.VWF 12:6128443:T:C
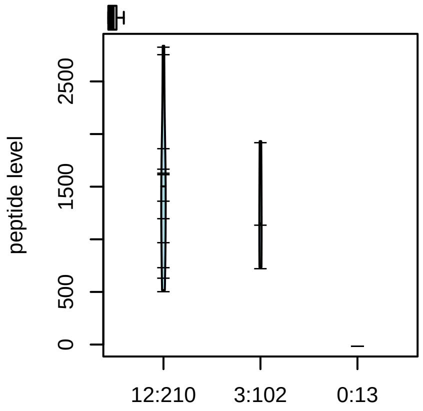

LQRIEDLPTMVTLGNSFLHKpc3_NP.2
p−min=0.27, pX=0.17, N=15

var.26.VWF 12:6128443:T:C
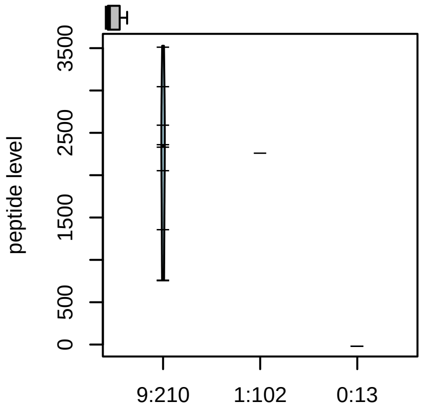

LQRIEDLPTMVTLGNSFLHKpc3_NP.3
p−min=0.1, pX=0.13, N=10

var.26.VWF 12:6128443:T:C
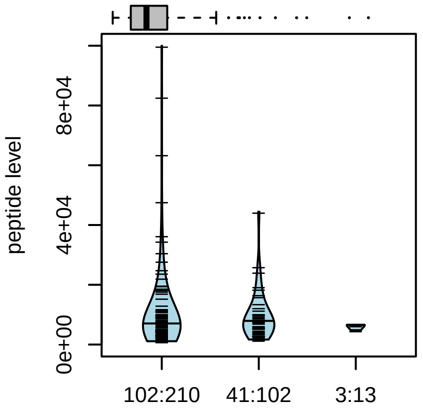

LQRIEDLPTMVTLGNSFLHKpc3_NP.4
p−min=0.081, pX=0.071, N=146

var.26.VWF 12:6128443:T:C
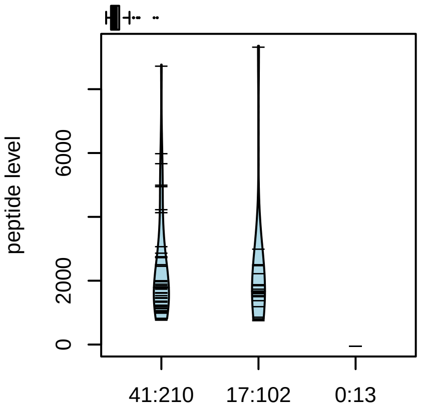

LQRIEDLPTMVTLGNSFLHKpc3_NP.5
p−maj=0.14, pX=0.18, N=58

var.26.VWF 12:6128443:T:C
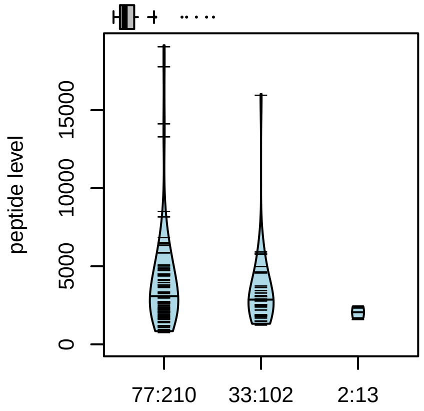

LQRIEDLPTMVTLGNSFLHKpc4_NP.1
p−maj=0.23, pX=0.065, N=112

var.26.VWF 12:6128443:T:C
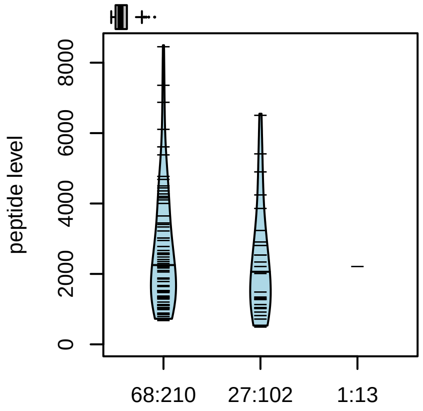

LQRIEDLPTMVTLGNSFLHKpc4_NP.2
p−maj=0.12, pX=0.043, N=96

var.26.VWF 12:6128443:T:C
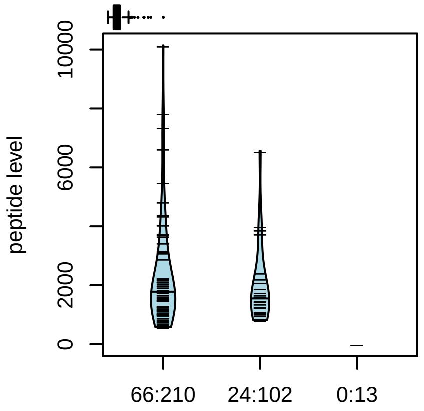

LQRIEDLPTMVTLGNSFLHKpc4_NP.3
p−maj=0.023, pX=0.02, N=90

var.26.VWF 12:6128443:T:C
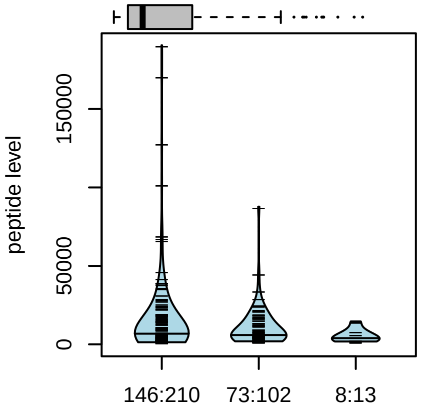

LQRIEDLPTMVTLGNSFLHKpc4_NP.4
p−maj=0.54, pX=0.076, N=227

var.26.VWF 12:6128443:T:C
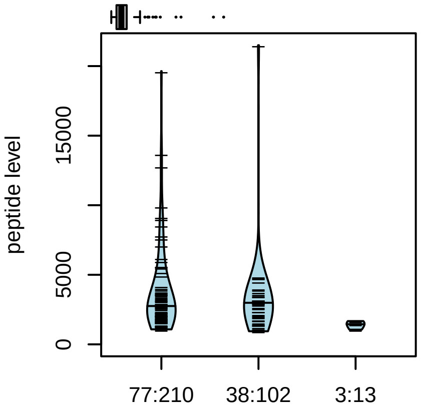

LQRIEDLPTMVTLGNSFLHKpc4_NP.5
p−maj=0.39, pX=0.21, N=118

var.26.VWF 12:6128443:T:C
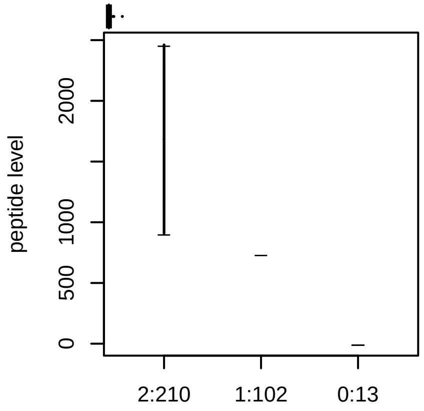

LSEAEFEVLKpc2_NP.1
p−maj=1, pX=0.57, N=3

var.26.VWF 12:6128443:T:C
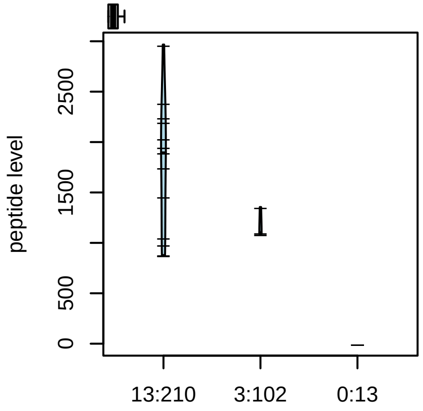

LSEAEFEVLKpc2_NP.2
p−min=0.19, pX=0.08, N=16

var.26.VWF 12:6128443:T:C
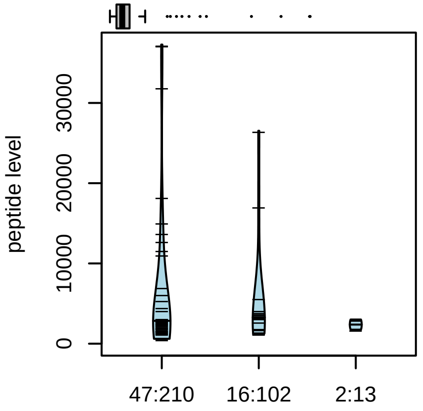

LSEAEFEVLKpc2_NP.4
p−min=0.19, pX=0.2, N=65

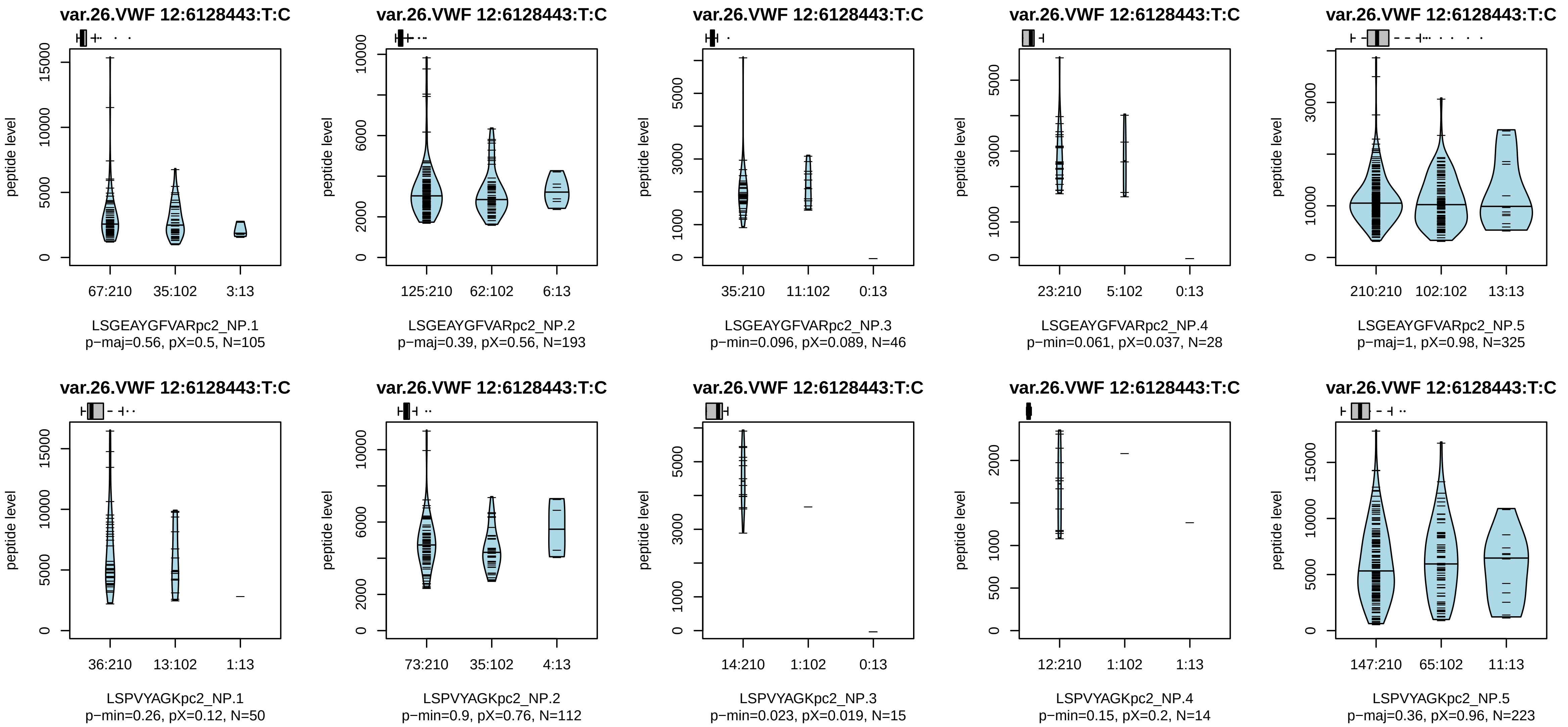
var.26.VWF 12:6128443:T:C
peptide level
67:210
35:102
3:13
LSGEAYGFVARpc2_NP.1
p−maj=0.56, pX=0.5, N=105
var.26.VWF 12:6128443:T:C
peptide level
125:210
62:102
6:13
LSGEAYGFVARpc2_NP.2
p−maj=0.39, pX=0.56, N=193
var.26.VWF 12:6128443:T:C
peptide level
35:210
11:102
0:13
LSGEAYGFVARpc2_NP.3
p−min=0.096, pX=0.089, N=46
var.26.VWF 12:6128443:T:C
peptide level
23:210
5:102
0:13
LSGEAYGFVARpc2_NP.4
p−min=0.061, pX=0.037, N=28
var.26.VWF 12:6128443:T:C
peptide level
210:210
102:102
13:13
LSGEAYGFVARpc2_NP.5
p−maj=1, pX=0.98, N=325
var.26.VWF 12:6128443:T:C
peptide level
36:210
13:102
1:13
LSPVYAGKpc2_NP.1
p−min=0.26, pX=0.12, N=50
var.26.VWF 12:6128443:T:C
peptide level
73:210
35:102
4:13
LSPVYAGKpc2_NP.2
p−min=0.9, pX=0.76, N=112
var.26.VWF 12:6128443:T:C
peptide level
14:210
1:102
0:13
LSPVYAGKpc2_NP.3
p−min=0.023, pX=0.019, N=15
var.26.VWF 12:6128443:T:C
peptide level
12:210
1:102
1:13
LSPVYAGKpc2_NP.4
p−min=0.15, pX=0.2, N=14
var.26.VWF 12:6128443:T:C
peptide level
147:210
65:102
11:13
LSPVYAGKpc2_NP.5
p−maj=0.36, pX=0.96, N=223

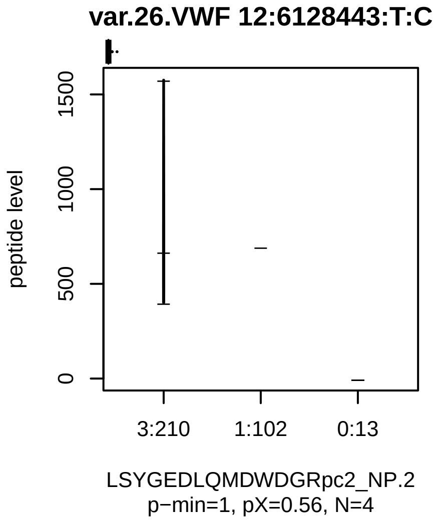
var.26.VWF 12:6128443:T:C
peptide level
0
500
1000
1500
3:210
1:102
0:13
LSYGEDLQMDWDGRpc2_NP.2
p−min=1, pX=0.56, N=4

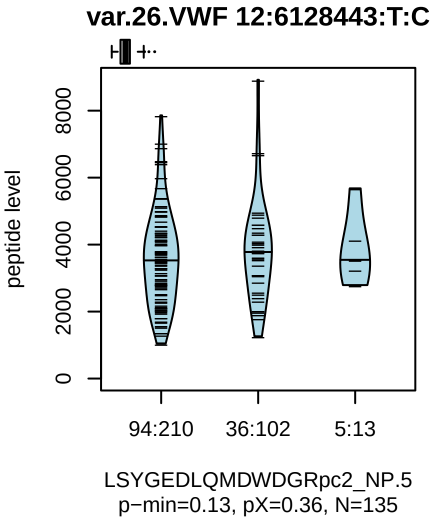
var.26.VWF 12:6128443:T:C
peptide level
0
2000
4000
6000
8000
94:210
36:102
5:13
LSYGEDLQMDWDGRpc2_NP.5
p−min=0.13, pX=0.36, N=135

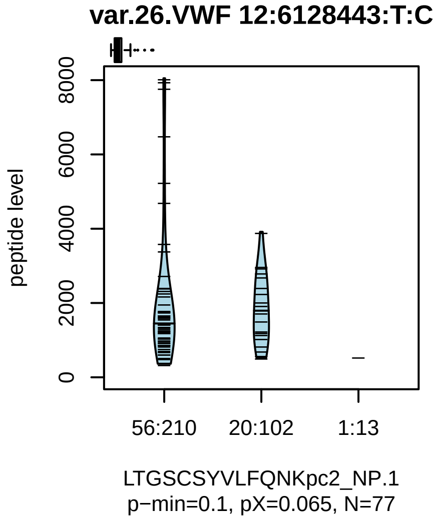
var.26.VWF 12:6128443:T:C
peptide level
0
2000
4000
6000
8000
56:210
20:102
1:13
LTGSCSYVLFQNKpc2_NP.1
p−min=0.1, pX=0.065, N=77

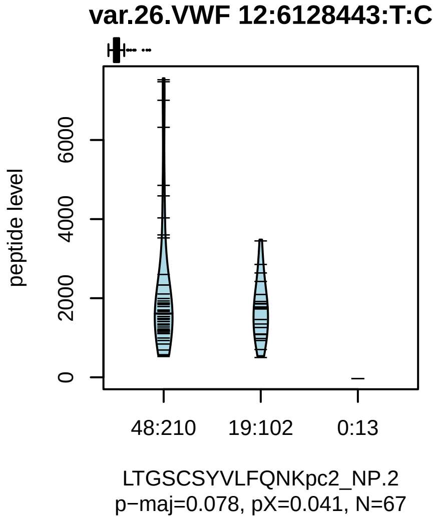
var.26.VWF 12:6128443:T:C
peptide level
0
2000
4000
6000
48:210
19:102
0:13
LTGSCSYVLFQNKpc2_NP.2
p−maj=0.078, pX=0.041, N=67

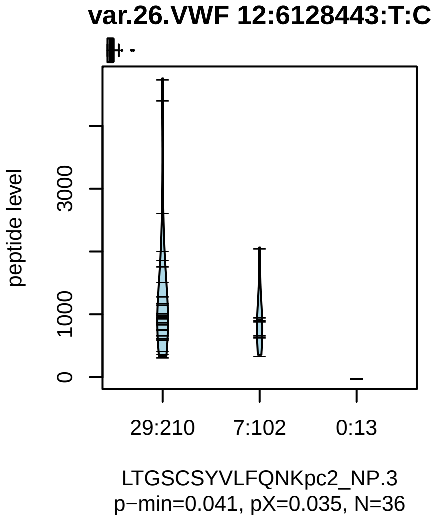
var.26.VWF 12:6128443:T:C
peptide level
0
1000
3000
29:210
7:102
0:13
LTGSCSYVLFQNKpc2_NP.3
p−min=0.041, pX=0.035, N=36

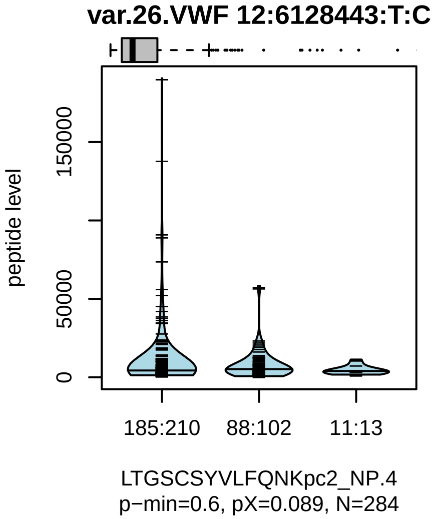
var.26.VWF 12:6128443:T:C
peptide level
0
50000
150000
185:210
88:102
11:13
LTGSCSYVLFQNKpc2_NP.4
p−min=0.6, pX=0.089, N=284

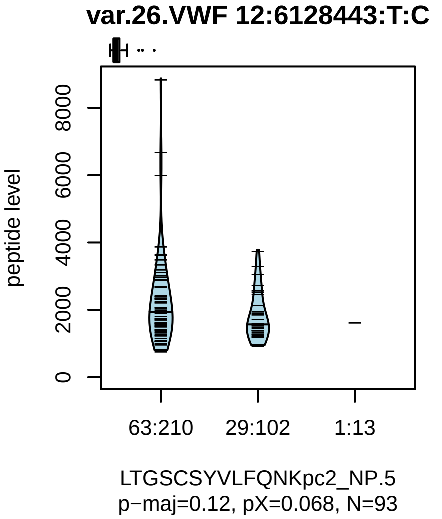
var.26.VWF 12:6128443:T:C
peptide level
0
2000
4000
6000
8000
63:210
29:102
1:13
LTGSCSYVLFQNKpc2_NP.5
p−maj=0.12, pX=0.068, N=93

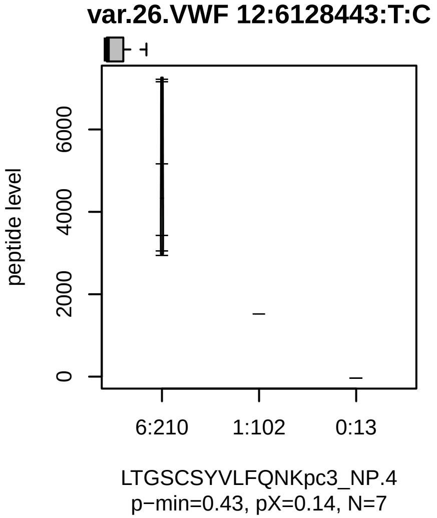
var.26.VWF 12:6128443:T:C
peptide level
0
2000
4000
6000
6:210
1:102
0:13
LTGSCSYVLFQNKpc3_NP.4
p−min=0.43, pX=0.14, N=7

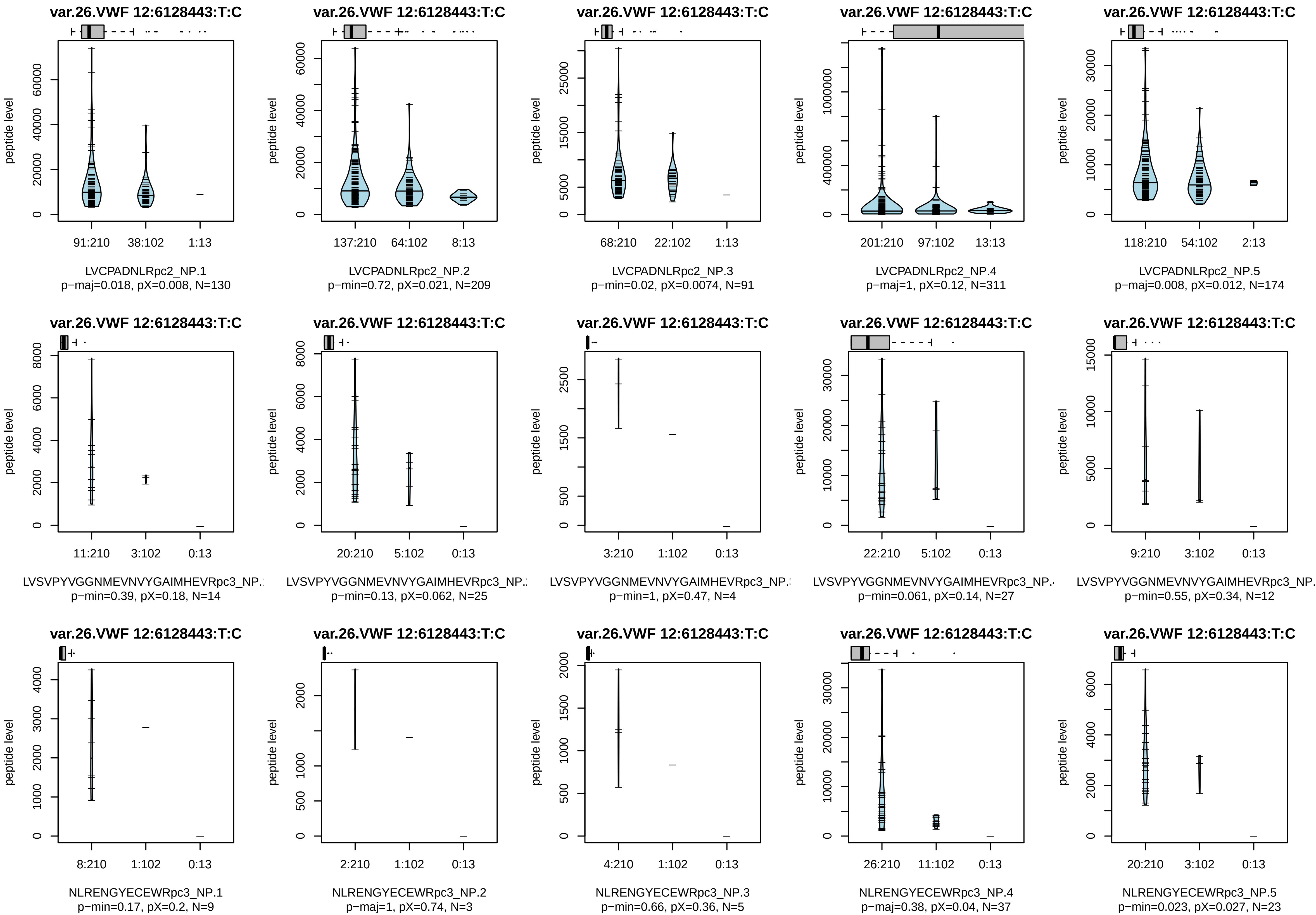
var.26.VWF 12:6128443:T:C
peptide level
91:210
38:102
1:13
LVCPADNLRpc2_NP.1
p−maj=0.018, pX=0.008, N=130
var.26.VWF 12:6128443:T:C
peptide level
137:210
64:102
8:13
LVCPADNLRpc2_NP.2
p−min=0.72, pX=0.021, N=209
var.26.VWF 12:6128443:T:C
peptide level
68:210
22:102
1:13
LVCPADNLRpc2_NP.3
p−min=0.02, pX=0.0074, N=91
var.26.VWF 12:6128443:T:C
peptide level
201:210
97:102
13:13
LVCPADNLRpc2_NP.4
p−maj=1, pX=0.12, N=311
var.26.VWF 12:6128443:T:C
peptide level
118:210
54:102
2:13
LVCPADNLRpc2_NP.5
p−maj=0.008, pX=0.012, N=174
var.26.VWF 12:6128443:T:C
peptide level
11:210
3:102
0:13
p−min=0.39, pX=0.18, N=14
var.26.VWF 12:6128443:T:C
peptide level
20:210
5:102
0:13
p−min=0.13, pX=0.062, N=25
var.26.VWF 12:6128443:T:C
peptide level
3:210
1:102
0:13
p−min=1, pX=0.47, N=4
var.26.VWF 12:6128443:T:C
peptide level
22:210
5:102
0:13
p−min=0.061, pX=0.14, N=27
var.26.VWF 12:6128443:T:C
peptide level
9:210
3:102
0:13
p−min=0.55, pX=0.34, N=12
var.26.VWF 12:6128443:T:C
peptide level
8:210
1:102
0:13
NLRENGYECEWRpc3_NP.1
p−min=0.17, pX=0.2, N=9
var.26.VWF 12:6128443:T:C
peptide level
2:210
1:102
0:13
NLRENGYECEWRpc3_NP.2
p−maj=1, pX=0.74, N=3
var.26.VWF 12:6128443:T:C
peptide level
4:210
1:102
0:13
NLRENGYECEWRpc3_NP.3
p−min=0.66, pX=0.36, N=5
var.26.VWF 12:6128443:T:C
peptide level
26:210
11:102
0:13
NLRENGYECEWRpc3_NP.4
p−maj=0.38, pX=0.04, N=37
var.26.VWF 12:6128443:T:C
peptide level
20:210
3:102
0:13
NLRENGYECEWRpc3_NP.5
p−min=0.023, pX=0.027, N=23

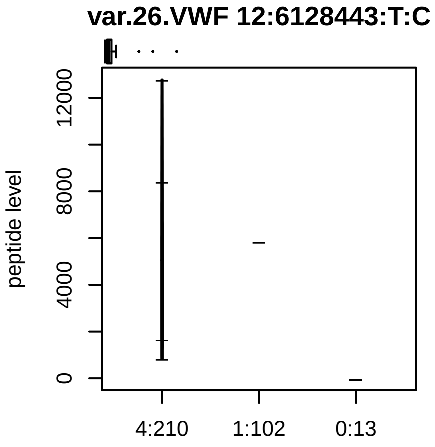

NSMVLDVAFVLEGSDKpc2_NP.4
p−min=0.66, pX=0.53, N=5

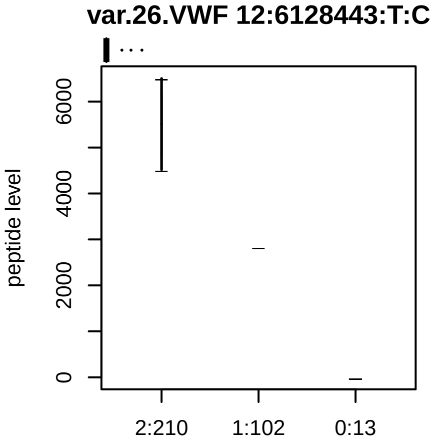

QCVSPCARpc2_NP.1
p−maj=1, pX=0.58, N=3

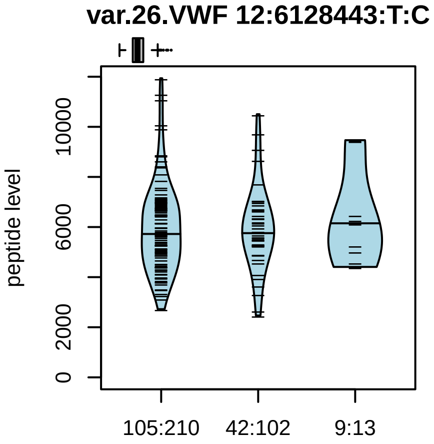

QCVSPCARpc2_NP.5
p−maj=0.16, pX=0.9, N=156

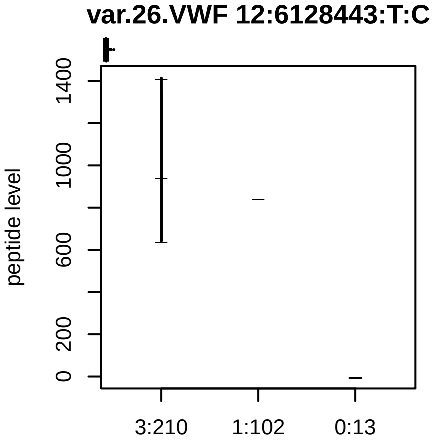

QHSDPCALNPRpc2_NP.1
p−min=1, pX=0.55, N=4

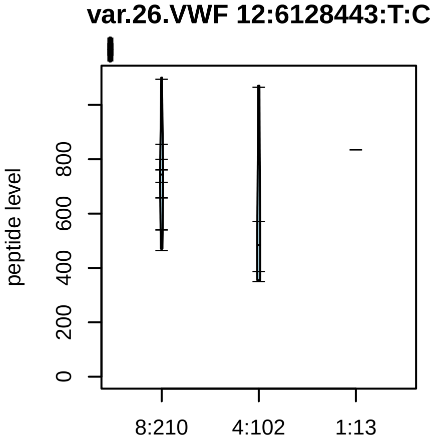

QHSDPCALNPRpc2_NP.2
p−maj=0.42, pX=0.76, N=13

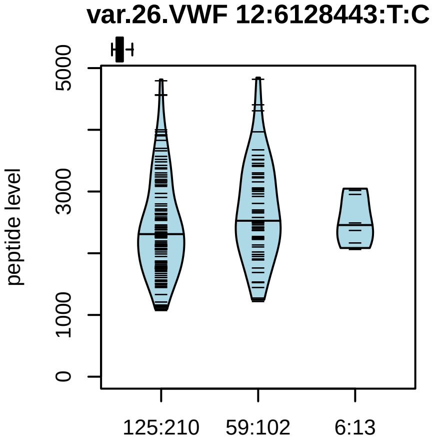

QHSDPCALNPRpc2_NP.5
p−maj=0.4, pX=0.98, N=190

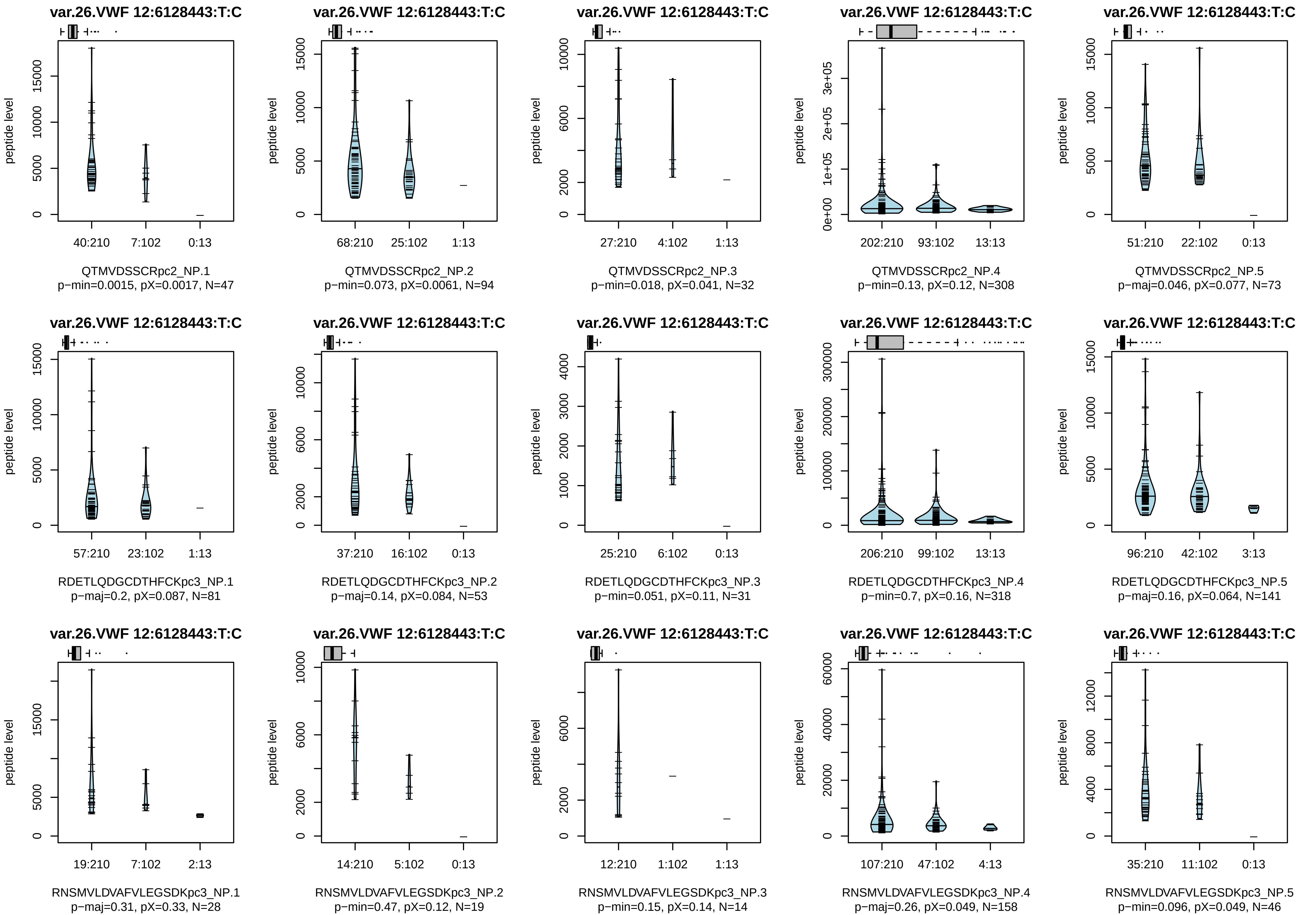
var.26.VWF 12:6128443:T:C
peptide level
40:210
7:102
0:13
QTMVDSSCRpc2_NP.1
p−min=0.0015, pX=0.0017, N=47
var.26.VWF 12:6128443:T:C
peptide level
68:210
25:102
1:13
QTMVDSSCRpc2_NP.2
p−min=0.073, pX=0.0061, N=94
var.26.VWF 12:6128443:T:C
peptide level
27:210
4:102
1:13
QTMVDSSCRpc2_NP.3
p−min=0.018, pX=0.041, N=32
var.26.VWF 12:6128443:T:C
peptide level
202:210
93:102
13:13
QTMVDSSCRpc2_NP.4
p−min=0.13, pX=0.12, N=308
var.26.VWF 12:6128443:T:C
peptide level
51:210
22:102
0:13
QTMVDSSCRpc2_NP.5
p−maj=0.046, pX=0.077, N=73
var.26.VWF 12:6128443:T:C
peptide level
57:210
23:102
1:13
RDETLQDGCDTHFCKpc3_NP.1
p−maj=0.2, pX=0.087, N=81
var.26.VWF 12:6128443:T:C
peptide level
37:210
16:102
0:13
RDETLQDGCDTHFCKpc3_NP.2
p−maj=0.14, pX=0.084, N=53
var.26.VWF 12:6128443:T:C
peptide level
25:210
6:102
0:13
RDETLQDGCDTHFCKpc3_NP.3
p−min=0.051, pX=0.11, N=31
var.26.VWF 12:6128443:T:C
peptide level
206:210
99:102
13:13
RDETLQDGCDTHFCKpc3_NP.4
p−min=0.7, pX=0.16, N=318
var.26.VWF 12:6128443:T:C
peptide level
96:210
42:102
3:13
RDETLQDGCDTHFCKpc3_NP.5
p−maj=0.16, pX=0.064, N=141
var.26.VWF 12:6128443:T:C
peptide level
19:210
7:102
2:13
RNSMVLDVAFVLEGSDKpc3_NP.1
p−maj=0.31, pX=0.33, N=28
var.26.VWF 12:6128443:T:C
peptide level
14:210
5:102
0:13
RNSMVLDVAFVLEGSDKpc3_NP.2
p−min=0.47, pX=0.12, N=19
var.26.VWF 12:6128443:T:C
peptide level
12:210
1:102
1:13
RNSMVLDVAFVLEGSDKpc3_NP.3
p−min=0.15, pX=0.14, N=14
var.26.VWF 12:6128443:T:C
peptide level
107:210
47:102
4:13
RNSMVLDVAFVLEGSDKpc3_NP.4
p−maj=0.26, pX=0.049, N=158
var.26.VWF 12:6128443:T:C
peptide level
35:210
11:102
0:13
RNSMVLDVAFVLEGSDKpc3_NP.5
p−min=0.096, pX=0.049, N=46

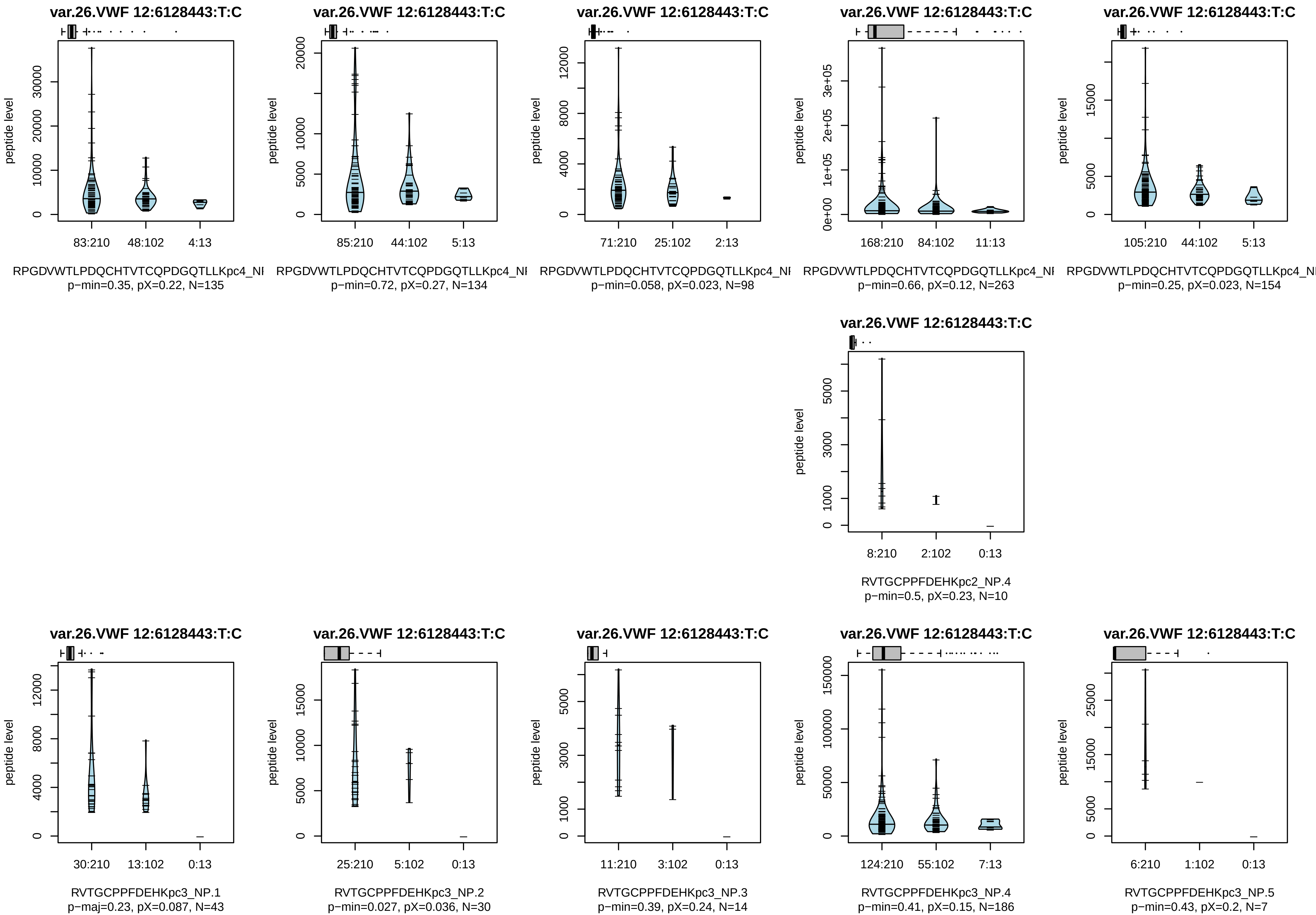
var.26.VWF 12:6128443:T:C
peptide level
83:210
48:102
4:13
RPGDVWTLPDQCHTVTCQPDGQTLLKpc4_NI
p−min=0.35, pX=0.22, N=135
var.26.VWF 12:6128443:T:C
peptide level
85:210
44:102
5:13
RPGDVWTLPDQCHTVTCQPDGQTLLKpc4_NI
p−min=0.72, pX=0.27, N=134
var.26.VWF 12:6128443:T:C
peptide level
71:210
25:102
2:13
RPGDVWTLPDQCHTVTCQPDGQTLLKpc4_NI
p−min=0.058, pX=0.023, N=98
var.26.VWF 12:6128443:T:C
peptide level
168:210
84:102
11:13
RPGDVWTLPDQCHTVTCQPDGQTLLKpc4_NI
p−min=0.66, pX=0.12, N=263
var.26.VWF 12:6128443:T:C
peptide level
105:210
44:102
5:13
RPGDVWTLPDQCHTVTCQPDGQTLLKpc4_NI
p−min=0.25, pX=0.023, N=154
var.26.VWF 12:6128443:T:C
peptide level
8:210
2:102
0:13
RVTGCPPFDEHKpc2_NP.4
p−min=0.5, pX=0.23, N=10
var.26.VWF 12:6128443:T:C
peptide level
30:210
13:102
0:13
RVTGCPPFDEHKpc3_NP.1
p−maj=0.23, pX=0.087, N=43
var.26.VWF 12:6128443:T:C
peptide level
25:210
5:102
0:13
RVTGCPPFDEHKpc3_NP.2
p−min=0.027, pX=0.036, N=30
var.26.VWF 12:6128443:T:C
peptide level
11:210
3:102
0:13
RVTGCPPFDEHKpc3_NP.3
p−min=0.39, pX=0.24, N=14
var.26.VWF 12:6128443:T:C
peptide level
124:210
55:102
7:13
RVTGCPPFDEHKpc3_NP.4
p−min=0.41, pX=0.15, N=186
var.26.VWF 12:6128443:T:C
peptide level
6:210
1:102
0:13
RVTGCPPFDEHKpc3_NP.5
p−min=0.43, pX=0.2, N=7

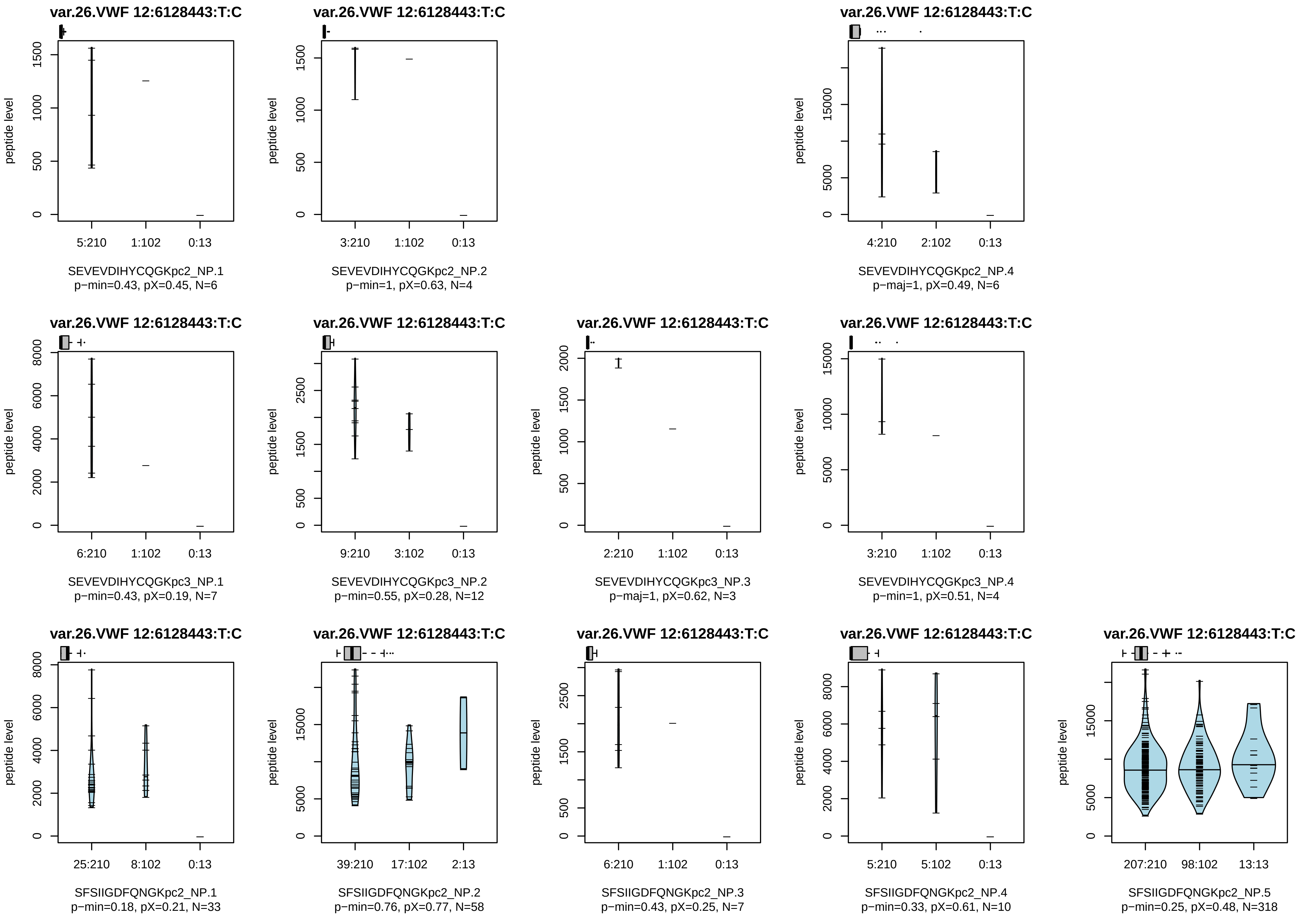
var.26.VWF 12:6128443:T:C
SEVEVDIHYCQGKpc2_NP.1
p−min=0.43, pX=0.45, N=6
5:210
1:102
0:13
var.26.VWF 12:6128443:T:C
SEVEVDIHYCQGKpc2_NP.2
p−min=1, pX=0.63, N=4
3:210
1:102
0:13
var.26.VWF 12:6128443:T:C
SEVEVDIHYCQGKpc2_NP.4
p−maj=1, pX=0.49, N=6
4:210
2:102
0:13
var.26.VWF 12:6128443:T:C
SEVEVDIHYCQGKpc3_NP.1
p−min=0.43, pX=0.19, N=7
6:210
1:102
0:13
var.26.VWF 12:6128443:T:C
SEVEVDIHYCQGKpc3_NP.2
p−min=0.55, pX=0.28, N=12
9:210
3:102
0:13
var.26.VWF 12:6128443:T:C
SEVEVDIHYCQGKpc3_NP.3
p−maj=1, pX=0.62, N=3
2:210
1:102
0:13
var.26.VWF 12:6128443:T:C
SEVEVDIHYCQGKpc3_NP.4
p−min=1, pX=0.51, N=4
3:210
1:102
0:13
var.26.VWF 12:6128443:T:C
SFSIIGDFQNGKpc2_NP.1
p−min=0.18, pX=0.21, N=33
25:210
8:102
0:13
var.26.VWF 12:6128443:T:C
SFSIIGDFQNGKpc2_NP.2
p−min=0.76, pX=0.77, N=58
39:210
17:102
2:13
var.26.VWF 12:6128443:T:C
SFSIIGDFQNGKpc2_NP.3
p−min=0.43, pX=0.25, N=7
6:210
1:102
0:13
var.26.VWF 12:6128443:T:C
SFSIIGDFQNGKpc2_NP.4
p−min=0.33, pX=0.61, N=10
5:210
5:102
0:13
var.26.VWF 12:6128443:T:C
SFSIIGDFQNGKpc2_NP.5
p−min=0.25, pX=0.48, N=318
207:210
98:102
13:13
peptide level

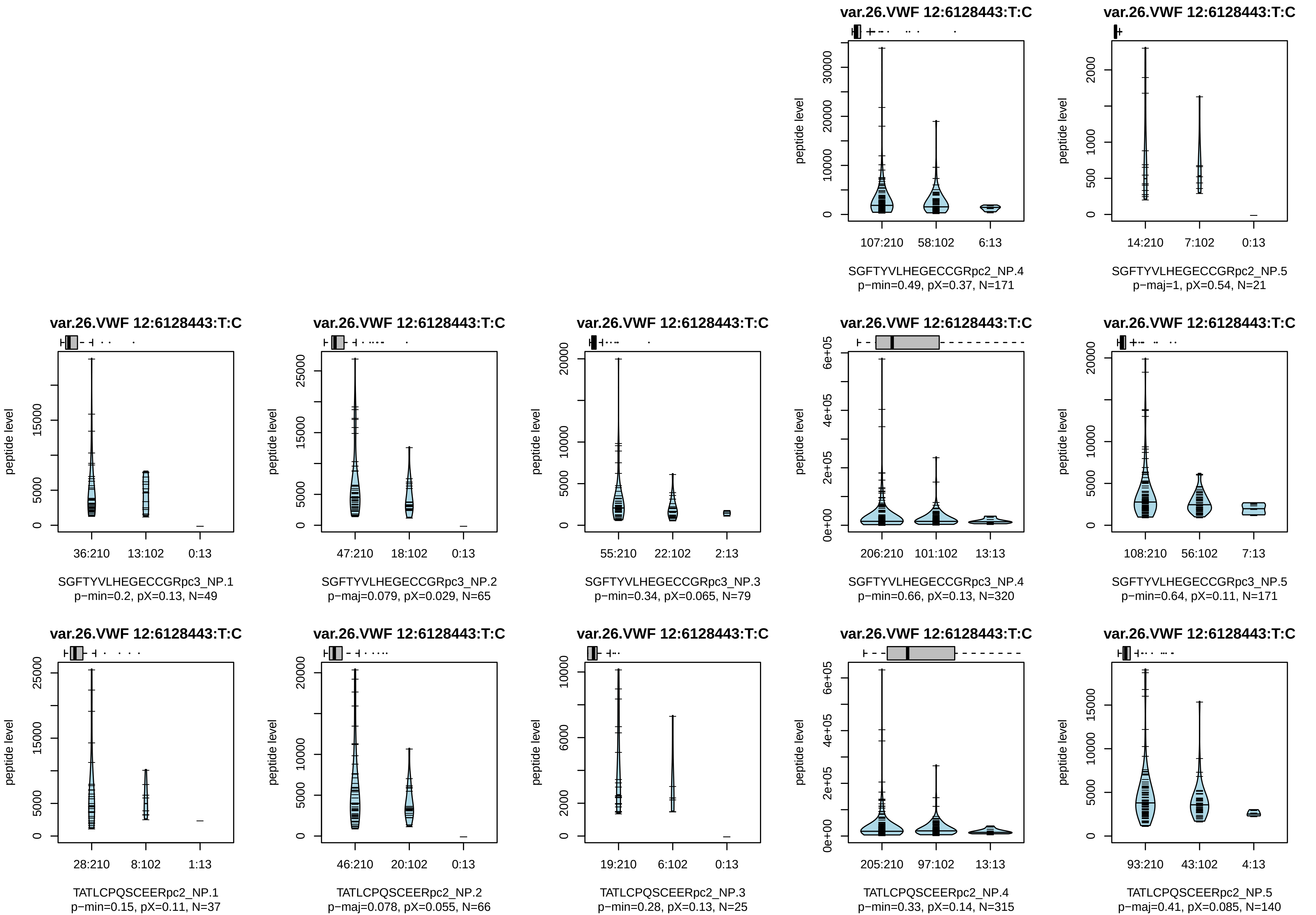

var.26.VWF 12:6128443:T:C
peptide level
107:210
58:102
6:13
SGFTYVLHEGECCGRpc2_NP.4
p−min=0.49, pX=0.37, N=171
var.26.VWF 12:6128443:T:C
peptide level
14:210
7:102
0:13
SGFTYVLHEGECCGRpc2_NP.5
p−maj=1, pX=0.54, N=21
var.26.VWF 12:6128443:T:C
peptide level
36:210
13:102
0:13
SGFTYVLHEGECCGRpc3_NP.1
p−min=0.2, pX=0.13, N=49
var.26.VWF 12:6128443:T:C
peptide level
47:210
18:102
0:13
SGFTYVLHEGECCGRpc3_NP.2
p−maj=0.079, pX=0.029, N=65
var.26.VWF 12:6128443:T:C
peptide level
55:210
22:102
2:13
SGFTYVLHEGECCGRpc3_NP.3
p−min=0.34, pX=0.065, N=79
var.26.VWF 12:6128443:T:C
peptide level
206:210
101:102
13:13
SGFTYVLHEGECCGRpc3_NP.4
p−min=0.66, pX=0.13, N=320
var.26.VWF 12:6128443:T:C
peptide level
108:210
56:102
7:13
SGFTYVLHEGECCGRpc3_NP.5
p−min=0.64, pX=0.11, N=171
var.26.VWF 12:6128443:T:C
peptide level
28:210
8:102
1:13
TATLCPQSCEERpc2_NP.1
p−min=0.15, pX=0.11, N=37
var.26.VWF 12:6128443:T:C
peptide level
46:210
20:102
0:13
TATLCPQSCEERpc2_NP.2
p−maj=0.078, pX=0.055, N=66
var.26.VWF 12:6128443:T:C
peptide level
19:210
6:102
0:13
TATLCPQSCEERpc2_NP.3
p−min=0.28, pX=0.13, N=25
var.26.VWF 12:6128443:T:C
peptide level
205:210
97:102
13:13
TATLCPQSCEERpc2_NP.4
p−min=0.33, pX=0.14, N=315
var.26.VWF 12:6128443:T:C
peptide level
93:210
43:102
4:13
TATLCPQSCEERpc2_NP.5
p−maj=0.41, pX=0.085, N=140

var.26.VWF 12:6128443:T:C

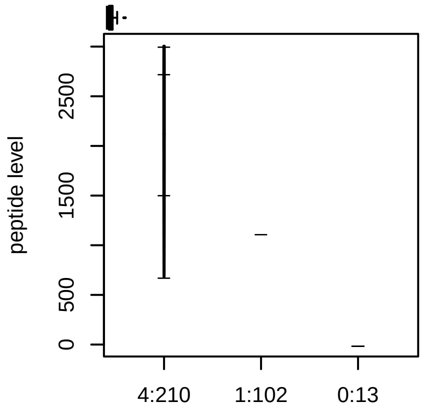

TCQSLHINEMCQERpc3_NP.1
p−min=0.66, pX=0.34, N=5

var.26.VWF 12:6128443:T:C

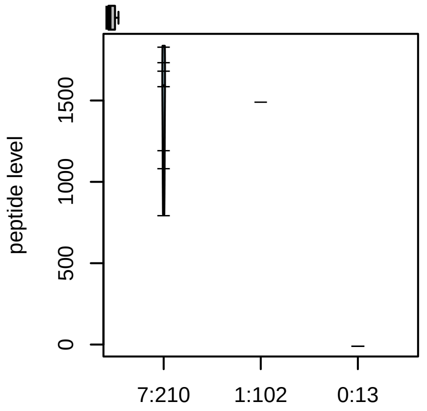

TCQSLHINEMCQERpc3_NP.2
p−min=0.27, pX=0.2, N=8

var.26.VWF 12:6128443:T:C

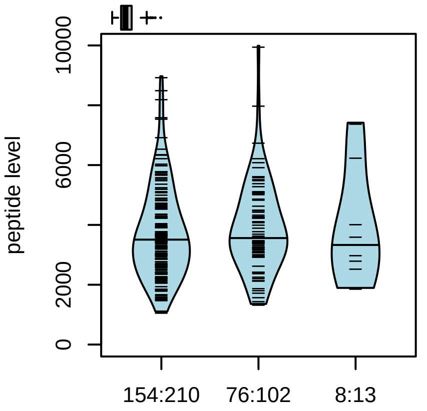

TCQSLHINEMCQERpc3_NP.5
p−maj=0.34, pX=0.87, N=238

var.26.VWF 12:6128443:T:C

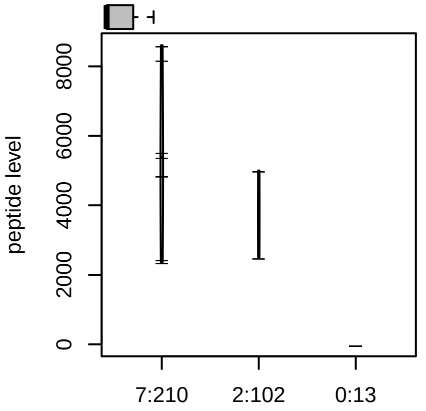

TLVQEWTVQRpc2_NP.4
p−min=0.5, pX=0.27, N=9

var.26.VWF 12:6128443:T:C

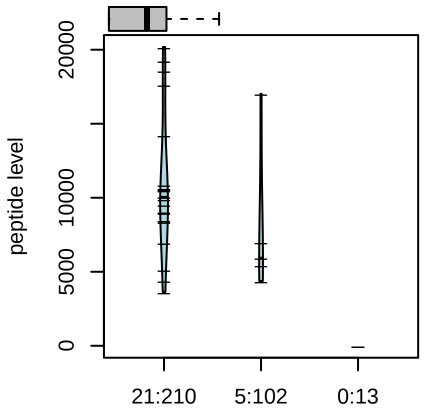

TNGVCVDWRpc2_NP.1
p−min=0.088, pX=0.037, N=26

var.26.VWF 12:6128443:T:C

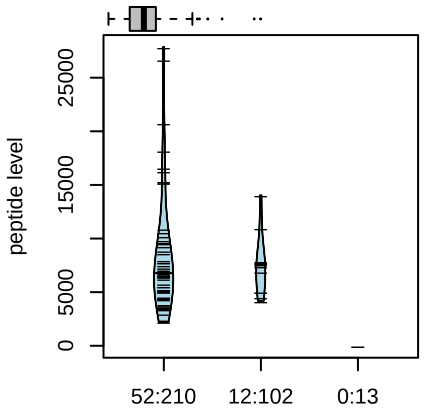

TNGVCVDWRpc2_NP.2
p−min=0.002, pX=0.0032, N=64

var.26.VWF 12:6128443:T:C

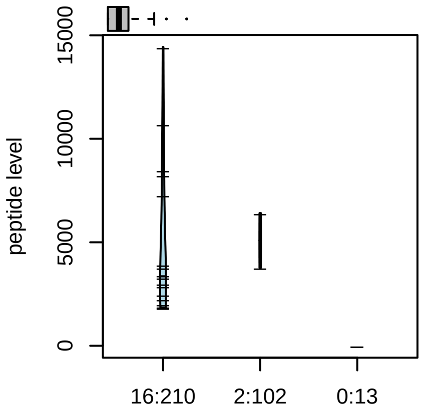

TNGVCVDWRpc2_NP.3
p−min=0.039, pX=0.076, N=18

var.26.VWF 12:6128443:T:C

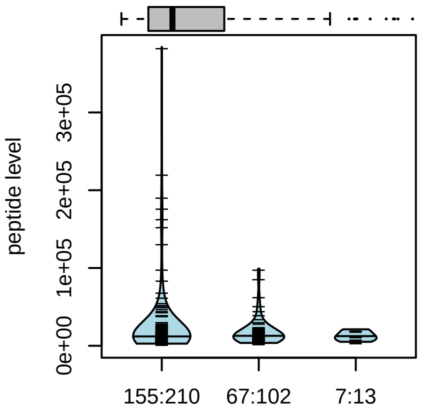

TNGVCVDWRpc2_NP.4
p−min=0.077, pX=0.051, N=229

var.26.VWF 12:6128443:T:C

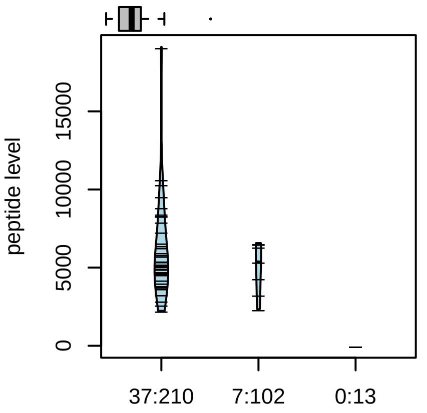

TNGVCVDWRpc2_NP.5
p−min=0.0036, pX=0.0037, N=44

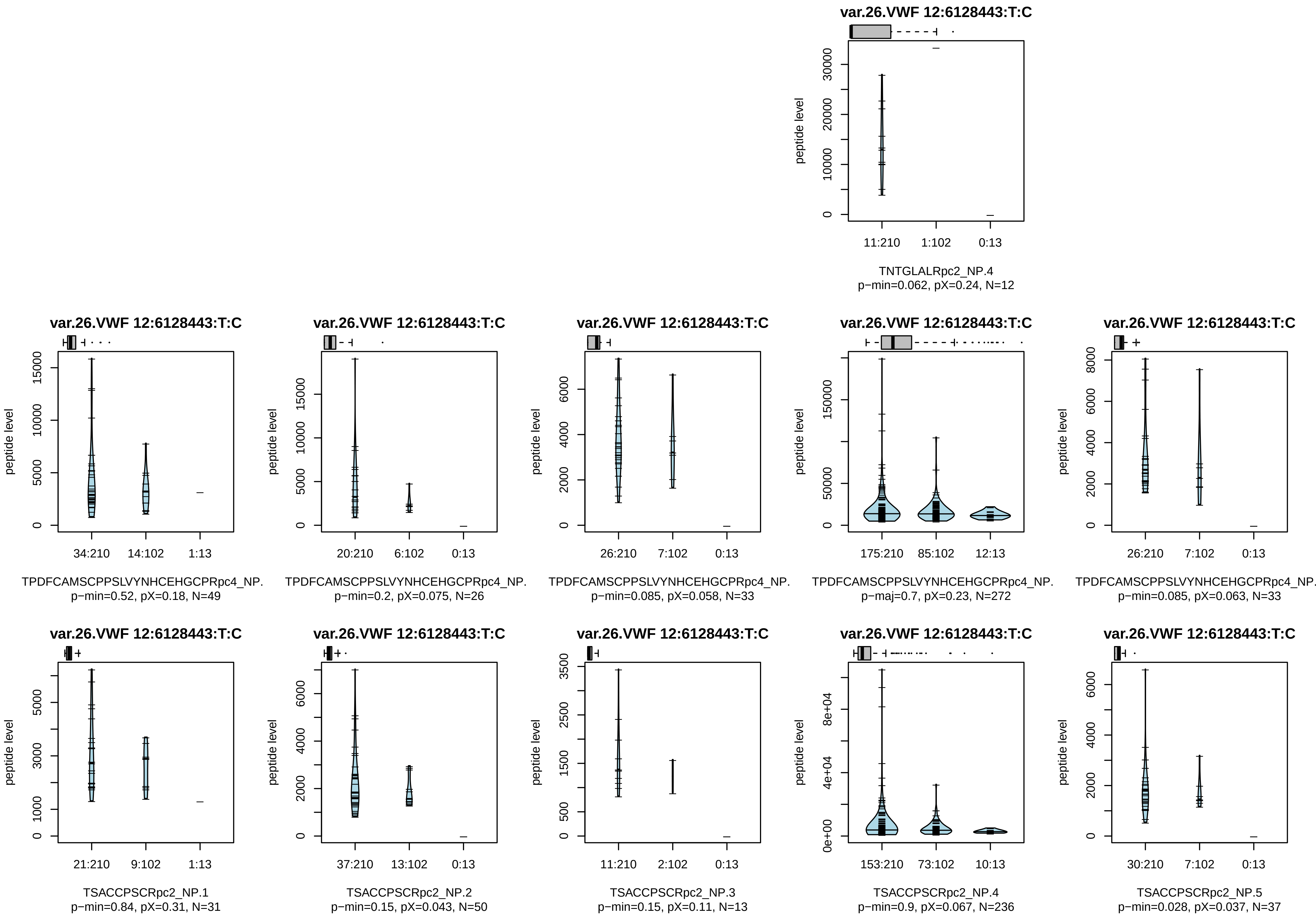
var.26.VWF 12:6128443:T:C
11:210
1:102
0:13
TNTGLALRpc2_NP.4
p−min=0.062, pX=0.24, N=12
var.26.VWF 12:6128443:T:C
34:210
14:102
1:13
TPDFCAMSCPPSLVYNHCEHGCPRpc4_NP.
p−min=0.52, pX=0.18, N=49
var.26.VWF 12:6128443:T:C
20:210
6:102
0:13
TPDFCAMSCPPSLVYNHCEHGCPRpc4_NP.
p−min=0.2, pX=0.075, N=26
var.26.VWF 12:6128443:T:C
26:210
7:102
0:13
TPDFCAMSCPPSLVYNHCEHGCPRpc4_NP.
p−min=0.085, pX=0.058, N=33
var.26.VWF 12:6128443:T:C
175:210
85:102
12:13
TPDFCAMSCPPSLVYNHCEHGCPRpc4_NP.
p−maj=0.7, pX=0.23, N=272
var.26.VWF 12:6128443:T:C
26:210
7:102
0:13
TPDFCAMSCPPSLVYNHCEHGCPRpc4_NP.
p−min=0.085, pX=0.063, N=33
var.26.VWF 12:6128443:T:C
21:210
9:102
1:13
TSACCPSCRpc2_NP.1
p−min=0.84, pX=0.31, N=31
var.26.VWF 12:6128443:T:C
37:210
13:102
0:13
TSACCPSCRpc2_NP.2
p−min=0.15, pX=0.043, N=50
var.26.VWF 12:6128443:T:C
11:210
2:102
0:13
TSACCPSCRpc2_NP.3
p−min=0.15, pX=0.11, N=13
var.26.VWF 12:6128443:T:C
153:210
73:102
10:13
TSACCPSCRpc2_NP.4
p−min=0.9, pX=0.067, N=236
var.26.VWF 12:6128443:T:C
30:210
7:102
0:13
TSACCPSCRpc2_NP.5
p−min=0.028, pX=0.037, N=37

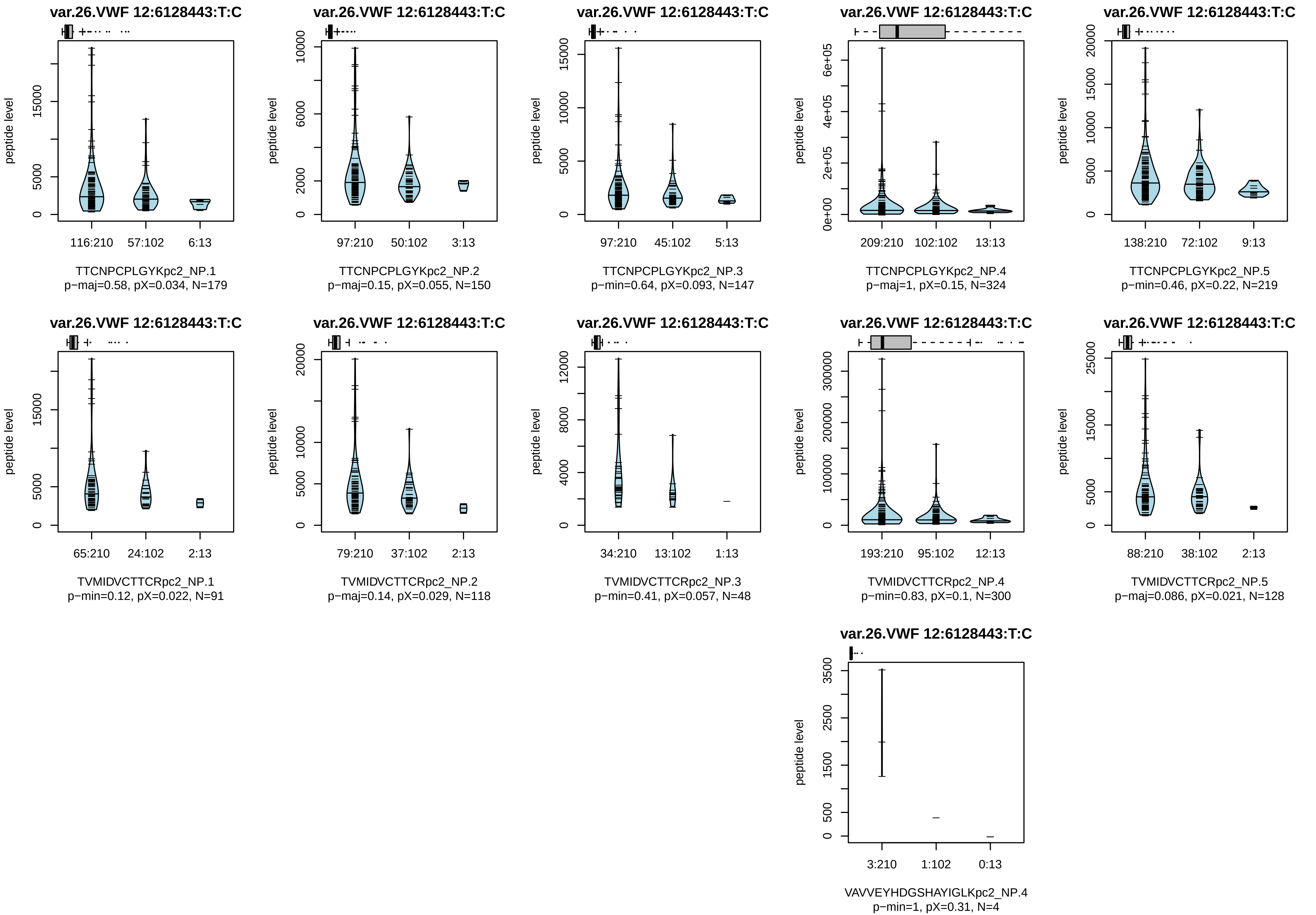
var.26.VWF 12:6128443:T:C
peptide level
116:210
57:102
6:13
TTCNPCPLGYKpc2_NP.1
p−maj=0.58, pX=0.034, N=179
var.26.VWF 12:6128443:T:C
peptide level
97:210
50:102
3:13
TTCNPCPLGYKpc2_NP.2
p−maj=0.15, pX=0.055, N=150
var.26.VWF 12:6128443:T:C
peptide level
97:210
45:102
5:13
TTCNPCPLGYKpc2_NP.3
p−min=0.64, pX=0.093, N=147
var.26.VWF 12:6128443:T:C
peptide level
209:210
102:102
13:13
TTCNPCPLGYKpc2_NP.4
p−maj=1, pX=0.15, N=324
var.26.VWF 12:6128443:T:C
peptide level
138:210
72:102
9:13
TTCNPCPLGYKpc2_NP.5
p−min=0.46, pX=0.22, N=219
var.26.VWF 12:6128443:T:C
peptide level
65:210
24:102
2:13
TVMIDVCTTCRpc2_NP.1
p−min=0.12, pX=0.022, N=91
var.26.VWF 12:6128443:T:C
peptide level
79:210
37:102
2:13
TVMIDVCTTCRpc2_NP.2
p−maj=0.14, pX=0.029, N=118
var.26.VWF 12:6128443:T:C
peptide level
34:210
13:102
1:13
TVMIDVCTTCRpc2_NP.3
p−min=0.41, pX=0.057, N=48
var.26.VWF 12:6128443:T:C
peptide level
193:210
95:102
12:13
TVMIDVCTTCRpc2_NP.4
p−min=0.83, pX=0.1, N=300
var.26.VWF 12:6128443:T:C
peptide level
88:210
38:102
2:13
TVMIDVCTTCRpc2_NP.5
p−maj=0.086, pX=0.021, N=128
var.26.VWF 12:6128443:T:C
peptide level
3:210
1:102
0:13
VAVVEYHDGSHAYIGLKpc2_NP.4
p−min=1, pX=0.31, N=4

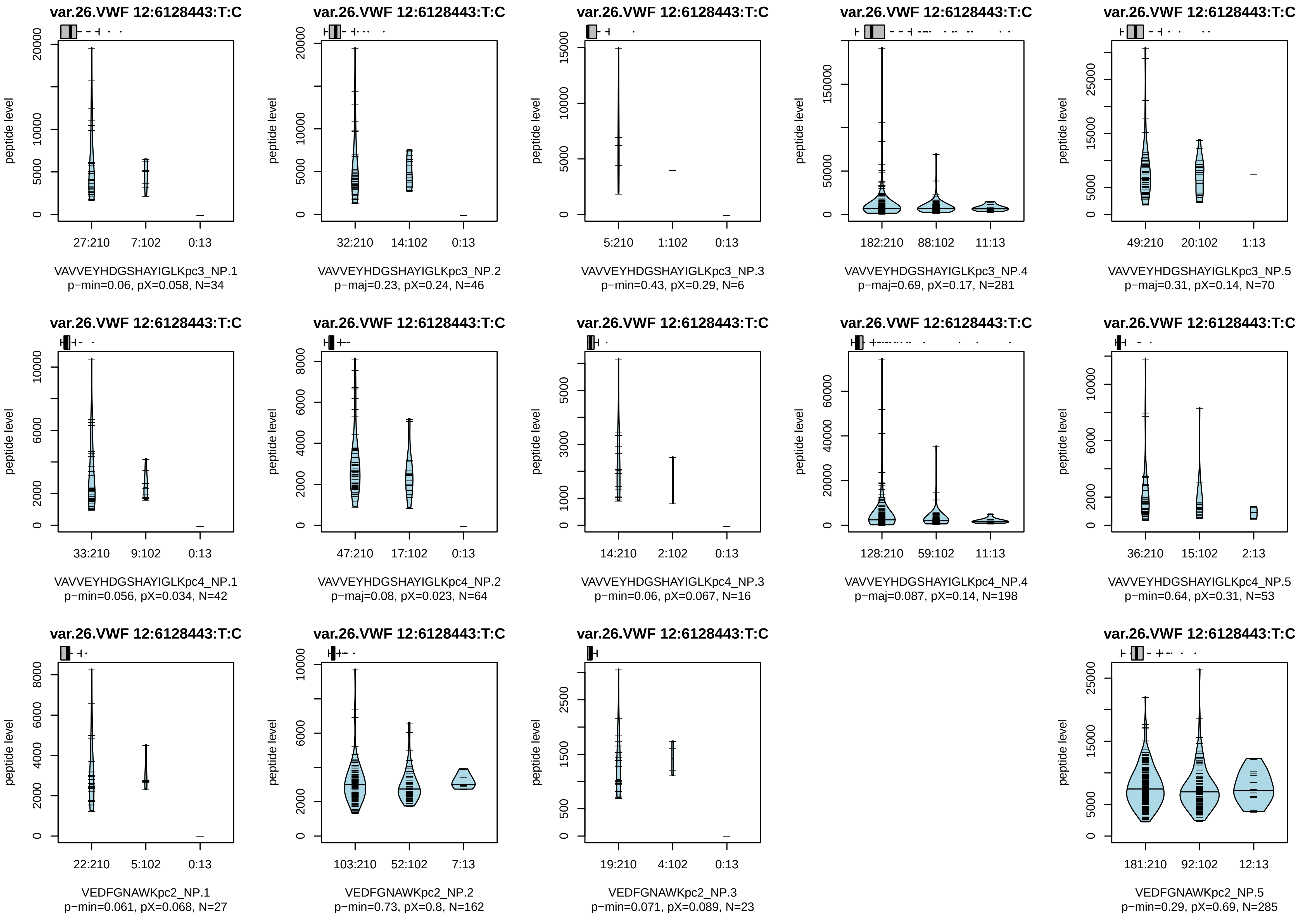
var.26.VWF 12:6128443:T:C
peptide level
27:210
7:102
0:13
VAVVEYHDGSHAYIGLKpc3_NP.1
p−min=0.06, pX=0.058, N=34
var.26.VWF 12:6128443:T:C
peptide level
32:210
14:102
0:13
VAVVEYHDGSHAYIGLKpc3_NP.2
p−maj=0.23, pX=0.24, N=46
var.26.VWF 12:6128443:T:C
peptide level
5:210
1:102
0:13
VAVVEYHDGSHAYIGLKpc3_NP.3
p−min=0.43, pX=0.29, N=6
var.26.VWF 12:6128443:T:C
peptide level
182:210
88:102
11:13
VAVVEYHDGSHAYIGLKpc3_NP.4
p−maj=0.69, pX=0.17, N=281
var.26.VWF 12:6128443:T:C
peptide level
49:210
20:102
1:13
VAVVEYHDGSHAYIGLKpc3_NP.5
p−maj=0.31, pX=0.14, N=70
var.26.VWF 12:6128443:T:C
peptide level
33:210
9:102
0:13
VAVVEYHDGSHAYIGLKpc4_NP.1
p−min=0.056, pX=0.034, N=42
var.26.VWF 12:6128443:T:C
peptide level
47:210
17:102
0:13
VAVVEYHDGSHAYIGLKpc4_NP.2
p−maj=0.08, pX=0.023, N=64
var.26.VWF 12:6128443:T:C
peptide level
14:210
2:102
0:13
VAVVEYHDGSHAYIGLKpc4_NP.3
p−min=0.06, pX=0.067, N=16
var.26.VWF 12:6128443:T:C
peptide level
128:210
59:102
11:13
VAVVEYHDGSHAYIGLKpc4_NP.4
p−maj=0.087, pX=0.14, N=198
var.26.VWF 12:6128443:T:C
peptide level
36:210
15:102
2:13
VAVVEYHDGSHAYIGLKpc4_NP.5
p−min=0.64, pX=0.31, N=53
var.26.VWF 12:6128443:T:C
peptide level
22:210
5:102
0:13
VEDFGNAWKpc2_NP.1
p−min=0.061, pX=0.068, N=27
var.26.VWF 12:6128443:T:C
peptide level
103:210
52:102
7:13
VEDFGNAWKpc2_NP.2
p−min=0.73, pX=0.8, N=162
var.26.VWF 12:6128443:T:C
peptide level
19:210
4:102
0:13
VEDFGNAWKpc2_NP.3
p−min=0.071, pX=0.089, N=23
var.26.VWF 12:6128443:T:C
peptide level
181:210
92:102
12:13
VEDFGNAWKpc2_NP.5
p−min=0.29, pX=0.69, N=285

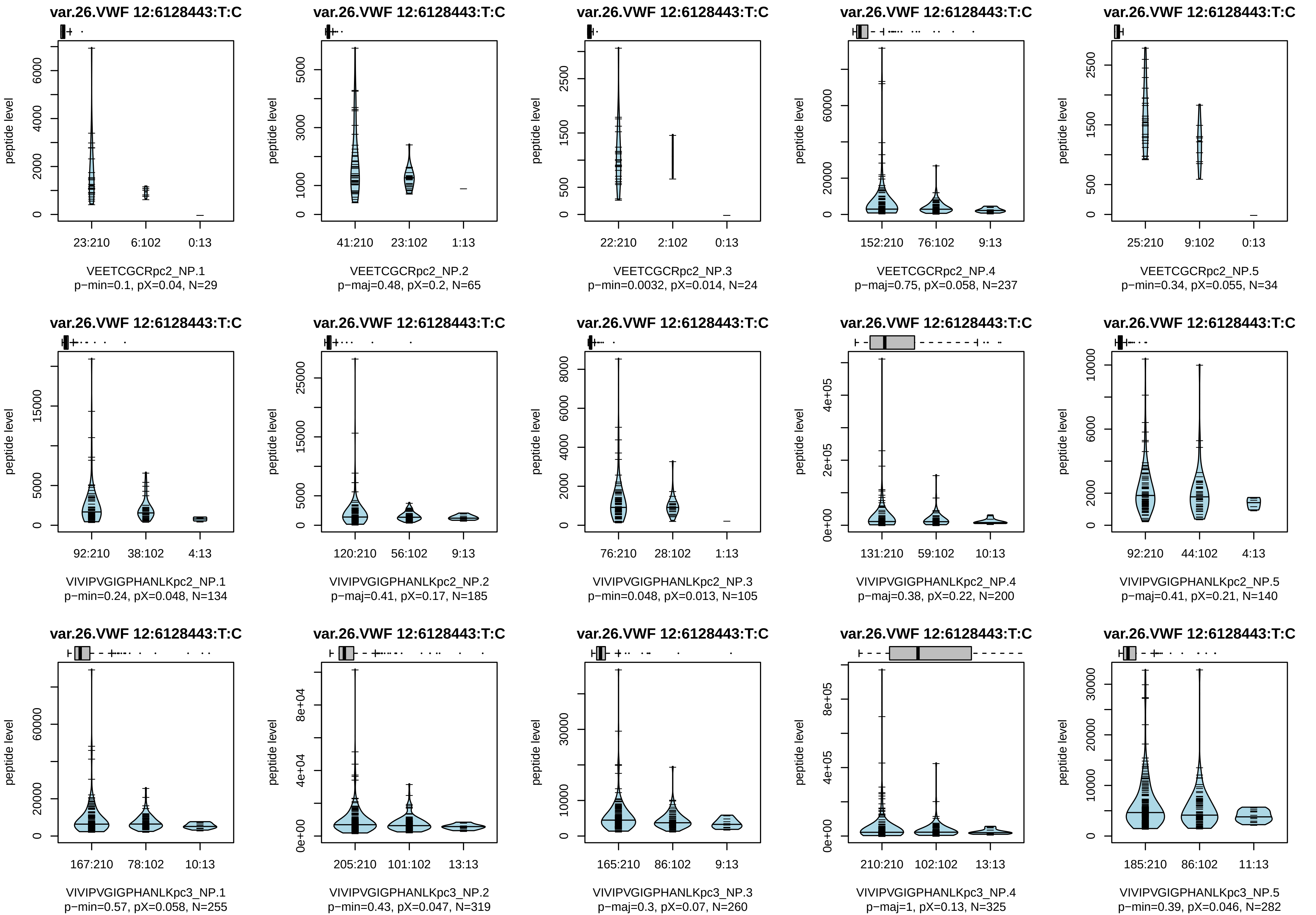
var.26.VWF 12:6128443:T:C
peptide level
23:210
6:102
0:13
VEETCGCRpc2_NP.1
p−min=0.1, pX=0.04, N=29
var.26.VWF 12:6128443:T:C
peptide level
41:210
23:102
1:13
VEETCGCRpc2_NP.2
p−maj=0.48, pX=0.2, N=65
var.26.VWF 12:6128443:T:C
peptide level
22:210
2:102
0:13
VEETCGCRpc2_NP.3
p−min=0.0032, pX=0.014, N=24
var.26.VWF 12:6128443:T:C
peptide level
152:210
76:102
9:13
VEETCGCRpc2_NP.4
p−maj=0.75, pX=0.058, N=237
var.26.VWF 12:6128443:T:C
peptide level
25:210
9:102
0:13
VEETCGCRpc2_NP.5
p−min=0.34, pX=0.055, N=34
var.26.VWF 12:6128443:T:C
peptide level
92:210
38:102
4:13
VIVIPVGIGPHANLKpc2_NP.1
p−min=0.24, pX=0.048, N=134
var.26.VWF 12:6128443:T:C
peptide level
120:210
56:102
9:13
VIVIPVGIGPHANLKpc2_NP.2
p−maj=0.41, pX=0.17, N=185
var.26.VWF 12:6128443:T:C
peptide level
76:210
28:102
1:13
VIVIPVGIGPHANLKpc2_NP.3
p−min=0.048, pX=0.013, N=105
var.26.VWF 12:6128443:T:C
peptide level
131:210
59:102
10:13
VIVIPVGIGPHANLKpc2_NP.4
p−maj=0.38, pX=0.22, N=200
var.26.VWF 12:6128443:T:C
peptide level
92:210
44:102
4:13
VIVIPVGIGPHANLKpc2_NP.5
p−maj=0.41, pX=0.21, N=140
var.26.VWF 12:6128443:T:C
peptide level
167:210
78:102
10:13
VIVIPVGIGPHANLKpc3_NP.1
p−min=0.57, pX=0.058, N=255
var.26.VWF 12:6128443:T:C
peptide level
205:210
101:102
13:13
VIVIPVGIGPHANLKpc3_NP.2
p−min=0.43, pX=0.047, N=319
var.26.VWF 12:6128443:T:C
peptide level
165:210
86:102
9:13
VIVIPVGIGPHANLKpc3_NP.3
p−maj=0.3, pX=0.07, N=260
var.26.VWF 12:6128443:T:C
peptide level
210:210
102:102
13:13
VIVIPVGIGPHANLKpc3_NP.4
p−maj=1, pX=0.13, N=325
var.26.VWF 12:6128443:T:C
peptide level
185:210
86:102
11:13
VIVIPVGIGPHANLKpc3_NP.5
p−min=0.39, pX=0.046, N=282

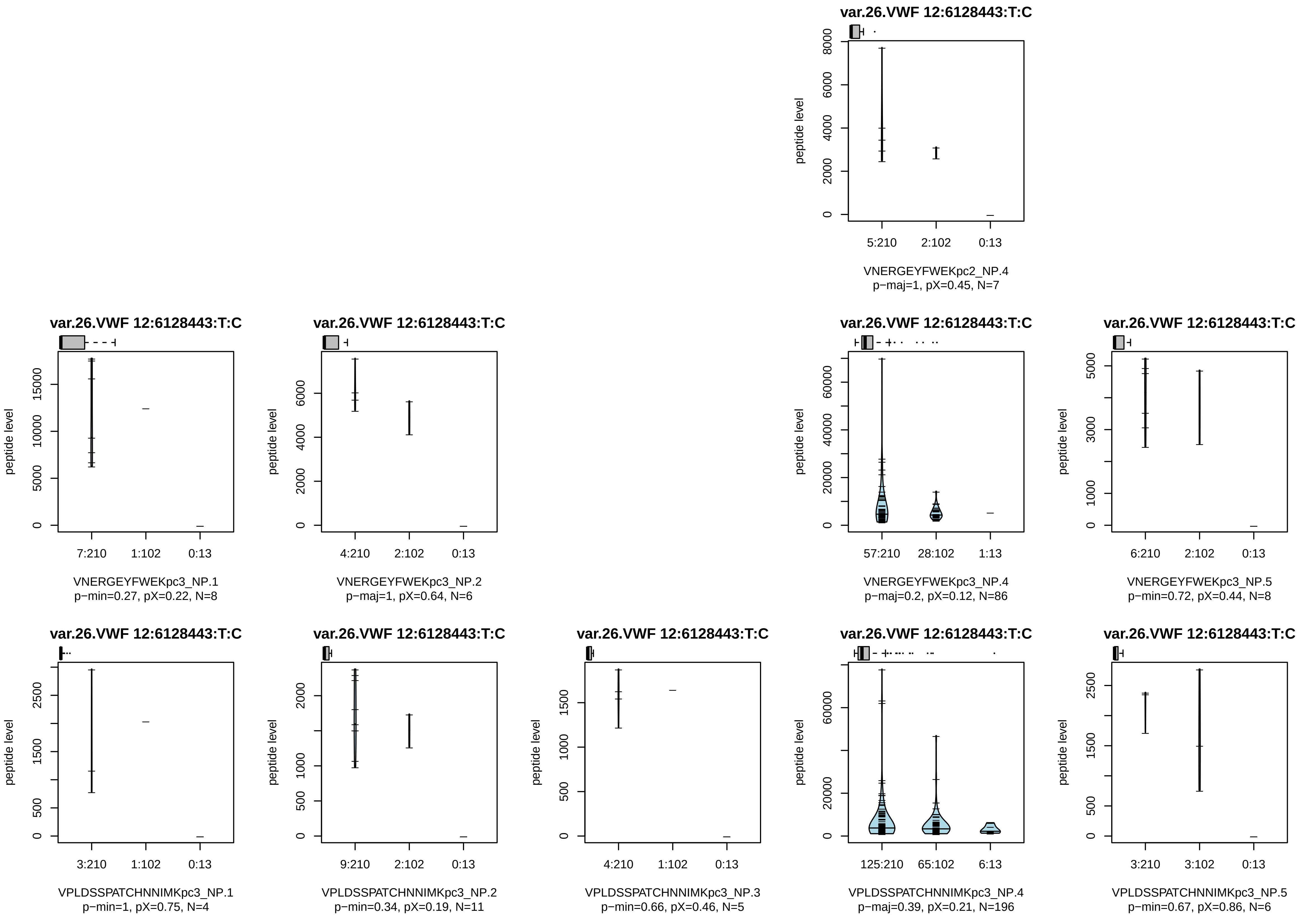
var.26.VWF 12:6128443:T:C
peptide level
5:210
2:102
0:13
VNERGEYFWEKpc2_NP.4
p−maj=1, pX=0.45, N=7
var.26.VWF 12:6128443:T:C
7:210
1:102
0:13
VNERGEYFWEKpc3_NP.1
p−min=0.27, pX=0.22, N=8
var.26.VWF 12:6128443:T:C
4:210
2:102
0:13
VNERGEYFWEKpc3_NP.2
p−maj=1, pX=0.64, N=6
var.26.VWF 12:6128443:T:C
57:210
28:102
1:13
VNERGEYFWEKpc3_NP.4
p−maj=0.2, pX=0.12, N=86
var.26.VWF 12:6128443:T:C
6:210
2:102
0:13
VNERGEYFWEKpc3_NP.5
p−min=0.72, pX=0.44, N=8
var.26.VWF 12:6128443:T:C
3:210
1:102
0:13
VPLDSSPATCHNNIMKpc3_NP.1
p−min=1, pX=0.75, N=4
var.26.VWF 12:6128443:T:C
9:210
2:102
0:13
VPLDSSPATCHNNIMKpc3_NP.2
p−min=0.34, pX=0.19, N=11
var.26.VWF 12:6128443:T:C
4:210
1:102
0:13
VPLDSSPATCHNNIMKpc3_NP.3
p−min=0.66, pX=0.46, N=5
var.26.VWF 12:6128443:T:C
125:210
65:102
6:13
VPLDSSPATCHNNIMKpc3_NP.4
p−maj=0.39, pX=0.21, N=196
var.26.VWF 12:6128443:T:C
3:210
3:102
0:13
VPLDSSPATCHNNIMKpc3_NP.5
p−min=0.67, pX=0.86, N=6

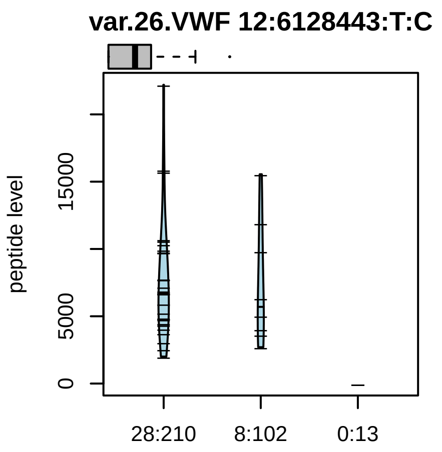

VSPPSCPPHRpc2_NP.2
p−min=0.097, pX=0.087, N=36

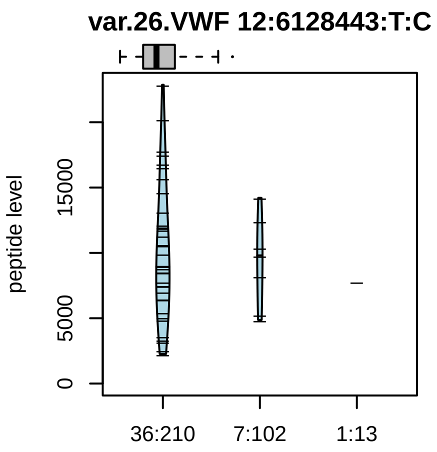

VSPPSCPPHRpc2_NP.3
p−min=0.011, pX=0.02, N=44

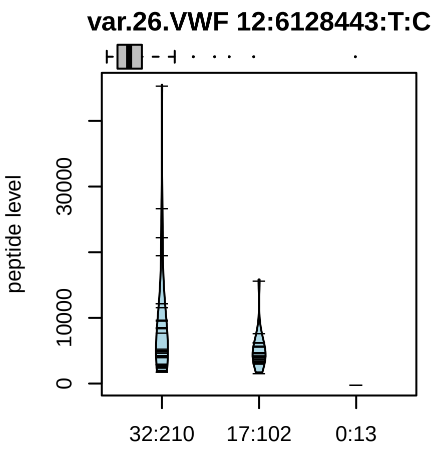

VSPPSCPPHRpc2_NP.4
p−maj=0.23, pX=0.18, N=49

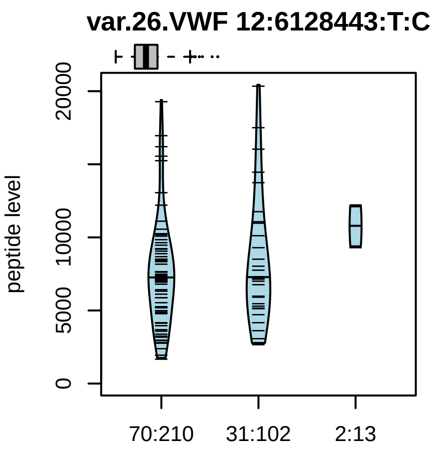

VSPPSCPPHRpc2_NP.5
p−maj=0.24, pX=0.73, N=103

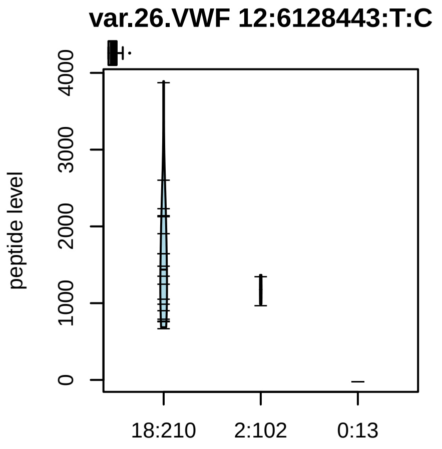

VSSQCADTRpc2_NP.2
p−min=0.015, pX=0.021, N=20

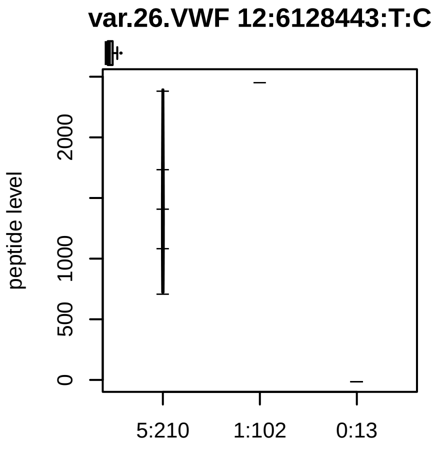

VSSQCADTRpc2_NP.3
p−min=0.43, pX=0.56, N=6

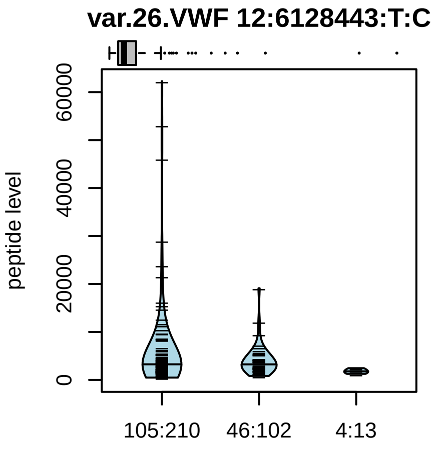

VSSQCADTRpc2_NP.4
p−maj=0.26, pX=0.039, N=155

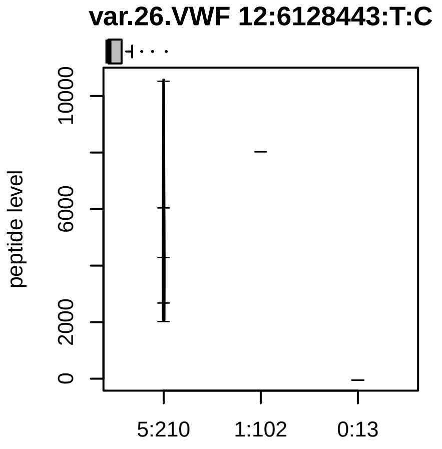

VTGCPPFDEHKpc2_NP.2
p−min=0.43, pX=0.55, N=6

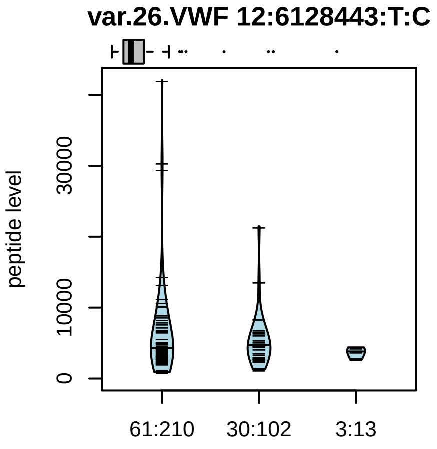

VTGCPPFDEHKpc2_NP.4
p−maj=0.76, pX=0.33, N=94

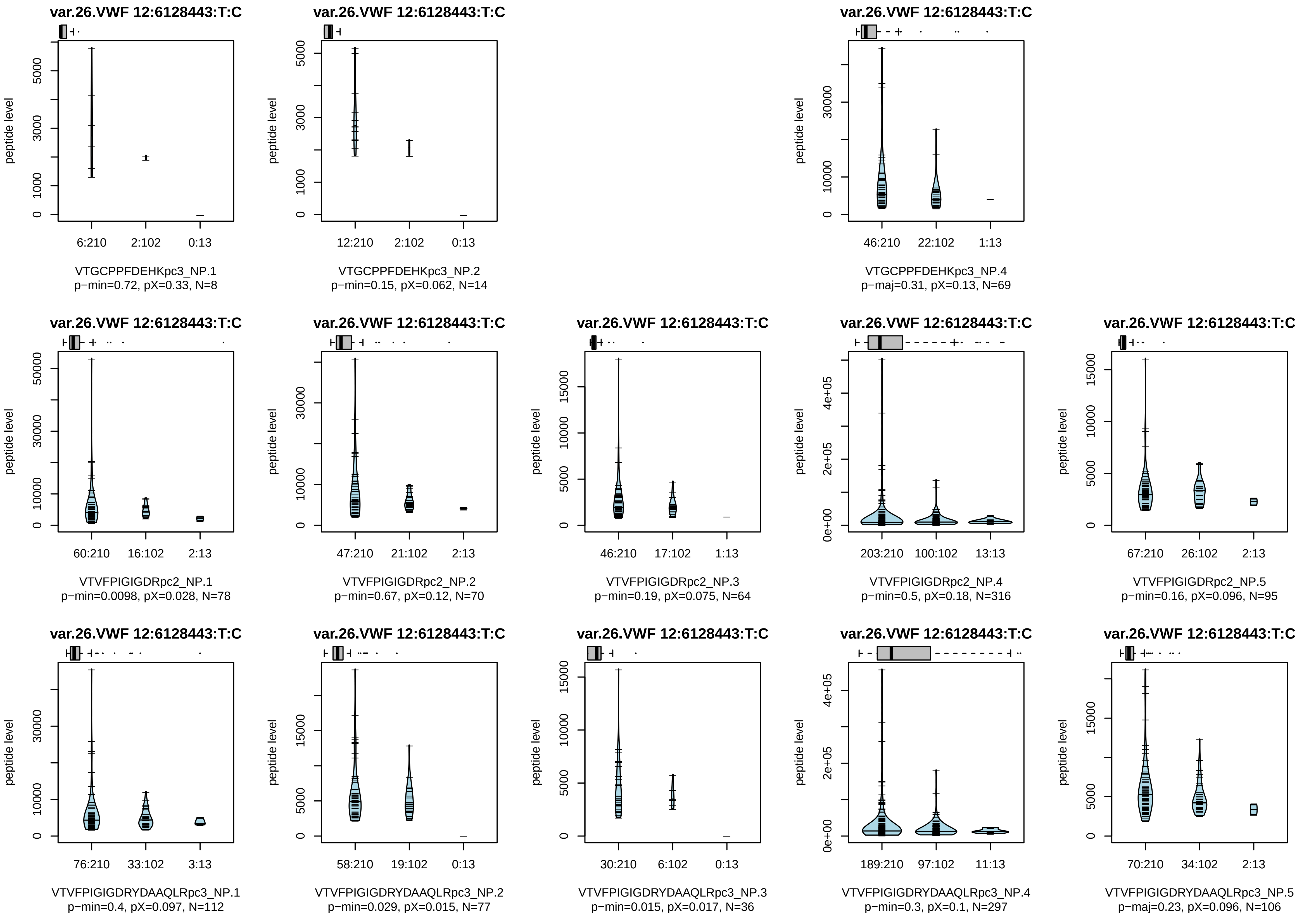
var.26.VWF 12:6128443:T:C
peptide level
6:210
2:102
0:13
VTGCPPFDEHKpc3_NP.1
p−min=0.72, pX=0.33, N=8
var.26.VWF 12:6128443:T:C
peptide level
12:210
2:102
0:13
VTGCPPFDEHKpc3_NP.2
p−min=0.15, pX=0.062, N=14
var.26.VWF 12:6128443:T:C
peptide level
46:210
22:102
1:13
VTGCPPFDEHKpc3_NP.4
p−maj=0.31, pX=0.13, N=69
var.26.VWF 12:6128443:T:C
peptide level
60:210
16:102
2:13
VTVFPIGIGDRpc2_NP.1
p−min=0.0098, pX=0.028, N=78
var.26.VWF 12:6128443:T:C
peptide level
47:210
21:102
2:13
VTVFPIGIGDRpc2_NP.2
p−min=0.67, pX=0.12, N=70
var.26.VWF 12:6128443:T:C
peptide level
46:210
17:102
1:13
VTVFPIGIGDRpc2_NP.3
p−min=0.19, pX=0.075, N=64
var.26.VWF 12:6128443:T:C
peptide level
203:210
100:102
13:13
VTVFPIGIGDRpc2_NP.4
p−min=0.5, pX=0.18, N=316
var.26.VWF 12:6128443:T:C
peptide level
67:210
26:102
2:13
VTVFPIGIGDRpc2_NP.5
p−min=0.16, pX=0.096, N=95
var.26.VWF 12:6128443:T:C
peptide level
76:210
33:102
3:13
VTVFPIGIGDRYDAAQLRpc3_NP.1
p−min=0.4, pX=0.097, N=112
var.26.VWF 12:6128443:T:C
peptide level
58:210
19:102
0:13
VTVFPIGIGDRYDAAQLRpc3_NP.2
p−min=0.029, pX=0.015, N=77
var.26.VWF 12:6128443:T:C
peptide level
30:210
6:102
0:13
VTVFPIGIGDRYDAAQLRpc3_NP.3
p−min=0.015, pX=0.017, N=36
var.26.VWF 12:6128443:T:C
peptide level
189:210
97:102
11:13
VTVFPIGIGDRYDAAQLRpc3_NP.4
p−min=0.3, pX=0.1, N=297
var.26.VWF 12:6128443:T:C
peptide level
70:210
34:102
2:13
VTVFPIGIGDRYDAAQLRpc3_NP.5
p−maj=0.23, pX=0.096, N=106

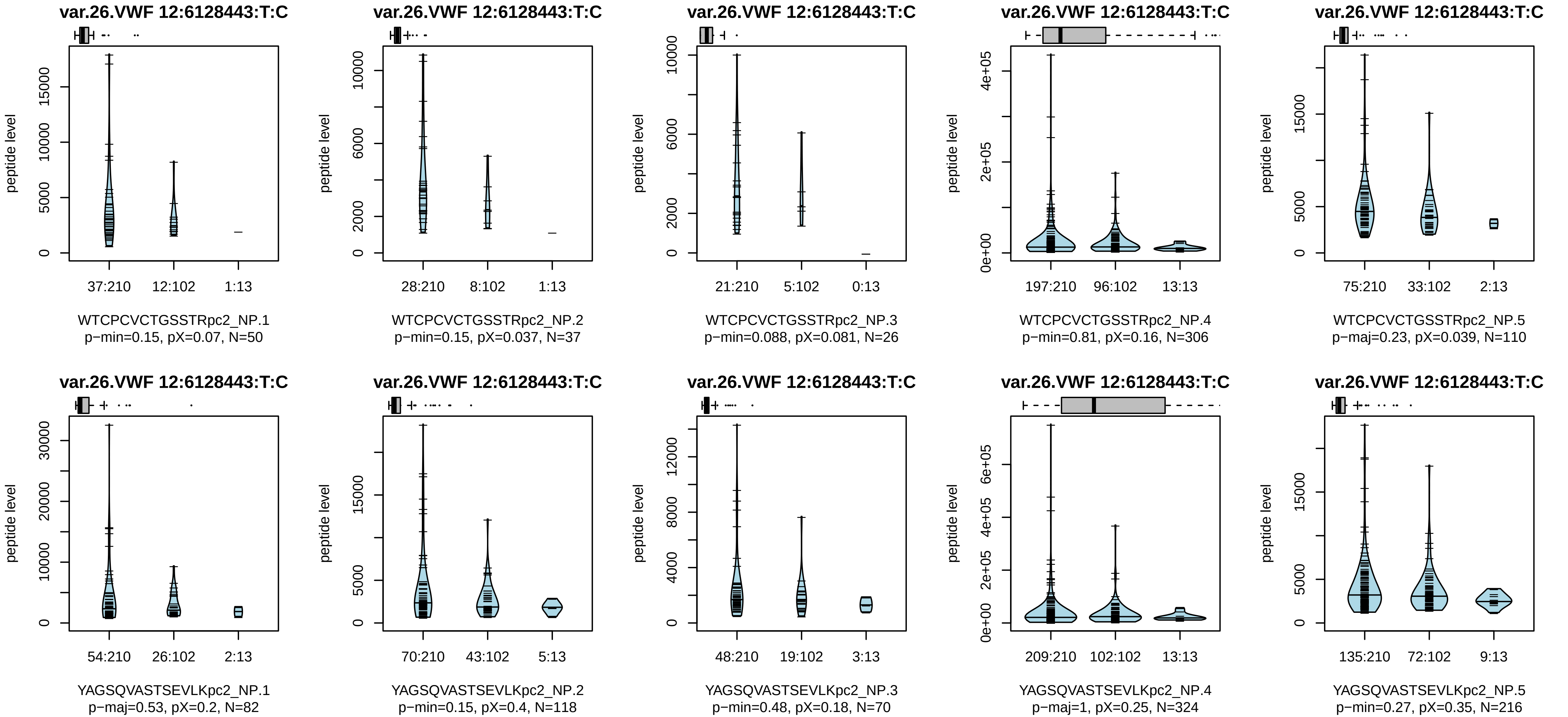
var.26.VWF 12:6128443:T:C
peptide level
37:210
12:102
1:13
WTCPCVCTGSSTRpc2_NP.1
p−min=0.15, pX=0.07, N=50
28:210
8:102
1:13
WTCPCVCTGSSTRpc2_NP.2
p−min=0.15, pX=0.037, N=37
21:210
5:102
0:13
WTCPCVCTGSSTRpc2_NP.3
p−min=0.088, pX=0.081, N=26
197:210
96:102
13:13
WTCPCVCTGSSTRpc2_NP.4
p−min=0.81, pX=0.16, N=306
75:210
33:102
2:13
WTCPCVCTGSSTRpc2_NP.5
p−maj=0.23, pX=0.039, N=110
54:210
26:102
2:13
YAGSQVASTSEVLKpc2_NP.1
p−maj=0.53, pX=0.2, N=82
70:210
43:102
5:13
YAGSQVASTSEVLKpc2_NP.2
p−min=0.15, pX=0.4, N=118
48:210
19:102
3:13
YAGSQVASTSEVLKpc2_NP.3
p−min=0.48, pX=0.18, N=70
209:210
102:102
13:13
YAGSQVASTSEVLKpc2_NP.4
p−maj=1, pX=0.25, N=324
135:210
72:102
9:13
YAGSQVASTSEVLKpc2_NP.5
p−min=0.27, pX=0.35, N=216

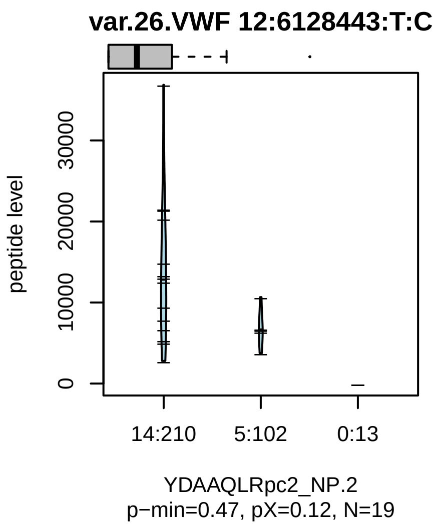
var.26.VWF 12:6128443:T:C
peptide level
14:210
5:102
0:13
YDAAQLRpc2_NP.2
p−min=0.47, pX=0.12, N=19

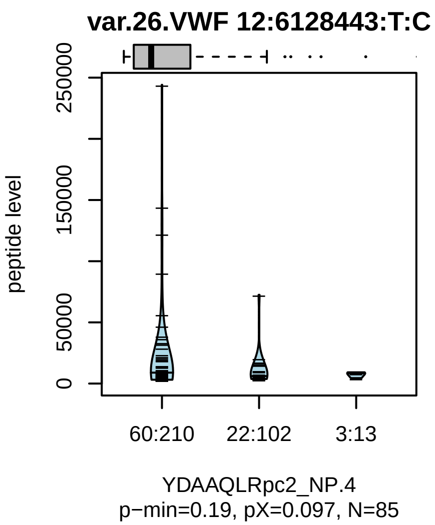
var.26.VWF 12:6128443:T:C
peptide level
60:210
22:102
3:13
YDAAQLRpc2_NP.4
p−min=0.19, pX=0.097, N=85

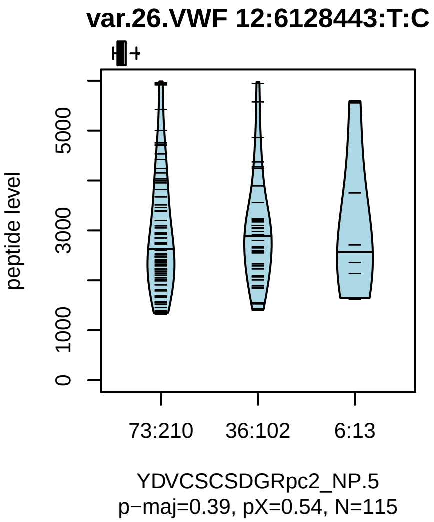
var.26.VWF 12:6128443:T:C
peptide level
73:210
36:102
6:13
YDVCSCSDGRpc2_NP.5
p−maj=0.39, pX=0.54, N=115

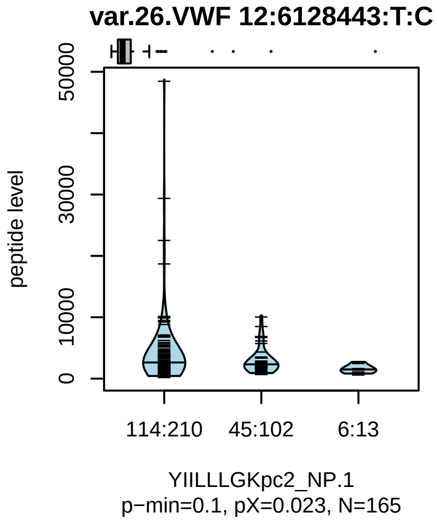
var.26.VWF 12:6128443:T:C
peptide level
114:210
45:102
6:13
YIILLLGKpc2_NP.1
p−min=0.1, pX=0.023, N=165

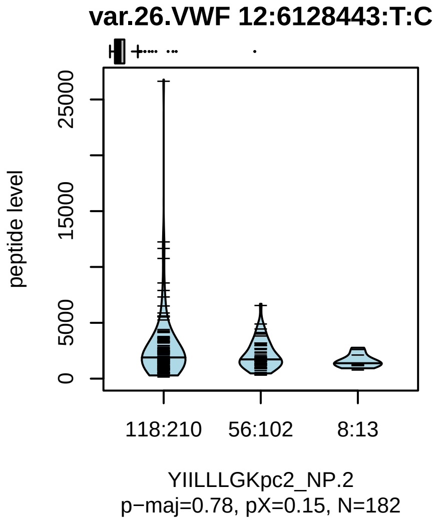
var.26.VWF 12:6128443:T:C
peptide level
118:210
56:102
8:13
YIILLLGKpc2_NP.2
p−maj=0.78, pX=0.15, N=182

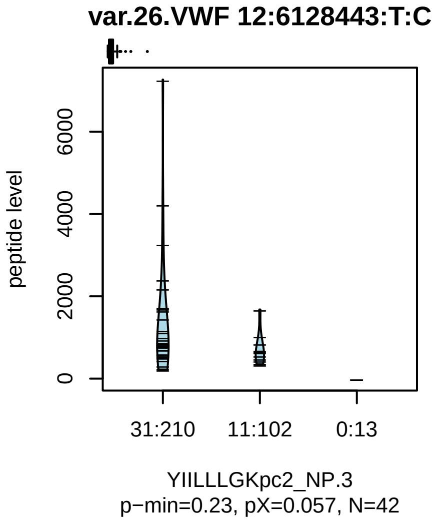
var.26.VWF 12:6128443:T:C
peptide level
31:210
11:102
0:13
YIILLLGKpc2_NP.3
p−min=0.23, pX=0.057, N=42

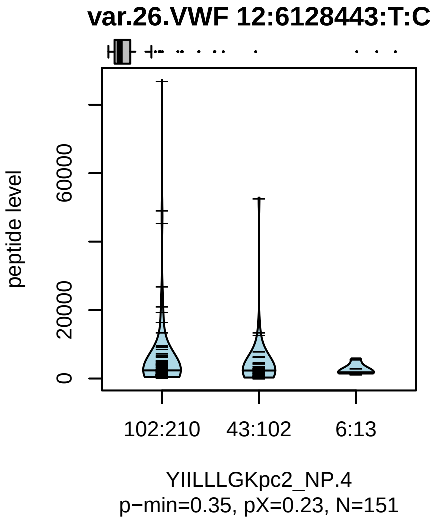
var.26.VWF 12:6128443:T:C
peptide level
102:210
43:102
6:13
YIILLLGKpc2_NP.4
p−min=0.35, pX=0.23, N=151

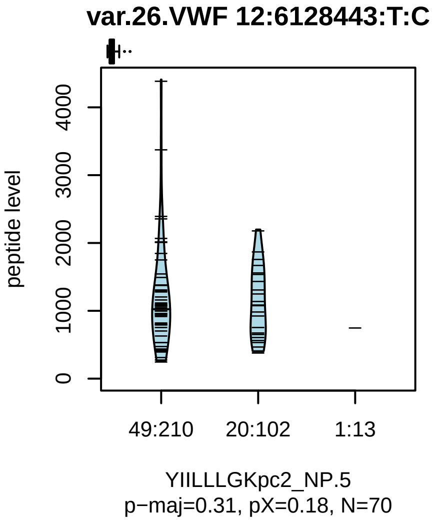
var.26.VWF 12:6128443:T:C
peptide level
49:210
20:102
1:13
YIILLLGKpc2_NP.5
p−maj=0.31, pX=0.18, N=70

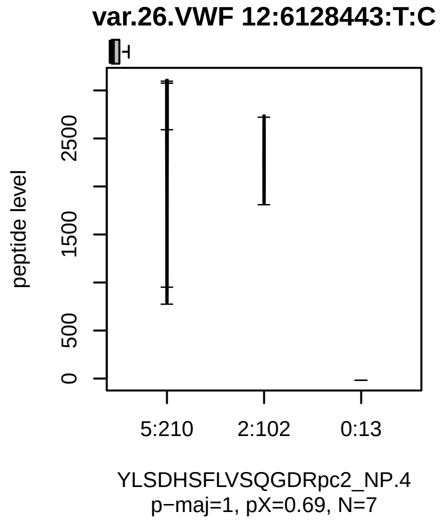
var.26.VWF 12:6128443:T:C
peptide level
0
500
1500
2500
5:210
2:102
0:13
YLSDHSFLVSQGDRpc2_NP.4
p−maj=1, pX=0.69, N=7

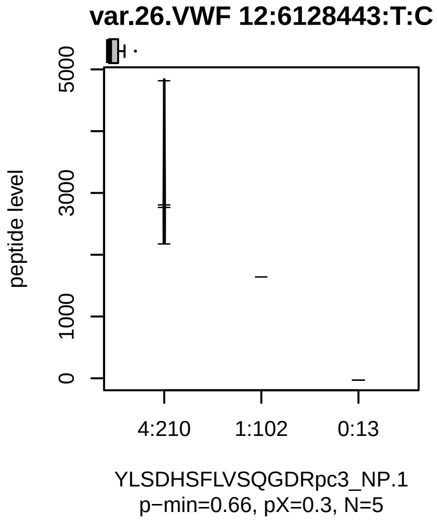
var.26.VWF 12:6128443:T:C
peptide level
0
1000
3000
5000
4:210
1:102
0:13
YLSDHSFLVSQGDRpc3_NP.1
p−min=0.66, pX=0.3, N=5

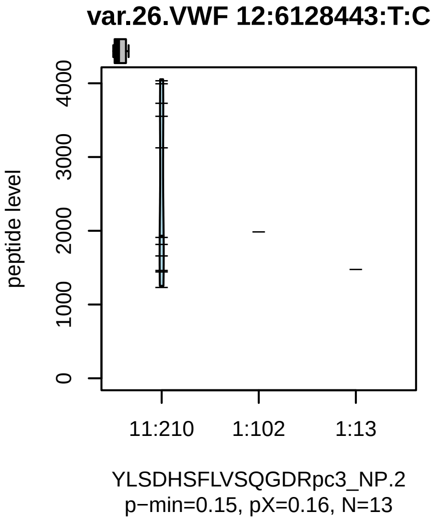
var.26.VWF 12:6128443:T:C
peptide level
0
1000
2000
3000
4000
11:210
1:102
1:13
YLSDHSFLVSQGDRpc3_NP.2
p−min=0.15, pX=0.16, N=13

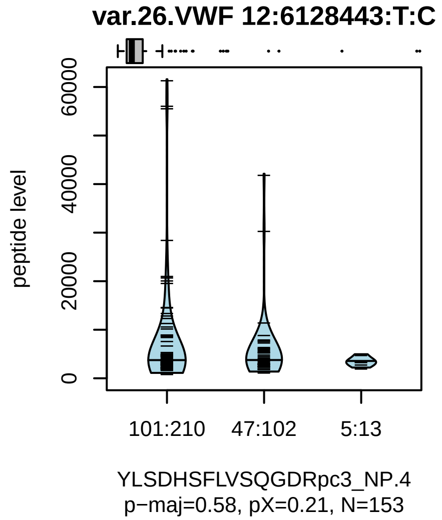
var.26.VWF 12:6128443:T:C
peptide level
0
20000
40000
60000
101:210
47:102
5:13
YLSDHSFLVSQGDRpc3_NP.4
p−maj=0.58, pX=0.21, N=153

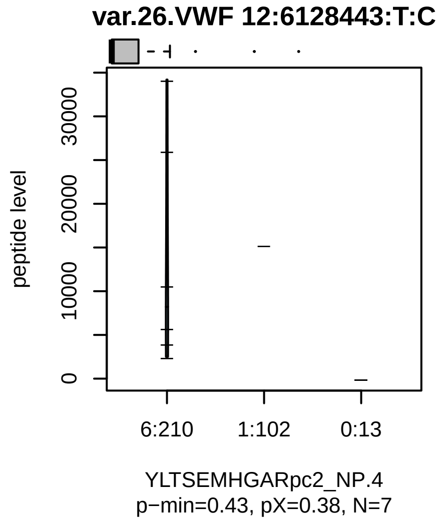
var.26.VWF 12:6128443:T:C
peptide level
0
10000
20000
30000
6:210
1:102
0:13
YLTSEMHGARpc2_NP.4
p−min=0.43, pX=0.38, N=7

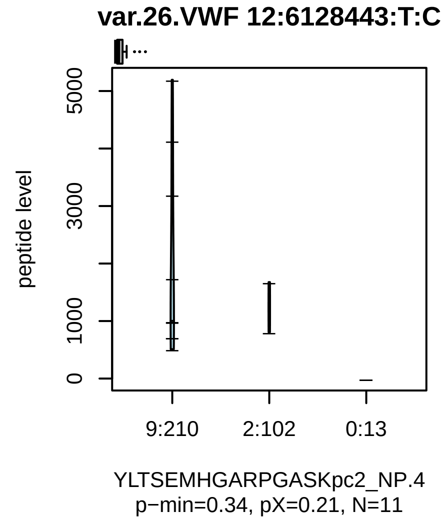
var.26.VWF 12:6128443:T:C
peptide level
0
1000
3000
5000
9:210
2:102
0:13
YLTSEMHGARPGASKpc2_NP.4
p−min=0.34, pX=0.21, N=11

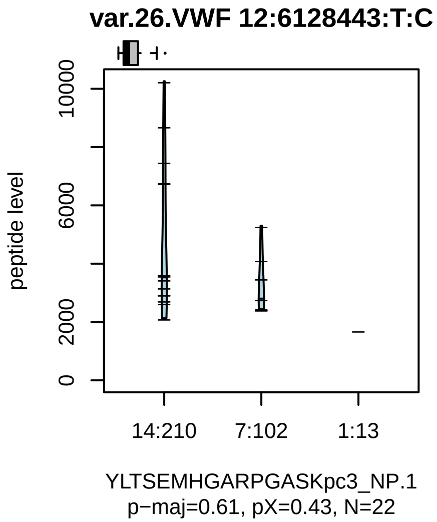
var.26.VWF 12:6128443:T:C
peptide level
0
2000
6000
10000
14:210
7:102
1:13
YLTSEMHGARPGASKpc3_NP.1
p−maj=0.61, pX=0.43, N=22

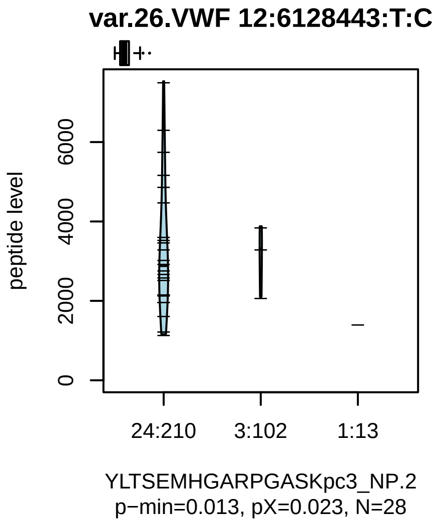
var.26.VWF 12:6128443:T:C
peptide level
0
2000
4000
6000
24:210
3:102
1:13
YLTSEMHGARPGASKpc3_NP.2
p−min=0.013, pX=0.023, N=28

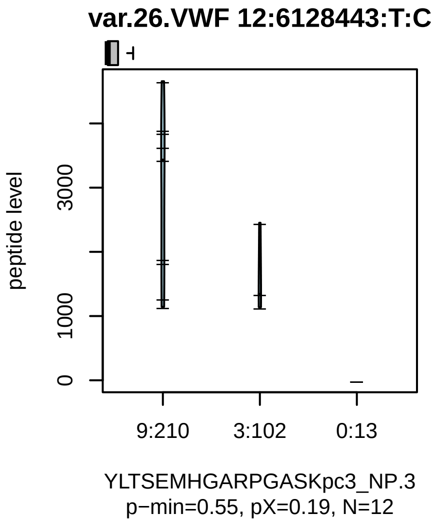
var.26.VWF 12:6128443:T:C
peptide level
0
1000
3000
9:210
3:102
0:13
YLTSEMHGARPGASKpc3_NP.3
p−min=0.55, pX=0.19, N=12

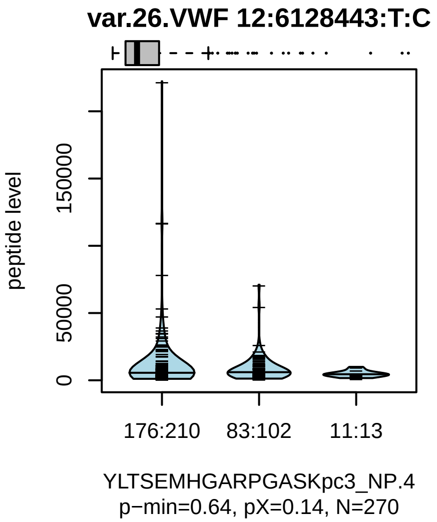
var.26.VWF 12:6128443:T:C
peptide level
0
50000
150000
176:210
83:102
11:13
YLTSEMHGARPGASKpc3_NP.4
p−min=0.64, pX=0.14, N=270

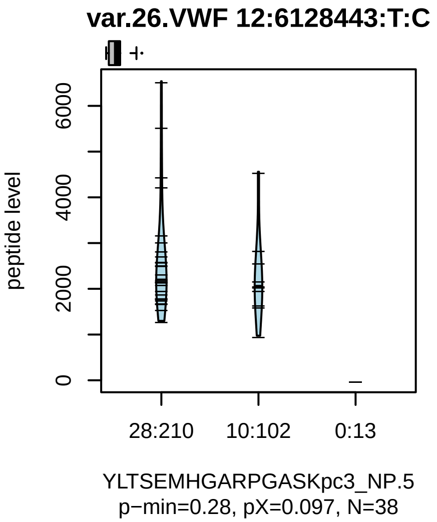
var.26.VWF 12:6128443:T:C
peptide level
0
2000
4000
6000
28:210
10:102
0:13
YLTSEMHGARPGASKpc3_NP.5
p−min=0.28, pX=0.097, N=38

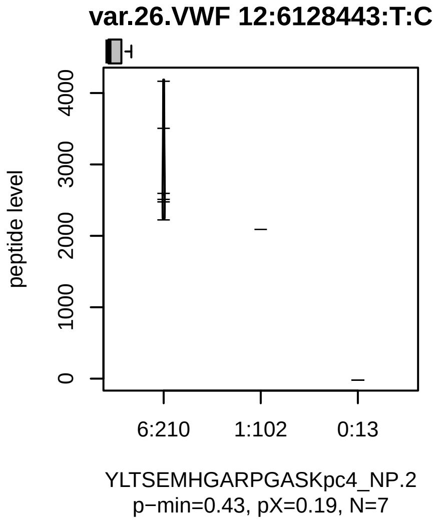
var.26.VWF 12:6128443:T:C
peptide level
0
1000
2000
3000
4000
6:210
1:102
0:13
YLTSEMHGARPGASKpc4_NP.2
p−min=0.43, pX=0.19, N=7

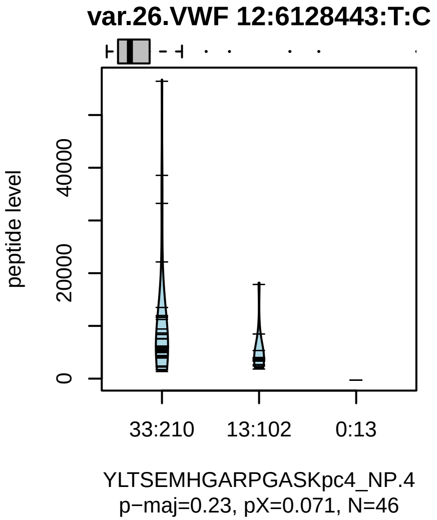
var.26.VWF 12:6128443:T:C
peptide level
0
20000
40000
33:210
13:102
0:13
YLTSEMHGARPGASKpc4_NP.4
p−maj=0.23, pX=0.071, N=46

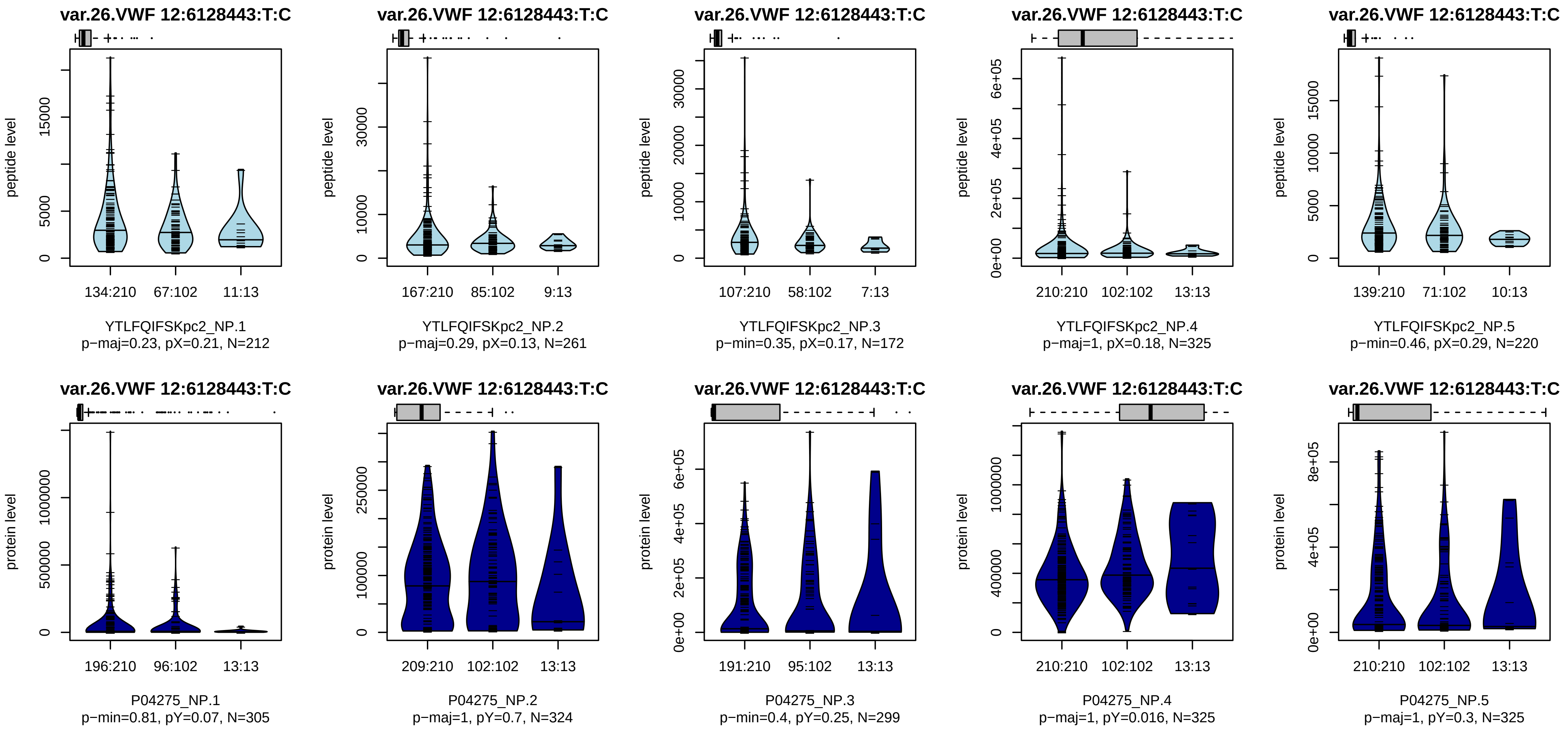
var.26.VWF 12:6128443:T:C
peptide level
134:210
67:102
11:13
YTLFQIFSKpc2_NP.1
p−maj=0.23, pX=0.21, N=212
var.26.VWF 12:6128443:T:C
peptide level
167:210
85:102
9:13
YTLFQIFSKpc2_NP.2
p−maj=0.29, pX=0.13, N=261
var.26.VWF 12:6128443:T:C
peptide level
107:210
58:102
7:13
YTLFQIFSKpc2_NP.3
p−min=0.35, pX=0.17, N=172
var.26.VWF 12:6128443:T:C
peptide level
210:210
102:102
13:13
YTLFQIFSKpc2_NP.4
p−maj=1, pX=0.18, N=325
var.26.VWF 12:6128443:T:C
peptide level
139:210
71:102
10:13
YTLFQIFSKpc2_NP.5
p−min=0.46, pX=0.29, N=220
var.26.VWF 12:6128443:T:C
protein level
196:210
96:102
13:13
P04275_NP.1
p−min=0.81, pY=0.07, N=305
var.26.VWF 12:6128443:T:C
protein level
209:210
102:102
13:13
P04275_NP.2
p−maj=1, pY=0.7, N=324
var.26.VWF 12:6128443:T:C
protein level
191:210
95:102
13:13
P04275_NP.3
p−min=0.4, pY=0.25, N=299
var.26.VWF 12:6128443:T:C
protein level
210:210
102:102
13:13
P04275_NP.4
p−maj=1, pY=0.016, N=325
var.26.VWF 12:6128443:T:C
protein level
210:210
102:102
13:13
P04275_NP.5
p−maj=1, pY=0.3, N=325

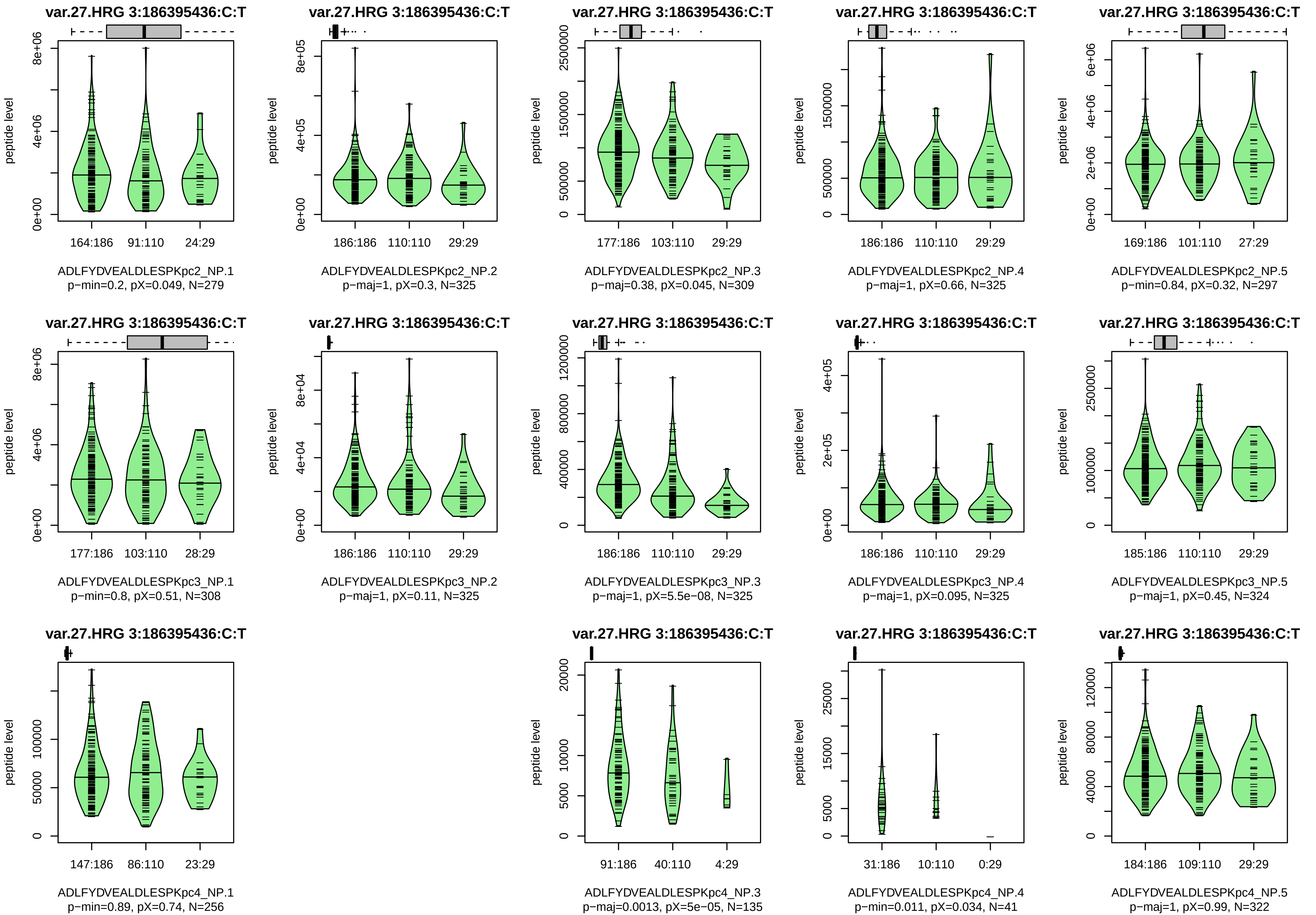
var.27.HRG 3:186395436:C:T
peptide level
164:186
91:110
24:29
ADLFYDVEALDLESPKpc2_NP.1
p−min=0.2, pX=0.049, N=279
var.27.HRG 3:186395436:C:T
peptide level
186:186
110:110
29:29
ADLFYDVEALDLESPKpc2_NP.2
p−maj=1, pX=0.3, N=325
var.27.HRG 3:186395436:C:T
peptide level
177:186
103:110
29:29
ADLFYDVEALDLESPKpc2_NP.3
p−maj=0.38, pX=0.045, N=309
var.27.HRG 3:186395436:C:T
peptide level
186:186
110:110
29:29
ADLFYDVEALDLESPKpc2_NP.4
p−maj=1, pX=0.66, N=325
var.27.HRG 3:186395436:C:T
peptide level
169:186
101:110
27:29
ADLFYDVEALDLESPKpc2_NP.5
p−min=0.84, pX=0.32, N=297
var.27.HRG 3:186395436:C:T
peptide level
177:186
103:110
28:29
ADLFYDVEALDLESPKpc3_NP.1
p−min=0.8, pX=0.51, N=308
var.27.HRG 3:186395436:C:T
peptide level
186:186
110:110
29:29
ADLFYDVEALDLESPKpc3_NP.2
p−maj=1, pX=0.11, N=325
var.27.HRG 3:186395436:C:T
peptide level
186:186
110:110
29:29
ADLFYDVEALDLESPKpc3_NP.3
p−maj=1, pX=5.5e−08, N=325
var.27.HRG 3:186395436:C:T
peptide level
186:186
110:110
29:29
ADLFYDVEALDLESPKpc3_NP.4
p−maj=1, pX=0.095, N=325
var.27.HRG 3:186395436:C:T
peptide level
185:186
110:110
29:29
ADLFYDVEALDLESPKpc3_NP.5
p−maj=1, pX=0.45, N=324
var.27.HRG 3:186395436:C:T
peptide level
147:186
86:110
23:29
ADLFYDVEALDLESPKpc4_NP.1
p−min=0.89, pX=0.74, N=256
var.27.HRG 3:186395436:C:T
peptide level
91:186
40:110
4:29
ADLFYDVEALDLESPKpc4_NP.3
p−maj=0.0013, pX=5e−05, N=135
var.27.HRG 3:186395436:C:T
peptide level
31:186
10:110
0:29
ADLFYDVEALDLESPKpc4_NP.4
p−min=0.011, pX=0.034, N=41
var.27.HRG 3:186395436:C:T
peptide level
184:186
109:110
29:29
ADLFYDVEALDLESPKpc4_NP.5
p−maj=1, pX=0.99, N=322

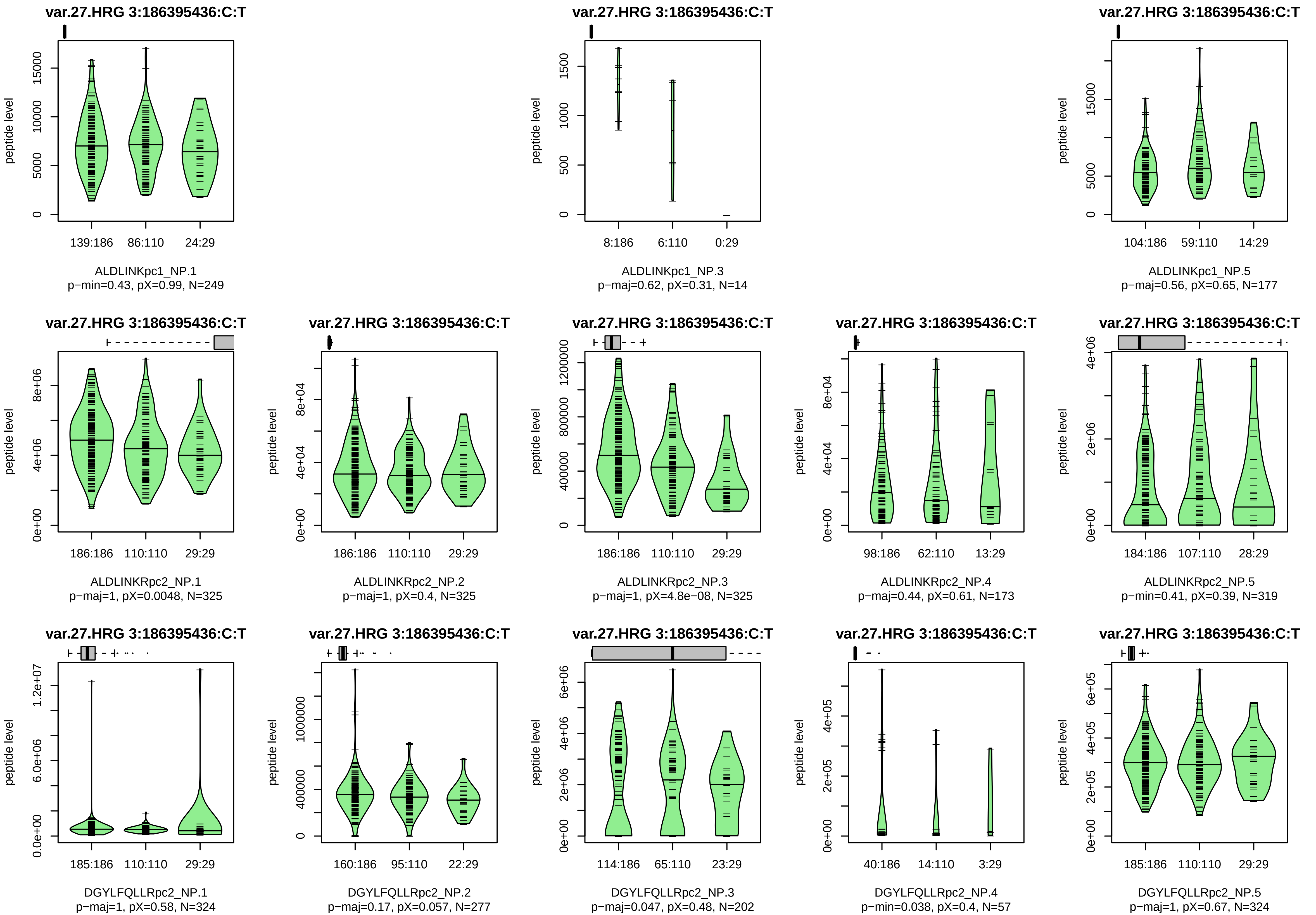
var.27.HRG 3:186395436:C:T
peptide level
139:186
86:110
24:29
ALDLINKpc1_NP.1
p−min=0.43, pX=0.99, N=249
var.27.HRG 3:186395436:C:T
peptide level
8:186
6:110
0:29
ALDLINKpc1_NP.3
p−maj=0.62, pX=0.31, N=14
var.27.HRG 3:186395436:C:T
peptide level
104:186
59:110
14:29
ALDLINKpc1_NP.5
p−maj=0.56, pX=0.65, N=177
var.27.HRG 3:186395436:C:T
peptide level
186:186
110:110
29:29
ALDLINKRpc2_NP.1
p−maj=1, pX=0.0048, N=325
var.27.HRG 3:186395436:C:T
peptide level
186:186
110:110
29:29
ALDLINKRpc2_NP.2
p−maj=1, pX=0.4, N=325
var.27.HRG 3:186395436:C:T
peptide level
186:186
110:110
29:29
ALDLINKRpc2_NP.3
p−maj=1, pX=4.8e−08, N=325
var.27.HRG 3:186395436:C:T
peptide level
98:186
62:110
13:29
ALDLINKRpc2_NP.4
p−maj=0.44, pX=0.61, N=173
var.27.HRG 3:186395436:C:T
peptide level
184:186
107:110
28:29
ALDLINKRpc2_NP.5
p−min=0.41, pX=0.39, N=319
var.27.HRG 3:186395436:C:T
peptide level
185:186
110:110
29:29
DGYLFQLLRpc2_NP.1
p−maj=1, pX=0.58, N=324
var.27.HRG 3:186395436:C:T
peptide level
160:186
95:110
22:29
DGYLFQLLRpc2_NP.2
p−maj=0.17, pX=0.057, N=277
var.27.HRG 3:186395436:C:T
peptide level
114:186
65:110
23:29
DGYLFQLLRpc2_NP.3
p−maj=0.047, pX=0.48, N=202
var.27.HRG 3:186395436:C:T
peptide level
40:186
14:110
3:29
DGYLFQLLRpc2_NP.4
p−min=0.038, pX=0.4, N=57
var.27.HRG 3:186395436:C:T
peptide level
185:186
110:110
29:29
DGYLFQLLRpc2_NP.5
p−maj=1, pX=0.67, N=324

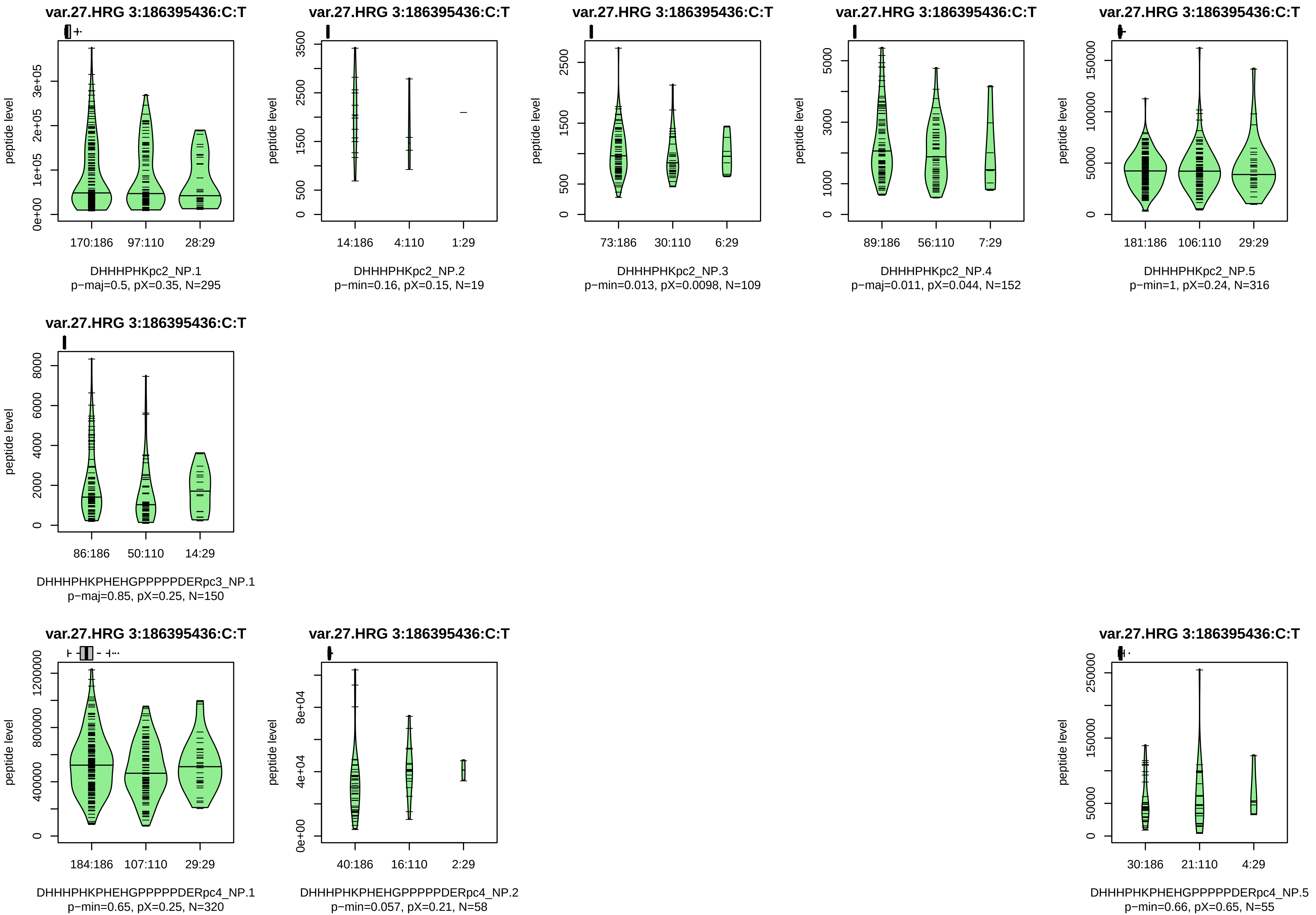
var.27.HRG 3:186395436:C:T
peptide level
170:186
97:110
28:29
DHHHPHKpc2_NP.1
p−maj=0.5, pX=0.35, N=295
var.27.HRG 3:186395436:C:T
peptide level
14:186
4:110
1:29
DHHHPHKpc2_NP.2
p−min=0.16, pX=0.15, N=19
var.27.HRG 3:186395436:C:T
peptide level
73:186
30:110
6:29
DHHHPHKpc2_NP.3
p−min=0.013, pX=0.0098, N=109
var.27.HRG 3:186395436:C:T
peptide level
89:186
56:110
7:29
DHHHPHKpc2_NP.4
p−maj=0.011, pX=0.044, N=152
var.27.HRG 3:186395436:C:T
peptide level
181:186
106:110
29:29
DHHHPHKpc2_NP.5
p−min=1, pX=0.24, N=316
var.27.HRG 3:186395436:C:T
peptide level
86:186
50:110
14:29
DHHHPHKPHEHGPPPPPDERpc3_NP.1
p−maj=0.85, pX=0.25, N=150
var.27.HRG 3:186395436:C:T
peptide level
184:186
107:110
29:29
DHHHPHKPHEHGPPPPPDERpc4_NP.1
p−min=0.65, pX=0.25, N=320
var.27.HRG 3:186395436:C:T
peptide level
40:186
16:110
2:29
DHHHPHKPHEHGPPPPPDERpc4_NP.2
p−min=0.057, pX=0.21, N=58
var.27.HRG 3:186395436:C:T
peptide level
30:186
21:110
4:29
DHHHPHKPHEHGPPPPPDERpc4_NP.5
p−min=0.66, pX=0.65, N=55

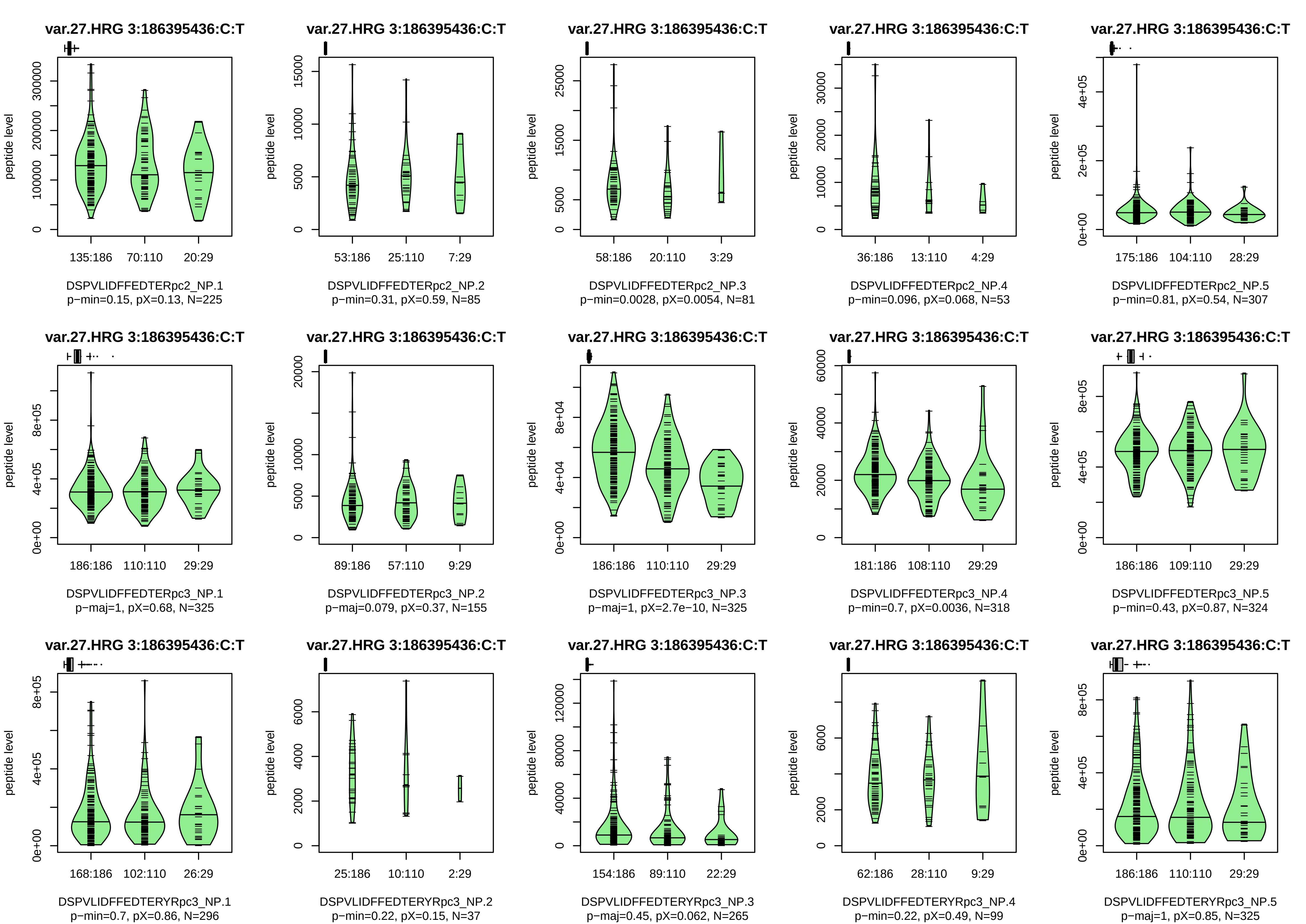
var.27.HRG 3:186395436:C:T
peptide level
135:186
70:110
20:29
DSPVLIDFFEDTERpc2_NP.1
p−min=0.15, pX=0.13, N=225
var.27.HRG 3:186395436:C:T
peptide level
53:186
25:110
7:29
DSPVLIDFFEDTERpc2_NP.2
p−min=0.31, pX=0.59, N=85
var.27.HRG 3:186395436:C:T
peptide level
58:186
20:110
3:29
DSPVLIDFFEDTERpc2_NP.3
p−min=0.0028, pX=0.0054, N=81
var.27.HRG 3:186395436:C:T
peptide level
36:186
13:110
4:29
DSPVLIDFFEDTERpc2_NP.4
p−min=0.096, pX=0.068, N=53
var.27.HRG 3:186395436:C:T
peptide level
175:186
104:110
28:29
DSPVLIDFFEDTERpc2_NP.5
p−min=0.81, pX=0.54, N=307
var.27.HRG 3:186395436:C:T
peptide level
186:186
110:110
29:29
DSPVLIDFFEDTERpc3_NP.1
p−maj=1, pX=0.68, N=325
var.27.HRG 3:186395436:C:T
peptide level
89:186
57:110
9:29
DSPVLIDFFEDTERpc3_NP.2
p−maj=0.079, pX=0.37, N=155
var.27.HRG 3:186395436:C:T
peptide level
186:186
110:110
29:29
DSPVLIDFFEDTERpc3_NP.3
p−maj=1, pX=2.7e−10, N=325
var.27.HRG 3:186395436:C:T
peptide level
181:186
108:110
29:29
DSPVLIDFFEDTERpc3_NP.4
p−min=0.7, pX=0.0036, N=318
var.27.HRG 3:186395436:C:T
peptide level
186:186
109:110
29:29
DSPVLIDFFEDTERpc3_NP.5
p−min=0.43, pX=0.87, N=324
var.27.HRG 3:186395436:C:T
peptide level
168:186
102:110
26:29
DSPVLIDFFEDTERYRpc3_NP.1
p−min=0.7, pX=0.86, N=296
var.27.HRG 3:186395436:C:T
peptide level
25:186
10:110
2:29
DSPVLIDFFEDTERYRpc3_NP.2
p−min=0.22, pX=0.15, N=37
var.27.HRG 3:186395436:C:T
peptide level
154:186
89:110
22:29
DSPVLIDFFEDTERYRpc3_NP.3
p−maj=0.45, pX=0.062, N=265
var.27.HRG 3:186395436:C:T
peptide level
62:186
28:110
9:29
DSPVLIDFFEDTERYRpc3_NP.4
p−min=0.22, pX=0.49, N=99
var.27.HRG 3:186395436:C:T
peptide level
186:186
110:110
29:29
DSPVLIDFFEDTERYRpc3_NP.5
p−maj=1, pX=0.85, N=325

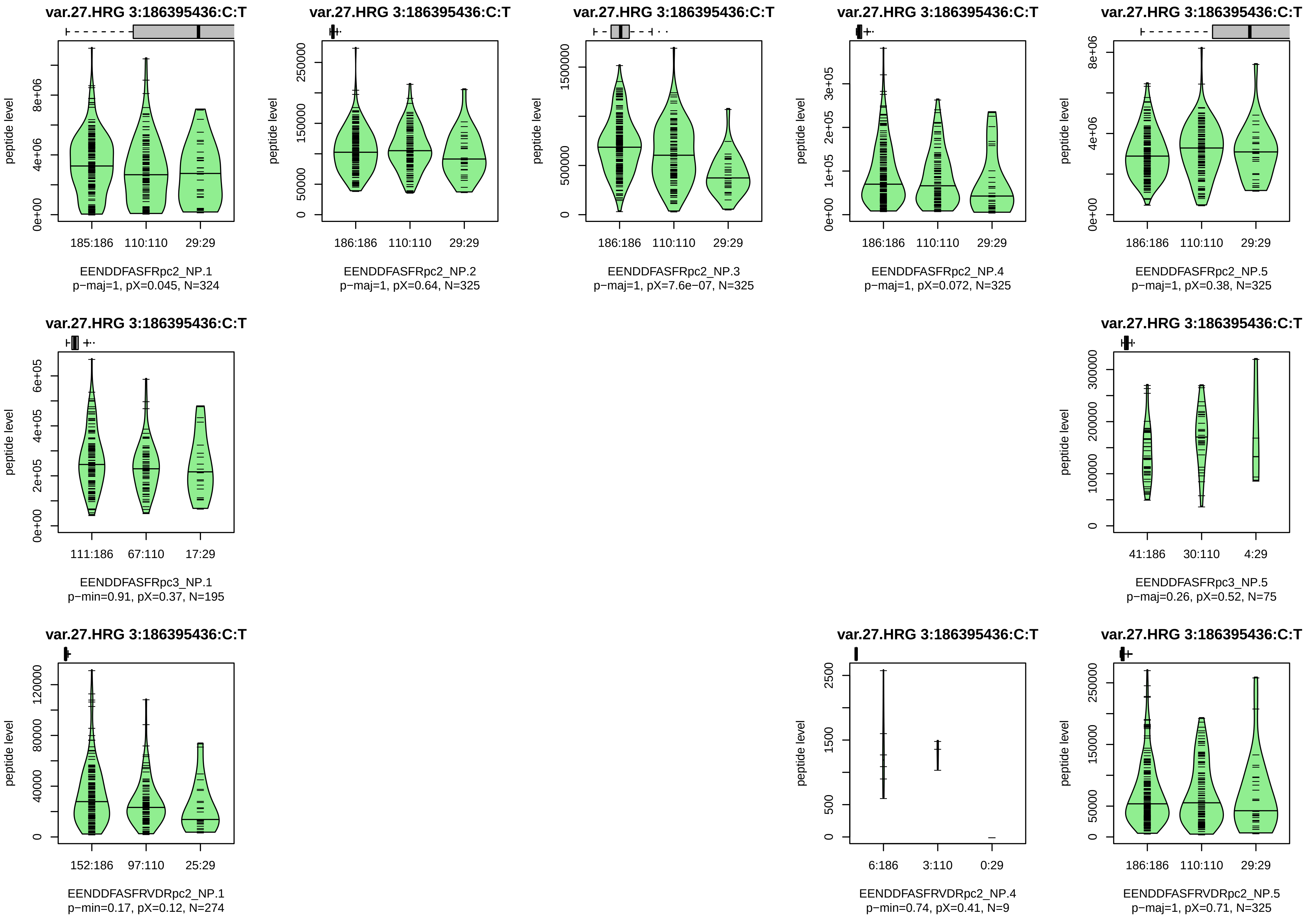
var.27.HRG 3:186395436:C:T
peptide level
185:186
110:110
29:29
EENDDFASFRpc2_NP.1
p−maj=1, pX=0.045, N=324
var.27.HRG 3:186395436:C:T
peptide level
186:186
110:110
29:29
EENDDFASFRpc2_NP.2
p−maj=1, pX=0.64, N=325
var.27.HRG 3:186395436:C:T
peptide level
186:186
110:110
29:29
EENDDFASFRpc2_NP.3
p−maj=1, pX=7.6e−07, N=325
var.27.HRG 3:186395436:C:T
peptide level
186:186
110:110
29:29
EENDDFASFRpc2_NP.4
p−maj=1, pX=0.072, N=325
var.27.HRG 3:186395436:C:T
peptide level
186:186
110:110
29:29
EENDDFASFRpc2_NP.5
p−maj=1, pX=0.38, N=325
var.27.HRG 3:186395436:C:T
peptide level
111:186
67:110
17:29
EENDDFASFRpc3_NP.1
p−min=0.91, pX=0.37, N=195
var.27.HRG 3:186395436:C:T
peptide level
41:186
30:110
4:29
EENDDFASFRpc3_NP.5
p−maj=0.26, pX=0.52, N=75
var.27.HRG 3:186395436:C:T
peptide level
152:186
97:110
25:29
EENDDFASFRVDRpc2_NP.1
p−min=0.17, pX=0.12, N=274
var.27.HRG 3:186395436:C:T
peptide level
6:186
3:110
0:29
EENDDFASFRVDRpc2_NP.4
p−min=0.74, pX=0.41, N=9
var.27.HRG 3:186395436:C:T
peptide level
186:186
110:110
29:29
EENDDFASFRVDRpc2_NP.5
p−maj=1, pX=0.71, N=325

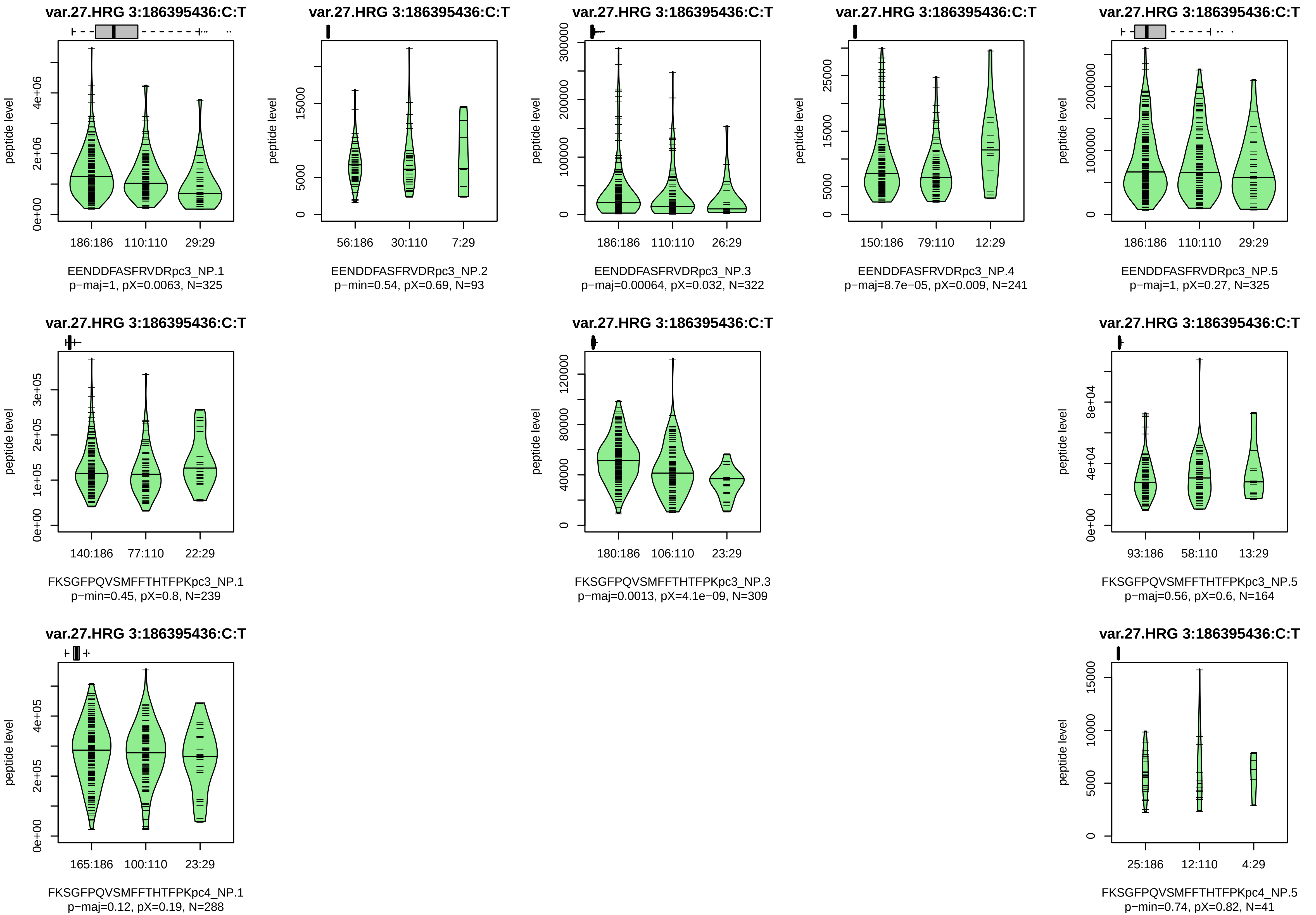
var.27.HRG 3:186395436:C:T
peptide level
186:186
110:110
29:29
EENDDFASFRVDRpc3_NP.1
p−maj=1, pX=0.0063, N=325
var.27.HRG 3:186395436:C:T
peptide level
56:186
30:110
7:29
EENDDFASFRVDRpc3_NP.2
p−min=0.54, pX=0.69, N=93
var.27.HRG 3:186395436:C:T
peptide level
186:186
110:110
26:29
EENDDFASFRVDRpc3_NP.3
p−maj=0.00064, pX=0.032, N=322
var.27.HRG 3:186395436:C:T
peptide level
150:186
79:110
12:29
EENDDFASFRVDRpc3_NP.4
p−maj=8.7e−05, pX=0.009, N=241
var.27.HRG 3:186395436:C:T
peptide level
186:186
110:110
29:29
EENDDFASFRVDRpc3_NP.5
p−maj=1, pX=0.27, N=325
var.27.HRG 3:186395436:C:T
peptide level
140:186
77:110
22:29
FKSGFPQVSMFFTHTFPKpc3_NP.1
p−min=0.45, pX=0.8, N=239
var.27.HRG 3:186395436:C:T
peptide level
180:186
106:110
23:29
FKSGFPQVSMFFTHTFPKpc3_NP.3
p−maj=0.0013, pX=4.1e−09, N=309
var.27.HRG 3:186395436:C:T
peptide level
93:186
58:110
13:29
FKSGFPQVSMFFTHTFPKpc3_NP.5
p−maj=0.56, pX=0.6, N=164
var.27.HRG 3:186395436:C:T
peptide level
165:186
100:110
23:29
FKSGFPQVSMFFTHTFPKpc4_NP.1
p−maj=0.12, pX=0.19, N=288
var.27.HRG 3:186395436:C:T
peptide level
25:186
12:110
4:29
FKSGFPQVSMFFTHTFPKpc4_NP.5
p−min=0.74, pX=0.82, N=41

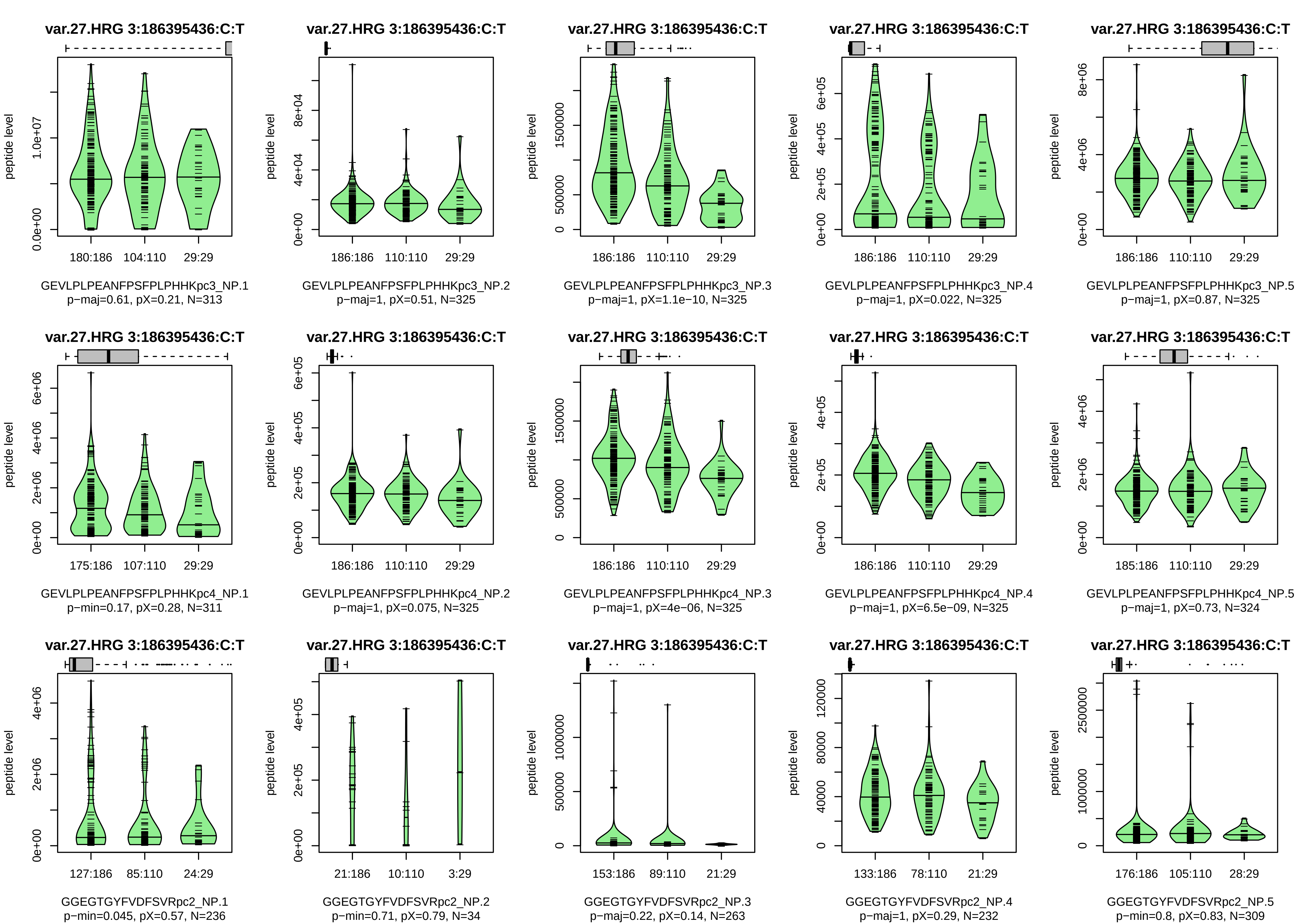
var.27.HRG 3:186395436:C:T
peptide level
180:186
104:110
29:29
GEVLPLPEANFPSFPLPHHKpc3_NP.1
p−maj=0.61, pX=0.21, N=313
var.27.HRG 3:186395436:C:T
186:186
110:110
29:29
GEVLPLPEANFPSFPLPHHKpc3_NP.2
p−maj=1, pX=0.51, N=325
var.27.HRG 3:186395436:C:T
GEVLPLPEANFPSFPLPHHKpc3_NP.3
p−maj=1, pX=1.1e−10, N=325
var.27.HRG 3:186395436:C:T
GEVLPLPEANFPSFPLPHHKpc3_NP.4
p−maj=1, pX=0.022, N=325
var.27.HRG 3:186395436:C:T
GEVLPLPEANFPSFPLPHHKpc3_NP.5
p−maj=1, pX=0.87, N=325
var.27.HRG 3:186395436:C:T
175:186
107:110
GEVLPLPEANFPSFPLPHHKpc4_NP.1
p−min=0.17, pX=0.28, N=311
var.27.HRG 3:186395436:C:T
GEVLPLPEANFPSFPLPHHKpc4_NP.2
p−maj=1, pX=0.075, N=325
var.27.HRG 3:186395436:C:T
GEVLPLPEANFPSFPLPHHKpc4_NP.3
p−maj=1, pX=4e−06, N=325
var.27.HRG 3:186395436:C:T
GEVLPLPEANFPSFPLPHHKpc4_NP.4
p−maj=1, pX=6.5e−09, N=325
var.27.HRG 3:186395436:C:T
185:186
GEVLPLPEANFPSFPLPHHKpc4_NP.5
p−maj=1, pX=0.73, N=324
var.27.HRG 3:186395436:C:T
127:186
85:110
24:29
GGEGTGYFVDFSVRpc2_NP.1
p−min=0.045, pX=0.57, N=236
var.27.HRG 3:186395436:C:T
21:186
10:110
3:29
GGEGTGYFVDFSVRpc2_NP.2
p−min=0.71, pX=0.79, N=34
var.27.HRG 3:186395436:C:T
153:186
89:110
21:29
GGEGTGYFVDFSVRpc2_NP.3
p−maj=0.22, pX=0.14, N=263
var.27.HRG 3:186395436:C:T
133:186
78:110
21:29
GGEGTGYFVDFSVRpc2_NP.4
p−maj=1, pX=0.29, N=232
var.27.HRG 3:186395436:C:T
176:186
105:110
28:29
GGEGTGYFVDFSVRpc2_NP.5
p−min=0.8, pX=0.83, N=309

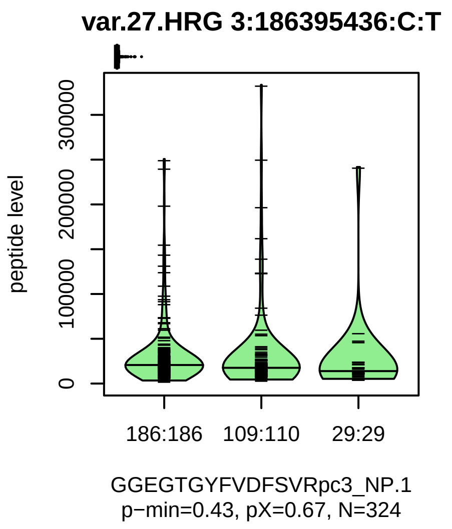
var.27.HRG 3:186395436:C:T
peptide level
0
100000
200000
300000
186:186
109:110
29:29
GGEGTGYFVDFSVRpc3_NP.1
p−min=0.43, pX=0.67, N=324

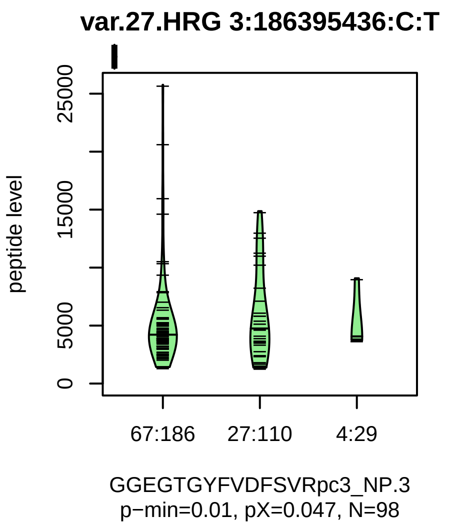
var.27.HRG 3:186395436:C:T
peptide level
0
5000
15000
25000
67:186
27:110
4:29
GGEGTGYFVDFSVRpc3_NP.3
p−min=0.01, pX=0.047, N=98

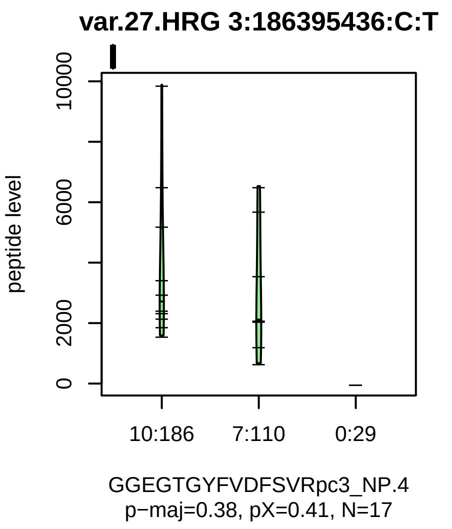
var.27.HRG 3:186395436:C:T
peptide level
0
2000
6000
10000
10:186
7:110
0:29
GGEGTGYFVDFSVRpc3_NP.4
p−maj=0.38, pX=0.41, N=17

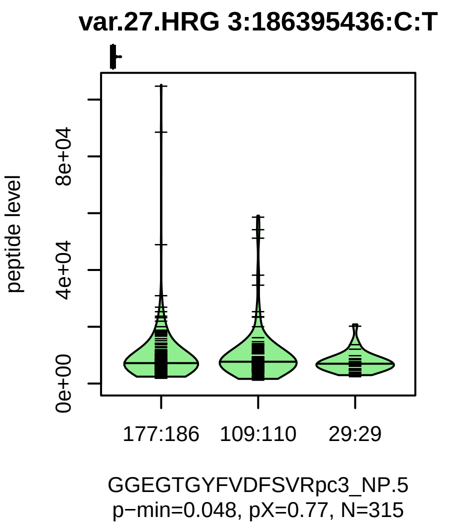
var.27.HRG 3:186395436:C:T
peptide level
0e+00
4e+04
8e+04
177:186
109:110
29:29
GGEGTGYFVDFSVRpc3_NP.5
p−min=0.048, pX=0.77, N=315

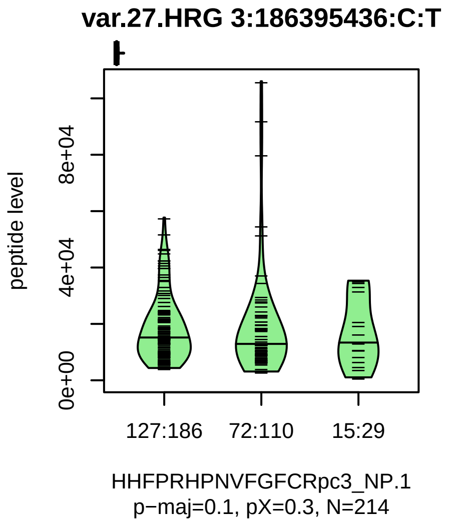
var.27.HRG 3:186395436:C:T
peptide level
0e+00
4e+04
8e+04
127:186
72:110
15:29
HHFPRHPNVFGFCRpc3_NP.1
p−maj=0.1, pX=0.3, N=214

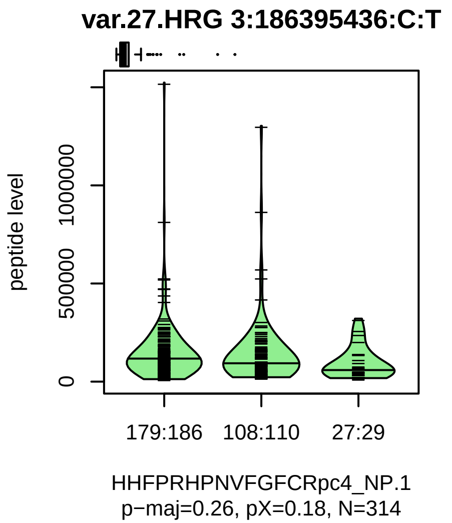
var.27.HRG 3:186395436:C:T
peptide level
0
500000
1000000
179:186
108:110
27:29
HHFPRHPNVFGFCRpc4_NP.1
p−maj=0.26, pX=0.18, N=314

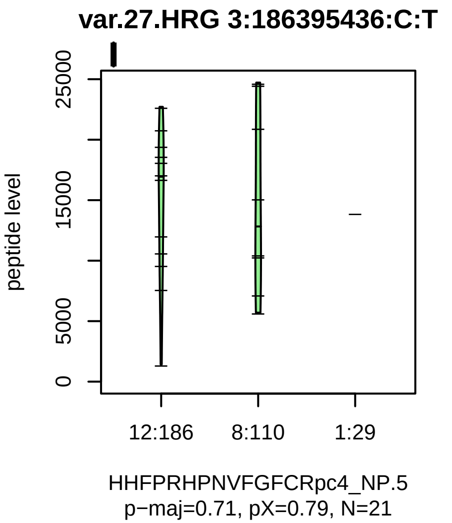
var.27.HRG 3:186395436:C:T
peptide level
0
5000
15000
25000
12:186
8:110
1:29
HHFPRHPNVFGFCRpc4_NP.5
p−maj=0.71, pX=0.79, N=21

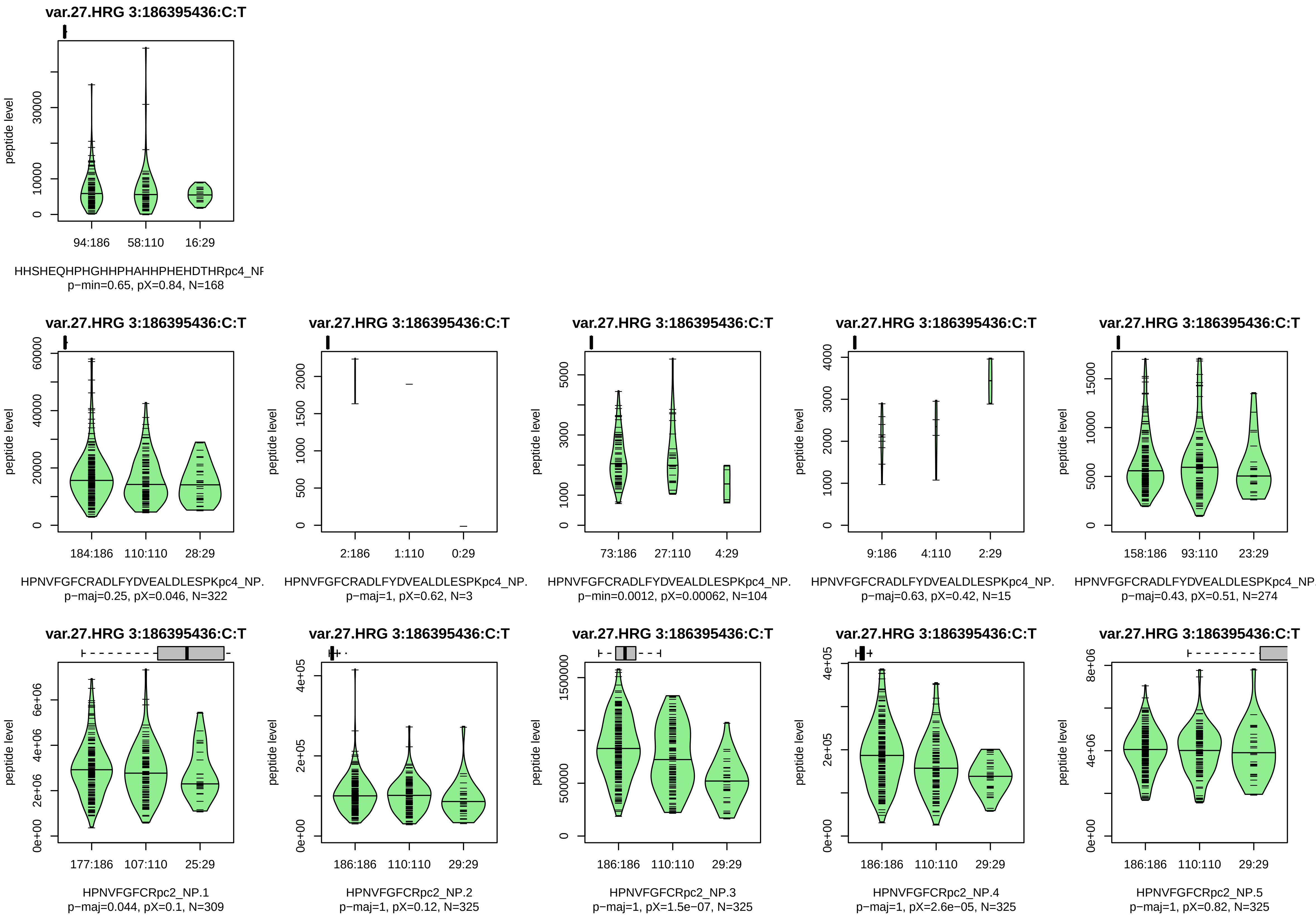

var.27.HRG 3:186395436:C:T
peptide level
94:186
58:110
16:29
HHSHEQHPHGHHPHAHHPHEHDTHRpc4_NF
p−min=0.65, pX=0.84, N=168
var.27.HRG 3:186395436:C:T
184:186
110:110
28:29
HPNVFGFCRADLFYDVEALDLESPKpc4_NP.
p−maj=0.25, pX=0.046, N=322
var.27.HRG 3:186395436:C:T
2:186
1:110
0:29
HPNVFGFCRADLFYDVEALDLESPKpc4_NP.
p−maj=1, pX=0.62, N=3
var.27.HRG 3:186395436:C:T
73:186
27:110
4:29
HPNVFGFCRADLFYDVEALDLESPKpc4_NP.
p−min=0.0012, pX=0.00062, N=104
var.27.HRG 3:186395436:C:T
9:186
4:110
2:29
HPNVFGFCRADLFYDVEALDLESPKpc4_NP.
p−maj=0.63, pX=0.42, N=15
var.27.HRG 3:186395436:C:T
158:186
93:110
23:29
HPNVFGFCRADLFYDVEALDLESPKpc4_NP
p−maj=0.43, pX=0.51, N=274
var.27.HRG 3:186395436:C:T
177:186
107:110
25:29
HPNVFGFCRpc2_NP.1
p−maj=0.044, pX=0.1, N=309
var.27.HRG 3:186395436:C:T
186:186
110:110
29:29
HPNVFGFCRpc2_NP.2
p−maj=1, pX=0.12, N=325
var.27.HRG 3:186395436:C:T
186:186
110:110
29:29
HPNVFGFCRpc2_NP.3
p−maj=1, pX=1.5e−07, N=325
var.27.HRG 3:186395436:C:T
186:186
110:110
29:29
HPNVFGFCRpc2_NP.4
p−maj=1, pX=2.6e−05, N=325
var.27.HRG 3:186395436:C:T
186:186
110:110
29:29
HPNVFGFCRpc2_NP.5
p−maj=1, pX=0.82, N=325

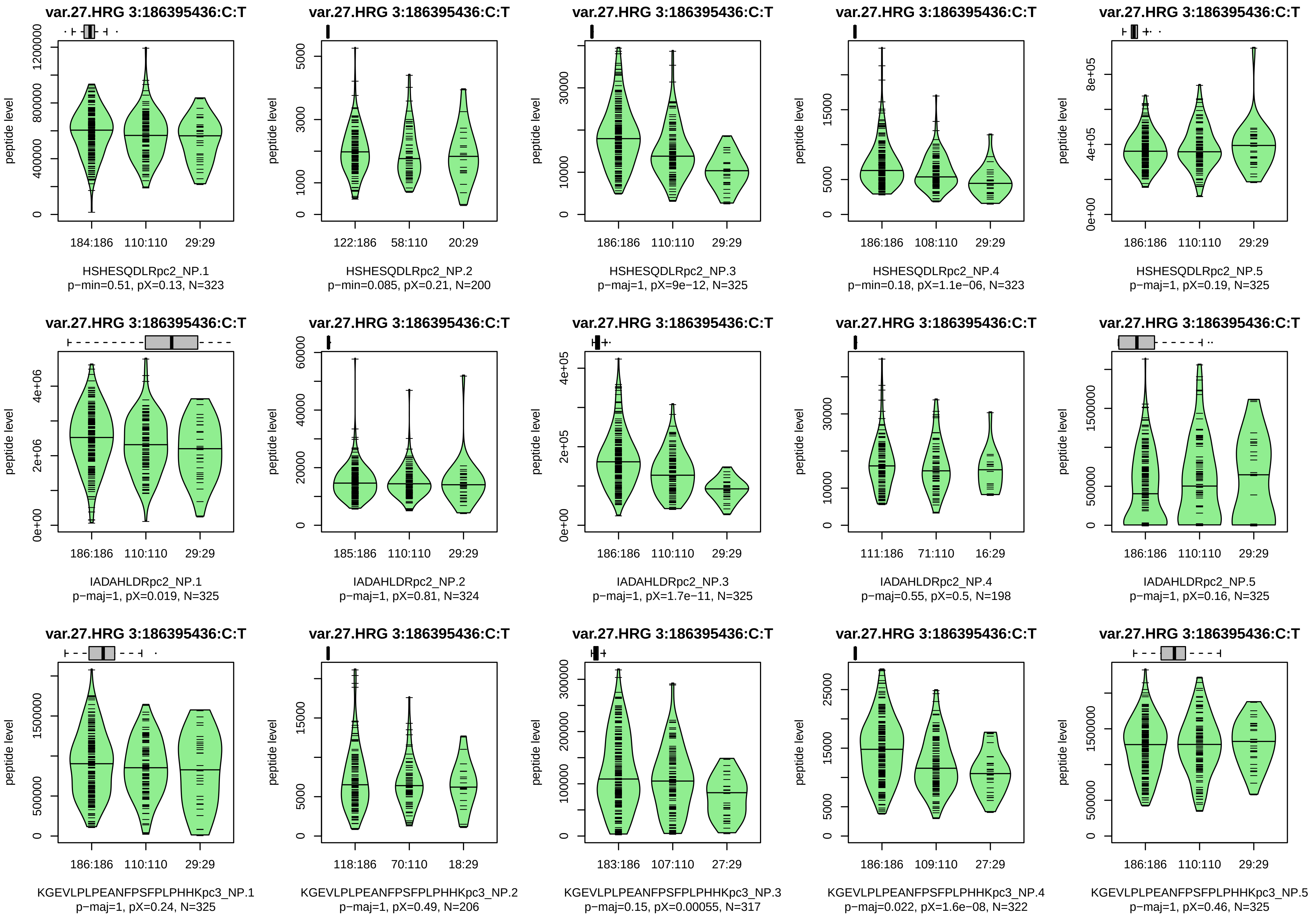
var.27.HRG 3:186395436:C:T
peptide level
0
400000
800000
1200000
184:186
110:110
29:29
HSHESQDLRpc2_NP.1
p−min=0.51, pX=0.13, N=323
var.27.HRG 3:186395436:C:T
peptide level
0
1000
3000
5000
122:186
58:110
20:29
HSHESQDLRpc2_NP.2
p−min=0.085, pX=0.21, N=200
var.27.HRG 3:186395436:C:T
peptide level
0
10000
30000
186:186
110:110
29:29
HSHESQDLRpc2_NP.3
p−maj=1, pX=9e−12, N=325
var.27.HRG 3:186395436:C:T
peptide level
0
5000
15000
186:186
108:110
29:29
HSHESQDLRpc2_NP.4
p−min=0.18, pX=1.1e−06, N=323
var.27.HRG 3:186395436:C:T
peptide level
0e+00
4e+05
8e+05
186:186
110:110
29:29
HSHESQDLRpc2_NP.5
p−maj=1, pX=0.19, N=325
var.27.HRG 3:186395436:C:T
peptide level
0e+00
2e+06
4e+06
186:186
110:110
29:29
IADAHLDRpc2_NP.1
p−maj=1, pX=0.019, N=325
var.27.HRG 3:186395436:C:T
peptide level
0
20000
40000
60000
185:186
110:110
29:29
IADAHLDRpc2_NP.2
p−maj=1, pX=0.81, N=324
var.27.HRG 3:186395436:C:T
peptide level
0e+00
2e+05
4e+05
186:186
110:110
29:29
IADAHLDRpc2_NP.3
p−maj=1, pX=1.7e−11, N=325
var.27.HRG 3:186395436:C:T
peptide level
0
10000
30000
111:186
71:110
16:29
IADAHLDRpc2_NP.4
p−maj=0.55, pX=0.5, N=198
var.27.HRG 3:186395436:C:T
peptide level
0
500000
1500000
186:186
110:110
29:29
IADAHLDRpc2_NP.5
p−maj=1, pX=0.16, N=325
var.27.HRG 3:186395436:C:T
peptide level
0
500000
1500000
186:186
110:110
29:29
KGEVLPLPEANFPSFPLPHHKpc3_NP.1
p−maj=1, pX=0.24, N=325
var.27.HRG 3:186395436:C:T
peptide level
0
5000
15000
118:186
70:110
18:29
KGEVLPLPEANFPSFPLPHHKpc3_NP.2
p−maj=1, pX=0.49, N=206
var.27.HRG 3:186395436:C:T
peptide level
0
100000
200000
300000
183:186
107:110
27:29
KGEVLPLPEANFPSFPLPHHKpc3_NP.3
p−maj=0.15, pX=0.00055, N=317
var.27.HRG 3:186395436:C:T
peptide level
0
5000
15000
25000
186:186
109:110
27:29
KGEVLPLPEANFPSFPLPHHKpc3_NP.4
p−maj=0.022, pX=1.6e−08, N=322
var.27.HRG 3:186395436:C:T
peptide level
0
500000
1500000
186:186
110:110
29:29
KGEVLPLPEANFPSFPLPHHKpc3_NP.5
p−maj=1, pX=0.46, N=325

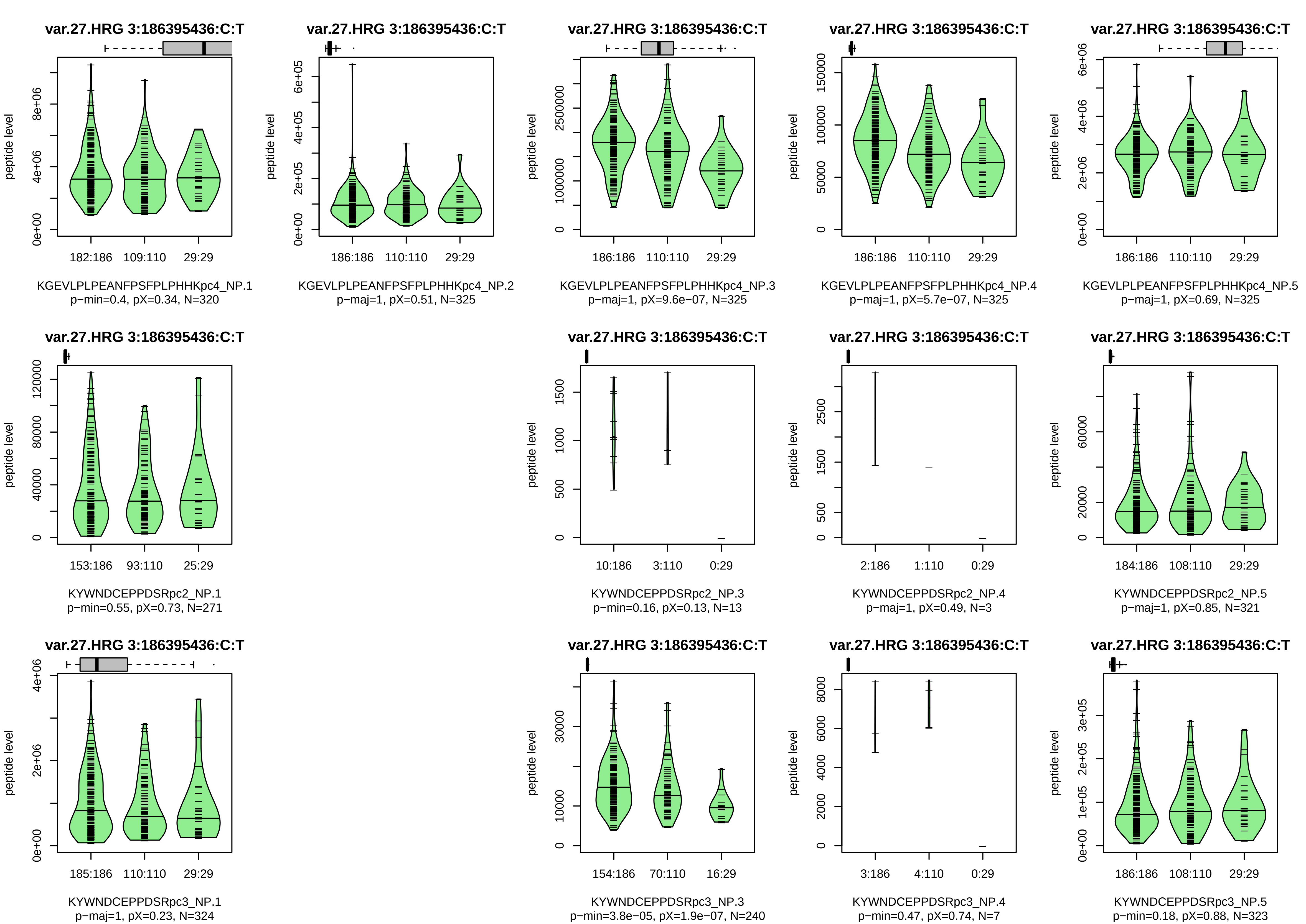
var.27.HRG 3:186395436:C:T
peptide level
182:186 109:110 29:29
KGEVLPLPEANFPSFPLPHHKpc4_NP.1
p−min=0.4, pX=0.34, N=320
var.27.HRG 3:186395436:C:T
peptide level
186:186 110:110 29:29
KGEVLPLPEANFPSFPLPHHKpc4_NP.2
p−maj=1, pX=0.51, N=325
var.27.HRG 3:186395436:C:T
peptide level
186:186 110:110 29:29
KGEVLPLPEANFPSFPLPHHKpc4_NP.3
p−maj=1, pX=9.6e−07, N=325
var.27.HRG 3:186395436:C:T
peptide level
186:186 110:110 29:29
KGEVLPLPEANFPSFPLPHHKpc4_NP.4
p−maj=1, pX=5.7e−07, N=325
var.27.HRG 3:186395436:C:T
peptide level
186:186 110:110 29:29
KGEVLPLPEANFPSFPLPHHKpc4_NP.5
p−maj=1, pX=0.69, N=325
var.27.HRG 3:186395436:C:T
peptide level
153:186 93:110 25:29
KYWNDCEPPDSRpc2_NP.1
p−min=0.55, pX=0.73, N=271
var.27.HRG 3:186395436:C:T
peptide level
10:186 3:110 0:29
KYWNDCEPPDSRpc2_NP.3
p−min=0.16, pX=0.13, N=13
var.27.HRG 3:186395436:C:T
peptide level
2:186 1:110 0:29
KYWNDCEPPDSRpc2_NP.4
p−maj=1, pX=0.49, N=3
var.27.HRG 3:186395436:C:T
peptide level
184:186 108:110 29:29
KYWNDCEPPDSRpc2_NP.5
p−maj=1, pX=0.85, N=321
var.27.HRG 3:186395436:C:T
peptide level
185:186 110:110 29:29
KYWNDCEPPDSRpc3_NP.1
p−maj=1, pX=0.23, N=324
var.27.HRG 3:186395436:C:T
peptide level
154:186 70:110 16:29
KYWNDCEPPDSRpc3_NP.3
p−min=3.8e−05, pX=1.9e−07, N=240
var.27.HRG 3:186395436:C:T
peptide level
3:186 4:110 0:29
KYWNDCEPPDSRpc3_NP.4
p−min=0.47, pX=0.74, N=7
var.27.HRG 3:186395436:C:T
peptide level
186:186 108:110 29:29
KYWNDCEPPDSRpc3_NP.5
p−min=0.18, pX=0.88, N=323

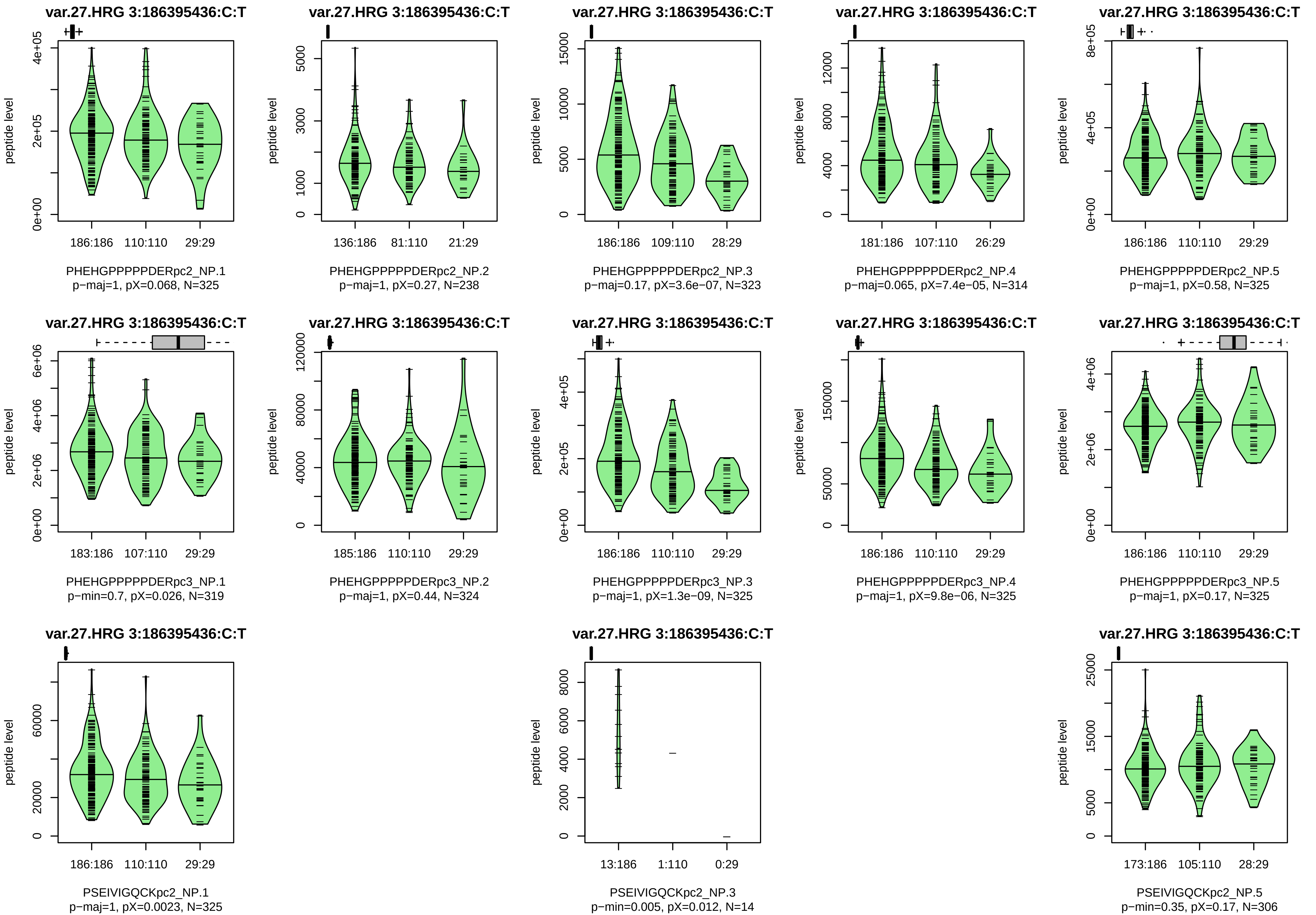
var.27.HRG 3:186395436:C:T
peptide level
186:186 110:110 29:29
PHEHGPPPPPDERpc2_NP.1
p−maj=1, pX=0.068, N=325
var.27.HRG 3:186395436:C:T
peptide level
136:186 81:110 21:29
PHEHGPPPPPDERpc2_NP.2
p−maj=1, pX=0.27, N=238
var.27.HRG 3:186395436:C:T
peptide level
186:186 109:110 28:29
PHEHGPPPPPDERpc2_NP.3
p−maj=0.17, pX=3.6e−07, N=323
var.27.HRG 3:186395436:C:T
peptide level
181:186 107:110 26:29
PHEHGPPPPPDERpc2_NP.4
p−maj=0.065, pX=7.4e−05, N=314
var.27.HRG 3:186395436:C:T
peptide level
186:186 110:110 29:29
PHEHGPPPPPDERpc2_NP.5
p−maj=1, pX=0.58, N=325
var.27.HRG 3:186395436:C:T
peptide level
183:186 107:110 29:29
PHEHGPPPPPDERpc3_NP.1
p−min=0.7, pX=0.026, N=319
var.27.HRG 3:186395436:C:T
peptide level
185:186 110:110 29:29
PHEHGPPPPPDERpc3_NP.2
p−maj=1, pX=0.44, N=324
var.27.HRG 3:186395436:C:T
peptide level
186:186 110:110 29:29
PHEHGPPPPPDERpc3_NP.3
p−maj=1, pX=1.3e−09, N=325
var.27.HRG 3:186395436:C:T
peptide level
186:186 110:110 29:29
PHEHGPPPPPDERpc3_NP.4
p−maj=1, pX=9.8e−06, N=325
var.27.HRG 3:186395436:C:T
peptide level
186:186 110:110 29:29
PHEHGPPPPPDERpc3_NP.5
p−maj=1, pX=0.17, N=325
var.27.HRG 3:186395436:C:T
peptide level
186:186 110:110 29:29
PSEIVIGQCKpc2_NP.1
p−maj=1, pX=0.0023, N=325
var.27.HRG 3:186395436:C:T
peptide level
13:186 1:110 0:29
PSEIVIGQCKpc2_NP.3
p−min=0.005, pX=0.012, N=14
var.27.HRG 3:186395436:C:T
peptide level
173:186 105:110 28:29
PSEIVIGQCKpc2_NP.5
p−min=0.35, pX=0.17, N=306

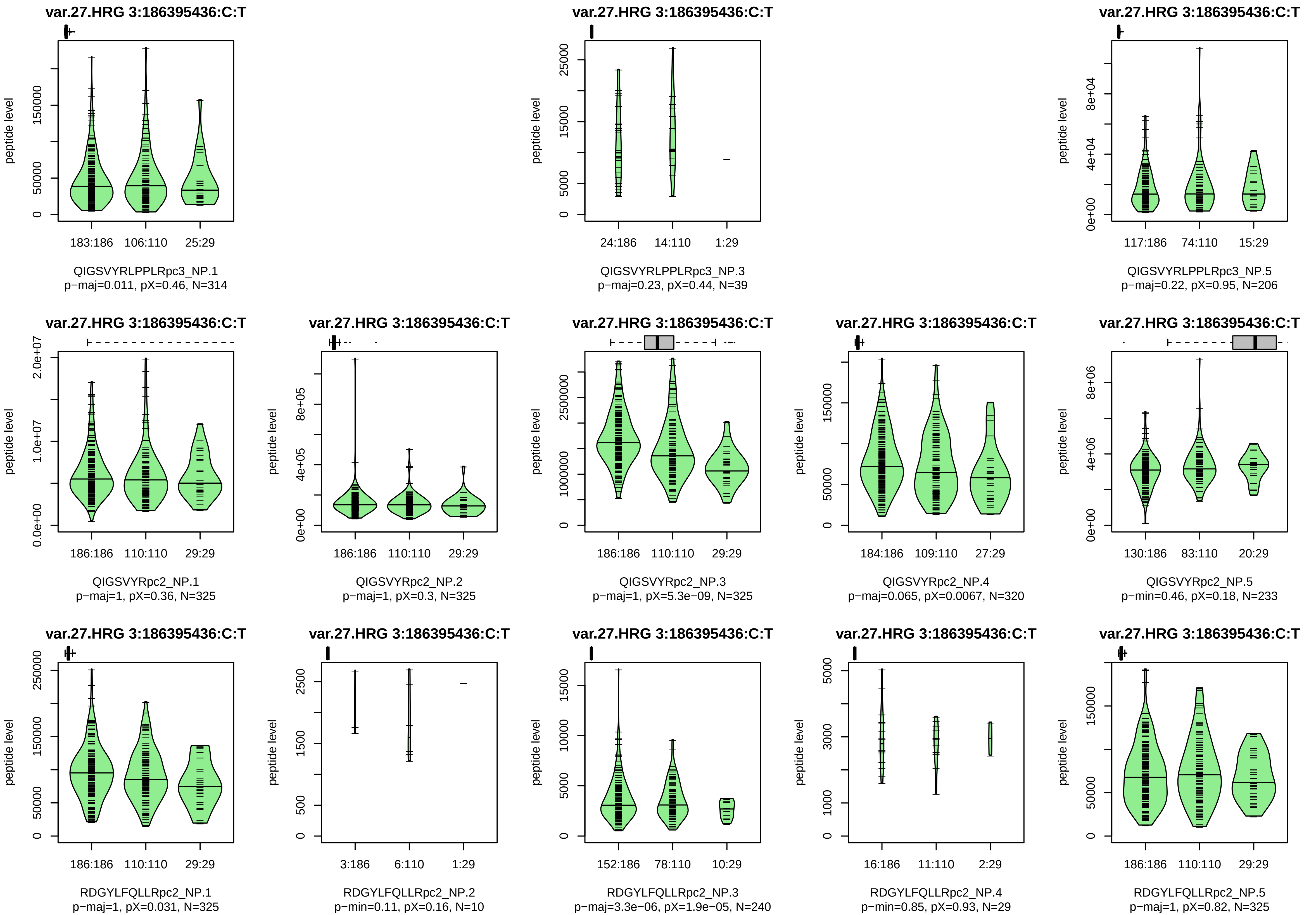
var.27.HRG 3:186395436:C:T
peptide level
183:186
106:110
25:29
QIGSVYRLPPLRpc3_NP.1
p−maj=0.011, pX=0.46, N=314
var.27.HRG 3:186395436:C:T
peptide level
24:186
14:110
1:29
QIGSVYRLPPLRpc3_NP.3
p−maj=0.23, pX=0.44, N=39
var.27.HRG 3:186395436:C:T
peptide level
117:186
74:110
15:29
QIGSVYRLPPLRpc3_NP.5
p−maj=0.22, pX=0.95, N=206
var.27.HRG 3:186395436:C:T
peptide level
186:186
110:110
29:29
QIGSVYRpc2_NP.1
p−maj=1, pX=0.36, N=325
var.27.HRG 3:186395436:C:T
peptide level
186:186
110:110
29:29
QIGSVYRpc2_NP.2
p−maj=1, pX=0.3, N=325
var.27.HRG 3:186395436:C:T
peptide level
186:186
110:110
29:29
QIGSVYRpc2_NP.3
p−maj=1, pX=5.3e−09, N=325
var.27.HRG 3:186395436:C:T
peptide level
184:186
109:110
27:29
QIGSVYRpc2_NP.4
p−maj=0.065, pX=0.0067, N=320
var.27.HRG 3:186395436:C:T
peptide level
130:186
83:110
20:29
QIGSVYRpc2_NP.5
p−min=0.46, pX=0.18, N=233
var.27.HRG 3:186395436:C:T
peptide level
186:186
110:110
29:29
RDGYLFQLLRpc2_NP.1
p−maj=1, pX=0.031, N=325
var.27.HRG 3:186395436:C:T
peptide level
3:186
6:110
1:29
RDGYLFQLLRpc2_NP.2
p−min=0.11, pX=0.16, N=10
var.27.HRG 3:186395436:C:T
peptide level
152:186
78:110
10:29
RDGYLFQLLRpc2_NP.3
p−maj=3.3e−06, pX=1.9e−05, N=240
var.27.HRG 3:186395436:C:T
peptide level
16:186
11:110
2:29
RDGYLFQLLRpc2_NP.4
p−min=0.85, pX=0.93, N=29
var.27.HRG 3:186395436:C:T
peptide level
186:186
110:110
29:29
RDGYLFQLLRpc2_NP.5
p−maj=1, pX=0.82, N=325

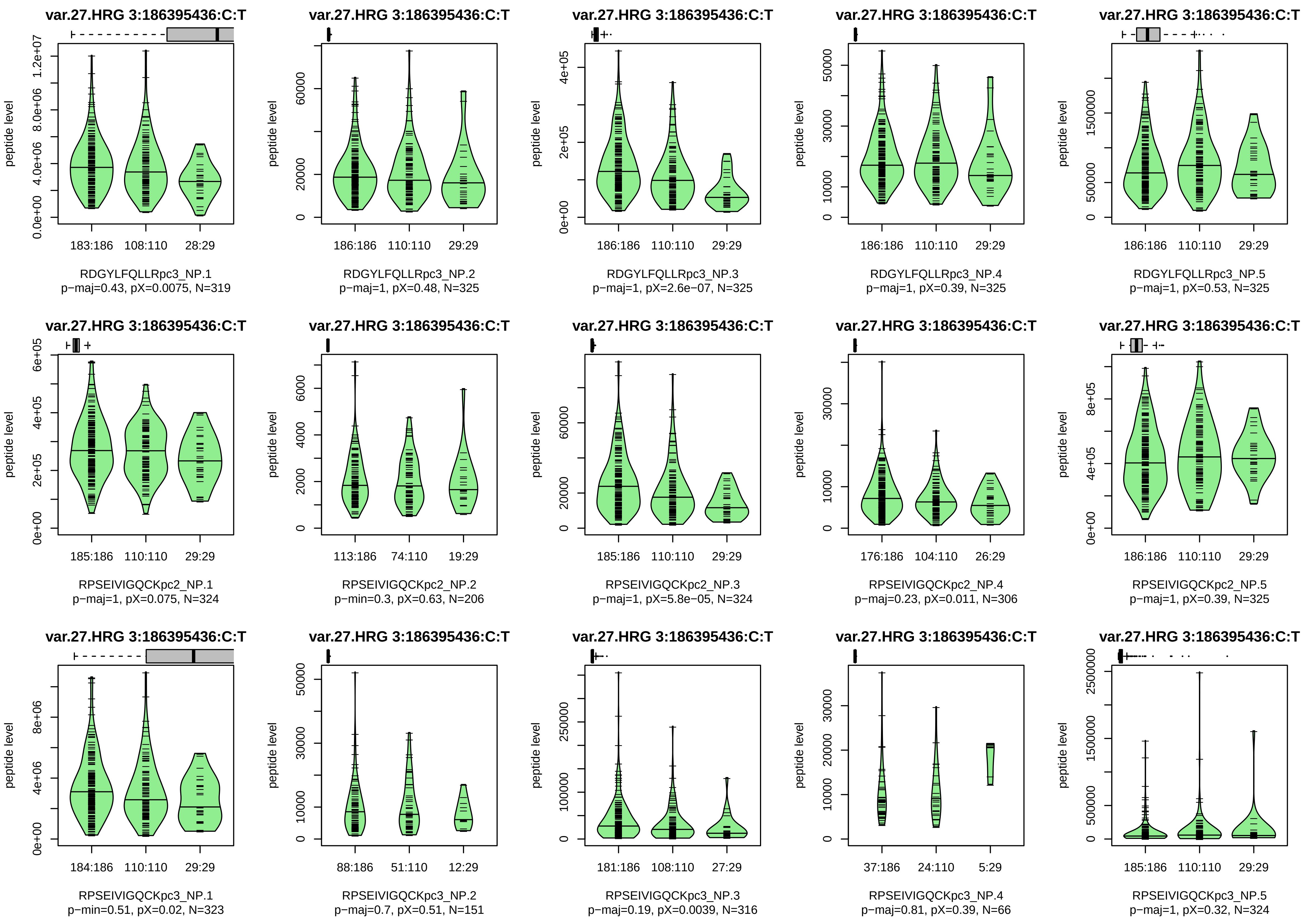
var.27.HRG 3:186395436:C:T
peptide level
183:186 108:110 28:29
RDGYLFQLLRpc3_NP.1
p−maj=0.43, pX=0.0075, N=319
var.27.HRG 3:186395436:C:T
186:186 110:110 29:29
RDGYLFQLLRpc3_NP.2
p−maj=1, pX=0.48, N=325
var.27.HRG 3:186395436:C:T
186:186 110:110 29:29
RDGYLFQLLRpc3_NP.3
p−maj=1, pX=2.6e−07, N=325
var.27.HRG 3:186395436:C:T
186:186 110:110 29:29
RDGYLFQLLRpc3_NP.4
p−maj=1, pX=0.39, N=325
var.27.HRG 3:186395436:C:T
186:186 110:110 29:29
RDGYLFQLLRpc3_NP.5
p−maj=1, pX=0.53, N=325
var.27.HRG 3:186395436:C:T
185:186 110:110 29:29
RPSEIVIGQCKpc2_NP.1
p−maj=1, pX=0.075, N=324
var.27.HRG 3:186395436:C:T
113:186 74:110 19:29
RPSEIVIGQCKpc2_NP.2
p−min=0.3, pX=0.63, N=206
var.27.HRG 3:186395436:C:T
185:186 110:110 29:29
RPSEIVIGQCKpc2_NP.3
p−maj=1, pX=5.8e−05, N=324
var.27.HRG 3:186395436:C:T
176:186 104:110 26:29
RPSEIVIGQCKpc2_NP.4
p−maj=0.23, pX=0.011, N=306
var.27.HRG 3:186395436:C:T
186:186 110:110 29:29
RPSEIVIGQCKpc2_NP.5
p−maj=1, pX=0.39, N=325
var.27.HRG 3:186395436:C:T
184:186 110:110 29:29
RPSEIVIGQCKpc3_NP.1
p−min=0.51, pX=0.02, N=323
var.27.HRG 3:186395436:C:T
88:186 51:110 12:29
RPSEIVIGQCKpc3_NP.2
p−maj=0.7, pX=0.51, N=151
var.27.HRG 3:186395436:C:T
181:186 108:110 27:29
RPSEIVIGQCKpc3_NP.3
p−maj=0.19, pX=0.0039, N=316
var.27.HRG 3:186395436:C:T
37:186 24:110 5:29
RPSEIVIGQCKpc3_NP.4
p−maj=0.81, pX=0.39, N=66
var.27.HRG 3:186395436:C:T
185:186 110:110 29:29
RPSEIVIGQCKpc3_NP.5
p−maj=1, pX=0.32, N=324

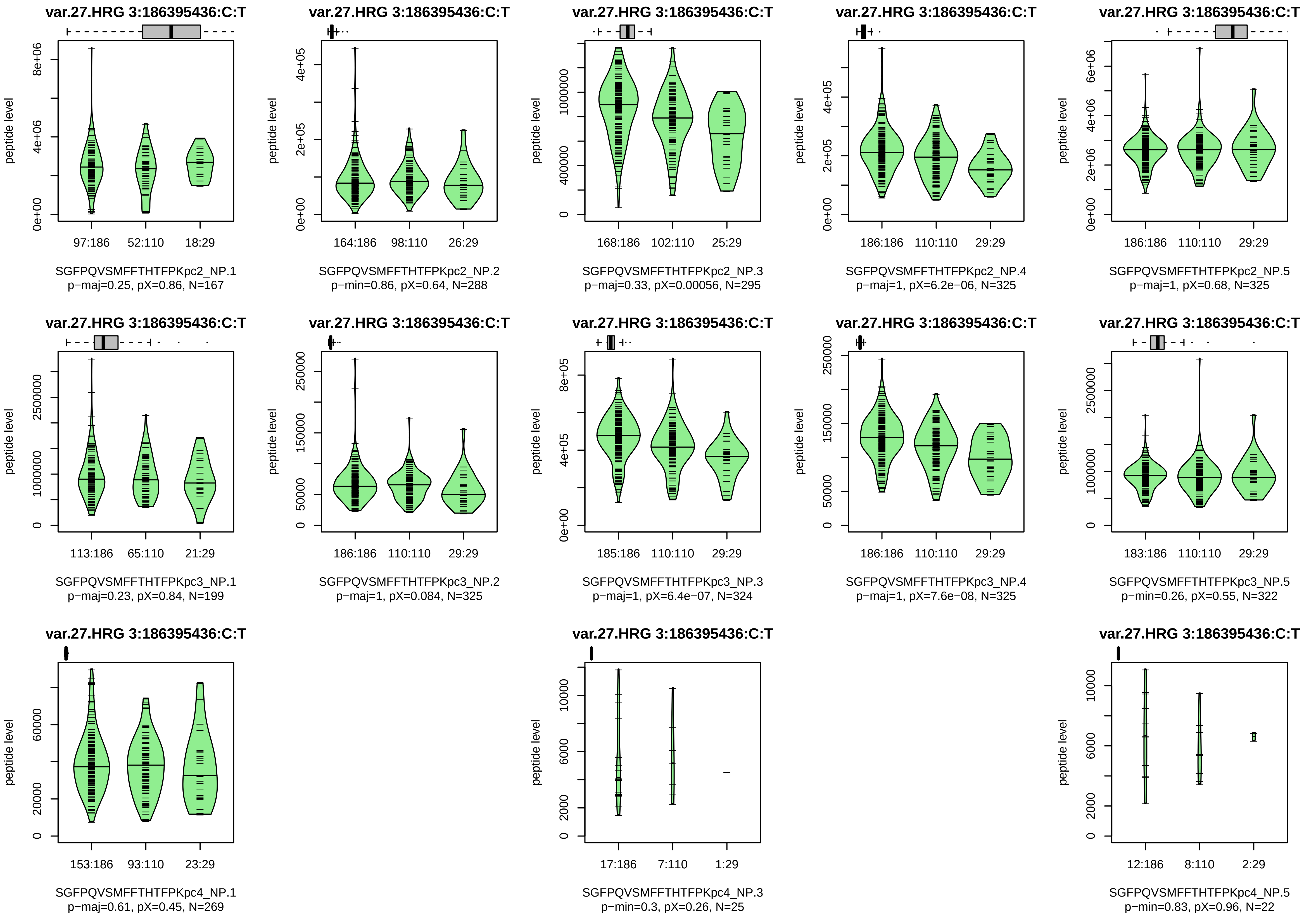
var.27.HRG 3:186395436:C:T
peptide level
97:186
52:110
18:29
SGFPQVSMFFTHTFPKpc2_NP.1
p−maj=0.25, pX=0.86, N=167
var.27.HRG 3:186395436:C:T
164:186
98:110
26:29
SGFPQVSMFFTHTFPKpc2_NP.2
p−min=0.86, pX=0.64, N=288
var.27.HRG 3:186395436:C:T
168:186
102:110
25:29
SGFPQVSMFFTHTFPKpc2_NP.3
p−maj=0.33, pX=0.00056, N=295
var.27.HRG 3:186395436:C:T
186:186
110:110
29:29
SGFPQVSMFFTHTFPKpc2_NP.4
p−maj=1, pX=6.2e−06, N=325
var.27.HRG 3:186395436:C:T
186:186
110:110
29:29
SGFPQVSMFFTHTFPKpc2_NP.5
p−maj=1, pX=0.68, N=325
var.27.HRG 3:186395436:C:T
113:186
65:110
21:29
SGFPQVSMFFTHTFPKpc3_NP.1
p−maj=0.23, pX=0.84, N=199
var.27.HRG 3:186395436:C:T
186:186
110:110
29:29
SGFPQVSMFFTHTFPKpc3_NP.2
p−maj=1, pX=0.084, N=325
var.27.HRG 3:186395436:C:T
185:186
110:110
29:29
SGFPQVSMFFTHTFPKpc3_NP.3
p−maj=1, pX=6.4e−07, N=324
var.27.HRG 3:186395436:C:T
186:186
110:110
29:29
SGFPQVSMFFTHTFPKpc3_NP.4
p−maj=1, pX=7.6e−08, N=325
var.27.HRG 3:186395436:C:T
183:186
110:110
29:29
SGFPQVSMFFTHTFPKpc3_NP.5
p−min=0.26, pX=0.55, N=322
var.27.HRG 3:186395436:C:T
153:186
93:110
23:29
SGFPQVSMFFTHTFPKpc4_NP.1
p−maj=0.61, pX=0.45, N=269
var.27.HRG 3:186395436:C:T
17:186
7:110
1:29
SGFPQVSMFFTHTFPKpc4_NP.3
p−min=0.3, pX=0.26, N=25
var.27.HRG 3:186395436:C:T
12:186
8:110
2:29
SGFPQVSMFFTHTFPKpc4_NP.5
p−min=0.83, pX=0.96, N=22

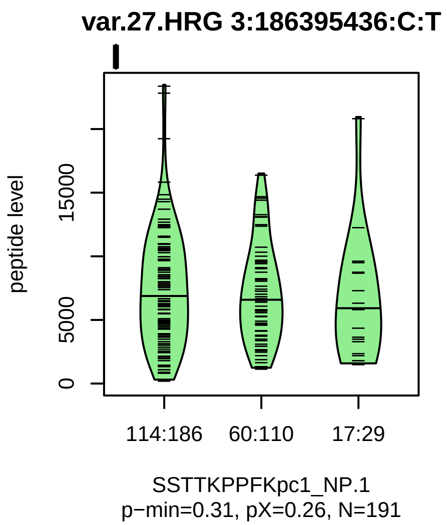

var.27.HRG 3:186395436:C:T
peptide level
0
5000
15000
114:186
60:110
17:29
SSTTKPPFKpc1_NP.1
p−min=0.31, pX=0.26, N=191

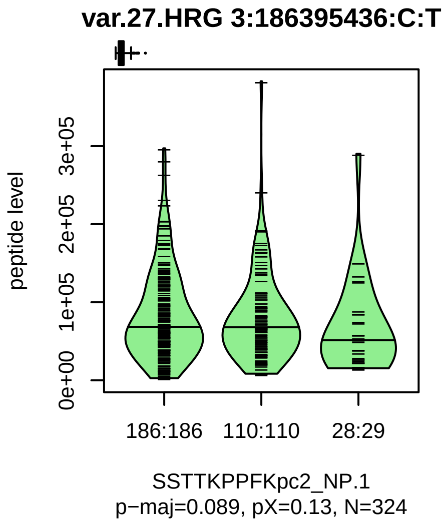

var.27.HRG 3:186395436:C:T
peptide level
0e+00
1e+05
2e+05
3e+05
186:186
110:110
28:29
SSTTKPPFKpc2_NP.1
p−maj=0.089, pX=0.13, N=324

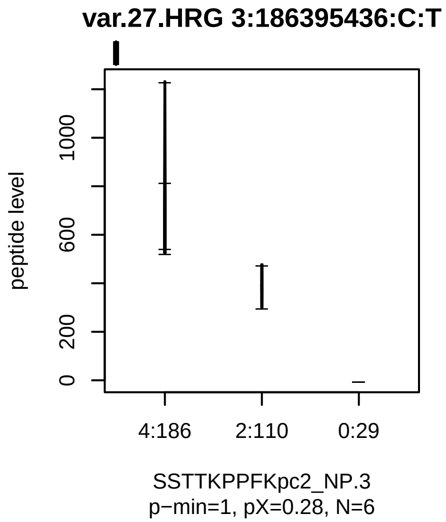

var.27.HRG 3:186395436:C:T
peptide level
0
200
600
1000
4:186
2:110
0:29
SSTTKPPFKpc2_NP.3
p−min=1, pX=0.28, N=6

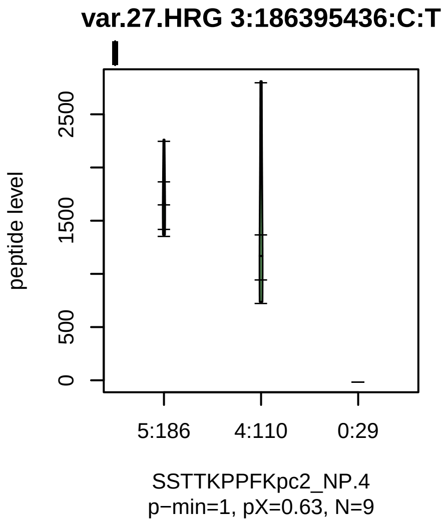

var.27.HRG 3:186395436:C:T
peptide level
0
500
1500
2500
5:186
4:110
0:29
SSTTKPPFKpc2_NP.4
p−min=1, pX=0.63, N=9

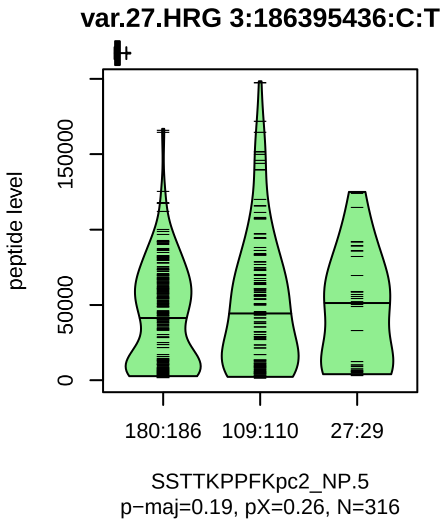

var.27.HRG 3:186395436:C:T
peptide level
0
50000
150000
180:186
109:110
27:29
SSTTKPPFKpc2_NP.5
p−maj=0.19, pX=0.26, N=316

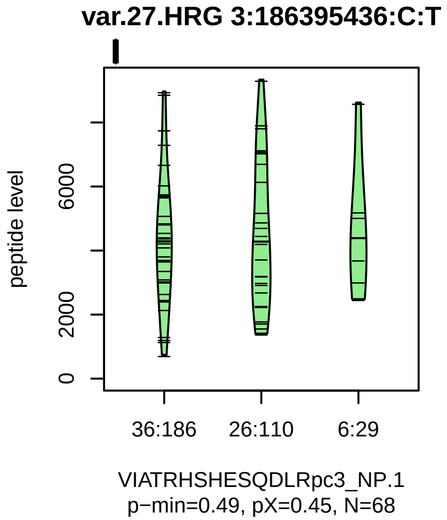

var.27.HRG 3:186395436:C:T
peptide level
0
2000
6000
36:186
26:110
6:29
VIATRHSHESQDLRpc3_NP.1
p−min=0.49, pX=0.45, N=68

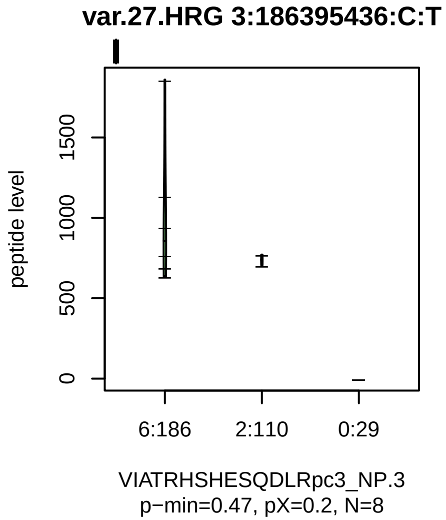

var.27.HRG 3:186395436:C:T
peptide level
0
500
1000
1500
6:186
2:110
0:29
VIATRHSHESQDLRpc3_NP.3
p−min=0.47, pX=0.2, N=8

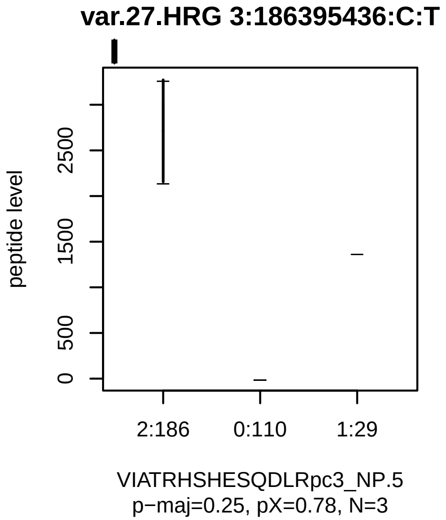

var.27.HRG 3:186395436:C:T
peptide level
0
500
1500
2500
2:186
0:110
1:29
VIATRHSHESQDLRpc3_NP.5
p−maj=0.25, pX=0.78, N=3

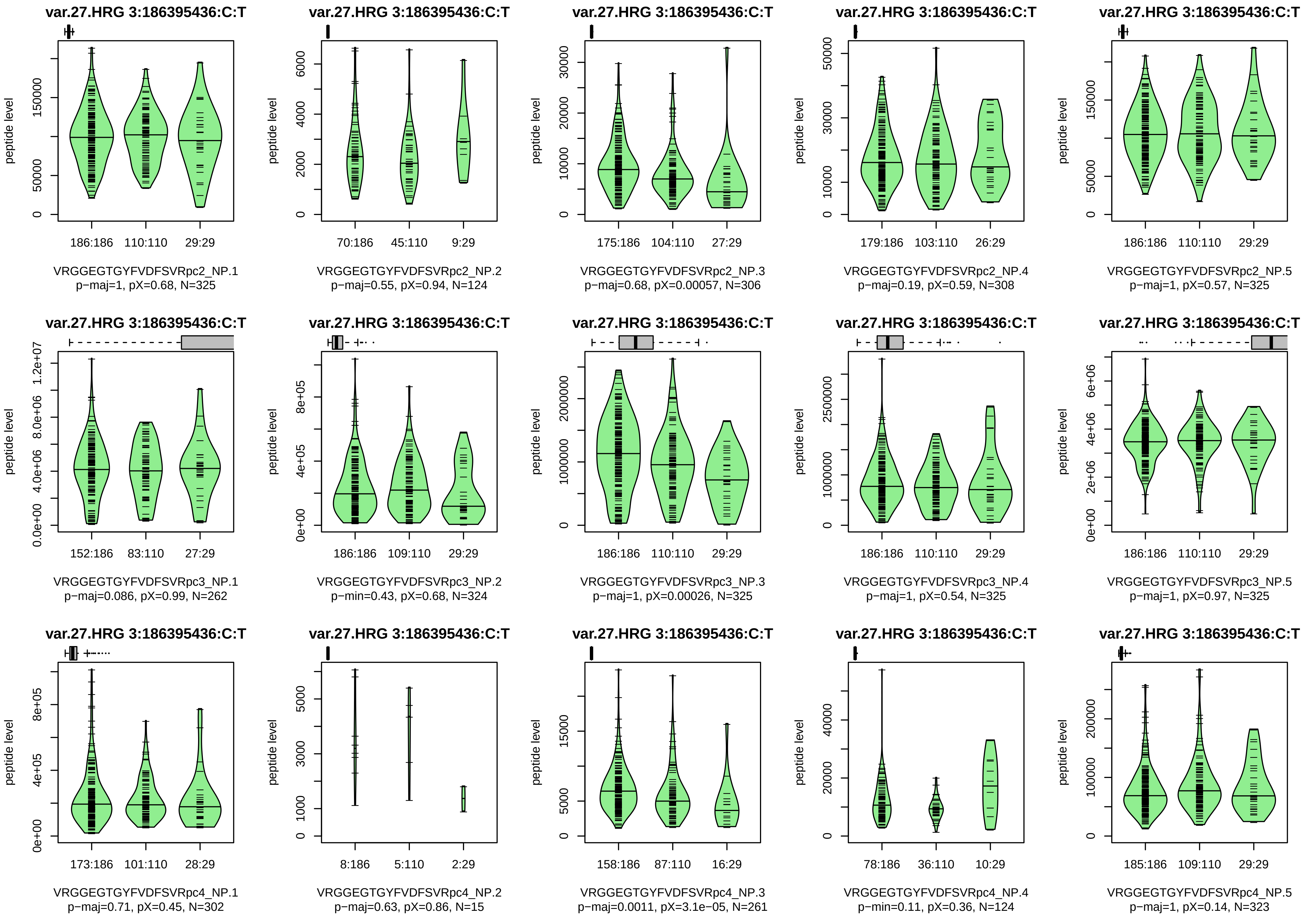
var.27.HRG 3:186395436:C:T
peptide level
186:186
110:110
29:29
VRGGEGTGYFVDFSVRpc2_NP.1
p−maj=1, pX=0.68, N=325
var.27.HRG 3:186395436:C:T
peptide level
70:186
45:110
9:29
VRGGEGTGYFVDFSVRpc2_NP.2
p−maj=0.55, pX=0.94, N=124
var.27.HRG 3:186395436:C:T
peptide level
175:186
104:110
27:29
VRGGEGTGYFVDFSVRpc2_NP.3
p−maj=0.68, pX=0.00057, N=306
var.27.HRG 3:186395436:C:T
peptide level
179:186
103:110
26:29
VRGGEGTGYFVDFSVRpc2_NP.4
p−maj=0.19, pX=0.59, N=308
var.27.HRG 3:186395436:C:T
peptide level
186:186
110:110
29:29
VRGGEGTGYFVDFSVRpc2_NP.5
p−maj=1, pX=0.57, N=325
var.27.HRG 3:186395436:C:T
peptide level
152:186
83:110
27:29
VRGGEGTGYFVDFSVRpc3_NP.1
p−maj=0.086, pX=0.99, N=262
var.27.HRG 3:186395436:C:T
peptide level
186:186
109:110
29:29
VRGGEGTGYFVDFSVRpc3_NP.2
p−min=0.43, pX=0.68, N=324
var.27.HRG 3:186395436:C:T
peptide level
186:186
110:110
29:29
VRGGEGTGYFVDFSVRpc3_NP.3
p−maj=1, pX=0.00026, N=325
var.27.HRG 3:186395436:C:T
peptide level
186:186
110:110
29:29
VRGGEGTGYFVDFSVRpc3_NP.4
p−maj=1, pX=0.54, N=325
var.27.HRG 3:186395436:C:T
peptide level
186:186
110:110
29:29
VRGGEGTGYFVDFSVRpc3_NP.5
p−maj=1, pX=0.97, N=325
var.27.HRG 3:186395436:C:T
peptide level
173:186
101:110
28:29
VRGGEGTGYFVDFSVRpc4_NP.1
p−maj=0.71, pX=0.45, N=302
var.27.HRG 3:186395436:C:T
peptide level
8:186
5:110
2:29
VRGGEGTGYFVDFSVRpc4_NP.2
p−maj=0.63, pX=0.86, N=15
var.27.HRG 3:186395436:C:T
peptide level
158:186
87:110
16:29
VRGGEGTGYFVDFSVRpc4_NP.3
p−maj=0.0011, pX=3.1e−05, N=261
var.27.HRG 3:186395436:C:T
peptide level
78:186
36:110
10:29
VRGGEGTGYFVDFSVRpc4_NP.4
p−min=0.11, pX=0.36, N=124
var.27.HRG 3:186395436:C:T
peptide level
185:186
109:110
29:29
VRGGEGTGYFVDFSVRpc4_NP.5
p−maj=1, pX=0.14, N=323

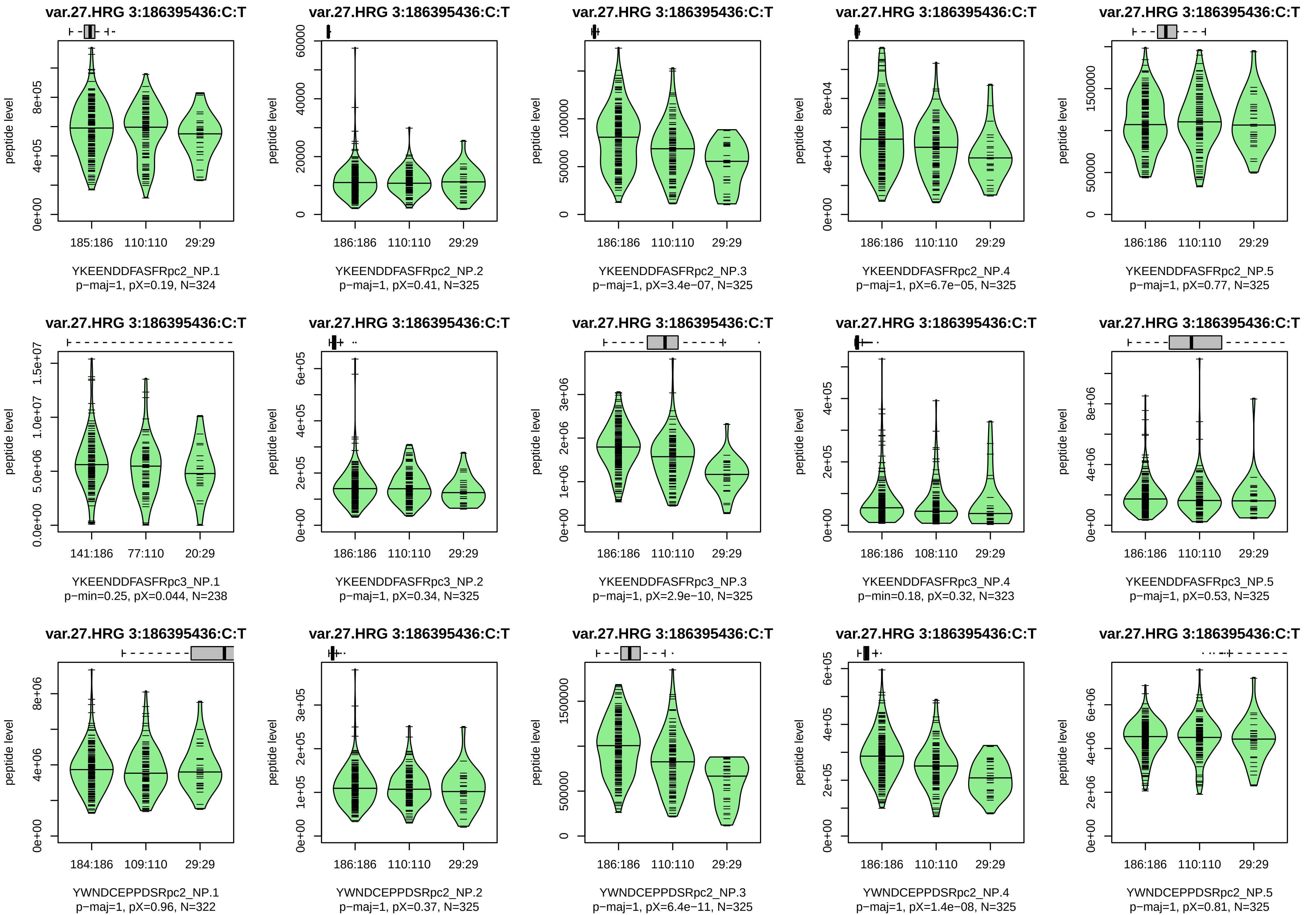
var.27.HRG 3:186395436:C:T
peptide level
185:186
110:110
29:29
YKEENDDFASFRpc2_NP.1
p−maj=1, pX=0.19, N=324
var.27.HRG 3:186395436:C:T
peptide level
186:186
110:110
29:29
YKEENDDFASFRpc2_NP.2
p−maj=1, pX=0.41, N=325
var.27.HRG 3:186395436:C:T
peptide level
186:186
110:110
29:29
YKEENDDFASFRpc2_NP.3
p−maj=1, pX=3.4e−07, N=325
var.27.HRG 3:186395436:C:T
peptide level
186:186
110:110
29:29
YKEENDDFASFRpc2_NP.4
p−maj=1, pX=6.7e−05, N=325
var.27.HRG 3:186395436:C:T
peptide level
186:186
110:110
29:29
YKEENDDFASFRpc2_NP.5
p−maj=1, pX=0.77, N=325
var.27.HRG 3:186395436:C:T
peptide level
141:186
77:110
20:29
YKEENDDFASFRpc3_NP.1
p−min=0.25, pX=0.044, N=238
var.27.HRG 3:186395436:C:T
peptide level
186:186
110:110
29:29
YKEENDDFASFRpc3_NP.2
p−maj=1, pX=0.34, N=325
var.27.HRG 3:186395436:C:T
peptide level
186:186
110:110
29:29
YKEENDDFASFRpc3_NP.3
p−maj=1, pX=2.9e−10, N=325
var.27.HRG 3:186395436:C:T
peptide level
186:186
108:110
29:29
YKEENDDFASFRpc3_NP.4
p−min=0.18, pX=0.32, N=323
var.27.HRG 3:186395436:C:T
peptide level
186:186
110:110
29:29
YKEENDDFASFRpc3_NP.5
p−maj=1, pX=0.53, N=325
var.27.HRG 3:186395436:C:T
peptide level
184:186
109:110
29:29
YWNDCEPPDSRpc2_NP.1
p−maj=1, pX=0.96, N=322
var.27.HRG 3:186395436:C:T
peptide level
186:186
110:110
29:29
YWNDCEPPDSRpc2_NP.2
p−maj=1, pX=0.37, N=325
var.27.HRG 3:186395436:C:T
peptide level
186:186
110:110
29:29
YWNDCEPPDSRpc2_NP.3
p−maj=1, pX=6.4e−11, N=325
var.27.HRG 3:186395436:C:T
peptide level
186:186
110:110
29:29
YWNDCEPPDSRpc2_NP.4
p−maj=1, pX=1.4e−08, N=325
var.27.HRG 3:186395436:C:T
peptide level
186:186
110:110
29:29
YWNDCEPPDSRpc2_NP.5
p−maj=1, pX=0.81, N=325

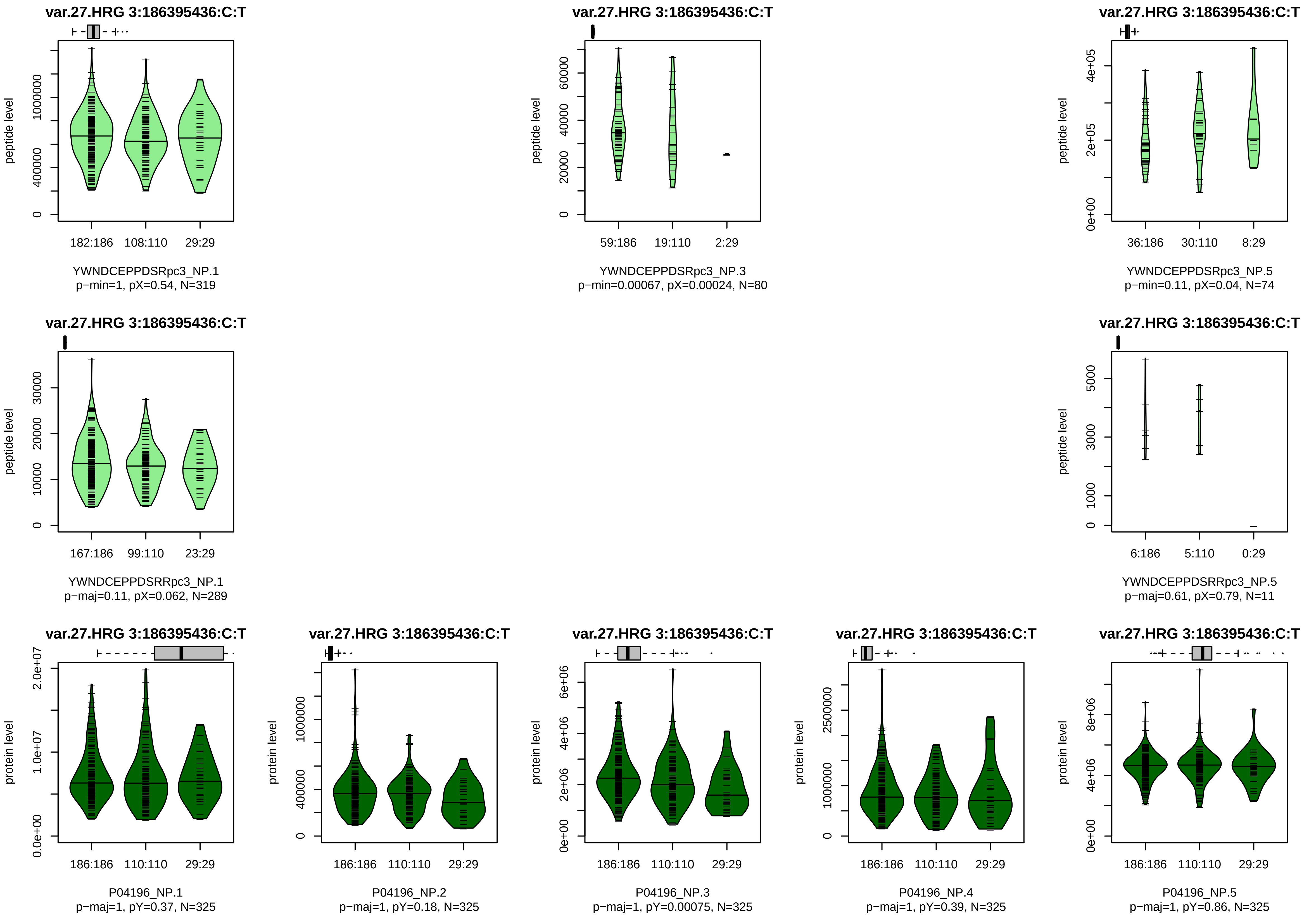
var.27.HRG 3:186395436:C:T
peptide level
182:186
108:110
29:29
YWNDCEPPDSRpc3_NP.1
p−min=1, pX=0.54, N=319
var.27.HRG 3:186395436:C:T
peptide level
59:186
19:110
2:29
YWNDCEPPDSRpc3_NP.3
p−min=0.00067, pX=0.00024, N=80
var.27.HRG 3:186395436:C:T
peptide level
36:186
30:110
8:29
YWNDCEPPDSRpc3_NP.5
p−min=0.11, pX=0.04, N=74
var.27.HRG 3:186395436:C:T
peptide level
167:186
99:110
23:29
YWNDCEPPDSRRpc3_NP.1
p−maj=0.11, pX=0.062, N=289
var.27.HRG 3:186395436:C:T
peptide level
6:186
5:110
0:29
YWNDCEPPDSRRpc3_NP.5
p−maj=0.61, pX=0.79, N=11
var.27.HRG 3:186395436:C:T
protein level
186:186
110:110
29:29
P04196_NP.1
p−maj=1, pY=0.37, N=325
var.27.HRG 3:186395436:C:T
protein level
186:186
110:110
29:29
P04196_NP.2
p−maj=1, pY=0.18, N=325
var.27.HRG 3:186395436:C:T
protein level
186:186
110:110
29:29
P04196_NP.3
p−maj=1, pY=0.00075, N=325
var.27.HRG 3:186395436:C:T
protein level
186:186
110:110
29:29
P04196_NP.4
p−maj=1, pY=0.39, N=325
var.27.HRG 3:186395436:C:T
protein level
186:186
110:110
29:29
P04196_NP.5
p−maj=1, pY=0.86, N=325

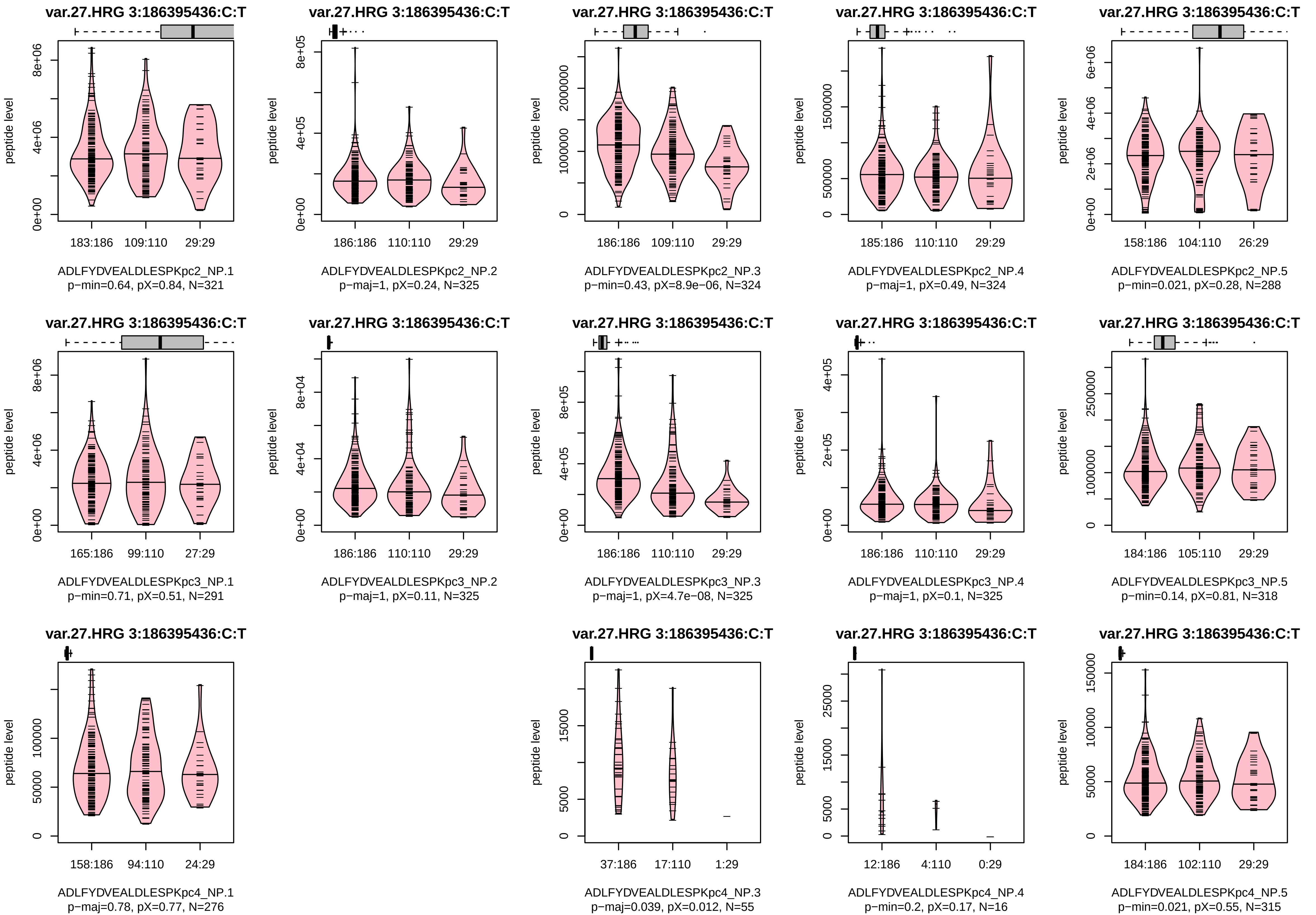

var.27.HRG 3:186395436:C:T
peptide level
183:186
109:110
29:29
ADLFYDVEALDLESPKpc2_NP.1
p−min=0.64, pX=0.84, N=321
var.27.HRG 3:186395436:C:T
peptide level
186:186
110:110
29:29
ADLFYDVEALDLESPKpc2_NP.2
p−maj=1, pX=0.24, N=325
var.27.HRG 3:186395436:C:T
peptide level
186:186
109:110
29:29
ADLFYDVEALDLESPKpc2_NP.3
p−min=0.43, pX=8.9e−06, N=324
var.27.HRG 3:186395436:C:T
peptide level
185:186
110:110
29:29
ADLFYDVEALDLESPKpc2_NP.4
p−maj=1, pX=0.49, N=324
var.27.HRG 3:186395436:C:T
peptide level
158:186
104:110
26:29
ADLFYDVEALDLESPKpc2_NP.5
p−min=0.021, pX=0.28, N=288
var.27.HRG 3:186395436:C:T
peptide level
165:186
99:110
27:29
ADLFYDVEALDLESPKpc3_NP.1
p−min=0.71, pX=0.51, N=291
var.27.HRG 3:186395436:C:T
peptide level
186:186
110:110
29:29
ADLFYDVEALDLESPKpc3_NP.2
p−maj=1, pX=0.11, N=325
var.27.HRG 3:186395436:C:T
peptide level
186:186
110:110
29:29
ADLFYDVEALDLESPKpc3_NP.3
p−maj=1, pX=4.7e−08, N=325
var.27.HRG 3:186395436:C:T
peptide level
186:186
110:110
29:29
ADLFYDVEALDLESPKpc3_NP.4
p−maj=1, pX=0.1, N=325
var.27.HRG 3:186395436:C:T
peptide level
184:186
105:110
29:29
ADLFYDVEALDLESPKpc3_NP.5
p−min=0.14, pX=0.81, N=318
var.27.HRG 3:186395436:C:T
peptide level
158:186
94:110
24:29
ADLFYDVEALDLESPKpc4_NP.1
p−maj=0.78, pX=0.77, N=276
var.27.HRG 3:186395436:C:T
peptide level
37:186
17:110
1:29
ADLFYDVEALDLESPKpc4_NP.3
p−maj=0.039, pX=0.012, N=55
var.27.HRG 3:186395436:C:T
peptide level
12:186
4:110
0:29
ADLFYDVEALDLESPKpc4_NP.4
p−min=0.2, pX=0.17, N=16
var.27.HRG 3:186395436:C:T
peptide level
184:186
102:110
29:29
ADLFYDVEALDLESPKpc4_NP.5
p−min=0.021, pX=0.55, N=315

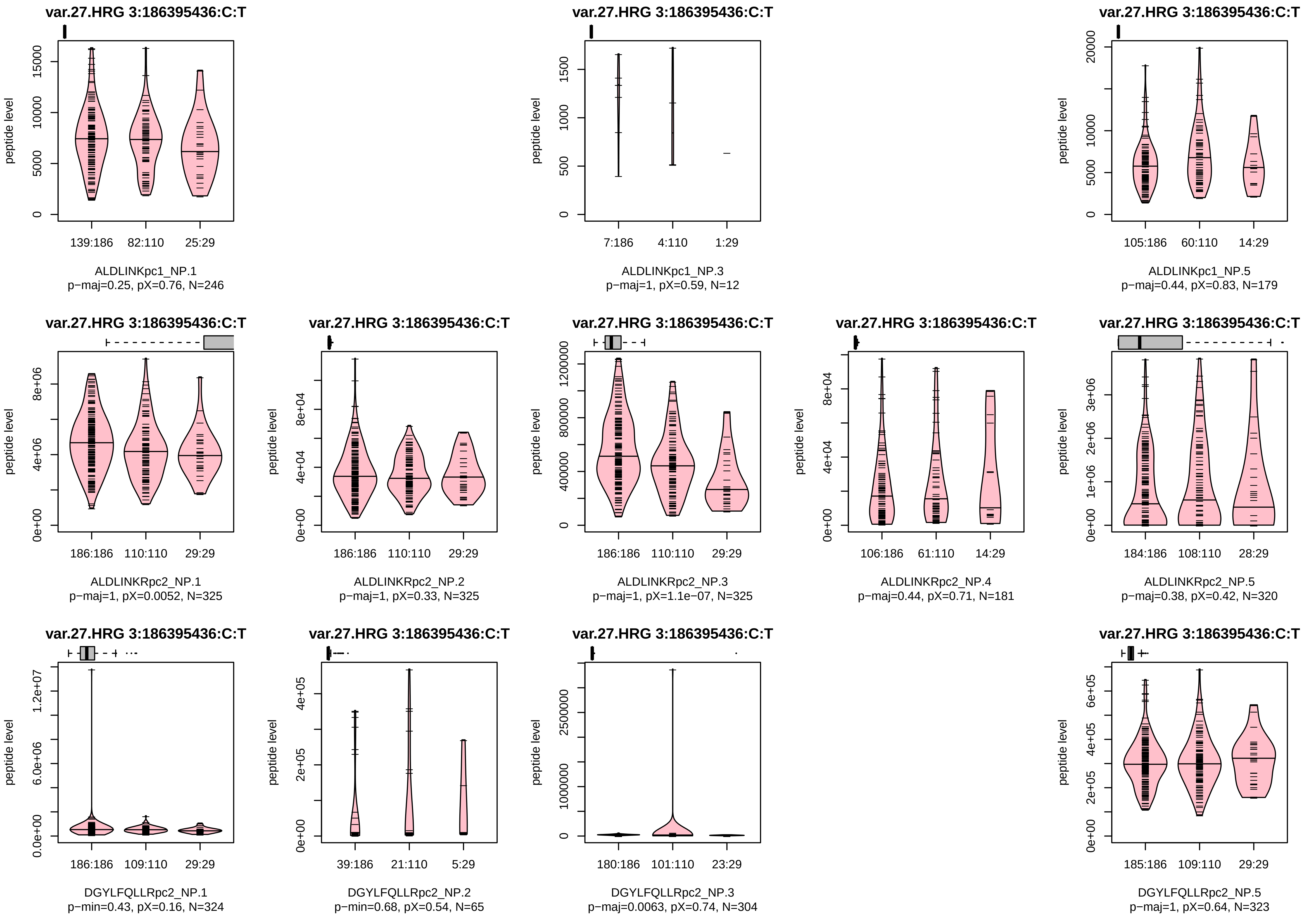
var.27.HRG 3:186395436:C:T
peptide level
139:186
82:110
25:29
ALDLINKpc1_NP.1
p−maj=0.25, pX=0.76, N=246
var.27.HRG 3:186395436:C:T
peptide level
7:186
4:110
1:29
ALDLINKpc1_NP.3
p−maj=1, pX=0.59, N=12
var.27.HRG 3:186395436:C:T
peptide level
105:186
60:110
14:29
ALDLINKpc1_NP.5
p−maj=0.44, pX=0.83, N=179
var.27.HRG 3:186395436:C:T
peptide level
186:186
110:110
29:29
ALDLINKRpc2_NP.1
p−maj=1, pX=0.0052, N=325
var.27.HRG 3:186395436:C:T
peptide level
186:186
110:110
29:29
ALDLINKRpc2_NP.2
p−maj=1, pX=0.33, N=325
var.27.HRG 3:186395436:C:T
peptide level
186:186
110:110
29:29
ALDLINKRpc2_NP.3
p−maj=1, pX=1.1e−07, N=325
var.27.HRG 3:186395436:C:T
peptide level
106:186
61:110
14:29
ALDLINKRpc2_NP.4
p−maj=0.44, pX=0.71, N=181
var.27.HRG 3:186395436:C:T
peptide level
184:186
108:110
28:29
ALDLINKRpc2_NP.5
p−maj=0.38, pX=0.42, N=320
var.27.HRG 3:186395436:C:T
peptide level
186:186
109:110
29:29
DGYLFQLLRpc2_NP.1
p−min=0.43, pX=0.16, N=324
var.27.HRG 3:186395436:C:T
peptide level
39:186
21:110
5:29
DGYLFQLLRpc2_NP.2
p−min=0.68, pX=0.54, N=65
var.27.HRG 3:186395436:C:T
peptide level
180:186
101:110
23:29
DGYLFQLLRpc2_NP.3
p−maj=0.0063, pX=0.74, N=304
var.27.HRG 3:186395436:C:T
peptide level
185:186
109:110
29:29
DGYLFQLLRpc2_NP.5
p−maj=1, pX=0.64, N=323

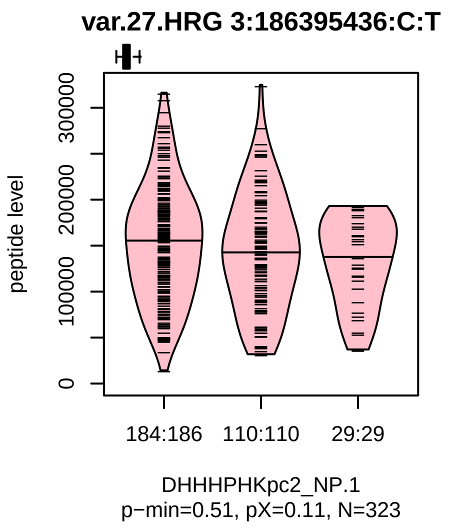
var.27.HRG 3:186395436:C:T
peptide level
0
100000
200000
300000
184:186
110:110
29:29
DHHHPHKpc2_NP.1
p−min=0.51, pX=0.11, N=323

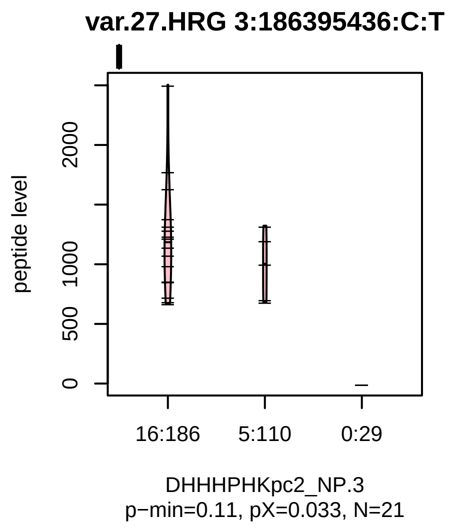
var.27.HRG 3:186395436:C:T
peptide level
0
500
1000
2000
16:186
5:110
0:29
DHHHPHKpc2_NP.3
p−min=0.11, pX=0.033, N=21

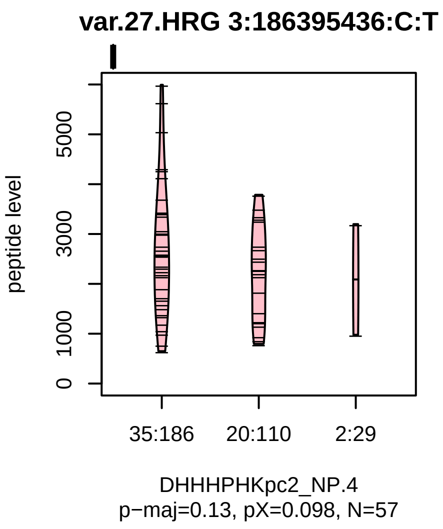
var.27.HRG 3:186395436:C:T
peptide level
0
1000
3000
5000
35:186
20:110
2:29
DHHHPHKpc2_NP.4
p−maj=0.13, pX=0.098, N=57

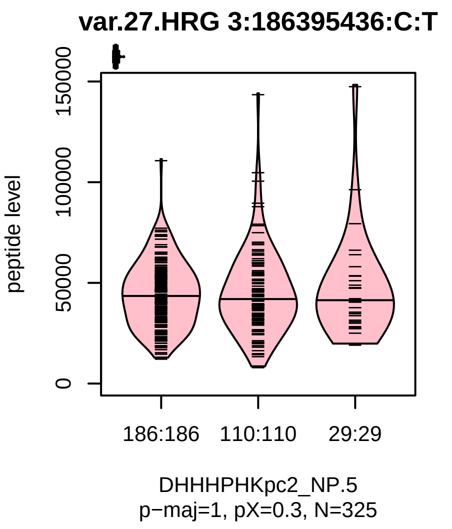
var.27.HRG 3:186395436:C:T
peptide level
0
50000
100000
150000
186:186
110:110
29:29
DHHHPHKpc2_NP.5
p−maj=1, pX=0.3, N=325

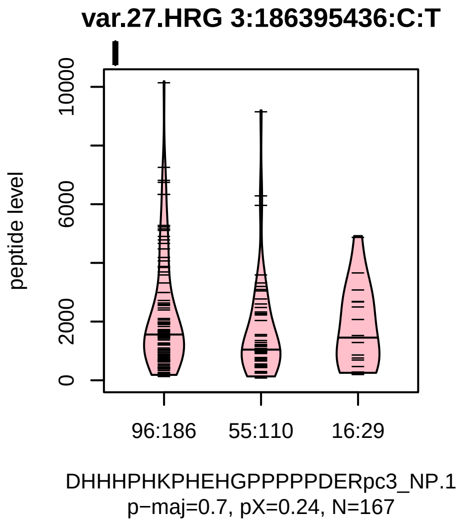
var.27.HRG 3:186395436:C:T
peptide level
0
2000
6000
10000
96:186
55:110
16:29
DHHHPHKPHEHGPPPPPDERpc3_NP.1
p−maj=0.7, pX=0.24, N=167

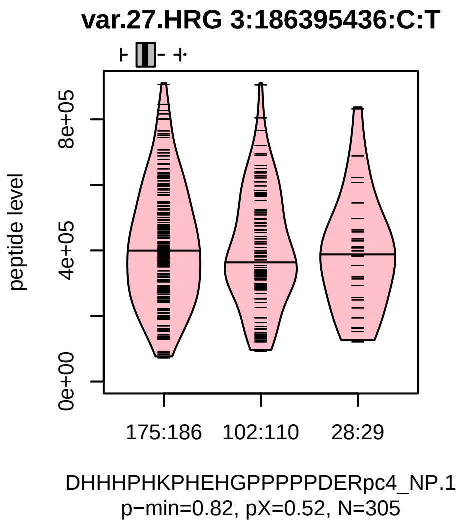
var.27.HRG 3:186395436:C:T
peptide level
0e+00
4e+05
8e+05
175:186
102:110
28:29
DHHHPHKPHEHGPPPPPDERpc4_NP.1
p−min=0.82, pX=0.52, N=305

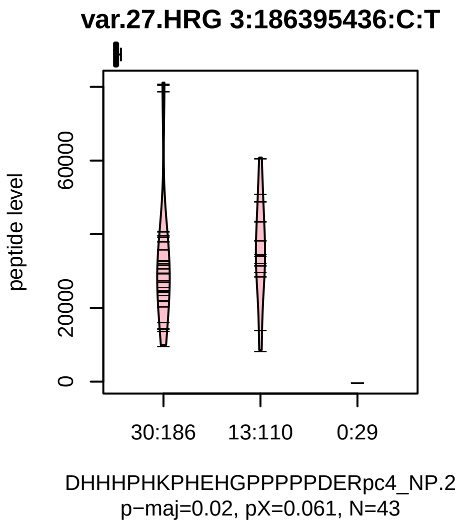
var.27.HRG 3:186395436:C:T
peptide level
0
20000
60000
30:186
13:110
0:29
DHHHPHKPHEHGPPPPPDERpc4_NP.2
p−maj=0.02, pX=0.061, N=43

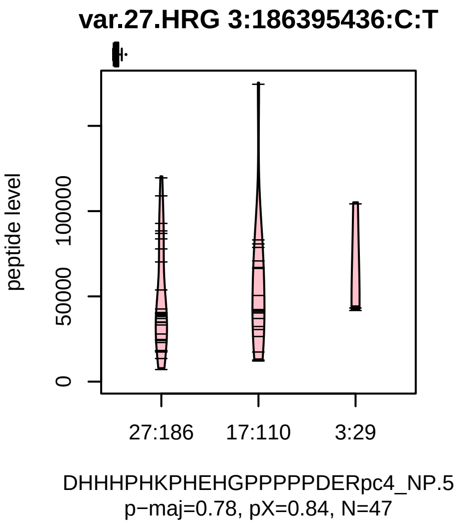
var.27.HRG 3:186395436:C:T
peptide level
0
50000
100000
27:186
17:110
3:29
DHHHPHKPHEHGPPPPPDERpc4_NP.5
p−maj=0.78, pX=0.84, N=47

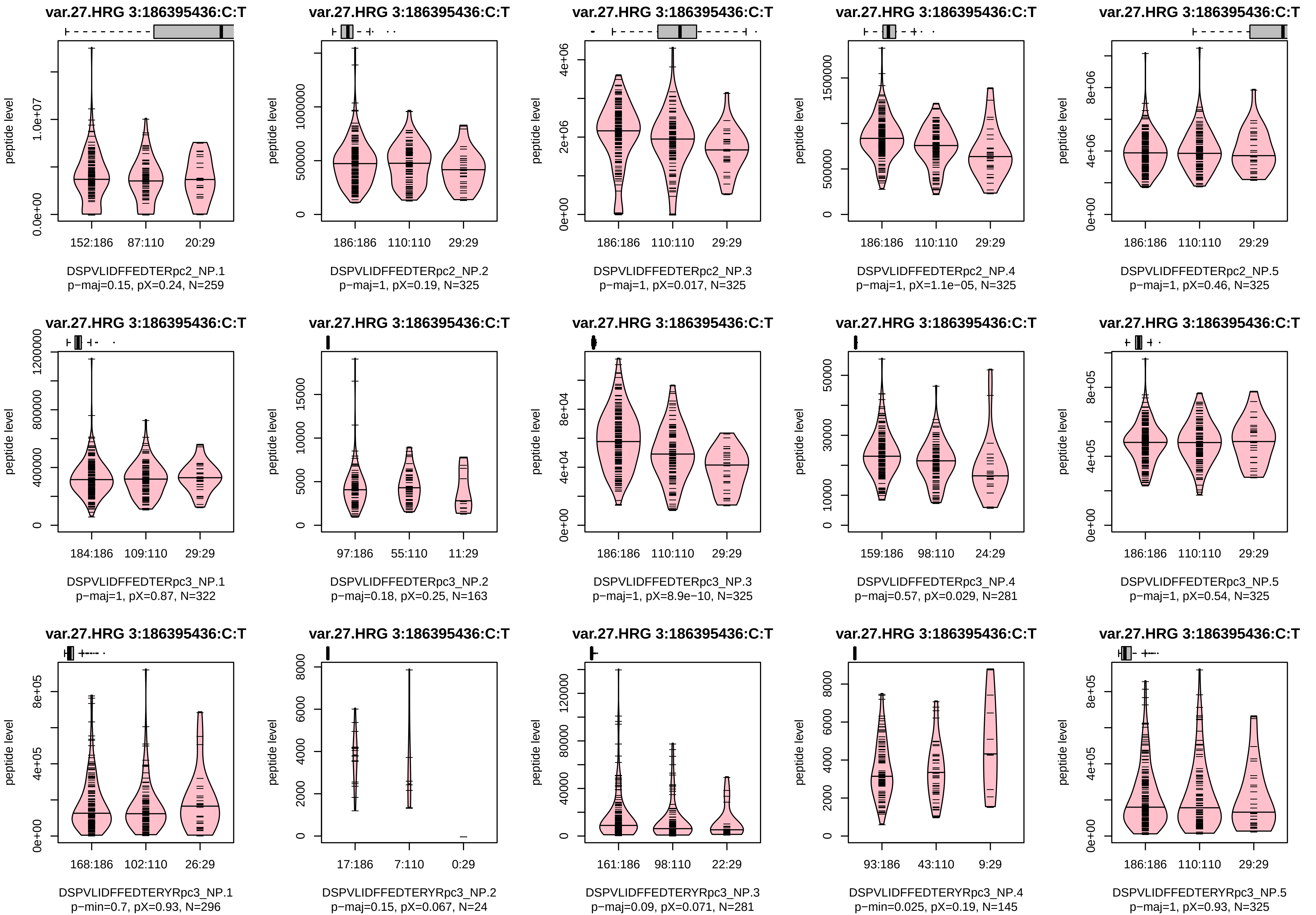

var.27.HRG 3:186395436:C:T
peptide level
0.0e+00
1.0e+07
152:186
87:110
20:29
DSPVLIDFFEDTERpc2_NP.1
p−maj=0.15, pX=0.24, N=259
var.27.HRG 3:186395436:C:T
peptide level
0
500000
1000000
186:186
110:110
29:29
DSPVLIDFFEDTERpc2_NP.2
p−maj=1, pX=0.19, N=325
var.27.HRG 3:186395436:C:T
peptide level
0e+00
2e+06
4e+06
186:186
110:110
29:29
DSPVLIDFFEDTERpc2_NP.3
p−maj=1, pX=0.017, N=325
var.27.HRG 3:186395436:C:T
peptide level
0
500000
1500000
186:186
110:110
29:29
DSPVLIDFFEDTERpc2_NP.4
p−maj=1, pX=1.1e−05, N=325
var.27.HRG 3:186395436:C:T
peptide level
0e+00
4e+06
8e+06
186:186
110:110
29:29
DSPVLIDFFEDTERpc2_NP.5
p−maj=1, pX=0.46, N=325
var.27.HRG 3:186395436:C:T
peptide level
0
400000
800000
1200000
184:186
109:110
29:29
DSPVLIDFFEDTERpc3_NP.1
p−maj=1, pX=0.87, N=322
var.27.HRG 3:186395436:C:T
peptide level
0
5000
10000
15000
97:186
55:110
11:29
DSPVLIDFFEDTERpc3_NP.2
p−maj=0.18, pX=0.25, N=163
var.27.HRG 3:186395436:C:T
peptide level
0e+00
4e+04
8e+04
186:186
110:110
29:29
DSPVLIDFFEDTERpc3_NP.3
p−maj=1, pX=8.9e−10, N=325
var.27.HRG 3:186395436:C:T
peptide level
0
10000
30000
50000
159:186
98:110
24:29
DSPVLIDFFEDTERpc3_NP.4
p−maj=0.57, pX=0.029, N=281
var.27.HRG 3:186395436:C:T
peptide level
0e+00
4e+05
8e+05
186:186
110:110
29:29
DSPVLIDFFEDTERpc3_NP.5
p−maj=1, pX=0.54, N=325
var.27.HRG 3:186395436:C:T
peptide level
0e+00
4e+05
8e+05
168:186
102:110
26:29
DSPVLIDFFEDTERYRpc3_NP.1
p−min=0.7, pX=0.93, N=296
var.27.HRG 3:186395436:C:T
peptide level
0
2000
4000
6000
8000
17:186
7:110
0:29
DSPVLIDFFEDTERYRpc3_NP.2
p−maj=0.15, pX=0.067, N=24
var.27.HRG 3:186395436:C:T
peptide level
0
40000
80000
120000
161:186
98:110
22:29
DSPVLIDFFEDTERYRpc3_NP.3
p−maj=0.09, pX=0.071, N=281
var.27.HRG 3:186395436:C:T
peptide level
0
2000
4000
6000
8000
93:186
43:110
9:29
DSPVLIDFFEDTERYRpc3_NP.4
p−min=0.025, pX=0.19, N=145
var.27.HRG 3:186395436:C:T
peptide level
0e+00
4e+05
8e+05
186:186
110:110
29:29
DSPVLIDFFEDTERYRpc3_NP.5
p−maj=1, pX=0.93, N=325

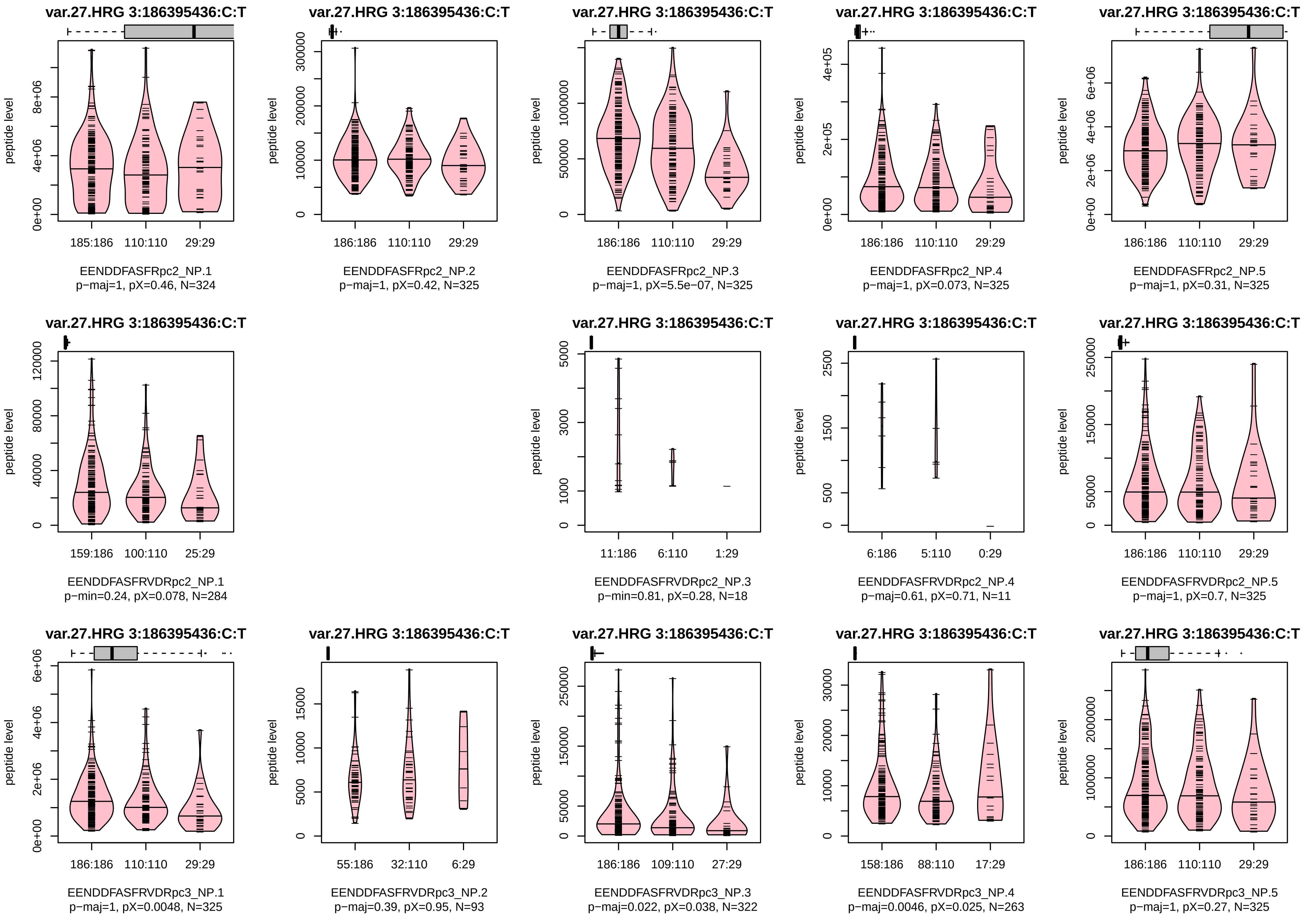
var.27.HRG 3:186395436:C:T
185:186 110:110 29:29
EENDDFASFRpc2_NP.1
p−maj=1, pX=0.46, N=324
var.27.HRG 3:186395436:C:T
186:186 110:110 29:29
EENDDFASFRpc2_NP.2
p−maj=1, pX=0.42, N=325
var.27.HRG 3:186395436:C:T
186:186 110:110 29:29
EENDDFASFRpc2_NP.3
p−maj=1, pX=5.5e−07, N=325
var.27.HRG 3:186395436:C:T
186:186 110:110 29:29
EENDDFASFRpc2_NP.4
p−maj=1, pX=0.073, N=325
var.27.HRG 3:186395436:C:T
186:186 110:110 29:29
EENDDFASFRpc2_NP.5
p−maj=1, pX=0.31, N=325
var.27.HRG 3:186395436:C:T
159:186 100:110 25:29
EENDDFASFRVDRpc2_NP.1
p−min=0.24, pX=0.078, N=284
var.27.HRG 3:186395436:C:T
11:186 6:110 1:29
EENDDFASFRVDRpc2_NP.3
p−min=0.81, pX=0.28, N=18
var.27.HRG 3:186395436:C:T
6:186 5:110 0:29
EENDDFASFRVDRpc2_NP.4
p−maj=0.61, pX=0.71, N=11
var.27.HRG 3:186395436:C:T
186:186 110:110 29:29
EENDDFASFRVDRpc2_NP.5
p−maj=1, pX=0.7, N=325
var.27.HRG 3:186395436:C:T
186:186 110:110 29:29
EENDDFASFRVDRpc3_NP.1
p−maj=1, pX=0.0048, N=325
var.27.HRG 3:186395436:C:T
55:186 32:110 6:29
EENDDFASFRVDRpc3_NP.2
p−maj=0.39, pX=0.95, N=93
var.27.HRG 3:186395436:C:T
186:186 109:110 27:29
EENDDFASFRVDRpc3_NP.3
p−maj=0.022, pX=0.038, N=322
var.27.HRG 3:186395436:C:T
158:186 88:110 17:29
EENDDFASFRVDRpc3_NP.4
p−maj=0.0046, pX=0.025, N=263
var.27.HRG 3:186395436:C:T
186:186 110:110 29:29
EENDDFASFRVDRpc3_NP.5
p−maj=1, pX=0.27, N=325
peptide level

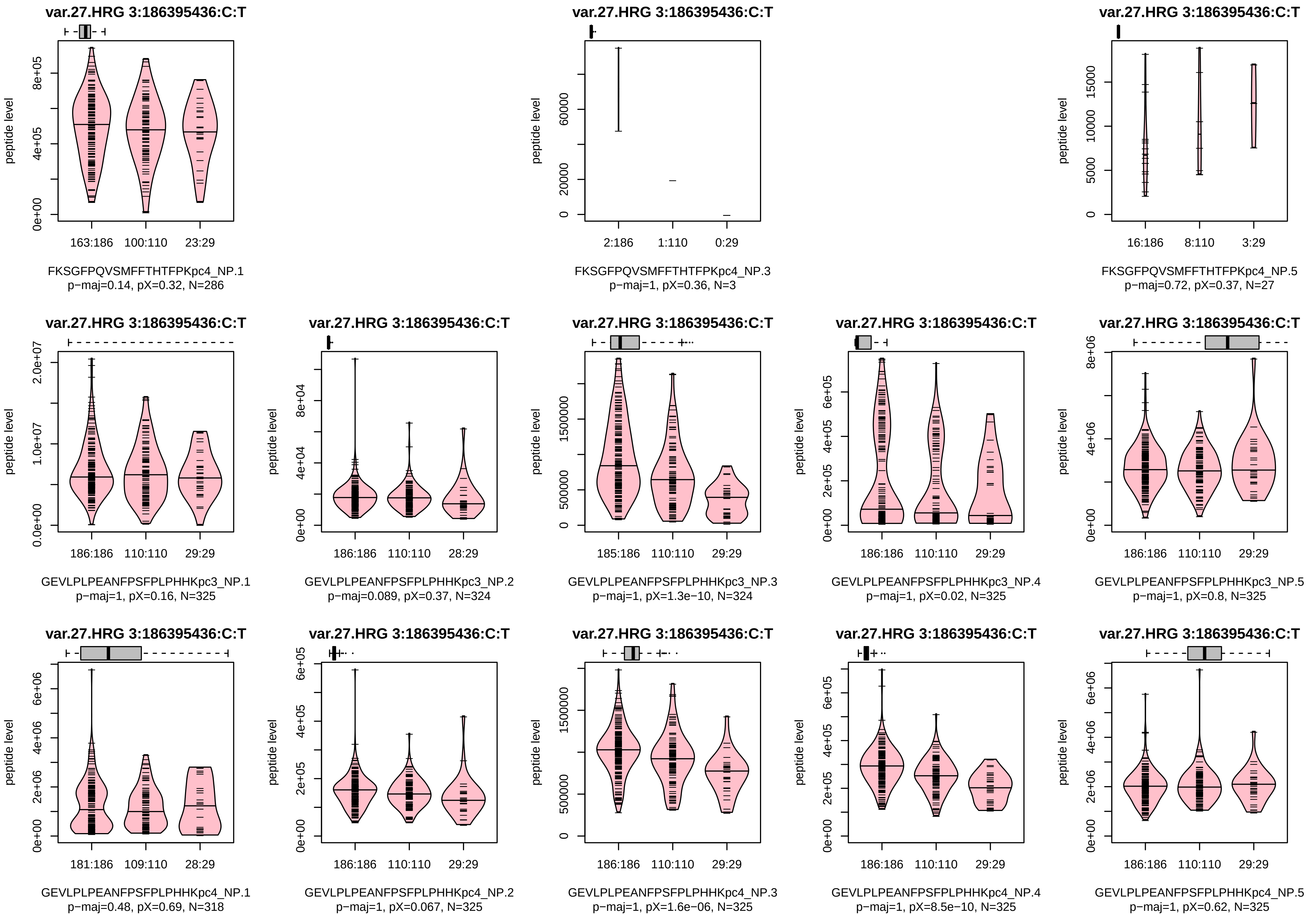
var.27.HRG 3:186395436:C:T
peptide level
0e+00
4e+05
8e+05
163:186
100:110
23:29
FKSGFPQVSMFFTHTFPKpc4_NP.1
p−maj=0.14, pX=0.32, N=286
var.27.HRG 3:186395436:C:T
peptide level
0
20000
60000
2:186
1:110
0:29
FKSGFPQVSMFFTHTFPKpc4_NP.3
p−maj=1, pX=0.36, N=3
var.27.HRG 3:186395436:C:T
peptide level
0
5000
10000
15000
16:186
8:110
3:29
FKSGFPQVSMFFTHTFPKpc4_NP.5
p−maj=0.72, pX=0.37, N=27
var.27.HRG 3:186395436:C:T
peptide level
0.0e+00
1.0e+07
2.0e+07
186:186
110:110
29:29
GEVLPLPEANFPSFPLPHHKpc3_NP.1
p−maj=1, pX=0.16, N=325
var.27.HRG 3:186395436:C:T
peptide level
0e+00
4e+04
8e+04
186:186
110:110
28:29
GEVLPLPEANFPSFPLPHHKpc3_NP.2
p−maj=0.089, pX=0.37, N=324
var.27.HRG 3:186395436:C:T
peptide level
0
500000
1500000
185:186
110:110
29:29
GEVLPLPEANFPSFPLPHHKpc3_NP.3
p−maj=1, pX=1.3e−10, N=324
var.27.HRG 3:186395436:C:T
peptide level
0e+00
2e+05
4e+05
6e+05
186:186
110:110
29:29
GEVLPLPEANFPSFPLPHHKpc3_NP.4
p−maj=1, pX=0.02, N=325
var.27.HRG 3:186395436:C:T
peptide level
0e+00
4e+06
8e+06
186:186
110:110
29:29
GEVLPLPEANFPSFPLPHHKpc3_NP.5
p−maj=1, pX=0.8, N=325
var.27.HRG 3:186395436:C:T
peptide level
0e+00
2e+06
4e+06
6e+06
181:186
109:110
28:29
GEVLPLPEANFPSFPLPHHKpc4_NP.1
p−maj=0.48, pX=0.69, N=318
var.27.HRG 3:186395436:C:T
peptide level
0e+00
2e+05
4e+05
6e+05
186:186
110:110
29:29
GEVLPLPEANFPSFPLPHHKpc4_NP.2
p−maj=1, pX=0.067, N=325
var.27.HRG 3:186395436:C:T
peptide level
0
500000
1500000
186:186
110:110
29:29
GEVLPLPEANFPSFPLPHHKpc4_NP.3
p−maj=1, pX=1.6e−06, N=325
var.27.HRG 3:186395436:C:T
peptide level
0e+00
2e+05
4e+05
6e+05
186:186
110:110
29:29
GEVLPLPEANFPSFPLPHHKpc4_NP.4
p−maj=1, pX=8.5e−10, N=325
var.27.HRG 3:186395436:C:T
peptide level
0e+00
2e+06
4e+06
6e+06
186:186
110:110
29:29
GEVLPLPEANFPSFPLPHHKpc4_NP.5
p−maj=1, pX=0.62, N=325

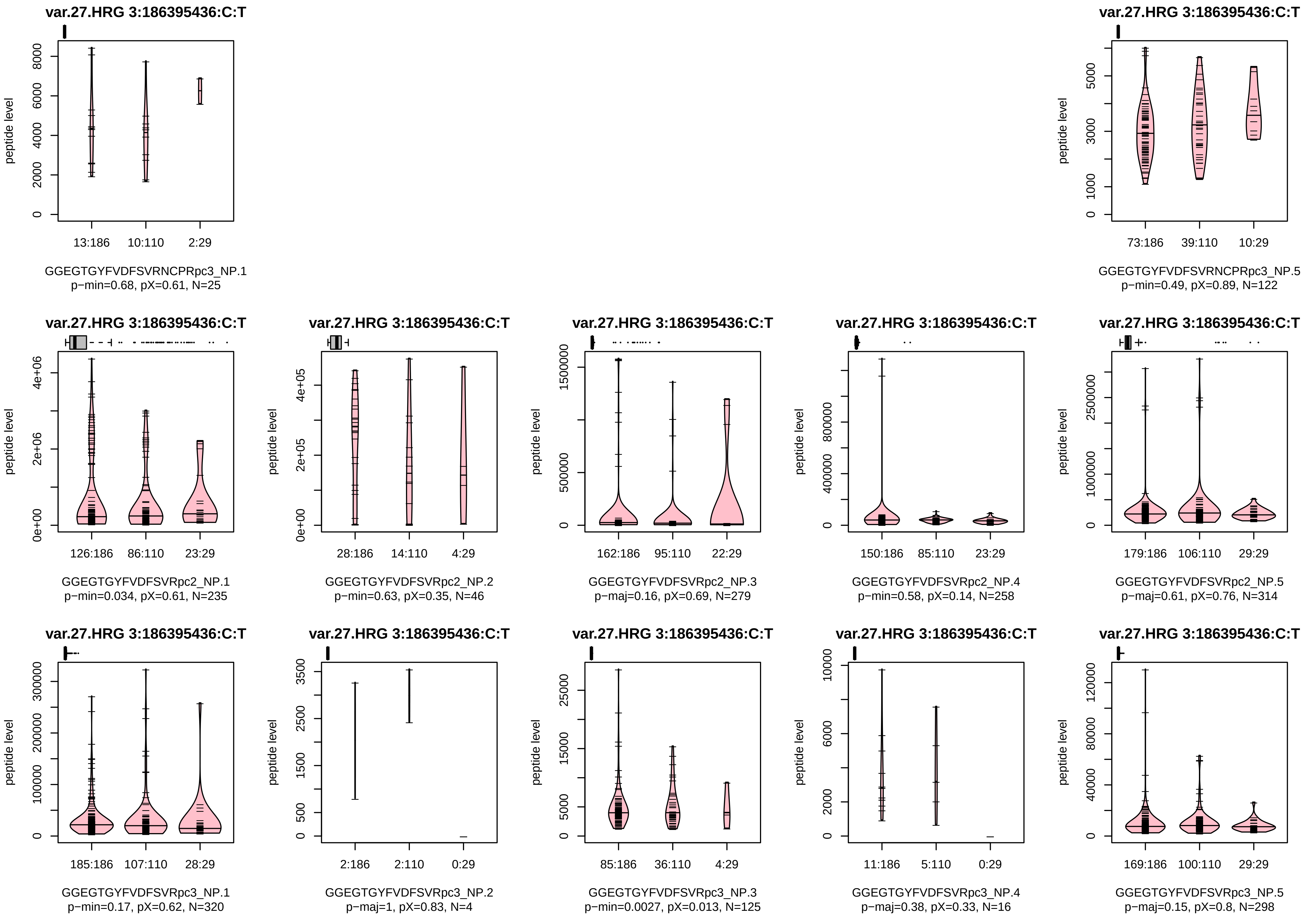
var.27.HRG 3:186395436:C:T
peptide level
13:186
10:110
2:29
GGEGTGYFVDFSVRNCPRpc3_NP.1
p−min=0.68, pX=0.61, N=25
var.27.HRG 3:186395436:C:T
peptide level
73:186
39:110
10:29
GGEGTGYFVDFSVRNCPRpc3_NP.5
p−min=0.49, pX=0.89, N=122
var.27.HRG 3:186395436:C:T
peptide level
126:186
86:110
23:29
GGEGTGYFVDFSVRpc2_NP.1
p−min=0.034, pX=0.61, N=235
var.27.HRG 3:186395436:C:T
peptide level
28:186
14:110
4:29
GGEGTGYFVDFSVRpc2_NP.2
p−min=0.63, pX=0.35, N=46
var.27.HRG 3:186395436:C:T
peptide level
162:186
95:110
22:29
GGEGTGYFVDFSVRpc2_NP.3
p−maj=0.16, pX=0.69, N=279
var.27.HRG 3:186395436:C:T
peptide level
150:186
85:110
23:29
GGEGTGYFVDFSVRpc2_NP.4
p−min=0.58, pX=0.14, N=258
var.27.HRG 3:186395436:C:T
peptide level
179:186
106:110
29:29
GGEGTGYFVDFSVRpc2_NP.5
p−maj=0.61, pX=0.76, N=314
var.27.HRG 3:186395436:C:T
peptide level
185:186
107:110
28:29
GGEGTGYFVDFSVRpc3_NP.1
p−min=0.17, pX=0.62, N=320
var.27.HRG 3:186395436:C:T
peptide level
2:186
2:110
0:29
GGEGTGYFVDFSVRpc3_NP.2
p−maj=1, pX=0.83, N=4
var.27.HRG 3:186395436:C:T
peptide level
85:186
36:110
4:29
GGEGTGYFVDFSVRpc3_NP.3
p−min=0.0027, pX=0.013, N=125
var.27.HRG 3:186395436:C:T
peptide level
11:186
5:110
0:29
GGEGTGYFVDFSVRpc3_NP.4
p−maj=0.38, pX=0.33, N=16
var.27.HRG 3:186395436:C:T
peptide level
169:186
100:110
29:29
GGEGTGYFVDFSVRpc3_NP.5
p−maj=0.15, pX=0.8, N=298

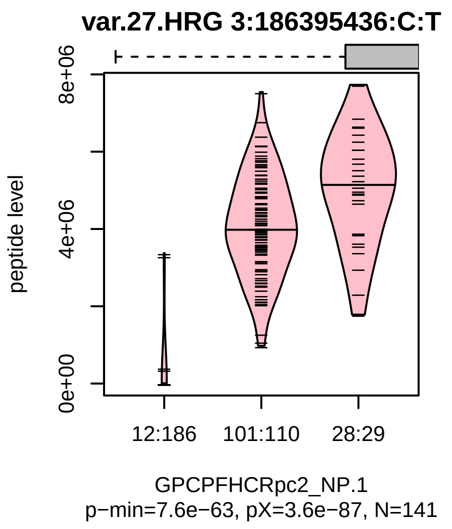
var.27.HRG 3:186395436:C:T
peptide level
0e+00
4e+06
8e+06
12:186
101:110
28:29
GPCPFHCRpc2_NP.1
p−min=7.6e−63, pX=3.6e−87, N=141

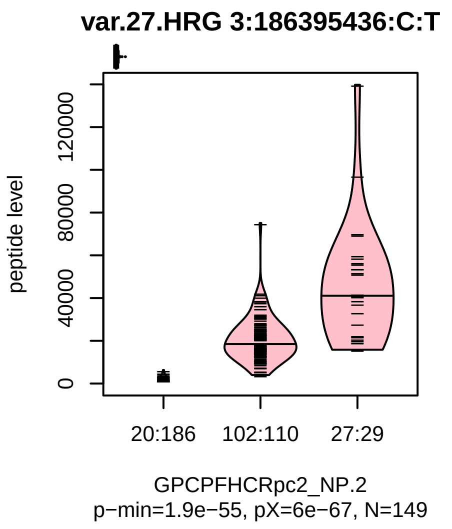
var.27.HRG 3:186395436:C:T
peptide level
0
40000
80000
120000
20:186
102:110
27:29
GPCPFHCRpc2_NP.2
p−min=1.9e−55, pX=6e−67, N=149

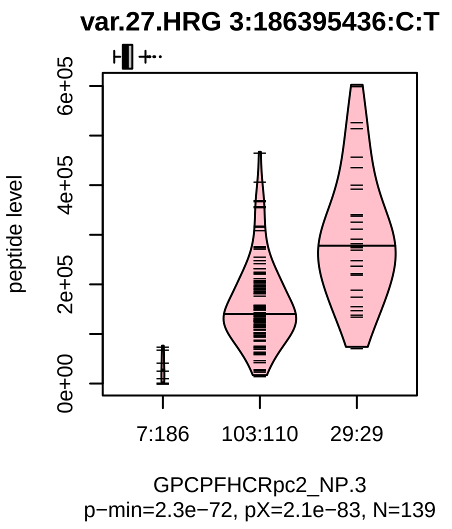
var.27.HRG 3:186395436:C:T
peptide level
0e+00
2e+05
4e+05
6e+05
7:186
103:110
29:29
GPCPFHCRpc2_NP.3
p−min=2.3e−72, pX=2.1e−83, N=139

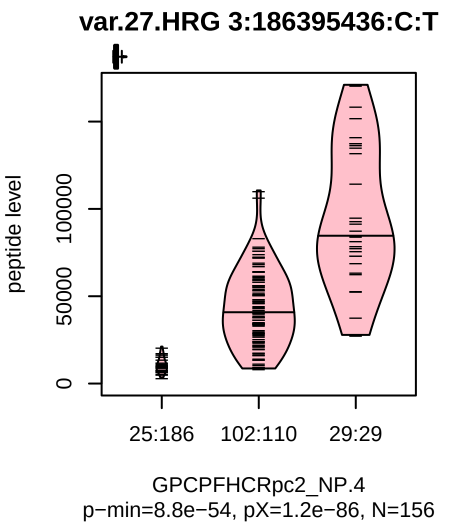
var.27.HRG 3:186395436:C:T
peptide level
0
50000
100000
25:186
102:110
29:29
GPCPFHCRpc2_NP.4
p−min=8.8e−54, pX=1.2e−86, N=156

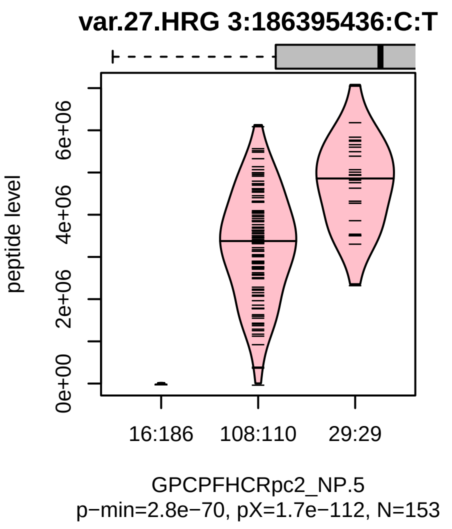
var.27.HRG 3:186395436:C:T
peptide level
0e+00
2e+06
4e+06
6e+06
16:186
108:110
29:29
GPCPFHCRpc2_NP.5
p−min=2.8e−70, pX=1.7e−112, N=153

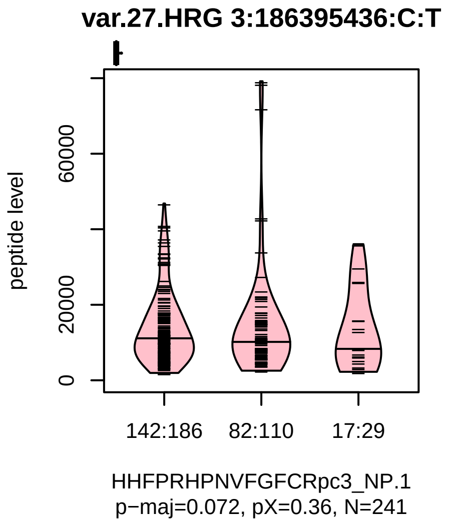
var.27.HRG 3:186395436:C:T
peptide level
0
20000
40000
60000
142:186
82:110
17:29
HHFPRHPNVFGFCRpc3_NP.1
p−maj=0.072, pX=0.36, N=241

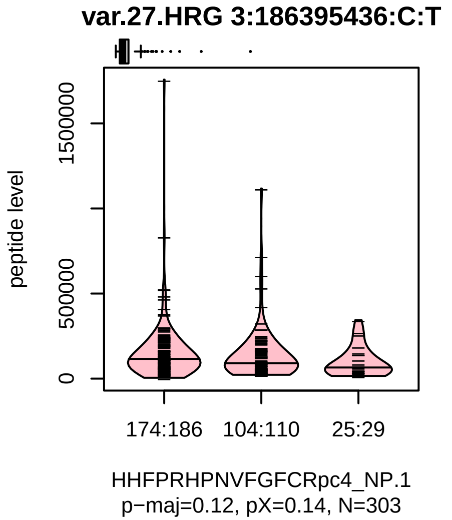
var.27.HRG 3:186395436:C:T
peptide level
0
500000
1000000
1500000
174:186
104:110
25:29
HHFPRHPNVFGFCRpc4_NP.1
p−maj=0.12, pX=0.14, N=303

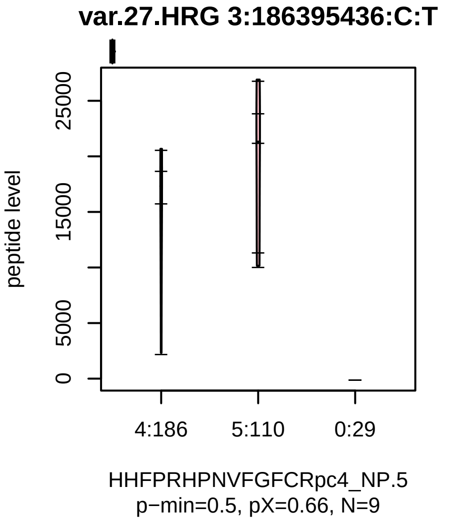
var.27.HRG 3:186395436:C:T
peptide level
0
5000
15000
25000
4:186
5:110
0:29
HHFPRHPNVFGFCRpc4_NP.5
p−min=0.5, pX=0.66, N=9

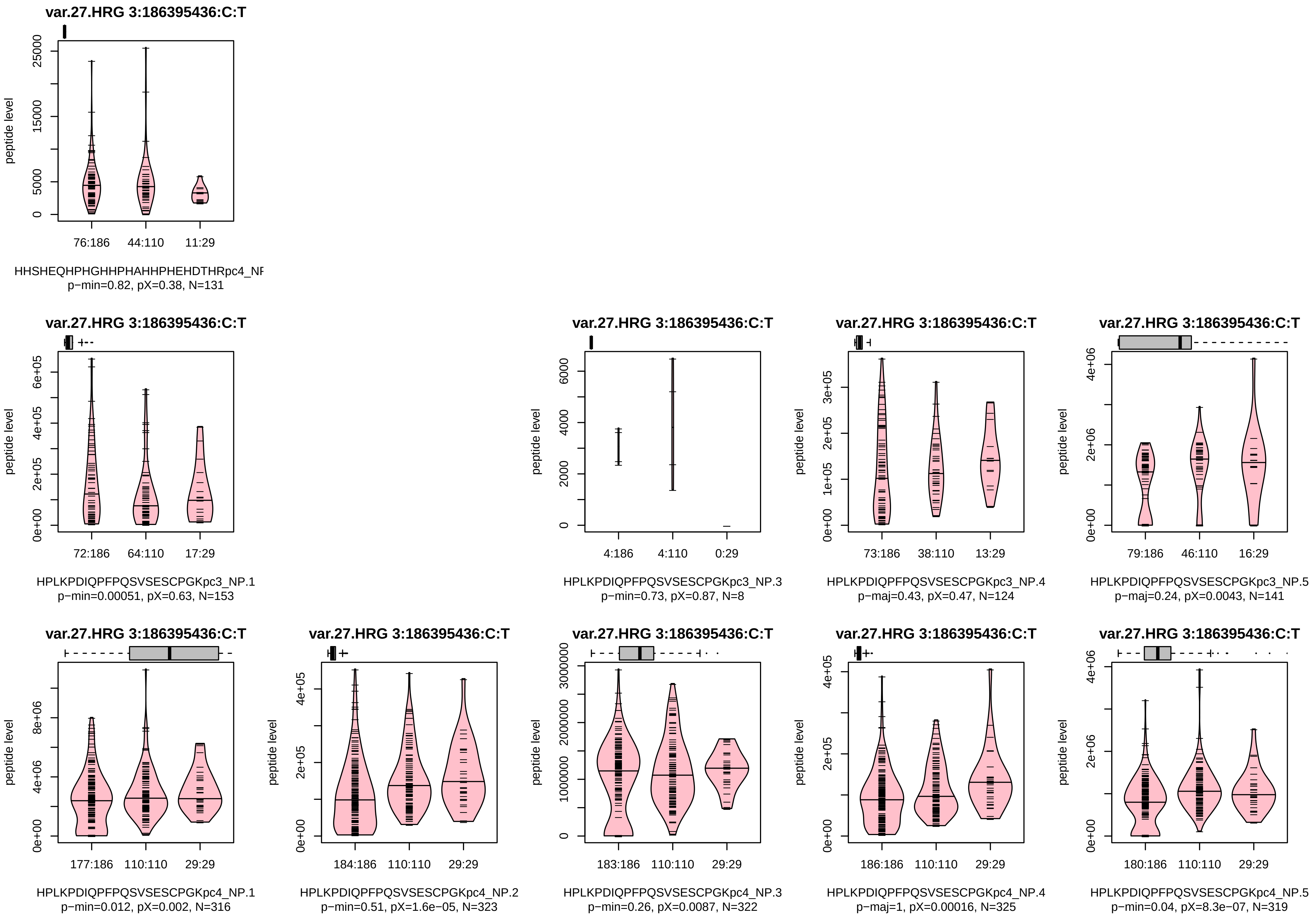
var.27.HRG 3:186395436:C:T
peptide level
76:186
44:110
11:29
HHSHEQHPHGHHPHAHHPHEHDTHRpc4_NF
p−min=0.82, pX=0.38, N=131
var.27.HRG 3:186395436:C:T
peptide level
72:186
64:110
17:29
HPLKPDIQPFPQSVSESCPGKpc3_NP.1
p−min=0.00051, pX=0.63, N=153
var.27.HRG 3:186395436:C:T
peptide level
4:186
4:110
0:29
HPLKPDIQPFPQSVSESCPGKpc3_NP.3
p−min=0.73, pX=0.87, N=8
var.27.HRG 3:186395436:C:T
peptide level
73:186
38:110
13:29
HPLKPDIQPFPQSVSESCPGKpc3_NP.4
p−maj=0.43, pX=0.47, N=124
var.27.HRG 3:186395436:C:T
peptide level
79:186
46:110
16:29
HPLKPDIQPFPQSVSESCPGKpc3_NP.5
p−maj=0.24, pX=0.0043, N=141
var.27.HRG 3:186395436:C:T
peptide level
177:186
110:110
29:29
HPLKPDIQPFPQSVSESCPGKpc4_NP.1
p−min=0.012, pX=0.002, N=316
var.27.HRG 3:186395436:C:T
peptide level
184:186
110:110
29:29
HPLKPDIQPFPQSVSESCPGKpc4_NP.2
p−min=0.51, pX=1.6e−05, N=323
var.27.HRG 3:186395436:C:T
peptide level
183:186
110:110
29:29
HPLKPDIQPFPQSVSESCPGKpc4_NP.3
p−min=0.26, pX=0.0087, N=322
var.27.HRG 3:186395436:C:T
peptide level
186:186
110:110
29:29
HPLKPDIQPFPQSVSESCPGKpc4_NP.4
p−maj=1, pX=0.00016, N=325
var.27.HRG 3:186395436:C:T
peptide level
180:186
110:110
29:29
HPLKPDIQPFPQSVSESCPGKpc4_NP.5
p−min=0.04, pX=8.3e−07, N=319

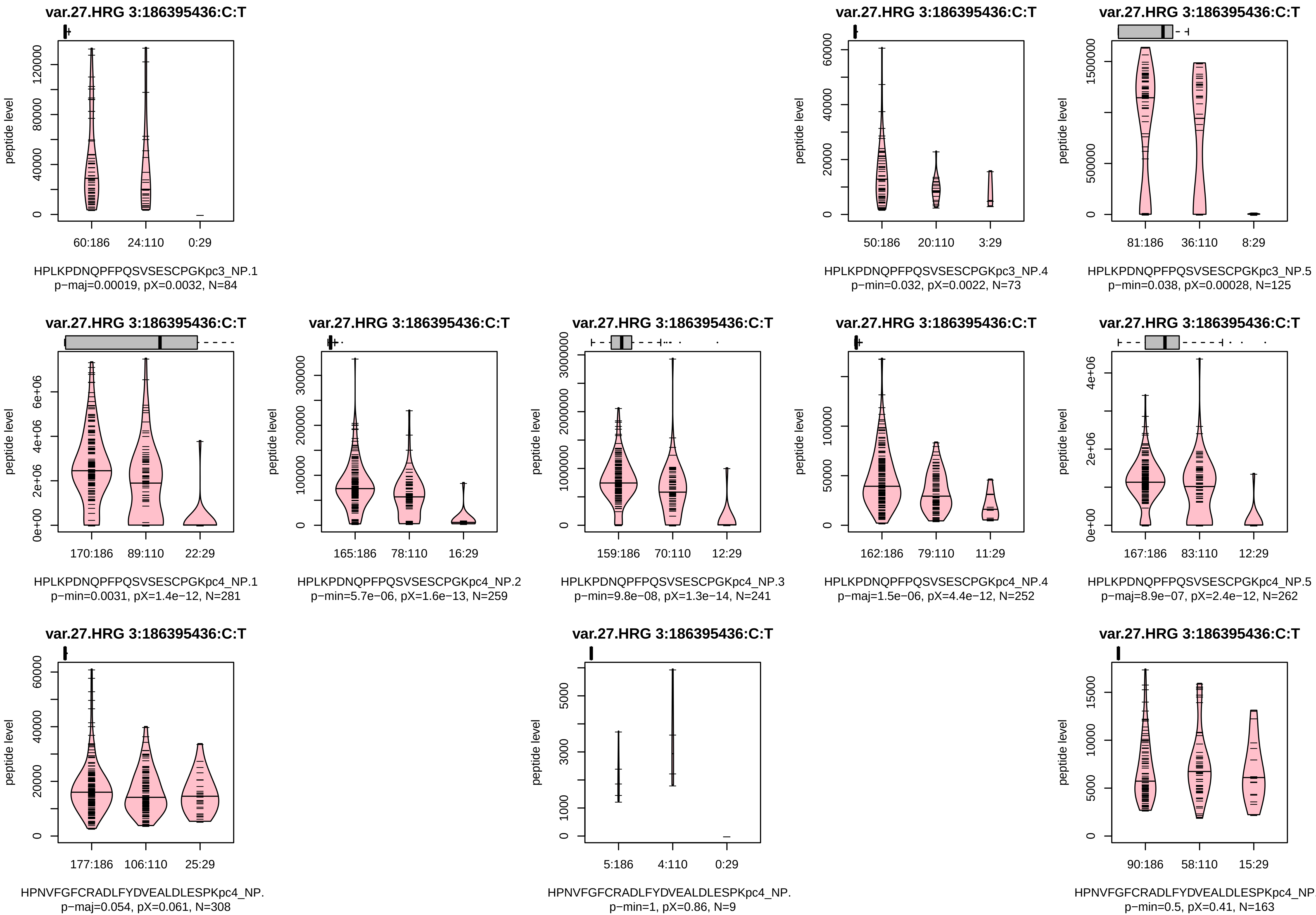
var.27.HRG 3:186395436:C:T
peptide level
0
40000
80000
120000
60:186
24:110
0:29
HPLKPDNQPFPQSVSESCPGKpc3_NP.1
p−maj=0.00019, pX=0.0032, N=84
var.27.HRG 3:186395436:C:T
peptide level
0
20000
40000
60000
50:186
20:110
3:29
HPLKPDNQPFPQSVSESCPGKpc3_NP.4
p−min=0.032, pX=0.0022, N=73
var.27.HRG 3:186395436:C:T
peptide level
0
500000
1500000
81:186
36:110
8:29
HPLKPDNQPFPQSVSESCPGKpc3_NP.5
p−min=0.038, pX=0.00028, N=125
var.27.HRG 3:186395436:C:T
peptide level
0e+00
2e+06
4e+06
6e+06
170:186
89:110
22:29
HPLKPDNQPFPQSVSESCPGKpc4_NP.1
p−min=0.0031, pX=1.4e−12, N=281
var.27.HRG 3:186395436:C:T
peptide level
0
100000
200000
300000
165:186
78:110
16:29
HPLKPDNQPFPQSVSESCPGKpc4_NP.2
p−min=5.7e−06, pX=1.6e−13, N=259
var.27.HRG 3:186395436:C:T
peptide level
0
1000000
2000000
3000000
159:186
70:110
12:29
HPLKPDNQPFPQSVSESCPGKpc4_NP.3
p−min=9.8e−08, pX=1.3e−14, N=241
var.27.HRG 3:186395436:C:T
peptide level
0
50000
100000
162:186
79:110
11:29
HPLKPDNQPFPQSVSESCPGKpc4_NP.4
p−maj=1.5e−06, pX=4.4e−12, N=252
var.27.HRG 3:186395436:C:T
peptide level
0e+00
2e+06
4e+06
167:186
83:110
12:29
HPLKPDNQPFPQSVSESCPGKpc4_NP.5
p−maj=8.9e−07, pX=2.4e−12, N=262
var.27.HRG 3:186395436:C:T
peptide level
0
20000
40000
60000
177:186
106:110
25:29
HPNVFGFCRADLFYDVEALDLESPKpc4_NP.
p−maj=0.054, pX=0.061, N=308
var.27.HRG 3:186395436:C:T
peptide level
0
1000
3000
5000
5:186
4:110
0:29
HPNVFGFCRADLFYDVEALDLESPKpc4_NP.
p−min=1, pX=0.86, N=9
var.27.HRG 3:186395436:C:T
peptide level
0
5000
10000
15000
90:186
58:110
15:29
HPNVFGFCRADLFYDVEALDLESPKpc4_NP.
p−min=0.5, pX=0.41, N=163

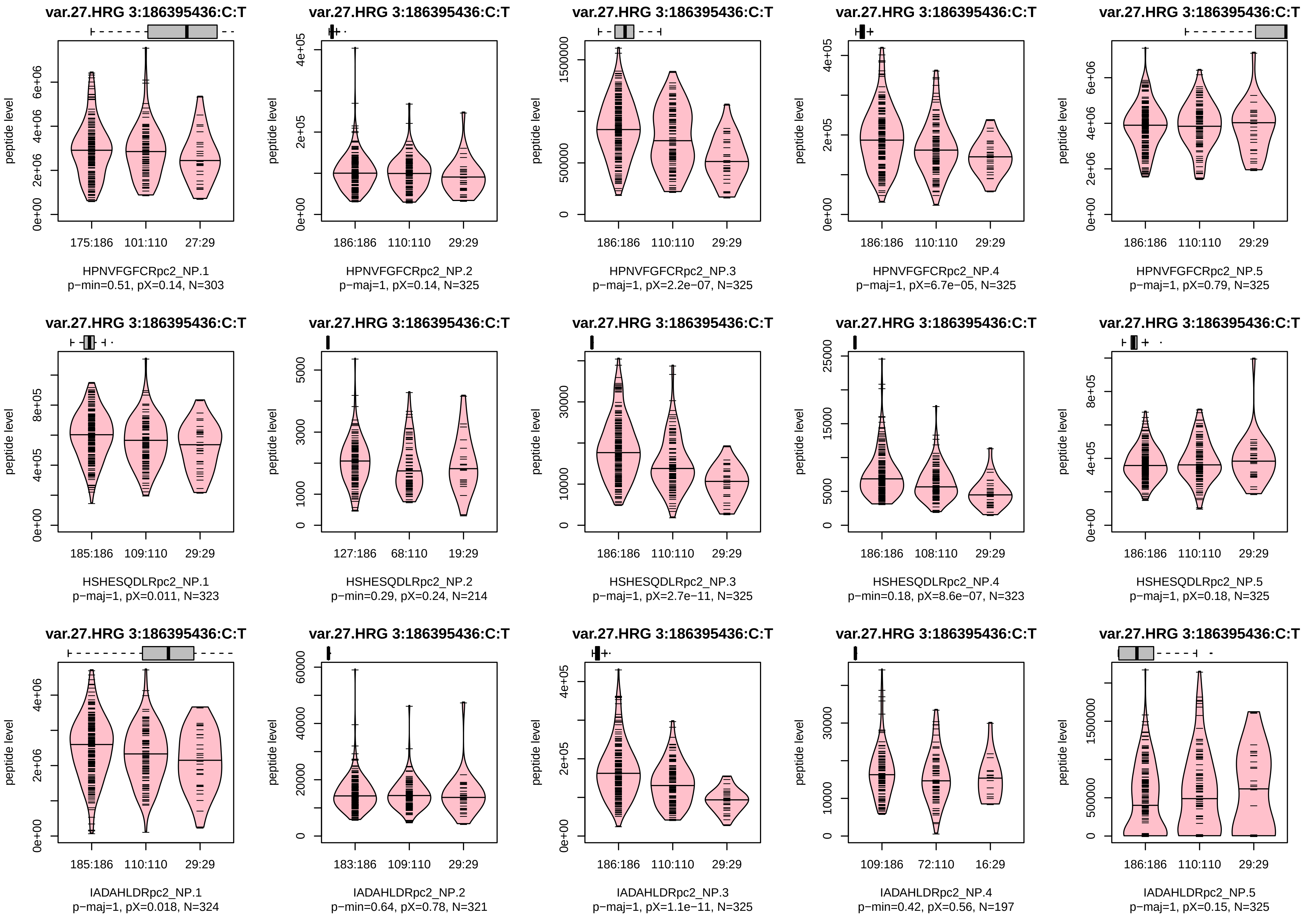
var.27.HRG 3:186395436:C:T
peptide level
175:186
101:110
27:29
HPNVFGFCRpc2_NP.1
p−min=0.51, pX=0.14, N=303
var.27.HRG 3:186395436:C:T
peptide level
186:186
110:110
29:29
HPNVFGFCRpc2_NP.2
p−maj=1, pX=0.14, N=325
var.27.HRG 3:186395436:C:T
peptide level
186:186
110:110
29:29
HPNVFGFCRpc2_NP.3
p−maj=1, pX=2.2e−07, N=325
var.27.HRG 3:186395436:C:T
peptide level
186:186
110:110
29:29
HPNVFGFCRpc2_NP.4
p−maj=1, pX=6.7e−05, N=325
var.27.HRG 3:186395436:C:T
peptide level
186:186
110:110
29:29
HPNVFGFCRpc2_NP.5
p−maj=1, pX=0.79, N=325
var.27.HRG 3:186395436:C:T
peptide level
185:186
109:110
29:29
HSHESQDLRpc2_NP.1
p−maj=1, pX=0.011, N=323
var.27.HRG 3:186395436:C:T
peptide level
127:186
68:110
19:29
HSHESQDLRpc2_NP.2
p−min=0.29, pX=0.24, N=214
var.27.HRG 3:186395436:C:T
peptide level
186:186
110:110
29:29
HSHESQDLRpc2_NP.3
p−maj=1, pX=2.7e−11, N=325
var.27.HRG 3:186395436:C:T
peptide level
186:186
108:110
29:29
HSHESQDLRpc2_NP.4
p−min=0.18, pX=8.6e−07, N=323
var.27.HRG 3:186395436:C:T
peptide level
186:186
110:110
29:29
HSHESQDLRpc2_NP.5
p−maj=1, pX=0.18, N=325
var.27.HRG 3:186395436:C:T
peptide level
185:186
110:110
29:29
IADAHLDRpc2_NP.1
p−maj=1, pX=0.018, N=324
var.27.HRG 3:186395436:C:T
peptide level
183:186
109:110
29:29
IADAHLDRpc2_NP.2
p−min=0.64, pX=0.78, N=321
var.27.HRG 3:186395436:C:T
peptide level
186:186
110:110
29:29
IADAHLDRpc2_NP.3
p−maj=1, pX=1.1e−11, N=325
var.27.HRG 3:186395436:C:T
peptide level
109:186
72:110
16:29
IADAHLDRpc2_NP.4
p−min=0.42, pX=0.56, N=197
var.27.HRG 3:186395436:C:T
peptide level
186:186
110:110
29:29
IADAHLDRpc2_NP.5
p−maj=1, pX=0.15, N=325

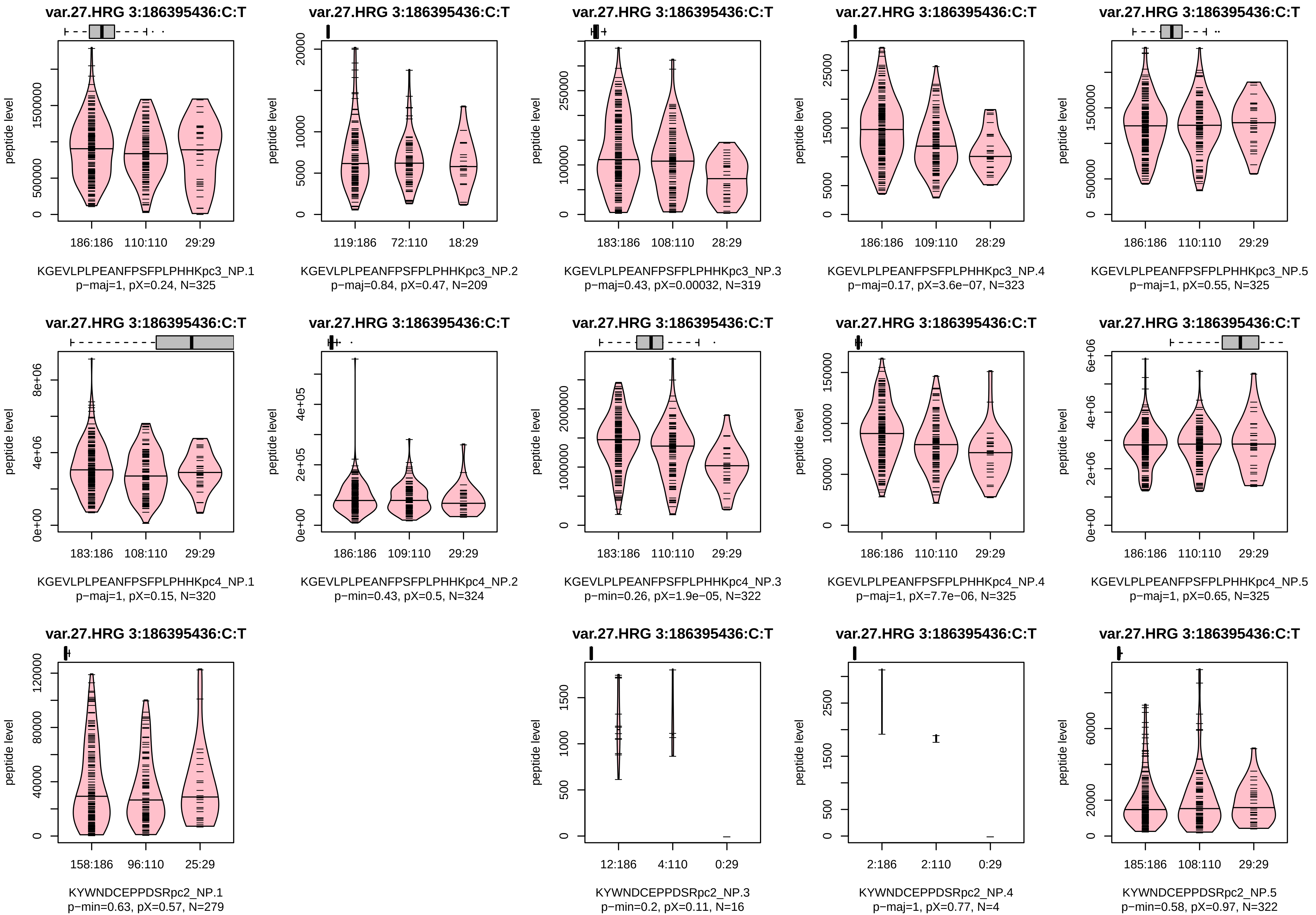
var.27.HRG 3:186395436:C:T
peptide level
186:186
110:110
29:29
KGEVLPLPEANFPSFPLPHHKpc3_NP.1
p−maj=1, pX=0.24, N=325
var.27.HRG 3:186395436:C:T
peptide level
119:186
72:110
18:29
KGEVLPLPEANFPSFPLPHHKpc3_NP.2
p−maj=0.84, pX=0.47, N=209
var.27.HRG 3:186395436:C:T
peptide level
183:186
108:110
28:29
KGEVLPLPEANFPSFPLPHHKpc3_NP.3
p−maj=0.43, pX=0.00032, N=319
var.27.HRG 3:186395436:C:T
peptide level
186:186
109:110
28:29
KGEVLPLPEANFPSFPLPHHKpc3_NP.4
p−maj=0.17, pX=3.6e−07, N=323
var.27.HRG 3:186395436:C:T
peptide level
186:186
110:110
29:29
KGEVLPLPEANFPSFPLPHHKpc3_NP.5
p−maj=1, pX=0.55, N=325
var.27.HRG 3:186395436:C:T
peptide level
183:186
108:110
29:29
KGEVLPLPEANFPSFPLPHHKpc4_NP.1
p−maj=1, pX=0.15, N=320
var.27.HRG 3:186395436:C:T
peptide level
186:186
109:110
29:29
KGEVLPLPEANFPSFPLPHHKpc4_NP.2
p−min=0.43, pX=0.5, N=324
var.27.HRG 3:186395436:C:T
peptide level
183:186
110:110
29:29
KGEVLPLPEANFPSFPLPHHKpc4_NP.3
p−min=0.26, pX=1.9e−05, N=322
var.27.HRG 3:186395436:C:T
peptide level
186:186
110:110
29:29
KGEVLPLPEANFPSFPLPHHKpc4_NP.4
p−maj=1, pX=7.7e−06, N=325
var.27.HRG 3:186395436:C:T
peptide level
186:186
110:110
29:29
KGEVLPLPEANFPSFPLPHHKpc4_NP.5
p−maj=1, pX=0.65, N=325
var.27.HRG 3:186395436:C:T
peptide level
158:186
96:110
25:29
KYWNDCEPPDSRpc2_NP.1
p−min=0.63, pX=0.57, N=279
var.27.HRG 3:186395436:C:T
peptide level
12:186
4:110
0:29
KYWNDCEPPDSRpc2_NP.3
p−min=0.2, pX=0.11, N=16
var.27.HRG 3:186395436:C:T
peptide level
2:186
2:110
0:29
KYWNDCEPPDSRpc2_NP.4
p−maj=1, pX=0.77, N=4
var.27.HRG 3:186395436:C:T
peptide level
185:186
108:110
29:29
KYWNDCEPPDSRpc2_NP.5
p−min=0.58, pX=0.97, N=322

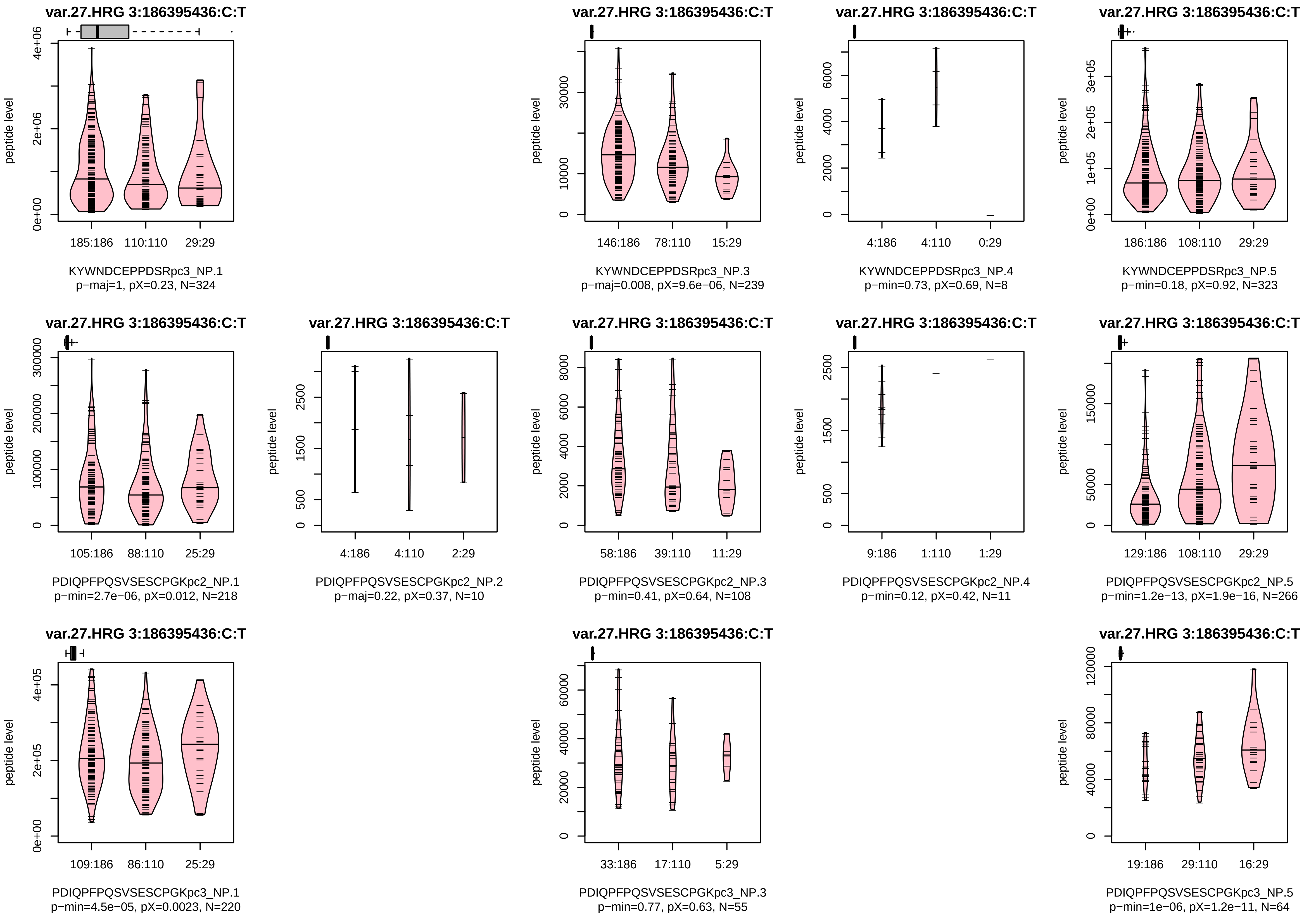
var.27.HRG 3:186395436:C:T
peptide level
185:186 110:110 29:29
KYWNDCEPPDSRpc3_NP.1
p−maj=1, pX=0.23, N=324
var.27.HRG 3:186395436:C:T
peptide level
146:186 78:110 15:29
KYWNDCEPPDSRpc3_NP.3
p−maj=0.008, pX=9.6e−06, N=239
var.27.HRG 3:186395436:C:T
peptide level
4:186 4:110 0:29
KYWNDCEPPDSRpc3_NP.4
p−min=0.73, pX=0.69, N=8
var.27.HRG 3:186395436:C:T
peptide level
186:186 108:110 29:29
KYWNDCEPPDSRpc3_NP.5
p−min=0.18, pX=0.92, N=323
var.27.HRG 3:186395436:C:T
peptide level
105:186 88:110 25:29
PDIQPFPQSVSESCPGKpc2_NP.1
p−min=2.7e−06, pX=0.012, N=218
var.27.HRG 3:186395436:C:T
peptide level
4:186 4:110 2:29
PDIQPFPQSVSESCPGKpc2_NP.2
p−maj=0.22, pX=0.37, N=10
var.27.HRG 3:186395436:C:T
peptide level
58:186 39:110 11:29
PDIQPFPQSVSESCPGKpc2_NP.3
p−min=0.41, pX=0.64, N=108
var.27.HRG 3:186395436:C:T
peptide level
9:186 1:110 1:29
PDIQPFPQSVSESCPGKpc2_NP.4
p−min=0.12, pX=0.42, N=11
var.27.HRG 3:186395436:C:T
peptide level
129:186 108:110 29:29
PDIQPFPQSVSESCPGKpc2_NP.5
p−min=1.2e−13, pX=1.9e−16, N=266
var.27.HRG 3:186395436:C:T
peptide level
109:186 86:110 25:29
PDIQPFPQSVSESCPGKpc3_NP.1
p−min=4.5e−05, pX=0.0023, N=220
var.27.HRG 3:186395436:C:T
peptide level
33:186 17:110 5:29
PDIQPFPQSVSESCPGKpc3_NP.3
p−min=0.77, pX=0.63, N=55
var.27.HRG 3:186395436:C:T
peptide level
19:186 29:110 16:29
PDIQPFPQSVSESCPGKpc3_NP.5
p−min=1e−06, pX=1.2e−11, N=64

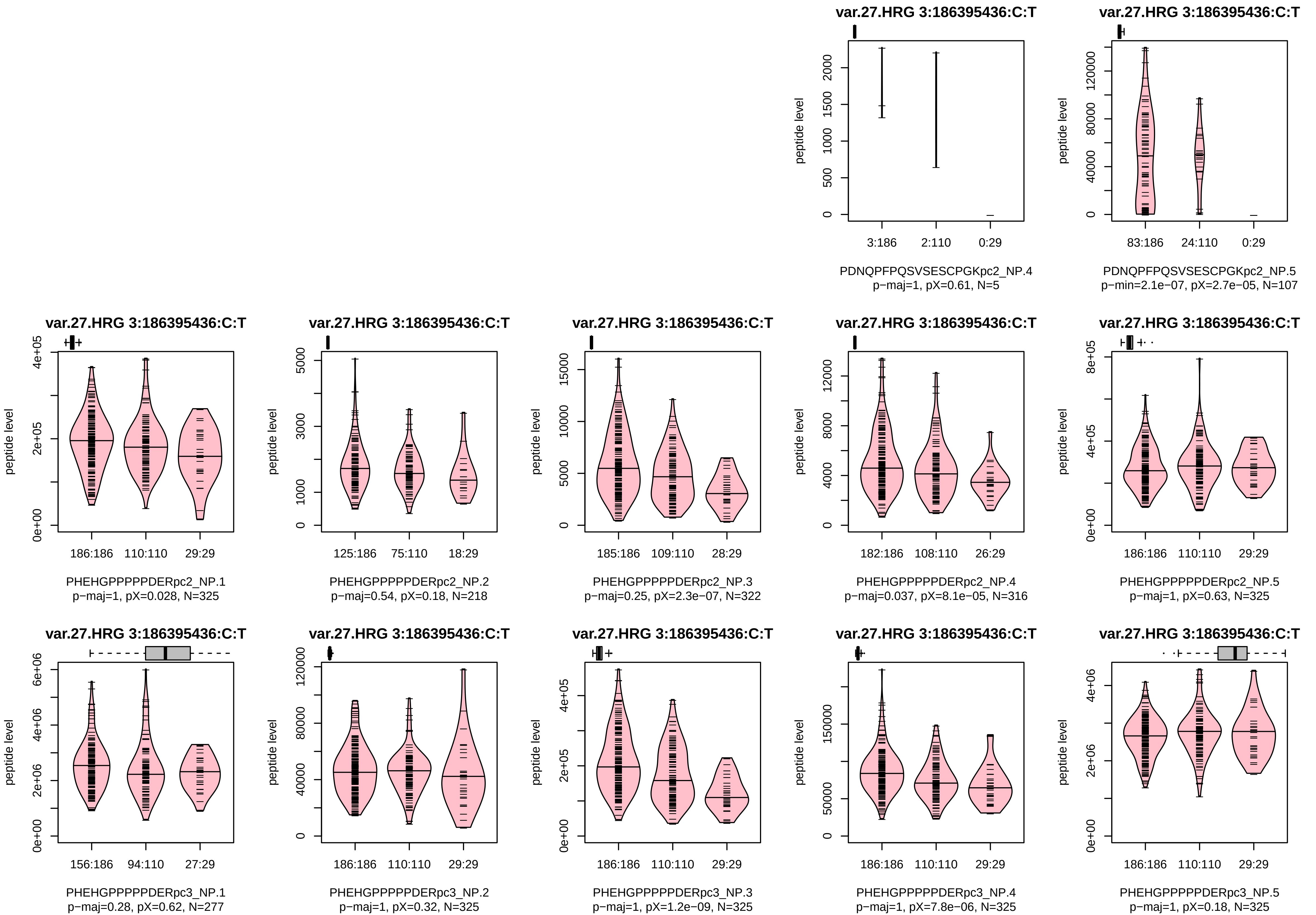
var.27.HRG 3:186395436:C:T
peptide level
3:186
2:110
0:29
PDNQPFPQSVSESCPGKpc2_NP.4
p−maj=1, pX=0.61, N=5
var.27.HRG 3:186395436:C:T
peptide level
83:186
24:110
0:29
PDNQPFPQSVSESCPGKpc2_NP.5
p−min=2.1e−07, pX=2.7e−05, N=107
var.27.HRG 3:186395436:C:T
peptide level
186:186
110:110
29:29
PHEHGPPPPPDERpc2_NP.1
p−maj=1, pX=0.028, N=325
var.27.HRG 3:186395436:C:T
peptide level
125:186
75:110
18:29
PHEHGPPPPPDERpc2_NP.2
p−maj=0.54, pX=0.18, N=218
var.27.HRG 3:186395436:C:T
peptide level
185:186
109:110
28:29
PHEHGPPPPPDERpc2_NP.3
p−maj=0.25, pX=2.3e−07, N=322
var.27.HRG 3:186395436:C:T
peptide level
182:186
108:110
26:29
PHEHGPPPPPDERpc2_NP.4
p−maj=0.037, pX=8.1e−05, N=316
var.27.HRG 3:186395436:C:T
peptide level
186:186
110:110
29:29
PHEHGPPPPPDERpc2_NP.5
p−maj=1, pX=0.63, N=325
var.27.HRG 3:186395436:C:T
peptide level
156:186
94:110
27:29
PHEHGPPPPPDERpc3_NP.1
p−maj=0.28, pX=0.62, N=277
var.27.HRG 3:186395436:C:T
peptide level
186:186
110:110
29:29
PHEHGPPPPPDERpc3_NP.2
p−maj=1, pX=0.32, N=325
var.27.HRG 3:186395436:C:T
peptide level
186:186
110:110
29:29
PHEHGPPPPPDERpc3_NP.3
p−maj=1, pX=1.2e−09, N=325
var.27.HRG 3:186395436:C:T
peptide level
186:186
110:110
29:29
PHEHGPPPPPDERpc3_NP.4
p−maj=1, pX=7.8e−06, N=325
var.27.HRG 3:186395436:C:T
peptide level
186:186
110:110
29:29
PHEHGPPPPPDERpc3_NP.5
p−maj=1, pX=0.18, N=325

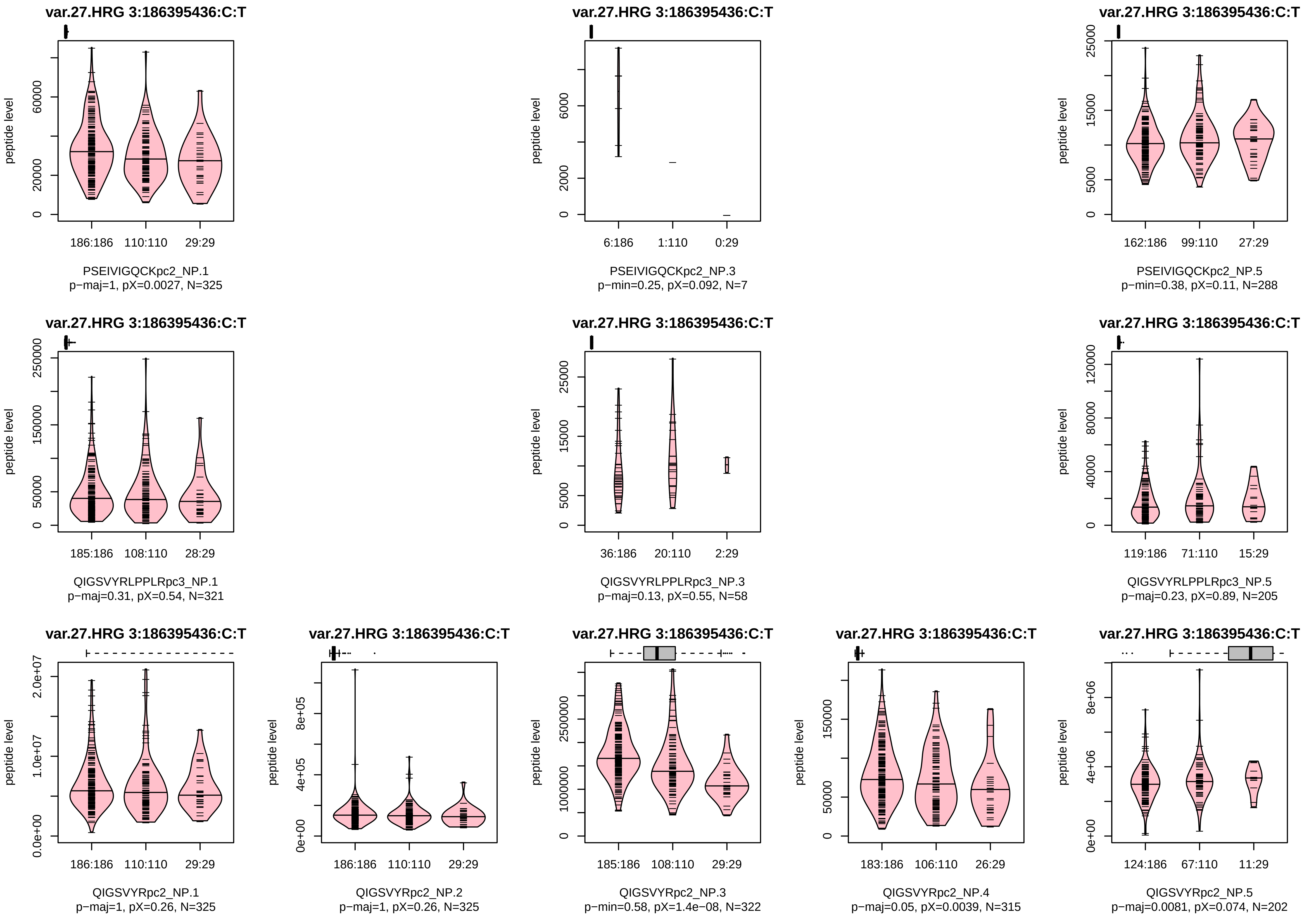
var.27.HRG 3:186395436:C:T
peptide level
186:186 110:110 29:29
PSEIVIGQCKpc2_NP.1
p−maj=1, pX=0.0027, N=325
var.27.HRG 3:186395436:C:T
peptide level
6:186 1:110 0:29
PSEIVIGQCKpc2_NP.3
p−min=0.25, pX=0.092, N=7
var.27.HRG 3:186395436:C:T
peptide level
162:186 99:110 27:29
PSEIVIGQCKpc2_NP.5
p−min=0.38, pX=0.11, N=288
var.27.HRG 3:186395436:C:T
peptide level
185:186 108:110 28:29
QIGSVYRLPPLRpc3_NP.1
p−maj=0.31, pX=0.54, N=321
var.27.HRG 3:186395436:C:T
peptide level
36:186 20:110 2:29
QIGSVYRLPPLRpc3_NP.3
p−maj=0.13, pX=0.55, N=58
var.27.HRG 3:186395436:C:T
peptide level
119:186 71:110 15:29
QIGSVYRLPPLRpc3_NP.5
p−maj=0.23, pX=0.89, N=205
var.27.HRG 3:186395436:C:T
peptide level
186:186 110:110 29:29
QIGSVYRpc2_NP.1
p−maj=1, pX=0.26, N=325
var.27.HRG 3:186395436:C:T
peptide level
186:186 110:110 29:29
QIGSVYRpc2_NP.2
p−maj=1, pX=0.26, N=325
var.27.HRG 3:186395436:C:T
peptide level
185:186 108:110 29:29
QIGSVYRpc2_NP.3
p−min=0.58, pX=1.4e−08, N=322
var.27.HRG 3:186395436:C:T
peptide level
183:186 106:110 26:29
QIGSVYRpc2_NP.4
p−maj=0.05, pX=0.0039, N=315
var.27.HRG 3:186395436:C:T
peptide level
124:186 67:110 11:29
QIGSVYRpc2_NP.5
p−maj=0.0081, pX=0.074, N=202

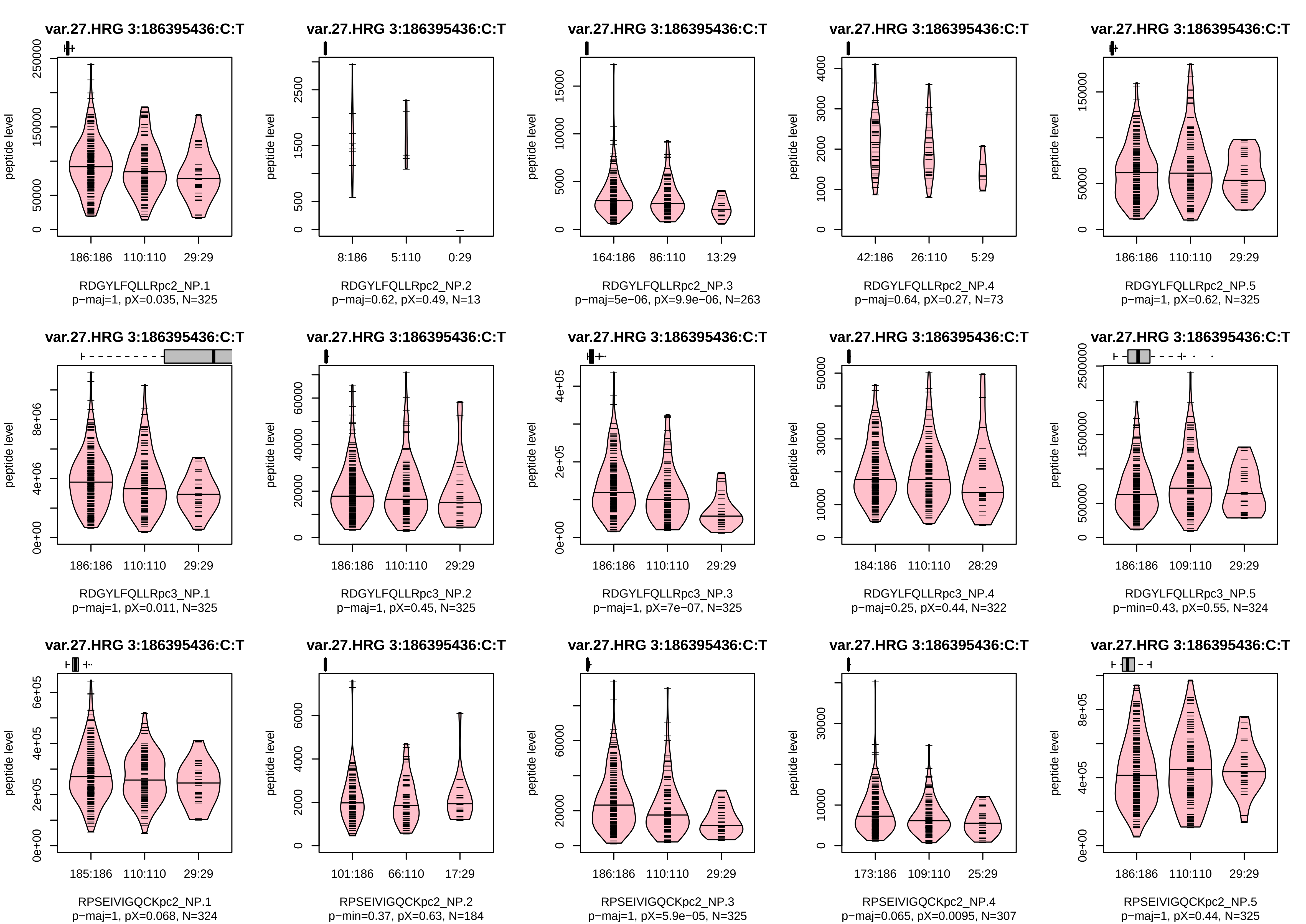
var.27.HRG 3:186395436:C:T
peptide level
186:186 110:110 29:29
RDGYLFQLLRpc2_NP.1
p−maj=1, pX=0.035, N=325
var.27.HRG 3:186395436:C:T
8:186 5:110 0:29
RDGYLFQLLRpc2_NP.2
p−maj=0.62, pX=0.49, N=13
var.27.HRG 3:186395436:C:T
164:186 86:110 13:29
RDGYLFQLLRpc2_NP.3
p−maj=5e−06, pX=9.9e−06, N=263
var.27.HRG 3:186395436:C:T
42:186 26:110 5:29
RDGYLFQLLRpc2_NP.4
p−maj=0.64, pX=0.27, N=73
var.27.HRG 3:186395436:C:T
186:186 110:110 29:29
RDGYLFQLLRpc2_NP.5
p−maj=1, pX=0.62, N=325
var.27.HRG 3:186395436:C:T
186:186 110:110 29:29
RDGYLFQLLRpc3_NP.1
p−maj=1, pX=0.011, N=325
var.27.HRG 3:186395436:C:T
186:186 110:110 29:29
RDGYLFQLLRpc3_NP.2
p−maj=1, pX=0.45, N=325
var.27.HRG 3:186395436:C:T
186:186 110:110 29:29
RDGYLFQLLRpc3_NP.3
p−maj=1, pX=7e−07, N=325
var.27.HRG 3:186395436:C:T
184:186 110:110 28:29
RDGYLFQLLRpc3_NP.4
p−maj=0.25, pX=0.44, N=322
var.27.HRG 3:186395436:C:T
186:186 109:110 29:29
RDGYLFQLLRpc3_NP.5
p−min=0.43, pX=0.55, N=324
var.27.HRG 3:186395436:C:T
185:186 110:110 29:29
RPSEIVIGQCKpc2_NP.1
p−maj=1, pX=0.068, N=324
var.27.HRG 3:186395436:C:T
101:186 66:110 17:29
RPSEIVIGQCKpc2_NP.2
p−min=0.37, pX=0.63, N=184
var.27.HRG 3:186395436:C:T
186:186 110:110 29:29
RPSEIVIGQCKpc2_NP.3
p−maj=1, pX=5.9e−05, N=325
var.27.HRG 3:186395436:C:T
173:186 109:110 25:29
RPSEIVIGQCKpc2_NP.4
p−maj=0.065, pX=0.0095, N=307
var.27.HRG 3:186395436:C:T
186:186 110:110 29:29
RPSEIVIGQCKpc2_NP.5
p−maj=1, pX=0.44, N=325

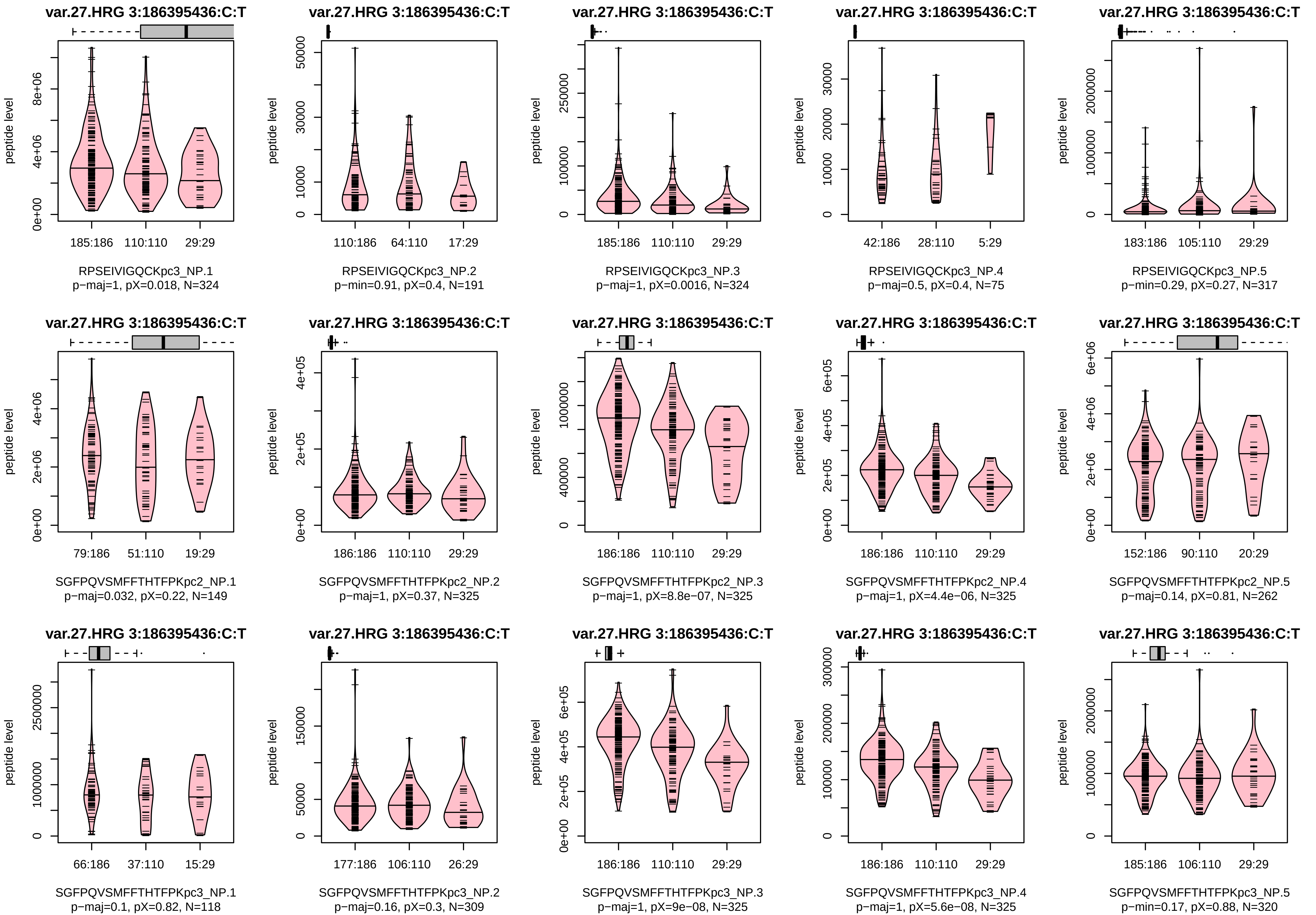
var.27.HRG 3:186395436:C:T
peptide level
0e+00
4e+06
8e+06
185:186
110:110
29:29
RPSEIVIGQCKpc3_NP.1
p−maj=1, pX=0.018, N=324
var.27.HRG 3:186395436:C:T
peptide level
0
10000
30000
50000
110:186
64:110
17:29
RPSEIVIGQCKpc3_NP.2
p−min=0.91, pX=0.4, N=191
var.27.HRG 3:186395436:C:T
peptide level
0
100000
250000
185:186
110:110
29:29
RPSEIVIGQCKpc3_NP.3
p−maj=1, pX=0.0016, N=324
var.27.HRG 3:186395436:C:T
peptide level
0
10000
20000
30000
42:186
28:110
5:29
RPSEIVIGQCKpc3_NP.4
p−maj=0.5, pX=0.4, N=75
var.27.HRG 3:186395436:C:T
peptide level
0
1000000
2000000
183:186
105:110
29:29
RPSEIVIGQCKpc3_NP.5
p−min=0.29, pX=0.27, N=317
var.27.HRG 3:186395436:C:T
peptide level
0e+00
2e+06
4e+06
79:186
51:110
19:29
SGFPQVSMFFTHTFPKpc2_NP.1
p−maj=0.032, pX=0.22, N=149
var.27.HRG 3:186395436:C:T
peptide level
0e+00
2e+05
4e+05
186:186
110:110
29:29
SGFPQVSMFFTHTFPKpc2_NP.2
p−maj=1, pX=0.37, N=325
var.27.HRG 3:186395436:C:T
peptide level
0
400000
1000000
186:186
110:110
29:29
SGFPQVSMFFTHTFPKpc2_NP.3
p−maj=1, pX=8.8e−07, N=325
var.27.HRG 3:186395436:C:T
peptide level
0e+00
2e+05
4e+05
6e+05
186:186
110:110
29:29
SGFPQVSMFFTHTFPKpc2_NP.4
p−maj=1, pX=4.4e−06, N=325
var.27.HRG 3:186395436:C:T
peptide level
0e+00
2e+06
4e+06
6e+06
152:186
90:110
20:29
SGFPQVSMFFTHTFPKpc2_NP.5
p−maj=0.14, pX=0.81, N=262
var.27.HRG 3:186395436:C:T
peptide level
0
1000000
2500000
66:186
37:110
15:29
SGFPQVSMFFTHTFPKpc3_NP.1
p−maj=0.1, pX=0.82, N=118
var.27.HRG 3:186395436:C:T
peptide level
0
50000
150000
177:186
106:110
26:29
SGFPQVSMFFTHTFPKpc3_NP.2
p−maj=0.16, pX=0.3, N=309
var.27.HRG 3:186395436:C:T
peptide level
0e+00
2e+05
4e+05
6e+05
186:186
110:110
29:29
SGFPQVSMFFTHTFPKpc3_NP.3
p−maj=1, pX=9e−08, N=325
var.27.HRG 3:186395436:C:T
peptide level
0
100000
200000
300000
186:186
110:110
29:29
SGFPQVSMFFTHTFPKpc3_NP.4
p−maj=1, pX=5.6e−08, N=325
var.27.HRG 3:186395436:C:T
peptide level
0
1000000
2000000
185:186
106:110
29:29
SGFPQVSMFFTHTFPKpc3_NP.5
p−min=0.17, pX=0.88, N=320

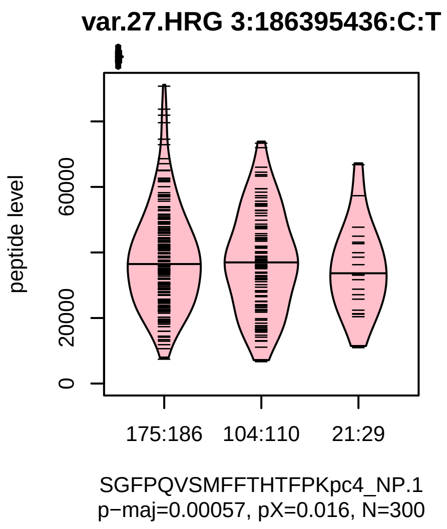
var.27.HRG 3:186395436:C:T
peptide level
0
20000
60000
175:186
104:110
21:29
SGFPQVSMFFTHTFPKpc4_NP.1
p−maj=0.00057, pX=0.016, N=300

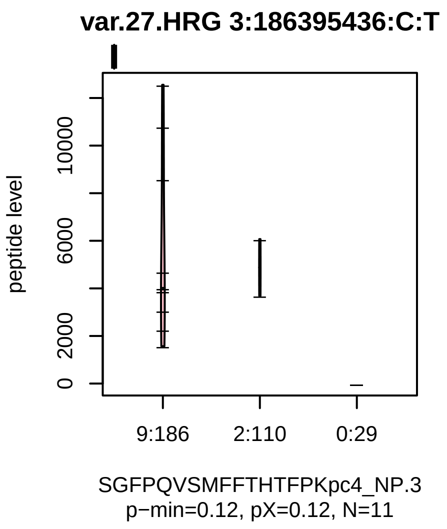
var.27.HRG 3:186395436:C:T
peptide level
0
2000
6000
10000
9:186
2:110
0:29
SGFPQVSMFFTHTFPKpc4_NP.3
p−min=0.12, pX=0.12, N=11

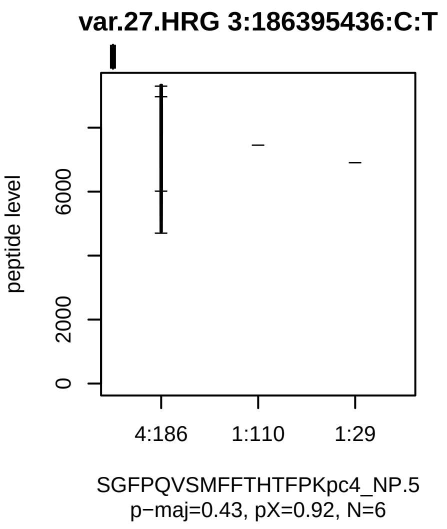
var.27.HRG 3:186395436:C:T
peptide level
0
2000
6000
4:186
1:110
1:29
SGFPQVSMFFTHTFPKpc4_NP.5
p−maj=0.43, pX=0.92, N=6

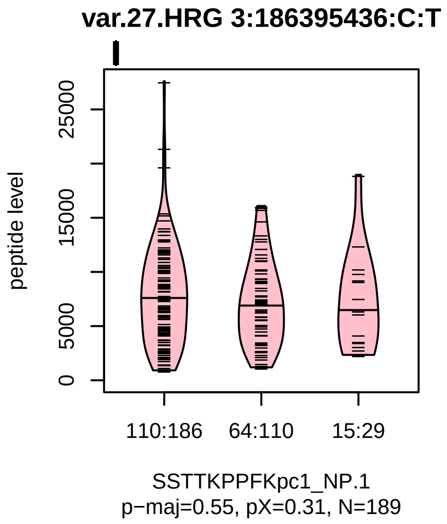
var.27.HRG 3:186395436:C:T
peptide level
0
5000
15000
25000
110:186
64:110
15:29
SSTTKPPFKpc1_NP.1
p−maj=0.55, pX=0.31, N=189

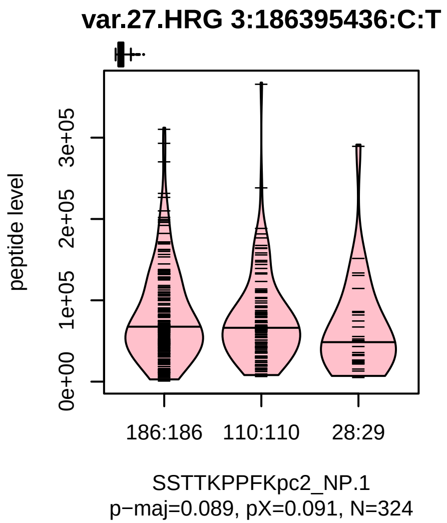
var.27.HRG 3:186395436:C:T
peptide level
0e+00
1e+05
2e+05
3e+05
186:186
110:110
28:29
SSTTKPPFKpc2_NP.1
p−maj=0.089, pX=0.091, N=324

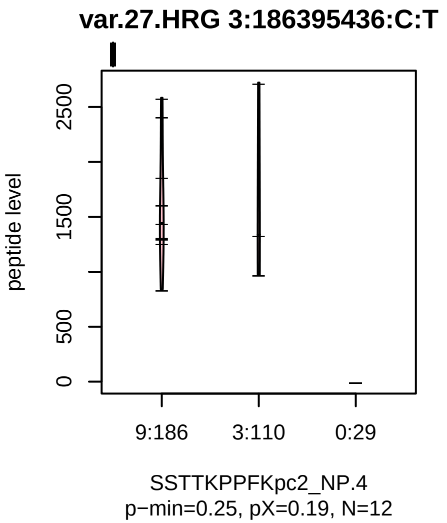
var.27.HRG 3:186395436:C:T
peptide level
0
500
1500
2500
9:186
3:110
0:29
SSTTKPPFKpc2_NP.4
p−min=0.25, pX=0.19, N=12

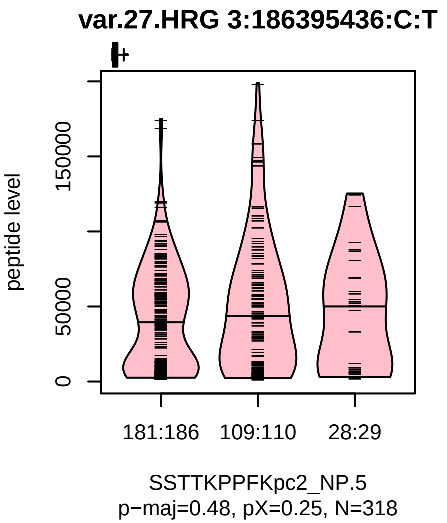
var.27.HRG 3:186395436:C:T
peptide level
0
50000
150000
181:186
109:110
28:29
SSTTKPPFKpc2_NP.5
p−maj=0.48, pX=0.25, N=318

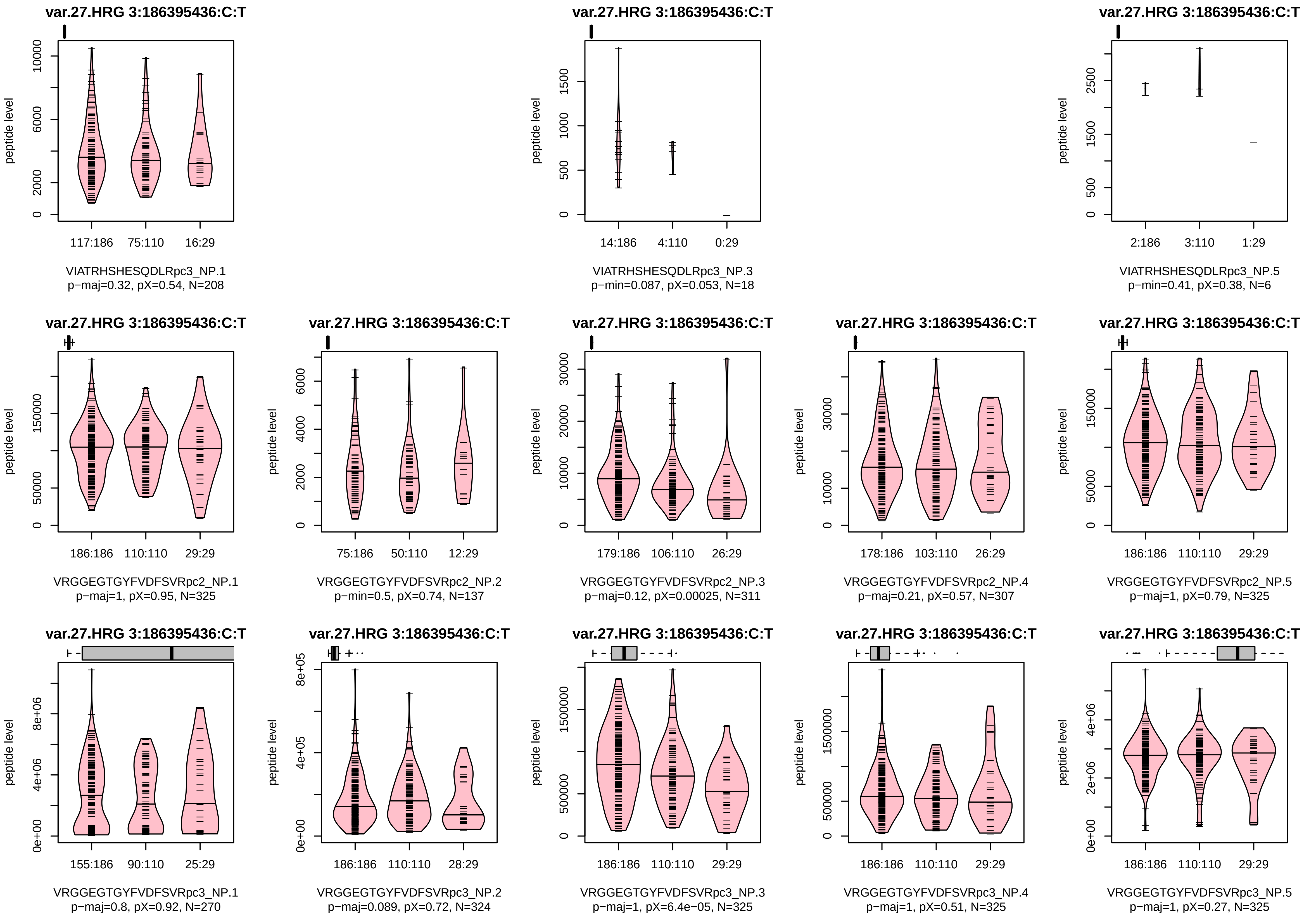
var.27.HRG 3:186395436:C:T
peptide level
117:186
75:110
16:29
VIATRHSHESQDLRpc3_NP.1
p−maj=0.32, pX=0.54, N=208
var.27.HRG 3:186395436:C:T
peptide level
14:186
4:110
0:29
VIATRHSHESQDLRpc3_NP.3
p−min=0.087, pX=0.053, N=18
var.27.HRG 3:186395436:C:T
peptide level
2:186
3:110
1:29
VIATRHSHESQDLRpc3_NP.5
p−min=0.41, pX=0.38, N=6
var.27.HRG 3:186395436:C:T
peptide level
186:186
110:110
29:29
VRGGEGTGYFVDFSVRpc2_NP.1
p−maj=1, pX=0.95, N=325
var.27.HRG 3:186395436:C:T
peptide level
75:186
50:110
12:29
VRGGEGTGYFVDFSVRpc2_NP.2
p−min=0.5, pX=0.74, N=137
var.27.HRG 3:186395436:C:T
peptide level
179:186
106:110
26:29
VRGGEGTGYFVDFSVRpc2_NP.3
p−maj=0.12, pX=0.00025, N=311
var.27.HRG 3:186395436:C:T
peptide level
178:186
103:110
26:29
VRGGEGTGYFVDFSVRpc2_NP.4
p−maj=0.21, pX=0.57, N=307
var.27.HRG 3:186395436:C:T
peptide level
186:186
110:110
29:29
VRGGEGTGYFVDFSVRpc2_NP.5
p−maj=1, pX=0.79, N=325
var.27.HRG 3:186395436:C:T
peptide level
155:186
90:110
25:29
VRGGEGTGYFVDFSVRpc3_NP.1
p−maj=0.8, pX=0.92, N=270
var.27.HRG 3:186395436:C:T
peptide level
186:186
110:110
28:29
VRGGEGTGYFVDFSVRpc3_NP.2
p−maj=0.089, pX=0.72, N=324
var.27.HRG 3:186395436:C:T
peptide level
186:186
110:110
29:29
VRGGEGTGYFVDFSVRpc3_NP.3
p−maj=1, pX=6.4e−05, N=325
var.27.HRG 3:186395436:C:T
peptide level
186:186
110:110
29:29
VRGGEGTGYFVDFSVRpc3_NP.4
p−maj=1, pX=0.51, N=325
var.27.HRG 3:186395436:C:T
peptide level
186:186
110:110
29:29
VRGGEGTGYFVDFSVRpc3_NP.5
p−maj=1, pX=0.27, N=325

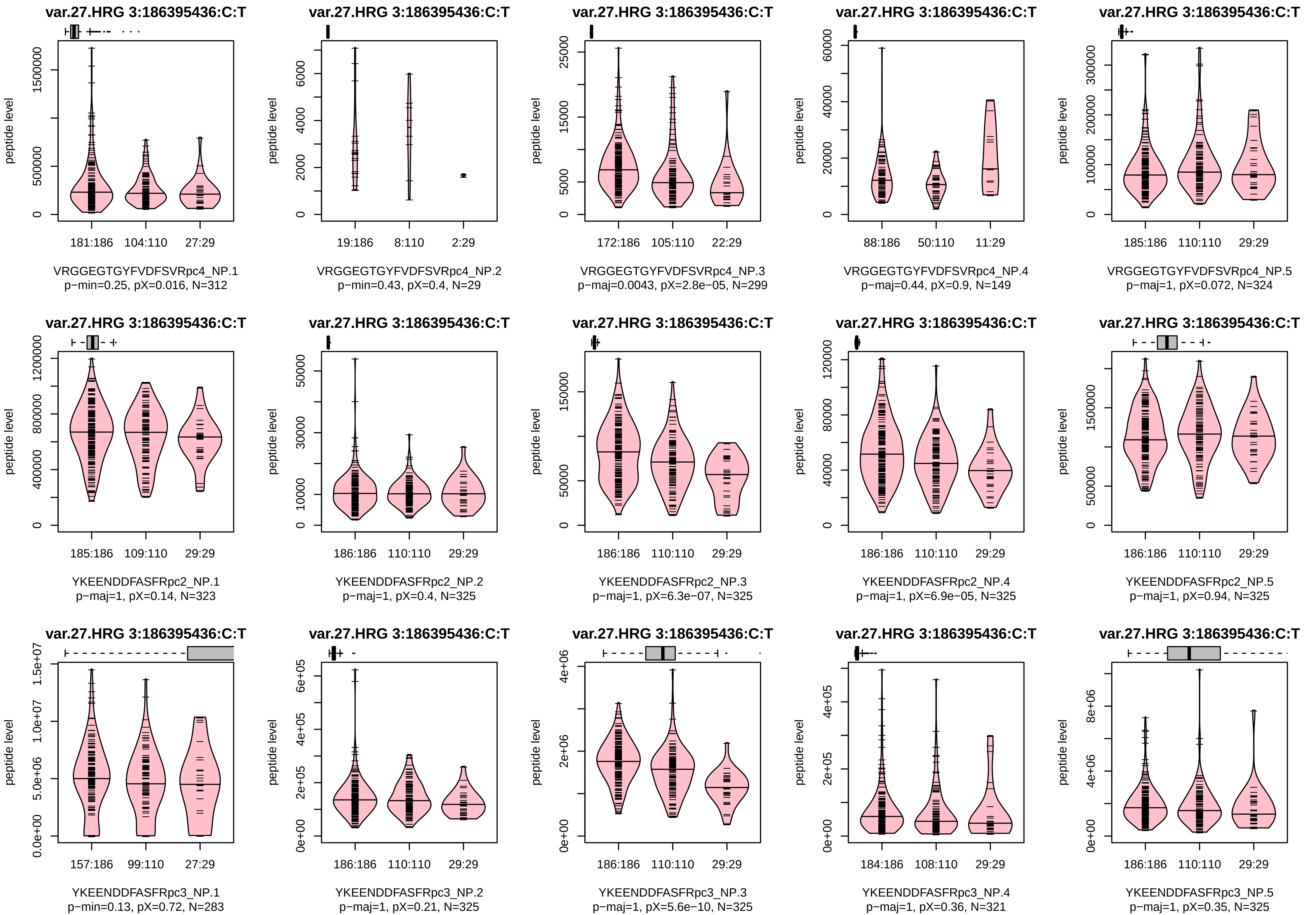
var.27.HRG 3:186395436:C:T
peptide level
181:186
104:110
27:29
VRGGEGTGYFVDFSVRpc4_NP.1
p−min=0.25, pX=0.016, N=312
var.27.HRG 3:186395436:C:T
peptide level
19:186
8:110
2:29
VRGGEGTGYFVDFSVRpc4_NP.2
p−min=0.43, pX=0.4, N=29
var.27.HRG 3:186395436:C:T
peptide level
172:186
105:110
22:29
VRGGEGTGYFVDFSVRpc4_NP.3
p−maj=0.0043, pX=2.8e−05, N=299
var.27.HRG 3:186395436:C:T
peptide level
88:186
50:110
11:29
VRGGEGTGYFVDFSVRpc4_NP.4
p−maj=0.44, pX=0.9, N=149
var.27.HRG 3:186395436:C:T
peptide level
185:186
110:110
29:29
VRGGEGTGYFVDFSVRpc4_NP.5
p−maj=1, pX=0.072, N=324
var.27.HRG 3:186395436:C:T
peptide level
185:186
109:110
29:29
YKEENDDFASFRpc2_NP.1
p−maj=1, pX=0.14, N=323
var.27.HRG 3:186395436:C:T
peptide level
186:186
110:110
29:29
YKEENDDFASFRpc2_NP.2
p−maj=1, pX=0.4, N=325
var.27.HRG 3:186395436:C:T
peptide level
186:186
110:110
29:29
YKEENDDFASFRpc2_NP.3
p−maj=1, pX=6.3e−07, N=325
var.27.HRG 3:186395436:C:T
peptide level
186:186
110:110
29:29
YKEENDDFASFRpc2_NP.4
p−maj=1, pX=6.9e−05, N=325
var.27.HRG 3:186395436:C:T
peptide level
186:186
110:110
29:29
YKEENDDFASFRpc2_NP.5
p−maj=1, pX=0.94, N=325
var.27.HRG 3:186395436:C:T
peptide level
157:186
99:110
27:29
YKEENDDFASFRpc3_NP.1
p−min=0.13, pX=0.72, N=283
var.27.HRG 3:186395436:C:T
peptide level
186:186
110:110
29:29
YKEENDDFASFRpc3_NP.2
p−maj=1, pX=0.21, N=325
var.27.HRG 3:186395436:C:T
peptide level
186:186
110:110
29:29
YKEENDDFASFRpc3_NP.3
p−maj=1, pX=5.6e−10, N=325
var.27.HRG 3:186395436:C:T
peptide level
184:186
108:110
29:29
YKEENDDFASFRpc3_NP.4
p−maj=1, pX=0.36, N=321
var.27.HRG 3:186395436:C:T
peptide level
186:186
110:110
29:29
YKEENDDFASFRpc3_NP.5
p−maj=1, pX=0.35, N=325

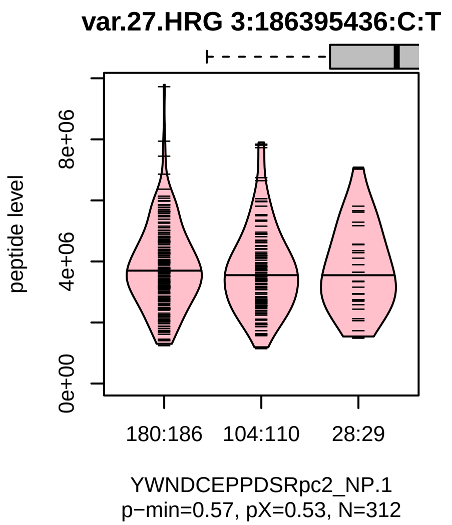
var.27.HRG 3:186395436:C:T
peptide level
0e+00
4e+06
8e+06
180:186
104:110
28:29
YWNDCEPPDSRpc2_NP.1
p−min=0.57, pX=0.53, N=312

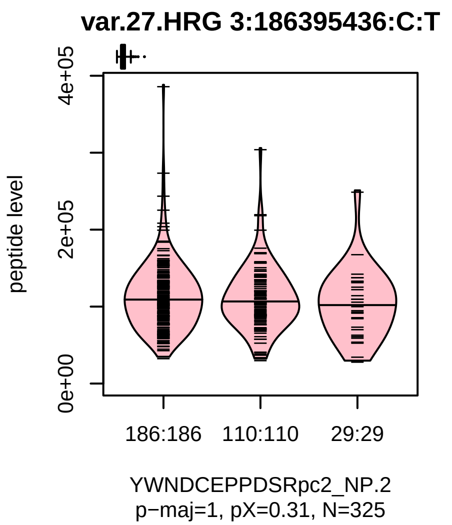
var.27.HRG 3:186395436:C:T
peptide level
0e+00
2e+05
4e+05
186:186
110:110
29:29
YWNDCEPPDSRpc2_NP.2
p−maj=1, pX=0.31, N=325

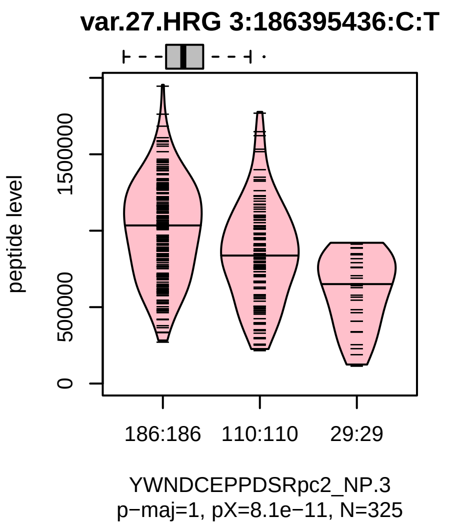
var.27.HRG 3:186395436:C:T
peptide level
0
500000
1500000
186:186
110:110
29:29
YWNDCEPPDSRpc2_NP.3
p−maj=1, pX=8.1e−11, N=325

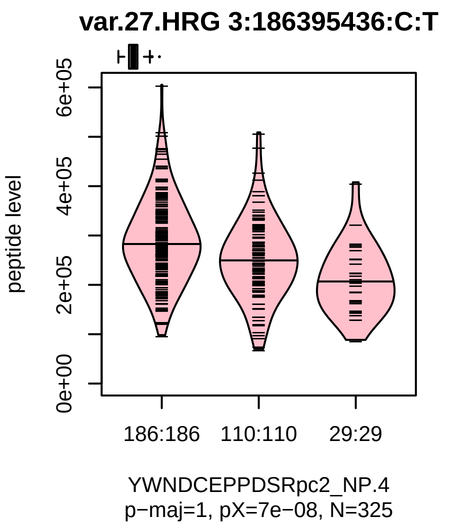
var.27.HRG 3:186395436:C:T
peptide level
0e+00
2e+05
4e+05
6e+05
186:186
110:110
29:29
YWNDCEPPDSRpc2_NP.4
p−maj=1, pX=7e−08, N=325

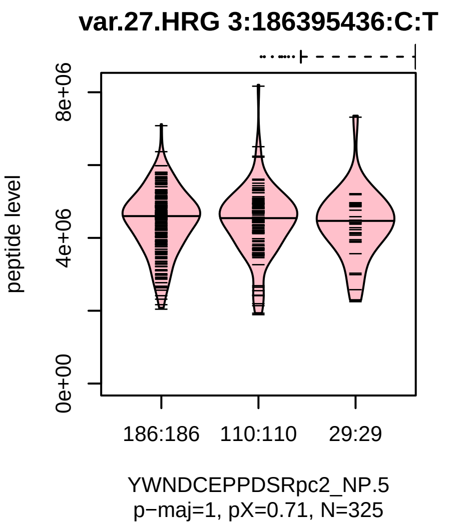
var.27.HRG 3:186395436:C:T
peptide level
0e+00
4e+06
8e+06
186:186
110:110
29:29
YWNDCEPPDSRpc2_NP.5
p−maj=1, pX=0.71, N=325

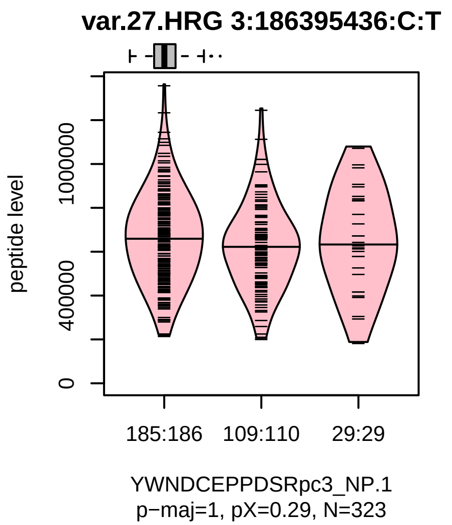
var.27.HRG 3:186395436:C:T
peptide level
0
400000
1000000
185:186
109:110
29:29
YWNDCEPPDSRpc3_NP.1
p−maj=1, pX=0.29, N=323

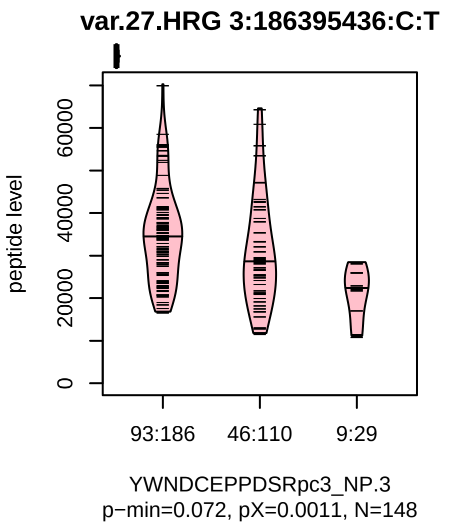
var.27.HRG 3:186395436:C:T
peptide level
0
20000
40000
60000
93:186
46:110
9:29
YWNDCEPPDSRpc3_NP.3
p−min=0.072, pX=0.0011, N=148

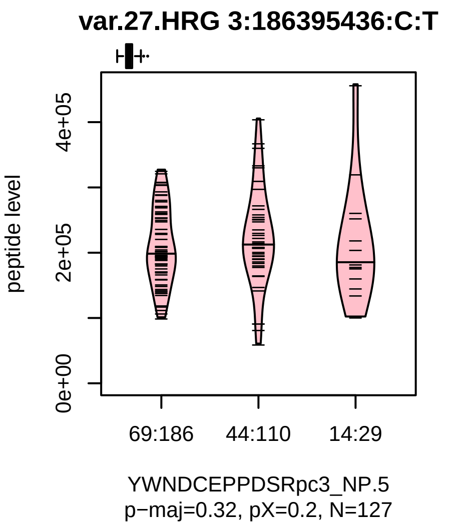
var.27.HRG 3:186395436:C:T
peptide level
0e+00
2e+05
4e+05
69:186
44:110
14:29
YWNDCEPPDSRpc3_NP.5
p−maj=0.32, pX=0.2, N=127

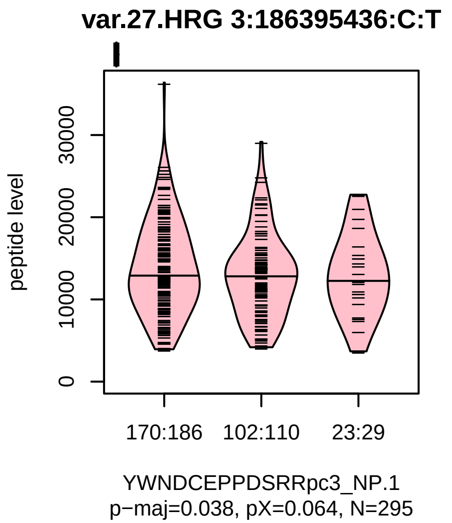
var.27.HRG 3:186395436:C:T
peptide level
0
10000
20000
30000
170:186
102:110
23:29
YWNDCEPPDSRRpc3_NP.1
p−maj=0.038, pX=0.064, N=295

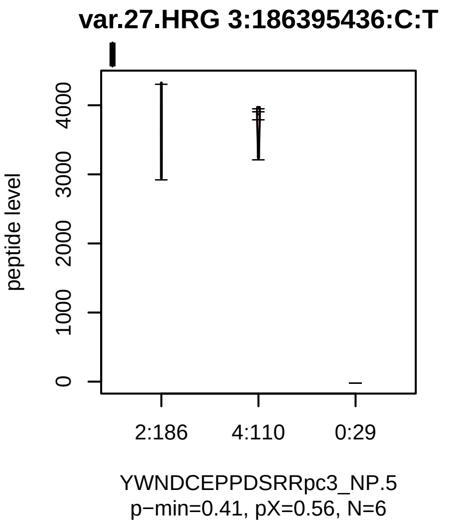
var.27.HRG 3:186395436:C:T
peptide level
0
1000
2000
3000
4000
2:186
4:110
0:29
YWNDCEPPDSRRpc3_NP.5
p−min=0.41, pX=0.56, N=6

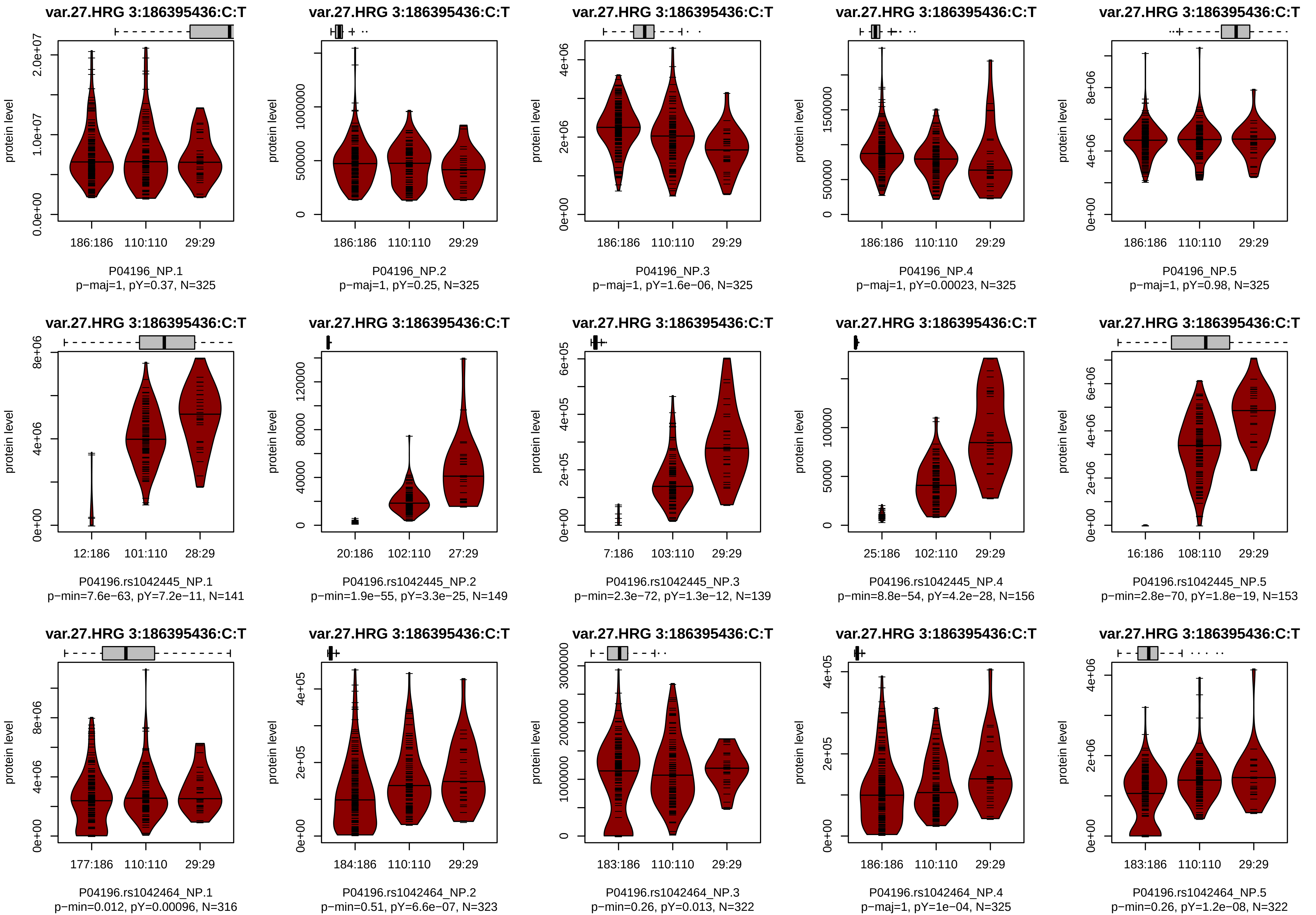

var.27.HRG 3:186395436:C:T
protein level
186:186
110:110
29:29
P04196_NP.1
p−maj=1, pY=0.37, N=325
var.27.HRG 3:186395436:C:T
186:186
110:110
29:29
P04196_NP.2
p−maj=1, pY=0.25, N=325
var.27.HRG 3:186395436:C:T
186:186
110:110
29:29
P04196_NP.3
p−maj=1, pY=1.6e−06, N=325
var.27.HRG 3:186395436:C:T
186:186
110:110
29:29
P04196_NP.4
p−maj=1, pY=0.00023, N=325
var.27.HRG 3:186395436:C:T
186:186
110:110
29:29
P04196_NP.5
p−maj=1, pY=0.98, N=325
var.27.HRG 3:186395436:C:T
12:186
101:110
28:29
P04196.rs1042445_NP.1
p−min=7.6e−63, pY=7.2e−11, N=141
var.27.HRG 3:186395436:C:T
20:186
102:110
27:29
P04196.rs1042445_NP.2
p−min=1.9e−55, pY=3.3e−25, N=149
var.27.HRG 3:186395436:C:T
7:186
103:110
29:29
P04196.rs1042445_NP.3
p−min=2.3e−72, pY=1.3e−12, N=139
var.27.HRG 3:186395436:C:T
25:186
102:110
29:29
P04196.rs1042445_NP.4
p−min=8.8e−54, pY=4.2e−28, N=156
var.27.HRG 3:186395436:C:T
16:186
108:110
29:29
P04196.rs1042445_NP.5
p−min=2.8e−70, pY=1.8e−19, N=153
var.27.HRG 3:186395436:C:T
177:186
110:110
29:29
P04196.rs1042464_NP.1
p−min=0.012, pY=0.00096, N=316
var.27.HRG 3:186395436:C:T
184:186
110:110
29:29
P04196.rs1042464_NP.2
p−min=0.51, pY=6.6e−07, N=323
var.27.HRG 3:186395436:C:T
183:186
110:110
29:29
P04196.rs1042464_NP.3
p−min=0.26, pY=0.013, N=322
var.27.HRG 3:186395436:C:T
186:186
110:110
29:29
P04196.rs1042464_NP.4
p−maj=1, pY=1e−04, N=325
var.27.HRG 3:186395436:C:T
183:186
110:110
29:29
P04196.rs1042464_NP.5
p−min=0.26, pY=1.2e−08, N=322

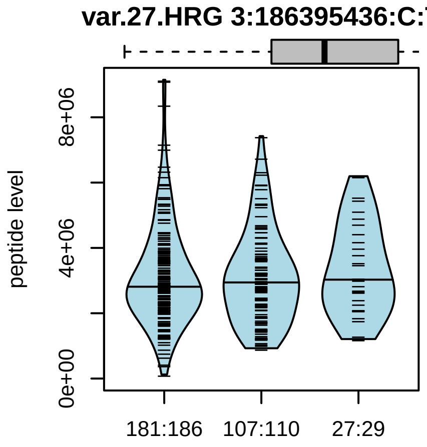

ADLFYDVEALDLESPKpc2_NP.1
p−maj=0.22, pX=0.99, N=315

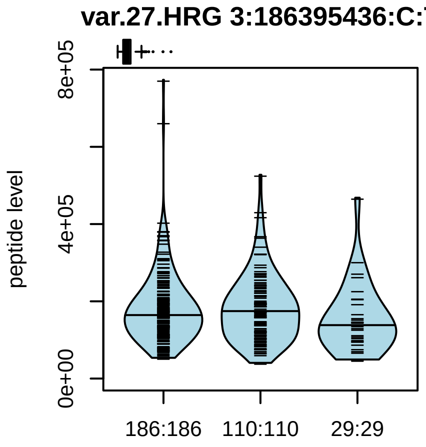

ADLFYDVEALDLESPKpc2_NP.2
p−maj=1, pX=0.29, N=325

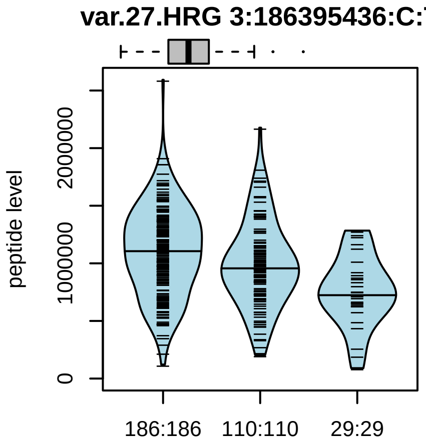

ADLFYDVEALDLESPKpc2_NP.3
p−maj=1, pX=5e−06, N=325

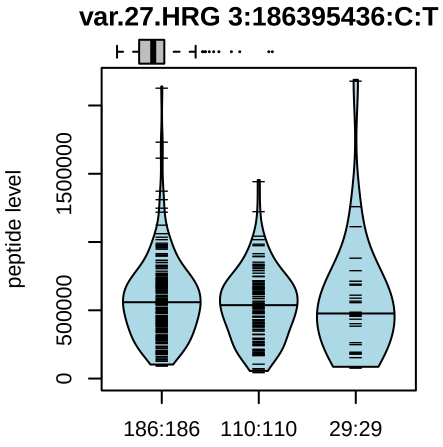

ADLFYDVEALDLESPKpc2_NP.4
p−maj=1, pX=0.42, N=325

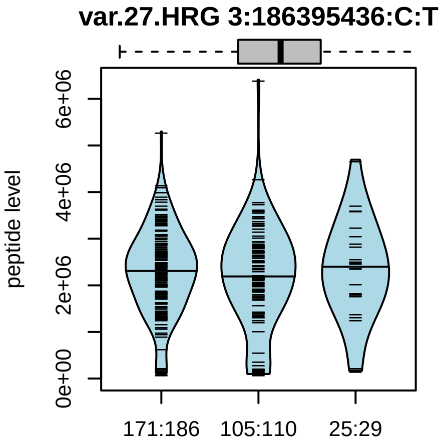

ADLFYDVEALDLESPKpc2_NP.5
p−maj=0.25, pX=0.71, N=301

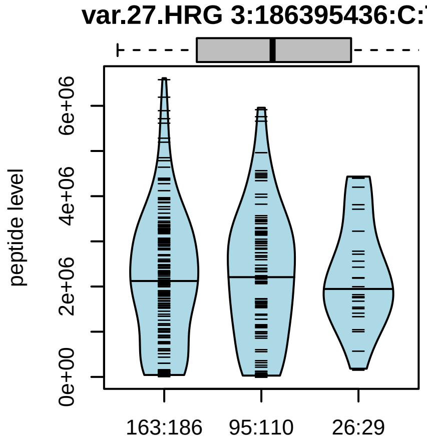

ADLFYDVEALDLESPKpc3_NP.1
p−min=0.87, pX=0.78, N=284

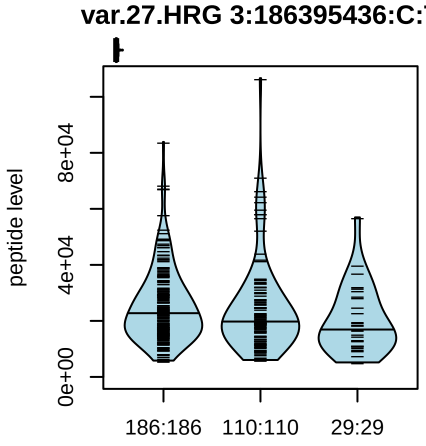

ADLFYDVEALDLESPKpc3_NP.2
p−maj=1, pX=0.12, N=325

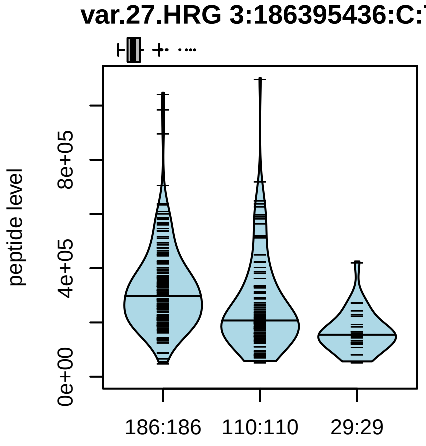

ADLFYDVEALDLESPKpc3_NP.3
p−maj=1, pX=6.2e−08, N=325

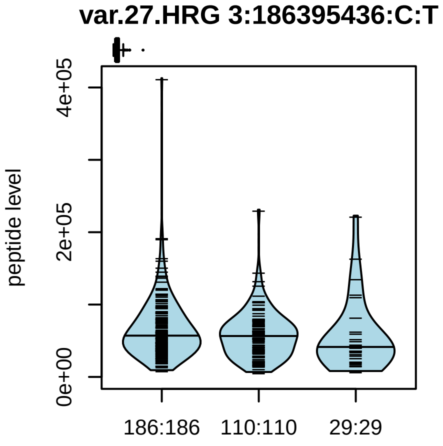

ADLFYDVEALDLESPKpc3_NP.4
p−maj=1, pX=0.075, N=325

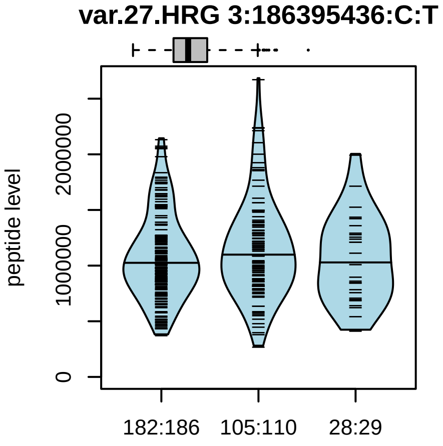

ADLFYDVEALDLESPKpc3_NP.5
p−min=0.34, pX=0.93, N=315

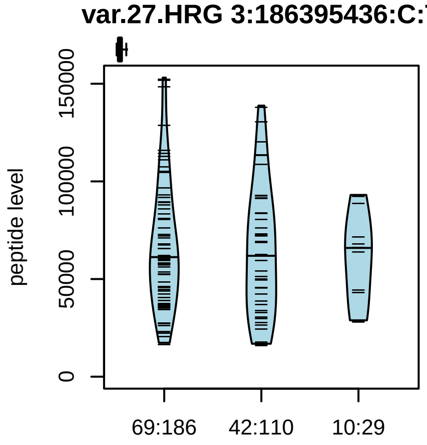

ADLFYDVEALDLESPKpc4_NP.1
p−maj=0.84, pX=0.65, N=121

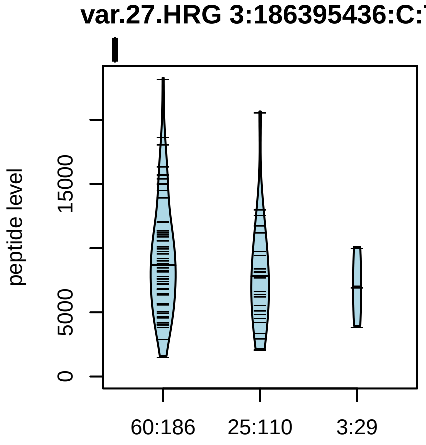

ADLFYDVEALDLESPKpc4_NP.3
p−min=0.017, pX=0.0022, N=88

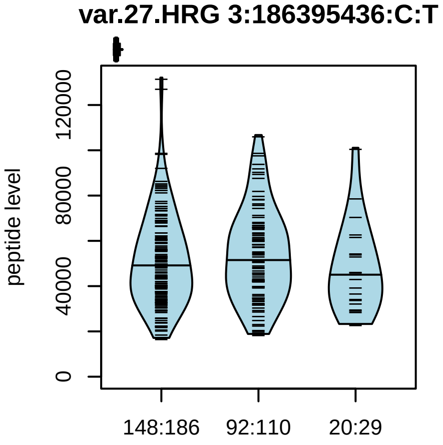

ADLFYDVEALDLESPKpc4_NP.5
p−maj=0.14, pX=0.73, N=260

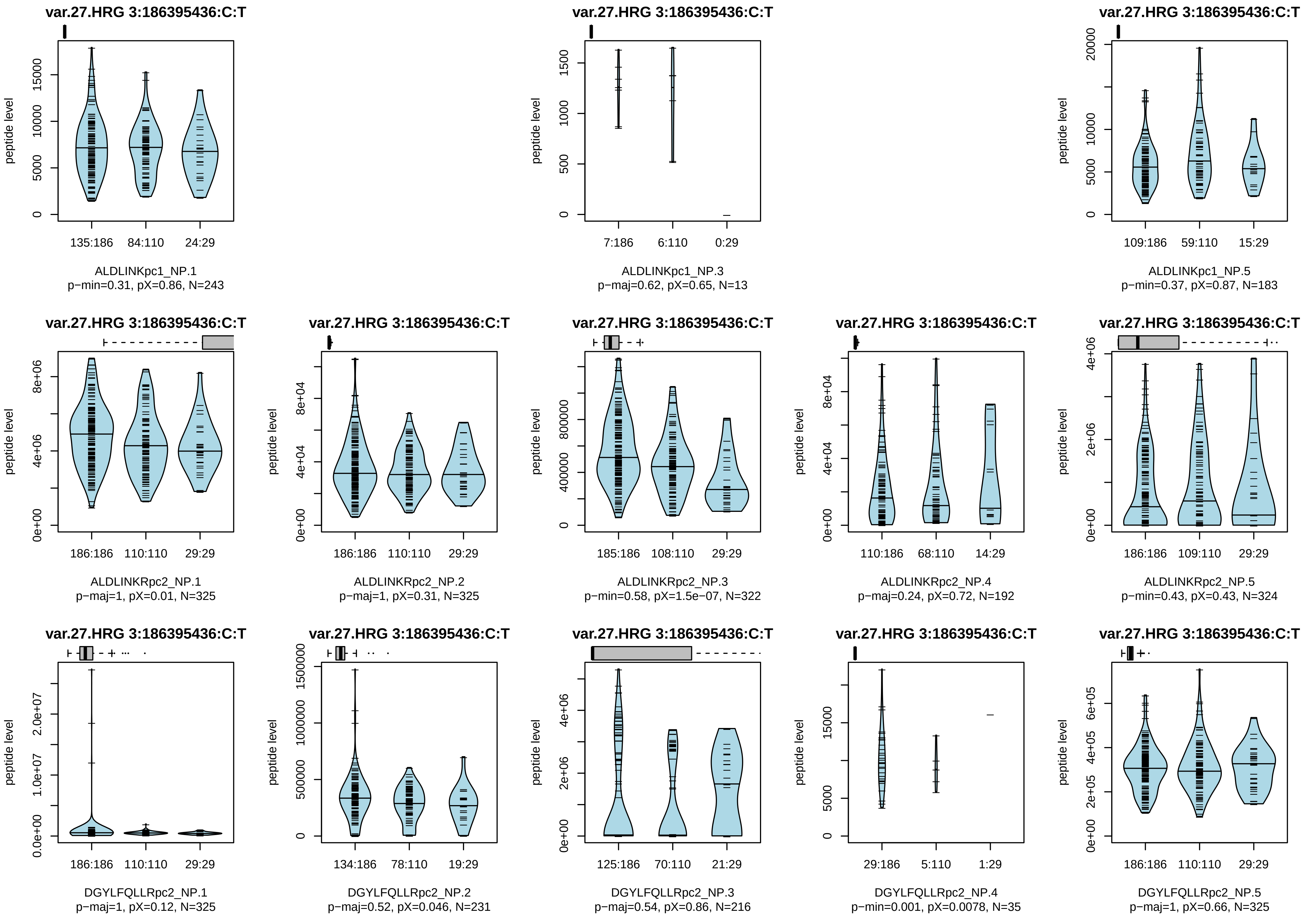
var.27.HRG 3:186395436:C:T
peptide level
135:186
84:110
24:29
ALDLINKpc1_NP.1
p−min=0.31, pX=0.86, N=243
var.27.HRG 3:186395436:C:T
peptide level
7:186
6:110
0:29
ALDLINKpc1_NP.3
p−maj=0.62, pX=0.65, N=13
var.27.HRG 3:186395436:C:T
peptide level
109:186
59:110
15:29
ALDLINKpc1_NP.5
p−min=0.37, pX=0.87, N=183
var.27.HRG 3:186395436:C:T
peptide level
186:186
110:110
29:29
ALDLINKRpc2_NP.1
p−maj=1, pX=0.01, N=325
var.27.HRG 3:186395436:C:T
peptide level
186:186
110:110
29:29
ALDLINKRpc2_NP.2
p−maj=1, pX=0.31, N=325
var.27.HRG 3:186395436:C:T
peptide level
185:186
108:110
29:29
ALDLINKRpc2_NP.3
p−min=0.58, pX=1.5e−07, N=322
var.27.HRG 3:186395436:C:T
peptide level
110:186
68:110
14:29
ALDLINKRpc2_NP.4
p−maj=0.24, pX=0.72, N=192
var.27.HRG 3:186395436:C:T
peptide level
186:186
109:110
29:29
ALDLINKRpc2_NP.5
p−min=0.43, pX=0.43, N=324
var.27.HRG 3:186395436:C:T
peptide level
186:186
110:110
29:29
DGYLFQLLRpc2_NP.1
p−maj=1, pX=0.12, N=325
var.27.HRG 3:186395436:C:T
peptide level
134:186
78:110
19:29
DGYLFQLLRpc2_NP.2
p−maj=0.52, pX=0.046, N=231
var.27.HRG 3:186395436:C:T
peptide level
125:186
70:110
21:29
DGYLFQLLRpc2_NP.3
p−maj=0.54, pX=0.86, N=216
var.27.HRG 3:186395436:C:T
peptide level
29:186
5:110
1:29
DGYLFQLLRpc2_NP.4
p−min=0.001, pX=0.0078, N=35
var.27.HRG 3:186395436:C:T
peptide level
186:186
110:110
29:29
DGYLFQLLRpc2_NP.5
p−maj=1, pX=0.66, N=325

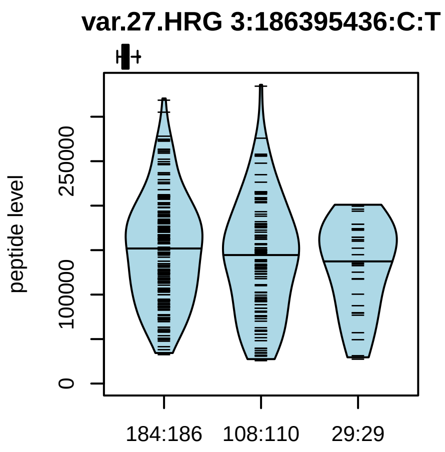

DHHHPHKpc2_NP.1
p−maj=1, pX=0.076, N=321

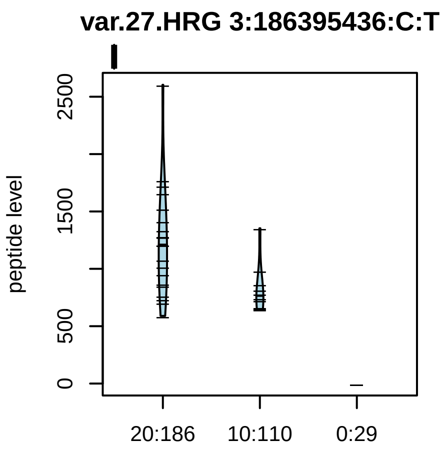

DHHHPHKpc2_NP.3
p−maj=0.091, pX=0.031, N=30

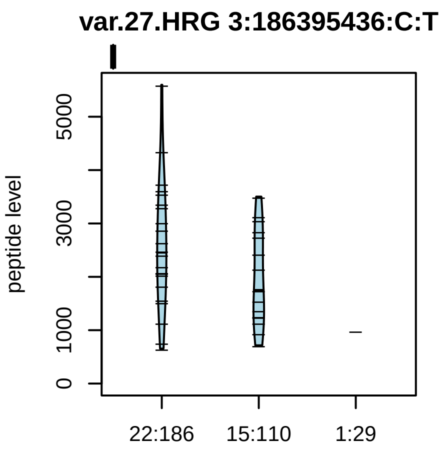

DHHHPHKpc2_NP.4
p−maj=0.22, pX=0.17, N=38

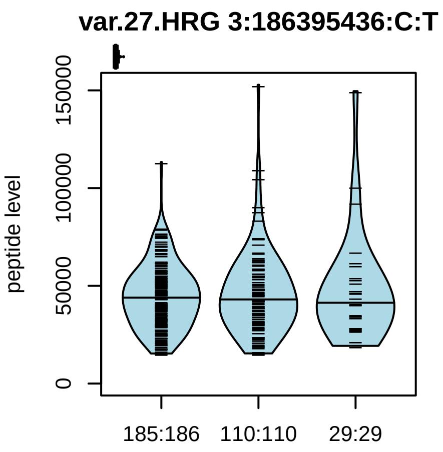

DHHHPHKpc2_NP.5
p−maj=1, pX=0.2, N=324

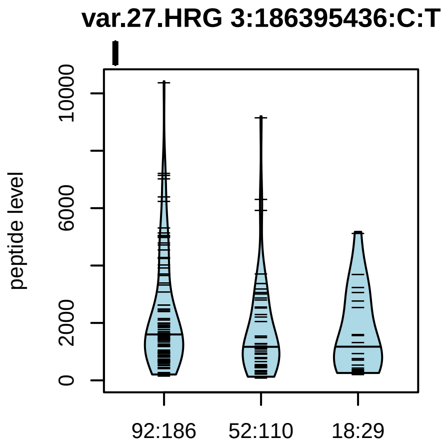

DHHHPHKPHEHGPPPPPDERpc3_NP.1
p−maj=0.18, pX=0.32, N=162

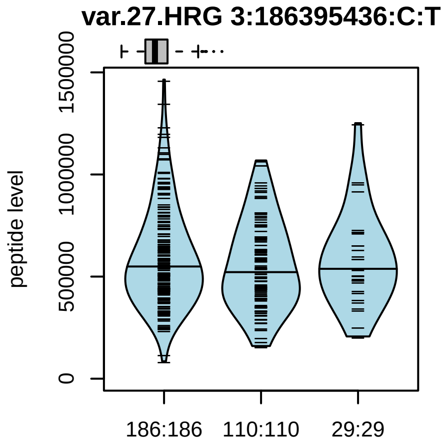

DHHHPHKPHEHGPPPPPDERpc4_NP.1
p−maj=1, pX=0.42, N=325

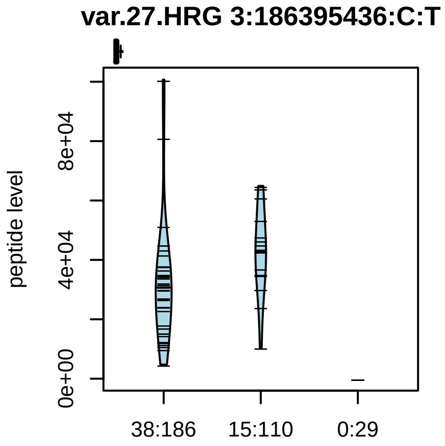

DHHHPHKPHEHGPPPPPDERpc4_NP.2
p−maj=0.0071, pX=0.067, N=53

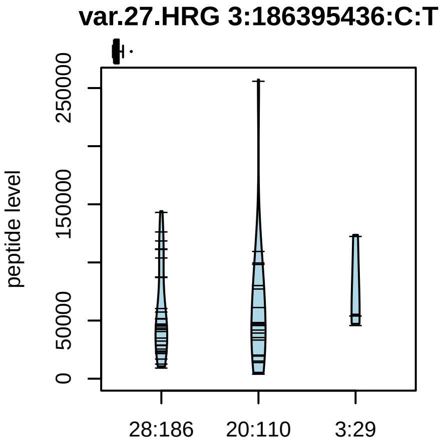

DHHHPHKPHEHGPPPPPDERpc4_NP.5
p−maj=0.59, pX=0.81, N=51

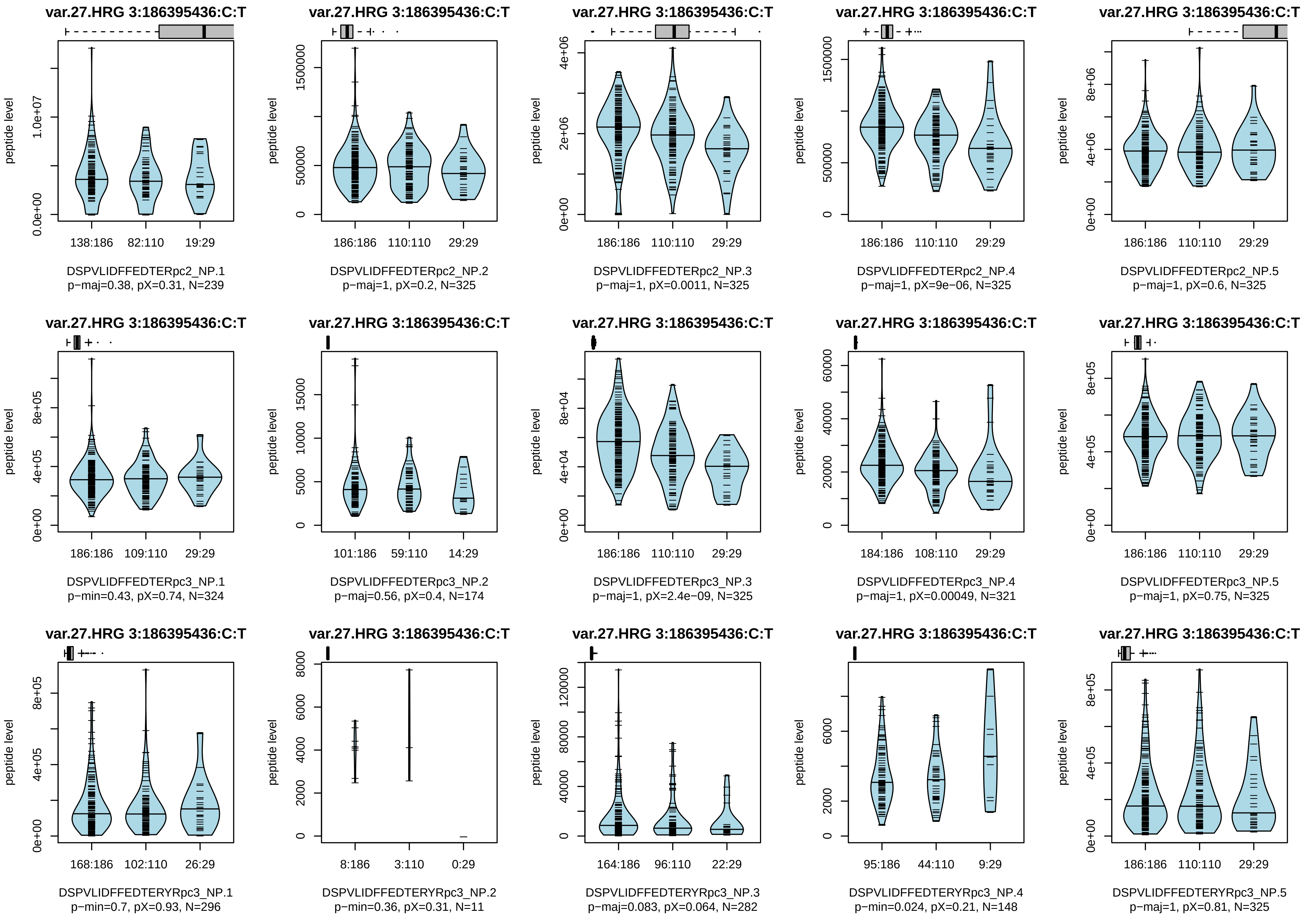

var.27.HRG 3:186395436:C:T
peptide level
0.0e+00
1.0e+07
138:186
82:110
19:29
DSPVLIDFFEDTERpc2_NP.1
p−maj=0.38, pX=0.31, N=239
var.27.HRG 3:186395436:C:T
peptide level
0
500000
1500000
186:186
110:110
29:29
DSPVLIDFFEDTERpc2_NP.2
p−maj=1, pX=0.2, N=325
var.27.HRG 3:186395436:C:T
peptide level
0e+00
2e+06
4e+06
186:186
110:110
29:29
DSPVLIDFFEDTERpc2_NP.3
p−maj=1, pX=0.0011, N=325
var.27.HRG 3:186395436:C:T
peptide level
0
500000
1500000
186:186
110:110
29:29
DSPVLIDFFEDTERpc2_NP.4
p−maj=1, pX=9e−06, N=325
var.27.HRG 3:186395436:C:T
peptide level
0e+00
4e+06
8e+06
186:186
110:110
29:29
DSPVLIDFFEDTERpc2_NP.5
p−maj=1, pX=0.6, N=325
var.27.HRG 3:186395436:C:T
peptide level
0e+00
4e+05
8e+05
186:186
109:110
29:29
DSPVLIDFFEDTERpc3_NP.1
p−min=0.43, pX=0.74, N=324
var.27.HRG 3:186395436:C:T
peptide level
0
5000
10000
15000
101:186
59:110
14:29
DSPVLIDFFEDTERpc3_NP.2
p−maj=0.56, pX=0.4, N=174
var.27.HRG 3:186395436:C:T
peptide level
0e+00
4e+04
8e+04
186:186
110:110
29:29
DSPVLIDFFEDTERpc3_NP.3
p−maj=1, pX=2.4e−09, N=325
var.27.HRG 3:186395436:C:T
peptide level
0
20000
40000
60000
184:186
108:110
29:29
DSPVLIDFFEDTERpc3_NP.4
p−maj=1, pX=0.00049, N=321
var.27.HRG 3:186395436:C:T
peptide level
0e+00
4e+05
8e+05
186:186
110:110
29:29
DSPVLIDFFEDTERpc3_NP.5
p−maj=1, pX=0.75, N=325
var.27.HRG 3:186395436:C:T
peptide level
0e+00
4e+05
8e+05
168:186
102:110
26:29
DSPVLIDFFEDTERYRpc3_NP.1
p−min=0.7, pX=0.93, N=296
var.27.HRG 3:186395436:C:T
peptide level
0
2000
4000
6000
8000
8:186
3:110
0:29
DSPVLIDFFEDTERYRpc3_NP.2
p−min=0.36, pX=0.31, N=11
var.27.HRG 3:186395436:C:T
peptide level
0
40000
80000
120000
164:186
96:110
22:29
DSPVLIDFFEDTERYRpc3_NP.3
p−maj=0.083, pX=0.064, N=282
var.27.HRG 3:186395436:C:T
peptide level
0
2000
6000
95:186
44:110
9:29
DSPVLIDFFEDTERYRpc3_NP.4
p−min=0.024, pX=0.21, N=148
var.27.HRG 3:186395436:C:T
peptide level
0e+00
4e+05
8e+05
186:186
110:110
29:29
DSPVLIDFFEDTERYRpc3_NP.5
p−maj=1, pX=0.81, N=325

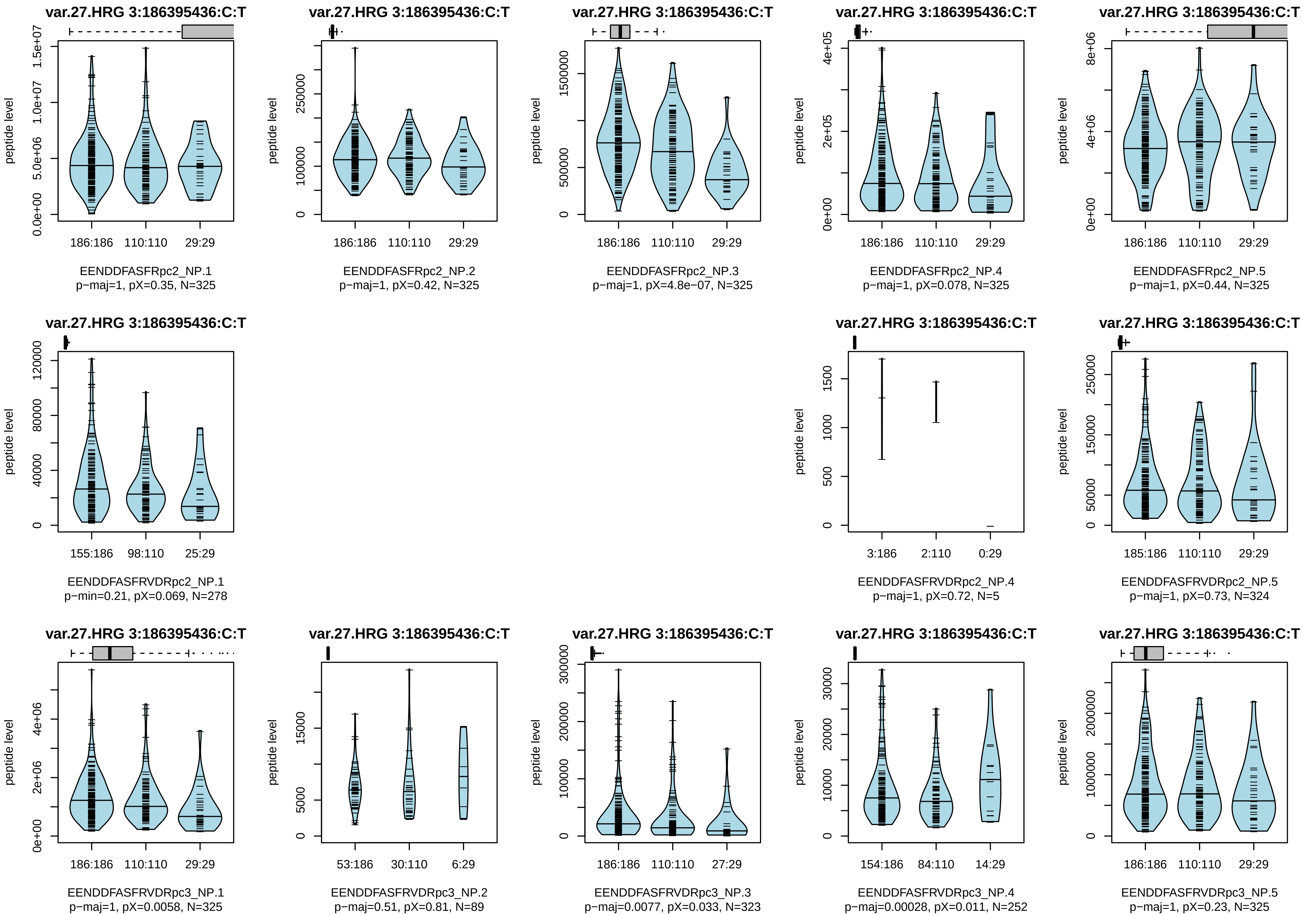
var.27.HRG 3:186395436:C:T
peptide level
186:186
110:110
29:29
EENDDFASFRpc2_NP.1
p−maj=1, pX=0.35, N=325
var.27.HRG 3:186395436:C:T
peptide level
186:186
110:110
29:29
EENDDFASFRpc2_NP.2
p−maj=1, pX=0.42, N=325
var.27.HRG 3:186395436:C:T
peptide level
186:186
110:110
29:29
EENDDFASFRpc2_NP.3
p−maj=1, pX=4.8e−07, N=325
var.27.HRG 3:186395436:C:T
peptide level
186:186
110:110
29:29
EENDDFASFRpc2_NP.4
p−maj=1, pX=0.078, N=325
var.27.HRG 3:186395436:C:T
peptide level
186:186
110:110
29:29
EENDDFASFRpc2_NP.5
p−maj=1, pX=0.44, N=325
var.27.HRG 3:186395436:C:T
peptide level
155:186
98:110
25:29
EENDDFASFRVDRpc2_NP.1
p−min=0.21, pX=0.069, N=278
var.27.HRG 3:186395436:C:T
peptide level
3:186
2:110
0:29
EENDDFASFRVDRpc2_NP.4
p−maj=1, pX=0.72, N=5
var.27.HRG 3:186395436:C:T
peptide level
185:186
110:110
29:29
EENDDFASFRVDRpc2_NP.5
p−maj=1, pX=0.73, N=324
var.27.HRG 3:186395436:C:T
peptide level
186:186
110:110
29:29
EENDDFASFRVDRpc3_NP.1
p−maj=1, pX=0.0058, N=325
var.27.HRG 3:186395436:C:T
peptide level
53:186
30:110
6:29
EENDDFASFRVDRpc3_NP.2
p−maj=0.51, pX=0.81, N=89
var.27.HRG 3:186395436:C:T
peptide level
186:186
110:110
27:29
EENDDFASFRVDRpc3_NP.3
p−maj=0.0077, pX=0.033, N=323
var.27.HRG 3:186395436:C:T
peptide level
154:186
84:110
14:29
EENDDFASFRVDRpc3_NP.4
p−maj=0.00028, pX=0.011, N=252
var.27.HRG 3:186395436:C:T
peptide level
186:186
110:110
29:29
EENDDFASFRVDRpc3_NP.5
p−maj=1, pX=0.23, N=325

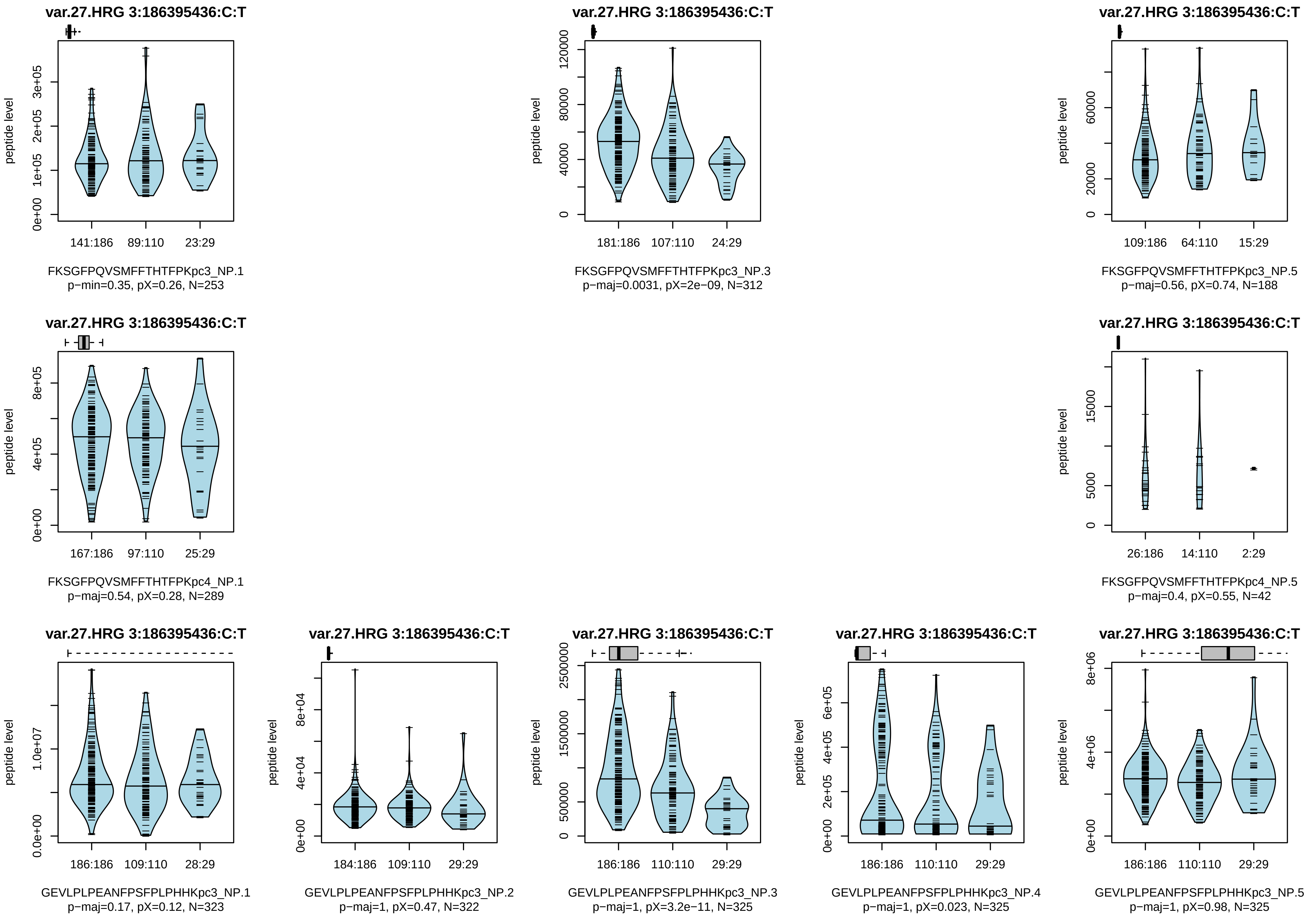
var.27.HRG 3:186395436:C:T
peptide level
0e+00
1e+05
2e+05
3e+05
141:186
89:110
23:29
FKSGFPQVSMFFTHTFPKpc3_NP.1
p−min=0.35, pX=0.26, N=253
var.27.HRG 3:186395436:C:T
peptide level
0
40000
80000
120000
181:186
107:110
24:29
FKSGFPQVSMFFTHTFPKpc3_NP.3
p−maj=0.0031, pX=2e−09, N=312
var.27.HRG 3:186395436:C:T
peptide level
0
20000
60000
109:186
64:110
15:29
FKSGFPQVSMFFTHTFPKpc3_NP.5
p−maj=0.56, pX=0.74, N=188
var.27.HRG 3:186395436:C:T
peptide level
0e+00
4e+05
8e+05
167:186
97:110
25:29
FKSGFPQVSMFFTHTFPKpc4_NP.1
p−maj=0.54, pX=0.28, N=289
var.27.HRG 3:186395436:C:T
peptide level
0
5000
15000
26:186
14:110
2:29
FKSGFPQVSMFFTHTFPKpc4_NP.5
p−maj=0.4, pX=0.55, N=42
var.27.HRG 3:186395436:C:T
peptide level
0.0e+00
1.0e+07
186:186
109:110
28:29
GEVLPLPEANFPSFPLPHHKpc3_NP.1
p−maj=0.17, pX=0.12, N=323
var.27.HRG 3:186395436:C:T
peptide level
0e+00
4e+04
8e+04
184:186
109:110
29:29
GEVLPLPEANFPSFPLPHHKpc3_NP.2
p−maj=1, pX=0.47, N=322
var.27.HRG 3:186395436:C:T
peptide level
0
500000
1500000
2500000
186:186
110:110
29:29
GEVLPLPEANFPSFPLPHHKpc3_NP.3
p−maj=1, pX=3.2e−11, N=325
var.27.HRG 3:186395436:C:T
peptide level
0e+00
2e+05
4e+05
6e+05
186:186
110:110
29:29
GEVLPLPEANFPSFPLPHHKpc3_NP.4
p−maj=1, pX=0.023, N=325
var.27.HRG 3:186395436:C:T
peptide level
0e+00
4e+06
8e+06
186:186
110:110
29:29
GEVLPLPEANFPSFPLPHHKpc3_NP.5
p−maj=1, pX=0.98, N=325

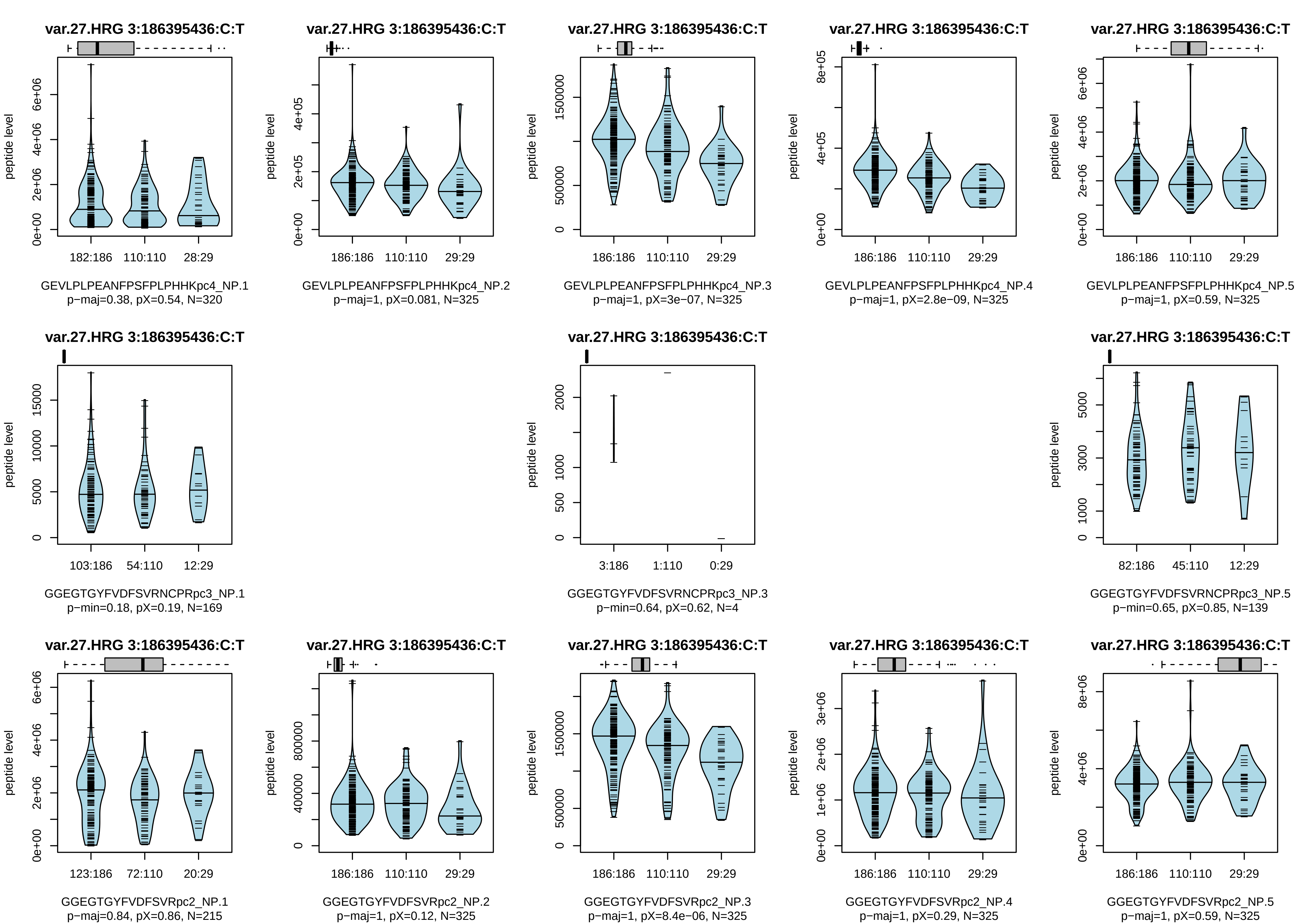
var.27.HRG 3:186395436:C:T
peptide level
182:186
110:110
28:29
GEVLPLPEANFPSFPLPHHKpc4_NP.1
p−maj=0.38, pX=0.54, N=320
var.27.HRG 3:186395436:C:T
186:186
110:110
29:29
GEVLPLPEANFPSFPLPHHKpc4_NP.2
p−maj=1, pX=0.081, N=325
var.27.HRG 3:186395436:C:T
GEVLPLPEANFPSFPLPHHKpc4_NP.3
p−maj=1, pX=3e−07, N=325
var.27.HRG 3:186395436:C:T
GEVLPLPEANFPSFPLPHHKpc4_NP.4
p−maj=1, pX=2.8e−09, N=325
var.27.HRG 3:186395436:C:T
GEVLPLPEANFPSFPLPHHKpc4_NP.5
p−maj=1, pX=0.59, N=325
var.27.HRG 3:186395436:C:T
103:186
54:110
12:29
GGEGTGYFVDFSVRNCPRpc3_NP.1
p−min=0.18, pX=0.19, N=169
var.27.HRG 3:186395436:C:T
3:186
1:110
0:29
GGEGTGYFVDFSVRNCPRpc3_NP.3
p−min=0.64, pX=0.62, N=4
var.27.HRG 3:186395436:C:T
82:186
45:110
12:29
GGEGTGYFVDFSVRNCPRpc3_NP.5
p−min=0.65, pX=0.85, N=139
var.27.HRG 3:186395436:C:T
123:186
72:110
20:29
GGEGTGYFVDFSVRpc2_NP.1
p−maj=0.84, pX=0.86, N=215
var.27.HRG 3:186395436:C:T
GGEGTGYFVDFSVRpc2_NP.2
p−maj=1, pX=0.12, N=325
var.27.HRG 3:186395436:C:T
GGEGTGYFVDFSVRpc2_NP.3
p−maj=1, pX=8.4e−06, N=325
var.27.HRG 3:186395436:C:T
GGEGTGYFVDFSVRpc2_NP.4
p−maj=1, pX=0.29, N=325
var.27.HRG 3:186395436:C:T
GGEGTGYFVDFSVRpc2_NP.5
p−maj=1, pX=0.59, N=325

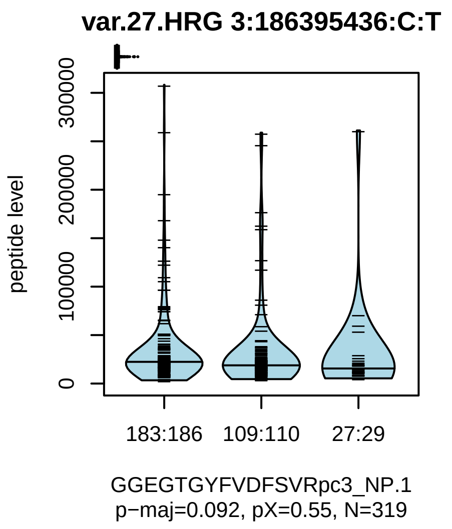
var.27.HRG 3:186395436:C:T
peptide level
183:186
109:110
27:29
GGEGTGYFVDFSVRpc3_NP.1
p−maj=0.092, pX=0.55, N=319

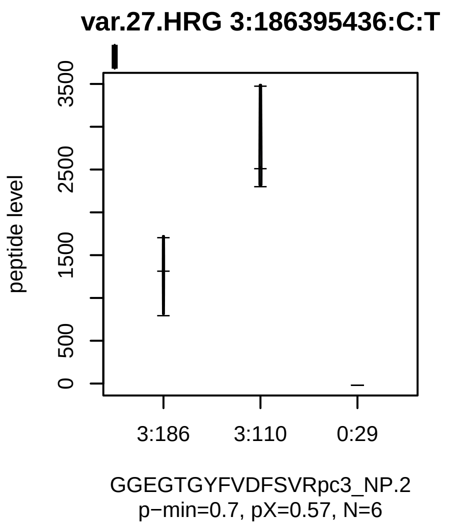
var.27.HRG 3:186395436:C:T
peptide level
3:186
3:110
0:29
GGEGTGYFVDFSVRpc3_NP.2
p−min=0.7, pX=0.57, N=6

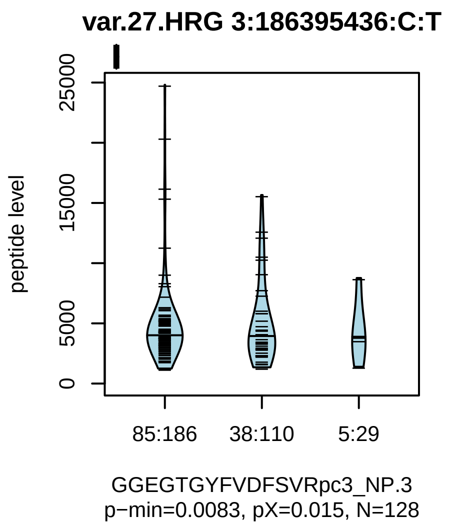
var.27.HRG 3:186395436:C:T
peptide level
85:186
38:110
5:29
GGEGTGYFVDFSVRpc3_NP.3
p−min=0.0083, pX=0.015, N=128

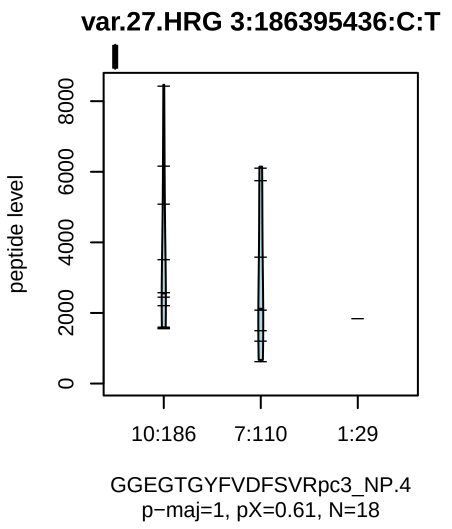
var.27.HRG 3:186395436:C:T
peptide level
10:186
7:110
1:29
GGEGTGYFVDFSVRpc3_NP.4
p−maj=1, pX=0.61, N=18

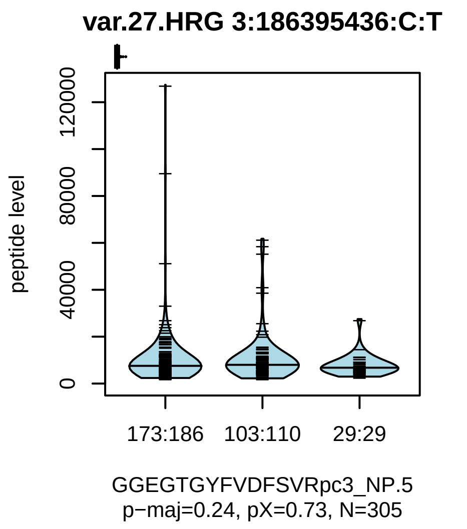
var.27.HRG 3:186395436:C:T
peptide level
173:186
103:110
29:29
GGEGTGYFVDFSVRpc3_NP.5
p−maj=0.24, pX=0.73, N=305

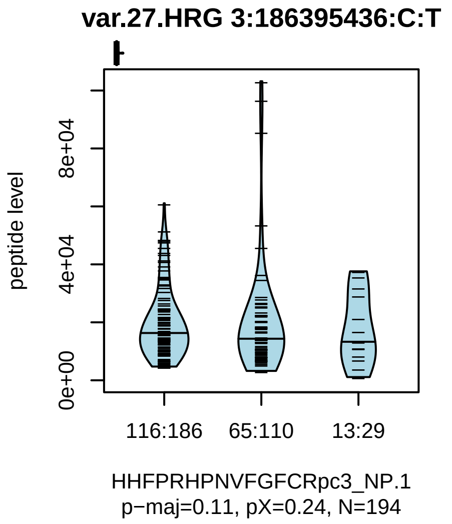
var.27.HRG 3:186395436:C:T
peptide level
116:186
65:110
13:29
HHFPRHPNVFGFCRpc3_NP.1
p−maj=0.11, pX=0.24, N=194

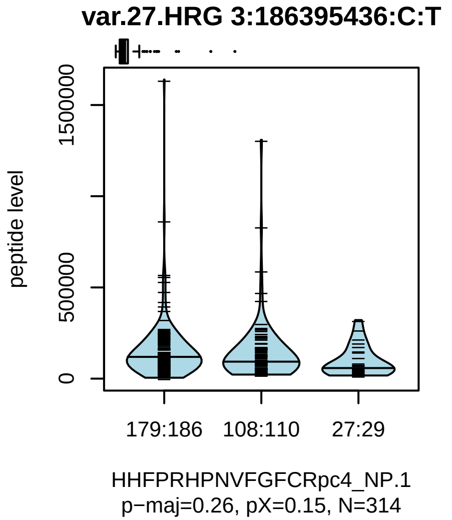
var.27.HRG 3:186395436:C:T
peptide level
179:186
108:110
27:29
HHFPRHPNVFGFCRpc4_NP.1
p−maj=0.26, pX=0.15, N=314

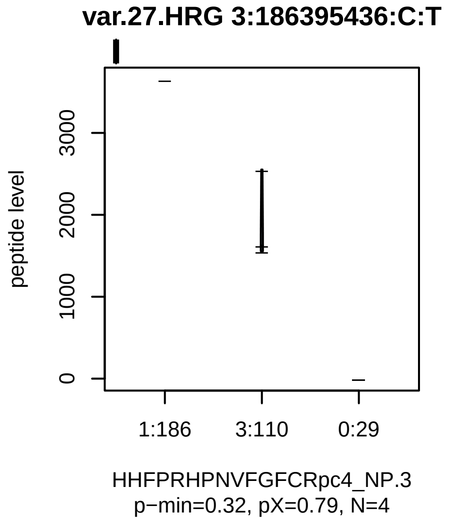
var.27.HRG 3:186395436:C:T
peptide level
1:186
3:110
0:29
HHFPRHPNVFGFCRpc4_NP.3
p−min=0.32, pX=0.79, N=4

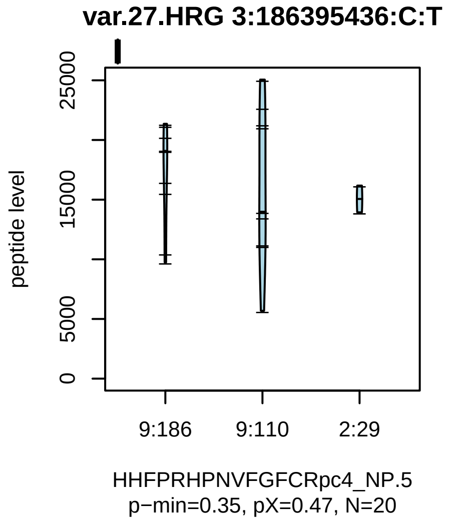
var.27.HRG 3:186395436:C:T
peptide level
9:186
9:110
2:29
HHFPRHPNVFGFCRpc4_NP.5
p−min=0.35, pX=0.47, N=20

var.27.HRG 3:186395436:C:T

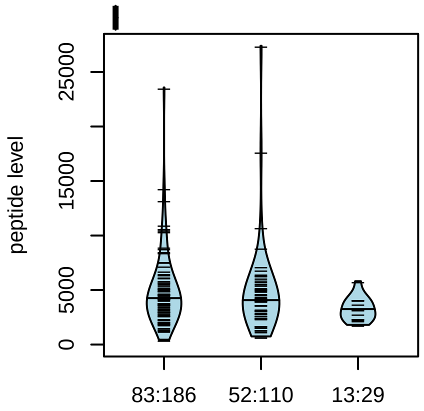

HHSHEQHPHGHHPHAHHPHEHDTHRpc4_NF
p−min=0.74, pX=0.47, N=148

var.27.HRG 3:186395436:C:T

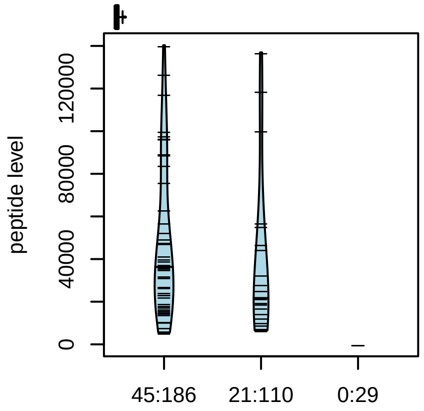

HPLKPDNQPFPQSVSESCPGKpc3_NP.1
p−maj=0.0013, pX=0.012, N=66

var.27.HRG 3:186395436:C:T

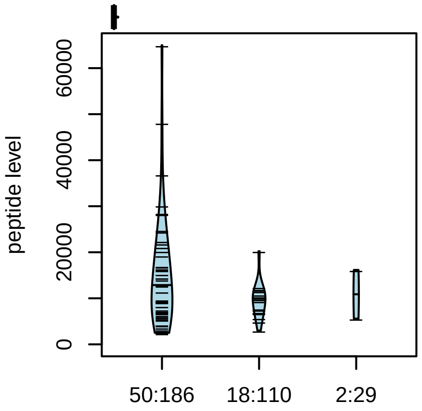

HPLKPDNQPFPQSVSESCPGKpc3_NP.4
p−min=0.0066, pX=0.0016, N=70

var.27.HRG 3:186395436:C:T

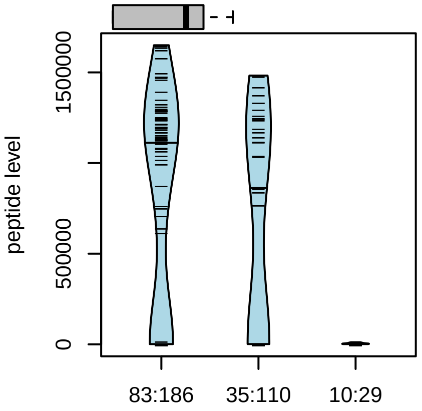

HPLKPDNQPFPQSVSESCPGKpc3_NP.5
p−min=0.029, pX=0.00017, N=128

var.27.HRG 3:186395436:C:T

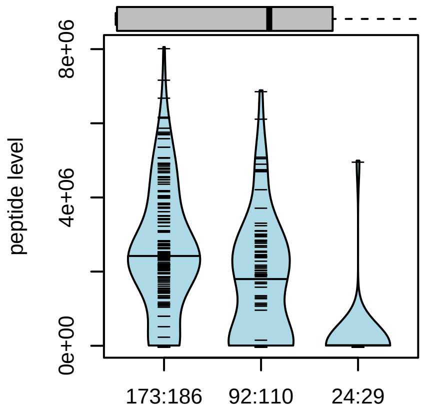

HPLKPDNQPFPQSVSESCPGKpc4_NP.1
p−min=0.0075, pX=1.8e−12, N=289

var.27.HRG 3:186395436:C:T

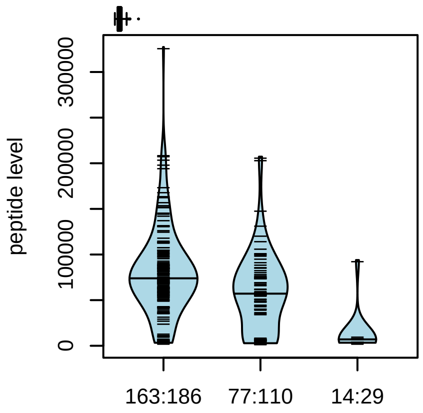

HPLKPDNQPFPQSVSESCPGKpc4_NP.2
p−min=2.8e−06, pX=2.6e−13, N=254

var.27.HRG 3:186395436:C:T

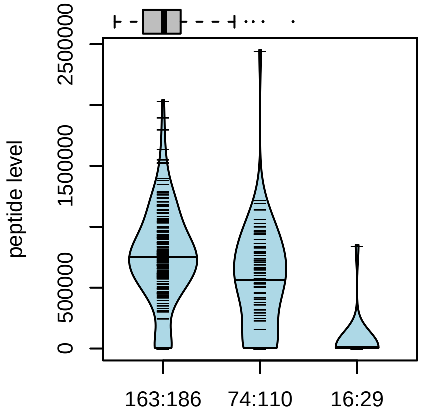

HPLKPDNQPFPQSVSESCPGKpc4_NP.3
p−min=1.5e−06, pX=9.3e−16, N=253

var.27.HRG 3:186395436:C:T

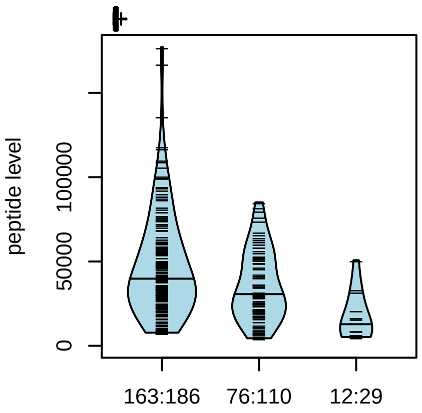

HPLKPDNQPFPQSVSESCPGKpc4_NP.4
p−min=2.8e−07, pX=1.1e−11, N=251

var.27.HRG 3:186395436:C:T

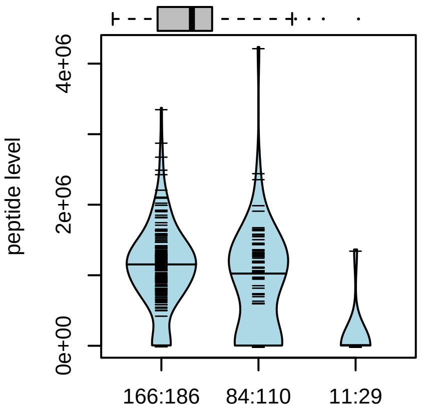

HPLKPDNQPFPQSVSESCPGKpc4_NP.5
p−maj=1.4e−07, pX=2.8e−12, N=261

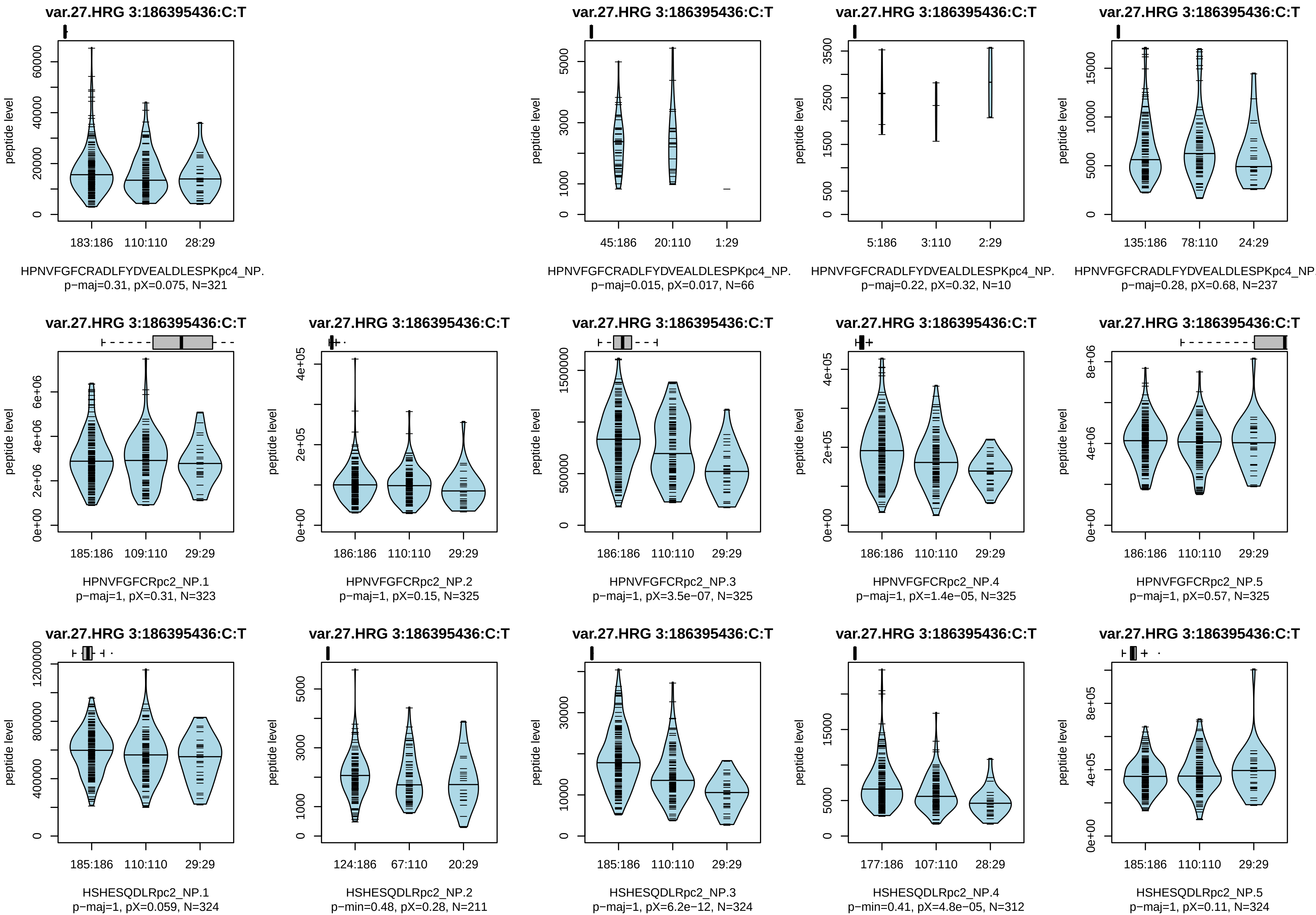
var.27.HRG 3:186395436:C:T
peptide level
183:186
110:110
28:29
HPNVFGFCRADLFYDVEALDLESPKpc4_NP.
p−maj=0.31, pX=0.075, N=321
var.27.HRG 3:186395436:C:T
peptide level
45:186
20:110
1:29
HPNVFGFCRADLFYDVEALDLESPKpc4_NP.
p−maj=0.015, pX=0.017, N=66
var.27.HRG 3:186395436:C:T
peptide level
5:186
3:110
2:29
HPNVFGFCRADLFYDVEALDLESPKpc4_NP.
p−maj=0.22, pX=0.32, N=10
var.27.HRG 3:186395436:C:T
peptide level
135:186
78:110
24:29
HPNVFGFCRADLFYDVEALDLESPKpc4_NP.
p−maj=0.28, pX=0.68, N=237
var.27.HRG 3:186395436:C:T
peptide level
185:186
109:110
29:29
HPNVFGFCRpc2_NP.1
p−maj=1, pX=0.31, N=323
var.27.HRG 3:186395436:C:T
peptide level
186:186
110:110
29:29
HPNVFGFCRpc2_NP.2
p−maj=1, pX=0.15, N=325
var.27.HRG 3:186395436:C:T
peptide level
186:186
110:110
29:29
HPNVFGFCRpc2_NP.3
p−maj=1, pX=3.5e−07, N=325
var.27.HRG 3:186395436:C:T
peptide level
186:186
110:110
29:29
HPNVFGFCRpc2_NP.4
p−maj=1, pX=1.4e−05, N=325
var.27.HRG 3:186395436:C:T
peptide level
186:186
110:110
29:29
HPNVFGFCRpc2_NP.5
p−maj=1, pX=0.57, N=325
var.27.HRG 3:186395436:C:T
peptide level
185:186
110:110
29:29
HSHESQDLRpc2_NP.1
p−maj=1, pX=0.059, N=324
var.27.HRG 3:186395436:C:T
peptide level
124:186
67:110
20:29
HSHESQDLRpc2_NP.2
p−min=0.48, pX=0.28, N=211
var.27.HRG 3:186395436:C:T
peptide level
185:186
110:110
29:29
HSHESQDLRpc2_NP.3
p−maj=1, pX=6.2e−12, N=324
var.27.HRG 3:186395436:C:T
peptide level
177:186
107:110
28:29
HSHESQDLRpc2_NP.4
p−min=0.41, pX=4.8e−05, N=312
var.27.HRG 3:186395436:C:T
peptide level
185:186
110:110
29:29
HSHESQDLRpc2_NP.5
p−maj=1, pX=0.11, N=324

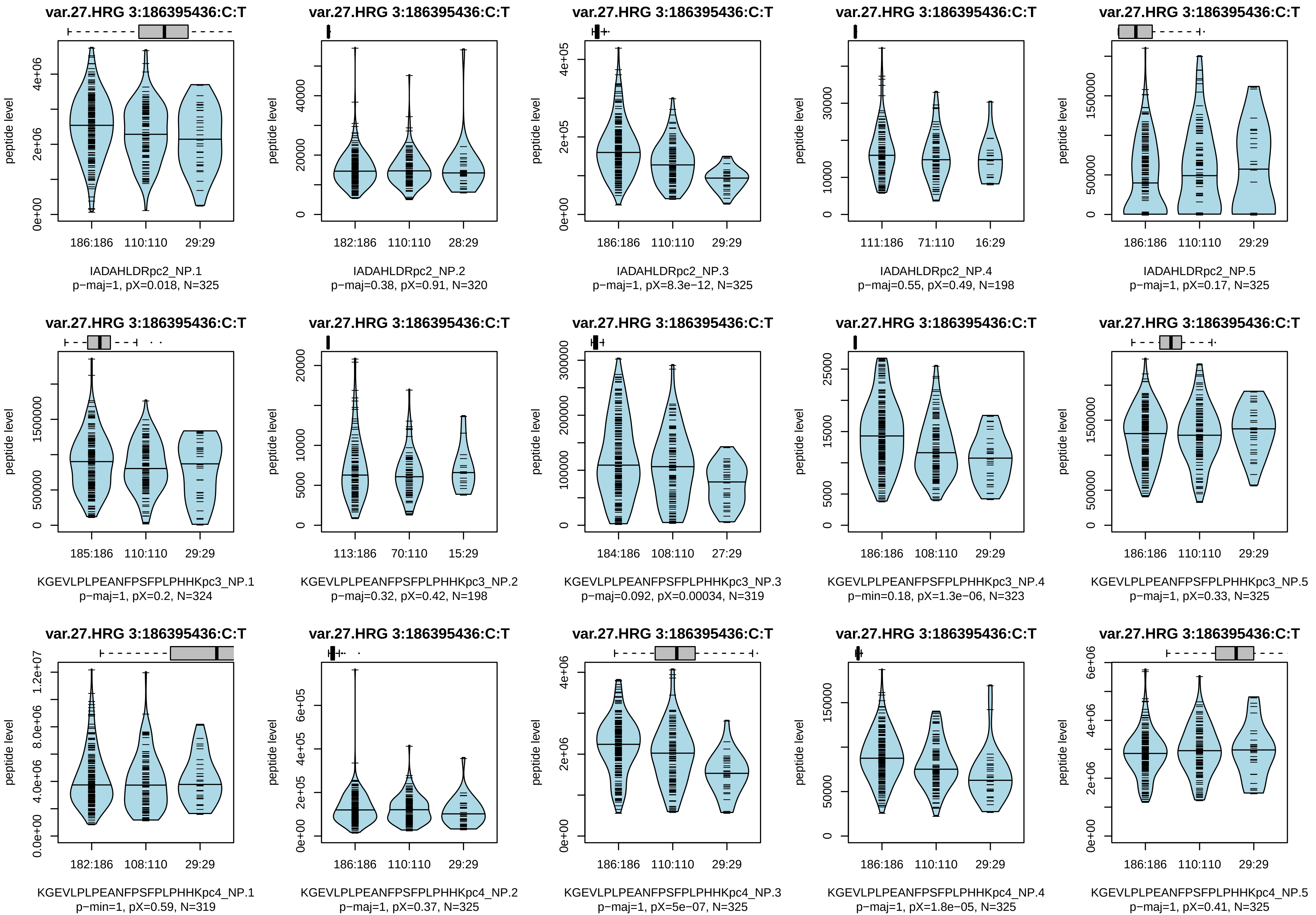

var.27.HRG 3:186395436:C:T
peptide level
0e+00
2e+06
4e+06
186:186
110:110
29:29
IADAHLDRpc2_NP.1
p−maj=1, pX=0.018, N=325
var.27.HRG 3:186395436:C:T
peptide level
0
20000
40000
182:186
110:110
28:29
IADAHLDRpc2_NP.2
p−maj=0.38, pX=0.91, N=320
var.27.HRG 3:186395436:C:T
peptide level
0e+00
2e+05
4e+05
186:186
110:110
29:29
IADAHLDRpc2_NP.3
p−maj=1, pX=8.3e−12, N=325
var.27.HRG 3:186395436:C:T
peptide level
0
10000
30000
111:186
71:110
16:29
IADAHLDRpc2_NP.4
p−maj=0.55, pX=0.49, N=198
var.27.HRG 3:186395436:C:T
peptide level
0
500000
1500000
186:186
110:110
29:29
IADAHLDRpc2_NP.5
p−maj=1, pX=0.17, N=325
var.27.HRG 3:186395436:C:T
peptide level
0
500000
1500000
185:186
110:110
29:29
KGEVLPLPEANFPSFPLPHHKpc3_NP.1
p−maj=1, pX=0.2, N=324
var.27.HRG 3:186395436:C:T
peptide level
0
5000
10000
20000
113:186
70:110
15:29
KGEVLPLPEANFPSFPLPHHKpc3_NP.2
p−maj=0.32, pX=0.42, N=198
var.27.HRG 3:186395436:C:T
peptide level
0
100000
200000
300000
184:186
108:110
27:29
KGEVLPLPEANFPSFPLPHHKpc3_NP.3
p−maj=0.092, pX=0.00034, N=319
var.27.HRG 3:186395436:C:T
peptide level
0
5000
15000
25000
186:186
108:110
29:29
KGEVLPLPEANFPSFPLPHHKpc3_NP.4
p−min=0.18, pX=1.3e−06, N=323
var.27.HRG 3:186395436:C:T
peptide level
0
500000
1500000
186:186
110:110
29:29
KGEVLPLPEANFPSFPLPHHKpc3_NP.5
p−maj=1, pX=0.33, N=325
var.27.HRG 3:186395436:C:T
peptide level
0.0e+00
4.0e+06
8.0e+06
1.2e+07
182:186
108:110
29:29
KGEVLPLPEANFPSFPLPHHKpc4_NP.1
p−min=1, pX=0.59, N=319
var.27.HRG 3:186395436:C:T
peptide level
0e+00
2e+05
4e+05
6e+05
186:186
110:110
29:29
KGEVLPLPEANFPSFPLPHHKpc4_NP.2
p−maj=1, pX=0.37, N=325
var.27.HRG 3:186395436:C:T
peptide level
0e+00
2e+06
4e+06
186:186
110:110
29:29
KGEVLPLPEANFPSFPLPHHKpc4_NP.3
p−maj=1, pX=5e−07, N=325
var.27.HRG 3:186395436:C:T
peptide level
0
50000
150000
186:186
110:110
29:29
KGEVLPLPEANFPSFPLPHHKpc4_NP.4
p−maj=1, pX=1.8e−05, N=325
var.27.HRG 3:186395436:C:T
peptide level
0e+00
2e+06
4e+06
6e+06
186:186
110:110
29:29
KGEVLPLPEANFPSFPLPHHKpc4_NP.5
p−maj=1, pX=0.41, N=325

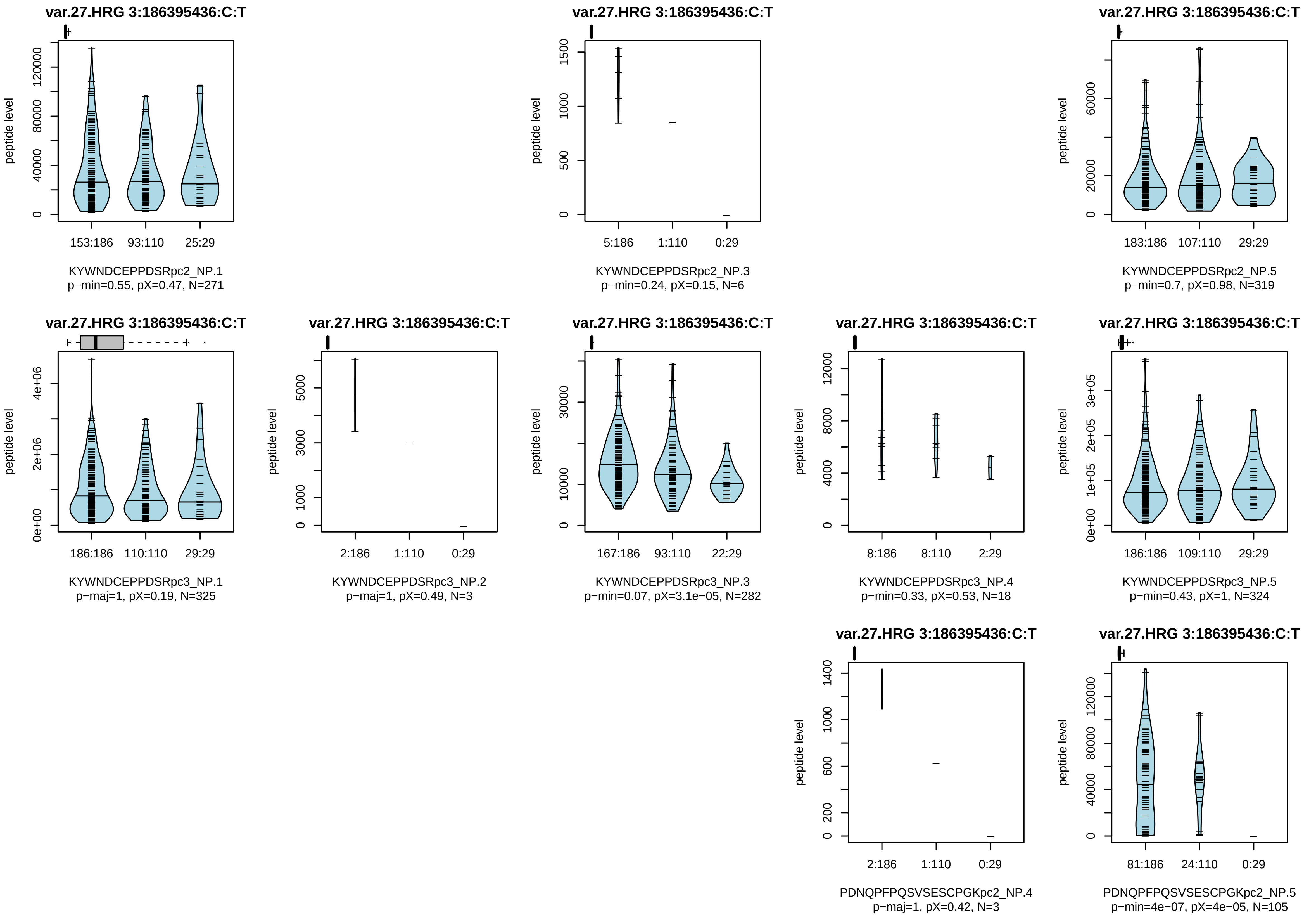

var.27.HRG 3:186395436:C:T
peptide level
153:186
93:110
25:29
KYWNDCEPPDSRpc2_NP.1
p−min=0.55, pX=0.47, N=271
var.27.HRG 3:186395436:C:T
peptide level
5:186
1:110
0:29
KYWNDCEPPDSRpc2_NP.3
p−min=0.24, pX=0.15, N=6
var.27.HRG 3:186395436:C:T
peptide level
183:186
107:110
29:29
KYWNDCEPPDSRpc2_NP.5
p−min=0.7, pX=0.98, N=319
var.27.HRG 3:186395436:C:T
peptide level
186:186
110:110
29:29
KYWNDCEPPDSRpc3_NP.1
p−maj=1, pX=0.19, N=325
var.27.HRG 3:186395436:C:T
peptide level
2:186
1:110
0:29
KYWNDCEPPDSRpc3_NP.2
p−maj=1, pX=0.49, N=3
var.27.HRG 3:186395436:C:T
peptide level
167:186
93:110
22:29
KYWNDCEPPDSRpc3_NP.3
p−min=0.07, pX=3.1e−05, N=282
var.27.HRG 3:186395436:C:T
peptide level
8:186
8:110
2:29
KYWNDCEPPDSRpc3_NP.4
p−min=0.33, pX=0.53, N=18
var.27.HRG 3:186395436:C:T
peptide level
186:186
109:110
29:29
KYWNDCEPPDSRpc3_NP.5
p−min=0.43, pX=1, N=324
var.27.HRG 3:186395436:C:T
peptide level
2:186
1:110
0:29
PDNQPFPQSVSESCPGKpc2_NP.4
p−maj=1, pX=0.42, N=3
var.27.HRG 3:186395436:C:T
peptide level
81:186
24:110
0:29
PDNQPFPQSVSESCPGKpc2_NP.5
p−min=4e−07, pX=4e−05, N=105

var.27.HRG 3:186395436:C:T

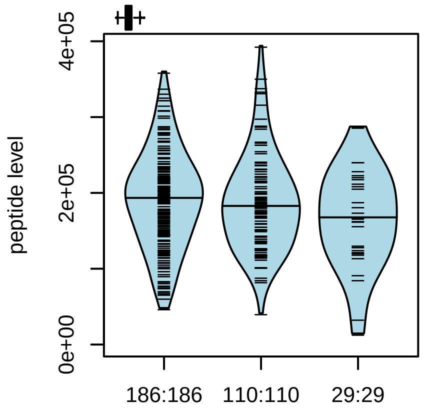

PHEHGPPPPPDERpc2_NP.1
p−maj=1, pX=0.074, N=325

var.27.HRG 3:186395436:C:T

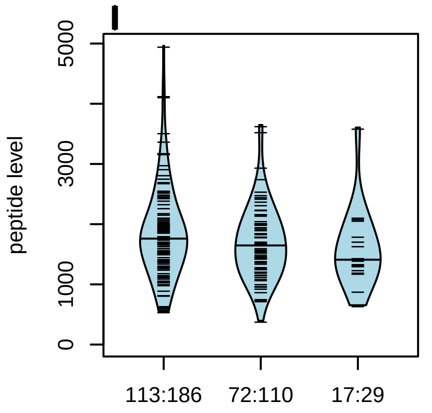

PHEHGPPPPPDERpc2_NP.2
p−min=0.57, pX=0.44, N=202

var.27.HRG 3:186395436:C:T

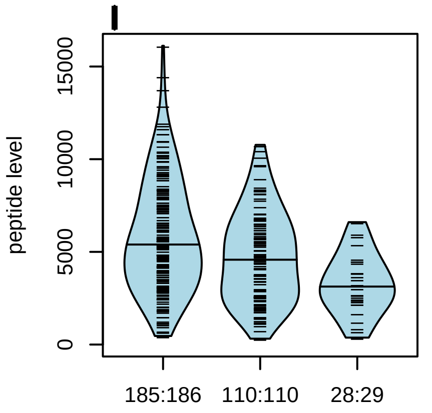

PHEHGPPPPPDERpc2_NP.3
p−maj=0.17, pX=3.8e−07, N=323

var.27.HRG 3:186395436:C:T

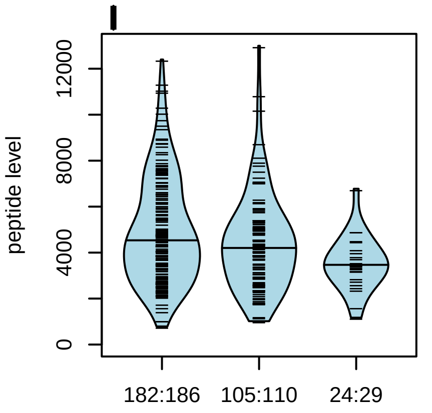

PHEHGPPPPPDERpc2_NP.4
p−maj=0.0045, pX=2.1e−05, N=311

var.27.HRG 3:186395436:C:T

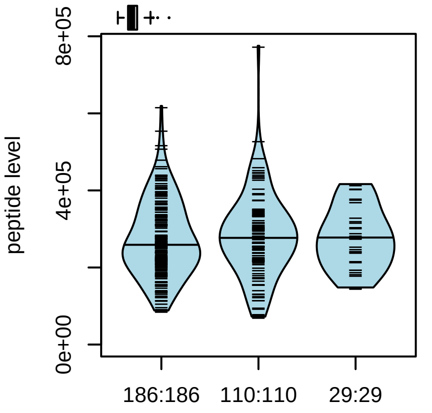

PHEHGPPPPPDERpc2_NP.5
p−maj=1, pX=0.64, N=325

var.27.HRG 3:186395436:C:T

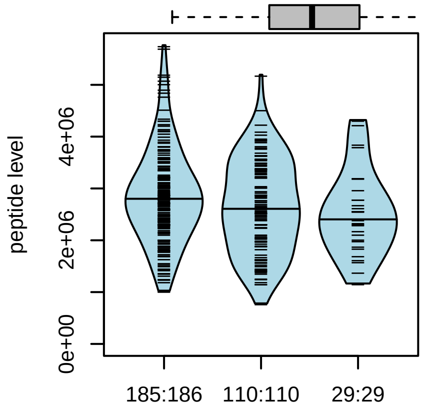

PHEHGPPPPPDERpc3_NP.1
p−maj=1, pX=0.027, N=324

var.27.HRG 3:186395436:C:T

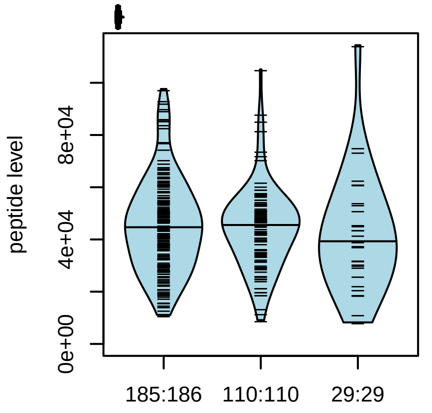

PHEHGPPPPPDERpc3_NP.2
p−maj=1, pX=0.37, N=324

var.27.HRG 3:186395436:C:T

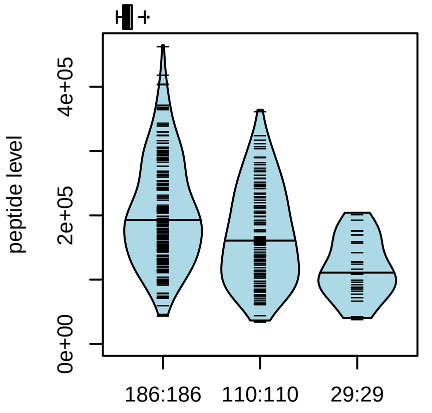

PHEHGPPPPPDERpc3_NP.3
p−maj=1, pX=6e−10, N=325

var.27.HRG 3:186395436:C:T

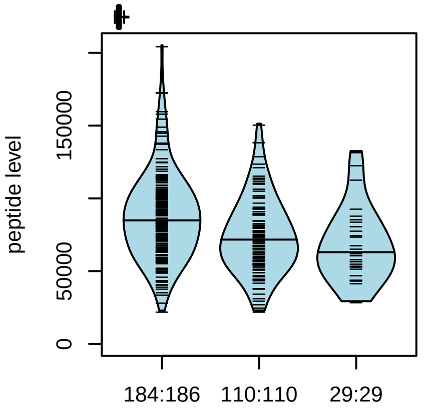

PHEHGPPPPPDERpc3_NP.4
p−min=0.51, pX=2.8e−05, N=323

var.27.HRG 3:186395436:C:T

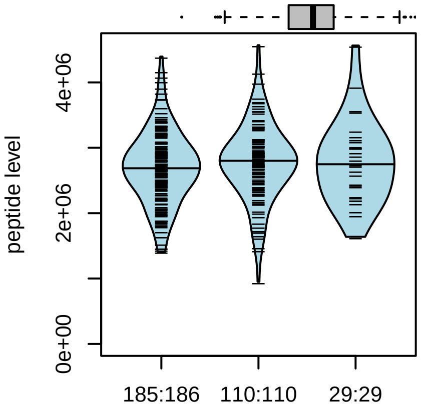

PHEHGPPPPPDERpc3_NP.5
p−maj=1, pX=0.17, N=324

var.27.HRG 3:186395436:C:T

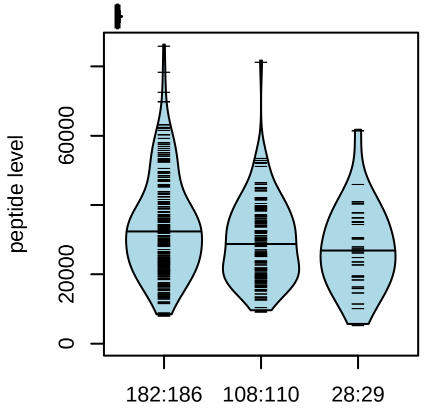

PSEIVIGQCKpc2_NP.1
p−maj=0.48, pX=0.003, N=318

var.27.HRG 3:186395436:C:T

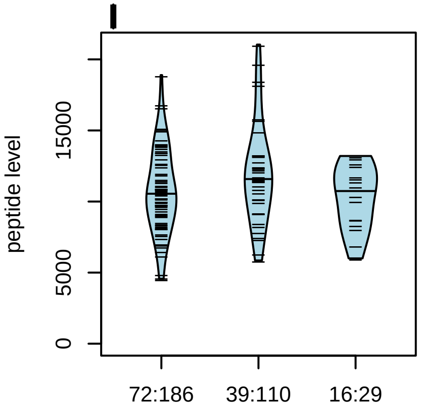

PSEIVIGQCKpc2_NP.5
p−maj=0.074, pX=0.31, N=127

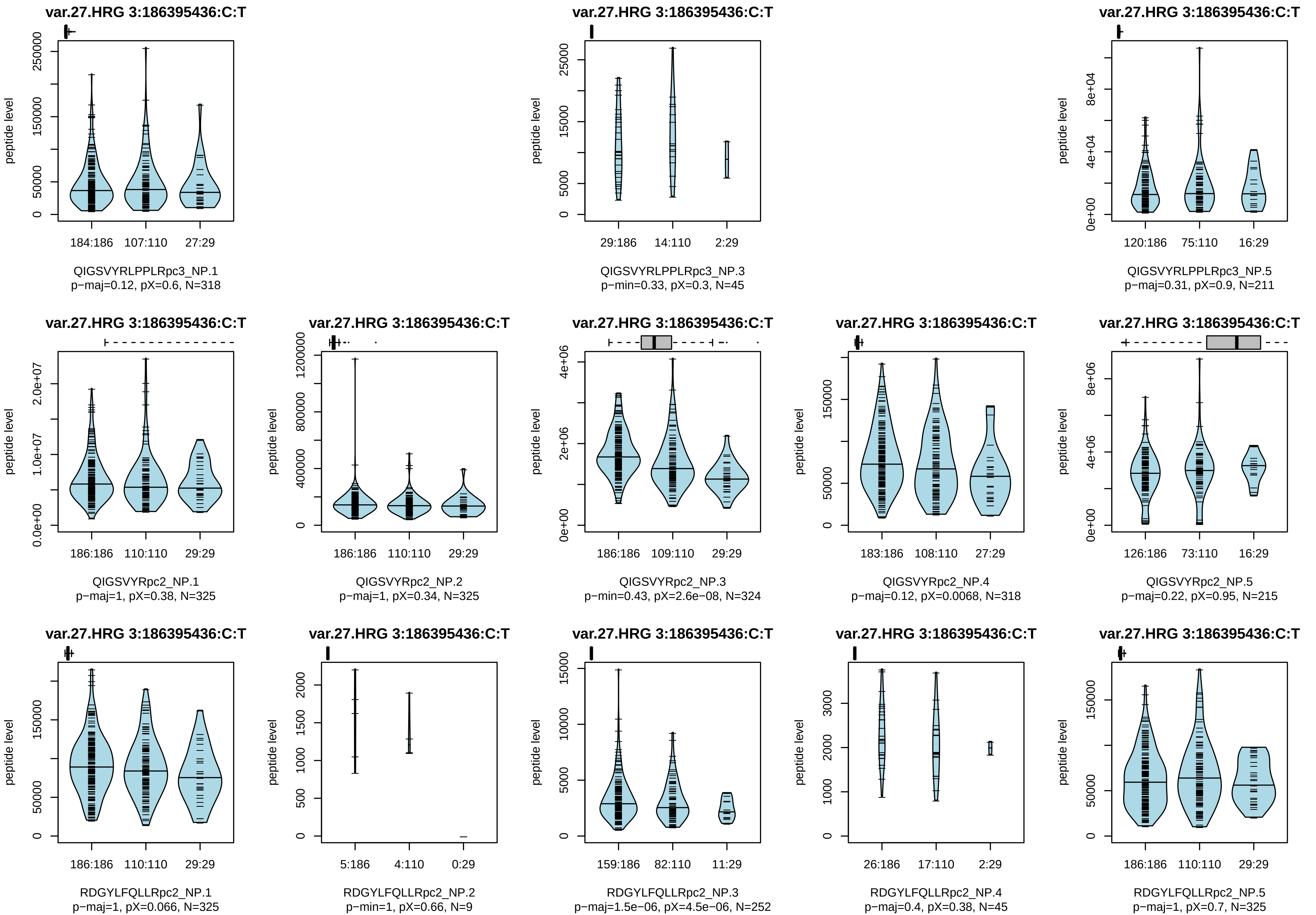

var.27.HRG 3:186395436:C:T
peptide level
184:186 107:110 27:29
QIGSVYRLPPLRpc3_NP.1
p−maj=0.12, pX=0.6, N=318
var.27.HRG 3:186395436:C:T
peptide level
29:186 14:110 2:29
QIGSVYRLPPLRpc3_NP.3
p−min=0.33, pX=0.3, N=45
var.27.HRG 3:186395436:C:T
peptide level
120:186 75:110 16:29
QIGSVYRLPPLRpc3_NP.5
p−maj=0.31, pX=0.9, N=211
var.27.HRG 3:186395436:C:T
peptide level
186:186 110:110 29:29
QIGSVYRpc2_NP.1
p−maj=1, pX=0.38, N=325
var.27.HRG 3:186395436:C:T
peptide level
186:186 110:110 29:29
QIGSVYRpc2_NP.2
p−maj=1, pX=0.34, N=325
var.27.HRG 3:186395436:C:T
peptide level
186:186 109:110 29:29
QIGSVYRpc2_NP.3
p−min=0.43, pX=2.6e−08, N=324
var.27.HRG 3:186395436:C:T
peptide level
183:186 108:110 27:29
QIGSVYRpc2_NP.4
p−maj=0.12, pX=0.0068, N=318
var.27.HRG 3:186395436:C:T
peptide level
126:186 73:110 16:29
QIGSVYRpc2_NP.5
p−maj=0.22, pX=0.95, N=215
var.27.HRG 3:186395436:C:T
peptide level
186:186 110:110 29:29
RDGYLFQLLRpc2_NP.1
p−maj=1, pX=0.066, N=325
var.27.HRG 3:186395436:C:T
peptide level
5:186 4:110 0:29
RDGYLFQLLRpc2_NP.2
p−min=1, pX=0.66, N=9
var.27.HRG 3:186395436:C:T
peptide level
159:186 82:110 11:29
RDGYLFQLLRpc2_NP.3
p−maj=1.5e−06, pX=4.5e−06, N=252
var.27.HRG 3:186395436:C:T
peptide level
26:186 17:110 2:29
RDGYLFQLLRpc2_NP.4
p−maj=0.4, pX=0.38, N=45
var.27.HRG 3:186395436:C:T
peptide level
186:186 110:110 29:29
RDGYLFQLLRpc2_NP.5
p−maj=1, pX=0.7, N=325

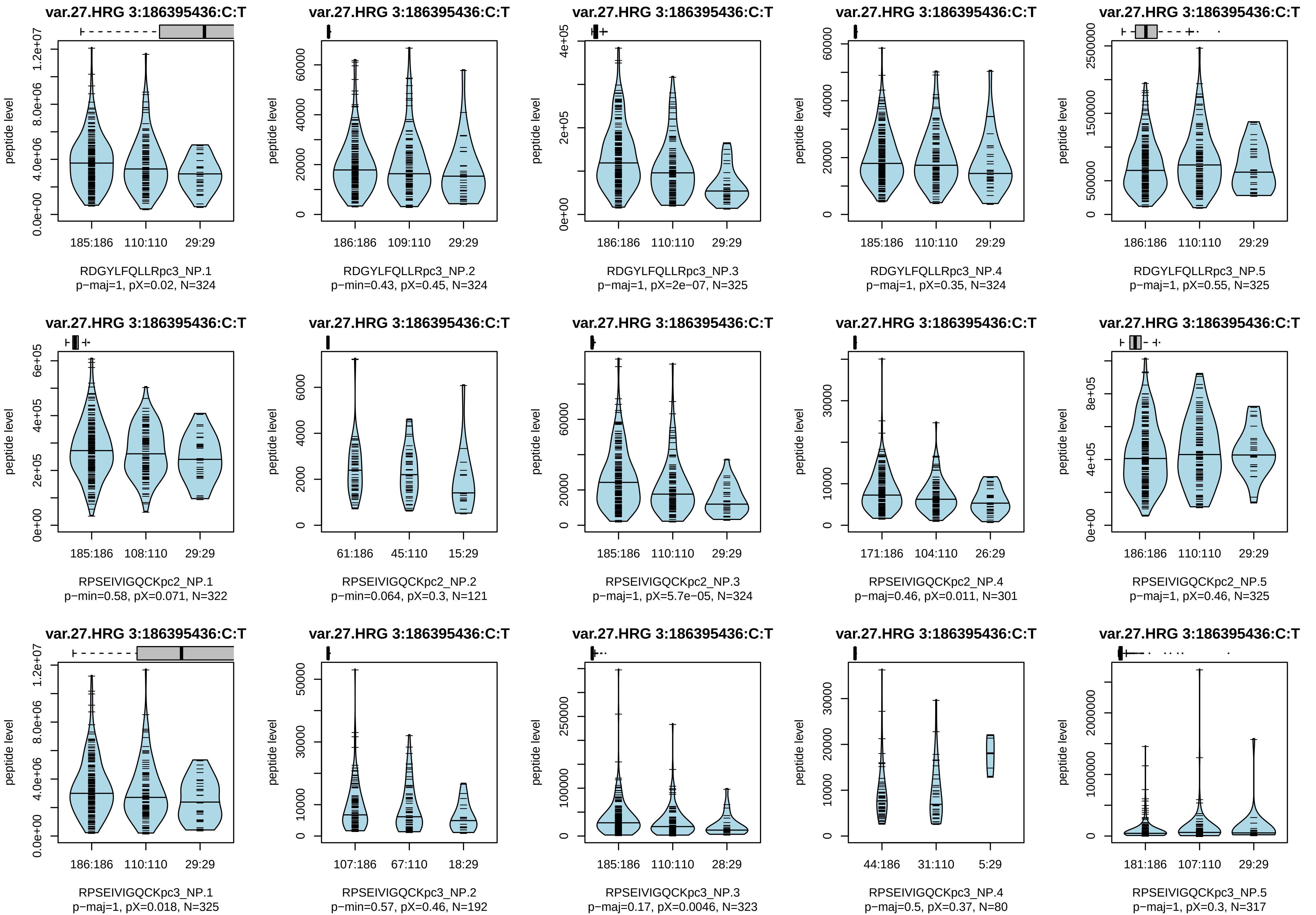
var.27.HRG 3:186395436:C:T
peptide level
185:186 110:110 29:29
RDGYLFQLLRpc3_NP.1
p−maj=1, pX=0.02, N=324
var.27.HRG 3:186395436:C:T
peptide level
186:186 109:110 29:29
RDGYLFQLLRpc3_NP.2
p−min=0.43, pX=0.45, N=324
var.27.HRG 3:186395436:C:T
peptide level
186:186 110:110 29:29
RDGYLFQLLRpc3_NP.3
p−maj=1, pX=2e−07, N=325
var.27.HRG 3:186395436:C:T
peptide level
185:186 110:110 29:29
RDGYLFQLLRpc3_NP.4
p−maj=1, pX=0.35, N=324
var.27.HRG 3:186395436:C:T
peptide level
186:186 110:110 29:29
RDGYLFQLLRpc3_NP.5
p−maj=1, pX=0.55, N=325
var.27.HRG 3:186395436:C:T
peptide level
185:186 108:110 29:29
RPSEIVIGQCKpc2_NP.1
p−min=0.58, pX=0.071, N=322
var.27.HRG 3:186395436:C:T
peptide level
61:186 45:110 15:29
RPSEIVIGQCKpc2_NP.2
p−min=0.064, pX=0.3, N=121
var.27.HRG 3:186395436:C:T
peptide level
185:186 110:110 29:29
RPSEIVIGQCKpc2_NP.3
p−maj=1, pX=5.7e−05, N=324
var.27.HRG 3:186395436:C:T
peptide level
171:186 104:110 26:29
RPSEIVIGQCKpc2_NP.4
p−maj=0.46, pX=0.011, N=301
var.27.HRG 3:186395436:C:T
peptide level
186:186 110:110 29:29
RPSEIVIGQCKpc2_NP.5
p−maj=1, pX=0.46, N=325
var.27.HRG 3:186395436:C:T
peptide level
186:186 110:110 29:29
RPSEIVIGQCKpc3_NP.1
p−maj=1, pX=0.018, N=325
var.27.HRG 3:186395436:C:T
peptide level
107:186 67:110 18:29
RPSEIVIGQCKpc3_NP.2
p−min=0.57, pX=0.46, N=192
var.27.HRG 3:186395436:C:T
peptide level
185:186 110:110 28:29
RPSEIVIGQCKpc3_NP.3
p−maj=0.17, pX=0.0046, N=323
var.27.HRG 3:186395436:C:T
peptide level
44:186 31:110 5:29
RPSEIVIGQCKpc3_NP.4
p−maj=0.5, pX=0.37, N=80
var.27.HRG 3:186395436:C:T
peptide level
181:186 107:110 29:29
RPSEIVIGQCKpc3_NP.5
p−maj=1, pX=0.3, N=317

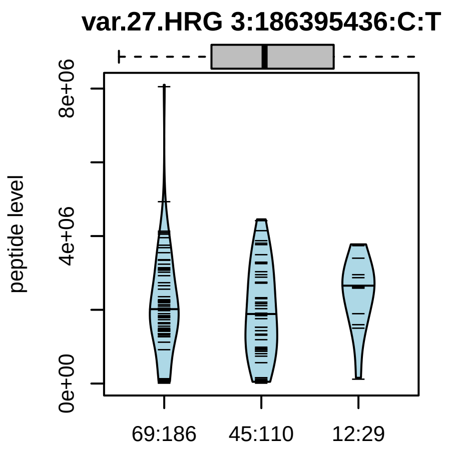

SGFPQVSMFFTHTFPKpc2_NP.1
p−min=0.49, pX=0.52, N=126

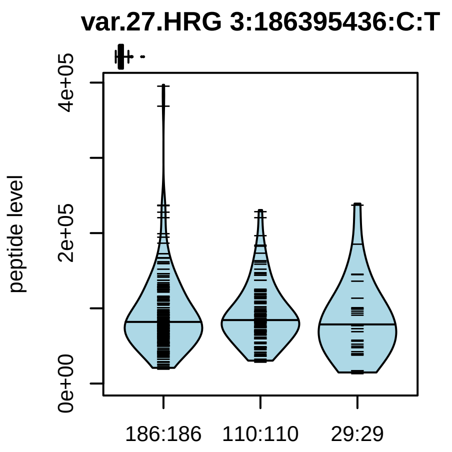

SGFPQVSMFFTHTFPKpc2_NP.2
p−maj=1, pX=0.45, N=325

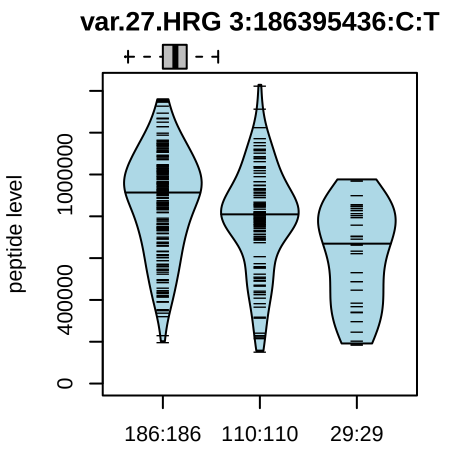

SGFPQVSMFFTHTFPKpc2_NP.3
p−maj=1, pX=1.9e−07, N=325

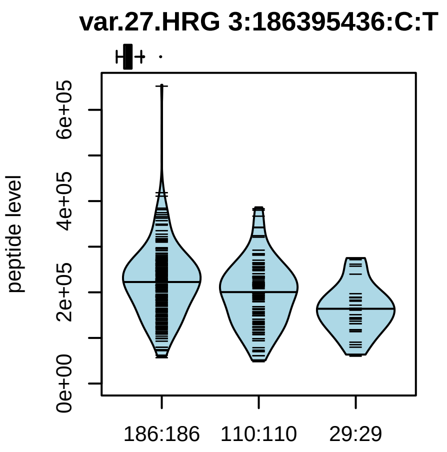

SGFPQVSMFFTHTFPKpc2_NP.4
p−maj=1, pX=6.3e−06, N=325

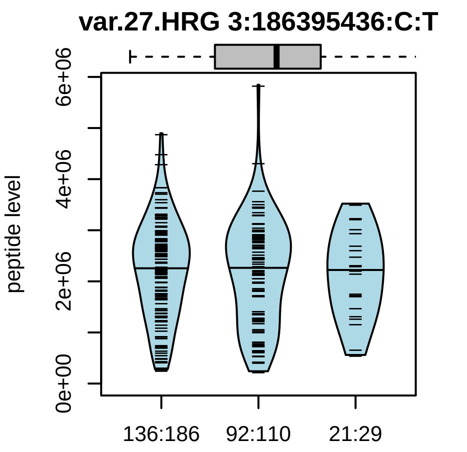

SGFPQVSMFFTHTFPKpc2_NP.5
p−min=0.087, pX=0.64, N=249

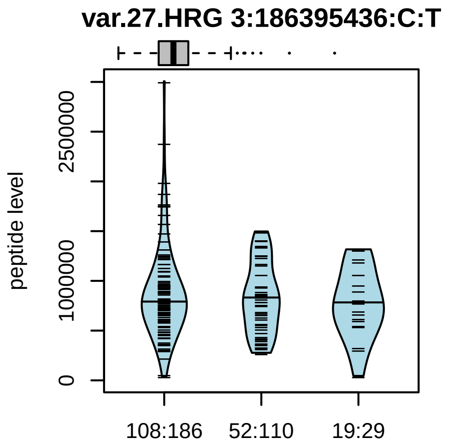

SGFPQVSMFFTHTFPKpc3_NP.1
p−min=0.22, pX=0.35, N=179

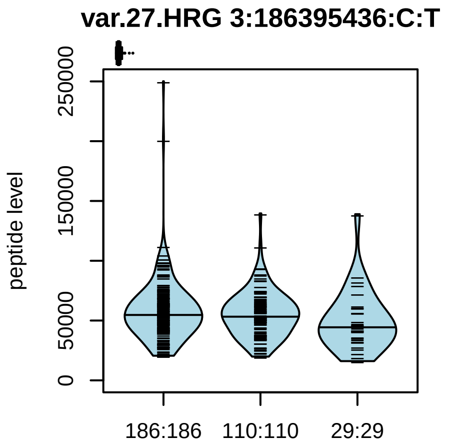

SGFPQVSMFFTHTFPKpc3_NP.2
p−maj=1, pX=0.085, N=325

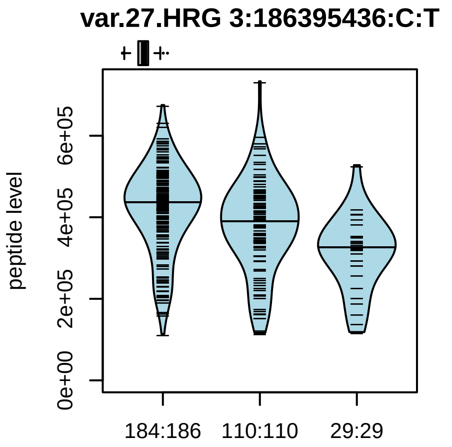

SGFPQVSMFFTHTFPKpc3_NP.3
p−min=0.51, pX=2.1e−06, N=323

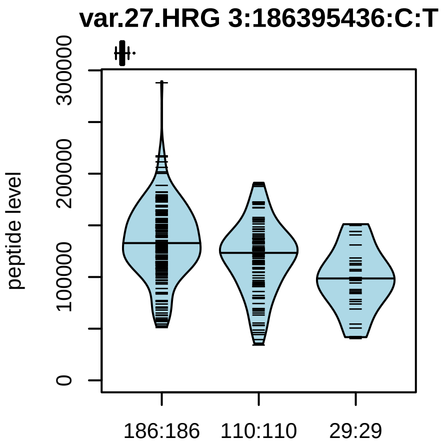

SGFPQVSMFFTHTFPKpc3_NP.4
p−maj=1, pX=4.8e−09, N=325

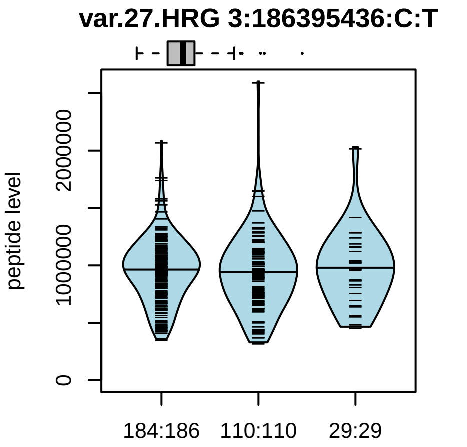

SGFPQVSMFFTHTFPKpc3_NP.5
p−min=0.51, pX=0.86, N=323

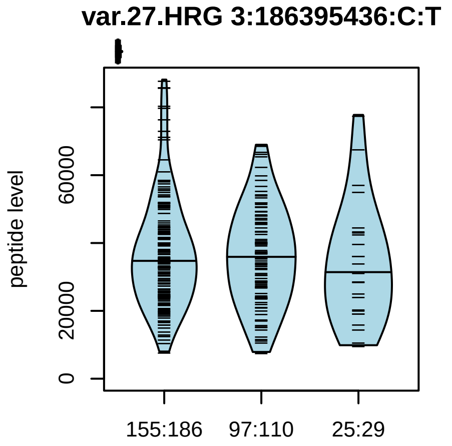

SGFPQVSMFFTHTFPKpc4_NP.1
p−min=0.34, pX=0.94, N=277

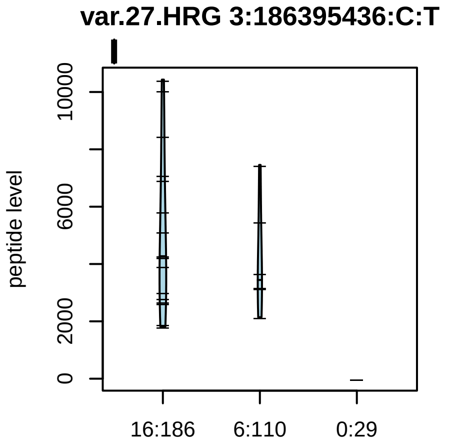

SGFPQVSMFFTHTFPKpc4_NP.3
p−min=0.18, pX=0.067, N=22

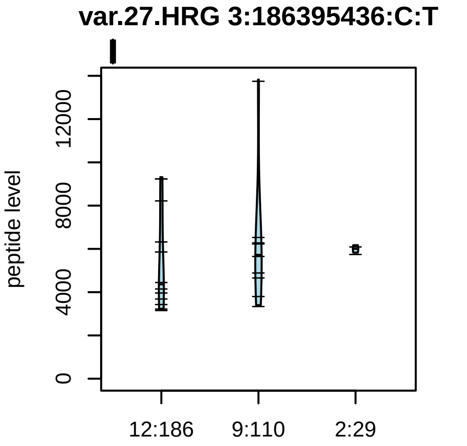

SGFPQVSMFFTHTFPKpc4_NP.5
p−min=0.67, pX=0.58, N=23

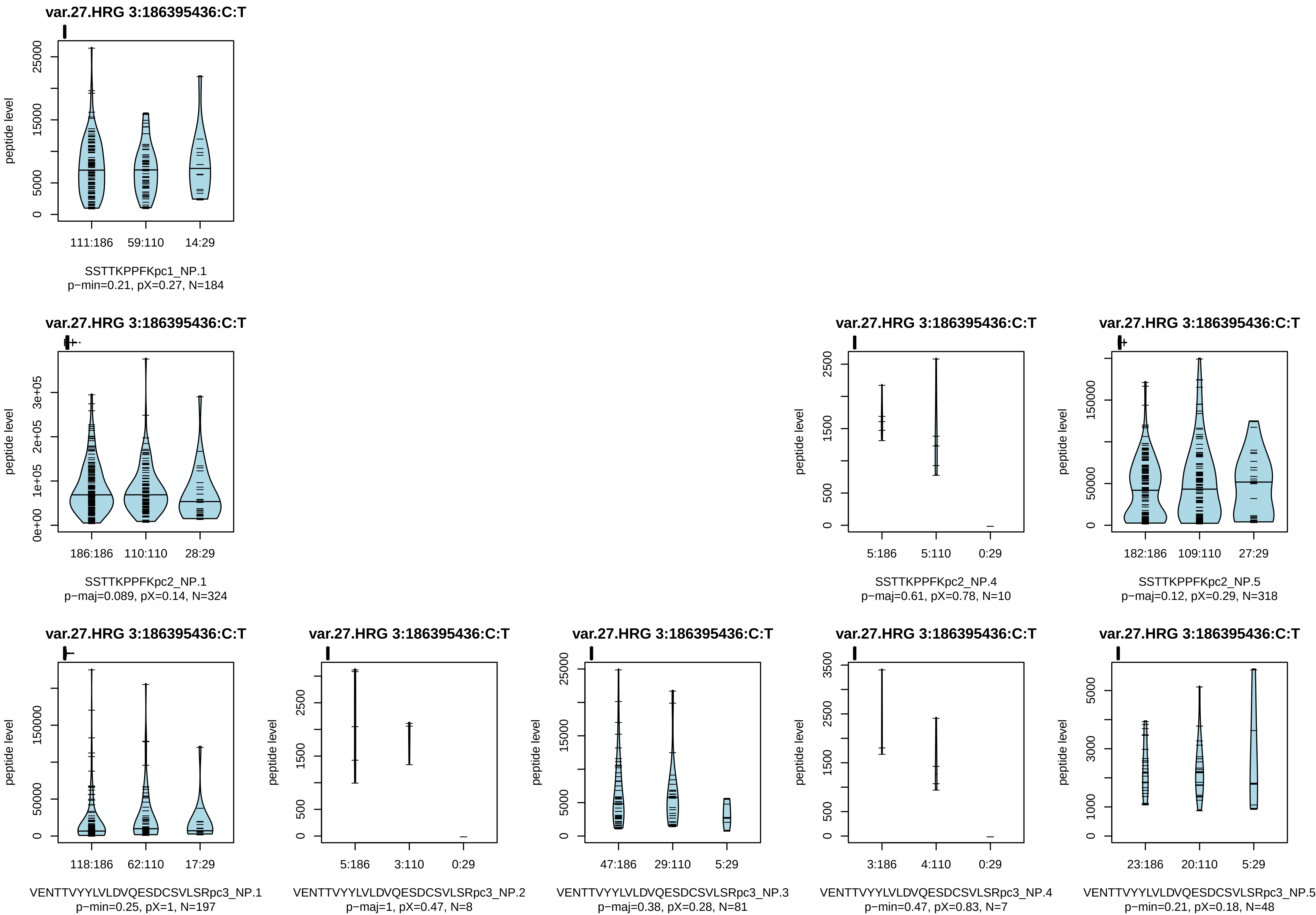
var.27.HRG 3:186395436:C:T
peptide level
111:186 59:110 14:29
SSTTKPPFKpc1_NP.1
p−min=0.21, pX=0.27, N=184
var.27.HRG 3:186395436:C:T
peptide level
186:186 110:110 28:29
SSTTKPPFKpc2_NP.1
p−maj=0.089, pX=0.14, N=324
var.27.HRG 3:186395436:C:T
peptide level
5:186 5:110 0:29
SSTTKPPFKpc2_NP.4
p−maj=0.61, pX=0.78, N=10
var.27.HRG 3:186395436:C:T
peptide level
182:186 109:110 27:29
SSTTKPPFKpc2_NP.5
p−maj=0.12, pX=0.29, N=318
var.27.HRG 3:186395436:C:T
peptide level
118:186 62:110 17:29
VENTTVYYLVLDVQESDCSVLSRpc3_NP.1
p−min=0.25, pX=1, N=197
var.27.HRG 3:186395436:C:T
peptide level
5:186 3:110 0:29
VENTTVYYLVLDVQESDCSVLSRpc3_NP.2
p−maj=1, pX=0.47, N=8
var.27.HRG 3:186395436:C:T
peptide level
47:186 29:110 5:29
VENTTVYYLVLDVQESDCSVLSRpc3_NP.3
p−maj=0.38, pX=0.28, N=81
var.27.HRG 3:186395436:C:T
peptide level
3:186 4:110 0:29
VENTTVYYLVLDVQESDCSVLSRpc3_NP.4
p−min=0.47, pX=0.83, N=7
var.27.HRG 3:186395436:C:T
peptide level
23:186 20:110 5:29
VENTTVYYLVLDVQESDCSVLSRpc3_NP.5
p−min=0.21, pX=0.18, N=48

var.27.HRG 3:186395436:C:T

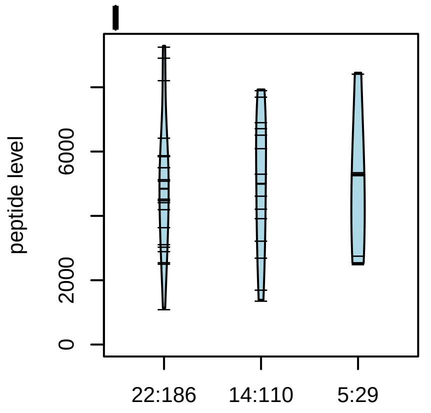

VIATRHSHESQDLRpc3_NP.1
p−maj=0.39, pX=0.53, N=41

var.27.HRG 3:186395436:C:T

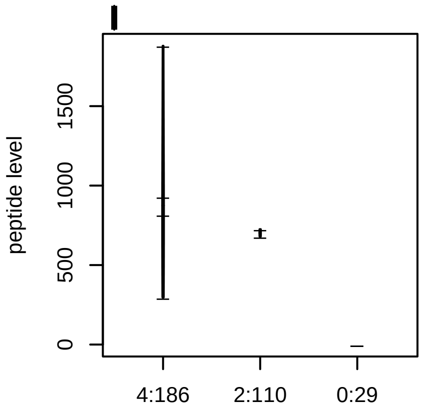

VIATRHSHESQDLRpc3_NP.3
p−min=1, pX=0.4, N=6

var.27.HRG 3:186395436:C:T

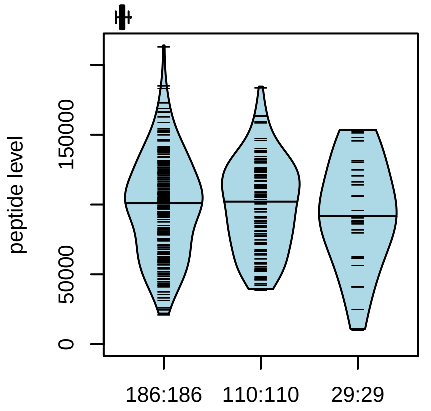

VRGGEGTGYFVDFSVRpc2_NP.1
p−maj=1, pX=0.68, N=325

var.27.HRG 3:186395436:C:T

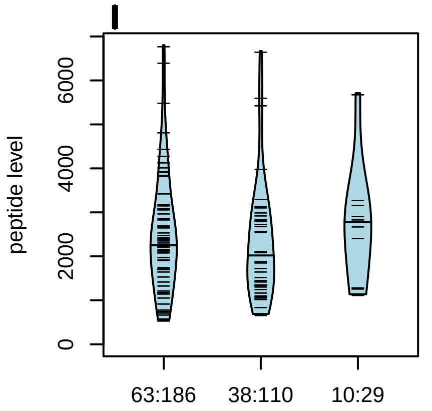

VRGGEGTGYFVDFSVRpc2_NP.2
p−min=0.91, pX=0.92, N=111

var.27.HRG 3:186395436:C:T

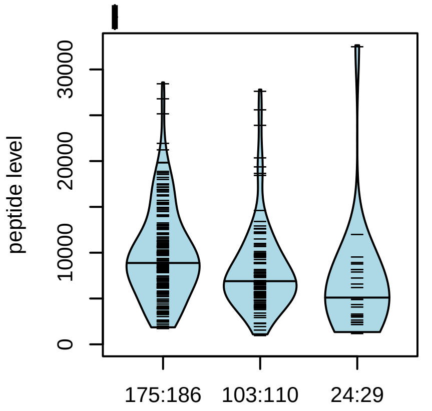

VRGGEGTGYFVDFSVRpc2_NP.3
p−maj=0.042, pX=0.00023, N=302

var.27.HRG 3:186395436:C:T

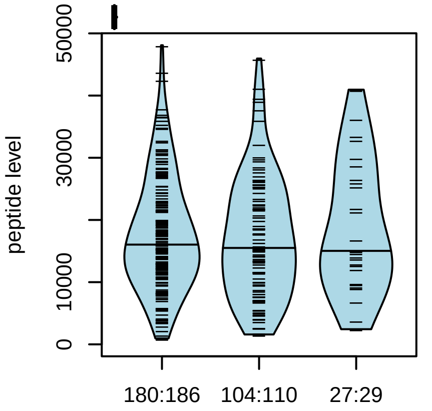

VRGGEGTGYFVDFSVRpc2_NP.4
p−min=0.28, pX=0.72, N=311

var.27.HRG 3:186395436:C:T

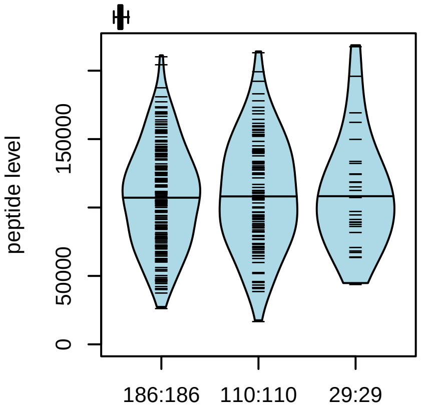

VRGGEGTGYFVDFSVRpc2_NP.5
p−maj=1, pX=0.62, N=325

var.27.HRG 3:186395436:C:T

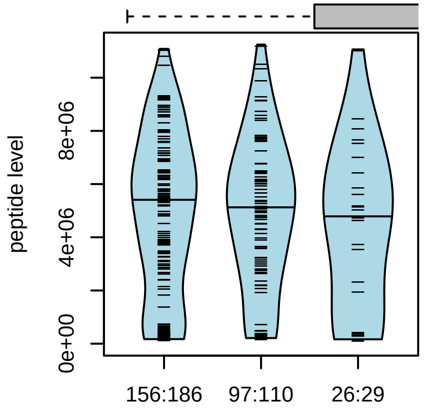

VRGGEGTGYFVDFSVRpc3_NP.1
p−min=0.26, pX=0.93, N=279

var.27.HRG 3:186395436:C:T

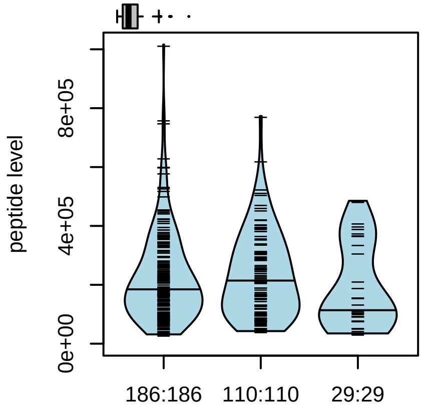

VRGGEGTGYFVDFSVRpc3_NP.2
p−maj=1, pX=0.52, N=325

var.27.HRG 3:186395436:C:T

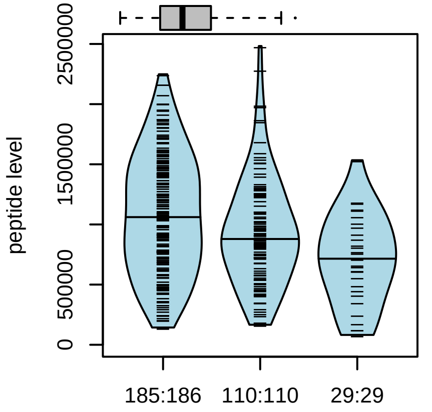

VRGGEGTGYFVDFSVRpc3_NP.3
p−maj=1, pX=6.1e−05, N=324

var.27.HRG 3:186395436:C:T

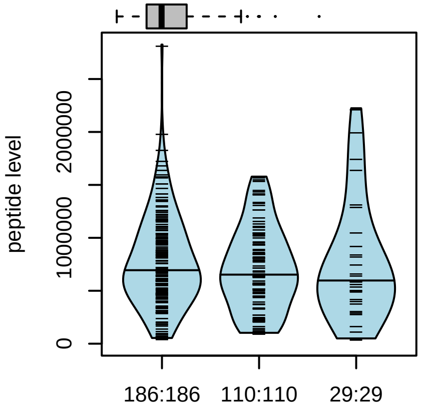

VRGGEGTGYFVDFSVRpc3_NP.4
p−maj=1, pX=0.5, N=325

var.27.HRG 3:186395436:C:T

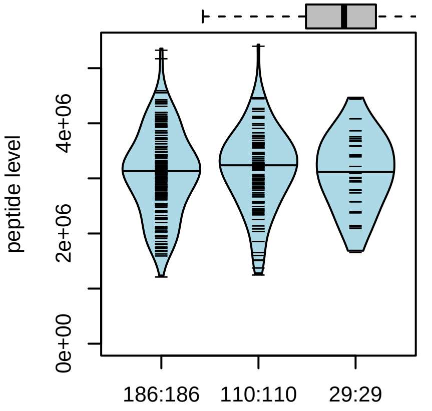

VRGGEGTGYFVDFSVRpc3_NP.5
p−maj=1, pX=0.59, N=325

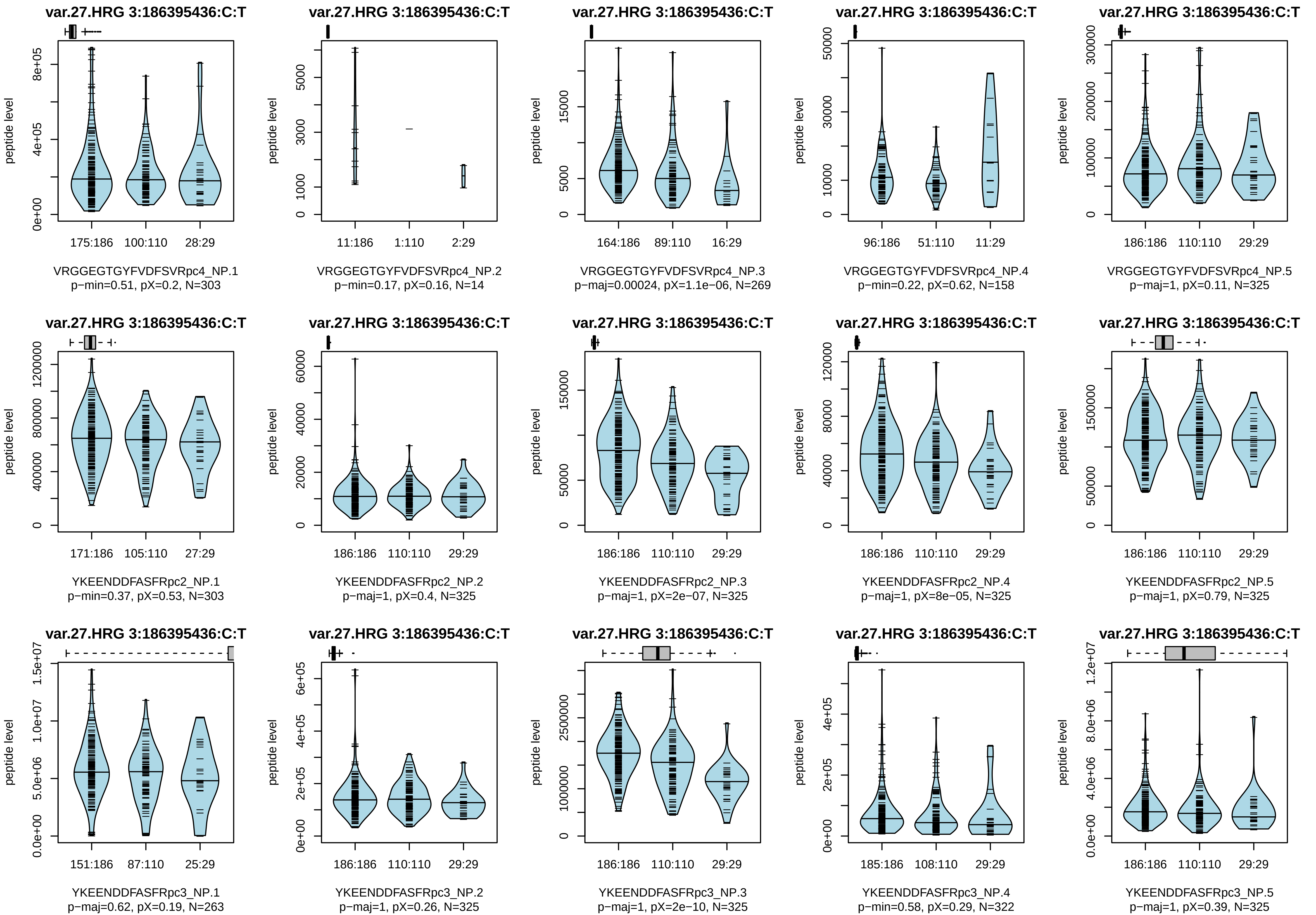
var.27.HRG 3:186395436:C:T
peptide level
175:186
100:110
28:29
VRGGEGTGYFVDFSVRpc4_NP.1
p−min=0.51, pX=0.2, N=303
var.27.HRG 3:186395436:C:T
peptide level
11:186
1:110
2:29
VRGGEGTGYFVDFSVRpc4_NP.2
p−min=0.17, pX=0.16, N=14
var.27.HRG 3:186395436:C:T
peptide level
164:186
89:110
16:29
VRGGEGTGYFVDFSVRpc4_NP.3
p−maj=0.00024, pX=1.1e−06, N=269
var.27.HRG 3:186395436:C:T
peptide level
96:186
51:110
11:29
VRGGEGTGYFVDFSVRpc4_NP.4
p−min=0.22, pX=0.62, N=158
var.27.HRG 3:186395436:C:T
peptide level
186:186
110:110
29:29
VRGGEGTGYFVDFSVRpc4_NP.5
p−maj=1, pX=0.11, N=325
var.27.HRG 3:186395436:C:T
peptide level
171:186
105:110
27:29
YKEENDDFASFRpc2_NP.1
p−min=0.37, pX=0.53, N=303
var.27.HRG 3:186395436:C:T
peptide level
186:186
110:110
29:29
YKEENDDFASFRpc2_NP.2
p−maj=1, pX=0.4, N=325
var.27.HRG 3:186395436:C:T
peptide level
186:186
110:110
29:29
YKEENDDFASFRpc2_NP.3
p−maj=1, pX=2e−07, N=325
var.27.HRG 3:186395436:C:T
peptide level
186:186
110:110
29:29
YKEENDDFASFRpc2_NP.4
p−maj=1, pX=8e−05, N=325
var.27.HRG 3:186395436:C:T
peptide level
186:186
110:110
29:29
YKEENDDFASFRpc2_NP.5
p−maj=1, pX=0.79, N=325
var.27.HRG 3:186395436:C:T
peptide level
151:186
87:110
25:29
YKEENDDFASFRpc3_NP.1
p−maj=0.62, pX=0.19, N=263
var.27.HRG 3:186395436:C:T
peptide level
186:186
110:110
29:29
YKEENDDFASFRpc3_NP.2
p−maj=1, pX=0.26, N=325
var.27.HRG 3:186395436:C:T
peptide level
186:186
110:110
29:29
YKEENDDFASFRpc3_NP.3
p−maj=1, pX=2e−10, N=325
var.27.HRG 3:186395436:C:T
peptide level
185:186
108:110
29:29
YKEENDDFASFRpc3_NP.4
p−min=0.58, pX=0.29, N=322
var.27.HRG 3:186395436:C:T
peptide level
186:186
110:110
29:29
YKEENDDFASFRpc3_NP.5
p−maj=1, pX=0.39, N=325

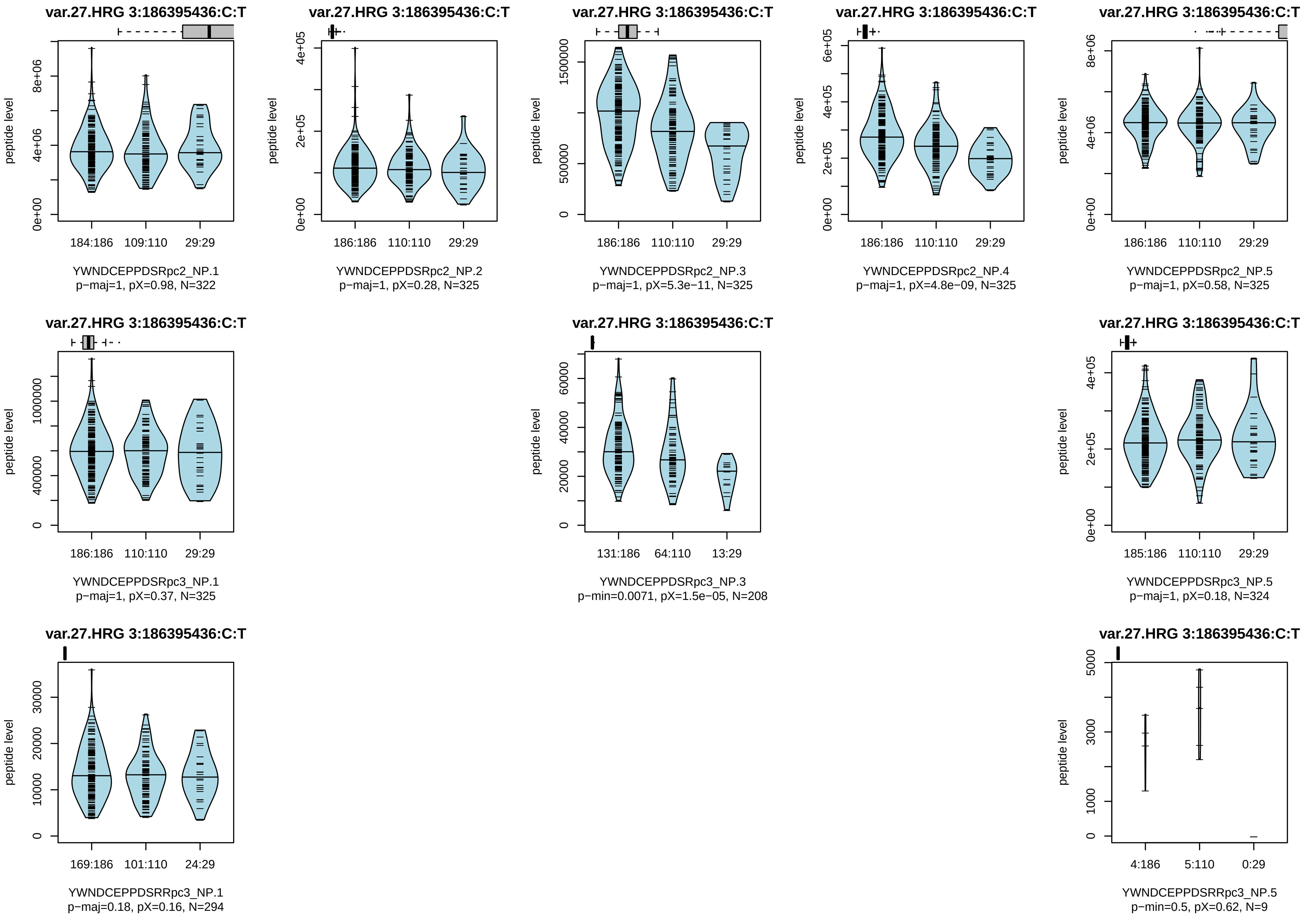
var.27.HRG 3:186395436:C:T
peptide level
184:186
109:110
29:29
YWNDCEPPDSRpc2_NP.1
p−maj=1, pX=0.98, N=322
var.27.HRG 3:186395436:C:T
186:186
110:110
29:29
YWNDCEPPDSRpc2_NP.2
p−maj=1, pX=0.28, N=325
var.27.HRG 3:186395436:C:T
186:186
110:110
29:29
YWNDCEPPDSRpc2_NP.3
p−maj=1, pX=5.3e−11, N=325
var.27.HRG 3:186395436:C:T
186:186
110:110
29:29
YWNDCEPPDSRpc2_NP.4
p−maj=1, pX=4.8e−09, N=325
var.27.HRG 3:186395436:C:T
186:186
110:110
29:29
YWNDCEPPDSRpc2_NP.5
p−maj=1, pX=0.58, N=325
var.27.HRG 3:186395436:C:T
186:186
110:110
29:29
YWNDCEPPDSRpc3_NP.1
p−maj=1, pX=0.37, N=325
var.27.HRG 3:186395436:C:T
131:186
64:110
13:29
YWNDCEPPDSRpc3_NP.3
p−min=0.0071, pX=1.5e−05, N=208
var.27.HRG 3:186395436:C:T
185:186
110:110
29:29
YWNDCEPPDSRpc3_NP.5
p−maj=1, pX=0.18, N=324
var.27.HRG 3:186395436:C:T
169:186
101:110
24:29
YWNDCEPPDSRRpc3_NP.1
p−maj=0.18, pX=0.16, N=294
var.27.HRG 3:186395436:C:T
4:186
5:110
0:29
YWNDCEPPDSRRpc3_NP.5
p−min=0.5, pX=0.62, N=9

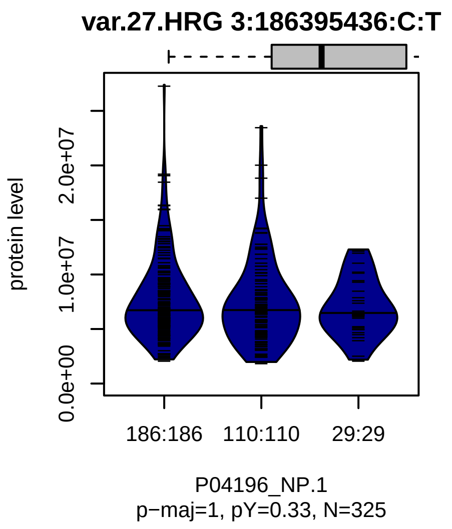
var.27.HRG 3:186395436:C:T
protein level
0.0e+00
1.0e+07
2.0e+07
186:186
110:110
29:29
P04196_NP.1
p−maj=1, pY=0.33, N=325

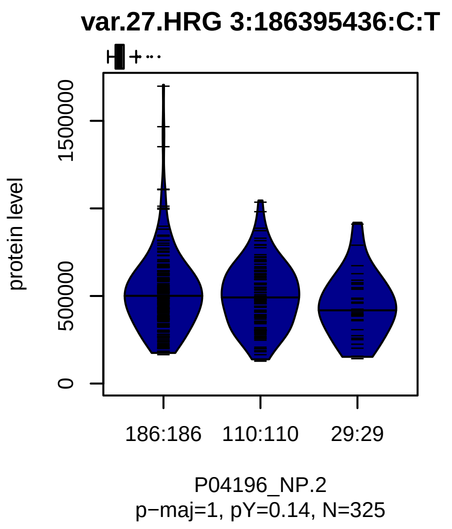
var.27.HRG 3:186395436:C:T
protein level
0
500000
1500000
186:186
110:110
29:29
P04196_NP.2
p−maj=1, pY=0.14, N=325

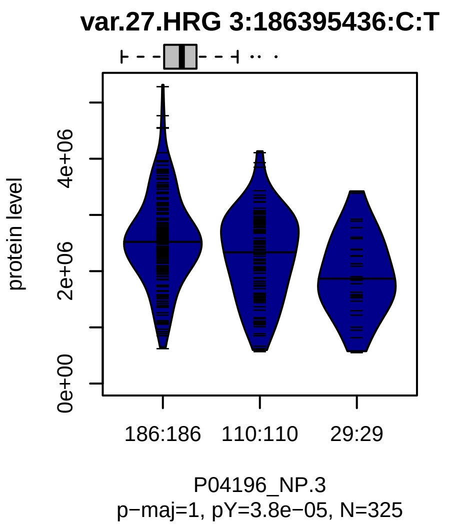
var.27.HRG 3:186395436:C:T
protein level
0e+00
2e+06
4e+06
186:186
110:110
29:29
P04196_NP.3
p−maj=1, pY=3.8e−05, N=325

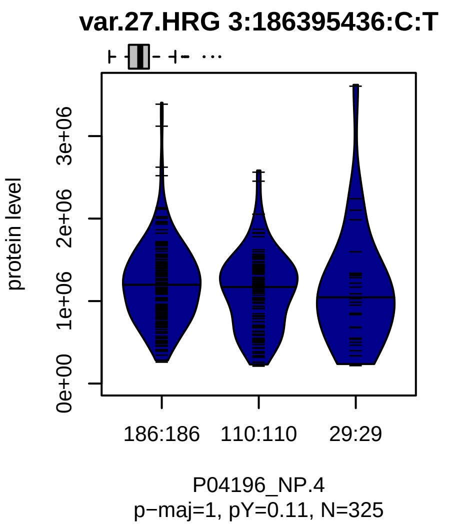
var.27.HRG 3:186395436:C:T
protein level
0e+00
1e+06
2e+06
3e+06
186:186
110:110
29:29
P04196_NP.4
p−maj=1, pY=0.11, N=325

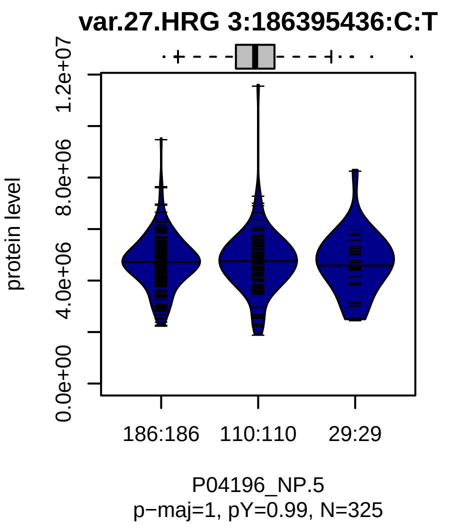
var.27.HRG 3:186395436:C:T
protein level
0.0e+00
4.0e+06
8.0e+06
1.2e+07
186:186
110:110
29:29
P04196_NP.5
p−maj=1, pY=0.99, N=325

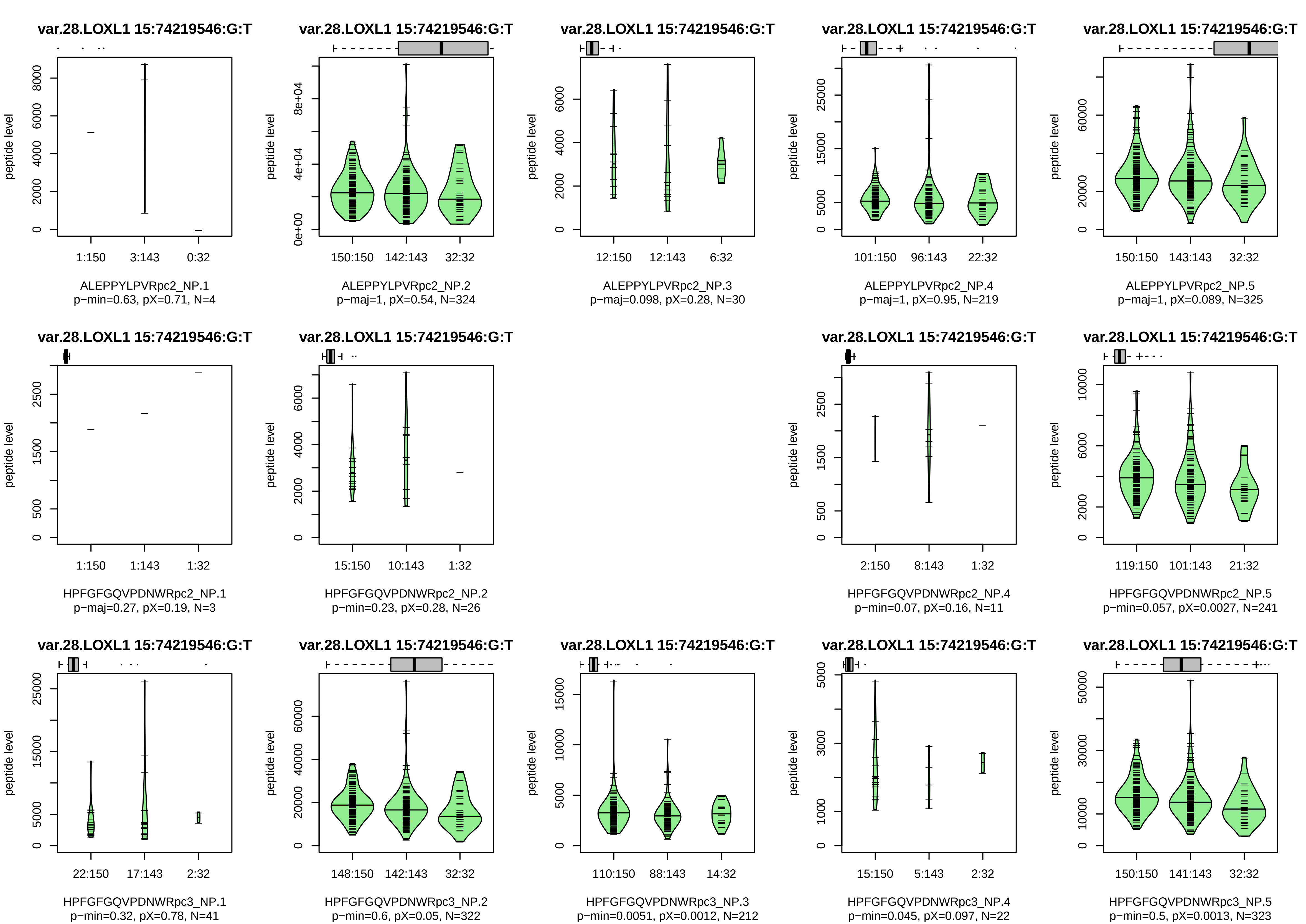
var.28.LOXL1 15:74219546:G:T
peptide level
0
2000
4000
6000
8000
1:150
3:143
0:32
ALEPPYLPVRpc2_NP.1
p−min=0.63, pX=0.71, N=4
var.28.LOXL1 15:74219546:G:T
peptide level
0e+00
4e+04
8e+04
150:150
142:143
32:32
ALEPPYLPVRpc2_NP.2
p−maj=1, pX=0.54, N=324
var.28.LOXL1 15:74219546:G:T
peptide level
0
2000
4000
6000
12:150
12:143
6:32
ALEPPYLPVRpc2_NP.3
p−maj=0.098, pX=0.28, N=30
var.28.LOXL1 15:74219546:G:T
peptide level
0
5000
15000
25000
101:150
96:143
22:32
ALEPPYLPVRpc2_NP.4
p−maj=1, pX=0.95, N=219
var.28.LOXL1 15:74219546:G:T
peptide level
0
20000
60000
150:150
143:143
32:32
ALEPPYLPVRpc2_NP.5
p−maj=1, pX=0.089, N=325
var.28.LOXL1 15:74219546:G:T
peptide level
0
500
1500
2500
1:150
1:143
1:32
HPFGFGQVPDNWRpc2_NP.1
p−maj=0.27, pX=0.19, N=3
var.28.LOXL1 15:74219546:G:T
peptide level
0
2000
4000
6000
15:150
10:143
1:32
HPFGFGQVPDNWRpc2_NP.2
p−min=0.23, pX=0.28, N=26
var.28.LOXL1 15:74219546:G:T
peptide level
0
500
1500
2500
2:150
8:143
1:32
HPFGFGQVPDNWRpc2_NP.4
p−min=0.07, pX=0.16, N=11
var.28.LOXL1 15:74219546:G:T
peptide level
0
2000
6000
10000
119:150
101:143
21:32
HPFGFGQVPDNWRpc2_NP.5
p−min=0.057, pX=0.0027, N=241
var.28.LOXL1 15:74219546:G:T
peptide level
0
5000
15000
25000
22:150
17:143
2:32
HPFGFGQVPDNWRpc3_NP.1
p−min=0.32, pX=0.78, N=41
var.28.LOXL1 15:74219546:G:T
peptide level
0
20000
40000
60000
148:150
142:143
32:32
HPFGFGQVPDNWRpc3_NP.2
p−min=0.6, pX=0.05, N=322
var.28.LOXL1 15:74219546:G:T
peptide level
0
5000
10000
15000
110:150
88:143
14:32
HPFGFGQVPDNWRpc3_NP.3
p−min=0.0051, pX=0.0012, N=212
var.28.LOXL1 15:74219546:G:T
peptide level
0
1000
3000
5000
15:150
5:143
2:32
HPFGFGQVPDNWRpc3_NP.4
p−min=0.045, pX=0.097, N=22
var.28.LOXL1 15:74219546:G:T
peptide level
0
10000
30000
50000
150:150
141:143
32:32
HPFGFGQVPDNWRpc3_NP.5
p−min=0.5, pX=0.0013, N=323

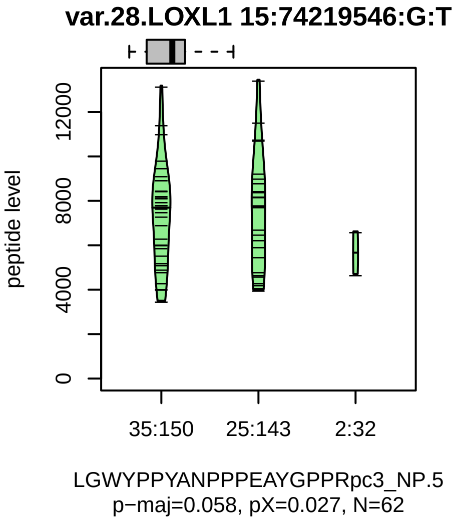
var.28.LOXL1 15:74219546:G:T
peptide level
0
4000
8000
12000
35:150
25:143
2:32
LGWYPPYANPPPEAYGPPRpc3_NP.5
p−maj=0.058, pX=0.027, N=62

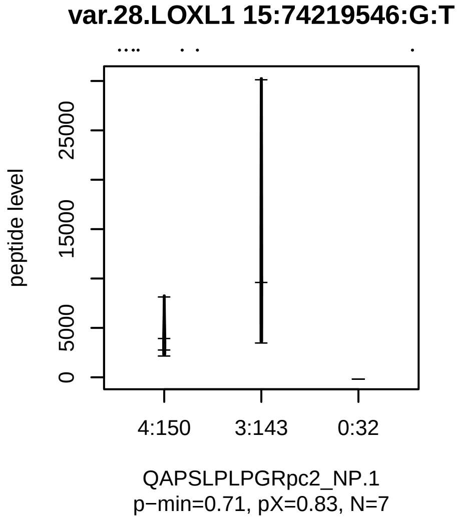
var.28.LOXL1 15:74219546:G:T
peptide level
0
5000
15000
25000
4:150
3:143
0:32
QAPSLPLPGRpc2_NP.1
p−min=0.71, pX=0.83, N=7

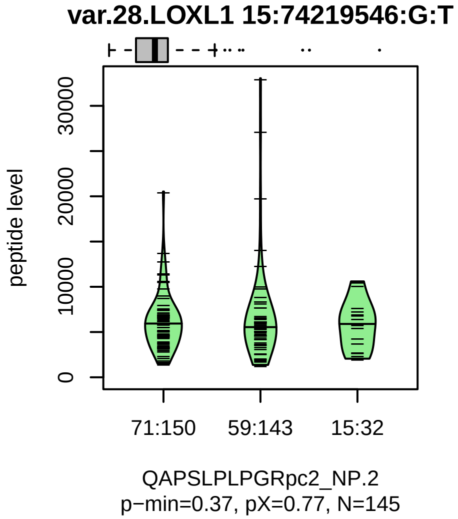
var.28.LOXL1 15:74219546:G:T
peptide level
0
10000
20000
30000
71:150
59:143
15:32
QAPSLPLPGRpc2_NP.2
p−min=0.37, pX=0.77, N=145

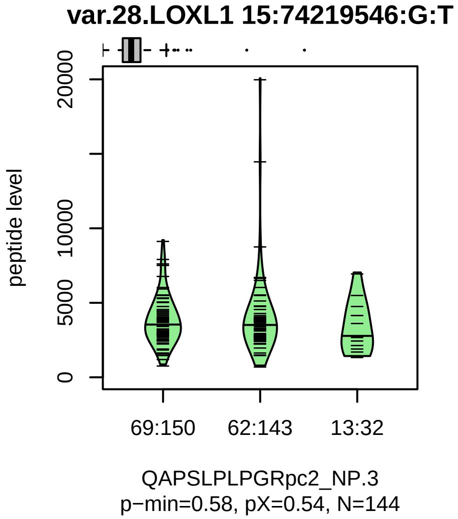
var.28.LOXL1 15:74219546:G:T
peptide level
0
5000
10000
20000
69:150
62:143
13:32
QAPSLPLPGRpc2_NP.3
p−min=0.58, pX=0.54, N=144

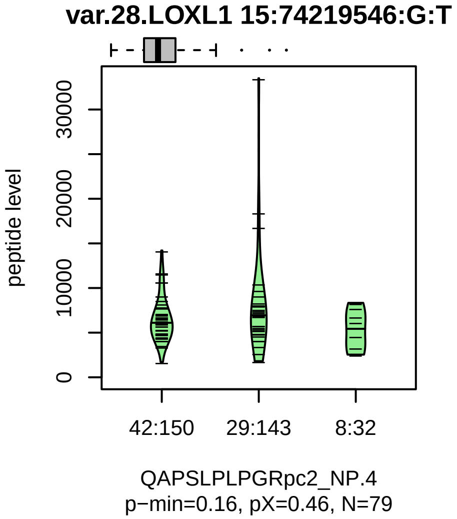
var.28.LOXL1 15:74219546:G:T
peptide level
0
10000
20000
30000
42:150
29:143
8:32
QAPSLPLPGRpc2_NP.4
p−min=0.16, pX=0.46, N=79

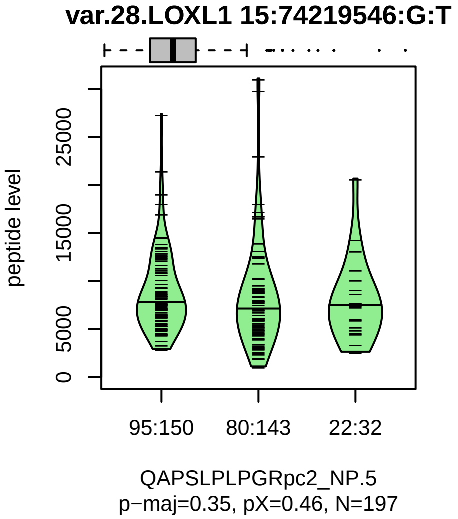
var.28.LOXL1 15:74219546:G:T
peptide level
0
5000
15000
25000
95:150
80:143
22:32
QAPSLPLPGRpc2_NP.5
p−maj=0.35, pX=0.46, N=197

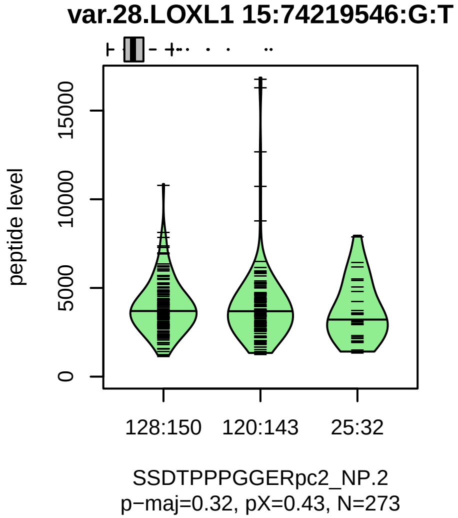
var.28.LOXL1 15:74219546:G:T
peptide level
0
5000
10000
15000
128:150
120:143
25:32
SSDTPPPGGERpc2_NP.2
p−maj=0.32, pX=0.43, N=273

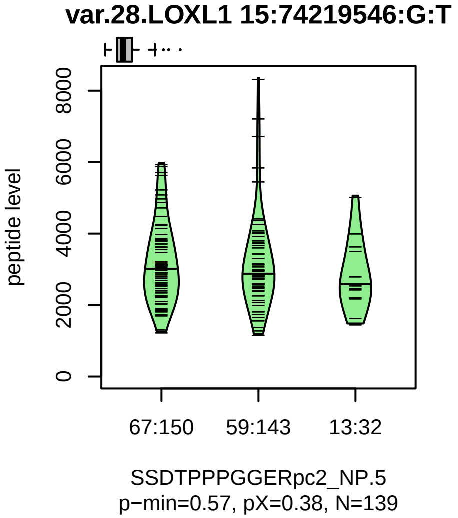
var.28.LOXL1 15:74219546:G:T
peptide level
0
2000
4000
6000
8000
67:150
59:143
13:32
SSDTPPPGGERpc2_NP.5
p−min=0.57, pX=0.38, N=139

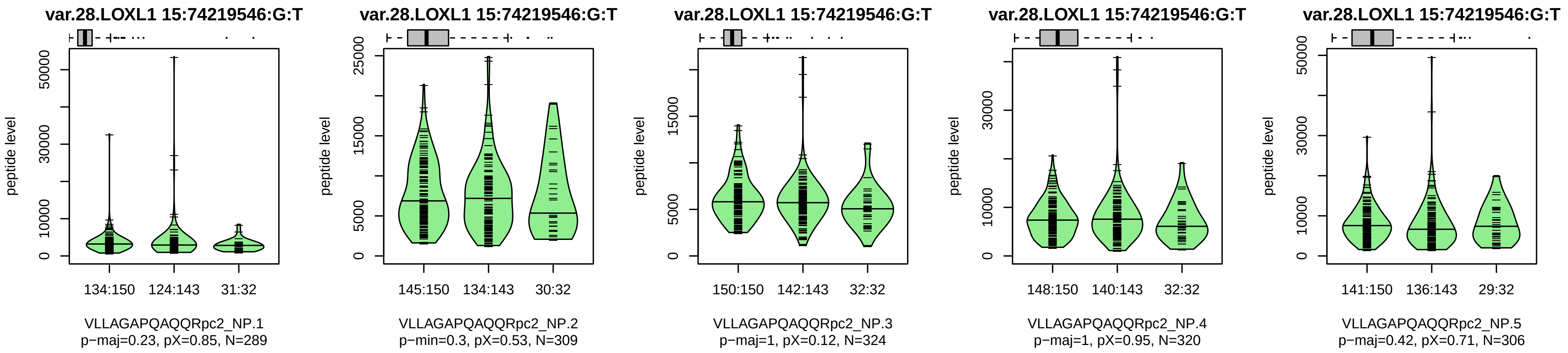

var.28.LOXL1 15:74219546:G:T
peptide level
134:150
124:143
31:32
VLLAGAPQAQQRpc2_NP.1
p−maj=0.23, pX=0.85, N=289
var.28.LOXL1 15:74219546:G:T
peptide level
145:150
134:143
30:32
VLLAGAPQAQQRpc2_NP.2
p−min=0.3, pX=0.53, N=309
var.28.LOXL1 15:74219546:G:T
peptide level
150:150
142:143
32:32
VLLAGAPQAQQRpc2_NP.3
p−maj=1, pX=0.12, N=324
var.28.LOXL1 15:74219546:G:T
peptide level
148:150
140:143
32:32
VLLAGAPQAQQRpc2_NP.4
p−maj=1, pX=0.95, N=320
var.28.LOXL1 15:74219546:G:T
peptide level
141:150
136:143
29:32
VLLAGAPQAQQRpc2_NP.5
p−maj=0.42, pX=0.71, N=306

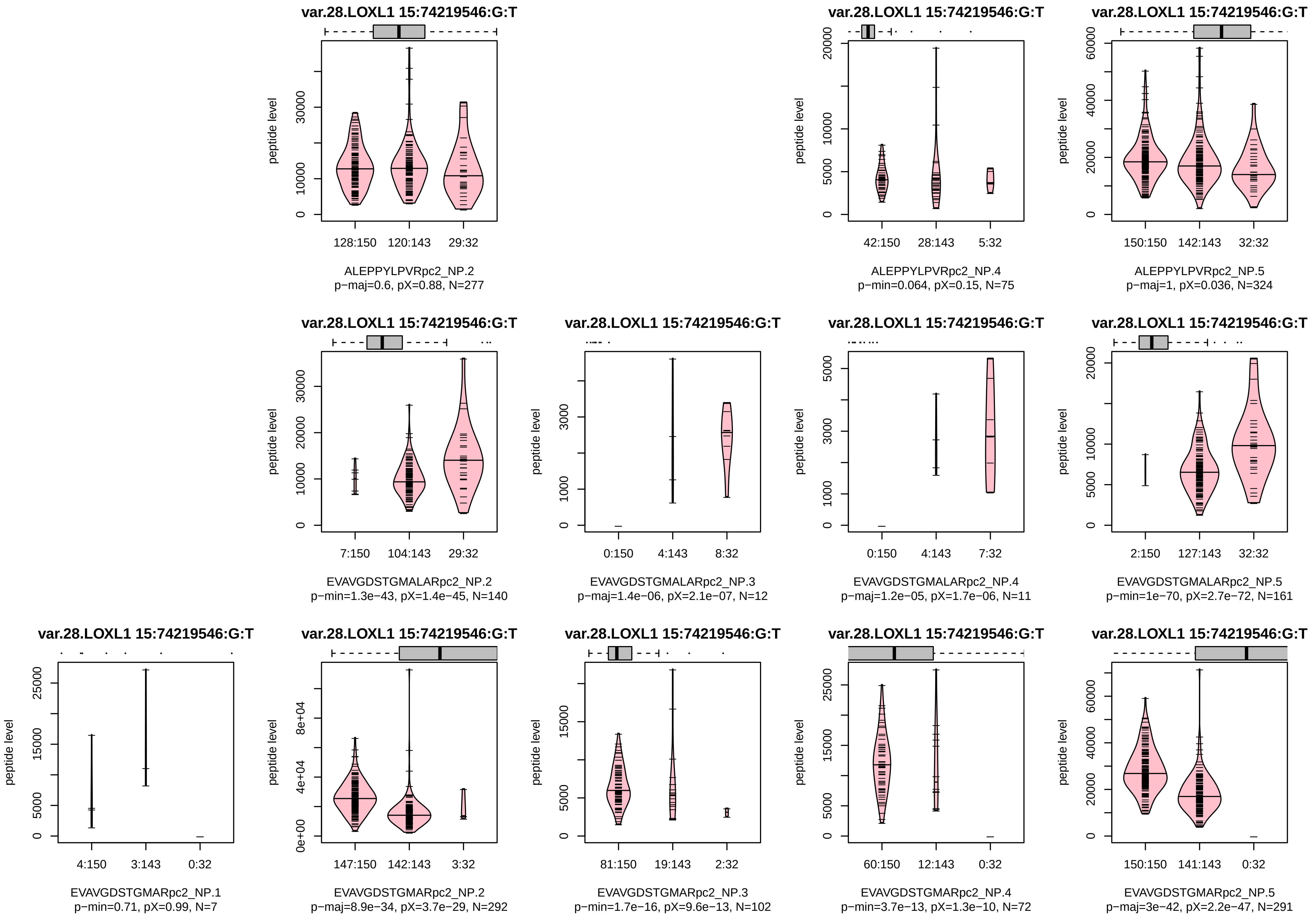
var.28.LOXL1 15:74219546:G:T
peptide level
128:150
120:143
29:32
ALEPPYLPVRpc2_NP.2
p−maj=0.6, pX=0.88, N=277
var.28.LOXL1 15:74219546:G:T
peptide level
42:150
28:143
5:32
ALEPPYLPVRpc2_NP.4
p−min=0.064, pX=0.15, N=75
var.28.LOXL1 15:74219546:G:T
peptide level
150:150
142:143
32:32
ALEPPYLPVRpc2_NP.5
p−maj=1, pX=0.036, N=324
var.28.LOXL1 15:74219546:G:T
peptide level
7:150
104:143
29:32
EVAVGDSTGMALARpc2_NP.2
p−min=1.3e−43, pX=1.4e−45, N=140
var.28.LOXL1 15:74219546:G:T
peptide level
0:150
4:143
8:32
EVAVGDSTGMALARpc2_NP.3
p−maj=1.4e−06, pX=2.1e−07, N=12
var.28.LOXL1 15:74219546:G:T
peptide level
0:150
4:143
7:32
EVAVGDSTGMALARpc2_NP.4
p−maj=1.2e−05, pX=1.7e−06, N=11
var.28.LOXL1 15:74219546:G:T
peptide level
2:150
127:143
32:32
EVAVGDSTGMALARpc2_NP.5
p−min=1e−70, pX=2.7e−72, N=161
var.28.LOXL1 15:74219546:G:T
peptide level
4:150
3:143
0:32
EVAVGDSTGMARpc2_NP.1
p−min=0.71, pX=0.99, N=7
var.28.LOXL1 15:74219546:G:T
peptide level
147:150
142:143
3:32
EVAVGDSTGMARpc2_NP.2
p−maj=8.9e−34, pX=3.7e−29, N=292
var.28.LOXL1 15:74219546:G:T
peptide level
81:150
19:143
2:32
EVAVGDSTGMARpc2_NP.3
p−min=1.7e−16, pX=9.6e−13, N=102
var.28.LOXL1 15:74219546:G:T
peptide level
60:150
12:143
0:32
EVAVGDSTGMARpc2_NP.4
p−min=3.7e−13, pX=1.3e−10, N=72
var.28.LOXL1 15:74219546:G:T
peptide level
150:150
141:143
0:32
EVAVGDSTGMARpc2_NP.5
p−maj=3e−42, pX=2.2e−47, N=291

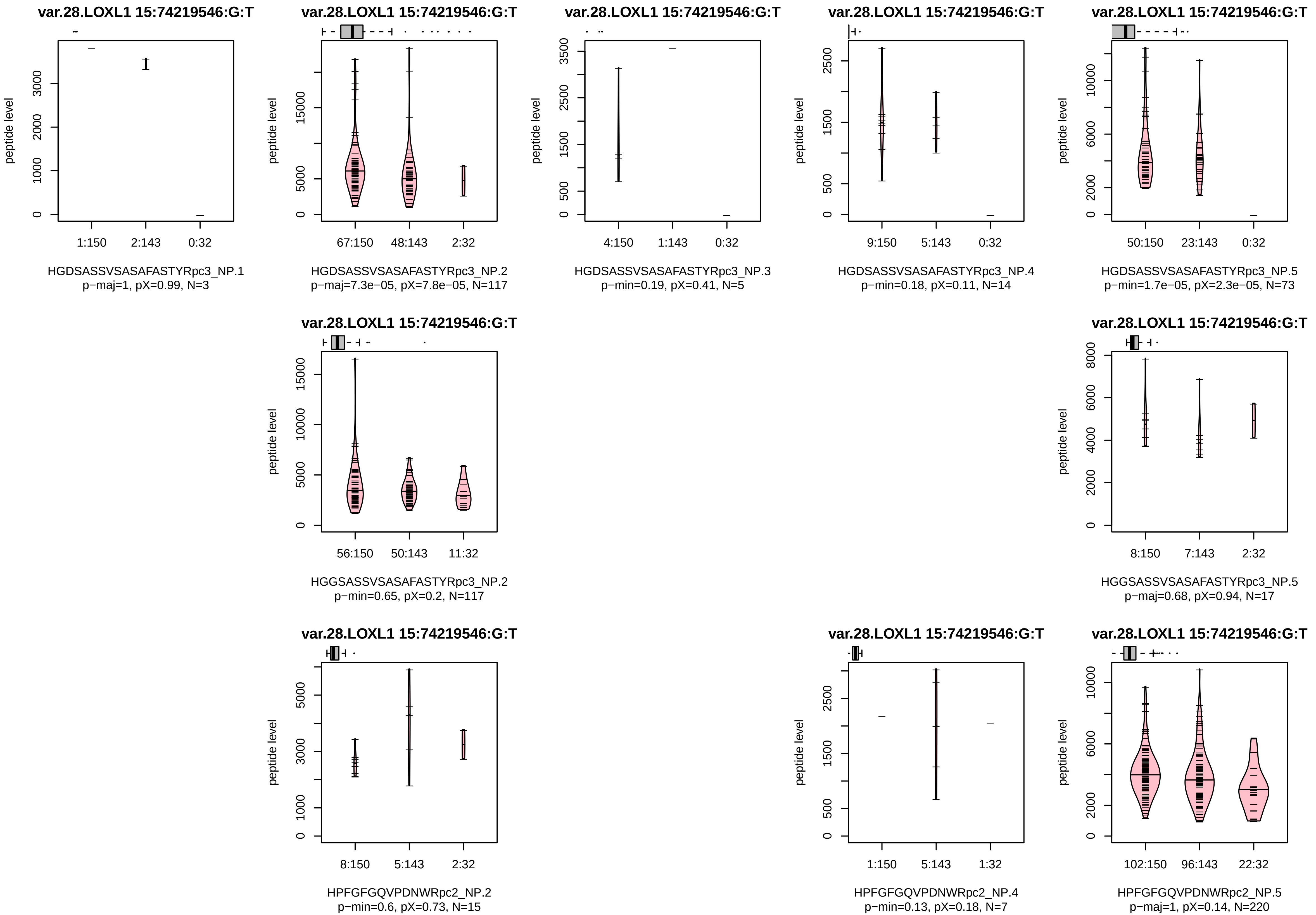

var.28.LOXL1 15:74219546:G:T
peptide level
1:150
2:143
0:32
HGDSASSVSASAFASTYRpc3_NP.1
p−maj=1, pX=0.99, N=3
var.28.LOXL1 15:74219546:G:T
peptide level
67:150
48:143
2:32
HGDSASSVSASAFASTYRpc3_NP.2
p−maj=7.3e−05, pX=7.8e−05, N=117
var.28.LOXL1 15:74219546:G:T
peptide level
4:150
1:143
0:32
HGDSASSVSASAFASTYRpc3_NP.3
p−min=0.19, pX=0.41, N=5
var.28.LOXL1 15:74219546:G:T
peptide level
9:150
5:143
0:32
HGDSASSVSASAFASTYRpc3_NP.4
p−min=0.18, pX=0.11, N=14
var.28.LOXL1 15:74219546:G:T
peptide level
50:150
23:143
0:32
HGDSASSVSASAFASTYRpc3_NP.5
p−min=1.7e−05, pX=2.3e−05, N=73
var.28.LOXL1 15:74219546:G:T
peptide level
56:150
50:143
11:32
HGGSASSVSASAFASTYRpc3_NP.2
p−min=0.65, pX=0.2, N=117
var.28.LOXL1 15:74219546:G:T
peptide level
8:150
7:143
2:32
HGGSASSVSASAFASTYRpc3_NP.5
p−maj=0.68, pX=0.94, N=17
var.28.LOXL1 15:74219546:G:T
peptide level
8:150
5:143
2:32
HPFGFGQVPDNWRpc2_NP.2
p−min=0.6, pX=0.73, N=15
var.28.LOXL1 15:74219546:G:T
peptide level
1:150
5:143
1:32
HPFGFGQVPDNWRpc2_NP.4
p−min=0.13, pX=0.18, N=7
var.28.LOXL1 15:74219546:G:T
peptide level
102:150
96:143
22:32
HPFGFGQVPDNWRpc2_NP.5
p−maj=1, pX=0.14, N=220

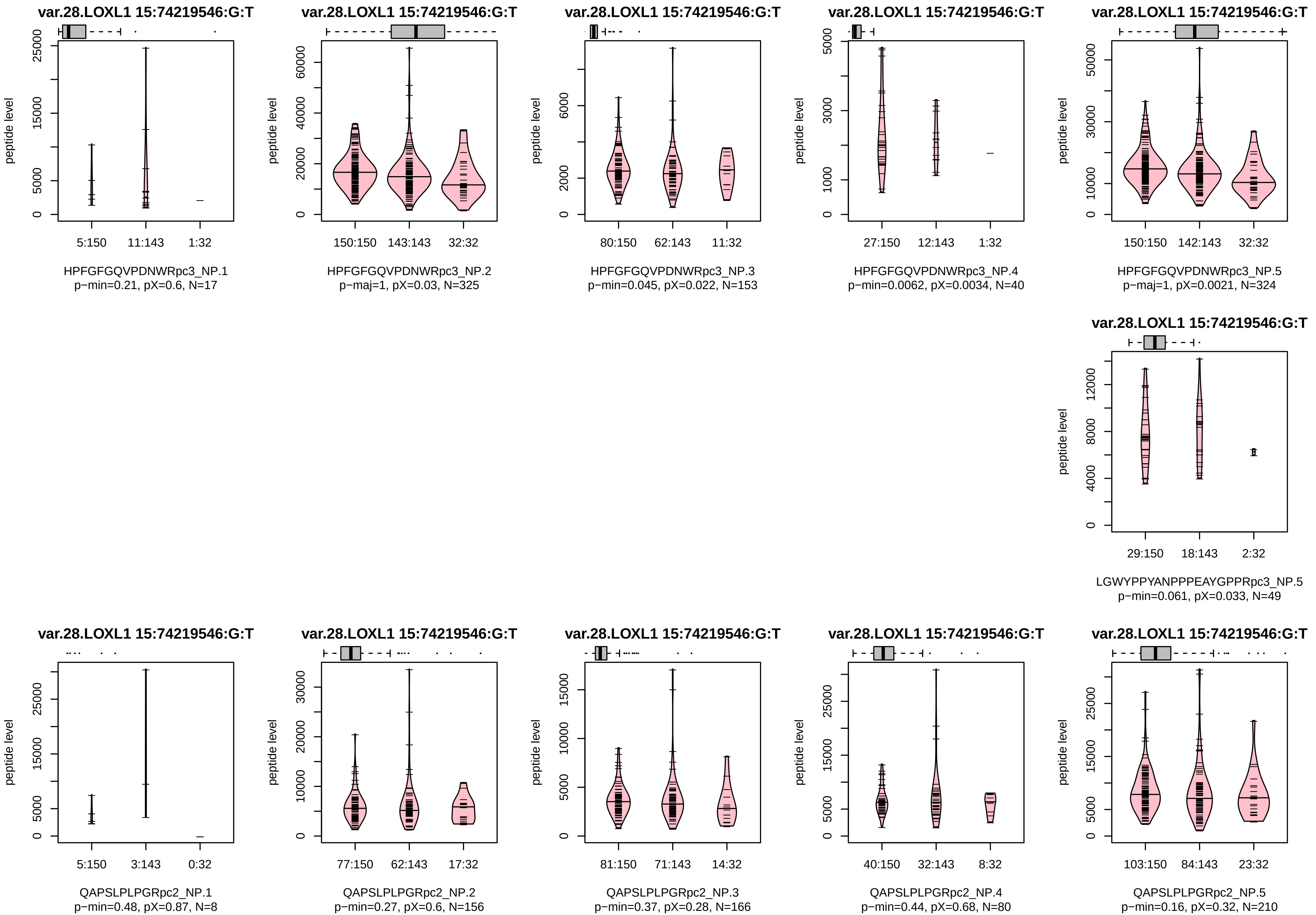

var.28.LOXL1 15:74219546:G:T
peptide level
5:150
11:143
1:32
HPFGFGQVPDNWRpc3_NP.1
p−min=0.21, pX=0.6, N=17
var.28.LOXL1 15:74219546:G:T
150:150
143:143
32:32
HPFGFGQVPDNWRpc3_NP.2
p−maj=1, pX=0.03, N=325
var.28.LOXL1 15:74219546:G:T
80:150
62:143
11:32
HPFGFGQVPDNWRpc3_NP.3
p−min=0.045, pX=0.022, N=153
var.28.LOXL1 15:74219546:G:T
27:150
12:143
1:32
HPFGFGQVPDNWRpc3_NP.4
p−min=0.0062, pX=0.0034, N=40
var.28.LOXL1 15:74219546:G:T
150:150
142:143
32:32
HPFGFGQVPDNWRpc3_NP.5
p−maj=1, pX=0.0021, N=324
var.28.LOXL1 15:74219546:G:T
29:150
18:143
2:32
LGWYPPYANPPPEAYGPPRpc3_NP.5
p−min=0.061, pX=0.033, N=49
var.28.LOXL1 15:74219546:G:T
5:150
3:143
0:32
QAPSLPLPGRpc2_NP.1
p−min=0.48, pX=0.87, N=8
var.28.LOXL1 15:74219546:G:T
77:150
62:143
17:32
QAPSLPLPGRpc2_NP.2
p−min=0.27, pX=0.6, N=156
var.28.LOXL1 15:74219546:G:T
81:150
71:143
14:32
QAPSLPLPGRpc2_NP.3
p−min=0.37, pX=0.28, N=166
var.28.LOXL1 15:74219546:G:T
40:150
32:143
8:32
QAPSLPLPGRpc2_NP.4
p−min=0.44, pX=0.68, N=80
var.28.LOXL1 15:74219546:G:T
103:150
84:143
23:32
QAPSLPLPGRpc2_NP.5
p−min=0.16, pX=0.32, N=210

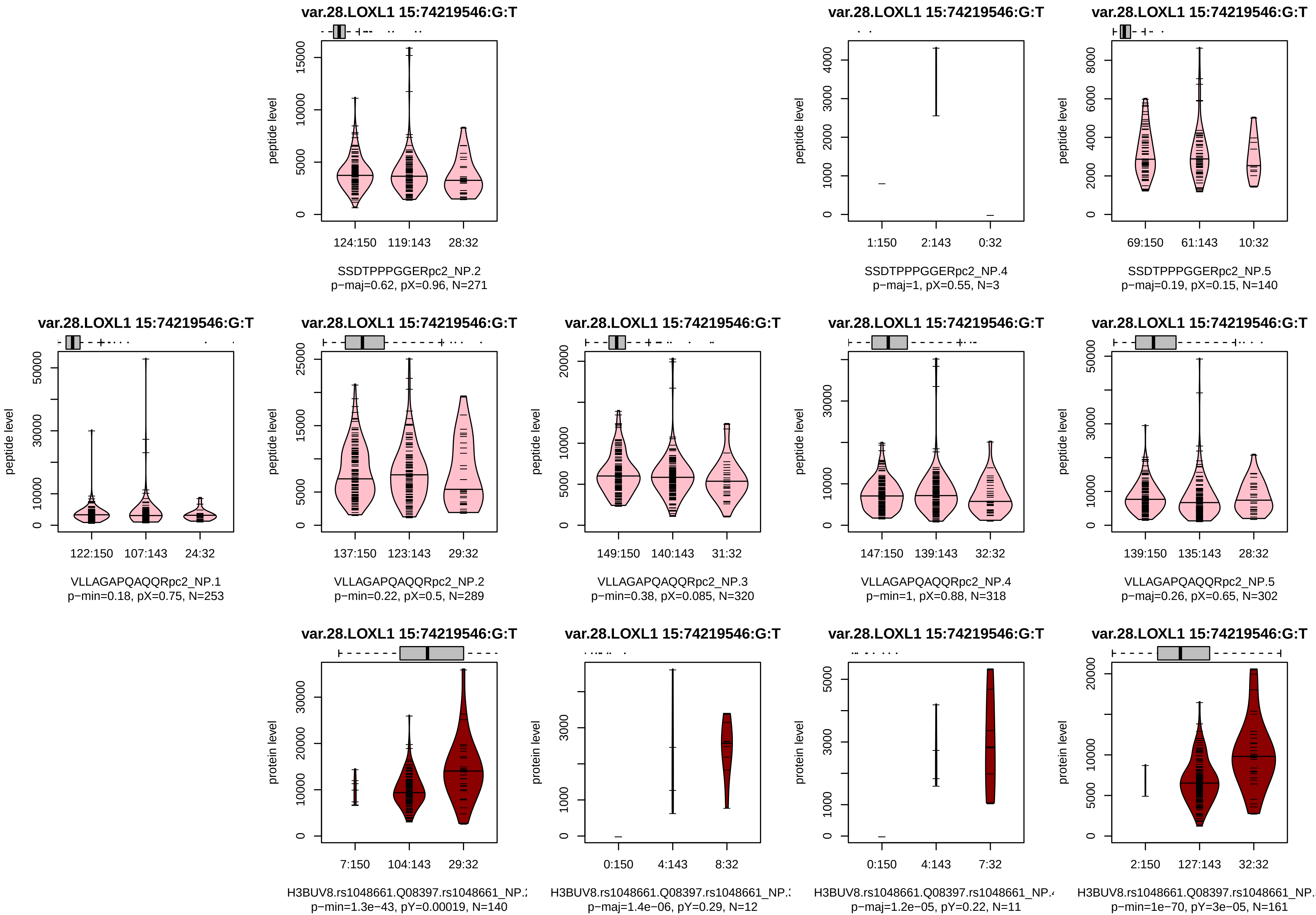
var.28.LOXL1 15:74219546:G:T
peptide level
124:150 119:143 28:32
SSDTPPPGGERpc2_NP.2
p−maj=0.62, pX=0.96, N=271
var.28.LOXL1 15:74219546:G:T
peptide level
1:150 2:143 0:32
SSDTPPPGGERpc2_NP.4
p−maj=1, pX=0.55, N=3
var.28.LOXL1 15:74219546:G:T
peptide level
69:150 61:143 10:32
SSDTPPPGGERpc2_NP.5
p−maj=0.19, pX=0.15, N=140
var.28.LOXL1 15:74219546:G:T
peptide level
122:150 107:143 24:32
VLLAGAPQAQQRpc2_NP.1
p−min=0.18, pX=0.75, N=253
var.28.LOXL1 15:74219546:G:T
peptide level
137:150 123:143 29:32
VLLAGAPQAQQRpc2_NP.2
p−min=0.22, pX=0.5, N=289
var.28.LOXL1 15:74219546:G:T
peptide level
149:150 140:143 31:32
VLLAGAPQAQQRpc2_NP.3
p−min=0.38, pX=0.085, N=320
var.28.LOXL1 15:74219546:G:T
peptide level
147:150 139:143 32:32
VLLAGAPQAQQRpc2_NP.4
p−min=1, pX=0.88, N=318
var.28.LOXL1 15:74219546:G:T
peptide level
139:150 135:143 28:32
VLLAGAPQAQQRpc2_NP.5
p−maj=0.26, pX=0.65, N=302
var.28.LOXL1 15:74219546:G:T
protein level
7:150 104:143 29:32
H3BUV8.rs1048661.Q08397.rs1048661_NP.
p−min=1.3e−43, pY=0.00019, N=140
var.28.LOXL1 15:74219546:G:T
protein level
0:150 4:143 8:32
H3BUV8.rs1048661.Q08397.rs1048661_NP.
p−maj=1.4e−06, pY=0.29, N=12
var.28.LOXL1 15:74219546:G:T
protein level
0:150 4:143 7:32
H3BUV8.rs1048661.Q08397.rs1048661_NP.
p−maj=1.2e−05, pY=0.22, N=11
var.28.LOXL1 15:74219546:G:T
protein level
2:150 127:143 32:32
H3BUV8.rs1048661.Q08397.rs1048661_NP.
p−min=1e−70, pY=3e−05, N=161

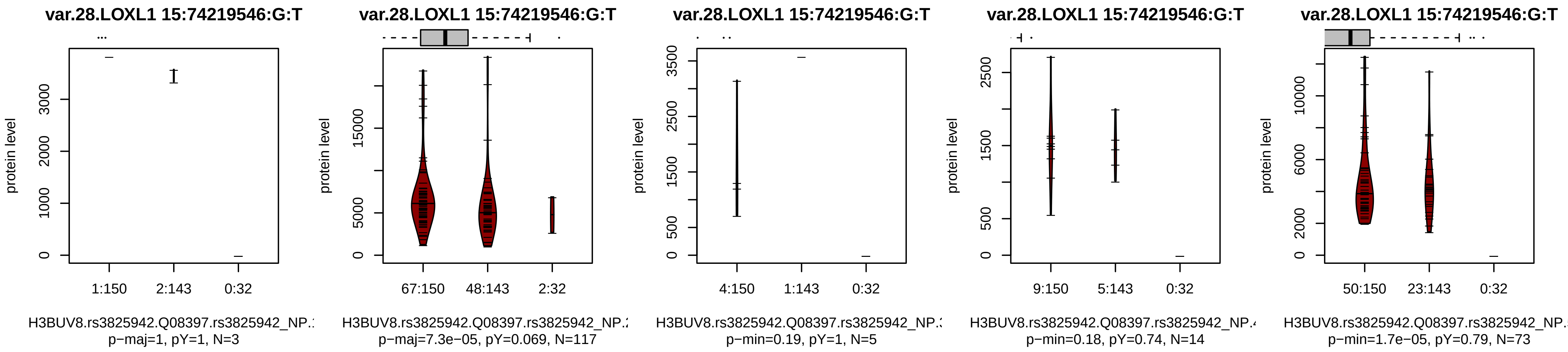
var.28.LOXL1 15:74219546:G:T
protein level
0
1000
2000
3000
1:150
2:143
0:32
H3BUV8.rs3825942.Q08397.rs3825942_NP.1
p−maj=1, pY=1, N=3
var.28.LOXL1 15:74219546:G:T
protein level
0
5000
15000
67:150
48:143
2:32
H3BUV8.rs3825942.Q08397.rs3825942_NP.2
p−maj=7.3e−05, pY=0.069, N=117
var.28.LOXL1 15:74219546:G:T
protein level
0
500
1500
2500
3500
4:150
1:143
0:32
H3BUV8.rs3825942.Q08397.rs3825942_NP.3
p−min=0.19, pY=1, N=5
var.28.LOXL1 15:74219546:G:T
protein level
0
500
1500
2500
9:150
5:143
0:32
H3BUV8.rs3825942.Q08397.rs3825942_NP.4
p−min=0.18, pY=0.74, N=14
var.28.LOXL1 15:74219546:G:T
protein level
0
2000
6000
10000
50:150
23:143
0:32
H3BUV8.rs3825942.Q08397.rs3825942_NP.5
p−min=1.7e−05, pY=0.79, N=73

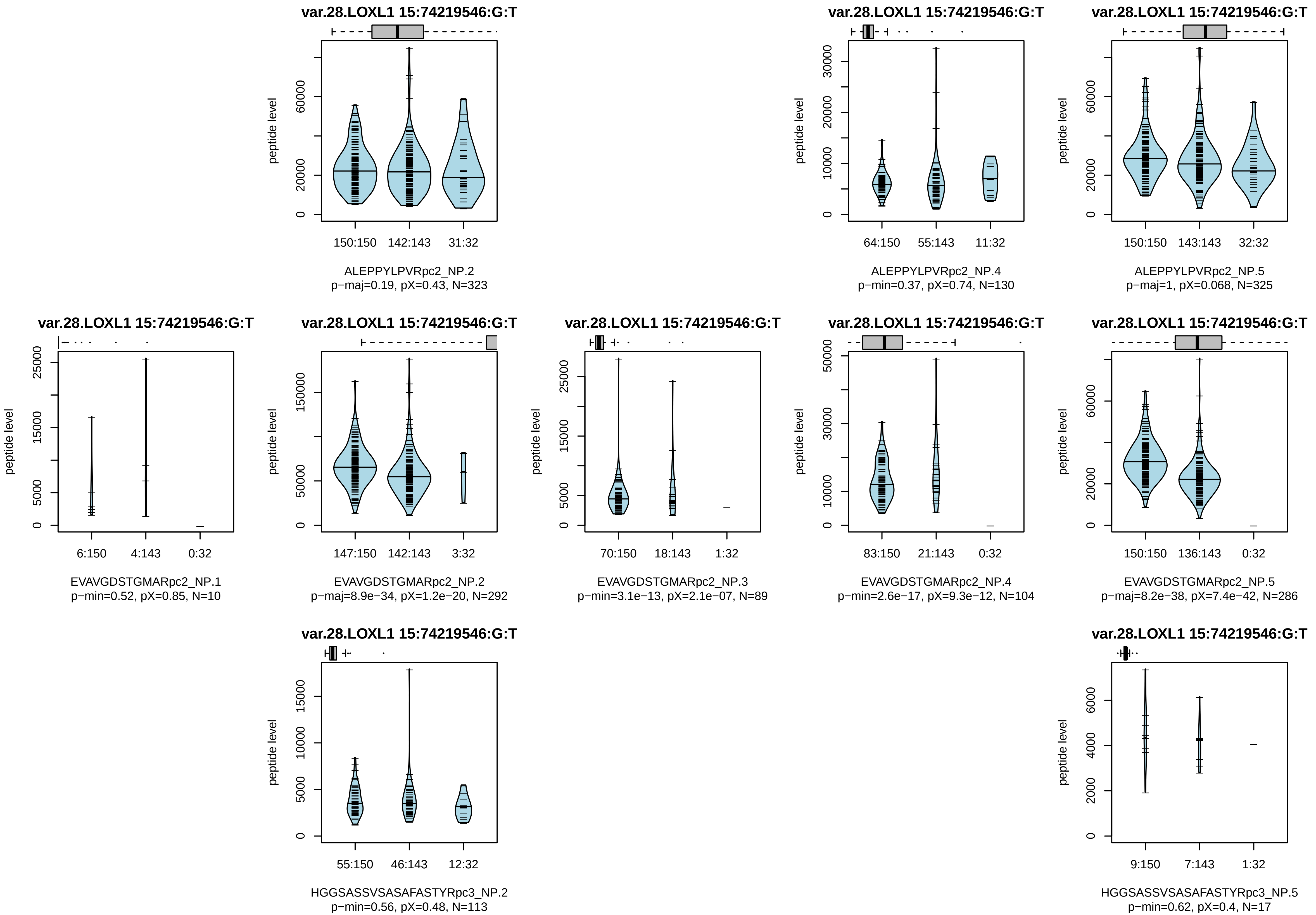
var.28.LOXL1 15:74219546:G:T
peptide level
150:150
142:143
31:32
ALEPPYLPVRpc2_NP.2
p−maj=0.19, pX=0.43, N=323
var.28.LOXL1 15:74219546:G:T
peptide level
64:150
55:143
11:32
ALEPPYLPVRpc2_NP.4
p−min=0.37, pX=0.74, N=130
var.28.LOXL1 15:74219546:G:T
peptide level
150:150
143:143
32:32
ALEPPYLPVRpc2_NP.5
p−maj=1, pX=0.068, N=325
var.28.LOXL1 15:74219546:G:T
peptide level
6:150
4:143
0:32
EVAVGDSTGMARpc2_NP.1
p−min=0.52, pX=0.85, N=10
var.28.LOXL1 15:74219546:G:T
peptide level
147:150
142:143
3:32
EVAVGDSTGMARpc2_NP.2
p−maj=8.9e−34, pX=1.2e−20, N=292
var.28.LOXL1 15:74219546:G:T
peptide level
70:150
18:143
1:32
EVAVGDSTGMARpc2_NP.3
p−min=3.1e−13, pX=2.1e−07, N=89
var.28.LOXL1 15:74219546:G:T
peptide level
83:150
21:143
0:32
EVAVGDSTGMARpc2_NP.4
p−min=2.6e−17, pX=9.3e−12, N=104
var.28.LOXL1 15:74219546:G:T
peptide level
150:150
136:143
0:32
EVAVGDSTGMARpc2_NP.5
p−maj=8.2e−38, pX=7.4e−42, N=286
var.28.LOXL1 15:74219546:G:T
peptide level
55:150
46:143
12:32
HGGSASSVSASAFASTYRpc3_NP.2
p−min=0.56, pX=0.48, N=113
var.28.LOXL1 15:74219546:G:T
peptide level
9:150
7:143
1:32
HGGSASSVSASAFASTYRpc3_NP.5
p−min=0.62, pX=0.4, N=17

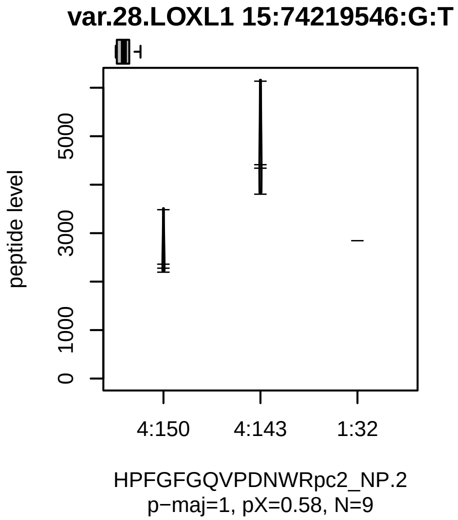
var.28.LOXL1 15:74219546:G:T
peptide level
0
1000
3000
5000
4:150
4:143
1:32
HPFGFGQVPDNWRpc2_NP.2
p−maj=1, pX=0.58, N=9

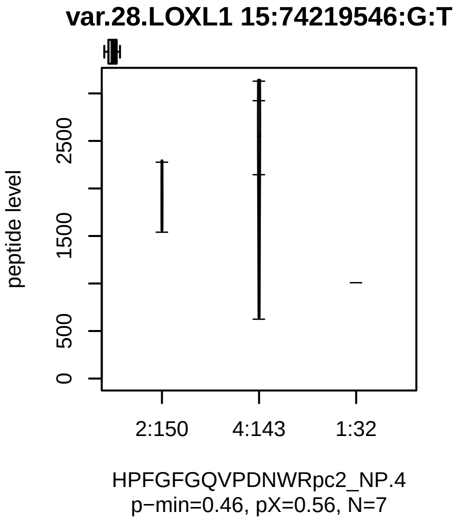
var.28.LOXL1 15:74219546:G:T
peptide level
0
500
1500
2500
2:150
4:143
1:32
HPFGFGQVPDNWRpc2_NP.4
p−min=0.46, pX=0.56, N=7

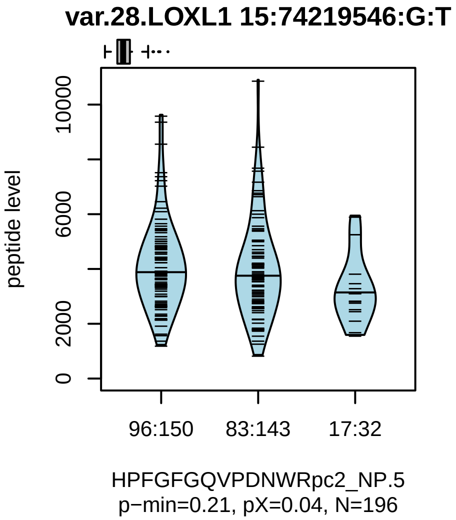
var.28.LOXL1 15:74219546:G:T
peptide level
0
2000
6000
10000
96:150
83:143
17:32
HPFGFGQVPDNWRpc2_NP.5
p−min=0.21, pX=0.04, N=196

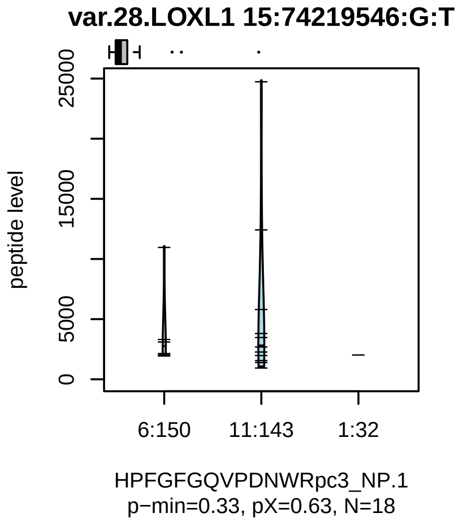
var.28.LOXL1 15:74219546:G:T
peptide level
0
5000
15000
25000
6:150
11:143
1:32
HPFGFGQVPDNWRpc3_NP.1
p−min=0.33, pX=0.63, N=18

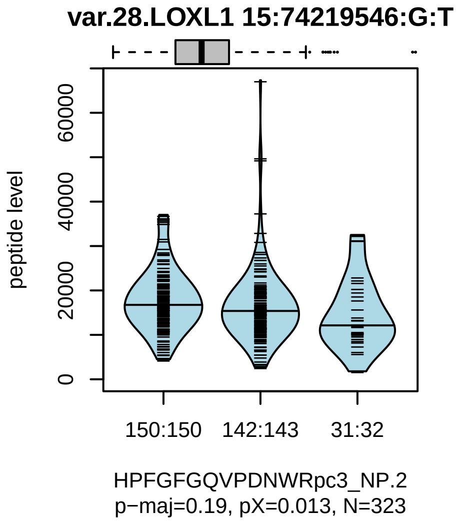
var.28.LOXL1 15:74219546:G:T
peptide level
0
20000
40000
60000
150:150
142:143
31:32
HPFGFGQVPDNWRpc3_NP.2
p−maj=0.19, pX=0.013, N=323

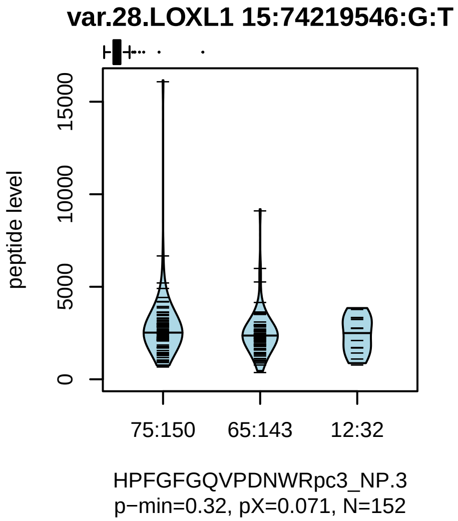
var.28.LOXL1 15:74219546:G:T
peptide level
0
5000
10000
15000
75:150
65:143
12:32
HPFGFGQVPDNWRpc3_NP.3
p−min=0.32, pX=0.071, N=152

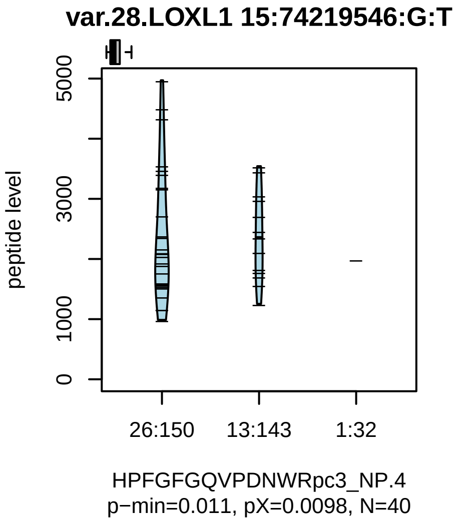
var.28.LOXL1 15:74219546:G:T
peptide level
0
1000
3000
5000
26:150
13:143
1:32
HPFGFGQVPDNWRpc3_NP.4
p−min=0.011, pX=0.0098, N=40

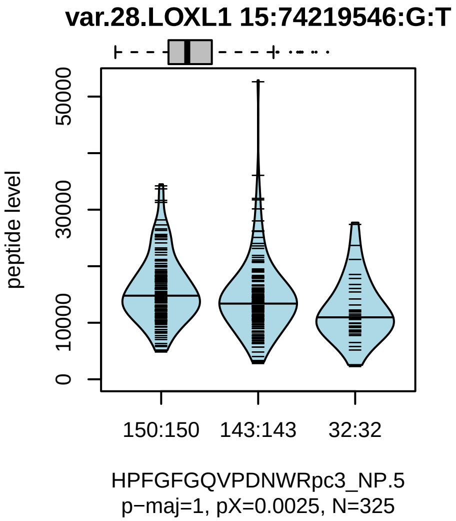
var.28.LOXL1 15:74219546:G:T
peptide level
10000
30000
50000
150:150
143:143
32:32
HPFGFGQVPDNWRpc3_NP.5
p−maj=1, pX=0.0025, N=325

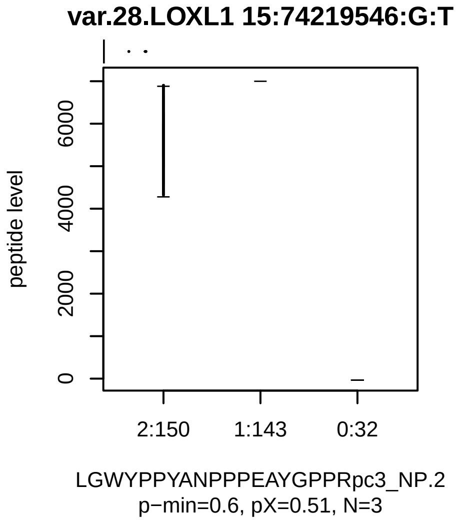
var.28.LOXL1 15:74219546:G:T
peptide level
0
2000
4000
6000
2:150
1:143
0:32
LGWYPPYANPPPEAYGPPRpc3_NP.2
p−min=0.6, pX=0.51, N=3

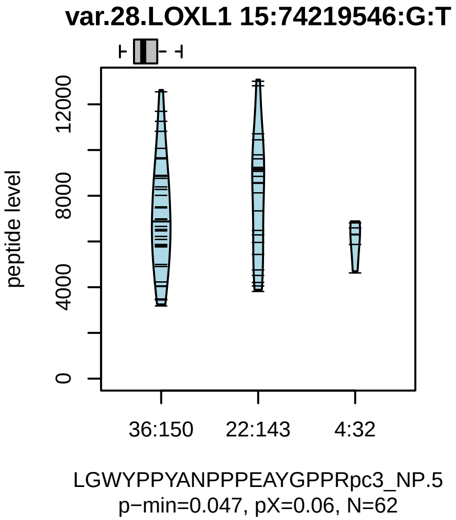
var.28.LOXL1 15:74219546:G:T
peptide level
0
4000
8000
12000
36:150
22:143
4:32
LGWYPPYANPPPEAYGPPRpc3_NP.5
p−min=0.047, pX=0.06, N=62

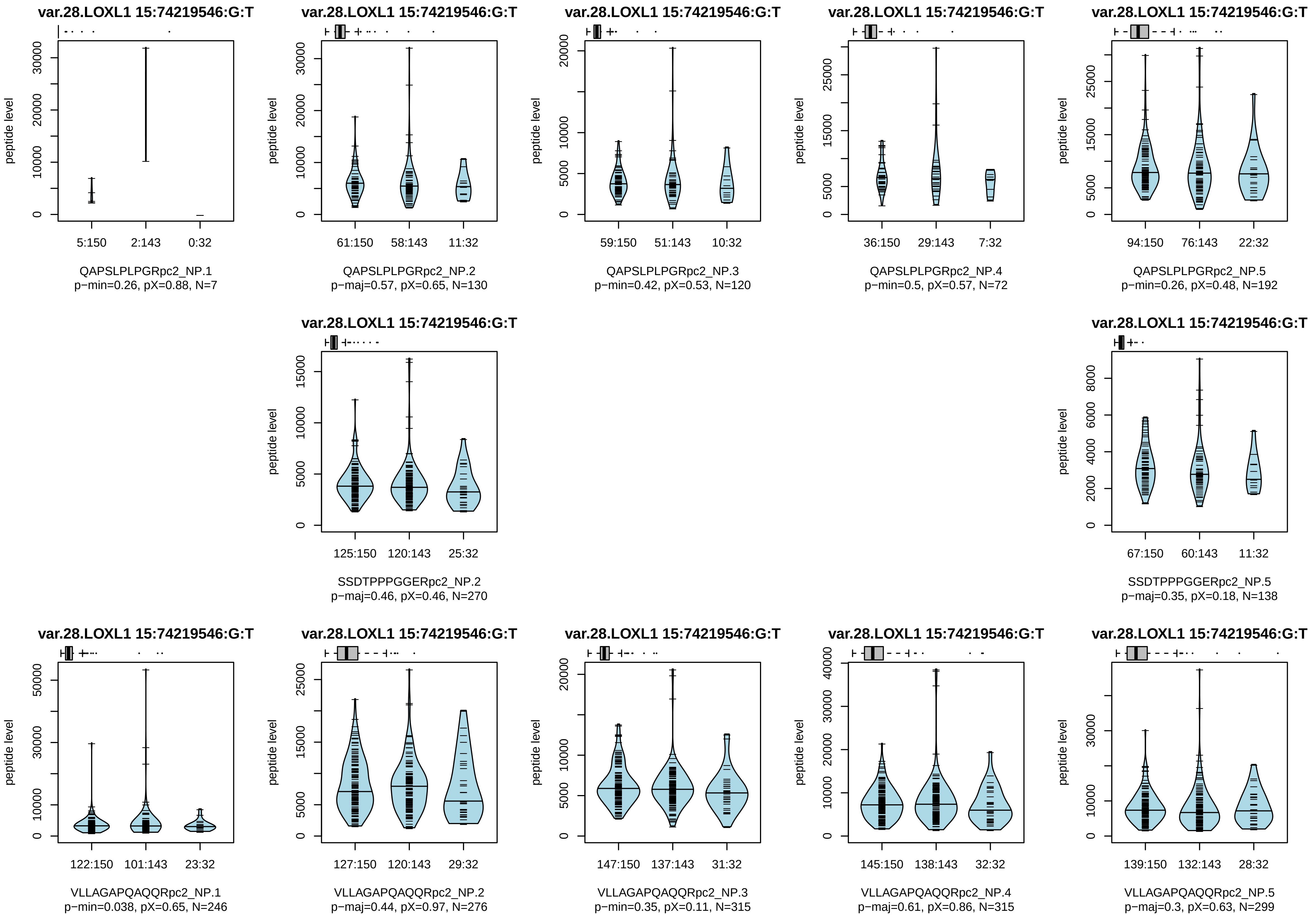
var.28.LOXL1 15:74219546:G:T
peptide level
5:150
2:143
0:32
QAPSLPLPGRpc2_NP.1
p−min=0.26, pX=0.88, N=7
var.28.LOXL1 15:74219546:G:T
61:150
58:143
11:32
QAPSLPLPGRpc2_NP.2
p−maj=0.57, pX=0.65, N=130
var.28.LOXL1 15:74219546:G:T
59:150
51:143
10:32
QAPSLPLPGRpc2_NP.3
p−min=0.42, pX=0.53, N=120
var.28.LOXL1 15:74219546:G:T
36:150
29:143
7:32
QAPSLPLPGRpc2_NP.4
p−min=0.5, pX=0.57, N=72
var.28.LOXL1 15:74219546:G:T
94:150
76:143
22:32
QAPSLPLPGRpc2_NP.5
p−min=0.26, pX=0.48, N=192
var.28.LOXL1 15:74219546:G:T
125:150
120:143
25:32
SSDTPPPGGERpc2_NP.2
p−maj=0.46, pX=0.46, N=270
var.28.LOXL1 15:74219546:G:T
67:150
60:143
11:32
SSDTPPPGGERpc2_NP.5
p−maj=0.35, pX=0.18, N=138
var.28.LOXL1 15:74219546:G:T
122:150
101:143
23:32
VLLAGAPQAQQRpc2_NP.1
p−min=0.038, pX=0.65, N=246
var.28.LOXL1 15:74219546:G:T
127:150
120:143
29:32
VLLAGAPQAQQRpc2_NP.2
p−maj=0.44, pX=0.97, N=276
var.28.LOXL1 15:74219546:G:T
147:150
137:143
31:32
VLLAGAPQAQQRpc2_NP.3
p−min=0.35, pX=0.11, N=315
var.28.LOXL1 15:74219546:G:T
145:150
138:143
32:32
VLLAGAPQAQQRpc2_NP.4
p−maj=0.61, pX=0.86, N=315
var.28.LOXL1 15:74219546:G:T
139:150
132:143
28:32
VLLAGAPQAQQRpc2_NP.5
p−maj=0.3, pX=0.63, N=299

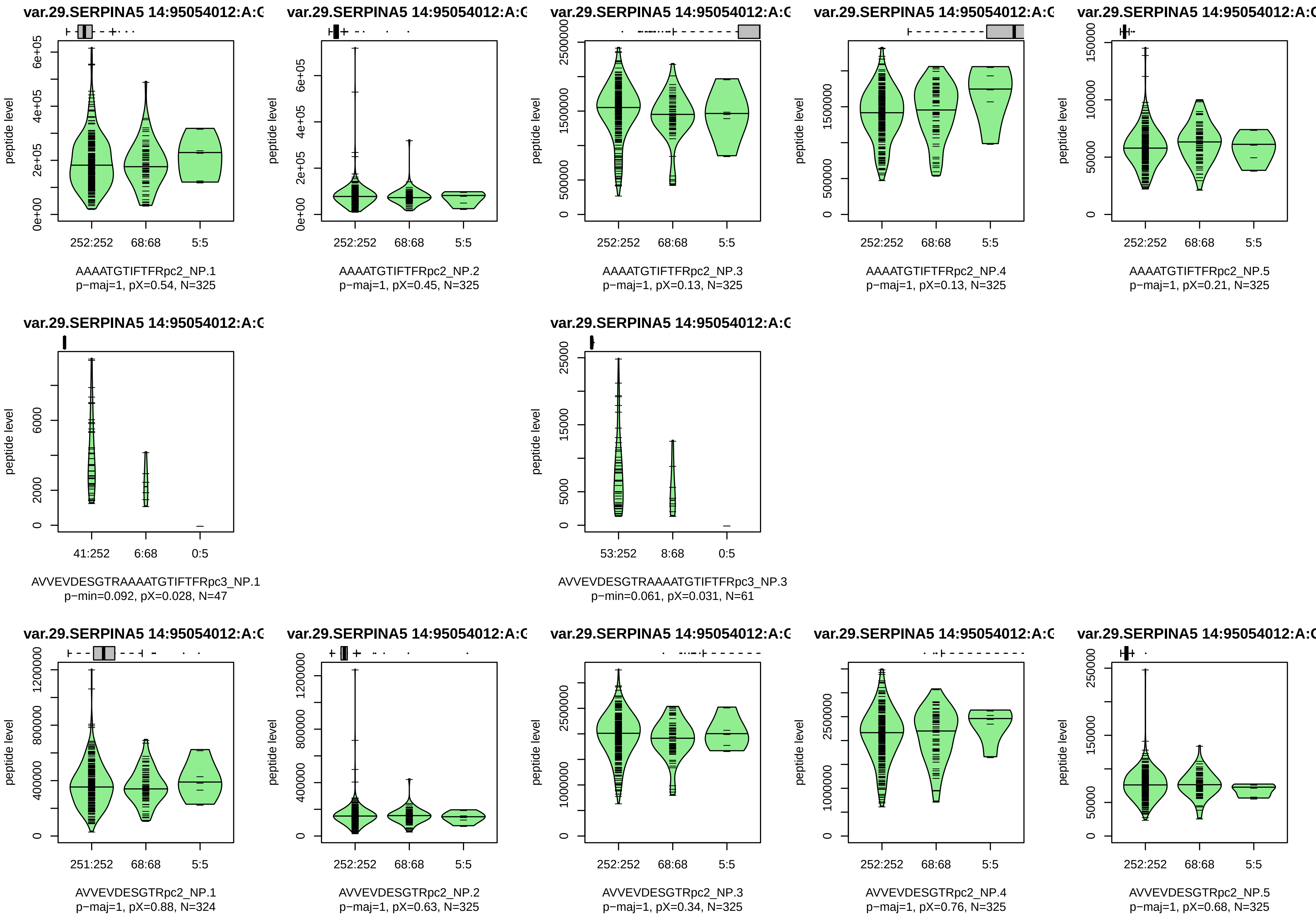

var.29.SERPINA5 14:95054012:A:G
peptide level
0e+00
2e+05
4e+05
6e+05
252:252
68:68
5:5
AAAATGTIFTFRpc2_NP.1
p−maj=1, pX=0.54, N=325
var.29.SERPINA5 14:95054012:A:G
peptide level
0e+00
2e+05
4e+05
6e+05
252:252
68:68
5:5
AAAATGTIFTFRpc2_NP.2
p−maj=1, pX=0.45, N=325
var.29.SERPINA5 14:95054012:A:G
peptide level
0
500000
1500000
2500000
252:252
68:68
5:5
AAAATGTIFTFRpc2_NP.3
p−maj=1, pX=0.13, N=325
var.29.SERPINA5 14:95054012:A:G
peptide level
0
500000
1500000
252:252
68:68
5:5
AAAATGTIFTFRpc2_NP.4
p−maj=1, pX=0.13, N=325
var.29.SERPINA5 14:95054012:A:G
peptide level
0
50000
100000
150000
252:252
68:68
5:5
AAAATGTIFTFRpc2_NP.5
p−maj=1, pX=0.21, N=325
var.29.SERPINA5 14:95054012:A:G
peptide level
0
2000
6000
41:252
6:68
0:5
AVVEVDESGTRAAAATGTIFTFRpc3_NP.1
p−min=0.092, pX=0.028, N=47
var.29.SERPINA5 14:95054012:A:G
peptide level
0
5000
15000
25000
53:252
8:68
0:5
AVVEVDESGTRAAAATGTIFTFRpc3_NP.3
p−min=0.061, pX=0.031, N=61
var.29.SERPINA5 14:95054012:A:G
peptide level
0
400000
800000
1200000
251:252
68:68
5:5
AVVEVDESGTRpc2_NP.1
p−maj=1, pX=0.88, N=324
var.29.SERPINA5 14:95054012:A:G
peptide level
0
400000
800000
1200000
252:252
68:68
5:5
AVVEVDESGTRpc2_NP.2
p−maj=1, pX=0.63, N=325
var.29.SERPINA5 14:95054012:A:G
peptide level
0
1000000
2500000
252:252
68:68
5:5
AVVEVDESGTRpc2_NP.3
p−maj=1, pX=0.34, N=325
var.29.SERPINA5 14:95054012:A:G
peptide level
0
1000000
2500000
252:252
68:68
5:5
AVVEVDESGTRpc2_NP.4
p−maj=1, pX=0.76, N=325
var.29.SERPINA5 14:95054012:A:G
peptide level
0
50000
150000
250000
252:252
68:68
5:5
AVVEVDESGTRpc2_NP.5
p−maj=1, pX=0.68, N=325

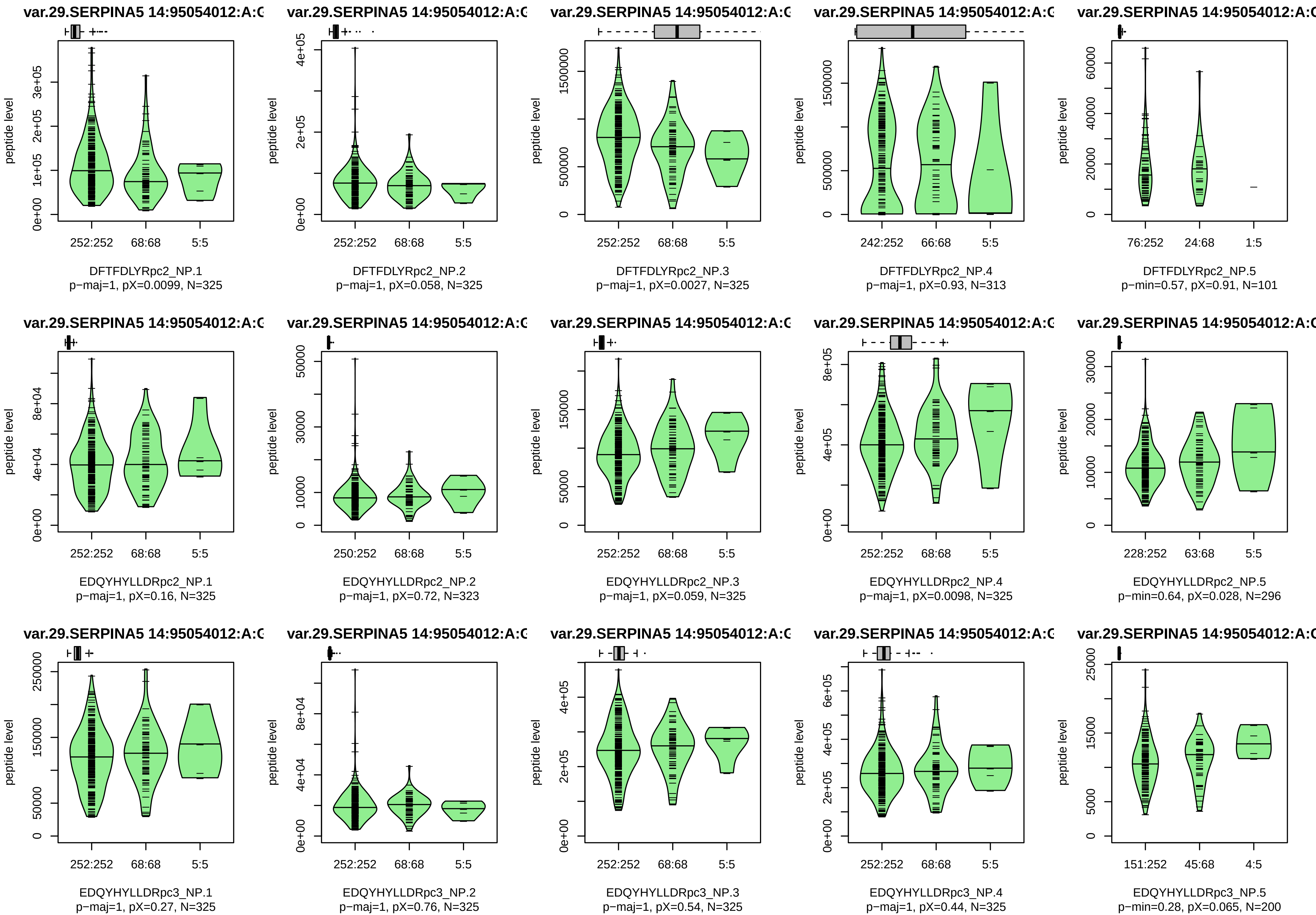

var.29.SERPINA5 14:95054012:A:G
peptide level
252:252
68:68
5:5
DFTFDLYRpc2_NP.1
p−maj=1, pX=0.0099, N=325
var.29.SERPINA5 14:95054012:A:G
peptide level
252:252
68:68
5:5
DFTFDLYRpc2_NP.2
p−maj=1, pX=0.058, N=325
var.29.SERPINA5 14:95054012:A:G
peptide level
252:252
68:68
5:5
DFTFDLYRpc2_NP.3
p−maj=1, pX=0.0027, N=325
var.29.SERPINA5 14:95054012:A:G
peptide level
242:252
66:68
5:5
DFTFDLYRpc2_NP.4
p−maj=1, pX=0.93, N=313
var.29.SERPINA5 14:95054012:A:G
peptide level
76:252
24:68
1:5
DFTFDLYRpc2_NP.5
p−min=0.57, pX=0.91, N=101
var.29.SERPINA5 14:95054012:A:G
peptide level
252:252
68:68
5:5
EDQYHYLLDRpc2_NP.1
p−maj=1, pX=0.16, N=325
var.29.SERPINA5 14:95054012:A:G
peptide level
250:252
68:68
5:5
EDQYHYLLDRpc2_NP.2
p−maj=1, pX=0.72, N=323
var.29.SERPINA5 14:95054012:A:G
peptide level
252:252
68:68
5:5
EDQYHYLLDRpc2_NP.3
p−maj=1, pX=0.059, N=325
var.29.SERPINA5 14:95054012:A:G
peptide level
252:252
68:68
5:5
EDQYHYLLDRpc2_NP.4
p−maj=1, pX=0.0098, N=325
var.29.SERPINA5 14:95054012:A:G
peptide level
228:252
63:68
5:5
EDQYHYLLDRpc2_NP.5
p−min=0.64, pX=0.028, N=296
var.29.SERPINA5 14:95054012:A:G
peptide level
252:252
68:68
5:5
EDQYHYLLDRpc3_NP.1
p−maj=1, pX=0.27, N=325
var.29.SERPINA5 14:95054012:A:G
peptide level
252:252
68:68
5:5
EDQYHYLLDRpc3_NP.2
p−maj=1, pX=0.76, N=325
var.29.SERPINA5 14:95054012:A:G
peptide level
252:252
68:68
5:5
EDQYHYLLDRpc3_NP.3
p−maj=1, pX=0.54, N=325
var.29.SERPINA5 14:95054012:A:G
peptide level
252:252
68:68
5:5
EDQYHYLLDRpc3_NP.4
p−maj=1, pX=0.44, N=325
var.29.SERPINA5 14:95054012:A:G
peptide level
151:252
45:68
4:5
EDQYHYLLDRpc3_NP.5
p−min=0.28, pX=0.065, N=200

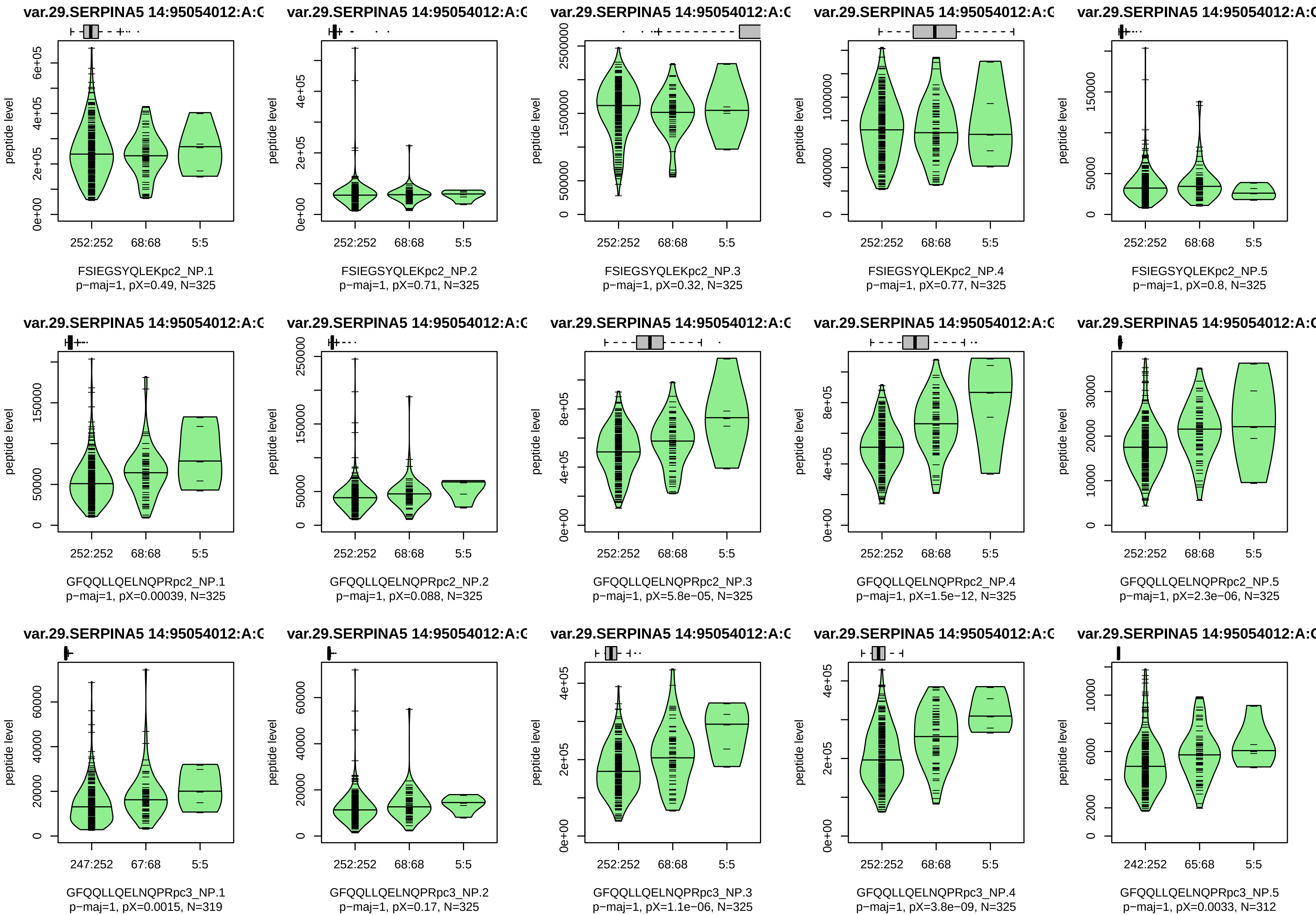
var.29.SERPINA5 14:95054012:A:
peptide level
252:252
68:68
5:5
FSIEGSYQLEKpc2_NP.1
p−maj=1, pX=0.49, N=325
FSIEGSYQLEKpc2_NP.2
p−maj=1, pX=0.71, N=325
FSIEGSYQLEKpc2_NP.3
p−maj=1, pX=0.32, N=325
FSIEGSYQLEKpc2_NP.4
p−maj=1, pX=0.77, N=325
FSIEGSYQLEKpc2_NP.5
p−maj=1, pX=0.8, N=325
GFQQLLQELNQPRpc2_NP.1
p−maj=1, pX=0.00039, N=325
GFQQLLQELNQPRpc2_NP.2
p−maj=1, pX=0.088, N=325
GFQQLLQELNQPRpc2_NP.3
p−maj=1, pX=5.8e−05, N=325
GFQQLLQELNQPRpc2_NP.4
p−maj=1, pX=1.5e−12, N=325
GFQQLLQELNQPRpc2_NP.5
p−maj=1, pX=2.3e−06, N=325
247:252
67:68
GFQQLLQELNQPRpc3_NP.1
p−maj=1, pX=0.0015, N=319
GFQQLLQELNQPRpc3_NP.2
p−maj=1, pX=0.17, N=325
GFQQLLQELNQPRpc3_NP.3
p−maj=1, pX=1.1e−06, N=325
GFQQLLQELNQPRpc3_NP.4
p−maj=1, pX=3.8e−09, N=325
242:252
65:68
GFQQLLQELNQPRpc3_NP.5
p−maj=1, pX=0.0033, N=312

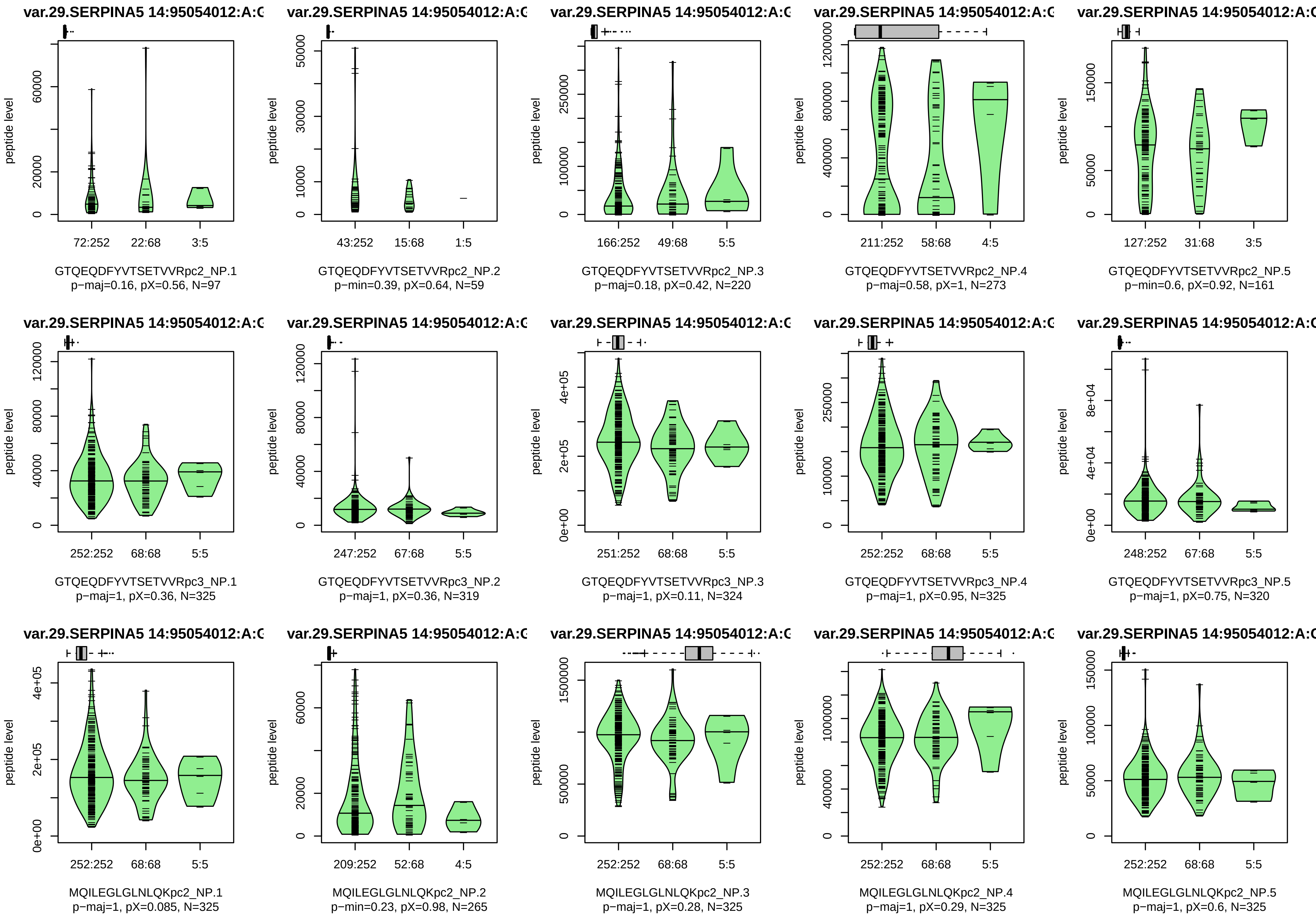
var.29.SERPINA5 14:95054012:A:G
peptide level
72:252
22:68
3:5
GTQEQDFYVTSETVVRpc2_NP.1
p−maj=0.16, pX=0.56, N=97
43:252
15:68
1:5
GTQEQDFYVTSETVVRpc2_NP.2
p−min=0.39, pX=0.64, N=59
166:252
49:68
5:5
GTQEQDFYVTSETVVRpc2_NP.3
p−maj=0.18, pX=0.42, N=220
211:252
58:68
4:5
GTQEQDFYVTSETVVRpc2_NP.4
p−maj=0.58, pX=1, N=273
127:252
31:68
3:5
GTQEQDFYVTSETVVRpc2_NP.5
p−min=0.6, pX=0.92, N=161
252:252
68:68
5:5
GTQEQDFYVTSETVVRpc3_NP.1
p−maj=1, pX=0.36, N=325
247:252
67:68
5:5
GTQEQDFYVTSETVVRpc3_NP.2
p−maj=1, pX=0.36, N=319
251:252
68:68
5:5
GTQEQDFYVTSETVVRpc3_NP.3
p−maj=1, pX=0.11, N=324
252:252
68:68
5:5
GTQEQDFYVTSETVVRpc3_NP.4
p−maj=1, pX=0.95, N=325
248:252
67:68
5:5
GTQEQDFYVTSETVVRpc3_NP.5
p−maj=1, pX=0.75, N=320
252:252
68:68
5:5
MQILEGLGLNLQKpc2_NP.1
p−maj=1, pX=0.085, N=325
209:252
52:68
4:5
MQILEGLGLNLQKpc2_NP.2
p−min=0.23, pX=0.98, N=265
252:252
68:68
5:5
MQILEGLGLNLQKpc2_NP.3
p−maj=1, pX=0.28, N=325
252:252
68:68
5:5
MQILEGLGLNLQKpc2_NP.4
p−maj=1, pX=0.29, N=325
252:252
68:68
5:5
MQILEGLGLNLQKpc2_NP.5
p−maj=1, pX=0.6, N=325

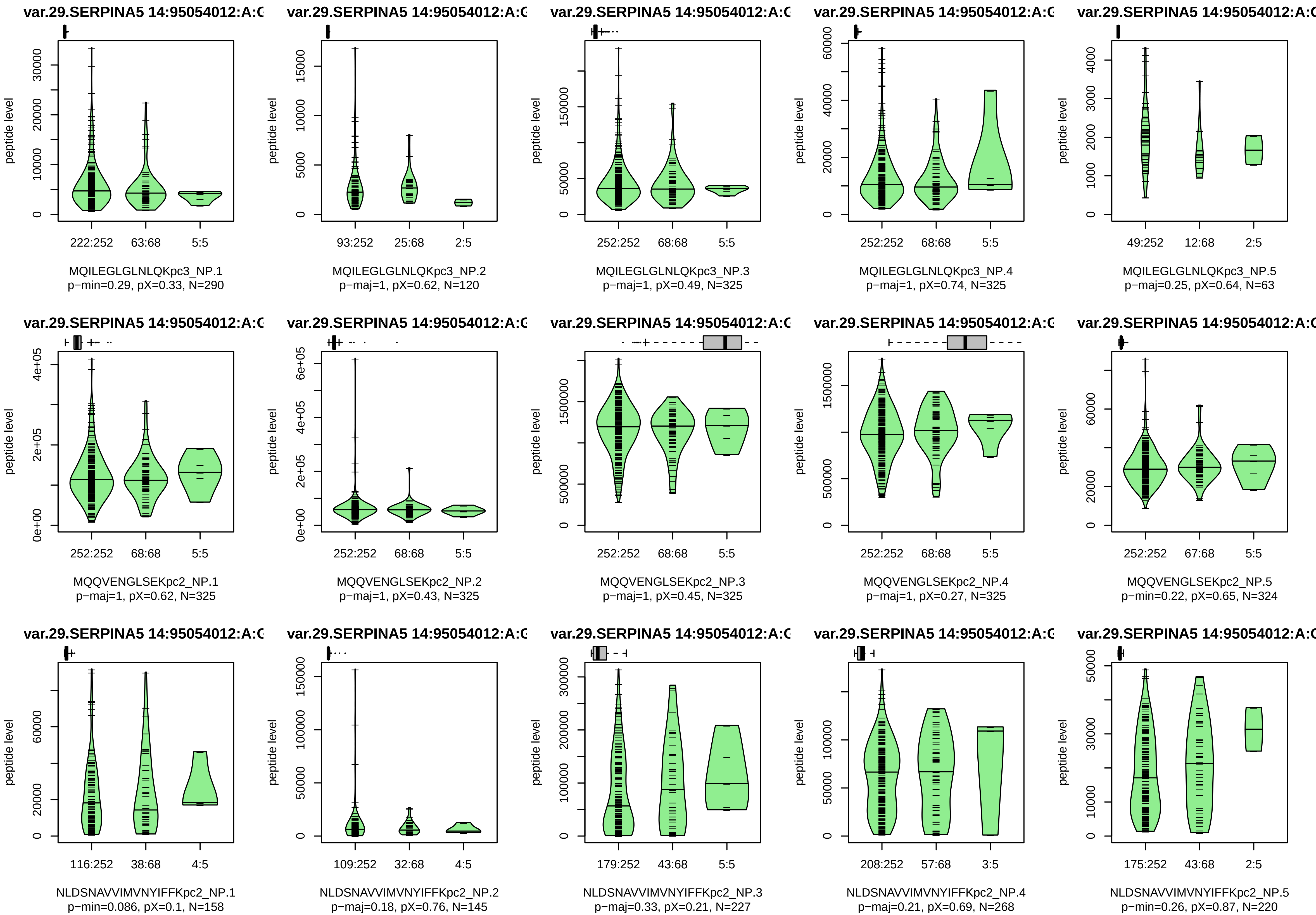
var.29.SERPINA5 14:95054012:A:G
peptide level
0
10000
20000
30000
222:252
63:68
5:5
MQILEGLGLNLQKpc3_NP.1
p−min=0.29, pX=0.33, N=290
var.29.SERPINA5 14:95054012:A:G
peptide level
0
5000
10000
15000
93:252
25:68
2:5
MQILEGLGLNLQKpc3_NP.2
p−maj=1, pX=0.62, N=120
var.29.SERPINA5 14:95054012:A:G
peptide level
0
50000
150000
252:252
68:68
5:5
MQILEGLGLNLQKpc3_NP.3
p−maj=1, pX=0.49, N=325
var.29.SERPINA5 14:95054012:A:G
peptide level
0
20000
40000
60000
252:252
68:68
5:5
MQILEGLGLNLQKpc3_NP.4
p−maj=1, pX=0.74, N=325
var.29.SERPINA5 14:95054012:A:G
peptide level
0
1000
2000
3000
4000
49:252
12:68
2:5
MQILEGLGLNLQKpc3_NP.5
p−maj=0.25, pX=0.64, N=63
var.29.SERPINA5 14:95054012:A:G
peptide level
0e+00
2e+05
4e+05
252:252
68:68
5:5
MQQVENGLSEKpc2_NP.1
p−maj=1, pX=0.62, N=325
var.29.SERPINA5 14:95054012:A:G
peptide level
0e+00
2e+05
4e+05
6e+05
252:252
68:68
5:5
MQQVENGLSEKpc2_NP.2
p−maj=1, pX=0.43, N=325
var.29.SERPINA5 14:95054012:A:G
peptide level
0
500000
1500000
252:252
68:68
5:5
MQQVENGLSEKpc2_NP.3
p−maj=1, pX=0.45, N=325
var.29.SERPINA5 14:95054012:A:G
peptide level
0
500000
1500000
252:252
68:68
5:5
MQQVENGLSEKpc2_NP.4
p−maj=1, pX=0.27, N=325
var.29.SERPINA5 14:95054012:A:G
peptide level
0
20000
60000
252:252
67:68
5:5
MQQVENGLSEKpc2_NP.5
p−min=0.22, pX=0.65, N=324
var.29.SERPINA5 14:95054012:A:G
peptide level
0
20000
60000
116:252
38:68
4:5
NLDSNAVVIMVNYIFFKpc2_NP.1
p−min=0.086, pX=0.1, N=158
var.29.SERPINA5 14:95054012:A:G
peptide level
0
50000
100000
150000
109:252
32:68
4:5
NLDSNAVVIMVNYIFFKpc2_NP.2
p−maj=0.18, pX=0.76, N=145
var.29.SERPINA5 14:95054012:A:G
peptide level
0
100000
200000
300000
179:252
43:68
5:5
NLDSNAVVIMVNYIFFKpc2_NP.3
p−maj=0.33, pX=0.21, N=227
var.29.SERPINA5 14:95054012:A:G
peptide level
0
50000
100000
208:252
57:68
3:5
NLDSNAVVIMVNYIFFKpc2_NP.4
p−maj=0.21, pX=0.69, N=268
var.29.SERPINA5 14:95054012:A:G
peptide level
0
10000
30000
50000
175:252
43:68
2:5
NLDSNAVVIMVNYIFFKpc2_NP.5
p−min=0.26, pX=0.87, N=220

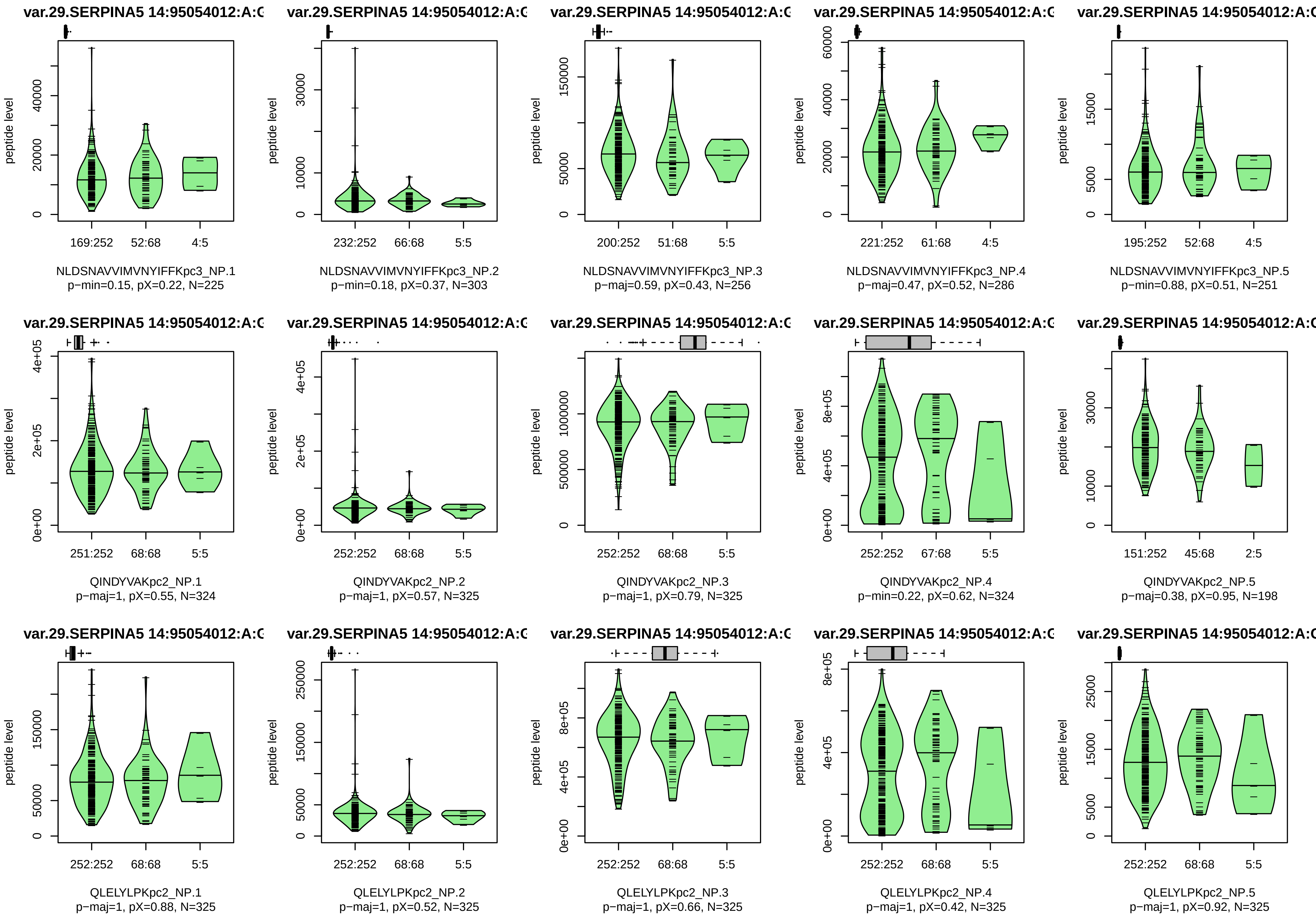
var.29.SERPINA5 14:95054012:A:G
peptide level
169:252
52:68
4:5
NLDSNAVVIMVNYIFFKpc3_NP.1
p−min=0.15, pX=0.22, N=225
232:252
66:68
5:5
NLDSNAVVIMVNYIFFKpc3_NP.2
p−min=0.18, pX=0.37, N=303
200:252
51:68
5:5
NLDSNAVVIMVNYIFFKpc3_NP.3
p−maj=0.59, pX=0.43, N=256
221:252
61:68
4:5
NLDSNAVVIMVNYIFFKpc3_NP.4
p−maj=0.47, pX=0.52, N=286
195:252
52:68
4:5
NLDSNAVVIMVNYIFFKpc3_NP.5
p−min=0.88, pX=0.51, N=251
251:252
68:68
5:5
QINDYVAKpc2_NP.1
p−maj=1, pX=0.55, N=324
252:252
68:68
5:5
QINDYVAKpc2_NP.2
p−maj=1, pX=0.57, N=325
252:252
68:68
5:5
QINDYVAKpc2_NP.3
p−maj=1, pX=0.79, N=325
252:252
67:68
5:5
QINDYVAKpc2_NP.4
p−min=0.22, pX=0.62, N=324
151:252
45:68
2:5
QINDYVAKpc2_NP.5
p−maj=0.38, pX=0.95, N=198
252:252
68:68
5:5
QLELYLPKpc2_NP.1
p−maj=1, pX=0.88, N=325
QLELYLPKpc2_NP.2
p−maj=1, pX=0.52, N=325
QLELYLPKpc2_NP.3
p−maj=1, pX=0.66, N=325
QLELYLPKpc2_NP.4
p−maj=1, pX=0.42, N=325
QLELYLPKpc2_NP.5
p−maj=1, pX=0.92, N=325

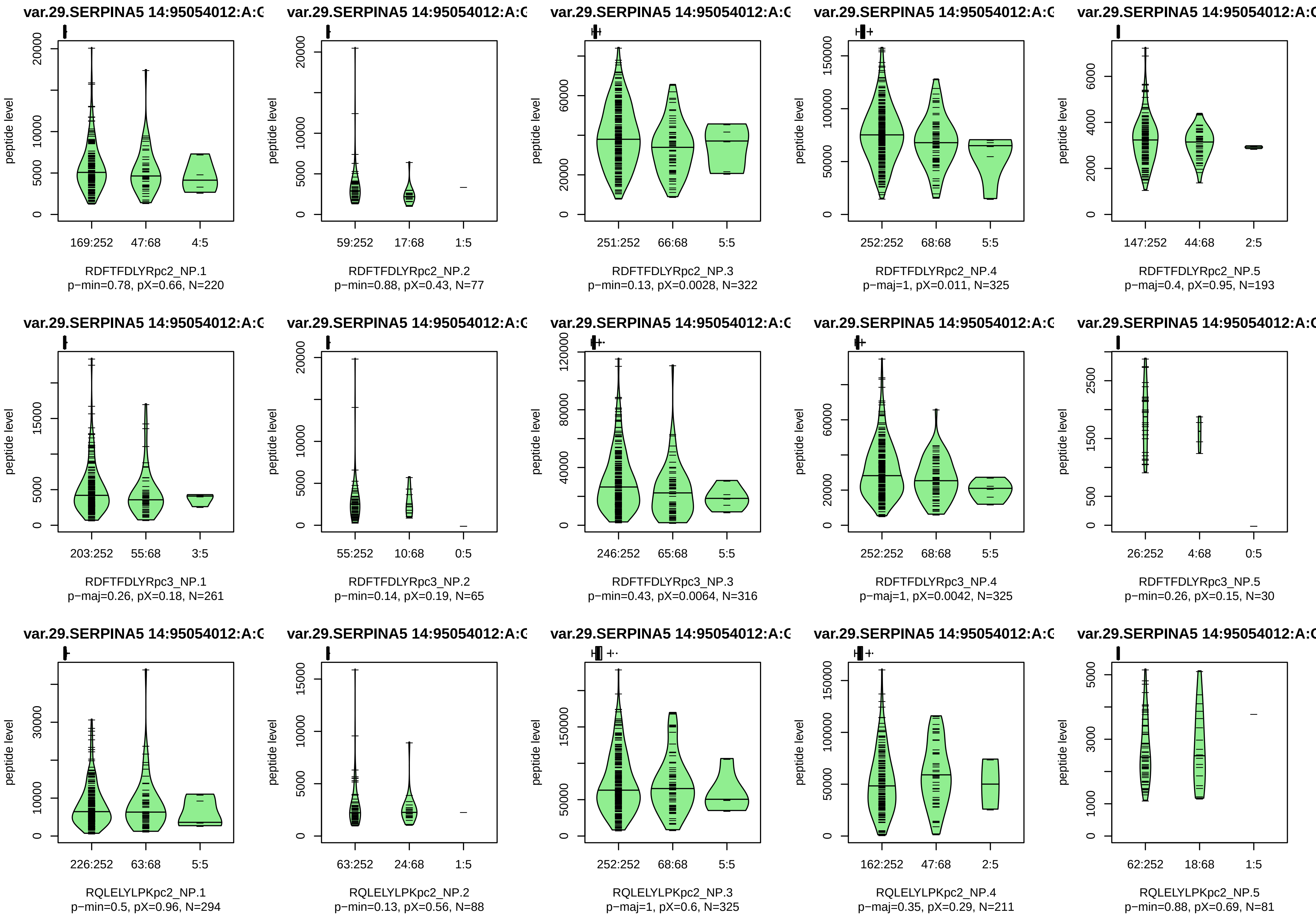
var.29.SERPINA5 14:95054012:A:G
peptide level
169:252
47:68
4:5
RDFTFDLYRpc2_NP.1
p−min=0.78, pX=0.66, N=220
var.29.SERPINA5 14:95054012:A:G
peptide level
59:252
17:68
1:5
RDFTFDLYRpc2_NP.2
p−min=0.88, pX=0.43, N=77
var.29.SERPINA5 14:95054012:A:G
peptide level
251:252
66:68
5:5
RDFTFDLYRpc2_NP.3
p−min=0.13, pX=0.0028, N=322
var.29.SERPINA5 14:95054012:A:G
peptide level
252:252
68:68
5:5
RDFTFDLYRpc2_NP.4
p−maj=1, pX=0.011, N=325
var.29.SERPINA5 14:95054012:A:G
peptide level
147:252
44:68
2:5
RDFTFDLYRpc2_NP.5
p−maj=0.4, pX=0.95, N=193
var.29.SERPINA5 14:95054012:A:G
peptide level
203:252
55:68
3:5
RDFTFDLYRpc3_NP.1
p−maj=0.26, pX=0.18, N=261
var.29.SERPINA5 14:95054012:A:G
peptide level
55:252
10:68
0:5
RDFTFDLYRpc3_NP.2
p−min=0.14, pX=0.19, N=65
var.29.SERPINA5 14:95054012:A:G
peptide level
246:252
65:68
5:5
RDFTFDLYRpc3_NP.3
p−min=0.43, pX=0.0064, N=316
var.29.SERPINA5 14:95054012:A:G
peptide level
252:252
68:68
5:5
RDFTFDLYRpc3_NP.4
p−maj=1, pX=0.0042, N=325
var.29.SERPINA5 14:95054012:A:G
peptide level
26:252
4:68
0:5
RDFTFDLYRpc3_NP.5
p−min=0.26, pX=0.15, N=30
var.29.SERPINA5 14:95054012:A:G
peptide level
226:252
63:68
5:5
RQLELYLPKpc2_NP.1
p−min=0.5, pX=0.96, N=294
var.29.SERPINA5 14:95054012:A:G
peptide level
63:252
24:68
1:5
RQLELYLPKpc2_NP.2
p−min=0.13, pX=0.56, N=88
var.29.SERPINA5 14:95054012:A:G
peptide level
252:252
68:68
5:5
RQLELYLPKpc2_NP.3
p−maj=1, pX=0.6, N=325
var.29.SERPINA5 14:95054012:A:G
peptide level
162:252
47:68
2:5
RQLELYLPKpc2_NP.4
p−maj=0.35, pX=0.29, N=211
var.29.SERPINA5 14:95054012:A:G
peptide level
62:252
18:68
1:5
RQLELYLPKpc2_NP.5
p−min=0.88, pX=0.69, N=81

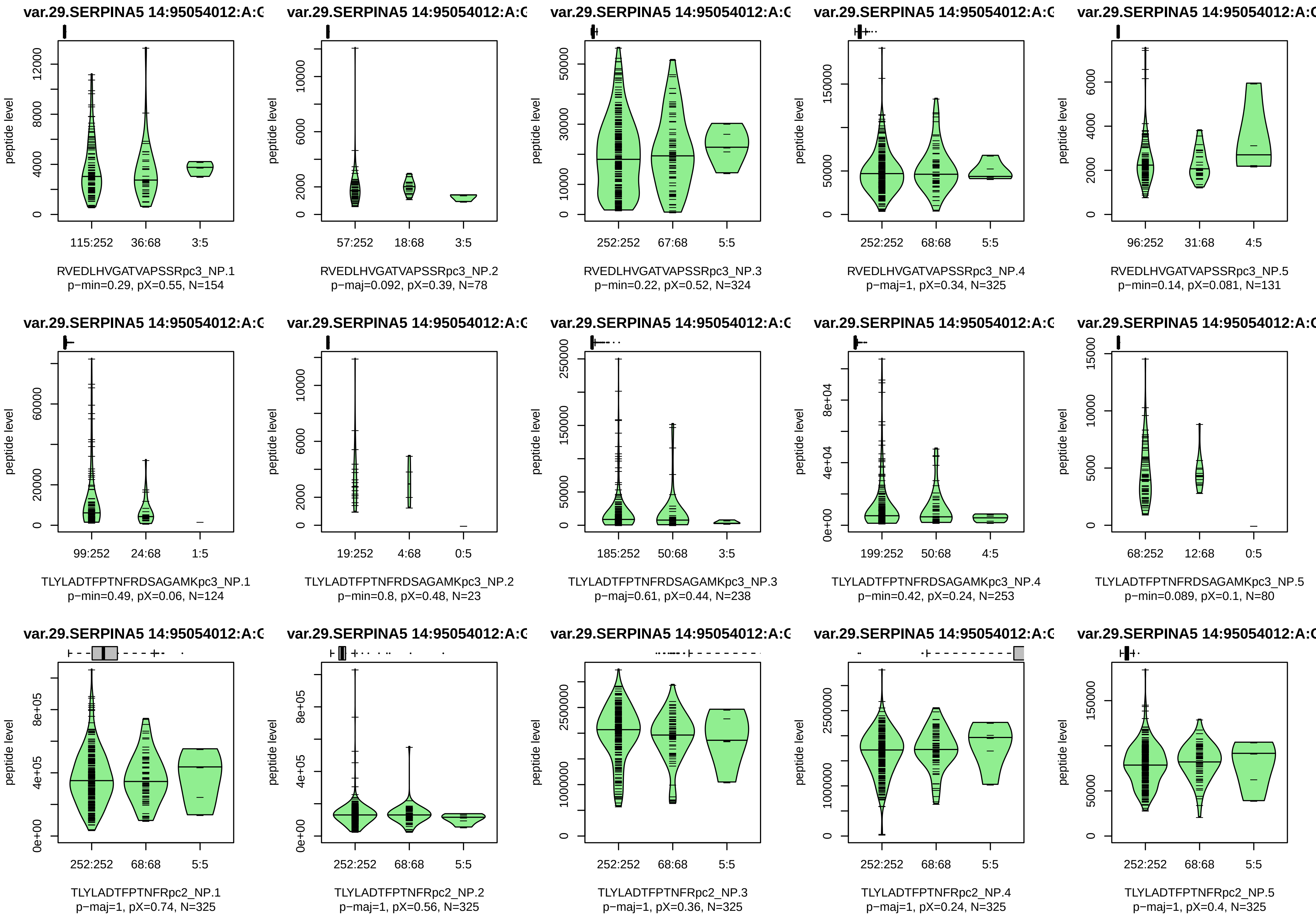
var.29.SERPINA5 14:95054012:A:G
peptide level
0
4000
8000
12000
115:252
36:68
3:5
RVEDLHVGATVAPSSRpc3_NP.1
p−min=0.29, pX=0.55, N=154
var.29.SERPINA5 14:95054012:A:G
peptide level
0
2000
6000
10000
57:252
18:68
3:5
RVEDLHVGATVAPSSRpc3_NP.2
p−maj=0.092, pX=0.39, N=78
var.29.SERPINA5 14:95054012:A:G
peptide level
0
10000
30000
50000
252:252
67:68
5:5
RVEDLHVGATVAPSSRpc3_NP.3
p−min=0.22, pX=0.52, N=324
var.29.SERPINA5 14:95054012:A:G
peptide level
0
50000
150000
252:252
68:68
5:5
RVEDLHVGATVAPSSRpc3_NP.4
p−maj=1, pX=0.34, N=325
var.29.SERPINA5 14:95054012:A:G
peptide level
0
2000
4000
6000
96:252
31:68
4:5
RVEDLHVGATVAPSSRpc3_NP.5
p−min=0.14, pX=0.081, N=131
var.29.SERPINA5 14:95054012:A:G
peptide level
0
20000
60000
99:252
24:68
1:5
TLYLADTFPTNFRDSAGAMKpc3_NP.1
p−min=0.49, pX=0.06, N=124
var.29.SERPINA5 14:95054012:A:G
peptide level
0
2000
6000
10000
19:252
4:68
0:5
TLYLADTFPTNFRDSAGAMKpc3_NP.2
p−min=0.8, pX=0.48, N=23
var.29.SERPINA5 14:95054012:A:G
peptide level
0
50000
150000
250000
185:252
50:68
3:5
TLYLADTFPTNFRDSAGAMKpc3_NP.3
p−maj=0.61, pX=0.44, N=238
var.29.SERPINA5 14:95054012:A:G
peptide level
0e+00
4e+04
8e+04
199:252
50:68
4:5
TLYLADTFPTNFRDSAGAMKpc3_NP.4
p−min=0.42, pX=0.24, N=253
var.29.SERPINA5 14:95054012:A:G
peptide level
0
5000
10000
15000
68:252
12:68
0:5
TLYLADTFPTNFRDSAGAMKpc3_NP.5
p−min=0.089, pX=0.1, N=80
var.29.SERPINA5 14:95054012:A:G
peptide level
0e+00
4e+05
8e+05
252:252
68:68
5:5
TLYLADTFPTNFRpc2_NP.1
p−maj=1, pX=0.74, N=325
var.29.SERPINA5 14:95054012:A:G
peptide level
0e+00
4e+05
8e+05
252:252
68:68
5:5
TLYLADTFPTNFRpc2_NP.2
p−maj=1, pX=0.56, N=325
var.29.SERPINA5 14:95054012:A:G
peptide level
0
1000000
2500000
252:252
68:68
5:5
TLYLADTFPTNFRpc2_NP.3
p−maj=1, pX=0.36, N=325
var.29.SERPINA5 14:95054012:A:G
peptide level
0
1000000
2500000
252:252
68:68
5:5
TLYLADTFPTNFRpc2_NP.4
p−maj=1, pX=0.24, N=325
var.29.SERPINA5 14:95054012:A:G
peptide level
0
50000
150000
252:252
68:68
5:5
TLYLADTFPTNFRpc2_NP.5
p−maj=1, pX=0.4, N=325

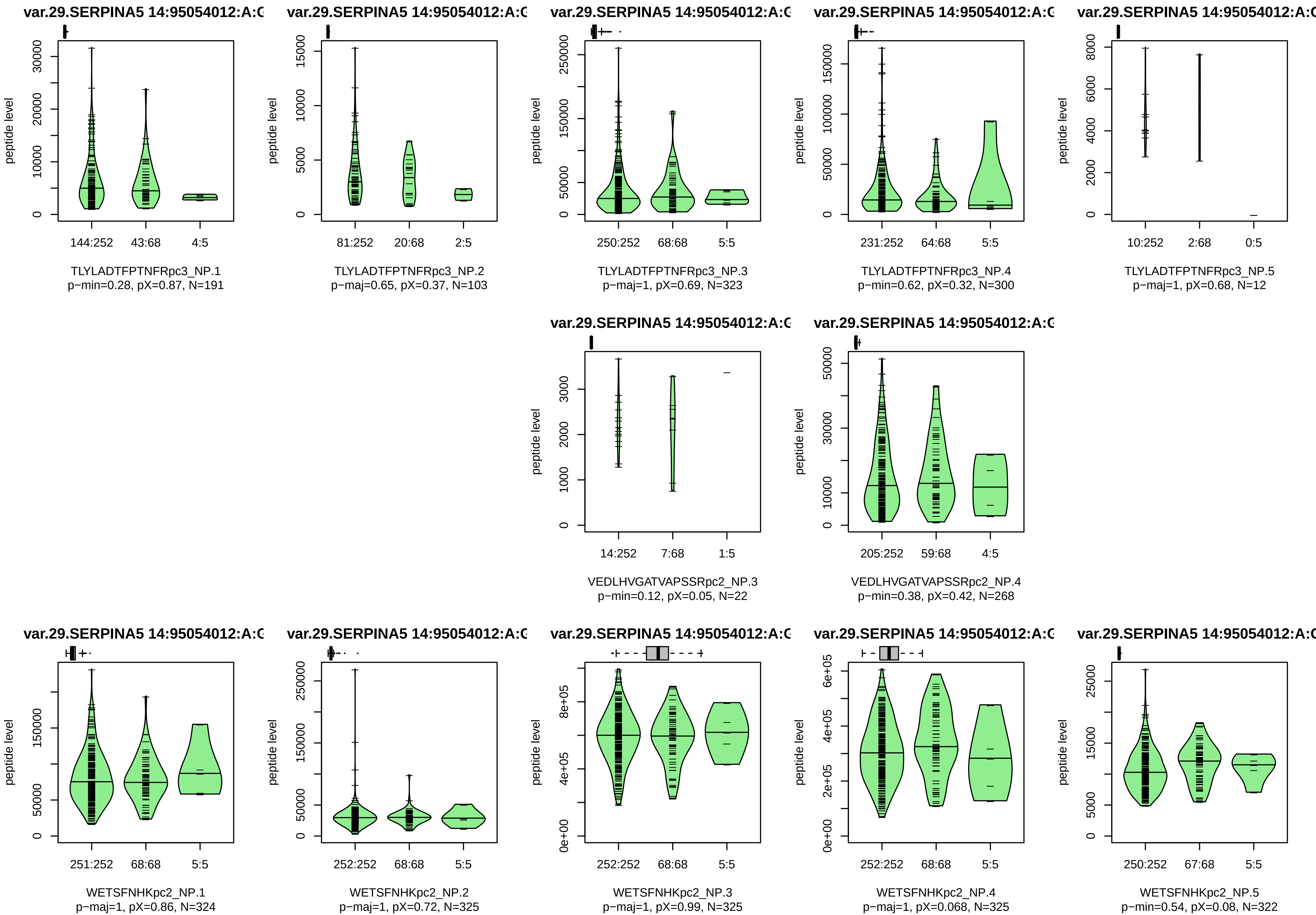

var.29.SERPINA5 14:95054012:A:G
peptide level
144:252
43:68
4:5
TLYLADTFPTNFRpc3_NP.1
p−min=0.28, pX=0.87, N=191
var.29.SERPINA5 14:95054012:A:G
peptide level
81:252
20:68
2:5
TLYLADTFPTNFRpc3_NP.2
p−maj=0.65, pX=0.37, N=103
var.29.SERPINA5 14:95054012:A:G
peptide level
250:252
68:68
5:5
TLYLADTFPTNFRpc3_NP.3
p−maj=1, pX=0.69, N=323
var.29.SERPINA5 14:95054012:A:G
peptide level
231:252
64:68
5:5
TLYLADTFPTNFRpc3_NP.4
p−min=0.62, pX=0.32, N=300
var.29.SERPINA5 14:95054012:A:G
peptide level
10:252
2:68
0:5
TLYLADTFPTNFRpc3_NP.5
p−maj=1, pX=0.68, N=12
var.29.SERPINA5 14:95054012:A:G
peptide level
14:252
7:68
1:5
VEDLHVGATVAPSSRpc2_NP.3
p−min=0.12, pX=0.05, N=22
var.29.SERPINA5 14:95054012:A:G
peptide level
205:252
59:68
4:5
VEDLHVGATVAPSSRpc2_NP.4
p−min=0.38, pX=0.42, N=268
var.29.SERPINA5 14:95054012:A:G
peptide level
251:252
68:68
5:5
WETSFNHKpc2_NP.1
p−maj=1, pX=0.86, N=324
var.29.SERPINA5 14:95054012:A:G
peptide level
252:252
68:68
5:5
WETSFNHKpc2_NP.2
p−maj=1, pX=0.72, N=325
var.29.SERPINA5 14:95054012:A:G
peptide level
252:252
68:68
5:5
WETSFNHKpc2_NP.3
p−maj=1, pX=0.99, N=325
var.29.SERPINA5 14:95054012:A:G
peptide level
252:252
68:68
5:5
WETSFNHKpc2_NP.4
p−maj=1, pX=0.068, N=325
var.29.SERPINA5 14:95054012:A:G
peptide level
250:252
67:68
5:5
WETSFNHKpc2_NP.5
p−min=0.54, pX=0.08, N=322

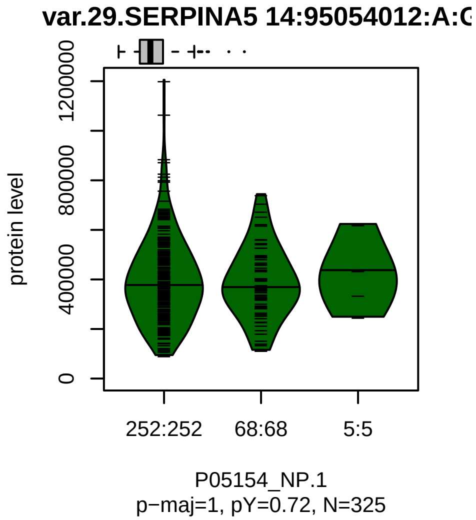

var.29.SERPINA5 14:95054012:A:G
protein level
252:252
68:68
5:5
P05154_NP.1
p−maj=1, pY=0.72, N=325

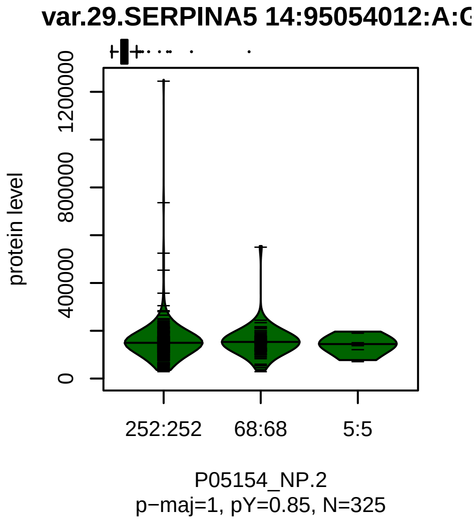

var.29.SERPINA5 14:95054012:A:G
protein level
252:252
68:68
5:5
P05154_NP.2
p−maj=1, pY=0.85, N=325

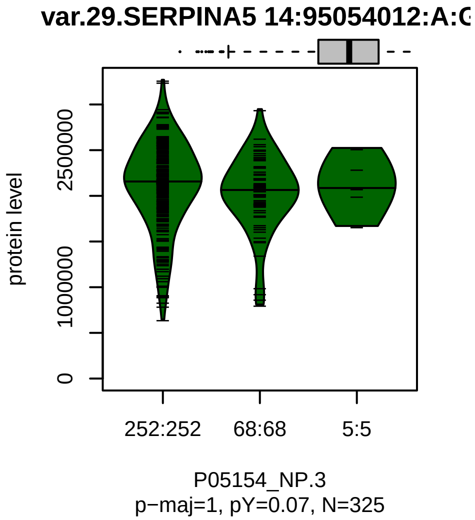

var.29.SERPINA5 14:95054012:A:G
protein level
252:252
68:68
5:5
P05154_NP.3
p−maj=1, pY=0.07, N=325

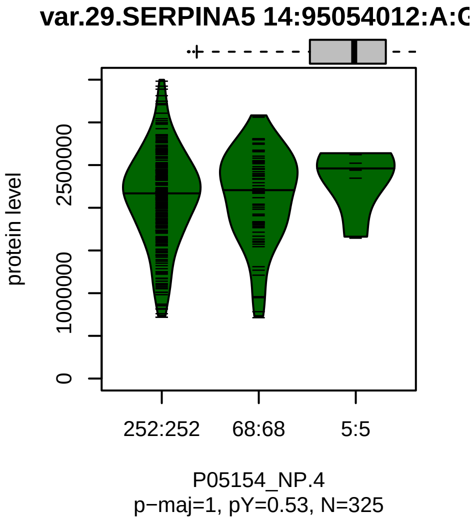

var.29.SERPINA5 14:95054012:A:G
protein level
252:252
68:68
5:5
P05154_NP.4
p−maj=1, pY=0.53, N=325

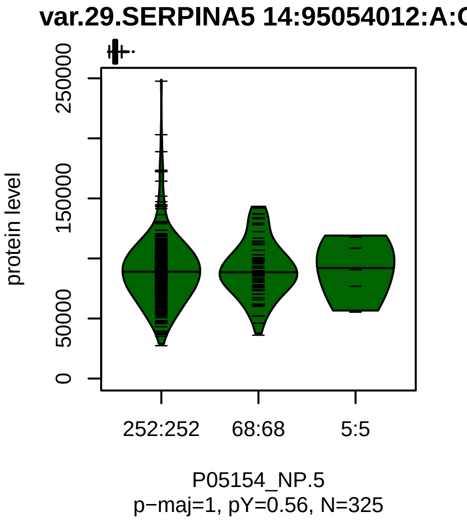

var.29.SERPINA5 14:95054012:A:G
protein level
252:252
68:68
5:5
P05154_NP.5
p−maj=1, pY=0.56, N=325

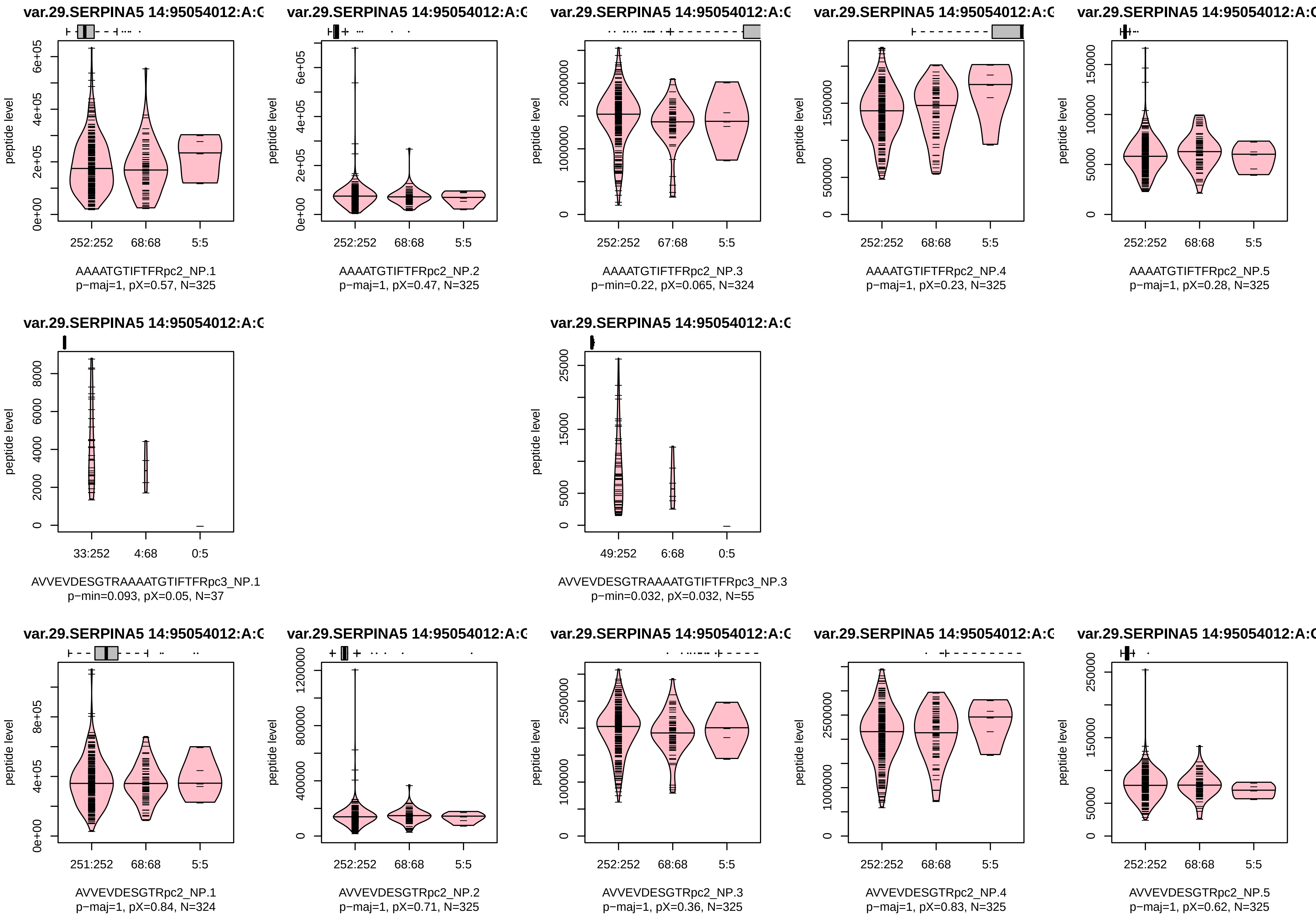
var.29.SERPINA5 14:95054012:A:G
peptide level
252:252
68:68
5:5
AAAATGTIFTFRpc2_NP.1
p−maj=1, pX=0.57, N=325
var.29.SERPINA5 14:95054012:A:G
252:252
68:68
5:5
AAAATGTIFTFRpc2_NP.2
p−maj=1, pX=0.47, N=325
var.29.SERPINA5 14:95054012:A:G
252:252
67:68
5:5
AAAATGTIFTFRpc2_NP.3
p−min=0.22, pX=0.065, N=324
var.29.SERPINA5 14:95054012:A:G
252:252
68:68
5:5
AAAATGTIFTFRpc2_NP.4
p−maj=1, pX=0.23, N=325
var.29.SERPINA5 14:95054012:A:G
252:252
68:68
5:5
AAAATGTIFTFRpc2_NP.5
p−maj=1, pX=0.28, N=325
var.29.SERPINA5 14:95054012:A:G
33:252
4:68
0:5
AVVEVDESGTRAAAATGTIFTFRpc3_NP.1
p−min=0.093, pX=0.05, N=37
var.29.SERPINA5 14:95054012:A:G
49:252
6:68
0:5
AVVEVDESGTRAAAATGTIFTFRpc3_NP.3
p−min=0.032, pX=0.032, N=55
var.29.SERPINA5 14:95054012:A:G
251:252
68:68
5:5
AVVEVDESGTRpc2_NP.1
p−maj=1, pX=0.84, N=324
var.29.SERPINA5 14:95054012:A:G
252:252
68:68
5:5
AVVEVDESGTRpc2_NP.2
p−maj=1, pX=0.71, N=325
var.29.SERPINA5 14:95054012:A:G
252:252
68:68
5:5
AVVEVDESGTRpc2_NP.3
p−maj=1, pX=0.36, N=325
var.29.SERPINA5 14:95054012:A:G
252:252
68:68
5:5
AVVEVDESGTRpc2_NP.4
p−maj=1, pX=0.83, N=325
var.29.SERPINA5 14:95054012:A:G
252:252
68:68
5:5
AVVEVDESGTRpc2_NP.5
p−maj=1, pX=0.62, N=325

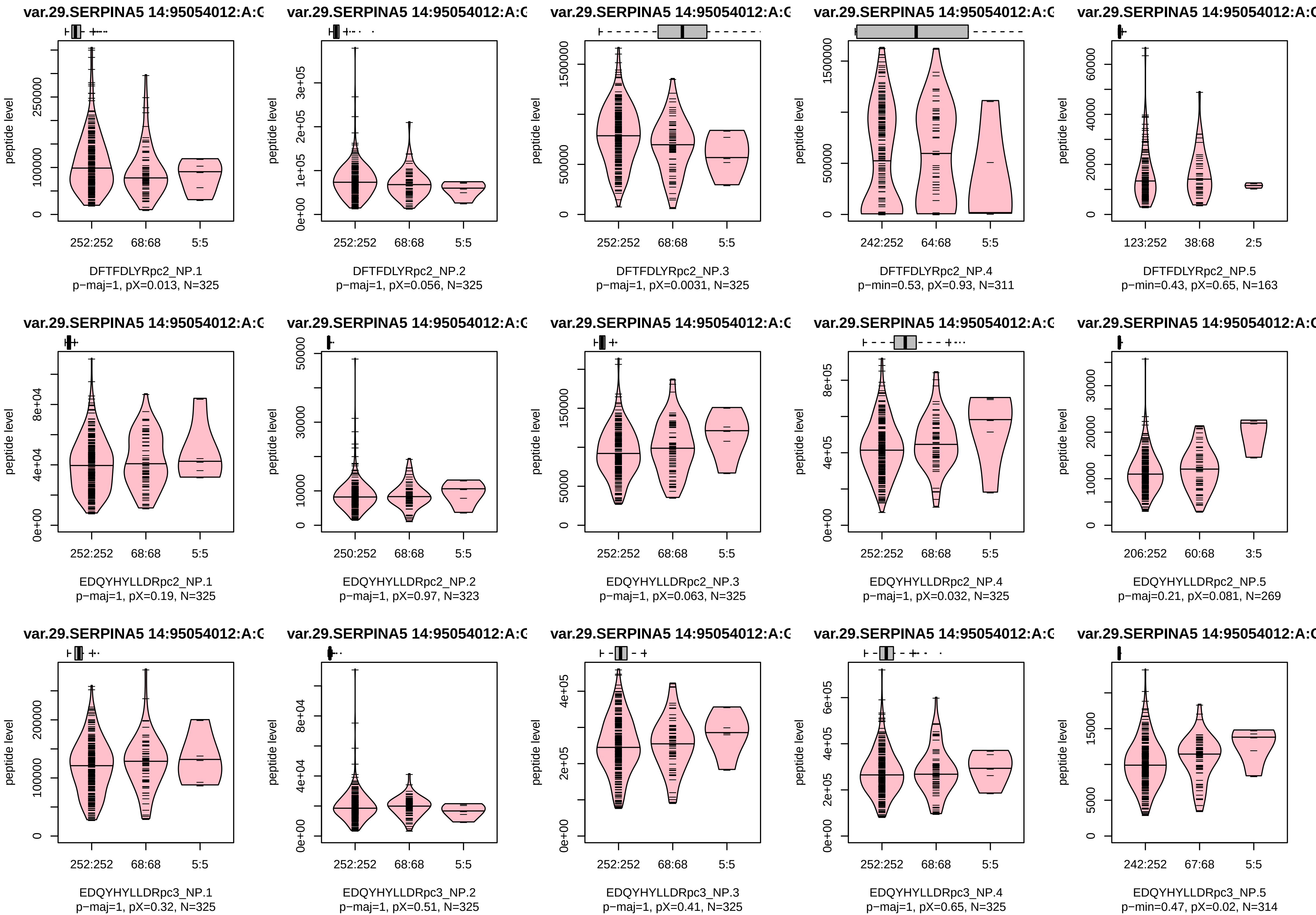

var.29.SERPINA5 14:95054012:A:G
peptide level
252:252
68:68
5:5
DFTFDLYRpc2_NP.1
p−maj=1, pX=0.013, N=325
var.29.SERPINA5 14:95054012:A:G
peptide level
252:252
68:68
5:5
DFTFDLYRpc2_NP.2
p−maj=1, pX=0.056, N=325
var.29.SERPINA5 14:95054012:A:G
peptide level
252:252
68:68
5:5
DFTFDLYRpc2_NP.3
p−maj=1, pX=0.0031, N=325
var.29.SERPINA5 14:95054012:A:G
peptide level
242:252
64:68
5:5
DFTFDLYRpc2_NP.4
p−min=0.53, pX=0.93, N=311
var.29.SERPINA5 14:95054012:A:G
peptide level
123:252
38:68
2:5
DFTFDLYRpc2_NP.5
p−min=0.43, pX=0.65, N=163
var.29.SERPINA5 14:95054012:A:G
peptide level
252:252
68:68
5:5
EDQYHYLLDRpc2_NP.1
p−maj=1, pX=0.19, N=325
var.29.SERPINA5 14:95054012:A:G
peptide level
250:252
68:68
5:5
[truncated: 584,883 more chars]
